# Safety Assessment of the CP4 EPSPS and NPTII Proteins in Eucalyptus

**DOI:** 10.1080/21645698.2023.2222436

**Published:** 2023-06-19

**Authors:** Dror Avisar, Shelly Azulay, Lorena Bombonato, Denise Carvalho, Heitor Dallapicolla, Carla de Souza, Anselmo dos Santos, Tatiane Dias, Maria Paula Galan, Milton Galvao, José Mateus Gonsalves, Esteban Gonzales, Rodrigo Graça, Sivan Livne, Reginaldo Mafia, Alexandre Manoeli, Mike May, Thaís Regina Drezza Menezes, Ana Cristina Pinheiro, Antonio Porto, Carolina Rocha, Ariane Schafer, Barry Schafer, Edival Zauza, William Silva

**Affiliations:** a R&D, FuturaGene Israel Ltd, Rehovot, Israel; b Suzano S.A. (FuturaGene - Biotech Division), Itapetininga, Brazil; c Suzano S.A. (Forest Management), SP, Brazil; d Schafer Scientific Solutions LLC, Indianapolis, USA; e W J Silva Consultoria Agricola S/C LTDA, Jardinópolis, Brazil

**Keywords:** CP4-EPSPS, eucalyptus, glyphosate-tolerance, genetically modified, saftey assesments

## Abstract

Glyphosate herbicide treatment is essential to sustainable Eucalyptus plantation management in Brazil. Eucalyptus is highly sensitive to glyphosate, and Suzano/FuturaGene has genetically modified eucalyptus to tolerate glyphosate, with the aim of both protecting eucalyptus trees from glyphosate application damage and improving weed management. This study presents the biosafety results of the glyphosate-tolerant eucalyptus event 751K032, which expresses the selection marker neomycin phosphotransferase II (NPTII) enzyme and CP4-EPSPS, a glyphosate-tolerant variant of plant 5-enolpyruvyl-shikimate−3-phosphate synthase enzyme. The transgenic genetically modified (GM) event 751K032 behaved in the plantations like conventional non-transgenic eucalyptus clone, FGN-K, and had no effects on arthropods and soil microorganisms. The engineered NPTII and CP4 EPSPS proteins were heat-labile, readily digestible, and according to the bioinformatics analyses, unlikely to cause an allergenic or toxic reaction in humans or animals. This assessment of the biosafety of the glyphosate-tolerant eucalyptus event 751K032 concludes that it is safe to be used for wood production.

## Introduction

The demand for round-wood is expected to double in the next decade from about 2 billion cubic meters to 4 billion cubic meters.^1^ To meet these needs, a quantum leap in sustainable production, trade and consumption of forest products is needed worldwide. Fast-growing eucalyptus plantations carry potential to meet increasing and diversifying wood demands whilst avoiding logging pressure on natural forests. More specifically, whilst the eucalyptus commercial plantation area represents only about 0.5% of the world’s forest cover (~22.57 million ha. globally^2^), it accounts for about 10% of the actual global round wood demands. This makes eucalyptus one of the most important species for future wood supply.

Brazil is currently the world leader in planted area of non-native eucalyptus plantations (22%), followed by China (20%) and India (17%).^3^ More importantly, Brazil is the leader in productivity, with an average mass accumulation of 40 m^3^ha^−1^ year^−1^. A decades-long history of investment in breeding and improvements in silvicultural practices in Brazil have also created a diverse clonal base of varieties that are highly adaptable to many biomes and relatively tolerant to biotic and abiotic stress.^4–8^ Long-term studies in Brazil have demonstrated that the eucalyptus plantations are highly sustainable, and very efficient at capturing CO_2_ and at low water consumption compared to other species. Furthermore, under the norms of the Brazilian Forest Code, companies must commit over 20% of the land on each farm to native forest renewal, making this industry one of the leaders in degraded land restoration.^9–11^

Weed competition is one of the main unresolved challenges in eucalyptus plantation management, significantly reducing yield and increasing operational costs. Weed-associated yield loss caused by direct competition for key resources such as water, light and minerals in other crops is estimated at 40%.^12^ Glyphosate is widely used for weed control in eucalyptus, and because plantation establishment is only permitted by law on degraded pasture lands in Brazil, where the presence of weeds is high, it is applied over the entire area before planting, to control coppice regrowth and to control weeds between planted rows. This herbicide acts on the enzyme 5-enolpyruvyl-shikimate−3-phosphate-synthase (EPSPS), which catalyzes the reaction between shikimate−3-phosphate (S3P) and phosphoenolpyruvate (PEP) to form 5-enolpyruvylshikimate−3-phosphate (EPSP) and phosphate. Inhibition of EPSPS blocks the synthesis of aromatic amino acids essential for synthesizing proteins and some secondary metabolites. EPSPS is present in microorganisms and all plants but not in animals and is the sole target of glyphosate in plants.^13,14^ Conventional eucalyptus is highly sensitive to glyphosate,^15,16^ such that during application, herbicide drift between planted rows can cause 15–100% damage to young plantlets, significantly reducing yield and harvesting efficiency.^3^

To overcome this challenge, Suzano S.A. with its subsidiary FuturaGene developed a genetically modified (GM) glyphosate-tolerant eucalyptus event, 751K032, which is a transformed *Eucalyptus urophylla* hybrid clone – FGN-K. It carries the glyphosate-tolerant EPSPS enzyme from *Agrobacterium tumefaciens* strain CP4 (CP4-EPSPS). This homologue has a low affinity for glyphosate but a high affinity for PEP,^17,18^ thereby serving as a bypass to the endogenous EPSPS whenever glyphosate is applied. The GM eucalyptus event 751K032 displays a high level of tolerance to glyphosate at the suggested application dosage. It remains unresponsive to the herbicide even when exposed to doses as high as double the recommended level (Figure S01).In addition, it is kanamycin-resistant, due to expression of the selectable marker gene neomycin phosphotransferase II – *nptII*,^19,20^ which is found in more than 120 commercial GM events worldwide [https://www.isaaa.org/gmapprovaldatabase/gene/default.asp?GeneID=

18&Gene=nptII].Similar herbicide tolerant products in other crops were first commercialized three decades ago and were one of the first and most widely used applications of modern biotechnology tools in advanced sustainable modern agriculture.^21^ The event was recently approved for commercial use by the National Technical Commission of Biosafety (CTNBio) of Brazil (Official Gazette DOU 214, Nov 16^th^, 2021 – Section 1, page 8), after extensive lab, greenhouse, and field biosafety studies.

This paper presents data obtained from various safety assessment studies of the eucalyptus event 751K032 under field and lab conditions and concludes that it is safe for the environment and wood production. The event was found to be similar to the conventional clone based on various analyses such as agronomic, morphological, reproductive characteristics, and chemical composition.^22^ Field studies over three years demonstrated that cultivation of the event had no adverse effects on non-target organisms including arthropods and soil microbiota, and a study evaluating the effects of 751K032 and non-GM FGN-K pollen on the bee species *Apis mellifera* and *Scaptotrigona bipunctata* larvae and adults, found no differences in mortality and survival rates.^23^ Moreover, the CP4-EPSPS and NPTII proteins were heat-labile, readily digestible, and according to the bioinformatics analyses, are unlikely to elicit allergenic reactions. Both the CP4-EPSPS protein^24^and^25^ and the NPTII protein^26^ are safe for human and animal consumption and exposure to the environment.

## Materials and Methods

### Field Design

Eucalyptus event 751K032 and the wild type (wt) control clone FGN-K were planted in four sites in Brazil in a randomized complete-block design: two in the State of São Paulo (SP), one in the State of Bahia (BA) and one in the State of Maranhão (MA). Planting design was in square plots of 16 plants each. Five square plots of each event/clone were planted in blocks randomly distributed in the field among other plots of unrelated clones that were not part of the experiments (Figure S02).

### Quantitation of CP4-EPSPS and NPTII Proteins

Samples of young leaves (one of the two smallest leaves at the tip of a secondary branch in the middle of the canopy), mature leaves (one of the fully developed leaves near the same branch attachment) and stems (of the same branch) were collected from eucalyptus event 751K032 and the control, conventional wild-type (wt) clone FGN-K, 6, 12 and 30 months after planting. Floral buds and pollen were collected during the first flowering. All samples were analyzed in three biological and three technical triplicates. All samples were ground, using a Thermo Scientific Tissue Lyser II, and then lyophilized for 96 h at −56°C, using a Labconco FreeZone freeze dryer.

NPTII levels were quantified using the Agdia NPTII enzyme-linked immunosorbent assay (ELISA) Kits (PSP 73,000), following the manufacturer’s protocol. Samples were extracted from 0.03 g lyophilized tissue with 3 mL Agdia PEB 1× Extraction Buffer and then diluted (1:4). Each plate contained a six-point standard curve, created with Agdia Protein Standard for neomycin phosphotransferase II (LST73000).

CP4-EPSPS levels were quantified using Envirologix QualiPlate Kits for CP4-EPSPS (AP 010 NW V10), following the manufacturer’s protocol. Samples were extracted from 0.03 g lyophilized tissue with 3 mL 1×-Tris borate buffer (Trisma base 100 mM; Na_2_B_4_O_7_x10H_2_O 100 mM; MgCl_2_x6H_2_O 5 mM; Tween−20 0,05% (v/v), pH 7.8). Samples from young leaves, mature leaves and stems were then diluted 1:300, 1:400 and 1:200, respectively. Each plate contained a seven-point standard curve, built with MyBiosource CP4-EPSPS Recombinant *Agrobacterium sp*. 3-phosphoshikimate 1-carboxyvinyltransferase-aroA (MBS1422940).

ELISA plates were read on a ThermoScientific Multiskan Sky Microplate Spectrophotometer plate reader (620 nm and 450 nm). Data were analyzed using the SkanIt RE Version 5.0 software.

### Arthropod Collection and Analysis

Twelve arthropod samplings were conducted at each of the four farms over a period of three years. The 751K032 event plots (S06) were compared to eucalyptus wt control FGN-K plots (S07). Five arthropod sampling methods were applied in 5 random repeats of 16 square plots: (1) A modified “beating sheet/net” in which the branches were shaken for 30 s inside a plastic bag (10 samples per plot). The branch shaking method was used only twice, at ages 6 and 9 months, before the trees were too high to shake the branches. This method effectively detects fewer active species in the leaves of eucalyptus trees, like hemipterans. (2) To count epigean species, pitfall traps (10 cm-diameter, 15 cm-high, filled with 5 cm insect-preserving solution (1–2% detergent and 4% formaldehyde) were placed at the center of each plot for 72 h. (3) To count flying species, adhesive cards (adhesive sheets of attractive yellow color, 14 × 23 cm – (ISCA Technologies, CA, USA), were placed at the center of each plot at the height of the treetops – for 72 h). (4) Soil collection (10 samples per plot, 10 cm in diameter, 5 cm in depth): A Berlese-Tüllgren funnel^27^ was used to extract organisms from soil. (5) Litter collection (5 samples per plot, 25 cm^2^): The Winkler extractor method^28,29^ was used to extract organisms from the litter.

Organisms obtained using the 5 collection methods were preserved in 70% ethanol and 5% glycerin solution for later analyses and classification. The classification into different taxa was done by comparison with reference collections or by consultation of specific literature.

The arthropod community was analyzed using the computer program DivEs (version 4.0), which determines the Shannon ecological diversity index (H’). The H’ index indicates the diversity of species, by considering the richness (number of species) and abundance of each species. The results obtained for H’ were submitted to analysis of variance and the means obtained were compared by the Agricolae package (version 1.3.5) for R language (version 4.2.1) using the Tukey’s Test, at 5% of significance.

### Microbial Community Analysis

Microbial diversity and density studies were performed 30 months after planting. Soil samples were collected using a clean auger to 15 cm depth, at locations that were cleared of all weed or plant residues. Samples were sealed in plastic bags and kept in ice and/or at 4°C for up to 72 h before analysis.

To evaluate the microbial density, 1.0 g of each soil sample was added to 10 ml phosphate-buffered saline (PBS) (NaCl 4 g, KCl 0.1 g, Na _4_ HPO _4_ 0.72 g, KH _2_ PO _4_ 0.12 g and pH 7.4 to 1 L of distilled water) and centrifuged at 140 rpm for 1 h. To quantify the bacterial community, 100 µL of various sample dilutions were then seeded on 5% TSB culture medium (Tryptone Soy Agar – HIMEDIA®: Hydrolyzed Casein 1.5%, Papaya Digested Soybean 0.5%, NaCl 0.5%, Agar 1.5%) supplemented with 50 g/ml Derosal (carbendazim – chemical group Benzimidazole) and incubated at 28°C for up to 24 h. To evaluate the fungal community, 100 µL of various sample dilutions were seeded on PDA culture medium (Potato Dextrose Agar – HIMEDIA®: Potato Infusion 20%, Dextrose 2.0%, Agar 1.5%) supplemented with 50 g/ml tetracycline, and incubated at 28°C for up to 96 h. After growth, microbial density was estimated and presented as Log_10_ of CFU/g soil. Statistical analysis was performed by ANOVA and means (5 biological replicates per treatment) were compared using the Tukey’s test.

To evaluate the microbial diversity, total DNA was extracted from 0.25 g soil, using the “DNeasyPowerSoil Kit®” (Qiagen), according to the manufacturer’s instructions. An Illumina DNA library was prepared as described.^30^ Bacterial 16S rRNA was amplified with the primers V3 and V4 (V3-341F: 5”-CC TAC GGG NGG CWG CAG−3‘ and V4-805 R: 5’-GAC TAC HVG GGT ATC TAA TCC− 3‘). Fungal rDNA (gene 5.8S rRNA partial, ITS2 and gene 28S rRNA partial) were amplified with the forward primer ITS3 (5’-GCA TCG ATG AAG AAC GCA GC−3‘) and reverse primer ITS4 (5’-TCC TCC GCT TAT TGA TAT GC−3”). Each sample was marked with a unique primer tag.^31,32^ NovaSeq reads (0.03 Mb/sample, 250PE) were analyzed using QIIME software,^33^ based on the SILVA 132 rRNA database,^34^ and evaluated for quality. The search parameters were based on similarity, where the greater the similarity, the lower the identified taxonomic level. Sequence similarity analysis was performed according to the system recommendations (domain >0%, phylum > 75%, class > 85%, order > 91%, family > 92%, genus > 95%, species > 97% and lineage = 100%). The alpha-diversity (Chao−1 richness estimator, Shannon-Wiener and Simpson diversity indices) of the microbial community in the different samples was calculated using the “alpha diversity” tool from the QIIME pipeline.

Principal coordinate analysis (PCoA) was used to compare groups of samples based on phylogenetic and count-based distance metrics.^35,36^

### Allergenicity, Toxicity and Digestibility

#### Bioinformatics Evaluation for Potential Allergenicity

The FAST amino acid sequences of NPTII and CP4-EPSPS proteins (Figure S03) were analyzed using the search tools of the COMPARE allergen database, which is managed by the HESI (Health and Environmental Sciences Institute) Protein Allergens, Toxins and Bioinformatics Committee (https://comparedatabase.org/about-compare-database). Three independent types of sequence comparisons were performed: Full-length sequence search, 80-mer sliding window and 8-mer FASTA searches^37,38^ (https://comparedatabase.org/process-development/).

#### Bioinformatics Evaluation for Potential Toxicity

Due to the lack of a protein toxin database and the stringency of criteria specified by CTNBio for the safety assessment of a newly expressed protein in GM crops, a conservative approach was adopted to evaluate potential NPTII and CP4-EPSPS protein toxicity. The amino acid sequences of NPTII and CP4-EPSPS proteins (Figure S03) were used in a sequence similarity search by BLASTP^39^ in both NCBI databases (https://blast.ncbi.nlm.nih.gov/Blast.cgi?PROGRAM=blastp&PAGE_TYPE=BlastSearch&LINK_LOC=blasthome) and the UniProtKB blast website (https://www.uniprot.org/blast), since these curated databases include all of the identified and known proteins. The results were sorted by the *E*-value, which represents the probability of the alignment occurring by chance. Typically, an *E*-value less than 0.0001 is most likely to indicate a meaningful biological similarity between two sequences.^40^

#### Simulated Gastric Fluid and Simulated Intestinal Fluid Digestibility

Susceptibility of proteins to degradation by pepsin in simulated gastric fluid (SGF) and by pancreatin in simulated intestinal (SIF) fluid, was evaluated as previously described.^41–43^ These assays are routinely used in the safety assessment of novel recombinant proteins.^44^ Standard SGF contains 0.32% pepsin at pH 1.2 and SIF contains 10 mg/mL pancreatin at pH 6.8.^45^ Digestion of a protein in SGF and SIF involve enzyme-catalyzed hydrolysis of the protein under acidic conditions under the same physiological conditions as those in the natural environment.

The recombinant CP4-EPSPS protein expressed in this study is approximately 59 kDa (47.6 kDa CP4-EPSPS plus 11.8 kDa SUMO/6× His tag) and the NPTII is ~ 29 kDa. A SUMO/His tag was added to the CP4-EPSPS protein to increase protein expression and solubility and to enable affinity-purification of the protein from microbial extracts.^46^ The digestion of the CP4-EPSPS, NPTII and control bovine serum albumin (BSA) and β-lac proteins was tested over time intervals of approximately 30 s, 1, 2, 4, 8, 16 and 32 min in a water bath set to 37°C. The recombinant CP4-EPSPS protein (MyBioSource Inc. Cat #: MBS1422940), the recombinant NPTII protein (Agdia Inc. Cat #: LST73000), negative control BSA [99% purity, sigma Cat#:A7638 Lot#:SCLB7618], and β-lactoglobulin (β -lac) [98% purity, sigma Cat#:L7880 Lot#:SLC6719] served as reference proteins. Bovine serum albumin (BSA) control is known to degrade rapidly in SGF and persist in SIF. β-lactoglobulin (β-lac) control is known to persist in SGF and degrade rapidly in SIF.^41,47^

The proteins were separated on a denaturing sodium dodecyl sulfate polyacrylamide gel (Bolt^TM^ 4–12% Bis-Tris Plus, Invitrogen Cat #: NW04122BOX) and then subjected to Western blotting. The NPTII protein was probed with a rabbit anti-NPTII polyclonal antibody (My BioSource Cat #: MBS534760) and the CP4-EPSPS protein was probed with a mouse anti-CP4-EPSPS antibody (My BioSource Cat #: MBS857729). Horseradish peroxidase (HRP)-conjugated goat anti-rabbit IgG (H+L) (Invitrogen, Cat #: A27036) and HRP-conjugated goat anti-mouse IgG (H+L) (Invitrogen, Cat #: A28177) antibodies were used as the secondary/detection antibodies.

Gel and blot images were captured using the iBright 1500 and iBright Analysis Software (version 4.0.0, Thermo Fisher Scientific).

## Thermostability Assessment

**NPTII**: The thermal stability of NPTII (150 ng/ml in 20 mM Tris-HCl, pH 8.0) was assessed by incubating samples at 0, 40, 60, 80, 90, 100, 110, 120, 130, 140, 150°C for 20 min. Samples were then centrifuge for 2 min at 14,000 RPM, 4°C. The soluble fractions were analyzed by both SDS-PAGE and with the Agdia NPTII ELISA Kit (PSP 73,000).

**CP4-EPSPS**: The thermal stability of CP4-EPSPS (300 ng/µL in 50 mM of HEPES, 16.6% glycerol) was assessed by incubating samples at 0, 50, 70, 80, 90, 100, 110, 130, 150°C for 20 min. Samples were then centrifuge for 2 min at 14,000 RPM, 4°C. The soluble fractions were analyzed by both SDS-PAGE and with the Envirologix QualiPlate ELISA Kit for CP4-EPSPS (AP 010 NW V10).

Image Lab software 6.1, BioRad was used for gel image capture and densitometry analyses.

## Results

### Expression Analysis of CP4 EPSPS and NPTII

The protein levels of both CP4-EPSPS and NPTII were analyzed in young leaves, mature leaves, stems, floral buds, and pollen samples of eucalyptus event 751K032 6, 12 and 30 months after planting. CP4-EPSPS content was measured with levels significantly higher than NPTII of approximately 20–400-fold (µg/g dry tissue) (Table 1). Due to the impact of environmental and seasonal factors on CP4-EPSPS expression in young and mature leaves during the growth stages, we also present the maximum measurement level in each sample that can be used as a reference for future studies. In pollen, CP4-EPSPS and NPTII concentrations were below the minimum detection limits of the kits used and thus were excluded from Table 1.

**Table 1.** Average concentration of CP4 EPSPS and NPTII proteins in Eucalyptus 751K032.

Tissue	Time After Planting	CP4-EPSPS			NPTII		
		(µg/g dry tissue)			(µg/g dry tissue)		
		Mean	SD	Max	Mean	SD	Max
Young Leaves	6 months	237.33	68.39	342.00	4.26	0.84	5.23
	12 months	67.73	31.65	105.00	3.63	0.51	4.47
	30 months	215.83	87.14	305.00	4.37	1.43	5.85
Mature Leaves	6 months	636.33	165.08	808.00	2.47	0.50	3.33
	12 months	1021.33	357.78	1520.00	2.71	0.79	3.50
	30 months	473.00	280.06	1020.00	3.36	0.62	4.10
Stems	6 months	154.13	53.43	226.00	1.28	0.12	1.49
	12 months	149.33	21.29	175.00	1.06	0.15	1.36
	30 months	123.25	30.92	173.00	1.31	0.32	1.77
Floral buds	Flowering	33.47	2.79	36.50	1.16	0.20	1.34

### Effect of Eucalyptus 751K032 on Arthropod Diversity

**Figure 1.**
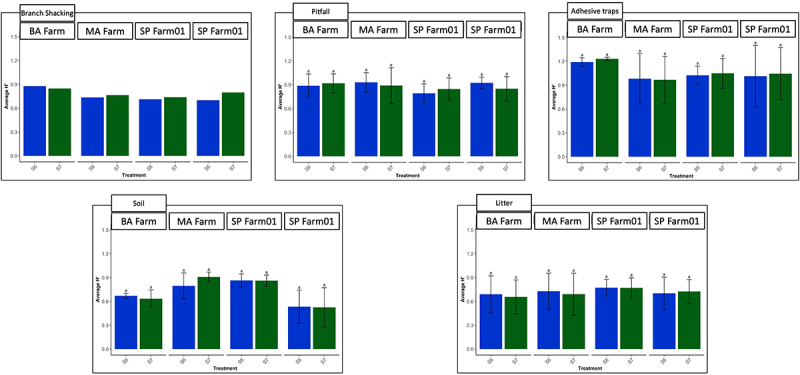
Arthropod richness and abundance studies. the Shannon ecological diversity index (H’) of richness and abundance of arthropod species shows no significant differences between the 751K032 plots and the wt FGN-K plots in all the farms and for all collection methods. Five specimen collection methods were applied in four farms over three years in 751K032 (S06) and wild type (wt) FGN-K (S07) plots: branch shacking, pitfalls, adhesive traps, soil and litter sampling. Samples were then analyzed in the laboratory for taxonomical identity and abundance. The “Branch shaking” collection method was applied only twice, due to the unreachable height of trees age 1 year and up, thus its average is presented without Tukey’s statistical test.

The Shannon ecological diversity index analysis showed no significant differences between the event 751K032 plots and the wt FGN-K plots in all the farms and for all collection methods (Figure 1). Eucalyptus event 751K032 did not significantly impact the arthropod populations in any of the tested regions in Brazil; the arthropod populations showed similar parameters whether they were sampled within event 751K032 plots or within nearby conventional FGN-K clone plots.

### Effect of Eucalyptus 751K032 on Microbial Community in the Soil

**Figure 2.**
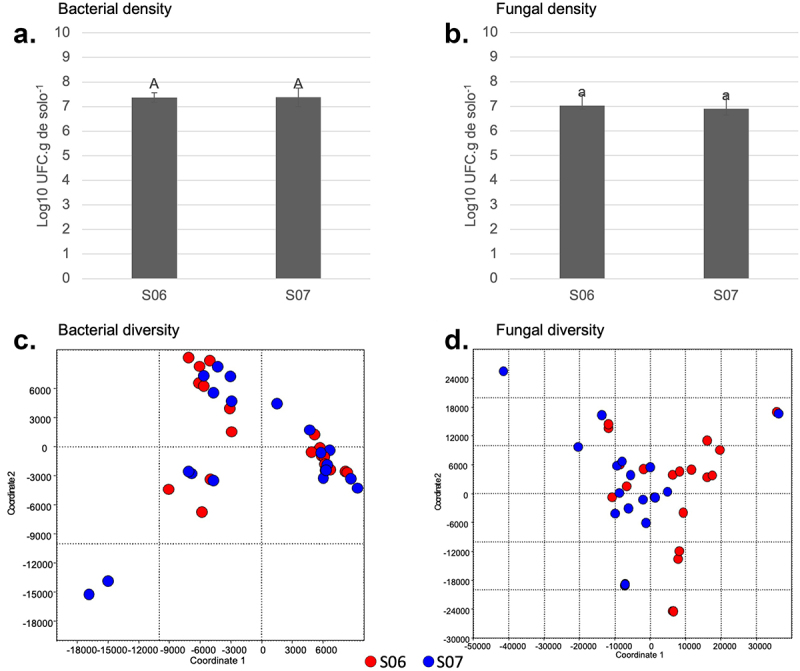
Comparison of microbial composition of soil samples. the soil samples were collected from 751K032 (S06) and wild type (wt) FGN-K (S07) plots 30 months after planting. Colony forming unit analyses were performed to evaluate microbial densities (a and b) and principal coordinate analysis (PCoA) to assess microbial population diversity (c and d). Both analyses indicated no significant difference between 751K032 and wt FGN-K samples.

Microbial studies performed 30 months after planting found no significant difference in the microbial density of bacteria and fungi (*p* > .05) of event 751K032 versus wt FGN-K plots (Figure 2a,b). The principal coordinate analysis (PCoA) (Figure 2c,d) found no correlation between the structuring of the soil microbial community and the cultivation of the genetically modified event 751K032 (S06) vs. wt FGN-K. Taken together, when compared to the wt FGN-K eucalyptus genotype, cultivation of event 751K032 did not significantly affect the soil microbial community.

### Toxicity, Allergenicity and Digestibility

#### Bioinformatics Evaluation for Potential Allergenicity and Toxicity

Allergenicity analyses showed no above-threshold (>35% identity over 80 amino acids (aa) or longer) sequence similarity to NPTII and CP4-EPSPS when running the 80-mer alignments (Tables S1 and S2). In addition, no contiguous 8 aa exact matches were identified when comparing the NPTII and CP4-EPSPS protein sequences with all of the known allergens in the COMPARE 2020 database (Tables S3 and S4). The full-length sequence search returned no sequence with an E-value smaller than 1.

The BLASTP search of the NPTII protein sequence against the NCBI nr and the UniProtKB databases returned 5000 (Table S5) and 699 alignments (Table S6), respectively. The proteins in these alignments fell into the following groups from different organisms: neomycin phosphotransferase, streptomycin 3’-kinase, aminoglycoside phosphotransferase or aminoglycoside phosphotransferase, phosphotransferase, streptomycin phosphotransferase, tetratricopeptide repeat protein, putative kanamycin kinase, putative phosphotransferase, predicted proteins and others (see details in Tables S5 and S6). A similar search with the CP4-EPSPS protein sequence returned 4987 (Table S7) and 1000 alignments (Table S8), respectively. The proteins involved in these alignments fell into the following groups from different organisms: EPSPS, phosphoshikimate 1-carboxyvinyltransferase, cytidylate kinase, unknown proteins, and hypothetical proteins. The phosphoshikimate 1-carboxyvinyltransferase catalyzes the transfer of the enolpyruvyl moiety of phosphoenolpyruvate (PEP) to the 5-hydroxyl of shikimate−3-phosphate (S3P) to produce enolpyruvyl shikimate−3-phosphate, inorganic phosphate and others (see details in Tables S7 and S8). None of the matched proteins were toxic to humans or animals. A search of the BLASTP output for “toxic,” “toxin,” “anti-nutrition,” “agglutinin,” “trypsin inhibitor,” and “protease inhibitor” returned no matches.

#### In Vitro*-Simulated Gastric and Intestinal Fluid Digestibility*

**Figure 3.**
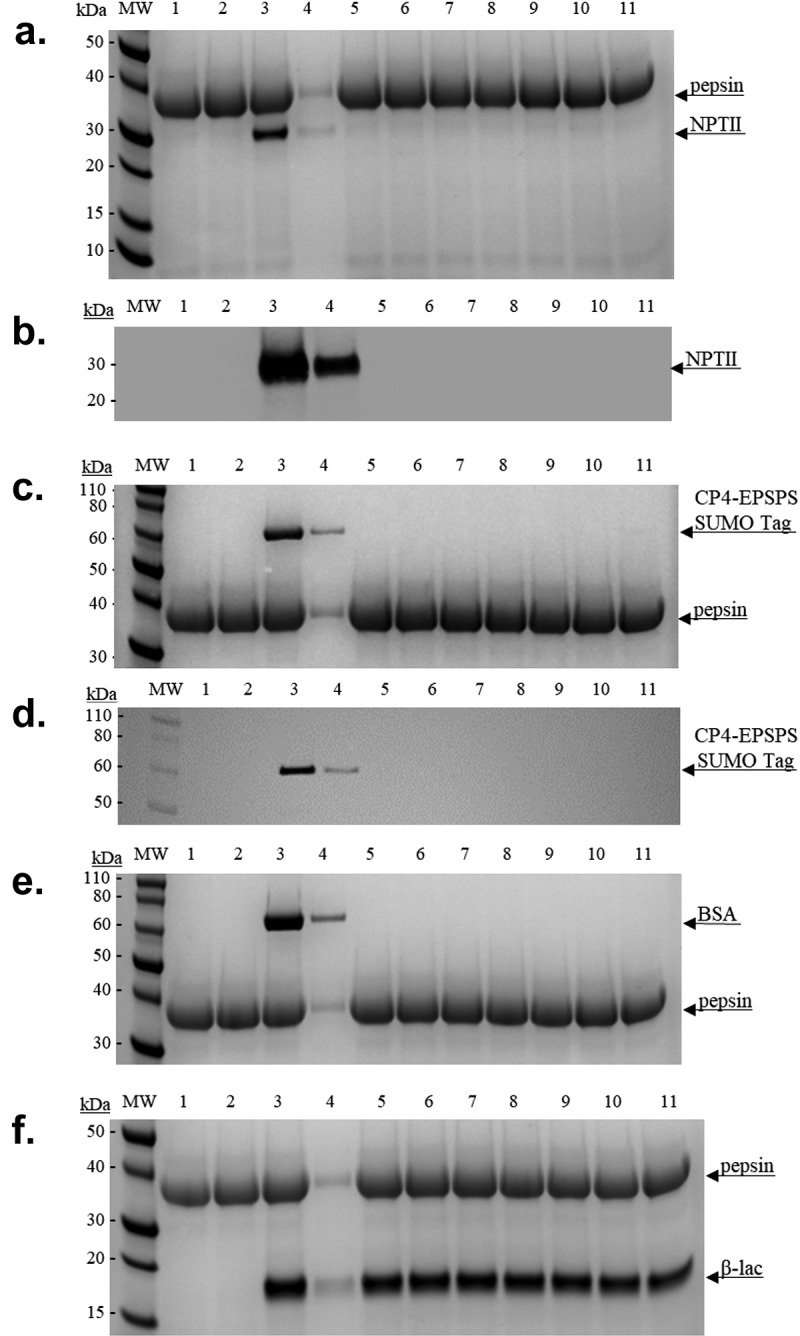
Simulated gastric fluid digestibility. Denaturing SDS-PAGE 4–12% and Coomassie blue and/or Western blot analyses of the simulated gastric fluid (SGF)-digested NPTII and CP4-EPEPS proteins and controls. Lanes (M) Novex Sharp Unstained Standard. (1) SGF Reagent Blank, 0-minute incubation. (2) SGF Reagent Blank, 32 minute incubation. (3) Neutralized sample. (4) Neutralized sample (1:10 dilution of amount in lane 3). Sample digestions: (5) 30 seconds. (6) 1 minute. (7) 2 minutes. (8) 4 minutes. (9) 8 minutes. (10) 16 minutes. (11) 32 minutes.

**Figure 4.**
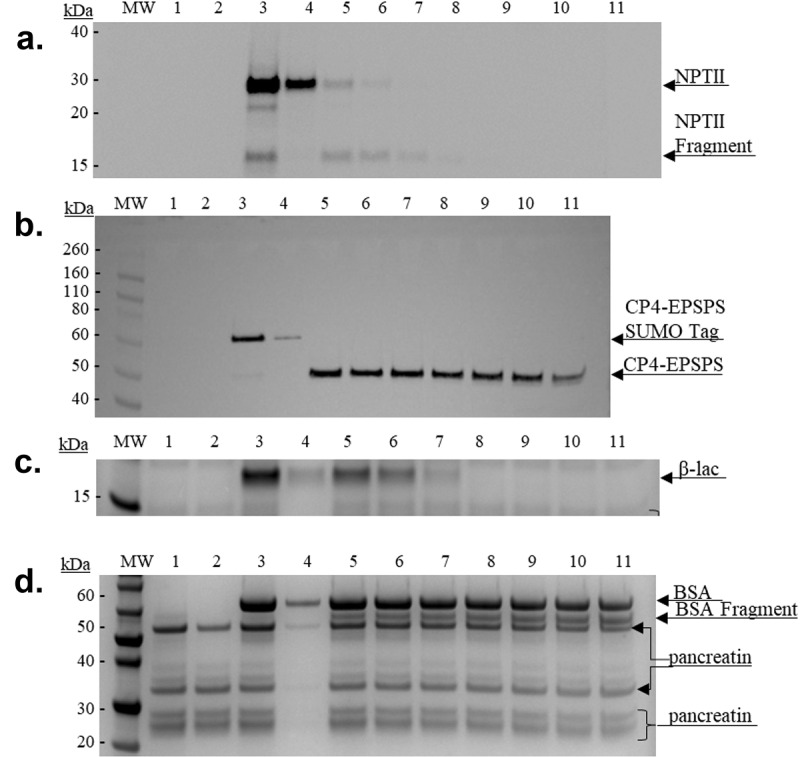
Simulated intestinal fluid digestibility. Denaturing SDS-PAGE 4–12% and with Coomassie blue and/or Western blot analyses of the simulated intestinal fluid(SIF)-digested NPTII and CP4-EPEPS proteins and controls. Lanes (M) Novex Sharp Unstained Standard. (1) SGF Reagent Blank, 0-minute incubation. (2) SGF Reagent Blank, 32 minute incubation. (3) Neutralized sample. (4) Neutralized sample (1:10 dilution of amount in lane 3). Sample digestions: (5) 30 seconds. (6) 1 minute. (7) 2 minutes. (8) 4 minutes. (9) 8 minutes. (10) 16 minutes. (11) 32 minutes.

Exposure of CP4-EPSPS and NPTII to SGF resulted in their full degradation within 30 s (Fig. 3a-d). The positive and negative controls responded as expected (Figure 3e,f), with β-lac remaining detectable for 32 min (the duration of the experiment) (Figure 3f, lane 11) and BSA undergoing full degradation within 30 s of exposure to the simulated gastric environment (Figure 3e, lane 5). Exposure of NPTII to SIF resulted in its full degradation within 2 min (Figure 4a). The SUMO tag peptide was fully digested by pancreatin within 30 s (Figure 4b). Exposure of CP4-EPSPS (48 kDa) to SIF resulted in 50% degradation within 32 min. The positive and negative controls responded as expected (Figure 4c,d), with BSA remaining detectable for 32 min (the duration of the experiment) (Figure 4d, lane 11) and β-lac undergoing full degradation within 4 min of exposure to the simulated intestinal environment (Figure 4c, lane 8).

a. NPTII protein digestion (M.W. ~29 kDa) 1 µg per lane.

b.NPTII protein digestion (M.W. ~29 kDa) 50 ng per lane.

c.Sumo-tagged CP4-EPSPS protein digestion (M.W. ~59 kDa) 1 µg per lane.

d.Sumo-tagged CP4-EPSPS protein digestion (M.W. ~59 kDa) 50 ng per lane.

e.BSA protein digestion (M.W. ~66 kDa) 1 µg per lane.

f.β-lac protein digestion (M.W. ~18 kDa) 1 µg per lane.

a. NPTII protein digestion (M.W. ~29 kDa) 50 ng per lane.

b.Sumo-tagged CP4-EPSPS protein digestion (M.W. ~59 kDa) 50 ng per lane.

c.Coomassie blue of β-lac protein digestion (M.W. ~66 kDa) 1 µg per lane.

d.Coomassie blue of BSA protein digestion (M.W. ~18 kDa) 1 µg per lane.

#### Thermostability of CP4-EPSPS and NPTII

In thermostability studies, NPTII was degraded at increasing temperatures, with pronounced degradation at temperatures higher than 120°C (Table 2, Figure S04 A). No degradation byproducts were identified by NPTII-specific antibodies. There was a gradual decrease in NPTII solubility at the tested temperatures, as determined by densitometry. NPTII immunoreactivity, as determined by reduced binding to ELISA plates, gradually decreased with the increase in temperature, reaching 0% immunoreactivity at 150°C (Table 2). Similarly, CP4-EPSPS was degraded as temperatures were increased, with protein degradation most pronounced at temperatures exceeding 130°C (Table 2, Figure S04 B). No degradation byproducts were identified by CP4-EPSPS-specific antibodies. There was a gradual decrease in solubility of the CP4-EPSPS protein at the tested temperatures, as determined by densitometry. CP4-EPSPS immunoreactivity was reduced following heating and dropped to 0% at temperatures of 70°C and above (Table 2).

**Table 2.** Densitometric Measurement (by SDS-PAGE) and Immunoreactivity (by ELISA) of the Effect of Heat Treatment on NPTII and CP4-EPSPS Proteins after 20 min exposure.

	Treatment (°C)	Densitometry (%)	Immunoreactivity (%)
NPTII	0	100	100
	40	92	79.4
	60	94	70.1
	80	95	68.3
	90	88	53.5
	100	84	43.9
	110	75	35.5
	120	56	34.2
	130	34	26.7
	140	4	12.9
	150	0	0
CP4-EPSPS	0	100	100
	50	84	87.8
	70	84	0
	80	80	0
	90	54	0
	100	72	0
	110	71	0
	130	54	0
	150	14	0

## Discussion

Transgenic events expressing the NPTII and CP4-EPSPS proteins were first deregulated in the U.S. in the 1990s. Today, more than 120 registered events contain NPTII and CP4-EPSPS (ISAAA.org, updated Dec. 2020). NPTII and CP4-EPSPS have a long history of safe use, in the environment and as food and feed, with safety data accumulated over 25 years.^44,48^ In addition, both proteins are expressed in events authorized for cultivation in the U.S. and Brazil (www.biotradestatus.com). Transgenic plants expressing NPTII and CP4-EPSPS have been cultivated commercially in the U.S. and other countries for over two decades. In fact, in 2007, the U.S. Environmental Protection Agency waived the requirement of a tolerance for the NPTII and CP4-EPSPS proteins in all plants (40 CFR § 174.521 and 40 CFR § 174.523). The decision was based on safety assessments of the proteins, including digestibility in simulated gastric/intestinal fluids, lack of homology to known allergens and protein toxins, and lack of toxicity as demonstrated by acute oral mouse gavage studies.

This work described biosafety evaluation experiments conducted on 751K032, the first glyphosate-tolerant eucalyptus event to be approved for commercial use in Brazil. While some of the biosafety tests and results presented in this research may seem redundant given the over 25 years of safe use of the traits reported, it is still relevant to conduct these analyses to verify the safety of genetically modified eucalyptus, which is a relatively new species to be modified. Furthermore, conducting these tests is necessary to meet current regulatory demands. Eucalyptus plantations are an integral part of Brazilian agriculture and are subject to changing environmental conditions and ecosystems, as discussed in the “Consensus Document on the Biology of Eucalyptus spp.”^49^ FuturaGene and Suzano continuously monitor these changes and make necessary adjustments to their biosafety tests in accordance with CTNBio’s normative.

Under protocols established by CTNBio, the safety assessment of the glyphosate-tolerant 751K032 eucalyptus event encompassed several factors, including expression of the NPTII and CP4 EPSPS proteins, stability of these enzymes under digestive conditions and heat, allergenic and toxicity and environmental impacts on arthropods and microorganisms in the field.

The evaluation of expression levels is an integral aspect of determining event identity, as it enables the description of transgene expression levels, which in turn provides insights into potential activity and environmental exposure based on the tissue and age of the organism. Differences in expression levels across various tissues can be explained by the specific activity of the promoter in each tissue and the varying purification efficiencies from different parts of the tree. The expression of CP4-EPSPS can vary significantly in young and mature leaves during the growth stages (6 to 12 to 30 months) due to environmental and seasonal factors, but the expression levels of both CP4-EPSPS and NPTII in event 751K032 were similar to those measured in other published commercial glyphosate-tolerant soybean and corn varieties^50,51^ thus sharing similar safety margins as the widely used food crops. The Max expression data can serve as a point of reference for comparison and for calculations related to environmental exposure.

In the process of developing genetically modified crops, one of the primary concerns is ensuring their safety for human consumption. An important step in this process is the evaluation of potential allergenicity and toxicity of the expressed protein even for modified eucalyptus which is not food nor feed. This is typically done through *in-silico* (computer-based) analysis, which is part of a weight-of-evidence approach to safety assessment. Allergenicity and toxicity analyses were performed on recent datasets (February 2022) and indicated that the proteins in question do not pose any significant risks to human health.

This indication is also supported by the protein digestibility assays which confirmed the findings of^48^and^44^from almost 30 years ago, which showed that both NPTII and CP4-EPSPS proteins are digestible in both the gastric and intestinal fluids. These findings provide assurance that the use of event 751K032 as a source of wood material is safe, and it can be safely released to the market for use in wood products.

Arthropods and soil microbes are significant organisms in the ecological balance, and in agricultural fields and plantations as well.^52,53^ They are highly susceptible to changes in crop, growth, and silviculture practices, and therefore, can be used as a “fingerprint” for identifying specific ecological scenarios in the field. In this context, they are important bioindicators. The study conducted on four farms aimed to investigate the effects of the genetically modified event 751K032 on the populations of arthropods and soil microbes in comparison to the non-modified wt clone FGN-K. The three-year study found no significant differences in the arthropod and soil microbial populations between the two plots. This suggests that the introduction of the genetically modified event did not cause any ecological changes, and it is safe for the environment.

In conclusion, according to the safety assessments conducted, the genetically modified eucalyptus event 751K032 is just as safe for humans, animals, and the environment as the conventional clone FGN-K. Additionally, since this GM eucalyptus is intended for producing fiber and wood products that are not for human or animal consumption, it poses no oral threat or danger to them. The introduction of the glyphosate-tolerant event 751K032 in genetically modified eucalyptus trees provides a targeted and effective approach to weed control, thus reducing competition and preventing unintended damage to young trees. Moreover, this modification increases yield, making it a valuable sustainable tool for wood production. Based on the biosafety results, it has been established that this genetically modified eucalyptus event is safe for wood production. However, to establish the best-integrated weed management and control program for eucalyptus plantations, further research is underway to develop additional glyphosate tolerance events and alternative herbicide modes of action tolerance traits.

## Supplementary Material

Supplemental Material
