# Supplementary material for: Safety Assessment of the CP4 EPSPS and NPTII Proteins in Eucalyptus
Source: GM Crops Food. 2023 Jun 19;14(1):1–14. doi: 10.1080/21645698.2023.2222436 (PMC10281460; doi:10.1080/21645698.2023.2222436)
Supplement: Supplemental Material [file KGMC_A_2222436_SM4294.zip › TableS01.docx]

Output of an 80 aa sliding-window FASTA search of the NPTII protein sequence against the COMPARE 2020 allergen database

```
A Recap of the Input and Significance Criteria Library:
COMPARE_2020.lib
Input Sequence:
>NPTII MIEQDGLHAGSPAAWVERLFGYDWAQQTIGCSDAAVFRLSAQGRPVLFVKTDLSGALNELQDEAARLSWLATTGVPCAAVLDVVTEAGRDWLLLGEV PGQDLLSSHLAPAEKVSIMADAMRRLHTLDPATCPFDHQAKHRIERARTRMEAGLVDQDDLDEEHQGLAPAELFARLKARMPDGEDLVVTHGDACLP NIMVENGRFSGFIDCGRLGVADRYQDIALATRDIAEELGGEWADRFLVLYGIAAPDSQRIAFYRLLDEFF
Input Parameters
E-value Search limit Word Size	Gap/Extension 10	2	10/2
Significance Limits
% Identity	Overlap Length
> 35	80
80-Mer Sliding Window FASTA Search Result
Query Window	Alignments Displaying 35% Identity in 80 amino acids 1:80	None
2:81	None
3:82	None
4:83	None
5:84	None
6:85	None
7:86	None
8:87	None
9:88	None
10:89 None
11:90 None
12:91 None
13:92 None
14:93 None
15:94 None
16:95 None
17:96 None
18:97 None
19:98 None
20:99 None
21:100 None
22:101 None
23:102 None
24:103 None
25:104 None
26:105 None
27:106 None
28:107 None
29:108 None
30:109 None
31:110 None
32:111 None
33:112 None
34:113 None
35:114 None
36:115 None
37:116 None
38:117 None
39:118 None
40:119 None
41:120 None
42:121 None
43:122 None
44:123 None
45:124 None
46:125 None
47:126 None
48:127 None
49:128 None

50:129 None
51:130 None
52:131 None
53:132 None
54:133 None
55:134 None
56:135 None
57:136 None
58:137 None
59:138 None
60:139 None
61:140 None
62:141 None
63:142 None
64:143 None
65:144 None
66:145 None
67:146 None
68:147 None
69:148 None
70:149 None
71:150 None
72:151 None
73:152 None
74:153 None
75:154 None
76:155 None
77:156 None
78:157 None
79:158 None
80:159 None
81:160 None
82:161 None
83:162 None
84:163 None
85:164 None
86:165 None
87:166 None
88:167 None
89:168 None
90:169 None
91:170 None
92:171 None
93:172 None
94:173 None
95:174 None
96:175 None
97:176 None
98:177 None
99:178 None
100:179 None
101:180 None
102:181 None
103:182 None
104:183 None
105:184 None
106:185 None
107:186 None
108:187 None
109:188 None
110:189 None
111:190 None
112:191 None
113:192 None
114:193 None
115:194 None
116:195 None
117:196 None
118:197 None
119:198 None
120:199 None
121:200 None

122:201 None
123:202 None
124:203 None
125:204 None
126:205 None
127:206 None
128:207 None
129:208 None
130:209 None
131:210 None
132:211 None
133:212 None
134:213 None
135:214 None
136:215 None
137:216 None
138:217 None
139:218 None
140:219 None
141:220 None
142:221 None
143:222 None
144:223 None
145:224 None
146:225 None
147:226 None
148:227 None
149:228 None
150:229 None
151:230 None
152:231 None
153:232 None
154:233 None
155:234 None
156:235 None
157:236 None
158:237 None
159:238 None
160:239 None
161:240 None
162:241 None
163:242 None
164:243 None
165:244 None
166:245 None
167:246 None
168:247 None
169:248 None
170:249 None
171:250 None
172:251 None
173:252 None
174:253 None
175:254 None
176:255 None
177:256 None
178:257 None
179:258 None
180:259 None
181:260 None
182:261 None
183:262 None
184:263 None
185:264 None
```
