# Supplementary material for: Safety Assessment of the CP4 EPSPS and NPTII Proteins in Eucalyptus
Source: GM Crops Food. 2023 Jun 19;14(1):1–14. doi: 10.1080/21645698.2023.2222436 (PMC10281460; doi:10.1080/21645698.2023.2222436)
Supplement: Supplemental Material [file KGMC_A_2222436_SM4294.zip › Tables02.docx]

Output of 80 (amino acid) aa sliding-window FASTA search of the CP4-EPSPS protein sequence against the COMPARE 2020 allergen database.

```
Library:
COMPARE_2020.lib
Input Sequence:
>CP4_EPSPS MLHGASSRPATARKSSGLSGTVRIPGDKSISHRSFMFGGLASGETRITGLLEGEDVINTGKAMQAMGARIRKEGDTW IIDGVGNGGLLAPEAPLDFGNAATGCRLTMGLVGVYDFDSTFIGDASLTKRPMGRVLNPLREMGVQVKSEDGDRLPV TLRGPKTPTPITYRVPMASAQVKSAVLLAGLNTPGITTVIEPIMTRDHTEKMLQGFGANLTVETDADGVRTIRLEGR GKLTGQVIDVPGDPSSTAFPLVAALLVPGSDVTILNVLMNPTRTGLILTLQEMGADIEVINPRLAGGEDVADLRVRS STLKGVTVPEDRAPSMIDEYPILAVAAAFAEGATVMNGLEELRVKESDRLSAVANGLKLNGVDCDEGETSLVVRGRP DGKGLGNASGAAVATHLDHRIAMSFLVMGLVSENPVTVDDATMIATSFPEFMDLMAGLGAKIELSDTKAA
Input Parameters
E-value Search limit	Word Size	Gap/Extension 10	2	10/2
Significance Limits
% Identity Overlap Length
> 35 80
80-Mer Sliding Window FASTA Search Result
Query Window	Alignments Displaying 35% Identity in 80 amino acids 1:80 None
2:81 None
3:82 None
4:83 None
5:84 None
6:85 None
7:86 None
8:87 None
9:88 None
10:89 None
11:90 None
12:91 None
13:92 None
14:93 None
15:94 None
16:95 None
17:96 None
18:97 None
19:98 None
20:99 None
21:100	None
22:101	None
23:102	None
24:103	None
25:104	None
26:105	None
27:106	None
28:107	None
29:108	None

31:110	None
32:111	None
33:112	None
34:113	None
35:114	None
36:115	None
37:116	None
38:117	None
39:118	None
40:119	None
41:120	None
42:121	None
43:122	None
44:123	None
45:124	None
46:125	None
47:126	None
48:127	None
49:128	None
50:129	None
51:130	None
52:131	None
53:132	None
54:133	None
55:134	None
56:135	None
57:136	None
58:137	None
59:138	None
60:139	None
61:140	None
62:141	None
63:142	None
64:143	None
65:144	None
66:145	None
67:146	None
68:147	None
69:148	None
70:149	None
71:150	None
72:151	None
73:152	None
74:153	None
75:154	None
76:155	None
77:156	None
78:157	None
79:158	None
80:159	None
81:160	None
82:161	None

85:164	None
86:165	None
87:166	None
88:167	None
89:168	None
90:169	None
91:170	None
92:171	None
93:172	None
94:173	None
95:174	None
96:175	None
97:176	None
98:177	None
99:178	None
100:179	None
101:180	None
102:181	None
103:182	None
104:183	None
105:184	None
106:185	None
107:186	None
108:187	None
109:188	None
110:189	None
111:190	None
112:191	None
113:192	None
114:193	None
115:194	None
116:195	None
117:196	None
118:197	None
119:198	None
120:199	None
121:200	None
122:201	None
123:202	None
124:203	None
125:204	None
126:205	None
127:206	None
128:207	None
129:208	None
130:209	None
131:210	None
132:211	None
133:212	None
134:213	None
135:214	None
136:215	None

139:218	None
140:219	None
141:220	None
142:221	None
143:222	None
144:223	None
145:224	None
146:225	None
147:226	None
148:227	None
149:228	None
150:229	None
151:230	None
152:231	None
153:232	None
154:233	None
155:234	None
156:235	None
157:236	None
158:237	None
159:238	None
160:239	None
161:240	None
162:241	None
163:242	None
164:243	None
165:244	None
166:245	None
167:246	None
168:247	None
169:248	None
170:249	None
171:250	None
172:251	None
173:252	None
174:253	None
175:254	None
176:255	None
177:256	None
178:257	None
179:258	None
180:259	None
181:260	None
182:261	None
183:262	None
184:263	None
185:264	None
186:265	None
187:266	None
188:267	None
189:268	None
190:269	None

193:272	None
194:273	None
195:274	None
196:275	None
197:276	None
198:277	None
199:278	None
200:279	None
201:280	None
202:281	None
203:282	None
204:283	None
205:284	None
206:285	None
207:286	None
208:287	None
209:288	None
210:289	None
211:290	None
212:291	None
213:292	None
214:293	None
215:294	None
216:295	None
217:296	None
218:297	None
219:298	None
220:299	None
221:300	None
222:301	None
223:302	None
224:303	None
225:304	None
226:305	None
227:306	None
228:307	None
229:308	None
230:309	None
231:310	None
232:311	None
233:312	None
234:313	None
235:314	None
236:315	None
237:316	None
238:317	None
239:318	None
240:319	None
241:320	None
242:321	None
243:322	None
244:323	None

247:326	None
248:327	None
249:328	None
250:329	None
251:330	None
252:331	None
253:332	None
254:333	None
255:334	None
256:335	None
257:336	None
258:337	None
259:338	None
260:339	None
261:340	None
262:341	None
263:342	None
264:343	None
265:344	None
266:345	None
267:346	None
268:347	None
269:348	None
270:349	None
271:350	None
272:351	None
273:352	None
274:353	None
275:354	None
276:355	None
277:356	None
278:357	None
279:358	None
280:359	None
281:360	None
282:361	None
283:362	None
284:363	None
285:364	None
286:365	None
287:366	None
288:367	None
289:368	None
290:369	None
291:370	None
292:371	None
293:372	None
294:373	None
295:374	None
296:375	None
297:376	None
298:377	None

301:380	None
302:381	None
303:382	None
304:383	None
305:384	None
306:385	None
307:386	None
308:387	None
309:388	None
310:389	None
311:390	None
312:391	None
313:392	None
314:393	None
315:394	None
316:395	None
317:396	None
318:397	None
319:398	None
320:399	None
321:400	None
322:401	None
323:402	None
324:403	None
325:404	None
326:405	None
327:406	None
328:407	None
329:408	None
330:409	None
331:410	None
332:411	None
333:412	None
334:413	None
335:414	None
336:415	None
337:416	None
338:417	None
339:418	None
340:419	None
341:420	None
342:421	None
343:422	None
344:423	None
345:424	None
346:425	None
347:426	None
348:427	None
349:428	None
350:429	None
351:430	None
352:431	None

355:434	None
356:435	None
357:436	None
358:437	None
359:438	None
360:439	None
361:440	None
362:441	None
363:442	None
364:443	None
365:444	None
366:445	None
367:446	None
368:447	None
369:448	None
370:449	None
371:450	None
372:451	None
373:452	None
374:453	None
375:454	None
376:455	None
```
