# Supplementary material for: Safety Assessment of the CP4 EPSPS and NPTII Proteins in Eucalyptus
Source: GM Crops Food. 2023 Jun 19;14(1):1–14. doi: 10.1080/21645698.2023.2222436 (PMC10281460; doi:10.1080/21645698.2023.2222436)
Supplement: Supplemental Material [file KGMC_A_2222436_SM4294.zip › TableS03.docx]

Output of search for an 8 aa exact match between the NPTII protein sequence and allergens in the COMPARE 2020 database

```
A Recap of the Input and Significance Criteria Library:
COMPARE_2020.lib
Input Sequence:
>NPTII MIEQDGLHAGSPAAWVERLFGYDWAQQTIGCSDAAVFRLSAQGRPVLFVKTDLSGALNELQDEAARLSWLATTGVPCAAVLDVVTEAGRDWLLLGEV PGQDLLSSHLAPAEKVSIMADAMRRLHTLDPATCPFDHQAKHRIERARTRMEAGLVDQDDLDEEHQGLAPAELFARLKARMPDGEDLVVTHGDACLP NIMVENGRFSGFIDCGRLGVADRYQDIALATRDIAEELGGEWADRFLVLYGIAAPDSQRIAFYRLLDEFF
Input Parameters
E-value Search limit Word Size	Gap/Extension 10	2	10/2
8-Mer FASTA Search Result
Query Window	Alignments Displaying 100% Identity in 8 amino acids 1:8	None
2:9	None
3:10	None
4:11	None
5:12	None
6:13	None
7:14	None
8:15	None
9:16	None
10:17 None
11:18 None
12:19 None
13:20 None
14:21 None
15:22 None
16:23 None
17:24 None
18:25 None
19:26 None
20:27 None
21:28 None
22:29 None
23:30 None
24:31 None
25:32 None
26:33 None
27:34 None
28:35 None
29:36 None
30:37 None
31:38 None
32:39 None
33:40 None
34:41 None
35:42 None
36:43 None
37:44 None
38:45 None
39:46 None
40:47 None
41:48 None
42:49 None
43:50 None
44:51 None
45:52 None
46:53 None
47:54 None
48:55 None
49:56 None
50:57 None
51:58 None

53:60 None
54:61 None
55:62 None
56:63 None
57:64 None
58:65 None
59:66 None
60:67 None
61:68 None
62:69 None
63:70 None
64:71 None
65:72 None
66:73 None
67:74 None
68:75 None
69:76 None
70:77 None
71:78 None
72:79 None
73:80 None
74:81 None
75:82 None
76:83 None
77:84 None
78:85 None
79:86 None
80:87 None
81:88 None
82:89 None
83:90 None
84:91 None
85:92 None
86:93 None
87:94 None
88:95 None
89:96 None
90:97 None
91:98 None
92:99 None
93:100 None
94:101 None
95:102 None
96:103 None
97:104 None
98:105 None
99:106 None
100:107 None
101:108 None
102:109 None
103:110 None
104:111 None
105:112 None
106:113 None
107:114 None
108:115 None
109:116 None
110:117 None
111:118 None
112:119 None
113:120 None
114:121 None
115:122 None
116:123 None
117:124 None
118:125 None
119:126 None
120:127 None
121:128 None
122:129 None
123:130 None

125:132 None
126:133 None
127:134 None
128:135 None
129:136 None
130:137 None
131:138 None
132:139 None
133:140 None
134:141 None
135:142 None
136:143 None
137:144 None
138:145 None
139:146 None
140:147 None
141:148 None
142:149 None
143:150 None
144:151 None
145:152 None
146:153 None
147:154 None
148:155 None
149:156 None
150:157 None
151:158 None
152:159 None
153:160 None
154:161 None
155:162 None
156:163 None
157:164 None
158:165 None
159:166 None
160:167 None
161:168 None
162:169 None
163:170 None
164:171 None
165:172 None
166:173 None
167:174 None
168:175 None
169:176 None
170:177 None
171:178 None
172:179 None
173:180 None
174:181 None
175:182 None
176:183 None
177:184 None
178:185 None
179:186 None
180:187 None
181:188 None
182:189 None
183:190 None
184:191 None
185:192 None
186:193 None
187:194 None
188:195 None
189:196 None
190:197 None
191:198 None
192:199 None
193:200 None
194:201 None
195:202 None

197:204 None
198:205 None
199:206 None
200:207 None
201:208 None
202:209 None
203:210 None
204:211 None
205:212 None
206:213 None
207:214 None
208:215 None
209:216 None
210:217 None
211:218 None
212:219 None
213:220 None
214:221 None
215:222 None
216:223 None
217:224 None
218:225 None
219:226 None
220:227 None
221:228 None
222:229 None
223:230 None
224:231 None
225:232 None
226:233 None
227:234 None
228:235 None
229:236 None
230:237 None
231:238 None
232:239 None
233:240 None
234:241 None
235:242 None
236:243 None
237:244 None
238:245 None
239:246 None
240:247 None
241:248 None
242:249 None
243:250 None
244:251 None
245:252 None
246:253 None
247:254 None
248:255 None
249:256 None
250:257 None
251:258 None
252:259 None
253:260 None
254:261 None
255:262 None
256:263 None
257:264 None
```
