# Supplementary material for: Safety Assessment of the CP4 EPSPS and NPTII Proteins in Eucalyptus
Source: GM Crops Food. 2023 Jun 19;14(1):1–14. doi: 10.1080/21645698.2023.2222436 (PMC10281460; doi:10.1080/21645698.2023.2222436)
Supplement: Supplemental Material [file KGMC_A_2222436_SM4294.zip › TableS04.docx]

Output of search for an 8 aa exact match between the CP4-EPSPS protein sequence and allergens in the COMPARE 2020 database.

```
A Recap of the Input and Significance Criteria Library:
COMPARE_2020.lib
Input Sequence:
>CP4_EPSPS MLHGASSRPATARKSSGLSGTVRIPGDKSISHRSFMFGGLASGETRITGLLEGEDVINTGKAMQAMGARIRKEGDTWIIDGVGNGGLLAPEAPLDFG NAATGCRLTMGLVGVYDFDSTFIGDASLTKRPMGRVLNPLREMGVQVKSEDGDRLPVTLRGPKTPTPITYRVPMASAQVKSAVLLAGLNTPGITTVI EPIMTRDHTEKMLQGFGANLTVETDADGVRTIRLEGRGKLTGQVIDVPGDPSSTAFPLVAALLVPGSDVTILNVLMNPTRTGLILTLQEMGADIEVI NPRLAGGEDVADLRVRSSTLKGVTVPEDRAPSMIDEYPILAVAAAFAEGATVMNGLEELRVKESDRLSAVANGLKLNGVDCDEGETSLVVRGRPDGK GLGNASGAAVATHLDHRIAMSFLVMGLVSENPVTVDDATMIATSFPEFMDLMAGLGAKIELSDTKAA
Input Parameters
E-value Search limit Word Size	Gap/Extension 10	2	10/2
8-Mer FASTA Search Result
Query Window	Alignments Displaying 100% Identity in 8 amino acids 1:8	None
2:9	None
3:10	None
4:11	None
5:12	None
6:13	None
7:14	None
8:15	None
9:16	None
10:17 None
11:18 None
12:19 None
13:20 None
14:21 None
15:22 None
16:23 None
17:24 None
18:25 None
19:26 None
20:27 None
21:28 None
22:29 None
23:30 None
24:31 None
25:32 None
26:33 None
27:34 None
28:35 None
29:36 None
30:37 None
31:38 None
32:39 None
33:40 None
34:41 None
35:42 None
36:43 None
37:44 None
38:45 None
39:46 None
40:47 None
41:48 None
42:49 None
43:50 None
44:51 None
45:52 None
46:53 None
47:54 None
48:55 None
49:56 None

51:58 None
52:59 None
53:60 None
54:61 None
55:62 None
56:63 None
57:64 None
58:65 None
59:66 None
60:67 None
61:68 None
62:69 None
63:70 None
64:71 None
65:72 None
66:73 None
67:74 None
68:75 None
69:76 None
70:77 None
71:78 None
72:79 None
73:80 None
74:81 None
75:82 None
76:83 None
77:84 None
78:85 None
79:86 None
80:87 None
81:88 None
82:89 None
83:90 None
84:91 None
85:92 None
86:93 None
87:94 None
88:95 None
89:96 None
90:97 None
91:98 None
92:99 None
93:100 None
94:101 None
95:102 None
96:103 None
97:104 None
98:105 None
99:106 None
100:107 None
101:108 None
102:109 None
103:110 None
104:111 None
105:112 None
106:113 None
107:114 None
108:115 None
109:116 None
110:117 None
111:118 None
112:119 None
113:120 None
114:121 None
115:122 None
116:123 None
117:124 None
118:125 None
119:126 None
120:127 None

123:130 None
124:131 None
125:132 None
126:133 None
127:134 None
128:135 None
129:136 None
130:137 None
131:138 None
132:139 None
133:140 None
134:141 None
135:142 None
136:143 None
137:144 None
138:145 None
139:146 None
140:147 None
141:148 None
142:149 None
143:150 None
144:151 None
145:152 None
146:153 None
147:154 None
148:155 None
149:156 None
150:157 None
151:158 None
152:159 None
153:160 None
154:161 None
155:162 None
156:163 None
157:164 None
158:165 None
159:166 None
160:167 None
161:168 None
162:169 None
163:170 None
164:171 None
165:172 None
166:173 None
167:174 None
168:175 None
169:176 None
170:177 None
171:178 None
172:179 None
173:180 None
174:181 None
175:182 None
176:183 None
177:184 None
178:185 None
179:186 None
180:187 None
181:188 None
182:189 None
183:190 None
184:191 None
185:192 None
186:193 None
187:194 None
188:195 None
189:196 None
190:197 None
191:198 None
192:199 None

195:202 None
196:203 None
197:204 None
198:205 None
199:206 None
200:207 None
201:208 None
202:209 None
203:210 None
204:211 None
205:212 None
206:213 None
207:214 None
208:215 None
209:216 None
210:217 None
211:218 None
212:219 None
213:220 None
214:221 None
215:222 None
216:223 None
217:224 None
218:225 None
219:226 None
220:227 None
221:228 None
222:229 None
223:230 None
224:231 None
225:232 None
226:233 None
227:234 None
228:235 None
229:236 None
230:237 None
231:238 None
232:239 None
233:240 None
234:241 None
235:242 None
236:243 None
237:244 None
238:245 None
239:246 None
240:247 None
241:248 None
242:249 None
243:250 None
244:251 None
245:252 None
246:253 None
247:254 None
248:255 None
249:256 None
250:257 None
251:258 None
252:259 None
253:260 None
254:261 None
255:262 None
256:263 None
257:264 None
258:265 None
259:266 None
260:267 None
261:268 None
262:269 None
263:270 None
264:271 None

267:274 None
268:275 None
269:276 None
270:277 None
271:278 None
272:279 None
273:280 None
274:281 None
275:282 None
276:283 None
277:284 None
278:285 None
279:286 None
280:287 None
281:288 None
282:289 None
283:290 None
284:291 None
285:292 None
286:293 None
287:294 None
288:295 None
289:296 None
290:297 None
291:298 None
292:299 None
293:300 None
294:301 None
295:302 None
296:303 None
297:304 None
298:305 None
299:306 None
300:307 None
301:308 None
302:309 None
303:310 None
304:311 None
305:312 None
306:313 None
307:314 None
308:315 None
309:316 None
310:317 None
311:318 None
312:319 None
313:320 None
314:321 None
315:322 None
316:323 None
317:324 None
318:325 None
319:326 None
320:327 None
321:328 None
322:329 None
323:330 None
324:331 None
325:332 None
326:333 None
327:334 None
328:335 None
329:336 None
330:337 None
331:338 None
332:339 None
333:340 None
334:341 None
335:342 None
336:343 None

339:346 None
340:347 None
341:348 None
342:349 None
343:350 None
344:351 None
345:352 None
346:353 None
347:354 None
348:355 None
349:356 None
350:357 None
351:358 None
352:359 None
353:360 None
354:361 None
355:362 None
356:363 None
357:364 None
358:365 None
359:366 None
360:367 None
361:368 None
362:369 None
363:370 None
364:371 None
365:372 None
366:373 None
367:374 None
368:375 None
369:376 None
370:377 None
371:378 None
372:379 None
373:380 None
374:381 None
375:382 None
376:383 None
377:384 None
378:385 None
379:386 None
380:387 None
381:388 None
382:389 None
383:390 None
384:391 None
385:392 None
386:393 None
387:394 None
388:395 None
389:396 None
390:397 None
391:398 None
392:399 None
393:400 None
394:401 None
395:402 None
396:403 None
397:404 None
398:405 None
399:406 None
400:407 None
401:408 None
402:409 None
403:410 None
404:411 None
405:412 None
406:413 None
407:414 None
408:415 None

411:418 None
412:419 None
413:420 None
414:421 None
415:422 None
416:423 None
417:424 None
418:425 None
419:426 None
420:427 None
421:428 None
422:429 None
423:430 None
424:431 None
425:432 None
426:433 None
427:434 None
428:435 None
429:436 None
430:437 None
431:438 None
432:439 None
433:440 None
434:441 None
435:442 None
436:443 None
437:444 None
438:445 None
439:446 None
440:447 None
441:448 None
442:449 None
443:450 None
444:451 None
445:452 None
446:453 None
447:454 None
448:455 None
```
