# Supplementary material for: Safety Assessment of the CP4 EPSPS and NPTII Proteins in Eucalyptus
Source: GM Crops Food. 2023 Jun 19;14(1):1–14. doi: 10.1080/21645698.2023.2222436 (PMC10281460; doi:10.1080/21645698.2023.2222436)
Supplement: Supplemental Material [file KGMC_A_2222436_SM4294.zip › TableS05.docx]

BLASTP output of NPTII protein sequence against the NCBI nr protein database

BLASTP 2.11.0+

Reference: Stephen F. Altschul, Thomas L. Madden, Alejandro

A. Schaffer, Jinghui Zhang, Zheng Zhang, Webb Miller, and David J. Lipman (1997), "Gapped BLAST and PSI-BLAST: a new generation of protein database search programs", Nucleic Acids Res. 25:3389-3402.

Reference for compositional score matrix adjustment: Stephen

F. Altschul, John C. Wootton, E. Michael Gertz, Richa Agarwala, Aleksandr Morgulis, Alejandro A. Schaffer, and Yi-Kuo Yu (2005) "Protein database searches using compositionally adjusted substitution matrices", FEBS J. 272:5101-5109.

RID: X2T587ZT013

Database: All non-redundant GenBank CDS translations+PDB+SwissProt+PIR+PRF excluding environmental samples from WGS projects

330,470,260 sequences; 119,575,496,927 total letters

| Query= NPTII |  | | |
| --- | --- | --- | --- |
| Length=264 |  |  |  |
| Sequences producing significant alignments: | Score (Bits) | E  Value | Max Ident |
| BAD00047.1 Fusion protein, Feo [Hepacivirus C] | 546 | 0.0 | 100% |
| AGU01702.1 firefly luciferase-neomycin phosphotransferase fusi... | 543 | 0.0 | 100% |
| AAT02530.1 lacZ/gfp/neomycin phosphotransferase fusion protein... | 542 | 0.0 | 100% |
| AAT02528.1 gfp/neomycin phosphotransferase fusion protein [Clo... | 542 | 0.0 | 100% |
| AIO05660.1 Rluc-ubiquitin-neo fusion protein [synthetic constr... | 541 | 0.0 | 100% |
| ABC61923.1 ubiquitin-NPTII fusion [Binary vector pBINPLUS/ARS] | 540 | 0.0 | 100% |
| AAB66883.1 fluorescent neomycin phosphotransferase fusion prot... | 540 | 0.0 | 100% |
| ANA50877.1 CodA-nptII [Cloning vector pCTAG7] | 539 | 0.0 | 100% |
| BAB32740.1 red-shift green fluorescent protein-fused neomycin ... | 539 | 0.0 | 100% |
| AII16593.1 GFPm2-NPTII fusion protein [Expression vector pT7 R... | 538 | 0.0 | 100% |
| ATN39931.1 PRK/NPTII fusion protein [synthetic construct] | 538 | 0.0 | 100% |
| AMB57377.1 UbiM-NptII [Binary vector pARS2] | 538 | 0.0 | 100% |
| ABI49756.1 renilla luciferase/neomycin phosphotransferase fusi... | 537 | 0.0 | 100% |
| CAB46914.1 core-neo fusion protein [Hepatitis C virus] [Hepati... | 537 | 0.0 | 100% |
| CAB46910.1 core-neo fusion protein [Hepatitis C virus] [Hepati... | 536 | 0.0 | 100% |
| AJF44503.1 Neo [Cloning vector pNZP1-SGR] | 536 | 0.0 | 100% |
| BAM38519.1 neomycin-kanamycin phosphotransferase type II [Hepa... | 536 | 0.0 | 100% |
| CAD21956.1 neomycin phosphotransferase [Hepatitis GB virus B] | 536 | 0.0 | 100% |
| ABO65261.1 GFPNeo fusion protein [Cloning vector pEFBOS-IRESGF... | 536 | 0.0 | 100% |
| AET75770.1 CodAnptII [Cloning vector pHUGE-Red] | 536 | 0.0 | 100% |
| ACH99098.1 Aph [Allelic replacement vector pJC84] | 535 | 0.0 | 100% |
| AAA72988.1 luciferase/kanamycin resistance protein [synthetic ... | 535 | 0.0 | 100% |
| WP_000572405.1 MULTISPECIES: aminoglycoside O-phosphotransfera... | 535 | 0.0 | 100% |
| AAD31025.1 aminoglycoside 3'-phosphotransferase [Cloning vecto... | 535 | 0.0 | 100% |
| AAG00918.1 neomycin phosphotransferase [Cloning vector pBIG] | 535 | 0.0 | 100% |
| WP_097474700.1 APH(3')-II family aminoglycoside O-phosphotrans... | 535 | 0.0 | 100% |
| ASU87773.1 NPTII [Ti plasmid binary vector pGSh16.1010] | 535 | 0.0 | 100% |
| WP_096935528.1 APH(3')-II family aminoglycoside O-phosphotrans... | 534 | 0.0 | 100% |
| WP_114741199.1 APH(3')-II family aminoglycoside O-phosphotrans... | 534 | 0.0 | 100% |
| EFB5119094.1 APH(3')-II family aminoglycoside O-phosphotransfe... | 534 | 0.0 | 100% |
| WP_171502934.1 MULTISPECIES: APH(3')-II family aminoglycoside ... | 534 | 0.0 | 100% |
| ABX39480.1 ATP synthase beta subunit/neomycin phosphotransfera... | 534 | 0.0 | 100% |
| QEQ90639.1 3xV5 tagged NeoR marker protein [Cloning vector p57... | 534 | 0.0 | 100% |
| AAO21931.1 aminoglycoside-3'-O-phosphotransferase [Expression ... | 534 | 0.0 | 99% |
| WP_165472676.1 APH(3')-II family aminoglycoside O-phosphotrans... | 533 | 0.0 | 100% |

| EEV6129758.1 APH(3')-II family aminoglycoside O-phosphotransfe... | 533 | 0.0 | 100% |
| --- | --- | --- | --- |
| EAY7200867.1 APH(3')-II family aminoglycoside O-phosphotransfe... | 533 | 0.0 | 100% |
| EFH5635854.1 APH(3')-II family aminoglycoside O-phosphotransfe... | 533 | 0.0 | 100% |
| WP_196744400.1 APH(3')-II family aminoglycoside O-phosphotrans... | 533 | 0.0 | 100% |
| WP_104730796.1 APH(3')-II family aminoglycoside O-phosphotrans... | 533 | 0.0 | 100% |
| EBO5400315.1 APH(3')-II family aminoglycoside O-phosphotransfe... | 533 | 0.0 | 100% |
| EDA3939420.1 APH(3')-II family aminoglycoside O-phosphotransfe... | 533 | 0.0 | 100% |
| EDX9152563.1 APH(3')-II family aminoglycoside O-phosphotransfe... | 533 | 0.0 | 100% |
| WP_163800230.1 APH(3')-II family aminoglycoside O-phosphotrans... | 533 | 0.0 | 100% |
| EGF4810723.1 APH(3')-II family aminoglycoside O-phosphotransfe... | 533 | 0.0 | 100% |
| WP_187790236.1 APH(3')-II family aminoglycoside O-phosphotrans... | 533 | 0.0 | 100% |
| AAA87574.1 neomycin resistance protein [Cloning vector pMC1neo] | 533 | 0.0 | 100% |
| WP_161571218.1 APH(3')-II family aminoglycoside O-phosphotrans... | 533 | 0.0 | 100% |
| WP_061182288.1 APH(3')-II family aminoglycoside O-phosphotrans... | 533 | 0.0 | 100% |
| WP_118142372.1 APH(3')-II family aminoglycoside O-phosphotrans... | 533 | 0.0 | 100% |
| WP_004614937.1 MULTISPECIES: APH(3')-II family aminoglycoside ... | 533 | 0.0 | 100% |
| WP_094545108.1 APH(3')-II family aminoglycoside O-phosphotrans... | 533 | 0.0 | 100% |
| QGM50382.1 NPTII (MO1) [Expression vector pHP81561] | 533 | 0.0 | 99% |
| AWV91929.1 Kan(r) [Cloning vector pFNK-202-qsc119] | 533 | 0.0 | 100% |
| AAD50549.1 aminoglycoside 3'-phosphotransferase mutant [synthe... | 533 | 0.0 | 100% |
| EBQ6784660.1 APH(3')-II family aminoglycoside O-phosphotransfe... | 533 | 0.0 | 100% |
| EEM0239697.1 APH(3')-II family aminoglycoside O-phosphotransfe... | 533 | 0.0 | 100% |
| AFN70907.1 neomycine phosphotransferase [Plant transformation ... | 533 | 0.0 | 100% |
| HAS1700150.1 TPA: APH(3')-II family aminoglycoside O-phosphotr... | 533 | 0.0 | 100% |
| EFB7215585.1 APH(3')-II family aminoglycoside O-phosphotransfe... | 533 | 0.0 | 100% |
| CAD27779.1 neomycin resistance protein [Cloning vector pTarg2] | 533 | 0.0 | 100% |
| EAO9809309.1 APH(3')-II family aminoglycoside O-phosphotransfe... | 533 | 0.0 | 100% |
| AAF82797.1 kanamycin resistance-conferring protein [synthetic ... | 533 | 0.0 | 100% |
| ABX65303.1 neomycin phosphotransferase [Cloning vector pSAM] | 533 | 0.0 | 100% |
| AFV73969.1 neomycin phosphotransferase [Cloning vector ATG-les... | 533 | 0.0 | 100% |
| AAF82796.1 kanamycin resistance-conferring protein [synthetic ... | 532 | 0.0 | 100% |
| ADP06893.1 NPTII [synthetic construct] [Nicotiana tabacum] | 532 | 0.0 | 100% |
| EEZ6492202.1 APH(3')-II family aminoglycoside O-phosphotransfe... | 532 | 0.0 | 100% |
| HAD8642335.1 TPA: APH(3')-II family aminoglycoside O-phosphotr... | 532 | 0.0 | 100% |
| EBW8244226.1 APH(3')-II family aminoglycoside O-phosphotransfe... | 532 | 0.0 | 100% |
| BAA21468.1 neomycin-kanamycin phosphotransferase [Cloning vect... | 532 | 0.0 | 100% |
| EEW8171270.1 APH(3')-II family aminoglycoside O-phosphotransfe... | 532 | 0.0 | 100% |
| EFC6378189.1 APH(3')-II family aminoglycoside O-phosphotransfe... | 532 | 0.0 | 100% |
| EDR5533633.1 APH(3')-II family aminoglycoside O-phosphotransfe... | 532 | 0.0 | 100% |
| ECW5825779.1 APH(3')-II family aminoglycoside O-phosphotransfe... | 532 | 0.0 | 100% |
| ECS5261092.1 APH(3')-II family aminoglycoside O-phosphotransfe... | 532 | 0.0 | 100% |
| AAL78958.1 neomycin phosphotransferase [Binary vector pLH9000] | 532 | 0.0 | 100% |
| EDJ1353951.1 APH(3') family aminoglycoside O-phosphotransferas... | 532 | 0.0 | 100% |
| WP_133425265.1 APH(3')-II family aminoglycoside O-phosphotrans... | 532 | 0.0 | 100% |
| ECM1697434.1 APH(3')-II family aminoglycoside O-phosphotransfe... | 532 | 0.0 | 100% |
| AAX14466.1 neomycin phosphotransferase [synthetic construct] | 532 | 0.0 | 100% |
| EBE1709735.1 APH(3')-II family aminoglycoside O-phosphotransfe... | 532 | 0.0 | 100% |
| AAA72847.1 neomycin phosphotransferase [unidentified cloning v... | 532 | 0.0 | 100% |
| AAC53631.1 neomycin phosphotransferase [Cloning vector pBSL99] | 532 | 0.0 | 100% |
| WP_150102527.1 APH(3')-II family aminoglycoside O-phosphotrans... | 532 | 0.0 | 100% |
| EDT4821918.1 APH(3')-II family aminoglycoside O-phosphotransfe... | 532 | 0.0 | 100% |
| ACV83807.1 NptII [Physcomitrella transformation vector pBS108-... | 532 | 0.0 | 99% |
| ABG81368.1 neomycin phosphotransferase II [Cloning vector pINT] | 531 | 0.0 | 100% |
| AAA72769.1 beta-glucuronidase:neomycin phosphotransferase II f... | 531 | 0.0 | 100% |
| AAK92455.1 PGK-neo [Retrofitting vector pRetroES] | 531 | 0.0 | 100% |
| PTB82200.1 APH(3')-II family aminoglycoside O-phosphotransfera... | 531 | 0.0 | 99% |
| WP_134413266.1 APH(3')-II family aminoglycoside O-phosphotrans... | 531 | 0.0 | 99% |
| ACH73255.1 AphII [Agrobacterium binary gap repair vector pGAP-Km] | 531 | 0.0 | 100% |
| BAL60567.1 neomycin phosphotransferase [Moss transformation ve... | 531 | 0.0 | 99% |
| WP_000572406.1 MULTISPECIES: APH(3')-II family aminoglycoside ... | 531 | 0.0 | 100% |
| ATG88232.1 neomycin phosphotransferase [Yeast dual vector pCtB1] | 531 | 0.0 | 100% |
| AAY29062.1 APH(3')-IIA [Cloning vector LUCTRAP-1] | 531 | 0.0 | 99% |
| QDG03204.1 kanamycin resistance protein [Cloning vector pGHKW4] | 530 | 0.0 | 100% |
| BBD52293.1 neomycin-kanamycin phosphotransferase [Cloning vect... | 530 | 0.0 | 99% |
| AGV76759.1 AphA [Cloning vector pAV012] | 530 | 0.0 | 100% |
| AQK76559.1 neomycin phosphotransferase [Cloning vector pL0A_0-1] | 530 | 0.0 | 99% |
| AOO95910.1 Meo [Cloning vector GAPTrapT2AIresMeo] | 530 | 0.0 | 100% |
| AMQ45842.1 neomycin-kanamycin phosphotransferase [Synthetic pl... | 530 | 0.0 | 99% |
| ALD65381.1 aminoglycoside phosphotransferase [Arthrobacter sp.... | 529 | 0.0 | 99% |
| WP_141989015.1 APH(3')-II family aminoglycoside O-phosphotrans... | 529 | 0.0 | 99% |
| AAL30400.1 neomycin phosphotransferase [Cloning vector pWS31] | 529 | 0.0 | 99% |
| AAA72821.1 heat-shock protein 82/neomycin phosphotransferase f... | 528 | 0.0 | 100% |

| WP_099526830.1 APH(3')-II family aminoglycoside O-phosphotrans... 528 | 0.0 | 99% |
| --- | --- | --- |
| WP_160187495.1 APH(3')-II family aminoglycoside O-phosphotrans... 528 | 0.0 | 100% |
| BAH70332.1 kanamycin resistance [synthetic construct] 528 | 0.0 | 99% |
| AII99780.1 nptII_A7120 [Broad host range vector vector pCVD012] 528 | 0.0 | 100% |
| EGJ3639417.1 APH(3')-II family aminoglycoside O-phosphotransfe... 528 | 0.0 | 100% |
| SJL88773.1 unnamed protein product [Vector pPNT] 528 | 0.0 | 99% |
| WP_128318427.1 APH(3')-II family aminoglycoside O-phosphotrans... 527 | 0.0 | 100% |
| AAK28132.1 aminoglycoside 3'-phosphotransferase [Cloning vecto... 525 | 0.0 | 98% |
| AAF85969.1 kanamycin resistance protein [synthetic construct] 524 | 0.0 | 100% |
| AAC15800.1 neomycin/kanamycin resistance protein [Eukaryotic e... 523 | 0.0 | 98% |
| AAQ05967.1 kanamycin resistance protein [Cloning vector pHells... 521 | 0.0 | 99% |
| WP_021018481.1 APH(3')-II family aminoglycoside O-phosphotrans... 516 | 0.0 | 96% |
| AHH29548.1 neomycin phosphotransferase II [Salmonella enterica... 508 | 0.0 | 100% |
| WP_109815304.1 APH(3')-II family aminoglycoside O-phosphotrans... 508 | 0.0 | 100% |
| EFN0055451.1 APH(3')-II family aminoglycoside O-phosphotransfe... 503 | 1e-179 | 100% |
| WP_057505145.1 APH(3')-II family aminoglycoside O-phosphotrans... 502 | 5e-179 | 93% |
| BAG12832.1 beta-geo-lessCpG [Exchangeable gene trap vector pU-... 535 | 3e-178 | 100% |
| AHB86974.1 beta-geo [PiggyBac insertional mutagenesis vector S... 535 | 3e-178 | 100% |
| AAB51768.1 beta-geo [synthetic construct] 534 | 4e-178 | 100% |
| AEL31639.1 beta-geo [Deletion homozygosity selection vector 1] 534 | 4e-178 | 100% |
| ADF55556.1 beta-geo [PiggyBac insertional mutagenesis vector S... 535 | 4e-178 | 100% |
| ACD62478.1 beta-geo [Gene trapping vector VICTR76] 534 | 5e-178 | 100% |
| ECS2458178.1 APH(3')-II family aminoglycoside O-phosphotransfe... 498 | 1e-177 | 100% |
| ADK39026.1 beta-geo [cloning vector pPNT] [Mus musculus] 533 | 2e-177 | 100% |
| CAA54105.1 lacZ neomycin phosphotransferase fusion protein [sy... 530 | 3e-176 | 100% |
| EBL6081130.1 APH(3')-II family aminoglycoside O-phosphotransfe... 494 | 5e-176 | 100% |
| WP_083563809.1 APH(3')-II family aminoglycoside O-phosphotrans... 494 | 7e-176 | 92% |
| WP_196524206.1 APH(3')-II family aminoglycoside O-phosphotrans... 492 | 2e-175 | 100% |
| EBR1815709.1 APH(3')-II family aminoglycoside O-phosphotransfe... 492 | 3e-175 | 100% |
| WP_031759051.1 MULTISPECIES: APH(3')-II family aminoglycoside ... 493 | 3e-175 | 92% |
| WP_100022224.1 APH(3')-II family aminoglycoside O-phosphotrans... 489 | 4e-174 | 100% |
| EDA9786694.1 APH(3')-II family aminoglycoside O-phosphotransfe... 489 | 6e-174 | 100% |
| HAL2320778.1 TPA: APH(3')-II family aminoglycoside O-phosphotr... 484 | 3e-172 | 100% |
| ABG21298.1 neomycin phosphotransferase [uncultured bacterium] 483 | 1e-171 | 99% |
| ABG21297.1 neomycin phosphotransferase [uncultured bacterium] 483 | 1e-171 | 99% |
| ABG21299.1 neomycin phosphotransferase [uncultured bacterium] 482 | 2e-171 | 99% |
| ARU77007.1 neomycin phosphotransferase II [synthetic construct] 482 | 3e-171 | 100% |
| WP_116003610.1 MULTISPECIES: APH(3')-II family aminoglycoside ... 483 | 3e-171 | 91% |
| ABG21300.1 neomycin phosphotransferase [uncultured bacterium] 481 | 4e-171 | 99% |
| AFM74288.1 aminoglycoside-3'-O-phosphotransferase [Riemerella ... 481 | 6e-171 | 100% |
| AFM74299.1 aminoglycoside-3'-O-phosphotransferase [Riemerella ... 480 | 1e-170 | 100% |
| ABG21302.1 neomycin phosphotransferase [uncultured bacterium] 480 | 1e-170 | 99% |
| ABG21296.1 neomycin phosphotransferase [uncultured bacterium] 479 | 3e-170 | 99% |
| AFM74301.1 aminoglycoside-3'-O-phosphotransferase [Riemerella ... 479 | 3e-170 | 100% |
| AFM74305.1 aminoglycoside-3'-O-phosphotransferase [Riemerella ... 479 | 3e-170 | 100% |
| AFM74291.1 aminoglycoside-3'-O-phosphotransferase [Riemerella ... 479 | 3e-170 | 100% |
| AFM74293.1 aminoglycoside-3'-O-phosphotransferase [Riemerella ... 479 | 4e-170 | 100% |
| AFM74319.1 aminoglycoside-3'-O-phosphotransferase [Riemerella ... 479 | 4e-170 | 100% |
| AFM74292.1 aminoglycoside-3'-O-phosphotransferase [Riemerella ... 478 | 7e-170 | 100% |
| AFM74320.1 aminoglycoside-3'-O-phosphotransferase [Riemerella ... 478 | 8e-170 | 100% |
| AFM74297.1 aminoglycoside-3'-O-phosphotransferase [Riemerella ... 478 | 8e-170 | 100% |
| AFM74304.1 aminoglycoside-3'-O-phosphotransferase [Riemerella ... 478 | 8e-170 | 100% |
| AFM74313.1 aminoglycoside-3'-O-phosphotransferase [Riemerella ... 478 | 9e-170 | 100% |
| AFM74298.1 aminoglycoside-3'-O-phosphotransferase [Riemerella ... 478 | 1e-169 | 99% |
| ABG21301.1 neomycin phosphotransferase [uncultured bacterium] 478 | 1e-169 | 98% |
| AFM74294.1 aminoglycoside-3'-O-phosphotransferase [Riemerella ... 477 | 3e-169 | 99% |
| AFM74316.1 aminoglycoside-3'-O-phosphotransferase [Riemerella ... 476 | 3e-169 | 99% |
| AFM74317.1 aminoglycoside-3'-O-phosphotransferase [Riemerella ... 476 | 5e-169 | 99% |
| AFM74296.1 aminoglycoside-3'-O-phosphotransferase [Riemerella ... 476 | 6e-169 | 99% |
| AFM74309.1 aminoglycoside-3'-O-phosphotransferase [Riemerella ... 476 | 6e-169 | 99% |
| AFM74311.1 aminoglycoside-3'-O-phosphotransferase [Riemerella ... 473 | 5e-168 | 99% |
| AFM74321.1 aminoglycoside-3'-O-phosphotransferase [Riemerella ... 473 | 1e-167 | 98% |
| AFM74290.1 aminoglycoside-3'-O-phosphotransferase [Riemerella ... 472 | 2e-167 | 98% |
| EDA9786412.1 APH(3')-II family aminoglycoside O-phosphotransfe... 466 | 2e-165 | 100% |
| WP_072712887.1 APH(3')-II family aminoglycoside O-phosphotrans... 465 | 7e-165 | 100% |
| WP_039697162.1 APH(3')-II family aminoglycoside O-phosphotrans... 464 | 2e-164 | 100% |
| ABO72539.1 neomycin phosphotransferase [Integrative expression... 459 | 3e-162 | 100% |
| EBB4175231.1 APH(3')-II family aminoglycoside O-phosphotransfe... 455 | 7e-161 | 100% |
| EBB4175234.1 APH(3')-II family aminoglycoside O-phosphotransfe... 454 | 1e-160 | 100% |
| EBB4175232.1 APH(3')-II family aminoglycoside O-phosphotransfe... 453 | 3e-160 | 100% |
| MHS33331.1 APH(3')-II family aminoglycoside O-phosphotransfera... 452 | 6e-160 | 100% |
| WP_109017674.1 APH(3')-II family aminoglycoside O-phosphotrans... 451 | 4e-159 | 100% |

| MHS33138.1 APH(3')-II family aminoglycoside O-phosphotransfera... | | | | | 437 | 4e-154 | 100% |
| --- | --- | --- | --- | --- | --- | --- | --- |
| EEC0615971.1 APH(3')-II family aminoglycoside O-phosphotransfe... | | | | | 436 | 1e-153 | 100% |
| EDA9634201.1 APH(3')-II family aminoglycoside O-phosphotransfe... | | | | | 435 | 5e-153 | 100% |
| WP_071211688.1 APH(3')-II family aminoglycoside O-phosphotrans... | | | | | 433 | 2e-152 | 99% |
| WP_192957320.1 APH(3')-II family aminoglycoside O-phosphotrans... | | | | | 432 | 4e-152 | 100% |
| EEZ8619431.1 APH(3')-II family aminoglycoside O-phosphotransfe... | | | | | 428 | 1e-150 | 100% |
| ABL86711.1 aminoglycoside phosphotransferase [Escherichia coli] | | | | | 415 | 3e-145 | 100% |
| WP_160518435.1 APH(3') family aminoglycoside O-phosphotransfer... | | | | | 414 | 7e-145 | 100% |
| HAA3509436.1 TPA: APH(3') family aminoglycoside O-phosphotrans... | | | | | 409 | 5e-143 | 100% |
| CCM44346.1 neomycin phosphotransferase [Cloning vector pSAWlox... | | | | | 405 | 2e-141 | 99% |
| EBB4180054.1 APH(3')-II family aminoglycoside O-phosphotransfe... | | | | | 394 | 3e-137 | 100% |
| WP_107804178.1 APH(3')-II family aminoglycoside O-phosphotrans... | | | | | 393 | 6e-136 | 73% |
| EBT1094324.1 APH(3')-II family aminoglycoside O-phosphotransfe... | | | | | 390 | 7e-136 | 100% |
| EBK1610741.1 APH(3')-II family aminoglycoside O-phosphotransfe... | | | | | 388 | 5e-135 | 100% |
| WP_148712105.1 APH(3')-II family aminoglycoside O-phosphotrans... | | | | | 386 | 3e-134 | 99% |
| AVX51082.1 Aminoglycoside 3'-phosphotransferase protein [Pseud... | | | | | 389 | 5e-134 | 72% |
| WP_000262467.1 MULTISPECIES: APH(3')-II family aminoglycoside ... | | | | | 388 | 7e-134 | 72% |
| EBR0922135.1 aminoglycoside 3'-phosphotransferase [Salmonella ... | | | | | 384 | 1e-133 | 100% |
| WP_188246630.1 phosphotransferase [Serratia marcescens] | | | | | 382 | 1e-132 | 100% |
| WP_039677830.1 aminoglycoside 3'-phosphotransferase [Enterobac... | | | | | 374 | 1e-129 | 99% |
| EDT1758707.1 aminoglycoside 3'-phosphotransferase [Salmonella ... | | | | | 370 | 4e-128 | 100% |
| AEP02953.1 neomycin phosphotransferase [Gene trapping and targ... | | | | | 365 | 4e-126 | 99% |
| EEM0239916.1 aminoglycoside 3'-phosphotransferase [Salmonella ... | | | | | 362 | 7e-125 | 99% |
| WP_071218897.1 aminoglycoside 3'-phosphotransferase [Acinetoba... | | | | | 361 | 2e-124 | 100% |
| WP_173190126.1 APH(3')-II family aminoglycoside O-phosphotrans... | | | | | 364 | 3e-124 | 67% |
| ANF07102.1 NPTII, partial [chloroplast transformation vector p... | | | | | 353 | 5e-121 | 100% |
| WP_031613531.1 aminoglycoside phosphotransferase APH(3') [Salm... | | | | | 351 | 1e-120 | 100% |
| WP_187588653.1 phosphotransferase [Arenibacter sp. BSSL-BM3] | | | | | 351 | 2e-120 | 99% |
| WP_033471608.1 aminoglycoside 3'-phosphotransferase [Bordetell... | | | | | 350 | 2e-120 | 100% |
| EPJ91417.1 aminoglycoside phosphotransferase [Pseudomonas sp. ... | | | | | 354 | 3e-120 | 68% |
| WP_033062581.1 MULTISPECIES: APH(3')-II family aminoglycoside ... | | | | | 352 | 7e-120 | 68% |
| EEX3345874.1 aminoglycoside 3'-phosphotransferase [Escherichia... | | | | | 349 | 7e-120 | 100% |
| WP_006239673.1 APH(3')-II family aminoglycoside O-phosphotrans... | | | | | 353 | 7e-120 | 67% |
| WP_139353145.1 APH(3')-II family aminoglycoside O-phosphotrans... | | | | | 351 | 6e-119 | 67% |
| QBB73052.1 aminoglycoside resistance protein [Streptococcus th... | | | | | 347 | 7e-119 | 100% |
| OOG85846.1 APH(3') family aminoglycoside O-phosphotransferase ... | | | | | 350 | 1e-118 | 67% |
| EBW8244220.1 aminoglycoside 3'-phosphotransferase [Salmonella ... | | | | | 345 | 2e-118 | 100% |
| WP_053180723.1 APH(3')-II family aminoglycoside O-phosphotrans... | | | | | 349 | 2e-118 | 67% |
| HAS1851170.1 TPA: phosphotransferase [Salmonella enterica subs... | | | | | 344 | 6e-118 | 100% |
| WP_029866945.1 APH(3')-II family aminoglycoside O-phosphotrans... | | | | | 346 | 3e-117 | 66% |
| WP_057193879.1 APH(3')-II family aminoglycoside O-phosphotrans... | | | | | 346 | 3e-117 | 65% |
| WP_186707530.1 APH(3')-II family aminoglycoside O-phosphotrans... | | | | | 343 | 4e-116 | 67% |
| EAO3620360.1 aminoglycoside 3'-phosphotransferase [Salmonella ... | | | | | 339 | 4e-116 | 100% |
| WP_122568535.1 APH(3')-II family aminoglycoside O-phosphotrans... | | | | | 342 | 1e-115 | 65% |
| WP_167657122.1 phosphotransferase [Salmonella enterica] | | | | | 337 | 6e-115 | 99% |
| WP_163858992.1 APH(3')-II family aminoglycoside O-phosphotrans... | | | | | 337 | 1e-113 | 64% |
| WP_019407658.1 APH(3')-II family aminoglycoside O-phosphotrans... | | | | | 336 | 3e-113 | 66% |
| WP_045632183.1 APH(3')-II family aminoglycoside O-phosphotrans... | | | | | 335 | 9e-113 | 66% |
| WP_125865759.1 APH(3')-II family aminoglycoside O-phosphotrans... | | | | | 335 | 9e-113 | 66% |
| WP_024031215.1 aminoglycoside 3'-phosphotransferase, partial [... WP_017245313.1 APH(3')-II family aminoglycoside O-phosphotrans... WP_183004849.1 APH(3')-II family aminoglycoside O-phosphotrans...  HAG18071.1 TPA: APH(3')-II family aminoglycoside O-phosphotran... | | | | | 330  333  333  333 | 1e-112  3e-112  3e-112  5e-112 | 99%  66%  60%  66% |
| HAO75576.1 TPA: APH(3')-II family aminoglycoside O-phosphotran... | | | | | 332 | 1e-111 | 66% |
| WP_084901678.1 | APH(3')-II | family | aminoglycoside | O-phosphotrans... | 332 | 1e-111 | 66% |
| WP_054093134.1 | APH(3')-II | family | aminoglycoside | O-phosphotrans... | 330 | 1e-110 | 68% |
| WP_045157838.1 | APH(3')-II | family | aminoglycoside | O-phosphotrans... | 329 | 1e-110 | 66% |
| WP_080970997.1 | APH(3')-II | family | aminoglycoside | O-phosphotrans... | 329 | 1e-110 | 64% |
| WP_174992756.1 | APH(3')-II | family | aminoglycoside | O-phosphotrans... | 330 | 2e-110 | 64% |
| WP_038659539.1 | APH(3')-II | family | aminoglycoside | O-phosphotrans... | 329 | 2e-110 | 66% |
| WP_122172600.1 | aminoglycoside 3'-phosphotransferase [Burkholde... | | | | 328 | 2e-110 | 65% |
| ALX16133.1 aminoglycoside phosphotransferase [Burkholderia cep... | | | | | 328 | 3e-110 | 64% |
| WP_156714764.1 APH(3')-II family aminoglycoside O-phosphotrans... | | | | | 328 | 4e-110 | 68% |
| VWC54586.1 aminoglycoside phosphotransferase APH(3') [Burkhold... | | | | | 328 | 5e-110 | 64% |
| WP_175997228.1 APH(3') family aminoglycoside O-phosphotransfer... | | | | | 328 | 5e-110 | 64% |
| WP_077683452.1 APH(3')-II family aminoglycoside O-phosphotrans... | | | | | 327 | 7e-110 | 67% |
| WP_043177745.1 APH(3')-II family aminoglycoside O-phosphotrans... | | | | | 327 | 8e-110 | 63% |
| WP_142918852.1 aminoglycoside 3'-phosphotransferase [Staphyloc... | | | | | 323 | 9e-110 | 100% |
| WP_155627211.1 aminoglycoside 3'-phosphotransferase [Burkholde... | | | | | 327 | 1e-109 | 63% |
| WP_176039657.1 APH(3') family aminoglycoside O-phosphotransfer... | | | | | 327 | 1e-109 | 64% |
| WP_125853427.1 APH(3')-II family aminoglycoside O-phosphotrans... | | | | | 327 | 2e-109 | 65% |
| QGW60326.1 hypothetical protein [bacterium] | | | | | 326 | 2e-109 | 67% |

| VBB16329.1 Aminoglycoside 3'-phosphotransferase,Aminoglycoside... | 327 | 2e-109 | 64% |
| --- | --- | --- | --- |
| WP_174978922.1 APH(3')-II family aminoglycoside O-phosphotrans... | 326 | 3e-109 | 64% |
| WP_174949184.1 APH(3') family aminoglycoside O-phosphotransfer... | 325 | 5e-109 | 64% |
| VWD16734.1 aminoglycoside phosphotransferase APH(3') [Burkhold... | 324 | 1e-108 | 64% |
| WP_137937931.1 APH(3')-II family aminoglycoside O-phosphotrans... | 324 | 1e-108 | 62% |
| WP_165881814.1 APH(3') family aminoglycoside O-phosphotransfer... | 320 | 1e-108 | 100% |
| WP_138300002.1 APH(3')-II family aminoglycoside O-phosphotrans... | 324 | 2e-108 | 67% |
| WP_182364673.1 APH(3') family aminoglycoside O-phosphotransfer... | 320 | 2e-108 | 100% |
| WP_175031633.1 APH(3') family aminoglycoside O-phosphotransfer... | 323 | 2e-108 | 64% |
| MBB1626375.1 APH(3') family aminoglycoside O-phosphotransferas... | 323 | 2e-108 | 63% |
| WP_167654213.1 APH(3') family aminoglycoside O-phosphotransfer... | 319 | 4e-108 | 100% |
| WP_175011774.1 APH(3') family aminoglycoside O-phosphotransfer... | 323 | 5e-108 | 64% |
| WP_057384224.1 APH(3')-II family aminoglycoside O-phosphotrans... | 322 | 8e-108 | 65% |
| WP_175004359.1 APH(3')-II family aminoglycoside O-phosphotrans... | 322 | 1e-107 | 63% |
| WP_176045972.1 APH(3') family aminoglycoside O-phosphotransfer... | 322 | 1e-107 | 63% |
| WP_175045087.1 APH(3') family aminoglycoside O-phosphotransfer... | 322 | 1e-107 | 64% |
| WP_089453407.1 APH(3')-II family aminoglycoside O-phosphotrans... | 322 | 1e-107 | 63% |
| VWB83275.1 aminoglycoside phosphotransferase APH(3') [Burkhold... | 322 | 1e-107 | 63% |
| WP_011355791.1 aminoglycoside 3'-phosphotransferase [Burkholde... | 322 | 1e-107 | 63% |
| KML48857.1 aminoglycoside phosphotransferase [Burkholderia cep... | 321 | 2e-107 | 67% |
| WP_069750481.1 aminoglycoside 3'-phosphotransferase [Burkholde... | 322 | 2e-107 | 64% |
| WP_174937040.1 APH(3') family aminoglycoside O-phosphotransfer... | 321 | 2e-107 | 63% |
| WP_174938983.1 APH(3') family aminoglycoside O-phosphotransfer... | 321 | 2e-107 | 63% |
| VWB66384.1 aminoglycoside phosphotransferase APH(3') [Burkhold... | 321 | 3e-107 | 63% |
| WP_174973015.1 APH(3')-II family aminoglycoside O-phosphotrans... | 320 | 8e-107 | 63% |
| CAB3970912.1 aminoglycoside phosphotransferase APH(3') [Burkho... | 319 | 2e-106 | 62% |
| WP_174960806.1 MULTISPECIES: APH(3') family aminoglycoside O-p... | 319 | 2e-106 | 62% |
| WP_174982902.1 APH(3') family aminoglycoside O-phosphotransfer... | 319 | 2e-106 | 63% |
| WP_175023006.1 APH(3')-II family aminoglycoside O-phosphotrans... | 319 | 2e-106 | 63% |
| WP_186248588.1 APH(3')-II family aminoglycoside O-phosphotrans... | 318 | 5e-106 | 62% |
| VWC44666.1 aminoglycoside phosphotransferase APH(3') [Burkhold... | 318 | 6e-106 | 61% |
| WP_122949180.1 aminoglycoside 3'-phosphotransferase [Burkholde... | 318 | 7e-106 | 61% |
| VWC03873.1 aminoglycoside phosphotransferase APH(3') [Burkhold... | 316 | 2e-105 | 62% |
| WP_192968248.1 APH(3') family aminoglycoside O-phosphotransfer... | 311 | 3e-105 | 100% |
| WP_174930574.1 APH(3') family aminoglycoside O-phosphotransfer... | 315 | 4e-105 | 63% |
| WP_174917470.1 APH(3') family aminoglycoside O-phosphotransfer... | 315 | 1e-104 | 62% |
| HAA1110234.1 TPA: APH(3') family aminoglycoside O-phosphotrans... | 310 | 2e-104 | 100% |
| WP_166954165.1 APH(3') family aminoglycoside O-phosphotransfer... | 314 | 2e-104 | 62% |
| ORT83030.1 APH(3') family aminoglycoside O-phosphotransferase ... | 313 | 5e-104 | 63% |
| WP_181211204.1 APH(3') family aminoglycoside O-phosphotransfer... | 308 | 8e-104 | 99% |
| KAF1038439.1 Aminoglycoside 3'-phosphotransferase [Burkholderi... | 311 | 1e-103 | 62% |
| WP_106409019.1 aminoglycoside 3'-phosphotransferase [Burkholde... | 310 | 5e-103 | 62% |
| WP_067650744.1 APH(3')-II family aminoglycoside O-phosphotrans... | 308 | 2e-102 | 63% |
| WP_175807858.1 APH(3') family aminoglycoside O-phosphotransfer... | 303 | 1e-100 | 62% |
| AIO44444.1 aminoglycoside 3'-phosphotransferase [Burkholderia ... | 303 | 2e-100 | 63% |
| KIS51351.1 aminoglycoside 3'-phosphotransferase [Burkholderia ... | 302 | 7e-100 | 63% |
| WP_181645687.1 APH(3')-II family aminoglycoside O-phosphotrans... | 301 | 2e-99 | 63% |
| RBL65130.1 APH(3') family aminoglycoside O-phosphotransferase ... | 296 | 2e-99 | 100% |
| CDN62684.1 Aminoglycoside 3'-phosphotransferase [Burkholderia ... | 301 | 3e-99 | 62% |
| ECZ5199065.1 phosphotransferase [Salmonella enterica subsp. en... | 296 | 4e-99 | 100% |
| WP_181159040.1 MULTISPECIES: APH(3')-II family aminoglycoside ... | 300 | 6e-99 | 62% |
| WP_034203382.1 APH(3')-II family aminoglycoside O-phosphotrans... | 299 | 6e-99 | 64% |
| EPZ90555.1 putative streptomycin 3''-kinase [Burkholderia ceno... | 300 | 6e-99 | 62% |
| WP_124550430.1 APH(3')-II family aminoglycoside O-phosphotrans... | 299 | 8e-99 | 64% |
| WP_124698428.1 aminoglycoside 3'-phosphotransferase [Burkholde... | 299 | 9e-99 | 61% |
| WP_043205170.1 APH(3')-II family aminoglycoside O-phosphotrans... | 299 | 1e-98 | 64% |
| WP_181365749.1 APH(3')-II family aminoglycoside O-phosphotrans... | 299 | 1e-98 | 62% |
| WP_077190840.1 MULTISPECIES: APH(3')-II family aminoglycoside ... | 298 | 2e-98 | 64% |
| WP_034195681.1 APH(3')-II family aminoglycoside O-phosphotrans... | 298 | 2e-98 | 64% |
| WP_059722735.1 MULTISPECIES: APH(3')-II family aminoglycoside ... | 298 | 2e-98 | 64% |
| WP_102898688.1 APH(3')-II family aminoglycoside O-phosphotrans... | 298 | 2e-98 | 64% |
| WP_125332658.1 APH(3')-II family aminoglycoside O-phosphotrans... | 298 | 3e-98 | 64% |
| WP_062885658.1 MULTISPECIES: APH(3')-II family aminoglycoside ... | 298 | 3e-98 | 64% |
| WP_167665805.1 phosphotransferase [Salmonella enterica] | 293 | 4e-98 | 99% |
| WP_111442483.1 APH(3')-II family aminoglycoside O-phosphotrans... | 298 | 6e-98 | 62% |
| WP_138808442.1 aminoglycoside 3'-phosphotransferase [Salmonell... | 292 | 8e-98 | 100% |
| WP_105848337.1 APH(3')-II family aminoglycoside O-phosphotrans... | 298 | 8e-98 | 62% |
| SOT42392.1 Aminoglycoside 3'-phosphotransferase [Burkholderia ... | 297 | 1e-97 | 62% |
| ALV60636.1 aminoglycoside phosphotransferase [Burkholderia cen... | 296 | 1e-97 | 64% |
| WP_077184043.1 APH(3')-II family aminoglycoside O-phosphotrans... | 296 | 2e-97 | 64% |
| AOK39449.1 aminoglycoside phosphotransferase [Burkholderia cen... | 295 | 3e-97 | 64% |
| EBV9530355.1 aminoglycoside 3'-phosphotransferase [Salmonella ... | 290 | 4e-97 | 100% |

| WP_091664058.1 APH(3')-II family aminoglycoside O-phosphotrans... | 294 | 1e-96 | 65% |
| --- | --- | --- | --- |
| WP_070638544.1 MULTISPECIES: aminoglycoside 3'-phosphotransfer... | 293 | 3e-96 | 61% |
| WP_141835329.1 aminoglycoside 3'-phosphotransferase [Herbaspir... | 293 | 3e-96 | 60% |
| EBK2638683.1 aminoglycoside 3'-phosphotransferase [Salmonella ... | 288 | 5e-96 | 100% |
| WP_175842659.1 APH(3') family aminoglycoside O-phosphotransfer... | 293 | 7e-96 | 61% |
| WP_048411529.1 APH(3')-II family aminoglycoside O-phosphotrans... | 292 | 7e-96 | 61% |
| WP_135471052.1 aminoglycoside 3'-phosphotransferase [Crenalkal... | 286 | 1e-95 | 100% |
| WP_153069972.1 aminoglycoside 3'-phosphotransferase [Escherich... | 285 | 3e-95 | 100% |
| WP_109329702.1 aminoglycoside 3'-phosphotransferase [Azospiril... | 290 | 5e-95 | 60% |
| WP_110782320.1 aminoglycoside 3'-phosphotransferase [Rhodopseu... | 290 | 7e-95 | 59% |
| WP_107143287.1 aminoglycoside 3'-phosphotransferase [Massilia ... | 288 | 2e-94 | 58% |
| WP_145875946.1 APH(3')-II family aminoglycoside O-phosphotrans... | 287 | 4e-94 | 67% |
| TWI47673.1 aminoglycoside 3'-phosphotransferase-2 [Massilia fl... | 287 | 6e-94 | 67% |
| WP_153886501.1 aminoglycoside 3'-phosphotransferase [Acinetoba... | 282 | 6e-94 | 100% |
| PTF36309.1 APH(3') family aminoglycoside O-phosphotransferase ... | 281 | 1e-93 | 100% |
| HAJ2624193.1 TPA: APH(3') family aminoglycoside O-phosphotrans... | 279 | 1e-92 | 100% |
| TGV08178.1 aminoglycoside 3'-phosphotransferase [Alcaligenacea... | 281 | 8e-92 | 55% |
| WP_166235037.1 APH(3') family aminoglycoside O-phosphotransfer... | 281 | 2e-91 | 59% |
| WP_046795547.1 aminoglycoside 3'-phosphotransferase [Rhizobium... | 280 | 2e-91 | 54% |
| WP_169434572.1 APH(3') family aminoglycoside O-phosphotransfer... | 280 | 2e-91 | 60% |
| WP_115731144.1 aminoglycoside 3'-phosphotransferase [Aminobact... | 280 | 3e-91 | 54% |
| WP_192315073.1 APH(3') family aminoglycoside O-phosphotransfer... | 280 | 4e-91 | 54% |
| WP_137932453.1 aminoglycoside 3'-phosphotransferase [Mesorhizo... | 280 | 4e-91 | 56% |
| PZQ17451.1 APH(3')-II family aminoglycoside O-phosphotransfera... | 279 | 8e-91 | 61% |
| WP_112935848.1 aminoglycoside 3'-phosphotransferase [Massilia ... | 278 | 1e-90 | 60% |
| WP_105378816.1 APH(3')-II family aminoglycoside O-phosphotrans... | 278 | 2e-90 | 66% |
| TXE23233.1 aminoglycoside 3'-phosphotransferase [[Empedobacter... | 278 | 3e-90 | 57% |
| WP_173517697.1 APH(3') family aminoglycoside O-phosphotransfer... | 275 | 2e-89 | 53% |
| APF39609.1 APH(3') family aminoglycoside O-phosphotransferase ... | 275 | 3e-89 | 57% |
| WP_082464773.1 MULTISPECIES: APH(3')-II family aminoglycoside ... | 275 | 4e-89 | 57% |
| WP_036238904.1 APH(3')-II family aminoglycoside O-phosphotrans... | 273 | 1e-88 | 65% |
| WP_052225993.1 APH(3')-II family aminoglycoside O-phosphotrans... | 274 | 1e-88 | 54% |
| MBB4019514.1 aminoglycoside 3'-phosphotransferase-2 [Chelatoco... | 273 | 1e-88 | 59% |
| WP_183318411.1 APH(3')-II family aminoglycoside O-phosphotrans... | 273 | 2e-88 | 59% |
| WP_166408899.1 APH(3') family aminoglycoside O-phosphotransfer... | 273 | 2e-88 | 55% |
| WP_143354576.1 aminoglycoside 3'-phosphotransferase [Erwinia a... | 267 | 3e-88 | 100% |
| OJU33800.1 APH(3') family aminoglycoside O-phosphotransferase ... | 272 | 6e-88 | 54% |
| WP_065774127.1 MULTISPECIES: aminoglycoside 3'-phosphotransfer... | 270 | 2e-87 | 54% |
| WP_019402260.1 MULTISPECIES: APH(3')-II family aminoglycoside ... | 270 | 3e-87 | 58% |
| WP_109578104.1 aminoglycoside 3'-phosphotransferase [Aminobact... | 269 | 5e-87 | 50% |
| BBF98342.1 aminoglycoside 3'-phosphotransferase, partial [synt... | 264 | 6e-87 | 99% |
| WP_026619993.1 aminoglycoside 3'-phosphotransferase [Ensifer s... | 268 | 1e-86 | 54% |
| WP_147849977.1 APH(3')-II family aminoglycoside O-phosphotrans... | 268 | 2e-86 | 56% |
| EFH3592115.1 phosphotransferase [Escherichia coli] | 263 | 2e-86 | 100% |
| WP_196687139.1 APH(3')-II family aminoglycoside O-phosphotrans... | 268 | 2e-86 | 54% |
| WP_195178681.1 APH(3') family aminoglycoside O-phosphotransfer... | 268 | 2e-86 | 55% |
| WP_125910168.1 APH(3')-II family aminoglycoside O-phosphotrans... | 267 | 3e-86 | 53% |
| WP_046120501.1 MULTISPECIES: aminoglycoside 3'-phosphotransfer... | 267 | 4e-86 | 53% |
| WP_082005961.1 aminoglycoside 3'-phosphotransferase [Ensifer s... | 269 | 5e-86 | 52% |
| WP_116274031.1 aminoglycoside 3'-phosphotransferase [Rhizobium... | 266 | 6e-86 | 52% |
| WP_182510979.1 APH(3') family aminoglycoside O-phosphotransfer... | 266 | 7e-86 | 52% |
| WP_026615559.1 aminoglycoside 3'-phosphotransferase [Ensifer a... | 266 | 9e-86 | 53% |
| MBA8903988.1 aminoglycoside 3'-phosphotransferase-2 [Phyllobac... | 266 | 1e-85 | 52% |
| WP_083335564.1 MULTISPECIES: aminoglycoside 3'-phosphotransfer... | 266 | 1e-85 | 52% |
| WP_057383888.1 APH(3')-II family aminoglycoside O-phosphotrans... | 266 | 1e-85 | 55% |
| OHV23727.1 APH(3') family aminoglycoside O-phosphotransferase ... | 265 | 1e-85 | 52% |
| WP_140734246.1 aminoglycoside 3'-phosphotransferase [Mesorhizo... | 266 | 1e-85 | 55% |
| WP_105735079.1 aminoglycoside 3'-phosphotransferase [Phyllobac... | 265 | 2e-85 | 54% |
| WP_049736257.1 aminoglycoside 3'-phosphotransferase [Rhizobium... | 265 | 2e-85 | 52% |
| WP_184344750.1 APH(3') family aminoglycoside O-phosphotransfer... | 265 | 2e-85 | 52% |
| WP_196658889.1 APH(3')-II family aminoglycoside O-phosphotrans... | 265 | 2e-85 | 53% |
| ETR77329.1 aminoglycoside 3'-phosphotransferase [Afipia sp. P5... | 265 | 2e-85 | 52% |
| PWV89634.1 aminoglycoside 3'-phosphotransferase-2 [Phyllobacte... | 265 | 2e-85 | 54% |
| MBG6825528.1 APH(3')-II family aminoglycoside O-phosphotransfe... | 265 | 2e-85 | 54% |
| WP_034468071.1 aminoglycoside 3'-phosphotransferase [Afipia sp... | 265 | 2e-85 | 52% |
| WP_140771434.1 aminoglycoside 3'-phosphotransferase [Mesorhizo... | 265 | 3e-85 | 53% |
| WP_033936220.1 APH(3')-II family aminoglycoside O-phosphotrans... | 265 | 3e-85 | 54% |
| PYF57585.1 aminoglycoside 3'-phosphotransferase-2 [Ensifer sp.... | 265 | 3e-85 | 54% |
| WP_110878793.1 aminoglycoside 3'-phosphotransferase [Ensifer s... | 265 | 3e-85 | 54% |
| WP_024925422.1 MULTISPECIES: aminoglycoside 3'-phosphotransfer... | 265 | 3e-85 | 55% |
| WP_163273106.1 APH(3') family aminoglycoside O-phosphotransfer... | 265 | 4e-85 | 55% |
| WP_034081652.1 APH(3')-II family aminoglycoside O-phosphotrans... | 265 | 4e-85 | 54% |

| NUS67715.1 aminoglycoside 3'-phosphotransferase [Ensifer adhae... | | | | | 264 | 5e-85 | 51% |
| --- | --- | --- | --- | --- | --- | --- | --- |
| WP_146777229.1 phosphotransferase [Pseudomonas sp. MWU12-2534b] | | | | | 259 | 6e-85 | 100% |
| MBG6543847.1 APH(3')-II family aminoglycoside O-phosphotransfe... | | | | | 264 | 7e-85 | 53% |
| WP_003124042.1 APH(3')-II family aminoglycoside O-phosphotrans... | | | | | 264 | 7e-85 | 53% |
| WP_192450931.1 APH(3') family aminoglycoside O-phosphotransfer... | | | | | 265 | 8e-85 | 53% |
| WP_112947666.1 aminoglycoside 3'-phosphotransferase [Sinorhizo... | | | | | 263 | 8e-85 | 54% |
| WP_116800860.1 APH(3')-II family aminoglycoside O-phosphotrans... | | | | | 264 | 9e-85 | 53% |
| WP_153517559.1 aminoglycoside 3'-phosphotransferase [Sinorhizo... | | | | | 263 | 1e-84 | 55% |
| WP_070336929.1 APH(3')-II family aminoglycoside O-phosphotrans... | | | | | 263 | 1e-84 | 53% |
| WP_126676646.1 APH(3')-II family aminoglycoside O-phosphotrans... | | | | | 263 | 1e-84 | 53% |
| WP_146722387.1 aminoglycoside 3'-phosphotransferase [Sinorhizo... | | | | | 263 | 1e-84 | 55% |
| WP_070656501.1 APH(3')-II family aminoglycoside O-phosphotrans... | | | | | 263 | 1e-84 | 53% |
| WP_096246790.1 APH(3')-II family aminoglycoside O-phosphotrans... | | | | | 263 | 1e-84 | 53% |
| WP_023518031.1 MULTISPECIES: APH(3')-II family aminoglycoside ... | | | | | 263 | 1e-84 | 53% |
| WP_035095871.1 aminoglycoside 3'-phosphotransferase [Devosia s... | | | | | 263 | 2e-84 | 54% |
| WP_003157056.1 | APH(3')-II | family | aminoglycoside | O-phosphotrans... | 263 | 2e-84 | 54% |
| WP_033969574.1 | APH(3')-II | family | aminoglycoside | O-phosphotrans... | 263 | 2e-84 | 53% |
| WP_149586404.1 | APH(3')-II | family | aminoglycoside | O-phosphotrans... | 263 | 2e-84 | 53% |
| WP_163802427.1 | APH(3')-II | family | aminoglycoside | O-phosphotrans... | 263 | 2e-84 | 53% |
| WP_124988352.1 | APH(3')-II | family | aminoglycoside | O-phosphotrans... | 263 | 2e-84 | 54% |
| WP_033977771.1 | APH(3')-II | family | aminoglycoside | O-phosphotrans... | 263 | 2e-84 | 53% |
| WP_109242846.1 | APH(3')-II | family | aminoglycoside | O-phosphotrans... | 263 | 2e-84 | 53% |
| WP_134622164.1 | APH(3')-II | family | aminoglycoside | O-phosphotrans... | 263 | 2e-84 | 53% |
| WP_196701535.1 | APH(3')-II | family | aminoglycoside | O-phosphotrans... | 263 | 2e-84 | 53% |
| WP_057382169.1 | APH(3')-II | family | aminoglycoside | O-phosphotrans... | 263 | 2e-84 | 53% |
| WP_096760010.1 | APH(3')-II | family | aminoglycoside | O-phosphotrans... | 263 | 2e-84 | 53% |
| WP_003137562.1 | MULTISPECIES: APH(3')-II family aminoglycoside ... | | | | 263 | 2e-84 | 53% |
| WP_182400191.1 | APH(3') family aminoglycoside O-phosphotransfer... | | | | 263 | 2e-84 | 54% |
| WP_003121928.1 | MULTISPECIES: APH(3')-II family aminoglycoside ... | | | | 263 | 2e-84 | 53% |
| WP_070748368.1 | MULTISPECIES: APH(3')-II family aminoglycoside ... | | | | 263 | 2e-84 | 53% |
| WP_003093441.1 | MULTISPECIES: APH(3')-II family aminoglycoside ... | | | | 263 | 2e-84 | 53% |
| WP_004345974.1 | MULTISPECIES: APH(3')-II family aminoglycoside ... | | | | 263 | 2e-84 | 53% |
| WP_124138621.1 | APH(3')-II family aminoglycoside O-phosphotrans... | | | | 263 | 2e-84 | 53% |
| WP_062842754.1 | APH(3')-II family aminoglycoside O-phosphotrans... | | | | 263 | 2e-84 | 53% |
| WP_116805535.1 | APH(3')-II family aminoglycoside O-phosphotrans... | | | | 263 | 2e-84 | 53% |
| WP_073669326.1 | APH(3')-II family aminoglycoside O-phosphotrans... | | | | 263 | 2e-84 | 53% |
| WP_077968675.1 | aminoglycoside 3'-phosphotransferase [Ensifer a... | | | | 263 | 2e-84 | 51% |
| MBG6562873.1 APH(3')-II family aminoglycoside O-phosphotransfe... | | | | | 263 | 2e-84 | 53% |
| WP_127527170.1 aminoglycoside 3'-phosphotransferase [Sinorhizo... | | | | | 262 | 3e-84 | 55% |
| WP_070623109.1 APH(3')-II family aminoglycoside O-phosphotrans... | | | | | 263 | 3e-84 | 53% |
| WP_033993831.1 APH(3')-II family aminoglycoside O-phosphotrans... | | | | | 263 | 3e-84 | 53% |
| WP_061360967.1 APH(3')-II family aminoglycoside O-phosphotrans... | | | | | 263 | 3e-84 | 53% |
| WP_035031015.1 aminoglycoside 3'-phosphotransferase [Devosia s... | | | | | 262 | 3e-84 | 54% |
| WP_010970345.1 MULTISPECIES: aminoglycoside 3'-phosphotransfer... | | | | | 262 | 3e-84 | 55% |
| WP_104665589.1 aminoglycoside 3'-phosphotransferase [Ensifer a... | | | | | 264 | 3e-84 | 51% |
| WP_023109641.1 APH(3')-II family aminoglycoside O-phosphotrans... | | | | | 263 | 3e-84 | 53% |
| WP_132555846.1 APH(3')-II family aminoglycoside O-phosphotrans... | | | | | 263 | 3e-84 | 53% |
| WP_058179574.1 APH(3')-II family aminoglycoside O-phosphotrans... | | | | | 262 | 3e-84 | 53% |
| MBG6828692.1 APH(3')-II family aminoglycoside O-phosphotransfe... | | | | | 262 | 3e-84 | 53% |
| WP_124131361.1 APH(3')-II family aminoglycoside O-phosphotrans... | | | | | 262 | 3e-84 | 53% |
| WP_061197678.1 APH(3')-II family aminoglycoside O-phosphotrans... | | | | | 262 | 3e-84 | 53% |
| WP_073649904.1 APH(3')-II family aminoglycoside O-phosphotrans... | | | | | 262 | 4e-84 | 53% |
| PLP56035.1 APH(3') family aminoglycoside O-phosphotransferase ... | | | | | 262 | 4e-84 | 53% |
| WP_093233146.1 aminoglycoside 3'-phosphotransferase [Sinorhizo... | | | | | 262 | 4e-84 | 53% |
| SDA69881.1 aminoglycoside 3'-phosphotransferase-2 [Sinorhizobi... | | | | | 262 | 4e-84 | 53% |
| WP_180883024.1 APH(3') family aminoglycoside O-phosphotransfer... | | | | | 262 | 4e-84 | 53% |
| WP_019371448.1 APH(3')-II family aminoglycoside O-phosphotrans... | | | | | 262 | 4e-84 | 53% |
| MBG6570794.1 APH(3')-II family aminoglycoside O-phosphotransfe... | | | | | 262 | 4e-84 | 53% |
| WP_003113011.1 MULTISPECIES: aminoglycoside O-phosphotransfera... | | | | | 262 | 4e-84 | 53% |
| WP_023105023.1 APH(3')-II family aminoglycoside O-phosphotrans... | | | | | 262 | 4e-84 | 53% |
| WP_023122977.1 APH(3')-II family aminoglycoside O-phosphotrans... | | | | | 262 | 4e-84 | 53% |
| WP_156414742.1 MULTISPECIES: aminoglycoside 3'-phosphotransfer... | | | | | 261 | 4e-84 | 53% |
| WP_127535131.1 aminoglycoside 3'-phosphotransferase [Sinorhizo... | | | | | 262 | 5e-84 | 55% |
| KRD50177.1 aminoglycoside phosphotransferase [Ensifer sp. Root... | | | | | 262 | 5e-84 | 53% |
| WP_083556162.1 APH(3')-II family aminoglycoside O-phosphotrans... | | | | | 262 | 5e-84 | 53% |
| WP_058018060.1 APH(3')-II family aminoglycoside O-phosphotrans... | | | | | 262 | 5e-84 | 53% |
| WP_074207167.1 APH(3')-II family aminoglycoside O-phosphotrans... | | | | | 262 | 6e-84 | 54% |
| WP_114232455.1 APH(3')-II family aminoglycoside O-phosphotrans... | | | | | 262 | 6e-84 | 53% |
| WP_196667399.1 APH(3')-II family aminoglycoside O-phosphotrans... | | | | | 262 | 6e-84 | 53% |
| WP_184699706.1 APH(3') family aminoglycoside O-phosphotransfer... | | | | | 261 | 6e-84 | 52% |
| WP_058181524.1 APH(3')-II family aminoglycoside O-phosphotrans... | | | | | 262 | 6e-84 | 53% |
| WP_070335920.1 APH(3')-II family aminoglycoside O-phosphotrans... | | | | | 262 | 6e-84 | 53% |

| WP_126859588.1 APH(3')-II family aminoglycoside O-phosphotrans... | 261 | 6e-84 | 53% |
| --- | --- | --- | --- |
| WP_023874917.1 APH(3')-II family aminoglycoside O-phosphotrans... | 261 | 6e-84 | 53% |
| WP_127719427.1 aminoglycoside 3'-phosphotransferase [Sinorhizo... | 261 | 7e-84 | 55% |
| WP_023108306.1 MULTISPECIES: APH(3')-II family aminoglycoside ... | 261 | 7e-84 | 53% |
| WP_134280349.1 APH(3')-II family aminoglycoside O-phosphotrans... | 261 | 7e-84 | 53% |
| WP_126622713.1 APH(3')-II family aminoglycoside O-phosphotrans... | 261 | 7e-84 | 53% |
| WP_143480337.1 APH(3')-II family aminoglycoside O-phosphotrans... | 261 | 7e-84 | 53% |
| MBG6313062.1 APH(3')-II family aminoglycoside O-phosphotransfe... | 261 | 7e-84 | 53% |
| WP_134481983.1 APH(3')-II family aminoglycoside O-phosphotrans... | 261 | 7e-84 | 53% |
| WP_058177127.1 APH(3')-II family aminoglycoside O-phosphotrans... | 261 | 7e-84 | 53% |
| WP_113399506.1 aminoglycoside 3'-phosphotransferase [Sinorhizo... | 261 | 7e-84 | 53% |
| WP_126118968.1 APH(3')-II family aminoglycoside O-phosphotrans... | 261 | 7e-84 | 53% |
| MBG6527585.1 APH(3')-II family aminoglycoside O-phosphotransfe... | 261 | 8e-84 | 53% |
| WP_172348945.1 APH(3') family aminoglycoside O-phosphotransfer... | 261 | 8e-84 | 53% |
| WP_031692397.1 APH(3')-II family aminoglycoside O-phosphotrans... | 261 | 8e-84 | 53% |
| KAA1182777.1 aminoglycoside 3'-phosphotransferase [Rhizobium t... | 261 | 8e-84 | 51% |
| MBG6702430.1 APH(3')-II family aminoglycoside O-phosphotransfe... | 261 | 8e-84 | 53% |
| WP_132710354.1 APH(3')-II family aminoglycoside O-phosphotrans... | 261 | 8e-84 | 53% |
| WP_153500606.1 aminoglycoside 3'-phosphotransferase [Sinorhizo... | 261 | 8e-84 | 54% |
| WP_132678652.1 APH(3')-II family aminoglycoside O-phosphotrans... | 261 | 8e-84 | 53% |
| SQC59007.1 aminoglycoside 3-N-acetyltransferase [Pseudomonas a... | 261 | 9e-84 | 53% |
| MBG7045669.1 APH(3')-II family aminoglycoside O-phosphotransfe... | 261 | 9e-84 | 53% |
| WP_188112553.1 APH(3') family aminoglycoside O-phosphotransfer... | 261 | 1e-83 | 51% |
| WP_130272335.1 aminoglycoside 3'-phosphotransferase [Phyllobac... | 261 | 1e-83 | 54% |
| WP_132565192.1 APH(3')-II family aminoglycoside O-phosphotrans... | 261 | 1e-83 | 53% |
| WP_116844549.1 APH(3')-II family aminoglycoside O-phosphotrans... | 261 | 1e-83 | 53% |
| WP_034022832.1 APH(3')-II family aminoglycoside O-phosphotrans... | 261 | 1e-83 | 53% |
| WP_034032241.1 APH(3')-II family aminoglycoside O-phosphotrans... | 261 | 1e-83 | 53% |
| WP_132659571.1 APH(3')-II family aminoglycoside O-phosphotrans... | 261 | 1e-83 | 53% |
| RZS79098.1 aminoglycoside 3'-phosphotransferase-2 [Phyllobacte... | 261 | 1e-83 | 54% |
| WP_125001236.1 APH(3')-II family aminoglycoside O-phosphotrans... | 261 | 1e-83 | 53% |
| MBG6964287.1 APH(3')-II family aminoglycoside O-phosphotransfe... | 261 | 1e-83 | 53% |
| MBG6484523.1 APH(3')-II family aminoglycoside O-phosphotransfe... | 261 | 1e-83 | 53% |
| MBG6557881.1 APH(3')-II family aminoglycoside O-phosphotransfe... | 261 | 1e-83 | 53% |
| WP_127583821.1 aminoglycoside 3'-phosphotransferase [Sinorhizo... | 260 | 1e-83 | 54% |
| WP_009313327.1 MULTISPECIES: APH(3')-II family aminoglycoside ... | 261 | 1e-83 | 53% |
| WP_196672556.1 APH(3')-II family aminoglycoside O-phosphotrans... | 261 | 1e-83 | 53% |
| WP_192568609.1 APH(3') family aminoglycoside O-phosphotransfer... | 261 | 2e-83 | 53% |
| WP_181855713.1 APH(3')-II family aminoglycoside O-phosphotrans... | 261 | 2e-83 | 53% |
| WP_058150946.1 APH(3')-II family aminoglycoside O-phosphotrans... | 261 | 2e-83 | 53% |
| WP_010793441.1 MULTISPECIES: APH(3')-II family aminoglycoside ... | 261 | 2e-83 | 53% |
| WP_024915527.1 APH(3')-II family aminoglycoside O-phosphotrans... | 261 | 2e-83 | 53% |
| WP_134288241.1 APH(3')-II family aminoglycoside O-phosphotrans... | 261 | 2e-83 | 53% |
| WP_003101274.1 MULTISPECIES: APH(3')-II family aminoglycoside ... | 261 | 2e-83 | 53% |
| WP_128695974.1 APH(3')-II family aminoglycoside O-phosphotrans... | 261 | 2e-83 | 53% |
| WP_083203601.1 aminoglycoside 3'-phosphotransferase [Ensifer a... | 260 | 2e-83 | 52% |
| WP_003132309.1 MULTISPECIES: aminoglycoside O-phosphotransfera... | 261 | 2e-83 | 53% |
| WP_128687719.1 APH(3')-II family aminoglycoside O-phosphotrans... | 261 | 2e-83 | 53% |
| ANK74915.1 APH(3') family aminoglycoside O-phosphotransferase ... | 260 | 2e-83 | 52% |
| WP_042931753.1 APH(3')-II family aminoglycoside O-phosphotrans... | 261 | 2e-83 | 53% |
| WP_196669784.1 APH(3')-II family aminoglycoside O-phosphotrans... | 261 | 2e-83 | 53% |
| WP_134536175.1 APH(3')-II family aminoglycoside O-phosphotrans... | 260 | 2e-83 | 53% |
| MBG7140394.1 APH(3')-II family aminoglycoside O-phosphotransfe... | 260 | 2e-83 | 53% |
| WP_034026268.1 APH(3')-II family aminoglycoside O-phosphotrans... | 260 | 2e-83 | 53% |
| WP_023097209.1 APH(3')-II family aminoglycoside O-phosphotrans... | 260 | 2e-83 | 53% |
| WP_121238749.1 APH(3')-II family aminoglycoside O-phosphotrans... | 260 | 2e-83 | 53% |
| WP_033951409.1 APH(3')-II family aminoglycoside O-phosphotrans... | 260 | 2e-83 | 53% |
| WP_196706147.1 APH(3')-II family aminoglycoside O-phosphotrans... | 260 | 2e-83 | 53% |
| WP_196660279.1 APH(3')-II family aminoglycoside O-phosphotrans... | 260 | 2e-83 | 53% |
| WP_144377070.1 aminoglycoside 3'-phosphotransferase [Mesorhizo... | 260 | 2e-83 | 52% |
| WP_196689763.1 APH(3')-II family aminoglycoside O-phosphotrans... | 260 | 2e-83 | 53% |
| WP_124125952.1 APH(3')-II family aminoglycoside O-phosphotrans... | 260 | 2e-83 | 53% |
| MBG6500747.1 APH(3')-II family aminoglycoside O-phosphotransfe... | 260 | 2e-83 | 53% |
| WP_031652465.1 APH(3')-II family aminoglycoside O-phosphotrans... | 260 | 2e-83 | 53% |
| WP_128666488.1 APH(3')-II family aminoglycoside O-phosphotrans... | 260 | 2e-83 | 53% |
| WP_058161625.1 APH(3')-II family aminoglycoside O-phosphotrans... | 260 | 2e-83 | 53% |
| WP_087587933.1 APH(3')-II family aminoglycoside O-phosphotrans... | 260 | 2e-83 | 53% |
| WP_124097390.1 APH(3')-II family aminoglycoside O-phosphotrans... | 260 | 2e-83 | 53% |
| WP_121290907.1 APH(3')-II family aminoglycoside O-phosphotrans... | 260 | 2e-83 | 53% |
| WP_057388031.1 APH(3')-II family aminoglycoside O-phosphotrans... | 260 | 2e-83 | 53% |
| WP_023118901.1 APH(3')-II family aminoglycoside O-phosphotrans... | 260 | 2e-83 | 53% |
| WP_124120135.1 APH(3')-II family aminoglycoside O-phosphotrans... | 260 | 2e-83 | 53% |

| WP_125884705.1 APH(3')-II family aminoglycoside O-phosphotrans... | 260 | 3e-83 | 53% |
| --- | --- | --- | --- |
| WP_094954585.1 APH(3')-II family aminoglycoside O-phosphotrans... | 260 | 3e-83 | 53% |
| WP_128552421.1 APH(3')-II family aminoglycoside O-phosphotrans... | 260 | 3e-83 | 53% |
| WP_171428883.1 APH(3')-II family aminoglycoside O-phosphotrans... | 260 | 3e-83 | 53% |
| WP_056577984.1 MULTISPECIES: aminoglycoside 3'-phosphotransfer... | 260 | 3e-83 | 56% |
| WP_196704556.1 APH(3')-II family aminoglycoside O-phosphotrans... | 260 | 3e-83 | 53% |
| MBG6633171.1 APH(3')-II family aminoglycoside O-phosphotransfe... | 260 | 3e-83 | 53% |
| WP_060627542.1 APH(3')-II family aminoglycoside O-phosphotrans... | 260 | 3e-83 | 53% |
| WP_196628099.1 APH(3')-II family aminoglycoside O-phosphotrans... | 260 | 3e-83 | 53% |
| MBG7159259.1 APH(3')-II family aminoglycoside O-phosphotransfe... | 260 | 3e-83 | 53% |
| MBG7397283.1 APH(3')-II family aminoglycoside O-phosphotransfe... | 260 | 3e-83 | 53% |
| WP_049950167.1 APH(3')-II family aminoglycoside O-phosphotrans... | 260 | 3e-83 | 54% |
| WP_181261834.1 APH(3')-II family aminoglycoside O-phosphotrans... | 259 | 3e-83 | 53% |
| WP_114230448.1 APH(3')-II family aminoglycoside O-phosphotrans... | 260 | 4e-83 | 53% |
| WP_126560154.1 APH(3')-II family aminoglycoside O-phosphotrans... | 259 | 4e-83 | 53% |
| WP_061361696.1 APH(3')-II family aminoglycoside O-phosphotrans... | 259 | 4e-83 | 53% |
| WP_082572993.1 MULTISPECIES: aminoglycoside 3'-phosphotransfer... | 260 | 4e-83 | 52% |
| WP_018429030.1 aminoglycoside 3'-phosphotransferase [Hoeflea s... | 259 | 4e-83 | 52% |
| MBG6944880.1 APH(3')-II family aminoglycoside O-phosphotransfe... | 259 | 4e-83 | 53% |
| MBG6453832.1 APH(3')-II family aminoglycoside O-phosphotransfe... | 259 | 4e-83 | 53% |
| WP_108084245.1 APH(3')-II family aminoglycoside O-phosphotrans... | 259 | 4e-83 | 53% |
| OKP80448.1 APH(3') family aminoglycoside O-phosphotransferase ... | 259 | 4e-83 | 52% |
| MBG7096971.1 APH(3')-II family aminoglycoside O-phosphotransfe... | 259 | 4e-83 | 53% |
| WP_153069973.1 MULTISPECIES: phosphotransferase [Proteobacteria] | 254 | 4e-83 | 100% |
| WP_124086737.1 APH(3')-II family aminoglycoside O-phosphotrans... | 259 | 5e-83 | 53% |
| WP_061193432.1 APH(3')-II family aminoglycoside O-phosphotrans... | 259 | 5e-83 | 53% |
| WP_023111352.1 APH(3')-II family aminoglycoside O-phosphotrans... | 259 | 5e-83 | 53% |
| WP_096220110.1 APH(3')-II family aminoglycoside O-phosphotrans... | 259 | 5e-83 | 53% |
| WP_134311622.1 APH(3')-II family aminoglycoside O-phosphotrans... | 259 | 5e-83 | 53% |
| WP_196747719.1 APH(3')-II family aminoglycoside O-phosphotrans... | 259 | 5e-83 | 53% |
| WP_196650023.1 APH(3')-II family aminoglycoside O-phosphotrans... | 259 | 5e-83 | 53% |
| WP_134384507.1 aminoglycoside 3'-phosphotransferase [Massilia ... | 259 | 5e-83 | 57% |
| WP_121327273.1 APH(3')-II family aminoglycoside O-phosphotrans... | 259 | 6e-83 | 53% |
| WP_196658176.1 APH(3')-II family aminoglycoside O-phosphotrans... | 259 | 6e-83 | 53% |
| WP_196710796.1 APH(3')-II family aminoglycoside O-phosphotrans... | 259 | 6e-83 | 53% |
| KSV61385.1 hypothetical protein N185_10185 [Sinorhizobium sp. ... | 261 | 6e-83 | 52% |
| WP_192441052.1 APH(3') family aminoglycoside O-phosphotransfer... | 260 | 7e-83 | 52% |
| WP_196622922.1 APH(3')-II family aminoglycoside O-phosphotrans... | 259 | 7e-83 | 53% |
| WP_050153044.1 APH(3')-II family aminoglycoside O-phosphotrans... | 259 | 7e-83 | 52% |
| WP_006224268.1 APH(3')-II family aminoglycoside O-phosphotrans... | 259 | 7e-83 | 52% |
| WP_003528565.1 aminoglycoside 3'-phosphotransferase [Sinorhizo... | 259 | 7e-83 | 54% |
| WP_033953215.1 APH(3')-II family aminoglycoside O-phosphotrans... | 259 | 8e-83 | 52% |
| WP_113135852.1 MULTISPECIES: aminoglycoside 3'-phosphotransfer... | 259 | 8e-83 | 53% |
| WP_132506357.1 APH(3')-II family aminoglycoside O-phosphotrans... | 259 | 8e-83 | 52% |
| WP_132654435.1 APH(3')-II family aminoglycoside O-phosphotrans... | 259 | 8e-83 | 53% |
| WP_034003798.1 APH(3')-II family aminoglycoside O-phosphotrans... | 259 | 9e-83 | 53% |
| WP_128684639.1 APH(3')-II family aminoglycoside O-phosphotrans... | 259 | 9e-83 | 53% |
| WP_132718772.1 APH(3')-II family aminoglycoside O-phosphotrans... | 259 | 1e-82 | 52% |
| WP_096067735.1 APH(3')-II family aminoglycoside O-phosphotrans... | 259 | 1e-82 | 53% |
| WP_127610550.1 aminoglycoside 3'-phosphotransferase [Sinorhizo... | 258 | 1e-82 | 54% |
| WP_196680207.1 APH(3')-II family aminoglycoside O-phosphotrans... | 258 | 1e-82 | 53% |
| WP_142918966.1 aminoglycoside 3'-phosphotransferase [Staphyloc... | 253 | 1e-82 | 98% |
| WP_023913265.1 APH(3')-II family aminoglycoside O-phosphotrans... | 258 | 1e-82 | 52% |
| WP_132632367.1 APH(3')-II family aminoglycoside O-phosphotrans... | 258 | 1e-82 | 53% |
| WP_058156211.1 APH(3')-II family aminoglycoside O-phosphotrans... | 258 | 1e-82 | 53% |
| WP_181851369.1 APH(3')-II family aminoglycoside O-phosphotrans... | 258 | 1e-82 | 53% |
| WP_134269618.1 APH(3')-II family aminoglycoside O-phosphotrans... | 258 | 1e-82 | 52% |
| WP_124082237.1 APH(3')-II family aminoglycoside O-phosphotrans... | 258 | 1e-82 | 52% |
| AMS41341.1 aminoglycoside phosphotransferase [Aminobacter amin... | 258 | 1e-82 | 53% |
| WP_033993413.1 APH(3')-II family aminoglycoside O-phosphotrans... | 258 | 1e-82 | 52% |
| WP_134885922.1 aminoglycoside 3'-phosphotransferase [Ensifer a... | 259 | 1e-82 | 52% |
| MBA8881929.1 aminoglycoside 3'-phosphotransferase-2 [Phyllobac... | 258 | 1e-82 | 52% |
| WP_121196160.1 APH(3')-II family aminoglycoside O-phosphotrans... | 258 | 1e-82 | 52% |
| WP_067966257.1 MULTISPECIES: aminoglycoside 3'-phosphotransfer... | 258 | 1e-82 | 53% |
| WP_143973340.1 aminoglycoside 3'-phosphotransferase [Mesorhizo... | 258 | 1e-82 | 56% |
| MBG6791069.1 APH(3')-II family aminoglycoside O-phosphotransfe... | 258 | 1e-82 | 53% |
| WP_181854418.1 APH(3')-II family aminoglycoside O-phosphotrans... | 258 | 1e-82 | 52% |
| WP_124171550.1 APH(3')-II family aminoglycoside O-phosphotrans... | 258 | 1e-82 | 53% |
| WP_132898834.1 APH(3')-II family aminoglycoside O-phosphotrans... | 258 | 2e-82 | 52% |
| WP_124127182.1 APH(3')-II family aminoglycoside O-phosphotrans... | 258 | 2e-82 | 53% |
| MBG7175407.1 APH(3')-II family aminoglycoside O-phosphotransfe... | 258 | 2e-82 | 52% |
| WP_038542852.1 aminoglycoside 3'-phosphotransferase [Neorhizob... | 258 | 2e-82 | 53% |

| WP_182552602.1 APH(3') family aminoglycoside O-phosphotransfer... | 258 | 2e-82 | 52% |
| --- | --- | --- | --- |
| WP_055970481.1 aminoglycoside 3'-phosphotransferase [Aminobact... | 258 | 2e-82 | 52% |
| WP_121366548.1 APH(3')-II family aminoglycoside O-phosphotrans... | 258 | 2e-82 | 53% |
| WP_176205607.1 phosphotransferase [Salmonella enterica] | 253 | 2e-82 | 100% |
| WP_043094975.1 APH(3')-II family aminoglycoside O-phosphotrans... | 258 | 2e-82 | 53% |
| MBG6663591.1 APH(3')-II family aminoglycoside O-phosphotransfe... | 258 | 2e-82 | 52% |
| WP_196733514.1 APH(3')-II family aminoglycoside O-phosphotrans... | 258 | 2e-82 | 52% |
| WP_058175559.1 APH(3')-II family aminoglycoside O-phosphotrans... | 258 | 2e-82 | 52% |
| WP_087851757.1 APH(3')-II family aminoglycoside O-phosphotrans... | 258 | 2e-82 | 52% |
| WP_033987203.1 APH(3')-II family aminoglycoside O-phosphotrans... | 258 | 2e-82 | 53% |
| WP_096796437.1 aminoglycoside 3'-phosphotransferase [Pseudomon... | 257 | 2e-82 | 52% |
| WP_196741451.1 APH(3')-II family aminoglycoside O-phosphotrans... | 258 | 2e-82 | 52% |
| WP_088936496.1 aminoglycoside 3'-phosphotransferase [Rhizobium... | 258 | 2e-82 | 51% |
| WP_196696260.1 APH(3')-II family aminoglycoside O-phosphotrans... | 258 | 2e-82 | 53% |
| WP_183262305.1 APH(3') family aminoglycoside O-phosphotransfer... | 258 | 2e-82 | 51% |
| WP_171959019.1 APH(3')-II family aminoglycoside O-phosphotrans... | 258 | 3e-82 | 53% |
| WP_090295468.1 MULTISPECIES: aminoglycoside 3'-phosphotransfer... | 258 | 3e-82 | 52% |
| WP_058142673.1 APH(3')-II family aminoglycoside O-phosphotrans... | 258 | 3e-82 | 53% |
| MRX32977.1 APH(3') family aminoglycoside O-phosphotransferase ... | 257 | 3e-82 | 53% |
| MBB4648332.1 aminoglycoside 3'-phosphotransferase-2 [Aminobact... | 258 | 3e-82 | 51% |
| WP_168296728.1 APH(3') family aminoglycoside O-phosphotransfer... | 257 | 3e-82 | 51% |
| WP_185910392.1 APH(3') family aminoglycoside O-phosphotransfer... | 257 | 3e-82 | 53% |
| WP_121305226.1 APH(3')-II family aminoglycoside O-phosphotrans... | 257 | 4e-82 | 53% |
| WP_027613036.1 MULTISPECIES: aminoglycoside 3'-phosphotransfer... | 257 | 4e-82 | 52% |
| WP_187588648.1 phosphotransferase [Arenibacter sp. BSSL-BM3] | 252 | 4e-82 | 99% |
| WP_033942278.1 APH(3')-II family aminoglycoside O-phosphotrans... | 257 | 4e-82 | 53% |
| WP_081278565.1 aminoglycoside 3'-phosphotransferase [Rhizobium... | 257 | 4e-82 | 50% |
| SNY16958.1 aminoglycoside 3'-phosphotransferase-2 [Pseudomonas... | 256 | 4e-82 | 52% |
| WP_129413823.1 aminoglycoside 3'-phosphotransferase [Mesorhizo... | 257 | 4e-82 | 51% |
| WP_074222317.1 APH(3')-II family aminoglycoside O-phosphotrans... | 257 | 5e-82 | 52% |
| WP_012484598.1 MULTISPECIES: aminoglycoside 3'-phosphotransfer... | 256 | 6e-82 | 51% |
| MBG6602928.1 APH(3')-II family aminoglycoside O-phosphotransfe... | 256 | 7e-82 | 53% |
| WP_112344283.1 MULTISPECIES: aminoglycoside 3'-phosphotransfer... | 256 | 7e-82 | 51% |
| OJY00856.1 APH(3') family aminoglycoside O-phosphotransferase ... | 256 | 8e-82 | 52% |
| WP_081279261.1 MULTISPECIES: aminoglycoside 3'-phosphotransfer... | 256 | 8e-82 | 50% |
| WP_108609794.1 aminoglycoside 3'-phosphotransferase [Aminobact... | 256 | 8e-82 | 51% |
| WP_183924477.1 APH(3') family aminoglycoside O-phosphotransfer... | 256 | 8e-82 | 51% |
| WP_115989559.1 aminoglycoside 3'-phosphotransferase [Pseudomon... | 256 | 8e-82 | 52% |
| WP_136959039.1 aminoglycoside 3'-phosphotransferase [Phreatoba... | 256 | 9e-82 | 54% |
| WP_092583030.1 aminoglycoside 3'-phosphotransferase [Rhizobium... | 256 | 1e-81 | 51% |
| RED81569.1 aminoglycoside 3'-phosphotransferase-2 [Pseudomonas... | 256 | 1e-81 | 52% |
| WP_184766830.1 APH(3') family aminoglycoside O-phosphotransfer... | 256 | 1e-81 | 52% |
| TAJ65700.1 aminoglycoside 3'-phosphotransferase [Variovorax sp.] | 256 | 1e-81 | 55% |
| WP_171663723.1 APH(3')-II family aminoglycoside O-phosphotrans... | 256 | 1e-81 | 52% |
| MBG6434212.1 APH(3')-II family aminoglycoside O-phosphotransfe... | 256 | 1e-81 | 53% |
| WP_060506553.1 aminoglycoside 3'-phosphotransferase [Rhizobium... | 256 | 1e-81 | 49% |
| WP_150774741.1 aminoglycoside 3'-phosphotransferase [Pseudomon... | 255 | 1e-81 | 51% |
| WP_126473451.1 APH(3')-II family aminoglycoside O-phosphotrans... | 256 | 2e-81 | 53% |
| WP_132661274.1 aminoglycoside 3'-phosphotransferase [Rhizobium... | 256 | 2e-81 | 52% |
| WP_177239337.1 APH(3') family aminoglycoside O-phosphotransfer... | 255 | 2e-81 | 55% |
| WP_172372335.1 APH(3') family aminoglycoside O-phosphotransfer... | 255 | 2e-81 | 55% |
| KQX44410.1 aminoglycoside phosphotransferase [Ensifer sp. Root... | 255 | 2e-81 | 52% |
| SFO53387.1 aminoglycoside 3'-phosphotransferase-2 [Variovorax ... | 255 | 2e-81 | 55% |
| WP_125896096.1 APH(3')-II family aminoglycoside O-phosphotrans... | 255 | 2e-81 | 53% |
| WP_033998715.1 APH(3')-II family aminoglycoside O-phosphotrans... | 255 | 3e-81 | 53% |
| WP_034025656.1 APH(3')-II family aminoglycoside O-phosphotrans... | 255 | 3e-81 | 53% |
| WP_023465172.1 MULTISPECIES: APH(3')-II family aminoglycoside ... | 255 | 3e-81 | 53% |
| WP_113251131.1 MULTISPECIES: aminoglycoside 3'-phosphotransfer... | 254 | 4e-81 | 51% |
| WP_196644764.1 APH(3')-II family aminoglycoside O-phosphotrans... | 254 | 4e-81 | 53% |
| WP_054310564.1 aminoglycoside 3'-phosphotransferase [Mesorhizo... | 254 | 4e-81 | 52% |
| WP_160040445.1 APH(3') family aminoglycoside O-phosphotransfer... | 254 | 4e-81 | 51% |
| WP_124133756.1 APH(3')-II family aminoglycoside O-phosphotrans... | 254 | 5e-81 | 53% |
| WP_127928220.1 aminoglycoside 3'-phosphotransferase [Pseudomon... | 254 | 5e-81 | 51% |
| WP_038403805.1 APH(3')-II family aminoglycoside O-phosphotrans... | 254 | 5e-81 | 52% |
| WP_082566533.1 MULTISPECIES: aminoglycoside 3'-phosphotransfer... | 255 | 6e-81 | 52% |
| WP_037078345.1 aminoglycoside 3'-phosphotransferase [Rhizobium... | 254 | 6e-81 | 51% |
| MBG6505398.1 APH(3')-II family aminoglycoside O-phosphotransfe... | 254 | 6e-81 | 52% |
| WP_115419083.1 aminoglycoside 3'-phosphotransferase [Ensifer s... | 254 | 7e-81 | 51% |
| WP_016733662.1 aminoglycoside 3'-phosphotransferase [Rhizobium... | 254 | 7e-81 | 50% |
| WP_097664897.1 aminoglycoside 3'-phosphotransferase [Rhizobium... | 254 | 7e-81 | 53% |
| WP_154350657.1 APH(3')-II family aminoglycoside O-phosphotrans... | 254 | 7e-81 | 52% |
| WP_160057641.1 APH(3') family aminoglycoside O-phosphotransfer... | 253 | 7e-81 | 51% |

| WP_123586670.1 aminoglycoside 3'-phosphotransferase [Pseudomon... | 253 | 7e-81 | 52% |
| --- | --- | --- | --- |
| WP_183836930.1 APH(3') family aminoglycoside O-phosphotransfer... | 254 | 7e-81 | 53% |
| WP_053500030.1 MULTISPECIES: APH(3')-II family aminoglycoside ... | 254 | 8e-81 | 53% |
| WP_033962597.1 APH(3')-II family aminoglycoside O-phosphotrans... | 254 | 8e-81 | 53% |
| WP_008126610.1 aminoglycoside 3'-phosphotransferase [Phyllobac... | 253 | 8e-81 | 53% |
| WP_149255094.1 aminoglycoside 3'-phosphotransferase [Labrys sp... | 253 | 1e-80 | 54% |
| WP_023129437.1 APH(3')-II family aminoglycoside O-phosphotrans... | 253 | 1e-80 | 53% |
| WP_032931420.1 MULTISPECIES: aminoglycoside 3'-phosphotransfer... | 253 | 1e-80 | 54% |
| WP_175121197.1 APH(3') family aminoglycoside O-phosphotransfer... | 253 | 1e-80 | 49% |
| CAB3651731.1 Aminoglycoside 3'-phosphotransferase [Achromobact... | 253 | 1e-80 | 49% |
| WP_031755390.1 APH(3')-II family aminoglycoside O-phosphotrans... | 253 | 1e-80 | 53% |
| WP_010911111.1 aminoglycoside 3'-phosphotransferase [Mesorhizo... | 253 | 1e-80 | 54% |
| WP_023092411.1 APH(3')-II family aminoglycoside O-phosphotrans... | 253 | 1e-80 | 53% |
| WP_164229544.1 APH(3')-II family aminoglycoside O-phosphotrans... | 253 | 1e-80 | 53% |
| WP_060511889.1 MULTISPECIES: aminoglycoside 3'-phosphotransfer... | 253 | 1e-80 | 49% |
| WP_137216867.1 aminoglycoside 3'-phosphotransferase [Pseudomon... | 253 | 1e-80 | 51% |
| WP_150751039.1 aminoglycoside 3'-phosphotransferase [Pseudomon... | 253 | 1e-80 | 50% |
| WP_183820553.1 APH(3') family aminoglycoside O-phosphotransfer... | 253 | 1e-80 | 50% |
| WP_132561683.1 aminoglycoside 3'-phosphotransferase [Rhizobium... | 253 | 1e-80 | 53% |
| WP_099495155.1 APH(3')-II family aminoglycoside O-phosphotrans... | 253 | 2e-80 | 53% |
| WP_145317576.1 aminoglycoside 3'-phosphotransferase [Rhizobium... | 253 | 2e-80 | 51% |
| WP_028355383.1 APH(3')-II family aminoglycoside O-phosphotrans... | 253 | 2e-80 | 57% |
| EJT05030.1 aminoglycoside phosphotransferase [Rhizobium sp. CC... | 252 | 2e-80 | 51% |
| WP_085742928.1 MULTISPECIES: aminoglycoside 3'-phosphotransfer... | 253 | 2e-80 | 50% |
| WP_100477982.1 APH(3')-II family aminoglycoside O-phosphotrans... | 253 | 2e-80 | 53% |
| WP_186449439.1 aminoglycoside 3'-phosphotransferase [Rhizobium... | 253 | 2e-80 | 51% |
| APO74403.1 aminoglycoside 3'-phosphotransferase protein [Rhizo... | 252 | 2e-80 | 51% |
| WP_003110134.1 APH(3')-II family aminoglycoside O-phosphotrans... | 253 | 2e-80 | 53% |
| WP_016562273.1 MULTISPECIES: APH(3')-II family aminoglycoside ... | 253 | 2e-80 | 53% |
| WP_051336470.1 aminoglycoside 3'-phosphotransferase [Rhizobium... | 252 | 2e-80 | 52% |
| WP_132552189.1 aminoglycoside 3'-phosphotransferase [Rhizobium... | 253 | 2e-80 | 51% |
| WP_081282797.1 aminoglycoside 3'-phosphotransferase [Rhizobium... | 252 | 2e-80 | 50% |
| WP_081284251.1 aminoglycoside 3'-phosphotransferase [Rhizobium... | 252 | 2e-80 | 50% |
| WP_057589670.1 aminoglycoside 3'-phosphotransferase [Variovora... | 252 | 3e-80 | 55% |
| WP_196776327.1 aminoglycoside 3'-phosphotransferase [Rhizobium... | 252 | 3e-80 | 51% |
| WP_077502189.1 aminoglycoside 3'-phosphotransferase [Sinorhizo... | 252 | 3e-80 | 51% |
| WP_194730113.1 APH(3') family aminoglycoside O-phosphotransfer... | 252 | 3e-80 | 51% |
| WP_022714890.1 aminoglycoside 3'-phosphotransferase [Rhizobium... | 252 | 3e-80 | 50% |
| WP_049413834.1 APH(3')-II family aminoglycoside O-phosphotrans... | 252 | 3e-80 | 53% |
| MBS79415.1 APH(3') family aminoglycoside O-phosphotransferase ... | 252 | 3e-80 | 55% |
| EJZ58787.1 Aminoglycoside phosphotransferase [Pseudomonas fluo... | 252 | 3e-80 | 51% |
| WP_023980230.1 MULTISPECIES: APH(3')-II family aminoglycoside ... | 252 | 3e-80 | 52% |
| MBG6629815.1 APH(3')-II family aminoglycoside O-phosphotransfe... | 252 | 3e-80 | 52% |
| WP_045122502.1 aminoglycoside 3'-phosphotransferase [Pseudomon... | 252 | 3e-80 | 51% |
| WP_088587125.1 APH(3')-II family aminoglycoside O-phosphotrans... | 252 | 3e-80 | 50% |
| WP_074067510.1 aminoglycoside 3'-phosphotransferase [Rhizobium... | 252 | 3e-80 | 51% |
| MBG6497053.1 APH(3')-II family aminoglycoside O-phosphotransfe... | 252 | 4e-80 | 53% |
| WP_103485884.1 aminoglycoside 3'-phosphotransferase [Pseudomon... | 252 | 4e-80 | 50% |
| WP_112772955.1 MULTISPECIES: aminoglycoside 3'-phosphotransfer... | 253 | 4e-80 | 52% |
| WP_090704353.1 aminoglycoside 3'-phosphotransferase [Beijerinc... | 252 | 4e-80 | 53% |
| WP_134967981.1 APH(3')-II family aminoglycoside O-phosphotrans... | 252 | 4e-80 | 53% |
| MBG6520829.1 APH(3')-II family aminoglycoside O-phosphotransfe... | 252 | 4e-80 | 53% |
| WP_164283431.1 APH(3')-II family aminoglycoside O-phosphotrans... | 252 | 4e-80 | 53% |
| WP_111096174.1 APH(3')-II family aminoglycoside O-phosphotrans... | 252 | 5e-80 | 53% |
| OWK24067.1 aminoglycoside phosphotransferase [Rhizobium yangli... | 251 | 6e-80 | 51% |
| RBL72727.1 APH(3') family aminoglycoside O-phosphotransferase ... | 251 | 6e-80 | 51% |
| WP_146546610.1 aminoglycoside 3'-phosphotransferase [Reyranell... | 251 | 6e-80 | 54% |
| WP_094806573.1 APH(3')-II family aminoglycoside O-phosphotrans... | 251 | 6e-80 | 57% |
| WP_164262363.1 APH(3')-II family aminoglycoside O-phosphotrans... | 251 | 6e-80 | 53% |
| WP_097604739.1 aminoglycoside 3'-phosphotransferase [Rhizobium... | 251 | 6e-80 | 52% |
| EBN0119945.1 aminoglycoside 3'-phosphotransferase [Salmonella ... | 246 | 7e-80 | 100% |
| WP_124200570.1 APH(3')-II family aminoglycoside O-phosphotrans... | 251 | 7e-80 | 52% |
| WP_007913110.1 MULTISPECIES: aminoglycoside 3'-phosphotransfer... | 251 | 7e-80 | 51% |
| VUO05133.1 Aminoglycoside 3'-phosphotransferase [Stenotrophomo... | 251 | 7e-80 | 53% |
| WP_143355935.1 aminoglycoside 3'-phosphotransferase [Escherich... | 246 | 8e-80 | 94% |
| WP_064684283.1 aminoglycoside 3'-phosphotransferase [Rhizobium... | 251 | 8e-80 | 52% |
| WP_058134659.1 APH(3')-II family aminoglycoside O-phosphotrans... | 251 | 9e-80 | 52% |
| WP_061194935.1 APH(3')-II family aminoglycoside O-phosphotrans... | 251 | 9e-80 | 52% |
| WP_096477536.1 aminoglycoside 3'-phosphotransferase [Rhizobium... | 251 | 9e-80 | 50% |
| WP_072168935.1 APH(3')-II family aminoglycoside O-phosphotrans... | 251 | 9e-80 | 53% |
| WP_081279126.1 aminoglycoside 3'-phosphotransferase [Rhizobium... | 251 | 9e-80 | 50% |
| WP_111129029.1 MULTISPECIES: APH(3')-II family aminoglycoside ... | 251 | 1e-79 | 53% |

| WP_095377702.1 APH(3')-II family aminoglycoside O-phosphotrans... | 251 | 1e-79 | 53% |
| --- | --- | --- | --- |
| WP_155029427.1 APH(3')-II family aminoglycoside O-phosphotrans... | 251 | 1e-79 | 53% |
| WP_065177500.1 MULTISPECIES: APH(3')-II family aminoglycoside ... | 251 | 1e-79 | 53% |
| CAB3817415.1 Aminoglycoside 3'-phosphotransferase [Achromobact... | 251 | 1e-79 | 48% |
| WP_027030648.1 aminoglycoside 3'-phosphotransferase [Mesorhizo... | 251 | 1e-79 | 51% |
| AJD40892.1 aminoglycoside 3'-phosphotransferase protein [Rhizo... | 251 | 1e-79 | 50% |
| WP_087005395.1 aminoglycoside 3'-phosphotransferase [Rhizobium... | 251 | 1e-79 | 52% |
| WP_025425025.1 MULTISPECIES: aminoglycoside 3'-phosphotransfer... | 251 | 1e-79 | 51% |
| WP_191575458.1 APH(3') family aminoglycoside O-phosphotransfer... | 251 | 1e-79 | 49% |
| WP_123376854.1 aminoglycoside 3'-phosphotransferase [Pseudomon... | 250 | 1e-79 | 51% |
| WP_171956004.1 APH(3')-II family aminoglycoside O-phosphotrans... | 251 | 1e-79 | 53% |
| WP_175183732.1 APH(3') family aminoglycoside O-phosphotransfer... | 250 | 1e-79 | 48% |
| WP_061477864.1 APH(3')-II family aminoglycoside O-phosphotrans... | 251 | 1e-79 | 53% |
| EGE56818.1 putative aminoglycoside 3'-phosphotransferase prote... | 250 | 1e-79 | 50% |
| WP_164093019.1 APH(3')-II family aminoglycoside O-phosphotrans... | 250 | 2e-79 | 53% |
| VUP59444.1 Aminoglycoside 3'-phosphotransferase [Stenotrophomo... | 250 | 2e-79 | 52% |
| WP_037117315.1 MULTISPECIES: aminoglycoside 3'-phosphotransfer... | 250 | 2e-79 | 50% |
| WP_173418213.1 APH(3') family aminoglycoside O-phosphotransfer... | 250 | 2e-79 | 50% |
| WP_097540934.1 aminoglycoside 3'-phosphotransferase [Rhizobium... | 250 | 2e-79 | 50% |
| WP_150652876.1 aminoglycoside 3'-phosphotransferase [Pseudomon... | 250 | 2e-79 | 50% |
| WP_077991802.1 MULTISPECIES: aminoglycoside 3'-phosphotransfer... | 250 | 2e-79 | 52% |
| WP_133940267.1 aminoglycoside 3'-phosphotransferase [Rhizobium... | 250 | 2e-79 | 50% |
| WP_095111988.1 aminoglycoside 3'-phosphotransferase [Pseudomon... | 249 | 2e-79 | 51% |
| WP_034476770.1 aminoglycoside 3'-phosphotransferase [Agrobacte... | 250 | 2e-79 | 50% |
| WP_188246262.1 APH(3')-II family aminoglycoside O-phosphotrans... | 250 | 2e-79 | 53% |
| WP_069139932.1 APH(3')-II family aminoglycoside O-phosphotrans... | 250 | 2e-79 | 53% |
| WP_005416290.1 APH(3')-II family aminoglycoside O-phosphotrans... | 250 | 2e-79 | 53% |
| WP_097578691.1 aminoglycoside 3'-phosphotransferase [Rhizobium... | 249 | 3e-79 | 52% |
| WP_099527006.1 APH(3')-II family aminoglycoside O-phosphotrans... | 250 | 3e-79 | 53% |
| WP_106795451.1 aminoglycoside 3'-phosphotransferase [Rhizobium... | 249 | 3e-79 | 52% |
| WP_173411002.1 APH(3') family aminoglycoside O-phosphotransfer... | 250 | 3e-79 | 51% |
| WP_188241290.1 APH(3')-II family aminoglycoside O-phosphotrans... | 250 | 3e-79 | 52% |
| WP_169269307.1 APH(3') family aminoglycoside O-phosphotransfer... | 250 | 3e-79 | 51% |
| WP_164222148.1 APH(3')-II family aminoglycoside O-phosphotrans... | 249 | 3e-79 | 53% |
| WP_174180146.1 APH(3') family aminoglycoside O-phosphotransfer... | 249 | 3e-79 | 49% |
| WP_099487410.1 APH(3')-II family aminoglycoside O-phosphotrans... | 249 | 4e-79 | 53% |
| WP_052212711.1 MULTISPECIES: aminoglycoside 3'-phosphotransfer... | 249 | 4e-79 | 51% |
| WP_196633161.1 APH(3')-II family aminoglycoside O-phosphotrans... | 249 | 4e-79 | 53% |
| WP_049396294.1 APH(3')-II family aminoglycoside O-phosphotrans... | 249 | 4e-79 | 53% |
| WP_138953620.1 APH(3')-II family aminoglycoside O-phosphotrans... | 249 | 5e-79 | 52% |
| WP_007960488.1 aminoglycoside 3'-phosphotransferase [Pseudomon... | 249 | 5e-79 | 51% |
| WP_134709277.1 APH(3')-II family aminoglycoside O-phosphotrans... | 249 | 5e-79 | 53% |
| WP_196627072.1 APH(3')-II family aminoglycoside O-phosphotrans... | 249 | 5e-79 | 52% |
| WP_175136224.1 APH(3') family aminoglycoside O-phosphotransfer... | 249 | 5e-79 | 48% |
| WP_196685833.1 APH(3')-II family aminoglycoside O-phosphotrans... | 249 | 6e-79 | 51% |
| ASW08172.1 APH(3') family aminoglycoside O-phosphotransferase ... | 249 | 6e-79 | 51% |
| WP_196664338.1 APH(3')-II family aminoglycoside O-phosphotrans... | 249 | 6e-79 | 53% |
| WP_180890604.1 APH(3')-II family aminoglycoside O-phosphotrans... | 249 | 6e-79 | 52% |
| CAB3814286.1 Aminoglycoside 3'-phosphotransferase [Achromobact... | 249 | 6e-79 | 48% |
| WP_039621131.1 aminoglycoside 3'-phosphotransferase [Rhizobium... | 249 | 6e-79 | 50% |
| WP_174718601.1 APH(3') family aminoglycoside O-phosphotransfer... | 249 | 6e-79 | 51% |
| WP_180845672.1 MULTISPECIES: APH(3')-II family aminoglycoside ... | 249 | 6e-79 | 53% |
| WP_088436185.1 APH(3')-II family aminoglycoside O-phosphotrans... | 249 | 7e-79 | 53% |
| WP_183812986.1 APH(3') family aminoglycoside O-phosphotransfer... | 249 | 7e-79 | 51% |
| WP_037194572.1 MULTISPECIES: aminoglycoside 3'-phosphotransfer... | 249 | 8e-79 | 51% |
| WP_132653716.1 MULTISPECIES: aminoglycoside 3'-phosphotransfer... | 249 | 8e-79 | 50% |
| WP_168842434.1 MULTISPECIES: APH(3') family aminoglycoside O-p... | 248 | 8e-79 | 49% |
| WP_049449629.1 APH(3')-II family aminoglycoside O-phosphotrans... | 248 | 9e-79 | 52% |
| WP_112411627.1 aminoglycoside 3'-phosphotransferase [Rhizobium... | 248 | 1e-78 | 50% |
| WP_069612848.1 aminoglycoside 3'-phosphotransferase [Rhizobium... | 248 | 1e-78 | 51% |
| WP_154833201.1 aminoglycoside 3'-phosphotransferase [Delftia t... | 248 | 1e-78 | 51% |
| WP_053462776.1 APH(3')-II family aminoglycoside O-phosphotrans... | 248 | 1e-78 | 52% |
| WP_122606987.1 aminoglycoside 3'-phosphotransferase [Pseudomon... | 248 | 1e-78 | 50% |
| WP_183013532.1 APH(3') family aminoglycoside O-phosphotransfer... | 248 | 1e-78 | 50% |
| WP_196710007.1 APH(3')-II family aminoglycoside O-phosphotrans... | 248 | 1e-78 | 53% |
| WP_092577247.1 aminoglycoside 3'-phosphotransferase [Rhizobium... | 248 | 1e-78 | 51% |
| WP_170251222.1 APH(3') family aminoglycoside O-phosphotransfer... | 248 | 1e-78 | 50% |
| VUR21701.1 Aminoglycoside 3'-phosphotransferase [Stenotrophomo... | 248 | 1e-78 | 52% |
| WP_142807817.1 APH(3')-II family aminoglycoside O-phosphotrans... | 248 | 1e-78 | 53% |
| WP_005409358.1 APH(3')-II family aminoglycoside O-phosphotrans... | 248 | 1e-78 | 53% |
| WP_175887290.1 APH(3') family aminoglycoside O-phosphotransfer... | 248 | 1e-78 | 50% |
| WP_019661354.1 APH(3')-II family aminoglycoside O-phosphotrans... | 248 | 1e-78 | 52% |

| RQT26395.1 aminoglycoside 3'-phosphotransferase [Burkholderia ... | 248 | 1e-78 | 50% |
| --- | --- | --- | --- |
| WP_060186137.1 aminoglycoside 3'-phosphotransferase [Alcaligen... | 248 | 2e-78 | 51% |
| WP_115635296.1 aminoglycoside 3'-phosphotransferase [Alcaligen... | 248 | 2e-78 | 52% |
| WP_049449616.1 APH(3')-II family aminoglycoside O-phosphotrans... | 248 | 2e-78 | 52% |
| OCP18229.1 APH(3') family aminoglycoside O-phosphotransferase ... | 248 | 2e-78 | 49% |
| HBJ70292.1 TPA: APH(3') family aminoglycoside O-phosphotransfe... | 248 | 2e-78 | 52% |
| NNH33211.1 aminoglycoside 3'-phosphotransferase [Rhizobium sp.... | 248 | 2e-78 | 50% |
| HCW19070.1 TPA: APH(3')-II family aminoglycoside O-phosphotran... | 248 | 2e-78 | 51% |
| WP_035268392.1 aminoglycoside 3'-phosphotransferase [Alcaligen... | 248 | 2e-78 | 52% |
| WP_137430814.1 aminoglycoside 3'-phosphotransferase [Alcaligen... | 248 | 2e-78 | 51% |
| WP_191862845.1 APH(3')-II family aminoglycoside O-phosphotrans... | 248 | 2e-78 | 52% |
| WP_065197025.1 APH(3')-II family aminoglycoside O-phosphotrans... | 248 | 2e-78 | 52% |
| WP_099589766.1 APH(3')-II family aminoglycoside O-phosphotrans... | 248 | 2e-78 | 53% |
| WP_083054316.1 aminoglycoside 3'-phosphotransferase [Alcaligen... | 247 | 2e-78 | 52% |
| PSD16950.1 APH(3')-II family aminoglycoside O-phosphotransfera... | 248 | 2e-78 | 52% |
| WP_083222448.1 MULTISPECIES: aminoglycoside 3'-phosphotransfer... | 248 | 2e-78 | 49% |
| NED64886.1 APH(3')-II family aminoglycoside O-phosphotransfera... | 248 | 2e-78 | 52% |
| VUN57646.1 Aminoglycoside 3'-phosphotransferase [Stenotrophomo... | 247 | 2e-78 | 52% |
| KRG41425.1 aminoglycoside phosphotransferase [[Pseudomonas] ge... | 247 | 2e-78 | 52% |
| WP_054171560.1 APH(3')-II family aminoglycoside O-phosphotrans... | 247 | 3e-78 | 50% |
| WP_057500939.1 APH(3')-II family aminoglycoside O-phosphotrans... | 247 | 3e-78 | 53% |
| WP_122477756.1 aminoglycoside 3'-phosphotransferase [Burkholde... | 248 | 3e-78 | 50% |
| WP_088451364.1 aminoglycoside 3'-phosphotransferase [Alcaligen... | 247 | 3e-78 | 52% |
| WP_060380027.1 APH(3')-II family aminoglycoside O-phosphotrans... | 247 | 3e-78 | 53% |
| WP_180868435.1 APH(3')-II family aminoglycoside O-phosphotrans... | 247 | 3e-78 | 52% |
| WP_047853731.1 MULTISPECIES: aminoglycoside 3'-phosphotransfer... | 247 | 3e-78 | 50% |
| WP_099529527.1 APH(3')-II family aminoglycoside O-phosphotrans... | 247 | 3e-78 | 52% |
| WP_154348935.1 APH(3')-II family aminoglycoside O-phosphotrans... | 247 | 3e-78 | 52% |
| WP_132667369.1 APH(3')-II family aminoglycoside O-phosphotrans... | 247 | 3e-78 | 52% |
| WP_124492982.1 aminoglycoside 3'-phosphotransferase [Burkholde... | 247 | 4e-78 | 50% |
| WP_126924258.1 aminoglycoside 3'-phosphotransferase [Rhizobium... | 247 | 4e-78 | 52% |
| WP_123532154.1 aminoglycoside 3'-phosphotransferase [Pseudomon... | 246 | 4e-78 | 50% |
| WP_111189369.1 APH(3')-II family aminoglycoside O-phosphotrans... | 247 | 4e-78 | 53% |
| WP_062606334.1 APH(3')-II family aminoglycoside O-phosphotrans... | 247 | 4e-78 | 53% |
| WP_057494509.1 APH(3')-II family aminoglycoside O-phosphotrans... | 247 | 4e-78 | 52% |
| ACI97686.1 Aminoglycoside 3'-phosphotransferase, putative [Rho... | 247 | 4e-78 | 56% |
| WP_171968861.1 APH(3')-II family aminoglycoside O-phosphotrans... | 247 | 4e-78 | 53% |
| WP_109338033.1 APH(3')-II family aminoglycoside O-phosphotrans... | 247 | 4e-78 | 53% |
| WP_041785050.1 aminoglycoside 3'-phosphotransferase [Rhodospir... | 246 | 4e-78 | 56% |
| WP_094001862.1 APH(3')-II family aminoglycoside O-phosphotrans... | 247 | 5e-78 | 52% |
| WP_105556691.1 APH(3')-II family aminoglycoside O-phosphotrans... | 247 | 5e-78 | 50% |
| WP_071305756.1 APH(3')-II family aminoglycoside O-phosphotrans... | 247 | 5e-78 | 51% |
| VUR53297.1 Aminoglycoside 3'-phosphotransferase [Stenotrophomo... | 247 | 5e-78 | 53% |
| WP_180884419.1 APH(3')-II family aminoglycoside O-phosphotrans... | 246 | 5e-78 | 52% |
| WP_184703200.1 APH(3') family aminoglycoside O-phosphotransfer... | 246 | 5e-78 | 48% |
| WP_111150888.1 APH(3')-II family aminoglycoside O-phosphotrans... | 246 | 5e-78 | 53% |
| WP_076738960.1 APH(3')-II family aminoglycoside O-phosphotrans... | 246 | 5e-78 | 52% |
| WP_124647922.1 aminoglycoside 3'-phosphotransferase [Burkholde... | 247 | 5e-78 | 50% |
| WP_134735669.1 APH(3')-II family aminoglycoside O-phosphotrans... | 246 | 5e-78 | 52% |
| WP_097628160.1 MULTISPECIES: aminoglycoside 3'-phosphotransfer... | 246 | 5e-78 | 52% |
| WP_121341917.1 APH(3')-II family aminoglycoside O-phosphotrans... | 246 | 6e-78 | 54% |
| VUL73972.1 Aminoglycoside 3'-phosphotransferase [Stenotrophomo... | 246 | 6e-78 | 50% |
| MBG6471870.1 APH(3')-II family aminoglycoside O-phosphotransfe... | 246 | 6e-78 | 52% |
| VUP53063.1 Aminoglycoside 3'-phosphotransferase [Stenotrophomo... | 247 | 6e-78 | 51% |
| WP_099478979.1 APH(3')-II family aminoglycoside O-phosphotrans... | 246 | 6e-78 | 53% |
| WP_099552881.1 APH(3')-II family aminoglycoside O-phosphotrans... | 246 | 6e-78 | 52% |
| VUM25775.1 Aminoglycoside 3'-phosphotransferase [Stenotrophomo... | 246 | 7e-78 | 52% |
| WP_059034263.1 APH(3')-II family aminoglycoside O-phosphotrans... | 246 | 8e-78 | 53% |
| WP_071228923.1 APH(3')-II family aminoglycoside O-phosphotrans... | 246 | 8e-78 | 52% |
| WP_034024097.1 aminoglycoside 3'-phosphotransferase [Pseudomon... | 246 | 8e-78 | 51% |
| VUK53317.1 Aminoglycoside 3'-phosphotransferase [Stenotrophomo... | 246 | 8e-78 | 50% |
| VUJ63248.1 Aminoglycoside 3'-phosphotransferase [Stenotrophomo... | 246 | 8e-78 | 52% |
| WP_164117190.1 APH(3')-II family aminoglycoside O-phosphotrans... | 246 | 9e-78 | 52% |
| WP_108270053.1 APH(3')-II family aminoglycoside O-phosphotrans... | 246 | 9e-78 | 53% |
| VUQ68611.1 Aminoglycoside 3'-phosphotransferase [Stenotrophomo... | 246 | 9e-78 | 52% |
| VUL89945.1 Aminoglycoside 3'-phosphotransferase [Stenotrophomo... | 246 | 1e-77 | 52% |
| WP_103302299.1 aminoglycoside 3'-phosphotransferase [Pseudomon... | 245 | 1e-77 | 50% |
| WP_012479999.1 aminoglycoside O-phosphotransferase APH(3')-IIc... | 246 | 1e-77 | 53% |
| WP_049298729.1 APH(3')-II family aminoglycoside O-phosphotrans... | 245 | 1e-77 | 53% |
| ECZ5199100.1 aminoglycoside 3'-phosphotransferase [Salmonella ... | 240 | 1e-77 | 100% |
| WP_003561190.1 aminoglycoside 3'-phosphotransferase [Rhizobium... | 246 | 1e-77 | 52% |
| WP_112334363.1 aminoglycoside 3'-phosphotransferase [Rhizobium... | 246 | 1e-77 | 50% |

| WP_126905890.1 MULTISPECIES: aminoglycoside 3'-phosphotransfer... | 245 | 1e-77 | 49% |
| --- | --- | --- | --- |
| WP_095190961.1 aminoglycoside 3'-phosphotransferase [Pseudomon... | 245 | 1e-77 | 49% |
| WP_021447065.1 aminoglycoside 3'-phosphotransferase [Alcaligen... | 245 | 1e-77 | 51% |
| MBG7135377.1 APH(3')-II family aminoglycoside O-phosphotransfe... | 246 | 1e-77 | 53% |
| WP_132717099.1 aminoglycoside 3'-phosphotransferase [Rhizobium... | 245 | 1e-77 | 49% |
| WP_187757379.1 APH(3')-II family aminoglycoside O-phosphotrans... | 245 | 1e-77 | 53% |
| WP_155001344.1 aminoglycoside 3'-phosphotransferase [Pseudomon... | 245 | 1e-77 | 49% |
| WP_189719573.1 APH(3')-II family aminoglycoside O-phosphotrans... | 245 | 2e-77 | 52% |
| WP_169448694.1 APH(3')-II family aminoglycoside O-phosphotrans... | 245 | 2e-77 | 52% |
| WP_164079688.1 APH(3')-II family aminoglycoside O-phosphotrans... | 245 | 2e-77 | 51% |
| WP_121503112.1 APH(3')-II family aminoglycoside O-phosphotrans... | 245 | 2e-77 | 52% |
| WP_150826461.1 MULTISPECIES: APH(3')-II family aminoglycoside ... | 245 | 2e-77 | 51% |
| WP_097047956.1 APH(3')-II family aminoglycoside O-phosphotrans... | 245 | 2e-77 | 51% |
| WP_180844097.1 APH(3')-II family aminoglycoside O-phosphotrans... | 245 | 2e-77 | 52% |
| WP_058982626.1 APH(3')-II family aminoglycoside O-phosphotrans... | 245 | 2e-77 | 52% |
| WP_176354535.1 APH(3') family aminoglycoside O-phosphotransfer... | 248 | 2e-77 | 49% |
| NIK80981.1 aminoglycoside 3'-phosphotransferase-2 [Rhizobium s... | 245 | 2e-77 | 48% |
| WP_113230447.1 aminoglycoside 3'-phosphotransferase [Rhizobium... | 245 | 2e-77 | 48% |
| VUP93328.1 Aminoglycoside 3'-phosphotransferase [Stenotrophomo... | 245 | 2e-77 | 52% |
| WP_043035050.1 APH(3')-II family aminoglycoside O-phosphotrans... | 245 | 2e-77 | 52% |
| WP_182267680.1 APH(3')-II family aminoglycoside O-phosphotrans... | 245 | 2e-77 | 51% |
| WP_153228883.1 aminoglycoside 3'-phosphotransferase [Alcaligen... | 244 | 2e-77 | 50% |
| WP_032129737.1 APH(3')-II family aminoglycoside O-phosphotrans... | 245 | 2e-77 | 51% |
| WP_088430213.1 APH(3')-II family aminoglycoside O-phosphotrans... | 245 | 2e-77 | 52% |
| MBD3740732.1 APH(3')-II family aminoglycoside O-phosphotransfe... | 245 | 2e-77 | 51% |
| WP_049436299.1 APH(3')-II family aminoglycoside O-phosphotrans... | 245 | 2e-77 | 50% |
| WP_134731417.1 APH(3')-II family aminoglycoside O-phosphotrans... | 245 | 2e-77 | 52% |
| WP_049445834.1 APH(3')-II family aminoglycoside O-phosphotrans... | 245 | 2e-77 | 51% |
| WP_064695926.1 aminoglycoside 3'-phosphotransferase [Rhizobium... | 245 | 2e-77 | 49% |
| WP_096763339.1 MULTISPECIES: aminoglycoside 3'-phosphotransfer... | 245 | 2e-77 | 50% |
| WP_065723058.1 APH(3')-II family aminoglycoside O-phosphotrans... | 245 | 2e-77 | 52% |
| WP_134958273.1 APH(3')-II family aminoglycoside O-phosphotrans... | 245 | 2e-77 | 51% |
| WP_164212623.1 APH(3')-II family aminoglycoside O-phosphotrans... | 245 | 2e-77 | 52% |
| WP_186450295.1 aminoglycoside 3'-phosphotransferase [Rhizobium... | 245 | 2e-77 | 49% |
| WP_033838442.1 aminoglycoside 3'-phosphotransferase [Mesorhizo... | 244 | 3e-77 | 51% |
| WP_184371171.1 APH(3') family aminoglycoside O-phosphotransfer... | 245 | 3e-77 | 49% |
| WP_125892918.1 APH(3')-II family aminoglycoside O-phosphotrans... | 245 | 3e-77 | 52% |
| WP_153451750.1 aminoglycoside 3'-phosphotransferase [Sinorhizo... | 244 | 3e-77 | 54% |
| WP_164158428.1 APH(3')-II family aminoglycoside O-phosphotrans... | 244 | 3e-77 | 52% |
| WP_111174387.1 APH(3')-II family aminoglycoside O-phosphotrans... | 244 | 3e-77 | 52% |
| WP_135967552.1 APH(3')-II family aminoglycoside O-phosphotrans... | 244 | 3e-77 | 51% |
| WP_004116317.1 aminoglycoside 3'-phosphotransferase [Rhizobium... | 244 | 3e-77 | 48% |
| WP_165710397.1 APH(3')-II family aminoglycoside O-phosphotrans... | 244 | 3e-77 | 52% |
| WP_085860095.1 MULTISPECIES: aminoglycoside 3'-phosphotransfer... | 244 | 4e-77 | 51% |
| WP_005413136.1 APH(3')-II family aminoglycoside O-phosphotrans... | 244 | 4e-77 | 52% |
| WP_173002436.1 MULTISPECIES: aminoglycoside 3'-phosphotransfer... | 244 | 4e-77 | 49% |
| WP_164086031.1 APH(3')-II family aminoglycoside O-phosphotrans... | 244 | 4e-77 | 52% |
| WP_099497621.1 APH(3')-II family aminoglycoside O-phosphotrans... | 244 | 4e-77 | 52% |
| VUN21478.1 Aminoglycoside 3'-phosphotransferase [Stenotrophomo... | 244 | 4e-77 | 52% |
| WP_128688214.1 APH(3')-II family aminoglycoside O-phosphotrans... | 244 | 4e-77 | 53% |
| WP_050984095.1 aminoglycoside 3'-phosphotransferase [Sinorhizo... | 244 | 4e-77 | 54% |
| WP_053442897.1 APH(3')-II family aminoglycoside O-phosphotrans... | 244 | 5e-77 | 52% |
| NYB77909.1 APH(3')-II family aminoglycoside O-phosphotransfera... | 244 | 5e-77 | 52% |
| WP_049421316.1 APH(3')-II family aminoglycoside O-phosphotrans... | 244 | 5e-77 | 51% |
| WP_154993524.1 aminoglycoside 3'-phosphotransferase [Pseudomon... | 244 | 5e-77 | 49% |
| WP_088025762.1 APH(3')-II family aminoglycoside O-phosphotrans... | 244 | 5e-77 | 51% |
| WP_092711567.1 aminoglycoside 3'-phosphotransferase [Rhizobium... | 244 | 5e-77 | 50% |
| WP_183912555.1 APH(3') family aminoglycoside O-phosphotransfer... | 244 | 5e-77 | 51% |
| WP_134619153.1 APH(3')-II family aminoglycoside O-phosphotrans... | 243 | 6e-77 | 53% |
| WP_096772015.1 aminoglycoside 3'-phosphotransferase [Rhizobium... | 244 | 6e-77 | 50% |
| WP_171952084.1 APH(3')-II family aminoglycoside O-phosphotrans... | 244 | 6e-77 | 52% |
| WP_164089385.1 APH(3')-II family aminoglycoside O-phosphotrans... | 244 | 6e-77 | 51% |
| WP_099559461.1 APH(3')-II family aminoglycoside O-phosphotrans... | 244 | 6e-77 | 51% |
| WP_168269291.1 APH(3') family aminoglycoside O-phosphotransfer... | 244 | 6e-77 | 50% |
| WP_180874612.1 APH(3')-II family aminoglycoside O-phosphotrans... | 244 | 6e-77 | 52% |
| VUN14167.1 Aminoglycoside 3'-phosphotransferase [Stenotrophomo... | 244 | 6e-77 | 52% |
| VUM57579.1 Aminoglycoside 3'-phosphotransferase [Stenotrophomo... | 244 | 7e-77 | 51% |
| VUL71239.1 Aminoglycoside 3'-phosphotransferase [Stenotrophomo... | 244 | 7e-77 | 52% |
| WP_086059731.1 aminoglycoside 3'-phosphotransferase [Alcaligen... | 243 | 7e-77 | 50% |
| VUK78760.1 Aminoglycoside 3'-phosphotransferase [Stenotrophomo... | 238 | 7e-77 | 100% |
| WP_150796062.1 aminoglycoside 3'-phosphotransferase [Pseudomon... | 243 | 7e-77 | 50% |
| WP_126928024.1 APH(3')-II family aminoglycoside O-phosphotrans... | 244 | 7e-77 | 52% |

| VUJ56063.1 Aminoglycoside 3'-phosphotransferase [Stenotrophomo... | 244 | 7e-77 | 51% |
| --- | --- | --- | --- |
| WP_154263322.1 APH(3')-II family aminoglycoside O-phosphotrans... | 244 | 8e-77 | 51% |
| WP_111103743.1 APH(3')-II family aminoglycoside O-phosphotrans... | 244 | 8e-77 | 52% |
| WP_119007869.1 APH(3')-II family aminoglycoside O-phosphotrans... | 243 | 8e-77 | 51% |
| WP_180864567.1 APH(3')-II family aminoglycoside O-phosphotrans... | 243 | 9e-77 | 50% |
| WP_034504136.1 aminoglycoside 3'-phosphotransferase [Agrobacte... | 243 | 9e-77 | 49% |
| WP_049471268.1 MULTISPECIES: APH(3')-II family aminoglycoside ... | 243 | 1e-76 | 51% |
| WP_064678486.1 aminoglycoside 3'-phosphotransferase [Pseudomon... | 243 | 1e-76 | 49% |
| WP_134545924.1 APH(3')-II family aminoglycoside O-phosphotrans... | 242 | 1e-76 | 55% |
| VUQ41399.1 Aminoglycoside 3'-phosphotransferase [Stenotrophomo... | 243 | 1e-76 | 51% |
| WP_125303934.1 aminoglycoside 3'-phosphotransferase [Alcaligen... | 243 | 1e-76 | 50% |
| WP_154352443.1 APH(3')-II family aminoglycoside O-phosphotrans... | 243 | 1e-76 | 51% |
| WP_043397129.1 APH(3')-II family aminoglycoside O-phosphotrans... | 243 | 1e-76 | 52% |
| PKA72529.1 aminoglycoside 3'-phosphotransferase-2 [Pseudomonas... | 243 | 1e-76 | 51% |
| WP_188249774.1 APH(3')-II family aminoglycoside O-phosphotrans... | 243 | 1e-76 | 51% |
| WP_149909139.1 APH(3')-II family aminoglycoside O-phosphotrans... | 243 | 1e-76 | 51% |
| NHC52627.1 APH(3') family aminoglycoside O-phosphotransferase ... | 243 | 1e-76 | 52% |
| VUR95505.1 Aminoglycoside 3'-phosphotransferase [Stenotrophomo... | 243 | 2e-76 | 51% |
| VUO61439.1 Aminoglycoside 3'-phosphotransferase [Stenotrophomo... | 243 | 2e-76 | 51% |
| WP_053249296.1 aminoglycoside 3'-phosphotransferase [Ensifer a... | 243 | 2e-76 | 49% |
| WP_183709535.1 MULTISPECIES: APH(3') family aminoglycoside O-p... | 243 | 2e-76 | 49% |
| WP_097602636.1 aminoglycoside 3'-phosphotransferase [Rhizobium... | 243 | 2e-76 | 49% |
| WP_196648334.1 APH(3')-II family aminoglycoside O-phosphotrans... | 243 | 2e-76 | 53% |
| WP_053504986.1 APH(3')-II family aminoglycoside O-phosphotrans... | 243 | 2e-76 | 51% |
| WP_100848512.1 aminoglycoside 3'-phosphotransferase [Pseudomon... | 242 | 2e-76 | 51% |
| VUR34649.1 Aminoglycoside 3'-phosphotransferase [Stenotrophomo... | 243 | 2e-76 | 51% |
| WP_107232491.1 MULTISPECIES: APH(3')-II family aminoglycoside ... | 243 | 2e-76 | 51% |
| WP_151591718.1 aminoglycoside 3'-phosphotransferase [Ochrobact... | 243 | 2e-76 | 49% |
| WP_075851030.1 aminoglycoside 3'-phosphotransferase [Rhizobium... | 243 | 2e-76 | 49% |
| WP_183870768.1 APH(3') family aminoglycoside O-phosphotransfer... | 243 | 2e-76 | 49% |
| WP_140026165.1 aminoglycoside 3'-phosphotransferase [Ochrobact... | 243 | 2e-76 | 49% |
| WP_100441621.1 APH(3')-II family aminoglycoside O-phosphotrans... | 243 | 2e-76 | 51% |
| WP_184454282.1 APH(3') family aminoglycoside O-phosphotransfer... | 242 | 2e-76 | 49% |
| WP_109089015.1 aminoglycoside 3'-phosphotransferase [Alcaligen... | 242 | 2e-76 | 50% |
| WP_038690273.1 aminoglycoside 3'-phosphotransferase [Rhizobium... | 242 | 2e-76 | 50% |
| WP_076015284.1 aminoglycoside 3'-phosphotransferase [Pseudomon... | 242 | 2e-76 | 51% |
| WP_154330475.1 APH(3')-II family aminoglycoside O-phosphotrans... | 243 | 2e-76 | 51% |
| WP_064238319.1 MULTISPECIES: APH(3')-II family aminoglycoside ... | 243 | 2e-76 | 51% |
| WP_135273319.1 APH(3')-II family aminoglycoside O-phosphotrans... | 242 | 2e-76 | 52% |
| WP_154976088.1 aminoglycoside 3'-phosphotransferase [Pseudomon... | 242 | 2e-76 | 49% |
| WP_074726005.1 APH(3')-II family aminoglycoside O-phosphotrans... | 242 | 2e-76 | 51% |
| WP_049410842.1 APH(3')-II family aminoglycoside O-phosphotrans... | 242 | 3e-76 | 51% |
| WP_180832258.1 APH(3')-II family aminoglycoside O-phosphotrans... | 242 | 3e-76 | 51% |
| WP_125437553.1 APH(3')-II family aminoglycoside O-phosphotrans... | 242 | 3e-76 | 52% |
| SDK55887.1 aminoglycoside 3'-phosphotransferase-2 [Stenotropho... | 243 | 3e-76 | 51% |
| KOE99731.1 aminoglycoside phosphotransferase [[Pseudomonas] ge... | 242 | 3e-76 | 52% |
| WP_180836928.1 APH(3')-II family aminoglycoside O-phosphotrans... | 242 | 3e-76 | 51% |
| WP_134808795.1 APH(3')-II family aminoglycoside O-phosphotrans... | 242 | 3e-76 | 51% |
| WP_021202874.1 APH(3')-II family aminoglycoside O-phosphotrans... | 242 | 3e-76 | 51% |
| WP_092850984.1 aminoglycoside 3'-phosphotransferase [Rhizobium... | 242 | 3e-76 | 49% |
| SCB32369.1 aminoglycoside 3'-phosphotransferase-2 [Rhizobium m... | 242 | 3e-76 | 49% |
| WP_164077566.1 APH(3')-II family aminoglycoside O-phosphotrans... | 242 | 3e-76 | 51% |
| WP_085737875.1 aminoglycoside 3'-phosphotransferase [Rhizobium... | 242 | 3e-76 | 50% |
| VUO84224.1 Aminoglycoside 3'-phosphotransferase [Stenotrophomo... | 243 | 3e-76 | 51% |
| VUK31393.1 Aminoglycoside 3'-phosphotransferase [Stenotrophomo... | 242 | 3e-76 | 51% |
| VUO71045.1 Aminoglycoside 3'-phosphotransferase [Stenotrophomo... | 243 | 3e-76 | 51% |
| ARM89293.1 aminoglycoside 3'-phosphotransferase protein [Rhizo... | 242 | 3e-76 | 50% |
| WP_111206790.1 MULTISPECIES: APH(3')-II family aminoglycoside ... | 242 | 3e-76 | 51% |
| WP_111687175.1 APH(3')-II family aminoglycoside O-phosphotrans... | 242 | 3e-76 | 52% |
| WP_077469385.1 aminoglycoside 3'-phosphotransferase [Rhizobium... | 242 | 4e-76 | 49% |
| WP_063689378.1 aminoglycoside 3'-phosphotransferase [Alcaligen... | 241 | 4e-76 | 50% |
| WP_075076796.1 APH(3')-II family aminoglycoside O-phosphotrans... | 242 | 4e-76 | 51% |
| WP_128096343.1 aminoglycoside 3'-phosphotransferase [Stenotrop... | 242 | 4e-76 | 51% |
| WP_064711498.1 aminoglycoside 3'-phosphotransferase [Rhizobium... | 241 | 4e-76 | 50% |
| WP_124379363.1 aminoglycoside 3'-phosphotransferase [Pseudomon... | 241 | 4e-76 | 52% |
| VUK70898.1 Aminoglycoside 3'-phosphotransferase [Stenotrophomo... | 242 | 4e-76 | 51% |
| WP_092748620.1 aminoglycoside 3'-phosphotransferase [Rhizobium... | 241 | 4e-76 | 50% |
| WP_168329919.1 APH(3') family aminoglycoside O-phosphotransfer... | 242 | 4e-76 | 49% |
| WP_166618453.1 APH(3') family aminoglycoside O-phosphotransfer... | 241 | 4e-76 | 50% |
| WP_019338462.1 APH(3')-II family aminoglycoside O-phosphotrans... | 242 | 4e-76 | 51% |
| WP_150787052.1 aminoglycoside 3'-phosphotransferase [Pseudomon... | 241 | 4e-76 | 49% |
| WP_033834473.1 APH(3')-II family aminoglycoside O-phosphotrans... | 241 | 5e-76 | 50% |

| WP_056789071.1 MULTISPECIES: aminoglycoside 3'-phosphotransfer... | 241 | 5e-76 | 52% |
| --- | --- | --- | --- |
| WP_049462051.1 APH(3')-II family aminoglycoside O-phosphotrans... | 241 | 5e-76 | 51% |
| WP_183740996.1 APH(3') family aminoglycoside O-phosphotransfer... | 241 | 5e-76 | 48% |
| WP_125428073.1 APH(3')-II family aminoglycoside O-phosphotrans... | 241 | 5e-76 | 51% |
| VUN67894.1 Aminoglycoside 3'-phosphotransferase [Stenotrophomo... | 241 | 5e-76 | 51% |
| WP_113750355.1 aminoglycoside 3'-phosphotransferase [Bordetell... | 241 | 6e-76 | 53% |
| WP_046430302.1 MULTISPECIES: APH(3')-II family aminoglycoside ... | 241 | 6e-76 | 51% |
| VUP55126.1 Aminoglycoside 3'-phosphotransferase [Stenotrophomo... | 241 | 6e-76 | 51% |
| WP_088673147.1 aminoglycoside 3'-phosphotransferase [Rhizobium... | 241 | 6e-76 | 48% |
| WP_106467838.1 APH(3')-II family aminoglycoside O-phosphotrans... | 241 | 6e-76 | 51% |
| EBO3241016.1 aminoglycoside 3'-phosphotransferase [Salmonella ... | 236 | 6e-76 | 100% |
| HBP04691.1 TPA: APH(3')-II family aminoglycoside O-phosphotran... | 241 | 6e-76 | 53% |
| WP_164688020.1 APH(3')-II family aminoglycoside O-phosphotrans... | 241 | 6e-76 | 51% |
| WP_049403183.1 APH(3')-II family aminoglycoside O-phosphotrans... | 241 | 6e-76 | 50% |
| WP_132809976.1 APH(3')-II family aminoglycoside O-phosphotrans... | 241 | 7e-76 | 51% |
| WP_100448432.1 MULTISPECIES: APH(3')-II family aminoglycoside ... | 241 | 7e-76 | 51% |
| WP_109369674.1 aminoglycoside 3'-phosphotransferase [Ochrobact... | 241 | 7e-76 | 49% |
| WP_171962387.1 APH(3') family aminoglycoside O-phosphotransfer... | 241 | 7e-76 | 53% |
| WP_164241085.1 APH(3')-II family aminoglycoside O-phosphotrans... | 241 | 7e-76 | 51% |
| WP_049483201.1 APH(3')-II family aminoglycoside O-phosphotrans... | 241 | 7e-76 | 51% |
| WP_049398550.1 APH(3')-II family aminoglycoside O-phosphotrans... | 241 | 7e-76 | 51% |
| VUO98825.1 Aminoglycoside 3'-phosphotransferase [Stenotrophomo... | 241 | 8e-76 | 51% |
| WP_136638745.1 APH(3')-II family aminoglycoside O-phosphotrans... | 241 | 8e-76 | 51% |
| WP_014646913.1 APH(3')-II family aminoglycoside O-phosphotrans... | 241 | 8e-76 | 50% |
| WP_124418410.1 aminoglycoside 3'-phosphotransferase [Pseudomon... | 241 | 9e-76 | 50% |
| WP_089042367.1 aminoglycoside 3'-phosphotransferase [Pseudomon... | 241 | 9e-76 | 52% |
| WP_142234634.1 APH(3')-II family aminoglycoside O-phosphotrans... | 241 | 9e-76 | 50% |
| WP_101765352.1 APH(3')-II family aminoglycoside O-phosphotrans... | 241 | 9e-76 | 51% |
| WP_037140469.1 MULTISPECIES: aminoglycoside 3'-phosphotransfer... | 241 | 1e-75 | 51% |
| RTY17322.1 APH(3')-II family aminoglycoside O-phosphotransfera... | 241 | 1e-75 | 51% |
| WP_038368729.1 aminoglycoside 3'-phosphotransferase [Pseudomon... | 240 | 1e-75 | 49% |
| WP_102357651.1 aminoglycoside 3'-phosphotransferase [Pseudomon... | 240 | 1e-75 | 50% |
| WP_178124262.1 APH(3') family aminoglycoside O-phosphotransfer... | 240 | 1e-75 | 51% |
| WP_177048671.1 APH(3') family aminoglycoside O-phosphotransfer... | 240 | 1e-75 | 52% |
| WP_133634335.1 aminoglycoside 3'-phosphotransferase [Pseudomon... | 240 | 1e-75 | 52% |
| PMQ09303.1 Aminoglycoside 3'-phosphotransferase [Pseudomonas s... | 240 | 1e-75 | 50% |
| WP_169918582.1 APH(3') family aminoglycoside O-phosphotransfer... | 240 | 1e-75 | 52% |
| WP_179676762.1 APH(3') family aminoglycoside O-phosphotransfer... | 240 | 1e-75 | 52% |
| WP_178122924.1 APH(3') family aminoglycoside O-phosphotransfer... | 239 | 1e-75 | 53% |
| WP_076813318.1 aminoglycoside 3'-phosphotransferase [Achromoba... | 240 | 1e-75 | 51% |
| WP_181461225.1 APH(3') family aminoglycoside O-phosphotransfer... | 240 | 1e-75 | 49% |
| WP_126412020.1 APH(3')-II family aminoglycoside O-phosphotrans... | 240 | 1e-75 | 50% |
| WP_168807345.1 APH(3') family aminoglycoside O-phosphotransfer... | 240 | 1e-75 | 48% |
| WP_196651072.1 APH(3')-II family aminoglycoside O-phosphotrans... | 240 | 1e-75 | 52% |
| WP_113210761.1 aminoglycoside 3'-phosphotransferase [Rhizobium... | 240 | 1e-75 | 47% |
| WP_183833487.1 APH(3') family aminoglycoside O-phosphotransfer... | 240 | 1e-75 | 49% |
| WP_119736845.1 aminoglycoside 3'-phosphotransferase [Pseudomon... | 240 | 1e-75 | 50% |
| WP_180877254.1 APH(3')-II family aminoglycoside O-phosphotrans... | 240 | 2e-75 | 51% |
| WP_045930681.1 aminoglycoside 3'-phosphotransferase [Alcaligen... | 240 | 2e-75 | 50% |
| VUK30170.1 Aminoglycoside 3'-phosphotransferase [Stenotrophomo... | 240 | 2e-75 | 52% |
| WP_106118318.1 MULTISPECIES: aminoglycoside 3'-phosphotransfer... | 240 | 2e-75 | 52% |
| WP_179609770.1 MULTISPECIES: APH(3') family aminoglycoside O-p... | 240 | 2e-75 | 50% |
| PXB81517.1 APH(3')-II family aminoglycoside O-phosphotransfera... | 239 | 2e-75 | 53% |
| WP_183825630.1 APH(3') family aminoglycoside O-phosphotransfer... | 240 | 2e-75 | 48% |
| WP_167862063.1 APH(3') family aminoglycoside O-phosphotransfer... | 240 | 2e-75 | 51% |
| NIL16929.1 aminoglycoside 3'-phosphotransferase [Pseudomonas s... | 240 | 2e-75 | 51% |
| WP_164110582.1 APH(3')-II family aminoglycoside O-phosphotrans... | 240 | 2e-75 | 50% |
| WP_111824560.1 aminoglycoside 3'-phosphotransferase [Achromoba... | 239 | 2e-75 | 50% |
| WP_109813910.1 APH(3')-II family aminoglycoside O-phosphotrans... | 240 | 2e-75 | 51% |
| WP_098950446.1 aminoglycoside 3'-phosphotransferase [Pseudomon... | 239 | 2e-75 | 52% |
| WP_164144310.1 APH(3')-II family aminoglycoside O-phosphotrans... | 240 | 2e-75 | 51% |
| WP_111519515.1 aminoglycoside 3'-phosphotransferase [Cupriavid... | 240 | 2e-75 | 49% |
| WP_177033361.1 APH(3') family aminoglycoside O-phosphotransfer... | 239 | 2e-75 | 49% |
| WP_174071801.1 APH(3') family aminoglycoside O-phosphotransfer... | 240 | 2e-75 | 48% |
| WP_102772570.1 aminoglycoside 3'-phosphotransferase [Achromoba... | 239 | 2e-75 | 50% |
| WP_177068170.1 APH(3') family aminoglycoside O-phosphotransfer... | 239 | 2e-75 | 52% |
| WP_049430707.1 APH(3')-II family aminoglycoside O-phosphotrans... | 239 | 3e-75 | 50% |
| PIB54121.1 aminoglycoside phosphotransferase [Pseudomonas sp. ... | 239 | 3e-75 | 50% |
| WP_106998377.1 aminoglycoside 3'-phosphotransferase [Rhizobium... | 239 | 3e-75 | 49% |
| VUM56806.1 Aminoglycoside 3'-phosphotransferase [Stenotrophomo... | 239 | 3e-75 | 50% |
| WP_153872430.1 aminoglycoside 3'-phosphotransferase [Pseudomon... | 239 | 3e-75 | 50% |
| VUL58411.1 Aminoglycoside 3'-phosphotransferase [Stenotrophomo... | 239 | 3e-75 | 51% |

| MRJ39784.1 aminoglycoside 3'-phosphotransferase [Pseudomonas h... | 239 | 3e-75 | 50% |
| --- | --- | --- | --- |
| WP_177010109.1 MULTISPECIES: APH(3') family aminoglycoside O-p... | 239 | 4e-75 | 52% |
| WP_094949446.1 aminoglycoside 3'-phosphotransferase [Pseudomon... | 239 | 4e-75 | 51% |
| MBA2130525.1 APH(3')-II family aminoglycoside O-phosphotransfe... | 239 | 4e-75 | 51% |
| WP_063474697.1 MULTISPECIES: aminoglycoside 3'-phosphotransfer... | 239 | 4e-75 | 50% |
| WP_168298873.1 APH(3') family aminoglycoside O-phosphotransfer... | 239 | 4e-75 | 50% |
| WP_064803955.1 MULTISPECIES: aminoglycoside 3'-phosphotransfer... | 239 | 4e-75 | 50% |
| WP_162089683.1 APH(3') family aminoglycoside O-phosphotransfer... | 239 | 4e-75 | 50% |
| VUO94157.1 Aminoglycoside 3'-phosphotransferase [Stenotrophomo... | 239 | 4e-75 | 51% |
| WP_051981070.1 aminoglycoside 3'-phosphotransferase [Rhizobium... | 239 | 4e-75 | 49% |
| PZX29283.1 aminoglycoside 3'-phosphotransferase-2 [Cupriavidus... | 240 | 4e-75 | 49% |
| WP_187517385.1 APH(3') family aminoglycoside O-phosphotransfer... | 239 | 4e-75 | 50% |
| WP_049440786.1 APH(3')-II family aminoglycoside O-phosphotrans... | 239 | 5e-75 | 50% |
| WP_169367546.1 APH(3') family aminoglycoside O-phosphotransfer... | 239 | 5e-75 | 49% |
| WP_049453184.1 APH(3')-II family aminoglycoside O-phosphotrans... | 239 | 5e-75 | 51% |
| WP_062802245.1 aminoglycoside 3'-phosphotransferase [Cupriavid... | 239 | 5e-75 | 49% |
| WP_134177166.1 aminoglycoside 3'-phosphotransferase [Pseudomon... | 239 | 5e-75 | 49% |
| WP_096144550.1 aminoglycoside 3'-phosphotransferase [Pseudomon... | 239 | 5e-75 | 51% |
| WP_014036950.1 APH(3')-II family aminoglycoside O-phosphotrans... | 239 | 5e-75 | 50% |
| WP_082900720.1 aminoglycoside 3'-phosphotransferase [Achromoba... | 239 | 6e-75 | 49% |
| WP_034128012.1 MULTISPECIES: aminoglycoside 3'-phosphotransfer... | 238 | 6e-75 | 52% |
| WP_053494449.1 APH(3')-II family aminoglycoside O-phosphotrans... | 239 | 6e-75 | 50% |
| HCL44825.1 TPA: APH(3')-II family aminoglycoside O-phosphotran... | 239 | 6e-75 | 51% |
| WP_153854967.1 APH(3')-II family aminoglycoside O-phosphotrans... | 239 | 6e-75 | 51% |
| VUK78988.1 Aminoglycoside 3'-phosphotransferase [Stenotrophomo... | 239 | 7e-75 | 51% |
| WP_154908635.1 aminoglycoside 3'-phosphotransferase [Pseudomon... | 238 | 7e-75 | 49% |
| WP_099602724.1 MULTISPECIES: aminoglycoside 3'-phosphotransfer... | 239 | 7e-75 | 50% |
| WP_086069558.1 aminoglycoside 3'-phosphotransferase [Alcaligen... | 238 | 8e-75 | 50% |
| WP_192729799.1 APH(3') family aminoglycoside O-phosphotransfer... | 238 | 8e-75 | 48% |
| WP_097592933.1 aminoglycoside 3'-phosphotransferase [Rhizobium... | 238 | 8e-75 | 49% |
| WP_162524937.1 APH(3') family aminoglycoside O-phosphotransfer... | 238 | 9e-75 | 50% |
| WP_183731358.1 MULTISPECIES: APH(3') family aminoglycoside O-p... | 238 | 9e-75 | 47% |
| WP_116665407.1 aminoglycoside 3'-phosphotransferase [Pseudomon... | 238 | 9e-75 | 50% |
| WP_164569310.1 APH(3') family aminoglycoside O-phosphotransfer... | 238 | 9e-75 | 48% |
| WP_153365147.1 aminoglycoside 3'-phosphotransferase [Rhizobium... | 238 | 9e-75 | 47% |
| WP_186527710.1 APH(3') family aminoglycoside O-phosphotransfer... | 238 | 1e-74 | 51% |
| WP_049457265.1 APH(3')-II family aminoglycoside O-phosphotrans... | 238 | 1e-74 | 50% |
| WP_078803729.1 aminoglycoside 3'-phosphotransferase [Pseudomon... | 238 | 1e-74 | 52% |
| WP_184469285.1 APH(3') family aminoglycoside O-phosphotransfer... | 238 | 1e-74 | 49% |
| WP_012510849.1 APH(3')-II family aminoglycoside O-phosphotrans... | 238 | 1e-74 | 51% |
| WP_183853295.1 APH(3') family aminoglycoside O-phosphotransfer... | 238 | 1e-74 | 50% |
| WP_090368739.1 aminoglycoside 3'-phosphotransferase [Pseudomon... | 238 | 1e-74 | 50% |
| HCT25163.1 TPA: APH(3')-II family aminoglycoside O-phosphotran... | 238 | 1e-74 | 49% |
| WP_047611780.1 MULTISPECIES: aminoglycoside 3'-phosphotransfer... | 238 | 1e-74 | 50% |
| OAE51465.1 APH(3') family aminoglycoside O-phosphotransferase ... | 238 | 1e-74 | 51% |
| WP_161550249.1 APH(3') family aminoglycoside O-phosphotransfer... | 237 | 1e-74 | 50% |
| WP_053451292.1 APH(3')-II family aminoglycoside O-phosphotrans... | 238 | 1e-74 | 50% |
| MBB3522437.1 aminoglycoside 3'-phosphotransferase-2 [Rhizobium... | 237 | 2e-74 | 52% |
| WP_081992951.1 aminoglycoside 3'-phosphotransferase [Rhizobium... | 237 | 2e-74 | 50% |
| WP_088681245.1 aminoglycoside 3'-phosphotransferase [Rhizobium... | 238 | 2e-74 | 49% |
| WP_037173068.1 aminoglycoside 3'-phosphotransferase [Rhizobium... | 238 | 2e-74 | 50% |
| WP_033534052.1 aminoglycoside 3'-phosphotransferase [Bordetell... | 237 | 2e-74 | 53% |
| WP_169412163.1 APH(3')-II family aminoglycoside O-phosphotrans... | 238 | 2e-74 | 49% |
| WP_110657968.1 aminoglycoside 3'-phosphotransferase [Pseudomon... | 237 | 2e-74 | 49% |
| EZP63091.1 aminoglycoside 3'-phosphotransferase [Pseudomonas s... | 237 | 2e-74 | 50% |
| WP_139977044.1 aminoglycoside 3'-phosphotransferase [Ochrobact... | 238 | 2e-74 | 49% |
| WP_034115878.1 aminoglycoside 3'-phosphotransferase [Pseudomon... | 237 | 2e-74 | 50% |
| WP_171955029.1 APH(3')-II family aminoglycoside O-phosphotrans... | 238 | 2e-74 | 51% |
| WP_132572665.1 aminoglycoside 3'-phosphotransferase [Rhizobium... | 237 | 2e-74 | 50% |
| WP_086082471.1 aminoglycoside 3'-phosphotransferase [Rhizobium... | 237 | 2e-74 | 49% |
| WP_063769464.1 aminoglycoside 3'-phosphotransferase [Rhizobium... | 237 | 2e-74 | 46% |
| WP_169377092.1 APH(3') family aminoglycoside O-phosphotransfer... | 237 | 2e-74 | 50% |
| WP_150725387.1 aminoglycoside 3'-phosphotransferase [Pseudomon... | 237 | 2e-74 | 48% |
| WP_047632349.1 aminoglycoside 3'-phosphotransferase [Rhizobium... | 237 | 2e-74 | 46% |
| VUN34047.1 Aminoglycoside 3'-phosphotransferase [Stenotrophomo... | 237 | 2e-74 | 51% |
| WP_006362677.1 APH(3')-II family aminoglycoside O-phosphotrans... | 237 | 2e-74 | 51% |
| WP_183608102.1 MULTISPECIES: APH(3') family aminoglycoside O-p... | 237 | 2e-74 | 49% |
| WP_081279372.1 aminoglycoside 3'-phosphotransferase [Rhizobium... | 237 | 2e-74 | 49% |
| WP_113485578.1 aminoglycoside 3'-phosphotransferase [Ochrobact... | 237 | 3e-74 | 48% |
| WP_168280451.1 APH(3') family aminoglycoside O-phosphotransfer... | 237 | 3e-74 | 49% |
| WP_169875627.1 APH(3') family aminoglycoside O-phosphotransfer... | 237 | 3e-74 | 50% |
| WP_150692702.1 aminoglycoside 3'-phosphotransferase [Pseudomon... | 237 | 3e-74 | 49% |

| WP_188489266.1 APH(3')-II family aminoglycoside O-phosphotrans... | 237 | 3e-74 | 50% |
| --- | --- | --- | --- |
| WP_100434525.1 APH(3')-II family aminoglycoside O-phosphotrans... | 237 | 3e-74 | 51% |
| WP_122763827.1 aminoglycoside 3'-phosphotransferase [Pseudomon... | 238 | 3e-74 | 50% |
| WP_081797851.1 aminoglycoside 3'-phosphotransferase [Pseudomon... | 238 | 3e-74 | 50% |
| WP_180859310.1 APH(3')-II family aminoglycoside O-phosphotrans... | 237 | 3e-74 | 50% |
| VUN64401.1 Aminoglycoside 3'-phosphotransferase [Stenotrophomo... | 237 | 3e-74 | 50% |
| WP_184393311.1 APH(3') family aminoglycoside O-phosphotransfer... | 237 | 4e-74 | 47% |
| WP_012758375.1 aminoglycoside 3'-phosphotransferase [Rhizobium... | 237 | 4e-74 | 49% |
| WP_159496572.1 APH(3') family aminoglycoside O-phosphotransfer... | 237 | 4e-74 | 51% |
| WP_112906620.1 aminoglycoside 3'-phosphotransferase [Rhizobium... | 236 | 4e-74 | 49% |
| WP_164161365.1 APH(3')-II family aminoglycoside O-phosphotrans... | 237 | 4e-74 | 50% |
| AXA39165.1 Phosphotransferase enzyme family protein [Rhizobium... | 237 | 4e-74 | 49% |
| WP_100449933.1 APH(3')-II family aminoglycoside O-phosphotrans... | 236 | 4e-74 | 49% |
| MBG6617281.1 APH(3')-II family aminoglycoside O-phosphotransfe... | 236 | 4e-74 | 51% |
| WP_063841661.1 aminoglycoside O-phosphotransferase APH(3')-IIc... | 236 | 5e-74 | 49% |
| WP_122753164.1 aminoglycoside 3'-phosphotransferase [Pseudomon... | 237 | 5e-74 | 50% |
| WP_177072071.1 APH(3') family aminoglycoside O-phosphotransfer... | 236 | 5e-74 | 50% |
| WP_150764162.1 aminoglycoside 3'-phosphotransferase [Pseudomon... | 236 | 5e-74 | 48% |
| KFC72561.1 Aminoglycoside 3'-phosphotransferase [Devosia sp. LC5] | 236 | 5e-74 | 48% |
| WP_180847242.1 APH(3')-II family aminoglycoside O-phosphotrans... | 236 | 6e-74 | 50% |
| WP_183847074.1 APH(3') family aminoglycoside O-phosphotransfer... | 236 | 6e-74 | 46% |
| WP_179611677.1 APH(3') family aminoglycoside O-phosphotransfer... | 236 | 6e-74 | 49% |
| WP_122756097.1 MULTISPECIES: aminoglycoside 3'-phosphotransfer... | 237 | 6e-74 | 50% |
| WP_087922356.1 APH(3')-II family aminoglycoside O-phosphotrans... | 236 | 6e-74 | 51% |
| WP_083263060.1 aminoglycoside 3'-phosphotransferase [Pseudomon... | 236 | 6e-74 | 51% |
| WP_179179902.1 APH(3') family aminoglycoside O-phosphotransfer... | 237 | 7e-74 | 50% |
| WP_088391815.1 aminoglycoside 3'-phosphotransferase [Rhizobium... | 236 | 7e-74 | 49% |
| WP_025517223.1 aminoglycoside 3'-phosphotransferase [Bordetell... | 236 | 7e-74 | 53% |
| WP_047711715.1 MULTISPECIES: aminoglycoside 3'-phosphotransfer... | 236 | 7e-74 | 51% |
| MBB3512525.1 aminoglycoside 3'-phosphotransferase-2 [Rhizobium... | 236 | 7e-74 | 49% |
| VUJ30207.1 Aminoglycoside 3'-phosphotransferase [Stenotrophomo... | 236 | 7e-74 | 50% |
| WP_035097084.1 aminoglycoside 3'-phosphotransferase [Devosia s... | 236 | 7e-74 | 48% |
| WP_044268061.1 MULTISPECIES: aminoglycoside 3'-phosphotransfer... | 236 | 7e-74 | 51% |
| WP_079212234.1 aminoglycoside 3'-phosphotransferase [Ochrobact... | 236 | 7e-74 | 48% |
| WP_122598441.1 aminoglycoside 3'-phosphotransferase [Pseudomon... | 236 | 7e-74 | 48% |
| WP_071632545.1 aminoglycoside 3'-phosphotransferase [Ochrobact... | 236 | 7e-74 | 50% |
| OEC55112.1 APH(3') family aminoglycoside O-phosphotransferase ... | 236 | 7e-74 | 51% |
| WP_105518872.1 MULTISPECIES: aminoglycoside 3'-phosphotransfer... | 236 | 8e-74 | 48% |
| WP_128410840.1 aminoglycoside 3'-phosphotransferase [Rhizobium... | 236 | 8e-74 | 51% |
| WP_131602251.1 aminoglycoside 3'-phosphotransferase [Rhizobium... | 236 | 8e-74 | 48% |
| EEV6129530.1 aminoglycoside 3'-phosphotransferase [Escherichia... | 230 | 8e-74 | 100% |
| WP_060754300.1 aminoglycoside 3'-phosphotransferase [Pseudomon... | 235 | 9e-74 | 49% |
| WP_169997266.1 APH(3') family aminoglycoside O-phosphotransfer... | 235 | 1e-73 | 50% |
| WP_125843480.1 aminoglycoside 3'-phosphotransferase [Rhizobium... | 236 | 1e-73 | 50% |
| WP_078827264.1 aminoglycoside 3'-phosphotransferase [Pseudomon... | 235 | 1e-73 | 49% |
| WP_110712074.1 APH(3')-II family aminoglycoside O-phosphotrans... | 236 | 1e-73 | 50% |
| RAK13720.1 aminoglycoside 3'-phosphotransferase-2 [Rhizobium e... | 235 | 1e-73 | 49% |
| WP_161904605.1 APH(3') family aminoglycoside O-phosphotransfer... | 235 | 1e-73 | 52% |
| OPB14905.1 APH(3') family aminoglycoside O-phosphotransferase ... | 236 | 1e-73 | 49% |
| WP_133934487.1 APH(3')-II family aminoglycoside O-phosphotrans... | 236 | 1e-73 | 50% |
| WP_181639817.1 APH(3') family aminoglycoside O-phosphotransfer... | 236 | 1e-73 | 50% |
| WP_171012445.1 MULTISPECIES: APH(3') family aminoglycoside O-p... | 235 | 1e-73 | 50% |
| TDV30503.1 aminoglycoside 3'-phosphotransferase-2 [Stenotropho... | 236 | 1e-73 | 50% |
| WP_003565362.1 aminoglycoside 3'-phosphotransferase [Rhizobium... | 235 | 1e-73 | 50% |
| WP_181459238.1 APH(3') family aminoglycoside O-phosphotransfer... | 235 | 1e-73 | 49% |
| MBG6489287.1 APH(3')-II family aminoglycoside O-phosphotransfe... | 235 | 1e-73 | 51% |
| WP_057960559.1 aminoglycoside 3'-phosphotransferase [Pseudomon... | 235 | 1e-73 | 50% |
| WP_100436182.1 APH(3')-II family aminoglycoside O-phosphotrans... | 235 | 1e-73 | 51% |
| WP_153675442.1 APH(3')-II family aminoglycoside O-phosphotrans... | 235 | 2e-73 | 50% |
| WP_112944398.1 aminoglycoside 3'-phosphotransferase [Rhizobium... | 234 | 2e-73 | 50% |
| WP_021492270.1 aminoglycoside 3'-phosphotransferase [Pseudomon... | 235 | 2e-73 | 50% |
| WP_053257859.1 MULTISPECIES: aminoglycoside 3'-phosphotransfer... | 235 | 2e-73 | 49% |
| WP_188265381.1 APH(3') family aminoglycoside O-phosphotransfer... | 235 | 2e-73 | 48% |
| WP_010207941.1 MULTISPECIES: aminoglycoside 3'-phosphotransfer... | 235 | 2e-73 | 50% |
| WP_178120376.1 APH(3') family aminoglycoside O-phosphotransfer... | 235 | 2e-73 | 50% |
| WP_130793266.1 aminoglycoside 3'-phosphotransferase [Rhizobium... | 235 | 2e-73 | 48% |
| WP_168255506.1 APH(3') family aminoglycoside O-phosphotransfer... | 235 | 2e-73 | 48% |
| OEC72174.1 APH(3') family aminoglycoside O-phosphotransferase ... | 235 | 2e-73 | 49% |
| WP_164046697.1 APH(3') family aminoglycoside O-phosphotransfer... | 235 | 2e-73 | 48% |
| WP_128399570.1 aminoglycoside 3'-phosphotransferase [Rhizobium... | 235 | 2e-73 | 50% |
| BBO51444.1 aminoglycoside phosphotransferase APH(3') [Stenotro... | 234 | 2e-73 | 50% |
| VUO51001.1 Aminoglycoside 3'-phosphotransferase [Stenotrophomo... | 235 | 2e-73 | 50% |

| WP_164036447.1 APH(3') family aminoglycoside O-phosphotransfer... | 235 | 2e-73 | 48% |
| --- | --- | --- | --- |
| WP_097620332.1 aminoglycoside 3'-phosphotransferase [Rhizobium... | 234 | 2e-73 | 50% |
| RMO69318.1 hypothetical protein ALQ35_00094 [Pseudomonas fluor... | 234 | 2e-73 | 49% |
| WP_053508945.1 APH(3')-II family aminoglycoside O-phosphotrans... | 235 | 2e-73 | 48% |
| WP_117310224.1 APH(3')-II family aminoglycoside O-phosphotrans... | 234 | 3e-73 | 51% |
| WP_003541597.1 aminoglycoside 3'-phosphotransferase [Rhizobium... | 234 | 3e-73 | 48% |
| WP_064321004.1 aminoglycoside 3'-phosphotransferase [Ochrobact... | 234 | 3e-73 | 48% |
| WP_171319617.1 APH(3') family aminoglycoside O-phosphotransfer... | 234 | 3e-73 | 48% |
| MBB3133522.1 aminoglycoside 3'-phosphotransferase-2 [Rhizobium... | 234 | 3e-73 | 50% |
| WP_095185815.1 aminoglycoside 3'-phosphotransferase [Pseudomon... | 235 | 3e-73 | 49% |
| WP_113205711.1 aminoglycoside 3'-phosphotransferase [Rhizobium... | 234 | 3e-73 | 46% |
| WP_177026589.1 APH(3') family aminoglycoside O-phosphotransfer... | 234 | 3e-73 | 50% |
| WP_093207658.1 aminoglycoside 3'-phosphotransferase [Pseudomon... | 234 | 4e-73 | 50% |
| WP_063028299.1 aminoglycoside 3'-phosphotransferase [Pseudomon... | 234 | 4e-73 | 50% |
| WP_085778857.1 aminoglycoside 3'-phosphotransferase [Rhizobium... | 234 | 5e-73 | 49% |
| VUM08569.1 Aminoglycoside 3'-phosphotransferase [Stenotrophomo... | 234 | 5e-73 | 50% |
| PWJ33797.1 aminoglycoside 3'-phosphotransferase-2 [Pseudomonas... | 230 | 5e-73 | 67% |
| VUN51095.1 Aminoglycoside 3'-phosphotransferase [Stenotrophomo... | 234 | 5e-73 | 51% |
| WP_153475161.1 aminoglycoside 3'-phosphotransferase [Pseudomon... | 233 | 5e-73 | 51% |
| WP_105163943.1 MULTISPECIES: APH(3')-II family aminoglycoside ... | 234 | 5e-73 | 51% |
| WP_003581044.1 aminoglycoside 3'-phosphotransferase [Rhizobium... | 234 | 5e-73 | 50% |
| WP_008083769.1 aminoglycoside 3'-phosphotransferase [Pseudomon... | 233 | 5e-73 | 49% |
| WP_040009711.1 APH(3')-II family aminoglycoside O-phosphotrans... | 234 | 6e-73 | 49% |
| WP_183706686.1 APH(3') family aminoglycoside O-phosphotransfer... | 233 | 6e-73 | 48% |
| SDT77001.1 aminoglycoside 3'-phosphotransferase-2 [Pseudomonas... | 234 | 6e-73 | 48% |
| WP_033045746.1 MULTISPECIES: aminoglycoside 3'-phosphotransfer... | 233 | 6e-73 | 48% |
| VUQ25678.1 Aminoglycoside 3'-phosphotransferase [Stenotrophomo... | 234 | 6e-73 | 48% |
| KWS01950.1 aminoglycoside phosphotransferase [Pseudomonas aeru... | 233 | 7e-73 | 51% |
| WP_104994738.1 aminoglycoside 3'-phosphotransferase [Pseudomon... | 233 | 7e-73 | 49% |
| MBB3356104.1 aminoglycoside 3'-phosphotransferase-2 [Rhizobium... | 233 | 7e-73 | 48% |
| WP_122680808.1 aminoglycoside 3'-phosphotransferase [Pseudomon... | 234 | 7e-73 | 50% |
| WP_159719090.1 phosphotransferase [Geminicoccus sp. CPCC 101082] | 230 | 7e-73 | 72% |
| WP_134583025.1 APH(3')-II family aminoglycoside O-phosphotrans... | 232 | 8e-73 | 54% |
| WP_025416699.1 MULTISPECIES: aminoglycoside 3'-phosphotransfer... | 233 | 9e-73 | 50% |
| WP_134545008.1 APH(3')-II family aminoglycoside O-phosphotrans... | 232 | 9e-73 | 53% |
| WP_164594720.1 APH(3') family aminoglycoside O-phosphotransfer... | 233 | 9e-73 | 49% |
| WP_017736805.1 aminoglycoside 3'-phosphotransferase [Pseudomon... | 233 | 9e-73 | 51% |
| WP_174052051.1 APH(3') family aminoglycoside O-phosphotransfer... | 233 | 9e-73 | 49% |
| WP_180831237.1 APH(3')-II family aminoglycoside O-phosphotrans... | 233 | 9e-73 | 51% |
| WP_127430200.1 MULTISPECIES: aminoglycoside 3'-phosphotransfer... | 233 | 9e-73 | 49% |
| WP_011426190.1 aminoglycoside 3'-phosphotransferase [Rhizobium... | 233 | 9e-73 | 48% |
| WP_130744708.1 aminoglycoside 3'-phosphotransferase [Rhizobium... | 233 | 9e-73 | 49% |
| WP_128593882.1 aminoglycoside 3'-phosphotransferase [Pseudomon... | 233 | 1e-72 | 49% |
| WP_017961406.1 aminoglycoside 3'-phosphotransferase [Rhizobium... | 233 | 1e-72 | 49% |
| WP_028219265.1 aminoglycoside 3'-phosphotransferase [Paraburkh... | 233 | 1e-72 | 49% |
| WP_130664889.1 aminoglycoside 3'-phosphotransferase [Rhizobium... | 233 | 1e-72 | 49% |
| WP_134832441.1 APH(3')-II family aminoglycoside O-phosphotrans... | 233 | 1e-72 | 51% |
| NNA65321.1 aminoglycoside 3'-phosphotransferase [Pseudomonas g... | 233 | 1e-72 | 50% |
| CEJ14297.1 Aminoglycoside 3'-phosphotransferase [bacterium YEK... | 252 | 1e-72 | 54% |
| WP_166478438.1 APH(3') family aminoglycoside O-phosphotransfer... | 233 | 1e-72 | 49% |
| WP_097594412.1 MULTISPECIES: aminoglycoside 3'-phosphotransfer... | 233 | 1e-72 | 49% |
| ECZ5199073.1 aminoglycoside 3'-phosphotransferase [Salmonella ... | 227 | 1e-72 | 100% |
| WP_145164176.1 aminoglycoside 3'-phosphotransferase [Pseudomon... | 232 | 2e-72 | 48% |
| WP_037099878.1 aminoglycoside 3'-phosphotransferase [Rhizobium... | 232 | 2e-72 | 46% |
| WP_080148981.1 APH(3')-II family aminoglycoside O-phosphotrans... | 233 | 2e-72 | 51% |
| MBC3289441.1 aminoglycoside 3'-phosphotransferase [Pseudomonas... | 232 | 2e-72 | 50% |
| WP_132227746.1 aminoglycoside 3'-phosphotransferase [Ochrobact... | 232 | 2e-72 | 48% |
| WP_165758433.1 MULTISPECIES: phosphotransferase [Bacteria] | 227 | 2e-72 | 100% |
| WP_057437966.1 aminoglycoside 3'-phosphotransferase [Pseudomon... | 232 | 2e-72 | 50% |
| WP_020921854.1 aminoglycoside 3'-phosphotransferase [Rhizobium... | 232 | 2e-72 | 48% |
| TCQ66921.1 aminoglycoside 3'-phosphotransferase-2 [Rhizobium s... | 232 | 3e-72 | 48% |
| EJK79442.1 aminoglycoside phosphotransferase [Rhizobium sp. AP16] | 232 | 3e-72 | 49% |
| WP_180835516.1 APH(3')-II family aminoglycoside O-phosphotrans... | 232 | 3e-72 | 51% |
| WP_141103976.1 aminoglycoside 3'-phosphotransferase [Rhizobium... | 232 | 3e-72 | 48% |
| EJZ19589.1 aminoglycoside phosphotransferase [Rhizobium sp. Pop5] | 231 | 3e-72 | 50% |
| WP_112812693.1 aminoglycoside 3'-phosphotransferase [Ochrobact... | 232 | 3e-72 | 47% |
| ERI28269.1 putative kanamycin kinase [Burkholderia cenocepacia... | 229 | 3e-72 | 66% |
| WP_024584558.1 MULTISPECIES: aminoglycoside 3'-phosphotransfer... | 231 | 3e-72 | 50% |
| WP_178118147.1 MULTISPECIES: APH(3') family aminoglycoside O-p... | 232 | 3e-72 | 50% |
| WP_064651882.1 aminoglycoside 3'-phosphotransferase [Rhizobium... | 232 | 3e-72 | 48% |
| WP_130787408.1 aminoglycoside 3'-phosphotransferase [Rhizobium... | 232 | 3e-72 | 49% |
| ODS85240.1 hypothetical protein ABS47_17220 [Devosia sp. SCN 6... | 231 | 3e-72 | 50% |

| WP_025874898.1 APH(3')-II family aminoglycoside O-phosphotrans... | 232 | 3e-72 | 51% |
| --- | --- | --- | --- |
| WP_134630749.1 APH(3')-II family aminoglycoside O-phosphotrans... | 231 | 3e-72 | 53% |
| WP_032976102.1 APH(3')-II family aminoglycoside O-phosphotrans... | 232 | 3e-72 | 50% |
| ANK86594.1 aminoglycoside 3'-phosphotransferase protein [Rhizo... | 231 | 4e-72 | 50% |
| WP_047524785.1 aminoglycoside 3'-phosphotransferase [Agrobacte... | 232 | 4e-72 | 48% |
| WP_057013188.1 aminoglycoside 3'-phosphotransferase [Pseudomon... | 231 | 4e-72 | 48% |
| KEA06263.1 aminoglycoside phosphotransferase [Agrobacterium rh... | 231 | 4e-72 | 48% |
| WP_127564570.1 APH(3')-II family aminoglycoside O-phosphotrans... | 231 | 4e-72 | 50% |
| WP_007876461.1 MULTISPECIES: aminoglycoside 3'-phosphotransfer... | 231 | 4e-72 | 47% |
| NMY23021.1 aminoglycoside 3'-phosphotransferase [Pseudomonas s... | 231 | 4e-72 | 50% |
| WP_059396536.1 aminoglycoside 3'-phosphotransferase [Pseudomon... | 231 | 5e-72 | 48% |
| WP_027683665.1 aminoglycoside 3'-phosphotransferase [Rhizobium... | 231 | 5e-72 | 50% |
| RXY82729.1 APH(3')-II family aminoglycoside O-phosphotransfera... | 231 | 5e-72 | 49% |
| OCJ11401.1 APH(3') family aminoglycoside O-phosphotransferase ... | 231 | 5e-72 | 47% |
| WP_083196756.1 aminoglycoside 3'-phosphotransferase [Rhizobium... | 231 | 5e-72 | 47% |
| WP_175007326.1 APH(3') family aminoglycoside O-phosphotransfer... | 232 | 5e-72 | 50% |
| WP_099783779.1 APH(3')-II family aminoglycoside O-phosphotrans... | 231 | 5e-72 | 51% |
| WP_130655940.1 MULTISPECIES: aminoglycoside 3'-phosphotransfer... | 231 | 5e-72 | 49% |
| WP_116406574.1 aminoglycoside 3'-phosphotransferase [Rhizobium... | 231 | 6e-72 | 50% |
| WP_178124602.1 APH(3') family aminoglycoside O-phosphotransfer... | 231 | 6e-72 | 50% |
| WP_049711654.1 aminoglycoside 3'-phosphotransferase [Pseudomon... | 231 | 6e-72 | 49% |
| WP_093819731.1 MULTISPECIES: APH(3')-II family aminoglycoside ... | 231 | 6e-72 | 50% |
| WP_012651898.1 MULTISPECIES: aminoglycoside 3'-phosphotransfer... | 231 | 6e-72 | 48% |
| WP_131712614.1 aminoglycoside 3'-phosphotransferase [Rhizobium... | 231 | 6e-72 | 49% |
| SET82618.1 aminoglycoside 3'-phosphotransferase-2 [Stenotropho... | 231 | 7e-72 | 50% |
| EED40215.1 aminoglycoside phosphotransferase [Stenotrophomonas... | 233 | 7e-72 | 49% |
| WP_163885145.1 APH(3') family aminoglycoside O-phosphotransfer... | 231 | 7e-72 | 49% |
| WP_112822001.1 aminoglycoside 3'-phosphotransferase [Rhizobium... | 232 | 7e-72 | 48% |
| WP_164568163.1 APH(3') family aminoglycoside O-phosphotransfer... | 231 | 7e-72 | 49% |
| OCX32333.1 APH(3') family aminoglycoside O-phosphotransferase ... | 230 | 8e-72 | 53% |
| WP_178123154.1 APH(3') family aminoglycoside O-phosphotransfer... | 230 | 8e-72 | 49% |
| WP_129419776.1 aminoglycoside 3'-phosphotransferase [Rhizobium... | 231 | 8e-72 | 49% |
| WP_024319578.1 MULTISPECIES: aminoglycoside 3'-phosphotransfer... | 231 | 8e-72 | 49% |
| WP_113136835.1 aminoglycoside 3'-phosphotransferase [Agrobacte... | 231 | 9e-72 | 48% |
| WP_017994770.1 aminoglycoside 3'-phosphotransferase [Rhizobium... | 231 | 9e-72 | 48% |
| AGC24326.1 aminoglycoside 3'-phosphotransferase [uncultured ba... | 225 | 1e-71 | 97% |
| WP_094463514.1 aminoglycoside 3'-phosphotransferase [Pannoniba... | 231 | 1e-71 | 48% |
| WP_088426498.1 aminoglycoside 3'-phosphotransferase [Pseudomon... | 230 | 1e-71 | 50% |
| EGE8715013.1 aminoglycoside 3'-phosphotransferase [Salmonella ... | 225 | 1e-71 | 100% |
| WP_164052816.1 APH(3') family aminoglycoside O-phosphotransfer... | 231 | 1e-71 | 48% |
| WP_151519166.1 aminoglycoside 3'-phosphotransferase [Rhizobium... | 230 | 1e-71 | 49% |
| OJH79456.1 APH(3') family aminoglycoside O-phosphotransferase ... | 230 | 1e-71 | 50% |
| WP_046069428.1 aminoglycoside 3'-phosphotransferase [Pseudomon... | 230 | 1e-71 | 48% |
| WP_174161345.1 APH(3') family aminoglycoside O-phosphotransfer... | 230 | 1e-71 | 48% |
| WP_134624307.1 APH(3')-II family aminoglycoside O-phosphotrans... | 229 | 1e-71 | 52% |
| WP_146777341.1 aminoglycoside 3'-phosphotransferase [Pseudomon... | 224 | 1e-71 | 99% |
| WP_168830842.1 APH(3') family aminoglycoside O-phosphotransfer... | 230 | 1e-71 | 49% |
| WP_174160011.1 APH(3') family aminoglycoside O-phosphotransfer... | 230 | 1e-71 | 48% |
| WP_099821324.1 APH(3')-II family aminoglycoside O-phosphotrans... | 230 | 2e-71 | 49% |
| WP_130820302.1 aminoglycoside 3'-phosphotransferase [Rhizobium... | 230 | 2e-71 | 49% |
| WP_049465703.1 APH(3')-II family aminoglycoside O-phosphotrans... | 230 | 2e-71 | 49% |
| WP_065696502.1 MULTISPECIES: aminoglycoside 3'-phosphotransfer... | 230 | 2e-71 | 48% |
| WP_097610829.1 aminoglycoside 3'-phosphotransferase [Rhizobium... | 230 | 2e-71 | 49% |
| WP_056847567.1 aminoglycoside 3'-phosphotransferase [Pseudomon... | 229 | 2e-71 | 48% |
| WP_047618433.1 aminoglycoside 3'-phosphotransferase [Rhizobium... | 230 | 2e-71 | 49% |
| WP_132790456.1 APH(3')-II family aminoglycoside O-phosphotrans... | 228 | 2e-71 | 53% |
| NMY86042.1 aminoglycoside 3'-phosphotransferase [Pseudomonas s... | 229 | 2e-71 | 48% |
| WP_130816123.1 aminoglycoside 3'-phosphotransferase [Rhizobium... | 230 | 2e-71 | 48% |
| WP_018074047.1 aminoglycoside 3'-phosphotransferase [Rhizobium... | 229 | 2e-71 | 48% |
| WP_182208801.1 APH(3') family aminoglycoside O-phosphotransfer... | 229 | 2e-71 | 49% |
| WP_177003203.1 APH(3') family aminoglycoside O-phosphotransfer... | 229 | 3e-71 | 46% |
| WP_064839857.1 MULTISPECIES: aminoglycoside 3'-phosphotransfer... | 229 | 3e-71 | 50% |
| VUK40843.1 Aminoglycoside 3'-phosphotransferase [Stenotrophomo... | 229 | 3e-71 | 50% |
| WP_110621581.1 aminoglycoside 3'-phosphotransferase [Pseudomon... | 229 | 3e-71 | 50% |
| WP_130713575.1 aminoglycoside 3'-phosphotransferase [Rhizobium... | 229 | 3e-71 | 48% |
| WP_160028615.1 APH(3') family aminoglycoside O-phosphotransfer... | 229 | 3e-71 | 49% |
| WP_144435734.1 APH(3')-II family aminoglycoside O-phosphotrans... | 228 | 3e-71 | 53% |
| WP_164591136.1 APH(3') family aminoglycoside O-phosphotransfer... | 229 | 3e-71 | 49% |
| NMY34054.1 aminoglycoside 3'-phosphotransferase [Pseudomonas s... | 229 | 4e-71 | 49% |
| WP_130700843.1 aminoglycoside 3'-phosphotransferase [Rhizobium... | 229 | 4e-71 | 49% |
| WP_075224493.1 aminoglycoside 3'-phosphotransferase [Rhizobium... | 229 | 4e-71 | 48% |
| VUI46630.1 Aminoglycoside 3'-phosphotransferase [Stenotrophomo... | 229 | 4e-71 | 50% |

| MBB3607310.1 aminoglycoside 3'-phosphotransferase-2 [Rhizobium... | 229 | 5e-71 | 48% |
| --- | --- | --- | --- |
| WP_184355745.1 APH(3') family aminoglycoside O-phosphotransfer... | 229 | 5e-71 | 48% |
| WP_065706208.1 aminoglycoside 3'-phosphotransferase [Agrobacte... | 229 | 5e-71 | 48% |
| WP_068967670.1 aminoglycoside 3'-phosphotransferase [Pseudomon... | 228 | 6e-71 | 48% |
| WP_151557617.1 aminoglycoside 3'-phosphotransferase [Ochrobact... | 228 | 6e-71 | 50% |
| WP_011652881.1 aminoglycoside 3'-phosphotransferase [Rhizobium... | 228 | 7e-71 | 48% |
| WP_184378328.1 APH(3') family aminoglycoside O-phosphotransfer... | 228 | 7e-71 | 49% |
| WP_124401814.1 aminoglycoside 3'-phosphotransferase [Pseudomon... | 228 | 7e-71 | 48% |
| WP_055996263.1 aminoglycoside 3'-phosphotransferase [Devosia s... | 228 | 7e-71 | 48% |
| WP_134660178.1 MULTISPECIES: aminoglycoside 3'-phosphotransfer... | 228 | 8e-71 | 50% |
| WP_174018517.1 APH(3') family aminoglycoside O-phosphotransfer... | 228 | 8e-71 | 48% |
| WP_097542116.1 MULTISPECIES: aminoglycoside 3'-phosphotransfer... | 228 | 9e-71 | 48% |
| WP_095015540.1 aminoglycoside 3'-phosphotransferase [Pseudomon... | 228 | 9e-71 | 46% |
| WP_055878021.1 aminoglycoside 3'-phosphotransferase [Devosia s... | 227 | 1e-70 | 50% |
| WP_028758339.1 APH(3') family aminoglycoside O-phosphotransfer... | 228 | 1e-70 | 49% |
| WP_180166886.1 APH(3')-II family aminoglycoside O-phosphotrans... | 228 | 1e-70 | 49% |
| VUJ20710.1 Aminoglycoside 3'-phosphotransferase [Stenotrophomo... | 228 | 1e-70 | 50% |
| WP_043046835.1 MULTISPECIES: aminoglycoside 3'-phosphotransfer... | 228 | 1e-70 | 47% |
| WP_176020920.1 APH(3')-II family aminoglycoside O-phosphotrans... | 228 | 1e-70 | 50% |
| WP_140226005.1 APH(3')-II family aminoglycoside O-phosphotrans... | 228 | 1e-70 | 50% |
| WP_164016515.1 APH(3') family aminoglycoside O-phosphotransfer... | 228 | 1e-70 | 50% |
| WP_108266479.1 APH(3')-II family aminoglycoside O-phosphotrans... | 228 | 1e-70 | 48% |
| WP_130836753.1 aminoglycoside 3'-phosphotransferase [Rhizobium... | 228 | 2e-70 | 48% |
| WP_178112191.1 APH(3') family aminoglycoside O-phosphotransfer... | 226 | 2e-70 | 50% |
| WP_081619848.1 aminoglycoside 3'-phosphotransferase [Rhizobium... | 228 | 2e-70 | 48% |
| WP_151678553.1 aminoglycoside 3'-phosphotransferase [Ochrobact... | 227 | 2e-70 | 50% |
| WP_182522244.1 APH(3') family aminoglycoside O-phosphotransfer... | 227 | 2e-70 | 50% |
| WP_126912230.1 aminoglycoside 3'-phosphotransferase [Rhizobium... | 227 | 2e-70 | 50% |
| WP_149064628.1 aminoglycoside 3'-phosphotransferase [Achromoba... | 227 | 2e-70 | 50% |
| RBL80703.1 APH(3') family aminoglycoside O-phosphotransferase ... | 227 | 2e-70 | 48% |
| WP_130675480.1 aminoglycoside 3'-phosphotransferase [Rhizobium... | 227 | 2e-70 | 48% |
| PYF97943.1 aminoglycoside 3'-phosphotransferase-2 [Pseudomonas... | 228 | 2e-70 | 46% |
| WP_130682114.1 aminoglycoside 3'-phosphotransferase [Rhizobium... | 227 | 2e-70 | 48% |
| WP_112965502.1 aminoglycoside 3'-phosphotransferase [Achromoba... | 227 | 2e-70 | 50% |
| WP_018484560.1 aminoglycoside 3'-phosphotransferase [Rhizobium... | 227 | 3e-70 | 48% |
| WP_178112907.1 APH(3') family aminoglycoside O-phosphotransfer... | 226 | 3e-70 | 51% |
| EGE8719732.1 phosphotransferase [Salmonella enterica subsp. en... | 221 | 3e-70 | 100% |
| WP_175217914.1 APH(3') family aminoglycoside O-phosphotransfer... | 226 | 3e-70 | 48% |
| WP_128408067.1 aminoglycoside 3'-phosphotransferase [Rhizobium... | 227 | 3e-70 | 48% |
| WP_110747070.1 aminoglycoside 3'-phosphotransferase [Pseudomon... | 226 | 3e-70 | 46% |
| WP_026159965.1 aminoglycoside 3'-phosphotransferase [Rhizobium... | 227 | 3e-70 | 50% |
| WP_172314414.1 APH(3') family aminoglycoside O-phosphotransfer... | 226 | 3e-70 | 50% |
| WP_130692321.1 aminoglycoside 3'-phosphotransferase [Rhizobium... | 227 | 3e-70 | 48% |
| WP_097596892.1 aminoglycoside 3'-phosphotransferase [Rhizobium... | 226 | 3e-70 | 49% |
| PBD23203.1 APH(3') family aminoglycoside O-phosphotransferase ... | 225 | 4e-70 | 54% |
| WP_074294492.1 aminoglycoside 3'-phosphotransferase [Paraburkh... | 226 | 5e-70 | 47% |
| WP_099842364.1 APH(3')-II family aminoglycoside O-phosphotrans... | 226 | 6e-70 | 49% |
| WP_175198993.1 APH(3') family aminoglycoside O-phosphotransfer... | 226 | 6e-70 | 49% |
| WP_104653456.1 aminoglycoside 3'-phosphotransferase [Achromoba... | 226 | 6e-70 | 49% |
| VUL12205.1 Aminoglycoside 3'-phosphotransferase [Stenotrophomo... | 226 | 6e-70 | 51% |
| WP_062945240.1 aminoglycoside 3'-phosphotransferase [Rhizobium... | 226 | 6e-70 | 49% |
| WP_175194959.1 APH(3') family aminoglycoside O-phosphotransfer... | 226 | 6e-70 | 48% |
| WP_078339890.1 MULTISPECIES: aminoglycoside 3'-phosphotransfer... | 226 | 7e-70 | 49% |
| VUS07180.1 Aminoglycoside 3'-phosphotransferase [Stenotrophomo... | 226 | 7e-70 | 51% |
| WP_088100669.1 APH(3')-II family aminoglycoside O-phosphotrans... | 226 | 7e-70 | 49% |
| WP_063640726.1 aminoglycoside 3'-phosphotransferase [Achromoba... | 226 | 7e-70 | 50% |
| WP_100472085.1 APH(3')-II family aminoglycoside O-phosphotrans... | 226 | 8e-70 | 51% |
| WP_183885934.1 MULTISPECIES: APH(3') family aminoglycoside O-p... | 226 | 9e-70 | 46% |
| WP_131718147.1 aminoglycoside 3'-phosphotransferase [Rhizobium... | 225 | 1e-69 | 48% |
| OJY50475.1 APH(3') family aminoglycoside O-phosphotransferase ... | 225 | 1e-69 | 48% |
| WP_033830952.1 APH(3')-II family aminoglycoside O-phosphotrans... | 225 | 1e-69 | 48% |
| NMY28622.1 aminoglycoside 3'-phosphotransferase [Pseudomonas s... | 225 | 1e-69 | 50% |
| OJU20640.1 APH(3') family aminoglycoside O-phosphotransferase ... | 225 | 1e-69 | 48% |
| WP_065276148.1 aminoglycoside 3'-phosphotransferase [Rhizobium... | 225 | 1e-69 | 49% |
| WP_170042965.1 APH(3') family aminoglycoside O-phosphotransfer... | 224 | 2e-69 | 50% |
| AIL06562.1 aminoglycoside 3'-phosphotransferase [Stenotrophomo... | 226 | 2e-69 | 52% |
| WP_183431584.1 APH(3') family aminoglycoside O-phosphotransfer... | 224 | 2e-69 | 49% |
| WP_100552361.1 MULTISPECIES: APH(3')-II family aminoglycoside ... | 224 | 2e-69 | 48% |
| WP_169956681.1 APH(3') family aminoglycoside O-phosphotransfer... | 224 | 2e-69 | 50% |
| WP_097630797.1 aminoglycoside 3'-phosphotransferase [Rhizobium... | 224 | 2e-69 | 48% |
| WP_057021686.1 aminoglycoside 3'-phosphotransferase [Pseudomon... | 224 | 3e-69 | 47% |
| WP_165378577.1 APH(3')-II family aminoglycoside O-phosphotrans... | 224 | 3e-69 | 47% |

| WP_151653436.1 aminoglycoside 3'-phosphotransferase [Ochrobact... | 224 | 3e-69 | 49% |
| --- | --- | --- | --- |
| WP_168757215.1 APH(3') family aminoglycoside O-phosphotransfer... | 224 | 3e-69 | 46% |
| WP_100465153.1 APH(3')-II family aminoglycoside O-phosphotrans... | 224 | 3e-69 | 48% |
| MBB4439444.1 aminoglycoside 3'-phosphotransferase-2 [Rhizobium... | 224 | 3e-69 | 49% |
| WP_184499584.1 APH(3') family aminoglycoside O-phosphotransfer... | 224 | 4e-69 | 49% |
| WP_168309889.1 APH(3') family aminoglycoside O-phosphotransfer... | 224 | 4e-69 | 50% |
| WP_003593187.1 aminoglycoside 3'-phosphotransferase [Rhizobium... | 224 | 4e-69 | 50% |
| ALV29849.1 hypothetical protein APZ00_03710 [Pannonibacter phr... | 223 | 5e-69 | 48% |
| WP_151648029.1 aminoglycoside 3'-phosphotransferase [Ochrobact... | 224 | 5e-69 | 51% |
| VVQ12645.1 Aminoglycoside 3'-phosphotransferase [Pseudomonas f... | 222 | 5e-69 | 51% |
| WP_082676116.1 aminoglycoside 3'-phosphotransferase [Pannoniba... | 224 | 7e-69 | 48% |
| WP_075674644.1 APH(3')-II family aminoglycoside O-phosphotrans... | 223 | 7e-69 | 51% |
| WP_187043687.1 APH(3') family aminoglycoside O-phosphotransfer... | 223 | 7e-69 | 48% |
| WP_166883610.1 APH(3') family aminoglycoside O-phosphotransfer... | 223 | 9e-69 | 49% |
| WP_025140955.1 aminoglycoside 3'-phosphotransferase [Achromoba... | 223 | 9e-69 | 48% |
| WP_134477092.1 APH(3')-II family aminoglycoside O-phosphotrans... | 221 | 1e-68 | 55% |
| TAH65435.1 aminoglycoside 3'-phosphotransferase [Rhodopseudomo... | 220 | 1e-68 | 59% |
| WP_121242531.1 APH(3')-II family aminoglycoside O-phosphotrans... | 221 | 1e-68 | 55% |
| WP_130750072.1 aminoglycoside 3'-phosphotransferase [Rhizobium... | 223 | 1e-68 | 49% |
| WP_010659386.1 MULTISPECIES: aminoglycoside 3'-phosphotransfer... | 223 | 1e-68 | 51% |
| WP_130678061.1 aminoglycoside 3'-phosphotransferase [Rhizobium... | 223 | 1e-68 | 49% |
| MBB3963573.1 aminoglycoside 3'-phosphotransferase-2 [Rhizobium... | 222 | 2e-68 | 48% |
| WP_134691667.1 aminoglycoside 3'-phosphotransferase [Pseudomon... | 222 | 2e-68 | 49% |
| WP_128405272.1 aminoglycoside 3'-phosphotransferase [Rhizobium... | 222 | 2e-68 | 49% |
| WP_180692009.1 APH(3') family aminoglycoside O-phosphotransfer... | 222 | 2e-68 | 50% |
| WP_164070069.1 APH(3') family aminoglycoside O-phosphotransfer... | 222 | 2e-68 | 49% |
| WP_089240634.1 MULTISPECIES: APH(3')-II family aminoglycoside ... | 222 | 2e-68 | 48% |
| WP_130809369.1 aminoglycoside 3'-phosphotransferase [Rhizobium... | 222 | 2e-68 | 49% |
| WP_171012773.1 MULTISPECIES: APH(3') family aminoglycoside O-p... | 221 | 3e-68 | 48% |
| WP_130669098.1 aminoglycoside 3'-phosphotransferase [Rhizobium... | 221 | 3e-68 | 48% |
| WP_125334334.1 aminoglycoside 3'-phosphotransferase [Ochrobact... | 221 | 4e-68 | 51% |
| WP_181292487.1 APH(3') family aminoglycoside O-phosphotransfer... | 221 | 4e-68 | 48% |
| QND13180.1 aminoglycoside 3'-phosphotransferase [Rhizobium leg... | 221 | 4e-68 | 49% |
| WP_105006838.1 aminoglycoside 3'-phosphotransferase [Rhizobium... | 221 | 5e-68 | 48% |
| WP_018495410.1 aminoglycoside 3'-phosphotransferase [Rhizobium... | 221 | 5e-68 | 47% |
| WP_138787242.1 aminoglycoside 3'-phosphotransferase [Ochrobact... | 221 | 6e-68 | 49% |
| KND16133.1 hypothetical protein ADZ37_24855 [Pannonibacter phr... | 220 | 6e-68 | 48% |
| MBD4643108.1 phosphotransferase [Xanthomonas citri pv. citri] | 215 | 7e-68 | 100% |
| WP_012558443.1 MULTISPECIES: aminoglycoside 3'-phosphotransfer... | 221 | 7e-68 | 49% |
| WP_105229609.1 aminoglycoside 3'-phosphotransferase [Serratia ... | 221 | 7e-68 | 48% |
| WP_137189305.1 aminoglycoside 3'-phosphotransferase [Stenotrop... | 221 | 8e-68 | 53% |
| WP_065280765.1 aminoglycoside 3'-phosphotransferase [Rhizobium... | 221 | 8e-68 | 49% |
| WP_151659721.1 aminoglycoside 3'-phosphotransferase [Brucella ... | 221 | 9e-68 | 47% |
| WP_171604139.1 APH(3') family aminoglycoside O-phosphotransfer... | 220 | 9e-68 | 49% |
| WP_123474145.1 aminoglycoside 3'-phosphotransferase [Pseudomon... | 220 | 1e-67 | 48% |
| WP_050475463.1 aminoglycoside 3'-phosphotransferase [Pannoniba... | 221 | 1e-67 | 47% |
| WP_121343744.1 APH(3')-II family aminoglycoside O-phosphotrans... | 219 | 1e-67 | 53% |
| VVN89869.1 Aminoglycoside 3'-phosphotransferase [Pseudomonas f... | 218 | 1e-67 | 50% |
| WP_183899408.1 APH(3') family aminoglycoside O-phosphotransfer... | 219 | 1e-67 | 48% |
| MBB4228490.1 aminoglycoside 3'-phosphotransferase-2 [Rhizobium... | 218 | 2e-67 | 52% |
| WP_111737790.1 aminoglycoside 3'-phosphotransferase [Serratia ... | 220 | 2e-67 | 47% |
| WP_126571138.1 aminoglycoside 3'-phosphotransferase [Pseudomon... | 219 | 2e-67 | 48% |
| WP_186393151.1 MULTISPECIES: APH(3') family aminoglycoside O-p... | 220 | 2e-67 | 47% |
| WP_163883482.1 APH(3') family aminoglycoside O-phosphotransfer... | 219 | 2e-67 | 49% |
| WP_160008050.1 APH(3') family aminoglycoside O-phosphotransfer... | 219 | 2e-67 | 49% |
| WP_120156637.1 aminoglycoside 3'-phosphotransferase [Achromoba... | 219 | 2e-67 | 46% |
| WP_069909550.1 aminoglycoside 3'-phosphotransferase [Devosia i... | 219 | 2e-67 | 47% |
| WP_020665282.1 aminoglycoside 3'-phosphotransferase [Rhizobium... | 219 | 2e-67 | 49% |
| MBB3914113.1 aminoglycoside 3'-phosphotransferase-2 [Rhizobium... | 218 | 2e-67 | 52% |
| WP_028732900.1 aminoglycoside 3'-phosphotransferase [Rhizobium... | 219 | 2e-67 | 49% |
| QAB44215.1 APH(3')-II family aminoglycoside O-phosphotransfera... | 219 | 2e-67 | 47% |
| WP_125299968.1 aminoglycoside 3'-phosphotransferase [Ochrobact... | 219 | 2e-67 | 50% |
| WP_130702805.1 aminoglycoside 3'-phosphotransferase [Rhizobium... | 219 | 3e-67 | 50% |
| WP_061321452.1 aminoglycoside 3'-phosphotransferase [Serratia ... | 219 | 3e-67 | 48% |
| WP_123195569.1 aminoglycoside 3'-phosphotransferase [Pannoniba... | 219 | 3e-67 | 47% |
| RYE46850.1 aminoglycoside 3'-phosphotransferase [Rhizobiales b... | 218 | 3e-67 | 48% |
| WP_130683495.1 aminoglycoside 3'-phosphotransferase [Rhizobium... | 219 | 4e-67 | 49% |
| WP_015670734.1 aminoglycoside 3'-phosphotransferase [Serratia ... | 219 | 4e-67 | 48% |
| WP_018446622.1 MULTISPECIES: aminoglycoside 3'-phosphotransfer... | 218 | 6e-67 | 49% |
| WP_122519181.1 aminoglycoside 3'-phosphotransferase [Pannoniba... | 219 | 6e-67 | 47% |
| WP_165881921.1 phosphotransferase [Bacillus amyloliquefaciens] | 213 | 7e-67 | 100% |
| WP_131713629.1 aminoglycoside 3'-phosphotransferase [Rhizobium... | 218 | 8e-67 | 48% |

| WP_105542543.1 aminoglycoside 3'-phosphotransferase [Ochrobact... | 218 | 8e-67 | 46% |
| --- | --- | --- | --- |
| WP_130652806.1 aminoglycoside 3'-phosphotransferase [Rhizobium... | 218 | 9e-67 | 49% |
| WP_179040431.1 APH(3') family aminoglycoside O-phosphotransfer... | 218 | 9e-67 | 50% |
| WP_164722840.1 APH(3') family aminoglycoside O-phosphotransfer... | 218 | 9e-67 | 48% |
| WP_193443508.1 APH(3') family aminoglycoside O-phosphotransfer... | 218 | 9e-67 | 50% |
| WP_029873345.1 aminoglycoside 3'-phosphotransferase [Rhizobium... | 218 | 1e-66 | 48% |
| WP_131710363.1 aminoglycoside 3'-phosphotransferase [Rhizobium... | 218 | 1e-66 | 50% |
| WP_028740701.1 aminoglycoside 3'-phosphotransferase [Rhizobium... | 218 | 1e-66 | 48% |
| VEI62228.1 Aminoglycoside 3'-phosphotransferase [Serratia rubi... | 218 | 1e-66 | 48% |
| WP_180193707.1 APH(3') family aminoglycoside O-phosphotransfer... | 218 | 1e-66 | 49% |
| WP_168258863.1 APH(3') family aminoglycoside O-phosphotransfer... | 218 | 1e-66 | 49% |
| WP_180694522.1 APH(3') family aminoglycoside O-phosphotransfer... | 218 | 1e-66 | 49% |
| ODT78407.1 hypothetical protein ABS76_23735 [Pelagibacterium s... | 217 | 1e-66 | 47% |
| WP_130717295.1 aminoglycoside 3'-phosphotransferase [Rhizobium... | 217 | 2e-66 | 49% |
| WP_168306992.1 APH(3') family aminoglycoside O-phosphotransfer... | 217 | 2e-66 | 49% |
| WP_018242972.1 aminoglycoside 3'-phosphotransferase [Rhizobium... | 217 | 2e-66 | 49% |
| WP_054782685.1 aminoglycoside 3'-phosphotransferase [Pseudovib... | 216 | 3e-66 | 44% |
| WP_055049030.1 aminoglycoside 3'-phosphotransferase [Devosia s... | 216 | 4e-66 | 48% |
| WP_152985223.1 APH(3')-II family aminoglycoside O-phosphotrans... | 215 | 5e-66 | 51% |
| WP_007824222.1 aminoglycoside 3'-phosphotransferase [Rhizobium... | 216 | 5e-66 | 45% |
| WP_130707821.1 aminoglycoside 3'-phosphotransferase [Rhizobium... | 216 | 6e-66 | 48% |
| WP_068880107.1 aminoglycoside 3'-phosphotransferase [Paramesor... | 216 | 6e-66 | 49% |
| KRG50969.1 aminoglycoside phosphotransferase [Stenotrophomonas... | 215 | 6e-66 | 51% |
| SEP37276.1 aminoglycoside 3'-phosphotransferase-2 [Rhodopseudo... | 214 | 7e-66 | 66% |
| WP_164578965.1 APH(3') family aminoglycoside O-phosphotransfer... | 215 | 9e-66 | 49% |
| WP_025395398.1 aminoglycoside 3'-phosphotransferase [Rhizobium... | 215 | 1e-65 | 48% |
| WP_138386972.1 aminoglycoside 3'-phosphotransferase [Rhizobium... | 215 | 1e-65 | 48% |
| WP_131645822.1 aminoglycoside 3'-phosphotransferase [Rhizobium... | 215 | 1e-65 | 49% |
| WP_191977660.1 APH(3') family aminoglycoside O-phosphotransfer... | 215 | 1e-65 | 47% |
| WP_051598845.1 aminoglycoside 3'-phosphotransferase [Stenotrop... | 215 | 1e-65 | 52% |
| WP_051423949.1 aminoglycoside 3'-phosphotransferase [Martelell... | 215 | 1e-65 | 48% |
| WP_054307635.1 aminoglycoside 3'-phosphotransferase [Serratia ... | 215 | 1e-65 | 47% |
| WP_171377537.1 APH(3') family aminoglycoside O-phosphotransfer... | 215 | 1e-65 | 49% |
| WP_168272164.1 APH(3') family aminoglycoside O-phosphotransfer... | 215 | 1e-65 | 47% |
| WP_128144180.1 aminoglycoside 3'-phosphotransferase [Serratia ... | 215 | 1e-65 | 47% |
| WP_130763681.1 aminoglycoside 3'-phosphotransferase [Rhizobium... | 214 | 2e-65 | 49% |
| WP_028619660.1 aminoglycoside 3'-phosphotransferase [Pseudomon... | 214 | 2e-65 | 49% |
| WP_027664173.1 aminoglycoside 3'-phosphotransferase [Rhizobium... | 214 | 2e-65 | 48% |
| WP_168344504.1 APH(3') family aminoglycoside O-phosphotransfer... | 214 | 2e-65 | 48% |
| WP_131624026.1 aminoglycoside 3'-phosphotransferase [Rhizobium... | 214 | 3e-65 | 48% |
| WP_068933999.1 aminoglycoside 3'-phosphotransferase [Pseudomon... | 214 | 3e-65 | 49% |
| WP_122684286.1 aminoglycoside 3'-phosphotransferase [Pseudomon... | 214 | 3e-65 | 49% |
| MAM12424.1 APH(3') family aminoglycoside O-phosphotransferase ... | 214 | 3e-65 | 48% |
| AHY59265.1 aminoglycoside phosphotransferase [Stenotrophomonas... | 213 | 4e-65 | 52% |
| WP_012091421.1 MULTISPECIES: aminoglycoside 3'-phosphotransfer... | 214 | 4e-65 | 49% |
| WP_064247973.1 aminoglycoside 3'-phosphotransferase [Rhizobium... | 213 | 5e-65 | 48% |
| WP_114216158.1 aminoglycoside 3'-phosphotransferase [Ochrobact... | 213 | 6e-65 | 49% |
| WP_182563457.1 APH(3') family aminoglycoside O-phosphotransfer... | 213 | 6e-65 | 50% |
| WP_131639562.1 aminoglycoside 3'-phosphotransferase [Rhizobium... | 213 | 7e-65 | 48% |
| WP_094514127.1 aminoglycoside 3'-phosphotransferase [Ochrobact... | 213 | 8e-65 | 49% |
| WP_168335748.1 APH(3') family aminoglycoside O-phosphotransfer... | 213 | 1e-64 | 48% |
| WP_151665089.1 MULTISPECIES: aminoglycoside 3'-phosphotransfer... | 213 | 1e-64 | 49% |
| WP_170283032.1 APH(3') family aminoglycoside O-phosphotransfer... | 213 | 1e-64 | 48% |
| WP_121190354.1 APH(3')-II family aminoglycoside O-phosphotrans... | 211 | 1e-64 | 54% |
| AII10432.1 hypothetical protein EP51_39775 [Rhodococcus opacus] | 212 | 2e-64 | 44% |
| WP_014286608.1 aminoglycoside 3'-phosphotransferase [Pseudovib... | 212 | 2e-64 | 45% |
| WP_128642533.1 aminoglycoside 3'-phosphotransferase [Rhodococc... | 212 | 2e-64 | 44% |
| WP_061345570.1 aminoglycoside 3'-phosphotransferase [Ochrobact... | 212 | 2e-64 | 49% |
| QFQ66234.1 kanamycin resistance protein [Cloning vector pMG_DV] | 209 | 3e-64 | 100% |
| WP_168287751.1 APH(3') family aminoglycoside O-phosphotransfer... | 211 | 3e-64 | 48% |
| WP_130721963.1 aminoglycoside 3'-phosphotransferase [Rhizobium... | 211 | 3e-64 | 48% |
| WP_151615449.1 aminoglycoside 3'-phosphotransferase [Ochrobact... | 211 | 3e-64 | 49% |
| WP_130725880.1 aminoglycoside 3'-phosphotransferase [Rhizobium... | 211 | 3e-64 | 48% |
| WP_131664641.1 aminoglycoside 3'-phosphotransferase [Rhizobium... | 211 | 3e-64 | 48% |
| WP_130705419.1 aminoglycoside 3'-phosphotransferase [Rhizobium... | 211 | 4e-64 | 48% |
| WP_121398844.1 APH(3') family aminoglycoside O-phosphotransfer... | 209 | 4e-64 | 55% |
| WP_168314090.1 APH(3') family aminoglycoside O-phosphotransfer... | 211 | 4e-64 | 48% |
| WP_033180233.1 aminoglycoside 3'-phosphotransferase [Rhizobium... | 211 | 7e-64 | 47% |
| WP_077978855.1 aminoglycoside 3'-phosphotransferase [Rhizobium... | 210 | 9e-64 | 48% |
| WP_100651805.1 MULTISPECIES: aminoglycoside 3'-phosphotransfer... | 210 | 1e-63 | 47% |
| WP_151570716.1 aminoglycoside 3'-phosphotransferase [Brucella ... | 210 | 1e-63 | 47% |
| WP_130672915.1 aminoglycoside 3'-phosphotransferase [Rhizobium... | 210 | 1e-63 | 47% |

| WP_130727229.1 aminoglycoside 3'-phosphotransferase [Rhizobium... | 210 | 1e-63 | 48% |
| --- | --- | --- | --- |
| MWW17397.1 APH(3') family aminoglycoside O-phosphotransferase ... | 204 | 1e-63 | 99% |
| WP_063881549.1 MULTISPECIES: aminoglycoside 3'-phosphotransfer... | 210 | 1e-63 | 47% |
| WP_142881699.1 phosphotransferase [Klebsiella pneumoniae] | 204 | 1e-63 | 100% |
| WP_140021495.1 aminoglycoside 3'-phosphotransferase [Ochrobact... | 209 | 2e-63 | 47% |
| WP_121985825.1 MULTISPECIES: aminoglycoside 3'-phosphotransfer... | 209 | 2e-63 | 47% |
| HHV70215.1 TPA: aminoglycoside 3'-phosphotransferase [Brucella... | 209 | 3e-63 | 47% |
| WP_082240525.1 aminoglycoside 3'-phosphotransferase [Pseudomon... | 210 | 3e-63 | 47% |
| WP_130712095.1 aminoglycoside 3'-phosphotransferase [Rhizobium... | 208 | 6e-63 | 48% |
| HAA6109508.1 TPA: APH(3') family aminoglycoside O-phosphotrans... | 202 | 7e-63 | 100% |
| WP_025090783.1 MULTISPECIES: aminoglycoside 3'-phosphotransfer... | 208 | 8e-63 | 47% |
| WP_183515984.1 MULTISPECIES: APH(3') family aminoglycoside O-p... | 208 | 8e-63 | 47% |
| AUZ17069.1 neomycin/kanamycin resistance protein [Cloning vect... | 202 | 9e-63 | 100% |
| WP_047353269.1 aminoglycoside 3'-phosphotransferase [Enterobac... | 207 | 1e-62 | 47% |
| WP_114439263.1 aminoglycoside 3'-phosphotransferase [Phyllobac... | 207 | 1e-62 | 47% |
| WP_006472209.1 aminoglycoside 3'-phosphotransferase [Brucella ... | 207 | 2e-62 | 47% |
| WP_048280827.1 APH(3')-II family aminoglycoside O-phosphotrans... | 204 | 4e-62 | 51% |
| WP_151657737.1 aminoglycoside 3'-phosphotransferase [Ochrobact... | 206 | 5e-62 | 46% |
| WP_151685828.1 aminoglycoside 3'-phosphotransferase [Brucella ... | 206 | 5e-62 | 47% |
| WP_176074517.1 APH(3') family aminoglycoside O-phosphotransfer... | 206 | 5e-62 | 47% |
| WP_095447270.1 aminoglycoside 3'-phosphotransferase [Ochrobact... | 205 | 1e-61 | 45% |
| WP_151093558.1 MULTISPECIES: aminoglycoside 3'-phosphotransfer... | 205 | 1e-61 | 45% |
| NYF92373.1 aminoglycoside phosphotransferase [Pseudomonas kunm... | 201 | 1e-61 | 69% |
| WP_134448409.1 APH(3') family aminoglycoside O-phosphotransfer... | 202 | 2e-61 | 55% |
| MBA3693045.1 aminoglycoside 3'-phosphotransferase [Acidobacter... | 204 | 2e-61 | 42% |
| EPD42350.1 aminoglycoside 3'-phosphotransferase [Delftia acido... | 204 | 2e-61 | 47% |
| WP_138330448.1 MULTISPECIES: aminoglycoside 3'-phosphotransfer... | 203 | 5e-61 | 48% |
| MBA4182512.1 aminoglycoside 3'-phosphotransferase [Acidobacter... | 202 | 1e-60 | 42% |
| ODT70698.1 hypothetical protein ABS75_11210 [Pelagibacterium s... | 202 | 1e-60 | 43% |
| MBA3785049.1 aminoglycoside 3'-phosphotransferase [Acidobacter... | 202 | 1e-60 | 42% |
| WP_022567995.1 aminoglycoside 3'-phosphotransferase [Brucella ... | 202 | 2e-60 | 46% |
| WP_021587130.1 MULTISPECIES: aminoglycoside 3'-phosphotransfer... | 202 | 2e-60 | 46% |
| MBF0678754.1 aminoglycoside 3'-phosphotransferase [Devosia sp.] | 201 | 2e-60 | 44% |
| EAX9587989.1 APH(3') family aminoglycoside O-phosphotransferas... | 196 | 2e-60 | 100% |
| MBA4121991.1 aminoglycoside 3'-phosphotransferase [Acidobacter... | 201 | 3e-60 | 42% |
| WP_134617906.1 APH(3') family aminoglycoside O-phosphotransfer... | 197 | 6e-60 | 57% |
| WP_159183489.1 phosphotransferase [Escherichia coli] | 195 | 7e-60 | 95% |
| WP_126152229.1 aminoglycoside 3'-phosphotransferase [Devosia s... | 200 | 7e-60 | 44% |
| NMA97209.1 aminoglycoside 3'-phosphotransferase [Phyllobacteri... | 200 | 8e-60 | 42% |
| HAW39147.1 TPA: APH(3') family aminoglycoside O-phosphotransfe... | 196 | 1e-59 | 62% |
| WP_017965206.1 aminoglycoside 3'-phosphotransferase [Rhizobium... | 199 | 2e-59 | 47% |
| WP_162119560.1 APH(3') family aminoglycoside O-phosphotransfer... | 199 | 3e-59 | 48% |
| WP_151215170.1 aminoglycoside 3'-phosphotransferase [Pseudomon... | 196 | 3e-59 | 50% |
| AWM26734.1 Aminoglycoside phosphotransferase [Sinorhizobium fr... | 195 | 3e-59 | 56% |
| HCH71410.1 TPA: APH(3') family aminoglycoside O-phosphotransfe... | 198 | 5e-59 | 45% |
| MBE0578874.1 aminoglycoside 3'-phosphotransferase [Devosia sp.] | 198 | 6e-59 | 47% |
| AKA87233.1 StrA [Escherichia coli] | 192 | 8e-59 | 81% |
| ODT50155.1 hypothetical protein ABS74_04310 [Pelagibacterium s... | 196 | 2e-58 | 43% |
| WP_156295949.1 phosphotransferase [Helicobacter ailurogastricus] | 190 | 3e-58 | 100% |
| RBL66504.1 APH(3') family aminoglycoside O-phosphotransferase ... | 190 | 4e-58 | 99% |
| WP_170263505.1 APH(3') family aminoglycoside O-phosphotransfer... | 196 | 4e-58 | 47% |
| KKB09646.1 hypothetical protein VE26_07165 [Devosia chinhatensis] | 195 | 4e-58 | 42% |
| WP_143847016.1 aminoglycoside 3'-phosphotransferase [Bacillus ... | 195 | 6e-58 | 41% |
| WP_160297817.1 APH(3') family aminoglycoside O-phosphotransfer... | 195 | 8e-58 | 42% |
| WP_104894285.1 MULTISPECIES: aminoglycoside 3'-phosphotransfer... | 194 | 1e-57 | 44% |
| WP_193728047.1 APH(3') family aminoglycoside O-phosphotransfer... | 194 | 1e-57 | 44% |
| WP_092424023.1 aminoglycoside 3'-phosphotransferase [Devosia c... | 194 | 1e-57 | 44% |
| WP_152359569.1 APH(3') family aminoglycoside O-phosphotransfer... | 191 | 1e-57 | 60% |
| WP_116588656.1 aminoglycoside 3'-phosphotransferase [Devosia s... | 194 | 1e-57 | 45% |
| SFV32771.1 aminoglycoside 3'-phosphotransferase-2 [Devosia cro... | 194 | 2e-57 | 44% |
| WP_136312324.1 APH(3') family aminoglycoside O-phosphotransfer... | 190 | 3e-57 | 57% |
| WP_134603729.1 APH(3') family aminoglycoside O-phosphotransfer... | 190 | 3e-57 | 57% |
| WP_056640379.1 aminoglycoside 3'-phosphotransferase [Paenibaci... | 193 | 4e-57 | 39% |
| KQN72961.1 hypothetical protein ASE94_10160 [Devosia sp. Leaf64] | 192 | 5e-57 | 43% |
| WP_157289229.1 APH(3') family aminoglycoside O-phosphotransfer... | 192 | 6e-57 | 44% |
| WP_082461255.1 MULTISPECIES: aminoglycoside 3'-phosphotransfer... | 192 | 7e-57 | 43% |
| MBA3599904.1 aminoglycoside 3'-phosphotransferase [Acidobacter... | 192 | 9e-57 | 41% |
| WP_082560937.1 aminoglycoside 3'-phosphotransferase [Devosia s... | 192 | 1e-56 | 42% |
| ODT75203.1 hypothetical protein ABS76_34820 [Pelagibacterium s... | 192 | 1e-56 | 42% |
| WP_143479004.1 APH(3') family aminoglycoside O-phosphotransfer... | 189 | 1e-56 | 59% |
| WP_135154599.1 aminoglycoside 3'-phosphotransferase [Cohnella ... | 191 | 2e-56 | 45% |
| WP_183309287.1 APH(3') family aminoglycoside O-phosphotransfer... | 191 | 4e-56 | 43% |

| WP_129889932.1 aminoglycoside 3'-phosphotransferase [Ktedonosp... | 191 | 4e-56 | 43% |
| --- | --- | --- | --- |
| WP_180211851.1 APH(3') family aminoglycoside O-phosphotransfer... | 190 | 4e-56 | 38% |
| WP_071975821.1 aminoglycoside 3'-phosphotransferase [Bacillus ... | 190 | 5e-56 | 38% |
| WP_158322518.1 APH(3') family aminoglycoside O-phosphotransfer... | 190 | 6e-56 | 43% |
| WP_194485670.1 APH(3') family aminoglycoside O-phosphotransfer... | 186 | 8e-56 | 61% |
| WP_194484020.1 APH(3') family aminoglycoside O-phosphotransfer... | 186 | 8e-56 | 61% |
| WP_067457548.1 aminoglycoside 3'-phosphotransferase [Devosia e... | 189 | 2e-55 | 41% |
| KFB16682.1 aminoglycoside 3'-phosphotransferase [Pseudomonas a... | 185 | 2e-55 | 60% |
| WP_172123425.1 MULTISPECIES: APH(3') family aminoglycoside O-p... | 188 | 4e-55 | 43% |
| WP_058005372.1 aminoglycoside 3'-phosphotransferase [Bacillus ... | 188 | 4e-55 | 38% |
| WP_035080955.1 aminoglycoside 3'-phosphotransferase [Devosia r... | 187 | 5e-55 | 41% |
| WP_164533960.1 APH(3') family aminoglycoside O-phosphotransfer... | 187 | 1e-54 | 44% |
| WP_143200830.1 APH(3') family aminoglycoside O-phosphotransfer... | 183 | 1e-54 | 61% |
| WP_127753209.1 aminoglycoside 3'-phosphotransferase [Devosia s... | 187 | 1e-54 | 43% |
| WP_092184822.1 aminoglycoside 3'-phosphotransferase [Devosia s... | 187 | 1e-54 | 45% |
| ODT68620.1 hypothetical protein ABS75_19880 [Pelagibacterium s... | 187 | 1e-54 | 42% |
| WP_194486977.1 APH(3') family aminoglycoside O-phosphotransfer... | 182 | 1e-54 | 62% |
| KWV84905.1 Aminoglycoside 3'-phosphotransferase [Pseudomonas f... | 183 | 1e-54 | 55% |
| WP_194517786.1 APH(3') family aminoglycoside O-phosphotransfer... | 182 | 1e-54 | 62% |
| WP_066325395.1 aminoglycoside 3'-phosphotransferase [Metabacil... | 186 | 2e-54 | 38% |
| SEQ39729.1 aminoglycoside 3'-phosphotransferase-2 [Devosia sp.... | 187 | 2e-54 | 45% |
| WP_151140455.1 aminoglycoside 3'-phosphotransferase [Pseudomon... | 182 | 3e-54 | 52% |
| WP_194493512.1 APH(3') family aminoglycoside O-phosphotransfer... | 181 | 6e-54 | 61% |
| WP_185653059.1 APH(3') family aminoglycoside O-phosphotransfer... | 184 | 8e-54 | 39% |
| WP_191772970.1 APH(3') family aminoglycoside O-phosphotransfer... | 184 | 9e-54 | 42% |
| KKB78126.1 hypothetical protein VW35_12425 [Devosia soli] | 184 | 9e-54 | 41% |
| NKJ04071.1 aminoglycoside phosphotransferase [Rhizobium sp. SG... | 181 | 1e-53 | 54% |
| WP_136777929.1 aminoglycoside 3'-phosphotransferase [Cohnella ... | 184 | 1e-53 | 41% |
| MBA1528844.1 APH(3') family aminoglycoside O-phosphotransferas... | 178 | 1e-53 | 99% |
| WP_052951697.1 aminoglycoside 3'-phosphotransferase [Devosia s... | 184 | 2e-53 | 41% |
| WP_028608462.1 aminoglycoside 3'-phosphotransferase [Paenibaci... | 183 | 3e-53 | 40% |
| WP_057304375.1 MULTISPECIES: aminoglycoside 3'-phosphotransfer... | 183 | 3e-53 | 43% |
| NLO66659.1 aminoglycoside 3'-phosphotransferase [Firmicutes ba... | 183 | 4e-53 | 42% |
| WP_097120109.1 aminoglycoside 3'-phosphotransferase [Pseudoxan... | 183 | 5e-53 | 40% |
| WP_046510676.1 aminoglycoside 3'-phosphotransferase [Bacillus ... | 182 | 6e-53 | 38% |
| MBF3185594.1 APH(3') family aminoglycoside O-phosphotransferas... | 178 | 8e-53 | 62% |
| NLJ03706.1 aminoglycoside 3'-phosphotransferase [Firmicutes ba... | 181 | 1e-52 | 41% |
| WP_145982041.1 phosphotransferase [Klebsiella pneumoniae] | 175 | 2e-52 | 97% |
| WP_170926365.1 aminoglycoside 3'-phosphotransferase [Devosia l... | 181 | 3e-52 | 39% |
| WP_090888411.1 aminoglycoside 3'-phosphotransferase [Bacillus ... | 181 | 4e-52 | 39% |
| WP_194511186.1 APH(3') family aminoglycoside O-phosphotransfer... | 176 | 4e-52 | 61% |
| MBA4170512.1 aminoglycoside 3'-phosphotransferase [Chloroflexi... | 180 | 4e-52 | 44% |
| CAA9383093.1 hypothetical protein AVDCRST_MAG74-517 [unculture... | 180 | 5e-52 | 39% |
| WP_182395839.1 APH(3') family aminoglycoside O-phosphotransfer... | 180 | 6e-52 | 39% |
| PLS82534.1 APH(3') family aminoglycoside O-phosphotransferase ... | 180 | 6e-52 | 41% |
| WP_057985134.1 MULTISPECIES: aminoglycoside 3'-phosphotransfer... | 179 | 7e-52 | 38% |
| HHT72965.1 TPA: aminoglycoside 3'-phosphotransferase [Firmicut... | 179 | 9e-52 | 40% |
| WP_076237826.1 aminoglycoside 3'-phosphotransferase [Paenibaci... | 179 | 9e-52 | 40% |
| KLU41077.1 hypothetical protein AA931_05140 [Peptococcaceae ba... | 179 | 9e-52 | 40% |
| WP_139262630.1 APH(3') family aminoglycoside O-phosphotransfer... | 175 | 1e-51 | 61% |
| HAK92458.1 TPA: APH(3')-I family aminoglycoside O-phosphotrans... | 179 | 1e-51 | 41% |
| AGB71857.1 aminoglycoside 3'-phosphotransferase [Rhizobium tro... | 176 | 2e-51 | 53% |
| KEA41287.1 aminoglycoside phosphotransferase [Pseudomonas aeru... | 175 | 2e-51 | 61% |
| NLM39531.1 aminoglycoside 3'-phosphotransferase [Firmicutes ba... | 179 | 2e-51 | 42% |
| WP_013157458.1 aminoglycoside 3'-phosphotransferase [Meiotherm... | 179 | 2e-51 | 41% |
| WP_005667286.1 APH(3')-I family aminoglycoside O-phosphotransf... | 179 | 2e-51 | 40% |
| WP_119276222.1 aminoglycoside 3'-phosphotransferase [Calidithe... | 178 | 4e-51 | 41% |
| WP_169193767.1 APH(3') family aminoglycoside O-phosphotransfer... | 177 | 4e-51 | 38% |
| MBB4646165.1 aminoglycoside 3'-phosphotransferase-1 [Massilia ... | 177 | 4e-51 | 40% |
| WP_049315769.1 APH(3') family aminoglycoside O-phosphotransfer... | 176 | 5e-51 | 49% |
| WP_184238703.1 APH(3')-I family aminoglycoside O-phosphotransf... | 177 | 5e-51 | 40% |
| WP_051304356.1 aminoglycoside 3'-phosphotransferase [Calidithe... | 177 | 6e-51 | 41% |
| MBC7340183.1 aminoglycoside 3'-phosphotransferase [Firmicutes ... | 177 | 8e-51 | 42% |
| WP_046172983.1 aminoglycoside 3'-phosphotransferase [Devosia p... | 177 | 9e-51 | 43% |
| WP_114383809.1 aminoglycoside 3'-phosphotransferase [Paenibaci... | 176 | 1e-50 | 41% |
| WP_185682867.1 APH(3') family aminoglycoside O-phosphotransfer... | 176 | 2e-50 | 42% |
| NLS78734.1 aminoglycoside 3'-phosphotransferase [Chloroflexi b... | 176 | 2e-50 | 45% |
| WP_110713708.1 APH(3') family aminoglycoside O-phosphotransfer... | 174 | 2e-50 | 49% |
| EBL6076229.1 phosphotransferase [Salmonella enterica subsp. en... | 169 | 3e-50 | 100% |
| AEA70131.1 Putative Kanamycin kinase [Pseudomonas brassicacear... | 171 | 3e-50 | 71% |
| WP_165261966.1 APH(3') family aminoglycoside O-phosphotransfer... | 176 | 4e-50 | 44% |
| WP_065851175.1 aminoglycoside 3'-phosphotransferase [Paenibaci... | 175 | 5e-50 | 37% |

| NHJ02489.1 aminoglycoside 3'-phosphotransferase [Candidatus He... | 175 | 5e-50 | 36% |
| --- | --- | --- | --- |
| WP_035163090.1 aminoglycoside 3'-phosphotransferase [Caloranae... | 175 | 5e-50 | 38% |
| WP_136219683.1 APH(3')-I family aminoglycoside O-phosphotransf... | 175 | 7e-50 | 40% |
| WP_167068329.1 APH(3') family aminoglycoside O-phosphotransfer... | 174 | 8e-50 | 37% |
| WP_119669449.1 aminoglycoside 3'-phosphotransferase [Fibrisoma... | 175 | 8e-50 | 42% |
| WP_158882850.1 APH(3') family aminoglycoside O-phosphotransfer... | 174 | 1e-49 | 44% |
| HHS54124.1 TPA: aminoglycoside 3'-phosphotransferase [Ktedonob... | 173 | 2e-49 | 40% |
| WP_107674814.1 APH(3')-I family aminoglycoside O-phosphotransf... | 174 | 2e-49 | 38% |
| WP_086820890.1 aminoglycoside 3'-phosphotransferase [Allokutzn... | 173 | 2e-49 | 42% |
| EPA97786.1 aminoglycoside phosphotransferase [Pseudomonas sp. ... | 170 | 2e-49 | 51% |
| NJO33785.1 aminoglycoside 3'-phosphotransferase [Rhodospirilla... | 170 | 2e-49 | 49% |
| WP_189422756.1 APH(3') family aminoglycoside O-phosphotransfer... | 172 | 4e-49 | 39% |
| GGM12209.1 aminoglycoside 3'-phosphotransferase [Deinococcus a... | 172 | 5e-49 | 41% |
| WP_123068927.1 APH(3')-I family aminoglycoside O-phosphotransf... | 172 | 5e-49 | 39% |
| WP_188904302.1 APH(3') family aminoglycoside O-phosphotransfer... | 172 | 5e-49 | 41% |
| WP_062104339.1 aminoglycoside 3'-phosphotransferase [Bacillus ... | 172 | 7e-49 | 39% |
| WP_073221298.1 APH(3')-I family aminoglycoside O-phosphotransf... | 172 | 8e-49 | 39% |
| WP_102126033.1 aminoglycoside 3'-phosphotransferase [Deinococc... | 172 | 8e-49 | 42% |
| WP_112925941.1 aminoglycoside O-phosphotransferase APH(3')-XV ... | 172 | 9e-49 | 39% |
| WP_039187979.1 APH(3')-I family aminoglycoside O-phosphotransf... | 172 | 9e-49 | 38% |
| WP_129728879.1 aminoglycoside 3'-phosphotransferase [Bacillus ... | 172 | 9e-49 | 38% |
| WP_015909235.1 aminoglycoside 3'-phosphotransferase [Chlorofle... | 172 | 9e-49 | 40% |
| ABY35717.1 aminoglycoside phosphotransferase [Chloroflexus aur... | 171 | 1e-48 | 40% |
| AYV46305.1 APH(3') family aminoglycoside O-phosphotransferase ... | 171 | 1e-48 | 45% |
| WP_153022992.1 phosphotransferase [Rhodanobacter thiooxydans] | 165 | 1e-48 | 99% |
| WP_163954285.1 APH(3') family aminoglycoside O-phosphotransfer... | 171 | 1e-48 | 40% |
| MPR13796.1 APH(3') family aminoglycoside O-phosphotransferase ... | 165 | 2e-48 | 100% |
| KJB97892.2 hypothetical protein TN43_24635 [Enterobacter horma... | 171 | 2e-48 | 39% |
| PZN20756.1 APH(3') family aminoglycoside O-phosphotransferase ... | 172 | 2e-48 | 45% |
| OCN27988.1 APH(3') family aminoglycoside O-phosphotransferase ... | 171 | 2e-48 | 39% |
| WP_019407932.1 MULTISPECIES: aminoglycoside O-phosphotransfera... | 171 | 2e-48 | 39% |
| HAS1853092.1 TPA: phosphotransferase [Salmonella enterica subs... | 164 | 2e-48 | 100% |
| WP_134487725.1 aminoglycoside O-phosphotransferase APH(3')-XV ... | 171 | 2e-48 | 39% |
| MBC7790567.1 aminoglycoside 3'-phosphotransferase [Anaerolinea... | 171 | 2e-48 | 40% |
| WP_053001269.1 aminoglycoside 3'-phosphotransferase [Kosmotoga... | 170 | 3e-48 | 36% |
| WP_032492579.1 aminoglycoside O-phosphotransferase APH(3')-XV ... | 171 | 3e-48 | 39% |
| WP_112955156.1 APH(3')-I family aminoglycoside O-phosphotransf... | 171 | 3e-48 | 38% |
| WP_033945914.1 MULTISPECIES: aminoglycoside O-phosphotransfera... | 170 | 3e-48 | 39% |
| WP_153004593.1 phosphotransferase [Pseudomonas sp. ICMP 19500] | 165 | 4e-48 | 81% |
| WP_080957134.1 aminoglycoside 3'-phosphotransferase [Pseudomon... | 167 | 6e-48 | 56% |
| AFR44075.1 aminoglycoside 3'-phosphotransferase [uncultured ba... | 163 | 6e-48 | 99% |
| WP_093316772.1 APH(3')-I family aminoglycoside O-phosphotransf... | 170 | 7e-48 | 40% |
| MBD4636481.1 phosphotransferase [Xanthomonas citri pv. citri] | 163 | 8e-48 | 100% |
| WP_140851573.1 APH(3')-I family aminoglycoside O-phosphotransf... | 169 | 8e-48 | 38% |
| WP_166897398.1 MULTISPECIES: APH(3')-I family aminoglycoside O... | 169 | 9e-48 | 39% |
| WP_185686080.1 APH(3') family aminoglycoside O-phosphotransfer... | 169 | 1e-47 | 41% |
| WP_114438885.1 APH(3')-I family aminoglycoside O-phosphotransf... | 169 | 1e-47 | 38% |
| QIQ10685.1 Aminoglycoside 3'-phosphotransferase [uncultured ba... | 169 | 2e-47 | 39% |
| WP_127597642.1 APH(3')-I family aminoglycoside O-phosphotransf... | 169 | 2e-47 | 38% |
| AKI98340.1 hypothetical protein IX53_08290 [Kosmotoga pacifica] | 168 | 2e-47 | 36% |
| WP_161407665.1 APH(3') family aminoglycoside O-phosphotransfer... | 168 | 2e-47 | 37% |
| WP_026792830.1 APH(3')-I family aminoglycoside O-phosphotransf... | 168 | 2e-47 | 40% |
| PWS05620.1 APH(3') family aminoglycoside O-phosphotransferase ... | 162 | 3e-47 | 100% |
| APT66224.1 AphA15 [Pseudomonas aeruginosa] | 168 | 3e-47 | 39% |
| WP_164039070.1 APH(3') family aminoglycoside O-phosphotransfer... | 168 | 3e-47 | 38% |
| WP_066276343.1 aminoglycoside 3'-phosphotransferase [Arthrobac... | 168 | 3e-47 | 40% |
| WP_088744577.1 MULTISPECIES: aminoglycoside O-phosphotransfera... | 168 | 3e-47 | 39% |
| MYD52066.1 aminoglycoside 3'-phosphotransferase [Dehalococcoid... | 167 | 3e-47 | 39% |
| PMP82804.1 aminoglycoside 3'-phosphotransferase [Chloroflexus ... | 167 | 4e-47 | 39% |
| WP_034067921.1 aminoglycoside O-phosphotransferase APH(3')-XV ... | 167 | 4e-47 | 39% |
| WP_128142502.1 APH(3')-I family aminoglycoside O-phosphotransf... | 167 | 5e-47 | 40% |
| WP_040005495.1 APH(3') family aminoglycoside O-phosphotransfer... | 167 | 5e-47 | 40% |
| WP_106135975.1 aminoglycoside 3'-phosphotransferase [Spirosoma... | 167 | 6e-47 | 38% |
| SCZ01064.1 aminoglycoside 3'-phosphotransferase-1 [Microvirga ... | 167 | 6e-47 | 39% |
| MXY45171.1 aminoglycoside 3'-phosphotransferase [Dehalococcoid... | 167 | 6e-47 | 39% |
| WP_167626694.1 phosphotransferase, partial [Paracoccus luteus] | 160 | 7e-47 | 100% |
| WP_151576255.1 APH(3')-I family aminoglycoside O-phosphotransf... | 167 | 7e-47 | 39% |
| CCH52391.1 aminoglycoside 3'-phosphotransferase [Fibrisoma lim... | 167 | 8e-47 | 40% |
| NLM68389.1 aminoglycoside 3'-phosphotransferase [Firmicutes ba... | 163 | 8e-47 | 51% |
| WP_091137294.1 APH(3')-I family aminoglycoside O-phosphotransf... | 167 | 1e-46 | 39% |
| WP_161038676.1 APH(3') family aminoglycoside O-phosphotransfer... | 167 | 1e-46 | 39% |
| PVM84386.1 APH(3') family aminoglycoside O-phosphotransferase ... | 167 | 1e-46 | 44% |

| WP_103080760.1 | aminoglycoside 3'-phosphotransferase [Pseudoclo... | 166 | 1e-46 | 38% |
| --- | --- | --- | --- | --- |
| WP_152249820.1 | APH(3')-I family aminoglycoside O-phosphotransf... | 166 | 1e-46 | 41% |
| WP_010891085.1 | aminoglycoside O-phosphotransferase APH(3')-Id ... | 166 | 1e-46 | 38% |
| WP_168019926.1 | MULTISPECIES: APH(3')-I family aminoglycoside O... | 166 | 2e-46 | 37% |
| WP_110888787.1 | aminoglycoside 3'-phosphotransferase [Deinococc... | 166 | 2e-46 | 39% |
| WP_085486582.1 | aminoglycoside 3'-phosphotransferase [Paraburkh... | 164 | 2e-46 | 43% |
| WP_139089894.1 | APH(3')-I family aminoglycoside O-phosphotransf... | 166 | 2e-46 | 39% |
| WP_168023184.1 | APH(3')-I family aminoglycoside O-phosphotransf... | 166 | 2e-46 | 37% |
| WP_146198061.1 | aminoglycoside 3'-phosphotransferase [Caulobact... | 167 | 2e-46 | 44% |
| WP_061834464.1 | aminoglycoside 3'-phosphotransferase [Bdellovib... | 166 | 2e-46 | 39% |
| WP_055788028.1 | MULTISPECIES: APH(3')-I family aminoglycoside O... | 166 | 2e-46 | 39% |
| WP_007663592.1 | APH(3')-I family aminoglycoside O-phosphotransf... | 166 | 3e-46 | 38% |
| WP_000463316.1 | aminoglycoside phosphotransferase APH(3') [Stap... | 159 | 3e-46 | 100% |
| WP_166803673.1 | phosphotransferase [Pseudomonas aeruginosa] | 162 | 3e-46 | 53% |
| RJL33085.1 aminoglycoside 3'-phosphotransferase [Streptosporan... | | 165 | 3e-46 | 44% |
| NMB97245.1 aminoglycoside 3'-phosphotransferase [Clostridiacea... | | 165 | 4e-46 | 37% |
| WP_129472096.1 APH(3')-I family aminoglycoside O-phosphotransf... | | 165 | 4e-46 | 40% |
| WP_129117629.1 aminoglycoside 3'-phosphotransferase [Deinococc... | | 165 | 4e-46 | 41% |
| NBV19426.1 APH(3')-I family aminoglycoside O-phosphotransferas... | | 165 | 4e-46 | 40% |
| WP_126351860.1 aminoglycoside 3'-phosphotransferase [Deinococc... | | 165 | 4e-46 | 39% |
| OJV94721.1 hypothetical protein BGO39_22155 [Chloroflexi bacte... | | 165 | 5e-46 | 40% |
| EAT6395399.1 APH(3')-I family aminoglycoside O-phosphotransfer... | | 165 | 5e-46 | 38% |
| MBC7974493.1 aminoglycoside 3'-phosphotransferase [Myxococcale... | | 164 | 6e-46 | 39% |
| WP_123314918.1 aminoglycoside 3'-phosphotransferase [Virgibaci... | | 164 | 6e-46 | 37% |
| WP_147164453.1 APH(3')-I family aminoglycoside O-phosphotransf... | | 164 | 7e-46 | 39% |
| WP_127458525.1 aminoglycoside 3'-phosphotransferase [Paenibaci... | | 164 | 8e-46 | 37% |
| WP_019424209.1 aminoglycoside 3'-phosphotransferase [Paenibaci... | | 164 | 9e-46 | 37% |
| MBD4156059.1 phosphotransferase [Xanthomonas citri pv. citri] | | 158 | 1e-45 | 100% |
| WP_190859275.1 APH(3') family aminoglycoside O-phosphotransfer... | | 164 | 1e-45 | 38% |
| WP_183984199.1 APH(3') family aminoglycoside O-phosphotransfer... | | 164 | 1e-45 | 41% |
| WP_090441999.1 APH(3')-I family aminoglycoside O-phosphotransf... | | 164 | 1e-45 | 38% |
| WP_110524776.1 MULTISPECIES: aminoglycoside 3'-phosphotransfer... | | 164 | 1e-45 | 38% |
| WP_111268057.1 APH(3')-I family aminoglycoside O-phosphotransf... | | 164 | 1e-45 | 40% |
| WP_185130053.1 APH(3') family aminoglycoside O-phosphotransfer... | | 164 | 2e-45 | 37% |
| WP_139404392.1 aminoglycoside 3'-phosphotransferase [Deinococc... | | 163 | 2e-45 | 42% |
| WP_112476829.1 MULTISPECIES: aminoglycoside 3'-phosphotransfer... | | 162 | 2e-45 | 43% |
| NPD87296.1 aminoglycoside 3'-phosphotransferase [Asgard group ... | | 163 | 2e-45 | 33% |
| WP_039682327.1 aminoglycoside 3'-phosphotransferase [Deinococc... | | 163 | 3e-45 | 42% |
| WP_179162406.1 APH(3') family aminoglycoside O-phosphotransfer... | | 163 | 3e-45 | 42% |
| WP_184136941.1 APH(3') family aminoglycoside O-phosphotransfer... | | 163 | 3e-45 | 41% |
| WP_126141999.1 aminoglycoside 3'-phosphotransferase [Paenibaci... | | 163 | 3e-45 | 38% |
| WP_194489247.1 phosphotransferase [Pseudomonas aeruginosa] | | 158 | 4e-45 | 59% |
| WP_188968196.1 APH(3') family aminoglycoside O-phosphotransfer... | | 162 | 4e-45 | 40% |
| WP_157319799.1 MULTISPECIES: APH(3') family aminoglycoside O-p... | | 162 | 4e-45 | 37% |
| PSD49961.1 APH(3')-II family aminoglycoside O-phosphotransfera... | | 158 | 5e-45 | 57% |
| WP_063977000.1 APH(3')-I family aminoglycoside O-phosphotransf... | | 162 | 5e-45 | 38% |
| WP_029483573.1 aminoglycoside 3'-phosphotransferase [Deinococc... | | 162 | 5e-45 | 40% |
| WP_156050644.1 aminoglycoside 3'-phosphotransferase [Allokutzn... | | 162 | 5e-45 | 41% |
| SDN31820.1 aminoglycoside 3'-phosphotransferase-2 [Allokutzner... | | 162 | 6e-45 | 41% |
| NUO39156.1 aminoglycoside 3'-phosphotransferase [Gemmatimonada... | | 162 | 6e-45 | 39% |
| NUP71962.1 aminoglycoside 3'-phosphotransferase [Gemmatimonada... | | 162 | 6e-45 | 39% |
| OWX75347.1 APH(3') family aminoglycoside O-phosphotransferase ... | | 162 | 7e-45 | 35% |
| WP_152871354.1 aminoglycoside 3'-phosphotransferase [Deinococc... | | 161 | 7e-45 | 41% |
| GHF77103.1 aminoglycoside 3'-phosphotransferase [Deinococcus f... | | 161 | 7e-45 | 42% |
| EFH5635597.1 phosphotransferase [Escherichia coli] | | 155 | 8e-45 | 100% |
| WP_070288732.1 aminoglycoside 3'-phosphotransferase [Janthinob... | | 162 | 1e-44 | 41% |
| WP_191241142.1 APH(3') family aminoglycoside O-phosphotransfer... | | 161 | 1e-44 | 41% |
| ODU39669.1 APH(3') family aminoglycoside O-phosphotransferase ... | | 159 | 1e-44 | 49% |
| WP_099394237.1 APH(3')-I family aminoglycoside O-phosphotransf... | | 161 | 1e-44 | 40% |
| WP_151847701.1 aminoglycoside 3'-phosphotransferase [Deinococc... | | 161 | 1e-44 | 40% |
| EBV9307222.1 APH(3')-I family aminoglycoside O-phosphotransfer... | | 161 | 1e-44 | 35% |
| WP_011039766.1 MULTISPECIES: aminoglycoside O-phosphotransfera... | | 161 | 1e-44 | 35% |
| WP_034779455.1 APH(3')-I family aminoglycoside O-phosphotransf... | | 161 | 1e-44 | 40% |
| WP_176392811.1 APH(3') family aminoglycoside O-phosphotransfer... | | 161 | 1e-44 | 36% |
| WP_102121849.1 APH(3')-I family aminoglycoside O-phosphotransf... | | 161 | 1e-44 | 44% |
| WP_073974069.1 APH(3')-I family aminoglycoside O-phosphotransf... | | 161 | 1e-44 | 36% |
| WP_064380413.1 APH(3')-I family aminoglycoside O-phosphotransf... | | 161 | 2e-44 | 35% |
| HEY32847.1 TPA: aminoglycoside 3'-phosphotransferase [Dehaloco... | | 160 | 2e-44 | 38% |
| WP_157176052.1 APH(3')-I family aminoglycoside O-phosphotransf... | | 161 | 2e-44 | 38% |
| WP_132026539.1 APH(3')-I family aminoglycoside O-phosphotransf... | | 161 | 2e-44 | 36% |
| WP_038424510.1 MULTISPECIES: APH(3')-I family aminoglycoside O... | | 161 | 2e-44 | 35% |
| WP_075787502.1 aminoglycoside 3'-phosphotransferase [Rhodovulu... | | 160 | 2e-44 | 37% |

| WP_101976100.1 MULTISPECIES: APH(3')-I family aminoglycoside O... | 161 | 2e-44 | 35% |
| --- | --- | --- | --- |
| ECM3652122.1 APH(3')-I family aminoglycoside O-phosphotransfer... | 161 | 2e-44 | 35% |
| WP_066785815.1 aminoglycoside 3'-phosphotransferase [Chlorofle... | 160 | 2e-44 | 40% |
| WP_075833822.1 aminoglycoside 3'-phosphotransferase [Deinococc... | 160 | 2e-44 | 40% |
| PKT22986.1 APH(3') family aminoglycoside O-phosphotransferase ... | 154 | 2e-44 | 100% |
| GGO37288.1 aminoglycoside 3'-phosphotransferase [Deinococcus h... | 159 | 2e-44 | 43% |
| WP_034386205.1 aminoglycoside 3'-phosphotransferase [Deinococc... | 160 | 2e-44 | 40% |
| EDR7968605.1 APH(3')-I family aminoglycoside O-phosphotransfer... | 160 | 3e-44 | 35% |
| ECS5455889.1 APH(3')-I family aminoglycoside O-phosphotransfer... | 160 | 3e-44 | 35% |
| HAI51689.1 TPA: aminoglycoside phosphotransferase APH(3') [Fir... | 160 | 3e-44 | 38% |
| RTL16629.1 aminoglycoside 3'-phosphotransferase [Burkholderial... | 160 | 3e-44 | 37% |
| ACO47191.2 putative kanamycin kinase (aminoglycoside 3-phospho... | 160 | 3e-44 | 39% |
| WP_074418740.1 APH(3')-I family aminoglycoside O-phosphotransf... | 160 | 3e-44 | 35% |
| WP_064407248.1 APH(3')-I family aminoglycoside O-phosphotransf... | 160 | 3e-44 | 35% |
| WP_086073932.1 APH(3')-I family aminoglycoside O-phosphotransf... | 160 | 4e-44 | 35% |
| WP_104889678.1 APH(3')-I family aminoglycoside O-phosphotransf... | 160 | 4e-44 | 35% |
| WP_184031453.1 APH(3') family aminoglycoside O-phosphotransfer... | 159 | 4e-44 | 39% |
| WP_049070618.1 APH(3')-I family aminoglycoside O-phosphotransf... | 160 | 4e-44 | 35% |
| EEM2987832.1 APH(3')-I family aminoglycoside O-phosphotransfer... | 160 | 4e-44 | 35% |
| EDQ0658162.1 APH(3')-I family aminoglycoside O-phosphotransfer... | 160 | 4e-44 | 35% |
| WP_041227266.1 aminoglycoside 3'-phosphotransferase [Deinococc... | 160 | 5e-44 | 39% |
| WP_134076764.1 aminoglycoside 3'-phosphotransferase [Rhodovulu... | 159 | 5e-44 | 37% |
| MBA4216309.1 APH(3') family aminoglycoside O-phosphotransferas... | 160 | 5e-44 | 37% |
| WP_022800547.1 aminoglycoside 3'-phosphotransferase [Deinococc... | 159 | 5e-44 | 41% |
| WP_039677907.1 APH(3')-I family aminoglycoside O-phosphotransf... | 160 | 5e-44 | 35% |
| WP_027463591.1 aminoglycoside 3'-phosphotransferase [Deinococc... | 159 | 5e-44 | 41% |
| WP_032719106.1 APH(3')-I family aminoglycoside O-phosphotransf... | 160 | 5e-44 | 35% |
| EDA6521876.1 APH(3')-I family aminoglycoside O-phosphotransfer... | 160 | 6e-44 | 35% |
| WP_189008423.1 APH(3') family aminoglycoside O-phosphotransfer... | 159 | 6e-44 | 40% |
| WP_195170753.1 APH(3') family aminoglycoside O-phosphotransfer... | 159 | 6e-44 | 35% |
| WP_133008730.1 aminoglycoside 3'-phosphotransferase [Deinococc... | 159 | 6e-44 | 41% |
| WP_161023984.1 APH(3')-I family aminoglycoside O-phosphotransf... | 159 | 6e-44 | 37% |
| EDM5836550.1 APH(3') family aminoglycoside O-phosphotransferas... | 160 | 6e-44 | 35% |
| WP_143850749.1 APH(3')-I family aminoglycoside O-phosphotransf... | 159 | 6e-44 | 35% |
| PHJ08806.1 hypothetical protein A5N81_25350 [Pseudomonas aerug... | 157 | 6e-44 | 47% |
| ABQ65196.1 kanamycin resistance protein [Cloning vector pIPKTA38] | 159 | 7e-44 | 35% |
| MBC4887153.1 APH(3')-I family aminoglycoside O-phosphotransfer... | 159 | 7e-44 | 35% |
| WP_188964655.1 APH(3') family aminoglycoside O-phosphotransfer... | 159 | 7e-44 | 40% |
| WP_026677339.1 aminoglycoside 3'-phosphotransferase [Fictibaci... | 159 | 7e-44 | 35% |
| EBS3149277.1 APH(3')-I family aminoglycoside O-phosphotransfer... | 159 | 7e-44 | 35% |
| EDA6517011.1 APH(3')-I family aminoglycoside O-phosphotransfer... | 159 | 7e-44 | 35% |
| EDB9128337.1 APH(3')-I family aminoglycoside O-phosphotransfer... | 159 | 7e-44 | 35% |
| WP_143439757.1 APH(3')-I family aminoglycoside O-phosphotransf... | 159 | 7e-44 | 35% |
| AAB63351.1 Tn903 aminoglycoside phosphotransferase [synthetic ... | 159 | 7e-44 | 35% |
| EAZ1769069.1 APH(3')-I family aminoglycoside O-phosphotransfer... | 159 | 8e-44 | 35% |
| HAD9928789.1 TPA: APH(3')-I family aminoglycoside O-phosphotra... | 159 | 8e-44 | 35% |
| EBL5403164.1 APH(3')-I family aminoglycoside O-phosphotransfer... | 159 | 8e-44 | 35% |
| EDA6517012.1 APH(3')-I family aminoglycoside O-phosphotransfer... | 159 | 8e-44 | 35% |
| WP_160552849.1 APH(3')-I family aminoglycoside O-phosphotransf... | 159 | 8e-44 | 35% |
| WP_171682970.1 APH(3') family aminoglycoside O-phosphotransfer... | 159 | 9e-44 | 35% |
| WP_196450895.1 APH(3') family aminoglycoside O-phosphotransfer... | 159 | 9e-44 | 44% |
| WP_176376159.1 APH(3')-I family aminoglycoside O-phosphotransf... | 159 | 9e-44 | 40% |
| WP_065286408.1 APH(3')-I family aminoglycoside O-phosphotransf... | 159 | 9e-44 | 35% |
| WP_160230712.1 MULTISPECIES: APH(3')-I family aminoglycoside O... | 159 | 9e-44 | 35% |
| TDX33575.1 aminoglycoside 3'-phosphotransferase-1 [Rhodovulum ... | 159 | 9e-44 | 37% |
| WP_182157931.1 APH(3')-I family aminoglycoside O-phosphotransf... | 159 | 9e-44 | 35% |
| WP_088182419.1 APH(3')-I family aminoglycoside O-phosphotransf... | 159 | 9e-44 | 37% |
| WP_035010642.1 APH(3')-I family aminoglycoside O-phosphotransf... | 159 | 9e-44 | 35% |
| GGJ88360.1 aminoglycoside 3'-phosphotransferase [Deinococcus a... | 159 | 9e-44 | 40% |
| AAS90620.1 aminoglycoside 3'-phosphotransferase [uncultured so... | 159 | 1e-43 | 41% |
| EDB0668341.1 APH(3')-I family aminoglycoside O-phosphotransfer... | 158 | 1e-43 | 35% |
| WP_011531371.1 aminoglycoside 3'-phosphotransferase [Deinococc... | 159 | 1e-43 | 41% |
| HAE2373734.1 TPA: APH(3')-I family aminoglycoside O-phosphotra... | 159 | 1e-43 | 35% |
| WP_177331748.1 APH(3')-I family aminoglycoside O-phosphotransf... | 159 | 1e-43 | 35% |
| WP_052803280.1 APH(3')-I family aminoglycoside O-phosphotransf... | 159 | 1e-43 | 35% |
| WP_042946206.1 MULTISPECIES: APH(3')-I family aminoglycoside O... | 159 | 1e-43 | 35% |
| MBB9645739.1 APH(3')-I family aminoglycoside O-phosphotransfer... | 159 | 1e-43 | 35% |
| EBK0357565.1 APH(3')-I family aminoglycoside O-phosphotransfer... | 158 | 1e-43 | 35% |
| EBS3149273.1 APH(3')-I family aminoglycoside O-phosphotransfer... | 159 | 1e-43 | 35% |
| WP_165322538.1 APH(3') family aminoglycoside O-phosphotransfer... | 159 | 1e-43 | 40% |
| WP_189116256.1 APH(3')-I family aminoglycoside O-phosphotransf... | 159 | 1e-43 | 35% |
| ECS4615632.1 APH(3')-I family aminoglycoside O-phosphotransfer... | 159 | 1e-43 | 35% |

| EBB0489202.1 APH(3')-I family aminoglycoside O-phosphotransfer... | 159 | 1e-43 | 35% |
| --- | --- | --- | --- |
| WP_001393661.1 APH(3')-I family aminoglycoside O-phosphotransf... | 159 | 1e-43 | 35% |
| EFH3572139.1 APH(3')-I family aminoglycoside O-phosphotransfer... | 158 | 1e-43 | 35% |
| WP_088758324.1 APH(3')-I family aminoglycoside O-phosphotransf... | 158 | 1e-43 | 35% |
| WP_058818663.1 APH(3')-I family aminoglycoside O-phosphotransf... | 158 | 1e-43 | 35% |
| WP_069199697.1 APH(3')-I family aminoglycoside O-phosphotransf... | 159 | 1e-43 | 35% |
| EBS3149281.1 APH(3')-I family aminoglycoside O-phosphotransfer... | 159 | 1e-43 | 35% |
| WP_094339573.1 APH(3')-I family aminoglycoside O-phosphotransf... | 159 | 1e-43 | 35% |
| WP_141108750.1 APH(3')-I family aminoglycoside O-phosphotransf... | 158 | 1e-43 | 35% |
| WP_137530630.1 MULTISPECIES: APH(3')-I family aminoglycoside O... | 158 | 1e-43 | 35% |
| EDD3697588.1 APH(3')-I family aminoglycoside O-phosphotransfer... | 159 | 1e-43 | 35% |
| WP_141170125.1 aminoglycoside 3'-phosphotransferase [Janthinob... | 159 | 1e-43 | 41% |
| WP_053273111.1 APH(3')-I family aminoglycoside O-phosphotransf... | 159 | 1e-43 | 35% |
| EAP0460802.1 APH(3')-I family aminoglycoside O-phosphotransfer... | 159 | 1e-43 | 35% |
| EBK4270314.1 APH(3')-I family aminoglycoside O-phosphotransfer... | 159 | 1e-43 | 35% |
| EEN3586250.1 APH(3')-I family aminoglycoside O-phosphotransfer... | 158 | 1e-43 | 35% |
| AKQ71012.1 aminoglycoside 3'-phosphotransferase [Cloning vecto... | 158 | 1e-43 | 35% |
| WP_138816636.1 APH(3')-I family aminoglycoside O-phosphotransf... | 159 | 1e-43 | 35% |
| AEC32941.1 aminoglycoside 3'-phosphotransferase-like protein [... | 159 | 1e-43 | 35% |
| EEA4668005.1 APH(3')-I family aminoglycoside O-phosphotransfer... | 159 | 1e-43 | 35% |
| WP_052803015.1 MULTISPECIES: APH(3')-I family aminoglycoside O... | 159 | 1e-43 | 35% |
| WP_138136781.1 APH(3')-I family aminoglycoside O-phosphotransf... | 159 | 1e-43 | 35% |
| EBK0357535.1 APH(3')-I family aminoglycoside O-phosphotransfer... | 158 | 1e-43 | 35% |
| EGI6886307.1 APH(3')-I family aminoglycoside O-phosphotransfer... | 159 | 1e-43 | 35% |
| WP_193157376.1 APH(3')-I family aminoglycoside O-phosphotransf... | 159 | 1e-43 | 35% |
| AAC64939.1 kanamycin 3'-phosphotransferase [Cloning vector p34... | 159 | 1e-43 | 35% |
| EDP9035216.1 APH(3')-I family aminoglycoside O-phosphotransfer... | 159 | 1e-43 | 35% |
| ECU1741619.1 APH(3')-I family aminoglycoside O-phosphotransfer... | 159 | 1e-43 | 35% |
| WP_196539003.1 APH(3')-I family aminoglycoside O-phosphotransf... | 158 | 1e-43 | 35% |
| WP_061595533.1 APH(3')-I family aminoglycoside O-phosphotransf... | 159 | 1e-43 | 35% |
| WP_061873476.1 APH(3')-I family aminoglycoside O-phosphotransf... | 158 | 1e-43 | 35% |
| HAG1797939.1 TPA: APH(3')-I family aminoglycoside O-phosphotra... | 159 | 1e-43 | 35% |
| AHZ08396.1 aminoglycoside 3'-phosphotransferase [Cloning vecto... | 159 | 1e-43 | 35% |
| WP_192481523.1 APH(3')-I family aminoglycoside O-phosphotransf... | 159 | 1e-43 | 35% |
| WP_097504369.1 APH(3')-I family aminoglycoside O-phosphotransf... | 159 | 1e-43 | 35% |
| WP_096198752.1 APH(3')-I family aminoglycoside O-phosphotransf... | 159 | 1e-43 | 35% |
| WP_184448168.1 APH(3') family aminoglycoside O-phosphotransfer... | 159 | 1e-43 | 40% |
| WP_061602544.1 APH(3')-I family aminoglycoside O-phosphotransf... | 159 | 1e-43 | 35% |
| EDG5578971.1 APH(3')-I family aminoglycoside O-phosphotransfer... | 159 | 1e-43 | 35% |
| WP_133050651.1 aminoglycoside 3'-phosphotransferase [Stenotrop... | 157 | 1e-43 | 40% |
| WP_125134846.1 APH(3')-I family aminoglycoside O-phosphotransf... | 159 | 1e-43 | 35% |
| WP_171931707.1 APH(3')-I family aminoglycoside O-phosphotransf... | 159 | 1e-43 | 35% |
| WP_096245197.1 APH(3')-I family aminoglycoside O-phosphotransf... | 159 | 1e-43 | 35% |
| EFH4548931.1 APH(3')-I family aminoglycoside O-phosphotransfer... | 159 | 2e-43 | 35% |
| WP_102381258.1 MULTISPECIES: APH(3')-I family aminoglycoside O... | 158 | 2e-43 | 35% |
| WP_000018326.1 MULTISPECIES: aminoglycoside O-phosphotransfera... | 159 | 2e-43 | 35% |
| ECS9584356.1 APH(3')-I family aminoglycoside O-phosphotransfer... | 159 | 2e-43 | 35% |
| EAM3772361.1 APH(3')-I family aminoglycoside O-phosphotransfer... | 159 | 2e-43 | 35% |
| WP_124042610.1 APH(3')-I family aminoglycoside O-phosphotransf... | 159 | 2e-43 | 35% |
| WP_063842098.1 aminoglycoside O-phosphotransferase APH(3')-Ia ... | 159 | 2e-43 | 35% |
| WP_070224065.1 MULTISPECIES: aminoglycoside 3'-phosphotransfer... | 159 | 2e-43 | 42% |
| EED9163438.1 APH(3')-I family aminoglycoside O-phosphotransfer... | 159 | 2e-43 | 35% |
| MHN88063.1 APH(3')-I family aminoglycoside O-phosphotransferas... | 159 | 2e-43 | 35% |
| WP_181596660.1 APH(3')-I family aminoglycoside O-phosphotransf... | 159 | 2e-43 | 36% |
| WP_113440062.1 APH(3')-I family aminoglycoside O-phosphotransf... | 159 | 2e-43 | 35% |
| EBB8601238.1 APH(3')-I family aminoglycoside O-phosphotransfer... | 158 | 2e-43 | 35% |
| VCY58942.1 aph(3')-Ic_1_X62115 [Escherichia coli] | 159 | 2e-43 | 35% |
| WP_063841683.1 aminoglycoside O-phosphotransferase APH(3')-Ia ... | 159 | 2e-43 | 35% |
| ECV4155626.1 APH(3')-I family aminoglycoside O-phosphotransfer... | 159 | 2e-43 | 35% |
| WP_170448081.1 APH(3')-I family aminoglycoside O-phosphotransf... | 159 | 2e-43 | 35% |
| EBI7534138.1 APH(3')-I family aminoglycoside O-phosphotransfer... | 159 | 2e-43 | 35% |
| KXI15614.1 phosphotransferase enzyme family protein [Corynebac... | 159 | 2e-43 | 35% |
| WP_107381798.1 MULTISPECIES: APH(3')-I family aminoglycoside O... | 159 | 2e-43 | 35% |
| WP_000018330.1 MULTISPECIES: aminoglycoside O-phosphotransfera... | 159 | 2e-43 | 35% |
| WP_087851980.1 MULTISPECIES: APH(3')-I family aminoglycoside O... | 158 | 2e-43 | 35% |
| WP_117066841.1 MULTISPECIES: APH(3')-I family aminoglycoside O... | 158 | 2e-43 | 35% |
| WP_000018321.1 MULTISPECIES: aminoglycoside O-phosphotransfera... | 158 | 2e-43 | 35% |
| WP_055823613.1 MULTISPECIES: APH(3')-I family aminoglycoside O... | 158 | 2e-43 | 38% |
| ECE5539112.1 APH(3')-I family aminoglycoside O-phosphotransfer... | 158 | 2e-43 | 35% |
| 4FEV_A Crystal structure of the aminoglycoside phosphotransfer... | 158 | 2e-43 | 35% |
| WP_180070904.1 APH(3')-I family aminoglycoside O-phosphotransf... | 158 | 2e-43 | 35% |
| AKP24019.1 aminoglycoside-O-phosphotransferase [Corynebacteriu... | 158 | 2e-43 | 35% |

| WP_191221477.1 APH(3')-I family aminoglycoside O-phosphotransf... | 158 | 2e-43 | 35% |
| --- | --- | --- | --- |
| WP_000338412.1 MULTISPECIES: APH(3')-I family aminoglycoside O... | 158 | 2e-43 | 35% |
| EDA1994633.1 APH(3')-I family aminoglycoside O-phosphotransfer... | 158 | 2e-43 | 35% |
| EFJ1719504.1 APH(3')-I family aminoglycoside O-phosphotransfer... | 158 | 2e-43 | 35% |
| WP_075290646.1 APH(3')-I family aminoglycoside O-phosphotransf... | 158 | 2e-43 | 35% |
| EDA6517014.1 APH(3')-I family aminoglycoside O-phosphotransfer... | 158 | 2e-43 | 35% |
| EBK6456868.1 APH(3')-I family aminoglycoside O-phosphotransfer... | 158 | 2e-43 | 35% |
| EEB3969119.1 APH(3')-I family aminoglycoside O-phosphotransfer... | 158 | 2e-43 | 35% |
| WP_052799555.1 MULTISPECIES: APH(3')-I family aminoglycoside O... | 158 | 2e-43 | 35% |
| EAB1549095.1 APH(3')-I family aminoglycoside O-phosphotransfer... | 158 | 2e-43 | 35% |
| EAM1884616.1 APH(3')-I family aminoglycoside O-phosphotransfer... | 158 | 2e-43 | 35% |
| EAW1527123.1 APH(3')-I family aminoglycoside O-phosphotransfer... | 158 | 2e-43 | 35% |
| EBQ4001585.1 APH(3')-I family aminoglycoside O-phosphotransfer... | 158 | 2e-43 | 35% |
| WP_082243125.1 APH(3')-I family aminoglycoside O-phosphotransf... | 158 | 2e-43 | 35% |
| WP_099588326.1 MULTISPECIES: APH(3')-I family aminoglycoside O... | 158 | 2e-43 | 35% |
| AWP40280.1 KanA [Cloning vector pANY6] | 158 | 2e-43 | 35% |
| OVY43518.1 APH(3') family aminoglycoside O-phosphotransferase ... | 158 | 2e-43 | 35% |
| WP_122633949.1 MULTISPECIES: APH(3')-I family aminoglycoside O... | 158 | 2e-43 | 35% |
| WP_039103497.1 MULTISPECIES: APH(3')-I family aminoglycoside O... | 158 | 2e-43 | 35% |
| WP_152919765.1 APH(3')-I family aminoglycoside O-phosphotransf... | 158 | 2e-43 | 35% |
| WP_189642068.1 APH(3') family aminoglycoside O-phosphotransfer... | 158 | 2e-43 | 39% |
| HAE3394755.1 TPA: APH(3')-I family aminoglycoside O-phosphotra... | 158 | 2e-43 | 35% |
| EDA6581017.1 APH(3')-I family aminoglycoside O-phosphotransfer... | 158 | 2e-43 | 35% |
| WP_071534618.1 APH(3')-I family aminoglycoside O-phosphotransf... | 158 | 2e-43 | 34% |
| EDK2857674.1 APH(3') family aminoglycoside O-phosphotransferas... | 158 | 2e-43 | 35% |
| EGF3737090.1 APH(3')-I family aminoglycoside O-phosphotransfer... | 158 | 2e-43 | 35% |
| EBL3577538.1 APH(3')-I family aminoglycoside O-phosphotransfer... | 158 | 2e-43 | 35% |
| EDP9035213.1 APH(3')-I family aminoglycoside O-phosphotransfer... | 158 | 2e-43 | 35% |
| WP_121926944.1 APH(3')-I family aminoglycoside O-phosphotransf... | 158 | 2e-43 | 35% |
| ARA91538.1 KanR [synthetic construct] | 158 | 2e-43 | 35% |
| WP_124064411.1 APH(3')-I family aminoglycoside O-phosphotransf... | 158 | 2e-43 | 35% |
| WP_000018323.1 MULTISPECIES: APH(3')-I family aminoglycoside O... | 158 | 2e-43 | 35% |
| ECT6011088.1 APH(3')-I family aminoglycoside O-phosphotransfer... | 158 | 2e-43 | 35% |
| EED3836227.1 APH(3')-I family aminoglycoside O-phosphotransfer... | 158 | 2e-43 | 35% |
| WP_149529085.1 APH(3')-I family aminoglycoside O-phosphotransf... | 158 | 2e-43 | 35% |
| ABV03164.1 Aph [Cloning vector pEN-Km] | 158 | 2e-43 | 35% |
| QGV92314.1 APH(3')-I family aminoglycoside O-phosphotransferas... | 158 | 2e-43 | 35% |
| EBK8391897.1 APH(3')-I family aminoglycoside O-phosphotransfer... | 158 | 2e-43 | 35% |
| EFL6736652.1 APH(3')-I family aminoglycoside O-phosphotransfer... | 158 | 2e-43 | 35% |
| EBS3149275.1 APH(3')-I family aminoglycoside O-phosphotransfer... | 158 | 2e-43 | 35% |
| WP_192473079.1 APH(3')-I family aminoglycoside O-phosphotransf... | 158 | 2e-43 | 35% |
| WP_113453250.1 APH(3')-I family aminoglycoside O-phosphotransf... | 158 | 2e-43 | 35% |
| WP_000018322.1 MULTISPECIES: APH(3')-I family aminoglycoside O... | 158 | 2e-43 | 35% |
| WP_159215366.1 APH(3')-I family aminoglycoside O-phosphotransf... | 158 | 2e-43 | 35% |
| AJI43668.1 neomycin phosphotransferase [C.glutamicum-E.coli sh... | 158 | 2e-43 | 35% |
| ASK37968.1 aph(3\\')-Ia [Klebsiella pneumoniae] | 159 | 2e-43 | 35% |
| RKW75845.1 APH(3')-I family aminoglycoside O-phosphotransferas... | 158 | 2e-43 | 35% |
| WP_038349982.1 APH(3')-I family aminoglycoside O-phosphotransf... | 158 | 2e-43 | 35% |
| WP_067060733.1 aminoglycoside 3'-phosphotransferase [Mitsuaria... | 158 | 2e-43 | 37% |
| WP_042896151.1 MULTISPECIES: aminoglycoside O-phosphotransfera... | 158 | 2e-43 | 35% |
| AAB60527.1 aminoglycoside 3'-phosphotransferase [Cloning vecto... | 158 | 2e-43 | 35% |
| WP_100006101.1 APH(3')-I family aminoglycoside O-phosphotransf... | 158 | 2e-43 | 35% |
| WP_086267546.1 APH(3')-I family aminoglycoside O-phosphotransf... | 158 | 2e-43 | 35% |
| WP_063090588.1 MULTISPECIES: APH(3')-I family aminoglycoside O... | 158 | 2e-43 | 35% |
| WP_051011623.1 APH(3')-I family aminoglycoside O-phosphotransf... | 157 | 2e-43 | 39% |
| WP_092037698.1 aminoglycoside 3'-phosphotransferase [Planifilu... | 157 | 2e-43 | 35% |
| EDP9035214.1 APH(3')-I family aminoglycoside O-phosphotransfer... | 158 | 2e-43 | 35% |
| AAA75562.1 kanamycin resistance protein [unidentified cloning ... | 158 | 2e-43 | 35% |
| EAZ2053794.1 APH(3')-I family aminoglycoside O-phosphotransfer... | 158 | 2e-43 | 35% |
| EBM0122125.1 APH(3')-I family aminoglycoside O-phosphotransfer... | 158 | 2e-43 | 35% |
| WP_089718955.1 APH(3')-I family aminoglycoside O-phosphotransf... | 158 | 2e-43 | 35% |
| WP_063842148.1 aminoglycoside O-phosphotransferase APH(3')-Ia ... | 158 | 2e-43 | 35% |
| WP_070282180.1 APH(3')-I family aminoglycoside O-phosphotransf... | 158 | 2e-43 | 39% |
| WP_010886714.1 aminoglycoside 3'-phosphotransferase [Deinococc... | 158 | 2e-43 | 39% |
| WP_117027390.1 APH(3')-I family aminoglycoside O-phosphotransf... | 158 | 2e-43 | 35% |
| WP_000018329.1 MULTISPECIES: aminoglycoside O-phosphotransfera... | 158 | 2e-43 | 35% |
| EEF3559792.1 APH(3')-I family aminoglycoside O-phosphotransfer... | 158 | 2e-43 | 35% |
| EEZ1474031.1 APH(3')-I family aminoglycoside O-phosphotransfer... | 158 | 2e-43 | 35% |
| WP_138679355.1 APH(3')-I family aminoglycoside O-phosphotransf... | 158 | 2e-43 | 35% |
| WP_012695470.1 MULTISPECIES: aminoglycoside O-phosphotransfera... | 158 | 2e-43 | 35% |
| WP_136637824.1 MULTISPECIES: APH(3')-I family aminoglycoside O... | 158 | 2e-43 | 35% |
| AAF71713.1 aminoglycoside 3'-phosphotransferase [Cloning vecto... | 158 | 2e-43 | 35% |

| WP_185143122.1 APH(3') family aminoglycoside O-phosphotransfer... | 158 | 3e-43 | 38% |
| --- | --- | --- | --- |
| WP_187491314.1 APH(3')-I family aminoglycoside O-phosphotransf... | 158 | 3e-43 | 35% |
| WP_049083576.1 MULTISPECIES: APH(3')-I family aminoglycoside O... | 158 | 3e-43 | 35% |
| EFC6595419.1 APH(3')-I family aminoglycoside O-phosphotransfer... | 158 | 3e-43 | 35% |
| WP_089640561.1 APH(3')-I family aminoglycoside O-phosphotransf... | 158 | 3e-43 | 35% |
| EEB5767695.1 APH(3')-I family aminoglycoside O-phosphotransfer... | 158 | 3e-43 | 35% |
| EEF3559795.1 APH(3')-I family aminoglycoside O-phosphotransfer... | 158 | 3e-43 | 35% |
| EDT8175324.1 APH(3')-I family aminoglycoside O-phosphotransfer... | 158 | 3e-43 | 35% |
| WP_117040329.1 APH(3')-I family aminoglycoside O-phosphotransf... | 158 | 3e-43 | 35% |
| EEG9915234.1 APH(3')-I family aminoglycoside O-phosphotransfer... | 158 | 3e-43 | 35% |
| ECU7010994.1 APH(3')-I family aminoglycoside O-phosphotransfer... | 158 | 3e-43 | 35% |
| WP_163585293.1 APH(3')-I family aminoglycoside O-phosphotransf... | 158 | 3e-43 | 35% |
| WP_027480238.1 aminoglycoside 3'-phosphotransferase [Deinococc... | 157 | 3e-43 | 39% |
| AHY21932.1 KanR [Cloning vector pSUMO3_ck5] | 158 | 3e-43 | 35% |
| WP_088238916.1 APH(3')-I family aminoglycoside O-phosphotransf... | 158 | 3e-43 | 35% |
| WP_175869731.1 APH(3')-I family aminoglycoside O-phosphotransf... | 158 | 3e-43 | 35% |
| EDN1255190.1 APH(3') family aminoglycoside O-phosphotransferas... | 157 | 3e-43 | 35% |
| EBA9288365.1 APH(3')-I family aminoglycoside O-phosphotransfer... | 158 | 3e-43 | 35% |
| WP_060204919.1 aminoglycoside 3'-phosphotransferase [Sporosarc... | 157 | 3e-43 | 35% |
| WP_014683755.1 aminoglycoside 3'-phosphotransferase [Deinococc... | 157 | 3e-43 | 40% |
| WP_094868296.1 APH(3')-I family aminoglycoside O-phosphotransf... | 158 | 3e-43 | 35% |
| EEU9697279.1 APH(3')-I family aminoglycoside O-phosphotransfer... | 158 | 3e-43 | 35% |
| HAN9482121.1 TPA: APH(3')-I family aminoglycoside O-phosphotra... | 158 | 3e-43 | 35% |
| EEQ8378944.1 APH(3')-I family aminoglycoside O-phosphotransfer... | 158 | 3e-43 | 35% |
| WP_075319812.1 APH(3')-I family aminoglycoside O-phosphotransf... | 158 | 3e-43 | 35% |
| EKF18778.1 aminoglycoside 3'-phosphotransferase [Nitratireduct... | 158 | 3e-43 | 39% |
| STR62551.1 aminoglycoside 3'-phosphotransferase [Klebsiella mi... | 158 | 3e-43 | 35% |
| EEM6546702.1 APH(3')-I family aminoglycoside O-phosphotransfer... | 158 | 3e-43 | 35% |
| EBG6769730.1 APH(3')-I family aminoglycoside O-phosphotransfer... | 158 | 3e-43 | 35% |
| HAI4895100.1 TPA: APH(3')-I family aminoglycoside O-phosphotra... | 158 | 3e-43 | 35% |
| HAE3394758.1 TPA: APH(3')-I family aminoglycoside O-phosphotra... | 158 | 3e-43 | 35% |
| WP_187751272.1 APH(3') family aminoglycoside O-phosphotransfer... | 157 | 3e-43 | 43% |
| WP_013815100.1 MULTISPECIES: APH(3')-I family aminoglycoside O... | 157 | 3e-43 | 35% |
| WP_080876968.1 APH(3')-I family aminoglycoside O-phosphotransf... | 157 | 3e-43 | 35% |
| ACA63833.1 aminoglycoside 3' phosphotransferase [Cloning vecto... | 157 | 3e-43 | 35% |
| EAP0460867.1 APH(3')-I family aminoglycoside O-phosphotransfer... | 157 | 3e-43 | 35% |
| WP_117058484.1 APH(3')-I family aminoglycoside O-phosphotransf... | 157 | 3e-43 | 35% |
| WP_012846873.1 APH(3')-I family aminoglycoside O-phosphotransf... | 157 | 4e-43 | 35% |
| WP_166888432.1 APH(3')-I family aminoglycoside O-phosphotransf... | 157 | 4e-43 | 36% |
| GDE99225.1 aminoglycoside 3'-phosphotransferase [Escherichia c... | 158 | 4e-43 | 35% |
| WP_099667182.1 APH(3')-I family aminoglycoside O-phosphotransf... | 157 | 4e-43 | 40% |
| WP_079898648.1 MULTISPECIES: APH(3')-I family aminoglycoside O... | 157 | 4e-43 | 35% |
| WP_163548854.1 APH(3')-I family aminoglycoside O-phosphotransf... | 157 | 4e-43 | 35% |
| EGJ0095411.1 APH(3')-I family aminoglycoside O-phosphotransfer... | 157 | 4e-43 | 35% |
| EEF3559793.1 APH(3')-I family aminoglycoside O-phosphotransfer... | 157 | 4e-43 | 35% |
| WP_140309047.1 APH(3')-I family aminoglycoside O-phosphotransf... | 157 | 4e-43 | 35% |
| ECU6198267.1 APH(3')-I family aminoglycoside O-phosphotransfer... | 157 | 4e-43 | 35% |
| WP_085521167.1 APH(3')-I family aminoglycoside O-phosphotransf... | 157 | 4e-43 | 35% |
| WP_140256072.1 APH(3')-I family aminoglycoside O-phosphotransf... | 157 | 4e-43 | 35% |
| WP_056302550.1 aminoglycoside 3'-phosphotransferase [Deinococc... | 157 | 4e-43 | 39% |
| WP_181819406.1 APH(3')-I family aminoglycoside O-phosphotransf... | 157 | 4e-43 | 36% |
| EBA4024683.1 APH(3')-I family aminoglycoside O-phosphotransfer... | 157 | 4e-43 | 35% |
| WP_181617043.1 APH(3')-I family aminoglycoside O-phosphotransf... | 157 | 5e-43 | 36% |
| EED3836225.1 APH(3')-I family aminoglycoside O-phosphotransfer... | 157 | 5e-43 | 35% |
| ECU6792898.1 APH(3')-I family aminoglycoside O-phosphotransfer... | 157 | 5e-43 | 35% |
| WP_189055478.1 APH(3') family aminoglycoside O-phosphotransfer... | 157 | 5e-43 | 39% |
| EAR2371734.1 APH(3')-I family aminoglycoside O-phosphotransfer... | 157 | 5e-43 | 35% |
| HAO3181046.1 TPA: APH(3')-I family aminoglycoside O-phosphotra... | 157 | 5e-43 | 35% |
| MBE5323309.1 APH(3')-I family aminoglycoside O-phosphotransfer... | 157 | 5e-43 | 35% |
| EBI7038103.1 APH(3')-I family aminoglycoside O-phosphotransfer... | 157 | 5e-43 | 35% |
| WP_135953134.1 APH(3')-I family aminoglycoside O-phosphotransf... | 157 | 5e-43 | 35% |
| EBK6456866.1 APH(3')-I family aminoglycoside O-phosphotransfer... | 157 | 5e-43 | 35% |
| WP_184643987.1 APH(3') family aminoglycoside O-phosphotransfer... | 157 | 5e-43 | 39% |
| WP_151096499.1 APH(3')-I family aminoglycoside O-phosphotransf... | 157 | 5e-43 | 40% |
| WP_160964368.1 MULTISPECIES: APH(3') family aminoglycoside O-p... | 157 | 5e-43 | 39% |
| ECK1859648.1 APH(3')-I family aminoglycoside O-phosphotransfer... | 157 | 6e-43 | 35% |
| EEE7318048.1 APH(3')-I family aminoglycoside O-phosphotransfer... | 157 | 6e-43 | 35% |
| WP_077406992.1 APH(3')-I family aminoglycoside O-phosphotransf... | 157 | 6e-43 | 41% |
| BCA49202.1 aminoglycoside 3'-phosphotransferase [Bacteroides t... | 157 | 6e-43 | 37% |
| WP_189930032.1 APH(3')-I family aminoglycoside O-phosphotransf... | 157 | 6e-43 | 37% |
| EBO3273847.1 APH(3')-I family aminoglycoside O-phosphotransfer... | 157 | 6e-43 | 35% |
| EFM0641110.1 APH(3')-I family aminoglycoside O-phosphotransfer... | 157 | 6e-43 | 35% |

| EBP6278974.1 APH(3')-I family aminoglycoside O-phosphotransfer... | 157 | 6e-43 | 35% |
| --- | --- | --- | --- |
| MBC7569787.1 aminoglycoside 3'-phosphotransferase [Spirosoma sp.] | 157 | 6e-43 | 38% |
| WP_010341196.1 aminoglycoside 3'-phosphotransferase [Xanthomon... | 157 | 6e-43 | 39% |
| WP_055527703.1 aminoglycoside 3'-phosphotransferase [Streptomy... | 157 | 7e-43 | 41% |
| EBL2053913.1 APH(3')-I family aminoglycoside O-phosphotransfer... | 157 | 7e-43 | 35% |
| EBS8133245.1 APH(3')-I family aminoglycoside O-phosphotransfer... | 157 | 7e-43 | 35% |
| WP_128696308.1 APH(3')-I family aminoglycoside O-phosphotransf... | 157 | 7e-43 | 35% |
| WP_149937515.1 MULTISPECIES: APH(3')-I family aminoglycoside O... | 157 | 7e-43 | 37% |
| WP_158208945.1 APH(3')-I family aminoglycoside O-phosphotransf... | 157 | 7e-43 | 35% |
| HAH0580820.1 TPA: APH(3')-I family aminoglycoside O-phosphotra... | 157 | 7e-43 | 35% |
| EAX4346306.1 APH(3')-I family aminoglycoside O-phosphotransfer... | 157 | 7e-43 | 35% |
| WP_152578380.1 APH(3')-I family aminoglycoside O-phosphotransf... | 157 | 7e-43 | 37% |
| WP_058179949.1 aminoglycoside 3'-phosphotransferase [Pseudomon... | 157 | 7e-43 | 40% |
| WP_033825274.1 aminoglycoside 3'-phosphotransferase [Kitasatos... | 157 | 7e-43 | 44% |
| WP_119672565.1 aminoglycoside 3'-phosphotransferase [Deinococc... | 156 | 7e-43 | 39% |
| WP_011205810.1 MULTISPECIES: aminoglycoside O-phosphotransfera... | 157 | 7e-43 | 37% |
| WP_117249202.1 MULTISPECIES: APH(3')-I family aminoglycoside O... | 157 | 7e-43 | 37% |
| WP_070255564.1 aminoglycoside 3'-phosphotransferase [Janthinob... | 157 | 7e-43 | 39% |
| EDG8698729.1 APH(3')-I family aminoglycoside O-phosphotransfer... | 157 | 7e-43 | 35% |
| WP_046877577.1 APH(3')-I family aminoglycoside O-phosphotransf... | 157 | 7e-43 | 35% |
| EFC5395134.1 APH(3')-I family aminoglycoside O-phosphotransfer... | 157 | 8e-43 | 35% |
| EBW5763190.1 APH(3')-I family aminoglycoside O-phosphotransfer... | 157 | 8e-43 | 35% |
| EEM2964977.1 APH(3')-I family aminoglycoside O-phosphotransfer... | 157 | 8e-43 | 35% |
| ABQ42699.1 NptI [Cloning vector pGreenII] | 157 | 8e-43 | 35% |
| WP_092606089.1 APH(3')-I family aminoglycoside O-phosphotransf... | 157 | 8e-43 | 39% |
| BAA84588.1 aminoglycoside phosphotransferase [Cloning vector p... | 157 | 8e-43 | 35% |
| EFO2106570.1 APH(3')-I family aminoglycoside O-phosphotransfer... | 157 | 8e-43 | 35% |
| WP_039090702.1 APH(3')-I family aminoglycoside O-phosphotransf... | 157 | 8e-43 | 35% |
| WP_167953503.1 APH(3') family aminoglycoside O-phosphotransfer... | 157 | 8e-43 | 38% |
| HAM7155053.1 TPA: APH(3')-I family aminoglycoside O-phosphotra... | 157 | 8e-43 | 35% |
| WP_109240361.1 APH(3')-I family aminoglycoside O-phosphotransf... | 157 | 9e-43 | 35% |
| MSQ31639.1 aminoglycoside 3'-phosphotransferase [Dehalococcoid... | 156 | 9e-43 | 36% |
| WP_181491349.1 APH(3')-I family aminoglycoside O-phosphotransf... | 157 | 9e-43 | 36% |
| WP_104774160.1 APH(3')-I family aminoglycoside O-phosphotransf... | 157 | 9e-43 | 35% |
| EGJ6193631.1 APH(3')-I family aminoglycoside O-phosphotransfer... | 157 | 9e-43 | 35% |
| WP_181695556.1 APH(3')-I family aminoglycoside O-phosphotransf... | 157 | 9e-43 | 37% |
| ECA4397846.1 APH(3')-I family aminoglycoside O-phosphotransfer... | 155 | 1e-42 | 35% |
| WP_115258349.1 MULTISPECIES: APH(3')-I family aminoglycoside O... | 156 | 1e-42 | 36% |
| WP_176384980.1 APH(3')-I family aminoglycoside O-phosphotransf... | 156 | 1e-42 | 39% |
| WP_117342872.1 MULTISPECIES: APH(3')-I family aminoglycoside O... | 156 | 1e-42 | 36% |
| WP_012477822.1 aminoglycoside O-phosphotransferase APH(3')-Ia ... | 156 | 1e-42 | 35% |
| EGO6620158.1 APH(3')-I family aminoglycoside O-phosphotransfer... | 156 | 1e-42 | 35% |
| WP_174349526.1 APH(3')-I family aminoglycoside O-phosphotransf... | 157 | 1e-42 | 37% |
| EAO4048312.1 APH(3')-I family aminoglycoside O-phosphotransfer... | 156 | 1e-42 | 35% |
| EBB4909900.1 APH(3')-I family aminoglycoside O-phosphotransfer... | 156 | 1e-42 | 35% |
| ECO0630965.1 APH(3')-I family aminoglycoside O-phosphotransfer... | 156 | 1e-42 | 35% |
| EEF4232897.1 APH(3')-I family aminoglycoside O-phosphotransfer... | 156 | 1e-42 | 35% |
| WP_096490079.1 APH(3')-I family aminoglycoside O-phosphotransf... | 156 | 1e-42 | 35% |
| MAU13815.1 APH(3') family aminoglycoside O-phosphotransferase ... | 156 | 1e-42 | 36% |
| WP_169551912.1 APH(3') family aminoglycoside O-phosphotransfer... | 156 | 1e-42 | 36% |
| WP_139845550.1 APH(3')-I family aminoglycoside O-phosphotransf... | 156 | 1e-42 | 35% |
| WP_032492145.1 MULTISPECIES: aminoglycoside O-phosphotransfera... | 156 | 1e-42 | 35% |
| WP_027480926.1 aminoglycoside 3'-phosphotransferase [Deinococc... | 156 | 1e-42 | 41% |
| WP_063119415.1 APH(3')-I family aminoglycoside O-phosphotransf... | 156 | 1e-42 | 35% |
| WP_126076806.1 APH(3')-I family aminoglycoside O-phosphotransf... | 156 | 1e-42 | 37% |
| WP_181695728.1 APH(3')-I family aminoglycoside O-phosphotransf... | 156 | 1e-42 | 35% |
| QED66339.1 APH(3')-I family aminoglycoside O-phosphotransferas... | 156 | 1e-42 | 35% |
| EFU0358342.1 APH(3')-I family aminoglycoside O-phosphotransfer... | 155 | 1e-42 | 35% |
| WP_104772212.1 APH(3')-I family aminoglycoside O-phosphotransf... | 155 | 1e-42 | 35% |
| EFU1153261.1 APH(3')-I family aminoglycoside O-phosphotransfer... | 156 | 1e-42 | 35% |
| WP_062773153.1 APH(3')-I family aminoglycoside O-phosphotransf... | 156 | 1e-42 | 37% |
| HAL1491056.1 TPA: APH(3')-I family aminoglycoside O-phosphotra... | 156 | 1e-42 | 35% |
| WP_181498247.1 APH(3')-I family aminoglycoside O-phosphotransf... | 156 | 1e-42 | 36% |
| WP_089649309.1 MULTISPECIES: APH(3')-I family aminoglycoside O... | 156 | 1e-42 | 35% |
| WP_157460068.1 APH(3') family aminoglycoside O-phosphotransfer... | 156 | 1e-42 | 40% |
| WP_181630889.1 APH(3')-I family aminoglycoside O-phosphotransf... | 156 | 1e-42 | 36% |
| WP_181689054.1 APH(3')-I family aminoglycoside O-phosphotransf... | 156 | 1e-42 | 36% |
| WP_101290188.1 APH(3')-I family aminoglycoside O-phosphotransf... | 156 | 1e-42 | 40% |
| WP_076571983.1 aminoglycoside 3'-phosphotransferase [Janthinob... | 156 | 2e-42 | 42% |
| WP_179505272.1 MULTISPECIES: APH(3')-I family aminoglycoside O... | 156 | 2e-42 | 36% |
| HAL9951080.1 TPA: APH(3')-I family aminoglycoside O-phosphotra... | 156 | 2e-42 | 35% |
| WP_101425723.1 aminoglycoside 3'-phosphotransferase [Streptomy... | 156 | 2e-42 | 41% |

| WP_181624403.1 APH(3')-I family aminoglycoside O-phosphotransf... | 156 | 2e-42 | 37% |
| --- | --- | --- | --- |
| WP_191695774.1 APH(3') family aminoglycoside O-phosphotransfer... | 154 | 2e-42 | 42% |
| WP_062159368.1 aminoglycoside 3'-phosphotransferase [Deinococc... | 155 | 2e-42 | 39% |
| EAM3772360.1 APH(3')-I family aminoglycoside O-phosphotransfer... | 156 | 2e-42 | 35% |
| EBH2609264.1 APH(3')-I family aminoglycoside O-phosphotransfer... | 156 | 2e-42 | 35% |
| EBH5563350.1 APH(3')-I family aminoglycoside O-phosphotransfer... | 155 | 2e-42 | 35% |
| WP_071323150.1 APH(3')-I family aminoglycoside O-phosphotransf... | 155 | 2e-42 | 38% |
| EAM3772362.1 APH(3')-I family aminoglycoside O-phosphotransfer... | 155 | 2e-42 | 35% |
| WP_166794113.1 phosphotransferase [Pseudomonas aeruginosa] | 151 | 2e-42 | 60% |
| EBC9078609.1 APH(3')-I family aminoglycoside O-phosphotransfer... | 155 | 2e-42 | 35% |
| WP_017870772.1 aminoglycoside 3'-phosphotransferase [Deinococc... | 155 | 2e-42 | 39% |
| WP_068842196.1 APH(3')-I family aminoglycoside O-phosphotransf... | 155 | 2e-42 | 35% |
| EAT0354937.1 APH(3')-I family aminoglycoside O-phosphotransfer... | 155 | 2e-42 | 35% |
| AYE54292.1 kanamycin resistance protein [synthetic construct] | 155 | 2e-42 | 35% |
| EFP0324113.1 APH(3')-I family aminoglycoside O-phosphotransfer... | 155 | 2e-42 | 35% |
| WP_152125938.1 aminoglycoside 3'-phosphotransferase [Rudanella... | 155 | 2e-42 | 38% |
| WP_153742217.1 APH(3')-I family aminoglycoside O-phosphotransf... | 155 | 2e-42 | 37% |
| ECS4615634.1 APH(3')-I family aminoglycoside O-phosphotransfer... | 155 | 2e-42 | 35% |
| WP_039246168.1 aminoglycoside 3'-phosphotransferase [Brevundim... | 155 | 2e-42 | 39% |
| WP_021263754.1 APH(3')-I family aminoglycoside O-phosphotransf... | 155 | 2e-42 | 38% |
| ECY8464165.1 APH(3')-I family aminoglycoside O-phosphotransfer... | 155 | 2e-42 | 35% |
| WP_153014771.1 phosphotransferase [Syntrophomonas wolfei] | 150 | 2e-42 | 89% |
| WP_168556951.1 APH(3')-I family aminoglycoside O-phosphotransf... | 155 | 3e-42 | 36% |
| WP_176443620.1 APH(3')-I family aminoglycoside O-phosphotransf... | 155 | 3e-42 | 35% |
| WP_167078513.1 APH(3')-I family aminoglycoside O-phosphotransf... | 155 | 3e-42 | 36% |
| WP_155439885.1 APH(3')-I family aminoglycoside O-phosphotransf... | 155 | 3e-42 | 40% |
| ECU7010996.1 APH(3')-I family aminoglycoside O-phosphotransfer... | 155 | 3e-42 | 35% |
| ECK1854979.1 APH(3')-I family aminoglycoside O-phosphotransfer... | 155 | 3e-42 | 35% |
| WP_099388366.1 aminoglycoside 3'-phosphotransferase [Janthinob... | 155 | 3e-42 | 39% |
| EEC7526824.1 APH(3')-I family aminoglycoside O-phosphotransfer... | 155 | 3e-42 | 35% |
| WP_071076389.1 aminoglycoside 3'-phosphotransferase [Janthinob... | 155 | 3e-42 | 39% |
| ECT6831072.1 APH(3')-I family aminoglycoside O-phosphotransfer... | 155 | 3e-42 | 35% |
| ECU7010997.1 APH(3')-I family aminoglycoside O-phosphotransfer... | 155 | 3e-42 | 35% |
| WP_169476118.1 MULTISPECIES: APH(3')-I family aminoglycoside O... | 155 | 3e-42 | 36% |
| EDS4179116.1 APH(3')-I family aminoglycoside O-phosphotransfer... | 155 | 3e-42 | 35% |
| WP_126053077.1 APH(3')-I family aminoglycoside O-phosphotransf... | 155 | 3e-42 | 35% |
| EFC86919.1 phosphotransferase enzyme family [Neisseria mucosa ... | 155 | 3e-42 | 35% |
| NLM49900.1 aminoglycoside 3'-phosphotransferase [Clostridiacea... | 155 | 3e-42 | 38% |
| WP_099861500.1 aminoglycoside 3'-phosphotransferase [Mitsuaria... | 155 | 3e-42 | 38% |
| MBG7016124.1 phosphotransferase [Pseudomonas aeruginosa] | 150 | 3e-42 | 61% |
| ECV0933940.1 APH(3')-I family aminoglycoside O-phosphotransfer... | 155 | 3e-42 | 35% |
| ADC79586.1 KnR [MISSA recipient vector TAC-RTL] | 155 | 3e-42 | 35% |
| EDL5342072.1 APH(3') family aminoglycoside O-phosphotransferas... | 155 | 3e-42 | 35% |
| EAY7223896.1 APH(3')-I family aminoglycoside O-phosphotransfer... | 155 | 3e-42 | 35% |
| WP_099130641.1 APH(3')-I family aminoglycoside O-phosphotransf... | 155 | 3e-42 | 35% |
| EFF1165230.1 APH(3')-I family aminoglycoside O-phosphotransfer... | 155 | 3e-42 | 35% |
| AAY29060.1 NPT1 [Cloning vector LUCTRAP-1] | 155 | 4e-42 | 35% |
| EDG2814514.1 APH(3')-I family aminoglycoside O-phosphotransfer... | 155 | 4e-42 | 35% |
| WP_195686495.1 APH(3')-I family aminoglycoside O-phosphotransf... | 155 | 4e-42 | 35% |
| WP_130021832.1 APH(3')-I family aminoglycoside O-phosphotransf... | 155 | 4e-42 | 37% |
| WP_110846552.1 MULTISPECIES: APH(3')-I family aminoglycoside O... | 155 | 4e-42 | 36% |
| WP_189073476.1 APH(3') family aminoglycoside O-phosphotransfer... | 155 | 4e-42 | 40% |
| ECV3502736.1 APH(3')-I family aminoglycoside O-phosphotransfer... | 155 | 4e-42 | 35% |
| WP_174367709.1 APH(3') family aminoglycoside O-phosphotransfer... | 155 | 4e-42 | 40% |
| WP_103473230.1 APH(3')-I family aminoglycoside O-phosphotransf... | 155 | 4e-42 | 35% |
| EBR1025959.1 APH(3')-I family aminoglycoside O-phosphotransfer... | 155 | 4e-42 | 35% |
| WP_154916547.1 APH(3')-I family aminoglycoside O-phosphotransf... | 155 | 4e-42 | 35% |
| WP_113417813.1 APH(3')-I family aminoglycoside O-phosphotransf... | 155 | 4e-42 | 35% |
| WP_149682973.1 APH(3')-I family aminoglycoside O-phosphotransf... | 155 | 4e-42 | 36% |
| WP_039856594.1 MULTISPECIES: APH(3')-I family aminoglycoside O... | 155 | 4e-42 | 35% |
| WP_023460056.1 APH(3')-I family aminoglycoside O-phosphotransf... | 155 | 4e-42 | 38% |
| WP_181506946.1 APH(3')-I family aminoglycoside O-phosphotransf... | 155 | 5e-42 | 36% |
| ECE0951219.1 APH(3')-I family aminoglycoside O-phosphotransfer... | 155 | 5e-42 | 35% |
| MBB5377589.1 kanamycin kinase/aminoglycoside 3'-phosphotransfe... | 154 | 5e-42 | 39% |
| QBA55272.1 KanR [Cloning vector L1611] | 155 | 5e-42 | 35% |
| MHZ55488.1 APH(3')-I family aminoglycoside O-phosphotransferas... | 155 | 5e-42 | 35% |
| WP_145657985.1 aminoglycoside 3'-phosphotransferase [Paenibaci... | 154 | 5e-42 | 36% |
| EDS8707362.1 APH(3')-I family aminoglycoside O-phosphotransfer... | 155 | 5e-42 | 35% |
| WP_135229202.1 aminoglycoside 3'-phosphotransferase [Deinococc... | 154 | 5e-42 | 38% |
| WP_078761272.1 aminoglycoside 3'-phosphotransferase [Marinacti... | 155 | 5e-42 | 42% |
| WP_187228179.1 APH(3')-I family aminoglycoside O-phosphotransf... | 154 | 6e-42 | 35% |
| GGR02743.1 aminoglycoside 3'-phosphotransferase [Deinococcus r... | 154 | 6e-42 | 37% |

| WP_114950149.1 APH(3')-I family aminoglycoside O-phosphotransf... | 154 | 6e-42 | 35% |
| --- | --- | --- | --- |
| EDW8525770.1 APH(3')-I family aminoglycoside O-phosphotransfer... | 154 | 6e-42 | 35% |
| HAF7291478.1 TPA: APH(3')-I family aminoglycoside O-phosphotra... | 154 | 7e-42 | 35% |
| WP_031942551.1 APH(3')-I family aminoglycoside O-phosphotransf... | 154 | 7e-42 | 35% |
| EDW8525776.1 APH(3')-I family aminoglycoside O-phosphotransfer... | 154 | 7e-42 | 35% |
| WP_184113343.1 APH(3') family aminoglycoside O-phosphotransfer... | 154 | 7e-42 | 38% |
| WP_092263534.1 aminoglycoside 3'-phosphotransferase [Deinococc... | 154 | 7e-42 | 39% |
| WP_160962816.1 MULTISPECIES: APH(3') family aminoglycoside O-p... | 154 | 8e-42 | 39% |
| WP_055762973.1 APH(3')-I family aminoglycoside O-phosphotransf... | 154 | 9e-42 | 38% |
| WP_104991765.1 aminoglycoside 3'-phosphotransferase [Deinococc... | 154 | 9e-42 | 38% |
| WP_137381770.1 APH(3')-I family aminoglycoside O-phosphotransf... | 154 | 9e-42 | 36% |
| EEV7391971.1 APH(3')-I family aminoglycoside O-phosphotransfer... | 153 | 1e-41 | 35% |
| EEX1006711.1 APH(3')-I family aminoglycoside O-phosphotransfer... | 153 | 1e-41 | 35% |
| WP_058974769.1 aminoglycoside 3'-phosphotransferase [Deinococc... | 154 | 1e-41 | 39% |
| WP_189088878.1 APH(3') family aminoglycoside O-phosphotransfer... | 154 | 1e-41 | 37% |
| WP_172739652.1 APH(3')-I family aminoglycoside O-phosphotransf... | 153 | 1e-41 | 35% |
| WP_078301665.1 aminoglycoside 3'-phosphotransferase [Deinococc... | 153 | 1e-41 | 40% |
| WP_155472109.1 APH(3')-I family aminoglycoside O-phosphotransf... | 154 | 1e-41 | 37% |
| WP_103310861.1 aminoglycoside 3'-phosphotransferase [Deinococc... | 153 | 1e-41 | 38% |
| WP_160975883.1 APH(3') family aminoglycoside O-phosphotransfer... | 153 | 1e-41 | 38% |
| PTS89660.1 APH(3')-I family aminoglycoside O-phosphotransferas... | 154 | 1e-41 | 37% |
| WP_181632184.1 APH(3')-I family aminoglycoside O-phosphotransf... | 154 | 1e-41 | 36% |
| WP_157181132.1 APH(3') family aminoglycoside O-phosphotransfer... | 154 | 1e-41 | 37% |
| WP_074351544.1 aminoglycoside 3'-phosphotransferase [Proteinib... | 153 | 1e-41 | 34% |
| WP_169112635.1 APH(3')-I family aminoglycoside O-phosphotransf... | 153 | 2e-41 | 35% |
| WP_170109648.1 aminoglycoside 3'-phosphotransferase [Melghirim... | 153 | 2e-41 | 37% |
| WP_120569343.1 APH(3')-V family aminoglycoside O-phosphotransf... | 153 | 2e-41 | 39% |
| EAX8865028.1 APH(3')-I family aminoglycoside O-phosphotransfer... | 153 | 2e-41 | 35% |
| WP_183485689.1 APH(3') family aminoglycoside O-phosphotransfer... | 153 | 2e-41 | 37% |
| HHW72905.1 TPA: aminoglycoside 3'-phosphotransferase [Firmicut... | 153 | 2e-41 | 36% |
| GGK96269.1 aminoglycoside 3'-phosphotransferase [Deinococcus r... | 153 | 2e-41 | 40% |
| WP_132910957.1 APH(3')-I family aminoglycoside O-phosphotransf... | 153 | 2e-41 | 39% |
| WP_096756908.1 MULTISPECIES: APH(3')-I family aminoglycoside O... | 153 | 2e-41 | 35% |
| WP_100271021.1 aminoglycoside 3'-phosphotransferase [Moraxella... | 153 | 2e-41 | 39% |
| SCG84372.1 aminoglycoside 3'-phosphotransferase [Proteiniborus... | 153 | 2e-41 | 34% |
| WP_034354575.1 aminoglycoside 3'-phosphotransferase [Deinococc... | 152 | 2e-41 | 38% |
| 4FEU_A Crystal structure of the aminoglycoside phosphotransfer... | 153 | 2e-41 | 35% |
| EIE42957.1 aminoglycoside phosphotransferase [Pseudomonas aeru... | 148 | 2e-41 | 63% |
| WP_145496586.1 APH(3')-I family aminoglycoside O-phosphotransf... | 153 | 2e-41 | 34% |
| WP_125657091.1 APH(3')-I family aminoglycoside O-phosphotransf... | 152 | 2e-41 | 39% |
| WP_121670054.1 aminoglycoside 3'-phosphotransferase [Janthinob... | 153 | 2e-41 | 41% |
| WP_126001632.1 aminoglycoside 3'-phosphotransferase [Sphingomo... | 153 | 2e-41 | 38% |
| WP_010891090.1 aminoglycoside O-phosphotransferase APH(3')-Ia ... | 153 | 3e-41 | 35% |
| WP_086378041.1 APH(3')-I family aminoglycoside O-phosphotransf... | 153 | 3e-41 | 34% |
| WP_056622893.1 APH(3')-I family aminoglycoside O-phosphotransf... | 152 | 3e-41 | 37% |
| WP_091720594.1 MULTISPECIES: aminoglycoside 3'-phosphotransfer... | 153 | 3e-41 | 38% |
| WP_125043361.1 aminoglycoside 3'-phosphotransferase [Streptomy... | 152 | 3e-41 | 39% |
| MBB3284505.1 aminoglycoside 3'-phosphotransferase-1 [Mitsuaria... | 153 | 3e-41 | 37% |
| WP_082468975.1 aminoglycoside 3'-phosphotransferase [Sciscione... | 151 | 3e-41 | 45% |
| MBA4090402.1 APH(3')-I family aminoglycoside O-phosphotransfer... | 152 | 3e-41 | 34% |
| WP_125051140.1 aminoglycoside 3'-phosphotransferase [Streptomy... | 152 | 3e-41 | 39% |
| WP_055757794.1 MULTISPECIES: APH(3')-I family aminoglycoside O... | 152 | 3e-41 | 39% |
| 4EJ7_A Crystal structure of the aminoglycoside phosphotransfer... | 153 | 3e-41 | 35% |
| WP_091453555.1 aminoglycoside 3'-phosphotransferase [Actinokin... | 153 | 3e-41 | 40% |
| WP_124454199.1 aminoglycoside 3'-phosphotransferase [Paucibact... | 154 | 3e-41 | 38% |
| WP_049715017.1 APH(3')-V family aminoglycoside O-phosphotransf... | 152 | 3e-41 | 40% |
| NLH88198.1 aminoglycoside 3'-phosphotransferase [Firmicutes ba... | 152 | 3e-41 | 38% |
| WP_063080027.1 APH(3')-I family aminoglycoside O-phosphotransf... | 152 | 3e-41 | 35% |
| WP_188446005.1 APH(3')-I family aminoglycoside O-phosphotransf... | 152 | 4e-41 | 39% |
| NMN06370.1 aminoglycoside 3'-phosphotransferase-1 [Novosphingo... | 154 | 4e-41 | 36% |
| RZI60294.1 APH(3')-I family aminoglycoside O-phosphotransferas... | 152 | 4e-41 | 40% |
| WP_086397576.1 APH(3')-I family aminoglycoside O-phosphotransf... | 152 | 4e-41 | 34% |
| EAR2847155.1 APH(3')-I family aminoglycoside O-phosphotransfer... | 151 | 4e-41 | 35% |
| WP_181636823.1 APH(3')-I family aminoglycoside O-phosphotransf... | 152 | 4e-41 | 36% |
| WP_189068217.1 APH(3') family aminoglycoside O-phosphotransfer... | 152 | 5e-41 | 40% |
| WP_088483196.1 aminoglycoside 3'-phosphotransferase [Pelomonas... | 152 | 5e-41 | 37% |
| GCD40688.1 putative streptomycin phosphotransferase [Streptomy... | 152 | 5e-41 | 39% |
| TFG09548.1 aminoglycoside 3'-phosphotransferase [Candidatus He... | 152 | 6e-41 | 32% |
| WP_056458163.1 APH(3')-I family aminoglycoside O-phosphotransf... | 152 | 6e-41 | 37% |
| WP_063842179.1 aminoglycoside O-phosphotransferase APH(3')-Vc ... | 152 | 6e-41 | 38% |
| WP_169578023.1 APH(3') family aminoglycoside O-phosphotransfer... | 151 | 6e-41 | 37% |
| VDM22420.1 unnamed protein product [Wuchereria bancrofti] | 157 | 7e-41 | 35% |

| WP_063842178.1 aminoglycoside O-phosphotransferase APH(3')-Vb ... | 151 | 7e-41 | 39% |
| --- | --- | --- | --- |
| MBA7967378.1 APH(3')-I family aminoglycoside O-phosphotransfer... | 152 | 7e-41 | 36% |
| WP_133030882.1 APH(3')-I family aminoglycoside O-phosphotransf... | 152 | 8e-41 | 39% |
| WP_182293082.1 APH(3')-I family aminoglycoside O-phosphotransf... | 152 | 8e-41 | 36% |
| WP_099748505.1 aminoglycoside 3'-phosphotransferase [Deinococc... | 151 | 8e-41 | 39% |
| WP_188086334.1 APH(3') family aminoglycoside O-phosphotransfer... | 151 | 8e-41 | 39% |
| WP_148620062.1 APH(3')-I family aminoglycoside O-phosphotransf... | 150 | 8e-41 | 35% |
| EGI4185596.1 APH(3')-I family aminoglycoside O-phosphotransfer... | 151 | 9e-41 | 34% |
| GCD32765.1 putative streptomycin phosphotransferase [Streptomy... | 152 | 9e-41 | 39% |
| WP_027460614.1 aminoglycoside 3'-phosphotransferase [Deinococc... | 151 | 1e-40 | 41% |
| TCP67412.1 aminoglycoside 3'-phosphotransferase-1 [Sphingomona... | 151 | 1e-40 | 39% |
| WP_034405396.1 aminoglycoside 3'-phosphotransferase [Deinococc... | 151 | 1e-40 | 41% |
| WP_196210549.1 APH(3')-I family aminoglycoside O-phosphotransf... | 151 | 1e-40 | 34% |
| WP_013615667.1 aminoglycoside 3'-phosphotransferase [Deinococc... | 151 | 1e-40 | 38% |
| MBG7016137.1 phosphotransferase [Pseudomonas aeruginosa] | 148 | 1e-40 | 47% |
| WP_086351200.1 APH(3')-I family aminoglycoside O-phosphotransf... | 151 | 1e-40 | 34% |
| WP_074284126.1 phosphotransferase [Pseudomonas aeruginosa] | 146 | 1e-40 | 63% |
| WP_162960074.1 phosphotransferase [Pseudomonas aeruginosa] | 146 | 1e-40 | 63% |
| WP_019010415.1 aminoglycoside 3'-phosphotransferase [Deinococc... | 150 | 1e-40 | 39% |
| WP_030420357.1 APH(3')-V family aminoglycoside O-phosphotransf... | 150 | 1e-40 | 39% |
| WP_048491483.1 aminoglycoside 3'-phosphotransferase [Xanthomon... | 151 | 2e-40 | 37% |
| WP_056402240.1 MULTISPECIES: APH(3')-I family aminoglycoside O... | 150 | 2e-40 | 36% |
| WP_119766423.1 aminoglycoside 3'-phosphotransferase [Deinococc... | 150 | 2e-40 | 37% |
| WP_056498673.1 APH(3')-I family aminoglycoside O-phosphotransf... | 150 | 2e-40 | 39% |
| WP_189066392.1 MULTISPECIES: APH(3') family aminoglycoside O-p... | 150 | 2e-40 | 38% |
| WP_084047809.1 aminoglycoside 3'-phosphotransferase [Deinococc... | 150 | 2e-40 | 38% |
| MBF2994896.1 phosphotransferase [Pseudomonas aeruginosa] | 145 | 2e-40 | 63% |
| WP_152240195.1 aminoglycoside 3'-phosphotransferase [Xanthomon... | 150 | 3e-40 | 37% |
| HAL6236955.1 TPA: APH(3')-I family aminoglycoside O-phosphotra... | 149 | 3e-40 | 35% |
| AEK22050.1 GST-(Pch PRP8 intein with G418r)-6xH [Cloning vecto... | 158 | 3e-40 | 35% |
| WP_134620804.1 phosphotransferase [Pseudomonas aeruginosa] | 147 | 3e-40 | 48% |
| WP_110831767.1 MULTISPECIES: aminoglycoside 3'-phosphotransfer... | 150 | 3e-40 | 38% |
| AEK22021.1 NLS-GFP-(Pch PRP8 intein with G418r)-FLAG [Cloning ... | 158 | 3e-40 | 35% |
| EFE8748784.1 APH(3')-I family aminoglycoside O-phosphotransfer... | 149 | 4e-40 | 34% |
| WP_196161291.1 APH(3') family aminoglycoside O-phosphotransfer... | 149 | 4e-40 | 34% |
| CAB3888505.1 Aminoglycoside 3'-phosphotransferase [Achromobact... | 145 | 4e-40 | 54% |
| WP_034156961.1 APH(3')-I family aminoglycoside O-phosphotransf... | 150 | 4e-40 | 36% |
| WP_107127056.1 aminoglycoside 3'-phosphotransferase [Streptomy... | 149 | 4e-40 | 39% |
| WP_152243707.1 aminoglycoside 3'-phosphotransferase [Xanthomon... | 150 | 4e-40 | 37% |
| PZT63089.1 APH(3')-I family aminoglycoside O-phosphotransferas... | 149 | 4e-40 | 34% |
| WP_185707393.1 APH(3')-I family aminoglycoside O-phosphotransf... | 149 | 4e-40 | 38% |
| WP_151954739.1 aminoglycoside 3'-phosphotransferase [Sphingomo... | 149 | 4e-40 | 36% |
| MBG6857568.1 phosphotransferase [Pseudomonas aeruginosa] | 147 | 4e-40 | 48% |
| WP_086140231.1 MULTISPECIES: aminoglycoside 3'-phosphotransfer... | 150 | 5e-40 | 40% |
| WP_181482651.1 MULTISPECIES: APH(3')-I family aminoglycoside O... | 149 | 5e-40 | 35% |
| KAB0787689.1 phosphotransferase [Pseudomonas aeruginosa] | 147 | 5e-40 | 46% |
| EEV9133211.1 APH(3')-I family aminoglycoside O-phosphotransfer... | 149 | 5e-40 | 34% |
| WP_103131028.1 aminoglycoside 3'-phosphotransferase [Deinococc... | 149 | 5e-40 | 37% |
| WP_031129630.1 MULTISPECIES: aminoglycoside O-phosphotransfera... | 149 | 6e-40 | 38% |
| EEV8110741.1 APH(3')-I family aminoglycoside O-phosphotransfer... | 149 | 6e-40 | 34% |
| WP_019588793.1 aminoglycoside 3'-phosphotransferase [Deinococc... | 149 | 6e-40 | 38% |
| WP_126640537.1 aminoglycoside 3'-phosphotransferase [Embleya h... | 149 | 6e-40 | 41% |
| WP_163582355.1 APH(3')-I family aminoglycoside O-phosphotransf... | 149 | 6e-40 | 34% |
| WP_189008625.1 APH(3') family aminoglycoside O-phosphotransfer... | 149 | 6e-40 | 36% |
| ASW31119.1 aminoglycoside 3'-phosphotransferase [Streptomyces ... | 149 | 7e-40 | 38% |
| MBG7016136.1 phosphotransferase [Pseudomonas aeruginosa] | 146 | 7e-40 | 46% |
| WP_182784716.1 phosphotransferase [Tessaracoccus sp. MC1627] | 144 | 8e-40 | 63% |
| WP_055667530.1 aminoglycoside 3'-phosphotransferase [Desnuesie... | 149 | 8e-40 | 36% |
| WP_155712959.1 APH(3')-I family aminoglycoside O-phosphotransf... | 149 | 8e-40 | 36% |
| WP_136391183.1 aminoglycoside 3'-phosphotransferase [Deinococc... | 149 | 9e-40 | 37% |
| RBP56417.1 phosphotransferase family enzyme [Herbinix hemicell... | 146 | 9e-40 | 44% |
| WP_103203518.1 aminoglycoside 3'-phosphotransferase [Herbinix ... | 146 | 9e-40 | 44% |
| WP_066726365.1 APH(3')-I family aminoglycoside O-phosphotransf... | 149 | 9e-40 | 36% |
| WP_121412134.1 phosphotransferase [Pseudomonas aeruginosa] | 146 | 9e-40 | 47% |
| WP_009877461.1 phosphotransferase [Pseudomonas aeruginosa] | 145 | 1e-39 | 47% |
| VCV94102.1 aph(3')-Ia_1_V00359 [Klebsiella pneumoniae] | 149 | 1e-39 | 34% |
| WP_193460522.1 APH(3')-V family aminoglycoside O-phosphotransf... | 148 | 1e-39 | 38% |
| WP_121584691.1 MULTISPECIES: APH(3')-I family aminoglycoside O... | 149 | 1e-39 | 35% |
| WP_067283051.1 aminoglycoside 3'-phosphotransferase [Mitsuaria... | 148 | 1e-39 | 39% |
| WP_017916342.1 MULTISPECIES: aminoglycoside 3'-phosphotransfer... | 148 | 1e-39 | 36% |
| WP_139175996.1 MULTISPECIES: APH(3')-I family aminoglycoside O... | 148 | 1e-39 | 42% |
| EGO4222555.1 APH(3')-I family aminoglycoside O-phosphotransfer... | 148 | 1e-39 | 34% |

| WP_085808352.1 APH(3')-I family aminoglycoside O-phosphotransf... | 148 | 2e-39 | 36% |
| --- | --- | --- | --- |
| WP_017912397.1 aminoglycoside 3'-phosphotransferase [Xanthomon... | 148 | 2e-39 | 37% |
| WP_111896400.1 APH(3')-I family aminoglycoside O-phosphotransf... | 147 | 2e-39 | 35% |
| GHC29644.1 aminoglycoside 3'-phosphotransferase [Streptomyces ... | 148 | 2e-39 | 38% |
| EFN9115607.1 APH(3')-I family aminoglycoside O-phosphotransfer... | 147 | 2e-39 | 34% |
| WP_038344436.1 APH(3')-I family aminoglycoside O-phosphotransf... | 147 | 2e-39 | 34% |
| EBV8854141.1 APH(3')-I family aminoglycoside O-phosphotransfer... | 148 | 2e-39 | 34% |
| EAY7079809.1 APH(3')-I family aminoglycoside O-phosphotransfer... | 148 | 2e-39 | 34% |
| WP_103057454.1 MULTISPECIES: APH(3')-I family aminoglycoside O... | 148 | 2e-39 | 34% |
| WP_026290584.1 APH(3')-V family aminoglycoside O-phosphotransf... | 148 | 2e-39 | 37% |
| HAL6237329.1 TPA: APH(3')-I family aminoglycoside O-phosphotra... | 148 | 2e-39 | 34% |
| WP_041231541.1 APH(3') family aminoglycoside O-phosphotransfer... | 147 | 2e-39 | 37% |
| WP_101482068.1 aminoglycoside 3'-phosphotransferase [Janthinob... | 148 | 2e-39 | 38% |
| WP_056001027.1 APH(3')-I family aminoglycoside O-phosphotransf... | 148 | 2e-39 | 35% |
| AFZ68185.1 aminoglycoside phosphotransferase [Deinococcus pera... | 147 | 2e-39 | 37% |
| WP_070311383.1 aminoglycoside 3'-phosphotransferase [Janthinob... | 148 | 2e-39 | 41% |
| WP_090787887.1 aminoglycoside 3'-phosphotransferase [Asanoa is... | 147 | 2e-39 | 39% |
| AEK22036.1 6xH-GFP-(Pch PRP8 intein with G418r)-FLAG [Cloning ... | 155 | 2e-39 | 35% |
| WP_051872746.1 aminoglycoside 3'-phosphotransferase [Chryseoba... | 147 | 3e-39 | 32% |
| HAL2865297.1 TPA: APH(3')-I family aminoglycoside O-phosphotra... | 147 | 3e-39 | 34% |
| WP_150477762.1 aminoglycoside 3'-phosphotransferase [Streptomy... | 147 | 3e-39 | 38% |
| WP_107137156.1 aminoglycoside 3'-phosphotransferase [Deinococc... | 147 | 3e-39 | 40% |
| WP_030781520.1 aminoglycoside 3'-phosphotransferase [Streptomy... | 147 | 3e-39 | 40% |
| WP_033450617.1 MULTISPECIES: APH(3')-I family aminoglycoside O... | 147 | 3e-39 | 36% |
| WP_089968899.1 aminoglycoside 3'-phosphotransferase [Lihuaxuel... | 147 | 3e-39 | 35% |
| WP_167482910.1 APH(3') family aminoglycoside O-phosphotransfer... | 145 | 3e-39 | 46% |
| TDY34729.1 aminoglycoside 3'-phosphotransferase-1 [Janthinobac... | 147 | 3e-39 | 39% |
| WP_134596205.1 phosphotransferase [Pseudomonas aeruginosa] | 144 | 4e-39 | 46% |
| WP_155298845.1 aminoglycoside 3'-phosphotransferase [Deinococc... | 147 | 4e-39 | 37% |
| WP_099410944.1 aminoglycoside 3'-phosphotransferase [Janthinob... | 147 | 4e-39 | 40% |
| WP_086356538.1 APH(3')-I family aminoglycoside O-phosphotransf... | 146 | 4e-39 | 35% |
| WP_133990168.1 aminoglycoside 3'-phosphotransferase [Janthinob... | 147 | 4e-39 | 39% |
| PZU75450.1 APH(3') family aminoglycoside O-phosphotransferase ... | 147 | 4e-39 | 40% |
| KAK50383.1 putative kanamycin kinase [Bordetella bronchiseptic... | 148 | 4e-39 | 36% |
| EBG3508176.1 APH(3')-I family aminoglycoside O-phosphotransfer... | 146 | 5e-39 | 34% |
| WP_153751122.1 aminoglycoside 3'-phosphotransferase [Xanthomon... | 147 | 5e-39 | 36% |
| WP_128501036.1 aminoglycoside 3'-phosphotransferase [Ornithoba... | 147 | 5e-39 | 32% |
| MTI48104.1 aminoglycoside 3'-phosphotransferase [Firmicutes ba... | 147 | 5e-39 | 32% |
| WP_186720932.1 APH(3') family aminoglycoside O-phosphotransfer... | 146 | 5e-39 | 41% |
| PKV45024.1 aminoglycoside 3'-phosphotransferase-1 [Janthinobac... | 147 | 5e-39 | 38% |
| OWQ47732.1 APH(3') family aminoglycoside O-phosphotransferase ... | 147 | 5e-39 | 36% |
| WP_161283699.1 APH(3')-I family aminoglycoside O-phosphotransf... | 146 | 5e-39 | 34% |
| WP_150411172.1 aminoglycoside 3'-phosphotransferase [Xanthomon... | 147 | 5e-39 | 36% |
| OLS32932.1 Aminoglycoside 3'-phosphotransferase [Candidatus He... | 146 | 6e-39 | 30% |
| WP_104915339.1 APH(3')-I family aminoglycoside O-phosphotransf... | 145 | 6e-39 | 34% |
| RYF61906.1 aminoglycoside 3'-phosphotransferase [Comamonadacea... | 147 | 6e-39 | 37% |
| WP_092814707.1 APH(3')-I family aminoglycoside O-phosphotransf... | 147 | 6e-39 | 35% |
| WP_095757910.1 APH(3')-V family aminoglycoside O-phosphotransf... | 146 | 7e-39 | 39% |
| EBR4615070.1 APH(3')-I family aminoglycoside O-phosphotransfer... | 145 | 7e-39 | 34% |
| WP_100652194.1 MULTISPECIES: APH(3')-I family aminoglycoside O... | 146 | 7e-39 | 37% |
| WP_071213050.1 APH(3')-I family aminoglycoside O-phosphotransf... | 145 | 7e-39 | 34% |
| WP_055774624.1 APH(3')-I family aminoglycoside O-phosphotransf... | 146 | 8e-39 | 36% |
| WP_160947626.1 MULTISPECIES: APH(3') family aminoglycoside O-p... | 146 | 8e-39 | 37% |
| WP_175617866.1 APH(3')-I family aminoglycoside O-phosphotransf... | 145 | 9e-39 | 34% |
| WP_058675408.1 APH(3')-I family aminoglycoside O-phosphotransf... | 145 | 9e-39 | 34% |
| EGO4222556.1 APH(3')-I family aminoglycoside O-phosphotransfer... | 146 | 9e-39 | 34% |
| WP_063842176.1 aminoglycoside O-phosphotransferase APH(3')-Va ... | 146 | 1e-38 | 38% |
| WP_152257122.1 aminoglycoside 3'-phosphotransferase [Janthinob... | 146 | 1e-38 | 40% |
| WP_065917911.1 aminoglycoside 3'-phosphotransferase [Lentzea g... | 145 | 1e-38 | 46% |
| PJD00553.1 APH(3') family aminoglycoside O-phosphotransferase ... | 145 | 2e-38 | 41% |
| HAL2865227.1 TPA: APH(3')-I family aminoglycoside O-phosphotra... | 144 | 2e-38 | 35% |
| WP_148828819.1 MULTISPECIES: aminoglycoside 3'-phosphotransfer... | 145 | 2e-38 | 36% |
| WP_038404948.1 aminoglycoside O-phosphotransferase APH(3')-Ib ... | 145 | 2e-38 | 36% |
| WP_051318280.1 aminoglycoside 3'-phosphotransferase [Cohnella ... | 142 | 2e-38 | 42% |
| WP_143215070.1 APH(3')-I family aminoglycoside O-phosphotransf... | 145 | 2e-38 | 34% |
| WP_172684309.1 APH(3')-I family aminoglycoside O-phosphotransf... | 145 | 2e-38 | 34% |
| WP_046843513.1 aminoglycoside 3'-phosphotransferase [Deinococc... | 145 | 2e-38 | 37% |
| WP_124900036.1 aminoglycoside 3'-phosphotransferase [Flavobact... | 145 | 2e-38 | 32% |
| HAE2751345.1 TPA: APH(3')-I family aminoglycoside O-phosphotra... | 145 | 2e-38 | 34% |
| WP_114259893.1 APH(3')-I family aminoglycoside O-phosphotransf... | 145 | 2e-38 | 34% |
| WP_158210470.1 APH(3') family aminoglycoside O-phosphotransfer... | 145 | 2e-38 | 32% |
| ACN66868.1 aminoglycoside 3'-phosphotransferase, truncated [Es... | 145 | 2e-38 | 34% |

| WP_166105832.1 APH(3')-I family aminoglycoside O-phosphotransf... | 145 | 3e-38 | 35% |
| --- | --- | --- | --- |
| CAJ85716.1 TPA: aminoglycoside 3'-phospho-transferase [Birming... | 145 | 3e-38 | 36% |
| SSW43920.1 aminoglycoside 3'-phosphotransferase aphA1 [Acineto... | 145 | 3e-38 | 34% |
| WP_086146417.1 aminoglycoside 3'-phosphotransferase [Janthinob... | 145 | 3e-38 | 41% |
| EDW8045156.1 APH(3')-I family aminoglycoside O-phosphotransfer... | 145 | 3e-38 | 34% |
| WP_056433730.1 APH(3')-I family aminoglycoside O-phosphotransf... | 145 | 3e-38 | 38% |
| WP_154138269.1 MULTISPECIES: aminoglycoside 3'-phosphotransfer... | 145 | 3e-38 | 32% |
| WP_116468961.1 aminoglycoside 3'-phosphotransferase [Sphingomo... | 145 | 3e-38 | 40% |
| WP_167476375.1 phosphotransferase [Nocardia arthritidis] | 144 | 3e-38 | 43% |
| WP_096236617.1 aminoglycoside 3'-phosphotransferase [Janthinob... | 145 | 3e-38 | 39% |
| WP_017909896.1 aminoglycoside 3'-phosphotransferase [Xanthomon... | 145 | 4e-38 | 37% |
| WP_133400368.1 APH(3')-I family aminoglycoside O-phosphotransf... | 144 | 4e-38 | 35% |
| WP_064013516.1 aminoglycoside 3'-phosphotransferase [Deinococc... | 144 | 5e-38 | 37% |
| WP_086018395.1 MULTISPECIES: aminoglycoside 3'-phosphotransfer... | 144 | 5e-38 | 40% |
| MBE2408242.1 APH(3')-I family aminoglycoside O-phosphotransfer... | 143 | 5e-38 | 34% |
| EEW8136590.1 APH(3')-I family aminoglycoside O-phosphotransfer... | 143 | 5e-38 | 34% |
| WP_126562519.1 APH(3')-I family aminoglycoside O-phosphotransf... | 144 | 6e-38 | 34% |
| PHV16501.1 APH(3') family aminoglycoside O-phosphotransferase ... | 144 | 6e-38 | 41% |
| MBB8913568.1 APH(3')-I family aminoglycoside O-phosphotransfer... | 143 | 6e-38 | 34% |
| WP_188138366.1 APH(3')-I family aminoglycoside O-phosphotransf... | 143 | 6e-38 | 34% |
| EFO6018403.1 APH(3')-I family aminoglycoside O-phosphotransfer... | 144 | 7e-38 | 35% |
| WP_152739290.1 phosphotransferase [Microvirga tunisiensis] | 137 | 7e-38 | 100% |
| WP_084559977.1 APH(3')-I family aminoglycoside O-phosphotransf... | 144 | 7e-38 | 35% |
| WP_188198796.1 APH(3')-I family aminoglycoside O-phosphotransf... | 144 | 7e-38 | 35% |
| WP_057067380.1 MULTISPECIES: APH(3')-I family aminoglycoside O... | 144 | 7e-38 | 34% |
| WP_063109385.1 aminoglycoside 3'-phosphotransferase [Escherich... | 145 | 8e-38 | 31% |
| WP_193787350.1 APH(3')-I family aminoglycoside O-phosphotransf... | 144 | 8e-38 | 34% |
| WP_073661347.1 phosphotransferase [Pseudomonas aeruginosa] | 139 | 8e-38 | 62% |
| WP_167819982.1 APH(3')-I family aminoglycoside O-phosphotransf... | 144 | 8e-38 | 34% |
| EGO7905753.1 APH(3')-I family aminoglycoside O-phosphotransfer... | 143 | 8e-38 | 35% |
| WP_152283204.1 aminoglycoside 3'-phosphotransferase [Janthinob... | 143 | 8e-38 | 41% |
| EGO7905894.1 APH(3')-I family aminoglycoside O-phosphotransfer... | 143 | 9e-38 | 35% |
| WP_162865541.1 APH(3') family aminoglycoside O-phosphotransfer... | 143 | 9e-38 | 39% |
| AXG99637.1 aminoglycoside 3'-phosphotransferase [Deinococcus w... | 143 | 9e-38 | 39% |
| EFM4248294.1 APH(3')-I family aminoglycoside O-phosphotransfer... | 143 | 1e-37 | 34% |
| WP_042227921.1 aminoglycoside O-phosphotransferase APH(3')-IVa... | 143 | 1e-37 | 34% |
| WP_188445339.1 APH(3') family aminoglycoside O-phosphotransfer... | 143 | 1e-37 | 36% |
| WP_009672468.1 MULTISPECIES: aminoglycoside O-phosphotransfera... | 143 | 1e-37 | 35% |
| WP_123267273.1 APH(3')-I family aminoglycoside O-phosphotransf... | 143 | 1e-37 | 33% |
| HAL7118015.1 TPA: APH(3')-I family aminoglycoside O-phosphotra... | 142 | 1e-37 | 34% |
| WP_114142246.1 APH(3')-I family aminoglycoside O-phosphotransf... | 142 | 1e-37 | 34% |
| EDP9035159.1 APH(3')-I family aminoglycoside O-phosphotransfer... | 142 | 1e-37 | 34% |
| WP_174575170.1 APH(3')-I family aminoglycoside O-phosphotransf... | 142 | 1e-37 | 34% |
| EAN5392160.1 APH(3')-I family aminoglycoside O-phosphotransfer... | 143 | 1e-37 | 35% |
| EFM4248130.1 APH(3')-I family aminoglycoside O-phosphotransfer... | 142 | 2e-37 | 34% |
| NNM04610.1 aminoglycoside 3'-phosphotransferase [Gemmatimonade... | 142 | 2e-37 | 39% |
| WP_161881748.1 APH(3') family aminoglycoside O-phosphotransfer... | 143 | 2e-37 | 39% |
| WP_071446303.1 APH(3')-I family aminoglycoside O-phosphotransf... | 143 | 2e-37 | 33% |
| HAL4277215.1 TPA: APH(3')-I family aminoglycoside O-phosphotra... | 142 | 2e-37 | 34% |
| WP_023124366.1 aminoglycoside 3'-phosphotransferase [Pseudomon... | 143 | 2e-37 | 39% |
| WP_047935614.1 APH(3')-I family aminoglycoside O-phosphotransf... | 142 | 2e-37 | 34% |
| WP_051668656.1 APH(3') family aminoglycoside O-phosphotransfer... | 142 | 2e-37 | 40% |
| WP_050614934.1 aminoglycoside 3'-phosphotransferase [Bacillus ... | 142 | 2e-37 | 35% |
| HAL1399580.1 TPA: APH(3')-I family aminoglycoside O-phosphotra... | 141 | 2e-37 | 34% |
| HAL4277477.1 TPA: APH(3')-I family aminoglycoside O-phosphotra... | 142 | 3e-37 | 34% |
| WP_038347152.1 aminoglycoside 3'-phosphotransferase [Acinetoba... | 141 | 3e-37 | 33% |
| WP_168844352.1 APH(3') family aminoglycoside O-phosphotransfer... | 142 | 3e-37 | 36% |
| WP_125521830.1 aminoglycoside O-phosphotransferase APH(3')-VII... | 142 | 3e-37 | 39% |
| WP_087878563.1 MULTISPECIES: APH(3')-I family aminoglycoside O... | 142 | 3e-37 | 33% |
| WP_019371160.1 MULTISPECIES: aminoglycoside 3'-phosphotransfer... | 142 | 3e-37 | 38% |
| WP_114259732.1 APH(3')-I family aminoglycoside O-phosphotransf... | 142 | 3e-37 | 34% |
| WP_021283646.1 aminoglycoside 3'-phosphotransferase [Clostridi... | 142 | 4e-37 | 31% |
| WP_060682146.1 APH(3')-I family aminoglycoside O-phosphotransf... | 142 | 4e-37 | 33% |
| WP_030183523.1 MULTISPECIES: aminoglycoside O-phosphotransfera... | 142 | 4e-37 | 39% |
| WP_073971717.1 APH(3')-I family aminoglycoside O-phosphotransf... | 141 | 4e-37 | 34% |
| EAP9786484.1 APH(3')-I family aminoglycoside O-phosphotransfer... | 141 | 4e-37 | 34% |
| WP_188844242.1 APH(3') family aminoglycoside O-phosphotransfer... | 142 | 4e-37 | 39% |
| WP_141654552.1 APH(3')-I family aminoglycoside O-phosphotransf... | 140 | 4e-37 | 34% |
| HAG5826889.1 TPA: APH(3')-I family aminoglycoside O-phosphotra... | 142 | 4e-37 | 33% |
| OYY66220.1 hypothetical protein B7Y49_03265 [Sphingomonas sp. ... | 141 | 4e-37 | 40% |
| AQN67803.1 aminoglycoside 3'-phosphotransferase VIII [Cloning ... | 142 | 5e-37 | 39% |
| HAL1399617.1 TPA: APH(3')-I family aminoglycoside O-phosphotra... | 141 | 5e-37 | 34% |

| WP_149859140.1 APH(3')-I family aminoglycoside O-phosphotransf... | 141 | 5e-37 | 34% |
| --- | --- | --- | --- |
| WP_048223939.1 MULTISPECIES: APH(3')-I family aminoglycoside O... | 142 | 5e-37 | 33% |
| KHH88456.1 aminoglycoside phosphotransferase Aph(3')-Ia [Esche... | 141 | 5e-37 | 34% |
| 4H05_A Crystal structure of aminoglycoside-3'-phosphotransfera... | 142 | 5e-37 | 39% |
| ECS3490810.1 APH(3')-I family aminoglycoside O-phosphotransfer... | 140 | 5e-37 | 34% |
| WP_194210866.1 APH(3')-I family aminoglycoside O-phosphotransf... | 142 | 5e-37 | 33% |
| WP_097313168.1 APH(3')-I family aminoglycoside O-phosphotransf... | 141 | 5e-37 | 34% |
| WP_057091773.1 MULTISPECIES: APH(3')-I family aminoglycoside O... | 141 | 5e-37 | 34% |
| PZO74441.1 APH(3')-I family aminoglycoside O-phosphotransferas... | 142 | 5e-37 | 36% |
| WP_136391921.1 APH(3')-I family aminoglycoside O-phosphotransf... | 142 | 5e-37 | 33% |
| WP_081681243.1 aminoglycoside 3'-phosphotransferase [Flectobac... | 141 | 6e-37 | 33% |
| WP_094899812.1 aminoglycoside 3'-phosphotransferase [Tissierel... | 141 | 6e-37 | 34% |
| OQW43014.1 hypothetical protein A4S12_05955 [Proteobacteria ba... | 141 | 6e-37 | 39% |
| WP_064753535.1 APH(3')-I family aminoglycoside O-phosphotransf... | 140 | 6e-37 | 34% |
| WP_148746359.1 APH(3')-I family aminoglycoside O-phosphotransf... | 140 | 6e-37 | 35% |
| WP_149607277.1 APH(3')-I family aminoglycoside O-phosphotransf... | 141 | 7e-37 | 33% |
| EFL1176934.1 APH(3')-I family aminoglycoside O-phosphotransfer... | 140 | 8e-37 | 35% |
| MBA3918677.1 APH(3') family aminoglycoside O-phosphotransferas... | 143 | 8e-37 | 39% |
| WP_048216288.1 MULTISPECIES: APH(3')-I family aminoglycoside O... | 141 | 8e-37 | 33% |
| WP_103015390.1 APH(3')-I family aminoglycoside O-phosphotransf... | 141 | 8e-37 | 33% |
| WP_109725602.1 aminoglycoside 3'-phosphotransferase [Ruminococ... | 141 | 9e-37 | 34% |
| WP_093150079.1 aminoglycoside 3'-phosphotransferase [Saccharop... | 141 | 9e-37 | 40% |
| WP_030630943.1 aminoglycoside O-phosphotransferase APH(3')-VII... | 141 | 9e-37 | 38% |
| EBF9424123.1 APH(3')-I family aminoglycoside O-phosphotransfer... | 140 | 9e-37 | 34% |
| EDW9496508.1 APH(3')-I family aminoglycoside O-phosphotransfer... | 140 | 1e-36 | 34% |
| WP_143719746.1 aminoglycoside 3'-phosphotransferase [Deinococc... | 141 | 1e-36 | 36% |
| WP_033035562.1 aminoglycoside O-phosphotransferase APH(3')-VII... | 141 | 1e-36 | 39% |
| WP_030375832.1 aminoglycoside O-phosphotransferase APH(3')-VII... | 140 | 1e-36 | 37% |
| WP_124872314.1 aminoglycoside 3'-phosphotransferase [Deinococc... | 141 | 1e-36 | 36% |
| SPN80502.1 Aminoglycoside phosphotransferase [Klebsiella pneum... | 139 | 1e-36 | 35% |
| EAY3373742.1 APH(3')-I family aminoglycoside O-phosphotransfer... | 139 | 1e-36 | 34% |
| WP_030646682.1 aminoglycoside O-phosphotransferase APH(3')-VII... | 140 | 1e-36 | 38% |
| WP_114140244.1 APH(3')-I family aminoglycoside O-phosphotransf... | 139 | 1e-36 | 35% |
| WP_141986995.1 aminoglycoside 3'-phosphotransferase [Sphingomo... | 140 | 1e-36 | 37% |
| WP_112380391.1 APH(3')-I family aminoglycoside O-phosphotransf... | 139 | 1e-36 | 34% |
| EAT1503845.1 APH(3')-I family aminoglycoside O-phosphotransfer... | 139 | 1e-36 | 35% |
| AGC70726.1 Aminoglycoside 3'-phosphotransferase AphA1-IAB [Aci... | 139 | 1e-36 | 35% |
| WP_013555510.1 aminoglycoside 3'-phosphotransferase [Deinococc... | 141 | 1e-36 | 39% |
| EEE8175964.1 APH(3')-I family aminoglycoside O-phosphotransfer... | 139 | 1e-36 | 35% |
| BAL48648.1 aminoglycoside 3'-phosphotransferase type 1 [Salmon... | 139 | 1e-36 | 35% |
| MBE2536075.1 APH(3')-I family aminoglycoside O-phosphotransfer... | 139 | 1e-36 | 35% |
| MBE82460.1 hypothetical protein [Gemmatimonadetes bacterium] | 140 | 1e-36 | 32% |
| WP_114140243.1 MULTISPECIES: APH(3')-I family aminoglycoside O... | 139 | 1e-36 | 35% |
| EDT8430686.1 APH(3')-I family aminoglycoside O-phosphotransfer... | 140 | 1e-36 | 34% |
| EFB3492438.1 APH(3')-I family aminoglycoside O-phosphotransfer... | 139 | 1e-36 | 35% |
| WP_156189469.1 APH(3')-I family aminoglycoside O-phosphotransf... | 140 | 2e-36 | 34% |
| WP_100298206.1 aminoglycoside 3'-phosphotransferase [Streptomy... | 140 | 2e-36 | 41% |
| EEZ9088188.1 APH(3')-I family aminoglycoside O-phosphotransfer... | 139 | 2e-36 | 34% |
| WP_141011060.1 APH(3')-I family aminoglycoside O-phosphotransf... | 139 | 2e-36 | 34% |
| ADF30268.1 aminoglycoside (3') phosphotransferase [Salmonella ... | 139 | 2e-36 | 36% |
| WP_088248937.1 aminoglycoside 3'-phosphotransferase [Deinococc... | 140 | 2e-36 | 39% |
| EAY3373719.1 APH(3')-I family aminoglycoside O-phosphotransfer... | 139 | 2e-36 | 35% |
| ARO93318.1 APH(3') family aminoglycoside O-phosphotransferase ... | 139 | 2e-36 | 35% |
| WP_106642949.1 APH(3')-I family aminoglycoside O-phosphotransf... | 139 | 2e-36 | 35% |
| ECE0951139.1 APH(3')-I family aminoglycoside O-phosphotransfer... | 140 | 2e-36 | 34% |
| EDU9028685.1 APH(3')-I family aminoglycoside O-phosphotransfer... | 139 | 2e-36 | 35% |
| WP_164168851.1 APH(3') family aminoglycoside O-phosphotransfer... | 140 | 2e-36 | 33% |
| HAJ6712856.1 TPA: APH(3')-I family aminoglycoside O-phosphotra... | 139 | 2e-36 | 35% |
| ECE5562644.1 phosphotransferase [Salmonella enterica] | 134 | 2e-36 | 100% |
| NEU04480.1 aminoglycoside 3'-phosphotransferase [Clostridium s... | 140 | 2e-36 | 34% |
| GHG31100.1 aminoglycoside 3'-phosphotransferase [Deinococcus i... | 140 | 2e-36 | 39% |
| AJP73089.1 aminoglycoside phosphotransferase [Sphingomonas hen... | 140 | 2e-36 | 36% |
| ADF30267.1 aminoglycoside (3') phosphotransferase [Escherichia... | 139 | 2e-36 | 36% |
| HAS1768415.1 TPA: APH(3')-I family aminoglycoside O-phosphotra... | 139 | 2e-36 | 34% |
| EFH5321783.1 APH(3')-I family aminoglycoside O-phosphotransfer... | 140 | 2e-36 | 34% |
| WP_053802359.1 aminoglycoside O-phosphotransferase APH(3')-VII... | 140 | 2e-36 | 39% |
| WP_112383194.1 APH(3')-I family aminoglycoside O-phosphotransf... | 140 | 2e-36 | 35% |
| TOL07500.1 APH(3') family aminoglycoside O-phosphotransferase ... | 139 | 2e-36 | 35% |
| WP_121529103.1 APH(3')-I family aminoglycoside O-phosphotransf... | 140 | 2e-36 | 33% |
| EFH3870022.1 APH(3')-I family aminoglycoside O-phosphotransfer... | 139 | 2e-36 | 34% |
| WP_183982643.1 APH(3') family aminoglycoside O-phosphotransfer... | 140 | 2e-36 | 41% |
| EFM0500134.1 APH(3')-I family aminoglycoside O-phosphotransfer... | 139 | 2e-36 | 34% |

| AEA08386.1 aminoglycoside phosphotransferase [Escherichia coli] | 139 | 2e-36 | 34% |
| --- | --- | --- | --- |
| ADF30265.1 aminoglycoside (3') phosphotransferase [Escherichia... | 139 | 2e-36 | 36% |
| WP_113945579.1 aminoglycoside 3'-phosphotransferase [Pseudochr... | 139 | 3e-36 | 34% |
| AOT62238.1 Aminoglycoside 3'-phosphotransferase [Streptomyces ... | 147 | 3e-36 | 38% |
| WP_047014552.1 MULTISPECIES: aminoglycoside 3'-phosphotransfer... | 140 | 3e-36 | 41% |
| WP_084331012.1 APH(3')-I family aminoglycoside O-phosphotransf... | 139 | 3e-36 | 36% |
| WP_195118479.1 phosphotransferase [Nocardia terpenica] | 139 | 3e-36 | 43% |
| WP_138556499.1 APH(3')-I family aminoglycoside O-phosphotransf... | 139 | 3e-36 | 36% |
| AMP52176.1 hypothetical protein [uncultured bacterium] | 133 | 3e-36 | 100% |
| EAU8986540.1 APH(3')-I family aminoglycoside O-phosphotransfer... | 139 | 3e-36 | 36% |
| WP_116964937.1 APH(3')-I family aminoglycoside O-phosphotransf... | 138 | 3e-36 | 36% |
| WP_147522608.1 APH(3')-I family aminoglycoside O-phosphotransf... | 139 | 3e-36 | 36% |
| WP_113981418.1 aminoglycoside 3'-phosphotransferase [Sphaerisp... | 139 | 3e-36 | 42% |
| WP_129950464.1 MULTISPECIES: APH(3')-I family aminoglycoside O... | 139 | 3e-36 | 36% |
| WP_104943014.1 aminoglycoside 3'-phosphotransferase [Porphyrob... | 139 | 3e-36 | 34% |
| WP_061994419.1 aminoglycoside 3'-phosphotransferase [Clostridi... | 139 | 4e-36 | 34% |
| WP_084488933.1 APH(3') family aminoglycoside O-phosphotransfer... | 142 | 4e-36 | 44% |
| WP_138704400.1 APH(3')-I family aminoglycoside O-phosphotransf... | 138 | 4e-36 | 36% |
| EEU9210890.1 APH(3')-I family aminoglycoside O-phosphotransfer... | 138 | 4e-36 | 36% |
| EBV4810653.1 APH(3')-I family aminoglycoside O-phosphotransfer... | 139 | 4e-36 | 36% |
| WP_136957034.1 APH(3')-I family aminoglycoside O-phosphotransf... | 139 | 4e-36 | 36% |
| EEN7811629.1 APH(3')-I family aminoglycoside O-phosphotransfer... | 138 | 4e-36 | 36% |
| WP_139846203.1 APH(3')-I family aminoglycoside O-phosphotransf... | 139 | 4e-36 | 36% |
| WP_096939791.1 APH(3')-I family aminoglycoside O-phosphotransf... | 139 | 4e-36 | 36% |
| WP_077228669.1 APH(3')-I family aminoglycoside O-phosphotransf... | 140 | 4e-36 | 36% |
| EFU7708135.1 APH(3')-I family aminoglycoside O-phosphotransfer... | 139 | 4e-36 | 36% |
| AAY27879.1 aminoglycoside-O-phosphotransferase VIII [synthetic... | 139 | 4e-36 | 39% |
| WP_138053290.1 APH(3')-I family aminoglycoside O-phosphotransf... | 139 | 4e-36 | 36% |
| WP_095998846.1 aminoglycoside 3'-phosphotransferase [Sphingomo... | 139 | 4e-36 | 36% |
| WP_050196407.1 APH(3')-I family aminoglycoside O-phosphotransf... | 139 | 4e-36 | 36% |
| EFO6018324.1 APH(3')-I family aminoglycoside O-phosphotransfer... | 139 | 4e-36 | 36% |
| EAN5392084.1 APH(3')-I family aminoglycoside O-phosphotransfer... | 139 | 4e-36 | 36% |
| WP_130122868.1 APH(3')-I family aminoglycoside O-phosphotransf... | 139 | 4e-36 | 36% |
| HAJ6926535.1 TPA: APH(3')-I family aminoglycoside O-phosphotra... | 138 | 5e-36 | 36% |
| WP_086011762.1 aminoglycoside 3'-phosphotransferase [Sphingomo... | 139 | 5e-36 | 36% |
| EAU8986742.1 APH(3')-I family aminoglycoside O-phosphotransfer... | 139 | 5e-36 | 36% |
| EAO4048243.1 APH(3')-I family aminoglycoside O-phosphotransfer... | 138 | 5e-36 | 36% |
| WP_193157375.1 APH(3')-I family aminoglycoside O-phosphotransf... | 139 | 5e-36 | 36% |
| EEI8652741.1 APH(3')-I family aminoglycoside O-phosphotransfer... | 138 | 5e-36 | 36% |
| XP_001893601.1 aminoglycoside 3'-phosphotransferase [Brugia ma... | 139 | 5e-36 | 36% |
| WP_099723128.1 APH(3')-I family aminoglycoside O-phosphotransf... | 138 | 5e-36 | 36% |
| EFU7708136.1 APH(3')-I family aminoglycoside O-phosphotransfer... | 139 | 5e-36 | 36% |
| AAR08152.1 aph [Cloning vector pPmr1] | 139 | 5e-36 | 39% |
| AAR08153.1 aph [Cloning vector pPmr2] | 139 | 5e-36 | 39% |
| EFL9372011.1 APH(3')-I family aminoglycoside O-phosphotransfer... | 138 | 5e-36 | 36% |
| WP_063102219.1 MULTISPECIES: APH(3')-I family aminoglycoside O... | 138 | 5e-36 | 36% |
| RBO92136.1 aminoglycoside 3'-phosphotransferase-1 [Pseudochrob... | 139 | 6e-36 | 34% |
| WP_125933716.1 aminoglycoside 3'-phosphotransferase [Streptomy... | 139 | 6e-36 | 41% |
| EER7520135.1 APH(3')-I family aminoglycoside O-phosphotransfer... | 138 | 6e-36 | 36% |
| EKH31232.1 aminoglycoside phosphotransferase [Escherichia coli... | 137 | 6e-36 | 34% |
| WP_140947450.1 APH(3')-I family aminoglycoside O-phosphotransf... | 137 | 6e-36 | 35% |
| WP_100675322.1 APH(3')-I family aminoglycoside O-phosphotransf... | 138 | 6e-36 | 36% |
| EGK3953196.1 APH(3')-I family aminoglycoside O-phosphotransfer... | 138 | 6e-36 | 36% |
| OJS63886.1 APH(3') family aminoglycoside O-phosphotransferase ... | 137 | 6e-36 | 34% |
| WP_103848183.1 APH(3')-I family aminoglycoside O-phosphotransf... | 138 | 6e-36 | 36% |
| WP_096892136.1 APH(3')-I family aminoglycoside O-phosphotransf... | 138 | 6e-36 | 36% |
| EFH1606711.1 APH(3')-I family aminoglycoside O-phosphotransfer... | 138 | 6e-36 | 36% |
| HAO7577486.1 TPA: APH(3')-I family aminoglycoside O-phosphotra... | 138 | 6e-36 | 36% |
| WP_066722314.1 aminoglycoside 3'-phosphotransferase [Sphingomo... | 139 | 6e-36 | 37% |
| WP_132392224.1 aminoglycoside 3'-phosphotransferase [Micromono... | 138 | 6e-36 | 41% |
| MBE82909.1 hypothetical protein [Gemmatimonadetes bacterium] | 139 | 7e-36 | 33% |
| EAW2954162.1 APH(3')-I family aminoglycoside O-phosphotransfer... | 137 | 7e-36 | 35% |
| AAR08154.1 aph [Cloning vector pPmr3] | 139 | 7e-36 | 39% |
| ECR9682479.1 APH(3')-I family aminoglycoside O-phosphotransfer... | 138 | 7e-36 | 36% |
| MGQ27207.1 APH(3')-I family aminoglycoside O-phosphotransferas... | 138 | 7e-36 | 36% |
| WP_020498418.1 aminoglycoside 3'-phosphotransferase [Sciscione... | 137 | 8e-36 | 42% |
| WP_167488729.1 phosphotransferase [Nocardia terpenica] | 138 | 8e-36 | 41% |
| PNO46862.1 APH(3') family aminoglycoside O-phosphotransferase ... | 137 | 8e-36 | 35% |
| VDB01903.1 aph(3')-Ia_1_V00359 [Klebsiella pneumoniae] | 138 | 8e-36 | 36% |
| WP_109827839.1 aminoglycoside 3'-phosphotransferase [Deinococc... | 139 | 8e-36 | 38% |
| RZI57569.1 aminoglycoside 3'-phosphotransferase [Rubrivivax sp.] | 139 | 9e-36 | 37% |
| WP_031191644.1 MULTISPECIES: aminoglycoside O-phosphotransfera... | 138 | 9e-36 | 37% |

| WP_185679039.1 APH(3') family aminoglycoside O-phosphotransfer... | 138 | 9e-36 | 40% |
| --- | --- | --- | --- |
| EOZ46318.1 putative kanamycin kinase [Klebsiella pneumoniae VA... | 137 | 9e-36 | 34% |
| AAK13425.1 aminoglycoside-3'-phosphotransferase [Promoter prob... | 137 | 1e-35 | 36% |
| OCV01369.1 APH(3') family aminoglycoside O-phosphotransferase ... | 137 | 1e-35 | 34% |
| WP_018347413.1 aminoglycoside 3'-phosphotransferase [Longispor... | 137 | 1e-35 | 40% |
| AVX34095.1 Aminoglycoside phosphotransferase [Klebsiella pneum... | 137 | 1e-35 | 34% |
| MBG6139583.1 aminoglycoside phosphotransferase [Longispora fulva] | 137 | 1e-35 | 41% |
| WP_112261473.1 aminoglycoside 3'-phosphotransferase [Lentzea t... | 137 | 1e-35 | 42% |
| WP_093351411.1 MULTISPECIES: aminoglycoside 3'-phosphotransfer... | 137 | 2e-35 | 40% |
| EFM2051035.1 aminoglycoside 3'-phosphotransferase [Escherichia... | 137 | 2e-35 | 36% |
| HAN87386.1 TPA: aminoglycoside 3'-phosphotransferase [Firmicut... | 135 | 2e-35 | 42% |
| WP_012920937.1 aminoglycoside 3'-phosphotransferase [Kribbella... | 137 | 2e-35 | 38% |
| KZM74910.1 aminoglycoside phosphotransferase [Nocardia terpenica] | 137 | 2e-35 | 42% |
| WP_053699462.1 aminoglycoside O-phosphotransferase APH(3')-VII... | 137 | 2e-35 | 37% |
| NKB69314.1 APH(3') family aminoglycoside O-phosphotransferase ... | 137 | 2e-35 | 37% |
| AFD33551.1 putative regulatory/resistance fusion protein [Stre... | 144 | 2e-35 | 39% |
| WP_067964639.1 aminoglycoside 3'-phosphotransferase [Nocardiop... | 137 | 2e-35 | 39% |
| WP_125510785.1 aminoglycoside 3'-phosphotransferase [Streptomy... | 137 | 2e-35 | 38% |
| WP_082871268.1 aminoglycoside 3'-phosphotransferase [Nocardia ... | 137 | 2e-35 | 42% |
| WP_191864884.1 APH(3')-I family aminoglycoside O-phosphotransf... | 137 | 2e-35 | 36% |
| WP_187162148.1 APH(3') family aminoglycoside O-phosphotransfer... | 135 | 2e-35 | 36% |
| WP_177221365.1 aminoglycoside 3'-phosphotransferase [Lentzea x... | 136 | 3e-35 | 45% |
| XP_011015936.1 PREDICTED: uncharacterized protein LOC105119490... | 136 | 3e-35 | 37% |
| WP_093940363.1 aminoglycoside 3'-phosphotransferase [Actinoall... | 137 | 4e-35 | 39% |
| WP_017413808.1 aminoglycoside 3'-phosphotransferase [Clostridi... | 137 | 4e-35 | 30% |
| WP_173329250.1 APH(3') family aminoglycoside O-phosphotransfer... | 136 | 4e-35 | 32% |
| WP_043078543.1 aminoglycoside 3'-phosphotransferase [Mycobacte... | 137 | 4e-35 | 37% |
| EER7671017.1 aminoglycoside 3'-phosphotransferase [Escherichia... | 135 | 4e-35 | 35% |
| MBE6900280.1 aminoglycoside 3'-phosphotransferase [Ruminococca... | 136 | 4e-35 | 37% |
| NLY44578.1 aminoglycoside 3'-phosphotransferase [Tissierella sp.] | 136 | 4e-35 | 33% |
| WP_145813422.1 aminoglycoside 3'-phosphotransferase [Kribbella... | 136 | 5e-35 | 40% |
| MBF3178390.1 phosphotransferase [Pseudomonas aeruginosa] | 131 | 5e-35 | 62% |
| MBE6893929.1 aminoglycoside 3'-phosphotransferase [Ruminococca... | 136 | 5e-35 | 32% |
| TWD84350.1 streptomycin 3'-kinase [Kribbella amoyensis] | 136 | 5e-35 | 40% |
| WP_063842177.1 aminoglycoside O-phosphotransferase APH(3')-Vb ... | 136 | 6e-35 | 38% |
| WP_074291024.1 phosphotransferase [Pseudomonas aeruginosa] | 131 | 6e-35 | 62% |
| WP_112245379.1 aminoglycoside 3'-phosphotransferase [Kribbella... | 136 | 6e-35 | 38% |
| KJI70652.1 aminoglycoside phosphotransferase [Enterobacter hor... | 135 | 6e-35 | 35% |
| WP_064631202.1 aminoglycoside 3'-phosphotransferase [Mycobacte... | 136 | 7e-35 | 37% |
| RSR57484.1 aminoglycoside 3'-phosphotransferase [Acinetobacter... | 134 | 7e-35 | 35% |
| WP_140960996.1 aminoglycoside 3'-phosphotransferase [Acinetoba... | 134 | 7e-35 | 35% |
| WP_027767066.1 aminoglycoside 3'-phosphotransferase [Streptomy... | 136 | 7e-35 | 41% |
| WP_063841674.1 aminoglycoside O-phosphotransferase APH(3')-IVa... | 136 | 7e-35 | 34% |
| WP_175573998.1 phosphotransferase [Pseudomonas aeruginosa] | 133 | 7e-35 | 49% |
| WP_136959044.1 aminoglycoside 3'-phosphotransferase [Phreatoba... | 134 | 7e-35 | 55% |
| WP_161358476.1 APH(3') family aminoglycoside O-phosphotransfer... | 136 | 8e-35 | 41% |
| WP_039671574.1 aminoglycoside 3'-phosphotransferase [Enterobac... | 134 | 8e-35 | 35% |
| ECI6722551.1 aminoglycoside 3'-phosphotransferase [Salmonella ... | 134 | 8e-35 | 35% |
| WP_052776689.1 APH(3')-I family aminoglycoside O-phosphotransf... | 134 | 8e-35 | 35% |
| prf\|\|0910185A transferase,aminoglycoside phospho [Bacillus cir... | 135 | 8e-35 | 34% |
| WP_050336696.1 aminoglycoside 3'-phosphotransferase [Klebsiell... | 134 | 8e-35 | 35% |
| WP_109688820.1 aminoglycoside 3'-phosphotransferase [Tumebacil... | 135 | 9e-35 | 32% |
| HAI8042793.1 TPA: aminoglycoside 3'-phosphotransferase [Escher... | 134 | 1e-34 | 34% |
| WP_173914212.1 APH(3') family aminoglycoside O-phosphotransfer... | 135 | 1e-34 | 32% |
| EDU9028648.1 APH(3')-I family aminoglycoside O-phosphotransfer... | 134 | 1e-34 | 35% |
| MBB5909982.1 aminoglycoside phosphotransferase [Actinoalloteic... | 136 | 1e-34 | 37% |
| WP_049406432.1 aminoglycoside 3'-phosphotransferase [Stenotrop... | 133 | 1e-34 | 44% |
| WP_026123491.1 aminoglycoside 3'-phosphotransferase [Nocardiop... | 135 | 1e-34 | 39% |
| SCL15251.1 kanamycin kinase [Micromonospora inyonensis] | 135 | 1e-34 | 35% |
| OJP86789.1 APH(3') family aminoglycoside O-phosphotransferase ... | 134 | 1e-34 | 34% |
| WP_091453436.1 aminoglycoside 3'-phosphotransferase [Micromono... | 135 | 1e-34 | 35% |
| WP_171259834.1 phosphotransferase [Acinetobacter baumannii] | 129 | 1e-34 | 100% |
| WP_060732357.1 aminoglycoside O-phosphotransferase APH(3')-VII... | 135 | 2e-34 | 37% |
| WP_111171390.1 aminoglycoside 3'-phosphotransferase [Desertiac... | 135 | 2e-34 | 38% |
| WP_173385122.1 APH(3') family aminoglycoside O-phosphotransfer... | 135 | 2e-34 | 32% |
| WP_132154252.1 aminoglycoside 3'-phosphotransferase [Kribbella... | 134 | 2e-34 | 38% |
| WP_030600485.1 MULTISPECIES: aminoglycoside O-phosphotransfera... | 135 | 2e-34 | 37% |
| EFS9893368.1 aminoglycoside 3'-phosphotransferase [Salmonella ... | 133 | 2e-34 | 35% |
| OYY78669.1 hypothetical protein B7Y43_05380 [Sphingomonas sp. ... | 134 | 2e-34 | 37% |
| EER7670999.1 aminoglycoside 3'-phosphotransferase [Escherichia... | 133 | 2e-34 | 35% |
| ECJ0937988.1 aminoglycoside 3'-phosphotransferase [Salmonella ... | 133 | 3e-34 | 35% |
| MBF3231000.1 phosphotransferase [Pseudomonas aeruginosa] | 129 | 3e-34 | 63% |

| AMJ37233.1 putative protein [uncultured bacterium] | 128 | 3e-34 | 100% |
| --- | --- | --- | --- |
| WP_017591742.1 aminoglycoside 3'-phosphotransferase [Nocardiop... | 134 | 4e-34 | 37% |
| WP_173369608.1 APH(3') family aminoglycoside O-phosphotransfer... | 134 | 4e-34 | 32% |
| WP_069853331.1 aminoglycoside 3'-phosphotransferase [Actinoall... | 134 | 4e-34 | 37% |
| WP_084445809.1 aminoglycoside 3'-phosphotransferase [Sphingomo... | 134 | 4e-34 | 36% |
| OAN58317.1 APH(3') family aminoglycoside O-phosphotransferase ... | 134 | 4e-34 | 36% |
| WP_173544458.1 APH(3') family aminoglycoside O-phosphotransfer... | 134 | 4e-34 | 32% |
| WP_181608226.1 phosphotransferase [Nonomuraea soli] | 137 | 5e-34 | 41% |
| WP_104091582.1 aminoglycoside 3'-phosphotransferase [Arthrobac... | 133 | 5e-34 | 33% |
| WP_132319058.1 aminoglycoside 3'-phosphotransferase [Kribbella... | 133 | 5e-34 | 37% |
| WP_184794803.1 phosphotransferase [Kribbella italica] | 133 | 5e-34 | 38% |
| EEZ0046766.1 aminoglycoside 3'-phosphotransferase [Escherichia... | 132 | 7e-34 | 35% |
| WP_171955344.1 APH(3') family aminoglycoside O-phosphotransfer... | 134 | 7e-34 | 37% |
| WP_141101373.1 aminoglycoside 3'-phosphotransferase [Acinetoba... | 132 | 8e-34 | 35% |
| WP_083750744.1 aminoglycoside 3'-phosphotransferase [Kribbella... | 133 | 8e-34 | 38% |
| ECC9684632.1 aminoglycoside 3'-phosphotransferase [Salmonella ... | 131 | 8e-34 | 35% |
| EGO7871620.1 aminoglycoside 3'-phosphotransferase [Escherichia... | 131 | 9e-34 | 35% |
| PWK82791.1 kanamycin kinase/aminoglycoside 3'-phosphotransfera... | 132 | 1e-33 | 47% |
| WP_109640141.1 aminoglycoside 3'-phosphotransferase [Lentzea d... | 132 | 1e-33 | 47% |
| ALI88676.1 kanamycin resistance protein [Brucella abortus] | 132 | 1e-33 | 35% |
| WP_146234864.1 phosphotransferase [Pseudomonas aeruginosa] | 128 | 1e-33 | 63% |
| EEN7811349.1 aminoglycoside 3'-phosphotransferase [Salmonella ... | 132 | 1e-33 | 35% |
| WP_111863535.1 aminoglycoside 3'-phosphotransferase [Actinomad... | 133 | 1e-33 | 38% |
| VCV93145.1 aph(3')-Ic_1_X62115 [Klebsiella pneumoniae] | 132 | 1e-33 | 35% |
| ONI73612.1 aminoglycoside 3'-phosphotransferase [Kribbella sp.... | 132 | 1e-33 | 38% |
| SFP66651.1 Phosphotransferase enzyme family protein [Sphingomo... | 130 | 1e-33 | 42% |
| OQW48635.1 hypothetical protein A4S16_05705 [Proteobacteria ba... | 132 | 2e-33 | 38% |
| MQA89568.1 APH(3') family aminoglycoside O-phosphotransferase ... | 133 | 2e-33 | 32% |
| WP_150246936.1 aminoglycoside 3'-phosphotransferase [Streptomy... | 132 | 2e-33 | 38% |
| WP_046681131.1 aminoglycoside 3'-phosphotransferase [Paenibaci... | 131 | 2e-33 | 35% |
| MZH02764.1 aminoglycoside 3'-phosphotransferase [Nitrospinae b... | 132 | 2e-33 | 32% |
| WP_168188754.1 APH(3') family aminoglycoside O-phosphotransfer... | 132 | 2e-33 | 36% |
| WP_112229593.1 aminoglycoside 3'-phosphotransferase [Lentzea a... | 131 | 2e-33 | 46% |
| RAL24048.1 aminoglycoside 3'-phosphotransferase [Thermoflavimi... | 132 | 2e-33 | 36% |
| WP_189255392.1 phosphotransferase [Lentzea flava] | 131 | 2e-33 | 45% |
| RAS62568.1 kanamycin kinase/aminoglycoside 3'-phosphotransfera... | 131 | 3e-33 | 46% |
| WP_161554099.1 APH(3') family aminoglycoside O-phosphotransfer... | 131 | 3e-33 | 35% |
| WP_083933363.1 aminoglycoside 3'-phosphotransferase [Kribbella... | 133 | 3e-33 | 39% |
| WP_188104449.1 APH(3') family aminoglycoside O-phosphotransfer... | 131 | 3e-33 | 38% |
| WP_189125947.1 phosphotransferase [Nonomuraea cavernae] | 131 | 3e-33 | 38% |
| MBE6930105.1 aminoglycoside 3'-phosphotransferase [Ruminococca... | 132 | 4e-33 | 36% |
| WP_130455359.1 aminoglycoside 3'-phosphotransferase [Streptomy... | 132 | 4e-33 | 40% |
| WP_018852513.1 aminoglycoside 3'-phosphotransferase [Streptomy... | 132 | 4e-33 | 40% |
| ECC9684601.1 aminoglycoside 3'-phosphotransferase [Salmonella ... | 130 | 4e-33 | 35% |
| WP_037741748.1 aminoglycoside 3'-phosphotransferase [Streptomy... | 131 | 4e-33 | 40% |
| WP_113613819.1 aminoglycoside 3'-phosphotransferase [Chitinoph... | 131 | 4e-33 | 34% |
| WP_103996040.1 aminoglycoside O-phosphotransferase APH(3')-IVa... | 131 | 5e-33 | 34% |
| WP_092774576.1 aminoglycoside 3'-phosphotransferase [Actinokin... | 130 | 5e-33 | 37% |
| WP_184669290.1 APH(3') family aminoglycoside O-phosphotransfer... | 130 | 5e-33 | 39% |
| WP_097062732.1 APH(3')-I family aminoglycoside O-phosphotransf... | 131 | 5e-33 | 40% |
| WP_115377085.1 aminoglycoside 3'-phosphotransferase [Sphingomo... | 131 | 5e-33 | 41% |
| TVZ88585.1 kanamycin kinase/aminoglycoside 3'-phosphotransfera... | 131 | 6e-33 | 40% |
| WP_195084016.1 APH(3') family aminoglycoside O-phosphotransfer... | 134 | 6e-33 | 41% |
| WP_186543190.1 APH(3') family aminoglycoside O-phosphotransfer... | 131 | 6e-33 | 40% |
| WP_184090413.1 APH(3') family aminoglycoside O-phosphotransfer... | 131 | 6e-33 | 34% |
| WP_104481443.1 aminoglycoside 3'-phosphotransferase [Actinokin... | 131 | 7e-33 | 37% |
| AOS61996.1 aminoglycoside phosphotransferase [Actinoalloteichu... | 130 | 7e-33 | 38% |
| WP_066706119.1 aminoglycoside 3'-phosphotransferase [Sphingomo... | 130 | 8e-33 | 42% |
| WP_018836270.1 MULTISPECIES: aminoglycoside 3'-phosphotransfer... | 131 | 8e-33 | 40% |
| WP_153759029.1 aminoglycoside 3'-phosphotransferase [Actinomar... | 130 | 1e-32 | 36% |
| WP_184844769.1 APH(3') family aminoglycoside O-phosphotransfer... | 130 | 1e-32 | 35% |
| WP_170176519.1 aminoglycoside 3'-phosphotransferase [Lentzea f... | 129 | 1e-32 | 46% |
| CAH60150.1 putative phosphotransferase [Streptomyces tenjimari... | 130 | 1e-32 | 36% |
| MBB5983708.1 aminoglycoside phosphotransferase [Kribbella solani] | 130 | 1e-32 | 35% |
| WP_121392666.1 aminoglycoside 3'-phosphotransferase [Actinokin... | 130 | 1e-32 | 36% |
| WP_003939835.1 aminoglycoside 3'-phosphotransferase [Rhodococc... | 130 | 1e-32 | 36% |
| WP_191094260.1 tetratricopeptide repeat protein [Nocardia colli] | 133 | 1e-32 | 40% |
| WP_078978824.1 aminoglycoside 3'-phosphotransferase [Embleya s... | 130 | 1e-32 | 41% |
| WP_170286412.1 aminoglycoside 3'-phosphotransferase [Micromono... | 130 | 1e-32 | 39% |
| WP_131353003.1 aminoglycoside 3'-phosphotransferase [Kribbella... | 129 | 2e-32 | 38% |
| KHX50299.1 aminoglycoside phosphotransferase [Acinetobacter ba... | 128 | 2e-32 | 34% |
| ETR59139.1 phosphotransferase enzyme family protein [Acinetoba... | 128 | 2e-32 | 34% |

| KAA8884602.1 tetratricopeptide repeat protein [Nocardia colli] | 133 | 2e-32 | 40% |
| --- | --- | --- | --- |
| WP_024439789.1 aminoglycoside 3'-phosphotransferase [Acinetoba... | 129 | 2e-32 | 35% |
| WP_132333296.1 aminoglycoside 3'-phosphotransferase [Kribbella... | 129 | 2e-32 | 36% |
| WP_193159497.1 APH(3') family aminoglycoside O-phosphotransfer... | 128 | 2e-32 | 35% |
| EFR5949010.1 aminoglycoside 3'-phosphotransferase [Salmonella ... | 128 | 2e-32 | 35% |
| WP_160592789.1 APH(3') family aminoglycoside O-phosphotransfer... | 129 | 2e-32 | 37% |
| ABI20448.1 truncated aminoglycoside-phosphotransferase [uncult... | 128 | 2e-32 | 35% |
| WP_082730454.1 aminoglycoside 3'-phosphotransferase [Sphingomo... | 131 | 2e-32 | 38% |
| EFH9753228.1 aminoglycoside 3'-phosphotransferase [Escherichia... | 128 | 2e-32 | 35% |
| WP_023560527.1 aminoglycoside 3'-phosphotransferase [Actinopla... | 128 | 2e-32 | 41% |
| WP_181890175.1 APH(3') family aminoglycoside O-phosphotransfer... | 127 | 2e-32 | 35% |
| WP_083863766.1 phosphotransferase [Nocardia exalbida] | 132 | 3e-32 | 40% |
| WP_131515023.1 aminoglycoside 3'-phosphotransferase [Kribbella... | 128 | 3e-32 | 38% |
| NMA62068.1 aminoglycoside 3'-phosphotransferase [Firmicutes ba... | 129 | 3e-32 | 35% |
| MBA3652971.1 aminoglycoside 3'-phosphotransferase [Actinobacte... | 129 | 3e-32 | 39% |
| WP_072034762.1 aminoglycoside 3'-phosphotransferase [Klebsiell... | 127 | 3e-32 | 35% |
| WP_131517120.1 aminoglycoside 3'-phosphotransferase [Kribbella... | 129 | 3e-32 | 38% |
| WP_134123342.1 aminoglycoside 3'-phosphotransferase [Kribbella... | 129 | 3e-32 | 38% |
| WP_086787052.1 aminoglycoside 3'-phosphotransferase [Crossiell... | 129 | 3e-32 | 41% |
| AAF71712.1 aminoglycoside 3'-phosphotransferase [Cloning vecto... | 127 | 3e-32 | 35% |
| REF95389.1 kanamycin kinase/aminoglycoside 3'-phosphotransfera... | 128 | 3e-32 | 37% |
| MBF3061302.1 phosphotransferase [Pseudomonas aeruginosa] | 124 | 3e-32 | 59% |
| WP_105615800.1 aminoglycoside 3'-phosphotransferase [Vallitale... | 129 | 4e-32 | 32% |
| WP_158281693.1 MULTISPECIES: aminoglycoside 3'-phosphotransfer... | 129 | 4e-32 | 31% |
| WP_028521182.1 aminoglycoside 3'-phosphotransferase [Ruminococ... | 129 | 4e-32 | 34% |
| WP_074303118.1 phosphotransferase [Pseudomonas aeruginosa] | 124 | 4e-32 | 66% |
| WP_130446463.1 aminoglycoside 3'-phosphotransferase [Kribbella... | 128 | 4e-32 | 39% |
| WP_184388699.1 APH(3') family aminoglycoside O-phosphotransfer... | 129 | 4e-32 | 39% |
| WP_171678809.1 phosphotransferase [Kribbella sandramycini] | 128 | 6e-32 | 39% |
| WP_108992919.1 aminoglycoside 3'-phosphotransferase [Paenibaci... | 128 | 6e-32 | 30% |
| WP_132208890.1 aminoglycoside 3'-phosphotransferase [Kribbella... | 128 | 6e-32 | 38% |
| WP_093298673.1 aminoglycoside 3'-phosphotransferase [Sphingomo... | 128 | 6e-32 | 37% |
| HDU43823.1 TPA: aminoglycoside 3'-phosphotransferase [Chlorofl... | 129 | 6e-32 | 33% |
| WP_168874695.1 APH(3') family aminoglycoside O-phosphotransfer... | 128 | 7e-32 | 36% |
| WP_164978027.1 aminoglycoside 3'-phosphotransferase [Geobacill... | 127 | 7e-32 | 34% |
| SCL39608.1 kanamycin kinase [Micromonospora pallida] | 128 | 8e-32 | 35% |
| WP_093621166.1 aminoglycoside 3'-phosphotransferase [Actinopla... | 127 | 8e-32 | 41% |
| WP_078673223.1 aminoglycoside 3'-phosphotransferase [Chitinoph... | 127 | 9e-32 | 37% |
| WP_110035759.1 tetratricopeptide repeat protein [Nocardia neoc... | 131 | 9e-32 | 40% |
| NYF92372.1 aminoglycoside phosphotransferase [Pseudomonas kunm... | 123 | 9e-32 | 64% |
| WP_067636336.1 tetratricopeptide repeat protein [Nocardia coub... | 131 | 1e-31 | 41% |
| WP_067492732.1 tetratricopeptide repeat protein [Nocardia igno... | 131 | 1e-31 | 41% |
| MBA3878379.1 APH(3') family aminoglycoside O-phosphotransferas... | 127 | 1e-31 | 37% |
| WP_147925344.1 aminoglycoside 3'-phosphotransferase [Quadrisph... | 127 | 1e-31 | 41% |
| WP_084499126.1 phosphotransferase [Nocardia gamkensis] | 130 | 1e-31 | 39% |
| WP_177320223.1 aminoglycoside 3'-phosphotransferase [Lentzea w... | 127 | 1e-31 | 43% |
| WP_187695854.1 phosphotransferase [Georgenia faecalis] | 127 | 1e-31 | 38% |
| EBI6767325.1 aminoglycoside 3'-phosphotransferase [Salmonella ... | 125 | 1e-31 | 35% |
| WP_063102474.1 aminoglycoside 3'-phosphotransferase [Escherich... | 126 | 1e-31 | 34% |
| NQE71323.1 Aminoglycoside 3'-phosphotransferase [Nocardia gamk... | 130 | 1e-31 | 39% |
| WP_084497735.1 phosphotransferase [Nocardia amamiensis] | 130 | 2e-31 | 40% |
| APX42445.1 aminoglycoside 3'-phosphotransferase [Acinetobacter... | 125 | 2e-31 | 34% |
| WP_148852057.1 phosphotransferase [Streptococcus pyogenes] | 124 | 2e-31 | 48% |
| WP_127573348.1 aminoglycoside 3'-phosphotransferase [Georgenia... | 128 | 2e-31 | 38% |
| VCX04805.1 aph(3')-Ic_1_X62115 [Escherichia coli] | 126 | 2e-31 | 35% |
| OEV08409.1 aminoglycoside phosphotransferase [Streptomyces nan... | 127 | 2e-31 | 39% |
| RDI64828.1 kanamycin kinase/aminoglycoside 3'-phosphotransfera... | 127 | 2e-31 | 40% |
| WP_052788994.1 MULTISPECIES: aminoglycoside 3'-phosphotransfer... | 125 | 2e-31 | 35% |
| WP_049406330.1 phosphotransferase [Stenotrophomonas maltophilia] | 122 | 2e-31 | 61% |
| WP_195125636.1 phosphotransferase [Nocardia puris] | 130 | 2e-31 | 41% |
| WP_116075762.1 aminoglycoside 3'-phosphotransferase [Asanoa fe... | 127 | 2e-31 | 37% |
| WP_084538225.1 phosphotransferase [Nocardia puris] | 130 | 2e-31 | 41% |
| WP_169225715.1 APH(3') family aminoglycoside O-phosphotransfer... | 127 | 2e-31 | 36% |
| WP_139712342.1 aminoglycoside 3'-phosphotransferase [Streptomy... | 126 | 2e-31 | 40% |
| WP_134003682.1 aminoglycoside 3'-phosphotransferase [Kribbella... | 127 | 2e-31 | 37% |
| WP_168802990.1 APH(3') family aminoglycoside O-phosphotransfer... | 127 | 2e-31 | 36% |
| EBQ6784656.1 APH(3') family aminoglycoside O-phosphotransferas... | 120 | 2e-31 | 100% |
| WP_079132624.1 aminoglycoside 3'-phosphotransferase [Streptomy... | 127 | 2e-31 | 39% |
| WP_101422413.1 APH(3'') family aminoglycoside O-phosphotransfe... | 127 | 2e-31 | 39% |
| KLD71911.1 aminoglycoside phosphotransferase [Xanthomonas pisi... | 127 | 2e-31 | 36% |
| WP_176967972.1 aminoglycoside 3'-phosphotransferase [Proteinib... | 127 | 2e-31 | 31% |
| MBA4013121.1 aminoglycoside 3'-phosphotransferase [Phenylobact... | 126 | 2e-31 | 39% |

| WP_166665481.1 phosphotransferase [Kribbella sp. VKM Ac-2527] | 126 | 3e-31 | 37% |
| --- | --- | --- | --- |
| WP_082875771.1 aminoglycoside 3'-phosphotransferase [Nocardia ... | 126 | 3e-31 | 40% |
| NLA06494.1 aminoglycoside 3'-phosphotransferase [Firmicutes ba... | 126 | 3e-31 | 34% |
| RYE99778.1 hypothetical protein EOO78_13415 [Oxalobacteraceae ... | 123 | 3e-31 | 63% |
| MPZ39534.1 APH(3') family aminoglycoside O-phosphotransferase ... | 127 | 3e-31 | 35% |
| NNM34024.1 aminoglycoside 3'-phosphotransferase [Gemmatimonade... | 127 | 3e-31 | 35% |
| RBO96549.1 kanamycin kinase [Nocardia puris] | 130 | 3e-31 | 41% |
| WP_161839731.1 APH(3') family aminoglycoside O-phosphotransfer... | 126 | 3e-31 | 33% |
| WP_083889512.1 tetratricopeptide repeat protein [Nocardia pneu... | 129 | 4e-31 | 39% |
| WP_046963058.1 aminoglycoside 3'-phosphotransferase [Xanthomon... | 127 | 4e-31 | 35% |
| WP_178127683.1 phosphotransferase [Nocardia cyriacigeorgica] | 125 | 4e-31 | 37% |
| WP_030675367.1 aminoglycoside 3'-phosphotransferase [Streptomy... | 126 | 4e-31 | 39% |
| WP_164369777.1 phosphotransferase [Streptomyces sp. SID14446] | 126 | 4e-31 | 39% |
| WP_194992226.1 phosphotransferase [Nocardia cyriacigeorgica] | 125 | 4e-31 | 37% |
| NEW26543.1 aminoglycoside 3'-phosphotransferase [Nocardia cyri... | 125 | 5e-31 | 37% |
| WP_167217641.1 phosphotransferase [Kribbella shirazensis] | 125 | 5e-31 | 38% |
| MBB4676750.1 kanamycin kinase/aminoglycoside 3'-phosphotransfe... | 125 | 5e-31 | 37% |
| TDO47800.1 streptomycin 3'-kinase [Kribbella sp. VKM Ac-2527] | 126 | 5e-31 | 37% |
| WP_166801749.1 phosphotransferase [Pseudomonas aeruginosa] | 120 | 5e-31 | 68% |
| WP_096613385.1 aminoglycoside 3'-phosphotransferase [Sphingomo... | 126 | 5e-31 | 39% |
| WP_185010644.1 APH(3') family aminoglycoside O-phosphotransfer... | 125 | 5e-31 | 37% |
| PZU14140.1 APH(3') family aminoglycoside O-phosphotransferase ... | 125 | 6e-31 | 34% |
| WP_176738592.1 APH(3') family aminoglycoside O-phosphotransfer... | 125 | 6e-31 | 37% |
| WP_137786202.1 aminoglycoside 3'-phosphotransferase [Sphingomo... | 125 | 6e-31 | 35% |
| WP_036723013.1 aminoglycoside 3'-phosphotransferase [Paenibaci... | 125 | 6e-31 | 31% |
| WP_093863642.1 aminoglycoside 3'-phosphotransferase [Streptomy... | 125 | 7e-31 | 37% |
| WP_101363327.1 aminoglycoside 3'-phosphotransferase [Xanthomon... | 126 | 8e-31 | 35% |
| WP_113701844.1 aminoglycoside 3'-phosphotransferase [Nonomurae... | 125 | 8e-31 | 39% |
| WP_148108058.1 aminoglycoside 3'-phosphotransferase [Streptomy... | 124 | 9e-31 | 39% |
| WP_195019680.1 phosphotransferase [Nocardia cyriacigeorgica] | 125 | 9e-31 | 37% |
| KYJ34089.1 aminoglycoside phosphotransferase [Salmonella enter... | 125 | 9e-31 | 34% |
| NUT90696.1 aminoglycoside 3'-phosphotransferase [Saccharothrix... | 124 | 9e-31 | 41% |
| SDT82450.1 kanamycin kinase/aminoglycoside 3'-phosphotransfera... | 125 | 9e-31 | 37% |
| WP_132150830.1 aminoglycoside 3'-phosphotransferase [Actinomad... | 125 | 9e-31 | 39% |
| WP_052793627.1 aminoglycoside 3'-phosphotransferase [Acinetoba... | 123 | 1e-30 | 35% |
| WP_066488254.1 aminoglycoside 3'-phosphotransferase [Sphingomo... | 125 | 1e-30 | 39% |
| GES13330.1 aminoglycoside O-phosphotransferase APH(3')-IIb [Ac... | 124 | 1e-30 | 38% |
| EEZ9267516.1 aminoglycoside 3'-phosphotransferase [Escherichia... | 123 | 1e-30 | 35% |
| WP_179266046.1 phosphotransferase [Asanoa hainanensis] | 122 | 1e-30 | 44% |
| WP_121220423.1 aminoglycoside 3'-phosphotransferase [Saccharot... | 124 | 1e-30 | 38% |
| WP_083511614.1 aminoglycoside 3'-phosphotransferase [Alicyclob... | 124 | 1e-30 | 33% |
| WP_131353922.1 aminoglycoside 3'-phosphotransferase [Kribbella... | 124 | 1e-30 | 37% |
| WP_088981628.1 aminoglycoside 3'-phosphotransferase [Micromono... | 125 | 1e-30 | 34% |
| WP_082364389.1 aminoglycoside 3'-phosphotransferase [Novosphin... | 125 | 1e-30 | 37% |
| WP_155358585.1 aminoglycoside 3'-phosphotransferase [Acrocarpo... | 124 | 1e-30 | 38% |
| RZU14296.1 streptomycin 3'-kinase [Kribbella soli] | 124 | 1e-30 | 39% |
| SCE97136.1 kanamycin kinase [Micromonospora echinospora] | 125 | 1e-30 | 34% |
| AAR98555.1 GntI [Micromonospora echinospora] | 125 | 1e-30 | 34% |
| WP_049566424.1 aminoglycoside 3'-phosphotransferase [Nonomurae... | 124 | 2e-30 | 37% |
| RKR24719.1 aminoglycoside 3'-phosphotransferase-1 [Sphingomona... | 124 | 2e-30 | 36% |
| WP_181696939.1 tetratricopeptide repeat protein [Nocardia sp. ... | 128 | 2e-30 | 40% |
| WP_116951507.1 aminoglycoside 3'-phosphotransferase [Jiangella... | 124 | 2e-30 | 40% |
| WP_186542827.1 APH(3') family aminoglycoside O-phosphotransfer... | 124 | 2e-30 | 34% |
| HAK8359886.1 TPA: aminoglycoside 3'-phosphotransferase [Salmon... | 122 | 2e-30 | 34% |
| WP_076156175.1 aminoglycoside 3'-phosphotransferase [Paenibaci... | 124 | 2e-30 | 30% |
| MAS38059.1 hypothetical protein [Anaerolineaceae bacterium] | 124 | 2e-30 | 34% |
| WP_130383104.1 aminoglycoside 3'-phosphotransferase [Kribbella... | 124 | 2e-30 | 39% |
| WP_067980783.1 tetratricopeptide repeat protein [Nocardia cais... | 127 | 2e-30 | 39% |
| WP_183772452.1 phosphotransferase [Phenylobacterium haematophi... | 124 | 2e-30 | 39% |
| WP_168860020.1 APH(3') family aminoglycoside O-phosphotransfer... | 124 | 2e-30 | 35% |
| RIL71582.1 APH(3') family aminoglycoside O-phosphotransferase ... | 117 | 3e-30 | 100% |
| HAI8935615.1 TPA: aminoglycoside 3'-phosphotransferase [Escher... | 122 | 3e-30 | 34% |
| PXX54633.1 kanamycin kinase [Nocardia tenerifensis] | 127 | 3e-30 | 40% |
| WP_080898286.1 APH(3'') family aminoglycoside O-phosphotransfe... | 124 | 3e-30 | 36% |
| ELQ83470.1 aminoglycoside phosphotransferase [Streptomyces rim... | 122 | 3e-30 | 39% |
| WP_040737017.1 tetratricopeptide repeat protein [Nocardia tene... | 127 | 3e-30 | 40% |
| OLE22789.1 aminoglycoside phosphotransferase APH(3') [Actinoba... | 123 | 3e-30 | 42% |
| WP_104588071.1 aminoglycoside 3'-phosphotransferase [Xanthomon... | 124 | 3e-30 | 35% |
| WP_009877462.1 aminoglycoside phosphotransferase APH(3') [Pseu... | 118 | 3e-30 | 70% |
| MBB3891137.1 aminoglycoside phosphotransferase [Phenylobacteri... | 124 | 3e-30 | 39% |
| WP_146358961.1 aminoglycoside 3'-phosphotransferase [Lentzea s... | 124 | 3e-30 | 42% |
| WP_054972947.1 aminoglycoside 3'-phosphotransferase [Paenibaci... | 124 | 4e-30 | 30% |

| WP_138447300.1 aminoglycoside 3'-phosphotransferase [Nocardia ... | 123 | 4e-30 | 40% |
| --- | --- | --- | --- |
| CNP00675.1 aminoglycoside 3'-phosphotransferase [Salmonella en... | 122 | 4e-30 | 34% |
| WP_081222323.1 aminoglycoside 3'-phosphotransferase [Streptomy... | 124 | 4e-30 | 37% |
| OMF83756.1 hypothetical protein BK146_32910 [Paenibacillus sp.... | 124 | 4e-30 | 30% |
| WP_138147029.1 aminoglycoside 3'-phosphotransferase [Acinetoba... | 121 | 4e-30 | 37% |
| EFB1701068.1 aminoglycoside 3'-phosphotransferase [Escherichia... | 121 | 4e-30 | 37% |
| WP_078616340.1 aminoglycoside 3'-phosphotransferase [Streptomy... | 124 | 4e-30 | 40% |
| WP_084466685.1 phosphotransferase [Nocardia arthritidis] | 126 | 4e-30 | 39% |
| WP_080943036.1 MULTISPECIES: aminoglycoside 3'-phosphotransfer... | 122 | 4e-30 | 39% |
| WP_162884194.1 APH(3') family aminoglycoside O-phosphotransfer... | 121 | 4e-30 | 37% |
| EDU5774928.1 aminoglycoside 3'-phosphotransferase [Salmonella ... | 122 | 4e-30 | 34% |
| WP_057524592.1 aminoglycoside 3'-phosphotransferase [Salmonell... | 121 | 4e-30 | 34% |
| WP_084581996.1 aminoglycoside 3'-phosphotransferase [Sphingomo... | 124 | 4e-30 | 36% |
| HAH5529195.1 TPA: aminoglycoside 3'-phosphotransferase [Escher... | 121 | 4e-30 | 37% |
| HAH5531112.1 TPA: aminoglycoside 3'-phosphotransferase [Escher... | 121 | 4e-30 | 37% |
| WP_139774317.1 aminoglycoside 3'-phosphotransferase [Salmonell... | 121 | 5e-30 | 37% |
| WP_164587667.1 phosphotransferase [Streptomyces sp. SID10815] | 123 | 5e-30 | 36% |
| AVE22111.1 Aminoglycoside phosphotransferase [Pseudomonas aeru... | 121 | 5e-30 | 34% |
| WP_119659018.1 aminoglycoside 3'-phosphotransferase [Jiangella... | 122 | 5e-30 | 41% |
| WP_148032831.1 aminoglycoside 3'-phosphotransferase [Nonomurae... | 122 | 5e-30 | 41% |
| SNS85446.1 kanamycin kinase/aminoglycoside 3'-phosphotransfera... | 120 | 5e-30 | 46% |
| SBL27554.1 aminoglycoside 3'-phosphotransferase [Klebsiella mi... | 121 | 5e-30 | 34% |
| EDJ0631423.1 APH(3') family aminoglycoside O-phosphotransferas... | 121 | 5e-30 | 37% |
| EBT7277217.1 aminoglycoside 3'-phosphotransferase [Salmonella ... | 121 | 5e-30 | 37% |
| WP_031975521.1 MULTISPECIES: aminoglycoside 3'-phosphotransfer... | 121 | 5e-30 | 34% |
| WP_091764248.1 aminoglycoside 3'-phosphotransferase [Blastococ... | 123 | 5e-30 | 39% |
| EFO5638188.1 aminoglycoside 3'-phosphotransferase [Escherichia... | 121 | 5e-30 | 37% |
| ACJ04635.1 phosphotransferase GTC17 [Micromonospora inyonensis] | 123 | 5e-30 | 34% |
| WP_132291351.1 aminoglycoside 3'-phosphotransferase [Kribbella... | 123 | 6e-30 | 36% |
| NUP50311.1 aminoglycoside 3'-phosphotransferase [Catenulispora... | 123 | 6e-30 | 37% |
| GGA37746.1 aminoglycoside 3'-phosphotransferase [Sphingomonas ... | 122 | 6e-30 | 35% |
| EGN4569121.1 aminoglycoside 3'-phosphotransferase [Salmonella ... | 121 | 6e-30 | 37% |
| WP_072297598.1 unnamed protein product | 121 | 6e-30 | 37% |
| WP_017537684.1 aminoglycoside 3'-phosphotransferase [Nocardiop... | 123 | 6e-30 | 38% |
| EFB7019667.1 aminoglycoside 3'-phosphotransferase [Salmonella ... | 121 | 6e-30 | 37% |
| EBB4213149.1 aminoglycoside 3'-phosphotransferase [Salmonella ... | 121 | 6e-30 | 37% |
| WP_100030936.1 aminoglycoside 3'-phosphotransferase [Escherich... | 121 | 6e-30 | 37% |
| WP_131460472.1 aminoglycoside 3'-phosphotransferase [Kribbella... | 123 | 6e-30 | 37% |
| HAS0712517.1 TPA: aminoglycoside 3'-phosphotransferase [Entero... | 121 | 6e-30 | 37% |
| EYI83950.1 aminoglycoside phosphotransferase [Salmonella enter... | 117 | 7e-30 | 100% |
| EFO5638290.1 aminoglycoside 3'-phosphotransferase [Escherichia... | 121 | 7e-30 | 37% |
| WP_084131325.1 aminoglycoside 3'-phosphotransferase [Actinocat... | 123 | 7e-30 | 40% |
| WP_107439308.1 aminoglycoside 3'-phosphotransferase [Streptomy... | 123 | 7e-30 | 37% |
| SDF10221.1 kanamycin kinase [Blastococcus sp. DSM 44268] | 123 | 7e-30 | 39% |
| WP_030408553.1 aminoglycoside 3'-phosphotransferase [Streptomy... | 123 | 7e-30 | 39% |
| WP_032122478.1 aminoglycoside 3'-phosphotransferase [Clostridi... | 123 | 7e-30 | 32% |
| EGQ5547569.1 aminoglycoside 3'-phosphotransferase [Salmonella ... | 120 | 7e-30 | 37% |
| WP_000530949.1 MULTISPECIES: aminoglycoside 3'-phosphotransfer... | 120 | 8e-30 | 37% |
| MBD4578037.1 APH(3') family aminoglycoside O-phosphotransferas... | 116 | 8e-30 | 100% |
| WP_046770091.1 aminoglycoside 3'-phosphotransferase [Jiangella... | 122 | 8e-30 | 40% |
| WP_111324629.1 aminoglycoside 3'-phosphotransferase [Thermospo... | 123 | 8e-30 | 36% |
| WP_063081063.1 aminoglycoside 3'-phosphotransferase [Escherich... | 120 | 8e-30 | 37% |
| WP_157555369.1 phosphotransferase [Nocardia crassostreae] | 121 | 9e-30 | 44% |
| WP_031175004.1 MULTISPECIES: aminoglycoside 3'-phosphotransfer... | 123 | 9e-30 | 39% |
| ECJ0937968.1 aminoglycoside 3'-phosphotransferase [Salmonella ... | 121 | 9e-30 | 34% |
| WP_049649986.1 aminoglycoside 3'-phosphotransferase [Kitasatos... | 123 | 1e-29 | 38% |
| SYL43168.1 aminoglycoside 3'-phosphotransferase [Klebsiella pn... | 121 | 1e-29 | 37% |
| WP_017555865.1 aminoglycoside 3'-phosphotransferase [Nocardiop... | 122 | 1e-29 | 38% |
| WP_100250246.1 aminoglycoside 3'-phosphotransferase [Escherich... | 120 | 1e-29 | 37% |
| WP_110818326.1 aminoglycoside 3'-phosphotransferase [Pseudomon... | 120 | 1e-29 | 41% |
| WP_146226922.1 MULTISPECIES: aminoglycoside 3'-phosphotransfer... | 119 | 1e-29 | 41% |
| WP_119994777.1 aminoglycoside 3'-phosphotransferase [Streptomy... | 122 | 1e-29 | 37% |
| BCL14533.1 aminoglycoside 3'-phosphotransferase [Micromonospor... | 122 | 1e-29 | 33% |
| WP_144973331.1 MULTISPECIES: aminoglycoside 3'-phosphotransfer... | 122 | 1e-29 | 31% |
| WP_133980050.1 aminoglycoside 3'-phosphotransferase [Kribbella... | 122 | 1e-29 | 35% |
| WP_132414174.1 aminoglycoside 3'-phosphotransferase [Kribbella... | 122 | 1e-29 | 35% |
| AVR63931.1 Aminoglycoside 3'-phosphotransferase [Escherichia c... | 119 | 1e-29 | 41% |
| WP_036699958.1 aminoglycoside 3'-phosphotransferase [Paenibaci... | 122 | 2e-29 | 30% |
| TXJ26811.1 aminoglycoside 3'-phosphotransferase [Chitinophagac... | 122 | 2e-29 | 32% |
| AEO09370.1 aminoglycoside (3') phosphotransferase [Escherichia... | 120 | 2e-29 | 35% |
| TWJ26582.1 kanamycin kinase [Micromonospora sagamiensis] | 122 | 2e-29 | 33% |
| WP_023273927.1 aminoglycoside 3'-phosphotransferase [Acinetoba... | 122 | 2e-29 | 31% |

| MBF3260288.1 phosphotransferase [Pseudomonas aeruginosa] | 116 | 2e-29 | 70% |
| --- | --- | --- | --- |
| WP_083880701.1 phosphotransferase [Nocardia araoensis] | 125 | 2e-29 | 43% |
| WP_134130738.1 aminoglycoside 3'-phosphotransferase [Kribbella... | 122 | 2e-29 | 36% |
| WP_089911813.1 aminoglycoside 3'-phosphotransferase [Lentzea a... | 121 | 2e-29 | 41% |
| EPD91711.1 hypothetical protein HMPREF1486_04670 [Streptomyces... | 122 | 2e-29 | 39% |
| WP_132102022.1 aminoglycoside 3'-phosphotransferase [Jiangella... | 121 | 2e-29 | 40% |
| WP_194836020.1 phosphotransferase [Nocardia sp. XZ_19_369] | 124 | 2e-29 | 39% |
| WP_137160840.1 aminoglycoside 3'-phosphotransferase [Blastococ... | 121 | 2e-29 | 41% |
| EEY3559616.1 aminoglycoside 3'-phosphotransferase [Escherichia... | 119 | 2e-29 | 37% |
| WP_167769109.1 phosphotransferase [Nocardia sp. CS682] | 124 | 2e-29 | 39% |
| WP_074002549.1 aminoglycoside 3'-phosphotransferase [Streptomy... | 122 | 2e-29 | 38% |
| WP_131988083.1 aminoglycoside 3'-phosphotransferase [Jiangella... | 120 | 3e-29 | 40% |
| WP_134160043.1 aminoglycoside 3'-phosphotransferase [Alicyclob... | 121 | 3e-29 | 32% |
| WP_107660735.1 phosphotransferase [Nocardia suismassiliense] | 125 | 3e-29 | 40% |
| WP_113703793.1 aminoglycoside 3'-phosphotransferase [Nonomurae... | 122 | 3e-29 | 34% |
| EEY3560375.1 aminoglycoside 3'-phosphotransferase [Escherichia... | 119 | 3e-29 | 37% |
| WP_055498058.1 aminoglycoside 3'-phosphotransferase [Streptomy... | 121 | 3e-29 | 38% |
| NUR59009.1 aminoglycoside 3'-phosphotransferase [Catenulispora... | 121 | 3e-29 | 35% |
| WP_035793651.1 aminoglycoside 3'-phosphotransferase [Kitasatos... | 121 | 3e-29 | 38% |
| WP_012859852.1 aminoglycoside 3'-phosphotransferase [Sebaldell... | 121 | 3e-29 | 30% |
| WP_159838313.1 APH(3') family aminoglycoside O-phosphotransfer... | 124 | 3e-29 | 42% |
| WP_185082336.1 phosphotransferase [Nonomuraea muscovyensis] | 120 | 3e-29 | 37% |
| WP_131295699.1 aminoglycoside 3'-phosphotransferase [Kribbella... | 121 | 3e-29 | 37% |
| WP_110768803.1 aminoglycoside 3'-phosphotransferase [Pseudomon... | 117 | 3e-29 | 42% |
| AQW48827.1 aminoglycoside phosphotransferase [Streptomyces hyg... | 123 | 4e-29 | 37% |
| WP_051784226.1 aminoglycoside 3'-phosphotransferase [Lentzea a... | 120 | 4e-29 | 43% |
| WP_184827348.1 APH(3') family aminoglycoside O-phosphotransfer... | 120 | 4e-29 | 40% |
| WP_062987995.1 tetratricopeptide repeat protein [Nocardia salm... | 124 | 4e-29 | 39% |
| WP_140367094.1 aminoglycoside 3'-phosphotransferase [Vibrio pa... | 119 | 4e-29 | 36% |
| WP_076189781.1 aminoglycoside 3'-phosphotransferase [Salmonell... | 120 | 4e-29 | 34% |
| QBS45691.1 aminoglycoside phosphotransferase APH(3') [Nocardia... | 124 | 4e-29 | 39% |
| OTT00321.1 APH(3') family aminoglycoside O-phosphotransferase ... | 119 | 4e-29 | 34% |
| TKJ20922.1 aminoglycoside phosphotransferase APH(3') [Blastoco... | 121 | 4e-29 | 41% |
| TDJ08224.1 aminoglycoside 3'-phosphotransferase [Deltaproteoba... | 120 | 4e-29 | 30% |
| WP_141858273.1 aminoglycoside 3'-phosphotransferase [Kribbella... | 120 | 4e-29 | 37% |
| AFR43495.1 aminoglycoside 3'-phosphotransferase [Salmonella en... | 119 | 5e-29 | 36% |
| WP_140408800.1 aminoglycoside 3'-phosphotransferase [Acinetoba... | 119 | 5e-29 | 34% |
| WP_139846315.1 aminoglycoside 3'-phosphotransferase [Acinetoba... | 119 | 5e-29 | 34% |
| AMP56023.1 hypothetical protein [uncultured bacterium] | 114 | 5e-29 | 100% |
| WP_194895346.1 phosphotransferase [Catenulispora sp. NF3] | 120 | 5e-29 | 36% |
| WP_063004506.1 tetratricopeptide repeat protein [Nocardia salm... | 124 | 5e-29 | 39% |
| WP_170989686.1 phosphotransferase [Kribbella sp. NEAU-THZ 27] | 120 | 6e-29 | 36% |
| WP_051358536.1 MULTISPECIES: aminoglycoside 3'-phosphotransfer... | 120 | 6e-29 | 32% |
| TKK81933.1 aminoglycoside 3'-phosphotransferase [Kribbella sp.... | 120 | 6e-29 | 36% |
| WP_032460838.1 aminoglycoside 3'-phosphotransferase [Streptoco... | 119 | 6e-29 | 33% |
| WP_078957228.1 aminoglycoside 3'-phosphotransferase [Streptomy... | 120 | 7e-29 | 35% |
| WP_117487004.1 aminoglycoside 3'-phosphotransferase [Kitasatos... | 120 | 7e-29 | 38% |
| MBE6144746.1 aminoglycoside 3'-phosphotransferase [Firmicutes ... | 120 | 7e-29 | 29% |
| WP_038089357.1 aminoglycoside 3'-phosphotransferase [Tumebacil... | 120 | 7e-29 | 34% |
| SUB53831.1 Aminoglycoside 3'-phosphotransferase [Nocardia bras... | 123 | 8e-29 | 38% |
| WP_073591145.1 aminoglycoside 3'-phosphotransferase [Anaerocol... | 120 | 8e-29 | 31% |
| WP_060475669.1 aminoglycoside 3'-phosphotransferase [Geobacill... | 120 | 9e-29 | 32% |
| WP_042256910.1 tetratricopeptide repeat protein [Nocardia bras... | 123 | 9e-29 | 38% |
| WP_084501669.1 tetratricopeptide repeat protein [Nocardia xish... | 123 | 9e-29 | 41% |
| ECL6242480.1 aminoglycoside O-phosphotransferase APH(3')-IIIa ... | 120 | 9e-29 | 32% |
| WP_195096139.1 APH(3') family aminoglycoside O-phosphotransfer... | 123 | 9e-29 | 42% |
| WP_186818774.1 phosphotransferase [Nocardia ninae] | 123 | 9e-29 | 42% |
| WP_051870780.1 MULTISPECIES: aminoglycoside 3'-phosphotransfer... | 120 | 1e-28 | 32% |
| WP_148984157.1 aminoglycoside 3'-phosphotransferase [Enterobac... | 119 | 1e-28 | 34% |
| WP_043654191.1 aminoglycoside phosphotransferase APH(3') [Noca... | 123 | 1e-28 | 39% |
| WP_101031679.1 MULTISPECIES: aminoglycoside O-phosphotransfera... | 120 | 1e-28 | 32% |
| WP_033971072.1 aminoglycoside phosphotransferase APH(3') [Pseu... | 117 | 1e-28 | 46% |
| OGO85899.1 hypothetical protein A2Y24_07180 [Clostridiales bac... | 120 | 1e-28 | 31% |
| WP_071929168.1 phosphotransferase [Nocardia mangyaensis] | 123 | 1e-28 | 41% |
| HAP6720278.1 TPA: aminoglycoside O-phosphotransferase APH(3')-... | 120 | 1e-28 | 32% |
| WP_025848408.1 aminoglycoside 3'-phosphotransferase [Paenibaci... | 120 | 1e-28 | 29% |
| CDR01453.1 phosphotransferase [Streptomyces iranensis] | 120 | 1e-28 | 35% |
| MBA2554891.1 aminoglycoside 3'-phosphotransferase [Geodermatop... | 120 | 1e-28 | 38% |
| AQW79398.1 KanR [Streptococcus pneumoniae] | 119 | 1e-28 | 32% |
| WP_186499682.1 APH(3') family aminoglycoside O-phosphotransfer... | 119 | 1e-28 | 33% |
| EFP7671791.1 aminoglycoside 3'-phosphotransferase [Shigella so... | 117 | 1e-28 | 38% |
| WP_063055485.1 tetratricopeptide repeat protein [Nocardia salm... | 122 | 1e-28 | 39% |

| WP_055503240.1 aminoglycoside 3'-phosphotransferase [Nonomurae... | 119 | 1e-28 | 41% |
| --- | --- | --- | --- |
| WP_020521244.1 aminoglycoside 3'-phosphotransferase [Catelligl... | 119 | 1e-28 | 32% |
| WP_084508227.1 phosphotransferase [Nocardia pseudovaccinii] | 122 | 1e-28 | 42% |
| WP_195149582.1 APH(3') family aminoglycoside O-phosphotransfer... | 122 | 1e-28 | 40% |
| EFU8609014.1 aminoglycoside 3'-phosphotransferase [Escherichia... | 116 | 1e-28 | 38% |
| WP_046727030.1 aminoglycoside 3'-phosphotransferase [Streptomy... | 119 | 2e-28 | 37% |
| EAI0748225.1 aminoglycoside O-phosphotransferase APH(3')-IIIa ... | 119 | 2e-28 | 32% |
| GEM42439.1 hypothetical protein NN4_69580 [Nocardia ninae NBRC... | 122 | 2e-28 | 42% |
| WP_152727845.1 MULTISPECIES: aminoglycoside 3'-phosphotransfer... | 119 | 2e-28 | 37% |
| WP_189084817.1 phosphotransferase [Agromyces mediolanus] | 119 | 2e-28 | 35% |
| WP_043680707.1 tetratricopeptide repeat protein [Nocardia vuln... | 122 | 2e-28 | 38% |
| EEU9590774.1 aminoglycoside 3'-phosphotransferase [Escherichia... | 116 | 2e-28 | 38% |
| WP_109527569.1 tetratricopeptide repeat protein [Nocardia sp. ... | 123 | 2e-28 | 45% |
| HHX80162.1 TPA: aminoglycoside 3'-phosphotransferase [Acholepl... | 119 | 2e-28 | 31% |
| WP_167978970.1 phosphotransferase [Lentzea indica] | 118 | 2e-28 | 42% |
| WP_189143277.1 phosphotransferase [Nonomuraea glycinis] | 119 | 2e-28 | 36% |
| WP_189024132.1 unnamed protein product | 122 | 2e-28 | 37% |
| WP_091099775.1 aminoglycoside 3'-phosphotransferase [Nonomurae... | 119 | 2e-28 | 41% |
| WP_142855012.1 aminoglycoside 3'-phosphotransferase [Salmonell... | 116 | 2e-28 | 38% |
| WP_077944264.1 aminoglycoside 3'-phosphotransferase [Salmonell... | 116 | 2e-28 | 38% |
| WP_181720548.1 APH(3') family aminoglycoside O-phosphotransfer... | 122 | 2e-28 | 42% |
| MBC7302054.1 tetratricopeptide repeat protein [Nocardia sp.] | 122 | 2e-28 | 41% |
| WP_101783164.1 aminoglycoside 3'-phosphotransferase [Nonomurae... | 119 | 2e-28 | 42% |
| WP_157245430.1 phosphotransferase [Nonomuraea typhae] | 122 | 2e-28 | 40% |
| OHV49240.1 hypothetical protein CgIS1_21425 [Frankia sp. CgIS1] | 118 | 2e-28 | 39% |
| WP_145737919.1 aminoglycoside 3'-phosphotransferase [Saccharop... | 118 | 2e-28 | 36% |
| WP_146882958.1 aminoglycoside 3'-phosphotransferase [Deinococc... | 119 | 2e-28 | 33% |
| WP_000331111.1 aminoglycoside 3'-phosphotransferase [Escherich... | 116 | 2e-28 | 38% |
| EDM9822576.1 APH(3') family aminoglycoside O-phosphotransferas... | 116 | 2e-28 | 38% |
| WP_129297428.1 aminoglycoside 3'-phosphotransferase [Streptomy... | 119 | 2e-28 | 37% |
| NQO08731.1 aminoglycoside O-phosphotransferase APH(3')-IIIa [S... | 119 | 2e-28 | 32% |
| WP_147135455.1 aminoglycoside 3'-phosphotransferase [Stackebra... | 119 | 2e-28 | 37% |
| RLA65161.1 hypothetical protein DRQ88_09715 [Epsilonproteobact... | 119 | 3e-28 | 31% |
| EFE5558596.1 aminoglycoside 3'-phosphotransferase [Escherichia... | 115 | 3e-28 | 38% |
| SFT10704.1 streptomycin 3-kinase [Streptomyces harbinensis] | 119 | 3e-28 | 38% |
| WP_012891566.1 aminoglycoside 3'-phosphotransferase [Streptosp... | 118 | 3e-28 | 36% |
| WP_140368102.1 aminoglycoside 3'-phosphotransferase [Vibrio pa... | 115 | 3e-28 | 38% |
| AIF29520.1 APH [synthetic construct] | 119 | 3e-28 | 32% |
| WP_164708935.1 phosphotransferase [Escherichia coli] | 115 | 3e-28 | 38% |
| HAH3517579.1 TPA: APH(3') family aminoglycoside O-phosphotrans... | 115 | 3e-28 | 38% |
| EES9957578.1 aminoglycoside 3'-phosphotransferase [Escherichia... | 115 | 3e-28 | 38% |
| EEQ96518.1 Aminoglycoside 3'-phosphotransferase [Ochrobactrum ... | 115 | 3e-28 | 54% |
| EFN8309472.1 aminoglycoside 3'-phosphotransferase [Escherichia... | 116 | 3e-28 | 38% |
| WP_138060532.1 aminoglycoside 3'-phosphotransferase [Acinetoba... | 116 | 3e-28 | 38% |
| WP_000298295.1 aminoglycoside 3'-phosphotransferase [Acinetoba... | 115 | 3e-28 | 38% |
| HAH3517501.1 TPA: APH(3') family aminoglycoside O-phosphotrans... | 115 | 3e-28 | 38% |
| EEU9590772.1 aminoglycoside 3'-phosphotransferase [Escherichia... | 116 | 3e-28 | 38% |
| AUV46756.1 hypothetical protein C2U43_29060 [Citrobacter freun... | 116 | 3e-28 | 38% |
| WP_140329235.1 aminoglycoside 3'-phosphotransferase [Vibrio pa... | 115 | 3e-28 | 38% |
| WP_138142393.1 aminoglycoside 3'-phosphotransferase [Acinetoba... | 116 | 3e-28 | 38% |
| EAI6092107.1 aminoglycoside O-phosphotransferase APH(3')-IIIa ... | 119 | 3e-28 | 32% |
| WP_138147086.1 aminoglycoside 3'-phosphotransferase [Acinetoba... | 116 | 3e-28 | 38% |
| ECI9710144.1 aminoglycoside 3'-phosphotransferase [Salmonella ... | 116 | 3e-28 | 38% |
| WP_138701489.1 aminoglycoside 3'-phosphotransferase [Streptoco... | 116 | 3e-28 | 38% |
| WP_142890817.1 aminoglycoside 3'-phosphotransferase [Klebsiell... | 116 | 3e-28 | 38% |
| AAA23017.1 3'-aminoglycoside phosphotransferase [Campylobacter... | 116 | 3e-28 | 38% |
| WP_176959813.1 aminoglycoside 3'-phosphotransferase [Lentzea j... | 117 | 3e-28 | 42% |
| WP_082061017.1 MULTISPECIES: aminoglycoside 3'-phosphotransfer... | 115 | 3e-28 | 38% |
| WP_173083302.1 phosphotransferase [Phytohabitans rumicis] | 118 | 3e-28 | 37% |
| EGJ8352427.1 aminoglycoside 3'-phosphotransferase [Salmonella ... | 116 | 4e-28 | 38% |
| WP_139846286.1 aminoglycoside 3'-phosphotransferase [Acinetoba... | 116 | 4e-28 | 38% |
| WP_146708654.1 aminoglycoside O-phosphotransferase APH(3')-III... | 118 | 4e-28 | 32% |
| ECO5633136.1 aminoglycoside O-phosphotransferase APH(3')-IIIa ... | 118 | 4e-28 | 32% |
| WP_093843937.1 APH(3'') family aminoglycoside O-phosphotransfe... | 119 | 4e-28 | 38% |
| NLT40748.1 aminoglycoside 3'-phosphotransferase [Clostridiales... | 118 | 4e-28 | 33% |
| WP_184507773.1 APH(3') family aminoglycoside O-phosphotransfer... | 118 | 4e-28 | 34% |
| NKE62335.1 aminoglycoside 3'-phosphotransferase [Lentzea indica] | 118 | 4e-28 | 42% |
| MBA3417768.1 aminoglycoside 3'-phosphotransferase [Geodermatop... | 118 | 4e-28 | 37% |
| WP_184293170.1 phosphotransferase [Nocardiopsis algeriensis] | 118 | 4e-28 | 36% |
| HBX87061.1 TPA: aminoglycoside O-phosphotransferase APH(3')-II... | 118 | 4e-28 | 32% |
| WP_194815621.1 phosphotransferase [Nocardia sp. XZ_19_385] | 115 | 4e-28 | 42% |
| SDF64609.1 kanamycin kinase [Blastococcus sp. DSM 44205] | 118 | 4e-28 | 38% |

| WP_084965627.1 phosphotransferase [Thermoactinospora rubra] | 121 | 4e-28 | 41% |
| --- | --- | --- | --- |
| CAB0622915.1 aminoglycoside O-phosphotransferase APH(3')-Ia [C... | 115 | 5e-28 | 38% |
| 2BKK_A Crystal structure of Aminoglycoside Phosphotransferase ... | 118 | 5e-28 | 32% |
| GFJ86770.1 putative phosphotransferase [Phytohabitans rumicis] | 118 | 5e-28 | 37% |
| WP_195468025.1 APH(3') family aminoglycoside O-phosphotransfer... | 118 | 5e-28 | 31% |
| TWG08006.1 kanamycin kinase [Saccharopolyspora dendranthemae] | 118 | 5e-28 | 36% |
| WP_155865000.1 aminoglycoside 3'-phosphotransferase [Gryllotal... | 117 | 5e-28 | 38% |
| WP_051415752.1 aminoglycoside 3'-phosphotransferase [Nocardiop... | 119 | 5e-28 | 34% |
| WP_162865184.1 aminoglycoside 3'-phosphotransferase [Frankia c... | 118 | 5e-28 | 38% |
| SOY29303.1 Aminoglycoside 3'-phosphotransferase [Acetatifactor... | 118 | 5e-28 | 32% |
| WP_084510809.1 phosphotransferase [Nocardia lijiangensis] | 121 | 5e-28 | 41% |
| EAK4451211.1 aminoglycoside O-phosphotransferase APH(3')-IIIa ... | 118 | 5e-28 | 32% |
| QNL14341.1 aminoglycoside 3'-phosphotransferase [synthetic con... | 118 | 5e-28 | 32% |
| WP_095022614.1 aminoglycoside 3'-phosphotransferase [Klebsiell... | 117 | 5e-28 | 34% |
| WP_140317689.1 aminoglycoside 3'-phosphotransferase [Vibrio pa... | 114 | 6e-28 | 39% |
| HAP4357125.1 TPA: aminoglycoside O-phosphotransferase APH(3')-... | 118 | 6e-28 | 32% |
| ECO5633380.1 aminoglycoside O-phosphotransferase APH(3')-IIIa ... | 118 | 6e-28 | 32% |
| KGE53928.1 phosphotransferase enzyme family protein [Streptoco... | 116 | 6e-28 | 33% |
| WP_011437749.1 MULTISPECIES: aminoglycoside 3'-phosphotransfer... | 118 | 6e-28 | 39% |
| EFU6049290.1 aminoglycoside 3'-phosphotransferase [Escherichia... | 115 | 6e-28 | 38% |
| ECP2987496.1 aminoglycoside O-phosphotransferase APH(3')-IIIa ... | 118 | 6e-28 | 32% |
| WP_084568461.1 MULTISPECIES: aminoglycoside 3'-phosphotransfer... | 114 | 6e-28 | 39% |
| EAL8994190.1 aminoglycoside O-phosphotransferase APH(3')-IIIa ... | 118 | 6e-28 | 32% |
| WP_133023100.1 aminoglycoside 3'-phosphotransferase [Streptomy... | 118 | 7e-28 | 37% |
| WP_146040018.1 aminoglycoside 3'-phosphotransferase [Acetatifa... | 118 | 7e-28 | 32% |
| WP_138714232.1 aminoglycoside 3'-phosphotransferase [Nonomurae... | 117 | 7e-28 | 36% |
| ECC2263526.1 aminoglycoside O-phosphotransferase APH(3')-IIIa ... | 118 | 7e-28 | 32% |
| WP_031874690.1 aminoglycoside O-phosphotransferase APH(3')-III... | 117 | 7e-28 | 32% |
| WP_195115233.1 APH(3') family aminoglycoside O-phosphotransfer... | 117 | 7e-28 | 37% |
| WP_176295407.1 aminoglycoside O-phosphotransferase APH(3')-III... | 117 | 7e-28 | 32% |
| CAA9210959.1 aminoglycoside 3'-phosphotransferase [uncultured ... | 118 | 7e-28 | 36% |
| WP_193616468.1 phosphotransferase [Modestobacter sp. I12A-02628] | 119 | 7e-28 | 37% |
| WP_196582009.1 aminoglycoside O-phosphotransferase APH(3')-III... | 117 | 7e-28 | 32% |
| MBE6709782.1 aminoglycoside 3'-phosphotransferase [Ruminococca... | 117 | 8e-28 | 36% |
| EDJ5511106.1 aminoglycoside O-phosphotransferase APH(3')-IIIa ... | 117 | 8e-28 | 32% |
| WP_031789272.1 aminoglycoside O-phosphotransferase APH(3')-III... | 117 | 8e-28 | 32% |
| WP_195043079.1 phosphotransferase [Nocardia beijingensis] | 120 | 8e-28 | 41% |
| QNL14344.1 aminoglycoside 3'-phosphotransferase [synthetic con... | 117 | 8e-28 | 32% |
| ACJ12745.1 NptII [Cloning vector pEX302] | 117 | 8e-28 | 32% |
| RJX26070.1 aminoglycoside 3'-phosphotransferase [Acholeplasma ... | 117 | 8e-28 | 30% |
| WP_127487237.1 aminoglycoside 3'-phosphotransferase [Paenibaci... | 117 | 8e-28 | 29% |
| ONH24731.1 hypothetical protein CcI156_15010 [Frankia sp. CcI156] | 117 | 8e-28 | 38% |
| ARM38994.1 aminoglycoside phosphotransferase [Streptococcus pn... | 117 | 8e-28 | 32% |
| WP_078875199.1 APH(3'') family aminoglycoside O-phosphotransfe... | 118 | 8e-28 | 37% |
| WP_130473351.1 aminoglycoside 3'-phosphotransferase [Amycolato... | 117 | 9e-28 | 39% |
| WP_195999628.1 APH(3') family aminoglycoside O-phosphotransfer... | 117 | 9e-28 | 30% |
| ECO5633383.1 aminoglycoside O-phosphotransferase APH(3')-IIIa ... | 117 | 9e-28 | 32% |
| WP_088796683.1 MULTISPECIES: aminoglycoside 3'-phosphotransfer... | 117 | 9e-28 | 36% |
| WP_160172372.1 APH(3') family aminoglycoside O-phosphotransfer... | 117 | 9e-28 | 39% |
| HAP5827104.1 TPA: aminoglycoside O-phosphotransferase APH(3')-... | 117 | 9e-28 | 32% |
| EAK1521743.1 aminoglycoside O-phosphotransferase APH(3')-IIIa ... | 117 | 9e-28 | 32% |
| WP_156755497.1 phosphotransferase [Actinokineospora sp. TRM65233] | 117 | 9e-28 | 36% |
| WP_111149630.1 aminoglycoside O-phosphotransferase APH(3')-III... | 117 | 9e-28 | 32% |
| WP_063841507.1 aminoglycoside O-phosphotransferase APH(3')-III... | 117 | 9e-28 | 32% |
| CAC8379508.1 aminoglycoside 3'-phosphotransferase [Staphylococ... | 117 | 9e-28 | 32% |
| ADN28038.1 aminoglycoside 3'-phosphotransferase [Streptococcus... | 117 | 9e-28 | 32% |
| WP_194924497.1 phosphotransferase [Catenulispora sp. NH11] | 117 | 9e-28 | 36% |
| AEO73307.1 aminoglycoside 3'-phosphotransferase type IIb [Pseu... | 112 | 9e-28 | 68% |
| WP_031859803.1 aminoglycoside O-phosphotransferase APH(3')-III... | 117 | 9e-28 | 32% |
| HAQ3015468.1 TPA: aminoglycoside O-phosphotransferase APH(3')-... | 117 | 9e-28 | 32% |
| WP_031860034.1 aminoglycoside O-phosphotransferase APH(3')-III... | 117 | 9e-28 | 32% |
| EAJ3815666.1 aminoglycoside O-phosphotransferase APH(3')-IIIa ... | 117 | 1e-27 | 32% |
| EAH8887986.1 aminoglycoside O-phosphotransferase APH(3')-IIIa ... | 117 | 1e-27 | 32% |
| ECZ3110123.1 aminoglycoside O-phosphotransferase APH(3')-IIIa ... | 117 | 1e-27 | 32% |
| CAA4002411.1 aminoglycoside 3'-phosphotransferase [Staphylococ... | 117 | 1e-27 | 32% |
| AHH29449.1 neomycin phosphotransferase III [Enterococcus faecium] | 117 | 1e-27 | 32% |
| WP_134105471.1 aminoglycoside 3'-phosphotransferase [Kribbella... | 117 | 1e-27 | 36% |
| WP_031912847.1 aminoglycoside O-phosphotransferase APH(3')-III... | 117 | 1e-27 | 32% |
| WP_083899494.1 tetratricopeptide repeat protein [Nocardia absc... | 120 | 1e-27 | 40% |
| HAQ0809818.1 TPA: aminoglycoside O-phosphotransferase APH(3')-... | 117 | 1e-27 | 32% |
| WP_142001221.1 aminoglycoside 3'-phosphotransferase [Amycolato... | 117 | 1e-27 | 34% |
| WP_149562878.1 APH(3'') family aminoglycoside O-phosphotransfe... | 118 | 1e-27 | 37% |

| ECX5382443.1 aminoglycoside O-phosphotransferase APH(3')-IIIa ... | 117 | 1e-27 | 32% |
| --- | --- | --- | --- |
| AHH29232.1 neomycin phosphotransferase III [Escherichia coli] | 117 | 1e-27 | 32% |
| MBE6630696.1 aminoglycoside 3'-phosphotransferase [Ruminococca... | 117 | 1e-27 | 36% |
| WP_185097354.1 aminoglycoside O-phosphotransferase APH(3')-III... | 117 | 1e-27 | 32% |
| HAR6794479.1 TPA: aminoglycoside O-phosphotransferase APH(3')-... | 117 | 1e-27 | 32% |
| WP_061573581.1 aminoglycoside O-phosphotransferase APH(3')-III... | 117 | 1e-27 | 32% |
| WP_187241748.1 APH(3') family aminoglycoside O-phosphotransfer... | 117 | 1e-27 | 42% |
| WP_103961158.1 aminoglycoside 3'-phosphotransferase [Nonomurae... | 117 | 1e-27 | 39% |
| HAQ9071068.1 TPA: aminoglycoside O-phosphotransferase APH(3')-... | 117 | 1e-27 | 32% |
| WP_184033656.1 APH(3') family aminoglycoside O-phosphotransfer... | 117 | 1e-27 | 33% |
| EAK2357370.1 aminoglycoside O-phosphotransferase APH(3')-IIIa ... | 117 | 1e-27 | 32% |
| HAP8967474.1 TPA: aminoglycoside O-phosphotransferase APH(3')-... | 117 | 1e-27 | 32% |
| WP_195032832.1 phosphotransferase [Nocardia abscessus] | 120 | 1e-27 | 40% |
| HAR6886887.1 TPA: aminoglycoside O-phosphotransferase APH(3')-... | 117 | 1e-27 | 32% |
| ECC6429716.1 aminoglycoside 3'-phosphotransferase [Salmonella ... | 114 | 1e-27 | 39% |
| WP_187092814.1 phosphotransferase [Nakamurella sp. PAMC28650] | 117 | 1e-27 | 36% |
| WP_086118478.1 aminoglycoside O-phosphotransferase APH(3')-III... | 117 | 1e-27 | 32% |
| AHH29445.1 neomycin phosphotransferase III [Enterococcus faecium] | 117 | 1e-27 | 32% |
| WP_084823721.1 phosphotransferase [Nocardia beijingensis] | 120 | 1e-27 | 50% |
| HAQ8903927.1 TPA: aminoglycoside O-phosphotransferase APH(3')-... | 117 | 1e-27 | 32% |
| WP_086096002.1 aminoglycoside O-phosphotransferase APH(3')-III... | 117 | 1e-27 | 32% |
| EAK5420891.1 aminoglycoside O-phosphotransferase APH(3')-IIIa ... | 117 | 1e-27 | 32% |
| WP_002360696.1 MULTISPECIES: aminoglycoside O-phosphotransfera... | 117 | 1e-27 | 32% |
| APO15809.1 aminoglycoside phosphotransferase [Streptococcus pn... | 117 | 1e-27 | 31% |
| EAK3587837.1 aminoglycoside O-phosphotransferase APH(3')-IIIa ... | 117 | 1e-27 | 32% |
| PWM21535.1 hypothetical protein DBX53_06020 [Clostridiales bac... | 117 | 1e-27 | 30% |
| WP_113996318.1 aminoglycoside O-phosphotransferase APH(3')-III... | 117 | 1e-27 | 32% |
| WP_001637127.1 MULTISPECIES: aminoglycoside O-phosphotransfera... | 117 | 1e-27 | 32% |
| WP_186980773.1 aminoglycoside O-phosphotransferase APH(3')-III... | 117 | 1e-27 | 32% |
| WP_133784827.1 aminoglycoside 3'-phosphotransferase [Kribbella... | 117 | 1e-27 | 36% |
| WP_065211529.1 aminoglycoside O-phosphotransferase APH(3')-III... | 117 | 1e-27 | 32% |
| RXE88330.1 aminoglycoside O-phosphotransferase APH(3')-IIIa [E... | 117 | 1e-27 | 32% |
| CBZ42060.1 aminoglycoside 3'-phosphotransferase [Streptococcus... | 117 | 1e-27 | 32% |
| WP_044119039.1 MULTISPECIES: aminoglycoside O-phosphotransfera... | 116 | 1e-27 | 32% |
| EVO61729.1 aminoglycoside 3'-phosphotransferase [Staphylococcu... | 117 | 1e-27 | 32% |
| ORT96555.1 hypothetical protein UK99_08820 [Frankia casuarinae] | 116 | 1e-27 | 38% |
| OFB39825.1 hypothetical protein Manayef4_20090 [Frankia sp. Cg... | 116 | 1e-27 | 38% |
| CAA4051729.1 aminoglycoside 3'-phosphotransferase [Staphylococ... | 117 | 1e-27 | 32% |
| EGQ1519538.1 aminoglycoside O-phosphotransferase APH(3')-IIIa ... | 117 | 1e-27 | 32% |
| OTU76632.1 APH(3') family aminoglycoside O-phosphotransferase ... | 115 | 1e-27 | 33% |
| WP_063841495.1 aminoglycoside O-phosphotransferase APH(3')-III... | 117 | 1e-27 | 32% |
| HAR2610503.1 TPA: APH(3') family aminoglycoside O-phosphotrans... | 117 | 1e-27 | 32% |
| CAA3928533.1 aminoglycoside 3'-phosphotransferase [Staphylococ... | 117 | 1e-27 | 32% |
| WP_140159896.1 aminoglycoside 3'-phosphotransferase [Acinetoba... | 115 | 1e-27 | 33% |
| WP_113835084.1 aminoglycoside 3'-phosphotransferase [Blastococ... | 117 | 1e-27 | 39% |
| WP_099506454.1 MULTISPECIES: aminoglycoside 3'-phosphotransfer... | 117 | 1e-27 | 35% |
| CAA4174215.1 aminoglycoside 3'-phosphotransferase [Staphylococ... | 117 | 1e-27 | 32% |
| WP_031173215.1 aminoglycoside 3'-phosphotransferase [Streptosp... | 116 | 1e-27 | 36% |
| WP_001096887.1 MULTISPECIES: aminoglycoside O-phosphotransfera... | 117 | 1e-27 | 32% |
| EAH4520482.1 aminoglycoside O-phosphotransferase APH(3')-IIIa ... | 117 | 1e-27 | 32% |
| KEZ37023.1 aminoglycoside phosphotransferase [Frankia sp. CeD] | 117 | 1e-27 | 39% |
| HHY69283.1 TPA: aminoglycoside 3'-phosphotransferase [Firmicut... | 117 | 1e-27 | 30% |
| WP_103150433.1 aminoglycoside O-phosphotransferase APH(3')-III... | 117 | 1e-27 | 32% |
| WP_032464674.1 aminoglycoside 3'-phosphotransferase [Streptoco... | 115 | 1e-27 | 33% |
| WP_170243249.1 aminoglycoside O-phosphotransferase APH(3')-III... | 117 | 1e-27 | 32% |
| EAH6354994.1 aminoglycoside O-phosphotransferase APH(3')-IIIa ... | 117 | 1e-27 | 32% |
| WP_123836036.1 aminoglycoside O-phosphotransferase APH(3')-III... | 117 | 2e-27 | 32% |
| EGQ3532118.1 aminoglycoside O-phosphotransferase APH(3')-IIIa ... | 117 | 2e-27 | 32% |
| AWV07369.1 hypothetical protein C9I47_1673 [Lysobacter maris] | 114 | 2e-27 | 41% |
| WP_086307091.1 aminoglycoside O-phosphotransferase APH(3')-III... | 117 | 2e-27 | 32% |
| WP_131344733.1 aminoglycoside 3'-phosphotransferase [Kribbella... | 117 | 2e-27 | 36% |
| CAA4051868.1 aminoglycoside 3'-phosphotransferase [Staphylococ... | 117 | 2e-27 | 32% |
| WP_194757636.1 phosphotransferase [Avibacterium paragallinarum] | 114 | 2e-27 | 36% |
| WP_050964892.1 aminoglycoside O-phosphotransferase APH(3')-III... | 117 | 2e-27 | 32% |
| WP_141029202.1 aminoglycoside 3'-phosphotransferase [Salmonell... | 114 | 2e-27 | 36% |
| WP_082006749.1 aminoglycoside 3'-phosphotransferase [Sphingomo... | 113 | 2e-27 | 40% |
| EAK5553005.1 aminoglycoside O-phosphotransferase APH(3')-IIIa ... | 117 | 2e-27 | 32% |
| NRA26289.1 aminoglycoside 3'-phosphotransferase [Opitutales ba... | 117 | 2e-27 | 31% |
| AQY75633.1 APH(3') family aminoglycoside O-phosphotransferase,... | 117 | 2e-27 | 31% |
| WP_111168121.1 aminoglycoside O-phosphotransferase APH(3')-III... | 117 | 2e-27 | 32% |
| EGB1229853.1 aminoglycoside O-phosphotransferase APH(3')-IIIa ... | 117 | 2e-27 | 31% |
| WP_152133423.1 aminoglycoside O-phosphotransferase APH(3')-III... | 117 | 2e-27 | 32% |

| WP_077251313.1 aminoglycoside 3'-phosphotransferase [Escherich... | 113 | 2e-27 | 38% |
| --- | --- | --- | --- |
| WP_031925451.1 aminoglycoside O-phosphotransferase APH(3')-III... | 117 | 2e-27 | 32% |
| QBC82936.1 aminoglycoside O-phosphotransferase APH(3')-Ia [Aci... | 114 | 2e-27 | 35% |
| EGD5505665.1 aminoglycoside O-phosphotransferase APH(3')-IIIa ... | 117 | 2e-27 | 32% |
| WP_070309734.1 aminoglycoside O-phosphotransferase APH(3')-III... | 117 | 2e-27 | 32% |
| MBD5479146.1 aminoglycoside 3'-phosphotransferase [Lachnospira... | 117 | 2e-27 | 31% |
| EDB0097308.1 aminoglycoside O-phosphotransferase APH(3')-IIIa ... | 117 | 2e-27 | 32% |
| HAR6756871.1 TPA: aminoglycoside O-phosphotransferase APH(3')-... | 116 | 2e-27 | 32% |
| NGS43944.1 aminoglycoside O-phosphotransferase APH(3')-IIIa [S... | 116 | 2e-27 | 32% |
| KFB03760.1 aminoglycoside phosphotransferase [Frankia sp. Allo2] | 117 | 2e-27 | 39% |
| WP_031784165.1 aminoglycoside O-phosphotransferase APH(3')-III... | 116 | 2e-27 | 32% |
| WP_175443427.1 APH(3') family aminoglycoside O-phosphotransfer... | 116 | 2e-27 | 38% |
| WP_056826622.1 MULTISPECIES: aminoglycoside 3'-phosphotransfer... | 116 | 2e-27 | 38% |
| WP_140087761.1 aminoglycoside 3'-phosphotransferase [Vibrio pa... | 114 | 2e-27 | 36% |
| CAD35325.1 kanamycin resistance protein [Campylobacter jejuni] | 116 | 2e-27 | 31% |
| EGQ3125045.1 aminoglycoside O-phosphotransferase APH(3')-IIIa ... | 116 | 2e-27 | 32% |
| MBD5511306.1 aminoglycoside 3'-phosphotransferase [Lachnospira... | 116 | 2e-27 | 31% |
| EEP3469576.1 aminoglycoside O-phosphotransferase APH(3')-IIIa ... | 116 | 2e-27 | 32% |
| WP_104847099.1 aminoglycoside O-phosphotransferase APH(3')-III... | 116 | 2e-27 | 32% |
| WP_033861728.1 aminoglycoside O-phosphotransferase APH(3')-III... | 116 | 2e-27 | 31% |
| EAJ1102904.1 aminoglycoside O-phosphotransferase APH(3')-IIIa ... | 116 | 2e-27 | 31% |
| SCJ62694.1 Aminoglycoside 3'-phosphotransferase [uncultured Fl... | 116 | 2e-27 | 32% |
| EGO2736042.1 aminoglycoside O-phosphotransferase APH(3')-IIIa ... | 116 | 2e-27 | 32% |
| WP_078874096.1 APH(3'') family aminoglycoside O-phosphotransfe... | 117 | 2e-27 | 36% |
| WP_025133653.1 aminoglycoside 3'-phosphotransferase [Leucobact... | 117 | 2e-27 | 36% |
| WP_048519415.1 aminoglycoside O-phosphotransferase APH(3')-III... | 116 | 2e-27 | 32% |
| WP_111037803.1 aminoglycoside O-phosphotransferase APH(3')-III... | 116 | 2e-27 | 32% |
| WP_078292476.1 MULTISPECIES: APH(3'') family aminoglycoside O-... | 116 | 2e-27 | 36% |
| WP_175637601.1 APH(3') family aminoglycoside O-phosphotransfer... | 116 | 2e-27 | 38% |
| PWL99139.1 aminoglycoside O-phosphotransferase APH(3')-IIIa [S... | 116 | 2e-27 | 30% |
| RDI65181.1 streptomycin 3'-kinase [Nocardia pseudobrasiliensis] | 116 | 2e-27 | 36% |
| HAP7019454.1 TPA: aminoglycoside O-phosphotransferase APH(3')-... | 116 | 3e-27 | 32% |
| ABA56503.1 aminoglycoside 3'-phosphotransferase [Salmonella en... | 114 | 3e-27 | 36% |
| EAH9996565.1 aminoglycoside O-phosphotransferase APH(3')-IIIa ... | 116 | 3e-27 | 32% |
| EAJ0793688.1 aminoglycoside O-phosphotransferase APH(3')-IIIa ... | 116 | 3e-27 | 32% |
| WP_166055201.1 phosphotransferase [Lentzea sp. NEAU-D13] | 115 | 3e-27 | 42% |
| CAA4804872.1 aminoglycoside 3'-phosphotransferase [Staphylococ... | 116 | 3e-27 | 32% |
| WP_114755704.1 APH(3'') family aminoglycoside O-phosphotransfe... | 116 | 3e-27 | 36% |
| WP_195065052.1 APH(3') family aminoglycoside O-phosphotransfer... | 119 | 3e-27 | 41% |
| WP_089518440.1 aminoglycoside O-phosphotransferase APH(3')-III... | 116 | 3e-27 | 31% |
| HAP7207273.1 TPA: aminoglycoside O-phosphotransferase APH(3')-... | 116 | 3e-27 | 32% |
| EAL7630422.1 aminoglycoside O-phosphotransferase APH(3')-IIIa ... | 116 | 3e-27 | 32% |
| WP_185070109.1 phosphotransferase [Nonomuraea jabiensis] | 115 | 3e-27 | 38% |
| WP_010724707.1 aminoglycoside O-phosphotransferase APH(3')-III... | 115 | 3e-27 | 32% |
| WP_165758272.1 APH(3') family aminoglycoside O-phosphotransfer... | 116 | 3e-27 | 38% |
| WP_053866202.1 aminoglycoside O-phosphotransferase APH(3')-III... | 116 | 3e-27 | 32% |
| QNN02186.1 neomycin phosphotransferase II [Cloning vector PjCA... | 116 | 3e-27 | 31% |
| CAA3910286.1 aminoglycoside 3'-phosphotransferase [Staphylococ... | 116 | 3e-27 | 32% |
| WP_057037386.1 aminoglycoside O-phosphotransferase APH(3')-III... | 116 | 3e-27 | 32% |
| QNR54945.1 aminoglycoside O-phosphotransferase APH(3')-IIIa [C... | 116 | 3e-27 | 31% |
| WP_061491638.1 aminoglycoside O-phosphotransferase APH(3')-III... | 116 | 3e-27 | 32% |
| EAH8139172.1 aminoglycoside O-phosphotransferase APH(3')-IIIa ... | 115 | 3e-27 | 32% |
| EAL4172436.1 aminoglycoside O-phosphotransferase APH(3')-IIIa ... | 116 | 3e-27 | 32% |
| WP_069106888.1 MULTISPECIES: aminoglycoside O-phosphotransfera... | 116 | 4e-27 | 32% |
| WP_105126012.1 aminoglycoside O-phosphotransferase APH(3')-III... | 116 | 4e-27 | 32% |
| EEZ8608893.1 aminoglycoside 3'-phosphotransferase [Escherichia... | 114 | 4e-27 | 33% |
| EAH9407082.1 aminoglycoside O-phosphotransferase APH(3')-IIIa ... | 115 | 4e-27 | 32% |
| EGQ1530571.1 aminoglycoside O-phosphotransferase APH(3')-IIIa ... | 115 | 4e-27 | 32% |
| WP_131498323.1 aminoglycoside 3'-phosphotransferase [Kribbella... | 115 | 4e-27 | 37% |
| EGI4180814.1 aminoglycoside 3'-phosphotransferase [Escherichia... | 114 | 4e-27 | 33% |
| EAK6388190.1 aminoglycoside O-phosphotransferase APH(3')-IIIa ... | 115 | 4e-27 | 32% |
| WP_194752152.1 APH(3') family aminoglycoside O-phosphotransfer... | 113 | 4e-27 | 36% |
| WP_082393195.1 tetratricopeptide repeat protein [Nocardia ariz... | 118 | 4e-27 | 40% |
| HBG08852.1 TPA: aminoglycoside 3'-phosphotransferase [Firmicut... | 112 | 4e-27 | 41% |
| TQM31607.1 kanamycin kinase [Nocardia bhagyanarayanae] | 119 | 4e-27 | 46% |
| HAP3694768.1 TPA: aminoglycoside O-phosphotransferase APH(3')-... | 115 | 4e-27 | 31% |
| WP_169040408.1 aminoglycoside O-phosphotransferase APH(3')-III... | 115 | 4e-27 | 31% |
| WP_110165078.1 aminoglycoside O-phosphotransferase APH(3')-III... | 115 | 4e-27 | 31% |
| WP_140054642.1 aminoglycoside 3'-phosphotransferase [Vibrio pa... | 112 | 4e-27 | 38% |
| EAI7488668.1 aminoglycoside O-phosphotransferase APH(3')-IIIa ... | 115 | 4e-27 | 31% |
| PZO88333.1 APH(3') family aminoglycoside O-phosphotransferase ... | 115 | 4e-27 | 35% |
| WP_195124127.1 APH(3'') family aminoglycoside O-phosphotransfe... | 115 | 4e-27 | 38% |

| WP_165122347.1 MULTISPECIES: APH(3') family aminoglycoside O-p... | 115 | 5e-27 | 32% |
| --- | --- | --- | --- |
| QHG79832.1 aminoglycoside O-phosphotransferase APH(3')-IIIa [S... | 115 | 5e-27 | 32% |
| WP_185757047.1 phosphotransferase [Nocardia bhagyanarayanae] | 119 | 5e-27 | 46% |
| WP_111011898.1 aminoglycoside O-phosphotransferase APH(3')-III... | 115 | 5e-27 | 31% |
| EAL6418954.1 aminoglycoside O-phosphotransferase APH(3')-IIIa ... | 115 | 5e-27 | 31% |
| EAK1715683.1 aminoglycoside O-phosphotransferase APH(3')-IIIa ... | 115 | 5e-27 | 31% |
| EGG1579012.1 aminoglycoside O-phosphotransferase APH(3')-IIIa ... | 115 | 5e-27 | 31% |
| HCJ31825.1 TPA: hypothetical protein [Firmicutes bacterium] | 115 | 5e-27 | 30% |
| WP_145780788.1 APH(3'') family aminoglycoside O-phosphotransfe... | 115 | 5e-27 | 37% |
| EAL2880164.1 aminoglycoside O-phosphotransferase APH(3')-IIIa ... | 115 | 5e-27 | 31% |
| EAI2324741.1 aminoglycoside O-phosphotransferase APH(3')-IIIa ... | 115 | 5e-27 | 31% |
| EAV9659810.1 aminoglycoside O-phosphotransferase APH(3')-IIIa ... | 115 | 5e-27 | 31% |
| TDZ90519.1 Aminoglycoside 3'-phosphotransferase [Mycobacteroid... | 115 | 5e-27 | 38% |
| EAJ7322571.1 aminoglycoside O-phosphotransferase APH(3')-IIIa ... | 115 | 5e-27 | 32% |
| QNO58682.1 aminoglycoside O-phosphotransferase [Campylobacter ... | 115 | 5e-27 | 31% |
| WP_031784349.1 aminoglycoside O-phosphotransferase APH(3')-III... | 115 | 6e-27 | 31% |
| WP_126406515.1 tetratricopeptide repeat protein [Pilimelia anu... | 118 | 6e-27 | 43% |
| WP_096150774.1 aminoglycoside 3'-phosphotransferase [Enterobac... | 112 | 6e-27 | 36% |
| WP_147033265.1 aminoglycoside 3'-phosphotransferase [Klebsiell... | 112 | 6e-27 | 38% |
| WP_176656666.1 APH(3'') family aminoglycoside O-phosphotransfe... | 115 | 6e-27 | 35% |
| AHF51365.1 kanR [synthetic construct] | 115 | 6e-27 | 31% |
| MVJ29645.1 aminoglycoside O-phosphotransferase APH(3')-IIIa [S... | 115 | 6e-27 | 31% |
| WP_081898980.1 aminoglycoside 3'-phosphotransferase [Herbidosp... | 116 | 6e-27 | 36% |
| MBA3419969.1 aminoglycoside 3'-phosphotransferase [Geodermatop... | 114 | 6e-27 | 39% |
| WP_196444399.1 phosphotransferase [Planomonospora sp. ID67723] | 114 | 6e-27 | 41% |
| MBF3325498.1 phosphotransferase [Pseudomonas aeruginosa] | 109 | 7e-27 | 68% |
| QGW04489.1 aminoglycoside 3'-phosphotransferase [Proteus cibar... | 115 | 7e-27 | 32% |
| TEA00468.1 Aminoglycoside 3'-phosphotransferase [Mycobacteroid... | 115 | 7e-27 | 38% |
| WP_019048055.1 aminoglycoside phosphotransferase APH(3') [Noca... | 118 | 7e-27 | 43% |
| WP_134206788.1 aminoglycoside 3'-phosphotransferase [Mycobacte... | 115 | 7e-27 | 38% |
| EAJ6812876.1 aminoglycoside O-phosphotransferase APH(3')-IIIa ... | 115 | 7e-27 | 31% |
| WP_139155756.1 MULTISPECIES: aminoglycoside 3'-phosphotransfer... | 112 | 7e-27 | 36% |
| WP_134087166.1 aminoglycoside 3'-phosphotransferase [Mycobacte... | 115 | 7e-27 | 38% |
| EAJ6089551.1 aminoglycoside O-phosphotransferase APH(3')-IIIa ... | 115 | 8e-27 | 31% |
| AHH29478.1 neomycin phosphotransferase III [Enterococcus faeca... | 114 | 8e-27 | 32% |
| WP_132114884.1 APH(3'') family aminoglycoside O-phosphotransfe... | 115 | 8e-27 | 35% |
| WP_110335380.1 APH(3'') family aminoglycoside O-phosphotransfe... | 115 | 8e-27 | 36% |
| MBE1469386.1 streptomycin 3'-kinase [Kibdelosporangium phytoha... | 115 | 8e-27 | 37% |
| WP_097247658.1 phosphotransferase [Nocardia amikacinitolerans] | 117 | 8e-27 | 46% |
| CAA9584662.1 hypothetical protein AVDCRST_MAG86-3323 [uncultur... | 112 | 8e-27 | 41% |
| AWT62855.1 aminoglycoside 3'-phosphotransferase [synthetic con... | 115 | 8e-27 | 32% |
| EDO6739734.1 aminoglycoside O-phosphotransferase APH(3')-IIIa ... | 115 | 8e-27 | 32% |
| AHH29417.1 neomycin phosphotransferase III [Enterococcus faeca... | 114 | 8e-27 | 32% |
| AHH29233.1 neomycin phosphotransferase III [Enterococcus faecium] | 114 | 9e-27 | 32% |
| AHH29446.1 neomycin phosphotransferase III [Enterococcus faeca... | 114 | 9e-27 | 32% |
| WP_157493435.1 APH(3') family aminoglycoside O-phosphotransfer... | 116 | 9e-27 | 38% |
| HAR5838686.1 TPA: aminoglycoside O-phosphotransferase APH(3')-... | 115 | 9e-27 | 32% |
| WP_125119336.1 aminoglycoside O-phosphotransferase APH(3')-III... | 115 | 9e-27 | 31% |
| CAA4043194.1 aminoglycoside 3'-phosphotransferase [Staphylococ... | 115 | 9e-27 | 32% |
| WP_132623805.1 aminoglycoside 3'-phosphotransferase [Saccharop... | 115 | 9e-27 | 36% |
| CAA9231760.1 weak similarity to aminoglycoside phosphotransfer... | 114 | 9e-27 | 37% |
| WP_054296836.1 APH(3'') family aminoglycoside O-phosphotransfe... | 115 | 1e-26 | 37% |
| TCO62827.1 streptomycin 3'-kinase [Actinocrispum wychmicini] | 115 | 1e-26 | 35% |
| WP_158584389.1 APH(3') family aminoglycoside O-phosphotransfer... | 114 | 1e-26 | 31% |
| CAA4162891.1 aminoglycoside 3'-phosphotransferase [Staphylococ... | 115 | 1e-26 | 31% |
| WP_061734663.1 aminoglycoside O-phosphotransferase APH(3')-III... | 114 | 1e-26 | 31% |
| WP_190215688.1 phosphotransferase [Kitasatospora indigofera] | 115 | 1e-26 | 35% |
| WP_033589219.1 aminoglycoside O-phosphotransferase APH(3')-III... | 114 | 1e-26 | 32% |
| HHU60333.1 TPA: aminoglycoside 3'-phosphotransferase [Natronin... | 114 | 1e-26 | 31% |
| VUP34563.1 Aminoglycoside 3'-phosphotransferase [Stenotrophomo... | 112 | 1e-26 | 44% |
| EGO8708928.1 aminoglycoside O-phosphotransferase APH(3')-IIIa ... | 114 | 1e-26 | 31% |
| WP_195109479.1 APH(3') family aminoglycoside O-phosphotransfer... | 114 | 1e-26 | 38% |
| WP_026022243.1 aminoglycoside 3'-phosphotransferase [Clostridi... | 112 | 1e-26 | 34% |
| SNY88776.1 kanamycin kinase [Nocardia amikacinitolerans] | 117 | 1e-26 | 46% |
| WP_067831704.1 aminoglycoside 3'-phosphotransferase [Actinomad... | 114 | 1e-26 | 38% |
| WP_047017491.1 APH(3'') family aminoglycoside O-phosphotransfe... | 114 | 1e-26 | 36% |
| WP_042285241.1 aminoglycoside 3'-phosphotransferase [Nocardiop... | 115 | 1e-26 | 34% |
| HAP8753309.1 TPA: aminoglycoside O-phosphotransferase APH(3')-... | 114 | 1e-26 | 32% |
| RHP36326.1 aminoglycoside 3'-phosphotransferase [Lachnotalea s... | 114 | 1e-26 | 31% |
| QCX20236.1 aminoglycoside O-phosphotransferase APH(3')-Ia [Esc... | 112 | 1e-26 | 36% |
| WP_116443248.1 aminoglycoside O-phosphotransferase APH(3')-III... | 114 | 1e-26 | 32% |
| WP_086037374.1 aminoglycoside O-phosphotransferase APH(3')-III... | 114 | 1e-26 | 32% |

| WP_101466605.1 phosphotransferase [Nocardia fluminea] | 117 | 1e-26 | 41% |
| --- | --- | --- | --- |
| WP_163187182.1 phosphotransferase [Cellulosimicrobium sp. SL-1] | 114 | 1e-26 | 37% |
| WP_031870106.1 aminoglycoside O-phosphotransferase APH(3')-III... | 114 | 1e-26 | 31% |
| WP_155560075.1 aminoglycoside O-phosphotransferase APH(3')-III... | 114 | 1e-26 | 31% |
| WP_072512560.1 aminoglycoside O-phosphotransferase APH(3')-III... | 114 | 1e-26 | 31% |
| HAQ4662027.1 TPA: aminoglycoside O-phosphotransferase APH(3')-... | 114 | 1e-26 | 31% |
| WP_113590176.1 aminoglycoside O-phosphotransferase APH(3')-III... | 114 | 1e-26 | 31% |
| CAA4171824.1 aminoglycoside 3'-phosphotransferase [Staphylococ... | 114 | 2e-26 | 31% |
| WP_097195953.1 aminoglycoside 3'-phosphotransferase [Blastococ... | 114 | 2e-26 | 36% |
| WP_049882886.1 MULTISPECIES: aminoglycoside O-phosphotransfera... | 114 | 2e-26 | 32% |
| WP_076097742.1 aminoglycoside 3'-phosphotransferase [Salmonell... | 113 | 2e-26 | 33% |
| OCI09314.1 APH(3') family aminoglycoside O-phosphotransferase ... | 114 | 2e-26 | 32% |
| EGQ1474457.1 aminoglycoside O-phosphotransferase APH(3')-IIIa ... | 114 | 2e-26 | 31% |
| WP_196496234.1 aminoglycoside O-phosphotransferase APH(3')-III... | 114 | 2e-26 | 31% |
| WP_086045164.1 aminoglycoside O-phosphotransferase APH(3')-III... | 114 | 2e-26 | 31% |
| WP_132177488.1 aminoglycoside 3'-phosphotransferase [Kribbella... | 114 | 2e-26 | 34% |
| GHE26148.1 aminoglycoside phosphotransferase APH(3') [Kitasato... | 115 | 2e-26 | 35% |
| CAA4053685.1 aminoglycoside 3'-phosphotransferase [Staphylococ... | 114 | 2e-26 | 31% |
| WP_125936591.1 APH(3'') family aminoglycoside O-phosphotransfe... | 114 | 2e-26 | 36% |
| NSQ73706.1 aminoglycoside O-phosphotransferase APH(3')-IIIa [E... | 114 | 2e-26 | 31% |
| WP_086045028.1 aminoglycoside O-phosphotransferase APH(3')-III... | 114 | 2e-26 | 31% |
| MBD5498824.1 aminoglycoside 3'-phosphotransferase [Lachnospira... | 114 | 2e-26 | 31% |
| WP_055984612.1 aminoglycoside 3'-phosphotransferase [Sphingomo... | 114 | 2e-26 | 36% |
| GAS89222.1 aminoglycoside 3'-phosphotransferase [Mycolicibacte... | 114 | 2e-26 | 38% |
| WP_174188380.1 tetratricopeptide repeat protein [Nocardia bard... | 117 | 2e-26 | 40% |
| WP_062829792.1 aminoglycoside 3'-phosphotransferase [Mycolicib... | 114 | 2e-26 | 38% |
| EGQ4172736.1 aminoglycoside O-phosphotransferase APH(3')-IIIa ... | 114 | 2e-26 | 31% |
| WP_055068576.1 MULTISPECIES: aminoglycoside 3'-phosphotransfer... | 114 | 2e-26 | 33% |
| WP_099790290.1 APH(3'') family aminoglycoside O-phosphotransfe... | 114 | 2e-26 | 36% |
| EGQ1530569.1 aminoglycoside O-phosphotransferase APH(3')-IIIa ... | 114 | 2e-26 | 30% |
| WP_195139111.1 APH(3') family aminoglycoside O-phosphotransfer... | 117 | 2e-26 | 40% |
| WP_030813850.1 aminoglycoside 3'-phosphotransferase [Streptomy... | 113 | 2e-26 | 39% |
| EGQ3843707.1 aminoglycoside O-phosphotransferase APH(3')-IIIa ... | 114 | 2e-26 | 31% |
| WP_159920268.1 MULTISPECIES: APH(3') family aminoglycoside O-p... | 116 | 2e-26 | 40% |
| NSR75352.1 aminoglycoside O-phosphotransferase APH(3')-IIIa [E... | 114 | 2e-26 | 31% |
| WP_064890262.1 aminoglycoside 3'-phosphotransferase [Mycolicib... | 114 | 2e-26 | 37% |
| KGM01491.1 aminoglycoside phosphotransferase [Cellulomonas cel... | 114 | 2e-26 | 38% |
| AHH29419.1 neomycin phosphotransferase III [Enterococcus faeca... | 114 | 2e-26 | 32% |
| SMG54884.1 Phosphotransferase enzyme family protein [Paraburkh... | 109 | 2e-26 | 45% |
| QEN99269.1 Aminoglycoside O-phosphotransferase APH(3')-Ia [mix... | 112 | 2e-26 | 36% |
| WP_144917456.1 aminoglycoside 3'-phosphotransferase [Klebsiell... | 112 | 2e-26 | 33% |
| WP_084317082.1 aminoglycoside 3'-phosphotransferase [Actinospi... | 113 | 2e-26 | 36% |
| WP_181580826.1 phosphotransferase [Nocardia sp. WCH-YHL-001] | 114 | 2e-26 | 42% |
| WP_031895205.1 aminoglycoside O-phosphotransferase APH(3')-III... | 114 | 2e-26 | 32% |
| WP_027768502.1 APH(3'') family aminoglycoside O-phosphotransfe... | 114 | 2e-26 | 36% |
| HAQ0220257.1 TPA: aminoglycoside O-phosphotransferase APH(3')-... | 114 | 2e-26 | 32% |
| WP_142346395.1 aminoglycoside 3'-phosphotransferase [Staphyloc... | 111 | 2e-26 | 37% |
| EGC7555114.1 aminoglycoside O-phosphotransferase APH(3')-IIIa ... | 114 | 2e-26 | 31% |
| EDO8956100.1 aminoglycoside O-phosphotransferase APH(3')-IIIa ... | 114 | 2e-26 | 31% |
| MBD3495274.1 aminoglycoside O-phosphotransferase APH(3')-IIIa ... | 114 | 2e-26 | 31% |
| WP_171440583.1 aminoglycoside O-phosphotransferase APH(3')-III... | 114 | 2e-26 | 31% |
| EAL0928475.1 aminoglycoside O-phosphotransferase APH(3')-IIIa ... | 114 | 2e-26 | 31% |
| HAP4152412.1 TPA: aminoglycoside O-phosphotransferase APH(3')-... | 114 | 2e-26 | 31% |
| QEN99288.1 Aminoglycoside O-phosphotransferase APH(3')-Ia [mix... | 111 | 2e-26 | 36% |
| WP_035843416.1 aminoglycoside 3'-phosphotransferase [Kitasatos... | 114 | 2e-26 | 35% |
| WP_069160157.1 phosphotransferase [Nocardia altamirensis] | 117 | 2e-26 | 42% |
| WP_013019321.1 APH(3'') family aminoglycoside O-phosphotransfe... | 114 | 2e-26 | 37% |
| CVI65624.1 Aminoglycoside 3'-phosphotransferase [Clostridiales... | 114 | 2e-26 | 29% |
| EAI4087095.1 aminoglycoside O-phosphotransferase APH(3')-IIIa ... | 114 | 2e-26 | 31% |
| WP_051931711.1 aminoglycoside 3'-phosphotransferase [Clostridi... | 113 | 2e-26 | 30% |
| WP_057488376.1 aminoglycoside O-phosphotransferase APH(3')-III... | 114 | 3e-26 | 31% |
| WP_081788811.1 aminoglycoside 3'-phosphotransferase [Candidatu... | 113 | 3e-26 | 37% |
| WP_123976779.1 aminoglycoside 3'-phosphotransferase [Streptomy... | 114 | 3e-26 | 36% |
| MXY47695.1 aminoglycoside 3'-phosphotransferase [Gemmatimonade... | 114 | 3e-26 | 32% |
| AAX53116.1 kanamycin resistance protein [Escherichia coli] | 111 | 3e-26 | 36% |
| EAI5807692.1 aminoglycoside O-phosphotransferase APH(3')-IIIa ... | 113 | 3e-26 | 31% |
| HBO22432.1 TPA: aminoglycoside O-phosphotransferase APH(3')-II... | 113 | 3e-26 | 35% |
| WP_018840698.1 APH(3'') family aminoglycoside O-phosphotransfe... | 113 | 3e-26 | 36% |
| WP_077944871.1 aminoglycoside 3'-phosphotransferase [Salmonell... | 110 | 3e-26 | 38% |
| AHH29412.1 neomycin phosphotransferase III [Enterococcus faeca... | 113 | 3e-26 | 31% |
| WP_030413323.1 aminoglycoside 3'-phosphotransferase [Streptomy... | 113 | 3e-26 | 37% |
| WP_064922125.1 MULTISPECIES: aminoglycoside 3'-phosphotransfer... | 113 | 4e-26 | 38% |

| WP_132890846.1 aminoglycoside 3'-phosphotransferase [Streptomy... | 113 | 4e-26 | 37% |
| --- | --- | --- | --- |
| AHH29485.1 neomycin phosphotransferase III [Enterococcus faeca... | 113 | 4e-26 | 31% |
| AUG75918.1 aminoglycoside phosphotransferase [Kitasatospora sp... | 113 | 4e-26 | 36% |
| WP_046283821.1 MULTISPECIES: aminoglycoside 3'-phosphotransfer... | 113 | 4e-26 | 37% |
| WP_132672804.1 aminoglycoside 3'-phosphotransferase [Saccharop... | 113 | 4e-26 | 36% |
| WP_018841969.1 APH(3'') family aminoglycoside O-phosphotransfe... | 113 | 4e-26 | 36% |
| WP_105503311.1 aminoglycoside O-phosphotransferase APH(3')-III... | 113 | 4e-26 | 32% |
| WP_195078959.1 tetratricopeptide repeat protein [Nocardia beij... | 115 | 4e-26 | 40% |
| WP_104820663.1 aminoglycoside 3'-phosphotransferase [Kitasatos... | 113 | 4e-26 | 36% |
| AHH29416.1 neomycin phosphotransferase III [Enterococcus faeca... | 112 | 4e-26 | 31% |
| WP_163608906.1 phosphotransferase [Modestobacter muralis] | 115 | 4e-26 | 37% |
| RRE00805.1 APH(3'') family aminoglycoside O-phosphotransferase... | 113 | 4e-26 | 36% |
| AHH29414.1 neomycin phosphotransferase III [Enterococcus faeca... | 112 | 4e-26 | 31% |
| WP_184585002.1 phosphotransferase [Lipingzhangella halophila] | 113 | 4e-26 | 37% |
| AHH29418.1 neomycin phosphotransferase III [Enterococcus faeca... | 112 | 4e-26 | 31% |
| HAP3637999.1 TPA: aminoglycoside O-phosphotransferase APH(3')-... | 112 | 4e-26 | 32% |
| MXX04130.1 aminoglycoside 3'-phosphotransferase [Gemmatimonade... | 113 | 4e-26 | 32% |
| CDH97911.2 AphA3 protein [Campylobacter coli] | 113 | 4e-26 | 31% |
| WP_071661646.1 aminoglycoside O-phosphotransferase APH(3')-III... | 113 | 4e-26 | 31% |
| EGO5129664.1 aminoglycoside O-phosphotransferase APH(3')-IIIa ... | 113 | 4e-26 | 31% |
| CCX49839.1 aminoglycoside phosphotransferase [Clostridium sp. ... | 113 | 4e-26 | 33% |
| WP_185715284.1 APH(3'') family aminoglycoside O-phosphotransfe... | 113 | 4e-26 | 36% |
| EIU14428.1 streptomycin 3''-kinase [Mycobacteroides abscessus ... | 113 | 5e-26 | 36% |
| CAA4055604.1 aminoglycoside 3'-phosphotransferase [Staphylococ... | 113 | 5e-26 | 31% |
| MBD5534840.1 aminoglycoside 3'-phosphotransferase [Lachnospira... | 113 | 5e-26 | 29% |
| WP_076051640.1 aminoglycoside 3'-phosphotransferase [Mycobacte... | 113 | 5e-26 | 37% |
| WP_031166105.1 APH(3'') family aminoglycoside O-phosphotransfe... | 113 | 5e-26 | 36% |
| EED6458634.1 aminoglycoside 3'-phosphotransferase [Salmonella ... | 111 | 5e-26 | 33% |
| TCR69478.1 kanamycin kinase [Streptomyces sp. BK674] | 113 | 5e-26 | 37% |
| WP_189226529.1 phosphotransferase [Saccharothrix coeruleofusca] | 113 | 5e-26 | 37% |
| RIT45006.1 APH(3'') family aminoglycoside O-phosphotransferase... | 112 | 5e-26 | 36% |
| WP_005105234.1 APH(3'') family aminoglycoside O-phosphotransfe... | 112 | 5e-26 | 36% |
| WP_005058584.1 APH(3'') family aminoglycoside O-phosphotransfe... | 112 | 5e-26 | 36% |
| WP_162293875.1 APH(3'') family aminoglycoside O-phosphotransfe... | 112 | 5e-26 | 37% |
| ACN38351.1 aminoglycoside phosphotransferase [Micromonospora i... | 113 | 5e-26 | 35% |
| WP_195109385.1 unnamed protein product | 115 | 6e-26 | 41% |
| WP_159754395.1 APH(3') family aminoglycoside O-phosphotransfer... | 112 | 6e-26 | 36% |
| EGQ0291835.1 aminoglycoside O-phosphotransferase APH(3')-IIIa ... | 112 | 6e-26 | 31% |
| WP_185062128.1 phosphotransferase [Pseudonocardia eucalypti] | 113 | 6e-26 | 37% |
| WP_084142805.1 aminoglycoside 3'-phosphotransferase [Cellulomo... | 113 | 6e-26 | 38% |
| WP_165703507.1 APH(3'') family aminoglycoside O-phosphotransfe... | 112 | 6e-26 | 36% |
| WP_005119838.1 APH(3'') family aminoglycoside O-phosphotransfe... | 112 | 6e-26 | 36% |
| WP_165694839.1 APH(3'') family aminoglycoside O-phosphotransfe... | 112 | 6e-26 | 36% |
| WP_158076830.1 APH(3') family aminoglycoside O-phosphotransfer... | 112 | 6e-26 | 31% |
| WP_165693748.1 APH(3'') family aminoglycoside O-phosphotransfe... | 112 | 6e-26 | 36% |
| WP_065152140.1 aminoglycoside 3'-phosphotransferase [Mycolicib... | 112 | 6e-26 | 37% |
| WP_194824303.1 tetratricopeptide repeat protein [Nocardia sp. ... | 115 | 6e-26 | 39% |
| WP_196077463.1 MULTISPECIES: APH(3') family aminoglycoside O-p... | 112 | 7e-26 | 32% |
| WP_139604938.1 aminoglycoside 3'-phosphotransferase [Paenibaci... | 112 | 7e-26 | 32% |
| WP_051426125.1 aminoglycoside 3'-phosphotransferase [Jiangella... | 112 | 8e-26 | 39% |
| WP_125520878.1 APH(3'') family aminoglycoside O-phosphotransfe... | 112 | 8e-26 | 38% |
| WP_090827123.1 phosphotransferase [Paenibacillus sp. yr247] | 107 | 8e-26 | 54% |
| MBE5802735.1 aminoglycoside 3'-phosphotransferase [Clostridial... | 112 | 8e-26 | 35% |
| WP_012890594.1 APH(3'') family aminoglycoside O-phosphotransfe... | 112 | 8e-26 | 36% |
| WP_165694033.1 APH(3'') family aminoglycoside O-phosphotransfe... | 112 | 8e-26 | 36% |
| PZQ61149.1 APH(3') family aminoglycoside O-phosphotransferase ... | 112 | 8e-26 | 36% |
| WP_127628297.1 APH(3'') family aminoglycoside O-phosphotransfe... | 112 | 8e-26 | 34% |
| WP_151774796.1 aminoglycoside 3'-phosphotransferase [Streptomy... | 112 | 8e-26 | 36% |
| WP_005096825.1 APH(3'') family aminoglycoside O-phosphotransfe... | 112 | 8e-26 | 36% |
| NUR26639.1 aminoglycoside 3'-phosphotransferase [Catenulispora... | 112 | 8e-26 | 35% |
| WP_184970806.1 phosphotransferase [Nonomuraea endophytica] | 115 | 8e-26 | 42% |
| WP_067460635.1 tetratricopeptide repeat protein [Nocardia alba] | 115 | 9e-26 | 41% |
| WP_018853508.1 APH(3'') family aminoglycoside O-phosphotransfe... | 112 | 9e-26 | 36% |
| EIT97899.1 streptomycin 3''-kinase [Mycobacteroides abscessus ... | 112 | 9e-26 | 36% |
| WP_184836875.1 phosphotransferase [Kribbella solani] | 112 | 9e-26 | 34% |
| WP_108297688.1 APH(3'') family aminoglycoside O-phosphotransfe... | 112 | 9e-26 | 37% |
| WP_022685897.1 aminoglycoside 3'-phosphotransferase [Sphingomo... | 112 | 9e-26 | 33% |
| ALA09310.1 neomycin phosphotransferase [Zea mays] | 106 | 9e-26 | 100% |
| NED74469.1 aminoglycoside 3'-phosphotransferase [Streptomyces ... | 112 | 9e-26 | 38% |
| WP_109551756.1 APH(3'') family aminoglycoside O-phosphotransfe... | 112 | 9e-26 | 35% |
| RBO93696.1 streptomycin 3'-kinase [Nocardia puris] | 112 | 1e-25 | 37% |
| SJL78961.1 unnamed protein product [Gateway entry vector pENTR... | 110 | 1e-25 | 33% |

| WP_176163387.1 APH(3'') family aminoglycoside O-phosphotransfe... | 112 | 1e-25 | 35% |
| --- | --- | --- | --- |
| WP_145898252.1 APH(3'') family aminoglycoside O-phosphotransfe... | 112 | 1e-25 | 36% |
| WP_184786613.1 phosphotransferase [Phytomonospora endophytica] | 112 | 1e-25 | 32% |
| WP_130456913.1 APH(3'') family aminoglycoside O-phosphotransfe... | 112 | 1e-25 | 36% |
| WP_159459717.1 APH(3') family aminoglycoside O-phosphotransfer... | 112 | 1e-25 | 33% |
| MBB2923338.1 kanamycin kinase [Cellulomonas cellasea] | 112 | 1e-25 | 38% |
| NLL33694.1 aminoglycoside 3'-phosphotransferase [Clostridiales... | 112 | 1e-25 | 31% |
| WP_037748895.1 APH(3'') family aminoglycoside O-phosphotransfe... | 112 | 1e-25 | 36% |
| WP_016891158.1 APH(3'') family aminoglycoside O-phosphotransfe... | 112 | 1e-25 | 36% |
| WP_184726393.1 phosphotransferase [Saccharopolyspora phatthalu... | 112 | 1e-25 | 35% |
| WP_084537261.1 APH(3'') family aminoglycoside O-phosphotransfe... | 112 | 1e-25 | 37% |
| QEN99306.1 Aminoglycoside O-phosphotransferase APH(3')-Ia [mix... | 109 | 1e-25 | 36% |
| WP_031884901.1 aminoglycoside O-phosphotransferase APH(3')-III... | 111 | 1e-25 | 32% |
| WP_074321895.1 APH(3'') family aminoglycoside O-phosphotransfe... | 112 | 1e-25 | 36% |
| WP_139324354.1 aminoglycoside 3'-phosphotransferase [Salmonell... | 110 | 1e-25 | 35% |
| WP_052538991.1 APH(3'') family aminoglycoside O-phosphotransfe... | 112 | 1e-25 | 36% |
| WP_084482753.1 phosphotransferase [Nocardia amikacinitolerans] | 114 | 1e-25 | 40% |
| MVJ93287.1 aminoglycoside O-phosphotransferase APH(3')-IIIa [S... | 111 | 1e-25 | 32% |
| WP_125531911.1 APH(3'') family aminoglycoside O-phosphotransfe... | 112 | 1e-25 | 38% |
| MBE5960687.1 aminoglycoside 3'-phosphotransferase [Lachnospira... | 112 | 1e-25 | 29% |
| WP_063103994.1 aminoglycoside 3'-phosphotransferase [Escherich... | 110 | 1e-25 | 33% |
| WP_077942037.1 aminoglycoside 3'-phosphotransferase [Salmonell... | 108 | 1e-25 | 38% |
| AHH29463.1 neomycin phosphotransferase III [Enterococcus faeca... | 111 | 1e-25 | 31% |
| AHH29465.1 neomycin phosphotransferase III [Enterococcus faeca... | 111 | 1e-25 | 32% |
| SIJ69597.1 Probable streptomycin phosphotransferase [Mycobacte... | 111 | 1e-25 | 36% |
| MBE5794979.1 aminoglycoside 3'-phosphotransferase [Clostridial... | 111 | 1e-25 | 33% |
| WP_005461291.1 aminoglycoside 3'-phosphotransferase [Saccharom... | 112 | 1e-25 | 36% |
| AHH29387.1 neomycin phosphotransferase III [Enterococcus faeca... | 111 | 1e-25 | 32% |
| WP_086151198.1 aminoglycoside 3'-phosphotransferase [Cellulosi... | 112 | 2e-25 | 36% |
| WP_165736828.1 APH(3'') family aminoglycoside O-phosphotransfe... | 111 | 2e-25 | 36% |
| WP_083898588.1 phosphotransferase [Nocardia vinacea] | 114 | 2e-25 | 41% |
| WP_048280811.1 phosphotransferase [Pseudomonas aeruginosa] | 106 | 2e-25 | 68% |
| WP_161111833.1 phosphotransferase [Nocardiopsis alba] | 112 | 2e-25 | 35% |
| RIR89737.1 APH(3'') family aminoglycoside O-phosphotransferase... | 111 | 2e-25 | 36% |
| WP_012066515.1 MULTISPECIES: APH(3'') family aminoglycoside O-... | 112 | 2e-25 | 34% |
| WP_050511936.1 APH(3'') family aminoglycoside O-phosphotransfe... | 112 | 2e-25 | 38% |
| WP_037160408.1 APH(3'') family aminoglycoside O-phosphotransfe... | 111 | 2e-25 | 34% |
| WP_062912330.1 APH(3'') family aminoglycoside O-phosphotransfe... | 111 | 2e-25 | 36% |
| WP_087080500.1 aminoglycoside 3'-phosphotransferase [Mycobacte... | 112 | 2e-25 | 37% |
| MBD3162401.1 phosphotransferase [Candidatus Latescibacteria ba... | 108 | 2e-25 | 39% |
| WP_085387629.1 MULTISPECIES: aminoglycoside 3'-phosphotransfer... | 112 | 2e-25 | 36% |
| SDG03398.1 kanamycin kinase [Cellulosimicrobium cellulans] | 112 | 2e-25 | 36% |
| WP_162293595.1 APH(3'') family aminoglycoside O-phosphotransfe... | 111 | 2e-25 | 36% |
| WP_152838210.1 aminoglycoside 3'-phosphotransferase [Rugamonas... | 111 | 2e-25 | 37% |
| WP_061266840.1 MULTISPECIES: aminoglycoside 3'-phosphotransfer... | 112 | 2e-25 | 36% |
| WP_132608846.1 APH(3'') family aminoglycoside O-phosphotransfe... | 111 | 2e-25 | 37% |
| WP_042423589.1 aminoglycoside 3'-phosphotransferase [Streptaci... | 111 | 2e-25 | 37% |
| MBA3249948.1 aminoglycoside 3'-phosphotransferase [Geodermatop... | 110 | 2e-25 | 37% |
| WP_165983521.1 APH(3') family aminoglycoside O-phosphotransfer... | 111 | 2e-25 | 31% |
| WP_162298534.1 APH(3'') family aminoglycoside O-phosphotransfe... | 111 | 2e-25 | 36% |
| WP_183296195.1 phosphotransferase [Cellulomonas cellasea] | 112 | 2e-25 | 38% |
| WP_162294297.1 APH(3'') family aminoglycoside O-phosphotransfe... | 111 | 2e-25 | 36% |
| WP_186376463.1 APH(3'') family aminoglycoside O-phosphotransfe... | 111 | 2e-25 | 36% |
| TDM40318.1 aminoglycoside 3'-phosphotransferase [Macrococcus g... | 111 | 2e-25 | 31% |
| WP_175008268.1 phosphotransferase [Cellulosimicrobium sp. TH-20] | 111 | 2e-25 | 36% |
| WP_051670491.1 aminoglycoside 3'-phosphotransferase [Bryobacte... | 110 | 2e-25 | 36% |
| KZM05000.1 hypothetical protein AN929_33105 [Pseudomonas aerug... | 106 | 2e-25 | 49% |
| WP_076986079.1 APH(3'') family aminoglycoside O-phosphotransfe... | 111 | 2e-25 | 35% |
| WP_096341199.1 aminoglycoside 3'-phosphotransferase, partial [... | 110 | 2e-25 | 32% |
| VDA12220.1 aph(3')-Ic_1_X62115 [Acinetobacter baumannii] | 109 | 2e-25 | 33% |
| WP_074243408.1 unnamed protein product | 111 | 2e-25 | 36% |
| WP_049945468.1 aminoglycoside O-phosphotransferase APH(3')-III... | 111 | 3e-25 | 31% |
| WP_170915078.1 MULTISPECIES: APH(3'') family aminoglycoside O-... | 110 | 3e-25 | 37% |
| MPZ67336.1 phosphotransferase [Pseudonocardiaceae bacterium] | 110 | 3e-25 | 35% |
| SDQ15783.1 kanamycin kinase [Quadrisphaera sp. DSM 44207] | 111 | 3e-25 | 39% |
| MBC7842441.1 aminoglycoside 3'-phosphotransferase [Gemmatimona... | 111 | 3e-25 | 31% |
| WP_185074692.1 APH(3'') family aminoglycoside O-phosphotransfe... | 111 | 3e-25 | 36% |
| MBF3307585.1 phosphotransferase [Pseudomonas aeruginosa] | 105 | 3e-25 | 55% |
| WP_144722630.1 aminoglycoside 3'-phosphotransferase [Cellulosi... | 111 | 3e-25 | 36% |
| WP_074332222.1 APH(3'') family aminoglycoside O-phosphotransfe... | 110 | 3e-25 | 36% |
| WP_068040460.1 APH(3'') family aminoglycoside O-phosphotransfe... | 111 | 3e-25 | 36% |
| RIR63789.1 APH(3'') family aminoglycoside O-phosphotransferase... | 110 | 3e-25 | 36% |

| AMP53507.1 hypothetical protein [uncultured bacterium] | 104 | 3e-25 | 100% |
| --- | --- | --- | --- |
| VDA75788.1 aph(3')-Ia_1_V00359 [Klebsiella pneumoniae] | 109 | 3e-25 | 33% |
| WP_088797444.1 MULTISPECIES: APH(3'') family aminoglycoside O-... | 111 | 3e-25 | 35% |
| WP_107608220.1 APH(3'') family aminoglycoside O-phosphotransfe... | 111 | 3e-25 | 32% |
| WP_052430837.1 aminoglycoside 3'-phosphotransferase [Robinsoni... | 110 | 3e-25 | 33% |
| WP_005088884.1 APH(3'') family aminoglycoside O-phosphotransfe... | 110 | 3e-25 | 36% |
| AHH29521.1 neomycin phosphotransferase III [Enterococcus faeca... | 110 | 3e-25 | 31% |
| WP_074232634.1 APH(3'') family aminoglycoside O-phosphotransfe... | 110 | 3e-25 | 36% |
| WP_049232627.1 APH(3'') family aminoglycoside O-phosphotransfe... | 110 | 3e-25 | 36% |
| WP_136964820.1 APH(3'') family aminoglycoside O-phosphotransfe... | 111 | 3e-25 | 36% |
| WP_050503690.1 APH(3'') family aminoglycoside O-phosphotransfe... | 111 | 3e-25 | 37% |
| WP_162295644.1 APH(3'') family aminoglycoside O-phosphotransfe... | 110 | 3e-25 | 36% |
| WP_037210411.1 APH(3'') family aminoglycoside O-phosphotransfe... | 110 | 3e-25 | 34% |
| WP_085989894.1 APH(3'') family aminoglycoside O-phosphotransfe... | 110 | 3e-25 | 36% |
| WP_025089389.1 APH(3'') family aminoglycoside O-phosphotransfe... | 110 | 3e-25 | 36% |
| WP_162293680.1 APH(3'') family aminoglycoside O-phosphotransfe... | 110 | 3e-25 | 36% |
| WP_188894049.1 phosphotransferase [Microlunatus endophyticus] | 110 | 3e-25 | 34% |
| WP_194520959.1 phosphotransferase [Cellulosimicrobium sp. JZ28] | 111 | 3e-25 | 36% |
| WP_005110890.1 APH(3'') family aminoglycoside O-phosphotransfe... | 110 | 3e-25 | 36% |
| WP_005114345.1 APH(3'') family aminoglycoside O-phosphotransfe... | 110 | 3e-25 | 36% |
| QNO58513.1 APH(3')-Ia [uncultured bacterium] | 108 | 4e-25 | 35% |
| RIS84275.1 APH(3'') family aminoglycoside O-phosphotransferase... | 110 | 4e-25 | 36% |
| WP_016893098.1 APH(3'') family aminoglycoside O-phosphotransfe... | 110 | 4e-25 | 36% |
| PVA60335.1 APH(3'') family aminoglycoside O-phosphotransferase... | 110 | 4e-25 | 36% |
| EFK5232600.1 aminoglycoside 3'-phosphotransferase [Escherichia... | 109 | 4e-25 | 33% |
| WP_162295563.1 APH(3'') family aminoglycoside O-phosphotransfe... | 110 | 4e-25 | 36% |
| WP_167488361.1 APH(3') family aminoglycoside O-phosphotransfer... | 110 | 4e-25 | 36% |
| WP_165692954.1 APH(3'') family aminoglycoside O-phosphotransfe... | 110 | 4e-25 | 36% |
| WP_091264228.1 aminoglycoside 3'-phosphotransferase [Alkalibac... | 110 | 4e-25 | 29% |
| WP_194906053.1 phosphotransferase [Catenulispora rubra] | 110 | 4e-25 | 35% |
| WP_067974981.1 aminoglycoside 3'-phosphotransferase [Mycolicib... | 110 | 4e-25 | 36% |
| WP_191986931.1 APH(3'') family aminoglycoside O-phosphotransfe... | 110 | 4e-25 | 35% |
| PTU56877.1 aminoglycoside phosphotransferase APH(3') [Sphaeris... | 110 | 4e-25 | 36% |
| WP_127659591.1 APH(3'') family aminoglycoside O-phosphotransfe... | 110 | 4e-25 | 34% |
| WP_063840676.1 aminoglycoside O-phosphotransferase APH(3'')-Ia... | 110 | 4e-25 | 38% |
| MRW83443.1 APH(3') family aminoglycoside O-phosphotransferase ... | 110 | 4e-25 | 36% |
| WP_162295286.1 APH(3'') family aminoglycoside O-phosphotransfe... | 110 | 5e-25 | 36% |
| WP_119553251.1 APH(3'') family aminoglycoside O-phosphotransfe... | 110 | 5e-25 | 35% |
| WP_183643736.1 phosphotransferase [Nonomuraea dietziae] | 110 | 5e-25 | 36% |
| RIS61649.1 APH(3'') family aminoglycoside O-phosphotransferase... | 110 | 5e-25 | 36% |
| WP_078336369.1 APH(3'') family aminoglycoside O-phosphotransfe... | 110 | 5e-25 | 35% |
| WP_082428419.1 aminoglycoside 3'-phosphotransferase [Pseudomon... | 110 | 5e-25 | 34% |
| WP_101380471.1 aminoglycoside 3'-phosphotransferase [Streptomy... | 110 | 5e-25 | 36% |
| WP_165702955.1 APH(3'') family aminoglycoside O-phosphotransfe... | 110 | 5e-25 | 36% |
| WP_017205247.1 APH(3'') family aminoglycoside O-phosphotransfe... | 110 | 5e-25 | 36% |
| WP_031826967.1 aminoglycoside O-phosphotransferase APH(3')-III... | 109 | 5e-25 | 31% |
| WP_166454806.1 APH(3') family aminoglycoside O-phosphotransfer... | 110 | 5e-25 | 36% |
| WP_129835641.1 APH(3'') family aminoglycoside O-phosphotransfe... | 110 | 5e-25 | 36% |
| WP_164217934.1 APH(3'') family aminoglycoside O-phosphotransfe... | 110 | 5e-25 | 37% |
| WP_165645498.1 APH(3'') family aminoglycoside O-phosphotransfe... | 110 | 5e-25 | 36% |
| WP_128229190.1 APH(3'') family aminoglycoside O-phosphotransfe... | 110 | 5e-25 | 33% |
| PVA36879.1 APH(3'') family aminoglycoside O-phosphotransferase... | 110 | 5e-25 | 36% |
| WP_087763728.1 MULTISPECIES: APH(3'') family aminoglycoside O-... | 110 | 6e-25 | 38% |
| WP_052377665.1 MULTISPECIES: aminoglycoside 3'-phosphotransfer... | 110 | 6e-25 | 33% |
| WP_050514113.1 MULTISPECIES: APH(3'') family aminoglycoside O-... | 110 | 6e-25 | 38% |
| TOH33970.1 APH(3') family aminoglycoside O-phosphotransferase ... | 107 | 6e-25 | 35% |
| WP_012230841.1 phosphotransferase [Candidatus Microthrix parvi... | 109 | 6e-25 | 35% |
| WP_030202653.1 aminoglycoside 3'-phosphotransferase [Streptomy... | 110 | 6e-25 | 38% |
| HBM98987.1 TPA: hypothetical protein [Ruminococcus sp.] | 107 | 6e-25 | 36% |
| WP_043655102.1 aminoglycoside 3'-phosphotransferase [Cellulosi... | 110 | 6e-25 | 36% |
| WP_188340299.1 phosphotransferase [Streptomyces sp. CRXT-Y-14] | 110 | 6e-25 | 40% |
| AHH29454.1 neomycin phosphotransferase III [Enterococcus faecium] | 109 | 6e-25 | 32% |
| WP_165616158.1 APH(3'') family aminoglycoside O-phosphotransfe... | 110 | 7e-25 | 36% |
| WP_162296149.1 APH(3'') family aminoglycoside O-phosphotransfe... | 110 | 7e-25 | 36% |
| WP_052099593.1 aminoglycoside 3'-phosphotransferase [Blautia p... | 109 | 7e-25 | 33% |
| WP_027992916.1 APH(3'') family aminoglycoside O-phosphotransfe... | 110 | 7e-25 | 32% |
| WP_074320350.1 APH(3'') family aminoglycoside O-phosphotransfe... | 110 | 7e-25 | 36% |
| EFF91122.1 kanamycin kinase [Streptomyces sp. e14] | 109 | 7e-25 | 38% |
| CPT97305.1 Probable streptomycin phosphotransferase [Mycobacte... | 110 | 7e-25 | 36% |
| WP_194982373.1 phosphotransferase [Nocardia cyriacigeorgica] | 109 | 7e-25 | 40% |
| WP_169438789.1 phosphotransferase [Streptomyces sp. GMY01] | 109 | 7e-25 | 38% |
| WP_084484119.1 phosphotransferase [Nocardia anaemiae] | 112 | 7e-25 | 40% |

| WP_165983392.1 APH(3') family aminoglycoside O-phosphotransfer... | 109 | 7e-25 | 30% |
| --- | --- | --- | --- |
| WP_090424355.1 MULTISPECIES: APH(3'') family aminoglycoside O-... | 109 | 7e-25 | 36% |
| WP_159271463.1 APH(3'') family aminoglycoside O-phosphotransfe... | 110 | 7e-25 | 35% |
| WP_062878951.1 APH(3'') family aminoglycoside O-phosphotransfe... | 110 | 7e-25 | 36% |
| WP_052524735.1 APH(3'') family aminoglycoside O-phosphotransfe... | 110 | 8e-25 | 36% |
| WP_194980337.1 phosphotransferase [Nocardia cyriacigeorgica] | 109 | 8e-25 | 40% |
| TDM45667.1 aminoglycoside 3'-phosphotransferase [Macrococcus g... | 109 | 8e-25 | 30% |
| WP_074363908.1 APH(3'') family aminoglycoside O-phosphotransfe... | 109 | 8e-25 | 36% |
| WP_195136373.1 tetratricopeptide repeat protein [Nocardia bras... | 112 | 8e-25 | 39% |
| WP_162145597.1 APH(3'') family aminoglycoside O-phosphotransfe... | 110 | 8e-25 | 32% |
| WP_089222361.1 aminoglycoside 3'-phosphotransferase [Streptomy... | 109 | 8e-25 | 37% |
| ELQ83814.1 aminoglycoside phosphotransferase [Streptomyces rim... | 110 | 8e-25 | 38% |
| WP_056811412.1 tetratricopeptide repeat protein [Nocardia sp. ... | 112 | 8e-25 | 39% |
| WP_028010479.1 APH(3'') family aminoglycoside O-phosphotransfe... | 110 | 8e-25 | 32% |
| WP_165663137.1 APH(3'') family aminoglycoside O-phosphotransfe... | 109 | 8e-25 | 36% |
| WP_129810389.1 APH(3'') family aminoglycoside O-phosphotransfe... | 110 | 8e-25 | 36% |
| WP_095745776.1 APH(3'') family aminoglycoside O-phosphotransfe... | 110 | 8e-25 | 35% |
| WP_182844556.1 phosphotransferase [Actinomadura namibiensis] | 109 | 8e-25 | 37% |
| ORA24579.1 APH(3'') family aminoglycoside O-phosphotransferase... | 109 | 9e-25 | 36% |
| WP_067776017.1 APH(3'') family aminoglycoside O-phosphotransfe... | 109 | 9e-25 | 35% |
| WP_165704763.1 APH(3'') family aminoglycoside O-phosphotransfe... | 109 | 9e-25 | 36% |
| MYX42345.1 phosphotransferase [Streptomyces sp. SID89] | 109 | 9e-25 | 38% |
| WP_003964117.1 MULTISPECIES: aminoglycoside O-phosphotransfera... | 110 | 9e-25 | 37% |
| WP_050504558.1 MULTISPECIES: APH(3'') family aminoglycoside O-... | 110 | 9e-25 | 38% |
| WP_194227552.1 phosphotransferase [Cellulosimicrobium cellulans] | 110 | 9e-25 | 35% |
| WP_005075456.1 APH(3'') family aminoglycoside O-phosphotransfe... | 109 | 9e-25 | 36% |
| PBC82150.1 streptomycin 3'-kinase [Streptomyces sp. 2321.6] | 109 | 9e-25 | 36% |
| WP_024454460.1 MULTISPECIES: APH(3'') family aminoglycoside O-... | 109 | 9e-25 | 37% |
| WP_195047653.1 phosphotransferase [Nocardia cyriacigeorgica] | 109 | 9e-25 | 40% |
| WP_179559847.1 phosphotransferase [Microbacterium sp. AK009] | 109 | 9e-25 | 35% |
| WP_050508721.1 APH(3'') family aminoglycoside O-phosphotransfe... | 110 | 1e-24 | 38% |
| WP_195092453.1 tetratricopeptide repeat protein [Nocardia bras... | 112 | 1e-24 | 39% |
| KGE57456.1 phosphotransferase enzyme family protein [Streptoco... | 107 | 1e-24 | 32% |
| WP_179936198.1 APH(3'') family aminoglycoside O-phosphotransfe... | 109 | 1e-24 | 37% |
| CPZ16266.1 Probable streptomycin phosphotransferase [Mycobacte... | 108 | 1e-24 | 38% |
| WP_049885644.1 aminoglycoside O-phosphotransferase APH(3')-III... | 109 | 1e-24 | 31% |
| WP_082871825.1 aminoglycoside 3'-phosphotransferase [Nocardia ... | 108 | 1e-24 | 35% |
| WP_165704390.1 APH(3'') family aminoglycoside O-phosphotransfe... | 109 | 1e-24 | 36% |
| WP_127657967.1 APH(3'') family aminoglycoside O-phosphotransfe... | 109 | 1e-24 | 32% |
| EFT5606734.1 aminoglycoside 3'-phosphotransferase [Escherichia... | 105 | 1e-24 | 39% |
| WP_130917186.1 aminoglycoside 3'-phosphotransferase [Nocardia ... | 108 | 1e-24 | 39% |
| WP_148169726.1 aminoglycoside 3'-phosphotransferase [Escherich... | 108 | 1e-24 | 33% |
| WP_129858362.1 APH(3'') family aminoglycoside O-phosphotransfe... | 109 | 1e-24 | 36% |
| WP_043260855.1 aminoglycoside 3'-phosphotransferase [Streptomy... | 109 | 1e-24 | 38% |
| WP_092861778.1 aminoglycoside 3'-phosphotransferase [Quadrisph... | 110 | 1e-24 | 39% |
| WP_168944330.1 phosphotransferase [Cellulosimicrobium aquatile] | 109 | 1e-24 | 36% |
| WP_067170103.1 aminoglycoside 3'-phosphotransferase [Microtetr... | 109 | 1e-24 | 38% |
| EBH8612176.1 aminoglycoside 3'-phosphotransferase [Salmonella ... | 105 | 1e-24 | 38% |
| WP_145904985.1 aminoglycoside 3'-phosphotransferase [Kitasatos... | 109 | 1e-24 | 37% |
| WP_165702401.1 APH(3'') family aminoglycoside O-phosphotransfe... | 109 | 1e-24 | 36% |
| WP_012377648.1 MULTISPECIES: aminoglycoside O-phosphotransfera... | 109 | 1e-24 | 38% |
| WP_104782762.1 APH(3'') family aminoglycoside O-phosphotransfe... | 109 | 1e-24 | 35% |
| WP_165696683.1 APH(3'') family aminoglycoside O-phosphotransfe... | 109 | 1e-24 | 36% |
| EAK1521749.1 aminoglycoside O-phosphotransferase APH(3')-IIIa ... | 109 | 1e-24 | 31% |
| VUP34548.1 Aminoglycoside 3'-phosphotransferase [Stenotrophomo... | 103 | 1e-24 | 63% |
| WP_063840677.1 aminoglycoside O-phosphotransferase APH(3'')-Ia... | 109 | 1e-24 | 38% |
| TMD53263.1 aminoglycoside 3'-phosphotransferase [Chloroflexi b... | 109 | 1e-24 | 35% |
| WP_013281618.1 aminoglycoside 3'-phosphotransferase [Butyrivib... | 109 | 1e-24 | 31% |
| WP_093565947.1 aminoglycoside O-phosphotransferase APH(3'')-Ia... | 109 | 1e-24 | 37% |
| PJD74539.1 kanamycin kinase [Enterobacter hormaechei] | 105 | 1e-24 | 38% |
| EET3986573.1 aminoglycoside 3'-phosphotransferase [Escherichia... | 105 | 1e-24 | 38% |
| WP_032449434.1 MULTISPECIES: aminoglycoside 3'-phosphotransfer... | 105 | 1e-24 | 39% |
| WP_084540336.1 MULTISPECIES: aminoglycoside 3'-phosphotransfer... | 105 | 1e-24 | 38% |
| HAP4790815.1 TPA: aminoglycoside O-phosphotransferase APH(3')-... | 108 | 1e-24 | 31% |
| WP_096501287.1 APH(3'') family aminoglycoside O-phosphotransfe... | 108 | 1e-24 | 35% |
| KGE55559.1 phosphotransferase enzyme family protein [Streptoco... | 107 | 1e-24 | 32% |
| WP_127516867.1 APH(3'') family aminoglycoside O-phosphotransfe... | 109 | 2e-24 | 32% |
| WP_064957078.1 APH(3'') family aminoglycoside O-phosphotransfe... | 108 | 2e-24 | 36% |
| WP_053700369.1 APH(3'') family aminoglycoside O-phosphotransfe... | 109 | 2e-24 | 38% |
| WP_031899839.1 aminoglycoside 3'-phosphotransferase [Staphyloc... | 107 | 2e-24 | 33% |
| WP_092965221.1 aminoglycoside 3'-phosphotransferase [Agromyces... | 108 | 2e-24 | 35% |
| GFE07530.1 putative streptomycin phosphotransferase [Streptomy... | 108 | 2e-24 | 36% |

| WP_149771726.1 MULTISPECIES: APH(3'') family aminoglycoside O-... | 109 | 2e-24 | 35% |
| --- | --- | --- | --- |
| WP_179766739.1 APH(3'') family aminoglycoside O-phosphotransfe... | 109 | 2e-24 | 38% |
| WP_160196330.1 APH(3') family aminoglycoside O-phosphotransfer... | 108 | 2e-24 | 28% |
| HAQ0955004.1 TPA: aminoglycoside O-phosphotransferase APH(3')-... | 108 | 2e-24 | 31% |
| SHR03763.1 Probable streptomycin phosphotransferase [Mycobacte... | 108 | 2e-24 | 37% |
| WP_031859716.1 aminoglycoside 3'-phosphotransferase [Staphyloc... | 107 | 2e-24 | 33% |
| MBE6949966.1 APH(3') family aminoglycoside O-phosphotransferas... | 111 | 2e-24 | 32% |
| SEP37268.1 aminoglycoside 3'-phosphotransferase-2 [Rhodopseudo... | 103 | 2e-24 | 74% |
| HAQ1816173.1 TPA: aminoglycoside O-phosphotransferase APH(3')-... | 108 | 2e-24 | 31% |
| WP_033049154.1 APH(3'') family aminoglycoside O-phosphotransfe... | 109 | 2e-24 | 32% |
| HAP6181828.1 TPA: aminoglycoside 3'-phosphotransferase [Entero... | 107 | 2e-24 | 33% |
| WP_084479105.1 aminoglycoside 3'-phosphotransferase [Nocardia ... | 108 | 2e-24 | 36% |
| WP_111180691.1 APH(3'') family aminoglycoside O-phosphotransfe... | 108 | 2e-24 | 36% |
| EHK74667.1 Aminoglycoside phosphotransferase [Sinorhizobium me... | 109 | 2e-24 | 32% |
| WP_091510760.1 aminoglycoside 3'-phosphotransferase [Amycolato... | 108 | 2e-24 | 36% |
| EFI3643107.1 aminoglycoside 3'-phosphotransferase [Escherichia... | 105 | 2e-24 | 38% |
| WP_053801698.1 APH(3'') family aminoglycoside O-phosphotransfe... | 109 | 2e-24 | 38% |
| HAP4791038.1 TPA: aminoglycoside O-phosphotransferase APH(3')-... | 108 | 2e-24 | 31% |
| WP_002409341.1 aminoglycoside O-phosphotransferase APH(3')-III... | 107 | 2e-24 | 33% |
| CFF91146.1 aminoglycoside 3'-phosphotransferase [Staphylococcu... | 107 | 2e-24 | 33% |
| WP_031926567.1 aminoglycoside 3'-phosphotransferase [Staphyloc... | 107 | 2e-24 | 33% |
| VDA76739.1 aph(3')-Ia_1_V00359 [Klebsiella pneumoniae] | 107 | 2e-24 | 33% |
| WP_034654480.1 aminoglycoside 3'-phosphotransferase [Cellulosi... | 108 | 2e-24 | 35% |
| WP_005079339.1 APH(3'') family aminoglycoside O-phosphotransfe... | 108 | 2e-24 | 36% |
| WP_130806432.1 aminoglycoside 3'-phosphotransferase [Senegalia... | 108 | 2e-24 | 28% |
| ECC6429654.1 aminoglycoside 3'-phosphotransferase [Salmonella ... | 105 | 2e-24 | 39% |
| WP_153022770.1 aminoglycoside 3'-phosphotransferase [Luteimicr... | 108 | 2e-24 | 36% |
| WP_195039892.1 phosphotransferase [Nocardia cyriacigeorgica] | 108 | 2e-24 | 40% |
| EAJ1960881.1 aminoglycoside 3'-phosphotransferase [Campylobact... | 107 | 2e-24 | 33% |
| WP_192783781.1 APH(3'') family aminoglycoside O-phosphotransfe... | 108 | 2e-24 | 36% |
| WP_060811600.1 aminoglycoside O-phosphotransferase APH(3')-III... | 108 | 2e-24 | 31% |
| WP_036538113.1 aminoglycoside 3'-phosphotransferase [Nocardia ... | 108 | 2e-24 | 39% |
| MIS62221.1 aminoglycoside 3'-phosphotransferase [Salmonella en... | 108 | 2e-24 | 35% |
| HAP8592835.1 TPA: aminoglycoside O-phosphotransferase APH(3')-... | 107 | 2e-24 | 33% |
| WP_087168971.1 aminoglycoside 3'-phosphotransferase [Drancourt... | 108 | 2e-24 | 32% |
| WP_142440803.1 aminoglycoside 3'-phosphotransferase [Escherich... | 107 | 2e-24 | 33% |
| WP_104359189.1 aminoglycoside 3'-phosphotransferase [Nocardia ... | 108 | 2e-24 | 40% |
| WP_165443328.1 aminoglycoside O-phosphotransferase APH(3')-III... | 110 | 2e-24 | 31% |
| WP_165640520.1 APH(3'') family aminoglycoside O-phosphotransfe... | 108 | 2e-24 | 36% |
| WP_031824769.1 aminoglycoside O-phosphotransferase APH(3')-III... | 107 | 2e-24 | 33% |
| WP_159477292.1 APH(3'') family aminoglycoside O-phosphotransfe... | 108 | 2e-24 | 36% |
| WP_174186651.1 APH(3'') family aminoglycoside O-phosphotransfe... | 108 | 2e-24 | 38% |
| WP_030594525.1 MULTISPECIES: APH(3'') family aminoglycoside O-... | 108 | 2e-24 | 37% |
| WP_129814553.1 APH(3'') family aminoglycoside O-phosphotransfe... | 108 | 2e-24 | 36% |
| WP_027747792.1 APH(3'') family aminoglycoside O-phosphotransfe... | 108 | 2e-24 | 36% |
| HAB3957682.1 TPA: aminoglycoside O-phosphotransferase APH(3'')... | 108 | 2e-24 | 35% |
| WP_031825816.1 aminoglycoside O-phosphotransferase APH(3')-III... | 107 | 2e-24 | 33% |
| MXZ76410.1 aminoglycoside 3'-phosphotransferase [Gemmatimonade... | 108 | 2e-24 | 31% |
| WP_167464157.1 tetratricopeptide repeat protein [Nocardia bras... | 111 | 2e-24 | 38% |
| AEO78182.1 streptomycin phosphotransferase A [Salmonella enter... | 108 | 2e-24 | 35% |
| EAY7223869.1 aminoglycoside O-phosphotransferase APH(3'')-Ib [... | 108 | 2e-24 | 35% |
| WP_195011564.1 phosphotransferase [Nocardia cyriacigeorgica] | 108 | 3e-24 | 40% |
| MYA76954.1 aminoglycoside 3'-phosphotransferase [Gemmatimonade... | 108 | 3e-24 | 32% |
| WP_030765638.1 MULTISPECIES: APH(3'') family aminoglycoside O-... | 108 | 3e-24 | 36% |
| ADO33189.1 aminoglycoside phosphotransferase APH (3')-IIIA [Cl... | 107 | 3e-24 | 33% |
| WP_130181023.1 aminoglycoside 3'-phosphotransferase [Blautia p... | 108 | 3e-24 | 32% |
| OTR22617.1 APH(3'') family aminoglycoside O-phosphotransferase... | 107 | 3e-24 | 38% |
| WP_071339474.1 MULTISPECIES: APH(3'') family aminoglycoside O-... | 108 | 3e-24 | 36% |
| WP_191495406.1 APH(3') family aminoglycoside O-phosphotransfer... | 107 | 3e-24 | 32% |
| WP_195730656.1 APH(3'') family aminoglycoside O-phosphotransfe... | 108 | 3e-24 | 37% |
| WP_009715727.1 APH(3'') family aminoglycoside O-phosphotransfe... | 108 | 3e-24 | 37% |
| QFU99691.1 Kanamycin kinase [Luteimicrobium xylanilyticum] | 108 | 3e-24 | 36% |
| KTT70024.1 hypothetical protein NS334_13455 [Sphingomonas endo... | 108 | 3e-24 | 36% |
| WP_118636657.1 aminoglycoside 3'-phosphotransferase [Ruminococ... | 108 | 3e-24 | 32% |
| PWM44392.1 aminoglycoside phosphotransferase APH(3') [Clostrid... | 108 | 3e-24 | 34% |
| SDB58150.1 aminoglycoside 3'-phosphotransferase-2 [Pseudomonas... | 103 | 3e-24 | 61% |
| TDZ77657.1 Aminoglycoside 3'-phosphotransferase [Mycobacteroid... | 108 | 3e-24 | 36% |
| VVO88799.1 hypothetical protein PS838_02197 [Pseudomonas fluor... | 105 | 3e-24 | 46% |
| WP_162297700.1 APH(3'') family aminoglycoside O-phosphotransfe... | 108 | 3e-24 | 36% |
| ASR39524.1 APH(3'') family aminoglycoside O-phosphotransferase... | 108 | 3e-24 | 34% |
| WP_191987737.1 APH(3'') family aminoglycoside O-phosphotransfe... | 108 | 3e-24 | 36% |
| RLA60914.1 hypothetical protein DRQ89_12145 [Epsilonproteobact... | 106 | 3e-24 | 34% |

| WP_117349556.1 APH(3'') family aminoglycoside O-phosphotransfe... | 108 | 4e-24 | 36% |
| --- | --- | --- | --- |
| EEZ5220991.1 aminoglycoside O-phosphotransferase APH(3'')-Ib [... | 108 | 4e-24 | 35% |
| WP_155349620.1 aminoglycoside 3'-phosphotransferase [Acrocarpo... | 107 | 4e-24 | 37% |
| WP_086370949.1 aminoglycoside 3'-phosphotransferase [Mycobacte... | 107 | 4e-24 | 36% |
| OTR00805.1 APH(3'') family aminoglycoside O-phosphotransferase... | 107 | 4e-24 | 38% |
| WP_163120628.1 phosphotransferase [Acinetobacter pittii] | 102 | 4e-24 | 61% |
| WP_150243784.1 APH(3'') family aminoglycoside O-phosphotransfe... | 108 | 4e-24 | 36% |
| WP_030696209.1 APH(3'') family aminoglycoside O-phosphotransfe... | 108 | 4e-24 | 37% |
| WP_086009099.1 APH(3'') family aminoglycoside O-phosphotransfe... | 107 | 4e-24 | 38% |
| WP_084501015.1 APH(3'') family aminoglycoside O-phosphotransfe... | 108 | 4e-24 | 37% |
| WP_005456047.1 APH(3'') family aminoglycoside O-phosphotransfe... | 108 | 4e-24 | 36% |
| WP_065039527.1 aminoglycoside 3'-phosphotransferase [Mycolicib... | 108 | 4e-24 | 35% |
| P0A3Y6.1 RecName: Full=Aminoglycoside 3'-phosphotransferase; A... | 108 | 4e-24 | 30% |
| WP_142846650.1 APH(3'') family aminoglycoside O-phosphotransfe... | 108 | 4e-24 | 35% |
| WP_005067977.1 aminoglycoside 3'-phosphotransferase [Mycobacte... | 107 | 4e-24 | 36% |
| TDZ85303.1 Aminoglycoside 3'-phosphotransferase [Mycobacteroid... | 107 | 4e-24 | 36% |
| KUJ68314.1 3'-kinase [Streptomyces albus subsp. albus] | 108 | 4e-24 | 34% |
| NLD79230.1 aminoglycoside 3'-phosphotransferase [Mollicutes ba... | 107 | 4e-24 | 28% |
| HAN5012832.1 TPA: aminoglycoside O-phosphotransferase APH(3'')... | 108 | 4e-24 | 35% |
| EEK3056580.1 aminoglycoside 3'-phosphotransferase [Salmonella ... | 106 | 4e-24 | 33% |
| WP_026235248.1 MULTISPECIES: APH(3'') family aminoglycoside O-... | 108 | 4e-24 | 36% |
| WP_047466020.1 APH(3'') family aminoglycoside O-phosphotransfe... | 108 | 4e-24 | 37% |
| WP_139073516.1 MULTISPECIES: aminoglycoside 3'-phosphotransfer... | 108 | 4e-24 | 37% |
| WP_058756469.1 aminoglycoside 3'-phosphotransferase [Sphingomo... | 107 | 4e-24 | 36% |
| EES0288101.1 aminoglycoside O-phosphotransferase APH(3'')-Ib [... | 108 | 4e-24 | 35% |
| WP_165338912.1 phosphotransferase [Streptomyces sp. YC419] | 107 | 4e-24 | 37% |
| WP_187061623.1 MULTISPECIES: APH(3'') family aminoglycoside O-... | 108 | 5e-24 | 35% |
| WP_165662826.1 APH(3'') family aminoglycoside O-phosphotransfe... | 107 | 5e-24 | 36% |
| WP_052466698.1 aminoglycoside 3'-phosphotransferase [Beduini m... | 107 | 5e-24 | 31% |
| WP_165262943.1 phosphotransferase [Streptomyces sp. HC44] | 107 | 5e-24 | 35% |
| NLH68404.1 phosphotransferase [Candidatus Microthrix parvicella] | 107 | 5e-24 | 34% |
| WP_162289239.1 phosphotransferase [Cellulosimicrobium cellulans] | 108 | 5e-24 | 36% |
| WP_134069797.1 APH(3'') family aminoglycoside O-phosphotransfe... | 107 | 5e-24 | 36% |
| TWF77487.1 streptomycin 3'-kinase [Pseudonocardia hierapolitana] | 107 | 5e-24 | 36% |
| WP_037075101.1 APH(3'') family aminoglycoside O-phosphotransfe... | 107 | 5e-24 | 34% |
| WP_146579726.1 APH(3'') family aminoglycoside O-phosphotransfe... | 108 | 5e-24 | 36% |
| WP_138403799.1 aminoglycoside 3'-phosphotransferase [Facklamia... | 107 | 5e-24 | 29% |
| WP_182784695.1 APH(3'') family aminoglycoside O-phosphotransfe... | 107 | 5e-24 | 35% |
| CAF34039.1 aminoglycoside-O-phosphoryl-transferase [Micromonos... | 107 | 5e-24 | 34% |
| WP_165617373.1 APH(3'') family aminoglycoside O-phosphotransfe... | 107 | 6e-24 | 36% |
| HCT76036.1 TPA: aminoglycoside phosphotransferase APH(3') [Mic... | 107 | 6e-24 | 32% |
| WP_147256659.1 APH(3'') family aminoglycoside O-phosphotransfe... | 107 | 6e-24 | 36% |
| WP_139666702.1 MULTISPECIES: aminoglycoside 3'-phosphotransfer... | 107 | 6e-24 | 34% |
| WP_106434970.1 MULTISPECIES: aminoglycoside 3'-phosphotransfer... | 107 | 6e-24 | 38% |
| WP_087252143.1 aminoglycoside 3'-phosphotransferase [Drancourt... | 107 | 6e-24 | 31% |
| WP_140149204.1 aminoglycoside 3'-phosphotransferase [Vibrio pa... | 104 | 6e-24 | 36% |
| WP_052850452.1 APH(3'') family aminoglycoside O-phosphotransfe... | 107 | 6e-24 | 34% |
| WP_091810279.1 APH(3'') family aminoglycoside O-phosphotransfe... | 108 | 6e-24 | 34% |
| WP_083911488.1 phosphotransferase [Nocardia takedensis] | 110 | 6e-24 | 38% |
| WP_013136946.1 MULTISPECIES: aminoglycoside O-phosphotransfera... | 107 | 6e-24 | 35% |
| WP_047180397.1 APH(3'') family aminoglycoside O-phosphotransfe... | 107 | 7e-24 | 37% |
| WP_189081087.1 phosphotransferase [Mangrovihabitans endophyticus] | 107 | 7e-24 | 35% |
| WP_109507164.1 APH(3'') family aminoglycoside O-phosphotransfe... | 107 | 7e-24 | 36% |
| EFH4982392.1 aminoglycoside O-phosphotransferase APH(3'')-Ib [... | 107 | 7e-24 | 35% |
| CCF64488.1 putative aminoglycoside 3'-O-phosphotransferase [No... | 107 | 7e-24 | 35% |
| WP_159798359.1 APH(3'') family aminoglycoside O-phosphotransfe... | 107 | 7e-24 | 38% |
| ECA9156492.1 aminoglycoside O-phosphotransferase APH(3'')-Ib [... | 107 | 7e-24 | 35% |
| WP_078863926.1 APH(3'') family aminoglycoside O-phosphotransfe... | 107 | 7e-24 | 35% |
| WP_031859755.1 aminoglycoside 3'-phosphotransferase [Staphyloc... | 105 | 7e-24 | 33% |
| WP_033239265.1 APH(3'') family aminoglycoside O-phosphotransfe... | 107 | 7e-24 | 37% |
| PXY29925.1 APH(3'') family aminoglycoside O-phosphotransferase... | 107 | 7e-24 | 36% |
| WP_141801883.1 aminoglycoside 3'-phosphotransferase [Nocardioi... | 107 | 7e-24 | 35% |
| EEI7390449.1 aminoglycoside O-phosphotransferase APH(3'')-Ib [... | 107 | 7e-24 | 35% |
| EBW5961451.1 aminoglycoside O-phosphotransferase APH(3'')-Ib [... | 107 | 7e-24 | 35% |
| OXL23102.1 aminoglycoside phosphotransferase APH(3') [Streptom... | 106 | 8e-24 | 36% |
| WP_031860225.1 aminoglycoside 3'-phosphotransferase [Staphyloc... | 105 | 8e-24 | 33% |
| WP_086072180.1 APH(3'') family aminoglycoside O-phosphotransfe... | 107 | 8e-24 | 34% |
| WP_148280916.1 aminoglycoside 3'-phosphotransferase [Nocardia ... | 107 | 8e-24 | 35% |
| WP_136129362.1 aminoglycoside O-phosphotransferase APH(3'')-Ib... | 107 | 8e-24 | 35% |
| SMC31222.1 kanamycin kinase [Ruminococcaceae bacterium] | 107 | 8e-24 | 32% |
| WP_095947132.1 APH(3'') family aminoglycoside O-phosphotransfe... | 107 | 8e-24 | 34% |
| MBC9925584.1 aminoglycoside O-phosphotransferase APH(3'')-Ib [... | 107 | 8e-24 | 35% |

| WP_078322531.1 APH(3'') family aminoglycoside O-phosphotransfe... | 107 | 8e-24 | 36% |
| --- | --- | --- | --- |
| HAH2286349.1 TPA: aminoglycoside O-phosphotransferase APH(3'')... | 107 | 8e-24 | 34% |
| MBB2181901.1 APH(3') family aminoglycoside O-phosphotransferas... | 110 | 8e-24 | 31% |
| WP_094463989.1 aminoglycoside 3'-phosphotransferase [Staphyloc... | 105 | 8e-24 | 33% |
| WP_087773868.1 APH(3'') family aminoglycoside O-phosphotransfe... | 107 | 8e-24 | 36% |
| WP_119104783.1 aminoglycoside 3'-phosphotransferase [Streptomy... | 107 | 8e-24 | 35% |
| WP_191835078.1 APH(3'') family aminoglycoside O-phosphotransfe... | 107 | 8e-24 | 37% |
| WP_083887644.1 phosphotransferase [Nocardia asiatica] | 109 | 8e-24 | 37% |
| WP_166617680.1 phosphotransferase [Kineococcus vitellinus] | 107 | 9e-24 | 35% |
| WP_031943936.1 MULTISPECIES: aminoglycoside O-phosphotransfera... | 107 | 9e-24 | 35% |
| EDL3417896.1 APH(3'') family aminoglycoside O-phosphotransfera... | 107 | 9e-24 | 35% |
| EFH3215500.1 aminoglycoside O-phosphotransferase APH(3'')-Ib [... | 107 | 9e-24 | 35% |
| KIF69288.1 3'-kinase [Streptomyces sp. AcH 505] | 107 | 9e-24 | 35% |
| WP_051440807.1 APH(3'') family aminoglycoside O-phosphotransfe... | 107 | 9e-24 | 33% |
| MBE6131462.1 aminoglycoside 3'-phosphotransferase [Erysipelotr... | 107 | 9e-24 | 31% |
| WP_129804628.1 APH(3'') family aminoglycoside O-phosphotransfe... | 107 | 9e-24 | 36% |
| WP_085147592.1 APH(3'') family aminoglycoside O-phosphotransfe... | 106 | 9e-24 | 37% |
| WP_065553273.1 aminoglycoside O-phosphotransferase APH(3'')-Ib... | 107 | 1e-23 | 35% |
| EEI9864677.1 aminoglycoside O-phosphotransferase APH(3'')-Ib [... | 107 | 1e-23 | 35% |
| ECY5643992.1 aminoglycoside O-phosphotransferase APH(3'')-Ib [... | 107 | 1e-23 | 35% |
| EDT9245544.1 aminoglycoside O-phosphotransferase APH(3'')-Ib [... | 107 | 1e-23 | 35% |
| WP_067849142.1 APH(3'') family aminoglycoside O-phosphotransfe... | 107 | 1e-23 | 37% |
| WP_180750366.1 APH(3'') family aminoglycoside O-phosphotransfe... | 107 | 1e-23 | 33% |
| SJZ89904.1 streptomycin 3-kinase [Marinactinospora thermotoler... | 107 | 1e-23 | 36% |
| WP_139703067.1 APH(3'') family aminoglycoside O-phosphotransfe... | 107 | 1e-23 | 34% |
| WP_031825931.1 aminoglycoside 3'-phosphotransferase [Staphyloc... | 105 | 1e-23 | 34% |
| WP_049978454.1 MULTISPECIES: APH(3'') family aminoglycoside O-... | 107 | 1e-23 | 35% |
| WP_166232741.1 APH(3'') family aminoglycoside O-phosphotransfe... | 107 | 1e-23 | 35% |
| WP_192969215.1 APH(3'') family aminoglycoside O-phosphotransfe... | 106 | 1e-23 | 35% |
| MBE5958998.1 aminoglycoside 3'-phosphotransferase [Lachnospira... | 107 | 1e-23 | 31% |
| WP_138374024.1 MULTISPECIES: phosphotransferase [Ruminococcaceae] | 104 | 1e-23 | 35% |
| WP_169442690.1 APH(3'') family aminoglycoside O-phosphotransfe... | 107 | 1e-23 | 35% |
| WP_161562186.1 APH(3'') family aminoglycoside O-phosphotransfe... | 107 | 1e-23 | 36% |
| AMP56031.1 hypothetical protein [uncultured bacterium] | 100 | 1e-23 | 100% |
| WP_070939979.1 APH(3'') family aminoglycoside O-phosphotransfe... | 106 | 1e-23 | 34% |
| EGF9610499.1 APH(3') family aminoglycoside O-phosphotransferas... | 100 | 1e-23 | 100% |
| EAC1561288.1 aminoglycoside O-phosphotransferase APH(3'')-Ib [... | 107 | 1e-23 | 35% |
| WP_116907199.1 MULTISPECIES: APH(3'') family aminoglycoside O-... | 107 | 1e-23 | 33% |
| WP_078761139.1 APH(3'') family aminoglycoside O-phosphotransfe... | 107 | 1e-23 | 36% |
| WP_129811142.1 APH(3'') family aminoglycoside O-phosphotransfe... | 107 | 1e-23 | 35% |
| WP_129290423.1 APH(3'') family aminoglycoside O-phosphotransfe... | 107 | 1e-23 | 35% |
| EBA2612496.1 aminoglycoside O-phosphotransferase APH(3'')-Ib [... | 107 | 1e-23 | 35% |
| WP_031861361.1 aminoglycoside 3'-phosphotransferase [Staphyloc... | 105 | 1e-23 | 34% |
| WP_020416808.1 APH(3'') family aminoglycoside O-phosphotransfe... | 106 | 1e-23 | 36% |
| WP_031890219.1 aminoglycoside 3'-phosphotransferase [Staphyloc... | 105 | 1e-23 | 34% |
| EBF2003766.1 aminoglycoside O-phosphotransferase APH(3'')-Ib [... | 107 | 1e-23 | 34% |
| WP_160887566.1 APH(3'') family aminoglycoside O-phosphotransfe... | 107 | 1e-23 | 37% |
| EBZ9341142.1 aminoglycoside O-phosphotransferase APH(3'')-Ib [... | 107 | 1e-23 | 34% |
| WP_127450139.1 APH(3'') family aminoglycoside O-phosphotransfe... | 107 | 1e-23 | 35% |
| WP_176646257.1 APH(3') family aminoglycoside O-phosphotransfer... | 106 | 1e-23 | 35% |
| KAF1024624.1 Aminoglycoside 3'-phosphotransferase [Acinetobact... | 106 | 1e-23 | 32% |
| HBV51886.1 TPA: hypothetical protein [Clostridiales bacterium] | 105 | 1e-23 | 30% |
| WP_031825111.1 aminoglycoside 3'-phosphotransferase [Staphyloc... | 105 | 1e-23 | 34% |
| WP_151488315.1 aminoglycoside O-phosphotransferase APH(3'')-Ib... | 106 | 1e-23 | 35% |
| WP_016889070.1 APH(3'') family aminoglycoside O-phosphotransfe... | 106 | 1e-23 | 36% |
| WP_195018276.1 phosphotransferase [Nocardia cyriacigeorgica] | 106 | 1e-23 | 38% |
| WP_031788723.1 aminoglycoside 3'-phosphotransferase [Staphyloc... | 105 | 1e-23 | 34% |
| PWM63641.1 hypothetical protein DBX63_02010 [Clostridia bacter... | 106 | 1e-23 | 34% |
| WP_179285692.1 phosphotransferase [Streptomyces sp. NBS 14/10] | 106 | 1e-23 | 36% |
| MBB9161612.1 aminoglycoside O-phosphotransferase APH(3'')-Ib [... | 106 | 1e-23 | 34% |
| EWO50491.1 aminoglycoside 3'-phosphotransferase [Staphylococcu... | 104 | 1e-23 | 34% |
| WP_111715937.1 aminoglycoside 3'-phosphotransferase [Macrococc... | 106 | 1e-23 | 32% |
| EFV6593084.1 aminoglycoside O-phosphotransferase APH(3'')-Ib [... | 106 | 1e-23 | 34% |
| HAQ3499409.1 TPA: aminoglycoside 3'-phosphotransferase [Entero... | 105 | 1e-23 | 34% |
| WP_194994934.1 phosphotransferase [Nocardia cyriacigeorgica] | 106 | 1e-23 | 38% |
| WP_093492736.1 MULTISPECIES: APH(3'') family aminoglycoside O-... | 107 | 1e-23 | 36% |
| WP_026125351.1 aminoglycoside 3'-phosphotransferase [Nocardiop... | 107 | 1e-23 | 34% |
| EAY6849827.1 aminoglycoside O-phosphotransferase APH(3'')-Ib [... | 106 | 1e-23 | 34% |
| WP_147875417.1 APH(3'') family aminoglycoside O-phosphotransfe... | 106 | 1e-23 | 34% |
| QCM14195.1 aminoglycoside O-phosphotransferase APH(3'')-Ib [Ag... | 106 | 1e-23 | 34% |
| WP_031793406.1 aminoglycoside 3'-phosphotransferase [Staphyloc... | 104 | 1e-23 | 34% |
| EEV7654018.1 aminoglycoside O-phosphotransferase APH(3'')-Ib [... | 106 | 1e-23 | 34% |

| ECR4734927.1 aminoglycoside O-phosphotransferase APH(3'')-Ib [... | 106 | 1e-23 | 34% |
| --- | --- | --- | --- |
| EEZ70711.1 putative kanamycin kinase [Neisseria cinerea ATCC 1... | 104 | 1e-23 | 32% |
| WP_100299358.1 APH(3'') family aminoglycoside O-phosphotransfe... | 106 | 2e-23 | 35% |
| ECS3585718.1 aminoglycoside O-phosphotransferase APH(3'')-Ib [... | 106 | 2e-23 | 35% |
| HAH1358803.1 TPA: aminoglycoside O-phosphotransferase APH(3'')... | 106 | 2e-23 | 35% |
| WP_025464697.1 MULTISPECIES: aminoglycoside O-phosphotransfera... | 106 | 2e-23 | 35% |
| EGO4149673.1 aminoglycoside O-phosphotransferase APH(3'')-Ib [... | 106 | 2e-23 | 34% |
| EUQ45910.1 aminoglycoside 3'-phosphotransferase [Staphylococcu... | 104 | 2e-23 | 34% |
| EFA8364160.1 aminoglycoside O-phosphotransferase APH(3'')-Ib [... | 106 | 2e-23 | 34% |
| EBQ1534850.1 aminoglycoside O-phosphotransferase APH(3'')-Ib [... | 106 | 2e-23 | 35% |
| EDN7036356.1 aminoglycoside O-phosphotransferase APH(3'')-Ib [... | 106 | 2e-23 | 34% |
| WP_181727088.1 aminoglycoside O-phosphotransferase APH(3'')-Ib... | 106 | 2e-23 | 34% |
| WP_147506969.1 aminoglycoside 3'-phosphotransferase [Acinetoba... | 105 | 2e-23 | 33% |
| WP_135122337.1 aminoglycoside 3'-phosphotransferase [Mycobacte... | 106 | 2e-23 | 36% |
| EFO2629716.1 aminoglycoside 3'-phosphotransferase [Escherichia... | 106 | 2e-23 | 34% |
| SEV89163.1 kanamycin kinase/aminoglycoside 3'-phosphotransfera... | 106 | 2e-23 | 31% |
| EBQ1757473.1 aminoglycoside O-phosphotransferase APH(3'')-Ib [... | 106 | 2e-23 | 34% |
| WP_138207730.1 aminoglycoside 3'-phosphotransferase [Haloimpat... | 106 | 2e-23 | 28% |
| VFT39859.1 aminoglycoside 3'-phosphotransferase type IIb [Pseu... | 100 | 2e-23 | 67% |
| EBV8922371.1 aminoglycoside O-phosphotransferase APH(3'')-Ib [... | 106 | 2e-23 | 34% |
| WP_140351020.1 aminoglycoside 3'-phosphotransferase [Vibrio pa... | 103 | 2e-23 | 35% |
| WP_014909082.1 aminoglycoside 3'-phosphotransferase [Nocardiop... | 106 | 2e-23 | 34% |
| WP_070269529.1 aminoglycoside 3'-phosphotransferase [Duganella... | 106 | 2e-23 | 36% |
| WP_030306913.1 MULTISPECIES: APH(3'') family aminoglycoside O-... | 106 | 2e-23 | 35% |
| EFQ0968071.1 aminoglycoside O-phosphotransferase APH(3'')-Ib [... | 106 | 2e-23 | 34% |
| EFW1053157.1 aminoglycoside O-phosphotransferase APH(3'')-Ib [... | 106 | 2e-23 | 34% |
| EJG22722.1 streptomycin 3''-kinase [Acinetobacter baumannii OI... | 106 | 2e-23 | 35% |
| WP_128472939.1 aminoglycoside O-phosphotransferase APH(3'')-Ib... | 106 | 2e-23 | 34% |
| WP_073746572.1 APH(3'') family aminoglycoside O-phosphotransfe... | 106 | 2e-23 | 36% |
| EDX5217043.1 aminoglycoside O-phosphotransferase APH(3'')-Ib [... | 106 | 2e-23 | 35% |
| WP_115942852.1 aminoglycoside 3'-phosphotransferase [Cellulosi... | 106 | 2e-23 | 36% |
| WP_096953406.1 aminoglycoside 3'-phosphotransferase [Escherich... | 105 | 2e-23 | 35% |
| EFW1053620.1 aminoglycoside O-phosphotransferase APH(3'')-Ib [... | 106 | 2e-23 | 34% |
| MBE6987662.1 aminoglycoside 3'-phosphotransferase [Ruminococca... | 106 | 2e-23 | 30% |
| WP_191974647.1 aminoglycoside O-phosphotransferase APH(3'')-Ib... | 106 | 2e-23 | 34% |
| ADU56649.1 StrA [Acinetobacter baumannii] | 106 | 2e-23 | 35% |
| GFH94987.1 aminoglycoside 3'-phosphotransferase [Lachnospirace... | 106 | 2e-23 | 32% |
| EFN4359587.1 aminoglycoside O-phosphotransferase APH(3'')-Ib [... | 106 | 2e-23 | 34% |
| EFQ0968903.1 aminoglycoside O-phosphotransferase APH(3'')-Ib [... | 106 | 2e-23 | 34% |
| EBA2612486.1 aminoglycoside O-phosphotransferase APH(3'')-Ib [... | 106 | 2e-23 | 35% |
| EDG8352341.1 aminoglycoside O-phosphotransferase APH(3'')-Ib [... | 106 | 2e-23 | 34% |
| EFI3735716.1 aminoglycoside O-phosphotransferase APH(3'')-Ib [... | 106 | 2e-23 | 34% |
| WP_134041469.1 MULTISPECIES: APH(3'') family aminoglycoside O-... | 106 | 2e-23 | 36% |
| WP_150218359.1 aminoglycoside O-phosphotransferase APH(3'')-Ib... | 106 | 2e-23 | 34% |
| EBP9143780.1 aminoglycoside O-phosphotransferase APH(3'')-Ib [... | 106 | 2e-23 | 34% |
| WP_077202330.1 aminoglycoside O-phosphotransferase APH(3'')-Ib... | 106 | 2e-23 | 34% |
| WP_134240859.1 aminoglycoside O-phosphotransferase APH(3'')-Ib... | 106 | 2e-23 | 35% |
| EBJ4452588.1 aminoglycoside O-phosphotransferase APH(3'')-Ib [... | 106 | 2e-23 | 34% |
| EFW9449062.1 aminoglycoside O-phosphotransferase APH(3'')-Ib [... | 106 | 2e-23 | 34% |
| WP_029899186.1 tetratricopeptide repeat protein [Nocardia bras... | 108 | 2e-23 | 38% |
| EAU2875402.1 aminoglycoside O-phosphotransferase APH(3'')-Ib [... | 106 | 2e-23 | 34% |
| EGO4144588.1 aminoglycoside O-phosphotransferase APH(3'')-Ib [... | 106 | 2e-23 | 34% |
| EFH6287909.1 aminoglycoside O-phosphotransferase APH(3'')-Ib [... | 106 | 2e-23 | 34% |
| WP_107810877.1 aminoglycoside O-phosphotransferase APH(3'')-Ib... | 106 | 2e-23 | 34% |
| ECT5108493.1 aminoglycoside O-phosphotransferase APH(3'')-Ib [... | 106 | 2e-23 | 34% |
| WP_152937817.1 aminoglycoside 3'-phosphotransferase [Escherich... | 104 | 2e-23 | 33% |
| EAW0990214.1 aminoglycoside O-phosphotransferase APH(3'')-Ib [... | 106 | 2e-23 | 35% |
| WP_027217738.1 aminoglycoside 3'-phosphotransferase [Butyrivib... | 106 | 2e-23 | 28% |
| ECP3706485.1 aminoglycoside O-phosphotransferase APH(3'')-Ib [... | 106 | 2e-23 | 34% |
| WP_183273594.1 MULTISPECIES: aminoglycoside O-phosphotransfera... | 106 | 2e-23 | 34% |
| HAS2536680.1 TPA: aminoglycoside O-phosphotransferase APH(3'')... | 105 | 2e-23 | 34% |
| EEB8351866.1 aminoglycoside O-phosphotransferase APH(3'')-Ib [... | 106 | 2e-23 | 34% |
| WP_093201840.1 APH(3'') family aminoglycoside O-phosphotransfe... | 106 | 2e-23 | 35% |
| MBE6992352.1 aminoglycoside 3'-phosphotransferase [Ruminococca... | 105 | 2e-23 | 31% |
| WP_150386761.1 aminoglycoside O-phosphotransferase APH(3'')-Ib... | 106 | 2e-23 | 34% |
| EFQ9904682.1 aminoglycoside O-phosphotransferase APH(3'')-Ib [... | 106 | 2e-23 | 34% |
| EED9113631.1 aminoglycoside O-phosphotransferase APH(3'')-Ib [... | 106 | 2e-23 | 34% |
| WP_166721156.1 aminoglycoside O-phosphotransferase APH(3'')-Ib... | 106 | 2e-23 | 34% |
| EFP8939540.1 aminoglycoside O-phosphotransferase APH(3'')-Ib [... | 106 | 2e-23 | 34% |
| EFG0792844.1 aminoglycoside O-phosphotransferase APH(3'')-Ib [... | 106 | 2e-23 | 34% |
| HAQ2074446.1 TPA: aminoglycoside O-phosphotransferase APH(3')-... | 105 | 2e-23 | 31% |
| WP_058998950.1 aminoglycoside O-phosphotransferase APH(3'')-Ib... | 106 | 2e-23 | 34% |

| WP_164397942.1 APH(3'') family aminoglycoside O-phosphotransfe... | 106 | 2e-23 | 36% |
| --- | --- | --- | --- |
| WP_141691643.1 tetratricopeptide repeat protein [Nocardia bras... | 108 | 2e-23 | 38% |
| WP_081373708.1 aminoglycoside 3'-phosphotransferase [Butyrivib... | 106 | 2e-23 | 28% |
| EDA9910874.1 aminoglycoside O-phosphotransferase APH(3'')-Ib [... | 106 | 2e-23 | 34% |
| KAE8430774.1 aminoglycoside O-phosphotransferase APH(3'')-Ib [... | 106 | 2e-23 | 34% |
| EFO3519687.1 aminoglycoside 3'-phosphotransferase [Escherichia... | 106 | 2e-23 | 34% |
| HAO2583819.1 TPA: aminoglycoside O-phosphotransferase APH(3'')... | 106 | 2e-23 | 34% |
| WP_130076399.1 aminoglycoside O-phosphotransferase APH(3'')-Ib... | 106 | 2e-23 | 34% |
| WP_053021300.1 aminoglycoside O-phosphotransferase APH(3'')-Ib... | 105 | 2e-23 | 34% |
| AEG54147.1 Aminoglycoside phosphotransferase [Sinorhizobium me... | 106 | 2e-23 | 32% |
| WP_071610874.1 aminoglycoside O-phosphotransferase APH(3'')-Ib... | 106 | 2e-23 | 34% |
| EAX5048664.1 aminoglycoside O-phosphotransferase APH(3'')-Ib [... | 106 | 2e-23 | 34% |
| EGO8785630.1 aminoglycoside O-phosphotransferase APH(3'')-Ib [... | 106 | 2e-23 | 34% |
| GDW48355.1 aminoglycoside 3'-phosphotransferase [Escherichia c... | 104 | 2e-23 | 33% |
| WP_184693474.1 APH(3'') family aminoglycoside O-phosphotransfe... | 106 | 2e-23 | 36% |
| EEW2006630.1 aminoglycoside O-phosphotransferase APH(3'')-Ib [... | 106 | 2e-23 | 34% |
| HAK9726795.1 TPA: aminoglycoside O-phosphotransferase APH(3'')... | 106 | 2e-23 | 34% |
| ECT6039756.1 aminoglycoside O-phosphotransferase APH(3'')-Ib [... | 106 | 2e-23 | 34% |
| WP_169134541.1 phosphotransferase [Streptomyces sp. HNM0575] | 105 | 2e-23 | 36% |
| WP_005453155.1 aminoglycoside 3'-phosphotransferase [Saccharom... | 106 | 2e-23 | 35% |
| HAM2542000.1 TPA: aminoglycoside O-phosphotransferase APH(3'')... | 106 | 2e-23 | 34% |
| AMJ34192.1 APH3'' [uncultured bacterium] | 106 | 2e-23 | 34% |
| HAN3152867.1 TPA: aminoglycoside O-phosphotransferase APH(3'')... | 106 | 2e-23 | 34% |
| WP_137463450.1 aminoglycoside O-phosphotransferase APH(3'')-Ib... | 106 | 2e-23 | 34% |
| ECT6011080.1 aminoglycoside O-phosphotransferase APH(3'')-Ib [... | 106 | 2e-23 | 34% |
| ECT2290873.1 aminoglycoside O-phosphotransferase APH(3'')-Ib [... | 106 | 2e-23 | 34% |
| HAN7263390.1 TPA: aminoglycoside O-phosphotransferase APH(3'')... | 106 | 2e-23 | 34% |
| EDW6896419.1 aminoglycoside O-phosphotransferase APH(3'')-Ib [... | 106 | 2e-23 | 34% |
| WP_164993359.1 APH(3') family aminoglycoside O-phosphotransfer... | 105 | 2e-23 | 30% |
| EBP8018940.1 aminoglycoside O-phosphotransferase APH(3'')-Ib [... | 106 | 2e-23 | 34% |
| WP_078318572.1 aminoglycoside O-phosphotransferase APH(3'')-Ib... | 106 | 2e-23 | 34% |
| WP_001082316.1 MULTISPECIES: aminoglycoside O-phosphotransfera... | 106 | 2e-23 | 34% |
| EAS8594833.1 aminoglycoside O-phosphotransferase APH(3'')-Ib [... | 106 | 2e-23 | 34% |
| ADI08729.1 phosphotransferase [Streptomyces bingchenggensis BC... | 105 | 2e-23 | 36% |
| WP_058670999.1 MULTISPECIES: aminoglycoside O-phosphotransfera... | 105 | 2e-23 | 34% |
| WP_125281635.1 MULTISPECIES: aminoglycoside O-phosphotransfera... | 105 | 2e-23 | 34% |
| WP_195071538.1 APH(3'') family aminoglycoside O-phosphotransfe... | 106 | 3e-23 | 35% |
| WP_027990129.1 APH(3'') family aminoglycoside O-phosphotransfe... | 106 | 3e-23 | 32% |
| HAS1328361.1 TPA: aminoglycoside O-phosphotransferase APH(3'')... | 105 | 3e-23 | 34% |
| MIG22053.1 aminoglycoside O-phosphotransferase APH(3'')-Ib [Sa... | 105 | 3e-23 | 34% |
| HAM8929179.1 TPA: aminoglycoside O-phosphotransferase APH(3'')... | 105 | 3e-23 | 34% |
| WP_070359049.1 aminoglycoside 3'-phosphotransferase [Duganella... | 105 | 3e-23 | 36% |
| WP_096305516.1 aminoglycoside O-phosphotransferase APH(3'')-Ib... | 105 | 3e-23 | 34% |
| EBA2612491.1 aminoglycoside O-phosphotransferase APH(3'')-Ib [... | 105 | 3e-23 | 34% |
| EBF4861292.1 aminoglycoside O-phosphotransferase APH(3'')-Ib [... | 105 | 3e-23 | 34% |
| GHH54019.1 aminoglycoside O-phosphotransferase APH(3')-IIIa [[... | 105 | 3e-23 | 33% |
| EFV7877367.1 aminoglycoside O-phosphotransferase APH(3'')-Ib [... | 105 | 3e-23 | 34% |
| EBT9685592.1 aminoglycoside O-phosphotransferase APH(3'')-Ib [... | 105 | 3e-23 | 34% |
| RXK18519.1 aminoglycoside 3'-phosphotransferase [Macrococcus s... | 105 | 3e-23 | 30% |
| ECS6465741.1 aminoglycoside O-phosphotransferase APH(3'')-Ib [... | 105 | 3e-23 | 34% |
| WP_000310978.1 MULTISPECIES: aminoglycoside O-phosphotransfera... | 105 | 3e-23 | 34% |
| EAW5289103.1 aminoglycoside O-phosphotransferase APH(3'')-Ib [... | 105 | 3e-23 | 34% |
| WP_112380388.1 aminoglycoside O-phosphotransferase APH(3'')-Ib... | 105 | 3e-23 | 34% |
| WP_103566516.1 aminoglycoside O-phosphotransferase APH(3'')-Ib... | 105 | 3e-23 | 34% |
| EFA7009755.1 aminoglycoside O-phosphotransferase APH(3'')-Ib [... | 105 | 3e-23 | 35% |
| WP_162145500.1 APH(3'') family aminoglycoside O-phosphotransfe... | 106 | 3e-23 | 32% |
| HAQ2074717.1 TPA: aminoglycoside O-phosphotransferase APH(3')-... | 105 | 3e-23 | 31% |
| EEW1585334.1 aminoglycoside O-phosphotransferase APH(3'')-Ib [... | 105 | 3e-23 | 34% |
| EEO8496344.1 aminoglycoside O-phosphotransferase APH(3'')-Ib [... | 105 | 3e-23 | 34% |
| EBF4832732.1 aminoglycoside O-phosphotransferase APH(3'')-Ib [... | 105 | 3e-23 | 34% |
| EET1312205.1 aminoglycoside O-phosphotransferase APH(3'')-Ib [... | 105 | 3e-23 | 34% |
| WP_159345254.1 aminoglycoside O-phosphotransferase APH(3'')-Ib... | 105 | 3e-23 | 34% |
| EBY8367178.1 aminoglycoside O-phosphotransferase APH(3'')-Ib [... | 105 | 3e-23 | 34% |
| WP_057963435.1 MULTISPECIES: APH(3'') family aminoglycoside O-... | 105 | 3e-23 | 35% |
| WP_107409975.1 aminoglycoside 3'-phosphotransferase [Streptomy... | 106 | 3e-23 | 34% |
| ECS6465856.1 aminoglycoside O-phosphotransferase APH(3'')-Ib [... | 105 | 3e-23 | 34% |
| HAD8642344.1 TPA: aminoglycoside O-phosphotransferase APH(3'')... | 105 | 3e-23 | 34% |
| WP_126738165.1 aminoglycoside O-phosphotransferase APH(3'')-Ib... | 105 | 3e-23 | 34% |
| HAH6057555.1 TPA: APH(3'') family aminoglycoside O-phosphotran... | 105 | 3e-23 | 34% |
| WP_086727023.1 aminoglycoside O-phosphotransferase APH(3'')-Ib... | 105 | 3e-23 | 34% |
| WP_103954827.1 aminoglycoside 3'-phosphotransferase [Nonomurae... | 105 | 3e-23 | 36% |
| EFW9449912.1 aminoglycoside O-phosphotransferase APH(3'')-Ib [... | 105 | 3e-23 | 34% |

| WP_135716750.1 aminoglycoside O-phosphotransferase APH(3'')-Ib... | 105 | 3e-23 | 34% |
| --- | --- | --- | --- |
| WP_186141663.1 aminoglycoside O-phosphotransferase APH(3'')-Ib... | 105 | 3e-23 | 34% |
| WP_021566288.1 MULTISPECIES: aminoglycoside O-phosphotransfera... | 105 | 3e-23 | 34% |
| ECJ7081468.1 aminoglycoside O-phosphotransferase APH(3'')-Ib [... | 105 | 3e-23 | 34% |
| ALJ64911.1 streptomycin 3''-kinase [Vibrio cholerae O1 str. KW3] | 105 | 3e-23 | 34% |
| EFH3912182.1 aminoglycoside O-phosphotransferase APH(3'')-Ib [... | 105 | 3e-23 | 34% |
| WP_080884645.1 aminoglycoside O-phosphotransferase APH(3'')-Ib... | 105 | 3e-23 | 34% |
| WP_096262150.1 aminoglycoside O-phosphotransferase APH(3'')-Ib... | 105 | 3e-23 | 34% |
| WP_029110665.1 APH(3'') family aminoglycoside O-phosphotransfe... | 105 | 3e-23 | 34% |
| MBD4503277.1 phosphotransferase [Xanthomonas citri pv. citri] | 100 | 3e-23 | 64% |
| HAN2194282.1 TPA: aminoglycoside O-phosphotransferase APH(3'')... | 105 | 3e-23 | 34% |
| EDI6902305.1 APH(3'') family aminoglycoside O-phosphotransfera... | 105 | 3e-23 | 34% |
| WP_099147762.1 MULTISPECIES: aminoglycoside O-phosphotransfera... | 105 | 3e-23 | 34% |
| EAY9148677.1 aminoglycoside O-phosphotransferase APH(3'')-Ib [... | 105 | 3e-23 | 34% |
| WP_079983178.1 aminoglycoside O-phosphotransferase APH(3'')-Ib... | 105 | 3e-23 | 34% |
| HAG9143999.1 TPA: aminoglycoside O-phosphotransferase APH(3'')... | 105 | 3e-23 | 34% |
| EGF3087595.1 aminoglycoside O-phosphotransferase APH(3'')-Ib [... | 105 | 3e-23 | 34% |
| EAW4265317.1 aminoglycoside O-phosphotransferase APH(3'')-Ib [... | 105 | 3e-23 | 34% |
| HAS2081434.1 TPA: aminoglycoside O-phosphotransferase APH(3'')... | 105 | 3e-23 | 34% |
| EEA9607116.1 aminoglycoside O-phosphotransferase APH(3'')-Ib [... | 105 | 3e-23 | 34% |
| WP_032428352.1 MULTISPECIES: aminoglycoside O-phosphotransfera... | 105 | 3e-23 | 34% |
| WP_074526573.1 aminoglycoside O-phosphotransferase APH(3'')-Ib... | 105 | 3e-23 | 34% |
| EFE3815897.1 aminoglycoside O-phosphotransferase APH(3'')-Ib [... | 105 | 3e-23 | 34% |
| WP_063735973.1 APH(3'') family aminoglycoside O-phosphotransfe... | 105 | 3e-23 | 38% |
| ECT7396448.1 aminoglycoside O-phosphotransferase APH(3'')-Ib [... | 105 | 3e-23 | 34% |
| EEX9167129.1 aminoglycoside O-phosphotransferase APH(3'')-Ib [... | 105 | 3e-23 | 34% |
| EBF7195725.1 aminoglycoside O-phosphotransferase APH(3'')-Ib [... | 105 | 3e-23 | 34% |
| EAA5403829.1 aminoglycoside 3'-phosphotransferase [Salmonella ... | 105 | 3e-23 | 34% |
| WP_159924223.1 MULTISPECIES: APH(3'') family aminoglycoside O-... | 105 | 3e-23 | 35% |
| EFN4364632.1 aminoglycoside O-phosphotransferase APH(3'')-Ib [... | 105 | 3e-23 | 34% |
| HAN7608807.1 TPA: aminoglycoside O-phosphotransferase APH(3'')... | 105 | 3e-23 | 34% |
| TAG96577.1 APH(3') family aminoglycoside O-phosphotransferase ... | 101 | 3e-23 | 40% |
| MBB5957929.1 streptomycin 3'-kinase [Saccharothrix tamanrasset... | 105 | 3e-23 | 36% |
| EFA8424256.1 aminoglycoside O-phosphotransferase APH(3'')-Ib [... | 105 | 3e-23 | 34% |
| EBF4047439.1 aminoglycoside O-phosphotransferase APH(3'')-Ib [... | 105 | 3e-23 | 34% |
| WP_084567673.1 aminoglycoside 3'-phosphotransferase [Salmonell... | 103 | 3e-23 | 33% |
| AFU03961.1 aminoglycoside 3'-O-phosphotransferase [Nocardia br... | 108 | 3e-23 | 38% |
| HAS1328363.1 TPA: aminoglycoside O-phosphotransferase APH(3'')... | 105 | 3e-23 | 34% |
| WP_104190285.1 aminoglycoside O-phosphotransferase APH(3'')-Ib... | 105 | 3e-23 | 34% |
| WP_024256776.1 MULTISPECIES: aminoglycoside O-phosphotransfera... | 105 | 3e-23 | 34% |
| WP_069627186.1 APH(3'') family aminoglycoside O-phosphotransfe... | 105 | 3e-23 | 35% |
| EBG7093885.1 aminoglycoside O-phosphotransferase APH(3'')-Ib [... | 105 | 3e-23 | 34% |
| WP_024840713.1 aminoglycoside 3'-phosphotransferase [Cellulosi... | 105 | 3e-23 | 36% |
| EGE2176197.1 aminoglycoside 3'-phosphotransferase [Escherichia... | 105 | 3e-23 | 34% |
| EED8932150.1 aminoglycoside O-phosphotransferase APH(3'')-Ib [... | 105 | 3e-23 | 34% |
| EFY5814297.1 aminoglycoside O-phosphotransferase APH(3'')-Ib [... | 105 | 3e-23 | 34% |
| EDM8556539.1 APH(3'') family aminoglycoside O-phosphotransfera... | 105 | 3e-23 | 34% |
| WP_106482781.1 aminoglycoside 3'-phosphotransferase [Acinetoba... | 103 | 3e-23 | 33% |
| EDM8486789.1 APH(3'') family aminoglycoside O-phosphotransfera... | 105 | 3e-23 | 34% |
| WP_123907540.1 aminoglycoside O-phosphotransferase APH(3'')-Ib... | 105 | 3e-23 | 34% |
| EFP7748945.1 aminoglycoside O-phosphotransferase APH(3'')-Ib [... | 105 | 3e-23 | 34% |
| EAY0253475.1 aminoglycoside O-phosphotransferase APH(3'')-Ib [... | 105 | 3e-23 | 34% |
| ECP5059875.1 APH(3'') family aminoglycoside O-phosphotransfera... | 105 | 3e-23 | 35% |
| EDQ1099524.1 aminoglycoside O-phosphotransferase APH(3'')-Ib [... | 105 | 3e-23 | 34% |
| AMJ34402.1 APH3'' [uncultured bacterium] | 105 | 3e-23 | 34% |
| HAH0150955.1 TPA: APH(3'') family aminoglycoside O-phosphotran... | 105 | 3e-23 | 34% |
| WP_099816052.1 aminoglycoside O-phosphotransferase APH(3'')-Ib... | 105 | 3e-23 | 35% |
| WP_136748370.1 aminoglycoside O-phosphotransferase APH(3'')-Ib... | 105 | 3e-23 | 34% |
| WP_063122853.1 MULTISPECIES: aminoglycoside O-phosphotransfera... | 105 | 3e-23 | 34% |
| HAE3585792.1 TPA: aminoglycoside O-phosphotransferase APH(3'')... | 105 | 3e-23 | 34% |
| WP_129942113.1 aminoglycoside O-phosphotransferase APH(3'')-Ib... | 105 | 3e-23 | 34% |
| EBA2875120.1 aminoglycoside O-phosphotransferase APH(3'')-Ib [... | 105 | 3e-23 | 34% |
| HAH1851644.1 TPA: aminoglycoside O-phosphotransferase APH(3'')... | 105 | 3e-23 | 34% |
| WP_070949638.1 APH(3'') family aminoglycoside O-phosphotransfe... | 105 | 3e-23 | 35% |
| EAX1241610.1 aminoglycoside O-phosphotransferase APH(3'')-Ib [... | 105 | 3e-23 | 34% |
| EFN5646607.1 aminoglycoside O-phosphotransferase APH(3'')-Ib [... | 105 | 3e-23 | 34% |
| EBD2098782.1 aminoglycoside O-phosphotransferase APH(3'')-Ib [... | 105 | 3e-23 | 34% |
| ECT2290869.1 aminoglycoside O-phosphotransferase APH(3'')-Ib [... | 105 | 3e-23 | 34% |
| EEK3075160.1 aminoglycoside O-phosphotransferase APH(3'')-Ib [... | 105 | 3e-23 | 34% |
| EDA4618481.1 aminoglycoside O-phosphotransferase APH(3'')-Ib [... | 105 | 3e-23 | 34% |
| WP_140298733.1 aminoglycoside 3'-phosphotransferase [Vibrio pa... | 102 | 3e-23 | 35% |
| WP_161185668.1 aminoglycoside O-phosphotransferase APH(3'')-Ia... | 105 | 3e-23 | 37% |

| WP_063739545.1 APH(3'') family aminoglycoside O-phosphotransfe... | 105 | 3e-23 | 36% |
| --- | --- | --- | --- |
| EDA9892011.1 aminoglycoside O-phosphotransferase APH(3'')-Ib [... | 105 | 3e-23 | 34% |
| WP_072664391.1 aminoglycoside O-phosphotransferase APH(3'')-Ib... | 105 | 3e-23 | 34% |
| WP_187959373.1 aminoglycoside O-phosphotransferase APH(3'')-Ib... | 105 | 3e-23 | 34% |
| EFL9504173.1 aminoglycoside O-phosphotransferase APH(3'')-Ib [... | 105 | 3e-23 | 34% |
| WP_128475656.1 aminoglycoside O-phosphotransferase APH(3'')-Ib... | 105 | 3e-23 | 34% |
| EDW9406108.1 aminoglycoside O-phosphotransferase APH(3'')-Ib [... | 105 | 3e-23 | 34% |
| WP_042962194.1 aminoglycoside O-phosphotransferase APH(3'')-Ib... | 105 | 3e-23 | 34% |
| WP_114140747.1 MULTISPECIES: aminoglycoside O-phosphotransfera... | 105 | 3e-23 | 34% |
| WP_064725876.1 MULTISPECIES: aminoglycoside O-phosphotransfera... | 105 | 3e-23 | 34% |
| EAA9574747.1 aminoglycoside O-phosphotransferase APH(3'')-Ib [... | 105 | 3e-23 | 34% |
| WP_113426899.1 aminoglycoside O-phosphotransferase APH(3'')-Ib... | 105 | 3e-23 | 34% |
| EFR5763656.1 aminoglycoside O-phosphotransferase APH(3'')-Ib [... | 105 | 3e-23 | 34% |
| WP_123188435.1 aminoglycoside 3'-phosphotransferase [Streptomy... | 105 | 3e-23 | 36% |
| WP_084043583.1 aminoglycoside 3'-phosphotransferase [Blautia s... | 105 | 3e-23 | 30% |
| HAI9592529.1 TPA: aminoglycoside O-phosphotransferase APH(3'')... | 105 | 3e-23 | 34% |
| EBI5659579.1 aminoglycoside O-phosphotransferase APH(3'')-Ib [... | 105 | 3e-23 | 34% |
| WP_001082319.1 MULTISPECIES: aminoglycoside O-phosphotransfera... | 105 | 3e-23 | 34% |
| EGK3938712.1 aminoglycoside O-phosphotransferase APH(3'')-Ib [... | 105 | 3e-23 | 34% |
| WP_070858948.1 aminoglycoside O-phosphotransferase APH(3'')-Ib... | 105 | 3e-23 | 34% |
| WP_080896127.1 aminoglycoside O-phosphotransferase APH(3'')-Ib... | 105 | 3e-23 | 34% |
| WP_122102652.1 APH(3'') family aminoglycoside O-phosphotransfe... | 105 | 3e-23 | 32% |
| WP_156195524.1 aminoglycoside O-phosphotransferase APH(3'')-Ib... | 105 | 3e-23 | 34% |
| ECW5775652.1 aminoglycoside O-phosphotransferase APH(3'')-Ib [... | 105 | 3e-23 | 34% |
| EBW6275602.1 aminoglycoside 3'-phosphotransferase [Salmonella ... | 105 | 3e-23 | 34% |
| WP_111779447.1 aminoglycoside O-phosphotransferase APH(3'')-Ib... | 105 | 3e-23 | 34% |
| EEK5505428.1 aminoglycoside O-phosphotransferase APH(3'')-Ib [... | 105 | 3e-23 | 34% |
| WP_085440772.1 aminoglycoside O-phosphotransferase APH(3'')-Ib... | 105 | 3e-23 | 34% |
| WP_061873689.1 aminoglycoside O-phosphotransferase APH(3'')-Ib... | 105 | 3e-23 | 34% |
| WP_039854955.1 aminoglycoside 3'-phosphotransferase [Neisseria... | 104 | 3e-23 | 32% |
| WP_087713575.1 aminoglycoside O-phosphotransferase APH(3'')-Ib... | 105 | 3e-23 | 34% |
| QAY18309.1 aminoglycoside phosphotransferase APH(3') [Vibrio c... | 106 | 3e-23 | 34% |
| EBC6586417.1 aminoglycoside O-phosphotransferase APH(3'')-Ib [... | 105 | 3e-23 | 34% |
| ECS3235080.1 aminoglycoside O-phosphotransferase APH(3'')-Ib [... | 105 | 3e-23 | 34% |
| EAX0408214.1 aminoglycoside O-phosphotransferase APH(3'')-Ib [... | 105 | 3e-23 | 34% |
| WP_076773815.1 aminoglycoside O-phosphotransferase APH(3'')-Ib... | 105 | 3e-23 | 34% |
| EAM9478048.1 aminoglycoside O-phosphotransferase APH(3'')-Ib [... | 105 | 3e-23 | 34% |
| WP_063074887.1 aminoglycoside O-phosphotransferase APH(3'')-Ib... | 105 | 3e-23 | 34% |
| EAW1159843.1 aminoglycoside 3'-phosphotransferase [Salmonella ... | 103 | 3e-23 | 33% |
| EEV4457857.1 aminoglycoside O-phosphotransferase APH(3'')-Ib [... | 105 | 3e-23 | 34% |
| WP_161272377.1 APH(3'') family aminoglycoside O-phosphotransfe... | 105 | 3e-23 | 34% |
| WP_136520666.1 aminoglycoside 3'-phosphotransferase [Cellulomo... | 105 | 3e-23 | 40% |
| EEA4872926.1 aminoglycoside O-phosphotransferase APH(3'')-Ib [... | 105 | 3e-23 | 34% |
| EFC2040057.1 aminoglycoside O-phosphotransferase APH(3'')-Ib [... | 105 | 3e-23 | 34% |
| WP_181716078.1 aminoglycoside O-phosphotransferase APH(3'')-Ib... | 105 | 3e-23 | 34% |
| BBJ45547.1 putative streptomycin phosphotransferase [Streptomy... | 105 | 3e-23 | 37% |
| EAX0966702.1 aminoglycoside O-phosphotransferase APH(3'')-Ib [... | 105 | 3e-23 | 34% |
| WP_112914691.1 aminoglycoside O-phosphotransferase APH(3'')-Ib... | 105 | 3e-23 | 34% |
| WP_136859699.1 aminoglycoside O-phosphotransferase APH(3'')-Ib... | 105 | 3e-23 | 34% |
| EFL3897723.1 aminoglycoside O-phosphotransferase APH(3'')-Ib [... | 105 | 3e-23 | 33% |
| HAO9790290.1 TPA: aminoglycoside O-phosphotransferase APH(3'')... | 105 | 3e-23 | 34% |
| EEV7142446.1 aminoglycoside O-phosphotransferase APH(3'')-Ib [... | 105 | 3e-23 | 34% |
| EGH1022399.1 aminoglycoside O-phosphotransferase APH(3'')-Ib [... | 105 | 3e-23 | 34% |
| EFD0501909.1 aminoglycoside O-phosphotransferase APH(3'')-Ib [... | 105 | 4e-23 | 34% |
| WP_052989108.1 aminoglycoside O-phosphotransferase APH(3'')-Ib... | 105 | 4e-23 | 34% |
| PTR09915.1 streptomycin 3'-kinase [Rhodobacter azotoformans] | 107 | 4e-23 | 35% |
| WP_181727023.1 aminoglycoside O-phosphotransferase APH(3'')-Ib... | 105 | 4e-23 | 34% |
| WP_047737029.1 aminoglycoside O-phosphotransferase APH(3'')-Ib... | 105 | 4e-23 | 34% |
| WP_072665756.1 aminoglycoside 3'-phosphotransferase [Escherich... | 103 | 4e-23 | 33% |
| WP_078177521.1 aminoglycoside O-phosphotransferase APH(3'')-Ib... | 105 | 4e-23 | 34% |
| HAN5226903.1 TPA: aminoglycoside O-phosphotransferase APH(3'')... | 105 | 4e-23 | 34% |
| EEE7009489.1 aminoglycoside O-phosphotransferase APH(3'')-Ib [... | 105 | 4e-23 | 34% |
| EDS5530029.1 aminoglycoside O-phosphotransferase APH(3'')-Ib [... | 105 | 4e-23 | 34% |
| HAD8168287.1 TPA: aminoglycoside O-phosphotransferase APH(3'')... | 105 | 4e-23 | 34% |
| WP_139942761.1 aminoglycoside O-phosphotransferase APH(3'')-Ib... | 105 | 4e-23 | 34% |
| WP_112005891.1 aminoglycoside O-phosphotransferase APH(3'')-Ib... | 105 | 4e-23 | 34% |
| EEG4979874.1 aminoglycoside O-phosphotransferase APH(3'')-Ib [... | 105 | 4e-23 | 34% |
| EAY2253115.1 aminoglycoside 3'-phosphotransferase [Salmonella ... | 105 | 4e-23 | 34% |
| WP_015060269.1 aminoglycoside O-phosphotransferase APH(3'')-Ib... | 105 | 4e-23 | 34% |
| WP_159312526.1 APH(3'') family aminoglycoside O-phosphotransfe... | 105 | 4e-23 | 35% |
| EAZ2475052.1 aminoglycoside O-phosphotransferase APH(3'')-Ib [... | 105 | 4e-23 | 34% |
| EAU2950379.1 aminoglycoside O-phosphotransferase APH(3'')-Ib [... | 105 | 4e-23 | 34% |

| EET0873090.1 aminoglycoside O-phosphotransferase APH(3'')-Ib [... | 105 | 4e-23 | 34% |
| --- | --- | --- | --- |
| WP_163434008.1 aminoglycoside O-phosphotransferase APH(3'')-Ib... | 105 | 4e-23 | 34% |
| ECR7205707.1 aminoglycoside O-phosphotransferase APH(3'')-Ib [... | 105 | 4e-23 | 34% |
| WP_134989845.1 aminoglycoside O-phosphotransferase APH(3'')-Ib... | 105 | 4e-23 | 34% |
| EFP7799684.1 aminoglycoside O-phosphotransferase APH(3'')-Ib [... | 105 | 4e-23 | 34% |
| EFH6641763.1 aminoglycoside O-phosphotransferase APH(3'')-Ib [... | 105 | 4e-23 | 34% |
| WP_096928990.1 aminoglycoside O-phosphotransferase APH(3'')-Ib... | 105 | 4e-23 | 34% |
| WP_075168336.1 aminoglycoside O-phosphotransferase APH(3'')-Ib... | 105 | 4e-23 | 34% |
| EAC1914186.1 aminoglycoside O-phosphotransferase APH(3'')-Ib [... | 105 | 4e-23 | 34% |
| MIB49184.1 aminoglycoside O-phosphotransferase APH(3'')-Ib [Es... | 105 | 4e-23 | 34% |
| EFY9292866.1 aminoglycoside O-phosphotransferase APH(3'')-Ib [... | 105 | 4e-23 | 34% |
| EBR5364808.1 aminoglycoside O-phosphotransferase APH(3'')-Ib [... | 105 | 4e-23 | 34% |
| ECT2290875.1 aminoglycoside O-phosphotransferase APH(3'')-Ib [... | 105 | 4e-23 | 34% |
| WP_109545041.1 aminoglycoside O-phosphotransferase APH(3'')-Ib... | 105 | 4e-23 | 34% |
| WP_148432646.1 aminoglycoside 3'-phosphotransferase [Nonomurae... | 105 | 4e-23 | 36% |
| WP_141029386.1 aminoglycoside 3'-phosphotransferase [Salmonell... | 102 | 4e-23 | 38% |
| WP_188141508.1 APH(3'') family aminoglycoside O-phosphotransfe... | 104 | 4e-23 | 35% |
| EDB4303456.1 aminoglycoside O-phosphotransferase APH(3'')-Ib [... | 105 | 4e-23 | 34% |
| WP_015060202.1 MULTISPECIES: aminoglycoside O-phosphotransfera... | 105 | 4e-23 | 34% |
| WP_135017269.1 MULTISPECIES: aminoglycoside O-phosphotransfera... | 105 | 4e-23 | 34% |
| EGH1022813.1 aminoglycoside O-phosphotransferase APH(3'')-Ib [... | 105 | 4e-23 | 34% |
| WP_079124535.1 APH(3'') family aminoglycoside O-phosphotransfe... | 105 | 4e-23 | 35% |
| EFI2822290.1 aminoglycoside O-phosphotransferase APH(3'')-Ib [... | 105 | 4e-23 | 34% |
| HAL2559773.1 TPA: aminoglycoside O-phosphotransferase APH(3'')... | 105 | 4e-23 | 34% |
| EAB1116365.1 aminoglycoside O-phosphotransferase APH(3'')-Ib [... | 105 | 4e-23 | 34% |
| HAD8829772.1 TPA: aminoglycoside O-phosphotransferase APH(3'')... | 105 | 4e-23 | 34% |
| WP_180075653.1 aminoglycoside O-phosphotransferase APH(3'')-Ib... | 105 | 4e-23 | 34% |
| WP_024261901.1 MULTISPECIES: aminoglycoside O-phosphotransfera... | 105 | 4e-23 | 34% |
| EAU1237160.1 aminoglycoside O-phosphotransferase APH(3'')-Ib [... | 105 | 4e-23 | 34% |
| MBA5728679.1 aminoglycoside 3'-phosphotransferase [Aerococcace... | 105 | 4e-23 | 28% |
| WP_193096989.1 aminoglycoside O-phosphotransferase APH(3'')-Ib... | 105 | 4e-23 | 34% |
| EEA6552100.1 aminoglycoside O-phosphotransferase APH(3'')-Ib [... | 105 | 4e-23 | 34% |
| HAL1360806.1 TPA: aminoglycoside O-phosphotransferase APH(3'')... | 105 | 4e-23 | 34% |
| HAM8813581.1 TPA: aminoglycoside O-phosphotransferase APH(3'')... | 105 | 4e-23 | 34% |
| WP_191436300.1 APH(3') family aminoglycoside O-phosphotransfer... | 105 | 4e-23 | 31% |
| WP_129806903.1 APH(3'') family aminoglycoside O-phosphotransfe... | 105 | 4e-23 | 35% |
| WP_104772264.1 aminoglycoside O-phosphotransferase APH(3'')-Ib... | 105 | 4e-23 | 34% |
| WP_075319815.1 aminoglycoside O-phosphotransferase APH(3'')-Ib... | 105 | 4e-23 | 33% |
| WP_172690366.1 aminoglycoside O-phosphotransferase APH(3'')-Ib... | 105 | 4e-23 | 34% |
| EDC6608680.1 aminoglycoside O-phosphotransferase APH(3'')-Ib [... | 105 | 4e-23 | 34% |
| EDC6617680.1 aminoglycoside O-phosphotransferase APH(3'')-Ib [... | 105 | 4e-23 | 34% |
| EAY6942740.1 aminoglycoside O-phosphotransferase APH(3'')-Ib [... | 105 | 4e-23 | 34% |
| EEZ5225590.1 aminoglycoside O-phosphotransferase APH(3'')-Ib [... | 105 | 4e-23 | 34% |
| WP_011270146.1 MULTISPECIES: aminoglycoside O-phosphotransfera... | 105 | 4e-23 | 34% |
| WP_090381073.1 APH(3'') family aminoglycoside O-phosphotransfe... | 105 | 4e-23 | 33% |
| HAJ5517709.1 TPA: aminoglycoside O-phosphotransferase APH(3'')... | 105 | 4e-23 | 34% |
| WP_096961647.1 aminoglycoside O-phosphotransferase APH(3'')-Ib... | 105 | 4e-23 | 34% |
| HAL2163126.1 TPA: aminoglycoside O-phosphotransferase APH(3'')... | 105 | 4e-23 | 34% |
| EBH9833461.1 aminoglycoside O-phosphotransferase APH(3'')-Ib [... | 105 | 4e-23 | 34% |
| TZE85633.1 aminoglycoside O-phosphotransferase APH(3'')-Ib [Es... | 105 | 4e-23 | 34% |
| WP_172692145.1 aminoglycoside O-phosphotransferase APH(3'')-Ib... | 105 | 4e-23 | 34% |
| WP_060635864.1 aminoglycoside O-phosphotransferase APH(3'')-Ib... | 105 | 4e-23 | 34% |
| EFZ6190393.1 aminoglycoside O-phosphotransferase APH(3'')-Ib [... | 105 | 4e-23 | 34% |
| ECK0882718.1 aminoglycoside O-phosphotransferase APH(3'')-Ib [... | 105 | 4e-23 | 34% |
| EBD7308296.1 aminoglycoside O-phosphotransferase APH(3'')-Ib [... | 105 | 4e-23 | 34% |
| ECR6725875.1 aminoglycoside O-phosphotransferase APH(3'')-Ib [... | 105 | 4e-23 | 34% |
| MIB64275.1 aminoglycoside O-phosphotransferase APH(3'')-Ib [Es... | 105 | 4e-23 | 34% |
| EFI2836798.1 aminoglycoside O-phosphotransferase APH(3'')-Ib [... | 105 | 4e-23 | 34% |
| WP_001082320.1 MULTISPECIES: aminoglycoside O-phosphotransfera... | 105 | 4e-23 | 34% |
| EFH5573420.1 aminoglycoside O-phosphotransferase APH(3'')-Ib [... | 105 | 4e-23 | 34% |
| EFH4755413.1 aminoglycoside O-phosphotransferase APH(3'')-Ib [... | 105 | 4e-23 | 34% |
| KIE94666.1 putative aminoglycoside phosphotransferase [Actinob... | 105 | 4e-23 | 34% |
| ECZ1763768.1 aminoglycoside O-phosphotransferase APH(3'')-Ib [... | 105 | 4e-23 | 34% |
| HAS2076524.1 TPA: aminoglycoside O-phosphotransferase APH(3'')... | 105 | 4e-23 | 34% |
| RKD78829.1 streptomycin 3'-kinase [Streptomyces sp. 2112.5] | 105 | 4e-23 | 37% |
| EGU58284.1 hypothetical protein VITU9109_06979 [Vibrio tubiash... | 105 | 4e-23 | 34% |
| WP_185253689.1 phosphotransferase [Nocardioides sp. KIGAM211] | 105 | 4e-23 | 39% |
| MBD4429513.1 aminoglycoside O-phosphotransferase APH(3'')-Ib [... | 105 | 4e-23 | 34% |
| ECY2236118.1 aminoglycoside O-phosphotransferase APH(3'')-Ib [... | 105 | 4e-23 | 34% |
| WP_159124589.1 MULTISPECIES: aminoglycoside O-phosphotransfera... | 105 | 4e-23 | 34% |
| WP_052935330.1 aminoglycoside O-phosphotransferase APH(3'')-Ib... | 105 | 4e-23 | 34% |
| EAI5189672.1 aminoglycoside 3'-phosphotransferase [Campylobact... | 103 | 4e-23 | 37% |

| EMD01457.1 StrA [Escherichia coli SEPT362] | 105 | 4e-23 | 34% |
| --- | --- | --- | --- |
| EBP7036431.1 aminoglycoside O-phosphotransferase APH(3'')-Ib [... | 105 | 4e-23 | 34% |
| WP_100423047.1 aminoglycoside 3'-phosphotransferase [Sediminih... | 105 | 4e-23 | 37% |
| WP_005826108.1 MULTISPECIES: aminoglycoside O-phosphotransfera... | 105 | 4e-23 | 34% |
| ECT2290871.1 aminoglycoside O-phosphotransferase APH(3'')-Ib [... | 105 | 4e-23 | 34% |
| EAX5048668.1 aminoglycoside O-phosphotransferase APH(3'')-Ib [... | 105 | 4e-23 | 34% |
| MBD5520481.1 aminoglycoside 3'-phosphotransferase [Lachnospira... | 105 | 4e-23 | 34% |
| WP_063569290.1 aminoglycoside O-phosphotransferase APH(3'')-Ib... | 105 | 4e-23 | 34% |
| WP_187089732.1 APH(3'') family aminoglycoside O-phosphotransfe... | 105 | 4e-23 | 35% |
| WP_105711229.1 aminoglycoside 3'-phosphotransferase [Microbact... | 105 | 4e-23 | 34% |
| EBF4856553.1 aminoglycoside O-phosphotransferase APH(3'')-Ib [... | 105 | 4e-23 | 34% |
| EAQ8351437.1 aminoglycoside O-phosphotransferase APH(3'')-Ib [... | 105 | 4e-23 | 34% |
| EFN3820348.1 aminoglycoside O-phosphotransferase APH(3'')-Ib [... | 105 | 4e-23 | 34% |
| EBP4718594.1 aminoglycoside O-phosphotransferase APH(3'')-Ib [... | 105 | 4e-23 | 34% |
| WP_117245190.1 aminoglycoside O-phosphotransferase APH(3'')-Ib... | 105 | 4e-23 | 34% |
| WP_131890213.1 aminoglycoside 3'-phosphotransferase [Jiangella... | 104 | 4e-23 | 39% |
| EDU0549675.1 aminoglycoside O-phosphotransferase APH(3'')-Ib [... | 105 | 4e-23 | 34% |
| WP_120366336.1 aminoglycoside O-phosphotransferase APH(3'')-Ib... | 105 | 4e-23 | 34% |
| EDJ3301538.1 APH(3'') family aminoglycoside O-phosphotransfera... | 105 | 4e-23 | 34% |
| EAU6648721.1 aminoglycoside O-phosphotransferase APH(3'')-Ib [... | 105 | 4e-23 | 34% |
| EFY7755648.1 aminoglycoside O-phosphotransferase APH(3'')-Ib [... | 105 | 4e-23 | 34% |
| ECV3054786.1 aminoglycoside O-phosphotransferase APH(3'')-Ib [... | 105 | 4e-23 | 34% |
| ECG7805352.1 aminoglycoside O-phosphotransferase APH(3'')-Ib [... | 105 | 4e-23 | 34% |
| WP_165241156.1 APH(3'') family aminoglycoside O-phosphotransfe... | 105 | 4e-23 | 35% |
| ECU7314596.1 aminoglycoside O-phosphotransferase APH(3'')-Ib [... | 105 | 4e-23 | 34% |
| WP_086589754.1 aminoglycoside O-phosphotransferase APH(3'')-Ib... | 105 | 4e-23 | 34% |
| EEZ3438769.1 aminoglycoside O-phosphotransferase APH(3'')-Ib [... | 105 | 4e-23 | 34% |
| EAY4638075.1 aminoglycoside O-phosphotransferase APH(3'')-Ib [... | 105 | 4e-23 | 34% |
| CSH44864.1 putative trasposase [Shigella sonnei] | 105 | 4e-23 | 34% |
| WP_003949093.1 MULTISPECIES: APH(3'') family aminoglycoside O-... | 105 | 4e-23 | 35% |
| KRJ53080.1 aminoglycoside phosphotransferase [Acinetobacter ba... | 105 | 4e-23 | 34% |
| WP_032008827.1 aminoglycoside O-phosphotransferase APH(3'')-Ib... | 105 | 4e-23 | 34% |
| EFA4297798.1 aminoglycoside O-phosphotransferase APH(3'')-Ib [... | 105 | 4e-23 | 34% |
| WP_112320274.1 aminoglycoside O-phosphotransferase APH(3'')-Ib... | 105 | 4e-23 | 34% |
| ECT6940575.1 aminoglycoside O-phosphotransferase APH(3'')-Ib [... | 105 | 4e-23 | 34% |
| EFW6294528.1 aminoglycoside O-phosphotransferase APH(3'')-Ib [... | 105 | 4e-23 | 34% |
| HAR0785633.1 TPA: aminoglycoside 3'-phosphotransferase [Entero... | 102 | 4e-23 | 37% |
| QDD59516.1 APH(3'') family aminoglycoside O-phosphotransferase... | 105 | 4e-23 | 36% |
| ECT2290879.1 aminoglycoside O-phosphotransferase APH(3'')-Ib [... | 105 | 4e-23 | 34% |
| WP_031942890.1 MULTISPECIES: aminoglycoside O-phosphotransfera... | 105 | 4e-23 | 34% |
| EBW5201398.1 aminoglycoside 3'-phosphotransferase [Salmonella ... | 105 | 4e-23 | 34% |
| WP_145991852.1 APH(3'') family aminoglycoside O-phosphotransfe... | 105 | 4e-23 | 35% |
| WP_156095027.1 APH(3'') family aminoglycoside O-phosphotransfe... | 105 | 4e-23 | 35% |
| SMC66566.1 kanamycin kinase [Clostridiales bacterium] | 105 | 4e-23 | 33% |
| WP_041915140.1 APH(3'') family aminoglycoside O-phosphotransfe... | 105 | 4e-23 | 33% |
| OLD19450.1 hypothetical protein AUJ01_05950 [Acidobacteria bac... | 103 | 4e-23 | 34% |
| WP_171468143.1 phosphotransferase [Cellulosimicrobium sp. 72-3] | 105 | 4e-23 | 35% |
| WP_081357115.1 aminoglycoside 3'-phosphotransferase [Butyrivib... | 105 | 4e-23 | 28% |
| EAX1441096.1 aminoglycoside O-phosphotransferase APH(3'')-Ib [... | 105 | 4e-23 | 34% |
| EAS4576141.1 aminoglycoside O-phosphotransferase APH(3'')-Ib [... | 105 | 4e-23 | 34% |
| ESA65784.1 streptomycin 3-kinase [Escherichia coli 110957] | 105 | 4e-23 | 34% |
| EAA8302931.1 aminoglycoside O-phosphotransferase APH(3'')-Ib [... | 105 | 4e-23 | 34% |
| TZD40859.1 aminoglycoside O-phosphotransferase APH(3'')-Ib [Es... | 105 | 5e-23 | 34% |
| ETQ17752.1 streptomycin 3''-kinase [Acinetobacter baumannii UH... | 105 | 5e-23 | 34% |
| ADX01900.1 StrA [Acinetobacter baumannii 1656-2] | 105 | 5e-23 | 34% |
| EIC33837.1 hypothetical protein SEEH1563_15484 [Salmonella ent... | 105 | 5e-23 | 34% |
| WP_114476071.1 aminoglycoside O-phosphotransferase APH(3'')-Ib... | 105 | 5e-23 | 34% |
| EFO3044675.1 aminoglycoside 3'-phosphotransferase [Escherichia... | 105 | 5e-23 | 34% |
| WP_112258014.1 aminoglycoside 3'-phosphotransferase [Phytoacti... | 105 | 5e-23 | 36% |
| EFJ86044.1 phosphotransferase enzyme family [Escherichia coli ... | 105 | 5e-23 | 34% |
| WP_070061098.1 APH(3'') family aminoglycoside O-phosphotransfe... | 105 | 5e-23 | 34% |
| EFO2366121.1 aminoglycoside 3'-phosphotransferase [Escherichia... | 105 | 5e-23 | 34% |
| MGH61120.1 aminoglycoside O-phosphotransferase APH(3'')-Ib [Es... | 105 | 5e-23 | 34% |
| EFX8829118.1 aminoglycoside O-phosphotransferase APH(3'')-Ib [... | 105 | 5e-23 | 34% |
| EAM9802872.1 aminoglycoside O-phosphotransferase APH(3'')-Ib [... | 105 | 5e-23 | 34% |
| AFK33002.1 StrA [Escherichia coli] | 105 | 5e-23 | 34% |
| ARR90282.1 StrA [Klebsiella pneumoniae] | 105 | 5e-23 | 34% |
| HAI6958740.1 TPA: aminoglycoside O-phosphotransferase APH(3'')... | 105 | 5e-23 | 34% |
| WP_110161512.1 aminoglycoside 3'-phosphotransferase [Staphyloc... | 103 | 5e-23 | 37% |
| WP_061807144.1 aminoglycoside O-phosphotransferase APH(3'')-Ib... | 105 | 5e-23 | 34% |
| WP_074417338.1 aminoglycoside O-phosphotransferase APH(3'')-Ib... | 105 | 5e-23 | 34% |
| EFI3735338.1 aminoglycoside O-phosphotransferase APH(3'')-Ib [... | 105 | 5e-23 | 34% |

| ECT6311050.1 aminoglycoside O-phosphotransferase APH(3'')-Ib [... | 105 | 5e-23 | 34% |
| --- | --- | --- | --- |
| HAD8661505.1 TPA: aminoglycoside O-phosphotransferase APH(3'')... | 105 | 5e-23 | 34% |
| EDC6979972.1 aminoglycoside O-phosphotransferase APH(3'')-Ib [... | 105 | 5e-23 | 34% |
| WP_120160148.1 aminoglycoside O-phosphotransferase APH(3'')-Ib... | 105 | 5e-23 | 34% |
| WP_149513347.1 APH(3'') family aminoglycoside O-phosphotransfe... | 105 | 5e-23 | 36% |
| WP_113621824.1 aminoglycoside O-phosphotransferase APH(3'')-Ib... | 105 | 5e-23 | 34% |
| EEO1732588.1 aminoglycoside O-phosphotransferase APH(3'')-Ib [... | 105 | 5e-23 | 34% |
| HAQ8675080.1 TPA: aminoglycoside 3'-phosphotransferase [Entero... | 102 | 5e-23 | 37% |
| WP_192937674.1 APH(3'') family aminoglycoside O-phosphotransfe... | 105 | 5e-23 | 33% |
| WP_175509433.1 APH(3'') family aminoglycoside O-phosphotransfe... | 105 | 5e-23 | 35% |
| EEH9132244.1 aminoglycoside O-phosphotransferase APH(3'')-Ib [... | 105 | 5e-23 | 35% |
| ADP21235.1 strepyomycin phosphotransferase A [Glaesserella par... | 105 | 5e-23 | 34% |
| EAJ8662456.1 aminoglycoside 3'-phosphotransferase [Campylobact... | 102 | 5e-23 | 37% |
| AMJ34218.1 APH3'' [uncultured bacterium] | 105 | 5e-23 | 34% |
| HAL2676999.1 TPA: aminoglycoside O-phosphotransferase APH(3'')... | 105 | 5e-23 | 34% |
| WP_129848779.1 APH(3'') family aminoglycoside O-phosphotransfe... | 105 | 5e-23 | 36% |
| EDL7150013.1 APH(3'') family aminoglycoside O-phosphotransfera... | 105 | 5e-23 | 34% |
| HAP3213726.1 TPA: aminoglycoside O-phosphotransferase APH(3'')... | 105 | 5e-23 | 34% |
| ECT7405496.1 aminoglycoside O-phosphotransferase APH(3'')-Ib [... | 105 | 5e-23 | 34% |
| WP_107102104.1 aminoglycoside 3'-phosphotransferase [Streptomy... | 105 | 5e-23 | 34% |
| ECP5059873.1 APH(3'') family aminoglycoside O-phosphotransfera... | 104 | 5e-23 | 35% |
| EBK5561128.1 aminoglycoside 3'-phosphotransferase [Salmonella ... | 105 | 5e-23 | 34% |
| EBD8868758.1 aminoglycoside O-phosphotransferase APH(3'')-Ib [... | 105 | 5e-23 | 34% |
| EYI42201.1 hypothetical protein SEEH1567_00017 [Salmonella ent... | 105 | 5e-23 | 34% |
| GEI12924.1 streptomycin phosphotransferase [Escherichia coli O... | 105 | 5e-23 | 34% |
| EDB8549715.1 aminoglycoside O-phosphotransferase APH(3'')-Ib [... | 105 | 5e-23 | 34% |
| QMS84739.1 aminoglycoside 3'-phosphotransferase [Firmicutes ba... | 105 | 5e-23 | 30% |
| QHU23929.1 aph(3')-1b [Klebsiella oxytoca] | 105 | 5e-23 | 34% |
| ETP76332.1 streptomycin 3''-kinase [Acinetobacter baumannii UH... | 105 | 5e-23 | 34% |
| EFS2282609.1 aminoglycoside O-phosphotransferase APH(3'')-Ib [... | 105 | 5e-23 | 34% |
| HAJ6597383.1 TPA: aminoglycoside O-phosphotransferase APH(3'')... | 105 | 5e-23 | 34% |
| WP_031861278.1 aminoglycoside 3'-phosphotransferase [Staphyloc... | 103 | 5e-23 | 37% |
| NMU86525.1 aminoglycoside O-phosphotransferase APH(3'')-Ib [Vi... | 105 | 5e-23 | 34% |
| PKZ39391.1 APH(3'') family aminoglycoside O-phosphotransferase... | 104 | 5e-23 | 35% |
| HAI3416744.1 TPA: aminoglycoside O-phosphotransferase APH(3'')... | 105 | 5e-23 | 34% |
| HAD8214484.1 TPA: aminoglycoside O-phosphotransferase APH(3'')... | 105 | 5e-23 | 34% |
| WP_156205483.1 APH(3'') family aminoglycoside O-phosphotransfe... | 105 | 5e-23 | 36% |
| EFX9615137.1 aminoglycoside O-phosphotransferase APH(3'')-Ib [... | 105 | 5e-23 | 34% |
| EBK5561188.1 aminoglycoside 3'-phosphotransferase [Salmonella ... | 105 | 5e-23 | 34% |
| WP_131193643.1 aminoglycoside O-phosphotransferase APH(3'')-Ib... | 105 | 5e-23 | 34% |
| EDK6678669.1 APH(3'') family aminoglycoside O-phosphotransfera... | 105 | 5e-23 | 34% |
| WP_049883138.1 aminoglycoside O-phosphotransferase APH(3')-III... | 104 | 5e-23 | 31% |
| EKA64012.1 streptomycin 3''-kinase [Acinetobacter baumannii WC... | 105 | 5e-23 | 34% |
| ECS3253687.1 aminoglycoside O-phosphotransferase APH(3'')-Ib [... | 105 | 5e-23 | 34% |
| EFF84395.1 phosphotransferase enzyme family [Acinetobacter hae... | 105 | 5e-23 | 34% |
| WP_044051771.1 aminoglycoside 3'-phosphotransferase [Enterococ... | 102 | 5e-23 | 37% |
| WP_043487319.1 MULTISPECIES: aminoglycoside 3'-phosphotransfer... | 104 | 5e-23 | 36% |
| EBO3034199.1 aminoglycoside O-phosphotransferase APH(3'')-Ib [... | 105 | 5e-23 | 34% |
| WP_026868823.1 APH(3'') family aminoglycoside O-phosphotransfe... | 105 | 5e-23 | 36% |
| WP_132880767.1 APH(3'') family aminoglycoside O-phosphotransfe... | 105 | 5e-23 | 34% |
| EBQ7827330.1 aminoglycoside O-phosphotransferase APH(3'')-Ib [... | 105 | 5e-23 | 34% |
| ECA9541474.1 aminoglycoside O-phosphotransferase APH(3'')-Ib [... | 105 | 5e-23 | 34% |
| EGC04972.1 phosphotransferase enzyme family protein [Escherich... | 105 | 5e-23 | 34% |
| WP_010890156.1 aminoglycoside O-phosphotransferase APH(3'')-Ib... | 105 | 5e-23 | 34% |
| WP_064882290.1 APH(3'') family aminoglycoside O-phosphotransfe... | 104 | 6e-23 | 36% |
| WP_043488497.1 MULTISPECIES: APH(3'') family aminoglycoside O-... | 105 | 6e-23 | 34% |
| EAN08500.1 Kanamycin kinase [Enterococcus faecium DO] | 102 | 6e-23 | 37% |
| AFW18064.1 StrA [[Pasteurella] aerogenes] | 105 | 6e-23 | 34% |
| EAM5211843.1 aminoglycoside O-phosphotransferase APH(3'')-Ib [... | 105 | 6e-23 | 34% |
| WP_143915017.1 aminoglycoside 3'-phosphotransferase [Aeromicro... | 104 | 6e-23 | 35% |
| PJJ73777.1 kanamycin kinase [Sediminihabitans luteus] | 105 | 6e-23 | 37% |
| HAH3672385.1 TPA: APH(3') family aminoglycoside O-phosphotrans... | 101 | 6e-23 | 38% |
| CRZ20108.1 Streptomycin 3''-kinase [Kingella kingae] | 105 | 6e-23 | 34% |
| WP_138409840.1 aminoglycoside O-phosphotransferase APH(3'')-Ib... | 105 | 6e-23 | 35% |
| WP_126713695.1 aminoglycoside O-phosphotransferase APH(3'')-Ib... | 105 | 6e-23 | 34% |
| EDD1686557.1 aminoglycoside O-phosphotransferase APH(3'')-Ib [... | 105 | 6e-23 | 34% |
| EFD4198784.1 aminoglycoside O-phosphotransferase APH(3'')-Ib [... | 105 | 6e-23 | 34% |
| EFH3823279.1 aminoglycoside O-phosphotransferase APH(3'')-Ib [... | 105 | 6e-23 | 34% |
| WP_088824730.1 aminoglycoside 3'-phosphotransferase [Staphyloc... | 102 | 6e-23 | 37% |
| WP_027931400.1 APH(3'') family aminoglycoside O-phosphotransfe... | 104 | 6e-23 | 35% |
| ECK5682689.1 aminoglycoside O-phosphotransferase APH(3')-IIIa ... | 104 | 6e-23 | 31% |
| ORG28215.1 APH(3') family aminoglycoside O-phosphotransferase ... | 102 | 6e-23 | 35% |

| WP_139793680.1 aminoglycoside 3'-phosphotransferase [Salmonell... | 102 | 6e-23 | 35% |
| --- | --- | --- | --- |
| WP_075950771.1 aminoglycoside 3'-phosphotransferase [Pseudonoc... | 105 | 6e-23 | 35% |
| WP_031860326.1 aminoglycoside 3'-phosphotransferase [Staphyloc... | 102 | 6e-23 | 37% |
| WP_030751707.1 aminoglycoside 3'-phosphotransferase [Streptomy... | 104 | 6e-23 | 35% |
| ECU3280503.1 aminoglycoside O-phosphotransferase APH(3'')-Ib [... | 105 | 6e-23 | 34% |
| EGJ2957404.1 aminoglycoside O-phosphotransferase APH(3'')-Ib [... | 105 | 6e-23 | 34% |
| WP_187171410.1 phosphotransferase [Enterococcus faecium] | 102 | 6e-23 | 37% |
| WP_126755962.1 aminoglycoside O-phosphotransferase APH(3'')-Ib... | 105 | 6e-23 | 33% |
| WP_101991691.1 aminoglycoside O-phosphotransferase APH(3'')-Ib... | 105 | 6e-23 | 34% |
| EBB4175237.1 APH(3') family aminoglycoside O-phosphotransferas... | 98.6 | 6e-23 | 100% |
| MYC16183.1 aminoglycoside 3'-phosphotransferase [Gemmatimonade... | 105 | 6e-23 | 33% |
| AIU94573.1 aminoglycoside 3'-phosphotransferase protein [Steno... | 104 | 6e-23 | 35% |
| WP_085105770.1 APH(3'') family aminoglycoside O-phosphotransfe... | 104 | 6e-23 | 37% |
| WP_180977739.1 phosphotransferase [Pseudomonas aeruginosa] | 100 | 6e-23 | 49% |
| ADI03324.1 aminoglycoside phosphotransferase [Streptomyces bin... | 105 | 6e-23 | 34% |
| EFB4375153.1 aminoglycoside O-phosphotransferase APH(3'')-Ib [... | 105 | 6e-23 | 34% |
| ECL5628228.1 aminoglycoside O-phosphotransferase APH(3'')-Ib [... | 105 | 6e-23 | 34% |
| AYU67953.1 aminoglycoside O-phosphotransferase APH(3'')-Ib [Es... | 105 | 6e-23 | 34% |
| EBO2306119.1 aminoglycoside O-phosphotransferase APH(3'')-Ib [... | 105 | 6e-23 | 34% |
| WP_117255756.1 aminoglycoside O-phosphotransferase APH(3'')-Ib... | 105 | 6e-23 | 34% |
| EEK1719278.1 aminoglycoside O-phosphotransferase APH(3'')-Ib [... | 105 | 6e-23 | 33% |
| EBI9757356.1 aminoglycoside 3'-phosphotransferase [Salmonella ... | 104 | 6e-23 | 34% |
| WP_103730907.1 MULTISPECIES: aminoglycoside 3'-phosphotransfer... | 104 | 6e-23 | 32% |
| WP_135458445.1 APH(3'') family aminoglycoside O-phosphotransfe... | 104 | 6e-23 | 36% |
| MBE5940615.1 aminoglycoside 3'-phosphotransferase [Lachnospira... | 105 | 6e-23 | 30% |
| WP_154579421.1 APH(3'') family aminoglycoside O-phosphotransfe... | 105 | 6e-23 | 35% |
| WP_060617748.1 aminoglycoside O-phosphotransferase APH(3'')-Ib... | 105 | 6e-23 | 34% |
| ARQ67179.1 APH(3'') family aminoglycoside O-phosphotransferase... | 103 | 7e-23 | 37% |
| EDT3185526.1 aminoglycoside O-phosphotransferase APH(3'')-Ib [... | 105 | 7e-23 | 34% |
| WP_165743684.1 aminoglycoside O-phosphotransferase APH(3'')-Ib... | 105 | 7e-23 | 34% |
| EVO36170.1 aminoglycoside 3'-phosphotransferase [Staphylococcu... | 102 | 7e-23 | 37% |
| ECS2212684.1 aminoglycoside O-phosphotransferase APH(3'')-Ib [... | 105 | 7e-23 | 34% |
| HBN84193.1 TPA: aminoglycoside O-phosphotransferase APH(3')-II... | 104 | 7e-23 | 30% |
| WP_135475557.1 aminoglycoside 3'-phosphotransferase [Candidatu... | 104 | 7e-23 | 30% |
| WP_097652217.1 aminoglycoside 3'-phosphotransferase [Candidatu... | 104 | 7e-23 | 30% |
| EAN7978725.1 aminoglycoside O-phosphotransferase APH(3'')-Ib [... | 105 | 7e-23 | 34% |
| WP_027744675.1 APH(3'') family aminoglycoside O-phosphotransfe... | 104 | 7e-23 | 36% |
| EAP2002514.1 aminoglycoside O-phosphotransferase APH(3'')-Ib [... | 104 | 7e-23 | 34% |
| EAJ3547834.1 aminoglycoside 3'-phosphotransferase [Campylobact... | 103 | 7e-23 | 33% |
| WP_195108282.1 APH(3'') family aminoglycoside O-phosphotransfe... | 104 | 7e-23 | 35% |
| EFH6016997.1 aminoglycoside O-phosphotransferase APH(3'')-Ib [... | 104 | 7e-23 | 34% |
| EFU7160520.1 aminoglycoside O-phosphotransferase APH(3'')-Ib [... | 104 | 7e-23 | 34% |
| EAC1831222.1 aminoglycoside O-phosphotransferase APH(3'')-Ib [... | 104 | 7e-23 | 34% |
| EBV6906561.1 aminoglycoside O-phosphotransferase APH(3'')-Ib [... | 104 | 7e-23 | 33% |
| ECY0199459.1 aminoglycoside O-phosphotransferase APH(3'')-Ib [... | 104 | 7e-23 | 34% |
| WP_140316779.1 aminoglycoside 3'-phosphotransferase [Vibrio pa... | 102 | 7e-23 | 34% |
| WP_030271137.1 aminoglycoside 3'-phosphotransferase [Streptomy... | 104 | 7e-23 | 38% |
| WP_161012399.1 APH(3') family aminoglycoside O-phosphotransfer... | 104 | 7e-23 | 33% |
| EFQ0547937.1 aminoglycoside O-phosphotransferase APH(3'')-Ib [... | 104 | 7e-23 | 34% |
| HAA1119538.1 TPA: aminoglycoside O-phosphotransferase APH(3'')... | 104 | 7e-23 | 34% |
| EBP2859844.1 aminoglycoside O-phosphotransferase APH(3'')-Ib [... | 104 | 7e-23 | 34% |
| EFH4755927.1 aminoglycoside O-phosphotransferase APH(3'')-Ib [... | 104 | 7e-23 | 34% |
| TOP99708.1 APH(3') family aminoglycoside O-phosphotransferase ... | 102 | 7e-23 | 35% |
| WP_164254497.1 APH(3'') family aminoglycoside O-phosphotransfe... | 104 | 7e-23 | 35% |
| HAP2654443.1 TPA: aminoglycoside O-phosphotransferase APH(3'')... | 104 | 7e-23 | 34% |
| WP_088814729.1 aminoglycoside 3'-phosphotransferase [Staphyloc... | 102 | 7e-23 | 37% |
| WP_167632632.1 phosphotransferase [Microbacterium oxydans] | 104 | 7e-23 | 33% |
| WP_036429537.1 APH(3'') family aminoglycoside O-phosphotransfe... | 104 | 7e-23 | 36% |
| WP_060505445.1 MULTISPECIES: APH(3'') family aminoglycoside O-... | 104 | 7e-23 | 35% |
| EEQ7721689.1 aminoglycoside O-phosphotransferase APH(3'')-Ib [... | 104 | 8e-23 | 34% |
| CDL12242.1 Aminoglycoside 3'-phosphotransferase [Klebsiella pn... | 105 | 8e-23 | 34% |
| TXI57913.1 APH(3'') family aminoglycoside O-phosphotransferase... | 104 | 8e-23 | 36% |
| EAK3381821.1 aminoglycoside 3'-phosphotransferase [Campylobact... | 102 | 8e-23 | 37% |
| HAQ1584029.1 TPA: aminoglycoside 3'-phosphotransferase [Entero... | 102 | 8e-23 | 37% |
| WP_193795737.1 aminoglycoside O-phosphotransferase APH(3'')-Ib... | 104 | 8e-23 | 34% |
| WP_027215618.1 aminoglycoside 3'-phosphotransferase [Butyrivib... | 104 | 8e-23 | 28% |
| WP_031885364.1 aminoglycoside 3'-phosphotransferase [Staphyloc... | 103 | 8e-23 | 34% |
| ECZ3208277.1 aminoglycoside O-phosphotransferase APH(3'')-Ib [... | 104 | 8e-23 | 34% |
| ECZ3208265.1 aminoglycoside O-phosphotransferase APH(3'')-Ib [... | 104 | 8e-23 | 34% |
| ECT4913297.1 aminoglycoside O-phosphotransferase APH(3'')-Ib [... | 104 | 8e-23 | 34% |
| OLC40273.1 hypothetical protein AUH43_26735 [Acidobacteria bac... | 103 | 8e-23 | 34% |
| WP_126725982.1 aminoglycoside O-phosphotransferase APH(3'')-Ib... | 104 | 8e-23 | 34% |

| MGT13564.1 aminoglycoside O-phosphotransferase APH(3'')-Ib [Es... | 104 | 8e-23 | 34% |
| --- | --- | --- | --- |
| WP_121408021.1 aminoglycoside 3'-phosphotransferase [Streptomy... | 104 | 8e-23 | 36% |
| WP_031884431.1 aminoglycoside 3'-phosphotransferase [Staphyloc... | 102 | 8e-23 | 37% |
| WP_001354041.1 unnamed protein product | 105 | 8e-23 | 34% |
| ECQ1688543.1 aminoglycoside O-phosphotransferase APH(3'')-Ib [... | 104 | 8e-23 | 34% |
| ECG5995795.1 aminoglycoside O-phosphotransferase APH(3'')-Ib [... | 104 | 8e-23 | 34% |
| OMA65880.1 APH(3') family aminoglycoside O-phosphotransferase ... | 102 | 8e-23 | 34% |
| ANI75976.1 Streptomycin Phosphotransferase StrA [Klebsiella pn... | 105 | 8e-23 | 34% |
| EFN5307717.1 aminoglycoside O-phosphotransferase APH(3'')-Ib [... | 104 | 8e-23 | 34% |
| WP_063809419.1 aminoglycoside O-phosphotransferase APH(3'')-Ib... | 104 | 8e-23 | 34% |
| HAP5217757.1 TPA: aminoglycoside 3'-phosphotransferase [Entero... | 102 | 8e-23 | 37% |
| EFM0542861.1 aminoglycoside O-phosphotransferase APH(3'')-Ib [... | 104 | 8e-23 | 34% |
| WP_095873724.1 aminoglycoside 3'-phosphotransferase [Streptomy... | 103 | 8e-23 | 37% |
| MYB56988.1 aminoglycoside 3'-phosphotransferase [Gemmatimonade... | 104 | 8e-23 | 32% |
| EBF3571372.1 aminoglycoside O-phosphotransferase APH(3'')-Ib [... | 104 | 8e-23 | 34% |
| EBD4024270.1 aminoglycoside O-phosphotransferase APH(3'')-Ib [... | 104 | 8e-23 | 34% |
| CCD32474.1 aminoglycoside resistance gene [Corynebacterium str... | 101 | 8e-23 | 35% |
| ORG68848.1 APH(3') family aminoglycoside O-phosphotransferase ... | 102 | 9e-23 | 35% |
| EWQ68838.1 aminoglycoside 3'-phosphotransferase [Staphylococcu... | 102 | 9e-23 | 37% |
| EFB9246457.1 aminoglycoside O-phosphotransferase APH(3'')-Ib [... | 104 | 9e-23 | 34% |
| WP_044352037.1 aminoglycoside O-phosphotransferase APH(3'')-Ib... | 104 | 9e-23 | 34% |
| EOS78194.1 hypothetical protein C819_00316 [Lachnospiraceae ba... | 104 | 9e-23 | 29% |
| WP_191244702.1 APH(3'') family aminoglycoside O-phosphotransfe... | 104 | 9e-23 | 34% |
| WP_128512421.1 APH(3'') family aminoglycoside O-phosphotransfe... | 104 | 9e-23 | 38% |
| WP_126469008.1 APH(3'') family aminoglycoside O-phosphotransfe... | 104 | 9e-23 | 34% |
| ECM7452106.1 aminoglycoside O-phosphotransferase APH(3'')-Ib [... | 104 | 9e-23 | 34% |
| EDB8336865.1 aminoglycoside O-phosphotransferase APH(3'')-Ib [... | 104 | 9e-23 | 34% |
| EEO0862501.1 aminoglycoside O-phosphotransferase APH(3'')-Ib [... | 104 | 9e-23 | 34% |
| WP_031889199.1 MULTISPECIES: aminoglycoside 3'-phosphotransfer... | 102 | 9e-23 | 37% |
| ECC6397664.1 aminoglycoside O-phosphotransferase APH(3'')-Ib [... | 104 | 9e-23 | 34% |
| HHX59765.1 TPA: aminoglycoside 3'-phosphotransferase [Epulopis... | 104 | 9e-23 | 28% |
| EBP5571218.1 aminoglycoside O-phosphotransferase APH(3'')-Ib [... | 104 | 9e-23 | 34% |
| EDX6720617.1 aminoglycoside O-phosphotransferase APH(3'')-Ib [... | 104 | 9e-23 | 34% |
| EEV2754937.1 aminoglycoside O-phosphotransferase APH(3'')-Ib [... | 104 | 9e-23 | 34% |
| EEW2362381.1 aminoglycoside O-phosphotransferase APH(3'')-Ib [... | 104 | 9e-23 | 34% |
| EFA9345932.1 aminoglycoside O-phosphotransferase APH(3'')-Ib [... | 104 | 9e-23 | 34% |
| HAH3855380.1 TPA: APH(3'') family aminoglycoside O-phosphotran... | 104 | 9e-23 | 34% |
| WP_184803370.1 phosphotransferase [Kribbella italica] | 103 | 9e-23 | 33% |
| AXN76033.1 Aminoglycoside phosphotransferase [uncultured bacte... | 102 | 9e-23 | 34% |
| EAA7790229.1 aminoglycoside O-phosphotransferase APH(3'')-Ib [... | 104 | 9e-23 | 34% |
| EAY6914511.1 aminoglycoside O-phosphotransferase APH(3'')-Ib [... | 104 | 9e-23 | 34% |
| MBD8943340.1 aminoglycoside 3'-phosphotransferase [Ruminococca... | 104 | 9e-23 | 31% |
| HAQ6149958.1 TPA: aminoglycoside 3'-phosphotransferase [Entero... | 102 | 9e-23 | 34% |
| WP_008447165.1 aminoglycoside 3'-phosphotransferase [Janthinob... | 104 | 9e-23 | 35% |
| OKH77769.1 3'-kinase [Mycobacterium sp. SWH-M3] | 104 | 9e-23 | 36% |
| WP_065542726.1 MULTISPECIES: aminoglycoside 3'-phosphotransfer... | 104 | 9e-23 | 30% |
| WP_006141398.1 aminoglycoside 3'-phosphotransferase [Streptomy... | 104 | 9e-23 | 36% |
| ECV2704363.1 aminoglycoside 3'-phosphotransferase [Salmonella ... | 104 | 9e-23 | 34% |
| EAP4022735.1 aminoglycoside O-phosphotransferase APH(3'')-Ib [... | 104 | 9e-23 | 34% |
| ADX01892.1 StrA [Acinetobacter baumannii 1656-2] | 105 | 9e-23 | 34% |
| WP_161097877.1 APH(3') family aminoglycoside O-phosphotransfer... | 104 | 9e-23 | 35% |
| WP_010794532.1 MULTISPECIES: aminoglycoside O-phosphotransfera... | 104 | 1e-22 | 34% |
| PBC78466.1 kanamycin kinase [Streptomyces sp. TLI_235] | 103 | 1e-22 | 37% |
| EFD1910437.1 aminoglycoside 3'-phosphotransferase [Escherichia... | 102 | 1e-22 | 33% |
| EBR1815721.1 aminoglycoside O-phosphotransferase APH(3'')-Ib [... | 104 | 1e-22 | 34% |
| QHY95566.1 Aminoglycoside 3'-phosphotransferase [Streptomyces ... | 104 | 1e-22 | 35% |
| ECS4832060.1 aminoglycoside O-phosphotransferase APH(3'')-Ib [... | 104 | 1e-22 | 34% |
| EEC3955844.1 aminoglycoside O-phosphotransferase APH(3'')-Ib [... | 104 | 1e-22 | 34% |
| WP_139157063.1 aminoglycoside 3'-phosphotransferase [Klebsiell... | 100 | 1e-22 | 38% |
| EEZ9620718.1 aminoglycoside O-phosphotransferase APH(3'')-Ib [... | 104 | 1e-22 | 34% |
| WP_113644746.1 APH(3'') family aminoglycoside O-phosphotransfe... | 104 | 1e-22 | 35% |
| ALP55317.1 aminoglycoside phosphotransferase [Klebsiella pneum... | 105 | 1e-22 | 34% |
| WP_093845688.1 MULTISPECIES: APH(3'') family aminoglycoside O-... | 104 | 1e-22 | 34% |
| WP_106239314.1 aminoglycoside 3'-phosphotransferase [Nonomurae... | 104 | 1e-22 | 37% |
| WP_047232546.1 aminoglycoside 3'-phosphotransferase [Cellulosi... | 104 | 1e-22 | 35% |
| WP_077532824.1 aminoglycoside 3'-phosphotransferase [Massilima... | 104 | 1e-22 | 34% |
| EFH5890628.1 aminoglycoside O-phosphotransferase APH(3'')-Ib [... | 104 | 1e-22 | 34% |
| WP_018895089.1 APH(3'') family aminoglycoside O-phosphotransfe... | 104 | 1e-22 | 34% |
| WP_186780855.1 APH(3'') family aminoglycoside O-phosphotransfe... | 104 | 1e-22 | 34% |
| WP_102399880.1 aminoglycoside 3'-phosphotransferase [Haloimpat... | 103 | 1e-22 | 28% |
| WP_064317233.1 aminoglycoside 3'-phosphotransferase [Cellulosi... | 104 | 1e-22 | 35% |
| EAY2946965.1 aminoglycoside O-phosphotransferase APH(3'')-Ib [... | 104 | 1e-22 | 34% |

| EFC7440378.1 aminoglycoside O-phosphotransferase APH(3'')-Ib [... | 104 | 1e-22 | 34% |
| --- | --- | --- | --- |
| WP_091741142.1 aminoglycoside 3'-phosphotransferase [Phenyloba... | 104 | 1e-22 | 34% |
| EDB4303451.1 aminoglycoside O-phosphotransferase APH(3'')-Ib [... | 104 | 1e-22 | 34% |
| EDB4303461.1 aminoglycoside O-phosphotransferase APH(3'')-Ib [... | 104 | 1e-22 | 34% |
| ECU6324924.1 aminoglycoside O-phosphotransferase APH(3'')-Ib [... | 103 | 1e-22 | 34% |
| EEZ2358520.1 aminoglycoside O-phosphotransferase APH(3'')-Ib [... | 104 | 1e-22 | 34% |
| EEI3333026.1 aminoglycoside O-phosphotransferase APH(3'')-Ib [... | 104 | 1e-22 | 34% |
| EBV6976590.1 aminoglycoside O-phosphotransferase APH(3'')-Ib [... | 104 | 1e-22 | 34% |
| WP_096043062.1 aminoglycoside O-phosphotransferase APH(3'')-Ib... | 104 | 1e-22 | 34% |
| WP_140976804.1 aminoglycoside O-phosphotransferase APH(3'')-Ib... | 104 | 1e-22 | 34% |
| WP_135069917.1 APH(3'') family aminoglycoside O-phosphotransfe... | 104 | 1e-22 | 36% |
| WP_185083137.1 phosphotransferase [Nonomuraea muscovyensis] | 104 | 1e-22 | 35% |
| HAO0815564.1 TPA: aminoglycoside O-phosphotransferase APH(3'')... | 103 | 1e-22 | 34% |
| ABG49218.1 hypothetical protein [Escherichia coli] | 105 | 1e-22 | 34% |
| EDL5332224.1 APH(3'') family aminoglycoside O-phosphotransfera... | 104 | 1e-22 | 34% |
| EEJ7641237.1 aminoglycoside 3'-phosphotransferase [Salmonella ... | 101 | 1e-22 | 35% |
| WP_097932595.1 MULTISPECIES: APH(3'') family aminoglycoside O-... | 104 | 1e-22 | 35% |
| HAN9135362.1 TPA: aminoglycoside O-phosphotransferase APH(3'')... | 104 | 1e-22 | 34% |
| WP_124288387.1 MULTISPECIES: APH(3'') family aminoglycoside O-... | 104 | 1e-22 | 34% |
| WP_084598723.1 aminoglycoside 3'-phosphotransferase [Actinopla... | 104 | 1e-22 | 36% |
| WP_123683179.1 APH(3'') family aminoglycoside O-phosphotransfe... | 103 | 1e-22 | 36% |
| ECJ7427007.1 aminoglycoside O-phosphotransferase APH(3'')-Ib [... | 104 | 1e-22 | 34% |
| MYD63012.1 aminoglycoside 3'-phosphotransferase [Gemmatimonade... | 103 | 1e-22 | 32% |
| WP_065552989.1 MULTISPECIES: aminoglycoside O-phosphotransfera... | 103 | 1e-22 | 34% |
| PYT27058.1 hypothetical protein DMG57_19560 [Acidobacteria bac... | 103 | 1e-22 | 35% |
| OLC83776.1 hypothetical protein AUH72_03520 [Acidobacteria bac... | 103 | 1e-22 | 34% |
| WP_100022253.1 APH(3'') family aminoglycoside O-phosphotransfe... | 103 | 1e-22 | 34% |
| PRX65908.1 kanamycin kinase [Nonomuraea fuscirosea] | 104 | 1e-22 | 37% |
| EDL5222072.1 APH(3'') family aminoglycoside O-phosphotransfera... | 104 | 1e-22 | 34% |
| ECG8710716.1 aminoglycoside O-phosphotransferase APH(3'')-Ib [... | 104 | 1e-22 | 34% |
| WP_064956332.1 aminoglycoside 3'-phosphotransferase [Microbact... | 103 | 1e-22 | 34% |
| WP_111779524.1 APH(3'') family aminoglycoside O-phosphotransfe... | 103 | 1e-22 | 34% |
| WP_032491780.1 aminoglycoside O-phosphotransferase APH(3')-III... | 103 | 1e-22 | 30% |
| HAG0113061.1 TPA: aminoglycoside O-phosphotransferase APH(3'')... | 103 | 1e-22 | 34% |
| EGC1091791.1 aminoglycoside O-phosphotransferase APH(3'')-Ib [... | 104 | 1e-22 | 34% |
| EGD4223870.1 aminoglycoside O-phosphotransferase APH(3'')-Ib [... | 104 | 1e-22 | 34% |
| EAX8225076.1 aminoglycoside O-phosphotransferase APH(3'')-Ib [... | 104 | 1e-22 | 34% |
| WP_172693114.1 aminoglycoside O-phosphotransferase APH(3'')-Ib... | 104 | 1e-22 | 34% |
| WP_137542665.1 aminoglycoside O-phosphotransferase APH(3'')-Ib... | 104 | 1e-22 | 34% |
| WP_032256586.1 MULTISPECIES: APH(3'') family aminoglycoside O-... | 103 | 1e-22 | 34% |
| EFB4185046.1 aminoglycoside O-phosphotransferase APH(3'')-Ib [... | 104 | 1e-22 | 34% |
| HAM7812110.1 TPA: aminoglycoside O-phosphotransferase APH(3'')... | 104 | 1e-22 | 34% |
| EFN3825342.1 aminoglycoside O-phosphotransferase APH(3'')-Ib [... | 104 | 1e-22 | 34% |
| EGO4026949.1 aminoglycoside O-phosphotransferase APH(3'')-Ib [... | 104 | 1e-22 | 34% |
| WP_126670881.1 aminoglycoside O-phosphotransferase APH(3'')-Ib... | 104 | 1e-22 | 34% |
| ECT7405499.1 aminoglycoside O-phosphotransferase APH(3'')-Ib [... | 104 | 1e-22 | 34% |
| EDT8841658.1 aminoglycoside O-phosphotransferase APH(3'')-Ib [... | 103 | 1e-22 | 34% |
| WP_057969235.1 MULTISPECIES: APH(3'') family aminoglycoside O-... | 103 | 1e-22 | 35% |
| EAT6318564.1 aminoglycoside O-phosphotransferase APH(3'')-Ib [... | 103 | 1e-22 | 34% |
| EBP6288416.1 aminoglycoside O-phosphotransferase APH(3'')-Ib [... | 103 | 1e-22 | 34% |
| HAJ4958632.1 TPA: aminoglycoside O-phosphotransferase APH(3'')... | 103 | 1e-22 | 34% |
| WP_060589430.1 MULTISPECIES: aminoglycoside O-phosphotransfera... | 103 | 1e-22 | 34% |
| ECC8129918.1 aminoglycoside O-phosphotransferase APH(3'')-Ib [... | 103 | 1e-22 | 34% |
| EFI2841528.1 aminoglycoside O-phosphotransferase APH(3'')-Ib [... | 103 | 1e-22 | 34% |
| MWQ77623.1 APH(3'') family aminoglycoside O-phosphotransferase... | 103 | 1e-22 | 34% |
| EEJ7641236.1 aminoglycoside 3'-phosphotransferase [Salmonella ... | 100 | 1e-22 | 35% |
| KAA9863231.1 aminoglycoside 3'-phosphotransferase [Escherichia... | 100 | 1e-22 | 38% |
| WP_001145206.1 APH(3'') family aminoglycoside O-phosphotransfe... | 103 | 1e-22 | 34% |
| EAX0750720.1 aminoglycoside O-phosphotransferase APH(3'')-Ib [... | 103 | 1e-22 | 34% |
| ECM5257286.1 aminoglycoside O-phosphotransferase APH(3'')-Ib [... | 103 | 1e-22 | 34% |
| WP_167416306.1 aminoglycoside O-phosphotransferase APH(3'')-Ib... | 103 | 1e-22 | 34% |
| EBD1331162.1 aminoglycoside O-phosphotransferase APH(3'')-Ib [... | 103 | 1e-22 | 34% |
| WP_065954107.1 aminoglycoside O-phosphotransferase APH(3'')-Ib... | 103 | 1e-22 | 34% |
| HAG5981653.1 TPA: aminoglycoside O-phosphotransferase APH(3'')... | 103 | 1e-22 | 34% |
| EEC4135775.1 aminoglycoside O-phosphotransferase APH(3'')-Ib [... | 103 | 1e-22 | 34% |
| ECI1878937.1 aminoglycoside O-phosphotransferase APH(3'')-Ib [... | 103 | 1e-22 | 34% |
| ACH85851.1 APH(3'')-Ib [Salmonella enterica subsp. enterica se... | 103 | 1e-22 | 34% |
| WP_032271369.1 MULTISPECIES: APH(3'') family aminoglycoside O-... | 103 | 1e-22 | 34% |
| HAO0815571.1 TPA: aminoglycoside O-phosphotransferase APH(3'')... | 103 | 1e-22 | 34% |
| EFG6162055.1 aminoglycoside O-phosphotransferase APH(3'')-Ib [... | 103 | 1e-22 | 34% |
| EFF5356040.1 aminoglycoside O-phosphotransferase APH(3'')-Ib [... | 103 | 1e-22 | 34% |
| WP_069556661.1 MULTISPECIES: APH(3'') family aminoglycoside O-... | 103 | 1e-22 | 34% |

| WP_059346078.1 aminoglycoside O-phosphotransferase APH(3'')-Ib... | 103 | 1e-22 | 34% |
| --- | --- | --- | --- |
| AUF80590.1 Streptomycin 3''-O-adenylyltransferase [Raoultella ... | 105 | 1e-22 | 34% |
| EAT1215141.1 APH(3'') family aminoglycoside O-phosphotransfera... | 103 | 1e-22 | 34% |
| WP_067964703.1 APH(3'') family aminoglycoside O-phosphotransfe... | 103 | 1e-22 | 40% |
| WP_023146014.1 MULTISPECIES: APH(3'') family aminoglycoside O-... | 103 | 1e-22 | 34% |
| HAD9997107.1 TPA: aminoglycoside O-phosphotransferase APH(3'')... | 103 | 1e-22 | 34% |
| WP_059214276.1 APH(3'') family aminoglycoside O-phosphotransfe... | 104 | 1e-22 | 35% |
| EBQ1757484.1 aminoglycoside O-phosphotransferase APH(3'')-Ib [... | 103 | 2e-22 | 34% |
| EEW1914442.1 aminoglycoside O-phosphotransferase APH(3'')-Ib [... | 103 | 2e-22 | 34% |
| WP_027754838.1 APH(3'') family aminoglycoside O-phosphotransfe... | 103 | 2e-22 | 35% |
| WP_049746691.1 APH(3'') family aminoglycoside O-phosphotransfe... | 103 | 2e-22 | 33% |
| EBM9395628.1 APH(3'') family aminoglycoside O-phosphotransfera... | 103 | 2e-22 | 34% |
| WP_053031821.1 APH(3'') family aminoglycoside O-phosphotransfe... | 103 | 2e-22 | 34% |
| WP_058624054.1 aminoglycoside 3'-phosphotransferase [Microbact... | 103 | 2e-22 | 35% |
| WP_135140823.1 APH(3'') family aminoglycoside O-phosphotransfe... | 103 | 2e-22 | 34% |
| ECX7722123.1 aminoglycoside O-phosphotransferase APH(3'')-Ib [... | 103 | 2e-22 | 34% |
| WP_142287369.1 APH(3'') family aminoglycoside O-phosphotransfe... | 103 | 2e-22 | 34% |
| WP_031178793.1 APH(3'') family aminoglycoside O-phosphotransfe... | 103 | 2e-22 | 34% |
| WP_100029583.1 APH(3'') family aminoglycoside O-phosphotransfe... | 103 | 2e-22 | 34% |
| AKT72273.1 StrA [Escherichia coli] | 105 | 2e-22 | 34% |
| WP_100885594.1 aminoglycoside 3'-phosphotransferase [Acinetoba... | 103 | 2e-22 | 30% |
| ECA3672553.1 APH(3'') family aminoglycoside O-phosphotransfera... | 103 | 2e-22 | 34% |
| WP_029387627.1 APH(3'') family aminoglycoside O-phosphotransfe... | 103 | 2e-22 | 36% |
| WP_052897455.1 APH(3'') family aminoglycoside O-phosphotransfe... | 103 | 2e-22 | 34% |
| CCD32475.1 aminoglycoside resistance gene [Corynebacterium str... | 100 | 2e-22 | 38% |
| WP_109208949.1 MULTISPECIES: aminoglycoside 3'-phosphotransfer... | 103 | 2e-22 | 35% |
| EAA0946183.1 APH(3'') family aminoglycoside O-phosphotransfera... | 103 | 2e-22 | 34% |
| OLE26707.1 aminoglycoside phosphotransferase APH(3') [Catenuli... | 103 | 2e-22 | 33% |
| WP_032251621.1 aminoglycoside O-phosphotransferase APH(3'')-Ib... | 103 | 2e-22 | 34% |
| ECT5066440.1 aminoglycoside O-phosphotransferase APH(3'')-Ib [... | 103 | 2e-22 | 34% |
| NPI81920.1 aminoglycoside O-phosphotransferase APH(3'')-Ib [Es... | 103 | 2e-22 | 34% |
| WP_027771127.1 APH(3'') family aminoglycoside O-phosphotransfe... | 103 | 2e-22 | 35% |
| WP_119230569.1 APH(3'') family aminoglycoside O-phosphotransfe... | 103 | 2e-22 | 34% |
| EBT8157357.1 aminoglycoside O-phosphotransferase APH(3'')-Ib [... | 103 | 2e-22 | 34% |
| WP_129310821.1 APH(3'') family aminoglycoside O-phosphotransfe... | 103 | 2e-22 | 34% |
| WP_114364131.1 aminoglycoside O-phosphotransferase APH(3'')-Ib... | 103 | 2e-22 | 34% |
| WP_119230475.1 APH(3'') family aminoglycoside O-phosphotransfe... | 103 | 2e-22 | 34% |
| WP_144824128.1 APH(3'') family aminoglycoside O-phosphotransfe... | 103 | 2e-22 | 34% |
| WP_152807375.1 aminoglycoside 3'-phosphotransferase [Rugamonas... | 103 | 2e-22 | 36% |
| TZF52619.1 APH(3'') family aminoglycoside O-phosphotransferase... | 103 | 2e-22 | 34% |
| CUJ57836.1 Aminoglycoside 3'-phosphotransferase [Achromobacter... | 105 | 2e-22 | 34% |
| MBF0687849.1 aminoglycoside 3'-phosphotransferase [Cellulomona... | 103 | 2e-22 | 33% |
| WP_140045479.1 APH(3'') family aminoglycoside O-phosphotransfe... | 103 | 2e-22 | 34% |
| ECU6081594.1 aminoglycoside O-phosphotransferase APH(3'')-Ib [... | 103 | 2e-22 | 34% |
| EFL9504177.1 APH(3'') family aminoglycoside O-phosphotransfera... | 103 | 2e-22 | 34% |
| WP_111886407.1 aminoglycoside O-phosphotransferase APH(3'')-Ib... | 103 | 2e-22 | 34% |
| WP_041542731.1 aminoglycoside phosphotransferase APH(3') [Cate... | 103 | 2e-22 | 34% |
| AFP25013.1 StrA [Escherichia coli] | 105 | 2e-22 | 34% |
| API82191.1 APH(3'') family aminoglycoside O-phosphotransferase... | 103 | 2e-22 | 34% |
| EFV0120442.1 aminoglycoside O-phosphotransferase APH(3'')-Ib [... | 103 | 2e-22 | 34% |
| ORY82264.1 aminoglycoside phosphotransferase [Neocallimastix c... | 103 | 2e-22 | 25% |
| WP_098409114.1 aminoglycoside 3'-phosphotransferase [Microbact... | 103 | 2e-22 | 33% |
| EEF4641166.1 aminoglycoside O-phosphotransferase APH(3'')-Ib [... | 103 | 2e-22 | 34% |
| EAV8884938.1 APH(3'') family aminoglycoside O-phosphotransfera... | 103 | 2e-22 | 34% |
| ECT7405502.1 aminoglycoside O-phosphotransferase APH(3'')-Ib [... | 103 | 2e-22 | 34% |
| EDB8336863.1 aminoglycoside O-phosphotransferase APH(3'')-Ib [... | 103 | 2e-22 | 34% |
| ECN0130933.1 aminoglycoside O-phosphotransferase APH(3'')-Ib [... | 103 | 2e-22 | 34% |
| WP_130515724.1 APH(3'') family aminoglycoside O-phosphotransfe... | 103 | 2e-22 | 34% |
| HCK10686.1 TPA: APH(3') family aminoglycoside O-phosphotransfe... | 100 | 2e-22 | 38% |
| WP_067166276.1 aminoglycoside 3'-phosphotransferase [Microbact... | 103 | 2e-22 | 36% |
| ECH7700575.1 aminoglycoside O-phosphotransferase APH(3'')-Ib [... | 103 | 2e-22 | 34% |
| EGO4584929.1 aminoglycoside O-phosphotransferase APH(3'')-Ib [... | 103 | 2e-22 | 34% |
| EAZ9329618.1 aminoglycoside 3'-phosphotransferase [Salmonella ... | 103 | 2e-22 | 34% |
| KZE88464.1 Aminoglycoside 3'-phosphotransferase [Microbacteriu... | 103 | 2e-22 | 36% |
| EBE3458258.1 APH(3'') family aminoglycoside O-phosphotransfera... | 103 | 2e-22 | 34% |
| WP_181779788.1 phosphotransferase [Pseudonocardia sp. CAP47R] | 102 | 2e-22 | 35% |
| PZR70066.1 aminoglycoside phosphotransferase APH(3') [Candidat... | 103 | 2e-22 | 34% |
| WP_063059107.1 aminoglycoside 3'-phosphotransferase [Nocardia ... | 103 | 2e-22 | 36% |
| MIS62225.1 aminoglycoside 3'-phosphotransferase [Salmonella en... | 103 | 2e-22 | 34% |
| WP_171497494.1 APH(3') family aminoglycoside O-phosphotransfer... | 103 | 2e-22 | 30% |
| WP_001145207.1 MULTISPECIES: APH(3'') family aminoglycoside O-... | 103 | 2e-22 | 34% |
| WP_129839370.1 APH(3'') family aminoglycoside O-phosphotransfe... | 103 | 2e-22 | 34% |

| MBA2394671.1 aminoglycoside 3'-phosphotransferase [Ktedonobact... | 103 | 2e-22 | 28% |
| --- | --- | --- | --- |
| WP_116755867.1 aminoglycoside 3'-phosphotransferase [Amnibacte... | 103 | 2e-22 | 36% |
| WP_052195018.1 APH(3'') family aminoglycoside O-phosphotransfe... | 103 | 2e-22 | 33% |
| OWP21557.1 aminoglycoside phosphotransferase APH(3') [Microbac... | 102 | 2e-22 | 33% |
| MXW05946.1 aminoglycoside 3'-phosphotransferase [Gemmatimonade... | 103 | 2e-22 | 31% |
| ACU75033.1 aminoglycoside phosphotransferase [Catenulispora ac... | 103 | 2e-22 | 34% |
| WP_195128190.1 tetratricopeptide repeat protein [Nocardia amam... | 105 | 2e-22 | 39% |
| WP_045253326.1 aminoglycoside 3'-phosphotransferase [Microbact... | 102 | 2e-22 | 34% |
| EBU3991153.1 APH(3'') family aminoglycoside O-phosphotransfera... | 103 | 2e-22 | 34% |
| WP_166909010.1 APH(3'') family aminoglycoside O-phosphotransfe... | 103 | 2e-22 | 34% |
| WP_074416959.1 aminoglycoside O-phosphotransferase APH(3'')-Ib... | 103 | 2e-22 | 34% |
| EBO6606554.1 aminoglycoside O-phosphotransferase APH(3'')-Ib [... | 103 | 2e-22 | 34% |
| ECK1616461.1 APH(3'') family aminoglycoside O-phosphotransfera... | 102 | 2e-22 | 34% |
| EAY7218947.1 APH(3'') family aminoglycoside O-phosphotransfera... | 102 | 2e-22 | 34% |
| WP_089801277.1 aminoglycoside 3'-phosphotransferase [Cellulomo... | 103 | 2e-22 | 34% |
| WP_001418834.1 APH(3'') family aminoglycoside O-phosphotransfe... | 103 | 2e-22 | 34% |
| KHJ53236.1 aminoglycoside phosphotransferase [Aureimonas altam... | 103 | 2e-22 | 33% |
| WP_193857648.1 APH(3'') family aminoglycoside O-phosphotransfe... | 103 | 2e-22 | 34% |
| WP_017794445.1 aminoglycoside 3'-phosphotransferase [Leucobact... | 103 | 2e-22 | 34% |
| WP_032490182.1 aminoglycoside O-phosphotransferase APH(3'')-Ib... | 103 | 2e-22 | 34% |
| EFU0831156.1 aminoglycoside O-phosphotransferase APH(3'')-Ib [... | 103 | 2e-22 | 34% |
| PIG58543.1 kanamycin kinase [Streptomyces sp. 1] | 103 | 2e-22 | 34% |
| EAX5269405.1 aminoglycoside O-phosphotransferase APH(3'')-Ib [... | 103 | 2e-22 | 34% |
| EBP5410153.1 APH(3'') family aminoglycoside O-phosphotransfera... | 102 | 2e-22 | 34% |
| EEL3407800.1 APH(3'') family aminoglycoside O-phosphotransfera... | 103 | 2e-22 | 34% |
| ECP5059869.1 APH(3'') family aminoglycoside O-phosphotransfera... | 102 | 2e-22 | 34% |
| WP_001082317.1 MULTISPECIES: aminoglycoside O-phosphotransfera... | 103 | 2e-22 | 34% |
| OJU44947.1 APH(3'') family aminoglycoside O-phosphotransferase... | 103 | 2e-22 | 35% |
| OBC17507.1 aminoglycoside phosphotransferase APH(3') [Mycobact... | 103 | 2e-22 | 37% |
| MSK53887.1 aminoglycoside O-phosphotransferase APH(3'')-Ib [Es... | 103 | 2e-22 | 34% |
| WP_119506059.1 aminoglycoside O-phosphotransferase APH(3'')-Ib... | 103 | 2e-22 | 34% |
| WP_190853987.1 APH(3'') family aminoglycoside O-phosphotransfe... | 103 | 2e-22 | 36% |
| EDL5222067.1 APH(3'') family aminoglycoside O-phosphotransfera... | 102 | 2e-22 | 34% |
| ECY3180707.1 APH(3'') family aminoglycoside O-phosphotransfera... | 103 | 2e-22 | 34% |
| WP_089613233.1 aminoglycoside O-phosphotransferase APH(3'')-Ib... | 103 | 2e-22 | 34% |
| EAC1383418.1 aminoglycoside O-phosphotransferase APH(3'')-Ib [... | 103 | 2e-22 | 34% |
| WP_033084919.1 APH(3'') family aminoglycoside O-phosphotransfe... | 102 | 2e-22 | 35% |
| WP_026939875.1 aminoglycoside 3'-phosphotransferase [Humibacte... | 103 | 2e-22 | 34% |
| HAM7518727.1 TPA: aminoglycoside O-phosphotransferase APH(3'')... | 103 | 2e-22 | 34% |
| BAX97632.1 putative streptomycin phosphotransferase [[Mycobact... | 102 | 2e-22 | 35% |
| WP_141899151.1 aminoglycoside 3'-phosphotransferase [Microbact... | 102 | 3e-22 | 33% |
| EGB1307091.1 aminoglycoside O-phosphotransferase APH(3'')-Ib [... | 103 | 3e-22 | 34% |
| WP_051540333.1 aminoglycoside 3'-phosphotransferase [Clostridi... | 103 | 3e-22 | 29% |
| HAH2400189.1 TPA: APH(3'') family aminoglycoside O-phosphotran... | 103 | 3e-22 | 34% |
| WP_162291785.1 MULTISPECIES: APH(3') family aminoglycoside O-p... | 103 | 3e-22 | 30% |
| WP_154378140.1 aminoglycoside 3'-phosphotransferase [Duganella... | 102 | 3e-22 | 35% |
| EDA9915578.1 aminoglycoside O-phosphotransferase APH(3'')-Ib [... | 103 | 3e-22 | 34% |
| WP_082944565.1 aminoglycoside 3'-phosphotransferase [Mycobacte... | 103 | 3e-22 | 37% |
| WP_168100489.1 phosphotransferase [Streptomyces sp. PLAI1-29] | 103 | 3e-22 | 36% |
| EDB8775185.1 aminoglycoside O-phosphotransferase APH(3'')-Ib [... | 103 | 3e-22 | 34% |
| WP_070930894.1 APH(3'') family aminoglycoside O-phosphotransfe... | 103 | 3e-22 | 35% |
| WP_042189037.1 APH(3'') family aminoglycoside O-phosphotransfe... | 103 | 3e-22 | 34% |
| WP_161668600.1 aminoglycoside O-phosphotransferase APH(3'')-Ib... | 103 | 3e-22 | 34% |
| EBK1711357.1 aminoglycoside O-phosphotransferase APH(3'')-Ib [... | 103 | 3e-22 | 34% |
| EFA4308055.1 aminoglycoside O-phosphotransferase APH(3'')-Ib [... | 103 | 3e-22 | 34% |
| WP_172407833.1 phosphotransferase [Microbacterium sp. AISO3] | 102 | 3e-22 | 33% |
| WP_042034167.1 APH(3'') family aminoglycoside O-phosphotransfe... | 102 | 3e-22 | 34% |
| WP_106914048.1 aminoglycoside O-phosphotransferase APH(3'')-Ib... | 103 | 3e-22 | 34% |
| MIE31771.1 APH(3'') family aminoglycoside O-phosphotransferase... | 102 | 3e-22 | 34% |
| WP_064317824.1 MULTISPECIES: APH(3'') family aminoglycoside O-... | 102 | 3e-22 | 35% |
| ABW39611.1 StrA [Escherichia coli] | 103 | 3e-22 | 34% |
| WP_156998137.1 phosphotransferase [Agromyces terreus] | 102 | 3e-22 | 36% |
| ECM7377432.1 aminoglycoside O-phosphotransferase APH(3'')-Ib [... | 103 | 3e-22 | 34% |
| MKV39193.1 aminoglycoside O-phosphotransferase APH(3'')-Ib [Sa... | 103 | 3e-22 | 34% |
| ECX3366908.1 aminoglycoside O-phosphotransferase APH(3'')-Ib [... | 103 | 3e-22 | 34% |
| ECV3054780.1 aminoglycoside O-phosphotransferase APH(3'')-Ib [... | 103 | 3e-22 | 34% |
| QIS37791.1 Aminoglycoside 3''-phosphotransferase [Salmonella e... | 105 | 3e-22 | 34% |
| EEK7994468.1 APH(3'') family aminoglycoside O-phosphotransfera... | 102 | 3e-22 | 34% |
| WP_075921940.1 APH(3'') family aminoglycoside O-phosphotransfe... | 102 | 3e-22 | 36% |
| ALIGNMENTS  >BAD00047.1 Fusion protein, Feo [Hepacivirus C] |  |  |  |

```
Length=832

Score = 546 bits (1407), Expect = 0.0, Method: Compositional matrix adjust. Identities = 264/264 (100%), Positives = 264/264 (100%), Gaps = 0/264 (0%)

Query 1	MIEQDGLHAGSPAAWVERLFGYDWAQQTIGCSDAAVFRLSAQGRPVLFVKTDLSGALNEL 60 MIEQDGLHAGSPAAWVERLFGYDWAQQTIGCSDAAVFRLSAQGRPVLFVKTDLSGALNEL
Sbjct 569 MIEQDGLHAGSPAAWVERLFGYDWAQQTIGCSDAAVFRLSAQGRPVLFVKTDLSGALNEL 628

Query 61	QDEAARLSWLATTGVPCAAVLDVVTEAGRDWLLLGEVPGQDLLSSHLAPAEKVSIMADAM 120 QDEAARLSWLATTGVPCAAVLDVVTEAGRDWLLLGEVPGQDLLSSHLAPAEKVSIMADAM
Sbjct 629 QDEAARLSWLATTGVPCAAVLDVVTEAGRDWLLLGEVPGQDLLSSHLAPAEKVSIMADAM 688

Query 121 RRLHTLDPATCPFDHQAKHRIERARTRMEAGLVDQDDLDEEHQGLAPAELFARLKARMPD 180 RRLHTLDPATCPFDHQAKHRIERARTRMEAGLVDQDDLDEEHQGLAPAELFARLKARMPD
Sbjct 689 RRLHTLDPATCPFDHQAKHRIERARTRMEAGLVDQDDLDEEHQGLAPAELFARLKARMPD 748

Query 181 GEDLVVTHGDACLPNIMVENGRFSGFIDCGRLGVADRYQDIALATRDIAEELGGEWADRF 240 GEDLVVTHGDACLPNIMVENGRFSGFIDCGRLGVADRYQDIALATRDIAEELGGEWADRF
Sbjct 749 GEDLVVTHGDACLPNIMVENGRFSGFIDCGRLGVADRYQDIALATRDIAEELGGEWADRF 808

Query 241 LVLYGIAAPDSQRIAFYRLLDEFF 264 LVLYGIAAPDSQRIAFYRLLDEFF
Sbjct 809 LVLYGIAAPDSQRIAFYRLLDEFF 832

>AGU01702.1 firefly luciferase-neomycin phosphotransferase fusion protein [Cloning vector pCRm-luc2-NEO]
Length=829

Score = 543 bits (1398), Expect = 0.0, Method: Compositional matrix adjust. Identities = 263/263 (100%), Positives = 263/263 (100%), Gaps = 0/263 (0%)

Query 2	IEQDGLHAGSPAAWVERLFGYDWAQQTIGCSDAAVFRLSAQGRPVLFVKTDLSGALNELQ 61 IEQDGLHAGSPAAWVERLFGYDWAQQTIGCSDAAVFRLSAQGRPVLFVKTDLSGALNELQ
Sbjct 567 IEQDGLHAGSPAAWVERLFGYDWAQQTIGCSDAAVFRLSAQGRPVLFVKTDLSGALNELQ 626

Query 62	DEAARLSWLATTGVPCAAVLDVVTEAGRDWLLLGEVPGQDLLSSHLAPAEKVSIMADAMR 121 DEAARLSWLATTGVPCAAVLDVVTEAGRDWLLLGEVPGQDLLSSHLAPAEKVSIMADAMR
Sbjct 627 DEAARLSWLATTGVPCAAVLDVVTEAGRDWLLLGEVPGQDLLSSHLAPAEKVSIMADAMR 686

Query 122 RLHTLDPATCPFDHQAKHRIERARTRMEAGLVDQDDLDEEHQGLAPAELFARLKARMPDG 181 RLHTLDPATCPFDHQAKHRIERARTRMEAGLVDQDDLDEEHQGLAPAELFARLKARMPDG
Sbjct 687 RLHTLDPATCPFDHQAKHRIERARTRMEAGLVDQDDLDEEHQGLAPAELFARLKARMPDG 746

Query 182 EDLVVTHGDACLPNIMVENGRFSGFIDCGRLGVADRYQDIALATRDIAEELGGEWADRFL 241 EDLVVTHGDACLPNIMVENGRFSGFIDCGRLGVADRYQDIALATRDIAEELGGEWADRFL
Sbjct 747 EDLVVTHGDACLPNIMVENGRFSGFIDCGRLGVADRYQDIALATRDIAEELGGEWADRFL 806

Query 242 VLYGIAAPDSQRIAFYRLLDEFF 264
VLYGIAAPDSQRIAFYRLLDEFF Sbjct 807 VLYGIAAPDSQRIAFYRLLDEFF 829

>AAT02530.1 lacZ/gfp/neomycin phosphotransferase fusion protein [Cloning vector pUC-gfp-neo]
Length=549

Score = 542 bits (1397), Expect = 0.0, Method: Compositional matrix adjust. Identities = 264/264 (100%), Positives = 264/264 (100%), Gaps = 0/264 (0%)

Query 1	MIEQDGLHAGSPAAWVERLFGYDWAQQTIGCSDAAVFRLSAQGRPVLFVKTDLSGALNEL 60 MIEQDGLHAGSPAAWVERLFGYDWAQQTIGCSDAAVFRLSAQGRPVLFVKTDLSGALNEL
Sbjct 286 MIEQDGLHAGSPAAWVERLFGYDWAQQTIGCSDAAVFRLSAQGRPVLFVKTDLSGALNEL 345

Query 61	QDEAARLSWLATTGVPCAAVLDVVTEAGRDWLLLGEVPGQDLLSSHLAPAEKVSIMADAM 120 QDEAARLSWLATTGVPCAAVLDVVTEAGRDWLLLGEVPGQDLLSSHLAPAEKVSIMADAM
Sbjct 346 QDEAARLSWLATTGVPCAAVLDVVTEAGRDWLLLGEVPGQDLLSSHLAPAEKVSIMADAM 405

Query 121 RRLHTLDPATCPFDHQAKHRIERARTRMEAGLVDQDDLDEEHQGLAPAELFARLKARMPD 180 RRLHTLDPATCPFDHQAKHRIERARTRMEAGLVDQDDLDEEHQGLAPAELFARLKARMPD
Sbjct 406 RRLHTLDPATCPFDHQAKHRIERARTRMEAGLVDQDDLDEEHQGLAPAELFARLKARMPD 465
```

| Query | 181 | GEDLVVTHGDACLPNIMVENGRFSGFIDCGRLGVADRYQDIALATRDIAEELGGEWADRF 240 |
| --- | --- | --- |
| Sbjct | 466 | GEDLVVTHGDACLPNIMVENGRFSGFIDCGRLGVADRYQDIALATRDIAEELGGEWADRF GEDLVVTHGDACLPNIMVENGRFSGFIDCGRLGVADRYQDIALATRDIAEELGGEWADRF 525 |
| Query | 241 | LVLYGIAAPDSQRIAFYRLLDEFF 264 |
| Sbjct | 526 | LVLYGIAAPDSQRIAFYRLLDEFF  LVLYGIAAPDSQRIAFYRLLDEFF 549 |

```
>AAT02528.1 gfp/neomycin phosphotransferase fusion protein [Cloning vector pUC-gfp-neo]
Length=527

Score = 542 bits (1396), Expect = 0.0, Method: Compositional matrix adjust. Identities = 264/264 (100%), Positives = 264/264 (100%), Gaps = 0/264 (0%)

Query 1	MIEQDGLHAGSPAAWVERLFGYDWAQQTIGCSDAAVFRLSAQGRPVLFVKTDLSGALNEL 60 MIEQDGLHAGSPAAWVERLFGYDWAQQTIGCSDAAVFRLSAQGRPVLFVKTDLSGALNEL
Sbjct 264 MIEQDGLHAGSPAAWVERLFGYDWAQQTIGCSDAAVFRLSAQGRPVLFVKTDLSGALNEL 323

Query 61	QDEAARLSWLATTGVPCAAVLDVVTEAGRDWLLLGEVPGQDLLSSHLAPAEKVSIMADAM 120 QDEAARLSWLATTGVPCAAVLDVVTEAGRDWLLLGEVPGQDLLSSHLAPAEKVSIMADAM
Sbjct 324 QDEAARLSWLATTGVPCAAVLDVVTEAGRDWLLLGEVPGQDLLSSHLAPAEKVSIMADAM 383

Query 121 RRLHTLDPATCPFDHQAKHRIERARTRMEAGLVDQDDLDEEHQGLAPAELFARLKARMPD 180 RRLHTLDPATCPFDHQAKHRIERARTRMEAGLVDQDDLDEEHQGLAPAELFARLKARMPD
Sbjct 384 RRLHTLDPATCPFDHQAKHRIERARTRMEAGLVDQDDLDEEHQGLAPAELFARLKARMPD 443

Query 181 GEDLVVTHGDACLPNIMVENGRFSGFIDCGRLGVADRYQDIALATRDIAEELGGEWADRF 240 GEDLVVTHGDACLPNIMVENGRFSGFIDCGRLGVADRYQDIALATRDIAEELGGEWADRF
Sbjct 444 GEDLVVTHGDACLPNIMVENGRFSGFIDCGRLGVADRYQDIALATRDIAEELGGEWADRF 503

Query 241 LVLYGIAAPDSQRIAFYRLLDEFF 264 LVLYGIAAPDSQRIAFYRLLDEFF
Sbjct 504 LVLYGIAAPDSQRIAFYRLLDEFF 527

>AIO05660.1 Rluc-ubiquitin-neo fusion protein [synthetic construct] AIO05662.1 Rluc-ubiquitin-neo fusion protein [synthetic construct]
Length=656

Score = 541 bits (1395), Expect = 0.0, Method: Compositional matrix adjust. Identities = 264/264 (100%), Positives = 264/264 (100%), Gaps = 0/264 (0%)

Query 1	MIEQDGLHAGSPAAWVERLFGYDWAQQTIGCSDAAVFRLSAQGRPVLFVKTDLSGALNEL 60 MIEQDGLHAGSPAAWVERLFGYDWAQQTIGCSDAAVFRLSAQGRPVLFVKTDLSGALNEL
Sbjct 393 MIEQDGLHAGSPAAWVERLFGYDWAQQTIGCSDAAVFRLSAQGRPVLFVKTDLSGALNEL 452

Query 61	QDEAARLSWLATTGVPCAAVLDVVTEAGRDWLLLGEVPGQDLLSSHLAPAEKVSIMADAM 120 QDEAARLSWLATTGVPCAAVLDVVTEAGRDWLLLGEVPGQDLLSSHLAPAEKVSIMADAM
Sbjct 453 QDEAARLSWLATTGVPCAAVLDVVTEAGRDWLLLGEVPGQDLLSSHLAPAEKVSIMADAM 512

Query 121 RRLHTLDPATCPFDHQAKHRIERARTRMEAGLVDQDDLDEEHQGLAPAELFARLKARMPD 180 RRLHTLDPATCPFDHQAKHRIERARTRMEAGLVDQDDLDEEHQGLAPAELFARLKARMPD
Sbjct 513 RRLHTLDPATCPFDHQAKHRIERARTRMEAGLVDQDDLDEEHQGLAPAELFARLKARMPD 572

Query 181 GEDLVVTHGDACLPNIMVENGRFSGFIDCGRLGVADRYQDIALATRDIAEELGGEWADRF 240 GEDLVVTHGDACLPNIMVENGRFSGFIDCGRLGVADRYQDIALATRDIAEELGGEWADRF
Sbjct 573 GEDLVVTHGDACLPNIMVENGRFSGFIDCGRLGVADRYQDIALATRDIAEELGGEWADRF 632

Query 241 LVLYGIAAPDSQRIAFYRLLDEFF 264 LVLYGIAAPDSQRIAFYRLLDEFF
Sbjct 633 LVLYGIAAPDSQRIAFYRLLDEFF 656

>ABC61923.1 ubiquitin-NPTII fusion [Binary vector pBINPLUS/ARS] Length=343

Score = 540 bits (1392), Expect = 0.0, Method: Compositional matrix adjust. Identities = 264/264 (100%), Positives = 264/264 (100%), Gaps = 0/264 (0%)
```

| Query | 1 | MIEQDGLHAGSPAAWVERLFGYDWAQQTIGCSDAAVFRLSAQGRPVLFVKTDLSGALNEL | 60 |
| --- | --- | --- | --- |
| Sbjct | 80 | MIEQDGLHAGSPAAWVERLFGYDWAQQTIGCSDAAVFRLSAQGRPVLFVKTDLSGALNEL MIEQDGLHAGSPAAWVERLFGYDWAQQTIGCSDAAVFRLSAQGRPVLFVKTDLSGALNEL | 139 |
| Query | 61 | QDEAARLSWLATTGVPCAAVLDVVTEAGRDWLLLGEVPGQDLLSSHLAPAEKVSIMADAM | 120 |
| Sbjct | 140 | QDEAARLSWLATTGVPCAAVLDVVTEAGRDWLLLGEVPGQDLLSSHLAPAEKVSIMADAM QDEAARLSWLATTGVPCAAVLDVVTEAGRDWLLLGEVPGQDLLSSHLAPAEKVSIMADAM | 199 |
| Query | 121 | RRLHTLDPATCPFDHQAKHRIERARTRMEAGLVDQDDLDEEHQGLAPAELFARLKARMPD | 180 |
| Sbjct | 200 | RRLHTLDPATCPFDHQAKHRIERARTRMEAGLVDQDDLDEEHQGLAPAELFARLKARMPD RRLHTLDPATCPFDHQAKHRIERARTRMEAGLVDQDDLDEEHQGLAPAELFARLKARMPD | 259 |
| Query | 181 | GEDLVVTHGDACLPNIMVENGRFSGFIDCGRLGVADRYQDIALATRDIAEELGGEWADRF | 240 |
| Sbjct | 260 | GEDLVVTHGDACLPNIMVENGRFSGFIDCGRLGVADRYQDIALATRDIAEELGGEWADRF GEDLVVTHGDACLPNIMVENGRFSGFIDCGRLGVADRYQDIALATRDIAEELGGEWADRF | 319 |
| Query  Sbjct | 241  320 | LVLYGIAAPDSQRIAFYRLLDEFF 264 LVLYGIAAPDSQRIAFYRLLDEFF LVLYGIAAPDSQRIAFYRLLDEFF 343 |  |

```
>AAB66883.1 fluorescent neomycin phosphotransferase fusion protein [synthetic construct]
Length=504

Score = 540 bits (1390), Expect = 0.0, Method: Compositional matrix adjust. Identities = 263/263 (100%), Positives = 263/263 (100%), Gaps = 0/263 (0%)

Query 2	IEQDGLHAGSPAAWVERLFGYDWAQQTIGCSDAAVFRLSAQGRPVLFVKTDLSGALNELQ 61 IEQDGLHAGSPAAWVERLFGYDWAQQTIGCSDAAVFRLSAQGRPVLFVKTDLSGALNELQ
Sbjct 242 IEQDGLHAGSPAAWVERLFGYDWAQQTIGCSDAAVFRLSAQGRPVLFVKTDLSGALNELQ 301

Query 62	DEAARLSWLATTGVPCAAVLDVVTEAGRDWLLLGEVPGQDLLSSHLAPAEKVSIMADAMR 121 DEAARLSWLATTGVPCAAVLDVVTEAGRDWLLLGEVPGQDLLSSHLAPAEKVSIMADAMR
Sbjct 302 DEAARLSWLATTGVPCAAVLDVVTEAGRDWLLLGEVPGQDLLSSHLAPAEKVSIMADAMR 361

Query 122 RLHTLDPATCPFDHQAKHRIERARTRMEAGLVDQDDLDEEHQGLAPAELFARLKARMPDG 181 RLHTLDPATCPFDHQAKHRIERARTRMEAGLVDQDDLDEEHQGLAPAELFARLKARMPDG
Sbjct 362 RLHTLDPATCPFDHQAKHRIERARTRMEAGLVDQDDLDEEHQGLAPAELFARLKARMPDG 421

Query 182 EDLVVTHGDACLPNIMVENGRFSGFIDCGRLGVADRYQDIALATRDIAEELGGEWADRFL 241 EDLVVTHGDACLPNIMVENGRFSGFIDCGRLGVADRYQDIALATRDIAEELGGEWADRFL
Sbjct 422 EDLVVTHGDACLPNIMVENGRFSGFIDCGRLGVADRYQDIALATRDIAEELGGEWADRFL 481

Query 242 VLYGIAAPDSQRIAFYRLLDEFF 264
VLYGIAAPDSQRIAFYRLLDEFF Sbjct 482 VLYGIAAPDSQRIAFYRLLDEFF 504

>ANA50877.1 CodA-nptII [Cloning vector pCTAG7]
AYG86907.1 bifunctional CodA-NPTII fusion protein [Cloning vector pCTAGII-GUSPlus] AYG86912.1 bifunctional CodA-NPTII fusion protein [Cloning vector pCTAG6]
Length=698

Score = 539 bits (1389), Expect = 0.0, Method: Compositional matrix adjust. Identities = 263/263 (100%), Positives = 263/263 (100%), Gaps = 0/263 (0%)

Query 2	IEQDGLHAGSPAAWVERLFGYDWAQQTIGCSDAAVFRLSAQGRPVLFVKTDLSGALNELQ 61 IEQDGLHAGSPAAWVERLFGYDWAQQTIGCSDAAVFRLSAQGRPVLFVKTDLSGALNELQ
Sbjct 436 IEQDGLHAGSPAAWVERLFGYDWAQQTIGCSDAAVFRLSAQGRPVLFVKTDLSGALNELQ 495

Query 62	DEAARLSWLATTGVPCAAVLDVVTEAGRDWLLLGEVPGQDLLSSHLAPAEKVSIMADAMR 121 DEAARLSWLATTGVPCAAVLDVVTEAGRDWLLLGEVPGQDLLSSHLAPAEKVSIMADAMR
Sbjct 496 DEAARLSWLATTGVPCAAVLDVVTEAGRDWLLLGEVPGQDLLSSHLAPAEKVSIMADAMR 555

Query 122 RLHTLDPATCPFDHQAKHRIERARTRMEAGLVDQDDLDEEHQGLAPAELFARLKARMPDG 181 RLHTLDPATCPFDHQAKHRIERARTRMEAGLVDQDDLDEEHQGLAPAELFARLKARMPDG
Sbjct 556 RLHTLDPATCPFDHQAKHRIERARTRMEAGLVDQDDLDEEHQGLAPAELFARLKARMPDG 615

Query 182 EDLVVTHGDACLPNIMVENGRFSGFIDCGRLGVADRYQDIALATRDIAEELGGEWADRFL 241 EDLVVTHGDACLPNIMVENGRFSGFIDCGRLGVADRYQDIALATRDIAEELGGEWADRFL
Sbjct 616 EDLVVTHGDACLPNIMVENGRFSGFIDCGRLGVADRYQDIALATRDIAEELGGEWADRFL 675

Query 242 VLYGIAAPDSQRIAFYRLLDEFF 264
VLYGIAAPDSQRIAFYRLLDEFF Sbjct 676 VLYGIAAPDSQRIAFYRLLDEFF 698

>BAB32740.1 red-shift green fluorescent protein-fused neomycin phosphotransferase [Cloning vector pLCPVRGNR104]
Length=506

Score = 539 bits (1388), Expect = 0.0, Method: Compositional matrix adjust. Identities = 263/263 (100%), Positives = 263/263 (100%), Gaps = 0/263 (0%)

Query 2	IEQDGLHAGSPAAWVERLFGYDWAQQTIGCSDAAVFRLSAQGRPVLFVKTDLSGALNELQ 61 IEQDGLHAGSPAAWVERLFGYDWAQQTIGCSDAAVFRLSAQGRPVLFVKTDLSGALNELQ
Sbjct 244 IEQDGLHAGSPAAWVERLFGYDWAQQTIGCSDAAVFRLSAQGRPVLFVKTDLSGALNELQ 303

Query 62	DEAARLSWLATTGVPCAAVLDVVTEAGRDWLLLGEVPGQDLLSSHLAPAEKVSIMADAMR 121 DEAARLSWLATTGVPCAAVLDVVTEAGRDWLLLGEVPGQDLLSSHLAPAEKVSIMADAMR
Sbjct 304 DEAARLSWLATTGVPCAAVLDVVTEAGRDWLLLGEVPGQDLLSSHLAPAEKVSIMADAMR 363

Query 122 RLHTLDPATCPFDHQAKHRIERARTRMEAGLVDQDDLDEEHQGLAPAELFARLKARMPDG 181 RLHTLDPATCPFDHQAKHRIERARTRMEAGLVDQDDLDEEHQGLAPAELFARLKARMPDG
Sbjct 364 RLHTLDPATCPFDHQAKHRIERARTRMEAGLVDQDDLDEEHQGLAPAELFARLKARMPDG 423

Query 182 EDLVVTHGDACLPNIMVENGRFSGFIDCGRLGVADRYQDIALATRDIAEELGGEWADRFL 241 EDLVVTHGDACLPNIMVENGRFSGFIDCGRLGVADRYQDIALATRDIAEELGGEWADRFL
Sbjct 424 EDLVVTHGDACLPNIMVENGRFSGFIDCGRLGVADRYQDIALATRDIAEELGGEWADRFL 483

Query 242 VLYGIAAPDSQRIAFYRLLDEFF 264
VLYGIAAPDSQRIAFYRLLDEFF Sbjct 484 VLYGIAAPDSQRIAFYRLLDEFF 506

>AII16593.1 GFPm2-NPTII fusion protein [Expression vector pT7 RNAP]
AII16596.1 green fluorescent protein-neomycin phosphotransferase II fusion protein [Expression vector pT7 RNAP_HR(eba175)]
AII16601.1 green fluorescent protein-neomycin phosphotransferase II fusion protein [Expression vector pT7 RNAP_HR(kahrp)]
Length=503

Score = 538 bits (1387), Expect = 0.0, Method: Compositional matrix adjust. Identities = 263/263 (100%), Positives = 263/263 (100%), Gaps = 0/263 (0%)

Query 2	IEQDGLHAGSPAAWVERLFGYDWAQQTIGCSDAAVFRLSAQGRPVLFVKTDLSGALNELQ 61 IEQDGLHAGSPAAWVERLFGYDWAQQTIGCSDAAVFRLSAQGRPVLFVKTDLSGALNELQ
Sbjct 241 IEQDGLHAGSPAAWVERLFGYDWAQQTIGCSDAAVFRLSAQGRPVLFVKTDLSGALNELQ 300

Query 62	DEAARLSWLATTGVPCAAVLDVVTEAGRDWLLLGEVPGQDLLSSHLAPAEKVSIMADAMR 121 DEAARLSWLATTGVPCAAVLDVVTEAGRDWLLLGEVPGQDLLSSHLAPAEKVSIMADAMR
Sbjct 301 DEAARLSWLATTGVPCAAVLDVVTEAGRDWLLLGEVPGQDLLSSHLAPAEKVSIMADAMR 360

Query 122 RLHTLDPATCPFDHQAKHRIERARTRMEAGLVDQDDLDEEHQGLAPAELFARLKARMPDG 181 RLHTLDPATCPFDHQAKHRIERARTRMEAGLVDQDDLDEEHQGLAPAELFARLKARMPDG
Sbjct 361 RLHTLDPATCPFDHQAKHRIERARTRMEAGLVDQDDLDEEHQGLAPAELFARLKARMPDG 420

Query 182 EDLVVTHGDACLPNIMVENGRFSGFIDCGRLGVADRYQDIALATRDIAEELGGEWADRFL 241 EDLVVTHGDACLPNIMVENGRFSGFIDCGRLGVADRYQDIALATRDIAEELGGEWADRFL
Sbjct 421 EDLVVTHGDACLPNIMVENGRFSGFIDCGRLGVADRYQDIALATRDIAEELGGEWADRFL 480

Query 242 VLYGIAAPDSQRIAFYRLLDEFF 264
VLYGIAAPDSQRIAFYRLLDEFF Sbjct 481 VLYGIAAPDSQRIAFYRLLDEFF 503

>ATN39931.1 PRK/NPTII fusion protein [synthetic construct] Length=595

Score = 538 bits (1386), Expect = 0.0, Method: Compositional matrix adjust. Identities = 263/263 (100%), Positives = 263/263 (100%), Gaps = 0/263 (0%)

Query 2	IEQDGLHAGSPAAWVERLFGYDWAQQTIGCSDAAVFRLSAQGRPVLFVKTDLSGALNELQ 61
```

| Sbjct | 333 | IEQDGLHAGSPAAWVERLFGYDWAQQTIGCSDAAVFRLSAQGRPVLFVKTDLSGALNELQ IEQDGLHAGSPAAWVERLFGYDWAQQTIGCSDAAVFRLSAQGRPVLFVKTDLSGALNELQ | 392 |
| --- | --- | --- | --- |
| Query | 62 | DEAARLSWLATTGVPCAAVLDVVTEAGRDWLLLGEVPGQDLLSSHLAPAEKVSIMADAMR | 121 |
| Sbjct | 393 | DEAARLSWLATTGVPCAAVLDVVTEAGRDWLLLGEVPGQDLLSSHLAPAEKVSIMADAMR DEAARLSWLATTGVPCAAVLDVVTEAGRDWLLLGEVPGQDLLSSHLAPAEKVSIMADAMR | 452 |
| Query | 122 | RLHTLDPATCPFDHQAKHRIERARTRMEAGLVDQDDLDEEHQGLAPAELFARLKARMPDG | 181 |
| Sbjct | 453 | RLHTLDPATCPFDHQAKHRIERARTRMEAGLVDQDDLDEEHQGLAPAELFARLKARMPDG RLHTLDPATCPFDHQAKHRIERARTRMEAGLVDQDDLDEEHQGLAPAELFARLKARMPDG | 512 |
| Query | 182 | EDLVVTHGDACLPNIMVENGRFSGFIDCGRLGVADRYQDIALATRDIAEELGGEWADRFL | 241 |
| Sbjct | 513 | EDLVVTHGDACLPNIMVENGRFSGFIDCGRLGVADRYQDIALATRDIAEELGGEWADRFL EDLVVTHGDACLPNIMVENGRFSGFIDCGRLGVADRYQDIALATRDIAEELGGEWADRFL | 572 |
| Query  Sbjct | 242  573 | VLYGIAAPDSQRIAFYRLLDEFF 264 VLYGIAAPDSQRIAFYRLLDEFF VLYGIAAPDSQRIAFYRLLDEFF 595 |  |

```
>AMB57377.1 UbiM-NptII [Binary vector pARS2] Length=343

Score = 538 bits (1385), Expect = 0.0, Method: Compositional matrix adjust. Identities = 263/263 (100%), Positives = 263/263 (100%), Gaps = 0/263 (0%)

Query 2	IEQDGLHAGSPAAWVERLFGYDWAQQTIGCSDAAVFRLSAQGRPVLFVKTDLSGALNELQ 61 IEQDGLHAGSPAAWVERLFGYDWAQQTIGCSDAAVFRLSAQGRPVLFVKTDLSGALNELQ
Sbjct 81	IEQDGLHAGSPAAWVERLFGYDWAQQTIGCSDAAVFRLSAQGRPVLFVKTDLSGALNELQ 140

Query 62	DEAARLSWLATTGVPCAAVLDVVTEAGRDWLLLGEVPGQDLLSSHLAPAEKVSIMADAMR 121 DEAARLSWLATTGVPCAAVLDVVTEAGRDWLLLGEVPGQDLLSSHLAPAEKVSIMADAMR
Sbjct 141 DEAARLSWLATTGVPCAAVLDVVTEAGRDWLLLGEVPGQDLLSSHLAPAEKVSIMADAMR 200

Query 122 RLHTLDPATCPFDHQAKHRIERARTRMEAGLVDQDDLDEEHQGLAPAELFARLKARMPDG 181 RLHTLDPATCPFDHQAKHRIERARTRMEAGLVDQDDLDEEHQGLAPAELFARLKARMPDG
Sbjct 201 RLHTLDPATCPFDHQAKHRIERARTRMEAGLVDQDDLDEEHQGLAPAELFARLKARMPDG 260

Query 182 EDLVVTHGDACLPNIMVENGRFSGFIDCGRLGVADRYQDIALATRDIAEELGGEWADRFL 241 EDLVVTHGDACLPNIMVENGRFSGFIDCGRLGVADRYQDIALATRDIAEELGGEWADRFL
Sbjct 261 EDLVVTHGDACLPNIMVENGRFSGFIDCGRLGVADRYQDIALATRDIAEELGGEWADRFL 320

Query 242 VLYGIAAPDSQRIAFYRLLDEFF 264
VLYGIAAPDSQRIAFYRLLDEFF Sbjct 321 VLYGIAAPDSQRIAFYRLLDEFF 343

>ABI49756.1 renilla luciferase/neomycin phosphotransferase fusion protein [CMV hRluc-neo Flexi Vector pF9A]
ACJ37464.1 hRluc-neo fusion protein [Cloning vector pmirGLO]
ACT53141.1 hRluc-neo fusion protein [Cloning vector pFN26A (BIND) hRluc-neo] ACT53145.1 hRluc-neo fusion protein [Cloning vector pBIND-ER (alpha)] ACT53149.1 hRluc-neo fusion protein [Cloning vector pBIND-GR]
AFU76705.1 hRluc-neo [Hepatitis C virus replicon 4a ED43-RlucNeo (R+I)]
AGU01697.1 Renilla luciferase-neomycin phosphotransferase fusion protein [Cloning vector pCRm-Rluc-NEO]
Length=591

Score = 537 bits (1384), Expect = 0.0, Method: Compositional matrix adjust. Identities = 263/263 (100%), Positives = 263/263 (100%), Gaps = 0/263 (0%)

Query 2	IEQDGLHAGSPAAWVERLFGYDWAQQTIGCSDAAVFRLSAQGRPVLFVKTDLSGALNELQ 61 IEQDGLHAGSPAAWVERLFGYDWAQQTIGCSDAAVFRLSAQGRPVLFVKTDLSGALNELQ
Sbjct 329 IEQDGLHAGSPAAWVERLFGYDWAQQTIGCSDAAVFRLSAQGRPVLFVKTDLSGALNELQ 388

Query 62	DEAARLSWLATTGVPCAAVLDVVTEAGRDWLLLGEVPGQDLLSSHLAPAEKVSIMADAMR 121 DEAARLSWLATTGVPCAAVLDVVTEAGRDWLLLGEVPGQDLLSSHLAPAEKVSIMADAMR
Sbjct 389 DEAARLSWLATTGVPCAAVLDVVTEAGRDWLLLGEVPGQDLLSSHLAPAEKVSIMADAMR 448

Query 122 RLHTLDPATCPFDHQAKHRIERARTRMEAGLVDQDDLDEEHQGLAPAELFARLKARMPDG 181 RLHTLDPATCPFDHQAKHRIERARTRMEAGLVDQDDLDEEHQGLAPAELFARLKARMPDG
Sbjct 449 RLHTLDPATCPFDHQAKHRIERARTRMEAGLVDQDDLDEEHQGLAPAELFARLKARMPDG 508
```

| Query | 182 | EDLVVTHGDACLPNIMVENGRFSGFIDCGRLGVADRYQDIALATRDIAEELGGEWADRFL 241 |
| --- | --- | --- |
| Sbjct | 509 | EDLVVTHGDACLPNIMVENGRFSGFIDCGRLGVADRYQDIALATRDIAEELGGEWADRFL EDLVVTHGDACLPNIMVENGRFSGFIDCGRLGVADRYQDIALATRDIAEELGGEWADRFL 568 |
| Query | 242 | VLYGIAAPDSQRIAFYRLLDEFF 264 |
| Sbjct | 569 | VLYGIAAPDSQRIAFYRLLDEFF  VLYGIAAPDSQRIAFYRLLDEFF 591 |

```
>CAB46914.1 core-neo fusion protein [Hepatitis C virus] [Hepatitis C virus replicon I389/NS2-3'UTR]
CAB46916.1 core-neo fusion protein [Hepatitis C virus] [Hepatitis C virus replicon I389/NS3-3'UTR]
Length=283

Score = 537 bits (1384), Expect = 0.0, Method: Compositional matrix adjust. Identities = 264/264 (100%), Positives = 264/264 (100%), Gaps = 0/264 (0%)

Query 1	MIEQDGLHAGSPAAWVERLFGYDWAQQTIGCSDAAVFRLSAQGRPVLFVKTDLSGALNEL 60 MIEQDGLHAGSPAAWVERLFGYDWAQQTIGCSDAAVFRLSAQGRPVLFVKTDLSGALNEL
Sbjct 20	MIEQDGLHAGSPAAWVERLFGYDWAQQTIGCSDAAVFRLSAQGRPVLFVKTDLSGALNEL 79

Query 61	QDEAARLSWLATTGVPCAAVLDVVTEAGRDWLLLGEVPGQDLLSSHLAPAEKVSIMADAM 120 QDEAARLSWLATTGVPCAAVLDVVTEAGRDWLLLGEVPGQDLLSSHLAPAEKVSIMADAM
Sbjct 80	QDEAARLSWLATTGVPCAAVLDVVTEAGRDWLLLGEVPGQDLLSSHLAPAEKVSIMADAM 139

Query 121 RRLHTLDPATCPFDHQAKHRIERARTRMEAGLVDQDDLDEEHQGLAPAELFARLKARMPD 180 RRLHTLDPATCPFDHQAKHRIERARTRMEAGLVDQDDLDEEHQGLAPAELFARLKARMPD
Sbjct 140 RRLHTLDPATCPFDHQAKHRIERARTRMEAGLVDQDDLDEEHQGLAPAELFARLKARMPD 199

Query 181 GEDLVVTHGDACLPNIMVENGRFSGFIDCGRLGVADRYQDIALATRDIAEELGGEWADRF 240 GEDLVVTHGDACLPNIMVENGRFSGFIDCGRLGVADRYQDIALATRDIAEELGGEWADRF
Sbjct 200 GEDLVVTHGDACLPNIMVENGRFSGFIDCGRLGVADRYQDIALATRDIAEELGGEWADRF 259

Query 241 LVLYGIAAPDSQRIAFYRLLDEFF 264 LVLYGIAAPDSQRIAFYRLLDEFF
Sbjct 260 LVLYGIAAPDSQRIAFYRLLDEFF 283

>CAB46910.1 core-neo fusion protein [Hepatitis C virus] [Hepatitis C virus replicon I377/NS2-3'UTR]
CAB46912.1 core-neo fusion protein [Hepatitis C virus] [Hepatitis C virus replicon I377/NS3-3'UTR]
Length=279

Score = 536 bits (1382), Expect = 0.0, Method: Compositional matrix adjust. Identities = 264/264 (100%), Positives = 264/264 (100%), Gaps = 0/264 (0%)

Query 1	MIEQDGLHAGSPAAWVERLFGYDWAQQTIGCSDAAVFRLSAQGRPVLFVKTDLSGALNEL 60 MIEQDGLHAGSPAAWVERLFGYDWAQQTIGCSDAAVFRLSAQGRPVLFVKTDLSGALNEL
Sbjct 16	MIEQDGLHAGSPAAWVERLFGYDWAQQTIGCSDAAVFRLSAQGRPVLFVKTDLSGALNEL 75

Query 61	QDEAARLSWLATTGVPCAAVLDVVTEAGRDWLLLGEVPGQDLLSSHLAPAEKVSIMADAM 120 QDEAARLSWLATTGVPCAAVLDVVTEAGRDWLLLGEVPGQDLLSSHLAPAEKVSIMADAM
Sbjct 76	QDEAARLSWLATTGVPCAAVLDVVTEAGRDWLLLGEVPGQDLLSSHLAPAEKVSIMADAM 135

Query 121 RRLHTLDPATCPFDHQAKHRIERARTRMEAGLVDQDDLDEEHQGLAPAELFARLKARMPD 180 RRLHTLDPATCPFDHQAKHRIERARTRMEAGLVDQDDLDEEHQGLAPAELFARLKARMPD
Sbjct 136 RRLHTLDPATCPFDHQAKHRIERARTRMEAGLVDQDDLDEEHQGLAPAELFARLKARMPD 195

Query 181 GEDLVVTHGDACLPNIMVENGRFSGFIDCGRLGVADRYQDIALATRDIAEELGGEWADRF 240 GEDLVVTHGDACLPNIMVENGRFSGFIDCGRLGVADRYQDIALATRDIAEELGGEWADRF
Sbjct 196 GEDLVVTHGDACLPNIMVENGRFSGFIDCGRLGVADRYQDIALATRDIAEELGGEWADRF 255

Query 241 LVLYGIAAPDSQRIAFYRLLDEFF 264 LVLYGIAAPDSQRIAFYRLLDEFF
Sbjct 256 LVLYGIAAPDSQRIAFYRLLDEFF 279

>AJF44503.1 Neo [Cloning vector pNZP1-SGR] Length=279

Score = 536 bits (1382), Expect = 0.0, Method: Compositional matrix adjust. Identities = 264/264 (100%), Positives = 264/264 (100%), Gaps = 0/264 (0%)

Query 1	MIEQDGLHAGSPAAWVERLFGYDWAQQTIGCSDAAVFRLSAQGRPVLFVKTDLSGALNEL 60 MIEQDGLHAGSPAAWVERLFGYDWAQQTIGCSDAAVFRLSAQGRPVLFVKTDLSGALNEL
Sbjct 16	MIEQDGLHAGSPAAWVERLFGYDWAQQTIGCSDAAVFRLSAQGRPVLFVKTDLSGALNEL 75

Query 61	QDEAARLSWLATTGVPCAAVLDVVTEAGRDWLLLGEVPGQDLLSSHLAPAEKVSIMADAM 120 QDEAARLSWLATTGVPCAAVLDVVTEAGRDWLLLGEVPGQDLLSSHLAPAEKVSIMADAM
Sbjct 76	QDEAARLSWLATTGVPCAAVLDVVTEAGRDWLLLGEVPGQDLLSSHLAPAEKVSIMADAM 135

Query 121 RRLHTLDPATCPFDHQAKHRIERARTRMEAGLVDQDDLDEEHQGLAPAELFARLKARMPD 180 RRLHTLDPATCPFDHQAKHRIERARTRMEAGLVDQDDLDEEHQGLAPAELFARLKARMPD
Sbjct 136 RRLHTLDPATCPFDHQAKHRIERARTRMEAGLVDQDDLDEEHQGLAPAELFARLKARMPD 195

Query 181 GEDLVVTHGDACLPNIMVENGRFSGFIDCGRLGVADRYQDIALATRDIAEELGGEWADRF 240 GEDLVVTHGDACLPNIMVENGRFSGFIDCGRLGVADRYQDIALATRDIAEELGGEWADRF
Sbjct 196 GEDLVVTHGDACLPNIMVENGRFSGFIDCGRLGVADRYQDIALATRDIAEELGGEWADRF 255

Query 241 LVLYGIAAPDSQRIAFYRLLDEFF 264 LVLYGIAAPDSQRIAFYRLLDEFF
Sbjct 256 LVLYGIAAPDSQRIAFYRLLDEFF 279

>BAM38519.1 neomycin-kanamycin phosphotransferase type II [Hepatitis C virus replicon pSGR-S310/A]
BAM38521.1 neomycin-kanamycin phosphotransferase type II [Hepatitis C virus replicon pSGR-S310/B]
Length=283

Score = 536 bits (1381), Expect = 0.0, Method: Compositional matrix adjust. Identities = 264/264 (100%), Positives = 264/264 (100%), Gaps = 0/264 (0%)

Query 1	MIEQDGLHAGSPAAWVERLFGYDWAQQTIGCSDAAVFRLSAQGRPVLFVKTDLSGALNEL 60 MIEQDGLHAGSPAAWVERLFGYDWAQQTIGCSDAAVFRLSAQGRPVLFVKTDLSGALNEL
Sbjct 20	MIEQDGLHAGSPAAWVERLFGYDWAQQTIGCSDAAVFRLSAQGRPVLFVKTDLSGALNEL 79

Query 61	QDEAARLSWLATTGVPCAAVLDVVTEAGRDWLLLGEVPGQDLLSSHLAPAEKVSIMADAM 120 QDEAARLSWLATTGVPCAAVLDVVTEAGRDWLLLGEVPGQDLLSSHLAPAEKVSIMADAM
Sbjct 80	QDEAARLSWLATTGVPCAAVLDVVTEAGRDWLLLGEVPGQDLLSSHLAPAEKVSIMADAM 139

Query 121 RRLHTLDPATCPFDHQAKHRIERARTRMEAGLVDQDDLDEEHQGLAPAELFARLKARMPD 180 RRLHTLDPATCPFDHQAKHRIERARTRMEAGLVDQDDLDEEHQGLAPAELFARLKARMPD
Sbjct 140 RRLHTLDPATCPFDHQAKHRIERARTRMEAGLVDQDDLDEEHQGLAPAELFARLKARMPD 199

Query 181 GEDLVVTHGDACLPNIMVENGRFSGFIDCGRLGVADRYQDIALATRDIAEELGGEWADRF 240 GEDLVVTHGDACLPNIMVENGRFSGFIDCGRLGVADRYQDIALATRDIAEELGGEWADRF
Sbjct 200 GEDLVVTHGDACLPNIMVENGRFSGFIDCGRLGVADRYQDIALATRDIAEELGGEWADRF 259

Query 241 LVLYGIAAPDSQRIAFYRLLDEFF 264 LVLYGIAAPDSQRIAFYRLLDEFF
Sbjct 260 LVLYGIAAPDSQRIAFYRLLDEFF 283

>CAD21956.1 neomycin phosphotransferase [Hepatitis GB virus B] Length=275

Score = 536 bits (1381), Expect = 0.0, Method: Compositional matrix adjust. Identities = 264/264 (100%), Positives = 264/264 (100%), Gaps = 0/264 (0%)

Query 1	MIEQDGLHAGSPAAWVERLFGYDWAQQTIGCSDAAVFRLSAQGRPVLFVKTDLSGALNEL 60 MIEQDGLHAGSPAAWVERLFGYDWAQQTIGCSDAAVFRLSAQGRPVLFVKTDLSGALNEL
Sbjct 12	MIEQDGLHAGSPAAWVERLFGYDWAQQTIGCSDAAVFRLSAQGRPVLFVKTDLSGALNEL 71

Query 61	QDEAARLSWLATTGVPCAAVLDVVTEAGRDWLLLGEVPGQDLLSSHLAPAEKVSIMADAM 120 QDEAARLSWLATTGVPCAAVLDVVTEAGRDWLLLGEVPGQDLLSSHLAPAEKVSIMADAM
Sbjct 72	QDEAARLSWLATTGVPCAAVLDVVTEAGRDWLLLGEVPGQDLLSSHLAPAEKVSIMADAM 131

Query 121 RRLHTLDPATCPFDHQAKHRIERARTRMEAGLVDQDDLDEEHQGLAPAELFARLKARMPD 180 RRLHTLDPATCPFDHQAKHRIERARTRMEAGLVDQDDLDEEHQGLAPAELFARLKARMPD
Sbjct 132 RRLHTLDPATCPFDHQAKHRIERARTRMEAGLVDQDDLDEEHQGLAPAELFARLKARMPD 191
```

| Query | 181 | GEDLVVTHGDACLPNIMVENGRFSGFIDCGRLGVADRYQDIALATRDIAEELGGEWADRF 240 |
| --- | --- | --- |
| Sbjct | 192 | GEDLVVTHGDACLPNIMVENGRFSGFIDCGRLGVADRYQDIALATRDIAEELGGEWADRF GEDLVVTHGDACLPNIMVENGRFSGFIDCGRLGVADRYQDIALATRDIAEELGGEWADRF 251 |
| Query | 241 | LVLYGIAAPDSQRIAFYRLLDEFF 264 |
| Sbjct | 252 | LVLYGIAAPDSQRIAFYRLLDEFF  LVLYGIAAPDSQRIAFYRLLDEFF 275 |

>ABO65261.1 GFPNeo fusion protein [Cloning vector pEFBOS-IRESGFPNeo] Length=507

Score = 536 bits (1380), Expect = 0.0, Method: Compositional matrix adjust.

| Identities | = 262/263 (99%), Positives = 262/263 (99%), Gaps = 0/263 (0%) |  |
| --- | --- | --- |
| Query 2 | IEQDGLHAGSPAAWVERLFGYDWAQQTIGCSDAAVFRLSAQGRPVLFVKTDLSGALNELQ | 61 |
| Sbjct 245 | IEQDGLHAGSPAAWVERLFGYDWAQQTIGCSDAAVFRLSAQGRPVLFVKTDLSGALNELQ IEQDGLHAGSPAAWVERLFGYDWAQQTIGCSDAAVFRLSAQGRPVLFVKTDLSGALNELQ | 304 |
| Query 62 | DEAARLSWLATTGVPCAAVLDVVTEAGRDWLLLGEVPGQDLLSSHLAPAEKVSIMADAMR | 121 |
| Sbjct 305 | DEAARLSWLATTGVPCAAVLDVVTEAGRDWLLLGEVPGQDLLSSHLAPAEKVSIMADAMR DEAARLSWLATTGVPCAAVLDVVTEAGRDWLLLGEVPGQDLLSSHLAPAEKVSIMADAMR | 364 |
| Query 122 | RLHTLDPATCPFDHQAKHRIERARTRMEAGLVDQDDLDEEHQGLAPAELFARLKARMPDG | 181 |
| Sbjct 365 | RLHTLDPATCPFDHQAKHRIERARTRMEAGLVDQDDLDEEHQGLAPAELFARLKARMPDG RLHTLDPATCPFDHQAKHRIERARTRMEAGLVDQDDLDEEHQGLAPAELFARLKARMPDG | 424 |
| Query 182 | EDLVVTHGDACLPNIMVENGRFSGFIDCGRLGVADRYQDIALATRDIAEELGGEWADRFL | 241 |
| Sbjct 425 | EDLVVTHGDACLPNIMVENGRFSGFIDCGRLGVADRYQDIALATRDIAEEL GEWADRFL EDLVVTHGDACLPNIMVENGRFSGFIDCGRLGVADRYQDIALATRDIAEELCGEWADRFL | 484 |
| Query 242  Sbjct 485 | VLYGIAAPDSQRIAFYRLLDEFF 264 VLYGIAAPDSQRIAFYRLLDEFF VLYGIAAPDSQRIAFYRLLDEFF 507 |  |

>AET75770.1 CodAnptII [Cloning vector pHUGE-Red] AET75779.1 CodAnptII [Cloning vector pHUGE-MtNFS] AET75791.1 CodAnptII [Cloning vector pHUGE-LjMtNFS]

Length=691

Score = 536 bits (1380), Expect = 0.0, Method: Compositional matrix adjust.

| Identities | = 261/262 (99%), Positives = 262/262 (100%), Gaps = 0/262 (0%) |  |
| --- | --- | --- |
| Query 3 | EQDGLHAGSPAAWVERLFGYDWAQQTIGCSDAAVFRLSAQGRPVLFVKTDLSGALNELQD | 62 |
| Sbjct 430 | EQDGLHAGSPAAWVERLFGYDWAQQTIGCSDAAVFRLSAQGRPVLFVKTDLSGALNELQD EQDGLHAGSPAAWVERLFGYDWAQQTIGCSDAAVFRLSAQGRPVLFVKTDLSGALNELQD | 489 |
| Query 63 | EAARLSWLATTGVPCAAVLDVVTEAGRDWLLLGEVPGQDLLSSHLAPAEKVSIMADAMRR | 122 |
| Sbjct 490 | EAARLSWLATTGVPCAAVLDVVTEAGRDWLLLGEVPGQDLLSSHLAPAEKVSIMADAMRR EAARLSWLATTGVPCAAVLDVVTEAGRDWLLLGEVPGQDLLSSHLAPAEKVSIMADAMRR | 549 |
| Query 123 | LHTLDPATCPFDHQAKHRIERARTRMEAGLVDQDDLDEEHQGLAPAELFARLKARMPDGE | 182 |
| Sbjct 550 | LHTLDPATCPFDHQAKHRIERARTRMEAGLVDQDDLDEEHQGLAPAELFARLKARMPDG+ LHTLDPATCPFDHQAKHRIERARTRMEAGLVDQDDLDEEHQGLAPAELFARLKARMPDGD | 609 |
| Query 183 | DLVVTHGDACLPNIMVENGRFSGFIDCGRLGVADRYQDIALATRDIAEELGGEWADRFLV | 242 |
| Sbjct 610 | DLVVTHGDACLPNIMVENGRFSGFIDCGRLGVADRYQDIALATRDIAEELGGEWADRFLV DLVVTHGDACLPNIMVENGRFSGFIDCGRLGVADRYQDIALATRDIAEELGGEWADRFLV | 669 |
| Query 243  Sbjct 670 | LYGIAAPDSQRIAFYRLLDEFF 264 LYGIAAPDSQRIAFYRLLDEFF LYGIAAPDSQRIAFYRLLDEFF 691 |  |

>ACH99098.1 Aph [Allelic replacement vector pJC84] Length=284

Score = 535 bits (1379), Expect = 0.0, Method: Compositional matrix adjust. Identities = 264/264 (100%), Positives = 264/264 (100%), Gaps = 0/264 (0%)

| Query | 1 | MIEQDGLHAGSPAAWVERLFGYDWAQQTIGCSDAAVFRLSAQGRPVLFVKTDLSGALNEL | 60 |
| --- | --- | --- | --- |
| Sbjct | 21 | MIEQDGLHAGSPAAWVERLFGYDWAQQTIGCSDAAVFRLSAQGRPVLFVKTDLSGALNEL MIEQDGLHAGSPAAWVERLFGYDWAQQTIGCSDAAVFRLSAQGRPVLFVKTDLSGALNEL | 80 |
| Query | 61 | QDEAARLSWLATTGVPCAAVLDVVTEAGRDWLLLGEVPGQDLLSSHLAPAEKVSIMADAM | 120 |
| Sbjct | 81 | QDEAARLSWLATTGVPCAAVLDVVTEAGRDWLLLGEVPGQDLLSSHLAPAEKVSIMADAM QDEAARLSWLATTGVPCAAVLDVVTEAGRDWLLLGEVPGQDLLSSHLAPAEKVSIMADAM | 140 |
| Query | 121 | RRLHTLDPATCPFDHQAKHRIERARTRMEAGLVDQDDLDEEHQGLAPAELFARLKARMPD | 180 |
| Sbjct | 141 | RRLHTLDPATCPFDHQAKHRIERARTRMEAGLVDQDDLDEEHQGLAPAELFARLKARMPD RRLHTLDPATCPFDHQAKHRIERARTRMEAGLVDQDDLDEEHQGLAPAELFARLKARMPD | 200 |
| Query | 181 | GEDLVVTHGDACLPNIMVENGRFSGFIDCGRLGVADRYQDIALATRDIAEELGGEWADRF | 240 |
| Sbjct | 201 | GEDLVVTHGDACLPNIMVENGRFSGFIDCGRLGVADRYQDIALATRDIAEELGGEWADRF GEDLVVTHGDACLPNIMVENGRFSGFIDCGRLGVADRYQDIALATRDIAEELGGEWADRF | 260 |
| Query  Sbjct | 241  261 | LVLYGIAAPDSQRIAFYRLLDEFF 264 LVLYGIAAPDSQRIAFYRLLDEFF LVLYGIAAPDSQRIAFYRLLDEFF 284 |  |

>AAA72988.1 luciferase/kanamycin resistance protein [synthetic construct] Length=821

Score = 535 bits (1379), Expect = 0.0, Method: Compositional matrix adjust. Identities = 259/259 (100%), Positives = 259/259 (100%), Gaps = 0/259 (0%)

Query 6	GLHAGSPAAWVERLFGYDWAQQTIGCSDAAVFRLSAQGRPVLFVKTDLSGALNELQDEAA 65 GLHAGSPAAWVERLFGYDWAQQTIGCSDAAVFRLSAQGRPVLFVKTDLSGALNELQDEAA

Sbjct 563 GLHAGSPAAWVERLFGYDWAQQTIGCSDAAVFRLSAQGRPVLFVKTDLSGALNELQDEAA 622

Query 66	RLSWLATTGVPCAAVLDVVTEAGRDWLLLGEVPGQDLLSSHLAPAEKVSIMADAMRRLHT 125 RLSWLATTGVPCAAVLDVVTEAGRDWLLLGEVPGQDLLSSHLAPAEKVSIMADAMRRLHT

Sbjct 623 RLSWLATTGVPCAAVLDVVTEAGRDWLLLGEVPGQDLLSSHLAPAEKVSIMADAMRRLHT 682

Query 126 LDPATCPFDHQAKHRIERARTRMEAGLVDQDDLDEEHQGLAPAELFARLKARMPDGEDLV 185 LDPATCPFDHQAKHRIERARTRMEAGLVDQDDLDEEHQGLAPAELFARLKARMPDGEDLV

Sbjct 683 LDPATCPFDHQAKHRIERARTRMEAGLVDQDDLDEEHQGLAPAELFARLKARMPDGEDLV 742

Query 186 VTHGDACLPNIMVENGRFSGFIDCGRLGVADRYQDIALATRDIAEELGGEWADRFLVLYG 245 VTHGDACLPNIMVENGRFSGFIDCGRLGVADRYQDIALATRDIAEELGGEWADRFLVLYG

Sbjct 743 VTHGDACLPNIMVENGRFSGFIDCGRLGVADRYQDIALATRDIAEELGGEWADRFLVLYG 802

Query 246 IAAPDSQRIAFYRLLDEFF 264

IAAPDSQRIAFYRLLDEFF Sbjct 803 IAAPDSQRIAFYRLLDEFF 821

>WP_000572405.1 MULTISPECIES: aminoglycoside O-phosphotransferase APH(3')-IIa [Bacteria]

P00552.1 RecName: Full=Aminoglycoside 3'-phosphotransferase; AltName: Full=APH(3')-II; Short=APH(3')II; AltName: Full=Kanamycin kinase,

type II; AltName: Full=Neomycin-kanamycin phosphotransferase type II [Klebsiella pneumoniae]

1ND4_A Crystal structure of aminoglycoside-3'-phosphotransferase-IIa [Klebsiella pneumoniae]

1ND4_B Crystal structure of aminoglycoside-3'-phosphotransferase-IIa [Klebsiella pneumoniae]

AAA03699.1 neomycin phosphotransferase [synthetic construct]

AAA56726.1 aminoglycoside 3'-phosphotransferase [unidentified cloning vector] AAA61621.1 aminoglycoside phosphotransferase [Cloning vector pKSM711] AAA61623.1 aminoglycoside phosphotransferase [Cloning vector pKSM713] AAA61626.1 aminoglycoside phosphotransferase [Cloning vector pKSM715] AAA72062.1 neomycin phosphotransferase [Retroviral vector pLN]

AAA72063.1 neomycin phosphotransferase [Retroviral vector pLNSX] AAA72064.1 neomycin phosphotransferase [Retroviral vector pLNCX] AAA72065.1 neomycin phosphotransferase [Retroviral vector pLXSN] AAA73424.1 kanamycin phosphotransferase [Cloning vector pMAKK76] AAA73460.1 neomycin-kanamycin phosphotransferase [Cloning vector pDO192] AAA73462.1 neomycin-kanamycin phosphotransferase [Cloning vector pDO193] AAA88781.1 neomycin phosphotransferase [Cloning vector pCI-neo] AAA96030.1 kanamycin acetyl transferase [Cloning vector pBRkanf1]

AAB00444.1 neomycin phosphotransferase [Plasmid pRL1063a] AAB00451.1 neomycin phosphotransferase [Plasmid pRL765]

AAB05990.1 kanamycin resistance determinant [Cloning vector pBBR1MCS-2] AAB18986.1 kanamycin acetyl transferase [Cloning vector pBRkanf4] AAB36665.1 aminoglycoside-3'-o-phosphotransferase [Expression vector pEXT22] AAB60865.1 aminoglycoside phosphotransferase [Shuttle cosmid vector pFD666] AAC16769.1 neomycin phosphotransferase [Retrotransposon vector pVLBAG] AAC16771.1 neomycin phosphotransferase [Retrotransposon vector pVLIBAG] AAC26829.1 aminoglycoside 3'-phosphotransferase [Cloning vector pWEB] AAC35766.1 neomycin phosphotransferase [Cloning vector pCD341]

AAC35777.1 neomycin phosphotransferase [Cloning vector pCD342] AAC43251.1 kanamycin phosphotransferase [Cloning vector Pk184] AAC48873.1 aminoglycoside 3'-phosphotransferase [Cloning vector pAL-Z] AAC53610.1 neomycin phosphotransferase [Cloning vector pBSL128] AAC53629.1 neomycin phosphotransferase [Cloning vector pBSL97] AAC53671.1 neomycin-kanamycin phosphotransferase [Cloning vector pSUM36] AAC53702.1 neomycin phosphotransferase [Cloning vector pBI101.2] AAC53704.1 neomycin phosphotransferase [Cloning vector pBI101.3] AAC53714.1 aminoglycoside 3'-phosphotransferase [Cloning vector pAL-F]

AAD28569.1 aminoglycoside-3'-O-phosphotransferase [xylene oxygenase expression vector pSPZ3]

AAD28571.1 aminoglycoside-3'-O-phosphotransferase [alk-system expression vector pSPZ4]

AAD49600.1 neomycin phosphotransferase [Cloning vector pUNI10] AAD49601.1 neomycin phosphotransferase [Cloning vector pUNI20] AAD49602.1 neomycin phosphotransferase [Cloning vector pUNI30] AAD50685.1 neomycin phosphotransferase [Cloning vector pUNI50] AAD50686.1 neomycin phosphotransferase [Cloning vector pUNI60] AAD50687.1 neomycin phosphotransferase [Cloning vector pUNI70] AAD50748.1 neomycin phosphotransferase [Cloning vector pUNI55] AAD50749.1 neomycin phosphotransferase [Cloning vector pUNI65] AAD50750.1 neomycin phosphotransferase [Cloning vector pUNI75] AAF13158.1 kanamicin-resistance protein [Integration vector pLOI2224] AAF13161.1 kanamicin-resistance protein [Integration vector pLOI2227]

AAF26422.1 neomycin phosphotransferase [Activation tagging vector pSKI074] AAF89184.1 neomycin phosphotransferase [Cloning Vector pACT]

AAG09774.1 aminoglycoside 3'-phosphotransferase [Vibrio cholerae] AAG29448.1 kanamycin phosphotransferase [Promoter probe vector pPROBE-NI] AAG29452.1 kanamycin phosphotransferase [Promoter probe vector pPROBE-NI'] AAG29456.1 kanamycin phosphotransferase [Promoter probe vector pPROBE-NT] AAG29460.1 kanamycin phosphotransferase [Promoter probe vector pPROBE-NT']

AAG29464.1 kanamycin phosphotransferase [Promoter probe vector pPROBE-gfp[tagless]] AAG29468.1 kanamycin phosphotransferase [Promoter probe vector pPROBE'-gfp[tagless]] AAG29472.1 kanamycin phosphotransferase [Promoter probe vector pPROBE-gfp[AAV]] AAG29476.1 kanamycin phosphotransferase [Promoter probe vector pPROBE'-gfp[AAV]] AAG29480.1 kanamycin phosphotransferase [Promoter probe vector pPROBE-gfp[ASV]] AAG29484.1 kanamycin phosphotransferase [Promoter probe vector pPROBE'-gfp[ASV]] AAG29488.1 kanamycin phosphotransferase [Promoter probe vector pPROBE-gfp[LVA]] AAG29492.1 kanamycin phosphotransferase [Promoter probe vector pPROBE'-gfp[LVA]] AAG48611.1 neomycin phosphotransferase [Retroviral vector NIT]

AAK94925.1 kanamycin phosphotransferase [Expression vector pKSUC1] AAK96248.1 kanamycin phosphotransferase [Expression vector pSTKST] AAL02035.1 Tn5 neomycin phosphotransferase [Template plasmid pKD4] AAL02037.1 Tn5 neomycin phosphotransferase [Template plasmid pKD13] AAL05849.1 neomycin phosphotransferase [Transposon vector phiMycoMarT7] AAL09170.1 aminoglycoside 3'-phosphotransferase [CRIM plasmid pAH125] AAL09172.1 aminoglycoside 3'-phosphotransferase [CRIM plasmid pLA2] AAL09843.1 kan [CRIM plasmid pAH55]

AAL09846.1 kan [CRIM plasmid pAH56] AAL09853.1 kan [CRIM plasmid pAH63] AAL09859.1 kan [CRIM plasmid pAH70] AAL09867.1 kan [CRIM plasmid pAH95] AAL09869.1 kan [CRIM plasmid pAH120] AAL09900.1 kan [CRIM plasmid pAH150]

AAL30402.1 neomycin phosphotransferase [Cloning vector pWS32] AAL38582.1 kanamycin resistance protein [Cloning vector pEC-K18mob2] AAL57861.1 neomycin phosphotransferase [Retroviral vector VLMB]

AAL61987.1 neomycin phosphotransferase AphII [Expression vector pANT1201]

AAL62335.1 neomycin/kanamycin-phosphotransferase-(type II) [Transformation vector pRLE6]

AAM28615.1 kanamycin resistance determinant [Cloning vector pBBRT] AAM82290.1 aminoglycoside 3'-phosphotransferase [Transposon delivery vector

pSC189]

AAM82567.1 kanamycin resistance [P-element cloning system vector pPUNIdelta] AAO12737.1 aminoglycoside 3'-phosphotransferase [CRIM plasmid pLA4] AAO12739.1 aminoglycoside 3'-phosphotransferase [CRIM plasmid pLA5] AAO12741.1 aminoglycoside 3'-phosphotransferase [CRIM plasmid pLA7] AAO12743.1 aminoglycoside 3'-phosphotransferase [CRIM plasmid pLA8] AAO12745.1 aminoglycoside 3'-phosphotransferase [CRIM plasmid pLA9] AAO12747.1 aminoglycoside 3'-phosphotransferase [CRIM plasmid pLZ31] AAO63174.1 aminoglycoside-3'-O-phosphotransferase [Cloning Vector pHRGFPGUS] AAO65197.1 Aph(3')-IIa [Shuttle expression vector pEC-XK99E]

AAO65205.1 Aph(3')-IIa [Expression vector pXK99E] AAO65208.1 Aph(3')-IIa [Expression vector pXK99CAT] AAO65216.1 Aph(3')-IIa [Shuttle vector pBHK18] AAO65222.1 Aph(3')-IIa [Shuttle vector pBHK18mob2] AAO65226.1 Aph(3')-IIa [Cloning vector pK19MECA2] AAO65230.1 Aph(3')-IIa [Shuttle vector pEC-K19MECA2] AAO65231.1 Aph(3')-IIa [Shuttle vector pBHK19MECA] AAO65233.1 Aph(3')-IIa [Shuttle vector pBHK19MECA2] AAO65976.1 Aph(3')-IIa [Cloning vector pK18PolyF2] AAO66594.1 Aph(3')-IIa [Cloning vector pK18mob2] AAO66595.1 Aph(3')-IIa [Cloning vector pK19mob2] AAO66606.1 Aph(3')-IIa [Shuttle vector pEC-K19mob2]

AAO92240.1 aminoglycoside-3'-O-phosphotransferase [Cloning vector pHR3-km] AAO92599.1 neomycin phosphotransferase [Shuttle vector pAM2770] AAP04589.1 aminoglycoside-3'-O-phosphotransferase [Cloning vector pHR50] AAP20227.1 neomycin phosphotransferase [Cloning vector pUNI51]

AAP21549.1 neomycin phosphotransferase II [Cloning vector pPLEX-505] AAP21550.1 neomycin phosphotransferase II [Cloning vector pPLEX-506] AAP21551.1 neomycin phosphotransferase II [Cloning vector pPLEX-3001] AAP21552.1 neomycin phosphotransferase II [Cloning vector pPLEX-3002] AAP21553.1 neomycin phosphotransferase II [Cloning vector pPLEX-3003] AAP21554.1 neomycin phosphotransferase II [Cloning vector pPLEX-3004] AAP21555.1 neomycin phosphotransferase II [Cloning vector pPLEX-501] AAP21556.1 neomycin phosphotransferase II [Cloning vector pPLEX-502] AAP21557.1 neomycin phosphotransferase II [Cloning vector pPLEX-4001] AAP21558.1 neomycin phosphotransferase II [Cloning vector pPLEX-4002] AAP21559.1 neomycin phosphotransferase II [Cloning vector pPLEX-4003] AAP21560.1 neomycin phosphotransferase II [Cloning vector pPLEX-4004] AAP31912.1 aminoglycoside-3'-O-phosphotransferase [Cloning vector pHRE1-km] AAP46043.1 neomycin phosphotransferase [Retrotransposon vector ELM 5] AAP46045.1 neomycin phosphotransferase [Retrotransposon vector MEL/ELM]

AAQ24373.1 kanamycin resistance factor [Escherichia coli/Mycobacteria shuttle vector pGB9.2]

AAR91730.1 neomycin phosphotransferase [Cloning vector psiSTRIKE Neomycin] AAS48584.1 neomycin phosphotransferase [Mammalian expression vector pTargeT] AAS78935.1 NptII [Binary vector pORE_E2]

AAS78943.1 NptII [Binary vector pORE_E4] AAS78947.1 NptII [Binary vector pORE_EX] AAS78953.1 NptII [Binary vector pORE_O2] AAS78959.1 NptII [Binary vector pORE_O4] AAS78966.1 NptII [Binary vector pORE_R1] AAS78970.1 NptII [Binary vector pORE_R2] AAS78974.1 NptII [Binary vector pORE_R3] AAS78978.1 NptII [Binary vector pORE_R4]

AAT02529.1 neomycin phosphotransferase [Cloning vector pUC-gfp-neo] AAT44338.1 kanamycin resistance protein [Shuttle vector pFNLTP1]

AAT44344.1 kanamycin resistance protein [Temperature-sensitive shuttle vector pFNLTP9]

AAT48416.1 aminoglycoside phosphotransferase [Cloning vector pSRalphaneo] AAT48429.1 neomycin phosphotransferase [Cloning vector pSRalphaneoR] AAT74894.1 neomycin phosphotransferase [Anabaena transfer vector pRL2665b] AAU06202.1 neomycin phosphotransferase [Shuttle cosmid vector pHZ1358] AAU44363.1 aminoglycoside phosphotransferase [Cloning vector pSW29] AAU44364.1 aminoglycoside phosphotransferase [Cloning vector pSW29T] AAU44621.1 kanamycin resistance protein [Cloning vector pJK95]

AAU94556.1 neomycin phosphotransferase [pGeneClip Neomycin Vector] AAW58937.1 kanamycin resistance [Cloning vector MuNXKan] AAX29984.1 KanR [Expression vector pFNK-101]

AAX29987.1 kanamycin resistance protein [Expression vector pINV-110] AAX31729.1 neomycin phosphotransferase [Cloning shuttle vector pSCR119] AAX31731.1 neomycin phosphotransferase [Shuttle reporter vector pSUN119] AAX63489.1 kanamycin resistance protein [Low threshold vector pLTSUB-302]

AAY99782.1 KanR [Insertion vector pWSMK-T] AAZ76380.1 AphA [Cloning vector pSCR001]

ABA01556.1 neomycin phosphotransferase [Transfection vector pC-PTP-NEO]

ABA41654.1 neomycin phosphotransferase [Firefly luciferase reporter vector pGL4.17[luc2/Neo]]

ABA41657.1 neomycin phosphotransferase [Firefly luciferase reporter vector pGL4.18[luc2P/Neo]]

ABA41660.1 neomycin phosphotransferase [Firefly luciferase reporter vector pGL4.19[luc2CP/Neo]]

ABA41672.1 neomycin phosphotransferase [Renilla luciferase reporter vector pGL4.79[hRluc/Neo]]

ABA41675.1 neomycin phosphotransferase [Renilla luciferase reporter vector pGL4.80[hRlucP/Neo]]

ABA41678.1 neomycin phosphotransferase [Renilla luciferase reporter vector pGL4.81[hRlucCP/Neo]]

ABB17064.1 neomycin phosphotransferase [Moss transformation vector pMBL5] ABB17066.1 neomycin phosphotransferase [Moss transformation vector pMBL6]

ABB59014.1 neomycin phosphotransferase [Himar1-delivery and mutagenesis vector pFNLTP16 H3]

ABB59950.1 NPTII [Gene trapping Ds/T-DNA vector pUR224NA] ABB59956.1 NPTII [Gene trapping Ds/T-DNA vector pUR224NB] ABD73289.1 NptII [Transposon mutagenesis vector pG18-STM]

ABF48725.1 kanamycin resistance protein [HIS3/URA3 reporter vector pH3U3] ABG01695.1 neomycin phosphotransferase [Flexi vector pFN10A (ACT)] ABG47802.1 neomycin phosphotransferase [Flexi vector pF5A CMV-neo] ABG47805.1 neomycin phosphotransferase [Flexi vector pF5K CMV-neo] ABG72756.1 Neo [Expression vector mce2]

ABG72759.1 Neo [Expression vector mce3] ABG72762.1 Neo [Expression vector mce4]

ABI64163.1 Neo [Keratinocyte expression vector phPK14H] ABI74451.1 Neo [Expression vector pcDNA3-hFIX]

ABK01318.1 neomyocin phosphotransferase [Cloning vector pCAMBIA5105]

ABK40096.1 neomycin phosphotransferase [Inducible protein expression vector pReg Neo]

ABK79307.1 kanamycin resistance [Cloning/transgenesis vector pC5-Kan]

ABK79308.1 kanamycin resistance [Cloning/transgenesis vector pC5-Kan_delta_SphI] ABM65100.1 neomycin phosphotransferase [Expression vector pUNIV]

ABM69035.1 kanamycin resistance protein [Cloning vector pBEO210] ABP57399.1 Kan [Gateway binary vector pKPFWG2]

ABP57404.1 Kan [Gateway binary vector pKPWGF2] ABS87321.1 NptII [Cloning vector pCPP5702] ABS87328.1 NptII [Cloning vector pCPP5250]

ABU42127.1 neomycin phosphotransferase [Cloning vector pDC8]

ABW35347.1 kanamycin resistance protein [Kanamycin resistance FRT vector pFKM2]

ABW35350.1 kanamycin resistance protein [Kanamycin resistance loxP vector pLKM1]

ABW35376.1 kanamycin resistance protein [Transposition vector pUC18T-mini-Tn7T-Km::loxP] ABW88015.1 neomycin phosphotransferase [Cloning vector pEASY-T1]

ABW94985.1 kanamycin resistance protein [Transposition vector pUC18T-mini-Tn7T-Km::FRT] ABX47151.1 neomycin phosphotransferase [Cloning vector pVMG-TnpR]

ABX47155.1 neomycin phosphotransferase [Cloning vector pVMGCRT85] ABY87906.1 aminoglycoside phosphotransferase [Cloning vector pSW4427T]

ACA52078.1 aminoglycoside 3'-phosphotransferase [Suicide vector pEX18Km-pheS] ACB45195.1 aminoglycoside 3'-phosphotransferase [Cloning vector pMQ118] ACB45218.1 aminoglycoside 3'-phosphotransferase [Cloning vector pMQ150] ACD47105.1 NeoTet [neomycin resistance cassette pNeo4]

ACE77053.1 neomycin phosphotransferase [Cloning vector pSMARTGC Blue] ACF04937.1 aminoglycoside phosphotransferase [Template plasmid pCLF4] ACF93713.1 Km R [Burkholderia suicide vector pMo130]

ACF93717.1 Km R [Burkholderia replicative vector pMo168]

ACH53167.1 Neomycin phosphotransferase [Firefly luciferase reporter vector pGL4.51[luc2/CMV/Neo]]

ACI42372.1 AphA [Suicide vector pMO90]

ACI42374.1 AphA [Insertional disruption vector pMO126] ACI42378.1 AphA [Replicative vector pMO79]

ACJ12806.1 NptII [Suicide vector pMQ200]

ACJ66844.1 neomycin phosphotransferase [Cloning vector pK18mobsacB]

ACK37847.1 neomycin phosphotransferase II [Himar1-delivery and mutagenesis vector pHBurk3]

ACK37850.1 neomycin phosphotransferase II [Himar1-delivery and mutagenesis vector pHBurk5]

ACL01113.1 Aac(6')-Ib* [Reporter vector p6851]

ACL35311.1 neomycin phosphotransferase [Binary vector pWY107] ACL97678.1 kanamycin resistance protein [Expression vector pHF5Ca] ACM16583.1 kanamycin resistance protein [Expression vector pHF5Na] ACO48265.1 kanamycin resistance protein [Cloning vector pNIGEL19] ACO48274.1 kanamycin resistance protein [Cloning vector pNIGEL18] ACO48890.1 neomycin phosphotransferase [Gene replacement vector pEXKm4] ACO50707.1 neomycin phosphotransferase [Cloning vector pDMK3] ACS28254.1 aminoglicoside phosphotransferase [Expression vector pPM2a] ACS35606.1 KanR marker [Allelic replacement vector pMQ236]

ACS73371.1 NptII [Gene replacement vector pEXKm5]

ACV96475.1 aminoglycoside 3'-phosphotransferase [Vibrio cholerae Mex1] ACX33126.1 neomycin phospotransferase [EIAV-based lentiviral vector] ACX42316.1 kanamycin resistance protein [Recombineering donor plasmid pDOC-H] ACX42319.1 kanamycin resistance protein [Recombineering donor plasmid pDOC-F] ACX42322.1 kanamycin resistance protein [Recombineering donor plasmid pDOC-P] ACX42326.1 kanamycin resistance protein [Recombineering donor plasmid pDOC-G] ACY78420.1 kanamycin resistance protein [T-vector pRNT4]

ADD84777.1 kanamycin resistance protein [Cloning vector pJW103] ADF80259.1 aminoglycoside phosphotransferase [Cloning vector pPM7g] ADG64877.1 AphA [Mycobacterial tetracycline-inducible vector pMEND-Lx]

ADJ00053.1 kanamycin phosphotransferase [Promoter probe vector pEvoGlowRed]

ADJ00055.1 kanamycin phosphotransferase [Mariner mini-transposon delivery vector pMaGlowRedKm]

ADJ00061.1 kanamycin phosphotransferase [Mariner mini-transposon delivery vector pMaTcGlow]

ADJ00066.1 kanamycin phosphotransferase [Mariner mini-transposon delivery vector pMaGlow]

ADJ00072.1 kanamycin phosphotransferase [Mariner mini-transposon delivery vector pMaEvo]

ADJ00078.1 kanamycin phosphotransferase [Reporter vector pGlowRed] ADJ58594.1 aminoglycoside-3'-O-phosphotransferase [Cloning vector pTAP6] ADO63844.1 kanamycin resistance [Cloning vector pKaKa1]

ADQ64039.1 neomycin phosphotransferase [mini Tn5 transposon vector pFAJ1819] ADR00352.1 aminoglycoside 3'-O-phosphotransferase [Shuttle vector pYS1R1] ADR65091.1 neomycin phosphotransferase [Giardia integration vector pcAUK-3HA-NEO]

ADV36644.1 aminoglycoside-3'-O-phosphotransferase II [RNA interference vector pIBB103]

ADZ46391.1 aminoglycoside 3'-phosphotransferase [Lux single copy cloning vector pMH30]

AEF32776.1 G418 drug marker protein [Artificial vector pJL517] AEF32779.1 G418 drug marker protein [Artificial vector pJL541] AEG76963.1 neomycin phosphotransferase II [Cloning vector pAmin82] AEG76964.1 neomycin phosphotransferase II [Cloning vector pAmin81] AEG76965.1 neomycin phosphotransferase II [Cloning vector pAmin78] AEG76966.1 neomycin phosphotransferase II [Cloning vector pAmin79] AEG76967.1 neomycin phosphotransferase II [Cloning vector pAmin80]

AEH59866.1 aminoglycoside 3-phosphotransferase [Gene deletion vector pEMG]

AEI59102.1 aminoglycoside 3'-phosphotransferase [Reporter gene-fusion vector pFU69]

AEJ38143.1 Kan(R) [Neurospora cloning vector pTH1067.9] AEJ38147.1 Kan(R) [Neurospora cloning vector pTH1108.2] AEJ38152.1 Kan(R) [Neurospora cloning vector pTH1111.1] AEJ38155.1 Kan(R) [Neurospora cloning vector pTH1112.8] AEJ38159.1 Kan(R) [Neurospora cloning vector pTH1117.12] AEJ38164.1 Kan(R) [Neurospora cloning vector pTH1123.1] AEJ38167.1 Kan(R) [Neurospora cloning vector pTH1124.1] AEJ82267.1 KAN-R [Cloning vector pCASP1::NLS:GFP:GUS] AEJ82270.1 KAN-R [Cloning vector pCASP2::NLS:GFP:GUS]

AEJ82295.1 KAN-R [Cloning vector pCASP1::CASP1:mTurquoise]

AEK70322.1 neomycin phosphotransferase [Flexi vector pFC27A HaloTag CMV-neo] AEK70323.1 neomycin phosphotransferase [Flexi vector pFC27K HaloTag CMV-neo] AEK70327.1 neomycin phosphotransferase [Flexi vector pFN28A HaloTag(R) CMV-neo] AEK70329.1 neomycin phosphotransferase [Flexi vector pFN28K HaloTag(R) CMV-neo] AEL31643.1 neomycin resistance protein [Cloning vector TNN]

AEO86687.1 neomycin phosphotransferase [Expression vector pLNHX_cHS4_650] AEQ28650.1 neomycin phosphotransferase [HaloTag CMV-neo vector pHTN] AEQ28653.1 neomycin phosphotransferase [HaloTag CMV-neo vector pHTC] AEW50181.1 kanamycin resistance protein [Expression vector pBBRKW2HTsLsM] AFC41193.1 nptII [Binary expression vector D3cpv-C]

AFC41196.1 nptII [Binary expression vector NES-YC3.6] AFC41199.1 nptII [Binary expression vector NLS-YC3.6]

AFC41202.1 nptII [Binary expression vector PM-YC3.6-LTI6b] AFC41205.1 nptII [Binary expression vector TP-D3cpv] AFC41208.1 nptII [Binary expression vector YC3.6-C] AFC41211.1 nptII [Binary expression vector YC3.6-N]

AFC75902.1 aminoglycoside-3'-O-phosphotransferase [Cloning vector pKfloxANTSAsc] AFC75904.1 aminoglycoside-3'-O-phosphotransferase [Cloning vector pKfloxANTSAsc-tsOri-pBR] AFC75906.1 aminoglycoside-3'-O-phosphotransferase [Cloning vector pRepAts-dnaA]

AFC76263.1 Aminoglycoside 3'-phosphotransferase [Cloning vector pNG1A] AFH41828.1 NPTII [Cloning vector pAK405]

AFK93530.1 aminoglycoside-3'-O-phosphotransferase [Suicide vector pJP5603]

AFM78095.1 kanamycin resistance protein [Tn7 transposase expression vector pSTNSK]

AFM78102.1 kanamycin resistance protein [Tn7 transposase expression vector pSTNSK-Cm]

AFM78110.1 neomycin phosphotransferase [Tn7 transposase expression vector pSTNSK-Tp]

AFN68289.1 KanR [Bi-directional promoter binary vector p2CABA] AFN88262.1 Neo resistance (Kan) [Cloning vector DH6-64] AFN88265.1 Neo resistance (Kan) [Cloning vector DH4-27] AFN88268.1 Neo resistance (Kan) [Cloning vector DH6-78] AFN88288.1 Neo Resistance (Kan) [Cloning vector DH7-18] AFN88292.1 Neo Resistance (Kan) [Cloning vector DH7-19] AFN88296.1 Neo Resistance (Kan) [Cloning vector DH7-20] AFN88300.1 Neo Resistance (Kan) [Cloning vector DH7-21] AFN88304.1 Neo Resistance (Kan) [Cloning vector DH7-22] AFN88308.1 Neo Resistance (Kan) [Cloning vector DH7-23] AFN88312.1 Neo resistance (Kan) [Cloning vector DH7-24] AFP65782.1 kanamycin resistance protein [Cloning vector pAJ63a] AFP67885.1 neomycin resistance protein [Cloning vector pamGFP+] AFP67888.1 neomycin resistance protein [Cloning vector pamGFP5'] AFP67892.1 neomycin resistance protein [Cloning vector phis-5GFP]

AFP67895.1 neomycin resistance protein [Cloning vector phis-5GFP5'] AFQ55251.1 Km resistance protein [Expression vector pJRD215] AFQ55264.1 Km resistance protein [URT reporter vector pKP55-M] AFQ55275.1 Km resistance protein [URT cloning vector pKP77]

AFQ97505.1 aminoglycoside phosphotransferase [Shuttle expression vector pTGR5]

AFR24711.1 Neomycin phosphotransferase [Cloning vector pGRG36pir-116-FKm] AFR53955.1 aminoglycoside phosphotransferase [Cloning vector pT2/Gene-Trap]

AFS32160.1 neomycin-kanamycin phosphotransferase type II [Cloning vector pJAK14-Blue]

AFS33190.1 neomycin phosphotransferase II [Episomal vector pSpiro-NptII-3xMYC-C] AFS33209.1 neomycin phosphotransferase II [Episomal vector pSpiro-NptII-ESP-C-SF-TAP]

AFU76702.1 neomycin phosphotransferase [Hepatitis C virus replicon 4a ED43-Neo (R+I)]

AFV53145.1 NptII [Plasmid vector pFKm4]

AFV73972.1 neomycin phosphotransferase [Cloning vector SV40p TV] AFW89950.1 aminoglycoside 3'-phosphotransferase [TREX vector pIC20H-RL] AFZ75333.1 kanamycin resistance protein [Cloning vector pKOS6b] AGG53915.1 KanR [Cloning vector pDAL649]

AGG56500.1 NptII [Binary vector pYBA-100] AGG56504.1 NptII [Binary vector pYBA-200] AGG56507.1 NptII [Binary vector pYBA-300] AGH89393.1 KanR [Integration vector pMD-TV]

AGI04940.1 NptII [Promoter capture vector pTn7oluxK3] AGI04946.1 NptII [Promoter capture vector pTn7tluxK3] AGI04952.1 NptII [Promoter capture vector pTn7xluxK3] AGI04958.1 NptII [Promoter capture vector pTn7oluxK4] AGI04972.1 NptII [Promoter capture vector pTn5.7luxK3] AGI04979.1 NptII [Promoter capture vector pTn5.7luxK6] AGI38107.1 NptII [Cloning vector pPOP6IPT]

AGI38112.1 NptII [Cloning vector pPOP6IRPDS]

AGL73306.1 NptII [Cloning vector pR6KT-miniTn7T-P1eGFP-FK] AGM33194.1 neomycin phosphotransferase [Cloning vector pFTCAPL-GW] AGO61963.1 neomycin phosphotransferase [Cloning vector pFTarget2-Tn7] AGO61970.1 neomycin phosphotransferase [Cloning vector pFTarget3-Tn7]

AGO96860.1 Tn5 neomycin phosphotransferase [Cloning vector pKIKOarsBKm] AGO96862.1 Tn5 neomycin phosphotransferase [Cloning vector pKIKOlacZKm] AGO96864.1 Tn5 neomycin phosphotransferase [Cloning vector pKIKOrbsARKm] AGS44633.1 NPTII [Ti plasmid binary vector pGH00.0126]

AGS44637.1 NPTII [Ti binary plasmid pGH00.0131]

AGV01072.1 kanamycin resistance protein [Cloning vector pSTn5-KM]

AGW21649.1 neomycin phosphotransferase [Cloning vector pTH1937] AHA61713.1 NptII [Cloning vector pPS3081]

AHA61716.1 NptII [Cloning vector pTn5.7luxK4] AHA61723.1 NptII [Cloning vector pTN5.7luxK5] AHA61736.1 NptII [Cloning vector pTn7APH1AluxK5] AHA61774.1 NptII [Cloning vector pLG107]

AHE38547.1 neomycin phosphotransferase II [Cloning vector pGGF007] AHH35069.1 neomycin phosphotransferase II [Cloning vector pTn7xK]

AHH41348.1 neomycin phosphotransferase [NanoLuc Luciferase Flexi Vector pFN31K]

AHH41354.1 neomycin phosphotransferase [NLuc CMV-Neo Flexi Vector pFC32K] AHH41366.1 neomycin phosphotransferase [HIF1A CMV-Neo Vector pNLF1] AHH41368.1 neomycin phosphotransferase [NRF2 CMV-Neo Vector pNLF1] AHI88627.1 NPTII [Ti plasmid binary vector pGZ12.0501]

AHJ09732.1 NptII [Binary vector pYBA-1131] AHJ09736.1 NptII [Binary vector pYBA-1132] AHJ09740.1 NptII [Binary vector pYBA-1133] AHJ09744.1 NptII [Binary vector pYBA-1134] AHJ09748.1 NptII [Binary vector pYBA-1135] AHJ09752.1 NptII [Binary vector pYBA-1136] AHJ09756.1 NptII [Binary vector pYBA-1137] AHJ09760.1 NptII [Binary vector pYBA-1138] AHJ09764.1 NptII [Binary vector pYBA-1151] AHJ09768.1 NptII [Binary vector pYBA-1152] AHJ09772.1 NptII [Binary vector pYBA-1153] AHJ09776.1 NptII [Binary vector pYBA-1154] AHJ09780.1 NptII [Binary vector pYBA-1155] AHJ09784.1 NptII [Binary vector pYBA-1156] AHJ09788.1 NptII [Binary vector pYBA-1157] AHJ09792.1 NptII [Binary vector pYBA-1158] AHJ09796.1 NptII [Binary vector pYBA-1121] AHJ09799.1 NptII [Binary vector pYBA-1122] AHJ09802.1 NptII [Binary vector pYBA-1123] AHJ09805.1 NptII [Binary vector pYBA-1124] AHJ09808.1 NptII [Binary vector pYBA-1125] AHJ09811.1 NptII [Binary vector pYBA-1126] AHJ09814.1 NptII [Binary vector pYBA-1127] AHJ09817.1 NptII [Binary vector pYBA-1128] AHL24864.1 NPTII [Binary Vector pGZ12.0108]

AHX24180.1 neomycin phosphotransferase [Cloning vector pAMNPT-MCS] AHZ10953.1 NptII [Integration vector pZR606]

AIC38373.1 aminoglycoside phosphotransferase [Cloning vector pGCD19] AID37296.1 aminoglycoside-3'-O-phosphotransferase [Enterococcus faecalis] AIE16111.1 neomycin phosphotransferase II [Hepatitis C virus replicon 5a

SA1/SG-neo]

AIE57568.1 kanamycin resistance protein [Cloning vector pC5-Kan P2A] AIQ78249.1 nptII [Cloning vector pPLV02_v2]

AIQ78253.1 nptII [Cloning vector pPLV04_v2] AIQ78259.1 nptII [Cloning vector pPLV06_v2] AIQ78265.1 nptII [Cloning vector pPLV08_v2] AIQ78271.1 nptII [Cloning vector pPLV17_v2] AIQ78280.1 nptII [Cloning vector pPLV20_v2]

AIR09514.1 NeoR [U1Ap-mCherry expression vector pSA906]

AIS76353.1 aminoglycoside-3'-O-phosphotransferase [Transposon donor vector pRZ101]

AIU94516.1 KanR [Cloning vector pECT3] AIU94518.1 KanR [Cloning vector pECM1] AIU94527.1 KanR [Cloning vector pECJ3]

AIZ09113.1 kanamycin phosphotransferase [Cloning vector pFF746] AJQ81212.1 NptII [Binary vector pYBA-1129]

AJQ81215.1 NptII [Binary vector pYBA-1139] AJQ81218.1 NptII [Binary vector pYBA-1159] AJS13697.1 Kan [Plasmid vector pMD.SV] AJS13699.1 Kan [Plasmid vector pMD.SD] AJS13704.1 Kan [Plasmid vector pMD.SD.Gad] AJZ74096.1 NptII [Binary vector pYBA-1351] AJZ74099.1 NptII [Binary vector pYBA-1352] AJZ74102.1 NptII [Binary vector pYBA-1353] AJZ74105.1 NptII [Binary vector pYBA-1354] AJZ74107.1 NptII [Binary vector pYBA-1355] AJZ74110.1 NptII [Binary vector pYBA-1356] AJZ74113.1 NptII [Binary vector pYBA-1357]

AJZ74116.1 NptII [Binary vector pYBA-1358] AJZ74119.1 NptII [Binary vector pYBA-1359]

AKA27930.1 neomycin phosphotransferase [PtGAUT12 RNAi binary vector pAGW641]

AKA27931.1 neomycin phosphotransferase [Binary RNAi Gateway destination vector pAGSM552]

AKA28276.1 aminoglycoside-3'-O-phosphotransferase [Transposon donor vector pRZ49]

AKA28278.1 aminoglycoside-3'-O-phosphotransferase [Transposon donor vector pRZ98]

AKA28280.1 aminoglycoside-3'-O-phosphotransferase [Transposon donor vector pRZ99]

AKA28282.1 aminoglycoside-3'-O-phosphotransferase [Transposon donor vector pRZ102]

AKA28284.1 aminoglycoside-3'-O-phosphotransferase [Transposon donor vector pRZ103]

AKA28286.1 aminoglycoside-3'-O-phosphotransferase [Transposon donor vector pRZ106]

AKA95285.1 NptII [Binary Vector pYBA-1321] AKA95289.1 NptII [Binary Vector pYBA-1322] AKA95293.1 NptII [Binary Vector pYBA-1323] AKA95297.1 NptII [Binary Vector pYBA-1324] AKA95301.1 NptII [Binary Vector pYBA-1325] AKA95305.1 NptII [Binary vector pYBA-1326] AKA95309.1 NptII [Binary vector pYBA-1327] AKA95313.1 NptII [Binary vector pYBA-1328] AKA95317.1 NptII [Binary vector pYBA-1329] AKA95321.1 NptII [Binary vector pYBA-1331] AKA95325.1 NptII [Binary vector pYBA-1332] AKA95329.1 NptII [Binary vector pYBA-1333] AKA95333.1 NptII [Binary vector pYBA-1334] AKA95337.1 NptII [Binary vector pYBA-1335] AKA95341.1 NptII [Binary vector pYBA-1336] AKA95345.1 NptII [Binary vector pYBA-1337] AKA95349.1 NptII [Binary vector pYBA-1338] AKA95353.1 NptII [Binary vector pYBA-1339] AKG55611.1 KanR [Integration vector pMD.TV.s.dy]

AKN19589.1 KmR [Cloning vector p17YFP(lox-Sm-E-Kn)-Tc] AKN19594.1 KmR [Cloning vector p17GFP(lox-Sm-E-Kn)-Tc] AKR52856.1 NPTII [Ti plasmid binary vector pGZ12.0224] AKR52861.1 NPTII [Ti plasmid binary vector pGZ12.0106] ALA09307.1 neomycin phosphotransferase [Zea mays] ALE71929.1 Aph [Cloning vector pFL8]

ALI16836.1 aminoglycoside phosphotransferase [Cloning vector pMG401]

ALJ52448.1 kanamycin neomycin resistance marker [translation factor module pTFM1]

ALO78651.1 aminoglycoside phosphotransferase [Mammalian expression vector pcDNA3-N-HA-LIC]

ALP32100.1 kanamycin resistance protein [Cloning vector pPasscode1] ALP32104.1 kanamycin resistance protein [Cloning vector pPasscode2] ALP32108.1 kanamycin resistance protein [Cloning vector pPasscode3] ALP32216.1 KanR [Cloning vector pBAC-LC]

ALP32218.1 KanR [Cloning vector pBAC-TC]

ALR84549.1 neomycin phosphotransferase [Cloning vector pNB20] ALR84550.1 neomycin phosphotransferase [Cloning vector pNB51] ALR84552.1 neomycin phosphotransferase [Cloning vector pNB90] ALR84554.1 neomycin phosphotransferase [Cloning vector pNB92] ALR84556.1 neomycin phosphotransferase [Cloning vector pNB95] ALR84559.1 neomycin phosphotransferase [Cloning vector pNB97] ALR84563.1 neomycin phosphotransferase [Cloning vector pNB102] ALS09705.1 kanamycin resistance protein [Cloning vector pYP247] ANA09464.1 TviD [Expression vector pMD.TV.SF2a]

ANA09483.1 KanR [Expression vector pMD.TV.SF3A]

ANA75345.1 neomycin phosphotransferase II [Cloning vector pXF20pemIK-GW] ANH10903.1 APH [Cloning vector pSLI-PfEHD2xFKBP]

ANH21023.1 NptII [Binary vector pYBA-1101] ANH21026.1 NptII [Binary vector pYBA-1102] ANH21029.1 NptII [Binary vector pYBA-1103] ANH21032.1 NptII [Binary vector pYBA-1104] ANH21035.1 NptII [Binary vector pYBA-1105] ANH21039.1 NptII [Binary vector pYBA-1201] ANH21042.1 NptII [Binary vector pYBA-1202] ANH21045.1 NptII [Binary vector pYBA-1203]

ANH21048.1 NptII [Binary vector pYBA-1204] ANH21051.1 NptII [Binary vector pYBA-1205] ANH21054.1 NptII [Binary vector pYBA-1301] ANH21057.1 NptII [Binary vector pYBA-1302] ANH21060.1 NptII [Binary vector pYBA-1303] ANH21063.1 NptII [Binary vector pYBA-1304] ANH21066.1 NptII [Binary vector pYBA-1305] ANZ72460.1 NeoR [Cloning vector pADNpcDNA3.1 KanR]

AOE47734.1 kanamicyn resistance protein [Cloning vector pGCD21] AOG18102.1 NeoR [Cloning vector pAAV2neo-1.3HBV(adr)] AOG18120.1 NeoR [Cloning vector pAAV2neo-1.3HBV(ayw)]

APD15312.1 aminoglycoside 3'-phosphotransferase [Cloning vector p34E-Km] APZ73801.1 kanamycin phosphotransferase [Cloning vector pKMAG] APZ73803.1 kanamycin phosphotransferase [Cloning vector pKMAG-C] AQN67808.1 NPTII [Binary vector pFH17]

AQX17846.1 KanR [Cloning vector pRI857-BAC] AQX17852.1 KanR [Cloning vector pRI857] AQZ26643.1 Aph [Cloning vector pDN211] ARE67152.1 KanR [Cloning vector pJS31]

ASG92105.1 NptII [Cloning vector pLX-B2-NptII-DsRED] ASG92121.1 NptII [Cloning vector pLX-B2-XT1-XT2-hCas9] ASJ77178.1 neomycin phosphotransferase [Binary vector pAGW633] ASK86114.1 kan [Expression vector pAC-crRNA-Km]

ASS85310.1 Kan/neoR [Cloning vector pPKm-118] ASV51141.1 NPTII [Ti plasmid binary vector pGZ13.0313]

ATN29907.1 NeoR/KanR [Cloning vector pCRII-attB-aTubCas9bTub-U63g4-dsRed-attB] ATN29910.1 NeoR/KanR [Cloning vector pCRII-attB-Rcd1rp-aTubCas9bTub-U63g4-DsRed-attB] ATN29913.1 NeoR/KanR [Cloning vector pCRII-tra4R-3xP3CFP-tra4L]

ATO98410.1 kanamycin resistance protein [Cloning vector pAH-mini-Mu(LER)-YK] ATO98419.1 kanamycin resistance protein [Cloning vector pAH-mini-Mu(LER)-YS] ATY38623.1 neomycin resistance protein [Cloning vector pHR-Ech_0230-tuf-aadA] ATY38626.1 neomycin resistance protein [Cloning vector pHR-Ech_0379-tuf-aadA] ATY38631.1 neomycin resistance protein [Cloning vector pHR-res-Ech_0379-Amtr-mCh-Gent] AUG68174.1 neomycin phosphotransferase [Expression vector pAGW570]

AUO29333.1 KanR [Binary vector pK7WG_ASX] AUO38280.1 Kan/neoR [Cloning vector pPKm-230] AUO38283.1 Kan/neoR [Cloning vector pPKm-102] AUO38286.1 Kan/neoR [Cloning vector pPKm-195] AVA19490.1 Kan/neoR [Cloning vector pPKm-292] AVA19492.1 Kan/neoR [Cloning vector pPKm-293]

AVE26327.1 aminoglycoside phosphotransferase from Tn5 [Binary vector pCNRNF3] AVE26333.1 aminoglycoside phosphotransferase from Tn5 [Binary vector pCNF3] AVE26338.1 aminoglycoside phosphotransferase from Tn5 [Binary vector pCNM3] AVN68412.1 kanamycin resistance protein [Expression vector pARKANI]

AVP12418.1 kanamycin resistance protein [E. coli-T. kodakarensis shuttle vector pTNAg]

AVP12423.1 kanamycin resistance protein [E. coli-T. kodakarensis shuttle vector pTNTrpE]

AVQ67768.1 neomycin phosphotransferase II [Chloroplast expression vector pCEV3]

AVQ67774.1 neomycin phosphotransferase II [Auxiliary vector pECnpt] AVR55009.1 aminoglycoside O-phosphotransferase [Cloning vector pTHSSe_04] AVR55158.1 aminoglycoside O-phosphotransferase [Cloning vector pTHSSe_55] AVR55194.1 aminoglycoside O-phosphotransferase [Cloning vector pTHSSe_70] AVR55199.1 aminoglycoside O-phosphotransferase [Cloning vector pTHSSe_71] AWK60330.1 neomycin phosphotransferase [Cloning vector pK18sB]

AWN56777.1 neomycin phosphotransferase II [Fungal transformation vector pKT300]

AWN56786.1 neomycin phosphotransferase II [Fungal transformation vector pKT303]

AWN56795.1 neomycin phosphotransferase II [Fungal transformation vector pKT311]

AWV91932.1 Kan(r) [Cloning vector pFNK-503-qscrsaL] AWV91942.1 Kan(r) [Cloning vector pFNK-804-lacO-lacI] AWV91943.1 Kan(r) [Cloning vector pFNK-806]

AXN70083.1 kanamycin resistance protein [Cloning vector pBBR1-rppA] AXN70086.1 kanamycin resistance protein [Cloning vector pBBR1-His-rppA] AYC12545.1 kanamycin resistance protein [Cloning Vector pCG6] AYC44195.1 kanamycin resistance protein [Cloning Vector pCG18] AYC44196.1 kanamycin resistance protein [Cloning Vector pCG18.glr1] AYC44197.1 kanamycin resistance protein [Cloning Vector pCG18.Hem] AYC44198.1 kanamycin resistance protein [Cloning Vector pCG18.HF]

AYC44199.1 kanamycin resistance protein [Cloning Vector pCG18.HlyA] AYC44200.1 kanamycin resistance protein [Cloning Vector pCG18.HlyC] AYM54603.1 Kan/neoR [Vector NanoLucNeo]

AYU58840.1 neomycin phosphotransferase [Cloning vector pEASY-tub/ble] AYU58843.1 neomycin phosphotransferase [Cloning vector pEASY-tub/aadA] AYU58846.1 neomycin phosphotransferase [Cloning vector pEASY-tub/hptII] AZB74553.1 AphA [Cloning vector pPS-BR]

AZP53433.1 aminoglycoside phosphotransferase from Tn5 [Vector pcDNA3.1-CMV.GFP] AZP53435.1 aminoglycoside phosphotransferase from Tn5 [Vector pLeu-tRNA.GFP(STOP)] AZP53438.1 aminoglycoside phosphotransferase from Tn5 [Vector pLeu-tRNA.LeuRS] AZP53441.1 aminoglycoside phosphotransferase from Tn5 [Vector AdProt.FLAG] AZP53444.1 aminoglycoside phosphotransferase from Tn5 [Vector (LoxP)2Term.AdProt] AZP53446.1 aminoglycoside phosphotransferase from Tn5 [Vector AdProt(STOP)] AZP56000.1 aminoglycoside phosphotransferase from Tn5 [Vector pcDNA3.1-KanFDEST]

EBK6339134.1 aminoglycoside O-phosphotransferase APH(3')-IIa [Salmonella enterica subsp. enterica serovar Typhi]

EBL6081020.1 aminoglycoside O-phosphotransferase APH(3')-IIa [Salmonella enterica subsp. enterica serovar Kentucky]

EBQ9816394.1 aminoglycoside O-phosphotransferase APH(3')-IIa [Salmonella enterica subsp. enterica serovar Agona]

EBS0352805.1 aminoglycoside O-phosphotransferase APH(3')-IIa [Salmonella enterica subsp. enterica serovar Java]

EBV7234941.1 aminoglycoside O-phosphotransferase APH(3')-IIa [Salmonella enterica subsp. enterica serovar Hadar]

EBZ1745573.1 aminoglycoside O-phosphotransferase APH(3')-IIa [Salmonella enterica subsp. enterica serovar Singapore]

ECB9282330.1 aminoglycoside O-phosphotransferase APH(3')-IIa [Salmonella enterica subsp. enterica serovar Bredeney]

ECD4200822.1 aminoglycoside O-phosphotransferase APH(3')-IIa [Salmonella enterica subsp. enterica serovar Muenchen]

ECF7431164.1 aminoglycoside O-phosphotransferase APH(3')-IIa [Salmonella enterica subsp. enterica serovar 4,[5],12:r:-]

ECJ4424321.1 aminoglycoside O-phosphotransferase APH(3')-IIa [Salmonella enterica subsp. enterica serovar Mbandaka]

ECS3566051.1 aminoglycoside O-phosphotransferase APH(3')-IIa [Salmonella enterica subsp. enterica serovar Liverpool]

ECS3609273.1 aminoglycoside O-phosphotransferase APH(3')-IIa [Salmonella enterica subsp. enterica serovar Brandenburg]

ECT6258768.1 aminoglycoside O-phosphotransferase APH(3')-IIa [Salmonella enterica subsp. enterica serovar Idikan]

ECX6282056.1 aminoglycoside O-phosphotransferase APH(3')-IIa [Salmonella enterica subsp. enterica serovar Rubislaw]

EDI4836280.1 aminoglycoside O-phosphotransferase APH(3')-IIa [Salmonella enterica subsp. enterica serovar Infantis]

EDQ7221572.1 aminoglycoside O-phosphotransferase APH(3')-IIa [Salmonella enterica subsp. enterica serovar Newport]

EDR5533379.1 aminoglycoside O-phosphotransferase APH(3')-IIa [Salmonella enterica subsp. enterica serovar 4,12:i:-]

EDR7498383.1 aminoglycoside O-phosphotransferase APH(3')-IIa [Salmonella enterica subsp. enterica serovar Kiambu]

EDT3033370.1 aminoglycoside O-phosphotransferase APH(3')-IIa [Salmonella enterica subsp. enterica serovar Montevideo]

EDT4821661.1 aminoglycoside O-phosphotransferase APH(3')-IIa [Salmonella enterica subsp. enterica serovar 4,[5],12:i:-]

EDU0572873.1 aminoglycoside O-phosphotransferase APH(3')-IIa [Salmonella enterica subsp. enterica serovar Putten]

EDV5070193.1 aminoglycoside O-phosphotransferase APH(3')-IIa [Salmonella enterica subsp. enterica serovar Ohio]

EDX4084298.1 aminoglycoside O-phosphotransferase APH(3')-IIa [Salmonella enterica subsp. enterica serovar Dublin str. CFSAN000516]

EEB4974905.1 aminoglycoside O-phosphotransferase APH(3')-IIa [Salmonella enterica subsp. enterica serovar Derby]

EEZ5762118.1 aminoglycoside O-phosphotransferase APH(3')-IIa [Escherichia coli O101]

EEZ6491669.1 aminoglycoside O-phosphotransferase APH(3')-IIa [Escherichia coli O156]

EEZ6622953.1 aminoglycoside O-phosphotransferase APH(3')-IIa [Escherichia coli O7]

EEZ8619321.1 aminoglycoside O-phosphotransferase APH(3')-IIa [Escherichia coli O160]

EEZ8703313.1 aminoglycoside O-phosphotransferase APH(3')-IIa [Escherichia coli O91]

EEZ9625377.1 aminoglycoside O-phosphotransferase APH(3')-IIa [Escherichia coli O32]

EEZ9850924.1 aminoglycoside O-phosphotransferase APH(3')-IIa [Escherichia coli O21]

EFA4120235.1 aminoglycoside O-phosphotransferase APH(3')-IIa [Escherichia coli O14]

EFA4125370.1 aminoglycoside O-phosphotransferase APH(3')-IIa [Escherichia coli O49:H9]

EFA4204701.1 aminoglycoside O-phosphotransferase APH(3')-IIa [Escherichia coli O2:H32]

EFN8672942.1 aminoglycoside O-phosphotransferase APH(3')-IIa [Escherichia coli O171]

EGC0951949.1 aminoglycoside O-phosphotransferase APH(3')-IIa [Salmonella enterica subsp. enterica serovar Uganda]

EGF4810518.1 aminoglycoside O-phosphotransferase APH(3')-IIa [Salmonella enterica subsp. enterica serovar Adelaide]

EMH95655.1 aminoglycoside 3'-phosphotransferase [Klebsiella pneumoniae RYC492]

MBE9013990.1 aminoglycoside O-phosphotransferase APH(3')-IIa [Pseudanabaenaceae cyanobacterium LEGE 13415]

PHX41535.1 aminoglycoside phosphotransferase [Pseudomonas sp. NZIPFR-PS5]

PWS23311.1 aminoglycoside O-phosphotransferase APH(3')-IIa [Enterococcus faecium]

QBC76174.1 kanamycin resistance protein [Cloning vector yTREX] QCD25067.1 neomycin phosphotransferase [Cloning vector pCMV-VEE-GFP] QCO31434.1 kanamycin resistance protein [Cloning vector pLS2] QCO31436.1 kanamycin resistance protein [Cloning vector pLB2] QCO69776.1 kanamycin resistance protein [Cloning vector pLBR2] QCO69779.1 kanamycin resistance protein [Cloning vector pLBS2] QCO69782.1 kanamycin resistance protein [Cloning vector pLSR2] QCO69798.1 kanamycin resistance protein [Cloning vector pLSRB2] QCO69802.1 kanamycin resistance protein [Cloning vector pLSRC2] QCO69806.1 kanamycin resistance protein [Cloning vector pLSRT2] QCO69826.1 kanamycin resistance protein [Cloning vector pLSRBC2] QCO69831.1 kanamycin resistance protein [Cloning vector pLSRBT2] QCO69836.1 kanamycin resistance protein [Cloning vector pLSRCT2] QCS27644.1 neomycin phosphotransferase [Broad host range vector pCON] QCS27647.1 neomycin phosphotransferase [Broad host range vector pIND] QDG03211.1 kanamycin resistance protein [Cloning vector pGHKW2] QDH44609.1 kanamycin resistance protein [Cloning vector pKill1] QDH44612.1 kanamycin resistance protein [Cloning vector pKill3]

QED89934.1 kanamycin resistance protein [Cloning vector pKW5_MB1Kan_TracrK_Spacer] QEG99046.1 neomycin phosphotransferase [Cloning vector pTrichoGate-20]

QFU20096.1 NeoR [Expression vector pVG182]

QFU20098.1 NeoR [Expression vector pVG312_vasa-DD2-Cas9] QFU20101.1 NeoR [Expression vector pVG303_nanos-Cas9] QFU20104.1 NeoR [Expression vector pVG186_y1-w2] QFU20107.1 NeoR [Expression vector pVG316_vasa-Cas9] QFU20110.1 NeoR [Expression vector pVG185_w2-y1] QFU20113.1 NeoR [Expression vector pVG307_truncated] QFU20116.1 NeoR [Expression vector pVMG47_truncated-L] QFU20118.1 NeoR [Expression vector pVMG48_truncated-R] QFU20121.1 NeoR [Expression vector pVG304_e1-y1]

QFU95558.1 NeoR/KanR [Cloning vector Master_Architecture_8916] QFU95562.1 NeoR/KanR [Cloning vector Master_Architecture_RbsR_KSL] QFU95566.1 NeoR/KanR [Cloning vector Master_Architecture_RbsR_HQN] QFU95617.1 NeoR/KanR [Cloning vector Rep_O1]

QFU95620.1 NeoR/KanR [Cloning vector Rep_Osym] QFU95623.1 NeoR/KanR [Cloning vector Rep_Ogta] QFU95626.1 NeoR/KanR [Cloning vector Rep_Ottg] QFU95629.1 NeoR/KanR [Cloning vector Rep_Otta] QFU95632.1 NeoR/KanR [Cloning vector Rep_Ogac] QFU95635.1 NeoR/KanR [Cloning vector Rep_Octt] QFU95638.1 NeoR/KanR [Cloning vector Rep_Oagg]

QFU95641.1 NeoR/KanR [Cloning vector Rep_Series_Otta_Osym] QGA67138.1 neomycin phosphotransferase [Cloning vector pVMG] QGA70028.1 kanamycin phosphotransferase [Promoter probe vector pNG1]

QGX42294.1 kanamycin/neomycin resistance protein [Expression vector pDU1] QGX42298.1 kanamycin/neomycin resistance protein [Expression vector pMTagBFP2-npt] QHD44352.1 kanamycin resistance protein [Cloning vector pABE]

QHW06081.1 neomycin phosphotransferase [YFV replicon vector] QIJ31329.1 neomycin phosphotransferase [Binary vector pK2GW7_HSP_FT] QJA16367.1 neo [Cloning vector pSimple-tub+eGFP]

QJA16369.1 neo [Cloning vector pEASY-tub/neo] QJE38017.1 KanR/neoR [Cloning vector pCG101] QJE38021.1 KanR/neoR [Cloning vector pCG102] QJE38025.1 KanR/neoR [Cloning vector pCG103] QJE38031.1 KanR/neoR [Cloning vector pCG301] QJE38035.1 KanR/neoR [Cloning vector pCG302] QJE38039.1 KanR/neoR [Cloning vector pCG303] QJE38045.1 KanR/neoR [Cloning vector pCG401] QJE38049.1 KanR/neoR [Cloning vector pCG402] QJE38053.1 KanR/neoR [Cloning vector pCG403] QJE38058.1 KanR [Cloning vector pZS2-200] QJX60210.1 KanR [Cloning vector pcDNA3.1_+] QJX60214.1 KanR [Cloning vector sh-AFP] QJX60215.1 KanR [Cloning vector sh-ctrl] QJX60216.1 KanR [Cloning vector sh-PPP2CA.dna] QJX60217.1 KanR [Cloning vector sh-AFP-PPP2CA]

QKU37008.1 Aph(3')-IIa [Shuttle expression vector pEC-XK99-Gold]

QKX95803.1 NeoR/KanR [Cloning vector pMAJIc] QKX95807.1 NeoR/KanR [Cloning vector pMAJIIc] QLY89016.1 NeoR/KanR [Cloning vector NAND_Reporter] QLY89019.1 NeoR/KanR [Cloning vector NORcp_Reporter] QLY89023.1 NeoR/KanR [Cloning vector XNOR_Reporter]

QLY89026.1 NeoR/KanR [Cloning vector O1(core)_Reporter] QLY89029.1 NeoR/KanR [Cloning vector Oagg(core)_Reporter] QLY89032.1 NeoR/KanR [Cloning vector Octt(core)_Reporter] QLY89035.1 NeoR/KanR [Cloning vector Ofru(core)_Reporter] QLY89038.1 NeoR/KanR [Cloning vector Ogac(core)_Reporter] QLY89041.1 NeoR/KanR [Cloning vector Ogta(core)_Reporter] QLY89044.1 NeoR/KanR [Cloning vector Orbs(prox)_Reporter] QLY89047.1 NeoR/KanR [Cloning vector Osym(core)_Reporter] QLY89050.1 NeoR/KanR [Cloning vector Otta(core)_Reporter] QLY89053.1 NeoR/KanR [Cloning vector Ottg(core)_Reporter] QMS43739.1 kanamycin resistance protein [Cloning vector pYI11] QMS43740.1 kanamycin resistance protein [Cloning vector pYI37] QMT62612.1 KanR [Cloning vector pgRNA-ccdB-1]

QMT62615.1 KanR [Cloning vector pgRNA-ccdB-2]

QNI18898.1 aminoglycoside 3'-phosphotransferase [Cloning vector pYTSK01K_0G7] QOD95421.1 NptII [Vector PmCloverBlaster-kan]

CAA07593.1 aminoglycoside phosphotransferase [Cloning vector pEH1] CAA71572.1 kanamycin marker [Cloning vector pKIL194]

CAA71574.1 kanamycin marker [Cloning vector pKIL194T]

CAB46258.1 neomycin phospho transferase [Cloning vector pYanni3] CAB54528.1 neomycin/kanamycin resistance protein [Cloning vector pKG1M] CAB62545.1 kanamycin resistance protein [Shuttle vector pMJ-A]

CAB93492.1 aminoglycoside 3'-phosphotransferase [Cloning vector pBRINT-TsKm] CAB93902.1 aminoglycoside 3'-pfosphotransferase [Artificial vector pLoxKan2] CAC14446.1 aminoglycoside 3'-phosphotransferase [Cloning vector pBRINTs-Kan2] CAC14457.1 aminoglycoside 3'-phosphotransferase [Cloning vector pBPSKan2] CAD21776.1 neomycin phosphotransferase [Trypanosoma brucei]

CAD68164.1 aminoglycoside 3'-phosphotransferase [Cloning vector pEUKA11] CAN84671.1 neomycin phosphotransferase [Transposon mutagenesis vector pMiET] CAQ57295.1 neomycin phosphotranferase [Trypanosoma brucei brucei]

SIW61222.1 aminoglycoside phosphotransferase from Tn5 [Trichophyton benhamiae] SJL86557.1 unnamed protein product [Mammalian vector pdcDNA-R4R3-dhCMV] SJL86566.1 unnamed protein product [Reporter vector pFAJ1819]

SJL86585.1 unnamed protein product [Mammalian expression vector pFB-Neo-E-hA20] SJL86595.1 unnamed protein product [Mammalian expression vector pcDNA3-RF-PITSLRE-Di-4mB] SJL86671.1 unnamed protein product [Mammalian vector pdcDNA-Myc]

SJL86940.1 unnamed protein product [Mammalian expression vector pCD-F-Bcl10] SJL86943.1 unnamed protein product [Mammalian expression vector pCD-MK] SJL86947.1 unnamed protein product [Mammalian expression vector pCD-A7M8K] SJL86951.1 unnamed protein product [Mammalian expression vector pCD-A7M8K-p20mut] SJL86966.1 unnamed protein product [Mammalian expression vector pCD-MK-p20mut] SJL86987.1 Neomycin [Mammalian expression vector pHM6-Cezanne(16-858)] SJL86990.1 unnamed protein product [Mammalian expression vector pcDNA3-hIRAK1-KD]

SJL86993.1 unnamed protein product [Mammalian expression vector pcDNA3-FLAG-hIRAK1-N-T66A] SJL86996.1 unnamed protein product [Mammalian expression vector pcDNA3-FLAG-hIRAK1-KD] SJL86999.1 unnamed protein product [Mammalian expression vector pcDNA3-hIRAK1]

SJL87002.1 unnamed protein product [Mammalian expression vector pcDNA3-hIRAK1-T66A] SJL87008.1 unnamed protein product [Mammalian expression vector pcDNA3.1-EGFP-mMyD88] SJL87017.1 unnamed protein product [Mammalian expression vector pCD-F-bio-MK] SJL87020.1 unnamed protein product [Mammalian expression vector pCD-F-bio-MK-E4A]

SJL87024.1 unnamed protein product [Mammalian expression vector pCD-F-HLH-MK-bioC] SJL87028.1 unnamed protein product [Mammalian expression vector pCD-F-HLH-MK-E4A-bio] SJL87125.1 unnamed protein product [Mammalian expression vector pcDNA3-di-RF-ODC] SJL87160.1 unnamed protein product [Mammalian expression vector pcDNA3-HA-IkappaBalpha-

superrepressor]

SJL87168.1 unnamed protein product [Mammalian expression vector pEF1hCASP-9-His] SJL87171.1 unnamed protein product [Mammalian expression vector pEF1hCASP-9S-His] SJL87175.1 unnamed protein product [Mammalian expression vector pcDNA3-RF-PITSLRE-Di-4] SJL87179.1 unnamed protein product [Mammalian expression vector pcDNA3-RF-PITSLRE-Di-4mF] SJL87183.1 unnamed protein product [Mammalian expression vector pcDNA3-RF-PITSLRE-Di-4mD] SJL87193.1 unnamed protein product [Mammalian expression vector pCD-Lck-Flag]

SJL87195.1 unnamed protein product [Mammalian expression vector pcDNA3.1-Flag] SJL87262.1 unnamed protein product [Vector pRP-neo]

SJL87374.1 unnamed protein product [Mammalian expression vector pRcRSVp65] SJL87377.1 unnamed protein product [Mammalian expression vector pRcRSVp65S311A] SJL87380.1 unnamed protein product [Mammalian expression vector pRcRSVp65S529A] SJL87383.1 unnamed protein product [Mammalian expression vector pRcRSVp65S276C] SJL87386.1 unnamed protein product [Mammalian expression vector pEF1crmA] SJL87389.1 unnamed protein product [Mammalian expression vector pEF1crmA-T291R] SJL87392.1 unnamed protein product [Mammalian expression vector pEF1hBCL2] SJL87395.1 unnamed protein product [Mammalian expression vector pEF1-hXIAP] SJL87428.1 unnamed protein product [Mammalian expression vector pcDNA3flag-Tax1BP1] SJL87457.1 unnamed protein product [Mammalian expression vector pEF1-hOMI] SJL87460.1 unnamed protein product [Mammalian expression vector pEF1-hOMI-S306A] SJL87463.1 unnamed protein product [Mammalian expression vector pEF1-AVPS-hOMI]

SJL87466.1 unnamed protein product [Mammalian expression vector pEF1-AVPS-hOMI-S306A]

SJL87522.1 unnamed protein product [Mammalian expression vector pEF1-2FKBP-link-hFADD] SJL87528.1 unnamed protein product [Mammalian expression vector pEF1-2FKBP-link-hFADD-DN] SJL87534.1 unnamed protein product [Mammalian expression vector pEF1-2FKBP-link-hFADD-DED] SJL87555.1 unnamed protein product [Mammalian expression vector pEF1-2FKBP-link-mCASP-8] SJL87587.1 unnamed protein product [Mammalian expression vector pHis-p65f2-286]

SJL87590.1 unnamed protein product [Mammalian expression vector pHis-p65] SJL87593.1 unnamed protein product [Mammalian expression vector pHis-p65f2-311] SJL87596.1 unnamed protein product [Mammalian expression vector pHis-p65f311-551] SJL87599.1 unnamed protein product [Mammalian expression vector pHis-p65f285-551] SJL87635.1 unnamed protein product [Mammalian vector pdcDNA-FLAG]

SJL87639.1 unnamed protein product [Mammalian vector pdcDNA-FLAGMyc] SJL87669.1 unnamed protein product [Mammalian expression vector pEF1-hRIP1]

SJL87672.1 unnamed protein product [Mammalian expression vector pEF1-mRIP-V5/His] SJL87675.1 unnamed protein product [Mammalian expression vector pEF1-mRIP] SJL87698.1 unnamed protein product [Mammalian expression vector pEF1mBID] SJL87724.1 unnamed protein product [Vector pREP9FLAG]

SJL87888.1 unnamed protein product [Mammalian vector pdcDNA-HA]

SJL87927.1 unnamed protein product [Mammalian expression vector pCDNA-mCASP-8] SJL87930.1 unnamed protein product [Mammalian expression vector pcDNAFLAGhRAIDDMyc] SJL87933.1 unnamed protein product [Mammalian expression vector pCDNA-B-catenin] SJL88063.1 unnamed protein product [Suicide vector pVIK107]

SJL88067.1 unnamed protein product [Suicide vector pVIK110] SJL88071.1 unnamed protein product [Suicide vector pVIK111] SJL88075.1 unnamed protein product [Suicide vector pVIK112] SJL88077.1 unnamed protein product [Suicide vector pVIK165]

SJL88127.1 unnamed protein product [Mammalian expression vector LNXRO2] SJL88129.1 unnamed protein product [Mammalian expression vector LNXCO3] SJL88131.1 unnamed protein product [Mammalian expression vector LNXCO4] SJL88146.1 unnamed protein product [Mammalian expression vector pEF1mtBIDHis] SJL88149.1 unnamed protein product [Mammalian expression vector pEF1mBIDHis] SJL88269.1 unnamed protein product [Mammalian expression vector pcDNA3mB7-1] SJL88272.1 unnamed protein product [Mammalian expression vector pcDNA3mB7-2]

SJL88474.1 unnamed protein product [Mammalian expression vector pRcCMVhuIKB-alpha] SJL88524.1 unnamed protein product [Vector pT7POL26]

SJL88849.1 unnamed protein product [Vector pDMIphoAE6L] SJL88903.1 unnamed protein product [Vector pDMI,1]

SJL89036.1 unnamed protein product [Mammalian expression vector pBM6DraA6] BAA23653.1 neomycin phosphotransferase [Cloning vector pAP3neo]

BAA78209.1 neomycine phosphotransferase [Dual-tagging gene trap vector pGT1] BAA94847.1 neo [Retroviral vector pCXneo]

BAC41724.1 aminoglycoside phosphotransferase [Retroviral vector pCX4neo]

BAD06941.1 neomysin resistance gene product [Hepatitis C virus replicon pSGR-JFH1]

BAD42593.1 aminoglycoside-3'-0-phosphotransferase [Cloning vector pHN267] BAF02355.1 aminoglycoside 3'-O-phosphotransferase [Shuttle vector pNV18] BAF02359.1 aminoglycoside 3'-O-phosphotransgerase [Shuttle vector pNV19]

BAF34892.1 neomycin-resistant gene [Hepatitis C virus full-length replicon pFGR-JFH1]

BAF46211.1 neomycin phosphotransferase [Moss transformation vector pTN80] BAF46213.1 neomycin phosphotransferase [Moss transformation vector pTN182] BAF46215.1 neomycin phosphotransferase [Moss transformation vector pTN85] BAH85870.1 neomycin phosphotransferase II [Gene silencing vector pSilent-Dual1] BAH86763.1 neomycin phosphotransferase [Mammalian expression vector pC[Delta]E-

chimUAAgaCAA21FLucH]

BAH86767.1 neomycin phosphotransferase [Mammalian expression vector pCInx-hRPSIVCAA21FLuc] BAI59712.1 neomycin phosphotransferase [Moss transformation vector pTN83]

BAJ04909.1 aminoglycoside-3'-0-phosphotransferase [Cloning vector pRTSK-sacB] BAL46488.1 neomycin-kanamycin phosphotransferase [Ti-curing vector pK18msr24A] BAL46513.1 neomycin-kanamycin phosphotransferase [Cloning vector pK18msr] BAL60569.1 neomycin phosphotransferase [Moss transformation vector pCTRN-NPTII

2]

BAM15663.1 neomycin phosphotransferase [Moss transformation vector pTN82]

BAM28682.1 neomycin-kanamycin phosphotransferase type II [Hepatitis C virus replicon pSGR-JFH2.1]

BAM28684.1 neomycin-kanamycin phosphotransferase type II [Hepatitis C virus replicon pSGR-JFH2.2]

BAN13546.1 neomycin phosphotransferase [Activation tagging vector pKANACK]

BAN21746.1 neomycin phosphotransferase [Suicide vector for unmarked mutagenesis pKFRT/FLP]

BAN28915.1 kanamycin phosphotransferase [Cloning vector pRMR6K-Km] BAN28923.1 kanamycin phosphotransferase [Cloning vector pRMTn-Km] BAP82231.1 aminoglycoside phosphotransferase [Expression vector pKU1021gps] BAP82235.1 aminoglycoside phosphotransferase [Expression vector pKU1021fps] BAP82239.1 aminoglycoside phosphotransferase [Expression vector pKU1021ggs]

BAQ08343.1 neomycin phosphotransferase I [Transposon delivery vector pKUT-Tn5-Km] BAU09068.1 neomycin phosphotransferase [Moss transformation vector p3xHA

NPTII]

BAU09070.1 neomycin phosphotransferase [Moss transformation vector p3xMyc NPTII]

BAU19378.1 neomycin phosphotransferase [Moss transformation vector pPGX6-km] BAX01901.1 aminoglycoside phosphotransferase [Cloning vector pNT562]

BAX25487.1 confers resistance to neomycin, kanamycin, and G418 [Cloning vector pK19]

BAX25489.1 confers resistance to neomycin, kanamycin, and G418 [Cloning vector pK19mob]

BAX25495.1 confers resistance to neomycin, kanamycin, and G418 [Cloning vector pK19mobsacB]

BBA12671.1 Tn5 aminoglycoside phosphotransferase [Mammalian expression vector BsrGI-MCS-pcDNA3.1]

BBA12673.1 Tn5 aminoglycoside phosphotransferase [Mammalian expression vector Flag-MCS-pcDNA3.1]

BBA12675.1 Tn5 aminoglycoside phosphotransferase [Mammalian expression vector Flag-EGFP-MCS-pcDNA3.1]

BBA12677.1 Tn5 aminoglycoside phosphotransferase [Mammalian expression vector Flag-mCherry-MCS-pcDNA3.1]

BBA12679.1 Tn5 aminoglycoside phosphotransferase [Mammalian expression vector Flag-tdTomato-MCS-pcDNA3.1]

BBA12681.1 Tn5 aminoglycoside phosphotransferase [Mammalian expression vector HA-MCS-pcDNA3.1]

BBA12683.1 Tn5 aminoglycoside phosphotransferase [Mammalian expression vector myc-MCS-pcDNA3.1]

BBA12685.1 Tn5 aminoglycoside phosphotransferase [Mammalian expression vector EGFP-MCS-pcDNA3.1]

BBA12687.1 Tn5 aminoglycoside phosphotransferase [Mammalian expression vector mCherry-MCS-pcDNA3.1]

BBA12689.1 Tn5 aminoglycoside phosphotransferase [Mammalian expression vector tdTomato-MCS-pcDNA3.1]

BBB86758.1 neo [Plasmid vector pSAP2-g-multi]

BBH54780.1 Neomycin phosphotransferase, resistance for Kanamycin [Expression vector pK7WG2-mitoTAL1-orf125]

BBH54784.1 Neomycin phosphotransferase, resistance for Kanamycin [Expression vector pK7WG2-mitoTAL2-orf125]

Length=264

Score = 535 bits (1378), Expect = 0.0, Method: Compositional matrix adjust. Identities = 264/264 (100%), Positives = 264/264 (100%), Gaps = 0/264 (0%)

Query 1	MIEQDGLHAGSPAAWVERLFGYDWAQQTIGCSDAAVFRLSAQGRPVLFVKTDLSGALNEL 60

| Sbjct | 1 | MIEQDGLHAGSPAAWVERLFGYDWAQQTIGCSDAAVFRLSAQGRPVLFVKTDLSGALNEL MIEQDGLHAGSPAAWVERLFGYDWAQQTIGCSDAAVFRLSAQGRPVLFVKTDLSGALNEL | 60 |
| --- | --- | --- | --- |
| Query | 61 | QDEAARLSWLATTGVPCAAVLDVVTEAGRDWLLLGEVPGQDLLSSHLAPAEKVSIMADAM | 120 |
| Sbjct | 61 | QDEAARLSWLATTGVPCAAVLDVVTEAGRDWLLLGEVPGQDLLSSHLAPAEKVSIMADAM QDEAARLSWLATTGVPCAAVLDVVTEAGRDWLLLGEVPGQDLLSSHLAPAEKVSIMADAM | 120 |
| Query | 121 | RRLHTLDPATCPFDHQAKHRIERARTRMEAGLVDQDDLDEEHQGLAPAELFARLKARMPD | 180 |
| Sbjct | 121 | RRLHTLDPATCPFDHQAKHRIERARTRMEAGLVDQDDLDEEHQGLAPAELFARLKARMPD RRLHTLDPATCPFDHQAKHRIERARTRMEAGLVDQDDLDEEHQGLAPAELFARLKARMPD | 180 |
| Query | 181 | GEDLVVTHGDACLPNIMVENGRFSGFIDCGRLGVADRYQDIALATRDIAEELGGEWADRF | 240 |
| Sbjct | 181 | GEDLVVTHGDACLPNIMVENGRFSGFIDCGRLGVADRYQDIALATRDIAEELGGEWADRF GEDLVVTHGDACLPNIMVENGRFSGFIDCGRLGVADRYQDIALATRDIAEELGGEWADRF | 240 |
| Query  Sbjct | 241  241 | LVLYGIAAPDSQRIAFYRLLDEFF 264 LVLYGIAAPDSQRIAFYRLLDEFF LVLYGIAAPDSQRIAFYRLLDEFF 264 |  |

```
>AAD31025.1 aminoglycoside 3'-phosphotransferase [Cloning vector pFBneo] AAG02189.1 neomycin/kanamycin resistance protein [Cloning vector pFB-ERV] ADV36888.1 aminoglycoside 3'-phosphotransferase [synthetic construct]
Length=269

Score = 535 bits (1378), Expect = 0.0, Method: Compositional matrix adjust. Identities = 264/264 (100%), Positives = 264/264 (100%), Gaps = 0/264 (0%)

Query 1	MIEQDGLHAGSPAAWVERLFGYDWAQQTIGCSDAAVFRLSAQGRPVLFVKTDLSGALNEL 60 MIEQDGLHAGSPAAWVERLFGYDWAQQTIGCSDAAVFRLSAQGRPVLFVKTDLSGALNEL
Sbjct 6	MIEQDGLHAGSPAAWVERLFGYDWAQQTIGCSDAAVFRLSAQGRPVLFVKTDLSGALNEL 65

Query 61	QDEAARLSWLATTGVPCAAVLDVVTEAGRDWLLLGEVPGQDLLSSHLAPAEKVSIMADAM 120 QDEAARLSWLATTGVPCAAVLDVVTEAGRDWLLLGEVPGQDLLSSHLAPAEKVSIMADAM
Sbjct 66	QDEAARLSWLATTGVPCAAVLDVVTEAGRDWLLLGEVPGQDLLSSHLAPAEKVSIMADAM 125

Query 121 RRLHTLDPATCPFDHQAKHRIERARTRMEAGLVDQDDLDEEHQGLAPAELFARLKARMPD 180 RRLHTLDPATCPFDHQAKHRIERARTRMEAGLVDQDDLDEEHQGLAPAELFARLKARMPD
Sbjct 126 RRLHTLDPATCPFDHQAKHRIERARTRMEAGLVDQDDLDEEHQGLAPAELFARLKARMPD 185

Query 181 GEDLVVTHGDACLPNIMVENGRFSGFIDCGRLGVADRYQDIALATRDIAEELGGEWADRF 240 GEDLVVTHGDACLPNIMVENGRFSGFIDCGRLGVADRYQDIALATRDIAEELGGEWADRF
Sbjct 186 GEDLVVTHGDACLPNIMVENGRFSGFIDCGRLGVADRYQDIALATRDIAEELGGEWADRF 245

Query 241 LVLYGIAAPDSQRIAFYRLLDEFF 264 LVLYGIAAPDSQRIAFYRLLDEFF
Sbjct 246 LVLYGIAAPDSQRIAFYRLLDEFF 269

>AAG00918.1 neomycin phosphotransferase [Cloning vector pBIG] AAG00920.1 neomycin phosphotransferase [Cloning vector pLITTLE] AAG00922.1 neomycin phosphotransferase [Cloning vector pTIKL] AAG00923.1 neomycin phosphotransferase [Cloning vector pDXA-GFP2] AAG00926.1 neomycin phosphotransferase [Cloning vector pDXA-FLAG] AAG00931.1 neomycin phosphotransferase [Cloning vector pTX-GFP] AAG00934.1 neomycin phosphotransferase [Cloning vector pTX-FLAG] ABK40467.1 neomycin phosphotransferase [Cloning vector EXP4(+)] ABK40469.1 neomycin phosphotransferase [Cloning vector EXP5(+)] ABK40471.1 neomycin phosphotransferase [Cloning vector pDV-NYFP] ABK40473.1 neomycin phosphotransferase [Cloning vector pDV-NTAP] ABK40475.1 neomycin phosphotransferase [Cloning vector pDV-CGFP] ABK40477.1 neomycin phosphotransferase [Cloning vector pDV-CYFP] ABK40479.1 neomycin phosphotransferase [Cloning vector pDV-CTAP] ABK40481.1 neomycin phosphotransferase [Cloning vector pDV-NTAP-NYFP] ABK40483.1 neomycin phosphotransferase [Cloning vector pDV-NYFP-CTAP] ABK40485.1 neomycin phosphotransferase [Cloning vector pDV-CGFP-CTAP] ABK40487.1 neomycin phosphotransferase [Cloning vector pDV-CYFP-CTAP] ABK40489.1 neomycin phosphotransferase [Cloning vector pDV-NTAP-CGFP] ABK40491.1 neomycin phosphotransferase [Cloning vector pDV-NTAP-CYFP] ACG80830.1 neomycin phosphotransferase [Expression vector pDM310] ACG80848.1 neomycin phosphotransferase [Expression vector pDM309] ACH81549.1 neomycin phosphotransferase [Expression vector pDM304]

ACH81555.1 neomycin phosphotransferase [Expression vector pDM320] AEX09392.1 NeoR [Cloning vector pTX-NLS-Cre]
CAD53291.1 kanamycin kinase [Cloning vector pDXA-CFP-MCS] CAD53294.1 kanamycin kinase [Cloning vector pDXA-YFP-MCS] CAD53297.1 kanamycin kinase [Cloning vector pDXA-YFP-NotI] CAD53300.1 kanamycin kinase [Cloning vector pDXA-MCS-YFP] CAD53303.1 kanamycin kinase [Cloning vector pDXA-3strep] CAD53307.1 kanamycin kinase [Cloning vector pDXA-CFP] CAD53310.1 kanamycin kinase [Cloning vector pDXA-yFP] CAD53313.1 kanamycin kinase [Cloning vector pDXA-3FLAG] CAD53315.1 kanamycin kinase [Cloning vector pDXA-GST]
Length=276

Score = 535 bits (1378), Expect = 0.0, Method: Compositional matrix adjust. Identities = 264/264 (100%), Positives = 264/264 (100%), Gaps = 0/264 (0%)

Query 1	MIEQDGLHAGSPAAWVERLFGYDWAQQTIGCSDAAVFRLSAQGRPVLFVKTDLSGALNEL 60 MIEQDGLHAGSPAAWVERLFGYDWAQQTIGCSDAAVFRLSAQGRPVLFVKTDLSGALNEL
Sbjct 13	MIEQDGLHAGSPAAWVERLFGYDWAQQTIGCSDAAVFRLSAQGRPVLFVKTDLSGALNEL 72

Query 61	QDEAARLSWLATTGVPCAAVLDVVTEAGRDWLLLGEVPGQDLLSSHLAPAEKVSIMADAM 120 QDEAARLSWLATTGVPCAAVLDVVTEAGRDWLLLGEVPGQDLLSSHLAPAEKVSIMADAM
Sbjct 73	QDEAARLSWLATTGVPCAAVLDVVTEAGRDWLLLGEVPGQDLLSSHLAPAEKVSIMADAM 132

Query 121 RRLHTLDPATCPFDHQAKHRIERARTRMEAGLVDQDDLDEEHQGLAPAELFARLKARMPD 180 RRLHTLDPATCPFDHQAKHRIERARTRMEAGLVDQDDLDEEHQGLAPAELFARLKARMPD
Sbjct 133 RRLHTLDPATCPFDHQAKHRIERARTRMEAGLVDQDDLDEEHQGLAPAELFARLKARMPD 192

Query 181 GEDLVVTHGDACLPNIMVENGRFSGFIDCGRLGVADRYQDIALATRDIAEELGGEWADRF 240 GEDLVVTHGDACLPNIMVENGRFSGFIDCGRLGVADRYQDIALATRDIAEELGGEWADRF
Sbjct 193 GEDLVVTHGDACLPNIMVENGRFSGFIDCGRLGVADRYQDIALATRDIAEELGGEWADRF 252

Query 241 LVLYGIAAPDSQRIAFYRLLDEFF 264 LVLYGIAAPDSQRIAFYRLLDEFF
Sbjct 253 LVLYGIAAPDSQRIAFYRLLDEFF 276

>WP_097474700.1 APH(3')-II family aminoglycoside O-phosphotransferase [Escherichia coli]
EGC4106884.1 APH(3')-II family aminoglycoside O-phosphotransferase [Escherichia coli]
EGC4107119.1 APH(3')-II family aminoglycoside O-phosphotransferase [Escherichia coli]
Length=264

Score = 535 bits (1377), Expect = 0.0, Method: Compositional matrix adjust.
```

| Identities | = 263/264 (99%), Positives = 264/264 (100%), Gaps = 0/264 (0%) |  |
| --- | --- | --- |
| Query 1 | MIEQDGLHAGSPAAWVERLFGYDWAQQTIGCSDAAVFRLSAQGRPVLFVKTDLSGALNEL | 60 |
| Sbjct 1 | MIEQDGLHAGSPAAWVERLFGYDWAQQTIGCSDAAVFRLSAQGRPVLFVKTDLSGALNEL MIEQDGLHAGSPAAWVERLFGYDWAQQTIGCSDAAVFRLSAQGRPVLFVKTDLSGALNEL | 60 |
| Query 61 | QDEAARLSWLATTGVPCAAVLDVVTEAGRDWLLLGEVPGQDLLSSHLAPAEKVSIMADAM | 120 |
| Sbjct 61 | QDEAARLSWLATTGVPCAAVLDVVTEAGRDWLLLGEVPGQDLLSSHLAPAEKVSIMADAM QDEAARLSWLATTGVPCAAVLDVVTEAGRDWLLLGEVPGQDLLSSHLAPAEKVSIMADAM | 120 |
| Query 121 | RRLHTLDPATCPFDHQAKHRIERARTRMEAGLVDQDDLDEEHQGLAPAELFARLKARMPD | 180 |
| Sbjct 121 | RR+HTLDPATCPFDHQAKHRIERARTRMEAGLVDQDDLDEEHQGLAPAELFARLKARMPD RRMHTLDPATCPFDHQAKHRIERARTRMEAGLVDQDDLDEEHQGLAPAELFARLKARMPD | 180 |
| Query 181 | GEDLVVTHGDACLPNIMVENGRFSGFIDCGRLGVADRYQDIALATRDIAEELGGEWADRF | 240 |
| Sbjct 181 | GEDLVVTHGDACLPNIMVENGRFSGFIDCGRLGVADRYQDIALATRDIAEELGGEWADRF GEDLVVTHGDACLPNIMVENGRFSGFIDCGRLGVADRYQDIALATRDIAEELGGEWADRF | 240 |
| Query 241  Sbjct 241 | LVLYGIAAPDSQRIAFYRLLDEFF 264 LVLYGIAAPDSQRIAFYRLLDEFF LVLYGIAAPDSQRIAFYRLLDEFF 264 |  |

>ASU87773.1 NPTII [Ti plasmid binary vector pGSh16.1010] ASU89325.1 NPTII [Ti plasmid binary vector pGSh16.1012] QGV13007.1 NPTII [Plant binary expression vector pDEL.ROS]

Length=264

Score = 535 bits (1377), Expect = 0.0, Method: Compositional matrix adjust.

| Identities | = 263/264 (99%), Positives = 264/264 (100%), Gaps = 0/264 (0%) |  |
| --- | --- | --- |
| Query 1 | MIEQDGLHAGSPAAWVERLFGYDWAQQTIGCSDAAVFRLSAQGRPVLFVKTDLSGALNEL | 60 |
| Sbjct 1 | M+EQDGLHAGSPAAWVERLFGYDWAQQTIGCSDAAVFRLSAQGRPVLFVKTDLSGALNEL MVEQDGLHAGSPAAWVERLFGYDWAQQTIGCSDAAVFRLSAQGRPVLFVKTDLSGALNEL | 60 |
| Query 61 | QDEAARLSWLATTGVPCAAVLDVVTEAGRDWLLLGEVPGQDLLSSHLAPAEKVSIMADAM | 120 |
| Sbjct 61 | QDEAARLSWLATTGVPCAAVLDVVTEAGRDWLLLGEVPGQDLLSSHLAPAEKVSIMADAM QDEAARLSWLATTGVPCAAVLDVVTEAGRDWLLLGEVPGQDLLSSHLAPAEKVSIMADAM | 120 |
| Query 121 | RRLHTLDPATCPFDHQAKHRIERARTRMEAGLVDQDDLDEEHQGLAPAELFARLKARMPD | 180 |
| Sbjct 121 | RRLHTLDPATCPFDHQAKHRIERARTRMEAGLVDQDDLDEEHQGLAPAELFARLKARMPD RRLHTLDPATCPFDHQAKHRIERARTRMEAGLVDQDDLDEEHQGLAPAELFARLKARMPD | 180 |
| Query 181 | GEDLVVTHGDACLPNIMVENGRFSGFIDCGRLGVADRYQDIALATRDIAEELGGEWADRF | 240 |
| Sbjct 181 | GEDLVVTHGDACLPNIMVENGRFSGFIDCGRLGVADRYQDIALATRDIAEELGGEWADRF GEDLVVTHGDACLPNIMVENGRFSGFIDCGRLGVADRYQDIALATRDIAEELGGEWADRF | 240 |
| Query 241  Sbjct 241 | LVLYGIAAPDSQRIAFYRLLDEFF 264 LVLYGIAAPDSQRIAFYRLLDEFF LVLYGIAAPDSQRIAFYRLLDEFF 264 |  |

>WP_096935528.1 APH(3')-II family aminoglycoside O-phosphotransferase [Escherichia coli]

Length=264

Score = 534 bits (1376), Expect = 0.0, Method: Compositional matrix adjust.

| Identities | = 263/264 (99%), Positives = 264/264 (100%), Gaps = 0/264 (0%) |  |
| --- | --- | --- |
| Query 1 | MIEQDGLHAGSPAAWVERLFGYDWAQQTIGCSDAAVFRLSAQGRPVLFVKTDLSGALNEL | 60 |
| Sbjct 1 | MIEQDGLHAGSPAAWVERLFGYDWAQQTIGCSDAAVFRLSAQGRPVLFVKTDLSGALNEL MIEQDGLHAGSPAAWVERLFGYDWAQQTIGCSDAAVFRLSAQGRPVLFVKTDLSGALNEL | 60 |
| Query 61 | QDEAARLSWLATTGVPCAAVLDVVTEAGRDWLLLGEVPGQDLLSSHLAPAEKVSIMADAM | 120 |
| Sbjct 61 | QDEAARLSWLATTGVPCAAVLDVVTEAGRDWLLLGEVPGQDLLSSHLAPAEKVSIMADAM QDEAARLSWLATTGVPCAAVLDVVTEAGRDWLLLGEVPGQDLLSSHLAPAEKVSIMADAM | 120 |
| Query 121 | RRLHTLDPATCPFDHQAKHRIERARTRMEAGLVDQDDLDEEHQGLAPAELFARLKARMPD | 180 |
| Sbjct 121 | RRLHTLDPATCPFDHQAKHRIERARTRMEAGLVDQDDLDEEHQGLAPAELFARLKARMP+ RRLHTLDPATCPFDHQAKHRIERARTRMEAGLVDQDDLDEEHQGLAPAELFARLKARMPN | 180 |
| Query 181 | GEDLVVTHGDACLPNIMVENGRFSGFIDCGRLGVADRYQDIALATRDIAEELGGEWADRF | 240 |
| Sbjct 181 | GEDLVVTHGDACLPNIMVENGRFSGFIDCGRLGVADRYQDIALATRDIAEELGGEWADRF GEDLVVTHGDACLPNIMVENGRFSGFIDCGRLGVADRYQDIALATRDIAEELGGEWADRF | 240 |
| Query 241  Sbjct 241 | LVLYGIAAPDSQRIAFYRLLDEFF 264 LVLYGIAAPDSQRIAFYRLLDEFF LVLYGIAAPDSQRIAFYRLLDEFF 264 |  |

>WP_114741199.1 APH(3')-II family aminoglycoside O-phosphotransferase [Mycolicibacterium moriokaense]

RDH73957.1 APH(3')-II family aminoglycoside O-phosphotransferase [Mycolicibacterium moriokaense]

Length=264

Score = 534 bits (1375), Expect = 0.0, Method: Compositional matrix adjust.

| Identities | = 263/264 (99%), Positives = 264/264 (100%), Gaps = 0/264 (0%) |  |
| --- | --- | --- |
| Query 1 | MIEQDGLHAGSPAAWVERLFGYDWAQQTIGCSDAAVFRLSAQGRPVLFVKTDLSGALNEL | 60 |
| Sbjct 1 | MIEQDGLHAGSPAAWVERLFGYDWAQQT+GCSDAAVFRLSAQGRPVLFVKTDLSGALNEL MIEQDGLHAGSPAAWVERLFGYDWAQQTLGCSDAAVFRLSAQGRPVLFVKTDLSGALNEL | 60 |
| Query 61 | QDEAARLSWLATTGVPCAAVLDVVTEAGRDWLLLGEVPGQDLLSSHLAPAEKVSIMADAM | 120 |
| Sbjct 61 | QDEAARLSWLATTGVPCAAVLDVVTEAGRDWLLLGEVPGQDLLSSHLAPAEKVSIMADAM QDEAARLSWLATTGVPCAAVLDVVTEAGRDWLLLGEVPGQDLLSSHLAPAEKVSIMADAM | 120 |
| Query 121 | RRLHTLDPATCPFDHQAKHRIERARTRMEAGLVDQDDLDEEHQGLAPAELFARLKARMPD | 180 |

| Sbjct | 121 | RRLHTLDPATCPFDHQAKHRIERARTRMEAGLVDQDDLDEEHQGLAPAELFARLKARMPD RRLHTLDPATCPFDHQAKHRIERARTRMEAGLVDQDDLDEEHQGLAPAELFARLKARMPD | 180 |
| --- | --- | --- | --- |
| Query | 181 | GEDLVVTHGDACLPNIMVENGRFSGFIDCGRLGVADRYQDIALATRDIAEELGGEWADRF | 240 |
| Sbjct | 181 | GEDLVVTHGDACLPNIMVENGRFSGFIDCGRLGVADRYQDIALATRDIAEELGGEWADRF GEDLVVTHGDACLPNIMVENGRFSGFIDCGRLGVADRYQDIALATRDIAEELGGEWADRF | 240 |
| Query  Sbjct | 241  241 | LVLYGIAAPDSQRIAFYRLLDEFF 264 LVLYGIAAPDSQRIAFYRLLDEFF LVLYGIAAPDSQRIAFYRLLDEFF 264 |  |

>EFB5119094.1 APH(3')-II family aminoglycoside O-phosphotransferase [Escherichia coli]

Length=264

Score = 534 bits (1375), Expect = 0.0, Method: Compositional matrix adjust.

| Identities | = 263/264 (99%), Positives = 264/264 (100%), Gaps = 0/264 (0%) |  |
| --- | --- | --- |
| Query 1 | MIEQDGLHAGSPAAWVERLFGYDWAQQTIGCSDAAVFRLSAQGRPVLFVKTDLSGALNEL | 60 |
| Sbjct 1 | MIEQDGLHAGSPAAWVERLFGYDWAQQTIGCSDAAVFRLSAQGRPVLF+KTDLSGALNEL MIEQDGLHAGSPAAWVERLFGYDWAQQTIGCSDAAVFRLSAQGRPVLFLKTDLSGALNEL | 60 |
| Query 61 | QDEAARLSWLATTGVPCAAVLDVVTEAGRDWLLLGEVPGQDLLSSHLAPAEKVSIMADAM | 120 |
| Sbjct 61 | QDEAARLSWLATTGVPCAAVLDVVTEAGRDWLLLGEVPGQDLLSSHLAPAEKVSIMADAM QDEAARLSWLATTGVPCAAVLDVVTEAGRDWLLLGEVPGQDLLSSHLAPAEKVSIMADAM | 120 |
| Query 121 | RRLHTLDPATCPFDHQAKHRIERARTRMEAGLVDQDDLDEEHQGLAPAELFARLKARMPD | 180 |
| Sbjct 121 | RRLHTLDPATCPFDHQAKHRIERARTRMEAGLVDQDDLDEEHQGLAPAELFARLKARMPD RRLHTLDPATCPFDHQAKHRIERARTRMEAGLVDQDDLDEEHQGLAPAELFARLKARMPD | 180 |
| Query 181 | GEDLVVTHGDACLPNIMVENGRFSGFIDCGRLGVADRYQDIALATRDIAEELGGEWADRF | 240 |
| Sbjct 181 | GEDLVVTHGDACLPNIMVENGRFSGFIDCGRLGVADRYQDIALATRDIAEELGGEWADRF GEDLVVTHGDACLPNIMVENGRFSGFIDCGRLGVADRYQDIALATRDIAEELGGEWADRF | 240 |
| Query 241  Sbjct 241 | LVLYGIAAPDSQRIAFYRLLDEFF 264 LVLYGIAAPDSQRIAFYRLLDEFF LVLYGIAAPDSQRIAFYRLLDEFF 264 |  |

>WP_171502934.1 MULTISPECIES: APH(3')-II family aminoglycoside O-phosphotransferase [Gammaproteobacteria]

AAV87649.1 kanamycin resistance gene [Flexi vector pF1K T7] AAV87655.1 kanamycin resistance gene [Flexi vector pFN2K (GST)] AAV87659.1 kanamycin resistance gene [Flexi vector pF4K (CMV)] AAX78238.1 kanamycin resistance [Flexi vector pF3K WG (BYDV)] AAZ81409.1 kanamycin resistance protein [pFN6K Flexi Vector] AAZ81413.1 kanamycin resistance protein [pFC7K (HQ) Flexi Vector] AAZ81417.1 kanamycin resistance protein [Cloning vector pFC8K]

ABK40094.1 aminoglycoside 3'-phosphotransferase [Mammalian expression vector pF12K RM Flexi]

ABV21786.1 aminoglycoside phosphotransferase [CMV Flexi Vector pFC14K (HaloTag 7)]

ABY48992.1 kanamycin resistance protein [CMVd1 Flexi Vector pFC15K (HaloTag 7)]

ABY48996.1 kanamycin resistance protein [CMVd2 Flexi Vector pFC16K (HaloTag 7)]

ABY49000.1 kanamycin resistance protein [CMVd3 Flexi Vector pFC17K (HaloTag 7)]

ACC85624.1 kanamycin resistance protein [T7 Flexi Vector pFN18K (HaloTag 7)]

ACC85628.1 kanamycin resistance protein [T7 SP6 Flexi Vector pFN19K (HaloTag 7)]

ACC85632.1 kanamycin resistance protein [T7 SP6 Flexi Vector pFC20K (HaloTag 7)]

ACF22985.1 kanamycin resistance protein [CMV Flexi Vector pFN21K (HaloTag 7)]

ACF22989.1 kanamycin resistance protein [CMVd1 Flexi Vector pFN22K (HaloTag 7)]

ACF22993.1 kanamycin resistance protein [CMVd2 Flexi Vector pFN23K (HaloTag 7)]

ACF22997.1 kanamycin resistance protein [CMVd3 Flexi Vector pFN24K (HaloTag

7)]

ACF93192.1 kanamycin resistance protein [Flexi vector pF25K ICE T7] AEX08656.1 kanamycin resistance protein [T7 Flexi Vector pFN29K His6HaloTag] AEX08657.1 kanamycin resistance protein [T7 Flexi Vector pFC30K His6HaloTag] AEZ51433.1 Neo [Cloning vector pTcR-HG-Neo-plus]

AEZ51435.1 Neo [Cloning vector pTcR-HG-Neo-minus] AEZ51453.1 Neo [Cloning vector pTcR-GA-Neo-plus] AEZ51455.1 Neo [Cloning vector pTcR-GA-Neo-minus] AEZ51475.1 Neo [Cloning vector pTREXL-Neo] AEZ51490.1 Neo [Cloning vector pTEXL-Neo] QIZ31018.1 neomycin [Cloning vector EF-1]

Length=264

Score = 534 bits (1375), Expect = 0.0, Method: Compositional matrix adjust.

| Identities | = 263/264 (99%), Positives = 264/264 (100%), Gaps = 0/264 (0%) |  |
| --- | --- | --- |
| Query 1 | MIEQDGLHAGSPAAWVERLFGYDWAQQTIGCSDAAVFRLSAQGRPVLFVKTDLSGALNEL | 60 |
| Sbjct 1 | M+EQDGLHAGSPAAWVERLFGYDWAQQTIGCSDAAVFRLSAQGRPVLFVKTDLSGALNEL MLEQDGLHAGSPAAWVERLFGYDWAQQTIGCSDAAVFRLSAQGRPVLFVKTDLSGALNEL | 60 |
| Query 61 | QDEAARLSWLATTGVPCAAVLDVVTEAGRDWLLLGEVPGQDLLSSHLAPAEKVSIMADAM | 120 |
| Sbjct 61 | QDEAARLSWLATTGVPCAAVLDVVTEAGRDWLLLGEVPGQDLLSSHLAPAEKVSIMADAM QDEAARLSWLATTGVPCAAVLDVVTEAGRDWLLLGEVPGQDLLSSHLAPAEKVSIMADAM | 120 |
| Query 121 | RRLHTLDPATCPFDHQAKHRIERARTRMEAGLVDQDDLDEEHQGLAPAELFARLKARMPD | 180 |
| Sbjct 121 | RRLHTLDPATCPFDHQAKHRIERARTRMEAGLVDQDDLDEEHQGLAPAELFARLKARMPD RRLHTLDPATCPFDHQAKHRIERARTRMEAGLVDQDDLDEEHQGLAPAELFARLKARMPD | 180 |
| Query 181 | GEDLVVTHGDACLPNIMVENGRFSGFIDCGRLGVADRYQDIALATRDIAEELGGEWADRF | 240 |
| Sbjct 181 | GEDLVVTHGDACLPNIMVENGRFSGFIDCGRLGVADRYQDIALATRDIAEELGGEWADRF GEDLVVTHGDACLPNIMVENGRFSGFIDCGRLGVADRYQDIALATRDIAEELGGEWADRF | 240 |
| Query 241  Sbjct 241 | LVLYGIAAPDSQRIAFYRLLDEFF 264 LVLYGIAAPDSQRIAFYRLLDEFF LVLYGIAAPDSQRIAFYRLLDEFF 264 |  |

```
>ABX39480.1 ATP synthase beta subunit/neomycin phosphotransferase II fusion [Plastid transformation vector pPRV145C]
ABX39481.1 ATP synthase beta subunit/neomycin phosphotransferase II fusion [Plastid transformation vector pPRV145D]
Length=278

Score = 534 bits (1375), Expect = 0.0, Method: Compositional matrix adjust. Identities = 263/263 (100%), Positives = 263/263 (100%), Gaps = 0/263 (0%)

Query 2	IEQDGLHAGSPAAWVERLFGYDWAQQTIGCSDAAVFRLSAQGRPVLFVKTDLSGALNELQ 61 IEQDGLHAGSPAAWVERLFGYDWAQQTIGCSDAAVFRLSAQGRPVLFVKTDLSGALNELQ
Sbjct 16	IEQDGLHAGSPAAWVERLFGYDWAQQTIGCSDAAVFRLSAQGRPVLFVKTDLSGALNELQ 75

Query 62	DEAARLSWLATTGVPCAAVLDVVTEAGRDWLLLGEVPGQDLLSSHLAPAEKVSIMADAMR 121 DEAARLSWLATTGVPCAAVLDVVTEAGRDWLLLGEVPGQDLLSSHLAPAEKVSIMADAMR
Sbjct 76	DEAARLSWLATTGVPCAAVLDVVTEAGRDWLLLGEVPGQDLLSSHLAPAEKVSIMADAMR 135

Query 122 RLHTLDPATCPFDHQAKHRIERARTRMEAGLVDQDDLDEEHQGLAPAELFARLKARMPDG 181 RLHTLDPATCPFDHQAKHRIERARTRMEAGLVDQDDLDEEHQGLAPAELFARLKARMPDG
Sbjct 136 RLHTLDPATCPFDHQAKHRIERARTRMEAGLVDQDDLDEEHQGLAPAELFARLKARMPDG 195

Query 182 EDLVVTHGDACLPNIMVENGRFSGFIDCGRLGVADRYQDIALATRDIAEELGGEWADRFL 241 EDLVVTHGDACLPNIMVENGRFSGFIDCGRLGVADRYQDIALATRDIAEELGGEWADRFL
Sbjct 196 EDLVVTHGDACLPNIMVENGRFSGFIDCGRLGVADRYQDIALATRDIAEELGGEWADRFL 255

Query 242 VLYGIAAPDSQRIAFYRLLDEFF 264
VLYGIAAPDSQRIAFYRLLDEFF Sbjct 256 VLYGIAAPDSQRIAFYRLLDEFF 278

>QEQ90639.1 3xV5 tagged NeoR marker protein [Cloning vector p57-V5+NeoR] Length=312

Score = 534 bits (1375), Expect = 0.0, Method: Compositional matrix adjust. Identities = 263/263 (100%), Positives = 263/263 (100%), Gaps = 0/263 (0%)
```

| Query | 2 | IEQDGLHAGSPAAWVERLFGYDWAQQTIGCSDAAVFRLSAQGRPVLFVKTDLSGALNELQ | 61 |
| --- | --- | --- | --- |
| Sbjct | 50 | IEQDGLHAGSPAAWVERLFGYDWAQQTIGCSDAAVFRLSAQGRPVLFVKTDLSGALNELQ IEQDGLHAGSPAAWVERLFGYDWAQQTIGCSDAAVFRLSAQGRPVLFVKTDLSGALNELQ | 109 |
| Query | 62 | DEAARLSWLATTGVPCAAVLDVVTEAGRDWLLLGEVPGQDLLSSHLAPAEKVSIMADAMR | 121 |
| Sbjct | 110 | DEAARLSWLATTGVPCAAVLDVVTEAGRDWLLLGEVPGQDLLSSHLAPAEKVSIMADAMR DEAARLSWLATTGVPCAAVLDVVTEAGRDWLLLGEVPGQDLLSSHLAPAEKVSIMADAMR | 169 |
| Query | 122 | RLHTLDPATCPFDHQAKHRIERARTRMEAGLVDQDDLDEEHQGLAPAELFARLKARMPDG | 181 |
| Sbjct | 170 | RLHTLDPATCPFDHQAKHRIERARTRMEAGLVDQDDLDEEHQGLAPAELFARLKARMPDG RLHTLDPATCPFDHQAKHRIERARTRMEAGLVDQDDLDEEHQGLAPAELFARLKARMPDG | 229 |
| Query | 182 | EDLVVTHGDACLPNIMVENGRFSGFIDCGRLGVADRYQDIALATRDIAEELGGEWADRFL | 241 |
| Sbjct | 230 | EDLVVTHGDACLPNIMVENGRFSGFIDCGRLGVADRYQDIALATRDIAEELGGEWADRFL EDLVVTHGDACLPNIMVENGRFSGFIDCGRLGVADRYQDIALATRDIAEELGGEWADRFL | 289 |
| Query  Sbjct | 242  290 | VLYGIAAPDSQRIAFYRLLDEFF 264 VLYGIAAPDSQRIAFYRLLDEFF VLYGIAAPDSQRIAFYRLLDEFF 312 |  |

>AAO21931.1 aminoglycoside-3'-O-phosphotransferase [Expression vector pYPX251] Length=264

Score = 534 bits (1375), Expect = 0.0, Method: Compositional matrix adjust.

| Identities | = 262/264 (99%), Positives = 264/264 (100%), Gaps = 0/264 (0%) |  |
| --- | --- | --- |
| Query 1 | MIEQDGLHAGSPAAWVERLFGYDWAQQTIGCSDAAVFRLSAQGRPVLFVKTDLSGALNEL | 60 |
| Sbjct 1 | M+EQDGLHAGSPAAWVERLFGYDWAQQTIGCSDAAVFRLSAQGRPVLFVKTDLSGALNEL MVEQDGLHAGSPAAWVERLFGYDWAQQTIGCSDAAVFRLSAQGRPVLFVKTDLSGALNEL | 60 |
| Query 61 | QDEAARLSWLATTGVPCAAVLDVVTEAGRDWLLLGEVPGQDLLSSHLAPAEKVSIMADAM | 120 |
| Sbjct 61 | QDEAARLSWLATTGVPCAAVLDVVTEAGRDWLLLGEVPGQDLLSSHLAPAEKVSIMADAM QDEAARLSWLATTGVPCAAVLDVVTEAGRDWLLLGEVPGQDLLSSHLAPAEKVSIMADAM | 120 |
| Query 121 | RRLHTLDPATCPFDHQAKHRIERARTRMEAGLVDQDDLDEEHQGLAPAELFARLKARMPD | 180 |
| Sbjct 121 | RRLHTLDPATCP+DHQAKHRIERARTRMEAGLVDQDDLDEEHQGLAPAELFARLKARMPD RRLHTLDPATCPYDHQAKHRIERARTRMEAGLVDQDDLDEEHQGLAPAELFARLKARMPD | 180 |
| Query 181 | GEDLVVTHGDACLPNIMVENGRFSGFIDCGRLGVADRYQDIALATRDIAEELGGEWADRF | 240 |
| Sbjct 181 | GEDLVVTHGDACLPNIMVENGRFSGFIDCGRLGVADRYQDIALATRDIAEELGGEWADRF GEDLVVTHGDACLPNIMVENGRFSGFIDCGRLGVADRYQDIALATRDIAEELGGEWADRF | 240 |
| Query 241  Sbjct 241 | LVLYGIAAPDSQRIAFYRLLDEFF 264 LVLYGIAAPDSQRIAFYRLLDEFF LVLYGIAAPDSQRIAFYRLLDEFF 264 |  |

>WP_165472676.1 APH(3')-II family aminoglycoside O-phosphotransferase [Escherichia coli]

Length=264

Score = 533 bits (1374), Expect = 0.0, Method: Compositional matrix adjust.

| Identities | = 263/264 (99%), Positives = 263/264 (99%), Gaps = 0/264 (0%) |  |
| --- | --- | --- |
| Query 1 | MIEQDGLHAGSPAAWVERLFGYDWAQQTIGCSDAAVFRLSAQGRPVLFVKTDLSGALNEL | 60 |
| Sbjct 1 | MIEQDGLHAGSPAAWVERLFGYDWAQQTIGCSDA VFRLSAQGRPVLFVKTDLSGALNEL MIEQDGLHAGSPAAWVERLFGYDWAQQTIGCSDATVFRLSAQGRPVLFVKTDLSGALNEL | 60 |
| Query 61 | QDEAARLSWLATTGVPCAAVLDVVTEAGRDWLLLGEVPGQDLLSSHLAPAEKVSIMADAM | 120 |
| Sbjct 61 | QDEAARLSWLATTGVPCAAVLDVVTEAGRDWLLLGEVPGQDLLSSHLAPAEKVSIMADAM QDEAARLSWLATTGVPCAAVLDVVTEAGRDWLLLGEVPGQDLLSSHLAPAEKVSIMADAM | 120 |
| Query 121 | RRLHTLDPATCPFDHQAKHRIERARTRMEAGLVDQDDLDEEHQGLAPAELFARLKARMPD | 180 |
| Sbjct 121 | RRLHTLDPATCPFDHQAKHRIERARTRMEAGLVDQDDLDEEHQGLAPAELFARLKARMPD RRLHTLDPATCPFDHQAKHRIERARTRMEAGLVDQDDLDEEHQGLAPAELFARLKARMPD | 180 |
| Query 181 | GEDLVVTHGDACLPNIMVENGRFSGFIDCGRLGVADRYQDIALATRDIAEELGGEWADRF | 240 |
| Sbjct 181 | GEDLVVTHGDACLPNIMVENGRFSGFIDCGRLGVADRYQDIALATRDIAEELGGEWADRF  GEDLVVTHGDACLPNIMVENGRFSGFIDCGRLGVADRYQDIALATRDIAEELGGEWADRF | 240 |

Query 241 LVLYGIAAPDSQRIAFYRLLDEFF 264 LVLYGIAAPDSQRIAFYRLLDEFF

Sbjct 241 LVLYGIAAPDSQRIAFYRLLDEFF 264

>EEV6129758.1 APH(3')-II family aminoglycoside O-phosphotransferase [Escherichia coli]

Length=264

Score = 533 bits (1374), Expect = 0.0, Method: Compositional matrix adjust.

| Identities | = 263/264 (99%), Positives = 263/264 (99%), Gaps = 0/264 (0%) |  |
| --- | --- | --- |
| Query 1 | MIEQDGLHAGSPAAWVERLFGYDWAQQTIGCSDAAVFRLSAQGRPVLFVKTDLSGALNEL | 60 |
| Sbjct 1 | MIEQDGLHAGSPAAWVERLFGYDWAQQTIGCSDAAVFRLSAQGRPVLFVKTDLSGALNEL MIEQDGLHAGSPAAWVERLFGYDWAQQTIGCSDAAVFRLSAQGRPVLFVKTDLSGALNEL | 60 |
| Query 61 | QDEAARLSWLATTGVPCAAVLDVVTEAGRDWLLLGEVPGQDLLSSHLAPAEKVSIMADAM | 120 |
| Sbjct 61 | QDEAARLSWLATTGVPCAAVLDVVTEAGRDWLLLGEVPGQDLLSSHLAPAEKVSIMADAM QDEAARLSWLATTGVPCAAVLDVVTEAGRDWLLLGEVPGQDLLSSHLAPAEKVSIMADAM | 120 |
| Query 121 | RRLHTLDPATCPFDHQAKHRIERARTRMEAGLVDQDDLDEEHQGLAPAELFARLKARMPD | 180 |
| Sbjct 121 | RRLHTLDPATCPFDHQAKHRIER RTRMEAGLVDQDDLDEEHQGLAPAELFARLKARMPD RRLHTLDPATCPFDHQAKHRIERTRTRMEAGLVDQDDLDEEHQGLAPAELFARLKARMPD | 180 |
| Query 181 | GEDLVVTHGDACLPNIMVENGRFSGFIDCGRLGVADRYQDIALATRDIAEELGGEWADRF | 240 |
| Sbjct 181 | GEDLVVTHGDACLPNIMVENGRFSGFIDCGRLGVADRYQDIALATRDIAEELGGEWADRF GEDLVVTHGDACLPNIMVENGRFSGFIDCGRLGVADRYQDIALATRDIAEELGGEWADRF | 240 |
| Query 241  Sbjct 241 | LVLYGIAAPDSQRIAFYRLLDEFF 264 LVLYGIAAPDSQRIAFYRLLDEFF LVLYGIAAPDSQRIAFYRLLDEFF 264 |  |

>EAY7200867.1 APH(3')-II family aminoglycoside O-phosphotransferase [Salmonella enterica]

Length=264

Score = 533 bits (1374), Expect = 0.0, Method: Compositional matrix adjust.

| Identities | = 263/264 (99%), Positives = 263/264 (99%), Gaps = 0/264 (0%) |  |
| --- | --- | --- |
| Query 1 | MIEQDGLHAGSPAAWVERLFGYDWAQQTIGCSDAAVFRLSAQGRPVLFVKTDLSGALNEL | 60 |
| Sbjct 1 | MIEQDGLHAGSPAAWVERLFGYDWAQQTIGCSDAAVFRLSAQGRPVLFVKTDLSGALNEL MIEQDGLHAGSPAAWVERLFGYDWAQQTIGCSDAAVFRLSAQGRPVLFVKTDLSGALNEL | 60 |
| Query 61 | QDEAARLSWLATTGVPCAAVLDVVTEAGRDWLLLGEVPGQDLLSSHLAPAEKVSIMADAM | 120 |
| Sbjct 61 | QDEAARLSWL TTGVPCAAVLDVVTEAGRDWLLLGEVPGQDLLSSHLAPAEKVSIMADAM QDEAARLSWLTTTGVPCAAVLDVVTEAGRDWLLLGEVPGQDLLSSHLAPAEKVSIMADAM | 120 |
| Query 121 | RRLHTLDPATCPFDHQAKHRIERARTRMEAGLVDQDDLDEEHQGLAPAELFARLKARMPD | 180 |
| Sbjct 121 | RRLHTLDPATCPFDHQAKHRIERARTRMEAGLVDQDDLDEEHQGLAPAELFARLKARMPD RRLHTLDPATCPFDHQAKHRIERARTRMEAGLVDQDDLDEEHQGLAPAELFARLKARMPD | 180 |
| Query 181 | GEDLVVTHGDACLPNIMVENGRFSGFIDCGRLGVADRYQDIALATRDIAEELGGEWADRF | 240 |
| Sbjct 181 | GEDLVVTHGDACLPNIMVENGRFSGFIDCGRLGVADRYQDIALATRDIAEELGGEWADRF GEDLVVTHGDACLPNIMVENGRFSGFIDCGRLGVADRYQDIALATRDIAEELGGEWADRF | 240 |
| Query 241  Sbjct 241 | LVLYGIAAPDSQRIAFYRLLDEFF 264 LVLYGIAAPDSQRIAFYRLLDEFF LVLYGIAAPDSQRIAFYRLLDEFF 264 |  |

```
>EFH5635854.1 APH(3')-II family aminoglycoside O-phosphotransferase [Escherichia coli]
Length=264

Score = 533 bits (1374), Expect = 0.0, Method: Compositional matrix adjust. Identities = 263/264 (99%), Positives = 264/264 (100%), Gaps = 0/264 (0%)

Query 1	MIEQDGLHAGSPAAWVERLFGYDWAQQTIGCSDAAVFRLSAQGRPVLFVKTDLSGALNEL 60 MIEQDGLHAGSPAAWVERLFGYDWAQQTIGCSDAAVFRLSAQGRPVLFVKTDLSGALNEL
Sbjct 1	MIEQDGLHAGSPAAWVERLFGYDWAQQTIGCSDAAVFRLSAQGRPVLFVKTDLSGALNEL 60
```

| Query | 61 | QDEAARLSWLATTGVPCAAVLDVVTEAGRDWLLLGEVPGQDLLSSHLAPAEKVSIMADAM | 120 |
| --- | --- | --- | --- |
| Sbjct | 61 | QDEAARLSWLATTGVPCAAVLDVVTEAGRDWLLLGEVPGQDLLSSHLAPAEKVSIMADAM QDEAARLSWLATTGVPCAAVLDVVTEAGRDWLLLGEVPGQDLLSSHLAPAEKVSIMADAM | 120 |
| Query | 121 | RRLHTLDPATCPFDHQAKHRIERARTRMEAGLVDQDDLDEEHQGLAPAELFARLKARMPD | 180 |
| Sbjct | 121 | RRLHTLDPATCPFDHQAKHRIERARTRMEAGLVDQDDLDEEHQGLAPAELFARLKARMPD RRLHTLDPATCPFDHQAKHRIERARTRMEAGLVDQDDLDEEHQGLAPAELFARLKARMPD | 180 |
| Query | 181 | GEDLVVTHGDACLPNIMVENGRFSGFIDCGRLGVADRYQDIALATRDIAEELGGEWADRF | 240 |
| Sbjct | 181 | G+DLVVTHGDACLPNIMVENGRFSGFIDCGRLGVADRYQDIALATRDIAEELGGEWADRF GKDLVVTHGDACLPNIMVENGRFSGFIDCGRLGVADRYQDIALATRDIAEELGGEWADRF | 240 |
| Query  Sbjct | 241  241 | LVLYGIAAPDSQRIAFYRLLDEFF 264 LVLYGIAAPDSQRIAFYRLLDEFF LVLYGIAAPDSQRIAFYRLLDEFF 264 |  |

>WP_196744400.1 APH(3')-II family aminoglycoside O-phosphotransferase [Pseudomonas aeruginosa]

MBG5567909.1 APH(3')-II family aminoglycoside O-phosphotransferase [Pseudomonas aeruginosa]

Length=264

Score = 533 bits (1374), Expect = 0.0, Method: Compositional matrix adjust.

| Identities | = 263/264 (99%), Positives = 263/264 (99%), Gaps = 0/264 (0%) |  |
| --- | --- | --- |
| Query 1 | MIEQDGLHAGSPAAWVERLFGYDWAQQTIGCSDAAVFRLSAQGRPVLFVKTDLSGALNEL | 60 |
| Sbjct 1 | MIEQDGLHAGSPAAWVERLFGYDWAQQTIGCSDAAVFRLSAQGRPVLFVKTDLSGALNEL MIEQDGLHAGSPAAWVERLFGYDWAQQTIGCSDAAVFRLSAQGRPVLFVKTDLSGALNEL | 60 |
| Query 61 | QDEAARLSWLATTGVPCAAVLDVVTEAGRDWLLLGEVPGQDLLSSHLAPAEKVSIMADAM | 120 |
| Sbjct 61 | QDEAARLSWLATT VPCAAVLDVVTEAGRDWLLLGEVPGQDLLSSHLAPAEKVSIMADAM QDEAARLSWLATTSVPCAAVLDVVTEAGRDWLLLGEVPGQDLLSSHLAPAEKVSIMADAM | 120 |
| Query 121 | RRLHTLDPATCPFDHQAKHRIERARTRMEAGLVDQDDLDEEHQGLAPAELFARLKARMPD | 180 |
| Sbjct 121 | RRLHTLDPATCPFDHQAKHRIERARTRMEAGLVDQDDLDEEHQGLAPAELFARLKARMPD RRLHTLDPATCPFDHQAKHRIERARTRMEAGLVDQDDLDEEHQGLAPAELFARLKARMPD | 180 |
| Query 181 | GEDLVVTHGDACLPNIMVENGRFSGFIDCGRLGVADRYQDIALATRDIAEELGGEWADRF | 240 |
| Sbjct 181 | GEDLVVTHGDACLPNIMVENGRFSGFIDCGRLGVADRYQDIALATRDIAEELGGEWADRF GEDLVVTHGDACLPNIMVENGRFSGFIDCGRLGVADRYQDIALATRDIAEELGGEWADRF | 240 |
| Query 241  Sbjct 241 | LVLYGIAAPDSQRIAFYRLLDEFF 264 LVLYGIAAPDSQRIAFYRLLDEFF LVLYGIAAPDSQRIAFYRLLDEFF 264 |  |

>WP_104730796.1 APH(3')-II family aminoglycoside O-phosphotransferase [Escherichia coli]

PPY57655.1 APH(3')-II family aminoglycoside O-phosphotransferase [Escherichia coli]

Length=264

Score = 533 bits (1374), Expect = 0.0, Method: Compositional matrix adjust.

| Identities | = 263/264 (99%), Positives = 263/264 (99%), Gaps = 0/264 (0%) |  |
| --- | --- | --- |
| Query 1 | MIEQDGLHAGSPAAWVERLFGYDWAQQTIGCSDAAVFRLSAQGRPVLFVKTDLSGALNEL | 60 |
| Sbjct 1 | MIEQDGLHAGSPAAWVERLFGYDWAQQTIGCSDAAVFRLSAQGRPVLFVKTDLS ALNEL MIEQDGLHAGSPAAWVERLFGYDWAQQTIGCSDAAVFRLSAQGRPVLFVKTDLSSALNEL | 60 |
| Query 61 | QDEAARLSWLATTGVPCAAVLDVVTEAGRDWLLLGEVPGQDLLSSHLAPAEKVSIMADAM | 120 |
| Sbjct 61 | QDEAARLSWLATTGVPCAAVLDVVTEAGRDWLLLGEVPGQDLLSSHLAPAEKVSIMADAM QDEAARLSWLATTGVPCAAVLDVVTEAGRDWLLLGEVPGQDLLSSHLAPAEKVSIMADAM | 120 |
| Query 121 | RRLHTLDPATCPFDHQAKHRIERARTRMEAGLVDQDDLDEEHQGLAPAELFARLKARMPD | 180 |
| Sbjct 121 | RRLHTLDPATCPFDHQAKHRIERARTRMEAGLVDQDDLDEEHQGLAPAELFARLKARMPD RRLHTLDPATCPFDHQAKHRIERARTRMEAGLVDQDDLDEEHQGLAPAELFARLKARMPD | 180 |
| Query 181 | GEDLVVTHGDACLPNIMVENGRFSGFIDCGRLGVADRYQDIALATRDIAEELGGEWADRF | 240 |
| Sbjct 181 | GEDLVVTHGDACLPNIMVENGRFSGFIDCGRLGVADRYQDIALATRDIAEELGGEWADRF  GEDLVVTHGDACLPNIMVENGRFSGFIDCGRLGVADRYQDIALATRDIAEELGGEWADRF | 240 |

Query 241 LVLYGIAAPDSQRIAFYRLLDEFF 264 LVLYGIAAPDSQRIAFYRLLDEFF

Sbjct 241 LVLYGIAAPDSQRIAFYRLLDEFF 264

>EBO5400315.1 APH(3')-II family aminoglycoside O-phosphotransferase [Salmonella enterica subsp. enterica serovar Heidelberg]

Length=264

Score = 533 bits (1374), Expect = 0.0, Method: Compositional matrix adjust.

| Identities | = 263/264 (99%), Positives = 263/264 (99%), Gaps = 0/264 (0%) |  |
| --- | --- | --- |
| Query 1 | MIEQDGLHAGSPAAWVERLFGYDWAQQTIGCSDAAVFRLSAQGRPVLFVKTDLSGALNEL | 60 |
| Sbjct 1 | MIEQDGLHAGSPAAWVERLFGYDWAQQTIGCSDAAVFRLSAQGRPVLFVKTDLSGALNEL MIEQDGLHAGSPAAWVERLFGYDWAQQTIGCSDAAVFRLSAQGRPVLFVKTDLSGALNEL | 60 |
| Query 61 | QDEAARLSWLATTGVPCAAVLDVVTEAGRDWLLLGEVPGQDLLSSHLAPAEKVSIMADAM | 120 |
| Sbjct 61 | QDEAARLSWLATTGVPCAAVLDVVTEAGRDWLLLGEVPGQDLLSSHLAPAEKVSIMADAM QDEAARLSWLATTGVPCAAVLDVVTEAGRDWLLLGEVPGQDLLSSHLAPAEKVSIMADAM | 120 |
| Query 121 | RRLHTLDPATCPFDHQAKHRIERARTRMEAGLVDQDDLDEEHQGLAPAELFARLKARMPD | 180 |
| Sbjct 121 | RRLHTLDPATCPFDHQ KHRIERARTRMEAGLVDQDDLDEEHQGLAPAELFARLKARMPD RRLHTLDPATCPFDHQTKHRIERARTRMEAGLVDQDDLDEEHQGLAPAELFARLKARMPD | 180 |
| Query 181 | GEDLVVTHGDACLPNIMVENGRFSGFIDCGRLGVADRYQDIALATRDIAEELGGEWADRF | 240 |
| Sbjct 181 | GEDLVVTHGDACLPNIMVENGRFSGFIDCGRLGVADRYQDIALATRDIAEELGGEWADRF GEDLVVTHGDACLPNIMVENGRFSGFIDCGRLGVADRYQDIALATRDIAEELGGEWADRF | 240 |
| Query 241  Sbjct 241 | LVLYGIAAPDSQRIAFYRLLDEFF 264 LVLYGIAAPDSQRIAFYRLLDEFF LVLYGIAAPDSQRIAFYRLLDEFF 264 |  |

>EDA3939420.1 APH(3')-II family aminoglycoside O-phosphotransferase [Salmonella enterica subsp. enterica serovar Heidelberg]

Length=264

Score = 533 bits (1374), Expect = 0.0, Method: Compositional matrix adjust.

| Identities | = 263/264 (99%), Positives = 264/264 (100%), Gaps = 0/264 (0%) |  |
| --- | --- | --- |
| Query 1 | MIEQDGLHAGSPAAWVERLFGYDWAQQTIGCSDAAVFRLSAQGRPVLFVKTDLSGALNEL | 60 |
| Sbjct 1 | MIEQDGLHAGSPAAWVERLFGYDWAQQTIGCSDAAVFRLSAQGRPVLFVKTDLSGALNEL MIEQDGLHAGSPAAWVERLFGYDWAQQTIGCSDAAVFRLSAQGRPVLFVKTDLSGALNEL | 60 |
| Query 61 | QDEAARLSWLATTGVPCAAVLDVVTEAGRDWLLLGEVPGQDLLSSHLAPAEKVSIMADAM | 120 |
| Sbjct 61 | QDEAARLSWLATTGVPCAAVLDVVTEAGRDWLLLGEVPGQDLLSSHLAPA+KVSIMADAM QDEAARLSWLATTGVPCAAVLDVVTEAGRDWLLLGEVPGQDLLSSHLAPAKKVSIMADAM | 120 |
| Query 121 | RRLHTLDPATCPFDHQAKHRIERARTRMEAGLVDQDDLDEEHQGLAPAELFARLKARMPD | 180 |
| Sbjct 121 | RRLHTLDPATCPFDHQAKHRIERARTRMEAGLVDQDDLDEEHQGLAPAELFARLKARMPD RRLHTLDPATCPFDHQAKHRIERARTRMEAGLVDQDDLDEEHQGLAPAELFARLKARMPD | 180 |
| Query 181 | GEDLVVTHGDACLPNIMVENGRFSGFIDCGRLGVADRYQDIALATRDIAEELGGEWADRF | 240 |
| Sbjct 181 | GEDLVVTHGDACLPNIMVENGRFSGFIDCGRLGVADRYQDIALATRDIAEELGGEWADRF GEDLVVTHGDACLPNIMVENGRFSGFIDCGRLGVADRYQDIALATRDIAEELGGEWADRF | 240 |
| Query 241  Sbjct 241 | LVLYGIAAPDSQRIAFYRLLDEFF 264 LVLYGIAAPDSQRIAFYRLLDEFF LVLYGIAAPDSQRIAFYRLLDEFF 264 |  |

```
>EDX9152563.1 APH(3')-II family aminoglycoside O-phosphotransferase [Salmonella enterica subsp. enterica serovar 4,[5],12:i:-]
Length=264

Score = 533 bits (1373), Expect = 0.0, Method: Compositional matrix adjust. Identities = 263/264 (99%), Positives = 263/264 (99%), Gaps = 0/264 (0%)

Query 1	MIEQDGLHAGSPAAWVERLFGYDWAQQTIGCSDAAVFRLSAQGRPVLFVKTDLSGALNEL 60 MIEQDGLHAGSPAAWVERLFGYDWAQQTIGCSDAAVFRLSAQGRPVLFV TDLSGALNEL
Sbjct 1	MIEQDGLHAGSPAAWVERLFGYDWAQQTIGCSDAAVFRLSAQGRPVLFVNTDLSGALNEL 60
```

| Query | 61 | QDEAARLSWLATTGVPCAAVLDVVTEAGRDWLLLGEVPGQDLLSSHLAPAEKVSIMADAM | 120 |
| --- | --- | --- | --- |
| Sbjct | 61 | QDEAARLSWLATTGVPCAAVLDVVTEAGRDWLLLGEVPGQDLLSSHLAPAEKVSIMADAM QDEAARLSWLATTGVPCAAVLDVVTEAGRDWLLLGEVPGQDLLSSHLAPAEKVSIMADAM | 120 |
| Query | 121 | RRLHTLDPATCPFDHQAKHRIERARTRMEAGLVDQDDLDEEHQGLAPAELFARLKARMPD | 180 |
| Sbjct | 121 | RRLHTLDPATCPFDHQAKHRIERARTRMEAGLVDQDDLDEEHQGLAPAELFARLKARMPD RRLHTLDPATCPFDHQAKHRIERARTRMEAGLVDQDDLDEEHQGLAPAELFARLKARMPD | 180 |
| Query | 181 | GEDLVVTHGDACLPNIMVENGRFSGFIDCGRLGVADRYQDIALATRDIAEELGGEWADRF | 240 |
| Sbjct | 181 | GEDLVVTHGDACLPNIMVENGRFSGFIDCGRLGVADRYQDIALATRDIAEELGGEWADRF GEDLVVTHGDACLPNIMVENGRFSGFIDCGRLGVADRYQDIALATRDIAEELGGEWADRF | 240 |
| Query  Sbjct | 241  241 | LVLYGIAAPDSQRIAFYRLLDEFF 264 LVLYGIAAPDSQRIAFYRLLDEFF LVLYGIAAPDSQRIAFYRLLDEFF 264 |  |

>WP_163800230.1 APH(3')-II family aminoglycoside O-phosphotransferase [Pantoea agglomerans]

AAC53707.1 neomycin phosphotransferase [Cloning vector pBI101] AAC53708.1 neomycin phosphotransferase [Cloning vector pBin19] AAC53711.1 neomycin phosphotransferse [Cloning vector pdeltagusBin19]

AAK08506.1 neomycin phosphoratransferase [Plant DNA excision vector pX6-GFP] AAK29771.1 kanamycin resistance protein [YTT vector B]

AAK29775.1 kanamycin resistance protein [YTT vector D] AAL92039.1 neomycin phosphotransferase II [Binary vector pBI121]

AAM19765.1 neomycin phosphotransferase [Cloning Vector pBANF-bar] AAR17784.1 neomycin phosphotransferase II [Cloning vector pZGA22] AAV51261.1 neomycin phosphotransferase II [Expression vector pBI121_pro] AAV97905.1 nptII [Kanamycin resistance plant expression vector pSAT4-nptII]

AAX97771.1 neomycin phosphotransferase II [Binary vector pBin19 35S YFP-attR] AAX97777.1 neomycin phosphotransferase II [Binary vector pBin19 35S attR-YFP] AAY25363.1 NptII [Cloning vector pSAT1A-ocsAocsP-nptII-ocsT]

AAY25389.1 NptII [Cloning vector pRCS2-ocs-nptII]

ABG29429.1 neomycin phosphotransferase [Reporter vector pBNP1] ABN59460.1 NptII [Binary vector pSITE-4CA]

ABN59464.1 NptII [Binary vector pSITE-3CA] ABN59468.1 NptII [Binary vector pSITE-2CA] ABN59472.1 NptII [Binary vector pSITE-4NA] ABN59476.1 NptII [Binary vector pSITE-2NB] ABN59480.1 NptII [Binary vector pSITE-1NB] ABN59484.1 NptII [Binary vector pSITE-4NB] ABN59488.1 NptII [Binary vector pSITE-2NA] ABN59491.1 NptII [Binary vector pSITE-0A] ABN59494.1 NptII [Binary vector pSITE-0B] ABN59498.1 NptII [Binary vector pSITE-1CA]

ABY41255.1 neomycin phosphotransferase II [Binary vector pBLNSC] ABY50072.1 neomycin phosphotransferase II [Binary vector pBLOXNPT] ABY50074.1 neomycin phosphotransferase II [Binary vector pBCRE] ABY83638.1 aminoglycoside-3'-O-phosphotransferase [Shuttle vector pZJY41]

ABY83641.1 aminoglycoside-3'-O-phosphotransferase [Shuttle vector pZJY156] ACI16380.1 neomycin phosphotransferase [Transformation vector pBISN1-IN] ACV49927.1 neomycin phosphotransferase II [Binary vector pEAQselectK] ACV49930.1 neomycin phosphotransferase II [Binary vector pEAQspecialK] ACV49934.1 neomycin phosphotransferase II [Binary vector pEAQspecialKm] ACV49939.1 neomycin phosphotransferase II [Binary vector pEAQ-HT] ACV49942.1 neomycin phosphotransferase II [Binary vector pEAQ-HT-DEST1] ACV49947.1 neomycin phosphotransferase II [Binary vector pEAQ-HT-DEST2] ACV49952.1 neomycin phosphotransferase II [Binary vector pEAQ-HT-DEST3] ADC42102.1 NptII [Cloning vector pTDuExAn6]

ADE08583.1 nptII [Binary vector pBIB-KAN]

ADE48813.1 NptII [Plant transformation vector pSITE BiFC nEYFP-N1] ADE48817.1 NptII [Plant transformation vector pSITE BiFC cEYFP-N1] ADE48821.1 NptII [Plant transformation vector pSITEII-7N1] ADE48825.1 NptII [Plant transformation vector pSITE BiFC nEYFP-C1] ADE48829.1 NptII [Plant transformation vector pSITE BiFC cEFYP C1] ADE48833.1 NptII [Plant transformation vector pSITEII-1C1] ADE48837.1 NptII [Plant transformation vector pSITEII-2C1] ADE48841.1 NptII [Plant transformation vector pSITEII-4C1] ADE48845.1 NptII [Plant transformation vector pSITEII-5C1] ADE48849.1 NptII [Plant transformation vector pSITEII-6C1] ADE48853.1 NptII [Plant transformation vector pSITEII-7C1]

ADE48857.1 NptII [Plant transformation vector pSITEII-8C1] ADM88876.1 neomycin phosphotransferase [Cloning vector pTcYFPN] ADM88877.1 neomycin phosphotransferase [Cloning vector pTcMYCN] ADM88878.1 neomycin phosphotransferase [Cloning vector pTcTAPN] ADM88879.1 neomycin phosphotransferase [Cloning vector pTcGFPN] ADM88881.1 neomycin phosphotransferase [Cloning vector pTcCFPN] ADM88882.1 neomycin phosphotransferase [Cloning vector pTc6HN]

ADN51986.1 neomycin phosphotransferase II [Binary vector pBI121-ELEMENTS] AGK36279.1 neomycin phosphotransferase [Expression vector pCL0143] AGK36282.1 neomycin phosphotransferase [Expression vector pCL0153] AGK36284.1 neomycin phosphotransferase [Expression vector pCL0154] AGK36286.1 neomycin phosphotransferase [Expression vector pCL0160] AGK36288.1 neomycin phosphotransferase [Expression vector pCL0161] AGO05979.1 neomycin phosphotransferase II [Gateway binary vector pGWB470] AGS44632.1 NPTII [Ti plasmid binary vector pGH00.0126]

AGS44636.1 NPTII [Ti binary plasmid pGH00.0131] AHI88625.1 NPTII [Ti plasmid binary vector pGZ12.0501] AHL24862.1 NPTII [Binary Vector pGZ12.0108]

AIG59962.1 NptII [Cloning vector pAM1956]

AKR52854.1 NPTII [Ti plasmid binary vector pGZ12.0224] AKR52859.1 NPTII [Ti plasmid binary vector pGZ12.0106]

AKT95044.1 neomycin phosphotransferase [Protein fusion vector pTcGFPN-CO 1.1]

AKT95045.1 neomycin phosphotransferase [Protein fusion vector pTcGFPN-NH 1.1]

AKT95046.1 neomycin phosphotransferase [Protein fusion vector pTcProtCN-CO 1.1]

AKT95047.1 neomycin phosphotransferase [Protein fusion vector pTcProtCN-NH 1.1]

AKT95048.1 neomycin phosphotransferase [Protein fusion vector pTcPTPN-CO 1.1]

AKT95049.1 neomycin phosphotransferase [Protein fusion vector pTcPTPN-NH 1.1]

AQT31662.1 NptII [synthetic construct] [Nicotiana benthamiana] AQZ40908.1 neomycin phosphotransferase II [Cloning vector pEff] ASV51143.1 NPTII [Ti plasmid binary vector pGZ13.0313] QDF60591.1 neomycin phosphotransferase II [Binary vector pBI-Xeg] QEY10702.1 neomycin phosphotransferase [Cloning vector pHRE-EV] QEY10704.1 neomycin phosphotransferase [Cloning vector pHREAC-EV]

QOE83628.1 neomycin phosphotransferase [Cloning vector pTcHAN-CO 2.0] QOE83629.1 neomycin phosphotransferase [Cloning vector pTcHAN-NH 2.0] QOE83630.1 neomycin phosphotransferase [Cloning vector pTcFlagN-CO 2.0] QOE83631.1 neomycin phosphotransferase [Cloning vector pTcFlagN-NH 2.0] QOE83632.1 neomycin phosphotransferase [Cloning vector pTcStrepN-CO 2.0] QOE83633.1 neomycin phosphotransferase [Cloning vector pTcStrepN-NH 2.0] QOE83634.1 neomycin phosphotransferase [Cloning vector pTcGFPN-CO 2.0] QOE83635.1 neomycin phosphotransferase [Cloning vector pTcGFPN-NH 2.0] QOE83636.1 neomycin phosphotransferase [Cloning vector pTcmKateN-CO 2.0] QOE83637.1 neomycin phosphotransferase [Cloning vector pTcmKateN-NH 2.0] QOE83638.1 neomycin phosphotransferase [Cloning vector pTcTy1N-CO 2.0] QOE83639.1 neomycin phosphotransferase [Cloning vector pTcTy1N-NH 2.0] QOE83708.1 neomycin phosphotransferase II [Binary vector pRATIO2151] QOE83714.1 neomycin phosphotransferase II [Binary vector pRATIO2131] QOE83721.1 neomycin phosphotransferase II [Binary vector pRATIO2112] QOE83728.1 neomycin phosphotransferase II [Binary vector pRATIO2214] QOE83735.1 neomycin phosphotransferase II [Binary vector pRATIO2251] QOE83742.1 neomycin phosphotransferase II [Binary vector pRATIO2231] QOE83749.1 neomycin phosphotransferase II [Binary vector pRATIO2212] QOE83756.1 neomycin phosphotransferase II [Binary vector pRATIO1151] QOE83763.1 neomycin phosphotransferase II [Binary vector pRATIO1251] QOE83770.1 neomycin phosphotransferase II [Binary vector pRATIO1131] QOE83777.1 neomycin phosphotransferase II [Binary vector pRATIO1112] QOE83784.1 neomycin phosphotransferase II [Binary vector pRATIO1212] QOE83791.1 neomycin phosphotransferase II [Binary vector pRATIO1267] QOE83798.1 neomycin phosphotransferase II [Binary vector pRATIO4214] QOE83804.1 neomycin phosphotransferase II [Binary vector pRATIO4231] QOE83810.1 neomycin phosphotransferase II [Binary vector pRATIO4212] QOE83816.1 neomycin phosphotransferase II [Binary vector pRATIO3212] QOE83822.1 neomycin phosphotransferase II [Binary vector pRATIO3267] CCD28593.1 neomicin phospho-transferase [Cloning vector pAJM-EPRV] BAF44946.1 neomycin phosphotransferase II [Gateway binary vector pGWB1] BAF44950.1 neomycin phosphotransferase II [Gateway binary vector pGWB2]

BAF44954.1 neomycin phosphotransferase II [Gateway binary vector pGWB3] BAF44959.1 neomycin phosphotransferase II [Gateway binary vector pGWB4] BAF44969.1 neomycin phosphotransferase II [Gateway binary vector pGWB6] BAF44973.1 neomycin phosphotransferase II [Gateway binary vector pGWB7] BAF44977.1 neomycin phosphotransferase II [Gateway binary vector pGWB8] BAF44981.1 neomycin phosphotransferase II [Gateway binary vector pGWB9] BAF44985.1 neomycin phosphotransferase II [Gateway binary vector pGWB10] BAF44989.1 neomycin phosphotransferase II [Gateway binary vector pGWB11] BAF44993.1 neomycin phosphotransferase II [Gateway binary vector pGWB12] BAF44997.1 neomycin phosphotransferase II [Gateway binary vector pGWB13] BAF45001.1 neomycin phosphotransferase II [Gateway binary vector pGWB14] BAF45005.1 neomycin phosphotransferase II [Gateway binary vector pGWB15] BAF45009.1 neomycin phosphotransferase II [Gateway binary vector pGWB16] BAF45013.1 neomycin phosphotransferase II [Gateway binary vector pGWB17] BAF45017.1 neomycin phosphotransferase II [Gateway binary vector pGWB18] BAF45021.1 neomycin phosphotransferase II [Gateway binary vector pGWB19] BAF45025.1 neomycin phosphotransferase II [Gateway binary vector pGWB20] BAF45029.1 neomycin phosphotransferase II [Gateway binary vector pGWB21] BAF45033.1 neomycin phosphotransferase II [Gateway binary vector pGWB22] BAF45037.1 neomycin phosphotransferase II [Gateway binary vector pGWB23] BAF45041.1 neomycin phosphotransferase II [Gateway binary vector pGWB24] BAF45045.1 neomycin phosphotransferase II [Gateway binary vector pGWB25] BAF45049.1 neomycin phosphotransferase II [Gateway binary vector pGWB26] BAF45053.1 neomycin phosphotransferase II [Gateway binary vector pGWB27] BAF45057.1 neomycin phosphotransferase II [Gateway binary vector pGWB28] BAF45061.1 neomycin phosphotransferase II [Gateway binary vector pGWB29] BAF45065.1 neomycin phosphotransferase II [Gateway binary vector pGWB35] BAF45070.1 neomycin phosphotransferase II [Gateway binary vector pGWB40] BAF45074.1 neomycin phosphotransferase II [Gateway binary vector pGWB41] BAF45078.1 neomycin phosphotransferase II [Gateway binary vector pGWB42] BAF45082.1 neomycin phosphotransferase II [Gateway binary vector pGWB43] BAF45086.1 neomycin phosphotransferase II [Gateway binary vector pGWB44] BAF45090.1 neomycin phosphotransferase II [Gateway binary vector pGWB45] BAF45094.1 neomycin phosphotransferase II [Gateway binary vector pGWB203] BAF45099.1 neomycin phosphotransferase II [Gateway binary vector pGWB204] BAF45104.1 neomycin phosphotransferase II [Gateway binary vector pGWB228] BAF45108.1 neomycin phosphotransferase II [Gateway binary vector pGWB235] BAF47387.1 neomycin phosphotransferase II [Gateway binary vector pGWB5] BAF47418.1 neomycin phosphotransferase II [Gateway binary vector pGWB401] BAF47421.1 neomycin phosphotransferase II [Gateway binary vector pGWB402] BAF47424.1 neomycin phosphotransferase II [Gateway binary vector pGWB402omega] BAF47428.1 neomycin phosphotransferase II [Gateway binary vector pGWB404] BAF47432.1 neomycin phosphotransferase II [Gateway binary vector pGWB405] BAF47435.1 neomycin phosphotransferase II [Gateway binary vector pGWB406] BAF47438.1 neomycin phosphotransferase II [Gateway binary vector pGWB407] BAF47441.1 neomycin phosphotransferase II [Gateway binary vector pGWB408] BAF47444.1 neomycin phosphotransferase II [Gateway binary vector pGWB409] BAF47447.1 neomycin phosphotransferase II [Gateway binary vector pGWB410] BAF47450.1 neomycin phosphotransferase II [Gateway binary vector pGWB411] BAF47453.1 neomycin phosphotransferase II [Gateway binary vector pGWB412] BAF47456.1 neomycin phosphotransferase II [Gateway binary vector pGWB413] BAF47459.1 neomycin phosphotransferase II [Gateway binary vector pGWB414] BAF47462.1 neomycin phosphotransferase II [Gateway binary vector pGWB415] BAF47465.1 neomycin phosphotransferase II [Gateway binary vector pGWB416] BAF47468.1 neomycin phosphotransferase II [Gateway binary vector pGWB417] BAF47471.1 neomycin phosphotransferase II [Gateway binary vector pGWB418] BAF47474.1 neomycin phosphotransferase II [Gateway binary vector pGWB419] BAF47477.1 neomycin phosphotransferase II [Gateway binary vector pGWB420] BAF47480.1 neomycin phosphotransferase II [Gateway binary vector pGWB421] BAF47483.1 neomycin phosphotransferase II [Gateway binary vector pGWB422] BAF47486.1 neomycin phosphotransferase II [Gateway binary vector pGWB423] BAF47489.1 neomycin phosphotransferase II [Gateway binary vector pGWB424] BAF47492.1 neomycin phosphotransferase II [Gateway binary vector pGWB425] BAF47495.1 neomycin phosphotransferase II [Gateway binary vector pGWB426] BAF47498.1 neomycin phosphotransferase II [Gateway binary vector pGWB427] BAF47501.1 neomycin phosphotransferase II [Gateway binary vector pGWB428] BAF47504.1 neomycin phosphotransferase II [Gateway binary vector pGWB429] BAF47508.1 neomycin phosphotransferase II [Gateway binary vector pGWB433] BAF47512.1 neomycin phosphotransferase II [Gateway binary vector pGWB435] BAF47515.1 neomycin phosphotransferase II [Gateway binary vector pGWB440] BAF47518.1 neomycin phosphotransferase II [Gateway binary vector pGWB441] BAF47521.1 neomycin phosphotransferase II [Gateway binary vector pGWB442]

BAF47524.1 neomycin phosphotransferase II [Gateway binary vector pGWB443] BAF47527.1 neomycin phosphotransferase II [Gateway binary vector pGWB444] BAF47530.1 neomycin phosphotransferase II [Gateway binary vector pGWB445] BAF47534.1 neomycin phosphotransferase II [Gateway binary vector pGWB450] BAF47538.1 neomycin phosphotransferase II [Gateway binary vector pGWB451] BAF47541.1 neomycin phosphotransferase II [Gateway binary vector pGWB452] BAF47545.1 neomycin phosphotransferase II [Gateway binary vector pGWB453] BAF47549.1 neomycin phosphotransferase II [Gateway binary vector pGWB454] BAF47552.1 neomycin phosphotransferase II [Gateway binary vector pGWB455] BAF91004.1 neomycin phosphotransferase II [Gateway binary vector R4pGWB401] BAF91008.1 neomycin phosphotransferase II [Gateway binary vector R4pGWB404] BAF91011.1 neomycin phosphotransferase II [Gateway binary vector R4pGWB407] BAF91014.1 neomycin phosphotransferase II [Gateway binary vector R4pGWB410] BAF91017.1 neomycin phosphotransferase II [Gateway binary vector R4pGWB413] BAF91020.1 neomycin phosphotransferase II [Gateway binary vector R4pGWB416] BAF91023.1 neomycin phosphotransferase II [Gateway binary vector R4pGWB419] BAF91026.1 neomycin phosphotransferase II [Gateway binary vector R4pGWB422] BAF91029.1 neomycin phosphotransferase II [Gateway binary vector R4pGWB425] BAF91032.1 neomycin phosphotransferase II [Gateway binary vector R4pGWB428] BAF91036.1 neomycin phosphotransferase II [Gateway binary vector R4pGWB433] BAF91040.1 neomycin phosphotransferase II [Gateway binary vector R4pGWB435] BAF91043.1 neomycin phosphotransferase II [Gateway binary vector R4pGWB440] BAF91046.1 neomycin phosphotransferase II [Gateway binary vector R4pGWB443] BAF91050.1 neomycin phosphotransferase II [Gateway binary vector R4pGWB450] BAF91054.1 neomycin phosphotransferase II [Gateway binary vector R4pGWB453] BAH24257.1 neomycin phosphotransferase II [Plant binary vector pIG121-Hm] BAI43858.1 neomycin phosphotransferase II [Gateway binary vector R4L1pGWB432] BAI43862.1 neomycin phosphotransferase II [Gateway binary vector R4L1pGWB433] BAI43866.1 neomycin phosphotransferase II [Gateway binary vector R4L1pGWB435] BAI43870.1 neomycin phosphotransferase II [Gateway binary vector R4L1pGWB440] BAI43874.1 neomycin phosphotransferase II [Gateway binary vector R4L1pGWB443] BAI43878.1 neomycin phosphotransferase II [Gateway binary vector R4L1pGWB450] BAI43882.1 neomycin phosphotransferase II [Gateway binary vector R4L1pGWB459] BAI67572.1 neomycin phosphotransferase II [Gateway binary vector pGWB459] BAI67576.1 neomycin phosphotransferase II [Gateway binary vector pGWB460] BAI67579.1 neomycin phosphotransferase II [Gateway binary vector pGWB461] BAI67594.1 neomycin phosphotransferase II [Gateway binary vector R4pGWB459] BAM62093.1 neomycin phosphotransferase II [Gateway binary vector pGWB480omega] BAM62097.1 neomycin phosphotransferase II [Gateway binary vector pGWB80] BAO59018.1 neomycin phosphotransferase [Activation tagging vector pKANAC2K] BAV38483.1 neomycin phosphotransferase II [Dual site Gateway binary vector

R4pGWB6401-MD8]

BAV38488.1 neomycin phosphotransferase II [Dual site Gateway binary vector R4pGWB6404-MD8]

BAV38492.1 neomycin phosphotransferase II [Dual site Gateway binary vector R4pGWB6407-MD8]

BAV38496.1 neomycin phosphotransferase II [Dual site Gateway binary vector R4pGWB6410-MD8]

BAV38500.1 neomycin phosphotransferase II [Dual site Gateway binary vector R4pGWB6413-MD8]

BAV38504.1 neomycin phosphotransferase II [Dual site Gateway binary vector R4pGWB6416-MD8]

BAV38508.1 neomycin phosphotransferase II [Dual site Gateway binary vector R4pGWB6419-MD8]

BAV38512.1 neomycin phosphotransferase II [Dual site Gateway binary vector R4pGWB6422-MD8]

BAV38516.1 neomycin phosphotransferase II [Dual site Gateway binary vector R4pGWB6425-MD8]

BAV38521.1 neomycin phosphotransferase II [Dual site Gateway binary vector R4pGWB6433-MD8]

BAV38526.1 neomycin phosphotransferase II [Dual site Gateway binary vector R4pGWB6435-MD8]

BAV38530.1 neomycin phosphotransferase II [Dual site Gateway binary vector R4pGWB6440-MD8]

BAV38534.1 neomycin phosphotransferase II [Dual site Gateway binary vector R4pGWB6443-MD8]

BAV38539.1 neomycin phosphotransferase II [Dual site Gateway binary vector R4pGWB6450-MD8]

BAV38544.1 neomycin phosphotransferase II [Dual site Gateway binary vector R4pGWB6453-MD8]

BAV38549.1 neomycin phosphotransferase II [Dual site Gateway binary vector R4pGWB6459-MD8]

BAV38554.1 neomycin phosphotransferase II [Dual site Gateway binary vector R4pGWB6400-MD8-NY2]

BAV38559.1 neomycin phosphotransferase II [Dual site Gateway binary vector R4pGWB6400-MD8-CY2]

BAV38802.1 neomycin phosphotransferase II [Gateway binary vector R5R6pGWB401] BAV44485.1 neomycin phosphotransferase II [Gateway vector pB4cCGGW] BAV44488.1 neomycin phosphotransferase II [Gateway vector pB4cRGW]

BAV44491.1 neomycin phosphotransferase II [Gateway vector pB4cYGW] BAV44494.1 neomycin phosphotransferase II [Gateway vector pB4nCGW] BAV44497.1 neomycin phosphotransferase II [Gateway vector pB4nGGW] BAV44500.1 neomycin phosphotransferase II [Gateway vector pB4nRGW] BAV44503.1 neomycin phosphotransferase II [Gateway vector pB4nYGW] BAV44507.1 neomycin phosphotransferase II [Gateway vector pB4GWcCG] BAV44510.1 neomycin phosphotransferase II [Gateway vector pB4GWcR] BAV44514.1 neomycin phosphotransferase II [Gateway vector pB4GWcY] BAV44518.1 neomycin phosphotransferase II [Gateway vector pB4GWnC] BAV44522.1 neomycin phosphotransferase II [Gateway vector pB4GWnG] BAV44526.1 neomycin phosphotransferase II [Gateway vector pB4GWnR] BAV44530.1 neomycin phosphotransferase II [Gateway vector pB4GWnY]

BAX01566.1 neomycin phosphotransferase II [Dual site Gateway binary vector pGWB6402-MD8-Pnos]

BAX01570.1 neomycin phosphotransferase II [Dual site Gateway binary vector pGWB6406-MD8-Pnos]

BAX01574.1 neomycin phosphotransferase II [Dual site Gateway binary vector pGWB6409-MD8-Pnos]

BAX01578.1 neomycin phosphotransferase II [Dual site Gateway binary vector pGWB6412-MD8-Pnos]

BAX01582.1 neomycin phosphotransferase II [Dual site Gateway binary vector pGWB6415-MD8-Pnos]

BAX01586.1 neomycin phosphotransferase II [Dual site Gateway binary vector pGWB6418-MD8-Pnos]

BAX01590.1 neomycin phosphotransferase II [Dual site Gateway binary vector pGWB6421-MD8-Pnos]

BAX01594.1 neomycin phosphotransferase II [Dual site Gateway binary vector pGWB6424-MD8-Pnos]

BAX01598.1 neomycin phosphotransferase II [Dual site Gateway binary vector pGWB6427-MD8-Pnos]

BAX01602.1 neomycin phosphotransferase II [Dual site Gateway binary vector pGWB6442-MD8-Pnos]

BAX01606.1 neomycin phosphotransferase II [Dual site Gateway binary vector pGWB6445-MD8-Pnos]

BAX01610.1 neomycin phosphotransferase II [Dual site Gateway binary vector pGWB6452-MD8-Pnos]

BAX01614.1 neomycin phosphotransferase II [Dual site Gateway binary vector pGWB6455-MD8-Pnos]

BAX01618.1 neomycin phosphotransferase II [Dual site Gateway binary vector pGWB6461-MD8-Pnos]

BAX01622.1 neomycin phosphotransferase II [Dual site Gateway binary vector pGWB6400-MD8-Pnos-NY0]

BAX01626.1 neomycin phosphotransferase II [Dual site Gateway binary vector pGWB6400-MD8-Pnos-CY0]

BAX01631.1 neomycin phosphotransferase II [Dual site Gateway binary vector pGWB6400-MD8-Pnos-NY2]

BAX01636.1 neomycin phosphotransferase II [Dual site Gateway binary vector pGWB6400-MD8-Pnos-CY2]

BBG75462.1 neomycin phosphotransferase II [Gateway binary vector R4L1pGWB462] BBG75466.1 neomycin phosphotransferase II [Gateway binary vector R4L1pGWB465] BBG75470.1 neomycin phosphotransferase II [Gateway binary vector R4L1pGWB468] BBG75474.1 neomycin phosphotransferase II [Gateway binary vector R4L1pGWB471] BBG75478.1 neomycin phosphotransferase II [Gateway binary vector R4L1pGWB485] BBG75482.1 neomycin phosphotransferase II [Gateway binary vector R4L1pGWB488] BBG75486.1 neomycin phosphotransferase II [Gateway binary vector R4L1pGWB491] BBG75574.1 neomycin phosphotransferase II [Gateway binary vector pGWB462] BBG75578.1 neomycin phosphotransferase II [Gateway binary vector pGWB465] BBG75582.1 neomycin phosphotransferase II [Gateway binary vector pGWB468] BBG75586.1 neomycin phosphotransferase II [Gateway binary vector pGWB471] BBG75590.1 neomycin phosphotransferase II [Gateway binary vector pGWB485] BBG75594.1 neomycin phosphotransferase II [Gateway binary vector pGWB488] BBG75598.1 neomycin phosphotransferase II [Gateway binary vector pGWB491] BBI25410.1 neomycin phosphotransferase II [Gateway binary vector pB4nVGW3] BBI25413.1 neomycin phosphotransferase II [Gateway binary vector pB4cVGW] BBI25416.1 neomycin phosphotransferase II [Gateway binary vector pB4GWnV3]

BBI25419.1 neomycin phosphotransferase II [Gateway binary vector pB4GWcV] Length=264

Score = 533 bits (1373), Expect = 0.0, Method: Compositional matrix adjust.

| Identities | = 263/264 (99%), Positives = 264/264 (100%), Gaps = 0/264 (0%) |  |
| --- | --- | --- |
| Query 1 | MIEQDGLHAGSPAAWVERLFGYDWAQQTIGCSDAAVFRLSAQGRPVLFVKTDLSGALNEL | 60 |
| Sbjct 1 | MIEQDGLHAGSPAAWVERLFGYDWAQQTIGCSDAAVFRLSAQGRPVLFVKTDLSGALNEL MIEQDGLHAGSPAAWVERLFGYDWAQQTIGCSDAAVFRLSAQGRPVLFVKTDLSGALNEL | 60 |
| Query 61 | QDEAARLSWLATTGVPCAAVLDVVTEAGRDWLLLGEVPGQDLLSSHLAPAEKVSIMADAM | 120 |
| Sbjct 61 | QDEAARLSWLATTGVPCAAVLDVVTEAGRDWLLLGEVPGQDLLSSHLAPAEKVSIMADAM QDEAARLSWLATTGVPCAAVLDVVTEAGRDWLLLGEVPGQDLLSSHLAPAEKVSIMADAM | 120 |
| Query 121 | RRLHTLDPATCPFDHQAKHRIERARTRMEAGLVDQDDLDEEHQGLAPAELFARLKARMPD | 180 |
| Sbjct 121 | RRLHTLDPATCPFDHQAKHRIERARTRMEAGLVDQDDLDEEHQGLAPAELFARLKARMPD RRLHTLDPATCPFDHQAKHRIERARTRMEAGLVDQDDLDEEHQGLAPAELFARLKARMPD | 180 |
| Query 181 | GEDLVVTHGDACLPNIMVENGRFSGFIDCGRLGVADRYQDIALATRDIAEELGGEWADRF | 240 |
| Sbjct 181 | G+DLVVTHGDACLPNIMVENGRFSGFIDCGRLGVADRYQDIALATRDIAEELGGEWADRF GDDLVVTHGDACLPNIMVENGRFSGFIDCGRLGVADRYQDIALATRDIAEELGGEWADRF | 240 |
| Query 241  Sbjct 241 | LVLYGIAAPDSQRIAFYRLLDEFF 264 LVLYGIAAPDSQRIAFYRLLDEFF LVLYGIAAPDSQRIAFYRLLDEFF 264 |  |

>EGF4810723.1 APH(3')-II family aminoglycoside O-phosphotransferase [Salmonella enterica subsp. enterica serovar Adelaide]

Length=264

Score = 533 bits (1373), Expect = 0.0, Method: Compositional matrix adjust.

| Identities | = 263/264 (99%), Positives = 264/264 (100%), Gaps = 0/264 (0%) |  |
| --- | --- | --- |
| Query 1 | MIEQDGLHAGSPAAWVERLFGYDWAQQTIGCSDAAVFRLSAQGRPVLFVKTDLSGALNEL | 60 |
| Sbjct 1 | MIEQDGLHAGSPAAWVERLFGYDWAQQTIGCSDAAVFRLSAQGRPVLFVKTDLSGALNEL MIEQDGLHAGSPAAWVERLFGYDWAQQTIGCSDAAVFRLSAQGRPVLFVKTDLSGALNEL | 60 |
| Query 61 | QDEAARLSWLATTGVPCAAVLDVVTEAGRDWLLLGEVPGQDLLSSHLAPAEKVSIMADAM | 120 |
| Sbjct 61 | QDEAARLSWLATTGVPCAAVLDVVTEAGRDWLLLGEVPGQDLLSSHLAPAEKVSIMADAM QDEAARLSWLATTGVPCAAVLDVVTEAGRDWLLLGEVPGQDLLSSHLAPAEKVSIMADAM | 120 |
| Query 121 | RRLHTLDPATCPFDHQAKHRIERARTRMEAGLVDQDDLDEEHQGLAPAELFARLKARMPD | 180 |
| Sbjct 121 | RRLHTLDPATCPFDHQAKHRIERARTRMEAGLVDQDDLDEEHQGLAPAELFARLKARMPD RRLHTLDPATCPFDHQAKHRIERARTRMEAGLVDQDDLDEEHQGLAPAELFARLKARMPD | 180 |
| Query 181 | GEDLVVTHGDACLPNIMVENGRFSGFIDCGRLGVADRYQDIALATRDIAEELGGEWADRF | 240 |
| Sbjct 181 | GEDLVVTHGDACLPNIMVENGRF+GFIDCGRLGVADRYQDIALATRDIAEELGGEWADRF GEDLVVTHGDACLPNIMVENGRFAGFIDCGRLGVADRYQDIALATRDIAEELGGEWADRF | 240 |
| Query 241  Sbjct 241 | LVLYGIAAPDSQRIAFYRLLDEFF 264 LVLYGIAAPDSQRIAFYRLLDEFF LVLYGIAAPDSQRIAFYRLLDEFF 264 |  |

>WP_187790236.1 APH(3')-II family aminoglycoside O-phosphotransferase [Salmonella sp. S005_59879]

Length=264

Score = 533 bits (1373), Expect = 0.0, Method: Compositional matrix adjust.

| Identities | = 263/264 (99%), Positives = 263/264 (99%), Gaps = 0/264 (0%) |  |
| --- | --- | --- |
| Query 1 | MIEQDGLHAGSPAAWVERLFGYDWAQQTIGCSDAAVFRLSAQGRPVLFVKTDLSGALNEL | 60 |
| Sbjct 1 | MIEQDGLHAGSPAAWVERLFGYDWAQQTIGCSDAAVFRLSAQGRPVLFVKTDLSGALNEL MIEQDGLHAGSPAAWVERLFGYDWAQQTIGCSDAAVFRLSAQGRPVLFVKTDLSGALNEL | 60 |
| Query 61 | QDEAARLSWLATTGVPCAAVLDVVTEAGRDWLLLGEVPGQDLLSSHLAPAEKVSIMADAM | 120 |
| Sbjct 61 | QDEAARLSWLATTGVPCAAVLDVVTEAGRDWLLLGEVPGQDLLSSHLAPAEKVSIM DAM QDEAARLSWLATTGVPCAAVLDVVTEAGRDWLLLGEVPGQDLLSSHLAPAEKVSIMVDAM | 120 |
| Query 121 | RRLHTLDPATCPFDHQAKHRIERARTRMEAGLVDQDDLDEEHQGLAPAELFARLKARMPD RRLHTLDPATCPFDHQAKHRIERARTRMEAGLVDQDDLDEEHQGLAPAELFARLKARMPD | 180 |

| Sbjct | 121 | RRLHTLDPATCPFDHQAKHRIERARTRMEAGLVDQDDLDEEHQGLAPAELFARLKARMPD | 180 |
| --- | --- | --- | --- |
| Query | 181 | GEDLVVTHGDACLPNIMVENGRFSGFIDCGRLGVADRYQDIALATRDIAEELGGEWADRF | 240 |
| Sbjct | 181 | GEDLVVTHGDACLPNIMVENGRFSGFIDCGRLGVADRYQDIALATRDIAEELGGEWADRF GEDLVVTHGDACLPNIMVENGRFSGFIDCGRLGVADRYQDIALATRDIAEELGGEWADRF | 240 |
| Query  Sbjct | 241  241 | LVLYGIAAPDSQRIAFYRLLDEFF 264 LVLYGIAAPDSQRIAFYRLLDEFF LVLYGIAAPDSQRIAFYRLLDEFF 264 |  |

>AAA87574.1 neomycin resistance protein [Cloning vector pMC1neo] AAA93304.1 neomycin resistance protein [Cloning vector pMC1neo-polyA] AAB49981.1 neomycin phosphotransferase [unidentified cloning vector] AAC08734.1 neomycin resistance protein [Expression vector pRIBOTEX] AAC12959.1 neomycin resistance protein [Cloning vector pOPRSVIMCS] AAC53677.1 neomycin phosphotransferase [Cloning vector pRAY-1] AAC53679.1 neomycin phosphotransferase [Cloning vector pRAY-2] AAD20228.1 neomycin phosphotransferase [Cloning vector pLXRN] AAD47089.1 neomycin phosphotransferase [Cloning vector pACN] AAK28133.1 neomycin phosphotransferase [Cloning vector pPGKneo-I] AAK28136.1 neomycin phosphotransferase [Cloning vector pPGKneo-II] ABR10609.1 neomycin phosphotransferase II [synthetic construct] ACC91772.1 neomycin resistance protein [Cloning vector pEGFPpA-ACN] ACC91776.1 neomycin resistance protein [Cloning vector pECFPpA-ACN] ACC91780.1 neomycin resistance protein [Cloning vector pAP-ACN] ACC91784.1 neomycin resistance protein [Cloning vector pnlslacZ-ACN] ACC91788.1 neomycin resistance protein [Cloning vector pEYFPpA-ACN] ACD62480.1 Neo [Gene trapping vector VICTR74]

ACD62481.1 Neo [Gene trapping vector VICTR48]

ADO40089.1 neomycin resistance protein [Knock-in vector FBDki] AEL00440.1 neomycin phosphotransferase [Cloning vector pHPmK3]

AEL00443.1 neomycin phosphotransferase [Cloning vector pMA_F3RoxNeoRoxLoxPFR] AGV76761.1 AphA [Cloning vector pAV001]

AGV76763.1 kanamycin resistance protein [Cloning vector pAV007] AMH38323.1 neomycin phosphotransferase [Cloning vector pRP-VL] AMH38324.1 neomycin phosphotransferase [Cloning vector pRP-LA] AMH38325.1 neomycin phosphotransferase [Cloning vector pRP-LB] AMH38326.1 neomycin phosphotransferase [Cloning vector pRP-M] AMH38327.1 neomycin phosphotransferase [Cloning vector pRP-H] AMH38328.1 neomycin phosphotransferase [Cloning vector pRP-VH] AMH38329.1 neomycin phosphotransferase [Cloning vector pRP-VL-GFP-N] AMH38330.1 neomycin phosphotransferase [Cloning vector pRP-LA-GFP-N] AMH38331.1 neomycin phosphotransferase [Cloning vector pRP-LB-GFP-N] AMH38332.1 neomycin phosphotransferase [Cloning vector pRP-M-GFP-N] AMH38333.1 neomycin phosphotransferase [Cloning vector pRP-H-GFP-N] AMH38334.1 neomycin phosphotransferase [Cloning vector pRP-VH-GFP-N] AMH38335.1 neomycin phosphotransferase [Cloning vector pRP-VL-GFP-C] AMH38336.1 neomycin phosphotransferase [Cloning vector pRP-LA-GFP-C] AMH38337.1 neomycin phosphotransferase [Cloning vector pRP-LB-GFP-C] AMH38338.1 neomycin phosphotransferase [Cloning vector pRP-M-GFP-C] AMH38339.1 neomycin phosphotransferase [Cloning vector pRP-H-GFP-C] AMH38340.1 neomycin phosphotransferase [Cloning vector pRP-VH-GFP-C] AWJ58490.1 neomycin phosphotransferase [Cloning vector pLXIN2]

AXR98588.1 neomycin phosphotransferase II [Cloning vector pR6Kamp-LAP(GFP)]

AXR98591.1 neomycin phosphotransferase II [Cloning vector ENCODE_CRISPR_GFP]

EER6597815.1 APH(3')-II family aminoglycoside O-phosphotransferase [Escherichia coli]

QOE77463.1 aminoglycoside 3-prime-phosphotransferase [Vector pDP002] QOE77466.1 aminoglycoside 3-prime-phosphotransferase [Vector pDP003] QOE77469.1 aminoglycoside 3-prime-phosphotransferase [Vector pDP004] CAF29073.1 neomycin resistance protein [Cloning vector pRAY-Cre]

BAF80810.1 neomycin phosphotransferase [Exchangeable gene trap vector pU-22] BAI50645.1 neomycin phosphotransferase [Targeting vector pTgKCNQ2] BAL04098.1 neomycin phosphotransferase [Gene trap vector pCMT-SAhygpA-NP21] BAL04102.1 neomycin phosphotransferase [Gene trap vector pCMT-SAhygpA-NP22]

BAL04106.1 neomycin phosphotransferase [Tol2 transposon-based gene trap vector pT2F2-SAhygpA-NP21]

BAL04110.1 neomycin phosphotransferase [Tol2 transposon-based gene trap vector pT2F2-SAhygpA-NP22]

BAL04114.1 neomycin phosphotransferase [Tol2 transposon-based gene trap vector pT2F2-SAhygpA-NP21-Rev]

BAS54040.1 neomycin resistance protein [Bi-allelically poly(A)-trapping (BPATrap) vector]

Length=267

Score = 533 bits (1373), Expect = 0.0, Method: Compositional matrix adjust. Identities = 263/263 (100%), Positives = 263/263 (100%), Gaps = 0/263 (0%)

Query 2	IEQDGLHAGSPAAWVERLFGYDWAQQTIGCSDAAVFRLSAQGRPVLFVKTDLSGALNELQ 61 IEQDGLHAGSPAAWVERLFGYDWAQQTIGCSDAAVFRLSAQGRPVLFVKTDLSGALNELQ

Sbjct 5	IEQDGLHAGSPAAWVERLFGYDWAQQTIGCSDAAVFRLSAQGRPVLFVKTDLSGALNELQ 64

Query 62	DEAARLSWLATTGVPCAAVLDVVTEAGRDWLLLGEVPGQDLLSSHLAPAEKVSIMADAMR 121 DEAARLSWLATTGVPCAAVLDVVTEAGRDWLLLGEVPGQDLLSSHLAPAEKVSIMADAMR

Sbjct 65	DEAARLSWLATTGVPCAAVLDVVTEAGRDWLLLGEVPGQDLLSSHLAPAEKVSIMADAMR 124

Query 122 RLHTLDPATCPFDHQAKHRIERARTRMEAGLVDQDDLDEEHQGLAPAELFARLKARMPDG 181 RLHTLDPATCPFDHQAKHRIERARTRMEAGLVDQDDLDEEHQGLAPAELFARLKARMPDG

Sbjct 125 RLHTLDPATCPFDHQAKHRIERARTRMEAGLVDQDDLDEEHQGLAPAELFARLKARMPDG 184

Query 182 EDLVVTHGDACLPNIMVENGRFSGFIDCGRLGVADRYQDIALATRDIAEELGGEWADRFL 241 EDLVVTHGDACLPNIMVENGRFSGFIDCGRLGVADRYQDIALATRDIAEELGGEWADRFL

Sbjct 185 EDLVVTHGDACLPNIMVENGRFSGFIDCGRLGVADRYQDIALATRDIAEELGGEWADRFL 244

Query 242 VLYGIAAPDSQRIAFYRLLDEFF 264

VLYGIAAPDSQRIAFYRLLDEFF Sbjct 245 VLYGIAAPDSQRIAFYRLLDEFF 267

>WP_161571218.1 APH(3')-II family aminoglycoside O-phosphotransferase [Vibrio cholerae]

QHQ89906.1 APH(3')-II family aminoglycoside O-phosphotransferase [Vibrio cholerae O1]

Length=264

Score = 533 bits (1373), Expect = 0.0, Method: Compositional matrix adjust.

| Identities | = 263/264 (99%), Positives = 264/264 (100%), Gaps = 0/264 (0%) |  |
| --- | --- | --- |
| Query 1 | MIEQDGLHAGSPAAWVERLFGYDWAQQTIGCSDAAVFRLSAQGRPVLFVKTDLSGALNEL | 60 |
| Sbjct 1 | MIEQDGLHAGSPAAWVERLFGYDWAQQTIGCSDAAVFRLSAQGRPVLFVKTDLSGALNEL MIEQDGLHAGSPAAWVERLFGYDWAQQTIGCSDAAVFRLSAQGRPVLFVKTDLSGALNEL | 60 |
| Query 61 | QDEAARLSWLATTGVPCAAVLDVVTEAGRDWLLLGEVPGQDLLSSHLAPAEKVSIMADAM | 120 |
| Sbjct 61 | QDEAARLSWLATTGVPCAAVLDVVTEAGRDWLLLGEVPGQDLLSSHLAPAEKVSIMADAM QDEAARLSWLATTGVPCAAVLDVVTEAGRDWLLLGEVPGQDLLSSHLAPAEKVSIMADAM | 120 |
| Query 121 | RRLHTLDPATCPFDHQAKHRIERARTRMEAGLVDQDDLDEEHQGLAPAELFARLKARMPD | 180 |
| Sbjct 121 | RRLHTLDPATCPFDHQAKHRIERARTRMEAGLVDQDDLDEEHQGLAPAELFARLKARMPD RRLHTLDPATCPFDHQAKHRIERARTRMEAGLVDQDDLDEEHQGLAPAELFARLKARMPD | 180 |
| Query 181 | GEDLVVTHGDACLPNIMVENGRFSGFIDCGRLGVADRYQDIALATRDIAEELGGEWADRF | 240 |
| Sbjct 181 | GEDLVVTHGDACLPNI+VENGRFSGFIDCGRLGVADRYQDIALATRDIAEELGGEWADRF GEDLVVTHGDACLPNIIVENGRFSGFIDCGRLGVADRYQDIALATRDIAEELGGEWADRF | 240 |
| Query 241  Sbjct 241 | LVLYGIAAPDSQRIAFYRLLDEFF 264 LVLYGIAAPDSQRIAFYRLLDEFF LVLYGIAAPDSQRIAFYRLLDEFF 264 |  |

```
>WP_061182288.1 APH(3')-II family aminoglycoside O-phosphotransferase [Escherichia coli]
AML00736.1 aminoglycoside phosphotransferase [Escherichia coli str. K-12 substr. MG1655]
KXU69933.1 aminoglycoside phosphotransferase [Escherichia coli] Length=264

Score = 533 bits (1373), Expect = 0.0, Method: Compositional matrix adjust. Identities = 263/264 (99%), Positives = 264/264 (100%), Gaps = 0/264 (0%)

Query 1	MIEQDGLHAGSPAAWVERLFGYDWAQQTIGCSDAAVFRLSAQGRPVLFVKTDLSGALNEL 60 MIEQDGLHAGSPAAWVERLFGYDWAQQTIGCSDAAVFRLSAQGRPVLFVKTDLSGALNEL
Sbjct 1	MIEQDGLHAGSPAAWVERLFGYDWAQQTIGCSDAAVFRLSAQGRPVLFVKTDLSGALNEL 60
```

| Query | 61 | QDEAARLSWLATTGVPCAAVLDVVTEAGRDWLLLGEVPGQDLLSSHLAPAEKVSIMADAM | 120 |
| --- | --- | --- | --- |
| Sbjct | 61 | QDEAARLSWLATTGVPCAAVLDVVTEAGRDWLLLGEVPGQDLLSSHLAPAEKVSIMADAM QDEAARLSWLATTGVPCAAVLDVVTEAGRDWLLLGEVPGQDLLSSHLAPAEKVSIMADAM | 120 |
| Query | 121 | RRLHTLDPATCPFDHQAKHRIERARTRMEAGLVDQDDLDEEHQGLAPAELFARLKARMPD | 180 |
| Sbjct | 121 | RRLHTLDPATCPFDHQAKHRIERARTRMEAGLVDQDDLDEEHQGLAPAELFARLKARMPD RRLHTLDPATCPFDHQAKHRIERARTRMEAGLVDQDDLDEEHQGLAPAELFARLKARMPD | 180 |
| Query | 181 | GEDLVVTHGDACLPNIMVENGRFSGFIDCGRLGVADRYQDIALATRDIAEELGGEWADRF | 240 |
| Sbjct | 181 | GEDLVVTHGDACLPNI+VENGRFSGFIDCGRLGVADRYQDIALATRDIAEELGGEWADRF GEDLVVTHGDACLPNIVVENGRFSGFIDCGRLGVADRYQDIALATRDIAEELGGEWADRF | 240 |
| Query  Sbjct | 241  241 | LVLYGIAAPDSQRIAFYRLLDEFF 264 LVLYGIAAPDSQRIAFYRLLDEFF LVLYGIAAPDSQRIAFYRLLDEFF 264 |  |

>WP_118142372.1 APH(3')-II family aminoglycoside O-phosphotransferase [Streptomyces clavuligerus]

AXU16473.1 APH(3')-II family aminoglycoside O-phosphotransferase [Streptomyces clavuligerus]

Length=264

Score = 533 bits (1373), Expect = 0.0, Method: Compositional matrix adjust.

| Identities | = 263/264 (99%), Positives = 263/264 (99%), Gaps = 0/264 (0%) |  |
| --- | --- | --- |
| Query 1 | MIEQDGLHAGSPAAWVERLFGYDWAQQTIGCSDAAVFRLSAQGRPVLFVKTDLSGALNEL | 60 |
| Sbjct 1 | MIEQDGLHAGSPAAWVERLFGYDWAQQTIGCSDAAVFRLSAQGRPVLFVKTDLSGALNEL MIEQDGLHAGSPAAWVERLFGYDWAQQTIGCSDAAVFRLSAQGRPVLFVKTDLSGALNEL | 60 |
| Query 61 | QDEAARLSWLATTGVPCAAVLDVVTEAGRDWLLLGEVPGQDLLSSHLAPAEKVSIMADAM | 120 |
| Sbjct 61 | QDEA RLSWLATTGVPCAAVLDVVTEAGRDWLLLGEVPGQDLLSSHLAPAEKVSIMADAM QDEAVRLSWLATTGVPCAAVLDVVTEAGRDWLLLGEVPGQDLLSSHLAPAEKVSIMADAM | 120 |
| Query 121 | RRLHTLDPATCPFDHQAKHRIERARTRMEAGLVDQDDLDEEHQGLAPAELFARLKARMPD | 180 |
| Sbjct 121 | RRLHTLDPATCPFDHQAKHRIERARTRMEAGLVDQDDLDEEHQGLAPAELFARLKARMPD RRLHTLDPATCPFDHQAKHRIERARTRMEAGLVDQDDLDEEHQGLAPAELFARLKARMPD | 180 |
| Query 181 | GEDLVVTHGDACLPNIMVENGRFSGFIDCGRLGVADRYQDIALATRDIAEELGGEWADRF | 240 |
| Sbjct 181 | GEDLVVTHGDACLPNIMVENGRFSGFIDCGRLGVADRYQDIALATRDIAEELGGEWADRF GEDLVVTHGDACLPNIMVENGRFSGFIDCGRLGVADRYQDIALATRDIAEELGGEWADRF | 240 |
| Query 241  Sbjct 241 | LVLYGIAAPDSQRIAFYRLLDEFF 264 LVLYGIAAPDSQRIAFYRLLDEFF LVLYGIAAPDSQRIAFYRLLDEFF 264 |  |

>WP_004614937.1 MULTISPECIES: APH(3')-II family aminoglycoside O-phosphotransferase [Bacteria]

AAA69543.1 neomycin phosphotransferase [Cloning vector pGFP-1] AAA69545.1 neomycin phosphotransferase [Cloning vector pGFP-N3] AAA69547.1 neomycin phosphotransferase [Cloning vector pGFP-C3] AAA69549.1 neomycin phosphotransferase [Cloning vector pGFP-N1] AAA69551.1 neomycin phosphotransferase [Cloning vector pGFP-C1] AAA69553.1 neomycin phosphotransferase [Cloning vector pGFP-C2] AAA69555.1 neomycin phosphotransferase [Cloning vector pGFP-N2] AAA79305.1 neomycin phosphotransferase [Cloning vector pRSGFP-C1] AAA79307.1 neomycin phosphotransferase [Cloning vector pS65T-C1] AAB02573.1 neomycin phosphotransferase [Cloning vector pEGFP-1] AAB02575.1 neomycin phosphotransferase [Cloning vector pEGFP-N1] AAB02577.1 neomycin phosphotransferase [Cloning vector pEGFP-C1] AAB08059.1 neomycin phosphotransferase [Cloning vector pEGFP-C2] AAB08061.1 neomycin phosphotransferase [Cloning vector pEGFP-C3] AAB08063.1 neomycin phosphotransferase [Cloning vector pEGFP-N2] AAB08065.1 neomycin phosphotransferase [Cloning vector pEGFP-N3] AAC23676.1 kanamycin resistance protein [Cloning vector pCMV-scriptEX] AAC25420.1 aminoglycoside phosphotransferase [Cloning vector pCMVTAG2a] AAC25421.1 aminoglycoside phosphotransferase [Cloning vector pCMVTAG2b] AAC25422.1 aminoglycoside phosphotransferase [Cloning vector pCMVTAG2c] AAC25426.1 aminoglycoside phosphotransferase [Cloning vector pCMVTAG3a] AAC25427.1 aminoglycoside phosphotransferase [Cloning vector pCMVTAG3b] AAC25428.1 aminoglycoside phosphotransferase [Cloning vector pCMVTAG3c]

AAC25429.1 aminoglycoside phosphotransferase [Cloning vector pCMVTAG4a] AAC26801.1 aminoglycoside aminotransferase [Cloning vector pCMVTAG4b] AAC26802.1 aminoglycoside aminotransferase [Cloning vector pCMVTAG4c] AAC26803.1 aminoglycoside aminotransferase [Cloning vector pCMVTAG5a] AAC26805.1 aminoglycoside aminotransferase [Cloning vector pCMVTAG5b] AAC26806.1 aminoglycoside aminotransferase [Cloning vector pCMVTAG5c] AAC34497.1 neomycin phosphotransferase [Cloning vector pFAC-dbd] AAC78279.1 kanamycin marker protein [Cloning vector pKILHIS-1] AAC78280.1 kanamycin marker protein [Cloning vector pKILHIS-2] AAC95153.1 neomycin phosphotransferase [Cloning vector pERV3] AAD42381.1 neomycin resistance [Cloning vector pCMV-BD]

AAK01721.1 neomycin phosphotransferase [AdEasy vector pShuttle]

AAL31968.1 kanamycin resistance protein [Expression vector pGA1] AAL31969.1 kanamycin resistance protein [Expression vector pGA2] AAL31970.1 kanamycin resistance protein [Expression vector pGA3] AAO20038.1 neomycin phosphotransferase [Expression vector pHis-TAT-GFP] AAO20040.1 neomycin phosphotransferase [Expression vector pHis-TAT-m-GFP] AAO20042.1 neomycin phosphotransferase [Expression vector pHis-GFP] AAO72327.1 neomycin phosphotransferase [synthetic construct]

AAQ06253.1 neomycin phosphotransferase [Cloning vector pCpG-Neo deltaCpG] AAS78490.1 Kan [Cloning vector pZero++ Kan]

AAY63959.1 kanamycin/neomycin resistance protein [Expression vector pEGFP-Pax9] ABG26757.1 neomycin/kanamycin resistance protein [Phagemid vector pBK-CMV] ABK62678.1 aminoglycoside 3'-phosphotransferase [ORF selection vector pSOS] ABZ91088.1 neomycin phosphotransferase [Episomal vector pMARS-mODC-AZ] ABZ91090.1 neomycin phosphotransferase [Episomal vector pMARS-mODC-ZA] ACM10012.1 Kan(R) [Neurospora knock-in vector p3xFLAG::hph::loxP]

ACM10014.1 Kan(R) [Neurospora knock-in vector p3xHA::hph::loxP] ACM10017.1 Kan(R) [Neurospora knock-in vector pGFP::hph::loxP] ACM10019.1 Kan(R) [Neurospora knock-in vector p13xMyc::hph::loxP] ACT68285.1 kanamycin resistance protein [Cloning vector pRama-31] ACT68288.1 kanamycin resistance protein [Cloning vector pRama-34] ACT68290.1 kanamycin resistance protein [Cloning vector pRama-35]

ACU30026.1 kanamycin resistance [Fusion vector pDsred-express::plac::prrn::pd1egfp] ACV85763.1 kanamycin resistance protein [Cloning vector pSpark III]

ADC42840.1 Neo [Cloning vector pMAZe]

ADH43302.1 kanamycin resistance protein [Acinetobacter baumannii expression vector pOT-RA]

ADR71678.1 NeoR [Retroviral Tet-shRNA expression vector TRMPV-Neo] ADR71693.1 NeoR [Retroviral Tet-shRNA expression vector TtTMPV-Neo] AEJ22721.1 NptII [Cloning vector pSOL8DCL2]

AEJ22725.1 NptII [Cloning vector pSOL9CAP] AEJ22730.1 NptII [Cloning vector pSOL8DC3]

AEJ82531.1 kanamycin-neomycin resistance protein [Cloning vector pEGPF-N2-NRSE4x] AEJ82533.1 kanamycin-neomycin resistance protein [Cloning vector pEGFP-N2-NRSE4x-ohne-CMV] AEJ89477.1 kanamycin resistance protein [Shuttle vector pSC-R1LambdaR2]

AEW43852.1 NeoR/KanR [Cloning vector pSI-AKR1] AEW43863.1 NeoR/KanR [Cloning vector pSI-AKR8] AEW43868.1 NeoR/KanR [Cloning vector pSI-AKR7]

AFV47155.1 neomycin phosphotransferase class II [Cloning vector pRESC800]

AGE44208.1 aminoglycoside 3'-phosphotransferase [Adenoviral vector pAdSh.SR(alpha).lacZ(Delta)CpG]

AGH70231.1 Kan/NeoR [Expression vector pPV472] AGI38099.1 NptII [Cloning vector pSOL9LHGRC] AGI38104.1 NptII [Cloning vector pSOL9LHGRP]

AHA35649.1 NeoR/KanR [Mammalian expression vector pKR43]

AHC69779.1 kanamycin resistance protein [Expression vector pMVAX1(c)] AHW49187.1 kanamycin resistance protein [Cloning vector pHD-Kan]

AHW81493.1 aminoglycoside 3'-phosphotransferase [Cloning vector pBullet-pm-n] AHW81499.1 aminoglycoside 3'-phosphotransferase [Cloning vector pBullet-er-c] AHW81505.1 aminoglycoside 3'-phosphotransferase [Cloning vector pBullet-cg-n] AHW81511.1 aminoglycoside 3'-phosphotransferase [Cloning vector pBullet-pm-c] AHW81517.1 aminoglycoside 3'-phosphotransferase [Cloning vector pBullet-er-n] AHW81523.1 aminoglycoside 3'-phosphotransferase [Cloning vector pBullet-vac-c] AHW81529.1 aminoglycoside 3'-phosphotransferase [Cloning vector pBullet-cyt-c] AHW81535.1 aminoglycoside 3'-phosphotransferase [Cloning vector pBullet-vac-n] AHW81541.1 aminoglycoside 3'-phosphotransferase [Cloning vector pBullet-cyt-n] AHW81547.1 aminoglycoside 3'-phosphotransferase [Cloning vector pBullet-end-n] AHW81553.1 aminoglycoside 3'-phosphotransferase [Cloning vector pBullet-tg-c] AHW81559.1 aminoglycoside 3'-phosphotransferase [Cloning vector pBullet-end-c] AHW81565.1 aminoglycoside 3'-phosphotransferase [Cloning vector pBullet-tg-n] AHW81571.1 aminoglycoside 3'-phosphotransferase [Cloning vector pBullet-cg-c]

AHY21924.1 KanR [Cloning vector pMK-RQ_MXAN_1527_1531exp] AHY21926.1 KanR [Cloning vector pMK-RQ_MXAN_1530-1529exp] AHZ55763.1 NptII [Cloning vector pSOL9DEF1]

AHZ55768.1 NptII [Cloning vector pSOL9DEF2]

AII99786.1 KanR [Broad host range vector vector pCVD005] AIZ65953.1 KanR/NeoR [Cloning vector pCAG-EYFP] AIZ65963.1 KanR/NeoR [Cloning vector pCMV-TALER1] AIZ65964.1 KanR/NeoR [Cloning vector pCMV-TALER2] AIZ65965.1 KanR/NeoR [Cloning vector pCMV-TALER4] AIZ65966.1 KanR/NeoR [Cloning vector pCMV-TALER5] AIZ65967.1 KanR/NeoR [Cloning vector pCMV-TALER9] AIZ65968.1 KanR/NeoR [Cloning vector pCMV-TALER10] AIZ65969.1 KanR/NeoR [Cloning vector pCMV-TALER11] AIZ65970.1 KanR/NeoR [Cloning vector pCMV-TALER12] AIZ65971.1 KanR/NeoR [Cloning vector pCMV-TALER13] AIZ65972.1 KanR/NeoR [Cloning vector pCMV-TALER14] AIZ65973.1 KanR/NeoR [Cloning vector pCMV-TALER15] AIZ65974.1 KanR/NeoR [Cloning vector pCMV-TALER16] AIZ65975.1 KanR/NeoR [Cloning vector pCMV-TALER17] AIZ65977.1 KanR/NeoR [Cloning vector pCMV-TALER19] AIZ65979.1 KanR/NeoR [Cloning vector pCMV-TALER21] AIZ65980.1 KanR/NeoR [Cloning vector pCMV-TALER22] AIZ65981.1 KanR/NeoR [Cloning vector pCMV-TALER23] AIZ65983.1 KanR/NeoR [Cloning vector pCMV-TALER26] AIZ65984.1 KanR/NeoR [Cloning vector pCMV-TALER29] AIZ65985.1 KanR/NeoR [Cloning vector pCMV-TALER30] AIZ65986.1 KanR/NeoR [Cloning vector pCMV-TALER31] AIZ65987.1 KanR/NeoR [Cloning vector pCMV-TALER32] AIZ65988.1 KanR/NeoR [Cloning vector pCMV-TALER35] AMM04546.1 AphA1 [Cloning vector pCntrlPIGver2]

AMM04550.1 neomycin-kanamycin phosphotransferase [Cloning vector pSplPIGver2]

ANB62415.1 kanamycin resistance protein [Cloning vector pPV576] ANN24999.1 neomycin resistance protein [Gallid alphaherpesvirus 1] AOU74537.1 kanamycin resistance protein [Cloning vector pTOPO-1705] AOU74540.1 kanamycin resistance protein [Cloning vector pTOPO-0705] APB62233.1 kanR [Cloning vector pYL15]

APB62235.1 kanR [Cloning vector pYL2]

AQQ16622.1 neomycin phosphotransferase class II [Cloning vector pRESC8AGO8] AQR57380.1 kanamycin/neomycin resistance protein [Cloning vector pGZ-DSB-SDSA] AUX13697.1 KanR [Cloning vector pLCP.CFH]

AUX13699.1 KanR [Cloning vector pNR.CFH]

AVR52678.1 aminoglycoside phosphotransferase [Cloning vector pCPC51] AVR52682.1 aminoglycoside phosphotransferase from Tn5 [Cloning vector pCPC52] EEA82270.1 kanamycin kinase [Tyzzerella nexilis DSM 1787]

MBF3325164.1 APH(3')-II family aminoglycoside O-phosphotransferase [Pseudomonas aeruginosa]

QAT92925.1 aminoglycoside phosphotransferase [Cloning vector pCPC53] QAT92929.1 aminoglycoside phosphotransferase [Cloning vector pCPC54] QBR34674.1 NeoR/KanR [Cloning vector pVAX1-BMP2]

QBR34676.1 NeoR/KanR [Cloning vector pVAX1-BMP7] QBR34678.1 NeoR/KanR [Cloning vector pVAX1-BMP2/7 (-)] QBR34681.1 NeoR/KanR [Cloning vector pVAX1-BMP2/7 (+)]

QBZ68792.1 aminoglycoside phosphotransferase from Tn5 [Expression vector pCMV-NP868R]

QDA77217.1 aminoglycoside phosphotransferase [Cloning vector pBM065] QDA77219.1 aminoglycoside phosphotransferase [Cloning vector pBM064] QDJ00486.1 neomycin phosphotransferase class II [Cloning vector pRESC8AGO4] QEG99024.1 kanamycine resistance [Cloning vector pTrichoGate-6]

QEG99029.1 kanamycine resistance [Cloning vector pTrichoGate-9] QEG99033.1 kanamycine resistance [Cloning vector pTrichoGate-12] QEG99042.1 kanamycine resistance [Cloning vector pTrichoGate-19]

QGN63598.1 APH(3')-II family aminoglycoside O-phosphotransferase [Xanthomonas oryzae pv. oryzae]

QHN70772.1 aminoglycoside phosphotransferase [Expression vector pMT-2] QHN70774.1 aminoglycoside phosphotransferase [Expression vector pMT-1] QID92304.1 kanamycin resistance protein [Cloning vector pSN-caSAT1] QJS06992.1 KanR [Cloning vector pXJ133]

QKX95812.1 aminoglycoside phosphotransferase [Expression vector pMT-3] QKX95815.1 aminoglycoside phosphotransferase [Expression vector pMT-4] QKX95818.1 aminoglycoside phosphotransferase [Expression vector pMT-5] QKX95821.1 aminoglycoside phosphotransferase [Expression vector pMT-6] QPB77382.1 Kan/neoR [Cloning vector pBoxCDGC_2xKMet_EGFP]

RYG70982.1 APH(3')-II family aminoglycoside O-phosphotransferase [Lentibacillus sp. SSKP1-9]

CAA71576.1 kanamycin marker [Cloning vector pZeRO-2T] CAD27780.1 kanamycin resistance protein [Cloning vector pTarg2]

CAL59733.1 kanamycin resistance gene [Cloning vector PGK/Tn5-sA/IRES/NLS-LacZpA] CDI44075.1 unnamed protein product [Cloning vector pTAL7A]

CDI44077.1 unnamed protein product [Cloning vector pTAL7B]

SJL86500.1 unnamed protein product [Mammalian expression vector pEGFP-p65] SJL86503.1 unnamed protein product [Mammalian expression vector pEYFP-p65] SJL86506.1 unnamed protein product [Mammalian expression vector pECFP-YFP] SJL86509.1 unnamed protein product [Mammalian expression vector pEYFP-p65-mDsRed] SJL86510.1 unnamed protein product [Mammalian expression vector pDsRed-N-GW] SJL86517.1 unnamed protein product [Mammalian vector pCFP-N-GW]

SJL86521.1 unnamed protein product [Vector pGFP-N-GW]

SJL86603.1 unnamed protein product [Mammalian expression vector pEGFP-N1-mito-TRAP1] SJL86616.1 unnamed protein product [Mammalian expression vector pEGFP-N1-TRAP1] SJL86629.1 unnamed protein product [Mammalian expression vector pEGFP-NLS] SJL86688.1 unnamed protein product [Mammalian expression vector pEGFP-LC3] SJL87099.1 unnamed protein product [Mammalian expression vector pPTB-HcRED] SJL87102.1 unnamed protein product [Mammalian expression vector pEGFP-hnRNPC1]

SJL87441.1 unnamed protein product [Mammalian expression vector pEGFPC2-haEctn(69-3454)] SJL87899.1 unnamed protein product [Diatom vector pDEST-EGFP]

SJL87918.1 unnamed protein product [Mammalian expression vector pEGFPC2-haNctn(39-2885)] SJL87921.1 unnamed protein product [Mammalian expression vector pEGFPC2-haTctn(179-2860)]

BAM10897.1 kanamycin resistance protein [Tetracycline-inducible expression vector p9xtetO-IE2mini(L)-DEST]

BAM10903.1 kanamycin resistance protein [Cloning vector pZErO-2/9xtetO-IE2mini(L)-hairpin-A3int- Gm(R)]

BAM36533.1 kanamycin resistance protein [Donor vector pZErOTM7ZeoGFPattB] BAV54115.1 neomycin/kanamycin resistance protein [Cloning vector pCAG-dCas9TET1CD]

BAV54117.1 neomycin/kanamycin resistance protein [Cloning vector pCAG-dCas9-10xGCN4_v4] BAV54119.1 neomycin/kanamycin resistance protein [Cloning vector pCAG-dCas9-5xPlat2AflD] BAV54121.1 neomycin/kanamycin resistance protein [Cloning vector pCAG-dCas9-3.5xSuper] BAV54123.1 neomycin/kanamycin resistance protein [Cloning vector pCAG-scFvGCN4sfGFPTET1CD] BAV54125.1 neomycin/kanamycin resistance protein [Cloning vector pPlatTET-gRNA2]

HAA6103496.1 APH(3') family aminoglycoside O-phosphotransferase [Listeria monocytogenes]

Length=264

Score = 533 bits (1373), Expect = 0.0, Method: Compositional matrix adjust.

| Identities | = 263/264 (99%), Positives = 263/264 (99%), Gaps = 0/264 (0%) |  |
| --- | --- | --- |
| Query 1 | MIEQDGLHAGSPAAWVERLFGYDWAQQTIGCSDAAVFRLSAQGRPVLFVKTDLSGALNEL | 60 |
| Sbjct 1 | MIEQDGLHAGSPAAWVERLFGYDWAQQTIGCSDAAVFRLSAQGRPVLFVKTDLSGALNEL MIEQDGLHAGSPAAWVERLFGYDWAQQTIGCSDAAVFRLSAQGRPVLFVKTDLSGALNEL | 60 |
| Query 61 | QDEAARLSWLATTGVPCAAVLDVVTEAGRDWLLLGEVPGQDLLSSHLAPAEKVSIMADAM | 120 |
| Sbjct 61 | QDEAARLSWLATTGVPCAAVLDVVTEAGRDWLLLGEVPGQDLLSSHLAPAEKVSIMADAM QDEAARLSWLATTGVPCAAVLDVVTEAGRDWLLLGEVPGQDLLSSHLAPAEKVSIMADAM | 120 |
| Query 121 | RRLHTLDPATCPFDHQAKHRIERARTRMEAGLVDQDDLDEEHQGLAPAELFARLKARMPD | 180 |
| Sbjct 121 | RRLHTLDPATCPFDHQAKHRIERARTRMEAGLVDQDDLDEEHQGLAPAELFARLKA MPD RRLHTLDPATCPFDHQAKHRIERARTRMEAGLVDQDDLDEEHQGLAPAELFARLKASMPD | 180 |
| Query 181 | GEDLVVTHGDACLPNIMVENGRFSGFIDCGRLGVADRYQDIALATRDIAEELGGEWADRF | 240 |
| Sbjct 181 | GEDLVVTHGDACLPNIMVENGRFSGFIDCGRLGVADRYQDIALATRDIAEELGGEWADRF GEDLVVTHGDACLPNIMVENGRFSGFIDCGRLGVADRYQDIALATRDIAEELGGEWADRF | 240 |
| Query 241  Sbjct 241 | LVLYGIAAPDSQRIAFYRLLDEFF 264 LVLYGIAAPDSQRIAFYRLLDEFF LVLYGIAAPDSQRIAFYRLLDEFF 264 |  |

>WP_094545108.1 APH(3')-II family aminoglycoside O-phosphotransferase [Klebsiella pneumoniae]

Length=264

Score = 533 bits (1373), Expect = 0.0, Method: Compositional matrix adjust. Identities = 263/264 (99%), Positives = 263/264 (99%), Gaps = 0/264 (0%)

Query 1	MIEQDGLHAGSPAAWVERLFGYDWAQQTIGCSDAAVFRLSAQGRPVLFVKTDLSGALNEL 60 MIEQDGLHAGSPAAWVERLFGYDWAQQTIGCSDAAVFRLSAQGRPVLFVKTDLSGALNEL

| Sbjct | 1 | MIEQDGLHAGSPAAWVERLFGYDWAQQTIGCSDAAVFRLSAQGRPVLFVKTDLSGALNEL | 60 |
| --- | --- | --- | --- |
| Query | 61 | QDEAARLSWLATTGVPCAAVLDVVTEAGRDWLLLGEVPGQDLLSSHLAPAEKVSIMADAM | 120 |
| Sbjct | 61 | QDEAARLSWLATTGVPCAAVLDVVTEAGRDWLLLGEVPGQDLLSSHLAPAEKVSIMADAM QDEAARLSWLATTGVPCAAVLDVVTEAGRDWLLLGEVPGQDLLSSHLAPAEKVSIMADAM | 120 |
| Query | 121 | RRLHTLDPATCPFDHQAKHRIERARTRMEAGLVDQDDLDEEHQGLAPAELFARLKARMPD | 180 |
| Sbjct | 121 | RRLHTLDPATCPFDHQAKHRIERARTRMEA LVDQDDLDEEHQGLAPAELFARLKARMPD RRLHTLDPATCPFDHQAKHRIERARTRMEAALVDQDDLDEEHQGLAPAELFARLKARMPD | 180 |
| Query | 181 | GEDLVVTHGDACLPNIMVENGRFSGFIDCGRLGVADRYQDIALATRDIAEELGGEWADRF | 240 |
| Sbjct | 181 | GEDLVVTHGDACLPNIMVENGRFSGFIDCGRLGVADRYQDIALATRDIAEELGGEWADRF GEDLVVTHGDACLPNIMVENGRFSGFIDCGRLGVADRYQDIALATRDIAEELGGEWADRF | 240 |
| Query  Sbjct | 241  241 | LVLYGIAAPDSQRIAFYRLLDEFF 264 LVLYGIAAPDSQRIAFYRLLDEFF LVLYGIAAPDSQRIAFYRLLDEFF 264 |  |

>QGM50382.1 NPTII (MO1) [Expression vector pHP81561] QIN53501.1 NPTII [Cloning vector 4-UBI:NPTII:PINII]

QJD07420.1 NPTII [Cloning vector pLGS1] QJD07429.1 NPTII [Cloning vector P2]

Length=264

Score = 533 bits (1373), Expect = 0.0, Method: Compositional matrix adjust.

| Identities | = 262/264 (99%), Positives = 264/264 (100%), Gaps = 0/264 (0%) |  |
| --- | --- | --- |
| Query 1 | MIEQDGLHAGSPAAWVERLFGYDWAQQTIGCSDAAVFRLSAQGRPVLFVKTDLSGALNEL | 60 |
| Sbjct 1 | M+EQDGLHAGSPAAWVERLFGYDWAQQTIGCSDAAVFRLSAQGRPVLFVKTDLSGALNEL MVEQDGLHAGSPAAWVERLFGYDWAQQTIGCSDAAVFRLSAQGRPVLFVKTDLSGALNEL | 60 |
| Query 61 | QDEAARLSWLATTGVPCAAVLDVVTEAGRDWLLLGEVPGQDLLSSHLAPAEKVSIMADAM | 120 |
| Sbjct 61 | QDEAARLSWLATTGVPCAAVLDVVTEAGRDWLLLGEVPGQDLLSSHLAPAEKVSIMADAM QDEAARLSWLATTGVPCAAVLDVVTEAGRDWLLLGEVPGQDLLSSHLAPAEKVSIMADAM | 120 |
| Query 121 | RRLHTLDPATCPFDHQAKHRIERARTRMEAGLVDQDDLDEEHQGLAPAELFARLKARMPD | 180 |
| Sbjct 121 | RRLHTLDPATCPFDHQAKHRIERARTRMEAGLVDQDDLDEEHQGLAPAELFARLKARMPD RRLHTLDPATCPFDHQAKHRIERARTRMEAGLVDQDDLDEEHQGLAPAELFARLKARMPD | 180 |
| Query 181 | GEDLVVTHGDACLPNIMVENGRFSGFIDCGRLGVADRYQDIALATRDIAEELGGEWADRF | 240 |
| Sbjct 181 | G+DLVVTHGDACLPNIMVENGRFSGFIDCGRLGVADRYQDIALATRDIAEELGGEWADRF GDDLVVTHGDACLPNIMVENGRFSGFIDCGRLGVADRYQDIALATRDIAEELGGEWADRF | 240 |
| Query 241  Sbjct 241 | LVLYGIAAPDSQRIAFYRLLDEFF 264 LVLYGIAAPDSQRIAFYRLLDEFF LVLYGIAAPDSQRIAFYRLLDEFF 264 |  |

>AWV91929.1 Kan(r) [Cloning vector pFNK-202-qsc119] Length=264

Score = 533 bits (1372), Expect = 0.0, Method: Compositional matrix adjust.

| Identities | = 263/264 (99%), Positives = 263/264 (99%), Gaps = 0/264 (0%) |  |
| --- | --- | --- |
| Query 1 | MIEQDGLHAGSPAAWVERLFGYDWAQQTIGCSDAAVFRLSAQGRPVLFVKTDLSGALNEL | 60 |
| Sbjct 1 | MIEQDGLHAGSPAAWVERLFGYDWAQQ IGCSDAAVFRLSAQGRPVLFVKTDLSGALNEL MIEQDGLHAGSPAAWVERLFGYDWAQQAIGCSDAAVFRLSAQGRPVLFVKTDLSGALNEL | 60 |
| Query 61 | QDEAARLSWLATTGVPCAAVLDVVTEAGRDWLLLGEVPGQDLLSSHLAPAEKVSIMADAM | 120 |
| Sbjct 61 | QDEAARLSWLATTGVPCAAVLDVVTEAGRDWLLLGEVPGQDLLSSHLAPAEKVSIMADAM QDEAARLSWLATTGVPCAAVLDVVTEAGRDWLLLGEVPGQDLLSSHLAPAEKVSIMADAM | 120 |
| Query 121 | RRLHTLDPATCPFDHQAKHRIERARTRMEAGLVDQDDLDEEHQGLAPAELFARLKARMPD | 180 |
| Sbjct 121 | RRLHTLDPATCPFDHQAKHRIERARTRMEAGLVDQDDLDEEHQGLAPAELFARLKARMPD RRLHTLDPATCPFDHQAKHRIERARTRMEAGLVDQDDLDEEHQGLAPAELFARLKARMPD | 180 |
| Query 181 | GEDLVVTHGDACLPNIMVENGRFSGFIDCGRLGVADRYQDIALATRDIAEELGGEWADRF | 240 |
| Sbjct 181 | GEDLVVTHGDACLPNIMVENGRFSGFIDCGRLGVADRYQDIALATRDIAEELGGEWADRF GEDLVVTHGDACLPNIMVENGRFSGFIDCGRLGVADRYQDIALATRDIAEELGGEWADRF | 240 |
| Query 241 | LVLYGIAAPDSQRIAFYRLLDEFF 264 |  |

LVLYGIAAPDSQRIAFYRLLDEFF Sbjct 241 LVLYGIAAPDSQRIAFYRLLDEFF 264

>AAD50549.1 aminoglycoside 3'-phosphotransferase mutant [synthetic construct] AAF65391.1 aminoglycoside phosphotransferase [Binary vector pCAMBIA-2200] AAF65396.1 neomycin phosphotransferase [Binary vector pCAMBIA-2201] AAF65400.1 neomycin phosphotransferase [Binary vector pCAMBIA-2300] AAF65403.1 neomycin phosphotransferase [Binary vector pCAMBIA-2301] ABY41244.1 neomycin phosphotransferase [Transformation vector pC23HC] ABY41247.1 neomycin phosphotransferase [Transformation vector pC23HG] ABY41250.1 neomycin phosphotransferase [Transformation vector pC23LB] ABY41253.1 neomycin phosphotransferase [Transformation vector pC23LG] ABY55041.1 neomycin phosphotransferase [Transformation vector pC23C] ACJ06733.1 kanamycin resistance protein [Cloning vector pRGK374]

ADD10760.1 neomycin phosphotransferase II [Binary vector pCOXS3-phiC31]

AEP19804.1 neomycin phosphotransferase II [Binary vector pPIPRA560] AEP19806.1 neomycin phosphotransferase II [Binary vector pPIPRA561] AFF61395.1 neomycin phosphotransferase II [Binary vector pCOXS3-Bxb1] AFI23678.1 neomycin phosphotransferase II [Cloning vector pPIPRA587] AFI23683.1 neomycin phosphotransferase II [Cloning vector pPIPRA589] AFI23688.1 neomycin phosphotransferase II [Cloning vector pPIPRA590] AFI93671.1 NptII [Binary vector pVCPGUS(I)23010]

AFI93676.1 NptII [Binary vector pVCPGUS23010] AFM76957.1 NPTII [Plant transformation vector pCAM-NOS] AFM76959.1 NPTII [Plant transformation vector pCAM-PROM]

AFM76963.1 NPTII [Plant transformation vector pCAM-AtSUC] AFM76965.1 NPTII [Plant transformation vector pCAM-RSs1] AFM76968.1 NPTII [Plant transformation vector pCAM-rolC] AFM76971.1 NPTII [Plant transformation vector pCAM-RTBV] AGU26662.1 kanamycin resistance protein [Binary vector pSiM24]

AII71482.1 neomycin phosphotransferase [standard reference vector pMCS] AKC88571.1 neomycin phosphotransferase [Binary vector PC-GW-Kan] AKE42810.1 neomycin phosphotransferase [Cloning vector pPIPRA540] AKE42812.1 neomycin phosphotransferase [Cloning vector pPIPRA542] AKE42815.1 neomycin phosphotransferase [Cloning vector pPIPRA719] AKE42818.1 neomycin phosphotransferase [Cloning vector pPIPRA728] AKE42827.1 neomycin phosphotransferase [Cloning vector pPIPRA726]

AKE81019.1 neomycin phosphotransferase II [Plant multiplex genome editing vector pYLCRISPR/Cas9P35s-N]

AMB57372.1 KanR [Binary vector pARS1]

ASK07509.1 neomycin phosphotransferase [Plant TransGene Stacking II system binary acceptor vector pYLTAC380N]

ASK07517.1 neomycin phosphotransferase [Plant TransGene Stacking II system marker-free donor vector pYLMF-N]

ASR91724.1 aminoglycoside phosphotransferase [Cloning vector pGapneoR3] ATV83153.1 aminoglycoside phosphotransferase [Expression vector pGapneoR12] AVD96840.1 neomycin phosphotransferase II [Vector pYLCRISPR/Cas9Pubi-N]

AWK49568.1 aminoglycoside phosphotransferase [Cloning vector GAANTRY P donor nptII]

QAA77509.1 NPTII [Plant Stable Expression Vector pRGE] QAA77512.1 NPTII [Plant Stable Expression Vector pRGEcHA] QAA77515.1 NPTII [Plant Stable Expression Vector pRGEcMyc] QAA77519.1 NPTII [Plant Stable Expression Vector pRGEcYFP] QAA77522.1 NPTII [Plant Stable Overexpression Vector pRGV] QAA77525.1 NPTII [Plant Stable Overexpression Vector pRGVcHA] QAA77528.1 NPTII [Plant Stable Overexpression Vector pRGVcMyc] QAA77532.1 NPTII [Plant Stable Overexpression Vector pRGVcGFP] QAA77536.1 NPTII [Plant Stable Overexpression Vector pRGVcRFP] QAA77544.1 NPTII [Plant Genome Editing Vector pRGCas9] QEG99091.1 neomycin phosphotransferase [Binary vector pCIP99]

CAP08926.1 neomycin phosphotransferase [Transformation vector pICH14313]

BAL49714.1 Kanamycin kinase, type II [Cloning vector pSolycp00001] BAL49717.1 Kanamycin kinase, type II [Cloning vector pSolycp00002] BAL49720.1 Kanamycin kinase, type II [Cloning vector pSolycp00003] BAL49723.1 Kanamycin kinase, type II [Cloning vector pSolycp00004] BAL49726.1 Kanamycin kinase, type II [Cloning vector pSolycp00005] BAL49729.1 Kanamycin kinase, type II [Cloning vector pSolycp00006] BAL49732.1 Kanamycin kinase, type II [Cloning vector pSolycp00007] BAL49735.1 Kanamycin kinase, type II [Cloning vector pSolycp00008] BAL49738.1 Kanamycin kinase, type II [Cloning vector pSolycp00009] BAL49741.1 Kanamycin kinase, type II [Cloning vector pSolycp00010]

BAL49744.1 Kanamycin kinase, type II [Cloning vector pSolycp00011] BAL49747.1 Kanamycin kinase, type II [Cloning vector pSolycp00012] BAL49750.1 Kanamycin kinase, type II [Cloning vector pSolycp00013] BAL49753.1 Kanamycin kinase, type II [Cloning vector pSolycp00014] BAL49756.1 Kanamycin kinase, type II [Cloning vector pSolycp00015] BAL49759.1 Kanamycin kinase, type II [Cloning vector pSolycp00016] BAL49762.1 Kanamycin kinase, type II [Cloning vector pSolycp00017] BAL49765.1 Kanamycin kinase, type II [Cloning vector pSolycp00018] BAL49768.1 Kanamycin kinase, type II [Cloning vector pSolycp00019] BAL49771.1 Kanamycin kinase, type II [Cloning vector pSolycp00020] BAL49774.1 Kanamycin kinase, type II [Cloning vector pSolycp00021] BAL49777.1 Kanamycin kinase, type II [Cloning vector pSolycp00022] BAL49780.1 Kanamycin kinase, type II [Cloning vector pSolycp00023] BAL49783.1 Kanamycin kinase, type II [Cloning vector pSolycp00024] BAL49786.1 Kanamycin kinase, type II [Cloning vector pSolycp00025] BAL49789.1 Kanamycin kinase, type II [Cloning vector pSolycp00026] BAL49792.1 Kanamycin kinase, type II [Cloning vector pSolycp00027] BAL49795.1 Kanamycin kinase, type II [Cloning vector pSolycp00028] BAL49798.1 Kanamycin kinase, type II [Cloning vector pSolycp00029] BAL49801.1 Kanamycin kinase, type II [Cloning vector pSolycp00030] BAL49804.1 Kanamycin kinase, type II [Cloning vector pSolycp00031] BAL49807.1 Kanamycin kinase, type II [Cloning vector pSolycp00032] BAS50009.1 neomycin phosphotransferase II [Binary vector pMpGWB400] BAS50012.1 neomycin phosphotransferase II [Binary vector pMpGWB401] BAS50015.1 neomycin phosphotransferase II [Binary vector pMpGWB402] BAS50018.1 neomycin phosphotransferase II [Binary vector pMpGWB403] BAS50022.1 neomycin phosphotransferase II [Binary vector pMpGWB404] BAS50026.1 neomycin phosphotransferase II [Binary vector pMpGWB405] BAS50030.1 neomycin phosphotransferase II [Binary vector pMpGWB406] BAS50034.1 neomycin phosphotransferase II [Binary vector pMpGWB407] BAS50038.1 neomycin phosphotransferase II [Binary vector pMpGWB408] BAS50041.1 neomycin phosphotransferase II [Binary vector pMpGWB409] BAS50044.1 neomycin phosphotransferase II [Binary vector pMpGWB410] BAS50047.1 neomycin phosphotransferase II [Binary vector pMpGWB411] BAS50051.1 neomycin phosphotransferase II [Binary vector pMpGWB412] BAS50055.1 neomycin phosphotransferase II [Binary vector pMpGWB413] BAS50059.1 neomycin phosphotransferase II [Binary vector pMpGWB414] BAS50063.1 neomycin phosphotransferase II [Binary vector pMpGWB415] BAS50067.1 neomycin phosphotransferase II [Binary vector pMpGWB416] BAS50070.1 neomycin phosphotransferase II [Binary vector pMpGWB417] BAS50073.1 neomycin phosphotransferase II [Binary vector pMpGWB418] BAS50076.1 neomycin phosphotransferase II [Binary vector pMpGWB419] BAS50080.1 neomycin phosphotransferase II [Binary vector pMpGWB420] BAS50084.1 neomycin phosphotransferase II [Binary vector pMpGWB421] BAS50088.1 neomycin phosphotransferase II [Binary vector pMpGWB422] BAS50092.1 neomycin phosphotransferase II [Binary vector pMpGWB423] BAS50096.1 neomycin phosphotransferase II [Binary vector pMpGWB424] BAS50100.1 neomycin phosphotransferase II [Binary vector pMpGWB425] BAS50104.1 neomycin phosphotransferase II [Binary vector pMpGWB426] BAS50108.1 neomycin phosphotransferase II [Binary vector pMpGWB427] BAS50112.1 neomycin phosphotransferase II [Binary vector pMpGWB428] BAS50116.1 neomycin phosphotransferase II [Binary vector pMpGWB429] BAS50120.1 neomycin phosphotransferase II [Binary vector pMpGWB430] BAS50124.1 neomycin phosphotransferase II [Binary vector pMpGWB431] BAS50127.1 neomycin phosphotransferase II [Binary vector pMpGWB432] BAS50131.1 neomycin phosphotransferase II [Binary vector pMpGWB433] BAS50135.1 neomycin phosphotransferase II [Binary vector pMpGWB434] BAS50139.1 neomycin phosphotransferase II [Binary vector pMpGWB435] BAS50143.1 neomycin phosphotransferase II [Binary vector pMpGWB436] BAT32769.1 neomycin phosphotransferase II [Cloning vector pFungiway3] BAT32772.1 neomycin phosphotransferase II [Cloning vector pFungiway4] BAT32782.1 neomycin phosphotransferase II [Cloning vector pFungiway6] BAT32786.1 neomycin phosphotransferase II [Cloning vector pFungiway7] BAT32792.1 neomycin phosphotransferase II [Cloning vector pFungiway8]

BAV01243.1 aminoglycoside phosphotransferase [Binary vector pEgP226-2A-gfbsd2]

BBD96373.1 neomycin phosphotransferase II [Gateway vector R4pMpGWB401] BBD96376.1 neomycin phosphotransferase II [Gateway vector R4pMpGWB404] BBD96379.1 neomycin phosphotransferase II [Gateway vector R4pMpGWB407] BBD96382.1 neomycin phosphotransferase II [Gateway vector R4pMpGWB409] BBD96385.1 neomycin phosphotransferase II [Gateway vector R4pMpGWB431] BBD96388.1 neomycin phosphotransferase II [Gateway vector R4pMpGWB439] BBD96391.1 neomycin phosphotransferase II [Gateway vector R4pMpGWB454]

BBD96394.1 neomycin phosphotransferase II [Gateway vector R4pMpGWB456] BBD96397.1 neomycin phosphotransferase II [Gateway vector R4pMpGWB457] BBD96400.1 neomycin phosphotransferase II [Gateway vector R4pMpGWB494] BBD96403.1 neomycin phosphotransferase II [Gateway vector R4pMpGWB495] BBD96478.1 neomycin phosphotransferase II [Gateway vector R4L1pMpGWB404] BBD96481.1 neomycin phosphotransferase II [Gateway vector R4L1pMpGWB407] BBD96484.1 neomycin phosphotransferase II [Gateway vector R4L1pMpGWB431] BBD96487.1 neomycin phosphotransferase II [Gateway vector R4L1pMpGWB432] BBD96490.1 neomycin phosphotransferase II [Gateway vector R4L1pMpGWB435] BBD96493.1 neomycin phosphotransferase II [Gateway vector R4L1pMpGWB439] BBD96496.1 neomycin phosphotransferase II [Gateway vector R4L1pMpGWB494] BBD96499.1 neomycin phosphotransferase II [Gateway vector R4L1pMpGWB495]

Length=265

Score = 533 bits (1372), Expect = 0.0, Method: Compositional matrix adjust. Identities = 263/263 (100%), Positives = 263/263 (100%), Gaps = 0/263 (0%)

Query 2	IEQDGLHAGSPAAWVERLFGYDWAQQTIGCSDAAVFRLSAQGRPVLFVKTDLSGALNELQ 61 IEQDGLHAGSPAAWVERLFGYDWAQQTIGCSDAAVFRLSAQGRPVLFVKTDLSGALNELQ

Sbjct 3	IEQDGLHAGSPAAWVERLFGYDWAQQTIGCSDAAVFRLSAQGRPVLFVKTDLSGALNELQ 62

Query 62	DEAARLSWLATTGVPCAAVLDVVTEAGRDWLLLGEVPGQDLLSSHLAPAEKVSIMADAMR 121 DEAARLSWLATTGVPCAAVLDVVTEAGRDWLLLGEVPGQDLLSSHLAPAEKVSIMADAMR

Sbjct 63	DEAARLSWLATTGVPCAAVLDVVTEAGRDWLLLGEVPGQDLLSSHLAPAEKVSIMADAMR 122

Query 122 RLHTLDPATCPFDHQAKHRIERARTRMEAGLVDQDDLDEEHQGLAPAELFARLKARMPDG 181 RLHTLDPATCPFDHQAKHRIERARTRMEAGLVDQDDLDEEHQGLAPAELFARLKARMPDG

Sbjct 123 RLHTLDPATCPFDHQAKHRIERARTRMEAGLVDQDDLDEEHQGLAPAELFARLKARMPDG 182

Query 182 EDLVVTHGDACLPNIMVENGRFSGFIDCGRLGVADRYQDIALATRDIAEELGGEWADRFL 241 EDLVVTHGDACLPNIMVENGRFSGFIDCGRLGVADRYQDIALATRDIAEELGGEWADRFL

Sbjct 183 EDLVVTHGDACLPNIMVENGRFSGFIDCGRLGVADRYQDIALATRDIAEELGGEWADRFL 242

Query 242 VLYGIAAPDSQRIAFYRLLDEFF 264

VLYGIAAPDSQRIAFYRLLDEFF Sbjct 243 VLYGIAAPDSQRIAFYRLLDEFF 265

>EBQ6784660.1 APH(3')-II family aminoglycoside O-phosphotransferase [Salmonella enterica]

Length=264

Score = 533 bits (1372), Expect = 0.0, Method: Compositional matrix adjust.

| Identities | = 263/264 (99%), Positives = 263/264 (99%), Gaps = 0/264 (0%) |  |
| --- | --- | --- |
| Query 1 | MIEQDGLHAGSPAAWVERLFGYDWAQQTIGCSDAAVFRLSAQGRPVLFVKTDLSGALNEL | 60 |
| Sbjct 1 | MIEQDGLHAGSPAAWVERLFGYDWAQQTIGCSDAAVFRLSAQGRPVLFVKTDLSGALNEL MIEQDGLHAGSPAAWVERLFGYDWAQQTIGCSDAAVFRLSAQGRPVLFVKTDLSGALNEL | 60 |
| Query 61 | QDEAARLSWLATTGVPCAAVLDVVTEAGRDWLLLGEVPGQDLLSSHLAPAEKVSIMADAM | 120 |
| Sbjct 61 | QDEAARLSWLATTGVPCAAVLDVVTEAGRDWLLLGEVPGQDLLSSHLAPAEKVSIMADAM QDEAARLSWLATTGVPCAAVLDVVTEAGRDWLLLGEVPGQDLLSSHLAPAEKVSIMADAM | 120 |
| Query 121 | RRLHTLDPATCPFDHQAKHRIERARTRMEAGLVDQDDLDEEHQGLAPAELFARLKARMPD | 180 |
| Sbjct 121 | RRLHTLDPATCPFDHQAKHRIERARTRMEAGLVDQDDLDEEHQGLAPAELFARLKARMPD RRLHTLDPATCPFDHQAKHRIERARTRMEAGLVDQDDLDEEHQGLAPAELFARLKARMPD | 180 |
| Query 181 | GEDLVVTHGDACLPNIMVENGRFSGFIDCGRLGVADRYQDIALATRDIAEELGGEWADRF | 240 |
| Sbjct 181 | GEDLVVTHGDACLPNIMVENGRFS FIDCGRLGVADRYQDIALATRDIAEELGGEWADRF GEDLVVTHGDACLPNIMVENGRFSEFIDCGRLGVADRYQDIALATRDIAEELGGEWADRF | 240 |
| Query 241  Sbjct 241 | LVLYGIAAPDSQRIAFYRLLDEFF 264 LVLYGIAAPDSQRIAFYRLLDEFF LVLYGIAAPDSQRIAFYRLLDEFF 264 |  |

>EEM0239697.1 APH(3')-II family aminoglycoside O-phosphotransferase [Salmonella enterica subsp. enterica serovar Typhimurium]

Length=264

Score = 533 bits (1372), Expect = 0.0, Method: Compositional matrix adjust. Identities = 263/264 (99%), Positives = 263/264 (99%), Gaps = 0/264 (0%)

| Query | 1 | MIEQDGLHAGSPAAWVERLFGYDWAQQTIGCSDAAVFRLSAQGRPVLFVKTDLSGALNEL | 60 |
| --- | --- | --- | --- |
| Sbjct | 1 | MIEQDGLHAGSPAAWVERLFGYDWAQQTIGCSDAAVFRLSAQGRPVLFVKTDLSGALNEL MIEQDGLHAGSPAAWVERLFGYDWAQQTIGCSDAAVFRLSAQGRPVLFVKTDLSGALNEL | 60 |
| Query | 61 | QDEAARLSWLATTGVPCAAVLDVVTEAGRDWLLLGEVPGQDLLSSHLAPAEKVSIMADAM | 120 |
| Sbjct | 61 | QDEAARLSWLATTGVPCAAVLDVVTEAGRDWLLLGEVPGQDLLSSHLAPAEKVSIMADAM QDEAARLSWLATTGVPCAAVLDVVTEAGRDWLLLGEVPGQDLLSSHLAPAEKVSIMADAM | 120 |
| Query | 121 | RRLHTLDPATCPFDHQAKHRIERARTRMEAGLVDQDDLDEEHQGLAPAELFARLKARMPD | 180 |
| Sbjct | 121 | RRLHTLDPATCPFDHQAKHRIERARTRMEAGLVDQDDLDEEHQGLAPAELFARLKARMPD RRLHTLDPATCPFDHQAKHRIERARTRMEAGLVDQDDLDEEHQGLAPAELFARLKARMPD | 180 |
| Query | 181 | GEDLVVTHGDACLPNIMVENGRFSGFIDCGRLGVADRYQDIALATRDIAEELGGEWADRF | 240 |
| Sbjct | 181 | GEDLVVTHGDACLPNIMVENGRFSGFIDCGRLGVAD YQDIALATRDIAEELGGEWADRF GEDLVVTHGDACLPNIMVENGRFSGFIDCGRLGVADHYQDIALATRDIAEELGGEWADRF | 240 |
| Query  Sbjct | 241  241 | LVLYGIAAPDSQRIAFYRLLDEFF 264 LVLYGIAAPDSQRIAFYRLLDEFF LVLYGIAAPDSQRIAFYRLLDEFF 264 |  |

```
>AFN70907.1 neomycine phosphotransferase [Plant transformation vector pAKE1::35S BpMADS4]
SJL87345.1 unnamed protein product [Plant expression vector pGSCZSOD32] Length=267

Score = 533 bits (1372), Expect = 0.0, Method: Compositional matrix adjust. Identities = 263/263 (100%), Positives = 263/263 (100%), Gaps = 0/263 (0%)

Query 2	IEQDGLHAGSPAAWVERLFGYDWAQQTIGCSDAAVFRLSAQGRPVLFVKTDLSGALNELQ 61 IEQDGLHAGSPAAWVERLFGYDWAQQTIGCSDAAVFRLSAQGRPVLFVKTDLSGALNELQ
Sbjct 5	IEQDGLHAGSPAAWVERLFGYDWAQQTIGCSDAAVFRLSAQGRPVLFVKTDLSGALNELQ 64

Query 62	DEAARLSWLATTGVPCAAVLDVVTEAGRDWLLLGEVPGQDLLSSHLAPAEKVSIMADAMR 121 DEAARLSWLATTGVPCAAVLDVVTEAGRDWLLLGEVPGQDLLSSHLAPAEKVSIMADAMR
Sbjct 65	DEAARLSWLATTGVPCAAVLDVVTEAGRDWLLLGEVPGQDLLSSHLAPAEKVSIMADAMR 124

Query 122 RLHTLDPATCPFDHQAKHRIERARTRMEAGLVDQDDLDEEHQGLAPAELFARLKARMPDG 181 RLHTLDPATCPFDHQAKHRIERARTRMEAGLVDQDDLDEEHQGLAPAELFARLKARMPDG
Sbjct 125 RLHTLDPATCPFDHQAKHRIERARTRMEAGLVDQDDLDEEHQGLAPAELFARLKARMPDG 184

Query 182 EDLVVTHGDACLPNIMVENGRFSGFIDCGRLGVADRYQDIALATRDIAEELGGEWADRFL 241 EDLVVTHGDACLPNIMVENGRFSGFIDCGRLGVADRYQDIALATRDIAEELGGEWADRFL
Sbjct 185 EDLVVTHGDACLPNIMVENGRFSGFIDCGRLGVADRYQDIALATRDIAEELGGEWADRFL 244

Query 242 VLYGIAAPDSQRIAFYRLLDEFF 264
VLYGIAAPDSQRIAFYRLLDEFF Sbjct 245 VLYGIAAPDSQRIAFYRLLDEFF 267

>HAS1700150.1 APH(3')-II family aminoglycoside O-phosphotransferase [Salmonella enterica subsp. enterica]
HAS1854174.1 APH(3')-II family aminoglycoside O-phosphotransferase [Salmonella enterica subsp. enterica]
HAS1913717.1 APH(3')-II family aminoglycoside O-phosphotransferase [Salmonella enterica subsp. enterica]
HAS1928602.1 APH(3')-II family aminoglycoside O-phosphotransferase [Salmonella enterica subsp. enterica]
HAS2006262.1 APH(3')-II family aminoglycoside O-phosphotransferase [Salmonella enterica subsp. enterica]
Length=264

Score = 533 bits (1372), Expect = 0.0, Method: Compositional matrix adjust. Identities = 263/264 (99%), Positives = 263/264 (99%), Gaps = 0/264 (0%)
```

| Query | 1 | MIEQDGLHAGSPAAWVERLFGYDWAQQTIGCSDAAVFRLSAQGRPVLFVKTDLSGALNEL | 60 |
| --- | --- | --- | --- |
| Sbjct | 1 | MIEQDGLHAGSPAAWVERLFGYDWAQQTIGCSDAAVFRLSAQGRPVLFVKTDLSGALNEL MIEQDGLHAGSPAAWVERLFGYDWAQQTIGCSDAAVFRLSAQGRPVLFVKTDLSGALNEL | 60 |
| Query | 61 | QDEAARLSWLATTGVPCAAVLDVVTEAGRDWLLLGEVPGQDLLSSHLAPAEKVSIMADAM QDEAARLSWLATTGVPCAAVLDVVTEAGRDWLLLGEVPGQDLLSSHLAPAEKVSIMADAM | 120 |

| Sbjct | 61 | QDEAARLSWLATTGVPCAAVLDVVTEAGRDWLLLGEVPGQDLLSSHLAPAEKVSIMADAM | 120 |
| --- | --- | --- | --- |
| Query | 121 | RRLHTLDPATCPFDHQAKHRIERARTRMEAGLVDQDDLDEEHQGLAPAELFARLKARMPD | 180 |
| Sbjct | 121 | RRLHTLDPATCPFDHQAKHRIERARTRMEAGLVDQDDLDEEHQGLAPAELFARLKAR PD RRLHTLDPATCPFDHQAKHRIERARTRMEAGLVDQDDLDEEHQGLAPAELFARLKARTPD | 180 |
| Query | 181 | GEDLVVTHGDACLPNIMVENGRFSGFIDCGRLGVADRYQDIALATRDIAEELGGEWADRF | 240 |
| Sbjct | 181 | GEDLVVTHGDACLPNIMVENGRFSGFIDCGRLGVADRYQDIALATRDIAEELGGEWADRF GEDLVVTHGDACLPNIMVENGRFSGFIDCGRLGVADRYQDIALATRDIAEELGGEWADRF | 240 |
| Query  Sbjct | 241  241 | LVLYGIAAPDSQRIAFYRLLDEFF 264 LVLYGIAAPDSQRIAFYRLLDEFF LVLYGIAAPDSQRIAFYRLLDEFF 264 |  |

>EFB7215585.1 APH(3')-II family aminoglycoside O-phosphotransferase [Escherichia coli]

EFB7215722.1 APH(3')-II family aminoglycoside O-phosphotransferase [Escherichia coli]

EFH2624102.1 APH(3')-II family aminoglycoside O-phosphotransferase [Escherichia coli]

EFH2624175.1 APH(3')-II family aminoglycoside O-phosphotransferase [Escherichia coli]

Length=264

Score = 533 bits (1372), Expect = 0.0, Method: Compositional matrix adjust.

| Identities | = 263/264 (99%), Positives = 263/264 (99%), Gaps = 0/264 (0%) |  |
| --- | --- | --- |
| Query 1 | MIEQDGLHAGSPAAWVERLFGYDWAQQTIGCSDAAVFRLSAQGRPVLFVKTDLSGALNEL | 60 |
| Sbjct 1 | MIEQDGLHAGSPAAWVERLFGYDWAQQTIGCSDAAVFRLSAQGRPVLFVKTDLSGALNEL MIEQDGLHAGSPAAWVERLFGYDWAQQTIGCSDAAVFRLSAQGRPVLFVKTDLSGALNEL | 60 |
| Query 61 | QDEAARLSWLATTGVPCAAVLDVVTEAGRDWLLLGEVPGQDLLSSHLAPAEKVSIMADAM | 120 |
| Sbjct 61 | QDEAARLSWLATTGVPCAAVLDVVTEAGRDWLLLGEVPGQDLLSS LAPAEKVSIMADAM QDEAARLSWLATTGVPCAAVLDVVTEAGRDWLLLGEVPGQDLLSSQLAPAEKVSIMADAM | 120 |
| Query 121 | RRLHTLDPATCPFDHQAKHRIERARTRMEAGLVDQDDLDEEHQGLAPAELFARLKARMPD | 180 |
| Sbjct 121 | RRLHTLDPATCPFDHQAKHRIERARTRMEAGLVDQDDLDEEHQGLAPAELFARLKARMPD RRLHTLDPATCPFDHQAKHRIERARTRMEAGLVDQDDLDEEHQGLAPAELFARLKARMPD | 180 |
| Query 181 | GEDLVVTHGDACLPNIMVENGRFSGFIDCGRLGVADRYQDIALATRDIAEELGGEWADRF | 240 |
| Sbjct 181 | GEDLVVTHGDACLPNIMVENGRFSGFIDCGRLGVADRYQDIALATRDIAEELGGEWADRF GEDLVVTHGDACLPNIMVENGRFSGFIDCGRLGVADRYQDIALATRDIAEELGGEWADRF | 240 |
| Query 241  Sbjct 241 | LVLYGIAAPDSQRIAFYRLLDEFF 264 LVLYGIAAPDSQRIAFYRLLDEFF LVLYGIAAPDSQRIAFYRLLDEFF 264 |  |

```
>CAD27779.1 neomycin resistance protein [Cloning vector pTarg2] Length=279

Score = 533 bits (1372), Expect = 0.0, Method: Compositional matrix adjust. Identities = 263/263 (100%), Positives = 263/263 (100%), Gaps = 0/263 (0%)

Query 2	IEQDGLHAGSPAAWVERLFGYDWAQQTIGCSDAAVFRLSAQGRPVLFVKTDLSGALNELQ 61 IEQDGLHAGSPAAWVERLFGYDWAQQTIGCSDAAVFRLSAQGRPVLFVKTDLSGALNELQ
Sbjct 17	IEQDGLHAGSPAAWVERLFGYDWAQQTIGCSDAAVFRLSAQGRPVLFVKTDLSGALNELQ 76

Query 62	DEAARLSWLATTGVPCAAVLDVVTEAGRDWLLLGEVPGQDLLSSHLAPAEKVSIMADAMR 121 DEAARLSWLATTGVPCAAVLDVVTEAGRDWLLLGEVPGQDLLSSHLAPAEKVSIMADAMR
Sbjct 77	DEAARLSWLATTGVPCAAVLDVVTEAGRDWLLLGEVPGQDLLSSHLAPAEKVSIMADAMR 136

Query 122 RLHTLDPATCPFDHQAKHRIERARTRMEAGLVDQDDLDEEHQGLAPAELFARLKARMPDG 181 RLHTLDPATCPFDHQAKHRIERARTRMEAGLVDQDDLDEEHQGLAPAELFARLKARMPDG
Sbjct 137 RLHTLDPATCPFDHQAKHRIERARTRMEAGLVDQDDLDEEHQGLAPAELFARLKARMPDG 196

Query 182 EDLVVTHGDACLPNIMVENGRFSGFIDCGRLGVADRYQDIALATRDIAEELGGEWADRFL 241 EDLVVTHGDACLPNIMVENGRFSGFIDCGRLGVADRYQDIALATRDIAEELGGEWADRFL
Sbjct 197 EDLVVTHGDACLPNIMVENGRFSGFIDCGRLGVADRYQDIALATRDIAEELGGEWADRFL 256

Query 242 VLYGIAAPDSQRIAFYRLLDEFF 264

VLYGIAAPDSQRIAFYRLLDEFF Sbjct 257 VLYGIAAPDSQRIAFYRLLDEFF 279

>EAO9809309.1 APH(3')-II family aminoglycoside O-phosphotransferase [Salmonella enterica]
Length=264

Score = 533 bits (1372), Expect = 0.0, Method: Compositional matrix adjust.
```

| Identities | = 263/264 (99%), Positives = 263/264 (99%), Gaps = 0/264 (0%) |  |
| --- | --- | --- |
| Query 1 | MIEQDGLHAGSPAAWVERLFGYDWAQQTIGCSDAAVFRLSAQGRPVLFVKTDLSGALNEL | 60 |
| Sbjct 1 | MIEQDGLHAGSPAAWV RLFGYDWAQQTIGCSDAAVFRLSAQGRPVLFVKTDLSGALNEL MIEQDGLHAGSPAAWVVRLFGYDWAQQTIGCSDAAVFRLSAQGRPVLFVKTDLSGALNEL | 60 |
| Query 61 | QDEAARLSWLATTGVPCAAVLDVVTEAGRDWLLLGEVPGQDLLSSHLAPAEKVSIMADAM | 120 |
| Sbjct 61 | QDEAARLSWLATTGVPCAAVLDVVTEAGRDWLLLGEVPGQDLLSSHLAPAEKVSIMADAM QDEAARLSWLATTGVPCAAVLDVVTEAGRDWLLLGEVPGQDLLSSHLAPAEKVSIMADAM | 120 |
| Query 121 | RRLHTLDPATCPFDHQAKHRIERARTRMEAGLVDQDDLDEEHQGLAPAELFARLKARMPD | 180 |
| Sbjct 121 | RRLHTLDPATCPFDHQAKHRIERARTRMEAGLVDQDDLDEEHQGLAPAELFARLKARMPD RRLHTLDPATCPFDHQAKHRIERARTRMEAGLVDQDDLDEEHQGLAPAELFARLKARMPD | 180 |
| Query 181 | GEDLVVTHGDACLPNIMVENGRFSGFIDCGRLGVADRYQDIALATRDIAEELGGEWADRF | 240 |
| Sbjct 181 | GEDLVVTHGDACLPNIMVENGRFSGFIDCGRLGVADRYQDIALATRDIAEELGGEWADRF GEDLVVTHGDACLPNIMVENGRFSGFIDCGRLGVADRYQDIALATRDIAEELGGEWADRF | 240 |
| Query 241  Sbjct 241 | LVLYGIAAPDSQRIAFYRLLDEFF 264 LVLYGIAAPDSQRIAFYRLLDEFF LVLYGIAAPDSQRIAFYRLLDEFF 264 |  |

>AAF82797.1 kanamycin resistance-conferring protein [synthetic construct] Length=288

Score = 533 bits (1372), Expect = 0.0, Method: Compositional matrix adjust.

| Identities | = 262/263 (99%), Positives = 263/263 (100%), Gaps = 0/263 (0%) |  |
| --- | --- | --- |
| Query 2 | IEQDGLHAGSPAAWVERLFGYDWAQQTIGCSDAAVFRLSAQGRPVLFVKTDLSGALNELQ | 61 |
| Sbjct 26 | IEQDGLHAGSPAAWVERLFGYDWAQQTIGCSDAAVFRLSAQGRPVLFVKTDLSGALNELQ IEQDGLHAGSPAAWVERLFGYDWAQQTIGCSDAAVFRLSAQGRPVLFVKTDLSGALNELQ | 85 |
| Query 62 | DEAARLSWLATTGVPCAAVLDVVTEAGRDWLLLGEVPGQDLLSSHLAPAEKVSIMADAMR | 121 |
| Sbjct 86 | DEAARLSWLATTGVPCAAVLDVVTEAGRDWLLLGEVPGQDLLSSHLAPAEKVSIMADAMR DEAARLSWLATTGVPCAAVLDVVTEAGRDWLLLGEVPGQDLLSSHLAPAEKVSIMADAMR | 145 |
| Query 122 | RLHTLDPATCPFDHQAKHRIERARTRMEAGLVDQDDLDEEHQGLAPAELFARLKARMPDG | 181 |
| Sbjct 146 | RLHTLDPATCP+DHQAKHRIERARTRMEAGLVDQDDLDEEHQGLAPAELFARLKARMPDG RLHTLDPATCPYDHQAKHRIERARTRMEAGLVDQDDLDEEHQGLAPAELFARLKARMPDG | 205 |
| Query 182 | EDLVVTHGDACLPNIMVENGRFSGFIDCGRLGVADRYQDIALATRDIAEELGGEWADRFL | 241 |
| Sbjct 206 | EDLVVTHGDACLPNIMVENGRFSGFIDCGRLGVADRYQDIALATRDIAEELGGEWADRFL EDLVVTHGDACLPNIMVENGRFSGFIDCGRLGVADRYQDIALATRDIAEELGGEWADRFL | 265 |
| Query 242  Sbjct 266 | VLYGIAAPDSQRIAFYRLLDEFF 264 VLYGIAAPDSQRIAFYRLLDEFF VLYGIAAPDSQRIAFYRLLDEFF 288 |  |

>ABX65303.1 neomycin phosphotransferase [Cloning vector pSAM] ABX65304.1 neomycin phosphotransferase [Cloning vector pTOR] ABX65306.1 neomycin phosphotransferase [Cloning vector pNICO] ABX65308.1 neomycin phosphotransferase [Cloning vector pNC-GFP]

Length=264

Score = 533 bits (1372), Expect = 0.0, Method: Compositional matrix adjust. Identities = 263/264 (99%), Positives = 263/264 (99%), Gaps = 0/264 (0%)

Query 1	MIEQDGLHAGSPAAWVERLFGYDWAQQTIGCSDAAVFRLSAQGRPVLFVKTDLSGALNEL 60 MIEQDGLHAGSPAAWVERLFGYDWAQQTIGCSDAAVFRLSAQGRPVLFVKTDLSGALNEL

Sbjct 1	MIEQDGLHAGSPAAWVERLFGYDWAQQTIGCSDAAVFRLSAQGRPVLFVKTDLSGALNEL 60

| Query | 61 | QDEAARLSWLATTGVPCAAVLDVVTEAGRDWLLLGEVPGQDLLSSHLAPAEKVSIMADAM | 120 |
| --- | --- | --- | --- |
| Sbjct | 61 | QDE ARLSWLATTGVPCAAVLDVVTEAGRDWLLLGEVPGQDLLSSHLAPAEKVSIMADAM QDEPARLSWLATTGVPCAAVLDVVTEAGRDWLLLGEVPGQDLLSSHLAPAEKVSIMADAM | 120 |
| Query | 121 | RRLHTLDPATCPFDHQAKHRIERARTRMEAGLVDQDDLDEEHQGLAPAELFARLKARMPD | 180 |
| Sbjct | 121 | RRLHTLDPATCPFDHQAKHRIERARTRMEAGLVDQDDLDEEHQGLAPAELFARLKARMPD RRLHTLDPATCPFDHQAKHRIERARTRMEAGLVDQDDLDEEHQGLAPAELFARLKARMPD | 180 |
| Query | 181 | GEDLVVTHGDACLPNIMVENGRFSGFIDCGRLGVADRYQDIALATRDIAEELGGEWADRF | 240 |
| Sbjct | 181 | GEDLVVTHGDACLPNIMVENGRFSGFIDCGRLGVADRYQDIALATRDIAEELGGEWADRF GEDLVVTHGDACLPNIMVENGRFSGFIDCGRLGVADRYQDIALATRDIAEELGGEWADRF | 240 |
| Query  Sbjct | 241  241 | LVLYGIAAPDSQRIAFYRLLDEFF 264 LVLYGIAAPDSQRIAFYRLLDEFF LVLYGIAAPDSQRIAFYRLLDEFF 264 |  |

>AFV73969.1 neomycin phosphotransferase [Cloning vector ATG-less TV] AFV73975.1 neomycin phosphotransferase [Cloning vector 3' EGFP TV] AFV73979.1 neomycin phosphotransferase [Cloning vector 5' EGFP reporter]

Length=264

Score = 533 bits (1372), Expect = 0.0, Method: Compositional matrix adjust.

| Identities | = 263/264 (99%), Positives = 263/264 (99%), Gaps = 0/264 (0%) |  |
| --- | --- | --- |
| Query 1 | MIEQDGLHAGSPAAWVERLFGYDWAQQTIGCSDAAVFRLSAQGRPVLFVKTDLSGALNEL | 60 |
| Sbjct 1 | M EQDGLHAGSPAAWVERLFGYDWAQQTIGCSDAAVFRLSAQGRPVLFVKTDLSGALNEL MAEQDGLHAGSPAAWVERLFGYDWAQQTIGCSDAAVFRLSAQGRPVLFVKTDLSGALNEL | 60 |
| Query 61 | QDEAARLSWLATTGVPCAAVLDVVTEAGRDWLLLGEVPGQDLLSSHLAPAEKVSIMADAM | 120 |
| Sbjct 61 | QDEAARLSWLATTGVPCAAVLDVVTEAGRDWLLLGEVPGQDLLSSHLAPAEKVSIMADAM QDEAARLSWLATTGVPCAAVLDVVTEAGRDWLLLGEVPGQDLLSSHLAPAEKVSIMADAM | 120 |
| Query 121 | RRLHTLDPATCPFDHQAKHRIERARTRMEAGLVDQDDLDEEHQGLAPAELFARLKARMPD | 180 |
| Sbjct 121 | RRLHTLDPATCPFDHQAKHRIERARTRMEAGLVDQDDLDEEHQGLAPAELFARLKARMPD RRLHTLDPATCPFDHQAKHRIERARTRMEAGLVDQDDLDEEHQGLAPAELFARLKARMPD | 180 |
| Query 181 | GEDLVVTHGDACLPNIMVENGRFSGFIDCGRLGVADRYQDIALATRDIAEELGGEWADRF | 240 |
| Sbjct 181 | GEDLVVTHGDACLPNIMVENGRFSGFIDCGRLGVADRYQDIALATRDIAEELGGEWADRF GEDLVVTHGDACLPNIMVENGRFSGFIDCGRLGVADRYQDIALATRDIAEELGGEWADRF | 240 |
| Query 241  Sbjct 241 | LVLYGIAAPDSQRIAFYRLLDEFF 264 LVLYGIAAPDSQRIAFYRLLDEFF LVLYGIAAPDSQRIAFYRLLDEFF 264 |  |

>AAF82796.1 kanamycin resistance-conferring protein [synthetic construct] Length=270

Score = 532 bits (1371), Expect = 0.0, Method: Compositional matrix adjust.

| Identities | = 262/263 (99%), Positives = 263/263 (100%), Gaps = 0/263 (0%) |  |
| --- | --- | --- |
| Query 2 | IEQDGLHAGSPAAWVERLFGYDWAQQTIGCSDAAVFRLSAQGRPVLFVKTDLSGALNELQ | 61 |
| Sbjct 8 | IEQDGLHAGSPAAWVERLFGYDWAQQTIGCSDAAVFRLSAQGRPVLFVKTDLSGALNELQ IEQDGLHAGSPAAWVERLFGYDWAQQTIGCSDAAVFRLSAQGRPVLFVKTDLSGALNELQ | 67 |
| Query 62 | DEAARLSWLATTGVPCAAVLDVVTEAGRDWLLLGEVPGQDLLSSHLAPAEKVSIMADAMR | 121 |
| Sbjct 68 | DEAARLSWLATTGVPCAAVLDVVTEAGRDWLLLGEVPGQDLLSSHLAPAEKVSIMADAMR DEAARLSWLATTGVPCAAVLDVVTEAGRDWLLLGEVPGQDLLSSHLAPAEKVSIMADAMR | 127 |
| Query 122 | RLHTLDPATCPFDHQAKHRIERARTRMEAGLVDQDDLDEEHQGLAPAELFARLKARMPDG | 181 |
| Sbjct 128 | RLHTLDPATCP+DHQAKHRIERARTRMEAGLVDQDDLDEEHQGLAPAELFARLKARMPDG RLHTLDPATCPYDHQAKHRIERARTRMEAGLVDQDDLDEEHQGLAPAELFARLKARMPDG | 187 |
| Query 182 | EDLVVTHGDACLPNIMVENGRFSGFIDCGRLGVADRYQDIALATRDIAEELGGEWADRFL | 241 |
| Sbjct 188 | EDLVVTHGDACLPNIMVENGRFSGFIDCGRLGVADRYQDIALATRDIAEELGGEWADRFL EDLVVTHGDACLPNIMVENGRFSGFIDCGRLGVADRYQDIALATRDIAEELGGEWADRFL | 247 |
| Query 242  Sbjct 248 | VLYGIAAPDSQRIAFYRLLDEFF 264 VLYGIAAPDSQRIAFYRLLDEFF VLYGIAAPDSQRIAFYRLLDEFF 270 |  |

```
>ADP06893.1 NPTII [synthetic construct] [Nicotiana tabacum] Length=274

Score = 532 bits (1371), Expect = 0.0, Method: Compositional matrix adjust. Identities = 263/263 (100%), Positives = 263/263 (100%), Gaps = 0/263 (0%)

Query 2	IEQDGLHAGSPAAWVERLFGYDWAQQTIGCSDAAVFRLSAQGRPVLFVKTDLSGALNELQ 61 IEQDGLHAGSPAAWVERLFGYDWAQQTIGCSDAAVFRLSAQGRPVLFVKTDLSGALNELQ
Sbjct 12	IEQDGLHAGSPAAWVERLFGYDWAQQTIGCSDAAVFRLSAQGRPVLFVKTDLSGALNELQ 71

Query 62	DEAARLSWLATTGVPCAAVLDVVTEAGRDWLLLGEVPGQDLLSSHLAPAEKVSIMADAMR 121 DEAARLSWLATTGVPCAAVLDVVTEAGRDWLLLGEVPGQDLLSSHLAPAEKVSIMADAMR
Sbjct 72	DEAARLSWLATTGVPCAAVLDVVTEAGRDWLLLGEVPGQDLLSSHLAPAEKVSIMADAMR 131

Query 122 RLHTLDPATCPFDHQAKHRIERARTRMEAGLVDQDDLDEEHQGLAPAELFARLKARMPDG 181 RLHTLDPATCPFDHQAKHRIERARTRMEAGLVDQDDLDEEHQGLAPAELFARLKARMPDG
Sbjct 132 RLHTLDPATCPFDHQAKHRIERARTRMEAGLVDQDDLDEEHQGLAPAELFARLKARMPDG 191

Query 182 EDLVVTHGDACLPNIMVENGRFSGFIDCGRLGVADRYQDIALATRDIAEELGGEWADRFL 241 EDLVVTHGDACLPNIMVENGRFSGFIDCGRLGVADRYQDIALATRDIAEELGGEWADRFL
Sbjct 192 EDLVVTHGDACLPNIMVENGRFSGFIDCGRLGVADRYQDIALATRDIAEELGGEWADRFL 251

Query 242 VLYGIAAPDSQRIAFYRLLDEFF 264
VLYGIAAPDSQRIAFYRLLDEFF Sbjct 252 VLYGIAAPDSQRIAFYRLLDEFF 274

>EEZ6492202.1 APH(3')-II family aminoglycoside O-phosphotransferase [Escherichia coli O156]
Length=264

Score = 532 bits (1371), Expect = 0.0, Method: Compositional matrix adjust.
```

| Identities | = 263/264 (99%), Positives = 263/264 (99%), Gaps = 0/264 (0%) |  |
| --- | --- | --- |
| Query 1 | MIEQDGLHAGSPAAWVERLFGYDWAQQTIGCSDAAVFRLSAQGRPVLFVKTDLSGALNEL | 60 |
| Sbjct 1 | MIEQDGLHAGSPAAWVERLFGYDWAQQTIGCSDAAVFRLSAQGRPVLFVKTDLSGALNEL MIEQDGLHAGSPAAWVERLFGYDWAQQTIGCSDAAVFRLSAQGRPVLFVKTDLSGALNEL | 60 |
| Query 61 | QDEAARLSWLATTGVPCAAVLDVVTEAGRDWLLLGEVPGQDLLSSHLAPAEKVSIMADAM | 120 |
| Sbjct 61 | QDEAARLSWLATTGVPCAAVLDV TEAGRDWLLLGEVPGQDLLSSHLAPAEKVSIMADAM QDEAARLSWLATTGVPCAAVLDVDTEAGRDWLLLGEVPGQDLLSSHLAPAEKVSIMADAM | 120 |
| Query 121 | RRLHTLDPATCPFDHQAKHRIERARTRMEAGLVDQDDLDEEHQGLAPAELFARLKARMPD | 180 |
| Sbjct 121 | RRLHTLDPATCPFDHQAKHRIERARTRMEAGLVDQDDLDEEHQGLAPAELFARLKARMPD RRLHTLDPATCPFDHQAKHRIERARTRMEAGLVDQDDLDEEHQGLAPAELFARLKARMPD | 180 |
| Query 181 | GEDLVVTHGDACLPNIMVENGRFSGFIDCGRLGVADRYQDIALATRDIAEELGGEWADRF | 240 |
| Sbjct 181 | GEDLVVTHGDACLPNIMVENGRFSGFIDCGRLGVADRYQDIALATRDIAEELGGEWADRF GEDLVVTHGDACLPNIMVENGRFSGFIDCGRLGVADRYQDIALATRDIAEELGGEWADRF | 240 |
| Query 241  Sbjct 241 | LVLYGIAAPDSQRIAFYRLLDEFF 264 LVLYGIAAPDSQRIAFYRLLDEFF LVLYGIAAPDSQRIAFYRLLDEFF 264 |  |

>HAD8642335.1 APH(3')-II family aminoglycoside O-phosphotransferase [Salmonella enterica]

Length=264

Score = 532 bits (1371), Expect = 0.0, Method: Compositional matrix adjust. Identities = 263/264 (99%), Positives = 263/264 (99%), Gaps = 0/264 (0%)

| Query | 1 | MIEQDGLHAGSPAAWVERLFGYDWAQQTIGCSDAAVFRLSAQGRPVLFVKTDLSGALNEL | 60 |
| --- | --- | --- | --- |
| Sbjct | 1 | MIEQDGLHAGSPAAWVERLFGYDWAQQTIGCSDAAVFRLSAQGRPVLFVKTDLSGALNEL MIEQDGLHAGSPAAWVERLFGYDWAQQTIGCSDAAVFRLSAQGRPVLFVKTDLSGALNEL | 60 |
| Query | 61 | QDEAARLSWLATTGVPCAAVLDVVTEAGRDWLLLGEVPGQDLLSSHLAPAEKVSIMADAM | 120 |
| Sbjct | 61 | QDEAARLSWLATTGVPCAAVLDVVTEAGRDWLLLGEV GQDLLSSHLAPAEKVSIMADAM QDEAARLSWLATTGVPCAAVLDVVTEAGRDWLLLGEVSGQDLLSSHLAPAEKVSIMADAM | 120 |
| Query | 121 | RRLHTLDPATCPFDHQAKHRIERARTRMEAGLVDQDDLDEEHQGLAPAELFARLKARMPD | 180 |

| Sbjct | 121 | RRLHTLDPATCPFDHQAKHRIERARTRMEAGLVDQDDLDEEHQGLAPAELFARLKARMPD RRLHTLDPATCPFDHQAKHRIERARTRMEAGLVDQDDLDEEHQGLAPAELFARLKARMPD | 180 |
| --- | --- | --- | --- |
| Query | 181 | GEDLVVTHGDACLPNIMVENGRFSGFIDCGRLGVADRYQDIALATRDIAEELGGEWADRF | 240 |
| Sbjct | 181 | GEDLVVTHGDACLPNIMVENGRFSGFIDCGRLGVADRYQDIALATRDIAEELGGEWADRF GEDLVVTHGDACLPNIMVENGRFSGFIDCGRLGVADRYQDIALATRDIAEELGGEWADRF | 240 |
| Query  Sbjct | 241  241 | LVLYGIAAPDSQRIAFYRLLDEFF 264 LVLYGIAAPDSQRIAFYRLLDEFF LVLYGIAAPDSQRIAFYRLLDEFF 264 |  |

>EBW8244226.1 APH(3')-II family aminoglycoside O-phosphotransferase [Salmonella enterica subsp. enterica serovar Heidelberg]

Length=264

Score = 532 bits (1371), Expect = 0.0, Method: Compositional matrix adjust.

| Identities | = 263/264 (99%), Positives = 263/264 (99%), Gaps = 0/264 (0%) |  |
| --- | --- | --- |
| Query 1 | MIEQDGLHAGSPAAWVERLFGYDWAQQTIGCSDAAVFRLSAQGRPVLFVKTDLSGALNEL | 60 |
| Sbjct 1 | MIEQDGLHAGSPAAWVERLFGYDWAQQTIGCSDAAVFRLSAQGRPVLFVKTDLSGALNEL MIEQDGLHAGSPAAWVERLFGYDWAQQTIGCSDAAVFRLSAQGRPVLFVKTDLSGALNEL | 60 |
| Query 61 | QDEAARLSWLATTGVPCAAVLDVVTEAGRDWLLLGEVPGQDLLSSHLAPAEKVSIMADAM | 120 |
| Sbjct 61 | QDEAARLSWLATTGVPCAAVLDVVTEAGRDWLLL EVPGQDLLSSHLAPAEKVSIMADAM QDEAARLSWLATTGVPCAAVLDVVTEAGRDWLLLDEVPGQDLLSSHLAPAEKVSIMADAM | 120 |
| Query 121 | RRLHTLDPATCPFDHQAKHRIERARTRMEAGLVDQDDLDEEHQGLAPAELFARLKARMPD | 180 |
| Sbjct 121 | RRLHTLDPATCPFDHQAKHRIERARTRMEAGLVDQDDLDEEHQGLAPAELFARLKARMPD RRLHTLDPATCPFDHQAKHRIERARTRMEAGLVDQDDLDEEHQGLAPAELFARLKARMPD | 180 |
| Query 181 | GEDLVVTHGDACLPNIMVENGRFSGFIDCGRLGVADRYQDIALATRDIAEELGGEWADRF | 240 |
| Sbjct 181 | GEDLVVTHGDACLPNIMVENGRFSGFIDCGRLGVADRYQDIALATRDIAEELGGEWADRF GEDLVVTHGDACLPNIMVENGRFSGFIDCGRLGVADRYQDIALATRDIAEELGGEWADRF | 240 |
| Query 241  Sbjct 241 | LVLYGIAAPDSQRIAFYRLLDEFF 264 LVLYGIAAPDSQRIAFYRLLDEFF LVLYGIAAPDSQRIAFYRLLDEFF 264 |  |

>BAA21468.1 neomycin-kanamycin phosphotransferase [Cloning vector pMW218] BAA21470.1 neomycin-kanamycin phosphotransferase [Cloning vector pMW219]

Length=264

Score = 532 bits (1371), Expect = 0.0, Method: Compositional matrix adjust.

| Identities | = 263/264 (99%), Positives = 263/264 (99%), Gaps = 0/264 (0%) |  |
| --- | --- | --- |
| Query 1 | MIEQDGLHAGSPAAWVERLFGYDWAQQTIGCSDAAVFRLSAQGRPVLFVKTDLSGALNEL | 60 |
| Sbjct 1 | MIEQDGLHAGSPAAWVERLFGYDWAQQTIGCSDAAVFRLSAQGRPVLFVKTDLSGALNEL MIEQDGLHAGSPAAWVERLFGYDWAQQTIGCSDAAVFRLSAQGRPVLFVKTDLSGALNEL | 60 |
| Query 61 | QDEAARLSWLATTGVPCAAVLDVVTEAGRDWLLLGEVPGQDLLSSHLAPAEKVSIMADAM | 120 |
| Sbjct 61 | QDEAARLSWLATTGVPCAAVLDV TEAGRDWLLLGEVPGQDLLSSHLAPAEKVSIMADAM QDEAARLSWLATTGVPCAAVLDVGTEAGRDWLLLGEVPGQDLLSSHLAPAEKVSIMADAM | 120 |
| Query 121 | RRLHTLDPATCPFDHQAKHRIERARTRMEAGLVDQDDLDEEHQGLAPAELFARLKARMPD | 180 |
| Sbjct 121 | RRLHTLDPATCPFDHQAKHRIERARTRMEAGLVDQDDLDEEHQGLAPAELFARLKARMPD RRLHTLDPATCPFDHQAKHRIERARTRMEAGLVDQDDLDEEHQGLAPAELFARLKARMPD | 180 |
| Query 181 | GEDLVVTHGDACLPNIMVENGRFSGFIDCGRLGVADRYQDIALATRDIAEELGGEWADRF | 240 |
| Sbjct 181 | GEDLVVTHGDACLPNIMVENGRFSGFIDCGRLGVADRYQDIALATRDIAEELGGEWADRF GEDLVVTHGDACLPNIMVENGRFSGFIDCGRLGVADRYQDIALATRDIAEELGGEWADRF | 240 |
| Query 241  Sbjct 241 | LVLYGIAAPDSQRIAFYRLLDEFF 264 LVLYGIAAPDSQRIAFYRLLDEFF LVLYGIAAPDSQRIAFYRLLDEFF 264 |  |

>EEW8171270.1 APH(3')-II family aminoglycoside O-phosphotransferase [Escherichia coli]

Length=264

Score = 532 bits (1371), Expect = 0.0, Method: Compositional matrix adjust.

| Identities | = 263/264 (99%), Positives = 263/264 (99%), Gaps = 0/264 (0%) |  |
| --- | --- | --- |
| Query 1 | MIEQDGLHAGSPAAWVERLFGYDWAQQTIGCSDAAVFRLSAQGRPVLFVKTDLSGALNEL | 60 |
| Sbjct 1 | MIEQDGLHAGSPAAWVERLFGYDWAQQTIGCSDAAVFRLSAQGRPVLFVKTDLSGALNEL MIEQDGLHAGSPAAWVERLFGYDWAQQTIGCSDAAVFRLSAQGRPVLFVKTDLSGALNEL | 60 |
| Query 61 | QDEAARLSWLATTGVPCAAVLDVVTEAGRDWLLLGEVPGQDLLSSHLAPAEKVSIMADAM | 120 |
| Sbjct 61 | QDEAARLSWLATTGVPCAAVLDVVTEAGRDWLLLGEVPGQDLLSSHLAPAEKVSIMADAM QDEAARLSWLATTGVPCAAVLDVVTEAGRDWLLLGEVPGQDLLSSHLAPAEKVSIMADAM | 120 |
| Query 121 | RRLHTLDPATCPFDHQAKHRIERARTRMEAGLVDQDDLDEEHQGLAPAELFARLKARMPD | 180 |
| Sbjct 121 | RRLHTLDPATCPFDHQAKHRIERARTRMEAGLVDQDDLDEEHQGLAPAE FARLKARMPD RRLHTLDPATCPFDHQAKHRIERARTRMEAGLVDQDDLDEEHQGLAPAEPFARLKARMPD | 180 |
| Query 181 | GEDLVVTHGDACLPNIMVENGRFSGFIDCGRLGVADRYQDIALATRDIAEELGGEWADRF | 240 |
| Sbjct 181 | GEDLVVTHGDACLPNIMVENGRFSGFIDCGRLGVADRYQDIALATRDIAEELGGEWADRF GEDLVVTHGDACLPNIMVENGRFSGFIDCGRLGVADRYQDIALATRDIAEELGGEWADRF | 240 |
| Query 241  Sbjct 241 | LVLYGIAAPDSQRIAFYRLLDEFF 264 LVLYGIAAPDSQRIAFYRLLDEFF LVLYGIAAPDSQRIAFYRLLDEFF 264 |  |

>EFC6378189.1 APH(3')-II family aminoglycoside O-phosphotransferase [Escherichia coli]

Length=264

Score = 532 bits (1371), Expect = 0.0, Method: Compositional matrix adjust.

| Identities | = 263/264 (99%), Positives = 263/264 (99%), Gaps = 0/264 (0%) |  |
| --- | --- | --- |
| Query 1 | MIEQDGLHAGSPAAWVERLFGYDWAQQTIGCSDAAVFRLSAQGRPVLFVKTDLSGALNEL | 60 |
| Sbjct 1 | MIEQDGLHAGSPAAWVERLFGYDWAQQTIGCSDAAVFRLSAQGRPVL VKTDLSGALNEL MIEQDGLHAGSPAAWVERLFGYDWAQQTIGCSDAAVFRLSAQGRPVLVVKTDLSGALNEL | 60 |
| Query 61 | QDEAARLSWLATTGVPCAAVLDVVTEAGRDWLLLGEVPGQDLLSSHLAPAEKVSIMADAM | 120 |
| Sbjct 61 | QDEAARLSWLATTGVPCAAVLDVVTEAGRDWLLLGEVPGQDLLSSHLAPAEKVSIMADAM QDEAARLSWLATTGVPCAAVLDVVTEAGRDWLLLGEVPGQDLLSSHLAPAEKVSIMADAM | 120 |
| Query 121 | RRLHTLDPATCPFDHQAKHRIERARTRMEAGLVDQDDLDEEHQGLAPAELFARLKARMPD | 180 |
| Sbjct 121 | RRLHTLDPATCPFDHQAKHRIERARTRMEAGLVDQDDLDEEHQGLAPAELFARLKARMPD RRLHTLDPATCPFDHQAKHRIERARTRMEAGLVDQDDLDEEHQGLAPAELFARLKARMPD | 180 |
| Query 181 | GEDLVVTHGDACLPNIMVENGRFSGFIDCGRLGVADRYQDIALATRDIAEELGGEWADRF | 240 |
| Sbjct 181 | GEDLVVTHGDACLPNIMVENGRFSGFIDCGRLGVADRYQDIALATRDIAEELGGEWADRF GEDLVVTHGDACLPNIMVENGRFSGFIDCGRLGVADRYQDIALATRDIAEELGGEWADRF | 240 |
| Query 241  Sbjct 241 | LVLYGIAAPDSQRIAFYRLLDEFF 264 LVLYGIAAPDSQRIAFYRLLDEFF LVLYGIAAPDSQRIAFYRLLDEFF 264 |  |

>EDR5533633.1 APH(3')-II family aminoglycoside O-phosphotransferase [Salmonella enterica subsp. enterica serovar 4,12:i:-]

Length=264

Score = 532 bits (1371), Expect = 0.0, Method: Compositional matrix adjust.

| Identities | = 263/264 (99%), Positives = 263/264 (99%), Gaps = 0/264 (0%) |  |
| --- | --- | --- |
| Query 1 | MIEQDGLHAGSPAAWVERLFGYDWAQQTIGCSDAAVFRLSAQGRPVLFVKTDLSGALNEL | 60 |
| Sbjct 1 | MIEQDGLHAGSPAAWVERLFGYDWAQQTIGCSDAAVFRLSAQGRPVLFVKTDLSGALNEL MIEQDGLHAGSPAAWVERLFGYDWAQQTIGCSDAAVFRLSAQGRPVLFVKTDLSGALNEL | 60 |
| Query 61 | QDEAARLSWLATTGVPCAAVLDVVTEAGRDWLLLGEVPGQDLLSSHLAPAEKVSIMADAM | 120 |
| Sbjct 61 | QDEAARLSWLATTGVPCAAVLDVVTEAGRDWLLLGEVPGQDLLSSHLAPAEKVSIMADAM QDEAARLSWLATTGVPCAAVLDVVTEAGRDWLLLGEVPGQDLLSSHLAPAEKVSIMADAM | 120 |
| Query 121 | RRLHTLDPATCPFDHQAKHRIERARTRMEAGLVDQDDLDEEHQGLAPAELFARLKARMPD | 180 |
| Sbjct 121 | RRLHTLDPATCPFDHQAKHRIERART MEAGLVDQDDLDEEHQGLAPAELFARLKARMPD RRLHTLDPATCPFDHQAKHRIERARTLMEAGLVDQDDLDEEHQGLAPAELFARLKARMPD | 180 |
| Query 181 | GEDLVVTHGDACLPNIMVENGRFSGFIDCGRLGVADRYQDIALATRDIAEELGGEWADRF | 240 |

| Sbjct | 181 | GEDLVVTHGDACLPNIMVENGRFSGFIDCGRLGVADRYQDIALATRDIAEELGGEWADRF GEDLVVTHGDACLPNIMVENGRFSGFIDCGRLGVADRYQDIALATRDIAEELGGEWADRF 240 |
| --- | --- | --- |
| Query | 241 | LVLYGIAAPDSQRIAFYRLLDEFF 264 |
| Sbjct | 241 | LVLYGIAAPDSQRIAFYRLLDEFF  LVLYGIAAPDSQRIAFYRLLDEFF 264 |

>ECW5825779.1 APH(3')-II family aminoglycoside O-phosphotransferase [Salmonella enterica subsp. enterica serovar Heidelberg]

Length=264

Score = 532 bits (1371), Expect = 0.0, Method: Compositional matrix adjust.

| Identities | = 263/264 (99%), Positives = 263/264 (99%), Gaps = 0/264 (0%) |  |
| --- | --- | --- |
| Query 1 | MIEQDGLHAGSPAAWVERLFGYDWAQQTIGCSDAAVFRLSAQGRPVLFVKTDLSGALNEL | 60 |
| Sbjct 1 | MIEQDGLHAGSPAAWVERLFGYDWAQQTIGCSDAAVFRLSAQGRPVLFVKTDLSGALNEL MIEQDGLHAGSPAAWVERLFGYDWAQQTIGCSDAAVFRLSAQGRPVLFVKTDLSGALNEL | 60 |
| Query 61 | QDEAARLSWLATTGVPCAAVLDVVTEAGRDWLLLGEVPGQDLLSSHLAPAEKVSIMADAM | 120 |
| Sbjct 61 | QDEAARLSWLATTGVPCAAVLDVVTEAGRDWLLLGEVPGQDLLSSHLAPAEKVSIMADAM QDEAARLSWLATTGVPCAAVLDVVTEAGRDWLLLGEVPGQDLLSSHLAPAEKVSIMADAM | 120 |
| Query 121 | RRLHTLDPATCPFDHQAKHRIERARTRMEAGLVDQDDLDEEHQGLAPAELFARLKARMPD | 180 |
| Sbjct 121 | RRLHTLDPATCPFDHQAKHRIERARTRMEAGLVDQDDLDEEHQGLAPAELFARLKARMPD RRLHTLDPATCPFDHQAKHRIERARTRMEAGLVDQDDLDEEHQGLAPAELFARLKARMPD | 180 |
| Query 181 | GEDLVVTHGDACLPNIMVENGRFSGFIDCGRLGVADRYQDIALATRDIAEELGGEWADRF | 240 |
| Sbjct 181 | GEDLVVTHGDACLPNIMVENGRFSGFIDCGRLGVADRYQDIALATRDIAEELGGEWADRF GEDLVVTHGDACLPNIMVENGRFSGFIDCGRLGVADRYQDIALATRDIAEELGGEWADRF | 240 |
| Query 241  Sbjct 241 | LVLYGIAAPDSQRIAFYRLLDEFF 264 LVLYGIAAPDSQRIAFYR LDEFF LVLYGIAAPDSQRIAFYRHLDEFF 264 |  |

>ECS5261092.1 APH(3')-II family aminoglycoside O-phosphotransferase [Salmonella enterica subsp. enterica serovar Heidelberg]

Length=264

Score = 532 bits (1371), Expect = 0.0, Method: Compositional matrix adjust.

| Identities | = 263/264 (99%), Positives = 263/264 (99%), Gaps = 0/264 (0%) |  |
| --- | --- | --- |
| Query 1 | MIEQDGLHAGSPAAWVERLFGYDWAQQTIGCSDAAVFRLSAQGRPVLFVKTDLSGALNEL | 60 |
| Sbjct 1 | MIEQDGLHAGSPAAWVERLFGYDWAQQTIGCSDAAVFRLSAQGRPVLFVKTDLSGALNE MIEQDGLHAGSPAAWVERLFGYDWAQQTIGCSDAAVFRLSAQGRPVLFVKTDLSGALNER | 60 |
| Query 61 | QDEAARLSWLATTGVPCAAVLDVVTEAGRDWLLLGEVPGQDLLSSHLAPAEKVSIMADAM | 120 |
| Sbjct 61 | QDEAARLSWLATTGVPCAAVLDVVTEAGRDWLLLGEVPGQDLLSSHLAPAEKVSIMADAM QDEAARLSWLATTGVPCAAVLDVVTEAGRDWLLLGEVPGQDLLSSHLAPAEKVSIMADAM | 120 |
| Query 121 | RRLHTLDPATCPFDHQAKHRIERARTRMEAGLVDQDDLDEEHQGLAPAELFARLKARMPD | 180 |
| Sbjct 121 | RRLHTLDPATCPFDHQAKHRIERARTRMEAGLVDQDDLDEEHQGLAPAELFARLKARMPD RRLHTLDPATCPFDHQAKHRIERARTRMEAGLVDQDDLDEEHQGLAPAELFARLKARMPD | 180 |
| Query 181 | GEDLVVTHGDACLPNIMVENGRFSGFIDCGRLGVADRYQDIALATRDIAEELGGEWADRF | 240 |
| Sbjct 181 | GEDLVVTHGDACLPNIMVENGRFSGFIDCGRLGVADRYQDIALATRDIAEELGGEWADRF GEDLVVTHGDACLPNIMVENGRFSGFIDCGRLGVADRYQDIALATRDIAEELGGEWADRF | 240 |
| Query 241  Sbjct 241 | LVLYGIAAPDSQRIAFYRLLDEFF 264 LVLYGIAAPDSQRIAFYRLLDEFF LVLYGIAAPDSQRIAFYRLLDEFF 264 |  |

>AAL78958.1 neomycin phosphotransferase [Binary vector pLH9000] AAL78963.1 neomycin phosphotransferase [Binary vector pLH9500] AAR20818.1 neomycin phosphotransferase [Binary vector pRE1] ABI30071.1 NPTII [Binary vector pMOA33]

AGJ84389.1 neomycin phosphotransferase II [Binary vector pRED-PT4exp] AGW32435.1 neomycin phosphotransferase II [Binary Vector pGCGi]

AIW65091.1 neomycin phosphotransferase II [Plant transformation vector p9-Dao-FLPi-HcrVf2] AKJ78230.1 neomycin phosphotransferase II [BiFC expression vector kanII-mRYCE]

AKJ78233.1 neomycin phosphotransferase II [BiFC expression vector kanII-mRYCE(R)] AKJ78242.1 neomycin phosphotransferase II [BiFC expression vector kanII-mVYCE] AKJ78245.1 neomycin phosphotransferase II [BiFC expression vector kanII-mVYCE(R)] AKJ78248.1 neomycin phosphotransferase II [BiFC expression vector kanII-triRV] AKJ78251.1 neomycin phosphotransferase II [BiFC expression vector kanII-triVR] AKJ78260.1 neomycin phosphotransferase II [BiFC expression vector kanII-pSmRYCE] AKJ78263.1 neomycin phosphotransferase II [BiFC expression vector kanII-pSmRYCE(R)] AKJ78266.1 neomycin phosphotransferase II [BiFC expression vector kanII-StriRV] AKJ78269.1 neomycin phosphotransferase II [BiFC expression vector kanII-StriVR]

Length=267

Score = 532 bits (1371), Expect = 0.0, Method: Compositional matrix adjust. Identities = 263/263 (100%), Positives = 263/263 (100%), Gaps = 0/263 (0%)

| Query | 2 | IEQDGLHAGSPAAWVERLFGYDWAQQTIGCSDAAVFRLSAQGRPVLFVKTDLSGALNELQ | 61 |
| --- | --- | --- | --- |
| Sbjct | 5 | IEQDGLHAGSPAAWVERLFGYDWAQQTIGCSDAAVFRLSAQGRPVLFVKTDLSGALNELQ IEQDGLHAGSPAAWVERLFGYDWAQQTIGCSDAAVFRLSAQGRPVLFVKTDLSGALNELQ | 64 |
| Query | 62 | DEAARLSWLATTGVPCAAVLDVVTEAGRDWLLLGEVPGQDLLSSHLAPAEKVSIMADAMR | 121 |
| Sbjct | 65 | DEAARLSWLATTGVPCAAVLDVVTEAGRDWLLLGEVPGQDLLSSHLAPAEKVSIMADAMR DEAARLSWLATTGVPCAAVLDVVTEAGRDWLLLGEVPGQDLLSSHLAPAEKVSIMADAMR | 124 |
| Query | 122 | RLHTLDPATCPFDHQAKHRIERARTRMEAGLVDQDDLDEEHQGLAPAELFARLKARMPDG | 181 |
| Sbjct | 125 | RLHTLDPATCPFDHQAKHRIERARTRMEAGLVDQDDLDEEHQGLAPAELFARLKARMPDG RLHTLDPATCPFDHQAKHRIERARTRMEAGLVDQDDLDEEHQGLAPAELFARLKARMPDG | 184 |
| Query | 182 | EDLVVTHGDACLPNIMVENGRFSGFIDCGRLGVADRYQDIALATRDIAEELGGEWADRFL | 241 |
| Sbjct | 185 | EDLVVTHGDACLPNIMVENGRFSGFIDCGRLGVADRYQDIALATRDIAEELGGEWADRFL EDLVVTHGDACLPNIMVENGRFSGFIDCGRLGVADRYQDIALATRDIAEELGGEWADRFL | 244 |
| Query  Sbjct | 242  245 | VLYGIAAPDSQRIAFYRLLDEFF 264 VLYGIAAPDSQRIAFYRLLDEFF VLYGIAAPDSQRIAFYRLLDEFF 267 |  |

>EDJ1353951.1 APH(3') family aminoglycoside O-phosphotransferase [Salmonella enterica]

Length=264

Score = 532 bits (1371), Expect = 0.0, Method: Compositional matrix adjust.

| Identities | = 263/264 (99%), Positives = 263/264 (99%), Gaps = 0/264 (0%) |  |
| --- | --- | --- |
| Query 1 | MIEQDGLHAGSPAAWVERLFGYDWAQQTIGCSDAAVFRLSAQGRPVLFVKTDLSGALNEL | 60 |
| Sbjct 1 | MIEQDGLHAGSPAAWVERLFGYDWAQQTIGCSDAAVFRLSAQGRPVLFVKTDLSGALNEL MIEQDGLHAGSPAAWVERLFGYDWAQQTIGCSDAAVFRLSAQGRPVLFVKTDLSGALNEL | 60 |
| Query 61 | QDEAARLSWLATTGVPCAAVLDVVTEAGRDWLLLGEVPGQDLLSSHLAPAEKVSIMADAM | 120 |
| Sbjct 61 | QDEAARLSWLATTGVPCAAVLDVVTEAGRDWLLLGEVPGQDLLSSHLAPAEKVSIMADAM QDEAARLSWLATTGVPCAAVLDVVTEAGRDWLLLGEVPGQDLLSSHLAPAEKVSIMADAM | 120 |
| Query 121 | RRLHTLDPATCPFDHQAKHRIERARTRMEAGLVDQDDLDEEHQGLAPAELFARLKARMPD | 180 |
| Sbjct 121 | RRLHTLDPATCPFDHQAKHRIERARTRMEAGLVDQDDLDEEHQGLAPAELFARLKARMPD RRLHTLDPATCPFDHQAKHRIERARTRMEAGLVDQDDLDEEHQGLAPAELFARLKARMPD | 180 |
| Query 181 | GEDLVVTHGDACLPNIMVENGRFSGFIDCGRLGVADRYQDIALATRDIAEELGGEWADRF | 240 |
| Sbjct 181 | GEDLVVTHGDACLPNIMVENGRFSGFIDCGRLGVADRYQDIALATRDIAEELGGEWADRF GEDLVVTHGDACLPNIMVENGRFSGFIDCGRLGVADRYQDIALATRDIAEELGGEWADRF | 240 |
| Query 241  Sbjct 241 | LVLYGIAAPDSQRIAFYRLLDEFF 264 LVLYGIAAPDSQRIAFYRLL EFF LVLYGIAAPDSQRIAFYRLLAEFF 264 |  |

>WP_133425265.1 APH(3')-II family aminoglycoside O-phosphotransferase [Escherichia coli]

QBO96418.1 APH(3')-II family aminoglycoside O-phosphotransferase [Escherichia coli]

Length=264

Score = 532 bits (1371), Expect = 0.0, Method: Compositional matrix adjust. Identities = 263/264 (99%), Positives = 263/264 (99%), Gaps = 0/264 (0%)

| Query | 1 | MIEQDGLHAGSPAAWVERLFGYDWAQQTIGCSDAAVFRLSAQGRPVLFVKTDLSGALNEL | 60 |
| --- | --- | --- | --- |
| Sbjct | 1 | MIEQDGLHAGSPAAWVERLFGYDWAQQTIGCSDAAVFRLSAQGRPVLFVKTDLSGALNEL MIEQDGLHAGSPAAWVERLFGYDWAQQTIGCSDAAVFRLSAQGRPVLFVKTDLSGALNEL | 60 |
| Query | 61 | QDEAARLSWLATTGVPCAAVLDVVTEAGRDWLLLGEVPGQDLLSSHLAPAEKVSIMADAM | 120 |
| Sbjct | 61 | QDEAARLSWLATTGVPCAAVLDVVTEAGRDWLL GEVPGQDLLSSHLAPAEKVSIMADAM QDEAARLSWLATTGVPCAAVLDVVTEAGRDWLLPGEVPGQDLLSSHLAPAEKVSIMADAM | 120 |
| Query | 121 | RRLHTLDPATCPFDHQAKHRIERARTRMEAGLVDQDDLDEEHQGLAPAELFARLKARMPD | 180 |
| Sbjct | 121 | RRLHTLDPATCPFDHQAKHRIERARTRMEAGLVDQDDLDEEHQGLAPAELFARLKARMPD RRLHTLDPATCPFDHQAKHRIERARTRMEAGLVDQDDLDEEHQGLAPAELFARLKARMPD | 180 |
| Query | 181 | GEDLVVTHGDACLPNIMVENGRFSGFIDCGRLGVADRYQDIALATRDIAEELGGEWADRF | 240 |
| Sbjct | 181 | GEDLVVTHGDACLPNIMVENGRFSGFIDCGRLGVADRYQDIALATRDIAEELGGEWADRF GEDLVVTHGDACLPNIMVENGRFSGFIDCGRLGVADRYQDIALATRDIAEELGGEWADRF | 240 |
| Query  Sbjct | 241  241 | LVLYGIAAPDSQRIAFYRLLDEFF 264 LVLYGIAAPDSQRIAFYRLLDEFF LVLYGIAAPDSQRIAFYRLLDEFF 264 |  |

>ECM1697434.1 APH(3')-II family aminoglycoside O-phosphotransferase [Salmonella enterica subsp. enterica serovar Typhimurium]

Length=264

Score = 532 bits (1371), Expect = 0.0, Method: Compositional matrix adjust.

| Identities | = 263/264 (99%), Positives = 263/264 (99%), Gaps = 0/264 (0%) |  |
| --- | --- | --- |
| Query 1 | MIEQDGLHAGSPAAWVERLFGYDWAQQTIGCSDAAVFRLSAQGRPVLFVKTDLSGALNEL | 60 |
| Sbjct 1 | MIEQDGLHAGSPAAWVERLFGYDWAQQTIGCSDAAVFRLSAQGRPVLFVKTDLSGALNEL MIEQDGLHAGSPAAWVERLFGYDWAQQTIGCSDAAVFRLSAQGRPVLFVKTDLSGALNEL | 60 |
| Query 61 | QDEAARLSWLATTGVPCAAVLDVVTEAGRDWLLLGEVPGQDLLSSHLAPAEKVSIMADAM | 120 |
| Sbjct 61 | QDEAARLSWLATTGVPCAAVLDVVTEAGRDWLLLGEVPGQDLLSSHLAPAEKVSIMADAM QDEAARLSWLATTGVPCAAVLDVVTEAGRDWLLLGEVPGQDLLSSHLAPAEKVSIMADAM | 120 |
| Query 121 | RRLHTLDPATCPFDHQAKHRIERARTRMEAGLVDQDDLDEEHQGLAPAELFARLKARMPD | 180 |
| Sbjct 121 | RRLHTLDPATCPFDHQAKHRIERARTRMEAGLVDQDDLDEEHQGLAPAELFARLKARMPD RRLHTLDPATCPFDHQAKHRIERARTRMEAGLVDQDDLDEEHQGLAPAELFARLKARMPD | 180 |
| Query 181 | GEDLVVTHGDACLPNIMVENGRFSGFIDCGRLGVADRYQDIALATRDIAEELGGEWADRF | 240 |
| Sbjct 181 | GEDLVVTHGDACLPNIMVENGRFSGFIDCGRLGVADRYQDIALATRDIAEELGGEWADRF GEDLVVTHGDACLPNIMVENGRFSGFIDCGRLGVADRYQDIALATRDIAEELGGEWADRF | 240 |
| Query 241  Sbjct 241 | LVLYGIAAPDSQRIAFYRLLDEFF 264 LVLYGIAAPDSQRIAFYR LDEFF LVLYGIAAPDSQRIAFYRPLDEFF 264 |  |

>AAX14466.1 neomycin phosphotransferase [synthetic construct] Length=267

Score = 532 bits (1370), Expect = 0.0, Method: Compositional matrix adjust.

| Identities | = 262/263 (99%), Positives = 263/263 (100%), Gaps = 0/263 (0%) |  |
| --- | --- | --- |
| Query 2 | IEQDGLHAGSPAAWVERLFGYDWAQQTIGCSDAAVFRLSAQGRPVLFVKTDLSGALNELQ | 61 |
| Sbjct 5 | IEQDGLHAGSPAAWVERLFGYDWAQQTIGCSDAAVFRLSAQGRPVLFVKTDLSGALNELQ IEQDGLHAGSPAAWVERLFGYDWAQQTIGCSDAAVFRLSAQGRPVLFVKTDLSGALNELQ | 64 |
| Query 62 | DEAARLSWLATTGVPCAAVLDVVTEAGRDWLLLGEVPGQDLLSSHLAPAEKVSIMADAMR | 121 |
| Sbjct 65 | DEAARLSWLATTGVPCAAVLDVVTEAGRDWLLLGEVPGQDLLSSHLAPAEKVSIMADAMR DEAARLSWLATTGVPCAAVLDVVTEAGRDWLLLGEVPGQDLLSSHLAPAEKVSIMADAMR | 124 |
| Query 122 | RLHTLDPATCPFDHQAKHRIERARTRMEAGLVDQDDLDEEHQGLAPAELFARLKARMPDG | 181 |
| Sbjct 125 | RLHTLDPATCPFDHQAKHRIERARTRMEAGLV+QDDLDEEHQGLAPAELFARLKARMPDG RLHTLDPATCPFDHQAKHRIERARTRMEAGLVNQDDLDEEHQGLAPAELFARLKARMPDG | 184 |
| Query 182 | EDLVVTHGDACLPNIMVENGRFSGFIDCGRLGVADRYQDIALATRDIAEELGGEWADRFL | 241 |
| Sbjct 185 | EDLVVTHGDACLPNIMVENGRFSGFIDCGRLGVADRYQDIALATRDIAEELGGEWADRFL EDLVVTHGDACLPNIMVENGRFSGFIDCGRLGVADRYQDIALATRDIAEELGGEWADRFL | 244 |
| Query 242 | VLYGIAAPDSQRIAFYRLLDEFF 264 |  |

VLYGIAAPDSQRIAFYRLLDEFF Sbjct 245 VLYGIAAPDSQRIAFYRLLDEFF 267

>EBE1709735.1 APH(3')-II family aminoglycoside O-phosphotransferase [Salmonella enterica]

Length=264

Score = 532 bits (1370), Expect = 0.0, Method: Compositional matrix adjust.

| Identities | = 263/264 (99%), Positives = 263/264 (99%), Gaps = 0/264 (0%) |  |
| --- | --- | --- |
| Query 1 | MIEQDGLHAGSPAAWVERLFGYDWAQQTIGCSDAAVFRLSAQGRPVLFVKTDLSGALNEL | 60 |
| Sbjct 1 | MIEQDGLHAGSPAAWVERLF YDWAQQTIGCSDAAVFRLSAQGRPVLFVKTDLSGALNEL MIEQDGLHAGSPAAWVERLFCYDWAQQTIGCSDAAVFRLSAQGRPVLFVKTDLSGALNEL | 60 |
| Query 61 | QDEAARLSWLATTGVPCAAVLDVVTEAGRDWLLLGEVPGQDLLSSHLAPAEKVSIMADAM | 120 |
| Sbjct 61 | QDEAARLSWLATTGVPCAAVLDVVTEAGRDWLLLGEVPGQDLLSSHLAPAEKVSIMADAM QDEAARLSWLATTGVPCAAVLDVVTEAGRDWLLLGEVPGQDLLSSHLAPAEKVSIMADAM | 120 |
| Query 121 | RRLHTLDPATCPFDHQAKHRIERARTRMEAGLVDQDDLDEEHQGLAPAELFARLKARMPD | 180 |
| Sbjct 121 | RRLHTLDPATCPFDHQAKHRIERARTRMEAGLVDQDDLDEEHQGLAPAELFARLKARMPD RRLHTLDPATCPFDHQAKHRIERARTRMEAGLVDQDDLDEEHQGLAPAELFARLKARMPD | 180 |
| Query 181 | GEDLVVTHGDACLPNIMVENGRFSGFIDCGRLGVADRYQDIALATRDIAEELGGEWADRF | 240 |
| Sbjct 181 | GEDLVVTHGDACLPNIMVENGRFSGFIDCGRLGVADRYQDIALATRDIAEELGGEWADRF GEDLVVTHGDACLPNIMVENGRFSGFIDCGRLGVADRYQDIALATRDIAEELGGEWADRF | 240 |
| Query 241  Sbjct 241 | LVLYGIAAPDSQRIAFYRLLDEFF 264 LVLYGIAAPDSQRIAFYRLLDEFF LVLYGIAAPDSQRIAFYRLLDEFF 264 |  |

>AAA72847.1 neomycin phosphotransferase [unidentified cloning vector] AAN05010.1 kanamycin resistance [Binary vector pGV4945]

AAN63047.1 kanamycin resistance protein [Binary vector pGV4939]

AVM80406.1 neomycin phosphotransferase II [synthetic construct] [Petunia x hybrida]

CAD10665.1 neomycine phosphotransferase [Binary vector pGV4042] CAD10667.1 neomycine phosphotransferase [Binary vector pGV4126] CAD10669.1 neomycine phosphotransferase [Binary vector pGV4128] CAD10671.1 neomycine phosphotransferase [Binary vector pGV4223]

Length=265

Score = 532 bits (1370), Expect = 0.0, Method: Compositional matrix adjust. Identities = 263/263 (100%), Positives = 263/263 (100%), Gaps = 0/263 (0%)

Query 2	IEQDGLHAGSPAAWVERLFGYDWAQQTIGCSDAAVFRLSAQGRPVLFVKTDLSGALNELQ 61 IEQDGLHAGSPAAWVERLFGYDWAQQTIGCSDAAVFRLSAQGRPVLFVKTDLSGALNELQ

Sbjct 3	IEQDGLHAGSPAAWVERLFGYDWAQQTIGCSDAAVFRLSAQGRPVLFVKTDLSGALNELQ 62

Query 62	DEAARLSWLATTGVPCAAVLDVVTEAGRDWLLLGEVPGQDLLSSHLAPAEKVSIMADAMR 121 DEAARLSWLATTGVPCAAVLDVVTEAGRDWLLLGEVPGQDLLSSHLAPAEKVSIMADAMR

Sbjct 63	DEAARLSWLATTGVPCAAVLDVVTEAGRDWLLLGEVPGQDLLSSHLAPAEKVSIMADAMR 122

Query 122 RLHTLDPATCPFDHQAKHRIERARTRMEAGLVDQDDLDEEHQGLAPAELFARLKARMPDG 181 RLHTLDPATCPFDHQAKHRIERARTRMEAGLVDQDDLDEEHQGLAPAELFARLKARMPDG

Sbjct 123 RLHTLDPATCPFDHQAKHRIERARTRMEAGLVDQDDLDEEHQGLAPAELFARLKARMPDG 182

Query 182 EDLVVTHGDACLPNIMVENGRFSGFIDCGRLGVADRYQDIALATRDIAEELGGEWADRFL 241 EDLVVTHGDACLPNIMVENGRFSGFIDCGRLGVADRYQDIALATRDIAEELGGEWADRFL

Sbjct 183 EDLVVTHGDACLPNIMVENGRFSGFIDCGRLGVADRYQDIALATRDIAEELGGEWADRFL 242

Query 242 VLYGIAAPDSQRIAFYRLLDEFF 264

VLYGIAAPDSQRIAFYRLLDEFF Sbjct 243 VLYGIAAPDSQRIAFYRLLDEFF 265

>AAC53631.1 neomycin phosphotransferase [Cloning vector pBSL99]

AII16599.1 neomycin phosphotransferase II [Expression vector pT7 RNAP_HR(eba175)] AII16602.1 neomycin phosphotransferase II [Expression vector pT7 RNAP_HR(kahrp)] AIW62893.1 neomycin phosphotransferase II [Expression vector pSG230]

AIW62896.1 neomycin phosphotransferase [Expression vector pSG231]

AIW62898.1 neomycin phosphotransferase II [Expression vector pSG252] AJW72788.1 aminoglycoside 3'-phosphotransferase [Cloning vector pSLS51] QOQ50724.1 neomycin phosphotransferase [Cloning vector pcDNA3-WSN-PA] QOQ50727.1 neomycin phosphotransferase [Cloning vector pcDNA3-WSN-PB1] QOQ50730.1 neomycin phosphotransferase [Cloning vector pcDNA3-WSN-PB2] QOQ50734.1 neomycin phosphotransferase [Cloning vector pGluc2-Nter-ERI1] QOQ50737.1 neomycin phosphotransferase [Cloning vector pGluc2-Nter-ERI1-noDE] QOQ50740.1 neomycin phosphotransferase [Cloning vector pGluc2-Nter-ERI1-R105A]

QOQ50743.1 neomycin phosphotransferase [Cloning vector pGluc2-Nter-ERI1-deltaSAP] QOQ50746.1 neomycin phosphotransferase [Cloning vector pIBA105-strep-ERI1] QOQ50749.1 neomycin phosphotransferase [Cloning vector pIBA105-strep-ERI1-noDE] QOQ50752.1 neomycin phosphotransferase [Cloning vector pIBA105-strep-ERI1-R105A] QOQ50755.1 neomycin phosphotransferase [Cloning vector pIBA105-strep-ERI1-deltaSAP] QOQ50757.1 neomycin phosphotransferase [Cloning vector pIBA105-strep-empty] QOQ50760.1 neomycin phosphotransferase [Cloning vector pIBA105-strep-mCherry] QOQ50763.1 neomycin phosphotransferase [Cloning vector pIBA105-strep-SLBP] QOQ50766.1 neomycin phosphotransferase [Cloning vector pCIneo-3xFLAG-SLBP]

Length=263

Score = 532 bits (1370), Expect = 0.0, Method: Compositional matrix adjust. Identities = 262/263 (99%), Positives = 263/263 (100%), Gaps = 0/263 (0%)

| Query | 2 | IEQDGLHAGSPAAWVERLFGYDWAQQTIGCSDAAVFRLSAQGRPVLFVKTDLSGALNELQ | 61 |
| --- | --- | --- | --- |
| Sbjct | 1 | +EQDGLHAGSPAAWVERLFGYDWAQQTIGCSDAAVFRLSAQGRPVLFVKTDLSGALNELQ MEQDGLHAGSPAAWVERLFGYDWAQQTIGCSDAAVFRLSAQGRPVLFVKTDLSGALNELQ | 60 |
| Query | 62 | DEAARLSWLATTGVPCAAVLDVVTEAGRDWLLLGEVPGQDLLSSHLAPAEKVSIMADAMR | 121 |
| Sbjct | 61 | DEAARLSWLATTGVPCAAVLDVVTEAGRDWLLLGEVPGQDLLSSHLAPAEKVSIMADAMR DEAARLSWLATTGVPCAAVLDVVTEAGRDWLLLGEVPGQDLLSSHLAPAEKVSIMADAMR | 120 |
| Query | 122 | RLHTLDPATCPFDHQAKHRIERARTRMEAGLVDQDDLDEEHQGLAPAELFARLKARMPDG | 181 |
| Sbjct | 121 | RLHTLDPATCPFDHQAKHRIERARTRMEAGLVDQDDLDEEHQGLAPAELFARLKARMPDG RLHTLDPATCPFDHQAKHRIERARTRMEAGLVDQDDLDEEHQGLAPAELFARLKARMPDG | 180 |
| Query | 182 | EDLVVTHGDACLPNIMVENGRFSGFIDCGRLGVADRYQDIALATRDIAEELGGEWADRFL | 241 |
| Sbjct | 181 | EDLVVTHGDACLPNIMVENGRFSGFIDCGRLGVADRYQDIALATRDIAEELGGEWADRFL EDLVVTHGDACLPNIMVENGRFSGFIDCGRLGVADRYQDIALATRDIAEELGGEWADRFL | 240 |
| Query  Sbjct | 242  241 | VLYGIAAPDSQRIAFYRLLDEFF 264 VLYGIAAPDSQRIAFYRLLDEFF VLYGIAAPDSQRIAFYRLLDEFF 263 |  |

>WP_150102527.1 APH(3')-II family aminoglycoside O-phosphotransferase [Klebsiella pneumoniae]

QER51539.1 APH(3')-II family aminoglycoside O-phosphotransferase [Klebsiella pneumoniae]

Length=264

Score = 532 bits (1370), Expect = 0.0, Method: Compositional matrix adjust.

| Identities | = 263/264 (99%), Positives = 263/264 (99%), Gaps = 0/264 (0%) |  |
| --- | --- | --- |
| Query 1 | MIEQDGLHAGSPAAWVERLFGYDWAQQTIGCSDAAVFRLSAQGRPVLFVKTDLSGALNEL | 60 |
| Sbjct 1 | MIEQDGLHAGSPAAWVERLFGYDWAQQTIGCSDAAVFRLSAQGRPVLFVKTDLSGALNEL MIEQDGLHAGSPAAWVERLFGYDWAQQTIGCSDAAVFRLSAQGRPVLFVKTDLSGALNEL | 60 |
| Query 61 | QDEAARLSWLATTGVPCAAVLDVVTEAGRDWLLLGEVPGQDLLSSHLAPAEKVSIMADAM | 120 |
| Sbjct 61 | QDEAARLSWLATTGVPCAAVLDVVTEAGRDWLLLGEVPGQDLLSSHLAPAEKVSIMADAM QDEAARLSWLATTGVPCAAVLDVVTEAGRDWLLLGEVPGQDLLSSHLAPAEKVSIMADAM | 120 |
| Query 121 | RRLHTLDPATCPFDHQAKHRIERARTRMEAGLVDQDDLDEEHQGLAPAELFARLKARMPD | 180 |
| Sbjct 121 | RRLHTLDPATCPFDHQAKHRIERARTRMEAGLVDQDDLDEEHQGLAPAELFA LKARMPD RRLHTLDPATCPFDHQAKHRIERARTRMEAGLVDQDDLDEEHQGLAPAELFAVLKARMPD | 180 |
| Query 181 | GEDLVVTHGDACLPNIMVENGRFSGFIDCGRLGVADRYQDIALATRDIAEELGGEWADRF | 240 |
| Sbjct 181 | GEDLVVTHGDACLPNIMVENGRFSGFIDCGRLGVADRYQDIALATRDIAEELGGEWADRF GEDLVVTHGDACLPNIMVENGRFSGFIDCGRLGVADRYQDIALATRDIAEELGGEWADRF | 240 |
| Query 241  Sbjct 241 | LVLYGIAAPDSQRIAFYRLLDEFF 264 LVLYGIAAPDSQRIAFYRLLDEFF LVLYGIAAPDSQRIAFYRLLDEFF 264 |  |

>EDT4821918.1 APH(3')-II family aminoglycoside O-phosphotransferase [Salmonella enterica subsp. enterica serovar 4,[5],12:i:-]

Length=264

Score = 532 bits (1370), Expect = 0.0, Method: Compositional matrix adjust.

| Identities | = 263/264 (99%), Positives = 263/264 (99%), Gaps = 0/264 (0%) |  |
| --- | --- | --- |
| Query 1 | MIEQDGLHAGSPAAWVERLFGYDWAQQTIGCSDAAVFRLSAQGRPVLFVKTDLSGALNEL | 60 |
| Sbjct 1 | MIEQDGLHAGSPAAWVERLFGYDWAQQTIGCSDAAVFRLSAQGRPVLFVKTDLSGALNEL MIEQDGLHAGSPAAWVERLFGYDWAQQTIGCSDAAVFRLSAQGRPVLFVKTDLSGALNEL | 60 |
| Query 61 | QDEAARLSWLATTGVPCAAVLDVVTEAGRDWLLLGEVPGQDLLSSHLAPAEKVSIMADAM | 120 |
| Sbjct 61 | QDEAARLSWLATTGVPCAAVLDVVTEAGRDWLLLGEVPGQDLLSSHLAPAEKVSIMADAM QDEAARLSWLATTGVPCAAVLDVVTEAGRDWLLLGEVPGQDLLSSHLAPAEKVSIMADAM | 120 |
| Query 121 | RRLHTLDPATCPFDHQAKHRIERARTRMEAGLVDQDDLDEEHQGLAPAELFARLKARMPD | 180 |
| Sbjct 121 | RRLHTLDPATCPFDHQAKHRIERARTRMEAGLVDQDDLDEEHQGLAPAELFARLKARMPD RRLHTLDPATCPFDHQAKHRIERARTRMEAGLVDQDDLDEEHQGLAPAELFARLKARMPD | 180 |
| Query 181 | GEDLVVTHGDACLPNIMVENGRFSGFIDCGRLGVADRYQDIALATRDIAEELGGEWADRF | 240 |
| Sbjct 181 | GEDLVVTHGDACLPNIMVENGRFSGFIDCGRLGVADRYQDIALATRDIAEELGGEWADRF GEDLVVTHGDACLPNIMVENGRFSGFIDCGRLGVADRYQDIALATRDIAEELGGEWADRF | 240 |
| Query 241  Sbjct 241 | LVLYGIAAPDSQRIAFYRLLDEFF 264 LVLY IAAPDSQRIAFYRLLDEFF LVLYVIAAPDSQRIAFYRLLDEFF 264 |  |

>ACV83807.1 NptII [Physcomitrella transformation vector pBS108-35SNPT-b]

AKE44003.1 NPTII [Physcomitrella transformation vector pHSP-NLS-mCherry-108-35SNPT] ALH22600.1 NPTII [Physcomitrella transformation vector pUbi-MCS-GFP-108-35SNPT]

Length=335

Score = 532 bits (1370), Expect = 0.0, Method: Compositional matrix adjust.

| Identities | = 261/263 (99%), Positives = 261/263 (99%), Gaps = 0/263 (0%) |  |
| --- | --- | --- |
| Query 2 | IEQDGLHAGSPAAWVERLFGYDWAQQTIGCSDAAVFRLSAQGRPVLFVKTDLSGALNELQ | 61 |
| Sbjct 73 | I DGLHAGSPAAWVERLFGYDWAQQTIGCSDAAVFRLSAQGRPVLFVKTDLSGALNELQ ISLDGLHAGSPAAWVERLFGYDWAQQTIGCSDAAVFRLSAQGRPVLFVKTDLSGALNELQ | 132 |
| Query 62 | DEAARLSWLATTGVPCAAVLDVVTEAGRDWLLLGEVPGQDLLSSHLAPAEKVSIMADAMR | 121 |
| Sbjct 133 | DEAARLSWLATTGVPCAAVLDVVTEAGRDWLLLGEVPGQDLLSSHLAPAEKVSIMADAMR DEAARLSWLATTGVPCAAVLDVVTEAGRDWLLLGEVPGQDLLSSHLAPAEKVSIMADAMR | 192 |
| Query 122 | RLHTLDPATCPFDHQAKHRIERARTRMEAGLVDQDDLDEEHQGLAPAELFARLKARMPDG | 181 |
| Sbjct 193 | RLHTLDPATCPFDHQAKHRIERARTRMEAGLVDQDDLDEEHQGLAPAELFARLKARMPDG RLHTLDPATCPFDHQAKHRIERARTRMEAGLVDQDDLDEEHQGLAPAELFARLKARMPDG | 252 |
| Query 182 | EDLVVTHGDACLPNIMVENGRFSGFIDCGRLGVADRYQDIALATRDIAEELGGEWADRFL | 241 |
| Sbjct 253 | EDLVVTHGDACLPNIMVENGRFSGFIDCGRLGVADRYQDIALATRDIAEELGGEWADRFL EDLVVTHGDACLPNIMVENGRFSGFIDCGRLGVADRYQDIALATRDIAEELGGEWADRFL | 312 |
| Query 242  Sbjct 313 | VLYGIAAPDSQRIAFYRLLDEFF 264 VLYGIAAPDSQRIAFYRLLDEFF VLYGIAAPDSQRIAFYRLLDEFF 335 |  |

>ABG81368.1 neomycin phosphotransferase II [Cloning vector pINT] Length=266

Score = 531 bits (1369), Expect = 0.0, Method: Compositional matrix adjust.

| Identities | = 262/263 (99%), Positives = 263/263 (100%), Gaps = 0/263 (0%) |  |
| --- | --- | --- |
| Query 2 | IEQDGLHAGSPAAWVERLFGYDWAQQTIGCSDAAVFRLSAQGRPVLFVKTDLSGALNELQ | 61 |
| Sbjct 4 | IEQDGLHAGSPAAWVERLFGYDWAQQTIGCSDAAVFRLSAQGRPVLFVKTDLSGALNELQ IEQDGLHAGSPAAWVERLFGYDWAQQTIGCSDAAVFRLSAQGRPVLFVKTDLSGALNELQ | 63 |
| Query 62 | DEAARLSWLATTGVPCAAVLDVVTEAGRDWLLLGEVPGQDLLSSHLAPAEKVSIMADAMR | 121 |
| Sbjct 64 | DEAARLSWLATTGVPCAAVLDVVTEAGRDWLLLGEVPGQDLLSSHLAPAEKVSIMADAMR  DEAARLSWLATTGVPCAAVLDVVTEAGRDWLLLGEVPGQDLLSSHLAPAEKVSIMADAMR | 123 |

| Query | 122 | RLHTLDPATCPFDHQAKHRIERARTRMEAGLVDQDDLDEEHQGLAPAELFARLKARMPDG | 181 |
| --- | --- | --- | --- |
| Sbjct | 124 | RLHTLDPATCPFDHQAKHRIERARTRMEAGLVDQDDLDEEHQGLAPAELFARLKARMPDG RLHTLDPATCPFDHQAKHRIERARTRMEAGLVDQDDLDEEHQGLAPAELFARLKARMPDG | 183 |
| Query | 182 | EDLVVTHGDACLPNIMVENGRFSGFIDCGRLGVADRYQDIALATRDIAEELGGEWADRFL | 241 |
| Sbjct | 184 | +DLVVTHGDACLPNIMVENGRFSGFIDCGRLGVADRYQDIALATRDIAEELGGEWADRFL DDLVVTHGDACLPNIMVENGRFSGFIDCGRLGVADRYQDIALATRDIAEELGGEWADRFL | 243 |
| Query  Sbjct | 242  244 | VLYGIAAPDSQRIAFYRLLDEFF 264 VLYGIAAPDSQRIAFYRLLDEFF VLYGIAAPDSQRIAFYRLLDEFF 266 |  |

```
>AAA72769.1 beta-glucuronidase:neomycin phosphotransferase II fusion protein, partial [synthetic construct]
Length=885

Score = 531 bits (1369), Expect = 0.0, Method: Compositional matrix adjust. Identities = 260/260 (100%), Positives = 260/260 (100%), Gaps = 0/260 (0%)

Query 5	DGLHAGSPAAWVERLFGYDWAQQTIGCSDAAVFRLSAQGRPVLFVKTDLSGALNELQDEA 64 DGLHAGSPAAWVERLFGYDWAQQTIGCSDAAVFRLSAQGRPVLFVKTDLSGALNELQDEA
Sbjct 626 DGLHAGSPAAWVERLFGYDWAQQTIGCSDAAVFRLSAQGRPVLFVKTDLSGALNELQDEA 685

Query 65	ARLSWLATTGVPCAAVLDVVTEAGRDWLLLGEVPGQDLLSSHLAPAEKVSIMADAMRRLH 124 ARLSWLATTGVPCAAVLDVVTEAGRDWLLLGEVPGQDLLSSHLAPAEKVSIMADAMRRLH
Sbjct 686 ARLSWLATTGVPCAAVLDVVTEAGRDWLLLGEVPGQDLLSSHLAPAEKVSIMADAMRRLH 745

Query 125 TLDPATCPFDHQAKHRIERARTRMEAGLVDQDDLDEEHQGLAPAELFARLKARMPDGEDL 184 TLDPATCPFDHQAKHRIERARTRMEAGLVDQDDLDEEHQGLAPAELFARLKARMPDGEDL
Sbjct 746 TLDPATCPFDHQAKHRIERARTRMEAGLVDQDDLDEEHQGLAPAELFARLKARMPDGEDL 805

Query 185 VVTHGDACLPNIMVENGRFSGFIDCGRLGVADRYQDIALATRDIAEELGGEWADRFLVLY 244 VVTHGDACLPNIMVENGRFSGFIDCGRLGVADRYQDIALATRDIAEELGGEWADRFLVLY
Sbjct 806 VVTHGDACLPNIMVENGRFSGFIDCGRLGVADRYQDIALATRDIAEELGGEWADRFLVLY 865

Query 245 GIAAPDSQRIAFYRLLDEFF 264
GIAAPDSQRIAFYRLLDEFF Sbjct 866 GIAAPDSQRIAFYRLLDEFF 885

>AAK92455.1 PGK-neo [Retrofitting vector pRetroES] ADB98018.1 Neo [Donor vector pTKIP-neo]
ADN52072.1 neomycin resistance [Targeting vector R26-Stop-HA3-ICA512-CCF] AEM37565.1 Neo [Cloning vector EN.Cherry]
AEM37567.1 Neo [Cloning vector EN.GFP] AEM37569.1 Neo [Cloning vector EN.CFP] AEM37571.1 Neo [Cloning vector EN.YFP] AEM37573.1 Neo [Cloning vector EN.Apple] AEM37575.1 Neo [Cloning vector NonEN.CFP] AEM37577.1 Neo [Cloning vector EN.CFP.bGbpA] AEM37579.1 Neo [Cloning vector EN.CFP.SV40]
AEO31554.1 kanamycin resistance protein [synthetic construct] AIA24531.1 neomycin resistance protein [Cloning vector pTVDll42AdVenus]
AIA24533.1 neomycin resistance protein [Cloning vector pTVDll42AEmerald] AKR53650.1 Neo [Donor vector pTKDP-neo]
ANW61889.1 NeoR [Cloning vector DBH-FLPo] ANW61892.1 NeoR [Cloning vector DBH-p2a-FLPo] AUS83930.1 Kan/Neo [Targeting vector dCas9_Rosa26]
AXB26958.1 neomycin-kanamycin phosphotransferase type II [Cloning vector H3.3K27M]
AYM26641.1 neo resistance protein [Cloning vector pTLD39]
AZP55982.1 aminoglycoside phosphotransferase from Tn5 [Vector R6K-kan-ccdB] AZP55995.1 aminoglycoside phosphotransferase from Tn5 [Vector pcDNA3.1-KanFDEST] AZP56108.1 aminoglycoside phosphotransferase from Tn5 [Vector AdEvolve-DEST] QCY41235.1 aminoglycoside phosphotransferase [Cloning vector pR6K-AKC] QCY41241.1 aminoglycoside phosphotransferase [Cloning vector pR6K-KCA] SJL86619.1 unnamed protein product [Vector pROSA26-DV1]
BAE48697.1 neomycin resistance protein [Cloning vector pSINTK] BAF36705.1 neomycin resistance protein [Expression vector pCXGFP-1]
BAL72059.1 neomycin resistance protein [Gene-trapping transposon vector pUPATrap-CTP2F]

BAL72063.1 neomycin resistance protein [Gene-trapping transposon vector pUPATrap-TMtt-01]
BAL72066.1 neomycin resistance protein [Gene-trapping transposon vector pUPATrap-TMtt-02]
BAL72069.1 neomycin resistance protein [Gene-trapping transposon vector pUPATrap-TMtt-03]
BAL72072.1 neomycin resistance protein [Gene-trapping transposon vector pUPATrap-TMtt-04]
BAL72075.1 neomycin resistance protein [Gene-trapping transposon vector pUPATrap-TMtt-05]
BAL72078.1 neomycin resistance protein [Gene-trapping transposon vector pUPATrap-TMtt-06]
BAL72081.1 neomycin resistance protein [Gene-trapping transposon vector pUPATrap-TMtt-07]
BAL72084.1 neomycin resistance protein [Gene-trapping transposon vector pUPATrap-TMtt-08]
BAL72087.1 neomycin resistance protein [Gene-trapping transposon vector pUPATrap-TMtt-09]
BAL72090.1 neomycin resistance protein [Gene-trapping transposon vector pUPATrap-TMtt-10]
BAL72093.1 neomycin resistance protein [Gene-trapping transposon vector pUPATrap-TMtt-11]
BAL72096.1 neomycin resistance protein [Gene-trapping transposon vector pUPATrap-TMtt-12]
BAL72099.1 neomycin resistance protein [Gene-trapping transposon vector pUPATrap-TMtt-13]
BAL72102.1 neomycin resistance protein [Gene-trapping transposon vector pUPATrap-TMtt-14]
BAL72105.1 neomycin resistance protein [Gene-trapping transposon vector pUPATrap-TMtt-15]
BAL72108.1 neomycin resistance protein [Gene-trapping transposon vector pUPATrap-TMat-01]
BAL72111.1 neomycin resistance protein [Gene-trapping transposon vector pUPATrap-TMat-02]
BAL72114.1 neomycin resistance protein [Gene-trapping transposon vector pUPATrap-TMat-03]
BAL72117.1 neomycin resistance protein [Gene-trapping transposon vector pUPATrap-TMat-04]
BAL72120.1 neomycin resistance protein [Gene-trapping transposon vector pUPATrap-TMat-05]
BAL72123.1 neomycin resistance protein [Gene-trapping transposon vector pUPATrap-TMat-06]
BAL72126.1 neomycin resistance protein [Gene-trapping transposon vector pUPATrap-TMat-07]
BAL72129.1 neomycin resistance protein [Gene-trapping transposon vector pUPATrap-TMat-08]
BAL72132.1 neomycin resistance protein [Gene-trapping transposon vector pUPATrap-TMat-09]
BAL72135.1 neomycin resistance protein [Gene-trapping transposon vector pUPATrap-TMat-10]
BAL72138.1 neomycin resistance protein [Gene-trapping transposon vector pUPATrap-TMat-11]
BAL72141.1 neomycin resistance protein [Gene-trapping transposon vector pUPATrap-TMat-12]
BAL72144.1 neomycin resistance protein [Gene-trapping transposon vector pUPATrap-TMat-13]
BAL72147.1 neomycin resistance protein [Gene-trapping transposon vector pUPATrap-TMat-14]
BAL72150.1 neomycin resistance protein [Gene-trapping transposon vector pUPATrap-TMat-15]
BAL72153.1 neomycin resistance protein [Gene-trapping retrovirus vector pUPATrap-CRV2]
BAS53447.1 neomycin resistance protein [Gene-trapping transposon vector psDT-01]
BAS53451.1 neomycin resistance protein [Gene-trapping transposon vector pdDT-01]
BAS53456.1 neomycin resistance protein [Gene-trapping transposon vector psDT176-01]
BAS53460.1 neomycin resistance protein [Gene-trapping transposon vector pdDT176-01]
BAX73945.1 aminoglycoside 3-phosphotransferase [Targeting vector pHPRT8.9-Neo2(-)] Length=267

Score = 531 bits (1369), Expect = 0.0, Method: Compositional matrix adjust.
```

| Identities | = 262/263 (99%), Positives = 263/263 (100%), Gaps = 0/263 (0%) |  |
| --- | --- | --- |
| Query 2 | IEQDGLHAGSPAAWVERLFGYDWAQQTIGCSDAAVFRLSAQGRPVLFVKTDLSGALNELQ | 61 |
| Sbjct 5 | IEQDGLHAGSPAAWVERLFGYDWAQQTIGCSDAAVFRLSAQGRPVLFVKTDLSGALNELQ IEQDGLHAGSPAAWVERLFGYDWAQQTIGCSDAAVFRLSAQGRPVLFVKTDLSGALNELQ | 64 |
| Query 62 | DEAARLSWLATTGVPCAAVLDVVTEAGRDWLLLGEVPGQDLLSSHLAPAEKVSIMADAMR | 121 |
| Sbjct 65 | DEAARLSWLATTGVPCAAVLDVVTEAGRDWLLLGEVPGQDLLSSHLAPAEKVSIMADAMR DEAARLSWLATTGVPCAAVLDVVTEAGRDWLLLGEVPGQDLLSSHLAPAEKVSIMADAMR | 124 |
| Query 122 | RLHTLDPATCPFDHQAKHRIERARTRMEAGLVDQDDLDEEHQGLAPAELFARLKARMPDG | 181 |
| Sbjct 125 | RLHTLDPATCPFDHQAKHRIERARTRMEAGLVDQDDLDEEHQGLAPAELFARLKARMPDG RLHTLDPATCPFDHQAKHRIERARTRMEAGLVDQDDLDEEHQGLAPAELFARLKARMPDG | 184 |
| Query 182 | EDLVVTHGDACLPNIMVENGRFSGFIDCGRLGVADRYQDIALATRDIAEELGGEWADRFL | 241 |
| Sbjct 185 | +DLVVTHGDACLPNIMVENGRFSGFIDCGRLGVADRYQDIALATRDIAEELGGEWADRFL DDLVVTHGDACLPNIMVENGRFSGFIDCGRLGVADRYQDIALATRDIAEELGGEWADRFL | 244 |
| Query 242  Sbjct 245 | VLYGIAAPDSQRIAFYRLLDEFF 264 VLYGIAAPDSQRIAFYRLLDEFF VLYGIAAPDSQRIAFYRLLDEFF 267 |  |

>PTB82200.1 APH(3')-II family aminoglycoside O-phosphotransferase [Alcanivorax venustensis]

Length=264

Score = 531 bits (1368), Expect = 0.0, Method: Compositional matrix adjust.

| Identities | = 262/264 (99%), Positives = 263/264 (99%), Gaps = 0/264 (0%) |  |
| --- | --- | --- |
| Query 1 | MIEQDGLHAGSPAAWVERLFGYDWAQQTIGCSDAAVFRLSAQGRPVLFVKTDLSGALNEL | 60 |
| Sbjct 1 | MIEQDGLHAGSPAAWVERLFGYDWAQQTIGCSDAAVFRLSAQGRPVLFVKTDLSGALNEL MIEQDGLHAGSPAAWVERLFGYDWAQQTIGCSDAAVFRLSAQGRPVLFVKTDLSGALNEL | 60 |
| Query 61 | QDEAARLSWLATTGVPCAAVLDVVTEAGRDWLLLGEVPGQDLLSSHLAPAEKVSIMADAM | 120 |
| Sbjct 61 | QDEAARLSWLATTGVPCAAVLDVVTEAGRDWLLLGEVPGQDLLSSHLAPAEKVSIMADAM QDEAARLSWLATTGVPCAAVLDVVTEAGRDWLLLGEVPGQDLLSSHLAPAEKVSIMADAM | 120 |
| Query 121 | RRLHTLDPATCPFDHQAKHRIERARTRMEAGLVDQDDLDEEHQGLAPAELFARLKARMPD | 180 |
| Sbjct 121 | RRLHTLDPATCPFDHQAKHRIERARTRMEAGLVDQDDLDEEHQGLAPAELFARLKARMPD RRLHTLDPATCPFDHQAKHRIERARTRMEAGLVDQDDLDEEHQGLAPAELFARLKARMPD | 180 |
| Query 181 | GEDLVVTHGDACLPNIMVENGRFSGFIDCGRLGVADRYQDIALATRDIAEELGGEWADRF | 240 |
| Sbjct 181 | G+DLVVTHGDACLPNIMVENGRFSGFIDCGRLGVADRYQDI LATRDIAEELGGEWADRF GDDLVVTHGDACLPNIMVENGRFSGFIDCGRLGVADRYQDIELATRDIAEELGGEWADRF | 240 |
| Query 241  Sbjct 241 | LVLYGIAAPDSQRIAFYRLLDEFF 264 LVLYGIAAPDSQRIAFYRLLDEFF LVLYGIAAPDSQRIAFYRLLDEFF 264 |  |

>WP_134413266.1 APH(3')-II family aminoglycoside O-phosphotransferase [Pseudomonas aeruginosa]

Length=264

Score = 531 bits (1367), Expect = 0.0, Method: Compositional matrix adjust.

| Identities | = 262/264 (99%), Positives = 262/264 (99%), Gaps = 0/264 (0%) |  |
| --- | --- | --- |
| Query 1 | MIEQDGLHAGSPAAWVERLFGYDWAQQTIGCSDAAVFRLSAQGRPVLFVKTDLSGALNEL | 60 |
| Sbjct 1 | MIEQDGLHAGSPAAWVERLFGYDWAQQTIGCSDAAVFRLSAQGRPVLFVKTDLSGALNEL MIEQDGLHAGSPAAWVERLFGYDWAQQTIGCSDAAVFRLSAQGRPVLFVKTDLSGALNEL | 60 |
| Query 61 | QDEAARLSWLATTGVPCAAVLDVVTEAGRDWLLLGEVPGQDLLSSHLAPAEKVSIMADAM | 120 |
| Sbjct 61 | QDEAARLSWLATTGVPCAAVLDVVTEAGRDWLLLGEVPGQDLLSSHLAPAEKVSIMADAM QDEAARLSWLATTGVPCAAVLDVVTEAGRDWLLLGEVPGQDLLSSHLAPAEKVSIMADAM | 120 |
| Query 121 | RRLHTLDPATCPFDHQAKHRIERARTRMEAGLVDQDDLDEEHQGLAPAELFARLKARMPD | 180 |
| Sbjct 121 | RRLHTLDPATCPFDHQAKHRIERARTRMEAGLVDQDDLDEEHQGLAPAELFARLKARMPD  RRLHTLDPATCPFDHQAKHRIERARTRMEAGLVDQDDLDEEHQGLAPAELFARLKARMPD | 180 |

| Query | 181 | GEDLVVTHGDACLPNIMVENGRFSGFIDCGRLGVADRYQDIALATRDIAEELGGEWADRF 240 |
| --- | --- | --- |
| Sbjct | 181 | GEDLVVTHGDACLPNIMVENGRFSGFIDCGRL VADRYQDIALATRDIAEELGGEWADRF GEDLVVTHGDACLPNIMVENGRFSGFIDCGRLDVADRYQDIALATRDIAEELGGEWADRF 240 |
| Query | 241 | LVLYGIAAPDSQRIAFYRLLDEFF 264 |
| Sbjct | 241 | LVLY IAAPDSQRIAFYRLLDEFF  LVLYSIAAPDSQRIAFYRLLDEFF 264 |

>ACH73255.1 AphII [Agrobacterium binary gap repair vector pGAP-Km] Length=265

Score = 531 bits (1367), Expect = 0.0, Method: Compositional matrix adjust.

| Identities | = 262/263 (99%), Positives = 262/263 (99%), Gaps = 0/263 (0%) |  |
| --- | --- | --- |
| Query 2 | IEQDGLHAGSPAAWVERLFGYDWAQQTIGCSDAAVFRLSAQGRPVLFVKTDLSGALNELQ | 61 |
| Sbjct 3 | IEQDGLHAGSPAAWVERLFGYDWAQQTIGCSDAAVFRLSAQGRPVLFVKTDLSGALNELQ IEQDGLHAGSPAAWVERLFGYDWAQQTIGCSDAAVFRLSAQGRPVLFVKTDLSGALNELQ | 62 |
| Query 62 | DEAARLSWLATTGVPCAAVLDVVTEAGRDWLLLGEVPGQDLLSSHLAPAEKVSIMADAMR | 121 |
| Sbjct 63 | DEAARLSWLATTGVPCAAVLDVVTEAGRDWLLLGEVPGQDLLSSHLAPAEKVSIMADAMR DEAARLSWLATTGVPCAAVLDVVTEAGRDWLLLGEVPGQDLLSSHLAPAEKVSIMADAMR | 122 |
| Query 122 | RLHTLDPATCPFDHQAKHRIERARTRMEAGLVDQDDLDEEHQGLAPAELFARLKARMPDG | 181 |
| Sbjct 123 | RLHTLDPATCPFDHQAKHRIERARTRMEAGLVDQDDLDEEHQGLAPAELFARLKARMPDG RLHTLDPATCPFDHQAKHRIERARTRMEAGLVDQDDLDEEHQGLAPAELFARLKARMPDG | 182 |
| Query 182 | EDLVVTHGDACLPNIMVENGRFSGFIDCGRLGVADRYQDIALATRDIAEELGGEWADRFL | 241 |
| Sbjct 183 | EDLVVTHGDACLPNIMVENGRFSGFIDCGRLGVADRYQDIALATRDIAEELGGEWADRFL EDLVVTHGDACLPNIMVENGRFSGFIDCGRLGVADRYQDIALATRDIAEELGGEWADRFL | 242 |
| Query 242  Sbjct 243 | VLYGIAAPDSQRIAFYRLLDEFF 264 VLYGIAAPDSQRIAFYR LDEFF VLYGIAAPDSQRIAFYRFLDEFF 265 |  |

>BAL60567.1 neomycin phosphotransferase [Moss transformation vector pGUS-NPTII-2] BBC20729.1 neomycin phosphotransferase [Moss transformation vector pGUSmutNPTII]

Length=264

Score = 531 bits (1367), Expect = 0.0, Method: Compositional matrix adjust.

| Identities | = 262/264 (99%), Positives = 262/264 (99%), Gaps = 0/264 (0%) |  |
| --- | --- | --- |
| Query 1 | MIEQDGLHAGSPAAWVERLFGYDWAQQTIGCSDAAVFRLSAQGRPVLFVKTDLSGALNEL | 60 |
| Sbjct 1 | MIEQDGLHAGSPAAWVERLFGYDWAQQTIGCSDAAVFRLSAQGRPVLFVKTDLSGALNEL MIEQDGLHAGSPAAWVERLFGYDWAQQTIGCSDAAVFRLSAQGRPVLFVKTDLSGALNEL | 60 |
| Query 61 | QDEAARLSWLATTGVPCAAVLDVVTEAGRDWLLLGEVPGQDLLSSHLAPAEKVSIMADAM | 120 |
| Sbjct 61 | QDEAARLSWLATTGVPCAAVLDVVTEAGRDWLLLGEVPGQDLLSSHLAPAEKVSIMADAM QDEAARLSWLATTGVPCAAVLDVVTEAGRDWLLLGEVPGQDLLSSHLAPAEKVSIMADAM | 120 |
| Query 121 | RRLHTLDPATCPFDHQAKHRIERARTRMEAGLVDQDDLDEEHQGLAPAELFARLKARMPD | 180 |
| Sbjct 121 | RRLHTLDPATCPFDHQAKHRIERARTRMEAGLVDQDDLDEEHQGLAP ELFA LKARMPD RRLHTLDPATCPFDHQAKHRIERARTRMEAGLVDQDDLDEEHQGLAPXELFAXLKARMPD | 180 |
| Query 181 | GEDLVVTHGDACLPNIMVENGRFSGFIDCGRLGVADRYQDIALATRDIAEELGGEWADRF | 240 |
| Sbjct 181 | GEDLVVTHGDACLPNIMVENGRFSGFIDCGRLGVADRYQDIALATRDIAEELGGEWADRF GEDLVVTHGDACLPNIMVENGRFSGFIDCGRLGVADRYQDIALATRDIAEELGGEWADRF | 240 |
| Query 241  Sbjct 241 | LVLYGIAAPDSQRIAFYRLLDEFF 264 LVLYGIAAPDSQRIAFYRLLDEFF LVLYGIAAPDSQRIAFYRLLDEFF 264 |  |

>WP_000572406.1 MULTISPECIES: APH(3')-II family aminoglycoside O-phosphotransferase [Bacteria]

ACE98755.1 aminoglycoside phosphotransferase [Rhodopseudomonas palustris TIE-1]

MBA2041495.1 APH(3')-II family aminoglycoside O-phosphotransferase [Escherichia coli]

Length=264

Score = 531 bits (1367), Expect = 0.0, Method: Compositional matrix adjust.

| Identities | = 263/264 (99%), Positives = 263/264 (99%), Gaps = 0/264 (0%) |  |
| --- | --- | --- |
| Query 1 | MIEQDGLHAGSPAAWVERLFGYDWAQQTIGCSDAAVFRLSAQGRPVLFVKTDLSGALNEL | 60 |
| Sbjct 1 | MIEQDGLHAGSPAAWVERLFGYDWAQQTIG SDAAVFRLSAQGRPVLFVKTDLSGALNEL MIEQDGLHAGSPAAWVERLFGYDWAQQTIGRSDAAVFRLSAQGRPVLFVKTDLSGALNEL | 60 |
| Query 61 | QDEAARLSWLATTGVPCAAVLDVVTEAGRDWLLLGEVPGQDLLSSHLAPAEKVSIMADAM | 120 |
| Sbjct 61 | QDEAARLSWLATTGVPCAAVLDVVTEAGRDWLLLGEVPGQDLLSSHLAPAEKVSIMADAM QDEAARLSWLATTGVPCAAVLDVVTEAGRDWLLLGEVPGQDLLSSHLAPAEKVSIMADAM | 120 |
| Query 121 | RRLHTLDPATCPFDHQAKHRIERARTRMEAGLVDQDDLDEEHQGLAPAELFARLKARMPD | 180 |
| Sbjct 121 | RRLHTLDPATCPFDHQAKHRIERARTRMEAGLVDQDDLDEEHQGLAPAELFARLKARMPD RRLHTLDPATCPFDHQAKHRIERARTRMEAGLVDQDDLDEEHQGLAPAELFARLKARMPD | 180 |
| Query 181 | GEDLVVTHGDACLPNIMVENGRFSGFIDCGRLGVADRYQDIALATRDIAEELGGEWADRF | 240 |
| Sbjct 181 | GEDLVVTHGDACLPNIMVENGRFSGFIDCGRLGVADRYQDIALATRDIAEELGGEWADRF GEDLVVTHGDACLPNIMVENGRFSGFIDCGRLGVADRYQDIALATRDIAEELGGEWADRF | 240 |
| Query 241  Sbjct 241 | LVLYGIAAPDSQRIAFYRLLDEFF 264 LVLYGIAAPDSQRIAFYRLLDEFF LVLYGIAAPDSQRIAFYRLLDEFF 264 |  |

>ATG88232.1 neomycin phosphotransferase [Yeast dual vector pCtB1]

ATG88234.1 neomycin phosphotransferase [Corynebacterium glutamicum overexpression vector pGS-Ex1]

ATG88236.1 neomycin phosphotransferase [Corynebacterium glutamicum shuttle vector pGS-Term]

Length=271

Score = 531 bits (1367), Expect = 0.0, Method: Compositional matrix adjust.

| Identities | = 262/263 (99%), Positives = 262/263 (99%), Gaps = 0/263 (0%) |  |
| --- | --- | --- |
| Query 2 | IEQDGLHAGSPAAWVERLFGYDWAQQTIGCSDAAVFRLSAQGRPVLFVKTDLSGALNELQ | 61 |
| Sbjct 9 | IEQDGLHAGSPAAWVERLFGYDWAQQTIGCSDAAVFRLSAQGRPVLFVKTDLSGALNELQ IEQDGLHAGSPAAWVERLFGYDWAQQTIGCSDAAVFRLSAQGRPVLFVKTDLSGALNELQ | 68 |
| Query 62 | DEAARLSWLATTGVPCAAVLDVVTEAGRDWLLLGEVPGQDLLSSHLAPAEKVSIMADAMR | 121 |
| Sbjct 69 | DEAARLSWLATTGVPCAAVLDVVTEAGRDWLLLGEVPGQDLLSSHLAPAEKVSIMADAMR DEAARLSWLATTGVPCAAVLDVVTEAGRDWLLLGEVPGQDLLSSHLAPAEKVSIMADAMR | 128 |
| Query 122 | RLHTLDPATCPFDHQAKHRIERARTRMEAGLVDQDDLDEEHQGLAPAELFARLKARMPDG | 181 |
| Sbjct 129 | RLHTLDPATCPFDHQAKHRIERARTRMEAGLVDQDDLDEEHQGLAPAELFARLKA MPDG RLHTLDPATCPFDHQAKHRIERARTRMEAGLVDQDDLDEEHQGLAPAELFARLKASMPDG | 188 |
| Query 182 | EDLVVTHGDACLPNIMVENGRFSGFIDCGRLGVADRYQDIALATRDIAEELGGEWADRFL | 241 |
| Sbjct 189 | EDLVVTHGDACLPNIMVENGRFSGFIDCGRLGVADRYQDIALATRDIAEELGGEWADRFL EDLVVTHGDACLPNIMVENGRFSGFIDCGRLGVADRYQDIALATRDIAEELGGEWADRFL | 248 |
| Query 242  Sbjct 249 | VLYGIAAPDSQRIAFYRLLDEFF 264 VLYGIAAPDSQRIAFYRLLDEFF VLYGIAAPDSQRIAFYRLLDEFF 271 |  |

>AAY29062.1 APH(3')-IIA [Cloning vector LUCTRAP-1] AAY29064.1 APH(3')-IIA [Cloning vector LUCTRAP-2] AAY33853.1 APH(3')-IIA [Cloning vector LUCTRAP-3(GW)] AAZ23213.1 APH(3')-IIA [Cloning vector LUCTRAP]

ABU39927.1 kanamycin resistance protein [Cloning vector pGreenII 0029] ABU39929.1 kanamycin resistance protein [Cloning vector pGreenII 0029 62-SK] AEG42735.1 NPTII [Cloning vector pPLV02]

AEG42739.1 NPTII [Cloning vector pPLV04] AEG42745.1 NPTII [Cloning vector pPLV06] AEG42751.1 NPTII [Cloning vector pPLV08] AEG42760.1 NPTII [Cloning vector pPLV11] AEG42769.1 NPTII [Cloning vector pPLV14] AEG42776.1 NPTII [Cloning vector pPLV17] AEG42785.1 NPTII [Cloning vector pPLV20] AEG42794.1 NPTII [Cloning vector pPLV23] AEG42802.1 NPTII [Cloning vector pPLV26] AEG42810.1 NPTII [Cloning vector pPLV30]

AEG42816.1 NPTII [Cloning vector pPLV33]

AKB90830.1 neomycin phosphotransferase II [Cloning vector pJF753] AKB90836.1 neomycin phosphotransferase II [Cloning vector pJF803] QEJ82236.1 NeoR/KanR [Plant binary expression vector pNC-BiFC-Ecc] QEJ82238.1 NeoR/KanR [Plant binary expression vector pNC-BiFC-Ecn] QEJ82240.1 NeoR/KanR [Plant binary expression vector pNC-BiFC-ED1] QEJ82242.1 NeoR/KanR [Plant binary expression vector pNC-BiFC-ED2] QEJ82244.1 NeoR/KanR [Plant binary expression vector pNC-BiFC-ED3] QEJ82246.1 NeoR/KanR [Plant binary expression vector pNC-BiFC-ED4] QEJ82248.1 NeoR/KanR [Plant binary expression vector pNC-BiFC-Enc] QEJ82250.1 NeoR/KanR [Plant binary expression vector pNC-BiFC-Enn] QEJ82265.1 NeoR/KanR [Plant binary expression vector pNC-Green0029] QEJ82268.1 NeoR/KanR [Plant binary expression vector pNC-Green0029-35S]

Length=264

Score = 531 bits (1367), Expect = 0.0, Method: Compositional matrix adjust. Identities = 262/264 (99%), Positives = 262/264 (99%), Gaps = 0/264 (0%)

| Query | 1 | MIEQDGLHAGSPAAWVERLFGYDWAQQTIGCSDAAVFRLSAQGRPVLFVKTDLSGALNEL | 60 |
| --- | --- | --- | --- |
| Sbjct | 1 | MIEQDGLHAGSPAAWVERLFGYDWAQQTIGCSDAAVFRLSAQGRPVLFVKTDLSGALNEL MIEQDGLHAGSPAAWVERLFGYDWAQQTIGCSDAAVFRLSAQGRPVLFVKTDLSGALNEL | 60 |
| Query | 61 | QDEAARLSWLATTGVPCAAVLDVVTEAGRDWLLLGEVPGQDLLSSHLAPAEKVSIMADAM | 120 |
| Sbjct | 61 | QDEAARLSWLATTGVPCAAVLDVVTEAGRDWLLLGEVPGQDLLSSHLAPAEKVSIMADAM QDEAARLSWLATTGVPCAAVLDVVTEAGRDWLLLGEVPGQDLLSSHLAPAEKVSIMADAM | 120 |
| Query | 121 | RRLHTLDPATCPFDHQAKHRIERARTRMEAGLVDQDDLDEEHQGLAPAELFARLKARMPD | 180 |
| Sbjct | 121 | RRLHTLDPATCPFDHQAKHRIER RTRMEAGLVDQDDLDEEHQGLAPAELFARLKARMPD RRLHTLDPATCPFDHQAKHRIERGRTRMEAGLVDQDDLDEEHQGLAPAELFARLKARMPD | 180 |
| Query | 181 | GEDLVVTHGDACLPNIMVENGRFSGFIDCGRLGVADRYQDIALATRDIAEELGGEWADRF | 240 |
| Sbjct | 181 | GEDLVVT GDACLPNIMVENGRFSGFIDCGRLGVADRYQDIALATRDIAEELGGEWADRF GEDLVVTQGDACLPNIMVENGRFSGFIDCGRLGVADRYQDIALATRDIAEELGGEWADRF | 240 |
| Query  Sbjct | 241  241 | LVLYGIAAPDSQRIAFYRLLDEFF 264 LVLYGIAAPDSQRIAFYRLLDEFF LVLYGIAAPDSQRIAFYRLLDEFF 264 |  |

```
>QDG03204.1 kanamycin resistance protein [Cloning vector pGHKW4] Length=293

Score = 530 bits (1366), Expect = 0.0, Method: Compositional matrix adjust. Identities = 260/260 (100%), Positives = 260/260 (100%), Gaps = 0/260 (0%)

Query 1	MIEQDGLHAGSPAAWVERLFGYDWAQQTIGCSDAAVFRLSAQGRPVLFVKTDLSGALNEL 60 MIEQDGLHAGSPAAWVERLFGYDWAQQTIGCSDAAVFRLSAQGRPVLFVKTDLSGALNEL
Sbjct 1	MIEQDGLHAGSPAAWVERLFGYDWAQQTIGCSDAAVFRLSAQGRPVLFVKTDLSGALNEL 60

Query 61	QDEAARLSWLATTGVPCAAVLDVVTEAGRDWLLLGEVPGQDLLSSHLAPAEKVSIMADAM 120 QDEAARLSWLATTGVPCAAVLDVVTEAGRDWLLLGEVPGQDLLSSHLAPAEKVSIMADAM
Sbjct 61	QDEAARLSWLATTGVPCAAVLDVVTEAGRDWLLLGEVPGQDLLSSHLAPAEKVSIMADAM 120

Query 121 RRLHTLDPATCPFDHQAKHRIERARTRMEAGLVDQDDLDEEHQGLAPAELFARLKARMPD 180 RRLHTLDPATCPFDHQAKHRIERARTRMEAGLVDQDDLDEEHQGLAPAELFARLKARMPD
Sbjct 121 RRLHTLDPATCPFDHQAKHRIERARTRMEAGLVDQDDLDEEHQGLAPAELFARLKARMPD 180

Query 181 GEDLVVTHGDACLPNIMVENGRFSGFIDCGRLGVADRYQDIALATRDIAEELGGEWADRF 240 GEDLVVTHGDACLPNIMVENGRFSGFIDCGRLGVADRYQDIALATRDIAEELGGEWADRF
Sbjct 181 GEDLVVTHGDACLPNIMVENGRFSGFIDCGRLGVADRYQDIALATRDIAEELGGEWADRF 240

Query 241 LVLYGIAAPDSQRIAFYRLL 260
LVLYGIAAPDSQRIAFYRLL Sbjct 241 LVLYGIAAPDSQRIAFYRLL 260

>BBD52293.1 neomycin-kanamycin phosphotransferase [Cloning vector pMW219-blaCTX-M-14] Length=264

Score = 530 bits (1366), Expect = 0.0, Method: Compositional matrix adjust. Identities = 262/264 (99%), Positives = 262/264 (99%), Gaps = 0/264 (0%)
```

| Query | 1 | MIEQDGLHAGSPAAWVERLFGYDWAQQTIGCSDAAVFRLSAQGRPVLFVKTDLSGALNEL | 60 |
| --- | --- | --- | --- |
| Sbjct | 1 | MIEQDGLHAGSPAAWVERLFGYDWAQQTIGCSDAAVFRLSAQGRPVLFVKTDLSGALNEL MIEQDGLHAGSPAAWVERLFGYDWAQQTIGCSDAAVFRLSAQGRPVLFVKTDLSGALNEL | 60 |
| Query | 61 | QDEAARLSWLATTGVPCAAVLDVVTEAGRDWLLLGEVPGQDLLSSHLAPAEKVSIMADAM | 120 |
| Sbjct | 61 | QDEAARLSWLATTGVPCAAVLDV TEAGRDWLLLGEVPGQDLLSSHLAPAEKVSIMADAM QDEAARLSWLATTGVPCAAVLDVGTEAGRDWLLLGEVPGQDLLSSHLAPAEKVSIMADAM | 120 |
| Query | 121 | RRLHTLDPATCPFDHQAKHRIERARTRMEAGLVDQDDLDEEHQGLAPAELFARLKARMPD | 180 |
| Sbjct | 121 | RRLHTLDPATCPFDHQAKHRIERARTRMEAGLVDQDDLDEEHQGLAPAELFARLKA MPD RRLHTLDPATCPFDHQAKHRIERARTRMEAGLVDQDDLDEEHQGLAPAELFARLKASMPD | 180 |
| Query | 181 | GEDLVVTHGDACLPNIMVENGRFSGFIDCGRLGVADRYQDIALATRDIAEELGGEWADRF | 240 |
| Sbjct | 181 | GEDLVVTHGDACLPNIMVENGRFSGFIDCGRLGVADRYQDIALATRDIAEELGGEWADRF GEDLVVTHGDACLPNIMVENGRFSGFIDCGRLGVADRYQDIALATRDIAEELGGEWADRF | 240 |
| Query  Sbjct | 241  241 | LVLYGIAAPDSQRIAFYRLLDEFF 264 LVLYGIAAPDSQRIAFYRLLDEFF LVLYGIAAPDSQRIAFYRLLDEFF 264 |  |

>AGV76759.1 AphA [Cloning vector pAV012] Length=267

Score = 530 bits (1366), Expect = 0.0, Method: Compositional matrix adjust.

| Identities | = 262/263 (99%), Positives = 262/263 (99%), Gaps = 0/263 (0%) |  |
| --- | --- | --- |
| Query 2 | IEQDGLHAGSPAAWVERLFGYDWAQQTIGCSDAAVFRLSAQGRPVLFVKTDLSGALNELQ | 61 |
| Sbjct 5 | IEQDGLHAGSPAAWVERLFGYDWAQQTIGCSDAAVFRLSAQGRPVLFVKTDLSGALNELQ IEQDGLHAGSPAAWVERLFGYDWAQQTIGCSDAAVFRLSAQGRPVLFVKTDLSGALNELQ | 64 |
| Query 62 | DEAARLSWLATTGVPCAAVLDVVTEAGRDWLLLGEVPGQDLLSSHLAPAEKVSIMADAMR | 121 |
| Sbjct 65 | DEAARLSWLATTGVPCAAVLDVVTEAGRDWLLLGEVPGQDLLSSHLAPAEKVSIMADAMR DEAARLSWLATTGVPCAAVLDVVTEAGRDWLLLGEVPGQDLLSSHLAPAEKVSIMADAMR | 124 |
| Query 122 | RLHTLDPATCPFDHQAKHRIERARTRMEAGLVDQDDLDEEHQGLAPAELFARLKARMPDG | 181 |
| Sbjct 125 | RLHTLDPATCPFDHQAKHRIERARTRMEAGLVDQDDLDEEHQGLAPAELFARLKARMP G RLHTLDPATCPFDHQAKHRIERARTRMEAGLVDQDDLDEEHQGLAPAELFARLKARMPGG | 184 |
| Query 182 | EDLVVTHGDACLPNIMVENGRFSGFIDCGRLGVADRYQDIALATRDIAEELGGEWADRFL | 241 |
| Sbjct 185 | EDLVVTHGDACLPNIMVENGRFSGFIDCGRLGVADRYQDIALATRDIAEELGGEWADRFL EDLVVTHGDACLPNIMVENGRFSGFIDCGRLGVADRYQDIALATRDIAEELGGEWADRFL | 244 |
| Query 242  Sbjct 245 | VLYGIAAPDSQRIAFYRLLDEFF 264 VLYGIAAPDSQRIAFYRLLDEFF VLYGIAAPDSQRIAFYRLLDEFF 267 |  |

>AQK76559.1 neomycin phosphotransferase [Cloning vector pL0A_0-1] AQK76561.1 neomycin phosphotransferase [Cloning vector pL0A_0-R] AQK76563.1 neomycin phosphotransferase [Cloning vector pL0A_1-2] AQK76565.1 neomycin phosphotransferase [Cloning vector pL0A_1-R] AQK76567.1 neomycin phosphotransferase [Cloning vector pL0A_2-3] AQK76569.1 neomycin phosphotransferase [Cloning vector pL0A_2-R] AQK76571.1 neomycin phosphotransferase [Cloning vector pL0A_3-4] AQK76573.1 neomycin phosphotransferase [Cloning vector pL0A_3-R] AQK76575.1 neomycin phosphotransferase [Cloning vector pL0A_4-R] AQK76577.1 neomycin phosphotransferase [Cloning vector pL0B_0-1] AQK76579.1 neomycin phosphotransferase [Cloning vector pL0B_0-R] AQK76581.1 neomycin phosphotransferase [Cloning vector pL0B_1-2] AQK76583.1 neomycin phosphotransferase [Cloning vector pL0B_1-R] AQK76585.1 neomycin phosphotransferase [Cloning vector pL0B_2-3] AQK76587.1 neomycin phosphotransferase [Cloning vector pL0B_2-R] AQK76589.1 neomycin phosphotransferase [Cloning vector pL0B_3-4] AQK76591.1 neomycin phosphotransferase [Cloning vector pL0B_3-R] AQK76593.1 neomycin phosphotransferase [Cloning vector pL0B_4-R] AQK76595.1 neomycin phosphotransferase [Cloning vector pL0C_0-1] AQK76597.1 neomycin phosphotransferase [Cloning vector pL0C_0-R] AQK76599.1 neomycin phosphotransferase [Cloning vector pL0C_1-2] AQK76601.1 neomycin phosphotransferase [Cloning vector pL0C_1-R] AQK76603.1 neomycin phosphotransferase [Cloning vector pL0C_2-3] AQK76605.1 neomycin phosphotransferase [Cloning vector pL0C_2-R]

AQK76607.1 neomycin phosphotransferase [Cloning vector pL0C_3-4] AQK76609.1 neomycin phosphotransferase [Cloning vector pL0C_3-R] AQK76611.1 neomycin phosphotransferase [Cloning vector pL0C_4-R] AQK76613.1 neomycin phosphotransferase [Cloning vector pL0D_0-1] AQK76615.1 neomycin phosphotransferase [Cloning vector pL0D_0-R] AQK76617.1 neomycin phosphotransferase [Cloning vector pL0D_1-2] AQK76619.1 neomycin phosphotransferase [Cloning vector pL0D_1-R] AQK76621.1 kanamycine resistance [Cloning vector pL0D_2-3] AQK76623.1 neomycin phosphotransferase [Cloning vector pL0D_2-R] AQK76625.1 neomycin phosphotransferase [Cloning vector pL0D_3-4] AQK76627.1 neomycin phosphotransferase [Cloning vector pL0D_3-R] AQK76629.1 neomycin phosphotransferase [Cloning vector pL0D_4-R] AQK76631.1 neomycin phosphotransferase [Cloning vector pL0E_0-1] AQK76633.1 neomycin phosphotransferase [Cloning vector pL0E_0-R] AQK76635.1 neomycin phosphotransferase [Cloning vector pL0E_1-2] AQK76637.1 neomycin phosphotransferase [Cloning vector pL0E_1-R] AQK76639.1 neomycin phosphotransferase [Cloning vector pL0E_2-3] AQK76641.1 neomycin phosphotransferase [Cloning vector pL0E_2-R] AQK76643.1 neomycin phosphotransferase [Cloning vector pL0E_3-4] AQK76645.1 neomycin phosphotransferase [Cloning vector pL0E_3-R] AQK76647.1 neomycin phosphotransferase [Cloning vector pL0E_4-R]

SJL86976.1 unnamed protein product [Yeast expression vector pGBKT7-Cezanne(17-858)]

SJL86980.1 unnamed protein product [Yeast expression vector pGBKT7-Cezanne(17-443)] SJL86984.1 unnamed protein product [Yeast expression vector pGBKT7-Cezanne(444-858)] SJL87903.1 unnamed protein product [Yeast vector pDEST-GBKT7]

Length=264

Score = 530 bits (1366), Expect = 0.0, Method: Compositional matrix adjust.

| Identities | = 262/264 (99%), Positives = 262/264 (99%), Gaps = 0/264 (0%) |  |
| --- | --- | --- |
| Query 1 | MIEQDGLHAGSPAAWVERLFGYDWAQQTIGCSDAAVFRLSAQGRPVLFVKTDLSGALNEL | 60 |
| Sbjct 1 | MIEQDGLHAGSPAAWVERLFGYDWAQQTIGCSDAAVFRLSAQGRPVLFVKTDLSGALNEL MIEQDGLHAGSPAAWVERLFGYDWAQQTIGCSDAAVFRLSAQGRPVLFVKTDLSGALNEL | 60 |
| Query 61 | QDEAARLSWLATTGVPCAAVLDVVTEAGRDWLLLGEVPGQDLLSSHLAPAEKVSIMADAM | 120 |
| Sbjct 61 | QDEAARLSWLATTGVPCAAVLDVVTEAGRDWLLLGEVPGQDLLSSHLAPAEKVSIMADAM QDEAARLSWLATTGVPCAAVLDVVTEAGRDWLLLGEVPGQDLLSSHLAPAEKVSIMADAM | 120 |
| Query 121 | RRLHTLDPATCPFDHQAKHRIERARTRMEAGLVDQDDLDEEHQGLAPAELFARLKARMPD | 180 |
| Sbjct 121 | RRLHTLDPATCPFDHQAKHRIERARTRMEAGLVDQDDLDEEHQGLAPAELFARLKA MPD RRLHTLDPATCPFDHQAKHRIERARTRMEAGLVDQDDLDEEHQGLAPAELFARLKASMPD | 180 |
| Query 181 | GEDLVVTHGDACLPNIMVENGRFSGFIDCGRLGVADRYQDIALATRDIAEELGGEWADRF | 240 |
| Sbjct 181 | GEDLVVTH DACLPNIMVENGRFSGFIDCGRLGVADRYQDIALATRDIAEELGGEWADRF GEDLVVTHDDACLPNIMVENGRFSGFIDCGRLGVADRYQDIALATRDIAEELGGEWADRF | 240 |
| Query 241  Sbjct 241 | LVLYGIAAPDSQRIAFYRLLDEFF 264 LVLYGIAAPDSQRIAFYRLLDEFF LVLYGIAAPDSQRIAFYRLLDEFF 264 |  |

>AOO95910.1 Meo [Cloning vector GAPTrapT2AIresMeo] Length=270

Score = 530 bits (1365), Expect = 0.0, Method: Compositional matrix adjust.

| Identities | = 262/263 (99%), Positives = 262/263 (99%), Gaps = 0/263 (0%) |  |
| --- | --- | --- |
| Query 2 | IEQDGLHAGSPAAWVERLFGYDWAQQTIGCSDAAVFRLSAQGRPVLFVKTDLSGALNELQ | 61 |
| Sbjct 8 | IEQDGLHAGSPAAWVERLFGYDWAQQTIGCSDAAVFRLSAQGRPVLFVKTDLSGALNELQ IEQDGLHAGSPAAWVERLFGYDWAQQTIGCSDAAVFRLSAQGRPVLFVKTDLSGALNELQ | 67 |
| Query 62 | DEAARLSWLATTGVPCAAVLDVVTEAGRDWLLLGEVPGQDLLSSHLAPAEKVSIMADAMR | 121 |
| Sbjct 68 | DEAARLSWLATTGVPCAAVLDVVTEAGRDWLLLGEVPGQDLLSSHLAPAEKVSIMADAMR DEAARLSWLATTGVPCAAVLDVVTEAGRDWLLLGEVPGQDLLSSHLAPAEKVSIMADAMR | 127 |
| Query 122 | RLHTLDPATCPFDHQAKHRIERARTRMEAGLVDQDDLDEEHQGLAPAELFARLKARMPDG | 181 |
| Sbjct 128 | RLHTLDPATCPFDHQAKHRIERARTRMEAGLVDQDDLDEEHQGLAPAELFARLKARMPDG RLHTLDPATCPFDHQAKHRIERARTRMEAGLVDQDDLDEEHQGLAPAELFARLKARMPDG | 187 |
| Query 182 | EDLVVTHGDACLPNIMVENGRFSGFIDCGRLGVADRYQDIALATRDIAEELGGEWADRFL | 241 |
| Sbjct 188 | EDLVVTHGDACLPNIMVENGRFSGFIDCGRLGVADRYQDIALATRDIAEEL GEWADRFL  EDLVVTHGDACLPNIMVENGRFSGFIDCGRLGVADRYQDIALATRDIAEELCGEWADRFL | 247 |

Query 242 VLYGIAAPDSQRIAFYRLLDEFF 264

VLYGIAAPDSQRIAFYRLLDEFF Sbjct 248 VLYGIAAPDSQRIAFYRLLDEFF 270

>AMQ45842.1 neomycin-kanamycin phosphotransferase [Synthetic plasmid pKI_Pdx1] AMQ45848.1 neomycin-kanamycin phosphotransferase [Synthetic plasmid pEGFPN1-Pdx1]

Length=263

Score = 530 bits (1364), Expect = 0.0, Method: Compositional matrix adjust.

| Identities | = 261/263 (99%), Positives = 262/263 (99%), Gaps = 0/263 (0%) |  |
| --- | --- | --- |
| Query 2 | IEQDGLHAGSPAAWVERLFGYDWAQQTIGCSDAAVFRLSAQGRPVLFVKTDLSGALNELQ | 61 |
| Sbjct 1 | +EQDGLHAGSPAAWVERLFGYDWAQQTIGCSDAAVFRLSAQGRPVLFVKTDLSGALNELQ MEQDGLHAGSPAAWVERLFGYDWAQQTIGCSDAAVFRLSAQGRPVLFVKTDLSGALNELQ | 60 |
| Query 62 | DEAARLSWLATTGVPCAAVLDVVTEAGRDWLLLGEVPGQDLLSSHLAPAEKVSIMADAMR | 121 |
| Sbjct 61 | DEAARLSWLATTGVPCAAVLDVVTEAGRDWLLLGEVPGQDLLSSHLAPAEKVSIMADAMR DEAARLSWLATTGVPCAAVLDVVTEAGRDWLLLGEVPGQDLLSSHLAPAEKVSIMADAMR | 120 |
| Query 122 | RLHTLDPATCPFDHQAKHRIERARTRMEAGLVDQDDLDEEHQGLAPAELFARLKARMPDG | 181 |
| Sbjct 121 | RLHTLDPATCPFDHQAKHRIERARTRMEAGLVDQDDLDEEHQGLAPAELFARLKA MPDG RLHTLDPATCPFDHQAKHRIERARTRMEAGLVDQDDLDEEHQGLAPAELFARLKASMPDG | 180 |
| Query 182 | EDLVVTHGDACLPNIMVENGRFSGFIDCGRLGVADRYQDIALATRDIAEELGGEWADRFL | 241 |
| Sbjct 181 | EDLVVTHGDACLPNIMVENGRFSGFIDCGRLGVADRYQDIALATRDIAEELGGEWADRFL EDLVVTHGDACLPNIMVENGRFSGFIDCGRLGVADRYQDIALATRDIAEELGGEWADRFL | 240 |
| Query 242  Sbjct 241 | VLYGIAAPDSQRIAFYRLLDEFF 264 VLYGIAAPDSQRIAFYRLLDEFF VLYGIAAPDSQRIAFYRLLDEFF 263 |  |

>ALD65381.1 aminoglycoside phosphotransferase [Arthrobacter sp. LS16] Length=264

Score = 529 bits (1363), Expect = 0.0, Method: Compositional matrix adjust.

| Identities | = 262/264 (99%), Positives = 262/264 (99%), Gaps = 0/264 (0%) |  |
| --- | --- | --- |
| Query 1 | MIEQDGLHAGSPAAWVERLFGYDWAQQTIGCSDAAVFRLSAQGRPVLFVKTDLSGALNEL | 60 |
| Sbjct 1 | MIEQDGLHAGSPAAWVERLFGYDWAQQTIGCSDAAVFRLSAQGR VLFVKTDLSGALNEL MIEQDGLHAGSPAAWVERLFGYDWAQQTIGCSDAAVFRLSAQGRLVLFVKTDLSGALNEL | 60 |
| Query 61 | QDEAARLSWLATTGVPCAAVLDVVTEAGRDWLLLGEVPGQDLLSSHLAPAEKVSIMADAM | 120 |
| Sbjct 61 | QDEAARLSWLATTGVPCAAVLDVVTEAGRDWLLLGEVPGQDLLSSHLAPAEKVSIMADAM QDEAARLSWLATTGVPCAAVLDVVTEAGRDWLLLGEVPGQDLLSSHLAPAEKVSIMADAM | 120 |
| Query 121 | RRLHTLDPATCPFDHQAKHRIERARTRMEAGLVDQDDLDEEHQGLAPAELFARLKARMPD | 180 |
| Sbjct 121 | RRLHTLDPATCPFDHQAKHRIERARTRMEAGLVDQDDLDEEHQGLAPAELFARLKA MPD RRLHTLDPATCPFDHQAKHRIERARTRMEAGLVDQDDLDEEHQGLAPAELFARLKASMPD | 180 |
| Query 181 | GEDLVVTHGDACLPNIMVENGRFSGFIDCGRLGVADRYQDIALATRDIAEELGGEWADRF | 240 |
| Sbjct 181 | GEDLVVTHGDACLPNIMVENGRFSGFIDCGRLGVADRYQDIALATRDIAEELGGEWADRF GEDLVVTHGDACLPNIMVENGRFSGFIDCGRLGVADRYQDIALATRDIAEELGGEWADRF | 240 |
| Query 241  Sbjct 241 | LVLYGIAAPDSQRIAFYRLLDEFF 264 LVLYGIAAPDSQRIAFYRLLDEFF LVLYGIAAPDSQRIAFYRLLDEFF 264 |  |

>WP_141989015.1 APH(3')-II family aminoglycoside O-phosphotransferase [Pseudomonas aeruginosa]

TQG79956.1 APH(3')-II family aminoglycoside O-phosphotransferase [Pseudomonas aeruginosa]

TQH08546.1 APH(3')-II family aminoglycoside O-phosphotransferase [Pseudomonas aeruginosa]

TQH18529.1 APH(3')-II family aminoglycoside O-phosphotransferase [Pseudomonas aeruginosa]

Length=264

Score = 529 bits (1362), Expect = 0.0, Method: Compositional matrix adjust.

| Identities | = 262/264 (99%), Positives = 262/264 (99%), Gaps = 0/264 (0%) |  |
| --- | --- | --- |
| Query 1 | MIEQDGLHAGSPAAWVERLFGYDWAQQTIGCSDAAVFRLSAQGRPVLFVKTDLSGALNEL | 60 |
| Sbjct 1 | MIEQDGLHAGSPAAWVERLFGYDWAQQTIG SDA VFRLSAQGRPVLFVKTDLSGALNEL MIEQDGLHAGSPAAWVERLFGYDWAQQTIGLSDADVFRLSAQGRPVLFVKTDLSGALNEL | 60 |
| Query 61 | QDEAARLSWLATTGVPCAAVLDVVTEAGRDWLLLGEVPGQDLLSSHLAPAEKVSIMADAM | 120 |
| Sbjct 61 | QDEAARLSWLATTGVPCAAVLDVVTEAGRDWLLLGEVPGQDLLSSHLAPAEKVSIMADAM QDEAARLSWLATTGVPCAAVLDVVTEAGRDWLLLGEVPGQDLLSSHLAPAEKVSIMADAM | 120 |
| Query 121 | RRLHTLDPATCPFDHQAKHRIERARTRMEAGLVDQDDLDEEHQGLAPAELFARLKARMPD | 180 |
| Sbjct 121 | RRLHTLDPATCPFDHQAKHRIERARTRMEAGLVDQDDLDEEHQGLAPAELFARLKARMPD RRLHTLDPATCPFDHQAKHRIERARTRMEAGLVDQDDLDEEHQGLAPAELFARLKARMPD | 180 |
| Query 181 | GEDLVVTHGDACLPNIMVENGRFSGFIDCGRLGVADRYQDIALATRDIAEELGGEWADRF | 240 |
| Sbjct 181 | GEDLVVTHGDACLPNIMVENGRFSGFIDCGRLGVADRYQDIALATRDIAEELGGEWADRF GEDLVVTHGDACLPNIMVENGRFSGFIDCGRLGVADRYQDIALATRDIAEELGGEWADRF | 240 |
| Query 241  Sbjct 241 | LVLYGIAAPDSQRIAFYRLLDEFF 264 LVLYGIAAPDSQRIAFYRLLDEFF LVLYGIAAPDSQRIAFYRLLDEFF 264 |  |

>AAL30400.1 neomycin phosphotransferase [Cloning vector pWS31] Length=264

Score = 529 bits (1362), Expect = 0.0, Method: Compositional matrix adjust.

| Identities | = 261/264 (99%), Positives = 262/264 (99%), Gaps = 0/264 (0%) |  |
| --- | --- | --- |
| Query 1 | MIEQDGLHAGSPAAWVERLFGYDWAQQTIGCSDAAVFRLSAQGRPVLFVKTDLSGALNEL | 60 |
| Sbjct 1 | MIEQDGLHAGSPAAWVERLFGYDWAQQTIGCSDAAVFRLSAQGRPVLFVKTDLSGALNEL MIEQDGLHAGSPAAWVERLFGYDWAQQTIGCSDAAVFRLSAQGRPVLFVKTDLSGALNEL | 60 |
| Query 61 | QDEAARLSWLATTGVPCAAVLDVVTEAGRDWLLLGEVPGQDLLSSHLAPAEKVSIMADAM | 120 |
| Sbjct 61 | QDEAARLSWLATTGVPCAAVLDVVTEAGRDWLLLGEVPGQDLLSSHLAPAEKVSI +AM QDEAARLSWLATTGVPCAAVLDVVTEAGRDWLLLGEVPGQDLLSSHLAPAEKVSIRLNAM | 120 |
| Query 121 | RRLHTLDPATCPFDHQAKHRIERARTRMEAGLVDQDDLDEEHQGLAPAELFARLKARMPD | 180 |
| Sbjct 121 | RRLHTLDPATCPFDHQAKHRIERARTRMEAGLVDQDDLDEEHQGLAPAELFARLKARMPD RRLHTLDPATCPFDHQAKHRIERARTRMEAGLVDQDDLDEEHQGLAPAELFARLKARMPD | 180 |
| Query 181 | GEDLVVTHGDACLPNIMVENGRFSGFIDCGRLGVADRYQDIALATRDIAEELGGEWADRF | 240 |
| Sbjct 181 | GEDLVVTHGDACLPNIMVENGRFSGFIDCGRLGVADRYQDIALATRDIAEELGGEWADRF GEDLVVTHGDACLPNIMVENGRFSGFIDCGRLGVADRYQDIALATRDIAEELGGEWADRF | 240 |
| Query 241  Sbjct 241 | LVLYGIAAPDSQRIAFYRLLDEFF 264 LVLYGIAAPDSQRIAFYRLLDEFF LVLYGIAAPDSQRIAFYRLLDEFF 264 |  |

```
>AAA72821.1 heat-shock protein 82/neomycin phosphotransferase fusion protein [Cloning vector pHS85]
Length=285

Score = 528 bits (1361), Expect = 0.0, Method: Compositional matrix adjust. Identities = 260/260 (100%), Positives = 260/260 (100%), Gaps = 0/260 (0%)

Query 5	DGLHAGSPAAWVERLFGYDWAQQTIGCSDAAVFRLSAQGRPVLFVKTDLSGALNELQDEA 64 DGLHAGSPAAWVERLFGYDWAQQTIGCSDAAVFRLSAQGRPVLFVKTDLSGALNELQDEA
Sbjct 26	DGLHAGSPAAWVERLFGYDWAQQTIGCSDAAVFRLSAQGRPVLFVKTDLSGALNELQDEA 85

Query 65	ARLSWLATTGVPCAAVLDVVTEAGRDWLLLGEVPGQDLLSSHLAPAEKVSIMADAMRRLH 124 ARLSWLATTGVPCAAVLDVVTEAGRDWLLLGEVPGQDLLSSHLAPAEKVSIMADAMRRLH
Sbjct 86	ARLSWLATTGVPCAAVLDVVTEAGRDWLLLGEVPGQDLLSSHLAPAEKVSIMADAMRRLH 145

Query 125 TLDPATCPFDHQAKHRIERARTRMEAGLVDQDDLDEEHQGLAPAELFARLKARMPDGEDL 184 TLDPATCPFDHQAKHRIERARTRMEAGLVDQDDLDEEHQGLAPAELFARLKARMPDGEDL
Sbjct 146 TLDPATCPFDHQAKHRIERARTRMEAGLVDQDDLDEEHQGLAPAELFARLKARMPDGEDL 205

Query 185 VVTHGDACLPNIMVENGRFSGFIDCGRLGVADRYQDIALATRDIAEELGGEWADRFLVLY 244 VVTHGDACLPNIMVENGRFSGFIDCGRLGVADRYQDIALATRDIAEELGGEWADRFLVLY
Sbjct 206 VVTHGDACLPNIMVENGRFSGFIDCGRLGVADRYQDIALATRDIAEELGGEWADRFLVLY 265

Query 245 GIAAPDSQRIAFYRLLDEFF 264
GIAAPDSQRIAFYRLLDEFF Sbjct 266 GIAAPDSQRIAFYRLLDEFF 285

>WP_099526830.1 APH(3')-II family aminoglycoside O-phosphotransferase [Pseudomonas sediminis]
PIA65714.1 APH(3') family aminoglycoside O-phosphotransferase [Pseudomonas sediminis]
Length=267

Score = 528 bits (1361), Expect = 0.0, Method: Compositional matrix adjust.
```

| Identities | = 261/263 (99%), Positives = 262/263 (99%), Gaps = 0/263 (0%) |  |
| --- | --- | --- |
| Query 2 | IEQDGLHAGSPAAWVERLFGYDWAQQTIGCSDAAVFRLSAQGRPVLFVKTDLSGALNELQ | 61 |
| Sbjct 5 | IEQDGLHAGSPAAWVERLFGYDWAQQTIGCSDAAVFRLSAQGRPVLFVKTDLSGALNELQ IEQDGLHAGSPAAWVERLFGYDWAQQTIGCSDAAVFRLSAQGRPVLFVKTDLSGALNELQ | 64 |
| Query 62 | DEAARLSWLATTGVPCAAVLDVVTEAGRDWLLLGEVPGQDLLSSHLAPAEKVSIMADAMR | 121 |
| Sbjct 65 | DEAARLSWLATTGVPCAAVLDVVTEAGRDWLLLGEVPGQDLLSSHLAPAEKVSIMADAMR DEAARLSWLATTGVPCAAVLDVVTEAGRDWLLLGEVPGQDLLSSHLAPAEKVSIMADAMR | 124 |
| Query 122 | RLHTLDPATCPFDHQAKHRIERARTRMEAGLVDQDDLDEEHQGLAPAELFARLKARMPDG | 181 |
| Sbjct 125 | RLHTLDPATCPFDHQAKHRIERARTRMEAGLVDQDDLDEEHQGLAPAELFARLKARMPDG RLHTLDPATCPFDHQAKHRIERARTRMEAGLVDQDDLDEEHQGLAPAELFARLKARMPDG | 184 |
| Query 182 | EDLVVTHGDACLPNIMVENGRFSGFIDCGRLGVADRYQDIALATRDIAEELGGEWADRFL | 241 |
| Sbjct 185 | +DLVVTHGDACLPNIMVENGRFSGFIDC RLGVADRYQDIALATRDIAEELGGEWADRFL DDLVVTHGDACLPNIMVENGRFSGFIDCVRLGVADRYQDIALATRDIAEELGGEWADRFL | 244 |
| Query 242  Sbjct 245 | VLYGIAAPDSQRIAFYRLLDEFF 264 VLYGIAAPDSQRIAFYRLLDEFF VLYGIAAPDSQRIAFYRLLDEFF 267 |  |

>WP_160187495.1 APH(3')-II family aminoglycoside O-phosphotransferase [Escherichia coli]

Length=263

Score = 528 bits (1361), Expect = 0.0, Method: Compositional matrix adjust.

| Identities | = 263/264 (99%), Positives = 263/264 (99%), Gaps = 1/264 (0%) |  |
| --- | --- | --- |
| Query 1 | MIEQDGLHAGSPAAWVERLFGYDWAQQTIGCSDAAVFRLSAQGRPVLFVKTDLSGALNEL | 60 |
| Sbjct 1 | MIEQDGLHAGSPAAWVERLFGYDWAQQTIGCSDAAVFRLSAQGRPVLFVKTDLSGALNEL MIEQDGLHAGSPAAWVERLFGYDWAQQTIGCSDAAVFRLSAQGRPVLFVKTDLSGALNEL | 60 |
| Query 61 | QDEAARLSWLATTGVPCAAVLDVVTEAGRDWLLLGEVPGQDLLSSHLAPAEKVSIMADAM | 120 |
| Sbjct 61 | Q EAARLSWLATTGVPCAAVLDVVTEAGRDWLLLGEVPGQDLLSSHLAPAEKVSIMADAM Q-EAARLSWLATTGVPCAAVLDVVTEAGRDWLLLGEVPGQDLLSSHLAPAEKVSIMADAM | 119 |
| Query 121 | RRLHTLDPATCPFDHQAKHRIERARTRMEAGLVDQDDLDEEHQGLAPAELFARLKARMPD | 180 |
| Sbjct 120 | RRLHTLDPATCPFDHQAKHRIERARTRMEAGLVDQDDLDEEHQGLAPAELFARLKARMPD RRLHTLDPATCPFDHQAKHRIERARTRMEAGLVDQDDLDEEHQGLAPAELFARLKARMPD | 179 |
| Query 181 | GEDLVVTHGDACLPNIMVENGRFSGFIDCGRLGVADRYQDIALATRDIAEELGGEWADRF | 240 |
| Sbjct 180 | GEDLVVTHGDACLPNIMVENGRFSGFIDCGRLGVADRYQDIALATRDIAEELGGEWADRF GEDLVVTHGDACLPNIMVENGRFSGFIDCGRLGVADRYQDIALATRDIAEELGGEWADRF | 239 |
| Query 241  Sbjct 240 | LVLYGIAAPDSQRIAFYRLLDEFF 264 LVLYGIAAPDSQRIAFYRLLDEFF LVLYGIAAPDSQRIAFYRLLDEFF 263 |  |

>BAH70332.1 kanamycin resistance [synthetic construct] Length=264

Score = 528 bits (1361), Expect = 0.0, Method: Compositional matrix adjust. Identities = 262/264 (99%), Positives = 262/264 (99%), Gaps = 0/264 (0%)

Query 1	MIEQDGLHAGSPAAWVERLFGYDWAQQTIGCSDAAVFRLSAQGRPVLFVKTDLSGALNEL 60 MIEQDGLHAGSPAAWVERLFGYDWAQQTIGCSDAAVFRLSAQGRPVLFVKTDLSGALNEL

| Sbjct | 1 | MIEQDGLHAGSPAAWVERLFGYDWAQQTIGCSDAAVFRLSAQGRPVLFVKTDLSGALNEL | 60 |
| --- | --- | --- | --- |
| Query | 61 | QDEAARLSWLATTGVPCAAVLDVVTEAGRDWLLLGEVPGQDLLSSHLAPAEKVSIMADAM | 120 |
| Sbjct | 61 | QDEAARLSWLATTGVPCAAVLDVVTEAGRDWLLLGEVPGQDLLSSHLAPAEKVSIMADAM QDEAARLSWLATTGVPCAAVLDVVTEAGRDWLLLGEVPGQDLLSSHLAPAEKVSIMADAM | 120 |
| Query | 121 | RRLHTLDPATCPFDHQAKHRIERARTRMEAGLVDQDDLDEEHQGLAPAELFARLKARMPD | 180 |
| Sbjct | 121 | RRLHTLDPATCPFDHQAKHRIERARTRMEAGLVDQDDLD EHQGLAPAELFARLKARMPD RRLHTLDPATCPFDHQAKHRIERARTRMEAGLVDQDDLDGEHQGLAPAELFARLKARMPD | 180 |
| Query | 181 | GEDLVVTHGDACLPNIMVENGRFSGFIDCGRLGVADRYQDIALATRDIAEELGGEWADRF | 240 |
| Sbjct | 181 | GEDLVVTHGDACLPNIMVENGRFSGFIDCGRLGVADRYQDIALATRDIA ELGGEWADRF GEDLVVTHGDACLPNIMVENGRFSGFIDCGRLGVADRYQDIALATRDIAGELGGEWADRF | 240 |
| Query  Sbjct | 241  241 | LVLYGIAAPDSQRIAFYRLLDEFF 264 LVLYGIAAPDSQRIAFYRLLDEFF LVLYGIAAPDSQRIAFYRLLDEFF 264 |  |

>AII99780.1 nptII_A7120 [Broad host range vector vector pCVD012] Length=263

Score = 528 bits (1361), Expect = 0.0, Method: Compositional matrix adjust.

| Identities | = 263/264 (99%), Positives = 263/264 (99%), Gaps = 1/264 (0%) |  |
| --- | --- | --- |
| Query 1 | MIEQDGLHAGSPAAWVERLFGYDWAQQTIGCSDAAVFRLSAQGRPVLFVKTDLSGALNEL | 60 |
| Sbjct 1 | MIEQDGLHAGSPAAWVERLFGYDWAQQTIGCSDAAVFRLSAQGRPVLFVKTDLSGALNEL MIEQDGLHAGSPAAWVERLFGYDWAQQTIGCSDAAVFRLSAQGRPVLFVKTDLSGALNEL | 60 |
| Query 61 | QDEAARLSWLATTGVPCAAVLDVVTEAGRDWLLLGEVPGQDLLSSHLAPAEKVSIMADAM | 120 |
| Sbjct 61 | QDEAARLSWLATTGVPCAAVLDVVTEAGRDWLLLGEVPGQDLLSSHLAPAEKVSIMADAM QDEAARLSWLATTGVPCAAVLDVVTEAGRDWLLLGEVPGQDLLSSHLAPAEKVSIMADAM | 120 |
| Query 121 | RRLHTLDPATCPFDHQAKHRIERARTRMEAGLVDQDDLDEEHQGLAPAELFARLKARMPD | 180 |
| Sbjct 121 | RRLHTLDPATCPFDHQAKHRIERARTRMEAGLVDQDDLDEEHQGLAPAELFARLKARMPD RRLHTLDPATCPFDHQAKHRIERARTRMEAGLVDQDDLDEEHQGLAPAELFARLKARMPD | 180 |
| Query 181 | GEDLVVTHGDACLPNIMVENGRFSGFIDCGRLGVADRYQDIALATRDIAEELGGEWADRF | 240 |
| Sbjct 181 | GEDLVVTHGDACLPNIMVENGRFSGFIDCGRLGVADRYQDIALATRDIAEEL GEWADRF GEDLVVTHGDACLPNIMVENGRFSGFIDCGRLGVADRYQDIALATRDIAEEL-GEWADRF | 239 |
| Query 241  Sbjct 240 | LVLYGIAAPDSQRIAFYRLLDEFF 264 LVLYGIAAPDSQRIAFYRLLDEFF LVLYGIAAPDSQRIAFYRLLDEFF 263 |  |

```
>EGJ3639417.1 APH(3')-II family aminoglycoside O-phosphotransferase [Escherichia coli]
Length=261

Score = 528 bits (1360), Expect = 0.0, Method: Compositional matrix adjust. Identities = 261/261 (100%), Positives = 261/261 (100%), Gaps = 0/261 (0%)

Query 1	MIEQDGLHAGSPAAWVERLFGYDWAQQTIGCSDAAVFRLSAQGRPVLFVKTDLSGALNEL 60 MIEQDGLHAGSPAAWVERLFGYDWAQQTIGCSDAAVFRLSAQGRPVLFVKTDLSGALNEL
Sbjct 1	MIEQDGLHAGSPAAWVERLFGYDWAQQTIGCSDAAVFRLSAQGRPVLFVKTDLSGALNEL 60

Query 61	QDEAARLSWLATTGVPCAAVLDVVTEAGRDWLLLGEVPGQDLLSSHLAPAEKVSIMADAM 120 QDEAARLSWLATTGVPCAAVLDVVTEAGRDWLLLGEVPGQDLLSSHLAPAEKVSIMADAM
Sbjct 61	QDEAARLSWLATTGVPCAAVLDVVTEAGRDWLLLGEVPGQDLLSSHLAPAEKVSIMADAM 120

Query 121 RRLHTLDPATCPFDHQAKHRIERARTRMEAGLVDQDDLDEEHQGLAPAELFARLKARMPD 180 RRLHTLDPATCPFDHQAKHRIERARTRMEAGLVDQDDLDEEHQGLAPAELFARLKARMPD
Sbjct 121 RRLHTLDPATCPFDHQAKHRIERARTRMEAGLVDQDDLDEEHQGLAPAELFARLKARMPD 180

Query 181 GEDLVVTHGDACLPNIMVENGRFSGFIDCGRLGVADRYQDIALATRDIAEELGGEWADRF 240 GEDLVVTHGDACLPNIMVENGRFSGFIDCGRLGVADRYQDIALATRDIAEELGGEWADRF
Sbjct 181 GEDLVVTHGDACLPNIMVENGRFSGFIDCGRLGVADRYQDIALATRDIAEELGGEWADRF 240

Query 241 LVLYGIAAPDSQRIAFYRLLD 261
LVLYGIAAPDSQRIAFYRLLD Sbjct 241 LVLYGIAAPDSQRIAFYRLLD 261


>SJL88773.1 unnamed protein product [Vector pPNT] SJL88810.1 unnamed protein product [Vector pPNEcad]
Length=264

Score = 528 bits (1359), Expect = 0.0, Method: Compositional matrix adjust.
```

| Identities | = 261/264 (99%), Positives = 261/264 (99%), Gaps = 0/264 (0%) |  |
| --- | --- | --- |
| Query 1 | MIEQDGLHAGSPAAWVERLFGYDWAQQTIGCSDAAVFRLSAQGRPVLFVKTDLSGALNEL | 60 |
| Sbjct 1 | M DGLHAGSPAAWVERLFGYDWAQQTIGCSDAAVFRLSAQGRPVLFVKTDLSGALNEL MGSADGLHAGSPAAWVERLFGYDWAQQTIGCSDAAVFRLSAQGRPVLFVKTDLSGALNEL | 60 |
| Query 61 | QDEAARLSWLATTGVPCAAVLDVVTEAGRDWLLLGEVPGQDLLSSHLAPAEKVSIMADAM | 120 |
| Sbjct 61 | QDEAARLSWLATTGVPCAAVLDVVTEAGRDWLLLGEVPGQDLLSSHLAPAEKVSIMADAM QDEAARLSWLATTGVPCAAVLDVVTEAGRDWLLLGEVPGQDLLSSHLAPAEKVSIMADAM | 120 |
| Query 121 | RRLHTLDPATCPFDHQAKHRIERARTRMEAGLVDQDDLDEEHQGLAPAELFARLKARMPD | 180 |
| Sbjct 121 | RRLHTLDPATCPFDHQAKHRIERARTRMEAGLVDQDDLDEEHQGLAPAELFARLKARMPD RRLHTLDPATCPFDHQAKHRIERARTRMEAGLVDQDDLDEEHQGLAPAELFARLKARMPD | 180 |
| Query 181 | GEDLVVTHGDACLPNIMVENGRFSGFIDCGRLGVADRYQDIALATRDIAEELGGEWADRF | 240 |
| Sbjct 181 | GEDLVVTHGDACLPNIMVENGRFSGFIDCGRLGVADRYQDIALATRDIAEELGGEWADRF GEDLVVTHGDACLPNIMVENGRFSGFIDCGRLGVADRYQDIALATRDIAEELGGEWADRF | 240 |
| Query 241  Sbjct 241 | LVLYGIAAPDSQRIAFYRLLDEFF 264 LVLYGIAAPDSQRIAFYRLLDEFF LVLYGIAAPDSQRIAFYRLLDEFF 264 |  |

>WP_128318427.1 APH(3')-II family aminoglycoside O-phosphotransferase [Aeromonas caviae]

RWS94068.1 APH(3')-II family aminoglycoside O-phosphotransferase [Aeromonas caviae]

Length=263

Score = 527 bits (1358), Expect = 0.0, Method: Compositional matrix adjust.

| Identities | = 260/261 (99%), Positives = 261/261 (100%), Gaps = 0/261 (0%) |  |
| --- | --- | --- |
| Query 4 | QDGLHAGSPAAWVERLFGYDWAQQTIGCSDAAVFRLSAQGRPVLFVKTDLSGALNELQDE | 63 |
| Sbjct 3 | +DGLHAGSPAAWVERLFGYDWAQQTIGCSDAAVFRLSAQGRPVLFVKTDLSGALNELQDE EDGLHAGSPAAWVERLFGYDWAQQTIGCSDAAVFRLSAQGRPVLFVKTDLSGALNELQDE | 62 |
| Query 64 | AARLSWLATTGVPCAAVLDVVTEAGRDWLLLGEVPGQDLLSSHLAPAEKVSIMADAMRRL | 123 |
| Sbjct 63 | AARLSWLATTGVPCAAVLDVVTEAGRDWLLLGEVPGQDLLSSHLAPAEKVSIMADAMRRL AARLSWLATTGVPCAAVLDVVTEAGRDWLLLGEVPGQDLLSSHLAPAEKVSIMADAMRRL | 122 |
| Query 124 | HTLDPATCPFDHQAKHRIERARTRMEAGLVDQDDLDEEHQGLAPAELFARLKARMPDGED | 183 |
| Sbjct 123 | HTLDPATCPFDHQAKHRIERARTRMEAGLVDQDDLDEEHQGLAPAELFARLKARMPDGED HTLDPATCPFDHQAKHRIERARTRMEAGLVDQDDLDEEHQGLAPAELFARLKARMPDGED | 182 |
| Query 184 | LVVTHGDACLPNIMVENGRFSGFIDCGRLGVADRYQDIALATRDIAEELGGEWADRFLVL | 243 |
| Sbjct 183 | LVVTHGDACLPNIMVENGRFSGFIDCGRLGVADRYQDIALATRDIAEELGGEWADRFLVL LVVTHGDACLPNIMVENGRFSGFIDCGRLGVADRYQDIALATRDIAEELGGEWADRFLVL | 242 |
| Query 244  Sbjct 243 | YGIAAPDSQRIAFYRLLDEFF 264 YGIAAPDSQRIAFYRLLDEFF YGIAAPDSQRIAFYRLLDEFF 263 |  |

>AAK28132.1 aminoglycoside 3'-phosphotransferase [Cloning vector pPGKneo-I] AAK28134.1 aminoglycoside 3'-phosphotransferase [Cloning vector pPGKneo-II] ACQ43937.1 aminoglycoside 3'-phosphotransferase [Cloning vector pHC02] ACQ43940.1 aminoglycoside 3'-phosphotransferase [Cloning vector pHC03] ACQ43943.1 aminoglycoside 3'-phosphotransferase [Cloning vector pHC04] AFI26420.1 KanR [Cloning vector pBACr-AraT7940_rbs9]

AGL73303.1 NptII [Cloning vector pCR2.1-sacB] AHA61709.1 NptII [Cloning vector pPS2305] AHA61711.1 NptII [Cloning vector pPS2836] APA32049.1 Kan/neoR [Synthetic plasmid pCore]

SJL87737.1 unnamed protein product [Plant expression vector pAS104] SJL88486.1 unnamed protein product [Vector pCRhBCL2]

Length=264

Score = 525 bits (1352), Expect = 0.0, Method: Compositional matrix adjust.

| Identities | = 260/264 (98%), Positives = 260/264 (98%), Gaps = 0/264 (0%) |  |
| --- | --- | --- |
| Query 1 | MIEQDGLHAGSPAAWVERLFGYDWAQQTIGCSDAAVFRLSAQGRPVLFVKTDLSGALNEL | 60 |
| Sbjct 1 | MIEQDGLHAGSPAAWVERLFGYDWAQQTIGCSDAAVFRLSAQGRPVLFVKTDLSGALNEL MIEQDGLHAGSPAAWVERLFGYDWAQQTIGCSDAAVFRLSAQGRPVLFVKTDLSGALNEL | 60 |
| Query 61 | QDEAARLSWLATTGVPCAAVLDVVTEAGRDWLLLGEVPGQDLLSSHLAPAEKVSIMADAM | 120 |
| Sbjct 61 | QDEAARLSWLATTGVPCAAVLDVVTEAGRDWLLLGEVPGQDLLSS LAPAEKVSIMADAM QDEAARLSWLATTGVPCAAVLDVVTEAGRDWLLLGEVPGQDLLSSRLAPAEKVSIMADAM | 120 |
| Query 121 | RRLHTLDPATCPFDHQAKHRIERARTRMEAGLVDQDDLDEEHQGLAPAELFARLKARMPD | 180 |
| Sbjct 121 | RRLHTLDPATCPFDHQAKHRIERARTRMEAGLVDQDDLDEEHQGLAPAELFARLKARMPD RRLHTLDPATCPFDHQAKHRIERARTRMEAGLVDQDDLDEEHQGLAPAELFARLKARMPD | 180 |
| Query 181 | GEDLVVTHGDACLPNIMVENGRFSGFIDCGRLGVADRYQDIALATRDIAEELGGEWADRF | 240 |
| Sbjct 181 | GEDLVV HGDACLPNIMVENGRFSGF DCGRLGVADRYQDIAL TRDIAEELGGEWADRF GEDLVVIHGDACLPNIMVENGRFSGFNDCGRLGVADRYQDIALDTRDIAEELGGEWADRF | 240 |
| Query 241  Sbjct 241 | LVLYGIAAPDSQRIAFYRLLDEFF 264 LVLYGIAAPDSQRIAFYRLLDEFF LVLYGIAAPDSQRIAFYRLLDEFF 264 |  |

>AAF85969.1 kanamycin resistance protein [synthetic construct] Length=263

Score = 524 bits (1349), Expect = 0.0, Method: Compositional matrix adjust.

| Identities | = 258/259 (99%), Positives = 259/259 (100%), Gaps = 0/259 (0%) |  |
| --- | --- | --- |
| Query 6 | GLHAGSPAAWVERLFGYDWAQQTIGCSDAAVFRLSAQGRPVLFVKTDLSGALNELQDEAA | 65 |
| Sbjct 5 | GLHAGSPAAWVERLFGYDWAQQTIGCSDAAVFRLSAQGRPVLFVKTDLSGALNELQDEAA GLHAGSPAAWVERLFGYDWAQQTIGCSDAAVFRLSAQGRPVLFVKTDLSGALNELQDEAA | 64 |
| Query 66 | RLSWLATTGVPCAAVLDVVTEAGRDWLLLGEVPGQDLLSSHLAPAEKVSIMADAMRRLHT | 125 |
| Sbjct 65 | RLSWLATTGVPCAAVLDVVTEAGRDWLLLGEVPGQDLLSSHLAPAEKVSIMADAMRRLHT RLSWLATTGVPCAAVLDVVTEAGRDWLLLGEVPGQDLLSSHLAPAEKVSIMADAMRRLHT | 124 |
| Query 126 | LDPATCPFDHQAKHRIERARTRMEAGLVDQDDLDEEHQGLAPAELFARLKARMPDGEDLV | 185 |
| Sbjct 125 | LDPATCP+DHQAKHRIERARTRMEAGLVDQDDLDEEHQGLAPAELFARLKARMPDGEDLV LDPATCPYDHQAKHRIERARTRMEAGLVDQDDLDEEHQGLAPAELFARLKARMPDGEDLV | 184 |
| Query 186 | VTHGDACLPNIMVENGRFSGFIDCGRLGVADRYQDIALATRDIAEELGGEWADRFLVLYG | 245 |
| Sbjct 185 | VTHGDACLPNIMVENGRFSGFIDCGRLGVADRYQDIALATRDIAEELGGEWADRFLVLYG VTHGDACLPNIMVENGRFSGFIDCGRLGVADRYQDIALATRDIAEELGGEWADRFLVLYG | 244 |
| Query 246  Sbjct 245 | IAAPDSQRIAFYRLLDEFF 264 IAAPDSQRIAFYRLLDEFF IAAPDSQRIAFYRLLDEFF 263 |  |

>AAC15800.1 neomycin/kanamycin resistance protein [Eukaryotic expression vector pCR3.1mBCL-XL]

SJL86525.1 unnamed protein product [Vector pGST-N-GW]

SJL86530.1 unnamed protein product [Mammalian vector pCR3-GW-GST-C] SJL86535.1 unnamed protein product [Mammalian vector pCR3-EGFPC-GW] SJL86540.1 unnamed protein product [Mammalian vector pCR3-ECFPC-GW] SJL86545.1 unnamed protein product [Mammalian vector pCR3-EYFPC-GW]

SJL86698.1 unnamed protein product [Mammalian expression vector pCR3-pro-IL-1B-D117A] SJL86701.1 unnamed protein product [Mammalian expression vector pCR3-pro-IL-1B-D108A] SJL86704.1 unnamed protein product [Mammalian expression vector pCR3-pro-IL-1B-D105A] SJL86707.1 unnamed protein product [Mammalian expression vector pCR3-pro-IL-1B] SJL87292.1 unnamed protein product [Mammalian expression vector pCR3-FLAGhRIP1] SJL88337.1 unnamed protein product [Mammalian expression vector pCR3VSV-CED-4] SJL88340.1 unnamed protein product [Mammalian expression vector pCR3FLAG-CED-3] SJL88343.1 unnamed protein product [Mammalian expression vector pCR3Flag-E8-FLIP]

Length=262

Score = 523 bits (1347), Expect = 0.0, Method: Compositional matrix adjust. Identities = 260/264 (98%), Positives = 261/264 (99%), Gaps = 2/264 (1%)

| Query | 1 | MIEQDGLHAGSPAAWVERLFGYDWAQQTIGCSDAAVFRLSAQGRPVLFVKTDLSGALNEL | 60 |
| --- | --- | --- | --- |
| Sbjct | 1 | MIEQDGLHAGSPAAWVERLFGYDWAQQTIGCSDAAVFRLSAQGRPVLFVKTDLSGALNEL MIEQDGLHAGSPAAWVERLFGYDWAQQTIGCSDAAVFRLSAQGRPVLFVKTDLSGALNEL | 60 |
| Query | 61 | QDEAARLSWLATTGVPCAAVLDVVTEAGRDWLLLGEVPGQDLLSSHLAPAEKVSIMADAM | 120 |
| Sbjct | 61 | QDEAARLSWLATTGVPCAAVLDVVTEAGRDWLLLGEVPGQDLLSSHLAPAEKVSIMADAM QDEAARLSWLATTGVPCAAVLDVVTEAGRDWLLLGEVPGQDLLSSHLAPAEKVSIMADAM | 120 |
| Query | 121 | RRLHTLDPATCPFDHQAKHRIERARTRMEAGLVDQDDLDEEHQGLAPAELFARLKARMPD | 180 |
| Sbjct | 121 | RRLHTLDPATCPFDHQAKHRIERARTRMEAGLVDQD D+EHQGLAPAELFARLKA MPD RRLHTLDPATCPFDHQAKHRIERARTRMEAGLVDQD--DQEHQGLAPAELFARLKASMPD | 178 |
| Query | 181 | GEDLVVTHGDACLPNIMVENGRFSGFIDCGRLGVADRYQDIALATRDIAEELGGEWADRF | 240 |
| Sbjct | 179 | GEDLVVTHGDACLPNIMVENGRFSGFIDCGRLGVADRYQDIALATRDIAEELGGEWADRF GEDLVVTHGDACLPNIMVENGRFSGFIDCGRLGVADRYQDIALATRDIAEELGGEWADRF | 238 |
| Query  Sbjct | 241  239 | LVLYGIAAPDSQRIAFYRLLDEFF 264 LVLYGIAAPDSQRIAFYRLLDEFF LVLYGIAAPDSQRIAFYRLLDEFF 262 |  |

>AAQ05967.1 kanamycin resistance protein [Cloning vector pHellsgate 8] AAV66537.1 neomycin phosphotransferase II [Binary vector pGA643] ACK58633.1 neophosphotransferase [Carica papaya]

ADG63448.1 neomycin phosphotransferase II [Binary vector pKM24KH] AGF68623.1 neomycin phosphotransferase II [RNAi silencing vector pCAPD] AMD82484.1 neomycin phosphotransferase [Binary vector pAPn]

CAC86252.1 kanomycin resistance protein [Cloning vector pHELLSGATE] CAY39269.1 nptII [Cloning vector pKIGW]

Length=273

Score = 521 bits (1343), Expect = 0.0, Method: Compositional matrix adjust.

| Identities | = 256/258 (99%), Positives = 257/258 (99%), Gaps = 0/258 (0%) |  |
| --- | --- | --- |
| Query 7 | LHAGSPAAWVERLFGYDWAQQTIGCSDAAVFRLSAQGRPVLFVKTDLSGALNELQDEAAR | 66 |
| Sbjct 16 | + AGSPAAWVERLFGYDWAQQTIGCSDAAVFRLSAQGRPVLFVKTDLSGALNELQDEAAR IRAGSPAAWVERLFGYDWAQQTIGCSDAAVFRLSAQGRPVLFVKTDLSGALNELQDEAAR | 75 |
| Query 67 | LSWLATTGVPCAAVLDVVTEAGRDWLLLGEVPGQDLLSSHLAPAEKVSIMADAMRRLHTL | 126 |
| Sbjct 76 | LSWLATTGVPCAAVLDVVTEAGRDWLLLGEVPGQDLLSSHLAPAEKVSIMADAMRRLHTL LSWLATTGVPCAAVLDVVTEAGRDWLLLGEVPGQDLLSSHLAPAEKVSIMADAMRRLHTL | 135 |
| Query 127 | DPATCPFDHQAKHRIERARTRMEAGLVDQDDLDEEHQGLAPAELFARLKARMPDGEDLVV | 186 |
| Sbjct 136 | DPATCPFDHQAKHRIERARTRMEAGLVDQDDLDEEHQGLAPAELFARLKARMPDGEDLVV DPATCPFDHQAKHRIERARTRMEAGLVDQDDLDEEHQGLAPAELFARLKARMPDGEDLVV | 195 |
| Query 187 | THGDACLPNIMVENGRFSGFIDCGRLGVADRYQDIALATRDIAEELGGEWADRFLVLYGI | 246 |
| Sbjct 196 | THGDACLPNIMVENGRFSGFIDCGRLGVADRYQDIALATRDIAEELGGEWADRFLVLYGI THGDACLPNIMVENGRFSGFIDCGRLGVADRYQDIALATRDIAEELGGEWADRFLVLYGI | 255 |
| Query 247  Sbjct 256 | AAPDSQRIAFYRLLDEFF 264 AAPDSQRIAFYRLLDEFF AAPDSQRIAFYRLLDEFF 273 |  |

>WP_021018481.1 APH(3')-II family aminoglycoside O-phosphotransferase [Pseudomonas aeruginosa]

AGL46257.1 aminoglycoside 3'-O-phosphotransferase [Pseudomonas aeruginosa PA96]

ORL54878.1 aminoglycoside phosphotransferase APH(3') [Pseudomonas aeruginosa] Length=264

Score = 516 bits (1329), Expect = 0.0, Method: Compositional matrix adjust. Identities = 253/264 (96%), Positives = 255/264 (97%), Gaps = 0/264 (0%)

| Query | 1 | MIEQDGLHAGSPAAWVERLFGYDWAQQTIGCSDAAVFRLSAQGRPVLFVKTDLSGALNEL | 60 |
| --- | --- | --- | --- |
| Sbjct | 1 | MIEQDGLHAGSPAAWVERLFGYDWAQQTIGCSDAAVFRLSAQGRP LFVKTDLSGALNEL MIEQDGLHAGSPAAWVERLFGYDWAQQTIGCSDAAVFRLSAQGRPALFVKTDLSGALNEL | 60 |
| Query | 61 | QDEAARLSWLATTGVPCAAVLDVVTEAGRDWLLLGEVPGQDLLSSHLAPAEKVSIMADAM QDEAARLSWLATT VPCAAVLDVVTEAGRDWLLLGEVPGQDLLSSHLAPAEKVSIMADA+ | 120 |

| Sbjct | 61 | QDEAARLSWLATTSVPCAAVLDVVTEAGRDWLLLGEVPGQDLLSSHLAPAEKVSIMADAL | 120 |
| --- | --- | --- | --- |
| Query | 121 | RRLHTLDPATCPFDHQAKHRIERARTRMEAGLVDQDDLDEEHQGLAPAELFARLKARMPD | 180 |
| Sbjct | 121 | RRLH LDP CPFDHQAKHR+ERAR RMEAGLVDQDDLDE HQGLAPAELFARL ARMPD RRLHALDPTACPFDHQAKHRVERARARMEAGLVDQDDLDEAHQGLAPAELFARLNARMPD | 180 |
| Query | 181 | GEDLVVTHGDACLPNIMVENGRFSGFIDCGRLGVADRYQDIALATRDIAEELGGEWADRF | 240 |
| Sbjct | 181 | GEDLVVTHGDACLPNIMVENGRFSGFIDCGRLGVADRYQDIALATRDIAEELGGEWADRF GEDLVVTHGDACLPNIMVENGRFSGFIDCGRLGVADRYQDIALATRDIAEELGGEWADRF | 240 |
| Query  Sbjct | 241  241 | LVLYGIAAPDSQRIAFYRLLDEFF 264 LVLYGI APDSQRIAFYRLLDEFF LVLYGIGAPDSQRIAFYRLLDEFF 264 |  |

```
>AHH29548.1 neomycin phosphotransferase II, partial [Salmonella enterica subsp. enterica serovar Typhimurium]
Length=251

Score = 508 bits (1308), Expect = 0.0, Method: Compositional matrix adjust. Identities = 251/251 (100%), Positives = 251/251 (100%), Gaps = 0/251 (0%)

Query 12	PAAWVERLFGYDWAQQTIGCSDAAVFRLSAQGRPVLFVKTDLSGALNELQDEAARLSWLA 71 PAAWVERLFGYDWAQQTIGCSDAAVFRLSAQGRPVLFVKTDLSGALNELQDEAARLSWLA
Sbjct 1	PAAWVERLFGYDWAQQTIGCSDAAVFRLSAQGRPVLFVKTDLSGALNELQDEAARLSWLA 60

Query 72	TTGVPCAAVLDVVTEAGRDWLLLGEVPGQDLLSSHLAPAEKVSIMADAMRRLHTLDPATC 131 TTGVPCAAVLDVVTEAGRDWLLLGEVPGQDLLSSHLAPAEKVSIMADAMRRLHTLDPATC
Sbjct 61	TTGVPCAAVLDVVTEAGRDWLLLGEVPGQDLLSSHLAPAEKVSIMADAMRRLHTLDPATC 120

Query 132 PFDHQAKHRIERARTRMEAGLVDQDDLDEEHQGLAPAELFARLKARMPDGEDLVVTHGDA 191 PFDHQAKHRIERARTRMEAGLVDQDDLDEEHQGLAPAELFARLKARMPDGEDLVVTHGDA
Sbjct 121 PFDHQAKHRIERARTRMEAGLVDQDDLDEEHQGLAPAELFARLKARMPDGEDLVVTHGDA 180

Query 192 CLPNIMVENGRFSGFIDCGRLGVADRYQDIALATRDIAEELGGEWADRFLVLYGIAAPDS 251 CLPNIMVENGRFSGFIDCGRLGVADRYQDIALATRDIAEELGGEWADRFLVLYGIAAPDS
Sbjct 181 CLPNIMVENGRFSGFIDCGRLGVADRYQDIALATRDIAEELGGEWADRFLVLYGIAAPDS 240

Query 252 QRIAFYRLLDE 262
QRIAFYRLLDE Sbjct 241 QRIAFYRLLDE 251

>WP_109815304.1 APH(3')-II family aminoglycoside O-phosphotransferase, partial [Stenotrophomonas maltophilia]
PWQ83851.1 APH(3')-II family aminoglycoside O-phosphotransferase, partial [Stenotrophomonas maltophilia]
Length=251

Score = 508 bits (1308), Expect = 0.0, Method: Compositional matrix adjust. Identities = 251/251 (100%), Positives = 251/251 (100%), Gaps = 0/251 (0%)

Query 1	MIEQDGLHAGSPAAWVERLFGYDWAQQTIGCSDAAVFRLSAQGRPVLFVKTDLSGALNEL 60 MIEQDGLHAGSPAAWVERLFGYDWAQQTIGCSDAAVFRLSAQGRPVLFVKTDLSGALNEL
Sbjct 1	MIEQDGLHAGSPAAWVERLFGYDWAQQTIGCSDAAVFRLSAQGRPVLFVKTDLSGALNEL 60

Query 61	QDEAARLSWLATTGVPCAAVLDVVTEAGRDWLLLGEVPGQDLLSSHLAPAEKVSIMADAM 120 QDEAARLSWLATTGVPCAAVLDVVTEAGRDWLLLGEVPGQDLLSSHLAPAEKVSIMADAM
Sbjct 61	QDEAARLSWLATTGVPCAAVLDVVTEAGRDWLLLGEVPGQDLLSSHLAPAEKVSIMADAM 120

Query 121 RRLHTLDPATCPFDHQAKHRIERARTRMEAGLVDQDDLDEEHQGLAPAELFARLKARMPD 180 RRLHTLDPATCPFDHQAKHRIERARTRMEAGLVDQDDLDEEHQGLAPAELFARLKARMPD
Sbjct 121 RRLHTLDPATCPFDHQAKHRIERARTRMEAGLVDQDDLDEEHQGLAPAELFARLKARMPD 180

Query 181 GEDLVVTHGDACLPNIMVENGRFSGFIDCGRLGVADRYQDIALATRDIAEELGGEWADRF 240 GEDLVVTHGDACLPNIMVENGRFSGFIDCGRLGVADRYQDIALATRDIAEELGGEWADRF
Sbjct 181 GEDLVVTHGDACLPNIMVENGRFSGFIDCGRLGVADRYQDIALATRDIAEELGGEWADRF 240

Query 241 LVLYGIAAPDS 251
LVLYGIAAPDS Sbjct 241 LVLYGIAAPDS 251

>EFN0055451.1 APH(3')-II family aminoglycoside O-phosphotransferase [Escherichia coli]
Length=249

Score = 503 bits (1295), Expect = 1e-179, Method: Compositional matrix adjust.
```

| Identities | = 248/249 (99%), Positives = 249/249 (100%), Gaps = 0/249 (0%) |  |
| --- | --- | --- |
| Query 16 | VERLFGYDWAQQTIGCSDAAVFRLSAQGRPVLFVKTDLSGALNELQDEAARLSWLATTGV | 75 |
| Sbjct 1 | +ERLFGYDWAQQTIGCSDAAVFRLSAQGRPVLFVKTDLSGALNELQDEAARLSWLATTGV MERLFGYDWAQQTIGCSDAAVFRLSAQGRPVLFVKTDLSGALNELQDEAARLSWLATTGV | 60 |
| Query 76 | PCAAVLDVVTEAGRDWLLLGEVPGQDLLSSHLAPAEKVSIMADAMRRLHTLDPATCPFDH | 135 |
| Sbjct 61 | PCAAVLDVVTEAGRDWLLLGEVPGQDLLSSHLAPAEKVSIMADAMRRLHTLDPATCPFDH PCAAVLDVVTEAGRDWLLLGEVPGQDLLSSHLAPAEKVSIMADAMRRLHTLDPATCPFDH | 120 |
| Query 136 | QAKHRIERARTRMEAGLVDQDDLDEEHQGLAPAELFARLKARMPDGEDLVVTHGDACLPN | 195 |
| Sbjct 121 | QAKHRIERARTRMEAGLVDQDDLDEEHQGLAPAELFARLKARMPDGEDLVVTHGDACLPN QAKHRIERARTRMEAGLVDQDDLDEEHQGLAPAELFARLKARMPDGEDLVVTHGDACLPN | 180 |
| Query 196 | IMVENGRFSGFIDCGRLGVADRYQDIALATRDIAEELGGEWADRFLVLYGIAAPDSQRIA | 255 |
| Sbjct 181 | IMVENGRFSGFIDCGRLGVADRYQDIALATRDIAEELGGEWADRFLVLYGIAAPDSQRIA IMVENGRFSGFIDCGRLGVADRYQDIALATRDIAEELGGEWADRFLVLYGIAAPDSQRIA | 240 |
| Query 256  Sbjct 241 | FYRLLDEFF 264 FYRLLDEFF FYRLLDEFF 249 |  |

>WP_057505145.1 APH(3')-II family aminoglycoside O-phosphotransferase [Stenotrophomonas nitritireducens]

KRG58636.1 aminoglycoside phosphotransferase [Stenotrophomonas nitritireducens] Length=264

Score = 502 bits (1293), Expect = 5e-179, Method: Compositional matrix adjust.

| Identities | = 246/264 (93%), Positives = 252/264 (95%), Gaps = 0/264 (0%) |  |
| --- | --- | --- |
| Query 1 | MIEQDGLHAGSPAAWVERLFGYDWAQQTIGCSDAAVFRLSAQGRPVLFVKTDLSGALNEL | 60 |
| Sbjct 1 | MIEQ GLHAGSP AWVERL GY WA QTIGCSDAAVFRLSAQGRPVLFVKTDLSGALNEL MIEQGGLHAGSPTAWVERLSGYVWAHQTIGCSDAAVFRLSAQGRPVLFVKTDLSGALNEL | 60 |
| Query 61 | QDEAARLSWLATTGVPCAAVLDVVTEAGRDWLLLGEVPGQDLLSSHLAPAEKVSIMADAM | 120 |
| Sbjct 61 | QDEAARLSWLATTGVPCA VLDVVTEAGRDWLLLGEVPGQDLLSSHLAPAEKVS+MADA+ QDEAARLSWLATTGVPCATVLDVVTEAGRDWLLLGEVPGQDLLSSHLAPAEKVSLMADAL | 120 |
| Query 121 | RRLHTLDPATCPFDHQAKHRIERARTRMEAGLVDQDDLDEEHQGLAPAELFARLKARMPD | 180 |
| Sbjct 121 | RRLH LDPA CPFDHQAKHR+ERAR RMEAGLVDQDDLDE HQGLAPAELFARLKAR+PD RRLHALDPAACPFDHQAKHRVERARARMEAGLVDQDDLDEAHQGLAPAELFARLKARVPD | 180 |
| Query 181 | GEDLVVTHGDACLPNIMVENGRFSGFIDCGRLGVADRYQDIALATRDIAEELGGEWADRF | 240 |
| Sbjct 181 | GEDLVVTHGDACLPNIMVENGRFSGFIDCGRLGVADRYQDIALATRDIAEELGGEWA+RF GEDLVVTHGDACLPNIMVENGRFSGFIDCGRLGVADRYQDIALATRDIAEELGGEWANRF | 240 |
| Query 241  Sbjct 241 | LVLYGIAAPDSQRIAFYRLLDEFF 264 LV YGIAAPDS RIAFYRLLDEF+ LVRYGIAAPDSHRIAFYRLLDEFY 264 |  |

>BAG12832.1 beta-geo-lessCpG [Exchangeable gene trap vector pU-21W] Length=1285

Score = 535 bits (1377), Expect = 3e-178, Method: Compositional matrix adjust. Identities = 263/263 (100%), Positives = 263/263 (100%), Gaps = 0/263 (0%)

| Query | 2 | IEQDGLHAGSPAAWVERLFGYDWAQQTIGCSDAAVFRLSAQGRPVLFVKTDLSGALNELQ | 61 |
| --- | --- | --- | --- |
| Sbjct | 1023 | IEQDGLHAGSPAAWVERLFGYDWAQQTIGCSDAAVFRLSAQGRPVLFVKTDLSGALNELQ IEQDGLHAGSPAAWVERLFGYDWAQQTIGCSDAAVFRLSAQGRPVLFVKTDLSGALNELQ | 1082 |
| Query | 62 | DEAARLSWLATTGVPCAAVLDVVTEAGRDWLLLGEVPGQDLLSSHLAPAEKVSIMADAMR | 121 |
| Sbjct | 1083 | DEAARLSWLATTGVPCAAVLDVVTEAGRDWLLLGEVPGQDLLSSHLAPAEKVSIMADAMR  DEAARLSWLATTGVPCAAVLDVVTEAGRDWLLLGEVPGQDLLSSHLAPAEKVSIMADAMR | 1142 |

| Query | 122 | RLHTLDPATCPFDHQAKHRIERARTRMEAGLVDQDDLDEEHQGLAPAELFARLKARMPDG | 181 |
| --- | --- | --- | --- |
| Sbjct | 1143 | RLHTLDPATCPFDHQAKHRIERARTRMEAGLVDQDDLDEEHQGLAPAELFARLKARMPDG RLHTLDPATCPFDHQAKHRIERARTRMEAGLVDQDDLDEEHQGLAPAELFARLKARMPDG | 1202 |
| Query | 182 | EDLVVTHGDACLPNIMVENGRFSGFIDCGRLGVADRYQDIALATRDIAEELGGEWADRFL | 241 |
| Sbjct | 1203 | EDLVVTHGDACLPNIMVENGRFSGFIDCGRLGVADRYQDIALATRDIAEELGGEWADRFL EDLVVTHGDACLPNIMVENGRFSGFIDCGRLGVADRYQDIALATRDIAEELGGEWADRFL | 1262 |
| Query  Sbjct | 242  1263 | VLYGIAAPDSQRIAFYRLLDEFF 264 VLYGIAAPDSQRIAFYRLLDEFF VLYGIAAPDSQRIAFYRLLDEFF 1285 |  |

```
>AHB86974.1 beta-geo [PiggyBac insertional mutagenesis vector Slingshot-PB] Length=1312

Score = 535 bits (1378), Expect = 3e-178, Method: Compositional matrix adjust. Identities = 263/263 (100%), Positives = 263/263 (100%), Gaps = 0/263 (0%)

Query 2	IEQDGLHAGSPAAWVERLFGYDWAQQTIGCSDAAVFRLSAQGRPVLFVKTDLSGALNELQ 61 IEQDGLHAGSPAAWVERLFGYDWAQQTIGCSDAAVFRLSAQGRPVLFVKTDLSGALNELQ
Sbjct 1050 IEQDGLHAGSPAAWVERLFGYDWAQQTIGCSDAAVFRLSAQGRPVLFVKTDLSGALNELQ 1109

Query 62	DEAARLSWLATTGVPCAAVLDVVTEAGRDWLLLGEVPGQDLLSSHLAPAEKVSIMADAMR 121 DEAARLSWLATTGVPCAAVLDVVTEAGRDWLLLGEVPGQDLLSSHLAPAEKVSIMADAMR
Sbjct 1110 DEAARLSWLATTGVPCAAVLDVVTEAGRDWLLLGEVPGQDLLSSHLAPAEKVSIMADAMR 1169

Query 122	RLHTLDPATCPFDHQAKHRIERARTRMEAGLVDQDDLDEEHQGLAPAELFARLKARMPDG 181 RLHTLDPATCPFDHQAKHRIERARTRMEAGLVDQDDLDEEHQGLAPAELFARLKARMPDG
Sbjct 1170 RLHTLDPATCPFDHQAKHRIERARTRMEAGLVDQDDLDEEHQGLAPAELFARLKARMPDG 1229

Query 182	EDLVVTHGDACLPNIMVENGRFSGFIDCGRLGVADRYQDIALATRDIAEELGGEWADRFL 241 EDLVVTHGDACLPNIMVENGRFSGFIDCGRLGVADRYQDIALATRDIAEELGGEWADRFL
Sbjct 1230 EDLVVTHGDACLPNIMVENGRFSGFIDCGRLGVADRYQDIALATRDIAEELGGEWADRFL 1289

Query 242	VLYGIAAPDSQRIAFYRLLDEFF 264
VLYGIAAPDSQRIAFYRLLDEFF Sbjct 1290 VLYGIAAPDSQRIAFYRLLDEFF 1312

>AAB51768.1 beta-geo [synthetic construct]
BAD98320.1 beta-geo [Exchangeable gene trap vector pU-18] BAD98321.1 beta-geo [Exchangeable gene trap vector pU-21] BAE48721.1 beta-geo [Exchangeable gene trap vector pU-Hachi] BAE93216.1 beta-geo [Exchangeable gene trap vector pU-21B] BAE93217.1 beta-geo [Exchangeable gene trap vector pU-21T]
Length=1293

Score = 534 bits (1376), Expect = 4e-178, Method: Compositional matrix adjust. Identities = 263/263 (100%), Positives = 263/263 (100%), Gaps = 0/263 (0%)
```

| Query | 2 | IEQDGLHAGSPAAWVERLFGYDWAQQTIGCSDAAVFRLSAQGRPVLFVKTDLSGALNELQ | 61 |
| --- | --- | --- | --- |
| Sbjct | 1031 | IEQDGLHAGSPAAWVERLFGYDWAQQTIGCSDAAVFRLSAQGRPVLFVKTDLSGALNELQ IEQDGLHAGSPAAWVERLFGYDWAQQTIGCSDAAVFRLSAQGRPVLFVKTDLSGALNELQ | 1090 |
| Query | 62 | DEAARLSWLATTGVPCAAVLDVVTEAGRDWLLLGEVPGQDLLSSHLAPAEKVSIMADAMR | 121 |
| Sbjct | 1091 | DEAARLSWLATTGVPCAAVLDVVTEAGRDWLLLGEVPGQDLLSSHLAPAEKVSIMADAMR DEAARLSWLATTGVPCAAVLDVVTEAGRDWLLLGEVPGQDLLSSHLAPAEKVSIMADAMR | 1150 |
| Query | 122 | RLHTLDPATCPFDHQAKHRIERARTRMEAGLVDQDDLDEEHQGLAPAELFARLKARMPDG | 181 |
| Sbjct | 1151 | RLHTLDPATCPFDHQAKHRIERARTRMEAGLVDQDDLDEEHQGLAPAELFARLKARMPDG RLHTLDPATCPFDHQAKHRIERARTRMEAGLVDQDDLDEEHQGLAPAELFARLKARMPDG | 1210 |
| Query | 182 | EDLVVTHGDACLPNIMVENGRFSGFIDCGRLGVADRYQDIALATRDIAEELGGEWADRFL | 241 |
| Sbjct | 1211 | EDLVVTHGDACLPNIMVENGRFSGFIDCGRLGVADRYQDIALATRDIAEELGGEWADRFL EDLVVTHGDACLPNIMVENGRFSGFIDCGRLGVADRYQDIALATRDIAEELGGEWADRFL | 1270 |
| Query  Sbjct | 242  1271 | VLYGIAAPDSQRIAFYRLLDEFF 264 VLYGIAAPDSQRIAFYRLLDEFF VLYGIAAPDSQRIAFYRLLDEFF 1293 |  |

>AEL31639.1 beta-geo [Deletion homozygosity selection vector 1] Length=1293

Score = 534 bits (1376), Expect = 4e-178, Method: Compositional matrix adjust. Identities = 263/263 (100%), Positives = 263/263 (100%), Gaps = 0/263 (0%)

| Query | 2 | IEQDGLHAGSPAAWVERLFGYDWAQQTIGCSDAAVFRLSAQGRPVLFVKTDLSGALNELQ | 61 |
| --- | --- | --- | --- |
| Sbjct | 1031 | IEQDGLHAGSPAAWVERLFGYDWAQQTIGCSDAAVFRLSAQGRPVLFVKTDLSGALNELQ IEQDGLHAGSPAAWVERLFGYDWAQQTIGCSDAAVFRLSAQGRPVLFVKTDLSGALNELQ | 1090 |
| Query | 62 | DEAARLSWLATTGVPCAAVLDVVTEAGRDWLLLGEVPGQDLLSSHLAPAEKVSIMADAMR | 121 |
| Sbjct | 1091 | DEAARLSWLATTGVPCAAVLDVVTEAGRDWLLLGEVPGQDLLSSHLAPAEKVSIMADAMR DEAARLSWLATTGVPCAAVLDVVTEAGRDWLLLGEVPGQDLLSSHLAPAEKVSIMADAMR | 1150 |
| Query | 122 | RLHTLDPATCPFDHQAKHRIERARTRMEAGLVDQDDLDEEHQGLAPAELFARLKARMPDG | 181 |
| Sbjct | 1151 | RLHTLDPATCPFDHQAKHRIERARTRMEAGLVDQDDLDEEHQGLAPAELFARLKARMPDG RLHTLDPATCPFDHQAKHRIERARTRMEAGLVDQDDLDEEHQGLAPAELFARLKARMPDG | 1210 |
| Query | 182 | EDLVVTHGDACLPNIMVENGRFSGFIDCGRLGVADRYQDIALATRDIAEELGGEWADRFL | 241 |
| Sbjct | 1211 | EDLVVTHGDACLPNIMVENGRFSGFIDCGRLGVADRYQDIALATRDIAEELGGEWADRFL EDLVVTHGDACLPNIMVENGRFSGFIDCGRLGVADRYQDIALATRDIAEELGGEWADRFL | 1270 |
| Query  Sbjct | 242  1271 | VLYGIAAPDSQRIAFYRLLDEFF 264 VLYGIAAPDSQRIAFYRLLDEFF VLYGIAAPDSQRIAFYRLLDEFF 1293 |  |

>ADF55556.1 beta-geo [PiggyBac insertional mutagenesis vector Slingshot-PB] Length=1311

Score = 535 bits (1377), Expect = 4e-178, Method: Compositional matrix adjust. Identities = 263/263 (100%), Positives = 263/263 (100%), Gaps = 0/263 (0%)

| Query | 2 | IEQDGLHAGSPAAWVERLFGYDWAQQTIGCSDAAVFRLSAQGRPVLFVKTDLSGALNELQ | 61 |
| --- | --- | --- | --- |
| Sbjct | 1049 | IEQDGLHAGSPAAWVERLFGYDWAQQTIGCSDAAVFRLSAQGRPVLFVKTDLSGALNELQ IEQDGLHAGSPAAWVERLFGYDWAQQTIGCSDAAVFRLSAQGRPVLFVKTDLSGALNELQ | 1108 |
| Query | 62 | DEAARLSWLATTGVPCAAVLDVVTEAGRDWLLLGEVPGQDLLSSHLAPAEKVSIMADAMR | 121 |
| Sbjct | 1109 | DEAARLSWLATTGVPCAAVLDVVTEAGRDWLLLGEVPGQDLLSSHLAPAEKVSIMADAMR DEAARLSWLATTGVPCAAVLDVVTEAGRDWLLLGEVPGQDLLSSHLAPAEKVSIMADAMR | 1168 |
| Query | 122 | RLHTLDPATCPFDHQAKHRIERARTRMEAGLVDQDDLDEEHQGLAPAELFARLKARMPDG | 181 |
| Sbjct | 1169 | RLHTLDPATCPFDHQAKHRIERARTRMEAGLVDQDDLDEEHQGLAPAELFARLKARMPDG RLHTLDPATCPFDHQAKHRIERARTRMEAGLVDQDDLDEEHQGLAPAELFARLKARMPDG | 1228 |
| Query | 182 | EDLVVTHGDACLPNIMVENGRFSGFIDCGRLGVADRYQDIALATRDIAEELGGEWADRFL | 241 |
| Sbjct | 1229 | EDLVVTHGDACLPNIMVENGRFSGFIDCGRLGVADRYQDIALATRDIAEELGGEWADRFL EDLVVTHGDACLPNIMVENGRFSGFIDCGRLGVADRYQDIALATRDIAEELGGEWADRFL | 1288 |
| Query  Sbjct | 242  1289 | VLYGIAAPDSQRIAFYRLLDEFF 264 VLYGIAAPDSQRIAFYRLLDEFF VLYGIAAPDSQRIAFYRLLDEFF 1311 |  |

>ACD62478.1 beta-geo [Gene trapping vector VICTR76] ACD62479.1 beta-geo [Gene trapping vector VICTR75]

Length=1297

Score = 534 bits (1376), Expect = 5e-178, Method: Compositional matrix adjust. Identities = 263/263 (100%), Positives = 263/263 (100%), Gaps = 0/263 (0%)

| Query | 2 | IEQDGLHAGSPAAWVERLFGYDWAQQTIGCSDAAVFRLSAQGRPVLFVKTDLSGALNELQ | 61 |
| --- | --- | --- | --- |
| Sbjct | 1035 | IEQDGLHAGSPAAWVERLFGYDWAQQTIGCSDAAVFRLSAQGRPVLFVKTDLSGALNELQ IEQDGLHAGSPAAWVERLFGYDWAQQTIGCSDAAVFRLSAQGRPVLFVKTDLSGALNELQ | 1094 |
| Query | 62 | DEAARLSWLATTGVPCAAVLDVVTEAGRDWLLLGEVPGQDLLSSHLAPAEKVSIMADAMR | 121 |
| Sbjct | 1095 | DEAARLSWLATTGVPCAAVLDVVTEAGRDWLLLGEVPGQDLLSSHLAPAEKVSIMADAMR DEAARLSWLATTGVPCAAVLDVVTEAGRDWLLLGEVPGQDLLSSHLAPAEKVSIMADAMR | 1154 |
| Query | 122 | RLHTLDPATCPFDHQAKHRIERARTRMEAGLVDQDDLDEEHQGLAPAELFARLKARMPDG | 181 |
| Sbjct | 1155 | RLHTLDPATCPFDHQAKHRIERARTRMEAGLVDQDDLDEEHQGLAPAELFARLKARMPDG  RLHTLDPATCPFDHQAKHRIERARTRMEAGLVDQDDLDEEHQGLAPAELFARLKARMPDG | 1214 |

| Query | 182 | EDLVVTHGDACLPNIMVENGRFSGFIDCGRLGVADRYQDIALATRDIAEELGGEWADRFL 241 |
| --- | --- | --- |
| Sbjct | 1215 | EDLVVTHGDACLPNIMVENGRFSGFIDCGRLGVADRYQDIALATRDIAEELGGEWADRFL EDLVVTHGDACLPNIMVENGRFSGFIDCGRLGVADRYQDIALATRDIAEELGGEWADRFL 1274 |
| Query | 242 | VLYGIAAPDSQRIAFYRLLDEFF 264 |
| Sbjct | 1275 | VLYGIAAPDSQRIAFYRLLDEFF  VLYGIAAPDSQRIAFYRLLDEFF 1297 |

```
>ECS2458178.1 APH(3')-II family aminoglycoside O-phosphotransferase [Salmonella enterica subsp. enterica serovar Heidelberg]
ECS2458181.1 APH(3')-II family aminoglycoside O-phosphotransferase [Salmonella enterica subsp. enterica serovar Heidelberg]
Length=246

Score = 498 bits (1283), Expect = 1e-177, Method: Compositional matrix adjust. Identities = 246/246 (100%), Positives = 246/246 (100%), Gaps = 0/246 (0%)

Query 19	LFGYDWAQQTIGCSDAAVFRLSAQGRPVLFVKTDLSGALNELQDEAARLSWLATTGVPCA 78 LFGYDWAQQTIGCSDAAVFRLSAQGRPVLFVKTDLSGALNELQDEAARLSWLATTGVPCA
Sbjct 1	LFGYDWAQQTIGCSDAAVFRLSAQGRPVLFVKTDLSGALNELQDEAARLSWLATTGVPCA 60

Query 79	AVLDVVTEAGRDWLLLGEVPGQDLLSSHLAPAEKVSIMADAMRRLHTLDPATCPFDHQAK 138 AVLDVVTEAGRDWLLLGEVPGQDLLSSHLAPAEKVSIMADAMRRLHTLDPATCPFDHQAK
Sbjct 61	AVLDVVTEAGRDWLLLGEVPGQDLLSSHLAPAEKVSIMADAMRRLHTLDPATCPFDHQAK 120

Query 139 HRIERARTRMEAGLVDQDDLDEEHQGLAPAELFARLKARMPDGEDLVVTHGDACLPNIMV 198 HRIERARTRMEAGLVDQDDLDEEHQGLAPAELFARLKARMPDGEDLVVTHGDACLPNIMV
Sbjct 121 HRIERARTRMEAGLVDQDDLDEEHQGLAPAELFARLKARMPDGEDLVVTHGDACLPNIMV 180

Query 199 ENGRFSGFIDCGRLGVADRYQDIALATRDIAEELGGEWADRFLVLYGIAAPDSQRIAFYR 258 ENGRFSGFIDCGRLGVADRYQDIALATRDIAEELGGEWADRFLVLYGIAAPDSQRIAFYR
Sbjct 181 ENGRFSGFIDCGRLGVADRYQDIALATRDIAEELGGEWADRFLVLYGIAAPDSQRIAFYR 240

Query 259 LLDEFF 264
LLDEFF Sbjct 241 LLDEFF 246

>ADK39026.1 beta-geo [cloning vector pPNT] [Mus musculus] Length=1297

Score = 533 bits (1372), Expect = 2e-177, Method: Compositional matrix adjust. Identities = 262/263 (99%), Positives = 263/263 (100%), Gaps = 0/263 (0%)

Query 2	IEQDGLHAGSPAAWVERLFGYDWAQQTIGCSDAAVFRLSAQGRPVLFVKTDLSGALNELQ 61 IEQDGLHAGSPAAWVERLFGYDWAQQTIGCSDAAVFRLSAQGRPVLFVKTDLSGALNELQ
Sbjct 1035 IEQDGLHAGSPAAWVERLFGYDWAQQTIGCSDAAVFRLSAQGRPVLFVKTDLSGALNELQ 1094

Query 62	DEAARLSWLATTGVPCAAVLDVVTEAGRDWLLLGEVPGQDLLSSHLAPAEKVSIMADAMR 121 DEAARLSWLATTGVPCAAVLDVVTEAGRDWLLLGEVPGQDLLSSHLAPAEKVSIMADAMR
Sbjct 1095 DEAARLSWLATTGVPCAAVLDVVTEAGRDWLLLGEVPGQDLLSSHLAPAEKVSIMADAMR 1154

Query 122	RLHTLDPATCPFDHQAKHRIERARTRMEAGLVDQDDLDEEHQGLAPAELFARLKARMPDG 181 RLHTLDPATCPFDHQAKHRIERARTRMEAGLVDQDDLDEEHQGLAPAELFARLKARMPDG
Sbjct 1155 RLHTLDPATCPFDHQAKHRIERARTRMEAGLVDQDDLDEEHQGLAPAELFARLKARMPDG 1214

Query 182	EDLVVTHGDACLPNIMVENGRFSGFIDCGRLGVADRYQDIALATRDIAEELGGEWADRFL 241
+DLVVTHGDACLPNIMVENGRFSGFIDCGRLGVADRYQDIALATRDIAEELGGEWADRFL Sbjct 1215 DDLVVTHGDACLPNIMVENGRFSGFIDCGRLGVADRYQDIALATRDIAEELGGEWADRFL 1274

Query 242	VLYGIAAPDSQRIAFYRLLDEFF 264
VLYGIAAPDSQRIAFYRLLDEFF Sbjct 1275 VLYGIAAPDSQRIAFYRLLDEFF 1297

>CAA54105.1 lacZ neomycin phosphotransferase fusion protein [synthetic construct] Length=1311

Score = 530 bits (1365), Expect = 3e-176, Method: Compositional matrix adjust. Identities = 260/260 (100%), Positives = 260/260 (100%), Gaps = 0/260 (0%)
```

| Query | 5 | DGLHAGSPAAWVERLFGYDWAQQTIGCSDAAVFRLSAQGRPVLFVKTDLSGALNELQDEA | 64 |
| --- | --- | --- | --- |
| Sbjct | 1052 | DGLHAGSPAAWVERLFGYDWAQQTIGCSDAAVFRLSAQGRPVLFVKTDLSGALNELQDEA DGLHAGSPAAWVERLFGYDWAQQTIGCSDAAVFRLSAQGRPVLFVKTDLSGALNELQDEA | 1111 |
| Query | 65 | ARLSWLATTGVPCAAVLDVVTEAGRDWLLLGEVPGQDLLSSHLAPAEKVSIMADAMRRLH | 124 |
| Sbjct | 1112 | ARLSWLATTGVPCAAVLDVVTEAGRDWLLLGEVPGQDLLSSHLAPAEKVSIMADAMRRLH ARLSWLATTGVPCAAVLDVVTEAGRDWLLLGEVPGQDLLSSHLAPAEKVSIMADAMRRLH | 1171 |
| Query | 125 | TLDPATCPFDHQAKHRIERARTRMEAGLVDQDDLDEEHQGLAPAELFARLKARMPDGEDL | 184 |
| Sbjct | 1172 | TLDPATCPFDHQAKHRIERARTRMEAGLVDQDDLDEEHQGLAPAELFARLKARMPDGEDL TLDPATCPFDHQAKHRIERARTRMEAGLVDQDDLDEEHQGLAPAELFARLKARMPDGEDL | 1231 |
| Query | 185 | VVTHGDACLPNIMVENGRFSGFIDCGRLGVADRYQDIALATRDIAEELGGEWADRFLVLY | 244 |
| Sbjct | 1232 | VVTHGDACLPNIMVENGRFSGFIDCGRLGVADRYQDIALATRDIAEELGGEWADRFLVLY VVTHGDACLPNIMVENGRFSGFIDCGRLGVADRYQDIALATRDIAEELGGEWADRFLVLY | 1291 |
| Query  Sbjct | 245  1292 | GIAAPDSQRIAFYRLLDEFF 264 GIAAPDSQRIAFYRLLDEFF GIAAPDSQRIAFYRLLDEFF 1311 |  |

```
>EBL6081130.1 APH(3')-II family aminoglycoside O-phosphotransferase [Salmonella enterica subsp. enterica serovar Kentucky]
Length=244

Score = 494 bits (1271), Expect = 5e-176, Method: Compositional matrix adjust. Identities = 244/244 (100%), Positives = 244/244 (100%), Gaps = 0/244 (0%)

Query 21	GYDWAQQTIGCSDAAVFRLSAQGRPVLFVKTDLSGALNELQDEAARLSWLATTGVPCAAV 80 GYDWAQQTIGCSDAAVFRLSAQGRPVLFVKTDLSGALNELQDEAARLSWLATTGVPCAAV
Sbjct 1	GYDWAQQTIGCSDAAVFRLSAQGRPVLFVKTDLSGALNELQDEAARLSWLATTGVPCAAV 60

Query 81	LDVVTEAGRDWLLLGEVPGQDLLSSHLAPAEKVSIMADAMRRLHTLDPATCPFDHQAKHR 140 LDVVTEAGRDWLLLGEVPGQDLLSSHLAPAEKVSIMADAMRRLHTLDPATCPFDHQAKHR
Sbjct 61	LDVVTEAGRDWLLLGEVPGQDLLSSHLAPAEKVSIMADAMRRLHTLDPATCPFDHQAKHR 120

Query 141 IERARTRMEAGLVDQDDLDEEHQGLAPAELFARLKARMPDGEDLVVTHGDACLPNIMVEN 200 IERARTRMEAGLVDQDDLDEEHQGLAPAELFARLKARMPDGEDLVVTHGDACLPNIMVEN
Sbjct 121 IERARTRMEAGLVDQDDLDEEHQGLAPAELFARLKARMPDGEDLVVTHGDACLPNIMVEN 180

Query 201 GRFSGFIDCGRLGVADRYQDIALATRDIAEELGGEWADRFLVLYGIAAPDSQRIAFYRLL 260 GRFSGFIDCGRLGVADRYQDIALATRDIAEELGGEWADRFLVLYGIAAPDSQRIAFYRLL
Sbjct 181 GRFSGFIDCGRLGVADRYQDIALATRDIAEELGGEWADRFLVLYGIAAPDSQRIAFYRLL 240

Query 261 DEFF 264
DEFF Sbjct 241 DEFF 244

>WP_083563809.1 APH(3')-II family aminoglycoside O-phosphotransferase [Pseudomonas aeruginosa]
ORE61650.1 APH(3') family aminoglycoside O-phosphotransferase [Pseudomonas aeruginosa]
Length=264

Score = 494 bits (1273), Expect = 7e-176, Method: Compositional matrix adjust.
```

| Identities | = 243/264 (92%), Positives = 249/264 (94%), Gaps = 0/264 (0%) |  |
| --- | --- | --- |
| Query 1 | MIEQDGLHAGSPAAWVERLFGYDWAQQTIGCSDAAVFRLSAQGRPVLFVKTDLSGALNEL | 60 |
| Sbjct 1 | MIEQDGLHAGSPAAW ERL GY WA QTIGCSDAAVFRLSA+GRP+LFVKTDLSGALNEL MIEQDGLHAGSPAAWEERLSGYVWAHQTIGCSDAAVFRLSAEGRPILFVKTDLSGALNEL | 60 |
| Query 61 | QDEAARLSWLATTGVPCAAVLDVVTEAGRDWLLLGEVPGQDLLSSHLAPAEKVSIMADAM | 120 |
| Sbjct 61 | QDEA RLSWLATTG+PC AVLDVVTEAGRDWLLLGEVPGQDLLSS LAPAEKVSIMADA+ QDEAVRLSWLATTGLPCPAVLDVVTEAGRDWLLLGEVPGQDLLSSRLAPAEKVSIMADAL | 120 |
| Query 121 | RRLHTLDPATCPFDHQAKHRIERARTRMEAGLVDQDDLDEEHQGLAPAELFARLKARMPD | 180 |
| Sbjct 121 | RRLH LDPA CPFDHQA+HR+ERAR RMEAGLVDQDDLDE HQGLAPAELFARL ARMPD RRLHALDPAACPFDHQARHRVERARARMEAGLVDQDDLDEAHQGLAPAELFARLNARMPD | 180 |
| Query 181 | GEDLVVTHGDACLPNIMVENGRFSGFIDCGRLGVADRYQDIALATRDIAEELGGEWADRF | 240 |

| Sbjct | 181 | GEDLVVTHGDACLPNIMVENGRFSGFIDCGRLGVADRYQDIALATRDIAEELGGEWADRF GEDLVVTHGDACLPNIMVENGRFSGFIDCGRLGVADRYQDIALATRDIAEELGGEWADRF 240 |
| --- | --- | --- |
| Query | 241 | LVLYGIAAPDSQRIAFYRLLDEFF 264 |
| Sbjct | 241 | LV YGI APDS RIAFYRLLDEFF  LVRYGIGAPDSHRIAFYRLLDEFF 264 |

>WP_196524206.1 APH(3')-II family aminoglycoside O-phosphotransferase, partial [Nostoc sp. BAE]

MBG1259778.1 APH(3')-II family aminoglycoside O-phosphotransferase [Nostoc sp. BAE]

Length=244

Score = 492 bits (1267), Expect = 2e-175, Method: Compositional matrix adjust.

| Identities | = 243/244 (99%), Positives = 243/244 (99%), Gaps = 0/244 (0%) |  |
| --- | --- | --- |
| Query 21 | GYDWAQQTIGCSDAAVFRLSAQGRPVLFVKTDLSGALNELQDEAARLSWLATTGVPCAAV | 80 |
| Sbjct 1 | GYDWAQQTIGCSDAAVFRLSAQGRPVLFVKTDLSGALNELQDEAARLSWLATTGVPCAAV GYDWAQQTIGCSDAAVFRLSAQGRPVLFVKTDLSGALNELQDEAARLSWLATTGVPCAAV | 60 |
| Query 81 | LDVVTEAGRDWLLLGEVPGQDLLSSHLAPAEKVSIMADAMRRLHTLDPATCPFDHQAKHR | 140 |
| Sbjct 61 | LDVVTEAGRDWLLLGEVPGQDLLSSHLAPAEKVSIMADAMRRLHTLDPATCPFDHQAKHR LDVVTEAGRDWLLLGEVPGQDLLSSHLAPAEKVSIMADAMRRLHTLDPATCPFDHQAKHR | 120 |
| Query 141 | IERARTRMEAGLVDQDDLDEEHQGLAPAELFARLKARMPDGEDLVVTHGDACLPNIMVEN | 200 |
| Sbjct 121 | IERARTRMEAGLVDQDDLDEEHQGLAPAELFARLKA MPDGEDLVVTHGDACLPNIMVEN IERARTRMEAGLVDQDDLDEEHQGLAPAELFARLKASMPDGEDLVVTHGDACLPNIMVEN | 180 |
| Query 201 | GRFSGFIDCGRLGVADRYQDIALATRDIAEELGGEWADRFLVLYGIAAPDSQRIAFYRLL | 260 |
| Sbjct 181 | GRFSGFIDCGRLGVADRYQDIALATRDIAEELGGEWADRFLVLYGIAAPDSQRIAFYRLL GRFSGFIDCGRLGVADRYQDIALATRDIAEELGGEWADRFLVLYGIAAPDSQRIAFYRLL | 240 |
| Query 261  Sbjct 241 | DEFF 264 DEFF  DEFF 244 |  |

```
>EBR1815709.1 APH(3')-II family aminoglycoside O-phosphotransferase [Salmonella enterica]
Length=243

Score = 492 bits (1266), Expect = 3e-175, Method: Compositional matrix adjust. Identities = 243/243 (100%), Positives = 243/243 (100%), Gaps = 0/243 (0%)

Query 22	YDWAQQTIGCSDAAVFRLSAQGRPVLFVKTDLSGALNELQDEAARLSWLATTGVPCAAVL 81 YDWAQQTIGCSDAAVFRLSAQGRPVLFVKTDLSGALNELQDEAARLSWLATTGVPCAAVL
Sbjct 1	YDWAQQTIGCSDAAVFRLSAQGRPVLFVKTDLSGALNELQDEAARLSWLATTGVPCAAVL 60

Query 82	DVVTEAGRDWLLLGEVPGQDLLSSHLAPAEKVSIMADAMRRLHTLDPATCPFDHQAKHRI 141 DVVTEAGRDWLLLGEVPGQDLLSSHLAPAEKVSIMADAMRRLHTLDPATCPFDHQAKHRI
Sbjct 61	DVVTEAGRDWLLLGEVPGQDLLSSHLAPAEKVSIMADAMRRLHTLDPATCPFDHQAKHRI 120

Query 142 ERARTRMEAGLVDQDDLDEEHQGLAPAELFARLKARMPDGEDLVVTHGDACLPNIMVENG 201 ERARTRMEAGLVDQDDLDEEHQGLAPAELFARLKARMPDGEDLVVTHGDACLPNIMVENG
Sbjct 121 ERARTRMEAGLVDQDDLDEEHQGLAPAELFARLKARMPDGEDLVVTHGDACLPNIMVENG 180

Query 202 RFSGFIDCGRLGVADRYQDIALATRDIAEELGGEWADRFLVLYGIAAPDSQRIAFYRLLD 261 RFSGFIDCGRLGVADRYQDIALATRDIAEELGGEWADRFLVLYGIAAPDSQRIAFYRLLD
Sbjct 181 RFSGFIDCGRLGVADRYQDIALATRDIAEELGGEWADRFLVLYGIAAPDSQRIAFYRLLD 240

Query 262 EFF 264
EFF Sbjct 241 EFF 243

>WP_031759051.1 MULTISPECIES: APH(3')-II family aminoglycoside O-phosphotransferase [Pseudomonas]
ALY45237.1 aminoglycoside phosphotransferase [Pseudomonas aeruginosa] KSH14834.1 aminoglycoside phosphotransferase APH(3') [Pseudomonas aeruginosa]
MBG6992437.1 APH(3')-II family aminoglycoside O-phosphotransferase [Pseudomonas aeruginosa]

PPB00369.1 APH(3')-II family aminoglycoside O-phosphotransferase [Pseudomonas aeruginosa]
PPB29793.1 APH(3')-II family aminoglycoside O-phosphotransferase [Pseudomonas aeruginosa]
Length=264

Score = 493 bits (1268), Expect = 3e-175, Method: Compositional matrix adjust.
```

| Identities | = 243/264 (92%), Positives = 247/264 (94%), Gaps = 0/264 (0%) |  |
| --- | --- | --- |
| Query 1 | MIEQDGLHAGSPAAWVERLFGYDWAQQTIGCSDAAVFRLSAQGRPVLFVKTDLSGALNEL | 60 |
| Sbjct 1 | MI QDGLHAG P AWVERL GYDWA QTIGCSDAAVFRLSAQGRP LFVKTDLSGALNEL MIGQDGLHAGPPDAWVERLSGYDWAHQTIGCSDAAVFRLSAQGRPALFVKTDLSGALNEL | 60 |
| Query 61 | QDEAARLSWLATTGVPCAAVLDVVTEAGRDWLLLGEVPGQDLLSSHLAPAEKVSIMADAM | 120 |
| Sbjct 61 | QDEAARLSWLATTGVPCAAVLDVVTEAGRDWLLLGEVPGQDLLSSHLAPAEKVSIMADA+ QDEAARLSWLATTGVPCAAVLDVVTEAGRDWLLLGEVPGQDLLSSHLAPAEKVSIMADAL | 120 |
| Query 121 | RRLHTLDPATCPFDHQAKHRIERARTRMEAGLVDQDDLDEEHQGLAPAELFARLKARMPD | 180 |
| Sbjct 121 | RRLH LDP CPFDH+AKHR+ERAR RMEAGLVDQDDLDE HQGLAPAELFARLKARMPD RRLHALDPTACPFDHRAKHRVERARMRMEAGLVDQDDLDEAHQGLAPAELFARLKARMPD | 180 |
| Query 181 | GEDLVVTHGDACLPNIMVENGRFSGFIDCGRLGVADRYQDIALATRDIAEELGGEWADRF | 240 |
| Sbjct 181 | GEDLVVTHGDACLPNIMVENGRFSGFIDCGRLGVADRYQDIALATRDIAEELGGEW +RF GEDLVVTHGDACLPNIMVENGRFSGFIDCGRLGVADRYQDIALATRDIAEELGGEWVNRF | 240 |
| Query 241  Sbjct 241 | LVLYGIAAPDSQRIAFYRLLDEFF 264 LV YGI DS RIAFYRLLDEFF LVRYGIGTLDSHRIAFYRLLDEFF 264 |  |

```
>WP_100022224.1 APH(3')-II family aminoglycoside O-phosphotransferase, partial [Escherichia coli]
Length=242

Score = 489 bits (1259), Expect = 4e-174, Method: Compositional matrix adjust. Identities = 242/242 (100%), Positives = 242/242 (100%), Gaps = 0/242 (0%)

Query 23	DWAQQTIGCSDAAVFRLSAQGRPVLFVKTDLSGALNELQDEAARLSWLATTGVPCAAVLD 82 DWAQQTIGCSDAAVFRLSAQGRPVLFVKTDLSGALNELQDEAARLSWLATTGVPCAAVLD
Sbjct 1	DWAQQTIGCSDAAVFRLSAQGRPVLFVKTDLSGALNELQDEAARLSWLATTGVPCAAVLD 60

Query 83	VVTEAGRDWLLLGEVPGQDLLSSHLAPAEKVSIMADAMRRLHTLDPATCPFDHQAKHRIE 142 VVTEAGRDWLLLGEVPGQDLLSSHLAPAEKVSIMADAMRRLHTLDPATCPFDHQAKHRIE
Sbjct 61	VVTEAGRDWLLLGEVPGQDLLSSHLAPAEKVSIMADAMRRLHTLDPATCPFDHQAKHRIE 120

Query 143 RARTRMEAGLVDQDDLDEEHQGLAPAELFARLKARMPDGEDLVVTHGDACLPNIMVENGR 202 RARTRMEAGLVDQDDLDEEHQGLAPAELFARLKARMPDGEDLVVTHGDACLPNIMVENGR
Sbjct 121 RARTRMEAGLVDQDDLDEEHQGLAPAELFARLKARMPDGEDLVVTHGDACLPNIMVENGR 180

Query 203 FSGFIDCGRLGVADRYQDIALATRDIAEELGGEWADRFLVLYGIAAPDSQRIAFYRLLDE 262 FSGFIDCGRLGVADRYQDIALATRDIAEELGGEWADRFLVLYGIAAPDSQRIAFYRLLDE
Sbjct 181 FSGFIDCGRLGVADRYQDIALATRDIAEELGGEWADRFLVLYGIAAPDSQRIAFYRLLDE 240

Query 263 FF 264
FF
Sbjct 241 FF 242

>EDA9786694.1 APH(3')-II family aminoglycoside O-phosphotransferase [Salmonella enterica subsp. enterica serovar Heidelberg]
Length=241

Score = 489 bits (1258), Expect = 6e-174, Method: Compositional matrix adjust. Identities = 241/241 (100%), Positives = 241/241 (100%), Gaps = 0/241 (0%)
```

| Query | 1 | MIEQDGLHAGSPAAWVERLFGYDWAQQTIGCSDAAVFRLSAQGRPVLFVKTDLSGALNEL | 60 |
| --- | --- | --- | --- |
| Sbjct | 1 | MIEQDGLHAGSPAAWVERLFGYDWAQQTIGCSDAAVFRLSAQGRPVLFVKTDLSGALNEL MIEQDGLHAGSPAAWVERLFGYDWAQQTIGCSDAAVFRLSAQGRPVLFVKTDLSGALNEL | 60 |
| Query | 61 | QDEAARLSWLATTGVPCAAVLDVVTEAGRDWLLLGEVPGQDLLSSHLAPAEKVSIMADAM | 120 |
| Sbjct | 61 | QDEAARLSWLATTGVPCAAVLDVVTEAGRDWLLLGEVPGQDLLSSHLAPAEKVSIMADAM  QDEAARLSWLATTGVPCAAVLDVVTEAGRDWLLLGEVPGQDLLSSHLAPAEKVSIMADAM | 120 |

| Query | 121 | RRLHTLDPATCPFDHQAKHRIERARTRMEAGLVDQDDLDEEHQGLAPAELFARLKARMPD | 180 |
| --- | --- | --- | --- |
| Sbjct | 121 | RRLHTLDPATCPFDHQAKHRIERARTRMEAGLVDQDDLDEEHQGLAPAELFARLKARMPD RRLHTLDPATCPFDHQAKHRIERARTRMEAGLVDQDDLDEEHQGLAPAELFARLKARMPD | 180 |
| Query | 181 | GEDLVVTHGDACLPNIMVENGRFSGFIDCGRLGVADRYQDIALATRDIAEELGGEWADRF | 240 |
| Sbjct | 181 | GEDLVVTHGDACLPNIMVENGRFSGFIDCGRLGVADRYQDIALATRDIAEELGGEWADRF GEDLVVTHGDACLPNIMVENGRFSGFIDCGRLGVADRYQDIALATRDIAEELGGEWADRF | 240 |
| Query  Sbjct | 241  241 | L 241 L  L 241 |  |

```
>HAL2320778.1 APH(3')-II family aminoglycoside O-phosphotransferase [Escherichia coli]
Length=240

Score = 484 bits (1247), Expect = 3e-172, Method: Compositional matrix adjust. Identities = 240/240 (100%), Positives = 240/240 (100%), Gaps = 0/240 (0%)

Query 25	AQQTIGCSDAAVFRLSAQGRPVLFVKTDLSGALNELQDEAARLSWLATTGVPCAAVLDVV 84 AQQTIGCSDAAVFRLSAQGRPVLFVKTDLSGALNELQDEAARLSWLATTGVPCAAVLDVV
Sbjct 1	AQQTIGCSDAAVFRLSAQGRPVLFVKTDLSGALNELQDEAARLSWLATTGVPCAAVLDVV 60

Query 85	TEAGRDWLLLGEVPGQDLLSSHLAPAEKVSIMADAMRRLHTLDPATCPFDHQAKHRIERA 144 TEAGRDWLLLGEVPGQDLLSSHLAPAEKVSIMADAMRRLHTLDPATCPFDHQAKHRIERA
Sbjct 61	TEAGRDWLLLGEVPGQDLLSSHLAPAEKVSIMADAMRRLHTLDPATCPFDHQAKHRIERA 120

Query 145 RTRMEAGLVDQDDLDEEHQGLAPAELFARLKARMPDGEDLVVTHGDACLPNIMVENGRFS 204 RTRMEAGLVDQDDLDEEHQGLAPAELFARLKARMPDGEDLVVTHGDACLPNIMVENGRFS
Sbjct 121 RTRMEAGLVDQDDLDEEHQGLAPAELFARLKARMPDGEDLVVTHGDACLPNIMVENGRFS 180

Query 205 GFIDCGRLGVADRYQDIALATRDIAEELGGEWADRFLVLYGIAAPDSQRIAFYRLLDEFF 264 GFIDCGRLGVADRYQDIALATRDIAEELGGEWADRFLVLYGIAAPDSQRIAFYRLLDEFF
Sbjct 181 GFIDCGRLGVADRYQDIALATRDIAEELGGEWADRFLVLYGIAAPDSQRIAFYRLLDEFF 240

>ABG21298.1 neomycin phosphotransferase, partial [uncultured bacterium] Length=242

Score = 483 bits (1243), Expect = 1e-171, Method: Compositional matrix adjust. Identities = 241/243 (99%), Positives = 241/243 (99%), Gaps = 1/243 (0%)
```

| Query | 3 | EQDGLHAGSPAAWVERLFGYDWAQQTIGCSDAAVFRLSAQGRPVLFVKTDLSGALNELQD | 62 |
| --- | --- | --- | --- |
| Sbjct | 1 | EQDGLHAGSPAAWVERLFGYDWAQQTIGCSDAAVFRLSAQGRPVLFVKTDLSGALNELQD EQDGLHAGSPAAWVERLFGYDWAQQTIGCSDAAVFRLSAQGRPVLFVKTDLSGALNELQD | 60 |
| Query | 63 | EAARLSWLATTGVPCAAVLDVVTEAGRDWLLLGEVPGQDLLSSHLAPAEKVSIMADAMRR | 122 |
| Sbjct | 61 | EAARLSWLATTGVPCAAVLDVVTEAGRDWLLLGEVPGQDLLSSHLAPAEKVSIMADAMRR EAARLSWLATTGVPCAAVLDVVTEAGRDWLLLGEVPGQDLLSSHLAPAEKVSIMADAMRR | 120 |
| Query | 123 | LHTLDPATCPFDHQAKHRIERARTRMEAGLVDQDDLDEEHQGLAPAELFARLKARMPDGE | 182 |
| Sbjct | 121 | LHTLDPATCPFDHQAKHRIERARTRMEAGLVDQDDLDEEHQGLAPAELFARLKARMPDGE LHTLDPATCPFDHQAKHRIERARTRMEAGLVDQDDLDEEHQGLAPAELFARLKARMPDGE | 180 |
| Query | 183 | DLVVTHGDACLPNIMVENGRFSGFIDCGRLGVADRYQDIALATRDIAEELGGEWADRFLV | 242 |
| Sbjct | 181 | DLV THGDACLPNIMVENGRFSGFIDCGRLGVADRYQDIALATRDIAEELGGEWADR LV DLVETHGDACLPNIMVENGRFSGFIDCGRLGVADRYQDIALATRDIAEELGGEWADR-LV | 239 |
| Query  Sbjct | 243  240 | LYG 245 LYG  LYG 242 |  |

>ABG21297.1 neomycin phosphotransferase, partial [uncultured bacterium] Length=242

Score = 483 bits (1242), Expect = 1e-171, Method: Compositional matrix adjust. Identities = 241/243 (99%), Positives = 241/243 (99%), Gaps = 1/243 (0%)

Query 3	EQDGLHAGSPAAWVERLFGYDWAQQTIGCSDAAVFRLSAQGRPVLFVKTDLSGALNELQD 62

| Sbjct | 1 | EQDGLHAGSPAAWVERLFGYDWAQQTIGCSDAAVFRLSAQGR VLFVKTDLSGALNELQD EQDGLHAGSPAAWVERLFGYDWAQQTIGCSDAAVFRLSAQGRTVLFVKTDLSGALNELQD | 60 |
| --- | --- | --- | --- |
| Query | 63 | EAARLSWLATTGVPCAAVLDVVTEAGRDWLLLGEVPGQDLLSSHLAPAEKVSIMADAMRR | 122 |
| Sbjct | 61 | EAARLSWLATTGVPCAAVLDVVTEAGRDWLLLGEVPGQDLLSSHLAPAEKVSIMADAMRR EAARLSWLATTGVPCAAVLDVVTEAGRDWLLLGEVPGQDLLSSHLAPAEKVSIMADAMRR | 120 |
| Query | 123 | LHTLDPATCPFDHQAKHRIERARTRMEAGLVDQDDLDEEHQGLAPAELFARLKARMPDGE | 182 |
| Sbjct | 121 | LHTLDPATCPFDHQAKHRIERARTRMEAGLVDQDDLDEEHQGLAPAELFARLKARMPDGE LHTLDPATCPFDHQAKHRIERARTRMEAGLVDQDDLDEEHQGLAPAELFARLKARMPDGE | 180 |
| Query | 183 | DLVVTHGDACLPNIMVENGRFSGFIDCGRLGVADRYQDIALATRDIAEELGGEWADRFLV | 242 |
| Sbjct | 181 | DLVVTHGDACLPNIMVENGRFSGFIDCGRLGVADRYQDIALATRDIAEELGGEWADR LV DLVVTHGDACLPNIMVENGRFSGFIDCGRLGVADRYQDIALATRDIAEELGGEWADR-LV | 239 |
| Query  Sbjct | 243  240 | LYG 245 LYG  LYG 242 |  |

>ABG21299.1 neomycin phosphotransferase, partial [uncultured bacterium] Length=242

Score = 482 bits (1241), Expect = 2e-171, Method: Compositional matrix adjust. Identities = 240/243 (99%), Positives = 241/243 (99%), Gaps = 1/243 (0%)

| Query | 3 | EQDGLHAGSPAAWVERLFGYDWAQQTIGCSDAAVFRLSAQGRPVLFVKTDLSGALNELQD | 62 |
| --- | --- | --- | --- |
| Sbjct | 1 | EQDGLHAGSPAAWVERLFGYDWAQQTIGCSDAAVFRLSAQGRPVLFVKTDLSGALNELQD EQDGLHAGSPAAWVERLFGYDWAQQTIGCSDAAVFRLSAQGRPVLFVKTDLSGALNELQD | 60 |
| Query | 63 | EAARLSWLATTGVPCAAVLDVVTEAGRDWLLLGEVPGQDLLSSHLAPAEKVSIMADAMRR | 122 |
| Sbjct | 61 | EAARLSWLATTGVPCAAVLDVVTEAGRDWLLLGEVPGQDLLSSHLAPAEKV IMADAMRR EAARLSWLATTGVPCAAVLDVVTEAGRDWLLLGEVPGQDLLSSHLAPAEKVPIMADAMRR | 120 |
| Query | 123 | LHTLDPATCPFDHQAKHRIERARTRMEAGLVDQDDLDEEHQGLAPAELFARLKARMPDGE | 182 |
| Sbjct | 121 | LHTLDPATCPF+HQAKHRIERARTRMEAGLVDQDDLDEEHQGLAPAELFARLKARMPDGE LHTLDPATCPFNHQAKHRIERARTRMEAGLVDQDDLDEEHQGLAPAELFARLKARMPDGE | 180 |
| Query | 183 | DLVVTHGDACLPNIMVENGRFSGFIDCGRLGVADRYQDIALATRDIAEELGGEWADRFLV | 242 |
| Sbjct | 181 | DLVVTHGDACLPNIMVENGRFSGFIDCGRLGVADRYQDIALATRDIAEELGGEWADR LV DLVVTHGDACLPNIMVENGRFSGFIDCGRLGVADRYQDIALATRDIAEELGGEWADR-LV | 239 |
| Query  Sbjct | 243  240 | LYG 245 LYG  LYG 242 |  |

```
>ARU77007.1 neomycin phosphotransferase II, partial [synthetic construct] Length=242

Score = 482 bits (1240), Expect = 3e-171, Method: Compositional matrix adjust. Identities = 238/238 (100%), Positives = 238/238 (100%), Gaps = 0/238 (0%)

Query 2	IEQDGLHAGSPAAWVERLFGYDWAQQTIGCSDAAVFRLSAQGRPVLFVKTDLSGALNELQ 61 IEQDGLHAGSPAAWVERLFGYDWAQQTIGCSDAAVFRLSAQGRPVLFVKTDLSGALNELQ
Sbjct 5	IEQDGLHAGSPAAWVERLFGYDWAQQTIGCSDAAVFRLSAQGRPVLFVKTDLSGALNELQ 64

Query 62	DEAARLSWLATTGVPCAAVLDVVTEAGRDWLLLGEVPGQDLLSSHLAPAEKVSIMADAMR 121 DEAARLSWLATTGVPCAAVLDVVTEAGRDWLLLGEVPGQDLLSSHLAPAEKVSIMADAMR
Sbjct 65	DEAARLSWLATTGVPCAAVLDVVTEAGRDWLLLGEVPGQDLLSSHLAPAEKVSIMADAMR 124

Query 122 RLHTLDPATCPFDHQAKHRIERARTRMEAGLVDQDDLDEEHQGLAPAELFARLKARMPDG 181 RLHTLDPATCPFDHQAKHRIERARTRMEAGLVDQDDLDEEHQGLAPAELFARLKARMPDG
Sbjct 125 RLHTLDPATCPFDHQAKHRIERARTRMEAGLVDQDDLDEEHQGLAPAELFARLKARMPDG 184

Query 182 EDLVVTHGDACLPNIMVENGRFSGFIDCGRLGVADRYQDIALATRDIAEELGGEWADR 239 EDLVVTHGDACLPNIMVENGRFSGFIDCGRLGVADRYQDIALATRDIAEELGGEWADR
Sbjct 185 EDLVVTHGDACLPNIMVENGRFSGFIDCGRLGVADRYQDIALATRDIAEELGGEWADR 242

>WP_116003610.1 MULTISPECIES: APH(3')-II family aminoglycoside O-phosphotransferase [unclassified Simplicispira]

PVY55494.1 aminoglycoside 3'-phosphotransferase-2 [Simplicispira sp. 125] REG16437.1 aminoglycoside 3'-phosphotransferase-2 [Simplicispira sp. 110]
Length=260

Score = 483 bits (1242), Expect = 3e-171, Method: Compositional matrix adjust.
```

| Identities | = 241/264 (91%), Positives = 245/264 (93%), Gaps = 4/264 (2%) |  |
| --- | --- | --- |
| Query 1 | MIEQDGLHAGSPAAWVERLFGYDWAQQTIGCSDAAVFRLSAQGRPVLFVKTDLSGALNEL | 60 |
| Sbjct 1 | MIEQDGLHAG PAAWVERL YDWA Q IGCS AAVFRLSAQGRPVLFVKTDLSGALNEL MIEQDGLHAGPPAAWVERLSSYDWAHQAIGCSHAAVFRLSAQGRPVLFVKTDLSGALNEL | 60 |
| Query 61 | QDEAARLSWLATTGVPCAAVLDVVTEAGRDWLLLGEVPGQDLLSSHLAPAEKVSIMADAM | 120 |
| Sbjct 61 | QDEAARLSWLATTGVPCA VLDVVTEAG DWLLLGEVPGQDLLSSHLAPAEKVS+MADA+ QDEAARLSWLATTGVPCATVLDVVTEAGTDWLLLGEVPGQDLLSSHLAPAEKVSLMADAL | 120 |
| Query 121 | RRLHTLDPATCPFDHQAKHRIERARTRMEAGLVDQDDLDEEHQGLAPAELFARLKARMPD | 180 |
| Sbjct 121 | RRLH LDPA CPFDHQAKHR+ERAR RMEAGLVDQDDLDE HQGLAPAELFARLKAR+PD RRLHALDPAACPFDHQAKHRVERARARMEAGLVDQDDLDEAHQGLAPAELFARLKARVPD | 180 |
| Query 181 | GEDLVVTHGDACLPNIMVENGRFSGFIDCGRLGVADRYQDIALATRDIAEELGGEWADRF | 240 |
| Sbjct 181 | GEDLV DACLPNIMVENGRFSGFIDCGRLGVADRYQDIALATRDIAEELGGEWADRF GEDLV DACLPNIMVENGRFSGFIDCGRLGVADRYQDIALATRDIAEELGGEWADRF | 236 |
| Query 241  Sbjct 237 | LVLYGIAAPDSQRIAFYRLLDEFF 264 LV YGI A DSQRIAFYRLLDEFF LVRYGIGASDSQRIAFYRLLDEFF 260 |  |

>ABG21300.1 neomycin phosphotransferase, partial [uncultured bacterium] Length=242

Score = 481 bits (1239), Expect = 4e-171, Method: Compositional matrix adjust. Identities = 241/243 (99%), Positives = 241/243 (99%), Gaps = 1/243 (0%)

| Query | 3 | EQDGLHAGSPAAWVERLFGYDWAQQTIGCSDAAVFRLSAQGRPVLFVKTDLSGALNELQD | 62 |
| --- | --- | --- | --- |
| Sbjct | 1 | EQDGLHAGSPAAWVERLFGYDWAQQTIGCSDAAVFRLSAQGRPVLFVKTDLSGALNELQD EQDGLHAGSPAAWVERLFGYDWAQQTIGCSDAAVFRLSAQGRPVLFVKTDLSGALNELQD | 60 |
| Query | 63 | EAARLSWLATTGVPCAAVLDVVTEAGRDWLLLGEVPGQDLLSSHLAPAEKVSIMADAMRR | 122 |
| Sbjct | 61 | EAARLSWLATTGVPCAAVLDVVTEAGRDWLLLGEVPGQDLLSSHLAPAEKVSIMADAMRR EAARLSWLATTGVPCAAVLDVVTEAGRDWLLLGEVPGQDLLSSHLAPAEKVSIMADAMRR | 120 |
| Query | 123 | LHTLDPATCPFDHQAKHRIERARTRMEAGLVDQDDLDEEHQGLAPAELFARLKARMPDGE | 182 |
| Sbjct | 121 | LHTLDPATCPFDHQAKHRIERARTRMEAGLVDQDDLDEEHQGLAPAELFARLKARMPDGE LHTLDPATCPFDHQAKHRIERARTRMEAGLVDQDDLDEEHQGLAPAELFARLKARMPDGE | 180 |
| Query | 183 | DLVVTHGDACLPNIMVENGRFSGFIDCGRLGVADRYQDIALATRDIAEELGGEWADRFLV | 242 |
| Sbjct | 181 | DLVVTHGDACLPNIMVE GRFSGFIDCGRLGVADRYQDIALATRDIAEELGGEWADR LV DLVVTHGDACLPNIMVEIGRFSGFIDCGRLGVADRYQDIALATRDIAEELGGEWADR-LV | 239 |
| Query  Sbjct | 243  240 | LYG 245 LYG  LYG 242 |  |

>AFM74288.1 aminoglycoside-3'-O-phosphotransferase, partial [Riemerella anatipestifer] AFM74289.1 aminoglycoside-3'-O-phosphotransferase, partial [Riemerella anatipestifer] AFM74295.1 aminoglycoside-3'-O-phosphotransferase, partial [Riemerella anatipestifer] AFM74300.1 aminoglycoside-3'-O-phosphotransferase, partial [Riemerella anatipestifer] AFM74302.1 aminoglycoside-3'-O-phosphotransferase, partial [Riemerella anatipestifer]

Length=238

Score = 481 bits (1238), Expect = 6e-171, Method: Compositional matrix adjust. Identities = 238/238 (100%), Positives = 238/238 (100%), Gaps = 0/238 (0%)

Query 23	DWAQQTIGCSDAAVFRLSAQGRPVLFVKTDLSGALNELQDEAARLSWLATTGVPCAAVLD 82 DWAQQTIGCSDAAVFRLSAQGRPVLFVKTDLSGALNELQDEAARLSWLATTGVPCAAVLD

Sbjct 1	DWAQQTIGCSDAAVFRLSAQGRPVLFVKTDLSGALNELQDEAARLSWLATTGVPCAAVLD 60

Query 83	VVTEAGRDWLLLGEVPGQDLLSSHLAPAEKVSIMADAMRRLHTLDPATCPFDHQAKHRIE 142 VVTEAGRDWLLLGEVPGQDLLSSHLAPAEKVSIMADAMRRLHTLDPATCPFDHQAKHRIE

Sbjct 61 VVTEAGRDWLLLGEVPGQDLLSSHLAPAEKVSIMADAMRRLHTLDPATCPFDHQAKHRIE 120

| Query | 143 | RARTRMEAGLVDQDDLDEEHQGLAPAELFARLKARMPDGEDLVVTHGDACLPNIMVENGR 202 |
| --- | --- | --- |
| Sbjct | 121 | RARTRMEAGLVDQDDLDEEHQGLAPAELFARLKARMPDGEDLVVTHGDACLPNIMVENGR RARTRMEAGLVDQDDLDEEHQGLAPAELFARLKARMPDGEDLVVTHGDACLPNIMVENGR 180 |
| Query | 203 | FSGFIDCGRLGVADRYQDIALATRDIAEELGGEWADRFLVLYGIAAPDSQRIAFYRLL 260 |
| Sbjct | 181 | FSGFIDCGRLGVADRYQDIALATRDIAEELGGEWADRFLVLYGIAAPDSQRIAFYRLL  FSGFIDCGRLGVADRYQDIALATRDIAEELGGEWADRFLVLYGIAAPDSQRIAFYRLL 238 |

>AFM74299.1 aminoglycoside-3'-O-phosphotransferase, partial [Riemerella anatipestifer] Length=238

Score = 480 bits (1236), Expect = 1e-170, Method: Compositional matrix adjust.

| Identities | | = 237/238 (99%), Positives = 238/238 (100%), Gaps = 0/238 (0%) |  |
| --- | --- | --- | --- |
| Query 23  Sbjct 1 | | DWAQQTIGCSDAAVFRLSAQGRPVLFVKTDLSGALNELQDEAARLSWLATTGVPCAAVLD DWAQQTIGCSDAAVFRLSAQGRPVLFVKTDLSGALNELQDEAARLSWLATTGVPCAAVLD DWAQQTIGCSDAAVFRLSAQGRPVLFVKTDLSGALNELQDEAARLSWLATTGVPCAAVLD | 82  60 |
| Query 83  Sbjct 61 | | VVTEAGRDWLLLGEVPGQDLLSSHLAPAEKVSIMADAMRRLHTLDPATCPFDHQAKHRIE VVTEAGRDWLLLGEVPGQDLLSSHLAPAEKVSIMADAMRRLHTLDPATCPFDHQAKHRIE VVTEAGRDWLLLGEVPGQDLLSSHLAPAEKVSIMADAMRRLHTLDPATCPFDHQAKHRIE | 142  120 |
| Query 143  Sbjct 121 | | RARTRMEAGLVDQDDLDEEHQGLAPAELFARLKARMPDGEDLVVTHGDACLPNIMVENGR RARTRMEAGLVDQDDLDEEHQGLAPAELFARLKARMPDGEDLVVTHGDACLPNIMVENGR RARTRMEAGLVDQDDLDEEHQGLAPAELFARLKARMPDGEDLVVTHGDACLPNIMVENGR | 202  180 |
| Query Sbjct | 203  181 | FSGFIDCGRLGVADRYQDIALATRDIAEELGGEWADRFLVLYGIAAPDSQRIAFYRLL 260 FSGFIDCGRLGVADRYQDIALATRDIAEELGGEWADRFLVLYGIAAPDSQR+AFYRLL FSGFIDCGRLGVADRYQDIALATRDIAEELGGEWADRFLVLYGIAAPDSQRVAFYRLL 238 | |

>ABG21302.1 neomycin phosphotransferase, partial [uncultured bacterium] Length=242

Score = 480 bits (1236), Expect = 1e-170, Method: Compositional matrix adjust.

| Identities | = 240/243 (99%), Positives = 240/243 (99%), Gaps = 1/243 (0%) |  |
| --- | --- | --- |
| Query 3 | EQDGLHAGSPAAWVERLFGYDWAQQTIGCSDAAVFRLSAQGRPVLFVKTDLSGALNELQD | 62 |
| Sbjct 1 | EQDGLHAGSPAAWVERLFGYDWAQQTIGCSDAAVFRLSAQGRPVLFVKTDLSGALNELQD EQDGLHAGSPAAWVERLFGYDWAQQTIGCSDAAVFRLSAQGRPVLFVKTDLSGALNELQD | 60 |
| Query 63 | EAARLSWLATTGVPCAAVLDVVTEAGRDWLLLGEVPGQDLLSSHLAPAEKVSIMADAMRR | 122 |
| Sbjct 61 | EAARLSWLATTGVPCAAVLDVVTEAGRDWLLLGEVPGQDLLSSHLAPAEKVSIMADAMRR EAARLSWLATTGVPCAAVLDVVTEAGRDWLLLGEVPGQDLLSSHLAPAEKVSIMADAMRR | 120 |
| Query 123 | LHTLDPATCPFDHQAKHRIERARTRMEAGLVDQDDLDEEHQGLAPAELFARLKARMPDGE | 182 |
| Sbjct 121 | LHTLDPATCPFD QAKHRIERARTRMEAGLVDQDDLDEEHQGLAPAELFARLKARMPDGE LHTLDPATCPFDRQAKHRIERARTRMEAGLVDQDDLDEEHQGLAPAELFARLKARMPDGE | 180 |
| Query 183 | DLVVTHGDACLPNIMVENGRFSGFIDCGRLGVADRYQDIALATRDIAEELGGEWADRFLV | 242 |
| Sbjct 181 | DLVVTHGDACLPNIMVENGRFSGF DCGRLGVADRYQDIALATRDIAEELGGEWADR LV DLVVTHGDACLPNIMVENGRFSGFNDCGRLGVADRYQDIALATRDIAEELGGEWADR-LV | 239 |
| Query 243  Sbjct 240 | LYG 245 LYG  LYG 242 |  |

>ABG21296.1 neomycin phosphotransferase, partial [uncultured bacterium] Length=242

Score = 479 bits (1234), Expect = 3e-170, Method: Compositional matrix adjust. Identities = 239/242 (99%), Positives = 240/242 (99%), Gaps = 1/242 (0%)

| Query | 4 | QDGLHAGSPAAWVERLFGYDWAQQTIGCSDAAVFRLSAQGRPVLFVKTDLSGALNELQDE | 63 |
| --- | --- | --- | --- |
| Sbjct | 2 | Q GLHAGSPAAWVERLFGYDWAQQTIGCSDAAVFRLSAQGRPVLFVKTDLSGALNELQDE QIGLHAGSPAAWVERLFGYDWAQQTIGCSDAAVFRLSAQGRPVLFVKTDLSGALNELQDE | 61 |
| Query | 64 | AARLSWLATTGVPCAAVLDVVTEAGRDWLLLGEVPGQDLLSSHLAPAEKVSIMADAMRRL AARLSWLATTGVPCAAVLDVVTEAGRDWLLLGEVPGQDLLSSHLAPAEKVSIMADAMRRL | 123 |

| Sbjct | 62 | AARLSWLATTGVPCAAVLDVVTEAGRDWLLLGEVPGQDLLSSHLAPAEKVSIMADAMRRL | 121 |
| --- | --- | --- | --- |
| Query | 124 | HTLDPATCPFDHQAKHRIERARTRMEAGLVDQDDLDEEHQGLAPAELFARLKARMPDGED | 183 |
| Sbjct | 122 | HTLDPATCPFDHQAKHRIERARTRMEAGLVDQDDLDEEHQGLAPAELFARLKARMPDGED HTLDPATCPFDHQAKHRIERARTRMEAGLVDQDDLDEEHQGLAPAELFARLKARMPDGED | 181 |
| Query | 184 | LVVTHGDACLPNIMVENGRFSGFIDCGRLGVADRYQDIALATRDIAEELGGEWADRFLVL | 243 |
| Sbjct | 182 | LVVTHGDACLPNIMVENGRFSGFIDCG+LGVADRYQDIALATRDIAEELGGEWADR LVL LVVTHGDACLPNIMVENGRFSGFIDCGQLGVADRYQDIALATRDIAEELGGEWADR-LVL | 240 |
| Query  Sbjct | 244  241 | YG 245 YG  YG 242 |  |

>AFM74301.1 aminoglycoside-3'-O-phosphotransferase, partial [Riemerella anatipestifer] Length=238

Score = 479 bits (1233), Expect = 3e-170, Method: Compositional matrix adjust.

| Identities | | = 237/238 (99%), Positives = 237/238 (99%), Gaps = 0/238 (0%) |  |
| --- | --- | --- | --- |
| Query 23  Sbjct 1 | | DWAQQTIGCSDAAVFRLSAQGRPVLFVKTDLSGALNELQDEAARLSWLATTGVPCAAVLD DWAQQTIGCSDAAVFRLSAQGRPVLFVKTDLSGALNELQDEAARLSWLATTGVPCAAVLD DWAQQTIGCSDAAVFRLSAQGRPVLFVKTDLSGALNELQDEAARLSWLATTGVPCAAVLD | 82  60 |
| Query 83  Sbjct 61 | | VVTEAGRDWLLLGEVPGQDLLSSHLAPAEKVSIMADAMRRLHTLDPATCPFDHQAKHRIE VVTEAGRDWLLLGEVPGQDLLSSHLAPAEKVSIMADAMRRLHTLDPATCPFDHQAKHRIE VVTEAGRDWLLLGEVPGQDLLSSHLAPAEKVSIMADAMRRLHTLDPATCPFDHQAKHRIE | 142  120 |
| Query 143  Sbjct 121 | | RARTRMEAGLVDQDDLDEEHQGLAPAELFARLKARMPDGEDLVVTHGDACLPNIMVENGR RARTRMEAGLVDQDDLDEEHQGLAPAELFARLKARMPDGEDLVVTHGDACLPNIMVENGR RARTRMEAGLVDQDDLDEEHQGLAPAELFARLKARMPDGEDLVVTHGDACLPNIMVENGR | 202  180 |
| Query Sbjct | 203  181 | FSGFIDCGRLGVADRYQDIALATRDIAEELGGEWADRFLVLYGIAAPDSQRIAFYRLL 260 FSGFIDCGRLG ADRYQDIALATRDIAEELGGEWADRFLVLYGIAAPDSQRIAFYRLL FSGFIDCGRLGAADRYQDIALATRDIAEELGGEWADRFLVLYGIAAPDSQRIAFYRLL 238 | |

>AFM74305.1 aminoglycoside-3'-O-phosphotransferase, partial [Riemerella anatipestifer] Length=238

Score = 479 bits (1233), Expect = 3e-170, Method: Compositional matrix adjust.

| Identities | | = 237/238 (99%), Positives = 237/238 (99%), Gaps = 0/238 (0%) |  |
| --- | --- | --- | --- |
| Query 23  Sbjct 1 | | DWAQQTIGCSDAAVFRLSAQGRPVLFVKTDLSGALNELQDEAARLSWLATTGVPCAAVLD DWAQQTIGCSDAAVFRLSAQGRPVLFVKTDLSGALNELQDEAARLSWLATTGVPCAAVLD DWAQQTIGCSDAAVFRLSAQGRPVLFVKTDLSGALNELQDEAARLSWLATTGVPCAAVLD | 82  60 |
| Query 83  Sbjct 61 | | VVTEAGRDWLLLGEVPGQDLLSSHLAPAEKVSIMADAMRRLHTLDPATCPFDHQAKHRIE VVTEAGRDWLLLGEVPGQDLLSSHLAPAEKVSIMADAMRRLHTLDPATCPFDHQAKHRIE VVTEAGRDWLLLGEVPGQDLLSSHLAPAEKVSIMADAMRRLHTLDPATCPFDHQAKHRIE | 142  120 |
| Query 143  Sbjct 121 | | RARTRMEAGLVDQDDLDEEHQGLAPAELFARLKARMPDGEDLVVTHGDACLPNIMVENGR RARTRMEAGLVDQDDLDEEHQGLAPAELFARLKARMPDGEDLVVTHGDACLPNIMVENGR RARTRMEAGLVDQDDLDEEHQGLAPAELFARLKARMPDGEDLVVTHGDACLPNIMVENGR | 202  180 |
| Query Sbjct | 203  181 | FSGFIDCGRLGVADRYQDIALATRDIAEELGGEWADRFLVLYGIAAPDSQRIAFYRLL 260 FSGFIDCGRLGVADRYQD ALATRDIAEELGGEWADRFLVLYGIAAPDSQRIAFYRLL FSGFIDCGRLGVADRYQDTALATRDIAEELGGEWADRFLVLYGIAAPDSQRIAFYRLL 238 | |

>AFM74291.1 aminoglycoside-3'-O-phosphotransferase, partial [Riemerella anatipestifer] Length=238

Score = 479 bits (1233), Expect = 3e-170, Method: Compositional matrix adjust.

| Identities | = 237/238 (99%), Positives = 237/238 (99%), Gaps = 0/238 (0%) |  |
| --- | --- | --- |
| Query 23 | DWAQQTIGCSDAAVFRLSAQGRPVLFVKTDLSGALNELQDEAARLSWLATTGVPCAAVLD | 82 |
| Sbjct 1 | DWAQQTIGCSDAAVFRLSAQGRP LFVKTDLSGALNELQDEAARLSWLATTGVPCAAVLD DWAQQTIGCSDAAVFRLSAQGRPALFVKTDLSGALNELQDEAARLSWLATTGVPCAAVLD | 60 |
| Query 83 | VVTEAGRDWLLLGEVPGQDLLSSHLAPAEKVSIMADAMRRLHTLDPATCPFDHQAKHRIE | 142 |

| Sbjct | 61 | VVTEAGRDWLLLGEVPGQDLLSSHLAPAEKVSIMADAMRRLHTLDPATCPFDHQAKHRIE VVTEAGRDWLLLGEVPGQDLLSSHLAPAEKVSIMADAMRRLHTLDPATCPFDHQAKHRIE | 120 |
| --- | --- | --- | --- |
| Query | 143 | RARTRMEAGLVDQDDLDEEHQGLAPAELFARLKARMPDGEDLVVTHGDACLPNIMVENGR | 202 |
| Sbjct | 121 | RARTRMEAGLVDQDDLDEEHQGLAPAELFARLKARMPDGEDLVVTHGDACLPNIMVENGR RARTRMEAGLVDQDDLDEEHQGLAPAELFARLKARMPDGEDLVVTHGDACLPNIMVENGR | 180 |
| Query | 203 | FSGFIDCGRLGVADRYQDIALATRDIAEELGGEWADRFLVLYGIAAPDSQRIAFYRLL | 260 |
| Sbjct | 181 | FSGFIDCGRLGVADRYQDIALATRDIAEELGGEWADRFLVLYGIAAPDSQRIAFYRLL  FSGFIDCGRLGVADRYQDIALATRDIAEELGGEWADRFLVLYGIAAPDSQRIAFYRLL | 238 |

```
>AFM74293.1 aminoglycoside-3'-O-phosphotransferase, partial [Riemerella anatipestifer] AFM74318.1 aminoglycoside-3'-O-phosphotransferase, partial [Riemerella anatipestifer]
Length=238

Score = 479 bits (1232), Expect = 4e-170, Method: Compositional matrix adjust. Identities = 237/238 (99%), Positives = 237/238 (99%), Gaps = 0/238 (0%)

Query 23	DWAQQTIGCSDAAVFRLSAQGRPVLFVKTDLSGALNELQDEAARLSWLATTGVPCAAVLD 82 DWAQQTIGCSDAAVFRLSAQGRPVLFVKTDLSGALNELQDEAARLSWLATTGVPCAAVLD
Sbjct 1	DWAQQTIGCSDAAVFRLSAQGRPVLFVKTDLSGALNELQDEAARLSWLATTGVPCAAVLD 60

Query 83	VVTEAGRDWLLLGEVPGQDLLSSHLAPAEKVSIMADAMRRLHTLDPATCPFDHQAKHRIE 142 VVTEAGRDWLLLGEVPGQDLLSSHLAPAEKVSIMADAMRRLHTLDPATCPFDHQAKHRIE
Sbjct 61	VVTEAGRDWLLLGEVPGQDLLSSHLAPAEKVSIMADAMRRLHTLDPATCPFDHQAKHRIE 120

Query 143 RARTRMEAGLVDQDDLDEEHQGLAPAELFARLKARMPDGEDLVVTHGDACLPNIMVENGR 202 RARTRMEAGLVDQDDLDEEHQGLAPAELFARLKA MPDGEDLVVTHGDACLPNIMVENGR
Sbjct 121 RARTRMEAGLVDQDDLDEEHQGLAPAELFARLKASMPDGEDLVVTHGDACLPNIMVENGR 180

Query 203 FSGFIDCGRLGVADRYQDIALATRDIAEELGGEWADRFLVLYGIAAPDSQRIAFYRLL 260 FSGFIDCGRLGVADRYQDIALATRDIAEELGGEWADRFLVLYGIAAPDSQRIAFYRLL
Sbjct 181 FSGFIDCGRLGVADRYQDIALATRDIAEELGGEWADRFLVLYGIAAPDSQRIAFYRLL 238

>AFM74319.1 aminoglycoside-3'-O-phosphotransferase, partial [Riemerella anatipestifer] Length=238

Score = 479 bits (1232), Expect = 4e-170, Method: Compositional matrix adjust. Identities = 237/238 (99%), Positives = 237/238 (99%), Gaps = 0/238 (0%)

Query 23	DWAQQTIGCSDAAVFRLSAQGRPVLFVKTDLSGALNELQDEAARLSWLATTGVPCAAVLD 82 DWAQQTIGCSDAAVFRLSAQGRPVLFVKTDLSGALNELQDEAARLSWLATTGVPCAAVLD
Sbjct 1	DWAQQTIGCSDAAVFRLSAQGRPVLFVKTDLSGALNELQDEAARLSWLATTGVPCAAVLD 60

Query 83	VVTEAGRDWLLLGEVPGQDLLSSHLAPAEKVSIMADAMRRLHTLDPATCPFDHQAKHRIE 142 VVTEAGRDWLLLGEVPGQDLLSSHLAPAEKVSIMADAMRRLHTLDPATCPFDHQAKHRIE
Sbjct 61	VVTEAGRDWLLLGEVPGQDLLSSHLAPAEKVSIMADAMRRLHTLDPATCPFDHQAKHRIE 120

Query 143 RARTRMEAGLVDQDDLDEEHQGLAPAELFARLKARMPDGEDLVVTHGDACLPNIMVENGR 202 RARTRMEAGLVDQDDLDEEHQGLAPAELFARLKARMPDGEDLVVTHGDACLPNIMVENGR
Sbjct 121 RARTRMEAGLVDQDDLDEEHQGLAPAELFARLKARMPDGEDLVVTHGDACLPNIMVENGR 180

Query 203 FSGFIDCGRLGVADRYQDIALATRDIAEELGGEWADRFLVLYGIAAPDSQRIAFYRLL 260 FSGFIDCGRLGVAD YQDIALATRDIAEELGGEWADRFLVLYGIAAPDSQRIAFYRLL
Sbjct 181 FSGFIDCGRLGVADHYQDIALATRDIAEELGGEWADRFLVLYGIAAPDSQRIAFYRLL 238

>AFM74292.1 aminoglycoside-3'-O-phosphotransferase, partial [Riemerella anatipestifer] Length=238

Score = 478 bits (1231), Expect = 7e-170, Method: Compositional matrix adjust.
```

| Identities | = 237/238 (99%), Positives = 237/238 (99%), Gaps = 0/238 (0%) |  |
| --- | --- | --- |
| Query 23 | DWAQQTIGCSDAAVFRLSAQGRPVLFVKTDLSGALNELQDEAARLSWLATTGVPCAAVLD | 82 |
| Sbjct 1 | DWAQQTIGCSDAAVFRL AQGRPVLFVKTDLSGALNELQDEAARLSWLATTGVPCAAVLD DWAQQTIGCSDAAVFRLPAQGRPVLFVKTDLSGALNELQDEAARLSWLATTGVPCAAVLD | 60 |
| Query 83 | VVTEAGRDWLLLGEVPGQDLLSSHLAPAEKVSIMADAMRRLHTLDPATCPFDHQAKHRIE | 142 |
| Sbjct 61 | VVTEAGRDWLLLGEVPGQDLLSSHLAPAEKVSIMADAMRRLHTLDPATCPFDHQAKHRIE  VVTEAGRDWLLLGEVPGQDLLSSHLAPAEKVSIMADAMRRLHTLDPATCPFDHQAKHRIE | 120 |

| Query | 143 | RARTRMEAGLVDQDDLDEEHQGLAPAELFARLKARMPDGEDLVVTHGDACLPNIMVENGR 202 |
| --- | --- | --- |
| Sbjct | 121 | RARTRMEAGLVDQDDLDEEHQGLAPAELFARLKARMPDGEDLVVTHGDACLPNIMVENGR RARTRMEAGLVDQDDLDEEHQGLAPAELFARLKARMPDGEDLVVTHGDACLPNIMVENGR 180 |
| Query | 203 | FSGFIDCGRLGVADRYQDIALATRDIAEELGGEWADRFLVLYGIAAPDSQRIAFYRLL 260 |
| Sbjct | 181 | FSGFIDCGRLGVADRYQDIALATRDIAEELGGEWADRFLVLYGIAAPDSQRIAFYRLL  FSGFIDCGRLGVADRYQDIALATRDIAEELGGEWADRFLVLYGIAAPDSQRIAFYRLL 238 |

>AFM74320.1 aminoglycoside-3'-O-phosphotransferase, partial [Riemerella anatipestifer] Length=238

Score = 478 bits (1230), Expect = 8e-170, Method: Compositional matrix adjust.

| Identities | | = 237/238 (99%), Positives = 237/238 (99%), Gaps = 0/238 (0%) |  |
| --- | --- | --- | --- |
| Query 23  Sbjct 1 | | DWAQQTIGCSDAAVFRLSAQGRPVLFVKTDLSGALNELQDEAARLSWLATTGVPCAAVLD DWAQQTIGCSDAAVFRLSAQGRPVLFVKTDLSGALNELQDEAARLSWLATTGVPCAAVL DWAQQTIGCSDAAVFRLSAQGRPVLFVKTDLSGALNELQDEAARLSWLATTGVPCAAVLY | 82  60 |
| Query 83  Sbjct 61 | | VVTEAGRDWLLLGEVPGQDLLSSHLAPAEKVSIMADAMRRLHTLDPATCPFDHQAKHRIE VVTEAGRDWLLLGEVPGQDLLSSHLAPAEKVSIMADAMRRLHTLDPATCPFDHQAKHRIE VVTEAGRDWLLLGEVPGQDLLSSHLAPAEKVSIMADAMRRLHTLDPATCPFDHQAKHRIE | 142  120 |
| Query 143  Sbjct 121 | | RARTRMEAGLVDQDDLDEEHQGLAPAELFARLKARMPDGEDLVVTHGDACLPNIMVENGR RARTRMEAGLVDQDDLDEEHQGLAPAELFARLKARMPDGEDLVVTHGDACLPNIMVENGR RARTRMEAGLVDQDDLDEEHQGLAPAELFARLKARMPDGEDLVVTHGDACLPNIMVENGR | 202  180 |
| Query Sbjct | 203  181 | FSGFIDCGRLGVADRYQDIALATRDIAEELGGEWADRFLVLYGIAAPDSQRIAFYRLL 260 FSGFIDCGRLGVADRYQDIALATRDIAEELGGEWADRFLVLYGIAAPDSQRIAFYRLL FSGFIDCGRLGVADRYQDIALATRDIAEELGGEWADRFLVLYGIAAPDSQRIAFYRLL 238 | |

>AFM74297.1 aminoglycoside-3'-O-phosphotransferase, partial [Riemerella anatipestifer] Length=238

Score = 478 bits (1230), Expect = 8e-170, Method: Compositional matrix adjust.

| Identities | | = 237/238 (99%), Positives = 237/238 (99%), Gaps = 0/238 (0%) |  |
| --- | --- | --- | --- |
| Query 23  Sbjct 1 | | DWAQQTIGCSDAAVFRLSAQGRPVLFVKTDLSGALNELQDEAARLSWLATTGVPCAAVLD DWAQQTIGCSDAAVFRLSAQGRPVLFVKTDLSGA NELQDEAARLSWLATTGVPCAAVLD DWAQQTIGCSDAAVFRLSAQGRPVLFVKTDLSGAPNELQDEAARLSWLATTGVPCAAVLD | 82  60 |
| Query 83  Sbjct 61 | | VVTEAGRDWLLLGEVPGQDLLSSHLAPAEKVSIMADAMRRLHTLDPATCPFDHQAKHRIE VVTEAGRDWLLLGEVPGQDLLSSHLAPAEKVSIMADAMRRLHTLDPATCPFDHQAKHRIE VVTEAGRDWLLLGEVPGQDLLSSHLAPAEKVSIMADAMRRLHTLDPATCPFDHQAKHRIE | 142  120 |
| Query 143  Sbjct 121 | | RARTRMEAGLVDQDDLDEEHQGLAPAELFARLKARMPDGEDLVVTHGDACLPNIMVENGR RARTRMEAGLVDQDDLDEEHQGLAPAELFARLKARMPDGEDLVVTHGDACLPNIMVENGR RARTRMEAGLVDQDDLDEEHQGLAPAELFARLKARMPDGEDLVVTHGDACLPNIMVENGR | 202  180 |
| Query Sbjct | 203  181 | FSGFIDCGRLGVADRYQDIALATRDIAEELGGEWADRFLVLYGIAAPDSQRIAFYRLL 260 FSGFIDCGRLGVADRYQDIALATRDIAEELGGEWADRFLVLYGIAAPDSQRIAFYRLL FSGFIDCGRLGVADRYQDIALATRDIAEELGGEWADRFLVLYGIAAPDSQRIAFYRLL 238 | |

>AFM74304.1 aminoglycoside-3'-O-phosphotransferase, partial [Riemerella anatipestifer] Length=238

Score = 478 bits (1230), Expect = 8e-170, Method: Compositional matrix adjust.

| Identities | = 237/238 (99%), Positives = 237/238 (99%), Gaps = 0/238 (0%) |  |
| --- | --- | --- |
| Query 23 | DWAQQTIGCSDAAVFRLSAQGRPVLFVKTDLSGALNELQDEAARLSWLATTGVPCAAVLD | 82 |
| Sbjct 1 | DWAQQTIGCSDAAVFRLSAQGRPVLFVKTDLSGALNELQDEAARLSWLATTGVPCAAVLD DWAQQTIGCSDAAVFRLSAQGRPVLFVKTDLSGALNELQDEAARLSWLATTGVPCAAVLD | 60 |
| Query 83 | VVTEAGRDWLLLGEVPGQDLLSSHLAPAEKVSIMADAMRRLHTLDPATCPFDHQAKHRIE | 142 |
| Sbjct 61 | VVTEAGRDW LLGEVPGQDLLSSHLAPAEKVSIMADAMRRLHTLDPATCPFDHQAKHRIE VVTEAGRDWPLLGEVPGQDLLSSHLAPAEKVSIMADAMRRLHTLDPATCPFDHQAKHRIE | 120 |
| Query 143 | RARTRMEAGLVDQDDLDEEHQGLAPAELFARLKARMPDGEDLVVTHGDACLPNIMVENGR RARTRMEAGLVDQDDLDEEHQGLAPAELFARLKARMPDGEDLVVTHGDACLPNIMVENGR | 202 |

| Sbjct | 121 | RARTRMEAGLVDQDDLDEEHQGLAPAELFARLKARMPDGEDLVVTHGDACLPNIMVENGR 180 |
| --- | --- | --- |
| Query | 203 | FSGFIDCGRLGVADRYQDIALATRDIAEELGGEWADRFLVLYGIAAPDSQRIAFYRLL 260 |
| Sbjct | 181 | FSGFIDCGRLGVADRYQDIALATRDIAEELGGEWADRFLVLYGIAAPDSQRIAFYRLL  FSGFIDCGRLGVADRYQDIALATRDIAEELGGEWADRFLVLYGIAAPDSQRIAFYRLL 238 |

>AFM74313.1 aminoglycoside-3'-O-phosphotransferase, partial [Riemerella anatipestifer] Length=238

Score = 478 bits (1230), Expect = 9e-170, Method: Compositional matrix adjust.

| Identities | | = 237/238 (99%), Positives = 237/238 (99%), Gaps = 0/238 (0%) |  |
| --- | --- | --- | --- |
| Query 23  Sbjct 1 | | DWAQQTIGCSDAAVFRLSAQGRPVLFVKTDLSGALNELQDEAARLSWLATTGVPCAAVLD DWAQQTIGCSDAAV RLSAQGRPVLFVKTDLSGALNELQDEAARLSWLATTGVPCAAVLD DWAQQTIGCSDAAVSRLSAQGRPVLFVKTDLSGALNELQDEAARLSWLATTGVPCAAVLD | 82  60 |
| Query 83  Sbjct 61 | | VVTEAGRDWLLLGEVPGQDLLSSHLAPAEKVSIMADAMRRLHTLDPATCPFDHQAKHRIE VVTEAGRDWLLLGEVPGQDLLSSHLAPAEKVSIMADAMRRLHTLDPATCPFDHQAKHRIE VVTEAGRDWLLLGEVPGQDLLSSHLAPAEKVSIMADAMRRLHTLDPATCPFDHQAKHRIE | 142  120 |
| Query 143  Sbjct 121 | | RARTRMEAGLVDQDDLDEEHQGLAPAELFARLKARMPDGEDLVVTHGDACLPNIMVENGR RARTRMEAGLVDQDDLDEEHQGLAPAELFARLKARMPDGEDLVVTHGDACLPNIMVENGR RARTRMEAGLVDQDDLDEEHQGLAPAELFARLKARMPDGEDLVVTHGDACLPNIMVENGR | 202  180 |
| Query Sbjct | 203  181 | FSGFIDCGRLGVADRYQDIALATRDIAEELGGEWADRFLVLYGIAAPDSQRIAFYRLL 260 FSGFIDCGRLGVADRYQDIALATRDIAEELGGEWADRFLVLYGIAAPDSQRIAFYRLL FSGFIDCGRLGVADRYQDIALATRDIAEELGGEWADRFLVLYGIAAPDSQRIAFYRLL 238 | |

>AFM74298.1 aminoglycoside-3'-O-phosphotransferase, partial [Riemerella anatipestifer] Length=238

Score = 478 bits (1229), Expect = 1e-169, Method: Compositional matrix adjust.

| Identities | | = 236/238 (99%), Positives = 237/238 (99%), Gaps = 0/238 (0%) |  |
| --- | --- | --- | --- |
| Query 23  Sbjct 1 | | DWAQQTIGCSDAAVFRLSAQGRPVLFVKTDLSGALNELQDEAARLSWLATTGVPCAAVLD DWAQQTIGCSDAAVFRLSAQGRPVLFVKTDLSGALNELQDEAARLSWLATTGVPCAAVLD DWAQQTIGCSDAAVFRLSAQGRPVLFVKTDLSGALNELQDEAARLSWLATTGVPCAAVLD | 82  60 |
| Query 83  Sbjct 61 | | VVTEAGRDWLLLGEVPGQDLLSSHLAPAEKVSIMADAMRRLHTLDPATCPFDHQAKHRIE VVTEAGRDWLLLGEVPGQDLLSSHLAPAEKVSIMADAMRRLHTLDPATCPFDHQAKHRIE VVTEAGRDWLLLGEVPGQDLLSSHLAPAEKVSIMADAMRRLHTLDPATCPFDHQAKHRIE | 142  120 |
| Query 143  Sbjct 121 | | RARTRMEAGLVDQDDLDEEHQGLAPAELFARLKARMPDGEDLVVTHGDACLPNIMVENGR RARTRMEAGLVDQDDL EEHQGLAPAELFARLKARMPDGEDLVVTHGDACLPNIMVENGR RARTRMEAGLVDQDDLGEEHQGLAPAELFARLKARMPDGEDLVVTHGDACLPNIMVENGR | 202  180 |
| Query Sbjct | 203  181 | FSGFIDCGRLGVADRYQDIALATRDIAEELGGEWADRFLVLYGIAAPDSQRIAFYRLL 260 FSGFIDCGRLGVADRYQD+ALATRDIAEELGGEWADRFLVLYGIAAPDSQRIAFYRLL FSGFIDCGRLGVADRYQDVALATRDIAEELGGEWADRFLVLYGIAAPDSQRIAFYRLL 238 | |

>ABG21301.1 neomycin phosphotransferase, partial [uncultured bacterium] Length=242

Score = 478 bits (1229), Expect = 1e-169, Method: Compositional matrix adjust.

| Identities | = 239/243 (98%), Positives = 240/243 (99%), Gaps = 1/243 (0%) |  |
| --- | --- | --- |
| Query 3 | EQDGLHAGSPAAWVERLFGYDWAQQTIGCSDAAVFRLSAQGRPVLFVKTDLSGALNELQD | 62 |
| Sbjct 1 | EQDGLHAGSPAAWVERLFGYDWAQQTIGCSDAAVFRLSA+GRPVLFVKTDLSGALNELQD EQDGLHAGSPAAWVERLFGYDWAQQTIGCSDAAVFRLSARGRPVLFVKTDLSGALNELQD | 60 |
| Query 63 | EAARLSWLATTGVPCAAVLDVVTEAGRDWLLLGEVPGQDLLSSHLAPAEKVSIMADAMRR | 122 |
| Sbjct 61 | EAARLSWLATTGVPCAAVLDVVTEAGRDWLLLGEVPGQDLLSSHLAPAEKVSIMADAMRR EAARLSWLATTGVPCAAVLDVVTEAGRDWLLLGEVPGQDLLSSHLAPAEKVSIMADAMRR | 120 |
| Query 123 | LHTLDPATCPFDHQAKHRIERARTRMEAGLVDQDDLDEEHQGLAPAELFARLKARMPDGE | 182 |
| Sbjct 121 | LHTLDPATCPFDHQAKHRIERARTRMEAGLVDQDDLDEEHQGLAPA LFA LKARMPDGE LHTLDPATCPFDHQAKHRIERARTRMEAGLVDQDDLDEEHQGLAPAGLFAGLKARMPDGE | 180 |
| Query 183 | DLVVTHGDACLPNIMVENGRFSGFIDCGRLGVADRYQDIALATRDIAEELGGEWADRFLV | 242 |

| Sbjct | 181 | DLVVTHGDACLPNIMVENGRFSGFIDCGRLGVADRYQDIALATRDIAEELGGEWADR LV DLVVTHGDACLPNIMVENGRFSGFIDCGRLGVADRYQDIALATRDIAEELGGEWADR-LV 239 |
| --- | --- | --- |
| Query | 243 | LYG 245 |
| Sbjct | 240 | LYG  LYG 242 |

>AFM74294.1 aminoglycoside-3'-O-phosphotransferase, partial [Riemerella anatipestifer] Length=238

Score = 477 bits (1227), Expect = 3e-169, Method: Compositional matrix adjust.

| Identities | | = 236/238 (99%), Positives = 237/238 (99%), Gaps = 0/238 (0%) |  |
| --- | --- | --- | --- |
| Query 23  Sbjct 1 | | DWAQQTIGCSDAAVFRLSAQGRPVLFVKTDLSGALNELQDEAARLSWLATTGVPCAAVLD DWAQQTIGCSDAAVFRLSAQGRPVLFVKTDLSGALNELQDEAARLSWLATTGVPCAAVLD DWAQQTIGCSDAAVFRLSAQGRPVLFVKTDLSGALNELQDEAARLSWLATTGVPCAAVLD | 82  60 |
| Query 83  Sbjct 61 | | VVTEAGRDWLLLGEVPGQDLLSSHLAPAEKVSIMADAMRRLHTLDPATCPFDHQAKHRIE VVTEAGRDWLLLGEVPGQDLLSSHLAPAEKVSIMADAMRRLHTLDPATCPFDHQAKHRIE VVTEAGRDWLLLGEVPGQDLLSSHLAPAEKVSIMADAMRRLHTLDPATCPFDHQAKHRIE | 142  120 |
| Query 143  Sbjct 121 | | RARTRMEAGLVDQDDLDEEHQGLAPAELFARLKARMPDGEDLVVTHGDACLPNIMVENGR RARTRMEAGLVDQDDLDEEHQGLAPAELFARLKAR PDGEDLVVTHGDACLPNI+VENGR RARTRMEAGLVDQDDLDEEHQGLAPAELFARLKARKPDGEDLVVTHGDACLPNIVVENGR | 202  180 |
| Query Sbjct | 203  181 | FSGFIDCGRLGVADRYQDIALATRDIAEELGGEWADRFLVLYGIAAPDSQRIAFYRLL 260 FSGFIDCGRLGVADRYQDIALATRDIAEELGGEWADRFLVLYGIAAPDSQRIAFYRLL FSGFIDCGRLGVADRYQDIALATRDIAEELGGEWADRFLVLYGIAAPDSQRIAFYRLL 238 | |

>AFM74316.1 aminoglycoside-3'-O-phosphotransferase, partial [Riemerella anatipestifer] Length=238

Score = 476 bits (1226), Expect = 3e-169, Method: Compositional matrix adjust.

| Identities | | = 236/238 (99%), Positives = 237/238 (99%), Gaps = 0/238 (0%) |  |
| --- | --- | --- | --- |
| Query 23  Sbjct 1 | | DWAQQTIGCSDAAVFRLSAQGRPVLFVKTDLSGALNELQDEAARLSWLATTGVPCAAVLD DWAQQTIGCSDAAVFRLSAQGRPVLFVKTDLSGALNELQDEAARLSWLATT VPCAAVLD DWAQQTIGCSDAAVFRLSAQGRPVLFVKTDLSGALNELQDEAARLSWLATTDVPCAAVLD | 82  60 |
| Query 83  Sbjct 61 | | VVTEAGRDWLLLGEVPGQDLLSSHLAPAEKVSIMADAMRRLHTLDPATCPFDHQAKHRIE VVTEAGRDWLLLGEVPGQDLLSSHLAPAEKVSIMADAMR+LHTLDPATCPFDHQAKHRIE VVTEAGRDWLLLGEVPGQDLLSSHLAPAEKVSIMADAMRQLHTLDPATCPFDHQAKHRIE | 142  120 |
| Query 143  Sbjct 121 | | RARTRMEAGLVDQDDLDEEHQGLAPAELFARLKARMPDGEDLVVTHGDACLPNIMVENGR RARTRMEAGLVDQDDLDEEHQGLAPAELFARLKARMPDGEDLVVTHGDACLPNIMVENGR RARTRMEAGLVDQDDLDEEHQGLAPAELFARLKARMPDGEDLVVTHGDACLPNIMVENGR | 202  180 |
| Query Sbjct | 203  181 | FSGFIDCGRLGVADRYQDIALATRDIAEELGGEWADRFLVLYGIAAPDSQRIAFYRLL 260 FSGFIDCGRLGVADRYQDIALATRDIAEELGGEWADRFLVLYGIAAPDSQRIAFYRLL FSGFIDCGRLGVADRYQDIALATRDIAEELGGEWADRFLVLYGIAAPDSQRIAFYRLL 238 | |

>AFM74317.1 aminoglycoside-3'-O-phosphotransferase, partial [Riemerella anatipestifer] AFM74323.1 aminoglycoside-3'-O-phosphotransferase, partial [Riemerella anatipestifer]

Length=238

Score = 476 bits (1225), Expect = 5e-169, Method: Compositional matrix adjust.

| Identities | = 236/238 (99%), Positives = 237/238 (99%), Gaps = 0/238 (0%) |  |
| --- | --- | --- |
| Query 23 | DWAQQTIGCSDAAVFRLSAQGRPVLFVKTDLSGALNELQDEAARLSWLATTGVPCAAVLD | 82 |
| Sbjct 1 | DWAQQTIGCSDAAVFRLSA+GRPVLFVKTDLSGALNE QDEAARLSWLATTGVPCAAVLD DWAQQTIGCSDAAVFRLSARGRPVLFVKTDLSGALNEPQDEAARLSWLATTGVPCAAVLD | 60 |
| Query 83 | VVTEAGRDWLLLGEVPGQDLLSSHLAPAEKVSIMADAMRRLHTLDPATCPFDHQAKHRIE | 142 |
| Sbjct 61 | VVTEAGRDWLLLGEVPGQDLLSSHLAPAEKVSIMADAMRRLHTLDPATCPFDHQAKHRIE VVTEAGRDWLLLGEVPGQDLLSSHLAPAEKVSIMADAMRRLHTLDPATCPFDHQAKHRIE | 120 |
| Query 143 | RARTRMEAGLVDQDDLDEEHQGLAPAELFARLKARMPDGEDLVVTHGDACLPNIMVENGR | 202 |
| Sbjct 121 | RARTRMEAGLVDQDDLDEEHQGLAPAELFARLKARMPDGEDLVVTHGDACLPNIMVENGR  RARTRMEAGLVDQDDLDEEHQGLAPAELFARLKARMPDGEDLVVTHGDACLPNIMVENGR | 180 |

```
Query 203 FSGFIDCGRLGVADRYQDIALATRDIAEELGGEWADRFLVLYGIAAPDSQRIAFYRLL 260 FSGFIDCGRLGVADRYQDIALATRDIAEELGGEWADRFLVLYGIAAPDSQRIAFYRLL
Sbjct 181 FSGFIDCGRLGVADRYQDIALATRDIAEELGGEWADRFLVLYGIAAPDSQRIAFYRLL 238

>AFM74296.1 aminoglycoside-3'-O-phosphotransferase, partial [Riemerella anatipestifer] Length=238

Score = 476 bits (1224), Expect = 6e-169, Method: Compositional matrix adjust. Identities = 236/238 (99%), Positives = 236/238 (99%), Gaps = 0/238 (0%)

Query 23	DWAQQTIGCSDAAVFRLSAQGRPVLFVKTDLSGALNELQDEAARLSWLATTGVPCAAVLD 82 DWAQQTIGCSDAAVFRL AQGRPVLFVKTDLSGALNELQDEAARLSWLATTGVPCAAVLD
Sbjct 1	DWAQQTIGCSDAAVFRLPAQGRPVLFVKTDLSGALNELQDEAARLSWLATTGVPCAAVLD 60

Query 83	VVTEAGRDWLLLGEVPGQDLLSSHLAPAEKVSIMADAMRRLHTLDPATCPFDHQAKHRIE 142 VVTEAGRDWLLLGEVPGQDLLSSHLAPAEKVSIMADAMRRLHTLDPATCPFDHQAKHRIE
Sbjct 61	VVTEAGRDWLLLGEVPGQDLLSSHLAPAEKVSIMADAMRRLHTLDPATCPFDHQAKHRIE 120

Query 143 RARTRMEAGLVDQDDLDEEHQGLAPAELFARLKARMPDGEDLVVTHGDACLPNIMVENGR 202 RARTRMEAGLVDQDDLDEEHQGLAPAELFARLKARMPDGED VVTHGDACLPNIMVENGR
Sbjct 121 RARTRMEAGLVDQDDLDEEHQGLAPAELFARLKARMPDGEDHVVTHGDACLPNIMVENGR 180

Query 203 FSGFIDCGRLGVADRYQDIALATRDIAEELGGEWADRFLVLYGIAAPDSQRIAFYRLL 260 FSGFIDCGRLGVADRYQDIALATRDIAEELGGEWADRFLVLYGIAAPDSQRIAFYRLL
Sbjct 181 FSGFIDCGRLGVADRYQDIALATRDIAEELGGEWADRFLVLYGIAAPDSQRIAFYRLL 238

>AFM74309.1 aminoglycoside-3'-O-phosphotransferase, partial [Riemerella anatipestifer] Length=238

Score = 476 bits (1224), Expect = 6e-169, Method: Compositional matrix adjust. Identities = 236/238 (99%), Positives = 236/238 (99%), Gaps = 0/238 (0%)

Query 23	DWAQQTIGCSDAAVFRLSAQGRPVLFVKTDLSGALNELQDEAARLSWLATTGVPCAAVLD 82 DWAQQTIGCSDAAVFRLSAQGRPVLFVKTDLSGALNELQDEAAR SWLATTGVPCAAVLD
Sbjct 1	DWAQQTIGCSDAAVFRLSAQGRPVLFVKTDLSGALNELQDEAARPSWLATTGVPCAAVLD 60

Query 83	VVTEAGRDWLLLGEVPGQDLLSSHLAPAEKVSIMADAMRRLHTLDPATCPFDHQAKHRIE 142 VVTEAGRDWLLLGEVPGQDLLSSHLAPAEKVSIMADAMRRLHTLDPATCPFDHQAKHRIE
Sbjct 61	VVTEAGRDWLLLGEVPGQDLLSSHLAPAEKVSIMADAMRRLHTLDPATCPFDHQAKHRIE 120

Query 143 RARTRMEAGLVDQDDLDEEHQGLAPAELFARLKARMPDGEDLVVTHGDACLPNIMVENGR 202 RARTRMEAGLVDQDDLDEEHQGLAPAELFARLKARMPDGEDLVVTHGDACLPNIMVENGR
Sbjct 121 RARTRMEAGLVDQDDLDEEHQGLAPAELFARLKARMPDGEDLVVTHGDACLPNIMVENGR 180

Query 203 FSGFIDCGRLGVADRYQDIALATRDIAEELGGEWADRFLVLYGIAAPDSQRIAFYRLL 260 FSGFIDCGRLGVADRYQD ALATRDIAEELGGEWADRFLVLYGIAAPDSQRIAFYRLL
Sbjct 181 FSGFIDCGRLGVADRYQDTALATRDIAEELGGEWADRFLVLYGIAAPDSQRIAFYRLL 238

>AFM74311.1 aminoglycoside-3'-O-phosphotransferase, partial [Riemerella anatipestifer] Length=238

Score = 473 bits (1218), Expect = 5e-168, Method: Compositional matrix adjust. Identities = 235/238 (99%), Positives = 235/238 (99%), Gaps = 0/238 (0%)

Query 23	DWAQQTIGCSDAAVFRLSAQGRPVLFVKTDLSGALNELQDEAARLSWLATTGVPCAAVLD 82 DWAQQTIGCSDAAVFRLSAQGRPVLFVKTDLSGALNELQDEAARLSWLATTGVPCAAVLD
Sbjct 1	DWAQQTIGCSDAAVFRLSAQGRPVLFVKTDLSGALNELQDEAARLSWLATTGVPCAAVLD 60

Query 83	VVTEAGRDWLLLGEVPGQDLLSSHLAPAEKVSIMADAMRRLHTLDPATCPFDHQAKHRIE 142 VVTEAGRDWLLLGEVPGQDL SHLAPAEKVSIMADAMRRLHTLDPATCPFDHQAKHRIE
Sbjct 61	VVTEAGRDWLLLGEVPGQDLRPSHLAPAEKVSIMADAMRRLHTLDPATCPFDHQAKHRIE 120

Query 143 RARTRMEAGLVDQDDLDEEHQGLAPAELFARLKARMPDGEDLVVTHGDACLPNIMVENGR 202 RARTRMEAGLVDQDDLDEEHQGLAPAELFARLKAR PDGEDLVVTHGDACLPNIMVENGR
Sbjct 121 RARTRMEAGLVDQDDLDEEHQGLAPAELFARLKARKPDGEDLVVTHGDACLPNIMVENGR 180

Query 203 FSGFIDCGRLGVADRYQDIALATRDIAEELGGEWADRFLVLYGIAAPDSQRIAFYRLL 260 FSGFIDCGRLGVADRYQDIALATRDIAEELGGEWADRFLVLYGIAAPDSQRIAFYRLL

Sbjct 181 FSGFIDCGRLGVADRYQDIALATRDIAEELGGEWADRFLVLYGIAAPDSQRIAFYRLL 238

>AFM74321.1 aminoglycoside-3'-O-phosphotransferase, partial [Riemerella anatipestifer] Length=238

Score = 473 bits (1216), Expect = 1e-167, Method: Compositional matrix adjust.
```

| Identities | | = 234/238 (98%), Positives = 234/238 (98%), Gaps = 0/238 (0%) |  |
| --- | --- | --- | --- |
| Query 23  Sbjct 1 | | DWAQQTIGCSDAAVFRLSAQGRPVLFVKTDLSGALNELQDEAARLSWLATTGVPCAAVLD DWAQQTIGCSDAAVFRLSAQGRPVLFVKTDLSGA NELQDEAARLSWLATTGVPCAAVLD DWAQQTIGCSDAAVFRLSAQGRPVLFVKTDLSGAPNELQDEAARLSWLATTGVPCAAVLD | 82  60 |
| Query 83  Sbjct 61 | | VVTEAGRDWLLLGEVPGQDLLSSHLAPAEKVSIMADAMRRLHTLDPATCPFDHQAKHRIE VVTEAGRDWLLLGEVPGQDLLSSHLAPAEKVSIM DAMRRLHTLDPATCPFDHQAKHRIE VVTEAGRDWLLLGEVPGQDLLSSHLAPAEKVSIMVDAMRRLHTLDPATCPFDHQAKHRIE | 142  120 |
| Query 143  Sbjct 121 | | RARTRMEAGLVDQDDLDEEHQGLAPAELFARLKARMPDGEDLVVTHGDACLPNIMVENGR RARTRMEAGLVDQDD DEEHQGLAPAELFARLKARMPDGEDLVVTHGDACLPNIMVENGR RARTRMEAGLVDQDDQDEEHQGLAPAELFARLKARMPDGEDLVVTHGDACLPNIMVENGR | 202  180 |
| Query Sbjct | 203  181 | FSGFIDCGRLGVADRYQDIALATRDIAEELGGEWADRFLVLYGIAAPDSQRIAFYRLL 260 FSGFIDCGRLGVADRYQDIALATRDIAEELG EWADRFLVLYGIAAPDSQRIAFYRLL FSGFIDCGRLGVADRYQDIALATRDIAEELGSEWADRFLVLYGIAAPDSQRIAFYRLL 238 | |

>AFM74290.1 aminoglycoside-3'-O-phosphotransferase, partial [Riemerella anatipestifer] Length=238

Score = 472 bits (1214), Expect = 2e-167, Method: Compositional matrix adjust.

| Identities | | = 234/238 (98%), Positives = 235/238 (99%), Gaps = 0/238 (0%) |  |
| --- | --- | --- | --- |
| Query 23  Sbjct 1 | | DWAQQTIGCSDAAVFRLSAQGRPVLFVKTDLSGALNELQDEAARLSWLATTGVPCAAVLD DWAQQTIGCSDAAVFRLSAQGRPVLFVKTDLSGALN LQDEAARLSWLATTGVPCAAVLD DWAQQTIGCSDAAVFRLSAQGRPVLFVKTDLSGALNGLQDEAARLSWLATTGVPCAAVLD | 82  60 |
| Query 83  Sbjct 61 | | VVTEAGRDWLLLGEVPGQDLLSSHLAPAEKVSIMADAMRRLHTLDPATCPFDHQAKHRIE VVTEAGRDWLLLGEVPGQDLLSSHLAPAEKVSIMADAMRR HTLDPATCPFDHQAKHRIE VVTEAGRDWLLLGEVPGQDLLSSHLAPAEKVSIMADAMRRQHTLDPATCPFDHQAKHRIE | 142  120 |
| Query 143  Sbjct 121 | | RARTRMEAGLVDQDDLDEEHQGLAPAELFARLKARMPDGEDLVVTHGDACLPNIMVENGR RARTRMEAGLVDQDDLDEEHQGLAPA+LFARLKARMPDGEDLVVT GDACLPNIMVENGR RARTRMEAGLVDQDDLDEEHQGLAPAKLFARLKARMPDGEDLVVTRGDACLPNIMVENGR | 202  180 |
| Query Sbjct | 203  181 | FSGFIDCGRLGVADRYQDIALATRDIAEELGGEWADRFLVLYGIAAPDSQRIAFYRLL 260 FSGFIDCGRLGVADRYQDIALATRDIAEELGGEWADRFLVLYGIAAPDSQRIAFYRLL FSGFIDCGRLGVADRYQDIALATRDIAEELGGEWADRFLVLYGIAAPDSQRIAFYRLL 238 | |

```
>EDA9786412.1 APH(3')-II family aminoglycoside O-phosphotransferase [Salmonella enterica subsp. enterica serovar Heidelberg]
Length=230

Score = 466 bits (1200), Expect = 2e-165, Method: Compositional matrix adjust. Identities = 230/230 (100%), Positives = 230/230 (100%), Gaps = 0/230 (0%)

Query 1	MIEQDGLHAGSPAAWVERLFGYDWAQQTIGCSDAAVFRLSAQGRPVLFVKTDLSGALNEL 60 MIEQDGLHAGSPAAWVERLFGYDWAQQTIGCSDAAVFRLSAQGRPVLFVKTDLSGALNEL
Sbjct 1	MIEQDGLHAGSPAAWVERLFGYDWAQQTIGCSDAAVFRLSAQGRPVLFVKTDLSGALNEL 60

Query 61	QDEAARLSWLATTGVPCAAVLDVVTEAGRDWLLLGEVPGQDLLSSHLAPAEKVSIMADAM 120 QDEAARLSWLATTGVPCAAVLDVVTEAGRDWLLLGEVPGQDLLSSHLAPAEKVSIMADAM
Sbjct 61	QDEAARLSWLATTGVPCAAVLDVVTEAGRDWLLLGEVPGQDLLSSHLAPAEKVSIMADAM 120

Query 121 RRLHTLDPATCPFDHQAKHRIERARTRMEAGLVDQDDLDEEHQGLAPAELFARLKARMPD 180 RRLHTLDPATCPFDHQAKHRIERARTRMEAGLVDQDDLDEEHQGLAPAELFARLKARMPD
Sbjct 121 RRLHTLDPATCPFDHQAKHRIERARTRMEAGLVDQDDLDEEHQGLAPAELFARLKARMPD 180

Query 181 GEDLVVTHGDACLPNIMVENGRFSGFIDCGRLGVADRYQDIALATRDIAE 230 GEDLVVTHGDACLPNIMVENGRFSGFIDCGRLGVADRYQDIALATRDIAE
Sbjct 181 GEDLVVTHGDACLPNIMVENGRFSGFIDCGRLGVADRYQDIALATRDIAE 230

>WP_072712887.1 APH(3')-II family aminoglycoside O-phosphotransferase, partial [Escherichia coli]
OJR83392.1 APH(3') family aminoglycoside O-phosphotransferase, partial [Escherichia coli]
Length=231

Score = 465 bits (1197), Expect = 7e-165, Method: Compositional matrix adjust. Identities = 231/231 (100%), Positives = 231/231 (100%), Gaps = 0/231 (0%)

Query 34	AAVFRLSAQGRPVLFVKTDLSGALNELQDEAARLSWLATTGVPCAAVLDVVTEAGRDWLL 93 AAVFRLSAQGRPVLFVKTDLSGALNELQDEAARLSWLATTGVPCAAVLDVVTEAGRDWLL
Sbjct 1	AAVFRLSAQGRPVLFVKTDLSGALNELQDEAARLSWLATTGVPCAAVLDVVTEAGRDWLL 60

Query 94	LGEVPGQDLLSSHLAPAEKVSIMADAMRRLHTLDPATCPFDHQAKHRIERARTRMEAGLV 153 LGEVPGQDLLSSHLAPAEKVSIMADAMRRLHTLDPATCPFDHQAKHRIERARTRMEAGLV
Sbjct 61	LGEVPGQDLLSSHLAPAEKVSIMADAMRRLHTLDPATCPFDHQAKHRIERARTRMEAGLV 120

Query 154 DQDDLDEEHQGLAPAELFARLKARMPDGEDLVVTHGDACLPNIMVENGRFSGFIDCGRLG 213 DQDDLDEEHQGLAPAELFARLKARMPDGEDLVVTHGDACLPNIMVENGRFSGFIDCGRLG
Sbjct 121 DQDDLDEEHQGLAPAELFARLKARMPDGEDLVVTHGDACLPNIMVENGRFSGFIDCGRLG 180

Query 214 VADRYQDIALATRDIAEELGGEWADRFLVLYGIAAPDSQRIAFYRLLDEFF 264 VADRYQDIALATRDIAEELGGEWADRFLVLYGIAAPDSQRIAFYRLLDEFF
Sbjct 181 VADRYQDIALATRDIAEELGGEWADRFLVLYGIAAPDSQRIAFYRLLDEFF 231

>WP_039697162.1 APH(3')-II family aminoglycoside O-phosphotransferase, partial [Enterobacter hormaechei]
KHM01029.1 aminoglycoside phosphotransferase, partial [Enterobacter hormaechei subsp. xiangfangensis]
Length=230

Score = 464 bits (1195), Expect = 2e-164, Method: Compositional matrix adjust.
```

| Identities | = 229/230 (99%), Positives = 229/230 (99%), Gaps = 0/230 (0%) |  |
| --- | --- | --- |
| Query 1 | MIEQDGLHAGSPAAWVERLFGYDWAQQTIGCSDAAVFRLSAQGRPVLFVKTDLSGALNEL | 60 |
| Sbjct 1 | MIEQDGLHAGSPAAWVERLFGYDWAQQTIGCSDAAVFRLSAQGRPVLFVKTDLSGALNEL MIEQDGLHAGSPAAWVERLFGYDWAQQTIGCSDAAVFRLSAQGRPVLFVKTDLSGALNEL | 60 |
| Query 61 | QDEAARLSWLATTGVPCAAVLDVVTEAGRDWLLLGEVPGQDLLSSHLAPAEKVSIMADAM | 120 |
| Sbjct 61 | QDEAARLSWLATTGVPCAAVLDVVTEAGRDWLLLGEVPGQDLLSSHLAPAEKVSIMADAM QDEAARLSWLATTGVPCAAVLDVVTEAGRDWLLLGEVPGQDLLSSHLAPAEKVSIMADAM | 120 |
| Query 121 | RRLHTLDPATCPFDHQAKHRIERARTRMEAGLVDQDDLDEEHQGLAPAELFARLKARMPD | 180 |
| Sbjct 121 | RRLHTLDPATCPFDHQAKHRIERARTRMEAGLVDQDDLDEEHQGLAPAELFARLKA MPD RRLHTLDPATCPFDHQAKHRIERARTRMEAGLVDQDDLDEEHQGLAPAELFARLKASMPD | 180 |
| Query 181  Sbjct 181 | GEDLVVTHGDACLPNIMVENGRFSGFIDCGRLGVADRYQDIALATRDIAE 230 GEDLVVTHGDACLPNIMVENGRFSGFIDCGRLGVADRYQDIALATRDIAE GEDLVVTHGDACLPNIMVENGRFSGFIDCGRLGVADRYQDIALATRDIAE 230 |  |

```
>ABO72539.1 neomycin phosphotransferase [Integrative expression vector pFPN] Length=243

Score = 459 bits (1181), Expect = 3e-162, Method: Compositional matrix adjust. Identities = 226/226 (100%), Positives = 226/226 (100%), Gaps = 0/226 (0%)

Query 1	MIEQDGLHAGSPAAWVERLFGYDWAQQTIGCSDAAVFRLSAQGRPVLFVKTDLSGALNEL 60 MIEQDGLHAGSPAAWVERLFGYDWAQQTIGCSDAAVFRLSAQGRPVLFVKTDLSGALNEL
Sbjct 1	MIEQDGLHAGSPAAWVERLFGYDWAQQTIGCSDAAVFRLSAQGRPVLFVKTDLSGALNEL 60

Query 61	QDEAARLSWLATTGVPCAAVLDVVTEAGRDWLLLGEVPGQDLLSSHLAPAEKVSIMADAM 120 QDEAARLSWLATTGVPCAAVLDVVTEAGRDWLLLGEVPGQDLLSSHLAPAEKVSIMADAM
Sbjct 61	QDEAARLSWLATTGVPCAAVLDVVTEAGRDWLLLGEVPGQDLLSSHLAPAEKVSIMADAM 120

Query 121 RRLHTLDPATCPFDHQAKHRIERARTRMEAGLVDQDDLDEEHQGLAPAELFARLKARMPD 180 RRLHTLDPATCPFDHQAKHRIERARTRMEAGLVDQDDLDEEHQGLAPAELFARLKARMPD
Sbjct 121 RRLHTLDPATCPFDHQAKHRIERARTRMEAGLVDQDDLDEEHQGLAPAELFARLKARMPD 180

Query 181 GEDLVVTHGDACLPNIMVENGRFSGFIDCGRLGVADRYQDIALATR 226

GEDLVVTHGDACLPNIMVENGRFSGFIDCGRLGVADRYQDIALATR Sbjct 181 GEDLVVTHGDACLPNIMVENGRFSGFIDCGRLGVADRYQDIALATR 226

>EBB4175231.1 APH(3')-II family aminoglycoside O-phosphotransferase [Salmonella enterica]
EBB4175233.1 APH(3')-II family aminoglycoside O-phosphotransferase [Salmonella enterica]
Length=224

Score = 455 bits (1170), Expect = 7e-161, Method: Compositional matrix adjust. Identities = 224/224 (100%), Positives = 224/224 (100%), Gaps = 0/224 (0%)

Query 1	MIEQDGLHAGSPAAWVERLFGYDWAQQTIGCSDAAVFRLSAQGRPVLFVKTDLSGALNEL 60 MIEQDGLHAGSPAAWVERLFGYDWAQQTIGCSDAAVFRLSAQGRPVLFVKTDLSGALNEL
Sbjct 1	MIEQDGLHAGSPAAWVERLFGYDWAQQTIGCSDAAVFRLSAQGRPVLFVKTDLSGALNEL 60

Query 61	QDEAARLSWLATTGVPCAAVLDVVTEAGRDWLLLGEVPGQDLLSSHLAPAEKVSIMADAM 120 QDEAARLSWLATTGVPCAAVLDVVTEAGRDWLLLGEVPGQDLLSSHLAPAEKVSIMADAM
Sbjct 61	QDEAARLSWLATTGVPCAAVLDVVTEAGRDWLLLGEVPGQDLLSSHLAPAEKVSIMADAM 120

Query 121 RRLHTLDPATCPFDHQAKHRIERARTRMEAGLVDQDDLDEEHQGLAPAELFARLKARMPD 180 RRLHTLDPATCPFDHQAKHRIERARTRMEAGLVDQDDLDEEHQGLAPAELFARLKARMPD
Sbjct 121 RRLHTLDPATCPFDHQAKHRIERARTRMEAGLVDQDDLDEEHQGLAPAELFARLKARMPD 180

Query 181 GEDLVVTHGDACLPNIMVENGRFSGFIDCGRLGVADRYQDIALA 224 GEDLVVTHGDACLPNIMVENGRFSGFIDCGRLGVADRYQDIALA
Sbjct 181 GEDLVVTHGDACLPNIMVENGRFSGFIDCGRLGVADRYQDIALA 224

>EBB4175234.1 APH(3')-II family aminoglycoside O-phosphotransferase [Salmonella enterica]
Length=224

Score = 454 bits (1168), Expect = 1e-160, Method: Compositional matrix adjust.
```

| Identities | = 223/224 (99%), Positives = 224/224 (100%), Gaps = 0/224 (0%) |  |
| --- | --- | --- |
| Query 1 | MIEQDGLHAGSPAAWVERLFGYDWAQQTIGCSDAAVFRLSAQGRPVLFVKTDLSGALNEL | 60 |
| Sbjct 1 | MIEQDGLHAGSPAAWVERLFGYDWAQQTIGCSDAAVFRLSAQGRPVLFVKTDLSGALNEL MIEQDGLHAGSPAAWVERLFGYDWAQQTIGCSDAAVFRLSAQGRPVLFVKTDLSGALNEL | 60 |
| Query 61 | QDEAARLSWLATTGVPCAAVLDVVTEAGRDWLLLGEVPGQDLLSSHLAPAEKVSIMADAM | 120 |
| Sbjct 61 | QDEAARLSWLATTGVPCAAVLDVVTEAGRDWLLLGEVPGQDLLSSHLAPAEKVSIMADAM QDEAARLSWLATTGVPCAAVLDVVTEAGRDWLLLGEVPGQDLLSSHLAPAEKVSIMADAM | 120 |
| Query 121 | RRLHTLDPATCPFDHQAKHRIERARTRMEAGLVDQDDLDEEHQGLAPAELFARLKARMPD | 180 |
| Sbjct 121 | RRLHTLDPATCPFDHQAKHRIERARTRMEAGLVDQDDLDEEHQGLAPAELF+RLKARMPD RRLHTLDPATCPFDHQAKHRIERARTRMEAGLVDQDDLDEEHQGLAPAELFSRLKARMPD | 180 |
| Query 181  Sbjct 181 | GEDLVVTHGDACLPNIMVENGRFSGFIDCGRLGVADRYQDIALA 224 GEDLVVTHGDACLPNIMVENGRFSGFIDCGRLGVADRYQDIALA GEDLVVTHGDACLPNIMVENGRFSGFIDCGRLGVADRYQDIALA 224 |  |

>EBB4175232.1 APH(3')-II family aminoglycoside O-phosphotransferase [Salmonella enterica]

Length=224

Score = 453 bits (1166), Expect = 3e-160, Method: Compositional matrix adjust.

| Identities | = 223/224 (99%), Positives = 223/224 (99%), Gaps = 0/224 (0%) |  |
| --- | --- | --- |
| Query 1 | MIEQDGLHAGSPAAWVERLFGYDWAQQTIGCSDAAVFRLSAQGRPVLFVKTDLSGALNEL | 60 |
| Sbjct 1 | MIEQDGLHAGSPAAWVERLFGYDWAQQTIGCSDAAVFRLSAQGRPVLFVKTDLSGALNEL MIEQDGLHAGSPAAWVERLFGYDWAQQTIGCSDAAVFRLSAQGRPVLFVKTDLSGALNEL | 60 |
| Query 61 | QDEAARLSWLATTGVPCAAVLDVVTEAGRDWLLLGEVPGQDLLSSHLAPAEKVSIMADAM | 120 |
| Sbjct 61 | QDEAARLSWLATTGVPCAAVLDVVTEAGRDWLLLGEVPGQDLLSSHLAPAEKVSIMADAM QDEAARLSWLATTGVPCAAVLDVVTEAGRDWLLLGEVPGQDLLSSHLAPAEKVSIMADAM | 120 |
| Query 121 | RRLHTLDPATCPFDHQAKHRIERARTRMEAGLVDQDDLDEEHQGLAPAELFARLKARMPD | 180 |
| Sbjct 121 | RRLHTLDPATCPFDHQAKHRIERARTRMEAGL DQDDLDEEHQGLAPAELFARLKARMPD  RRLHTLDPATCPFDHQAKHRIERARTRMEAGLADQDDLDEEHQGLAPAELFARLKARMPD | 180 |

```
Query 181 GEDLVVTHGDACLPNIMVENGRFSGFIDCGRLGVADRYQDIALA 224 GEDLVVTHGDACLPNIMVENGRFSGFIDCGRLGVADRYQDIALA
Sbjct 181 GEDLVVTHGDACLPNIMVENGRFSGFIDCGRLGVADRYQDIALA 224

>MHS33331.1 APH(3')-II family aminoglycoside O-phosphotransferase [Escherichia coli]
Length=222

Score = 452 bits (1164), Expect = 6e-160, Method: Compositional matrix adjust. Identities = 222/222 (100%), Positives = 222/222 (100%), Gaps = 0/222 (0%)

Query 1	MIEQDGLHAGSPAAWVERLFGYDWAQQTIGCSDAAVFRLSAQGRPVLFVKTDLSGALNEL 60 MIEQDGLHAGSPAAWVERLFGYDWAQQTIGCSDAAVFRLSAQGRPVLFVKTDLSGALNEL
Sbjct 1	MIEQDGLHAGSPAAWVERLFGYDWAQQTIGCSDAAVFRLSAQGRPVLFVKTDLSGALNEL 60

Query 61	QDEAARLSWLATTGVPCAAVLDVVTEAGRDWLLLGEVPGQDLLSSHLAPAEKVSIMADAM 120 QDEAARLSWLATTGVPCAAVLDVVTEAGRDWLLLGEVPGQDLLSSHLAPAEKVSIMADAM
Sbjct 61	QDEAARLSWLATTGVPCAAVLDVVTEAGRDWLLLGEVPGQDLLSSHLAPAEKVSIMADAM 120

Query 121 RRLHTLDPATCPFDHQAKHRIERARTRMEAGLVDQDDLDEEHQGLAPAELFARLKARMPD 180 RRLHTLDPATCPFDHQAKHRIERARTRMEAGLVDQDDLDEEHQGLAPAELFARLKARMPD
Sbjct 121 RRLHTLDPATCPFDHQAKHRIERARTRMEAGLVDQDDLDEEHQGLAPAELFARLKARMPD 180

Query 181 GEDLVVTHGDACLPNIMVENGRFSGFIDCGRLGVADRYQDIA 222 GEDLVVTHGDACLPNIMVENGRFSGFIDCGRLGVADRYQDIA
Sbjct 181 GEDLVVTHGDACLPNIMVENGRFSGFIDCGRLGVADRYQDIA 222

>WP_109017674.1 APH(3')-II family aminoglycoside O-phosphotransferase, partial [Klebsiella pneumoniae]
Length=222

Score = 451 bits (1159), Expect = 4e-159, Method: Compositional matrix adjust.
```

| Identities | = 221/222 (99%), Positives = 221/222 (99%), Gaps = 0/222 (0%) |  |
| --- | --- | --- |
| Query 1 | MIEQDGLHAGSPAAWVERLFGYDWAQQTIGCSDAAVFRLSAQGRPVLFVKTDLSGALNEL | 60 |
| Sbjct 1 | MIEQDGLHAGSPAAWVERLFGYDWAQQTIGCSDAAVFRLSAQGRPVLFVKTDLSGALNEL MIEQDGLHAGSPAAWVERLFGYDWAQQTIGCSDAAVFRLSAQGRPVLFVKTDLSGALNEL | 60 |
| Query 61 | QDEAARLSWLATTGVPCAAVLDVVTEAGRDWLLLGEVPGQDLLSSHLAPAEKVSIMADAM | 120 |
| Sbjct 61 | QDEAARLSWLATTGVPCAAVLDVVTEAGRDWLLLGEVPGQDLLSSHLAPAEKVSIMADAM QDEAARLSWLATTGVPCAAVLDVVTEAGRDWLLLGEVPGQDLLSSHLAPAEKVSIMADAM | 120 |
| Query 121 | RRLHTLDPATCPFDHQAKHRIERARTRMEAGLVDQDDLDEEHQGLAPAELFARLKARMPD | 180 |
| Sbjct 121 | RRLHTLDPATCPFDHQAKHRIERARTRMEAGLVDQDDLDEEHQGLAPAELFARLKA MPD RRLHTLDPATCPFDHQAKHRIERARTRMEAGLVDQDDLDEEHQGLAPAELFARLKASMPD | 180 |
| Query 181  Sbjct 181 | GEDLVVTHGDACLPNIMVENGRFSGFIDCGRLGVADRYQDIA 222 GEDLVVTHGDACLPNIMVENGRFSGFIDCGRLGVADRYQDIA GEDLVVTHGDACLPNIMVENGRFSGFIDCGRLGVADRYQDIA 222 |  |

```
>MHS33138.1 APH(3')-II family aminoglycoside O-phosphotransferase [Escherichia coli]
Length=215

Score = 437 bits (1125), Expect = 4e-154, Method: Compositional matrix adjust. Identities = 215/215 (100%), Positives = 215/215 (100%), Gaps = 0/215 (0%)

Query 1	MIEQDGLHAGSPAAWVERLFGYDWAQQTIGCSDAAVFRLSAQGRPVLFVKTDLSGALNEL 60 MIEQDGLHAGSPAAWVERLFGYDWAQQTIGCSDAAVFRLSAQGRPVLFVKTDLSGALNEL
Sbjct 1	MIEQDGLHAGSPAAWVERLFGYDWAQQTIGCSDAAVFRLSAQGRPVLFVKTDLSGALNEL 60

Query 61	QDEAARLSWLATTGVPCAAVLDVVTEAGRDWLLLGEVPGQDLLSSHLAPAEKVSIMADAM 120 QDEAARLSWLATTGVPCAAVLDVVTEAGRDWLLLGEVPGQDLLSSHLAPAEKVSIMADAM
Sbjct 61	QDEAARLSWLATTGVPCAAVLDVVTEAGRDWLLLGEVPGQDLLSSHLAPAEKVSIMADAM 120

Query 121 RRLHTLDPATCPFDHQAKHRIERARTRMEAGLVDQDDLDEEHQGLAPAELFARLKARMPD 180 RRLHTLDPATCPFDHQAKHRIERARTRMEAGLVDQDDLDEEHQGLAPAELFARLKARMPD
Sbjct 121 RRLHTLDPATCPFDHQAKHRIERARTRMEAGLVDQDDLDEEHQGLAPAELFARLKARMPD 180

Query 181 GEDLVVTHGDACLPNIMVENGRFSGFIDCGRLGVA 215 GEDLVVTHGDACLPNIMVENGRFSGFIDCGRLGVA
Sbjct 181 GEDLVVTHGDACLPNIMVENGRFSGFIDCGRLGVA 215

>EEC0615971.1 APH(3')-II family aminoglycoside O-phosphotransferase [Salmonella enterica subsp. enterica]
EFC6378195.1 APH(3')-II family aminoglycoside O-phosphotransferase [Escherichia coli]
Length=216

Score = 436 bits (1121), Expect = 1e-153, Method: Compositional matrix adjust. Identities = 216/216 (100%), Positives = 216/216 (100%), Gaps = 0/216 (0%)

Query 49	VKTDLSGALNELQDEAARLSWLATTGVPCAAVLDVVTEAGRDWLLLGEVPGQDLLSSHLA 108 VKTDLSGALNELQDEAARLSWLATTGVPCAAVLDVVTEAGRDWLLLGEVPGQDLLSSHLA
Sbjct 1	VKTDLSGALNELQDEAARLSWLATTGVPCAAVLDVVTEAGRDWLLLGEVPGQDLLSSHLA 60

Query 109 PAEKVSIMADAMRRLHTLDPATCPFDHQAKHRIERARTRMEAGLVDQDDLDEEHQGLAPA 168 PAEKVSIMADAMRRLHTLDPATCPFDHQAKHRIERARTRMEAGLVDQDDLDEEHQGLAPA
Sbjct 61	PAEKVSIMADAMRRLHTLDPATCPFDHQAKHRIERARTRMEAGLVDQDDLDEEHQGLAPA 120

Query 169 ELFARLKARMPDGEDLVVTHGDACLPNIMVENGRFSGFIDCGRLGVADRYQDIALATRDI 228 ELFARLKARMPDGEDLVVTHGDACLPNIMVENGRFSGFIDCGRLGVADRYQDIALATRDI
Sbjct 121 ELFARLKARMPDGEDLVVTHGDACLPNIMVENGRFSGFIDCGRLGVADRYQDIALATRDI 180

Query 229 AEELGGEWADRFLVLYGIAAPDSQRIAFYRLLDEFF 264 AEELGGEWADRFLVLYGIAAPDSQRIAFYRLLDEFF
Sbjct 181 AEELGGEWADRFLVLYGIAAPDSQRIAFYRLLDEFF 216

>EDA9634201.1 APH(3')-II family aminoglycoside O-phosphotransferase [Salmonella enterica subsp. enterica serovar Heidelberg]
EDA9634204.1 APH(3')-II family aminoglycoside O-phosphotransferase [Salmonella enterica subsp. enterica serovar Heidelberg]
Length=215

Score = 435 bits (1118), Expect = 5e-153, Method: Compositional matrix adjust. Identities = 215/215 (100%), Positives = 215/215 (100%), Gaps = 0/215 (0%)

Query 50	KTDLSGALNELQDEAARLSWLATTGVPCAAVLDVVTEAGRDWLLLGEVPGQDLLSSHLAP 109 KTDLSGALNELQDEAARLSWLATTGVPCAAVLDVVTEAGRDWLLLGEVPGQDLLSSHLAP
Sbjct 1	KTDLSGALNELQDEAARLSWLATTGVPCAAVLDVVTEAGRDWLLLGEVPGQDLLSSHLAP 60

Query 110 AEKVSIMADAMRRLHTLDPATCPFDHQAKHRIERARTRMEAGLVDQDDLDEEHQGLAPAE 169 AEKVSIMADAMRRLHTLDPATCPFDHQAKHRIERARTRMEAGLVDQDDLDEEHQGLAPAE
Sbjct 61	AEKVSIMADAMRRLHTLDPATCPFDHQAKHRIERARTRMEAGLVDQDDLDEEHQGLAPAE 120

Query 170 LFARLKARMPDGEDLVVTHGDACLPNIMVENGRFSGFIDCGRLGVADRYQDIALATRDIA 229 LFARLKARMPDGEDLVVTHGDACLPNIMVENGRFSGFIDCGRLGVADRYQDIALATRDIA
Sbjct 121 LFARLKARMPDGEDLVVTHGDACLPNIMVENGRFSGFIDCGRLGVADRYQDIALATRDIA 180

Query 230 EELGGEWADRFLVLYGIAAPDSQRIAFYRLLDEFF 264 EELGGEWADRFLVLYGIAAPDSQRIAFYRLLDEFF
Sbjct 181 EELGGEWADRFLVLYGIAAPDSQRIAFYRLLDEFF 215

>WP_071211688.1 APH(3')-II family aminoglycoside O-phosphotransferase, partial [Acinetobacter baumannii]
OIF85589.1 APH(3') family aminoglycoside O-phosphotransferase, partial [Acinetobacter baumannii]
Length=218

Score = 433 bits (1113), Expect = 2e-152, Method: Compositional matrix adjust. Identities = 215/218 (99%), Positives = 215/218 (99%), Gaps = 0/218 (0%)
```

| Query | 47 | LFVKTDLSGALNELQDEAARLSWLATTGVPCAAVLDVVTEAGRDWLLLGEVPGQDLLSSH | 106 |
| --- | --- | --- | --- |
|  |  | LF K LSGALNELQDEAARLSWLATTGVPCAAVLDVVTEAGRDWLLLGEVPGQDLLSSH |  |
| Sbjct | 1 | LFFKIYLSGALNELQDEAARLSWLATTGVPCAAVLDVVTEAGRDWLLLGEVPGQDLLSSH | 60 |
| Query | 107 | LAPAEKVSIMADAMRRLHTLDPATCPFDHQAKHRIERARTRMEAGLVDQDDLDEEHQGLA | 166 |

| Sbjct | 61 | LAPAEKVSIMADAMRRLHTLDPATCPFDHQAKHRIERARTRMEAGLVDQDDLDEEHQGLA LAPAEKVSIMADAMRRLHTLDPATCPFDHQAKHRIERARTRMEAGLVDQDDLDEEHQGLA | 120 |
| --- | --- | --- | --- |
| Query | 167 | PAELFARLKARMPDGEDLVVTHGDACLPNIMVENGRFSGFIDCGRLGVADRYQDIALATR | 226 |
| Sbjct | 121 | PAELFARLKARMPDGEDLVVTHGDACLPNIMVENGRFSGFIDCGRLGVADRYQDIALATR PAELFARLKARMPDGEDLVVTHGDACLPNIMVENGRFSGFIDCGRLGVADRYQDIALATR | 180 |
| Query  Sbjct | 227  181 | DIAEELGGEWADRFLVLYGIAAPDSQRIAFYRLLDEFF 264 DIAEELGGEWADRFLVLYGIAAPDSQRIAFYRLLDEFF DIAEELGGEWADRFLVLYGIAAPDSQRIAFYRLLDEFF 218 |  |

```
>WP_192957320.1 APH(3')-II family aminoglycoside O-phosphotransferase, partial [Acinetobacter baumannii]
Length=212

Score = 432 bits (1111), Expect = 4e-152, Method: Compositional matrix adjust. Identities = 212/212 (100%), Positives = 212/212 (100%), Gaps = 0/212 (0%)

Query 1	MIEQDGLHAGSPAAWVERLFGYDWAQQTIGCSDAAVFRLSAQGRPVLFVKTDLSGALNEL 60 MIEQDGLHAGSPAAWVERLFGYDWAQQTIGCSDAAVFRLSAQGRPVLFVKTDLSGALNEL
Sbjct 1	MIEQDGLHAGSPAAWVERLFGYDWAQQTIGCSDAAVFRLSAQGRPVLFVKTDLSGALNEL 60

Query 61	QDEAARLSWLATTGVPCAAVLDVVTEAGRDWLLLGEVPGQDLLSSHLAPAEKVSIMADAM 120 QDEAARLSWLATTGVPCAAVLDVVTEAGRDWLLLGEVPGQDLLSSHLAPAEKVSIMADAM
Sbjct 61	QDEAARLSWLATTGVPCAAVLDVVTEAGRDWLLLGEVPGQDLLSSHLAPAEKVSIMADAM 120

Query 121 RRLHTLDPATCPFDHQAKHRIERARTRMEAGLVDQDDLDEEHQGLAPAELFARLKARMPD 180 RRLHTLDPATCPFDHQAKHRIERARTRMEAGLVDQDDLDEEHQGLAPAELFARLKARMPD
Sbjct 121 RRLHTLDPATCPFDHQAKHRIERARTRMEAGLVDQDDLDEEHQGLAPAELFARLKARMPD 180

Query 181 GEDLVVTHGDACLPNIMVENGRFSGFIDCGRL 212 GEDLVVTHGDACLPNIMVENGRFSGFIDCGRL
Sbjct 181 GEDLVVTHGDACLPNIMVENGRFSGFIDCGRL 212

>EEZ8619431.1 APH(3')-II family aminoglycoside O-phosphotransferase [Escherichia coli O160]
HAD8748146.1 APH(3')-II family aminoglycoside O-phosphotransferase [Salmonella enterica]
Length=212

Score = 428 bits (1101), Expect = 1e-150, Method: Compositional matrix adjust. Identities = 212/212 (100%), Positives = 212/212 (100%), Gaps = 0/212 (0%)

Query 53	LSGALNELQDEAARLSWLATTGVPCAAVLDVVTEAGRDWLLLGEVPGQDLLSSHLAPAEK 112 LSGALNELQDEAARLSWLATTGVPCAAVLDVVTEAGRDWLLLGEVPGQDLLSSHLAPAEK
Sbjct 1	LSGALNELQDEAARLSWLATTGVPCAAVLDVVTEAGRDWLLLGEVPGQDLLSSHLAPAEK 60

Query 113 VSIMADAMRRLHTLDPATCPFDHQAKHRIERARTRMEAGLVDQDDLDEEHQGLAPAELFA 172 VSIMADAMRRLHTLDPATCPFDHQAKHRIERARTRMEAGLVDQDDLDEEHQGLAPAELFA
Sbjct 61	VSIMADAMRRLHTLDPATCPFDHQAKHRIERARTRMEAGLVDQDDLDEEHQGLAPAELFA 120

Query 173 RLKARMPDGEDLVVTHGDACLPNIMVENGRFSGFIDCGRLGVADRYQDIALATRDIAEEL 232 RLKARMPDGEDLVVTHGDACLPNIMVENGRFSGFIDCGRLGVADRYQDIALATRDIAEEL
Sbjct 121 RLKARMPDGEDLVVTHGDACLPNIMVENGRFSGFIDCGRLGVADRYQDIALATRDIAEEL 180

Query 233 GGEWADRFLVLYGIAAPDSQRIAFYRLLDEFF 264 GGEWADRFLVLYGIAAPDSQRIAFYRLLDEFF
Sbjct 181 GGEWADRFLVLYGIAAPDSQRIAFYRLLDEFF 212

>ABL86711.1 aminoglycoside phosphotransferase, partial [Escherichia coli] Length=206

Score = 415 bits (1066), Expect = 3e-145, Method: Compositional matrix adjust. Identities = 206/206 (100%), Positives = 206/206 (100%), Gaps = 0/206 (0%)

Query 38	RLSAQGRPVLFVKTDLSGALNELQDEAARLSWLATTGVPCAAVLDVVTEAGRDWLLLGEV 97 RLSAQGRPVLFVKTDLSGALNELQDEAARLSWLATTGVPCAAVLDVVTEAGRDWLLLGEV
Sbjct 1	RLSAQGRPVLFVKTDLSGALNELQDEAARLSWLATTGVPCAAVLDVVTEAGRDWLLLGEV 60
```

| Query | 98 | PGQDLLSSHLAPAEKVSIMADAMRRLHTLDPATCPFDHQAKHRIERARTRMEAGLVDQDD | 157 |
| --- | --- | --- | --- |
| Sbjct | 61 | PGQDLLSSHLAPAEKVSIMADAMRRLHTLDPATCPFDHQAKHRIERARTRMEAGLVDQDD PGQDLLSSHLAPAEKVSIMADAMRRLHTLDPATCPFDHQAKHRIERARTRMEAGLVDQDD | 120 |
| Query | 158 | LDEEHQGLAPAELFARLKARMPDGEDLVVTHGDACLPNIMVENGRFSGFIDCGRLGVADR | 217 |
| Sbjct | 121 | LDEEHQGLAPAELFARLKARMPDGEDLVVTHGDACLPNIMVENGRFSGFIDCGRLGVADR LDEEHQGLAPAELFARLKARMPDGEDLVVTHGDACLPNIMVENGRFSGFIDCGRLGVADR | 180 |
| Query  Sbjct | 218  181 | YQDIALATRDIAEELGGEWADRFLVL 243 YQDIALATRDIAEELGGEWADRFLVL YQDIALATRDIAEELGGEWADRFLVL 206 |  |

>WP_160518435.1 APH(3') family aminoglycoside O-phosphotransferase, partial [Pseudomonas aeruginosa]

MWW62436.1 APH(3') family aminoglycoside O-phosphotransferase [Pseudomonas aeruginosa]

Length=204

Score = 414 bits (1063), Expect = 7e-145, Method: Compositional matrix adjust.

| Identities | = 203/204 (99%), Positives = 204/204 (100%), Gaps = 0/204 (0%) |  |
| --- | --- | --- |
| Query 2 | IEQDGLHAGSPAAWVERLFGYDWAQQTIGCSDAAVFRLSAQGRPVLFVKTDLSGALNELQ | 61 |
| Sbjct 1 | +EQDGLHAGSPAAWVERLFGYDWAQQTIGCSDAAVFRLSAQGRPVLFVKTDLSGALNELQ MEQDGLHAGSPAAWVERLFGYDWAQQTIGCSDAAVFRLSAQGRPVLFVKTDLSGALNELQ | 60 |
| Query 62 | DEAARLSWLATTGVPCAAVLDVVTEAGRDWLLLGEVPGQDLLSSHLAPAEKVSIMADAMR | 121 |
| Sbjct 61 | DEAARLSWLATTGVPCAAVLDVVTEAGRDWLLLGEVPGQDLLSSHLAPAEKVSIMADAMR DEAARLSWLATTGVPCAAVLDVVTEAGRDWLLLGEVPGQDLLSSHLAPAEKVSIMADAMR | 120 |
| Query 122 | RLHTLDPATCPFDHQAKHRIERARTRMEAGLVDQDDLDEEHQGLAPAELFARLKARMPDG | 181 |
| Sbjct 121 | RLHTLDPATCPFDHQAKHRIERARTRMEAGLVDQDDLDEEHQGLAPAELFARLKARMPDG RLHTLDPATCPFDHQAKHRIERARTRMEAGLVDQDDLDEEHQGLAPAELFARLKARMPDG | 180 |
| Query 182  Sbjct 181 | EDLVVTHGDACLPNIMVENGRFSG 205 EDLVVTHGDACLPNIMVENGRFSG EDLVVTHGDACLPNIMVENGRFSG 204 |  |

>HAA3509436.1 APH(3') family aminoglycoside O-phosphotransferase [Listeria monocytogenes]

Length=202

Score = 409 bits (1050), Expect = 5e-143, Method: Compositional matrix adjust.

| Identities | = 201/202 (99%), Positives = 201/202 (99%), Gaps = 0/202 (0%) |  |
| --- | --- | --- |
| Query 1 | MIEQDGLHAGSPAAWVERLFGYDWAQQTIGCSDAAVFRLSAQGRPVLFVKTDLSGALNEL | 60 |
| Sbjct 1 | MIEQDGLHAGSPAAWVERLFGYDWAQQTIGCSDAAVFRLSAQGRPVLFVKTDLSGALNEL MIEQDGLHAGSPAAWVERLFGYDWAQQTIGCSDAAVFRLSAQGRPVLFVKTDLSGALNEL | 60 |
| Query 61 | QDEAARLSWLATTGVPCAAVLDVVTEAGRDWLLLGEVPGQDLLSSHLAPAEKVSIMADAM | 120 |
| Sbjct 61 | QDEAARLSWLATTGVPCAAVLDVVTEAGRDWLLLGEVPGQDLLSSHLAPAEKVSIMADAM QDEAARLSWLATTGVPCAAVLDVVTEAGRDWLLLGEVPGQDLLSSHLAPAEKVSIMADAM | 120 |
| Query 121 | RRLHTLDPATCPFDHQAKHRIERARTRMEAGLVDQDDLDEEHQGLAPAELFARLKARMPD | 180 |
| Sbjct 121 | RRLHTLDPATCPFDHQAKHRIERARTRMEAGLVDQDDLDEEHQGLAPAELFARLKA MPD RRLHTLDPATCPFDHQAKHRIERARTRMEAGLVDQDDLDEEHQGLAPAELFARLKASMPD | 180 |
| Query 181  Sbjct 181 | GEDLVVTHGDACLPNIMVENGR 202 GEDLVVTHGDACLPNIMVENGR GEDLVVTHGDACLPNIMVENGR 202 |  |

```
>CCM44346.1 neomycin phosphotransferase [Cloning vector pSAWloxP-K] Length=220

Score = 405 bits (1042), Expect = 2e-141, Method: Compositional matrix adjust. Identities = 200/203 (99%), Positives = 200/203 (99%), Gaps = 0/203 (0%)

Query 1	MIEQDGLHAGSPAAWVERLFGYDWAQQTIGCSDAAVFRLSAQGRPVLFVKTDLSGALNEL 60 MIEQDGLHAGSPAAWVERLFGYDWAQQTIGCSDAAVFRLSAQGRPVLFVKTDLSGALNEL
Sbjct 1	MIEQDGLHAGSPAAWVERLFGYDWAQQTIGCSDAAVFRLSAQGRPVLFVKTDLSGALNEL 60
```

| Query | 61 | QDEAARLSWLATTGVPCAAVLDVVTEAGRDWLLLGEVPGQDLLSSHLAPAEKVSIMADAM | 120 |
| --- | --- | --- | --- |
| Sbjct | 61 | QDEAARLSWLATTGVPCAAVLDVVTEAGRDWLLLGEVPGQDLLSSHLAPAEKVSIMADAM QDEAARLSWLATTGVPCAAVLDVVTEAGRDWLLLGEVPGQDLLSSHLAPAEKVSIMADAM | 120 |
| Query | 121 | RRLHTLDPATCPFDHQAKHRIERARTRMEAGLVDQDDLDEEHQGLAPAELFARLKARMPD | 180 |
| Sbjct | 121 | RRLHTLDPATCPFDHQAKHRIERARTRMEAGLVDQDDLDEEHQGLAPAELFARLKARMPD RRLHTLDPATCPFDHQAKHRIERARTRMEAGLVDQDDLDEEHQGLAPAELFARLKARMPD | 180 |
| Query  Sbjct | 181  181 | GEDLVVTHGDACLPNIMVENGRF 203 GEDLVVTHGDACLPNIMVE F GEDLVVTHGDACLPNIMVEMAAF 203 |  |

```
>EBB4180054.1 APH(3')-II family aminoglycoside O-phosphotransferase [Salmonella enterica]
Length=194

Score = 394 bits (1012), Expect = 3e-137, Method: Compositional matrix adjust. Identities = 194/194 (100%), Positives = 194/194 (100%), Gaps = 0/194 (0%)

Query 71	ATTGVPCAAVLDVVTEAGRDWLLLGEVPGQDLLSSHLAPAEKVSIMADAMRRLHTLDPAT 130 ATTGVPCAAVLDVVTEAGRDWLLLGEVPGQDLLSSHLAPAEKVSIMADAMRRLHTLDPAT
Sbjct 1	ATTGVPCAAVLDVVTEAGRDWLLLGEVPGQDLLSSHLAPAEKVSIMADAMRRLHTLDPAT 60

Query 131 CPFDHQAKHRIERARTRMEAGLVDQDDLDEEHQGLAPAELFARLKARMPDGEDLVVTHGD 190 CPFDHQAKHRIERARTRMEAGLVDQDDLDEEHQGLAPAELFARLKARMPDGEDLVVTHGD
Sbjct 61	CPFDHQAKHRIERARTRMEAGLVDQDDLDEEHQGLAPAELFARLKARMPDGEDLVVTHGD 120

Query 191 ACLPNIMVENGRFSGFIDCGRLGVADRYQDIALATRDIAEELGGEWADRFLVLYGIAAPD 250 ACLPNIMVENGRFSGFIDCGRLGVADRYQDIALATRDIAEELGGEWADRFLVLYGIAAPD
Sbjct 121 ACLPNIMVENGRFSGFIDCGRLGVADRYQDIALATRDIAEELGGEWADRFLVLYGIAAPD 180

Query 251 SQRIAFYRLLDEFF 264
SQRIAFYRLLDEFF Sbjct 181 SQRIAFYRLLDEFF 194

>WP_107804178.1 APH(3')-II family aminoglycoside O-phosphotransferase [Nitrosomonas oligotropha]
PTQ72046.1 aminoglycoside 3'-phosphotransferase-2 [Nitrosomonas oligotropha] Length=264

Score = 393 bits (1010), Expect = 6e-136, Method: Compositional matrix adjust.
```

| Identities | | = 192/264 (73%), Positives = 213/264 (81%), Gaps = 0/264 (0%) |  |
| --- | --- | --- | --- |
| Query 1  Sbjct 1 | | MIEQDGLHAGSPAAWVERLFGYDWAQQTIGCSDAAVFRLSAQGRPVLFVKTDLSGALNEL M+E+D GSPAAW L GY W QQTIGCS+A VFRL A G+P LFVKT+ + L+EL MLEKDKFTTGSPAAWKVTLAGYRWIQQTIGCSEATVFRLDALGKPTLFVKTEPASPLSEL | 60  60 |
| Query 61  Sbjct 61 | | QDEAARLSWLATTGVPCAAVLDVVTEAGRDWLLLGEVPGQDLLSSHLAPAEKVSIMADAM QDEAARL WLAT G+ CA VLD EAGRDWLLL VPG++LL + L P +KV+IMADA+ QDEAARLRWLATVGLSCAQVLDSANEAGRDWLLLNVVPGENLLLASLDPVDKVTIMADAL | 120  120 |
| Query 121  Sbjct 121 | | RRLHTLDPATCPFDHQAKHRIERARTRMEAGLVDQDDLDEEHQGLAPAELFARLKARMPD RRLH LDP TCPFDH+ +HRIERAR R+EAGLVDQDDLDEEHQGL PA+LFARL+A P RRLHQLDPGTCPFDHRVRHRIERARDRIEAGLVDQDDLDEEHQGLEPAKLFARLRAHKPT | 180  180 |
| Query 181 | | GEDLVVTHGDACLPNIMVENGRFSGFIDCGRLGVADRYQDIALATRDIAEELGGEWADRF EDLVVTHGDACLPNIMVENGRFSGFIDCGRLGVADRYQD+ALATRDIAEELG EW | 240 |
| Sbjct | 181 | TEDLVVTHGDACLPNIMVENGRFSGFIDCGRLGVADRYQDLALATRDIAEELGDEWIKPL | 240 |
| Query  Sbjct | 241  241 | LVLYGIAAPDSQRIAFYRLLDEFF 264 LV YGI D R AFYRLLDEF+ LVQYGINDLDPDRTAFYRLLDEFY 264 |  |

```
>EBT1094324.1 APH(3')-II family aminoglycoside O-phosphotransferase [Salmonella enterica]
Length=192

Score = 390 bits (1003), Expect = 7e-136, Method: Compositional matrix adjust.

Identities = 192/192 (100%), Positives = 192/192 (100%), Gaps = 0/192 (0%)

Query 73	TGVPCAAVLDVVTEAGRDWLLLGEVPGQDLLSSHLAPAEKVSIMADAMRRLHTLDPATCP 132 TGVPCAAVLDVVTEAGRDWLLLGEVPGQDLLSSHLAPAEKVSIMADAMRRLHTLDPATCP
Sbjct 1	TGVPCAAVLDVVTEAGRDWLLLGEVPGQDLLSSHLAPAEKVSIMADAMRRLHTLDPATCP 60

Query 133 FDHQAKHRIERARTRMEAGLVDQDDLDEEHQGLAPAELFARLKARMPDGEDLVVTHGDAC 192 FDHQAKHRIERARTRMEAGLVDQDDLDEEHQGLAPAELFARLKARMPDGEDLVVTHGDAC
Sbjct 61	FDHQAKHRIERARTRMEAGLVDQDDLDEEHQGLAPAELFARLKARMPDGEDLVVTHGDAC 120

Query 193 LPNIMVENGRFSGFIDCGRLGVADRYQDIALATRDIAEELGGEWADRFLVLYGIAAPDSQ 252 LPNIMVENGRFSGFIDCGRLGVADRYQDIALATRDIAEELGGEWADRFLVLYGIAAPDSQ
Sbjct 121 LPNIMVENGRFSGFIDCGRLGVADRYQDIALATRDIAEELGGEWADRFLVLYGIAAPDSQ 180

Query 253 RIAFYRLLDEFF 264
RIAFYRLLDEFF Sbjct 181 RIAFYRLLDEFF 192

>EBK1610741.1 APH(3')-II family aminoglycoside O-phosphotransferase [Salmonella enterica subsp. enterica serovar Heidelberg]
HAJ6816649.1 APH(3')-II family aminoglycoside O-phosphotransferase [Escherichia coli]
Length=191

Score = 388 bits (997), Expect = 5e-135, Method: Compositional matrix adjust. Identities = 191/191 (100%), Positives = 191/191 (100%), Gaps = 0/191 (0%)

Query 74	GVPCAAVLDVVTEAGRDWLLLGEVPGQDLLSSHLAPAEKVSIMADAMRRLHTLDPATCPF 133 GVPCAAVLDVVTEAGRDWLLLGEVPGQDLLSSHLAPAEKVSIMADAMRRLHTLDPATCPF
Sbjct 1	GVPCAAVLDVVTEAGRDWLLLGEVPGQDLLSSHLAPAEKVSIMADAMRRLHTLDPATCPF 60

Query 134 DHQAKHRIERARTRMEAGLVDQDDLDEEHQGLAPAELFARLKARMPDGEDLVVTHGDACL 193 DHQAKHRIERARTRMEAGLVDQDDLDEEHQGLAPAELFARLKARMPDGEDLVVTHGDACL
Sbjct 61	DHQAKHRIERARTRMEAGLVDQDDLDEEHQGLAPAELFARLKARMPDGEDLVVTHGDACL 120

Query 194 PNIMVENGRFSGFIDCGRLGVADRYQDIALATRDIAEELGGEWADRFLVLYGIAAPDSQR 253 PNIMVENGRFSGFIDCGRLGVADRYQDIALATRDIAEELGGEWADRFLVLYGIAAPDSQR
Sbjct 121 PNIMVENGRFSGFIDCGRLGVADRYQDIALATRDIAEELGGEWADRFLVLYGIAAPDSQR 180

Query 254 IAFYRLLDEFF 264
IAFYRLLDEFF Sbjct 181 IAFYRLLDEFF 191

>WP_148712105.1 APH(3')-II family aminoglycoside O-phosphotransferase, partial [Escherichia coli]
TXQ23221.1 APH(3')-II family aminoglycoside O-phosphotransferase, partial [Escherichia coli]
Length=191

Score = 386 bits (992), Expect = 3e-134, Method: Compositional matrix adjust.
```

| Identities | = 190/191 (99%), Positives = 190/191 (99%), Gaps = 0/191 (0%) |  |
| --- | --- | --- |
| Query 74 | GVPCAAVLDVVTEAGRDWLLLGEVPGQDLLSSHLAPAEKVSIMADAMRRLHTLDPATCPF | 133 |
| Sbjct 1 | GVPCAAVLDVVTEAGRDWLLLGEVPGQDLLSSHLAPAEKVSIMADAMRRLHTLDPATCPF GVPCAAVLDVVTEAGRDWLLLGEVPGQDLLSSHLAPAEKVSIMADAMRRLHTLDPATCPF | 60 |
| Query 134 | DHQAKHRIERARTRMEAGLVDQDDLDEEHQGLAPAELFARLKARMPDGEDLVVTHGDACL | 193 |
| Sbjct 61 | DHQAKHRIERARTRMEAGLVDQDDLDEEHQGLAPAELFARLKA MPDGEDLVVTHGDACL DHQAKHRIERARTRMEAGLVDQDDLDEEHQGLAPAELFARLKASMPDGEDLVVTHGDACL | 120 |
| Query 194 | PNIMVENGRFSGFIDCGRLGVADRYQDIALATRDIAEELGGEWADRFLVLYGIAAPDSQR | 253 |
| Sbjct 121 | PNIMVENGRFSGFIDCGRLGVADRYQDIALATRDIAEELGGEWADRFLVLYGIAAPDSQR PNIMVENGRFSGFIDCGRLGVADRYQDIALATRDIAEELGGEWADRFLVLYGIAAPDSQR | 180 |
| Query 254  Sbjct 181 | IAFYRLLDEFF 264 IAFYRLLDEFF IAFYRLLDEFF 191 |  |

>AVX51082.1 Aminoglycoside 3'-phosphotransferase protein [Pseudomonas aeruginosa]

Length=264

Score = 389 bits (998), Expect = 5e-134, Method: Compositional matrix adjust.

| Identities | = 189/264 (72%), Positives = 215/264 (81%), Gaps = 0/264 (0%) |  |
| --- | --- | --- |
| Query 1 | MIEQDGLHAGSPAAWVERLFGYDWAQQTIGCSDAAVFRLSAQGRPVLFVKTDLSGALNEL | 60 |
| Sbjct 1 | M+ + + P W+E+L YDWAQQ GCS+A+VFR++A G+ LFVKT+ G L+EL MVYAECMSVQIPTPWMEKLSAYDWAQQITGCSEASVFRVAAPGKSTLFVKTEPGGPLSEL | 60 |
| Query 61 | QDEAARLSWLATTGVPCAAVLDVVTEAGRDWLLLGEVPGQDLLSSHLAPAEKVSIMADAM | 120 |
| Sbjct 61 | QDEAARL WLATTG+PCA VLD EAGRDWLLL VPG+DLLSS + P EKV+IMAD + QDEAARLRWLATTGLPCAQVLDAAHEAGRDWLLLSAVPGEDLLSSPIDPVEKVTIMADVL | 120 |
| Query 121 | RRLHTLDPATCPFDHQAKHRIERARTRMEAGLVDQDDLDEEHQGLAPAELFARLKARMPD | 180 |
| Sbjct 121 | R+LH LDP TCPFDH+A+HRIERAR RM+AGLVDQDDLD+EHQ L PAELFARL+AR P RQLHGLDPVTCPFDHRAEHRIERARARMQAGLVDQDDLDDEHQELEPAELFARLQARGPA | 180 |
| Query 181 | GEDLVVTHGDACLPNIMVENGRFSGFIDCGRLGVADRYQDIALATRDIAEELGGEWADRF | 240 |
| Sbjct 181 | EDLVVTHGDACLPNIMVENGRFSGFIDCGRLGVADRYQD+ALATRDIAEELG EW F YEDLVVTHGDACLPNIMVENGRFSGFIDCGRLGVADRYQDLALATRDIAEELGDEWVQPF | 240 |
| Query 241  Sbjct 241 | LVLYGIAAPDSQRIAFYRLLDEFF 264 L YGI D R+AFYRLLDEF+ LDRYGIGRLDPDRVAFYRLLDEFY 264 |  |

>WP_000262467.1 MULTISPECIES: APH(3')-II family aminoglycoside O-phosphotransferase [Gammaproteobacteria]

AIE66270.1 aminoglycoside phosphotransferase [Enterobacter cloacae ECNIH2] AIX76121.1 aminoglycoside phosphotransferase [Pantoea sp. PSNIH2]

POU39252.1 APH(3')-II family aminoglycoside O-phosphotransferase [Pantoea sp. PSNIH5]

POU57862.1 APH(3')-II family aminoglycoside O-phosphotransferase [Pantoea sp. PSNIH4]

POY65297.1 APH(3')-II family aminoglycoside O-phosphotransferase [Pantoea sp. PSNIH3]

HAN2782566.1 APH(3')-II family aminoglycoside O-phosphotransferase [Escherichia coli O25b:H4-ST131]

Length=264

Score = 388 bits (997), Expect = 7e-134, Method: Compositional matrix adjust.

| Identities | = 189/264 (72%), Positives = 216/264 (82%), Gaps = 0/264 (0%) |  |
| --- | --- | --- |
| Query 1 | MIEQDGLHAGSPAAWVERLFGYDWAQQTIGCSDAAVFRLSAQGRPVLFVKTDLSGALNEL | 60 |
| Sbjct 1 | M+ + + P W+E+L YDWAQQT GCS+A+VFR +A G+ LFVKT+ G L+EL MVYGECMSVQIPTPWMEKLSAYDWAQQTTGCSEASVFRGAAPGKSTLFVKTEPGGPLSEL | 60 |
| Query 61 | QDEAARLSWLATTGVPCAAVLDVVTEAGRDWLLLGEVPGQDLLSSHLAPAEKVSIMADAM | 120 |
| Sbjct 61 | QDEAARL WLATTG+PCA VLD EAGRDWLLL VPG+DLLSS + P EKV+I+AD + QDEAARLRWLATTGLPCAQVLDAAHEAGRDWLLLSAVPGEDLLSSPIDPVEKVTIIADVL | 120 |
| Query 121 | RRLHTLDPATCPFDHQAKHRIERARTRMEAGLVDQDDLDEEHQGLAPAELFARLKARMPD | 180 |
| Sbjct 121 | R+LH LDP TCPFDH+A+HRIERAR RM+AGLVDQDDLD+EHQGL PAELFARL+AR P RQLHGLDPVTCPFDHRAEHRIERARARMQAGLVDQDDLDDEHQGLEPAELFARLQARGPA | 180 |
| Query 181 | GEDLVVTHGDACLPNIMVENGRFSGFIDCGRLGVADRYQDIALATRDIAEELGGEWADRF | 240 |
| Sbjct 181 | EDLVVTHGDACLPNIMVENGRFSGFIDCGRLGVADRYQD+ALATRD+AEELG EW F YEDLVVTHGDACLPNIMVENGRFSGFIDCGRLGVADRYQDLALATRDVAEELGDEWVQPF | 240 |
| Query 241  Sbjct 241 | LVLYGIAAPDSQRIAFYRLLDEFF 264 L YGI D R+AFYRLLDEF+ LDRYGIGRLDPDRVAFYRLLDEFY 264 |  |

>EBR0922135.1 aminoglycoside 3'-phosphotransferase [Salmonella enterica] Length=189

Score = 384 bits (987), Expect = 1e-133, Method: Compositional matrix adjust. Identities = 189/189 (100%), Positives = 189/189 (100%), Gaps = 0/189 (0%)

Query 76	PCAAVLDVVTEAGRDWLLLGEVPGQDLLSSHLAPAEKVSIMADAMRRLHTLDPATCPFDH 135 PCAAVLDVVTEAGRDWLLLGEVPGQDLLSSHLAPAEKVSIMADAMRRLHTLDPATCPFDH

| Sbjct | 1 | PCAAVLDVVTEAGRDWLLLGEVPGQDLLSSHLAPAEKVSIMADAMRRLHTLDPATCPFDH | 60 |
| --- | --- | --- | --- |
| Query | 136 | QAKHRIERARTRMEAGLVDQDDLDEEHQGLAPAELFARLKARMPDGEDLVVTHGDACLPN | 195 |
| Sbjct | 61 | QAKHRIERARTRMEAGLVDQDDLDEEHQGLAPAELFARLKARMPDGEDLVVTHGDACLPN QAKHRIERARTRMEAGLVDQDDLDEEHQGLAPAELFARLKARMPDGEDLVVTHGDACLPN | 120 |
| Query | 196 | IMVENGRFSGFIDCGRLGVADRYQDIALATRDIAEELGGEWADRFLVLYGIAAPDSQRIA | 255 |
| Sbjct | 121 | IMVENGRFSGFIDCGRLGVADRYQDIALATRDIAEELGGEWADRFLVLYGIAAPDSQRIA IMVENGRFSGFIDCGRLGVADRYQDIALATRDIAEELGGEWADRFLVLYGIAAPDSQRIA | 180 |
| Query  Sbjct | 256  181 | FYRLLDEFF 264 FYRLLDEFF FYRLLDEFF 189 |  |

```
>WP_188246630.1 phosphotransferase, partial [Serratia marcescens] Length=189

Score = 382 bits (981), Expect = 1e-132, Method: Compositional matrix adjust. Identities = 189/189 (100%), Positives = 189/189 (100%), Gaps = 0/189 (0%)

Query 1	MIEQDGLHAGSPAAWVERLFGYDWAQQTIGCSDAAVFRLSAQGRPVLFVKTDLSGALNEL 60 MIEQDGLHAGSPAAWVERLFGYDWAQQTIGCSDAAVFRLSAQGRPVLFVKTDLSGALNEL
Sbjct 1	MIEQDGLHAGSPAAWVERLFGYDWAQQTIGCSDAAVFRLSAQGRPVLFVKTDLSGALNEL 60

Query 61	QDEAARLSWLATTGVPCAAVLDVVTEAGRDWLLLGEVPGQDLLSSHLAPAEKVSIMADAM 120 QDEAARLSWLATTGVPCAAVLDVVTEAGRDWLLLGEVPGQDLLSSHLAPAEKVSIMADAM
Sbjct 61	QDEAARLSWLATTGVPCAAVLDVVTEAGRDWLLLGEVPGQDLLSSHLAPAEKVSIMADAM 120

Query 121 RRLHTLDPATCPFDHQAKHRIERARTRMEAGLVDQDDLDEEHQGLAPAELFARLKARMPD 180 RRLHTLDPATCPFDHQAKHRIERARTRMEAGLVDQDDLDEEHQGLAPAELFARLKARMPD
Sbjct 121 RRLHTLDPATCPFDHQAKHRIERARTRMEAGLVDQDDLDEEHQGLAPAELFARLKARMPD 180

Query 181 GEDLVVTHG 189
GEDLVVTHG Sbjct 181 GEDLVVTHG 189

>WP_039677830.1 aminoglycoside 3'-phosphotransferase, partial [Enterobacter hormaechei]
KHM22697.1 aminoglycoside phosphotransferase, partial [Enterobacter hormaechei subsp. xiangfangensis]
Length=187

Score = 374 bits (961), Expect = 1e-129, Method: Compositional matrix adjust.
```

| Identities | = 185/187 (99%), Positives = 185/187 (99%), Gaps = 0/187 (0%) |  |
| --- | --- | --- |
| Query 58 | NELQDEAARLSWLATTGVPCAAVLDVVTEAGRDWLLLGEVPGQDLLSSHLAPAEKVSIMA | 117 |
| Sbjct 1 | N LQDEAARLSWLATTGVPCAAVLDVVTEAGRDWLLLGEVPGQDLLSSHLAPAEKVSIMA NGLQDEAARLSWLATTGVPCAAVLDVVTEAGRDWLLLGEVPGQDLLSSHLAPAEKVSIMA | 60 |
| Query 118 | DAMRRLHTLDPATCPFDHQAKHRIERARTRMEAGLVDQDDLDEEHQGLAPAELFARLKAR | 177 |
| Sbjct 61 | DAMRRLHTLDPATCPFDHQAKHRIERARTRMEAGLVDQDDLDEEHQGLAPAELFARLKA DAMRRLHTLDPATCPFDHQAKHRIERARTRMEAGLVDQDDLDEEHQGLAPAELFARLKAS | 120 |
| Query 178 | MPDGEDLVVTHGDACLPNIMVENGRFSGFIDCGRLGVADRYQDIALATRDIAEELGGEWA | 237 |
| Sbjct 121 | MPDGEDLVVTHGDACLPNIMVENGRFSGFIDCGRLGVADRYQDIALATRDIAEELGGEWA MPDGEDLVVTHGDACLPNIMVENGRFSGFIDCGRLGVADRYQDIALATRDIAEELGGEWA | 180 |
| Query 238  Sbjct 181 | DRFLVLY 244 DRFLVLY DRFLVLY 187 |  |

>EDT1758707.1 aminoglycoside 3'-phosphotransferase [Salmonella enterica subsp. enterica serovar Enteritidis]

Length=182

Score = 370 bits (951), Expect = 4e-128, Method: Compositional matrix adjust. Identities = 182/182 (100%), Positives = 182/182 (100%), Gaps = 0/182 (0%)

Query 83	VVTEAGRDWLLLGEVPGQDLLSSHLAPAEKVSIMADAMRRLHTLDPATCPFDHQAKHRIE 142 VVTEAGRDWLLLGEVPGQDLLSSHLAPAEKVSIMADAMRRLHTLDPATCPFDHQAKHRIE

| Sbjct | 1 | VVTEAGRDWLLLGEVPGQDLLSSHLAPAEKVSIMADAMRRLHTLDPATCPFDHQAKHRIE | 60 |
| --- | --- | --- | --- |
| Query | 143 | RARTRMEAGLVDQDDLDEEHQGLAPAELFARLKARMPDGEDLVVTHGDACLPNIMVENGR | 202 |
| Sbjct | 61 | RARTRMEAGLVDQDDLDEEHQGLAPAELFARLKARMPDGEDLVVTHGDACLPNIMVENGR RARTRMEAGLVDQDDLDEEHQGLAPAELFARLKARMPDGEDLVVTHGDACLPNIMVENGR | 120 |
| Query | 203 | FSGFIDCGRLGVADRYQDIALATRDIAEELGGEWADRFLVLYGIAAPDSQRIAFYRLLDE | 262 |
| Sbjct | 121 | FSGFIDCGRLGVADRYQDIALATRDIAEELGGEWADRFLVLYGIAAPDSQRIAFYRLLDE FSGFIDCGRLGVADRYQDIALATRDIAEELGGEWADRFLVLYGIAAPDSQRIAFYRLLDE | 180 |
| Query  Sbjct | 263  181 | FF 264 FF  FF 182 |  |

>AEP02953.1 neomycin phosphotransferase, partial [Gene trapping and targeting vector pLTM330]

Length=180

Score = 365 bits (937), Expect = 4e-126, Method: Compositional matrix adjust.

| Identities | = 179/180 (99%), Positives = 180/180 (100%), Gaps = 0/180 (0%) |  |
| --- | --- | --- |
| Query 85 | TEAGRDWLLLGEVPGQDLLSSHLAPAEKVSIMADAMRRLHTLDPATCPFDHQAKHRIERA | 144 |
| Sbjct 1 | TEAGRDWLLLGEVPGQDLLSSHLAPAEKVSIMADAMRRLHTLDPATCPFDHQAKHRIERA TEAGRDWLLLGEVPGQDLLSSHLAPAEKVSIMADAMRRLHTLDPATCPFDHQAKHRIERA | 60 |
| Query 145 | RTRMEAGLVDQDDLDEEHQGLAPAELFARLKARMPDGEDLVVTHGDACLPNIMVENGRFS | 204 |
| Sbjct 61 | RTRMEAGLVDQDDLDEEHQGLAPAELFARLKARMPDG+DLVVTHGDACLPNIMVENGRFS RTRMEAGLVDQDDLDEEHQGLAPAELFARLKARMPDGDDLVVTHGDACLPNIMVENGRFS | 120 |
| Query 205 | GFIDCGRLGVADRYQDIALATRDIAEELGGEWADRFLVLYGIAAPDSQRIAFYRLLDEFF | 264 |
| Sbjct 121 | GFIDCGRLGVADRYQDIALATRDIAEELGGEWADRFLVLYGIAAPDSQRIAFYRLLDEFF  GFIDCGRLGVADRYQDIALATRDIAEELGGEWADRFLVLYGIAAPDSQRIAFYRLLDEFF | 180 |

>EEM0239916.1 aminoglycoside 3'-phosphotransferase [Salmonella enterica subsp. enterica serovar Typhimurium]

Length=179

Score = 362 bits (929), Expect = 7e-125, Method: Compositional matrix adjust.

| Identities | | = 178/179 (99%), Positives = 178/179 (99%), Gaps = 0/179 (0%) |  |
| --- | --- | --- | --- |
| Query 86  Sbjct 1 | | EAGRDWLLLGEVPGQDLLSSHLAPAEKVSIMADAMRRLHTLDPATCPFDHQAKHRIERAR EAGRDWLLLGEVPGQDLLSSHLAPAEKVSIMADAMRRLHTLDPATCPFDHQAKHRIERAR EAGRDWLLLGEVPGQDLLSSHLAPAEKVSIMADAMRRLHTLDPATCPFDHQAKHRIERAR | 145  60 |
| Query 146  Sbjct 61 | | TRMEAGLVDQDDLDEEHQGLAPAELFARLKARMPDGEDLVVTHGDACLPNIMVENGRFSG TRMEAGLVDQDDLDEEHQGLAPAELFARLKARMPDGEDLVVTHGDACLPNIMVENGRFSG TRMEAGLVDQDDLDEEHQGLAPAELFARLKARMPDGEDLVVTHGDACLPNIMVENGRFSG | 205  120 |
| Query Sbjct | 206  121 | FIDCGRLGVADRYQDIALATRDIAEELGGEWADRFLVLYGIAAPDSQRIAFYRLLDEFF 264 FIDCGRLGVAD YQDIALATRDIAEELGGEWADRFLVLYGIAAPDSQRIAFYRLLDEFF FIDCGRLGVADHYQDIALATRDIAEELGGEWADRFLVLYGIAAPDSQRIAFYRLLDEFF 179 | |

>WP_071218897.1 aminoglycoside 3'-phosphotransferase, partial [Acinetobacter baumannii]

OIB77404.1 APH(3') family aminoglycoside O-phosphotransferase, partial [Acinetobacter baumannii]

Length=177

Score = 361 bits (926), Expect = 2e-124, Method: Compositional matrix adjust. Identities = 177/177 (100%), Positives = 177/177 (100%), Gaps = 0/177 (0%)

| Query | 88 | GRDWLLLGEVPGQDLLSSHLAPAEKVSIMADAMRRLHTLDPATCPFDHQAKHRIERARTR | 147 |
| --- | --- | --- | --- |
| Sbjct | 1 | GRDWLLLGEVPGQDLLSSHLAPAEKVSIMADAMRRLHTLDPATCPFDHQAKHRIERARTR GRDWLLLGEVPGQDLLSSHLAPAEKVSIMADAMRRLHTLDPATCPFDHQAKHRIERARTR | 60 |
| Query | 148 | MEAGLVDQDDLDEEHQGLAPAELFARLKARMPDGEDLVVTHGDACLPNIMVENGRFSGFI | 207 |
| Sbjct | 61 | MEAGLVDQDDLDEEHQGLAPAELFARLKARMPDGEDLVVTHGDACLPNIMVENGRFSGFI  MEAGLVDQDDLDEEHQGLAPAELFARLKARMPDGEDLVVTHGDACLPNIMVENGRFSGFI | 120 |

Query 208 DCGRLGVADRYQDIALATRDIAEELGGEWADRFLVLYGIAAPDSQRIAFYRLLDEFF 264 DCGRLGVADRYQDIALATRDIAEELGGEWADRFLVLYGIAAPDSQRIAFYRLLDEFF

Sbjct 121 DCGRLGVADRYQDIALATRDIAEELGGEWADRFLVLYGIAAPDSQRIAFYRLLDEFF 177

>WP_173190126.1 APH(3')-II family aminoglycoside O-phosphotransferase [Mesorhizobium sediminum]

NRC54315.1 APH(3')-II family aminoglycoside O-phosphotransferase [Mesorhizobium sediminum]

NRC56983.1 APH(3')-II family aminoglycoside O-phosphotransferase [Mesorhizobium sediminum]

Length=264

Score = 364 bits (934), Expect = 3e-124, Method: Compositional matrix adjust.

| Identities | = 176/264 (67%), Positives = 212/264 (80%), Gaps = 0/264 (0%) |  |
| --- | --- | --- |
| Query 1 | MIEQDGLHAGSPAAWVERLFGYDWAQQTIGCSDAAVFRLSAQGRPVLFVKTDLSGALNEL | 60 |
| Sbjct 1 | M+E++ + +P AW E+L Y W QQT+G S+AAVFR+ A GR VLFVKT+ +G L+EL MLEEERVLTQAPMAWKEKLASYTWTQQTVGGSEAAVFRVEAPGRRVLFVKTEPAGLLSEL | 60 |
| Query 61 | QDEAARLSWLATTGVPCAAVLDVVTEAGRDWLLLGEVPGQDLLSSHLAPAEKVSIMADAM | 120 |
| Sbjct 61 | Q+EAARL WL+TTG+PCA VL V + RDWLLL VPG++LLS+ + EKV++MA + QEEAARLRWLSTTGLPCAQVLAEVHQQERDWLLLSAVPGENLLSASMGAEEKVAMMAAVL | 120 |
| Query 121 | RRLHTLDPATCPFDHQAKHRIERARTRMEAGLVDQDDLDEEHQGLAPAELFARLKARMPD | 180 |
| Sbjct 121 | R+LH LDP TCPFDH+A+HRIE AR RMEAGLVDQDDLD+EHQGL P ELFARL+AR P RQLHQLDPTTCPFDHRAEHRIELARARMEAGLVDQDDLDDEHQGLGPTELFARLQARKPL | 180 |
| Query 181 | GEDLVVTHGDACLPNIMVENGRFSGFIDCGRLGVADRYQDIALATRDIAEELGGEWADRF | 240 |
| Sbjct 181 | E+LVVTHGDACLPN+MVE+GRFSG IDCGRLG+ADRYQD+AL TRD+AEELG EW F RENLVVTHGDACLPNVMVESGRFSGVIDCGRLGLADRYQDLALTTRDVAEELGREWVKPF | 240 |
| Query 241  Sbjct 241 | LVLYGIAAPDSQRIAFYRLLDEFF 264 L YGI+ DS R AFYRLLDEF+ LGHYGISDFDSDRAAFYRLLDEFY 264 |  |

```
>ANF07102.1 NPTII, partial [chloroplast transformation vector pPRV111A::neoSTLS2] [Nicotiana tabacum]
Length=186

Score = 353 bits (905), Expect = 5e-121, Method: Compositional matrix adjust. Identities = 175/175 (100%), Positives = 175/175 (100%), Gaps = 0/175 (0%)

Query 2	IEQDGLHAGSPAAWVERLFGYDWAQQTIGCSDAAVFRLSAQGRPVLFVKTDLSGALNELQ 61 IEQDGLHAGSPAAWVERLFGYDWAQQTIGCSDAAVFRLSAQGRPVLFVKTDLSGALNELQ
Sbjct 12	IEQDGLHAGSPAAWVERLFGYDWAQQTIGCSDAAVFRLSAQGRPVLFVKTDLSGALNELQ 71

Query 62	DEAARLSWLATTGVPCAAVLDVVTEAGRDWLLLGEVPGQDLLSSHLAPAEKVSIMADAMR 121 DEAARLSWLATTGVPCAAVLDVVTEAGRDWLLLGEVPGQDLLSSHLAPAEKVSIMADAMR
Sbjct 72	DEAARLSWLATTGVPCAAVLDVVTEAGRDWLLLGEVPGQDLLSSHLAPAEKVSIMADAMR 131

Query 122 RLHTLDPATCPFDHQAKHRIERARTRMEAGLVDQDDLDEEHQGLAPAELFARLKA 176 RLHTLDPATCPFDHQAKHRIERARTRMEAGLVDQDDLDEEHQGLAPAELFARLKA
Sbjct 132 RLHTLDPATCPFDHQAKHRIERARTRMEAGLVDQDDLDEEHQGLAPAELFARLKA 186

>WP_031613531.1 aminoglycoside phosphotransferase APH(3'), partial [Salmonella enterica]
EFH5635805.1 aminoglycoside phosphotransferase APH(3') [Escherichia coli]
EYI77040.1 aminoglycoside phosphotransferase, partial [Salmonella enterica subsp. enterica serovar Heidelberg str. CVM24359]
Length=174

Score = 351 bits (901), Expect = 1e-120, Method: Compositional matrix adjust. Identities = 174/174 (100%), Positives = 174/174 (100%), Gaps = 0/174 (0%)
```

| Query | 1 | MIEQDGLHAGSPAAWVERLFGYDWAQQTIGCSDAAVFRLSAQGRPVLFVKTDLSGALNEL | 60 |
| --- | --- | --- | --- |
|  |  | MIEQDGLHAGSPAAWVERLFGYDWAQQTIGCSDAAVFRLSAQGRPVLFVKTDLSGALNEL |  |
| Sbjct | 1 | MIEQDGLHAGSPAAWVERLFGYDWAQQTIGCSDAAVFRLSAQGRPVLFVKTDLSGALNEL | 60 |
| Query | 61 | QDEAARLSWLATTGVPCAAVLDVVTEAGRDWLLLGEVPGQDLLSSHLAPAEKVSIMADAM | 120 |

| Sbjct | 61 | QDEAARLSWLATTGVPCAAVLDVVTEAGRDWLLLGEVPGQDLLSSHLAPAEKVSIMADAM QDEAARLSWLATTGVPCAAVLDVVTEAGRDWLLLGEVPGQDLLSSHLAPAEKVSIMADAM 120 |
| --- | --- | --- |
| Query | 121 | RRLHTLDPATCPFDHQAKHRIERARTRMEAGLVDQDDLDEEHQGLAPAELFARL 174 |
| Sbjct | 121 | RRLHTLDPATCPFDHQAKHRIERARTRMEAGLVDQDDLDEEHQGLAPAELFARL  RRLHTLDPATCPFDHQAKHRIERARTRMEAGLVDQDDLDEEHQGLAPAELFARL 174 |

>WP_187588653.1 phosphotransferase, partial [Arenibacter sp. BSSL-BM3] MBC8770654.1 phosphotransferase [Arenibacter sp. BSSL-BM3]

Length=177

Score = 351 bits (900), Expect = 2e-120, Method: Compositional matrix adjust.

| Identities | = 174/176 (99%), Positives = 175/176 (99%), Gaps = 0/176 (0%) |  |
| --- | --- | --- |
| Query 1 | MIEQDGLHAGSPAAWVERLFGYDWAQQTIGCSDAAVFRLSAQGRPVLFVKTDLSGALNEL | 60 |
| Sbjct 1 | MIEQDGLHAGSPAAWVERLFGYDWAQQTIGCSDAAVFRLSAQGRPVLFVKTDLSGALNEL MIEQDGLHAGSPAAWVERLFGYDWAQQTIGCSDAAVFRLSAQGRPVLFVKTDLSGALNEL | 60 |
| Query 61 | QDEAARLSWLATTGVPCAAVLDVVTEAGRDWLLLGEVPGQDLLSSHLAPAEKVSIMADAM | 120 |
| Sbjct 61 | QDEAARLSWLATTGVPCAAVLDVVTEAGRDWLLLGEVPGQDLLSSHLAPAEKVSIMADAM QDEAARLSWLATTGVPCAAVLDVVTEAGRDWLLLGEVPGQDLLSSHLAPAEKVSIMADAM | 120 |
| Query 121  Sbjct 121 | RRLHTLDPATCPFDHQAKHRIERARTRMEAGLVDQDDLDEEHQGLAPAELFARLKA 176 RRLHTLDPATCPFDHQAKHRIERARTRMEAGLVDQDDLDEEHQGLAPAELFAR +A RRLHTLDPATCPFDHQAKHRIERARTRMEAGLVDQDDLDEEHQGLAPAELFARPQA 176 |  |

```
>WP_033471608.1 aminoglycoside 3'-phosphotransferase, partial [Bordetella bronchiseptica] EFB5119115.1 aminoglycoside 3'-phosphotransferase [Escherichia coli]
EFB5119118.1 aminoglycoside 3'-phosphotransferase [Escherichia coli] KAK79461.1 putative kanamycin kinase, partial [Bordetella bronchiseptica
CA90 BB02]
Length=172

Score = 350 bits (899), Expect = 2e-120, Method: Compositional matrix adjust. Identities = 172/172 (100%), Positives = 172/172 (100%), Gaps = 0/172 (0%)

Query 93	LLGEVPGQDLLSSHLAPAEKVSIMADAMRRLHTLDPATCPFDHQAKHRIERARTRMEAGL 152 LLGEVPGQDLLSSHLAPAEKVSIMADAMRRLHTLDPATCPFDHQAKHRIERARTRMEAGL
Sbjct 1	LLGEVPGQDLLSSHLAPAEKVSIMADAMRRLHTLDPATCPFDHQAKHRIERARTRMEAGL 60

Query 153 VDQDDLDEEHQGLAPAELFARLKARMPDGEDLVVTHGDACLPNIMVENGRFSGFIDCGRL 212 VDQDDLDEEHQGLAPAELFARLKARMPDGEDLVVTHGDACLPNIMVENGRFSGFIDCGRL
Sbjct 61	VDQDDLDEEHQGLAPAELFARLKARMPDGEDLVVTHGDACLPNIMVENGRFSGFIDCGRL 120

Query 213 GVADRYQDIALATRDIAEELGGEWADRFLVLYGIAAPDSQRIAFYRLLDEFF 264 GVADRYQDIALATRDIAEELGGEWADRFLVLYGIAAPDSQRIAFYRLLDEFF
Sbjct 121 GVADRYQDIALATRDIAEELGGEWADRFLVLYGIAAPDSQRIAFYRLLDEFF 172

>EPJ91417.1 aminoglycoside phosphotransferase [Pseudomonas sp. CFII68] SEE81366.1 aminoglycoside 3'-phosphotransferase-2 [Pseudomonas kilonensis]
Length=273

Score = 354 bits (908), Expect = 3e-120, Method: Compositional matrix adjust.
```

| Identities | = 173/256 (68%), Positives = 200/256 (78%), Gaps = 0/256 (0%) |  |
| --- | --- | --- |
| Query 9 | AGSPAAWVERLFGYDWAQQTIGCSDAAVFRLSAQGRPVLFVKTDLSGALNELQDEAARLS | 68 |
| Sbjct 18 | A P+ W ER + W T+GCSDAAVFRL A G P LFVKT+ +GAL+EL+DEAARL AKLPSGWRERFKHHSWRPMTVGCSDAAVFRLDAPGAPALFVKTEPAGALSELRDEAARLR | 77 |
| Query 69 | WLATTGVPCAAVLDVVTEAGRDWLLLGEVPGQDLLSSHLAPAEKVSIMADAMRRLHTLDP | 128 |
| Sbjct 78 | WLA+TG PCA VLD E RDWLLL VPG DLLS+ + A KV++MADA+R+LH LDP WLASTGTPCAHVLDEAHETDRDWLLLSAVPGVDLLSAPIDEAAKVNVMADALRQLHQLDP | 137 |
| Query 129 | ATCPFDHQAKHRIERARTRMEAGLVDQDDLDEEHQGLAPAELFARLKARMPDGEDLVVTH | 188 |
| Sbjct 138 | A CPFDH A+HRI +AR RMEAGLVDQ++LDEEHQGLAPA LF +L R P +DLVVTH ADCPFDHSARHRIAKARARMEAGLVDQEELDEEHQGLAPALLFEKLLNRQPAEQDLVVTH | 197 |
| Query 189 | GDACLPNIMVENGRFSGFIDCGRLGVADRYQDIALATRDIAEELGGEWADRFLVLYGIAA GDAC+PNI+V+NGRF+GFIDCGRLGVADRYQD+ALATRDIAEELG +W FL Y + | 248 |

| Sbjct | 198 | GDACMPNIIVDNGRFTGFIDCGRLGVADRYQDLALATRDIAEELGEQWVAPFLRRYAVLE 257 |
| --- | --- | --- |
| Query | 249 | PDSQRIAFYRLLDEFF 264 |
| Sbjct | 258 | D RI FYRLLDEFF  ADQDRIYFYRLLDEFF 273 |

>WP_033062581.1 MULTISPECIES: APH(3')-II family aminoglycoside O-phosphotransferase [Pseudomonas]

Length=258

Score = 352 bits (904), Expect = 7e-120, Method: Compositional matrix adjust.

| Identities | | = 173/256 (68%), Positives = 200/256 (78%), Gaps = 0/256 (0%) |  |
| --- | --- | --- | --- |
| Query 9  Sbjct 3 | | AGSPAAWVERLFGYDWAQQTIGCSDAAVFRLSAQGRPVLFVKTDLSGALNELQDEAARLS A P+ W ER + W T+GCSDAAVFRL A G P LFVKT+ +GAL+EL+DEAARL AKLPSGWRERFKHHSWRPMTVGCSDAAVFRLDAPGAPALFVKTEPAGALSELRDEAARLR | 68  62 |
| Query 69  Sbjct 63 | | WLATTGVPCAAVLDVVTEAGRDWLLLGEVPGQDLLSSHLAPAEKVSIMADAMRRLHTLDP WLA+TG PCA VLD E RDWLLL VPG DLLS+ + A KV++MADA+R+LH LDP WLASTGTPCAHVLDEAHETDRDWLLLSAVPGVDLLSAPIDEAAKVNVMADALRQLHQLDP | 128  122 |
| Query 129  Sbjct 123 | | ATCPFDHQAKHRIERARTRMEAGLVDQDDLDEEHQGLAPAELFARLKARMPDGEDLVVTH A CPFDH A+HRI +AR RMEAGLVDQ++LDEEHQGLAPA LF +L R P +DLVVTH ADCPFDHSARHRIAKARARMEAGLVDQEELDEEHQGLAPALLFEKLLNRQPAEQDLVVTH | 188  182 |
| Query 189  Sbjct 183 | | GDACLPNIMVENGRFSGFIDCGRLGVADRYQDIALATRDIAEELGGEWADRFLVLYGIAA GDAC+PNI+V+NGRF+GFIDCGRLGVADRYQD+ALATRDIAEELG +W FL Y + GDACMPNIIVDNGRFTGFIDCGRLGVADRYQDLALATRDIAEELGEQWVAPFLRRYAVLE | 248  242 |
| Query 249 | | PDSQRIAFYRLLDEFF 264 D RI FYRLLDEFF |  |
| Sbjct | 243 | ADQDRIYFYRLLDEFF 258 | |

```
>EEX3345874.1 aminoglycoside 3'-phosphotransferase [Escherichia coli] Length=171

Score = 349 bits (896), Expect = 7e-120, Method: Compositional matrix adjust. Identities = 171/171 (100%), Positives = 171/171 (100%), Gaps = 0/171 (0%)

Query 94	LGEVPGQDLLSSHLAPAEKVSIMADAMRRLHTLDPATCPFDHQAKHRIERARTRMEAGLV 153 LGEVPGQDLLSSHLAPAEKVSIMADAMRRLHTLDPATCPFDHQAKHRIERARTRMEAGLV
Sbjct 1	LGEVPGQDLLSSHLAPAEKVSIMADAMRRLHTLDPATCPFDHQAKHRIERARTRMEAGLV 60

Query 154 DQDDLDEEHQGLAPAELFARLKARMPDGEDLVVTHGDACLPNIMVENGRFSGFIDCGRLG 213 DQDDLDEEHQGLAPAELFARLKARMPDGEDLVVTHGDACLPNIMVENGRFSGFIDCGRLG
Sbjct 61	DQDDLDEEHQGLAPAELFARLKARMPDGEDLVVTHGDACLPNIMVENGRFSGFIDCGRLG 120

Query 214 VADRYQDIALATRDIAEELGGEWADRFLVLYGIAAPDSQRIAFYRLLDEFF 264 VADRYQDIALATRDIAEELGGEWADRFLVLYGIAAPDSQRIAFYRLLDEFF
Sbjct 121 VADRYQDIALATRDIAEELGGEWADRFLVLYGIAAPDSQRIAFYRLLDEFF 171

>WP_006239673.1 APH(3')-II family aminoglycoside O-phosphotransferase [Saccharomonospora xinjiangensis]
EID55500.1 aminoglycoside phosphotransferase [Saccharomonospora xinjiangensis XJ-54]
Length=263

Score = 353 bits (905), Expect = 7e-120, Method: Compositional matrix adjust. Identities = 170/253 (67%), Positives = 203/253 (80%), Gaps = 0/253 (0%)
```

| Query | 12 | PAAWVERLFGYDWAQQTIGCSDAAVFRLSAQGRPVLFVKTDLSGALNELQDEAARLSWLA | 71 |
| --- | --- | --- | --- |
| Sbjct | 11 | P AW ERL Y+W QTIGCS+AAV+RL ++G+P LF+KT+ +G L+EL EAARL WL+ PKAWKERLTSYEWVLQTIGCSEAAVYRLDSEGKPSLFIKTEPAGPLSELPGEAARLRWLS | 70 |
| Query | 72 | TTGVPCAAVLDVVTEAGRDWLLLGEVPGQDLLSSHLAPAEKVSIMADAMRRLHTLDPATC | 131 |
| Sbjct | 71 | T VPCA VLD V EAG+ WLLL VPG+DLLS+ + PA KV+I+A A+ LH LDP TC MTEVPCADVLDEVREAGQHWLLLSAVPGRDLLSASIDPASKVTILAQALHHLHRLDPTTC | 130 |
| Query | 132 | PFDHQAKHRIERARTRMEAGLVDQDDLDEEHQGLAPAELFARLKARMPDGEDLVVTHGDA | 191 |

PFDH+A+HR+E+AR R++AGLVDQDDLDEE+Q +	+LF L+AR P EDLVVTHGDA

| Sbjct | 131 | PFDHRAEHRVEQARARLDAGLVDQDDLDEENQNVELDDLFTELRARKPRREDLVVTHGDA | 190 |
| --- | --- | --- | --- |
| Query | 192 | CLPNIMVENGRFSGFIDCGRLGVADRYQDIALATRDIAEELGGEWADRFLVLYGIAAPDS | 251 |
| Sbjct | 191 | CLPNIMVE GRFSGFIDCGRLG+ADRYQD+ALATRDIAEELG +W FL YGI D CLPNIMVEKGRFSGFIDCGRLGLADRYQDVALATRDIAEELGEKWVPLFLDQYGIPELDG | 250 |
| Query  Sbjct | 252  251 | QRIAFYRLLDEFF 264  +RIAFYRLLDEF+  ERIAFYRLLDEFY 263 |  |

>WP_139353145.1 APH(3')-II family aminoglycoside O-phosphotransferase [Pseudomonas sp. A25(2017)]

Length=273

Score = 351 bits (900), Expect = 6e-119, Method: Compositional matrix adjust.

| Identities | | = 171/256 (67%), Positives = 198/256 (77%), Gaps = 0/256 (0%) |  |
| --- | --- | --- | --- |
| Query 9  Sbjct 18 | | AGSPAAWVERLFGYDWAQQTIGCSDAAVFRLSAQGRPVLFVKTDLSGALNELQDEAARLS A P+ W ER + W +GCSDAAVFRL A G P LFVKT+ +G L+EL+DEAARL AKLPSGWRERFKHHSWRPMAVGCSDAAVFRLDAPGSPALFVKTEPAGVLSELRDEAARLR | 68  77 |
| Query 69  Sbjct 78 | | WLATTGVPCAAVLDVVTEAGRDWLLLGEVPGQDLLSSHLAPAEKVSIMADAMRRLHTLDP WLA+TG PCA VLD E RDWLLL VPG DLLS+ + A KV++MADA+R+LH LDP WLASTGTPCAHVLDEAHETDRDWLLLSAVPGVDLLSAPIDEAAKVNVMADALRQLHQLDP | 128  137 |
| Query 129  Sbjct 138 | | ATCPFDHQAKHRIERARTRMEAGLVDQDDLDEEHQGLAPAELFARLKARMPDGEDLVVTH A CPFDH A+HRI +AR RMEAGLVDQ++LDEEHQGLAPA LF +L R P +DLVVTH ADCPFDHSARHRIAKARARMEAGLVDQEELDEEHQGLAPALLFEKLLNRQPAEQDLVVTH | 188  197 |
| Query 189  Sbjct 198 | | GDACLPNIMVENGRFSGFIDCGRLGVADRYQDIALATRDIAEELGGEWADRFLVLYGIAA GDAC+PNI+V+NGRF+GFIDCGRLGVADRYQD+ALATRDIAEELG +W FL Y + GDACMPNIIVDNGRFTGFIDCGRLGVADRYQDLALATRDIAEELGEQWVAPFLRRYAVLE | 248  257 |
| Query 249 | | PDSQRIAFYRLLDEFF 264 D RI FYRLLDEFF |  |
| Sbjct | 258 | ADQDRIYFYRLLDEFF 273 | |

```
>QBB73052.1 aminoglycoside resistance protein, partial [Streptococcus thermophilus] Length=172

Score = 347 bits (889), Expect = 7e-119, Method: Compositional matrix adjust. Identities = 172/172 (100%), Positives = 172/172 (100%), Gaps = 0/172 (0%)

Query 23	DWAQQTIGCSDAAVFRLSAQGRPVLFVKTDLSGALNELQDEAARLSWLATTGVPCAAVLD 82 DWAQQTIGCSDAAVFRLSAQGRPVLFVKTDLSGALNELQDEAARLSWLATTGVPCAAVLD
Sbjct 1	DWAQQTIGCSDAAVFRLSAQGRPVLFVKTDLSGALNELQDEAARLSWLATTGVPCAAVLD 60

Query 83	VVTEAGRDWLLLGEVPGQDLLSSHLAPAEKVSIMADAMRRLHTLDPATCPFDHQAKHRIE 142 VVTEAGRDWLLLGEVPGQDLLSSHLAPAEKVSIMADAMRRLHTLDPATCPFDHQAKHRIE
Sbjct 61	VVTEAGRDWLLLGEVPGQDLLSSHLAPAEKVSIMADAMRRLHTLDPATCPFDHQAKHRIE 120

Query 143 RARTRMEAGLVDQDDLDEEHQGLAPAELFARLKARMPDGEDLVVTHGDACLP 194 RARTRMEAGLVDQDDLDEEHQGLAPAELFARLKARMPDGEDLVVTHGDACLP
Sbjct 121 RARTRMEAGLVDQDDLDEEHQGLAPAELFARLKARMPDGEDLVVTHGDACLP 172

>OOG85846.1 APH(3') family aminoglycoside O-phosphotransferase [Pseudomonas sp. A25(2017)]
Length=258

Score = 350 bits (897), Expect = 1e-118, Method: Compositional matrix adjust. Identities = 171/256 (67%), Positives = 198/256 (77%), Gaps = 0/256 (0%)
```

| Query | 9 | AGSPAAWVERLFGYDWAQQTIGCSDAAVFRLSAQGRPVLFVKTDLSGALNELQDEAARLS | 68 |
| --- | --- | --- | --- |
| Sbjct | 3 | A P+ W ER + W +GCSDAAVFRL A G P LFVKT+ +G L+EL+DEAARL AKLPSGWRERFKHHSWRPMAVGCSDAAVFRLDAPGSPALFVKTEPAGVLSELRDEAARLR | 62 |
| Query | 69 | WLATTGVPCAAVLDVVTEAGRDWLLLGEVPGQDLLSSHLAPAEKVSIMADAMRRLHTLDP WLA+TG PCA VLD E RDWLLL VPG DLLS+ + A KV++MADA+R+LH LDP | 128 |

| Sbjct | 63 | WLASTGTPCAHVLDEAHETDRDWLLLSAVPGVDLLSAPIDEAAKVNVMADALRQLHQLDP | 122 |
| --- | --- | --- | --- |
| Query | 129 | ATCPFDHQAKHRIERARTRMEAGLVDQDDLDEEHQGLAPAELFARLKARMPDGEDLVVTH | 188 |
| Sbjct | 123 | A CPFDH A+HRI +AR RMEAGLVDQ++LDEEHQGLAPA LF +L R P +DLVVTH ADCPFDHSARHRIAKARARMEAGLVDQEELDEEHQGLAPALLFEKLLNRQPAEQDLVVTH | 182 |
| Query | 189 | GDACLPNIMVENGRFSGFIDCGRLGVADRYQDIALATRDIAEELGGEWADRFLVLYGIAA | 248 |
| Sbjct | 183 | GDAC+PNI+V+NGRF+GFIDCGRLGVADRYQD+ALATRDIAEELG +W FL Y + GDACMPNIIVDNGRFTGFIDCGRLGVADRYQDLALATRDIAEELGEQWVAPFLRRYAVLE | 242 |
| Query | 249 | PDSQRIAFYRLLDEFF 264 D RI FYRLLDEFF |  |
| Sbjct | 243 | ADQDRIYFYRLLDEFF 258 | |

```
>EBW8244220.1 aminoglycoside 3'-phosphotransferase [Salmonella enterica subsp. enterica serovar Heidelberg]
Length=169

Score = 345 bits (886), Expect = 2e-118, Method: Compositional matrix adjust. Identities = 169/169 (100%), Positives = 169/169 (100%), Gaps = 0/169 (0%)

Query 96	EVPGQDLLSSHLAPAEKVSIMADAMRRLHTLDPATCPFDHQAKHRIERARTRMEAGLVDQ 155 EVPGQDLLSSHLAPAEKVSIMADAMRRLHTLDPATCPFDHQAKHRIERARTRMEAGLVDQ
Sbjct 1	EVPGQDLLSSHLAPAEKVSIMADAMRRLHTLDPATCPFDHQAKHRIERARTRMEAGLVDQ 60

Query 156 DDLDEEHQGLAPAELFARLKARMPDGEDLVVTHGDACLPNIMVENGRFSGFIDCGRLGVA 215 DDLDEEHQGLAPAELFARLKARMPDGEDLVVTHGDACLPNIMVENGRFSGFIDCGRLGVA
Sbjct 61	DDLDEEHQGLAPAELFARLKARMPDGEDLVVTHGDACLPNIMVENGRFSGFIDCGRLGVA 120

Query 216 DRYQDIALATRDIAEELGGEWADRFLVLYGIAAPDSQRIAFYRLLDEFF 264 DRYQDIALATRDIAEELGGEWADRFLVLYGIAAPDSQRIAFYRLLDEFF
Sbjct 121 DRYQDIALATRDIAEELGGEWADRFLVLYGIAAPDSQRIAFYRLLDEFF 169

>WP_053180723.1 APH(3')-II family aminoglycoside O-phosphotransferase [Pseudomonas kilonensis]
Length=258

Score = 349 bits (895), Expect = 2e-118, Method: Compositional matrix adjust.
```

| Identities | | = 171/256 (67%), Positives = 199/256 (78%), Gaps = 0/256 (0%) |  |
| --- | --- | --- | --- |
| Query 9  Sbjct 3 | | AGSPAAWVERLFGYDWAQQTIGCSDAAVFRLSAQGRPVLFVKTDLSGALNELQDEAARLS A P+ W ER + W T+GCSDAAVFRL A G P LFVKT+ +G L+EL+DEAARL AKLPSGWRERFKHHSWRPMTVGCSDAAVFRLDAPGAPALFVKTEPAGLLSELRDEAARLR | 68  62 |
| Query 69  Sbjct 63 | | WLATTGVPCAAVLDVVTEAGRDWLLLGEVPGQDLLSSHLAPAEKVSIMADAMRRLHTLDP WLA+TG PCA VLD E RDWLLL VPG DLLS+ + A KV++MADA+R+LH LDP WLASTGTPCAHVLDEAHETDRDWLLLSAVPGVDLLSAPIDEAAKVNVMADALRQLHQLDP | 128  122 |
| Query 129  Sbjct 123 | | ATCPFDHQAKHRIERARTRMEAGLVDQDDLDEEHQGLAPAELFARLKARMPDGEDLVVTH A CPFDH A+HRI +AR RMEAGLVDQ++LDEEHQGLAPA LF +L R P +DLVVTH ADCPFDHSARHRIAKARARMEAGLVDQEELDEEHQGLAPALLFEKLLNRQPAEQDLVVTH | 188  182 |
| Query 189  Sbjct 183 | | GDACLPNIMVENGRFSGFIDCGRLGVADRYQDIALATRDIAEELGGEWADRFLVLYGIAA GDAC+PNI+V+NGRF+GFIDCGRLGVADRYQD+ALATRDIAEELG +W FL Y + GDACMPNIIVDNGRFTGFIDCGRLGVADRYQDLALATRDIAEELGEQWVAPFLRRYAVLE | 248  242 |
| Query 249 | | PDSQRIAFYRLLDEFF 264 D RI FY+LLDEFF |  |
| Sbjct | 243 | ADQDRIYFYQLLDEFF 258 | |

>HAS1851170.1 phosphotransferase [Salmonella enterica subsp. enterica] HAS1912904.1 phosphotransferase [Salmonella enterica subsp. enterica] HAS1928516.1 phosphotransferase [Salmonella enterica subsp. enterica]

Length=170

Score = 344 bits (883), Expect = 6e-118, Method: Compositional matrix adjust. Identities = 170/170 (100%), Positives = 170/170 (100%), Gaps = 0/170 (0%)

Query 1	MIEQDGLHAGSPAAWVERLFGYDWAQQTIGCSDAAVFRLSAQGRPVLFVKTDLSGALNEL 60

| Sbjct | 1 | MIEQDGLHAGSPAAWVERLFGYDWAQQTIGCSDAAVFRLSAQGRPVLFVKTDLSGALNEL MIEQDGLHAGSPAAWVERLFGYDWAQQTIGCSDAAVFRLSAQGRPVLFVKTDLSGALNEL | 60 |
| --- | --- | --- | --- |
| Query | 61 | QDEAARLSWLATTGVPCAAVLDVVTEAGRDWLLLGEVPGQDLLSSHLAPAEKVSIMADAM | 120 |
| Sbjct | 61 | QDEAARLSWLATTGVPCAAVLDVVTEAGRDWLLLGEVPGQDLLSSHLAPAEKVSIMADAM QDEAARLSWLATTGVPCAAVLDVVTEAGRDWLLLGEVPGQDLLSSHLAPAEKVSIMADAM | 120 |
| Query  Sbjct | 121  121 | RRLHTLDPATCPFDHQAKHRIERARTRMEAGLVDQDDLDEEHQGLAPAEL 170 RRLHTLDPATCPFDHQAKHRIERARTRMEAGLVDQDDLDEEHQGLAPAEL RRLHTLDPATCPFDHQAKHRIERARTRMEAGLVDQDDLDEEHQGLAPAEL 170 |  |

>WP_029866945.1 APH(3')-II family aminoglycoside O-phosphotransferase [Pseudomonas kilonensis]

Length=258

Score = 346 bits (887), Expect = 3e-117, Method: Compositional matrix adjust.

| Identities | | = 169/256 (66%), Positives = 198/256 (77%), Gaps = 0/256 (0%) |  |
| --- | --- | --- | --- |
| Query 9  Sbjct 3 | | AGSPAAWVERLFGYDWAQQTIGCSDAAVFRLSAQGRPVLFVKTDLSGALNELQDEAARLS A P+ W ER + W T+GCSDAAVFRL A G P LFVKT+ +G L+EL+DEAARL AKLPSGWRERFKHHSWRPMTVGCSDAAVFRLDAPGSPALFVKTEPAGVLSELRDEAARLR | 68  62 |
| Query 69  Sbjct 63 | | WLATTGVPCAAVLDVVTEAGRDWLLLGEVPGQDLLSSHLAPAEKVSIMADAMRRLHTLDP WLA+TG PCA VLD E RDWLLL VPG DLLS+ + A KV++MADA+R+LH LDP WLASTGTPCAHVLDEAHETDRDWLLLSAVPGVDLLSAPIDEAAKVNVMADALRQLHQLDP | 128  122 |
| Query 129  Sbjct 123 | | ATCPFDHQAKHRIERARTRMEAGLVDQDDLDEEHQGLAPAELFARLKARMPDGEDLVVTH A CPFDH A+HRI +AR +MEAGLVDQ++LDEEH GLAPA LF +L R P +DLVVTH ADCPFDHSARHRIAKARAQMEAGLVDQEELDEEHLGLAPALLFEKLLNRQPAEQDLVVTH | 188  182 |
| Query 189  Sbjct 183 | | GDACLPNIMVENGRFSGFIDCGRLGVADRYQDIALATRDIAEELGGEWADRFLVLYGIAA GDAC+PNI+V+NGRF+GFIDCGRLGVADRYQD+ALATRDIAEELG +W FL Y + GDACMPNIIVDNGRFTGFIDCGRLGVADRYQDLALATRDIAEELGEQWVAPFLRRYAVLE | 248  242 |
| Query 249 | | PDSQRIAFYRLLDEFF 264 D R+ FYRLLDEFF |  |
| Sbjct | 243 | ADQDRVYFYRLLDEFF 258 | |

>WP_057193879.1 APH(3')-II family aminoglycoside O-phosphotransferase [Bosea sp. Root483D1]

KRE11829.1 aminoglycoside phosphotransferase [Bosea sp. Root483D1] Length=257

Score = 346 bits (887), Expect = 3e-117, Method: Compositional matrix adjust.

| Identities | | = 166/254 (65%), Positives = 200/254 (79%), Gaps = 0/254 (0%) |  |
| --- | --- | --- | --- |
| Query 11  Sbjct 4 | | SPAAWVERLFGYDWAQQTIGCSDAAVFRLSAQGRPVLFVKTDLSGALNELQDEAARLSWL  +P AW R+ Y W QQT+GCSDA VFRL G+P LFVKT+ + ++E+ DE RL WL APEAWKGRIAEYSWVQQTLGCSDAGVFRLVGNGKPPLFVKTEPASPMSEVPDEGNRLRWL | 70  63 |
| Query 71  Sbjct 64 | | ATTGVPCAAVLDVVTEAGRDWLLLGEVPGQDLLSSHLAPAEKVSIMADAMRRLHTLDPAT A+ G+P A V+D +EAGR WLLL VPG DLLS+ L P +K+ ++ADA+R LH LD A+ ASAGIPGAQVIDEASEAGRHWLLLSAVPGLDLLSASLEPLKKMEVIADALRLLHRLDTAS | 130  123 |
| Query 131  Sbjct 124 | | CPFDHQAKHRIERARTRMEAGLVDQDDLDEEHQGLAPAELFARLKARMPDGEDLVVTHGD CPFDH+A+HRI+RA+ RM AGLVD+DDLDEEHQGL P +LFARLKAR P EDLVVTHGD CPFDHRAEHRIQRAQARMLAGLVDEDDLDEEHQGLLPIDLFARLKARQPTHEDLVVTHGD | 190  183 |
| Query 191  Sbjct 184 | | ACLPNIMVENGRFSGFIDCGRLGVADRYQDIALATRDIAEELGGEWADRFLVLYGIAAPD ACLPNIMV+ G+FSGFIDCGRLGVADRYQD+AL TRDIAEELG ++ FL YG+A D ACLPNIMVDGGKFSGFIDCGRLGVADRYQDLALTTRDIAEELGDDFVKPFLERYGVAHLD | 250  243 |
| Query 251 | | SQRIAFYRLLDEFF 264 R+AFYRLLDEF+ |  |
| Sbjct | 244 | PDRVAFYRLLDEFY 257 | |

>WP_186707530.1 APH(3')-II family aminoglycoside O-phosphotransferase [Pseudomonas sp. SWRI12]

MBC3391607.1 APH(3')-II family aminoglycoside O-phosphotransferase [Pseudomonas

sp. SWRI12]

Length=258

Score = 343 bits (879), Expect = 4e-116, Method: Compositional matrix adjust.

| Identities | | = 169/253 (67%), Positives = 196/253 (77%), Gaps = 0/253 (0%) |  |
| --- | --- | --- | --- |
| Query 12  Sbjct 6 | | PAAWVERLFGYDWAQQTIGCSDAAVFRLSAQGRPVLFVKTDLSGALNELQDEAARLSWLA P+ W ERL + W T+GCSDAAVFRL A G P +F+KT+ +GALNEL+DEAARL WLA PSRWRERLKHHSWQPMTVGCSDAAVFRLDAPGAPAVFIKTEPAGALNELRDEAARLRWLA | 71  65 |
| Query 72  Sbjct 66 | | TTGVPCAAVLDVVTEAGRDWLLLGEVPGQDLLSSHLAPAEKVSIMADAMRRLHTLDPATC  +TG PCA LD EA RDWLLL VPG DLLS+ + A KV++MA A+R+LH LDP C STGTPCAHALDEAHEADRDWLLLSAVPGVDLLSAPIDEATKVNVMAGALRQLHQLDPTDC | 131  125 |
| Query 132  Sbjct 126 | | PFDHQAKHRIERARTRMEAGLVDQDDLDEEHQGLAPAELFARLKARMPDGEDLVVTHGDA PFDH+A HRI +AR RME GLVDQ++LDEEH+GLA A LF RL R P +DLVVTHGDA PFDHRAHHRITKARARMETGLVDQEELDEEHRGLALALLFERLLNRQPAEQDLVVTHGDA | 191  185 |
| Query 192  Sbjct 186 | | CLPNIMVENGRFSGFIDCGRLGVADRYQDIALATRDIAEELGGEWADRFLVLYGIAAPDS CLPNI+V+NGRF+GFIDCGRLGVADRYQD+ALATRDIAE LG +W FL YG+ D CLPNIIVDNGRFTGFIDCGRLGVADRYQDLALATRDIAEALGEQWVAPFLRRYGVIEIDQ | 251  245 |
| Query 252 | | QRIAFYRLLDEFF 264 RI FYRLLDEFF |  |
| Sbjct | 246 | DRIYFYRLLDEFF 258 | |

```
>EAO3620360.1 aminoglycoside 3'-phosphotransferase [Salmonella enterica] EAO3620363.1 aminoglycoside 3'-phosphotransferase [Salmonella enterica] HAD8642362.1 aminoglycoside 3'-phosphotransferase [Salmonella enterica]
Length=166

Score = 339 bits (870), Expect = 4e-116, Method: Compositional matrix adjust. Identities = 166/166 (100%), Positives = 166/166 (100%), Gaps = 0/166 (0%)

Query 99	GQDLLSSHLAPAEKVSIMADAMRRLHTLDPATCPFDHQAKHRIERARTRMEAGLVDQDDL 158 GQDLLSSHLAPAEKVSIMADAMRRLHTLDPATCPFDHQAKHRIERARTRMEAGLVDQDDL
Sbjct 1	GQDLLSSHLAPAEKVSIMADAMRRLHTLDPATCPFDHQAKHRIERARTRMEAGLVDQDDL 60

Query 159 DEEHQGLAPAELFARLKARMPDGEDLVVTHGDACLPNIMVENGRFSGFIDCGRLGVADRY 218 DEEHQGLAPAELFARLKARMPDGEDLVVTHGDACLPNIMVENGRFSGFIDCGRLGVADRY
Sbjct 61	DEEHQGLAPAELFARLKARMPDGEDLVVTHGDACLPNIMVENGRFSGFIDCGRLGVADRY 120

Query 219 QDIALATRDIAEELGGEWADRFLVLYGIAAPDSQRIAFYRLLDEFF 264 QDIALATRDIAEELGGEWADRFLVLYGIAAPDSQRIAFYRLLDEFF
Sbjct 121 QDIALATRDIAEELGGEWADRFLVLYGIAAPDSQRIAFYRLLDEFF 166

>WP_122568535.1 APH(3')-II family aminoglycoside O-phosphotransferase [Pseudomonas viridiflava]
Length=258

Score = 342 bits (876), Expect = 1e-115, Method: Compositional matrix adjust.
```

| Identities | = 166/256 (65%), Positives = 196/256 (77%), Gaps = 0/256 (0%) |  |
| --- | --- | --- |
| Query 9 | AGSPAAWVERLFGYDWAQQTIGCSDAAVFRLSAQGRPVLFVKTDLSGALNELQDEAARLS | 68 |
| Sbjct 3 | A P++W +R + W Q T+GCSDAAVFRL+A G P LF+KT+ +G L+EL+DEAARL AKLPSSWRQRFKHHSWQQMTVGCSDAAVFRLNAFGAPALFIKTEPAGPLSELRDEAARLR | 62 |
| Query 69 | WLATTGVPCAAVLDVVTEAGRDWLLLGEVPGQDLLSSHLAPAEKVSIMADAMRRLHTLDP | 128 |
| Sbjct 63 | WLA+TG PCA VLD + RDWLLL VPG DLLS+ + KV+IMADA+R+LH LDP WLASTGTPCAHVLDEAHDTDRDWLLLSAVPGVDLLSAPIDAVVKVNIMADALRQLHQLDP | 122 |
| Query 129 | ATCPFDHQAKHRIERARTRMEAGLVDQDDLDEEHQGLAPAELFARLKARMPDGEDLVVTH | 188 |
| Sbjct 123 | CPFDH HRI +AR RMEAGLVDQ++LDEEHQGLAP LF +L R P ++LVVTH VDCPFDHSPSHRIAKARARMEAGLVDQEELDEEHQGLAPDLLFEKLLNRQPAEQNLVVTH | 182 |
| Query 189 | GDACLPNIMVENGRFSGFIDCGRLGVADRYQDIALATRDIAEELGGEWADRFLVLYGIAA | 248 |
| Sbjct 183 | GDACLPNI+V+NGRF+GFIDCGRLGVADRYQD+AL TRDIAEELG +W FL Y + GDACLPNIIVDNGRFTGFIDCGRLGVADRYQDLALTTRDIAEELGEQWVAPFLRRYAVLE | 242 |
| Query 249 | PDSQRIAFYRLLDEFF 264 |  |

D RI FYRLLDEFF Sbjct 243 VDQDRIYFYRLLDEFF 258

>WP_167657122.1 phosphotransferase, partial [Salmonella enterica] NJH55163.1 phosphotransferase [Salmonella enterica subsp. enterica serovar

Anatum] Length=167

Score = 337 bits (863), Expect = 6e-115, Method: Compositional matrix adjust.

| Identities | = 166/167 (99%), Positives = 166/167 (99%), Gaps = 0/167 (0%) |  |
| --- | --- | --- |
| Query 42 | QGRPVLFVKTDLSGALNELQDEAARLSWLATTGVPCAAVLDVVTEAGRDWLLLGEVPGQD | 101 |
| Sbjct 1 | QGRPVLFVKTDLSGALNELQDEAARLSWLATTGVPCAAVLDVVTEAGRDWLLLGEVPGQD QGRPVLFVKTDLSGALNELQDEAARLSWLATTGVPCAAVLDVVTEAGRDWLLLGEVPGQD | 60 |
| Query 102 | LLSSHLAPAEKVSIMADAMRRLHTLDPATCPFDHQAKHRIERARTRMEAGLVDQDDLDEE | 161 |
| Sbjct 61 | LLSSHLAPAEKVSIMADAMRRL TLDPATCPFDHQAKHRIERARTRMEAGLVDQDDLDEE LLSSHLAPAEKVSIMADAMRRLRTLDPATCPFDHQAKHRIERARTRMEAGLVDQDDLDEE | 120 |
| Query 162  Sbjct 121 | HQGLAPAELFARLKARMPDGEDLVVTHGDACLPNIMVENGRFSGFID 208 HQGLAPAELFARLKARMPDGEDLVVTHGDACLPNIMVENGRFSGFID HQGLAPAELFARLKARMPDGEDLVVTHGDACLPNIMVENGRFSGFID 167 |  |

>WP_163858992.1 APH(3')-II family aminoglycoside O-phosphotransferase [Pseudomonas fluorescens]

QIB08915.1 APH(3')-II family aminoglycoside O-phosphotransferase [Pseudomonas fluorescens]

Length=258

Score = 337 bits (863), Expect = 1e-113, Method: Compositional matrix adjust.

| Identities | | = 165/256 (64%), Positives = 194/256 (76%), Gaps = 0/256 (0%) |  |
| --- | --- | --- | --- |
| Query 9  Sbjct 3 | | AGSPAAWVERLFGYDWAQQTIGCSDAAVFRLSAQGRPVLFVKTDLSGALNELQDEAARLS A P+ W +R + W T GCSDAAVFRL A G P LF+KT+ +G L+EL+DEAARL AKLPSDWQQRFKHHSWHPITGGCSDAAVFRLDAPGTPALFIKTEPAGPLSELRDEAARLR | 68  62 |
| Query 69  Sbjct 63 | | WLATTGVPCAAVLDVVTEAGRDWLLLGEVPGQDLLSSHLAPAEKVSIMADAMRRLHTLDP WLA+TG PCA VLD RDWLLL VPG DLLS+ + A KV+++ADA+R+LH LDP WLASTGTPCAHVLDEAHTTDRDWLLLSAVPGVDLLSAPIGEAAKVNVIADALRQLHQLDP | 128  122 |
| Query 129  Sbjct 123 | | ATCPFDHQAKHRIERARTRMEAGLVDQDDLDEEHQGLAPAELFARLKARMPDGEDLVVTH CPFDH A+HRI +AR RMEAGLVD + LDEEHQGLAPA LF +L R P +DLVVTH VDCPFDHSARHRIAKARARMEAGLVDHEALDEEHQGLAPALLFEKLLNRQPAEQDLVVTH | 188  182 |
| Query 189  Sbjct 183 | | GDACLPNIMVENGRFSGFIDCGRLGVADRYQDIALATRDIAEELGGEWADRFLVLYGIAA GDAC+PN++V+NGRF+GFIDCGRLGVADRYQD+ALATRDIAEELG +W FL Y + GDACMPNVIVDNGRFTGFIDCGRLGVADRYQDLALATRDIAEELGEQWVAPFLQRYAMLE | 248  242 |
| Query 249 | | PDSQRIAFYRLLDEFF 264 D RI FYRLLDEFF |  |
| Sbjct | 243 | ADQDRIYFYRLLDEFF 258 | |

>WP_019407658.1 APH(3')-II family aminoglycoside O-phosphotransferase [Pseudomonas stutzeri]

MTI89819.1 APH(3')-II family aminoglycoside O-phosphotransferase [Pseudomonas stutzeri]

OCX55296.1 APH(3') family aminoglycoside O-phosphotransferase [Pseudomonas stutzeri]

Length=264

Score = 336 bits (861), Expect = 3e-113, Method: Compositional matrix adjust. Identities = 168/253 (66%), Positives = 198/253 (78%), Gaps = 0/253 (0%)

| Query | 12 | PAAWVERLFGYDWAQQTIGCSDAAVFRLSAQGRPVLFVKTDLSGALNELQDEAARLSWLA | 71 |
| --- | --- | --- | --- |
| Sbjct | 12 | PAAW RL GY W Q GCSDAAVFRL+A GRP LFVK++ S L EL DEAARL WLA PAAWRPRLAGYQWTLQEQGCSDAAVFRLAAPGRPPLFVKSEPSDPLGELPDEAARLCWLA | 71 |
| Query | 72 | TTGVPCAAVLDVVTEAGRDWLLLGEVPGQDLLSSHLAPAEKVSIMADAMRRLHTLDPATC  +TG+ CA V++ ++E GRDWLLLG VPG++L +S L PA+KV+IMADA+R LH LDP +C | 131 |

| Sbjct | 72 | STGIACAPVVEAISEGGRDWLLLGAVPGENLSTSSLEPAQKVAIMADALRMLHALDPNSC | 131 |
| --- | --- | --- | --- |
| Query | 132 | PFDHQAKHRIERARTRMEAGLVDQDDLDEEHQGLAPAELFARLKARMPDGEDLVVTHGDA | 191 |
| Sbjct | 132 | PFDH+A++R+ RAR RM+AG+VD DD D++H+GL+ +LF LK +MP EDLVVTHGDA PFDHRAEYRVRRARARMDAGMVDDDDFDDDHRGLSSPQLFGLLKTQMPAVEDLVVTHGDA | 191 |
| Query | 192 | CLPNIMVENGRFSGFIDCGRLGVADRYQDIALATRDIAEELGGEWADRFLVLYGIAAPDS | 251 |
| Sbjct | 192 | CLPN MV+ GR SG IDCGRLGVADRYQD+ALA RDI EELG EW FL YGIAA D CLPNFMVDRGRLSGLIDCGRLGVADRYQDLALACRDITEELGEEWVQAFLACYGIAALDL | 251 |
| Query | 252 | QRIAFYRLLDEFF 264 R FYRLLDEFF |  |
| Sbjct | 252 | ARARFYRLLDEFF 264 | |

>WP_045632183.1 APH(3')-II family aminoglycoside O-phosphotransferase [Pseudomonas stutzeri]

OWG39688.1 APH(3') family aminoglycoside O-phosphotransferase [Pseudomonas stutzeri]

Length=264

Score = 335 bits (858), Expect = 9e-113, Method: Compositional matrix adjust.

| Identities | | = 168/253 (66%), Positives = 198/253 (78%), Gaps = 0/253 (0%) |  |
| --- | --- | --- | --- |
| Query 12  Sbjct 12 | | PAAWVERLFGYDWAQQTIGCSDAAVFRLSAQGRPVLFVKTDLSGALNELQDEAARLSWLA PAAW RL GY W Q GCS+AAVFRLSA GRP LFVK++ S L EL DEAARL WLA PAAWRPRLAGYQWTLQEQGCSNAAVFRLSAPGRPPLFVKSEPSDPLGELPDEAARLCWLA | 71  71 |
| Query 72  Sbjct 72 | | TTGVPCAAVLDVVTEAGRDWLLLGEVPGQDLLSSHLAPAEKVSIMADAMRRLHTLDPATC  +TG+ CA V++ ++E GRDWLLLG VPG++L +S L PA+KV+IMADA+R LH LDP +C STGIACAPVVEAISEGGRDWLLLGAVPGENLSTSSLEPAQKVAIMADALRMLHALDPNSC | 131  131 |
| Query 132  Sbjct 132 | | PFDHQAKHRIERARTRMEAGLVDQDDLDEEHQGLAPAELFARLKARMPDGEDLVVTHGDA PFDH+A++R+ RAR RM+AG+VD DD D++H+GL+ +LFA LK + P EDLVVTHGDA PFDHRAEYRVRRARARMDAGMVDDDDFDDDHRGLSSPQLFALLKTQRPAVEDLVVTHGDA | 191  191 |
| Query 192  Sbjct 192 | | CLPNIMVENGRFSGFIDCGRLGVADRYQDIALATRDIAEELGGEWADRFLVLYGIAAPDS CLPN MV+ GR SG IDCGRLGVADRYQD+ALA RDI EELG EW FL YGIAA D CLPNFMVDRGRLSGLIDCGRLGVADRYQDLALACRDITEELGEEWVQAFLACYGIAALDL | 251  251 |
| Query 252 | | QRIAFYRLLDEFF 264 R FYRLLDEFF |  |
| Sbjct | 252 | ARARFYRLLDEFF 264 | |

>WP_125865759.1 APH(3')-II family aminoglycoside O-phosphotransferase [Pseudomonas stutzeri]

RRV30391.1 APH(3')-II family aminoglycoside O-phosphotransferase [Pseudomonas stutzeri]

RRV74721.1 APH(3')-II family aminoglycoside O-phosphotransferase [Pseudomonas stutzeri]

RRV80903.1 APH(3')-II family aminoglycoside O-phosphotransferase [Pseudomonas stutzeri]

Length=264

Score = 335 bits (858), Expect = 9e-113, Method: Compositional matrix adjust.

| Identities | = 168/253 (66%), Positives = 198/253 (78%), Gaps = 0/253 (0%) |  |
| --- | --- | --- |
| Query 12 | PAAWVERLFGYDWAQQTIGCSDAAVFRLSAQGRPVLFVKTDLSGALNELQDEAARLSWLA | 71 |
| Sbjct 12 | PAAW RL GY W Q GCSDAAVFRL+A GRP LFVK++ S L EL DEAARL WLA PAAWRPRLAGYQWTLQEQGCSDAAVFRLAAPGRPPLFVKSEPSDPLGELPDEAARLCWLA | 71 |
| Query 72 | TTGVPCAAVLDVVTEAGRDWLLLGEVPGQDLLSSHLAPAEKVSIMADAMRRLHTLDPATC | 131 |
| Sbjct 72 | +TG+ CA V++ ++E GRDWLLLG VPG++L +S L PA+KV+IMADA+R LH LDP +C STGIACAPVVEAISEGGRDWLLLGAVPGENLSTSSLEPAQKVAIMADALRMLHALDPNSC | 131 |
| Query 132 | PFDHQAKHRIERARTRMEAGLVDQDDLDEEHQGLAPAELFARLKARMPDGEDLVVTHGDA | 191 |
| Sbjct 132 | PFDH+A++R+ RAR RM+AG+VD DD D++H+GL+ +LFA LK + P EDLVVTHGDA PFDHRAEYRVRRARARMDAGMVDDDDFDDDHRGLSSPQLFALLKTQRPAVEDLVVTHGDA | 191 |
| Query 192 | CLPNIMVENGRFSGFIDCGRLGVADRYQDIALATRDIAEELGGEWADRFLVLYGIAAPDS CLPN MV+ GR SG IDCGRLGVADRYQD+ALA RDI EELG EW FL YGIAA D | 251 |

| Sbjct | 192 | CLPNFMVDRGRLSGLIDCGRLGVADRYQDLALACRDITEELGEEWVQAFLACYGIAALDL 251 |
| --- | --- | --- |
| Query | 252 | QRIAFYRLLDEFF 264 |
| Sbjct | 252 | R FYRLLDEFF  ARARFYRLLDEFF 264 |

>WP_024031215.1 aminoglycoside 3'-phosphotransferase, partial [Neobacillus vireti] ETI65905.1 aminoglycoside phosphotransferase, partial [Bacillus vireti LMG

21834]

Length=164

Score = 330 bits (847), Expect = 1e-112, Method: Compositional matrix adjust.

| Identities | = 162/164 (99%), Positives = 162/164 (99%), Gaps = 0/164 (0%) |  |
| --- | --- | --- |
| Query 58 | NELQDEAARLSWLATTGVPCAAVLDVVTEAGRDWLLLGEVPGQDLLSSHLAPAEKVSIMA | 117 |
| Sbjct 1 | NELQDEAARLSWLATTGVPCAAVLDVVTEAGRDWLLLGEVPGQDLLSSHLAPAEKVSIMA NELQDEAARLSWLATTGVPCAAVLDVVTEAGRDWLLLGEVPGQDLLSSHLAPAEKVSIMA | 60 |
| Query 118 | DAMRRLHTLDPATCPFDHQAKHRIERARTRMEAGLVDQDDLDEEHQGLAPAELFARLKAR | 177 |
| Sbjct 61 | DAMRRLHTLDPATCPFDHQAKHRIERARTRMEAGLVDQDDLDEEHQGLAPAELFARLKA DAMRRLHTLDPATCPFDHQAKHRIERARTRMEAGLVDQDDLDEEHQGLAPAELFARLKAS | 120 |
| Query 178  Sbjct 121 | MPDGEDLVVTHGDACLPNIMVENGRFSGFIDCGRLGVADRYQDI 221 MPDGEDLVVTHGDACLPNIMVENGRFSGFIDCGRLGVADRYQ I MPDGEDLVVTHGDACLPNIMVENGRFSGFIDCGRLGVADRYQGI 164 |  |

>WP_017245313.1 APH(3')-II family aminoglycoside O-phosphotransferase [Pseudomonas stutzeri]

AWL01952.1 APH(3')-II family aminoglycoside O-phosphotransferase [Pseudomonas stutzeri]

MPS59655.1 APH(3')-II family aminoglycoside O-phosphotransferase [Pseudomonas sp.]

NNJ35884.1 APH(3')-II family aminoglycoside O-phosphotransferase [Pseudomonas stutzeri]

Length=264

Score = 333 bits (855), Expect = 3e-112, Method: Compositional matrix adjust.

| Identities | | = 167/253 (66%), Positives = 198/253 (78%), Gaps = 0/253 (0%) |  |
| --- | --- | --- | --- |
| Query 12  Sbjct 12 | | PAAWVERLFGYDWAQQTIGCSDAAVFRLSAQGRPVLFVKTDLSGALNELQDEAARLSWLA PAAW RL GY W Q GCS+AAVFRL+A GRP LFVK++ S L EL DEAARL WLA PAAWRPRLAGYQWTLQEQGCSNAAVFRLAAPGRPPLFVKSEPSDPLGELPDEAARLCWLA | 71  71 |
| Query 72  Sbjct 72 | | TTGVPCAAVLDVVTEAGRDWLLLGEVPGQDLLSSHLAPAEKVSIMADAMRRLHTLDPATC  +TG+ CA V++ ++E GRDWLLLG VPG++L +S L PA+KV+IMADA+R LH LDP +C STGIACAPVVEAISEGGRDWLLLGAVPGENLSTSSLEPAQKVAIMADALRMLHALDPNSC | 131  131 |
| Query 132  Sbjct 132 | | PFDHQAKHRIERARTRMEAGLVDQDDLDEEHQGLAPAELFARLKARMPDGEDLVVTHGDA PFDH+A++R+ RAR RM+AG+VD DD D++H+GL+ +LFA LK + P EDLVVTHGDA PFDHRAEYRVRRARARMDAGMVDDDDFDDDHRGLSSPQLFALLKTQRPAVEDLVVTHGDA | 191  191 |
| Query 192  Sbjct 192 | | CLPNIMVENGRFSGFIDCGRLGVADRYQDIALATRDIAEELGGEWADRFLVLYGIAAPDS CLPN MV+ GR SG IDCGRLGVADRYQD+ALA RDI EELG EW FL YGIAA D CLPNFMVDRGRLSGLIDCGRLGVADRYQDLALACRDITEELGEEWVQAFLACYGIAALDL | 251  251 |
| Query 252 | | QRIAFYRLLDEFF 264 R FYRLLDEFF |  |
| Sbjct | 252 | ARARFYRLLDEFF 264 | |

```
>WP_183004849.1 APH(3')-II family aminoglycoside O-phosphotransferase [Achromobacter sp. UMC71]
Length=264

Score = 333 bits (854), Expect = 3e-112, Method: Compositional matrix adjust. Identities = 159/264 (60%), Positives = 192/264 (73%), Gaps = 0/264 (0%)

Query 1	MIEQDGLHAGSPAAWVERLFGYDWAQQTIGCSDAAVFRLSAQGRPVLFVKTDLSGALNEL 60 M +	+ +P AW	L YDW +QT GCSDAAVF+L A RP LF+KT+ + L+EL
Sbjct 1	MPDNTDFNPSAPTAWRNTLASYDWTRQTAGCSDAAVFQLQAPNRPTLFLKTEPASPLSEL 60
```

| Query | 61 | QDEAARLSWLATTGVPCAAVLDVVTEAGRDWLLLGEVPGQDLLSSHLAPAEKVSIMADAM | 120 |
| --- | --- | --- | --- |
| Sbjct | 61 | Q+EAARL WL TG+PCA VLD+ EAGRDW+LL VPG DL S+ L PA+KV+I+A+ + QNEAARLRWLGQTGIPCAQVLDLAHEAGRDWMLLNAVPGTDLHSAPLPPADKVAILAEVL | 120 |
| Query | 121 | RRLHTLDPATCPFDHQAKHRIERARTRMEAGLVDQDDLDEEHQGLAPAELFARLKARMPD | 180 |
| Sbjct | 121 | RRLH++DP TCPFD +A RIE AR R+ AG VD DD DE+H+ L P LFA L+ P RRLHSVDPKTCPFDQRAARRIEHARARLAAGRVDTDDFDEDHRALTPTALFANLEQTRPR | 180 |
| Query | 181 | GEDLVVTHGDACLPNIMVENGRFSGFIDCGRLGVADRYQDIALATRDIAEELGGEWADRF | 240 |
| Sbjct | 181 | E VVTHGDACLPNIMV+ GR SG IDCGRLG+ADRYQD+ALA RDI + LG EW + F DEAPVVTHGDACLPNIMVDQGRVSGLIDCGRLGLADRYQDLALAARDIGDTLGPEWVNPF | 240 |
| Query  Sbjct | 241  241 | LVLYGIAAPDSQRIAFYRLLDEFF 264 L YGI PD +R+ FYRLLDEFF LDRYGIDRPDRERLKFYRLLDEFF 264 |  |

>HAG18071.1 APH(3')-II family aminoglycoside O-phosphotransferase [Pseudomonas sp.]

Length=264

Score = 333 bits (853), Expect = 5e-112, Method: Compositional matrix adjust.

| Identities | | = 168/253 (66%), Positives = 196/253 (77%), Gaps = 0/253 (0%) |  |
| --- | --- | --- | --- |
| Query 12  Sbjct 12 | | PAAWVERLFGYDWAQQTIGCSDAAVFRLSAQGRPVLFVKTDLSGALNELQDEAARLSWLA PAAW RL GY W Q CSDAAVFRL+A GRP LFVK++ S L EL DEAARL WLA PAAWRPRLAGYQWTLQEQACSDAAVFRLAAPGRPPLFVKSEPSDPLGELPDEAARLCWLA | 71  71 |
| Query 72  Sbjct 72 | | TTGVPCAAVLDVVTEAGRDWLLLGEVPGQDLLSSHLAPAEKVSIMADAMRRLHTLDPATC  +TG+ CA V+D ++E GRDWLLLG VPG +L +S L PA+KV+IMADA+R LH LDP +C STGIACAPVVDAISEGGRDWLLLGAVPGANLSTSSLEPAQKVAIMADALRMLHALDPNSC | 131  131 |
| Query 132  Sbjct 132 | | PFDHQAKHRIERARTRMEAGLVDQDDLDEEHQGLAPAELFARLKARMPDGEDLVVTHGDA PFDH+A++R+ RAR RM+AG+VD DD D++H+GL+ +LFA LK + P EDLVVTHGDA PFDHRAEYRVRRARARMDAGMVDDDDFDDDHRGLSSPQLFALLKTQRPAVEDLVVTHGDA | 191  191 |
| Query 192  Sbjct 192 | | CLPNIMVENGRFSGFIDCGRLGVADRYQDIALATRDIAEELGGEWADRFLVLYGIAAPDS CLPN MV+ GR SG IDCGRLGVADRYQD+ALA RDI EELG EW FL YGIAA D CLPNFMVDRGRLSGLIDCGRLGVADRYQDLALACRDITEELGEEWVQAFLACYGIAALDL | 251  251 |
| Query 252 | | QRIAFYRLLDEFF 264 R FYRLLDEFF |  |
| Sbjct | 252 | ARARFYRLLDEFF 264 | |

>HAO75576.1 APH(3')-II family aminoglycoside O-phosphotransferase [Pseudomonas sp.]

Length=264

Score = 332 bits (851), Expect = 1e-111, Method: Compositional matrix adjust.

| Identities | | = 167/253 (66%), Positives = 196/253 (77%), Gaps = 0/253 (0%) |  |
| --- | --- | --- | --- |
| Query 12  Sbjct 12 | | PAAWVERLFGYDWAQQTIGCSDAAVFRLSAQGRPVLFVKTDLSGALNELQDEAARLSWLA PAAW RL GY W Q GCSDAAVFRL+A GRP LFVK++ S L EL DEAARL WLA PAAWRPRLAGYQWTLQEQGCSDAAVFRLAAPGRPSLFVKSEPSDPLGELPDEAARLCWLA | 71  71 |
| Query 72  Sbjct 72 | | TTGVPCAAVLDVVTEAGRDWLLLGEVPGQDLLSSHLAPAEKVSIMADAMRRLHTLDPATC  +TG+ CA V+ ++E GRDWLLLG VPG++L +S L PA+KV+IMADA+R LH LDP +C STGIACAPVVAAISEGGRDWLLLGAVPGENLSTSSLEPAQKVAIMADALRMLHALDPNSC | 131  131 |
| Query 132  Sbjct 132 | | PFDHQAKHRIERARTRMEAGLVDQDDLDEEHQGLAPAELFARLKARMPDGEDLVVTHGDA PFDH+A++R+ RAR RM+AG+VD DD D++H+GL+ +LFA LK + P EDLVVTHGDA PFDHRAEYRVRRARARMDAGMVDDDDFDDDHRGLSSPQLFAVLKTQRPAVEDLVVTHGDA | 191  191 |
| Query 192  Sbjct 192 | | CLPNIMVENGRFSGFIDCGRLGVADRYQDIALATRDIAEELGGEWADRFLVLYGIAAPDS CLPN M + GR SG IDCGRLGVADRYQD+ALA RDI EELG EW FL YGIAA D CLPNFMADRGRLSGLIDCGRLGVADRYQDLALACRDITEELGEEWVQAFLACYGIAALDL | 251  251 |
| Query 252 | | QRIAFYRLLDEFF 264 R FYRLLDEFF |  |
| Sbjct | 252 | ARARFYRLLDEFF 264 | |

>WP_084901678.1 APH(3')-II family aminoglycoside O-phosphotransferase [Pseudomonas stutzeri]

OSO73547.1 APH(3') family aminoglycoside O-phosphotransferase [Pseudomonas stutzeri]

CAD2255179.1 Aminoglycoside 3'-phosphotransferase [Pseudomonas stutzeri] Length=264

Score = 332 bits (850), Expect = 1e-111, Method: Compositional matrix adjust.

| Identities | | = 167/253 (66%), Positives = 196/253 (77%), Gaps = 0/253 (0%) |  |
| --- | --- | --- | --- |
| Query 12  Sbjct 12 | | PAAWVERLFGYDWAQQTIGCSDAAVFRLSAQGRPVLFVKTDLSGALNELQDEAARLSWLA PA W RL GY W Q GCSDAAVFRL+A GRP LFVK++ S L EL DEAARL WLA PATWRPRLAGYQWTLQEQGCSDAAVFRLAAPGRPPLFVKSEPSDPLGELPDEAARLCWLA | 71  71 |
| Query 72  Sbjct 72 | | TTGVPCAAVLDVVTEAGRDWLLLGEVPGQDLLSSHLAPAEKVSIMADAMRRLHTLDPATC  +TG+ CA V++ ++E GRDWLLLG VPG++L +S L PA+KV+IMADA+R LH LDP +C STGIACAPVVEAISEGGRDWLLLGAVPGENLSTSSLEPAQKVAIMADALRMLHALDPNSC | 131  131 |
| Query 132  Sbjct 132 | | PFDHQAKHRIERARTRMEAGLVDQDDLDEEHQGLAPAELFARLKARMPDGEDLVVTHGDA PFDH+A++R+ RAR RM+AG+VD DD D++H+GL+ +LFA LK + P EDLVVTHGDA PFDHRAEYRVRRARARMDAGMVDDDDFDDDHRGLSSPQLFALLKTQRPAVEDLVVTHGDA | 191  191 |
| Query 192  Sbjct 192 | | CLPNIMVENGRFSGFIDCGRLGVADRYQDIALATRDIAEELGGEWADRFLVLYGIAAPDS CLPN MV GR SG IDCGRLGVADRYQD+ALA RDI EELG EW FL YGIAA D CLPNFMVGRGRLSGLIDCGRLGVADRYQDLALACRDITEELGEEWVQAFLACYGIAALDL | 251  251 |
| Query 252 | | QRIAFYRLLDEFF 264 R FYRLLDEFF |  |
| Sbjct | 252 | ARARFYRLLDEFF 264 | |

>WP_054093134.1 APH(3')-II family aminoglycoside O-phosphotransferase [Pseudomonas stutzeri]

Length=264

Score = 330 bits (845), Expect = 1e-110, Method: Compositional matrix adjust.

| Identities | = 172/254 (68%), Positives = 195/254 (77%), Gaps = 0/254 (0%) |  |
| --- | --- | --- |
| Query 11 | SPAAWVERLFGYDWAQQTIGCSDAAVFRLSAQGRPVLFVKTDLSGALNELQDEAARLSWL | 70 |
| Sbjct 11 | SPAAW L GY WA Q GCS AAVFRL+A G+P LFVK++ S L EL EAARL WL SPAAWRSLLAGYQWALQKQGCSGAAVFRLTASGQPSLFVKSEPSDPLCELLGEAARLRWL | 70 |
| Query 71 | ATTGVPCAAVLDVVTEAGRDWLLLGEVPGQDLLSSHLAPAEKVSIMADAMRRLHTLDPAT | 130 |
| Sbjct 71 | TG+PCA V+DV++E GRDWLL+GEVPG DLLSS L P +KV+IMADA+R LH LD +T ERTGIPCARVIDVLSEGGRDWLLMGEVPGVDLLSSSLDPEKKVAIMADAVRMLHELDSST | 130 |
| Query 131 | CPFDHQAKHRIERARTRMEAGLVDQDDLDEEHQGLAPAELFARLKARMPDGEDLVVTHGD | 190 |
| Sbjct 131 | CPFDH+A+ RI RA+ RM+AGLVD DD D++H+GL+ EL ARL + P EDLVVTHGD CPFDHRAELRIHRAQARMDAGLVDDDDFDDDHRGLSSNELLARLHEQRPAVEDLVVTHGD | 190 |
| Query 191 | ACLPNIMVENGRFSGFIDCGRLGVADRYQDIALATRDIAEELGGEWADRFLVLYGIAAPD | 250 |
| Sbjct 191 | ACLPNIMVE GRFSG IDCGRLGVADRYQD+AL RDI EELG EW FL YGI D ACLPNIMVEGGRFSGLIDCGRLGVADRYQDLALTCRDIGEELGEEWVPPFLERYGIEVFD | 250 |
| Query 251  Sbjct 251 | SQRIAFYRLLDEFF 264 S R FYRLLDEFF SARARFYRLLDEFF 264 |  |

>WP_045157838.1 APH(3')-II family aminoglycoside O-phosphotransferase [Pseudomonas stutzeri]

RRV82461.1 APH(3')-II family aminoglycoside O-phosphotransferase [Pseudomonas stutzeri]

RRV90690.1 APH(3')-II family aminoglycoside O-phosphotransferase [Pseudomonas stutzeri]

RRV92351.1 APH(3')-II family aminoglycoside O-phosphotransferase [Pseudomonas stutzeri]

RRV96008.1 APH(3')-II family aminoglycoside O-phosphotransferase [Pseudomonas stutzeri]

Length=264

Score = 329 bits (844), Expect = 1e-110, Method: Compositional matrix adjust.

| Identities | | = 166/253 (66%), Positives = 196/253 (77%), Gaps = 0/253 (0%) |  |
| --- | --- | --- | --- |
| Query 12  Sbjct 12 | | PAAWVERLFGYDWAQQTIGCSDAAVFRLSAQGRPVLFVKTDLSGALNELQDEAARLSWLA PAAW RL GY W Q GCS A+VFRL+A GRP LFVK++ S L EL DEAARL WLA PAAWRPRLAGYQWTLQEQGCSGASVFRLAAPGRPPLFVKSEPSDPLGELPDEAARLCWLA | 71  71 |
| Query 72  Sbjct 72 | | TTGVPCAAVLDVVTEAGRDWLLLGEVPGQDLLSSHLAPAEKVSIMADAMRRLHTLDPATC  +TG+ CA V++ ++E GRDWLLLG VPG++L +S L PA+KV+IMADA+R LH LDP C STGIACAPVVEAISEGGRDWLLLGAVPGENLSTSSLEPAQKVAIMADALRMLHALDPNGC | 131  131 |
| Query 132  Sbjct 132 | | PFDHQAKHRIERARTRMEAGLVDQDDLDEEHQGLAPAELFARLKARMPDGEDLVVTHGDA PFDH+A++R+ RAR RM+AG+VD DD D++H+GL+ +LFA LK + P EDLVVTHGDA PFDHRAEYRVRRARARMDAGMVDDDDFDDDHRGLSSPQLFALLKTQRPAVEDLVVTHGDA | 191  191 |
| Query 192  Sbjct 192 | | CLPNIMVENGRFSGFIDCGRLGVADRYQDIALATRDIAEELGGEWADRFLVLYGIAAPDS CLPN MV+ GR SG IDCGRLGVADRYQD+ALA RDI EELG EW FL YGIAA D CLPNFMVDRGRLSGLIDCGRLGVADRYQDLALACRDITEELGEEWVQAFLACYGIAALDL | 251  251 |
| Query 252 | | QRIAFYRLLDEFF 264 R FYRLLDEFF |  |
| Sbjct | 252 | ARARFYRLLDEFF 264 | |

>WP_080970997.1 APH(3')-II family aminoglycoside O-phosphotransferase [Burkholderia cepacia]

Length=264

Score = 329 bits (844), Expect = 1e-110, Method: Compositional matrix adjust.

| Identities | | = 170/264 (64%), Positives = 193/264 (73%), Gaps = 0/264 (0%) |  |
| --- | --- | --- | --- |
| Query 1  Sbjct 1 | | MIEQDGLHAGSPAAWVERLFGYDWAQQTIGCSDAAVFRLSAQGRPVLFVKTDLSGALNEL M + D PAAW L G W +QT G SDAAVFRL A+ FVKT+ +G L EL MSDDDKFWNDVPAAWKPMLAGCRWTRQTDGQSDAAVFRLDARDGSRRFVKTEPAGPLGEL | 60  60 |
| Query 61  Sbjct 61 | | QDEAARLSWLATTGVPCAAVLDVVTEAGRDWLLLGEVPGQDLLSSHLAPAEKVSIMADAM DEAARL WL T+G+P A VLDVV+ AGR+W+LL V G++L + LAPAEKV+IMADA+ NDEAARLRWLDTSGLPGAQVLDVVSAAGREWMLLSAVRGENLDMAQLAPAEKVAIMADAL | 120  120 |
| Query 121  Sbjct 121 | | RRLHTLDPATCPFDHQAKHRIERARTRMEAGLVDQDDLDEEHQGLAPAELFARLKARMPD R LH LDPATCPFDH A HRIERAR RM AGLVD+D+LD + G+ LFARL A P RALHRLDPATCPFDHGAAHRIERARARMHAGLVDEDNLDGTNTGVPLDALFARLLAHRPS | 180  180 |
| Query 181  Sbjct 181 | | GEDLVVTHGDACLPNIMVENGRFSGFIDCGRLGVADRYQDIALATRDIAEELGGEWADRF ED VVTHGDACLPN+M +NGRFSGFIDCGRLGVADRYQD+ALA RDI +LG EW RF TEDRVVTHGDACLPNLMADNGRFSGFIDCGRLGVADRYQDLALAARDIEADLGSEWVTRF | 240  240 |
| Query | 241 LVLYGIAAPDSQRIAFYRLLDEFF 264 | | |
|  |  | YGI PD RIAFYRLLDEFF |  |
| Sbjct | 241 | FTRYGIEHPDPDRIAFYRLLDEFF 264 | |

>WP_174992756.1 APH(3')-II family aminoglycoside O-phosphotransferase [Burkholderia arboris]

VWB63241.1 aminoglycoside phosphotransferase APH(3') [Burkholderia arboris] Length=284

Score = 330 bits (845), Expect = 2e-110, Method: Compositional matrix adjust.

| Identities | = 169/264 (64%), Positives = 195/264 (74%), Gaps = 0/264 (0%) |  |
| --- | --- | --- |
| Query 1 | MIEQDGLHAGSPAAWVERLFGYDWAQQTIGCSDAAVFRLSAQGRPVLFVKTDLSGALNEL | 60 |
| Sbjct 21 | +I+ D L P AW RL G W +QT G SDAAVFRL A+ FVKT +G L EL VIDNDPLGNEVPMAWQARLAGCRWTRQTDGQSDAAVFRLDARDGSRRFVKTAPAGPLGEL | 80 |
| Query 61 | QDEAARLSWLATTGVPCAAVLDVVTEAGRDWLLLGEVPGQDLLSSHLAPAEKVSIMADAM | 120 |
| Sbjct 81 | +DEAARL WLA+TG+P A VLDV + AGR+W+LL V G +L ++ LAPAEKV+IMADA+ RDEAARLRWLASTGLPGAQVLDVASAAGREWMLLSAVRGDNLETAQLAPAEKVAIMADAL | 140 |
| Query 121 | RRLHTLDPATCPFDHQAKHRIERARTRMEAGLVDQDDLDEEHQGLAPAELFARLKARMPD | 180 |
| Sbjct 141 | R LH LDPATCPFDH A HRIERAR RM AGL D+D+LD+ + G+ LFARL A P  RALHRLDPATCPFDHGAAHRIERARARMHAGLADEDNLDDTNAGVPLDALFARLLAHRPS | 200 |

Query 181 GEDLVVTHGDACLPNIMVENGRFSGFIDCGRLGVADRYQDIALATRDIAEELGGEWADRF 240

| Sbjct | 201 | ED VVTHGDACLPN+M ++GRFSGFIDCGRLGVADRYQD+ALA RDI +LG EW RF TEDRVVTHGDACLPNLMADDGRFSGFIDCGRLGVADRYQDLALAARDIEADLGSEWVARF 260 |
| --- | --- | --- |
| Query | 241 | LVLYGIAAPDSQRIAFYRLLDEFF 264 |
| Sbjct | 261 | YGI PD RIAFYRLLDEFF  FTRYGIEHPDPDRIAFYRLLDEFF 284 |

>WP_038659539.1 APH(3')-II family aminoglycoside O-phosphotransferase [Pseudomonas stutzeri]

AHY42791.1 aminoglycoside phosphotransferase [Pseudomonas stutzeri] Length=264

Score = 329 bits (843), Expect = 2e-110, Method: Compositional matrix adjust.

| Identities | | = 168/253 (66%), Positives = 194/253 (77%), Gaps = 0/253 (0%) |  |
| --- | --- | --- | --- |
| Query 12  Sbjct 12 | | PAAWVERLFGYDWAQQTIGCSDAAVFRLSAQGRPVLFVKTDLSGALNELQDEAARLSWLA P AW +L GY WA Q GCS AAVFRL+A G+P LFVK++ S L EL DEA RL WL PVAWRSQLAGYHWALQDQGCSGAAVFRLTASGQPSLFVKSEPSDPLCELLDEAVRLRWLE | 71  71 |
| Query 72  Sbjct 72 | | TTGVPCAAVLDVVTEAGRDWLLLGEVPGQDLLSSHLAPAEKVSIMADAMRRLHTLDPATC TG+PCA +D ++EAGRDWLLLGEVPG+DLLSS L P KV+IMAD +R LHTLDP+TC RTGIPCARAIDALSEAGRDWLLLGEVPGEDLLSSSLDPERKVTIMADVVRMLHTLDPSTC | 131  131 |
| Query 132  Sbjct 132 | | PFDHQAKHRIERARTRMEAGLVDQDDLDEEHQGLAPAELFARLKARMPDGEDLVVTHGDA PFDH+A+ RI RA+ RM+AGLVD DD D++H+GL+ EL +RL + P EDLVVTHGDA PFDHRAELRIHRAQARMDAGLVDDDDFDDDHRGLSSGELLSRLHEQRPAVEDLVVTHGDA | 191  191 |
| Query 192  Sbjct 192 | | CLPNIMVENGRFSGFIDCGRLGVADRYQDIALATRDIAEELGGEWADRFLVLYGIAAPDS CLPN+MVE GRFSG IDCGRLGVADRYQD+AL RDIAEELG EW FL YGI D CLPNVMVEGGRFSGLIDCGRLGVADRYQDLALTCRDIAEELGEEWVPPFLERYGIEVLDL | 251  251 |
| Query 252 | | QRIAFYRLLDEFF 264 R FYRLLDEF+ |  |
| Sbjct | 252 | ARARFYRLLDEFY 264 | |

>WP_122172600.1 aminoglycoside 3'-phosphotransferase [Burkholderia stabilis] Length=264

Score = 328 bits (842), Expect = 2e-110, Method: Compositional matrix adjust.

| Identities | | = 171/264 (65%), Positives = 194/264 (73%), Gaps = 0/264 (0%) |  |
| --- | --- | --- | --- |
| Query 1  Sbjct 1 | | MIEQDGLHAGSPAAWVERLFGYDWAQQTIGCSDAAVFRLSAQGRPVLFVKTDLSGALNEL MI++D L PAAW L G W +QT G SDAAVFRL A+ FVKT+ +G L EL MIDKDKLCNDVPAAWTATLAGCRWTRQTDGQSDAAVFRLDARDGSRRFVKTEPAGPLGEL | 60  60 |
| Query 61  Sbjct 61 | | QDEAARLSWLATTGVPCAAVLDVVTEAGRDWLLLGEVPGQDLLSSHLAPAEKVSIMADAM QDEAARL WLA TGVP A VLDVV+ AGR+W+LL V G++L ++ LAPA KV+IMADA+ QDEAARLRWLAATGVPGARVLDVVSTAGREWMLLSAVRGENLETARLAPAGKVTIMADAL | 120  120 |
| Query 121  Sbjct 121 | | RRLHTLDPATCPFDHQAKHRIERARTRMEAGLVDQDDLDEEHQGLAPAELFARLKARMPD R LH LDPA CPFDH A HRIERAR RM AGLVD+D+LD+ G+ LFARL+A P RALHRLDPAACPFDHGAAHRIERARARMNAGLVDEDNLDDSGTGVPLRALFARLQAHRPP | 180  180 |
| Query 181  Sbjct 181 | | GEDLVVTHGDACLPNIMVENGRFSGFIDCGRLGVADRYQDIALATRDIAEELGGEWADRF ED VVTHGDACLPN MV+ FSGFIDCGRLGVADRYQD+ALATRDI +LG EW F IEDKVVTHGDACLPNFMVDEEAFSGFIDCGRLGVADRYQDLALATRDIEADLGSEWVAPF | 240  240 |
| Query 241 | | LVLYGIAAPDSQRIAFYRLLDEFF 264 YGI PD RIAFYRLLDEFF |  |
| Sbjct | 241 | FARYGIEHPDPDRIAFYRLLDEFF 264 | |

>ALX16133.1 aminoglycoside phosphotransferase [Burkholderia cepacia JBK9] Length=264

Score = 328 bits (841), Expect = 3e-110, Method: Compositional matrix adjust. Identities = 168/264 (64%), Positives = 193/264 (73%), Gaps = 0/264 (0%)

| Query | 1 | MIEQDGLHAGSPAAWVERLFGYDWAQQTIGCSDAAVFRLSAQGRPVLFVKTDLSGALNEL | 60 |
| --- | --- | --- | --- |
| Sbjct | 1 | MI+ D L P AW RL G W +QT G SDA VFRL A+ FVKT +G L EL MIDNDPLGKDVPVAWQARLAGCRWTRQTDGQSDAVVFRLDARDGSRRFVKTAPAGPLGEL | 60 |
| Query | 61 | QDEAARLSWLATTGVPCAAVLDVVTEAGRDWLLLGEVPGQDLLSSHLAPAEKVSIMADAM | 120 |
| Sbjct | 61 | +DEAARL WLA+TG+P A VLDV + AGR+W+LL V G +L ++ LAPAEKV+IMADA+ RDEAARLRWLASTGLPGAQVLDVASAAGREWMLLSAVRGDNLETAQLAPAEKVAIMADAL | 120 |
| Query | 121 | RRLHTLDPATCPFDHQAKHRIERARTRMEAGLVDQDDLDEEHQGLAPAELFARLKARMPD | 180 |
| Sbjct | 121 | R LH LDPA CPFDH A HRIERAR RM AGL D+D+LD+ + G+ LFARL A P RALHRLDPAACPFDHGAAHRIERARARMHAGLADEDNLDDTNAGVPLDALFARLLAHRPS | 180 |
| Query | 181 | GEDLVVTHGDACLPNIMVENGRFSGFIDCGRLGVADRYQDIALATRDIAEELGGEWADRF | 240 |
| Sbjct | 181 | ED VVTHGDACLPN+M ++GRFSGFIDCGRLGVADRYQD+ALA RDI +LG EW RF TEDRVVTHGDACLPNLMADDGRFSGFIDCGRLGVADRYQDLALAARDIEADLGSEWVARF | 240 |
| Query | 241 | LVLYGIAAPDSQRIAFYRLLDEFF 264 YGI PD RIAFYRLLDEFF |  |
| Sbjct | 241 | FTRYGIEHPDPDRIAFYRLLDEFF 264 | |

>WP_156714764.1 APH(3')-II family aminoglycoside O-phosphotransferase [Pseudomonas stutzeri]

QGW19991.1 APH(3')-II family aminoglycoside O-phosphotransferase [Pseudomonas stutzeri]

Length=264

Score = 328 bits (841), Expect = 4e-110, Method: Compositional matrix adjust.

| Identities | = 172/254 (68%), Positives = 194/254 (76%), Gaps = 0/254 (0%) |  |
| --- | --- | --- |
| Query 11 | SPAAWVERLFGYDWAQQTIGCSDAAVFRLSAQGRPVLFVKTDLSGALNELQDEAARLSWL | 70 |
| Sbjct 11 | SPAAW L GY WA Q GCS AAVFRL A G+P LFVK++ S L EL EAARL WL SPAAWRSLLAGYQWALQKQGCSGAAVFRLIASGQPSLFVKSEPSDPLCELLGEAARLRWL | 70 |
| Query 71 | ATTGVPCAAVLDVVTEAGRDWLLLGEVPGQDLLSSHLAPAEKVSIMADAMRRLHTLDPAT | 130 |
| Sbjct 71 | TG+PCA V+DV++E GRDWLL+GEVPG DLLSS L P +KV+IMADA+R LH LD +T ERTGIPCARVIDVLSEGGRDWLLMGEVPGVDLLSSSLDPEKKVAIMADAVRMLHELDSST | 130 |
| Query 131 | CPFDHQAKHRIERARTRMEAGLVDQDDLDEEHQGLAPAELFARLKARMPDGEDLVVTHGD | 190 |
| Sbjct 131 | CPFDH+A+ RI RA+ RM+AGLVD DD D++H+GL+ EL ARL + P EDLVVTHGD CPFDHRAELRIHRAQARMDAGLVDDDDFDDDHRGLSSNELLARLHEQRPAVEDLVVTHGD | 190 |
| Query 191 | ACLPNIMVENGRFSGFIDCGRLGVADRYQDIALATRDIAEELGGEWADRFLVLYGIAAPD | 250 |
| Sbjct 191 | ACLPNIMVE GRFSG IDCGRLGVADRYQD+AL RDI EELG EW FL YGI D ACLPNIMVEGGRFSGLIDCGRLGVADRYQDLALTCRDIGEELGEEWVPPFLERYGIEVFD | 250 |
| Query 251  Sbjct 251 | SQRIAFYRLLDEFF 264 S R FYRLLDEFF SARARFYRLLDEFF 264 |  |

>VWC54586.1 aminoglycoside phosphotransferase APH(3') [Burkholderia lata] Length=264

Score = 328 bits (840), Expect = 5e-110, Method: Compositional matrix adjust.

| Identities | = 169/264 (64%), Positives = 194/264 (73%), Gaps = 0/264 (0%) |  |
| --- | --- | --- |
| Query 1 | MIEQDGLHAGSPAAWVERLFGYDWAQQTIGCSDAAVFRLSAQGRPVLFVKTDLSGALNEL | 60 |
| Sbjct 1 | MI+++ L PA W L W +QT G SDAAVFRL AQ FVKT+ +G L EL MIDKERLCIEVPATWNATLADCRWTRQTEGQSDAAVFRLDAQDGSCRFVKTEPAGPLGEL | 60 |
| Query 61 | QDEAARLSWLATTGVPCAAVLDVVTEAGRDWLLLGEVPGQDLLSSHLAPAEKVSIMADAM | 120 |
| Sbjct 61 | DEAARL WLATTG P A VL+V + AGR+W+LL V G++L + L PAEKV+I+ADA+ SDEAARLRWLATTGSPGAQVLEVASAAGREWMLLSAVCGENLEVAPLGPAEKVAIIADAL | 120 |
| Query 121 | RRLHTLDPATCPFDHQAKHRIERARTRMEAGLVDQDDLDEEHQGLAPAELFARLKARMPD | 180 |
| Sbjct 121 | R LH LDPATCPFDH A+ RI RAR RM AGLVD+D+LDE + G++ ELFARL+A P RALHRLDPATCPFDHGAESRIARARARMNAGLVDEDNLDEANMGVSLDELFARLRASRPS | 180 |
| Query 181 | GEDLVVTHGDACLPNIMVENGRFSGFIDCGRLGVADRYQDIALATRDIAEELGGEWADRF | 240 |
| Sbjct 181 | ED VVTHGDACLPN MVENGRFSGFIDCGRLGVADRYQD+ALA RDI +LG EW F  IEDRVVTHGDACLPNFMVENGRFSGFIDCGRLGVADRYQDLALAARDIEADLGSEWVAPF | 240 |

Query 241 LVLYGIAAPDSQRIAFYRLLDEFF 264

YGI PD RIAFYRLLDEFF Sbjct 241 FTRYGIEQPDPGRIAFYRLLDEFF 264

>WP_175997228.1 APH(3') family aminoglycoside O-phosphotransferase [Burkholderia stabilis]

Length=264

Score = 328 bits (840), Expect = 5e-110, Method: Compositional matrix adjust.

| Identities | | = 170/264 (64%), Positives = 193/264 (73%), Gaps = 0/264 (0%) |  |
| --- | --- | --- | --- |
| Query 1  Sbjct 1 | | MIEQDGLHAGSPAAWVERLFGYDWAQQTIGCSDAAVFRLSAQGRPVLFVKTDLSGALNEL MI++D L PAAW L G W +QT G SDAAVFRL A+ VKT+ +G L EL MIDKDKLCNDVPAAWTATLAGCRWTRQTDGQSDAAVFRLDARDGSRRLVKTEPAGPLGEL | 60  60 |
| Query 61  Sbjct 61 | | QDEAARLSWLATTGVPCAAVLDVVTEAGRDWLLLGEVPGQDLLSSHLAPAEKVSIMADAM QDEAARL WLA TGVP A VLDV + AGR+W+LL V G++L ++ LAPA KV+IMADA+ QDEAARLRWLAATGVPGARVLDVTSTAGREWVLLSAVRGENLETARLAPAAKVAIMADAL | 120  120 |
| Query 121  Sbjct 121 | | RRLHTLDPATCPFDHQAKHRIERARTRMEAGLVDQDDLDEEHQGLAPAELFARLKARMPD R LH LDPATCPFDH A HRIERAR RM AGLVD+D+LD+ G+ LFARL+A P RALHRLDPATCPFDHGAAHRIERARVRMNAGLVDEDNLDDSSTGVPLRALFARLQAHRPP | 180  180 |
| Query 181  Sbjct 181 | | GEDLVVTHGDACLPNIMVENGRFSGFIDCGRLGVADRYQDIALATRDIAEELGGEWADRF ED VVTHGDACLPN MV+ FSGFIDCGRLGVADRYQD+ALATRDI +LG EW F IEDKVVTHGDACLPNFMVDEEAFSGFIDCGRLGVADRYQDLALATRDIEADLGSEWVAPF | 240  240 |
| Query 241 | | LVLYGIAAPDSQRIAFYRLLDEFF 264 YGI PD RIAFYRLLDEFF |  |
| Sbjct | 241 | FARYGIEHPDPDRIAFYRLLDEFF 264 | |

>WP_077683452.1 APH(3')-II family aminoglycoside O-phosphotransferase [Pseudomonas stutzeri]

OOE09788.1 APH(3') family aminoglycoside O-phosphotransferase [Pseudomonas stutzeri]

Length=264

Score = 327 bits (839), Expect = 7e-110, Method: Compositional matrix adjust.

| Identities | = 171/254 (67%), Positives = 194/254 (76%), Gaps = 0/254 (0%) |  |
| --- | --- | --- |
| Query 11 | SPAAWVERLFGYDWAQQTIGCSDAAVFRLSAQGRPVLFVKTDLSGALNELQDEAARLSWL | 70 |
| Sbjct 11 | SPAAW L GY WA Q GCS AAVFRL A G+P LFVK++ S L EL EAARL WL SPAAWRSLLAGYQWALQKQGCSGAAVFRLIASGQPSLFVKSEPSDPLCELLGEAARLRWL | 70 |
| Query 71 | ATTGVPCAAVLDVVTEAGRDWLLLGEVPGQDLLSSHLAPAEKVSIMADAMRRLHTLDPAT | 130 |
| Sbjct 71 | TG+PCA V+DV++E GRDWLL+GEVPG DLLSS L P +KV+IMADA+R LH LD +T ERTGIPCARVIDVLSEGGRDWLLMGEVPGVDLLSSSLDPEKKVAIMADAVRMLHELDSST | 130 |
| Query 131 | CPFDHQAKHRIERARTRMEAGLVDQDDLDEEHQGLAPAELFARLKARMPDGEDLVVTHGD | 190 |
| Sbjct 131 | CPFDH+A+ RI RA+ RM+AGLVD DD D++H+GL+ EL ARL + P EDLVVTHGD CPFDHRAELRIHRAQARMDAGLVDDDDFDDDHRGLSSNELLARLHEQRPAVEDLVVTHGD | 190 |
| Query 191 | ACLPNIMVENGRFSGFIDCGRLGVADRYQDIALATRDIAEELGGEWADRFLVLYGIAAPD | 250 |
| Sbjct 191 | ACLPN+MVE GRFSG IDCGRLGVADRYQD+AL RDI EELG EW FL YGI D ACLPNVMVEGGRFSGLIDCGRLGVADRYQDLALTCRDIGEELGEEWVPPFLERYGIEVFD | 250 |
| Query 251  Sbjct 251 | SQRIAFYRLLDEFF 264 S R FYRLLDEFF SARARFYRLLDEFF 264 |  |

>WP_043177745.1 APH(3')-II family aminoglycoside O-phosphotransferase [Burkholderia cepacia]

KER73809.1 aminoglycoside phosphotransferase [Burkholderia cepacia] Length=264

Score = 327 bits (839), Expect = 8e-110, Method: Compositional matrix adjust. Identities = 167/264 (63%), Positives = 193/264 (73%), Gaps = 0/264 (0%)

| Query | 1 | MIEQDGLHAGSPAAWVERLFGYDWAQQTIGCSDAAVFRLSAQGRPVLFVKTDLSGALNEL | 60 |
| --- | --- | --- | --- |
| Sbjct | 1 | MI+ D L PA W L W +QT G SDAAVFRL A+ F+KT+++G L EL MIDNDKLCIEVPATWKAALADARWTRQTEGQSDAAVFRLDARDGSRRFIKTEVAGPLGEL | 60 |
| Query | 61 | QDEAARLSWLATTGVPCAAVLDVVTEAGRDWLLLGEVPGQDLLSSHLAPAEKVSIMADAM | 120 |
| Sbjct | 61 | +DEAARL WLATTG+P A VLDV + AGR+W+LL V G++L + LAPA KV+IMADA+ RDEAARLRWLATTGLPGAQVLDVASAAGREWMLLSAVRGENLEVAQLAPAAKVAIMADAL | 120 |
| Query | 121 | RRLHTLDPATCPFDHQAKHRIERARTRMEAGLVDQDDLDEEHQGLAPAELFARLKARMPD | 180 |
| Sbjct | 121 | R LH LDPATCPFDH RIERAR RM AGLVD+D+LD+ + G+ ELF RL+A P RELHRLDPATCPFDHGVASRIERARARMHAGLVDEDNLDDANTGVPIDELFVRLQASRPS | 180 |
| Query | 181 | GEDLVVTHGDACLPNIMVENGRFSGFIDCGRLGVADRYQDIALATRDIAEELGGEWADRF | 240 |
| Sbjct | 181 | ED VVTHGDACLPN MVE+GRFSGFIDCGRLGVADRYQD+ALA RDI +LG EW F IEDKVVTHGDACLPNFMVEDGRFSGFIDCGRLGVADRYQDLALAARDIEADLGSEWVAPF | 240 |
| Query | 241 | LVLYGIAAPDSQRIAFYRLLDEFF 264 YGI PD RIAFYRLLDEFF |  |
| Sbjct | 241 | FARYGIGQPDPDRIAFYRLLDEFF 264 | |

```
>WP_142918852.1 aminoglycoside 3'-phosphotransferase, partial [Staphylococcus cohnii]
EBS8169925.1 aminoglycoside 3'-phosphotransferase [Salmonella enterica]
RIL71411.1 aminoglycoside 3'-phosphotransferase, partial [Staphylococcus cohnii]
Length=158

Score = 323 bits (828), Expect = 9e-110, Method: Compositional matrix adjust. Identities = 158/158 (100%), Positives = 158/158 (100%), Gaps = 0/158 (0%)

Query 107 LAPAEKVSIMADAMRRLHTLDPATCPFDHQAKHRIERARTRMEAGLVDQDDLDEEHQGLA 166 LAPAEKVSIMADAMRRLHTLDPATCPFDHQAKHRIERARTRMEAGLVDQDDLDEEHQGLA
Sbjct 1	LAPAEKVSIMADAMRRLHTLDPATCPFDHQAKHRIERARTRMEAGLVDQDDLDEEHQGLA 60

Query 167 PAELFARLKARMPDGEDLVVTHGDACLPNIMVENGRFSGFIDCGRLGVADRYQDIALATR 226 PAELFARLKARMPDGEDLVVTHGDACLPNIMVENGRFSGFIDCGRLGVADRYQDIALATR
Sbjct 61	PAELFARLKARMPDGEDLVVTHGDACLPNIMVENGRFSGFIDCGRLGVADRYQDIALATR 120

Query 227 DIAEELGGEWADRFLVLYGIAAPDSQRIAFYRLLDEFF 264 DIAEELGGEWADRFLVLYGIAAPDSQRIAFYRLLDEFF
Sbjct 121 DIAEELGGEWADRFLVLYGIAAPDSQRIAFYRLLDEFF 158

>WP_155627211.1 aminoglycoside 3'-phosphotransferase [Burkholderia cepacia] Length=284

Score = 327 bits (839), Expect = 1e-109, Method: Compositional matrix adjust.
```

| Identities | | = 167/264 (63%), Positives = 193/264 (73%), Gaps = 0/264 (0%) |  |
| --- | --- | --- | --- |
| Query 1  Sbjct 21 | | MIEQDGLHAGSPAAWVERLFGYDWAQQTIGCSDAAVFRLSAQGRPVLFVKTDLSGALNEL  +I+ D L P AW RL G W +QT G SDA VFRL A+ FVKT +G L EL VIDNDPLGKDVPVAWQARLAGCRWTRQTDGQSDAVVFRLDARDGSRRFVKTAPAGPLGEL | 60  80 |
| Query 61  Sbjct 81 | | QDEAARLSWLATTGVPCAAVLDVVTEAGRDWLLLGEVPGQDLLSSHLAPAEKVSIMADAM  +DEAARL WLA+TG+P A VLDV + AGR+W+LL V G +L ++ LAPAEKV+IMADA+ RDEAARLRWLASTGLPGAQVLDVASAAGREWMLLSAVRGDNLETAQLAPAEKVAIMADAL | 120  140 |
| Query 121  Sbjct 141 | | RRLHTLDPATCPFDHQAKHRIERARTRMEAGLVDQDDLDEEHQGLAPAELFARLKARMPD R LH LDPA CPFDH A HRIERAR RM AGL D+D+LD+ + G+ LFARL A P RALHRLDPAACPFDHGAAHRIERARARMHAGLADEDNLDDTNAGVPLDALFARLLAHRPS | 180  200 |
| Query 181  Sbjct 201 | | GEDLVVTHGDACLPNIMVENGRFSGFIDCGRLGVADRYQDIALATRDIAEELGGEWADRF ED VVTHGDACLPN+M ++GRFSGFIDCGRLGVADRYQD+ALA RDI +LG EW RF TEDRVVTHGDACLPNLMADDGRFSGFIDCGRLGVADRYQDLALAARDIEADLGSEWVARF | 240  260 |
| Query 241 | | LVLYGIAAPDSQRIAFYRLLDEFF 264 YGI PD RIAFYRLLDEFF |  |
| Sbjct | 261 | FTRYGIEHPDPDRIAFYRLLDEFF 284 | |

>WP_176039657.1 APH(3') family aminoglycoside O-phosphotransferase [Burkholderia

stabilis] Length=264

Score = 327 bits (837), Expect = 1e-109, Method: Compositional matrix adjust.

| Identities | | = 170/264 (64%), Positives = 193/264 (73%), Gaps = 0/264 (0%) |  |
| --- | --- | --- | --- |
| Query 1  Sbjct 1 | | MIEQDGLHAGSPAAWVERLFGYDWAQQTIGCSDAAVFRLSAQGRPVLFVKTDLSGALNEL MI++D L PAAW L G W +QT G SDAAVFRL A+ FVKT+ +G L EL MIDKDKLCNDVPAAWTATLAGCRWTRQTDGQSDAAVFRLDARDGSRRFVKTEPAGPLGEL | 60  60 |
| Query 61  Sbjct 61 | | QDEAARLSWLATTGVPCAAVLDVVTEAGRDWLLLGEVPGQDLLSSHLAPAEKVSIMADAM QDEAARL WLA TGVP A VLDV + AGR+W+LL V G++L ++ LAPA KV+IMADA+ QDEAARLRWLAATGVPGARVLDVASTAGREWMLLSAVRGENLETARLAPAGKVTIMADAL | 120  120 |
| Query 121  Sbjct 121 | | RRLHTLDPATCPFDHQAKHRIERARTRMEAGLVDQDDLDEEHQGLAPAELFARLKARMPD R LH LDPA CPFDH A HRIERAR RM AGLVD+D+LD+ G+ LFARL+A P RALHRLDPAACPFDHGAAHRIERARARMNAGLVDEDNLDDSSTGVPLWVLFARLQAHRPP | 180  180 |
| Query 181  Sbjct 181 | | GEDLVVTHGDACLPNIMVENGRFSGFIDCGRLGVADRYQDIALATRDIAEELGGEWADRF ED VVTHGDACLPN MV+ FSGFIDCGRLGVADRYQD+ALATRDI +LG EW F IEDKVVTHGDACLPNFMVDEEAFSGFIDCGRLGVADRYQDLALATRDIEADLGSEWVAPF | 240  240 |
| Query 241 | | LVLYGIAAPDSQRIAFYRLLDEFF 264 YGI PD RIAFYRLLDEFF |  |
| Sbjct | 241 | FARYGIEHPDPDRIAFYRLLDEFF 264 | |

>WP_125853427.1 APH(3')-II family aminoglycoside O-phosphotransferase [Pseudomonas stutzeri]

RRW44677.1 APH(3')-II family aminoglycoside O-phosphotransferase [Pseudomonas stutzeri]

Length=264

Score = 327 bits (837), Expect = 2e-109, Method: Compositional matrix adjust.

| Identities | | = 165/253 (65%), Positives = 195/253 (77%), Gaps = 0/253 (0%) |  |
| --- | --- | --- | --- |
| Query 12  Sbjct 12 | | PAAWVERLFGYDWAQQTIGCSDAAVFRLSAQGRPVLFVKTDLSGALNELQDEAARLSWLA PAAW RL GY W Q GCS A+VFRL+A GRP LFVK++ S L EL DEAARL WLA PAAWRPRLAGYQWTLQEQGCSGASVFRLAAPGRPPLFVKSEPSDPLGELPDEAARLCWLA | 71  71 |
| Query 72  Sbjct 72 | | TTGVPCAAVLDVVTEAGRDWLLLGEVPGQDLLSSHLAPAEKVSIMADAMRRLHTLDPATC  +TG+ CA V++ ++E GRDWLLLG VPG++L +S L PA+KV+IMADA+R LH LDP C STGIACAPVVEAISEGGRDWLLLGAVPGENLSTSSLEPAQKVAIMADALRMLHALDPNGC | 131  131 |
| Query 132  Sbjct 132 | | PFDHQAKHRIERARTRMEAGLVDQDDLDEEHQGLAPAELFARLKARMPDGEDLVVTHGDA PFDH+A++R+ RAR RM+AG+VD DD D++H+GL+ +LFA LK + P EDLVVTH DA PFDHRAEYRVRRARARMDAGMVDDDDFDDDHRGLSSPQLFALLKTQRPAVEDLVVTHRDA | 191  191 |
| Query 192  Sbjct 192 | | CLPNIMVENGRFSGFIDCGRLGVADRYQDIALATRDIAEELGGEWADRFLVLYGIAAPDS CLPN MV+ GR SG IDCGRLGVADRYQD+ALA RDI EELG EW FL YGIAA D CLPNFMVDRGRLSGLIDCGRLGVADRYQDLALACRDITEELGEEWVQAFLACYGIAALDL | 251  251 |
| Query 252 | | QRIAFYRLLDEFF 264 R FYRLLDEFF |  |
| Sbjct | 252 | ARARFYRLLDEFF 264 | |

>QGW60326.1 hypothetical protein [bacterium] Length=264

Score = 326 bits (836), Expect = 2e-109, Method: Compositional matrix adjust.

| Identities | = 170/254 (67%), Positives = 193/254 (76%), Gaps = 0/254 (0%) |  |
| --- | --- | --- |
| Query 11 | SPAAWVERLFGYDWAQQTIGCSDAAVFRLSAQGRPVLFVKTDLSGALNELQDEAARLSWL | 70 |
| Sbjct 11 | SPAAW L GY WA Q GCS AAVFRL A G+P LFVK++ S L EL EAARL WL SPAAWRSLLAGYQWALQKQGCSGAAVFRLIASGQPSLFVKSEPSDPLCELLGEAARLRWL | 70 |
| Query 71 | ATTGVPCAAVLDVVTEAGRDWLLLGEVPGQDLLSSHLAPAEKVSIMADAMRRLHTLDPAT | 130 |
| Sbjct 71 | TG+PCA V+D ++E GRDWLL+GEVPG DLLSS L P +KV+IMADA+R LH LD +T ERTGIPCARVIDALSEGGRDWLLMGEVPGVDLLSSSLDPEKKVAIMADAVRMLHVLDSST | 130 |
| Query 131 | CPFDHQAKHRIERARTRMEAGLVDQDDLDEEHQGLAPAELFARLKARMPDGEDLVVTHGD | 190 |

| Sbjct | 131 | CPFDH+A+ RI RA+ RM+AGLVD DD D++H+GL+ EL ARL + P EDLVVTHGD CPFDHRAELRIHRAQARMDAGLVDDDDFDDDHRGLSSNELLARLHEQRPAVEDLVVTHGD | 190 |
| --- | --- | --- | --- |
| Query | 191 | ACLPNIMVENGRFSGFIDCGRLGVADRYQDIALATRDIAEELGGEWADRFLVLYGIAAPD | 250 |
| Sbjct | 191 | ACLPNIMVE GRFSG IDCGRLGVADRYQD+AL RDI EELG EW FL YG+ D ACLPNIMVEGGRFSGLIDCGRLGVADRYQDLALTCRDIGEELGEEWVPPFLERYGVEVFD | 250 |
| Query  Sbjct | 251  251 | SQRIAFYRLLDEFF 264 S R FYRLLDEFF SARARFYRLLDEFF 264 |  |

>VBB16329.1 Aminoglycoside 3'-phosphotransferase,Aminoglycoside phosphotransferase,Phosphotransferase

enzyme family [Burkholderia stabilis] Length=294

Score = 327 bits (839), Expect = 2e-109, Method: Compositional matrix adjust.

| Identities | | = 170/264 (64%), Positives = 194/264 (73%), Gaps = 0/264 (0%) |  |
| --- | --- | --- | --- |
| Query 1  Sbjct 31 | | MIEQDGLHAGSPAAWVERLFGYDWAQQTIGCSDAAVFRLSAQGRPVLFVKTDLSGALNEL  +I++D L PAAW L G W +QT G SDAAVFRL A+ FVKT+ +G L EL VIDKDKLCNDVPAAWTATLAGCRWTRQTDGQSDAAVFRLDARDGSRRFVKTEPAGPLGEL | 60  90 |
| Query 61  Sbjct 91 | | QDEAARLSWLATTGVPCAAVLDVVTEAGRDWLLLGEVPGQDLLSSHLAPAEKVSIMADAM QDEAARL WLA TGVP A VLDVV+ AGR+W+LL V G++L ++ LAPA KV+IMADA+ QDEAARLRWLAATGVPGARVLDVVSTAGREWMLLSAVRGENLETARLAPAGKVTIMADAL | 120  150 |
| Query 121  Sbjct 151 | | RRLHTLDPATCPFDHQAKHRIERARTRMEAGLVDQDDLDEEHQGLAPAELFARLKARMPD R LH LDPA CPFDH A HRIERAR RM AGLVD+D+LD+ G+ LFARL+A P RALHRLDPAACPFDHGAAHRIERARARMNAGLVDEDNLDDSGTGVPLRALFARLQAHRPP | 180  210 |
| Query 181  Sbjct 211 | | GEDLVVTHGDACLPNIMVENGRFSGFIDCGRLGVADRYQDIALATRDIAEELGGEWADRF ED VVTHGDACLPN MV+ FSGFIDCGRLGVADRYQD+ALATRDI +LG EW F IEDKVVTHGDACLPNFMVDEEAFSGFIDCGRLGVADRYQDLALATRDIEADLGSEWVAPF | 240  270 |
| Query 241 | | LVLYGIAAPDSQRIAFYRLLDEFF 264 YGI PD RIAFYRLLDEFF |  |
| Sbjct | 271 | FARYGIEHPDPDRIAFYRLLDEFF 294 | |

>WP_174978922.1 APH(3')-II family aminoglycoside O-phosphotransferase [Burkholderia lata]

Length=275

Score = 326 bits (836), Expect = 3e-109, Method: Compositional matrix adjust.

| Identities | | = 168/264 (64%), Positives = 194/264 (73%), Gaps = 0/264 (0%) |  |
| --- | --- | --- | --- |
| Query 1  Sbjct 12 | | MIEQDGLHAGSPAAWVERLFGYDWAQQTIGCSDAAVFRLSAQGRPVLFVKTDLSGALNEL  +I+++ L PA W L W +QT G SDAAVFRL AQ FVKT+ +G L EL VIDKERLCIEVPATWNATLADCRWTRQTEGQSDAAVFRLDAQDGSCRFVKTEPAGPLGEL | 60  71 |
| Query 61  Sbjct 72 | | QDEAARLSWLATTGVPCAAVLDVVTEAGRDWLLLGEVPGQDLLSSHLAPAEKVSIMADAM DEAARL WLATTG P A VL+V + AGR+W+LL V G++L + L PAEKV+I+ADA+ SDEAARLRWLATTGSPGAQVLEVASAAGREWMLLSAVCGENLEVAPLGPAEKVAIIADAL | 120  131 |
| Query 121  Sbjct 132 | | RRLHTLDPATCPFDHQAKHRIERARTRMEAGLVDQDDLDEEHQGLAPAELFARLKARMPD R LH LDPATCPFDH A+ RI RAR RM AGLVD+D+LDE + G++ ELFARL+A P RALHRLDPATCPFDHGAESRIARARARMNAGLVDEDNLDEANMGVSLDELFARLRASRPS | 180  191 |
| Query 181  Sbjct 192 | | GEDLVVTHGDACLPNIMVENGRFSGFIDCGRLGVADRYQDIALATRDIAEELGGEWADRF ED VVTHGDACLPN MVENGRFSGFIDCGRLGVADRYQD+ALA RDI +LG EW F IEDRVVTHGDACLPNFMVENGRFSGFIDCGRLGVADRYQDLALAARDIEADLGSEWVAPF | 240  251 |
| Query | 241 LVLYGIAAPDSQRIAFYRLLDEFF 264 | | |
|  |  | YGI PD RIAFYRLLDEFF |  |
| Sbjct | 252 | FTRYGIEQPDPGRIAFYRLLDEFF 275 | |

>WP_174949184.1 APH(3') family aminoglycoside O-phosphotransferase [Burkholderia lata]

VWC55392.1 aminoglycoside phosphotransferase APH(3') [Burkholderia lata]

Length=264

Score = 325 bits (833), Expect = 5e-109, Method: Compositional matrix adjust.

| Identities | = 170/264 (64%), Positives = 193/264 (73%), Gaps = 0/264 (0%) |  |
| --- | --- | --- |
| Query 1 | MIEQDGLHAGSPAAWVERLFGYDWAQQTIGCSDAAVFRLSAQGRPVLFVKTDLSGALNEL | 60 |
| Sbjct 1 | MI+ D L PA W L W QQT G SDAAVFRL A+ FVKT+ +G L EL MIDNDKLCIEVPATWKAALADCRWTQQTEGQSDAAVFRLDARDGSHRFVKTEPAGPLGEL | 60 |
| Query 61 | QDEAARLSWLATTGVPCAAVLDVVTEAGRDWLLLGEVPGQDLLSSHLAPAEKVSIMADAM | 120 |
| Sbjct 61 | DEAARL WLATTG+P A VLDV + AGR+W+LLG V G++L + L PAEKV+I+ADA+ SDEAARLRWLATTGLPGAQVLDVASAAGREWMLLGAVRGENLEVARLGPAEKVAIVADAL | 120 |
| Query 121 | RRLHTLDPATCPFDHQAKHRIERARTRMEAGLVDQDDLDEEHQGLAPAELFARLKARMPD | 180 |
| Sbjct 121 | R LH LDPATCPFDH RIERAR RM GLVD+ +LD+ + G+ ELFARL+A P RALHRLDPATCPFDHGVASRIERARARMNEGLVDEGNLDDANTGVPLDELFARLQASRPL | 180 |
| Query 181 | GEDLVVTHGDACLPNIMVENGRFSGFIDCGRLGVADRYQDIALATRDIAEELGGEWADRF | 240 |
| Sbjct 181 | ED VVTHGDACLPN MVE+GRFSGFIDCGRLGVADRYQD+ALATRDI +LG EW F IEDRVVTHGDACLPNFMVEDGRFSGFIDCGRLGVADRYQDLALATRDIEADLGSEWIASF | 240 |
| Query 241  Sbjct 241 | LVLYGIAAPDSQRIAFYRLLDEFF 264 L YGI PD RIAFYRLLDEFF LARYGIDQPDPGRIAFYRLLDEFF 264 |  |

>VWD16734.1 aminoglycoside phosphotransferase APH(3') [Burkholderia lata] Length=264

Score = 324 bits (831), Expect = 1e-108, Method: Compositional matrix adjust.

| Identities | | = 168/264 (64%), Positives = 193/264 (73%), Gaps = 0/264 (0%) |  |
| --- | --- | --- | --- |
| Query 1  Sbjct 1 | | MIEQDGLHAGSPAAWVERLFGYDWAQQTIGCSDAAVFRLSAQGRPVLFVKTDLSGALNEL MI+++ L PA W L W QQT G SDAAVFRL AQ FVKT+ +G L EL MIDRERLCNEVPATWNATLADCRWTQQTEGQSDAAVFRLDAQDGSCRFVKTEPAGPLGEL | 60  60 |
| Query 61  Sbjct 61 | | QDEAARLSWLATTGVPCAAVLDVVTEAGRDWLLLGEVPGQDLLSSHLAPAEKVSIMADAM DEAARL WLATTG+P A VL+V + AGR+W+LL V G +L + L PAEKV+I+ADA+ SDEAARLRWLATTGLPGAQVLEVASAAGREWMLLSAVCGANLEVAPLGPAEKVAIIADAL | 120  120 |
| Query 121  Sbjct 121 | | RRLHTLDPATCPFDHQAKHRIERARTRMEAGLVDQDDLDEEHQGLAPAELFARLKARMPD R LH LDPATCPFDH A+ RI RAR RM AGLVD+D+LD+ + G+ ELFARL+A P RALHRLDPATCPFDHGAESRIARARARMNAGLVDEDNLDDANMGVPVDELFARLRASRPS | 180  180 |
| Query 181  Sbjct 181 | | GEDLVVTHGDACLPNIMVENGRFSGFIDCGRLGVADRYQDIALATRDIAEELGGEWADRF ED VVTHGDACLPN MVE+GRFSGFIDCGRLGVADRYQD+ALA RDI +LG EW F IEDRVVTHGDACLPNFMVEDGRFSGFIDCGRLGVADRYQDLALAARDIEADLGSEWVAPF | 240  240 |
| Query | 241 LVLYGIAAPDSQRIAFYRLLDEFF 264 | | |
|  |  | YGI PD RIAFYRLLDEFF |  |
| Sbjct | 241 | FARYGIEQPDPGRIAFYRLLDEFF 264 | |

>WP_137937931.1 APH(3')-II family aminoglycoside O-phosphotransferase [Chitinivorax sp. B]

Length=260

Score = 324 bits (830), Expect = 1e-108, Method: Compositional matrix adjust.

| Identities | = 158/253 (62%), Positives = 191/253 (75%), Gaps = 1/253 (0%) |  |
| --- | --- | --- |
| Query 12 | PAAWVERLFGYDWAQQTIGCSDAAVFRLSAQGRPVLFVKTDLSGALNELQDEAARLSWLA | 71 |
| Sbjct 9 | P+ W +L GY W +QT+GCSDAAVFRL++ +LFVKT+ +G L+EL+DEAARL WL PSVWCSQLDGYTWTRQTVGCSDAAVFRLTSPNNAILFVKTEPAGPLSELRDEAARLRWLN | 68 |
| Query 72 | TTGVPCAAVLDVVTEAGRDWLLLGEVPGQDLLSSHLAPAEKVSIMADAMRRLHTLDPATC | 131 |
| Sbjct 69 | TTG+ CA VL V E+G+DWLLL VPG DL S+ L PA V+IMA A+R+LH LD A+C TTGITCAQVLSEVHESGQDWLLLSAVPGTDLFSTQLTPANTVAIMAAALRKLHQLDIASC | 128 |
| Query 132 | PFDHQAKHRIERARTRMEAGLVDQDDLDEEHQGLAPAELFARLKARMPDGEDLVVTHGDA | 191 |
| Sbjct 129 | PFDHQA RIERA RM AGLVD DDLD+ +QG +P L+ +L P ED VVTHGDA  PFDHQATIRIERALARMMAGLVDHDDLDDSNQGQSPEALYQQLHTLKPSIEDWVVTHGDA | 188 |

| Query | 192 | CLPNIMVENGRFSGFIDCGRLGVADRYQDIALATRDIAEELGGEWADRFLVLYGIAAPDS 251 |
| --- | --- | --- |
| Sbjct | 189 | C+PN+MV++ F+GFIDCGRLGVADR+QD+ALA RDIAEELG +W D FL YG+ D CMPNLMVDSSGFTGFIDCGRLGVADRHQDLALAVRDIAEELGEQWIDPFLDHYGMQL-DP 247 |
| Query | 252 | QRIAFYRLLDEFF 264 |
| Sbjct | 248 | R AFYRLLDEFF  NRAAFYRLLDEFF 260 |

```
>WP_165881814.1 APH(3') family aminoglycoside O-phosphotransferase, partial [Bacillus amyloliquefaciens]
NHN19445.1 aminoglycoside 3'-phosphotransferase [Bacillus amyloliquefaciens] HAL1648762.1 aminoglycoside 3'-phosphotransferase [Escherichia coli] HAL1648764.1 aminoglycoside 3'-phosphotransferase [Escherichia coli]
Length=156

Score = 320 bits (820), Expect = 1e-108, Method: Compositional matrix adjust. Identities = 156/156 (100%), Positives = 156/156 (100%), Gaps = 0/156 (0%)

Query 109 PAEKVSIMADAMRRLHTLDPATCPFDHQAKHRIERARTRMEAGLVDQDDLDEEHQGLAPA 168 PAEKVSIMADAMRRLHTLDPATCPFDHQAKHRIERARTRMEAGLVDQDDLDEEHQGLAPA
Sbjct 1	PAEKVSIMADAMRRLHTLDPATCPFDHQAKHRIERARTRMEAGLVDQDDLDEEHQGLAPA 60

Query 169 ELFARLKARMPDGEDLVVTHGDACLPNIMVENGRFSGFIDCGRLGVADRYQDIALATRDI 228 ELFARLKARMPDGEDLVVTHGDACLPNIMVENGRFSGFIDCGRLGVADRYQDIALATRDI
Sbjct 61	ELFARLKARMPDGEDLVVTHGDACLPNIMVENGRFSGFIDCGRLGVADRYQDIALATRDI 120

Query 229 AEELGGEWADRFLVLYGIAAPDSQRIAFYRLLDEFF 264 AEELGGEWADRFLVLYGIAAPDSQRIAFYRLLDEFF
Sbjct 121 AEELGGEWADRFLVLYGIAAPDSQRIAFYRLLDEFF 156

>WP_138300002.1 APH(3')-II family aminoglycoside O-phosphotransferase [Pseudomonas stutzeri]
QCT96808.1 APH(3')-II family aminoglycoside O-phosphotransferase [Pseudomonas stutzeri]
Length=264

Score = 324 bits (830), Expect = 2e-108, Method: Compositional matrix adjust.
```

| Identities | = 169/254 (67%), Positives = 193/254 (76%), Gaps = 0/254 (0%) |  |
| --- | --- | --- |
| Query 11 | SPAAWVERLFGYDWAQQTIGCSDAAVFRLSAQGRPVLFVKTDLSGALNELQDEAARLSWL | 70 |
| Sbjct 11 | SPAAW L GY WA Q GCS AAVFRL+A G+P LFVK++ S L EL EAARL WL SPAAWRSLLAGYQWALQKQGCSGAAVFRLTASGQPSLFVKSEPSDPLCELLGEAARLRWL | 70 |
| Query 71 | ATTGVPCAAVLDVVTEAGRDWLLLGEVPGQDLLSSHLAPAEKVSIMADAMRRLHTLDPAT | 130 |
| Sbjct 71 | TG+PCA V+D ++E GRDWLLLGEVPG DLLSS L P +KV+IMADA+R LH LD +T ERTGIPCARVIDALSEGGRDWLLLGEVPGVDLLSSSLDPEKKVAIMADAVRMLHELDSST | 130 |
| Query 131 | CPFDHQAKHRIERARTRMEAGLVDQDDLDEEHQGLAPAELFARLKARMPDGEDLVVTHGD | 190 |
| Sbjct 131 | CPFDH+A+ RI RA+ RM+AGLVD DD D++H+GL+ EL ARL + P EDLVVTHGD CPFDHRAELRIHRAQARMDAGLVDDDDFDDDHRGLSSNELLARLHEQRPAVEDLVVTHGD | 190 |
| Query 191 | ACLPNIMVENGRFSGFIDCGRLGVADRYQDIALATRDIAEELGGEWADRFLVLYGIAAPD | 250 |
| Sbjct 191 | ACLPN+MVE G FSG IDCGRLGVADRYQD+AL RDI EELG EW FL YG+ D ACLPNVMVEGGCFSGLIDCGRLGVADRYQDLALTCRDIGEELGEEWVPPFLERYGVEVFD | 250 |
| Query 251  Sbjct 251 | SQRIAFYRLLDEFF 264 S R FYRLLDEFF SARARFYRLLDEFF 264 |  |

>WP_182364673.1 APH(3') family aminoglycoside O-phosphotransferase, partial [Staphylococcus schleiferi]

MBA8775271.1 APH(3') family aminoglycoside O-phosphotransferase [Staphylococcus schleiferi subsp. coagulans]

Length=156

Score = 320 bits (819), Expect = 2e-108, Method: Compositional matrix adjust. Identities = 156/156 (100%), Positives = 156/156 (100%), Gaps = 0/156 (0%)

Query 103 LSSHLAPAEKVSIMADAMRRLHTLDPATCPFDHQAKHRIERARTRMEAGLVDQDDLDEEH 162

| Sbjct | 1 | LSSHLAPAEKVSIMADAMRRLHTLDPATCPFDHQAKHRIERARTRMEAGLVDQDDLDEEH LSSHLAPAEKVSIMADAMRRLHTLDPATCPFDHQAKHRIERARTRMEAGLVDQDDLDEEH | 60 |
| --- | --- | --- | --- |
| Query | 163 | QGLAPAELFARLKARMPDGEDLVVTHGDACLPNIMVENGRFSGFIDCGRLGVADRYQDIA | 222 |
| Sbjct | 61 | QGLAPAELFARLKARMPDGEDLVVTHGDACLPNIMVENGRFSGFIDCGRLGVADRYQDIA QGLAPAELFARLKARMPDGEDLVVTHGDACLPNIMVENGRFSGFIDCGRLGVADRYQDIA | 120 |
| Query  Sbjct | 223  121 | LATRDIAEELGGEWADRFLVLYGIAAPDSQRIAFYR 258 LATRDIAEELGGEWADRFLVLYGIAAPDSQRIAFYR LATRDIAEELGGEWADRFLVLYGIAAPDSQRIAFYR 156 |  |

>WP_175031633.1 APH(3') family aminoglycoside O-phosphotransferase [Burkholderia lata]

VWB57032.1 aminoglycoside phosphotransferase APH(3') [Burkholderia lata] Length=264

Score = 323 bits (829), Expect = 2e-108, Method: Compositional matrix adjust.

| Identities | | = 169/264 (64%), Positives = 191/264 (72%), Gaps = 0/264 (0%) |  |
| --- | --- | --- | --- |
| Query 1  Sbjct 1 | | MIEQDGLHAGSPAAWVERLFGYDWAQQTIGCSDAAVFRLSAQGRPVLFVKTDLSGALNEL MI+ D L PAAW L G W +QT G SDAAVFRL A+ FVKT+ +G L EL MIDNDKLCIEVPAAWQAALTGCRWTRQTDGQSDAAVFRLDARDGSRRFVKTEPAGPLGEL | 60  60 |
| Query 61  Sbjct 61 | | QDEAARLSWLATTGVPCAAVLDVVTEAGRDWLLLGEVPGQDLLSSHLAPAEKVSIMADAM DEAARL WLATTG+P A VLDV + AGR+WLLL V G++L + L P EKV+I+ADA+ SDEAARLRWLATTGLPGAQVLDVASSAGREWLLLSAVRGENLEVARLGPGEKVAIVADAL | 120  120 |
| Query 121  Sbjct 121 | | RRLHTLDPATCPFDHQAKHRIERARTRMEAGLVDQDDLDEEHQGLAPAELFARLKARMPD R LH LDPATCPFDH A HRIERA RM GLVD+D+LD + G+ LF RL+A P RALHRLDPATCPFDHGAAHRIERAGARMNEGLVDEDNLDAANTGVPLDALFVRLQASRPL | 180  180 |
| Query 181  Sbjct 181 | | GEDLVVTHGDACLPNIMVENGRFSGFIDCGRLGVADRYQDIALATRDIAEELGGEWADRF ED VVTHGDACLPN MVE+G FSGFIDCGRLGVADRYQD+ALATRDI +LG EW F SEDKVVTHGDACLPNFMVESGCFSGFIDCGRLGVADRYQDLALATRDIEADLGSEWVTPF | 240  240 |
| Query | 241 LVLYGIAAPDSQRIAFYRLLDEFF 264 | | |
|  |  | V YGI PD RIAFYRLLDEFF |  |
| Sbjct | 241 | FVRYGIEHPDPDRIAFYRLLDEFF 264 | |

>MBB1626375.1 APH(3') family aminoglycoside O-phosphotransferase [Achromobacter sp. UMC71]

Length=246

Score = 323 bits (827), Expect = 2e-108, Method: Compositional matrix adjust.

| Identities | = 154/246 (63%), Positives = 185/246 (75%), Gaps = 0/246 (0%) |  |
| --- | --- | --- |
| Query 19 | LFGYDWAQQTIGCSDAAVFRLSAQGRPVLFVKTDLSGALNELQDEAARLSWLATTGVPCA | 78 |
| Sbjct 1 | + YDW +QT GCSDAAVF+L A RP LF+KT+ + L+ELQ+EAARL WL TG+PCA MASYDWTRQTAGCSDAAVFQLQAPNRPTLFLKTEPASPLSELQNEAARLRWLGQTGIPCA | 60 |
| Query 79 | AVLDVVTEAGRDWLLLGEVPGQDLLSSHLAPAEKVSIMADAMRRLHTLDPATCPFDHQAK | 138 |
| Sbjct 61 | VLD+ EAGRDW+LL VPG DL S+ L PA+KV+I+A+ +RRLH++DP TCPFD +A QVLDLAHEAGRDWMLLNAVPGTDLHSAPLPPADKVAILAEVLRRLHSVDPKTCPFDQRAA | 120 |
| Query 139 | HRIERARTRMEAGLVDQDDLDEEHQGLAPAELFARLKARMPDGEDLVVTHGDACLPNIMV | 198 |
| Sbjct 121 | RIE AR R+ AG VD DD DE+H+ L P LFA L+ P E VVTHGDACLPNIMV RRIEHARARLAAGRVDTDDFDEDHRALTPTALFANLEQTRPRDEAPVVTHGDACLPNIMV | 180 |
| Query 199 | ENGRFSGFIDCGRLGVADRYQDIALATRDIAEELGGEWADRFLVLYGIAAPDSQRIAFYR | 258 |
| Sbjct 181 | + GR SG IDCGRLG+ADRYQD+ALA RDI + LG EW + FL YGI PD +R+ FYR DQGRVSGLIDCGRLGLADRYQDLALAARDIGDTLGPEWVNPFLDRYGIDRPDRERLKFYR | 240 |
| Query 259  Sbjct 241 | LLDEFF 264 LLDEFF LLDEFF 246 |  |

```
>WP_167654213.1 APH(3') family aminoglycoside O-phosphotransferase, partial [Salmonella enterica]
NJH41538.1 APH(3') family aminoglycoside O-phosphotransferase [Salmonella

enterica subsp. enterica serovar Anatum] Length=156

Score = 319 bits (817), Expect = 4e-108, Method: Compositional matrix adjust. Identities = 156/156 (100%), Positives = 156/156 (100%), Gaps = 0/156 (0%)

Query 69	WLATTGVPCAAVLDVVTEAGRDWLLLGEVPGQDLLSSHLAPAEKVSIMADAMRRLHTLDP 128 WLATTGVPCAAVLDVVTEAGRDWLLLGEVPGQDLLSSHLAPAEKVSIMADAMRRLHTLDP
Sbjct 1	WLATTGVPCAAVLDVVTEAGRDWLLLGEVPGQDLLSSHLAPAEKVSIMADAMRRLHTLDP 60

Query 129 ATCPFDHQAKHRIERARTRMEAGLVDQDDLDEEHQGLAPAELFARLKARMPDGEDLVVTH 188 ATCPFDHQAKHRIERARTRMEAGLVDQDDLDEEHQGLAPAELFARLKARMPDGEDLVVTH
Sbjct 61	ATCPFDHQAKHRIERARTRMEAGLVDQDDLDEEHQGLAPAELFARLKARMPDGEDLVVTH 120

Query 189 GDACLPNIMVENGRFSGFIDCGRLGVADRYQDIALA 224 GDACLPNIMVENGRFSGFIDCGRLGVADRYQDIALA
Sbjct 121 GDACLPNIMVENGRFSGFIDCGRLGVADRYQDIALA 156

>WP_175011774.1 APH(3') family aminoglycoside O-phosphotransferase [Burkholderia lata]
VWC88620.1 aminoglycoside phosphotransferase APH(3') [Burkholderia lata] Length=264

Score = 323 bits (827), Expect = 5e-108, Method: Compositional matrix adjust.
```

| Identities | | = 170/264 (64%), Positives = 195/264 (74%), Gaps = 0/264 (0%) |  |
| --- | --- | --- | --- |
| Query 1  Sbjct 1 | | MIEQDGLHAGSPAAWVERLFGYDWAQQTIGCSDAAVFRLSAQGRPVLFVKTDLSGALNEL MI+ D L PAAW L G W +QT G SDAAVFRL A+ FVKT+ +G L EL MIDNDKLCIEVPAAWKVALAGCRWTRQTEGQSDAAVFRLDARDGSRWFVKTEPAGPLGEL | 60  60 |
| Query 61  Sbjct 61 | | QDEAARLSWLATTGVPCAAVLDVVTEAGRDWLLLGEVPGQDLLSSHLAPAEKVSIMADAM DEAARL WLATTG+P A VLDV + AGR+WLLL V G++L + L PAEKV+I+ADA+ SDEAARLRWLATTGLPGAQVLDVTSSAGREWLLLSAVRGENLELARLGPAEKVAIIADAL | 120  120 |
| Query 121  Sbjct 121 | | RRLHTLDPATCPFDHQAKHRIERARTRMEAGLVDQDDLDEEHQGLAPAELFARLKARMPD R LH LDPATCPF H A RIERAR R+ AGLVD+D+LD+ + G+ ELFARL+A P RALHRLDPATCPFGHGAASRIERARARISAGLVDEDNLDDANTGVPLDELFARLQASRPL | 180  180 |
| Query 181  Sbjct 181 | | GEDLVVTHGDACLPNIMVENGRFSGFIDCGRLGVADRYQDIALATRDIAEELGGEWADRF ED VVTHGDACLPN MVE+G FSGFIDCGRLGVADRYQD+ALATRDI +LG EW F VEDRVVTHGDACLPNFMVESGCFSGFIDCGRLGVADRYQDLALATRDIEADLGREWVAPF | 240  240 |
| Query | 241 LVLYGIAAPDSQRIAFYRLLDEFF 264 | | |
|  |  | V YGI PD RIAFYR+LDEFF |  |
| Sbjct | 241 | FVRYGIEHPDPDRIAFYRVLDEFF 264 | |

>WP_057384224.1 APH(3')-II family aminoglycoside O-phosphotransferase [Pseudomonas aeruginosa]

KSE29962.1 aminoglycoside 3'-phosphotransferase [Pseudomonas aeruginosa] Length=264

Score = 322 bits (825), Expect = 8e-108, Method: Compositional matrix adjust.

| Identities | = 165/253 (65%), Positives = 191/253 (75%), Gaps = 0/253 (0%) |  |
| --- | --- | --- |
| Query 12 | PAAWVERLFGYDWAQQTIGCSDAAVFRLSAQGRPVLFVKTDLSGALNELQDEAARLSWLA | 71 |
| Sbjct 12 | P AW L GY WA Q GCS AAVFRL+A G+P LFVK++ S L EL DEAARL WL PVAWRSLLVGYHWALQDQGCSGAAVFRLTASGKPSLFVKSEPSDPLGELLDEAARLRWLE | 71 |
| Query 72 | TTGVPCAAVLDVVTEAGRDWLLLGEVPGQDLLSSHLAPAEKVSIMADAMRRLHTLDPATC | 131 |
| Sbjct 72 | TG+PCA V+D ++E GRDWLLLGEVPG+DLLSS L P KV+IMAD +R LH LDP TC RTGIPCARVIDALSEGGRDWLLLGEVPGEDLLSSSLDPERKVTIMADVVRMLHALDPFTC | 131 |
| Query 132 | PFDHQAKHRIERARTRMEAGLVDQDDLDEEHQGLAPAELFARLKARMPDGEDLVVTHGDA | 191 |
| Sbjct 132 | PFDH+ + RI RA+ RM+AGLVD DD D++H GL+ EL +RL + P EDLVVTHGDA PFDHRVELRIHRAQARMDAGLVDDDDFDDDHVGLSAGELLSRLHEQRPVFEDLVVTHGDA | 191 |
| Query 192 | CLPNIMVENGRFSGFIDCGRLGVADRYQDIALATRDIAEELGGEWADRFLVLYGIAAPDS | 251 |
| Sbjct 192 | CLPN++V+ RFSG IDCGRLGVADRYQD+AL RDIAEELG EW FL YGI A D  CLPNVIVDEDRFSGLIDCGRLGVADRYQDLALTCRDIAEELGEEWVQVFLRRYGIEALDP | 251 |

Query 252 QRIAFYRLLDEFF 264

R +FYRLLDEF+ Sbjct 252 ARASFYRLLDEFY 264

>WP_175004359.1 APH(3')-II family aminoglycoside O-phosphotransferase [Burkholderia lata]

Length=275

Score = 322 bits (826), Expect = 1e-107, Method: Compositional matrix adjust.

| Identities | | = 167/264 (63%), Positives = 193/264 (73%), Gaps = 0/264 (0%) |  |
| --- | --- | --- | --- |
| Query 1  Sbjct 12 | | MIEQDGLHAGSPAAWVERLFGYDWAQQTIGCSDAAVFRLSAQGRPVLFVKTDLSGALNEL  +I+++ L PA W L W QQT G SDAAVFRL AQ FVKT+ +G L EL VIDRERLCNEVPATWNATLADCRWTQQTEGQSDAAVFRLDAQDGSCRFVKTEPAGPLGEL | 60  71 |
| Query 61  Sbjct 72 | | QDEAARLSWLATTGVPCAAVLDVVTEAGRDWLLLGEVPGQDLLSSHLAPAEKVSIMADAM DEAARL WLATTG+P A VL+V + AGR+W+LL V G +L + L PAEKV+I+ADA+ SDEAARLRWLATTGLPGAQVLEVASAAGREWMLLSAVCGANLEVAPLGPAEKVAIIADAL | 120  131 |
| Query 121  Sbjct 132 | | RRLHTLDPATCPFDHQAKHRIERARTRMEAGLVDQDDLDEEHQGLAPAELFARLKARMPD R LH LDPATCPFDH A+ RI RAR RM AGLVD+D+LD+ + G+ ELFARL+A P RALHRLDPATCPFDHGAESRIARARARMNAGLVDEDNLDDANMGVPVDELFARLRASRPS | 180  191 |
| Query 181  Sbjct 192 | | GEDLVVTHGDACLPNIMVENGRFSGFIDCGRLGVADRYQDIALATRDIAEELGGEWADRF ED VVTHGDACLPN MVE+GRFSGFIDCGRLGVADRYQD+ALA RDI +LG EW F IEDRVVTHGDACLPNFMVEDGRFSGFIDCGRLGVADRYQDLALAARDIEADLGSEWVAPF | 240  251 |
| Query | 241 LVLYGIAAPDSQRIAFYRLLDEFF 264 | | |
|  |  | YGI PD RIAFYRLLDEFF |  |
| Sbjct | 252 | FARYGIEQPDPGRIAFYRLLDEFF 275 | |

>WP_176045972.1 APH(3') family aminoglycoside O-phosphotransferase [Burkholderia sp. BCC1644]

Length=264

Score = 322 bits (825), Expect = 1e-107, Method: Compositional matrix adjust.

| Identities | | = 167/264 (63%), Positives = 195/264 (74%), Gaps = 0/264 (0%) |  |
| --- | --- | --- | --- |
| Query 1  Sbjct 1 | | MIEQDGLHAGSPAAWVERLFGYDWAQQTIGCSDAAVFRLSAQGRPVLFVKTDLSGALNEL MI+ D L PAAW L G W +QT G SDAAVFRL A+ FVKT+ +G L EL MIDNDPLGKEVPAAWQASLAGCRWTRQTDGQSDAAVFRLDARDGSCRFVKTEPAGPLGEL | 60  60 |
| Query 61  Sbjct 61 | | QDEAARLSWLATTGVPCAAVLDVVTEAGRDWLLLGEVPGQDLLSSHLAPAEKVSIMADAM  +DEAARL WLATTGVP A VLDV + AGR+W+LL V G +L + LAPA KV+I+ADA+ RDEAARLRWLATTGVPGAQVLDVASAAGREWMLLSAVRGDNLELARLAPASKVTIVADAL | 120  120 |
| Query 121  Sbjct 121 | | RRLHTLDPATCPFDHQAKHRIERARTRMEAGLVDQDDLDEEHQGLAPAELFARLKARMPD R LH LDPATCPFDH+A RI RAR R++AG VD+ +LD+ + G+ +LFARL A P RALHRLDPATCPFDHRAALRIGRARARIDAGRVDEGNLDDANTGVPLDKLFARLLAHRPS | 180  180 |
| Query 181  Sbjct 181 | | GEDLVVTHGDACLPNIMVENGRFSGFIDCGRLGVADRYQDIALATRDIAEELGGEWADRF ED VVTHGDACLPNIMV++GRFSGFIDCGRLGVADRYQD+ALA RDI +LG EW F TEDRVVTHGDACLPNIMVDDGRFSGFIDCGRLGVADRYQDLALAARDIEADLGSEWVAPF | 240  240 |
| Query 241 | | LVLYGIAAPDSQRIAFYRLLDEFF 264 YGIA PD +RI FYRLLDEFF |  |
| Sbjct | 241 | FTRYGIAHPDPERITFYRLLDEFF 264 | |

```
>WP_175045087.1 APH(3') family aminoglycoside O-phosphotransferase [Burkholderia lata]
VWD00025.1 aminoglycoside phosphotransferase APH(3') [Burkholderia lata] Length=264

Score = 322 bits (825), Expect = 1e-107, Method: Compositional matrix adjust. Identities = 169/264 (64%), Positives = 192/264 (73%), Gaps = 0/264 (0%)

Query 1	MIEQDGLHAGSPAAWVERLFGYDWAQQTIGCSDAAVFRLSAQGRPVLFVKTDLSGALNEL 60 MI+ D L	PA W	L	W QQT G SDAAVFRL A+	FVK + +G L EL
Sbjct 1	MIDNDKLCIEVPATWKAALADCRWTQQTEGQSDAAVFRLDARDGSRRFVKIEPAGPLGEL 60
```

| Query | 61 | QDEAARLSWLATTGVPCAAVLDVVTEAGRDWLLLGEVPGQDLLSSHLAPAEKVSIMADAM | 120 |
| --- | --- | --- | --- |
| Sbjct | 61 | DEAARL WLATTG+P A VLDV + AGR+W+LLG V G++L + L PAEKV+I+ADA+ SDEAARLRWLATTGLPGAQVLDVASAAGREWMLLGAVRGENLEVARLGPAEKVAIIADAL | 120 |
| Query | 121 | RRLHTLDPATCPFDHQAKHRIERARTRMEAGLVDQDDLDEEHQGLAPAELFARLKARMPD | 180 |
| Sbjct | 121 | R LH LDPATCPFDH A RIERAR RM GLVD+ +LD+ + G+ ELFARL+A P RALHRLDPATCPFDHGAASRIERARARMNEGLVDEGNLDDANTGVPLDELFARLQASRPL | 180 |
| Query | 181 | GEDLVVTHGDACLPNIMVENGRFSGFIDCGRLGVADRYQDIALATRDIAEELGGEWADRF | 240 |
| Sbjct | 181 | ED VVTHGDACLPN MVENG FSGFIDCGRLGVADRYQD+ALATRDI +LG +W F IEDWVVTHGDACLPNFMVENGCFSGFIDCGRLGVADRYQDLALATRDIEADLGSQWIAPF | 240 |
| Query | 241 | LVLYGIAAPDSQRIAFYRLLDEFF 264 V YGI PD RIAFYRLLDEFF |  |
| Sbjct | 241 | FVRYGIEDPDPDRIAFYRLLDEFF 264 | |

>WP_089453407.1 APH(3')-II family aminoglycoside O-phosphotransferase [Burkholderia sp. AU17325]

OXI37018.1 APH(3') family aminoglycoside O-phosphotransferase [Burkholderia sp. AU17325]

Length=275

Score = 322 bits (826), Expect = 1e-107, Method: Compositional matrix adjust.

| Identities | | = 166/264 (63%), Positives = 190/264 (72%), Gaps = 0/264 (0%) |  |
| --- | --- | --- | --- |
| Query 1  Sbjct 12 | | MIEQDGLHAGSPAAWVERLFGYDWAQQTIGCSDAAVFRLSAQGRPVLFVKTDLSGALNEL  +I+ D L PA W L W QQT G SDAAVFRL A+ F+KT+ G L EL VIDNDKLCIEVPATWKAALADAQWTQQTEGQSDAAVFRLDARDGSRRFIKTEPVGPLGEL | 60  71 |
| Query 61  Sbjct 72 | | QDEAARLSWLATTGVPCAAVLDVVTEAGRDWLLLGEVPGQDLLSSHLAPAEKVSIMADAM  +DEAARL WL TTG+P A VLDV + AGR+W+LL V G++L + L PA KV+IMADA+ RDEAARLRWLVTTGLPGAQVLDVASAAGREWMLLSAVCGENLEVAQLVPAAKVAIMADAL | 120  131 |
| Query 121  Sbjct 132 | | RRLHTLDPATCPFDHQAKHRIERARTRMEAGLVDQDDLDEEHQGLAPAELFARLKARMPD R LH LDPATCPFDH RIERAR RM AGLVD+D+LD+ + G+ ELF RL+A P RGLHRLDPATCPFDHGVASRIERARARMHAGLVDEDNLDDANTGVPIDELFGRLQASRPS | 180  191 |
| Query 181  Sbjct 192 | | GEDLVVTHGDACLPNIMVENGRFSGFIDCGRLGVADRYQDIALATRDIAEELGGEWADRF ED VVTHGDACLPN MVE+GRFSGFIDCGRLGVADRYQD+ALA RDI +LG EW F IEDKVVTHGDACLPNFMVEDGRFSGFIDCGRLGVADRYQDLALAARDIEADLGSEWIAPF | 240  251 |
| Query 241 | | LVLYGIAAPDSQRIAFYRLLDEFF 264 V YGI PD RIAFYRLLDEFF |  |
| Sbjct | 252 | FVRYGIGQPDPDRIAFYRLLDEFF 275 | |

>VWB83275.1 aminoglycoside phosphotransferase APH(3') [Burkholderia lata] VWC32108.1 aminoglycoside phosphotransferase APH(3') [Burkholderia lata]

Length=264

Score = 322 bits (824), Expect = 1e-107, Method: Compositional matrix adjust.

| Identities | = 166/264 (63%), Positives = 194/264 (73%), Gaps = 0/264 (0%) |  |
| --- | --- | --- |
| Query 1 | MIEQDGLHAGSPAAWVERLFGYDWAQQTIGCSDAAVFRLSAQGRPVLFVKTDLSGALNEL | 60 |
| Sbjct 1 | MI+++ L PA W L W +QT G SDAAVFRL AQ FVKT+ +G L EL MIDKERLCIEVPATWNATLADCRWTRQTEGQSDAAVFRLDAQDGSCRFVKTEPAGPLGEL | 60 |
| Query 61 | QDEAARLSWLATTGVPCAAVLDVVTEAGRDWLLLGEVPGQDLLSSHLAPAEKVSIMADAM | 120 |
| Sbjct 61 | +DEAARL WLATTG+P A VL+ + AGR+W+LL V G++L + L PAEKV+I+ADA+ RDEAARLRWLATTGLPGAQVLEAASAAGREWMLLSAVCGENLEVAPLGPAEKVAIIADAL | 120 |
| Query 121 | RRLHTLDPATCPFDHQAKHRIERARTRMEAGLVDQDDLDEEHQGLAPAELFARLKARMPD | 180 |
| Sbjct 121 | R LH LDPATCPFDH A+ RI RAR RM AGLVD+D+LD+ + G+ ELFARL+A P RALHRLDPATCPFDHGAESRIARARARMNAGLVDEDNLDDANMGVPLDELFARLRASRPS | 180 |
| Query 181 | GEDLVVTHGDACLPNIMVENGRFSGFIDCGRLGVADRYQDIALATRDIAEELGGEWADRF | 240 |
| Sbjct 181 | ED VVTHGDACLPN MVE+GRFSGFIDCGRLGVADRYQD+ALA RDI +LG EW F IEDRVVTHGDACLPNFMVEDGRFSGFIDCGRLGVADRYQDLALAARDIEADLGSEWVAPF | 240 |
| Query 241 | LVLYGIAAPDSQRIAFYRLLDEFF 264 |  |

YGI PD RIAFYRLLDEFF Sbjct 241 FARYGIEQPDPGRIAFYRLLDEFF 264

>WP_011355791.1 aminoglycoside 3'-phosphotransferase [Burkholderia lata] ABB12308.1 Aminoglycoside phosphotransferase [Burkholderia lata]

Length=264

Score = 322 bits (824), Expect = 1e-107, Method: Compositional matrix adjust.

| Identities | | = 167/264 (63%), Positives = 191/264 (72%), Gaps = 0/264 (0%) |  |
| --- | --- | --- | --- |
| Query 1  Sbjct 1 | | MIEQDGLHAGSPAAWVERLFGYDWAQQTIGCSDAAVFRLSAQGRPVLFVKTDLSGALNEL MI+ D L P W L W QQT G SDAAVFRL A+ FVKT+ +G L EL MIDNDKLCIEVPVTWKAALADCGWTQQTEGQSDAAVFRLDARDGSRRFVKTEPAGPLGEL | 60  60 |
| Query 61  Sbjct 61 | | QDEAARLSWLATTGVPCAAVLDVVTEAGRDWLLLGEVPGQDLLSSHLAPAEKVSIMADAM DEAARL WLATTG+P A VLDV + AGR+W+LL V G++L + L PA KV+I+ADA+ SDEAARLRWLATTGLPGAQVLDVASAAGREWMLLSAVRGENLEVARLGPAAKVAIIADAL | 120  120 |
| Query 121  Sbjct 121 | | RRLHTLDPATCPFDHQAKHRIERARTRMEAGLVDQDDLDEEHQGLAPAELFARLKARMPD R LH LDP TCPFDH A RIERAR RM GLVD+D+LD+ + G+ ELFARL+A P RALHRLDPITCPFDHGAASRIERARARMNEGLVDEDNLDDANTGVPLDELFARLQASRPL | 180  180 |
| Query 181  Sbjct 181 | | GEDLVVTHGDACLPNIMVENGRFSGFIDCGRLGVADRYQDIALATRDIAEELGGEWADRF ED VVTHGDACLPN MVE+GRFSGFIDCGRLGVADRYQD+ALATRDI +LG EW F IEDRVVTHGDACLPNFMVEDGRFSGFIDCGRLGVADRYQDLALATRDIEADLGSEWVAPF | 240  240 |
| Query | 241 LVLYGIAAPDSQRIAFYRLLDEFF 264 | | |
|  |  | YGI PD+ RIAFYRLLDEFF |  |
| Sbjct | 241 | FARYGIKQPDAGRIAFYRLLDEFF 264 | |

>KML48857.1 aminoglycoside phosphotransferase [Burkholderia cepacia] Length=247

Score = 321 bits (822), Expect = 2e-107, Method: Compositional matrix adjust.

| Identities | = 164/246 (67%), Positives = 186/246 (76%), Gaps = 0/246 (0%) |  |
| --- | --- | --- |
| Query 19 | LFGYDWAQQTIGCSDAAVFRLSAQGRPVLFVKTDLSGALNELQDEAARLSWLATTGVPCA | 78 |
| Sbjct 2 | L G W +QT G SDAAVFRL A+ FVKT+ +G L EL DEAARL WL T+G+P A LAGCRWTRQTDGQSDAAVFRLDARDGSRRFVKTEPAGPLGELNDEAARLRWLDTSGLPGA | 61 |
| Query 79 | AVLDVVTEAGRDWLLLGEVPGQDLLSSHLAPAEKVSIMADAMRRLHTLDPATCPFDHQAK | 138 |
| Sbjct 62 | VLDVV+ AGR+W+LL V G++L + LAPAEKV+IMADA+R LH LDPATCPFDH A QVLDVVSAAGREWMLLSAVRGENLDMAQLAPAEKVAIMADALRALHRLDPATCPFDHGAA | 121 |
| Query 139 | HRIERARTRMEAGLVDQDDLDEEHQGLAPAELFARLKARMPDGEDLVVTHGDACLPNIMV | 198 |
| Sbjct 122 | HRIERAR RM AGLVD+D+LD + G+ LFARL A P ED VVTHGDACLPN+M HRIERARARMHAGLVDEDNLDGTNTGVPLDALFARLLAHRPSTEDRVVTHGDACLPNLMA | 181 |
| Query 199 | ENGRFSGFIDCGRLGVADRYQDIALATRDIAEELGGEWADRFLVLYGIAAPDSQRIAFYR | 258 |
| Sbjct 182 | +NGRFSGFIDCGRLGVADRYQD+ALA RDI +LG EW RF YGI PD RIAFYR DNGRFSGFIDCGRLGVADRYQDLALAARDIEADLGSEWVTRFFTRYGIEHPDPDRIAFYR | 241 |
| Query 259  Sbjct 242 | LLDEFF 264 LLDEFF LLDEFF 247 |  |

>WP_069750481.1 aminoglycoside 3'-phosphotransferase [Burkholderia stabilis]

AOR72393.1 APH(3') family aminoglycoside O-phosphotransferase [Burkholderia stabilis]

Length=264

Score = 322 bits (824), Expect = 2e-107, Method: Compositional matrix adjust. Identities = 169/264 (64%), Positives = 192/264 (73%), Gaps = 0/264 (0%)

| Query | 1 | MIEQDGLHAGSPAAWVERLFGYDWAQQTIGCSDAAVFRLSAQGRPVLFVKTDLSGALNEL | 60 |
| --- | --- | --- | --- |
|  |  | MI++D L PAAW L G W +QT G SDA VFRL A+ FVKT+ +G L EL |  |
| Sbjct | 1 | MIDKDKLCNDVPAAWTATLAGCRWTRQTDGQSDAVVFRLDARDGSRRFVKTEPAGPLGEL | 60 |
| Query | 61 | QDEAARLSWLATTGVPCAAVLDVVTEAGRDWLLLGEVPGQDLLSSHLAPAEKVSIMADAM | 120 |

| Sbjct | 61 | QDEAARL LA TGVP A VLDVV+ AGR+W+LL V G++L ++ LAPA KV+IMADA+ QDEAARLRRLAATGVPGARVLDVVSTAGREWMLLSAVRGENLETARLAPAGKVTIMADAL | 120 |
| --- | --- | --- | --- |
| Query | 121 | RRLHTLDPATCPFDHQAKHRIERARTRMEAGLVDQDDLDEEHQGLAPAELFARLKARMPD | 180 |
| Sbjct | 121 | R LH LDPA CPFDH A HRIERAR RM AGLVD+D+LD+ G+ LFARL+A P RALHRLDPAACPFDHGAAHRIERARARMNAGLVDEDNLDDSSTGVPLWVLFARLQAHRPP | 180 |
| Query | 181 | GEDLVVTHGDACLPNIMVENGRFSGFIDCGRLGVADRYQDIALATRDIAEELGGEWADRF | 240 |
| Sbjct | 181 | ED VVTHGDACLPN MV+ FSGFIDCGRLGVADRYQD+ALATRDI +LG EW F IEDKVVTHGDACLPNFMVDEEAFSGFIDCGRLGVADRYQDLALATRDIEADLGSEWVAPF | 240 |
| Query | 241 | LVLYGIAAPDSQRIAFYRLLDEFF 264 YGI PD RIAFYRLLDEFF |  |
| Sbjct | 241 | FARYGIEHPDPDRIAFYRLLDEFF 264 | |

>WP_174937040.1 APH(3') family aminoglycoside O-phosphotransferase [Burkholderia lata]

VWB12103.1 aminoglycoside phosphotransferase APH(3') [Burkholderia lata] Length=264

Score = 321 bits (823), Expect = 2e-107, Method: Compositional matrix adjust.

| Identities | | = 167/264 (63%), Positives = 192/264 (73%), Gaps = 0/264 (0%) |  |
| --- | --- | --- | --- |
| Query 1  Sbjct 1 | | MIEQDGLHAGSPAAWVERLFGYDWAQQTIGCSDAAVFRLSAQGRPVLFVKTDLSGALNEL MI+ D L PA W L W QQT G SDAAVFRL A+ FVKT+ +G L+EL MIDNDKLCIEVPATWKAALADCRWTQQTEGQSDAAVFRLDARDGSRRFVKTEPAGPLSEL | 60  60 |
| Query 61  Sbjct 61 | | QDEAARLSWLATTGVPCAAVLDVVTEAGRDWLLLGEVPGQDLLSSHLAPAEKVSIMADAM DEAARL WL+TTG+P A VLDV + AGR+W+LL V G++L + L PA KV+I+ADA+ GDEAARLHWLSTTGLPGAQVLDVASAAGREWMLLSAVSGENLEVARLGPAAKVAIIADAL | 120  120 |
| Query 121  Sbjct 121 | | RRLHTLDPATCPFDHQAKHRIERARTRMEAGLVDQDDLDEEHQGLAPAELFARLKARMPD R LH LDPATCPFDH A RIERAR RM GLVD+ +LD+ + G+ ELFARL+A P RALHRLDPATCPFDHGAASRIERARARMNEGLVDEGNLDDANTGVPLDELFARLQASRPL | 180  180 |
| Query 181  Sbjct 181 | | GEDLVVTHGDACLPNIMVENGRFSGFIDCGRLGVADRYQDIALATRDIAEELGGEWADRF ED VVTHGDACLPN MVE+GRFSGFIDCGRLGVADRYQD+ALATRDI +LG EW F IEDRVVTHGDACLPNFMVEDGRFSGFIDCGRLGVADRYQDLALATRDIEADLGSEWVAPF | 240  240 |
| Query | 241 LVLYGIAAPDSQRIAFYRLLDEFF 264 | | |
|  |  | YGI PD RIAFYRLLDEFF |  |
| Sbjct | 241 | FARYGIEQPDPGRIAFYRLLDEFF 264 | |

>WP_174938983.1 APH(3') family aminoglycoside O-phosphotransferase [Burkholderia lata]

VWM09544.1 aminoglycoside phosphotransferase APH(3') [Burkholderia lata] Length=264

Score = 321 bits (822), Expect = 2e-107, Method: Compositional matrix adjust.

| Identities | | = 167/264 (63%), Positives = 192/264 (73%), Gaps = 0/264 (0%) |  |
| --- | --- | --- | --- |
| Query 1  Sbjct 1 | | MIEQDGLHAGSPAAWVERLFGYDWAQQTIGCSDAAVFRLSAQGRPVLFVKTDLSGALNEL MI+ D L PA W L W QQT G SDAAVFRL A+ FVKT+ +G L+EL MIDNDKLCIEVPATWKAALADCRWTQQTEGQSDAAVFRLDARDGSRRFVKTEPAGPLSEL | 60  60 |
| Query 61  Sbjct 61 | | QDEAARLSWLATTGVPCAAVLDVVTEAGRDWLLLGEVPGQDLLSSHLAPAEKVSIMADAM DEAARL WL+TTG+P A VLDV + AGR+W+LL V G++L + L PA KV+I+ADA+ SDEAARLHWLSTTGLPGAQVLDVASAAGREWMLLSAVRGENLEVARLGPAAKVAIIADAL | 120  120 |
| Query 121  Sbjct 121 | | RRLHTLDPATCPFDHQAKHRIERARTRMEAGLVDQDDLDEEHQGLAPAELFARLKARMPD R LH LDPATCPFDH A RIERAR RM GLVD+ +LD+ + G+ ELFARL+A P RALHRLDPATCPFDHGAASRIERARARMSEGLVDEGNLDDANTGVPLDELFARLQASRPL | 180  180 |
| Query 181  Sbjct 181 | | GEDLVVTHGDACLPNIMVENGRFSGFIDCGRLGVADRYQDIALATRDIAEELGGEWADRF ED VVTHGDACLPN MVE+GRFSGFIDCGRLGVADRYQD+ALATRDI +LG EW F IEDRVVTHGDACLPNFMVEDGRFSGFIDCGRLGVADRYQDLALATRDIEADLGSEWVAPF | 240  240 |
| Query | 241 LVLYGIAAPDSQRIAFYRLLDEFF 264 | | |
|  |  | YGI PD RIAFYRLLDEFF |  |
| Sbjct | 241 | FARYGIEQPDPGRIAFYRLLDEFF 264 | |

>VWB66384.1 aminoglycoside phosphotransferase APH(3') [Burkholderia lata] Length=264

Score = 321 bits (822), Expect = 3e-107, Method: Compositional matrix adjust.

| Identities | | = 166/264 (63%), Positives = 190/264 (72%), Gaps = 0/264 (0%) |  |
| --- | --- | --- | --- |
| Query 1  Sbjct 1 | | MIEQDGLHAGSPAAWVERLFGYDWAQQTIGCSDAAVFRLSAQGRPVLFVKTDLSGALNEL MI+ D L PA W L W +QT G SDAAVFRL A+ F+KT+ +G L EL MIDNDKLCIEVPATWKAALADARWTRQTEGQSDAAVFRLDARDGSRRFIKTEPAGPLGEL | 60  60 |
| Query 61  Sbjct 61 | | QDEAARLSWLATTGVPCAAVLDVVTEAGRDWLLLGEVPGQDLLSSHLAPAEKVSIMADAM  +DEAARL WL T G+P A VLDV + AGR+W+LL V G++L + LAPA KV+IMADA+ RDEAARLHWLTTAGLPGAQVLDVASAAGREWMLLSAVRGENLEVAQLAPAAKVAIMADAL | 120  120 |
| Query 121  Sbjct 121 | | RRLHTLDPATCPFDHQAKHRIERARTRMEAGLVDQDDLDEEHQGLAPAELFARLKARMPD R LH LDPATCPFDH A RIERAR RM A LVD+D+LD+ + G+ ELFARL A P RELHRLDPATCPFDHGAASRIERARARMHARLVDEDNLDDANTGVPIDELFARLHASRPS | 180  180 |
| Query 181  Sbjct 181 | | GEDLVVTHGDACLPNIMVENGRFSGFIDCGRLGVADRYQDIALATRDIAEELGGEWADRF ED VVTHGDACLPN MVE+GRFSGFIDCGRLGVADRYQD+ALA RDI +LG EW F IEDKVVTHGDACLPNFMVEDGRFSGFIDCGRLGVADRYQDLALAARDIEADLGSEWIAPF | 240  240 |
| Query 241 | | LVLYGIAAPDSQRIAFYRLLDEFF 264 YGI PD RIAFYRLLDEFF |  |
| Sbjct | 241 | FARYGIGQPDPDRIAFYRLLDEFF 264 | |

>WP_174973015.1 APH(3')-II family aminoglycoside O-phosphotransferase [Burkholderia lata]

Length=275

Score = 320 bits (820), Expect = 8e-107, Method: Compositional matrix adjust.

| Identities | | = 165/264 (63%), Positives = 194/264 (73%), Gaps = 0/264 (0%) |  |
| --- | --- | --- | --- |
| Query 1  Sbjct 12 | | MIEQDGLHAGSPAAWVERLFGYDWAQQTIGCSDAAVFRLSAQGRPVLFVKTDLSGALNEL  +I+++ L PA W L W +QT G SDAAVFRL AQ FVKT+ +G L EL VIDKERLCIEVPATWNATLADCRWTRQTEGQSDAAVFRLDAQDGSCRFVKTEPAGPLGEL | 60  71 |
| Query 61  Sbjct 72 | | QDEAARLSWLATTGVPCAAVLDVVTEAGRDWLLLGEVPGQDLLSSHLAPAEKVSIMADAM  +DEAARL WLATTG+P A VL+ + AGR+W+LL V G++L + L PAEKV+I+ADA+ RDEAARLRWLATTGLPGAQVLEAASAAGREWMLLSAVCGENLEVAPLGPAEKVAIIADAL | 120  131 |
| Query 121  Sbjct 132 | | RRLHTLDPATCPFDHQAKHRIERARTRMEAGLVDQDDLDEEHQGLAPAELFARLKARMPD R LH LDPATCPFDH A+ RI RAR RM AGLVD+D+LD+ + G+ ELFARL+A P RALHRLDPATCPFDHGAESRIARARARMNAGLVDEDNLDDANMGVPLDELFARLRASRPS | 180  191 |
| Query 181  Sbjct 192 | | GEDLVVTHGDACLPNIMVENGRFSGFIDCGRLGVADRYQDIALATRDIAEELGGEWADRF ED VVTHGDACLPN MVE+GRFSGFIDCGRLGVADRYQD+ALA RDI +LG EW F IEDRVVTHGDACLPNFMVEDGRFSGFIDCGRLGVADRYQDLALAARDIEADLGSEWVAPF | 240  251 |
| Query 241 | | LVLYGIAAPDSQRIAFYRLLDEFF 264 YGI PD RIAFYRLLDEFF |  |
| Sbjct | 252 | FARYGIEQPDPGRIAFYRLLDEFF 275 | |

>CAB3970912.1 aminoglycoside phosphotransferase APH(3') [Burkholderia lata] VWD15569.1 aminoglycoside phosphotransferase APH(3') [Burkholderia lata] VWD34612.1 aminoglycoside phosphotransferase APH(3') [Burkholderia lata]

Length=277

Score = 319 bits (818), Expect = 2e-106, Method: Compositional matrix adjust.

| Identities | = 163/264 (62%), Positives = 191/264 (72%), Gaps = 0/264 (0%) |  |
| --- | --- | --- |
| Query 1 | MIEQDGLHAGSPAAWVERLFGYDWAQQTIGCSDAAVFRLSAQGRPVLFVKTDLSGALNEL | 60 |
| Sbjct 14 | +I+ D PA W L W +QT G SDAAVFRL A+ F+KT+ +G L EL VIDNDKFCIEVPATWKAALADGRWTRQTEGQSDAAVFRLDARDGSRRFIKTEPAGPLGEL | 73 |
| Query 61 | QDEAARLSWLATTGVPCAAVLDVVTEAGRDWLLLGEVPGQDLLSSHLAPAEKVSIMADAM | 120 |
| Sbjct 74 | +DEAARL WLATTG+P A VLDV + AGR+W+LL V G++L + LAPA KV+I+AD +  RDEAARLRWLATTGLPGAQVLDVASAAGREWMLLSAVRGENLEVAQLAPAAKVAIIADVL | 133 |

| Query | 121 | RRLHTLDPATCPFDHQAKHRIERARTRMEAGLVDQDDLDEEHQGLAPAELFARLKARMPD | 180 |
| --- | --- | --- | --- |
| Sbjct | 134 | R LH LDPATCPFDH A RIERAR RM AG VD+ +LD+ + G+ ELFARL+A P RALHRLDPATCPFDHGAASRIERARARMHAGRVDESNLDDANTGVPLDELFARLQASRPS | 193 |
| Query | 181 | GEDLVVTHGDACLPNIMVENGRFSGFIDCGRLGVADRYQDIALATRDIAEELGGEWADRF | 240 |
| Sbjct | 194 | ED VVTHGDACLPN MVE+GRFSGFIDCGRLGV+DRYQD+ALA RDI +LGGEW F SEDRVVTHGDACLPNFMVEDGRFSGFIDCGRLGVSDRYQDLALAARDIEADLGGEWVTPF | 253 |
| Query | 241 | LVLYGIAAPDSQRIAFYRLLDEFF 264 YGI PD RIAFYRLLDEFF |  |
| Sbjct | 254 | FARYGIEHPDPDRIAFYRLLDEFF 277 | |

>WP_174960806.1 MULTISPECIES: APH(3') family aminoglycoside O-phosphotransferase [Burkholderia]

Length=275

Score = 319 bits (818), Expect = 2e-106, Method: Compositional matrix adjust.

| Identities | | = 163/264 (62%), Positives = 191/264 (72%), Gaps = 0/264 (0%) |  |
| --- | --- | --- | --- |
| Query 1  Sbjct 12 | | MIEQDGLHAGSPAAWVERLFGYDWAQQTIGCSDAAVFRLSAQGRPVLFVKTDLSGALNEL  +I+ D PA W L W +QT G SDAAVFRL A+ F+KT+ +G L EL VIDNDKFCIEVPATWKAALADGRWTRQTEGQSDAAVFRLDARDGSRRFIKTEPAGPLGEL | 60  71 |
| Query 61  Sbjct 72 | | QDEAARLSWLATTGVPCAAVLDVVTEAGRDWLLLGEVPGQDLLSSHLAPAEKVSIMADAM  +DEAARL WLATTG+P A VLDV + AGR+W+LL V G++L + LAPA KV+I+AD + RDEAARLRWLATTGLPGAQVLDVASAAGREWMLLSAVRGENLEVAQLAPAAKVAIIADVL | 120  131 |
| Query 121  Sbjct 132 | | RRLHTLDPATCPFDHQAKHRIERARTRMEAGLVDQDDLDEEHQGLAPAELFARLKARMPD R LH LDPATCPFDH A RIERAR RM AG VD+ +LD+ + G+ ELFARL+A P RALHRLDPATCPFDHGAASRIERARARMHAGRVDESNLDDANTGVPLDELFARLQASRPS | 180  191 |
| Query 181  Sbjct 192 | | GEDLVVTHGDACLPNIMVENGRFSGFIDCGRLGVADRYQDIALATRDIAEELGGEWADRF ED VVTHGDACLPN MVE+GRFSGFIDCGRLGV+DRYQD+ALA RDI +LGGEW F SEDRVVTHGDACLPNFMVEDGRFSGFIDCGRLGVSDRYQDLALAARDIEADLGGEWVTPF | 240  251 |
| Query 241 | | LVLYGIAAPDSQRIAFYRLLDEFF 264 YGI PD RIAFYRLLDEFF |  |
| Sbjct | 252 | FARYGIEHPDPDRIAFYRLLDEFF 275 | |

>WP_174982902.1 APH(3') family aminoglycoside O-phosphotransferase [Burkholderia lata]

VWB29905.1 aminoglycoside phosphotransferase APH(3') [Burkholderia lata] Length=264

Score = 319 bits (817), Expect = 2e-106, Method: Compositional matrix adjust.

| Identities | | = 166/264 (63%), Positives = 190/264 (72%), Gaps = 0/264 (0%) |  |
| --- | --- | --- | --- |
| Query 1  Sbjct 1 | | MIEQDGLHAGSPAAWVERLFGYDWAQQTIGCSDAAVFRLSAQGRPVLFVKTDLSGALNEL MI+ D L PA W L W QQT G SDAAVFRL A+ FVKT+ +G L+EL MIDNDKLCIEVPATWKAALADCRWTQQTEGQSDAAVFRLDARDGSRRFVKTEPAGPLSEL | 60  60 |
| Query 61  Sbjct 61 | | QDEAARLSWLATTGVPCAAVLDVVTEAGRDWLLLGEVPGQDLLSSHLAPAEKVSIMADAM DEAARL WL+TTG+P A VLDV + AGR+W+LL V G++L + L PA KV+I+ADA+ SDEAARLHWLSTTGLPGAQVLDVASAAGREWMLLSAVSGENLEVARLGPAAKVAIIADAL | 120  120 |
| Query 121  Sbjct 121 | | RRLHTLDPATCPFDHQAKHRIERARTRMEAGLVDQDDLDEEHQGLAPAELFARLKARMPD R LH LDPA CPFDH A RIERAR RM GLVD +LD+ + G+ ELFARL+A P RALHRLDPAACPFDHGAASRIERARARMNEGLVDDGNLDDANTGVPLDELFARLQASRPL | 180  180 |
| Query 181  Sbjct 181 | | GEDLVVTHGDACLPNIMVENGRFSGFIDCGRLGVADRYQDIALATRDIAEELGGEWADRF ED VVTHGDACLPN MVE+GRFSGFIDCGRLGVADRYQD+ALATRDI +LG EW F IEDRVVTHGDACLPNFMVEDGRFSGFIDCGRLGVADRYQDLALATRDIEADLGSEWVAPF | 240  240 |
| Query | 241 LVLYGIAAPDSQRIAFYRLLDEFF 264 | | |
|  |  | YGI PD RIAFYRLLDEFF |  |
| Sbjct | 241 | FARYGIEQPDPGRIAFYRLLDEFF 264 | |

>WP_175023006.1 APH(3')-II family aminoglycoside O-phosphotransferase [Burkholderia

lata] Length=275

Score = 319 bits (817), Expect = 2e-106, Method: Compositional matrix adjust.

| Identities | | = 165/264 (63%), Positives = 190/264 (72%), Gaps = 0/264 (0%) |  |
| --- | --- | --- | --- |
| Query 1  Sbjct 12 | | MIEQDGLHAGSPAAWVERLFGYDWAQQTIGCSDAAVFRLSAQGRPVLFVKTDLSGALNEL  +I+ D L PA W L W +QT G SDAAVFRL A+ F+KT+ +G L EL VIDNDKLCIEVPATWKAALADARWTRQTEGQSDAAVFRLDARDGSRRFIKTEPAGPLGEL | 60  71 |
| Query 61  Sbjct 72 | | QDEAARLSWLATTGVPCAAVLDVVTEAGRDWLLLGEVPGQDLLSSHLAPAEKVSIMADAM  +DEAARL WL T G+P A VLDV + AGR+W+LL V G++L + LAPA KV+IMADA+ RDEAARLHWLTTAGLPGAQVLDVASAAGREWMLLSAVRGENLEVAQLAPAAKVAIMADAL | 120  131 |
| Query 121  Sbjct 132 | | RRLHTLDPATCPFDHQAKHRIERARTRMEAGLVDQDDLDEEHQGLAPAELFARLKARMPD R LH LDPATCPFDH A RIERAR RM A LVD+D+LD+ + G+ ELFARL A P RELHRLDPATCPFDHGAASRIERARARMHARLVDEDNLDDANTGVPIDELFARLHASRPS | 180  191 |
| Query 181  Sbjct 192 | | GEDLVVTHGDACLPNIMVENGRFSGFIDCGRLGVADRYQDIALATRDIAEELGGEWADRF ED VVTHGDACLPN MVE+GRFSGFIDCGRLGVADRYQD+ALA RDI +LG EW F IEDKVVTHGDACLPNFMVEDGRFSGFIDCGRLGVADRYQDLALAARDIEADLGSEWIAPF | 240  251 |
| Query 241 | | LVLYGIAAPDSQRIAFYRLLDEFF 264 YGI PD RIAFYRLLDEFF |  |
| Sbjct | 252 | FARYGIGQPDPDRIAFYRLLDEFF 275 | |

>WP_186248588.1 APH(3')-II family aminoglycoside O-phosphotransferase [Burkholderia sp. AU31652]

Length=275

Score = 318 bits (815), Expect = 5e-106, Method: Compositional matrix adjust.

| Identities | | = 164/264 (62%), Positives = 189/264 (72%), Gaps = 0/264 (0%) |  |
| --- | --- | --- | --- |
| Query 1  Sbjct 12 | | MIEQDGLHAGSPAAWVERLFGYDWAQQTIGCSDAAVFRLSAQGRPVLFVKTDLSGALNEL  +I+ D L P W L W +QT G SDAAVFRL A+ F+KT+ +G L EL VIDNDKLCIEVPETWKAELADARWTRQTEGQSDAAVFRLDARDGSRRFIKTEPAGPLGEL | 60  71 |
| Query 61  Sbjct 72 | | QDEAARLSWLATTGVPCAAVLDVVTEAGRDWLLLGEVPGQDLLSSHLAPAEKVSIMADAM  +DEAARL WL T G+P A VLDV + AGR+W+LL V G++L + LAPA KV+IMADA+ RDEAARLHWLTTAGLPGAQVLDVASAAGREWMLLSAVRGENLEVAQLAPAAKVAIMADAL | 120  131 |
| Query 121  Sbjct 132 | | RRLHTLDPATCPFDHQAKHRIERARTRMEAGLVDQDDLDEEHQGLAPAELFARLKARMPD R LH LDPATCPFDH A RIERAR RM A LVD+D+LD+ + G+ ELFARL A P RELHRLDPATCPFDHGAASRIERARARMHARLVDEDNLDDANTGVPIDELFARLHASRPS | 180  191 |
| Query 181  Sbjct 192 | | GEDLVVTHGDACLPNIMVENGRFSGFIDCGRLGVADRYQDIALATRDIAEELGGEWADRF ED VVTHGDACLPN MVE+GRFSGFIDCGRLGVADRYQD+ALA RDI +LG EW F IEDKVVTHGDACLPNFMVEDGRFSGFIDCGRLGVADRYQDLALAARDIEADLGSEWIAPF | 240  251 |
| Query 241 | | LVLYGIAAPDSQRIAFYRLLDEFF 264 YGI PD RIAFYRLLDEFF |  |
| Sbjct | 252 | FARYGIGQPDPDRIAFYRLLDEFF 275 | |

>VWC44666.1 aminoglycoside phosphotransferase APH(3') [Burkholderia lata] Length=277

Score = 318 bits (814), Expect = 6e-106, Method: Compositional matrix adjust.

| Identities | = 162/264 (61%), Positives = 190/264 (72%), Gaps = 0/264 (0%) |  |
| --- | --- | --- |
| Query 1 | MIEQDGLHAGSPAAWVERLFGYDWAQQTIGCSDAAVFRLSAQGRPVLFVKTDLSGALNEL | 60 |
| Sbjct 14 | +I+ D PA W L W +QT G SDAAVFRL A+ F+KT+ +G L EL VIDNDKFCIEVPATWKAALADGRWTRQTEGQSDAAVFRLDARDGSRRFIKTEPAGPLGEL | 73 |
| Query 61 | QDEAARLSWLATTGVPCAAVLDVVTEAGRDWLLLGEVPGQDLLSSHLAPAEKVSIMADAM | 120 |
| Sbjct 74 | +DEAARL WLATTG+P A VLDV + AGR+W+LL V G++L + LAPA KV+I+AD + RDEAARLRWLATTGLPGAQVLDVASAAGREWMLLSAVRGENLEVAQLAPAAKVAIIADVL | 133 |
| Query 121 | RRLHTLDPATCPFDHQAKHRIERARTRMEAGLVDQDDLDEEHQGLAPAELFARLKARMPD | 180 |
| Sbjct 134 | R LH LDPATCPFDH A RIERAR RM AG VD+ +LD+ + G+ ELF RL+A P  RALHRLDPATCPFDHGAASRIERARARMHAGRVDESNLDDANTGVPLDELFTRLQASRPS | 193 |

Query 181 GEDLVVTHGDACLPNIMVENGRFSGFIDCGRLGVADRYQDIALATRDIAEELGGEWADRF 240

| Sbjct | 194 | ED VVTHGDACLPN MVE+GRFSGFIDCGRLGV+DRYQD+ALA RDI +LGGEW F SEDRVVTHGDACLPNFMVEDGRFSGFIDCGRLGVSDRYQDLALAARDIEADLGGEWVTPF 253 |
| --- | --- | --- |
| Query | 241 | LVLYGIAAPDSQRIAFYRLLDEFF 264 |
| Sbjct | 254 | YGI PD RIAFYRLLDEFF  FARYGIEHPDPDRIAFYRLLDEFF 277 |

>WP_122949180.1 aminoglycoside 3'-phosphotransferase [Burkholderia lata]

AYQ42885.1 APH(3') family aminoglycoside O-phosphotransferase [Burkholderia lata]

Length=275

Score = 318 bits (814), Expect = 7e-106, Method: Compositional matrix adjust.

| Identities | | = 162/264 (61%), Positives = 190/264 (72%), Gaps = 0/264 (0%) |  |
| --- | --- | --- | --- |
| Query 1  Sbjct 12 | | MIEQDGLHAGSPAAWVERLFGYDWAQQTIGCSDAAVFRLSAQGRPVLFVKTDLSGALNEL  +I+ D PA W L W +QT G SDAAVFRL A+ F+KT+ +G L EL VIDNDKFCIEVPATWKAALADGRWTRQTEGQSDAAVFRLDARDGSRRFIKTEPAGPLGEL | 60  71 |
| Query 61  Sbjct 72 | | QDEAARLSWLATTGVPCAAVLDVVTEAGRDWLLLGEVPGQDLLSSHLAPAEKVSIMADAM  +DEAARL WLATTG+P A VLDV + AGR+W+LL V G++L + LAPA KV+I+AD + RDEAARLRWLATTGLPGAQVLDVASAAGREWMLLSAVRGENLEVAQLAPAAKVAIIADVL | 120  131 |
| Query 121  Sbjct 132 | | RRLHTLDPATCPFDHQAKHRIERARTRMEAGLVDQDDLDEEHQGLAPAELFARLKARMPD R LH LDPATCPFDH A RIERAR RM AG VD+ +LD+ + G+ ELF RL+A P RALHRLDPATCPFDHGAASRIERARARMHAGRVDESNLDDANTGVPLDELFTRLQASRPS | 180  191 |
| Query 181  Sbjct 192 | | GEDLVVTHGDACLPNIMVENGRFSGFIDCGRLGVADRYQDIALATRDIAEELGGEWADRF ED VVTHGDACLPN MVE+GRFSGFIDCGRLGV+DRYQD+ALA RDI +LGGEW F SEDRVVTHGDACLPNFMVEDGRFSGFIDCGRLGVSDRYQDLALAARDIEADLGGEWVTPF | 240  251 |
| Query 241 | | LVLYGIAAPDSQRIAFYRLLDEFF 264 YGI PD RIAFYRLLDEFF |  |
| Sbjct | 252 | FARYGIEHPDPDRIAFYRLLDEFF 275 | |

>VWC03873.1 aminoglycoside phosphotransferase APH(3') [Burkholderia lata] Length=264

Score = 316 bits (810), Expect = 2e-105, Method: Compositional matrix adjust.

| Identities | | = 164/264 (62%), Positives = 192/264 (73%), Gaps = 0/264 (0%) |  |
| --- | --- | --- | --- |
| Query 1  Sbjct 1 | | MIEQDGLHAGSPAAWVERLFGYDWAQQTIGCSDAAVFRLSAQGRPVLFVKTDLSGALNEL MI+++ L PA W L W +QT G SDAAVFRL AQ FVKT+ +G L EL MIDKERLCIEVPATWNATLADCRWTRQTEGQSDAAVFRLDAQDGSCRFVKTEPAGPLGEL | 60  60 |
| Query 61  Sbjct 61 | | QDEAARLSWLATTGVPCAAVLDVVTEAGRDWLLLGEVPGQDLLSSHLAPAEKVSIMADAM DEAARL WLATTG+P A VL+V + AGR+W+LL V G++L + L PAEKV+I+ADA+ SDEAARLRWLATTGLPGAQVLEVASAAGREWMLLSAVCGENLEVAPLGPAEKVAIIADAL | 120  120 |
| Query 121  Sbjct 121 | | RRLHTLDPATCPFDHQAKHRIERARTRMEAGLVDQDDLDEEHQGLAPAELFARLKARMPD R LH LDP TCPFDH A+ RI RAR RM AGLVD+D+LD+ + G+ ELFA+L A P RALHRLDPTTCPFDHGAESRIARARARMNAGLVDEDNLDDANMGVPLDELFAKLWASRPS | 180  180 |
| Query 181  Sbjct 181 | | GEDLVVTHGDACLPNIMVENGRFSGFIDCGRLGVADRYQDIALATRDIAEELGGEWADRF ED VVTHGDACLPN MVE+GRFSGFIDCGRLGVADRYQD+ALA RDI +LG EW F IEDRVVTHGDACLPNFMVEDGRFSGFIDCGRLGVADRYQDLALAARDIEADLGSEWVAPF | 240  240 |
| Query | 241 LVLYGIAAPDSQRIAFYRLLDEFF 264 | | |
|  |  | YGI PD RIAF+RLLDEFF |  |
| Sbjct | 241 | FARYGIEQPDPGRIAFHRLLDEFF 264 | |

>WP_192968248.1 APH(3') family aminoglycoside O-phosphotransferase, partial [Escherichia coli]

Length=152

Score = 311 bits (798), Expect = 3e-105, Method: Compositional matrix adjust. Identities = 152/152 (100%), Positives = 152/152 (100%), Gaps = 0/152 (0%)

| Query | 71 | ATTGVPCAAVLDVVTEAGRDWLLLGEVPGQDLLSSHLAPAEKVSIMADAMRRLHTLDPAT | 130 |
| --- | --- | --- | --- |
| Sbjct | 1 | ATTGVPCAAVLDVVTEAGRDWLLLGEVPGQDLLSSHLAPAEKVSIMADAMRRLHTLDPAT ATTGVPCAAVLDVVTEAGRDWLLLGEVPGQDLLSSHLAPAEKVSIMADAMRRLHTLDPAT | 60 |
| Query | 131 | CPFDHQAKHRIERARTRMEAGLVDQDDLDEEHQGLAPAELFARLKARMPDGEDLVVTHGD | 190 |
| Sbjct | 61 | CPFDHQAKHRIERARTRMEAGLVDQDDLDEEHQGLAPAELFARLKARMPDGEDLVVTHGD CPFDHQAKHRIERARTRMEAGLVDQDDLDEEHQGLAPAELFARLKARMPDGEDLVVTHGD | 120 |
| Query  Sbjct | 191  121 | ACLPNIMVENGRFSGFIDCGRLGVADRYQDIA 222 ACLPNIMVENGRFSGFIDCGRLGVADRYQDIA ACLPNIMVENGRFSGFIDCGRLGVADRYQDIA 152 |  |

>WP_174930574.1 APH(3') family aminoglycoside O-phosphotransferase [Burkholderia lata]

VWB46198.1 aminoglycoside phosphotransferase APH(3') [Burkholderia lata] Length=264

Score = 315 bits (807), Expect = 4e-105, Method: Compositional matrix adjust.

| Identities | | = 165/264 (63%), Positives = 189/264 (72%), Gaps = 0/264 (0%) |  |
| --- | --- | --- | --- |
| Query 1  Sbjct 1 | | MIEQDGLHAGSPAAWVERLFGYDWAQQTIGCSDAAVFRLSAQGRPVLFVKTDLSGALNEL MI+ D L P W L W QQT G SDAAVFRL A+ FVKT+ +G L EL MIDNDKLCIEVPVTWKAALADCRWTQQTEGQSDAAVFRLDARDGSRRFVKTEPAGPLGEL | 60  60 |
| Query 61  Sbjct 61 | | QDEAARLSWLATTGVPCAAVLDVVTEAGRDWLLLGEVPGQDLLSSHLAPAEKVSIMADAM DEAARL WLATTG+P A VLDV + AGR+W+LL V G++L + L PA KV+I+ADA+ SDEAARLRWLATTGLPGAQVLDVASAAGREWMLLSAVRGENLEVARLGPAAKVAIIADAL | 120  120 |
| Query 121  Sbjct 121 | | RRLHTLDPATCPFDHQAKHRIERARTRMEAGLVDQDDLDEEHQGLAPAELFARLKARMPD R LH LDP TCPFDH A RIERAR RM GLVD+D+LD+ + G+ LFARL+A P RALHRLDPITCPFDHGAVSRIERARARMNEGLVDEDNLDDANTGVPLDGLFARLQASRPL | 180  180 |
| Query 181  Sbjct 181 | | GEDLVVTHGDACLPNIMVENGRFSGFIDCGRLGVADRYQDIALATRDIAEELGGEWADRF ED VVTHGDACLPN MVE+G FSGFIDCGRLGVADRYQD+ALATRDI +LG EW F IEDRVVTHGDACLPNFMVEDGCFSGFIDCGRLGVADRYQDLALATRDIEADLGSEWVAPF | 240  240 |
| Query | 241 LVLYGIAAPDSQRIAFYRLLDEFF 264 | | |
|  |  | YGI PD+ RIAFYRLLDEFF |  |
| Sbjct | 241 | FARYGIKQPDAGRIAFYRLLDEFF 264 | |

>WP_174917470.1 APH(3') family aminoglycoside O-phosphotransferase [Burkholderia lata]

Length=275

Score = 315 bits (806), Expect = 1e-104, Method: Compositional matrix adjust.

| Identities | | = 163/264 (62%), Positives = 192/264 (73%), Gaps = 0/264 (0%) |  |
| --- | --- | --- | --- |
| Query 1  Sbjct 12 | | MIEQDGLHAGSPAAWVERLFGYDWAQQTIGCSDAAVFRLSAQGRPVLFVKTDLSGALNEL  +I+++ L PA W L W +QT G SDAAVFRL AQ FVKT+ +G L EL VIDKERLCIEVPATWNATLADCRWTRQTEGQSDAAVFRLDAQDGSCRFVKTEPAGPLGEL | 60  71 |
| Query 61  Sbjct 72 | | QDEAARLSWLATTGVPCAAVLDVVTEAGRDWLLLGEVPGQDLLSSHLAPAEKVSIMADAM DEAARL WLATTG+P A VL+V + AGR+W+LL V G++L + L PAEKV+I+ADA+ SDEAARLRWLATTGLPGAQVLEVASAAGREWMLLSAVCGENLEVAPLGPAEKVAIIADAL | 120  131 |
| Query 121  Sbjct 132 | | RRLHTLDPATCPFDHQAKHRIERARTRMEAGLVDQDDLDEEHQGLAPAELFARLKARMPD R LH LDP TCPFDH A+ RI RAR RM AGLVD+D+LD+ + G+ ELFA+L A P RALHRLDPTTCPFDHGAESRIARARARMNAGLVDEDNLDDANMGVPLDELFAKLWASRPS | 180  191 |
| Query 181  Sbjct 192 | | GEDLVVTHGDACLPNIMVENGRFSGFIDCGRLGVADRYQDIALATRDIAEELGGEWADRF ED VVTHGDACLPN MVE+GRFSGFIDCGRLGVADRYQD+ALA RDI +LG EW F IEDRVVTHGDACLPNFMVEDGRFSGFIDCGRLGVADRYQDLALAARDIEADLGSEWVAPF | 240  251 |
| Query | 241 LVLYGIAAPDSQRIAFYRLLDEFF 264 | | |
|  |  | YGI PD RIAF+RLLDEFF |  |
| Sbjct | 252 | FARYGIEQPDPGRIAFHRLLDEFF 275 | |

```
>HAA1110234.1 APH(3') family aminoglycoside O-phosphotransferase [Salmonella

enterica] Length=151

Score = 310 bits (793), Expect = 2e-104, Method: Compositional matrix adjust. Identities = 151/151 (100%), Positives = 151/151 (100%), Gaps = 0/151 (0%)

Query 114 SIMADAMRRLHTLDPATCPFDHQAKHRIERARTRMEAGLVDQDDLDEEHQGLAPAELFAR 173 SIMADAMRRLHTLDPATCPFDHQAKHRIERARTRMEAGLVDQDDLDEEHQGLAPAELFAR
Sbjct 1	SIMADAMRRLHTLDPATCPFDHQAKHRIERARTRMEAGLVDQDDLDEEHQGLAPAELFAR 60

Query 174 LKARMPDGEDLVVTHGDACLPNIMVENGRFSGFIDCGRLGVADRYQDIALATRDIAEELG 233 LKARMPDGEDLVVTHGDACLPNIMVENGRFSGFIDCGRLGVADRYQDIALATRDIAEELG
Sbjct 61	LKARMPDGEDLVVTHGDACLPNIMVENGRFSGFIDCGRLGVADRYQDIALATRDIAEELG 120

Query 234 GEWADRFLVLYGIAAPDSQRIAFYRLLDEFF 264 GEWADRFLVLYGIAAPDSQRIAFYRLLDEFF
Sbjct 121 GEWADRFLVLYGIAAPDSQRIAFYRLLDEFF 151

>WP_166954165.1 APH(3') family aminoglycoside O-phosphotransferase [Burkholderia sp. D-99]
NHV25836.1 aminoglycoside 3'-phosphotransferase [Burkholderia sp. D-99] Length=278

Score = 314 bits (805), Expect = 2e-104, Method: Compositional matrix adjust.
```

| Identities | | = 164/264 (62%), Positives = 190/264 (72%), Gaps = 0/264 (0%) |  |
| --- | --- | --- | --- |
| Query 1  Sbjct 15 | | MIEQDGLHAGSPAAWVERLFGYDWAQQTIGCSDAAVFRLSAQGRPVLFVKTDLSGALNEL  +I+ D L P W W +QT G SDAAVFRL A+ FVKT+ +G L EL VIDNDKLCIEVPVTWKAAWADCRWTRQTEGQSDAAVFRLDARDGSRRFVKTEPAGPLAEL | 60  74 |
| Query 61  Sbjct 75 | | QDEAARLSWLATTGVPCAAVLDVVTEAGRDWLLLGEVPGQDLLSSHLAPAEKVSIMADAM DEAARL WL TTG+P A VLDV + AGR+W+LL V G++L + L PAEKV+I+ADA+ SDEAARLRWLETTGLPGAQVLDVASAAGREWMLLSAVYGENLEVARLGPAEKVAIIADAL | 120  134 |
| Query 121  Sbjct 135 | | RRLHTLDPATCPFDHQAKHRIERARTRMEAGLVDQDDLDEEHQGLAPAELFARLKARMPD LH L+PATCPFDH A RIERAR RM AGLVD+D+LD+ + G+ ELFARL A P  CALHRLEPATCPFDHGAAFRIERARARMNAGLVDEDNLDDANTGVQLDELFARLLASRPS | 180  194 |
| Query 181  Sbjct 195 | | GEDLVVTHGDACLPNIMVENGRFSGFIDCGRLGVADRYQDIALATRDIAEELGGEWADRF ED VVTHGDACLPN MVE+GRFSGFIDCGRLGVADRYQD+ALA RDI +LGGEW F IEDRVVTHGDACLPNFMVEDGRFSGFIDCGRLGVADRYQDLALAARDIEADLGGEWVAPF | 240  254 |
| Query | 241 LVLYGIAAPDSQRIAFYRLLDEFF 264 | | |
|  |  | YGI PD +RIAFYRLLDEFF |  |
| Sbjct | 255 | FARYGIERPDPRRIAFYRLLDEFF 278 | |

>ORT83030.1 APH(3') family aminoglycoside O-phosphotransferase [Burkholderia puraquae]

CAB3745846.1 Aminoglycoside 3'-phosphotransferase [Burkholderia puraquae] Length=264

Score = 313 bits (801), Expect = 5e-104, Method: Compositional matrix adjust.

| Identities | = 165/264 (63%), Positives = 190/264 (72%), Gaps = 0/264 (0%) |  |
| --- | --- | --- |
| Query 1 | MIEQDGLHAGSPAAWVERLFGYDWAQQTIGCSDAAVFRLSAQGRPVLFVKTDLSGALNEL | 60 |
| Sbjct 1 | MI+ D L PAAW L W +QT G SDA VFRL A+ FVK + +G L EL MIDNDKLCIEMPAAWKAALADCRWMRQTDGQSDALVFRLDARDGTRRFVKIEPAGPLAEL | 60 |
| Query 61 | QDEAARLSWLATTGVPCAAVLDVVTEAGRDWLLLGEVPGQDLLSSHLAPAEKVSIMADAM | 120 |
| Sbjct 61 | DEAARL WLATTG+P A VLDV + AGR+W+LL V G++L + L PA KV+I+ADA+ SDEAARLRWLATTGLPGARVLDVASAAGREWMLLSAVRGENLEVARLGPAAKVAIIADAL | 120 |
| Query 121 | RRLHTLDPATCPFDHQAKHRIERARTRMEAGLVDQDDLDEEHQGLAPAELFARLKARMPD | 180 |
| Sbjct 121 | R LH LDPATCPFDH A RIERA RM AGLVD D+LD+ + G++ ELFARL+A P RALHRLDPATCPFDHGAASRIERACARMSAGLVDADNLDDANTGVSLDELFARLQASRPL | 180 |
| Query 181 | GEDLVVTHGDACLPNIMVENGRFSGFIDCGRLGVADRYQDIALATRDIAEELGGEWADRF | 240 |
| Sbjct 181 | E VVTHGDACLPN MVE+GRFSGFIDCGRLGVADRYQD+ALA RDI +LG EW F  IEGRVVTHGDACLPNFMVEDGRFSGFIDCGRLGVADRYQDLALAARDIEVDLGDEWVAPF | 240 |

Query 241 LVLYGIAAPDSQRIAFYRLLDEFF 264

YGI PD +RIAFYRLLDEFF Sbjct 241 FARYGIEQPDQERIAFYRLLDEFF 264

>WP_181211204.1 APH(3') family aminoglycoside O-phosphotransferase, partial [Escherichia coli]

MBA1779079.1 aminoglycoside 3'-phosphotransferase [Escherichia coli] Length=151

Score = 308 bits (788), Expect = 8e-104, Method: Compositional matrix adjust.

| Identities | = 150/151 (99%), Positives = 150/151 (99%), Gaps = 0/151 (0%) |  |
| --- | --- | --- |
| Query 114 | SIMADAMRRLHTLDPATCPFDHQAKHRIERARTRMEAGLVDQDDLDEEHQGLAPAELFAR | 173 |
| Sbjct 1 | SIMADAMRRLHTLDPATCPFDHQAKHRIERARTRMEAGLVDQDDLDEEHQGLAPAELFAR SIMADAMRRLHTLDPATCPFDHQAKHRIERARTRMEAGLVDQDDLDEEHQGLAPAELFAR | 60 |
| Query 174 | LKARMPDGEDLVVTHGDACLPNIMVENGRFSGFIDCGRLGVADRYQDIALATRDIAEELG | 233 |
| Sbjct 61 | LKA MPDGEDLVVTHGDACLPNIMVENGRFSGFIDCGRLGVADRYQDIALATRDIAEELG LKASMPDGEDLVVTHGDACLPNIMVENGRFSGFIDCGRLGVADRYQDIALATRDIAEELG | 120 |
| Query 234  Sbjct 121 | GEWADRFLVLYGIAAPDSQRIAFYRLLDEFF 264 GEWADRFLVLYGIAAPDSQRIAFYRLLDEFF GEWADRFLVLYGIAAPDSQRIAFYRLLDEFF 151 |  |

>KAF1038439.1 Aminoglycoside 3'-phosphotransferase [Burkholderia lata] Length=264

Score = 311 bits (798), Expect = 1e-103, Method: Compositional matrix adjust.

| Identities | | = 163/264 (62%), Positives = 188/264 (71%), Gaps = 0/264 (0%) |  |
| --- | --- | --- | --- |
| Query 1  Sbjct 1 | | MIEQDGLHAGSPAAWVERLFGYDWAQQTIGCSDAAVFRLSAQGRPVLFVKTDLSGALNEL MI+ D L P W L W QQT G SDAAVFRL A+ FVKT+ +G L EL MIDNDKLCIEVPVTWKAALADCRWTQQTEGQSDAAVFRLDARDGSRRFVKTEPAGPLGEL | 60  60 |
| Query 61  Sbjct 61 | | QDEAARLSWLATTGVPCAAVLDVVTEAGRDWLLLGEVPGQDLLSSHLAPAEKVSIMADAM DEAARL WLAT+G+P A VLDV + AGR+W+LL V G +L + L PA KV+I+ADA+ SDEAARLRWLATSGLPGAQVLDVASAAGREWMLLSAVRGDNLEVARLGPAAKVAIIADAL | 120  120 |
| Query 121  Sbjct 121 | | RRLHTLDPATCPFDHQAKHRIERARTRMEAGLVDQDDLDEEHQGLAPAELFARLKARMPD R LH LDP TCPFDH A RIERAR R+ GLVD+ +LD+ + G+ ELFARL+A P RALHRLDPITCPFDHGAASRIERARARISEGLVDERNLDDANTGVPLDELFARLQASRPL | 180  180 |
| Query 181  Sbjct 181 | | GEDLVVTHGDACLPNIMVENGRFSGFIDCGRLGVADRYQDIALATRDIAEELGGEWADRF ED VVTHGDACLPN MVE+G FSGFIDCGRLGVADRYQD+ALATRDI +LG EW F IEDRVVTHGDACLPNFMVEDGLFSGFIDCGRLGVADRYQDLALATRDIEADLGSEWVAPF | 240  240 |
| Query | 241 LVLYGIAAPDSQRIAFYRLLDEFF 264 | | |
|  |  | YGI PD+ RIAFYRLLDEFF |  |
| Sbjct | 241 | FARYGIEQPDAGRIAFYRLLDEFF 264 | |

>WP_106409019.1 aminoglycoside 3'-phosphotransferase [Burkholderia puraquae] Length=275

Score = 310 bits (795), Expect = 5e-103, Method: Compositional matrix adjust.

| Identities | = 164/264 (62%), Positives = 190/264 (72%), Gaps = 0/264 (0%) |  |
| --- | --- | --- |
| Query 1 | MIEQDGLHAGSPAAWVERLFGYDWAQQTIGCSDAAVFRLSAQGRPVLFVKTDLSGALNEL | 60 |
| Sbjct 12 | +I+ D L PAAW L W +QT G SDA VFRL A+ FVK + +G L EL VIDNDKLCIEMPAAWKAALADCRWMRQTDGQSDALVFRLDARDGTRRFVKIEPAGPLAEL | 71 |
| Query 61 | QDEAARLSWLATTGVPCAAVLDVVTEAGRDWLLLGEVPGQDLLSSHLAPAEKVSIMADAM | 120 |
| Sbjct 72 | DEAARL WLATTG+P A VLDV + AGR+W+LL V G++L + L PA KV+I+ADA+ SDEAARLRWLATTGLPGARVLDVASAAGREWMLLSAVRGENLEVARLGPAAKVAIIADAL | 131 |
| Query 121 | RRLHTLDPATCPFDHQAKHRIERARTRMEAGLVDQDDLDEEHQGLAPAELFARLKARMPD | 180 |
| Sbjct 132 | R LH LDPATCPFDH A RIERA RM AGLVD D+LD+ + G++ ELFARL+A P RALHRLDPATCPFDHGAASRIERACARMSAGLVDADNLDDANTGVSLDELFARLQASRPL | 191 |
| Query 181 | GEDLVVTHGDACLPNIMVENGRFSGFIDCGRLGVADRYQDIALATRDIAEELGGEWADRF | 240 |

| Sbjct | 192 | E VVTHGDACLPN MVE+GRFSGFIDCGRLGVADRYQD+ALA RDI +LG EW F IEGRVVTHGDACLPNFMVEDGRFSGFIDCGRLGVADRYQDLALAARDIEVDLGDEWVAPF 251 |
| --- | --- | --- |
| Query | 241 | LVLYGIAAPDSQRIAFYRLLDEFF 264 |
| Sbjct | 252 | YGI PD +RIAFYRLLDEFF  FARYGIEQPDQERIAFYRLLDEFF 275 |

>WP_067650744.1 APH(3')-II family aminoglycoside O-phosphotransferase [Dokdonella koreensis]

ANB19643.1 Neomycin phosphotransferase [Dokdonella koreensis DS-123] Length=263

Score = 308 bits (790), Expect = 2e-102, Method: Compositional matrix adjust.

| Identities | | = 159/253 (63%), Positives = 178/253 (70%), Gaps = 1/253 (0%) |  |
| --- | --- | --- | --- |
| Query 12  Sbjct 12 | | PAAWVERLFGYDWAQQTIGCSDAAVFRLSAQGRPVLFVKTDLSGALNELQDEAARLSWLA PA W E L GY QQTIGCSDAAVFR A GRP LF+KT+++ EL+DEA RL WLA PARWRETLEGYAVEQQTIGCSDAAVFRGVAPGRPTLFIKTEVADRFAELRDEALRLRWLA | 71  71 |
| Query 72  Sbjct 72 | | TTGVPCAAVLDVVTEAGRDWLLLGEVPGQDLLSSHLAPAEKVSIMADAMRRLHTLDPATC TTG PCA VLD V RDWLLL VPG+DL SS L P V I ADA+R LH +DPATC TTGRPCAGVLDQVLGPERDWLLLSAVPGRDLASSALGPETSVRIAADALRDLHRIDPATC | 131  131 |
| Query 132  Sbjct 132 | | PFDHQAKHRIERARTRMEAGLVDQDDLDEEHQGLAPAELFARLKARMPDGEDLVVTHGDA PFDH RI A R+EAG VD DD DE H+G +P L ARL+A P ED VV HGDA PFDHAVDRRIALAAARLEAGRVDADDFDEVHRGASPTALLARLRASRPAVEDRVVAHGDA | 191  191 |
| Query 192  Sbjct 192 | | CLPNIMVENGRFSGFIDCGRLGVADRYQDIALATRDIAEELGGEWADRFLVLYGIAAPDS CL N++ + GRF+GFIDCGRLGVADRYQD+ALA RDIAE G W FL YG+ PD CLSNLVADKGRFAGFIDCGRLGVADRYQDLALAARDIAETFGEAWVGPFLARYGV-TPDP | 251  250 |
| Query 252 | | QRIAFYRLLDEFF 264 R+AFYRLLDEFF |  |
| Sbjct | 251 | GRLAFYRLLDEFF 263 | |

>WP_175807858.1 APH(3') family aminoglycoside O-phosphotransferase [Burkholderia cenocepacia]

Length=254

Score = 303 bits (777), Expect = 1e-100, Method: Compositional matrix adjust.

| Identities | = 158/253 (62%), Positives = 184/253 (73%), Gaps = 0/253 (0%) |  |
| --- | --- | --- |
| Query 12 | PAAWVERLFGYDWAQQTIGCSDAAVFRLSAQGRPVLFVKTDLSGALNELQDEAARLSWLA | 71 |
| Sbjct 2 | P AW L G W +QT G SDAAVFRL A+ FVK + +G L EL DEAARL WLA PMAWQATLAGCRWTRQTDGQSDAAVFRLDARDGSRRFVKIEPAGPLGELPDEAARLRWLA | 61 |
| Query 72 | TTGVPCAAVLDVVTEAGRDWLLLGEVPGQDLLSSHLAPAEKVSIMADAMRRLHTLDPATC | 131 |
| Sbjct 62 | TTG+P A VLDV AGRDW+LL V G ++ + LAP KV ++ADA+R LH LDPA C TTGLPGARVLDVAAAAGRDWMLLSAVRGDNIAVAPLAPDAKVMLVADALRALHRLDPAAC | 121 |
| Query 132 | PFDHQAKHRIERARTRMEAGLVDQDDLDEEHQGLAPAELFARLKARMPDGEDLVVTHGDA | 191 |
| Sbjct 122 | FDH A RIERAR R++AGLVD+D+LD+ + + +FARL A+ P+ ED VVTHGDA LFDHGAALRIERARARIDAGLVDEDNLDDANTRVPLDTIFARLLAQRPENEDRVVTHGDA | 181 |
| Query 192 | CLPNIMVENGRFSGFIDCGRLGVADRYQDIALATRDIAEELGGEWADRFLVLYGIAAPDS | 251 |
| Sbjct 182 | CLPN MVE+GRFSGFIDCGRLGVADRYQD+ALA+RDI ELG EW F YGI PD CLPNFMVEDGRFSGFIDCGRLGVADRYQDLALASRDIEAELGSEWVAPFFARYGIEHPDP | 241 |
| Query 252  Sbjct 242 | QRIAFYRLLDEFF 264  +RIAFYRLLDEFF  ERIAFYRLLDEFF 254 |  |

>AIO44444.1 aminoglycoside 3'-phosphotransferase [Burkholderia cepacia] KGC00941.1 aminoglycoside 3'-phosphotransferase [Burkholderia cepacia]

Length=264

Score = 303 bits (777), Expect = 2e-100, Method: Compositional matrix adjust. Identities = 165/264 (63%), Positives = 194/264 (73%), Gaps = 0/264 (0%)

| Query | 1 | MIEQDGLHAGSPAAWVERLFGYDWAQQTIGCSDAAVFRLSAQGRPVLFVKTDLSGALNEL | 60 |
| --- | --- | --- | --- |
| Sbjct | 1 | M + D L P W RL G W +QT G SDAAVFRL A+ FVKT+ +G L+EL MTDNDPLINDMPTTWQARLAGCRWTRQTDGQSDAAVFRLDARDGSRRFVKTEPAGPLDEL | 60 |
| Query | 61 | QDEAARLSWLATTGVPCAAVLDVVTEAGRDWLLLGEVPGQDLLSSHLAPAEKVSIMADAM | 120 |
| Sbjct | 61 | DEA RL WLATTGVP A VLDV + AGR+WLLL V G +L + LAP KV+I+ADA+ PDEAVRLRWLATTGVPGARVLDVASAAGREWLLLSAVRGDNLAVASLAPETKVTIVADAL | 120 |
| Query | 121 | RRLHTLDPATCPFDHQAKHRIERARTRMEAGLVDQDDLDEEHQGLAPAELFARLKARMPD | 180 |
| Sbjct | 121 | R LH LDPATCPFDH A RI RAR R++AGLVD+D+LD+ + G+ ++FARL A+ P RALHRLDPATCPFDHGAARRIARARARIDAGLVDEDNLDDANTGMPLDKMFARLLAQRPA | 180 |
| Query | 181 | GEDLVVTHGDACLPNIMVENGRFSGFIDCGRLGVADRYQDIALATRDIAEELGGEWADRF | 240 |
| Sbjct | 181 | ED VVTHGDACLPN MV++GRFSGFIDCGRLGVADRYQD+ALA+RDI +LG EW F  IEDGVVTHGDACLPNFMVDDGRFSGFIDCGRLGVADRYQDLALASRDIEADLGSEWVAPF | 240 |
| Query | 241 LVLYGIAAPDSQRIAFYRLLDEFF 264 | | |
|  |  | V YGI PD +RIAFYRLLDEFF |  |
| Sbjct | 241 | FVRYGIERPDPERIAFYRLLDEFF 264 | |

>KIS51351.1 aminoglycoside 3'-phosphotransferase [Burkholderia cepacia] QNN06717.1 aminoglycoside 3'-phosphotransferase [Burkholderia cenocepacia] CAR54785.1 aminoglycoside 3'-phosphotransferase [Burkholderia cenocepacia

J2315]

SPV04683.1 aminoglycoside 3'-phosphotransferase [Burkholderia cenocepacia] SPV06468.1 aminoglycoside 3'-phosphotransferase [Burkholderia cenocepacia]

Length=264

Score = 302 bits (773), Expect = 7e-100, Method: Compositional matrix adjust.

| Identities | | = 165/264 (63%), Positives = 192/264 (73%), Gaps = 0/264 (0%) |  |
| --- | --- | --- | --- |
| Query 1  Sbjct 1 | | MIEQDGLHAGSPAAWVERLFGYDWAQQTIGCSDAAVFRLSAQGRPVLFVKTDLSGALNEL M + D L P W RL G W +QT G SDAAVFRL A+ FVKT+ +G L EL MTDNDPLINDMPTTWQARLAGCRWTRQTDGQSDAAVFRLDARDGSRRFVKTEPAGPLGEL | 60  60 |
| Query 61  Sbjct 61 | | QDEAARLSWLATTGVPCAAVLDVVTEAGRDWLLLGEVPGQDLLSSHLAPAEKVSIMADAM DEA RL WLATTGVP A VLDV + AGR+WLLL V G +L + LAP KV+I+ADA+ PDEAVRLRWLATTGVPGARVLDVASAAGREWLLLSAVRGDNLAVASLAPETKVTIVADAL | 120  120 |
| Query 121  Sbjct 121 | | RRLHTLDPATCPFDHQAKHRIERARTRMEAGLVDQDDLDEEHQGLAPAELFARLKARMPD R LH LDPATCPFDH A RI RAR R++AGLVD+D+LD+ + G+ +FARL A+ P RALHRLDPATCPFDHGAARRIARARARIDAGLVDEDNLDDANTGMPLDTMFARLLAQRPA | 180  180 |
| Query 181  Sbjct 181 | | GEDLVVTHGDACLPNIMVENGRFSGFIDCGRLGVADRYQDIALATRDIAEELGGEWADRF ED VVTHGDACLPN MV++GRFSGFIDCGRLGVADRYQD+ALA+RDI +LG EW F IEDGVVTHGDACLPNFMVDDGRFSGFIDCGRLGVADRYQDLALASRDIEADLGSEWVAPF | 240  240 |
| Query | 241 LVLYGIAAPDSQRIAFYRLLDEFF 264 | | |
|  |  | V YGI PD +RIAFYRLLDEFF |  |
| Sbjct | 241 | FVRYGIERPDPERIAFYRLLDEFF 264 | |

>WP_181645687.1 APH(3')-II family aminoglycoside O-phosphotransferase [Burkholderia cenocepacia]

SPU96742.1 aminoglycoside 3'-phosphotransferase [Burkholderia cenocepacia] Length=264

Score = 301 bits (770), Expect = 2e-99, Method: Compositional matrix adjust.

| Identities | = 165/264 (63%), Positives = 191/264 (72%), Gaps = 0/264 (0%) |  |
| --- | --- | --- |
| Query 1 | MIEQDGLHAGSPAAWVERLFGYDWAQQTIGCSDAAVFRLSAQGRPVLFVKTDLSGALNEL | 60 |
| Sbjct 1 | M + D L P W L G W +QT G SDAAVFRL A+ FVKT+ +G L EL MTDNDPLINDMPTTWQATLAGCRWTRQTDGQSDAAVFRLDARDGSRRFVKTEPAGPLGEL | 60 |
| Query 61 | QDEAARLSWLATTGVPCAAVLDVVTEAGRDWLLLGEVPGQDLLSSHLAPAEKVSIMADAM | 120 |
| Sbjct 61 | DEA RL WLATTGVP A VLDV + AGR+WLLL V G +L + LAP KV+I+ADA+ PDEAVRLRWLATTGVPGARVLDVASAAGREWLLLSAVRGDNLAVASLAPETKVTIVADAL | 120 |
| Query 121 | RRLHTLDPATCPFDHQAKHRIERARTRMEAGLVDQDDLDEEHQGLAPAELFARLKARMPD | 180 |
| Sbjct 121 | R LH LDPATCPFDH A RI RAR R++AGLVD+DDLD+ + G+ +FARL A+ P  RALHRLDPATCPFDHGAARRIARARARIDAGLVDEDDLDDANTGMPLDTMFARLLAQRPA | 180 |

Query 181 GEDLVVTHGDACLPNIMVENGRFSGFIDCGRLGVADRYQDIALATRDIAEELGGEWADRF 240

| Sbjct | 181 | ED VVTHGDACLPN MV++GRFSGFIDCGRLGVADRYQD+ALA+RDI +LG EW F IEDGVVTHGDACLPNFMVDDGRFSGFIDCGRLGVADRYQDLALASRDIEADLGSEWVAPF 240 |
| --- | --- | --- |
| Query | 241 | LVLYGIAAPDSQRIAFYRLLDEFF 264 |
| Sbjct | 241 | V YGI PD +RIAFYRLLDEFF  FVRYGIERPDPERIAFYRLLDEFF 264 |

```
>RBL65130.1 APH(3') family aminoglycoside O-phosphotransferase, partial [Pseudomonas sp. MWU13-2625]
Length=146

Score = 296 bits (758), Expect = 2e-99, Method: Compositional matrix adjust. Identities = 146/146 (100%), Positives = 146/146 (100%), Gaps = 0/146 (0%)

Query 14	AWVERLFGYDWAQQTIGCSDAAVFRLSAQGRPVLFVKTDLSGALNELQDEAARLSWLATT 73 AWVERLFGYDWAQQTIGCSDAAVFRLSAQGRPVLFVKTDLSGALNELQDEAARLSWLATT
Sbjct 1	AWVERLFGYDWAQQTIGCSDAAVFRLSAQGRPVLFVKTDLSGALNELQDEAARLSWLATT 60

Query 74	GVPCAAVLDVVTEAGRDWLLLGEVPGQDLLSSHLAPAEKVSIMADAMRRLHTLDPATCPF 133 GVPCAAVLDVVTEAGRDWLLLGEVPGQDLLSSHLAPAEKVSIMADAMRRLHTLDPATCPF
Sbjct 61	GVPCAAVLDVVTEAGRDWLLLGEVPGQDLLSSHLAPAEKVSIMADAMRRLHTLDPATCPF 120

Query 134 DHQAKHRIERARTRMEAGLVDQDDLD 159 DHQAKHRIERARTRMEAGLVDQDDLD
Sbjct 121 DHQAKHRIERARTRMEAGLVDQDDLD 146

>CDN62684.1 Aminoglycoside 3'-phosphotransferase [Burkholderia cenocepacia H111]
Length=284

Score = 301 bits (771), Expect = 3e-99, Method: Compositional matrix adjust.
```

| Identities | | = 164/264 (62%), Positives = 194/264 (73%), Gaps = 0/264 (0%) |  |
| --- | --- | --- | --- |
| Query 1  Sbjct 21 | | MIEQDGLHAGSPAAWVERLFGYDWAQQTIGCSDAAVFRLSAQGRPVLFVKTDLSGALNEL  + + D L P W RL G W +QT G SDAAVFRL A+ FVKT+ +G L+EL VTDNDPLINDMPTTWQARLAGCRWTRQTDGQSDAAVFRLDARDGSRRFVKTEPAGPLDEL | 60  80 |
| Query 61  Sbjct 81 | | QDEAARLSWLATTGVPCAAVLDVVTEAGRDWLLLGEVPGQDLLSSHLAPAEKVSIMADAM DEA RL WLATTGVP A VLDV + AGR+WLLL V G +L + LAP KV+I+ADA+ PDEAVRLRWLATTGVPGARVLDVASAAGREWLLLSAVRGDNLAVASLAPETKVTIVADAL | 120  140 |
| Query 121  Sbjct 141 | | RRLHTLDPATCPFDHQAKHRIERARTRMEAGLVDQDDLDEEHQGLAPAELFARLKARMPD R LH LDPATCPFDH A RI RAR R++AGLVD+D+LD+ + G+ ++FARL A+ P RALHRLDPATCPFDHGAARRIARARARIDAGLVDEDNLDDANTGVPLDKMFARLLAQRPA | 180  200 |
| Query 181  Sbjct 201 | | GEDLVVTHGDACLPNIMVENGRFSGFIDCGRLGVADRYQDIALATRDIAEELGGEWADRF ED VVTHGDACLPN MV++GRFSGFIDCGRLGVADRYQD+ALA+RDI +LG EW F IEDGVVTHGDACLPNFMVDDGRFSGFIDCGRLGVADRYQDLALASRDIEADLGSEWVAPF | 240  260 |
| Query | 241 LVLYGIAAPDSQRIAFYRLLDEFF 264 | | |
|  |  | V YGI PD +RIAFYRLLDEFF |  |
| Sbjct | 261 | FVRYGIERPDPERIAFYRLLDEFF 284 | |

>ECZ5199065.1 phosphotransferase [Salmonella enterica subsp. enterica serovar Enteritidis]

Length=145

Score = 296 bits (757), Expect = 4e-99, Method: Compositional matrix adjust. Identities = 145/145 (100%), Positives = 145/145 (100%), Gaps = 0/145 (0%)

| Query | 1 | MIEQDGLHAGSPAAWVERLFGYDWAQQTIGCSDAAVFRLSAQGRPVLFVKTDLSGALNEL | 60 |
| --- | --- | --- | --- |
| Sbjct | 1 | MIEQDGLHAGSPAAWVERLFGYDWAQQTIGCSDAAVFRLSAQGRPVLFVKTDLSGALNEL MIEQDGLHAGSPAAWVERLFGYDWAQQTIGCSDAAVFRLSAQGRPVLFVKTDLSGALNEL | 60 |
| Query | 61 | QDEAARLSWLATTGVPCAAVLDVVTEAGRDWLLLGEVPGQDLLSSHLAPAEKVSIMADAM | 120 |
| Sbjct | 61 | QDEAARLSWLATTGVPCAAVLDVVTEAGRDWLLLGEVPGQDLLSSHLAPAEKVSIMADAM  QDEAARLSWLATTGVPCAAVLDVVTEAGRDWLLLGEVPGQDLLSSHLAPAEKVSIMADAM | 120 |

Query 121 RRLHTLDPATCPFDHQAKHRIERAR 145 RRLHTLDPATCPFDHQAKHRIERAR

Sbjct 121 RRLHTLDPATCPFDHQAKHRIERAR 145

>WP_181159040.1 MULTISPECIES: APH(3')-II family aminoglycoside O-phosphotransferase [Burkholderia]

PZW94158.1 aminoglycoside 3'-phosphotransferase-2 [Burkholderia sp. 28_3] RAS42658.1 aminoglycoside 3'-phosphotransferase-2 [Burkholderia cenocepacia] TDV87110.1 aminoglycoside 3'-phosphotransferase-2 [Burkholderia cenocepacia] CAB5163415.1 aminoglycoside 3'-phosphotransferase [Burkholderia cenocepacia]

Length=264

Score = 300 bits (767), Expect = 6e-99, Method: Compositional matrix adjust.

| Identities | | = 164/264 (62%), Positives = 191/264 (72%), Gaps = 0/264 (0%) |  |
| --- | --- | --- | --- |
| Query 1  Sbjct 1 | | MIEQDGLHAGSPAAWVERLFGYDWAQQTIGCSDAAVFRLSAQGRPVLFVKTDLSGALNEL M + D L P W L G W +QT G SDAAVFRL A+ FVKT+ +G L EL MTDNDPLINDMPTTWQATLAGCRWTRQTDGQSDAAVFRLDARDGSRRFVKTEPAGPLGEL | 60  60 |
| Query 61  Sbjct 61 | | QDEAARLSWLATTGVPCAAVLDVVTEAGRDWLLLGEVPGQDLLSSHLAPAEKVSIMADAM DEA RL WLATTGVP A VLDV + AGR+WLLL V G +L + LAP KV+I+ADA+ PDEAVRLRWLATTGVPGARVLDVASAAGREWLLLSAVRGDNLAVASLAPETKVTIVADAL | 120  120 |
| Query 121  Sbjct 121 | | RRLHTLDPATCPFDHQAKHRIERARTRMEAGLVDQDDLDEEHQGLAPAELFARLKARMPD R LH LDPATCPFDH A RI RAR R++AGLVD+D+LD+ + G+ +FARL A+ P RALHRLDPATCPFDHGAARRIARARARIDAGLVDEDNLDDANTGMPLDTMFARLLAQRPA | 180  180 |
| Query 181  Sbjct 181 | | GEDLVVTHGDACLPNIMVENGRFSGFIDCGRLGVADRYQDIALATRDIAEELGGEWADRF ED VVTHGDACLPN MV++GRFSGFIDCGRLGVADRYQD+ALA+RDI +LG EW F IEDGVVTHGDACLPNFMVDDGRFSGFIDCGRLGVADRYQDLALASRDIEADLGSEWVAPF | 240  240 |
| Query | 241 LVLYGIAAPDSQRIAFYRLLDEFF 264 | | |
|  |  | V YGI PD +RIAFYRLLDEFF |  |
| Sbjct | 241 | FVRYGIERPDPERIAFYRLLDEFF 264 | |

>WP_034203382.1 APH(3')-II family aminoglycoside O-phosphotransferase [Burkholderia cepacia]

Length=254

Score = 299 bits (766), Expect = 6e-99, Method: Compositional matrix adjust.

| Identities | = 162/253 (64%), Positives = 190/253 (75%), Gaps = 0/253 (0%) |  |
| --- | --- | --- |
| Query 12 | PAAWVERLFGYDWAQQTIGCSDAAVFRLSAQGRPVLFVKTDLSGALNELQDEAARLSWLA | 71 |
| Sbjct 2 | P W RL G W +QT G SDAAVFRL A+ FVKT+ +G L+EL DEA RL WLA PTTWQARLAGCRWTRQTDGQSDAAVFRLDARDGSRRFVKTEPAGPLDELPDEAVRLRWLA | 61 |
| Query 72 | TTGVPCAAVLDVVTEAGRDWLLLGEVPGQDLLSSHLAPAEKVSIMADAMRRLHTLDPATC | 131 |
| Sbjct 62 | TTGVP A VLDV + AGR+WLLL V G +L + LAP KV+I+ADA+R LH LDPATC TTGVPGARVLDVASAAGREWLLLSAVRGDNLAVASLAPETKVTIVADALRALHRLDPATC | 121 |
| Query 132 | PFDHQAKHRIERARTRMEAGLVDQDDLDEEHQGLAPAELFARLKARMPDGEDLVVTHGDA | 191 |
| Sbjct 122 | PFDH A RI RAR R++AGLVD+D+LD+ + G+ ++FARL A+ P ED VVTHGDA PFDHGAARRIARARARIDAGLVDEDNLDDANTGMPLDKMFARLLAQRPAIEDGVVTHGDA | 181 |
| Query 192 | CLPNIMVENGRFSGFIDCGRLGVADRYQDIALATRDIAEELGGEWADRFLVLYGIAAPDS | 251 |
| Sbjct 182 | CLPN MV++GRFSGFIDCGRLGVADRYQD+ALA+RDI +LG EW F V YGI PD CLPNFMVDDGRFSGFIDCGRLGVADRYQDLALASRDIEADLGSEWVAPFFVRYGIERPDP | 241 |
| Query 252  Sbjct 242 | QRIAFYRLLDEFF 264  +RIAFYRLLDEFF  ERIAFYRLLDEFF 254 |  |

>EPZ90555.1 putative streptomycin 3''-kinase [Burkholderia cenocepacia K56-2Valvano] Length=284

Score = 300 bits (769), Expect = 6e-99, Method: Compositional matrix adjust. Identities = 164/264 (62%), Positives = 192/264 (73%), Gaps = 0/264 (0%)

| Query | 1 | MIEQDGLHAGSPAAWVERLFGYDWAQQTIGCSDAAVFRLSAQGRPVLFVKTDLSGALNEL | 60 |
| --- | --- | --- | --- |
| Sbjct | 21 | + + D L P W RL G W +QT G SDAAVFRL A+ FVKT+ +G L EL VTDNDPLINDMPTTWQARLAGCRWTRQTDGQSDAAVFRLDARDGSRRFVKTEPAGPLGEL | 80 |
| Query | 61 | QDEAARLSWLATTGVPCAAVLDVVTEAGRDWLLLGEVPGQDLLSSHLAPAEKVSIMADAM | 120 |
| Sbjct | 81 | DEA RL WLATTGVP A VLDV + AGR+WLLL V G +L + LAP KV+I+ADA+ PDEAVRLRWLATTGVPGARVLDVASAAGREWLLLSAVRGDNLAVASLAPETKVTIVADAL | 140 |
| Query | 121 | RRLHTLDPATCPFDHQAKHRIERARTRMEAGLVDQDDLDEEHQGLAPAELFARLKARMPD | 180 |
| Sbjct | 141 | R LH LDPATCPFDH A RI RAR R++AGLVD+D+LD+ + G+ +FARL A+ P RALHRLDPATCPFDHGAARRIARARARIDAGLVDEDNLDDANTGMPLDTMFARLLAQRPA | 200 |
| Query | 181 | GEDLVVTHGDACLPNIMVENGRFSGFIDCGRLGVADRYQDIALATRDIAEELGGEWADRF | 240 |
| Sbjct | 201 | ED VVTHGDACLPN MV++GRFSGFIDCGRLGVADRYQD+ALA+RDI +LG EW F  IEDGVVTHGDACLPNFMVDDGRFSGFIDCGRLGVADRYQDLALASRDIEADLGSEWVAPF | 260 |
| Query | 241 LVLYGIAAPDSQRIAFYRLLDEFF 264 | | |
|  |  | V YGI PD +RIAFYRLLDEFF |  |
| Sbjct | 261 | FVRYGIERPDPERIAFYRLLDEFF 284 | |

>WP_124550430.1 APH(3')-II family aminoglycoside O-phosphotransferase [Burkholderia cenocepacia]

RQU02680.1 APH(3')-II family aminoglycoside O-phosphotransferase [Burkholderia cenocepacia]

RQU58312.1 APH(3')-II family aminoglycoside O-phosphotransferase [Burkholderia cenocepacia]

Length=254

Score = 299 bits (765), Expect = 8e-99, Method: Compositional matrix adjust.

| Identities | = 162/253 (64%), Positives = 189/253 (75%), Gaps = 0/253 (0%) |  |
| --- | --- | --- |
| Query 12 | PAAWVERLFGYDWAQQTIGCSDAAVFRLSAQGRPVLFVKTDLSGALNELQDEAARLSWLA | 71 |
| Sbjct 2 | P W+ RL G W +QT G SDAAVFRL A+ FVKT+ +G L EL DEA RL WLA PTTWLARLAGCRWTRQTDGQSDAAVFRLDARDGSRRFVKTEPAGPLGELPDEAVRLRWLA | 61 |
| Query 72 | TTGVPCAAVLDVVTEAGRDWLLLGEVPGQDLLSSHLAPAEKVSIMADAMRRLHTLDPATC | 131 |
| Sbjct 62 | TTGVP A VLDV + AGR+WLLL V G +L + LAP KV+I+ADA+R LH LDPATC TTGVPGARVLDVASAAGREWLLLSAVRGDNLAVASLAPETKVTIVADALRALHRLDPATC | 121 |
| Query 132 | PFDHQAKHRIERARTRMEAGLVDQDDLDEEHQGLAPAELFARLKARMPDGEDLVVTHGDA | 191 |
| Sbjct 122 | PFDH A RI RAR R++AGLVD+D+LD+ + G+ +FARL A+ P ED VVTHGDA PFDHGAARRIARARARIDAGLVDEDNLDDANTGVPLDTMFARLLAQRPAIEDGVVTHGDA | 181 |
| Query 192 | CLPNIMVENGRFSGFIDCGRLGVADRYQDIALATRDIAEELGGEWADRFLVLYGIAAPDS | 251 |
| Sbjct 182 | CLPN MV++GRFSGFIDCGRLGVADRYQD+ALA+RDI +LG EW F V YGI PD CLPNFMVDDGRFSGFIDCGRLGVADRYQDLALASRDIEADLGSEWVAPFFVRYGIERPDP | 241 |
| Query 252  Sbjct 242 | QRIAFYRLLDEFF 264  +RIAFYRLLDEFF  ERIAFYRLLDEFF 254 |  |

>WP_124698428.1 aminoglycoside 3'-phosphotransferase [Burkholderia cenocepacia] RQV12984.1 aminoglycoside 3'-phosphotransferase [Burkholderia cenocepacia]

Length=254

Score = 299 bits (765), Expect = 9e-99, Method: Compositional matrix adjust.

| Identities | = 154/253 (61%), Positives = 183/253 (72%), Gaps = 0/253 (0%) |  |
| --- | --- | --- |
| Query 12 | PAAWVERLFGYDWAQQTIGCSDAAVFRLSAQGRPVLFVKTDLSGALNELQDEAARLSWLA | 71 |
| Sbjct 2 | P AW L G W +QT G SDAAVFRL A+ F+KT+ +G L EL DEA RL WLA PVAWQATLAGCRWTRQTDGQSDAAVFRLDARDGSRRFLKTEPAGPLGELPDEAERLRWLA | 61 |
| Query 72 | TTGVPCAAVLDVVTEAGRDWLLLGEVPGQDLLSSHLAPAEKVSIMADAMRRLHTLDPATC | 131 |
| Sbjct 62 | TTG+P A VLDV E RDW+LL V G ++ + LAP KV ++ADA+R LH LDPA C TTGLPGARVLDVAAEEERDWMLLSAVRGDNIAVAPLAPDAKVMLVADALRALHRLDPAAC | 121 |
| Query 132 | PFDHQAKHRIERARTRMEAGLVDQDDLDEEHQGLAPAELFARLKARMPDGEDLVVTHGDA | 191 |
| Sbjct 122 | PFDH A RIERAR R++AGLVD+D+LD+ + + +FARL A+ P+ ED VVTHGDA  PFDHGAALRIERARARIDAGLVDEDNLDDANTRVPLDTIFARLLAQRPENEDRVVTHGDA | 181 |

| Query | 192 | CLPNIMVENGRFSGFIDCGRLGVADRYQDIALATRDIAEELGGEWADRFLVLYGIAAPDS 251 |
| --- | --- | --- |
| Sbjct | 182 | CLPN+MVE+GRFSGFIDCGRLGVADRYQD+ LA+ DI +LG EW F YGI PD CLPNLMVEDGRFSGFIDCGRLGVADRYQDLELASCDIEADLGSEWVSPFFARYGIEHPDP 241 |
| Query | 252 | QRIAFYRLLDEFF 264 |
| Sbjct | 242 | +RIAFYRLLDEFF  ERIAFYRLLDEFF 254 |

>WP_043205170.1 APH(3')-II family aminoglycoside O-phosphotransferase [Burkholderia cenocepacia]

Length=254

Score = 299 bits (765), Expect = 1e-98, Method: Compositional matrix adjust.

| Identities | = 162/253 (64%), Positives = 190/253 (75%), Gaps = 0/253 (0%) |  |
| --- | --- | --- |
| Query 12 | PAAWVERLFGYDWAQQTIGCSDAAVFRLSAQGRPVLFVKTDLSGALNELQDEAARLSWLA | 71 |
| Sbjct 2 | P W RL G W +QT G SDAAVFRL A+ FVKT+ +G L+EL DEA RL WLA PTTWQARLAGCRWTRQTDGQSDAAVFRLDARDGSRRFVKTEPAGPLDELPDEAVRLRWLA | 61 |
| Query 72 | TTGVPCAAVLDVVTEAGRDWLLLGEVPGQDLLSSHLAPAEKVSIMADAMRRLHTLDPATC | 131 |
| Sbjct 62 | TTGVP A VLDV + AGR+WLLL V G +L + LAP KV+I+ADA+R LH LDPATC TTGVPGARVLDVASAAGREWLLLSAVRGDNLAVASLAPETKVTIVADALRALHRLDPATC | 121 |
| Query 132 | PFDHQAKHRIERARTRMEAGLVDQDDLDEEHQGLAPAELFARLKARMPDGEDLVVTHGDA | 191 |
| Sbjct 122 | PFDH A RI RAR R++AGLVD+D+LD+ + G+ ++FARL A+ P ED VVTHGDA PFDHGAARRIARARARIDAGLVDEDNLDDANTGVPLDKMFARLLAQRPAIEDGVVTHGDA | 181 |
| Query 192 | CLPNIMVENGRFSGFIDCGRLGVADRYQDIALATRDIAEELGGEWADRFLVLYGIAAPDS | 251 |
| Sbjct 182 | CLPN MV++GRFSGFIDCGRLGVADRYQD+ALA+RDI +LG EW F V YGI PD CLPNFMVDDGRFSGFIDCGRLGVADRYQDLALASRDIEADLGSEWVAPFFVRYGIERPDP | 241 |
| Query 252  Sbjct 242 | QRIAFYRLLDEFF 264  +RIAFYRLLDEFF  ERIAFYRLLDEFF 254 |  |

>WP_181365749.1 APH(3')-II family aminoglycoside O-phosphotransferase [Burkholderia cenocepacia]

Length=264

Score = 299 bits (765), Expect = 1e-98, Method: Compositional matrix adjust.

| Identities | | = 164/264 (62%), Positives = 191/264 (72%), Gaps = 0/264 (0%) |  |
| --- | --- | --- | --- |
| Query 1  Sbjct 1 | | MIEQDGLHAGSPAAWVERLFGYDWAQQTIGCSDAAVFRLSAQGRPVLFVKTDLSGALNEL M + D L P W L G W +QT G SDAAVFRL A+ FVKT+ +G L EL MTDNDPLINDMPTTWQATLAGCRWTRQTDGQSDAAVFRLDARDGSRRFVKTEPAGPLGEL | 60  60 |
| Query 61  Sbjct 61 | | QDEAARLSWLATTGVPCAAVLDVVTEAGRDWLLLGEVPGQDLLSSHLAPAEKVSIMADAM DEA RL WLATTGVP A VLDV + AGR+WLLL V G +L + LAP KV+I+ADA+ PDEAVRLRWLATTGVPGARVLDVASAAGREWLLLSAVRGDNLAVASLAPETKVTIVADAL | 120  120 |
| Query 121  Sbjct 121 | | RRLHTLDPATCPFDHQAKHRIERARTRMEAGLVDQDDLDEEHQGLAPAELFARLKARMPD R LH LDPATCPFDH A RI RAR R++AGLVD+D+LD+ + G+ +FARL A+ P RALHRLDPATCPFDHGAARRIARARARIDAGLVDEDNLDDANTGVPLDTMFARLLAQRPA | 180  180 |
| Query 181  Sbjct 181 | | GEDLVVTHGDACLPNIMVENGRFSGFIDCGRLGVADRYQDIALATRDIAEELGGEWADRF ED VVTHGDACLPN MV++GRFSGFIDCGRLGVADRYQD+ALA+RDI +LG EW F IEDGVVTHGDACLPNFMVDDGRFSGFIDCGRLGVADRYQDLALASRDIEADLGSEWVAPF | 240  240 |
| Query | 241 LVLYGIAAPDSQRIAFYRLLDEFF 264 | | |
|  |  | V YGI PD +RIAFYRLLDEFF |  |
| Sbjct | 241 | FVRYGIERPDPERIAFYRLLDEFF 264 | |

>WP_077190840.1 MULTISPECIES: APH(3')-II family aminoglycoside O-phosphotransferase [Burkholderia]

AQQ40025.1 APH(3') family aminoglycoside O-phosphotransferase [Burkholderia cenocepacia]

MBG0875894.1 APH(3')-II family aminoglycoside O-phosphotransferase [Burkholderia sp. 9775_39]

MBG0883901.1 APH(3')-II family aminoglycoside O-phosphotransferase [Burkholderia

sp. 9773_38]

ONV18597.1 APH(3') family aminoglycoside O-phosphotransferase [Burkholderia cenocepacia]

ONV29237.1 APH(3') family aminoglycoside O-phosphotransferase [Burkholderia cenocepacia]

Length=254

Score = 298 bits (763), Expect = 2e-98, Method: Compositional matrix adjust.

| Identities | = 162/253 (64%), Positives = 189/253 (75%), Gaps = 0/253 (0%) |  |
| --- | --- | --- |
| Query 12 | PAAWVERLFGYDWAQQTIGCSDAAVFRLSAQGRPVLFVKTDLSGALNELQDEAARLSWLA | 71 |
| Sbjct 2 | P W RL G W +QT G SDAAVFRL A+ FVKT+ +G L EL DEA RL WLA PTTWQARLAGCRWTRQTDGQSDAAVFRLDARDGSRRFVKTEPAGPLGELPDEAVRLRWLA | 61 |
| Query 72 | TTGVPCAAVLDVVTEAGRDWLLLGEVPGQDLLSSHLAPAEKVSIMADAMRRLHTLDPATC | 131 |
| Sbjct 62 | TTGVP A VLDV + AGR+WLLL V G +L + LAP KV+I+ADA+R LH LDPATC TTGVPGARVLDVASAAGREWLLLSAVRGDNLAVASLAPETKVTIVADALRALHRLDPATC | 121 |
| Query 132 | PFDHQAKHRIERARTRMEAGLVDQDDLDEEHQGLAPAELFARLKARMPDGEDLVVTHGDA | 191 |
| Sbjct 122 | PFDH A RI RAR R++AGLVD+D+LD+ + G+ ++FARL A+ P ED VVTHGDA PFDHGAARRIARARARIDAGLVDEDNLDDANTGVPLDKMFARLLAQRPAIEDGVVTHGDA | 181 |
| Query 192 | CLPNIMVENGRFSGFIDCGRLGVADRYQDIALATRDIAEELGGEWADRFLVLYGIAAPDS | 251 |
| Sbjct 182 | CLPN MV++GRFSGFIDCGRLGVADRYQD+ALA+RDI +LG EW F V YGI PD CLPNFMVDDGRFSGFIDCGRLGVADRYQDLALASRDIEADLGSEWVAPFFVRYGIERPDP | 241 |
| Query 252  Sbjct 242 | QRIAFYRLLDEFF 264  +RIAFYRLLDEFF  ERIAFYRLLDEFF 254 |  |

>WP_034195681.1 APH(3')-II family aminoglycoside O-phosphotransferase [Burkholderia cenocepacia]

AMU14246.1 APH(3') family aminoglycoside O-phosphotransferase [Burkholderia cenocepacia]

KKI81223.1 aminoglycoside phosphotransferase [Burkholderia cenocepacia]

NGO92545.1 APH(3') family aminoglycoside O-phosphotransferase [Burkholderia cenocepacia]

ONR60418.1 aminoglycoside phosphotransferase APH(3') [Burkholderia cenocepacia] ONR64510.1 aminoglycoside phosphotransferase APH(3') [Burkholderia cenocepacia]

Length=254

Score = 298 bits (763), Expect = 2e-98, Method: Compositional matrix adjust.

| Identities | = 162/253 (64%), Positives = 188/253 (74%), Gaps = 0/253 (0%) |  |
| --- | --- | --- |
| Query 12 | PAAWVERLFGYDWAQQTIGCSDAAVFRLSAQGRPVLFVKTDLSGALNELQDEAARLSWLA | 71 |
| Sbjct 2 | P W RL G W +QT G SDAAVFRL A+ FVKT+ +G L EL DEA RL WLA PTTWQARLAGCRWTRQTDGQSDAAVFRLDARDGSRRFVKTEPAGPLGELPDEAVRLRWLA | 61 |
| Query 72 | TTGVPCAAVLDVVTEAGRDWLLLGEVPGQDLLSSHLAPAEKVSIMADAMRRLHTLDPATC | 131 |
| Sbjct 62 | TTGVP A VLDV + AGR+WLLL V G +L + LAP KV+I+ADA+R LH LDPATC TTGVPGARVLDVASAAGREWLLLSAVRGDNLAVASLAPETKVTIVADALRALHRLDPATC | 121 |
| Query 132 | PFDHQAKHRIERARTRMEAGLVDQDDLDEEHQGLAPAELFARLKARMPDGEDLVVTHGDA | 191 |
| Sbjct 122 | PFDH A RI RAR R++AGLVD+D+LD+ + G+ +FARL A+ P ED VVTHGDA PFDHGAARRIARARARIDAGLVDEDNLDDANTGMPLDTMFARLLAQRPAIEDGVVTHGDA | 181 |
| Query 192 | CLPNIMVENGRFSGFIDCGRLGVADRYQDIALATRDIAEELGGEWADRFLVLYGIAAPDS | 251 |
| Sbjct 182 | CLPN MV++GRFSGFIDCGRLGVADRYQD+ALA+RDI +LG EW F V YGI PD CLPNFMVDDGRFSGFIDCGRLGVADRYQDLALASRDIEADLGSEWVAPFFVRYGIERPDP | 241 |
| Query 252  Sbjct 242 | QRIAFYRLLDEFF 264  +RIAFYRLLDEFF  ERIAFYRLLDEFF 254 |  |

>WP_059722735.1 MULTISPECIES: APH(3')-II family aminoglycoside O-phosphotransferase [Burkholderia]

KVF51859.1 aminoglycoside phosphotransferase [Burkholderia cenocepacia]

MBG0866703.1 APH(3')-II family aminoglycoside O-phosphotransferase [Burkholderia sp. 9779_493]

Length=254

Score = 298 bits (763), Expect = 2e-98, Method: Compositional matrix adjust.

| Identities | = 162/253 (64%), Positives = 187/253 (74%), Gaps = 0/253 (0%) |  |
| --- | --- | --- |
| Query 12 | PAAWVERLFGYDWAQQTIGCSDAAVFRLSAQGRPVLFVKTDLSGALNELQDEAARLSWLA | 71 |
| Sbjct 2 | P W RL G W +QT G SDAAVFRL A+ FVKT+ +G L EL DEA RL WLA PTTWQARLAGCRWTRQTDGQSDAAVFRLDARDGSRRFVKTEPAGPLGELPDEAVRLRWLA | 61 |
| Query 72 | TTGVPCAAVLDVVTEAGRDWLLLGEVPGQDLLSSHLAPAEKVSIMADAMRRLHTLDPATC | 131 |
| Sbjct 62 | TTGVP A VLDV + AGR+WLLL V G +L + LAP KV+I+ADA+R LH LDPATC TTGVPGARVLDVASAAGREWLLLSAVRGDNLAVASLAPETKVTIVADALRALHRLDPATC | 121 |
| Query 132 | PFDHQAKHRIERARTRMEAGLVDQDDLDEEHQGLAPAELFARLKARMPDGEDLVVTHGDA | 191 |
| Sbjct 122 | PFDH A RI RAR R++AGLVD+D+LD + G+ +FARL A+ P ED VVTHGDA PFDHGAARRIARARARIDAGLVDEDNLDNANTGMPLDTMFARLLAQRPAIEDGVVTHGDA | 181 |
| Query 192 | CLPNIMVENGRFSGFIDCGRLGVADRYQDIALATRDIAEELGGEWADRFLVLYGIAAPDS | 251 |
| Sbjct 182 | CLPN MV++GRFSGFIDCGRLGVADRYQD+ALA+RDI +LG EW F V YGI PD CLPNFMVDDGRFSGFIDCGRLGVADRYQDLALASRDIEADLGSEWVAPFFVRYGIERPDP | 241 |
| Query 252  Sbjct 242 | QRIAFYRLLDEFF 264  +RIAFYRLLDEFF  ERIAFYRLLDEFF 254 |  |

>WP_102898688.1 APH(3')-II family aminoglycoside O-phosphotransferase [Burkholderia cenocepacia]

PNF08731.1 APH(3')-II family aminoglycoside O-phosphotransferase [Burkholderia cenocepacia]

Length=254

Score = 298 bits (763), Expect = 2e-98, Method: Compositional matrix adjust.

| Identities | = 162/253 (64%), Positives = 189/253 (75%), Gaps = 0/253 (0%) |  |
| --- | --- | --- |
| Query 12 | PAAWVERLFGYDWAQQTIGCSDAAVFRLSAQGRPVLFVKTDLSGALNELQDEAARLSWLA | 71 |
| Sbjct 2 | P W RL G W +QT G SDAAVFRL A+ FVKT+ +G L EL DEA RL WLA PTTWQARLAGCRWTRQTDGQSDAAVFRLDARDGSRRFVKTEPAGPLGELPDEAVRLRWLA | 61 |
| Query 72 | TTGVPCAAVLDVVTEAGRDWLLLGEVPGQDLLSSHLAPAEKVSIMADAMRRLHTLDPATC | 131 |
| Sbjct 62 | TTGVP A VLDV + AGR+WLLL V G +L + LAP KV+I+ADA+R LH LDPATC TTGVPGARVLDVASAAGREWLLLSAVRGDNLAVASLAPETKVTIVADALRALHRLDPATC | 121 |
| Query 132 | PFDHQAKHRIERARTRMEAGLVDQDDLDEEHQGLAPAELFARLKARMPDGEDLVVTHGDA | 191 |
| Sbjct 122 | PFDH A RI RAR R++AGLVD+D+LD+ + G+ ++FARL A+ P ED VVTHGDA PFDHGAARRIARARARIDAGLVDEDNLDDANAGVPLDKMFARLLAQRPAIEDGVVTHGDA | 181 |
| Query 192 | CLPNIMVENGRFSGFIDCGRLGVADRYQDIALATRDIAEELGGEWADRFLVLYGIAAPDS | 251 |
| Sbjct 182 | CLPN MV++GRFSGFIDCGRLGVADRYQD+ALA+RDI +LG EW F V YGI PD CLPNFMVDDGRFSGFIDCGRLGVADRYQDLALASRDIEADLGSEWVAPFFVRYGIERPDP | 241 |
| Query 252  Sbjct 242 | QRIAFYRLLDEFF 264  +RIAFYRLLDEFF  ERIAFYRLLDEFF 254 |  |

>WP_125332658.1 APH(3')-II family aminoglycoside O-phosphotransferase [Burkholderia cenocepacia]

RSB88871.1 APH(3')-II family aminoglycoside O-phosphotransferase [Burkholderia cenocepacia]

Length=254

Score = 298 bits (762), Expect = 3e-98, Method: Compositional matrix adjust.

| Identities | = 161/253 (64%), Positives = 188/253 (74%), Gaps = 0/253 (0%) |  |
| --- | --- | --- |
| Query 12 | PAAWVERLFGYDWAQQTIGCSDAAVFRLSAQGRPVLFVKTDLSGALNELQDEAARLSWLA | 71 |
| Sbjct 2 | P W RL G W +QT G SDAAVFRL A+ FVKT+ +G L EL DEA RL WLA PTTWQARLAGCRWTRQTDGQSDAAVFRLDARDGSRRFVKTEPAGPLGELPDEAVRLRWLA | 61 |
| Query 72 | TTGVPCAAVLDVVTEAGRDWLLLGEVPGQDLLSSHLAPAEKVSIMADAMRRLHTLDPATC | 131 |
| Sbjct 62 | TTGVP A VLDV + AGR+WLLL V G +L + LAP KV+I+ADA+R LH LDPATC  TTGVPGARVLDVASAAGREWLLLSAVRGDNLAVASLAPETKVTIVADALRALHRLDPATC | 121 |

| Query | 132 | PFDHQAKHRIERARTRMEAGLVDQDDLDEEHQGLAPAELFARLKARMPDGEDLVVTHGDA | 191 |
| --- | --- | --- | --- |
| Sbjct | 122 | PFDH A RI RAR R++AGLVD+D+LD+ + G+ +F+RL A+ P ED VVTHGDA PFDHGAARRIARARARIDAGLVDEDNLDDANTGMPLDTMFSRLLAQRPAIEDRVVTHGDA | 181 |
| Query | 192 | CLPNIMVENGRFSGFIDCGRLGVADRYQDIALATRDIAEELGGEWADRFLVLYGIAAPDS | 251 |
| Sbjct | 182 | CLPN MV++GRFSGFIDCGRLGVADRYQD+ALA+RDI +LG EW F V YGI PD CLPNFMVDDGRFSGFIDCGRLGVADRYQDLALASRDIEADLGSEWVAPFFVRYGIERPDP | 241 |
| Query  Sbjct | 252  242 | QRIAFYRLLDEFF 264  +RIAFYRLLDEFF  ERIAFYRLLDEFF 254 |  |

>WP_062885658.1 MULTISPECIES: APH(3')-II family aminoglycoside O-phosphotransferase [Burkholderia cepacia complex]

AMU10500.1 APH(3') family aminoglycoside O-phosphotransferase [Burkholderia cenocepacia]

ONJ03977.1 aminoglycoside 3'-phosphotransferase [Burkholderia cenocepacia] ONJ23701.1 aminoglycoside 3'-phosphotransferase [Burkholderia cenocepacia] ONP27246.1 aminoglycoside 3'-phosphotransferase [Burkholderia cenocepacia] ONP32383.1 aminoglycoside 3'-phosphotransferase [Burkholderia cenocepacia]

Length=254

Score = 298 bits (762), Expect = 3e-98, Method: Compositional matrix adjust.

| Identities | = 162/253 (64%), Positives = 188/253 (74%), Gaps = 0/253 (0%) |  |
| --- | --- | --- |
| Query 12 | PAAWVERLFGYDWAQQTIGCSDAAVFRLSAQGRPVLFVKTDLSGALNELQDEAARLSWLA | 71 |
| Sbjct 2 | P W RL G W +QT G SDAAVFRL A+ FVKT+ +G L EL DEA RL WLA PTTWQARLAGCRWTRQTDGQSDAAVFRLDARDGSRRFVKTEPAGPLGELPDEAVRLRWLA | 61 |
| Query 72 | TTGVPCAAVLDVVTEAGRDWLLLGEVPGQDLLSSHLAPAEKVSIMADAMRRLHTLDPATC | 131 |
| Sbjct 62 | TTGVP A VLDV + AGR+WLLL V G +L + LAP KV+I+ADA+R LH LDPATC TTGVPGARVLDVASAAGREWLLLSAVRGDNLAVASLAPETKVTIVADALRALHRLDPATC | 121 |
| Query 132 | PFDHQAKHRIERARTRMEAGLVDQDDLDEEHQGLAPAELFARLKARMPDGEDLVVTHGDA | 191 |
| Sbjct 122 | PFDH A RI RAR R++AGLVD+D+LD+ + G+ +FARL A+ P ED VVTHGDA PFDHGAARRIARARARIDAGLVDEDNLDDANTGVPLDTMFARLLAQRPAIEDGVVTHGDA | 181 |
| Query 192 | CLPNIMVENGRFSGFIDCGRLGVADRYQDIALATRDIAEELGGEWADRFLVLYGIAAPDS | 251 |
| Sbjct 182 | CLPN MV++GRFSGFIDCGRLGVADRYQD+ALA+RDI +LG EW F V YGI PD CLPNFMVDDGRFSGFIDCGRLGVADRYQDLALASRDIEADLGSEWVAPFFVRYGIERPDP | 241 |
| Query 252  Sbjct 242 | QRIAFYRLLDEFF 264  +RIAFYRLLDEFF  ERIAFYRLLDEFF 254 |  |

>WP_167665805.1 phosphotransferase, partial [Salmonella enterica] NJG73832.1 phosphotransferase [Salmonella enterica subsp. enterica serovar

Anatum] Length=146

Score = 293 bits (750), Expect = 4e-98, Method: Compositional matrix adjust.

| Identities | = 145/146 (99%), Positives = 145/146 (99%), Gaps = 0/146 (0%) |  |
| --- | --- | --- |
| Query 22 | YDWAQQTIGCSDAAVFRLSAQGRPVLFVKTDLSGALNELQDEAARLSWLATTGVPCAAVL | 81 |
| Sbjct 1 | YDWAQQTIGCSDAAVFRLSAQGRPVLFVKTDLSGALNELQDEAARLSWLATTGVPCAAVL YDWAQQTIGCSDAAVFRLSAQGRPVLFVKTDLSGALNELQDEAARLSWLATTGVPCAAVL | 60 |
| Query 82 | DVVTEAGRDWLLLGEVPGQDLLSSHLAPAEKVSIMADAMRRLHTLDPATCPFDHQAKHRI | 141 |
| Sbjct 61 | DVVTEAGRDWLLLG VPGQDLLSSHLAPAEKVSIMADAMRRLHTLDPATCPFDHQAKHRI DVVTEAGRDWLLLGGVPGQDLLSSHLAPAEKVSIMADAMRRLHTLDPATCPFDHQAKHRI | 120 |
| Query 142  Sbjct 121 | ERARTRMEAGLVDQDDLDEEHQGLAP 167 ERARTRMEAGLVDQDDLDEEHQGLAP ERARTRMEAGLVDQDDLDEEHQGLAP 146 |  |

>WP_111442483.1 APH(3')-II family aminoglycoside O-phosphotransferase [Burkholderia cenocepacia]

Length=284

Score = 298 bits (763), Expect = 6e-98, Method: Compositional matrix adjust.

| Identities | | = 163/264 (62%), Positives = 191/264 (72%), Gaps = 0/264 (0%) |  |
| --- | --- | --- | --- |
| Query 1  Sbjct 21 | | MIEQDGLHAGSPAAWVERLFGYDWAQQTIGCSDAAVFRLSAQGRPVLFVKTDLSGALNEL  + + D L P W L G W +QT G SDAAVFRL A+ FVKT+ +G L EL VTDNDPLINDMPTTWQATLAGCRWTRQTDGQSDAAVFRLDARDGSRRFVKTEPAGPLGEL | 60  80 |
| Query 61  Sbjct 81 | | QDEAARLSWLATTGVPCAAVLDVVTEAGRDWLLLGEVPGQDLLSSHLAPAEKVSIMADAM DEA RL WLATTGVP A VLDV + AGR+WLLL V G +L + LAP KV+I+ADA+ PDEAVRLRWLATTGVPGARVLDVASAAGREWLLLSAVRGDNLAVASLAPETKVTIVADAL | 120  140 |
| Query 121  Sbjct 141 | | RRLHTLDPATCPFDHQAKHRIERARTRMEAGLVDQDDLDEEHQGLAPAELFARLKARMPD R LH LDPATCPFDH A RI RAR R++AGLVD+D+LD+ + G+ +FARL A+ P RALHRLDPATCPFDHGAARRIARARARIDAGLVDEDNLDDANTGMPLDTMFARLLAQRPA | 180  200 |
| Query 181  Sbjct 201 | | GEDLVVTHGDACLPNIMVENGRFSGFIDCGRLGVADRYQDIALATRDIAEELGGEWADRF ED VVTHGDACLPN MV++GRFSGFIDCGRLGVADRYQD+ALA+RDI +LG EW F IEDGVVTHGDACLPNFMVDDGRFSGFIDCGRLGVADRYQDLALASRDIEADLGSEWVAPF | 240  260 |
| Query | 241 LVLYGIAAPDSQRIAFYRLLDEFF 264 | | |
|  |  | V YGI PD +RIAFYRLLDEFF |  |
| Sbjct | 261 | FVRYGIERPDPERIAFYRLLDEFF 284 | |

```
>WP_138808442.1 aminoglycoside 3'-phosphotransferase, partial [Salmonella enterica]
TMW10081.1 aminoglycoside 3'-phosphotransferase, partial [Salmonella enterica subsp. enterica serovar Kentucky]
Length=142

Score = 292 bits (748), Expect = 8e-98, Method: Compositional matrix adjust. Identities = 142/142 (100%), Positives = 142/142 (100%), Gaps = 0/142 (0%)

Query 123 LHTLDPATCPFDHQAKHRIERARTRMEAGLVDQDDLDEEHQGLAPAELFARLKARMPDGE 182 LHTLDPATCPFDHQAKHRIERARTRMEAGLVDQDDLDEEHQGLAPAELFARLKARMPDGE
Sbjct 1	LHTLDPATCPFDHQAKHRIERARTRMEAGLVDQDDLDEEHQGLAPAELFARLKARMPDGE 60

Query 183 DLVVTHGDACLPNIMVENGRFSGFIDCGRLGVADRYQDIALATRDIAEELGGEWADRFLV 242 DLVVTHGDACLPNIMVENGRFSGFIDCGRLGVADRYQDIALATRDIAEELGGEWADRFLV
Sbjct 61	DLVVTHGDACLPNIMVENGRFSGFIDCGRLGVADRYQDIALATRDIAEELGGEWADRFLV 120

Query 243 LYGIAAPDSQRIAFYRLLDEFF 264
LYGIAAPDSQRIAFYRLLDEFF Sbjct 121 LYGIAAPDSQRIAFYRLLDEFF 142

>WP_105848337.1 APH(3')-II family aminoglycoside O-phosphotransferase [Burkholderia cenocepacia]
ARF87755.1 aminoglycoside 3'-phosphotransferase [Burkholderia cenocepacia]
NDV73281.1 APH(3')-II family aminoglycoside O-phosphotransferase [Burkholderia cenocepacia]
PRF89833.1 APH(3')-II family aminoglycoside O-phosphotransferase [Burkholderia cenocepacia]
PRG74771.1 APH(3')-II family aminoglycoside O-phosphotransferase [Burkholderia cenocepacia]
Length=284

Score = 298 bits (762), Expect = 8e-98, Method: Compositional matrix adjust.
```

| Identities | = 163/264 (62%), Positives = 191/264 (72%), Gaps = 0/264 (0%) |  |
| --- | --- | --- |
| Query 1 | MIEQDGLHAGSPAAWVERLFGYDWAQQTIGCSDAAVFRLSAQGRPVLFVKTDLSGALNEL | 60 |
| Sbjct 21 | + + D L P W L G W +QT G SDAAVFRL A+ FVKT+ +G L EL VTDNDPLINDMPTTWQATLAGCRWTRQTDGQSDAAVFRLDARDGSRRFVKTEPAGPLGEL | 80 |
| Query 61 | QDEAARLSWLATTGVPCAAVLDVVTEAGRDWLLLGEVPGQDLLSSHLAPAEKVSIMADAM | 120 |
| Sbjct 81 | DEA RL WLATTGVP A VLDV + AGR+WLLL V G +L + LAP KV+I+ADA+ PDEAVRLRWLATTGVPGARVLDVASAAGREWLLLSAVRGDNLAVASLAPETKVTIVADAL | 140 |
| Query 121 | RRLHTLDPATCPFDHQAKHRIERARTRMEAGLVDQDDLDEEHQGLAPAELFARLKARMPD | 180 |
| Sbjct 141 | R LH LDPATCPFDH A RI RAR R++AGLVD+D+LD+ + G+ +FARL A+ P RALHRLDPATCPFDHGAARRIARARARIDAGLVDEDNLDDANTGMPLDTMFARLLAQRPA | 200 |
| Query 181 | GEDLVVTHGDACLPNIMVENGRFSGFIDCGRLGVADRYQDIALATRDIAEELGGEWADRF | 240 |

| Sbjct | 201 | ED VVTHGDACLPN MV++GRFSGFIDCGRLGVADRYQD+ALA+RDI +LG EW F IEDGVVTHGDACLPNFMVDDGRFSGFIDCGRLGVADRYQDLALASRDIEADLGSEWVAPF 260 |
| --- | --- | --- |
| Query | 241 | LVLYGIAAPDSQRIAFYRLLDEFF 264 |
| Sbjct | 261 | V YGI PD +RIAFYRLLDEFF  FVRYGIERPDPERIAFYRLLDEFF 284 |

>SOT42392.1 Aminoglycoside 3'-phosphotransferase [Burkholderia cenocepacia] Length=284

Score = 297 bits (761), Expect = 1e-97, Method: Compositional matrix adjust.

| Identities | | = 163/264 (62%), Positives = 191/264 (72%), Gaps = 0/264 (0%) |  |
| --- | --- | --- | --- |
| Query 1  Sbjct 21 | | MIEQDGLHAGSPAAWVERLFGYDWAQQTIGCSDAAVFRLSAQGRPVLFVKTDLSGALNEL  + + D L P W L G W +QT G SDAAVFRL A+ FVKT+ +G L EL VTDNDPLINDMPTTWQATLAGCRWTRQTDGQSDAAVFRLDARDGSRRFVKTEPAGPLGEL | 60  80 |
| Query 61  Sbjct 81 | | QDEAARLSWLATTGVPCAAVLDVVTEAGRDWLLLGEVPGQDLLSSHLAPAEKVSIMADAM DEA RL WLATTGVP A VLDV + AGR+WLLL V G +L + LAP KV+I+ADA+ PDEAVRLRWLATTGVPGARVLDVASAAGREWLLLSAVRGDNLAVASLAPETKVTIVADAL | 120  140 |
| Query 121  Sbjct 141 | | RRLHTLDPATCPFDHQAKHRIERARTRMEAGLVDQDDLDEEHQGLAPAELFARLKARMPD R LH LDPATCPFDH A RI RAR R++AGLVD+D+LD+ + G+ +FARL A+ P RALHRLDPATCPFDHGAARRIARARARIDAGLVDEDNLDDANTGVPLDTMFARLLAQRPA | 180  200 |
| Query 181  Sbjct 201 | | GEDLVVTHGDACLPNIMVENGRFSGFIDCGRLGVADRYQDIALATRDIAEELGGEWADRF ED VVTHGDACLPN MV++GRFSGFIDCGRLGVADRYQD+ALA+RDI +LG EW F IEDGVVTHGDACLPNFMVDDGRFSGFIDCGRLGVADRYQDLALASRDIEADLGSEWVAPF | 240  260 |
| Query | 241 LVLYGIAAPDSQRIAFYRLLDEFF 264 | | |
|  |  | V YGI PD +RIAFYRLLDEFF |  |
| Sbjct | 261 | FVRYGIERPDPERIAFYRLLDEFF 284 | |

>ALV60636.1 aminoglycoside phosphotransferase [Burkholderia cenocepacia] AQQ17693.1 aminoglycoside 3'-phosphotransferase [Burkholderia cenocepacia] AQQ47860.1 aminoglycoside 3'-phosphotransferase [Burkholderia cenocepacia] KWF19731.1 aminoglycoside phosphotransferase [Burkholderia cenocepacia] ONI98197.1 aminoglycoside 3'-phosphotransferase [Burkholderia cenocepacia]

Length=254

Score = 296 bits (757), Expect = 1e-97, Method: Compositional matrix adjust.

| Identities | = 161/253 (64%), Positives = 187/253 (74%), Gaps = 0/253 (0%) |  |
| --- | --- | --- |
| Query 12 | PAAWVERLFGYDWAQQTIGCSDAAVFRLSAQGRPVLFVKTDLSGALNELQDEAARLSWLA | 71 |
| Sbjct 2 | P W L G W +QT G SDAAVFRL A+ FVKT+ +G L EL DEA RL WLA PTTWQATLAGCRWTRQTDGQSDAAVFRLDARDGSRRFVKTEPAGPLGELPDEAVRLRWLA | 61 |
| Query 72 | TTGVPCAAVLDVVTEAGRDWLLLGEVPGQDLLSSHLAPAEKVSIMADAMRRLHTLDPATC | 131 |
| Sbjct 62 | TTGVP A VLDV + AGR+WLLL V G +L + LAP KV+I+ADA+R LH LDPATC TTGVPGARVLDVASAAGREWLLLSAVRGDNLAVASLAPETKVTIVADALRALHRLDPATC | 121 |
| Query 132 | PFDHQAKHRIERARTRMEAGLVDQDDLDEEHQGLAPAELFARLKARMPDGEDLVVTHGDA | 191 |
| Sbjct 122 | PFDH A RI RAR R++AGLVD+D+LD+ + G+ +FARL A+ P ED VVTHGDA PFDHGAARRIARARARIDAGLVDEDNLDDANTGMPLDTMFARLLAQRPAIEDGVVTHGDA | 181 |
| Query 192 | CLPNIMVENGRFSGFIDCGRLGVADRYQDIALATRDIAEELGGEWADRFLVLYGIAAPDS | 251 |
| Sbjct 182 | CLPN MV++GRFSGFIDCGRLGVADRYQD+ALA+RDI +LG EW F V YGI PD CLPNFMVDDGRFSGFIDCGRLGVADRYQDLALASRDIEADLGSEWVAPFFVRYGIERPDP | 241 |
| Query 252  Sbjct 242 | QRIAFYRLLDEFF 264  +RIAFYRLLDEFF  ERIAFYRLLDEFF 254 |  |

>WP_077184043.1 APH(3')-II family aminoglycoside O-phosphotransferase [Burkholderia cenocepacia]

ONZ05525.1 APH(3') family aminoglycoside O-phosphotransferase [Burkholderia cenocepacia]

ONZ17809.1 APH(3') family aminoglycoside O-phosphotransferase [Burkholderia cenocepacia]

ONZ23319.1 APH(3') family aminoglycoside O-phosphotransferase [Burkholderia cenocepacia]

ONZ23430.1 APH(3') family aminoglycoside O-phosphotransferase [Burkholderia cenocepacia]

ONZ53223.1 APH(3') family aminoglycoside O-phosphotransferase [Burkholderia cenocepacia]

Length=254

Score = 296 bits (757), Expect = 2e-97, Method: Compositional matrix adjust.

| Identities | = 161/253 (64%), Positives = 187/253 (74%), Gaps = 0/253 (0%) |  |
| --- | --- | --- |
| Query 12 | PAAWVERLFGYDWAQQTIGCSDAAVFRLSAQGRPVLFVKTDLSGALNELQDEAARLSWLA | 71 |
| Sbjct 2 | P W RL G W +QT G SDAAVFRL A+ FVKT+ +G L EL DEA RL WLA PTTWQARLAGCRWTRQTDGQSDAAVFRLDARDGSRRFVKTEPAGPLGELPDEAVRLRWLA | 61 |
| Query 72 | TTGVPCAAVLDVVTEAGRDWLLLGEVPGQDLLSSHLAPAEKVSIMADAMRRLHTLDPATC | 131 |
| Sbjct 62 | TTGVP A VLDV + AGR+WLLL V G +L + LAP KV+I+ADA+R LH LDPATC TTGVPGARVLDVASAAGREWLLLSAVRGDNLAVASLAPETKVTIVADALRALHRLDPATC | 121 |
| Query 132 | PFDHQAKHRIERARTRMEAGLVDQDDLDEEHQGLAPAELFARLKARMPDGEDLVVTHGDA | 191 |
| Sbjct 122 | PFDH A RI RAR R++AGLVD+ +LD+ + G+ +FARL A+ P ED VVTHGDA PFDHGAARRIARARARIDAGLVDEGNLDDANTGMPLDTMFARLLAQRPAIEDGVVTHGDA | 181 |
| Query 192 | CLPNIMVENGRFSGFIDCGRLGVADRYQDIALATRDIAEELGGEWADRFLVLYGIAAPDS | 251 |
| Sbjct 182 | CLPN MV++GRFSGFIDCGRLGVADRYQD+ALA+RDI +LG EW F V YGI PD CLPNFMVDDGRFSGFIDCGRLGVADRYQDLALASRDIEADLGSEWVAPFFVRYGIERPDP | 241 |
| Query 252  Sbjct 242 | QRIAFYRLLDEFF 264  +RIAFYRLLDEFF  ERIAFYRLLDEFF 254 |  |

>AOK39449.1 aminoglycoside phosphotransferase [Burkholderia cenocepacia] KWF50091.1 aminoglycoside phosphotransferase [Burkholderia cenocepacia]

Length=254

Score = 295 bits (755), Expect = 3e-97, Method: Compositional matrix adjust.

| Identities | = 161/253 (64%), Positives = 187/253 (74%), Gaps = 0/253 (0%) |  |
| --- | --- | --- |
| Query 12 | PAAWVERLFGYDWAQQTIGCSDAAVFRLSAQGRPVLFVKTDLSGALNELQDEAARLSWLA | 71 |
| Sbjct 2 | P W L G W +QT G SDAAVFRL A+ FVKT+ +G L EL DEA RL WLA PTTWQATLAGCRWTRQTDGQSDAAVFRLDARDGSRRFVKTEPAGPLGELPDEAVRLRWLA | 61 |
| Query 72 | TTGVPCAAVLDVVTEAGRDWLLLGEVPGQDLLSSHLAPAEKVSIMADAMRRLHTLDPATC | 131 |
| Sbjct 62 | TTGVP A VLDV + AGR+WLLL V G +L + LAP KV+I+ADA+R LH LDPATC TTGVPGARVLDVASAAGREWLLLSAVRGDNLAVASLAPETKVTIVADALRALHRLDPATC | 121 |
| Query 132 | PFDHQAKHRIERARTRMEAGLVDQDDLDEEHQGLAPAELFARLKARMPDGEDLVVTHGDA | 191 |
| Sbjct 122 | PFDH A RI RAR R++AGLVD+D+LD+ + G+ +FARL A+ P ED VVTHGDA PFDHGAARRIARARARIDAGLVDEDNLDDANTGVPLDTMFARLLAQRPAIEDGVVTHGDA | 181 |
| Query 192 | CLPNIMVENGRFSGFIDCGRLGVADRYQDIALATRDIAEELGGEWADRFLVLYGIAAPDS | 251 |
| Sbjct 182 | CLPN MV++GRFSGFIDCGRLGVADRYQD+ALA+RDI +LG EW F V YGI PD CLPNFMVDDGRFSGFIDCGRLGVADRYQDLALASRDIEADLGSEWVAPFFVRYGIERPDP | 241 |
| Query 252  Sbjct 242 | QRIAFYRLLDEFF 264  +RIAFYRLLDEFF  ERIAFYRLLDEFF 254 |  |

>EBV9530355.1 aminoglycoside 3'-phosphotransferase [Salmonella enterica subsp. enterica serovar Heidelberg]

Length=141

Score = 290 bits (743), Expect = 4e-97, Method: Compositional matrix adjust. Identities = 141/141 (100%), Positives = 141/141 (100%), Gaps = 0/141 (0%)

| Query | 124 | HTLDPATCPFDHQAKHRIERARTRMEAGLVDQDDLDEEHQGLAPAELFARLKARMPDGED | 183 |
| --- | --- | --- | --- |
|  |  | HTLDPATCPFDHQAKHRIERARTRMEAGLVDQDDLDEEHQGLAPAELFARLKARMPDGED |  |
| Sbjct | 1 | HTLDPATCPFDHQAKHRIERARTRMEAGLVDQDDLDEEHQGLAPAELFARLKARMPDGED | 60 |
| Query | 184 | LVVTHGDACLPNIMVENGRFSGFIDCGRLGVADRYQDIALATRDIAEELGGEWADRFLVL | 243 |

| Sbjct | 61 | LVVTHGDACLPNIMVENGRFSGFIDCGRLGVADRYQDIALATRDIAEELGGEWADRFLVL LVVTHGDACLPNIMVENGRFSGFIDCGRLGVADRYQDIALATRDIAEELGGEWADRFLVL 120 |
| --- | --- | --- |
| Query | 244 | YGIAAPDSQRIAFYRLLDEFF 264 |
| Sbjct | 121 | YGIAAPDSQRIAFYRLLDEFF  YGIAAPDSQRIAFYRLLDEFF 141 |

>WP_091664058.1 APH(3')-II family aminoglycoside O-phosphotransferase [Massilia sp. PDC64]

SDD91258.1 aminoglycoside 3'-phosphotransferase-2 [Massilia sp. PDC64] Length=255

Score = 294 bits (752), Expect = 1e-96, Method: Compositional matrix adjust.

| Identities | = 166/254 (65%), Positives = 197/254 (78%), Gaps = 1/254 (0%) |  |
| --- | --- | --- |
| Query 11 | SPAAWVERLFGYDWAQQTIGCSDAAVFRLSAQGRPVLFVKTDLSGALNELQDEAARLSWL | 70 |
| Sbjct 3 | +P+AW RL GY W +Q IGCS+A VFRL A RPVLF K + SG L EL+DEAARL WL TPSAWKTRLAGYRWTRQDIGCSEADVFRLDAPERPVLFAKFEPSGPLAELRDEAARLRWL | 62 |
| Query 71 | ATTGVPCAAVLDVVTEAGRDWLLLGEVPGQDLLSSHLAPAEKVSIMADAMRRLHTLDPAT | 130 |
| Sbjct 63 | A TG+PCA VL ++ G DWLLLG VPG+DLL++ LAPAE V +A A+R LH LDPA AGTGIPCAPVLAEASDDGHDWLLLGAVPGRDLLAAGLAPAEVVEHLAAALRALHALDPAA | 122 |
| Query 131 | CPFDHQAKHRIERARTRMEAGLVDQDDLDEEHQGLAPAELFARLKARMPDGEDLVVTHGD | 190 |
| Sbjct 123 | CPFDH+A RIERAR RMEAGLVD+DDLD+ H+G+APA LF RL A+ P EDLVVTHGD CPFDHRAARRIERARARMEAGLVDEDDLDDGHRGIAPAVLFDRLSAQAPQREDLVVTHGD | 182 |
| Query 191 | ACLPNIMVENGRFSGFIDCGRLGVADRYQDIALATRDIAEELGGEWADRFLVLYGIAAPD | 250 |
| Sbjct 183 | ACLPN++V++ +G+IDCGRLGVADR+QD+ALATRDIAEELG EW FL YG D ACLPNLIVDDSGGTGWIDCGRLGVADRWQDLALATRDIAEELGEEWVAPFLAAYGTTF-D | 241 |
| Query 251  Sbjct 242 | SQRIAFYRLLDEFF 264  + + +FYRLLDEFF  AAKSSFYRLLDEFF 255 |  |

>WP_070638544.1 MULTISPECIES: aminoglycoside 3'-phosphotransferase [Kocuria] OFK07658.1 APH(3') family aminoglycoside O-phosphotransferase [Kocuria sp.

HMSC066H03]

PKZ39392.1 aminoglycoside 3'-phosphotransferase [Kocuria rhizophila] Length=261

Score = 293 bits (749), Expect = 3e-96, Method: Compositional matrix adjust.

| Identities | | = 154/253 (61%), Positives = 179/253 (71%), Gaps = 0/253 (0%) |  |
| --- | --- | --- | --- |
| Query 12  Sbjct 9 | | PAAWVERLFGYDWAQQTIGCSDAAVFRLSAQGRPVLFVKTDLSGALNELQDEAARLSWLA P W + L GY W QT G SDAAVFRL++ VLFVKT+ L EL EAARL WL PITWKQELTGYSWELQTTGGSDAAVFRLTSPDGDVLFVKTEPVRCLGELPGEAARLRWLT | 71  68 |
| Query 72  Sbjct 69 | | TTGVPCAAVLDVVTEAGRDWLLLGEVPGQDLLSSHLAPAEKVSIMADAMRRLHTLDPATC TTGVPCA VLD +T GR WLLL +VPG DL S+ + PA K I A A+RRLHTL +C TTGVPCARVLDEITSTGRHWLLLDDVPGNDLSSAPIEPAAKARIAAKALRRLHTLMTDSC | 131  128 |
| Query 132  Sbjct 129 | | PFDHQAKHRIERARTRMEAGLVDQDDLDEEHQGLAPAELFARLKARMPDGEDLVVTHGDA PFDH+ HRIE+AR R E+GLVD +D D+EH+ P+EL A L+ P EDLVVTHGDA PFDHRVSHRIEQARARWESGLVDTNDFDDEHRDRDPSELLAMLQGLRPVHEDLVVTHGDA | 191  188 |
| Query 192  Sbjct 189 | | CLPNIMVENGRFSGFIDCGRLGVADRYQDIALATRDIAEELGGEWADRFLVLYGIAAPDS CL N++V+ RFSGFIDCGRLGVADR+QD+ALATRDIAE+LG W FL YGI D CLSNLIVDEDRFSGFIDCGRLGVADRHQDLALATRDIAEQLGQSWVPLFLDQYGIEDLDP | 251  248 |
| Query 252 | | QRIAFYRLLDEFF 264 R FYRL+DEFF |  |
| Sbjct | 249 | ARTRFYRLMDEFF 261 | |

>WP_141835329.1 aminoglycoside 3'-phosphotransferase [Herbaspirillum sp. SJZ107] TQK04911.1 aminoglycoside 3'-phosphotransferase-2 [Herbaspirillum sp. SJZ107]

Length=274

Score = 293 bits (750), Expect = 3e-96, Method: Compositional matrix adjust.

| Identities | = 154/256 (60%), Positives = 185/256 (72%), Gaps = 4/256 (2%) |  |
| --- | --- | --- |
| Query 12 | PAAWVERLFGYDWAQQTIGCSDAAVFRLSAQGRPVLFVKTDLSGALNELQDEAARLSWLA | 71 |
| Sbjct 20 | PA W RL GY W + GCS A+VFRL G+P L++KT+ +G L EL EA RL WLA PAGWRARLQGYRWTEALEGCSAASVFRLDTPGQPSLYLKTEPAGLLAELPGEAERLRWLA | 79 |
| Query 72 | TTGVPCAAVLDVVTEAGRDWLLLGEVPGQDLLSSHLAPAEKVSIMADAMRRLHTLDPATC | 131 |
| Sbjct 80 | G+PCA VL EAGR W+LLGE+ G++L + PA KV +MA A+R LH LD A C AAGIPCAPVLGWEQEAGRYWMLLGELRGRNLEDAAFDPACKVRLMAAALRSLHALDVAGC | 139 |
| Query 132 | PFDHQAKHRIERARTRMEAGLVDQDDLDEEHQGLAPAELFARLKARMPDGEDLVVTHGDA | 191 |
| Sbjct 140 | PFDH+A RI A R+ AGLVD+DDLD+ H+GLAPA+LFARL+A+ P EDLVVTHGDA PFDHRAALRIGHAEARLRAGLVDEDDLDDAHRGLAPADLFARLQAQAPAREDLVVTHGDA | 199 |
| Query 192 | CLPNIMV---ENGRFSGFIDCGRLGVADRYQDIALATRDIAEELGGEWADRFLVLYGIAA | 248 |
| Sbjct 200 | CLPN++V ++ R SG+IDCGRLGVADR+QD+ALATRDI E LG EW FL YG A CLPNLIVQHEQHERISGWIDCGRLGVADRWQDLALATRDIGEVLGPEWVAPFLREYG-AQ | 258 |
| Query 249  Sbjct 259 | PDSQRIAFYRLLDEFF 264 PD R +FYRLLDEFF PDPARTSFYRLLDEFF 274 |  |

```
>EBK2638683.1 aminoglycoside 3'-phosphotransferase [Salmonella enterica subsp. enterica serovar Heidelberg]
Length=140

Score = 288 bits (736), Expect = 5e-96, Method: Compositional matrix adjust. Identities = 140/140 (100%), Positives = 140/140 (100%), Gaps = 0/140 (0%)

Query 125 TLDPATCPFDHQAKHRIERARTRMEAGLVDQDDLDEEHQGLAPAELFARLKARMPDGEDL 184 TLDPATCPFDHQAKHRIERARTRMEAGLVDQDDLDEEHQGLAPAELFARLKARMPDGEDL
Sbjct 1	TLDPATCPFDHQAKHRIERARTRMEAGLVDQDDLDEEHQGLAPAELFARLKARMPDGEDL 60

Query 185 VVTHGDACLPNIMVENGRFSGFIDCGRLGVADRYQDIALATRDIAEELGGEWADRFLVLY 244 VVTHGDACLPNIMVENGRFSGFIDCGRLGVADRYQDIALATRDIAEELGGEWADRFLVLY
Sbjct 61	VVTHGDACLPNIMVENGRFSGFIDCGRLGVADRYQDIALATRDIAEELGGEWADRFLVLY 120

Query 245 GIAAPDSQRIAFYRLLDEFF 264
GIAAPDSQRIAFYRLLDEFF Sbjct 121 GIAAPDSQRIAFYRLLDEFF 140

>WP_175842659.1 APH(3') family aminoglycoside O-phosphotransferase [Burkholderia arboris]
Length=284

Score = 293 bits (749), Expect = 7e-96, Method: Compositional matrix adjust.
```

| Identities | | = 162/264 (61%), Positives = 190/264 (72%), Gaps = 0/264 (0%) |  |
| --- | --- | --- | --- |
| Query 1  Sbjct 21 | | MIEQDGLHAGSPAAWVERLFGYDWAQQTIGCSDAAVFRLSAQGRPVLFVKTDLSGALNEL  +I+ D L P AW L G W +QT G SDAAVFRL A+ FVKT+ +G L EL VIDNDPLCKEVPVAWRATLAGCRWTRQTDGQSDAAVFRLDARDGSSRFVKTEPAGPLGEL | 60  80 |
| Query 61  Sbjct 81 | | QDEAARLSWLATTGVPCAAVLDVVTEAGRDWLLLGEVPGQDLLSSHLAPAEKVSIMADAM  +DEAARL WLA TG+P A VLDV + AGR+W+LL V G +L + LAPA KV+I+ADA+ RDEAARLRWLAATGLPGAQVLDVASAAGREWMLLSAVRGDNLEMAPLAPAVKVTILADAL | 120  140 |
| Query 121  Sbjct 141 | | RRLHTLDPATCPFDHQAKHRIERARTRMEAGLVDQDDLDEEHQGLAPAELFARLKARMPD R LH LDPA CPFDH+A RI RAR R++AGLVD+D+LD+ + G+ LFA L A P RALHRLDPAACPFDHRAALRIARARARIDAGLVDEDNLDDANTGVPLDVLFATLLAHRPA | 180  200 |
| Query 181  Sbjct 201 | | GEDLVVTHGDACLPNIMVENGRFSGFIDCGRLGVADRYQDIALATRDIAEELGGEWADRF ED VVTHGDACLPNIMV+ G FSGFIDCGRLGVADRYQD+ALA RDI +LG EW F TEDRVVTHGDACLPNIMVDEGGFSGFIDCGRLGVADRYQDLALAARDIEADLGSEWVAPF | 240  260 |
| Query 241 | | LVLYGIAAPDSQRIAFYRLLDEFF 264 YGI PD +RIAFYRLLDEFF |  |
| Sbjct | 261 | FAQYGIEHPDPERIAFYRLLDEFF 284 | |

>WP_048411529.1 APH(3')-II family aminoglycoside O-phosphotransferase [Chromobacterium

sp. LK1]

KMN30805.1 aminoglycoside phosphotransferase [Chromobacterium sp. LK1] Length=262

Score = 292 bits (747), Expect = 7e-96, Method: Compositional matrix adjust.

| Identities | = 161/264 (61%), Positives = 185/264 (70%), Gaps = 2/264 (1%) |  |
| --- | --- | --- |
| Query 1 | MIEQDGLHAGSPAAWVERLFGYDWAQQTIGCSDAAVFRLSAQGRPVLFVKTDLSGALNEL | 60 |
| Sbjct 1 | M +QD + PAAW ERL GY W +Q G S+AAVFRL A G+P LF KT+ +G L EL MTQQDNTLS-PPAAWRERLDGYAWTRQNDGDSNAAVFRLQAPGQPALFAKTEAAGPLAEL | 59 |
| Query 61 | QDEAARLSWLATTGVPCAAVLDVVTEAGRDWLLLGEVPGQDLLSSHLAPAEKVSIMADAM | 120 |
| Sbjct 60 | E ARL WLA G+PCA VLD AGRDW LL VPG++LL L P V IMADA+ DGEIARLRWLADNGLPCAQVLDATRCAGRDWALLSAVPGKNLLDCGLEPEATVRIMADAL | 119 |
| Query 121 | RRLHTLDPATCPFDHQAKHRIERARTRMEAGLVDQDDLDEEHQGLAPAELFARLKARMPD | 180 |
| Sbjct 120 | LH L A CPFDH+A RI AR R+EAG VD +D DEE G PA +FA+L A P LCLHRLPVAGCPFDHRADARIAAARQRLEAGCVDAEDFDEERLGQTPAAVFAQLLASRPQ | 179 |
| Query 181 | GEDLVVTHGDACLPNIMVENGRFSGFIDCGRLGVADRYQDIALATRDIAEELGGEWADRF | 240 |
| Sbjct 180 | EDLV+THGDACLPN++ E+GRFSGFIDCGRLGVADR+QD+ALA RDIA ELG W F HEDLVLTHGDACLPNLLAEDGRFSGFIDCGRLGVADRHQDLALAARDIATELGPRWVSPF | 239 |
| Query 241  Sbjct 240 | LVLYGIAAPDSQRIAFYRLLDEFF 264 L+ YGI PD RIAFY+LLDEFF LLRYGI-QPDPSRIAFYQLLDEFF 262 |  |

```
>WP_135471052.1 aminoglycoside 3'-phosphotransferase, partial [Crenalkalicoccus roseus]
Length=139

Score = 286 bits (733), Expect = 1e-95, Method: Compositional matrix adjust. Identities = 139/139 (100%), Positives = 139/139 (100%), Gaps = 0/139 (0%)

Query 119 AMRRLHTLDPATCPFDHQAKHRIERARTRMEAGLVDQDDLDEEHQGLAPAELFARLKARM 178 AMRRLHTLDPATCPFDHQAKHRIERARTRMEAGLVDQDDLDEEHQGLAPAELFARLKARM
Sbjct 1	AMRRLHTLDPATCPFDHQAKHRIERARTRMEAGLVDQDDLDEEHQGLAPAELFARLKARM 60

Query 179 PDGEDLVVTHGDACLPNIMVENGRFSGFIDCGRLGVADRYQDIALATRDIAEELGGEWAD 238 PDGEDLVVTHGDACLPNIMVENGRFSGFIDCGRLGVADRYQDIALATRDIAEELGGEWAD
Sbjct 61	PDGEDLVVTHGDACLPNIMVENGRFSGFIDCGRLGVADRYQDIALATRDIAEELGGEWAD 120

Query 239 RFLVLYGIAAPDSQRIAFY 257
RFLVLYGIAAPDSQRIAFY Sbjct 121 RFLVLYGIAAPDSQRIAFY 139

>WP_153069972.1 aminoglycoside 3'-phosphotransferase, partial [Escherichia coli] Length=139

Score = 285 bits (730), Expect = 3e-95, Method: Compositional matrix adjust. Identities = 139/139 (100%), Positives = 139/139 (100%), Gaps = 0/139 (0%)

Query 126 LDPATCPFDHQAKHRIERARTRMEAGLVDQDDLDEEHQGLAPAELFARLKARMPDGEDLV 185 LDPATCPFDHQAKHRIERARTRMEAGLVDQDDLDEEHQGLAPAELFARLKARMPDGEDLV
Sbjct 1	LDPATCPFDHQAKHRIERARTRMEAGLVDQDDLDEEHQGLAPAELFARLKARMPDGEDLV 60

Query 186 VTHGDACLPNIMVENGRFSGFIDCGRLGVADRYQDIALATRDIAEELGGEWADRFLVLYG 245 VTHGDACLPNIMVENGRFSGFIDCGRLGVADRYQDIALATRDIAEELGGEWADRFLVLYG
Sbjct 61	VTHGDACLPNIMVENGRFSGFIDCGRLGVADRYQDIALATRDIAEELGGEWADRFLVLYG 120

Query 246 IAAPDSQRIAFYRLLDEFF 264
IAAPDSQRIAFYRLLDEFF Sbjct 121 IAAPDSQRIAFYRLLDEFF 139

>WP_109329702.1 aminoglycoside 3'-phosphotransferase [Azospirillum thermophilum]
AWK87946.1 APH(3') family aminoglycoside O-phosphotransferase [Azospirillum thermophilum]
Length=268

Score = 290 bits (741), Expect = 5e-95, Method: Compositional matrix adjust.
```

| Identities | | = 152/254 (60%), Positives = 176/254 (69%), Gaps = 2/254 (1%) |  |
| --- | --- | --- | --- |
| Query 12  Sbjct 16 | | PAAWVERLFGYDWAQQTIGCSDAAVFRLSAQGRPVLFVKTDLSGALNELQDEAARLSWLA P W ERL GY W +QTIGCS AAVFRL A RP LFVKT+ +G L EL+DE AR+ WL PVGWAERLAGYRWTRQTIGCSGAAVFRLEATDRPPLFVKTEPAGPLAELRDETARMRWLE | 71  75 |
| Query 72  Sbjct 76 | | TTGVPCAAVLDVVTEAGRDWLLLGEVPGQDLLSSHLA-PAEKVSIMADAMRRLHTLDPAT T GVP V+D A RDWLL+ V G DL S PA V + A+A+R LH +D A TVGVPAPRVIDAAATADRDWLLMTAVAGHDLASEQRPEPARIVRLAAEALRSLHRIDRAA | 130  135 |
| Query 131  Sbjct 136 | | CPFDHQAKHRIERARTRMEAGLVDQDDLDEEHQGLAPAELFARLKARMPDGEDLVVTHGD CPFDH A RI AR RMEAGLV+++D + + G ELFA L A P EDLVVTHGD CPFDHSAGVRIAHARARMEAGLVEEEDYEGKEDGRTVGELFALLVAGRPRTEDLVVTHGD | 190  195 |
| Query 191  Sbjct 196 | | ACLPNIMVENGRFSGFIDCGRLGVADRYQDIALATRDIAEELGGEWADRFLVLYGIAAPD ACLPN+M E+G F+GFID GRLG+ADR+QD+ALATRDIAEELG W FL YGIAA D ACLPNLMAEDGAFTGFIDVGRLGIADRHQDLALATRDIAEELGEAWIAPFLDRYGIAA-D | 250  254 |
| Query 251 | | SQRIAFYRLLDEFF 264  ++ FYRLLDEFF |  |
| Sbjct | 255 | PAKMRFYRLLDEFF 268 | |

>WP_110782320.1 aminoglycoside 3'-phosphotransferase [Rhodopseudomonas faecalis] PYF01165.1 aminoglycoside 3'-phosphotransferase-2 [Rhodopseudomonas faecalis]

Length=281

Score = 290 bits (742), Expect = 7e-95, Method: Compositional matrix adjust.

| Identities | | = 149/253 (59%), Positives = 178/253 (70%), Gaps = 1/253 (0%) |  |
| --- | --- | --- | --- |
| Query 12  Sbjct 30 | | PAAWVERLFGYDWAQQTIGCSDAAVFRLSAQGRPVLFVKTDLSGALNELQDEAARLSWLA PAAW R+ G DW + T GCS+AAVFRL+A RP L+VKT S EL EAARL WL+ PAAWHCRIDGLDWTRATTGCSEAAVFRLAAPDRPTLYVKTAPSQPCGELAGEAARLRWLS | 71  89 |
| Query 72  Sbjct 90 | | TTGVPCAAVLDVVTEAGRDWLLLGEVPGQDLLSSHLAPAEKVSIMADAMRRLHTLDPATC  +TG+PCA VL RDWLLL VPG+DL ++ L+P E V++ A A+R+LH LD +C STGLPCAQVLASTRTNDRDWLLLSAVPGRDLSAAELSPRELVTLFAGALRQLHALDAGSC | 131  149 |
| Query 132  Sbjct 150 | | PFDHQAKHRIERARTRMEAGLVDQDDLDEEHQGLAPAELFARLKARMPDGEDLVVTHGDA PFDH A RI AR R+ AGLVD +D D+E+ G+ PAEL +L A+ P VVTHGDA PFDHSADQRIALARERLAAGLVDPEDFDQENVGVPPAELLQQLVAQQPAVTAPVVTHGDA | 191  209 |
| Query 192  Sbjct 210 | | CLPNIMVENGRFSGFIDCGRLGVADRYQDIALATRDIAEELGGEWADRFLVLYGIAAPDS CLPN+M E GRF+GFIDCGRLGVADR+QD+ALATRDIA ELG W FL YG D CLPNLMAEAGRFTGFIDCGRLGVADRHQDLALATRDIAAELGEGWVGPFLNSYGQPL-DW | 251  268 |
| Query 252 | | QRIAFYRLLDEFF 264  + AFYRLLDEFF |  |
| Sbjct | 269 | NKTAFYRLLDEFF 281 | |

>WP_107143287.1 aminoglycoside 3'-phosphotransferase [Massilia armeniaca]

AVR97948.1 APH(3') family aminoglycoside O-phosphotransferase [Massilia armeniaca]

Length=264

Score = 288 bits (737), Expect = 2e-94, Method: Compositional matrix adjust.

| Identities | = 153/265 (58%), Positives = 182/265 (69%), Gaps = 7/265 (3%) |  |
| --- | --- | --- |
| Query 5 | DGLHAGSPAAWVERLFGYDWAQQTIGCSDAAVFRL--SAQGRPVLFVKTDLSGALNELQD | 62 |
| Sbjct 2 | D L P W ERL GY Q+ GCSDAAV R +A G P F+KT +G L ELQD DELDTTLPPTWRERLQGYAIVPQSGGCSDAAVLRAIPAAGGAPTFFIKTAAAGPLAELQD | 61 |
| Query 63 | EAARLSWLATTGVPCAAVLDVVTEAGRDWLLLGEVPGQDLLSSHLAPAEKVSIMADAMRR | 122 |
| Sbjct 62 | EAARL WLA +G+ CA V+D T G+DWLLLG VPG +L +++ PA V++MA A+R EAARLRWLAQSGIACAPVIDTATTPGQDWLLLGAVPGTNL--TNVDPARAVAVMATALRT | 119 |
| Query 123 | LHTLDPATCPFDHQAKHRIERARTRMEAGLVDQDDLDEEHQGLAPAELFARLKARMPDGE | 182 |
| Sbjct 120 | LH LD CPFDH+A RI A R++AGLVD DD DE++ G AP EL A L R P E  LHALDVRECPFDHRADVRIGHALARLDAGLVDADDFDEDNAGCAPGELAALLAERKPLHE | 179 |

| Query | 183 | DLVVTHGDACLPNIMVENGRFSGFIDCGRLGVADRYQDIALATRDIAEELGGEWADRFLV 242 |
| --- | --- | --- |
| Sbjct | 180 | DLVVTHGDACLPN++ +G F+GFIDCGRLGVADR+QD+ALA RDI EELG +W + FL DLVVTHGDACLPNVLAADGTFAGFIDCGRLGVADRHQDLALAVRDIGEELGEQWVEPFLA 239 |
| Query | 243 | LYGIAAP---DSQRIAFYRLLDEFF 264 |
| Sbjct | 240 | Y AP D+ R AFYRLLDEFF  QYFAPAPIAFDAGRAAFYRLLDEFF 264 |

>WP_145875946.1 APH(3')-II family aminoglycoside O-phosphotransferase [Massilia flava]

QGZ43001.1 APH(3')-II family aminoglycoside O-phosphotransferase [Massilia flava]

Length=257

Score = 287 bits (734), Expect = 4e-94, Method: Compositional matrix adjust.

| Identities | | = 169/253 (67%), Positives = 193/253 (76%), Gaps = 1/253 (0%) |  |
| --- | --- | --- | --- |
| Query 12  Sbjct 6 | | PAAWVERLFGYDWAQQTIGCSDAAVFRLSAQGRPVLFVKTDLSGALNELQDEAARLSWLA PAAW ERL GY A GCS+A+V+RL A GRP LFVKT+ + AL+EL+DEAARL WLA PAAWRERLAGYACAAVDTGCSEASVYRLQAPGRPTLFVKTEEASALSELRDEAARLRWLA | 71  65 |
| Query 72  Sbjct 66 | | TTGVPCAAVLDVVTEAGRDWLLLGEVPGQDLLSSHLAPAEKVSIMADAMRRLHTLDPATC G+ CAAVLD T G WLLLGEVPG DLL++ L P KV ++A+A+R LH LDPA C GAGIACAAVLDEATADGHHWLLLGEVPGHDLLAADLPPRAKVRVLAEALRVLHALDPAGC | 131  125 |
| Query 132  Sbjct 126 | | PFDHQAKHRIERARTRMEAGLVDQDDLDEEHQGLAPAELFARLKARMPDGEDLVVTHGDA PFDH A RI RAR RMEAGLVD+DDLD+EH G+ PA LFA L+ R P EDLVVTHGDA PFDHGAAQRIARARARMEAGLVDEDDLDDEHAGMDPASLFALLEERRPAREDLVVTHGDA | 191  185 |
| Query 192  Sbjct 186 | | CLPNIMVENGRFSGFIDCGRLGVADRYQDIALATRDIAEELGGEWADRFLVLYGIAAPDS CLPN++V+ G +G IDCGRLGVADR+QD+ALATRDIAEELG EW D FL YGIA D CLPNVIVQGGALAGIIDCGRLGVADRWQDLALATRDIAEELGDEWVDVFLADYGIAL-DP | 251  244 |
| Query 252 | | QRIAFYRLLDEFF 264 R FYRLLDEFF |  |
| Sbjct | 245 | DRCRFYRLLDEFF 257 | |

>TWI47673.1 aminoglycoside 3'-phosphotransferase-2 [Massilia flava] Length=258

Score = 287 bits (734), Expect = 6e-94, Method: Compositional matrix adjust.

| Identities | | = 169/253 (67%), Positives = 193/253 (76%), Gaps = 1/253 (0%) |  |
| --- | --- | --- | --- |
| Query 12  Sbjct 7 | | PAAWVERLFGYDWAQQTIGCSDAAVFRLSAQGRPVLFVKTDLSGALNELQDEAARLSWLA PAAW ERL GY A GCS+A+V+RL A GRP LFVKT+ + AL+EL+DEAARL WLA PAAWRERLAGYACAAVDTGCSEASVYRLQAPGRPTLFVKTEEASALSELRDEAARLRWLA | 71  66 |
| Query 72  Sbjct 67 | | TTGVPCAAVLDVVTEAGRDWLLLGEVPGQDLLSSHLAPAEKVSIMADAMRRLHTLDPATC G+ CAAVLD T G WLLLGEVPG DLL++ L P KV ++A+A+R LH LDPA C GAGIACAAVLDEATADGHHWLLLGEVPGHDLLAADLPPRAKVRVLAEALRVLHALDPAGC | 131  126 |
| Query 132  Sbjct 127 | | PFDHQAKHRIERARTRMEAGLVDQDDLDEEHQGLAPAELFARLKARMPDGEDLVVTHGDA PFDH A RI RAR RMEAGLVD+DDLD+EH G+ PA LFA L+ R P EDLVVTHGDA PFDHGAAQRIARARARMEAGLVDEDDLDDEHAGMDPASLFALLEERRPAREDLVVTHGDA | 191  186 |
| Query 192  Sbjct 187 | | CLPNIMVENGRFSGFIDCGRLGVADRYQDIALATRDIAEELGGEWADRFLVLYGIAAPDS CLPN++V+ G +G IDCGRLGVADR+QD+ALATRDIAEELG EW D FL YGIA D CLPNVIVQGGALAGIIDCGRLGVADRWQDLALATRDIAEELGDEWVDVFLADYGIAL-DP | 251  245 |
| Query 252 | | QRIAFYRLLDEFF 264 R FYRLLDEFF |  |
| Sbjct | 246 | DRCRFYRLLDEFF 258 | |

```
>WP_153886501.1 aminoglycoside 3'-phosphotransferase, partial [Acinetobacter baumannii]
EAQ5285245.1 aminoglycoside 3'-phosphotransferase [Salmonella enterica] Length=137

Score = 282 bits (722), Expect = 6e-94, Method: Compositional matrix adjust.

Identities = 137/137 (100%), Positives = 137/137 (100%), Gaps = 0/137 (0%)

Query 128 PATCPFDHQAKHRIERARTRMEAGLVDQDDLDEEHQGLAPAELFARLKARMPDGEDLVVT 187 PATCPFDHQAKHRIERARTRMEAGLVDQDDLDEEHQGLAPAELFARLKARMPDGEDLVVT
Sbjct 1	PATCPFDHQAKHRIERARTRMEAGLVDQDDLDEEHQGLAPAELFARLKARMPDGEDLVVT 60

Query 188 HGDACLPNIMVENGRFSGFIDCGRLGVADRYQDIALATRDIAEELGGEWADRFLVLYGIA 247 HGDACLPNIMVENGRFSGFIDCGRLGVADRYQDIALATRDIAEELGGEWADRFLVLYGIA
Sbjct 61	HGDACLPNIMVENGRFSGFIDCGRLGVADRYQDIALATRDIAEELGGEWADRFLVLYGIA 120

Query 248 APDSQRIAFYRLLDEFF 264
APDSQRIAFYRLLDEFF Sbjct 121 APDSQRIAFYRLLDEFF 137

>PTF36309.1 APH(3') family aminoglycoside O-phosphotransferase, partial [Staphylococcus epidermidis]
Length=138

Score = 281 bits (720), Expect = 1e-93, Method: Compositional matrix adjust. Identities = 138/138 (100%), Positives = 138/138 (100%), Gaps = 0/138 (0%)

Query 1	MIEQDGLHAGSPAAWVERLFGYDWAQQTIGCSDAAVFRLSAQGRPVLFVKTDLSGALNEL 60 MIEQDGLHAGSPAAWVERLFGYDWAQQTIGCSDAAVFRLSAQGRPVLFVKTDLSGALNEL
Sbjct 1	MIEQDGLHAGSPAAWVERLFGYDWAQQTIGCSDAAVFRLSAQGRPVLFVKTDLSGALNEL 60

Query 61	QDEAARLSWLATTGVPCAAVLDVVTEAGRDWLLLGEVPGQDLLSSHLAPAEKVSIMADAM 120 QDEAARLSWLATTGVPCAAVLDVVTEAGRDWLLLGEVPGQDLLSSHLAPAEKVSIMADAM
Sbjct 61	QDEAARLSWLATTGVPCAAVLDVVTEAGRDWLLLGEVPGQDLLSSHLAPAEKVSIMADAM 120

Query 121 RRLHTLDPATCPFDHQAK 138
RRLHTLDPATCPFDHQAK Sbjct 121 RRLHTLDPATCPFDHQAK 138

>HAJ2624193.1 APH(3') family aminoglycoside O-phosphotransferase [Escherichia coli]
HAJ2624258.1 APH(3') family aminoglycoside O-phosphotransferase [Escherichia coli]
Length=137

Score = 279 bits (714), Expect = 1e-92, Method: Compositional matrix adjust. Identities = 137/137 (100%), Positives = 137/137 (100%), Gaps = 0/137 (0%)

Query 1	MIEQDGLHAGSPAAWVERLFGYDWAQQTIGCSDAAVFRLSAQGRPVLFVKTDLSGALNEL 60 MIEQDGLHAGSPAAWVERLFGYDWAQQTIGCSDAAVFRLSAQGRPVLFVKTDLSGALNEL
Sbjct 1	MIEQDGLHAGSPAAWVERLFGYDWAQQTIGCSDAAVFRLSAQGRPVLFVKTDLSGALNEL 60

Query 61	QDEAARLSWLATTGVPCAAVLDVVTEAGRDWLLLGEVPGQDLLSSHLAPAEKVSIMADAM 120 QDEAARLSWLATTGVPCAAVLDVVTEAGRDWLLLGEVPGQDLLSSHLAPAEKVSIMADAM
Sbjct 61	QDEAARLSWLATTGVPCAAVLDVVTEAGRDWLLLGEVPGQDLLSSHLAPAEKVSIMADAM 120

Query 121 RRLHTLDPATCPFDHQA 137
RRLHTLDPATCPFDHQA Sbjct 121 RRLHTLDPATCPFDHQA 137

>TGV08178.1 aminoglycoside 3'-phosphotransferase [Alcaligenaceae bacterium 429]
Length=267

Score = 281 bits (720), Expect = 8e-92, Method: Compositional matrix adjust.
```

| Identities | = 138/253 (55%), Positives = 174/253 (69%), Gaps = 0/253 (0%) |  |
| --- | --- | --- |
| Query 12 | PAAWVERLFGYDWAQQTIGCSDAAVFRLSAQGRPVLFVKTDLSGALNELQDEAARLSWLA | 71 |
| Sbjct 15 | PAAW + Y W QT GCSDAAV+RL A G +F+KT+ A EL DE RL WLA PAAWQSQFAAYSWTAQTEGCSDAAVYRLEAPGHDTVFIKTERMHANAELTDEEIRLRWLA | 74 |
| Query 72 | TTGVPCAAVLDVVTEAGRDWLLLGEVPGQDLLSSHLAPAEKVSIMADAMRRLHTLDPATC | 131 |
| Sbjct 75 | GV C V+D + R WLLL V G+DL S+ L EKV +MA A+R LH L A C  AQGVSCPIVIDAHYQPERGWLLLSAVAGKDLYSASLPAHEKVKMMALALRDLHQLGVAHC | 134 |

| Query | 132 | PFDHQAKHRIERARTRMEAGLVDQDDLDEEHQGLAPAELFARLKARMPDGEDLVVTHGDA | 191 |
| --- | --- | --- | --- |
| Sbjct | 135 | PFDH A R+ A RM+AGL++ DD+D+E+QGL+ +LF +L A P ED V+THGDA PFDHNASQRVAHANARMQAGLIELDDVDDENQGLSVEQLFQKLLATQPLQEDKVLTHGDA | 194 |
| Query | 192 | CLPNIMVENGRFSGFIDCGRLGVADRYQDIALATRDIAEELGGEWADRFLVLYGIAAPDS | 251 |
| Sbjct | 195 | CLPN M E+G +G+IDCGRLGVADRYQD+ALA RDI+++LG E+ FL YG+A D+ CLPNFMAEDGVLTGYIDCGRLGVADRYQDLALAIRDISDDLGAEYVPLFLQHYGLAQLDT | 254 |
| Query  Sbjct | 252  255 | QRIAFYRLLDEFF 264  +++AFY+LLDEFF  EKVAFYKLLDEFF 267 |  |

>WP_166235037.1 APH(3') family aminoglycoside O-phosphotransferase [Propioniciclava sp. HDW11]

QIK73840.1 aminoglycoside 3'-phosphotransferase [Propioniciclava sp. HDW11] Length=261

Score = 281 bits (718), Expect = 2e-91, Method: Compositional matrix adjust.

| Identities | | = 149/253 (59%), Positives = 175/253 (69%), Gaps = 0/253 (0%) |  |
| --- | --- | --- | --- |
| Query 12  Sbjct 9 | | PAAWVERLFGYDWAQQTIGCSDAAVFRLSAQGRPVLFVKTDLSGALNELQDEAARLSWLA P W + L GY W QT G SDAAVFRL++ VLFVKT+ L EL EAARL WL PITWKQELTGYSWELQTTGGSDAAVFRLTSPDGDVLFVKTEPVRCLGELPGEAARLRWLT | 71  68 |
| Query 72  Sbjct 69 | | TTGVPCAAVLDVVTEAGRDWLLLGEVPGQDLLSSHLAPAEKVSIMADAMRRLHTLDPATC TTGVPCA VLD +T GR W+LL +VPG DL S+ + PA K I A A+R LHTL +C TTGVPCARVLDEITATGRHWMLLDDVPGNDLSSAPIEPAAKARIAAKALRWLHTLMTDSC | 131  128 |
| Query 132  Sbjct 129 | | PFDHQAKHRIERARTRMEAGLVDQDDLDEEHQGLAPAELFARLKARMPDGEDLVVTHGDA PFDH+ HRIE+A R EAGLVD DD D+EH+ P+EL L+ P EDL+VTHGDA PFDHRVSHRIEQACARWEAGLVDTDDFDDEHRDRDPSELLVMLQRLRPVHEDLIVTHGDA | 191  188 |
| Query 192  Sbjct 189 | | CLPNIMVENGRFSGFIDCGRLGVADRYQDIALATRDIAEELGGEWADRFLVLYGIAAPDS CL N++V+ RFSGFIDCGRLG+ADR+QD+ALATRDIAE+LG W FL YGI D CLSNLIVDEDRFSGFIDCGRLGLADRHQDLALATRDIAEQLGQSWVPLFLDQYGIEDLDP | 251  248 |
| Query 252 | | QRIAFYRLLDEFF 264 R FYRL+DEF |  |
| Sbjct | 249 | ARTRFYRLMDEFL 261 | |

>WP_046795547.1 aminoglycoside 3'-phosphotransferase [Rhizobium sp. LC145] KKX29586.1 aminoglycoside phosphotransferase [Rhizobium sp. LC145] TKT68914.1 aminoglycoside 3'-phosphotransferase [Rhizobiaceae bacterium

LC148]

Length=264

Score = 280 bits (717), Expect = 2e-91, Method: Compositional matrix adjust.

| Identities | | = 138/254 (54%), Positives = 179/254 (70%), Gaps = 1/254 (0%) |  |
| --- | --- | --- | --- |
| Query 12  Sbjct 11 | | PAAWVERLFGYDWAQQTIGCSDAAVFRLSAQGRPVLFVKTDLSGALNELQDEAARLSWLA PA RL GYDW Q +G S A+VFRL A RP LF+K + +G EL DEA RL WLA PANLQARLAGYDWQQNMLGRSSASVFRLEAADRPTLFLKHEPAGPFTELPDEALRLRWLA | 71  70 |
| Query 72  Sbjct 71 | | TTGVPCAAVLDVVTEAGRDWLLLGEVPGQDLLSSH-LAPAEKVSIMADAMRRLHTLDPAT G+PCAAV+ +A R+WLLL V G+DL S+ ++ A++V+ +ADA+RRLH+LD AT AQGIPCAAVVAEAQDAERNWLLLTAVEGEDLASADGISAADRVTFLADALRRLHSLDIAT | 130  130 |
| Query 131  Sbjct 131 | | CPFDHQAKHRIERARTRMEAGLVDQDDLDEEHQGLAPAELFARLKARMPDGEDLVVTHGD CPFDH+ ++R+ R RM+AG+VD+ D D+E G + EL+ L+ P EDLVVTHGD CPFDHRLENRLPHVRARMQAGVVDESDFDDERIGRSSEELYRELETLKPANEDLVVTHGD | 190  190 |
| Query 191  Sbjct 191 | | ACLPNIMVENGRFSGFIDCGRLGVADRYQDIALATRDIAEELGGEWADRFLVLYGIAAPD ACLPNIM + F+GFIDC RLG+ADR+QDIAL+ R I LG EW FL LYG+ +PD ACLPNIMADPRGFTGFIDCARLGLADRHQDIALSCRSIRYNLGQEWVQPFLDLYGLPSPD | 250  250 |
| Query | 251 SQRIAFYRLLDEFF 264 | | |
|  |  | ++++YRLLDEFF |  |
| Sbjct | 251 | PAKLSYYRLLDEFF 264 | |

>WP_169434572.1 APH(3') family aminoglycoside O-phosphotransferase [Duganella sp. GN2-R2]

QJD99609.1 aminoglycoside 3'-phosphotransferase [Duganella sp. GN2-R2] Length=263

Score = 280 bits (717), Expect = 2e-91, Method: Compositional matrix adjust.

| Identities | | = 151/253 (60%), Positives = 180/253 (71%), Gaps = 1/253 (0%) |  |
| --- | --- | --- | --- |
| Query 12  Sbjct 12 | | PAAWVERLFGYDWAQQTIGCSDAAVFRLSAQGRPVLFVKTDLSGALNELQDEAARLSWLA P +W L G W +Q GCS+A V+RL A GRP LFVK + +G L+EL DEAARL WL PQSWRASLAGCRWTRQETGCSEAQVWRLDAPGRPALFVKAEPAGPLSELADEAARLRWLG | 71  71 |
| Query 72  Sbjct 72 | | TTGVPCAAVLDVVTEAGRDWLLLGEVPGQDLLSSHLAPAEKVSIMADAMRRLHTLDPATC  +G+PCA V AG DWLLLG VPG DL +S + P V+++A A+R LH LDPA C ASGIPCAPVRGEAHHAGHDWLLLGAVPGSDLEASGIDPGAGVALLAGALRALHALDPAGC | 131  131 |
| Query 132  Sbjct 132 | | PFDHQAKHRIERARTRMEAGLVDQDDLDEEHQGLAPAELFARLKARMPDGEDLVVTHGDA PFDH+A RIERA R++AGLVD DDLD+ H+GLAP E ARL+A+ P E+ VV HGDA PFDHRAALRIERADARLQAGLVDTDDLDQGHRGLAPVEPLARLRAQAPLREEPVVAHGDA | 191  191 |
| Query 192  Sbjct 192 | | CLPNIMVENGRFSGFIDCGRLGVADRYQDIALATRDIAEELGGEWADRFLVLYGIAAPDS CL N +V GRF+G+IDCGRLGVADR+QD+ALATRDI + G EW FL YG AAPD CLANAIVNEGRFNGWIDCGRLGVADRWQDLALATRDIGDTWGEEWVAPFLRAYG-AAPDP | 251  250 |
| Query 252 | | QRIAFYRLLDEFF 264 R FYRLLDEFF |  |
| Sbjct | 251 | ARNGFYRLLDEFF 263 | |

>WP_115731144.1 aminoglycoside 3'-phosphotransferase [Aminobacter aminovorans] TCS29024.1 aminoglycoside 3'-phosphotransferase-2 [Aminobacter aminovorans] SUU88877.1 Aminoglycoside 3'-phosphotransferase [Aminobacter aminovorans]

Length=264

Score = 280 bits (717), Expect = 3e-91, Method: Compositional matrix adjust.

| Identities | | = 137/253 (54%), Positives = 174/253 (69%), Gaps = 0/253 (0%) |  |
| --- | --- | --- | --- |
| Query 12  Sbjct 12 | | PAAWVERLFGYDWAQQTIGCSDAAVFRLSAQGRPVLFVKTDLSGALNELQDEAARLSWLA PA+ E L GY W +QTIG SDA VF+L+A G+PVLF+KT+LSG EL DE ARL WL PASLREALAGYSWDRQTIGHSDAGVFKLTANGKPVLFLKTELSGKFAELPDEVARLRWLG | 71  71 |
| Query 72  Sbjct 72 | | TTGVPCAAVLDVVTEAGRDWLLLGEVPGQDLLSSHLAPAEKVSIMADAMRRLHTLDPATC G+PC V T AG WLL+ V G+DL+S +AP + ++IMADA+RRLH LD ATC NQGIPCPEVFVFETHAGHHWLLMTAVAGRDLVSQEMAPDKAIAIMADALRRLHALDVATC | 131  131 |
| Query 132  Sbjct 132 | | PFDHQAKHRIERARTRMEAGLVDQDDLDEEHQGLAPAELFARLKARMPDGEDLVVTHGDA PFDH+ RI A+ R EAG VD+ D D+E G ++F L AR P E LVVTHGDA PFDHRLGQRIAVAKARAEAGEVDEYDFDDERLGQTAQQVFGELLARRPASETLVVTHGDA | 191  191 |
| Query 192  Sbjct 192 | | CLPNIMVENGRFSGFIDCGRLGVADRYQDIALATRDIAEELGGEWADRFLVLYGIAAPDS  +PN + + GRF+GF+DC RLGVAD +QD+ALA I LG EW + FL LYG+ D+  SMPNFVADGGRFTGFVDCSRLGVADLHQDLALACWSIHFNLGEEWIEPFLALYGLPEADA | 251  251 |
| Query | 252 QRIAFYRLLDEFF 264 | | |
|  |  | ++++Y+LLDEFF |  |
| Sbjct | 252 | AKLSYYQLLDEFF 264 | |

>WP_192315073.1 APH(3') family aminoglycoside O-phosphotransferase [Aminobacter sp. SR38]

QOF70550.1 aminoglycoside 3'-phosphotransferase [Aminobacter sp. SR38] Length=264

Score = 280 bits (715), Expect = 4e-91, Method: Compositional matrix adjust. Identities = 137/253 (54%), Positives = 174/253 (69%), Gaps = 0/253 (0%)

| Query | 12 | PAAWVERLFGYDWAQQTIGCSDAAVFRLSAQGRPVLFVKTDLSGALNELQDEAARLSWLA | 71 |
| --- | --- | --- | --- |
| Sbjct | 12 | PA+ E L GY W +QTIG SDA VF+L+A G+PVLF+KT+LSG EL DE ARL WL PASLREALAGYSWDRQTIGHSDAGVFKLTANGKPVLFLKTELSGKFAELPDEVARLRWLG | 71 |
| Query | 72 | TTGVPCAAVLDVVTEAGRDWLLLGEVPGQDLLSSHLAPAEKVSIMADAMRRLHTLDPATC G+PC V T AG WLL+ V G+DL+S +AP + ++IMADA+RRLH LD ATC | 131 |

| Sbjct | 72 | NHGIPCPEVFVFETHAGHHWLLMTAVAGRDLVSQEMAPDKAIAIMADALRRLHALDVATC | 131 |
| --- | --- | --- | --- |
| Query | 132 | PFDHQAKHRIERARTRMEAGLVDQDDLDEEHQGLAPAELFARLKARMPDGEDLVVTHGDA | 191 |
| Sbjct | 132 | PFDH+ RI A+ R EAG VD+ D D+E G ++F L AR P E LVVTHGDA PFDHRLGQRIAVAKARAEAGEVDEYDFDDERLGQTAQQVFGELLARRPASETLVVTHGDA | 191 |
| Query | 192 | CLPNIMVENGRFSGFIDCGRLGVADRYQDIALATRDIAEELGGEWADRFLVLYGIAAPDS | 251 |
| Sbjct | 192 | +PN + + GRF+GF+DC RLGVAD +QD+ALA I LG EW + FL LYG+ D+  SMPNFVADGGRFTGFVDCSRLGVADLHQDLALACWSIHFNLGEEWIEPFLALYGLPEADA | 251 |
| Query | 252 QRIAFYRLLDEFF 264 | | |
|  |  | ++++Y+LLDEFF |  |
| Sbjct | 252 | AKLSYYQLLDEFF 264 | |

>WP_137932453.1 aminoglycoside 3'-phosphotransferase [Mesorhizobium sp. 3P27G6] Length=262

Score = 280 bits (715), Expect = 4e-91, Method: Compositional matrix adjust.

| Identities | | = 141/253 (56%), Positives = 171/253 (68%), Gaps = 0/253 (0%) |  |
| --- | --- | --- | --- |
| Query 12  Sbjct 10 | | PAAWVERLFGYDWAQQTIGCSDAAVFRLSAQGRPVLFVKTDLSGALNELQDEAARLSWLA P + +RL Y W +QTIG S A VFRL A G+P LF+K + SG EL DEAARL WLA PRGFRDRLSAYAWHRQTIGQSQAGVFRLVADGKPTLFLKCEQSGPFAELADEAARLRWLA | 71  69 |
| Query 72  Sbjct 70 | | TTGVPCAAVLDVVTEAGRDWLLLGEVPGQDLLSSHLAPAEKVSIMADAMRRLHTLDPATC GV C V+D+ + AG DWLL+ V G+DL S+ + PA+ V IM A+R LH LD + C GQGVACPDVIDLESHAGHDWLLMSAVAGEDLASAVIDPADVVEIMVGALRELHALDTSAC | 131  129 |
| Query 132  Sbjct 130 | | PFDHQAKHRIERARTRMEAGLVDQDDLDEEHQGLAPAELFARLKARMPDGEDLVVTHGDA PFDH+ + RI AR RMEAG VD+ D D+E QG A+ FA L A P EDLVVTHGDA PFDHRIEGRIATARARMEAGEVDESDFDDERQGRTAADAFAELIALRPQAEDLVVTHGDA | 191  189 |
| Query 192  Sbjct 190 | | CLPNIMVENGRFSGFIDCGRLGVADRYQDIALATRDIAEELGGEWADRFLVLYGIAAPDS CLPN M GRFSGFIDCGRLGVADR+QD+ALA R I LG W F+ YG+ + CLPNFMAAEGRFSGFIDCGRLGVADRHQDLALACRSIRHNLGEAWVKPFMERYGLPQAEP | 251  249 |
| Query 252 | | QRIAFYRLLDEFF 264  ++++YRLLDEFF |  |
| Sbjct | 250 | AKLSWYRLLDEFF 262 | |

>PZQ17451.1 APH(3')-II family aminoglycoside O-phosphotransferase [Rhodanobacter denitrificans]

Length=256

Score = 279 bits (713), Expect = 8e-91, Method: Compositional matrix adjust.

| Identities | | = 154/253 (61%), Positives = 183/253 (72%), Gaps = 1/253 (0%) |  |
| --- | --- | --- | --- |
| Query 12  Sbjct 5 | | PAAWVERLFGYDWAQQTIGCSDAAVFRLSAQGRPVLFVKTDLSGALNELQDEAARLSWLA PA W L GY+ +QTIGCSDAA FRLSA GR FVKT+ +G + ++EAARL WL PARWRASLAGYEAERQTIGCSDAATFRLSAPGRETRFVKTERAGPFADPREEAARLPWLG | 71  64 |
| Query 72  Sbjct 65 | | TTGVPCAAVLDVVTEAGRDWLLLGEVPGQDLLSSHLAPAEKVSIMADAMRRLHTLDPATC G+PCA V+D V + RDWLLL +PG+DL S+ LAPA+ V I ADA+R LH LDPATC AAGIPCAGVIDTVFGSRRDWLLLSALPGRDLASAGLAPADVVRIAADALRALHRLDPATC | 131  124 |
| Query 132  Sbjct 125 | | PFDHQAKHRIERARTRMEAGLVDQDDLDEEHQGLAPAELFARLKARMPDGEDLVVTHGDA PFD + + R++ A R+ AG VD D D++H+ LAPA L ARL+A P EDLVVTHGDA PFDQRVERRLDAAEARLHAGKVDAADFDDDHRELAPAALLARLRAARPAVEDLVVTHGDA | 191  184 |
| Query 192  Sbjct 185 | | CLPNIMVENGRFSGFIDCGRLGVADRYQDIALATRDIAEELGGEWADRFLVLYGIAAPDS CLPN++VE GRFSGFIDC RLGVADR+QD+ALA RDIA G EW FL YGIAA D CLPNLIVEAGRFSGFIDCARLGVADRHQDLALAARDIASRFGAEWVAPFLDRYGIAA-DP | 251  243 |
| Query 252 | | QRIAFYRLLDEFF 264 R+ FYR LDEFF |  |
| Sbjct | 244 | GRLGFYRALDEFF 256 | |

>WP_112935848.1 aminoglycoside 3'-phosphotransferase [Massilia sp. YMA4] AXA89789.1 APH(3') family aminoglycoside O-phosphotransferase [Massilia

sp. YMA4]

Length=243

Score = 278 bits (710), Expect = 1e-90, Method: Compositional matrix adjust.

| Identities | = 146/242 (60%), Positives = 178/242 (74%), Gaps = 6/242 (2%) |  |
| --- | --- | --- |
| Query 27 | QTIGCSDAAVFRLSA-QGRPVLFVKTDLSGALNELQDEAARLSWLATTGVPCAAVLDVVT | 85 |
| Sbjct 4 | T GCS+A+V+R++ PV F+K++ + L EL EAARL WLA GVPCA V+D VT HTGGCSEASVWRVAQLDYPPVWFIKSEPATPLAELPGEAARLRWLAQAGVPCAPVVDTVT | 63 |
| Query 86 | EAGRDWLLLGEVPGQDLLSSHLAPAEKVSIMADAMRRLHTLDPATCPFDHQAKHRIERAR | 145 |
| Sbjct 64 | A + WLLLG VPG DL + + PA+ V++MADA+RRLH LDP CPFDH+A+ RI A TADQHWLLLGAVPGHDL--TGVEPAQAVTVMADALRRLHALDPRDCPFDHRAQVRIGHAL | 121 |
| Query 146 | TRMEAGLVDQDDLDEEHQGLAPAELFARLKARMPDGEDLVVTHGDACLPNIMVENGRFSG | 205 |
| Sbjct 122 | R++AGLVD DD DE++ G AP+EL A L R P EDLVVTHGDACLPN++ ++G FSG ARLDAGLVDADDFDEDNAGCAPSELAALLAERKPAQEDLVVTHGDACLPNVLAQDGEFSG | 181 |
| Query 206 | FIDCGRLGVADRYQDIALATRDIAEELGGEWADRFLVLY---GIAAPDSQRIAFYRLLDE | 262 |
| Sbjct 182 | FIDCGRLGVADR+QD+ALA RDI EELG EW + FLV Y G A D+ R AFYRLLDE FIDCGRLGVADRHQDLALAVRDIGEELGEEWVEPFLVRYFAPGPVAFDAGRAAFYRLLDE | 241 |
| Query 263  Sbjct 242 | FF 264 FF  FF 243 |  |

>WP_105378816.1 APH(3')-II family aminoglycoside O-phosphotransferase [Massilia phosphatilytica]

PQO95272.1 APH(3')-II family aminoglycoside O-phosphotransferase [Massilia phosphatilytica]

Length=255

Score = 278 bits (710), Expect = 2e-90, Method: Compositional matrix adjust.

| Identities | = 168/254 (66%), Positives = 198/254 (78%), Gaps = 1/254 (0%) |  |
| --- | --- | --- |
| Query 11 | SPAAWVERLFGYDWAQQTIGCSDAAVFRLSAQGRPVLFVKTDLSGALNELQDEAARLSWL | 70 |
| Sbjct 3 | +PAAW RL GY W +Q IGCS+A VFRL A G+PVLF K + +G L EL+DE RL WL TPAAWKTRLAGYRWTRQDIGCSEADVFRLDAPGQPVLFAKFEPAGPLAELRDEGERLRWL | 62 |
| Query 71 | ATTGVPCAAVLDVVTEAGRDWLLLGEVPGQDLLSSHLAPAEKVSIMADAMRRLHTLDPAT | 130 |
| Sbjct 63 | A TG+PCA VLD +E G DWLLLG VPG+DLL++ L P V +A A+R LH LDPAT AGTGIPCAPVLDAASEDGTDWLLLGAVPGRDLLAAGLEPGVAVERLAAALRALHALDPAT | 122 |
| Query 131 | CPFDHQAKHRIERARTRMEAGLVDQDDLDEEHQGLAPAELFARLKARMPDGEDLVVTHGD | 190 |
| Sbjct 123 | CPFDH+A RIERARTRM+AGLVD+DDLD+EHQG+APA LFARL A+ P EDLVVTHGD CPFDHRAARRIERARTRMDAGLVDEDDLDDEHQGMAPAALFARLAAQAPRSEDLVVTHGD | 182 |
| Query 191 | ACLPNIMVENGRFSGFIDCGRLGVADRYQDIALATRDIAEELGGEWADRFLVLYGIAAPD | 250 |
| Sbjct 183 | ACLPN++V+ +G+IDCGRLGVADR+QD+ALATRDIAEELG EW FL YG A D ACLPNLIVDAAGRTGWIDCGRLGVADRWQDLALATRDIAEELGEEWVAPFLDAYG-ATFD | 241 |
| Query 251  Sbjct 242 | SQRIAFYRLLDEFF 264  + + +FYRLLDEFF  AAKSSFYRLLDEFF 255 |  |

>TXE23233.1 aminoglycoside 3'-phosphotransferase [[Empedobacter] haloabium] Length=264

Score = 278 bits (710), Expect = 3e-90, Method: Compositional matrix adjust.

| Identities | = 147/257 (57%), Positives = 179/257 (70%), Gaps = 6/257 (2%) |  |
| --- | --- | --- |
| Query 12 | PAAWVERLFGYDWAQQTIGCSDAAVFRLS-AQGRPVLFVKTDLSGALNELQDEAARLSWL | 70 |
| Sbjct 10 | P W ERL Y T GCS+A+V+R++ PV F+K++ + L EL EA RL WL PNTWHERLERYVVQPHTGGCSEASVWRVAQPDNAPVWFIKSEPATPLAELPGEAVRLRWL | 69 |
| Query 71 | ATTGVPCAAVLDVVTEAGRDWLLLGEVPGQDLLSSHLAPAEKVSIMADAMRRLHTLDPAT | 130 |
| Sbjct 70 | A GVPCA V+D VT A + WLLLG VPG DL + + PA+ V+++ADA+RRLH LDP AQAGVPCAPVVDTVTAADQHWLLLGAVPGHDL--TGVEPAQAVAVLADALRRLHALDPRE | 127 |
| Query 131 | CPFDHQAKHRIERARTRMEAGLVDQDDLDEEHQGLAPAELFARLKARMPDGEDLVVTHGD | 190 |

| Sbjct | 128 | CPFDH+A+ RI A R++AGLVD DD DE++ G AP EL A L R P EDLVVTHGD CPFDHRAQVRIGHALARLDAGLVDADDFDEDNAGCAPGELAALLAERKPAQEDLVVTHGD | 187 |
| --- | --- | --- | --- |
| Query | 191 | ACLPNIMVENGRFSGFIDCGRLGVADRYQDIALATRDIAEELGGEWADRFLVLY---GIA | 247 |
| Sbjct | 188 | ACLPN++ ++G FSGFIDCGRLGVADR+QD+ALA RDI E LG W + FL Y G ACLPNVLAQDGEFSGFIDCGRLGVADRHQDLALAVRDIGEALGEAWVEPFLARYFAPGPV | 247 |
| Query  Sbjct | 248  248 | APDSQRIAFYRLLDEFF 264 A D+ R AFYRLLDEFF AFDAGRAAFYRLLDEFF 264 |  |

>WP_173517697.1 APH(3') family aminoglycoside O-phosphotransferase [Ensifer sesbaniae] NRQ15973.1 Aminoglycoside 3'-phosphotransferase [Ensifer sesbaniae]

Length=263

Score = 275 bits (704), Expect = 2e-89, Method: Compositional matrix adjust.

| Identities | | = 137/257 (53%), Positives = 172/257 (67%), Gaps = 0/257 (0%) |  |
| --- | --- | --- | --- |
| Query 8  Sbjct 7 | | HAGSPAAWVERLFGYDWAQQTIGCSDAAVFRLSAQGRPVLFVKTDLSGALNELQDEAARL H P ++ + GYDW + T+G SD+++F L A RP+L +K +++G E DEAARL HLELPDNFLPLVAGYDWNRDTLGQSDSSIFLLQAPDRPMLVLKIEVAGPFGEFVDEAARL | 67  66 |
| Query 68  Sbjct 67 | | SWLATTGVPCAAVLDVVTEAGRDWLLLGEVPGQDLLSSHLAPAEKVSIMADAMRRLHTLD WLA+ G+PC V+ E +WLLL V G DL S+ +P ++++I+ADA++RLH LD DWLASQGMPCPRVVARAFETETNWLLLQAVEGTDLASTTRSPRDRITILADALKRLHALD | 127  126 |
| Query 128  Sbjct 127 | | PATCPFDHQAKHRIERARTRMEAGLVDQDDLDEEHQGLAPAELFARLKARMPDGEDLVVT  + CPFDH+ RI ARTR EAGLVD+DD DE G +LFA L+AR+P VVT VSGCPFDHRIDKRIALARTRTEAGLVDEDDFDETRLGRTAVDLFAELQARVPSTGTRVVT | 187  186 |
| Query 188  Sbjct 187 | | HGDACLPNIMVENGRFSGFIDCGRLGVADRYQDIALATRDIAEELGGEWADRFLVLYGIA HGDACLPNI+ NGR SGFIDCGRLGVADRYQDIALA R I LG EW + FL YGI HGDACLPNIIENNGRLSGFIDCGRLGVADRYQDIALACRSITNNLGEEWVEPFLTAYGIN | 247  246 |
| Query 248 | | APDSQRIAFYRLLDEFF 264 D + FY LLDEFF |  |
| Sbjct | 247 | EDDPDKRDFYCLLDEFF 263 | |

>APF39609.1 APH(3') family aminoglycoside O-phosphotransferase [Chelatococcus daeguensis]

KZE29369.1 aminoglycoside phosphotransferase [Chelatococcus daeguensis] CUA87612.1 Aminoglycoside phosphotransferase [Chelatococcus sambhunathii]

Length=265

Score = 275 bits (703), Expect = 3e-89, Method: Compositional matrix adjust.

| Identities | | = 146/254 (57%), Positives = 178/254 (70%), Gaps = 2/254 (1%) |  |
| --- | --- | --- | --- |
| Query 12  Sbjct 13 | | PAAWVERLFGYDWAQQTIGCSDAAVFRLSAQGRPVLFVKTDLSGALNELQDEAARLSWLA P AWV L GY QTIGCS+AAV+RL A GRP LFVK + G L EL EAARL WLA PRAWVGDLAGYACRPQTIGCSEAAVYRLEAHGRPTLFVKAEPVGPLCELPGEAARLRWLA | 71  72 |
| Query 72  Sbjct 73 | | TTGVPCAAVLDVVTEAGRDWLLLGEVPGQDLLSSH-LAPAEKVSIMADAMRRLHTLDPAT G+ C VL EA WLL+ V G+DL SS LAP + V+I A+A+R LH L T GQGIACPRVLAEAEEAQCCWLLMSAVAGRDLASSTDLAPGQIVTIAAEALRGLHALAART | 130  132 |
| Query 131  Sbjct 133 | | CPFDHQAKHRIERARTRMEAGLVDQDDLDEEHQGLAPAELFARLKARMPDGEDLVVTHGD CPFDH + RI ARTRM+AGLVD++D D+E +G + A+LF L + P+GEDLVVTHGD CPFDHGLRPRIALARTRMDAGLVDEEDFDDERRGRSAADLFNDLLSSCPEGEDLVVTHGD | 190  192 |
| Query 191  Sbjct 193 | | ACLPNIMVENGRFSGFIDCGRLGVADRYQDIALATRDIAEELGGEWADRFLVLYGIAAPD ACLPN++ ++GRF+GF+DCGRLGVADR+QD+ALA+ I LG +W + FL YG D ACLPNLLAQDGRFTGFVDCGRLGVADRHQDLALASWSIRHNLGEDWVEPFLRHYG-GTVD | 250  251 |
| Query | 251 SQRIAFYRLLDEFF 264 | | |
|  |  | R+AFYRLLDEFF |  |
| Sbjct | 252 | PDRLAFYRLLDEFF 265 | |

>WP_082464773.1 MULTISPECIES: APH(3')-II family aminoglycoside O-phosphotransferase [Chelatococcus]

Length=258

Score = 275 bits (702), Expect = 4e-89, Method: Compositional matrix adjust.

| Identities | | = 146/254 (57%), Positives = 178/254 (70%), Gaps = 2/254 (1%) |  |
| --- | --- | --- | --- |
| Query 12  Sbjct 6 | | PAAWVERLFGYDWAQQTIGCSDAAVFRLSAQGRPVLFVKTDLSGALNELQDEAARLSWLA P AWV L GY QTIGCS+AAV+RL A GRP LFVK + G L EL EAARL WLA PRAWVGDLAGYACRPQTIGCSEAAVYRLEAHGRPTLFVKAEPVGPLCELPGEAARLRWLA | 71  65 |
| Query 72  Sbjct 66 | | TTGVPCAAVLDVVTEAGRDWLLLGEVPGQDLLSSH-LAPAEKVSIMADAMRRLHTLDPAT G+ C VL EA WLL+ V G+DL SS LAP + V+I A+A+R LH L T GQGIACPRVLAEAEEAQCCWLLMSAVAGRDLASSTDLAPGQIVTIAAEALRGLHALAART | 130  125 |
| Query 131  Sbjct 126 | | CPFDHQAKHRIERARTRMEAGLVDQDDLDEEHQGLAPAELFARLKARMPDGEDLVVTHGD CPFDH + RI ARTRM+AGLVD++D D+E +G + A+LF L + P+GEDLVVTHGD CPFDHGLRPRIALARTRMDAGLVDEEDFDDERRGRSAADLFNDLLSSCPEGEDLVVTHGD | 190  185 |
| Query 191  Sbjct 186 | | ACLPNIMVENGRFSGFIDCGRLGVADRYQDIALATRDIAEELGGEWADRFLVLYGIAAPD ACLPN++ ++GRF+GF+DCGRLGVADR+QD+ALA+ I LG +W + FL YG D ACLPNLLAQDGRFTGFVDCGRLGVADRHQDLALASWSIRHNLGEDWVEPFLRHYG-GTVD | 250  244 |
| Query | 251 SQRIAFYRLLDEFF 264 | | |
|  |  | R+AFYRLLDEFF |  |
| Sbjct | 245 | PDRLAFYRLLDEFF 258 | |

>WP_036238904.1 APH(3')-II family aminoglycoside O-phosphotransferase [Massilia sp. JS1662]

KGF79087.1 aminoglycoside phosphotransferase [Massilia sp. JS1662] Length=255

Score = 273 bits (699), Expect = 1e-88, Method: Compositional matrix adjust.

| Identities | = 164/254 (65%), Positives = 193/254 (76%), Gaps = 1/254 (0%) |  |
| --- | --- | --- |
| Query 11 | SPAAWVERLFGYDWAQQTIGCSDAAVFRLSAQGRPVLFVKTDLSGALNELQDEAARLSWL | 70 |
| Sbjct 3 | +P AW RL GY W +Q IGCS+A VFRL A RP+LF K + +G L EL+DE RL WL TPTAWKTRLAGYRWTRQDIGCSEADVFRLDAPDRPILFAKFEPAGPLAELRDEGERLRWL | 62 |
| Query 71 | ATTGVPCAAVLDVVTEAGRDWLLLGEVPGQDLLSSHLAPAEKVSIMADAMRRLHTLDPAT | 130 |
| Sbjct 63 | A TG+PCA VLD TE G DWLLLG VPG+DLL++ L PA V +A A+R LH LDPAT AGTGIPCAPVLDTATEDGHDWLLLGAVPGRDLLAAGLEPAVAVERLAAALRALHALDPAT | 122 |
| Query 131 | CPFDHQAKHRIERARTRMEAGLVDQDDLDEEHQGLAPAELFARLKARMPDGEDLVVTHGD | 190 |
| Sbjct 123 | CPFDH+A RIERAR RMEAGLVD+DDLD+EHQG+APA LF RL A+ P EDLVVTHGD CPFDHRAARRIERARARMEAGLVDEDDLDDEHQGMAPAALFDRLAAQAPRSEDLVVTHGD | 182 |
| Query 191 | ACLPNIMVENGRFSGFIDCGRLGVADRYQDIALATRDIAEELGGEWADRFLVLYGIAAPD | 250 |
| Sbjct 183 | ACLPN++V+ +G+IDCGRLGVADR+QD+ALATRDIA+ELG W FL YG D ACLPNLIVDAAGRTGWIDCGRLGVADRWQDLALATRDIADELGEAWVVPFLAAYGTTF-D | 241 |
| Query 251  Sbjct 242 | SQRIAFYRLLDEFF 264  + + +FYRLLDEFF  AAKSSFYRLLDEFF 255 |  |

>WP_052225993.1 APH(3')-II family aminoglycoside O-phosphotransferase [Mesorhizobium sp. F7]

Length=272

Score = 274 bits (701), Expect = 1e-88, Method: Compositional matrix adjust. Identities = 137/254 (54%), Positives = 172/254 (68%), Gaps = 2/254 (1%)

| Query | 12 | PAAWVERLFGYDWAQQTIGCSDAAVFRLSAQGRPVLFVKTDLSGALNELQDEAARLSWLA | 71 |
| --- | --- | --- | --- |
| Sbjct | 20 | P W L GY +QT+GCS AAVFRL A GRP LFVKT+ +G E+ DE ARL WLA PKTWTTDLAGYACERQTVGCSGAAVFRLEASGRPTLFVKTEKAGPFGEVPDEIARLRWLA | 79 |
| Query | 72 | TTGVPCAAVLDVVTEAGRDWLLLGEVPGQDLLSS-HLAPAEKVSIMADAMRRLHTLDPAT | 130 |
| Sbjct | 80 | G+ C VL + RDWLL+ VPG+DL SS L P + V+I A A+RRLH L+ ANGISCPRVLSEEHQEQRDWLLMSAVPGRDLASSPELEPGQIVAITAGALRRLHALEAGA | 139 |
| Query | 131 | CPFDHQAKHRIERARTRMEAGLVDQDDLDEEHQGLAPAELFARLKARMPDGEDLVVTHGD CPFDH+ RI A+ R+EAG+VD+ D D+E G A++FA L R P GED+VVTHGD | 190 |

| Sbjct | 140 | CPFDHRLDIRIALAKARLEAGVVDEADFDDERTGRTAADVFAELVVRRPAGEDIVVTHGD | 199 |
| --- | --- | --- | --- |
| Query | 191 | ACLPNIMVENGRFSGFIDCGRLGVADRYQDIALATRDIAEELGGEWADRFLVLYGIAAPD | 250 |
| Sbjct | 200 | ACLPN++ +GRF+GF+DC RLG+ADRYQD+ALA+ I LG W + FL YG D ACLPNLLARHGRFTGFVDCARLGIADRYQDLALASWSIRFNLGEAWVEPFLHRYG-GEID | 258 |
| Query | 251 | SQRIAFYRLLDEFF 264  +R+A+YR+LDEFF |  |
| Sbjct | 259 | PERLAYYRMLDEFF 272 | |

>MBB4019514.1 aminoglycoside 3'-phosphotransferase-2 [Chelatococcus caeni] Length=265

Score = 273 bits (699), Expect = 1e-88, Method: Compositional matrix adjust.

| Identities | | = 149/254 (59%), Positives = 175/254 (69%), Gaps = 2/254 (1%) |  |
| --- | --- | --- | --- |
| Query 12  Sbjct 13 | | PAAWVERLFGYDWAQQTIGCSDAAVFRLSAQGRPVLFVKTDLSGALNELQDEAARLSWLA P AW L GY QTIGCS+AAVFRL A+GRP LFVK + G L EL DE ARL WLA PRAWAADLSGYACRPQTIGCSEAAVFRLEAKGRPTLFVKAEPVGPLCELPDEIARLRWLA | 71  72 |
| Query 72  Sbjct 73 | | TTGVPCAAVLDVVTEAGRDWLLLGEVPGQDLLSS-HLAPAEKVSIMADAMRRLHTLDPAT V C VL EA R W+L+ V G+DL SS LAP V+I+A+A+R LH LD EQDVACPRVLAEAEEAQRRWVLMSAVDGRDLASSPDLAPERIVAIVAEALRGLHALDAHA | 130  132 |
| Query 131  Sbjct 133 | | CPFDHQAKHRIERARTRMEAGLVDQDDLDEEHQGLAPAELFARLKARMPDGEDLVVTHGD CPFDH+ + RI AR RMEAGLVD+ D DEE G + A+LF L A DGEDLVVTHGD CPFDHRLEGRIALARARMEAGLVDEADFDEERLGRSAADLFEDLLAGRLDGEDLVVTHGD | 190  192 |
| Query 191  Sbjct 193 | | ACLPNIMVENGRFSGFIDCGRLGVADRYQDIALATRDIAEELGGEWADRFLVLYGIAAPD ACLPN++ + GRF+GF+DCGRLGVADR+QD+ALA+ I LG +W + FL YG A D ACLPNLLAQAGRFTGFVDCGRLGVADRHQDLALASWSIRHNLGDDWVEPFLRCYG-GAVD | 250  251 |
| Query | 251 SQRIAFYRLLDEFF 264 | | |
|  |  | R+AFYRLLDEFF |  |
| Sbjct | 252 | PDRLAFYRLLDEFF 265 | |

>WP_183318411.1 APH(3')-II family aminoglycoside O-phosphotransferase [Chelatococcus caeni]

Length=258

Score = 273 bits (698), Expect = 2e-88, Method: Compositional matrix adjust.

| Identities | | = 149/254 (59%), Positives = 175/254 (69%), Gaps = 2/254 (1%) |  |
| --- | --- | --- | --- |
| Query 12  Sbjct 6 | | PAAWVERLFGYDWAQQTIGCSDAAVFRLSAQGRPVLFVKTDLSGALNELQDEAARLSWLA P AW L GY QTIGCS+AAVFRL A+GRP LFVK + G L EL DE ARL WLA PRAWAADLSGYACRPQTIGCSEAAVFRLEAKGRPTLFVKAEPVGPLCELPDEIARLRWLA | 71  65 |
| Query 72  Sbjct 66 | | TTGVPCAAVLDVVTEAGRDWLLLGEVPGQDLLSS-HLAPAEKVSIMADAMRRLHTLDPAT V C VL EA R W+L+ V G+DL SS LAP V+I+A+A+R LH LD EQDVACPRVLAEAEEAQRRWVLMSAVDGRDLASSPDLAPERIVAIVAEALRGLHALDAHA | 130  125 |
| Query 131  Sbjct 126 | | CPFDHQAKHRIERARTRMEAGLVDQDDLDEEHQGLAPAELFARLKARMPDGEDLVVTHGD CPFDH+ + RI AR RMEAGLVD+ D DEE G + A+LF L A DGEDLVVTHGD CPFDHRLEGRIALARARMEAGLVDEADFDEERLGRSAADLFEDLLAGRLDGEDLVVTHGD | 190  185 |
| Query 191  Sbjct 186 | | ACLPNIMVENGRFSGFIDCGRLGVADRYQDIALATRDIAEELGGEWADRFLVLYGIAAPD ACLPN++ + GRF+GF+DCGRLGVADR+QD+ALA+ I LG +W + FL YG A D ACLPNLLAQAGRFTGFVDCGRLGVADRHQDLALASWSIRHNLGDDWVEPFLRCYG-GAVD | 250  244 |
| Query | 251 SQRIAFYRLLDEFF 264 | | |
|  |  | R+AFYRLLDEFF |  |
| Sbjct | 245 | PDRLAFYRLLDEFF 258 | |

>WP_166408899.1 APH(3') family aminoglycoside O-phosphotransferase [Paenalcaligenes suwonensis]

NHC62350.1 aminoglycoside 3'-phosphotransferase [Paenalcaligenes suwonensis] Length=267

Score = 273 bits (698), Expect = 2e-88, Method: Compositional matrix adjust.

| Identities | = 138/253 (55%), Positives = 168/253 (66%), Gaps = 0/253 (0%) |  |
| --- | --- | --- |
| Query 12 | PAAWVERLFGYDWAQQTIGCSDAAVFRLSAQGRPVLFVKTDLSGALNELQDEAARLSWLA | 71 |
| Sbjct 15 | P W L Y W QT GCSDAAVFRL A G+ LF+KT+ A EL DE RL WLA PTDWQALLAPYRWRAQTEGCSDAAVFRLEAIGQNTLFLKTERMHAHAELADEETRLRWLA | 74 |
| Query 72 | TTGVPCAAVLDVVTEAGRDWLLLGEVPGQDLLSSHLAPAEKVSIMADAMRRLHTLDPATC | 131 |
| Sbjct 75 | G+ C AV+ ++ R WLLL V G ++ SS L A+ V ++A A++ LH + A C AQGLACPAVMASHFQSERGWLLLSAVAGNNIYSSALPAAQAVPLIAHALKALHQVTAANC | 134 |
| Query 132 | PFDHQAKHRIERARTRMEAGLVDQDDLDEEHQGLAPAELFARLKARMPDGEDLVVTHGDA | 191 |
| Sbjct 135 | PFDH A RI A+ R++AGLV DD+DEE+QGL +LF +L + P ED+VVTHGDA PFDHSAAVRITHAQARLQAGLVLLDDVDEENQGLTAEQLFQKLVSLQPKSEDMVVTHGDA | 194 |
| Query 192 | CLPNIMVENGRFSGFIDCGRLGVADRYQDIALATRDIAEELGGEWADRFLVLYGIAAPDS | 251 |
| Sbjct 195 | CLPN M ENG F+G+IDCGRLGVADRYQDIALA RDI + G + F+ YG+A D CLPNFMAENGTFTGYIDCGRLGVADRYQDIALALRDIHADFGSAYVPLFMEHYGLATLDE | 254 |
| Query 252  Sbjct 255 | QRIAFYRLLDEFF 264 QRIAFY LLDEFF QRIAFYTLLDEFF 267 |  |

```
>WP_143354576.1 aminoglycoside 3'-phosphotransferase, partial [Erwinia amylovora] Length=131

Score = 267 bits (683), Expect = 3e-88, Method: Compositional matrix adjust. Identities = 131/131 (100%), Positives = 131/131 (100%), Gaps = 0/131 (0%)

Query 134 DHQAKHRIERARTRMEAGLVDQDDLDEEHQGLAPAELFARLKARMPDGEDLVVTHGDACL 193 DHQAKHRIERARTRMEAGLVDQDDLDEEHQGLAPAELFARLKARMPDGEDLVVTHGDACL
Sbjct 1	DHQAKHRIERARTRMEAGLVDQDDLDEEHQGLAPAELFARLKARMPDGEDLVVTHGDACL 60

Query 194 PNIMVENGRFSGFIDCGRLGVADRYQDIALATRDIAEELGGEWADRFLVLYGIAAPDSQR 253 PNIMVENGRFSGFIDCGRLGVADRYQDIALATRDIAEELGGEWADRFLVLYGIAAPDSQR
Sbjct 61	PNIMVENGRFSGFIDCGRLGVADRYQDIALATRDIAEELGGEWADRFLVLYGIAAPDSQR 120

Query 254 IAFYRLLDEFF 264
IAFYRLLDEFF Sbjct 121 IAFYRLLDEFF 131

>OJU33800.1 APH(3') family aminoglycoside O-phosphotransferase [Alphaproteobacteria bacterium 65-37]
Length=264

Score = 272 bits (695), Expect = 6e-88, Method: Compositional matrix adjust.
```

| Identities | = 143/265 (54%), Positives = 174/265 (66%), Gaps = 2/265 (1%) |  |
| --- | --- | --- |
| Query 1 | MIEQDGLHAGSPAAWVERLFGYDWAQQTIGCSDAAVFRLSAQGRPVLFVKTDLSGALNEL | 60 |
| Sbjct 1 | M + G+ P AW L Y W Q IGCS AAVFRL A+GRP LFVK + +GAL EL MGARSGIPFDVPRAWQADLADYAWTAQEIGCSAAAVFRLDARGRPPLFVKIEPAGALEEL | 60 |
| Query 61 | QDEAARLSWLATTGVPCAAVLDVVTEAGRDWLLLGEVPGQDLLSS-HLAPAEKVSIMADA | 119 |
| Sbjct 61 | EA RL WLA+ GV C + ++G++WLL+ + G DL SS L + V I A A PSEALRLRWLASQGVACPTIQREAIDSGQNWLLISALAGHDLASSPKLGADQIVEITARA | 120 |
| Query 120 | MRRLHTLDPATCPFDHQAKHRIERARTRMEAGLVDQDDLDEEHQGLAPAELFARLKARMP | 179 |
| Sbjct 121 | +RRLH LD +CPFDH R+E AR R+EAG VD+ D D E +G +LF L AR P LRRLHELDIVSCPFDHGIDRRLEDARARVEAGAVDESDFDAEREGQTARDLFEELLARRP | 180 |
| Query 180 | DGEDLVVTHGDACLPNIMVENGRFSGFIDCGRLGVADRYQDIALATRDIAEELGGEWADR | 239 |
| Sbjct 181 | GEDLVVTHGDACLPN++ + GRFSGFIDC RLGVADR+QD++LA R I LG W + RGEDLVVTHGDACLPNLIADQGRFSGFIDCSRLGVADRHQDLSLAARSIRSNLGDPWGET | 240 |
| Query 240  Sbjct 241 | FLVLYGIAAPDSQRIAFYRLLDEFF 264 FL LYG D +R+AFY+LLDEFF FLRLYG-GPVDPERLAFYQLLDEFF 264 |  |

>WP_065774127.1 MULTISPECIES: aminoglycoside 3'-phosphotransferase [unclassified Ensifer]

OCP11174.1 APH(3') family aminoglycoside O-phosphotransferase [Ensifer sp. LC14]

OCP12825.1 APH(3') family aminoglycoside O-phosphotransferase [Ensifer sp. LC13]

OCP13505.1 APH(3') family aminoglycoside O-phosphotransferase [Ensifer sp. LC11]

OCP34268.1 APH(3') family aminoglycoside O-phosphotransferase [Ensifer sp. LC499]

Length=261

Score = 270 bits (691), Expect = 2e-87, Method: Compositional matrix adjust.

| Identities | = 132/244 (54%), Positives = 165/244 (68%), Gaps = 0/244 (0%) |  |
| --- | --- | --- |
| Query 21 | GYDWAQQTIGCSDAAVFRLSAQGRPVLFVKTDLSGALNELQDEAARLSWLATTGVPCAAV | 80 |
| Sbjct 18 | GYDW + T+G SD++++ L A GRP+L +K + +G E DEA RL WL + GVPC + GYDWNRDTLGQSDSSIYLLKAAGRPMLVLKVEAAGPFGEFADEATRLDWLTSQGVPCPQI | 77 |
| Query 81 | LDVVTEAGRDWLLLGEVPGQDLLSSHLAPAEKVSIMADAMRRLHTLDPATCPFDHQAKHR | 140 |
| Sbjct 78 | L A +WLLL V G DL S+ L+P +++ I+A+A++RLH LD A CPFDH+ R LARAFGAETNWLLLQAVEGTDLASTTLSPKDQIVILANALKRLHALDVAACPFDHRIDKR | 137 |
| Query 141 | IERARTRMEAGLVDQDDLDEEHQGLAPAELFARLKARMPDGEDLVVTHGDACLPNIMVEN | 200 |
| Sbjct 138 | I A R +AGL+D+ D DE G A+LF L+AR+P + LVVTHGDACLPNI+ N IAIASARTQAGLIDESDFDEARLGRTAADLFTELQARVPSTKVLVVTHGDACLPNIIESN | 197 |
| Query 201 | GRFSGFIDCGRLGVADRYQDIALATRDIAEELGGEWADRFLVLYGIAAPDSQRIAFYRLL | 260 |
| Sbjct 198 | GRF+GFIDC RLGVAD YQDIALA R IA LG EW FL LYGI D Q+ FY LL GRFAGFIDCSRLGVADPYQDIALACRSIAHNLGEEWVSPFLTLYGIDKDDRQKRDFYCLL | 257 |
| Query 261  Sbjct 258 | DEFF 264 DEFF  DEFF 261 |  |

>WP_019402260.1 MULTISPECIES: APH(3')-II family aminoglycoside O-phosphotransferase [unclassified Chelatococcus]

ALA17721.1 aminoglycoside phosphotransferase [Chelatococcus sp. CO-6] Length=258

Score = 270 bits (690), Expect = 3e-87, Method: Compositional matrix adjust.

| Identities | | = 148/254 (58%), Positives = 174/254 (69%), Gaps = 2/254 (1%) |  |
| --- | --- | --- | --- |
| Query 12  Sbjct 6 | | PAAWVERLFGYDWAQQTIGCSDAAVFRLSAQGRPVLFVKTDLSGALNELQDEAARLSWLA P AW L Y QTIGCS+AAVFRL A+GRP LFVK + G L EL DE ARL WLA PRAWAADLSDYACRPQTIGCSEAAVFRLEAKGRPTLFVKAEPVGPLCELPDEIARLRWLA | 71  65 |
| Query 72  Sbjct 66 | | TTGVPCAAVLDVVTEAGRDWLLLGEVPGQDLLSS-HLAPAEKVSIMADAMRRLHTLDPAT V C VL EA R W+L+ V G+DL SS LAP V+I+A+A+R LH LD GQDVACPRVLAEAEEAQRHWVLMSAVDGRDLASSPDLAPERIVAIVAEALRGLHALDAHA | 130  125 |
| Query 131  Sbjct 126 | | CPFDHQAKHRIERARTRMEAGLVDQDDLDEEHQGLAPAELFARLKARMPDGEDLVVTHGD CPFDH+ + RI AR RMEAGLVD+ D DEE G + A+LF L A DGEDLVVTHGD CPFDHRLEGRIALARARMEAGLVDEADFDEERLGRSAADLFEDLLAGRLDGEDLVVTHGD | 190  185 |
| Query 191  Sbjct 186 | | ACLPNIMVENGRFSGFIDCGRLGVADRYQDIALATRDIAEELGGEWADRFLVLYGIAAPD ACLPN++ + GRF+GF+DCGRLGVADR+QD+ALA+ I LG +W + FL YG A D ACLPNLLAQAGRFTGFVDCGRLGVADRHQDLALASWSIRHNLGDDWVEPFLRCYG-GAVD | 250  244 |
| Query | 251 SQRIAFYRLLDEFF 264 | | |
|  |  | R+AFYRLLDEFF |  |
| Sbjct | 245 | PDRLAFYRLLDEFF 258 | |

>WP_109578104.1 aminoglycoside 3'-phosphotransferase [Aminobacter sp. AP02] PWK63887.1 aminoglycoside 3'-phosphotransferase-2 [Aminobacter sp. AP02]

Length=264

Score = 269 bits (688), Expect = 5e-87, Method: Compositional matrix adjust. Identities = 132/264 (50%), Positives = 173/264 (66%), Gaps = 0/264 (0%)

Query 1	MIEQDGLHAGSPAAWVERLFGYDWAQQTIGCSDAAVFRLSAQGRPVLFVKTDLSGALNEL 60 M +	L	PA+	L GYDW +QTIG S A VFRL A +P LF+K + SG	EL

| Sbjct | 1 | MTQHSALETDLPASLRGALAGYDWQRQTIGASGAGVFRLEAACKPALFLKCEESGPFAEL | 60 |
| --- | --- | --- | --- |
| Query | 61 | QDEAARLSWLATTGVPCAAVLDVVTEAGRDWLLLGEVPGQDLLSSHLAPAEKVSIMADAM | 120 |
| Sbjct | 61 | DE ARL WL G+PC V+ T G +WLL+ + G+DL+S AP + ++IMA+A+ PDEVARLRWLGAQGIPCPEVIVFETHGGHNWLLMSALGGRDLVSQETAPDKAIAIMAEAL | 120 |
| Query | 121 | RRLHTLDPATCPFDHQAKHRIERARTRMEAGLVDQDDLDEEHQGLAPAELFARLKARMPD | 180 |
| Sbjct | 121 | R LH LD +TCPFDH+ RI A+TRMEAG++D+DD DEE G + +F ++ R P RTLHALDISTCPFDHRLAARIALAKTRMEAGIIDEDDFDEEWLGQSAETVFEQVLLRRPT | 180 |
| Query | 181 | GEDLVVTHGDACLPNIMVENGRFSGFIDCGRLGVADRYQDIALATRDIAEELGGEWADRF | 240 |
| Sbjct | 181 | E+LVVTHGDA +PN + E RF+GFIDC RLGVADR+QD+ +A I LG EW F SEELVVTHGDASMPNFIAEGERFTGFIDCSRLGVADRHQDLGIACWSIHLNLGPEWIAPF | 240 |
| Query  Sbjct | 241  241 | LVLYGIAAPDSQRIAFYRLLDEFF 264 L LYG+ D+ R+++Y+LLDEFF LALYGLPEADAARLSYYQLLDEFF 264 |  |

>BBF98342.1 aminoglycoside 3'-phosphotransferase, partial [synthetic construct] [Mus musculus]

Length=130

Score = 264 bits (675), Expect = 6e-87, Method: Compositional matrix adjust.

| Identities | = 129/130 (99%), Positives = 130/130 (100%), Gaps = 0/130 (0%) |  |
| --- | --- | --- |
| Query 135 | HQAKHRIERARTRMEAGLVDQDDLDEEHQGLAPAELFARLKARMPDGEDLVVTHGDACLP | 194 |
| Sbjct 1 | HQAKHRIERARTRMEAGLVDQDDLDEEHQGLAPAELFARLKARMPDG+DLVVTHGDACLP HQAKHRIERARTRMEAGLVDQDDLDEEHQGLAPAELFARLKARMPDGDDLVVTHGDACLP | 60 |
| Query 195 | NIMVENGRFSGFIDCGRLGVADRYQDIALATRDIAEELGGEWADRFLVLYGIAAPDSQRI | 254 |
| Sbjct 61 | NIMVENGRFSGFIDCGRLGVADRYQDIALATRDIAEELGGEWADRFLVLYGIAAPDSQRI NIMVENGRFSGFIDCGRLGVADRYQDIALATRDIAEELGGEWADRFLVLYGIAAPDSQRI | 120 |
| Query 255  Sbjct 121 | AFYRLLDEFF 264 AFYRLLDEFF AFYRLLDEFF 130 |  |

>WP_026619993.1 aminoglycoside 3'-phosphotransferase [Ensifer sp. WSM1721] Length=261

Score = 268 bits (686), Expect = 1e-86, Method: Compositional matrix adjust.

| Identities | = 136/253 (54%), Positives = 171/253 (68%), Gaps = 0/253 (0%) |  |
| --- | --- | --- |
| Query 12 | PAAWVERLFGYDWAQQTIGCSDAAVFRLSAQGRPVLFVKTDLSGALNELQDEAARLSWLA | 71 |
| Sbjct 9 | P+ + +L GY + + T+G S + V L + RP L +K + +G +EL DEAARL WLA PSGFRLQLSGYSFERDTLGRSASTVLLLESDNRPRLILKHEKAGRFSELNDEAARLQWLA | 68 |
| Query 72 | TTGVPCAAVLDVVTEAGRDWLLLGEVPGQDLLSSHLAPAEKVSIMADAMRRLHTLDPATC | 131 |
| Sbjct 69 | G+PC VL G+ WLLL V G DL SS L PA++++I+AD +RRLH LDPA C RQGIPCPTVLCNADHQGQSWLLLQAVEGIDLGSSPLPPAKRIAILADGLRRLHQLDPAAC | 128 |
| Query 132 | PFDHQAKHRIERARTRMEAGLVDQDDLDEEHQGLAPAELFARLKARMPDGEDLVVTHGDA | 191 |
| Sbjct 129 | PFD + K I AR RMEAGLVD+ D D+E +G A +ELF L +R P E LVVTHGDA PFDRRLKTSIAAARARMEAGLVDESDFDDERKGRAASELFDELLSRRPKDERLVVTHGDA | 188 |
| Query 192 | CLPNIMVENGRFSGFIDCGRLGVADRYQDIALATRDIAEELGGEWADRFLVLYGIAAPDS | 251 |
| Sbjct 189 | CLPNI+ GRF+GFIDCGRLGVAD +QDIALA R IA LG EW FL YG+ + D CLPNIIAAGGRFAGFIDCGRLGVADLHQDIALACRSIAYNLGEEWVSPFLAQYGVHSADP | 248 |
| Query 252  Sbjct 249 | QRIAFYRLLDEFF 264  +++ +Y LLDEFF  EKLFYYCLLDEFF 261 |  |

>WP_147849977.1 APH(3')-II family aminoglycoside O-phosphotransferase [Enhydrobacter sp. CC-CFT640]

TXL72356.1 APH(3')-II family aminoglycoside O-phosphotransferase [Enhydrobacter sp. CC-CFT640]

Length=263

Score = 268 bits (685), Expect = 2e-86, Method: Compositional matrix adjust.

| Identities | = 142/255 (56%), Positives = 174/255 (68%), Gaps = 2/255 (1%) |  |
| --- | --- | --- |
| Query 11 | SPAAWVERLFGYDWAQQTIGCSDAAVFRLSAQGRPVLFVKTDLSGALNELQDEAARLSWL | 70 |
| Sbjct 10 | +P AW L GY W +QTIG SDA VFRL A+GRP LF KT+ +G +EL EAARL WL TPRAWQSDLQGYAWHRQTIGHSDALVFRLEAEGRPTLFAKTEPAGPFSELPGEAARLRWL | 69 |
| Query 71 | ATTGVPCAAVLDVVTEAGRDWLLLGEVPGQDLLSS-HLAPAEKVSIMADAMRRLHTLDPA | 129 |
| Sbjct 70 | A+ V A VL + GR WLL VPG+DL SS L P+ V++ A A+R LH +D A ASRHVAAARVLAEALDEGRSWLLTTAVPGRDLASSAQLEPSRIVALAAGALRDLHRIDIA | 129 |
| Query 130 | TCPFDHQAKHRIERARTRMEAGLVDQDDLDEEHQGLAPAELFARLKARMPDGEDLVVTHG | 189 |
| Sbjct 130 | TCPFDH+ + RI +AR R+ AG+VD+ D D+E G ++L L AR P GEDL VTHG TCPFDHRLQQRIAQARARVAAGVVDESDFDDERLGRTASDLVEELTARRPQGEDLAVTHG | 189 |
| Query 190 | DACLPNIMVENGRFSGFIDCGRLGVADRYQDIALATRDIAEELGGEWADRFLVLYGIAAP | 249 |
| Sbjct 190 | DACLPN++V+ G F+GFIDCGRLGVADR+QD+ALA I LG EW + FL YG A DACLPNLLVDAGLFTGFIDCGRLGVADRHQDLALACWSIRYNLGEEWTEPFLRHYG-GAI | 248 |
| Query 250  Sbjct 249 | DSQRIAFYRLLDEFF 264 D ++ FYRLLDEFF DRGKLDFYRLLDEFF 263 |  |

```
>EFH3592115.1 phosphotransferase [Escherichia coli] Length=129

Score = 263 bits (672), Expect = 2e-86, Method: Compositional matrix adjust. Identities = 129/129 (100%), Positives = 129/129 (100%), Gaps = 0/129 (0%)

Query 136 QAKHRIERARTRMEAGLVDQDDLDEEHQGLAPAELFARLKARMPDGEDLVVTHGDACLPN 195 QAKHRIERARTRMEAGLVDQDDLDEEHQGLAPAELFARLKARMPDGEDLVVTHGDACLPN
Sbjct 1	QAKHRIERARTRMEAGLVDQDDLDEEHQGLAPAELFARLKARMPDGEDLVVTHGDACLPN 60

Query 196 IMVENGRFSGFIDCGRLGVADRYQDIALATRDIAEELGGEWADRFLVLYGIAAPDSQRIA 255 IMVENGRFSGFIDCGRLGVADRYQDIALATRDIAEELGGEWADRFLVLYGIAAPDSQRIA
Sbjct 61	IMVENGRFSGFIDCGRLGVADRYQDIALATRDIAEELGGEWADRFLVLYGIAAPDSQRIA 120

Query 256 FYRLLDEFF 264
FYRLLDEFF Sbjct 121 FYRLLDEFF 129

>WP_196687139.1 APH(3')-II family aminoglycoside O-phosphotransferase [Pseudomonas aeruginosa]
MBG4677001.1 APH(3')-II family aminoglycoside O-phosphotransferase [Pseudomonas aeruginosa]
Length=268

Score = 268 bits (685), Expect = 2e-86, Method: Compositional matrix adjust.
```

| Identities | | = 139/258 (54%), Positives = 171/258 (66%), Gaps = 5/258 (2%) |  |
| --- | --- | --- | --- |
| Query 11  Sbjct 12 | | SPAAWVERLFGYDWAQQTIGCSDAAVFRLSAQGRPVLFVKTDLSGALNELQDEAARLSWL  +P+ W + L GY W Q GCS A V RL A RP LFVK ++ A EL E ARL WL APSTWADYLAGYRWRGQGEGCSAATVHRLEAARRPTLFVKQEVLSAHAELPAEIARLRWL | 70  71 |
| Query 71  Sbjct 72 | | ATTGVPCAAVLDVVTEAGRDWLLLGEVPGQDLLS SHLAPAEKVSIMADAMRRLHTL  G+ C VL+ GR WLL+ VPG L + L P V ++A A+RRLH L HGAGIDCPQVLNETQSDGRQWLLMSAVPGDTLSALAQRGELEPERLVRLVAAALRRLHDL | 126  131 |
| Query 127  Sbjct 132 | | DPATCPFDHQAKHRIERARTRMEAGLVDQDDLDEEHQGLAPAELFARLKARMPDGEDLVV DPA CPFDH+ + R++ R R+EAGLVD+ D D++H+G + EL+ L AR P GEDLVV DPAACPFDHRLERRLDTVRQRVEAGLVDEADFDDDHRGRSATELYRLLLARRPAGEDLVV | 186  191 |
| Query 187  Sbjct 192 | | THGDACLPNIMVENGRFSGFIDCGRLGVADRYQDIALATRDIAEELGGEWADRFLVLYGI HGDACLPN++ E RFSGFIDCGRLGVADR+QD+ALA RDI ELG WA+ FLV YG AHGDACLPNLLAEGRRFSGFIDCGRLGVADRHQDLALAARDIEAELGAAWAEAFLVEYG- | 246  250 |
| Query | 247 AAPDSQRIAFYRLLDEFF 264 | | |
|  |  | D +R+A++RLLDEFF |  |
| Sbjct | 251 | GDIDGERLAYFRLLDEFF 268 | |

>WP_195178681.1 APH(3') family aminoglycoside O-phosphotransferase [Mesorhizobium sp. INR15]

QPC91256.1 APH(3') family aminoglycoside O-phosphotransferase [Mesorhizobium sp. INR15]

Length=264

Score = 268 bits (684), Expect = 2e-86, Method: Compositional matrix adjust.

| Identities | = 135/245 (55%), Positives = 166/245 (68%), Gaps = 1/245 (0%) |  |
| --- | --- | --- |
| Query 21 | GYDWAQQTIGCSDAAVFRLSAQGRPVLFVKTDLSGALNELQDEAARLSWLATTGVPCAAV | 80 |
| Sbjct 20 | G+ W +QTIG S A VFRL A+G+P LF+K + G EL DEAARL WL + C V GHGWNRQTIGQSQAGVFRLEAKGKPTLFLKIEPGGPFAELADEAARLRWLGRQDIDCPEV | 79 |
| Query 81 | LDVVTEAGRDWLLLGEVPGQDLLSSH-LAPAEKVSIMADAMRRLHTLDPATCPFDHQAKH | 139 |
| Sbjct 80 | + T AG DWLL+ VPG+DL+S+ + A + IMADA+RRLH LD + CPFDH IAFETHAGHDWLLMSAVPGRDLVSAQPIDAATSIGIMADALRRLHALDISACPFDHSLDR | 139 |
| Query 140 | RIERARTRMEAGLVDQDDLDEEHQGLAPAELFARLKARMPDGEDLVVTHGDACLPNIMVE | 199 |
| Sbjct 140 | RI AR RMEAG VD+ D D+E G A+ FA L+A+ PD EDLVVTHGDAC+PN MV RIAIARARMEAGEVDETDFDDERLGWTAAKAFAELQAQKPDSEDLVVTHGDACMPNFMVA | 199 |
| Query 200 | NGRFSGFIDCGRLGVADRYQDIALATRDIAEELGGEWADRFLVLYGIAAPDSQRIAFYRL | 259 |
| Sbjct 200 | + RFSGFIDCGRLG+ADR+QD+ALA I LG W FL YG + D ++++YRL DNRFSGFIDCGRLGIADRHQDLALACWSIRYNLGEAWEKPFLERYGSSGVDPGKLSWYRL | 259 |
| Query 260  Sbjct 260 | LDEFF 264 LDEFF LDEFF 264 |  |

>WP_125910168.1 APH(3')-II family aminoglycoside O-phosphotransferase [Pseudomonas aeruginosa]

RRY44101.1 APH(3')-II family aminoglycoside O-phosphotransferase [Pseudomonas aeruginosa]

Length=268

Score = 267 bits (683), Expect = 3e-86, Method: Compositional matrix adjust.

| Identities | | = 138/258 (53%), Positives = 172/258 (67%), Gaps = 5/258 (2%) |  |
| --- | --- | --- | --- |
| Query 11  Sbjct 12 | | SPAAWVERLFGYDWAQQTIGCSDAAVFRLSAQGRPVLFVKTDLSGALNELQDEAARLSWL  +P+ W + L GY W Q GCS A V RL A+ RP LFVK ++ A EL E ARL WL APSTWADYLAGYRWRGQGEGCSAATVHRLEAERRPTLFVKQEVLSAHAELPAEIARLRWL | 70  71 |
| Query 71  Sbjct 72 | | ATTGVPCAAVLDVVTEAGRDWLLLGEVPGQDLLS SHLAPAEKVSIMADAMRRLHTL  G+ C VL+ GR WLL+ VPG L + L P V ++A A+RRLH L HGAGIDCPQVLNETQSDGRQWLLMSAVPGDTLSALAQRGELEPERLVRLVAAALRRLHDL | 126  131 |
| Query 127  Sbjct 132 | | DPATCPFDHQAKHRIERARTRMEAGLVDQDDLDEEHQGLAPAELFARLKARMPDGEDLVV DPA CPFDH+ + R++ R R+EAGLVD+ D D++H+G + EL+ L+AR P EDLVV DPAACPFDHRLERRLDTVRQRVEAGLVDEADFDDDHRGRSATELYRLLRARRPAVEDLVV | 186  191 |
| Query 187  Sbjct 192 | | THGDACLPNIMVENGRFSGFIDCGRLGVADRYQDIALATRDIAEELGGEWADRFLVLYGI HGDACLPN++ E RFSGFIDCGRLGVADR+QD+ALA RDI ELG WA+ FLV YG AHGDACLPNLLAEGRRFSGFIDCGRLGVADRHQDLALAARDIEAELGAAWAEAFLVEYG- | 246  250 |
| Query | 247 AAPDSQRIAFYRLLDEFF 264 | | |
|  |  | D +R+A++RLLDEFF |  |
| Sbjct | 251 | GDIDGERLAYFRLLDEFF 268 | |

```
>WP_046120501.1 MULTISPECIES: aminoglycoside 3'-phosphotransferase [Sinorhizobium/Ensifer group]
Length=261

Score = 267 bits (682), Expect = 4e-86, Method: Compositional matrix adjust. Identities = 134/253 (53%), Positives = 169/253 (67%), Gaps = 0/253 (0%)

Query 12	PAAWVERLFGYDWAQQTIGCSDAAVFRLSAQGRPVLFVKTDLSGALNELQDEAARLSWLA 71 P + +L GY + + T+G S + V L + RP L +K + +G +EL DEAARL WLA
Sbjct 9	PPGFRLQLSGYSFERDTLGRSASTVVLLESDNRPRLILKHEKAGRFSELNDEAARLRWLA 68
```

| Query | 72 | TTGVPCAAVLDVVTEAGRDWLLLGEVPGQDLLSSHLAPAEKVSIMADAMRRLHTLDPATC | 131 |
| --- | --- | --- | --- |
| Sbjct | 69 | G+PC VL G+ WLLL V G DL SS L PAE+++I+AD +RRLH LDPA C RQGIPCPTVLGSADHEGQSWLLLQAVEGIDLASSPLPPAERIAILADGLRRLHQLDPAAC | 128 |
| Query | 132 | PFDHQAKHRIERARTRMEAGLVDQDDLDEEHQGLAPAELFARLKARMPDGEDLVVTHGDA | 191 |
| Sbjct | 129 | PFD + K I AR RMEAGLVD+ D D+E +G A +ELF + + P E LVV HGDA PFDRRLKTSIAAARARMEAGLVDESDFDDEREGRAASELFGEVLSLKPKDERLVVAHGDA | 188 |
| Query | 192 | CLPNIMVENGRFSGFIDCGRLGVADRYQDIALATRDIAEELGGEWADRFLVLYGIAAPDS | 251 |
| Sbjct | 189 | CLPNI+ GRF+GFIDCGRLGVAD +QDIALA R IA +G EW FL YG+ + D CLPNIIAAGGRFAGFIDCGRLGVADLHQDIALACRSIAYNVGEEWVSPFLAQYGLQSADQ | 248 |
| Query  Sbjct | 252  249 | QRIAFYRLLDEFF 264  ++++FY LLDEFF  EKLSFYCLLDEFF 261 |  |

>WP_082005961.1 aminoglycoside 3'-phosphotransferase [Ensifer sp. ZNC0028] Length=317

Score = 269 bits (687), Expect = 5e-86, Method: Compositional matrix adjust.

| Identities | | = 134/257 (52%), Positives = 171/257 (67%), Gaps = 0/257 (0%) |  |
| --- | --- | --- | --- |
| Query 8  Sbjct 61 | | HAGSPAAWVERLFGYDWAQQTIGCSDAAVFRLSAQGRPVLFVKTDLSGALNELQDEAARL H PA + + GY W + +G SD+++F L A RP+L +K + +G NE DEAARL HPELPAQFHPFVAGYQWNRDLLGQSDSSIFLLKAPDRPMLVLKLEEAGPFNEFVDEAARL | 67  120 |
| Query 68  Sbjct 121 | | SWLATTGVPCAAVLDVVTEAGRDWLLLGEVPGQDLLSSHLAPAEKVSIMADAMRRLHTLD WLA+ VPC V+ A +WLLL V G DL S+ L+P +++ I+ADA++RLH LD DWLASKDVPCPRVIARAFGAQTNWLLLQAVEGTDLASTTLSPKDQIVILADALKRLHALD | 127  180 |
| Query 128  Sbjct 181 | | PATCPFDHQAKHRIERARTRMEAGLVDQDDLDEEHQGLAPAELFARLKARMPDGEDLVVT A CPFDH+ + R+ A+ R +AG++D+ D DE G A+LFA L+ARMP LVVT FANCPFDHRIEKRVAIAKARTQAGMIDESDFDETRLGRTAADLFAELQARMPQAGKLVVT | 187  240 |
| Query 188  Sbjct 241 | | HGDACLPNIMVENGRFSGFIDCGRLGVADRYQDIALATRDIAEELGGEWADRFLVLYGIA HGDACLPNI+ +NGRFSGFIDC RLGVAD YQDIALA R +A LG EW FL YGI HGDACLPNIIEKNGRFSGFIDCSRLGVADPYQDIALACRSVAYNLGEEWVQPFLTRYGID | 247  300 |
| Query 248 | | APDSQRIAFYRLLDEFF 264 D ++ FY LLDEFF |  |
| Sbjct | 301 | KDDPEKRDFYCLLDEFF 317 | |

```
>WP_116274031.1 aminoglycoside 3'-phosphotransferase [Rhizobium leguminosarum]
RFB91824.1 APH(3') family aminoglycoside O-phosphotransferase [Rhizobium leguminosarum bv. trifolii]
RFB92341.1 APH(3') family aminoglycoside O-phosphotransferase [Rhizobium leguminosarum bv. trifolii]
Length=261

Score = 266 bits (681), Expect = 6e-86, Method: Compositional matrix adjust. Identities = 132/254 (52%), Positives = 173/254 (68%), Gaps = 1/254 (0%)

Query 12	PAAWVERLFGYDWAQQTIGCSDAAVFRLSAQGRPVLFVKTDLSGALNELQDEAARLSWLA 71 PAA ERL GY + + T+G S A+VFRL +G P L++K + +G	EL DEAARL+WL
Sbjct 8	PAALAERLSGYRFERDTLGRSTASVFRLEGEGLPALYLKVETAGPFGELADEAARLAWLK 67

Query 72	TTGVPCAAVLDVVTEAGRDWLLLGEVPGQDLLS-SHLAPAEKVSIMADAMRRLHTLDPAT 130 T G+PC V+	++ GR+WLL+G +PG DL S S L P +V ++A A+ RLH L A+
Sbjct 68	TAGLPCPEVIARDSDGGRNWLLIGALPGNDLASASALTPLARVELLAAALSRLHDLPIAS 127

Query 131 CPFDHQAKHRIERARTRMEAGLVDQDDLDEEHQGLAPAELFARLKARMPDGEDLVVTHGD 190 CPFDH+ ++RI A+ RM+AG+VD+ D DE	G + A LFA L++R P EDLVVTHGD
Sbjct 128 CPFDHRLENRIAAAKARMQAGIVDETDFDETRLGKSAATLFAELESRRPGREDLVVTHGD 187

Query 191 ACLPNIMVENGRFSGFIDCGRLGVADRYQDIALATRDIAEELGGEWADRFLVLYGIAAPD 250 ACLPN +	FSG++DC RLGVADR+QDIALA R IA	G	FL YG+ PD
Sbjct 188 ACLPNFIASAEGFSGYVDCSRLGVADRHQDIALACRSIAHNFGAALVQPFLDRYGLTLPD 247

Query 251 SQRIAFYRLLDEFF 264
R+ +++LLDEFF

Sbjct 248 PARLDYFQLLDEFF 261

>WP_182510979.1 APH(3') family aminoglycoside O-phosphotransferase [Phyllobacterium sp. P30BS-XVII]
Length=257

Score = 266 bits (681), Expect = 7e-86, Method: Compositional matrix adjust.
```

| Identities | = 127/245 (52%), Positives = 164/245 (67%), Gaps = 1/245 (0%) |  |
| --- | --- | --- |
| Query 21 | GYDWAQQTIGCSDAAVFRLSAQGRPVLFVKTDLSGALNELQDEAARLSWLATTGVPCAAV | 80 |
| Sbjct 13 | GY W + +G SDA VF+L A G P LF+KT+L EL DE ARL WLA +PC + GYSWRRDELGRSDAQVFKLEASGLPQLFIKTELISPYAELPDEVARLQWLALQDMPCPRI | 72 |
| Query 81 | LDVVTEAGRDWLLLGEVPGQDLLSS-HLAPAEKVSIMADAMRRLHTLDPATCPFDHQAKH | 139 |
| Sbjct 73 | L + AG WLL+ + G DL S+ L+ ++ I A A+ +LH LDP CPF+H+ LFAGSHAGHYWLLMSALAGADLASADTLSVESRIHIFAGALHQLHALDPTACPFNHRLDR | 132 |
| Query 140 | RIERARTRMEAGLVDQDDLDEEHQGLAPAELFARLKARMPDGEDLVVTHGDACLPNIMVE | 199 |
| Sbjct 133 | RI+ A+ RM+AGLVD+ D D++ G ELF +L A+ P+ ED+VVTHGDACLPN + E RIKAAKARMKAGLVDETDFDDDQLGRTATELFCKLLAKQPEEEDIVVTHGDACLPNFIAE | 192 |
| Query 200 | NGRFSGFIDCGRLGVADRYQDIALATRDIAEELGGEWADRFLVLYGIAAPDSQRIAFYRL | 259 |
| Sbjct 193 | NG FSGFIDC RLG+ADRYQDIALA R + + GG+W FL YG+ PD ++A+YRL NGAFSGFIDCARLGIADRYQDIALACRSVTDNFGGQWVHVFLDFYGLKTPDPAKLAYYRL | 252 |
| Query 260  Sbjct 253 | LDEFF 264 LDEFF LDEFF 257 |  |

>WP_026615559.1 aminoglycoside 3'-phosphotransferase [Ensifer aridi] Length=261

Score = 266 bits (680), Expect = 9e-86, Method: Compositional matrix adjust.

| Identities | = 134/253 (53%), Positives = 169/253 (67%), Gaps = 0/253 (0%) |  |
| --- | --- | --- |
| Query 12 | PAAWVERLFGYDWAQQTIGCSDAAVFRLSAQGRPVLFVKTDLSGALNELQDEAARLSWLA | 71 |
| Sbjct 9 | P + +L GY + + T+G S + V L + RP L +K + +G +EL DEAARL WLA PPGFRLQLSGYSFERDTLGRSASTVVLLESDNRPRLILKHEKAGRFSELNDEAARLRWLA | 68 |
| Query 72 | TTGVPCAAVLDVVTEAGRDWLLLGEVPGQDLLSSHLAPAEKVSIMADAMRRLHTLDPATC | 131 |
| Sbjct 69 | G+PC VL G+ WLLL V G DL SS L PAE+++I+AD +RRLH LDPA C RQGIPCPTVLGSADHEGQSWLLLQAVEGIDLASSPLPPAERIAILADGLRRLHQLDPAAC | 128 |
| Query 132 | PFDHQAKHRIERARTRMEAGLVDQDDLDEEHQGLAPAELFARLKARMPDGEDLVVTHGDA | 191 |
| Sbjct 129 | PFD + K I AR RMEAGLVD+ D D+E +G A +ELF + + P E LVV HGDA PFDLRLKTSIAAARARMEAGLVDESDFDDEREGRAASELFGEVLSLKPKDERLVVAHGDA | 188 |
| Query 192 | CLPNIMVENGRFSGFIDCGRLGVADRYQDIALATRDIAEELGGEWADRFLVLYGIAAPDS | 251 |
| Sbjct 189 | CLPNI+ GRF+GFIDCGRLGVAD +QDIALA R IA +G EW FL YG+ + D CLPNIIAAGGRFAGFIDCGRLGVADLHQDIALACRSIAYNVGEEWVSPFLAQYGLQSADQ | 248 |
| Query 252  Sbjct 249 | QRIAFYRLLDEFF 264  ++++FY LLDEFF  EKLSFYCLLDEFF 261 |  |

>MBA8903988.1 aminoglycoside 3'-phosphotransferase-2 [Phyllobacterium sp. P30BS-XVII] Length=267

Score = 266 bits (680), Expect = 1e-85, Method: Compositional matrix adjust. Identities = 127/245 (52%), Positives = 164/245 (67%), Gaps = 1/245 (0%)

| Query | 21 | GYDWAQQTIGCSDAAVFRLSAQGRPVLFVKTDLSGALNELQDEAARLSWLATTGVPCAAV | 80 |
| --- | --- | --- | --- |
| Sbjct | 23 | GY W + +G SDA VF+L A G P LF+KT+L EL DE ARL WLA +PC + GYSWRRDELGRSDAQVFKLEASGLPQLFIKTELISPYAELPDEVARLQWLALQDMPCPRI | 82 |
| Query | 81 | LDVVTEAGRDWLLLGEVPGQDLLSS-HLAPAEKVSIMADAMRRLHTLDPATCPFDHQAKH | 139 |
| Sbjct | 83 | L + AG WLL+ + G DL S+ L+ ++ I A A+ +LH LDP CPF+H+  LFAGSHAGHYWLLMSALAGADLASADTLSVESRIHIFAGALHQLHALDPTACPFNHRLDR | 142 |

| Query | 140 | RIERARTRMEAGLVDQDDLDEEHQGLAPAELFARLKARMPDGEDLVVTHGDACLPNIMVE | 199 |
| --- | --- | --- | --- |
| Sbjct | 143 | RI+ A+ RM+AGLVD+ D D++ G ELF +L A+ P+ ED+VVTHGDACLPN + E RIKAAKARMKAGLVDETDFDDDQLGRTATELFCKLLAKQPEEEDIVVTHGDACLPNFIAE | 202 |
| Query | 200 | NGRFSGFIDCGRLGVADRYQDIALATRDIAEELGGEWADRFLVLYGIAAPDSQRIAFYRL | 259 |
| Sbjct | 203 | NG FSGFIDC RLG+ADRYQDIALA R + + GG+W FL YG+ PD ++A+YRL NGAFSGFIDCARLGIADRYQDIALACRSVTDNFGGQWVHVFLDFYGLKTPDPAKLAYYRL | 262 |
| Query  Sbjct | 260  263 | LDEFF 264 LDEFF LDEFF 267 |  |

>WP_083335564.1 MULTISPECIES: aminoglycoside 3'-phosphotransferase [unclassified Rhizobium]

RVU13495.1 aminoglycoside 3'-phosphotransferase [Rhizobium sp. RMa-01] Length=261

Score = 266 bits (679), Expect = 1e-85, Method: Compositional matrix adjust.

| Identities | | = 133/254 (52%), Positives = 171/254 (67%), Gaps = 1/254 (0%) |  |
| --- | --- | --- | --- |
| Query 12  Sbjct 8 | | PAAWVERLFGYDWAQQTIGCSDAAVFRLSAQGRPVLFVKTDLSGALNELQDEAARLSWLA P A ERL GY + + T+G S A+VFRL +G PVL++K + +G EL DEA RL WL PGALAERLSGYRFERDTLGRSTASVFRLEGEGLPVLYLKVEAAGPFGELADEAVRLGWLK | 71  67 |
| Query 72  Sbjct 68 | | TTGVPCAAVLDVVTEAGRDWLLLGEVPGQDLLS-SHLAPAEKVSIMADAMRRLHTLDPAT T G+PC V+ ++ R+WLL+G +PG DL S S L P +V ++A A+ RLH L A+ TAGLPCPEVIARESDGRRNWLLIGALPGNDLASASALTPLVRVELLAAALSRLHDLPIAS | 130  127 |
| Query 131  Sbjct 128 | | CPFDHQAKHRIERARTRMEAGLVDQDDLDEEHQGLAPAELFARLKARMPDGEDLVVTHGD CPFDH+ ++R+ A+ RM+AG+VD+ D DE G + A LFA LK+R PD EDLVVTHGD CPFDHRLENRVATAKARMQAGIVDETDFDETRLGKSAATLFAELKSRRPDREDLVVTHGD | 190  187 |
| Query 191  Sbjct 188 | | ACLPNIMVENGRFSGFIDCGRLGVADRYQDIALATRDIAEELGGEWADRFLVLYGIAAPD ACLPN + FSG+IDC RLGVADR+QDIALA R IA G FL YG+ PD ACLPNFVASAEGFSGYIDCSRLGVADRHQDIALACRSIAYNFGEVLVQPFLDRYGLTMPD | 250  247 |
| Query 251 | | SQRIAFYRLLDEFF 264 R+ ++RLLDEFF |  |
| Sbjct | 248 | PARLDYFRLLDEFF 261 | |

>WP_057383888.1 APH(3')-II family aminoglycoside O-phosphotransferase [Pseudomonas aeruginosa]

KQJ57170.1 aminoglycoside phosphotransferase [Pseudomonas aeruginosa] SUC91518.1 aminoglycoside 3-N-acetyltransferase [Pseudomonas aeruginosa]

Length=262

Score = 266 bits (679), Expect = 1e-85, Method: Compositional matrix adjust.

| Identities | | = 139/254 (55%), Positives = 170/254 (67%), Gaps = 3/254 (1%) |  |
| --- | --- | --- | --- |
| Query 11  Sbjct 12 | | SPAAWVERLFGYDWAQQTIGCSDAAVFRLSAQGRPVLFVKTDLSGALNELQDEAARLSWL  +P+ W + L GY W Q GCS A V RL A RP LFVK ++ A EL E ARL WL APSTWADYLAGYRWRGQGEGCSAATVHRLEAARRPTLFVKQEVLSAHAELPAEIARLRWL | 70  71 |
| Query 71 | | ATTGVPCAAVLDVVTEAGRDWLLLGEVPGQDLLSSHLAPAEKVSIMADAMRRLHTLDPAT G+ C VL+ GR WLL+ VPG L S LAP V ++A A+RRLH LDPA | 130 |
| Sbjct | 72 | HGAGIDCPQVLNETQSDGRQWLLMSAVPGDTL--SALAPERLVRLVAAALRRLHDLDPAA | 129 |
| Query | 131 | CPFDHQAKHRIERARTRMEAGLVDQDDLDEEHQGLAPAELFARLKARMPDGEDLVVTHGD | 190 |
| Sbjct | 130 | CPFDH+ + R++ R R+EAGLVD+ D D++H+G + EL+ L R P EDLVV HGD CPFDHRLERRLDTVRQRVEAGLVDEADFDDDHRGRSATELYRLLLDRRPAVEDLVVAHGD | 189 |
| Query | 191 | ACLPNIMVENGRFSGFIDCGRLGVADRYQDIALATRDIAEELGGEWADRFLVLYGIAAPD | 250 |
| Sbjct | 190 | ACLPN++ E RFSGFIDCGRLGVADR+QD+ALA RDI ELG WA+ FLV YG D ACLPNLLAEGRRFSGFIDCGRLGVADRHQDLALAARDIEAELGAAWAEAFLVEYG-GDID | 248 |
| Query | 251 | SQRIAFYRLLDEFF 264  +R+A++RLLDEFF |  |
| Sbjct | 249 | GERLAYFRLLDEFF 262 | |

>OHV23727.1 APH(3') family aminoglycoside O-phosphotransferase [Rhizobium sp. RSm-3]

Length=255

Score = 265 bits (678), Expect = 1e-85, Method: Compositional matrix adjust.

| Identities | | = 133/254 (52%), Positives = 171/254 (67%), Gaps = 1/254 (0%) |  |
| --- | --- | --- | --- |
| Query 12  Sbjct 2 | | PAAWVERLFGYDWAQQTIGCSDAAVFRLSAQGRPVLFVKTDLSGALNELQDEAARLSWLA P A ERL GY + + T+G S A+VFRL +G PVL++K + +G EL DEA RL WL PGALAERLSGYRFERDTLGRSTASVFRLEGEGLPVLYLKVEAAGPFGELADEAVRLGWLK | 71  61 |
| Query 72  Sbjct 62 | | TTGVPCAAVLDVVTEAGRDWLLLGEVPGQDLLS-SHLAPAEKVSIMADAMRRLHTLDPAT T G+PC V+ ++ R+WLL+G +PG DL S S L P +V ++A A+ RLH L A+ TAGLPCPEVIARESDGRRNWLLIGALPGNDLASASALTPLVRVELLAAALSRLHDLPIAS | 130  121 |
| Query 131  Sbjct 122 | | CPFDHQAKHRIERARTRMEAGLVDQDDLDEEHQGLAPAELFARLKARMPDGEDLVVTHGD CPFDH+ ++R+ A+ RM+AG+VD+ D DE G + A LFA LK+R PD EDLVVTHGD CPFDHRLENRVATAKARMQAGIVDETDFDETRLGKSAATLFAELKSRRPDREDLVVTHGD | 190  181 |
| Query 191  Sbjct 182 | | ACLPNIMVENGRFSGFIDCGRLGVADRYQDIALATRDIAEELGGEWADRFLVLYGIAAPD ACLPN + FSG+IDC RLGVADR+QDIALA R IA G FL YG+ PD ACLPNFVASAEGFSGYIDCSRLGVADRHQDIALACRSIAYNFGEVLVQPFLDRYGLTMPD | 250  241 |
| Query 251 | | SQRIAFYRLLDEFF 264 R+ ++RLLDEFF |  |
| Sbjct | 242 | PARLDYFRLLDEFF 255 | |

>WP_140734246.1 aminoglycoside 3'-phosphotransferase [Mesorhizobium sp. B2-3-4] TPM32892.1 aminoglycoside 3'-phosphotransferase [Mesorhizobium sp. B2-3-4]

Length=264

Score = 266 bits (679), Expect = 1e-85, Method: Compositional matrix adjust.

| Identities | = 135/247 (55%), Positives = 164/247 (66%), Gaps = 1/247 (0%) |  |
| --- | --- | --- |
| Query 19 | LFGYDWAQQTIGCSDAAVFRLSAQGRPVLFVKTDLSGALNELQDEAARLSWLATTGVPCA | 78 |
| Sbjct 18 | + GY W +QTIG S A VFRL A +P LF+K + SG L EL DEAARL WL G+ C MSGYRWRRQTIGRSQAGVFRLEAGDKPALFLKIEESGPLAELADEAARLRWLGGQGIVCP | 77 |
| Query 79 | AVLDVVTEAGRDWLLLGEVPGQDLLSS-HLAPAEKVSIMADAMRRLHTLDPATCPFDHQA | 137 |
| Sbjct 78 | V+ ++AGR WLL+ +PG+DL S+ L P + IMA A+R LH LD +CPFDH+ EVIGFESDAGRHWLLMSAIPGRDLASARQLHPTIAIDIMAGALRELHALDTGSCPFDHRI | 137 |
| Query 138 | KHRIERARTRMEAGLVDQDDLDEEHQGLAPAELFARLKARMPDGEDLVVTHGDACLPNIM | 197 |
| Sbjct 138 | + RI AR RMEAG VD+ D D+E QG E+FA L A P EDLVVTHGDAC+PN M ERRIATARARMEAGEVDESDFDDERQGRTATEIFAELVALRPVAEDLVVTHGDACMPNFM | 197 |
| Query 198 | VENGRFSGFIDCGRLGVADRYQDIALATRDIAEELGGEWADRFLVLYGIAAPDSQRIAFY | 257 |
| Sbjct 198 | GRFSGFIDCGRLGVADR+QD+ALA I LG W FL YG + ++++Y AAEGRFSGFIDCGRLGVADRHQDLALACWSIRHNLGQAWVKPFLERYGPPRAEPAKLSWY | 257 |
| Query 258  Sbjct 258 | RLLDEFF 264 RLLDEFF RLLDEFF 264 |  |

>WP_105735079.1 aminoglycoside 3'-phosphotransferase [Phyllobacterium myrsinacearum]

PRD51522.1 APH(3') family aminoglycoside O-phosphotransferase [Phyllobacterium myrsinacearum]

Length=257

Score = 265 bits (678), Expect = 2e-85, Method: Compositional matrix adjust. Identities = 132/245 (54%), Positives = 165/245 (67%), Gaps = 1/245 (0%)

| Query | 21 | GYDWAQQTIGCSDAAVFRLSAQGRPVLFVKTDLSGALNELQDEAARLSWLATTGVPCAAV | 80 |
| --- | --- | --- | --- |
| Sbjct | 13 | GY W++ +GCSDA VF L +G P LF+K + EL DEAARL WL++ + C V GYSWSRDALGCSDAQVFMLQGEGLPQLFIKVEAVSPYGELPDEAARLRWLSSRHMLCPDV | 72 |
| Query | 81 | LDVVTEAGRDWLLLGEVPGQDLLSS-HLAPAEKVSIMADAMRRLHTLDPATCPFDHQAKH | 139 |
| Sbjct | 73 | L A R WLL+ VPG+DL S+ L+ ++ I+A A+R+LH LDPATCPFDH+  LFEGAHASRFWLLMSGVPGKDLASADSLSIETRIRILAGALRQLHALDPATCPFDHRLDM | 132 |

| Query | 140 | RIERARTRMEAGLVDQDDLDEEHQGLAPAELFARLKARMPDGEDLVVTHGDACLPNIMVE | 199 |
| --- | --- | --- | --- |
| Sbjct | 133 | RIE AR RM+AGLVD+ D D + G +ELF+RL A P D+VVTHGD CLPN M E RIEAARARMQAGLVDETDFDSDMLGKTASELFSRLVAERPGTADVVVTHGDGCLPNFMAE | 192 |
| Query | 200 | NGRFSGFIDCGRLGVADRYQDIALATRDIAEELGGEWADRFLVLYGIAAPDSQRIAFYRL | 259 |
| Sbjct | 193 | NG F+G+IDC RLG+ADRYQDIALA R IA+ G EW FL YG+ D ++A+YRL NGIFTGYIDCARLGLADRYQDIALACRSIADNFGEEWVRPFLDCYGLVQADPAKLAYYRL | 252 |
| Query  Sbjct | 260  253 | LDEFF 264 LDEFF LDEFF 257 |  |

>WP_049736257.1 aminoglycoside 3'-phosphotransferase [Rhizobium ecuadorense] Length=261

Score = 265 bits (678), Expect = 2e-85, Method: Compositional matrix adjust.

| Identities | | = 132/254 (52%), Positives = 171/254 (67%), Gaps = 1/254 (0%) |  |
| --- | --- | --- | --- |
| Query 12  Sbjct 8 | | PAAWVERLFGYDWAQQTIGCSDAAVFRLSAQGRPVLFVKTDLSGALNELQDEAARLSWLA P A ERL GY + + +G S A+VFRL +G PVL++K + +G EL DEA RL WL PGALAERLSGYRFERDALGRSTASVFRLEGEGLPVLYLKVEAAGPFGELADEAVRLGWLK | 71  67 |
| Query 72  Sbjct 68 | | TTGVPCAAVLDVVTEAGRDWLLLGEVPGQDLLS-SHLAPAEKVSIMADAMRRLHTLDPAT T G+PC V+ ++ GR+WLL+G +PG DL S S L P +V ++A A+ RLH L A+ TAGLPCPEVIARESDGGRNWLLIGALPGNDLASASALTPLARVELLAAALSRLHDLPIAS | 130  127 |
| Query 131  Sbjct 128 | | CPFDHQAKHRIERARTRMEAGLVDQDDLDEEHQGLAPAELFARLKARMPDGEDLVVTHGD CPFDH+ ++RI A+ RM+AG+VD+ D DE G + A LFA L++R PD EDLVVTHGD CPFDHRLENRIAAAKARMQAGIVDETDFDETRLGKSAATLFAELESRRPDREDLVVTHGD | 190  187 |
| Query 191  Sbjct 188 | | ACLPNIMVENGRFSGFIDCGRLGVADRYQDIALATRDIAEELGGEWADRFLVLYGIAAPD ACLPN + FSG+IDC RLGVADR+QDIALA R IA G FL YG+ PD ACLPNFVASAEGFSGYIDCSRLGVADRHQDIALACRSIAHNFGEALVQPFLDRYGLTMPD | 250  247 |
| Query 251 | | SQRIAFYRLLDEFF 264 R+ +++LLDEFF |  |
| Sbjct | 248 | PARLHYFQLLDEFF 261 | |

>WP_184344750.1 APH(3') family aminoglycoside O-phosphotransferase [Rhizobium aethiopicum]

Length=261

Score = 265 bits (678), Expect = 2e-85, Method: Compositional matrix adjust.

| Identities | | = 132/254 (52%), Positives = 173/254 (68%), Gaps = 1/254 (0%) |  |
| --- | --- | --- | --- |
| Query 12  Sbjct 8 | | PAAWVERLFGYDWAQQTIGCSDAAVFRLSAQGRPVLFVKTDLSGALNELQDEAARLSWLA P A ERL GY + + T+G S A+VFRL +G PVL++K + +G EL DEAA+L WL PGALAERLSGYRFERDTLGRSTASVFRLEGEGLPVLYLKVEAAGPFGELADEAAKLDWLK | 71  67 |
| Query 72  Sbjct 68 | | TTGVPCAAVLDVVTEAGRDWLLLGEVPGQDLLS-SHLAPAEKVSIMADAMRRLHTLDPAT  +G+PC V+ ++ GR+WLL+G +PG DL S S L P +V ++A A+ RLH L A+ ASGLPCPEVIAWESDGGRNWLLIGALPGNDLASASALTPLARVELLAAALSRLHHLPIAS | 130  127 |
| Query 131  Sbjct 128 | | CPFDHQAKHRIERARTRMEAGLVDQDDLDEEHQGLAPAELFARLKARMPDGEDLVVTHGD CPFDH+ ++RI A+ RM+AG+VD+ D DE G + A LFA L++R PD EDLVVTHGD CPFDHRLENRIAAAKARMQAGIVDETDFDETRLGKSAATLFAELESRRPDREDLVVTHGD | 190  187 |
| Query 191  Sbjct 188 | | ACLPNIMVENGRFSGFIDCGRLGVADRYQDIALATRDIAEELGGEWADRFLVLYGIAAPD ACLPN + FSG+IDC RLGVADR+QDIALA R IA G FL YG+ PD ACLPNFVASAEGFSGYIDCSRLGVADRHQDIALACRSIAHNFGEALVQPFLDRYGLTMPD | 250  247 |
| Query 251 | | SQRIAFYRLLDEFF 264 R+ +++LLDEFF |  |
| Sbjct | 248 | PARLHYFQLLDEFF 261 | |

>WP_196658889.1 APH(3')-II family aminoglycoside O-phosphotransferase [Pseudomonas aeruginosa]

MBG4980754.1 APH(3')-II family aminoglycoside O-phosphotransferase [Pseudomonas aeruginosa]

Length=268

Score = 265 bits (678), Expect = 2e-85, Method: Compositional matrix adjust.

| Identities | | = 138/258 (53%), Positives = 170/258 (66%), Gaps = 5/258 (2%) |  |
| --- | --- | --- | --- |
| Query 11  Sbjct 12 | | SPAAWVERLFGYDWAQQTIGCSDAAVFRLSAQGRPVLFVKTDLSGALNELQDEAARLSWL  +P+ W + L GY W Q GCS A V RL A RP LFVK ++ A EL E ARL WL APSTWADYLAGYRWRGQGEGCSAATVHRLEAARRPTLFVKQEVLSAHAELPAEIARLRWL | 70  71 |
| Query 71  Sbjct 72 | | ATTGVPCAAVLDVVTEAGRDWLLLGEVPGQDLLS SHLAPAEKVSIMADAMRRLHTL  G+ C VL+ GR WLL+ VPG L + L P V ++A A+RRLH L HGAGIDCPQVLNETQSDGRQWLLMSAVPGDTLSALAQCGELEPGRLVRLVAAALRRLHDL | 126  131 |
| Query 127  Sbjct 132 | | DPATCPFDHQAKHRIERARTRMEAGLVDQDDLDEEHQGLAPAELFARLKARMPDGEDLVV DPA CPFDH+ + R++ R R+EAGLVD+ D D++H+G + EL+ L R P GEDLVV DPAACPFDHRLERRLDTVRQRVEAGLVDEADFDDDHRGRSATELYRLLLDRRPAGEDLVV | 186  191 |
| Query 187  Sbjct 192 | | THGDACLPNIMVENGRFSGFIDCGRLGVADRYQDIALATRDIAEELGGEWADRFLVLYGI HGDACLPN++ E RFSGFIDCGRLGVADR+QD+ALA RDI ELG WA+ FLV YG AHGDACLPNLLAEGRRFSGFIDCGRLGVADRHQDLALAARDIEAELGAAWAEAFLVEYG- | 246  250 |
| Query | 247 AAPDSQRIAFYRLLDEFF 264 | | |
|  |  | D +R+A++RLLDEFF |  |
| Sbjct | 251 | GDIDGERLAYFRLLDEFF 268 | |

>ETR77329.1 aminoglycoside 3'-phosphotransferase [Afipia sp. P52-10] Length=258

Score = 265 bits (677), Expect = 2e-85, Method: Compositional matrix adjust.

| Identities | | = 133/254 (52%), Positives = 167/254 (66%), Gaps = 2/254 (1%) |  |
| --- | --- | --- | --- |
| Query 12  Sbjct 6 | | PAAWVERLFGYDWAQQTIGCSDAAVFRLSAQGRPVLFVKTDLSGALNELQDEAARLSWLA P W + L Y W QQTIG S AAVFRL A G+P LFVKT+ G N L EA RL WL PPTWTQELAHYAWRQQTIGRSGAAVFRLEANGQPTLFVKTEQRGGFNTLPAEAERLRWLG | 71  65 |
| Query 72  Sbjct 66 | | TTGVPCAAVLDVVTEAGRDWLLLGEVPGQDLLSS-HLAPAEKVSIMADAMRRLHTLDPAT TG+PC A+L + R+WLL +PG+DL SS HLAP + V+I ADA+R LH L P+ ETGIPCPAILKETRDGRRNWLLTTAIPGRDLASSPHLAPEQIVAIAADALRDLHRLAPSG | 130  125 |
| Query 131  Sbjct 126 | | CPFDHQAKHRIERARTRMEAGLVDQDDLDEEHQGLAPAELFARLKARMPDGEDLVVTHGD CPFDH+ + + A R +AGLVD+ D DE G + +LF L R PD EDLVV HGD CPFDHRLERALAEAEARTQAGLVDETDFDEPRLGRSAHDLFEELLRRRPDREDLVVAHGD | 190  185 |
| Query 191  Sbjct 186 | | ACLPNIMVENGRFSGFIDCGRLGVADRYQDIALATRDIAEELGGEWADRFLVLYGIAAPD LPN++ E GRFSGF+DC RLGVAD+++D+A+A+ + LG W FL YG A PD PSLPNLLAEAGRFSGFVDCARLGVADQHRDLAIASSSVRYNLGEAWVAPFLTRYG-AKPD | 250  244 |
| Query 251 | | SQRIAFYRLLDEFF 264  +R+ +Y+LLDEFF |  |
| Sbjct | 245 | PERMDYYQLLDEFF 258 | |

>PWV89634.1 aminoglycoside 3'-phosphotransferase-2 [Phyllobacterium myrsinacearum] RZU99775.1 aminoglycoside 3'-phosphotransferase-2 [Phyllobacterium myrsinacearum]

Length=267

Score = 265 bits (678), Expect = 2e-85, Method: Compositional matrix adjust.

| Identities | = 132/245 (54%), Positives = 165/245 (67%), Gaps = 1/245 (0%) |  |
| --- | --- | --- |
| Query 21 | GYDWAQQTIGCSDAAVFRLSAQGRPVLFVKTDLSGALNELQDEAARLSWLATTGVPCAAV | 80 |
| Sbjct 23 | GY W++ +GCSDA VF L +G P LF+K + EL DEAARL WL++ + C V GYSWSRDALGCSDAQVFMLQGEGLPQLFIKVEAVSPYGELPDEAARLRWLSSRHMLCPDV | 82 |
| Query 81 | LDVVTEAGRDWLLLGEVPGQDLLSS-HLAPAEKVSIMADAMRRLHTLDPATCPFDHQAKH | 139 |
| Sbjct 83 | L A R WLL+ VPG+DL S+ L+ ++ I+A A+R+LH LDPATCPFDH+ LFEGAHASRFWLLMSGVPGKDLASADSLSIETRIRILAGALRQLHALDPATCPFDHRLDM | 142 |
| Query 140 | RIERARTRMEAGLVDQDDLDEEHQGLAPAELFARLKARMPDGEDLVVTHGDACLPNIMVE | 199 |
| Sbjct 143 | RIE AR RM+AGLVD+ D D + G +ELF+RL A P D+VVTHGD CLPN M E  RIEAARARMQAGLVDETDFDSDMLGKTASELFSRLVAERPGTADVVVTHGDGCLPNFMAE | 202 |

| Query | 200 | NGRFSGFIDCGRLGVADRYQDIALATRDIAEELGGEWADRFLVLYGIAAPDSQRIAFYRL 259 |
| --- | --- | --- |
| Sbjct | 203 | NG F+G+IDC RLG+ADRYQDIALA R IA+ G EW FL YG+ D ++A+YRL NGIFTGYIDCARLGLADRYQDIALACRSIADNFGEEWVRPFLDCYGLVQADPAKLAYYRL 262 |
| Query | 260 | LDEFF 264 |
| Sbjct | 263 | LDEFF  LDEFF 267 |

>MBG6825528.1 APH(3')-II family aminoglycoside O-phosphotransferase [Pseudomonas aeruginosa]

Length=268

Score = 265 bits (678), Expect = 2e-85, Method: Compositional matrix adjust.

| Identities | | = 139/258 (54%), Positives = 170/258 (66%), Gaps = 5/258 (2%) |  |
| --- | --- | --- | --- |
| Query 11  Sbjct 12 | | SPAAWVERLFGYDWAQQTIGCSDAAVFRLSAQGRPVLFVKTDLSGALNELQDEAARLSWL  +PA W + L GY W Q GCS A V RL A RP LFVK ++ A EL E ARL WL APATWADYLAGYRWRGQGEGCSAATVHRLEAARRPTLFVKQEVLSAHAELPAEIARLRWL | 70  71 |
| Query 71  Sbjct 72 | | ATTGVPCAAVLDVVTEAGRDWLLLGEVPGQDLLS SHLAPAEKVSIMADAMRRLHTL  G+ C VL+ GR WLL+ VPG L + L P V ++A A+RRLH L HGAGIDCPQVLNETQSDGRQWLLMSAVPGDTLSALAQRGELEPERLVRLVAAALRRLHDL | 126  131 |
| Query 127  Sbjct 132 | | DPATCPFDHQAKHRIERARTRMEAGLVDQDDLDEEHQGLAPAELFARLKARMPDGEDLVV DPA CPFDH+ + R++ R R+EAGLVD+ D D++H+G + EL+ L AR P EDLVV DPAACPFDHRLERRLDTVRQRVEAGLVDEADFDDDHRGRSATELYRLLLARRPAVEDLVV | 186  191 |
| Query 187  Sbjct 192 | | THGDACLPNIMVENGRFSGFIDCGRLGVADRYQDIALATRDIAEELGGEWADRFLVLYGI HGDACLPN++ E RFSGFIDCGRLGVADR+QD+ALA RDI ELG WA+ FLV YG AHGDACLPNLLAEGRRFSGFIDCGRLGVADRHQDLALAARDIEAELGAAWAEAFLVEYG- | 246  250 |
| Query | 247 AAPDSQRIAFYRLLDEFF 264 | | |
|  |  | D +R+A++RLLDEFF |  |
| Sbjct | 251 | GDIDGERLAYFRLLDEFF 268 | |

>WP_034468071.1 aminoglycoside 3'-phosphotransferase [Afipia sp. P52-10] Length=267

Score = 265 bits (678), Expect = 2e-85, Method: Compositional matrix adjust.

| Identities | | = 133/254 (52%), Positives = 167/254 (66%), Gaps = 2/254 (1%) |  |
| --- | --- | --- | --- |
| Query 12  Sbjct 15 | | PAAWVERLFGYDWAQQTIGCSDAAVFRLSAQGRPVLFVKTDLSGALNELQDEAARLSWLA P W + L Y W QQTIG S AAVFRL A G+P LFVKT+ G N L EA RL WL PPTWTQELAHYAWRQQTIGRSGAAVFRLEANGQPTLFVKTEQRGGFNTLPAEAERLRWLG | 71  74 |
| Query 72  Sbjct 75 | | TTGVPCAAVLDVVTEAGRDWLLLGEVPGQDLLSS-HLAPAEKVSIMADAMRRLHTLDPAT TG+PC A+L + R+WLL +PG+DL SS HLAP + V+I ADA+R LH L P+ ETGIPCPAILKETRDGRRNWLLTTAIPGRDLASSPHLAPEQIVAIAADALRDLHRLAPSG | 130  134 |
| Query 131  Sbjct 135 | | CPFDHQAKHRIERARTRMEAGLVDQDDLDEEHQGLAPAELFARLKARMPDGEDLVVTHGD CPFDH+ + + A R +AGLVD+ D DE G + +LF L R PD EDLVV HGD CPFDHRLERALAEAEARTQAGLVDETDFDEPRLGRSAHDLFEELLRRRPDREDLVVAHGD | 190  194 |
| Query 191  Sbjct 195 | | ACLPNIMVENGRFSGFIDCGRLGVADRYQDIALATRDIAEELGGEWADRFLVLYGIAAPD LPN++ E GRFSGF+DC RLGVAD+++D+A+A+ + LG W FL YG A PD PSLPNLLAEAGRFSGFVDCARLGVADQHRDLAIASSSVRYNLGEAWVAPFLTRYG-AKPD | 250  253 |
| Query 251 | | SQRIAFYRLLDEFF 264  +R+ +Y+LLDEFF |  |
| Sbjct | 254 | PERMDYYQLLDEFF 267 | |

>WP_140771434.1 aminoglycoside 3'-phosphotransferase [Mesorhizobium sp. B2-1-3A] TPM97601.1 aminoglycoside 3'-phosphotransferase [Mesorhizobium sp. B2-1-3A]

Length=262

Score = 265 bits (677), Expect = 3e-85, Method: Compositional matrix adjust. Identities = 135/253 (53%), Positives = 168/253 (66%), Gaps = 0/253 (0%)

Query 12	PAAWVERLFGYDWAQQTIGCSDAAVFRLSAQGRPVLFVKTDLSGALNELQDEAARLSWLA 71

| Sbjct | 10 | P RL GY W +QTIG S A VFRL A + LF+K + +G EL DEAARL WLA PQELKNRLPGYRWHRQTIGRSQAGVFRLVADDKSALFLKCERNGPFAELADEAARLRWLA | 69 |
| --- | --- | --- | --- |
| Query | 72 | TTGVPCAAVLDVVTEAGRDWLLLGEVPGQDLLSSHLAPAEKVSIMADAMRRLHTLDPATC | 131 |
| Sbjct | 70 | G+ C V+ + + AG DWLL+ V G+DL S+ + PA+ + IMA A+R LH LD +C GQGIACPDVIALESHAGHDWLLMSAVAGEDLASAPIDPADVIEIMASALRDLHALDIGSC | 129 |
| Query | 132 | PFDHQAKHRIERARTRMEAGLVDQDDLDEEHQGLAPAELFARLKARMPDGEDLVVTHGDA | 191 |
| Sbjct | 130 | PFDH+ RI AR+RMEAG VD+ D D+E QG A+ FA L+A P EDLVV HGDA PFDHRLFRRIAAARSRMEAGEVDESDFDDERQGWTTAKAFAELEALRPTTEDLVVAHGDA | 189 |
| Query | 192 | CLPNIMVENGRFSGFIDCGRLGVADRYQDIALATRDIAEELGGEWADRFLVLYGIAAPDS | 251 |
| Sbjct | 190 | CLPN+M GRFSGFIDCGRLGVADR+QD+ALA I +G W + FL YG + CLPNVMAARGRFSGFIDCGRLGVADRHQDLALACSSIRYNIGETWIEPFLERYGPPQAEP | 249 |
| Query | 252 | QRIAFYRLLDEFF 264  ++++YRLLDEFF |  |
| Sbjct | 250 | AKLSWYRLLDEFF 262 | |

>WP_033936220.1 APH(3')-II family aminoglycoside O-phosphotransferase [Pseudomonas aeruginosa]

Length=268

Score = 265 bits (677), Expect = 3e-85, Method: Compositional matrix adjust.

| Identities | | = 139/258 (54%), Positives = 170/258 (66%), Gaps = 5/258 (2%) |  |
| --- | --- | --- | --- |
| Query 11  Sbjct 12 | | SPAAWVERLFGYDWAQQTIGCSDAAVFRLSAQGRPVLFVKTDLSGALNELQDEAARLSWL  +PA W + L GY W Q GCS A V RL A RP LFVK ++ A EL E ARL WL APATWADYLAGYRWRGQGEGCSAATVHRLEAARRPTLFVKQEVLSAHAELPAEIARLRWL | 70  71 |
| Query 71  Sbjct 72 | | ATTGVPCAAVLDVVTEAGRDWLLLGEVPGQDLLS SHLAPAEKVSIMADAMRRLHTL  G+ C VL+ GR WLL+ VPG L + L P V ++A A+RRLH L HGAGIDCPQVLNETQSDGRQWLLMSAVPGDTLSALAQRGELEPERLVRLVAAALRRLHDL | 126  131 |
| Query 127  Sbjct 132 | | DPATCPFDHQAKHRIERARTRMEAGLVDQDDLDEEHQGLAPAELFARLKARMPDGEDLVV DPA CPFDH+ + R++ R R+EAGLVD+ D D++H+G + EL+ L R P EDLVV DPAACPFDHRLERRLDTVRQRVEAGLVDEADFDDDHRGRSATELYRLLLDRRPAVEDLVV | 186  191 |
| Query 187  Sbjct 192 | | THGDACLPNIMVENGRFSGFIDCGRLGVADRYQDIALATRDIAEELGGEWADRFLVLYGI HGDACLPN++ E RFSGFIDCGRLGVADR+QD+ALATRDI ELG WA+ FLV YG AHGDACLPNLLAEGRRFSGFIDCGRLGVADRHQDLALATRDIEAELGAAWAEAFLVEYG- | 246  250 |
| Query | 247 AAPDSQRIAFYRLLDEFF 264 | | |
|  |  | D +R+A++RLLDEFF |  |
| Sbjct | 251 | GDIDGERLAYFRLLDEFF 268 | |

>PYF57585.1 aminoglycoside 3'-phosphotransferase-2 [Ensifer sp. AP48] Length=263

Score = 265 bits (677), Expect = 3e-85, Method: Compositional matrix adjust.

| Identities | = 131/244 (54%), Positives = 167/244 (68%), Gaps = 0/244 (0%) |  |
| --- | --- | --- |
| Query 21 | GYDWAQQTIGCSDAAVFRLSAQGRPVLFVKTDLSGALNELQDEAARLSWLATTGVPCAAV | 80 |
| Sbjct 20 | GYDW T+G SD++++ L A RP+L +K + +G +E DEA RL WLA+ G+PC V GYDWNHDTLGQSDSSIYLLKAPDRPMLVLKVEAAGPFSEFVDEAERLDWLASQGMPCPRV | 79 |
| Query 81 | LDVVTEAGRDWLLLGEVPGQDLLSSHLAPAEKVSIMADAMRRLHTLDPATCPFDHQAKHR | 140 |
| Sbjct 80 | + +A +WLLL V G DL S+ L+P +++ I+ADA++RLH LD A CPFDH+ + R IARAFDAETNWLLLQAVEGTDLASTTLSPKDRIVILADALKRLHGLDVADCPFDHRIEKR | 139 |
| Query 141 | IERARTRMEAGLVDQDDLDEEHQGLAPAELFARLKARMPDGEDLVVTHGDACLPNIMVEN | 200 |
| Sbjct 140 | I A R EAGL+D+DD DE G A+LFA+L+AR+P LV+THGDACLPNI+ +N IAIAGARTEAGLIDEDDFDETRLGRTAADLFAKLRARVPSIGKLVITHGDACLPNIIEKN | 199 |
| Query 201 | GRFSGFIDCGRLGVADRYQDIALATRDIAEELGGEWADRFLVLYGIAAPDSQRIAFYRLL | 260 |
| Sbjct 200 | GRFSGFIDC RLGVAD YQDIALA IA LG EW FL YGI D ++ Y LL GRFSGFIDCSRLGVADPYQDIALACHSIAHNLGEEWVQPFLARYGIDRDDPEKRDLYCLL | 259 |
| Query 261 | DEFF 264 DEFF |  |

Sbjct 260 DEFF 263

>WP_110878793.1 aminoglycoside 3'-phosphotransferase [Ensifer sp. AP48] Length=254

Score = 265 bits (676), Expect = 3e-85, Method: Compositional matrix adjust.

| Identities | = 131/244 (54%), Positives = 167/244 (68%), Gaps = 0/244 (0%) |  |
| --- | --- | --- |
| Query 21 | GYDWAQQTIGCSDAAVFRLSAQGRPVLFVKTDLSGALNELQDEAARLSWLATTGVPCAAV | 80 |
| Sbjct 11 | GYDW T+G SD++++ L A RP+L +K + +G +E DEA RL WLA+ G+PC V GYDWNHDTLGQSDSSIYLLKAPDRPMLVLKVEAAGPFSEFVDEAERLDWLASQGMPCPRV | 70 |
| Query 81 | LDVVTEAGRDWLLLGEVPGQDLLSSHLAPAEKVSIMADAMRRLHTLDPATCPFDHQAKHR | 140 |
| Sbjct 71 | + +A +WLLL V G DL S+ L+P +++ I+ADA++RLH LD A CPFDH+ + R IARAFDAETNWLLLQAVEGTDLASTTLSPKDRIVILADALKRLHGLDVADCPFDHRIEKR | 130 |
| Query 141 | IERARTRMEAGLVDQDDLDEEHQGLAPAELFARLKARMPDGEDLVVTHGDACLPNIMVEN | 200 |
| Sbjct 131 | I A R EAGL+D+DD DE G A+LFA+L+AR+P LV+THGDACLPNI+ +N IAIAGARTEAGLIDEDDFDETRLGRTAADLFAKLRARVPSIGKLVITHGDACLPNIIEKN | 190 |
| Query 201 | GRFSGFIDCGRLGVADRYQDIALATRDIAEELGGEWADRFLVLYGIAAPDSQRIAFYRLL | 260 |
| Sbjct 191 | GRFSGFIDC RLGVAD YQDIALA IA LG EW FL YGI D ++ Y LL GRFSGFIDCSRLGVADPYQDIALACHSIAHNLGEEWVQPFLARYGIDRDDPEKRDLYCLL | 250 |
| Query 261  Sbjct 251 | DEFF 264 DEFF  DEFF 254 |  |

>WP_024925422.1 MULTISPECIES: aminoglycoside 3'-phosphotransferase [Mesorhizobium] NYD61658.1 aminoglycoside 3'-phosphotransferase-2 [Mesorhizobium sp. YL-MPnR-2016]

OCX24776.1 APH(3') family aminoglycoside O-phosphotransferase [Mesorhizobium hungaricum]

Length=265

Score = 265 bits (677), Expect = 3e-85, Method: Compositional matrix adjust.

| Identities | = 139/254 (55%), Positives = 169/254 (67%), Gaps = 1/254 (0%) |  |
| --- | --- | --- |
| Query 12 | PAAWVERLFGYDWAQQTIGCSDAAVFRLSAQGRPVLFVKTDLSGALNELQDEAARLSWLA | 71 |
| Sbjct 12 | P A + GY W + T+G SDA VFRL A RP LF+K + SG EL E RL WLA PPAIRAMIAGYAWDRDTLGHSDAGVFRLEATDRPRLFLKVEESGGHAELPAEVERLRWLA | 71 |
| Query 72 | TTGVPCAAVLDVVTEAGRDWLLLGEVPGQDLLSSHLAPAEK-VSIMADAMRRLHTLDPAT | 130 |
| Sbjct 72 | GV C VL T AGRDWLL+ +PG+DL+S+ AEK +++MA A+R LH LD + GQGVACPEVLAFETHAGRDWLLMSAMPGRDLVSAGPLSAEKAIAVMAGALRNLHALDIRS | 131 |
| Query 131 | CPFDHQAKHRIERARTRMEAGLVDQDDLDEEHQGLAPAELFARLKARMPDGEDLVVTHGD | 190 |
| Sbjct 132 | CPFDH+ +HRI AR +EAGLVD++D D+E G ++F + A P EDLVVTHGD CPFDHRLEHRIADARAHLEAGLVDEEDFDDERMGRTADDVFGDVIALRPSSEDLVVTHGD | 191 |
| Query 191 | ACLPNIMVENGRFSGFIDCGRLGVADRYQDIALATRDIAEELGGEWADRFLVLYGIAAPD | 250 |
| Sbjct 192 | ACLPN MV+ RFSGFIDCGRLGVADR QD+ALA I LG +W D FL YG D ACLPNFMVDGDRFSGFIDCGRLGVADRCQDLALACWSIHHNLGPQWVDPFLAHYGAPDID | 251 |
| Query 251  Sbjct 252 | SQRIAFYRLLDEFF 264 S ++A+YRLLDEFF SGKLAYYRLLDEFF 265 |  |

>WP_163273106.1 APH(3') family aminoglycoside O-phosphotransferase [Chelativorans sp. UJN715]

Length=266

Score = 265 bits (676), Expect = 4e-85, Method: Compositional matrix adjust. Identities = 140/254 (55%), Positives = 167/254 (66%), Gaps = 2/254 (1%)

| Query | 12 | PAAWVERLFGYDWAQQTIGCSDAAVFRLSAQGRPVLFVKTDLSGALNELQDEAARLSWLA | 71 |
| --- | --- | --- | --- |
|  |  | PA+++ L GY W QTIG SDAAV+RL RP LFVKT+ + L EL EA+RL WL |  |
| Sbjct | 14 | PASFMAELRGYSWVLQTIGRSDAAVYRLEDGDRPTLFVKTERTHPLGELAGEASRLRWLR | 73 |
| Query | 72 | TTGVPCAAVLDVVTEAGRDWLLLGEVPGQDLLSSHLAPAEK-VSIMADAMRRLHTLDPAT | 130 |

| Sbjct | 74 | V C +L E GR WLL+ VPG+DL SS AE+ V I ADA+ RLH L+ A ERPVACPEILAEAAEDGRAWLLMSAVPGRDLASSPELSAERIVEIAADALHRLHQLNVAD | 133 |
| --- | --- | --- | --- |
| Query | 131 | CPFDHQAKHRIERARTRMEAGLVDQDDLDEEHQGLAPAELFARLKARMPDGEDLVVTHGD | 190 |
| Sbjct | 134 | CPFDH RI AR RMEAGLVD++D D E G +LF L R P E VV HGD CPFDHTLDRRIALARLRMEAGLVDEEDFDTERLGRTARQLFGELLERRPSDEVPVVAHGD | 193 |
| Query | 191 | ACLPNIMVENGRFSGFIDCGRLGVADRYQDIALATRDIAEELGGEWADRFLVLYGIAAPD | 250 |
| Sbjct | 194 | ACLPN++ NG F+GF+DCGRLG+ADR+QD+ALA R IA LGG+W + F YG A PD ACLPNLLAHNGHFTGFVDCGRLGMADRHQDLALAGRSIAHNLGGQWVEPFFRCYG-AEPD | 252 |
| Query | 251 | SQRIAFYRLLDEFF 264 R+A+YRLLDEFF |  |
| Sbjct | 253 | LDRLAYYRLLDEFF 266 | |

>WP_034081652.1 APH(3')-II family aminoglycoside O-phosphotransferase [Pseudomonas aeruginosa]

VTS38879.1 aminoglycoside 3'-phosphotransferase [Streptococcus dysgalactiae subsp. equisimilis]

KAB0746425.1 APH(3')-II family aminoglycoside O-phosphotransferase [Pseudomonas aeruginosa]

KPD27184.1 aminoglycoside phosphotransferase [Pseudomonas aeruginosa] KQB32424.1 aminoglycoside phosphotransferase [Pseudomonas aeruginosa] KRU84305.1 aminoglycoside phosphotransferase [Pseudomonas aeruginosa]

Length=268

Score = 265 bits (677), Expect = 4e-85, Method: Compositional matrix adjust.

| Identities | | = 140/258 (54%), Positives = 170/258 (66%), Gaps = 5/258 (2%) |  |
| --- | --- | --- | --- |
| Query 11  Sbjct 12 | | SPAAWVERLFGYDWAQQTIGCSDAAVFRLSAQGRPVLFVKTDLSGALNELQDEAARLSWL  +PAAW + L GY W Q GCS AAV RL A RP LFVK + EL DE ARL WL APAAWADYLSGYRWQAQREGCSAAAVHRLDAARRPTLFVKHEALSEQAELPDEIARLRWL | 70  71 |
| Query 71  Sbjct 72 | | ATTGVPCAAVLDVVTEAGRDWLLLGEVPGQDLLS SHLAPAEKVSIMADAMRRLHTL  G+ C VL+ GR WLL+ V G L + L P V ++A A+RRLH L HGAGIDCPQVLNETQGGGRQWLLMSAVAGDTLSALAQRGELEPERLVRLVAAALRRLHDL | 126  131 |
| Query 127  Sbjct 132 | | DPATCPFDHQAKHRIERARTRMEAGLVDQDDLDEEHQGLAPAELFARLKARMPDGEDLVV DPA CPFDH+ + R+E R R+EAGLV++ D D+EH+G + AEL+ L R P EDLVV DPAACPFDHRLERRLESVRRRVEAGLVEETDFDDEHRGRSAAELYRLLLDRRPAVEDLVV | 186  191 |
| Query 187  Sbjct 192 | | THGDACLPNIMVENGRFSGFIDCGRLGVADRYQDIALATRDIAEELGGEWADRFLVLYGI THGDACLPN++ E RFSGFIDCGRLGVADR+QD+ALA RDI +LG WA+ FL YG THGDACLPNLLAEGRRFSGFIDCGRLGVADRHQDLALAARDIETDLGAPWAEAFLEAYG- | 246  250 |
| Query 247 | | AAPDSQRIAFYRLLDEFF 264 D +R+A++RLLDEFF |  |
| Sbjct | 251 | GDIDGERLAYFRLLDEFF 268 | |

>NUS67715.1 aminoglycoside 3'-phosphotransferase [Ensifer adhaerens] Length=263

Score = 264 bits (675), Expect = 5e-85, Method: Compositional matrix adjust.

| Identities | = 131/257 (51%), Positives = 173/257 (67%), Gaps = 0/257 (0%) |  |
| --- | --- | --- |
| Query 8 | HAGSPAAWVERLFGYDWAQQTIGCSDAAVFRLSAQGRPVLFVKTDLSGALNELQDEAARL | 67 |
| Sbjct 7 | H P + + GY W + +G SD+++F L A RP+L +K + +G +E DEAARL HPELPDPFRPLVVGYQWNRDLLGQSDSSIFLLKAPDRPMLVLKLEEAGPFSEFVDEAARL | 66 |
| Query 68 | SWLATTGVPCAAVLDVVTEAGRDWLLLGEVPGQDLLSSHLAPAEKVSIMADAMRRLHTLD | 127 |
| Sbjct 67 | WLA+ +PC V+ A +WLLL V G DL S+ L+P +++ I+ADA+++LH LD DWLASKDMPCPQVIARAFGAETNWLLLQAVEGTDLASTTLSPKDQIVILADALKQLHALD | 126 |
| Query 128 | PATCPFDHQAKHRIERARTRMEAGLVDQDDLDEEHQGLAPAELFARLKARMPDGEDLVVT | 187 |
| Sbjct 127 | A CPFDH+ + RI A+ R +AG++D+ D DE G + A+LFA L+ARMP +LVVT VANCPFDHRIEKRIAIAKARTQAGVIDESDFDETRLGRSAADLFAELQARMPRSGELVVT | 186 |
| Query 188 | HGDACLPNIMVENGRFSGFIDCGRLGVADRYQDIALATRDIAEELGGEWADRFLVLYGIA | 247 |
| Sbjct 187 | HGDACLPNI+ +NGRFSGFIDC RLGVAD YQDIALA R IA LG EW FL YG+  HGDACLPNIIEKNGRFSGFIDCSRLGVADPYQDIALACRSIAHNLGEEWIQPFLTRYGLD | 246 |

```
Query 248 APDSQRIAFYRLLDEFF 264
D+++ FY LLDEFF Sbjct 247 NDDAEKRDFYCLLDEFF 263

>WP_146777229.1 phosphotransferase, partial [Pseudomonas sp. MWU12-2534b]
RBJ77043.1 APH(3') family aminoglycoside O-phosphotransferase, partial [Pseudomonas sp. MWU12-2534b]
Length=128

Score = 259 bits (662), Expect = 6e-85, Method: Compositional matrix adjust. Identities = 128/128 (100%), Positives = 128/128 (100%), Gaps = 0/128 (0%)

Query 1	MIEQDGLHAGSPAAWVERLFGYDWAQQTIGCSDAAVFRLSAQGRPVLFVKTDLSGALNEL 60 MIEQDGLHAGSPAAWVERLFGYDWAQQTIGCSDAAVFRLSAQGRPVLFVKTDLSGALNEL
Sbjct 1	MIEQDGLHAGSPAAWVERLFGYDWAQQTIGCSDAAVFRLSAQGRPVLFVKTDLSGALNEL 60

Query 61	QDEAARLSWLATTGVPCAAVLDVVTEAGRDWLLLGEVPGQDLLSSHLAPAEKVSIMADAM 120 QDEAARLSWLATTGVPCAAVLDVVTEAGRDWLLLGEVPGQDLLSSHLAPAEKVSIMADAM
Sbjct 61	QDEAARLSWLATTGVPCAAVLDVVTEAGRDWLLLGEVPGQDLLSSHLAPAEKVSIMADAM 120

Query 121 RRLHTLDP 128
RRLHTLDP Sbjct 121 RRLHTLDP 128

>MBG6543847.1 APH(3')-II family aminoglycoside O-phosphotransferase [Pseudomonas aeruginosa]
Length=268

Score = 264 bits (675), Expect = 7e-85, Method: Compositional matrix adjust.
```

| Identities | | = 138/258 (53%), Positives = 170/258 (66%), Gaps = 5/258 (2%) |  |
| --- | --- | --- | --- |
| Query 11  Sbjct 12 | | SPAAWVERLFGYDWAQQTIGCSDAAVFRLSAQGRPVLFVKTDLSGALNELQDEAARLSWL  +P+ W + L GY W Q GCS A V RL A RP LFVK ++ A EL E ARL WL APSTWADYLAGYRWRGQGEGCSAATVHRLEAARRPPLFVKQEVLSAHAELPAEIARLRWL | 70  71 |
| Query 71  Sbjct 72 | | ATTGVPCAAVLDVVTEAGRDWLLLGEVPGQDLLS SHLAPAEKVSIMADAMRRLHTL  G+ C VL+ GR WLL+ VPG L + L P V ++A A+RRLH L HGAGIDCPQVLNETQSDGRQWLLMSAVPGDTLSALAQRGELEPERLVRLVAAALRRLHDL | 126  131 |
| Query 127  Sbjct 132 | | DPATCPFDHQAKHRIERARTRMEAGLVDQDDLDEEHQGLAPAELFARLKARMPDGEDLVV DPA CPFDH+ + R++ R R+EAGLVD+ D D++H+G + EL+ L R P GEDLVV DPAACPFDHRLERRLDTVRQRVEAGLVDEADFDDDHRGRSATELYRLLLDRRPAGEDLVV | 186  191 |
| Query 187  Sbjct 192 | | THGDACLPNIMVENGRFSGFIDCGRLGVADRYQDIALATRDIAEELGGEWADRFLVLYGI HGDACLPN++ E RFSGFIDCGRLGVADR+QD+ALA RDI ELG WA+ FLV YG AHGDACLPNLLAEGRRFSGFIDCGRLGVADRHQDLALAARDIEAELGAAWAEAFLVEYG- | 246  250 |
| Query | 247 AAPDSQRIAFYRLLDEFF 264 | | |
|  |  | D +R+A++RLLDEFF |  |
| Sbjct | 251 | GDIDGERLAYFRLLDEFF 268 | |

>WP_003124042.1 APH(3')-II family aminoglycoside O-phosphotransferase [Pseudomonas aeruginosa]

EKA58864.1 aminoglycoside 3'-phosphotransferase type IIb [Pseudomonas aeruginosa ATCC 25324]

Length=268

Score = 264 bits (675), Expect = 7e-85, Method: Compositional matrix adjust. Identities = 138/258 (53%), Positives = 170/258 (66%), Gaps = 5/258 (2%)

| Query | 11 | SPAAWVERLFGYDWAQQTIGCSDAAVFRLSAQGRPVLFVKTDLSGALNELQDEAARLSWL | 70 |
| --- | --- | --- | --- |
| Sbjct | 12 | +P+ W + L GY W Q GCS A V RL RP LFVK ++ A EL E ARL WL APSTWADYLAGYRWRGQGEGCSAATVHRLEVARRPTLFVKQEVLSAHAELPAEIARLRWL | 71 |
| Query | 71 | ATTGVPCAAVLDVVTEAGRDWLLLGEVPGQDLLS SHLAPAEKVSIMADAMRRLHTL | 126 |
| Sbjct | 72 | G+ C VL+ GR WLL+ VPG L + LAPA V ++A A+RRLH L  HGAGIDCPQVLNETQSDGRQWLLMSAVPGDTLSALAQRGELAPARLVRLVAAALRRLHDL | 131 |

| Query | 127 | DPATCPFDHQAKHRIERARTRMEAGLVDQDDLDEEHQGLAPAELFARLKARMPDGEDLVV | 186 |
| --- | --- | --- | --- |
| Sbjct | 132 | DPA CPFDH+ + R++ R R+EAGLVD+ D D++H+G + EL+ L R P EDLVV DPAACPFDHRLERRLDTVRQRVEAGLVDEADFDDDHRGRSATELYRLLLDRRPAVEDLVV | 191 |
| Query | 187 | THGDACLPNIMVENGRFSGFIDCGRLGVADRYQDIALATRDIAEELGGEWADRFLVLYGI | 246 |
| Sbjct | 192 | HGDACLPN++ E RFSGFIDCGRLGVADR+QD+ALA RDI ELG WA+ FLV YG  AHGDACLPNLLAEGRRFSGFIDCGRLGVADRHQDLALAARDIEAELGAAWAEAFLVEYG- | 250 |
| Query | 247 AAPDSQRIAFYRLLDEFF 264 | | |
|  |  | D +R+A++RLLDEFF |  |
| Sbjct | 251 | GDIDGERLAYFRLLDEFF 268 | |

>WP_192450931.1 APH(3') family aminoglycoside O-phosphotransferase [Ensifer sp. ENS09]

MBD9652413.1 aminoglycoside 3'-phosphotransferase [Ensifer sp. ENS09] Length=309

Score = 265 bits (678), Expect = 8e-85, Method: Compositional matrix adjust.

| Identities | | = 135/257 (53%), Positives = 170/257 (66%), Gaps = 0/257 (0%) |  |
| --- | --- | --- | --- |
| Query 8  Sbjct 53 | | HAGSPAAWVERLFGYDWAQQTIGCSDAAVFRLSAQGRPVLFVKTDLSGALNELQDEAARL H PA + + GY W + +G SD+++F L A RP+L +K + +G E DEAARL HPQLPAQFHPFVDGYQWNRDLLGQSDSSIFLLKAPDRPMLVLKLEEAGPFGEFVDEAARL | 67  112 |
| Query 68  Sbjct 113 | | SWLATTGVPCAAVLDVVTEAGRDWLLLGEVPGQDLLSSHLAPAEKVSIMADAMRRLHTLD WLA+ VPC V+ A +WLLL V G DL S+ L+P ++V I+ADA++RLH LD DWLASKDVPCPQVIARSFGAQTNWLLLQAVEGTDLASTTLSPKDQVVILADALKRLHALD | 127  172 |
| Query 128  Sbjct 173 | | PATCPFDHQAKHRIERARTRMEAGLVDQDDLDEEHQGLAPAELFARLKARMPDGEDLVVT A CPFDH+ + RI A+ R +AG++D+ D DE G A+LFA L+ARMP LVVT IANCPFDHRIEKRIAIAKARTQAGVIDESDFDETRLGRTAADLFAELQARMPQAGKLVVT | 187  232 |
| Query 188  Sbjct 233 | | HGDACLPNIMVENGRFSGFIDCGRLGVADRYQDIALATRDIAEELGGEWADRFLVLYGIA HGDACLPNI+ +NG FSGFIDC RLGVAD YQDIALA R +A LG EW FL YGI HGDACLPNIIEKNGLFSGFIDCSRLGVADPYQDIALACRSVAYNLGEEWVQPFLTRYGID | 247  292 |
| Query 248 | | APDSQRIAFYRLLDEFF 264 DS++ FY LLDEFF |  |
| Sbjct | 293 | KDDSEKRDFYCLLDEFF 309 | |

>WP_112947666.1 aminoglycoside 3'-phosphotransferase [Sinorhizobium sp. Sb3] KSV84581.1 hypothetical protein N183_12205 [Sinorhizobium sp. Sb3]

Length=254

Score = 263 bits (673), Expect = 8e-85, Method: Compositional matrix adjust.

| Identities | = 131/244 (54%), Positives = 166/244 (68%), Gaps = 0/244 (0%) |  |
| --- | --- | --- |
| Query 21 | GYDWAQQTIGCSDAAVFRLSAQGRPVLFVKTDLSGALNELQDEAARLSWLATTGVPCAAV | 80 |
| Sbjct 11 | GYDW T+G SD++++ L A RP+L +K + +G +E DEA RL WLA+ G+PC V GYDWNHDTLGQSDSSIYLLKAPDRPMLVLKVEAAGPFSEFVDEAERLDWLASQGMPCPRV | 70 |
| Query 81 | LDVVTEAGRDWLLLGEVPGQDLLSSHLAPAEKVSIMADAMRRLHTLDPATCPFDHQAKHR | 140 |
| Sbjct 71 | + +A +WLLL V G DL S+ L+P +++ I+ADA++RLH LD A CPFDH+ + R IARAFDAETNWLLLQAVEGTDLASTTLSPKDRIVILADALKRLHGLDVADCPFDHRIEKR | 130 |
| Query 141 | IERARTRMEAGLVDQDDLDEEHQGLAPAELFARLKARMPDGEDLVVTHGDACLPNIMVEN | 200 |
| Sbjct 131 | I A R EAGL+D+DD DE G A+LFA L+AR+P LV+THGDACLPNI+ +N IAIAGARTEAGLIDEDDFDETRLGRTAADLFAELRARVPSIGKLVITHGDACLPNIIEKN | 190 |
| Query 201 | GRFSGFIDCGRLGVADRYQDIALATRDIAEELGGEWADRFLVLYGIAAPDSQRIAFYRLL | 260 |
| Sbjct 191 | GRFSGFIDC RLGVAD YQDIALA IA LG EW FL YGI D ++ Y LL GRFSGFIDCSRLGVADPYQDIALACHSIAHNLGEEWVQPFLARYGIDRDDPEKRDLYCLL | 250 |
| Query 261  Sbjct 251 | DEFF 264 DEFF  DEFF 254 |  |

>WP_116800860.1 APH(3')-II family aminoglycoside O-phosphotransferase [Pseudomonas aeruginosa]

NQA24934.1 APH(3')-II family aminoglycoside O-phosphotransferase [Pseudomonas aeruginosa]

Length=268

Score = 264 bits (674), Expect = 9e-85, Method: Compositional matrix adjust.

| Identities | | = 138/258 (53%), Positives = 170/258 (66%), Gaps = 5/258 (2%) |  |
| --- | --- | --- | --- |
| Query 11  Sbjct 12 | | SPAAWVERLFGYDWAQQTIGCSDAAVFRLSAQGRPVLFVKTDLSGALNELQDEAARLSWL  +PA W + L GY W Q GCS A V RL A+ RP LFVK ++ A EL E ARL WL APATWADYLAGYRWRGQGEGCSAATVHRLEAERRPTLFVKQEVLSAHAELPAEIARLRWL | 70  71 |
| Query 71  Sbjct 72 | | ATTGVPCAAVLDVVTEAGRDWLLLGEVPGQDLLS SHLAPAEKVSIMADAMRRLHTL  G+ C VL+ GR WLL+ VPG L + L P V ++A A+RRLH L HGAGIDCPQVLNETQSDGRQWLLMSAVPGDTLSALAQRGELEPERLVRLVAAALRRLHDL | 126  131 |
| Query 127  Sbjct 132 | | DPATCPFDHQAKHRIERARTRMEAGLVDQDDLDEEHQGLAPAELFARLKARMPDGEDLVV DPA CPFDH+ + R++ R R+EAGLVD+ D D++H+G + EL+ L R P EDLVV DPAACPFDHRLERRLDTVRQRVEAGLVDEADFDDDHRGRSATELYRLLLDRRPAVEDLVV | 186  191 |
| Query 187  Sbjct 192 | | THGDACLPNIMVENGRFSGFIDCGRLGVADRYQDIALATRDIAEELGGEWADRFLVLYGI HGDACLPN++ E RFSGFIDCGRLGVADR+QD+ALA RDI ELG WA+ FLV YG AHGDACLPNLLAEGRRFSGFIDCGRLGVADRHQDLALAARDIEAELGAAWAEAFLVEYG- | 246  250 |
| Query | 247 AAPDSQRIAFYRLLDEFF 264 | | |
|  |  | D +R+A++RLLDEFF |  |
| Sbjct | 251 | GDIDGERLAYFRLLDEFF 268 | |

>WP_153517559.1 aminoglycoside 3'-phosphotransferase [Sinorhizobium meliloti]

MQU96279.1 APH(3') family aminoglycoside O-phosphotransferase [Sinorhizobium meliloti]

MQV15377.1 APH(3') family aminoglycoside O-phosphotransferase [Sinorhizobium meliloti]

Length=261

Score = 263 bits (673), Expect = 1e-84, Method: Compositional matrix adjust.

| Identities | = 138/253 (55%), Positives = 163/253 (64%), Gaps = 0/253 (0%) |  |
| --- | --- | --- |
| Query 12 | PAAWVERLFGYDWAQQTIGCSDAAVFRLSAQGRPVLFVKTDLSGALNELQDEAARLSWLA | 71 |
| Sbjct 9 | P A+ L GY + + +G S + +F L AQ RP L +K + SG EL EAARL WLA PPAFRGLLSGYGFERDALGQSASTIFLLKAQSRPFLILKHEPSGPFTELTGEAARLHWLA | 68 |
| Query 72 | TTGVPCAAVLDVVTEAGRDWLLLGEVPGQDLLSSHLAPAEKVSIMADAMRRLHTLDPATC | 131 |
| Sbjct 69 | +PC VL AG WLLL G DL S L AE+++I+A+A+RRLH LDPA C AQDMPCPRVLGHDDHAGETWLLLEAAEGMDLASCRLPHAERIAILAEALRRLHRLDPAAC | 128 |
| Query 132 | PFDHQAKHRIERARTRMEAGLVDQDDLDEEHQGLAPAELFARLKARMPDGEDLVVTHGDA | 191 |
| Sbjct 129 | PFD + I A+ RMEAGLVD+ D DEE QG ELF L AR P E LVVTHGDA PFDRRLTASIAAAKARMEAGLVDESDFDEERQGRQTRELFDELLARRPKEEQLVVTHGDA | 188 |
| Query 192 | CLPNIMVENGRFSGFIDCGRLGVADRYQDIALATRDIAEELGGEWADRFLVLYGIAAPDS | 251 |
| Sbjct 189 | CLPN + +FSGFIDCGRLGVAD YQD+ALA I LG EW FL YG+AAPD CLPNFIAAGNQFSGFIDCGRLGVADIYQDLALACWSITHNLGEEWVAPFLARYGLAAPDP | 248 |
| Query 252  Sbjct 249 | QRIAFYRLLDEFF 264  +++AFYRLLDEFF  EKLAFYRLLDEFF 261 |  |

>WP_070336929.1 APH(3')-II family aminoglycoside O-phosphotransferase [Pseudomonas aeruginosa]

Length=268

Score = 263 bits (673), Expect = 1e-84, Method: Compositional matrix adjust. Identities = 138/258 (53%), Positives = 170/258 (66%), Gaps = 5/258 (2%)

| Query | 11 | SPAAWVERLFGYDWAQQTIGCSDAAVFRLSAQGRPVLFVKTDLSGALNELQDEAARLSWL | 70 |
| --- | --- | --- | --- |
| Sbjct | 12 | +PA W + L GY W Q GCS A V RL A RP LFVK ++ A EL E ARL WL APATWADYLAGYRWRGQGEGCSAATVHRLEAARRPTLFVKQEVLSAHAELPAEIARLRWL | 71 |
| Query | 71 | ATTGVPCAAVLDVVTEAGRDWLLLGEVPGQDLLS SHLAPAEKVSIMADAMRRLHTL  G+ C VL+ GR WLL+ VPG L + + L P V ++A A+RRLH L | 126 |

| Sbjct | 72 | HGAGIDCPQVLNETQSDGRQWLLMSAVPGDTLSALAQRAELEPERLVRLVAAALRRLHDL | 131 |
| --- | --- | --- | --- |
| Query | 127 | DPATCPFDHQAKHRIERARTRMEAGLVDQDDLDEEHQGLAPAELFARLKARMPDGEDLVV | 186 |
| Sbjct | 132 | DPA CPFDH+ + R++ R R+EAGLVD+ D D++H+G + EL+ L R P EDLVV DPAACPFDHRLERRLDTVRQRVEAGLVDEADFDDDHRGRSATELYRLLLDRRPAVEDLVV | 191 |
| Query | 187 | THGDACLPNIMVENGRFSGFIDCGRLGVADRYQDIALATRDIAEELGGEWADRFLVLYGI | 246 |
| Sbjct | 192 | HGDACLPN++ E RFSGFIDCGRLGVADR+QD+ALA RDI ELG WA+ FLV YG  AHGDACLPNLLAEGRRFSGFIDCGRLGVADRHQDLALAARDIEAELGAAWAEAFLVEYG- | 250 |
| Query | 247 AAPDSQRIAFYRLLDEFF 264 | | |
|  |  | D +R+A++RLLDEFF |  |
| Sbjct | 251 | GDIDGERLAYFRLLDEFF 268 | |

>WP_126676646.1 APH(3')-II family aminoglycoside O-phosphotransferase [Pseudomonas aeruginosa]

RUC04631.1 APH(3')-II family aminoglycoside O-phosphotransferase [Pseudomonas aeruginosa]

Length=268

Score = 263 bits (673), Expect = 1e-84, Method: Compositional matrix adjust.

| Identities | | = 138/258 (53%), Positives = 169/258 (66%), Gaps = 5/258 (2%) |  |
| --- | --- | --- | --- |
| Query 11  Sbjct 12 | | SPAAWVERLFGYDWAQQTIGCSDAAVFRLSAQGRPVLFVKTDLSGALNELQDEAARLSWL  +PA W + L GY W Q GCS A V RL A RP LFVK ++ A EL E ARL WL APATWADYLAGYRWRGQGEGCSAATVHRLEAARRPTLFVKQEVLSAHAELPAEIARLRWL | 70  71 |
| Query 71  Sbjct 72 | | ATTGVPCAAVLDVVTEAGRDWLLLGEVPGQDLLS SHLAPAEKVSIMADAMRRLHTL  G+ C VL+ GR WLL+ VPG L + L P V ++A A+RRLH L HGAGIDCPQVLNETQSDGRQWLLMSAVPGDTLSALAQRGELEPERLVRLVAAALRRLHDL | 126  131 |
| Query 127  Sbjct 132 | | DPATCPFDHQAKHRIERARTRMEAGLVDQDDLDEEHQGLAPAELFARLKARMPDGEDLVV DPA CPFDH+ + R++ R R+EAGLVD+ D D++H+G + EL+ L R P EDLVV DPAACPFDHRLERRLDTVRQRVEAGLVDEADFDDDHRGRSATELYRLLLDRRPAVEDLVV | 186  191 |
| Query 187  Sbjct 192 | | THGDACLPNIMVENGRFSGFIDCGRLGVADRYQDIALATRDIAEELGGEWADRFLVLYGI HGDACLPN++ E RFSGFIDCGRLGVADR+QD+ALA RDI ELG WA+ FLV YG AHGDACLPNLLAEGRRFSGFIDCGRLGVADRHQDLALAARDIEAELGAAWAEAFLVEYG- | 246  250 |
| Query | 247 AAPDSQRIAFYRLLDEFF 264 | | |
|  |  | D +R+A++RLLDEFF |  |
| Sbjct | 251 | GDIDGERLAYFRLLDEFF 268 | |

>WP_146722387.1 aminoglycoside 3'-phosphotransferase [Sinorhizobium meliloti] RVH00035.1 aminoglycoside 3'-phosphotransferase [Sinorhizobium meliloti] RVH64027.1 aminoglycoside 3'-phosphotransferase [Sinorhizobium meliloti]

Length=261

Score = 263 bits (672), Expect = 1e-84, Method: Compositional matrix adjust.

| Identities | = 139/253 (55%), Positives = 163/253 (64%), Gaps = 0/253 (0%) |  |
| --- | --- | --- |
| Query 12 | PAAWVERLFGYDWAQQTIGCSDAAVFRLSAQGRPVLFVKTDLSGALNELQDEAARLSWLA | 71 |
| Sbjct 9 | P A+ L GY + + +G S + +F L AQ RP L +K + SG EL EAARL WLA PPAFRGLLSGYGFERDALGQSASTIFLLKAQSRPFLILKHEPSGPFTELTGEAARLHWLA | 68 |
| Query 72 | TTGVPCAAVLDVVTEAGRDWLLLGEVPGQDLLSSHLAPAEKVSIMADAMRRLHTLDPATC | 131 |
| Sbjct 69 | +PC VL AG WLLL G DL S L AE+++I+A+A+RRLH LDPA C AQDMPCPRVLGHDDHAGETWLLLEAAEGMDLASCRLPHAERIAILAEALRRLHRLDPAAC | 128 |
| Query 132 | PFDHQAKHRIERARTRMEAGLVDQDDLDEEHQGLAPAELFARLKARMPDGEDLVVTHGDA | 191 |
| Sbjct 129 | PFD + I A+ RMEAGLVD+ D DEE QG ELF L AR P E LVVTHGDA PFDRRLTASIAAAKARMEAGLVDESDFDEERQGRQTRELFDELLARRPKEEQLVVTHGDA | 188 |
| Query 192 | CLPNIMVENGRFSGFIDCGRLGVADRYQDIALATRDIAEELGGEWADRFLVLYGIAAPDS | 251 |
| Sbjct 189 | CLPN + +FSGFIDCGRLGVAD YQDIALA I LG EW FL YG+AAPD CLPNFIAAGNQFSGFIDCGRLGVADIYQDIALACWSITHNLGEEWVAPFLDRYGLAAPDP | 248 |
| Query 252  Sbjct 249 | QRIAFYRLLDEFF 264  +++AFYRLLDEFF  EKLAFYRLLDEFF 261 |  |

>WP_070656501.1 APH(3')-II family aminoglycoside O-phosphotransferase [Pseudomonas aeruginosa]

OFJ88501.1 APH(3') family aminoglycoside O-phosphotransferase [Pseudomonas aeruginosa]

Length=267

Score = 263 bits (673), Expect = 1e-84, Method: Compositional matrix adjust.

| Identities | | = 137/258 (53%), Positives = 170/258 (66%), Gaps = 5/258 (2%) |  |
| --- | --- | --- | --- |
| Query 11  Sbjct 11 | | SPAAWVERLFGYDWAQQTIGCSDAAVFRLSAQGRPVLFVKTDLSGALNELQDEAARLSWL  +P+ W + L GY W Q GCS A V RL A+ RP LFVK ++ A EL E ARL WL APSTWADYLAGYRWRGQGEGCSAATVHRLEAERRPTLFVKQEVLSAHAELPAEIARLRWL | 70  70 |
| Query 71  Sbjct 71 | | ATTGVPCAAVLDVVTEAGRDWLLLGEVPGQDLLS SHLAPAEKVSIMADAMRRLHTL  G+ C VL+ GR WLL+ VPG L + L P V ++A A+RRLH L HGAGIDCPQVLNETQSDGRQWLLMSAVPGDTLSALAQRGELEPERLVRLVAAALRRLHDL | 126  130 |
| Query 127  Sbjct 131 | | DPATCPFDHQAKHRIERARTRMEAGLVDQDDLDEEHQGLAPAELFARLKARMPDGEDLVV DPA CPFDH+ + R++ R R+EAGLVD+ D D++H+G + EL+ L R P EDLVV DPAACPFDHRLERRLDTVRQRVEAGLVDEADFDDDHRGRSATELYLLLLDRRPAVEDLVV | 186  190 |
| Query 187  Sbjct 191 | | THGDACLPNIMVENGRFSGFIDCGRLGVADRYQDIALATRDIAEELGGEWADRFLVLYGI HGDACLPN++ E RFSGFIDCGRLGVADR+QD+ALA RDI ELG WA+ FLV YG AHGDACLPNLLAEGRRFSGFIDCGRLGVADRHQDLALAARDIEAELGAAWAEAFLVEYG- | 246  249 |
| Query | 247 AAPDSQRIAFYRLLDEFF 264 | | |
|  |  | D +R+A++RLLDEFF |  |
| Sbjct | 250 | GDIDGERLAYFRLLDEFF 267 | |

>WP_096246790.1 APH(3')-II family aminoglycoside O-phosphotransferase [Pseudomonas aeruginosa]

NPZ13701.1 APH(3')-II family aminoglycoside O-phosphotransferase [Pseudomonas aeruginosa]

PBV37646.1 APH(3') family aminoglycoside O-phosphotransferase [Pseudomonas aeruginosa]

Length=268

Score = 263 bits (673), Expect = 1e-84, Method: Compositional matrix adjust.

| Identities | | = 138/258 (53%), Positives = 169/258 (66%), Gaps = 5/258 (2%) |  |
| --- | --- | --- | --- |
| Query 11  Sbjct 12 | | SPAAWVERLFGYDWAQQTIGCSDAAVFRLSAQGRPVLFVKTDLSGALNELQDEAARLSWL  +PA W + L GY W Q GCS A V RL A RP LFVK ++ A EL E ARL WL APATWTDYLAGYRWRGQGEGCSAATVHRLEAARRPTLFVKQEVLSAHAELPAEIARLRWL | 70  71 |
| Query 71  Sbjct 72 | | ATTGVPCAAVLDVVTEAGRDWLLLGEVPGQDLLS SHLAPAEKVSIMADAMRRLHTL  G+ C VL+ GR WLL+ VPG L + L P V ++A A+RRLH L HGAGIDCPQVLNETQSDGRQWLLMSAVPGDTLSALAQRGELEPERLVRLVAAALRRLHDL | 126  131 |
| Query 127  Sbjct 132 | | DPATCPFDHQAKHRIERARTRMEAGLVDQDDLDEEHQGLAPAELFARLKARMPDGEDLVV DPA CPFDH+ + R++ R R+EAGLVD+ D D++H+G + EL+ L R P EDLVV DPAACPFDHRLERRLDTVRQRVEAGLVDEADFDDDHRGRSATELYRLLLDRRPAVEDLVV | 186  191 |
| Query 187  Sbjct 192 | | THGDACLPNIMVENGRFSGFIDCGRLGVADRYQDIALATRDIAEELGGEWADRFLVLYGI HGDACLPN++ E RFSGFIDCGRLGVADR+QD+ALA RDI ELG WA+ FLV YG AHGDACLPNLLAEGRRFSGFIDCGRLGVADRHQDLALAARDIEAELGAAWAEAFLVEYG- | 246  250 |
| Query | 247 AAPDSQRIAFYRLLDEFF 264 | | |
|  |  | D +R+A++RLLDEFF |  |
| Sbjct | 251 | GDIDGERLAYFRLLDEFF 268 | |

>WP_023518031.1 MULTISPECIES: APH(3')-II family aminoglycoside O-phosphotransferase [Pseudomonas]

KEA21858.1 aminoglycoside phosphotransferase [Pseudomonas aeruginosa C1913C] AHA17963.1 aminoglycoside phosphotransferase [Pseudomonas aeruginosa PA1] AHA23762.1 Aminoglycoside 3'-phosphotransferase type IIb [Pseudomonas aeruginosa

PA1R]

ALE46674.1 aminoglycoside phosphotransferase [Pseudomonas aeruginosa] AWZ94281.1 phosphotransferase enzyme family protein [Pseudomonas aeruginosa]

Length=268

Score = 263 bits (673), Expect = 1e-84, Method: Compositional matrix adjust.

| Identities | | = 138/258 (53%), Positives = 169/258 (66%), Gaps = 5/258 (2%) |  |
| --- | --- | --- | --- |
| Query 11  Sbjct 12 | | SPAAWVERLFGYDWAQQTIGCSDAAVFRLSAQGRPVLFVKTDLSGALNELQDEAARLSWL  +PA W + L GY W Q GCS A V RL A RP LFVK ++ A EL E ARL WL APATWADYLAGYRWRGQGEGCSAATVHRLEAARRPTLFVKQEVLSAHAELPAEIARLRWL | 70  71 |
| Query 71  Sbjct 72 | | ATTGVPCAAVLDVVTEAGRDWLLLGEVPGQDLLS SHLAPAEKVSIMADAMRRLHTL  G+ C VL+ GR WLL+ VPG L + L P V ++A A+RRLH L HGAGIDCPQVLNETQSDGRQWLLMSAVPGDTLSALAQRGELEPGRLVRLVAAALRRLHDL | 126  131 |
| Query 127  Sbjct 132 | | DPATCPFDHQAKHRIERARTRMEAGLVDQDDLDEEHQGLAPAELFARLKARMPDGEDLVV DPA CPFDH+ + R++ R R+EAGLVD+ D D++H+G + EL+ L R P EDLVV DPAACPFDHRLERRLDTVRQRVEAGLVDEADFDDDHRGRSATELYRLLLDRRPAVEDLVV | 186  191 |
| Query 187  Sbjct 192 | | THGDACLPNIMVENGRFSGFIDCGRLGVADRYQDIALATRDIAEELGGEWADRFLVLYGI HGDACLPN++ E RFSGFIDCGRLGVADR+QD+ALA RDI ELG WA+ FLV YG AHGDACLPNLLAEGRRFSGFIDCGRLGVADRHQDLALAARDIEAELGAAWAEAFLVEYG- | 246  250 |
| Query | 247 AAPDSQRIAFYRLLDEFF 264 | | |
|  |  | D +R+A++RLLDEFF |  |
| Sbjct | 251 | GDIDGERLAYFRLLDEFF 268 | |

>WP_035095871.1 aminoglycoside 3'-phosphotransferase [Devosia sp. 17-2-E-8] KFL24893.1 aminoglycoside phosphotransferase [Devosia sp. 17-2-E-8]

Length=264

Score = 263 bits (672), Expect = 2e-84, Method: Compositional matrix adjust.

| Identities | | = 139/259 (54%), Positives = 169/259 (65%), Gaps = 3/259 (1%) |  |
| --- | --- | --- | --- |
| Query 8  Sbjct 7 | | HAGS-PAAWVERLFGYDWAQQTIGCSDAAVFRLSAQGRPVLFVKTDLSGALNELQDEAAR HA + P W + L GY+W QTIG S A VFRL+A P LFVKT+ +EL+ EA R HASNLPPGWPD-LSGYEWVPQTIGQSGAMVFRLTAPQGPALFVKTESVSPHSELEGEAQR | 66  65 |
| Query 67  Sbjct 66 | | LSWLATTGVPCAAVLDVVTEAGRDWLLLGEVPGQDLLSS-HLAPAEKVSIMADAMRRLHT L WLA+ P VL GR WLL+ V G DL SS L+P+ + I A ++R LH LEWLASVDQPAPRVLGTAQHLGRQWLLMTGVEGLDLASSPDLSPSRIIEICATSLRALHA | 125  125 |
| Query 126  Sbjct 126 | | LDPATCPFDHQAKHRIERARTRMEAGLVDQDDLDEEHQGLAPAELFARLKARMPDGEDLV  +D A+CPFDH+ + AR R++AGLVD+ D D+ H G+ L+ARL A P+ EDLV IDVASCPFDHRLDTTLATARERLDAGLVDETDFDDAHLGMPARTLYARLLAERPETEDLV | 185  185 |
| Query 186  Sbjct 186 | | VTHGDACLPNIMVENGRFSGFIDCGRLGVADRYQDIALATRDIAEELGGEWADRFLVLYG VTHGDACLPN M + FSGFIDCGRLGVADRYQD+ALATR IA LG W + FL YG VTHGDACLPNFMASSREFSGFIDCGRLGVADRYQDLALATRSIAFNLGQVWVEPFLTAYG | 245  245 |
| Query 246 | | IAAPDSQRIAFYRLLDEFF 264  + PD RI +YRLLDEFF |  |
| Sbjct | 246 | ASQPDRARIGYYRLLDEFF 264 | |

```
>WP_003157056.1 APH(3')-II family aminoglycoside O-phosphotransferase [Pseudomonas aeruginosa]
KFF36384.1 aminoglycoside phosphotransferase [Pseudomonas aeruginosa VRFPA01] ABR86926.1 aminoglycoside 3-phosphotransferase type IIb [Pseudomonas aeruginosa
PA7]
AVK03675.1 phosphotransferase enzyme family protein [Pseudomonas aeruginosa]
AVR66199.1 APH(3')-II family aminoglycoside O-phosphotransferase [Pseudomonas aeruginosa]
AWE89163.1 phosphotransferase enzyme family protein [Pseudomonas aeruginosa] Length=268

Score = 263 bits (673), Expect = 2e-84, Method: Compositional matrix adjust. Identities = 139/258 (54%), Positives = 169/258 (66%), Gaps = 5/258 (2%)

Query 11	SPAAWVERLFGYDWAQQTIGCSDAAVFRLSAQGRPVLFVKTDLSGALNELQDEAARLSWL 70
+PAAW + L GY W Q GCS AAV RL A RP LFVK +	EL DE ARL WL Sbjct 12	APAAWADYLSGYRWQAQREGCSAAAVHRLDAARRPTLFVKHEALSEQAELPDEIARLRWL 71
```

| Query | 71 | ATTGVPCAAVLDVVTEAGRDWLLLGEVPGQDLLS SHLAPAEKVSIMADAMRRLHTL | 126 |
| --- | --- | --- | --- |
| Sbjct | 72 | G+ C VL+ GR WLL+ V G L + L P V ++A A+RRLH L HGAGIDCPQVLNETQGGGRQWLLMSAVAGDTLSALAQRGELEPERLVRLVAAALRRLHDL | 131 |
| Query | 127 | DPATCPFDHQAKHRIERARTRMEAGLVDQDDLDEEHQGLAPAELFARLKARMPDGEDLVV | 186 |
| Sbjct | 132 | DPA CPFDH+ + R+E R R+EAGLV++ D D+EH+G + EL+ L R P EDLVV DPAACPFDHRLERRLESVRRRVEAGLVEETDFDDEHRGRSAVELYRLLLDRRPAVEDLVV | 191 |
| Query | 187 | THGDACLPNIMVENGRFSGFIDCGRLGVADRYQDIALATRDIAEELGGEWADRFLVLYGI | 246 |
| Sbjct | 192 | THGDACLPN++ E RFSGFIDCGRLGVADR+QD+ALA RDI +LG WA+ FL YG THGDACLPNLLAEGRRFSGFIDCGRLGVADRHQDLALAARDIETDLGAPWAEAFLEAYG- | 250 |
| Query | 247 | AAPDSQRIAFYRLLDEFF 264 D +R+A++RLLDEFF |  |
| Sbjct | 251 | GDIDGERLAYFRLLDEFF 268 | |

>WP_033969574.1 APH(3')-II family aminoglycoside O-phosphotransferase [Pseudomonas aeruginosa]

KSS20900.1 aminoglycoside phosphotransferase APH(3') [Pseudomonas aeruginosa]

MBG5302359.1 APH(3')-II family aminoglycoside O-phosphotransferase [Pseudomonas aeruginosa]

NNB82886.1 APH(3')-II family aminoglycoside O-phosphotransferase [Pseudomonas aeruginosa]

QBL16260.1 APH(3')-II family aminoglycoside O-phosphotransferase [Pseudomonas aeruginosa]

RIZ35999.1 aminoglycoside O-phosphotransferase APH(3')-IIb [Pseudomonas aeruginosa]

Length=268

Score = 263 bits (673), Expect = 2e-84, Method: Compositional matrix adjust.

| Identities | | = 138/258 (53%), Positives = 169/258 (66%), Gaps = 5/258 (2%) |  |
| --- | --- | --- | --- |
| Query 11  Sbjct 12 | | SPAAWVERLFGYDWAQQTIGCSDAAVFRLSAQGRPVLFVKTDLSGALNELQDEAARLSWL  +PA W + L GY W Q GCS A V RL A RP LFVK ++ A EL E ARL WL APATWADYLAGYRWRGQGEGCSAATVHRLEAARRPTLFVKQEVLSAHAELPAEIARLRWL | 70  71 |
| Query 71  Sbjct 72 | | ATTGVPCAAVLDVVTEAGRDWLLLGEVPGQDLLS SHLAPAEKVSIMADAMRRLHTL  G+ C VL+ GR WLL+ VPG L + L P V ++A A+RRLH L HGAGIDCPQVLNETQSDGRQWLLMSAVPGDTLSALAQCGELEPGRLVRLVAAALRRLHDL | 126  131 |
| Query 127  Sbjct 132 | | DPATCPFDHQAKHRIERARTRMEAGLVDQDDLDEEHQGLAPAELFARLKARMPDGEDLVV DPA CPFDH+ + R++ R R+EAGLVD+ D D++H+G + EL+ L R P EDLVV DPAACPFDHRLERRLDTVRQRVEAGLVDEADFDDDHRGRSATELYRLLLDRRPAVEDLVV | 186  191 |
| Query 187  Sbjct 192 | | THGDACLPNIMVENGRFSGFIDCGRLGVADRYQDIALATRDIAEELGGEWADRFLVLYGI HGDACLPN++ E RFSGFIDCGRLGVADR+QD+ALA RDI ELG WA+ FLV YG AHGDACLPNLLAEGRRFSGFIDCGRLGVADRHQDLALAARDIEAELGAAWAEAFLVEYG- | 246  250 |
| Query | 247 AAPDSQRIAFYRLLDEFF 264 | | |
|  |  | D +R+A++RLLDEFF |  |
| Sbjct | 251 | GDIDGERLAYFRLLDEFF 268 | |

>WP_149586404.1 APH(3')-II family aminoglycoside O-phosphotransferase [Pseudomonas aeruginosa]

QEK84358.1 APH(3')-II family aminoglycoside O-phosphotransferase [Pseudomonas aeruginosa]

Length=268

Score = 263 bits (673), Expect = 2e-84, Method: Compositional matrix adjust. Identities = 137/258 (53%), Positives = 169/258 (66%), Gaps = 5/258 (2%)

| Query | 11 | SPAAWVERLFGYDWAQQTIGCSDAAVFRLSAQGRPVLFVKTDLSGALNELQDEAARLSWL | 70 |
| --- | --- | --- | --- |
| Sbjct | 12 | +P+ W + L GY W Q GCS A V RL A RP LFVK ++ L EL E ARL WL APSTWADYLAGYRWRGQGEGCSAATVHRLEAARRPTLFVKQEVLSVLAELPAEIARLRWL | 71 |
| Query | 71 | ATTGVPCAAVLDVVTEAGRDWLLLGEVPGQDLLS SHLAPAEKVSIMADAMRRLHTL | 126 |
| Sbjct | 72 | G+ C VL+ GR WLL+ VPG L + L P V ++A A+RRLH L HGAGIDCPQVLNETQSDGRQWLLMSAVPGDTLSALAQRGELEPERLVRLVAAALRRLHDL | 131 |
| Query | 127 | DPATCPFDHQAKHRIERARTRMEAGLVDQDDLDEEHQGLAPAELFARLKARMPDGEDLVV | 186 |

| Sbjct | 132 | DPA CPFDH+ + R++ R R+EAGLVD+ D D++H+G + EL+ L R P EDLVV DPAACPFDHRLERRLDTVRQRVEAGLVDEADFDDDHRGRSATELYRLLLDRRPAVEDLVV | 191 |
| --- | --- | --- | --- |
| Query | 187 | THGDACLPNIMVENGRFSGFIDCGRLGVADRYQDIALATRDIAEELGGEWADRFLVLYGI | 246 |
| Sbjct | 192 | HGDACLPN++ E RFSGFIDCGRLGVADR+QD+ALA RDI ELG WA+ FLV YG  AHGDACLPNLLAEGRRFSGFIDCGRLGVADRHQDLALAARDIEAELGAAWAEAFLVEYG- | 250 |
| Query | 247 AAPDSQRIAFYRLLDEFF 264 | | |
|  |  | D +R+A++RLLDEFF |  |
| Sbjct | 251 | GDIDGERLAYFRLLDEFF 268 | |

>WP_163802427.1 APH(3')-II family aminoglycoside O-phosphotransferase [Pseudomonas aeruginosa]

NDU60219.1 APH(3')-II family aminoglycoside O-phosphotransferase [Pseudomonas aeruginosa]

Length=267

Score = 263 bits (672), Expect = 2e-84, Method: Compositional matrix adjust.

| Identities | | = 138/258 (53%), Positives = 169/258 (66%), Gaps = 5/258 (2%) |  |
| --- | --- | --- | --- |
| Query 11  Sbjct 11 | | SPAAWVERLFGYDWAQQTIGCSDAAVFRLSAQGRPVLFVKTDLSGALNELQDEAARLSWL  +PA W + L GY W Q GCS A V RL A RP LFVK ++ A EL E ARL WL APATWADYLAGYRWRGQGEGCSAATVHRLEAARRPTLFVKQEVLSAHAELPAEIARLRWL | 70  70 |
| Query 71  Sbjct 71 | | ATTGVPCAAVLDVVTEAGRDWLLLGEVPGQDLLS SHLAPAEKVSIMADAMRRLHTL  G+ C VL+ GR WLL+ VPG L + L P V ++A A+RRLH L HGAGIDCPQVLNETQSDGRQWLLMSAVPGDTLSALAQRGELEPERLVRLVAAALRRLHDL | 126  130 |
| Query 127  Sbjct 131 | | DPATCPFDHQAKHRIERARTRMEAGLVDQDDLDEEHQGLAPAELFARLKARMPDGEDLVV DPA CPFDH+ + R++ R R+EAGLVD+ D D++H+G + EL+ L R P EDLVV DPAACPFDHRLERRLDTVRQRVEAGLVDEADFDDDHRGRSATELYRLLLDRRPAVEDLVV | 186  190 |
| Query 187  Sbjct 191 | | THGDACLPNIMVENGRFSGFIDCGRLGVADRYQDIALATRDIAEELGGEWADRFLVLYGI HGDACLPN++ E RFSGFIDCGRLGVADR+QD+ALA RDI ELG WA+ FLV YG AHGDACLPNLLAEGRRFSGFIDCGRLGVADRHQDLALAARDIEAELGAAWAEAFLVEYG- | 246  249 |
| Query | 247 AAPDSQRIAFYRLLDEFF 264 | | |
|  |  | D +R+A++RLLDEFF |  |
| Sbjct | 250 | GDIDGERLAYFRLLDEFF 267 | |

>WP_124988352.1 APH(3')-II family aminoglycoside O-phosphotransferase [Pseudomonas aeruginosa]

RRJ00780.1 APH(3')-II family aminoglycoside O-phosphotransferase [Pseudomonas aeruginosa]

Length=268

Score = 263 bits (672), Expect = 2e-84, Method: Compositional matrix adjust.

| Identities | = 139/259 (54%), Positives = 172/259 (66%), Gaps = 7/259 (3%) |  |
| --- | --- | --- |
| Query 11 | SPAAWVERLFGYDWAQQTIGCSDAAVFRLSAQGRPVLFVKTDLSGALNELQDEAARLSWL | 70 |
| Sbjct 12 | +P+ W + L GY W Q GCS A V RL A+ RP LFVK ++ A EL E ARL WL APSTWADYLAGYRWRGQGEGCSAATVHRLEAERRPTLFVKQEVLSAHAELPAEIARLRWL | 71 |
| Query 71 | ATTGVPCAAVLDVVTEAGRDWLLLGEVPGQDLLSS HLAPAEKVSIMADAMRRLHT | 125 |
| Sbjct 72 | G+ C VL+ GR WLL+ VPG D LS+ L P V ++A A+RRLH HGAGIDCPQVLNETQSDGRQWLLMSAVPG-DALSALAQRGELEPERLVRLVAAALRRLHD | 130 |
| Query 126 | LDPATCPFDHQAKHRIERARTRMEAGLVDQDDLDEEHQGLAPAELFARLKARMPDGEDLV | 185 |
| Sbjct 131 | LDPA CPFDH+ + R++ R R+EAGLVD+ D D++H+G + EL+ L R P EDLV LDPAACPFDHRLERRLDTVRQRVEAGLVDEADFDDDHRGRSATELYRLLLDRRPAVEDLV | 190 |
| Query 186 | VTHGDACLPNIMVENGRFSGFIDCGRLGVADRYQDIALATRDIAEELGGEWADRFLVLYG | 245 |
| Sbjct 191 | V HGDACLPN++ E RFSGFIDCGRLGVADR+QD+ALA RDI ELG WA+ FLV YG VAHGDACLPNLLAEGRRFSGFIDCGRLGVADRHQDLALAARDIEAELGAAWAEAFLVEYG | 250 |
| Query 246  Sbjct 251 | IAAPDSQRIAFYRLLDEFF 264 D +R+A++RLLDEFF  -GDIDGERLAYFRLLDEFF 268 |  |

>WP_033977771.1 APH(3')-II family aminoglycoside O-phosphotransferase [Pseudomonas aeruginosa]

Length=268

Score = 263 bits (672), Expect = 2e-84, Method: Compositional matrix adjust.

| Identities | | = 138/258 (53%), Positives = 170/258 (66%), Gaps = 5/258 (2%) |  |
| --- | --- | --- | --- |
| Query 11  Sbjct 12 | | SPAAWVERLFGYDWAQQTIGCSDAAVFRLSAQGRPVLFVKTDLSGALNELQDEAARLSWL  +PA W + L GY W Q GCS A V RL A RP LFVK ++ A EL E ARL WL APATWDDYLAGYRWRGQGEGCSAATVHRLEAARRPTLFVKQEVLSAHAELPAEIARLRWL | 70  71 |
| Query 71  Sbjct 72 | | ATTGVPCAAVLDVVTEAGRDWLLLGEVPGQDLLS SHLAPAEKVSIMADAMRRLHTL  G+ C VL+ GR WLL+ VPG L + L P V ++A A+RRLH L HGAGIDCPQVLNETQSDGRQWLLMSAVPGDTLSALAQRGELEPGRLVRLVAAALRRLHDL | 126  131 |
| Query 127  Sbjct 132 | | DPATCPFDHQAKHRIERARTRMEAGLVDQDDLDEEHQGLAPAELFARLKARMPDGEDLVV DPA CPFDH+ + R++ R R+EAGLVD+ D D++H+G + +EL+ L R P EDLVV DPAACPFDHRLERRLDTVRQRVEAGLVDEADFDDDHRGRSASELYRLLLDRRPAVEDLVV | 186  191 |
| Query 187  Sbjct 192 | | THGDACLPNIMVENGRFSGFIDCGRLGVADRYQDIALATRDIAEELGGEWADRFLVLYGI HGDACLPN++ E RFSGFIDCGRLGVADR+QD+ALA RDI ELG WA+ FLV YG AHGDACLPNLLAEGRRFSGFIDCGRLGVADRHQDLALAARDIEAELGAAWAEAFLVEYG- | 246  250 |
| Query | 247 AAPDSQRIAFYRLLDEFF 264 | | |
|  |  | D +R+A++RLLDEFF |  |
| Sbjct | 251 | GDIDGERLAYFRLLDEFF 268 | |

>WP_109242846.1 APH(3')-II family aminoglycoside O-phosphotransferase [Pseudomonas aeruginosa]

Length=268

Score = 263 bits (672), Expect = 2e-84, Method: Compositional matrix adjust.

| Identities | | = 138/258 (53%), Positives = 169/258 (66%), Gaps = 5/258 (2%) |  |
| --- | --- | --- | --- |
| Query 11  Sbjct 12 | | SPAAWVERLFGYDWAQQTIGCSDAAVFRLSAQGRPVLFVKTDLSGALNELQDEAARLSWL  +PA W + L GY W Q GCS A V RL A RP LFVK ++ A EL E ARL WL APATWADYLAGYRWQGQGEGCSAATVHRLEAARRPTLFVKQEVLSAHAELPAEIARLRWL | 70  71 |
| Query 71  Sbjct 72 | | ATTGVPCAAVLDVVTEAGRDWLLLGEVPGQDLLS SHLAPAEKVSIMADAMRRLHTL  G+ C VL+ GR WLL+ VPG L + L P V ++A A+RRLH L HGAGIDCPQVLNETQSDGRQWLLMSAVPGDTLSALAQRGELEPERLVRLVAAALRRLHDL | 126  131 |
| Query 127  Sbjct 132 | | DPATCPFDHQAKHRIERARTRMEAGLVDQDDLDEEHQGLAPAELFARLKARMPDGEDLVV DPA CPFDH+ + R++ R R+EAGLVD+ D D++H+G + EL+ L R P EDLVV DPAACPFDHRLERRLDTVRQRVEAGLVDEADFDDDHRGRSATELYRLLLDRRPAVEDLVV | 186  191 |
| Query 187  Sbjct 192 | | THGDACLPNIMVENGRFSGFIDCGRLGVADRYQDIALATRDIAEELGGEWADRFLVLYGI HGDACLPN++ E RFSGFIDCGRLGVADR+QD+ALA RDI ELG WA+ FLV YG AHGDACLPNLLAEGRRFSGFIDCGRLGVADRHQDLALAARDIEAELGAAWAEAFLVEYG- | 246  250 |
| Query | 247 AAPDSQRIAFYRLLDEFF 264 | | |
|  |  | D +R+A++RLLDEFF |  |
| Sbjct | 251 | GDIDGERLAYFRLLDEFF 268 | |

>WP_134622164.1 APH(3')-II family aminoglycoside O-phosphotransferase [Pseudomonas aeruginosa]

Length=267

Score = 263 bits (672), Expect = 2e-84, Method: Compositional matrix adjust. Identities = 137/258 (53%), Positives = 170/258 (66%), Gaps = 5/258 (2%)

| Query | 11 | SPAAWVERLFGYDWAQQTIGCSDAAVFRLSAQGRPVLFVKTDLSGALNELQDEAARLSWL | 70 |
| --- | --- | --- | --- |
| Sbjct | 11 | +P+ W + L GY W Q GCS A V RL A+ RP LFVK ++ A EL E ARL WL APSTWADYLAGYRWRGQGEGCSAATVHRLEAERRPTLFVKQEVLSAHAELPAEIARLRWL | 70 |
| Query | 71 | ATTGVPCAAVLDVVTEAGRDWLLLGEVPGQDLLS SHLAPAEKVSIMADAMRRLHTL | 126 |
| Sbjct | 71 | G+ C VL+ GR WLL+ VPG L + L P V ++A A+RRLH L HGAGIDCPQVLNETQSDGRQWLLMSAVPGDTLSALAQRGELEPERLVRLVAAALRRLHDL | 130 |
| Query | 127 | DPATCPFDHQAKHRIERARTRMEAGLVDQDDLDEEHQGLAPAELFARLKARMPDGEDLVV | 186 |

| Sbjct | 131 | DPA CPFDH+ + R++ R R+EAGLVD+ D D++H+G + EL+ L R P EDLVV DPAACPFDHRLERRLDTVRQRVEAGLVDEADFDDDHRGRSATELYRLLLDRRPAVEDLVV | 190 |
| --- | --- | --- | --- |
| Query | 187 | THGDACLPNIMVENGRFSGFIDCGRLGVADRYQDIALATRDIAEELGGEWADRFLVLYGI | 246 |
| Sbjct | 191 | HGDACLPN++ E RFSGFIDCGRLGVADR+QD+ALA RDI ELG WA+ FLV YG  AHGDACLPNLLAEGRRFSGFIDCGRLGVADRHQDLALAARDIEAELGAAWAEAFLVEYG- | 249 |
| Query | 247 AAPDSQRIAFYRLLDEFF 264 | | |
|  |  | D +R+A++RLLDEFF |  |
| Sbjct | 250 | GDIDGERLAYFRLLDEFF 267 | |

>WP_196701535.1 APH(3')-II family aminoglycoside O-phosphotransferase [Pseudomonas aeruginosa]

MBG4656051.1 APH(3')-II family aminoglycoside O-phosphotransferase [Pseudomonas aeruginosa]

Length=267

Score = 263 bits (672), Expect = 2e-84, Method: Compositional matrix adjust.

| Identities | | = 137/258 (53%), Positives = 170/258 (66%), Gaps = 5/258 (2%) |  |
| --- | --- | --- | --- |
| Query 11  Sbjct 11 | | SPAAWVERLFGYDWAQQTIGCSDAAVFRLSAQGRPVLFVKTDLSGALNELQDEAARLSWL  +P+ W + L GY W Q GCS A V RL A+ RP LFVK ++ A EL E ARL WL APSTWADYLAGYRWRGQGEGCSAATVHRLEAERRPTLFVKQEVLSAHAELPAEIARLRWL | 70  70 |
| Query 71  Sbjct 71 | | ATTGVPCAAVLDVVTEAGRDWLLLGEVPGQDLLS SHLAPAEKVSIMADAMRRLHTL  G+ C VL+ GR WLL+ VPG L + L P V ++A A+RRLH L HGAGIDCPQVLNETQSDGRQWLLMSAVPGDTLSALAQRGELEPERLVRLVAAALRRLHDL | 126  130 |
| Query 127  Sbjct 131 | | DPATCPFDHQAKHRIERARTRMEAGLVDQDDLDEEHQGLAPAELFARLKARMPDGEDLVV DPA CPFDH+ + R++ R R+EAGLVD+ D D++H+G + EL+ L R P EDLVV DPAACPFDHRLERRLDTVRQRVEAGLVDEADFDDDHRGRSATELYRLLLDRRPAVEDLVV | 186  190 |
| Query 187  Sbjct 191 | | THGDACLPNIMVENGRFSGFIDCGRLGVADRYQDIALATRDIAEELGGEWADRFLVLYGI HGDACLPN++ E RFSGFIDCGRLGVADR+QD+A A RDIA ELG WA+ FLV YG AHGDACLPNLLAEGRRFSGFIDCGRLGVADRHQDLARAARDIAAELGAAWAEAFLVEYG- | 246  249 |
| Query | 247 AAPDSQRIAFYRLLDEFF 264 | | |
|  |  | D +R+A++RLLDEFF |  |
| Sbjct | 250 | GDIDGERLAYFRLLDEFF 267 | |

>WP_057382169.1 APH(3')-II family aminoglycoside O-phosphotransferase [Pseudomonas aeruginosa]

Length=268

Score = 263 bits (672), Expect = 2e-84, Method: Compositional matrix adjust.

| Identities | | = 138/258 (53%), Positives = 169/258 (66%), Gaps = 5/258 (2%) |  |
| --- | --- | --- | --- |
| Query 11  Sbjct 12 | | SPAAWVERLFGYDWAQQTIGCSDAAVFRLSAQGRPVLFVKTDLSGALNELQDEAARLSWL  +PA W + L GY W Q GCS A V RL A RP LFVK ++ A EL E ARL WL APATWADYLAGYRWRGQGEGCSAATVHRLEAARRPTLFVKQEVLSAHAELPAEIARLRWL | 70  71 |
| Query 71  Sbjct 72 | | ATTGVPCAAVLDVVTEAGRDWLLLGEVPGQDLLS SHLAPAEKVSIMADAMRRLHTL  G+ C VL+ GR WLL+ VPG L + L P V ++A A+RRLH L HGAGIDCPQVLNETQSDGRQWLLMSAVPGDTLSALAQRGELEPEHLVRLVAAALRRLHDL | 126  131 |
| Query 127  Sbjct 132 | | DPATCPFDHQAKHRIERARTRMEAGLVDQDDLDEEHQGLAPAELFARLKARMPDGEDLVV DPA CPFDH+ + R++ R R+EAGLVD+ D D++H+G + EL+ L R P EDLVV DPAACPFDHRLERRLDTVRQRVEAGLVDEADFDDDHRGRSATELYRLLLDRRPAVEDLVV | 186  191 |
| Query 187  Sbjct 192 | | THGDACLPNIMVENGRFSGFIDCGRLGVADRYQDIALATRDIAEELGGEWADRFLVLYGI HGDACLPN++ E RFSGFIDCGRLGVADR+QD+ALA RDI ELG WA+ FLV YG AHGDACLPNLLAEGRRFSGFIDCGRLGVADRHQDLALAARDIEAELGAAWAEAFLVEYG- | 246  250 |
| Query | 247 AAPDSQRIAFYRLLDEFF 264 | | |
|  |  | D +R+A++RLLDEFF |  |
| Sbjct | 251 | GDIDGERLAYFRLLDEFF 268 | |

>WP_096760010.1 APH(3')-II family aminoglycoside O-phosphotransferase [Pseudomonas aeruginosa]

PCK44367.1 APH(3') family aminoglycoside O-phosphotransferase [Pseudomonas aeruginosa]

Length=268

Score = 263 bits (672), Expect = 2e-84, Method: Compositional matrix adjust.

| Identities | | = 137/258 (53%), Positives = 170/258 (66%), Gaps = 5/258 (2%) |  |
| --- | --- | --- | --- |
| Query 11  Sbjct 12 | | SPAAWVERLFGYDWAQQTIGCSDAAVFRLSAQGRPVLFVKTDLSGALNELQDEAARLSWL  +P+ W + L GY W Q GCS A V RL A+ RP LFVK ++ A EL E ARL WL APSTWADYLAGYRWRGQGEGCSAATVHRLEAERRPTLFVKQEVLSAHAELPAEIARLRWL | 70  71 |
| Query 71  Sbjct 72 | | ATTGVPCAAVLDVVTEAGRDWLLLGEVPGQDLLS SHLAPAEKVSIMADAMRRLHTL  G+ C VL+ GR WLL+ VPG L + L P V ++A A+RRLH L HGAGIDCPQVLNETQSDGRQWLLMSAVPGDTLSALXQRGELEPERLVRLVAAALRRLHDL | 126  131 |
| Query 127  Sbjct 132 | | DPATCPFDHQAKHRIERARTRMEAGLVDQDDLDEEHQGLAPAELFARLKARMPDGEDLVV DPA CPFDH+ + R++ R R+EAGLVD+ D D++H+G + EL+ L R P EDLVV DPAACPFDHRLERRLDTVRQRVEAGLVDEADFDDDHRGRSATELYRLLLDRRPAVEDLVV | 186  191 |
| Query 187  Sbjct 192 | | THGDACLPNIMVENGRFSGFIDCGRLGVADRYQDIALATRDIAEELGGEWADRFLVLYGI HGDACLPN++ E RFSGFIDCGRLGVADR+QD+ALA RDI ELG WA+ FLV YG AHGDACLPNLLAEGRRFSGFIDCGRLGVADRHQDLALAARDIEAELGAAWAEAFLVEYG- | 246  250 |
| Query | 247 AAPDSQRIAFYRLLDEFF 264 | | |
|  |  | D +R+A++RLLDEFF |  |
| Sbjct | 251 | GDIDGERLAYFRLLDEFF 268 | |

>WP_003137562.1 MULTISPECIES: APH(3')-II family aminoglycoside O-phosphotransferase [Pseudomonas]

SAJ27922.1 Aminoglycoside 3'-phosphotransferase [Enterobacter cloacae] ABJ13389.1 aminoglycoside 3'-phosphotransferase type IIb [Pseudomonas aeruginosa

UCBPP-PA14]

AHC63495.1 aminoglycoside 3'-phosphotransferase [Pseudomonas aeruginosa LES431]

AHK81791.1 aminoglycoside 3'-phosphotransferase [Pseudomonas aeruginosa LESlike5]

AHK87690.1 aminoglycoside 3'-phosphotransferase [Pseudomonas aeruginosa LESlike7]

Length=267

Score = 263 bits (672), Expect = 2e-84, Method: Compositional matrix adjust.

| Identities | | = 137/258 (53%), Positives = 170/258 (66%), Gaps = 5/258 (2%) |  |
| --- | --- | --- | --- |
| Query 11  Sbjct 11 | | SPAAWVERLFGYDWAQQTIGCSDAAVFRLSAQGRPVLFVKTDLSGALNELQDEAARLSWL  +P+ W + L GY W Q GCS A V RL A+ RP LFVK ++ A EL E ARL WL APSTWADYLAGYRWRGQGEGCSAATVHRLEAERRPTLFVKQEVLSAHAELPAEIARLRWL | 70  70 |
| Query 71  Sbjct 71 | | ATTGVPCAAVLDVVTEAGRDWLLLGEVPGQDLLS SHLAPAEKVSIMADAMRRLHTL  G+ C VL+ GR WLL+ VPG L + L P V ++A A+RRLH L HGAGIDCPQVLNETQSDGRQWLLMSAVPGDTLSALAQRGELEPERLVRLVAAALRRLHDL | 126  130 |
| Query 127  Sbjct 131 | | DPATCPFDHQAKHRIERARTRMEAGLVDQDDLDEEHQGLAPAELFARLKARMPDGEDLVV DPA CPFDH+ + R++ R R+EAGLVD+ D D++H+G + EL+ L R P EDLVV DPAACPFDHRLERRLDTVRQRVEAGLVDEADFDDDHRGRSATELYRLLLDRRPAVEDLVV | 186  190 |
| Query 187  Sbjct 191 | | THGDACLPNIMVENGRFSGFIDCGRLGVADRYQDIALATRDIAEELGGEWADRFLVLYGI HGDACLPN++ E RFSGFIDCGRLGVADR+QD+ALA RDI ELG WA+ FLV YG AHGDACLPNLLAEGRRFSGFIDCGRLGVADRHQDLALAARDIEAELGAAWAEAFLVEYG- | 246  249 |
| Query | 247 AAPDSQRIAFYRLLDEFF 264 | | |
|  |  | D +R+A++RLLDEFF |  |
| Sbjct | 250 | GDIDGERLAYFRLLDEFF 267 | |

>WP_182400191.1 APH(3') family aminoglycoside O-phosphotransferase [Devosia sp. D6-9]

QMV01893.1 APH(3') family aminoglycoside O-phosphotransferase [Devosia sp. D6-9]

Length=264

Score = 263 bits (671), Expect = 2e-84, Method: Compositional matrix adjust.

| Identities | | = 139/259 (54%), Positives = 168/259 (65%), Gaps = 3/259 (1%) |  |
| --- | --- | --- | --- |
| Query 8  Sbjct 7 | | HAGS-PAAWVERLFGYDWAQQTIGCSDAAVFRLSAQGRPVLFVKTDLSGALNELQDEAAR HA + P W + L GY+W QTIG S A VFRL+A P LFVKT+ +EL+ EA R HASNLPPGWPD-LSGYEWVPQTIGQSGAMVFRLTAPQGPALFVKTESVSPHSELEGEAHR | 66  65 |
| Query 67  Sbjct 66 | | LSWLATTGVPCAAVLDVVTEAGRDWLLLGEVPGQDLLSS-HLAPAEKVSIMADAMRRLHT L WLA+ P VL GR WLL+ V G DL SS L+P+ + I A ++R LH LEWLASVDQPAPRVLGTAQHLGRQWLLMTGVEGLDLASSPDLSPSRIIEICATSLRALHA | 125  125 |
| Query 126  Sbjct 126 | | LDPATCPFDHQAKHRIERARTRMEAGLVDQDDLDEEHQGLAPAELFARLKARMPDGEDLV  +D +CPFDH+ + AR R++AGLVD+ D D+ H G+ L+ARL A P+ EDLV IDVVSCPFDHRLDTTLATARERLDAGLVDETDFDDAHLGMPARTLYARLLAERPEAEDLV | 185  185 |
| Query 186  Sbjct 186 | | VTHGDACLPNIMVENGRFSGFIDCGRLGVADRYQDIALATRDIAEELGGEWADRFLVLYG VTHGDACLPN M FSGFIDCGRLGVADRYQD+ALATR IA LG WA+ FL YG VTHGDACLPNFMASGREFSGFIDCGRLGVADRYQDLALATRSIAFNLGQVWAEPFLTAYG | 245  245 |
| Query 246 | | IAAPDSQRIAFYRLLDEFF 264  + PD RI +YRLLDEFF |  |
| Sbjct | 246 | ASQPDRARIGYYRLLDEFF 264 | |

>WP_003121928.1 MULTISPECIES: APH(3')-II family aminoglycoside O-phosphotransferase [Pseudomonas]

KEA31245.1 aminoglycoside phosphotransferase [Pseudomonas aeruginosa C0324C] SCY57580.1 Aminoglycoside 3'-phosphotransferase [Acinetobacter baumannii] AHH52946.1 aminoglycoside 3'-phosphotransferase [Pseudomonas aeruginosa

YL84]

ALY35289.1 aminoglycoside phosphotransferase APH(3') [Pseudomonas aeruginosa] ALY87165.1 aminoglycoside phosphotransferase APH(3') [Pseudomonas aeruginosa]

Length=268

Score = 263 bits (672), Expect = 2e-84, Method: Compositional matrix adjust.

| Identities | | = 138/258 (53%), Positives = 169/258 (66%), Gaps = 5/258 (2%) |  |
| --- | --- | --- | --- |
| Query 11  Sbjct 12 | | SPAAWVERLFGYDWAQQTIGCSDAAVFRLSAQGRPVLFVKTDLSGALNELQDEAARLSWL  +PA W + L GY W Q GCS A V RL A RP LFVK ++ A EL E ARL WL APATWADYLAGYRWRGQGEGCSAATVHRLEAARRPTLFVKQEVLSAHAELPAEIARLRWL | 70  71 |
| Query 71  Sbjct 72 | | ATTGVPCAAVLDVVTEAGRDWLLLGEVPGQDLLS SHLAPAEKVSIMADAMRRLHTL  G+ C VL+ GR WLL+ VPG L + L P V ++A A+RRLH L HGAGIDCPQVLNETQSDGRQWLLMSAVPGDTLSALAQRGELEPERLVRLVAAALRRLHDL | 126  131 |
| Query 127  Sbjct 132 | | DPATCPFDHQAKHRIERARTRMEAGLVDQDDLDEEHQGLAPAELFARLKARMPDGEDLVV DPA CPFDH+ + R++ R R+EAGLVD+ D D++H+G + EL+ L R P EDLVV DPAACPFDHRLERRLDTVRQRVEAGLVDEADFDDDHRGRSATELYRLLLDRRPAVEDLVV | 186  191 |
| Query 187  Sbjct 192 | | THGDACLPNIMVENGRFSGFIDCGRLGVADRYQDIALATRDIAEELGGEWADRFLVLYGI HGDACLPN++ E RFSGFIDCGRLGVADR+QD+ALA RDI ELG WA+ FLV YG AHGDACLPNLLAEGRRFSGFIDCGRLGVADRHQDLALAARDIEAELGAAWAEAFLVEYG- | 246  250 |
| Query | 247 AAPDSQRIAFYRLLDEFF 264 | | |
|  |  | D +R+A++RLLDEFF |  |
| Sbjct | 251 | GDIDGERLAYFRLLDEFF 268 | |

```
>WP_070748368.1 MULTISPECIES: APH(3')-II family aminoglycoside O-phosphotransferase [Pseudomonas]
OFR46471.1 APH(3') family aminoglycoside O-phosphotransferase [Pseudomonas sp. HMSC066A08]
RUE49477.1 APH(3')-II family aminoglycoside O-phosphotransferase [Pseudomonas aeruginosa]
Length=268

Score = 263 bits (672), Expect = 2e-84, Method: Compositional matrix adjust. Identities = 138/258 (53%), Positives = 169/258 (66%), Gaps = 5/258 (2%)

Query 11	SPAAWVERLFGYDWAQQTIGCSDAAVFRLSAQGRPVLFVKTDLSGALNELQDEAARLSWL 70
+PA W + L GY W Q GCS A V RL A RP LFVK ++ A EL E ARL WL Sbjct 12	APATWADYLAGYRWRGQGEGCSAATVHRLEAARRPTLFVKQEVLSAHAELPAEIARLRWL 71
```

| Query | 71 | ATTGVPCAAVLDVVTEAGRDWLLLGEVPGQDLLS SHLAPAEKVSIMADAMRRLHTL | 126 |
| --- | --- | --- | --- |
| Sbjct | 72 | G+ C VL+ GR WLL+ VPG L + L P V ++A A+RRLH L HGAGIDCPQVLNETQSDGRQWLLMSAVPGDTLSALAQRGELEPGRLVRLVAAALRRLHDL | 131 |
| Query | 127 | DPATCPFDHQAKHRIERARTRMEAGLVDQDDLDEEHQGLAPAELFARLKARMPDGEDLVV | 186 |
| Sbjct | 132 | DPA CPFDH+ R++ R R+EAGLVD+ D D++H+G + +EL+ L R P EDLVV DPAACPFDHRLGRRLDTVRQRVEAGLVDEADFDDDHRGRSASELYRLLLDRRPAVEDLVV | 191 |
| Query | 187 | THGDACLPNIMVENGRFSGFIDCGRLGVADRYQDIALATRDIAEELGGEWADRFLVLYGI | 246 |
| Sbjct | 192 | HGDACLPN++ E RFSGFIDCGRLGVADR+QD+ALA RDI ELG WA+ FLV YG  AHGDACLPNLLAEGRRFSGFIDCGRLGVADRHQDLALAARDIEAELGAAWAEAFLVEYG- | 250 |
| Query | 247 AAPDSQRIAFYRLLDEFF 264 | | |
|  |  | D +R+A++RLLDEFF |  |
| Sbjct | 251 | GDIDGERLAYFRLLDEFF 268 | |

>WP_003093441.1 MULTISPECIES: APH(3')-II family aminoglycoside O-phosphotransferase [Pseudomonas]

ARG85007.1 aminoglycoside 3'-phosphotransferase type IIb [Pseudomonas aeruginosa]

AUA75400.1 APH(3')-II family aminoglycoside O-phosphotransferase [Pseudomonas aeruginosa]

AUB00025.1 APH(3')-II family aminoglycoside O-phosphotransferase [Pseudomonas aeruginosa]

AVJ96447.1 phosphotransferase enzyme family protein [Pseudomonas aeruginosa] AVK12276.1 phosphotransferase enzyme family protein [Pseudomonas aeruginosa]

Length=268

Score = 263 bits (672), Expect = 2e-84, Method: Compositional matrix adjust.

| Identities | | = 137/258 (53%), Positives = 170/258 (66%), Gaps = 5/258 (2%) |  |
| --- | --- | --- | --- |
| Query 11  Sbjct 12 | | SPAAWVERLFGYDWAQQTIGCSDAAVFRLSAQGRPVLFVKTDLSGALNELQDEAARLSWL  +P+ W + L GY W Q GCS A V RL A+ RP LFVK ++ A EL E ARL WL APSTWADYLAGYRWRGQGEGCSAATVHRLEAERRPTLFVKQEVLSAHAELPAEIARLRWL | 70  71 |
| Query 71  Sbjct 72 | | ATTGVPCAAVLDVVTEAGRDWLLLGEVPGQDLLS SHLAPAEKVSIMADAMRRLHTL  G+ C VL+ GR WLL+ VPG L + L P V ++A A+RRLH L HGAGIDCPQVLNETQSDGRQWLLMSAVPGDTLSALAQRGELEPERLVRLVAAALRRLHDL | 126  131 |
| Query 127  Sbjct 132 | | DPATCPFDHQAKHRIERARTRMEAGLVDQDDLDEEHQGLAPAELFARLKARMPDGEDLVV DPA CPFDH+ + R++ R R+EAGLVD+ D D++H+G + EL+ L R P EDLVV DPAACPFDHRLERRLDTVRQRVEAGLVDEADFDDDHRGRSATELYRLLLDRRPAVEDLVV | 186  191 |
| Query 187  Sbjct 192 | | THGDACLPNIMVENGRFSGFIDCGRLGVADRYQDIALATRDIAEELGGEWADRFLVLYGI HGDACLPN++ E RFSGFIDCGRLGVADR+QD+ALA RDI ELG WA+ FLV YG AHGDACLPNLLAEGRRFSGFIDCGRLGVADRHQDLALAARDIEAELGAAWAEAFLVEYG- | 246  250 |
| Query | 247 AAPDSQRIAFYRLLDEFF 264 | | |
|  |  | D +R+A++RLLDEFF |  |
| Sbjct | 251 | GDIDGERLAYFRLLDEFF 268 | |

>WP_004345974.1 MULTISPECIES: APH(3')-II family aminoglycoside O-phosphotransferase [Pseudomonas]

EAZ54448.1 aminoglycoside 3'-phosphotransferase type IIb [Pseudomonas aeruginosa C3719]

EOQ77368.1 aminoglycoside 3-N-acetyltransferase [Pseudomonas aeruginosa VRFPA02]

KEA18376.1 aminoglycoside phosphotransferase [Pseudomonas aeruginosa C2159M] CDI92604.1 aminoglycoside 3-N-acetyltransferase [Pseudomonas aeruginosa

PA38182]

SSU22139.1 aminoglycoside 3'-phosphotransferase aphA1 [Acinetobacter baumannii] Length=268

Score = 263 bits (672), Expect = 2e-84, Method: Compositional matrix adjust.

| Identities | = 138/258 (53%), Positives = 169/258 (66%), Gaps = 5/258 (2%) |  |
| --- | --- | --- |
| Query 11 | SPAAWVERLFGYDWAQQTIGCSDAAVFRLSAQGRPVLFVKTDLSGALNELQDEAARLSWL | 70 |
| Sbjct 12 | +PA W + L GY W Q GCS A V RL A RP LFVK ++ A EL E ARL WL APATWADYLAGYRWRGQGEGCSAATVHRLEAARRPTLFVKKEVLSAHAELPAEIARLRWL | 71 |
| Query 71 | ATTGVPCAAVLDVVTEAGRDWLLLGEVPGQDLLS SHLAPAEKVSIMADAMRRLHTL | 126 |

| Sbjct | 72 | G+ C VL+ GR WLL+ VPG L + L P V ++A A+RRLH L HGAGIDCPQVLNETQSDGRQWLLMSAVPGDTLSALAQRGELEPERLVRLVAAALRRLHDL | 131 |
| --- | --- | --- | --- |
| Query | 127 | DPATCPFDHQAKHRIERARTRMEAGLVDQDDLDEEHQGLAPAELFARLKARMPDGEDLVV | 186 |
| Sbjct | 132 | DPA CPFDH+ + R++ R R+EAGLVD+ D D++H+G + EL+ L R P EDLVV DPAACPFDHRLERRLDTVRQRVEAGLVDEADFDDDHRGRSATELYRLLLDRRPAVEDLVV | 191 |
| Query | 187 | THGDACLPNIMVENGRFSGFIDCGRLGVADRYQDIALATRDIAEELGGEWADRFLVLYGI | 246 |
| Sbjct | 192 | HGDACLPN++ E RFSGFIDCGRLGVADR+QD+ALA RDI ELG WA+ FLV YG  AHGDACLPNLLAEGRRFSGFIDCGRLGVADRHQDLALAARDIEAELGAAWAEAFLVEYG- | 250 |
| Query | 247 AAPDSQRIAFYRLLDEFF 264 | | |
|  |  | D +R+A++RLLDEFF |  |
| Sbjct | 251 | GDIDGERLAYFRLLDEFF 268 | |

>WP_124138621.1 APH(3')-II family aminoglycoside O-phosphotransferase [Pseudomonas aeruginosa]

RPY33083.1 APH(3') family aminoglycoside O-phosphotransferase [Pseudomonas aeruginosa]

RPY47248.1 APH(3') family aminoglycoside O-phosphotransferase [Pseudomonas aeruginosa]

Length=268

Score = 263 bits (671), Expect = 2e-84, Method: Compositional matrix adjust.

| Identities | | = 138/258 (53%), Positives = 169/258 (66%), Gaps = 5/258 (2%) |  |
| --- | --- | --- | --- |
| Query 11  Sbjct 12 | | SPAAWVERLFGYDWAQQTIGCSDAAVFRLSAQGRPVLFVKTDLSGALNELQDEAARLSWL  +PA W + L GY W Q GCS A V RL A RP LFVK ++ A EL E ARL WL APATWADYLAGYRWRGQGEGCSAATVHRLEAARRPTLFVKQEVLSAHAELPAEIARLRWL | 70  71 |
| Query 71  Sbjct 72 | | ATTGVPCAAVLDVVTEAGRDWLLLGEVPGQDLLS SHLAPAEKVSIMADAMRRLHTL  G+ C VL+ GR WLL+ VPG L + L P V ++A A+RRLH L HGAGIDCPQVLNETQSDGRQWLLMSAVPGDTLSALAQCGELEPGRLVRLVAAALRRLHDL | 126  131 |
| Query 127  Sbjct 132 | | DPATCPFDHQAKHRIERARTRMEAGLVDQDDLDEEHQGLAPAELFARLKARMPDGEDLVV DPA CPFDH+ R++ R R+EAGLVD+ D D++H+G + +EL+ L R P EDLVV DPAACPFDHRLGRRLDTVRQRVEAGLVDEADFDDDHRGRSASELYRLLLDRRPAVEDLVV | 186  191 |
| Query 187  Sbjct 192 | | THGDACLPNIMVENGRFSGFIDCGRLGVADRYQDIALATRDIAEELGGEWADRFLVLYGI HGDACLPN++ E RFSGFIDCGRLGVADR+QD+ALA RDI ELG WA+ FLV YG AHGDACLPNLLAEGRRFSGFIDCGRLGVADRHQDLALAARDIEAELGAAWAEAFLVEYG- | 246  250 |
| Query | 247 AAPDSQRIAFYRLLDEFF 264 | | |
|  |  | D +R+A++RLLDEFF |  |
| Sbjct | 251 | GDIDGERLAYFRLLDEFF 268 | |

>WP_062842754.1 APH(3')-II family aminoglycoside O-phosphotransferase [Pseudomonas aeruginosa]

KYO75012.1 Aminoglycoside 3'-phosphotransferase [Pseudomonas aeruginosa]

RTR51640.1 APH(3')-II family aminoglycoside O-phosphotransferase [Pseudomonas aeruginosa]

TGA89300.1 APH(3')-II family aminoglycoside O-phosphotransferase [Pseudomonas aeruginosa]

Length=268

Score = 263 bits (671), Expect = 2e-84, Method: Compositional matrix adjust.

| Identities | = 138/258 (53%), Positives = 168/258 (65%), Gaps = 5/258 (2%) |  |
| --- | --- | --- |
| Query 11 | SPAAWVERLFGYDWAQQTIGCSDAAVFRLSAQGRPVLFVKTDLSGALNELQDEAARLSWL | 70 |
| Sbjct 12 | +PA W + L GY W Q GCS A V RL A RP LFVK ++ A EL E ARL WL APATWADYLAGYRWRGQGEGCSAATVHRLEAARRPTLFVKQEVLSAHAELPAEIARLRWL | 71 |
| Query 71 | ATTGVPCAAVLDVVTEAGRDWLLLGEVPGQDLLS SHLAPAEKVSIMADAMRRLHTL | 126 |
| Sbjct 72 | G+ C VL+ GR WLL+ VPG L + L P V ++A A+RRLH L HGAGIDCPQVLNETQSDGRQWLLMSAVPGDTLSALAQRGELEPERLVRLVAAALRRLHDL | 131 |
| Query 127 | DPATCPFDHQAKHRIERARTRMEAGLVDQDDLDEEHQGLAPAELFARLKARMPDGEDLVV | 186 |
| Sbjct 132 | DPA CPFDH + R++ R R+EAGLVD+ D D++H+G + EL+ L R P EDLVV  DPAACPFDHHLERRLDTVRQRVEAGLVDEADFDDDHRGRSATELYRLLLDRRPAVEDLVV | 191 |

Query 187 THGDACLPNIMVENGRFSGFIDCGRLGVADRYQDIALATRDIAEELGGEWADRFLVLYGI 246

| Sbjct | 192 | HGDACLPN++ E RFSGFIDCGRLGVADR+QD+ALA RDI ELG WA+ FLV YG AHGDACLPNLLAEGRRFSGFIDCGRLGVADRHQDLALAARDIEAELGAAWAEAFLVEYG- 250 |
| --- | --- | --- |
| Query | 247 | AAPDSQRIAFYRLLDEFF 264 |
| Sbjct | 251 | D +R+A++RLLDEFF  GDIDGERLAYFRLLDEFF 268 |

>WP_116805535.1 APH(3')-II family aminoglycoside O-phosphotransferase [Pseudomonas aeruginosa]

MBG5591627.1 APH(3')-II family aminoglycoside O-phosphotransferase [Pseudomonas aeruginosa]

NQC38776.1 APH(3')-II family aminoglycoside O-phosphotransferase [Pseudomonas aeruginosa]

Length=268

Score = 263 bits (671), Expect = 2e-84, Method: Compositional matrix adjust.

| Identities | | = 138/258 (53%), Positives = 169/258 (66%), Gaps = 5/258 (2%) |  |
| --- | --- | --- | --- |
| Query 11  Sbjct 12 | | SPAAWVERLFGYDWAQQTIGCSDAAVFRLSAQGRPVLFVKTDLSGALNELQDEAARLSWL  +PA W + L GY W Q GCS A V RL A RP LFVK ++ A EL E ARL WL APATWADYLAGYRWRGQGEGCSAATVHRLEAARRPTLFVKQEVLSAHAELPAEIARLRWL | 70  71 |
| Query 71  Sbjct 72 | | ATTGVPCAAVLDVVTEAGRDWLLLGEVPGQDLLS SHLAPAEKVSIMADAMRRLHTL  G+ C VL+ GR WLL+ VPG L + L P V ++A A+RRLH L HGAGIDCPQVLNETQSDGRQWLLMSAVPGDTLSALAQRGELEPERLVRLVAAALRRLHDL | 126  131 |
| Query 127  Sbjct 132 | | DPATCPFDHQAKHRIERARTRMEAGLVDQDDLDEEHQGLAPAELFARLKARMPDGEDLVV DPA CPFDH+ + R++ R R+EAGLVD+ D D++H+G + EL+ L R P EDLVV DPAACPFDHRLERRLDTVRQRVEAGLVDEADFDDDHRGRSATELYRLLLDRCPAVEDLVV | 186  191 |
| Query 187  Sbjct 192 | | THGDACLPNIMVENGRFSGFIDCGRLGVADRYQDIALATRDIAEELGGEWADRFLVLYGI HGDACLPN++ E RFSGFIDCGRLGVADR+QD+ALA RDI ELG WA+ FLV YG AHGDACLPNLLAEGRRFSGFIDCGRLGVADRHQDLALAARDIEAELGAAWAEAFLVEYG- | 246  250 |
| Query | 247 AAPDSQRIAFYRLLDEFF 264 | | |
|  |  | D +R+A++RLLDEFF |  |
| Sbjct | 251 | GDIDGERLAYFRLLDEFF 268 | |

>WP_073669326.1 APH(3')-II family aminoglycoside O-phosphotransferase [Pseudomonas aeruginosa]

MBG5092224.1 APH(3')-II family aminoglycoside O-phosphotransferase [Pseudomonas aeruginosa]

PBW92694.1 APH(3')-II family aminoglycoside O-phosphotransferase [Pseudomonas aeruginosa]

RPM52989.1 APH(3')-II family aminoglycoside O-phosphotransferase [Pseudomonas aeruginosa]

RQC86308.1 APH(3')-II family aminoglycoside O-phosphotransferase [Pseudomonas aeruginosa]

RUI27281.1 APH(3')-II family aminoglycoside O-phosphotransferase [Pseudomonas aeruginosa]

Length=268

Score = 263 bits (671), Expect = 2e-84, Method: Compositional matrix adjust.

| Identities | = 137/258 (53%), Positives = 169/258 (66%), Gaps = 5/258 (2%) |  |
| --- | --- | --- |
| Query 11 | SPAAWVERLFGYDWAQQTIGCSDAAVFRLSAQGRPVLFVKTDLSGALNELQDEAARLSWL | 70 |
| Sbjct 12 | +P+ W + L GY W Q GCS A V RL A RP LFVK ++ A EL E ARL WL APSTWADYLAGYRWRGQGEGCSAATVHRLEAARRPTLFVKQEVLSAHAELPAEIARLRWL | 71 |
| Query 71 | ATTGVPCAAVLDVVTEAGRDWLLLGEVPGQDLLS SHLAPAEKVSIMADAMRRLHTL | 126 |
| Sbjct 72 | G+ C VL+ GR WLL+ VPG L + L P V ++A A+RRLH L HGAGIDCPQVLNETQSDGRQWLLMSAVPGNTLSALAQRDELEPERLVRLVAAALRRLHDL | 131 |
| Query 127 | DPATCPFDHQAKHRIERARTRMEAGLVDQDDLDEEHQGLAPAELFARLKARMPDGEDLVV | 186 |
| Sbjct 132 | DPA CPFDH+ + R++ R R+EAGLVD+ D D++H+G + EL+ L R P EDLVV DPAACPFDHRLERRLDTVRQRVEAGLVDEADFDDDHRGRSATELYRLLLDRRPAVEDLVV | 191 |
| Query 187 | THGDACLPNIMVENGRFSGFIDCGRLGVADRYQDIALATRDIAEELGGEWADRFLVLYGI HGDACLPN++ E RFSGFIDCGRLGVADR+QD+ALA RDI ELG WA+ FLV YG | 246 |

| Sbjct | 192 | AHGDACLPNLLAEGRRFSGFIDCGRLGVADRHQDLALAARDIEAELGAAWAEAFLVEYG- 250 |
| --- | --- | --- |
| Query | 247 | AAPDSQRIAFYRLLDEFF 264 |
| Sbjct | 251 | D +R+A++RLLDEFF  GDIDGERLAYFRLLDEFF 268 |

>WP_077968675.1 aminoglycoside 3'-phosphotransferase [Ensifer adhaerens] Length=263

Score = 263 bits (671), Expect = 2e-84, Method: Compositional matrix adjust.

| Identities | | = 132/257 (51%), Positives = 169/257 (66%), Gaps = 0/257 (0%) |  |
| --- | --- | --- | --- |
| Query 8  Sbjct 7 | | HAGSPAAWVERLFGYDWAQQTIGCSDAAVFRLSAQGRPVLFVKTDLSGALNELQDEAARL H P + + GY W + +G SD+++F L A RP+L +K + + +E DEAARL HPELPDQFRPLVAGYQWNRDLLGQSDSSIFLLKAPDRPMLVLKLEAAEPFSEFVDEAARL | 67  66 |
| Query 68  Sbjct 67 | | SWLATTGVPCAAVLDVVTEAGRDWLLLGEVPGQDLLSSHLAPAEKVSIMADAMRRLHTLD WL + GVPC V+ A +WLLL V G DL S+ L+P +++ I+ADA++RLH LD DWLVSEGVPCPRVVARAFGAEANWLLLQAVEGTDLASTTLSPKDQIVILADALKRLHALD | 127  126 |
| Query 128  Sbjct 127 | | PATCPFDHQAKHRIERARTRMEAGLVDQDDLDEEHQGLAPAELFARLKARMPDGEDLVVT A CPFDH+ + RI A+ R +AG++D+ D DE G A+LFA L+ARMP LVVT IANCPFDHRIEKRISIAKARTKAGVIDESDFDETRLGRTAADLFAELQARMPRTGKLVVT | 187  186 |
| Query 188  Sbjct 187 | | HGDACLPNIMVENGRFSGFIDCGRLGVADRYQDIALATRDIAEELGGEWADRFLVLYGIA HGDACLPNI+ +NGRFSGFIDC RLGVAD YQD ALA R IA LG EW FL YGI HGDACLPNIIEKNGRFSGFIDCSRLGVADPYQDTALACRSIAYNLGEEWVQPFLTRYGIE | 247  246 |
| Query 248 | | APDSQRIAFYRLLDEFF 264 D+++ FY LLDEFF |  |
| Sbjct | 247 | KDDAEKRDFYCLLDEFF 263 | |

>MBG6562873.1 APH(3')-II family aminoglycoside O-phosphotransferase [Pseudomonas aeruginosa]

Length=268

Score = 263 bits (671), Expect = 2e-84, Method: Compositional matrix adjust.

| Identities | | = 138/258 (53%), Positives = 169/258 (66%), Gaps = 5/258 (2%) |  |
| --- | --- | --- | --- |
| Query 11  Sbjct 12 | | SPAAWVERLFGYDWAQQTIGCSDAAVFRLSAQGRPVLFVKTDLSGALNELQDEAARLSWL  +PA W + L GY W Q GCS A V RL A+ RP LFVK ++ A EL E ARL WL APATWADYLAGYRWRGQGEGCSAATVHRLEAERRPTLFVKQEVLSAHAELPAEIARLRWL | 70  71 |
| Query 71  Sbjct 72 | | ATTGVPCAAVLDVVTEAGRDWLLLGEVPGQDLLS SHLAPAEKVSIMADAMRRLHTL  G+ C VL+ GR WLL+ VPG L + L P V ++A A+RRLH L HGAGIDCPQVLNETQSDGRQWLLMSAVPGATLSALAQRGELEPERLVRLVAAALRRLHDL | 126  131 |
| Query 127  Sbjct 132 | | DPATCPFDHQAKHRIERARTRMEAGLVDQDDLDEEHQGLAPAELFARLKARMPDGEDLVV DPA CPFDH+ + R++ R R EAGLVD+ D D++H+G + EL+ L R P EDLVV DPAACPFDHRLERRLDTVRQRGEAGLVDEADFDDDHRGRSATELYRLLLDRRPAVEDLVV | 186  191 |
| Query 187  Sbjct 192 | | THGDACLPNIMVENGRFSGFIDCGRLGVADRYQDIALATRDIAEELGGEWADRFLVLYGI HGDACLPN++ E RFSGFIDCGRLGVADR+QD+ALA RDI ELG WA+ FLV YG AHGDACLPNLLAEGRRFSGFIDCGRLGVADRHQDLALAARDIEAELGAAWAEAFLVEYG- | 246  250 |
| Query | 247 AAPDSQRIAFYRLLDEFF 264 | | |
|  |  | D +R+A++RLLDEFF |  |
| Sbjct | 251 | GDIDGERLAYFRLLDEFF 268 | |

```
>WP_127527170.1 aminoglycoside 3'-phosphotransferase [Sinorhizobium meliloti] RVJ50613.1 aminoglycoside 3'-phosphotransferase [Sinorhizobium meliloti]
Length=261

Score = 262 bits (670), Expect = 3e-84, Method: Compositional matrix adjust. Identities = 138/253 (55%), Positives = 163/253 (64%), Gaps = 0/253 (0%)

Query 12	PAAWVERLFGYDWAQQTIGCSDAAVFRLSAQGRPVLFVKTDLSGALNELQDEAARLSWLA 71 P A+	L GY + + +G S + +F L AQ RP L +K + SG	EL EAARL WLA
Sbjct 9	PPAFRGLLSGYGFERDALGQSASTIFLLKAQSRPFLILKHEPSGPFTELTGEAARLHWLA 68
```

| Query | 72 | TTGVPCAAVLDVVTEAGRDWLLLGEVPGQDLLSSHLAPAEKVSIMADAMRRLHTLDPATC | 131 |
| --- | --- | --- | --- |
| Sbjct | 69 | +PC VL AG WLLL G DL S L AE+++I+A+A+RRLH LDPA C AQDMPCPIVLGHDDHAGETWLLLEAAEGMDLASCRLPHAERIAILAEALRRLHRLDPAAC | 128 |
| Query | 132 | PFDHQAKHRIERARTRMEAGLVDQDDLDEEHQGLAPAELFARLKARMPDGEDLVVTHGDA | 191 |
| Sbjct | 129 | PFD + I A+ RMEAGLVD+ D DEE QG ELF L AR P E LVVTHGDA PFDRRLTASIAAAKARMEAGLVDESDFDEERQGRQTRELFDELLARRPKEEQLVVTHGDA | 188 |
| Query | 192 | CLPNIMVENGRFSGFIDCGRLGVADRYQDIALATRDIAEELGGEWADRFLVLYGIAAPDS | 251 |
| Sbjct | 189 | CLPN + +FSGFIDCGRLGVAD YQD+ALA I LG EW FL YG+AAPD CLPNFIAAGNQFSGFIDCGRLGVADIYQDLALACWSITHNLGEEWVAPFLDRYGLAAPDP | 248 |
| Query  Sbjct | 252  249 | QRIAFYRLLDEFF 264  +++AFYRLLDEFF  EKLAFYRLLDEFF 261 |  |

>WP_070623109.1 APH(3')-II family aminoglycoside O-phosphotransferase [Pseudomonas aeruginosa]

AXL69585.1 Aminoglycoside 3'-phosphotransferase [Pseudomonas aeruginosa]

KAA5570153.1 APH(3')-II family aminoglycoside O-phosphotransferase [Pseudomonas aeruginosa]

OFS69629.1 APH(3') family aminoglycoside O-phosphotransferase [Pseudomonas aeruginosa]

RUB24608.1 APH(3')-II family aminoglycoside O-phosphotransferase [Pseudomonas aeruginosa]

Length=268

Score = 263 bits (671), Expect = 3e-84, Method: Compositional matrix adjust.

| Identities | | = 138/258 (53%), Positives = 169/258 (66%), Gaps = 5/258 (2%) |  |
| --- | --- | --- | --- |
| Query 11  Sbjct 12 | | SPAAWVERLFGYDWAQQTIGCSDAAVFRLSAQGRPVLFVKTDLSGALNELQDEAARLSWL  +PA W + L GY W Q GCS A V RL A RP LFVK ++ A EL E ARL WL APATWADYLAGYRWRGQGEGCSAATVHRLEAARRPTLFVKQEVLSAHAELPAEIARLRWL | 70  71 |
| Query 71  Sbjct 72 | | ATTGVPCAAVLDVVTEAGRDWLLLGEVPGQDLLS SHLAPAEKVSIMADAMRRLHTL  G+ C VL+ GR WLL+ VPG L + L P V ++A A+RRLH L HGAGIDCPQVLNETQSDGRQWLLMSAVPGDTLSALAQRGELEPERLVRLVAAALRRLHDL | 126  131 |
| Query 127  Sbjct 132 | | DPATCPFDHQAKHRIERARTRMEAGLVDQDDLDEEHQGLAPAELFARLKARMPDGEDLVV DPA CPFDH+ + R++ R R+EAGLVD+ D D++H+G + EL+ L R P EDLVV DPAACPFDHRLERRLDTVRHRVEAGLVDEADFDDDHRGRSATELYRLLLDRRPAVEDLVV | 186  191 |
| Query 187  Sbjct 192 | | THGDACLPNIMVENGRFSGFIDCGRLGVADRYQDIALATRDIAEELGGEWADRFLVLYGI HGDACLPN++ E RFSGFIDCGRLGVADR+QD+ALA RDI ELG WA+ FLV YG AHGDACLPNLLAEGRRFSGFIDCGRLGVADRHQDLALAARDIEAELGAAWAEAFLVEYG- | 246  250 |
| Query | 247 AAPDSQRIAFYRLLDEFF 264 | | |
|  |  | D +R+A++RLLDEFF |  |
| Sbjct | 251 | GDIDGERLAYFRLLDEFF 268 | |

>WP_033993831.1 APH(3')-II family aminoglycoside O-phosphotransferase [Pseudomonas aeruginosa]

Length=268

Score = 263 bits (671), Expect = 3e-84, Method: Compositional matrix adjust.

| Identities | = 138/258 (53%), Positives = 169/258 (66%), Gaps = 5/258 (2%) |  |
| --- | --- | --- |
| Query 11 | SPAAWVERLFGYDWAQQTIGCSDAAVFRLSAQGRPVLFVKTDLSGALNELQDEAARLSWL | 70 |
| Sbjct 12 | +PA W + L GY W Q GCS A V RL A RP LFVK ++ A EL E ARL WL APATWADYLAGYRWRGQGEGCSAATVHRLEAARRPTLFVKQEVLSAHAELPAEIARLRWL | 71 |
| Query 71 | ATTGVPCAAVLDVVTEAGRDWLLLGEVPGQDLLS SHLAPAEKVSIMADAMRRLHTL | 126 |
| Sbjct 72 | G+ C VL+ GR WLL+ VPG L + L P V ++A A+RRLH L HGAGIDCPQVLNETQSDGRQWLLMSAVPGDTLSALAQRGELEPERLVRLVAAALRRLHDL | 131 |
| Query 127 | DPATCPFDHQAKHRIERARTRMEAGLVDQDDLDEEHQGLAPAELFARLKARMPDGEDLVV | 186 |
| Sbjct 132 | DPA CPFDH+ + R++ R R+EAGLVD+ D D++H+G + EL+ L R P EDLVV  DPAACPFDHRLERRLDTVRQRVEAGLVDEADFDDDHRGRSATELYRLLLDRRPVVEDLVV | 191 |

Query 187 THGDACLPNIMVENGRFSGFIDCGRLGVADRYQDIALATRDIAEELGGEWADRFLVLYGI 246

| Sbjct | 192 | HGDACLPN++ E RFSGFIDCGRLGVADR+QD+ALA RDI ELG WA+ FLV YG AHGDACLPNLLAEGRRFSGFIDCGRLGVADRHQDLALAARDIEAELGAAWAEAFLVEYG- 250 |
| --- | --- | --- |
| Query | 247 | AAPDSQRIAFYRLLDEFF 264 |
| Sbjct | 251 | D +R+A++RLLDEFF  GDIDGERLAYFRLLDEFF 268 |

>WP_061360967.1 APH(3')-II family aminoglycoside O-phosphotransferase [Pseudomonas aeruginosa]

KXE25235.1 aminoglycoside phosphotransferase [Pseudomonas aeruginosa] Length=268

Score = 263 bits (671), Expect = 3e-84, Method: Compositional matrix adjust.

| Identities | | = 137/258 (53%), Positives = 169/258 (66%), Gaps = 5/258 (2%) |  |
| --- | --- | --- | --- |
| Query 11  Sbjct 12 | | SPAAWVERLFGYDWAQQTIGCSDAAVFRLSAQGRPVLFVKTDLSGALNELQDEAARLSWL  +PA W + L GY W Q GCS A + RL A RP LFVK ++ A EL E ARL WL APATWADYLAGYRWRGQGEGCSAATIHRLEAARRPTLFVKQEVLSAHAELPAEIARLRWL | 70  71 |
| Query 71  Sbjct 72 | | ATTGVPCAAVLDVVTEAGRDWLLLGEVPGQDLLS SHLAPAEKVSIMADAMRRLHTL  G+ C VL+ GR WLL+ VPG L + L P V ++A A+RRLH L HGAGIDCPQVLNETQSDGRQWLLMSAVPGDTLSALAQRGELEPERLVRLVAAALRRLHDL | 126  131 |
| Query 127  Sbjct 132 | | DPATCPFDHQAKHRIERARTRMEAGLVDQDDLDEEHQGLAPAELFARLKARMPDGEDLVV DPA CPFDH+ + R++ R R+EAGLVD+ D D++H+G + EL+ L R P EDLVV DPAACPFDHRLERRLDTVRQRVEAGLVDEADFDDDHRGRSATELYRLLLDRRPAVEDLVV | 186  191 |
| Query 187  Sbjct 192 | | THGDACLPNIMVENGRFSGFIDCGRLGVADRYQDIALATRDIAEELGGEWADRFLVLYGI HGDACLPN++ E RFSGFIDCGRLGVADR+QD+ALA RDI ELG WA+ FLV YG AHGDACLPNLLAEGRRFSGFIDCGRLGVADRHQDLALAARDIEAELGAAWAEAFLVEYG- | 246  250 |
| Query | 247 AAPDSQRIAFYRLLDEFF 264 | | |
|  |  | D +R+A++RLLDEFF |  |
| Sbjct | 251 | GDIDGERLAYFRLLDEFF 268 | |

>WP_035031015.1 aminoglycoside 3'-phosphotransferase [Devosia sp. DBB001] CDP51049.1 Aminoglycoside phosphotransferase [Devosia sp. DBB001]

Length=264

Score = 262 bits (670), Expect = 3e-84, Method: Compositional matrix adjust.

| Identities | = 139/259 (54%), Positives = 167/259 (64%), Gaps = 3/259 (1%) |  |
| --- | --- | --- |
| Query 8 | HAGS-PAAWVERLFGYDWAQQTIGCSDAAVFRLSAQGRPVLFVKTDLSGALNELQDEAAR | 66 |
| Sbjct 7 | HA + P W + L GY+W QTIG S A VFRL+A P LFVKT+ +EL+ EA R HASNLPPGWPD-LSGYEWVPQTIGQSGAMVFRLTAPQGPALFVKTESVSPHSELEGEAHR | 65 |
| Query 67 | LSWLATTGVPCAAVLDVVTEAGRDWLLLGEVPGQDLLSS-HLAPAEKVSIMADAMRRLHT | 125 |
| Sbjct 66 | L WLA P VL GR WLL+ V G DL SS L+P+ + I A ++R LH LEWLAGVDQPAPRVLGTAQHLGRQWLLMTGVDGLDLASSPDLSPSRIIEICATSLRALHA | 125 |
| Query 126 | LDPATCPFDHQAKHRIERARTRMEAGLVDQDDLDEEHQGLAPAELFARLKARMPDGEDLV | 185 |
| Sbjct 126 | +D A+CPFDH+ + AR R++AGLVD+ D D+ H G+ L+ARL P+ EDLV IDVASCPFDHRLDTTLATARERLDAGLVDETDFDDAHLGMPARTLYARLLTERPEAEDLV | 185 |
| Query 186 | VTHGDACLPNIMVENGRFSGFIDCGRLGVADRYQDIALATRDIAEELGGEWADRFLVLYG | 245 |
| Sbjct 186 | VTHGDACLPN M FSGFIDCGRLGVADRYQD+ALATR IA LG WA+ FL YG VTHGDACLPNFMASGREFSGFIDCGRLGVADRYQDLALATRSIAFNLGQVWAEPFLTAYG | 245 |
| Query 246  Sbjct 246 | IAAPDSQRIAFYRLLDEFF 264  + PD RI +YRLLDEFF  VGQPDRARIGYYRLLDEFF 264 |  |

>WP_010970345.1 MULTISPECIES: aminoglycoside 3'-phosphotransferase [Rhizobiales] TWA96915.1 aminoglycoside 3'-phosphotransferase-2 [Ensifer sp. SEMIA 134] TWB32823.1 aminoglycoside 3'-phosphotransferase-2 [Ensifer sp. SEMIA 135] AEG05724.1 aminoglycoside phosphotransferase [Sinorhizobium meliloti BL225C] AEG54760.1 aminoglycoside phosphotransferase [Sinorhizobium meliloti AK83] AEH80414.1 putative aminoglycoside 3'-phosphotransferase protein [Sinorhizobium

meliloti SM11] Length=261

Score = 262 bits (670), Expect = 3e-84, Method: Compositional matrix adjust.

| Identities | = 138/253 (55%), Positives = 163/253 (64%), Gaps = 0/253 (0%) |  |
| --- | --- | --- |
| Query 12 | PAAWVERLFGYDWAQQTIGCSDAAVFRLSAQGRPVLFVKTDLSGALNELQDEAARLSWLA | 71 |
| Sbjct 9 | P A+ L GY + + +G S + +F L AQ RP L +K + SG EL EAARL WLA PPAFRGLLSGYGFERDALGQSASTIFLLKAQSRPFLILKHEPSGPFTELTGEAARLHWLA | 68 |
| Query 72 | TTGVPCAAVLDVVTEAGRDWLLLGEVPGQDLLSSHLAPAEKVSIMADAMRRLHTLDPATC | 131 |
| Sbjct 69 | +PC VL AG WLLL G DL S L AE+++I+A+A+RRLH LDPA C AQDMPCPRVLGHDDHAGETWLLLEAAEGMDLASCRLPHAERIAILAEALRRLHRLDPAAC | 128 |
| Query 132 | PFDHQAKHRIERARTRMEAGLVDQDDLDEEHQGLAPAELFARLKARMPDGEDLVVTHGDA | 191 |
| Sbjct 129 | PFD + I A+ RMEAGLVD+ D DEE QG ELF L AR P E LVVTHGDA PFDRRLTASIAAAKARMEAGLVDESDFDEERQGRQTRELFDELLARRPKEEQLVVTHGDA | 188 |
| Query 192 | CLPNIMVENGRFSGFIDCGRLGVADRYQDIALATRDIAEELGGEWADRFLVLYGIAAPDS | 251 |
| Sbjct 189 | CLPN + +FSGFIDCGRLGVAD YQD+ALA I LG EW FL YG+AAPD CLPNFIAAGNQFSGFIDCGRLGVADIYQDLALACWSITHNLGEEWVAPFLDRYGLAAPDP | 248 |
| Query 252  Sbjct 249 | QRIAFYRLLDEFF 264  +++AFYRLLDEFF  EKLAFYRLLDEFF 261 |  |

>WP_104665589.1 aminoglycoside 3'-phosphotransferase [Ensifer adhaerens] Length=308

Score = 264 bits (674), Expect = 3e-84, Method: Compositional matrix adjust.

| Identities | | = 132/257 (51%), Positives = 171/257 (67%), Gaps = 0/257 (0%) |  |
| --- | --- | --- | --- |
| Query 8  Sbjct 52 | | HAGSPAAWVERLFGYDWAQQTIGCSDAAVFRLSAQGRPVLFVKTDLSGALNELQDEAARL H P + + GY W + +G SD+++F L A RP+L +K + +G +E DEAARL HPELPDPFRPLVAGYQWNRDLLGQSDSSIFLLKAPDRPMLVLKVEEAGPFSEFVDEAARL | 67  111 |
| Query 68  Sbjct 112 | | SWLATTGVPCAAVLDVVTEAGRDWLLLGEVPGQDLLSSHLAPAEKVSIMADAMRRLHTLD WLA+ G+PC V+ +A +WLLL V G DL S+ L+P +++ I+ADA++RLH LD DWLASKGMPCPRVIARAFDAETNWLLLQAVEGTDLASATLSPKDQIVILADALKRLHALD | 127  171 |
| Query 128  Sbjct 172 | | PATCPFDHQAKHRIERARTRMEAGLVDQDDLDEEHQGLAPAELFARLKARMPDGEDLVVT ATCPFDH+ + RI A+ R AG++D+ D DE G A+LFA L+AR P + LVVT VATCPFDHRVEKRIAIAKARTCAGVIDESDFDETRLGRTAADLFAELQARKPQADTLVVT | 187  231 |
| Query 188  Sbjct 232 | | HGDACLPNIMVENGRFSGFIDCGRLGVADRYQDIALATRDIAEELGGEWADRFLVLYGIA HGDACLPNI+ +NGRFSGFIDC RLGVAD QDIALA R IA LG EW FL YG+ HGDACLPNIIEKNGRFSGFIDCSRLGVADPCQDIALACRSIAYNLGEEWVQPFLTRYGVD | 247  291 |
| Query 248 | | APDSQRIAFYRLLDEFF 264 D ++ FY LLDEFF |  |
| Sbjct | 292 | KDDPKKRDFYCLLDEFF 308 | |

>WP_023109641.1 APH(3')-II family aminoglycoside O-phosphotransferase [Pseudomonas aeruginosa]

ERY70032.1 aminoglycoside 3'-phosphotransferase type IIB [Pseudomonas aeruginosa BL01]

KSM31993.1 aminoglycoside phosphotransferase APH(3') [Pseudomonas aeruginosa]

MBG6284551.1 APH(3')-II family aminoglycoside O-phosphotransferase [Pseudomonas aeruginosa]

MBG6404514.1 APH(3')-II family aminoglycoside O-phosphotransferase [Pseudomonas aeruginosa]

NTT40729.1 APH(3')-II family aminoglycoside O-phosphotransferase [Pseudomonas aeruginosa]

Length=268

Score = 263 bits (671), Expect = 3e-84, Method: Compositional matrix adjust. Identities = 137/258 (53%), Positives = 169/258 (66%), Gaps = 5/258 (2%)

Query 11	SPAAWVERLFGYDWAQQTIGCSDAAVFRLSAQGRPVLFVKTDLSGALNELQDEAARLSWL 70

+P+ W + L GY W Q GCS A V RL A RP LFVK ++ A EL E ARL WL

| Sbjct | 12 | APSTWADYLAGYRWRGQGEGCSAATVHRLEAARRPTLFVKQEVLSAHAELPAEIARLRWL | 71 |
| --- | --- | --- | --- |
| Query | 71 | ATTGVPCAAVLDVVTEAGRDWLLLGEVPGQDLLS SHLAPAEKVSIMADAMRRLHTL | 126 |
| Sbjct | 72 | G+ C VL+ GR WLL+ VPG L + L P V ++A A+RRLH L HGAGIDCPQVLNETQSDGRQWLLMSAVPGDTLSALAQRGELEPGRLVRLVAAALRRLHDL | 131 |
| Query | 127 | DPATCPFDHQAKHRIERARTRMEAGLVDQDDLDEEHQGLAPAELFARLKARMPDGEDLVV | 186 |
| Sbjct | 132 | DPA CPFDH+ + R++ R R+EAGLVD+ D D++H+G + EL+ L R P EDLVV DPAACPFDHRLERRLDTVRQRVEAGLVDEADFDDDHRGRSATELYRLLLDRRPAVEDLVV | 191 |
| Query | 187 | THGDACLPNIMVENGRFSGFIDCGRLGVADRYQDIALATRDIAEELGGEWADRFLVLYGI | 246 |
| Sbjct | 192 | HGDACLPN++ E RFSGFIDCGRLGVADR+QD+ALA RDI ELG WA+ FLV YG  AHGDACLPNLLAEGRRFSGFIDCGRLGVADRHQDLALAARDIEAELGAAWAEAFLVEYG- | 250 |
| Query | 247 AAPDSQRIAFYRLLDEFF 264 | | |
|  |  | D +R+A++RLLDEFF |  |
| Sbjct | 251 | GDIDGERLAYFRLLDEFF 268 | |

>WP_132555846.1 APH(3')-II family aminoglycoside O-phosphotransferase [Pseudomonas aeruginosa]

Length=268

Score = 263 bits (671), Expect = 3e-84, Method: Compositional matrix adjust.

| Identities | | = 137/258 (53%), Positives = 170/258 (66%), Gaps = 5/258 (2%) |  |
| --- | --- | --- | --- |
| Query 11  Sbjct 12 | | SPAAWVERLFGYDWAQQTIGCSDAAVFRLSAQGRPVLFVKTDLSGALNELQDEAARLSWL  +P+ W + L GY W Q GCS A V RL A+ RP LFVK ++ A EL E ARL WL APSTWADYLAGYRWRGQGEGCSAATVHRLEAERRPTLFVKQEVLXAHAELPAEIARLRWL | 70  71 |
| Query 71  Sbjct 72 | | ATTGVPCAAVLDVVTEAGRDWLLLGEVPGQDLLS SHLAPAEKVSIMADAMRRLHTL  G+ C VL+ GR WLL+ VPG L + L P V ++A A+RRLH L HGAGIDCPQVLNETQSDGRQWLLMSAVPGDTLSALAQRGELEPERLVRLVAAALRRLHDL | 126  131 |
| Query 127  Sbjct 132 | | DPATCPFDHQAKHRIERARTRMEAGLVDQDDLDEEHQGLAPAELFARLKARMPDGEDLVV DPA CPFDH+ + R++ R R+EAGLVD+ D D++H+G + EL+ L R P EDLVV DPAACPFDHRLERRLDTVRQRVEAGLVDEADFDDDHRGRSATELYRLLLDRRPAVEDLVV | 186  191 |
| Query 187  Sbjct 192 | | THGDACLPNIMVENGRFSGFIDCGRLGVADRYQDIALATRDIAEELGGEWADRFLVLYGI HGDACLPN++ E RFSGFIDCGRLGVADR+QD+ALA RDI ELG WA+ FLV YG AHGDACLPNLLAEGRRFSGFIDCGRLGVADRHQDLALAARDIEAELGAAWAEAFLVEYG- | 246  250 |
| Query | 247 AAPDSQRIAFYRLLDEFF 264 | | |
|  |  | D +R+A++RLLDEFF |  |
| Sbjct | 251 | GDIDGERLAYFRLLDEFF 268 | |

>WP_058179574.1 APH(3')-II family aminoglycoside O-phosphotransferase [Pseudomonas aeruginosa]

KSJ02085.1 aminoglycoside 3'-phosphotransferase [Pseudomonas aeruginosa]

MBG7460320.1 APH(3')-II family aminoglycoside O-phosphotransferase [Pseudomonas aeruginosa]

RTU31798.1 APH(3')-II family aminoglycoside O-phosphotransferase [Pseudomonas aeruginosa]

Length=268

Score = 262 bits (670), Expect = 3e-84, Method: Compositional matrix adjust.

| Identities | = 138/258 (53%), Positives = 170/258 (66%), Gaps = 5/258 (2%) |  |
| --- | --- | --- |
| Query 11 | SPAAWVERLFGYDWAQQTIGCSDAAVFRLSAQGRPVLFVKTDLSGALNELQDEAARLSWL | 70 |
| Sbjct 12 | +PA W + L GY W Q GCS A V RL A RP LFVK ++ A EL E ARL WL APATWDDYLAGYRWRGQGEGCSAATVHRLEAARRPTLFVKQEVLSAHAELPAEIARLRWL | 71 |
| Query 71 | ATTGVPCAAVLDVVTEAGRDWLLLGEVPGQDLLS SHLAPAEKVSIMADAMRRLHTL | 126 |
| Sbjct 72 | G+ C VL+ GR WLL+ VPG L + L P V ++A A+RRLH L HGAGIDCPQVLNETQSDGRQWLLMSAVPGDTLSALAQCGELEPGRLVRLVAAALRRLHDL | 131 |
| Query 127 | DPATCPFDHQAKHRIERARTRMEAGLVDQDDLDEEHQGLAPAELFARLKARMPDGEDLVV | 186 |
| Sbjct 132 | DPA CPFDH+ + R++ R R+EAGLVD+ D D++H+G + +EL+ L R P EDLVV DPAACPFDHRLERRLDTVRQRVEAGLVDEADFDDDHRGRSASELYRLLLDRRPAVEDLVV | 191 |
| Query 187 | THGDACLPNIMVENGRFSGFIDCGRLGVADRYQDIALATRDIAEELGGEWADRFLVLYGI | 246 |

| Sbjct | 192 | HGDACLPN++ E RFSGFIDCGRLGVADR+QD+ALA RDI ELG WA+ FLV YG AHGDACLPNLLAEGRRFSGFIDCGRLGVADRHQDLALAARDIEAELGAAWAEAFLVEYG- 250 |
| --- | --- | --- |
| Query | 247 | AAPDSQRIAFYRLLDEFF 264 |
| Sbjct | 251 | D +R+A++RLLDEFF  GDIDGERLAYFRLLDEFF 268 |

>MBG6828692.1 APH(3')-II family aminoglycoside O-phosphotransferase [Pseudomonas aeruginosa]

Length=268

Score = 262 bits (670), Expect = 3e-84, Method: Compositional matrix adjust.

| Identities | | = 137/258 (53%), Positives = 169/258 (66%), Gaps = 5/258 (2%) |  |
| --- | --- | --- | --- |
| Query 11  Sbjct 12 | | SPAAWVERLFGYDWAQQTIGCSDAAVFRLSAQGRPVLFVKTDLSGALNELQDEAARLSWL  +P+ W + L GY W Q GCS A V RL A RP LFVK ++ A EL E ARL WL APSTWADYLAGYRWRGQGEGCSAATVHRLEAARRPTLFVKQEVLSAHAELPAEIARLRWL | 70  71 |
| Query 71  Sbjct 72 | | ATTGVPCAAVLDVVTEAGRDWLLLGEVPGQDLLS SHLAPAEKVSIMADAMRRLHTL  G+ C VL+ GR WLL+ VPG L + L P V ++A A+RRLH L HGAGIDCPQVLNETQSDGRQWLLMSAVPGDTLSALAQCGELEPGRLVRLVAAALRRLHDL | 126  131 |
| Query 127  Sbjct 132 | | DPATCPFDHQAKHRIERARTRMEAGLVDQDDLDEEHQGLAPAELFARLKARMPDGEDLVV DPA CPFDH+ + R++ R R+EAGLVD+ D D++H+G + EL+ L R P EDLVV DPAACPFDHRLERRLDTVRQRVEAGLVDEADFDDDHRGRSATELYRLLLDRRPAVEDLVV | 186  191 |
| Query 187  Sbjct 192 | | THGDACLPNIMVENGRFSGFIDCGRLGVADRYQDIALATRDIAEELGGEWADRFLVLYGI HGDACLPN++ E RFSGFIDCGRLGVADR+QD+ALA RDI ELG WA+ FLV YG AHGDACLPNLLAEGRRFSGFIDCGRLGVADRHQDLALAARDIEAELGAAWAEAFLVEYG- | 246  250 |
| Query | 247 AAPDSQRIAFYRLLDEFF 264 | | |
|  |  | D +R+A++RLLDEFF |  |
| Sbjct | 251 | GDIDGERLAYFRLLDEFF 268 | |

>WP_124131361.1 APH(3')-II family aminoglycoside O-phosphotransferase [Pseudomonas aeruginosa]

RPX45536.1 APH(3') family aminoglycoside O-phosphotransferase [Pseudomonas aeruginosa]

Length=268

Score = 262 bits (670), Expect = 3e-84, Method: Compositional matrix adjust.

| Identities | | = 138/258 (53%), Positives = 168/258 (65%), Gaps = 5/258 (2%) |  |
| --- | --- | --- | --- |
| Query 11  Sbjct 12 | | SPAAWVERLFGYDWAQQTIGCSDAAVFRLSAQGRPVLFVKTDLSGALNELQDEAARLSWL  +PA W + L GY W Q GCS A V RL A RP LFVK ++ A EL E ARL WL APATWADYLAGYRWRGQGEGCSAATVHRLEAARRPTLFVKQEVLSAHAELPAEIARLRWL | 70  71 |
| Query 71  Sbjct 72 | | ATTGVPCAAVLDVVTEAGRDWLLLGEVPGQDL LSSHLAPAEKVSIMADAMRRLHTL  G+ C VL+ GR WLL+ VPG L L P V ++A A+RRLH L HGAGIDCPQVLNETQSDGRQWLLMSAVPGDTLSXLAQRGELEPERLVRLVAAALRRLHDL | 126  131 |
| Query 127  Sbjct 132 | | DPATCPFDHQAKHRIERARTRMEAGLVDQDDLDEEHQGLAPAELFARLKARMPDGEDLVV DPA CPFDH+ + R++ R R+EAGLVD+ D D++H+G + EL+ L R P EDLVV DPAACPFDHRLERRLDTVRQRVEAGLVDEADFDDDHRGRSATELYRLLLDRCPAVEDLVV | 186  191 |
| Query 187  Sbjct 192 | | THGDACLPNIMVENGRFSGFIDCGRLGVADRYQDIALATRDIAEELGGEWADRFLVLYGI HGDACLPN++ E RFSGFIDCGRLGVADR+QD+ALA RDI ELG WA+ FLV YG AHGDACLPNLLAEGRRFSGFIDCGRLGVADRHQDLALAARDIEAELGAAWAEAFLVEYG- | 246  250 |
| Query | 247 AAPDSQRIAFYRLLDEFF 264 | | |
|  |  | D +R+A++RLLDEFF |  |
| Sbjct | 251 | GDIDGERLAYFRLLDEFF 268 | |

>WP_061197678.1 APH(3')-II family aminoglycoside O-phosphotransferase [Pseudomonas aeruginosa]

KXF23803.1 aminoglycoside phosphotransferase [Pseudomonas aeruginosa] Length=268

Score = 262 bits (670), Expect = 3e-84, Method: Compositional matrix adjust.

| Identities | | = 138/258 (53%), Positives = 169/258 (66%), Gaps = 5/258 (2%) |  |
| --- | --- | --- | --- |
| Query 11  Sbjct 12 | | SPAAWVERLFGYDWAQQTIGCSDAAVFRLSAQGRPVLFVKTDLSGALNELQDEAARLSWL  +PA W + L GY W Q GCS A V RL A RP LFVK ++ A EL E ARL WL APATWADYLAGYRWRGQGEGCSAATVHRLEAARRPTLFVKQEVLSAHAELPAEIARLRWL | 70  71 |
| Query 71  Sbjct 72 | | ATTGVPCAAVLDVVTEAGRDWLLLGEVPGQDLLS SHLAPAEKVSIMADAMRRLHTL  G+ C VL+ GR WLL+ VPG L + L P V ++A A+RRLH L HGAGIDCPQVLNETQSDGRQWLLMSAVPGGTLSALAQRGELEPERLVRLVAAALRRLHDL | 126  131 |
| Query 127  Sbjct 132 | | DPATCPFDHQAKHRIERARTRMEAGLVDQDDLDEEHQGLAPAELFARLKARMPDGEDLVV DPA CPFDH+ + R++ R R+EAGLVD+ D D++H+G + EL+ L R P EDLVV DPAACPFDHRLERRLDTVRQRVEAGLVDEADFDDDHRGRSATELYRLLLDRRPAVEDLVV | 186  191 |
| Query 187  Sbjct 192 | | THGDACLPNIMVENGRFSGFIDCGRLGVADRYQDIALATRDIAEELGGEWADRFLVLYGI HGDACLPN++ E RFSGFIDCGRLGVADR+QD+ALA RDI ELG WA+ FLV YG AHGDACLPNLLAEGRRFSGFIDCGRLGVADRHQDLALAARDIEAELGAAWAEAFLVEYG- | 246  250 |
| Query | 247 AAPDSQRIAFYRLLDEFF 264 | | |
|  |  | D +R+A++RLLDEFF |  |
| Sbjct | 251 | GDIDGERLAYFRLLDEFF 268 | |

>WP_073649904.1 APH(3')-II family aminoglycoside O-phosphotransferase [Pseudomonas aeruginosa]

Length=268

Score = 262 bits (670), Expect = 4e-84, Method: Compositional matrix adjust.

| Identities | | = 138/258 (53%), Positives = 170/258 (66%), Gaps = 5/258 (2%) |  |
| --- | --- | --- | --- |
| Query 11  Sbjct 12 | | SPAAWVERLFGYDWAQQTIGCSDAAVFRLSAQGRPVLFVKTDLSGALNELQDEAARLSWL  +PA W + L GY W Q GCS A V RL A RP LFVK ++ A EL E ARL WL APATWDDYLAGYRWRGQGEGCSAATVHRLEAARRPTLFVKQEVLSAHAELPAEIARLRWL | 70  71 |
| Query 71  Sbjct 72 | | ATTGVPCAAVLDVVTEAGRDWLLLGEVPGQDLLS SHLAPAEKVSIMADAMRRLHTL  G+ C VL+ GR WLL+ VPG L + L P V ++A A+RRLH L HGAGIDCPQVLNETQSDGRQWLLMSAVPGDTLSALAQRGELEPGRLVRLVAAALRRLHDL | 126  131 |
| Query 127  Sbjct 132 | | DPATCPFDHQAKHRIERARTRMEAGLVDQDDLDEEHQGLAPAELFARLKARMPDGEDLVV DPA CPFDH+ + R++ R R+EAGLVD+ D D++H+G + +EL+ L R P EDLVV DPAACPFDHRLERRLDTVRQRVEAGLVDEADFDDDHRGRSASELYRLLLDRRPAVEDLVV | 186  191 |
| Query 187  Sbjct 192 | | THGDACLPNIMVENGRFSGFIDCGRLGVADRYQDIALATRDIAEELGGEWADRFLVLYGI HGDACLPN++ E RFSGFIDCGRLGVADR+QD+ALA RDI ELG WA+ FLV YG AHGDACLPNLLAEGRRFSGFIDCGRLGVADRHQDLALAARDIEAELGAAWAEAFLVEYG- | 246  250 |
| Query | 247 AAPDSQRIAFYRLLDEFF 264 | | |
|  |  | D +R+A++RLLDEFF |  |
| Sbjct | 251 | GDIDGERLAYFRLLDEFF 268 | |

>PLP56035.1 APH(3') family aminoglycoside O-phosphotransferase [Mesorhizobium loti]

Length=269

Score = 262 bits (670), Expect = 4e-84, Method: Compositional matrix adjust.

| Identities | = 135/254 (53%), Positives = 169/254 (67%), Gaps = 1/254 (0%) |  |
| --- | --- | --- |
| Query 12 | PAAWVERLFGYDWAQQTIGCSDAAVFRLSAQGRPVLFVKTDLSGALNELQDEAARLSWLA | 71 |
| Sbjct 16 | P ++E + GY QQT+G S+A VF+L A +P LF+K D +G EL E RL WL PQPFLELVGGYSSLQQTVGLSNAGVFKLEAADKPSLFLKIDEAGNFAELPAEVERLRWLG | 75 |
| Query 72 | TTGVPCAAVLDVVTEAGRDWLLLGEVPGQDLLSSHLAPAEK-VSIMADAMRRLHTLDPAT | 130 |
| Sbjct 76 | G+ C VL R+WLL+ +PG+DL S+ PAE V+IMA A+R LH LD + QQGISCPEVLAFEAHGDRNWLLMSAMPGRDLESASSLPAETTVTIMASALRTLHALDVRS | 135 |
| Query 131 | CPFDHQAKHRIERARTRMEAGLVDQDDLDEEHQGLAPAELFARLKARMPDGEDLVVTHGD | 190 |
| Sbjct 136 | CPFDH+ RI A+ RMEAGLVD++D D E QG ++FA L A P EDLVVTHGD CPFDHRLDRRIADAKARMEAGLVDEEDFDGERQGRTARDVFADLIALRPASEDLVVTHGD | 195 |
| Query 191 | ACLPNIMVENGRFSGFIDCGRLGVADRYQDIALATRDIAEELGGEWADRFLVLYGIAAPD ACLPN MV+ RFSGFIDCGRLGVADR QD+ALA R I ++LG +W FL LYG + D | 250 |

| Sbjct | 196 | ACLPNFMVDADRFSGFIDCGRLGVADRCQDLALACRSIHDDLGEDWIAPFLALYGKSDTD 255 |
| --- | --- | --- |
| Query | 251 | SQRIAFYRLLDEFF 264 |
| Sbjct | 256 | ++A+Y+LLDEFF  PLKLAYYQLLDEFF 269 |

>WP_093233146.1 aminoglycoside 3'-phosphotransferase [Sinorhizobium sp. NFACC03] Length=254

Score = 262 bits (669), Expect = 4e-84, Method: Compositional matrix adjust.

| Identities | = 130/244 (53%), Positives = 166/244 (68%), Gaps = 0/244 (0%) |  |
| --- | --- | --- |
| Query 21 | GYDWAQQTIGCSDAAVFRLSAQGRPVLFVKTDLSGALNELQDEAARLSWLATTGVPCAAV | 80 |
| Sbjct 11 | GYDW T+G SD++++ L A RP+L +K + +G +E DEA RL WLA+ G+PC V GYDWNHDTLGQSDSSIYLLKAPDRPMLVLKVEAAGPFSEFVDEAERLDWLASQGMPCPRV | 70 |
| Query 81 | LDVVTEAGRDWLLLGEVPGQDLLSSHLAPAEKVSIMADAMRRLHTLDPATCPFDHQAKHR | 140 |
| Sbjct 71 | + +A +WLLL V G DL S+ L+P +++ I+ADA++RLH LD A CPFDH+ + R IARAFDAETNWLLLQAVEGTDLASTTLSPKDRIVILADALKRLHGLDVADCPFDHRIEKR | 130 |
| Query 141 | IERARTRMEAGLVDQDDLDEEHQGLAPAELFARLKARMPDGEDLVVTHGDACLPNIMVEN | 200 |
| Sbjct 131 | I A R EAGL+D+DD DE G A+LFA L+AR+P LV+THGDACLPNI+ +N IAIAGARTEAGLIDEDDFDETRLGRTAADLFAELRARVPSIGKLVITHGDACLPNIIEKN | 190 |
| Query 201 | GRFSGFIDCGRLGVADRYQDIALATRDIAEELGGEWADRFLVLYGIAAPDSQRIAFYRLL | 260 |
| Sbjct 191 | GRFSGFIDC +LGVAD YQDIALA IA LG EW FL YGI D ++ Y LL GRFSGFIDCSQLGVADPYQDIALACHSIAHNLGEEWVQPFLARYGIDRDDPEKRDLYCLL | 250 |
| Query 261  Sbjct 251 | DEFF 264 DEFF  DEFF 254 |  |

>SDA69881.1 aminoglycoside 3'-phosphotransferase-2 [Sinorhizobium sp. NFACC03] Length=263

Score = 262 bits (669), Expect = 4e-84, Method: Compositional matrix adjust.

| Identities | = 130/244 (53%), Positives = 166/244 (68%), Gaps = 0/244 (0%) |  |
| --- | --- | --- |
| Query 21 | GYDWAQQTIGCSDAAVFRLSAQGRPVLFVKTDLSGALNELQDEAARLSWLATTGVPCAAV | 80 |
| Sbjct 20 | GYDW T+G SD++++ L A RP+L +K + +G +E DEA RL WLA+ G+PC V GYDWNHDTLGQSDSSIYLLKAPDRPMLVLKVEAAGPFSEFVDEAERLDWLASQGMPCPRV | 79 |
| Query 81 | LDVVTEAGRDWLLLGEVPGQDLLSSHLAPAEKVSIMADAMRRLHTLDPATCPFDHQAKHR | 140 |
| Sbjct 80 | + +A +WLLL V G DL S+ L+P +++ I+ADA++RLH LD A CPFDH+ + R IARAFDAETNWLLLQAVEGTDLASTTLSPKDRIVILADALKRLHGLDVADCPFDHRIEKR | 139 |
| Query 141 | IERARTRMEAGLVDQDDLDEEHQGLAPAELFARLKARMPDGEDLVVTHGDACLPNIMVEN | 200 |
| Sbjct 140 | I A R EAGL+D+DD DE G A+LFA L+AR+P LV+THGDACLPNI+ +N IAIAGARTEAGLIDEDDFDETRLGRTAADLFAELRARVPSIGKLVITHGDACLPNIIEKN | 199 |
| Query 201 | GRFSGFIDCGRLGVADRYQDIALATRDIAEELGGEWADRFLVLYGIAAPDSQRIAFYRLL | 260 |
| Sbjct 200 | GRFSGFIDC +LGVAD YQDIALA IA LG EW FL YGI D ++ Y LL GRFSGFIDCSQLGVADPYQDIALACHSIAHNLGEEWVQPFLARYGIDRDDPEKRDLYCLL | 259 |
| Query 261  Sbjct 260 | DEFF 264 DEFF  DEFF 263 |  |

```
>WP_180883024.1 APH(3') family aminoglycoside O-phosphotransferase [Mesorhizobium loti]
Length=264

Score = 262 bits (669), Expect = 4e-84, Method: Compositional matrix adjust. Identities = 135/254 (53%), Positives = 169/254 (67%), Gaps = 1/254 (0%)

Query 12	PAAWVERLFGYDWAQQTIGCSDAAVFRLSAQGRPVLFVKTDLSGALNELQDEAARLSWLA 71 P ++E + GY	QQT+G S+A VF+L A +P LF+K D +G	EL E RL WL
Sbjct 11	PQPFLELVGGYSSLQQTVGLSNAGVFKLEAADKPSLFLKIDEAGNFAELPAEVERLRWLG 70
```

| Query | 72 | TTGVPCAAVLDVVTEAGRDWLLLGEVPGQDLLSSHLAPAEK-VSIMADAMRRLHTLDPAT | 130 |
| --- | --- | --- | --- |
| Sbjct | 71 | G+ C VL R+WLL+ +PG+DL S+ PAE V+IMA A+R LH LD + QQGISCPEVLAFEAHGDRNWLLMSAMPGRDLESASSLPAETTVTIMASALRTLHALDVRS | 130 |
| Query | 131 | CPFDHQAKHRIERARTRMEAGLVDQDDLDEEHQGLAPAELFARLKARMPDGEDLVVTHGD | 190 |
| Sbjct | 131 | CPFDH+ RI A+ RMEAGLVD++D D E QG ++FA L A P EDLVVTHGD CPFDHRLDRRIADAKARMEAGLVDEEDFDGERQGRTARDVFADLIALRPASEDLVVTHGD | 190 |
| Query | 191 | ACLPNIMVENGRFSGFIDCGRLGVADRYQDIALATRDIAEELGGEWADRFLVLYGIAAPD | 250 |
| Sbjct | 191 | ACLPN MV+ RFSGFIDCGRLGVADR QD+ALA R I ++LG +W FL LYG + D ACLPNFMVDADRFSGFIDCGRLGVADRCQDLALACRSIHDDLGEDWIAPFLALYGKSDTD | 250 |
| Query | 251 | SQRIAFYRLLDEFF 264  ++A+Y+LLDEFF |  |
| Sbjct | 251 | PLKLAYYQLLDEFF 264 | |

>WP_019371448.1 APH(3')-II family aminoglycoside O-phosphotransferase [Pseudomonas aeruginosa]

KSP19575.1 aminoglycoside 3'-phosphotransferase [Pseudomonas aeruginosa] KXC13923.1 aminoglycoside phosphotransferase [Pseudomonas aeruginosa] KXC17517.1 aminoglycoside phosphotransferase [Pseudomonas aeruginosa] KXC29686.1 aminoglycoside phosphotransferase [Pseudomonas aeruginosa] KXC31041.1 aminoglycoside phosphotransferase [Pseudomonas aeruginosa]

Length=267

Score = 262 bits (670), Expect = 4e-84, Method: Compositional matrix adjust.

| Identities | | = 137/258 (53%), Positives = 169/258 (66%), Gaps = 5/258 (2%) |  |
| --- | --- | --- | --- |
| Query 11  Sbjct 11 | | SPAAWVERLFGYDWAQQTIGCSDAAVFRLSAQGRPVLFVKTDLSGALNELQDEAARLSWL  +P+ W + L GY W Q GCS A V RL A RP LFVK ++ A EL E ARL WL APSTWADYLAGYRWRGQGEGCSAATVHRLEAARRPTLFVKQEVLSAHAELPAEIARLRWL | 70  70 |
| Query 71  Sbjct 71 | | ATTGVPCAAVLDVVTEAGRDWLLLGEVPGQDLLS SHLAPAEKVSIMADAMRRLHTL  G+ C VL+ GR WLL+ VPG L + L P V ++A A+RRLH L HGAGIDCPQVLNETQSDGRQWLLMSAVPGDTLSALAQRGELEPERLVRLVAAALRRLHDL | 126  130 |
| Query 127  Sbjct 131 | | DPATCPFDHQAKHRIERARTRMEAGLVDQDDLDEEHQGLAPAELFARLKARMPDGEDLVV DPA CPFDH+ + R++ R R+EAGLVD+ D D++H+G + EL+ L R P EDLVV DPAACPFDHRLERRLDTVRQRVEAGLVDEADFDDDHRGRSATELYRLLLDRRPAVEDLVV | 186  190 |
| Query 187  Sbjct 191 | | THGDACLPNIMVENGRFSGFIDCGRLGVADRYQDIALATRDIAEELGGEWADRFLVLYGI HGDACLPN++ E RFSGFIDCGRLGVADR+QD+ALA RDI ELG WA+ FLV YG AHGDACLPNLLAEGRRFSGFIDCGRLGVADRHQDLALAARDIEAELGAAWAEAFLVEYG- | 246  249 |
| Query | 247 AAPDSQRIAFYRLLDEFF 264 | | |
|  |  | D +R+A++RLLDEFF |  |
| Sbjct | 250 | GDIDGERLAYFRLLDEFF 267 | |

>MBG6570794.1 APH(3')-II family aminoglycoside O-phosphotransferase [Pseudomonas aeruginosa]

Length=268

Score = 262 bits (670), Expect = 4e-84, Method: Compositional matrix adjust.

| Identities | = 136/258 (53%), Positives = 169/258 (66%), Gaps = 5/258 (2%) |  |
| --- | --- | --- |
| Query 11 | SPAAWVERLFGYDWAQQTIGCSDAAVFRLSAQGRPVLFVKTDLSGALNELQDEAARLSWL | 70 |
| Sbjct 12 | +P+ W + L GY W Q GCS A V RL RP LFVK ++ A EL E ARL WL APSTWADYLAGYRWRGQGEGCSAATVHRLEVARRPTLFVKQEVLSAHAELPAEIARLRWL | 71 |
| Query 71 | ATTGVPCAAVLDVVTEAGRDWLLLGEVPGQDLLS SHLAPAEKVSIMADAMRRLHTL | 126 |
| Sbjct 72 | G+ C VL+ GR WLL+ VPG L + L P V ++A A+RRLH L HGAGIDCPQVLNETQSDGRQWLLMSAVPGDTLSALAQRGELEPERLVRLVAAALRRLHDL | 131 |
| Query 127 | DPATCPFDHQAKHRIERARTRMEAGLVDQDDLDEEHQGLAPAELFARLKARMPDGEDLVV | 186 |
| Sbjct 132 | DPA CPFDH+ + R++ R R+EAGLVD+ D D++H+G + EL+ L+AR P EDLVV DPAACPFDHRLERRLDTVRQRVEAGLVDEADFDDDHRGRSATELYRLLRARRPAVEDLVV | 191 |
| Query 187 | THGDACLPNIMVENGRFSGFIDCGRLGVADRYQDIALATRDIAEELGGEWADRFLVLYGI | 246 |
| Sbjct 192 | HGDACLPN++ E RFSGFIDCGRLGVA R+QD+ALA RDI ELG WA+ FLV YG  AHGDACLPNLLAEGRRFSGFIDCGRLGVAARHQDLALAARDIEAELGAAWAEAFLVEYG- | 250 |

Query 247 AAPDSQRIAFYRLLDEFF 264

D +R+A++RLLDEFF Sbjct 251 GDIDGERLAYFRLLDEFF 268

>WP_003113011.1 MULTISPECIES: aminoglycoside O-phosphotransferase APH(3')-IIb [Pseudomonas]

NP_252808.1 aminoglycoside 3-N-acetyltransferase [Pseudomonas aeruginosa PAO1]

ESR72708.1 aminoglycoside 3'-phosphotransferase [Pseudomonas aeruginosa VRFPA05]

EVT84343.1 aminoglycoside 3'-phosphotransferase [Pseudomonas aeruginosa VRFPA09]

SST08892.1 aminoglycoside 3'-phosphotransferase aphA1 [Acinetobacter baumannii] AAG07506.1 aminoglycoside 3'-phosphotransferase type IIb [Pseudomonas aeruginosa

PAO1]

AGI79746.1 aminoglycoside 3-N-acetyltransferase [Pseudomonas aeruginosa B136-33]

Length=268

Score = 262 bits (670), Expect = 4e-84, Method: Compositional matrix adjust.

| Identities | | = 137/258 (53%), Positives = 169/258 (66%), Gaps = 5/258 (2%) |  |
| --- | --- | --- | --- |
| Query 11  Sbjct 12 | | SPAAWVERLFGYDWAQQTIGCSDAAVFRLSAQGRPVLFVKTDLSGALNELQDEAARLSWL  +P+ W + L GY W Q GCS A V RL A RP LFVK ++ A EL E ARL WL APSTWADYLAGYRWRGQGEGCSAATVHRLEAARRPTLFVKQEVLSAHAELPAEIARLRWL | 70  71 |
| Query 71  Sbjct 72 | | ATTGVPCAAVLDVVTEAGRDWLLLGEVPGQDLLS SHLAPAEKVSIMADAMRRLHTL  G+ C VL+ GR WLL+ VPG L + L P V ++A A+RRLH L HGAGIDCPQVLNETQSDGRQWLLMSAVPGDTLSALAQRGELEPERLVRLVAAALRRLHDL | 126  131 |
| Query 127  Sbjct 132 | | DPATCPFDHQAKHRIERARTRMEAGLVDQDDLDEEHQGLAPAELFARLKARMPDGEDLVV DPA CPFDH+ + R++ R R+EAGLVD+ D D++H+G + EL+ L R P EDLVV DPAACPFDHRLERRLDTVRQRVEAGLVDEADFDDDHRGRSATELYRLLLDRRPAVEDLVV | 186  191 |
| Query 187  Sbjct 192 | | THGDACLPNIMVENGRFSGFIDCGRLGVADRYQDIALATRDIAEELGGEWADRFLVLYGI HGDACLPN++ E RFSGFIDCGRLGVADR+QD+ALA RDI ELG WA+ FLV YG AHGDACLPNLLAEGRRFSGFIDCGRLGVADRHQDLALAARDIEAELGAAWAEAFLVEYG- | 246  250 |
| Query | 247 AAPDSQRIAFYRLLDEFF 264 | | |
|  |  | D +R+A++RLLDEFF |  |
| Sbjct | 251 | GDIDGERLAYFRLLDEFF 268 | |

>WP_023105023.1 APH(3')-II family aminoglycoside O-phosphotransferase [Pseudomonas aeruginosa]

AHW69309.1 aminoglycoside 3'-phosphotransferase type IIb [Pseudomonas aeruginosa PA96]

ERY48040.1 aminoglycoside 3'-phosphotransferase type IIB [Pseudomonas aeruginosa BL05]

KSC21690.1 aminoglycoside phosphotransferase APH(3') [Pseudomonas aeruginosa] KSC95150.1 aminoglycoside phosphotransferase APH(3') [Pseudomonas aeruginosa] KSH26531.1 aminoglycoside phosphotransferase APH(3') [Pseudomonas aeruginosa]

Length=268

Score = 262 bits (670), Expect = 4e-84, Method: Compositional matrix adjust.

| Identities | = 138/258 (53%), Positives = 169/258 (66%), Gaps = 5/258 (2%) |  |
| --- | --- | --- |
| Query 11 | SPAAWVERLFGYDWAQQTIGCSDAAVFRLSAQGRPVLFVKTDLSGALNELQDEAARLSWL | 70 |
| Sbjct 12 | +PA W + L GY W Q GCS A V RL A RP LFVK ++ A EL E ARL WL APATWADYLAGYCWRGQGEGCSAATVHRLEAARRPTLFVKQEVLSAHAELPAEIARLRWL | 71 |
| Query 71 | ATTGVPCAAVLDVVTEAGRDWLLLGEVPGQDLLS SHLAPAEKVSIMADAMRRLHTL | 126 |
| Sbjct 72 | G+ C VL+ GR WLL+ VPG L + L P V ++A A+RRLH L HGAGIDCPQVLNETQSDGRQWLLMSAVPGDTLSALAQRGELEPERLVRLVAAALRRLHDL | 131 |
| Query 127 | DPATCPFDHQAKHRIERARTRMEAGLVDQDDLDEEHQGLAPAELFARLKARMPDGEDLVV | 186 |
| Sbjct 132 | DPA CPFDH+ + R++ R R+EAGLVD+ D D++H+G + EL+ L R P EDLVV DPAACPFDHRLERRLDTVRQRVEAGLVDEADFDDDHRGRSATELYRLLLDRRPAVEDLVV | 191 |
| Query 187 | THGDACLPNIMVENGRFSGFIDCGRLGVADRYQDIALATRDIAEELGGEWADRFLVLYGI | 246 |

| Sbjct | 192 | HGDACLPN++ E RFSGFIDCGRLGVADR+QD+ALA RDI ELG WA+ FLV YG AHGDACLPNLLAEGRRFSGFIDCGRLGVADRHQDLALAARDIEAELGAAWAEAFLVEYG- 250 |
| --- | --- | --- |
| Query | 247 | AAPDSQRIAFYRLLDEFF 264 |
| Sbjct | 251 | D +R+A++RLLDEFF  GDIDGERLAYFRLLDEFF 268 |

>WP_023122977.1 APH(3')-II family aminoglycoside O-phosphotransferase [Pseudomonas aeruginosa]

AHC74962.1 Aminoglycoside 3'-phosphotransferase type IIb [Pseudomonas aeruginosa SCV20265]

ERY88490.1 aminoglycoside 3'-phosphotransferase type IIB [Pseudomonas aeruginosa BWHPSA008]

MBG4628369.1 APH(3')-II family aminoglycoside O-phosphotransferase [Pseudomonas aeruginosa]

MBG5639959.1 APH(3')-II family aminoglycoside O-phosphotransferase [Pseudomonas aeruginosa]

MBG6338344.1 APH(3')-II family aminoglycoside O-phosphotransferase [Pseudomonas aeruginosa]

Length=268

Score = 262 bits (670), Expect = 4e-84, Method: Compositional matrix adjust.

| Identities | | = 137/258 (53%), Positives = 169/258 (66%), Gaps = 5/258 (2%) |  |
| --- | --- | --- | --- |
| Query 11  Sbjct 12 | | SPAAWVERLFGYDWAQQTIGCSDAAVFRLSAQGRPVLFVKTDLSGALNELQDEAARLSWL  +P+ W + L GY W Q GCS A V RL A RP LFVK ++ A EL E ARL WL APSTWADYLAGYRWRGQGEGCSAATVHRLEAARRPTLFVKQEVLSAHAELPAEIARLRWL | 70  71 |
| Query 71  Sbjct 72 | | ATTGVPCAAVLDVVTEAGRDWLLLGEVPGQDLLS SHLAPAEKVSIMADAMRRLHTL  G+ C VL+ GR WLL+ VPG L + L P V ++A A+RRLH L HGAGIDCPQVLNETQSDGRQWLLMSAVPGDTLSALAQRGELEPERLVRLVAAALRRLHDL | 126  131 |
| Query 127  Sbjct 132 | | DPATCPFDHQAKHRIERARTRMEAGLVDQDDLDEEHQGLAPAELFARLKARMPDGEDLVV DPA CPFDH+ + R++ R R+EAGLVD+ D D++H+G + EL+ L R P EDLVV DPAACPFDHRLERRLDTVRQRVEAGLVDEADFDDDHRGRSATELYRLLLDRCPAVEDLVV | 186  191 |
| Query 187  Sbjct 192 | | THGDACLPNIMVENGRFSGFIDCGRLGVADRYQDIALATRDIAEELGGEWADRFLVLYGI HGDACLPN++ E RFSGFIDCGRLGVADR+QD+ALA RDI ELG WA+ FLV YG AHGDACLPNLLAEGRRFSGFIDCGRLGVADRHQDLALAARDIEAELGAAWAEAFLVEYG- | 246  250 |
| Query | 247 AAPDSQRIAFYRLLDEFF 264 | | |
|  |  | D +R+A++RLLDEFF |  |
| Sbjct | 251 | GDIDGERLAYFRLLDEFF 268 | |

>WP_156414742.1 MULTISPECIES: aminoglycoside 3'-phosphotransferase [unclassified Ensifer]

MBD9509195.1 aminoglycoside 3'-phosphotransferase [Ensifer sp. ENS10] Length=254

Score = 261 bits (668), Expect = 4e-84, Method: Compositional matrix adjust.

| Identities | = 129/244 (53%), Positives = 164/244 (67%), Gaps = 0/244 (0%) |  |
| --- | --- | --- |
| Query 21 | GYDWAQQTIGCSDAAVFRLSAQGRPVLFVKTDLSGALNELQDEAARLSWLATTGVPCAAV | 80 |
| Sbjct 11 | GYDW T+G SD++++ L A RP+L +K + +G +E DEA RL WLA+ G+PC GYDWNHDTLGQSDSSIYLLKAPDRPMLVLKVEAAGPFSEFVDEAERLDWLASQGMPCPRA | 70 |
| Query 81 | LDVVTEAGRDWLLLGEVPGQDLLSSHLAPAEKVSIMADAMRRLHTLDPATCPFDHQAKHR | 140 |
| Sbjct 71 | + +A +WLLL V G DL S+ L+P +++ I+ DA++RLH LD A CPFDH+ + R IARAFDAETNWLLLQAVEGTDLASTTLSPKDRIVILVDALKRLHGLDVADCPFDHRIEKR | 130 |
| Query 141 | IERARTRMEAGLVDQDDLDEEHQGLAPAELFARLKARMPDGEDLVVTHGDACLPNIMVEN | 200 |
| Sbjct 131 | I A R EAGL+D+DD DE G A+LFA L+AR+P LV+THGDACLPNI+ +N IAIAGARTEAGLIDEDDFDETRLGRTAADLFAELRARVPSISKLVITHGDACLPNIIEKN | 190 |
| Query 201 | GRFSGFIDCGRLGVADRYQDIALATRDIAEELGGEWADRFLVLYGIAAPDSQRIAFYRLL | 260 |
| Sbjct 191 | GRFSGFIDC RLGVAD YQDIALA IA LG EW FL YGI D ++ Y LL GRFSGFIDCSRLGVADPYQDIALACHSIAHNLGEEWVQPFLARYGIDRDDPEKRDLYCLL | 250 |
| Query 261 | DEFF 264 DEFF |  |

Sbjct 251 DEFF 254

>WP_127535131.1 aminoglycoside 3'-phosphotransferase [Sinorhizobium meliloti]

MQV27886.1 APH(3') family aminoglycoside O-phosphotransferase [Sinorhizobium meliloti]

RVH64592.1 aminoglycoside 3'-phosphotransferase [Sinorhizobium meliloti] RVK67553.1 aminoglycoside 3'-phosphotransferase [Sinorhizobium meliloti]

Length=261

Score = 262 bits (669), Expect = 5e-84, Method: Compositional matrix adjust.

| Identities | = 138/253 (55%), Positives = 163/253 (64%), Gaps = 0/253 (0%) |  |
| --- | --- | --- |
| Query 12 | PAAWVERLFGYDWAQQTIGCSDAAVFRLSAQGRPVLFVKTDLSGALNELQDEAARLSWLA | 71 |
| Sbjct 9 | P A+ L GY + + +G S + +F L AQ RP L +K + SG EL EAARL WLA PPAFRGLLSGYGFERDALGQSASTIFLLKAQSRPFLILKHEPSGPFTELTGEAARLHWLA | 68 |
| Query 72 | TTGVPCAAVLDVVTEAGRDWLLLGEVPGQDLLSSHLAPAEKVSIMADAMRRLHTLDPATC | 131 |
| Sbjct 69 | +PC VL AG WLLL G DL S L AE+++I+A+A+RRLH LDPA C AQDMPCPRVLGHDDHAGETWLLLEAAEGMDLASCRLPHAERIAILAEALRRLHRLDPAAC | 128 |
| Query 132 | PFDHQAKHRIERARTRMEAGLVDQDDLDEEHQGLAPAELFARLKARMPDGEDLVVTHGDA | 191 |
| Sbjct 129 | PFD + I A+ RMEAGLVD+ D DEE QG ELF L AR P E LVVTHGDA PFDRRLTASIAAAKARMEAGLVDESDFDEERQGRQTRELFDELLARRPKEEQLVVTHGDA | 188 |
| Query 192 | CLPNIMVENGRFSGFIDCGRLGVADRYQDIALATRDIAEELGGEWADRFLVLYGIAAPDS | 251 |
| Sbjct 189 | CLPN + +FSGFIDCGRLGVAD YQD+ALA I LG EW FL YG+AAPD CLPNFIAAGNQFSGFIDCGRLGVADIYQDLALACWSITHNLGEEWVVPFLDRYGLAAPDP | 248 |
| Query 252  Sbjct 249 | QRIAFYRLLDEFF 264  +++AFYRLLDEFF  EKLAFYRLLDEFF 261 |  |

>KRD50177.1 aminoglycoside phosphotransferase [Ensifer sp. Root278] Length=263

Score = 262 bits (669), Expect = 5e-84, Method: Compositional matrix adjust.

| Identities | = 129/244 (53%), Positives = 164/244 (67%), Gaps = 0/244 (0%) |  |
| --- | --- | --- |
| Query 21 | GYDWAQQTIGCSDAAVFRLSAQGRPVLFVKTDLSGALNELQDEAARLSWLATTGVPCAAV | 80 |
| Sbjct 20 | GYDW T+G SD++++ L A RP+L +K + +G +E DEA RL WLA+ G+PC GYDWNHDTLGQSDSSIYLLKAPDRPMLVLKVEAAGPFSEFVDEAERLDWLASQGMPCPRA | 79 |
| Query 81 | LDVVTEAGRDWLLLGEVPGQDLLSSHLAPAEKVSIMADAMRRLHTLDPATCPFDHQAKHR | 140 |
| Sbjct 80 | + +A +WLLL V G DL S+ L+P +++ I+ DA++RLH LD A CPFDH+ + R IARAFDAETNWLLLQAVEGTDLASTTLSPKDRIVILVDALKRLHGLDVADCPFDHRIEKR | 139 |
| Query 141 | IERARTRMEAGLVDQDDLDEEHQGLAPAELFARLKARMPDGEDLVVTHGDACLPNIMVEN | 200 |
| Sbjct 140 | I A R EAGL+D+DD DE G A+LFA L+AR+P LV+THGDACLPNI+ +N IAIAGARTEAGLIDEDDFDETRLGRTAADLFAELRARVPSISKLVITHGDACLPNIIEKN | 199 |
| Query 201 | GRFSGFIDCGRLGVADRYQDIALATRDIAEELGGEWADRFLVLYGIAAPDSQRIAFYRLL | 260 |
| Sbjct 200 | GRFSGFIDC RLGVAD YQDIALA IA LG EW FL YGI D ++ Y LL GRFSGFIDCSRLGVADPYQDIALACHSIAHNLGEEWVQPFLARYGIDRDDPEKRDLYCLL | 259 |
| Query 261  Sbjct 260 | DEFF 264 DEFF  DEFF 263 |  |

>WP_083556162.1 APH(3')-II family aminoglycoside O-phosphotransferase [Pseudomonas aeruginosa]

AWQ87064.1 phosphotransferase enzyme family protein [Pseudomonas aeruginosa]

MBG4273187.1 APH(3')-II family aminoglycoside O-phosphotransferase [Pseudomonas aeruginosa]

MBG4396048.1 APH(3')-II family aminoglycoside O-phosphotransferase [Pseudomonas aeruginosa]

MUI60777.1 APH(3')-II family aminoglycoside O-phosphotransferase [Pseudomonas aeruginosa]

ORE50269.1 aminoglycoside O-phosphotransferase APH(3')-IIb [Pseudomonas aeruginosa]

Length=268

Score = 262 bits (669), Expect = 5e-84, Method: Compositional matrix adjust.

| Identities | | = 138/258 (53%), Positives = 169/258 (66%), Gaps = 5/258 (2%) |  |
| --- | --- | --- | --- |
| Query 11  Sbjct 12 | | SPAAWVERLFGYDWAQQTIGCSDAAVFRLSAQGRPVLFVKTDLSGALNELQDEAARLSWL  +PA W + L GY W Q GCS A V RL A RP LFVK ++ A EL E ARL WL APATWDDYLAGYRWRGQGEGCSAATVHRLEAARRPTLFVKQEVLSAHAELPAEIARLRWL | 70  71 |
| Query 71  Sbjct 72 | | ATTGVPCAAVLDVVTEAGRDWLLLGEVPGQDLLS SHLAPAEKVSIMADAMRRLHTL  G+ C VL+ GR WLL+ VPG L + L P V ++A A+RRLH L HGAGIDCPQVLNETQSDGRQWLLMSAVPGDTLSALAQCGELEPGRLVRLVAAALRRLHDL | 126  131 |
| Query 127  Sbjct 132 | | DPATCPFDHQAKHRIERARTRMEAGLVDQDDLDEEHQGLAPAELFARLKARMPDGEDLVV DPA CPFDH+ + R++ R R+EAGLVD+ D D++H+G + EL+ L R P EDLVV DPAACPFDHRLERRLDTVRQRVEAGLVDEADFDDDHRGRSATELYRLLLDRRPAVEDLVV | 186  191 |
| Query 187  Sbjct 192 | | THGDACLPNIMVENGRFSGFIDCGRLGVADRYQDIALATRDIAEELGGEWADRFLVLYGI HGDACLPN++ E RFSGFIDCGRLGVADR+QD+ALA RDI ELG WA+ FLV YG AHGDACLPNLLAEGRRFSGFIDCGRLGVADRHQDLALAARDIEAELGAAWAEAFLVEYG- | 246  250 |
| Query | 247 AAPDSQRIAFYRLLDEFF 264 | | |
|  |  | D +R+A++RLLDEFF |  |
| Sbjct | 251 | GDIDGERLAYFRLLDEFF 268 | |

>WP_058018060.1 APH(3')-II family aminoglycoside O-phosphotransferase [Pseudomonas aeruginosa]

KRU91689.1 aminoglycoside phosphotransferase [Pseudomonas aeruginosa] KRU99989.1 aminoglycoside phosphotransferase [Pseudomonas aeruginosa] SQC95072.1 aminoglycoside 3-N-acetyltransferase [Pseudomonas aeruginosa]

Length=268

Score = 262 bits (669), Expect = 5e-84, Method: Compositional matrix adjust.

| Identities | | = 138/258 (53%), Positives = 169/258 (66%), Gaps = 5/258 (2%) |  |
| --- | --- | --- | --- |
| Query 11  Sbjct 12 | | SPAAWVERLFGYDWAQQTIGCSDAAVFRLSAQGRPVLFVKTDLSGALNELQDEAARLSWL  +PA W + L GY W Q GCS A V RL A RP LFVK ++ A EL E ARL WL APATWDDYLAGYRWRGQGEGCSAATVHRLEAAWRPTLFVKQEVLSAHAELPAEIARLRWL | 70  71 |
| Query 71  Sbjct 72 | | ATTGVPCAAVLDVVTEAGRDWLLLGEVPGQDLLS SHLAPAEKVSIMADAMRRLHTL  G+ C VL+ GR WLL+ VPG L + L P V ++A A+RRLH L HGAGIDCPQVLNETQSDGRQWLLMSAVPGDTLSALAQRGELEPGRLVRLVAAALRRLHDL | 126  131 |
| Query 127  Sbjct 132 | | DPATCPFDHQAKHRIERARTRMEAGLVDQDDLDEEHQGLAPAELFARLKARMPDGEDLVV DPA CPFDH+ + R++ R R+EAGLVD+ D D++H+G + EL+ L R P EDLVV DPAACPFDHRLERRLDTVRQRVEAGLVDEADFDDDHRGRSATELYRLLLDRRPAVEDLVV | 186  191 |
| Query 187  Sbjct 192 | | THGDACLPNIMVENGRFSGFIDCGRLGVADRYQDIALATRDIAEELGGEWADRFLVLYGI HGDACLPN++ E RFSGFIDCGRLGVADR+QD+ALA RDI ELG WA+ FLV YG AHGDACLPNLLAEGRRFSGFIDCGRLGVADRHQDLALAARDIEAELGAAWAEAFLVEYG- | 246  250 |
| Query | 247 AAPDSQRIAFYRLLDEFF 264 | | |
|  |  | D +R+A++RLLDEFF |  |
| Sbjct | 251 | GDIDGERLAYFRLLDEFF 268 | |

>WP_074207167.1 APH(3')-II family aminoglycoside O-phosphotransferase [Pseudomonas aeruginosa]

NPT00417.1 APH(3')-II family aminoglycoside O-phosphotransferase [Pseudomonas aeruginosa]

QKE94855.1 APH(3')-II family aminoglycoside O-phosphotransferase [Pseudomonas aeruginosa]

RTV51228.1 APH(3')-II family aminoglycoside O-phosphotransferase [Pseudomonas aeruginosa]

Length=268

Score = 262 bits (669), Expect = 6e-84, Method: Compositional matrix adjust. Identities = 139/259 (54%), Positives = 171/259 (66%), Gaps = 7/259 (3%)

Query 11	SPAAWVERLFGYDWAQQTIGCSDAAVFRLSAQGRPVLFVKTDLSGALNELQDEAARLSWL 70

+P+ W + L GY W Q GCS A V RL A RP LFVK ++ A EL E ARL WL

| Sbjct | 12 | APSTWADYLAGYRWRGQGEGCSAATVHRLEAARRPTLFVKQEVLSAHAELPAEIARLRWL | 71 |
| --- | --- | --- | --- |
| Query | 71 | ATTGVPCAAVLDVVTEAGRDWLLLGEVPGQDLLSS HLAPAEKVSIMADAMRRLHT | 125 |
| Sbjct | 72 | G+ C VL+ GR WLL+ VPG D LS+ L P V ++A A+RRLH HGAGIDCPQVLNETQSDGRQWLLMSAVPG-DTLSALAQRDELEPERLVRLVAAALRRLHD | 130 |
| Query | 126 | LDPATCPFDHQAKHRIERARTRMEAGLVDQDDLDEEHQGLAPAELFARLKARMPDGEDLV | 185 |
| Sbjct | 131 | LDPA CPFDH+ + R++ R R+EAGLVD+ D D++H+G + EL+ L R P EDLV LDPAACPFDHRLERRLDTVRQRVEAGLVDEADFDDDHRGRSATELYRLLLDRRPAVEDLV | 190 |
| Query | 186 | VTHGDACLPNIMVENGRFSGFIDCGRLGVADRYQDIALATRDIAEELGGEWADRFLVLYG | 245 |
| Sbjct | 191 | V HGDACLPN++ E RFSGFIDCGRLGVADR+QD+ALA RDI ELG WA+ FLV YG VAHGDACLPNLLAEGRRFSGFIDCGRLGVADRHQDLALAARDIEAELGAAWAEAFLVEYG | 250 |
| Query  Sbjct | 246  251 | IAAPDSQRIAFYRLLDEFF 264 D +R+A++RLLDEFF  -GDIDGERLAYFRLLDEFF 268 |  |

>WP_114232455.1 APH(3')-II family aminoglycoside O-phosphotransferase [Pseudomonas aeruginosa]

MBF8799024.1 APH(3')-II family aminoglycoside O-phosphotransferase [Pseudomonas aeruginosa]

MBG6713155.1 APH(3')-II family aminoglycoside O-phosphotransferase [Pseudomonas aeruginosa]

MBG7427137.1 APH(3')-II family aminoglycoside O-phosphotransferase [Pseudomonas aeruginosa]

RCM90274.1 Aminoglycoside 3'-phosphotransferase [Pseudomonas aeruginosa]

RPX35529.1 APH(3') family aminoglycoside O-phosphotransferase [Pseudomonas aeruginosa]

Length=268

Score = 262 bits (669), Expect = 6e-84, Method: Compositional matrix adjust.

| Identities | | = 138/258 (53%), Positives = 169/258 (66%), Gaps = 5/258 (2%) |  |
| --- | --- | --- | --- |
| Query 11  Sbjct 12 | | SPAAWVERLFGYDWAQQTIGCSDAAVFRLSAQGRPVLFVKTDLSGALNELQDEAARLSWL  +PA W + L GY W Q GCS A V RL A RP LFVK ++ A EL E ARL WL APATWDDYLAGYRWRGQGEGCSAATVHRLEAARRPTLFVKQEVLSAHAELPAEIARLRWL | 70  71 |
| Query 71  Sbjct 72 | | ATTGVPCAAVLDVVTEAGRDWLLLGEVPGQDLLS SHLAPAEKVSIMADAMRRLHTL  G+ C VL+ GR WLL+ VPG L + L P V ++A A+RRLH L HGAGIDCPQVLNETQSDGRQWLLMSAVPGDTLSALAQRGELEPGRLVRLVAAALRRLHDL | 126  131 |
| Query 127  Sbjct 132 | | DPATCPFDHQAKHRIERARTRMEAGLVDQDDLDEEHQGLAPAELFARLKARMPDGEDLVV DPA CPFDH+ + R++ R R+EAGLVD+ D D++H+G + EL+ L R P EDLVV DPAACPFDHRLERRLDTVRQRVEAGLVDEADFDDDHRGRSATELYRLLLDRRPAVEDLVV | 186  191 |
| Query 187  Sbjct 192 | | THGDACLPNIMVENGRFSGFIDCGRLGVADRYQDIALATRDIAEELGGEWADRFLVLYGI HGDACLPN++ E RFSGFIDCGRLGVADR+QD+ALA RDI ELG WA+ FLV YG AHGDACLPNLLAEGRRFSGFIDCGRLGVADRHQDLALAARDIEAELGAAWAEAFLVEYG- | 246  250 |
| Query | 247 AAPDSQRIAFYRLLDEFF 264 | | |
|  |  | D +R+A++RLLDEFF |  |
| Sbjct | 251 | GDIDGERLAYFRLLDEFF 268 | |

>WP_196667399.1 APH(3')-II family aminoglycoside O-phosphotransferase [Pseudomonas aeruginosa]

MBG4640312.1 APH(3')-II family aminoglycoside O-phosphotransferase [Pseudomonas aeruginosa]

Length=267

Score = 262 bits (669), Expect = 6e-84, Method: Compositional matrix adjust. Identities = 137/258 (53%), Positives = 169/258 (66%), Gaps = 5/258 (2%)

| Query | 11 | SPAAWVERLFGYDWAQQTIGCSDAAVFRLSAQGRPVLFVKTDLSGALNELQDEAARLSWL | 70 |
| --- | --- | --- | --- |
| Sbjct | 11 | +P+ W + L GY W Q GCS A V RL A+ RP LFVK ++ A EL E ARL WL APSTWADYLAGYRWRGQGEGCSAATVHRLEAERRPTLFVKQEVLSAHAELPAEIARLRWL | 70 |
| Query | 71 | ATTGVPCAAVLDVVTEAGRDWLLLGEVPGQDLLS SHLAPAEKVSIMADAMRRLHTL | 126 |
| Sbjct | 71 | G+ C VL+ GR WLL+ VPG L + L P V ++A A+RRLH L  HGAGIDCPQVLNETQSDGRQWLLMSAVPGDTLSALAQRGELEPERLVRLVAAALRRLHDL | 130 |

| Query | 127 | DPATCPFDHQAKHRIERARTRMEAGLVDQDDLDEEHQGLAPAELFARLKARMPDGEDLVV | 186 |
| --- | --- | --- | --- |
| Sbjct | 131 | DPA CPFDH+ + R++ R R EAGLVD+ D D++H+G + EL+ L R P EDLVV DPAACPFDHRLERRLDTVRQRGEAGLVDEADFDDDHRGRSATELYRLLLDRRPAVEDLVV | 190 |
| Query | 187 | THGDACLPNIMVENGRFSGFIDCGRLGVADRYQDIALATRDIAEELGGEWADRFLVLYGI | 246 |
| Sbjct | 191 | HGDACLPN++ E RFSGFIDCGRLGVADR+QD+ALA RDI ELG WA+ FLV YG  AHGDACLPNLLAEGRRFSGFIDCGRLGVADRHQDLALAARDIEAELGAAWAEAFLVEYG- | 249 |
| Query | 247 AAPDSQRIAFYRLLDEFF 264 | | |
|  |  | D +R+A++RLLDEFF |  |
| Sbjct | 250 | GDIDGERLAYFRLLDEFF 267 | |

>WP_184699706.1 APH(3') family aminoglycoside O-phosphotransferase [Aminobacter aganoensis]

MBB6355003.1 aminoglycoside 3'-phosphotransferase-2 [Aminobacter aganoensis] Length=264

Score = 261 bits (668), Expect = 6e-84, Method: Compositional matrix adjust.

| Identities | = 136/264 (52%), Positives = 173/264 (66%), Gaps = 0/264 (0%) |  |
| --- | --- | --- |
| Query 1 | MIEQDGLHAGSPAAWVERLFGYDWAQQTIGCSDAAVFRLSAQGRPVLFVKTDLSGALNEL | 60 |
| Sbjct 1 | M + + PA+ E L GYDW +QTIG S A VFRL A G+P LF+KT+ +G EL MTQHPAIEIDLPASLREALAGYDWQRQTIGASGAGVFRLEASGKPTLFLKTEETGQFAEL | 60 |
| Query 61 | QDEAARLSWLATTGVPCAAVLDVVTEAGRDWLLLGEVPGQDLLSSHLAPAEKVSIMADAM | 120 |
| Sbjct 61 | DE ARL WL + G+PC AVL G +WLL+ V G DL++ +AP ++IMADA+ PDEVARLRWLGSQGIPCPAVLAFEAHGGHNWLLMSAVAGHDLVTQEMAPDRAIAIMADAL | 120 |
| Query 121 | RRLHTLDPATCPFDHQAKHRIERARTRMEAGLVDQDDLDEEHQGLAPAELFARLKARMPD | 180 |
| Sbjct 121 | RRLH LD A CPFDH RI A+ R +AG VD+DD D+E G + ++F L AR P RRLHALDAAACPFDHSLGPRIALAKARADAGEVDEDDFDDERLGQSAQQVFEELLARRPA | 180 |
| Query 181 | GEDLVVTHGDACLPNIMVENGRFSGFIDCGRLGVADRYQDIALATRDIAEELGGEWADRF | 240 |
| Sbjct 181 | E LVVTHGDA +PN M + GRF+GFIDC RLGVAD +QD+ALA I LG EW F SETLVVTHGDASMPNFMADGGRFTGFIDCSRLGVADLHQDLALACWSIHVNLGEEWIAPF | 240 |
| Query 241  Sbjct 241 | LVLYGIAAPDSQRIAFYRLLDEFF 264 L LYG+ D+ ++++Y+LLDEFF LALYGLPQADTAKLSYYQLLDEFF 264 |  |

>WP_058181524.1 APH(3')-II family aminoglycoside O-phosphotransferase [Pseudomonas aeruginosa]

KSE40957.1 aminoglycoside 3'-phosphotransferase [Pseudomonas aeruginosa] Length=268

Score = 262 bits (669), Expect = 6e-84, Method: Compositional matrix adjust.

| Identities | | = 137/258 (53%), Positives = 169/258 (66%), Gaps = 5/258 (2%) |  |
| --- | --- | --- | --- |
| Query 11  Sbjct 12 | | SPAAWVERLFGYDWAQQTIGCSDAAVFRLSAQGRPVLFVKTDLSGALNELQDEAARLSWL  +P+ W + L GY W Q GCS A V RL A RP LFVK ++ A EL E ARL WL APSTWADYLAGYRWRGQGEGCSAATVHRLEAARRPTLFVKQEVLSAHAELPAEIARLRWL | 70  71 |
| Query 71  Sbjct 72 | | ATTGVPCAAVLDVVTEAGRDWLLLGEVPGQDLLS SHLAPAEKVSIMADAMRRLHTL  G+ C VL+ GR WLL+ VPG L + L P V ++A A+RRLH L HGAGIDCPQVLNETQSDGRQWLLMSAVPGDTLSALAQRGELEPERLVRLVAVALRRLHDL | 126  131 |
| Query 127  Sbjct 132 | | DPATCPFDHQAKHRIERARTRMEAGLVDQDDLDEEHQGLAPAELFARLKARMPDGEDLVV DPA CPFDH+ + R++ R R+EAGLVD+ D D++H+G + EL+ L R P EDLVV DPAACPFDHRLERRLDTVRQRVEAGLVDEADFDDDHRGRSATELYRLLLDRRPAVEDLVV | 186  191 |
| Query 187  Sbjct 192 | | THGDACLPNIMVENGRFSGFIDCGRLGVADRYQDIALATRDIAEELGGEWADRFLVLYGI HGDACLPN++ E RFSGFIDCGRLGVADR+QD+ALA RDI ELG WA+ FLV YG AHGDACLPNLLAEGRRFSGFIDCGRLGVADRHQDLALAARDIEAELGAAWAEAFLVEYG- | 246  250 |
| Query | 247 AAPDSQRIAFYRLLDEFF 264 | | |
|  |  | D +R+A++RLLDEFF |  |
| Sbjct | 251 | GDIDGERLAYFRLLDEFF 268 | |

>WP_070335920.1 APH(3')-II family aminoglycoside O-phosphotransferase [Pseudomonas aeruginosa]

Length=268

Score = 262 bits (669), Expect = 6e-84, Method: Compositional matrix adjust.

| Identities | | = 137/258 (53%), Positives = 169/258 (66%), Gaps = 5/258 (2%) |  |
| --- | --- | --- | --- |
| Query 11  Sbjct 12 | | SPAAWVERLFGYDWAQQTIGCSDAAVFRLSAQGRPVLFVKTDLSGALNELQDEAARLSWL  +PA W + L GY W Q GCS A V RL A RP LFVK ++ A EL + ARL WL APATWADYLAGYRWRGQGEGCSAATVHRLEAARRPTLFVKQEVLSAHAELPAQIARLRWL | 70  71 |
| Query 71  Sbjct 72 | | ATTGVPCAAVLDVVTEAGRDWLLLGEVPGQDLLS SHLAPAEKVSIMADAMRRLHTL  G+ C VL+ GR WLL+ VPG L + L P V ++A A+RRLH L HGAGIDCPQVLNETQSDGRQWLLMSAVPGDTLSALAQRGELEPERLVRLVAAALRRLHDL | 126  131 |
| Query 127  Sbjct 132 | | DPATCPFDHQAKHRIERARTRMEAGLVDQDDLDEEHQGLAPAELFARLKARMPDGEDLVV DPA CPFDH+ + R++ R R+EAGLVD+ D D++H+G + EL+ L R P EDLVV DPAACPFDHRLERRLDTVRQRVEAGLVDEADFDDDHRGRSATELYRLLLDRRPAVEDLVV | 186  191 |
| Query 187  Sbjct 192 | | THGDACLPNIMVENGRFSGFIDCGRLGVADRYQDIALATRDIAEELGGEWADRFLVLYGI HGDACLPN++ E RFSGFIDCGRLGVADR+QD+ALA RDI ELG WA+ FLV YG AHGDACLPNLLAEGRRFSGFIDCGRLGVADRHQDLALAARDIEAELGAAWAEAFLVEYG- | 246  250 |
| Query | 247 AAPDSQRIAFYRLLDEFF 264 | | |
|  |  | D +R+A++RLLDEFF |  |
| Sbjct | 251 | GDIDGERLAYFRLLDEFF 268 | |

>WP_126859588.1 APH(3')-II family aminoglycoside O-phosphotransferase [Pseudomonas aeruginosa]

NPW04685.1 APH(3')-II family aminoglycoside O-phosphotransferase [Pseudomonas aeruginosa]

RUC22159.1 APH(3')-II family aminoglycoside O-phosphotransferase [Pseudomonas aeruginosa]

RUC35162.1 APH(3')-II family aminoglycoside O-phosphotransferase [Pseudomonas aeruginosa]

Length=268

Score = 261 bits (668), Expect = 6e-84, Method: Compositional matrix adjust.

| Identities | | = 137/258 (53%), Positives = 169/258 (66%), Gaps = 5/258 (2%) |  |
| --- | --- | --- | --- |
| Query 11  Sbjct 12 | | SPAAWVERLFGYDWAQQTIGCSDAAVFRLSAQGRPVLFVKTDLSGALNELQDEAARLSWL  +P+ W + L GY W Q GCS A V RL A RP LFVK ++ A EL E ARL WL APSTWADYLAGYRWRGQGEGCSAATVHRLEAARRPTLFVKQEVLSAHAELPAEIARLRWL | 70  71 |
| Query 71  Sbjct 72 | | ATTGVPCAAVLDVVTEAGRDWLLLGEVPGQDLLS SHLAPAEKVSIMADAMRRLHTL  G+ C VL+ GR WLL+ VPG L + L P V ++A A+RRLH L HGAGIDCPQVLNETQSDGRQWLLMSAVPGDTLSALAQRGELEPERLVRLVAVALRRLHDL | 126  131 |
| Query 127  Sbjct 132 | | DPATCPFDHQAKHRIERARTRMEAGLVDQDDLDEEHQGLAPAELFARLKARMPDGEDLVV DPA CPFDH+ + R++ R R+EAGLVD+ D D++H+G + EL+ L R P EDLVV DPAACPFDHRLERRLDTVRQRVEAGLVDEADFDDDHRGRSATELYRLLLDRRPAVEDLVV | 186  191 |
| Query 187  Sbjct 192 | | THGDACLPNIMVENGRFSGFIDCGRLGVADRYQDIALATRDIAEELGGEWADRFLVLYGI HGDACLPN++ E RFSGFIDCGRLGVADR+QD+ALA RDI ELG WA+ FLV YG AHGDACLPNLLAEGRRFSGFIDCGRLGVADRHQDLALAARDIEAELGAAWAEAFLVEYG- | 246  250 |
| Query | 247 AAPDSQRIAFYRLLDEFF 264 | | |
|  |  | D +R+A++RLLDEFF |  |
| Sbjct | 251 | GDIDGERLAYFRLLDEFF 268 | |

>WP_023874917.1 APH(3')-II family aminoglycoside O-phosphotransferase [Pseudomonas aeruginosa]

AHB54091.1 aminoglycoside 3'-phosphotransferase [Pseudomonas aeruginosa MTB-1]

RUE23328.1 APH(3')-II family aminoglycoside O-phosphotransferase [Pseudomonas aeruginosa]

Length=268

Score = 261 bits (668), Expect = 6e-84, Method: Compositional matrix adjust. Identities = 137/258 (53%), Positives = 169/258 (66%), Gaps = 5/258 (2%)

| Query | 11 | SPAAWVERLFGYDWAQQTIGCSDAAVFRLSAQGRPVLFVKTDLSGALNELQDEAARLSWL | 70 |
| --- | --- | --- | --- |
| Sbjct | 12 | +P+ W + L GY W Q GCS A V RL A RP LFVK ++ A EL E ARL WL APSTWADYLAGYRWRGQGEGCSAATVHRLEAARRPTLFVKQEVLSAHAELPAEIARLRWL | 71 |
| Query | 71 | ATTGVPCAAVLDVVTEAGRDWLLLGEVPGQDLLS SHLAPAEKVSIMADAMRRLHTL | 126 |
| Sbjct | 72 | G+ C VL+ GR WLL+ VPG L + L P V ++A A+RRLH L HGAGIDCPQVLNETQSDGRQWLLMSAVPGDTLSALAQRGELEPERLVRLVAAALRRLHDL | 131 |
| Query | 127 | DPATCPFDHQAKHRIERARTRMEAGLVDQDDLDEEHQGLAPAELFARLKARMPDGEDLVV | 186 |
| Sbjct | 132 | DPA CPFDH+ + R++ R R+EAGLVD+ D D++H+G + EL+ L R P EDLVV DPAACPFDHRLERRLDTVRQRVEAGLVDEADFDDDHRGRSATELYRLLLDRRPAVEDLVV | 191 |
| Query | 187 | THGDACLPNIMVENGRFSGFIDCGRLGVADRYQDIALATRDIAEELGGEWADRFLVLYGI | 246 |
| Sbjct | 192 | HGDACLPN++ E RFSGFIDCGRLGVADR+QD+ALA RDI ELG WA+ FLV YG  AHGDACLPNLLAEGRRFSGFIDCGRLGVADRHQDLALAARDIEAELGAAWAEDFLVEYG- | 250 |
| Query | 247 AAPDSQRIAFYRLLDEFF 264 | | |
|  |  | D +R+A++RLLDEFF |  |
| Sbjct | 251 | GDIDGERLAYFRLLDEFF 268 | |

>WP_127719427.1 aminoglycoside 3'-phosphotransferase [Sinorhizobium meliloti] RVQ24775.1 aminoglycoside 3'-phosphotransferase [Sinorhizobium meliloti] RVQ52021.1 aminoglycoside 3'-phosphotransferase [Sinorhizobium meliloti]

Length=247

Score = 261 bits (666), Expect = 7e-84, Method: Compositional matrix adjust.

| Identities | = 136/246 (55%), Positives = 160/246 (65%), Gaps = 0/246 (0%) |  |
| --- | --- | --- |
| Query 19 | LFGYDWAQQTIGCSDAAVFRLSAQGRPVLFVKTDLSGALNELQDEAARLSWLATTGVPCA | 78 |
| Sbjct 2 | L GY + + +G S + +F L AQ RP L +K + SG EL EAARL WLA +PC LSGYGFERDALGQSASTIFLLKAQSRPFLILKHEPSGPFTELTGEAARLHWLAAQDMPCP | 61 |
| Query 79 | AVLDVVTEAGRDWLLLGEVPGQDLLSSHLAPAEKVSIMADAMRRLHTLDPATCPFDHQAK | 138 |
| Sbjct 62 | VL AG WLLL G DL S L AE+++I+A+A+RRLH LDPA CPFD + RVLGHDDHAGETWLLLEAAEGMDLASCRLPHAERIAILAEALRRLHRLDPAACPFDRRLT | 121 |
| Query 139 | HRIERARTRMEAGLVDQDDLDEEHQGLAPAELFARLKARMPDGEDLVVTHGDACLPNIMV | 198 |
| Sbjct 122 | I A+ RMEAGLVD+ D DEE QG ELF L AR P E LVVTHGDACLPN + ASIAAAKARMEAGLVDESDFDEERQGRQTRELFDELLARRPKEEQLVVTHGDACLPNFIA | 181 |
| Query 199 | ENGRFSGFIDCGRLGVADRYQDIALATRDIAEELGGEWADRFLVLYGIAAPDSQRIAFYR | 258 |
| Sbjct 182 | +FSGFIDCGRLGVAD YQD+ALA I LG EW FL YG+AAPD +++AFYR AGNQFSGFIDCGRLGVADIYQDLALACWSITHNLGEEWVAPFLDRYGLAAPDPEKLAFYR | 241 |
| Query 259  Sbjct 242 | LLDEFF 264 LLDEFF LLDEFF 247 |  |

>WP_023108306.1 MULTISPECIES: APH(3')-II family aminoglycoside O-phosphotransferase [Pseudomonas]

AZZ15787.1 aminoglycoside phosphotransferase APH(3') [Pseudomonas aeruginosa] ERV66767.1 aminoglycoside 3'-phosphotransferase type IIB [Pseudomonas aeruginosa

BL04]

KSD37688.1 aminoglycoside phosphotransferase APH(3') [Pseudomonas aeruginosa] KSE15471.1 aminoglycoside phosphotransferase APH(3') [Pseudomonas aeruginosa] KSE84561.1 aminoglycoside phosphotransferase APH(3') [Pseudomonas aeruginosa]

Length=268

Score = 261 bits (668), Expect = 7e-84, Method: Compositional matrix adjust.

| Identities | = 137/258 (53%), Positives = 168/258 (65%), Gaps = 5/258 (2%) |  |
| --- | --- | --- |
| Query 11 | SPAAWVERLFGYDWAQQTIGCSDAAVFRLSAQGRPVLFVKTDLSGALNELQDEAARLSWL | 70 |
| Sbjct 12 | +P+ W + L GY W Q GCS A V RL A RP LFVK ++ A EL E ARL WL APSTWADYLAGYRWRGQGEGCSAATVHRLEAARRPTLFVKQEVLSAHAELPAEIARLRWL | 71 |
| Query 71 | ATTGVPCAAVLDVVTEAGRDWLLLGEVPGQDLLS SHLAPAEKVSIMADAMRRLHTL | 126 |
| Sbjct 72 | G+ C VL+ GR WLL+ VPG L + L P V ++A A+RRLH L  HGAGIDCPQVLNETQSDGRQWLLMSAVPGDTLSALAQRGELEPERLVRLVAAALRRLHDL | 131 |

| Query | 127 | DPATCPFDHQAKHRIERARTRMEAGLVDQDDLDEEHQGLAPAELFARLKARMPDGEDLVV | 186 |
| --- | --- | --- | --- |
| Sbjct | 132 | DPA CPFDH+ + R++ R R EAGLVD+ D D++H+G + EL+ L R P EDLVV DPAACPFDHRLERRLDTVRQRAEAGLVDEADFDDDHRGRSATELYRLLLDRRPAVEDLVV | 191 |
| Query | 187 | THGDACLPNIMVENGRFSGFIDCGRLGVADRYQDIALATRDIAEELGGEWADRFLVLYGI | 246 |
| Sbjct | 192 | HGDACLPN++ E RFSGFIDCGRLGVADR+QD+ALA RDI ELG WA+ FLV YG  AHGDACLPNLLAEGRRFSGFIDCGRLGVADRHQDLALAARDIEAELGAAWAEAFLVEYG- | 250 |
| Query | 247 AAPDSQRIAFYRLLDEFF 264 | | |
|  |  | D +R+A++RLLDEFF |  |
| Sbjct | 251 | GDIDGERLAYFRLLDEFF 268 | |

>WP_134280349.1 APH(3')-II family aminoglycoside O-phosphotransferase [Pseudomonas aeruginosa]

TEC92794.1 APH(3')-II family aminoglycoside O-phosphotransferase [Pseudomonas aeruginosa]

Length=268

Score = 261 bits (668), Expect = 7e-84, Method: Compositional matrix adjust.

| Identities | | = 136/258 (53%), Positives = 168/258 (65%), Gaps = 5/258 (2%) |  |
| --- | --- | --- | --- |
| Query 11  Sbjct 12 | | SPAAWVERLFGYDWAQQTIGCSDAAVFRLSAQGRPVLFVKTDLSGALNELQDEAARLSWL  +P+ W + L GY W Q GCS A V RL RP LFVK ++ A EL E ARL WL APSTWADYLAGYRWRGQGEGCSAATVHRLEVARRPTLFVKQEVLSAHTELPAEIARLRWL | 70  71 |
| Query 71  Sbjct 72 | | ATTGVPCAAVLDVVTEAGRDWLLLGEVPGQDLLS SHLAPAEKVSIMADAMRRLHTL  G+ C VL+ GR WLL+ VPG L + L P V ++A A+RRLH L HGAGIDCPQVLNETQSDGRQWLLMSAVPGDTLSALAQRGELEPERLVRLVAAALRRLHDL | 126  131 |
| Query 127  Sbjct 132 | | DPATCPFDHQAKHRIERARTRMEAGLVDQDDLDEEHQGLAPAELFARLKARMPDGEDLVV DPA CPFDH+ + R++ R R+EAGLVD+ D D++H+G + EL+ L R P EDLVV DPAACPFDHRLERRLDTVRQRVEAGLVDEADFDDDHRGRSATELYRLLLDRRPAVEDLVV | 186  191 |
| Query 187  Sbjct 192 | | THGDACLPNIMVENGRFSGFIDCGRLGVADRYQDIALATRDIAEELGGEWADRFLVLYGI HGDACLPN++ E RFSGFIDCGRLGVADR+QD+ALA RDI ELG WA+ FLV YG AHGDACLPNLLAEGRRFSGFIDCGRLGVADRHQDLALAARDIEAELGAAWAEAFLVEYG- | 246  250 |
| Query | 247 AAPDSQRIAFYRLLDEFF 264 | | |
|  |  | D +R+A++RLLDEFF |  |
| Sbjct | 251 | GDIDGERLAYFRLLDEFF 268 | |

>WP_126622713.1 APH(3')-II family aminoglycoside O-phosphotransferase [Pseudomonas aeruginosa]

MBG4415103.1 APH(3')-II family aminoglycoside O-phosphotransferase [Pseudomonas aeruginosa]

RTW69116.1 APH(3')-II family aminoglycoside O-phosphotransferase [Pseudomonas aeruginosa]

RUD81296.1 APH(3')-II family aminoglycoside O-phosphotransferase [Pseudomonas aeruginosa]

RUI81967.1 APH(3')-II family aminoglycoside O-phosphotransferase [Pseudomonas aeruginosa]

Length=268

Score = 261 bits (668), Expect = 7e-84, Method: Compositional matrix adjust.

| Identities | = 137/258 (53%), Positives = 168/258 (65%), Gaps = 5/258 (2%) |  |
| --- | --- | --- |
| Query 11 | SPAAWVERLFGYDWAQQTIGCSDAAVFRLSAQGRPVLFVKTDLSGALNELQDEAARLSWL | 70 |
| Sbjct 12 | +PA W + L GY W Q GCS A V RL RP LFVK ++ A EL E ARL WL APATWADYLAGYRWRGQGEGCSAATVHRLEVARRPTLFVKQEVLSAHAELPAEIARLRWL | 71 |
| Query 71 | ATTGVPCAAVLDVVTEAGRDWLLLGEVPGQDLLS SHLAPAEKVSIMADAMRRLHTL | 126 |
| Sbjct 72 | G+ C VL+ GR WLL+ VPG L + L P V ++A A+RRLH L HGAGIDCPQVLNETQSDGRQWLLMSAVPGDTLSALAQRGELEPERLVRLVAAALRRLHDL | 131 |
| Query 127 | DPATCPFDHQAKHRIERARTRMEAGLVDQDDLDEEHQGLAPAELFARLKARMPDGEDLVV | 186 |
| Sbjct 132 | DPA CPFDH+ + R++ R R+EAGLVD+ D D++H+G + EL+ L R P EDLVV DPAACPFDHRLERRLDTVRQRVEAGLVDEADFDDDHRGRSATELYRLLLDRRPAVEDLVV | 191 |
| Query 187 | THGDACLPNIMVENGRFSGFIDCGRLGVADRYQDIALATRDIAEELGGEWADRFLVLYGI HGDACLPN++ E RFSGFIDCGRLGVADR+QD+ALA RDI ELG WA+ FLV YG | 246 |

| Sbjct | 192 | AHGDACLPNLLAEGRRFSGFIDCGRLGVADRHQDLALAARDIEAELGAAWAEAFLVEYG- 250 |
| --- | --- | --- |
| Query | 247 | AAPDSQRIAFYRLLDEFF 264 |
| Sbjct | 251 | D +R+A++RLLDEFF  GDIDGERLAYFRLLDEFF 268 |

>WP_143480337.1 APH(3')-II family aminoglycoside O-phosphotransferase [Pseudomonas aeruginosa]

TRO73902.1 APH(3')-II family aminoglycoside O-phosphotransferase [Pseudomonas aeruginosa]

Length=268

Score = 261 bits (668), Expect = 7e-84, Method: Compositional matrix adjust.

| Identities | | = 137/258 (53%), Positives = 168/258 (65%), Gaps = 5/258 (2%) |  |
| --- | --- | --- | --- |
| Query 11  Sbjct 12 | | SPAAWVERLFGYDWAQQTIGCSDAAVFRLSAQGRPVLFVKTDLSGALNELQDEAARLSWL  +PA W + L GY W Q GCS A V RL A RP LFVK ++ A EL E ARL WL APATWADYLAGYRWRGQGEGCSAATVHRLEAARRPTLFVKQEVLSAHAELPAEIARLRWL | 70  71 |
| Query 71  Sbjct 72 | | ATTGVPCAAVLDVVTEAGRDWLLLGEVPGQDLLS SHLAPAEKVSIMADAMRRLHTL  G+ C VL+ GR WLL+ VPG L + L P V ++A A+RRLH L HGAGIDCPQVLNETQSDGRQWLLMSAVPGDTLSALAQRGELEPERLVRLVAAALRRLHDL | 126  131 |
| Query 127  Sbjct 132 | | DPATCPFDHQAKHRIERARTRMEAGLVDQDDLDEEHQGLAPAELFARLKARMPDGEDLVV DPA CPFDH+ + R++ R R+EAGLVD+ D D++H+G + EL+ L R P EDLVV DPAACPFDHRLERRLDTVRQRVEAGLVDEADFDDDHRGRSATELYRLLLDRRPAVEDLVV | 186  191 |
| Query 187  Sbjct 192 | | THGDACLPNIMVENGRFSGFIDCGRLGVADRYQDIALATRDIAEELGGEWADRFLVLYGI HGDACLPN++ E RFSGFIDCGRLGVADR+QD+AL RDI ELG WA+ FLV YG AHGDACLPNLLAEGRRFSGFIDCGRLGVADRHQDLALVARDIEAELGAAWAEAFLVEYG- | 246  250 |
| Query | 247 AAPDSQRIAFYRLLDEFF 264 | | |
|  |  | D +R+A++RLLDEFF |  |
| Sbjct | 251 | GDIDGERLAYFRLLDEFF 268 | |

>MBG6313062.1 APH(3')-II family aminoglycoside O-phosphotransferase [Pseudomonas aeruginosa]

Length=268

Score = 261 bits (668), Expect = 7e-84, Method: Compositional matrix adjust.

| Identities | | = 137/258 (53%), Positives = 168/258 (65%), Gaps = 5/258 (2%) |  |
| --- | --- | --- | --- |
| Query 11  Sbjct 12 | | SPAAWVERLFGYDWAQQTIGCSDAAVFRLSAQGRPVLFVKTDLSGALNELQDEAARLSWL  +PA W + L GY W Q GCS A V RL A RP LFVK ++ EL E ARL WL APATWADYLAGYRWRGQGEGCSAATVHRLEAARRPTLFVKQEVLSVHAELPAEIARLRWL | 70  71 |
| Query 71  Sbjct 72 | | ATTGVPCAAVLDVVTEAGRDWLLLGEVPGQDLLS SHLAPAEKVSIMADAMRRLHTL  G+ C VL+ GR WLL+ VPG L + L P V ++A A+RRLH L HGAGIDCPQVLNETQSDGRQWLLMSAVPGDTLSALAQRGELEPERLVRLVAAALRRLHDL | 126  131 |
| Query 127  Sbjct 132 | | DPATCPFDHQAKHRIERARTRMEAGLVDQDDLDEEHQGLAPAELFARLKARMPDGEDLVV DPA CPFDH+ + R++ R R+EAGLVD+ D D++H+G + EL+ L R P EDLVV DPAACPFDHRLERRLDTVRQRVEAGLVDEADFDDDHRGRSATELYRLLLDRRPAVEDLVV | 186  191 |
| Query 187  Sbjct 192 | | THGDACLPNIMVENGRFSGFIDCGRLGVADRYQDIALATRDIAEELGGEWADRFLVLYGI HGDACLPN++ E RFSGFIDCGRLGVADR+QD+ALA RDI ELG WA+ FLV YG AHGDACLPNLLAEGRRFSGFIDCGRLGVADRHQDLALAARDIEAELGAAWAEAFLVEYG- | 246  250 |
| Query | 247 AAPDSQRIAFYRLLDEFF 264 | | |
|  |  | D +R+A++RLLDEFF |  |
| Sbjct | 251 | GDIDGERLAYFRLLDEFF 268 | |

>WP_134481983.1 APH(3')-II family aminoglycoside O-phosphotransferase [Pseudomonas aeruginosa]

Length=268

Score = 261 bits (668), Expect = 7e-84, Method: Compositional matrix adjust. Identities = 137/258 (53%), Positives = 169/258 (66%), Gaps = 5/258 (2%)

| Query | 11 | SPAAWVERLFGYDWAQQTIGCSDAAVFRLSAQGRPVLFVKTDLSGALNELQDEAARLSWL | 70 |
| --- | --- | --- | --- |
| Sbjct | 12 | +P+ W + L GY W Q GCS A V RL A RP LFVK ++ A EL E ARL WL APSTWADYLAGYRWRGQGEGCSAATVHRLEAARRPTLFVKQEVLSAHAELPAEIARLRWL | 71 |
| Query | 71 | ATTGVPCAAVLDVVTEAGRDWLLLGEVPGQDLLS SHLAPAEKVSIMADAMRRLHTL | 126 |
| Sbjct | 72 | G+ C VL+ GR WLL+ VPG L + L P V ++A A+RRLH L HGAGIDCPQVLNETQSDGRQWLLMSAVPGGTLSALAQRGELEPERLVRLVAAALRRLHDL | 131 |
| Query | 127 | DPATCPFDHQAKHRIERARTRMEAGLVDQDDLDEEHQGLAPAELFARLKARMPDGEDLVV | 186 |
| Sbjct | 132 | DPA CPFDH+ + R++ R R+EAGLVD+ D D++H+G + EL+ L R P EDLVV DPAACPFDHRLERRLDTVRQRVEAGLVDEADFDDDHRGRSATELYRLLLDRRPAVEDLVV | 191 |
| Query | 187 | THGDACLPNIMVENGRFSGFIDCGRLGVADRYQDIALATRDIAEELGGEWADRFLVLYGI | 246 |
| Sbjct | 192 | HGDACLPN++ E RFSGFIDCGRLGVADR+QD+ALA RDI ELG WA+ FLV YG  AHGDACLPNLLAEGRRFSGFIDCGRLGVADRHQDLALAARDIEAELGAAWAEAFLVEYG- | 250 |
| Query | 247 AAPDSQRIAFYRLLDEFF 264 | | |
|  |  | D +R+A++RLLDEFF |  |
| Sbjct | 251 | GDIDGERLAYFRLLDEFF 268 | |

>WP_058177127.1 APH(3')-II family aminoglycoside O-phosphotransferase [Pseudomonas aeruginosa]

KSF86723.1 aminoglycoside 3'-phosphotransferase [Pseudomonas aeruginosa] Length=268

Score = 261 bits (668), Expect = 7e-84, Method: Compositional matrix adjust.

| Identities | | = 138/258 (53%), Positives = 168/258 (65%), Gaps = 5/258 (2%) |  |
| --- | --- | --- | --- |
| Query 11  Sbjct 12 | | SPAAWVERLFGYDWAQQTIGCSDAAVFRLSAQGRPVLFVKTDLSGALNELQDEAARLSWL  +PA W + L GY W Q GCS A V RL A RP LFVK ++ A EL E ARL WL APATWADYLAGYRWRGQGEGCSAATVHRLEAARRPTLFVKQEVLSAHAELPAEIARLRWL | 70  71 |
| Query 71  Sbjct 72 | | ATTGVPCAAVLDVVTEAGRDWLLLGEVPGQDLLS SHLAPAEKVSIMADAMRRLHTL  G+ C VL+ GR WLL+ VPG L + L P V ++A A+RRLH L HGAGIDCPQVLNETQSDGRQWLLMSAVPGDTLSALAQRGELEPERLVRLVAAALRRLHDL | 126  131 |
| Query 127  Sbjct 132 | | DPATCPFDHQAKHRIERARTRMEAGLVDQDDLDEEHQGLAPAELFARLKARMPDGEDLVV DPA CPFDH+ + R++ R R+EAGLVD+ D D +H+G + EL+ L R P EDLVV DPAACPFDHRLERRLDTVRQRVEAGLVDEADFDGDHRGRSATELYRLLLDRRPAVEDLVV | 186  191 |
| Query 187  Sbjct 192 | | THGDACLPNIMVENGRFSGFIDCGRLGVADRYQDIALATRDIAEELGGEWADRFLVLYGI HGDACLPN++ E RFSGFIDCGRLGVADR+QD+ALA RDI ELG WA+ FLV YG AHGDACLPNLLAEGRRFSGFIDCGRLGVADRHQDLALAARDIEAELGAAWAEAFLVEYG- | 246  250 |
| Query | 247 AAPDSQRIAFYRLLDEFF 264 | | |
|  |  | D +R+A++RLLDEFF |  |
| Sbjct | 251 | GDIDGERLAYFRLLDEFF 268 | |

>WP_113399506.1 aminoglycoside 3'-phosphotransferase [Sinorhizobium sp. GL28] KSV93759.1 hypothetical protein N184_05340 [Sinorhizobium sp. GL28]

Length=254

Score = 261 bits (667), Expect = 7e-84, Method: Compositional matrix adjust.

| Identities | = 130/244 (53%), Positives = 165/244 (68%), Gaps = 0/244 (0%) |  |
| --- | --- | --- |
| Query 21 | GYDWAQQTIGCSDAAVFRLSAQGRPVLFVKTDLSGALNELQDEAARLSWLATTGVPCAAV | 80 |
| Sbjct 11 | GYDW T+G S ++++ L A RP+L +K + +G +E DEA RL WLA+ G+PC V GYDWNHDTLGQSHSSIYLLKAPDRPMLVLKVEAAGPFSEFVDEAERLDWLASQGMPCPRV | 70 |
| Query 81 | LDVVTEAGRDWLLLGEVPGQDLLSSHLAPAEKVSIMADAMRRLHTLDPATCPFDHQAKHR | 140 |
| Sbjct 71 | + +A +WLLL V G DL S+ L+P +++ I+ADA++RLH LD A CPFDH+ + R IARAFDAETNWLLLQAVEGTDLASTTLSPKDRIVILADALKRLHGLDVADCPFDHRIEKR | 130 |
| Query 141 | IERARTRMEAGLVDQDDLDEEHQGLAPAELFARLKARMPDGEDLVVTHGDACLPNIMVEN | 200 |
| Sbjct 131 | I A R EAGL+D+DD DE G A+LFA L+AR+P LV+THGDACLPNI+ +N IAIAGARTEAGLIDEDDFDETRLGRTAADLFAELRARVPSIGKLVITHGDACLPNIIEKN | 190 |
| Query 201 | GRFSGFIDCGRLGVADRYQDIALATRDIAEELGGEWADRFLVLYGIAAPDSQRIAFYRLL | 260 |
| Sbjct 191 | GRFSGFIDC RLGVAD YQDIALA IA LG EW FL YGI D ++ Y LL  GRFSGFIDCSRLGVADPYQDIALACHSIAHNLGEEWVQPFLARYGIDRDDPEKRDLYCLL | 250 |

Query 261 DEFF 264

DEFF Sbjct 251 DEFF 254

>WP_126118968.1 APH(3')-II family aminoglycoside O-phosphotransferase [Pseudomonas aeruginosa]

RTB36446.1 APH(3')-II family aminoglycoside O-phosphotransferase [Pseudomonas aeruginosa]

RTC27800.1 APH(3')-II family aminoglycoside O-phosphotransferase [Pseudomonas aeruginosa]

RTC52845.1 APH(3')-II family aminoglycoside O-phosphotransferase [Pseudomonas aeruginosa]

Length=268

Score = 261 bits (668), Expect = 7e-84, Method: Compositional matrix adjust.

| Identities | | = 137/258 (53%), Positives = 169/258 (66%), Gaps = 5/258 (2%) |  |
| --- | --- | --- | --- |
| Query 11  Sbjct 12 | | SPAAWVERLFGYDWAQQTIGCSDAAVFRLSAQGRPVLFVKTDLSGALNELQDEAARLSWL  +P+ W + L GY W Q GCS A V RL A RP LFVK ++ A EL E ARL WL APSTWADYLAGYRWRGQGEGCSAATVHRLEAARRPTLFVKQEVLSAHAELPAEIARLRWL | 70  71 |
| Query 71  Sbjct 72 | | ATTGVPCAAVLDVVTEAGRDWLLLGEVPGQDLLS SHLAPAEKVSIMADAMRRLHTL  G+ C VL+ GR WLL+ VPG L + L P V ++A A+RRLH L HGAGIDCPQVLNETQSDGRQWLLMSAVPGDTLSALAQRGELEPERLVRLVAAALRRLHDL | 126  131 |
| Query 127  Sbjct 132 | | DPATCPFDHQAKHRIERARTRMEAGLVDQDDLDEEHQGLAPAELFARLKARMPDGEDLVV DPA CPFDH+ + R++ R R+EAGLVD+ D D++H+G + EL+ L R P EDLVV DPAACPFDHRLERRLDIVRQRVEAGLVDEADFDDDHRGRSATELYRLLLDRRPAVEDLVV | 186  191 |
| Query 187  Sbjct 192 | | THGDACLPNIMVENGRFSGFIDCGRLGVADRYQDIALATRDIAEELGGEWADRFLVLYGI HGDACLPN++ E RFSGFIDCGRLGVADR+QD+ALA RDI ELG WA+ FLV YG AHGDACLPNLLAEGRRFSGFIDCGRLGVADRHQDLALAARDIEAELGAAWAEAFLVEYG- | 246  250 |
| Query | 247 AAPDSQRIAFYRLLDEFF 264 | | |
|  |  | D +R+A++RLLDEFF |  |
| Sbjct | 251 | GDIDGERLAYFRLLDEFF 268 | |

>MBG6527585.1 APH(3')-II family aminoglycoside O-phosphotransferase [Pseudomonas aeruginosa]

Length=268

Score = 261 bits (668), Expect = 8e-84, Method: Compositional matrix adjust.

| Identities | | = 137/258 (53%), Positives = 168/258 (65%), Gaps = 5/258 (2%) |  |
| --- | --- | --- | --- |
| Query 11  Sbjct 12 | | SPAAWVERLFGYDWAQQTIGCSDAAVFRLSAQGRPVLFVKTDLSGALNELQDEAARLSWL  +P+ W L GY W Q GCS A V RL A RP LFVK ++ A EL E ARL WL APSTWAAYLAGYRWRGQGEGCSAATVHRLEAARRPTLFVKQEVLSAHAELPAEIARLRWL | 70  71 |
| Query 71  Sbjct 72 | | ATTGVPCAAVLDVVTEAGRDWLLLGEVPGQDLLS SHLAPAEKVSIMADAMRRLHTL  G+ C VL+ GR WLL+ VPG L + L P V ++A A+RRLH L HGAGIDCPQVLNETQSDGRQWLLMSAVPGDTLSALAQRGELEPERLVRLVAAALRRLHDL | 126  131 |
| Query 127  Sbjct 132 | | DPATCPFDHQAKHRIERARTRMEAGLVDQDDLDEEHQGLAPAELFARLKARMPDGEDLVV DPA CPFDH+ + R++ R R+EAGLVD+ D D++H+G + EL+ L R P EDLVV DPAACPFDHRLERRLDTVRQRVEAGLVDEADFDDDHRGRSATELYRLLLDRRPAVEDLVV | 186  191 |
| Query 187  Sbjct 192 | | THGDACLPNIMVENGRFSGFIDCGRLGVADRYQDIALATRDIAEELGGEWADRFLVLYGI HGDACLPN++ E RFSGFIDCGRLGVADR+QD+ALA RDI ELG WA+ FLV YG AHGDACLPNLLAEGRRFSGFIDCGRLGVADRHQDLALAARDIEAELGAAWAEAFLVEYG- | 246  250 |
| Query | 247 AAPDSQRIAFYRLLDEFF 264 | | |
|  |  | D +R+A++RLLDEFF |  |
| Sbjct | 251 | GDIDGERLAYFRLLDEFF 268 | |

>WP_172348945.1 APH(3') family aminoglycoside O-phosphotransferase [Mesorhizobium sp. NZP2298]

QKC95343.1 aminoglycoside 3'-phosphotransferase [Mesorhizobium sp. NZP2298] Length=261

Score = 261 bits (667), Expect = 8e-84, Method: Compositional matrix adjust.

| Identities | | = 135/253 (53%), Positives = 168/253 (66%), Gaps = 1/253 (0%) |  |
| --- | --- | --- | --- |
| Query 12  Sbjct 10 | | PAAWVERLFGYDWAQQTIGCSDAAVFRLSAQGRPVLFVKTDLSGALNELQDEAARLSWLA P RL GY W +QTIG S A VFRL A +P LF+K + +G EL DEAARL WLA PRELKNRLSGYRWHRQTIGKSKAGVFRLVADDKPALFLKCERNGPFAELADEAARLRWLA | 71  69 |
| Query 72  Sbjct 70 | | TTGVPCAAVLDVVTEAGRDWLLLGEVPGQDLLSSHLAPAEKVSIMADAMRRLHTLDPATC G+ C V+ + + AG DWLL+ V G+DL S+ + PA+ + IMA A+R LHTLD +C GQGIACPDVIALESHAGHDWLLMSAVAGEDLASAPIDPADVIEIMASALRDLHTLDIGSC | 131  129 |
| Query 132  Sbjct 130 | | PFDHQAKHRIERARTRMEAGLVDQDDLDEEHQGLAPAELFARLKARMPDGEDLVVTHGDA PFDH+ RI AR+RMEAG VD+ D D+E QG A+ FA L+A P EDLVV HGDA PFDHRLFRRIAAARSRMEAGDVDESDFDDERQGWTAAKAFAELEALRPTTEDLVVAHGDA | 191  189 |
| Query 192  Sbjct 190 | | CLPNIMVENGRFSGFIDCGRLGVADRYQDIALATRDIAEELGGEWADRFLVLYGIAAPDS CLPN+M FSGFIDCGRLGVADR+QD+ALA I +G W + FL YG + CLPNVMAARDSFSGFIDCGRLGVADRHQDLALACSSIRYNIGETWIEPFLERYG-PPQEP | 251  248 |
| Query 252 | | QRIAFYRLLDEFF 264  ++++YRLLDEFF |  |
| Sbjct | 249 | AKLSWYRLLDEFF 261 | |

>WP_031692397.1 APH(3')-II family aminoglycoside O-phosphotransferase [Pseudomonas aeruginosa]

EZP06800.1 aminoglycoside 3'-phosphotransferase type IIB [Pseudomonas aeruginosa BWH054]

KSD25299.1 aminoglycoside phosphotransferase APH(3') [Pseudomonas aeruginosa] KSF23689.1 aminoglycoside phosphotransferase APH(3') [Pseudomonas aeruginosa] KSH43749.1 aminoglycoside phosphotransferase APH(3') [Pseudomonas aeruginosa]

MBG7301282.1 APH(3')-II family aminoglycoside O-phosphotransferase [Pseudomonas aeruginosa]

Length=268

Score = 261 bits (668), Expect = 8e-84, Method: Compositional matrix adjust.

| Identities | | = 136/258 (53%), Positives = 169/258 (66%), Gaps = 5/258 (2%) |  |
| --- | --- | --- | --- |
| Query 11  Sbjct 12 | | SPAAWVERLFGYDWAQQTIGCSDAAVFRLSAQGRPVLFVKTDLSGALNELQDEAARLSWL  +P+ W + L GY W Q GCS A V RL A RP LFVK ++ A EL E ARL WL APSTWADYLAGYRWRGQGEGCSAATVHRLEAARRPTLFVKQEVLSAHAELPAEIARLRWL | 70  71 |
| Query 71  Sbjct 72 | | ATTGVPCAAVLDVVTEAGRDWLLLGEVPGQDLLS SHLAPAEKVSIMADAMRRLHTL  G+ C VL+ GR WLL+ VPG L + L P V ++A A+RRLH L HGAGIDCPQVLNETQSDGRQWLLMSAVPGDTLSALAQRGELEPERLVRLVAAALRRLHDL | 126  131 |
| Query 127  Sbjct 132 | | DPATCPFDHQAKHRIERARTRMEAGLVDQDDLDEEHQGLAPAELFARLKARMPDGEDLVV DPA CPFDH+ + R++ R R+EAGLVD+ D D++H+G + EL+ L R P EDLVV DPAACPFDHRLERRLDTVRQRVEAGLVDEADFDDDHRGRSATELYRLLLDRRPAVEDLVV | 186  191 |
| Query 187  Sbjct 192 | | THGDACLPNIMVENGRFSGFIDCGRLGVADRYQDIALATRDIAEELGGEWADRFLVLYGI HGDACLPN++ E RFSGFIDCGRLGVADR+QD+ALA RDI E+G WA+ FLV YG AHGDACLPNLLAEGRRFSGFIDCGRLGVADRHQDLALAARDIEAEIGAAWAEAFLVEYG- | 246  250 |
| Query | 247 AAPDSQRIAFYRLLDEFF 264 | | |
|  |  | D +R+A++RLLDEFF |  |
| Sbjct | 251 | GDIDGERLAYFRLLDEFF 268 | |

>KAA1182777.1 aminoglycoside 3'-phosphotransferase [Rhizobium tropici] Length=267

Score = 261 bits (668), Expect = 8e-84, Method: Compositional matrix adjust. Identities = 130/254 (51%), Positives = 170/254 (67%), Gaps = 1/254 (0%)

| Query | 12 | PAAWVERLFGYDWAQQTIGCSDAAVFRLSAQGRPVLFVKTDLSGALNELQDEAARLSWLA | 71 |
| --- | --- | --- | --- |
| Sbjct | 14 | P + L GY+W Q +G S A VFRL A GR +++KT+L L EL DE ARL WLA PMRLRDALAGYEWRQDALGRSSAHVFRLEADGRAPVYLKTELVDPLGELTDEVARLRWLA | 73 |
| Query | 72 | TTGVPCAAVLDVVTEAGRDWLLLGEVPGQDLLSSHLAP-AEKVSIMADAMRRLHTLDPAT  + G+ C V+ ++ R+WLL+ +PG DL+S L P E+V ++A+A+RRLH +D A | 130 |

| Sbjct | 74 | SQGLACPDVIAHESDGEREWLLMSALPGSDLVSEELTPPVERVRLLANALRRLHAIDIAA | 133 |
| --- | --- | --- | --- |
| Query | 131 | CPFDHQAKHRIERARTRMEAGLVDQDDLDEEHQGLAPAELFARLKARMPDGEDLVVTHGD | 190 |
| Sbjct | 134 | CPFDH+ ++RIE AR R+ AG VD+DD DE G + +LF L+A+ P ED VVTHGD CPFDHRLENRIETARARLLAGHVDEDDFDETRLGRSAEDLFRELEAKRPSTEDFVVTHGD | 193 |
| Query | 191 | ACLPNIMVENGRFSGFIDCGRLGVADRYQDIALATRDIAEELGGEWADRFLVLYGIAAPD | 250 |
| Sbjct | 194 | ACLPN M + RFSGFIDCGRLG+ADR+QDIAL I G E FL +YG P ACLPNFMADGDRFSGFIDCGRLGIADRHQDIALVCGSIEYNFGEELVAEFLKVYGNFEPK | 253 |
| Query  Sbjct | 251  254 | SQRIAFYRLLDEFF 264  ++++A+YRLLDEFF  AEKMAYYRLLDEFF 267 |  |

>MBG6702430.1 APH(3')-II family aminoglycoside O-phosphotransferase [Pseudomonas aeruginosa]

MBG7219278.1 APH(3')-II family aminoglycoside O-phosphotransferase [Pseudomonas aeruginosa]

MBG7457710.1 APH(3')-II family aminoglycoside O-phosphotransferase [Pseudomonas aeruginosa]

Length=268

Score = 261 bits (668), Expect = 8e-84, Method: Compositional matrix adjust.

| Identities | | = 137/258 (53%), Positives = 169/258 (66%), Gaps = 5/258 (2%) |  |
| --- | --- | --- | --- |
| Query 11  Sbjct 12 | | SPAAWVERLFGYDWAQQTIGCSDAAVFRLSAQGRPVLFVKTDLSGALNELQDEAARLSWL  +PA W + L GY W Q GCS A V RL A RP LFVK ++ A EL E ARL WL APATWADYLAGYRWRGQGEGCSAATVHRLEAARRPTLFVKQEVLSAHAELPAEIARLRWL | 70  71 |
| Query 71  Sbjct 72 | | ATTGVPCAAVLDVVTEAGRDWLLLGEVPGQDLLS SHLAPAEKVSIMADAMRRLHTL  G+ C VL+ GR WLL+ VPG L + L P V ++A A+RRLH L HGAGIDCPQVLNETQSDGRQWLLMSAVPGDTLSALAQRGELEPERLVRLVAAALRRLHDL | 126  131 |
| Query 127  Sbjct 132 | | DPATCPFDHQAKHRIERARTRMEAGLVDQDDLDEEHQGLAPAELFARLKARMPDGEDLVV DPA CPFDH+ + R++ R R++AGLVD+ D D++H+G + EL+ L R P EDLVV DPAACPFDHRLERRLDTVRQRVDAGLVDEADFDDDHRGRSATELYRLLLDRRPAVEDLVV | 186  191 |
| Query 187  Sbjct 192 | | THGDACLPNIMVENGRFSGFIDCGRLGVADRYQDIALATRDIAEELGGEWADRFLVLYGI HGDACLPN++ E RFSGFIDCGRLGVADR+QD+ALA RDI ELG WA+ FLV YG AHGDACLPNLLAEGRRFSGFIDCGRLGVADRHQDLALAARDIEAELGAAWAEAFLVEYG- | 246  250 |
| Query | 247 AAPDSQRIAFYRLLDEFF 264 | | |
|  |  | D +R+A++RLLDEFF |  |
| Sbjct | 251 | GDIDGERLAYFRLLDEFF 268 | |

```
>WP_132710354.1 APH(3')-II family aminoglycoside O-phosphotransferase [Pseudomonas aeruginosa]
Length=268

Score = 261 bits (668), Expect = 8e-84, Method: Compositional matrix adjust. Identities = 138/258 (53%), Positives = 170/258 (66%), Gaps = 5/258 (2%)

Query 11	SPAAWVERLFGYDWAQQTIGCSDAAVFRLSAQGRPVLFVKTDLSGALNELQDEAARLSWL 70
+PA W + L GY W Q GCS A V RL A RP LFVK ++ A EL E ARL WL Sbjct 12	APATWDDYLAGYRWRGQGEGCSAATVHRLEAARRPTLFVKQEVLSAHAELPAEIARLRWL 71

Query 71	ATTGVPCAAVLDVVTEAGRDWLLLGEVPGQDLLS----SHLAPAEKVSIMADAMRRLHTL 126 G+ C VL+	GR WLL+ VPG L +	L P	V ++A A+RRLH L
Sbjct 72	HGAGIDCPQVLNETQSDGRQWLLMSAVPGDTLSALAQCGELEPGRLVRLVAAALRRLHDL 131

Query 127 DPATCPFDHQAKHRIERARTRMEAGLVDQDDLDEEHQGLAPAELFARLKARMPDGEDLVV 186 DPA CPFDH+ + R++ R R+EAGLVD+ D D++H+G + +EL+ L R P EDLVV
Sbjct 132 DPAACPFDHRLERRLDTVRQRVEAGLVDEADFDDDHRGRSASELYRLLLDRRPAVEDLVV 191

Query 187 THGDACLPNIMVENGRFSGFIDCGRLGVADRYQDIALATRDIAEELGGEWADRFLVLYGI 246 HGDACLPN++ E RFSGFIDCGRLGVADR+QD+ALA RDI ELG WA+ FLV YG
Sbjct 192 AHGDACLPNLLAEGRRFSGFIDCGRLGVADRHQDLALAARDIEAELGPAWAEAFLVEYG- 250

Query 247 AAPDSQRIAFYRLLDEFF 264
D +R+A++RLLDEFF

Sbjct 251 GDIDGERLAYFRLLDEFF 268

>WP_153500606.1 aminoglycoside 3'-phosphotransferase [Sinorhizobium meliloti]
MQX43918.1 APH(3') family aminoglycoside O-phosphotransferase [Sinorhizobium meliloti]
Length=261

Score = 261 bits (667), Expect = 8e-84, Method: Compositional matrix adjust.
```

| Identities | = 137/253 (54%), Positives = 162/253 (64%), Gaps = 0/253 (0%) |  |
| --- | --- | --- |
| Query 12 | PAAWVERLFGYDWAQQTIGCSDAAVFRLSAQGRPVLFVKTDLSGALNELQDEAARLSWLA | 71 |
| Sbjct 9 | P A+ L GY + + +G S + +F L AQ RP L +K + SG EL EAARL WLA PPAFRGLLSGYGFERDALGQSASTIFLLKAQSRPFLILKHEPSGPFTELTGEAARLHWLA | 68 |
| Query 72 | TTGVPCAAVLDVVTEAGRDWLLLGEVPGQDLLSSHLAPAEKVSIMADAMRRLHTLDPATC | 131 |
| Sbjct 69 | +PC VL AG WLLL G D S L AE+++I+A+A+RRLH LDPA C AQDMPCPRVLGHDDHAGETWLLLEAAEGMDFASCRLPHAERIAILAEALRRLHRLDPAAC | 128 |
| Query 132 | PFDHQAKHRIERARTRMEAGLVDQDDLDEEHQGLAPAELFARLKARMPDGEDLVVTHGDA | 191 |
| Sbjct 129 | PFD + I A+ RMEAGLVD+ D DEE QG ELF L AR P E LVVTHGDA PFDRRLTASIAAAKARMEAGLVDESDFDEERQGRQTRELFDELLARRPKEEQLVVTHGDA | 188 |
| Query 192 | CLPNIMVENGRFSGFIDCGRLGVADRYQDIALATRDIAEELGGEWADRFLVLYGIAAPDS | 251 |
| Sbjct 189 | CLPN + +FSGFIDCGRLGVAD YQD+ALA I LG EW FL YG+AAPD CLPNFIAAGNQFSGFIDCGRLGVADIYQDLALACWSITHNLGEEWVAPFLDRYGLAAPDP | 248 |
| Query 252  Sbjct 249 | QRIAFYRLLDEFF 264  +++AFYRLLDEFF  EKLAFYRLLDEFF 261 |  |

>WP_132678652.1 APH(3')-II family aminoglycoside O-phosphotransferase [Pseudomonas aeruginosa]

Length=267

Score = 261 bits (668), Expect = 8e-84, Method: Compositional matrix adjust.

| Identities | | = 136/258 (53%), Positives = 169/258 (66%), Gaps = 5/258 (2%) |  |
| --- | --- | --- | --- |
| Query 11  Sbjct 11 | | SPAAWVERLFGYDWAQQTIGCSDAAVFRLSAQGRPVLFVKTDLSGALNELQDEAARLSWL  +P+ W + L GY W Q GCS A V RL A+ RP LFVK ++ A EL E ARL WL APSTWADYLAGYRWRGQGEGCSAATVHRLEAERRPTLFVKQEVLSAHAELPAEIARLRWL | 70  70 |
| Query 71  Sbjct 71 | | ATTGVPCAAVLDVVTEAGRDWLLLGEVPGQDLLS SHLAPAEKVSIMADAMRRLHTL  G+ C VL+ GR WLL+ VPG L + L P V ++A A+RRLH L HGAGIDCPQVLNETQSDGRQWLLMSAVPGDTLSALAQRGELEPERLVRLVAAALRRLHDL | 126  130 |
| Query 127  Sbjct 131 | | DPATCPFDHQAKHRIERARTRMEAGLVDQDDLDEEHQGLAPAELFARLKARMPDGEDLVV DPA CPFDH+ + R++ R R+EAGLVD+ D D++H+G + EL+ L R P EDLVV DPAACPFDHRLERRLDTVRQRVEAGLVDEADFDDDHRGRSATELYRLLLDRRPAVEDLVV | 186  190 |
| Query 187  Sbjct 191 | | THGDACLPNIMVENGRFSGFIDCGRLGVADRYQDIALATRDIAEELGGEWADRFLVLYGI HGDACLPN++ E RFSGFIDCGRLGVADR+QD+A A RDI ELG WA+ FLV YG AHGDACLPNLLAEGRRFSGFIDCGRLGVADRHQDLAXAARDIEAELGAAWAEAFLVEYG- | 246  249 |
| Query | 247 AAPDSQRIAFYRLLDEFF 264 | | |
|  |  | D +R+A++RLLDEFF |  |
| Sbjct | 250 | GDIDGERLAYFRLLDEFF 267 | |

>SQC59007.1 aminoglycoside 3-N-acetyltransferase [Pseudomonas aeruginosa] Length=268

Score = 261 bits (668), Expect = 9e-84, Method: Compositional matrix adjust.

| Identities | = 137/258 (53%), Positives = 168/258 (65%), Gaps = 5/258 (2%) |  |
| --- | --- | --- |
| Query 11 | SPAAWVERLFGYDWAQQTIGCSDAAVFRLSAQGRPVLFVKTDLSGALNELQDEAARLSWL | 70 |
| Sbjct 12 | +P+ W + L GY W Q GCS A V RL A RP LFVK ++ A EL E ARL WL APSTWADYLAGYRWRGQGEGCSAATVHRLEAARRPTLFVKQEVLSAHAELPAEIARLRWL | 71 |
| Query 71 | ATTGVPCAAVLDVVTEAGRDWLLLGEVPGQDLLS SHLAPAEKVSIMADAMRRLHTL  G+ C VL+ GR WLL+ VPG L L P V ++A A+RRLH L | 126 |

| Sbjct | 72 | HGAGIDCPQVLNETQSDGRQWLLMSAVPGDTLSELAQRGELEPERLVRLVAAALRRLHDL | 131 |
| --- | --- | --- | --- |
| Query | 127 | DPATCPFDHQAKHRIERARTRMEAGLVDQDDLDEEHQGLAPAELFARLKARMPDGEDLVV | 186 |
| Sbjct | 132 | DPA CPFDH+ + R++ R R+EAGLVD+ D D++H+G + EL+ L R P EDLVV DPAACPFDHRLERRLDTVRQRVEAGLVDEADFDDDHRGRSATELYRLLLDRRPAVEDLVV | 191 |
| Query | 187 | THGDACLPNIMVENGRFSGFIDCGRLGVADRYQDIALATRDIAEELGGEWADRFLVLYGI | 246 |
| Sbjct | 192 | HGDACLPN++ E RFSGFIDCGRLGVADR+QD+ALA RDI ELG WA+ FLV YG  AHGDACLPNLLAEGRRFSGFIDCGRLGVADRHQDLALAARDIEAELGAAWAEAFLVEYG- | 250 |
| Query | 247 AAPDSQRIAFYRLLDEFF 264 | | |
|  |  | D +R+A++RLLDEFF |  |
| Sbjct | 251 | GDIDGERLAYFRLLDEFF 268 | |

>MBG7045669.1 APH(3')-II family aminoglycoside O-phosphotransferase [Pseudomonas aeruginosa]

Length=268

Score = 261 bits (668), Expect = 9e-84, Method: Compositional matrix adjust.

| Identities | | = 138/258 (53%), Positives = 168/258 (65%), Gaps = 5/258 (2%) |  |
| --- | --- | --- | --- |
| Query 11  Sbjct 12 | | SPAAWVERLFGYDWAQQTIGCSDAAVFRLSAQGRPVLFVKTDLSGALNELQDEAARLSWL  +PA W + L GY W Q GCS A V RL A RP LFVK ++ A EL E ARL WL APATWADYLAGYRWRGQGEGCSAATVHRLEAARRPTLFVKQEVLSAHAELPAEIARLRWL | 70  71 |
| Query 71  Sbjct 72 | | ATTGVPCAAVLDVVTEAGRDWLLLGEVPGQDLLS SHLAPAEKVSIMADAMRRLHTL  G+ C VL+ GR WLL+ VPG L + L P V ++A A+RRLH L HGAGIDCPQVLNETQSDGRQWLLMSAVPGDTLSALAQRGELEPERLVRLVAAALRRLHDL | 126  131 |
| Query 127  Sbjct 132 | | DPATCPFDHQAKHRIERARTRMEAGLVDQDDLDEEHQGLAPAELFARLKARMPDGEDLVV DPA CPFDH+ + R + R R+EAGLVD+ D D++H+G + EL+ L R P EDLVV DPAACPFDHRLERRQDTVRQRVEAGLVDEADFDDDHRGRSATELYRLLLDRRPAVEDLVV | 186  191 |
| Query 187  Sbjct 192 | | THGDACLPNIMVENGRFSGFIDCGRLGVADRYQDIALATRDIAEELGGEWADRFLVLYGI HGDACLPN++ E RFSGFIDCGRLGVADR+QD+ALA RDI ELG WA+ FLV YG AHGDACLPNLLAEGRRFSGFIDCGRLGVADRHQDLALAARDIEAELGAAWAEAFLVEYG- | 246  250 |
| Query | 247 AAPDSQRIAFYRLLDEFF 264 | | |
|  |  | D +R+A++RLLDEFF |  |
| Sbjct | 251 | GDIDGERLAYFRLLDEFF 268 | |

>WP_188112553.1 APH(3') family aminoglycoside O-phosphotransferase [Rhizobium tropici]

Length=266

Score = 261 bits (667), Expect = 1e-83, Method: Compositional matrix adjust.

| Identities | = 130/254 (51%), Positives = 170/254 (67%), Gaps = 1/254 (0%) |  |
| --- | --- | --- |
| Query 12 | PAAWVERLFGYDWAQQTIGCSDAAVFRLSAQGRPVLFVKTDLSGALNELQDEAARLSWLA | 71 |
| Sbjct 13 | P + L GY+W Q +G S A VFRL A GR +++KT+L L EL DE ARL WLA PMRLRDALAGYEWRQDALGRSSAHVFRLEADGRAPVYLKTELVDPLGELTDEVARLRWLA | 72 |
| Query 72 | TTGVPCAAVLDVVTEAGRDWLLLGEVPGQDLLSSHLAP-AEKVSIMADAMRRLHTLDPAT | 130 |
| Sbjct 73 | + G+ C V+ ++ R+WLL+ +PG DL+S L P E+V ++A+A+RRLH +D A SQGLACPDVIAHESDGEREWLLMSALPGSDLVSEELTPPVERVRLLANALRRLHAIDIAA | 132 |
| Query 131 | CPFDHQAKHRIERARTRMEAGLVDQDDLDEEHQGLAPAELFARLKARMPDGEDLVVTHGD | 190 |
| Sbjct 133 | CPFDH+ ++RIE AR R+ AG VD+DD DE G + +LF L+A+ P ED VVTHGD CPFDHRLENRIETARARLLAGHVDEDDFDETRLGRSAEDLFRELEAKRPSTEDFVVTHGD | 192 |
| Query 191 | ACLPNIMVENGRFSGFIDCGRLGVADRYQDIALATRDIAEELGGEWADRFLVLYGIAAPD | 250 |
| Sbjct 193 | ACLPN M + RFSGFIDCGRLG+ADR+QDIAL I G E FL +YG P ACLPNFMADGDRFSGFIDCGRLGIADRHQDIALVCGSIEYNFGEELVAEFLKVYGNFEPK | 252 |
| Query 251  Sbjct 253 | SQRIAFYRLLDEFF 264  ++++A+YRLLDEFF  AEKMAYYRLLDEFF 266 |  |

>WP_130272335.1 aminoglycoside 3'-phosphotransferase [Phyllobacterium myrsinacearum]

Length=257

Score = 261 bits (666), Expect = 1e-83, Method: Compositional matrix adjust.

| Identities | = 133/245 (54%), Positives = 164/245 (67%), Gaps = 1/245 (0%) |  |
| --- | --- | --- |
| Query 21 | GYDWAQQTIGCSDAAVFRLSAQGRPVLFVKTDLSGALNELQDEAARLSWLATTGVPCAAV | 80 |
| Sbjct 13 | GY W++ +GCSDA VF L +G P LFVK + EL DEAARL WL++ + C V GYSWSRDALGCSDAQVFMLQGEGLPHLFVKVEAVSPYGELPDEAARLRWLSSRHMLCPDV | 72 |
| Query 81 | LDVVTEAGRDWLLLGEVPGQDLLSS-HLAPAEKVSIMADAMRRLHTLDPATCPFDHQAKH | 139 |
| Sbjct 73 | L A R WLL VPG+DL S+ L+ ++ I+A A+R+LH LD ATCPFDH+ LFEGAHASRFWLLTSGVPGKDLASADSLSIETRIRILAGALRQLHALDLATCPFDHRLDV | 132 |
| Query 140 | RIERARTRMEAGLVDQDDLDEEHQGLAPAELFARLKARMPDGEDLVVTHGDACLPNIMVE | 199 |
| Sbjct 133 | RIE AR RM+AGLVD+ D D + G +ELF+RL A P D+VVTHGDACLPN M E RIEAARARMQAGLVDETDFDSDMLGKTVSELFSRLVAERPGTADVVVTHGDACLPNFMAE | 192 |
| Query 200 | NGRFSGFIDCGRLGVADRYQDIALATRDIAEELGGEWADRFLVLYGIAAPDSQRIAFYRL | 259 |
| Sbjct 193 | NG F+G+IDC RLG+ADRYQDIALA R IA+ G EW FL YG+ D ++A+YRL NGIFTGYIDCARLGLADRYQDIALACRSIADNFGEEWVRPFLDCYGLVQADPAKLAYYRL | 252 |
| Query 260  Sbjct 253 | LDEFF 264 LDEFF LDEFF 257 |  |

>WP_132565192.1 APH(3')-II family aminoglycoside O-phosphotransferase [Pseudomonas aeruginosa]

Length=268

Score = 261 bits (667), Expect = 1e-83, Method: Compositional matrix adjust.

| Identities | | = 136/258 (53%), Positives = 169/258 (66%), Gaps = 5/258 (2%) |  |
| --- | --- | --- | --- |
| Query 11  Sbjct 12 | | SPAAWVERLFGYDWAQQTIGCSDAAVFRLSAQGRPVLFVKTDLSGALNELQDEAARLSWL  +P+ W + L GY W Q GCS A V RL A+ RP LFVK ++ A EL E ARL WL APSTWADYLAGYRWRGQGEGCSAATVHRLEAERRPTLFVKQEVLSAHAELPAEIARLRWL | 70  71 |
| Query 71  Sbjct 72 | | ATTGVPCAAVLDVVTEAGRDWLLLGEVPGQDLLS SHLAPAEKVSIMADAMRRLHTL  G+ C VL+ GR WLL+ VPG L + L P V ++A A+RRLH L HGAGIDCPQVLNETQSDGRQWLLMSAVPGDTLSALAQRGELEPERLVRLVAAALRRLHDL | 126  131 |
| Query 127  Sbjct 132 | | DPATCPFDHQAKHRIERARTRMEAGLVDQDDLDEEHQGLAPAELFARLKARMPDGEDLVV DPA CPFDH+ + R++ R R+EAGLVD+ D D++H+G + EL+ L R P EDLVV DPAACPFDHRLERRLDTVRQRVEAGLVDEADFDDDHRGRSATELYRLLLDRRPAVEDLVV | 186  191 |
| Query 187  Sbjct 192 | | THGDACLPNIMVENGRFSGFIDCGRLGVADRYQDIALATRDIAEELGGEWADRFLVLYGI HGDACLPN++ E RFSGFIDCGRLGVADR+QD+A A RDI ELG WA+ FLV YG AHGDACLPNLLAEGRRFSGFIDCGRLGVADRHQDLAXAARDIEAELGAAWAEAFLVEYG- | 246  250 |
| Query | 247 AAPDSQRIAFYRLLDEFF 264 | | |
|  |  | D +R+A++RLLDEFF |  |
| Sbjct | 251 | GDIDGERLAYFRLLDEFF 268 | |

>WP_116844549.1 APH(3')-II family aminoglycoside O-phosphotransferase [Pseudomonas aeruginosa]

NPY03979.1 APH(3')-II family aminoglycoside O-phosphotransferase [Pseudomonas aeruginosa]

RTT34831.1 APH(3')-II family aminoglycoside O-phosphotransferase [Pseudomonas aeruginosa]

RUB78232.1 APH(3')-II family aminoglycoside O-phosphotransferase [Pseudomonas aeruginosa]

Length=268

Score = 261 bits (667), Expect = 1e-83, Method: Compositional matrix adjust. Identities = 137/258 (53%), Positives = 169/258 (66%), Gaps = 5/258 (2%)

| Query | 11 | SPAAWVERLFGYDWAQQTIGCSDAAVFRLSAQGRPVLFVKTDLSGALNELQDEAARLSWL | 70 |
| --- | --- | --- | --- |
|  |  | +PA W + L GY W Q GCS A V RL A +P LFVK ++ A EL E ARL WL |  |
| Sbjct | 12 | APATWADYLAGYRWRGQGEGCSAATVHRLEAARQPTLFVKQEVLSAHAELPAEIARLRWL | 71 |
| Query | 71 | ATTGVPCAAVLDVVTEAGRDWLLLGEVPGQDLLS SHLAPAEKVSIMADAMRRLHTL | 126 |

| Sbjct | 72 | G+ C VL+ GR WLL+ VPG L + L P V ++A A+RRLH L HGAGIDCPQVLNETQSDGRQWLLMSAVPGDTLSALAQRGELEPERLVRLVAAALRRLHDL | 131 |
| --- | --- | --- | --- |
| Query | 127 | DPATCPFDHQAKHRIERARTRMEAGLVDQDDLDEEHQGLAPAELFARLKARMPDGEDLVV | 186 |
| Sbjct | 132 | DPA CPFDH+ + R++ R R+EAGLVD+ D D++H+G + EL+ L R P EDLVV DPAACPFDHRLERRLDTVRQRVEAGLVDEADFDDDHRGRSATELYRLLLDRRPAVEDLVV | 191 |
| Query | 187 | THGDACLPNIMVENGRFSGFIDCGRLGVADRYQDIALATRDIAEELGGEWADRFLVLYGI | 246 |
| Sbjct | 192 | HGDACLPN++ E RFSGFIDCGRLGVADR+QD+ALA RDI ELG WA+ FLV YG  AHGDACLPNLLAEGRRFSGFIDCGRLGVADRHQDLALAARDIEAELGAAWAEAFLVEYG- | 250 |
| Query | 247 AAPDSQRIAFYRLLDEFF 264 | | |
|  |  | D +R+A++RLLDEFF |  |
| Sbjct | 251 | GDIDGERLAYFRLLDEFF 268 | |

>WP_034022832.1 APH(3')-II family aminoglycoside O-phosphotransferase [Pseudomonas aeruginosa]

MBA6427859.1 APH(3')-II family aminoglycoside O-phosphotransferase [Pseudomonas aeruginosa]

MBG4704441.1 APH(3')-II family aminoglycoside O-phosphotransferase [Pseudomonas aeruginosa]

MBG5315719.1 APH(3')-II family aminoglycoside O-phosphotransferase [Pseudomonas aeruginosa]

MBG5376877.1 APH(3')-II family aminoglycoside O-phosphotransferase [Pseudomonas aeruginosa]

MBG5450226.1 APH(3')-II family aminoglycoside O-phosphotransferase [Pseudomonas aeruginosa]

Length=268

Score = 261 bits (667), Expect = 1e-83, Method: Compositional matrix adjust.

| Identities | | = 136/258 (53%), Positives = 168/258 (65%), Gaps = 5/258 (2%) |  |
| --- | --- | --- | --- |
| Query 11  Sbjct 12 | | SPAAWVERLFGYDWAQQTIGCSDAAVFRLSAQGRPVLFVKTDLSGALNELQDEAARLSWL  +P+ W + L GY W Q GCS A V RL RP LFVK ++ A EL E ARL WL APSTWADYLAGYRWRGQGEGCSAATVHRLEVARRPTLFVKQEVLSAHAELPAEIARLRWL | 70  71 |
| Query 71  Sbjct 72 | | ATTGVPCAAVLDVVTEAGRDWLLLGEVPGQDLLS SHLAPAEKVSIMADAMRRLHTL  G+ C VL+ GR WLL+ VPG L + L P V ++A A+RRLH L HGAGIDCPQVLNETQSDGRQWLLMSAVPGDTLSALAQRGELEPERLVRLVAAALRRLHDL | 126  131 |
| Query 127  Sbjct 132 | | DPATCPFDHQAKHRIERARTRMEAGLVDQDDLDEEHQGLAPAELFARLKARMPDGEDLVV DPA CPFDH+ + R++ R R+EAGLVD+ D D++H+G + EL+ L R P EDLVV DPAACPFDHRLERRLDTVRQRVEAGLVDEADFDDDHRGRSATELYRLLLDRRPAVEDLVV | 186  191 |
| Query 187  Sbjct 192 | | THGDACLPNIMVENGRFSGFIDCGRLGVADRYQDIALATRDIAEELGGEWADRFLVLYGI HGDACLPN++ E RFSGFIDCGRLGVADR+QD+ALA RDI ELG WA+ FLV YG AHGDACLPNLLAEGRRFSGFIDCGRLGVADRHQDLALAARDIEAELGAAWAEAFLVEYG- | 246  250 |
| Query | 247 AAPDSQRIAFYRLLDEFF 264 | | |
|  |  | D +R+A++RLLDEFF |  |
| Sbjct | 251 | GDIDGERLAYFRLLDEFF 268 | |

>WP_034032241.1 APH(3')-II family aminoglycoside O-phosphotransferase [Pseudomonas aeruginosa]

Length=268

Score = 261 bits (667), Expect = 1e-83, Method: Compositional matrix adjust.

| Identities | = 136/258 (53%), Positives = 168/258 (65%), Gaps = 5/258 (2%) |  |
| --- | --- | --- |
| Query 11 | SPAAWVERLFGYDWAQQTIGCSDAAVFRLSAQGRPVLFVKTDLSGALNELQDEAARLSWL | 70 |
| Sbjct 12 | +P+ W + L GY W Q GCS A V RL RP LFVK ++ A EL E ARL WL APSTWADYLAGYRWRGQGEGCSAATVHRLEVARRPTLFVKQEVLSAHAELPAEIARLRWL | 71 |
| Query 71 | ATTGVPCAAVLDVVTEAGRDWLLLGEVPGQDLLS SHLAPAEKVSIMADAMRRLHTL | 126 |
| Sbjct 72 | G+ C VL+ GR WLL+ VPG L + L P V ++A A+RRLH L HGAGIDCPQVLNETQSDGRQWLLMSTVPGDTLSALAQRGELEPERLVRLVAAALRRLHDL | 131 |
| Query 127 | DPATCPFDHQAKHRIERARTRMEAGLVDQDDLDEEHQGLAPAELFARLKARMPDGEDLVV | 186 |
| Sbjct 132 | DPA CPFDH+ + R++ R R+EAGLVD+ D D++H+G + EL+ L R P EDLVV  DPAACPFDHRLERRLDTVRQRVEAGLVDEADFDDDHRGRSATELYRLLLDRRPAVEDLVV | 191 |

Query 187 THGDACLPNIMVENGRFSGFIDCGRLGVADRYQDIALATRDIAEELGGEWADRFLVLYGI 246

| Sbjct | 192 | HGDACLPN++ E RFSGFIDCGRLGVADR+QD+ALA RDI ELG WA+ FLV YG AHGDACLPNLLAEGRRFSGFIDCGRLGVADRHQDLALAARDIEAELGAAWAEAFLVEYG- 250 |
| --- | --- | --- |
| Query | 247 | AAPDSQRIAFYRLLDEFF 264 |
| Sbjct | 251 | D +R+A++RLLDEFF  GDIDGERLAYFRLLDEFF 268 |

>WP_132659571.1 APH(3')-II family aminoglycoside O-phosphotransferase [Pseudomonas aeruginosa]

Length=268

Score = 261 bits (666), Expect = 1e-83, Method: Compositional matrix adjust.

| Identities | = 138/259 (53%), Positives = 171/259 (66%), Gaps = 7/259 (3%) |  |
| --- | --- | --- |
| Query 11 | SPAAWVERLFGYDWAQQTIGCSDAAVFRLSAQGRPVLFVKTDLSGALNELQDEAARLSWL | 70 |
| Sbjct 12 | +P+ W + L GY W Q GCS A V RL A RP LFVK ++ A EL E ARL WL APSTWADYLAGYRWRGQGEGCSAATVHRLEAARRPTLFVKQEVLSAHAELPAEIARLRWL | 71 |
| Query 71 | ATTGVPCAAVLDVVTEAGRDWLLLGEVPGQDLLSS HLAPAEKVSIMADAMRRLHT | 125 |
| Sbjct 72 | G+ C VL+ GR WLL+ +PG D LS+ L P V ++A A+RRLH HGAGIDCPQVLNETQSDGRQWLLMSAMPG-DTLSALAQRDELEPERLVRLVAAALRRLHD | 130 |
| Query 126 | LDPATCPFDHQAKHRIERARTRMEAGLVDQDDLDEEHQGLAPAELFARLKARMPDGEDLV | 185 |
| Sbjct 131 | LDPA CPFDH+ + R++ R R+EAGLVD+ D D++H+G + EL+ L R P EDLV LDPAACPFDHRLERRLDTVRQRVEAGLVDEADFDDDHRGRSATELYRLLLDRRPXVEDLV | 190 |
| Query 186 | VTHGDACLPNIMVENGRFSGFIDCGRLGVADRYQDIALATRDIAEELGGEWADRFLVLYG | 245 |
| Sbjct 191 | V HGDACLPN++ E RFSGFIDCGRLGVADR+QD+ALA RDI ELG WA+ FLV YG VAHGDACLPNLLAEGRRFSGFIDCGRLGVADRHQDLALAARDIEAELGAAWAEAFLVEYG | 250 |
| Query 246  Sbjct 251 | IAAPDSQRIAFYRLLDEFF 264 D +R+A++RLLDEFF  -GDIDGERLAYFRLLDEFF 268 |  |

>RZS79098.1 aminoglycoside 3'-phosphotransferase-2 [Phyllobacterium myrsinacearum] Length=267

Score = 261 bits (666), Expect = 1e-83, Method: Compositional matrix adjust.

| Identities | = 133/245 (54%), Positives = 164/245 (67%), Gaps = 1/245 (0%) |  |
| --- | --- | --- |
| Query 21 | GYDWAQQTIGCSDAAVFRLSAQGRPVLFVKTDLSGALNELQDEAARLSWLATTGVPCAAV | 80 |
| Sbjct 23 | GY W++ +GCSDA VF L +G P LFVK + EL DEAARL WL++ + C V GYSWSRDALGCSDAQVFMLQGEGLPHLFVKVEAVSPYGELPDEAARLRWLSSRHMLCPDV | 82 |
| Query 81 | LDVVTEAGRDWLLLGEVPGQDLLSS-HLAPAEKVSIMADAMRRLHTLDPATCPFDHQAKH | 139 |
| Sbjct 83 | L A R WLL VPG+DL S+ L+ ++ I+A A+R+LH LD ATCPFDH+ LFEGAHASRFWLLTSGVPGKDLASADSLSIETRIRILAGALRQLHALDLATCPFDHRLDV | 142 |
| Query 140 | RIERARTRMEAGLVDQDDLDEEHQGLAPAELFARLKARMPDGEDLVVTHGDACLPNIMVE | 199 |
| Sbjct 143 | RIE AR RM+AGLVD+ D D + G +ELF+RL A P D+VVTHGDACLPN M E RIEAARARMQAGLVDETDFDSDMLGKTVSELFSRLVAERPGTADVVVTHGDACLPNFMAE | 202 |
| Query 200 | NGRFSGFIDCGRLGVADRYQDIALATRDIAEELGGEWADRFLVLYGIAAPDSQRIAFYRL | 259 |
| Sbjct 203 | NG F+G+IDC RLG+ADRYQDIALA R IA+ G EW FL YG+ D ++A+YRL NGIFTGYIDCARLGLADRYQDIALACRSIADNFGEEWVRPFLDCYGLVQADPAKLAYYRL | 262 |
| Query 260  Sbjct 263 | LDEFF 264 LDEFF LDEFF 267 |  |

>WP_125001236.1 APH(3')-II family aminoglycoside O-phosphotransferase [Pseudomonas aeruginosa]

RRI94353.1 APH(3')-II family aminoglycoside O-phosphotransferase [Pseudomonas aeruginosa]

Length=268

Score = 261 bits (666), Expect = 1e-83, Method: Compositional matrix adjust.

| Identities | | = 137/258 (53%), Positives = 169/258 (66%), Gaps = 5/258 (2%) |  |
| --- | --- | --- | --- |
| Query 11  Sbjct 12 | | SPAAWVERLFGYDWAQQTIGCSDAAVFRLSAQGRPVLFVKTDLSGALNELQDEAARLSWL  +P+ W + L GY W Q GCS A V RL A RP LFVK ++ A EL E ARL WL APSTWADYLAGYRWRGQGEGCSAATVRRLEAARRPTLFVKQEVLSAHAELPAEIARLRWL | 70  71 |
| Query 71  Sbjct 72 | | ATTGVPCAAVLDVVTEAGRDWLLLGEVPGQDLLS SHLAPAEKVSIMADAMRRLHTL  G+ C VL+ GR WLL+ VPG L + L P V ++A A+RRLH L HGAGIDCPQVLNETQSDGRQWLLMSAVPGDTLSALAQRGELEPERLVRLVAVALRRLHDL | 126  131 |
| Query 127  Sbjct 132 | | DPATCPFDHQAKHRIERARTRMEAGLVDQDDLDEEHQGLAPAELFARLKARMPDGEDLVV DPA CPFDH+ + R++ R R+EAGLVD+ D D++H+G + EL+ L R P EDLVV DPAACPFDHRLERRLDTVRQRVEAGLVDEADFDDDHRGRSATELYRLLLDRRPAVEDLVV | 186  191 |
| Query 187  Sbjct 192 | | THGDACLPNIMVENGRFSGFIDCGRLGVADRYQDIALATRDIAEELGGEWADRFLVLYGI HGDACLPN++ E RFSGFIDCGRLGVADR+QD+ALA RDI ELG WA+ FLV YG AHGDACLPNLLAEGRRFSGFIDCGRLGVADRHQDLALAARDIEAELGAAWAEAFLVEYG- | 246  250 |
| Query | 247 AAPDSQRIAFYRLLDEFF 264 | | |
|  |  | D +R+A++RLLDEFF |  |
| Sbjct | 251 | GDIDGERLAYFRLLDEFF 268 | |

>MBG6964287.1 APH(3')-II family aminoglycoside O-phosphotransferase [Pseudomonas aeruginosa]

Length=268

Score = 261 bits (666), Expect = 1e-83, Method: Compositional matrix adjust.

| Identities | | = 136/258 (53%), Positives = 168/258 (65%), Gaps = 5/258 (2%) |  |
| --- | --- | --- | --- |
| Query 11  Sbjct 12 | | SPAAWVERLFGYDWAQQTIGCSDAAVFRLSAQGRPVLFVKTDLSGALNELQDEAARLSWL  +P+ W + L GY W Q GCS A V RL RP LFVK ++ A EL E ARL WL APSTWADYLAGYRWRGQGEGCSAATVHRLEVARRPTLFVKQEVLSAHAELPAEIARLRWL | 70  71 |
| Query 71  Sbjct 72 | | ATTGVPCAAVLDVVTEAGRDWLLLGEVPGQDLLS SHLAPAEKVSIMADAMRRLHTL  G+ C VL+ AGR WLL+ VPG L + L P V ++A A+RRLH L HGAGIDCPQVLNETQSAGRQWLLMSAVPGDTLSALAQRGELEPERLVRLVAAALRRLHDL | 126  131 |
| Query 127  Sbjct 132 | | DPATCPFDHQAKHRIERARTRMEAGLVDQDDLDEEHQGLAPAELFARLKARMPDGEDLVV DPA CPFDH+ + R++ R R+EAGLVD+ D D++H+ + EL+ L R P EDLVV DPAACPFDHRLERRLDTVRQRVEAGLVDEADFDDDHRSRSATELYRLLLDRRPAVEDLVV | 186  191 |
| Query 187  Sbjct 192 | | THGDACLPNIMVENGRFSGFIDCGRLGVADRYQDIALATRDIAEELGGEWADRFLVLYGI HGDACLPN++ E RFSGFIDCGRLGVADR+QD+ALA RDI ELG WA+ FLV YG AHGDACLPNLLAEGRRFSGFIDCGRLGVADRHQDLALAARDIEAELGAAWAEAFLVEYG- | 246  250 |
| Query | 247 AAPDSQRIAFYRLLDEFF 264 | | |
|  |  | D +R+A++RLLDEFF |  |
| Sbjct | 251 | GDIDGERLAYFRLLDEFF 268 | |

>MBG6484523.1 APH(3')-II family aminoglycoside O-phosphotransferase [Pseudomonas aeruginosa]

Length=268

Score = 261 bits (666), Expect = 1e-83, Method: Compositional matrix adjust.

| Identities | = 137/258 (53%), Positives = 168/258 (65%), Gaps = 5/258 (2%) |  |
| --- | --- | --- |
| Query 11 | SPAAWVERLFGYDWAQQTIGCSDAAVFRLSAQGRPVLFVKTDLSGALNELQDEAARLSWL | 70 |
| Sbjct 12 | +P+ W + L GY W Q GCS A V RL A RP LFVK ++ EL E ARL WL APSTWADYLAGYRWRGQGEGCSAATVHRLEAARRPTLFVKQEVLSVHAELPAEIARLRWL | 71 |
| Query 71 | ATTGVPCAAVLDVVTEAGRDWLLLGEVPGQDLLS SHLAPAEKVSIMADAMRRLHTL | 126 |
| Sbjct 72 | G+ C VL+ GR WLL+ VPG L + L PA V +A A+RRLH L HGAGIDCPQVLNETQSDGRQWLLMSAVPGDTLSALAQRGELEPARLVRRVAAALRRLHDL | 131 |
| Query 127 | DPATCPFDHQAKHRIERARTRMEAGLVDQDDLDEEHQGLAPAELFARLKARMPDGEDLVV | 186 |
| Sbjct 132 | DPA CPFDH+ + R++ R R+EAGLVD+ D D++H+G + EL+ L R P EDLVV DPAACPFDHRLERRLDTVRQRVEAGLVDEADFDDDHRGRSATELYRLLLDRRPAVEDLVV | 191 |
| Query 187 | THGDACLPNIMVENGRFSGFIDCGRLGVADRYQDIALATRDIAEELGGEWADRFLVLYGI HGDACLPN++ E RFSGFIDCGRLGVADR+QD+ALA RDI ELG WA+ FLV YG | 246 |

| Sbjct | 192 | AHGDACLPNLLAEGRRFSGFIDCGRLGVADRHQDLALAARDIEAELGAAWAEAFLVEYG- 250 |
| --- | --- | --- |
| Query | 247 | AAPDSQRIAFYRLLDEFF 264 |
| Sbjct | 251 | D +R+A++RLLDEFF  GDIDGERLAYFRLLDEFF 268 |

>MBG6557881.1 APH(3')-II family aminoglycoside O-phosphotransferase [Pseudomonas aeruginosa]

Length=268

Score = 261 bits (666), Expect = 1e-83, Method: Compositional matrix adjust.

| Identities | | = 137/258 (53%), Positives = 168/258 (65%), Gaps = 5/258 (2%) |  |
| --- | --- | --- | --- |
| Query 11  Sbjct 12 | | SPAAWVERLFGYDWAQQTIGCSDAAVFRLSAQGRPVLFVKTDLSGALNELQDEAARLSWL  +PA W + L GY W Q GCS A V RL A RP LFVK ++ A EL E ARL WL APATWADYLAGYRWRGQGEGCSAATVHRLEAARRPTLFVKQEVLSAHAELPAEIARLRWL | 70  71 |
| Query 71  Sbjct 72 | | ATTGVPCAAVLDVVTEAGRDWLLLGEVPGQDLLS SHLAPAEKVSIMADAMRRLHTL  G+ C VL+ GR WLL+ VPG L + L P V ++A A+RRLH L HGAGIDCPQVLNETQSDGRQWLLMSAVPGDTLSALAQRGELEPERVVRLVAAALRRLHDL | 126  131 |
| Query 127  Sbjct 132 | | DPATCPFDHQAKHRIERARTRMEAGLVDQDDLDEEHQGLAPAELFARLKARMPDGEDLVV DPA CPFDH+ + R++ R R+EAGLVD+ D D++H+G + EL+ L R P EDLVV DPAACPFDHRLERRLDTVRQRVEAGLVDEADFDDDHRGRSATELYRLLLDRRPAVEDLVV | 186  191 |
| Query 187  Sbjct 192 | | THGDACLPNIMVENGRFSGFIDCGRLGVADRYQDIALATRDIAEELGGEWADRFLVLYGI HGDACLPN++ E R SGFIDCGRLGVADR+QD+ALA RDI ELG WA+ FLV YG AHGDACLPNLLAEGRRVSGFIDCGRLGVADRHQDLALAARDIEAELGAAWAEAFLVEYG- | 246  250 |
| Query | 247 AAPDSQRIAFYRLLDEFF 264 | | |
|  |  | D +R+A++RLLDEFF |  |
| Sbjct | 251 | GDIDGERLAYFRLLDEFF 268 | |

>WP_127583821.1 aminoglycoside 3'-phosphotransferase [Sinorhizobium meliloti] RVK32272.1 aminoglycoside 3'-phosphotransferase [Sinorhizobium meliloti]

Length=261

Score = 260 bits (665), Expect = 1e-83, Method: Compositional matrix adjust.

| Identities | = 137/253 (54%), Positives = 162/253 (64%), Gaps = 0/253 (0%) |  |
| --- | --- | --- |
| Query 12 | PAAWVERLFGYDWAQQTIGCSDAAVFRLSAQGRPVLFVKTDLSGALNELQDEAARLSWLA | 71 |
| Sbjct 9 | P A+ L GY + + +G S + +F L AQ RP L +K + SG EL EAARL WLA PPAFRGLLSGYGFERDALGQSASTIFLLKAQSRPFLILKHEPSGPFTELTGEAARLHWLA | 68 |
| Query 72 | TTGVPCAAVLDVVTEAGRDWLLLGEVPGQDLLSSHLAPAEKVSIMADAMRRLHTLDPATC | 131 |
| Sbjct 69 | +PC VL AG WLLL G DL S L E+++I+A+A+RRLH LDPA C AQDMPCPRVLGHDDHAGETWLLLEAAEGMDLASCRLPHPERIAILAEALRRLHRLDPAAC | 128 |
| Query 132 | PFDHQAKHRIERARTRMEAGLVDQDDLDEEHQGLAPAELFARLKARMPDGEDLVVTHGDA | 191 |
| Sbjct 129 | PFD + I A+ RMEAGLVD+ D DEE QG ELF L AR P E LVVTHGDA PFDRRLTASIAAAKARMEAGLVDESDFDEERQGRQTRELFDELLARRPKEEQLVVTHGDA | 188 |
| Query 192 | CLPNIMVENGRFSGFIDCGRLGVADRYQDIALATRDIAEELGGEWADRFLVLYGIAAPDS | 251 |
| Sbjct 189 | CLPN + +FSGFIDCGRLGVAD YQD+ALA I LG EW FL YG+AAPD CLPNFIAAGNQFSGFIDCGRLGVADIYQDLALACWSITHNLGEEWVAPFLDRYGLAAPDP | 248 |
| Query 252  Sbjct 249 | QRIAFYRLLDEFF 264  +++AFYRLLDEFF  EKLAFYRLLDEFF 261 |  |

>WP_009313327.1 MULTISPECIES: APH(3')-II family aminoglycoside O-phosphotransferase [Pseudomonas]

EHF16061.1 hypothetical protein HMPREF1030_00093 [Pseudomonas aeruginosa] KSQ80567.1 aminoglycoside phosphotransferase APH(3') [Pseudomonas aeruginosa]

QKQ61732.1 APH(3')-II family aminoglycoside O-phosphotransferase [Pseudomonas sp. FDAARGOS_761]

RPU82370.1 APH(3')-II family aminoglycoside O-phosphotransferase [Pseudomonas aeruginosa]

SUD11127.1 aminoglycoside 3-N-acetyltransferase [Pseudomonas aeruginosa]

Length=268

Score = 261 bits (666), Expect = 1e-83, Method: Compositional matrix adjust.

| Identities | = 138/259 (53%), Positives = 170/259 (66%), Gaps = 7/259 (3%) |  |
| --- | --- | --- |
| Query 11 | SPAAWVERLFGYDWAQQTIGCSDAAVFRLSAQGRPVLFVKTDLSGALNELQDEAARLSWL | 70 |
| Sbjct 12 | +P+ W + L GY W Q GCS A V RL A RP LFVK ++ EL E ARL WL APSTWADYLAGYRWRGQGEGCSAATVHRLEAARRPTLFVKQEVLSVHAELPAEIARLRWL | 71 |
| Query 71 | ATTGVPCAAVLDVVTEAGRDWLLLGEVPGQDLLSS HLAPAEKVSIMADAMRRLHT | 125 |
| Sbjct 72 | G+ C VL+ GR WLL+ VPG D LS+ L P V ++A A+RRLH HGAGIDCPQVLNETQSDGRQWLLMSAVPG-DTLSALAQRDELEPERLVRLVAAALRRLHD | 130 |
| Query 126 | LDPATCPFDHQAKHRIERARTRMEAGLVDQDDLDEEHQGLAPAELFARLKARMPDGEDLV | 185 |
| Sbjct 131 | LDPA CPFDH+ + R++ R R+EAGLVD+ D D++H+G + EL+ L R P EDLV LDPAACPFDHRLERRLDTVRQRVEAGLVDEADFDDDHRGRSATELYRLLLDRRPAVEDLV | 190 |
| Query 186 | VTHGDACLPNIMVENGRFSGFIDCGRLGVADRYQDIALATRDIAEELGGEWADRFLVLYG | 245 |
| Sbjct 191 | V HGDACLPN++ E RFSGFIDCGRLGVADR+QD+ALA RDI ELG WA+ FLV YG VAHGDACLPNLLAEGRRFSGFIDCGRLGVADRHQDLALAARDIEAELGATWAEAFLVEYG | 250 |
| Query 246  Sbjct 251 | IAAPDSQRIAFYRLLDEFF 264 D +R+A++RLLDEFF  -GDIDGERLAYFRLLDEFF 268 |  |

>WP_196672556.1 APH(3')-II family aminoglycoside O-phosphotransferase [Pseudomonas aeruginosa]

MBG4835268.1 APH(3')-II family aminoglycoside O-phosphotransferase [Pseudomonas aeruginosa]

Length=267

Score = 261 bits (666), Expect = 1e-83, Method: Compositional matrix adjust.

| Identities | | = 137/258 (53%), Positives = 168/258 (65%), Gaps = 5/258 (2%) |  |
| --- | --- | --- | --- |
| Query 11  Sbjct 11 | | SPAAWVERLFGYDWAQQTIGCSDAAVFRLSAQGRPVLFVKTDLSGALNELQDEAARLSWL  +P+ W + L GY W Q GCS A V RL A RP LFVK ++ A EL E ARL WL APSTWADYLAGYRWRGQGEGCSAATVHRLEAARRPTLFVKQEVLSAHAELPAEIARLRWL | 70  70 |
| Query 71  Sbjct 71 | | ATTGVPCAAVLDVVTEAGRDWLLLGEVPGQDLLS SHLAPAEKVSIMADAMRRLHTL  G+ C VL+ GR WLL+ VPG L + L P V ++A A+RRLH L HGAGIDCPQVLNETQSDGRQWLLMSAVPGDTLSALAQRGELEPERLVRLVAAALRRLHDL | 126  130 |
| Query 127  Sbjct 131 | | DPATCPFDHQAKHRIERARTRMEAGLVDQDDLDEEHQGLAPAELFARLKARMPDGEDLVV DPA CPFDH+ + R++ R R EAGLVD+ D D++H+G + EL+ L R P EDLVV DPAACPFDHRLERRLDTVRQRGEAGLVDEADFDDDHRGRSATELYRLLLDRRPAVEDLVV | 186  190 |
| Query 187  Sbjct 191 | | THGDACLPNIMVENGRFSGFIDCGRLGVADRYQDIALATRDIAEELGGEWADRFLVLYGI HGDACLPN++ E RFSGFIDCGRLGVADR+QD+ALA RDI ELG WA+ FLV YG AHGDACLPNLLAEGRRFSGFIDCGRLGVADRHQDLALAARDIEAELGAAWAEAFLVEYG- | 246  249 |
| Query | 247 AAPDSQRIAFYRLLDEFF 264 | | |
|  |  | D +R+A++RLLDEFF |  |
| Sbjct | 250 | GDIDGERLAYFRLLDEFF 267 | |

>WP_192568609.1 APH(3') family aminoglycoside O-phosphotransferase [Aminobacter carboxidus]

MBE1207862.1 aminoglycoside 3'-phosphotransferase [Aminobacter carboxidus] Length=264

Score = 261 bits (666), Expect = 2e-83, Method: Compositional matrix adjust. Identities = 134/253 (53%), Positives = 173/253 (68%), Gaps = 0/253 (0%)

| Query | 12 | PAAWVERLFGYDWAQQTIGCSDAAVFRLSAQGRPVLFVKTDLSGALNELQDEAARLSWLA | 71 |
| --- | --- | --- | --- |
| Sbjct | 12 | PA+ L GYDW QQTIG SDA VF+L+A G+P LF+KT++SG EL DE RL WL PASLCLALAGYDWQQQTIGHSDAGVFKLAAGGKPTLFLKTEVSGDFAELPDEIVRLRWLG | 71 |
| Query | 72 | TTGVPCAAVLDVVTEAGRDWLLLGEVPGQDLLSSHLAPAEKVSIMADAMRRLHTLDPATC | 131 |
| Sbjct | 72 | + G+PC V+ T AG +WLL+ V G+DL+S P E ++IMA+A+RRLH LD A C  SQGIPCPEVIVYETHAGHNWLLMSAVAGRDLVSQQSEPGEAIAIMAEALRRLHALDIAAC | 131 |

| Query | 132 | PFDHQAKHRIERARTRMEAGLVDQDDLDEEHQGLAPAELFARLKARMPDGEDLVVTHGDA | 191 |
| --- | --- | --- | --- |
| Sbjct | 132 | PFDH+ RI A+ R +AG VD+DD D+E G ++F +L AR P E LVVTHGDA PFDHRLGQRIAVAKARADAGEVDEDDFDDERLGQTAQQVFEQLVARRPVSETLVVTHGDA | 191 |
| Query | 192 | CLPNIMVENGRFSGFIDCGRLGVADRYQDIALATRDIAEELGGEWADRFLVLYGIAAPDS | 251 |
| Sbjct | 192 | +PN + + GRF+GFIDC RLGVAD +QD+ALA I LG EW FL LYG+ D+  SMPNFIADGGRFTGFIDCSRLGVADLHQDLALACWSIQYNLGEEWVAPFLALYGLPEADA | 251 |
| Query | 252 QRIAFYRLLDEFF 264 | | |
|  |  | ++++Y+LLDEFF |  |
| Sbjct | 252 | NKLSYYQLLDEFF 264 | |

>WP_181855713.1 APH(3')-II family aminoglycoside O-phosphotransferase [Pseudomonas aeruginosa]

MBA4945898.1 APH(3')-II family aminoglycoside O-phosphotransferase [Pseudomonas aeruginosa]

Length=268

Score = 261 bits (666), Expect = 2e-83, Method: Compositional matrix adjust.

| Identities | | = 137/258 (53%), Positives = 168/258 (65%), Gaps = 5/258 (2%) |  |
| --- | --- | --- | --- |
| Query 11  Sbjct 12 | | SPAAWVERLFGYDWAQQTIGCSDAAVFRLSAQGRPVLFVKTDLSGALNELQDEAARLSWL  +PA W + L GY W Q GCS A V RL A RP LFVK ++ A EL E ARL WL APATWADYLAGYRWRGQGEGCSAATVHRLEAARRPTLFVKQEVLSAHAELPAEIARLRWL | 70  71 |
| Query 71  Sbjct 72 | | ATTGVPCAAVLDVVTEAGRDWLLLGEVPGQDLLS SHLAPAEKVSIMADAMRRLHTL  G+ C VL+ GR WLL+ VPG L + L P V ++A A+RRLH L HGAGIDCPQVLNETQSDGRQWLLMSAVPGDTLSALAQRGELEPERLVRLVAAALRRLHDL | 126  131 |
| Query 127  Sbjct 132 | | DPATCPFDHQAKHRIERARTRMEAGLVDQDDLDEEHQGLAPAELFARLKARMPDGEDLVV DPA CPFDH+ + R++ R R+EAGLVD+ D D++H+G + EL+ R P EDLVV DPAACPFDHRLERRLDTVRQRVEAGLVDEADFDDDHRGRSATELYRLQLDRRPAVEDLVV | 186  191 |
| Query 187  Sbjct 192 | | THGDACLPNIMVENGRFSGFIDCGRLGVADRYQDIALATRDIAEELGGEWADRFLVLYGI HGDACLPN++ E RFSGFIDCGRLGVADR+QD+ALA RDI ELG WA+ FLV YG AHGDACLPNLLAEGRRFSGFIDCGRLGVADRHQDLALAARDIEAELGAAWAEAFLVEYG- | 246  250 |
| Query | 247 AAPDSQRIAFYRLLDEFF 264 | | |
|  |  | D +R+A++RLLDEFF |  |
| Sbjct | 251 | GDIDGERLAYFRLLDEFF 268 | |

>WP_058150946.1 APH(3')-II family aminoglycoside O-phosphotransferase [Pseudomonas aeruginosa]

KSH21538.1 aminoglycoside 3'-phosphotransferase [Pseudomonas aeruginosa] Length=267

Score = 261 bits (666), Expect = 2e-83, Method: Compositional matrix adjust.

| Identities | | = 136/258 (53%), Positives = 169/258 (66%), Gaps = 5/258 (2%) |  |
| --- | --- | --- | --- |
| Query 11  Sbjct 11 | | SPAAWVERLFGYDWAQQTIGCSDAAVFRLSAQGRPVLFVKTDLSGALNELQDEAARLSWL  +P+ W + L GY W Q GCS A V RL A RP LFVK ++ A EL E ARL WL APSTWADYLAGYRWRGQGEGCSAATVHRLEAARRPTLFVKQEVLSAHAELPAEIARLRWL | 70  70 |
| Query 71  Sbjct 71 | | ATTGVPCAAVLDVVTEAGRDWLLLGEVPGQDLLS SHLAPAEKVSIMADAMRRLHTL  G+ C VL+ GR WLL+ VPG L + L P V ++A A+RRLH L HGAGIDCPQVLNETQSDGRQWLLMSAVPGDTLSALAQRGELEPERLVRLVAAALRRLHDL | 126  130 |
| Query 127  Sbjct 131 | | DPATCPFDHQAKHRIERARTRMEAGLVDQDDLDEEHQGLAPAELFARLKARMPDGEDLVV DPA CPFDH+ + R++ R R+EAGLVD+ D D++H+G + EL+ L R P EDLVV DPAACPFDHRLERRLDTVRQRVEAGLVDEADFDDDHRGRSATELYRLLLDRRPAVEDLVV | 186  190 |
| Query 187  Sbjct 191 | | THGDACLPNIMVENGRFSGFIDCGRLGVADRYQDIALATRDIAEELGGEWADRFLVLYGI HGDACLPN++ E RFSGFIDCGRLGVADR+QD+ALA RDI ELG WA+ FLV YG AHGDACLPNLLAEGRRFSGFIDCGRLGVADRHQDLALAARDIEAELGAAWAEAFLVEYG- | 246  249 |
| Query | 247 AAPDSQRIAFYRLLDEFF 264 | | |
|  |  | D +++A++RLLDEFF |  |
| Sbjct | 250 | GDIDGEQLAYFRLLDEFF 267 | |

>WP_010793441.1 MULTISPECIES: APH(3')-II family aminoglycoside O-phosphotransferase [Pseudomonas]

EQL39006.1 aminoglycoside 3'-phosphotransferase [Pseudomonas aeruginosa VRFPA03]

ALZ15551.1 aminoglycoside phosphotransferase APH(3') [Pseudomonas aeruginosa]

AON72662.1 APH(3') family aminoglycoside O-phosphotransferase [Pseudomonas aeruginosa]

ASP04166.1 aminoglycoside phosphotransferase APH(3') [Pseudomonas aeruginosa] ASP13932.1 aminoglycoside phosphotransferase APH(3') [Pseudomonas aeruginosa]

Length=268

Score = 261 bits (666), Expect = 2e-83, Method: Compositional matrix adjust.

| Identities | | = 136/258 (53%), Positives = 168/258 (65%), Gaps = 5/258 (2%) |  |
| --- | --- | --- | --- |
| Query 11  Sbjct 12 | | SPAAWVERLFGYDWAQQTIGCSDAAVFRLSAQGRPVLFVKTDLSGALNELQDEAARLSWL  +P+ W + L GY W Q GCS A V RL A RP LFVK ++ EL E ARL WL APSTWADYLAGYRWRGQGEGCSAATVHRLEAARRPTLFVKQEVLSVHAELPAEIARLRWL | 70  71 |
| Query 71  Sbjct 72 | | ATTGVPCAAVLDVVTEAGRDWLLLGEVPGQDLLS SHLAPAEKVSIMADAMRRLHTL  G+ C VL+ GR WLL+ VPG L + L P V ++A A+RRLH L HGAGIDCPQVLNETQSDGRQWLLMSAVPGDTLSALAQRGELEPERLVRLVAAALRRLHDL | 126  131 |
| Query 127  Sbjct 132 | | DPATCPFDHQAKHRIERARTRMEAGLVDQDDLDEEHQGLAPAELFARLKARMPDGEDLVV DPA CPFDH+ + R++ R R+EAGLVD+ D D++H+G + EL+ L R P EDLVV DPAACPFDHRLERRLDTVRQRVEAGLVDEADFDDDHRGRSATELYRLLLDRRPAVEDLVV | 186  191 |
| Query 187  Sbjct 192 | | THGDACLPNIMVENGRFSGFIDCGRLGVADRYQDIALATRDIAEELGGEWADRFLVLYGI HGDACLPN++ E RFSGFIDCGRLGVADR+QD+ALA RDI ELG WA+ FLV YG AHGDACLPNLLAEGRRFSGFIDCGRLGVADRHQDLALAARDIEAELGAAWAEAFLVEYG- | 246  250 |
| Query | 247 AAPDSQRIAFYRLLDEFF 264 | | |
|  |  | D +R+A++RLLDEFF |  |
| Sbjct | 251 | GDIDGERLAYFRLLDEFF 268 | |

>WP_024915527.1 APH(3')-II family aminoglycoside O-phosphotransferase [Pseudomonas aeruginosa]

KSD39641.1 aminoglycoside 3'-phosphotransferase [Pseudomonas aeruginosa]

NTS94858.1 APH(3')-II family aminoglycoside O-phosphotransferase [Pseudomonas aeruginosa]

RPW62934.1 APH(3')-II family aminoglycoside O-phosphotransferase [Pseudomonas aeruginosa]

RUA98053.1 APH(3')-II family aminoglycoside O-phosphotransferase [Pseudomonas aeruginosa]

Length=268

Score = 261 bits (666), Expect = 2e-83, Method: Compositional matrix adjust.

| Identities | | = 137/258 (53%), Positives = 168/258 (65%), Gaps = 5/258 (2%) |  |
| --- | --- | --- | --- |
| Query 11  Sbjct 12 | | SPAAWVERLFGYDWAQQTIGCSDAAVFRLSAQGRPVLFVKTDLSGALNELQDEAARLSWL  +P W + L GY W Q GCS A V RL A RP LFVK ++ A EL E ARL WL APDTWADYLAGYRWRGQGEGCSAATVHRLEAARRPTLFVKQEVLSAHAELPAEIARLRWL | 70  71 |
| Query 71  Sbjct 72 | | ATTGVPCAAVLDVVTEAGRDWLLLGEVPGQDLLS SHLAPAEKVSIMADAMRRLHTL  G+ C VL+ GR WLL+ VPG L + L P V ++A A+RRLH L HGAGIDCPQVLNETQSDGRQWLLMSAVPGDTLSALAQRGELEPERLVRLVAAALRRLHDL | 126  131 |
| Query 127  Sbjct 132 | | DPATCPFDHQAKHRIERARTRMEAGLVDQDDLDEEHQGLAPAELFARLKARMPDGEDLVV DPA CPFDH+ + R++ R R+EAGLVD+ D D++H+G + EL+ L R P EDLVV DPAACPFDHRLERRLDTVRQRVEAGLVDEADFDDDHRGRSATELYRLLLDRRPAVEDLVV | 186  191 |
| Query 187  Sbjct 192 | | THGDACLPNIMVENGRFSGFIDCGRLGVADRYQDIALATRDIAEELGGEWADRFLVLYGI HGDACLPN++ E RFSGFIDCGRLGVADR+QD+ALA RDI ELG WA+ FLV YG AHGDACLPNLLAEGRRFSGFIDCGRLGVADRHQDLALAARDIEAELGATWAEAFLVEYG- | 246  250 |
| Query | 247 AAPDSQRIAFYRLLDEFF 264 | | |
|  |  | D +R+A++RLLDEFF |  |
| Sbjct | 251 | GDIDGERLAYFRLLDEFF 268 | |

>WP_134288241.1 APH(3')-II family aminoglycoside O-phosphotransferase [Pseudomonas aeruginosa]

TEH88786.1 APH(3')-II family aminoglycoside O-phosphotransferase [Pseudomonas aeruginosa]

TEH94700.1 APH(3')-II family aminoglycoside O-phosphotransferase [Pseudomonas aeruginosa]

TEI06196.1 APH(3')-II family aminoglycoside O-phosphotransferase [Pseudomonas aeruginosa]

Length=268

Score = 261 bits (666), Expect = 2e-83, Method: Compositional matrix adjust.

| Identities | | = 136/258 (53%), Positives = 168/258 (65%), Gaps = 5/258 (2%) |  |
| --- | --- | --- | --- |
| Query 11  Sbjct 12 | | SPAAWVERLFGYDWAQQTIGCSDAAVFRLSAQGRPVLFVKTDLSGALNELQDEAARLSWL  +P+ W + L GY W Q GCS A V RL A RP LFVK ++ A EL E ARL WL APSTWADYLAGYRWRGQGEGCSAATVHRLEAARRPTLFVKQEVLSAHAELPAEIARLRWL | 70  71 |
| Query 71  Sbjct 72 | | ATTGVPCAAVLDVVTEAGRDWLLLGEVPGQDLLS SHLAPAEKVSIMADAMRRLHTL  G+ C VL+ GR WLL+ VPG L + L P V ++A A+RRLH L HGAGIDCPQVLNETQSDGRQWLLMSAVPGDTLSALAQRGELEPERLVRLVAAALRRLHDL | 126  131 |
| Query 127  Sbjct 132 | | DPATCPFDHQAKHRIERARTRMEAGLVDQDDLDEEHQGLAPAELFARLKARMPDGEDLVV DPA CPFDH+ + R++ R R+E GLVD+ D D++H+G + EL+ L R P EDLVV DPAACPFDHRLERRLDTVRQRVEVGLVDEADFDDDHRGRSATELYRLLLDRRPAVEDLVV | 186  191 |
| Query 187  Sbjct 192 | | THGDACLPNIMVENGRFSGFIDCGRLGVADRYQDIALATRDIAEELGGEWADRFLVLYGI HGDACLPN++ E RFSGFIDCGRLGVADR+QD+ALA RDI ELG WA+ FLV YG AHGDACLPNLLAEGRRFSGFIDCGRLGVADRHQDLALAARDIEAELGAAWAEAFLVEYG- | 246  250 |
| Query | 247 AAPDSQRIAFYRLLDEFF 264 | | |
|  |  | D +R+A++RLLDEFF |  |
| Sbjct | 251 | GDIDGERLAYFRLLDEFF 268 | |

>WP_003101274.1 MULTISPECIES: APH(3')-II family aminoglycoside O-phosphotransferase [Pseudomonas]

QFZ62750.1 APH(3')-II family aminoglycoside O-phosphotransferase [Pseudomonas aeruginosa PA99]

SVJ68373.1 aminoglycoside 3'-phosphotransferase [Klebsiella pneumoniae] VUO30596.1 Aminoglycoside 3'-phosphotransferase [Stenotrophomonas maltophilia]

AGV65482.1 aminoglycoside 3'-phosphotransferase [Pseudomonas aeruginosa c7447m]

AKQ17688.1 aminoglycoside phosphotransferase [Pseudomonas aeruginosa] Length=268

Score = 261 bits (666), Expect = 2e-83, Method: Compositional matrix adjust.

| Identities | | = 136/258 (53%), Positives = 168/258 (65%), Gaps = 5/258 (2%) |  |
| --- | --- | --- | --- |
| Query 11  Sbjct 12 | | SPAAWVERLFGYDWAQQTIGCSDAAVFRLSAQGRPVLFVKTDLSGALNELQDEAARLSWL  +P+ W + L GY W Q GCS A V RL RP LFVK ++ A EL E ARL WL APSTWADYLAGYRWRGQGEGCSAATVHRLEVARRPTLFVKQEVLSAHAELPAEIARLRWL | 70  71 |
| Query 71  Sbjct 72 | | ATTGVPCAAVLDVVTEAGRDWLLLGEVPGQDLLS SHLAPAEKVSIMADAMRRLHTL  G+ C VL+ GR WLL+ VPG L + L P V ++A A+RRLH L HGAGIDCPQVLNETQSDGRQWLLMSAVPGDTLSALAQRGELEPERLVRLVAAALRRLHDL | 126  131 |
| Query 127  Sbjct 132 | | DPATCPFDHQAKHRIERARTRMEAGLVDQDDLDEEHQGLAPAELFARLKARMPDGEDLVV DPA CPFDH+ + R++ R R+EAGLVD+ D D++H+G + EL+ L R P EDLVV DPAACPFDHRLERRLDTVRQRVEAGLVDEADFDDDHRGRSATELYRLLLDRRPAVEDLVV | 186  191 |
| Query 187  Sbjct 192 | | THGDACLPNIMVENGRFSGFIDCGRLGVADRYQDIALATRDIAEELGGEWADRFLVLYGI HGDACLPN++ E RFSGFIDCGRLGVADR+QD+ALA RDI ELG WA+ FLV YG AHGDACLPNLLAEGRRFSGFIDCGRLGVADRHQDLALAARDIEAELGAAWAEAFLVEYG- | 246  250 |
| Query | 247 AAPDSQRIAFYRLLDEFF 264 | | |
|  |  | D +R+A++RLLDEFF |  |
| Sbjct | 251 | GDIDGERLAYFRLLDEFF 268 | |

>WP_128695974.1 APH(3')-II family aminoglycoside O-phosphotransferase [Pseudomonas aeruginosa]

RUI19715.1 APH(3')-II family aminoglycoside O-phosphotransferase [Pseudomonas aeruginosa]

Length=268

Score = 261 bits (666), Expect = 2e-83, Method: Compositional matrix adjust.

| Identities | | = 136/258 (53%), Positives = 168/258 (65%), Gaps = 5/258 (2%) |  |
| --- | --- | --- | --- |
| Query 11  Sbjct 12 | | SPAAWVERLFGYDWAQQTIGCSDAAVFRLSAQGRPVLFVKTDLSGALNELQDEAARLSWL  +P+ W + L GY W Q GCS A V RL A RP LFVK ++ A EL E ARL WL APSTWADYLAGYRWRGQGEGCSAATVHRLEAARRPTLFVKQEVLSAHAELPAEIARLRWL | 70  71 |
| Query 71  Sbjct 72 | | ATTGVPCAAVLDVVTEAGRDWLLLGEVPGQDLLS SHLAPAEKVSIMADAMRRLHTL  G+ C VL+ GR WLL+ VPG L + L P V ++A A+RRLH L HGAGIDCPQVLNETQSDGRQWLLMSAVPGDTLSALAQRGELEPERLVRLVAAALRRLHDL | 126  131 |
| Query 127  Sbjct 132 | | DPATCPFDHQAKHRIERARTRMEAGLVDQDDLDEEHQGLAPAELFARLKARMPDGEDLVV DPA CPFDH+ + R++ R R+EAGLVD+ D D++H+G + EL+ L R P EDLVV DPAACPFDHRLERRLDTVRQRVEAGLVDEADFDDDHRGRSATELYRLLLDRRPAVEDLVV | 186  191 |
| Query 187  Sbjct 192 | | THGDACLPNIMVENGRFSGFIDCGRLGVADRYQDIALATRDIAEELGGEWADRFLVLYGI HGDACLPN++ E RFSGFIDCGRLGVADR+QD+ALA RDI ELG W + FLV YG AHGDACLPNLLAEGRRFSGFIDCGRLGVADRHQDLALAARDIEAELGAAWVEAFLVEYG- | 246  250 |
| Query | 247 AAPDSQRIAFYRLLDEFF 264 | | |
|  |  | D +R+A++RLLDEFF |  |
| Sbjct | 251 | GDIDGERLAYFRLLDEFF 268 | |

>WP_083203601.1 aminoglycoside 3'-phosphotransferase [Ensifer adhaerens] Length=263

Score = 260 bits (665), Expect = 2e-83, Method: Compositional matrix adjust.

| Identities | = 131/253 (52%), Positives = 168/253 (66%), Gaps = 0/253 (0%) |  |
| --- | --- | --- |
| Query 12 | PAAWVERLFGYDWAQQTIGCSDAAVFRLSAQGRPVLFVKTDLSGALNELQDEAARLSWLA | 71 |
| Sbjct 11 | P ++ + GY W + +G SD+++F L A RP+L +K + +G +E DE RL WLA PHEFLSLVAGYQWNRDLLGQSDSSIFLLKAPDRPMLVLKVEAAGPFSEFVDEGERLDWLA | 70 |
| Query 72 | TTGVPCAAVLDVVTEAGRDWLLLGEVPGQDLLSSHLAPAEKVSIMADAMRRLHTLDPATC | 131 |
| Sbjct 71 | + G+PC ++ A +WLLL V G DL S++L+P +++ I+ADA++ LH LD A C SQGMPCPRMIARAFGAETNWLLLQAVEGTDLASTNLSPKDRIVILADALKCLHGLDVADC | 130 |
| Query 132 | PFDHQAKHRIERARTRMEAGLVDQDDLDEEHQGLAPAELFARLKARMPDGEDLVVTHGDA | 191 |
| Sbjct 131 | PFDH+ + RI AR R EAGL+D+DD DE G A+LFA L+AR+P LVVTHGDA PFDHRIEKRIAIARARTEAGLIDEDDFDEARLGRTAADLFAELQARVPSTGKLVVTHGDA | 190 |
| Query 192 | CLPNIMVENGRFSGFIDCGRLGVADRYQDIALATRDIAEELGGEWADRFLVLYGIAAPDS | 251 |
| Sbjct 191 | CLPNI+ NGRFSGFIDC RLGVAD YQDIALA IA LG EW FL YGI D CLPNIIERNGRFSGFIDCSRLGVADLYQDIALACHSIAHSLGEEWVQPFLARYGIDRDDP | 250 |
| Query 252  Sbjct 251 | QRIAFYRLLDEFF 264  ++ FY LLDEFF  EKRDFYCLLDEFF 263 |  |

```
>WP_003132309.1 MULTISPECIES: aminoglycoside O-phosphotransferase APH(3')-IIb [Pseudomonas]
AIL00452.1 aminoglycoside phosphotransferase [Pseudomonas aeruginosa VRFPA04]
APB63361.1 aminoglycoside O-phosphotransferase APH(3')-IIb [Pseudomonas aeruginosa]
ARI89521.1 aminoglycoside O-phosphotransferase APH(3')-IIb [Pseudomonas aeruginosa]
ARI95956.1 aminoglycoside O-phosphotransferase APH(3')-IIb [Pseudomonas aeruginosa]
ASA13587.1 aminoglycoside O-phosphotransferase APH(3')-IIb [Pseudomonas aeruginosa]
Length=268

Score = 261 bits (666), Expect = 2e-83, Method: Compositional matrix adjust. Identities = 138/259 (53%), Positives = 171/259 (66%), Gaps = 7/259 (3%)

Query 11	SPAAWVERLFGYDWAQQTIGCSDAAVFRLSAQGRPVLFVKTDLSGALNELQDEAARLSWL 70
+P+ W + L GY W Q GCS A V RL A RP LFVK ++ A EL E ARL WL Sbjct 12	APSTWADYLAGYRWRGQGEGCSAATVHRLEAARRPTLFVKQEVLSAHAELPAEIARLRWL 71
```

| Query | 71 | ATTGVPCAAVLDVVTEAGRDWLLLGEVPGQDLLSS HLAPAEKVSIMADAMRRLHT | 125 |
| --- | --- | --- | --- |
| Sbjct | 72 | G+ C VL+ GR WLL+ +PG D LS+ L P V ++A A+RRLH HGAGIDCPQVLNETQSDGRQWLLMSAMPG-DTLSALAQRDELEPERLVRLVAAALRRLHD | 130 |
| Query | 126 | LDPATCPFDHQAKHRIERARTRMEAGLVDQDDLDEEHQGLAPAELFARLKARMPDGEDLV | 185 |
| Sbjct | 131 | LDPA CPFDH+ + R++ R R+EAGLVD+ D D++H+G + EL+ L R P EDLV LDPAACPFDHRLERRLDTVRQRVEAGLVDEADFDDDHRGRSATELYRLLLDRRPAVEDLV | 190 |
| Query | 186 | VTHGDACLPNIMVENGRFSGFIDCGRLGVADRYQDIALATRDIAEELGGEWADRFLVLYG | 245 |
| Sbjct | 191 | V HGDACLPN++ E RFSGFIDCGRLGVADR+QD+ALA RDI ELG WA+ FLV YG VAHGDACLPNLLAEGRRFSGFIDCGRLGVADRHQDLALAARDIEAELGAAWAEAFLVEYG | 250 |
| Query  Sbjct | 246  251 | IAAPDSQRIAFYRLLDEFF 264 D +R+A++RLLDEFF  -GDIDGERLAYFRLLDEFF 268 |  |

>WP_128687719.1 APH(3')-II family aminoglycoside O-phosphotransferase [Pseudomonas aeruginosa]

RUH70060.1 APH(3')-II family aminoglycoside O-phosphotransferase [Pseudomonas aeruginosa]

Length=268

Score = 261 bits (666), Expect = 2e-83, Method: Compositional matrix adjust.

| Identities | | = 136/258 (53%), Positives = 169/258 (66%), Gaps = 5/258 (2%) |  |
| --- | --- | --- | --- |
| Query 11  Sbjct 12 | | SPAAWVERLFGYDWAQQTIGCSDAAVFRLSAQGRPVLFVKTDLSGALNELQDEAARLSWL  +P+ W + L GY W Q GCS A V RL A RP LFVK ++ A EL E ARL WL APSTWADYLAGYRWRGQGEGCSAATVHRLEAARRPTLFVKQEVLSAHAELPAEIARLRWL | 70  71 |
| Query 71  Sbjct 72 | | ATTGVPCAAVLDVVTEAGRDWLLLGEVPGQDLLS SHLAPAEKVSIMADAMRRLHTL  G+ C VL+ GR WLL+ VPG L + L P V ++A A+RRLH L HGAGIDCPQVLNETQSDGRQWLLMSAVPGDTLSALAQRGELEPERLVRLVAAALRRLHDL | 126  131 |
| Query 127  Sbjct 132 | | DPATCPFDHQAKHRIERARTRMEAGLVDQDDLDEEHQGLAPAELFARLKARMPDGEDLVV DPA CPFDH+ + R++ R R+EAGLVD+ D D++H+G + EL+ L + P EDLVV DPAACPFDHRLERRLDTVRQRVEAGLVDEADFDDDHRGRSATELYRLLLDQRPAVEDLVV | 186  191 |
| Query 187  Sbjct 192 | | THGDACLPNIMVENGRFSGFIDCGRLGVADRYQDIALATRDIAEELGGEWADRFLVLYGI HGDACLPN++ E RFSGFIDCGRLGVADR+QD+ALA RDI ELG WA+ FLV YG AHGDACLPNLLAEGRRFSGFIDCGRLGVADRHQDLALAARDIEAELGAAWAEAFLVEYG- | 246  250 |
| Query | 247 AAPDSQRIAFYRLLDEFF 264 | | |
|  |  | D +R+A++RLLDEFF |  |
| Sbjct | 251 | GDIDGERLAYFRLLDEFF 268 | |

>ANK74915.1 APH(3') family aminoglycoside O-phosphotransferase [Ensifer adhaerens] KQX10201.1 aminoglycoside phosphotransferase [Ensifer sp. Root423]

KQZ43121.1 aminoglycoside phosphotransferase [Ensifer sp. Root558] Length=263

Score = 260 bits (665), Expect = 2e-83, Method: Compositional matrix adjust.

| Identities | = 128/244 (52%), Positives = 167/244 (68%), Gaps = 0/244 (0%) |  |
| --- | --- | --- |
| Query 21 | GYDWAQQTIGCSDAAVFRLSAQGRPVLFVKTDLSGALNELQDEAARLSWLATTGVPCAAV | 80 |
| Sbjct 20 | GY W + +G SD+++F L A RP+L +K + +G +E DEAARL WLA+ +PC V GYQWNRDLLGQSDSSIFLLKAPDRPMLVLKLEEAGPFSEFVDEAARLDWLASKDLPCPRV | 79 |
| Query 81 | LDVVTEAGRDWLLLGEVPGQDLLSSHLAPAEKVSIMADAMRRLHTLDPATCPFDHQAKHR | 140 |
| Sbjct 80 | + A +WLLL V G DL S+ L+P +++ I+ADA+++LH LD A CPFDH+ + R IARAFGAETNWLLLQAVQGTDLASTTLSPKDQIVILADALKQLHALDIANCPFDHRIEER | 139 |
| Query 141 | IERARTRMEAGLVDQDDLDEEHQGLAPAELFARLKARMPDGEDLVVTHGDACLPNIMVEN | 200 |
| Sbjct 140 | I A+ R +AG++D+ D DE G A+LFA L+AR+P +LVVTHGDACLPNI+ +N IAIAKARTQAGVIDESDFDETRLGRTAADLFAELQARVPQPGELVVTHGDACLPNIIEKN | 199 |
| Query 201 | GRFSGFIDCGRLGVADRYQDIALATRDIAEELGGEWADRFLVLYGIAAPDSQRIAFYRLL | 260 |
| Sbjct 200 | GRFSGFIDC RLGVAD YQDIALA R I LG EW FL YG+ DS++ FY LL GRFSGFIDCSRLGVADPYQDIALACRSIVHNLGEEWVQPFLTRYGLDKDDSEKRDFYCLL | 259 |
| Query 261 | DEFF 264 |  |

DEFF Sbjct 260 DEFF 263

>WP_042931753.1 APH(3')-II family aminoglycoside O-phosphotransferase [Pseudomonas aeruginosa]

OYP28682.1 aminoglycoside 3'-phosphotransferase [Pseudomonas aeruginosa] Length=268

Score = 261 bits (666), Expect = 2e-83, Method: Compositional matrix adjust.

| Identities | | = 136/258 (53%), Positives = 168/258 (65%), Gaps = 5/258 (2%) |  |
| --- | --- | --- | --- |
| Query 11  Sbjct 12 | | SPAAWVERLFGYDWAQQTIGCSDAAVFRLSAQGRPVLFVKTDLSGALNELQDEAARLSWL  +P+ W + L GY W Q GCS A V RL RP LFVK ++ A EL E ARL WL APSTWADYLAGYRWRGQGEGCSAATVHRLEVARRPTLFVKQEVLSAHAELPAEIARLRWL | 70  71 |
| Query 71  Sbjct 72 | | ATTGVPCAAVLDVVTEAGRDWLLLGEVPGQDLLS SHLAPAEKVSIMADAMRRLHTL  G+ C VL+ GR WLL+ VPG L + L P V ++A A+RRLH L HGAGIDCPQVLNETQSDGRQWLLMSAVPGDTLSALAQRGELEPERLVRLVAAALRRLHDL | 126  131 |
| Query 127  Sbjct 132 | | DPATCPFDHQAKHRIERARTRMEAGLVDQDDLDEEHQGLAPAELFARLKARMPDGEDLVV DPA CPFDH+ + R++ R R+EAGLVD+ D D++H+G + EL+ L R P EDLVV DPAACPFDHRLERRLDTVRQRVEAGLVDEADFDDDHRGRSATELYRLLLDRRPAVEDLVV | 186  191 |
| Query 187  Sbjct 192 | | THGDACLPNIMVENGRFSGFIDCGRLGVADRYQDIALATRDIAEELGGEWADRFLVLYGI HGDACLPN++ E RFSGFIDCGRLGVADR+QD+ALA RDI ELG WA+ FLV YG AHGDACLPNLLAEGRRFSGFIDCGRLGVADRHQDLALAARDIEAELGAAWAEAFLVEYG- | 246  250 |
| Query | 247 AAPDSQRIAFYRLLDEFF 264 | | |
|  |  | D +R+A++RLLDEFF |  |
| Sbjct | 251 | GDIDGERLAYFRLLDEFF 268 | |

>WP_196669784.1 APH(3')-II family aminoglycoside O-phosphotransferase [Pseudomonas aeruginosa]

MBG4419660.1 APH(3')-II family aminoglycoside O-phosphotransferase [Pseudomonas aeruginosa]

Length=268

Score = 261 bits (666), Expect = 2e-83, Method: Compositional matrix adjust.

| Identities | | = 137/258 (53%), Positives = 168/258 (65%), Gaps = 5/258 (2%) |  |
| --- | --- | --- | --- |
| Query 11  Sbjct 12 | | SPAAWVERLFGYDWAQQTIGCSDAAVFRLSAQGRPVLFVKTDLSGALNELQDEAARLSWL  +PA W + L GY W Q GCS A V RL A RP LFVK ++ A EL E ARL WL APATWADYLAGYRWRGQGEGCSAATVHRLEAARRPTLFVKQEVLSAHAELPAEIARLRWL | 70  71 |
| Query 71  Sbjct 72 | | ATTGVPCAAVLDVVTEAGRDWLLLGEVPGQDLLS SHLAPAEKVSIMADAMRRLHTL  G+ C VL+ GR WLL+ VPG L + L P V ++A A+RRLH L HGAGIDCPQVLNETQSDGRQWLLMSAVPGDTLSALAQRGELEPERLVRLVAAALRRLHDL | 126  131 |
| Query 127  Sbjct 132 | | DPATCPFDHQAKHRIERARTRMEAGLVDQDDLDEEHQGLAPAELFARLKARMPDGEDLVV DPA CPFDH+ + R++ R R+EAGLVD+ D D++H+G + EL+ L R P EDLVV DPAACPFDHRLERRLDTVRQRVEAGLVDEADFDDDHRGRSATELYRLLLDRRPAVEDLVV | 186  191 |
| Query 187  Sbjct 192 | | THGDACLPNIMVENGRFSGFIDCGRLGVADRYQDIALATRDIAEELGGEWADRFLVLYGI HGDACLPN++ E RFSGFIDCGRLGVADR+QD+ALA RDI E G WA+ FLV YG AHGDACLPNLLAEGRRFSGFIDCGRLGVADRHQDLALAARDIEAERGAAWAEAFLVEYG- | 246  250 |
| Query | 247 AAPDSQRIAFYRLLDEFF 264 | | |
|  |  | D +R+A++RLLDEFF |  |
| Sbjct | 251 | GDIDGERLAYFRLLDEFF 268 | |

```
>WP_134536175.1 APH(3')-II family aminoglycoside O-phosphotransferase [Pseudomonas aeruginosa]
Length=261

Score = 260 bits (665), Expect = 2e-83, Method: Compositional matrix adjust. Identities = 136/258 (53%), Positives = 168/258 (65%), Gaps = 5/258 (2%)

Query 11	SPAAWVERLFGYDWAQQTIGCSDAAVFRLSAQGRPVLFVKTDLSGALNELQDEAARLSWL 70
+P+ W + L GY W Q GCS A V RL A RP LFVK ++	EL E ARL WL
```

| Sbjct | 5 | APSTWADYLAGYRWRGQGEGCSAATVHRLEAARRPTLFVKQEVLSVHAELPAEIARLRWL | 64 |
| --- | --- | --- | --- |
| Query | 71 | ATTGVPCAAVLDVVTEAGRDWLLLGEVPGQDLLS SHLAPAEKVSIMADAMRRLHTL | 126 |
| Sbjct | 65 | G+ C VL+ GR WLL+ VPG L + L P V ++A A+RRLH L HGAGIDCPQVLNETQSDGRQWLLMSAVPGDTLSALAQRGELEPERLVRLVAAALRRLHDL | 124 |
| Query | 127 | DPATCPFDHQAKHRIERARTRMEAGLVDQDDLDEEHQGLAPAELFARLKARMPDGEDLVV | 186 |
| Sbjct | 125 | DPA CPFDH+ + R++ R R+EAGLVD+ D D++H+G + EL+ L R P EDLVV DPAACPFDHRLERRLDTVRQRVEAGLVDEADFDDDHRGRSATELYRLLLDRRPAVEDLVV | 184 |
| Query | 187 | THGDACLPNIMVENGRFSGFIDCGRLGVADRYQDIALATRDIAEELGGEWADRFLVLYGI | 246 |
| Sbjct | 185 | HGDACLPN++ E RFSGFIDCGRLGVADR+QD+ALA RDI ELG WA+ FLV YG  AHGDACLPNLLAEGRRFSGFIDCGRLGVADRHQDLALAARDIEAELGAAWAEAFLVEYG- | 243 |
| Query | 247 AAPDSQRIAFYRLLDEFF 264 | | |
|  |  | D +R+A++RLLDEFF |  |
| Sbjct | 244 | GDIDGERLAYFRLLDEFF 261 | |

>MBG7140394.1 APH(3')-II family aminoglycoside O-phosphotransferase [Pseudomonas aeruginosa]

Length=268

Score = 260 bits (665), Expect = 2e-83, Method: Compositional matrix adjust.

| Identities | | = 136/258 (53%), Positives = 168/258 (65%), Gaps = 5/258 (2%) |  |
| --- | --- | --- | --- |
| Query 11  Sbjct 12 | | SPAAWVERLFGYDWAQQTIGCSDAAVFRLSAQGRPVLFVKTDLSGALNELQDEAARLSWL  +P+ W + L GY W Q GCS A V RL RP LFVK ++ A EL E ARL WL APSTWADYLAGYRWRGQGEGCSAATVHRLEVARRPTLFVKQEVLSAHAELPAEIARLRWL | 70  71 |
| Query 71  Sbjct 72 | | ATTGVPCAAVLDVVTEAGRDWLLLGEVPGQDLLS SHLAPAEKVSIMADAMRRLHTL  G+ C VL+ GR WLL+ VPG L + L P V ++A A+RRLH L HGAGIDCPQVLNETQSDGRQWLLMSAVPGDTLSALAQRGELEPERLVRLVAAALRRLHDL | 126  131 |
| Query 127  Sbjct 132 | | DPATCPFDHQAKHRIERARTRMEAGLVDQDDLDEEHQGLAPAELFARLKARMPDGEDLVV DPA CPFDH+ + R++ R R+EAGLVD+ D D++H+G + EL+ L R P EDLVV DPAACPFDHRLERRLDTVRQRVEAGLVDEADFDDDHRGRSATELYRLLLDRRPAVEDLVV | 186  191 |
| Query 187  Sbjct 192 | | THGDACLPNIMVENGRFSGFIDCGRLGVADRYQDIALATRDIAEELGGEWADRFLVLYGI HGDACLPN++ E RFSGFIDCGRLGVADR+QD+ALA RDI ELG WA+ FLV YG AHGDACLPNLLAEGRRFSGFIDCGRLGVADRHQDLALAARDIEAELGAAWAEAFLVEYG- | 246  250 |
| Query | 247 AAPDSQRIAFYRLLDEFF 264 | | |
|  |  | D +R+A++RLLDEFF |  |
| Sbjct | 251 | GDIDGERLAYFRLLDEFF 268 | |

>WP_034026268.1 APH(3')-II family aminoglycoside O-phosphotransferase [Pseudomonas aeruginosa]

ORE36455.1 aminoglycoside 3'-phosphotransferase [Pseudomonas aeruginosa] Length=268

Score = 260 bits (665), Expect = 2e-83, Method: Compositional matrix adjust.

| Identities | = 138/259 (53%), Positives = 170/259 (66%), Gaps = 7/259 (3%) |  |
| --- | --- | --- |
| Query 11 | SPAAWVERLFGYDWAQQTIGCSDAAVFRLSAQGRPVLFVKTDLSGALNELQDEAARLSWL | 70 |
| Sbjct 12 | +P+ W + L GY W Q GCS A V RL A RP LFVK ++ EL E ARL WL APSTWADYLAGYRWRGQGEGCSAATVHRLEAARRPTLFVKQEVLSVHAELPAEIARLRWL | 71 |
| Query 71 | ATTGVPCAAVLDVVTEAGRDWLLLGEVPGQDLLSS HLAPAEKVSIMADAMRRLHT | 125 |
| Sbjct 72 | G+ C VL+ GR WLL+ VPG D LS+ L P V ++A A+RRLH HGAGIDCPQVLNETQSDGRQWLLMSAVPG-DTLSALAQRDELEPERLVRLVAAALRRLHD | 130 |
| Query 126 | LDPATCPFDHQAKHRIERARTRMEAGLVDQDDLDEEHQGLAPAELFARLKARMPDGEDLV | 185 |
| Sbjct 131 | LDPA CPFDH+ + R++ R R+EAGLVD+ D D++H+G + EL+ L R P EDLV LDPAACPFDHRLERRLDTVRQRVEAGLVDEADFDDDHRGRSATELYRLLLDRRPAVEDLV | 190 |
| Query 186 | VTHGDACLPNIMVENGRFSGFIDCGRLGVADRYQDIALATRDIAEELGGEWADRFLVLYG | 245 |
| Sbjct 191 | V HGDACLPN++ E RFSGFIDCGRLGVADR+QD+ALA RDI ELG WA+ FLV YG VAHGDACLPNLLAEGRRFSGFIDCGRLGVADRHQDLALAARDIEAELGAAWAEAFLVEYG | 250 |
| Query 246 | IAAPDSQRIAFYRLLDEFF 264 |  |

D +R+A++RLLDEFF Sbjct 251 -GDIDGERLAYFRLLDEFF 268

>WP_023097209.1 APH(3')-II family aminoglycoside O-phosphotransferase [Pseudomonas aeruginosa]

ERV19223.1 aminoglycoside 3'-phosphotransferase type IIB [Pseudomonas aeruginosa BL18]

KSD23432.1 aminoglycoside 3'-phosphotransferase [Pseudomonas aeruginosa]

MBA5325605.1 APH(3')-II family aminoglycoside O-phosphotransferase [Pseudomonas aeruginosa]

MBG6668125.1 APH(3')-II family aminoglycoside O-phosphotransferase [Pseudomonas aeruginosa]

RPW09509.1 APH(3')-II family aminoglycoside O-phosphotransferase [Pseudomonas aeruginosa]

Length=268

Score = 260 bits (665), Expect = 2e-83, Method: Compositional matrix adjust.

| Identities | | = 136/258 (53%), Positives = 169/258 (66%), Gaps = 5/258 (2%) |  |
| --- | --- | --- | --- |
| Query 11  Sbjct 12 | | SPAAWVERLFGYDWAQQTIGCSDAAVFRLSAQGRPVLFVKTDLSGALNELQDEAARLSWL  +P+ W + L GY W + GCS A V RL A RP LFVK ++ A EL E ARL WL APSTWADYLAGYRWRGEGEGCSAATVHRLEAARRPTLFVKQEVLSAHAELPAEIARLRWL | 70  71 |
| Query 71  Sbjct 72 | | ATTGVPCAAVLDVVTEAGRDWLLLGEVPGQDLLS SHLAPAEKVSIMADAMRRLHTL  G+ C VL+ GR WLL+ VPG L + L P V ++A A+RRLH L HGAGIDCPQVLNETQSDGRQWLLMSAVPGDTLSALAQRGELEPERLVRLVAAALRRLHDL | 126  131 |
| Query 127  Sbjct 132 | | DPATCPFDHQAKHRIERARTRMEAGLVDQDDLDEEHQGLAPAELFARLKARMPDGEDLVV DPA CPFDH+ + R++ R R+EAGLVD+ D D++H+G + EL+ L R P EDLVV DPAACPFDHRLERRLDTVRQRVEAGLVDEADFDDDHRGRSATELYRLLLDRRPAVEDLVV | 186  191 |
| Query 187  Sbjct 192 | | THGDACLPNIMVENGRFSGFIDCGRLGVADRYQDIALATRDIAEELGGEWADRFLVLYGI HGDACLPN++ E RFSGFIDCGRLGVADR+QD+ALA RDI ELG WA+ FLV YG AHGDACLPNLLAEGRRFSGFIDCGRLGVADRHQDLALAARDIEAELGAAWAEAFLVEYG- | 246  250 |
| Query | 247 AAPDSQRIAFYRLLDEFF 264 | | |
|  |  | D +R+A++RLLDEFF |  |
| Sbjct | 251 | GDIDGERLAYFRLLDEFF 268 | |

>WP_121238749.1 APH(3')-II family aminoglycoside O-phosphotransferase [Pseudomonas aeruginosa]

Length=268

Score = 260 bits (665), Expect = 2e-83, Method: Compositional matrix adjust.

| Identities | | = 136/258 (53%), Positives = 168/258 (65%), Gaps = 5/258 (2%) |  |
| --- | --- | --- | --- |
| Query 11  Sbjct 12 | | SPAAWVERLFGYDWAQQTIGCSDAAVFRLSAQGRPVLFVKTDLSGALNELQDEAARLSWL  +P+ W + L GY W Q GCS A V RL RP LFVK ++ A EL E ARL WL APSTWADYLAGYRWRGQGEGCSAATVHRLEVARRPTLFVKQEVLSAHAELPAEIARLRWL | 70  71 |
| Query 71  Sbjct 72 | | ATTGVPCAAVLDVVTEAGRDWLLLGEVPGQDLLS SHLAPAEKVSIMADAMRRLHTL  G+ C VL+ GR WLL+ VPG L + L P V ++A A+RRLH L HGAGIDCPQVLNETQSDGRQWLLMSAVPGDTLSALAQRGELEPERLVRLVAAALRRLHDL | 126  131 |
| Query 127  Sbjct 132 | | DPATCPFDHQAKHRIERARTRMEAGLVDQDDLDEEHQGLAPAELFARLKARMPDGEDLVV DPA CPFDH+ + R++ R R+EAGLVD+ D D++H+G + EL+ L R P EDLVV DPAACPFDHRLERRLDTVRQRVEAGLVDEADFDDDHRGRSATELYRLLLDRRPAVEDLVV | 186  191 |
| Query 187  Sbjct 192 | | THGDACLPNIMVENGRFSGFIDCGRLGVADRYQDIALATRDIAEELGGEWADRFLVLYGI HGDACLPN++ E RFSGFIDCGRLGVADR+QD+ALA RDI ELG WA+ FLV YG AHGDACLPNLLAEGRRFSGFIDCGRLGVADRHQDLALAARDIEAELGAAWAEVFLVEYG- | 246  250 |
| Query | 247 AAPDSQRIAFYRLLDEFF 264 | | |
|  |  | D +R+A++RLLDEFF |  |
| Sbjct | 251 | GDIDGERLAYFRLLDEFF 268 | |

>WP_033951409.1 APH(3')-II family aminoglycoside O-phosphotransferase [Pseudomonas aeruginosa]

Length=268

Score = 260 bits (665), Expect = 2e-83, Method: Compositional matrix adjust.

| Identities | | = 136/258 (53%), Positives = 168/258 (65%), Gaps = 5/258 (2%) |  |
| --- | --- | --- | --- |
| Query 11  Sbjct 12 | | SPAAWVERLFGYDWAQQTIGCSDAAVFRLSAQGRPVLFVKTDLSGALNELQDEAARLSWL  +PA W + L GY W Q GCS A V RL A RP LFVK ++ A EL E ARL WL APATWADYLAGYRWRGQGEGCSAATVHRLEAARRPTLFVKQEVLSAHAELPAEIARLRWL | 70  71 |
| Query 71  Sbjct 72 | | ATTGVPCAAVLDVVTEAGRDWLLLGEVPGQDLLS SHLAPAEKVSIMADAMRRLHTL  + C VL+ GR WLL+ VPG L + L P V ++A A+RRLH L HGASIDCPQVLNETQSDGRQWLLMSAVPGDTLSALAQRGELEPERLVRLVAAALRRLHDL | 126  131 |
| Query 127  Sbjct 132 | | DPATCPFDHQAKHRIERARTRMEAGLVDQDDLDEEHQGLAPAELFARLKARMPDGEDLVV DPA CPFDH+ + R++ R R+EAGLVD+ D D++H+G + EL+ L R P EDLVV DPAACPFDHRLERRLDTVRQRVEAGLVDEADFDDDHRGRSATELYRLLLDRRPAVEDLVV | 186  191 |
| Query 187  Sbjct 192 | | THGDACLPNIMVENGRFSGFIDCGRLGVADRYQDIALATRDIAEELGGEWADRFLVLYGI HGDACLPN++ E RFSGFIDCGRLGVADR+QD+ALA RD+ ELG WA+ FLV YG AHGDACLPNLLAEGRRFSGFIDCGRLGVADRHQDLALAARDVEAELGAAWAEAFLVEYG- | 246  250 |
| Query | 247 AAPDSQRIAFYRLLDEFF 264 | | |
|  |  | D +R+A++RLLDEFF |  |
| Sbjct | 251 | GDIDGERLAYFRLLDEFF 268 | |

>WP_196706147.1 APH(3')-II family aminoglycoside O-phosphotransferase [Pseudomonas aeruginosa]

MBG4934272.1 APH(3')-II family aminoglycoside O-phosphotransferase [Pseudomonas aeruginosa]

Length=267

Score = 260 bits (665), Expect = 2e-83, Method: Compositional matrix adjust.

| Identities | | = 136/258 (53%), Positives = 169/258 (66%), Gaps = 5/258 (2%) |  |
| --- | --- | --- | --- |
| Query 11  Sbjct 11 | | SPAAWVERLFGYDWAQQTIGCSDAAVFRLSAQGRPVLFVKTDLSGALNELQDEAARLSWL  +P+ W + L GY W Q GCS A V RL A+ RP LFVK ++ A EL E ARL WL APSTWADYLAGYRWRGQGEGCSAATVHRLEAERRPTLFVKQEVLSAHAELPAEIARLRWL | 70  70 |
| Query 71  Sbjct 71 | | ATTGVPCAAVLDVVTEAGRDWLLLGEVPGQDLLS SHLAPAEKVSIMADAMRRLHTL  G+ C VL+ GR WLL+ VPG L + L P V ++A A+RRLH L HGAGIDCPQVLNETQSDGRQWLLMSAVPGDTLSALAQRGELEPERLVRLVAAALRRLHDL | 126  130 |
| Query 127  Sbjct 131 | | DPATCPFDHQAKHRIERARTRMEAGLVDQDDLDEEHQGLAPAELFARLKARMPDGEDLVV DPA CPFDH+ + R++ R R+EAGLVD+ D D++H+G + EL+ L R P EDLV DPAACPFDHRLERRLDTVRQRVEAGLVDEADFDDDHRGRSATELYRLLLDRRPAVEDLVG | 186  190 |
| Query 187  Sbjct 191 | | THGDACLPNIMVENGRFSGFIDCGRLGVADRYQDIALATRDIAEELGGEWADRFLVLYGI HGDACLPN++ E RFSGFIDCGRLGVADR+QD+ALA RDI ELG WA+ FLV YG AHGDACLPNLLAEGRRFSGFIDCGRLGVADRHQDLALAARDIEAELGAAWAEAFLVEYG- | 246  249 |
| Query | 247 AAPDSQRIAFYRLLDEFF 264 | | |
|  |  | D +R+A++RLLDEFF |  |
| Sbjct | 250 | GDIDGERLAYFRLLDEFF 267 | |

>WP_196660279.1 APH(3')-II family aminoglycoside O-phosphotransferase [Pseudomonas aeruginosa]

MBG4732214.1 APH(3')-II family aminoglycoside O-phosphotransferase [Pseudomonas aeruginosa]

Length=267

Score = 260 bits (665), Expect = 2e-83, Method: Compositional matrix adjust. Identities = 136/258 (53%), Positives = 169/258 (66%), Gaps = 5/258 (2%)

| Query | 11 | SPAAWVERLFGYDWAQQTIGCSDAAVFRLSAQGRPVLFVKTDLSGALNELQDEAARLSWL | 70 |
| --- | --- | --- | --- |
| Sbjct | 11 | +P+ W + L GY W Q GCS A V RL A+ RP LFVK ++ A EL E ARL WL APSTWADYLAGYRWRGQGEGCSAATVHRLEAERRPTLFVKQEVLSAHAELPAEIARLRWL | 70 |
| Query | 71 | ATTGVPCAAVLDVVTEAGRDWLLLGEVPGQDLLS SHLAPAEKVSIMADAMRRLHTL | 126 |
| Sbjct | 71 | G+ C VL+ GR WLL+ VPG L + L P V ++A A+RRLH L  HGAGIDCPQVLNETQSDGRQWLLMSAVPGDTLSALAQRGELEPERLVRLVAAALRRLHDL | 130 |

| Query | 127 | DPATCPFDHQAKHRIERARTRMEAGLVDQDDLDEEHQGLAPAELFARLKARMPDGEDLVV | 186 |
| --- | --- | --- | --- |
| Sbjct | 131 | DPA CPFDH+ + R++ R R+EAGLVD+ D D++H+G + EL+ L R P EDLVV DPAACPFDHRLERRLDTVRQRVEAGLVDEADFDDDHRGRSATELYRLLLDRRPAVEDLVV | 190 |
| Query | 187 | THGDACLPNIMVENGRFSGFIDCGRLGVADRYQDIALATRDIAEELGGEWADRFLVLYGI | 246 |
| Sbjct | 191 | HGDACLPN++ E RFSGFIDCGRLG ADR+QD+ALA RDI ELG WA+ FLV YG  AHGDACLPNLLAEGRRFSGFIDCGRLGGADRHQDLALAARDIEAELGAAWAEAFLVEYG- | 249 |
| Query | 247 AAPDSQRIAFYRLLDEFF 264 | | |
|  |  | D +R+A++RLLDEFF |  |
| Sbjct | 250 | GDIDGERLAYFRLLDEFF 267 | |

>WP_144377070.1 aminoglycoside 3'-phosphotransferase [Mesorhizobium amorphae] Length=260

Score = 260 bits (664), Expect = 2e-83, Method: Compositional matrix adjust.

| Identities | | = 131/254 (52%), Positives = 168/254 (66%), Gaps = 1/254 (0%) |  |
| --- | --- | --- | --- |
| Query 12  Sbjct 7 | | PAAWVERLFGYDWAQQTIGCSDAAVFRLSAQGRPVLFVKTDLSGALNELQDEAARLSWLA P + E + GY QQT G SDA VF+L A G+ LF+K D + E+ EA RL WLA PLPFPELVIGYGARQQTTGLSDAGVFKLDAPGKSSLFLKVDDANGFAEVPAEAERLRWLA | 71  66 |
| Query 72  Sbjct 67 | | TTGVPCAAVLDVVTEAGRDWLLLGEVPGQDLLSSHLAPAEK-VSIMADAMRRLHTLDPAT G+PC VL GR+WLL+ +PG+DL S+ PAE ++++A A+R LH LD + AQGIPCPEVLAFAVHGGRNWLLMSALPGKDLESAGPLPAETTIAMIAGALRTLHALDVHS | 130  126 |
| Query 131  Sbjct 127 | | CPFDHQAKHRIERARTRMEAGLVDQDDLDEEHQGLAPAELFARLKARMPDGEDLVVTHGD CPFDH+ + RI AR R+EAGL+D+++ D E G ++FA L A P EDLVVTHGD CPFDHRLERRIADARARLEAGLIDEENFDGERLGQTAQQVFADLVALRPTSEDLVVTHGD | 190  186 |
| Query 191  Sbjct 187 | | ACLPNIMVENGRFSGFIDCGRLGVADRYQDIALATRDIAEELGGEWADRFLVLYGIAAPD ACLPN+MV+ GRFSGFIDCGRLGVADR QD+AL R I + G EW FL LYG D ACLPNLMVDGGRFSGFIDCGRLGVADRCQDLALVCRSIRADFGEEWIAPFLALYGKPDAD | 250  246 |
| Query 251 | | SQRIAFYRLLDEFF 264  ++++Y+LLDEFF |  |
| Sbjct | 247 | PAKLSYYQLLDEFF 260 | |

>WP_196689763.1 APH(3')-II family aminoglycoside O-phosphotransferase [Pseudomonas aeruginosa]

MBG4585184.1 APH(3')-II family aminoglycoside O-phosphotransferase [Pseudomonas aeruginosa]

Length=268

Score = 260 bits (665), Expect = 2e-83, Method: Compositional matrix adjust.

| Identities | | = 136/258 (53%), Positives = 168/258 (65%), Gaps = 5/258 (2%) |  |
| --- | --- | --- | --- |
| Query 11  Sbjct 12 | | SPAAWVERLFGYDWAQQTIGCSDAAVFRLSAQGRPVLFVKTDLSGALNELQDEAARLSWL  +P+ W + L GY W Q GCS A V RL RP LFVK ++ A EL E ARL WL APSTWADYLAGYRWRGQGEGCSAATVHRLEVARRPTLFVKQEVLSAHAELPAEIARLRWL | 70  71 |
| Query 71  Sbjct 72 | | ATTGVPCAAVLDVVTEAGRDWLLLGEVPGQDLLS SHLAPAEKVSIMADAMRRLHTL  G+ C VL+ GR WLL+ VPG L + L P V ++A A+RRLH L HGAGIDCPQVLNETQSDGRQWLLMSAVPGDTLSALAQRGELEPERLVRLVAAALRRLHDL | 126  131 |
| Query 127  Sbjct 132 | | DPATCPFDHQAKHRIERARTRMEAGLVDQDDLDEEHQGLAPAELFARLKARMPDGEDLVV DPA CP DH+ + R++ R R+EAGLVD+ D D++H+G + EL+ L AR P EDLVV DPAACPVDHRLERRLDTVRQRVEAGLVDEADFDDDHRGRSATELYRLLLARRPAVEDLVV | 186  191 |
| Query 187  Sbjct 192 | | THGDACLPNIMVENGRFSGFIDCGRLGVADRYQDIALATRDIAEELGGEWADRFLVLYGI HGDACLPN++ E RFSGFIDCGRLGVADR+QD+ALA RDI ELG WA+ FLV YG AHGDACLPNLLAEGRRFSGFIDCGRLGVADRHQDLALAARDIEAELGAAWAEAFLVEYG- | 246  250 |
| Query | 247 AAPDSQRIAFYRLLDEFF 264 | | |
|  |  | D +R+A++RLLDEFF |  |
| Sbjct | 251 | GDIDGERLAYFRLLDEFF 268 | |

>WP_124125952.1 APH(3')-II family aminoglycoside O-phosphotransferase [Pseudomonas aeruginosa]

RPT47314.1 APH(3') family aminoglycoside O-phosphotransferase [Pseudomonas aeruginosa]

Length=268

Score = 260 bits (665), Expect = 2e-83, Method: Compositional matrix adjust.

| Identities | | = 136/258 (53%), Positives = 168/258 (65%), Gaps = 5/258 (2%) |  |
| --- | --- | --- | --- |
| Query 11  Sbjct 12 | | SPAAWVERLFGYDWAQQTIGCSDAAVFRLSAQGRPVLFVKTDLSGALNELQDEAARLSWL  +P+ W + L GY W Q GCS A V RL RP LFVK ++ A EL E ARL WL APSTWADYLAGYRWRGQGEGCSAATVHRLEVARRPTLFVKQEVLSAHAELPAEIARLRWL | 70  71 |
| Query 71  Sbjct 72 | | ATTGVPCAAVLDVVTEAGRDWLLLGEVPGQDLLS SHLAPAEKVSIMADAMRRLHTL  G+ C VL+ GR WLL+ VPG L + L P V ++A A+RRLH L HGAGIDCPQVLNETQSDGRQWLLMSAVPGDTLSALAQRGELEPGRLVRLVAAALRRLHDL | 126  131 |
| Query 127  Sbjct 132 | | DPATCPFDHQAKHRIERARTRMEAGLVDQDDLDEEHQGLAPAELFARLKARMPDGEDLVV DPA CPFDH+ + R++ R R+EAGLVD+ D D++H+G + EL+ L R P EDLVV DPAGCPFDHRLERRLDTVRQRVEAGLVDEADFDDDHRGRSATELYRLLLDRRPVVEDLVV | 186  191 |
| Query 187  Sbjct 192 | | THGDACLPNIMVENGRFSGFIDCGRLGVADRYQDIALATRDIAEELGGEWADRFLVLYGI HGDACLPN++ E RFSGFIDCGRLGVADR+QD+ALA RDI ELG WA+ FLV YG AHGDACLPNLLAEGRRFSGFIDCGRLGVADRHQDLALAARDIEAELGAAWAEAFLVEYG- | 246  250 |
| Query | 247 AAPDSQRIAFYRLLDEFF 264 | | |
|  |  | D +R+A++RLLDEFF |  |
| Sbjct | 251 | GDIDGERLAYFRLLDEFF 268 | |

>MBG6500747.1 APH(3')-II family aminoglycoside O-phosphotransferase [Pseudomonas aeruginosa]

Length=268

Score = 260 bits (665), Expect = 2e-83, Method: Compositional matrix adjust.

| Identities | | = 136/258 (53%), Positives = 168/258 (65%), Gaps = 5/258 (2%) |  |
| --- | --- | --- | --- |
| Query 11  Sbjct 12 | | SPAAWVERLFGYDWAQQTIGCSDAAVFRLSAQGRPVLFVKTDLSGALNELQDEAARLSWL  +P+ W + L GY W Q GCS A V RL RP LFVK ++ A EL E ARL WL APSTWADYLAGYRWRGQGEGCSAATVHRLEVARRPTLFVKQEVLSAPAELPAEIARLRWL | 70  71 |
| Query 71  Sbjct 72 | | ATTGVPCAAVLDVVTEAGRDWLLLGEVPGQDLLS SHLAPAEKVSIMADAMRRLHTL  G+ C VL+ GR WLL+ VPG L + L P V ++A A+RRLH L HGAGIDCPQVLNETQSDGRQWLLMSAVPGDTLSALAQRGELEPERLVRLVAAALRRLHDL | 126  131 |
| Query 127  Sbjct 132 | | DPATCPFDHQAKHRIERARTRMEAGLVDQDDLDEEHQGLAPAELFARLKARMPDGEDLVV DPA CPFDH+ + R++ R R+EAGLVD+ D D++H+G + EL+ L R P EDLVV DPAACPFDHRLERRLDTVRQRVEAGLVDEADFDDDHRGRSATELYRLLLDRRPAVEDLVV | 186  191 |
| Query 187  Sbjct 192 | | THGDACLPNIMVENGRFSGFIDCGRLGVADRYQDIALATRDIAEELGGEWADRFLVLYGI HGDACLPN++ E RFSGFIDCGRLGVADR+QD+ALA RDI ELG WA+ FLV YG AHGDACLPNLLAEGRRFSGFIDCGRLGVADRHQDLALAARDIEAELGAAWAEAFLVEYG- | 246  250 |
| Query | 247 AAPDSQRIAFYRLLDEFF 264 | | |
|  |  | D +R+A++RLLDEFF |  |
| Sbjct | 251 | GDIDGERLAYFRLLDEFF 268 | |

>WP_031652465.1 APH(3')-II family aminoglycoside O-phosphotransferase [Pseudomonas aeruginosa]

AXR09437.1 APH(3')-II family aminoglycoside O-phosphotransferase [Pseudomonas aeruginosa]

EZO79538.1 aminoglycoside 3'-phosphotransferase type IIB [Pseudomonas aeruginosa BWH057]

EZO85336.1 aminoglycoside 3'-phosphotransferase type IIB [Pseudomonas aeruginosa BWH056]

KAB0754575.1 APH(3')-II family aminoglycoside O-phosphotransferase [Pseudomonas aeruginosa]

MBG4224631.1 APH(3')-II family aminoglycoside O-phosphotransferase [Pseudomonas aeruginosa]

Length=268

Score = 260 bits (665), Expect = 2e-83, Method: Compositional matrix adjust. Identities = 136/258 (53%), Positives = 168/258 (65%), Gaps = 5/258 (2%)

| Query | 11 | SPAAWVERLFGYDWAQQTIGCSDAAVFRLSAQGRPVLFVKTDLSGALNELQDEAARLSWL | 70 |
| --- | --- | --- | --- |
| Sbjct | 12 | +P+ W + L GY W Q GCS A V RL RP LFVK ++ A EL E ARL WL APSTWADYLAGYRWRGQGEGCSAATVHRLEVARRPTLFVKQEVLSAHAELPAEIARLRWL | 71 |
| Query | 71 | ATTGVPCAAVLDVVTEAGRDWLLLGEVPGQDLLS SHLAPAEKVSIMADAMRRLHTL | 126 |
| Sbjct | 72 | G+ C VL+ GR WLL+ VPG L + L P V ++A A+RRLH L HGAGIDCPQVLNETQSDGRQWLLMSAVPGDTLSALAQRGELEPERLVRLVAAALRRLHDL | 131 |
| Query | 127 | DPATCPFDHQAKHRIERARTRMEAGLVDQDDLDEEHQGLAPAELFARLKARMPDGEDLVV | 186 |
| Sbjct | 132 | DPA CPFDH+ + R++ R R+EAGLVD+ D D++H+G + EL+ L R P EDLVV DPAACPFDHRLERRLDTVRQRVEAGLVDEADFDDDHRGRSATELYRLLLDRRPVVEDLVV | 191 |
| Query | 187 | THGDACLPNIMVENGRFSGFIDCGRLGVADRYQDIALATRDIAEELGGEWADRFLVLYGI | 246 |
| Sbjct | 192 | HGDACLPN++ E RFSGFIDCGRLGVADR+QD+ALA RDI ELG WA+ FLV YG  AHGDACLPNLLAEGRRFSGFIDCGRLGVADRHQDLALAARDIEAELGAAWAEAFLVEYG- | 250 |
| Query | 247 AAPDSQRIAFYRLLDEFF 264 | | |
|  |  | D +R+A++RLLDEFF |  |
| Sbjct | 251 | GDIDGERLAYFRLLDEFF 268 | |

>WP_128666488.1 APH(3')-II family aminoglycoside O-phosphotransferase [Pseudomonas aeruginosa]

KAA5667092.1 APH(3')-II family aminoglycoside O-phosphotransferase [Pseudomonas aeruginosa]

MBG4922801.1 APH(3')-II family aminoglycoside O-phosphotransferase [Pseudomonas aeruginosa]

RUI42441.1 APH(3')-II family aminoglycoside O-phosphotransferase [Pseudomonas aeruginosa]

Length=268

Score = 260 bits (665), Expect = 2e-83, Method: Compositional matrix adjust.

| Identities | | = 136/258 (53%), Positives = 168/258 (65%), Gaps = 5/258 (2%) |  |
| --- | --- | --- | --- |
| Query 11  Sbjct 12 | | SPAAWVERLFGYDWAQQTIGCSDAAVFRLSAQGRPVLFVKTDLSGALNELQDEAARLSWL  +P+ W + L GY W Q GCS A V RL RP LFVK ++ A EL E ARL WL APSTWADYLAGYRWRGQGEGCSAATVHRLEVARRPTLFVKQEVLSAHAELPAEIARLRWL | 70  71 |
| Query 71  Sbjct 72 | | ATTGVPCAAVLDVVTEAGRDWLLLGEVPGQDLLS SHLAPAEKVSIMADAMRRLHTL  G+ C VL+ GR WLL+ VPG L + L P V ++A A+RRLH L HGAGIDCPQVLNETQSDGRQWLLMSAVPGDTLSALAQRGELEPERLVRLVAAALRRLHDL | 126  131 |
| Query 127  Sbjct 132 | | DPATCPFDHQAKHRIERARTRMEAGLVDQDDLDEEHQGLAPAELFARLKARMPDGEDLVV DPA CPFDH+ + R++ R R+EAGLVD+ D D++H+G + EL+ L R P EDLVV DPAACPFDHRLERRLDTVRQRVEAGLVDEVDFDDDHRGRSATELYRLLLDRRPAVEDLVV | 186  191 |
| Query 187  Sbjct 192 | | THGDACLPNIMVENGRFSGFIDCGRLGVADRYQDIALATRDIAEELGGEWADRFLVLYGI HGDACLPN++ E RFSGFIDCGRLGVADR+QD+ALA RDI ELG WA+ FLV YG AHGDACLPNLLAEGRRFSGFIDCGRLGVADRHQDLALAARDIEAELGAAWAEAFLVEYG- | 246  250 |
| Query | 247 AAPDSQRIAFYRLLDEFF 264 | | |
|  |  | D +R+A++RLLDEFF |  |
| Sbjct | 251 | GDIDGERLAYFRLLDEFF 268 | |

>WP_058161625.1 APH(3')-II family aminoglycoside O-phosphotransferase [Pseudomonas aeruginosa]

KSK01026.1 aminoglycoside 3'-phosphotransferase [Pseudomonas aeruginosa] Length=268

Score = 260 bits (665), Expect = 2e-83, Method: Compositional matrix adjust. Identities = 136/258 (53%), Positives = 168/258 (65%), Gaps = 5/258 (2%)

| Query | 11 | SPAAWVERLFGYDWAQQTIGCSDAAVFRLSAQGRPVLFVKTDLSGALNELQDEAARLSWL | 70 |
| --- | --- | --- | --- |
| Sbjct | 12 | +P+ W + L GY W Q GCS A V RL RP LFVK ++ A EL E ARL WL APSTWADYLAGYRWRGQGEGCSAATVHRLEVARRPTLFVKQEVLSAHAELPAEIARLRWL | 71 |
| Query | 71 | ATTGVPCAAVLDVVTEAGRDWLLLGEVPGQDLLS SHLAPAEKVSIMADAMRRLHTL | 126 |
| Sbjct | 72 | G+ C VL+ GR WLL+ VPG L + L P V ++A A+RRLH L  HGAGIDCPQVLNETQSDGRQWLLMSAVPGDTLSALAQRGELEPERLVRLVAAALRRLHDL | 131 |

| Query | 127 | DPATCPFDHQAKHRIERARTRMEAGLVDQDDLDEEHQGLAPAELFARLKARMPDGEDLVV | 186 |
| --- | --- | --- | --- |
| Sbjct | 132 | DPA CPFDH+ + R++ R R+EAGLVD+ D D++H+G + EL+ L R P EDLVV DPAACPFDHRLERRLDTVRQRVEAGLVDEADFDDDHRGRSATELYRLLLDRRPAVEDLVV | 191 |
| Query | 187 | THGDACLPNIMVENGRFSGFIDCGRLGVADRYQDIALATRDIAEELGGEWADRFLVLYGI | 246 |
| Sbjct | 192 | HGDACLPN++ E RFSGFIDCGRLGVADR+QD+ALA RDI ELG WA+ FLV YG  AHGDACLPNLLAEGWRFSGFIDCGRLGVADRHQDLALAARDIEAELGAAWAEAFLVEYG- | 250 |
| Query | 247 AAPDSQRIAFYRLLDEFF 264 | | |
|  |  | D +R+A++RLLDEFF |  |
| Sbjct | 251 | GDIDGERLAYFRLLDEFF 268 | |

>WP_087587933.1 APH(3')-II family aminoglycoside O-phosphotransferase [uncultured bacterium]

APR72113.1 3'-aminoglycoside phosphotransfrase [uncultured bacterium] APR72114.1 3'-aminoglycoside phosphotransfrase [synthetic construct]

Length=267

Score = 260 bits (665), Expect = 2e-83, Method: Compositional matrix adjust.

| Identities | = 130/245 (53%), Positives = 164/245 (67%), Gaps = 1/245 (0%) |  |
| --- | --- | --- |
| Query 21 | GYDWAQQTIGCSDAAVFRLSAQGRPVLFVKTDLSGALNELQDEAARLSWLATTGVPCAAV | 80 |
| Sbjct 23 | GY W + +GCSDA VF L +G P LF+K + EL DEA RL WL++ +PC V GYSWNRDALGCSDAQVFMLQGEGLPRLFIKVEAVSPYGELPDEAVRLRWLSSRDMPCPDV | 82 |
| Query 81 | LDVVTEAGRDWLLLGEVPGQDLLSS-HLAPAEKVSIMADAMRRLHTLDPATCPFDHQAKH | 139 |
| Sbjct 83 | L AGR WLL+ VPG+DL S+ L+ ++ I A A+R+LH LDPATCPFDH+ LFEGAHAGRFWLLMSGVPGEDLASAGSLSIETRIRIFAGALRQLHALDPATCPFDHRLDG | 142 |
| Query 140 | RIERARTRMEAGLVDQDDLDEEHQGLAPAELFARLKARMPDGEDLVVTHGDACLPNIMVE | 199 |
| Sbjct 143 | RIE AR RM+AGLVD+ D D++ G ++LF+RL A P D+VVTHGDACLPN M + RIEAARARMQAGLVDETDFDDDMLGKTTSDLFSRLAAEKPMVGDVVVTHGDACLPNFMAD | 202 |
| Query 200 | NGRFSGFIDCGRLGVADRYQDIALATRDIAEELGGEWADRFLVLYGIAAPDSQRIAFYRL | 259 |
| Sbjct 203 | G F+G+IDCGRLG+ADRYQDI LA R IA+ G E FL YG+ D ++A+YRL KGIFTGYIDCGRLGLADRYQDIGLACRSIADNFGDERVKLFLDCYGLMQADPAKLAYYRL | 262 |
| Query 260  Sbjct 263 | LDEFF 264 LDEFF LDEFF 267 |  |

>WP_124097390.1 APH(3')-II family aminoglycoside O-phosphotransferase [Pseudomonas aeruginosa]

RPN92854.1 APH(3') family aminoglycoside O-phosphotransferase [Pseudomonas aeruginosa]

Length=268

Score = 260 bits (665), Expect = 2e-83, Method: Compositional matrix adjust.

| Identities | | = 137/258 (53%), Positives = 168/258 (65%), Gaps = 5/258 (2%) |  |
| --- | --- | --- | --- |
| Query 11  Sbjct 12 | | SPAAWVERLFGYDWAQQTIGCSDAAVFRLSAQGRPVLFVKTDLSGALNELQDEAARLSWL  +PA W + L GY W Q GCS A V RL A RP LFVK ++ A EL E ARL WL APATWADYLAGYRWRGQGEGCSAATVHRLEAARRPTLFVKQEVLSAHAELPAEIARLRWL | 70  71 |
| Query 71  Sbjct 72 | | ATTGVPCAAVLDVVTEAGRDWLLLGEVPGQDLLS SHLAPAEKVSIMADAMRRLHTL  G+ C VL+ GR WLL+ VPG L + L P V ++A A+RRLH L HGAGIDCPQVLNETQSDGRQWLLMSAVPGDTLSALAQRGELEPERLVRLVAAALRRLHDL | 126  131 |
| Query 127  Sbjct 132 | | DPATCPFDHQAKHRIERARTRMEAGLVDQDDLDEEHQGLAPAELFARLKARMPDGEDLVV DPA CPFDH+ + R++ R R+EAGLVD+ D++H+G + EL+ L R P EDLVV DPAACPFDHRLERRLDTVRQRVEAGLVDEAGFDDDHRGRSATELYRLLLDRRPAVEDLVV | 186  191 |
| Query 187  Sbjct 192 | | THGDACLPNIMVENGRFSGFIDCGRLGVADRYQDIALATRDIAEELGGEWADRFLVLYGI HGDACLPN++ E RFSGFIDCGRLGVADR+QD+ALA RDI ELG WA+ FLV YG AHGDACLPNLLAEGRRFSGFIDCGRLGVADRHQDMALAARDIEAELGAAWAEAFLVEYG- | 246  250 |
| Query | 247 AAPDSQRIAFYRLLDEFF 264 | | |
|  |  | D +R+A++RLLDEFF |  |
| Sbjct | 251 | GDIDGERLAYFRLLDEFF 268 | |

>WP_121290907.1 APH(3')-II family aminoglycoside O-phosphotransferase [Pseudomonas aeruginosa]

Length=268

Score = 260 bits (665), Expect = 2e-83, Method: Compositional matrix adjust.

| Identities | | = 137/258 (53%), Positives = 168/258 (65%), Gaps = 5/258 (2%) |  |
| --- | --- | --- | --- |
| Query 11  Sbjct 12 | | SPAAWVERLFGYDWAQQTIGCSDAAVFRLSAQGRPVLFVKTDLSGALNELQDEAARLSWL  +PA W + L GY W Q GCS A V RL A RP LFVK ++ A EL E ARL WL APATWDDYLAGYRWRGQGEGCSAATVHRLEAARRPTLFVKQEVLSAHAELPAEIARLRWL | 70  71 |
| Query 71  Sbjct 72 | | ATTGVPCAAVLDVVTEAGRDWLLLGEVPGQDLLS SHLAPAEKVSIMADAMRRLHTL  G+ C VL+ GR WLL+ VPG L + L P V ++A A+RRLH L HGAGIDCPQVLNETQSDGRQWLLMSAVPGDTLSALAQRGELEPGRLVRLVAAALRRLHDL | 126  131 |
| Query 127  Sbjct 132 | | DPATCPFDHQAKHRIERARTRMEAGLVDQDDLDEEHQGLAPAELFARLKARMPDGEDLVV DPA CPFDH+ + R++ R R+EAGLVD+ D D++H+G + EL+ L R P EDLV DPAACPFDHRLERRLDTVRQRVEAGLVDEADFDDDHRGRSATELYRLLLDRRPAVEDLVA | 186  191 |
| Query 187  Sbjct 192 | | THGDACLPNIMVENGRFSGFIDCGRLGVADRYQDIALATRDIAEELGGEWADRFLVLYGI HGDACLPN++ E RFSGFIDCGRLGVADR+QD+ALA RDI ELG WA+ FLV YG AHGDACLPNLLAEGRRFSGFIDCGRLGVADRHQDLALAARDIEAELGAAWAEAFLVEYG- | 246  250 |
| Query | 247 AAPDSQRIAFYRLLDEFF 264 | | |
|  |  | D +R+A++RLLDEFF |  |
| Sbjct | 251 | GDIDGERLAYFRLLDEFF 268 | |

>WP_057388031.1 APH(3')-II family aminoglycoside O-phosphotransferase [Pseudomonas aeruginosa]

RUB28293.1 APH(3')-II family aminoglycoside O-phosphotransferase [Pseudomonas aeruginosa]

Length=268

Score = 260 bits (665), Expect = 2e-83, Method: Compositional matrix adjust.

| Identities | | = 137/258 (53%), Positives = 168/258 (65%), Gaps = 5/258 (2%) |  |
| --- | --- | --- | --- |
| Query 11  Sbjct 12 | | SPAAWVERLFGYDWAQQTIGCSDAAVFRLSAQGRPVLFVKTDLSGALNELQDEAARLSWL  +PA W + L GY W Q GCS A V RL A RP LFVK ++ A EL E ARL WL APATWDDYLAGYRWRGQGEGCSAATVHRLEAARRPTLFVKQEVLSAHAELPAEIARLRWL | 70  71 |
| Query 71  Sbjct 72 | | ATTGVPCAAVLDVVTEAGRDWLLLGEVPGQDLLS SHLAPAEKVSIMADAMRRLHTL  G+ C VL+ GR WLL+ VPG L + L P V ++A A+RRLH L HGAGIDCPQVLNETQSDGRQWLLMSAVPGDTLSALAQRGELEPGRLVCLVAAALRRLHDL | 126  131 |
| Query 127  Sbjct 132 | | DPATCPFDHQAKHRIERARTRMEAGLVDQDDLDEEHQGLAPAELFARLKARMPDGEDLVV DPA CPFDH+ + R++ R R+EAGLVD+ D D++H+G + EL+ L R P EDLV DPAACPFDHRLERRLDTVRQRVEAGLVDEADFDDDHRGRSATELYRLLLDRRPAVEDLVA | 186  191 |
| Query 187  Sbjct 192 | | THGDACLPNIMVENGRFSGFIDCGRLGVADRYQDIALATRDIAEELGGEWADRFLVLYGI HGDACLPN++ E RFSGFIDCGRLGVADR+QD+ALA RDI ELG WA+ FLV YG AHGDACLPNLLAEGRRFSGFIDCGRLGVADRHQDLALAARDIEAELGAAWAEAFLVEYG- | 246  250 |
| Query | 247 AAPDSQRIAFYRLLDEFF 264 | | |
|  |  | D +R+A++RLLDEFF |  |
| Sbjct | 251 | GDIDGERLAYFRLLDEFF 268 | |

>WP_023118901.1 APH(3')-II family aminoglycoside O-phosphotransferase [Pseudomonas aeruginosa]

AON05129.1 aminoglycoside O-phosphotransferase APH(3')-IIb [Pseudomonas aeruginosa]

AON11118.1 aminoglycoside O-phosphotransferase APH(3')-IIb [Pseudomonas aeruginosa]

AON17106.1 aminoglycoside O-phosphotransferase APH(3')-IIb [Pseudomonas aeruginosa]

AON23113.1 aminoglycoside O-phosphotransferase APH(3')-IIb [Pseudomonas aeruginosa]

AON29101.1 aminoglycoside O-phosphotransferase APH(3')-IIb [Pseudomonas aeruginosa]

Length=268

Score = 260 bits (665), Expect = 2e-83, Method: Compositional matrix adjust.

| Identities | | = 137/258 (53%), Positives = 168/258 (65%), Gaps = 5/258 (2%) |  |
| --- | --- | --- | --- |
| Query 11  Sbjct 12 | | SPAAWVERLFGYDWAQQTIGCSDAAVFRLSAQGRPVLFVKTDLSGALNELQDEAARLSWL  +PA W + L GY W Q GCS A V RL A RP LFVK ++ A EL E ARL WL APATWADYLAGYRWRGQGEGCSAATVHRLEAARRPTLFVKQEVLSAHAELPAEIARLRWL | 70  71 |
| Query 71  Sbjct 72 | | ATTGVPCAAVLDVVTEAGRDWLLLGEVPGQDLLS SHLAPAEKVSIMADAMRRLHTL  G+ C VL+ GR WLL+ VPG L + L P V ++A A+RRLH L HGAGIDCPQVLNETQSDGRQWLLMSAVPGDTLSALAQRGELEPERLVRLVAAALRRLHDL | 126  131 |
| Query 127  Sbjct 132 | | DPATCPFDHQAKHRIERARTRMEAGLVDQDDLDEEHQGLAPAELFARLKARMPDGEDLVV DPA CPFDH+ + R++ R R+EAGLVD+ D++H+G + EL+ L R P EDLVV DPAACPFDHRLERRLDTVRQRVEAGLVDEAGFDDDHRGRSATELYRLLLDRRPAVEDLVV | 186  191 |
| Query 187  Sbjct 192 | | THGDACLPNIMVENGRFSGFIDCGRLGVADRYQDIALATRDIAEELGGEWADRFLVLYGI HGDACLPN++ E RFSGFIDCGRLGVADR+QD+ALA RDI ELG WA+ FLV YG AHGDACLPNLLAEGRRFSGFIDCGRLGVADRHQDLALAARDIEAELGAAWAEAFLVEYG- | 246  250 |
| Query | 247 AAPDSQRIAFYRLLDEFF 264 | | |
|  |  | D +R+A++RLLDEFF |  |
| Sbjct | 251 | GDIDGERLAYFRLLDEFF 268 | |

>WP_124120135.1 APH(3')-II family aminoglycoside O-phosphotransferase [Pseudomonas aeruginosa]

RPS61219.1 APH(3') family aminoglycoside O-phosphotransferase [Pseudomonas aeruginosa]

RQF70435.1 APH(3') family aminoglycoside O-phosphotransferase [Pseudomonas aeruginosa]

Length=268

Score = 260 bits (665), Expect = 2e-83, Method: Compositional matrix adjust.

| Identities | | = 137/258 (53%), Positives = 168/258 (65%), Gaps = 5/258 (2%) |  |
| --- | --- | --- | --- |
| Query 11  Sbjct 12 | | SPAAWVERLFGYDWAQQTIGCSDAAVFRLSAQGRPVLFVKTDLSGALNELQDEAARLSWL  +PA W + L GY W Q GCS A V RL A RP LFVK ++ A EL E ARL WL APATWDDYLAGYRWRGQGEGCSAATVHRLEAARRPTLFVKQEVLSAHAELPAEIARLRWL | 70  71 |
| Query 71  Sbjct 72 | | ATTGVPCAAVLDVVTEAGRDWLLLGEVPGQDLLS SHLAPAEKVSIMADAMRRLHTL  G+ C VL+ GR WLL+ VPG L + L P V ++A A+RRLH L HGAGIDCPQVLNETQSDGRQWLLMSAVPGDTLSALAQRGELEPGRLVRLVAAALRRLHDL | 126  131 |
| Query 127  Sbjct 132 | | DPATCPFDHQAKHRIERARTRMEAGLVDQDDLDEEHQGLAPAELFARLKARMPDGEDLVV DPA CPFDH+ + R++ R R+EAGLVD+ D D++H+G + EL+ L R P EDLV DPAACPFDHRLERRLDTVRQRVEAGLVDEADFDDDHRGRSATELYRLLLDRRPAVEDLVA | 186  191 |
| Query 187  Sbjct 192 | | THGDACLPNIMVENGRFSGFIDCGRLGVADRYQDIALATRDIAEELGGEWADRFLVLYGI HGDACLPN++ E RFSGFIDCGRLGVADR+QD+ALA RDI ELG WA+ FLV YG AHGDACLPNMLAEGRRFSGFIDCGRLGVADRHQDLALAARDIEAELGAAWAEAFLVEYG- | 246  250 |
| Query | 247 AAPDSQRIAFYRLLDEFF 264 | | |
|  |  | D +R+A++RLLDEFF |  |
| Sbjct | 251 | GDIDGERLAYFRLLDEFF 268 | |

>WP_125884705.1 APH(3')-II family aminoglycoside O-phosphotransferase [Pseudomonas aeruginosa]

RRW98240.1 APH(3')-II family aminoglycoside O-phosphotransferase [Pseudomonas aeruginosa]

Length=268

Score = 260 bits (664), Expect = 3e-83, Method: Compositional matrix adjust. Identities = 137/258 (53%), Positives = 169/258 (66%), Gaps = 5/258 (2%)

| Query | 11 | SPAAWVERLFGYDWAQQTIGCSDAAVFRLSAQGRPVLFVKTDLSGALNELQDEAARLSWL | 70 |
| --- | --- | --- | --- |
| Sbjct | 12 | +P+ W + L GY W Q GCS A V RL A RP LFVK ++ A EL E ARL WL APSTWADYLAGYRWRGQGEGCSAATVHRLEAARRPPLFVKQEVLSAHAELPAEIARLRWL | 71 |
| Query | 71 | ATTGVPCAAVLDVVTEAGRDWLLLGEVPGQDLLS SHLAPAEKVSIMADAMRRLHTL  G+ C VL+ GR WLL+ VPG L + L P V ++A A+RRLH L | 126 |

| Sbjct | 72 | HGAGIDCPQVLNEPQRDGRQWLLMSAVPGDTLSALAQRGELEPGRLVRLVAAALRRLHDL | 131 |
| --- | --- | --- | --- |
| Query | 127 | DPATCPFDHQAKHRIERARTRMEAGLVDQDDLDEEHQGLAPAELFARLKARMPDGEDLVV | 186 |
| Sbjct | 132 | DPA CPFDH+ + R++ R R+EAGLVD+ D D++H+G + EL+ L R P EDLVV DPAACPFDHRLERRLDTVRQRVEAGLVDEADFDDDHRGRSATELYRLLLDRRPAVEDLVV | 191 |
| Query | 187 | THGDACLPNIMVENGRFSGFIDCGRLGVADRYQDIALATRDIAEELGGEWADRFLVLYGI | 246 |
| Sbjct | 192 | HGDACLPN++ E RFSGFIDCGRLGVADR+QD+ALA RDI ELG WA+ FLV YG  AHGDACLPNLLAEGRRFSGFIDCGRLGVADRHQDLALAARDIEAELGAAWAEAFLVEYG- | 250 |
| Query | 247 AAPDSQRIAFYRLLDEFF 264 | | |
|  |  | D +R+A++RLLDEFF |  |
| Sbjct | 251 | GDIDGERLAYFRLLDEFF 268 | |

>WP_094954585.1 APH(3')-II family aminoglycoside O-phosphotransferase [Pseudomonas aeruginosa]

NPT06378.1 APH(3')-II family aminoglycoside O-phosphotransferase [Pseudomonas aeruginosa]

OZO28245.1 APH(3') family aminoglycoside O-phosphotransferase [Pseudomonas aeruginosa]

RPW42889.1 APH(3')-II family aminoglycoside O-phosphotransferase [Pseudomonas aeruginosa]

RUG57024.1 APH(3')-II family aminoglycoside O-phosphotransferase [Pseudomonas aeruginosa]

Length=268

Score = 260 bits (664), Expect = 3e-83, Method: Compositional matrix adjust.

| Identities | | = 136/258 (53%), Positives = 168/258 (65%), Gaps = 5/258 (2%) |  |
| --- | --- | --- | --- |
| Query 11  Sbjct 12 | | SPAAWVERLFGYDWAQQTIGCSDAAVFRLSAQGRPVLFVKTDLSGALNELQDEAARLSWL  +P+ W + L GY W Q GCS A V RL A RP LFVK ++ A EL E ARL WL APSTWADYLAGYRWRGQGEGCSAATVHRLEAARRPTLFVKQEVLSAHAELPAEIARLRWL | 70  71 |
| Query 71  Sbjct 72 | | ATTGVPCAAVLDVVTEAGRDWLLLGEVPGQDLLS SHLAPAEKVSIMADAMRRLHTL  G+ C VL+ GR WLL+ VPG L + L P V ++A A+RRLH L HGAGIDCPQVLNETQSDGRQWLLMSAVPGDTLSALAQRGELEPERLVRLVAAALRRLHDL | 126  131 |
| Query 127  Sbjct 132 | | DPATCPFDHQAKHRIERARTRMEAGLVDQDDLDEEHQGLAPAELFARLKARMPDGEDLVV DPA CP DH+ + R++ R R+EAGLVD+ D D++H+G + EL+ L R P EDLVV DPAACPLDHRLERRLDTVRQRVEAGLVDEADFDDDHRGRSATELYRLLLDRRPAVEDLVV | 186  191 |
| Query 187  Sbjct 192 | | THGDACLPNIMVENGRFSGFIDCGRLGVADRYQDIALATRDIAEELGGEWADRFLVLYGI HGDACLPN++ E RFSGFIDCGRLGVADR+QD+ALA RDI ELG WA+ FLV YG AHGDACLPNLLAEGRRFSGFIDCGRLGVADRHQDLALAARDIEAELGAAWAEAFLVEYG- | 246  250 |
| Query | 247 AAPDSQRIAFYRLLDEFF 264 | | |
|  |  | D +R+A++RLLDEFF |  |
| Sbjct | 251 | GDIDGERLAYFRLLDEFF 268 | |

>WP_128552421.1 APH(3')-II family aminoglycoside O-phosphotransferase [Pseudomonas aeruginosa]

RMK29654.1 aminoglycoside O-phosphotransferase APH(3')-IIb [Pseudomonas aeruginosa]

SQK85795.1 aminoglycoside 3-N-acetyltransferase [Pseudomonas aeruginosa] Length=268

Score = 260 bits (664), Expect = 3e-83, Method: Compositional matrix adjust.

| Identities | = 136/258 (53%), Positives = 168/258 (65%), Gaps = 5/258 (2%) |  |
| --- | --- | --- |
| Query 11 | SPAAWVERLFGYDWAQQTIGCSDAAVFRLSAQGRPVLFVKTDLSGALNELQDEAARLSWL | 70 |
| Sbjct 12 | +P+ W + L GY W Q GCS A V RL A RP LFVK ++ A EL E ARL WL APSTWADYLAGYRWRGQGEGCSAATVHRLEAARRPTLFVKQEVLSAHAELPAEIARLRWL | 71 |
| Query 71 | ATTGVPCAAVLDVVTEAGRDWLLLGEVPGQDLLS SHLAPAEKVSIMADAMRRLHTL | 126 |
| Sbjct 72 | G+ C VL+ GR WLL+ VPG L + L P V ++A A+RRLH L HGAGIDCPQVLNETQSDGRQWLLMSAVPGDTLSALAQRGELEPERLVRLVAAALRRLHDL | 131 |
| Query 127 | DPATCPFDHQAKHRIERARTRMEAGLVDQDDLDEEHQGLAPAELFARLKARMPDGEDLVV | 186 |
| Sbjct 132 | DPA CPFDH+ + R++ R R+EAGLVD+ D D++H+G + EL+ L R P EDLVV  DPAACPFDHRLERRLDTVRQRVEAGLVDEADFDDDHRGRSATELYRLLLDRRPAVEDLVV | 191 |

Query 187 THGDACLPNIMVENGRFSGFIDCGRLGVADRYQDIALATRDIAEELGGEWADRFLVLYGI 246

| Sbjct | 192 | HGDACLPN++ E RFSGFIDCGRLGVADR+QD+ALA RDI ELG WA+ LV YG AHGDACLPNLLAEGRRFSGFIDCGRLGVADRHQDLALAARDIEAELGAAWAEALLVEYG- 250 |
| --- | --- | --- |
| Query | 247 | AAPDSQRIAFYRLLDEFF 264 |
| Sbjct | 251 | D +R+A++RLLDEFF  GDIDGERLAYFRLLDEFF 268 |

>WP_171428883.1 APH(3')-II family aminoglycoside O-phosphotransferase [Pseudomonas aeruginosa]

Length=268

Score = 260 bits (664), Expect = 3e-83, Method: Compositional matrix adjust.

| Identities | | = 137/258 (53%), Positives = 168/258 (65%), Gaps = 5/258 (2%) |  |
| --- | --- | --- | --- |
| Query 11  Sbjct 12 | | SPAAWVERLFGYDWAQQTIGCSDAAVFRLSAQGRPVLFVKTDLSGALNELQDEAARLSWL  +PA W + L GY W Q GCS A V RL A RP LFVK ++ A EL E ARL WL APATWADYLAGYRWRGQGEGCSAATVHRLEAARRPTLFVKQEVLSAHAELPAEIARLRWL | 70  71 |
| Query 71  Sbjct 72 | | ATTGVPCAAVLDVVTEAGRDWLLLGEVPGQDLLS SHLAPAEKVSIMADAMRRLHTL  G+ C V + GR WLL+ VPG L + L P V ++A A+RRLH L HGAGIDCPQVPNETQSDGRQWLLMSAVPGDTLSALAQRGELEPERLVRLVAAALRRLHDL | 126  131 |
| Query 127  Sbjct 132 | | DPATCPFDHQAKHRIERARTRMEAGLVDQDDLDEEHQGLAPAELFARLKARMPDGEDLVV DPA CPFDH+ + R++ R R+EAGLVD+ D D++H+G + EL+ L R P EDLVV DPAACPFDHRLERRLDTVRQRVEAGLVDEADFDDDHRGRSATELYRLLLDRRPAVEDLVV | 186  191 |
| Query 187  Sbjct 192 | | THGDACLPNIMVENGRFSGFIDCGRLGVADRYQDIALATRDIAEELGGEWADRFLVLYGI HGDACLPN++ E RFSGFIDCGRLGVADR+QD+ALA RDI ELG WA+ FLV YG AHGDACLPNLLAEGRRFSGFIDCGRLGVADRHQDLALAARDIEAELGAAWAEAFLVEYG- | 246  250 |
| Query | 247 AAPDSQRIAFYRLLDEFF 264 | | |
|  |  | D +R+A++RLLDEFF |  |
| Sbjct | 251 | GDIDGERLAYFRLLDEFF 268 | |

>WP_056577984.1 MULTISPECIES: aminoglycoside 3'-phosphotransferase [Mesorhizobium] KRB19154.1 aminoglycoside phosphotransferase [Mesorhizobium sp. Root172]

OBQ70906.1 APH(3') family aminoglycoside O-phosphotransferase [Mesorhizobium loti]

QKC69710.1 aminoglycoside 3'-phosphotransferase [Mesorhizobium loti] Length=262

Score = 260 bits (664), Expect = 3e-83, Method: Compositional matrix adjust.

| Identities | = 138/248 (56%), Positives = 173/248 (70%), Gaps = 0/248 (0%) |  |
| --- | --- | --- |
| Query 17 | ERLFGYDWAQQTIGCSDAAVFRLSAQGRPVLFVKTDLSGALNELQDEAARLSWLATTGVP | 76 |
| Sbjct 15 | RL GY W +QTIG S A VFRL A +P LF+K + +G EL DEAARL WLA G+ NRLSGYRWHRQTIGRSQAGVFRLVADDKPALFLKCERNGPFAELADEAARLRWLAGQGIA | 74 |
| Query 77 | CAAVLDVVTEAGRDWLLLGEVPGQDLLSSHLAPAEKVSIMADAMRRLHTLDPATCPFDHQ | 136 |
| Sbjct 75 | C V+ + + AG DWLL+ V G+DL S+ + PA+ + IMA A+R LH LD +CPFDH+ CPDVIAMESHAGHDWLLMSAVAGEDLASAPIDPADVIEIMASALRDLHALDIGSCPFDHR | 134 |
| Query 137 | AKHRIERARTRMEAGLVDQDDLDEEHQGLAPAELFARLKARMPDGEDLVVTHGDACLPNI | 196 |
| Sbjct 135 | RI AR+RMEAG+VD+DD D+E QG +PA+ FA L+A P EDLVVTHGDACLPN+ LFRRIAAARSRMEAGVVDEDDFDDERQGWSPAKAFAELEALRPASEDLVVTHGDACLPNV | 194 |
| Query 197 | MVENGRFSGFIDCGRLGVADRYQDIALATRDIAEELGGEWADRFLVLYGIAAPDSQRIAF | 256 |
| Sbjct 195 | M GRFSGFIDCGRLGVADR+QD+ALA I +G W + FL YG + ++++ MAARGRFSGFIDCGRLGVADRHQDLALACWSIRYNIGETWIEPFLERYGPPQAEPAKLSW | 254 |
| Query 257  Sbjct 255 | YRLLDEFF 264 YRLLDEFF YRLLDEFF 262 |  |

>WP_196704556.1 APH(3')-II family aminoglycoside O-phosphotransferase [Pseudomonas aeruginosa]

MBG4752424.1 APH(3')-II family aminoglycoside O-phosphotransferase [Pseudomonas

aeruginosa] Length=268

Score = 260 bits (664), Expect = 3e-83, Method: Compositional matrix adjust.

| Identities | | = 136/258 (53%), Positives = 168/258 (65%), Gaps = 5/258 (2%) |  |
| --- | --- | --- | --- |
| Query 11  Sbjct 12 | | SPAAWVERLFGYDWAQQTIGCSDAAVFRLSAQGRPVLFVKTDLSGALNELQDEAARLSWL  +P+ W + L GY W Q GCS A V RL RP LFVK ++ A EL E ARL WL APSTWADYLAGYRWRGQGEGCSAATVHRLEVARRPTLFVKQEVLSAHAELPAEIARLRWL | 70  71 |
| Query 71  Sbjct 72 | | ATTGVPCAAVLDVVTEAGRDWLLLGEVPGQDLLS SHLAPAEKVSIMADAMRRLHTL  G+ C VL+ GR WLL+ VPG L + L P V ++A A+RRLH L HGAGIDCPQVLNETQSDGRQWLLMSAVPGDTLSALAQRGELEPERLVRLVAAALRRLHDL | 126  131 |
| Query 127  Sbjct 132 | | DPATCPFDHQAKHRIERARTRMEAGLVDQDDLDEEHQGLAPAELFARLKARMPDGEDLVV DPA CPFDH+ + R++ R R+EAGLVD+ D D++H+G + EL+ L R P EDLVV DPAACPFDHRLERRLDTERQRVEAGLVDEADFDDDHRGRSATELYRLLLDRRPAVEDLVV | 186  191 |
| Query 187  Sbjct 192 | | THGDACLPNIMVENGRFSGFIDCGRLGVADRYQDIALATRDIAEELGGEWADRFLVLYGI HGDACLPN++ E RFSGFIDCGRLGVADR+QD+ALA RDI ELG WA+ FLV YG AHGDACLPNLLAEGRRFSGFIDCGRLGVADRHQDLALAARDIEAELGAAWAEAFLVEYG- | 246  250 |
| Query | 247 AAPDSQRIAFYRLLDEFF 264 | | |
|  |  | D +R+A++RLLDEFF |  |
| Sbjct | 251 | GDIDGERLAYFRLLDEFF 268 | |

>MBG6633171.1 APH(3')-II family aminoglycoside O-phosphotransferase [Pseudomonas aeruginosa]

Length=268

Score = 260 bits (664), Expect = 3e-83, Method: Compositional matrix adjust.

| Identities | | = 137/258 (53%), Positives = 168/258 (65%), Gaps = 5/258 (2%) |  |
| --- | --- | --- | --- |
| Query 11  Sbjct 12 | | SPAAWVERLFGYDWAQQTIGCSDAAVFRLSAQGRPVLFVKTDLSGALNELQDEAARLSWL  +PA W + L GY W Q GCS A V RL A RP LFVK ++ A EL E ARL WL APATWADYLAGYRWRGQGEGCSAATVHRLEAARRPTLFVKQEVLSAHAELPAEIARLRWL | 70  71 |
| Query 71  Sbjct 72 | | ATTGVPCAAVLDVVTEAGRDWLLLGEVPGQDLLS SHLAPAEKVSIMADAMRRLHTL  G+ C VL+ GR WLL+ VPG L + L P V ++A A+RRLH L HGAGIDCPQVLNETPSDGRQWLLMSAVPGDTLSALAQRGELEPERLVRLVAAALRRLHDL | 126  131 |
| Query 127  Sbjct 132 | | DPATCPFDHQAKHRIERARTRMEAGLVDQDDLDEEHQGLAPAELFARLKARMPDGEDLVV DPA CPFDH+ + R++ R R+EAGLVD+ D++H+G + EL+ L R P EDLVV DPAACPFDHRLERRLDTVRQRVEAGLVDEAGFDDDHRGRSATELYRLLLDRRPAVEDLVV | 186  191 |
| Query 187  Sbjct 192 | | THGDACLPNIMVENGRFSGFIDCGRLGVADRYQDIALATRDIAEELGGEWADRFLVLYGI HGDACLPN++ E RFSGFIDCGRLGVADR+QD+ALA RDI ELG WA+ FLV YG AHGDACLPNLLAEGRRFSGFIDCGRLGVADRHQDLALAARDIEAELGAAWAEAFLVEYG- | 246  250 |
| Query | 247 AAPDSQRIAFYRLLDEFF 264 | | |
|  |  | D +R+A++RLLDEFF |  |
| Sbjct | 251 | GDIDGERLAYFRLLDEFF 268 | |

>WP_060627542.1 APH(3')-II family aminoglycoside O-phosphotransferase [Pseudomonas aeruginosa]

ALP56572.1 aminoglycoside phosphotransferase [Pseudomonas aeruginosa]

MBG4826660.1 APH(3')-II family aminoglycoside O-phosphotransferase [Pseudomonas aeruginosa]

MBG6719037.1 APH(3')-II family aminoglycoside O-phosphotransferase [Pseudomonas aeruginosa]

Length=268

Score = 260 bits (664), Expect = 3e-83, Method: Compositional matrix adjust. Identities = 136/258 (53%), Positives = 168/258 (65%), Gaps = 5/258 (2%)

| Query | 11 | SPAAWVERLFGYDWAQQTIGCSDAAVFRLSAQGRPVLFVKTDLSGALNELQDEAARLSWL | 70 |
| --- | --- | --- | --- |
|  |  | +P+ W + L GY W Q GCS A V RL A RP LFVK ++ A EL E ARL WL |  |
| Sbjct | 12 | APSTWADYLAGYRWRGQGEGCSAATVHRLEAARRPTLFVKQEVLSAHAELPAEIARLRWL | 71 |
| Query | 71 | ATTGVPCAAVLDVVTEAGRDWLLLGEVPGQDLLS SHLAPAEKVSIMADAMRRLHTL | 126 |

| Sbjct | 72 | G+ C VL+ GR WLL+ VPG L + L P V ++A A+RRLH L HGAGIDCPQVLNETQSDGRQWLLMSAVPGDTLSALAQRGELEPERLVRLVAAALRRLHDL | 131 |
| --- | --- | --- | --- |
| Query | 127 | DPATCPFDHQAKHRIERARTRMEAGLVDQDDLDEEHQGLAPAELFARLKARMPDGEDLVV | 186 |
| Sbjct | 132 | DPA CPFDH+ + R++ R R+EAGLVD+ D D++H+G + EL+ L R P EDLVV DPAACPFDHRLERRLDTVRQRVEAGLVDEADFDDDHRGRSATELYRLLLDRRPAVEDLVV | 191 |
| Query | 187 | THGDACLPNIMVENGRFSGFIDCGRLGVADRYQDIALATRDIAEELGGEWADRFLVLYGI | 246 |
| Sbjct | 192 | HGDACLPN++ E RFSGFIDCGRLGVADR+QD+ALA RDI ELG WA+ FLV YG  AHGDACLPNLLAEGRRFSGFIDCGRLGVADRHQDLALAARDIEAELGAAWAEAFLVEYG- | 250 |
| Query | 247 AAPDSQRIAFYRLLDEFF 264 | | |
|  |  | D +R+A++R LDEFF |  |
| Sbjct | 251 | GDIDGERLAYFRRLDEFF 268 | |

>WP_196628099.1 APH(3')-II family aminoglycoside O-phosphotransferase [Pseudomonas aeruginosa]

MBG4887013.1 APH(3')-II family aminoglycoside O-phosphotransferase [Pseudomonas aeruginosa]

Length=268

Score = 260 bits (664), Expect = 3e-83, Method: Compositional matrix adjust.

| Identities | | = 136/258 (53%), Positives = 168/258 (65%), Gaps = 5/258 (2%) |  |
| --- | --- | --- | --- |
| Query 11  Sbjct 12 | | SPAAWVERLFGYDWAQQTIGCSDAAVFRLSAQGRPVLFVKTDLSGALNELQDEAARLSWL  +P+ W + L GY W Q GCS A V RL RP LFVK ++ A EL E ARL WL APSTWADYLAGYRWRGQGEGCSAATVHRLEVARRPTLFVKQEVLSAHAELPAEIARLRWL | 70  71 |
| Query 71  Sbjct 72 | | ATTGVPCAAVLDVVTEAGRDWLLLGEVPGQDLLS SHLAPAEKVSIMADAMRRLHTL  G+ C VL+ GR WLL+ VPG L + L P V ++A A+RRLH L HGAGIDCPQVLNETQSDGRQWLLMSAVPGDTLSALAQRGELEPERLVRLVAAALRRLHDL | 126  131 |
| Query 127  Sbjct 132 | | DPATCPFDHQAKHRIERARTRMEAGLVDQDDLDEEHQGLAPAELFARLKARMPDGEDLVV DPA CPFDH+ + R++ R R+EAGLVD+ D D++H+G + EL+ L R P EDLVV DPAACPFDHRLERRLDTVRQRVEAGLVDEADFDDDHRGRSAPELYRLLLDRRPAVEDLVV | 186  191 |
| Query 187  Sbjct 192 | | THGDACLPNIMVENGRFSGFIDCGRLGVADRYQDIALATRDIAEELGGEWADRFLVLYGI HGDACLPN++ E RFSGFIDCGRLGVADR+QD+ALA RDI ELG WA+ FLV YG AHGDACLPNLLAEGRRFSGFIDCGRLGVADRHQDLALAARDIEAELGAAWAEAFLVEYG- | 246  250 |
| Query | 247 AAPDSQRIAFYRLLDEFF 264 | | |
|  |  | D +R+A++RLLDEFF |  |
| Sbjct | 251 | GDIDGERLAYFRLLDEFF 268 | |

>MBG7159259.1 APH(3')-II family aminoglycoside O-phosphotransferase [Pseudomonas aeruginosa]

Length=268

Score = 260 bits (664), Expect = 3e-83, Method: Compositional matrix adjust.

| Identities | | = 136/258 (53%), Positives = 168/258 (65%), Gaps = 5/258 (2%) |  |
| --- | --- | --- | --- |
| Query 11  Sbjct 12 | | SPAAWVERLFGYDWAQQTIGCSDAAVFRLSAQGRPVLFVKTDLSGALNELQDEAARLSWL  +P+ W + L GY W Q GCS A V RL A RP LFVK ++ A EL E ARL WL APSTWADYLAGYRWRGQGEGCSAATVHRLEAARRPTLFVKQEVLSAHAELPAEIARLRWL | 70  71 |
| Query 71  Sbjct 72 | | ATTGVPCAAVLDVVTEAGRDWLLLGEVPGQDLLS SHLAPAEKVSIMADAMRRLHTL  G+ C VL+ GR WLL+ VPG L + L P V ++A A+RRLH L HGAGIDCPQVLNETQSDGRQWLLMSAVPGDTLSALAQRGELEPERLVRLVAAALRRLHDL | 126  131 |
| Query 127  Sbjct 132 | | DPATCPFDHQAKHRIERARTRMEAGLVDQDDLDEEHQGLAPAELFARLKARMPDGEDLVV DPA CPFDH+ + R++ R R+EAG VD+ D D++H+G + EL+ L R P EDLVV DPAACPFDHRLERRLDTVRQRVEAGRVDEADFDDDHRGRSATELYRLLLDRRPAVEDLVV | 186  191 |
| Query 187  Sbjct 192 | | THGDACLPNIMVENGRFSGFIDCGRLGVADRYQDIALATRDIAEELGGEWADRFLVLYGI HGDACLPN++ E RFSGFIDCGRLGVADR+QD+ALA RDI ELG WA+ FLV YG AHGDACLPNLLAEGRRFSGFIDCGRLGVADRHQDLALAARDIEAELGAAWAEAFLVEYG- | 246  250 |
| Query | 247 AAPDSQRIAFYRLLDEFF 264 | | |
|  |  | D +R+A++RLLDEFF |  |
| Sbjct | 251 | GDIDGERLAYFRLLDEFF 268 | |

>MBG7397283.1 APH(3')-II family aminoglycoside O-phosphotransferase [Pseudomonas aeruginosa]

Length=268

Score = 260 bits (664), Expect = 3e-83, Method: Compositional matrix adjust.

| Identities | | = 136/258 (53%), Positives = 168/258 (65%), Gaps = 5/258 (2%) |  |
| --- | --- | --- | --- |
| Query 11  Sbjct 12 | | SPAAWVERLFGYDWAQQTIGCSDAAVFRLSAQGRPVLFVKTDLSGALNELQDEAARLSWL  +P+ W + L GY W Q GCS A V RL A RP LFVK ++ A EL E ARL WL APSTWADYLAGYRWRGQGEGCSAATVHRLEAARRPTLFVKQEVLSAHAELPAEIARLRWL | 70  71 |
| Query 71  Sbjct 72 | | ATTGVPCAAVLDVVTEAGRDWLLLGEVPGQDLLS SHLAPAEKVSIMADAMRRLHTL  G+ C VL+ GR WLL+ VPG L + L P V ++A A+RRLH L HGAGIDCPQVLNETQSDGRQWLLMSAVPGDTLSALAQRGELEPERLVRLVAAALRRLHDL | 126  131 |
| Query 127  Sbjct 132 | | DPATCPFDHQAKHRIERARTRMEAGLVDQDDLDEEHQGLAPAELFARLKARMPDGEDLVV DPA CPFDH+ + R++ R R+EAGLVD+ D D++H+G + EL+ L R P EDLVV DPAACPFDHRLERRLDTVRQRVEAGLVDEADFDDDHRGRSATELYRLLLDRRPAVEDLVV | 186  191 |
| Query 187  Sbjct 192 | | THGDACLPNIMVENGRFSGFIDCGRLGVADRYQDIALATRDIAEELGGEWADRFLVLYGI HGDACLPN++ E RFSGFIDCGRLGVADR+QD+A A RDI ELG WA+ FLV YG AHGDACLPNLLAEGRRFSGFIDCGRLGVADRHQDLARAARDIEAELGAAWAEAFLVEYG- | 246  250 |
| Query | 247 AAPDSQRIAFYRLLDEFF 264 | | |
|  |  | D +R+A++RLLDEFF |  |
| Sbjct | 251 | GDIDGERLAYFRLLDEFF 268 | |

>WP_049950167.1 APH(3')-II family aminoglycoside O-phosphotransferase [Pseudomonas aeruginosa]

EYT97873.1 Aminoglycoside 3'-phosphotransferase type IIb [Pseudomonas aeruginosa PA99]

KSN10151.1 aminoglycoside 3'-phosphotransferase [Pseudomonas aeruginosa]

MBG5564234.1 APH(3')-II family aminoglycoside O-phosphotransferase [Pseudomonas aeruginosa]

NQB68843.1 APH(3')-II family aminoglycoside O-phosphotransferase [Pseudomonas aeruginosa]

OKQ98069.1 aminoglycoside O-phosphotransferase APH(3')-IIb [Pseudomonas aeruginosa]

Length=268

Score = 260 bits (664), Expect = 3e-83, Method: Compositional matrix adjust.

| Identities | | = 137/256 (54%), Positives = 167/256 (65%), Gaps = 5/256 (2%) |  |
| --- | --- | --- | --- |
| Query 13  Sbjct 14 | | AAWVERLFGYDWAQQTIGCSDAAVFRLSAQGRPVLFVKTDLSGALNELQDEAARLSWLAT A W + L GY W Q GCS A V RL A RP LFVK ++ A EL E ARL WL ATWADYLAGYRWRGQGEGCSAATVHRLEAARRPTLFVKQEVLSAHAELPAEIARLRWLHG | 72  73 |
| Query 73  Sbjct 74 | | TGVPCAAVLDVVTEAGRDWLLLGEVPGQDLLS SHLAPAEKVSIMADAMRRLHTLDP  G+ C VL+ GR WLL+ VPG L + L P V ++A A+RRLH LDP AGIDCPQVLNETQSDGRQWLLMSAVPGDTLSALAQRGELEPERLVRLVAAALRRLHDLDP | 128  133 |
| Query 129  Sbjct 134 | | ATCPFDHQAKHRIERARTRMEAGLVDQDDLDEEHQGLAPAELFARLKARMPDGEDLVVTH A CPFDH+ + R++ R R+EAGLVD+ D D++H+G + EL+ L R P EDLVV H AACPFDHRLERRLDTVRQRVEAGLVDEADFDDDHRGRSATELYRLLLDRRPAVEDLVVAH | 188  193 |
| Query 189  Sbjct 194 | | GDACLPNIMVENGRFSGFIDCGRLGVADRYQDIALATRDIAEELGGEWADRFLVLYGIAA GDACLPN++ E RFSGFIDCGRLGVADR+QD+ALA RDI ELG WA+ FLV YG GDACLPNLLAEGRRFSGFIDCGRLGVADRHQDLALAARDIEAELGAAWAEAFLVEYG-GD | 248  252 |
| Query 249 | | PDSQRIAFYRLLDEFF 264 D +R+A++RLLDEFF |  |
| Sbjct | 253 | IDGERLAYFRLLDEFF 268 | |

>WP_181261834.1 APH(3')-II family aminoglycoside O-phosphotransferase [Pseudomonas aeruginosa]

Length=264

Score = 259 bits (663), Expect = 3e-83, Method: Compositional matrix adjust.

| Identities | = 135/255 (53%), Positives = 169/255 (66%), Gaps = 2/255 (1%) |  |
| --- | --- | --- |
| Query 11 | SPAAWVERLFGYDWAQQTIGCSDAAVFRLSAQGRPVLFVKTDLSGALNELQDEAARLSWL | 70 |
| Sbjct 11 | +P+ W + L GY W Q GCS A V RL A+ RP LFVK ++ A EL E ARL WL APSTWADYLAGYRWRGQGEGCSAATVHRLEAERRPTLFVKQEVLSAHAELPAEIARLRWL | 70 |
| Query 71 | ATTGVPCAAVLDVVTEAGRDWLLLGEVPGQDLLS-SHLAPAEKVSIMADAMRRLHTLDPA | 129 |
| Sbjct 71 | G+ C VL+ GR WLL+ VPG L + + E ++A A+RRLH LDPA HGAGIDCPQVLNETQSDGRQWLLMSAVPGDTLSALAQRGELEPERLVAAALRRLHDLDPA | 130 |
| Query 130 | TCPFDHQAKHRIERARTRMEAGLVDQDDLDEEHQGLAPAELFARLKARMPDGEDLVVTHG | 189 |
| Sbjct 131 | CPFDH+ + R++ R R+EAGLVD+ D D++H+G + EL+ L R P EDLVV HG ACPFDHRLERRLDTVRQRVEAGLVDEADFDDDHRGRSATELYRLLLDRRPAVEDLVVAHG | 190 |
| Query 190 | DACLPNIMVENGRFSGFIDCGRLGVADRYQDIALATRDIAEELGGEWADRFLVLYGIAAP | 249 |
| Sbjct 191 | DACLPN++ E RFSGFIDCGRLGVADR+QD+ALA RDI ELG WA+ FLV YG DACLPNLLAEGRRFSGFIDCGRLGVADRHQDLALAARDIEAELGAAWAEAFLVEYG-GDI | 249 |
| Query 250  Sbjct 250 | DSQRIAFYRLLDEFF 264 D +R+A++RLLDEFF DGERLAYFRLLDEFF 264 |  |

>WP_114230448.1 APH(3')-II family aminoglycoside O-phosphotransferase [Pseudomonas aeruginosa]

RCM50244.1 Aminoglycoside 3'-phosphotransferase [Pseudomonas aeruginosa] Length=268

Score = 260 bits (664), Expect = 4e-83, Method: Compositional matrix adjust.

| Identities | | = 136/258 (53%), Positives = 169/258 (66%), Gaps = 5/258 (2%) |  |
| --- | --- | --- | --- |
| Query 11  Sbjct 12 | | SPAAWVERLFGYDWAQQTIGCSDAAVFRLSAQGRPVLFVKTDLSGALNELQDEAARLSWL  +P+ W + L GY W Q GCS A V RL A+ RP LFVK ++ A EL E ARL WL APSTWADYLAGYRWRGQGEGCSAATVHRLEAERRPTLFVKQEVLSAHAELPAEIARLRWL | 70  71 |
| Query 71  Sbjct 72 | | ATTGVPCAAVLDVVTEAGRDWLLLGEVPGQDLLS SHLAPAEKVSIMADAMRRLHTL  G+ C VL+ GR WLL+ VPG L + L P V ++A A+RRLH L HGAGIDCPQVLNETQSDGRQWLLMSAVPGDTLSALAQRGELEPERLVRLVAAALRRLHDL | 126  131 |
| Query 127  Sbjct 132 | | DPATCPFDHQAKHRIERARTRMEAGLVDQDDLDEEHQGLAPAELFARLKARMPDGEDLVV DPA CPFDH+ + R++ R R+EAGLVD+ D D++H+G + EL+ L R P EDLVV DPAACPFDHRLERRLDTVRQRVEAGLVDEADFDDDHRGRSATELYRLLLDRRPAVEDLVV | 186  191 |
| Query 187  Sbjct 192 | | THGDACLPNIMVENGRFSGFIDCGRLGVADRYQDIALATRDIAEELGGEWADRFLVLYGI HGDACLPN++ E RFSGFIDCGRLGVADR+QD+ALA RDI ELG WA+ FLV YG AHGDACLPNLLAEGRRFSGFIDCGRLGVADRHQDLALAARDIEAELGAAWAEAFLVEYG- | 246  250 |
| Query | 247 AAPDSQRIAFYRLLDEFF 264 | | |
|  |  | D + +A++RLLDEFF |  |
| Sbjct | 251 | GDIDGEWLAYFRLLDEFF 268 | |

>WP_126560154.1 APH(3')-II family aminoglycoside O-phosphotransferase [Pseudomonas aeruginosa]

RTX49576.1 APH(3')-II family aminoglycoside O-phosphotransferase [Pseudomonas aeruginosa]

Length=268

Score = 259 bits (663), Expect = 4e-83, Method: Compositional matrix adjust.

| Identities | = 136/258 (53%), Positives = 168/258 (65%), Gaps = 5/258 (2%) |  |
| --- | --- | --- |
| Query 11 | SPAAWVERLFGYDWAQQTIGCSDAAVFRLSAQGRPVLFVKTDLSGALNELQDEAARLSWL | 70 |
| Sbjct 12 | +P+ W + L GY W Q GCS A V RL A RP LFVK ++ A EL E ARL WL APSTWADYLAGYRWRGQGEGCSAATVHRLEAARRPTLFVKQEVLSAHAELPAEIARLRWL | 71 |
| Query 71 | ATTGVPCAAVLDVVTEAGRDWLLLGEVPGQDLLS SHLAPAEKVSIMADAMRRLHTL | 126 |
| Sbjct 72 | G+ C VL+ GR WLL+ VPG L + L P V ++A A+RRLH L HGAGIDCPQVLNETQSDGRQWLLMSAVPGDTLSALAQRGELEPERLVRLVAAALRRLHDL | 131 |
| Query 127 | DPATCPFDHQAKHRIERARTRMEAGLVDQDDLDEEHQGLAPAELFARLKARMPDGEDLVV | 186 |
| Sbjct 132 | DPA CPFDH+ + R++ R+EAGLVD+ D D++H+G + EL+ L R P EDLVV  DPAACPFDHRLERRLDTVHQRVEAGLVDEADFDDDHRGRSATELYRLLLDRRPAVEDLVV | 191 |

Query 187 THGDACLPNIMVENGRFSGFIDCGRLGVADRYQDIALATRDIAEELGGEWADRFLVLYGI 246

| Sbjct | 192 | HGDACLPN++ E RFSGFIDCGRLGVADR+QD+ALA RDI ELG WA+ FLV YG AHGDACLPNLLAEGRRFSGFIDCGRLGVADRHQDLALAARDIEAELGAAWAEAFLVEYG- 250 |
| --- | --- | --- |
| Query | 247 | AAPDSQRIAFYRLLDEFF 264 |
| Sbjct | 251 | D +R+A++RLLDEFF  GDIDGERLAYFRLLDEFF 268 |

>WP_061361696.1 APH(3')-II family aminoglycoside O-phosphotransferase [Pseudomonas aeruginosa]

KXE83728.1 aminoglycoside phosphotransferase [Pseudomonas aeruginosa] Length=268

Score = 259 bits (663), Expect = 4e-83, Method: Compositional matrix adjust.

| Identities | | = 136/258 (53%), Positives = 167/258 (65%), Gaps = 5/258 (2%) |  |
| --- | --- | --- | --- |
| Query 11  Sbjct 12 | | SPAAWVERLFGYDWAQQTIGCSDAAVFRLSAQGRPVLFVKTDLSGALNELQDEAARLSWL  +P+ W + L GY W Q GCS A V RL RP LFVK ++ A EL E ARL WL APSTWADYLAGYRWRGQGEGCSAATVHRLEVARRPTLFVKQEVLSAHAELPAEIARLRWL | 70  71 |
| Query 71  Sbjct 72 | | ATTGVPCAAVLDVVTEAGRDWLLLGEVPGQDLLS SHLAPAEKVSIMADAMRRLHTL  G+ C VL+ GR WLL+ VPG L + L P V ++A A+RRLH L HGAGIDCPQVLNETQSDGRQWLLMSAVPGDTLSALAQRGELEPERLVRLVAAALRRLHDL | 126  131 |
| Query 127  Sbjct 132 | | DPATCPFDHQAKHRIERARTRMEAGLVDQDDLDEEHQGLAPAELFARLKARMPDGEDLVV DPA CPFDH+ + R++ R R+EAGLVD+ D D++H+G EL+ L R P EDLVV DPAACPFDHRLERRLDTVRQRVEAGLVDEADFDDDHRGRGATELYRLLLDRRPAVEDLVV | 186  191 |
| Query 187  Sbjct 192 | | THGDACLPNIMVENGRFSGFIDCGRLGVADRYQDIALATRDIAEELGGEWADRFLVLYGI HGDACLPN++ E RFSGFIDCGRLGVADR+QD+ALA RDI ELG WA+ FLV YG AHGDACLPNLLAEGRRFSGFIDCGRLGVADRHQDLALAARDIEAELGAAWAEAFLVEYG- | 246  250 |
| Query | 247 AAPDSQRIAFYRLLDEFF 264 | | |
|  |  | D +R+A++RLLDEFF |  |
| Sbjct | 251 | GDIDGERLAYFRLLDEFF 268 | |

>WP_082572993.1 MULTISPECIES: aminoglycoside 3'-phosphotransferase [Sinorhizobium/Ensifer group]

OWZ92823.1 APH(3') family aminoglycoside O-phosphotransferase [Sinorhizobium sp. LM21]

KDP71322.1 aminoglycoside phosphotransferase [Ensifer adhaerens] MBD9497413.1 aminoglycoside 3'-phosphotransferase [Ensifer sp. ENS01] MBD9521382.1 aminoglycoside 3'-phosphotransferase [Ensifer sp. ENS02] QHG71933.1 aminoglycoside 3'-phosphotransferase [Ensifer adhaerens]

Length=290

Score = 260 bits (665), Expect = 4e-83, Method: Compositional matrix adjust.

| Identities | = 128/244 (52%), Positives = 167/244 (68%), Gaps = 0/244 (0%) |  |
| --- | --- | --- |
| Query 21 | GYDWAQQTIGCSDAAVFRLSAQGRPVLFVKTDLSGALNELQDEAARLSWLATTGVPCAAV | 80 |
| Sbjct 47 | GY W + +G SD+++F L A RP+L +K + +G +E DEAARL WLA+ +PC V GYQWNRDLLGQSDSSIFLLKAPDRPMLVLKLEEAGPFSEFVDEAARLDWLASKDLPCPRV | 106 |
| Query 81 | LDVVTEAGRDWLLLGEVPGQDLLSSHLAPAEKVSIMADAMRRLHTLDPATCPFDHQAKHR | 140 |
| Sbjct 107 | + A +WLLL V G DL S+ L+P +++ I+ADA+++LH LD A CPFDH+ + R IARAFGAETNWLLLQAVQGTDLASTTLSPKDQIVILADALKQLHALDIANCPFDHRIEER | 166 |
| Query 141 | IERARTRMEAGLVDQDDLDEEHQGLAPAELFARLKARMPDGEDLVVTHGDACLPNIMVEN | 200 |
| Sbjct 167 | I A+ R +AG++D+ D DE G A+LFA L+AR+P +LVVTHGDACLPNI+ +N IAIAKARTQAGVIDESDFDETRLGRTAADLFAELQARVPQPGELVVTHGDACLPNIIEKN | 226 |
| Query 201 | GRFSGFIDCGRLGVADRYQDIALATRDIAEELGGEWADRFLVLYGIAAPDSQRIAFYRLL | 260 |
| Sbjct 227 | GRFSGFIDC RLGVAD YQDIALA R I LG EW FL YG+ DS++ FY LL GRFSGFIDCSRLGVADPYQDIALACRSIVHNLGEEWVQPFLTRYGLDKDDSEKRDFYCLL | 286 |
| Query 261  Sbjct 287 | DEFF 264 DEFF  DEFF 290 |  |

>WP_018429030.1 aminoglycoside 3'-phosphotransferase [Hoeflea sp. 108] Length=264

Score = 259 bits (663), Expect = 4e-83, Method: Compositional matrix adjust.

| Identities | | = 132/253 (52%), Positives = 172/253 (68%), Gaps = 0/253 (0%) |  |
| --- | --- | --- | --- |
| Query 12  Sbjct 12 | | PAAWVERLFGYDWAQQTIGCSDAAVFRLSAQGRPVLFVKTDLSGALNELQDEAARLSWLA P + + L GY W++QTIG S+A VFRL A+G+PVLF+KT+ +G EL DE RLSWL PGSLRDALSGYGWSRQTIGASNAGVFRLEAEGQPVLFLKTEEAGPFAELPDEVTRLSWLT | 71  71 |
| Query 72  Sbjct 72 | | TTGVPCAAVLDVVTEAGRDWLLLGEVPGQDLLSSHLAPAEKVSIMADAMRRLHTLDPATC  + G+PC L T G WLL+ +PG DL+S + +++MA A+RRLH LD A+C SQGIPCPEALRFETHGGHHWLLMSALPGGDLVSQEMPAVGAIAVMAGALRRLHALDIASC | 131  131 |
| Query 132  Sbjct 132 | | PFDHQAKHRIERARTRMEAGLVDQDDLDEEHQGLAPAELFARLKARMPDGEDLVVTHGDA PFDH+ +HRI A+ R+EAG VD++D D+E +G FA L A P EDLVVTHGDA PFDHRLEHRIALAQRRVEAGEVDEEDFDDEREGRTAESAFAELLATRPASEDLVVTHGDA | 191  191 |
| Query 192  Sbjct 192 | | CLPNIMVENGRFSGFIDCGRLGVADRYQDIALATRDIAEELGGEWADRFLVLYGIAAPDS C PN + +GRF+GFIDC RLGVADR+QD+ALA I LG W + FL YG+ D+ CQPNYIAADGRFAGFIDCARLGVADRHQDLALACWSITHNLGKAWVEPFLAAYGMPDADA | 251  251 |
| Query 252 | | QRIAFYRLLDEFF 264  ++A+YRLLDEFF |  |
| Sbjct | 252 | GKLAYYRLLDEFF 264 | |

>MBG6944880.1 APH(3')-II family aminoglycoside O-phosphotransferase [Pseudomonas aeruginosa]

Length=268

Score = 259 bits (663), Expect = 4e-83, Method: Compositional matrix adjust.

| Identities | | = 137/258 (53%), Positives = 168/258 (65%), Gaps = 5/258 (2%) |  |
| --- | --- | --- | --- |
| Query 11  Sbjct 12 | | SPAAWVERLFGYDWAQQTIGCSDAAVFRLSAQGRPVLFVKTDLSGALNELQDEAARLSWL  +PA W + L GY W Q G S A V RL A RP LFVK ++ A EL E ARL WL APATWADYLAGYRWRGQGEGSSAATVHRLEAARRPTLFVKQEVLSAHAELPAEIARLRWL | 70  71 |
| Query 71  Sbjct 72 | | ATTGVPCAAVLDVVTEAGRDWLLLGEVPGQDLLS SHLAPAEKVSIMADAMRRLHTL  G+ C VL+ GR WLL+ VPG L + L P V ++A A+RRLH L HGAGIDCPQVLNETQSDGRQWLLMSAVPGDTLSALAQRGELEPERLVRLVAAALRRLHDL | 126  131 |
| Query 127  Sbjct 132 | | DPATCPFDHQAKHRIERARTRMEAGLVDQDDLDEEHQGLAPAELFARLKARMPDGEDLVV DPA CPFDH+ + R++ R R+EAGLVD+ D D++H+G + EL+ L R P EDLVV DPAACPFDHRLERRLDTVRQRVEAGLVDEADFDDDHRGRSATELYRLLLDRRPAVEDLVV | 186  191 |
| Query 187  Sbjct 192 | | THGDACLPNIMVENGRFSGFIDCGRLGVADRYQDIALATRDIAEELGGEWADRFLVLYGI HGDACLPN++ E RFSGFIDCGRLGVADR+QD+ALA RDI ELG WA+ FLV YG AHGDACLPNLLAEGRRFSGFIDCGRLGVADRHQDLALAARDIEAELGAAWAEAFLVEYG- | 246  250 |
| Query | 247 AAPDSQRIAFYRLLDEFF 264 | | |
|  |  | D +R+A++RLLDEFF |  |
| Sbjct | 251 | GDIDGERLAYFRLLDEFF 268 | |

>MBG6453832.1 APH(3')-II family aminoglycoside O-phosphotransferase [Pseudomonas aeruginosa]

Length=268

Score = 259 bits (663), Expect = 4e-83, Method: Compositional matrix adjust. Identities = 137/258 (53%), Positives = 167/258 (65%), Gaps = 5/258 (2%)

| Query | 11 | SPAAWVERLFGYDWAQQTIGCSDAAVFRLSAQGRPVLFVKTDLSGALNELQDEAARLSWL | 70 |
| --- | --- | --- | --- |
| Sbjct | 12 | +P+ W + L GY W Q GCS A V RL A RP LFVK ++ A EL E ARL WL APSTWADYLAGYRWRGQGEGCSAATVHRLEAARRPTLFVKQEVLSAHAELPAEIARLRWL | 71 |
| Query | 71 | ATTGVPCAAVLDVVTEAGRDWLLLGEVPGQDLLS SHLAPAEKVSIMADAMRRLHTL | 126 |
| Sbjct | 72 | G+ C VL+ GR WLL+ VPG L + L P V ++A A+RRLH L HGAGIDCPQVLNETQSDGRQWLLMSAVPGDTLSALAQRGELEPERLVRLVAAALRRLHDL | 131 |
| Query | 127 | DPATCPFDHQAKHRIERARTRMEAGLVDQDDLDEEHQGLAPAELFARLKARMPDGEDLVV | 186 |

| Sbjct | 132 | DPA CPFDH+ + R++ R R+EAGLVD+ D D+ H+G + EL+ L R P EDLVV DPAACPFDHRLERRLDTVRQRVEAGLVDEADFDDAHRGRSATELYRLLLDRRPAVEDLVV | 191 |
| --- | --- | --- | --- |
| Query | 187 | THGDACLPNIMVENGRFSGFIDCGRLGVADRYQDIALATRDIAEELGGEWADRFLVLYGI | 246 |
| Sbjct | 192 | HGDACLPN++ E RFSGFIDCGRLGVADR+QD ALA RDI ELG WA+ FLV YG  AHGDACLPNLLAEGRRFSGFIDCGRLGVADRHQDRALAARDIEAELGAAWAEAFLVEYG- | 250 |
| Query | 247 AAPDSQRIAFYRLLDEFF 264 | | |
|  |  | D +R+A++RLLDEFF |  |
| Sbjct | 251 | GDIDGERLAYFRLLDEFF 268 | |

>WP_108084245.1 APH(3')-II family aminoglycoside O-phosphotransferase [Pseudomonas aeruginosa]

PTZ95412.1 APH(3')-II family aminoglycoside O-phosphotransferase [Pseudomonas aeruginosa]

RQG24351.1 APH(3') family aminoglycoside O-phosphotransferase [Pseudomonas aeruginosa]

RUB32264.1 APH(3')-II family aminoglycoside O-phosphotransferase [Pseudomonas aeruginosa]

Length=268

Score = 259 bits (663), Expect = 4e-83, Method: Compositional matrix adjust.

| Identities | | = 137/258 (53%), Positives = 168/258 (65%), Gaps = 5/258 (2%) |  |
| --- | --- | --- | --- |
| Query 11  Sbjct 12 | | SPAAWVERLFGYDWAQQTIGCSDAAVFRLSAQGRPVLFVKTDLSGALNELQDEAARLSWL  +PA W + L GY W Q GCS A V RL A RP LFVK ++ A EL E ARL WL APATWADYLAGYRWRGQGEGCSAATVHRLEAARRPTLFVKQEVLSAHAELPAEIARLRWL | 70  71 |
| Query 71  Sbjct 72 | | ATTGVPCAAVLDVVTEAGRDWLLLGEVPGQDLLS SHLAPAEKVSIMADAMRRLHTL  G+ C VL+ GR WLL+ VPG L + L P V ++A A+RRLH L HGAGIDCPQVLNETQSDGRQWLLMSAVPGDTLSALAQRGELEPERLVRLVAAALRRLHDL | 126  131 |
| Query 127  Sbjct 132 | | DPATCPFDHQAKHRIERARTRMEAGLVDQDDLDEEHQGLAPAELFARLKARMPDGEDLVV DPA CPFDH+ + R++ R R+EAGLVD+ D D++H+G + EL+ L R EDLVV DPAACPFDHRLERRLDTVRQRVEAGLVDEADFDDDHRGRSATELYRLLLDRCLAVEDLVV | 186  191 |
| Query 187  Sbjct 192 | | THGDACLPNIMVENGRFSGFIDCGRLGVADRYQDIALATRDIAEELGGEWADRFLVLYGI HGDACLPN++ E RFSGFIDCGRLGVADR+QD+ALA RDI ELG WA+ FLV YG AHGDACLPNLLAEGRRFSGFIDCGRLGVADRHQDLALAARDIEAELGATWAEAFLVEYG- | 246  250 |
| Query | 247 AAPDSQRIAFYRLLDEFF 264 | | |
|  |  | D +R+A++RLLDEFF |  |
| Sbjct | 251 | GDIDGERLAYFRLLDEFF 268 | |

>OKP80448.1 APH(3') family aminoglycoside O-phosphotransferase [Ensifer adhaerens] Length=263

Score = 259 bits (662), Expect = 4e-83, Method: Compositional matrix adjust.

| Identities | = 128/244 (52%), Positives = 167/244 (68%), Gaps = 0/244 (0%) |  |
| --- | --- | --- |
| Query 21 | GYDWAQQTIGCSDAAVFRLSAQGRPVLFVKTDLSGALNELQDEAARLSWLATTGVPCAAV | 80 |
| Sbjct 20 | GY W + +G S++++F L A RP+L +K + +G +E DEAARL WLA+ VPC V GYQWNRDLLGQSNSSIFLLKAPDRPMLVLKLEEAGPFSEFVDEAARLDWLASKDVPCPRV | 79 |
| Query 81 | LDVVTEAGRDWLLLGEVPGQDLLSSHLAPAEKVSIMADAMRRLHTLDPATCPFDHQAKHR | 140 |
| Sbjct 80 | + A +WLLL V G DL S+ L+P +++ I+ADA+++LH LD A CPFDH+ + R IARAFGAETNWLLLQAVQGTDLASTTLSPKDQIVILADALKQLHALDIANCPFDHRIEVR | 139 |
| Query 141 | IERARTRMEAGLVDQDDLDEEHQGLAPAELFARLKARMPDGEDLVVTHGDACLPNIMVEN | 200 |
| Sbjct 140 | I A+ R +AG++D+ D DE G A+LFA L+AR+P +LVVTHGDACLPNI+ +N IAIAKARTQAGVIDESDFDETRLGRTAADLFAELQARVPQPGELVVTHGDACLPNIIEKN | 199 |
| Query 201 | GRFSGFIDCGRLGVADRYQDIALATRDIAEELGGEWADRFLVLYGIAAPDSQRIAFYRLL | 260 |
| Sbjct 200 | GRFSGFIDC RLGVAD YQDIALA R I LG EW FL YG+ DS++ FY LL GRFSGFIDCSRLGVADPYQDIALACRSIVHNLGEEWVQPFLTRYGLDKDDSEKRDFYCLL | 259 |
| Query 261  Sbjct 260 | DEFF 264 DEFF  DEFF 263 |  |

>MBG7096971.1 APH(3')-II family aminoglycoside O-phosphotransferase [Pseudomonas aeruginosa]

Length=268

Score = 259 bits (663), Expect = 4e-83, Method: Compositional matrix adjust.

| Identities | | = 136/258 (53%), Positives = 167/258 (65%), Gaps = 5/258 (2%) |  |
| --- | --- | --- | --- |
| Query 11  Sbjct 12 | | SPAAWVERLFGYDWAQQTIGCSDAAVFRLSAQGRPVLFVKTDLSGALNELQDEAARLSWL  +P+ W + L GY W Q GCS A V RL RP LFVK ++ A EL E ARL WL APSTWADYLAGYRWRGQGEGCSAATVHRLEVARRPTLFVKQEVLSAHAELPAEIARLRWL | 70  71 |
| Query 71  Sbjct 72 | | ATTGVPCAAVLDVVTEAGRDWLLLGEVPGQDLLS SHLAPAEKVSIMADAMRRLHTL  G+ C VL+ GR WLL+ VPG L + L P V ++A A+RRLH L HGAGIDCPQVLNETQSDGRQWLLMSAVPGDTLSALAQRGELEPERLVRLVAAALRRLHDL | 126  131 |
| Query 127  Sbjct 132 | | DPATCPFDHQAKHRIERARTRMEAGLVDQDDLDEEHQGLAPAELFARLKARMPDGEDLVV DPA CPFDH+ + R++ R R EAGLVD+ D D++H+G + EL+ L R P EDLVV DPAACPFDHRLERRLDTVRQRGEAGLVDEADFDDDHRGRSATELYRLLLDRRPAVEDLVV | 186  191 |
| Query 187  Sbjct 192 | | THGDACLPNIMVENGRFSGFIDCGRLGVADRYQDIALATRDIAEELGGEWADRFLVLYGI HGDACLPN++ E RFSGFIDCGRLGVADR+QD+ALA RDI ELG WA+ FLV YG AHGDACLPNLLAEGRRFSGFIDCGRLGVADRHQDLALAARDIEAELGAAWAEAFLVEYG- | 246  250 |
| Query | 247 AAPDSQRIAFYRLLDEFF 264 | | |
|  |  | D +R+A++RLLDEFF |  |
| Sbjct | 251 | GDIDGERLAYFRLLDEFF 268 | |

```
>WP_153069973.1 MULTISPECIES: phosphotransferase, partial [Proteobacteria] Length=126

Score = 254 bits (649), Expect = 4e-83, Method: Compositional matrix adjust. Identities = 126/126 (100%), Positives = 126/126 (100%), Gaps = 0/126 (0%)

Query 1	MIEQDGLHAGSPAAWVERLFGYDWAQQTIGCSDAAVFRLSAQGRPVLFVKTDLSGALNEL 60 MIEQDGLHAGSPAAWVERLFGYDWAQQTIGCSDAAVFRLSAQGRPVLFVKTDLSGALNEL
Sbjct 1	MIEQDGLHAGSPAAWVERLFGYDWAQQTIGCSDAAVFRLSAQGRPVLFVKTDLSGALNEL 60

Query 61	QDEAARLSWLATTGVPCAAVLDVVTEAGRDWLLLGEVPGQDLLSSHLAPAEKVSIMADAM 120 QDEAARLSWLATTGVPCAAVLDVVTEAGRDWLLLGEVPGQDLLSSHLAPAEKVSIMADAM
Sbjct 61	QDEAARLSWLATTGVPCAAVLDVVTEAGRDWLLLGEVPGQDLLSSHLAPAEKVSIMADAM 120

Query 121 RRLHTL 126
RRLHTL Sbjct 121 RRLHTL 126

>WP_124086737.1 APH(3')-II family aminoglycoside O-phosphotransferase [Pseudomonas aeruginosa]
RPM89373.1 APH(3') family aminoglycoside O-phosphotransferase [Pseudomonas aeruginosa]
Length=268

Score = 259 bits (663), Expect = 5e-83, Method: Compositional matrix adjust.
```

| Identities | = 136/258 (53%), Positives = 168/258 (65%), Gaps = 5/258 (2%) |  |
| --- | --- | --- |
| Query 11 | SPAAWVERLFGYDWAQQTIGCSDAAVFRLSAQGRPVLFVKTDLSGALNELQDEAARLSWL | 70 |
| Sbjct 12 | +P+ W + L GY W Q GCS A V RL A P LFVK ++ A EL E ARL WL APSTWADYLAGYRWRGQGEGCSAATVHRLEAARXPTLFVKQEVLSAHAELPAEIARLRWL | 71 |
| Query 71 | ATTGVPCAAVLDVVTEAGRDWLLLGEVPGQDLLS SHLAPAEKVSIMADAMRRLHTL | 126 |
| Sbjct 72 | G+ C VL+ GR WLL+ VPG L + L P V ++A A+RRLH L HGAGIDCPQVLNETQSDGRQWLLMSAVPGDTLSALAQRGELEPERLVRLVAAALRRLHDL | 131 |
| Query 127 | DPATCPFDHQAKHRIERARTRMEAGLVDQDDLDEEHQGLAPAELFARLKARMPDGEDLVV | 186 |
| Sbjct 132 | DPA CPFDH+ + R++ R R+EAGLVD+ D D++H+G + EL+ L R P EDLVV DPAACPFDHRLERRLDTVRQRVEAGLVDEADFDDDHRGRSATELYRLLLDRRPAVEDLVV | 191 |
| Query 187 | THGDACLPNIMVENGRFSGFIDCGRLGVADRYQDIALATRDIAEELGGEWADRFLVLYGI | 246 |
| Sbjct 192 | HGDACLPN++ E RFSGFIDCGRLGVADR+QD+ALA RDI ELG WA+ FLV YG  AHGDACLPNLLAEGRRFSGFIDCGRLGVADRHQDLALAARDIEAELGAAWAEAFLVEYG- | 250 |

Query 247 AAPDSQRIAFYRLLDEFF 264

D +R+A++RLLDEFF Sbjct 251 GDIDGERLAYFRLLDEFF 268

>WP_061193432.1 APH(3')-II family aminoglycoside O-phosphotransferase [Pseudomonas aeruginosa]

KXF05758.1 aminoglycoside phosphotransferase [Pseudomonas aeruginosa] Length=268

Score = 259 bits (663), Expect = 5e-83, Method: Compositional matrix adjust.

| Identities | | = 136/258 (53%), Positives = 168/258 (65%), Gaps = 5/258 (2%) |  |
| --- | --- | --- | --- |
| Query 11  Sbjct 12 | | SPAAWVERLFGYDWAQQTIGCSDAAVFRLSAQGRPVLFVKTDLSGALNELQDEAARLSWL  +P+ W + L GY W Q GCS A V RL A RP LFVK ++ A EL E ARL WL APSTWADYLAGYRWRGQGEGCSAATVHRLEAARRPTLFVKQEVLSAHAELPAEIARLRWL | 70  71 |
| Query 71  Sbjct 72 | | ATTGVPCAAVLDVVTEAGRDWLLLGEVPGQDLLS SHLAPAEKVSIMADAMRRLHTL  G+ C VL+ GR WLL+ V G L + L P V ++A A+RRLH L HGAGIDCPQVLNETQSDGRQWLLMSAVSGDTLSALAQRGELEPERLVRLVAAALRRLHDL | 126  131 |
| Query 127  Sbjct 132 | | DPATCPFDHQAKHRIERARTRMEAGLVDQDDLDEEHQGLAPAELFARLKARMPDGEDLVV DPA CPFDH+ + R++ R R+EAGLVD+ D D++H+G + EL+ L R P EDLVV DPAACPFDHRLERRLDTVRQRVEAGLVDEADFDDDHRGRSATELYRLLLDRRPAVEDLVV | 186  191 |
| Query 187  Sbjct 192 | | THGDACLPNIMVENGRFSGFIDCGRLGVADRYQDIALATRDIAEELGGEWADRFLVLYGI HGDACLPN++ E RFSGFIDCGRLGVADR+QD+ALA RDI ELG WA+ FLV YG AHGDACLPNLLAEGRRFSGFIDCGRLGVADRHQDLALAARDIEAELGAAWAEAFLVEYG- | 246  250 |
| Query | 247 AAPDSQRIAFYRLLDEFF 264 | | |
|  |  | D +R+A++RLLDEFF |  |
| Sbjct | 251 | GDIDGERLAYFRLLDEFF 268 | |

>WP_023111352.1 APH(3')-II family aminoglycoside O-phosphotransferase [Pseudomonas aeruginosa]

ERW01809.1 aminoglycoside 3'-phosphotransferase type IIB [Pseudomonas aeruginosa BWHPSA026]

ETU78001.1 aminoglycoside 3'-phosphotransferase type IIB [Pseudomonas aeruginosa PS50]

KRU53793.1 aminoglycoside phosphotransferase [Pseudomonas aeruginosa]

MBD3151485.1 APH(3')-II family aminoglycoside O-phosphotransferase [Pseudomonas aeruginosa]

MUH80159.1 APH(3')-II family aminoglycoside O-phosphotransferase [Pseudomonas aeruginosa]

Length=268

Score = 259 bits (663), Expect = 5e-83, Method: Compositional matrix adjust.

| Identities | | = 136/258 (53%), Positives = 169/258 (66%), Gaps = 5/258 (2%) |  |
| --- | --- | --- | --- |
| Query 11  Sbjct 12 | | SPAAWVERLFGYDWAQQTIGCSDAAVFRLSAQGRPVLFVKTDLSGALNELQDEAARLSWL  +P+ W + L GY W Q GCS A V RL A+ RP LFVK ++ A EL E ARL WL APSTWADYLAGYRWRGQGEGCSAATVHRLEAERRPTLFVKQEVLSAHAELPAEIARLRWL | 70  71 |
| Query 71  Sbjct 72 | | ATTGVPCAAVLDVVTEAGRDWLLLGEVPGQDLLS SHLAPAEKVSIMADAMRRLHTL  G+ C VL+ GR WLL+ V G L + L P V ++A A+RRLH L HGAGIDCPQVLNETQSDGRQWLLMSAVLGDTLSALAQRGELEPERLVRLVAAALRRLHDL | 126  131 |
| Query 127  Sbjct 132 | | DPATCPFDHQAKHRIERARTRMEAGLVDQDDLDEEHQGLAPAELFARLKARMPDGEDLVV DPA CPFDH+ + R++ R R+EAGLVD+ D D++H+G + EL+ L R P EDLVV DPAACPFDHRLERRLDTVRQRVEAGLVDEADFDDDHRGRSATELYRLLLDRRPAVEDLVV | 186  191 |
| Query 187  Sbjct 192 | | THGDACLPNIMVENGRFSGFIDCGRLGVADRYQDIALATRDIAEELGGEWADRFLVLYGI HGDACLPN++ E RFSGFIDCGRLGVADR+QD+ALA RDI ELG WA+ FLV YG AHGDACLPNLLAEGRRFSGFIDCGRLGVADRHQDLALAARDIEAELGAAWAEAFLVEYG- | 246  250 |
| Query | 247 AAPDSQRIAFYRLLDEFF 264 | | |
|  |  | D +R+A++RLLDEFF |  |
| Sbjct | 251 | GDIDGERLAYFRLLDEFF 268 | |

>WP_096220110.1 APH(3')-II family aminoglycoside O-phosphotransferase [Pseudomonas aeruginosa]

PBX22065.1 APH(3') family aminoglycoside O-phosphotransferase [Pseudomonas aeruginosa]

PBX27920.1 APH(3') family aminoglycoside O-phosphotransferase [Pseudomonas aeruginosa]

Length=268

Score = 259 bits (663), Expect = 5e-83, Method: Compositional matrix adjust.

| Identities | | = 136/258 (53%), Positives = 168/258 (65%), Gaps = 5/258 (2%) |  |
| --- | --- | --- | --- |
| Query 11  Sbjct 12 | | SPAAWVERLFGYDWAQQTIGCSDAAVFRLSAQGRPVLFVKTDLSGALNELQDEAARLSWL  +P+ W + L GY W Q GCS A V RL A RP LFVK ++ A EL E ARL WL APSTWADYLAGYRWRGQGEGCSAATVHRLEAARRPTLFVKQEVLSAHAELPAEIARLRWL | 70  71 |
| Query 71  Sbjct 72 | | ATTGVPCAAVLDVVTEAGRDWLLLGEVPGQDLLS SHLAPAEKVSIMADAMRRLHTL  G+ C VL+ GR WLL+ VPG L + L P V ++A A+RRLH L HGAGIDCPQVLNETQSDGRQWLLMSAVPGDTLSALAQRGELEPGRLVRLVAAALRRLHDL | 126  131 |
| Query 127  Sbjct 132 | | DPATCPFDHQAKHRIERARTRMEAGLVDQDDLDEEHQGLAPAELFARLKARMPDGEDLVV DPA CPFDH+ + R++ R R+EAGLVD+ D D++H+G + EL+ L R P EDLVV DPAACPFDHRLERRLDTVRQRVEAGLVDEADFDDDHRGRSATELYRLLLDRRPAVEDLVV | 186  191 |
| Query 187  Sbjct 192 | | THGDACLPNIMVENGRFSGFIDCGRLGVADRYQDIALATRDIAEELGGEWADRFLVLYGI HGDACL N++ E RFSGFIDCGRLGVADR+QD+ALA RDI ELG WA+ FLV YG AHGDACLQNLLAEGRRFSGFIDCGRLGVADRHQDLALAARDIEAELGAAWAEAFLVEYG- | 246  250 |
| Query | 247 AAPDSQRIAFYRLLDEFF 264 | | |
|  |  | D +R+A++RLLDEFF |  |
| Sbjct | 251 | GDIDGERLAYFRLLDEFF 268 | |

>WP_134311622.1 APH(3')-II family aminoglycoside O-phosphotransferase [Pseudomonas aeruginosa]

TEG63048.1 APH(3')-II family aminoglycoside O-phosphotransferase [Pseudomonas aeruginosa]

Length=268

Score = 259 bits (663), Expect = 5e-83, Method: Compositional matrix adjust.

| Identities | | = 136/258 (53%), Positives = 168/258 (65%), Gaps = 5/258 (2%) |  |
| --- | --- | --- | --- |
| Query 11  Sbjct 12 | | SPAAWVERLFGYDWAQQTIGCSDAAVFRLSAQGRPVLFVKTDLSGALNELQDEAARLSWL  +P+ W + L GY W Q GCS A V RL A RP LFVK ++ A EL E ARL WL APSTWADYLAGYRWRGQGEGCSAATVHRLEAARRPTLFVKQEVLSAHAELPAEIARLRWL | 70  71 |
| Query 71  Sbjct 72 | | ATTGVPCAAVLDVVTEAGRDWLLLGEVPGQDLLS SHLAPAEKVSIMADAMRRLHTL  G+ C VL+ GR WLL+ VPG L + L P V ++A A+RRLH L HGAGIDCPQVLNETQSDGRQWLLMSAVPGDTLSALAQRGELEPERLVRLVAAALRRLHDL | 126  131 |
| Query 127  Sbjct 132 | | DPATCPFDHQAKHRIERARTRMEAGLVDQDDLDEEHQGLAPAELFARLKARMPDGEDLVV DPA CPFDH+ + R++ R R+EAGLV + D D++H+G + EL+ L R P EDLVV DPAACPFDHRLERRLDTVRQRVEAGLVXEADFDDDHRGRSATELYRLLLDRRPAVEDLVV | 186  191 |
| Query 187  Sbjct 192 | | THGDACLPNIMVENGRFSGFIDCGRLGVADRYQDIALATRDIAEELGGEWADRFLVLYGI HGDACLPN++ E RFSGFIDCGRLGVADR+QD+ALA RDI ELG WA+ FLV YG AHGDACLPNLLAEGRRFSGFIDCGRLGVADRHQDLALAARDIEAELGAAWAEAFLVEYG- | 246  250 |
| Query | 247 AAPDSQRIAFYRLLDEFF 264 | | |
|  |  | D +R+A++RLLDEFF |  |
| Sbjct | 251 | GDIDGERLAYFRLLDEFF 268 | |

>WP_196747719.1 APH(3')-II family aminoglycoside O-phosphotransferase [Pseudomonas aeruginosa]

MBG5171935.1 APH(3')-II family aminoglycoside O-phosphotransferase [Pseudomonas aeruginosa]

Length=268

Score = 259 bits (662), Expect = 5e-83, Method: Compositional matrix adjust. Identities = 136/258 (53%), Positives = 168/258 (65%), Gaps = 5/258 (2%)

Query 11	SPAAWVERLFGYDWAQQTIGCSDAAVFRLSAQGRPVLFVKTDLSGALNELQDEAARLSWL 70

| Sbjct | 12 | +P+ W + L GY W Q GCS A V RL A RP LFVK ++ A EL E ARL WL APSTWADYLAGYRWRGQGEGCSAATVHRLEAARRPTLFVKQEVLSAHAELPAEIARLRWL | 71 |
| --- | --- | --- | --- |
| Query | 71 | ATTGVPCAAVLDVVTEAGRDWLLLGEVPGQDLLS SHLAPAEKVSIMADAMRRLHTL | 126 |
| Sbjct | 72 | G+ C VL+ GR WLL+ V G L + L P V ++A A+RRLH L HGAGIDCPQVLNETQSDGRQWLLMSAVQGDTLSALAQRGELEPERLVRLVAAALRRLHDL | 131 |
| Query | 127 | DPATCPFDHQAKHRIERARTRMEAGLVDQDDLDEEHQGLAPAELFARLKARMPDGEDLVV | 186 |
| Sbjct | 132 | DPA CPFDH+ + R++ R R+EAGLVD+ D D++H+G + EL+ L R P EDLVV DPAACPFDHRLERRLDTVRQRVEAGLVDEADFDDDHRGRSATELYRLLLDRRPAVEDLVV | 191 |
| Query | 187 | THGDACLPNIMVENGRFSGFIDCGRLGVADRYQDIALATRDIAEELGGEWADRFLVLYGI | 246 |
| Sbjct | 192 | HGDACLPN++ E RFSGFIDCGRLGVADR+QD+ALA RDI ELG WA+ FLV YG  AHGDACLPNLLAEGRRFSGFIDCGRLGVADRHQDLALAARDIEAELGAAWAEAFLVEYG- | 250 |
| Query | 247 AAPDSQRIAFYRLLDEFF 264 | | |
|  |  | D +R+A++RLLDEFF |  |
| Sbjct | 251 | GDIDGERLAYFRLLDEFF 268 | |

>WP_196650023.1 APH(3')-II family aminoglycoside O-phosphotransferase [Pseudomonas aeruginosa]

MBG4469080.1 APH(3')-II family aminoglycoside O-phosphotransferase [Pseudomonas aeruginosa]

Length=268

Score = 259 bits (662), Expect = 5e-83, Method: Compositional matrix adjust.

| Identities | | = 136/258 (53%), Positives = 167/258 (65%), Gaps = 5/258 (2%) |  |
| --- | --- | --- | --- |
| Query 11  Sbjct 12 | | SPAAWVERLFGYDWAQQTIGCSDAAVFRLSAQGRPVLFVKTDLSGALNELQDEAARLSWL  +P+ W + L GY W Q GCS A V RL A RP LFVK ++ EL E ARL WL APSTWADYLAGYRWRGQGEGCSAATVHRLEAARRPTLFVKQEVLSVHAELPAEIARLRWL | 70  71 |
| Query 71  Sbjct 72 | | ATTGVPCAAVLDVVTEAGRDWLLLGEVPGQDLLS SHLAPAEKVSIMADAMRRLHTL  G+ C VL GR WLL+ VPG L + L P V ++A A+RRLH L HGAGIDCPQVLTETQSDGRQWLLMSAVPGDTLSALAQRGELEPERLVRLVAAALRRLHDL | 126  131 |
| Query 127  Sbjct 132 | | DPATCPFDHQAKHRIERARTRMEAGLVDQDDLDEEHQGLAPAELFARLKARMPDGEDLVV DPA CPFDH+ + R++ R R+EAGLVD+ D D++H+G + EL+ L R P EDLVV DPAACPFDHRLERRLDTVRQRVEAGLVDEADFDDDHRGRSATELYRLLLDRRPAVEDLVV | 186  191 |
| Query 187  Sbjct 192 | | THGDACLPNIMVENGRFSGFIDCGRLGVADRYQDIALATRDIAEELGGEWADRFLVLYGI HGDACLPN++ E RFSGFIDCGRLGVADR+QD+ALA RDI ELG WA+ FLV YG AHGDACLPNLLAEGRRFSGFIDCGRLGVADRHQDLALAARDIEAELGAAWAEAFLVEYG- | 246  250 |
| Query | 247 AAPDSQRIAFYRLLDEFF 264 | | |
|  |  | D +R+A++RLLDEFF |  |
| Sbjct | 251 | GDIDGERLAYFRLLDEFF 268 | |

>WP_134384507.1 aminoglycoside 3'-phosphotransferase [Massilia plicata] QBQ36222.1 aminoglycoside 3'-phosphotransferase [Massilia plicata]

GGY76818.1 aminoglycoside O-phosphotransferase APH(3')-IIb [Massilia plicata] Length=264

Score = 259 bits (662), Expect = 5e-83, Method: Compositional matrix adjust.

| Identities | = 146/258 (57%), Positives = 177/258 (69%), Gaps = 7/258 (3%) |  |
| --- | --- | --- |
| Query 12 | PAAWVERLFGYDWAQQTIGCSDAAVFRL--SAQGRPVLFVKTDLSGALNELQDEAARLSW | 69 |
| Sbjct 9 | P W ERL G+ Q+ GCS AAV+RL +A G P F+KT+ + L EL DEAARL W PGPWRERLQGFTIVTQSGGCSAAAVYRLESAAGGAPRFFIKTEPADVLAELADEAARLRW | 68 |
| Query 70 | LATTGVPCAAVLDVVTEAGRDWLLLGEVPGQDLLSSHLAPAEKVSIMADAMRRLHTLDPA | 129 |
| Sbjct 69 | +A G+PC VLD+ T G WLLLG VPG +L + + P + V+++ADA+RRLH LDP VAAAGIPCPPVLDMTTVDGLHWLLLGAVPGANL--TDVPPEQSVAVLADALRRLHVLDPR | 126 |
| Query 130 | TCPFDHQAKHRIERARTRMEAGLVDQDDLDEEHQGLAPAELFARLKARMPDGEDLVVTHG | 189 |
| Sbjct 127 | CPFDH+A RIERA R+ AGLVD+DD D+++ G EL A L AR P EDLVVTHG DCPFDHRASGRIERALARLAAGLVDEDDFDDDNAGRTAGELAALLAARKPRDEDLVVTHG | 186 |
| Query 190 | DACLPNIMVENGRFSGFIDCGRLGVADRYQDIALATRDIAEELGGEWADRFLVLYGIAAP DACLPN++ G F+GFIDCGRLGV DR+QD+ LA RDIAEELG +W FL Y P | 249 |

| Sbjct | 187 | DACLPNVLASGGVFAGFIDCGRLGVGDRHQDLVLAVRDIAEELGEQWIAPFLQRYFAPEP 246 |
| --- | --- | --- |
| Query | 250 | ---DSQRIAFYRLLDEFF 264 |
| Sbjct | 247 | D+ R AFYRLLDEFF  IGFDAGRAAFYRLLDEFF 264 |

>WP_121327273.1 APH(3')-II family aminoglycoside O-phosphotransferase [Pseudomonas aeruginosa]

Length=268

Score = 259 bits (662), Expect = 6e-83, Method: Compositional matrix adjust.

| Identities | | = 136/258 (53%), Positives = 168/258 (65%), Gaps = 5/258 (2%) |  |
| --- | --- | --- | --- |
| Query 11  Sbjct 12 | | SPAAWVERLFGYDWAQQTIGCSDAAVFRLSAQGRPVLFVKTDLSGALNELQDEAARLSWL  +P+ W + L GY W Q GCS A V RL A RP LFVK ++ A EL E ARL WL APSTWADYLAGYRWRGQGEGCSAATVHRLEAARRPTLFVKQEVLSAHAELPAEIARLRWL | 70  71 |
| Query 71  Sbjct 72 | | ATTGVPCAAVLDVVTEAGRDWLLLGEVPGQDLLS SHLAPAEKVSIMADAMRRLHTL  + C VL+ GR WLL+ VPG L + L P V ++A A+RRLH L HGASIDCPQVLNETQSDGRQWLLMSAVPGDTLSALAQRGELEPERLVRLVAAALRRLHDL | 126  131 |
| Query 127  Sbjct 132 | | DPATCPFDHQAKHRIERARTRMEAGLVDQDDLDEEHQGLAPAELFARLKARMPDGEDLVV DPA CPFDH+ + R++ R R+EAGLVD+ D D++H+G + EL+ L R P EDLVV DPAACPFDHRLERRLDIVRQRVEAGLVDEADFDDDHRGRSATELYRLLLDRRPAVEDLVV | 186  191 |
| Query 187  Sbjct 192 | | THGDACLPNIMVENGRFSGFIDCGRLGVADRYQDIALATRDIAEELGGEWADRFLVLYGI HGDACLPN++ E RFSGFIDCGRLGVADR+QD+ALA RDI ELG WA+ FLV YG AHGDACLPNLLAEGRRFSGFIDCGRLGVADRHQDLALAARDIEAELGAAWAEAFLVEYG- | 246  250 |
| Query | 247 AAPDSQRIAFYRLLDEFF 264 | | |
|  |  | D +R+A++RLLDEFF |  |
| Sbjct | 251 | GDIDGERLAYFRLLDEFF 268 | |

>WP_196658176.1 APH(3')-II family aminoglycoside O-phosphotransferase [Pseudomonas aeruginosa]

MBG4616309.1 APH(3')-II family aminoglycoside O-phosphotransferase [Pseudomonas aeruginosa]

Length=268

Score = 259 bits (662), Expect = 6e-83, Method: Compositional matrix adjust.

| Identities | | = 137/258 (53%), Positives = 167/258 (65%), Gaps = 5/258 (2%) |  |
| --- | --- | --- | --- |
| Query 11  Sbjct 12 | | SPAAWVERLFGYDWAQQTIGCSDAAVFRLSAQGRPVLFVKTDLSGALNELQDEAARLSWL  +PA W + L GY W Q GCS A V RL A RP LFVK ++ A EL E ARL WL APATWADYLAGYRWRGQGEGCSAATVHRLEAARRPTLFVKQEVLSAHAELPAEIARLRWL | 70  71 |
| Query 71  Sbjct 72 | | ATTGVPCAAVLDVVTEAGRDWLLLGEVPGQDLLS SHLAPAEKVSIMADAMRRLHTL  G+ C VL+ GR WLL+ VPG L + L P V ++A A+RRLH L HGAGIDCPQVLNETQSDGRQWLLMSAVPGDTLSALAQRGELEPERLVRLVAAALRRLHDL | 126  131 |
| Query 127  Sbjct 132 | | DPATCPFDHQAKHRIERARTRMEAGLVDQDDLDEEHQGLAPAELFARLKARMPDGEDLVV DPA CPFDH+ + R++ R R EAGLVD+ D++H+G + EL+ L R P EDLVV DPAACPFDHRLERRLDTVRQRGEAGLVDEAGFDDDHRGRSATELYRLLLDRRPAVEDLVV | 186  191 |
| Query 187  Sbjct 192 | | THGDACLPNIMVENGRFSGFIDCGRLGVADRYQDIALATRDIAEELGGEWADRFLVLYGI HGDACLPN++ E RFSGFIDCGRLGVADR+QD+ALA RDI ELG WA+ FLV YG AHGDACLPNLLAEGRRFSGFIDCGRLGVADRHQDLALAARDIEAELGAAWAEAFLVEYG- | 246  250 |
| Query | 247 AAPDSQRIAFYRLLDEFF 264 | | |
|  |  | D +R+A++RLLDEFF |  |
| Sbjct | 251 | GDIDGERLAYFRLLDEFF 268 | |

>WP_196710796.1 APH(3')-II family aminoglycoside O-phosphotransferase [Pseudomonas aeruginosa]

MBG4762674.1 APH(3')-II family aminoglycoside O-phosphotransferase [Pseudomonas aeruginosa]

Length=268

Score = 259 bits (662), Expect = 6e-83, Method: Compositional matrix adjust.

| Identities | = 138/259 (53%), Positives = 170/259 (66%), Gaps = 7/259 (3%) |  |
| --- | --- | --- |
| Query 11 | SPAAWVERLFGYDWAQQTIGCSDAAVFRLSAQGRPVLFVKTDLSGALNELQDEAARLSWL | 70 |
| Sbjct 12 | +P+ W + L GY W Q GCS A V RL A RP LFVK ++ A EL E ARL WL APSTWADYLAGYRWRGQGEGCSAATVHRLEAARRPTLFVKQEVLSAHAELPAEIARLRWL | 71 |
| Query 71 | ATTGVPCAAVLDVVTEAGRDWLLLGEVPGQDLLSS HLAPAEKVSIMADAMRRLHT | 125 |
| Sbjct 72 | G+ C VL+ GR WLL+ +PG D LS+ L P V ++A A+RRLH HGAGIDCPQVLNETQSDGRQWLLMSAMPG-DTLSALAQRDELEPERLVRLVAAALRRLHD | 130 |
| Query 126 | LDPATCPFDHQAKHRIERARTRMEAGLVDQDDLDEEHQGLAPAELFARLKARMPDGEDLV | 185 |
| Sbjct 131 | LDPA CPFDH+ + R++ R R EAGLVD+ D D++H+G + EL+ L R P EDLV LDPAACPFDHRLERRLDTVRQRGEAGLVDEADFDDDHRGRSATELYRLLLDRRPAVEDLV | 190 |
| Query 186 | VTHGDACLPNIMVENGRFSGFIDCGRLGVADRYQDIALATRDIAEELGGEWADRFLVLYG | 245 |
| Sbjct 191 | V HGDACLPN++ E RFSGFIDCGRLGVADR+QD+ALA RDI ELG WA+ FLV YG VAHGDACLPNLLAEGRRFSGFIDCGRLGVADRHQDLALAARDIEAELGAAWAEAFLVEYG | 250 |
| Query 246  Sbjct 251 | IAAPDSQRIAFYRLLDEFF 264 D +R+A++RLLDEFF  -GDIDGERLAYFRLLDEFF 268 |  |

>KSV61385.1 hypothetical protein N185_10185 [Sinorhizobium sp. GW3] Length=314

Score = 261 bits (666), Expect = 6e-83, Method: Compositional matrix adjust.

| Identities | = 128/244 (52%), Positives = 167/244 (68%), Gaps = 0/244 (0%) |  |
| --- | --- | --- |
| Query 21 | GYDWAQQTIGCSDAAVFRLSAQGRPVLFVKTDLSGALNELQDEAARLSWLATTGVPCAAV | 80 |
| Sbjct 71 | GY W + +G SD+++F L A RP+L +K + +G +E DEAARL WLA+ +PC V GYQWNRDLLGQSDSSIFLLKAPDRPMLVLKLEEAGPFSEFVDEAARLDWLASKDLPCPRV | 130 |
| Query 81 | LDVVTEAGRDWLLLGEVPGQDLLSSHLAPAEKVSIMADAMRRLHTLDPATCPFDHQAKHR | 140 |
| Sbjct 131 | + A +WLLL V G DL S+ L+P +++ I+ADA+++LH LD A CPFDH+ + R IARAFGAETNWLLLQAVQGTDLASTTLSPKDQIVILADALKQLHALDIANCPFDHRIEER | 190 |
| Query 141 | IERARTRMEAGLVDQDDLDEEHQGLAPAELFARLKARMPDGEDLVVTHGDACLPNIMVEN | 200 |
| Sbjct 191 | I A+ R +AG++D+ D DE G A+LFA L+AR+P +LVVTHGDACLPNI+ +N IAIAKARTQAGVIDESDFDETRLGRTAADLFAELQARVPQPGELVVTHGDACLPNIIEKN | 250 |
| Query 201 | GRFSGFIDCGRLGVADRYQDIALATRDIAEELGGEWADRFLVLYGIAAPDSQRIAFYRLL | 260 |
| Sbjct 251 | GRFSGFIDC RLGVAD YQDIALA R I LG EW FL YG+ DS++ FY LL GRFSGFIDCSRLGVADPYQDIALACRSIVHNLGEEWVQPFLTRYGLDKDDSEKRDFYCLL | 310 |
| Query 261  Sbjct 311 | DEFF 264 DEFF  DEFF 314 |  |

>WP_192441052.1 APH(3') family aminoglycoside O-phosphotransferase [Ensifer sp. ENS04]

MBD9540074.1 aminoglycoside 3'-phosphotransferase [Ensifer sp. ENS04] Length=290

Score = 260 bits (664), Expect = 7e-83, Method: Compositional matrix adjust.

| Identities | = 128/244 (52%), Positives = 166/244 (68%), Gaps = 0/244 (0%) |  |
| --- | --- | --- |
| Query 21 | GYDWAQQTIGCSDAAVFRLSAQGRPVLFVKTDLSGALNELQDEAARLSWLATTGVPCAAV | 80 |
| Sbjct 47 | GY W + +G SD+ +F L A RP+L +K + +G +E DEAARL WLA+ +PC V GYQWNRDLLGQSDSGIFLLKAPDRPMLVLKLEEAGPFSEFVDEAARLDWLASKDLPCPRV | 106 |
| Query 81 | LDVVTEAGRDWLLLGEVPGQDLLSSHLAPAEKVSIMADAMRRLHTLDPATCPFDHQAKHR | 140 |
| Sbjct 107 | + A +WLLL V G DL S+ L+P +++ I+ADA+++LH LD A CPFDH+ + R IARAFGAETNWLLLQAVQGTDLASTTLSPKDQIVILADALKQLHALDIANCPFDHRIEER | 166 |
| Query 141 | IERARTRMEAGLVDQDDLDEEHQGLAPAELFARLKARMPDGEDLVVTHGDACLPNIMVEN | 200 |
| Sbjct 167 | I A+ R +AG++D+ D DE G A+LFA L+AR+P +LVVTHGDACLPNI+ +N IAIAKARTQAGVIDESDFDETRLGRTAADLFAELQARVPQPGELVVTHGDACLPNIIEKN | 226 |
| Query 201 | GRFSGFIDCGRLGVADRYQDIALATRDIAEELGGEWADRFLVLYGIAAPDSQRIAFYRLL GRFSGFIDC RLGVAD YQDIALA R I LG EW FL YG+ DS++ FY LL | 260 |

| Sbjct | 227 | GRFSGFIDCSRLGVADPYQDIALACRSIVHNLGEEWVQPFLTRYGLDKDDSEKRDFYCLL 286 |
| --- | --- | --- |
| Query | 261 | DEFF 264 |
| Sbjct | 287 | DEFF  DEFF 290 |

>WP_196622922.1 APH(3')-II family aminoglycoside O-phosphotransferase [Pseudomonas aeruginosa]

MBG4633600.1 APH(3')-II family aminoglycoside O-phosphotransferase [Pseudomonas aeruginosa]

Length=268

Score = 259 bits (662), Expect = 7e-83, Method: Compositional matrix adjust.

| Identities | | = 137/258 (53%), Positives = 167/258 (65%), Gaps = 5/258 (2%) |  |
| --- | --- | --- | --- |
| Query 11  Sbjct 12 | | SPAAWVERLFGYDWAQQTIGCSDAAVFRLSAQGRPVLFVKTDLSGALNELQDEAARLSWL  +PA W + L GY W Q GCS A V RL A RP LFVK ++ A EL E ARL WL APATWADYLAGYRWRGQGEGCSAATVHRLEAARRPTLFVKQEVLSAHAELPAEIARLRWL | 70  71 |
| Query 71  Sbjct 72 | | ATTGVPCAAVLDVVTEAGRDWLLLGEVPGQDLLS SHLAPAEKVSIMADAMRRLHTL  G+ C VL+ GR WLL+ VPG L + L P V ++A A+RRLH L HGAGIDCPQVLNETQSDGRQWLLMSAVPGDTLSALAQRGELEPERLVRLVAAALRRLHDL | 126  131 |
| Query 127  Sbjct 132 | | DPATCPFDHQAKHRIERARTRMEAGLVDQDDLDEEHQGLAPAELFARLKARMPDGEDLVV DPA CPFDH+ + R++ R R+EAGLVD+ D D++H+G + EL+ L R P EDLVV DPAACPFDHRLERRLDTVRQRVEAGLVDEADFDDDHRGRSATELYRLLLDRRPAVEDLVV | 186  191 |
| Query 187  Sbjct 192 | | THGDACLPNIMVENGRFSGFIDCGRLGVADRYQDIALATRDIAEELGGEWADRFLVLYGI HGDACLPN+ E R SGFIDCGRLGVADR+QD+ALA RDI ELG WA+ FLV YG AHGDACLPNLRAEGRRVSGFIDCGRLGVADRHQDLALAARDIEAELGAAWAEAFLVEYG- | 246  250 |
| Query | 247 AAPDSQRIAFYRLLDEFF 264 | | |
|  |  | D +R+A++RLLDEFF |  |
| Sbjct | 251 | GDIDGERLAYFRLLDEFF 268 | |

>WP_050153044.1 APH(3')-II family aminoglycoside O-phosphotransferase [Pseudomonas aeruginosa]

Length=268

Score = 259 bits (662), Expect = 7e-83, Method: Compositional matrix adjust.

| Identities | | = 135/258 (52%), Positives = 168/258 (65%), Gaps = 5/258 (2%) |  |
| --- | --- | --- | --- |
| Query 11  Sbjct 12 | | SPAAWVERLFGYDWAQQTIGCSDAAVFRLSAQGRPVLFVKTDLSGALNELQDEAARLSWL  +P+ W + L GY W Q GCS A V RL A+ RP LFVK ++ A EL E ARL WL APSTWADYLAGYRWRGQGEGCSAATVHRLEAERRPTLFVKQEVLSAHAELPAEIARLRWL | 70  71 |
| Query 71  Sbjct 72 | | ATTGVPCAAVLDVVTEAGRDWLLLGEVPGQDLLS SHLAPAEKVSIMADAMRRLHTL  G+ C VL+ GR WLL+ VPG L + L P V ++A A+RRLH L HGAGIDCPQVLNETQSDGRQWLLMSAVPGDTLSALAQRGELEPERLVRLVAAALRRLHDL | 126  131 |
| Query 127  Sbjct 132 | | DPATCPFDHQAKHRIERARTRMEAGLVDQDDLDEEHQGLAPAELFARLKARMPDGEDLVV DPA CPFDH+ + R++ R R+EAGLVD+ D D++H+G + EL+ L R P EDLVV DPAACPFDHRLERRLDTVRQRVEAGLVDEADFDDDHRGRSATELYRLLLDRRPAVEDLVV | 186  191 |
| Query 187  Sbjct 192 | | THGDACLPNIMVENGRFSGFIDCGRLGVADRYQDIALATRDIAEELGGEWADRFLVLYGI HGDACLPN++ E RFSGFIDCGRLG DR+QD+ALA RDI ELG WA+ FLV YG AHGDACLPNLLAEGRRFSGFIDCGRLGGGDRHQDLALAARDIEAELGAAWAEAFLVEYG- | 246  250 |
| Query | 247 AAPDSQRIAFYRLLDEFF 264 | | |
|  |  | D +R+A++RLLDEFF |  |
| Sbjct | 251 | GDIDGERLAYFRLLDEFF 268 | |

>WP_006224268.1 APH(3')-II family aminoglycoside O-phosphotransferase [Achromobacter marplatensis]

EJO31456.1 Aminoglycoside 3'-phosphotransferase [Achromobacter marplatensis] Length=266

Score = 259 bits (661), Expect = 7e-83, Method: Compositional matrix adjust. Identities = 131/253 (52%), Positives = 168/253 (66%), Gaps = 1/253 (0%)

| Query | 12 | PAAWVERLFGYDWAQQTIGCSDAAVFRLSAQGRPVLFVKTDLSGALNELQDEAARLSWLA | 71 |
| --- | --- | --- | --- |
| Sbjct | 15 | PA W + QQ+IG S A VFR+ G+P LF+K++ + ++NEL DE RL WL PAKWRDGFAHASIEQQSIGESRADVFRVRRPGQPDLFLKSESASSVNELPDEINRLRWLM | 74 |
| Query | 72 | TTGVPCAAVLDVVTEAGRDWLLLGEVPGQDLLSSHLAPAEKVSIMADAMRRLHTLDPATC | 131 |
| Sbjct | 75 | G P VLD VTE R WLL+ + GQDL S+ L PA+ + ++A A+R LH L A C QVGQPAPHVLDTVTEGARHWLLMTALRGQDLASAALPPAKVIGVLATALRDLHRLPVAAC | 134 |
| Query | 132 | PFDHQAKHRIERARTRMEAGLVDQDDLDEEHQGLAPAELFARLKARMPDGEDLVVTHGDA | 191 |
| Sbjct | 135 | PFDH+ + R+ RAR ++AGLVD+ D D+E G + ++FA L + P D VVTHGDA PFDHRLETRLARARLHVDAGLVDEADFDDERLGRSTNQVFAELLSNRPAQHDFVVTHGDA | 194 |
| Query | 192 | CLPNIMVENGRFSGFIDCGRLGVADRYQDIALATRDIAEELGGEWADRFLVLYGIAAPDS | 251 |
| Sbjct | 195 | CLPN MV+ RF+GFIDCGRLG+ADR+QD+ALA R IA +G EW F YG+ APD+ CLPNFMVDGNRFTGFIDCGRLGIADRFQDLALAARSIARNVGEEWVAPFFRKYGV-APDA | 253 |
| Query  Sbjct | 252  254 | QRIAFYRLLDEFF 264 QRI FY LLDEFF QRIKFYCLLDEFF 266 |  |

>WP_003528565.1 aminoglycoside 3'-phosphotransferase [Sinorhizobium meliloti] ASQ02696.1 aminoglycoside phosphotransferase APH(3') [Sinorhizobium meliloti] EHK77660.1 aminoglycoside phosphotransferase [Sinorhizobium meliloti CCNWSX0020]

MQU68488.1 APH(3') family aminoglycoside O-phosphotransferase [Sinorhizobium meliloti]

MQV34775.1 APH(3') family aminoglycoside O-phosphotransferase [Sinorhizobium meliloti]

MQV42039.1 APH(3') family aminoglycoside O-phosphotransferase [Sinorhizobium meliloti]

Length=261

Score = 259 bits (661), Expect = 7e-83, Method: Compositional matrix adjust.

| Identities | = 137/253 (54%), Positives = 161/253 (64%), Gaps = 0/253 (0%) |  |
| --- | --- | --- |
| Query 12 | PAAWVERLFGYDWAQQTIGCSDAAVFRLSAQGRPVLFVKTDLSGALNELQDEAARLSWLA | 71 |
| Sbjct 9 | P A+ L GY + + +G S + +F L AQ RP L +K + SG EL EAARL WLA PPAFRGLLSGYGFERDALGQSASTIFLLKAQSRPFLILKHEPSGPFAELTGEAARLHWLA | 68 |
| Query 72 | TTGVPCAAVLDVVTEAGRDWLLLGEVPGQDLLSSHLAPAEKVSIMADAMRRLHTLDPATC | 131 |
| Sbjct 69 | +PC VL AG WLLL G DL S L AE+++I+A+A+RRLH LDPA C AQDMPCPRVLGHDDHAGETWLLLEAAEGMDLASCRLPHAERIAILAEALRRLHRLDPAAC | 128 |
| Query 132 | PFDHQAKHRIERARTRMEAGLVDQDDLDEEHQGLAPAELFARLKARMPDGEDLVVTHGDA | 191 |
| Sbjct 129 | PFD + I A RMEAGLVD+ D DEE QG ELF L AR P E LVVTHGDA PFDRRLTDSIAAAEARMEAGLVDESDFDEERQGRQTRELFDELLARRPKEEQLVVTHGDA | 188 |
| Query 192 | CLPNIMVENGRFSGFIDCGRLGVADRYQDIALATRDIAEELGGEWADRFLVLYGIAAPDS | 251 |
| Sbjct 189 | CLPN + +FSGFIDCGRLGVAD YQD+AL I LG EW FL YG+AAPD CLPNFIAAGNQFSGFIDCGRLGVADIYQDLALVCWSITHNLGEEWVAPFLDRYGLAAPDP | 248 |
| Query 252  Sbjct 249 | QRIAFYRLLDEFF 264  +++AFYRLLDEFF  EKLAFYRLLDEFF 261 |  |

>WP_033953215.1 APH(3')-II family aminoglycoside O-phosphotransferase [Pseudomonas aeruginosa]

KSG39893.1 aminoglycoside phosphotransferase APH(3') [Pseudomonas aeruginosa]

MBG7193740.1 APH(3')-II family aminoglycoside O-phosphotransferase [Pseudomonas aeruginosa]

NPS51418.1 APH(3')-II family aminoglycoside O-phosphotransferase [Pseudomonas aeruginosa]

RMJ53902.1 aminoglycoside phosphotransferase APH(3') [Pseudomonas aeruginosa]

RPL42938.1 APH(3')-II family aminoglycoside O-phosphotransferase [Pseudomonas aeruginosa]

Length=268

Score = 259 bits (661), Expect = 8e-83, Method: Compositional matrix adjust. Identities = 135/258 (52%), Positives = 167/258 (65%), Gaps = 5/258 (2%)

| Query | 11 | SPAAWVERLFGYDWAQQTIGCSDAAVFRLSAQGRPVLFVKTDLSGALNELQDEAARLSWL | 70 |
| --- | --- | --- | --- |
| Sbjct | 12 | +P+ W + L GY W Q GCS A V RL A RP LFVK ++ EL E ARL WL APSTWADYLAGYRWRGQGEGCSAATVHRLEAARRPTLFVKQEVLSVHAELPAEIARLRWL | 71 |
| Query | 71 | ATTGVPCAAVLDVVTEAGRDWLLLGEVPGQDLLS SHLAPAEKVSIMADAMRRLHTL | 126 |
| Sbjct | 72 | G+ C VL+ GR WLL+ VPG L + L P V ++A A+RRLH L HGAGIDCPQVLNETQSDGRQWLLMSAVPGDTLSALAQRGELEPERLVRLVAAALRRLHDL | 131 |
| Query | 127 | DPATCPFDHQAKHRIERARTRMEAGLVDQDDLDEEHQGLAPAELFARLKARMPDGEDLVV | 186 |
| Sbjct | 132 | DP CPFDH+ + R++ R R+EAGLVD+ D D++H+G + EL+ L R P EDLVV DPVACPFDHRLERRLDTVRQRVEAGLVDEADFDDDHRGRSATELYRLLLDRRPAVEDLVV | 191 |
| Query | 187 | THGDACLPNIMVENGRFSGFIDCGRLGVADRYQDIALATRDIAEELGGEWADRFLVLYGI | 246 |
| Sbjct | 192 | HGDACLPN++ E RFSGFIDCGRLGVADR+QD+ALA RDI ELG WA+ FLV YG  AHGDACLPNLLAEGRRFSGFIDCGRLGVADRHQDLALAARDIEAELGAAWAEDFLVEYG- | 250 |
| Query | 247 AAPDSQRIAFYRLLDEFF 264 | | |
|  |  | D +R+A++RLLDEFF |  |
| Sbjct | 251 | GDIDGERLAYFRLLDEFF 268 | |

>WP_113135852.1 MULTISPECIES: aminoglycoside 3'-phosphotransferase [unclassified Ensifer]

KSV72903.1 hypothetical protein N182_28660 [Sinorhizobium sp. GL2] MBD9558368.1 aminoglycoside 3'-phosphotransferase [Ensifer sp. ENS03] MBD9569312.1 aminoglycoside 3'-phosphotransferase [Ensifer sp. ENS08]

Length=290

Score = 259 bits (663), Expect = 8e-83, Method: Compositional matrix adjust.

| Identities | = 129/244 (53%), Positives = 166/244 (68%), Gaps = 0/244 (0%) |  |
| --- | --- | --- |
| Query 21 | GYDWAQQTIGCSDAAVFRLSAQGRPVLFVKTDLSGALNELQDEAARLSWLATTGVPCAAV | 80 |
| Sbjct 47 | GY W + +G S +++F L A RP+L +K + +G +E DEAARL WLA+ VPC V GYQWNRDLLGQSGSSIFLLKAPDRPMLVLKLEEAGLFSEFVDEAARLDWLASKDVPCPRV | 106 |
| Query 81 | LDVVTEAGRDWLLLGEVPGQDLLSSHLAPAEKVSIMADAMRRLHTLDPATCPFDHQAKHR | 140 |
| Sbjct 107 | + A +WLLL V G DL SS L+P +++ I+ADA+++LH LD A CPFDH+ + R IARAFGAETNWLLLQAVQGTDLASSTLSPKDQIVILADALKQLHALDIANCPFDHRIEER | 166 |
| Query 141 | IERARTRMEAGLVDQDDLDEEHQGLAPAELFARLKARMPDGEDLVVTHGDACLPNIMVEN | 200 |
| Sbjct 167 | I A+ R +AG++D+ D DE G A+LFA L+AR+P +LVVTHGDACLPNI+ +N IAIAKARTQAGVIDESDFDETRLGRTAADLFAELQARVPQPGELVVTHGDACLPNIIEKN | 226 |
| Query 201 | GRFSGFIDCGRLGVADRYQDIALATRDIAEELGGEWADRFLVLYGIAAPDSQRIAFYRLL | 260 |
| Sbjct 227 | GRFSGFIDC RLGVAD YQDIALA R I LG EW FL YG+ DS++ FY LL GRFSGFIDCSRLGVADPYQDIALACRSIVHNLGEEWVQPFLTRYGLDKDDSEKRDFYCLL | 286 |
| Query 261  Sbjct 287 | DEFF 264 DEFF  DEFF 290 |  |

>WP_132506357.1 APH(3')-II family aminoglycoside O-phosphotransferase [Pseudomonas aeruginosa]

Length=268

Score = 259 bits (661), Expect = 8e-83, Method: Compositional matrix adjust.

| Identities | = 135/258 (52%), Positives = 167/258 (65%), Gaps = 5/258 (2%) |  |
| --- | --- | --- |
| Query 11 | SPAAWVERLFGYDWAQQTIGCSDAAVFRLSAQGRPVLFVKTDLSGALNELQDEAARLSWL | 70 |
| Sbjct 12 | +P+ W + L GY W Q GCS A V RL RP LFVK ++ A EL E ARL WL APSTWADYLAGYRWRGQGEGCSAATVHRLEVARRPTLFVKQEVLSAHAELPAEIARLRWL | 71 |
| Query 71 | ATTGVPCAAVLDVVTEAGRDWLLLGEVPGQDLLS SHLAPAEKVSIMADAMRRLHTL | 126 |
| Sbjct 72 | G+ C VL+ GR WLL+ VPG L + L P V ++A A+RRLH L HGAGIDCPQVLNETQSDGRQWLLMSAVPGDTLSALAQRGELEPERLVRLVAAALRRLHDL | 131 |
| Query 127 | DPATCPFDHQAKHRIERARTRMEAGLVDQDDLDEEHQGLAPAELFARLKARMPDGEDLVV | 186 |
| Sbjct 132 | DPA CPFDH+ + R++ R R+EAGLVD+ D D++H+G + EL+ L R P EDLVV DPAACPFDHRLERRLDTVRQRVEAGLVDEADFDDDHRGRSATELYRLLLDRRPAVEDLVV | 191 |
| Query 187 | THGDACLPNIMVENGRFSGFIDCGRLGVADRYQDIALATRDIAEELGGEWADRFLVLYGI | 246 |

| Sbjct | 192 | HGDACLPN++ E RFSGFIDCGRLGVADR+QD+ALA RDI ELG WA+ FLV YG AHGDACLPNLLAEGRRFSGFIDCGRLGVADRHQDLALAARDIEAELGAAWAEAFLVEYG- 250 |
| --- | --- | --- |
| Query | 247 | AAPDSQRIAFYRLLDEFF 264 |
| Sbjct | 251 | D +R+ ++RLLDEFF  GDIDGERLXYFRLLDEFF 268 |

>WP_132654435.1 APH(3')-II family aminoglycoside O-phosphotransferase [Pseudomonas aeruginosa]

Length=268

Score = 259 bits (661), Expect = 8e-83, Method: Compositional matrix adjust.

| Identities | | = 137/258 (53%), Positives = 168/258 (65%), Gaps = 5/258 (2%) |  |
| --- | --- | --- | --- |
| Query 11  Sbjct 12 | | SPAAWVERLFGYDWAQQTIGCSDAAVFRLSAQGRPVLFVKTDLSGALNELQDEAARLSWL  +PA W + L GY W Q GCS A V RL A RP LFVK ++ A EL E ARL L APATWADYLAGYRWRGQGEGCSAATVHRLEAARRPTLFVKKEVLSAHAELPAEIARLRXL | 70  71 |
| Query 71  Sbjct 72 | | ATTGVPCAAVLDVVTEAGRDWLLLGEVPGQDLLS SHLAPAEKVSIMADAMRRLHTL  G+ C VL+ GR WLL+ VPG L + L P V ++A A+RRLH L HGAGIDCPQVLNETQSDGRQWLLMSAVPGDTLSALAQRGELEPERLVRLVAAALRRLHDL | 126  131 |
| Query 127  Sbjct 132 | | DPATCPFDHQAKHRIERARTRMEAGLVDQDDLDEEHQGLAPAELFARLKARMPDGEDLVV DPA CPFDH+ + R++ R R+EAGLVD+ D D++H+G + EL+ L R P EDLVV DPAACPFDHRLERRLDTVRQRVEAGLVDEADFDDDHRGRSATELYRLLLDRRPAVEDLVV | 186  191 |
| Query 187  Sbjct 192 | | THGDACLPNIMVENGRFSGFIDCGRLGVADRYQDIALATRDIAEELGGEWADRFLVLYGI HGDACLPN++ E RFSGFIDCGRLGVADR+QD+ALA RDI ELG WA+ FLV YG AHGDACLPNLLAEGRRFSGFIDCGRLGVADRHQDLALAARDIEAELGAAWAEAFLVEYG- | 246  250 |
| Query | 247 AAPDSQRIAFYRLLDEFF 264 | | |
|  |  | D +R+A++RLLDEFF |  |
| Sbjct | 251 | GDIDGERLAYFRLLDEFF 268 | |

>WP_034003798.1 APH(3')-II family aminoglycoside O-phosphotransferase [Pseudomonas aeruginosa]

Length=268

Score = 259 bits (661), Expect = 9e-83, Method: Compositional matrix adjust.

| Identities | | = 137/258 (53%), Positives = 168/258 (65%), Gaps = 5/258 (2%) |  |
| --- | --- | --- | --- |
| Query 11  Sbjct 12 | | SPAAWVERLFGYDWAQQTIGCSDAAVFRLSAQGRPVLFVKTDLSGALNELQDEAARLSWL  +PA W + L GY W Q GCS A V RL A RP LFVK ++ A EL E ARL WL APATWADYLAGYRWRGQGEGCSAATVHRLEAARRPTLFVKQEVLSAHAELPAEIARLRWL | 70  71 |
| Query 71  Sbjct 72 | | ATTGVPCAAVLDVVTEAGRDWLLLGEVPGQDLLS SHLAPAEKVSIMADAMRRLHTL  G+ C VL+ GR WLL+ VPG L + L P V ++A A+RRLH L HGAGIDCPQVLNETQSDGRQWLLMSAVPGDTLSALAQRGELEPGRLVRLVAAALRRLHDL | 126  131 |
| Query 127  Sbjct 132 | | DPATCPFDHQAKHRIERARTRMEAGLVDQDDLDEEHQGLAPAELFARLKARMPDGEDLVV DPA PFDH+ + R++ R R+EAGLVD+ D D++H+G + EL+ L R P EDLVV DPAARPFDHRLERRLDTVRQRVEAGLVDEADFDDDHRGRSATELYRLLLDRRPAVEDLVV | 186  191 |
| Query 187  Sbjct 192 | | THGDACLPNIMVENGRFSGFIDCGRLGVADRYQDIALATRDIAEELGGEWADRFLVLYGI HGDACLPN++ E RFSGFIDCGRLGVADR+QD+ALA RDI ELG WA+ FLV YG AHGDACLPNLLAEGRRFSGFIDCGRLGVADRHQDLALAARDIEAELGAAWAEAFLVEYG- | 246  250 |
| Query | 247 AAPDSQRIAFYRLLDEFF 264 | | |
|  |  | D +R+A++RLLDEFF |  |
| Sbjct | 251 | GDIDGERLAYFRLLDEFF 268 | |

>WP_128684639.1 APH(3')-II family aminoglycoside O-phosphotransferase [Pseudomonas aeruginosa]

RUE94121.1 APH(3')-II family aminoglycoside O-phosphotransferase [Pseudomonas aeruginosa]

Length=268

Score = 259 bits (661), Expect = 9e-83, Method: Compositional matrix adjust. Identities = 137/258 (53%), Positives = 168/258 (65%), Gaps = 5/258 (2%)

| Query | 11 | SPAAWVERLFGYDWAQQTIGCSDAAVFRLSAQGRPVLFVKTDLSGALNELQDEAARLSWL | 70 |
| --- | --- | --- | --- |
| Sbjct | 12 | +PA W L GY W +Q GCS A V RL A RP LFVK ++ A EL E ARL WL APATWDAYLAGYRWRRQGEGCSAATVHRLEAARRPTLFVKQEVLSAHAELPAEIARLRWL | 71 |
| Query | 71 | ATTGVPCAAVLDVVTEAGRDWLLLGEVPGQDLLS SHLAPAEKVSIMADAMRRLHTL | 126 |
| Sbjct | 72 | G+ C VL+ G WLL+ VPG L + L P V ++A A+RRLH L HGAGIDCPQVLNETQSDGWQWLLMSAVPGDTLSALAQCGELEPGRLVRLVAAALRRLHDL | 131 |
| Query | 127 | DPATCPFDHQAKHRIERARTRMEAGLVDQDDLDEEHQGLAPAELFARLKARMPDGEDLVV | 186 |
| Sbjct | 132 | DPA CPFDH+ + R++ R R+EAGLVD+ D D++H+G + EL+ L R P EDLVV DPAACPFDHRLERRLDTVRQRVEAGLVDEADFDDDHRGRSATELYRLLLDRRPAVEDLVV | 191 |
| Query | 187 | THGDACLPNIMVENGRFSGFIDCGRLGVADRYQDIALATRDIAEELGGEWADRFLVLYGI | 246 |
| Sbjct | 192 | HGDACLPN++ E RFSGFIDCGRLGVADR+QD+ALA RDI ELG WA+ FLV YG  AHGDACLPNLLAEGRRFSGFIDCGRLGVADRHQDLALAARDIEAELGAAWAEAFLVEYG- | 250 |
| Query | 247 AAPDSQRIAFYRLLDEFF 264 | | |
|  |  | D +R+A++RLLDEFF |  |
| Sbjct | 251 | GDIDGERLAYFRLLDEFF 268 | |

>WP_132718772.1 APH(3')-II family aminoglycoside O-phosphotransferase [Pseudomonas aeruginosa]

Length=268

Score = 259 bits (661), Expect = 1e-82, Method: Compositional matrix adjust.

| Identities | | = 135/258 (52%), Positives = 167/258 (65%), Gaps = 5/258 (2%) |  |
| --- | --- | --- | --- |
| Query 11  Sbjct 12 | | SPAAWVERLFGYDWAQQTIGCSDAAVFRLSAQGRPVLFVKTDLSGALNELQDEAARLSWL  +P+ W + L GY W Q GCS A V RL RP LFVK ++ A EL E ARL WL APSTWADYLAGYRWRGQGEGCSAATVHRLEVARRPTLFVKQEVLSAHAELPAEIARLRWL | 70  71 |
| Query 71  Sbjct 72 | | ATTGVPCAAVLDVVTEAGRDWLLLGEVPGQDLLS SHLAPAEKVSIMADAMRRLHTL  G+ C VL+ GR WLL+ VPG L + L P V ++A A+RRLH L HGAGIDCPQVLNETQSDGRQWLLMSAVPGDTLSALAQRGELEPERLVRLVAAALRRLHDL | 126  131 |
| Query 127  Sbjct 132 | | DPATCPFDHQAKHRIERARTRMEAGLVDQDDLDEEHQGLAPAELFARLKARMPDGEDLVV DPA CPFDH+ + R++ R R+EAGLVD+ D D++H+G + EL+ L R P EDLVV DPAACPFDHRLERRLDTVRQRVEAGLVDEADFDDDHRGRSATELYRLLLDRRPAVEDLVV | 186  191 |
| Query 187  Sbjct 192 | | THGDACLPNIMVENGRFSGFIDCGRLGVADRYQDIALATRDIAEELGGEWADRFLVLYGI HGDACLPN++ E RFSGFIDCGRLGVADR+QD+A A RDI ELG WA+ FLV YG AHGDACLPNLLAEGRRFSGFIDCGRLGVADRHQDLAXAARDIEAELGAAWAEAFLVEYG- | 246  250 |
| Query | 247 AAPDSQRIAFYRLLDEFF 264 | | |
|  |  | D +R+A++RLLDEFF |  |
| Sbjct | 251 | GDIDGERLAYFRLLDEFF 268 | |

>WP_096067735.1 APH(3')-II family aminoglycoside O-phosphotransferase [Pseudomonas aeruginosa]

PBL34961.1 aminoglycoside O-phosphotransferase APH(3')-IIb [Pseudomonas aeruginosa]

PBN05842.1 aminoglycoside O-phosphotransferase APH(3')-IIb [Pseudomonas aeruginosa]

PBN09521.1 aminoglycoside O-phosphotransferase APH(3')-IIb [Pseudomonas aeruginosa]

PBN10821.1 aminoglycoside O-phosphotransferase APH(3')-IIb [Pseudomonas aeruginosa]

PBN26246.1 aminoglycoside O-phosphotransferase APH(3')-IIb [Pseudomonas aeruginosa]

Length=268

Score = 259 bits (661), Expect = 1e-82, Method: Compositional matrix adjust.

| Identities | = 136/258 (53%), Positives = 168/258 (65%), Gaps = 5/258 (2%) |  |
| --- | --- | --- |
| Query 11 | SPAAWVERLFGYDWAQQTIGCSDAAVFRLSAQGRPVLFVKTDLSGALNELQDEAARLSWL | 70 |
| Sbjct 12 | +P+ W + L GY W Q GCS A V RL A RP LFVK ++ A EL E ARL WL APSTWADYLAGYRWRGQGEGCSAATVHRLEAARRPTLFVKQEVLSAHAELPAEIARLRWL | 71 |
| Query 71 | ATTGVPCAAVLDVVTEAGRDWLLLGEVPGQDLLS SHLAPAEKVSIMADAMRRLHTL | 126 |

| Sbjct | 72 | G+ C VL+ GR WLL+ VPG L + L P V ++A A+RRLH L HGAGIDCPQVLNETQSDGRQWLLMSAVPGDTLSALAQRGELEPERLVRLVAAALRRLHDL | 131 |
| --- | --- | --- | --- |
| Query | 127 | DPATCPFDHQAKHRIERARTRMEAGLVDQDDLDEEHQGLAPAELFARLKARMPDGEDLVV | 186 |
| Sbjct | 132 | DPA CPFDH+ + R++ R R+EAGLVD+ D D++H+G + EL+ L R P EDLVV DPAACPFDHRLERRLDTVRQRVEAGLVDEADFDDDHRGRSATELYRLLLDRCPAVEDLVV | 191 |
| Query | 187 | THGDACLPNIMVENGRFSGFIDCGRLGVADRYQDIALATRDIAEELGGEWADRFLVLYGI | 246 |
| Sbjct | 192 | HGDA LPN++ E RFSGFIDCGRLGVADR+QD+ALA RDI ELG WA+ FLV YG  AHGDASLPNLLAEGRRFSGFIDCGRLGVADRHQDLALAARDIEAELGAAWAEAFLVEYG- | 250 |
| Query | 247 AAPDSQRIAFYRLLDEFF 264 | | |
|  |  | D +R+A++RLLDEFF |  |
| Sbjct | 251 | GDIDGERLAYFRLLDEFF 268 | |

>WP_127610550.1 aminoglycoside 3'-phosphotransferase [Sinorhizobium meliloti] RVH05251.1 aminoglycoside 3'-phosphotransferase [Sinorhizobium meliloti]

Length=261

Score = 258 bits (660), Expect = 1e-82, Method: Compositional matrix adjust.

| Identities | = 136/252 (54%), Positives = 162/252 (64%), Gaps = 0/252 (0%) |  |
| --- | --- | --- |
| Query 12 | PAAWVERLFGYDWAQQTIGCSDAAVFRLSAQGRPVLFVKTDLSGALNELQDEAARLSWLA | 71 |
| Sbjct 9 | P A+ L GY + + +G S + +F L AQ RP L +K + SG EL EAARL WLA PPAFRGLLSGYGFERDALGQSASTIFLLKAQSRPFLILKHEPSGPFTELTGEAARLHWLA | 68 |
| Query 72 | TTGVPCAAVLDVVTEAGRDWLLLGEVPGQDLLSSHLAPAEKVSIMADAMRRLHTLDPATC | 131 |
| Sbjct 69 | +PC VL AG WLLL G DL S L AE+++I+A+A+RRLH LDPA C AQDMPCPRVLGHDDHAGETWLLLEAAEGMDLASCRLPHAERIAILAEALRRLHRLDPAAC | 128 |
| Query 132 | PFDHQAKHRIERARTRMEAGLVDQDDLDEEHQGLAPAELFARLKARMPDGEDLVVTHGDA | 191 |
| Sbjct 129 | PFD + I A+ RMEAGLVD+ D DEE QG ELF L AR P E LVVTHGDA PFDRRLTASIAAAKARMEAGLVDESDFDEERQGRQTRELFDELLARRPKEEQLVVTHGDA | 188 |
| Query 192 | CLPNIMVENGRFSGFIDCGRLGVADRYQDIALATRDIAEELGGEWADRFLVLYGIAAPDS | 251 |
| Sbjct 189 | CLPN + +FSGFIDCGRLGVAD Y+D+ALA I LG EW FL YG+AAPD CLPNFIAAGNQFSGFIDCGRLGVADIYKDLALACWSITHNLGEEWVAPFLDRYGLAAPDP | 248 |
| Query 252  Sbjct 249 | QRIAFYRLLDEF 263  +++AFYRLLDEF  EKLAFYRLLDEF 260 |  |

>WP_196680207.1 APH(3')-II family aminoglycoside O-phosphotransferase [Pseudomonas aeruginosa]

MBG4103453.1 APH(3')-II family aminoglycoside O-phosphotransferase [Pseudomonas aeruginosa]

MBG4685202.1 APH(3')-II family aminoglycoside O-phosphotransferase [Pseudomonas aeruginosa]

Length=265

Score = 258 bits (660), Expect = 1e-82, Method: Compositional matrix adjust.

| Identities | = 135/255 (53%), Positives = 168/255 (66%), Gaps = 2/255 (1%) |  |
| --- | --- | --- |
| Query 11 | SPAAWVERLFGYDWAQQTIGCSDAAVFRLSAQGRPVLFVKTDLSGALNELQDEAARLSWL | 70 |
| Sbjct 12 | +P+ W + L GY W Q GCS A V RL A RP LFVK ++ A EL E ARL WL APSTWADYLAGYRWRGQGEGCSAATVHRLEAARRPTLFVKQEVLSAHAELPAEIARLRWL | 71 |
| Query 71 | ATTGVPCAAVLDVVTEAGRDWLLLGEVPGQDLLS-SHLAPAEKVSIMADAMRRLHTLDPA | 129 |
| Sbjct 72 | G+ C VL+ GR WLL+ VPG L + + E ++A A+RRLH LDPA HGAGIDCPQVLNETQSDGRQWLLMSAVPGDTLSALAQRGELEPERLVAAALRRLHDLDPA | 131 |
| Query 130 | TCPFDHQAKHRIERARTRMEAGLVDQDDLDEEHQGLAPAELFARLKARMPDGEDLVVTHG | 189 |
| Sbjct 132 | CPFDH+ + R++ R R+EAGLVD+ D D++H+G + EL+ L R P EDLVV HG ACPFDHRLERRLDTVRQRVEAGLVDEADFDDDHRGRSATELYRLLLDRRPAVEDLVVAHG | 191 |
| Query 190 | DACLPNIMVENGRFSGFIDCGRLGVADRYQDIALATRDIAEELGGEWADRFLVLYGIAAP | 249 |
| Sbjct 192 | DACLPN++ E RFSGFIDCGRLGVADR+QD+ALA RDI ELG WA+ FLV YG DACLPNLLAEGRRFSGFIDCGRLGVADRHQDLALAARDIEAELGAAWAEAFLVEYG-GDI | 250 |
| Query 250 | DSQRIAFYRLLDEFF 264 |  |

D +R+A++RLLDEFF Sbjct 251 DGERLAYFRLLDEFF 265

>WP_142918966.1 aminoglycoside 3'-phosphotransferase, partial [Staphylococcus cohnii]

RIL81816.1 aminoglycoside 3'-phosphotransferase, partial [Staphylococcus cohnii]

Length=127

Score = 253 bits (647), Expect = 1e-82, Method: Compositional matrix adjust.

| Identities | = 125/127 (98%), Positives = 125/127 (98%), Gaps = 0/127 (0%) |  |
| --- | --- | --- |
| Query 138 | KHRIERARTRMEAGLVDQDDLDEEHQGLAPAELFARLKARMPDGEDLVVTHGDACLPNIM | 197 |
| Sbjct 1 | KHRIERARTRMEAGLVDQDDLDEEHQGLAPAELFARLKARMPDGEDLVVTHGDACLP IM KHRIERARTRMEAGLVDQDDLDEEHQGLAPAELFARLKARMPDGEDLVVTHGDACLPTIM | 60 |
| Query 198 | VENGRFSGFIDCGRLGVADRYQDIALATRDIAEELGGEWADRFLVLYGIAAPDSQRIAFY | 257 |
| Sbjct 61 | VENGRFSG IDCGRLGVADRYQDIALATRDIAEELGGEWADRFLVLYGIAAPDSQRIAFY VENGRFSGCIDCGRLGVADRYQDIALATRDIAEELGGEWADRFLVLYGIAAPDSQRIAFY | 120 |
| Query 258  Sbjct 121 | RLLDEFF 264 RLLDEFF RLLDEFF 127 |  |

>WP_023913265.1 APH(3')-II family aminoglycoside O-phosphotransferase [Pseudomonas aeruginosa]

ETD73199.1 aminoglycoside 3'-phosphotransferase [Pseudomonas aeruginosa VRFPA06]

KRV15884.1 aminoglycoside phosphotransferase [Pseudomonas aeruginosa] KSF61439.1 aminoglycoside phosphotransferase APH(3') [Pseudomonas aeruginosa] KSQ81290.1 aminoglycoside phosphotransferase APH(3') [Pseudomonas aeruginosa]

MZZ15663.1 APH(3')-II family aminoglycoside O-phosphotransferase [Pseudomonas aeruginosa]

Length=268

Score = 258 bits (660), Expect = 1e-82, Method: Compositional matrix adjust.

| Identities | | = 135/258 (52%), Positives = 167/258 (65%), Gaps = 5/258 (2%) |  |
| --- | --- | --- | --- |
| Query 11  Sbjct 12 | | SPAAWVERLFGYDWAQQTIGCSDAAVFRLSAQGRPVLFVKTDLSGALNELQDEAARLSWL  +P+ W + L GY W Q GCS A V RL RP LFVK ++ A EL E ARL WL APSTWADYLAGYRWRGQGEGCSAATVHRLEVARRPTLFVKQEVLSAHAELPAEIARLRWL | 70  71 |
| Query 71  Sbjct 72 | | ATTGVPCAAVLDVVTEAGRDWLLLGEVPGQDLLS SHLAPAEKVSIMADAMRRLHTL  G+ C VL+ GR WLL+ VPG L + L P V ++A A+RRLH L HGAGIDCPQVLNETQSDGRQWLLMSAVPGDTLSALAQRGELEPERLVRLVAAALRRLHDL | 126  131 |
| Query 127  Sbjct 132 | | DPATCPFDHQAKHRIERARTRMEAGLVDQDDLDEEHQGLAPAELFARLKARMPDGEDLVV DPA CPFDH+ + R++ R R+EAGLVD+ D D++H+ + EL+ L R P EDLVV DPAACPFDHRLERRLDTVRQRVEAGLVDEADFDDDHRSRSATELYRLLLDRRPAVEDLVV | 186  191 |
| Query 187  Sbjct 192 | | THGDACLPNIMVENGRFSGFIDCGRLGVADRYQDIALATRDIAEELGGEWADRFLVLYGI HGDACLPN++ E RFSGFIDCGRLGVADR+QD+ALA RDI ELG WA+ FLV YG AHGDACLPNLLAEGRRFSGFIDCGRLGVADRHQDLALAARDIEAELGAAWAEAFLVEYG- | 246  250 |
| Query | 247 AAPDSQRIAFYRLLDEFF 264 | | |
|  |  | D +R+A++RLLDEFF |  |
| Sbjct | 251 | GDIDGERLAYFRLLDEFF 268 | |

```
>WP_132632367.1 APH(3')-II family aminoglycoside O-phosphotransferase [Pseudomonas aeruginosa]
Length=268

Score = 258 bits (660), Expect = 1e-82, Method: Compositional matrix adjust. Identities = 136/258 (53%), Positives = 168/258 (65%), Gaps = 5/258 (2%)

Query 11	SPAAWVERLFGYDWAQQTIGCSDAAVFRLSAQGRPVLFVKTDLSGALNELQDEAARLSWL 70
+P+ W + L GY W Q GCS A V RL A RP LFVK ++ A EL E ARL WL Sbjct 12	APSTWADYLAGYRWRGQGEGCSAATVHRLEAARRPTLFVKQEVLSAHAELPAEIARLRWL 71
```

| Query | 71 | ATTGVPCAAVLDVVTEAGRDWLLLGEVPGQDLLS SHLAPAEKVSIMADAMRRLHTL | 126 |
| --- | --- | --- | --- |
| Sbjct | 72 | G+ C VL+ GR WLL+ VPG L + L P V ++A A+RRL L HGAGIDCPQVLNETQSDGRQWLLMSAVPGDTLSALAQRGELEPERLVRLVAAALRRLXDL | 131 |
| Query | 127 | DPATCPFDHQAKHRIERARTRMEAGLVDQDDLDEEHQGLAPAELFARLKARMPDGEDLVV | 186 |
| Sbjct | 132 | DPA CPFDH+ + R++ R R+EAGLVD+ D D++H+G + EL+ L R P EDLVV DPAACPFDHRLERRLDTVRQRVEAGLVDEADFDDDHRGRSATELYRLLLDRRPAVEDLVV | 191 |
| Query | 187 | THGDACLPNIMVENGRFSGFIDCGRLGVADRYQDIALATRDIAEELGGEWADRFLVLYGI | 246 |
| Sbjct | 192 | HGDACLPN++ E RFSGFIDCGRLGVADR+QD+ALA RDI ELG WA+ FLV YG  AHGDACLPNLLAEGRRFSGFIDCGRLGVADRHQDLALAARDIEAELGAAWAEAFLVEYG- | 250 |
| Query | 247 AAPDSQRIAFYRLLDEFF 264 | | |
|  |  | D +R+A++RLLDEFF |  |
| Sbjct | 251 | GDIDGERLAYFRLLDEFF 268 | |

>WP_058156211.1 APH(3')-II family aminoglycoside O-phosphotransferase [Pseudomonas aeruginosa]

KSG76302.1 aminoglycoside 3'-phosphotransferase [Pseudomonas aeruginosa] Length=268

Score = 258 bits (660), Expect = 1e-82, Method: Compositional matrix adjust.

| Identities | | = 136/258 (53%), Positives = 168/258 (65%), Gaps = 5/258 (2%) |  |
| --- | --- | --- | --- |
| Query 11  Sbjct 12 | | SPAAWVERLFGYDWAQQTIGCSDAAVFRLSAQGRPVLFVKTDLSGALNELQDEAARLSWL  +P+ W + L GY W Q GCS A V RL A RP LFVK ++ A EL E ARL WL APSTWADYLAGYRWRGQGEGCSAATVHRLEAARRPTLFVKQEVLSAHAELPAEIARLRWL | 70  71 |
| Query 71  Sbjct 72 | | ATTGVPCAAVLDVVTEAGRDWLLLGEVPGQDLLS SHLAPAEKVSIMADAMRRLHTL  G+ C VL+ GR WLL+ VPG L + L P V ++A A+RRLH L HGAGIDCPQVLNETQSDGRQWLLMSAVPGDTLSALAQRGELEPERLVRLVAAALRRLHDL | 126  131 |
| Query 127  Sbjct 132 | | DPATCPFDHQAKHRIERARTRMEAGLVDQDDLDEEHQGLAPAELFARLKARMPDGEDLVV DPA CPFDH+ + R++ R R+EAGLVD+ D D++H+G + EL+ L R EDLVV DPAACPFDHRLERRLDTVRQRVEAGLVDEADFDDDHRGRSATELYRLLLDRCLAVEDLVV | 186  191 |
| Query 187  Sbjct 192 | | THGDACLPNIMVENGRFSGFIDCGRLGVADRYQDIALATRDIAEELGGEWADRFLVLYGI HGDACLPN++ E RFSGFIDCGRLGVADR+QD+ALA RDI ELG WA+ FLV YG AHGDACLPNLLAEGRRFSGFIDCGRLGVADRHQDLALAARDIEAELGAAWAEAFLVEYG- | 246  250 |
| Query | 247 AAPDSQRIAFYRLLDEFF 264 | | |
|  |  | D +R+A++RLLDEFF |  |
| Sbjct | 251 | GDIDGERLAYFRLLDEFF 268 | |

>WP_181851369.1 APH(3')-II family aminoglycoside O-phosphotransferase [Pseudomonas aeruginosa]

MBA4995522.1 APH(3')-II family aminoglycoside O-phosphotransferase [Pseudomonas aeruginosa]

Length=268

Score = 258 bits (660), Expect = 1e-82, Method: Compositional matrix adjust.

| Identities | | = 137/258 (53%), Positives = 168/258 (65%), Gaps = 5/258 (2%) |  |
| --- | --- | --- | --- |
| Query 11  Sbjct 12 | | SPAAWVERLFGYDWAQQTIGCSDAAVFRLSAQGRPVLFVKTDLSGALNELQDEAARLSWL  +PA W + L GY W Q GCS A V RL A RP LFVK ++ A EL E ARL WL APATWDDYLAGYRWRGQGEGCSAATVHRLEAARRPTLFVKQEVLSAHAELPAEIARLRWL | 70  71 |
| Query 71  Sbjct 72 | | ATTGVPCAAVLDVVTEAGRDWLLLGEVPGQDLLS SHLAPAEKVSIMADAMRRLHTL  G+ C VL+ GR WLL+ V G L + L P V ++A A+RRLH L HGAGIDCPQVLNETQSDGRQWLLMSAVLGDTLSALAQRGELEPGRLVRLVAAALRRLHDL | 126  131 |
| Query 127  Sbjct 132 | | DPATCPFDHQAKHRIERARTRMEAGLVDQDDLDEEHQGLAPAELFARLKARMPDGEDLVV DPA CPFDH+ + R++ R R+EAGLVD+ D D++H+G + EL+ L R P EDLVV DPAACPFDHRLERRLDTVRQRVEAGLVDEADFDDDHRGRSATELYRLLLDRRPAVEDLVV | 186  191 |
| Query 187  Sbjct 192 | | THGDACLPNIMVENGRFSGFIDCGRLGVADRYQDIALATRDIAEELGGEWADRFLVLYGI HGDACLPN++ E RFSGFIDCGRLGVADR+QD+ALA RDI ELG WA+ FLV YG AHGDACLPNLLAEGRRFSGFIDCGRLGVADRHQDLALAARDIEAELGAAWAEAFLVEYG- | 246  250 |
| Query | 247 AAPDSQRIAFYRLLDEFF 264 | | |

D +R+A++RLLDEFF Sbjct 251 GDIDGERLAYFRLLDEFF 268

>WP_134269618.1 APH(3')-II family aminoglycoside O-phosphotransferase [Pseudomonas aeruginosa]

TEL89006.1 APH(3')-II family aminoglycoside O-phosphotransferase [Pseudomonas aeruginosa]

TEM07811.1 APH(3')-II family aminoglycoside O-phosphotransferase [Pseudomonas aeruginosa]

TEM08605.1 APH(3')-II family aminoglycoside O-phosphotransferase [Pseudomonas aeruginosa]

TEM12519.1 APH(3')-II family aminoglycoside O-phosphotransferase [Pseudomonas aeruginosa]

TEM24550.1 APH(3')-II family aminoglycoside O-phosphotransferase [Pseudomonas aeruginosa]

Length=270

Score = 258 bits (660), Expect = 1e-82, Method: Compositional matrix adjust.

| Identities | = 137/261 (52%), Positives = 170/261 (65%), Gaps = 8/261 (3%) |  |
| --- | --- | --- |
| Query 11 | SPAAWVERLFGYDWAQQTIGCSDAAVFRLSAQGRPVLFVKTDLSGALNELQDEAARLSWL | 70 |
| Sbjct 11 | +P+ W + L GY W Q GCS A V RL A+ RP LFVK ++ A EL E ARL WL APSTWADYLAGYRWRGQGEGCSAATVHRLEAERRPTLFVKQEVLSAHAELPAEIARLRWL | 70 |
| Query 71 | ATTGVPCAAVLDVVTEAGRDWLLLGEVPGQDLLS----SHLAP---AEKVSIMADAMRRL | 123 |
| Sbjct 71 | G+ C VL+ GR WLL+ VPG L + L P V ++A A+RRL HGAGIDCPQVLNETQSDGRQWLLMSAVPGDTLSALAQRGELEPERLVRLVRLVAAALRRL | 130 |
| Query 124 | HTLDPATCPFDHQAKHRIERARTRMEAGLVDQDDLDEEHQGLAPAELFARLKARMPDGED | 183 |
| Sbjct 131 | H LDPA CPFDH+ + R++ R R+EAGLVD+ D D++H+G + EL+ L R P ED HDLDPAACPFDHRLERRLDTVRQRVEAGLVDEADFDDDHRGRSATELYRLLLDRRPAVED | 190 |
| Query 184 | LVVTHGDACLPNIMVENGRFSGFIDCGRLGVADRYQDIALATRDIAEELGGEWADRFLVL | 243 |
| Sbjct 191 | LVV HGDACLPN++ E RFSGFIDCGRLGVADR+QD+ALA RDI ELG WA+ FLV LVVAHGDACLPNLLAEGRRFSGFIDCGRLGVADRHQDLALAARDIEAELGAAWAEAFLVE | 250 |
| Query 244  Sbjct 251 | YGIAAPDSQRIAFYRLLDEFF 264 YG D +R+A++RLLDEFF  YG-GDIDGERLAYFRLLDEFF 270 |  |

>WP_124082237.1 APH(3')-II family aminoglycoside O-phosphotransferase [Pseudomonas aeruginosa]

RPP07562.1 APH(3') family aminoglycoside O-phosphotransferase [Pseudomonas aeruginosa]

Length=268

Score = 258 bits (660), Expect = 1e-82, Method: Compositional matrix adjust.

| Identities | | = 135/258 (52%), Positives = 167/258 (65%), Gaps = 5/258 (2%) |  |
| --- | --- | --- | --- |
| Query 11  Sbjct 12 | | SPAAWVERLFGYDWAQQTIGCSDAAVFRLSAQGRPVLFVKTDLSGALNELQDEAARLSWL  +P+ W + L GY W Q GCS A V RL RP LFVK ++ A EL E ARL WL APSTWADYLAGYRWRGQGEGCSAATVHRLEVARRPTLFVKQEVLSAHAELPAEIARLRWL | 70  71 |
| Query 71  Sbjct 72 | | ATTGVPCAAVLDVVTEAGRDWLLLGEVPGQDLLS SHLAPAEKVSIMADAMRRLHTL  G+ C VL+ GR WLL+ VPG L + L P V ++A A+RRLH L HGAGIDCPQVLNETQSDGRQWLLMSAVPGDTLSALAQRGELEPERLVRLVAAALRRLHDL | 126  131 |
| Query 127  Sbjct 132 | | DPATCPFDHQAKHRIERARTRMEAGLVDQDDLDEEHQGLAPAELFARLKARMPDGEDLVV DPA CPFDH+ + R++ R R+EAGLVD+ D D++H+G + EL+ L R P EDLVV DPAACPFDHRLERRLDTVRQRVEAGLVDEADFDDDHRGRSATELYRLLLDRRPAVEDLVV | 186  191 |
| Query 187  Sbjct 192 | | THGDACLPNIMVENGRFSGFIDCGRLGVADRYQDIALATRDIAEELGGEWADRFLVLYGI HGDACLPN++ E RFSGFIDCGRLGVADR+QD+ALA RDI ELG WA+ FL YG AHGDACLPNLLAEGRRFSGFIDCGRLGVADRHQDLALAARDIEAELGAAWAEAFLXEYG- | 246  250 |
| Query | 247 AAPDSQRIAFYRLLDEFF 264 | | |
|  |  | D +R+A++RLLDEFF |  |
| Sbjct | 251 | GDIDGERLAYFRLLDEFF 268 | |

>AMS41341.1 aminoglycoside phosphotransferase [Aminobacter aminovorans] MBA8904592.1 aminoglycoside 3'-phosphotransferase-2 [Aminobacter ciceronei] MBA9018370.1 aminoglycoside 3'-phosphotransferase-2 [Aminobacter ciceronei] MBB3707908.1 aminoglycoside 3'-phosphotransferase-2 [Aminobacter aminovorans]

Length=265

Score = 258 bits (660), Expect = 1e-82, Method: Compositional matrix adjust.

| Identities | | = 134/254 (53%), Positives = 168/254 (66%), Gaps = 1/254 (0%) |  |
| --- | --- | --- | --- |
| Query 12  Sbjct 12 | | PAAWVERLFGYDWAQQTIGCSDAAVFRLSAQGRPVLFVKTDLSGALNELQDEAARLSWLA PA E L Y W +QTIG S+A VF+L+A G+P LF+K + +G EL DE ARL WL PALLREALASYSWDRQTIGHSNAGVFKLAAAGKPTLFLKAEATGDFAELPDEVARLRWLG | 71  71 |
| Query 72  Sbjct 72 | | TTGVPCAAVLDVVTEAGRDWLLLGEVPGQDLLSSHLAPAEKVSIMADAMRRLHTLDPATC  +PC VL T AG WLL+ V GQDL+S + P + V+IMA+A+RRLH LD A+C GQDIPCPEVLAFETHAGTHWLLMTAVAGQDLVSHEVPPEQAVAIMANALRRLHALDIASC | 131  131 |
| Query 132  Sbjct 132 | | PFDHQAKHRIERARTRMEAGLVDQDDLDEEHQGLAPAELFARLKARMPDG-EDLVVTHGD PFDH+ RIE ARTRM AG+VD +D D+E G +F +L AR P G EDLVVTHGD PFDHRLSARIEVARTRMVAGVVDVEDFDDEWLGQPAEVVFEQLLARRPAGKEDLVVTHGD | 190  191 |
| Query 191  Sbjct 192 | | ACLPNIMVENGRFSGFIDCGRLGVADRYQDIALATRDIAEELGGEWADRFLVLYGIAAPD A +PN + + GRFSGFIDC RLGVADR QD+ + I LG +W FL YG+ + ASMPNFIADGGRFSGFIDCSRLGVADRCQDLGILCWSIHFNLGQQWVAPFLERYGLPDAE | 250  251 |
| Query 251 | | SQRIAFYRLLDEFF 264 R+A+Y+LLDEFF |  |
| Sbjct | 252 | PARLAYYQLLDEFF 265 | |

>WP_033993413.1 APH(3')-II family aminoglycoside O-phosphotransferase [Pseudomonas aeruginosa]

Length=268

Score = 258 bits (660), Expect = 1e-82, Method: Compositional matrix adjust.

| Identities | | = 135/258 (52%), Positives = 168/258 (65%), Gaps = 5/258 (2%) |  |
| --- | --- | --- | --- |
| Query 11  Sbjct 12 | | SPAAWVERLFGYDWAQQTIGCSDAAVFRLSAQGRPVLFVKTDLSGALNELQDEAARLSWL  +P+ W + L G+ W Q GCS A V RL RP LFVK ++ A EL E ARL WL APSTWADYLAGHRWRGQGEGCSAATVHRLEVARRPTLFVKQEVLSAHAELPAEIARLRWL | 70  71 |
| Query 71  Sbjct 72 | | ATTGVPCAAVLDVVTEAGRDWLLLGEVPGQDLLS SHLAPAEKVSIMADAMRRLHTL  G+ C VL+ GR WLL+ VPG L + L P V ++A A+RRLH L HGAGIDCPQVLNETQSDGRQWLLMSAVPGDTLSALAQRGELEPERLVRLVAAALRRLHDL | 126  131 |
| Query 127  Sbjct 132 | | DPATCPFDHQAKHRIERARTRMEAGLVDQDDLDEEHQGLAPAELFARLKARMPDGEDLVV DPA CPFDH+ + R++ R R+EAGLVD+ D D++H+G + EL+ L R P EDLVV DPAACPFDHRLERRLDTVRQRVEAGLVDEADFDDDHRGRSATELYRLLLDRRPAVEDLVV | 186  191 |
| Query 187  Sbjct 192 | | THGDACLPNIMVENGRFSGFIDCGRLGVADRYQDIALATRDIAEELGGEWADRFLVLYGI HGDACLPN++ E RFSGFIDCGRLGVADR+QD+ALA RDI ELG WA+ FLV YG AHGDACLPNLLAEGRRFSGFIDCGRLGVADRHQDLALAARDIEAELGAAWAEAFLVEYG- | 246  250 |
| Query | 247 AAPDSQRIAFYRLLDEFF 264 | | |
|  |  | D +R+A++RLLDEFF |  |
| Sbjct | 251 | GDIDGERLAYFRLLDEFF 268 | |

>WP_134885922.1 aminoglycoside 3'-phosphotransferase [Ensifer adhaerens] Length=290

Score = 259 bits (662), Expect = 1e-82, Method: Compositional matrix adjust. Identities = 128/244 (52%), Positives = 167/244 (68%), Gaps = 0/244 (0%)

| Query | 21 | GYDWAQQTIGCSDAAVFRLSAQGRPVLFVKTDLSGALNELQDEAARLSWLATTGVPCAAV | 80 |
| --- | --- | --- | --- |
| Sbjct | 47 | GY W + +G S++++F L A RP+L +K + +G +E DEAARL WLA+ VPC V GYQWNRDLLGQSNSSIFLLKAPDRPMLVLKLEEAGPFSEFVDEAARLDWLASKDVPCPRV | 106 |
| Query | 81 | LDVVTEAGRDWLLLGEVPGQDLLSSHLAPAEKVSIMADAMRRLHTLDPATCPFDHQAKHR | 140 |
| Sbjct | 107 | + A +WLLL V G DL S+ L+P +++ I+ADA+++LH LD A CPFDH+ + R  IARAFGAETNWLLLQAVQGTDLASTTLSPKDQIVILADALKQLHALDIANCPFDHRIEVR | 166 |

| Query | 141 | IERARTRMEAGLVDQDDLDEEHQGLAPAELFARLKARMPDGEDLVVTHGDACLPNIMVEN | 200 |
| --- | --- | --- | --- |
| Sbjct | 167 | I A+ R +AG++D+ D DE G A+LFA L+AR+P +LVVTHGDACLPNI+ +N IAIAKARTQAGVIDESDFDETRLGRTAADLFAELQARVPQPGELVVTHGDACLPNIIEKN | 226 |
| Query | 201 | GRFSGFIDCGRLGVADRYQDIALATRDIAEELGGEWADRFLVLYGIAAPDSQRIAFYRLL | 260 |
| Sbjct | 227 | GRFSGFIDC RLGVAD YQDIALA R I LG EW FL YG+ DS++ FY LL GRFSGFIDCSRLGVADPYQDIALACRSIVHNLGEEWVQPFLTRYGLDKDDSEKRDFYCLL | 286 |
| Query  Sbjct | 261  287 | DEFF 264 DEFF  DEFF 290 |  |

>MBA8881929.1 aminoglycoside 3'-phosphotransferase-2 [Phyllobacterium myrsinacearum] Length=267

Score = 258 bits (660), Expect = 1e-82, Method: Compositional matrix adjust.

| Identities | = 127/245 (52%), Positives = 162/245 (66%), Gaps = 1/245 (0%) |  |
| --- | --- | --- |
| Query 21 | GYDWAQQTIGCSDAAVFRLSAQGRPVLFVKTDLSGALNELQDEAARLSWLATTGVPCAAV | 80 |
| Sbjct 23 | GY W + +GCSDA VF L + P LF+K + EL DE ARL WL++ +PC + GYSWNRDELGCSDAQVFLLQGERLPRLFIKVETVSPYGELPDEIARLRWLSSCSLPCPDI | 82 |
| Query 81 | LDVVTEAGRDWLLLGEVPGQDLLSSHLAPAE-KVSIMADAMRRLHTLDPATCPFDHQAKH | 139 |
| Sbjct 83 | + AG WLL+ VPG DL S+ P E +V I+ADA+R+LH LDP+TCPFDH+ IFDGIHAGHHWLLMSAVPGTDLASADSVPVEPRVRILADALRQLHALDPSTCPFDHRLDM | 142 |
| Query 140 | RIERARTRMEAGLVDQDDLDEEHQGLAPAELFARLKARMPDGEDLVVTHGDACLPNIMVE | 199 |
| Sbjct 143 | RIE A R++AGLVD+ D D++ G AELF RL + P D+VVTHGDACLPN M + RIEAAGARVQAGLVDETDFDDDMLGETAAELFTRLVSEKPAVGDVVVTHGDACLPNFMAD | 202 |
| Query 200 | NGRFSGFIDCGRLGVADRYQDIALATRDIAEELGGEWADRFLVLYGIAAPDSQRIAFYRL | 259 |
| Sbjct 203 | G F+G+IDC RLG+ADRYQDIALA R I++ G EW FL YG+ D ++A+YRL KGIFTGYIDCARLGLADRYQDIALACRSISDNFGEEWVRPFLDCYGLKHADPAKLAYYRL | 262 |
| Query 260  Sbjct 263 | LDEFF 264 LDEFF LDEFF 267 |  |

>WP_121196160.1 APH(3')-II family aminoglycoside O-phosphotransferase [Pseudomonas aeruginosa]

Length=268

Score = 258 bits (660), Expect = 1e-82, Method: Compositional matrix adjust.

| Identities | | = 135/258 (52%), Positives = 167/258 (65%), Gaps = 5/258 (2%) |  |
| --- | --- | --- | --- |
| Query 11  Sbjct 12 | | SPAAWVERLFGYDWAQQTIGCSDAAVFRLSAQGRPVLFVKTDLSGALNELQDEAARLSWL  +P+ W + L GY W Q GCS A V RL RP LFVK ++ A EL E ARL WL APSTWADYLAGYRWRGQGEGCSAATVHRLEVARRPTLFVKQEVLSAHAELPAEIARLRWL | 70  71 |
| Query 71  Sbjct 72 | | ATTGVPCAAVLDVVTEAGRDWLLLGEVPGQDLLS SHLAPAEKVSIMADAMRRLHTL  G+ C VL+ GR WLL+ VPG L + L P V ++A A+RRLH L HGAGIDCPQVLNETQSDGRQWLLMSAVPGDTLSALAQRGELEPERLVRLVAAALRRLHDL | 126  131 |
| Query 127  Sbjct 132 | | DPATCPFDHQAKHRIERARTRMEAGLVDQDDLDEEHQGLAPAELFARLKARMPDGEDLVV DPA CPFDH+ + R++ R+EAGLVD+ D D++H+G + EL+ L R P EDLVV DPAACPFDHRLERRLDTVHQRVEAGLVDEADFDDDHRGRSATELYRLLLDRRPAVEDLVV | 186  191 |
| Query 187  Sbjct 192 | | THGDACLPNIMVENGRFSGFIDCGRLGVADRYQDIALATRDIAEELGGEWADRFLVLYGI HGDACLPN++ E RFSGFIDCGRLGVADR+QD+ALA RDI ELG WA+ FLV YG AHGDACLPNLLAEGRRFSGFIDCGRLGVADRHQDLALAARDIEAELGAAWAEAFLVEYG- | 246  250 |
| Query | 247 AAPDSQRIAFYRLLDEFF 264 | | |
|  |  | D +R+A++RLLDEFF |  |
| Sbjct | 251 | GDIDGERLAYFRLLDEFF 268 | |

>WP_067966257.1 MULTISPECIES: aminoglycoside 3'-phosphotransferase [Aminobacter] Length=257

Score = 258 bits (659), Expect = 1e-82, Method: Compositional matrix adjust.

| Identities | | = 134/254 (53%), Positives = 168/254 (66%), Gaps = 1/254 (0%) |  |
| --- | --- | --- | --- |
| Query 12  Sbjct 4 | | PAAWVERLFGYDWAQQTIGCSDAAVFRLSAQGRPVLFVKTDLSGALNELQDEAARLSWLA PA E L Y W +QTIG S+A VF+L+A G+P LF+K + +G EL DE ARL WL PALLREALASYSWDRQTIGHSNAGVFKLAAAGKPTLFLKAEATGDFAELPDEVARLRWLG | 71  63 |
| Query 72  Sbjct 64 | | TTGVPCAAVLDVVTEAGRDWLLLGEVPGQDLLSSHLAPAEKVSIMADAMRRLHTLDPATC  +PC VL T AG WLL+ V GQDL+S + P + V+IMA+A+RRLH LD A+C GQDIPCPEVLAFETHAGTHWLLMTAVAGQDLVSHEVPPEQAVAIMANALRRLHALDIASC | 131  123 |
| Query 132  Sbjct 124 | | PFDHQAKHRIERARTRMEAGLVDQDDLDEEHQGLAPAELFARLKARMPDG-EDLVVTHGD PFDH+ RIE ARTRM AG+VD +D D+E G +F +L AR P G EDLVVTHGD PFDHRLSARIEVARTRMVAGVVDVEDFDDEWLGQPAEVVFEQLLARRPAGKEDLVVTHGD | 190  183 |
| Query 191  Sbjct 184 | | ACLPNIMVENGRFSGFIDCGRLGVADRYQDIALATRDIAEELGGEWADRFLVLYGIAAPD A +PN + + GRFSGFIDC RLGVADR QD+ + I LG +W FL YG+ + ASMPNFIADGGRFSGFIDCSRLGVADRCQDLGILCWSIHFNLGQQWVAPFLERYGLPDAE | 250  243 |
| Query 251 | | SQRIAFYRLLDEFF 264 R+A+Y+LLDEFF |  |
| Sbjct | 244 | PARLAYYQLLDEFF 257 | |

>WP_143973340.1 aminoglycoside 3'-phosphotransferase [Mesorhizobium intechi] TSE13131.1 aminoglycoside 3'-phosphotransferase [Mesorhizobium intechi]

Length=263

Score = 258 bits (659), Expect = 1e-82, Method: Compositional matrix adjust.

| Identities | = 137/244 (56%), Positives = 167/244 (68%), Gaps = 0/244 (0%) |  |
| --- | --- | --- |
| Query 21 | GYDWAQQTIGCSDAAVFRLSAQGRPVLFVKTDLSGALNELQDEAARLSWLATTGVPCAAV | 80 |
| Sbjct 20 | GY W +QTIG S A VFRL+A G+P LF+K + +G E DEAARL WLA GV C V GYRWHRQTIGQSRAGVFRLAADGKPTLFLKCECNGPFAERADEAARLRWLAGQGVACPDV | 79 |
| Query 81 | LDVVTEAGRDWLLLGEVPGQDLLSSHLAPAEKVSIMADAMRRLHTLDPATCPFDHQAKHR | 140 |
| Sbjct 80 | + + + AG DWLL+ V G+DL S+ + PA+ + IMA A+R LH LD +CPFDH+ R IALESHAGHDWLLMSAVAGEDLASAAIDPADAIGIMAVALRDLHALDIRSCPFDHRLFRR | 139 |
| Query 141 | IERARTRMEAGLVDQDDLDEEHQGLAPAELFARLKARMPDGEDLVVTHGDACLPNIMVEN | 200 |
| Sbjct 140 | I AR RME G VD+ D DEE QG AE+FA L A P EDLVV+HGDACLPN+M IAAARARMEGGEVDESDFDEERQGRTAAEVFAALLALRPATEDLVVSHGDACLPNVMAAE | 199 |
| Query 201 | GRFSGFIDCGRLGVADRYQDIALATRDIAEELGGEWADRFLVLYGIAAPDSQRIAFYRLL | 260 |
| Sbjct 200 | GRFSGFIDCGRLGVADR+QD+ALA R I LG W + FL YG + ++++YRLL GRFSGFIDCGRLGVADRHQDLALACRSIGYNLGEAWIEPFLEQYGPPEAEPAKLSWYRLL | 259 |
| Query 261  Sbjct 260 | DEFF 264 DEFF  DEFF 263 |  |

>MBG6791069.1 APH(3')-II family aminoglycoside O-phosphotransferase [Pseudomonas aeruginosa]

Length=267

Score = 258 bits (659), Expect = 1e-82, Method: Compositional matrix adjust.

| Identities | = 136/258 (53%), Positives = 169/258 (66%), Gaps = 5/258 (2%) |  |
| --- | --- | --- |
| Query 11 | SPAAWVERLFGYDWAQQTIGCSDAAVFRLSAQGRPVLFVKTDLSGALNELQDEAARLSWL | 70 |
| Sbjct 11 | +P+ W + L GY W Q GCS A V RL A+ RP LFVK ++ A EL E ARL WL APSTWADYLAGYRWRGQGEGCSAATVHRLEAERRPTLFVKQEVLSAHAELPAEIARLRWL | 70 |
| Query 71 | ATTGVPCAAVLDVVTEAGRDWLLLGEVPGQDLLS SHLAPAEKVSIMADAMRRLHTL | 126 |
| Sbjct 71 | G+ C VL+ GR WLL+ VPG L + L P V ++A A+RRLH L HGAGIDCPQVLNETQSDGRQWLLMSAVPGDTLSALAQRGELEPERLVRLVAAALRRLHDL | 130 |
| Query 127 | DPATCPFDHQAKHRIERARTRMEAGLVDQDDLDEEHQGLAPAELFARLKARMPDGEDLVV | 186 |
| Sbjct 131 | DPA CPFDH+ + R++ R R+EAGLVD+ D D++H+G + EL+ L R P EDLVV DPAACPFDHRLERRLDTVRQRVEAGLVDEADFDDDHRGRSATELYRLLLDRRPAVEDLVV | 190 |
| Query 187 | THGDACLPNIMVENGRFSGFIDCGRLGVADRYQDIALATRDIAEELGGEWADRFLVLYGI HGDACLPN++ E RFSGFIDCGRLGVADR+Q +ALA RDI ELGG A+ FLV YG | 246 |

| Sbjct | 191 | AHGDACLPNLLAEGRRFSGFIDCGRLGVADRHQALALAARDIEAELGGAGAEAFLVEYG- 249 |
| --- | --- | --- |
| Query | 247 | AAPDSQRIAFYRLLDEFF 264 |
| Sbjct | 250 | D +R+A++RLLDEFF  GDIDGERLAYFRLLDEFF 267 |

>WP_181854418.1 APH(3')-II family aminoglycoside O-phosphotransferase [Pseudomonas aeruginosa]

MBA5045633.1 APH(3')-II family aminoglycoside O-phosphotransferase [Pseudomonas aeruginosa]

Length=268

Score = 258 bits (660), Expect = 1e-82, Method: Compositional matrix adjust.

| Identities | | = 135/258 (52%), Positives = 167/258 (65%), Gaps = 5/258 (2%) |  |
| --- | --- | --- | --- |
| Query 11  Sbjct 12 | | SPAAWVERLFGYDWAQQTIGCSDAAVFRLSAQGRPVLFVKTDLSGALNELQDEAARLSWL  +P+ W + L GY W Q GCS A V RL A RP LFVK ++ EL E ARL WL APSTWADYLAGYRWRGQGEGCSAATVHRLEAARRPTLFVKQEVLSVHAELPAEIARLRWL | 70  71 |
| Query 71  Sbjct 72 | | ATTGVPCAAVLDVVTEAGRDWLLLGEVPGQDLLS SHLAPAEKVSIMADAMRRLHTL  G+ C VL+ GR WLL+ VPG L + L P V ++A A+RRLH L HGAGIDCPQVLNETQSDGRQWLLMSAVPGDTLSALAQRGELEPERLVRLVAAALRRLHDL | 126  131 |
| Query 127  Sbjct 132 | | DPATCPFDHQAKHRIERARTRMEAGLVDQDDLDEEHQGLAPAELFARLKARMPDGEDLVV DPA CPFDH+ + R++ R R+EAGLVD+ D D++H+G + EL+ L R P EDLVV DPAACPFDHRLERRLDTVRQRVEAGLVDEADFDDDHRGRSATELYRLLLDRRPAVEDLVV | 186  191 |
| Query 187  Sbjct 192 | | THGDACLPNIMVENGRFSGFIDCGRLGVADRYQDIALATRDIAEELGGEWADRFLVLYGI HGDACLPN++ E RFSGFIDCGRLGVADR+QD+ALA RDI ELG WA+ LV YG AHGDACLPNLLAEGRRFSGFIDCGRLGVADRHQDLALAARDIEAELGAAWAEAILVEYG- | 246  250 |
| Query | 247 AAPDSQRIAFYRLLDEFF 264 | | |
|  |  | D +R+A++RLLDEFF |  |
| Sbjct | 251 | GDIDGERLAYFRLLDEFF 268 | |

>WP_124171550.1 APH(3')-II family aminoglycoside O-phosphotransferase [Pseudomonas aeruginosa]

RQC31295.1 APH(3') family aminoglycoside O-phosphotransferase [Pseudomonas aeruginosa]

Length=268

Score = 258 bits (660), Expect = 1e-82, Method: Compositional matrix adjust.

| Identities | | = 136/258 (53%), Positives = 167/258 (65%), Gaps = 5/258 (2%) |  |
| --- | --- | --- | --- |
| Query 11  Sbjct 12 | | SPAAWVERLFGYDWAQQTIGCSDAAVFRLSAQGRPVLFVKTDLSGALNELQDEAARLSWL  +P+ W + L GY W Q GCS A V RL A RP LFVK ++ A EL E ARL WL APSTWADYLAGYRWRGQGEGCSAATVHRLEAARRPTLFVKQEVLSAHAELPAEIARLRWL | 70  71 |
| Query 71  Sbjct 72 | | ATTGVPCAAVLDVVTEAGRDWLLLGEVPGQDLLS SHLAPAEKVSIMADAMRRLHTL  G+ C VL+ GR WLL+ VPG L + L P V ++A A+ RLH L HGAGIDCPQVLNETQSDGRQWLLMSAVPGDTLSALAQRGELEPERLVRLVAAALXRLHDL | 126  131 |
| Query 127  Sbjct 132 | | DPATCPFDHQAKHRIERARTRMEAGLVDQDDLDEEHQGLAPAELFARLKARMPDGEDLVV DPA CPFDH+ + R++ R R+EAGLVD+ D D++H+G + EL+ L R P EDLVV DPAACPFDHRLERRLDTVRQRVEAGLVDEADFDDDHRGRSATELYRLLLDRRPAVEDLVV | 186  191 |
| Query 187  Sbjct 192 | | THGDACLPNIMVENGRFSGFIDCGRLGVADRYQDIALATRDIAEELGGEWADRFLVLYGI HGDACLPN++ E RFSGFIDCGRLGVADR+QD+ALA RDI ELG WA+ FLV YG AHGDACLPNLLAEGRRFSGFIDCGRLGVADRHQDLALAARDIEAELGAAWAEAFLVEYG- | 246  250 |
| Query | 247 AAPDSQRIAFYRLLDEFF 264 | | |
|  |  | D +R A++RLLDEFF |  |
| Sbjct | 251 | GDIDGERXAYFRLLDEFF 268 | |

>WP_132898834.1 APH(3')-II family aminoglycoside O-phosphotransferase [Pseudomonas aeruginosa]

Length=268

Score = 258 bits (659), Expect = 2e-82, Method: Compositional matrix adjust.

| Identities | | = 135/258 (52%), Positives = 167/258 (65%), Gaps = 5/258 (2%) |  |
| --- | --- | --- | --- |
| Query 11  Sbjct 12 | | SPAAWVERLFGYDWAQQTIGCSDAAVFRLSAQGRPVLFVKTDLSGALNELQDEAARLSWL  +P+ W + L GY W GCS A V RL RP LFVK ++ A EL E ARL WL APSTWADYLAGYRWRGXGEGCSAATVHRLEVARRPTLFVKQEVLSAHAELPAEIARLRWL | 70  71 |
| Query 71  Sbjct 72 | | ATTGVPCAAVLDVVTEAGRDWLLLGEVPGQDLLS SHLAPAEKVSIMADAMRRLHTL  G+ C VL+ GR WLL+ VPG L + L P V ++A A+RRLH L HGAGIDCPQVLNETQSDGRQWLLMSAVPGDTLSALAQRGELEPERLVRLVAAALRRLHDL | 126  131 |
| Query 127  Sbjct 132 | | DPATCPFDHQAKHRIERARTRMEAGLVDQDDLDEEHQGLAPAELFARLKARMPDGEDLVV DPA CPFDH+ + R++ R R+EAGLVD+ D D++H+G + EL+ L R P EDLVV DPAACPFDHRLERRLDTVRQRVEAGLVDEADFDDDHRGRSATELYRLLLDRRPAVEDLVV | 186  191 |
| Query 187  Sbjct 192 | | THGDACLPNIMVENGRFSGFIDCGRLGVADRYQDIALATRDIAEELGGEWADRFLVLYGI HGDACLPN++ E RFSGFIDCGRLGVADR+QD+ALA RDI ELG WA+ FLV YG AHGDACLPNLLAEGRRFSGFIDCGRLGVADRHQDLALAARDIEAELGAAWAEAFLVEYG- | 246  250 |
| Query | 247 AAPDSQRIAFYRLLDEFF 264 | | |
|  |  | D +R+A++RLLDEFF |  |
| Sbjct | 251 | GDIDGERLAYFRLLDEFF 268 | |

>WP_124127182.1 APH(3')-II family aminoglycoside O-phosphotransferase [Pseudomonas aeruginosa]

RPV99655.1 APH(3') family aminoglycoside O-phosphotransferase [Pseudomonas aeruginosa]

Length=268

Score = 258 bits (659), Expect = 2e-82, Method: Compositional matrix adjust.

| Identities | | = 136/258 (53%), Positives = 167/258 (65%), Gaps = 5/258 (2%) |  |
| --- | --- | --- | --- |
| Query 11  Sbjct 12 | | SPAAWVERLFGYDWAQQTIGCSDAAVFRLSAQGRPVLFVKTDLSGALNELQDEAARLSWL  +P W + L GY W Q GCS A V RL A R LFVK ++ A EL E ARL WL APDTWADYLAGYRWRGQGEGCSAATVHRLEAARRSTLFVKQEVLSAHAELPAEIARLRWL | 70  71 |
| Query 71  Sbjct 72 | | ATTGVPCAAVLDVVTEAGRDWLLLGEVPGQDLLS SHLAPAEKVSIMADAMRRLHTL  G+ C VL+ GR WLL+ VPG L + L P V ++A A+RRLH L HGAGIDCPQVLNETQSDGRQWLLMSAVPGDTLSALAQRGELEPERLVRLVAAALRRLHDL | 126  131 |
| Query 127  Sbjct 132 | | DPATCPFDHQAKHRIERARTRMEAGLVDQDDLDEEHQGLAPAELFARLKARMPDGEDLVV DPA CPFDH+ + R++ R R+EAGLVD+ D D++H+G + EL+ L R P EDLVV DPAVCPFDHRLERRLDTVRQRVEAGLVDEADFDDDHRGRSATELYRLLLDRRPAVEDLVV | 186  191 |
| Query 187  Sbjct 192 | | THGDACLPNIMVENGRFSGFIDCGRLGVADRYQDIALATRDIAEELGGEWADRFLVLYGI HGDACLPN++ E RFSGFIDCGRLGVADR+QD+ALA RDI ELG WA+ FLV YG AHGDACLPNLLAEGRRFSGFIDCGRLGVADRHQDLALAARDIEAELGATWAEAFLVEYG- | 246  250 |
| Query | 247 AAPDSQRIAFYRLLDEFF 264 | | |
|  |  | D +R+A++RLLDEFF |  |
| Sbjct | 251 | GDIDGERLAYFRLLDEFF 268 | |

>MBG7175407.1 APH(3')-II family aminoglycoside O-phosphotransferase [Pseudomonas aeruginosa]

Length=268

Score = 258 bits (659), Expect = 2e-82, Method: Compositional matrix adjust.

| Identities | = 135/258 (52%), Positives = 166/258 (64%), Gaps = 5/258 (2%) |  |
| --- | --- | --- |
| Query 11 | SPAAWVERLFGYDWAQQTIGCSDAAVFRLSAQGRPVLFVKTDLSGALNELQDEAARLSWL | 70 |
| Sbjct 12 | +P+ W + L GY W Q GCS A V RL A RP LFVK ++ EL E ARL WL APSTWADYLAGYRWRGQGEGCSAATVHRLEAARRPTLFVKQEVLSVHAELPAEIARLRWL | 71 |
| Query 71 | ATTGVPCAAVLDVVTEAGRDWLLLGEVPGQDLLS SHLAPAEKVSIMADAMRRLHTL | 126 |
| Sbjct 72 | G+ C VL+ GR WLL+ VPG L + L P V ++A A+RRLH L HGAGIDCPQVLNETQSDGRQWLLMSAVPGDTLSALAQRGELEPERLVRLVAAALRRLHDL | 131 |
| Query 127 | DPATCPFDHQAKHRIERARTRMEAGLVDQDDLDEEHQGLAPAELFARLKARMPDGEDLVV | 186 |
| Sbjct 132 | DP CPFDH+ R++ R R+EAGLVD+ D D++H+G + EL+ L R P EDLVV  DPVACPFDHRLDRRLDTVRQRVEAGLVDEADFDDDHRGRSATELYRLLLDRRPAVEDLVV | 191 |

Query 187 THGDACLPNIMVENGRFSGFIDCGRLGVADRYQDIALATRDIAEELGGEWADRFLVLYGI 246

| Sbjct | 192 | HGDACLPN++ E RFSGFIDCGRLGVADR+QD+ALA RDI ELG WA+ FLV YG AHGDACLPNLLAEGRRFSGFIDCGRLGVADRHQDLALAARDIEAELGAAWAEDFLVEYG- 250 |
| --- | --- | --- |
| Query | 247 | AAPDSQRIAFYRLLDEFF 264 |
| Sbjct | 251 | D +R+A++RLLDEFF  GDIDGERLAYFRLLDEFF 268 |

>WP_038542852.1 aminoglycoside 3'-phosphotransferase [Neorhizobium galegae]

CDN54033.1 Kanamycin kinase [Neorhizobium galegae bv. officinalis bv. officinalis str. HAMBI 1141]

Length=260

Score = 258 bits (658), Expect = 2e-82, Method: Compositional matrix adjust.

| Identities | | = 133/253 (53%), Positives = 169/253 (67%), Gaps = 1/253 (0%) |  |
| --- | --- | --- | --- |
| Query 12  Sbjct 9 | | PAAWVERLFGYDWAQQTIGCSDAAVFRLSAQGRPVLFVKTDLSGALNELQDEAARLSWLA P AW ERL G+ IG S A+V RL+A V FVKT+ +L EAARL WL PEAWRERLGGHAVQDIMIGRSAASVHRLTAADGEVFFVKTEAVSPFADLPGEAARLRWLG | 71  68 |
| Query 72  Sbjct 69 | | TTGVPCAAVLDVVTEAGRDWLLLGEVPGQDLLSSHLAPAEKVSIMADAMRRLHTLDPATC T+G+PC V+D +TE R WLLL VPG +L+ ++L P + V I+A A+ RLH+L+P +C TSGIPCPIVIDEITEEARHWLLLSAVPGSNLVEANLPPEDIVEIVAQALHRLHSLEPGSC | 131  128 |
| Query 132  Sbjct 129 | | PFDHQAKHRIERARTRMEAGLVDQDDLDEEHQGLAPAELFARLKARMPDGEDLVVTHGDA PFDH+ RIE ARTR+EAGLVD+DD D E G A+L RL+ P ED+VV HGDA PFDHRVVARIELARTRVEAGLVDEDDFDAERLGTPIADLIRRLEKGRPAIEDMVVAHGDA | 191  188 |
| Query 192  Sbjct 189 | | CLPNIMVENGRFSGFIDCGRLGVADRYQDIALATRDIAEELGGEWADRFLVLYGIAAPDS CLPN++ + G FSGF+DCGRLGV+DRYQD+ALA I LG W + FL+ YG D CLPNLIADRGIFSGFVDCGRLGVSDRYQDLALAQHSIRYNLGSPWDEIFLIRYG-GDIDR | 251  247 |
| Query 252 | | QRIAFYRLLDEFF 264  +A++RLLDEFF |  |
| Sbjct | 248 | DCLAWFRLLDEFF 260 | |

>WP_182552602.1 APH(3') family aminoglycoside O-phosphotransferase [Phyllobacterium myrsinacearum]

Length=263

Score = 258 bits (659), Expect = 2e-82, Method: Compositional matrix adjust.

| Identities | = 127/245 (52%), Positives = 162/245 (66%), Gaps = 1/245 (0%) |  |
| --- | --- | --- |
| Query 21 | GYDWAQQTIGCSDAAVFRLSAQGRPVLFVKTDLSGALNELQDEAARLSWLATTGVPCAAV | 80 |
| Sbjct 19 | GY W + +GCSDA VF L + P LF+K + EL DE ARL WL++ +PC + GYSWNRDELGCSDAQVFLLQGERLPRLFIKVETVSPYGELPDEIARLRWLSSCSLPCPDI | 78 |
| Query 81 | LDVVTEAGRDWLLLGEVPGQDLLSSHLAPAE-KVSIMADAMRRLHTLDPATCPFDHQAKH | 139 |
| Sbjct 79 | + AG WLL+ VPG DL S+ P E +V I+ADA+R+LH LDP+TCPFDH+ IFDGIHAGHHWLLMSAVPGTDLASADSVPVEPRVRILADALRQLHALDPSTCPFDHRLDM | 138 |
| Query 140 | RIERARTRMEAGLVDQDDLDEEHQGLAPAELFARLKARMPDGEDLVVTHGDACLPNIMVE | 199 |
| Sbjct 139 | RIE A R++AGLVD+ D D++ G AELF RL + P D+VVTHGDACLPN M + RIEAAGARVQAGLVDETDFDDDMLGETAAELFTRLVSEKPAVGDVVVTHGDACLPNFMAD | 198 |
| Query 200 | NGRFSGFIDCGRLGVADRYQDIALATRDIAEELGGEWADRFLVLYGIAAPDSQRIAFYRL | 259 |
| Sbjct 199 | G F+G+IDC RLG+ADRYQDIALA R I++ G EW FL YG+ D ++A+YRL KGIFTGYIDCARLGLADRYQDIALACRSISDNFGEEWVRPFLDCYGLKHADPAKLAYYRL | 258 |
| Query 260  Sbjct 259 | LDEFF 264 LDEFF LDEFF 263 |  |

>WP_055970481.1 aminoglycoside 3'-phosphotransferase [Aminobacter sp. DSM 101952] KQU76637.1 aminoglycoside phosphotransferase [Aminobacter sp. DSM 101952] MBB5229750.1 aminoglycoside 3'-phosphotransferase-2 [Aminobacter sp. DSM 101952]

Length=264

Score = 258 bits (659), Expect = 2e-82, Method: Compositional matrix adjust.

| Identities | = 136/264 (52%), Positives = 172/264 (65%), Gaps = 0/264 (0%) |  |
| --- | --- | --- |
| Query 1 | MIEQDGLHAGSPAAWVERLFGYDWAQQTIGCSDAAVFRLSAQGRPVLFVKTDLSGALNEL | 60 |
| Sbjct 1 | M + + PA+ E L GYDW +QTIG S A VFRL A G+P LF+KT+ +G EL MTQHPAIEIDLPASLREALAGYDWQRQTIGASGAGVFRLEASGKPTLFLKTEETGQFAEL | 60 |
| Query 61 | QDEAARLSWLATTGVPCAAVLDVVTEAGRDWLLLGEVPGQDLLSSHLAPAEKVSIMADAM | 120 |
| Sbjct 61 | DE ARL WL + G+PC AVL G +WLL+ V G DL++ AP ++IMADA+ PDEVARLRWLDSQGIPCPAVLAFEAHGGHNWLLMSAVAGHDLVTQEAAPDRAIAIMADAL | 120 |
| Query 121 | RRLHTLDPATCPFDHQAKHRIERARTRMEAGLVDQDDLDEEHQGLAPAELFARLKARMPD | 180 |
| Sbjct 121 | RRLH LD A CPFDH RI A+ R +AG VD+DD D+E G + ++F L AR P RRLHALDAAACPFDHGLGPRIALAKARADAGEVDEDDFDDERLGKSAQQVFEELLARRPA | 180 |
| Query 181 | GEDLVVTHGDACLPNIMVENGRFSGFIDCGRLGVADRYQDIALATRDIAEELGGEWADRF | 240 |
| Sbjct 181 | E LVVTHGDA +PN M + GRF+GFIDC RLGVAD +QD+ALA I LG EW F SETLVVTHGDASMPNFMADGGRFTGFIDCSRLGVADLHQDLALACWSIHLNLGEEWIAPF | 240 |
| Query 241  Sbjct 241 | LVLYGIAAPDSQRIAFYRLLDEFF 264 L LYG+ D+ ++++Y+LLDEFF LALYGLPQADTAKLSYYQLLDEFF 264 |  |

>WP_121366548.1 APH(3')-II family aminoglycoside O-phosphotransferase [Pseudomonas aeruginosa]

QIZ28259.1 APH(3')-II family aminoglycoside O-phosphotransferase [Pseudomonas aeruginosa]

RPM78775.1 APH(3') family aminoglycoside O-phosphotransferase [Pseudomonas aeruginosa]

RPS00818.1 APH(3') family aminoglycoside O-phosphotransferase [Pseudomonas aeruginosa]

RQI00773.1 APH(3') family aminoglycoside O-phosphotransferase [Pseudomonas aeruginosa]

RUJ60352.1 APH(3')-II family aminoglycoside O-phosphotransferase [Pseudomonas aeruginosa]

Length=268

Score = 258 bits (659), Expect = 2e-82, Method: Compositional matrix adjust.

| Identities | | = 136/258 (53%), Positives = 167/258 (65%), Gaps = 5/258 (2%) |  |
| --- | --- | --- | --- |
| Query 11  Sbjct 12 | | SPAAWVERLFGYDWAQQTIGCSDAAVFRLSAQGRPVLFVKTDLSGALNELQDEAARLSWL  +P W + L GY W Q GCS A V RL A R LFVK ++ A EL E ARL WL APDTWADYLAGYRWRGQGEGCSAATVHRLEAARRSTLFVKQEVLSAHAELPAEIARLRWL | 70  71 |
| Query 71  Sbjct 72 | | ATTGVPCAAVLDVVTEAGRDWLLLGEVPGQDLLS SHLAPAEKVSIMADAMRRLHTL  G+ C VL+ GR WLL+ VPG L + L P V ++A A+RRLH L HGAGIDCPQVLNETQSDGRQWLLMSAVPGDTLSALAQRGELEPERLVRLVAAALRRLHDL | 126  131 |
| Query 127  Sbjct 132 | | DPATCPFDHQAKHRIERARTRMEAGLVDQDDLDEEHQGLAPAELFARLKARMPDGEDLVV DPA CPFDH+ + R++ R R+EAGLVD+ D D++H+G + EL+ L R P EDLVV DPAACPFDHRLERRLDTVRQRVEAGLVDEADFDDDHRGRSATELYRLLLDRRPAVEDLVV | 186  191 |
| Query 187  Sbjct 192 | | THGDACLPNIMVENGRFSGFIDCGRLGVADRYQDIALATRDIAEELGGEWADRFLVLYGI HGDACLPN++ E RFSGFIDCGRLGVADR+QD+ALA RDI ELG WA+ FLV YG AHGDACLPNLLAEGRRFSGFIDCGRLGVADRHQDLALAARDIEAELGATWAEAFLVEYG- | 246  250 |
| Query | 247 AAPDSQRIAFYRLLDEFF 264 | | |
|  |  | D +R+A++RLLDEFF |  |
| Sbjct | 251 | GDIDGERLAYFRLLDEFF 268 | |

>WP_176205607.1 phosphotransferase, partial [Salmonella enterica] Length=125

Score = 253 bits (645), Expect = 2e-82, Method: Compositional matrix adjust. Identities = 125/125 (100%), Positives = 125/125 (100%), Gaps = 0/125 (0%)

| Query | 1 | MIEQDGLHAGSPAAWVERLFGYDWAQQTIGCSDAAVFRLSAQGRPVLFVKTDLSGALNEL | 60 |
| --- | --- | --- | --- |
|  |  | MIEQDGLHAGSPAAWVERLFGYDWAQQTIGCSDAAVFRLSAQGRPVLFVKTDLSGALNEL |  |
| Sbjct | 1 | MIEQDGLHAGSPAAWVERLFGYDWAQQTIGCSDAAVFRLSAQGRPVLFVKTDLSGALNEL | 60 |
| Query | 61 | QDEAARLSWLATTGVPCAAVLDVVTEAGRDWLLLGEVPGQDLLSSHLAPAEKVSIMADAM | 120 |

| Sbjct | 61 | QDEAARLSWLATTGVPCAAVLDVVTEAGRDWLLLGEVPGQDLLSSHLAPAEKVSIMADAM QDEAARLSWLATTGVPCAAVLDVVTEAGRDWLLLGEVPGQDLLSSHLAPAEKVSIMADAM 120 |
| --- | --- | --- |
| Query | 121 | RRLHT 125 |
| Sbjct | 121 | RRLHT  RRLHT 125 |

>WP_043094975.1 APH(3')-II family aminoglycoside O-phosphotransferase [Pseudomonas aeruginosa]

Length=271

Score = 258 bits (659), Expect = 2e-82, Method: Compositional matrix adjust.

| Identities | = 138/261 (53%), Positives = 169/261 (65%), Gaps = 8/261 (3%) |  |
| --- | --- | --- |
| Query 11 | SPAAWVERLFGYDWAQQTIGCSDAAVFRLSAQGRPVLFVKTDLSGALNELQDEAARLSWL | 70 |
| Sbjct 12 | +PA W + L GY W Q GCS A V RL A RP LFVK ++ A EL E ARL WL APATWADYLAGYRWRGQGEGCSAATVHRLEAARRPTLFVKQEVLSAHAELPAEIARLRWL | 71 |
| Query 71 | ATTGVPCAAVLDVVTEAGRDWLLLGEVPGQDLLS----SHLAP---AEKVSIMADAMRRL | 123 |
| Sbjct 72 | G+ C VL+ GR WLL+ VPG L + L P V ++A A+RRL HGAGIDCPQVLNETQSDGRQWLLMSAVPGDTLSALAQRGELEPERLVRLVRLVAAALRRL | 131 |
| Query 124 | HTLDPATCPFDHQAKHRIERARTRMEAGLVDQDDLDEEHQGLAPAELFARLKARMPDGED | 183 |
| Sbjct 132 | H LDPA CPFDH+ + R++ R R+EAGLVD+ D D++H+G + EL+ L R P ED HDLDPAACPFDHRLERRLDTVRQRVEAGLVDEADFDDDHRGRSATELYRLLLDRRPAVED | 191 |
| Query 184 | LVVTHGDACLPNIMVENGRFSGFIDCGRLGVADRYQDIALATRDIAEELGGEWADRFLVL | 243 |
| Sbjct 192 | LVV HGDACLPN++ E RFSGFIDCGRLGVADR+QD+ALA RDI ELG WA+ FLV LVVAHGDACLPNLLAEGRRFSGFIDCGRLGVADRHQDLALAARDIEAELGAAWAEAFLVE | 251 |
| Query 244  Sbjct 252 | YGIAAPDSQRIAFYRLLDEFF 264 YG D +R+A++RLLDEFF  YG-GDIDGERLAYFRLLDEFF 271 |  |

>MBG6663591.1 APH(3')-II family aminoglycoside O-phosphotransferase [Pseudomonas aeruginosa]

Length=268

Score = 258 bits (659), Expect = 2e-82, Method: Compositional matrix adjust.

| Identities | | = 135/258 (52%), Positives = 167/258 (65%), Gaps = 5/258 (2%) |  |
| --- | --- | --- | --- |
| Query 11  Sbjct 12 | | SPAAWVERLFGYDWAQQTIGCSDAAVFRLSAQGRPVLFVKTDLSGALNELQDEAARLSWL  +P+ W + L GY W Q GCS A V RL A RP LFVK ++ A EL E ARL WL APSTWADYLAGYRWRGQGEGCSAATVHRLEAARRPTLFVKQEVLSAHAELPAEIARLRWL | 70  71 |
| Query 71  Sbjct 72 | | ATTGVPCAAVLDVVTEAGRDWLLLGEVPGQDLLS SHLAPAEKVSIMADAMRRLHTL  G+ C VL+ GR WLL+ VPG L + L P V ++A A+RRLH L HGAGIDCPQVLNETQSDGRQWLLMSAVPGDTLSALAQRGELEPERLVRLVAAALRRLHDL | 126  131 |
| Query 127  Sbjct 132 | | DPATCPFDHQAKHRIERARTRMEAGLVDQDDLDEEHQGLAPAELFARLKARMPDGEDLVV DPA CPFDH+ + R++ R R+EAGLVD+ D D++H+G + EL+ L R P EDLVV DPAACPFDHRLERRLDTVRQRVEAGLVDEADFDDDHRGRSATELYRLLLDRRPAVEDLVV | 186  191 |
| Query 187  Sbjct 192 | | THGDACLPNIMVENGRFSGFIDCGRLGVADRYQDIALATRDIAEELGGEWADRFLVLYGI HGDACLPN++ E RFSGFIDCGR G ADR+QD+ALA RDI ELG WA+ FLV YG AHGDACLPNLLAEGRRFSGFIDCGRRGAADRHQDLALAARDIEAELGAAWAEAFLVEYG- | 246  250 |
| Query | 247 AAPDSQRIAFYRLLDEFF 264 | | |
|  |  | D +R+A++RLLDEFF |  |
| Sbjct | 251 | GDIDGERLAYFRLLDEFF 268 | |

>WP_196733514.1 APH(3')-II family aminoglycoside O-phosphotransferase [Pseudomonas aeruginosa]

MBG5280764.1 APH(3')-II family aminoglycoside O-phosphotransferase [Pseudomonas aeruginosa]

MBG5519596.1 APH(3')-II family aminoglycoside O-phosphotransferase [Pseudomonas aeruginosa]

Length=268

Score = 258 bits (659), Expect = 2e-82, Method: Compositional matrix adjust.

| Identities | | = 135/258 (52%), Positives = 167/258 (65%), Gaps = 5/258 (2%) |  |
| --- | --- | --- | --- |
| Query 11  Sbjct 12 | | SPAAWVERLFGYDWAQQTIGCSDAAVFRLSAQGRPVLFVKTDLSGALNELQDEAARLSWL  +P+ W + L GY W Q GCS A V RL RP LFVK ++ A EL E ARL WL APSTWADYLAGYRWRGQGEGCSAATVHRLEVARRPTLFVKQEVLSAHAELPAEIARLRWL | 70  71 |
| Query 71  Sbjct 72 | | ATTGVPCAAVLDVVTEAGRDWLLLGEVPGQDLLS SHLAPAEKVSIMADAMRRLHTL  G+ C VL+ GR WLL+ VPG L + L P V ++A A+RRLH L HGAGIDCPQVLNETQSDGRQWLLMSAVPGDTLSALAQRGELEPERLVRLVAAALRRLHDL | 126  131 |
| Query 127  Sbjct 132 | | DPATCPFDHQAKHRIERARTRMEAGLVDQDDLDEEHQGLAPAELFARLKARMPDGEDLVV DPA CPFDH+ + R++ R R+EAGLV + D D++H+G + EL+ L R P EDLVV DPAACPFDHRLERRLDTVRQRVEAGLVGEADFDDDHRGRSATELYRLLLDRRPAVEDLVV | 186  191 |
| Query 187  Sbjct 192 | | THGDACLPNIMVENGRFSGFIDCGRLGVADRYQDIALATRDIAEELGGEWADRFLVLYGI HGDACLPN++ E RFSGFIDCGRLGVADR+QD+ALA RDI ELG WA+ FLV YG AHGDACLPNLLAEGRRFSGFIDCGRLGVADRHQDLALAARDIEAELGAAWAEAFLVEYG- | 246  250 |
| Query | 247 AAPDSQRIAFYRLLDEFF 264 | | |
|  |  | D +R+A++RLLDEFF |  |
| Sbjct | 251 | GDIDGERLAYFRLLDEFF 268 | |

>WP_058175559.1 APH(3')-II family aminoglycoside O-phosphotransferase [Pseudomonas aeruginosa]

KSP15319.1 aminoglycoside 3'-phosphotransferase [Pseudomonas aeruginosa]

MQH01086.1 APH(3')-II family aminoglycoside O-phosphotransferase [Pseudomonas aeruginosa]

Length=268

Score = 258 bits (659), Expect = 2e-82, Method: Compositional matrix adjust.

| Identities | | = 135/258 (52%), Positives = 167/258 (65%), Gaps = 5/258 (2%) |  |
| --- | --- | --- | --- |
| Query 11  Sbjct 12 | | SPAAWVERLFGYDWAQQTIGCSDAAVFRLSAQGRPVLFVKTDLSGALNELQDEAARLSWL  +P+ W + L GY W Q GCS A V RL RP LFVK ++ A EL E ARL WL APSTWADYLAGYRWRGQGEGCSAATVHRLEVARRPTLFVKQEVLSAHAELPAEIARLRWL | 70  71 |
| Query 71  Sbjct 72 | | ATTGVPCAAVLDVVTEAGRDWLLLGEVPGQDLLS SHLAPAEKVSIMADAMRRLHTL  G+ C VL+ GR WLL+ VPG L + L P V ++A A+RRLH L HGAGIDCPQVLNETQSDGRQWLLMSAVPGDTLSALAQRGELEPERLVRLVAAALRRLHDL | 126  131 |
| Query 127  Sbjct 132 | | DPATCPFDHQAKHRIERARTRMEAGLVDQDDLDEEHQGLAPAELFARLKARMPDGEDLVV DPA CPFDH+ + R++ R R+EAGLVD+ D++H+G + EL+ L R P EDLVV DPAACPFDHRLERRLDTVRQRVEAGLVDEAGFDDDHRGRSATELYRLLLDRRPAVEDLVV | 186  191 |
| Query 187  Sbjct 192 | | THGDACLPNIMVENGRFSGFIDCGRLGVADRYQDIALATRDIAEELGGEWADRFLVLYGI HGDACLPN++ E RFSGFIDCGRLGVADR+QD+ALA RDI ELG WA+ FLV YG AHGDACLPNLLAEGRRFSGFIDCGRLGVADRHQDLALAARDIEAELGAAWAEAFLVEYG- | 246  250 |
| Query | 247 AAPDSQRIAFYRLLDEFF 264 | | |
|  |  | D +R+A++RLLDEFF |  |
| Sbjct | 251 | GDIDGERLAYFRLLDEFF 268 | |

```
>WP_087851757.1 APH(3')-II family aminoglycoside O-phosphotransferase [Pseudomonas aeruginosa]
OVY93776.1 APH(3') family aminoglycoside O-phosphotransferase [Pseudomonas aeruginosa]
RPO48729.1 APH(3')-II family aminoglycoside O-phosphotransferase [Pseudomonas aeruginosa]
RPO60018.1 APH(3')-II family aminoglycoside O-phosphotransferase [Pseudomonas aeruginosa]
Length=268

Score = 258 bits (659), Expect = 2e-82, Method: Compositional matrix adjust. Identities = 135/258 (52%), Positives = 167/258 (65%), Gaps = 5/258 (2%)

Query 11	SPAAWVERLFGYDWAQQTIGCSDAAVFRLSAQGRPVLFVKTDLSGALNELQDEAARLSWL 70
+P+ W + L GY W	GCS A V RL A RP LFVK ++	EL E ARL WL Sbjct 12	APSTWADYLAGYRWRGLGEGCSAATVHRLEAARRPTLFVKQEVLSVHAELPAEIARLRWL 71
```

| Query | 71 | ATTGVPCAAVLDVVTEAGRDWLLLGEVPGQDLLS SHLAPAEKVSIMADAMRRLHTL | 126 |
| --- | --- | --- | --- |
| Sbjct | 72 | G+ C VL+ GR WLL+ VPG L + L P V ++A A+RRLH L HGAGIDCPQVLNETQSDGRQWLLMSAVPGDTLSALAQRGELEPERLVRLVAAALRRLHDL | 131 |
| Query | 127 | DPATCPFDHQAKHRIERARTRMEAGLVDQDDLDEEHQGLAPAELFARLKARMPDGEDLVV | 186 |
| Sbjct | 132 | DPA CPFDH+ + R++ R R+EAGLVD+ D D++H+G + EL+ L R P EDLVV DPAACPFDHRLERRLDTVRQRVEAGLVDEADFDDDHRGRSATELYRLLLDRRPAVEDLVV | 191 |
| Query | 187 | THGDACLPNIMVENGRFSGFIDCGRLGVADRYQDIALATRDIAEELGGEWADRFLVLYGI | 246 |
| Sbjct | 192 | HGDACLPN++ E RFSGFIDCGRLGVADR+QD+ALA RDI ELG WA+ FLV YG  AHGDACLPNLLAEGRRFSGFIDCGRLGVADRHQDLALAARDIEAELGAAWAEAFLVEYG- | 250 |
| Query | 247 AAPDSQRIAFYRLLDEFF 264 | | |
|  |  | D +R+A++RLLDEFF |  |
| Sbjct | 251 | GDIDGERLAYFRLLDEFF 268 | |

>WP_033987203.1 APH(3')-II family aminoglycoside O-phosphotransferase [Pseudomonas aeruginosa]

Length=268

Score = 258 bits (659), Expect = 2e-82, Method: Compositional matrix adjust.

| Identities | | = 136/258 (53%), Positives = 168/258 (65%), Gaps = 5/258 (2%) |  |
| --- | --- | --- | --- |
| Query 11  Sbjct 12 | | SPAAWVERLFGYDWAQQTIGCSDAAVFRLSAQGRPVLFVKTDLSGALNELQDEAARLSWL  +P+ + L GY W Q GCS A V RL A RP LFVK ++ A EL E ARL WL APSTLADYLAGYRWRGQGEGCSAATVHRLEAARRPTLFVKQEVLSAHAELPAEIARLRWL | 70  71 |
| Query 71  Sbjct 72 | | ATTGVPCAAVLDVVTEAGRDWLLLGEVPGQDLLS SHLAPAEKVSIMADAMRRLHTL  G+ C VL+ GR WLL+ VPG L + L P V ++A A+RRLH L HGAGIDCPQVLNETQSDGRQWLLMSAVPGDTLSALAQRGELEPERLVRLVAAALRRLHDL | 126  131 |
| Query 127  Sbjct 132 | | DPATCPFDHQAKHRIERARTRMEAGLVDQDDLDEEHQGLAPAELFARLKARMPDGEDLVV DPA CPFDH+ + R++ R R+EAGLVD+ D D++H+G + EL+ L R P EDLVV DPAACPFDHRLERRLDTVRQRVEAGLVDEADFDDDHRGRSATELYRLLLDRRPAVEDLVV | 186  191 |
| Query 187  Sbjct 192 | | THGDACLPNIMVENGRFSGFIDCGRLGVADRYQDIALATRDIAEELGGEWADRFLVLYGI HGDACLPN++ E RFSGFIDCGRLGVADR+QD+ALA RDI ELG WA+ FLV YG AHGDACLPNLLAEGRRFSGFIDCGRLGVADRHQDLALAARDIEAELGAAWAEAFLVEYG- | 246  250 |
| Query | 247 AAPDSQRIAFYRLLDEFF 264 | | |
|  |  | D +R+A++RLLDEFF |  |
| Sbjct | 251 | GDIDGERLAYFRLLDEFF 268 | |

>WP_096796437.1 aminoglycoside 3'-phosphotransferase [Pseudomonas fluorescens]

PCM48793.1 APH(3') family aminoglycoside O-phosphotransferase [Pseudomonas fluorescens]

Length=256

Score = 257 bits (657), Expect = 2e-82, Method: Compositional matrix adjust.

| Identities | | = 133/254 (52%), Positives = 169/254 (67%), Gaps = 2/254 (1%) |  |
| --- | --- | --- | --- |
| Query 12  Sbjct 4 | | PAAWVERLFGYDWAQQTIGCSDAAVFRLSAQGRPVLFVKTDLSGALNELQDEAARLSWLA P W +R + QQTIG S A VFRL+ G LFVK++ + EL E RL WLA PQTWRQRFADIRYEQQTIGESRADVFRLTPAGSDPLFVKSEPRISFAELPGEVQRLRWLA | 71  63 |
| Query 72  Sbjct 64 | | TTGVPCAAVLDVVTEAGRDWLLLGEVPGQDLLSS-HLAPAEKVSIMADAMRRLHTLDPAT  +P A VLD V E+ R WLL+ VPG+DL SS HLAP + VS+ A+A+R LH LD EQDLPAARVLDSVEESERFWLLMTAVPGRDLASSEHLAPQQIVSLAANALRTLHALDIKN | 130  123 |
| Query 131  Sbjct 124 | | CPFDHQAKHRIERARTRMEAGLVDQDDLDEEHQGLAPAELFARLKARMPDGEDLVVTHGD CPFDH+ +I AR + AGL+D++D D+ G + +F ++ A PD EDLVVTHGD CPFDHRLDAKIALAREHLLAGLIDEEDFDDSRLGQSAEAVFEQMLAARPDSEDLVVTHGD | 190  183 |
| Query 191  Sbjct 184 | | ACLPNIMVENGRFSGFIDCGRLGVADRYQDIALATRDIAEELGGEWADRFLVLYGIAAPD ACLPN++ +NGRFSGF+DCGRLGVADR+QD+ALA IA LG +W F YG+ PD ACLPNLLADNGRFSGFVDCGRLGVADRFQDLALAAHSIAYNLGEQWLPVFFEAYGV-QPD | 250  242 |
| Query | 251 SQRIAFYRLLDEFF 264 | | |
|  |  | ++IAFY+LLDEFF |  |
| Sbjct | 243 | QRKIAFYQLLDEFF 256 | |

>WP_196741451.1 APH(3')-II family aminoglycoside O-phosphotransferase [Pseudomonas aeruginosa]

MBG5743344.1 APH(3')-II family aminoglycoside O-phosphotransferase [Pseudomonas aeruginosa]

Length=268

Score = 258 bits (658), Expect = 2e-82, Method: Compositional matrix adjust.

| Identities | | = 135/258 (52%), Positives = 167/258 (65%), Gaps = 5/258 (2%) |  |
| --- | --- | --- | --- |
| Query 11  Sbjct 12 | | SPAAWVERLFGYDWAQQTIGCSDAAVFRLSAQGRPVLFVKTDLSGALNELQDEAARLSWL  +P+ W + L GY W Q GCS A V RL RP LFVK ++ A EL E ARL WL APSTWADYLAGYRWRGQGEGCSAATVHRLEVARRPTLFVKQEVLSAHAELPAEIARLRWL | 70  71 |
| Query 71  Sbjct 72 | | ATTGVPCAAVLDVVTEAGRDWLLLGEVPGQDLLS SHLAPAEKVSIMADAMRRLHTL  G+ C VL+ GR WLL+ VPG L + L P V ++A A+RRLH L HGAGIDCPQVLNETQSDGRQWLLMSAVPGDTLSALAQRGELEPERLVRLVAAALRRLHDL | 126  131 |
| Query 127  Sbjct 132 | | DPATCPFDHQAKHRIERARTRMEAGLVDQDDLDEEHQGLAPAELFARLKARMPDGEDLVV DPA CPFDH+ + R++ R R+EAGLVD+ D D++H+G + EL+ L R P EDLVV DPAACPFDHRLERRLDTVRQRVEAGLVDEADFDDDHRGRSATELYRLLLDRRPAVEDLVV | 186  191 |
| Query 187  Sbjct 192 | | THGDACLPNIMVENGRFSGFIDCGRLGVADRYQDIALATRDIAEELGGEWADRFLVLYGI HGDACLPN++ E RFSGFIDCGRLGVA R+QD+ALA RDI ELG WA+ FLV YG AHGDACLPNLLAEGRRFSGFIDCGRLGVAARHQDLALAARDIEAELGAAWAEAFLVEYG- | 246  250 |
| Query | 247 AAPDSQRIAFYRLLDEFF 264 | | |
|  |  | D +R+A++RLLDEFF |  |
| Sbjct | 251 | GDIDGERLAYFRLLDEFF 268 | |

>WP_088936496.1 aminoglycoside 3'-phosphotransferase [Rhizobium sp. N122] OWV90551.1 aminoglycoside phosphotransferase [Rhizobium sp. N122]

Length=261

Score = 258 bits (658), Expect = 2e-82, Method: Compositional matrix adjust.

| Identities | | = 129/254 (51%), Positives = 167/254 (66%), Gaps = 1/254 (0%) |  |
| --- | --- | --- | --- |
| Query 12  Sbjct 8 | | PAAWVERLFGYDWAQQTIGCSDAAVFRLSAQGRPVLFVKTDLSGALNELQDEAARLSWLA PAA ERL GY + + T+G S A+VFRL +G P L++K + +G EL DEAARL WL PAALAERLSGYRFERDTLGRSTASVFRLEGEGLPALYLKVETAGPFGELADEAARLGWLK | 71  67 |
| Query 72  Sbjct 68 | | TTGVPCAAVLDVVTEAGRDWLLLGEVPGQDLLS-SHLAPAEKVSIMADAMRRLHTLDPAT T G+PC V+ ++ G +WLL+G +PG DL S S L P +V ++A A+ RLH L + TAGLPCPEVIARDSDGGHNWLLIGALPGNDLASASALTPLARVELLAAALSRLHDLPISA | 130  127 |
| Query 131  Sbjct 128 | | CPFDHQAKHRIERARTRMEAGLVDQDDLDEEHQGLAPAELFARLKARMPDGEDLVVTHGD CPFDH+ ++RI A+ RM+AG+VD+ D DE G + LFA L++R P EDLVVTHGD CPFDHRLENRIAAAKARMQAGIVDETDFDETRLGKSAEALFAELESRRPGREDLVVTHGD | 190  187 |
| Query 191  Sbjct 188 | | ACLPNIMVENGRFSGFIDCGRLGVADRYQDIALATRDIAEELGGEWADRFLVLYGIAAPD ACLPN + FSG+IDC RLGVADRYQDIALA R I G FL YG+ PD ACLPNFIASAEGFSGYIDCSRLGVADRYQDIALACRSITYNFGEALVRPFLDRYGMTKPD | 250  247 |
| Query 251 | | SQRIAFYRLLDEFF 264  + +++LLDEFF |  |
| Sbjct | 248 | PATLHYFQLLDEFF 261 | |

```
>WP_196696260.1 APH(3')-II family aminoglycoside O-phosphotransferase [Pseudomonas aeruginosa]
MBG4315657.1 APH(3')-II family aminoglycoside O-phosphotransferase [Pseudomonas aeruginosa]
Length=268

Score = 258 bits (658), Expect = 2e-82, Method: Compositional matrix adjust. Identities = 136/258 (53%), Positives = 168/258 (65%), Gaps = 5/258 (2%)

Query 11	SPAAWVERLFGYDWAQQTIGCSDAAVFRLSAQGRPVLFVKTDLSGALNELQDEAARLSWL 70
+P+ W + L GY W Q GCS A V RL A RP LFVK ++ A EL E ARL WL Sbjct 12	APSTWADYLAGYRWRGQGEGCSAATVHRLEAARRPTLFVKQEVLSAHAELPAEIARLRWL 71
```

| Query | 71 | ATTGVPCAAVLDVVTEAGRDWLLLGEVPGQDLLS SHLAPAEKVSIMADAMRRLHTL | 126 |
| --- | --- | --- | --- |
| Sbjct | 72 | G+ C VL+ GR WLL+ VPG L + L P V ++A A+RRLH L HGAGIDCPQVLNETQSDGRQWLLMSAVPGDTLSALAQRGELEPERLVRLVAAALRRLHDL | 131 |
| Query | 127 | DPATCPFDHQAKHRIERARTRMEAGLVDQDDLDEEHQGLAPAELFARLKARMPDGEDLVV | 186 |
| Sbjct | 132 | DPA CPFDH+ + R++ R R+EAGLVD+ D D++H+G + EL+ L R P EDLVV DPAACPFDHRLERRLDTVRQRVEAGLVDEADFDDDHRGRSATELYRLLLDRRPAVEDLVV | 191 |
| Query | 187 | THGDACLPNIMVENGRFSGFIDCGRLGVADRYQDIALATRDIAEELGGEWADRFLVLYGI | 246 |
| Sbjct | 192 | HGDACLPN++ E RFSGFIDCGRLGVADR+QD+ALA RDI ELG A+ FLV YG  AHGDACLPNLLAEGRRFSGFIDCGRLGVADRHQDLALAARDIEAELGAARAEAFLVEYG- | 250 |
| Query | 247 AAPDSQRIAFYRLLDEFF 264 | | |
|  |  | D +R+A++RLLDEFF |  |
| Sbjct | 251 | GDIDGERLAYFRLLDEFF 268 | |

>WP_183262305.1 APH(3') family aminoglycoside O-phosphotransferase [Aminobacter niigataensis]

Length=264

Score = 258 bits (658), Expect = 2e-82, Method: Compositional matrix adjust.

| Identities | = 135/264 (51%), Positives = 173/264 (66%), Gaps = 0/264 (0%) |  |
| --- | --- | --- |
| Query 1 | MIEQDGLHAGSPAAWVERLFGYDWAQQTIGCSDAAVFRLSAQGRPVLFVKTDLSGALNEL | 60 |
| Sbjct 1 | M + + P+ E L GYDW QQTIG S A+VFRL A G+ LF+KT+ +G EL MTQHSTVEIDLPSTLREALAGYDWQQQTIGASGASVFRLEASGKSTLFLKTEETGDFAEL | 60 |
| Query 61 | QDEAARLSWLATTGVPCAAVLDVVTEAGRDWLLLGEVPGQDLLSSHLAPAEKVSIMADAM | 120 |
| Sbjct 61 | DE ARL WL GVPC V+ T AG WLL+ +PG DL++ + AE ++I+ADA+ SDEVARLRWLGGQGVPCPEVIVFETHAGLHWLLMTALPGGDLVTQQVPAAEAIAIIADAL | 120 |
| Query 121 | RRLHTLDPATCPFDHQAKHRIERARTRMEAGLVDQDDLDEEHQGLAPAELFARLKARMPD | 180 |
| Sbjct 121 | RRLH LD A+CPFDH+ RI AR RME G+VD +D D+E G + +F ++ AR P RRLHALDIASCPFDHRLSRRIPLARARMETGVVDVEDFDDEWLGQSAETVFEQVLARRPA | 180 |
| Query 181 | GEDLVVTHGDACLPNIMVENGRFSGFIDCGRLGVADRYQDIALATRDIAEELGGEWADRF | 240 |
| Sbjct 181 | E+LVVTHGDA +PN + E GRF+GFIDC RLGVADRYQD+ + I LG EW F SEELVVTHGDASMPNFIAEGGRFTGFIDCSRLGVADRYQDLGILCWSIHLNLGPEWVAPF | 240 |
| Query 241  Sbjct 241 | LVLYGIAAPDSQRIAFYRLLDEFF 264 L LYG+ D R+++Y+LLDEFF LELYGLPDADPARLSYYQLLDEFF 264 |  |

>WP_171959019.1 APH(3')-II family aminoglycoside O-phosphotransferase [Pseudomonas aeruginosa]

NPW34258.1 APH(3')-II family aminoglycoside O-phosphotransferase [Pseudomonas aeruginosa]

Length=265

Score = 258 bits (658), Expect = 3e-82, Method: Compositional matrix adjust.

| Identities | = 136/255 (53%), Positives = 168/255 (66%), Gaps = 2/255 (1%) |  |
| --- | --- | --- |
| Query 11 | SPAAWVERLFGYDWAQQTIGCSDAAVFRLSAQGRPVLFVKTDLSGALNELQDEAARLSWL | 70 |
| Sbjct 12 | +PA W + L GY W Q GCS A V RL A RP LFVK ++ A EL E ARL WL APATWDDYLAGYRWRGQGEGCSAATVHRLEAARRPTLFVKQEVLSAHAELPAEIARLRWL | 71 |
| Query 71 | ATTGVPCAAVLDVVTEAGRDWLLLGEVPGQDLLS-SHLAPAEKVSIMADAMRRLHTLDPA | 129 |
| Sbjct 72 | G+ C VL+ GR WLL+ VPG L + + E ++A A+RRLH LDPA HGAGIDCPQVLNETQSDGRQWLLMSAVPGDTLSALAQCGELEPGRLVAAALRRLHDLDPA | 131 |
| Query 130 | TCPFDHQAKHRIERARTRMEAGLVDQDDLDEEHQGLAPAELFARLKARMPDGEDLVVTHG | 189 |
| Sbjct 132 | CPFDH+ R++ R R+EAGLVD+ D D++H+G + +EL+ L R P EDLVV HG ACPFDHRLGRRLDTVRQRVEAGLVDEADFDDDHRGRSASELYRLLLDRRPAVEDLVVAHG | 191 |
| Query 190 | DACLPNIMVENGRFSGFIDCGRLGVADRYQDIALATRDIAEELGGEWADRFLVLYGIAAP | 249 |
| Sbjct 192 | DACLPN++ E RFSGFIDCGRLGVADR+QD+ALA RDI ELG WA+ FLV YG DACLPNLLAEGRRFSGFIDCGRLGVADRHQDLALAARDIEAELGAAWAEAFLVEYG-GDI | 250 |
| Query 250 | DSQRIAFYRLLDEFF 264 |  |

D +R+A++RLLDEFF Sbjct 251 DGERLAYFRLLDEFF 265

>WP_090295468.1 MULTISPECIES: aminoglycoside 3'-phosphotransferase [Ensifer] MBD9593523.1 aminoglycoside 3'-phosphotransferase [Ensifer sp. ENS05] MBD9640784.1 aminoglycoside 3'-phosphotransferase [Ensifer sp. ENS07] PYE73290.1 aminoglycoside 3'-phosphotransferase-2 [Ensifer adhaerens] SDL93482.1 aminoglycoside 3'-phosphotransferase-2 [Ensifer sp. YR511]

Length=290

Score = 258 bits (660), Expect = 3e-82, Method: Compositional matrix adjust.

| Identities | = 127/244 (52%), Positives = 166/244 (68%), Gaps = 0/244 (0%) |  |
| --- | --- | --- |
| Query 21 | GYDWAQQTIGCSDAAVFRLSAQGRPVLFVKTDLSGALNELQDEAARLSWLATTGVPCAAV | 80 |
| Sbjct 47 | GY W + +G SD+++F L A RP+L +K + +G +E DEAARL WLA+ +PC V GYQWNRDLLGQSDSSIFLLKAPDRPMLVLKLEEAGPFSEFVDEAARLDWLASKDLPCPRV | 106 |
| Query 81 | LDVVTEAGRDWLLLGEVPGQDLLSSHLAPAEKVSIMADAMRRLHTLDPATCPFDHQAKHR | 140 |
| Sbjct 107 | + A +WLLL V G DL S+ L+P +++ I+ADA+++LH LD A CPFDH+ + R IARAFGAETNWLLLQAVQGTDLASTTLSPKDQIVILADALKQLHALDIANCPFDHRIEER | 166 |
| Query 141 | IERARTRMEAGLVDQDDLDEEHQGLAPAELFARLKARMPDGEDLVVTHGDACLPNIMVEN | 200 |
| Sbjct 167 | I A+ R +AG++D+ D DE G A+LFA L+A +P +LVVTHGDACLPNI+ +N IAIAKARTQAGVIDESDFDETRLGRTAADLFAELQAHVPQPGELVVTHGDACLPNIIEKN | 226 |
| Query 201 | GRFSGFIDCGRLGVADRYQDIALATRDIAEELGGEWADRFLVLYGIAAPDSQRIAFYRLL | 260 |
| Sbjct 227 | GRFSGFIDC RLGVAD YQDIALA R I LG EW FL YG+ DS++ FY LL GRFSGFIDCSRLGVADPYQDIALACRSIVHNLGEEWVQPFLTRYGLDKDDSEKRDFYCLL | 286 |
| Query 261  Sbjct 287 | DEFF 264 DEFF  DEFF 290 |  |

>WP_058142673.1 APH(3')-II family aminoglycoside O-phosphotransferase [Pseudomonas aeruginosa]

KSO05457.1 aminoglycoside 3'-phosphotransferase [Pseudomonas aeruginosa]

MBG4185677.1 APH(3')-II family aminoglycoside O-phosphotransferase [Pseudomonas aeruginosa]

Length=268

Score = 258 bits (658), Expect = 3e-82, Method: Compositional matrix adjust.

| Identities | | = 136/258 (53%), Positives = 168/258 (65%), Gaps = 5/258 (2%) |  |
| --- | --- | --- | --- |
| Query 11  Sbjct 12 | | SPAAWVERLFGYDWAQQTIGCSDAAVFRLSAQGRPVLFVKTDLSGALNELQDEAARLSWL  +P+ W + L GY W Q GCS A V RL A RP LFVK ++ A EL E ARL WL APSTWADYLAGYRWRGQGEGCSAATVHRLEAARRPTLFVKQEVLSAHAELPAEIARLRWL | 70  71 |
| Query 71  Sbjct 72 | | ATTGVPCAAVLDVVTEAGRDWLLLGEVPGQDLLS SHLAPAEKVSIMADAMRRLHTL  G+ VL+ GR WLL+ VPG L + L P V ++A A+RRLH L HGAGIDWPQVLNETQSDGRQWLLMSAVPGDTLSALAQRGELEPERLVRLVAAALRRLHDL | 126  131 |
| Query 127  Sbjct 132 | | DPATCPFDHQAKHRIERARTRMEAGLVDQDDLDEEHQGLAPAELFARLKARMPDGEDLVV DPA CPFDH+ + R++ R R+EAGLVD+ D D++H+G + EL+ L R P EDLVV DPAACPFDHRLERRLDTVRQRVEAGLVDEADFDDDHRGRSATELYRLLLDRRPAVEDLVV | 186  191 |
| Query 187  Sbjct 192 | | THGDACLPNIMVENGRFSGFIDCGRLGVADRYQDIALATRDIAEELGGEWADRFLVLYGI HGDACLPN++ E RFSGFIDCGRLGVADR+QD+ALA RDI ELG WA+ FLV YG AHGDACLPNLLAEGRRFSGFIDCGRLGVADRHQDLALAARDIEAELGAAWAEAFLVEYG- | 246  250 |
| Query | 247 AAPDSQRIAFYRLLDEFF 264 | | |
|  |  | D +R+A++RLLDEFF |  |
| Sbjct | 251 | GDIDGERLAYFRLLDEFF 268 | |

>MRX32977.1 APH(3') family aminoglycoside O-phosphotransferase [Aminobacter sp. MDW-2]

Length=265

Score = 257 bits (657), Expect = 3e-82, Method: Compositional matrix adjust. Identities = 134/254 (53%), Positives = 168/254 (66%), Gaps = 1/254 (0%)

| Query | 12 | PAAWVERLFGYDWAQQTIGCSDAAVFRLSAQGRPVLFVKTDLSGALNELQDEAARLSWLA | 71 |
| --- | --- | --- | --- |
| Sbjct | 12 | PA E L Y W +QTIG S+A VF+L+A G+P LF+K + +G EL DE ARL WL PALLREALASYSWDRQTIGHSNAGVFKLAAAGKPTLFLKAEATGDFAELPDEVARLRWLD | 71 |
| Query | 72 | TTGVPCAAVLDVVTEAGRDWLLLGEVPGQDLLSSHLAPAEKVSIMADAMRRLHTLDPATC | 131 |
| Sbjct | 72 | +PC VL T AG WLL+ V GQDL+S + P + V+IMA+A+RRLH LD A+C GQDIPCPEVLAFETHAGTHWLLMTAVAGQDLVSHEVPPEQAVAIMANALRRLHALDIASC | 131 |
| Query | 132 | PFDHQAKHRIERARTRMEAGLVDQDDLDEEHQGLAPAELFARLKARMPDG-EDLVVTHGD | 190 |
| Sbjct | 132 | PFDH+ RIE ARTRM AG+VD +D D+E G +F +L AR P G EDLVVTHGD PFDHRLSARIEVARTRMVAGVVDVEDFDDEWLGQPAEVVFEQLLARRPAGKEDLVVTHGD | 191 |
| Query | 191 | ACLPNIMVENGRFSGFIDCGRLGVADRYQDIALATRDIAEELGGEWADRFLVLYGIAAPD | 250 |
| Sbjct | 192 | A +PN + + GRFSGFIDC RLGVADR QD+ + I LG +W FL YG+ + ASMPNFIADGGRFSGFIDCSRLGVADRCQDLGILCWSIHFNLGQQWVAPFLERYGLPDAE | 251 |
| Query | 251 | SQRIAFYRLLDEFF 264 R+A+Y+LLDEFF |  |
| Sbjct | 252 | PARLAYYQLLDEFF 265 | |

>MBB4648332.1 aminoglycoside 3'-phosphotransferase-2 [Aminobacter niigataensis] Length=270

Score = 258 bits (658), Expect = 3e-82, Method: Compositional matrix adjust.

| Identities | = 135/264 (51%), Positives = 173/264 (66%), Gaps = 0/264 (0%) |  |
| --- | --- | --- |
| Query 1 | MIEQDGLHAGSPAAWVERLFGYDWAQQTIGCSDAAVFRLSAQGRPVLFVKTDLSGALNEL | 60 |
| Sbjct 7 | M + + P+ E L GYDW QQTIG S A+VFRL A G+ LF+KT+ +G EL MTQHSTVEIDLPSTLREALAGYDWQQQTIGASGASVFRLEASGKSTLFLKTEETGDFAEL | 66 |
| Query 61 | QDEAARLSWLATTGVPCAAVLDVVTEAGRDWLLLGEVPGQDLLSSHLAPAEKVSIMADAM | 120 |
| Sbjct 67 | DE ARL WL GVPC V+ T AG WLL+ +PG DL++ + AE ++I+ADA+ SDEVARLRWLGGQGVPCPEVIVFETHAGLHWLLMTALPGGDLVTQQVPAAEAIAIIADAL | 126 |
| Query 121 | RRLHTLDPATCPFDHQAKHRIERARTRMEAGLVDQDDLDEEHQGLAPAELFARLKARMPD | 180 |
| Sbjct 127 | RRLH LD A+CPFDH+ RI AR RME G+VD +D D+E G + +F ++ AR P RRLHALDIASCPFDHRLSRRIPLARARMETGVVDVEDFDDEWLGQSAETVFEQVLARRPA | 186 |
| Query 181 | GEDLVVTHGDACLPNIMVENGRFSGFIDCGRLGVADRYQDIALATRDIAEELGGEWADRF | 240 |
| Sbjct 187 | E+LVVTHGDA +PN + E GRF+GFIDC RLGVADRYQD+ + I LG EW F SEELVVTHGDASMPNFIAEGGRFTGFIDCSRLGVADRYQDLGILCWSIHLNLGPEWVAPF | 246 |
| Query 241  Sbjct 247 | LVLYGIAAPDSQRIAFYRLLDEFF 264 L LYG+ D R+++Y+LLDEFF LELYGLPDADPARLSYYQLLDEFF 270 |  |

>WP_168296728.1 APH(3') family aminoglycoside O-phosphotransferase [Rhizobium leguminosarum]

NKL49135.1 APH(3') family aminoglycoside O-phosphotransferase [Rhizobium leguminosarum bv. viciae]

Length=261

Score = 257 bits (656), Expect = 3e-82, Method: Compositional matrix adjust.

| Identities | = 130/254 (51%), Positives = 171/254 (67%), Gaps = 1/254 (0%) |  |
| --- | --- | --- |
| Query 12 | PAAWVERLFGYDWAQQTIGCSDAAVFRLSAQGRPVLFVKTDLSGALNELQDEAARLSWLA | 71 |
| Sbjct 8 | PAA ERL GY + + +G S A+VFRL +G P L++K + +G EL DEAARLSWL PAALGERLSGYRFERDALGRSAASVFRLEGEGLPALYLKVEAAGPFGELADEAARLSWLK | 67 |
| Query 72 | TTGVPCAAVLDVVTEAGRDWLLLGEVPGQDLLS-SHLAPAEKVSIMADAMRRLHTLDPAT | 130 |
| Sbjct 68 | G+PC AV+ ++ RDWLL+ +PG DL S S L P +V ++A A+ LH L A+ AVGLPCPAVIARESDGERDWLLISALPGTDLASASALTPLTRVELLAAALSDLHDLSIAS | 127 |
| Query 131 | CPFDHQAKHRIERARTRMEAGLVDQDDLDEEHQGLAPAELFARLKARMPDGEDLVVTHGD | 190 |
| Sbjct 128 | CPFDH+ + R+ A+ RM+AG+VD++D D G + A LFA L P EDLVV HGD CPFDHRLEKRLSVAKARMQAGIVDEEDFDAARLGQSAASLFAELTRLRPGDEDLVVAHGD | 187 |
| Query 191 | ACLPNIMVENGRFSGFIDCGRLGVADRYQDIALATRDIAEELGGEWADRFLVLYGIAAPD ACLPN + +G+FSG+IDC RLGVADR+QDIALA R IA LG FL YG+AAPD | 250 |

| Sbjct | 188 | ACLPNFVASDGQFSGYIDCSRLGVADRHQDIALACRSIASNLGEALIRPFLDRYGLAAPD 247 |
| --- | --- | --- |
| Query | 251 | SQRIAFYRLLDEFF 264 |
| Sbjct | 248 | + ++ +++LLDEFF  AAKLHYFQLLDEFF 261 |

>WP_185910392.1 APH(3') family aminoglycoside O-phosphotransferase [Aminobacter sp. MDW-2]

QNH37139.1 aminoglycoside 3'-phosphotransferase [Aminobacter sp. MDW-2] Length=257

Score = 257 bits (656), Expect = 3e-82, Method: Compositional matrix adjust.

| Identities | | = 134/254 (53%), Positives = 168/254 (66%), Gaps = 1/254 (0%) |  |
| --- | --- | --- | --- |
| Query 12  Sbjct 4 | | PAAWVERLFGYDWAQQTIGCSDAAVFRLSAQGRPVLFVKTDLSGALNELQDEAARLSWLA PA E L Y W +QTIG S+A VF+L+A G+P LF+K + +G EL DE ARL WL PALLREALASYSWDRQTIGHSNAGVFKLAAAGKPTLFLKAEATGDFAELPDEVARLRWLD | 71  63 |
| Query 72  Sbjct 64 | | TTGVPCAAVLDVVTEAGRDWLLLGEVPGQDLLSSHLAPAEKVSIMADAMRRLHTLDPATC  +PC VL T AG WLL+ V GQDL+S + P + V+IMA+A+RRLH LD A+C GQDIPCPEVLAFETHAGTHWLLMTAVAGQDLVSHEVPPEQAVAIMANALRRLHALDIASC | 131  123 |
| Query 132  Sbjct 124 | | PFDHQAKHRIERARTRMEAGLVDQDDLDEEHQGLAPAELFARLKARMPDG-EDLVVTHGD PFDH+ RIE ARTRM AG+VD +D D+E G +F +L AR P G EDLVVTHGD PFDHRLSARIEVARTRMVAGVVDVEDFDDEWLGQPAEVVFEQLLARRPAGKEDLVVTHGD | 190  183 |
| Query 191  Sbjct 184 | | ACLPNIMVENGRFSGFIDCGRLGVADRYQDIALATRDIAEELGGEWADRFLVLYGIAAPD A +PN + + GRFSGFIDC RLGVADR QD+ + I LG +W FL YG+ + ASMPNFIADGGRFSGFIDCSRLGVADRCQDLGILCWSIHFNLGQQWVAPFLERYGLPDAE | 250  243 |
| Query 251 | | SQRIAFYRLLDEFF 264 R+A+Y+LLDEFF |  |
| Sbjct | 244 | PARLAYYQLLDEFF 257 | |

>WP_121305226.1 APH(3')-II family aminoglycoside O-phosphotransferase [Pseudomonas aeruginosa]

TEC50395.1 APH(3')-II family aminoglycoside O-phosphotransferase [Pseudomonas aeruginosa]

Length=268

Score = 257 bits (657), Expect = 4e-82, Method: Compositional matrix adjust.

| Identities | | = 136/258 (53%), Positives = 167/258 (65%), Gaps = 5/258 (2%) |  |
| --- | --- | --- | --- |
| Query 11  Sbjct 12 | | SPAAWVERLFGYDWAQQTIGCSDAAVFRLSAQGRPVLFVKTDLSGALNELQDEAARLSWL  +P W + L GY W Q GCS A V RL A R LFVK ++ A EL E ARL WL APDTWADYLAGYRWRGQGEGCSAATVHRLEAARRLTLFVKQEVLSAHAELPAEIARLRWL | 70  71 |
| Query 71  Sbjct 72 | | ATTGVPCAAVLDVVTEAGRDWLLLGEVPGQDLLS SHLAPAEKVSIMADAMRRLHTL  G+ C VL+ GR WLL+ VPG L + L P V ++A A+RRLH L HGAGIDCPQVLNETQSDGRQWLLMSAVPGDTLSALAQRGELEPERLVRLVAAALRRLHDL | 126  131 |
| Query 127  Sbjct 132 | | DPATCPFDHQAKHRIERARTRMEAGLVDQDDLDEEHQGLAPAELFARLKARMPDGEDLVV DPA CPFDH+ + R++ R R+EAGLVD+ D D++H+G + EL+ L R P EDLVV DPAACPFDHRLERRLDTVRQRVEAGLVDEADFDDDHRGRSATELYRLLLDRRPAVEDLVV | 186  191 |
| Query 187  Sbjct 192 | | THGDACLPNIMVENGRFSGFIDCGRLGVADRYQDIALATRDIAEELGGEWADRFLVLYGI HGDACLPN++ E RFSGFIDCGRLGVADR+QD+ALA RDI ELG WA+ FLV YG AHGDACLPNLLAEGRRFSGFIDCGRLGVADRHQDLALAARDIEAELGATWAEAFLVEYG- | 246  250 |
| Query | 247 AAPDSQRIAFYRLLDEFF 264 | | |
|  |  | D +R+A++RLLDEFF |  |
| Sbjct | 251 | GDIDGERLAYFRLLDEFF 268 | |

>WP_027613036.1 MULTISPECIES: aminoglycoside 3'-phosphotransferase [Pseudomonas] KAA8740463.1 aminoglycoside 3'-phosphotransferase [Pseudomonas koreensis] MBB6153270.1 aminoglycoside 3'-phosphotransferase-2 [Pseudomonas sp. JAI115] POA23980.1 aminoglycoside 3'-phosphotransferase [Pseudomonas sp. GW456-12-10-14-LB3] POA42806.1 aminoglycoside 3'-phosphotransferase [Pseudomonas sp. GW456-12-10-14-LB1]

Length=256

Score = 257 bits (656), Expect = 4e-82, Method: Compositional matrix adjust.

| Identities | | = 133/254 (52%), Positives = 168/254 (66%), Gaps = 2/254 (1%) |  |
| --- | --- | --- | --- |
| Query 12  Sbjct 4 | | PAAWVERLFGYDWAQQTIGCSDAAVFRLSAQGRPVLFVKTDLSGALNELQDEAARLSWLA P W +R + QQTIG S A VFRL+ G LFVK++ + EL E RL WLA PQTWRQRFADIRYEQQTIGESRADVFRLTPAGSDPLFVKSEPRISFAELPGEVQRLRWLA | 71  63 |
| Query 72  Sbjct 64 | | TTGVPCAAVLDVVTEAGRDWLLLGEVPGQDLLSS-HLAPAEKVSIMADAMRRLHTLDPAT  +P A VLD V E+ R WLL+ VPG+DL SS HLAP + VS+ A+A+R LH LD EQDLPAARVLDSVEESERFWLLMTAVPGRDLASSEHLAPQQIVSLAANALRTLHALDIKN | 130  123 |
| Query 131  Sbjct 124 | | CPFDHQAKHRIERARTRMEAGLVDQDDLDEEHQGLAPAELFARLKARMPDGEDLVVTHGD CPFDH+ +I AR + AGL+D++D D+ G + +F ++ A P EDLVVTHGD CPFDHRLDAKIALAREHLLAGLIDEEDFDDSRLGQSAEAVFEQMLAARPHSEDLVVTHGD | 190  183 |
| Query 191  Sbjct 184 | | ACLPNIMVENGRFSGFIDCGRLGVADRYQDIALATRDIAEELGGEWADRFLVLYGIAAPD ACLPN++ +NGRFSGF+DCGRLGVADR+QD+ALA IA LG +W F YG+ PD ACLPNLLADNGRFSGFVDCGRLGVADRFQDLALAAHSIAYNLGEQWLPVFFEAYGV-QPD | 250  242 |
| Query | 251 SQRIAFYRLLDEFF 264 | | |
|  |  | Q+IAFY+LLDEFF |  |
| Sbjct | 243 | QQKIAFYQLLDEFF 256 | |

>WP_187588648.1 phosphotransferase, partial [Arenibacter sp. BSSL-BM3] MBC8770650.1 aminoglycoside 3'-phosphotransferase [Arenibacter sp. BSSL-BM3]

Length=125

Score = 252 bits (643), Expect = 4e-82, Method: Compositional matrix adjust.

| Identities | = 124/125 (99%), Positives = 124/125 (99%), Gaps = 0/125 (0%) |  |
| --- | --- | --- |
| Query 140 | RIERARTRMEAGLVDQDDLDEEHQGLAPAELFARLKARMPDGEDLVVTHGDACLPNIMVE | 199 |
| Sbjct 1 | RIERARTRMEAGLVDQDDLDEEHQGLAPAELFARLKA MPDGEDLVVTHGDACLPNIMVE RIERARTRMEAGLVDQDDLDEEHQGLAPAELFARLKASMPDGEDLVVTHGDACLPNIMVE | 60 |
| Query 200 | NGRFSGFIDCGRLGVADRYQDIALATRDIAEELGGEWADRFLVLYGIAAPDSQRIAFYRL | 259 |
| Sbjct 61 | NGRFSGFIDCGRLGVADRYQDIALATRDIAEELGGEWADRFLVLYGIAAPDSQRIAFYRL NGRFSGFIDCGRLGVADRYQDIALATRDIAEELGGEWADRFLVLYGIAAPDSQRIAFYRL | 120 |
| Query 260  Sbjct 121 | LDEFF 264 LDEFF LDEFF 125 |  |

>WP_033942278.1 APH(3')-II family aminoglycoside O-phosphotransferase [Pseudomonas aeruginosa]

Length=268

Score = 257 bits (656), Expect = 4e-82, Method: Compositional matrix adjust.

| Identities | | = 136/258 (53%), Positives = 167/258 (65%), Gaps = 5/258 (2%) |  |
| --- | --- | --- | --- |
| Query 11  Sbjct 12 | | SPAAWVERLFGYDWAQQTIGCSDAAVFRLSAQGRPVLFVKTDLSGALNELQDEAARLSWL  +PA W + L GY W Q GCS A V RL A P LFVK ++ A EL E ARL WL APATWADYLAGYRWRGQGEGCSAATVHRLEAARWPTLFVKQEVLSAHAELPAEIARLRWL | 70  71 |
| Query 71  Sbjct 72 | | ATTGVPCAAVLDVVTEAGRDWLLLGEVPGQDLLS SHLAPAEKVSIMADAMRRLHTL  G+ C VL+ GR WLL+ VPG L + L P V ++A A+RRLH L HGAGIDCPQVLNETQSDGRQWLLMSAVPGDTLSALAQRGELEPERLVRLVAAALRRLHDL | 126  131 |
| Query 127  Sbjct 132 | | DPATCPFDHQAKHRIERARTRMEAGLVDQDDLDEEHQGLAPAELFARLKARMPDGEDLVV DPA CPFDH+ + R++ R R+EAGLVD+ D++H+G + EL+ L R P EDLVV DPAACPFDHRLERRLDTVRQRVEAGLVDEAGFDDDHRGRSATELYRLLLDRRPAVEDLVV | 186  191 |
| Query 187  Sbjct 192 | | THGDACLPNIMVENGRFSGFIDCGRLGVADRYQDIALATRDIAEELGGEWADRFLVLYGI HGDACLPN++ E RFSGFIDCGRLGVADR+QD+ALA RDI ELG WA+ FLV YG AHGDACLPNLLAEGRRFSGFIDCGRLGVADRHQDLALAARDIEAELGAAWAEAFLVEYG- | 246  250 |
| Query | 247 AAPDSQRIAFYRLLDEFF 264 | | |
|  |  | D +R+A++RLLDEFF |  |
| Sbjct | 251 | GDIDGERLAYFRLLDEFF 268 | |

>WP_081278565.1 aminoglycoside 3'-phosphotransferase [Rhizobium phaseoli] ANL73030.1 aminoglycoside 3'-phosphotransferase protein [Rhizobium phaseoli]

Length=261

Score = 257 bits (656), Expect = 4e-82, Method: Compositional matrix adjust.

| Identities | = 128/254 (50%), Positives = 171/254 (67%), Gaps = 1/254 (0%) |  |
| --- | --- | --- |
| Query 12 | PAAWVERLFGYDWAQQTIGCSDAAVFRLSAQGRPVLFVKTDLSGALNELQDEAARLSWLA | 71 |
| Sbjct 8 | PAA ERL GY + + +G S A+VFRL +G P L++K + +G EL DEAARLSWL PAALGERLSGYRFERDALGRSAASVFRLQGEGLPALYLKVEAAGPFGELADEAARLSWLK | 67 |
| Query 72 | TTGVPCAAVLDVVTEAGRDWLLLGEVPGQDLLS-SHLAPAEKVSIMADAMRRLHTLDPAT | 130 |
| Sbjct 68 | G+PC V+ ++ R+WLL+G +PG DL+S S L P +V ++A A+ LH L A+ AAGLPCPEVIARESDGERNWLLIGALPGADLVSASALTPMARVELLAAALSDLHDLPIAS | 127 |
| Query 131 | CPFDHQAKHRIERARTRMEAGLVDQDDLDEEHQGLAPAELFARLKARMPDGEDLVVTHGD | 190 |
| Sbjct 128 | CPFDH+ ++R+ A+ RM+AG+VD++D D G + A LF L P EDLVVTHGD CPFDHRLENRLPIAKVRMQAGIVDEEDFDAARLGKSAAALFIDLMRLRPGNEDLVVTHGD | 187 |
| Query 191 | ACLPNIMVENGRFSGFIDCGRLGVADRYQDIALATRDIAEELGGEWADRFLVLYGIAAPD | 250 |
| Sbjct 188 | ACLPN + NG+FSG+IDC RLGVADR+QDIALA R IA G FL YG+ APD ACLPNFVASNGKFSGYIDCSRLGVADRHQDIALACRSIASNFGEALIRPFLERYGLTAPD | 247 |
| Query 251  Sbjct 248 | SQRIAFYRLLDEFF 264  + ++ +++LLDEFF  ATKLHYFQLLDEFF 261 |  |

>SNY16958.1 aminoglycoside 3'-phosphotransferase-2 [Pseudomonas sp. LAMO17WK12:I5] SNY46783.1 aminoglycoside 3'-phosphotransferase-2 [Pseudomonas sp. LAMO17WK12:I6]

Length=258

Score = 256 bits (655), Expect = 4e-82, Method: Compositional matrix adjust.

| Identities | | = 133/254 (52%), Positives = 168/254 (66%), Gaps = 2/254 (1%) |  |
| --- | --- | --- | --- |
| Query 12  Sbjct 6 | | PAAWVERLFGYDWAQQTIGCSDAAVFRLSAQGRPVLFVKTDLSGALNELQDEAARLSWLA P W +R + QQTIG S A VFRL+ G LFVK++ + EL E RL WLA PQTWRQRFADIRYEQQTIGESRADVFRLTPAGSDPLFVKSEPRISFAELPGEVQRLRWLA | 71  65 |
| Query 72  Sbjct 66 | | TTGVPCAAVLDVVTEAGRDWLLLGEVPGQDLLSS-HLAPAEKVSIMADAMRRLHTLDPAT  +P A VLD V E+ R WLL+ VPG+DL SS HLAP + VS+ A+A+R LH LD EQDLPAARVLDSVEESERFWLLMTAVPGRDLASSEHLAPQQIVSLAANALRTLHALDIKN | 130  125 |
| Query 131  Sbjct 126 | | CPFDHQAKHRIERARTRMEAGLVDQDDLDEEHQGLAPAELFARLKARMPDGEDLVVTHGD CPFDH+ +I AR + AGL+D++D D+ G + +F ++ A P EDLVVTHGD CPFDHRLDAKIALAREHLLAGLIDEEDFDDSRLGQSAEAVFEQMLAARPHSEDLVVTHGD | 190  185 |
| Query 191  Sbjct 186 | | ACLPNIMVENGRFSGFIDCGRLGVADRYQDIALATRDIAEELGGEWADRFLVLYGIAAPD ACLPN++ +NGRFSGF+DCGRLGVADR+QD+ALA IA LG +W F YG+ PD ACLPNLLADNGRFSGFVDCGRLGVADRFQDLALAAHSIAYNLGEQWLPVFFEAYGV-QPD | 250  244 |
| Query | 251 SQRIAFYRLLDEFF 264 | | |
|  |  | Q+IAFY+LLDEFF |  |
| Sbjct | 245 | QQKIAFYQLLDEFF 258 | |

>WP_129413823.1 aminoglycoside 3'-phosphotransferase [Mesorhizobium sp. Pch-S]

QAZ44894.1 APH(3') family aminoglycoside O-phosphotransferase [Mesorhizobium sp. Pch-S]

Length=260

Score = 257 bits (656), Expect = 4e-82, Method: Compositional matrix adjust. Identities = 130/254 (51%), Positives = 167/254 (66%), Gaps = 1/254 (0%)

| Query | 12 | PAAWVERLFGYDWAQQTIGCSDAAVFRLSAQGRPVLFVKTDLSGALNELQDEAARLSWLA | 71 |
| --- | --- | --- | --- |
| Sbjct | 7 | P + E + GY QQ G SDA VF+L A G+ LF+K D + E+ EA RL WLA PLPFPELVIGYGARQQMTGLSDAGVFKLDAPGKSSLFLKIDDANGFAEVPAEAERLRWLA | 66 |
| Query | 72 | TTGVPCAAVLDVVTEAGRDWLLLGEVPGQDLLSSHLAPAEK-VSIMADAMRRLHTLDPAT | 130 |
| Sbjct | 67 | G+ C VL GR+WLL+ +PG+DL S+ PAE ++++A A+R LH LD +  AQGISCPEVLAFAVHGGRNWLLMSALPGKDLESAGPLPAETTIAMIAGALRTLHALDVHS | 126 |

| Query | 131 | CPFDHQAKHRIERARTRMEAGLVDQDDLDEEHQGLAPAELFARLKARMPDGEDLVVTHGD | 190 |
| --- | --- | --- | --- |
| Sbjct | 127 | CPFDH+ + RI AR R+EAGL+D++D D E G ++FA L A P EDLVVTHGD CPFDHRLERRIADARARLEAGLIDEEDFDGERLGQTAQQVFADLVALRPTSEDLVVTHGD | 186 |
| Query | 191 | ACLPNIMVENGRFSGFIDCGRLGVADRYQDIALATRDIAEELGGEWADRFLVLYGIAAPD | 250 |
| Sbjct | 187 | ACLPN+MV+ GRFSGFIDCGRLGVADR QD+AL R I ++ G EW FL LYG D ACLPNLMVDGGRFSGFIDCGRLGVADRCQDLALVCRSIRDDFGEEWIAPFLALYGKPDAD | 246 |
| Query | 251 | SQRIAFYRLLDEFF 264  ++++Y+LLDEFF |  |
| Sbjct | 247 | PAKLSYYQLLDEFF 260 | |

>WP_074222317.1 APH(3')-II family aminoglycoside O-phosphotransferase [Pseudomonas aeruginosa]

MBG6611218.1 APH(3')-II family aminoglycoside O-phosphotransferase [Pseudomonas aeruginosa]

RTX26247.1 APH(3')-II family aminoglycoside O-phosphotransferase [Pseudomonas aeruginosa]

Length=268

Score = 257 bits (656), Expect = 5e-82, Method: Compositional matrix adjust.

| Identities | | = 135/258 (52%), Positives = 167/258 (65%), Gaps = 5/258 (2%) |  |
| --- | --- | --- | --- |
| Query 11  Sbjct 12 | | SPAAWVERLFGYDWAQQTIGCSDAAVFRLSAQGRPVLFVKTDLSGALNELQDEAARLSWL  +P+ W + L GY W Q GCS A V RL A RP LFVK ++ EL E ARL WL APSTWADYLAGYRWRGQGEGCSAATVHRLEAARRPTLFVKQEVLSVHAELPAEIARLRWL | 70  71 |
| Query 71  Sbjct 72 | | ATTGVPCAAVLDVVTEAGRDWLLLGEVPGQDLLS SHLAPAEKVSIMADAMRRLHTL  G+ C VL+ GR WLL+ VPG L + L P V ++A A+RRLH L HGAGIDCPQVLNETQSDGRQWLLMSAVPGDTLSALAQRGELEPERLVRLVAAALRRLHDL | 126  131 |
| Query 127  Sbjct 132 | | DPATCPFDHQAKHRIERARTRMEAGLVDQDDLDEEHQGLAPAELFARLKARMPDGEDLVV DPA CPFDH+ + R++ R R+EAGLVD+ D D++H+G + EL+ L R EDLVV DPAACPFDHRLERRLDTVRQRVEAGLVDEADFDDDHRGRSATELYRLLLDRCLAVEDLVV | 186  191 |
| Query 187  Sbjct 192 | | THGDACLPNIMVENGRFSGFIDCGRLGVADRYQDIALATRDIAEELGGEWADRFLVLYGI HGDACLPN++ E RFSGFIDCGRLGVADR+QD+ALA RDI ELG WA+ FLV YG AHGDACLPNLLAEGRRFSGFIDCGRLGVADRHQDLALAARDIEAELGAAWAEAFLVEYG- | 246  250 |
| Query | 247 AAPDSQRIAFYRLLDEFF 264 | | |
|  |  | D +R+A++RLLDEFF |  |
| Sbjct | 251 | GDIDGERLAYFRLLDEFF 268 | |

>WP_012484598.1 MULTISPECIES: aminoglycoside 3'-phosphotransferase [Rhizobium] NKF39155.1 aminoglycoside 3'-phosphotransferase [Rhizobium phaseoli]

ACE92059.1 putative aminoglycoside 3'-phosphotransferase protein [Rhizobium etli CIAT 652]

ANL41469.1 aminoglycoside 3'-phosphotransferase protein [Rhizobium phaseoli] ANL54174.1 aminoglycoside 3'-phosphotransferase protein [Rhizobium phaseoli] ANL60456.1 aminoglycoside 3'-phosphotransferase protein [Rhizobium phaseoli]

Length=261

Score = 256 bits (655), Expect = 6e-82, Method: Compositional matrix adjust.

| Identities | = 129/254 (51%), Positives = 170/254 (67%), Gaps = 1/254 (0%) |  |
| --- | --- | --- |
| Query 12 | PAAWVERLFGYDWAQQTIGCSDAAVFRLSAQGRPVLFVKTDLSGALNELQDEAARLSWLA | 71 |
| Sbjct 8 | PAA ERL GY + + +G S A+VFRL +G P L++K + +G EL DEAARLSWL PAALGERLSGYRFERDALGRSAASVFRLEGEGLPALYLKVEAAGPFGELADEAARLSWLK | 67 |
| Query 72 | TTGVPCAAVLDVVTEAGRDWLLLGEVPGQDLLS-SHLAPAEKVSIMADAMRRLHTLDPAT | 130 |
| Sbjct 68 | G+PC V+ ++ R+WLL+G +PG DL+S S L P +V ++A A+ LH L A+ AAGLPCPDVIARESDGERNWLLIGALPGADLVSASALTPMARVELLAAALSDLHDLPIAS | 127 |
| Query 131 | CPFDHQAKHRIERARTRMEAGLVDQDDLDEEHQGLAPAELFARLKARMPDGEDLVVTHGD | 190 |
| Sbjct 128 | CPFDH+ ++R+ A+ RMEAG+VD++D D G A LF L P EDLVVTHGD CPFDHRLENRLPTAKARMEAGIVDEEDFDAARLGKNAAALFDDLMRLRPGNEDLVVTHGD | 187 |
| Query 191 | ACLPNIMVENGRFSGFIDCGRLGVADRYQDIALATRDIAEELGGEWADRFLVLYGIAAPD ACLPN + NG+FSG+IDC RLGVADR+QDIALA R IA G FL YG+ APD | 250 |

| Sbjct | 188 | ACLPNFVASNGKFSGYIDCSRLGVADRHQDIALACRSIASNFGEALIRPFLERYGLTAPD 247 |
| --- | --- | --- |
| Query | 251 | SQRIAFYRLLDEFF 264 |
| Sbjct | 248 | + ++ +++LLDEFF  ATKLHYFQLLDEFF 261 |

>MBG6602928.1 APH(3')-II family aminoglycoside O-phosphotransferase [Pseudomonas aeruginosa]

Length=268

Score = 256 bits (655), Expect = 7e-82, Method: Compositional matrix adjust.

| Identities | | = 136/258 (53%), Positives = 166/258 (64%), Gaps = 5/258 (2%) |  |
| --- | --- | --- | --- |
| Query 11  Sbjct 12 | | SPAAWVERLFGYDWAQQTIGCSDAAVFRLSAQGRPVLFVKTDLSGALNELQDEAARLSWL  +P+ W + L GY W Q GCS A V RL A RP LFVK ++ EL E ARL WL APSTWADYLAGYRWRGQGEGCSAATVHRLEAARRPTLFVKQEVLSVHAELPAEIARLRWL | 70  71 |
| Query 71  Sbjct 72 | | ATTGVPCAAVLDVVTEAGRDWLLLGEVPGQDLLS SHLAPAEKVSIMADAMRRLHTL  G+ C VL+ GR WLL+ VPG L + L P V + A A+RRLH L HGAGIDCPQVLNETQSDGRQWLLMSAVPGDTLSALAQRGELEPERLVRLGAAALRRLHEL | 126  131 |
| Query 127  Sbjct 132 | | DPATCPFDHQAKHRIERARTRMEAGLVDQDDLDEEHQGLAPAELFARLKARMPDGEDLVV DPA CPFDH+ + R++ R R+EAGLVD+ D D++H+G + EL+ L R P EDLVV DPAACPFDHRLERRLDTVRQRVEAGLVDEADFDDDHRGRSATELYRLLLDRRPAVEDLVV | 186  191 |
| Query 187  Sbjct 192 | | THGDACLPNIMVENGRFSGFIDCGRLGVADRYQDIALATRDIAEELGGEWADRFLVLYGI HGDACLPN++ E RFSGFIDCGRLGVADR+QD+ALA RDI ELG WA+ FLV YG AHGDACLPNLLAEGRRFSGFIDCGRLGVADRHQDLALAARDIEAELGAAWAEAFLVEYG- | 246  250 |
| Query | 247 AAPDSQRIAFYRLLDEFF 264 | | |
|  |  | D +R+A +RLLDEFF |  |
| Sbjct | 251 | GDIDGERLADFRLLDEFF 268 | |

>WP_112344283.1 MULTISPECIES: aminoglycoside 3'-phosphotransferase [Rhizobium]

RAX38914.1 APH(3') family aminoglycoside O-phosphotransferase [Rhizobium tropici]

TWE98748.1 aminoglycoside 3'-phosphotransferase-2 [Rhizobium miluonense] Length=266

Score = 256 bits (655), Expect = 7e-82, Method: Compositional matrix adjust.

| Identities | | = 129/254 (51%), Positives = 168/254 (66%), Gaps = 1/254 (0%) |  |
| --- | --- | --- | --- |
| Query 12  Sbjct 13 | | PAAWVERLFGYDWAQQTIGCSDAAVFRLSAQGRPVLFVKTDLSGALNELQDEAARLSWLA P + L GY+W Q +G S A VFRL + GR +++KT+L+ L EL E ARL WLA PTRLRDALAGYEWRQDALGHSSAHVFRLESDGRAPVYLKTELADPLGELAGEVARLRWLA | 71  72 |
| Query 72  Sbjct 73 | | TTGVPCAAVLDVVTEAGRDWLLLGEVPGQDLLSSH-LAPAEKVSIMADAMRRLHTLDPAT  + G+ C V+ + R+WLL+ +PG DL+S+ L P E+V ++ADA+RRLH++D A SQGLACPDVITHEKDGEREWLLMSALPGSDLVSAERLQPLERVRLLADALRRLHSVDIAG | 130  132 |
| Query 131  Sbjct 133 | | CPFDHQAKHRIERARTRMEAGLVDQDDLDEEHQGLAPAELFARLKARMPDGEDLVVTHGD CPFDH+ +RI AR RM AG VD+DD D+ G + +LF L+A+ P ED VVTHGD CPFDHRLGNRIAEARARMLAGRVDEDDFDDARLGRSAEDLFRELEAKRPSAEDFVVTHGD | 190  192 |
| Query 191  Sbjct 193 | | ACLPNIMVENGRFSGFIDCGRLGVADRYQDIALATRDIAEELGGEWADRFLVLYGIAAPD ACLPN M + GRFSG+IDCGRLG+ADR+QDIALA I G FL YG P ACLPNFMADGGRFSGYIDCGRLGIADRHQDIALACGSIEYNFGKGLVFEFLKAYGNFEPQ | 250  252 |
| Query 251 | | SQRIAFYRLLDEFF 264  +++AFYRLLDEFF |  |
| Sbjct | 253 | PEKMAFYRLLDEFF 266 | |

>OJY00856.1 APH(3') family aminoglycoside O-phosphotransferase [Rhizobiales bacterium 62-17]

Length=263

Score = 256 bits (654), Expect = 8e-82, Method: Compositional matrix adjust. Identities = 133/254 (52%), Positives = 165/254 (65%), Gaps = 2/254 (1%)

| Query | 12 | PAAWVERLFGYDWAQQTIGCSDAAVFRLSAQGRPVLFVKTDLSGALNELQDEAARLSWLA | 71 |
| --- | --- | --- | --- |
| Sbjct | 11 | P +W L GY W QQTIG S AA++RL A G+P LFVKT+ + A EL DEA RL WL PPSWQSDLAGYRWTQQTIGRSTAAIYRLEASGKPTLFVKTEPADAFGELPDEARRLRWLR | 70 |
| Query | 72 | TTGVPCAAVLDVVTEAGRDWLLLGEVPGQDLLSS-HLAPAEKVSIMADAMRRLHTLDPAT | 130 |
| Sbjct | 71 | + C VL+ + R+WLL+ +PGQDL SS HL + V+I ADA+R LH LD + ACDIACPDVLNESRDGARNWLLMQALPGQDLASSPHLTAEQIVTIAADALRDLHRLDASA | 130 |
| Query | 131 | CPFDHQAKHRIERARTRMEAGLVDQDDLDEEHQGLAPAELFARLKARMPDGEDLVVTHGD | 190 |
| Sbjct | 131 | CPFD + + RI A RM AG+VD+ D D+E G +++F L R P EDLVVTHGD CPFDQRLERRIAAAEARMTAGVVDESDFDDERLGWPVSKVFEELVRRRPAREDLVVTHGD | 190 |
| Query | 191 | ACLPNIMVENGRFSGFIDCGRLGVADRYQDIALATRDIAEELGGEWADRFLVLYGIAAPD | 250 |
| Sbjct | 191 | ACLPN+M RF+GFIDC RLGVADR+QD+AL + I LG W FL YG A D ACLPNLMATANRFTGFIDCARLGVADRHQDLALTSWSIHYNLGENWIAPFLRRYGAQA-D | 249 |
| Query  Sbjct | 251  250 | SQRIAFYRLLDEFF 264  + ++FYRLLDEFF  ADLLSFYRLLDEFF 263 |  |

>WP_081279261.1 MULTISPECIES: aminoglycoside 3'-phosphotransferase [Rhizobium] ANM05270.1 aminoglycoside 3'-phosphotransferase protein [Rhizobium phaseoli]

Length=261

Score = 256 bits (654), Expect = 8e-82, Method: Compositional matrix adjust.

| Identities | = 128/254 (50%), Positives = 171/254 (67%), Gaps = 1/254 (0%) |  |
| --- | --- | --- |
| Query 12 | PAAWVERLFGYDWAQQTIGCSDAAVFRLSAQGRPVLFVKTDLSGALNELQDEAARLSWLA | 71 |
| Sbjct 8 | PAA ERL GY + + +G S A+VFRL +G P L++K + +G EL DEAARLSWL PAALGERLSGYRFERDALGRSAASVFRLQGEGLPALYLKVEAAGPFGELADEAARLSWLK | 67 |
| Query 72 | TTGVPCAAVLDVVTEAGRDWLLLGEVPGQDLLS-SHLAPAEKVSIMADAMRRLHTLDPAT | 130 |
| Sbjct 68 | G+PC V+ ++ R+WLL+G +PG DL+S S L P +V ++A A+ LH L A+ AAGLPCPDVIAHESDGERNWLLIGALPGADLVSASALTPMARVELLAAALSDLHDLPIAS | 127 |
| Query 131 | CPFDHQAKHRIERARTRMEAGLVDQDDLDEEHQGLAPAELFARLKARMPDGEDLVVTHGD | 190 |
| Sbjct 128 | CPFDH+ ++R+ A+ RM+AG+VD++D D G + A LF L P EDLVVTHGD CPFDHRLENRLPIAKVRMQAGIVDEEDFDAARLGKSAAALFIDLMRLRPGNEDLVVTHGD | 187 |
| Query 191 | ACLPNIMVENGRFSGFIDCGRLGVADRYQDIALATRDIAEELGGEWADRFLVLYGIAAPD | 250 |
| Sbjct 188 | ACLPN + NG+FSG+IDC RLGVADR+QDIALA R IA G FL YG+ APD ACLPNFVASNGKFSGYIDCSRLGVADRHQDIALACRSIASNFGEALIRPFLERYGLTAPD | 247 |
| Query 251  Sbjct 248 | SQRIAFYRLLDEFF 264  + ++ +++LLDEFF  ATKLHYFQLLDEFF 261 |  |

>WP_108609794.1 aminoglycoside 3'-phosphotransferase [Aminobacter sp. MSH1] AWC22947.1 Aminoglycoside 3'-phosphotransferase [Aminobacter sp. MSH1]

Length=264

Score = 256 bits (654), Expect = 8e-82, Method: Compositional matrix adjust.

| Identities | = 135/264 (51%), Positives = 174/264 (66%), Gaps = 0/264 (0%) |  |
| --- | --- | --- |
| Query 1 | MIEQDGLHAGSPAAWVERLFGYDWAQQTIGCSDAAVFRLSAQGRPVLFVKTDLSGALNEL | 60 |
| Sbjct 1 | M + + P+ E L GYDW +QTIG S A+VFRL A G+ LF+KT+ +G EL MTQHSTVEIDLPSTLREALAGYDWQRQTIGASGASVFRLEASGKSTLFLKTEETGDFAEL | 60 |
| Query 61 | QDEAARLSWLATTGVPCAAVLDVVTEAGRDWLLLGEVPGQDLLSSHLAPAEKVSIMADAM | 120 |
| Sbjct 61 | DE ARL WL GVPC V+ T AG WLL+ +PG DL++ + AE ++I+ADA+ PDEVARLRWLGGQGVPCPEVIVFETHAGLHWLLMTALPGGDLVTQEVPAAEAIAIIADAL | 120 |
| Query 121 | RRLHTLDPATCPFDHQAKHRIERARTRMEAGLVDQDDLDEEHQGLAPAELFARLKARMPD | 180 |
| Sbjct 121 | RRLH LD A+CPFDH+ RI AR RMEAG+VD +D D+E G + +F ++ AR P RRLHALDIASCPFDHRLSRRIPLARARMEAGVVDVEDFDDEWLGQSAETVFEQVLARRPA | 180 |
| Query 181 | GEDLVVTHGDACLPNIMVENGRFSGFIDCGRLGVADRYQDIALATRDIAEELGGEWADRF | 240 |
| Sbjct 181 | E+LVVTHGDA +PN + E GRF+GFIDC RLGVADRYQD+ + I LG EW F  SEELVVTHGDASMPNFIAEGGRFTGFIDCSRLGVADRYQDLGILCWSIHLNLGPEWVAPF | 240 |

Query 241 LVLYGIAAPDSQRIAFYRLLDEFF 264 L LYG+	D R+++Y+LLDEFF

Sbjct 241 LELYGLPDADPARLSYYQLLDEFF 264

>WP_183924477.1 APH(3') family aminoglycoside O-phosphotransferase [Rhizobium mongolense]

MBB4273979.1 aminoglycoside 3'-phosphotransferase-2 [Rhizobium mongolense] Length=260

Score = 256 bits (654), Expect = 8e-82, Method: Compositional matrix adjust.

| Identities | = 125/244 (51%), Positives = 160/244 (66%), Gaps = 1/244 (0%) |  |
| --- | --- | --- |
| Query 22 | YDWAQQTIGCSDAAVFRLSAQGRPVLFVKTDLSGALNELQDEAARLSWLATTGVPCAAVL | 81 |
| Sbjct 17 | ++W Q +GCS A VFRL+ GR F+K +++G EL EA RL WL G+ C +V+ HEWKQDDLGCSAAEVFRLAEGGRTRFFLKGEIAGPFAELPLEAERLLWLGEQGLQCPSVV | 76 |
| Query 82 | DVVTEAGRDWLLLGEVPGQDLLSSHLAP-AEKVSIMADAMRRLHTLDPATCPFDHQAKHR | 140 |
| Sbjct 77 | + + GR+WLLL +PG DL SS L +++ I+A A+RRLH LDP TCPFDH+ R ALESHGGRNWLLLSALPGSDLASSKLTTDRQRIEILAAALRRLHELDPETCPFDHRLTAR | 136 |
| Query 141 | IERARTRMEAGLVDQDDLDEEHQGLAPAELFARLKARMPDGEDLVVTHGDACLPNIMVEN | 200 |
| Sbjct 137 | + A+ RMEAG VD+ D DEE Q A LFA L+A PD D VV HGDACLPN M LRLAKARMEAGAVDESDFDEERQNATAASLFAYLEANQPDVTDPVVAHGDACLPNFMTHE | 196 |
| Query 201 | GRFSGFIDCGRLGVADRYQDIALATRDIAEELGGEWADRFLVLYGIAAPDSQRIAFYRLL | 260 |
| Sbjct 197 | G+FSG++DCGRLGVADRYQDIALA R I +G W FL YG++ ++A+Y +L GQFSGYVDCGRLGVADRYQDIALALRSIRYNVGEAWVRHFLDCYGLSGISDAKVAYYMVL | 256 |
| Query 261  Sbjct 257 | DEFF 264 DEFF  DEFF 260 |  |

>WP_115989559.1 aminoglycoside 3'-phosphotransferase [Pseudomonas sp. 424] Length=256

Score = 256 bits (653), Expect = 8e-82, Method: Compositional matrix adjust.

| Identities | | = 133/254 (52%), Positives = 168/254 (66%), Gaps = 2/254 (1%) |  |
| --- | --- | --- | --- |
| Query 12  Sbjct 4 | | PAAWVERLFGYDWAQQTIGCSDAAVFRLSAQGRPVLFVKTDLSGALNELQDEAARLSWLA P W +R + QQTIG S A VFRL+ G LFVK++ + EL E RL WLA PQTWRQRFADIRYEQQTIGESRADVFRLTPAGSDPLFVKSEPRISFAELPGEVQRLRWLA | 71  63 |
| Query 72  Sbjct 64 | | TTGVPCAAVLDVVTEAGRDWLLLGEVPGQDLLSS-HLAPAEKVSIMADAMRRLHTLDPAT  +P A VLD V E+ R WLL+ VPG+DL SS HLAP + VS+ A+A+R LH LD EQDLPAARVLDSVEESERFWLLMTAVPGRDLASSEHLAPQQIVSLAANALRTLHALDIKH | 130  123 |
| Query 131  Sbjct 124 | | CPFDHQAKHRIERARTRMEAGLVDQDDLDEEHQGLAPAELFARLKARMPDGEDLVVTHGD CPFDH+ +I AR + AGL+D++D D+ G + +F ++ A P EDLVVTHGD CPFDHRLDAKIALAREHLLAGLIDEEDFDDSRLGQSAEAVFEQMLAARPHSEDLVVTHGD | 190  183 |
| Query 191  Sbjct 184 | | ACLPNIMVENGRFSGFIDCGRLGVADRYQDIALATRDIAEELGGEWADRFLVLYGIAAPD ACLPN++ +NGRFSGF+DCGRLGVADR+QD+ALA IA LG +W F YG+ PD ACLPNLLADNGRFSGFVDCGRLGVADRFQDLALAAHSIAYNLGEQWLPVFFEAYGV-QPD | 250  242 |
| Query | 251 SQRIAFYRLLDEFF 264 | | |
|  |  | Q+IAFY+LLDEFF |  |
| Sbjct | 243 | QQKIAFYQLLDEFF 256 | |

```
>WP_136959039.1 aminoglycoside 3'-phosphotransferase [Phreatobacter stygius] QCI63582.1 aminoglycoside 3'-phosphotransferase [Phreatobacter stygius]
Length=265

Score = 256 bits (654), Expect = 9e-82, Method: Compositional matrix adjust. Identities = 144/265 (54%), Positives = 169/265 (64%), Gaps = 1/265 (0%)

Query 1	MIEQDGLHAGSPAAWVERLFGYDWAQQTIGCSDAAVFRLSAQGRPVLFVKTDLSGALNEL 60 M E D +	P AW ERL Y W QTIG SDAAVFRL A GRP+LF K +	L EL
Sbjct 1	MPEADIVQPTIPPAWAERLGDYAWLPQTIGRSDAAVFRLEAGGRPILFAKAEAIRPLAEL 60
```

| Query | 61 | QDEAARLSWLATTGVPCAAVLDVVTEAGRDWLLLGEVPGQDLLSS-HLAPAEKVSIMADA | 119 |
| --- | --- | --- | --- |
| Sbjct | 61 | E ARL WLAT GV C VL AG WLL+ +PG DL S+ + PA +V I A A PGEIARLGWLATRGVACPRVLGTHEHAGWHWLLMSALPGPDLASAADMPPARRVEIAAGA | 120 |
| Query | 120 | MRRLHTLDPATCPFDHQAKHRIERARTRMEAGLVDQDDLDEEHQGLAPAELFARLKARMP | 179 |
| Sbjct | 121 | +RRLH LD A CPFDH+ HR+ AR RM AGLVD+ D D+E G +L+ +L A P LRRLHGLDTADCPFDHRLDHRLAAARARMAAGLVDETDFDDERLGRPLPDLYRQLIADRP | 180 |
| Query | 180 | DGEDLVVTHGDACLPNIMVENGRFSGFIDCGRLGVADRYQDIALATRDIAEELGGEWADR | 239 |
| Sbjct | 181 | E LVVTHGDACLPN++ F+GFIDCGRLG+ADRYQD+ALA+ I LG W PSEMLVVTHGDACLPNLIAGETGFAGFIDCGRLGLADRYQDLALASWSITYNLGAAWVAP | 240 |
| Query  Sbjct | 240  241 | FLVLYGIAAPDSQRIAFYRLLDEFF 264 F YG+ PD R+A+YRLLDEFF FFGHYGLPEPDPARLAYYRLLDEFF 265 |  |

>WP_092583030.1 aminoglycoside 3'-phosphotransferase [Rhizobium loessense] SCW27649.1 aminoglycoside 3'-phosphotransferase-2 [Rhizobium loessense]

Length=260

Score = 256 bits (653), Expect = 1e-81, Method: Compositional matrix adjust.

| Identities | = 124/244 (51%), Positives = 160/244 (66%), Gaps = 1/244 (0%) |  |
| --- | --- | --- |
| Query 22 | YDWAQQTIGCSDAAVFRLSAQGRPVLFVKTDLSGALNELQDEAARLSWLATTGVPCAAVL | 81 |
| Sbjct 17 | ++W + +GCS A VFRL+ GR F+K +++G EL EA RL WL G+ C +V+ HEWKKDELGCSSAEVFRLAEGGRTRFFLKGEIAGPFAELPLEAERLLWLGEQGLQCPSVV | 76 |
| Query 82 | DVVTEAGRDWLLLGEVPGQDLLSSHLAP-AEKVSIMADAMRRLHTLDPATCPFDHQAKHR | 140 |
| Sbjct 77 | + + GR+WLLL +PG DL SS L +++ I+A A+RRLH LDP TCPFDH+ R ALESHEGRNWLLLSALPGSDLASSKLTTDRQRIEILAAAIRRLHELDPQTCPFDHRLTAR | 136 |
| Query 141 | IERARTRMEAGLVDQDDLDEEHQGLAPAELFARLKARMPDGEDLVVTHGDACLPNIMVEN | 200 |
| Sbjct 137 | + A+ RMEAG VD+ D DEE Q A LFA L+A PD D VV HGDACLPN M LRLAKARMEAGAVDESDFDEERQNATAASLFAYLEANQPDVTDPVVAHGDACLPNFMTHE | 196 |
| Query 201 | GRFSGFIDCGRLGVADRYQDIALATRDIAEELGGEWADRFLVLYGIAAPDSQRIAFYRLL | 260 |
| Sbjct 197 | G+FSG++DCGRLGVADRYQDIALA R I +G W FL YG++ ++A+Y +L GQFSGYVDCGRLGVADRYQDIALALRSIRYNVGQAWVRHFLDCYGLSGISDAKVAYYMVL | 256 |
| Query 261  Sbjct 257 | DEFF 264 DEFF  DEFF 260 |  |

>RED81569.1 aminoglycoside 3'-phosphotransferase-2 [Pseudomonas sp. 424] Length=258

Score = 256 bits (653), Expect = 1e-81, Method: Compositional matrix adjust.

| Identities | | = 133/254 (52%), Positives = 168/254 (66%), Gaps = 2/254 (1%) |  |
| --- | --- | --- | --- |
| Query 12  Sbjct 6 | | PAAWVERLFGYDWAQQTIGCSDAAVFRLSAQGRPVLFVKTDLSGALNELQDEAARLSWLA P W +R + QQTIG S A VFRL+ G LFVK++ + EL E RL WLA PQTWRQRFADIRYEQQTIGESRADVFRLTPAGSDPLFVKSEPRISFAELPGEVQRLRWLA | 71  65 |
| Query 72  Sbjct 66 | | TTGVPCAAVLDVVTEAGRDWLLLGEVPGQDLLSS-HLAPAEKVSIMADAMRRLHTLDPAT  +P A VLD V E+ R WLL+ VPG+DL SS HLAP + VS+ A+A+R LH LD EQDLPAARVLDSVEESERFWLLMTAVPGRDLASSEHLAPQQIVSLAANALRTLHALDIKH | 130  125 |
| Query 131  Sbjct 126 | | CPFDHQAKHRIERARTRMEAGLVDQDDLDEEHQGLAPAELFARLKARMPDGEDLVVTHGD CPFDH+ +I AR + AGL+D++D D+ G + +F ++ A P EDLVVTHGD CPFDHRLDAKIALAREHLLAGLIDEEDFDDSRLGQSAEAVFEQMLAARPHSEDLVVTHGD | 190  185 |
| Query 191  Sbjct 186 | | ACLPNIMVENGRFSGFIDCGRLGVADRYQDIALATRDIAEELGGEWADRFLVLYGIAAPD ACLPN++ +NGRFSGF+DCGRLGVADR+QD+ALA IA LG +W F YG+ PD ACLPNLLADNGRFSGFVDCGRLGVADRFQDLALAAHSIAYNLGEQWLPVFFEAYGV-QPD | 250  244 |
| Query | 251 SQRIAFYRLLDEFF 264 | | |
|  |  | Q+IAFY+LLDEFF |  |
| Sbjct | 245 | QQKIAFYQLLDEFF 258 | |

>WP_184766830.1 APH(3') family aminoglycoside O-phosphotransferase [Aminobacter lissarensis]

MBB6464208.1 aminoglycoside 3'-phosphotransferase-2 [Aminobacter lissarensis] Length=267

Score = 256 bits (654), Expect = 1e-81, Method: Compositional matrix adjust.

| Identities | | = 132/256 (52%), Positives = 171/256 (67%), Gaps = 3/256 (1%) |  |
| --- | --- | --- | --- |
| Query 12  Sbjct 12 | | PAAWVERLFGYDWAQQTIGCSDAAVFRLSAQGRPVLFVKTDLSGALNELQDEAARLSWLA PA+ L GYDW QQTIG SDA VF+L+A G+P LF+KT++SG EL DE RL WL PASLCLALAGYDWQQQTIGHSDAGVFKLAAGGKPTLFLKTEVSGDFAELPDEIVRLRWLG | 71  71 |
| Query 72  Sbjct 72 | | TTGVPCAAVLDVVTEAGRDWLLLGEVPGQDLLSSHLAPAEKVSIMADAMRRLHTLDPATC  + G+PC V+ T AG +WLL+ V G+DL+S P E ++IMA+A+RRLH LD A C SQGIPCPEVIVYETHAGHNWLLMSAVAGRDLVSQQSEPGEAIAIMAEALRRLHALDIAAC | 131  131 |
| Query 132  Sbjct 132 | | PFDHQAKHRIERARTRMEAGLVDQDDLDEEHQ---GLAPAELFARLKARMPDGEDLVVTH PFDH+ RI A+ R +AG VD+ D D+ G ++F +L AR P E LVVTH PFDHRLGQRIAVAKARADAGEVDEVDEDDFDDERLGQTAQQVFEQLMARRPVSETLVVTH | 188  191 |
| Query 189  Sbjct 192 | | GDACLPNIMVENGRFSGFIDCGRLGVADRYQDIALATRDIAEELGGEWADRFLVLYGIAA GDA +PN + + GRF+GFIDC RLGVAD +QD+ALA I LG EW FL LYG+ GDASMPNFIADGGRFTGFIDCSRLGVADLHQDLALACWSIQYNLGEEWVAPFLALYGLPE | 248  251 |
| Query 249 | | PDSQRIAFYRLLDEFF 264 D+ ++++Y+LLDEFF |  |
| Sbjct | 252 | ADANKLSYYQLLDEFF 267 | |

>TAJ65700.1 aminoglycoside 3'-phosphotransferase [Variovorax sp.] Length=259

Score = 256 bits (653), Expect = 1e-81, Method: Compositional matrix adjust.

| Identities | = 140/253 (55%), Positives = 163/253 (64%), Gaps = 1/253 (0%) |  |
| --- | --- | --- |
| Query 12 | PAAWVERLFGYDWAQQTIGCSDAAVFRLSAQGRPVLFVKTDLSGALNELQDEAARLSWLA | 71 |
| Sbjct 8 | P AW RL W +QTIG S A VFR+ A +LF+K++ L EL E ARL WLA PRAWRARLADAHWQRQTIGESGADVFRIEAADGALLFLKSEPVAPLAELPGEIARLEWLA | 67 |
| Query 72 | TTGVPCAAVLDVVTEAGRDWLLLGEVPGQDLLSSHLAPAEKVSIMADAMRRLHTLDPATC | 131 |
| Sbjct 68 | +P AVLD E R WLL+ VPG+DL S PAE V MADA+R LH + ATC RQDLPAPAVLDQALEHDRHWLLMSAVPGRDLASGPFRPAEIVVHMADALRLLHRVPVATC | 127 |
| Query 132 | PFDHQAKHRIERARTRMEAGLVDQDDLDEEHQGLAPAELFARLKARMPDGEDLVVTHGDA | 191 |
| Sbjct 128 | PFDH+ HRIE A R AGLVD+ D D+E G +LF RL+A P EDLVVTHGDA PFDHRLAHRIEDAHVRAVAGLVDESDFDDERAGWTALDLFERLRATRPAHEDLVVTHGDA | 187 |
| Query 192 | CLPNIMVENGRFSGFIDCGRLGVADRYQDIALATRDIAEELGGEWADRFLVLYGIAAPDS | 251 |
| Sbjct 188 | CLPN+M F+GFIDCGR+GVADR+QD+ALA R I LG W F YGI APD+ CLPNLMATADGFTGFIDCGRVGVADRWQDLALAARSIGFNLGEGWTAPFFERYGI-APDA | 246 |
| Query 252  Sbjct 247 | QRIAFYRLLDEFF 264  +RIAFY LLDEFF  ERIAFYCLLDEFF 259 |  |

>WP_171663723.1 APH(3')-II family aminoglycoside O-phosphotransferase [Achromobacter deleyi]

NOK26462.1 APH(3')-II family aminoglycoside O-phosphotransferase [Achromobacter deleyi]

Length=263

Score = 256 bits (653), Expect = 1e-81, Method: Compositional matrix adjust. Identities = 131/254 (52%), Positives = 171/254 (67%), Gaps = 2/254 (1%)

| Query | 12 | PAAWVERLFGYDWAQQTIGCSDAAVFRLSAQGRPVLFVKTDLSGALNELQDEAARLSWLA | 71 |
| --- | --- | --- | --- |
| Sbjct | 11 | PA W + G QQ+IG S A VFR+ G+P LF+K++ +G EL DE RL W+A PAQWRDEFVGALIEQQSIGESRAQVFRIRRAGQPDLFLKSEAAGTACELPDEIERLQWMA | 70 |
| Query | 72 | TTGVPCAAVLDVVTEAGRDWLLLGEVPGQDLLS-SHLAPAEKVSIMADAMRRLHTLDPAT | 130 |
| Sbjct | 71 | G+P AVLDV TE R WLL+ + GQDL S +HL P+ + +MA A+R LH L A  RHGLPAPAVLDVATEQERHWLLMSAIAGQDLASATHLPPSRVIGLMAAALRALHQLPVAQ | 130 |

| Query | 131 | CPFDHQAKHRIERARTRMEAGLVDQDDLDEEHQGLAPAELFARLKARMPDGEDLVVTHGD | 190 |
| --- | --- | --- | --- |
| Sbjct | 131 | CPFDH+ + R+ AR ++AGLVD++D D+E GL A+ +A+L++ P DLVV HGD CPFDHRLELRMAAARQAVDAGLVDEEDFDDERLGLTAADAYAQLQSTQPATHDLVVAHGD | 190 |
| Query | 191 | ACLPNIMVENGRFSGFIDCGRLGVADRYQDIALATRDIAEELGGEWADRFLVLYGIAAPD | 250 |
| Sbjct | 191 | ACLPN M ++ F+G+IDCGRLGV+DR+QD+ALA R IA LG EW F YG+ APD ACLPNFMAQDQVFTGYIDCGRLGVSDRHQDLALAARSIARNLGQEWVAPFFSAYGM-APD | 249 |
| Query | 251 | SQRIAFYRLLDEFF 264  +R+AFY LLDEFF |  |
| Sbjct | 250 | ERRLAFYCLLDEFF 263 | |

>MBG6434212.1 APH(3')-II family aminoglycoside O-phosphotransferase [Pseudomonas aeruginosa]

Length=268

Score = 256 bits (653), Expect = 1e-81, Method: Compositional matrix adjust.

| Identities | | = 137/258 (53%), Positives = 166/258 (64%), Gaps = 5/258 (2%) |  |
| --- | --- | --- | --- |
| Query 11  Sbjct 12 | | SPAAWVERLFGYDWAQQTIGCSDAAVFRLSAQGRPVLFVKTDLSGALNELQDEAARLSWL  +PA W + L GY W Q GCS A V RL A RP LFVK ++ A EL E ARL L APATWADYLAGYRWRGQGEGCSAAPVHRLEAARRPTLFVKQEVLSAHAELPAEIARLRGL | 70  71 |
| Query 71  Sbjct 72 | | ATTGVPCAAVLDVVTEAGRDWLLLGEVPGQDLLS SHLAPAEKVSIMADAMRRLHTL  G+ C VL+ GR WLL+ VPG L + L P V ++A A+RRLH L HGAGIDCPQVLNETQSDGRQWLLMSAVPGDTLSALAQRGELEPERLVRLVAAALRRLHDL | 126  131 |
| Query 127  Sbjct 132 | | DPATCPFDHQAKHRIERARTRMEAGLVDQDDLDEEHQGLAPAELFARLKARMPDGEDLVV DPA CPFDH+ R++ R R+EAGLVD+ D D++H+G + EL+ L R P EDLVV DPAACPFDHRLARRLDTVRQRVEAGLVDEADFDDDHRGRSATELYRLLLDRRPAVEDLVV | 186  191 |
| Query 187  Sbjct 192 | | THGDACLPNIMVENGRFSGFIDCGRLGVADRYQDIALATRDIAEELGGEWADRFLVLYGI HGDACLPN+ E RFSGFIDCGRLGVADR+QD+ALA RDI ELG WA+ FLV YG AHGDACLPNLRAEGRRFSGFIDCGRLGVADRHQDLALAARDIEAELGAAWAEAFLVEYG- | 246  250 |
| Query | 247 AAPDSQRIAFYRLLDEFF 264 | | |
|  |  | D +R+A++RLLDEFF |  |
| Sbjct | 251 | GDIDGERLAYFRLLDEFF 268 | |

>WP_060506553.1 aminoglycoside 3'-phosphotransferase [Rhizobium sp. Root1204] KQV29219.1 aminoglycoside phosphotransferase [Rhizobium sp. Root1204]

Length=266

Score = 256 bits (653), Expect = 1e-81, Method: Compositional matrix adjust.

| Identities | | = 124/253 (49%), Positives = 165/253 (65%), Gaps = 0/253 (0%) |  |
| --- | --- | --- | --- |
| Query 12  Sbjct 14 | | PAAWVERLFGYDWAQQTIGCSDAAVFRLSAQGRPVLFVKTDLSGALNELQDEAARLSWLA P W RL Y + + +G S A VF+L G+P LFVK + +G EL DEA RL WLA PPEWRIRLSSYHFERDEVGQSAAGVFKLVGDGKPDLFVKCEPAGPFGELPDEATRLLWLA | 71  73 |
| Query 72  Sbjct 74 | | TTGVPCAAVLDVVTEAGRDWLLLGEVPGQDLLSSHLAPAEKVSIMADAMRRLHTLDPATC G+ C V+ G++WLLL +PG+DL + + P + + I+A A++ LH +D TC GAGIACPNVVAPDVHDGQNWLLLSAIPGRDLSVAPITPQQCIEILATALQLLHAIDIRTC | 131  133 |
| Query 132  Sbjct 134 | | PFDHQAKHRIERARTRMEAGLVDQDDLDEEHQGLAPAELFARLKARMPDGEDLVVTHGDA PFD R+ A+ RMEAG VD+DD DE + +LF L+ + PDG DLVVTHGDA PFDQSLSGRLALAKARMEAGEVDEDDFDEARLDQSAEDLFQLLETQKPDGADLVVTHGDA | 191  193 |
| Query 192  Sbjct 194 | | CLPNIMVENGRFSGFIDCGRLGVADRYQDIALATRDIAEELGGEWADRFLVLYGIAAPDS CLPN++ ++GRFSGFIDC RLGVADRYQD+ALA R + ++ G W FL LYG+ PD CLPNLLAQDGRFSGFIDCARLGVADRYQDLALAYRSVCDDFGEAWGQAFLDLYGVPQPDR | 251  253 |
| Query 252 | | QRIAFYRLLDEFF 264  ++A+Y+LLDEFF |  |
| Sbjct | 254 | AKLAYYQLLDEFF 266 | |

>WP_150774741.1 aminoglycoside 3'-phosphotransferase [Pseudomonas fluorescens] VVM74614.1 Aminoglycoside 3'-phosphotransferase [Pseudomonas fluorescens]

Length=256

Score = 255 bits (652), Expect = 1e-81, Method: Compositional matrix adjust.

| Identities | | = 130/254 (51%), Positives = 168/254 (66%), Gaps = 2/254 (1%) |  |
| --- | --- | --- | --- |
| Query 12  Sbjct 4 | | PAAWVERLFGYDWAQQTIGCSDAAVFRLSAQGRPVLFVKTDLSGALNELQDEAARLSWLA PAAW + G QQTIG S A V+R++ G FVKT+ EL E RL W+A PAAWRSQFAGAAIEQQTIGESRADVWRITPAGAAPQFVKTEQVMTWGELPGEVQRLRWMA | 71  63 |
| Query 72  Sbjct 64 | | TTGVPCAAVLDVVTEAGRDWLLLGEVPGQDLLSS-HLAPAEKVSIMADAMRRLHTLDPAT T +P VLD T+A +WLL+ VPG+DL SS HL+P + V++ A A+R LH + + TQDLPAPRVLDTTTQAQHNWLLMTAVPGRDLASSEHLSPEQIVALAAQALRTLHAVAIDS | 130  123 |
| Query 131  Sbjct 124 | | CPFDHQAKHRIERARTRMEAGLVDQDDLDEEHQGLAPAELFARLKARMPDGEDLVVTHGD CPFDH + +I RAR + AGL+D++D D+ G + +F ++ A P EDLVVTHGD CPFDHSLEQKITRAREHLLAGLIDEEDFDDSRIGQSAEAVFQQMLATRPTQEDLVVTHGD | 190  183 |
| Query 191  Sbjct 184 | | ACLPNIMVENGRFSGFIDCGRLGVADRYQDIALATRDIAEELGGEWADRFLVLYGIAAPD ACLPN++ NGRFSGF+DCGRLGVADRYQD+ALA IA+ LG +W F +YG+ PD ACLPNLLAANGRFSGFVDCGRLGVADRYQDLALAAHSIADNLGAQWLPLFFEVYGV-EPD | 250  242 |
| Query 251 | | SQRIAFYRLLDEFF 264 QRIAFY+LLDEFF |  |
| Sbjct | 243 | QQRIAFYQLLDEFF 256 | |

>WP_126473451.1 APH(3')-II family aminoglycoside O-phosphotransferase [Pseudomonas aeruginosa]

MBG7120693.1 APH(3')-II family aminoglycoside O-phosphotransferase [Pseudomonas aeruginosa]

RTT22831.1 APH(3')-II family aminoglycoside O-phosphotransferase [Pseudomonas aeruginosa]

Length=268

Score = 256 bits (653), Expect = 2e-81, Method: Compositional matrix adjust.

| Identities | | = 138/258 (53%), Positives = 168/258 (65%), Gaps = 5/258 (2%) |  |
| --- | --- | --- | --- |
| Query 11  Sbjct 12 | | SPAAWVERLFGYDWAQQTIGCSDAAVFRLSAQGRPVLFVKTDLSGALNELQDEAARLSWL  +PA W + L GY W Q GCS A V RL A RP LFVK ++ A EL E ARL WL APATWADYLAGYRWRGQGEGCSAATVHRLEAARRPTLFVKQEVLSAHAELPAEIARLRWL | 70  71 |
| Query 71  Sbjct 72 | | ATTGVPCAAVLDVVTEAGRDWLLLGEVPGQDLLS SHLAPAEKVSIMADAMRRLHTL  G+ C VL+ GR WLL+ VPG L + L P V ++A A+RRLH L HGAGIDCPQVLNETQSDGRQWLLMSAVPGDTLSALAQRGELEPERLVRLVAAALRRLHDL | 126  131 |
| Query 127  Sbjct 132 | | DPATCPFDHQAKHRIERARTRMEAGLVDQDDLDEEHQGLAPAELFARLKARMPDGEDLVV DPA CPFDH+ + R++ R R+EAGLVD+ D D++H+G + EL+ L R P EDLVV DPAACPFDHRLERRLDTVRQRVEAGLVDEADFDDDHRGRSATELYRLLLDRRPAVEDLVV | 186  191 |
| Query 187  Sbjct 192 | | THGDACLPNIMVENGRFSGFIDCGRLGVADRYQDIALATRDIAEELGGEWADRFLVLYGI HGDACLPN++ E RFSGFIDCGRLGVADR+QD+ALA RDI ELG WA FLV YG AHGDACLPNLLAEGRRFSGFIDCGRLGVADRHQDLALAARDIEAELGAAWAAAFLVEYG- | 246  250 |
| Query | 247 AAPDSQRIAFYRLLDEFF 264 | | |
|  |  | D +R+A++RLLDEFF |  |
| Sbjct | 251 | GDIDGERLAYFRLLDEFF 268 | |

>WP_132661274.1 aminoglycoside 3'-phosphotransferase [Rhizobium azibense] TCU23142.1 aminoglycoside 3'-phosphotransferase-2 [Rhizobium azibense]

Length=270

Score = 256 bits (653), Expect = 2e-81, Method: Compositional matrix adjust. Identities = 126/244 (52%), Positives = 162/244 (66%), Gaps = 1/244 (0%)

| Query | 22 | YDWAQQTIGCSDAAVFRLSAQGRPVLFVKTDLSGALNELQDEAARLSWLATTGVPCAAVL | 81 |
| --- | --- | --- | --- |
| Sbjct | 27 | ++W Q +GCS AAVFRL+ GR F+K +++G EL EA RL WLA G+ C +V+ HEWRQVDLGCSSAAVFRLAEGGRTQFFLKGEIAGPFAELPLEAERLLWLAEQGLACPSVV | 86 |
| Query | 82 | DVVTEAGRDWLLLGEVPGQDLLSSHLAP-AEKVSIMADAMRRLHTLDPATCPFDHQAKHR | 140 |
| Sbjct | 87 | + + GR+WLLL + G+DL SS P +++ I+A A+RRLH LDPA CPFDH+ R  ALESHEGRNWLLLSALDGKDLASSRSTPDGQRIEILAAALRRLHELDPANCPFDHRLTVR | 146 |

| Query | 141 | IERARTRMEAGLVDQDDLDEEHQGLAPAELFARLKARMPDGEDLVVTHGDACLPNIMVEN | 200 |
| --- | --- | --- | --- |
| Sbjct | 147 | + A+ RMEAG VD+ D DEE A LFA L+A PD D VV HGDACLPN M LRLAKARMEAGAVDESDFDEERLNATAASLFAYLEANQPDVTDPVVAHGDACLPNFMTHE | 206 |
| Query | 201 | GRFSGFIDCGRLGVADRYQDIALATRDIAEELGGEWADRFLVLYGIAAPDSQRIAFYRLL | 260 |
| Sbjct | 207 | G+FSG++DCGRLGVADRYQDIALA R I +G W FL YG++ D ++A+Y +L GQFSGYVDCGRLGVADRYQDIALALRSIRYNVGQTWVQHFLDCYGLSGIDDAKVAYYVVL | 266 |
| Query  Sbjct | 261  267 | DEFF 264 DEFF  DEFF 270 |  |

>WP_177239337.1 APH(3') family aminoglycoside O-phosphotransferase [Variovorax sp. PDC80]

Length=256

Score = 255 bits (651), Expect = 2e-81, Method: Compositional matrix adjust.

| Identities | = 139/253 (55%), Positives = 164/253 (65%), Gaps = 1/253 (0%) |  |
| --- | --- | --- |
| Query 12 | PAAWVERLFGYDWAQQTIGCSDAAVFRLSAQGRPVLFVKTDLSGALNELQDEAARLSWLA | 71 |
| Sbjct 5 | P AW RL W +QTIG S A VFR+ A +LF+K++ L EL E ARL+WLA PHAWRARLADAHWRRQTIGESGADVFRIEAAHGELLFLKSEPVAPLAELPGEIARLAWLA | 64 |
| Query 72 | TTGVPCAAVLDVVTEAGRDWLLLGEVPGQDLLSSHLAPAEKVSIMADAMRRLHTLDPATC | 131 |
| Sbjct 65 | +P AVLD E R WLL+ VPG+DL S PAE V MADA+R LH + ATC RQDLPAPAVLDQALEHDRHWLLMSAVPGRDLASGPFGPAEIVVHMADALRLLHRVPVATC | 124 |
| Query 132 | PFDHQAKHRIERARTRMEAGLVDQDDLDEEHQGLAPAELFARLKARMPDGEDLVVTHGDA | 191 |
| Sbjct 125 | PFDH+ +RIE A R AGLVD+ D D+E G +LF RL+A P EDLVVTHGDA PFDHRLAYRIEDAHARAVAGLVDESDFDDERAGWTALDLFERLRATRPASEDLVVTHGDA | 184 |
| Query 192 | CLPNIMVENGRFSGFIDCGRLGVADRYQDIALATRDIAEELGGEWADRFLVLYGIAAPDS | 251 |
| Sbjct 185 | CLPN+M F+GFIDCGR+GVADR+QD+ALA R I LG W F YGI APD+ CLPNLMATADGFTGFIDCGRIGVADRWQDLALAARSIGFNLGEGWTAPFFERYGI-APDA | 243 |
| Query 252  Sbjct 244 | QRIAFYRLLDEFF 264  +RIAFY LLDEFF  ERIAFYCLLDEFF 256 |  |

>WP_172372335.1 APH(3') family aminoglycoside O-phosphotransferase [Mesorhizobium sp. NZP2234]

QKC88998.1 aminoglycoside 3'-phosphotransferase [Mesorhizobium sp. NZP2234] Length=262

Score = 255 bits (651), Expect = 2e-81, Method: Compositional matrix adjust.

| Identities | = 136/248 (55%), Positives = 172/248 (69%), Gaps = 0/248 (0%) |  |
| --- | --- | --- |
| Query 17 | ERLFGYDWAQQTIGCSDAAVFRLSAQGRPVLFVKTDLSGALNELQDEAARLSWLATTGVP | 76 |
| Sbjct 15 | RL GY W +QTIG S A VFRL A +P LF+K + +G EL DEAARL WLA G+ NRLSGYRWHRQTIGRSQAGVFRLVADDKPALFLKCERNGPFAELADEAARLRWLAGQGIA | 74 |
| Query 77 | CAAVLDVVTEAGRDWLLLGEVPGQDLLSSHLAPAEKVSIMADAMRRLHTLDPATCPFDHQ | 136 |
| Sbjct 75 | C V+ + + AG DWLL+ V G+DL S+ + PA+ + IMA A+R LH LD +CPFDH+ CPDVIAMESHAGHDWLLMSAVAGEDLASAPIDPADVIEIMASALRDLHALDIGSCPFDHR | 134 |
| Query 137 | AKHRIERARTRMEAGLVDQDDLDEEHQGLAPAELFARLKARMPDGEDLVVTHGDACLPNI | 196 |
| Sbjct 135 | RI AR+RMEAG+VD+DD D+E QG + A+ FA L+A P EDLVVTHGDACLPN+ LFRRIAAARSRMEAGVVDEDDFDDERQGWSAAKAFAELEALRPASEDLVVTHGDACLPNV | 194 |
| Query 197 | MVENGRFSGFIDCGRLGVADRYQDIALATRDIAEELGGEWADRFLVLYGIAAPDSQRIAF | 256 |
| Sbjct 195 | M GRFSGFIDCGRLGVADR+QD+ALA I +G W + FL YG + ++++ MAARGRFSGFIDCGRLGVADRHQDLALACWSIRYNIGETWIEPFLERYGPRQAEPAKLSW | 254 |
| Query 257  Sbjct 255 | YRLLDEFF 264 YR+LDEFF YRVLDEFF 262 |  |

>KQX44410.1 aminoglycoside phosphotransferase [Ensifer sp. Root1298]

KQX73560.1 aminoglycoside phosphotransferase [Ensifer sp. Root1312] KRC16420.1 aminoglycoside phosphotransferase [Ensifer sp. Root74] KRD70295.1 aminoglycoside phosphotransferase [Ensifer sp. Root954]

Length=263

Score = 255 bits (651), Expect = 2e-81, Method: Compositional matrix adjust.

| Identities | = 127/244 (52%), Positives = 165/244 (68%), Gaps = 0/244 (0%) |  |
| --- | --- | --- |
| Query 21 | GYDWAQQTIGCSDAAVFRLSAQGRPVLFVKTDLSGALNELQDEAARLSWLATTGVPCAAV | 80 |
| Sbjct 20 | GY W + +G S++++F L A RP+L +K + +G +E DEAARL WLA+ VPC V GYQWNRDLLGQSNSSIFLLKAPDRPMLVLKLEEAGPFSEFVDEAARLDWLASKDVPCPRV | 79 |
| Query 81 | LDVVTEAGRDWLLLGEVPGQDLLSSHLAPAEKVSIMADAMRRLHTLDPATCPFDHQAKHR | 140 |
| Sbjct 80 | + A +WLLL V G DL SS L+P +++ I+ADA+++L LD CPFDH+ + R IARAFGAETNWLLLQAVQGTDLASSTLSPKDRIVILADALKQLCALDITNCPFDHRIEGR | 139 |
| Query 141 | IERARTRMEAGLVDQDDLDEEHQGLAPAELFARLKARMPDGEDLVVTHGDACLPNIMVEN | 200 |
| Sbjct 140 | I A+ R +AG++D+ D DE G A+LFA L+AR+P +LVVTHGDACLPNI+ +N IAIAKARTQAGVIDESDFDETRLGRTAADLFAELQARVPRTGELVVTHGDACLPNIIEKN | 199 |
| Query 201 | GRFSGFIDCGRLGVADRYQDIALATRDIAEELGGEWADRFLVLYGIAAPDSQRIAFYRLL | 260 |
| Sbjct 200 | GRFSGFIDC RLGVAD YQDIALA R I LG EW FL YG+ DS++ FY LL GRFSGFIDCSRLGVADPYQDIALACRSIVHNLGEEWVQPFLTRYGLDKDDSEKRDFYCLL | 259 |
| Query 261  Sbjct 260 | DEFF 264 DEFF  DEFF 263 |  |

>SFO53387.1 aminoglycoside 3'-phosphotransferase-2 [Variovorax sp. PDC80] Length=259

Score = 255 bits (651), Expect = 2e-81, Method: Compositional matrix adjust.

| Identities | = 139/253 (55%), Positives = 164/253 (65%), Gaps = 1/253 (0%) |  |
| --- | --- | --- |
| Query 12 | PAAWVERLFGYDWAQQTIGCSDAAVFRLSAQGRPVLFVKTDLSGALNELQDEAARLSWLA | 71 |
| Sbjct 8 | P AW RL W +QTIG S A VFR+ A +LF+K++ L EL E ARL+WLA PHAWRARLADAHWRRQTIGESGADVFRIEAAHGELLFLKSEPVAPLAELPGEIARLAWLA | 67 |
| Query 72 | TTGVPCAAVLDVVTEAGRDWLLLGEVPGQDLLSSHLAPAEKVSIMADAMRRLHTLDPATC | 131 |
| Sbjct 68 | +P AVLD E R WLL+ VPG+DL S PAE V MADA+R LH + ATC RQDLPAPAVLDQALEHDRHWLLMSAVPGRDLASGPFGPAEIVVHMADALRLLHRVPVATC | 127 |
| Query 132 | PFDHQAKHRIERARTRMEAGLVDQDDLDEEHQGLAPAELFARLKARMPDGEDLVVTHGDA | 191 |
| Sbjct 128 | PFDH+ +RIE A R AGLVD+ D D+E G +LF RL+A P EDLVVTHGDA PFDHRLAYRIEDAHARAVAGLVDESDFDDERAGWTALDLFERLRATRPASEDLVVTHGDA | 187 |
| Query 192 | CLPNIMVENGRFSGFIDCGRLGVADRYQDIALATRDIAEELGGEWADRFLVLYGIAAPDS | 251 |
| Sbjct 188 | CLPN+M F+GFIDCGR+GVADR+QD+ALA R I LG W F YGI APD+ CLPNLMATADGFTGFIDCGRIGVADRWQDLALAARSIGFNLGEGWTAPFFERYGI-APDA | 246 |
| Query 252  Sbjct 247 | QRIAFYRLLDEFF 264  +RIAFY LLDEFF  ERIAFYCLLDEFF 259 |  |

>WP_125896096.1 APH(3')-II family aminoglycoside O-phosphotransferase [Pseudomonas aeruginosa]

RRX98718.1 APH(3')-II family aminoglycoside O-phosphotransferase [Pseudomonas aeruginosa]

Length=268

Score = 255 bits (651), Expect = 2e-81, Method: Compositional matrix adjust. Identities = 136/259 (53%), Positives = 168/259 (65%), Gaps = 7/259 (3%)

| Query | 11 | SPAAWVERLFGYDWAQQTIGCSDAAVFRLSAQGRPVLFVKTDLSGALNELQDEAARLSWL | 70 |
| --- | --- | --- | --- |
| Sbjct | 12 | +P+ W + L GY W Q GCS A V RL RP LFVK ++ A EL E ARL WL APSTWADYLAGYRWRGQGEGCSAATVHRLEVARRPTLFVKQEVLSAHAELPAEIARLRWL | 71 |
| Query | 71 | ATTGVPCAAVLDVVTEAGRDWLLLGEVPGQDLLSS HLAPAEKVSIMADAMRRLHT | 125 |
| Sbjct | 72 | G+ C VL+ GR WLL+ VP D LS+ L P V ++A A+RRLH  HGAGIDCPQVLNETQSDGRQWLLMSAVP-WDTLSALAQRGELEPERLVRLVAAALRRLHD | 130 |

| Query | 126 | LDPATCPFDHQAKHRIERARTRMEAGLVDQDDLDEEHQGLAPAELFARLKARMPDGEDLV | 185 |
| --- | --- | --- | --- |
| Sbjct | 131 | LDPA CPFDH+ + R++ R R+EAGLVD+ D D++H+ + EL+ L R P EDLV LDPAACPFDHRLERRLDTVRQRVEAGLVDEADFDDDHRSRSATELYRLLLDRRPAVEDLV | 190 |
| Query | 186 | VTHGDACLPNIMVENGRFSGFIDCGRLGVADRYQDIALATRDIAEELGGEWADRFLVLYG | 245 |
| Sbjct | 191 | V HGDACLPN++ E RFSGFIDCGRLGVADR+QD+ALA RDI ELG WA+ FLV YG VAHGDACLPNLLAEGRRFSGFIDCGRLGVADRHQDLALAARDIEAELGAAWAEAFLVEYG | 250 |
| Query  Sbjct | 246  251 | IAAPDSQRIAFYRLLDEFF 264 D +R+A++RLLDEFF  -GDIDGERLAYFRLLDEFF 268 |  |

>WP_033998715.1 APH(3')-II family aminoglycoside O-phosphotransferase [Pseudomonas aeruginosa]

KSJ79348.1 aminoglycoside phosphotransferase APH(3') [Pseudomonas aeruginosa]

MBA5145295.1 APH(3')-II family aminoglycoside O-phosphotransferase [Pseudomonas aeruginosa]

MBF8387562.1 APH(3')-II family aminoglycoside O-phosphotransferase [Pseudomonas aeruginosa]

MBG4163637.1 APH(3')-II family aminoglycoside O-phosphotransferase [Pseudomonas aeruginosa]

MBG4198846.1 APH(3')-II family aminoglycoside O-phosphotransferase [Pseudomonas aeruginosa]

Length=268

Score = 255 bits (651), Expect = 3e-81, Method: Compositional matrix adjust.

| Identities | | = 137/258 (53%), Positives = 168/258 (65%), Gaps = 5/258 (2%) |  |
| --- | --- | --- | --- |
| Query 11  Sbjct 12 | | SPAAWVERLFGYDWAQQTIGCSDAAVFRLSAQGRPVLFVKTDLSGALNELQDEAARLSWL  +P+ W + L GY W Q GCS A V RL A RP LFVK ++ A EL E ARL WL APSTWADYLAGYRWRGQGEGCSAATVHRLEAARRPTLFVKQEVLSAHAELPAEIARLRWL | 70  71 |
| Query 71  Sbjct 72 | | ATTGVPCAAVLDVVTEAGRDWLLLGEVPGQDLLS SHLAPAEKVSIMADAMRRLHTL  G+ C VL+ GR WLL+ VPG L + L P V ++A A+RRLH L HGAGIDCPQVLNETQSDGRQWLLMSAVPGDTLSALAQRGELEPERLVRLVAAALRRLHDL | 126  131 |
| Query 127  Sbjct 132 | | DPATCPFDHQAKHRIERARTRMEAGLVDQDDLDEEHQGLAPAELFARLKARMPDGEDLVV DPA CPFDH+ + R++ R R+EAGLVD+ D D++H+G + EL+ L R P EDLVV DPAACPFDHRLERRLDTVRQRVEAGLVDEADFDDDHRGRSATELYRLLLDRRPAVEDLVV | 186  191 |
| Query 187  Sbjct 192 | | THGDACLPNIMVENGRFSGFIDCGRLGVADRYQDIALATRDIAEELGGEWADRFLVLYGI HGDACLPN++ E RFSGFIDCGRLGVADR+QD+ALA RDI ELG WA FLV YG AHGDACLPNLLAEGRRFSGFIDCGRLGVADRHQDLALAARDIEAELGAAWAAAFLVEYG- | 246  250 |
| Query | 247 AAPDSQRIAFYRLLDEFF 264 | | |
|  |  | D +R+A++RLLDEFF |  |
| Sbjct | 251 | GDIDGERLAYFRLLDEFF 268 | |

>WP_034025656.1 APH(3')-II family aminoglycoside O-phosphotransferase [Pseudomonas aeruginosa]

Length=268

Score = 255 bits (651), Expect = 3e-81, Method: Compositional matrix adjust.

| Identities | = 137/258 (53%), Positives = 168/258 (65%), Gaps = 5/258 (2%) |  |
| --- | --- | --- |
| Query 11 | SPAAWVERLFGYDWAQQTIGCSDAAVFRLSAQGRPVLFVKTDLSGALNELQDEAARLSWL | 70 |
| Sbjct 12 | +P+ W + L GY W Q GCS A V RL A RP LFVK ++ A EL E ARL WL APSTWADYLAGYRWRGQGEGCSAATVHRLEAARRPTLFVKQEVLSAHAELPAEIARLRWL | 71 |
| Query 71 | ATTGVPCAAVLDVVTEAGRDWLLLGEVPGQDLLS SHLAPAEKVSIMADAMRRLHTL | 126 |
| Sbjct 72 | G+ C VL+ GR WLL+ VPG L + L P V ++A A+RRLH L HGAGIDCPQVLNETQSDGRQWLLMSAVPGDTLSALAQRGELEPERLVRLVAAALRRLHDL | 131 |
| Query 127 | DPATCPFDHQAKHRIERARTRMEAGLVDQDDLDEEHQGLAPAELFARLKARMPDGEDLVV | 186 |
| Sbjct 132 | DPA CPFDH+ + R++ R R+EAGLVD+ D D++H+G + EL+ L R P EDLVV DPAACPFDHRLERRLDTVRQRVEAGLVDEADFDDDHRGRSATELYRLLLDRRPAVEDLVV | 191 |
| Query 187 | THGDACLPNIMVENGRFSGFIDCGRLGVADRYQDIALATRDIAEELGGEWADRFLVLYGI HGDACLPN++ E RFSGFIDCGRLGVADR+QD+ALA RDI ELG WA FLV YG | 246 |

| Sbjct | 192 | AHGDACLPNLLAEGRRFSGFIDCGRLGVADRHQDLALAARDIEAELGAAWAAAFLVEYG- 250 |
| --- | --- | --- |
| Query | 247 | AAPDSQRIAFYRLLDEFF 264 |
| Sbjct | 251 | D +R+A++RLLDEFF  GDIDGERLAYFRLLDEFF 268 |

>WP_023465172.1 MULTISPECIES: APH(3')-II family aminoglycoside O-phosphotransferase [Pseudomonas]

AMA35319.1 aminoglycoside phosphotransferase [Pseudomonas aeruginosa DHS01] AWE86682.1 phosphotransferase enzyme family protein [Pseudomonas aeruginosa]

AWR46332.1 APH(3')-II family aminoglycoside O-phosphotransferase [Pseudomonas aeruginosa]

ESZ80538.1 aminoglycoside 3'-phosphotransferase [Pseudomonas aeruginosa DHS29]

ETV10962.1 aminoglycoside 3'-phosphotransferase type IIB [Pseudomonas aeruginosa BWHPSA045]

Length=268

Score = 255 bits (651), Expect = 3e-81, Method: Compositional matrix adjust.

| Identities | | = 137/258 (53%), Positives = 168/258 (65%), Gaps = 5/258 (2%) |  |
| --- | --- | --- | --- |
| Query 11  Sbjct 12 | | SPAAWVERLFGYDWAQQTIGCSDAAVFRLSAQGRPVLFVKTDLSGALNELQDEAARLSWL  +P+ W + L GY W Q GCS A V RL A RP LFVK ++ A EL E ARL WL APSTWADYLAGYRWRGQGEGCSAATVHRLEAARRPTLFVKQEVLSAHAELPAEIARLRWL | 70  71 |
| Query 71  Sbjct 72 | | ATTGVPCAAVLDVVTEAGRDWLLLGEVPGQDLLS SHLAPAEKVSIMADAMRRLHTL  G+ C VL+ GR WLL+ VPG L + L P V ++A A+RRLH L HGAGIDCPQVLNETQSDGRQWLLMSAVPGDTLSALVQRGELEPERLVRLVAAALRRLHDL | 126  131 |
| Query 127  Sbjct 132 | | DPATCPFDHQAKHRIERARTRMEAGLVDQDDLDEEHQGLAPAELFARLKARMPDGEDLVV DPA CPFDH+ + R++ R R+EAGLVD+ D D++H+G + EL+ L R P EDLVV DPAACPFDHRLERRLDTVRQRVEAGLVDEADFDDDHRGRSATELYRLLLDRRPAVEDLVV | 186  191 |
| Query 187  Sbjct 192 | | THGDACLPNIMVENGRFSGFIDCGRLGVADRYQDIALATRDIAEELGGEWADRFLVLYGI HGDACLPN++ E RFSGFIDCGRLGVADR+QD+ALA RDI ELG WA FLV YG AHGDACLPNLLAEGRRFSGFIDCGRLGVADRHQDLALAARDIEAELGAAWAAAFLVEYG- | 246  250 |
| Query | 247 AAPDSQRIAFYRLLDEFF 264 | | |
|  |  | D +R+A++RLLDEFF |  |
| Sbjct | 251 | GDIDGERLAYFRLLDEFF 268 | |

>WP_113251131.1 MULTISPECIES: aminoglycoside 3'-phosphotransferase [unclassified Rhizobium]

MBB3291269.1 aminoglycoside 3'-phosphotransferase-2 [Rhizobium sp. BK252] MBB3405995.1 aminoglycoside 3'-phosphotransferase-2 [Rhizobium sp. BK289] MBB3418596.1 aminoglycoside 3'-phosphotransferase-2 [Rhizobium sp. BK284] MBB3486459.1 aminoglycoside 3'-phosphotransferase-2 [Rhizobium sp. BK347] MBB3492792.1 aminoglycoside 3'-phosphotransferase-2 [Rhizobium sp. BK338]

Length=266

Score = 254 bits (650), Expect = 4e-81, Method: Compositional matrix adjust.

| Identities | = 129/254 (51%), Positives = 167/254 (66%), Gaps = 1/254 (0%) |  |
| --- | --- | --- |
| Query 12 | PAAWVERLFGYDWAQQTIGCSDAAVFRLSAQGRPVLFVKTDLSGALNELQDEAARLSWLA | 71 |
| Sbjct 13 | P + L GY+W Q +G S A VFRL A GR +++KT+L+ L EL E ARL WLA PTRLRDALTGYEWRQDALGHSSAHVFRLEADGRAPVYLKTELADPLGELAGEVARLRWLA | 72 |
| Query 72 | TTGVPCAAVLDVVTEAGRDWLLLGEVPGQDLLSSH-LAPAEKVSIMADAMRRLHTLDPAT | 130 |
| Sbjct 73 | + G+ C V+ + R+WLL+ +PG DL+S+ L P E+V ++ADA+RRLH++D A SQGLACPDVITHEKDGEREWLLMSALPGSDLVSAERLQPLERVRLLADALRRLHSVDIAG | 132 |
| Query 131 | CPFDHQAKHRIERARTRMEAGLVDQDDLDEEHQGLAPAELFARLKARMPDGEDLVVTHGD | 190 |
| Sbjct 133 | CPFDH+ +RI AR RM AG VD+DD D+ G + +LF L+A+ P ED VVTHGD CPFDHRLGNRIAEARARMLAGRVDEDDFDDARLGRSAEDLFRELEAKRPSAEDFVVTHGD | 192 |
| Query 191 | ACLPNIMVENGRFSGFIDCGRLGVADRYQDIALATRDIAEELGGEWADRFLVLYGIAAPD | 250 |
| Sbjct 193 | ACLPN M + RFSG+IDCGRLG+ADR+QDIALA I G FL YG P ACLPNFMADGDRFSGYIDCGRLGIADRHQDIALACGSIEYNFGKGLVFEFLKAYGNFEPQ | 252 |
| Query 251 | SQRIAFYRLLDEFF 264 |  |

+++AFYRLLDEFF Sbjct 253 PEKMAFYRLLDEFF 266

>WP_196644764.1 APH(3')-II family aminoglycoside O-phosphotransferase [Pseudomonas aeruginosa]

MBG4688081.1 APH(3')-II family aminoglycoside O-phosphotransferase [Pseudomonas aeruginosa]

Length=268

Score = 254 bits (650), Expect = 4e-81, Method: Compositional matrix adjust.

| Identities | | = 136/258 (53%), Positives = 165/258 (64%), Gaps = 5/258 (2%) |  |
| --- | --- | --- | --- |
| Query 11  Sbjct 12 | | SPAAWVERLFGYDWAQQTIGCSDAAVFRLSAQGRPVLFVKTDLSGALNELQDEAARLSWL  +PA W + L GY W Q GCS A V RL A RP LFVK ++ A EL E ARL WL APATWADYLAGYRWRGQGEGCSAATVHRLEAARRPTLFVKQEVLSAHAELPAEIARLRWL | 70  71 |
| Query 71  Sbjct 72 | | ATTGVPCAAVLDVVTEAGRDWLLLGEVPGQDLLS SHLAPAEKVSIMADAMRRLHTL  G+ C VL+ GR WLL+ VPG L + L P V ++A A+RRLH L HGAGIDCPQVLNAPQSDGRQWLLMSAVPGDTLSALAQRGELEPERLVRLVAAALRRLHDL | 126  131 |
| Query 127  Sbjct 132 | | DPATCPFDHQAKHRIERARTRMEAGLVDQDDLDEEHQGLAPAELFARLKARMPDGEDLVV DPA CPFDH+ + R + R R+EAGLVD+ D D++H+G + E + L R P EDLVV DPAACPFDHRLERRRDTVRPRVEAGLVDEADFDDDHRGRSATERYRLLLDRRPAVEDLVV | 186  191 |
| Query 187  Sbjct 192 | | THGDACLPNIMVENGRFSGFIDCGRLGVADRYQDIALATRDIAEELGGEWADRFLVLYGI HGDACLPN++ E RFSGFIDCGRLGVA R QD+ALA RDI ELG WA+ FLV YG AHGDACLPNLLAEGRRFSGFIDCGRLGVAARRQDLALAARDIEAELGAAWAEAFLVEYG- | 246  250 |
| Query | 247 AAPDSQRIAFYRLLDEFF 264 | | |
|  |  | D +R+A++RLLDEFF |  |
| Sbjct | 251 | GDIDGERLAYFRLLDEFF 268 | |

>WP_054310564.1 aminoglycoside 3'-phosphotransferase [Mesorhizobium sp. 1M-11] Length=265

Score = 254 bits (649), Expect = 4e-81, Method: Compositional matrix adjust.

| Identities | | = 132/254 (52%), Positives = 164/254 (65%), Gaps = 1/254 (0%) |  |
| --- | --- | --- | --- |
| Query 12  Sbjct 12 | | PAAWVERLFGYDWAQQTIGCSDAAVFRLSAQGRPVLFVKTDLSGALNELQDEAARLSWLA PA R+ GY W + T+G SDA VF+L +P LF+K + + EL E RL WL PAVIRTRVDGYAWDRDTLGHSDAGVFKLEVTDKPSLFLKIEEADGFGELPAEVERLRWLG | 71  71 |
| Query 72  Sbjct 72 | | TTGVPCAAVLDVVTEAGRDWLLLGEVPGQDLLSSHLAPAEK-VSIMADAMRRLHTLDPAT G+ C VL AGR+WLL+ + G+DL+S+ PAEK +++MA A+R LH LD + HQGITCPEVLAFEVHAGRNWLLMSAISGKDLVSAGPLPAEKTIAVMARALRTLHALDIRS | 130  131 |
| Query 131  Sbjct 132 | | CPFDHQAKHRIERARTRMEAGLVDQDDLDEEHQGLAPAELFARLKARMPDGEDLVVTHGD CPFDH+ RI AR RMEAGLVD++D D+E G ++FA L A P EDLVV HGD CPFDHRLDRRIADARARMEAGLVDEEDFDDERLGQTAQDVFADLIALRPADEDLVVAHGD | 190  191 |
| Query 191  Sbjct 192 | | ACLPNIMVENGRFSGFIDCGRLGVADRYQDIALATRDIAEELGGEWADRFLVLYGIAAPD ACLPN MV+ RFSGFIDCGRLGVADR QD+AL+ I LG EW FL LYG D ACLPNFMVDGDRFSGFIDCGRLGVADRCQDLALSCWSIRHNLGEEWIAPFLTLYGTPDAD | 250  251 |
| Query 251 | | SQRIAFYRLLDEFF 264  ++A+YRLLDEFF |  |
| Sbjct | 252 | PAKLAYYRLLDEFF 265 | |

>WP_160040445.1 APH(3') family aminoglycoside O-phosphotransferase [Pseudomonas sp. S49]

QHF51181.1 APH(3') family aminoglycoside O-phosphotransferase [Pseudomonas sp. S49]

Length=256

Score = 254 bits (649), Expect = 4e-81, Method: Compositional matrix adjust. Identities = 129/254 (51%), Positives = 168/254 (66%), Gaps = 2/254 (1%)

Query 12	PAAWVERLFGYDWAQQTIGCSDAAVFRLSAQGRPVLFVKTDLSGALNELQDEAARLSWLA 71 PAAW + G	QQTIG S A V+R++ G	FVKT+	EL E RL W+A

| Sbjct | 4 | PAAWRSQFAGAAIEQQTIGESRADVWRITPAGAAPQFVKTEQVMTWGELPGEVQRLRWMA | 63 |
| --- | --- | --- | --- |
| Query | 72 | TTGVPCAAVLDVVTEAGRDWLLLGEVPGQDLLSS-HLAPAEKVSIMADAMRRLHTLDPAT | 130 |
| Sbjct | 64 | T +P VLD T+A +WLL+ VPG+DL SS HL+P + V++ A A+R LH + + TQDLPAPRVLDTTTQAQHNWLLMTAVPGRDLASSEHLSPEQIVALAAQALRTLHAVAIDS | 123 |
| Query | 131 | CPFDHQAKHRIERARTRMEAGLVDQDDLDEEHQGLAPAELFARLKARMPDGEDLVVTHGD | 190 |
| Sbjct | 124 | CPFDH + +I RAR + AGL+D++D D+ G + +F ++ A P+ EDLVVTHGD CPFDHSLEQKITRAREHLLAGLIDEEDFDDSRIGQSAEAVFQQMLAVRPEHEDLVVTHGD | 183 |
| Query | 191 | ACLPNIMVENGRFSGFIDCGRLGVADRYQDIALATRDIAEELGGEWADRFLVLYGIAAPD | 250 |
| Sbjct | 184 | ACLPN++ NGRFSGF+DCGRLGVADRYQD+ALA I + LG +W F +YG+ PD ACLPNLLAANGRFSGFVDCGRLGVADRYQDLALAAHSITDNLGAQWLPLFFEVYGV-EPD | 242 |
| Query | 251 | SQRIAFYRLLDEFF 264 QRIAFY+LLDEFF |  |
| Sbjct | 243 | QQRIAFYQLLDEFF 256 | |

>WP_124133756.1 APH(3')-II family aminoglycoside O-phosphotransferase [Pseudomonas aeruginosa]

RPX94640.1 APH(3') family aminoglycoside O-phosphotransferase [Pseudomonas aeruginosa]

Length=268

Score = 254 bits (650), Expect = 5e-81, Method: Compositional matrix adjust.

| Identities | | = 137/258 (53%), Positives = 168/258 (65%), Gaps = 5/258 (2%) |  |
| --- | --- | --- | --- |
| Query 11  Sbjct 12 | | SPAAWVERLFGYDWAQQTIGCSDAAVFRLSAQGRPVLFVKTDLSGALNELQDEAARLSWL  +P+ W + L GY W Q GCS A V RL A RP LFVK ++ A EL E ARL WL APSTWADYLAGYRWRGQGEGCSVATVHRLEAARRPTLFVKQEVLSAHAELPAEIARLRWL | 70  71 |
| Query 71  Sbjct 72 | | ATTGVPCAAVLDVVTEAGRDWLLLGEVPGQDLLS SHLAPAEKVSIMADAMRRLHTL  G+ C VL+ GR WLL+ VPG L + L P V ++A A+RRLH L HGAGIDCPQVLNETQSDGRQWLLMSAVPGDTLSALAQRGELEPERLVRLVAAALRRLHDL | 126  131 |
| Query 127  Sbjct 132 | | DPATCPFDHQAKHRIERARTRMEAGLVDQDDLDEEHQGLAPAELFARLKARMPDGEDLVV DPA CPFDH+ + R++ R R+EAGLVD+ D D++H+G + EL+ L R P EDLVV DPAACPFDHRLERRLDTVRQRVEAGLVDEADFDDDHRGRSATELYRLLLDRRPAVEDLVV | 186  191 |
| Query 187  Sbjct 192 | | THGDACLPNIMVENGRFSGFIDCGRLGVADRYQDIALATRDIAEELGGEWADRFLVLYGI HGDACLPN++ E RFSGFIDCGRLGVADR+QD+ALA RDI ELG WA FLV YG AHGDACLPNLLAEGRRFSGFIDCGRLGVADRHQDLALAARDIEAELGAAWAAAFLVEYG- | 246  250 |
| Query | 247 AAPDSQRIAFYRLLDEFF 264 | | |
|  |  | D +R+A++RLLDEFF |  |
| Sbjct | 251 | GDIDGERLAYFRLLDEFF 268 | |

>WP_127928220.1 aminoglycoside 3'-phosphotransferase [Pseudomonas sp. RU47]

AZZ76685.1 APH(3') family aminoglycoside O-phosphotransferase [Pseudomonas sp. RU47]

Length=256

Score = 254 bits (648), Expect = 5e-81, Method: Compositional matrix adjust.

| Identities | = 129/254 (51%), Positives = 167/254 (66%), Gaps = 2/254 (1%) |  |
| --- | --- | --- |
| Query 12 | PAAWVERLFGYDWAQQTIGCSDAAVFRLSAQGRPVLFVKTDLSGALNELQDEAARLSWLA | 71 |
| Sbjct 4 | PAAW + G QQTIG S A V+R++ G FVKT+ EL E RL W+A PAAWRSQFAGAAIEQQTIGESRADVWRITPAGAAPQFVKTEQVMTWGELPGEVQRLRWMA | 63 |
| Query 72 | TTGVPCAAVLDVVTEAGRDWLLLGEVPGQDLLSS-HLAPAEKVSIMADAMRRLHTLDPAT | 130 |
| Sbjct 64 | T +P VLD T+A +WLL+ VPG+DL SS HL+P + V++ A A+R LH + + TQDLPAPRVLDTTTQAQHNWLLMTAVPGRDLASSEHLSPEQIVALAAQALRTLHAVAIDS | 123 |
| Query 131 | CPFDHQAKHRIERARTRMEAGLVDQDDLDEEHQGLAPAELFARLKARMPDGEDLVVTHGD | 190 |
| Sbjct 124 | CPFDH + +I RAR + AGL+D++D D+ G + +F ++ A P EDLVVTHGD CPFDHSLEQKITRAREHLLAGLIDEEDFDDSRIGQSAEAVFQQMLATRPTQEDLVVTHGD | 183 |
| Query 191 | ACLPNIMVENGRFSGFIDCGRLGVADRYQDIALATRDIAEELGGEWADRFLVLYGIAAPD | 250 |
| Sbjct 184 | ACLPN++ NGRFSGF+DCGRLGVADRYQD+ALA I + LG +W F +YG+ PD  ACLPNLLAANGRFSGFVDCGRLGVADRYQDLALAAHSITDNLGAQWLPLFFEVYGV-EPD | 242 |

Query 251 SQRIAFYRLLDEFF 264

QRIAFY+LLDEFF Sbjct 243 QQRIAFYQLLDEFF 256

>WP_038403805.1 APH(3')-II family aminoglycoside O-phosphotransferase [Pseudomonas aeruginosa]

AID75315.1 aminoglycoside 3-N-acetyltransferase [Pseudomonas aeruginosa PAO1H2O]

Length=268

Score = 254 bits (649), Expect = 5e-81, Method: Compositional matrix adjust.

| Identities | | = 134/258 (52%), Positives = 166/258 (64%), Gaps = 5/258 (2%) |  |
| --- | --- | --- | --- |
| Query 11  Sbjct 12 | | SPAAWVERLFGYDWAQQTIGCSDAAVFRLSAQGRPVLFVKTDLSGALNELQDEAARLSWL  +P+ W + L GY W Q GCS A V RL RP LFVK ++ A EL E ARL WL APSTWADYLAGYRWRGQGEGCSAATVHRLEXARRPTLFVKQEVLSAHAELPAEIARLRWL | 70  71 |
| Query 71  Sbjct 72 | | ATTGVPCAAVLDVVTEAGRDWLLLGEVPGQDLLS SHLAPAEKVSIMADAMRRLHTL  G+ C VL+ GR WLL+ VPG L + L P V ++A A+RRLH L HGAGIDCPQVLNETQSDGRQWLLMSAVPGDTLSALAQRGELEPERLVRLVAAALRRLHDL | 126  131 |
| Query 127  Sbjct 132 | | DPATCPFDHQAKHRIERARTRMEAGLVDQDDLDEEHQGLAPAELFARLKARMPDGEDLVV DPA CPFDH+ + R++ R R+EAGLVD+ D ++H+G + EL+ L R P EDLVV DPAACPFDHRLERRLDTVRQRVEAGLVDEADFXDDHRGRSATELYRLLLDRRPAVEDLVV | 186  191 |
| Query 187  Sbjct 192 | | THGDACLPNIMVENGRFSGFIDCGRLGVADRYQDIALATRDIAEELGGEWADRFLVLYGI GDACLPN++ E RFSGFIDCGRLGVADR+QD+ALA RDI ELG WA+ FLV YG  AXGDACLPNLLAEGRRFSGFIDCGRLGVADRHQDLALAARDIEAELGAAWAEAFLVEYG- | 246  250 |
| Query | 247 AAPDSQRIAFYRLLDEFF 264 | | |
|  |  | D +R+A++RLLDEFF |  |
| Sbjct | 251 | GDIDGERLAYFRLLDEFF 268 | |

>WP_082566533.1 MULTISPECIES: aminoglycoside 3'-phosphotransferase [unclassified Ensifer]

Length=290

Score = 255 bits (651), Expect = 6e-81, Method: Compositional matrix adjust.

| Identities | = 127/244 (52%), Positives = 165/244 (68%), Gaps = 0/244 (0%) |  |
| --- | --- | --- |
| Query 21 | GYDWAQQTIGCSDAAVFRLSAQGRPVLFVKTDLSGALNELQDEAARLSWLATTGVPCAAV | 80 |
| Sbjct 47 | GY W + +G S++++F L A RP+L +K + +G +E DEAARL WLA+ VPC V GYQWNRDLLGQSNSSIFLLKAPDRPMLVLKLEEAGPFSEFVDEAARLDWLASKDVPCPRV | 106 |
| Query 81 | LDVVTEAGRDWLLLGEVPGQDLLSSHLAPAEKVSIMADAMRRLHTLDPATCPFDHQAKHR | 140 |
| Sbjct 107 | + A +WLLL V G DL SS L+P +++ I+ADA+++L LD CPFDH+ + R IARAFGAETNWLLLQAVQGTDLASSTLSPKDRIVILADALKQLCALDITNCPFDHRIEGR | 166 |
| Query 141 | IERARTRMEAGLVDQDDLDEEHQGLAPAELFARLKARMPDGEDLVVTHGDACLPNIMVEN | 200 |
| Sbjct 167 | I A+ R +AG++D+ D DE G A+LFA L+AR+P +LVVTHGDACLPNI+ +N IAIAKARTQAGVIDESDFDETRLGRTAADLFAELQARVPRTGELVVTHGDACLPNIIEKN | 226 |
| Query 201 | GRFSGFIDCGRLGVADRYQDIALATRDIAEELGGEWADRFLVLYGIAAPDSQRIAFYRLL | 260 |
| Sbjct 227 | GRFSGFIDC RLGVAD YQDIALA R I LG EW FL YG+ DS++ FY LL GRFSGFIDCSRLGVADPYQDIALACRSIVHNLGEEWVQPFLTRYGLDKDDSEKRDFYCLL | 286 |
| Query 261  Sbjct 287 | DEFF 264 DEFF  DEFF 290 |  |

>WP_037078345.1 aminoglycoside 3'-phosphotransferase [Rhizobium sp. CCGE 510] Length=262

Score = 254 bits (648), Expect = 6e-81, Method: Compositional matrix adjust. Identities = 135/265 (51%), Positives = 173/265 (65%), Gaps = 4/265 (2%)

Query 1	MIEQDGLHAGSPAAWVERLFGYDWAQQTIGCSDAAVFRLSAQGRPVLFVKTDLSGALNEL 60 M	GL AGS A	RL GY + + T+G S A+VFRL +G PVL++K + +G	EL

| Sbjct | 1 | MTFSGGLPAGSLEA---RLSGYRFERDTLGRSTASVFRLEGEGLPVLYLKVEEAGPFGEL | 57 |
| --- | --- | --- | --- |
| Query | 61 | QDEAARLSWLATTGVPCAAVLDVVTEAGRDWLLLGEVPGQDLLS-SHLAPAEKVSIMADA | 119 |
| Sbjct | 58 | DEAARLSWL +G+PC V+ ++ GR+ LL+ +PG DL S S L P +V ++A A ADEAARLSWLKASGLPCPDVIARESDGGRNRLLINALPGTDLASASALTPLARVELLAAA | 117 |
| Query | 120 | MRRLHTLDPATCPFDHQAKHRIERARTRMEAGLVDQDDLDEEHQGLAPAELFARLKARMP | 179 |
| Sbjct | 118 | + RLH L ++CPFDH+ + RI A+ RM+AG+VD+ D E G + LFA L++R LSRLHRLPISSCPFDHRLESRIAAAKARMQAGIVDETDFGETRLGKSAEALFAELESRRL | 177 |
| Query | 180 | DGEDLVVTHGDACLPNIMVENGRFSGFIDCGRLGVADRYQDIALATRDIAEELGGEWADR | 239 |
| Sbjct | 178 | EDLVVTHGDACLPN + FSG+IDC RLGVADRYQDIALA R I G R GREDLVVTHGDACLPNFIASAEGFSGYIDCSRLGVADRYQDIALACRSIGYNFGEALVQR | 237 |
| Query  Sbjct | 240  238 | FLVLYGIAAPDSQRIAFYRLLDEFF 264 FL YG+AAPD R+ +Y+LLDEFF FLDRYGMAAPDPARLDYYQLLDEFF 262 |  |

>MBG6505398.1 APH(3')-II family aminoglycoside O-phosphotransferase [Pseudomonas aeruginosa]

Length=268

Score = 254 bits (649), Expect = 6e-81, Method: Compositional matrix adjust.

| Identities | | = 136/260 (52%), Positives = 167/260 (64%), Gaps = 9/260 (3%) |  |
| --- | --- | --- | --- |
| Query 11  Sbjct 12 | | SPAAWVERLFGYDWAQQTIGCSDAAVFRLSAQGRPVLFVKTDLSGALNELQDEAARLSWL  +PA W + L GY W Q GCS A V RL A RP LFVK ++ A E E ARL WL APATWADYLAGYRWRGQGEGCSAATVHRLEAARRPTLFVKQEVLSAHAERPAEIARLRWL | 70  71 |
| Query 71  Sbjct 72 | | ATTGVPCAAVLDVVTEAGRDWLLLGEVPGQDLLS SHLAPAEKVSIMADAMRRLHTL  G+ C VL+ GR WLL+ VPG L + L P V ++A A+RRLH L HGAGIDCPQVLNETQSDGRQWLLMSAVPGDTLSALAQRGELEPERLVRLVAAALRRLHDL | 126  131 |
| Query 127  Sbjct 132 | | DPATCPFDHQAKHRIERARTRMEAGLVDQDDLDEEHQGLAPAELFARLKARMPDGEDLVV DPA CPFDH+ + R++ R R+EAG VD+ D D++H+G + EL+ L R P EDLVV DPAACPFDHRLERRLDTVRQRVEAGRVDEADFDDDHRGRSATELYRLLLDRRPAVEDLVV | 186  191 |
| Query 187  Sbjct 192 | | THGDACLPNIMVENGRFSGFIDCGRLGVADRYQDIALATRDIAEELGGEWADRFLVLYG- HGDACLPN++ E RFSGFIDCGR GVADR+QD+ALA RDI ELG WA+ FLV YG AHGDACLPNLLAEGRRFSGFIDCGRRGVADRHQDLALAARDIEAELGAAWAEAFLVEYGG | 245  251 |
| Query | 246 -IAAPDSQRIAFYRLLDEFF 264 | | |
|  |  | IA +R+A++RLLDEFF |  |
| Sbjct | 252 | DIA---GERLAYFRLLDEFF 268 | |

```
>WP_115419083.1 aminoglycoside 3'-phosphotransferase [Ensifer sp. M14] RDL49851.1 Aminoglycoside 3'-phosphotransferase [Ensifer sp. M14]
Length=263

Score = 254 bits (648), Expect = 7e-81, Method: Compositional matrix adjust. Identities = 130/253 (51%), Positives = 163/253 (64%), Gaps = 0/253 (0%)

Query 12	PAAWVERLFGYDWAQQTIGCSDAAVFRLSAQGRPVLFVKTDLSGALNELQDEAARLSWLA 71 P +	L GY W + ++G S ++VF	A+ R L +K + SG +EL DEA RL WL
Sbjct 11	PPGFAPLLKGYSWRKDSLGQSASSVFLFDAEARTPLVLKVEPSGPFDELVDEAERLEWLG 70

Query 72	TTGVPCAAVLDVVTEAGRDWLLLGEVPGQDLLSSHLAPAEKVSIMADAMRRLHTLDPATC 131 G+PC V+	A R+WLL+ V G DL SS LA +E+V ++A+A+RR+H LD	C
Sbjct 71	KQGMPCPRVIARDEYARRNWLLMEAVDGVDLASSSLAASEQVVLLAEAVRRMHALDVEAC 130

Query 132 PFDHQAKHRIERARTRMEAGLVDQDDLDEEHQGLAPAELFARLKARMPDGEDLVVTHGDA 191 PFDH+	R+ AR R EA L+D D DEE G	ELF L+A++P E LVVTHGDA
Sbjct 131 PFDHRVDARVAIARLRAEADLIDVTDFDEERLGKTAEELFVELQAKIPQEEKLVVTHGDA 190

Query 192 CLPNIMVENGRFSGFIDCGRLGVADRYQDIALATRDIAEELGGEWADRFLVLYGIAAPDS 251 CLPNI+V +GRF+GFIDC RLGVAD YQD+AL R IA LG EW	FL LYG+	D
Sbjct 191 CLPNIIVRDGRFAGFIDCSRLGVADPYQDLALTCRSIAYNLGEEWVQPFLDLYGVRRADP 250

Query 252 QRIAFYRLLDEFF 264
R+ FY LLDEFF

Sbjct 251 DRLDFYCLLDEFF 263

>WP_016733662.1 aminoglycoside 3'-phosphotransferase [Rhizobium phaseoli] KKZ86252.1 aminoglycoside 3'-phosphotransferase [Rhizobium phaseoli Ch24-10] RDJ07400.1 aminoglycoside phosphotransferase APH(3') [Rhizobium phaseoli] RDJ10844.1 aminoglycoside phosphotransferase APH(3') [Rhizobium phaseoli]
Length=261

Score = 254 bits (648), Expect = 7e-81, Method: Compositional matrix adjust.
```

| Identities | = 127/254 (50%), Positives = 170/254 (67%), Gaps = 1/254 (0%) |  |
| --- | --- | --- |
| Query 12 | PAAWVERLFGYDWAQQTIGCSDAAVFRLSAQGRPVLFVKTDLSGALNELQDEAARLSWLA | 71 |
| Sbjct 8 | PAA ERL GY + + +G S A+VFRL +G P L++K + +G EL DEAARLSWL PAALGERLSGYRFERDALGRSAASVFRLEGEGLPALYLKVEAAGPFGELADEAARLSWLK | 67 |
| Query 72 | TTGVPCAAVLDVVTEAGRDWLLLGEVPGQDLLS-SHLAPAEKVSIMADAMRRLHTLDPAT | 130 |
| Sbjct 68 | G+PC V+ ++ R+WLL+G +PG DL+S S L P +V ++A A+ LH L A+ AAGLPCPDVIARESDGERNWLLIGALPGADLVSASALTPMARVELLAAALSDLHDLPIAS | 127 |
| Query 131 | CPFDHQAKHRIERARTRMEAGLVDQDDLDEEHQGLAPAELFARLKARMPDGEDLVVTHGD | 190 |
| Sbjct 128 | CPFDH+ ++R+ A+ RM+AG+VD++D D G + A LF L P EDLVVTHGD CPFDHRLENRLPTAKARMQAGIVDEEDFDAARLGKSAAALFDDLMRLRPGNEDLVVTHGD | 187 |
| Query 191 | ACLPNIMVENGRFSGFIDCGRLGVADRYQDIALATRDIAEELGGEWADRFLVLYGIAAPD | 250 |
| Sbjct 188 | ACLPN + G+FSG+IDC RLGVADR+QDIALA R IA G FL YG+ APD ACLPNFVASTGKFSGYIDCSRLGVADRHQDIALACRSIASNFGEALIRPFLERYGLTAPD | 247 |
| Query 251  Sbjct 248 | SQRIAFYRLLDEFF 264  + ++ +++LLDEFF  ATKLHYFQLLDEFF 261 |  |

>WP_097664897.1 aminoglycoside 3'-phosphotransferase [Rhizobium sp. M1]

PDT10664.1 APH(3') family aminoglycoside O-phosphotransferase [Rhizobium sp. M1]

Length=261

Score = 254 bits (648), Expect = 7e-81, Method: Compositional matrix adjust.

| Identities | | = 134/254 (53%), Positives = 169/254 (67%), Gaps = 1/254 (0%) |  |
| --- | --- | --- | --- |
| Query 12  Sbjct 8 | | PAAWVERLFGYDWAQQTIGCSDAAVFRLSAQGRPVLFVKTDLSGALNELQDEAARLSWLA PAA ERL GY + + +G S A+VFRL +G P L++K + +G EL DEAARL WL PAALGERLSGYRFERDALGRSAASVFRLEGEGLPALYLKVEAAGPFGELADEAARLGWLK | 71  67 |
| Query 72  Sbjct 68 | | TTGVPCAAVLDVVTEAGRDWLLLGEVPGQDLLS-SHLAPAEKVSIMADAMRRLHTLDPAT G+PC V+ ++ RDWLL+G +PG DL S S L P +V ++A A+ LH L A+ AAGLPCPDVIARESDGERDWLLIGALPGTDLASASALTPLARVELLAAALADLHHLPIAS | 130  127 |
| Query 131  Sbjct 128 | | CPFDHQAKHRIERARTRMEAGLVDQDDLDEEHQGLAPAELFARLKARMPDGEDLVVTHGD CPFDH+ + R+ AR RM+AG+VD+ D DE G + LFA L+ R PD EDLVVTHGD CPFDHRLEKRLPTARARMQAGIVDETDFDETRLGKSAETLFAELEGRRPDREDLVVTHGD | 190  187 |
| Query 191  Sbjct 188 | | ACLPNIMVENGRFSGFIDCGRLGVADRYQDIALATRDIAEELGGEWADRFLVLYGIAAPD ACLPN + G+FSG+IDC RLGVADRYQDIALA R IA G FL YG+ APD ACLPNFVASGGQFSGYIDCSRLGVADRYQDIALACRSIAYNFGAALVQPFLDRYGLTAPD | 250  247 |
| Query 251 | | SQRIAFYRLLDEFF 264 R+ +Y+LLDEFF |  |
| Sbjct | 248 | PARLDYYQLLDEFF 261 | |

>WP_154350657.1 APH(3')-II family aminoglycoside O-phosphotransferase [Stenotrophomonas maltophilia]

QGL80226.1 APH(3')-II family aminoglycoside O-phosphotransferase [Stenotrophomonas maltophilia]

QNG76886.1 aminoglycoside O-phosphotransferase APH(3')-IIc [Stenotrophomonas maltophilia]

VUP57418.1 Aminoglycoside 3'-phosphotransferase [Stenotrophomonas maltophilia] Length=269

Score = 254 bits (649), Expect = 7e-81, Method: Compositional matrix adjust.

| Identities | | = 137/262 (52%), Positives = 171/262 (65%), Gaps = 3/262 (1%) |  |
| --- | --- | --- | --- |
| Query 5  Sbjct 9 | | DGLHAGSPAAWVERLFGYDWAQQTIGCSDAAVFRLSAQGRPVLFVKTDLSGALNELQDEA DGL P AW E L +Q+IG S A V R+ G+ F+K+++ A +EL DE DGLPLPLPRAWQEALADARIERQSIGVSRADVARVHRPGQADAFLKSEVIDAFSELGDEI | 64  68 |
| Query 65  Sbjct 69 | | ARLSWLATTGVPCAAVLDVVTEAGRDWLLLGEVPGQDLLSS-HLAPAEKVSIMADAMRRL RL WL G+P V+ EAGR WLL+ +PG+DL SS LAP + V ++ADA+RRL TRLRWLQAQGLPAPTVIATAEEAGRRWLLMSALPGRDLASSPELAPQQLVELLADALRRL | 123  128 |
| Query 124  Sbjct 129 | | HTLDPATCPFDHQAKHRIERARTRMEAGLVDQDDLDEEHQGLAPAELFARLKARMPDGED H L A CPFD + R++ A R+EAGLVD DD D+E G +P ++FA+L + PD ED HVLPVAACPFDQRLASRLQAAAARVEAGLVDADDFDDERLGQSPQQVFAQLCSTRPDHED | 183  188 |
| Query 184  Sbjct 189 | | LVVTHGDACLPNIMVENGRFSGFIDCGRLGVADRYQDIALATRDIAEELG-GEWADRFLV LVV+HGDACLPN+MV GRFSGFIDCGRLGVADRYQD+ALA R +A G W LVVSHGDACLPNLMVAEGRFSGFIDCGRLGVADRYQDLALAARSLAHNFGDTRWVTPLFQ | 242  248 |
| Query 243 | | LYGIAAPDSQRIAFYRLLDEFF 264 YG A D +R+AFYRLLDEFF |  |
| Sbjct | 249 | RYGAVA-DERRLAFYRLLDEFF 269 | |

>WP_160057641.1 APH(3') family aminoglycoside O-phosphotransferase [Pseudomonas sp. R84]

QHC98720.1 APH(3') family aminoglycoside O-phosphotransferase [Pseudomonas sp. R84]

Length=256

Score = 253 bits (647), Expect = 7e-81, Method: Compositional matrix adjust.

| Identities | | = 130/254 (51%), Positives = 168/254 (66%), Gaps = 2/254 (1%) |  |
| --- | --- | --- | --- |
| Query 12  Sbjct 4 | | PAAWVERLFGYDWAQQTIGCSDAAVFRLSAQGRPVLFVKTDLSGALNELQDEAARLSWLA PAAW + G QQTIG S A V+R++ G FVKT+L EL E RL W+A PAAWRSQFAGAAIEQQTIGESRADVWRITPAGAAPQFVKTELVMTWGELPGEVQRLRWMA | 71  63 |
| Query 72  Sbjct 64 | | TTGVPCAAVLDVVTEAGRDWLLLGEVPGQDLLSS-HLAPAEKVSIMADAMRRLHTLDPAT T +P VLD T+ +WLL+ V G+DL SS HL+P + V++ A+A+R LHT+ + TQDLPAPRVLDTTTQTQHNWLLMTAVAGRDLASSEHLSPQQIVTLAAEALRALHTVAIES | 130  123 |
| Query 131  Sbjct 124 | | CPFDHQAKHRIERARTRMEAGLVDQDDLDEEHQGLAPAELFARLKARMPDGEDLVVTHGD CPFDH + +I AR + AGL+D++D D+ G + E+F +L A P EDLVVTHGD CPFDHSLEAKIRLAREHLLAGLIDEEDFDDSRLGQSAEEVFQQLLATRPSQEDLVVTHGD | 190  183 |
| Query 191  Sbjct 184 | | ACLPNIMVENGRFSGFIDCGRLGVADRYQDIALATRDIAEELGGEWADRFLVLYGIAAPD ACLPN++ NGRFSGF+DCGRLGVADRYQD+ALA I + LG +W F +YG+ PD ACLPNLLAANGRFSGFVDCGRLGVADRYQDLALAAHSITDNLGAQWLPLFFEVYGV-EPD | 250  242 |
| Query 251 | | SQRIAFYRLLDEFF 264 QRIAFY+LLDEFF |  |
| Sbjct | 243 | LQRIAFYQLLDEFF 256 | |

>WP_123586670.1 aminoglycoside 3'-phosphotransferase [Pseudomonas fluorescens]

RON91754.1 APH(3') family aminoglycoside O-phosphotransferase [Pseudomonas fluorescens]

Length=256

Score = 253 bits (647), Expect = 7e-81, Method: Compositional matrix adjust.

| Identities | = 132/254 (52%), Positives = 165/254 (65%), Gaps = 2/254 (1%) |  |
| --- | --- | --- |
| Query 12 | PAAWVERLFGYDWAQQTIGCSDAAVFRLSAQGRPVLFVKTDLSGALNELQDEAARLSWLA | 71 |
| Sbjct 4 | P W +R + QQTIG S A VFRL+ G LFVK++ + EL E RL WLA PQTWRQRFADIRYEQQTIGESRADVFRLTPAGSDPLFVKSEPRISFAELPGEVQRLRWLA | 63 |
| Query 72 | TTGVPCAAVLDVVTEAGRDWLLLGEVPGQDLLSS-HLAPAEKVSIMADAMRRLHTLDPAT | 130 |
| Sbjct 64 | VP A VLD R WLL+ VPG+DL SS HLAP + VS+ A A+R LH LD KQDVPAARVLDSTEAHDRYWLLMTAVPGRDLASSEHLAPQQIVSLAAHALRTLHALDIKN | 123 |
| Query 131 | CPFDHQAKHRIERARTRMEAGLVDQDDLDEEHQGLAPAELFARLKARMPDGEDLVVTHGD | 190 |
| Sbjct 124 | CPFDH+ +I AR + AGL+D++D D+ G + +F ++ A PD EDLVVTHGD  CPFDHRLDAKIALAREHLLAGLIDEEDFDDSRLGQSAEAVFEQMLAARPDSEDLVVTHGD | 183 |

| Query | 191 | ACLPNIMVENGRFSGFIDCGRLGVADRYQDIALATRDIAEELGGEWADRFLVLYGIAAPD 250 |
| --- | --- | --- |
| Sbjct | 184 | ACLPN++ +NGRFSGF+DCGRLGV+DR+QD+ALA IA LG +W F YG+ PD ACLPNLLADNGRFSGFVDCGRLGVSDRFQDLALAAHSIAYNLGEQWLPVFFEAYGV-QPD 242 |
| Query | 251 | SQRIAFYRLLDEFF 264 |
| Sbjct | 243 | Q+IAFY+LLDEFF  QQKIAFYQLLDEFF 256 |

>WP_183836930.1 APH(3') family aminoglycoside O-phosphotransferase [Rhizobium etli]

MBB4477479.1 aminoglycoside 3'-phosphotransferase-2 [Rhizobium etli] MBB4533311.1 aminoglycoside 3'-phosphotransferase-2 [Rhizobium etli]

Length=261

Score = 254 bits (648), Expect = 7e-81, Method: Compositional matrix adjust.

| Identities | | = 134/254 (53%), Positives = 172/254 (68%), Gaps = 1/254 (0%) |  |
| --- | --- | --- | --- |
| Query 12  Sbjct 8 | | PAAWVERLFGYDWAQQTIGCSDAAVFRLSAQGRPVLFVKTDLSGALNELQDEAARLSWLA PAA ERL GY + + +G S A+VFRL +GRP L++K + +GA EL DEAARL WL PAALDERLSGYRFERDALGRSAASVFRLEGEGRPALYLKVEAAGAFGELADEAARLGWLK | 71  67 |
| Query 72  Sbjct 68 | | TTGVPCAAVLDVVTEAGRDWLLLGEVPGQDLLS-SHLAPAEKVSIMADAMRRLHTLDPAT G+PC V+ ++ RDWLL+G +PG DL S S L P +V ++A A+ LH L A+ AAGLPCPDVIARESDGERDWLLIGALPGTDLASASALTPLTRVELLAAALADLHHLPIAS | 130  127 |
| Query 131  Sbjct 128 | | CPFDHQAKHRIERARTRMEAGLVDQDDLDEEHQGLAPAELFARLKARMPDGEDLVVTHGD CPFDH+ + R+ AR RM+AG+VD+ D DE G + LFA L+ + PD EDLVVTHGD CPFDHRLEKRLPAARARMQAGIVDETDFDETRLGKSAETLFAELEGQRPDREDLVVTHGD | 190  187 |
| Query 191  Sbjct 188 | | ACLPNIMVENGRFSGFIDCGRLGVADRYQDIALATRDIAEELGGEWADRFLVLYGIAAPD ACLPN + +G+FSG+IDC RLGVADRYQD+ALA R IA G FL YG+ APD ACLPNFVASDGQFSGYIDCSRLGVADRYQDLALACRSIAYNFGAALVQPFLDRYGLTAPD | 250  247 |
| Query 251 | | SQRIAFYRLLDEFF 264 R+ +Y+LLDEFF |  |
| Sbjct | 248 | PTRLDYYQLLDEFF 261 | |

>WP_053500030.1 MULTISPECIES: APH(3')-II family aminoglycoside O-phosphotransferase [Stenotrophomonas]

KOQ62340.1 aminoglycoside phosphotransferase [Stenotrophomonas maltophilia]

OFU99259.1 APH(3') family aminoglycoside O-phosphotransferase [Stenotrophomonas sp. HMSC10F07]

Length=267

Score = 254 bits (648), Expect = 8e-81, Method: Compositional matrix adjust.

| Identities | = 135/255 (53%), Positives = 168/255 (66%), Gaps = 3/255 (1%) |  |
| --- | --- | --- |
| Query 12 | PAAWVERLFGYDWAQQTIGCSDAAVFRLSAQGRPVLFVKTDLSGALNELQDEAARLSWLA | 71 |
| Sbjct 14 | P AW + L G +Q++G S A V R+ GRP F+K++L A +EL DE ARL WL PLAWQKALAGARIERQSMGVSRADVARVLRPGRPDAFLKSELIDAFSELGDEIARLRWLQ | 73 |
| Query 72 | TTGVPCAAVLDVVTEAGRDWLLLGEVPGQDLLSS-HLAPAEKVSIMADAMRRLHTLDPAT | 130 |
| Sbjct 74 | G P V+ EAGR WLL+ +PG+DL SS LAP ++V I+ADA+R LH L A AQGQPAPTVIATAEEAGRHWLLMSALPGRDLASSPELAPQQRVEILADALRGLHALPVAA | 133 |
| Query 131 | CPFDHQAKHRIERARTRMEAGLVDQDDLDEEHQGLAPAELFARLKARMPDGEDLVVTHGD | 190 |
| Sbjct 134 | CPFD + R+ A+ R+EAG VD DD D+E G +P ++FA+L + PD EDLVV+HGD CPFDQRLASRLPAAQARVEAGRVDADDFDDERLGQSPQQVFAQLCSTRPDHEDLVVSHGD | 193 |
| Query 191 | ACLPNIMVENGRFSGFIDCGRLGVADRYQDIALATRDIAEELG-GEWADRFLVLYGIAAP | 249 |
| Sbjct 194 | ACLPN+MV GRFSGFIDCGRLGVADRYQD+ALA R + G W YG A ACLPNLMVAEGRFSGFIDCGRLGVADRYQDLALAARSLVHNFGDTRWVTPLFQRYGAVA- | 252 |
| Query 250  Sbjct 253 | DSQRIAFYRLLDEFF 264 D +R+AFYRLLDEFF DERRLAFYRLLDEFF 267 |  |

>WP_033962597.1 APH(3')-II family aminoglycoside O-phosphotransferase [Pseudomonas

aeruginosa] Length=267

Score = 254 bits (648), Expect = 8e-81, Method: Compositional matrix adjust.

| Identities | | = 137/258 (53%), Positives = 168/258 (65%), Gaps = 6/258 (2%) |  |
| --- | --- | --- | --- |
| Query 11  Sbjct 12 | | SPAAWVERLFGYDWAQQTIGCSDAAVFRLSAQGRPVLFVKTDLSGALNELQDEAARLSWL  +PA W + L GY W Q GCS A V RL A RP LFVK ++ A EL E ARL WL APATWADYLAGYRWRGQGEGCSAATVHRLEAARRPTLFVKQEVLSAHAELPAEIARLRWL | 70  71 |
| Query 71  Sbjct 72 | | ATTGVPCAAVLDVVTEAGRDWLLLGEVPGQDLLS SHLAPAEKVSIMADAMRRLHTL  G+ C VL+ GR WLL+ VPG L + L P V ++A A+RRLH L HGAGIDCPQVLNETQSDGRQWLLMSAVPGDTLSALAQRGELEPERLVRLVAAALRRLHDL | 126  131 |
| Query 127  Sbjct 132 | | DPATCPFDHQAKHRIERARTRMEAGLVDQDDLDEEHQGLAPAELFARLKARMPDGEDLVV DPA PFDH+ + R++ R R+EAGLVD+ D D++H+G + EL+ L R P EDLVV DPAG-PFDHRLERRLDTVRQRVEAGLVDEADFDDDHRGRSATELYRLLLDRRPAVEDLVV | 186  190 |
| Query 187  Sbjct 191 | | THGDACLPNIMVENGRFSGFIDCGRLGVADRYQDIALATRDIAEELGGEWADRFLVLYGI HGDACLPN++ E RFSGFIDCGRLGVADR+QD+ALA RDI ELG WA+ FLV YG AHGDACLPNLLAEGRRFSGFIDCGRLGVADRHQDLALAARDIEAELGAAWAEAFLVEYG- | 246  249 |
| Query | 247 AAPDSQRIAFYRLLDEFF 264 | | |
|  |  | D +R+A++RLLDEFF |  |
| Sbjct | 250 | GDIDGERLAYFRLLDEFF 267 | |

>WP_008126610.1 aminoglycoside 3'-phosphotransferase [Phyllobacterium sp. YR531] EJN01620.1 aminoglycoside phosphotransferase [Phyllobacterium sp. YR531]

Length=262

Score = 253 bits (647), Expect = 8e-81, Method: Compositional matrix adjust.

| Identities | = 134/254 (53%), Positives = 162/254 (64%), Gaps = 4/254 (2%) |  |
| --- | --- | --- |
| Query 12 | PAAWVERLFGYDWAQQTIGCSDAAVFRLSAQGRPVLFVKTDLSGALNELQDEAARLSWLA | 71 |
| Sbjct 12 | P W RL Y W QTIG S A VFRL A G P LFVK++++G EL DE +RL WLA PPDWRGRLAHYGWTAQTIGESGALVFRLEASGLPTLFVKSEIAGEFGELPDEISRLRWLA | 71 |
| Query 72 | TTGVPCAAVLDVVTEAGRDWLLLGEVPGQDLLSSHLAPAEK-VSIMADAMRRLHTLDPAT | 130 |
| Sbjct 72 | G+ VLD + GR WLL+ +PG+DL S PAE V I A +++ LH+LD A GQGIAAPKVLDQTEDNGRIWLLMSALPGRDLASDKEIPAENIVRIAAQSLKDLHSLDIAH | 131 |
| Query 131 | CPFDHQAKHRIERARTRMEAGLVDQDDLDEEHQGLAPAELFARLKARMPDGEDLVVTHGD | 190 |
| Sbjct 132 | CPF+H +RI A RM A VD+ D DE G AELF RL DGEDLVVTHGD CPFNHTLSNRIPVAHARMIANQVDESDFDE--VGPTAAELFERLLKLRSDGEDLVVTHGD | 189 |
| Query 191 | ACLPNIMVENGRFSGFIDCGRLGVADRYQDIALATRDIAEELGGEWADRFLVLYGIAAPD | 250 |
| Sbjct 190 | ACLPNI+ + F+GF+DC RLG+ADRYQD+ALA+R I LG EW + F YG+ D ACLPNILSDGSVFTGFVDCARLGIADRYQDLALASRSIEHNLGVEWVEPFFRSYGVDM-D | 248 |
| Query 251  Sbjct 249 | SQRIAFYRLLDEFF 264 S R+ +YRLLDEFF SSRVEYYRLLDEFF 262 |  |

>WP_149255094.1 aminoglycoside 3'-phosphotransferase [Labrys sp. KNU-23] QEN90929.1 aminoglycoside 3'-phosphotransferase [Labrys sp. KNU-23]

Length=263

Score = 253 bits (647), Expect = 1e-80, Method: Compositional matrix adjust. Identities = 137/255 (54%), Positives = 161/255 (63%), Gaps = 4/255 (2%)

| Query | 12 | PAAWVERLFGYDWAQQTIGCSDAAVFRLSAQGRPVLFVKTDLSGALNELQDEAARLSWLA | 71 |
| --- | --- | --- | --- |
| Sbjct | 11 | PA W L GY W Q+IG S A VFRL A RP LFVKT+ L EL DE RL WL PADWRRDLAGYGWKAQSIGASGAGVFRLEAPDRPDLFVKTEELSPLGELPDEGPRLDWLG | 70 |
| Query | 72 | TTGVPCAAVLDVVTEAGRDWLLLGEVPGQDLLSSH--LAPAEKVSIMADAMRRLHTLDPA | 129 |
| Sbjct | 71 | G+PC L GR WLL+ +PG +LSS LAP + V I ADA+R LH LD ARGIPCPKRLAETRHDGRHWLLMSAIPGT-ILSSQAGLAPEQVVDIAADALRHLHALDRT | 129 |
| Query | 130 | TCPFDHQAKHRIERARTRMEAGLVDQDDLDEEHQGLAPAELFARLKARMPDGEDLVVTHG CPFDH+A RI A R++AGLVD++D DEE G A++FA L P EDLVVTHG | 189 |

| Sbjct | 130 | GCPFDHRADSRIALAEARVKAGLVDEEDFDEERAGQTAADVFAELSPLRPRQEDLVVTHG | 189 |
| --- | --- | --- | --- |
| Query | 190 | DACLPNIMVENGRFSGFIDCGRLGVADRYQDIALATRDIAEELGGEWADRFLVLYGIAAP | 249 |
| Sbjct | 190 | DA L N++ + FSGFIDC R G+ADR+QD+ALATR I + G +RFL YGI P DATLENLLADKDVFSGFIDCARAGLADRHQDLALATRSIGDHFGPTQVERFLARYGI-VP | 248 |
| Query  Sbjct | 250  249 | DSQRIAFYRLLDEFF 264 D QR+AFY LLDEFF DPQRMAFYLLLDEFF 263 |  |

>WP_023129437.1 APH(3')-II family aminoglycoside O-phosphotransferase [Pseudomonas aeruginosa]

ERZ29678.1 aminoglycoside 3'-phosphotransferase type IIB [Pseudomonas aeruginosa CF5]

Length=268

Score = 253 bits (647), Expect = 1e-80, Method: Compositional matrix adjust.

| Identities | | = 136/258 (53%), Positives = 168/258 (65%), Gaps = 5/258 (2%) |  |
| --- | --- | --- | --- |
| Query 11  Sbjct 12 | | SPAAWVERLFGYDWAQQTIGCSDAAVFRLSAQGRPVLFVKTDLSGALNELQDEAARLSWL  +P+ W + L GY W Q GCS A V RL A RP LFVK ++ A EL E ARL WL APSTWADYLAGYRWRGQGEGCSAATVHRLEAARRPTLFVKQEVLSAHAELPAEIARLRWL | 70  71 |
| Query 71  Sbjct 72 | | ATTGVPCAAVLDVVTEAGRDWLLLGEVPGQDLLS SHLAPAEKVSIMADAMRRLHTL  G+ C VL+ GR WLL+ VPG L + L P V ++A A+RRLH L HGAGIDCPQVLNETQSDGRQWLLMSAVPGDTLSALAQRGELEPERLVRLVAAALRRLHDL | 126  131 |
| Query 127  Sbjct 132 | | DPATCPFDHQAKHRIERARTRMEAGLVDQDDLDEEHQGLAPAELFARLKARMPDGEDLVV DPA CPFDH+ + R++ R R+EAGLV++ D D++H+G + EL+ L R P EDLVV DPAACPFDHRLERRLDTVRQRVEAGLVNEADFDDDHRGRSATELYRLLLDRRPAVEDLVV | 186  191 |
| Query 187  Sbjct 192 | | THGDACLPNIMVENGRFSGFIDCGRLGVADRYQDIALATRDIAEELGGEWADRFLVLYGI HGDACLPN++ E RFSGFIDCGRLGVADR+QD+ALA RDI ELG WA FLV YG AHGDACLPNLLAEGRRFSGFIDCGRLGVADRHQDLALAARDIEAELGAAWAAAFLVEYG- | 246  250 |
| Query | 247 AAPDSQRIAFYRLLDEFF 264 | | |
|  |  | D +R+A++RLLDEFF |  |
| Sbjct | 251 | GDIDGERLAYFRLLDEFF 268 | |

>WP_032931420.1 MULTISPECIES: aminoglycoside 3'-phosphotransferase [Mesorhizobium] MBE1706390.1 aminoglycoside 3'-phosphotransferase [Mesorhizobium japonicum] MBE1715099.1 aminoglycoside 3'-phosphotransferase [Mesorhizobium japonicum]

MUT21687.1 APH(3') family aminoglycoside O-phosphotransferase [Mesorhizobium japonicum]

MUT27538.1 APH(3') family aminoglycoside O-phosphotransferase [Mesorhizobium japonicum]

OBP74168.1 APH(3') family aminoglycoside O-phosphotransferase [Mesorhizobium loti]

Length=263

Score = 253 bits (647), Expect = 1e-80, Method: Compositional matrix adjust.

| Identities | = 137/255 (54%), Positives = 168/255 (66%), Gaps = 0/255 (0%) |  |
| --- | --- | --- |
| Query 10 | GSPAAWVERLFGYDWAQQTIGCSDAAVFRLSAQGRPVLFVKTDLSGALNELQDEAARLSW | 69 |
| Sbjct 9 | G P + + GY W +QTIG S A VFRL+A GRP LF+K + SG EL DEAARL W GLPNGLRQLVSGYRWHRQTIGQSQAGVFRLAADGRPALFLKCERSGPFAELADEAARLRW | 68 |
| Query 70 | LATTGVPCAAVLDVVTEAGRDWLLLGEVPGQDLLSSHLAPAEKVSIMADAMRRLHTLDPA | 129 |
| Sbjct 69 | L GV C V+ + + AG DWLL+ V G+DL S+ + A+ + IMA A+R LH +D LTGQGVACPDVIALESHAGYDWLLMSAVAGEDLASAAIDQADVIGIMASALRDLHAIDIR | 128 |
| Query 130 | TCPFDHQAKHRIERARTRMEAGLVDQDDLDEEHQGLAPAELFARLKARMPDGEDLVVTHG | 189 |
| Sbjct 129 | +CPFDH+ RI AR RME G VD+ D DEE QG AE+FA L+A P EDLVVTHG SCPFDHRLIRRIAAARARMEGGQVDESDFDEERQGRTAAEVFAELEALRPATEDLVVTHG | 188 |
| Query 190 | DACLPNIMVENGRFSGFIDCGRLGVADRYQDIALATRDIAEELGGEWADRFLVLYGIAAP | 249 |
| Sbjct 189 | DACLPN+M G FSGFIDCGRLGVADR+QD+ALA I LG W + FL Y DACLPNVMTAGGGFSGFIDCGRLGVADRHQDLALACWSIRYNLGQVWIEPFLKRYEPPET | 248 |
| Query 250 | DSQRIAFYRLLDEFF 264 |  |

+ ++++YRLLDEFF Sbjct 249 EPAKLSWYRLLDEFF 263

>WP_175121197.1 APH(3') family aminoglycoside O-phosphotransferase [Achromobacter animicus]

Length=263

Score = 253 bits (647), Expect = 1e-80, Method: Compositional matrix adjust.

| Identities | | = 129/264 (49%), Positives = 166/264 (63%), Gaps = 1/264 (0%) |  |
| --- | --- | --- | --- |
| Query 1  Sbjct 1 | | MIEQDGLHAGSPAAWVERLFGYDWAQQTIGCSDAAVFRLSAQGRPVLFVKTDLSGALNEL M Q +H P W RL G QQ+IG S A V+R+ + +P ++K++ +G L EL MHAQYSIHDSLPLQWQPRLAGARLEQQSIGESRADVYRIRSANQPDRYLKSEHTGTLPEL | 60  60 |
| Query 61  Sbjct 61 | | QDEAARLSWLATTGVPCAAVLDVVTEAGRDWLLLGEVPGQDLLSSHLAPAEKVSIMADAM DE RL W+A G+PC V+D R WLL+ V GQDL S+ L PA+ ++IMADA+ PDEIERLQWMAAHGLPCPTVIDAAATPTRHWLLMSAVAGQDLASAGLPPAQVIAIMADAL | 120  120 |
| Query 121  Sbjct 121 | | RRLHTLDPATCPFDHQAKHRIERARTRMEAGLVDQDDLDEEHQGLAPAELFARLKARMPD RLH + CPFDH + RI AR ++AGLVD+ D D+E G + FA L A P HRLHCIPIDQCPFDHTLERRIAEARRALDAGLVDESDFDDERIGRTAEDAFAELLATRPT | 180  180 |
| Query 181  Sbjct 181 | | GEDLVVTHGDACLPNIMVENGRFSGFIDCGRLGVADRYQDIALATRDIAEELGGEWADRF DLVV HGDACLPN M + G F+G+IDCGRLGV+DR+QD+ALA R I LG EW + F THDLVVAHGDACLPNFMADGGVFTGYIDCGRLGVSDRHQDLALAARSIERNLGREWIEPF | 240  240 |
| Query 241 | | LVLYGIAAPDSQRIAFYRLLDEFF 264 YG+ PD +R+AFY LLDEFF |  |
| Sbjct | 241 | FRSYGV-EPDDRRLAFYCLLDEFF 263 | |

>CAB3651731.1 Aminoglycoside 3'-phosphotransferase [Achromobacter animicus] Length=266

Score = 253 bits (647), Expect = 1e-80, Method: Compositional matrix adjust.

| Identities | | = 129/264 (49%), Positives = 166/264 (63%), Gaps = 1/264 (0%) |  |
| --- | --- | --- | --- |
| Query 1  Sbjct 4 | | MIEQDGLHAGSPAAWVERLFGYDWAQQTIGCSDAAVFRLSAQGRPVLFVKTDLSGALNEL M Q +H P W RL G QQ+IG S A V+R+ + +P ++K++ +G L EL MHAQYSIHDSLPLQWQPRLAGARLEQQSIGESRADVYRIRSANQPDRYLKSEHTGTLPEL | 60  63 |
| Query 61  Sbjct 64 | | QDEAARLSWLATTGVPCAAVLDVVTEAGRDWLLLGEVPGQDLLSSHLAPAEKVSIMADAM DE RL W+A G+PC V+D R WLL+ V GQDL S+ L PA+ ++IMADA+ PDEIERLQWMAAHGLPCPTVIDAAATPTRHWLLMSAVAGQDLASAGLPPAQVIAIMADAL | 120  123 |
| Query 121  Sbjct 124 | | RRLHTLDPATCPFDHQAKHRIERARTRMEAGLVDQDDLDEEHQGLAPAELFARLKARMPD RLH + CPFDH + RI AR ++AGLVD+ D D+E G + FA L A P HRLHCIPIDQCPFDHTLERRIAEARRALDAGLVDESDFDDERIGRTAEDAFAELLATRPT | 180  183 |
| Query 181  Sbjct 184 | | GEDLVVTHGDACLPNIMVENGRFSGFIDCGRLGVADRYQDIALATRDIAEELGGEWADRF DLVV HGDACLPN M + G F+G+IDCGRLGV+DR+QD+ALA R I LG EW + F THDLVVAHGDACLPNFMADGGVFTGYIDCGRLGVSDRHQDLALAARSIERNLGREWIEPF | 240  243 |
| Query 241 | | LVLYGIAAPDSQRIAFYRLLDEFF 264 YG+ PD +R+AFY LLDEFF |  |
| Sbjct | 244 | FRSYGV-EPDDRRLAFYCLLDEFF 266 | |

```
>WP_031755390.1 APH(3')-II family aminoglycoside O-phosphotransferase [Pseudomonas aeruginosa]
EZO55884.1 aminoglycoside 3'-phosphotransferase type IIB [Pseudomonas aeruginosa PS75]
TEC83494.1 APH(3')-II family aminoglycoside O-phosphotransferase [Pseudomonas aeruginosa]
Length=268

Score = 253 bits (647), Expect = 1e-80, Method: Compositional matrix adjust. Identities = 136/258 (53%), Positives = 167/258 (65%), Gaps = 5/258 (2%)

Query 11	SPAAWVERLFGYDWAQQTIGCSDAAVFRLSAQGRPVLFVKTDLSGALNELQDEAARLSWL 70
+P+ W + L GY W Q GCS A V RL	RP LFVK ++ A EL E ARL WL
```

| Sbjct | 12 | APSTWADYLAGYRWRGQGEGCSAATVHRLEVARRPTLFVKQEVLSAHAELPAEIARLRWL | 71 |
| --- | --- | --- | --- |
| Query | 71 | ATTGVPCAAVLDVVTEAGRDWLLLGEVPGQDLLS SHLAPAEKVSIMADAMRRLHTL | 126 |
| Sbjct | 72 | G+ C VL+ GR WLL+ VPG L + L P V ++A A+RRLH L HGAGIDCPQVLNETQSDGRQWLLMSAVPGDTLSALAQRGELEPERLVRLVAAALRRLHDL | 131 |
| Query | 127 | DPATCPFDHQAKHRIERARTRMEAGLVDQDDLDEEHQGLAPAELFARLKARMPDGEDLVV | 186 |
| Sbjct | 132 | DPA CPFDH+ + R++ R R+EAGLVD+ D D++H+G + EL+ L R P EDLVV DPAACPFDHRLERRLDTVRQRVEAGLVDEADFDDDHRGRSATELYRLLLDRRPAVEDLVV | 191 |
| Query | 187 | THGDACLPNIMVENGRFSGFIDCGRLGVADRYQDIALATRDIAEELGGEWADRFLVLYGI | 246 |
| Sbjct | 192 | HGDACLPN++ E RFSGFIDCGRLGVADR+QD+ALA RDI ELG WA FLV YG  AHGDACLPNLLAEGRRFSGFIDCGRLGVADRHQDLALAARDIEAELGAAWAAAFLVEYG- | 250 |
| Query | 247 AAPDSQRIAFYRLLDEFF 264 | | |
|  |  | D +R+A++RLLDEFF |  |
| Sbjct | 251 | GDIDGERLAYFRLLDEFF 268 | |

>WP_010911111.1 aminoglycoside 3'-phosphotransferase [Mesorhizobium japonicum] BAB49762.1 kanamycin resistance protein [Mesorhizobium japonicum MAFF 303099]

Length=263

Score = 253 bits (646), Expect = 1e-80, Method: Compositional matrix adjust.

| Identities | = 137/255 (54%), Positives = 168/255 (66%), Gaps = 0/255 (0%) |  |
| --- | --- | --- |
| Query 10 | GSPAAWVERLFGYDWAQQTIGCSDAAVFRLSAQGRPVLFVKTDLSGALNELQDEAARLSW | 69 |
| Sbjct 9 | G P + + GY W +QTIG S A VFRL+A GRP LF+K + SG EL DEAARL W GLPNGLRQLVSGYRWHRQTIGQSQAGVFRLAADGRPALFLKCERSGPFAELADEAARLRW | 68 |
| Query 70 | LATTGVPCAAVLDVVTEAGRDWLLLGEVPGQDLLSSHLAPAEKVSIMADAMRRLHTLDPA | 129 |
| Sbjct 69 | L GV C V+ + + AG DWLL+ V G+DL S+ + A+ + IMA A+R LH +D LTGQGVACPDVIALESHAGYDWLLMSAVAGEDLASAAIDQADVIGIMASALRDLHAIDIR | 128 |
| Query 130 | TCPFDHQAKHRIERARTRMEAGLVDQDDLDEEHQGLAPAELFARLKARMPDGEDLVVTHG | 189 |
| Sbjct 129 | +CPFDH+ RI AR RME G VD+ D DEE QG AE+FA L+A P EDLVVTHG SCPFDHRLIRRIAAARARMEGGQVDESDFDEERQGRTDAEVFAELEALRPATEDLVVTHG | 188 |
| Query 190 | DACLPNIMVENGRFSGFIDCGRLGVADRYQDIALATRDIAEELGGEWADRFLVLYGIAAP | 249 |
| Sbjct 189 | DACLPN+M G FSGFIDCGRLGVADR+QD+ALA I LG W + FL Y DACLPNVMTAGGGFSGFIDCGRLGVADRHQDLALACWSIRYNLGQVWIEPFLKRYEPPET | 248 |
| Query 250  Sbjct 249 | DSQRIAFYRLLDEFF 264  + ++++YRLLDEFF  EPAKLSWYRLLDEFF 263 |  |

>WP_023092411.1 APH(3')-II family aminoglycoside O-phosphotransferase [Pseudomonas aeruginosa]

ERX92507.1 aminoglycoside 3'-phosphotransferase type IIB [Pseudomonas aeruginosa BL25]

KSP69662.1 aminoglycoside 3'-phosphotransferase [Pseudomonas aeruginosa] KSQ03027.1 aminoglycoside 3'-phosphotransferase [Pseudomonas aeruginosa] KSR65772.1 aminoglycoside 3'-phosphotransferase [Pseudomonas aeruginosa]

MBA4878524.1 APH(3')-II family aminoglycoside O-phosphotransferase [Pseudomonas aeruginosa]

Length=268

Score = 253 bits (647), Expect = 1e-80, Method: Compositional matrix adjust.

| Identities | = 136/258 (53%), Positives = 168/258 (65%), Gaps = 5/258 (2%) |  |
| --- | --- | --- |
| Query 11 | SPAAWVERLFGYDWAQQTIGCSDAAVFRLSAQGRPVLFVKTDLSGALNELQDEAARLSWL | 70 |
| Sbjct 12 | +P+ W + L GY W Q GCS A V RL A RP LFVK ++ A EL E ARL WL APSTWADYLVGYRWRGQGEGCSAATVHRLEAARRPTLFVKQEVLSAHAELPAEIARLRWL | 71 |
| Query 71 | ATTGVPCAAVLDVVTEAGRDWLLLGEVPGQDLLS SHLAPAEKVSIMADAMRRLHTL | 126 |
| Sbjct 72 | G+ C VL+ GR WLL+ VPG L + L P V ++A A+RRLH L HGAGIDCPQVLNETQSDGRQWLLMSAVPGDTLSALAQRGELEPERLVRLVAAALRRLHDL | 131 |
| Query 127 | DPATCPFDHQAKHRIERARTRMEAGLVDQDDLDEEHQGLAPAELFARLKARMPDGEDLVV | 186 |
| Sbjct 132 | DPA CPFDH+ + R++ R R+EAGLVD+ D D++H+G + EL+ L R EDLVV  DPAACPFDHRLERRLDTVRQRVEAGLVDEADFDDDHRGRSATELYRLLLDRRLAVEDLVV | 191 |

Query 187 THGDACLPNIMVENGRFSGFIDCGRLGVADRYQDIALATRDIAEELGGEWADRFLVLYGI 246

| Sbjct | 192 | HGDACLPN++ E RFSGFIDCGRLGVADR+QD+ALA RDI ELG WA+ FLV YG AHGDACLPNLLAEGRRFSGFIDCGRLGVADRHQDLALAARDIEAELGAAWAEAFLVEYG- 250 |
| --- | --- | --- |
| Query | 247 | AAPDSQRIAFYRLLDEFF 264 |
| Sbjct | 251 | D +R+A++RLLDEFF  GDIDGERLAYFRLLDEFF 268 |

>WP_164229544.1 APH(3')-II family aminoglycoside O-phosphotransferase [Stenotrophomonas maltophilia]

Length=267

Score = 253 bits (647), Expect = 1e-80, Method: Compositional matrix adjust.

| Identities | = 135/255 (53%), Positives = 167/255 (65%), Gaps = 3/255 (1%) |  |
| --- | --- | --- |
| Query 12 | PAAWVERLFGYDWAQQTIGCSDAAVFRLSAQGRPVLFVKTDLSGALNELQDEAARLSWLA | 71 |
| Sbjct 14 | P AW + L +Q++G S A V R+ GRP FVK++L A +EL DE ARL WL PLAWQKALADARIERQSMGVSRADVARVQRPGRPDAFVKSELIDAFSELGDEIARLRWLQ | 73 |
| Query 72 | TTGVPCAAVLDVVTEAGRDWLLLGEVPGQDLLSS-HLAPAEKVSIMADAMRRLHTLDPAT | 130 |
| Sbjct 74 | G P V+ EAGR WLL+ +PG+DL SS LAP ++V I+ADA+R LH L A AQGQPAPTVIATAEEAGRRWLLMSALPGRDLASSPELAPQQRVEILADALRGLHALPVAA | 133 |
| Query 131 | CPFDHQAKHRIERARTRMEAGLVDQDDLDEEHQGLAPAELFARLKARMPDGEDLVVTHGD | 190 |
| Sbjct 134 | CPFD + R+ A+ R+EAG VD DD D+E G +P ++FA+L + PD EDLVV+HGD CPFDQRLASRLPAAQARVEAGRVDADDFDDERLGQSPQQVFAQLCSNRPDHEDLVVSHGD | 193 |
| Query 191 | ACLPNIMVENGRFSGFIDCGRLGVADRYQDIALATRDIAEELG-GEWADRFLVLYGIAAP | 249 |
| Sbjct 194 | ACLPN+MV GRFSGFIDCGRLGVADRYQD+ALA R + G W YG A ACLPNLMVAEGRFSGFIDCGRLGVADRYQDLALAARSLVHNFGDTRWVTPLFQRYGAVA- | 252 |
| Query 250  Sbjct 253 | DSQRIAFYRLLDEFF 264 D +R+AFYRLLDEFF DERRLAFYRLLDEFF 267 |  |

>WP_060511889.1 MULTISPECIES: aminoglycoside 3'-phosphotransferase [unclassified Rhizobium]

KQY03735.1 aminoglycoside phosphotransferase [Rhizobium sp. Root1334] KRC00373.1 aminoglycoside phosphotransferase [Rhizobium sp. Root73]

Length=266

Score = 253 bits (646), Expect = 1e-80, Method: Compositional matrix adjust.

| Identities | | = 123/253 (49%), Positives = 164/253 (65%), Gaps = 0/253 (0%) |  |
| --- | --- | --- | --- |
| Query 12  Sbjct 14 | | PAAWVERLFGYDWAQQTIGCSDAAVFRLSAQGRPVLFVKTDLSGALNELQDEAARLSWLA P W R Y + + +G S A VF+L G+P LFVK + +G EL DEA RL WLA PPEWRIRPSSYHFERDEVGQSAAGVFKLVGDGKPDLFVKCEPAGPFGELPDEATRLLWLA | 71  73 |
| Query 72  Sbjct 74 | | TTGVPCAAVLDVVTEAGRDWLLLGEVPGQDLLSSHLAPAEKVSIMADAMRRLHTLDPATC G+ C V+ G++WLLL +PG+DL + + P + + I+A A++ LH +D TC GAGIACPNVVAPDVHDGQNWLLLSAIPGRDLSVAPITPQQCIEILATALQLLHAIDIRTC | 131  133 |
| Query 132  Sbjct 134 | | PFDHQAKHRIERARTRMEAGLVDQDDLDEEHQGLAPAELFARLKARMPDGEDLVVTHGDA PFD R+ A+ RMEAG VD+DD DE + +LF L+ + PDG DLVVTHGDA PFDQSLSGRLALAKARMEAGEVDEDDFDEARLDQSAEDLFQLLETQKPDGADLVVTHGDA | 191  193 |
| Query 192  Sbjct 194 | | CLPNIMVENGRFSGFIDCGRLGVADRYQDIALATRDIAEELGGEWADRFLVLYGIAAPDS CLPN++ ++GRFSGFIDC RLGVADRYQD+ALA R + ++ G W FL LYG+ PD CLPNLLAQDGRFSGFIDCARLGVADRYQDLALAYRSVCDDFGEAWGQAFLDLYGVPQPDR | 251  253 |
| Query 252 | | QRIAFYRLLDEFF 264  ++A+Y+LLDEFF |  |
| Sbjct | 254 | AKLAYYQLLDEFF 266 | |

>WP_137216867.1 aminoglycoside 3'-phosphotransferase [Pseudomonas koreensis]

TKJ87480.1 APH(3') family aminoglycoside O-phosphotransferase [Pseudomonas koreensis]

Length=256

Score = 253 bits (645), Expect = 1e-80, Method: Compositional matrix adjust.

| Identities | | = 130/254 (51%), Positives = 167/254 (66%), Gaps = 2/254 (1%) |  |
| --- | --- | --- | --- |
| Query 12  Sbjct 4 | | PAAWVERLFGYDWAQQTIGCSDAAVFRLSAQGRPVLFVKTDLSGALNELQDEAARLSWLA PAAW + G QQTIG S A V+R++ G FVKT+ EL E RL WLA PAAWRSQFAGAAIEQQTIGESRADVWRITPAGAAPQFVKTEQVMTWGELPGEVQRLRWLA | 71  63 |
| Query 72  Sbjct 64 | | TTGVPCAAVLDVVTEAGRDWLLLGEVPGQDLLSS-HLAPAEKVSIMADAMRRLHTLDPAT T +P VLD T+A +WLL+ VPG+DL SS HL+P + V++ A A+R LH + + TQDLPAPRVLDTTTQAQHNWLLMTAVPGRDLASSEHLSPEQIVALAAQALRTLHAVAIDS | 130  123 |
| Query 131  Sbjct 124 | | CPFDHQAKHRIERARTRMEAGLVDQDDLDEEHQGLAPAELFARLKARMPDGEDLVVTHGD CPFDH + +I RAR + AGL+D++D D+ G + +F ++ A P EDLVVTHGD CPFDHSLEQKITRAREHLLAGLIDEEDFDDSRIGQSAEAVFQQMLATRPTQEDLVVTHGD | 190  183 |
| Query 191  Sbjct 184 | | ACLPNIMVENGRFSGFIDCGRLGVADRYQDIALATRDIAEELGGEWADRFLVLYGIAAPD ACLPN++ NGRFSGF+DCGRLGVADRYQD+ALA I + LG +W F +YG+ PD ACLPNLLAANGRFSGFVDCGRLGVADRYQDLALAAHSITDNLGEQWLPLFFEVYGV-EPD | 250  242 |
| Query 251 | | SQRIAFYRLLDEFF 264 QRIAFY+LLDEFF |  |
| Sbjct | 243 | LQRIAFYQLLDEFF 256 | |

>WP_150751039.1 aminoglycoside 3'-phosphotransferase [Pseudomonas fluorescens] VVQ33863.1 Aminoglycoside 3'-phosphotransferase [Pseudomonas fluorescens]

Length=256

Score = 253 bits (645), Expect = 1e-80, Method: Compositional matrix adjust.

| Identities | | = 128/254 (50%), Positives = 166/254 (65%), Gaps = 2/254 (1%) |  |
| --- | --- | --- | --- |
| Query 12  Sbjct 4 | | PAAWVERLFGYDWAQQTIGCSDAAVFRLSAQGRPVLFVKTDLSGALNELQDEAARLSWLA PAAW + G QQTIG S A V+R++ G FVKT+ EL E RL W+A PAAWRSQFAGAAIEQQTIGESRADVWRITPAGAAPQFVKTEQVMTWGELPGEVQRLRWMA | 71  63 |
| Query 72  Sbjct 64 | | TTGVPCAAVLDVVTEAGRDWLLLGEVPGQDLLSS-HLAPAEKVSIMADAMRRLHTLDPAT T +P VLD T+A +WLL+ VPG+DL SS HL+P + V++ A A+R LH + + TQDLPAPRVLDTTTQAQHNWLLMTAVPGRDLASSEHLSPEQIVALAAQALRTLHAVAIDS | 130  123 |
| Query 131  Sbjct 124 | | CPFDHQAKHRIERARTRMEAGLVDQDDLDEEHQGLAPAELFARLKARMPDGEDLVVTHGD CPFDH + +I RAR + AGL+D++D D+ G + +F ++ P EDLVVTHGD CPFDHSLEQKITRAREHLLAGLIDEEDFDDSRIGQSAEAVFQQMLVTRPTQEDLVVTHGD | 190  183 |
| Query 191  Sbjct 184 | | ACLPNIMVENGRFSGFIDCGRLGVADRYQDIALATRDIAEELGGEWADRFLVLYGIAAPD ACLPN++ NGRFSGF+DCGRLGVADRYQD+ALA I + LG +W F +YG+ PD ACLPNLLAANGRFSGFVDCGRLGVADRYQDLALAAHSITDNLGAQWLPLFFEVYGV-EPD | 250  242 |
| Query 251 | | SQRIAFYRLLDEFF 264 QRIAFY+LLDEFF |  |
| Sbjct | 243 | QQRIAFYQLLDEFF 256 | |

>WP_183820553.1 APH(3') family aminoglycoside O-phosphotransferase [Rhizobium pisi]

Length=261

Score = 253 bits (646), Expect = 1e-80, Method: Compositional matrix adjust.

| Identities | = 128/254 (50%), Positives = 170/254 (67%), Gaps = 1/254 (0%) |  |
| --- | --- | --- |
| Query 12 | PAAWVERLFGYDWAQQTIGCSDAAVFRLSAQGRPVLFVKTDLSGALNELQDEAARLSWLA | 71 |
| Sbjct 8 | PAA ERL GY + + +G S A+VFRL +G P L++K + +G EL DEAARLSWL PAALGERLSGYRFERDALGRSGASVFRLQGEGLPALYLKVEAAGPFGELADEAARLSWLK | 67 |
| Query 72 | TTGVPCAAVLDVVTEAGRDWLLLGEVPGQDLLS-SHLAPAEKVSIMADAMRRLHTLDPAT | 130 |
| Sbjct 68 | G+PC V+ ++ R+WLL+G +PG DL S S L P +V ++A A+ LH L A+ AVGLPCPDVIARESDGERNWLLIGALPGTDLASASALTPLTRVELLAAALSDLHDLPIAS | 127 |
| Query 131 | CPFDHQAKHRIERARTRMEAGLVDQDDLDEEHQGLAPAELFARLKARMPDGEDLVVTHGD | 190 |
| Sbjct 128 | CPFDH+ ++R+ A+ RM+AG+VD++D D G + A LF L P E+LVVTHGD  CPFDHRLENRLPIAKARMQAGIVDEEDFDAARLGKSAAALFIELMRLRPGDEELVVTHGD | 187 |

| Query | 191 | ACLPNIMVENGRFSGFIDCGRLGVADRYQDIALATRDIAEELGGEWADRFLVLYGIAAPD 250 |
| --- | --- | --- |
| Sbjct | 188 | ACLPN + NG+FSG+IDC RLGVADR+QDIALA R IA LG FL YG+ APD ACLPNFVASNGQFSGYIDCSRLGVADRHQDIALACRSIASNLGEALIRPFLDRYGLTAPD 247 |
| Query | 251 | SQRIAFYRLLDEFF 264 |
| Sbjct | 248 | ++ +++LLDEFF  PAKLDYFQLLDEFF 261 |

>WP_132561683.1 aminoglycoside 3'-phosphotransferase [Rhizobium sullae] TCU17387.1 aminoglycoside 3'-phosphotransferase-2 [Rhizobium sullae]

Length=260

Score = 253 bits (646), Expect = 1e-80, Method: Compositional matrix adjust.

| Identities | = 129/244 (53%), Positives = 159/244 (65%), Gaps = 1/244 (0%) |  |
| --- | --- | --- |
| Query 22 | YDWAQQTIGCSDAAVFRLSAQGRPVLFVKTDLSGALNELQDEAARLSWLATTGVPCAAVL | 81 |
| Sbjct 17 | Y+W Q +GCS A VF L+ G+ F+K +++G EL EA RL WLA G+PC +V+ YEWKQDDLGCSSADVFVLAEGGQKRFFLKGEIAGPFAELPLEAERLLWLAKQGMPCPSVV | 76 |
| Query 82 | DVVTEAGRDWLLLGEVPGQDLLSSH-LAPAEKVSIMADAMRRLHTLDPATCPFDHQAKHR | 140 |
| Sbjct 77 | + + R+WLLL +PG DL SS A A + I+A A+RRLH LDP TCPFDH R AMESHEDRNWLLLNALPGSDLASSKSTADARCIEILAAALRRLHELDPETCPFDHGLTAR | 136 |
| Query 141 | IERARTRMEAGLVDQDDLDEEHQGLAPAELFARLKARMPDGEDLVVTHGDACLPNIMVEN | 200 |
| Sbjct 137 | + AR RM+AG VD+DD D+E Q A LFA L+A PD D VV HGDACLPN M LRLARARMDAGAVDEDDFDDERQSETAASLFAYLEASQPDVTDAVVAHGDACLPNFMTHE | 196 |
| Query 201 | GRFSGFIDCGRLGVADRYQDIALATRDIAEELGGEWADRFLVLYGIAAPDSQRIAFYRLL | 260 |
| Sbjct 197 | GRFSG+IDCGRLGVADRYQDIALA R I LG FL YG++ D ++A+Y +L GRFSGYIDCGRLGVADRYQDIALAMRSIRYNLGEAGVRHFLDCYGLSGIDDAKVAYYLVL | 256 |
| Query 261  Sbjct 257 | DEFF 264 DEFF  DEFF 260 |  |

>WP_099495155.1 APH(3')-II family aminoglycoside O-phosphotransferase [Stenotrophomonas maltophilia]

Length=267

Score = 253 bits (646), Expect = 2e-80, Method: Compositional matrix adjust.

| Identities | = 136/255 (53%), Positives = 167/255 (65%), Gaps = 3/255 (1%) |  |
| --- | --- | --- |
| Query 12 | PAAWVERLFGYDWAQQTIGCSDAAVFRLSAQGRPVLFVKTDLSGALNELQDEAARLSWLA | 71 |
| Sbjct 14 | P AW + L +Q++G S A V R+ GRP FVK++L A +EL DE ARL WL PLAWQKALADARIERQSMGVSRADVARVLRPGRPDAFVKSELIDAFSELGDEIARLRWLQ | 73 |
| Query 72 | TTGVPCAAVLDVVTEAGRDWLLLGEVPGQDLLSS-HLAPAEKVSIMADAMRRLHTLDPAT | 130 |
| Sbjct 74 | G P V+ EAGR WLL+ +PG+DL SS LAP ++V I+ADA+R LH L A AQGQPAPTVIATAEEAGRRWLLMSALPGRDLASSPELAPQQRVEILADALRGLHALPVAA | 133 |
| Query 131 | CPFDHQAKHRIERARTRMEAGLVDQDDLDEEHQGLAPAELFARLKARMPDGEDLVVTHGD | 190 |
| Sbjct 134 | CPFD + R+ A+ R+EAGLVD DD D+E G +P ++FA+L + PD EDLVV+HGD CPFDQRLASRLPAAQARVEAGLVDADDFDDERLGQSPQQVFAQLCSTRPDHEDLVVSHGD | 193 |
| Query 191 | ACLPNIMVENGRFSGFIDCGRLGVADRYQDIALATRDIAEELG-GEWADRFLVLYGIAAP | 249 |
| Sbjct 194 | ACLPN+MV GRFSGFIDCGRLGVADRYQD+ALA R + G W YG A ACLPNLMVAEGRFSGFIDCGRLGVADRYQDLALAARSLVHNFGDTRWVTPLFQRYGAVA- | 252 |
| Query 250  Sbjct 253 | DSQRIAFYRLLDEFF 264 D R+AFYRLLDEFF DECRLAFYRLLDEFF 267 |  |

>WP_145317576.1 aminoglycoside 3'-phosphotransferase [Rhizobium sp. ERR 922] Length=267

Score = 253 bits (646), Expect = 2e-80, Method: Compositional matrix adjust. Identities = 127/247 (51%), Positives = 167/247 (68%), Gaps = 1/247 (0%)

Query 19	LFGYDWAQQTIGCSDAAVFRLSAQGRPVLFVKTDLSGALNELQDEAARLSWLATTGVPCA 78

| Sbjct | 21 | L GY+W Q +G S A VFRL A GR +++KT+++ L EL E ARL WLA+ G C LAGYEWRQDALGRSSAHVFRLEADGRAPVYLKTEVTDPLGELAGEVARLRWLASQGFACP | 80 |
| --- | --- | --- | --- |
| Query | 79 | AVLDVVTEAGRDWLLLGEVPGQDLLSSH-LAPAEKVSIMADAMRRLHTLDPATCPFDHQA | 137 |
| Sbjct | 81 | ++ + R+WLL+ +PG D +S+ L P E+V ++ADA+RRLH +D A CPFDH+ DIIAHEKDGEREWLLMSALPGSDFVSAEWLQPLERVRLLADALRRLHAIDIAACPFDHRL | 140 |
| Query | 138 | KHRIERARTRMEAGLVDQDDLDEEHQGLAPAELFARLKARMPDGEDLVVTHGDACLPNIM | 197 |
| Sbjct | 141 | ++RI AR + AG VD+DD DE G + A+LF L+A+ P EDLVVTHGDACLPN + ENRIAAARAHLIAGRVDEDDFDEARLGRSAADLFRELQAKRPPTEDLVVTHGDACLPNFV | 200 |
| Query | 198 | VENGRFSGFIDCGRLGVADRYQDIALATRDIAEELGGEWADRFLVLYGIAAPDSQRIAFY | 257 |
| Sbjct | 201 | V+ RFSG+IDCGRLGVADR+QDIALA IA GGE FL YG ++++ +Y VDGDRFSGYIDCGRLGVADRHQDIALACGSIAHNFGGELVGEFLKAYGDFELQAEKMTYY | 260 |
| Query  Sbjct | 258  261 | RLLDEFF 264 RLLDEFF RLLDEFF 267 |  |

>WP_028355383.1 APH(3')-II family aminoglycoside O-phosphotransferase [Bordetella petrii]

Length=264

Score = 253 bits (646), Expect = 2e-80, Method: Compositional matrix adjust.

| Identities | = 146/254 (57%), Positives = 175/254 (69%), Gaps = 2/254 (1%) |  |
| --- | --- | --- |
| Query 12 | PAAWVERLFGYDWAQQTIGCSDAAVFRLSAQGRPVLFVKTDLSGALNELQDEAARLSWLA | 71 |
| Sbjct 12 | P W + L GY WA Q IG SDAAVFRL A GRP L+VKT+ +G+L EL E RL WLA PPDWRDALAGYAWAAQDIGRSDAAVFRLEASGRPPLYVKTETAGSLAELPGEVLRLRWLA | 71 |
| Query 72 | TTGVPCAAVLDVVTEAGRDWLLLGEVPGQDLLSS-HLAPAEKVSIMADAMRRLHTLDPAT | 130 |
| Sbjct 72 | G+ C VL AGR WLL+ VPG+DL S+ L A V+I A A+R LH LDPA QQGIDCPRVLRTAQAAGRHWLLMSAVPGRDLASTPELPAARIVAIAAAALRALHGLDPAG | 131 |
| Query 131 | CPFDHQAKHRIERARTRMEAGLVDQDDLDEEHQGLAPAELFARLKARMPDGEDLVVTHGD | 190 |
| Sbjct 132 | CPFDH+ RIE AR R++AGLVD+ D D+E G + A+ +A L A P EDLVVTHGD CPFDHRRAVRIEHARARVQAGLVDESDFDDERMGQSAAQAYAALCAGQPAHEDLVVTHGD | 191 |
| Query 191 | ACLPNIMVENGRFSGFIDCGRLGVADRYQDIALATRDIAEELGGEWADRFLVLYGIAAPD | 250 |
| Sbjct 192 | ACLPN++ +GRFSGF+DCGRLGVADR+QD+ALA I LG +WA FL YG D ACLPNLLAVDGRFSGFVDCGRLGVADRHQDLALAAWSIEYNLGAQWAQPFLDAYG-GPVD | 250 |
| Query 251  Sbjct 251 | SQRIAFYRLLDEFF 264  ++R+AFYRLLDEFF  TERLAFYRLLDEFF 264 |  |

>EJT05030.1 aminoglycoside phosphotransferase [Rhizobium sp. CCGE 510] Length=256

Score = 252 bits (644), Expect = 2e-80, Method: Compositional matrix adjust.

| Identities | = 131/255 (51%), Positives = 170/255 (67%), Gaps = 2/255 (1%) |  |
| --- | --- | --- |
| Query 12 | PAAWVE-RLFGYDWAQQTIGCSDAAVFRLSAQGRPVLFVKTDLSGALNELQDEAARLSWL | 70 |
| Sbjct 2 | PA +E RL GY + + T+G S A+VFRL +G PVL++K + +G EL DEAARLSWL PAGSLEARLSGYRFERDTLGRSTASVFRLEGEGLPVLYLKVEEAGPFGELADEAARLSWL | 61 |
| Query 71 | ATTGVPCAAVLDVVTEAGRDWLLLGEVPGQDLLS-SHLAPAEKVSIMADAMRRLHTLDPA | 129 |
| Sbjct 62 | +G+PC V+ ++ GR+ LL+ +PG DL S S L P +V ++A A+ RLH L + KASGLPCPDVIARESDGGRNRLLINALPGTDLASASALTPLARVELLAAALSRLHRLPIS | 121 |
| Query 130 | TCPFDHQAKHRIERARTRMEAGLVDQDDLDEEHQGLAPAELFARLKARMPDGEDLVVTHG | 189 |
| Sbjct 122 | +CPFDH+ + RI A+ RM+AG+VD+ D E G + LFA L++R EDLVVTHG SCPFDHRLESRIAAAKARMQAGIVDETDFGETRLGKSAEALFAELESRRLGREDLVVTHG | 181 |
| Query 190 | DACLPNIMVENGRFSGFIDCGRLGVADRYQDIALATRDIAEELGGEWADRFLVLYGIAAP | 249 |
| Sbjct 182 | DACLPN + FSG+IDC RLGVADRYQDIALA R I G RFL YG+AAP DACLPNFIASAEGFSGYIDCSRLGVADRYQDIALACRSIGYNFGEALVQRFLDRYGMAAP | 241 |
| Query 250 | DSQRIAFYRLLDEFF 264 D R+ +Y+LLDEFF |  |

Sbjct 242 DPARLDYYQLLDEFF 256

>WP_085742928.1 MULTISPECIES: aminoglycoside 3'-phosphotransferase [Rhizobium] ARM13251.1 aminoglycoside 3'-phosphotransferase protein [Rhizobium phaseoli

Brasil 5] Length=261

Score = 253 bits (645), Expect = 2e-80, Method: Compositional matrix adjust.

| Identities | | = 127/254 (50%), Positives = 170/254 (67%), Gaps = 1/254 (0%) |  |
| --- | --- | --- | --- |
| Query 12  Sbjct 8 | | PAAWVERLFGYDWAQQTIGCSDAAVFRLSAQGRPVLFVKTDLSGALNELQDEAARLSWLA PAA+ ERL GY + + +G S A+VFRL +G P L++K + +G EL DEAARLSWL PAAFGERLSGYRFERDALGRSAASVFRLQGEGLPALYLKVEAAGPFGELADEAARLSWLK | 71  67 |
| Query 72  Sbjct 68 | | TTGVPCAAVLDVVTEAGRDWLLLGEVPGQDLLS-SHLAPAEKVSIMADAMRRLHTLDPAT G+PC V+ ++ R+WLL+ +PG DL+S S L P +V ++A A+ LH L A+ AVGLPCPDVIARESDGERNWLLINALPGADLVSASALTPLTRVELLAAALSDLHDLPIAS | 130  127 |
| Query 131  Sbjct 128 | | CPFDHQAKHRIERARTRMEAGLVDQDDLDEEHQGLAPAELFARLKARMPDGEDLVVTHGD CPFDH+ ++R+ A+ RM+AG+VD++D D G + A LF L P EDLVVTHGD CPFDHRLENRLPTAKARMQAGIVDEEDFDAARLGKSAAALFDDLMRLRPGKEDLVVTHGD | 190  187 |
| Query 191  Sbjct 188 | | ACLPNIMVENGRFSGFIDCGRLGVADRYQDIALATRDIAEELGGEWADRFLVLYGIAAPD ACLPN + NG+FSG+IDC RLGVADR+QDIALA R IA G FL YG+ APD ACLPNFVASNGQFSGYIDCSRLGVADRHQDIALACRSIASNFGEALIRPFLDRYGLTAPD | 250  247 |
| Query 251 | | SQRIAFYRLLDEFF 264  ++ +++LLDEFF |  |
| Sbjct | 248 | PAKLHYFQLLDEFF 261 | |

>WP_100477982.1 APH(3')-II family aminoglycoside O-phosphotransferase [Stenotrophomonas maltophilia]

PJL72800.1 APH(3') family aminoglycoside O-phosphotransferase [Stenotrophomonas maltophilia]

Length=267

Score = 253 bits (645), Expect = 2e-80, Method: Compositional matrix adjust.

| Identities | = 135/255 (53%), Positives = 167/255 (65%), Gaps = 3/255 (1%) |  |
| --- | --- | --- |
| Query 12 | PAAWVERLFGYDWAQQTIGCSDAAVFRLSAQGRPVLFVKTDLSGALNELQDEAARLSWLA | 71 |
| Sbjct 14 | P AW + L +Q++G S A V R+ GRP FVK++L A +EL DE ARL WL PLAWQKALADARIERQSMGVSRADVARVQRPGRPDAFVKSELIDAFSELGDEIARLRWLQ | 73 |
| Query 72 | TTGVPCAAVLDVVTEAGRDWLLLGEVPGQDLLSS-HLAPAEKVSIMADAMRRLHTLDPAT | 130 |
| Sbjct 74 | G P V+ EAGR WLL+ +PG+DL SS LAP ++V I+ADA+R LH L A AQGQPAPTVIATAEEAGRRWLLMSALPGRDLASSPELAPQQRVQILADALRGLHALPVAA | 133 |
| Query 131 | CPFDHQAKHRIERARTRMEAGLVDQDDLDEEHQGLAPAELFARLKARMPDGEDLVVTHGD | 190 |
| Sbjct 134 | CPFD + R+ A+ R+EAGLVD DD D+E G +P ++FA+L + PD ED+VV+HGD CPFDQRLASRLPVAQARVEAGLVDADDFDDERLGQSPQQVFAQLCSTRPDHEDMVVSHGD | 193 |
| Query 191 | ACLPNIMVENGRFSGFIDCGRLGVADRYQDIALATRDIAEELG-GEWADRFLVLYGIAAP | 249 |
| Sbjct 194 | ACLPN+MV GRFSGFIDCGRLGVADRYQD+ALA R + G W YG A ACLPNLMVAEGRFSGFIDCGRLGVADRYQDLALAARSLVHNFGDTRWVTPLFQRYGAVA- | 252 |
| Query 250  Sbjct 253 | DSQRIAFYRLLDEFF 264 D R+AFYRLLDEFF DECRLAFYRLLDEFF 267 |  |

>WP_186449439.1 aminoglycoside 3'-phosphotransferase [Rhizobium sp. ERR 942] TWB61421.1 aminoglycoside 3'-phosphotransferase-2 [Rhizobium sp. ERR 922] TWC04347.1 aminoglycoside 3'-phosphotransferase-2 [Rhizobium sp. ERR 942]

Length=266

Score = 253 bits (645), Expect = 2e-80, Method: Compositional matrix adjust. Identities = 127/247 (51%), Positives = 167/247 (68%), Gaps = 1/247 (0%)

Query 19	LFGYDWAQQTIGCSDAAVFRLSAQGRPVLFVKTDLSGALNELQDEAARLSWLATTGVPCA 78 L GY+W Q +G S A VFRL A GR +++KT+++ L EL E ARL WLA+ G C

| Sbjct | 20 | LAGYEWRQDALGRSSAHVFRLEADGRAPVYLKTEVTDPLGELAGEVARLRWLASQGFACP | 79 |
| --- | --- | --- | --- |
| Query | 79 | AVLDVVTEAGRDWLLLGEVPGQDLLSSH-LAPAEKVSIMADAMRRLHTLDPATCPFDHQA | 137 |
| Sbjct | 80 | ++ + R+WLL+ +PG D +S+ L P E+V ++ADA+RRLH +D A CPFDH+ DIIAHEKDGEREWLLMSALPGSDFVSAEWLQPLERVRLLADALRRLHAIDIAACPFDHRL | 139 |
| Query | 138 | KHRIERARTRMEAGLVDQDDLDEEHQGLAPAELFARLKARMPDGEDLVVTHGDACLPNIM | 197 |
| Sbjct | 140 | ++RI AR + AG VD+DD DE G + A+LF L+A+ P EDLVVTHGDACLPN + ENRIAAARAHLIAGRVDEDDFDEARLGRSAADLFRELQAKRPPTEDLVVTHGDACLPNFV | 199 |
| Query | 198 | VENGRFSGFIDCGRLGVADRYQDIALATRDIAEELGGEWADRFLVLYGIAAPDSQRIAFY | 257 |
| Sbjct | 200 | V+ RFSG+IDCGRLGVADR+QDIALA IA GGE FL YG ++++ +Y VDGDRFSGYIDCGRLGVADRHQDIALACGSIAHNFGGELVGEFLKAYGDFELQAEKMTYY | 259 |
| Query  Sbjct | 258  260 | RLLDEFF 264 RLLDEFF RLLDEFF 266 |  |

>APO74403.1 aminoglycoside 3'-phosphotransferase protein [Rhizobium etli 8C-3]

Length=260

Score = 252 bits (644), Expect = 2e-80, Method: Compositional matrix adjust.

| Identities | = 125/244 (51%), Positives = 161/244 (66%), Gaps = 1/244 (0%) |  |
| --- | --- | --- |
| Query 22 | YDWAQQTIGCSDAAVFRLSAQGRPVLFVKTDLSGALNELQDEAARLSWLATTGVPCAAVL | 81 |
| Sbjct 17 | ++W Q +GCS AAVF L+ GR F+K +++G EL EA RL WLA G+ C +V+ HEWRQVDLGCSSAAVFGLAEGGRTQFFLKGEIAGPFAELPLEAERLLWLAEQGLACPSVV | 76 |
| Query 82 | DVVTEAGRDWLLLGEVPGQDLLSSHLAP-AEKVSIMADAMRRLHTLDPATCPFDHQAKHR | 140 |
| Sbjct 77 | + + GR+WLLL + G+DL SS P +++ I+A A+RRLH LDPA CPFDH+ R ALESHEGRNWLLLSALDGKDLASSRSTPDGQRIEILAAALRRLHELDPANCPFDHRLTVR | 136 |
| Query 141 | IERARTRMEAGLVDQDDLDEEHQGLAPAELFARLKARMPDGEDLVVTHGDACLPNIMVEN | 200 |
| Sbjct 137 | + A+ RMEAG VD+ D DEE A LFA L+A PD D VV HGDACLPN M LRLAKARMEAGAVDESDFDEERLNATAASLFAYLEANEPDVTDPVVAHGDACLPNFMTHE | 196 |
| Query 201 | GRFSGFIDCGRLGVADRYQDIALATRDIAEELGGEWADRFLVLYGIAAPDSQRIAFYRLL | 260 |
| Sbjct 197 | G+FSG++DCGRLGVADRYQDIALA R I +G W FL YG++ D ++A+Y +L GQFSGYVDCGRLGVADRYQDIALALRSIRYNVGQTWVQHFLDCYGLSGIDDAKVAYYVVL | 256 |
| Query 261  Sbjct 257 | DEFF 264 DEFF  DEFF 260 |  |

>WP_003110134.1 APH(3')-II family aminoglycoside O-phosphotransferase [Pseudomonas aeruginosa]

ALS12958.1 aminoglycoside phosphotransferase [Pseudomonas aeruginosa] ERY67846.1 aminoglycoside 3'-phosphotransferase type IIB [Pseudomonas aeruginosa

BL03]

ESQ68486.1 aminoglycoside 3'-phosphotransferase [Pseudomonas aeruginosa HB15]

KAJ07628.1 aminoglycoside 3'-phosphotransferase [Pseudomonas aeruginosa ID4365]

KKJ47609.1 aminoglycoside phosphotransferase [Pseudomonas aeruginosa MRSN 317]

Length=268

Score = 253 bits (645), Expect = 2e-80, Method: Compositional matrix adjust.

| Identities | = 136/258 (53%), Positives = 168/258 (65%), Gaps = 5/258 (2%) |  |
| --- | --- | --- |
| Query 11 | SPAAWVERLFGYDWAQQTIGCSDAAVFRLSAQGRPVLFVKTDLSGALNELQDEAARLSWL | 70 |
| Sbjct 12 | +P+ W + L GY W Q GCS A V RL A RP LFVK ++ A EL E ARL WL APSTWADYLAGYRWRGQGEGCSAATVHRLEAARRPTLFVKQEVLSAHAELPAEIARLRWL | 71 |
| Query 71 | ATTGVPCAAVLDVVTEAGRDWLLLGEVPGQDLLS SHLAPAEKVSIMADAMRRLHTL | 126 |
| Sbjct 72 | G+ C VL+ GR WLL+ VPG L + L P V ++A A+RRLH L HGAGIDCPQVLNETQSDGRQWLLMSAVPGDTLSALAQRGELEPERLVRLVAAALRRLHDL | 131 |
| Query 127 | DPATCPFDHQAKHRIERARTRMEAGLVDQDDLDEEHQGLAPAELFARLKARMPDGEDLVV | 186 |

| Sbjct | 132 | DPA CPFDH+ + R++ R R+EAGLVD+ D D++H+G + EL+ L R EDLVV DPAACPFDHRLERRLDTVRQRVEAGLVDEADFDDDHRGRSATELYRLLLDRRLAVEDLVV | 191 |
| --- | --- | --- | --- |
| Query | 187 | THGDACLPNIMVENGRFSGFIDCGRLGVADRYQDIALATRDIAEELGGEWADRFLVLYGI | 246 |
| Sbjct | 192 | HGDACLPN++ E RFSGFIDCGRLGVADR+QD+ALA RDI ELG WA+ FLV YG  AHGDACLPNLLAEGRRFSGFIDCGRLGVADRHQDLALAARDIEAELGAAWAEAFLVEYG- | 250 |
| Query | 247 AAPDSQRIAFYRLLDEFF 264 | | |
|  |  | D +R+A++RLLDEFF |  |
| Sbjct | 251 | GDIDGERLAYFRLLDEFF 268 | |

>WP_016562273.1 MULTISPECIES: APH(3')-II family aminoglycoside O-phosphotransferase [Pseudomonas]

AGO41643.1 aminoglycoside 3'-phosphotransferase [Pseudomonas aeruginosa RP73]

ALY49795.1 aminoglycoside phosphotransferase APH(3') [Pseudomonas aeruginosa] KSG68221.1 aminoglycoside phosphotransferase APH(3') [Pseudomonas aeruginosa] KSI43101.1 aminoglycoside phosphotransferase APH(3') [Pseudomonas aeruginosa] KUI83324.1 aminoglycoside phosphotransferase [Pseudomonas aeruginosa]

Length=268

Score = 253 bits (645), Expect = 2e-80, Method: Compositional matrix adjust.

| Identities | | = 136/258 (53%), Positives = 166/258 (64%), Gaps = 5/258 (2%) |  |
| --- | --- | --- | --- |
| Query 11  Sbjct 12 | | SPAAWVERLFGYDWAQQTIGCSDAAVFRLSAQGRPVLFVKTDLSGALNELQDEAARLSWL  +P+ W + L GY W Q GCS A V RL A RP LFVK ++ EL E ARL WL APSTWADYLAGYRWRGQGEGCSAATVHRLEAARRPTLFVKQEVLSVHAELPAEIARLRWL | 70  71 |
| Query 71  Sbjct 72 | | ATTGVPCAAVLDVVTEAGRDWLLLGEVPGQDL LSSHLAPAEKVSIMADAMRRLHTL  G+ C VL+ GR WLL+ VPG L L P V ++A A+RRLH L HGAGIDCPQVLNETQSDGRQWLLMSAVPGDTLSVLAQRGELEPERLVRLVAAALRRLHDL | 126  131 |
| Query 127  Sbjct 132 | | DPATCPFDHQAKHRIERARTRMEAGLVDQDDLDEEHQGLAPAELFARLKARMPDGEDLVV DPA CPFDH+ + R++ R R+EAGLVD+ D D++H+G + EL+ L R P EDLVV DPAACPFDHRLERRLDTVRQRVEAGLVDEADFDDDHRGRSATELYRLLLDRRPAVEDLVV | 186  191 |
| Query 187  Sbjct 192 | | THGDACLPNIMVENGRFSGFIDCGRLGVADRYQDIALATRDIAEELGGEWADRFLVLYGI HGDACLPN++ E RFSGFIDCGRLGVADR+QD+ALA RDI ELG WA FLV YG AHGDACLPNLLAEGRRFSGFIDCGRLGVADRHQDLALAARDIEAELGAAWAAAFLVEYG- | 246  250 |
| Query | 247 AAPDSQRIAFYRLLDEFF 264 | | |
|  |  | D +R+A++RLLDEFF |  |
| Sbjct | 251 | GDIDGERLAYFRLLDEFF 268 | |

>WP_051336470.1 aminoglycoside 3'-phosphotransferase [Rhizobium sullae] PKA41353.1 aminoglycoside 3'-phosphotransferase [Rhizobium sullae]

Length=260

Score = 252 bits (644), Expect = 2e-80, Method: Compositional matrix adjust.

| Identities | = 128/244 (52%), Positives = 159/244 (65%), Gaps = 1/244 (0%) |  |
| --- | --- | --- |
| Query 22 | YDWAQQTIGCSDAAVFRLSAQGRPVLFVKTDLSGALNELQDEAARLSWLATTGVPCAAVL | 81 |
| Sbjct 17 | Y+W Q +GCS A VF L+ G+ F+K +++G EL EA RL WLA G+PC +V+ YEWKQDDLGCSSADVFVLAEGGQKRFFLKGEMAGPFAELPLEAERLLWLAKQGMPCPSVV | 76 |
| Query 82 | DVVTEAGRDWLLLGEVPGQDLLSSH-LAPAEKVSIMADAMRRLHTLDPATCPFDHQAKHR | 140 |
| Sbjct 77 | + + R+WLLL +PG DL SS A A + I+A A+RRLH LDP TCPFDH R AMESHEDRNWLLLNALPGSDLASSKSTADARCIEILAAALRRLHELDPETCPFDHGLTAR | 136 |
| Query 141 | IERARTRMEAGLVDQDDLDEEHQGLAPAELFARLKARMPDGEDLVVTHGDACLPNIMVEN | 200 |
| Sbjct 137 | + AR RM+AG VD+DD D+E Q A LFA L+A PD D VV HGDACLPN M LRLARARMDAGAVDEDDFDKERQSETVASLFAYLEASQPDVTDAVVAHGDACLPNFMTHE | 196 |
| Query 201 | GRFSGFIDCGRLGVADRYQDIALATRDIAEELGGEWADRFLVLYGIAAPDSQRIAFYRLL | 260 |
| Sbjct 197 | GRFSG++DCGRLGVADRYQDIALA R I LG FL YG++ D ++A+Y +L GRFSGYVDCGRLGVADRYQDIALAMRSIRYNLGEAGVRHFLDCYGLSGIDDAKVAYYLVL | 256 |
| Query 261  Sbjct 257 | DEFF 264 DEFF  DEFF 260 |  |

>WP_132552189.1 aminoglycoside 3'-phosphotransferase [Rhizobium azibense] TCU36720.1 aminoglycoside 3'-phosphotransferase-2 [Rhizobium azibense]

Length=270

Score = 253 bits (645), Expect = 2e-80, Method: Compositional matrix adjust.

| Identities | = 125/244 (51%), Positives = 161/244 (66%), Gaps = 1/244 (0%) |  |
| --- | --- | --- |
| Query 22 | YDWAQQTIGCSDAAVFRLSAQGRPVLFVKTDLSGALNELQDEAARLSWLATTGVPCAAVL | 81 |
| Sbjct 27 | ++W Q +GCS AAVF L+ GR F+K +++G EL EA RL WLA G+ C +V+ HEWRQVDLGCSSAAVFGLAEGGRTQFFLKGEIAGPFAELPLEAERLLWLAEQGLACPSVV | 86 |
| Query 82 | DVVTEAGRDWLLLGEVPGQDLLSSHLAP-AEKVSIMADAMRRLHTLDPATCPFDHQAKHR | 140 |
| Sbjct 87 | + + GR+WLLL + G+DL SS P +++ I+A A+RRLH LDPA CPFDH+ R ALESHEGRNWLLLSALDGKDLASSRSTPDGQRIEILAAALRRLHELDPANCPFDHRLTVR | 146 |
| Query 141 | IERARTRMEAGLVDQDDLDEEHQGLAPAELFARLKARMPDGEDLVVTHGDACLPNIMVEN | 200 |
| Sbjct 147 | + A+ RMEAG VD+ D DEE A LFA L+A PD D VV HGDACLPN M LRLAKARMEAGAVDESDFDEERLNATAASLFAYLEANQPDVTDPVVAHGDACLPNFMTHE | 206 |
| Query 201 | GRFSGFIDCGRLGVADRYQDIALATRDIAEELGGEWADRFLVLYGIAAPDSQRIAFYRLL | 260 |
| Sbjct 207 | G+FSG++DCGRLGVADRYQDIALA R I +G W FL YG++ D ++A+Y +L GQFSGYVDCGRLGVADRYQDIALALRSIRYNVGQTWVQHFLDCYGLSGIDDAKVAYYVVL | 266 |
| Query 261  Sbjct 267 | DEFF 264 DEFF  DEFF 270 |  |

>WP_081282797.1 aminoglycoside 3'-phosphotransferase [Rhizobium phaseoli] ANL66623.1 aminoglycoside 3'-phosphotransferase protein [Rhizobium phaseoli] ANL79436.1 aminoglycoside 3'-phosphotransferase protein [Rhizobium phaseoli]

Length=261

Score = 252 bits (644), Expect = 2e-80, Method: Compositional matrix adjust.

| Identities | = 127/254 (50%), Positives = 169/254 (67%), Gaps = 1/254 (0%) |  |
| --- | --- | --- |
| Query 12 | PAAWVERLFGYDWAQQTIGCSDAAVFRLSAQGRPVLFVKTDLSGALNELQDEAARLSWLA | 71 |
| Sbjct 8 | PAA ERL GY + + +G S A+VFRL +G P L++K + +G EL DEAARLSWL PAALGERLSGYRFERDALGRSAASVFRLEGEGLPALYLKVEAAGPFGELADEAARLSWLK | 67 |
| Query 72 | TTGVPCAAVLDVVTEAGRDWLLLGEVPGQDLLS-SHLAPAEKVSIMADAMRRLHTLDPAT | 130 |
| Sbjct 68 | T +PC V+ ++ R+WLL+ +PG DL S S L P +V ++A A+ LH L A+ TVDLPCPDVIARESDGERNWLLINALPGTDLASASALTPLTRVELLAAALSDLHDLPIAS | 127 |
| Query 131 | CPFDHQAKHRIERARTRMEAGLVDQDDLDEEHQGLAPAELFARLKARMPDGEDLVVTHGD | 190 |
| Sbjct 128 | CPFDH+ ++R+ A+ RM+AG+VD++D D G + A LF L P EDLVVTHGD CPFDHRLENRLPIAKVRMQAGIVDEEDFDAARLGKSAAALFIDLMRLRPGNEDLVVTHGD | 187 |
| Query 191 | ACLPNIMVENGRFSGFIDCGRLGVADRYQDIALATRDIAEELGGEWADRFLVLYGIAAPD | 250 |
| Sbjct 188 | ACLPN + NG+FSG+IDC RLGVADR+QDIALA R IA G FL YG+ APD ACLPNFVASNGKFSGYIDCSRLGVADRHQDIALACRSIASNFGEALIRPFLERYGLTAPD | 247 |
| Query 251  Sbjct 248 | SQRIAFYRLLDEFF 264  + ++ +++LLDEFF  ATKLHYFQLLDEFF 261 |  |

>WP_081284251.1 aminoglycoside 3'-phosphotransferase [Rhizobium phaseoli] ANL28905.1 aminoglycoside 3'-phosphotransferase protein [Rhizobium phaseoli]

Length=261

Score = 252 bits (644), Expect = 2e-80, Method: Compositional matrix adjust. Identities = 127/254 (50%), Positives = 169/254 (67%), Gaps = 1/254 (0%)

| Query | 12 | PAAWVERLFGYDWAQQTIGCSDAAVFRLSAQGRPVLFVKTDLSGALNELQDEAARLSWLA | 71 |
| --- | --- | --- | --- |
| Sbjct | 8 | PAA ERL GY + + +G S A+VFRL +G P LF+K + +G EL DEAARLSWL PAALGERLSGYRFERDALGRSAASVFRLQGEGLPALFLKVEAAGPFGELADEAARLSWLK | 67 |
| Query | 72 | TTGVPCAAVLDVVTEAGRDWLLLGEVPGQDLLS-SHLAPAEKVSIMADAMRRLHTLDPAT T +PC V+ ++ R+WLL+ +PG DL+S S L P +V ++A A+ LH L A+ | 130 |

| Sbjct | 68 | TFDLPCPEVIARESDGERNWLLINALPGADLVSASALTPLTRVELLAAALSDLHDLPIAS | 127 |
| --- | --- | --- | --- |
| Query | 131 | CPFDHQAKHRIERARTRMEAGLVDQDDLDEEHQGLAPAELFARLKARMPDGEDLVVTHGD | 190 |
| Sbjct | 128 | CPFDH+ ++R+ A+ RM+AG+VD++D D G + A LF L P EDLVVTHGD CPFDHRLENRLPTAKARMQAGIVDEEDFDAARLGKSAAALFDDLMRLRPGNEDLVVTHGD | 187 |
| Query | 191 | ACLPNIMVENGRFSGFIDCGRLGVADRYQDIALATRDIAEELGGEWADRFLVLYGIAAPD | 250 |
| Sbjct | 188 | ACLPN + NG+FSG+IDC RLGVADR+QDIALA R IA G FL YG+ PD ACLPNFVASNGKFSGYIDCSRLGVADRHQDIALACRSIASNFGEALIRPFLDRYGLTVPD | 247 |
| Query  Sbjct | 251  248 | SQRIAFYRLLDEFF 264  + ++ +++LLDEFF  ATKLHYFQLLDEFF 261 |  |

>WP_057589670.1 aminoglycoside 3'-phosphotransferase [Variovorax paradoxus] KPU94008.1 aminoglycoside phosphotransferase [Variovorax paradoxus] KPV08006.1 aminoglycoside phosphotransferase [Variovorax paradoxus] KPV11802.1 aminoglycoside phosphotransferase [Variovorax paradoxus] KPV23580.1 aminoglycoside phosphotransferase [Variovorax paradoxus] KPV36585.1 aminoglycoside phosphotransferase [Variovorax paradoxus]

Length=261

Score = 252 bits (644), Expect = 3e-80, Method: Compositional matrix adjust.

| Identities | = 138/253 (55%), Positives = 161/253 (64%), Gaps = 1/253 (0%) |  |
| --- | --- | --- |
| Query 12 | PAAWVERLFGYDWAQQTIGCSDAAVFRLSAQGRPVLFVKTDLSGALNELQDEAARLSWLA | 71 |
| Sbjct 10 | P AW RL W +QTIG S A VFR+ A +LF+K++ L EL E ARL WLA PHAWRARLADAHWQRQTIGESGADVFRIEAADGELLFLKSEPVAPLAELPGEIARLEWLA | 69 |
| Query 72 | TTGVPCAAVLDVVTEAGRDWLLLGEVPGQDLLSSHLAPAEKVSIMADAMRRLHTLDPATC | 131 |
| Sbjct 70 | +P AVLD E R WLL+ VPG+DL S P E V MADA+R LH + ATC RQDLPAPAVLDQALEHDRHWLLMSAVPGRDLASGPFRPGEIVVHMADALRLLHRVPVATC | 129 |
| Query 132 | PFDHQAKHRIERARTRMEAGLVDQDDLDEEHQGLAPAELFARLKARMPDGEDLVVTHGDA | 191 |
| Sbjct 130 | PFDH+ RIE A R AGLVD+ D D+E G +LF RL+A P EDLVVTHGDA PFDHRLAQRIEDAHARAVAGLVDESDFDDERAGWTALDLFERLRATRPASEDLVVTHGDA | 189 |
| Query 192 | CLPNIMVENGRFSGFIDCGRLGVADRYQDIALATRDIAEELGGEWADRFLVLYGIAAPDS | 251 |
| Sbjct 190 | CLPN+M F+GFIDCGR+GVADR+QD+ALA R I LG W F YGI APD+ CLPNLMATADGFTGFIDCGRVGVADRWQDLALAARSIGFNLGEAWTAPFFERYGI-APDA | 248 |
| Query 252  Sbjct 249 | QRIAFYRLLDEFF 264  +RIAFY LLDEFF  ERIAFYCLLDEFF 261 |  |

>WP_196776327.1 aminoglycoside 3'-phosphotransferase [Rhizobium etli] Length=262

Score = 252 bits (644), Expect = 3e-80, Method: Compositional matrix adjust.

| Identities | = 125/244 (51%), Positives = 161/244 (66%), Gaps = 1/244 (0%) |  |
| --- | --- | --- |
| Query 22 | YDWAQQTIGCSDAAVFRLSAQGRPVLFVKTDLSGALNELQDEAARLSWLATTGVPCAAVL | 81 |
| Sbjct 19 | ++W Q +GCS AAVF L+ GR F+K +++G EL EA RL WLA G+ C +V+ HEWRQVDLGCSSAAVFGLAEGGRTQFFLKGEIAGPFAELPLEAERLLWLAEQGLACPSVV | 78 |
| Query 82 | DVVTEAGRDWLLLGEVPGQDLLSSHLAP-AEKVSIMADAMRRLHTLDPATCPFDHQAKHR | 140 |
| Sbjct 79 | + + GR+WLLL + G+DL SS P +++ I+A A+RRLH LDPA CPFDH+ R ALESHEGRNWLLLSALDGKDLASSRSTPDGQRIEILAAALRRLHELDPANCPFDHRLTVR | 138 |
| Query 141 | IERARTRMEAGLVDQDDLDEEHQGLAPAELFARLKARMPDGEDLVVTHGDACLPNIMVEN | 200 |
| Sbjct 139 | + A+ RMEAG VD+ D DEE A LFA L+A PD D VV HGDACLPN M LRLAKARMEAGAVDESDFDEERLNATAASLFAYLEANEPDVTDPVVAHGDACLPNFMTHE | 198 |
| Query 201 | GRFSGFIDCGRLGVADRYQDIALATRDIAEELGGEWADRFLVLYGIAAPDSQRIAFYRLL | 260 |
| Sbjct 199 | G+FSG++DCGRLGVADRYQDIALA R I +G W FL YG++ D ++A+Y +L GQFSGYVDCGRLGVADRYQDIALALRSIRYNVGQTWVQHFLDCYGLSGIDDAKVAYYVVL | 258 |
| Query 261  Sbjct 259 | DEFF 264 DEFF  DEFF 262 |  |

>WP_077502189.1 aminoglycoside 3'-phosphotransferase [Sinorhizobium sp. A49]

OOG70053.1 APH(3') family aminoglycoside O-phosphotransferase [Sinorhizobium sp. A49]

Length=263

Score = 252 bits (644), Expect = 3e-80, Method: Compositional matrix adjust.

| Identities | | = 129/253 (51%), Positives = 163/253 (64%), Gaps = 0/253 (0%) |  |
| --- | --- | --- | --- |
| Query 12  Sbjct 11 | | PAAWVERLFGYDWAQQTIGCSDAAVFRLSAQGRPVLFVKTDLSGALNELQDEAARLSWLA P + L GY W + ++G S ++VF A+ R L +K + SG +EL DEA RL WL PPGFAPLLKGYSWRKDSLGQSASSVFLFDAEARTPLVLKVEPSGPFDELVDEAERLEWLG | 71  70 |
| Query 72  Sbjct 71 | | TTGVPCAAVLDVVTEAGRDWLLLGEVPGQDLLSSHLAPAEKVSIMADAMRRLHTLDPATC G+PC V+ A R+WLL+ V G DL SS LA +E+V ++A+A+RR+H LD C KQGMPCPRVIARDEYARRNWLLMEAVDGVDLASSSLAASEQVVLLAEAVRRMHALDVEAC | 131  130 |
| Query 132  Sbjct 131 | | PFDHQAKHRIERARTRMEAGLVDQDDLDEEHQGLAPAELFARLKARMPDGEDLVVTHGDA PFDH+ R+ AR R EA L+D D D+E G ELF L+A++P E LVVTHGDA PFDHRVDARVAIARLRAEADLIDVTDFDKERLGKTAEELFVELQAKIPQEEKLVVTHGDA | 191  190 |
| Query 192  Sbjct 191 | | CLPNIMVENGRFSGFIDCGRLGVADRYQDIALATRDIAEELGGEWADRFLVLYGIAAPDS CLPNI+V +GRF+GFIDC RLGVAD YQD+AL R IA LG EW FL LYG+ D CLPNIIVRDGRFAGFIDCSRLGVADPYQDLALTCRSIAYNLGEEWVQPFLDLYGVRRADP | 251  250 |
| Query 252 | | QRIAFYRLLDEFF 264 R+ FY LLDEFF |  |
| Sbjct | 251 | DRLDFYCLLDEFF 263 | |

>WP_194730113.1 APH(3') family aminoglycoside O-phosphotransferase [Rhizobium sp. 007]

QPB21954.1 aminoglycoside 3'-phosphotransferase [Rhizobium sp. 007] Length=260

Score = 252 bits (644), Expect = 3e-80, Method: Compositional matrix adjust.

| Identities | = 124/244 (51%), Positives = 160/244 (66%), Gaps = 1/244 (0%) |  |
| --- | --- | --- |
| Query 22 | YDWAQQTIGCSDAAVFRLSAQGRPVLFVKTDLSGALNELQDEAARLSWLATTGVPCAAVL | 81 |
| Sbjct 17 | ++W Q +GCS A VFRL+ GR F+K +++G EL EA RL WLA G+ C +V+ HEWKQDDLGCSSADVFRLAEGGRTRFFLKGEIAGPFAELPLEAERLLWLAEQGLQCPSVV | 76 |
| Query 82 | DVVTEAGRDWLLLGEVPGQDLLSSHLAP-AEKVSIMADAMRRLHTLDPATCPFDHQAKHR | 140 |
| Sbjct 77 | + + GR+WLLL + G DL S L +++ I+A A+RRLH LDP TCPFDH+ R ALESHEGRNWLLLSALAGSDLASCKLTTDRQRIEILAAALRRLHELDPETCPFDHRLTAR | 136 |
| Query 141 | IERARTRMEAGLVDQDDLDEEHQGLAPAELFARLKARMPDGEDLVVTHGDACLPNIMVEN | 200 |
| Sbjct 137 | + A+ RMEAG VD+ D DEE Q A LFA L+A PD D VV HGDACLPN M LRLAKARMEAGAVDESDFDEERQNATAASLFAYLEANQPDVTDPVVAHGDACLPNFMTHE | 196 |
| Query 201 | GRFSGFIDCGRLGVADRYQDIALATRDIAEELGGEWADRFLVLYGIAAPDSQRIAFYRLL | 260 |
| Sbjct 197 | G+FSG++DCGRLGVADRYQDIALA R I +G W FL YG++ + ++A+Y +L GQFSGYVDCGRLGVADRYQDIALALRSIRYNVGEAWVRHFLDCYGLSGINDGKVAYYMVL | 256 |
| Query 261  Sbjct 257 | DEFF 264 DEFF  DEFF 260 |  |

```
>WP_022714890.1 aminoglycoside 3'-phosphotransferase [Rhizobium mongolense] TVZ64374.1 aminoglycoside 3'-phosphotransferase-2 [Rhizobium mongolense
USDA 1844]
Length=260

Score = 252 bits (643), Expect = 3e-80, Method: Compositional matrix adjust. Identities = 123/244 (50%), Positives = 158/244 (65%), Gaps = 1/244 (0%)

Query 22	YDWAQQTIGCSDAAVFRLSAQGRPVLFVKTDLSGALNELQDEAARLSWLATTGVPCAAVL 81
++W Q +GCS A VFRL+ GR	F+K +++G	EL EA RL WL	G+ C +V+ Sbjct 17	HEWKQDDLGCSSAEVFRLAEGGRTRFFLKGEIAGPFAELPLEAERLLWLGEQGLQCPSVV 76
```

| Query | 82 | DVVTEAGRDWLLLGEVPGQDLLSSHLAP-AEKVSIMADAMRRLHTLDPATCPFDHQAKHR | 140 |
| --- | --- | --- | --- |
| Sbjct | 77 | + + GR+WLLL +PG DL SS L +++ I+A A+RRLH LDP TCPFDH+ R ALESHEGRNWLLLSALPGSDLASSKLTTDRQRIEILAAALRRLHELDPETCPFDHRLTAR | 136 |
| Query | 141 | IERARTRMEAGLVDQDDLDEEHQGLAPAELFARLKARMPDGEDLVVTHGDACLPNIMVEN | 200 |
| Sbjct | 137 | + A+ RMEAG VD+ D DEE Q A LFA L+A P D VV HGDACLPN M LRLAKARMEAGAVDESDFDEERQNATAASLFAYLEANQPYVTDPVVAHGDACLPNFMTHE | 196 |
| Query | 201 | GRFSGFIDCGRLGVADRYQDIALATRDIAEELGGEWADRFLVLYGIAAPDSQRIAFYRLL | 260 |
| Sbjct | 197 | G FSG++DCGRLGVADRYQDIALA R I +G W FL YG++ ++A+Y +L GHFSGYVDCGRLGVADRYQDIALALRSIRYNVGEAWVRHFLDCYGLSGISDAKVAYYMVL | 256 |
| Query  Sbjct | 261  257 | DEFF 264 DEF+  DEFY 260 |  |

>WP_049413834.1 APH(3')-II family aminoglycoside O-phosphotransferase [Stenotrophomonas maltophilia]

MBA0400083.1 APH(3')-II family aminoglycoside O-phosphotransferase [Stenotrophomonas maltophilia]

PZT10794.1 APH(3') family aminoglycoside O-phosphotransferase [Stenotrophomonas maltophilia]

QNA95663.1 APH(3')-II family aminoglycoside O-phosphotransferase [Stenotrophomonas maltophilia]

VUQ19087.1 Aminoglycoside 3'-phosphotransferase [Stenotrophomonas maltophilia] Length=267

Score = 252 bits (644), Expect = 3e-80, Method: Compositional matrix adjust.

| Identities | = 136/255 (53%), Positives = 167/255 (65%), Gaps = 3/255 (1%) |  |
| --- | --- | --- |
| Query 12 | PAAWVERLFGYDWAQQTIGCSDAAVFRLSAQGRPVLFVKTDLSGALNELQDEAARLSWLA | 71 |
| Sbjct 14 | P AW + L +Q++G S A V R+ GRP FVK++L A +EL DE ARL WL PLAWQKALADARIERQSMGVSRADVARVLRPGRPDAFVKSELIDAFSELGDEIARLRWLQ | 73 |
| Query 72 | TTGVPCAAVLDVVTEAGRDWLLLGEVPGQDLLSS-HLAPAEKVSIMADAMRRLHTLDPAT | 130 |
| Sbjct 74 | G P V+ EAGR WLL+ +PG+DL SS LAP ++V I+ADA+R LH L A AQGQPAPTVIATAEEAGRRWLLMSALPGRDLASSPELAPQQRVQILADALRGLHALPVAA | 133 |
| Query 131 | CPFDHQAKHRIERARTRMEAGLVDQDDLDEEHQGLAPAELFARLKARMPDGEDLVVTHGD | 190 |
| Sbjct 134 | CPFD + R+ A+ R+EAGLVD DD D+E G +P ++FA+L + PD EDLVV+HGD CPFDQRLASRLPVAQARVEAGLVDADDFDDERLGQSPQQVFAQLCSTRPDHEDLVVSHGD | 193 |
| Query 191 | ACLPNIMVENGRFSGFIDCGRLGVADRYQDIALATRDIAEELG-GEWADRFLVLYGIAAP | 249 |
| Sbjct 194 | ACLPN+MV GRFSGFIDCGRLGVADRYQD+ALA R + G W YG A ACLPNLMVAEGRFSGFIDCGRLGVADRYQDLALAARSLVHNFGDTRWVTPLFQRYGAVA- | 252 |
| Query 250  Sbjct 253 | DSQRIAFYRLLDEFF 264 D R+AFYRLLDEFF DECRLAFYRLLDEFF 267 |  |

>MBS79415.1 APH(3') family aminoglycoside O-phosphotransferase [Variovorax sp.]

Length=259

Score = 252 bits (643), Expect = 3e-80, Method: Compositional matrix adjust.

| Identities | = 138/253 (55%), Positives = 161/253 (64%), Gaps = 1/253 (0%) |  |
| --- | --- | --- |
| Query 12 | PAAWVERLFGYDWAQQTIGCSDAAVFRLSAQGRPVLFVKTDLSGALNELQDEAARLSWLA | 71 |
| Sbjct 8 | P AW RL W +QTIG S A VFR+ +LF+K++ L EL E ARL WLA PHAWRARLADAHWQRQTIGESGADVFRIETADGELLFLKSEPVAPLAELPGEIARLEWLA | 67 |
| Query 72 | TTGVPCAAVLDVVTEAGRDWLLLGEVPGQDLLSSHLAPAEKVSIMADAMRRLHTLDPATC | 131 |
| Sbjct 68 | +P AVLD E R WLL+ VPG+DL S PAE V MADA+R LH + ATC RQDLPAPAVLDQALEHDRHWLLMSGVPGRDLASGPFRPAEIVVHMADALRLLHRVPVATC | 127 |
| Query 132 | PFDHQAKHRIERARTRMEAGLVDQDDLDEEHQGLAPAELFARLKARMPDGEDLVVTHGDA | 191 |
| Sbjct 128 | PFDH+ RIE A R AGLVD+ D D+E G +LF RL+A P EDLVVTHGDA PFDHRLAQRIEDAHARAVAGLVDESDFDDERAGWTALDLFERLRATRPANEDLVVTHGDA | 187 |
| Query 192 | CLPNIMVENGRFSGFIDCGRLGVADRYQDIALATRDIAEELGGEWADRFLVLYGIAAPDS | 251 |

CLPN+M	F+GFIDCGR+GVADR+QD+ALA R I	LG W	F	YGI APD+

| Sbjct | 188 | CLPNLMATADGFTGFIDCGRVGVADRWQDLALAARSIGFNLGEAWTAPFFERYGI-APDA 246 |
| --- | --- | --- |
| Query | 252 | QRIAFYRLLDEFF 264 |
| Sbjct | 247 | +RIAFY LLDEFF  ERIAFYCLLDEFF 259 |

>EJZ58787.1 Aminoglycoside phosphotransferase [Pseudomonas fluorescens R124] Length=258

Score = 252 bits (643), Expect = 3e-80, Method: Compositional matrix adjust.

| Identities | | = 129/254 (51%), Positives = 168/254 (66%), Gaps = 2/254 (1%) |  |
| --- | --- | --- | --- |
| Query 12  Sbjct 6 | | PAAWVERLFGYDWAQQTIGCSDAAVFRLSAQGRPVLFVKTDLSGALNELQDEAARLSWLA P W +R + QQTIG S A VFRL+ G LFVK++ + EL E RL WLA PQTWRQRFADTRYEQQTIGESRADVFRLTPAGSDPLFVKSEPRFSFAELPGEVQRLRWLA | 71  65 |
| Query 72  Sbjct 66 | | TTGVPCAAVLDVVTEAGRDWLLLGEVPGQDLLSS-HLAPAEKVSIMADAMRRLHTLDPAT  +P A VLD V E+ R WLL+ VPG+DL SS HL P + VS+ A+A+R LH +D EQDLPAARVLDSVEESARFWLLMTAVPGRDLASSEHLTPQQIVSLAANALRTLHAIDIED | 130  125 |
| Query 131  Sbjct 126 | | CPFDHQAKHRIERARTRMEAGLVDQDDLDEEHQGLAPAELFARLKARMPDGEDLVVTHGD CPFDH+ +I AR + AGL+D++D D+ G + +F ++ A PD EDLVVTHGD CPFDHRLDAKIALAREHLLAGLIDEEDFDDSRLGQSAEAVFEQMLAARPDSEDLVVTHGD | 190  185 |
| Query 191  Sbjct 186 | | ACLPNIMVENGRFSGFIDCGRLGVADRYQDIALATRDIAEELGGEWADRFLVLYGIAAPD ACLPN++V++GRFSGF+DCGRLGV+DR+QD+AL IA LG +W F YG+ PD ACLPNLLVDHGRFSGFVDCGRLGVSDRFQDLALTAHSIAYNLGEQWVPAFFEAYGV-QPD | 250  244 |
| Query | 251 SQRIAFYRLLDEFF 264 | | |
|  |  | ++IAFY+LLDEFF |  |
| Sbjct | 245 | LRKIAFYQLLDEFF 258 | |

>WP_023980230.1 MULTISPECIES: APH(3')-II family aminoglycoside O-phosphotransferase [Pseudomonas]

ANA71188.1 APH(3') family aminoglycoside O-phosphotransferase [Pseudomonas aeruginosa]

ETD55626.1 aminoglycoside 3'-phosphotransferase [Pseudomonas aeruginosa VRFPA07]

EZP07683.1 aminoglycoside 3'-phosphotransferase type IIB [Pseudomonas aeruginosa BWH050]

KAA5590106.1 APH(3')-II family aminoglycoside O-phosphotransferase [Pseudomonas aeruginosa]

KAA5615537.1 APH(3')-II family aminoglycoside O-phosphotransferase [Pseudomonas aeruginosa]

Length=268

Score = 252 bits (644), Expect = 3e-80, Method: Compositional matrix adjust.

| Identities | | = 135/258 (52%), Positives = 168/258 (65%), Gaps = 5/258 (2%) |  |
| --- | --- | --- | --- |
| Query 11  Sbjct 12 | | SPAAWVERLFGYDWAQQTIGCSDAAVFRLSAQGRPVLFVKTDLSGALNELQDEAARLSWL  +P+ W + L GY W Q GCS A V RL A RP LFVK ++ A EL E ARL WL APSTWADYLVGYRWRGQGEGCSAATVHRLEAARRPTLFVKQEVLSAHAELPAEIARLRWL | 70  71 |
| Query 71  Sbjct 72 | | ATTGVPCAAVLDVVTEAGRDWLLLGEVPGQDLLS SHLAPAEKVSIMADAMRRLHTL  G+ C VL+ GR WLL+ VPG L + L P V ++A A+RRLH L HGAGIDCPQVLNETQSDGRQWLLMSAVPGDTLSALAQRGELEPERLVRLVAAALRRLHDL | 126  131 |
| Query 127  Sbjct 132 | | DPATCPFDHQAKHRIERARTRMEAGLVDQDDLDEEHQGLAPAELFARLKARMPDGEDLVV DPA CPFDH+ + R++ R R+EAGLVD+ D D++H+G + EL+ L R E+LVV DPAACPFDHRLERRLDTVRQRVEAGLVDEADFDDDHRGRSATELYRLLLDRRLAVENLVV | 186  191 |
| Query 187  Sbjct 192 | | THGDACLPNIMVENGRFSGFIDCGRLGVADRYQDIALATRDIAEELGGEWADRFLVLYGI HGDACLPN++ E RFSGFIDCGRLGVADR+QD+ALA RDI ELG WA+ FLV YG AHGDACLPNLLAEGRRFSGFIDCGRLGVADRHQDLALAARDIEAELGAAWAEAFLVEYG- | 246  250 |
| Query | 247 AAPDSQRIAFYRLLDEFF 264 | | |
|  |  | D +R+A++RLLDEFF |  |
| Sbjct | 251 | GDIDGERLAYFRLLDEFF 268 | |

>MBG6629815.1 APH(3')-II family aminoglycoside O-phosphotransferase [Pseudomonas aeruginosa]

Length=268

Score = 252 bits (644), Expect = 3e-80, Method: Compositional matrix adjust.

| Identities | | = 134/258 (52%), Positives = 165/258 (64%), Gaps = 5/258 (2%) |  |
| --- | --- | --- | --- |
| Query 11  Sbjct 12 | | SPAAWVERLFGYDWAQQTIGCSDAAVFRLSAQGRPVLFVKTDLSGALNELQDEAARLSWL  +P+ W + L GY W Q GCS A V RL A RP LFVK ++ A EL E ARL WL APSTWADYLAGYRWRGQGEGCSAATVHRLEAARRPTLFVKQEVLSAHAELPAEIARLRWL | 70  71 |
| Query 71  Sbjct 72 | | ATTGVPCAAVLDVVTEAGRDWLLLGEVPGQDLLS SHLAPAEKVSIMADAMRRLHTL  G+ C VL+ GR WLL+ VPG L + L P V ++A A+RRLH L HGAGIDCPQVLNETQSDGRQWLLMSAVPGDTLSALAQRGELEPERLVRLVAAALRRLHDL | 126  131 |
| Query 127  Sbjct 132 | | DPATCPFDHQAKHRIERARTRMEAGLVDQDDLDEEHQGLAPAELFARLKARMPDGEDLVV DPA CPFDH+ + R++ R R+EAGLVD+ D D++H+G + EL+ L R P EDLVV DPAACPFDHRLERRLDTVRQRVEAGLVDEADFDDDHRGRSATELYRLLLDRRPAVEDLVV | 186  191 |
| Query 187  Sbjct 192 | | THGDACLPNIMVENGRFSGFIDCGRLGVADRYQDIALATRDIAEELGGEWADRFLVLYGI HGDACLPN++ E RFSGFIDCGRLGVADR D ALA RDI + G WA+ FLV YG AHGDACLPNLLAEGRRFSGFIDCGRLGVADRPPDRALAARDIEADRGAAWAEAFLVEYG- | 246  250 |
| Query | 247 AAPDSQRIAFYRLLDEFF 264 | | |
|  |  | D +R+A++RLLDEFF |  |
| Sbjct | 251 | GDIDGERLAYFRLLDEFF 268 | |

>WP_045122502.1 aminoglycoside 3'-phosphotransferase [Pseudomonas fluorescens] Length=256

Score = 252 bits (643), Expect = 3e-80, Method: Compositional matrix adjust.

| Identities | | = 129/254 (51%), Positives = 168/254 (66%), Gaps = 2/254 (1%) |  |
| --- | --- | --- | --- |
| Query 12  Sbjct 4 | | PAAWVERLFGYDWAQQTIGCSDAAVFRLSAQGRPVLFVKTDLSGALNELQDEAARLSWLA P W +R + QQTIG S A VFRL+ G LFVK++ + EL E RL WLA PQTWRQRFADTRYEQQTIGESRADVFRLTPAGSDPLFVKSEPRFSFAELPGEVQRLRWLA | 71  63 |
| Query 72  Sbjct 64 | | TTGVPCAAVLDVVTEAGRDWLLLGEVPGQDLLSS-HLAPAEKVSIMADAMRRLHTLDPAT  +P A VLD V E+ R WLL+ VPG+DL SS HL P + VS+ A+A+R LH +D EQDLPAARVLDSVEESARFWLLMTAVPGRDLASSEHLTPQQIVSLAANALRTLHAIDIED | 130  123 |
| Query 131  Sbjct 124 | | CPFDHQAKHRIERARTRMEAGLVDQDDLDEEHQGLAPAELFARLKARMPDGEDLVVTHGD CPFDH+ +I AR + AGL+D++D D+ G + +F ++ A PD EDLVVTHGD CPFDHRLDAKIALAREHLLAGLIDEEDFDDSRLGQSAEAVFEQMLAARPDSEDLVVTHGD | 190  183 |
| Query 191  Sbjct 184 | | ACLPNIMVENGRFSGFIDCGRLGVADRYQDIALATRDIAEELGGEWADRFLVLYGIAAPD ACLPN++V++GRFSGF+DCGRLGV+DR+QD+AL IA LG +W F YG+ PD ACLPNLLVDHGRFSGFVDCGRLGVSDRFQDLALTAHSIAYNLGEQWVPAFFEAYGV-QPD | 250  242 |
| Query | 251 SQRIAFYRLLDEFF 264 | | |
|  |  | ++IAFY+LLDEFF |  |
| Sbjct | 243 | LRKIAFYQLLDEFF 256 | |

>WP_088587125.1 APH(3')-II family aminoglycoside O-phosphotransferase [Achromobacter marplatensis]

OWT72009.1 APH(3') family aminoglycoside O-phosphotransferase [Achromobacter marplatensis]

RBP24727.1 aminoglycoside 3'-phosphotransferase-2 [Achromobacter marplatensis] CAB3624820.1 Aminoglycoside 3'-phosphotransferase [Achromobacter marplatensis]

Length=266

Score = 252 bits (644), Expect = 3e-80, Method: Compositional matrix adjust. Identities = 127/253 (50%), Positives = 167/253 (66%), Gaps = 1/253 (0%)

| Query | 12 | PAAWVERLFGYDWAQQTIGCSDAAVFRLSAQGRPVLFVKTDLSGALNELQDEAARLSWLA | 71 |
| --- | --- | --- | --- |
|  |  | PA W + QQ+IG S A V+R+ +G+P LF+K++L+ +NEL DE ARL WL |  |
| Sbjct | 15 | PAKWRDGFAHASIEQQSIGESRADVYRVRRRGQPDLFLKSELTSVVNELPDEIARLRWLR | 74 |
| Query | 72 | TTGVPCAAVLDVVTEAGRDWLLLGEVPGQDLLSSHLAPAEKVSIMADAMRRLHTLDPATC | 131 |

G P	VLD V + GR WLL+ + G DL S+ L P + + ++A A+R LH +	+C

| Sbjct | 75 | QVGQPAPTVLDSVDDGGRHWLLMTALEGCDLASATLPPGQVIGLLAAALRNLHRVPTESC | 134 |
| --- | --- | --- | --- |
| Query | 132 | PFDHQAKHRIERARTRMEAGLVDQDDLDEEHQGLAPAELFARLKARMPDGEDLVVTHGDA | 191 |
| Sbjct | 135 | PFDH+ RI A+ R++AGLVD+ D D+E G + ++ A L + P DLVVTHGDA PFDHRLAPRIADAKRRVDAGLVDEADFDDERVGQSAGQVLAELLSTCPTAHDLVVTHGDA | 194 |
| Query | 192 | CLPNIMVENGRFSGFIDCGRLGVADRYQDIALATRDIAEELGGEWADRFLVLYGIAAPDS | 251 |
| Sbjct | 195 | CLPN MV+ RF+GFIDCGRLG+ADR+QD+ALA R IA +G EW F YG+ APD+ CLPNFMVDGNRFTGFIDCGRLGIADRFQDLALAARSIARNVGEEWVAPFFREYGV-APDA | 253 |
| Query  Sbjct | 252  254 | QRIAFYRLLDEFF 264 QRI FY LLDEFF QRIKFYCLLDEFF 266 |  |

>WP_074067510.1 aminoglycoside 3'-phosphotransferase [Rhizobium gallicum] APO67228.1 aminoglycoside 3'-phosphotransferase protein [Rhizobium gallicum]

Length=260

Score = 252 bits (643), Expect = 3e-80, Method: Compositional matrix adjust.

| Identities | = 124/244 (51%), Positives = 159/244 (65%), Gaps = 1/244 (0%) |  |
| --- | --- | --- |
| Query 22 | YDWAQQTIGCSDAAVFRLSAQGRPVLFVKTDLSGALNELQDEAARLSWLATTGVPCAAVL | 81 |
| Sbjct 17 | ++W Q +GCS A VFRL+ GR F+K +++G EL EA RL WLA G+ C +V+ HEWKQDDLGCSSADVFRLAEGGRTRFFLKGEIAGPFAELPLEAERLLWLAEQGLQCPSVV | 76 |
| Query 82 | DVVTEAGRDWLLLGEVPGQDLLSSHLAP-AEKVSIMADAMRRLHTLDPATCPFDHQAKHR | 140 |
| Sbjct 77 | + + GR+WLLL + G DL S L +++ I+A A+RRLH LDP TCPFDH+ R ALESHEGRNWLLLSALAGSDLASCKLTTDRQRIEILAAALRRLHELDPETCPFDHRLTAR | 136 |
| Query 141 | IERARTRMEAGLVDQDDLDEEHQGLAPAELFARLKARMPDGEDLVVTHGDACLPNIMVEN | 200 |
| Sbjct 137 | + A+ RMEAG VD+ D DEE Q A LFA L A PD D VV HGDACLPN M LRLAKARMEAGAVDESDFDEERQNATAASLFAYLDANQPDVTDPVVAHGDACLPNFMTHE | 196 |
| Query 201 | GRFSGFIDCGRLGVADRYQDIALATRDIAEELGGEWADRFLVLYGIAAPDSQRIAFYRLL | 260 |
| Sbjct 197 | G+FSG++DCGRLGVADRYQDIALA R I +G W FL YG++ + ++A+Y +L GQFSGYVDCGRLGVADRYQDIALALRSIRYNVGEAWVRHFLDCYGLSGINDAKVAYYMVL | 256 |
| Query 261  Sbjct 257 | DEFF 264 DEFF  DEFF 260 |  |

>MBG6497053.1 APH(3')-II family aminoglycoside O-phosphotransferase [Pseudomonas aeruginosa]

Length=268

Score = 252 bits (644), Expect = 4e-80, Method: Compositional matrix adjust.

| Identities | | = 136/258 (53%), Positives = 167/258 (65%), Gaps = 5/258 (2%) |  |
| --- | --- | --- | --- |
| Query 11  Sbjct 12 | | SPAAWVERLFGYDWAQQTIGCSDAAVFRLSAQGRPVLFVKTDLSGALNELQDEAARLSWL  +P+ W + L GY W Q GCS A V RL A RP LFVK ++ A EL E ARL WL APSTWADYLAGYRWRGQGEGCSAATVHRLEAARRPTLFVKQEVLSAHAELPAEIARLRWL | 70  71 |
| Query 71  Sbjct 72 | | ATTGVPCAAVLDVVTEAGRDWLLLGEVPGQDLLS SHLAPAEKVSIMADAMRRLHTL  G+ C VL+ GR WLL+ VPG L + L P V ++A A+RRLH L HGAGIDCPQVLNETQSDGRQWLLMSAVPGDTLSALVQRGELEPERLVRLVAAALRRLHDL | 126  131 |
| Query 127  Sbjct 132 | | DPATCPFDHQAKHRIERARTRMEAGLVDQDDLDEEHQGLAPAELFARLKARMPDGEDLVV DPA CPFDH+ + R++ R R+EAGLVD+ D D++H+G + EL+ L R P EDLVV DPAACPFDHRLERRLDTVRQRVEAGLVDEADFDDDHRGRSATELYRLLLDRRPAVEDLVV | 186  191 |
| Query 187  Sbjct 192 | | THGDACLPNIMVENGRFSGFIDCGRLGVADRYQDIALATRDIAEELGGEWADRFLVLYGI HGDACLPN++ E RFSGFIDCGRLGVA R+QD+ALA RDI ELG WA FLV YG AHGDACLPNLLAEGRRFSGFIDCGRLGVAARHQDLALAARDIEAELGAAWAAAFLVEYG- | 246  250 |
| Query | 247 AAPDSQRIAFYRLLDEFF 264 | | |
|  |  | D +R+A++RLLDEFF |  |
| Sbjct | 251 | GDIDGERLAYFRLLDEFF 268 | |

>WP_103485884.1 aminoglycoside 3'-phosphotransferase [Pseudomonas sp. MWU12-2115]

RBC01497.1 APH(3') family aminoglycoside O-phosphotransferase [Pseudomonas sp. MWU12-2115]

Length=256

Score = 252 bits (643), Expect = 4e-80, Method: Compositional matrix adjust.

| Identities | | = 128/254 (50%), Positives = 169/254 (67%), Gaps = 2/254 (1%) |  |
| --- | --- | --- | --- |
| Query 12  Sbjct 4 | | PAAWVERLFGYDWAQQTIGCSDAAVFRLSAQGRPVLFVKTDLSGALNELQDEAARLSWLA P W +R + QQTIG S A VFRL+ G LFVK++ + +EL E RL WLA PQTWRQRFADTRYEQQTIGESRADVFRLTPAGSAPLFVKSEPRISFSELPGEVQRLRWLA | 71  63 |
| Query 72  Sbjct 64 | | TTGVPCAAVLDVVTEAGRDWLLLGEVPGQDLLSS-HLAPAEKVSIMADAMRRLHTLDPAT  +P A VLD V E+ R WLL+ VPG+DL SS HL P + VS+ A+A+R LH ++ EQDLPAARVLDSVEESERFWLLMTAVPGRDLASSEHLTPQQIVSLAANALRTLHAINIED | 130  123 |
| Query 131  Sbjct 124 | | CPFDHQAKHRIERARTRMEAGLVDQDDLDEEHQGLAPAELFARLKARMPDGEDLVVTHGD CPFDH+ +I AR + AGL+D++D D+ G + LF ++ A PD EDLVVTHGD CPFDHRLDAKIALAREHLLAGLIDEEDFDDSRLGQSAKALFEQMLAARPDSEDLVVTHGD | 190  183 |
| Query 191  Sbjct 184 | | ACLPNIMVENGRFSGFIDCGRLGVADRYQDIALATRDIAEELGGEWADRFLVLYGIAAPD ACLPN++V++GRFSGF+DCGRLGV+DR+QD+AL IA LG +W F YG+ PD ACLPNLLVDHGRFSGFVDCGRLGVSDRFQDLALTAHSIAYNLGEQWVPAFFEAYGV-QPD | 250  242 |
| Query | 251 SQRIAFYRLLDEFF 264 | | |
|  |  | +++AFY+LLDEFF |  |
| Sbjct | 243 | LRKVAFYQLLDEFF 256 | |

>WP_112772955.1 MULTISPECIES: aminoglycoside 3'-phosphotransferase [Ensifer] MBD9627453.1 aminoglycoside 3'-phosphotransferase [Ensifer sp. ENS06] RAS02587.1 aminoglycoside 3'-phosphotransferase-2 [Ensifer adhaerens]

Length=289

Score = 253 bits (646), Expect = 4e-80, Method: Compositional matrix adjust.

| Identities | = 128/244 (52%), Positives = 165/244 (68%), Gaps = 1/244 (0%) |  |
| --- | --- | --- |
| Query 21 | GYDWAQQTIGCSDAAVFRLSAQGRPVLFVKTDLSGALNELQDEAARLSWLATTGVPCAAV | 80 |
| Sbjct 47 | GY W + +G SD+++F L A RP+L +K + +G +E DEAARL WLA+ VPC V GYQWNRDLLGQSDSSIFLLKAPDRPMLVLKLEEAGPFSEFVDEAARLDWLASKDVPCPRV | 106 |
| Query 81 | LDVVTEAGRDWLLLGEVPGQDLLSSHLAPAEKVSIMADAMRRLHTLDPATCPFDHQAKHR | 140 |
| Sbjct 107 | + A +WLLL V G DL S+ L+P +++ I+ADA+++LH LD A CPFDH+ + R IARAFGAETNWLLLQAVQGTDLASTTLSPKDQIVILADALKQLHALDIANCPFDHRIEVR | 166 |
| Query 141 | IERARTRMEAGLVDQDDLDEEHQGLAPAELFARLKARMPDGEDLVVTHGDACLPNIMVEN | 200 |
| Sbjct 167 | I A+ R +AG++ + D DE G A+LFA L+AR P +LVVTHGDACLPNI+ +N IAIAKARTQAGVI-ESDFDETRLGRTTADLFAELQAREPQPGELVVTHGDACLPNIIEKN | 225 |
| Query 201 | GRFSGFIDCGRLGVADRYQDIALATRDIAEELGGEWADRFLVLYGIAAPDSQRIAFYRLL | 260 |
| Sbjct 226 | GRFSGFIDC RLGVAD YQDIALA R I LG EW FL YG+ DS++ FY LL GRFSGFIDCSRLGVADPYQDIALACRSIVHNLGEEWVQPFLTRYGLDKDDSEKRDFYCLL | 285 |
| Query 261  Sbjct 286 | DEFF 264 DEFF  DEFF 289 |  |

>WP_090704353.1 aminoglycoside 3'-phosphotransferase [Beijerinckia sp. 28-YEA-48] SEC66534.1 aminoglycoside 3'-phosphotransferase-2 [Beijerinckia sp. 28-YEA-48]

Length=266

Score = 252 bits (643), Expect = 4e-80, Method: Compositional matrix adjust. Identities = 135/254 (53%), Positives = 164/254 (65%), Gaps = 2/254 (1%)

| Query | 12 | PAAWVERLFGYDWAQQTIGCSDAAVFRLSAQGRPVLFVKTDLSGALNELQDEAARLSWLA | 71 |
| --- | --- | --- | --- |
| Sbjct | 14 | P W + L GY W QTIG S A VFRL A RP FVK + + A EL EAARL WLA PPHWRDDLAGYAWTTQTIGRSSATVFRLQADKRPSFFVKIEPADASGELLAEAARLRWLA | 73 |
| Query | 72 | TTGVPCAAVLDVVTEAGRDWLLLGEVPGQDLLSS-HLAPAEKVSIMADAMRRLHTLDPAT | 130 |
| Sbjct | 74 | +G+ C V + ++ R+WLL +PG+DL SS HLA + V+I ADA+R LH LDP+  ASGIACPQVENTSSDGQRNWLLTRALPGRDLASSPHLAAEQIVTIAADALRDLHRLDPSP | 133 |

| Query | 131 | CPFDHQAKHRIERARTRMEAGLVDQDDLDEEHQGLAPAELFARLKARMPDGEDLVVTHGD | 190 |
| --- | --- | --- | --- |
| Sbjct | 134 | CPFD + RI A RM AGLVD+ D D+E G +FA L R P EDLVVTHGD CPFDQRLDSRIAAAEARMRAGLVDESDFDDERLGQPAHAVFADLLRRRPAQEDLVVTHGD | 193 |
| Query | 191 | ACLPNIMVENGRFSGFIDCGRLGVADRYQDIALATRDIAEELGGEWADRFLVLYGIAAPD | 250 |
| Sbjct | 194 | ACLPN+M + RFSGF+DC RLGVADR+QD+ALA+ I LG W FL YG+ A D ACLPNLMADGERFSGFVDCARLGVADRHQDLALASWSIRYNLGQAWIAPFLQRYGLRA-D | 252 |
| Query | 251 | SQRIAFYRLLDEFF 264  + + FYRLLD+FF |  |
| Sbjct | 253 | PELLDFYRLLDDFF 266 | |

>WP_134967981.1 APH(3')-II family aminoglycoside O-phosphotransferase [Stenotrophomonas maltophilia]

PSD20830.1 APH(3')-II family aminoglycoside O-phosphotransferase [Stenotrophomonas maltophilia]

Length=267

Score = 252 bits (643), Expect = 4e-80, Method: Compositional matrix adjust.

| Identities | = 134/255 (53%), Positives = 167/255 (65%), Gaps = 3/255 (1%) |  |
| --- | --- | --- |
| Query 12 | PAAWVERLFGYDWAQQTIGCSDAAVFRLSAQGRPVLFVKTDLSGALNELQDEAARLSWLA | 71 |
| Sbjct 14 | P AW + L +Q++G S A V R+ QGRP F+K++L A +EL DE ARL WL PLAWQKALADARIERQSMGVSRADVARVLRQGRPDAFLKSELIDAFSELGDEIARLRWLQ | 73 |
| Query 72 | TTGVPCAAVLDVVTEAGRDWLLLGEVPGQDLLSS-HLAPAEKVSIMADAMRRLHTLDPAT | 130 |
| Sbjct 74 | G P V+ E GR WLL+ +PG+DL SS LAP ++V I+ADA+R LH L A AQGQPAPTVIATAEETGRRWLLMSALPGRDLASSPELAPQQRVEILADALRGLHALPVAA | 133 |
| Query 131 | CPFDHQAKHRIERARTRMEAGLVDQDDLDEEHQGLAPAELFARLKARMPDGEDLVVTHGD | 190 |
| Sbjct 134 | CPFD + R+ A+ R+EAG VD DD D+E G +P ++FA+L + PD EDLVV+HGD CPFDQRLASRLPAAQARVEAGRVDADDFDDERLGQSPQQVFAQLCSTRPDHEDLVVSHGD | 193 |
| Query 191 | ACLPNIMVENGRFSGFIDCGRLGVADRYQDIALATRDIAEELG-GEWADRFLVLYGIAAP | 249 |
| Sbjct 194 | ACLPN+MV GRFSGFIDCGRLGVADRYQD+ALA R + G W YG A ACLPNLMVAEGRFSGFIDCGRLGVADRYQDLALAARSLVHNFGDTRWVTPLFQRYGAVA- | 252 |
| Query 250  Sbjct 253 | DSQRIAFYRLLDEFF 264 D +R+AFYRLLDEFF DERRLAFYRLLDEFF 267 |  |

>MBG6520829.1 APH(3')-II family aminoglycoside O-phosphotransferase [Pseudomonas aeruginosa]

Length=268

Score = 252 bits (643), Expect = 4e-80, Method: Compositional matrix adjust.

| Identities | | = 136/258 (53%), Positives = 167/258 (65%), Gaps = 5/258 (2%) |  |
| --- | --- | --- | --- |
| Query 11  Sbjct 12 | | SPAAWVERLFGYDWAQQTIGCSDAAVFRLSAQGRPVLFVKTDLSGALNELQDEAARLSWL  +P+ W + L GY W Q GCS A V RL A RP LFVK ++ A EL E ARL WL APSTWADYLAGYRWRGQGEGCSAATVHRLEAARRPTLFVKQEVLSAHAELPAEIARLRWL | 70  71 |
| Query 71  Sbjct 72 | | ATTGVPCAAVLDVVTEAGRDWLLLGEVPGQDLLS SHLAPAEKVSIMADAMRRLHTL  G+ C VL+ GR WLL+ VPG L + L P ++A A+RRLH L HGAGIDCPQVLNETQSDGRQWLLMSAVPGDTLSALVQRGELEPERLGRLVAAALRRLHDL | 126  131 |
| Query 127  Sbjct 132 | | DPATCPFDHQAKHRIERARTRMEAGLVDQDDLDEEHQGLAPAELFARLKARMPDGEDLVV DPA CPFDH+ + R++ R R+EAGLVD+ D D++H+G + EL+ L R P EDLVV DPAACPFDHRLERRLDTVRQRVEAGLVDEADFDDDHRGRSATELYRLLLDRRPAVEDLVV | 186  191 |
| Query 187  Sbjct 192 | | THGDACLPNIMVENGRFSGFIDCGRLGVADRYQDIALATRDIAEELGGEWADRFLVLYGI HGDACLPN++ E RFSGFIDCGRLGVADR+QD+ALA RDI ELG WA FLV YG AHGDACLPNLLAEGRRFSGFIDCGRLGVADRHQDLALAARDIEAELGAAWAAAFLVEYG- | 246  250 |
| Query | 247 AAPDSQRIAFYRLLDEFF 264 | | |
|  |  | D +R+A++RLLDEFF |  |
| Sbjct | 251 | GDIDGERLAYFRLLDEFF 268 | |

>WP_164283431.1 APH(3')-II family aminoglycoside O-phosphotransferase [Stenotrophomonas maltophilia]

Length=267

Score = 252 bits (643), Expect = 4e-80, Method: Compositional matrix adjust.

| Identities | = 136/255 (53%), Positives = 167/255 (65%), Gaps = 3/255 (1%) |  |
| --- | --- | --- |
| Query 12 | PAAWVERLFGYDWAQQTIGCSDAAVFRLSAQGRPVLFVKTDLSGALNELQDEAARLSWLA | 71 |
| Sbjct 14 | P AW + L +Q++G S A V R+ QGRP FVK++L A +EL DE ARL WL PLAWQKALADARIERQSMGVSRADVARVLRQGRPDAFVKSELIDAFSELGDEIARLRWLQ | 73 |
| Query 72 | TTGVPCAAVLDVVTEAGRDWLLLGEVPGQDLLSS-HLAPAEKVSIMADAMRRLHTLDPAT | 130 |
| Sbjct 74 | G P V+ EAGR WLL+ +PG+DL SS LAP ++V I+ADA+R LH L A AQGQPAPTVIATAEEAGRRWLLMSALPGRDLASSPELAPQQRVQILADALRGLHALPVAA | 133 |
| Query 131 | CPFDHQAKHRIERARTRMEAGLVDQDDLDEEHQGLAPAELFARLKARMPDGEDLVVTHGD | 190 |
| Sbjct 134 | CPFD + R+ A+ R+EAGLVD DD D+E G +P ++FA+L + PD EDLVV+HGD CPFDQRLASRLPVAQARVEAGLVDADDFDDERLGQSPQQVFAQLCSTRPDHEDLVVSHGD | 193 |
| Query 191 | ACLPNIMVENGRFSGFIDCGRLGVADRYQDIALATRDIAEEL-GGEWADRFLVLYGIAAP | 249 |
| Sbjct 194 | ACLPN+MV GRFSGFIDCGRLGVADRYQD+ALA R + W YG A ACLPNLMVAEGRFSGFIDCGRLGVADRYQDLALAARSLVHNFDDTRWVTPLFQRYGAVA- | 252 |
| Query 250  Sbjct 253 | DSQRIAFYRLLDEFF 264 D R+AFYRLLDEFF DECRLAFYRLLDEFF 267 |  |

>WP_111096174.1 APH(3')-II family aminoglycoside O-phosphotransferase [Stenotrophomonas maltophilia]

PZS83707.1 APH(3') family aminoglycoside O-phosphotransferase [Stenotrophomonas maltophilia]

PZT19599.1 APH(3') family aminoglycoside O-phosphotransferase [Stenotrophomonas maltophilia]

Length=267

Score = 252 bits (643), Expect = 5e-80, Method: Compositional matrix adjust.

| Identities | = 135/255 (53%), Positives = 167/255 (65%), Gaps = 3/255 (1%) |  |
| --- | --- | --- |
| Query 12 | PAAWVERLFGYDWAQQTIGCSDAAVFRLSAQGRPVLFVKTDLSGALNELQDEAARLSWLA | 71 |
| Sbjct 14 | P AW + L +Q++G S A V R+ GRP FVK++L A +EL DE ARL WL PLAWQKALADARIERQSMGVSRADVARVQRPGRPDAFVKSELIDAFSELGDEIARLRWLQ | 73 |
| Query 72 | TTGVPCAAVLDVVTEAGRDWLLLGEVPGQDLLSS-HLAPAEKVSIMADAMRRLHTLDPAT | 130 |
| Sbjct 74 | G P V+ EAGR WLL+ +PG+DL SS LAP ++V I+ADA+R LH L A AQGQPAPTVIATAEEAGRRWLLMSALPGRDLASSPELAPQQRVQILADALRGLHALPVAA | 133 |
| Query 131 | CPFDHQAKHRIERARTRMEAGLVDQDDLDEEHQGLAPAELFARLKARMPDGEDLVVTHGD | 190 |
| Sbjct 134 | CPFD + R+ A+ R+EAGLVD DD D+E G +P ++FA+L + PD E+LVV+HGD CPFDQRLASRLPVAQARVEAGLVDADDFDDERLGQSPQQVFAQLCSTRPDHEELVVSHGD | 193 |
| Query 191 | ACLPNIMVENGRFSGFIDCGRLGVADRYQDIALATRDIAEELG-GEWADRFLVLYGIAAP | 249 |
| Sbjct 194 | ACLPN+MV GRFSGFIDCGRLGVADRYQD+ALA R + G W YG A ACLPNLMVAEGRFSGFIDCGRLGVADRYQDLALAARSLVHNFGDTRWVTPLFQRYGAVA- | 252 |
| Query 250  Sbjct 253 | DSQRIAFYRLLDEFF 264 D R+AFYRLLDEFF DECRLAFYRLLDEFF 267 |  |

>OWK24067.1 aminoglycoside phosphotransferase [Rhizobium yanglingense] Length=262

Score = 251 bits (642), Expect = 6e-80, Method: Compositional matrix adjust.

| Identities | = 124/244 (51%), Positives = 159/244 (65%), Gaps = 1/244 (0%) |  |
| --- | --- | --- |
| Query 22 | YDWAQQTIGCSDAAVFRLSAQGRPVLFVKTDLSGALNELQDEAARLSWLATTGVPCAAVL | 81 |
| Sbjct 19 | ++W Q +GCS A VFRL+ GR F+K +++G EL EA RL WLA G+ C +V+ HEWKQDDLGCSSADVFRLAEGGRTRFFLKGEIAGPFAELPLEAERLLWLAEQGLQCPSVV | 78 |
| Query 82 | DVVTEAGRDWLLLGEVPGQDLLSSHLAP-AEKVSIMADAMRRLHTLDPATCPFDHQAKHR  + + GR+WLLL + G DL S L +++ I+A A+RRLH LDP TCPFDH+ R | 140 |

| Sbjct | 79 | ALESHEGRNWLLLSALAGSDLASCKLTTDRQRIEILAAALRRLHELDPETCPFDHRLTAR | 138 |
| --- | --- | --- | --- |
| Query | 141 | IERARTRMEAGLVDQDDLDEEHQGLAPAELFARLKARMPDGEDLVVTHGDACLPNIMVEN | 200 |
| Sbjct | 139 | + A+ RMEAG VD+ D DEE Q A LFA L A PD D VV HGDACLPN M LRLAKARMEAGAVDESDFDEERQNATAASLFAYLDANQPDVTDPVVAHGDACLPNFMAHE | 198 |
| Query | 201 | GRFSGFIDCGRLGVADRYQDIALATRDIAEELGGEWADRFLVLYGIAAPDSQRIAFYRLL | 260 |
| Sbjct | 199 | G+FSG++DCGRLGVADRYQDIALA R I +G W FL YG++ + ++A+Y +L GQFSGYVDCGRLGVADRYQDIALALRSIRYNVGEAWVRHFLDCYGLSGINDAKVAYYMVL | 258 |
| Query  Sbjct | 261  259 | DEFF 264 DEFF  DEFF 262 |  |

>RBL72727.1 APH(3') family aminoglycoside O-phosphotransferase [Pseudomonas sp. MWU13-2625]

Length=256

Score = 251 bits (641), Expect = 6e-80, Method: Compositional matrix adjust.

| Identities | | = 129/254 (51%), Positives = 168/254 (66%), Gaps = 2/254 (1%) |  |
| --- | --- | --- | --- |
| Query 12  Sbjct 4 | | PAAWVERLFGYDWAQQTIGCSDAAVFRLSAQGRPVLFVKTDLSGALNELQDEAARLSWLA P W +R + QQTIG S A VFRL+ G LFVK++ + EL E RL WLA PQTWRQRFADTRYEQQTIGESRADVFRLTPAGSAPLFVKSEPRFSFAELPGEVQRLRWLA | 71  63 |
| Query 72  Sbjct 64 | | TTGVPCAAVLDVVTEAGRDWLLLGEVPGQDLLSS-HLAPAEKVSIMADAMRRLHTLDPAT  +P A VLD V E+ R WLL+ VPG+DL SS HL P + VS+ A+A+R LH ++ EQDLPAARVLDSVEESQRFWLLMTAVPGRDLASSEHLTPQQIVSLAANALRTLHAINIED | 130  123 |
| Query 131  Sbjct 124 | | CPFDHQAKHRIERARTRMEAGLVDQDDLDEEHQGLAPAELFARLKARMPDGEDLVVTHGD CPFDH+ +I AR + AGL+D++D D+ G + LF ++ A PD EDLVVTHGD CPFDHRLDAKIALAREHLLAGLIDEEDFDDSRLGQSAEVLFEQMLAARPDSEDLVVTHGD | 190  183 |
| Query 191  Sbjct 184 | | ACLPNIMVENGRFSGFIDCGRLGVADRYQDIALATRDIAEELGGEWADRFLVLYGIAAPD ACLPN++V++GRFSGF+DCGRLGV+DR+QD+AL IA LG +W F YG+ PD ACLPNLLVDHGRFSGFVDCGRLGVSDRFQDLALTAHSIAYNLGEQWVPAFFEAYGV-QPD | 250  242 |
| Query | 251 SQRIAFYRLLDEFF 264 | | |
|  |  | ++IAFY+LLDEFF |  |
| Sbjct | 243 | LRKIAFYQLLDEFF 256 | |

>WP_146546610.1 aminoglycoside 3'-phosphotransferase [Reyranella sp. CPCC 100927] TWS97041.1 aminoglycoside 3'-phosphotransferase [Reyranella sp. CPCC 100927]

Length=264

Score = 251 bits (642), Expect = 6e-80, Method: Compositional matrix adjust.

| Identities | | = 137/256 (54%), Positives = 164/256 (64%), Gaps = 3/256 (1%) |  |
| --- | --- | --- | --- |
| Query 12  Sbjct 9 | | PAAWV-ERLFGYDWAQQTIGCSDAAVFRLSAQGRPVLFVKTDLSGALNELQDEAARLSWL P AW +RL + W+QQTIG S AAVFRL+A VLF KT+ EL EA RL WL PPAWRRQRLASHRWSQQTIGRSAAAVFRLTADDGTVLFAKTEPIDPFGELTGEAERLRWL | 70  68 |
| Query 71  Sbjct 69 | | ATTGVPCAAVLDVVTEAGRDWLLLGEVPGQDLLSS-HLAPAEKVSIMADAMRRLHTLDPA G+ C VL + A R WLL+ + G DL SS HL+P+ V++ A A+R LH LD A GAQGIACPRVLAADSTADRHWLLISALGGHDLASSPHLSPSAIVALTATALRGLHALDIA | 129  128 |
| Query 130  Sbjct 129 | | TCPFDHQAKHRIERARTRMEAGLVDQDDLDEEHQGLAPAELFARLKARMPDG-EDLVVTH CPFDH+ RI AR R+ A +VD D D+E +G +F L +R P EDLVVTH RCPFDHRLDRRIAMARERVTACIVDDSDFDDERRGRTATSVFEELMSRRPAAAEDLVVTH | 188  188 |
| Query 189  Sbjct 189 | | GDACLPNIMVENGRFSGFIDCGRLGVADRYQDIALATRDIAEELGGEWADRFLVLYGIAA GDACLPN+M + GRFSGFIDCGRLGVADRYQDIALA I LG W FL YG+ GDACLPNLMADAGRFSGFIDCGRLGVADRYQDIALACWSITYNLGAAWVAPFLQHYGLPD | 248  248 |
| Query 249 | | PDSQRIAFYRLLDEFF 264 D+ R+A+YRLLDEFF |  |
| Sbjct | 249 | ADADRLAYYRLLDEFF 264 | |

>WP_094806573.1 APH(3')-II family aminoglycoside O-phosphotransferase [Bordetella

genomosp. 2]

OZI78376.1 APH(3') family aminoglycoside O-phosphotransferase [Bordetella genomosp. 2]

Length=264

Score = 251 bits (642), Expect = 6e-80, Method: Compositional matrix adjust.

| Identities | = 146/254 (57%), Positives = 174/254 (69%), Gaps = 2/254 (1%) |  |
| --- | --- | --- |
| Query 12 | PAAWVERLFGYDWAQQTIGCSDAAVFRLSAQGRPVLFVKTDLSGALNELQDEAARLSWLA | 71 |
| Sbjct 12 | P W + L GY WA Q IG SDAAVFRL A GRP L+VKT+ +G L EL E RL WLA PPDWRDALAGYAWAAQDIGRSDAAVFRLEASGRPPLYVKTETAGRLAELPGEVLRLRWLA | 71 |
| Query 72 | TTGVPCAAVLDVVTEAGRDWLLLGEVPGQDLLSS-HLAPAEKVSIMADAMRRLHTLDPAT | 130 |
| Sbjct 72 | G+ C VL AGR WLL+ VPG+DL S+ L A V+I A A+R LH LDPA QQGIDCPRVLRTAQAAGRHWLLMSAVPGRDLASTPELPAARIVAIAAAALRALHGLDPAG | 131 |
| Query 131 | CPFDHQAKHRIERARTRMEAGLVDQDDLDEEHQGLAPAELFARLKARMPDGEDLVVTHGD | 190 |
| Sbjct 132 | CPFDH+ RIE AR R++AGLVD+ D D+E G + A+ +A L A P EDLVVTHGD CPFDHRRAVRIEHARARVQAGLVDESDFDDERMGQSAAQAYAALCAGQPAHEDLVVTHGD | 191 |
| Query 191 | ACLPNIMVENGRFSGFIDCGRLGVADRYQDIALATRDIAEELGGEWADRFLVLYGIAAPD | 250 |
| Sbjct 192 | ACLPN++ +GRFSGF+DCGRLGVADR+QD+ALA I LG +WA FL YG D ACLPNLLAVDGRFSGFVDCGRLGVADRHQDLALAAWSIEYNLGAQWAQPFLDAYG-GPVD | 250 |
| Query 251  Sbjct 251 | SQRIAFYRLLDEFF 264  ++R+AFYRLLDEFF  TERLAFYRLLDEFF 264 |  |

>WP_164262363.1 APH(3')-II family aminoglycoside O-phosphotransferase [Stenotrophomonas maltophilia]

Length=267

Score = 251 bits (642), Expect = 6e-80, Method: Compositional matrix adjust.

| Identities | = 134/255 (53%), Positives = 166/255 (65%), Gaps = 3/255 (1%) |  |
| --- | --- | --- |
| Query 12 | PAAWVERLFGYDWAQQTIGCSDAAVFRLSAQGRPVLFVKTDLSGALNELQDEAARLSWLA | 71 |
| Sbjct 14 | P AW + L +Q++G S A V R+ GRP FVK++L A +EL DE ARL WL PLAWQKALADARIERQSMGVSRADVARVQRPGRPDAFVKSELIDAFSELGDEIARLRWLQ | 73 |
| Query 72 | TTGVPCAAVLDVVTEAGRDWLLLGEVPGQDLLSS-HLAPAEKVSIMADAMRRLHTLDPAT | 130 |
| Sbjct 74 | G P V+ E GR WLL+ +PG+DL SS LAP ++V I+ADA+R LH L A AQGQPAPTVIATAEEGGRHWLLMSALPGRDLASSPELAPQQRVEILADALRGLHALPVAA | 133 |
| Query 131 | CPFDHQAKHRIERARTRMEAGLVDQDDLDEEHQGLAPAELFARLKARMPDGEDLVVTHGD | 190 |
| Sbjct 134 | CPFD + R+ A+ R+EAG VD DD D+E G +P ++FA+L + PD EDLVV+HGD CPFDQRLASRLPAAQARVEAGRVDADDFDDERLGQSPQQVFAQLCSTRPDHEDLVVSHGD | 193 |
| Query 191 | ACLPNIMVENGRFSGFIDCGRLGVADRYQDIALATRDIAEELG-GEWADRFLVLYGIAAP | 249 |
| Sbjct 194 | ACLPN+MV GRFSGFIDCGRLGVADRYQD+ALA R + G W YG A ACLPNLMVAEGRFSGFIDCGRLGVADRYQDLALAARSLVHNFGDTRWVTPLFQRYGAVA- | 252 |
| Query 250  Sbjct 253 | DSQRIAFYRLLDEFF 264 D +R+AFYRLLDEFF DERRLAFYRLLDEFF 267 |  |

>WP_097604739.1 aminoglycoside 3'-phosphotransferase [Rhizobium sp. M10]

PDT38599.1 APH(3') family aminoglycoside O-phosphotransferase [Rhizobium sp. M10]

Length=261

Score = 251 bits (641), Expect = 6e-80, Method: Compositional matrix adjust. Identities = 133/254 (52%), Positives = 169/254 (67%), Gaps = 1/254 (0%)

| Query | 12 | PAAWVERLFGYDWAQQTIGCSDAAVFRLSAQGRPVLFVKTDLSGALNELQDEAARLSWLA | 71 |
| --- | --- | --- | --- |
| Sbjct | 8 | PAA ERL GY + + +G S A+VFRL +G P L++K + +G EL DEAARL WL PAALGERLSGYRFERDALGRSAASVFRLEGEGLPALYLKVEAAGPFGELADEAARLGWLK | 67 |
| Query | 72 | TTGVPCAAVLDVVTEAGRDWLLLGEVPGQDLLS-SHLAPAEKVSIMADAMRRLHTLDPAT | 130 |
| Sbjct | 68 | G+PC V+ ++ RDWLL+G +PG DL S S L P +V ++A A+ LH L A+  AAGLPCPDVIARESDGERDWLLIGALPGTDLASASALTPLTRVELLAAALADLHHLPIAS | 127 |

| Query | 131 | CPFDHQAKHRIERARTRMEAGLVDQDDLDEEHQGLAPAELFARLKARMPDGEDLVVTHGD | 190 |
| --- | --- | --- | --- |
| Sbjct | 128 | CPFDH+ + R+ AR RM+AG+VD+ D DE G + LFA L+ R PD EDLVVTHGD CPFDHRLEKRLPTARARMQAGIVDETDFDETRLGKSAETLFAELEGRRPDREDLVVTHGD | 187 |
| Query | 191 | ACLPNIMVENGRFSGFIDCGRLGVADRYQDIALATRDIAEELGGEWADRFLVLYGIAAPD | 250 |
| Sbjct | 188 | ACLPN + G+FSG+IDC RLGVADRYQD+ALA R IA G FL YG+ APD ACLPNFVASGGQFSGYIDCSRLGVADRYQDLALACRSIAYNFGAALVPSFLDRYGLTAPD | 247 |
| Query | 251 | SQRIAFYRLLDEFF 264 R+ +Y+LLDEFF |  |
| Sbjct | 248 | PARLDYYQLLDEFF 261 | |

```
>EBN0119945.1 aminoglycoside 3'-phosphotransferase [Salmonella enterica subsp. enterica serovar Heidelberg]
Length=121

Score = 246 bits (628), Expect = 7e-80, Method: Compositional matrix adjust. Identities = 121/121 (100%), Positives = 121/121 (100%), Gaps = 0/121 (0%)

Query 144 ARTRMEAGLVDQDDLDEEHQGLAPAELFARLKARMPDGEDLVVTHGDACLPNIMVENGRF 203 ARTRMEAGLVDQDDLDEEHQGLAPAELFARLKARMPDGEDLVVTHGDACLPNIMVENGRF
Sbjct 1	ARTRMEAGLVDQDDLDEEHQGLAPAELFARLKARMPDGEDLVVTHGDACLPNIMVENGRF 60

Query 204 SGFIDCGRLGVADRYQDIALATRDIAEELGGEWADRFLVLYGIAAPDSQRIAFYRLLDEF 263 SGFIDCGRLGVADRYQDIALATRDIAEELGGEWADRFLVLYGIAAPDSQRIAFYRLLDEF
Sbjct 61	SGFIDCGRLGVADRYQDIALATRDIAEELGGEWADRFLVLYGIAAPDSQRIAFYRLLDEF 120

Query 264 F 264
F
Sbjct 121 F 121

>WP_124200570.1 APH(3')-II family aminoglycoside O-phosphotransferase [Pseudomonas aeruginosa]
RQB64105.1 APH(3') family aminoglycoside O-phosphotransferase [Pseudomonas aeruginosa]
Length=268

Score = 251 bits (642), Expect = 7e-80, Method: Compositional matrix adjust.
```

| Identities | | = 133/258 (52%), Positives = 165/258 (64%), Gaps = 5/258 (2%) |  |
| --- | --- | --- | --- |
| Query 11  Sbjct 12 | | SPAAWVERLFGYDWAQQTIGCSDAAVFRLSAQGRPVLFVKTDLSGALNELQDEAARLSWL  +P+ W + L GY W Q GCS A V RL A RP LFVK ++ A EL E ARL WL APSTWADYLAGYRWRGQGEGCSAATVHRLDAARRPTLFVKQEVLSAHAELPAEIARLRWL | 70  71 |
| Query 71  Sbjct 72 | | ATTGVPCAAVLDVVTEAGRDWLLLGEVPGQDLLS SHLAPAEKVSIMADAMRRLHTL  G+ C VL+ GR WLL+ VPG L + P V ++A A+RRLH L HGAGIDCPQVLNETQSDGRQWLLMSAVPGDTLSALAQRGEREPERLVRLVAAALRRLHDL | 126  131 |
| Query 127  Sbjct 132 | | DPATCPFDHQAKHRIERARTRMEAGLVDQDDLDEEHQGLAPAELFARLKARMPDGEDLVV DPA CPFDH+ + R+E R R+EAGLVD+ D D++++G + L+ L P EDLVV DPAACPFDHRLERRLETVRQRVEAGLVDEADFDDDYRGRSATALYRLLLEWRPAVEDLVV | 186  191 |
| Query 187  Sbjct 192 | | THGDACLPNIMVENGRFSGFIDCGRLGVADRYQDIALATRDIAEELGGEWADRFLVLYGI HGDACLPN++ E RFSGFIDCGRLGVADR+QD+ALA RDI ELG WA+ FLV YG AHGDACLPNLLAEGRRFSGFIDCGRLGVADRHQDLALAARDIEAELGAAWAEAFLVEYG- | 246  250 |
| Query | 247 AAPDSQRIAFYRLLDEFF 264 | | |
|  |  | D +R+A++RLLDEF |  |
| Sbjct | 251 | GDIDGERLAYFRLLDEFL 268 | |

>WP_007913110.1 MULTISPECIES: aminoglycoside 3'-phosphotransferase [unclassified Pseudomonas]

EJM03772.1 aminoglycoside phosphotransferase [Pseudomonas sp. GM16] EJM34858.1 aminoglycoside phosphotransferase [Pseudomonas sp. GM24]

Length=256

Score = 251 bits (641), Expect = 7e-80, Method: Compositional matrix adjust. Identities = 130/254 (51%), Positives = 167/254 (66%), Gaps = 2/254 (1%)

| Query | 12 | PAAWVERLFGYDWAQQTIGCSDAAVFRLSAQGRPVLFVKTDLSGALNELQDEAARLSWLA | 71 |
| --- | --- | --- | --- |
| Sbjct | 4 | PAAW + G QQTIG S A V+R++ G FVKT+ EL E RL W+A PAAWRSQFAGAAIEQQTIGESRADVWRITPAGAAPQFVKTEQVMTWGELPGEVQRLRWMA | 63 |
| Query | 72 | TTGVPCAAVLDVVTEAGRDWLLLGEVPGQDLLSS-HLAPAEKVSIMADAMRRLHTLDPAT | 130 |
| Sbjct | 64 | T +P VLD T+A +WLL+ V G+DL SS HL+P + V++ A A+R LH + + TQDLPAPRVLDTTTQAQHNWLLMTAVAGRDLASSEHLSPQQIVTLAAQALRALHAVAIDS | 123 |
| Query | 131 | CPFDHQAKHRIERARTRMEAGLVDQDDLDEEHQGLAPAELFARLKARMPDGEDLVVTHGD | 190 |
| Sbjct | 124 | CPFDH + +I RAR + AGL+D++D D+ G + E+F +L A P EDLVVTHGD CPFDHCLEQKITRAREHLLAGLIDEEDFDDSRLGQSAEEVFEQLLATRPAHEDLVVTHGD | 183 |
| Query | 191 | ACLPNIMVENGRFSGFIDCGRLGVADRYQDIALATRDIAEELGGEWADRFLVLYGIAAPD | 250 |
| Sbjct | 184 | ACLPN++ NGRFSGF+DCGRLGVADRYQD+ALA I + LG +W F +YG+ PD ACLPNLLAANGRFSGFVDCGRLGVADRYQDLALAAHSITDNLGEQWLPLFFEVYGV-EPD | 242 |
| Query | 251 | SQRIAFYRLLDEFF 264 QRIAFY+LLDEFF |  |
| Sbjct | 243 | QQRIAFYQLLDEFF 256 | |

>VUO05133.1 Aminoglycoside 3'-phosphotransferase [Stenotrophomonas maltophilia] Length=267

Score = 251 bits (642), Expect = 7e-80, Method: Compositional matrix adjust.

| Identities | = 134/255 (53%), Positives = 167/255 (65%), Gaps = 3/255 (1%) |  |
| --- | --- | --- |
| Query 12 | PAAWVERLFGYDWAQQTIGCSDAAVFRLSAQGRPVLFVKTDLSGALNELQDEAARLSWLA | 71 |
| Sbjct 14 | P AW + L +Q++G S A V R+ GRP F+K++L A +EL DE ARL WL PLAWQKALADARIERQSMGVSRADVARVLRPGRPDAFLKSELIDAFSELGDEIARLRWLQ | 73 |
| Query 72 | TTGVPCAAVLDVVTEAGRDWLLLGEVPGQDLLSS-HLAPAEKVSIMADAMRRLHTLDPAT | 130 |
| Sbjct 74 | G P V+ EAGR WLL+ +PG+DL SS LAP ++V I+ADA+R LH L A AQGQPAPTVIATAEEAGRHWLLMSALPGRDLASSPELAPQQRVEILADALRGLHALPVAA | 133 |
| Query 131 | CPFDHQAKHRIERARTRMEAGLVDQDDLDEEHQGLAPAELFARLKARMPDGEDLVVTHGD | 190 |
| Sbjct 134 | CPFD + R+ A+ R+EAG VD DD D+E G +P ++FA+L + PD EDLVV+HGD CPFDQRLASRLPAAQARVEAGRVDADDFDDERLGQSPQQVFAQLCSNRPDHEDLVVSHGD | 193 |
| Query 191 | ACLPNIMVENGRFSGFIDCGRLGVADRYQDIALATRDIAEELG-GEWADRFLVLYGIAAP | 249 |
| Sbjct 194 | ACLPN+MV GRFSGFIDCGRLGVADRYQD+ALA R + G W YG A ACLPNLMVAEGRFSGFIDCGRLGVADRYQDLALAARSLVHNFGDTRWVTPLFQRYGAVA- | 252 |
| Query 250  Sbjct 253 | DSQRIAFYRLLDEFF 264 D +R+AFYRLLDEFF DERRLAFYRLLDEFF 267 |  |

>WP_143355935.1 aminoglycoside 3'-phosphotransferase, partial [Escherichia coli] RRB98047.1 aminoglycoside 3'-phosphotransferase, partial [Escherichia coli]

Length=129

Score = 246 bits (628), Expect = 8e-80, Method: Compositional matrix adjust.

| Identities | = 121/129 (94%), Positives = 121/129 (94%), Gaps = 0/129 (0%) |  |
| --- | --- | --- |
| Query 116 | MADAMRRLHTLDPATCPFDHQAKHRIERARTRMEAGLVDQDDLDEEHQGLAPAELFARLK | 175 |
| Sbjct 1 | MAD MR HTLD A C FDHQ KHRIERART MEAGLVDQDDLDEEHQGLAPAELFARLK MADKMRWQHTLDTANCQFDHQEKHRIERARTGMEAGLVDQDDLDEEHQGLAPAELFARLK | 60 |
| Query 176 | ARMPDGEDLVVTHGDACLPNIMVENGRFSGFIDCGRLGVADRYQDIALATRDIAEELGGE | 235 |
| Sbjct 61 | ARMPDGEDLVVTHGDACLPNIMVENGRFSGFIDCGRLGVADRYQDIALATRDIAEELGGE ARMPDGEDLVVTHGDACLPNIMVENGRFSGFIDCGRLGVADRYQDIALATRDIAEELGGE | 120 |
| Query 236  Sbjct 121 | WADRFLVLY 244 WADRFLVLY WADRFLVLY 129 |  |

>WP_064684283.1 aminoglycoside 3'-phosphotransferase [Rhizobium bangladeshense] Length=261

Score = 251 bits (641), Expect = 8e-80, Method: Compositional matrix adjust.

| Identities | | = 131/254 (52%), Positives = 170/254 (67%), Gaps = 1/254 (0%) |  |
| --- | --- | --- | --- |
| Query 12  Sbjct 8 | | PAAWVERLFGYDWAQQTIGCSDAAVFRLSAQGRPVLFVKTDLSGALNELQDEAARLSWLA PAA+ ERL GY + + +G S +VFRL +G P L++K + +G EL DEAARL WL PAAFAERLSGYRFERDALGRSATSVFRLEGEGLPALYLKVEAAGPFGELADEAARLGWLK | 71  67 |
| Query 72  Sbjct 68 | | TTGVPCAAVLDVVTEAGRDWLLLGEVPGQDLLS-SHLAPAEKVSIMADAMRRLHTLDPAT G+PC V+ ++ R+WLL+G +PG DL S S L P +V ++A A+ LH L A+ AAGLPCPDVIARESDGERNWLLIGALPGTDLASASALTPLTRVELLAAALADLHHLPIAS | 130  127 |
| Query 131  Sbjct 128 | | CPFDHQAKHRIERARTRMEAGLVDQDDLDEEHQGLAPAELFARLKARMPDGEDLVVTHGD CPFDH+ + R+ AR RM+AG+VD+ D DE G + LFA L+ R PD EDLVVTHGD CPFDHRLEKRLPTARARMQAGIVDETDFDETRLGKSAETLFAELEGRRPDREDLVVTHGD | 190  187 |
| Query 191  Sbjct 188 | | ACLPNIMVENGRFSGFIDCGRLGVADRYQDIALATRDIAEELGGEWADRFLVLYGIAAPD ACLPN + +G+FSG+IDC RLGVADRYQD+ALA R IA G FL YG+ APD ACLPNFVASDGQFSGYIDCSRLGVADRYQDLALACRSIAYNFGAALVQPFLDRYGLTAPD | 250  247 |
| Query 251 | | SQRIAFYRLLDEFF 264 R+ +Y+LLDEFF |  |
| Sbjct | 248 | PARLDYYQLLDEFF 261 | |

>WP_058134659.1 APH(3')-II family aminoglycoside O-phosphotransferase [Pseudomonas aeruginosa]

KSS22734.1 aminoglycoside 3'-phosphotransferase [Pseudomonas aeruginosa] OPF28091.1 aminoglycoside 3-N-acetyltransferase [Pseudomonas aeruginosa

P47]

OPF29072.1 aminoglycoside 3-N-acetyltransferase [Pseudomonas aeruginosa P37]

OPF29731.1 aminoglycoside 3-N-acetyltransferase [Pseudomonas aeruginosa SD9]

OPF44803.1 aminoglycoside 3-N-acetyltransferase [Pseudomonas aeruginosa P49]

Length=268

Score = 251 bits (641), Expect = 9e-80, Method: Compositional matrix adjust.

| Identities | | = 135/258 (52%), Positives = 167/258 (65%), Gaps = 5/258 (2%) |  |
| --- | --- | --- | --- |
| Query 11  Sbjct 12 | | SPAAWVERLFGYDWAQQTIGCSDAAVFRLSAQGRPVLFVKTDLSGALNELQDEAARLSWL  +P+ W + L GY W Q GCS A V RL A RP LFVK ++ EL E ARL WL APSTWADYLAGYRWRGQGEGCSAATVHRLEAARRPTLFVKQEVLSVHAELPAEIARLRWL | 70  71 |
| Query 71  Sbjct 72 | | ATTGVPCAAVLDVVTEAGRDWLLLGEVPGQDLLS SHLAPAEKVSIMADAMRRLHTL  G+ C VL+ GR WLL+ VPG L + L P V ++A A+RRLH L HGAGIDCPQVLNETQSDGRQWLLMSAVPGDTLSALAQRGELEPERLVRLVAAALRRLHDL | 126  131 |
| Query 127  Sbjct 132 | | DPATCPFDHQAKHRIERARTRMEAGLVDQDDLDEEHQGLAPAELFARLKARMPDGEDLVV DPA CPFDH+ + R++ R R+EAGLVD+ D D++H+G + EL+ L R EDLVV DPAACPFDHRLERRLDTVRQRVEAGLVDEADFDDDHRGRSATELYRLLLDRRLAVEDLVV | 186  191 |
| Query 187  Sbjct 192 | | THGDACLPNIMVENGRFSGFIDCGRLGVADRYQDIALATRDIAEELGGEWADRFLVLYGI HGDACLPN++ E RFSGFIDCGRLGVADR+QD+ALA RDI ELG WA+ FLV YG AHGDACLPNLLAEGRRFSGFIDCGRLGVADRHQDLALAARDIEAELGAAWAEAFLVEYG- | 246  250 |
| Query | 247 AAPDSQRIAFYRLLDEFF 264 | | |
|  |  | D +R+A++RLLDEFF |  |
| Sbjct | 251 | GDIDGERLAYFRLLDEFF 268 | |

```
>WP_061194935.1 APH(3')-II family aminoglycoside O-phosphotransferase [Pseudomonas aeruginosa]
KXC50509.1 aminoglycoside phosphotransferase [Pseudomonas aeruginosa] Length=268

Score = 251 bits (641), Expect = 9e-80, Method: Compositional matrix adjust. Identities = 135/258 (52%), Positives = 167/258 (65%), Gaps = 5/258 (2%)

Query 11	SPAAWVERLFGYDWAQQTIGCSDAAVFRLSAQGRPVLFVKTDLSGALNELQDEAARLSWL 70
+P+ W + L GY W Q GCS A V RL	RP LFVK ++ A EL E ARL WL Sbjct 12	APSTWADYLAGYRWRGQGEGCSAATVHRLEVARRPTLFVKQEVLSAHAELPAEIARLRWL 71
```

| Query | 71 | ATTGVPCAAVLDVVTEAGRDWLLLGEVPGQDLLS SHLAPAEKVSIMADAMRRLHTL | 126 |
| --- | --- | --- | --- |
| Sbjct | 72 | G+ C VL+ GR WLL+ VPG L + L P V ++A A+RRLH L HGAGIDCPQVLNETQSDGRQWLLMSAVPGDTLSALAQRGELEPERLVRLVAAALRRLHDL | 131 |
| Query | 127 | DPATCPFDHQAKHRIERARTRMEAGLVDQDDLDEEHQGLAPAELFARLKARMPDGEDLVV | 186 |
| Sbjct | 132 | DPA CPFDH+ + R++ R R+EAGLVD+ D D++H+G + EL+ L R EDLVV DPAACPFDHRLERRLDTVRQRVEAGLVDEADFDDDHRGRSATELYRLLLDRRLAVEDLVV | 191 |
| Query | 187 | THGDACLPNIMVENGRFSGFIDCGRLGVADRYQDIALATRDIAEELGGEWADRFLVLYGI | 246 |
| Sbjct | 192 | HGDACLPN++ E RFSGFIDCGRLGVADR+QD+ALA RDI ELG WA+ FLV YG  AHGDACLPNLLAEGRRFSGFIDCGRLGVADRHQDLALAARDIEAELGAAWAEAFLVEYG- | 250 |
| Query | 247 AAPDSQRIAFYRLLDEFF 264 | | |
|  |  | D +R+A++RLLDEFF |  |
| Sbjct | 251 | GDIDGERLAYFRLLDEFF 268 | |

>WP_096477536.1 aminoglycoside 3'-phosphotransferase [Rhizobium phaseoli]

PCD67941.1 APH(3') family aminoglycoside O-phosphotransferase [Rhizobium phaseoli]

Length=261

Score = 251 bits (640), Expect = 9e-80, Method: Compositional matrix adjust.

| Identities | | = 126/254 (50%), Positives = 167/254 (66%), Gaps = 1/254 (0%) |  |
| --- | --- | --- | --- |
| Query 12  Sbjct 8 | | PAAWVERLFGYDWAQQTIGCSDAAVFRLSAQGRPVLFVKTDLSGALNELQDEAARLSWLA P A ERL GY + + +G S A+VFRL +G P L++K + +G EL DE ARLSWL PTALGERLSGYRFERDALGRSAASVFRLEGEGLPALYLKVEAAGPFGELADETARLSWLK | 71  67 |
| Query 72  Sbjct 68 | | TTGVPCAAVLDVVTEAGRDWLLLGEVPGQDLLS-SHLAPAEKVSIMADAMRRLHTLDPAT G+PC V+ ++ RDWLL+G +PG DL S S L P +V ++A A+ LH L A+ AAGLPCPEVIARESDGERDWLLIGALPGTDLASASALTPLTRVELLAAALSDLHDLPIAS | 130  127 |
| Query 131  Sbjct 128 | | CPFDHQAKHRIERARTRMEAGLVDQDDLDEEHQGLAPAELFARLKARMPDGEDLVVTHGD CPFDH+ ++R+ A+ RM+AG+VD++D D G + A LF L P E+LVVTHGD CPFDHRLENRLPTAKARMQAGIVDEEDFDAARLGKSAAALFIELMRLRPGDEELVVTHGD | 190  187 |
| Query 191  Sbjct 188 | | ACLPNIMVENGRFSGFIDCGRLGVADRYQDIALATRDIAEELGGEWADRFLVLYGIAAPD ACLPN + NG+FSG+IDC RLGVADR+QDIALA R IA G FL YG+ APD ACLPNFVASNGQFSGYIDCSRLGVADRHQDIALACRSIASNFGEALIRPFLDRYGLTAPD | 250  247 |
| Query 251 | | SQRIAFYRLLDEFF 264  ++ +++LLDEFF |  |
| Sbjct | 248 | PAKLHYFQLLDEFF 261 | |

>WP_072168935.1 APH(3')-II family aminoglycoside O-phosphotransferase [Stenotrophomonas maltophilia]

OWQ72850.1 APH(3')-II family aminoglycoside O-phosphotransferase [Stenotrophomonas maltophilia]

PZT36182.1 APH(3') family aminoglycoside O-phosphotransferase [Stenotrophomonas maltophilia]

CRX70206.1 unnamed protein product [Stenotrophomonas maltophilia]

VUR18166.1 Aminoglycoside 3'-phosphotransferase [Stenotrophomonas maltophilia] Length=267

Score = 251 bits (641), Expect = 9e-80, Method: Compositional matrix adjust.

| Identities | = 134/255 (53%), Positives = 167/255 (65%), Gaps = 3/255 (1%) |  |
| --- | --- | --- |
| Query 12 | PAAWVERLFGYDWAQQTIGCSDAAVFRLSAQGRPVLFVKTDLSGALNELQDEAARLSWLA | 71 |
| Sbjct 14 | P AW + L +Q++G S A V R+ GRP F+K++L A +EL DE ARL WL PLAWQKALADARIERQSMGVSRADVARVLRPGRPDAFLKSELIDAFSELGDEIARLRWLQ | 73 |
| Query 72 | TTGVPCAAVLDVVTEAGRDWLLLGEVPGQDLLSS-HLAPAEKVSIMADAMRRLHTLDPAT | 130 |
| Sbjct 74 | G P V+ EAGR WLL+ +PG+DL SS LAP ++V I+ADA+R LH L A AQGQPAPTVIATAEEAGRHWLLMSALPGRDLASSPELAPQQRVEILADALRGLHALPVAA | 133 |
| Query 131 | CPFDHQAKHRIERARTRMEAGLVDQDDLDEEHQGLAPAELFARLKARMPDGEDLVVTHGD | 190 |
| Sbjct 134 | CPFD + R+ A+ R+EAG VD DD D+E G +P ++FA+L + PD EDLVV+HGD  CPFDQRLASRLPAAQARVEAGRVDADDFDDERLGQSPQQVFAQLCSTRPDHEDLVVSHGD | 193 |

| Query | 191 | ACLPNIMVENGRFSGFIDCGRLGVADRYQDIALATRDIAEELG-GEWADRFLVLYGIAAP 249 |
| --- | --- | --- |
| Sbjct | 194 | ACLPN+MV GRFSGFIDCGRLGVADRYQD+ALA R + G W YG A ACLPNLMVSEGRFSGFIDCGRLGVADRYQDLALAARSLVHNFGDTRWVTPLFQRYGAVA- 252 |
| Query | 250 | DSQRIAFYRLLDEFF 264 |
| Sbjct | 253 | D +R+AFYRLLDEFF  DERRLAFYRLLDEFF 267 |

>WP_081279126.1 aminoglycoside 3'-phosphotransferase [Rhizobium phaseoli] ANL47726.1 aminoglycoside 3'-phosphotransferase protein [Rhizobium phaseoli]

Length=261

Score = 251 bits (640), Expect = 9e-80, Method: Compositional matrix adjust.

| Identities | | = 126/254 (50%), Positives = 169/254 (67%), Gaps = 1/254 (0%) |  |
| --- | --- | --- | --- |
| Query 12  Sbjct 8 | | PAAWVERLFGYDWAQQTIGCSDAAVFRLSAQGRPVLFVKTDLSGALNELQDEAARLSWLA PAA ERL GY + + +G S A+VFRL +G P L++K + +G EL DEAARLSWL PAALGERLSGYRFERDALGRSGASVFRLQGEGLPALYLKVEAAGPFGELADEAARLSWLK | 71  67 |
| Query 72  Sbjct 68 | | TTGVPCAAVLDVVTEAGRDWLLLGEVPGQDLLS-SHLAPAEKVSIMADAMRRLHTLDPAT G+PC V+ ++ R+WLL+ +PG DL+S S L P +V ++A A+ LH L A+ AVGLPCPDVIARESDGERNWLLINALPGADLVSASALTPLTRVELLAAALSDLHDLPVAS | 130  127 |
| Query 131  Sbjct 128 | | CPFDHQAKHRIERARTRMEAGLVDQDDLDEEHQGLAPAELFARLKARMPDGEDLVVTHGD CP+DH+ ++R+ A+ RM+AG+VD++D D G + A LF L P EDLVVTHGD CPYDHRLENRLPTAKARMQAGIVDEEDFDAARLGKSAAALFDDLMRLRPGKEDLVVTHGD | 190  187 |
| Query 191  Sbjct 188 | | ACLPNIMVENGRFSGFIDCGRLGVADRYQDIALATRDIAEELGGEWADRFLVLYGIAAPD ACLPN + NG+FSG+IDC RLGVADR+QDIALA R IA G FL YG+ APD ACLPNFVASNGQFSGYIDCSRLGVADRHQDIALACRSIASNFGEALIRPFLDRYGLTAPD | 250  247 |
| Query 251 | | SQRIAFYRLLDEFF 264  ++ +++LLDEFF |  |
| Sbjct | 248 | PAKLHYFQLLDEFF 261 | |

>WP_111129029.1 MULTISPECIES: APH(3')-II family aminoglycoside O-phosphotransferase [Stenotrophomonas]

AYA90518.1 APH(3')-II family aminoglycoside O-phosphotransferase [Stenotrophomonas sp. Pemsol]

PZS93407.1 APH(3') family aminoglycoside O-phosphotransferase [Stenotrophomonas maltophilia]

PZT15462.1 APH(3') family aminoglycoside O-phosphotransferase [Stenotrophomonas maltophilia]

PZT38565.1 APH(3') family aminoglycoside O-phosphotransferase [Stenotrophomonas maltophilia]

Length=267

Score = 251 bits (641), Expect = 1e-79, Method: Compositional matrix adjust.

| Identities | = 134/255 (53%), Positives = 167/255 (65%), Gaps = 3/255 (1%) |  |
| --- | --- | --- |
| Query 12 | PAAWVERLFGYDWAQQTIGCSDAAVFRLSAQGRPVLFVKTDLSGALNELQDEAARLSWLA | 71 |
| Sbjct 14 | P AW + L +Q++G S A V R+ GRP F+K++L A +EL DE ARL WL PLAWQKALADARIERQSMGVSRADVARVLRPGRPDAFLKSELIDAFSELGDEIARLRWLQ | 73 |
| Query 72 | TTGVPCAAVLDVVTEAGRDWLLLGEVPGQDLLSS-HLAPAEKVSIMADAMRRLHTLDPAT | 130 |
| Sbjct 74 | G P V+ EAGR WLL+ +PG+DL SS LAP ++V I+ADA+R LH L A AQGQPAPTVIATAEEAGRHWLLMSALPGRDLASSAELAPQQRVEILADALRGLHALPVAA | 133 |
| Query 131 | CPFDHQAKHRIERARTRMEAGLVDQDDLDEEHQGLAPAELFARLKARMPDGEDLVVTHGD | 190 |
| Sbjct 134 | CPFD + R+ A+ R+EAG VD DD D+E G +P ++FA+L + PD EDLVV+HGD CPFDQRLASRLPAAQARVEAGRVDADDFDDERLGQSPQQVFAQLCSTRPDHEDLVVSHGD | 193 |
| Query 191 | ACLPNIMVENGRFSGFIDCGRLGVADRYQDIALATRDIAEELG-GEWADRFLVLYGIAAP | 249 |
| Sbjct 194 | ACLPN+MV GRFSGFIDCGRLGVADRYQD+ALA R + G W YG A ACLPNLMVAEGRFSGFIDCGRLGVADRYQDLALAARSLVHNFGDTRWVTPLFQRYGAVA- | 252 |
| Query 250  Sbjct 253 | DSQRIAFYRLLDEFF 264 D +R+AFYRLLDEFF DERRLAFYRLLDEFF 267 |  |

>WP_095377702.1 APH(3')-II family aminoglycoside O-phosphotransferase [Stenotrophomonas maltophilia]

PAM72276.1 APH(3') family aminoglycoside O-phosphotransferase [Stenotrophomonas maltophilia]

PSD18959.1 APH(3')-II family aminoglycoside O-phosphotransferase [Stenotrophomonas maltophilia]

PZT24000.1 APH(3') family aminoglycoside O-phosphotransferase [Stenotrophomonas maltophilia]

VUQ38991.1 Aminoglycoside 3'-phosphotransferase [Stenotrophomonas maltophilia] Length=267

Score = 251 bits (641), Expect = 1e-79, Method: Compositional matrix adjust.

| Identities | = 134/255 (53%), Positives = 167/255 (65%), Gaps = 3/255 (1%) |  |
| --- | --- | --- |
| Query 12 | PAAWVERLFGYDWAQQTIGCSDAAVFRLSAQGRPVLFVKTDLSGALNELQDEAARLSWLA | 71 |
| Sbjct 14 | P AW + L +Q++G S A V R+ GRP F+K++L A +EL DE ARL WL PLAWQKALADARIERQSMGVSRADVARVLRPGRPDAFLKSELIDAFSELGDEIARLRWLQ | 73 |
| Query 72 | TTGVPCAAVLDVVTEAGRDWLLLGEVPGQDLLSS-HLAPAEKVSIMADAMRRLHTLDPAT | 130 |
| Sbjct 74 | G P V+ EAGR WLL+ +PG+DL SS LAP ++V I+ADA+R LH L A AQGQPAPTVIATAEEAGRHWLLMSALPGRDLASSPELAPQQRVEILADALRGLHALPVAA | 133 |
| Query 131 | CPFDHQAKHRIERARTRMEAGLVDQDDLDEEHQGLAPAELFARLKARMPDGEDLVVTHGD | 190 |
| Sbjct 134 | CPFD + R+ A+ R+EAG VD DD D+E G +P ++FA+L + PD EDLVV+HGD CPFDQRLASRLPAAQARVEAGRVDADDFDDERLGQSPQQVFAQLCSTRPDHEDLVVSHGD | 193 |
| Query 191 | ACLPNIMVENGRFSGFIDCGRLGVADRYQDIALATRDIAEELG-GEWADRFLVLYGIAAP | 249 |
| Sbjct 194 | ACLPN+MV GRFSGFIDCGRLGVADRYQD+ALA R + G W YG A ACLPNLMVAEGRFSGFIDCGRLGVADRYQDLALAARSLVHNFGDTRWVTPLFQRYGAVA- | 252 |
| Query 250  Sbjct 253 | DSQRIAFYRLLDEFF 264 D +R+AFYRLLDEFF DERRLAFYRLLDEFF 267 |  |

>WP_155029427.1 APH(3')-II family aminoglycoside O-phosphotransferase [Stenotrophomonas maltophilia]

QGL88510.1 APH(3')-II family aminoglycoside O-phosphotransferase [Stenotrophomonas maltophilia]

Length=267

Score = 251 bits (640), Expect = 1e-79, Method: Compositional matrix adjust.

| Identities | = 134/255 (53%), Positives = 166/255 (65%), Gaps = 3/255 (1%) |  |
| --- | --- | --- |
| Query 12 | PAAWVERLFGYDWAQQTIGCSDAAVFRLSAQGRPVLFVKTDLSGALNELQDEAARLSWLA | 71 |
| Sbjct 14 | P AW E L +Q+IG S A V R+ G+P FVK+++ A +EL DE RL WL PRAWQEALADARIERQSIGVSRADVARVQQPGQPDAFVKSEVIDAFSELGDEITRLRWLQ | 73 |
| Query 72 | TTGVPCAAVLDVVTEAGRDWLLLGEVPGQDLLSS-HLAPAEKVSIMADAMRRLHTLDPAT | 130 |
| Sbjct 74 | G+P V+ EAGR WLL+ +PG+DL SS LAP + V ++ADA+RRLH L A AQGLPAPTVIATAEEAGRRWLLMSALPGRDLASSPELAPQQLVELLADALRRLHVLPVAA | 133 |
| Query 131 | CPFDHQAKHRIERARTRMEAGLVDQDDLDEEHQGLAPAELFARLKARMPDGEDLVVTHGD | 190 |
| Sbjct 134 | CPFD + R+E A+ R+EA LVD DD D+E G +P ++FA L + PD EDLVV+HGD CPFDQRLASRLEAAQARVEASLVDADDFDDERLGQSPQQVFAELCSTRPDHEDLVVSHGD | 193 |
| Query 191 | ACLPNIMVENGRFSGFIDCGRLGVADRYQDIALATRDIAEELG-GEWADRFLVLYGIAAP | 249 |
| Sbjct 194 | ACLPN+M GRFSGFIDCGRLGVADRYQD+ALA R + G W YG A ACLPNLMAVEGRFSGFIDCGRLGVADRYQDLALAARSLVHNFGDTRWVAPLFQRYG-ALV | 252 |
| Query 250  Sbjct 253 | DSQRIAFYRLLDEFF 264 D +R+AFYRLLDEFF DERRLAFYRLLDEFF 267 |  |

>WP_065177500.1 MULTISPECIES: APH(3')-II family aminoglycoside O-phosphotransferase [Stenotrophomonas]

CRR49904.1 Aminoglycoside 3'-phosphotransferase [Pseudomonas aeruginosa]

OBU55074.1 APH(3') family aminoglycoside O-phosphotransferase [Stenotrophomonas maltophilia]

OBU59237.1 APH(3') family aminoglycoside O-phosphotransferase [Stenotrophomonas

maltophilia]

PSD25160.1 APH(3')-II family aminoglycoside O-phosphotransferase [Stenotrophomonas maltophilia]

QCZ97052.1 APH(3')-II family aminoglycoside O-phosphotransferase [Stenotrophomonas sp. pho]

Length=267

Score = 251 bits (640), Expect = 1e-79, Method: Compositional matrix adjust.

| Identities | = 134/255 (53%), Positives = 167/255 (65%), Gaps = 3/255 (1%) |  |
| --- | --- | --- |
| Query 12 | PAAWVERLFGYDWAQQTIGCSDAAVFRLSAQGRPVLFVKTDLSGALNELQDEAARLSWLA | 71 |
| Sbjct 14 | P AW + L +Q++G S A V R+ GRP F+K++L A +EL DE ARL WL PLAWQKALADARIERQSMGVSRADVARVLRPGRPDAFLKSELIDAFSELGDEIARLRWLQ | 73 |
| Query 72 | TTGVPCAAVLDVVTEAGRDWLLLGEVPGQDLLSS-HLAPAEKVSIMADAMRRLHTLDPAT | 130 |
| Sbjct 74 | G P V+ EAGR WLL+ +PG+DL SS LAP ++V I+ADA+R LH L A AQGQPAPTVIATAEEAGRHWLLMSALPGRDLASSPELAPQQRVEILADALRGLHALPVAA | 133 |
| Query 131 | CPFDHQAKHRIERARTRMEAGLVDQDDLDEEHQGLAPAELFARLKARMPDGEDLVVTHGD | 190 |
| Sbjct 134 | CPFD + R+ A+ R+EAG VD DD D+E G +P ++FA+L + PD EDLVV+HGD CPFDQRLASRLPAAQARVEAGRVDADDFDDERLGQSPQQVFAQLCSTRPDHEDLVVSHGD | 193 |
| Query 191 | ACLPNIMVENGRFSGFIDCGRLGVADRYQDIALATRDIAEELG-GEWADRFLVLYGIAAP | 249 |
| Sbjct 194 | ACLPN+MV GRFSGFIDCGRLGVADRYQD+ALA R + G W YG A ACLPNLMVAEGRFSGFIDCGRLGVADRYQDLALAARSLVHNFGDTRWVTPLFQRYGAVA- | 252 |
| Query 250  Sbjct 253 | DSQRIAFYRLLDEFF 264 D +R+AFYRLLDEFF DERRLAFYRLLDEFF 267 |  |

>CAB3817415.1 Aminoglycoside 3'-phosphotransferase [Achromobacter animicus] Length=266

Score = 251 bits (640), Expect = 1e-79, Method: Compositional matrix adjust.

| Identities | | = 128/264 (48%), Positives = 165/264 (63%), Gaps = 1/264 (0%) |  |
| --- | --- | --- | --- |
| Query 1  Sbjct 4 | | MIEQDGLHAGSPAAWVERLFGYDWAQQTIGCSDAAVFRLSAQGRPVLFVKTDLSGALNEL M Q +H P W RL G QQ+IG S A V+R+ + +P ++K++ +G L EL MHAQYSIHDSLPLQWQPRLAGARLEQQSIGESRADVYRIRSANQPDRYLKSEHTGTLPEL | 60  63 |
| Query 61  Sbjct 64 | | QDEAARLSWLATTGVPCAAVLDVVTEAGRDWLLLGEVPGQDLLSSHLAPAEKVSIMADAM DE RL W+A G+PC V+D R WLL+ V GQDL S+ L PA+ ++IMADA+ PDEIERLQWMAAHGLPCPTVIDAAATPTRHWLLMSAVAGQDLASAGLPPAQVIAIMADAL | 120  123 |
| Query 121  Sbjct 124 | | RRLHTLDPATCPFDHQAKHRIERARTRMEAGLVDQDDLDEEHQGLAPAELFARLKARMPD RLH + CPFDH + RI AR ++AGLVD+ D D + G + FA L A P HRLHCIPIDQCPFDHTLERRIAEARRALDAGLVDESDFDYDRIGRTAEDAFAELLATRPT | 180  183 |
| Query 181  Sbjct 184 | | GEDLVVTHGDACLPNIMVENGRFSGFIDCGRLGVADRYQDIALATRDIAEELGGEWADRF DLVV HGDACLPN M + G F+G+IDCGRLGV+DR+QD+ALA R I LG EW + F THDLVVAHGDACLPNFMADGGVFTGYIDCGRLGVSDRHQDLALAARSIERNLGREWIEPF | 240  243 |
| Query 241 | | LVLYGIAAPDSQRIAFYRLLDEFF 264 YG+ PD +R+AFY LLDEFF |  |
| Sbjct | 244 | FRSYGV-EPDDRRLAFYCLLDEFF 266 | |

>WP_027030648.1 aminoglycoside 3'-phosphotransferase [Mesorhizobium loti] QKD02267.1 aminoglycoside 3'-phosphotransferase [Mesorhizobium loti R88b]

Length=262

Score = 251 bits (640), Expect = 1e-79, Method: Compositional matrix adjust. Identities = 127/248 (51%), Positives = 168/248 (68%), Gaps = 1/248 (0%)

| Query | 18 | RLFGYDWAQQTIGCSDAAVFRLSAQGRPVLFVKTDLSGALNELQDEAARLSWLATTGVPC | 77 |
| --- | --- | --- | --- |
| Sbjct | 15 | R+ GY W++QTIG S+A VFRL+ +P LF+K + +G E+ E ARL WLA G+ C RIGGYHWSRQTIGRSNAGVFRLTGDNKPSLFLKIEEAGLFAEVAAEEARLRWLAGQGIAC | 74 |
| Query | 78 | AAVLDVVTEAGRDWLLLGEVPGQDLLSSHLA-PAEKVSIMADAMRRLHTLDPATCPFDHQ | 136 |
| Sbjct | 75 | A++ AGR WLL+ +PGQDL S+ A V+IMADA+R LH +D +CPFDH+  PAIIAFEHHAGRHWLLMTALPGQDLASAGATDAASSVAIMADALRELHAIDAGSCPFDHR | 134 |

| Query | 137 | AKHRIERARTRMEAGLVDQDDLDEEHQGLAPAELFARLKARMPDGEDLVVTHGDACLPNI | 196 |
| --- | --- | --- | --- |
| Sbjct | 135 | +HRI AR R+EAG VD+ D D+E QG+ AE FA+L A P +D+VVTHGDAC+P+ LQHRIAEARARLEAGTVDESDFDDERQGMTAAEAFAQLAALKPASQDIVVTHGDACMPDF | 194 |
| Query | 197 | MVENGRFSGFIDCGRLGVADRYQDIALATRDIAEELGGEWADRFLVLYGIAAPDSQRIAF | 256 |
| Sbjct | 195 | + + RFSGF+DCGRLG+ADR+QD+ALA I +G W FL YG D R+++ VAASKRFSGFVDCGRLGLADRHQDLALACWSIRHNIGERWVQPFLDRYGFIGVDPSRLSY | 254 |
| Query  Sbjct | 257  255 | YRLLDEFF 264 YRLLDEFF YRLLDEFF 262 |  |

>AJD40892.1 aminoglycoside 3'-phosphotransferase protein [Rhizobium gallicum bv. gallicum R602]

Length=260

Score = 251 bits (640), Expect = 1e-79, Method: Compositional matrix adjust.

| Identities | = 123/244 (50%), Positives = 161/244 (66%), Gaps = 1/244 (0%) |  |
| --- | --- | --- |
| Query 22 | YDWAQQTIGCSDAAVFRLSAQGRPVLFVKTDLSGALNELQDEAARLSWLATTGVPCAAVL | 81 |
| Sbjct 17 | ++W + +GCS A VFRL+ GR F+K +++G EL EA RL WLA G+ C +V+ HEWKRDDLGCSSADVFRLAEAGRARYFLKGEIAGPFAELPLEAERLLWLAEQGLQCPSVV | 76 |
| Query 82 | DVVTEAGRDWLLLGEVPGQDLLSSHLAP-AEKVSIMADAMRRLHTLDPATCPFDHQAKHR | 140 |
| Sbjct 77 | + + GR+WLLL + G DL SS L +++ I+A A+RRLH LDP TCPFDH+ R ALESHEGRNWLLLSALSGSDLASSKLTTDRQRIEILAAALRRLHELDPETCPFDHRLTAR | 136 |
| Query 141 | IERARTRMEAGLVDQDDLDEEHQGLAPAELFARLKARMPDGEDLVVTHGDACLPNIMVEN | 200 |
| Sbjct 137 | + A+ RMEAG VD+ D DEE Q A LFA L+A PD D VV HGDACLPN + LRLAKARMEAGAVDEGDFDEERQNATAASLFAYLEAHQPDVIDPVVAHGDACLPNFLTHE | 196 |
| Query 201 | GRFSGFIDCGRLGVADRYQDIALATRDIAEELGGEWADRFLVLYGIAAPDSQRIAFYRLL | 260 |
| Sbjct 197 | G+F G++DCGRLGVADRYQDIALA R I +G EW FL YG++ + ++A+Y +L GQFGGYVDCGRLGVADRYQDIALALRSIRYNVGEEWVRHFLDCYGLSGINDAKVAYYMVL | 256 |
| Query 261  Sbjct 257 | DEFF 264 DEFF  DEFF 260 |  |

>WP_087005395.1 aminoglycoside 3'-phosphotransferase [Rhizobium sullae] Length=260

Score = 251 bits (640), Expect = 1e-79, Method: Compositional matrix adjust.

| Identities | = 128/244 (52%), Positives = 157/244 (64%), Gaps = 1/244 (0%) |  |
| --- | --- | --- |
| Query 22 | YDWAQQTIGCSDAAVFRLSAQGRPVLFVKTDLSGALNELQDEAARLSWLATTGVPCAAVL | 81 |
| Sbjct 17 | Y+W Q +GCS A VF L+ G+ F+K +++G EL EA RL WLA G+PC +V+ YEWKQDDLGCSSADVFVLAEGGQKRFFLKGEMAGPFAELPLEAERLLWLAKQGMPCPSVV | 76 |
| Query 82 | DVVTEAGRDWLLLGEVPGQDLLSSH-LAPAEKVSIMADAMRRLHTLDPATCPFDHQAKHR | 140 |
| Sbjct 77 | + + R+WLLL +PG DL SS A A + I+ A+RRLH L+P TCPFDH R AMESHEDRNWLLLNALPGSDLASSKSTADARCIEILVAALRRLHELNPETCPFDHGLTAR | 136 |
| Query 141 | IERARTRMEAGLVDQDDLDEEHQGLAPAELFARLKARMPDGEDLVVTHGDACLPNIMVEN | 200 |
| Sbjct 137 | + AR RM+AG VD DD DEE Q A LFA L+A PD D VV HGDACLPN M LRLARARMDAGAVDADDFDEERQSETVASLFAYLEASQPDVTDAVVAHGDACLPNFMTHE | 196 |
| Query 201 | GRFSGFIDCGRLGVADRYQDIALATRDIAEELGGEWADRFLVLYGIAAPDSQRIAFYRLL | 260 |
| Sbjct 197 | GRFSG+IDCGRLGVADRYQDIALA R I LG FL YG++ D ++A+Y +L GRFSGYIDCGRLGVADRYQDIALAMRSIRYNLGEAGVRHFLDCYGLSGIDDAKVAYYLVL | 256 |
| Query 261  Sbjct 257 | DEFF 264 DEFF  DEFF 260 |  |

>WP_025425025.1 MULTISPECIES: aminoglycoside 3'-phosphotransferase [Ensifer] KQU77296.1 aminoglycoside phosphotransferase [Ensifer sp. Root31] KQW34344.1 aminoglycoside phosphotransferase [Ensifer sp. Root1252]

KQW56133.1 aminoglycoside phosphotransferase [Ensifer sp. Root127] KQY61500.1 aminoglycoside phosphotransferase [Ensifer sp. Root142] KRC55213.1 aminoglycoside phosphotransferase [Ensifer sp. Root231]

Length=263

Score = 251 bits (640), Expect = 1e-79, Method: Compositional matrix adjust.

| Identities | = 129/253 (51%), Positives = 165/253 (65%), Gaps = 0/253 (0%) |  |
| --- | --- | --- |
| Query 12 | PAAWVERLFGYDWAQQTIGCSDAAVFRLSAQGRPVLFVKTDLSGALNELQDEAARLSWLA | 71 |
| Sbjct 11 | P L GYD + ++G SD++VF A+ RP L +K + +G +EL DEA RL WL PPTLAPLLAGYDCRKDSLGQSDSSVFLFDAKDRPPLVLKVEEAGPFDELVDEAERLEWLG | 70 |
| Query 72 | TTGVPCAAVLDVVTEAGRDWLLLGEVPGQDLLSSHLAPAEKVSIMADAMRRLHTLDPATC | 131 |
| Sbjct 71 | G+PC V+ A ++WLL+ V G DL SS LA +E+V ++A+A+RR+H LD C KQGMPCPRVVARDVYAQKNWLLMEAVEGIDLASSTLAASEQVVLLAEALRRMHALDVDAC | 130 |
| Query 132 | PFDHQAKHRIERARTRMEAGLVDQDDLDEEHQGLAPAELFARLKARMPDGEDLVVTHGDA | 191 |
| Sbjct 131 | PFDH+ R+ AR R EA LVD+ D D+E G ELF L+AR+P + VVTHGDA PFDHRIGARVAIARLRAEADLVDETDFDDERLGKTADELFVELQARIPQEGEPVVTHGDA | 190 |
| Query 192 | CLPNIMVENGRFSGFIDCGRLGVADRYQDIALATRDIAEELGGEWADRFLVLYGIAAPDS | 251 |
| Sbjct 191 | CLPNI+V +GRF+GFIDC RLGVAD YQD+AL R IA LG EW FL LYGI D CLPNIIVRDGRFAGFIDCSRLGVADPYQDLALTCRSIAYNLGEEWVQPFLDLYGIRRADP | 250 |
| Query 252  Sbjct 251 | QRIAFYRLLDEFF 264  +++ FY LLDEFF  EKLGFYCLLDEFF 263 |  |

>WP_191575458.1 APH(3') family aminoglycoside O-phosphotransferase [Achromobacter insolitus]

Length=263

Score = 251 bits (640), Expect = 1e-79, Method: Compositional matrix adjust.

| Identities | | = 129/264 (49%), Positives = 166/264 (63%), Gaps = 1/264 (0%) |  |
| --- | --- | --- | --- |
| Query 1  Sbjct 1 | | MIEQDGLHAGSPAAWVERLFGYDWAQQTIGCSDAAVFRLSAQGRPVLFVKTDLSGALNEL M Q +H P W RL G QQ+IG S A V+R+ + +P ++K++ +G EL MHAQYSIHDSLPLQWQPRLAGARLEQQSIGESRADVYRIRSANQPDRYLKSEHTGTQPEL | 60  60 |
| Query 61  Sbjct 61 | | QDEAARLSWLATTGVPCAAVLDVVTEAGRDWLLLGEVPGQDLLSSHLAPAEKVSIMADAM DE RL W+A G+PC V+D V R WLL+ V GQDL S+ L PA+ ++IMADA+ PDEIERLRWMAAHGLPCPTVIDAVATPTRHWLLMSAVAGQDLASAGLPPAQVIAIMADAL | 120  120 |
| Query 121  Sbjct 121 | | RRLHTLDPATCPFDHQAKHRIERARTRMEAGLVDQDDLDEEHQGLAPAELFARLKARMPD RLH + CPFDH + RI AR ++AGLVD+ D D+E G + FA L A P HRLHRIPIDQCPFDHTLEPRIAEARRALDAGLVDESDFDDERIGRTAEDAFAELLATRPT | 180  180 |
| Query 181  Sbjct 181 | | GEDLVVTHGDACLPNIMVENGRFSGFIDCGRLGVADRYQDIALATRDIAEELGGEWADRF DLVV HGDACLPN M + G F+G+IDCGRLGV+DR+QD+ALA R I LG EW + F THDLVVAHGDACLPNFMADGGVFTGYIDCGRLGVSDRHQDLALAARSIERNLGREWIEPF | 240  240 |
| Query 241 | | LVLYGIAAPDSQRIAFYRLLDEFF 264 YG+ PD +R+AFY LLDEFF |  |
| Sbjct | 241 | FRGYGV-EPDDRRLAFYCLLDEFF 263 | |

>WP_123376854.1 aminoglycoside 3'-phosphotransferase [Pseudomonas fluorescens]

RON89105.1 APH(3') family aminoglycoside O-phosphotransferase [Pseudomonas fluorescens]

Length=256

Score = 250 bits (639), Expect = 1e-79, Method: Compositional matrix adjust.

| Identities | = 129/254 (51%), Positives = 167/254 (66%), Gaps = 2/254 (1%) |  |
| --- | --- | --- |
| Query 12 | PAAWVERLFGYDWAQQTIGCSDAAVFRLSAQGRPVLFVKTDLSGALNELQDEAARLSWLA | 71 |
| Sbjct 4 | PAAW + G QQTIG S A V+R++ G FVKT+ EL E RL W+A PAAWRSQFAGAAIEQQTIGESRADVWRITPAGAAPQFVKTEQVMTWGELPGEVQRLRWMA | 63 |
| Query 72 | TTGVPCAAVLDVVTEAGRDWLLLGEVPGQDLLSS-HLAPAEKVSIMADAMRRLHTLDPAT | 130 |
| Sbjct 64 | T +P VLD T+A +WLL+ V G+DL SS HL+P + V++ A A+R LH + +  TQDLPAPRVLDTTTQAQHNWLLMTAVAGRDLASSEHLSPQQIVTLAAQALRALHAVAIDS | 123 |

| Query | 131 | CPFDHQAKHRIERARTRMEAGLVDQDDLDEEHQGLAPAELFARLKARMPDGEDLVVTHGD | 190 |
| --- | --- | --- | --- |
| Sbjct | 124 | CPFDH + +I RAR + AGL+D++D D+ G + E+F +L A P EDLVVTHGD CPFDHCLEQKITRAREHLLAGLIDEEDFDDSRLGQSAEEVFEQLLATRPAHEDLVVTHGD | 183 |
| Query | 191 | ACLPNIMVENGRFSGFIDCGRLGVADRYQDIALATRDIAEELGGEWADRFLVLYGIAAPD | 250 |
| Sbjct | 184 | ACLPN++ NGRFSGF+DCGRLGVADRYQD+ALA I + LG +W F +YG+ PD ACLPNLLAANGRFSGFVDCGRLGVADRYQDLALAAHSITDNLGEQWLPLFFEVYGV-EPD | 242 |
| Query | 251 | SQRIAFYRLLDEFF 264 QRIAFY++LDEFF |  |
| Sbjct | 243 | QQRIAFYQMLDEFF 256 | |

>WP_171956004.1 APH(3')-II family aminoglycoside O-phosphotransferase [Stenotrophomonas maltophilia]

Length=267

Score = 251 bits (640), Expect = 1e-79, Method: Compositional matrix adjust.

| Identities | = 134/255 (53%), Positives = 167/255 (65%), Gaps = 3/255 (1%) |  |
| --- | --- | --- |
| Query 12 | PAAWVERLFGYDWAQQTIGCSDAAVFRLSAQGRPVLFVKTDLSGALNELQDEAARLSWLA | 71 |
| Sbjct 14 | P AW + L +Q++G S A V R+ GRP F+K++L A +EL DE ARL WL PLAWQKALADARIERQSMGVSRADVARVLRPGRPDAFLKSELIDAFSELGDEIARLCWLQ | 73 |
| Query 72 | TTGVPCAAVLDVVTEAGRDWLLLGEVPGQDLLSS-HLAPAEKVSIMADAMRRLHTLDPAT | 130 |
| Sbjct 74 | G P V+ EAGR WLL+ +PG+DL SS LAP ++V I+ADA+R LH L A AQGQPAPTVIATAEEAGRHWLLMSALPGRDLASSPELAPQQRVEILADALRGLHALPVAA | 133 |
| Query 131 | CPFDHQAKHRIERARTRMEAGLVDQDDLDEEHQGLAPAELFARLKARMPDGEDLVVTHGD | 190 |
| Sbjct 134 | CPFD + R+ A+ R+EAG VD DD D+E G +P ++FA+L + PD EDLVV+HGD CPFDQRLASRLPAAQARVEAGRVDADDFDDERLGQSPQQVFAQLCSTRPDHEDLVVSHGD | 193 |
| Query 191 | ACLPNIMVENGRFSGFIDCGRLGVADRYQDIALATRDIAEELG-GEWADRFLVLYGIAAP | 249 |
| Sbjct 194 | ACLPN+MV GRFSGFIDCGRLGVADRYQD+ALA R + G W YG A ACLPNLMVAEGRFSGFIDCGRLGVADRYQDLALAARSLVHNFGDTRWVTPLFQRYGAVA- | 252 |
| Query 250  Sbjct 253 | DSQRIAFYRLLDEFF 264 D +R+AFYRLLDEFF DERRLAFYRLLDEFF 267 |  |

>WP_175183732.1 APH(3') family aminoglycoside O-phosphotransferase [Achromobacter animicus]

Length=263

Score = 250 bits (639), Expect = 1e-79, Method: Compositional matrix adjust.

| Identities | | = 128/264 (48%), Positives = 165/264 (63%), Gaps = 1/264 (0%) |  |
| --- | --- | --- | --- |
| Query 1  Sbjct 1 | | MIEQDGLHAGSPAAWVERLFGYDWAQQTIGCSDAAVFRLSAQGRPVLFVKTDLSGALNEL M Q +H P W RL G QQ+IG S A V+R+ + +P ++K++ +G L EL MHAQYSIHDSLPLQWQPRLAGARLEQQSIGESRADVYRIRSANQPDRYLKSEHTGTLPEL | 60  60 |
| Query 61  Sbjct 61 | | QDEAARLSWLATTGVPCAAVLDVVTEAGRDWLLLGEVPGQDLLSSHLAPAEKVSIMADAM DE RL W+A G+PC V+D R WLL+ V GQDL S+ L PA+ ++IMADA+ PDEIERLQWMAAHGLPCPTVIDAAATPTRHWLLMSAVAGQDLASAGLPPAQVIAIMADAL | 120  120 |
| Query 121  Sbjct 121 | | RRLHTLDPATCPFDHQAKHRIERARTRMEAGLVDQDDLDEEHQGLAPAELFARLKARMPD RLH + CPFDH + RI AR ++AGLVD+ D D + G + FA L A P HRLHCIPIDQCPFDHTLERRIAEARRALDAGLVDESDFDYDRIGRTAEDAFAELLATRPT | 180  180 |
| Query 181  Sbjct 181 | | GEDLVVTHGDACLPNIMVENGRFSGFIDCGRLGVADRYQDIALATRDIAEELGGEWADRF DLVV HGDACLPN M + G F+G+IDCGRLGV+DR+QD+ALA R I LG EW + F THDLVVAHGDACLPNFMADGGVFTGYIDCGRLGVSDRHQDLALAARSIERNLGREWIEPF | 240  240 |
| Query 241 | | LVLYGIAAPDSQRIAFYRLLDEFF 264 YG+ PD +R+AFY LLDEFF |  |
| Sbjct | 241 | FRSYGV-EPDDRRLAFYCLLDEFF 263 | |

>WP_061477864.1 APH(3')-II family aminoglycoside O-phosphotransferase [Stenotrophomonas sp. DDT-1]

KXU98973.1 aminoglycoside phosphotransferase [Stenotrophomonas sp. DDT-1] Length=267

Score = 251 bits (640), Expect = 1e-79, Method: Compositional matrix adjust.

| Identities | = 134/255 (53%), Positives = 167/255 (65%), Gaps = 3/255 (1%) |  |
| --- | --- | --- |
| Query 12 | PAAWVERLFGYDWAQQTIGCSDAAVFRLSAQGRPVLFVKTDLSGALNELQDEAARLSWLA | 71 |
| Sbjct 14 | P AW + L +Q++G S A V R+ QGRP F+K++L A +EL DE ARL WL PLAWQKALADARIERQSMGVSRADVARVLRQGRPDAFLKSELIDAFSELGDEIARLRWLQ | 73 |
| Query 72 | TTGVPCAAVLDVVTEAGRDWLLLGEVPGQDLLSS-HLAPAEKVSIMADAMRRLHTLDPAT | 130 |
| Sbjct 74 | G P V+ EAGR WLL+ +PG+DL SS LAP ++V I+ADA+R LH L A AQGQPAPTVIATAEEAGRRWLLMSALPGRDLASSPELAPQQRVEILADALRGLHALPVAA | 133 |
| Query 131 | CPFDHQAKHRIERARTRMEAGLVDQDDLDEEHQGLAPAELFARLKARMPDGEDLVVTHGD | 190 |
| Sbjct 134 | CPFD + R+ A+ R+EAG VD DD D+E G +P ++FA+L + PD EDLVV+HGD CPFDQRLASRLPAAQARVEAGRVDADDFDDERLGQSPQQVFAQLCSTRPDHEDLVVSHGD | 193 |
| Query 191 | ACLPNIMVENGRFSGFIDCGRLGVADRYQDIALATRDIAEEL-GGEWADRFLVLYGIAAP | 249 |
| Sbjct 194 | ACLPN+MV GRFSGFIDCGRLGVADRYQD+ALA R + W YG A ACLPNLMVAEGRFSGFIDCGRLGVADRYQDLALAARSLVHNFDDTRWVTPLFQRYGAVA- | 252 |
| Query 250  Sbjct 253 | DSQRIAFYRLLDEFF 264 D +R+AFYRLLDEFF DERRLAFYRLLDEFF 267 |  |

>EGE56818.1 putative aminoglycoside 3'-phosphotransferase protein [Rhizobium etli CNPAF512]

Length=255

Score = 250 bits (638), Expect = 1e-79, Method: Compositional matrix adjust.

| Identities | | = 126/254 (50%), Positives = 168/254 (66%), Gaps = 1/254 (0%) |  |
| --- | --- | --- | --- |
| Query 12  Sbjct 2 | | PAAWVERLFGYDWAQQTIGCSDAAVFRLSAQGRPVLFVKTDLSGALNELQDEAARLSWLA PAA ERL GY + + +G S A+VFRL +G P L++K + +G EL DEAARLSWL PAALGERLSGYRFERDALGRSAASVFRLEGEGLPALYLKVEAAGPFGELADEAARLSWLK | 71  61 |
| Query 72  Sbjct 62 | | TTGVPCAAVLDVVTEAGRDWLLLGEVPGQDLLS-SHLAPAEKVSIMADAMRRLHTLDPAT G+PC V+ ++ R+WLL+ +PG DL S S L P +V ++A A+ LH L A+ AVGLPCPDVIARESDGERNWLLINALPGTDLASASALTPLTRVELLAAALSDLHDLPIAS | 130  121 |
| Query 131  Sbjct 122 | | CPFDHQAKHRIERARTRMEAGLVDQDDLDEEHQGLAPAELFARLKARMPDGEDLVVTHGD CPFDH+ ++R+ A+ RM+AG+VD++D D G + A LF L P E+LVVTHGD CPFDHRLENRLPTAKARMQAGIVDEEDFDAARLGKSAAALFIELMRLRPGDEELVVTHGD | 190  181 |
| Query 191  Sbjct 182 | | ACLPNIMVENGRFSGFIDCGRLGVADRYQDIALATRDIAEELGGEWADRFLVLYGIAAPD ACLPN + NG+FSG+IDC RLGVADR+QDIALA R IA G FL YG+ APD ACLPNFVASNGQFSGYIDCSRLGVADRHQDIALACRSIASNFGEALIRPFLDRYGLTAPD | 250  241 |
| Query 251 | | SQRIAFYRLLDEFF 264  ++ +++LLDEFF |  |
| Sbjct | 242 | PAKLHYFQLLDEFF 255 | |

>WP_164093019.1 APH(3')-II family aminoglycoside O-phosphotransferase [Stenotrophomonas maltophilia]

Length=267

Score = 250 bits (639), Expect = 2e-79, Method: Compositional matrix adjust.

| Identities | = 134/255 (53%), Positives = 167/255 (65%), Gaps = 3/255 (1%) |  |
| --- | --- | --- |
| Query 12 | PAAWVERLFGYDWAQQTIGCSDAAVFRLSAQGRPVLFVKTDLSGALNELQDEAARLSWLA | 71 |
| Sbjct 14 | P AW + L +Q++G S A V R+ GRP F+K++L A +EL DE ARL WL PLAWEKALADARIERQSMGVSRADVARVLRPGRPDAFLKSELIDAFSELGDEIARLRWLQ | 73 |
| Query 72 | TTGVPCAAVLDVVTEAGRDWLLLGEVPGQDLLSS-HLAPAEKVSIMADAMRRLHTLDPAT | 130 |
| Sbjct 74 | G P V+ EAGR WLL+ +PG+DL SS LAP ++V I+ADA+R LH L A AQGQPAPTVIATAEEAGRHWLLMSALPGRDLASSPELAPQQRVEILADALRGLHALPVAA | 133 |
| Query 131 | CPFDHQAKHRIERARTRMEAGLVDQDDLDEEHQGLAPAELFARLKARMPDGEDLVVTHGD CPFD + R+ A+ R+EAG VD DD D+E G +P ++FA+L + PD EDLVV+HGD | 190 |

| Sbjct | 134 | CPFDQRLASRLPAAQARVEAGRVDADDFDDERLGQSPQQVFAQLCSTRPDHEDLVVSHGD | 193 |
| --- | --- | --- | --- |
| Query | 191 | ACLPNIMVENGRFSGFIDCGRLGVADRYQDIALATRDIAEELG-GEWADRFLVLYGIAAP | 249 |
| Sbjct | 194 | ACLPN+MV GRFSGFIDCGRLGVADRYQD+ALA R + G W YG A ACLPNLMVAEGRFSGFIDCGRLGVADRYQDLALAARSLVHNFGDTRWVTPLFQRYGAVA- | 252 |
| Query  Sbjct | 250  253 | DSQRIAFYRLLDEFF 264 D +R+AFYRLLDEFF DERRLAFYRLLDEFF 267 |  |

>VUP59444.1 Aminoglycoside 3'-phosphotransferase [Stenotrophomonas maltophilia] Length=269

Score = 250 bits (639), Expect = 2e-79, Method: Compositional matrix adjust.

| Identities | | = 137/262 (52%), Positives = 170/262 (65%), Gaps = 3/262 (1%) |  |
| --- | --- | --- | --- |
| Query 5  Sbjct 9 | | DGLHAGSPAAWVERLFGYDWAQQTIGCSDAAVFRLSAQGRPVLFVKTDLSGALNELQDEA DGL P AW E L +Q+IG S A V R+ G+P FVK+++ A +EL DE DGLPLPLPWAWQEALADARVERQSIGVSRADVARVQRPGQPDAFVKSEVIDAFSELGDEI | 64  68 |
| Query 65  Sbjct 69 | | ARLSWLATTGVPCAAVLDVVTEAGRDWLLLGEVPGQDLLSS-HLAPAEKVSIMADAMRRL ARL WL G P V+ EAGR WLL+ +PG+DL SS LAP + V ++ADA+R L ARLRWLQEQGQPAPTVIATAEEAGRRWLLMSALPGRDLASSPELAPQQLVELLADALRGL | 123  128 |
| Query 124  Sbjct 129 | | HTLDPATCPFDHQAKHRIERARTRMEAGLVDQDDLDEEHQGLAPAELFARLKARMPDGED H L A CPFD + +++ A+ R+EAGLVD DD D+E G +P ++FA L + PD ED HALPVAACPFDQRLHLQLQAAQARVEAGLVDADDFDDERLGQSPQQVFAELCSTRPDHED | 183  188 |
| Query 184  Sbjct 189 | | LVVTHGDACLPNIMVENGRFSGFIDCGRLGVADRYQDIALATRDIAEELG-GEWADRFLV LVV+HGDACLPN+MV GRFSGFIDCGRLGVADRYQD+ALA R + G W LVVSHGDACLPNLMVVEGRFSGFIDCGRLGVADRYQDLALAARSLVHNFGDTRWVAPLFQ | 242  248 |
| Query 243 | | LYGIAAPDSQRIAFYRLLDEFF 264 YG A D +R+AFYRLLDEFF |  |
| Sbjct | 249 | RYGAVA-DERRLAFYRLLDEFF 269 | |

>WP_037117315.1 MULTISPECIES: aminoglycoside 3'-phosphotransferase [Rhizobium] Length=261

Score = 250 bits (639), Expect = 2e-79, Method: Compositional matrix adjust.

| Identities | | = 126/254 (50%), Positives = 168/254 (66%), Gaps = 1/254 (0%) |  |
| --- | --- | --- | --- |
| Query 12  Sbjct 8 | | PAAWVERLFGYDWAQQTIGCSDAAVFRLSAQGRPVLFVKTDLSGALNELQDEAARLSWLA PAA ERL GY + + +G S A+VFRL +G P L++K + +G EL DEAARLSWL PAALGERLSGYRFERDALGRSAASVFRLEGEGLPALYLKVEAAGPFGELADEAARLSWLK | 71  67 |
| Query 72  Sbjct 68 | | TTGVPCAAVLDVVTEAGRDWLLLGEVPGQDLLS-SHLAPAEKVSIMADAMRRLHTLDPAT G+PC V+ ++ R+WLL+ +PG DL S S L P +V ++A A+ LH L A+ AVGLPCPDVIARESDGERNWLLINALPGTDLASASALTPLTRVELLAAALSDLHDLPIAS | 130  127 |
| Query 131  Sbjct 128 | | CPFDHQAKHRIERARTRMEAGLVDQDDLDEEHQGLAPAELFARLKARMPDGEDLVVTHGD CPFDH+ ++R+ A+ RM+AG+VD++D D G + A LF L P E+LVVTHGD CPFDHRLENRLPTAKARMQAGIVDEEDFDAARLGKSAAALFIELMRLRPGDEELVVTHGD | 190  187 |
| Query 191  Sbjct 188 | | ACLPNIMVENGRFSGFIDCGRLGVADRYQDIALATRDIAEELGGEWADRFLVLYGIAAPD ACLPN + NG+FSG+IDC RLGVADR+QDIALA R IA G FL YG+ APD ACLPNFVASNGQFSGYIDCSRLGVADRHQDIALACRSIASNFGEALIRPFLDRYGLTAPD | 250  247 |
| Query 251 | | SQRIAFYRLLDEFF 264  ++ +++LLDEFF |  |
| Sbjct | 248 | PAKLHYFQLLDEFF 261 | |

>WP_173418213.1 APH(3') family aminoglycoside O-phosphotransferase [Ensifer adhaerens] NRP16839.1 Aminoglycoside 3'-phosphotransferase [Ensifer adhaerens]

Length=263

Score = 250 bits (639), Expect = 2e-79, Method: Compositional matrix adjust. Identities = 127/253 (50%), Positives = 161/253 (64%), Gaps = 0/253 (0%)

| Query | 12 | PAAWVERLFGYDWAQQTIGCSDAAVFRLSAQGRPVLFVKTDLSGALNELQDEAARLSWLA | 71 |
| --- | --- | --- | --- |
| Sbjct | 11 | P + L GY W ++G S ++VF A+ RP L +K + S +EL DEA+RL WL PPGFAPLLNGYSWRMDSLGQSASSVFLFDAEARPPLVLKVEQSSPFDELVDEASRLEWLG | 70 |
| Query | 72 | TTGVPCAAVLDVVTEAGRDWLLLGEVPGQDLLSSHLAPAEKVSIMADAMRRLHTLDPATC | 131 |
| Sbjct | 71 | G+PC V+ A R+WLL+ V G DL SS LA +E+V ++A+A+RR+H LD C KQGMPCPRVIAREVYADRNWLLMEAVDGVDLASSTLAASEQVVLLAEAVRRMHALDAEAC | 130 |
| Query | 132 | PFDHQAKHRIERARTRMEAGLVDQDDLDEEHQGLAPAELFARLKARMPDGEDLVVTHGDA | 191 |
| Sbjct | 131 | PFDH+ RI AR R EA LVD D DE G ELF L+A++P LVVTHGDA PFDHRVGARIAIARLRAEADLVDVTDFDEARLGKTAEELFVELQAKIPQEGKLVVTHGDA | 190 |
| Query | 192 | CLPNIMVENGRFSGFIDCGRLGVADRYQDIALATRDIAEELGGEWADRFLVLYGIAAPDS | 251 |
| Sbjct | 191 | CLPN++ +GRF+GFIDC RLGVAD YQD+AL R IA LG EW FL LYG+ D CLPNVIARDGRFAGFIDCSRLGVADPYQDLALTCRSIAHNLGEEWVQPFLDLYGVRRADP | 250 |
| Query  Sbjct | 252  251 | QRIAFYRLLDEFF 264  +++ FY LLDEFF  EKLDFYCLLDEFF 263 |  |

>WP_097540934.1 aminoglycoside 3'-phosphotransferase [Rhizobium phaseoli]

PDS74550.1 APH(3') family aminoglycoside O-phosphotransferase [Rhizobium phaseoli]

Length=261

Score = 250 bits (638), Expect = 2e-79, Method: Compositional matrix adjust.

| Identities | | = 127/254 (50%), Positives = 168/254 (66%), Gaps = 1/254 (0%) |  |
| --- | --- | --- | --- |
| Query 12  Sbjct 8 | | PAAWVERLFGYDWAQQTIGCSDAAVFRLSAQGRPVLFVKTDLSGALNELQDEAARLSWLA PAA ERL GY + + +G S A+VFRL +G P L++K + G EL DEAARLSWL PAALGERLSGYRFERDALGRSAASVFRLQGEGLPALYLKVEAVGPFGELADEAARLSWLK | 71  67 |
| Query 72  Sbjct 68 | | TTGVPCAAVLDVVTEAGRDWLLLGEVPGQDLLS-SHLAPAEKVSIMADAMRRLHTLDPAT G+PC V+ ++ RDWLL+ +PG DL+S S L P +V ++A A+ LH L A+ AVGLPCPEVIARESDGERDWLLINALPGADLVSASALTPLTRVELLAAALSDLHDLPIAS | 130  127 |
| Query 131  Sbjct 128 | | CPFDHQAKHRIERARTRMEAGLVDQDDLDEEHQGLAPAELFARLKARMPDGEDLVVTHGD CPFDH+ ++R+ A+ RM+AG+VD++D D G + A LF L P E+LVVTHGD CPFDHRLENRLPIAKVRMQAGIVDEEDFDAARLGKSAAALFIDLMRLRPGNENLVVTHGD | 190  187 |
| Query 191  Sbjct 188 | | ACLPNIMVENGRFSGFIDCGRLGVADRYQDIALATRDIAEELGGEWADRFLVLYGIAAPD ACLPN + NG+FSG+IDC RLGVADR+QDIALA R IA G FL YG+A PD ACLPNFVASNGQFSGYIDCSRLGVADRHQDIALACRSIAYNFGEALIRPFLDRYGLADPD | 250  247 |
| Query 251 | | SQRIAFYRLLDEFF 264  ++ +++LLDEFF |  |
| Sbjct | 248 | PAKLHYFQLLDEFF 261 | |

>WP_150652876.1 aminoglycoside 3'-phosphotransferase [Pseudomonas fluorescens] VVN46063.1 Aminoglycoside 3'-phosphotransferase [Pseudomonas fluorescens]

Length=256

Score = 250 bits (638), Expect = 2e-79, Method: Compositional matrix adjust.

| Identities | = 128/254 (50%), Positives = 167/254 (66%), Gaps = 2/254 (1%) |  |
| --- | --- | --- |
| Query 12 | PAAWVERLFGYDWAQQTIGCSDAAVFRLSAQGRPVLFVKTDLSGALNELQDEAARLSWLA | 71 |
| Sbjct 4 | PAAW + G QQTIG S A V+R++ G FVKT+ EL E RL W+A PAAWRSQFAGAAIEQQTIGESRADVWRITPAGAAPQFVKTEQVMTWGELPGEVQRLRWMA | 63 |
| Query 72 | TTGVPCAAVLDVVTEAGRDWLLLGEVPGQDLLSS-HLAPAEKVSIMADAMRRLHTLDPAT | 130 |
| Sbjct 64 | T +P VLD TEA +WLL+ VPG+DL SS HL+P + V+++A+A+R LH + THDLPAPRVLDTATEAEHNWLLMTAVPGRDLASSEHLSPQQIVTLVAEALRTLHAVAIIN | 123 |
| Query 131 | CPFDHQAKHRIERARTRMEAGLVDQDDLDEEHQGLAPAELFARLKARMPDGEDLVVTHGD | 190 |
| Sbjct 124 | CPFDH+ + +I RAR + AGL+D+ D D+ G + ++F ++ P EDLVVTHGD CPFDHRLEQKITRAREHLLAGLIDEQDFDDSRLGQSAEDVFQQMLDARPAHEDLVVTHGD | 183 |
| Query 191 | ACLPNIMVENGRFSGFIDCGRLGVADRYQDIALATRDIAEELGGEWADRFLVLYGIAAPD | 250 |
| Sbjct 184 | ACLPN++ NG FSGF+DCGRLGVADRYQD+ALA I + LG +W F +YG+ PD  ACLPNLLAANGGFSGFVDCGRLGVADRYQDLALAAHSITDNLGAQWLPLFFEVYGV-EPD | 242 |

Query 251 SQRIAFYRLLDEFF 264

QRIAFY+LLDEFF Sbjct 243 QQRIAFYQLLDEFF 256

>WP_077991802.1 MULTISPECIES: aminoglycoside 3'-phosphotransferase [Rhizobium] ARQ59240.1 aminoglycoside 3'-phosphotransferase protein [Rhizobium sp. Kim5] RSB91822.1 aminoglycoside 3'-phosphotransferase [Rhizobium sophoriradicis]

Length=261

Score = 250 bits (638), Expect = 2e-79, Method: Compositional matrix adjust.

| Identities | | = 131/254 (52%), Positives = 169/254 (67%), Gaps = 1/254 (0%) |  |
| --- | --- | --- | --- |
| Query 12  Sbjct 8 | | PAAWVERLFGYDWAQQTIGCSDAAVFRLSAQGRPVLFVKTDLSGALNELQDEAARLSWLA PAA ERL GY + + +G S A+VFRL +G P L++K + +G EL DEAARLSWL PAALGERLTGYRFERDALGRSAASVFRLEGEGLPALYLKVEAAGPFGELADEAARLSWLK | 71  67 |
| Query 72  Sbjct 68 | | TTGVPCAAVLDVVTEAGRDWLLLGEVPGQDLLS-SHLAPAEKVSIMADAMRRLHTLDPAT G+PC V+ ++ R+WLL+G +PG +L S S L P +V ++A A+ LH L + AAGLPCPDVIARESDGKRNWLLIGALPGTNLASASALTPLARVELLAAALADLHHLPITS | 130  127 |
| Query 131  Sbjct 128 | | CPFDHQAKHRIERARTRMEAGLVDQDDLDEEHQGLAPAELFARLKARMPDGEDLVVTHGD CPFDH+ + R+ A RM+AG+VD+ D DE G + LFA L+ R PD EDLVVTHGD CPFDHRLEKRLPAAGARMQAGIVDETDFDETRLGKSAETLFAELEGRRPDREDLVVTHGD | 190  187 |
| Query 191  Sbjct 188 | | ACLPNIMVENGRFSGFIDCGRLGVADRYQDIALATRDIAEELGGEWADRFLVLYGIAAPD ACLPN + G+FSG+IDC RLGVADRYQDIALA R IA+ G FL YG+ APD ACLPNFVASGGQFSGYIDCSRLGVADRYQDIALACRSIADNFGAALVQPFLDRYGLTAPD | 250  247 |
| Query 251 | | SQRIAFYRLLDEFF 264 R+ +Y+LLDEFF |  |
| Sbjct | 248 | PARLDYYQLLDEFF 261 | |

>WP_133940267.1 aminoglycoside 3'-phosphotransferase [Rhizobium azibense] Length=268

Score = 250 bits (639), Expect = 2e-79, Method: Compositional matrix adjust.

| Identities | | = 124/247 (50%), Positives = 162/247 (66%), Gaps = 1/247 (0%) |  |
| --- | --- | --- | --- |
| Query 19  Sbjct 22 | | LFGYDWAQQTIGCSDAAVFRLSAQGRPVLFVKTDLSGALNELQDEAARLSWLATTGVPCA L ++W + +GCS A VFRL+ GR F+K +++G EL EA RL WLA G+ C LSQHEWKRDDLGCSSADVFRLAEAGRARYFLKGEIAGPFAELPLEAERLLWLAEQGLQCP | 78  81 |
| Query 79  Sbjct 82 | | AVLDVVTEAGRDWLLLGEVPGQDLLSSHLAP-AEKVSIMADAMRRLHTLDPATCPFDHQA  +V+ + + GR+WLLL + G DL SS L +++ I+A A+RRLH LDP TCPFDH+ SVVALESHEGRNWLLLSALSGSDLASSKLTTDRQRIEILAAALRRLHELDPETCPFDHRL | 137  141 |
| Query 138  Sbjct 142 | | KHRIERARTRMEAGLVDQDDLDEEHQGLAPAELFARLKARMPDGEDLVVTHGDACLPNIM R+ A+ RMEAG VD+ D DEE Q A LFA L+A PD D VV HGDACLPN + TARLRLAKARMEAGAVDEGDFDEERQNATAASLFAYLEAHQPDVIDPVVAHGDACLPNFL | 197  201 |
| Query 198  Sbjct 202 | | VENGRFSGFIDCGRLGVADRYQDIALATRDIAEELGGEWADRFLVLYGIAAPDSQRIAFY G+F G++DCGRLGVADRYQDIALA R I +G EW FL YG++ + ++A+Y THEGQFGGYVDCGRLGVADRYQDIALALRSIRYNVGEEWVRHFLDCYGLSGINDAKVAYY | 257  261 |
| Query 258 | | RLLDEFF 264  +LDEFF |  |
| Sbjct | 262 | MVLDEFF 268 | |

```
>WP_095111988.1 aminoglycoside 3'-phosphotransferase [Pseudomonas sp. Irchel 3H7]
Length=256

Score = 249 bits (637), Expect = 2e-79, Method: Compositional matrix adjust. Identities = 129/254 (51%), Positives = 166/254 (65%), Gaps = 2/254 (1%)

Query 12	PAAWVERLFGYDWAQQTIGCSDAAVFRLSAQGRPVLFVKTDLSGALNELQDEAARLSWLA 71 PAAW + G	QQTIG S A V+R++ G	FVKT+	EL E RL W+
Sbjct 4	PAAWRSQFAGAAIEQQTIGESRADVWRITPAGAAPQFVKTEQVMTWGELPGEVQRLRWMT 63
```

| Query | 72 | TTGVPCAAVLDVVTEAGRDWLLLGEVPGQDLLSS-HLAPAEKVSIMADAMRRLHTLDPAT | 130 |
| --- | --- | --- | --- |
| Sbjct | 64 | T +P VLD T+A +WLL+ V G+DL SS HL+P + V++ A A+R LH + + TQDLPAPRVLDTTTQAQHNWLLMTAVAGRDLASSEHLSPQQIVTLAAQALRALHAVAIDS | 123 |
| Query | 131 | CPFDHQAKHRIERARTRMEAGLVDQDDLDEEHQGLAPAELFARLKARMPDGEDLVVTHGD | 190 |
| Sbjct | 124 | CPFDH + +I RAR + AGL+D++D D+ G + E+F +L A P EDLVVTHGD CPFDHCLEQKITRAREHLLAGLIDEEDFDDSRLGQSAEEVFEQLLATRPAHEDLVVTHGD | 183 |
| Query | 191 | ACLPNIMVENGRFSGFIDCGRLGVADRYQDIALATRDIAEELGGEWADRFLVLYGIAAPD | 250 |
| Sbjct | 184 | ACLPN++ NGRFSGF+DCGRLGVADRYQD+ALA I + LG +W F +YG+ PD ACLPNLLAANGRFSGFVDCGRLGVADRYQDLALAAHSITDNLGEQWLPLFFEVYGV-EPD | 242 |
| Query | 251 | SQRIAFYRLLDEFF 264 QRIAFY+LLDEFF |  |
| Sbjct | 243 | QQRIAFYQLLDEFF 256 | |

>WP_034476770.1 aminoglycoside 3'-phosphotransferase [Agrobacterium rhizogenes] Length=266

Score = 250 bits (638), Expect = 2e-79, Method: Compositional matrix adjust.

| Identities | | = 128/254 (50%), Positives = 169/254 (67%), Gaps = 1/254 (0%) |  |
| --- | --- | --- | --- |
| Query 12  Sbjct 13 | | PAAWVERLFGYDWAQQTIGCSDAAVFRLSAQGRPVLFVKTDLSGALNELQDEAARLSWLA PA + L GY+W Q +G S A VFRL A+GRP +++KT++ L EL E +RL WLA PARLRDLLAGYNWLQDALGRSTAHVFRLEAKGRPPVYLKTEIIHPLGELGGEVSRLRWLA | 71  72 |
| Query 72  Sbjct 73 | | TTGVPCAAVLDVVTEAGRDWLLLGEVPGQDLLSS-HLAPAEKVSIMADAMRRLHTLDPAT  + G+ C V+ + R+WLL+ +PG DL+S LAP E+V ++ADA+RRLH LD A+ SEGIACPQVIAHEADERREWLLMSALPGADLVSGDRLAPIERVRMLADALRRLHGLDIAS | 130  132 |
| Query 131  Sbjct 133 | | CPFDHQAKHRIERARTRMEAGLVDQDDLDEEHQGLAPAELFARLKARMPDGEDLVVTHGD CPFDH+ ++RI AR R+ AGLVD+DD D E G + +LF L+ + P EDLVVTHGD CPFDHRLENRIAEARARLLAGLVDEDDFDGERLGRSAQDLFRELEEQRPRTEDLVVTHGD | 190  192 |
| Query 191  Sbjct 193 | | ACLPNIMVENGRFSGFIDCGRLGVADRYQDIALATRDIAEELGGEWADRFLVLYGIAAPD ACLPN + + G FSG+IDCGRL VADR+QD+ALA I G FL YG + ACLPNFIADGGSFSGYIDCGRLSVADRHQDMALACGSIERNFGEASVAEFLKAYGHFELN | 250  252 |
| Query 251 | | SQRIAFYRLLDEFF 264  ++ A+YRLLDEFF |  |
| Sbjct | 253 | PEKAAYYRLLDEFF 266 | |

>WP_188246262.1 APH(3')-II family aminoglycoside O-phosphotransferase [Stenotrophomonas maltophilia]

Length=267

Score = 250 bits (638), Expect = 2e-79, Method: Compositional matrix adjust.

| Identities | = 135/255 (53%), Positives = 167/255 (65%), Gaps = 3/255 (1%) |  |
| --- | --- | --- |
| Query 12 | PAAWVERLFGYDWAQQTIGCSDAAVFRLSAQGRPVLFVKTDLSGALNELQDEAARLSWLA | 71 |
| Sbjct 14 | P AW E L +Q+IG S A V R+ G+P FVK+++ A +EL DE ARL WL PWAWQEALADARVERQSIGVSRADVARVQRPGQPDAFVKSEVIDAFSELGDEIARLRWLQ | 73 |
| Query 72 | TTGVPCAAVLDVVTEAGRDWLLLGEVPGQDLLSS-HLAPAEKVSIMADAMRRLHTLDPAT | 130 |
| Sbjct 74 | G P V+ EAGR WLL+ +PG+DL SS LAP + V ++ADA+R LH L A EQGQPAPTVIATAEEAGRRWLLMSALPGRDLASSPELAPQQLVELLADALRGLHALPLAA | 133 |
| Query 131 | CPFDHQAKHRIERARTRMEAGLVDQDDLDEEHQGLAPAELFARLKARMPDGEDLVVTHGD | 190 |
| Sbjct 134 | CPFD + R++ A+ R+EAGLVD DD D+E G +P ++FA L + PD EDLVV+HGD CPFDQRLASRLQAAQARVEAGLVDADDFDDERLGQSPQQVFAELCSTRPDHEDLVVSHGD | 193 |
| Query 191 | ACLPNIMVENGRFSGFIDCGRLGVADRYQDIALATRDIAEELG-GEWADRFLVLYGIAAP | 249 |
| Sbjct 194 | ACLPN+MV GRFSGFIDCGRLGVADRYQD+ALA R + G W YG A ACLPNLMVVEGRFSGFIDCGRLGVADRYQDLALAARSLVHNFGDTRWVAPLFQRYGAVA- | 252 |
| Query 250  Sbjct 253 | DSQRIAFYRLLDEFF 264 D +R+AFYRLLDEFF DERRLAFYRLLDEFF 267 |  |

>WP_069139932.1 APH(3')-II family aminoglycoside O-phosphotransferase [Stenotrophomonas maltophilia]

PZT08352.1 APH(3') family aminoglycoside O-phosphotransferase [Stenotrophomonas maltophilia]

Length=267

Score = 250 bits (638), Expect = 2e-79, Method: Compositional matrix adjust.

| Identities | = 134/255 (53%), Positives = 166/255 (65%), Gaps = 3/255 (1%) |  |
| --- | --- | --- |
| Query 12 | PAAWVERLFGYDWAQQTIGCSDAAVFRLSAQGRPVLFVKTDLSGALNELQDEAARLSWLA | 71 |
| Sbjct 14 | P AW + L +Q++G S A V R+ GRP F+K++L A +EL DE ARL WL PLAWQKALADARIERQSMGVSRADVARVLRPGRPDAFLKSELIDAFSELGDEIARLRWLQ | 73 |
| Query 72 | TTGVPCAAVLDVVTEAGRDWLLLGEVPGQDLLSS-HLAPAEKVSIMADAMRRLHTLDPAT | 130 |
| Sbjct 74 | G P V+ EAGR WLL+ +PG+DL SS LAP ++V I+ADA+R LH L A AQGQPAPTVIATAEEAGRHWLLMSALPGRDLASSPELAPQQRVEILADALRGLHALPVAA | 133 |
| Query 131 | CPFDHQAKHRIERARTRMEAGLVDQDDLDEEHQGLAPAELFARLKARMPDGEDLVVTHGD | 190 |
| Sbjct 134 | CPFD + R+ A+ R+EAG VD DD D+E G P ++FA+L + PD EDLVV+HGD CPFDQRLASRLPAAQARVEAGRVDADDFDDERLGQCPQQVFAQLCSTRPDHEDLVVSHGD | 193 |
| Query 191 | ACLPNIMVENGRFSGFIDCGRLGVADRYQDIALATRDIAEELG-GEWADRFLVLYGIAAP | 249 |
| Sbjct 194 | ACLPN+MV GRFSGFIDCGRLGVADRYQD+ALA R + G W YG A ACLPNLMVAEGRFSGFIDCGRLGVADRYQDLALAARSLVHNFGDTRWVTPLFQRYGAVA- | 252 |
| Query 250  Sbjct 253 | DSQRIAFYRLLDEFF 264 D +R+AFYRLLDEFF DERRLAFYRLLDEFF 267 |  |

>WP_005416290.1 APH(3')-II family aminoglycoside O-phosphotransferase [Stenotrophomonas maltophilia]

EMI50317.1 aminoglycoside 3'-phosphotransferase [Stenotrophomonas maltophilia AU12-09]

Length=267

Score = 250 bits (638), Expect = 2e-79, Method: Compositional matrix adjust.

| Identities | = 134/255 (53%), Positives = 167/255 (65%), Gaps = 3/255 (1%) |  |
| --- | --- | --- |
| Query 12 | PAAWVERLFGYDWAQQTIGCSDAAVFRLSAQGRPVLFVKTDLSGALNELQDEAARLSWLA | 71 |
| Sbjct 14 | P AW + L +Q++G S A V R+ GRP F+K++L A +EL DE ARL WL PLAWQKALADARIERQSMGVSRADVARVLRPGRPDAFLKSELIDAFSELGDEIARLRWLQ | 73 |
| Query 72 | TTGVPCAAVLDVVTEAGRDWLLLGEVPGQDLLSS-HLAPAEKVSIMADAMRRLHTLDPAT | 130 |
| Sbjct 74 | G P V+ EAGR WLL+ +PG+DL SS LAP ++V I+ADA+R LH L A AQGQPAPTVIATAEEAGRHWLLMSALPGRDLASSPELAPQQRVEILADALRGLHALPVAA | 133 |
| Query 131 | CPFDHQAKHRIERARTRMEAGLVDQDDLDEEHQGLAPAELFARLKARMPDGEDLVVTHGD | 190 |
| Sbjct 134 | CPFD + R+ A+ R+EAG VD DD D+E G +P ++FA+L + PD EDLVV+HGD CPFDQRLASRLPAAQARVEAGRVDADDFDDERLGQSPQQVFAQLCSTRPDHEDLVVSHGD | 193 |
| Query 191 | ACLPNIMVENGRFSGFIDCGRLGVADRYQDIALATRDIAEELG-GEWADRFLVLYGIAAP | 249 |
| Sbjct 194 | ACLPN+MV GRFSGFIDCGRLGVADRYQD+ALA R + G W YG A ACLPNLMVAEGRFSGFIDCGRLGVADRYQDLALAARSLVHNSGDTRWVTPLFQRYGAVA- | 252 |
| Query 250  Sbjct 253 | DSQRIAFYRLLDEFF 264 D +R+AFYRLLDEFF DERRLAFYRLLDEFF 267 |  |

```
>WP_097578691.1 aminoglycoside 3'-phosphotransferase [Rhizobium sp. H4]
PDV90163.1 APH(3') family aminoglycoside O-phosphotransferase [Rhizobium sp. H4]
Length=261

Score = 249 bits (637), Expect = 3e-79, Method: Compositional matrix adjust. Identities = 132/254 (52%), Positives = 168/254 (66%), Gaps = 1/254 (0%)

Query 12	PAAWVERLFGYDWAQQTIGCSDAAVFRLSAQGRPVLFVKTDLSGALNELQDEAARLSWLA 71 PAA ERL GY + + +G S A+VFRL +G P L++K + +G	EL DEAARL WL
Sbjct 8	PAALGERLSGYRFERDALGRSAASVFRLEGEGLPALYLKVEAAGPFGELADEAARLGWLK 67
```

| Query | 72 | TTGVPCAAVLDVVTEAGRDWLLLGEVPGQDLLS-SHLAPAEKVSIMADAMRRLHTLDPAT | 130 |
| --- | --- | --- | --- |
| Sbjct | 68 | G+PC V+ ++ RDWLL+G +PG DL S S L P +V ++A A+ LH L A+ AAGLPCPDVIARESDGERDWLLIGALPGTDLASASALTPLARVELLAAALADLHHLPIAS | 127 |
| Query | 131 | CPFDHQAKHRIERARTRMEAGLVDQDDLDEEHQGLAPAELFARLKARMPDGEDLVVTHGD | 190 |
| Sbjct | 128 | CPFDH+ + R+ A RM+AG+VD+ D DE G + LFA L+ R PD EDLVV HGD CPFDHRLEKRLPTAGARMQAGIVDETDFDETRLGKSAETLFAELEGRRPDREDLVVAHGD | 187 |
| Query | 191 | ACLPNIMVENGRFSGFIDCGRLGVADRYQDIALATRDIAEELGGEWADRFLVLYGIAAPD | 250 |
| Sbjct | 188 | ACLPN + G+FSG+IDC RLGVADRYQDIALA R IA+ G FL YG+ APD ACLPNFVASGGQFSGYIDCSRLGVADRYQDIALACRSIADNFGAALVQPFLDRYGLTAPD | 247 |
| Query | 251 | SQRIAFYRLLDEFF 264 R+ +Y+LLDEFF |  |
| Sbjct | 248 | PARLDYYQLLDEFF 261 | |

>WP_099527006.1 APH(3')-II family aminoglycoside O-phosphotransferase [Stenotrophomonas maltophilia]

Length=267

Score = 250 bits (638), Expect = 3e-79, Method: Compositional matrix adjust.

| Identities | = 135/255 (53%), Positives = 167/255 (65%), Gaps = 3/255 (1%) |  |
| --- | --- | --- |
| Query 12 | PAAWVERLFGYDWAQQTIGCSDAAVFRLSAQGRPVLFVKTDLSGALNELQDEAARLSWLA | 71 |
| Sbjct 14 | P AW E L +Q+IG S A V R+ G+P FVK+++ A +EL DE ARL WL PWAWQEALADARVERQSIGVSRADVARVQRPGQPDAFVKSEVIDAFSELGDEIARLRWLQ | 73 |
| Query 72 | TTGVPCAAVLDVVTEAGRDWLLLGEVPGQDLLSS-HLAPAEKVSIMADAMRRLHTLDPAT | 130 |
| Sbjct 74 | G P V+ EAGR WLL+ +PG+DL SS LAP + V ++ADA+R LH L A EQGQPAPTVIATAEEAGRRWLLMSALPGRDLASSPELAPQQLVELLADALRGLHALPVAA | 133 |
| Query 131 | CPFDHQAKHRIERARTRMEAGLVDQDDLDEEHQGLAPAELFARLKARMPDGEDLVVTHGD | 190 |
| Sbjct 134 | CPFD + R++ A+ R+EAGLVD DD D+E G +P ++FA L + PD EDLVV+HGD CPFDQRLVSRLQAAQARVEAGLVDADDFDDERLGQSPQQVFAELCSTRPDHEDLVVSHGD | 193 |
| Query 191 | ACLPNIMVENGRFSGFIDCGRLGVADRYQDIALATRDIAEELG-GEWADRFLVLYGIAAP | 249 |
| Sbjct 194 | ACLPN+MV GRFSGFIDCGRLGVADRYQD+ALA R + G W YG A ACLPNLMVVEGRFSGFIDCGRLGVADRYQDLALAARSLVHNFGDTRWVAPLFQRYGAVA- | 252 |
| Query 250  Sbjct 253 | DSQRIAFYRLLDEFF 264 D +R+AFYRLLDEFF DERRLAFYRLLDEFF 267 |  |

>WP_106795451.1 aminoglycoside 3'-phosphotransferase [Rhizobium sp. H4] Length=261

Score = 249 bits (637), Expect = 3e-79, Method: Compositional matrix adjust.

| Identities | | = 131/254 (52%), Positives = 169/254 (67%), Gaps = 1/254 (0%) |  |
| --- | --- | --- | --- |
| Query 12  Sbjct 8 | | PAAWVERLFGYDWAQQTIGCSDAAVFRLSAQGRPVLFVKTDLSGALNELQDEAARLSWLA PAA ERL GY + + +G S A+VFRL +G P L++K + +G EL DEAARLSWL PAALGERLSGYRFERDALGRSAASVFRLEGEGLPALYLKVEAAGPFGELADEAARLSWLK | 71  67 |
| Query 72  Sbjct 68 | | TTGVPCAAVLDVVTEAGRDWLLLGEVPGQDLLS-SHLAPAEKVSIMADAMRRLHTLDPAT G+PC V+ ++ R+WLL+G +PG +L S S L P +V ++A A+ LH L + AAGLPCLDVIARESDGKRNWLLIGALPGTNLASASALTPLARVELLAAALADLHHLPITS | 130  127 |
| Query 131  Sbjct 128 | | CPFDHQAKHRIERARTRMEAGLVDQDDLDEEHQGLAPAELFARLKARMPDGEDLVVTHGD CPFDH+ + R+ A RM+AG+VD+ D DE G + LFA L+ R PD EDLVVTHGD CPFDHRLEKRLPAAGARMQAGIVDETDFDETRLGKSAETLFAELEGRRPDREDLVVTHGD | 190  187 |
| Query 191  Sbjct 188 | | ACLPNIMVENGRFSGFIDCGRLGVADRYQDIALATRDIAEELGGEWADRFLVLYGIAAPD ACLPN + G+FSG+IDC RLGVADRYQDIALA R IA+ G FL YG+ APD ACLPNFVASGGQFSGYIDCSRLGVADRYQDIALACRSIADNFGAALVQPFLDRYGLTAPD | 250  247 |
| Query 251 | | SQRIAFYRLLDEFF 264 R+ +Y+LLDEFF |  |
| Sbjct | 248 | PARLDYYQLLDEFF 261 | |

>WP_173411002.1 APH(3') family aminoglycoside O-phosphotransferase [Ensifer adhaerens] NRP86189.1 Aminoglycoside 3'-phosphotransferase [Ensifer adhaerens]

Length=266

Score = 250 bits (638), Expect = 3e-79, Method: Compositional matrix adjust.

| Identities | = 130/254 (51%), Positives = 168/254 (66%), Gaps = 1/254 (0%) |  |
| --- | --- | --- |
| Query 12 | PAAWVERLFGYDWAQQTIGCSDAAVFRLSAQGRPVLFVKTDLSGALNELQDEAARLSWLA | 71 |
| Sbjct 13 | PA + L GYDW +G S A VFRL A GR +++KT++ +L EL E +RL WLA PARLRDLLAGYDWRPAELGRSPAHVFRLEAVGRAPIYLKTEIIHSLGELAGEVSRLRWLA | 72 |
| Query 72 | TTGVPCAAVLDVVTEAGRDWLLLGEVPGQDLLSS-HLAPAEKVSIMADAMRRLHTLDPAT | 130 |
| Sbjct 73 | + G+ C V+ T+ GR+WLL+ +PG DL+S+ L P E+V I+A A+RRLH LD AT SKGLACPEVIAHETDGGREWLLMSALPGTDLVSNDRLPPIERVRILAAALRRLHGLDIAT | 132 |
| Query 131 | CPFDHQAKHRIERARTRMEAGLVDQDDLDEEHQGLAPAELFARLKARMPDGEDLVVTHGD | 190 |
| Sbjct 133 | CPFDH+ ++RI A+ R+ AG VD+DD D+ G + +LF L+ R P EDLVVTHGD CPFDHRLENRIADAKARLLAGCVDEDDFDDARLGRSAQDLFRELEERRPKTEDLVVTHGD | 192 |
| Query 191 | ACLPNIMVENGRFSGFIDCGRLGVADRYQDIALATRDIAEELGGEWADRFLVLYGIAAPD | 250 |
| Sbjct 193 | ACLPN + G SG+IDCGRLGVADR+QDIALA IA G + FL YG ACLPNFVAHEGSISGYIDCGRLGVADRHQDIALACGSIARNFGEDLVADFLKAYGNFEMH | 252 |
| Query 251  Sbjct 253 | SQRIAFYRLLDEFF 264 S++ A+YRLLDEFF SEKAAYYRLLDEFF 266 |  |

>WP_188241290.1 APH(3')-II family aminoglycoside O-phosphotransferase [Stenotrophomonas maltophilia]

Length=267

Score = 250 bits (638), Expect = 3e-79, Method: Compositional matrix adjust.

| Identities | = 133/255 (52%), Positives = 167/255 (65%), Gaps = 3/255 (1%) |  |
| --- | --- | --- |
| Query 12 | PAAWVERLFGYDWAQQTIGCSDAAVFRLSAQGRPVLFVKTDLSGALNELQDEAARLSWLA | 71 |
| Sbjct 14 | P AW + L +Q++G S A V R+ GRP F+K++L A +EL DE ARL WL PLAWQKALADARIERQSMGVSRADVARVLRPGRPDAFLKSELIDAFSELGDEIARLRWLQ | 73 |
| Query 72 | TTGVPCAAVLDVVTEAGRDWLLLGEVPGQDLLSS-HLAPAEKVSIMADAMRRLHTLDPAT | 130 |
| Sbjct 74 | G P V+ EAGR WLL+ +PG+DL SS LAP ++V I+ADA+R LH L A AQGQPAPTVIATAEEAGRHWLLMSALPGRDLASSPELAPQQRVEILADALRGLHALPVAA | 133 |
| Query 131 | CPFDHQAKHRIERARTRMEAGLVDQDDLDEEHQGLAPAELFARLKARMPDGEDLVVTHGD | 190 |
| Sbjct 134 | CPFD + R+ A+ R+EAG VD DD D+E G +P ++FA+L + PD EDLVV+HGD CPFDQRLASRLPAAQARVEAGRVDADDFDDERLGQSPQQVFAQLCSTRPDHEDLVVSHGD | 193 |
| Query 191 | ACLPNIMVENGRFSGFIDCGRLGVADRYQDIALATRDIAEELG-GEWADRFLVLYGIAAP | 249 |
| Sbjct 194 | AC+PN+MV GRFSGFIDCGRLGVADRYQD+ALA R + G W YG A ACVPNLMVSEGRFSGFIDCGRLGVADRYQDLALAARSLVHNFGDTRWVTPLFQRYGAVA- | 252 |
| Query 250  Sbjct 253 | DSQRIAFYRLLDEFF 264 D +R+AFYRLLDEFF DERRLAFYRLLDEFF 267 |  |

>WP_169269307.1 APH(3') family aminoglycoside O-phosphotransferase [Achromobacter sp. Bel]

NMK44854.1 aminoglycoside 3'-phosphotransferase [Achromobacter sp. Bel] Length=266

Score = 250 bits (638), Expect = 3e-79, Method: Compositional matrix adjust. Identities = 128/253 (51%), Positives = 167/253 (66%), Gaps = 1/253 (0%)

| Query | 12 | PAAWVERLFGYDWAQQTIGCSDAAVFRLSAQGRPVLFVKTDLSGALNELQDEAARLSWLA | 71 |
| --- | --- | --- | --- |
| Sbjct | 15 | PA W E QQ+IG S A VFR+ +P LF+K++ +GA++EL DE RL WL PAKWREGFTHASIEQQSIGESRADVFRVRRPNQPDLFLKSERAGAVDELPDEIDRLRWLR | 74 |
| Query | 72 | TTGVPCAAVLDVVTEAGRDWLLLGEVPGQDLLSSHLAPAEKVSIMADAMRRLHTLDPATC | 131 |
| Sbjct | 75 | G P VLD E GR WLL+ + G+DL ++ L PA+ + +A A+R LH L A+C  QVGQPAPHVLDTAMEGGRHWLLMTALLGRDLATAALPPAQVIGALAMALRNLHRLPIASC | 134 |

| Query | 132 | PFDHQAKHRIERARTRMEAGLVDQDDLDEEHQGLAPAELFARLKARMPDGEDLVVTHGDA | 191 |
| --- | --- | --- | --- |
| Sbjct | 135 | PFDH+ + RI +A+ ++AGLVD+ D D+E G + ++ A L + P DLVVTHGDA PFDHRLEARIPQAKRHVDAGLVDETDFDDERLGQSARQVLAELLSSRPTAHDLVVTHGDA | 194 |
| Query | 192 | CLPNIMVENGRFSGFIDCGRLGVADRYQDIALATRDIAEELGGEWADRFLVLYGIAAPDS | 251 |
| Sbjct | 195 | CLPN MV+ +F+GFIDCGRLG+ADR+QD+ALA R IA +G EW F YGI APD+ CLPNFMVDGHQFTGFIDCGRLGIADRFQDLALAARSIARNMGKEWVTPFFHEYGI-APDA | 253 |
| Query  Sbjct | 252  254 | QRIAFYRLLDEFF 264 QRI FY LLDEFF QRIRFYCLLDEFF 266 |  |

>WP_164222148.1 APH(3')-II family aminoglycoside O-phosphotransferase [Stenotrophomonas maltophilia]

Length=267

Score = 249 bits (637), Expect = 3e-79, Method: Compositional matrix adjust.

| Identities | = 135/255 (53%), Positives = 167/255 (65%), Gaps = 3/255 (1%) |  |
| --- | --- | --- |
| Query 12 | PAAWVERLFGYDWAQQTIGCSDAAVFRLSAQGRPVLFVKTDLSGALNELQDEAARLSWLA | 71 |
| Sbjct 14 | P AW E L +Q+IG S A V R+ G+P FVK+++ A +EL DE ARL WL PWAWQEALADARVERQSIGVSRADVARVQRPGQPDAFVKSEVIDAFSELGDEIARLRWLQ | 73 |
| Query 72 | TTGVPCAAVLDVVTEAGRDWLLLGEVPGQDLLSS-HLAPAEKVSIMADAMRRLHTLDPAT | 130 |
| Sbjct 74 | G P V+ EAGR WLL+ +PG+DL SS LAP + V ++ADA+R LH L A EQGQPAPTVIATAEEAGRRWLLMSALPGRDLASSPELAPQQLVELLADALRGLHALPLAA | 133 |
| Query 131 | CPFDHQAKHRIERARTRMEAGLVDQDDLDEEHQGLAPAELFARLKARMPDGEDLVVTHGD | 190 |
| Sbjct 134 | CPFD + R++ A+ R+EAGLVD DD D+E G +P ++FA L + PD EDLVV+HGD CPFDQRLHLRLQAAQARVEAGLVDADDFDDERLGQSPQQVFAELCSTRPDHEDLVVSHGD | 193 |
| Query 191 | ACLPNIMVENGRFSGFIDCGRLGVADRYQDIALATRDIAEELG-GEWADRFLVLYGIAAP | 249 |
| Sbjct 194 | ACLPN+MV GRFSGFIDCGRLGVADRYQD+ALA R + G W YG A ACLPNLMVVEGRFSGFIDCGRLGVADRYQDLALAARSLVHNFGDTRWVAPLFQCYGAVA- | 252 |
| Query 250  Sbjct 253 | DSQRIAFYRLLDEFF 264 D +R+AFYRLLDEFF DERRLAFYRLLDEFF 267 |  |

>WP_174180146.1 APH(3') family aminoglycoside O-phosphotransferase [Agrobacterium rhizogenes]

NTJ67317.1 aminoglycoside 3'-phosphotransferase [Agrobacterium rhizogenes] NTJ77161.1 aminoglycoside 3'-phosphotransferase [Agrobacterium rhizogenes]

Length=266

Score = 249 bits (637), Expect = 3e-79, Method: Compositional matrix adjust.

| Identities | | = 126/259 (49%), Positives = 170/259 (66%), Gaps = 1/259 (0%) |  |
| --- | --- | --- | --- |
| Query 7  Sbjct 8 | | LHAGSPAAWVERLFGYDWAQQTIGCSDAAVFRLSAQGRPVLFVKTDLSGALNELQDEAAR  + P + L GY+W Q +G S A VFRL A GR +++KT+ L EL E AR METSIPVRLRDALAGYEWRQDALGRSSAHVFRLEADGRAPVYLKTEPVDPLGELAGEVAR | 66  67 |
| Query 67  Sbjct 68 | | LSWLATTGVPCAAVLDVVTEAGRDWLLLGEVPGQDLLSS-HLAPAEKVSIMADAMRRLHT L WL + G+ C V+ E R+WLL+ +PG+DL+S+ L P E+V ++ADA+RRLH LQWLTSRGLACPEVIAHEQEDEREWLLMSALPGRDLVSAARLQPIERVRLLADALRRLHA | 125  127 |
| Query 126  Sbjct 128 | | LDPATCPFDHQAKHRIERARTRMEAGLVDQDDLDEEHQGLAPAELFARLKARMPDGEDLV  +D A CPFDH+ ++RI AR R+ AG VD++D DE G + +LF L+A+ P ED V VDIAACPFDHRLENRIGAARARLLAGRVDENDFDEARLGRSTEDLFRELEAKRPSTEDFV | 185  187 |
| Query 186  Sbjct 188 | | VTHGDACLPNIMVENGRFSGFIDCGRLGVADRYQDIALATRDIAEELGGEWADRFLVLYG VTHGDACLPN +V+ RFSG+IDCGRLG+ADR+QDIALA I G E FL +YG VTHGDACLPNFVVDGDRFSGYIDCGRLGIADRHQDIALACGSIEYNFGEELVAEFLKVYG | 245  247 |
| Query 246 | | IAAPDSQRIAFYRLLDEFF 264 P ++++A+Y+LLDEFF |  |
| Sbjct | 248 | DFEPKAEKMAYYQLLDEFF 266 | |

>WP_099487410.1 APH(3')-II family aminoglycoside O-phosphotransferase [Stenotrophomonas maltophilia]

Length=267

Score = 249 bits (637), Expect = 4e-79, Method: Compositional matrix adjust.

| Identities | = 134/255 (53%), Positives = 166/255 (65%), Gaps = 3/255 (1%) |  |
| --- | --- | --- |
| Query 12 | PAAWVERLFGYDWAQQTIGCSDAAVFRLSAQGRPVLFVKTDLSGALNELQDEAARLSWLA | 71 |
| Sbjct 14 | P AW + L +Q++G S A V R+ GRP FVK++L A +EL DE ARL WL PLAWQKALADARIERQSMGVSRADVARVLRPGRPDAFVKSELIDAFSELGDEIARLRWLQ | 73 |
| Query 72 | TTGVPCAAVLDVVTEAGRDWLLLGEVPGQDLLSS-HLAPAEKVSIMADAMRRLHTLDPAT | 130 |
| Sbjct 74 | G P V+ EAGR WLL+ +PG+DL SS LAP ++V ++A A+R LH L A AQGQPAPTVIATAEEAGRRWLLMSALPGRDLASSPELAPQQRVQVLAGALRGLHALPVAA | 133 |
| Query 131 | CPFDHQAKHRIERARTRMEAGLVDQDDLDEEHQGLAPAELFARLKARMPDGEDLVVTHGD | 190 |
| Sbjct 134 | CPFD + R+ A+ R+EAGLVD DD D+E G +P ++FA+L + PD EDLVV+HGD CPFDQRLASRLPAAQARVEAGLVDADDFDDERLGQSPQQVFAQLCSTRPDHEDLVVSHGD | 193 |
| Query 191 | ACLPNIMVENGRFSGFIDCGRLGVADRYQDIALATRDIAEELG-GEWADRFLVLYGIAAP | 249 |
| Sbjct 194 | ACLPN+MV GRFSGFIDCGRLGVADRYQD+ALA R + G W YG A ACLPNLMVAEGRFSGFIDCGRLGVADRYQDLALAARSLVHNFGDTRWVTPLFQRYGAVA- | 252 |
| Query 250  Sbjct 253 | DSQRIAFYRLLDEFF 264 D R+AFYRLLDEFF DECRLAFYRLLDEFF 267 |  |

>WP_052212711.1 MULTISPECIES: aminoglycoside 3'-phosphotransferase [Rhizobium] Length=266

Score = 249 bits (637), Expect = 4e-79, Method: Compositional matrix adjust.

| Identities | = 130/254 (51%), Positives = 168/254 (66%), Gaps = 1/254 (0%) |  |
| --- | --- | --- |
| Query 12 | PAAWVERLFGYDWAQQTIGCSDAAVFRLSAQGRPVLFVKTDLSGALNELQDEAARLSWLA | 71 |
| Sbjct 13 | PA + L GYDW +G S A VFRL A GR +++KT++ L+EL E +RL WLA PARLRDLLAGYDWRPAELGRSPAHVFRLEAVGRAPIYLKTEIIHPLSELAGEVSRLRWLA | 72 |
| Query 72 | TTGVPCAAVLDVVTEAGRDWLLLGEVPGQDLLSS-HLAPAEKVSIMADAMRRLHTLDPAT | 130 |
| Sbjct 73 | + G+ C V+ T+ GR+WLL+ +PG DL+S+ L P E+V I+A A+RRLH LD AT SKGLACPEVIAHETDGGREWLLMSALPGTDLVSNDRLPPIERVRILAAALRRLHGLDIAT | 132 |
| Query 131 | CPFDHQAKHRIERARTRMEAGLVDQDDLDEEHQGLAPAELFARLKARMPDGEDLVVTHGD | 190 |
| Sbjct 133 | CPFDH+ ++RI A+ R+ AG VD+DD D+ G + +LF L+ R P EDLVVTHGD CPFDHRLENRIADAKARLLAGCVDEDDFDDARLGRSAQDLFRELEERRPKTEDLVVTHGD | 192 |
| Query 191 | ACLPNIMVENGRFSGFIDCGRLGVADRYQDIALATRDIAEELGGEWADRFLVLYGIAAPD | 250 |
| Sbjct 193 | ACLPN + G SG+IDCGRLGVADR+QDIALA IA G + FL YG ACLPNFVAHEGSISGYIDCGRLGVADRHQDIALACGSIARNFGEDLVADFLKAYGNFEMH | 252 |
| Query 251  Sbjct 253 | SQRIAFYRLLDEFF 264 S++ A+YRLLDEFF SEKAAYYRLLDEFF 266 |  |

>WP_196633161.1 APH(3')-II family aminoglycoside O-phosphotransferase [Pseudomonas aeruginosa]

MBG5058830.1 APH(3')-II family aminoglycoside O-phosphotransferase [Pseudomonas aeruginosa]

Length=267

Score = 249 bits (637), Expect = 4e-79, Method: Compositional matrix adjust. Identities = 136/258 (53%), Positives = 169/258 (66%), Gaps = 5/258 (2%)

| Query | 11 | SPAAWVERLFGYDWAQQTIGCSDAAVFRLSAQGRPVLFVKTDLSGALNELQDEAARLSWL | 70 |
| --- | --- | --- | --- |
| Sbjct | 11 | +P+ W + L GY W Q GCS A V RL A+ RP LFVK ++ A EL E ARL WL APSTWADYLAGYRWRGQGEGCSAATVHRLEAERRPTLFVKQEVLSAHAELPAEIARLRWL | 70 |
| Query | 71 | ATTGVPCAAVLDVVTEAGRDWLLLGEVPGQDLLS SHLAPAEKVSIMADAMRRLHTL | 126 |
| Sbjct | 71 | G+ C VL+ GR WLL+ VPG L + L P V ++A A+RRLH L  HGAGIDCPQVLNETQSDGRQWLLMSAVPGDTLSALAQRGELEPERLVRLVAAALRRLHDL | 130 |

| Query | 127 | DPATCPFDHQAKHRIERARTRMEAGLVDQDDLDEEHQGLAPAELFARLKARMPDGEDLVV | 186 |
| --- | --- | --- | --- |
| Sbjct | 131 | DPA CPFDH+ + R++ R R+EAGLVD+ D D++H+G + EL+ L R P EDLVV DPAACPFDHRLERRLDTVRQRVEAGLVDEADFDDDHRGRSATELYRLLLDRRPAVEDLVV | 190 |
| Query | 187 | THGDACLPNIMVENGRFSGFIDCGRLGVADRYQDIALATRDIAEELGGEWADRFLVLYGI | 246 |
| Sbjct | 191 | HGDACLPN++ E RFSGFIDCGRLGVADR+QD+ALA RDI ELG A+ FLV YG  AHGDACLPNLLAEGRRFSGFIDCGRLGVADRHQDLALAARDIEAELGAAGAEAFLVEYG- | 249 |
| Query | 247 AAPDSQRIAFYRLLDEFF 264 | | |
|  |  | D +R+A++RLLDEFF |  |
| Sbjct | 250 | GDIDGERLAYFRLLDEFF 267 | |

>WP_049396294.1 APH(3')-II family aminoglycoside O-phosphotransferase [Stenotrophomonas maltophilia]

MBA0337813.1 APH(3')-II family aminoglycoside O-phosphotransferase [Stenotrophomonas maltophilia]

MBA0541295.1 APH(3')-II family aminoglycoside O-phosphotransferase [Stenotrophomonas maltophilia]

QGM10342.1 APH(3')-II family aminoglycoside O-phosphotransferase [Stenotrophomonas maltophilia]

QNG73844.1 aminoglycoside O-phosphotransferase APH(3')-IIc [Stenotrophomonas maltophilia]

REC83769.1 APH(3')-II family aminoglycoside O-phosphotransferase [Stenotrophomonas maltophilia]

Length=267

Score = 249 bits (637), Expect = 4e-79, Method: Compositional matrix adjust.

| Identities | = 135/255 (53%), Positives = 167/255 (65%), Gaps = 3/255 (1%) |  |
| --- | --- | --- |
| Query 12 | PAAWVERLFGYDWAQQTIGCSDAAVFRLSAQGRPVLFVKTDLSGALNELQDEAARLSWLA | 71 |
| Sbjct 14 | P AW E L +Q+IG S A V R+ G+P FVK+++ A +EL DE ARL WL PWAWQEALADARVERQSIGVSRADVARVQRPGQPDAFVKSEVIDAFSELGDEIARLRWLQ | 73 |
| Query 72 | TTGVPCAAVLDVVTEAGRDWLLLGEVPGQDLLSS-HLAPAEKVSIMADAMRRLHTLDPAT | 130 |
| Sbjct 74 | G P V+ EAGR WLL+ +PG+DL SS LAP + V ++ADA+R LH L A EQGQPAPTVIATAEEAGRRWLLMSALPGRDLASSPELAPQQLVELLADALRGLHALPLAA | 133 |
| Query 131 | CPFDHQAKHRIERARTRMEAGLVDQDDLDEEHQGLAPAELFARLKARMPDGEDLVVTHGD | 190 |
| Sbjct 134 | CPFD + R++ A+ R+EAGLVD DD D+E G +P ++FA L + PD EDLVV+HGD CPFDQRLHLRLQAAQARVEAGLVDADDFDDERLGQSPQQVFAELCSTRPDHEDLVVSHGD | 193 |
| Query 191 | ACLPNIMVENGRFSGFIDCGRLGVADRYQDIALATRDIAEELG-GEWADRFLVLYGIAAP | 249 |
| Sbjct 194 | ACLPN+MV GRFSGFIDCGRLGVADRYQD+ALA R + G W YG A ACLPNLMVVEGRFSGFIDCGRLGVADRYQDLALAARSLVHNFGDTRWVAPLFQRYGAVA- | 252 |
| Query 250  Sbjct 253 | DSQRIAFYRLLDEFF 264 D +R+AFYRLLDEFF DERRLAFYRLLDEFF 267 |  |

>WP_138953620.1 APH(3')-II family aminoglycoside O-phosphotransferase [Stenotrophomonas maltophilia]

TIK68913.1 APH(3')-II family aminoglycoside O-phosphotransferase [Stenotrophomonas maltophilia]

TIK71164.1 APH(3')-II family aminoglycoside O-phosphotransferase [Stenotrophomonas maltophilia]

Length=267

Score = 249 bits (636), Expect = 5e-79, Method: Compositional matrix adjust.

| Identities | = 133/255 (52%), Positives = 166/255 (65%), Gaps = 3/255 (1%) |  |
| --- | --- | --- |
| Query 12 | PAAWVERLFGYDWAQQTIGCSDAAVFRLSAQGRPVLFVKTDLSGALNELQDEAARLSWLA | 71 |
| Sbjct 14 | P AW + L +Q++G S A V R+ GRP F+K++L A +EL DE ARL WL PLAWQKALADARIERQSMGVSRADVARVLRPGRPDAFLKSELIDAFSELGDEIARLRWLQ | 73 |
| Query 72 | TTGVPCAAVLDVVTEAGRDWLLLGEVPGQDLLSS-HLAPAEKVSIMADAMRRLHTLDPAT | 130 |
| Sbjct 74 | G P V+ E GR WLL+ +PG+DL SS LAP ++V I+ADA+R LH L A AQGQPAPTVIATAEEGGRHWLLMSALPGRDLASSPELAPQQRVEILADALRGLHALPVAA | 133 |
| Query 131 | CPFDHQAKHRIERARTRMEAGLVDQDDLDEEHQGLAPAELFARLKARMPDGEDLVVTHGD CPFD + R+ A+ R+EAG VD DD D+E G +P ++FA+L + PD EDLVV+HGD | 190 |

| Sbjct | 134 | CPFDQRLASRLPAAQARVEAGRVDADDFDDERLGQSPQQVFAQLCSTRPDHEDLVVSHGD | 193 |
| --- | --- | --- | --- |
| Query | 191 | ACLPNIMVENGRFSGFIDCGRLGVADRYQDIALATRDIAEELG-GEWADRFLVLYGIAAP | 249 |
| Sbjct | 194 | ACLPN+MV GRFSGFIDCGRLGVADRYQD+ALA R + G W YG A ACLPNLMVAEGRFSGFIDCGRLGVADRYQDLALAARSLVHNFGDTRWVTPLFQRYGAVA- | 252 |
| Query  Sbjct | 250  253 | DSQRIAFYRLLDEFF 264 D +R+AFYRLLDEFF DERRLAFYRLLDEFF 267 |  |

>WP_007960488.1 aminoglycoside 3'-phosphotransferase [Pseudomonas sp. GM30] EUB85194.1 aminoglycoside phosphotransferase [Pseudomonas sp. GM30]

Length=256

Score = 249 bits (635), Expect = 5e-79, Method: Compositional matrix adjust.

| Identities | | = 130/254 (51%), Positives = 164/254 (65%), Gaps = 2/254 (1%) |  |
| --- | --- | --- | --- |
| Query 12  Sbjct 4 | | PAAWVERLFGYDWAQQTIGCSDAAVFRLSAQGRPVLFVKTDLSGALNELQDEAARLSWLA P W +R + QQTIG S A VFRL+ G LFVK++ + EL E RL WLA PPTWRQRFADIRYEQQTIGASRADVFRLTPAGSDPLFVKSEPRISFAELPGEVQRLRWLA | 71  63 |
| Query 72  Sbjct 64 | | TTGVPCAAVLDVVTEAGRDWLLLGEVPGQDLLSS-HLAPAEKVSIMADAMRRLHTLDPAT VP A VLD R WLL+ VPG+DL SS LAP + VS+ A A+R LH LD EQDVPAARVLDSTEAHDRYWLLMTAVPGRDLASSGDLAPQQIVSLAAHALRTLHALDIKN | 130  123 |
| Query 131  Sbjct 124 | | CPFDHQAKHRIERARTRMEAGLVDQDDLDEEHQGLAPAELFARLKARMPDGEDLVVTHGD CPFDH+ +I AR + AGL+D++D D+ G + +F ++ A PD EDLVVTHGD CPFDHRLDAKIALAREHLLAGLIDEEDFDDSRLGQSAEAVFEQMLAARPDSEDLVVTHGD | 190  183 |
| Query 191  Sbjct 184 | | ACLPNIMVENGRFSGFIDCGRLGVADRYQDIALATRDIAEELGGEWADRFLVLYGIAAPD ACLPN++ +NGRFSGF+DCGRLGV+DR+QD+ALA IA LG +W F YG+ PD ACLPNLLADNGRFSGFVDCGRLGVSDRFQDLALAAHSIAYNLGEQWLPVFFEAYGV-QPD | 250  242 |
| Query 251 | | SQRIAFYRLLDEFF 264  ++IAFY+LLDEFF |  |
| Sbjct | 243 | QRKIAFYQLLDEFF 256 | |

>WP_134709277.1 APH(3')-II family aminoglycoside O-phosphotransferase [Stenotrophomonas maltophilia]

Length=267

Score = 249 bits (636), Expect = 5e-79, Method: Compositional matrix adjust.

| Identities | = 134/255 (53%), Positives = 166/255 (65%), Gaps = 3/255 (1%) |  |
| --- | --- | --- |
| Query 12 | PAAWVERLFGYDWAQQTIGCSDAAVFRLSAQGRPVLFVKTDLSGALNELQDEAARLSWLA | 71 |
| Sbjct 14 | P AW + L +Q++G S A V R+ GRP FVK++L A +EL DE ARL WL PLAWQKALADARIERQSMGVSRADVARVLRPGRPDAFVKSELIDAFSELGDEIARLRWLQ | 73 |
| Query 72 | TTGVPCAAVLDVVTEAGRDWLLLGEVPGQDLLSS-HLAPAEKVSIMADAMRRLHTLDPAT | 130 |
| Sbjct 74 | G P V+ EAGR WLL+ +PG+DL SS LAP ++V ++A A+R LH L A AQGQPAPTVIATAEEAGRRWLLMSALPGRDLASSPELAPQQRVQVLAGALRGLHALPVAA | 133 |
| Query 131 | CPFDHQAKHRIERARTRMEAGLVDQDDLDEEHQGLAPAELFARLKARMPDGEDLVVTHGD | 190 |
| Sbjct 134 | CPFD + R+ A+ R+EAGLVD DD D+E G +P ++FA+L + PD EDLVV+HGD CPFDQRLASRLPVAQARVEAGLVDADDFDDERLGQSPQQVFAQLCSTRPDHEDLVVSHGD | 193 |
| Query 191 | ACLPNIMVENGRFSGFIDCGRLGVADRYQDIALATRDIAEELG-GEWADRFLVLYGIAAP | 249 |
| Sbjct 194 | ACLPN+MV GRFSGFIDCGRLGVADRYQD+ALA R + G W YG A ACLPNLMVAEGRFSGFIDCGRLGVADRYQDLALAARSLVHNFGDTRWVTPLFQRYGAVA- | 252 |
| Query 250  Sbjct 253 | DSQRIAFYRLLDEFF 264 D R+AFYRLLDEFF DECRLAFYRLLDEFF 267 |  |

>WP_196627072.1 APH(3')-II family aminoglycoside O-phosphotransferase, partial [Pseudomonas aeruginosa]

MBG4121893.1 APH(3')-II family aminoglycoside O-phosphotransferase [Pseudomonas aeruginosa]

Length=261

Score = 249 bits (635), Expect = 5e-79, Method: Compositional matrix adjust.

| Identities | | = 131/251 (52%), Positives = 162/251 (65%), Gaps = 5/251 (2%) |  |
| --- | --- | --- | --- |
| Query 11  Sbjct 12 | | SPAAWVERLFGYDWAQQTIGCSDAAVFRLSAQGRPVLFVKTDLSGALNELQDEAARLSWL  +PA W + L GY W Q GCS A V RL A RP LFVK ++ A EL E ARL WL APATWADYLAGYRWRGQGEGCSAATVHRLEAARRPTLFVKQEVLSAHAELPAEIARLRWL | 70  71 |
| Query 71  Sbjct 72 | | ATTGVPCAAVLDVVTEAGRDWLLLGEVPGQDLLS SHLAPAEKVSIMADAMRRLHTL  G+ C VL+ GR WLL+ VPG L + L P V ++A A+RRLH L HGAGIDCPQVLNETQSDGRQWLLMSAVPGDTLSALAQRGELEPERLVRLVAAALRRLHDL | 126  131 |
| Query 127  Sbjct 132 | | DPATCPFDHQAKHRIERARTRMEAGLVDQDDLDEEHQGLAPAELFARLKARMPDGEDLVV DPA CPFDH+ + R++ R R+EAGLVD+ D D++H+G + EL+ L R P EDLVV DPAACPFDHRLERRLDTVRQRVEAGLVDEADFDDDHRGRSATELYRLLLDRRPAVEDLVV | 186  191 |
| Query 187  Sbjct 192 | | THGDACLPNIMVENGRFSGFIDCGRLGVADRYQDIALATRDIAEELGGEWADRFLVLYGI HGDACLPN++ E RFSGFIDCGRLGVADR+QD+ALA RDI ELG WA+ FLV YG AHGDACLPNLLAEGRRFSGFIDCGRLGVADRHQDLALAARDIEAELGAAWAEAFLVEYG- | 246  250 |
| Query | 247 AAPDSQRIAFY 257 | | |
|  |  | D +R+A++ |  |
| Sbjct | 251 | GDIDGERLAYF 261 | |

>WP_175136224.1 APH(3') family aminoglycoside O-phosphotransferase [Achromobacter animicus]

Length=263

Score = 249 bits (635), Expect = 5e-79, Method: Compositional matrix adjust.

| Identities | | = 127/264 (48%), Positives = 165/264 (63%), Gaps = 1/264 (0%) |  |
| --- | --- | --- | --- |
| Query 1  Sbjct 1 | | MIEQDGLHAGSPAAWVERLFGYDWAQQTIGCSDAAVFRLSAQGRPVLFVKTDLSGALNEL M Q +H P W RL G QQ+IG S A V+R+ + +P ++K++ +G L EL MHAQYSVHDSLPLQWQPRLAGARLEQQSIGESRADVYRIRSANQPDRYLKSEHTGTLPEL | 60  60 |
| Query 61  Sbjct 61 | | QDEAARLSWLATTGVPCAAVLDVVTEAGRDWLLLGEVPGQDLLSSHLAPAEKVSIMADAM DE RL W+A G+PC V+D R WLL+ V GQDL S+ L PA+ ++IMADA+ PDEIERLQWMAAHGLPCPTVIDAAATPTRHWLLMSAVAGQDLASAGLPPAQVIAIMADAL | 120  120 |
| Query 121  Sbjct 121 | | RRLHTLDPATCPFDHQAKHRIERARTRMEAGLVDQDDLDEEHQGLAPAELFARLKARMPD RLH + CPFDH + RI AR ++AGLVD+ D D+E G + FA + A P HRLHCIPIDQCPFDHTLERRIAEARRALDAGLVDESDFDDERIGRTAEDAFAEVLATRPT | 180  180 |
| Query 181  Sbjct 181 | | GEDLVVTHGDACLPNIMVENGRFSGFIDCGRLGVADRYQDIALATRDIAEELGGEWADRF DLVV HGDACLPN M + F+G+IDCGRLGV+DR+QD+ALA R I LG EW + F THDLVVAHGDACLPNFMADGDVFTGYIDCGRLGVSDRHQDLALAARSIERNLGREWIEPF | 240  240 |
| Query 241 | | LVLYGIAAPDSQRIAFYRLLDEFF 264 YG+ PD +R+AFY LLDEFF |  |
| Sbjct | 241 | FRSYGV-EPDDRRLAFYCLLDEFF 263 | |

>WP_196685833.1 APH(3')-II family aminoglycoside O-phosphotransferase [Pseudomonas aeruginosa]

MBG4622275.1 APH(3')-II family aminoglycoside O-phosphotransferase [Pseudomonas aeruginosa]

Length=268

Score = 249 bits (636), Expect = 6e-79, Method: Compositional matrix adjust. Identities = 131/258 (51%), Positives = 163/258 (63%), Gaps = 5/258 (2%)

| Query | 11 | SPAAWVERLFGYDWAQQTIGCSDAAVFRLSAQGRPVLFVKTDLSGALNELQDEAARLSWL | 70 |
| --- | --- | --- | --- |
| Sbjct | 12 | +P+ W + L GY W Q GCS A V RL A RP LFVK ++ EL E ARL WL APSTWADYLAGYRWRGQGEGCSAATVHRLEAARRPTLFVKQEVLSVHAELPAEIARLRWL | 71 |
| Query | 71 | ATTGVPCAAVLDVVTEAGRDWLLLGEVPGQDLLS SHLAPAEKVSIMADAMRRLHTL | 126 |
| Sbjct | 72 | G+ C VL+ GR WLL+ VPG L + L P V ++A A+RRLH L HGAGIDCPQVLNETQSDGRQWLLMSAVPGDTLSALAQRGELEPERLVRLVAAALRRLHDL | 131 |
| Query | 127 | DPATCPFDHQAKHRIERARTRMEAGLVDQDDLDEEHQGLAPAELFARLKARMPDGEDLVV DPA CPFDH+ + R++ R R+EAGLVD+ D D++H+G + EL+ L AR P EDLVV | 186 |

| Sbjct | 132 | DPAACPFDHRLERRLDTVRQRVEAGLVDEADFDDDHRGRSATELYRLLLARRPAVEDLVV | 191 |
| --- | --- | --- | --- |
| Query | 187 | THGDACLPNIMVENGRFSGFIDCGRLGVADRYQDIALATRDIAEELGGEWADRFLVLYGI | 246 |
| Sbjct | 192 | HGDACLPN++ E RF GFIDCGR G ADR+QD+A A RD E G WA+ FLV YG  AHGDACLPNLLAEGRRFRGFIDCGRRGGADRHQDLARAARDSEAERGAAWAEAFLVEYG- | 250 |
| Query | 247 AAPDSQRIAFYRLLDEFF 264 | | |
|  |  | D +R+A++RLLDEFF |  |
| Sbjct | 251 | GDIDGERLAYFRLLDEFF 268 | |

>ASW08172.1 APH(3') family aminoglycoside O-phosphotransferase [Rhizobium sp. 11515TR]

Length=267

Score = 249 bits (636), Expect = 6e-79, Method: Compositional matrix adjust.

| Identities | = 126/247 (51%), Positives = 164/247 (66%), Gaps = 1/247 (0%) |  |
| --- | --- | --- |
| Query 19 | LFGYDWAQQTIGCSDAAVFRLSAQGRPVLFVKTDLSGALNELQDEAARLSWLATTGVPCA | 78 |
| Sbjct 21 | L GY+W Q +G S A VFRL A GR +++KT+L+ AL EL E ARL WLA+ G+ C LAGYEWRQDGLGRSSAHVFRLEADGRAPVYLKTELADALGELAGEVARLRWLASHGLACP | 80 |
| Query 79 | AVLDVVTEAGRDWLLLGEVPGQDLLSSH-LAPAEKVSIMADAMRRLHTLDPATCPFDHQA | 137 |
| Sbjct 81 | V+ T+ R+WLL+ +PG DL+S+ + P E+V ++ADA+RRLH +D A CPFDH+ EVIAYETDGEREWLLMSALPGSDLVSAERVQPLERVQLLADALRRLHAVDIAACPFDHRL | 140 |
| Query 138 | KHRIERARTRMEAGLVDQDDLDEEHQGLAPAELFARLKARMPDGEDLVVTHGDACLPNIM | 197 |
| Sbjct 141 | + RI A+ + AG VD+DD DE G +LF L P EDLVVTHGDACLPN M ESRIGTAKAHLLAGRVDEDDFDETRLGRTAEDLFRELAETRPPKEDLVVTHGDACLPNFM | 200 |
| Query 198 | VENGRFSGFIDCGRLGVADRYQDIALATRDIAEELGGEWADRFLVLYGIAAPDSQRIAFY | 257 |
| Sbjct 201 | + +FSG+IDCGRLGVADR+QDIALA I G E FL YG P ++++A+Y ADGPKFSGYIDCGRLGVADRHQDIALACGSIEHNFGEELVAEFLKAYGDFEPQAEKMAYY | 260 |
| Query 258  Sbjct 261 | RLLDEFF 264  +LLDEFF  QLLDEFF 267 |  |

>WP_196664338.1 APH(3')-II family aminoglycoside O-phosphotransferase [Pseudomonas aeruginosa]

MBG4894555.1 APH(3')-II family aminoglycoside O-phosphotransferase [Pseudomonas aeruginosa]

Length=268

Score = 249 bits (636), Expect = 6e-79, Method: Compositional matrix adjust.

| Identities | | = 137/258 (53%), Positives = 168/258 (65%), Gaps = 5/258 (2%) |  |
| --- | --- | --- | --- |
| Query 11  Sbjct 12 | | SPAAWVERLFGYDWAQQTIGCSDAAVFRLSAQGRPVLFVKTDLSGALNELQDEAARLSWL  +PA W + L GY W Q GCS A V RL A RP LFVK ++ A EL E ARL WL APATWADYLAGYRWRGQGEGCSAATVHRLEAARRPTLFVKQEVLSAHAELPAEIARLRWL | 70  71 |
| Query 71  Sbjct 72 | | ATTGVPCAAVLDVVTEAGRDWLLLGEVPGQDLLS SHLAPAEKVSIMADAMRRLHTL  G+ C VL+ GR WLL+ VPG L + L P V ++A A+RRLH L HGAGIDCPQVLNETQSDGRQWLLMSAVPGDTLSALAQRGELEPERLVRLVAAALRRLHDL | 126  131 |
| Query 127  Sbjct 132 | | DPATCPFDHQAKHRIERARTRMEAGLVDQDDLDEEHQGLAPAELFARLKARMPDGEDLVV DPA CPFDH+ + R++ R R+EAGLVD+ D D++H+G + EL+ L R P EDLVV DPAACPFDHRLERRLDTVRQRVEAGLVDEADFDDDHRGRSATELYRLLLDRRPAVEDLVV | 186  191 |
| Query 187  Sbjct 192 | | THGDACLPNIMVENGRFSGFIDCGRLGVADRYQDIALATRDIAEELGGEWADRFLVLYGI HGDACLPN++ E RFSGFIDCGRLGVADR+QD+ALA RDI ELG A+ FLV YG AHGDACLPNLLAEGRRFSGFIDCGRLGVADRHQDLALAARDIEAELGAAGAEAFLVEYG- | 246  250 |
| Query | 247 AAPDSQRIAFYRLLDEFF 264 | | |
|  |  | D +R+A++RLLDEFF |  |
| Sbjct | 251 | GDIDGERLAYFRLLDEFF 268 | |

>WP_180890604.1 APH(3')-II family aminoglycoside O-phosphotransferase [Stenotrophomonas maltophilia]

MBA0272901.1 APH(3')-II family aminoglycoside O-phosphotransferase [Stenotrophomonas

maltophilia] Length=267

Score = 249 bits (636), Expect = 6e-79, Method: Compositional matrix adjust.

| Identities | = 133/255 (52%), Positives = 166/255 (65%), Gaps = 3/255 (1%) |  |
| --- | --- | --- |
| Query 12 | PAAWVERLFGYDWAQQTIGCSDAAVFRLSAQGRPVLFVKTDLSGALNELQDEAARLSWLA | 71 |
| Sbjct 14 | P AW + L +Q++G S A V R+ GRP F+K++L A +EL DE ARL WL PLAWQKALADARIERQSMGVSRADVARVLRPGRPDAFLKSELIDAFSELGDEIARLRWLQ | 73 |
| Query 72 | TTGVPCAAVLDVVTEAGRDWLLLGEVPGQDLLSS-HLAPAEKVSIMADAMRRLHTLDPAT | 130 |
| Sbjct 74 | G P V+ EAGR WLL+ +PG+DL SS LAP ++V I+ADA+R LH L A AQGQPAPTVIATAEEAGRHWLLMSALPGRDLASSPELAPQQRVEILADALRGLHALPVAA | 133 |
| Query 131 | CPFDHQAKHRIERARTRMEAGLVDQDDLDEEHQGLAPAELFARLKARMPDGEDLVVTHGD | 190 |
| Sbjct 134 | CPFD + R+ A+ R+EAG VD DD D+E G +P ++FA+L + PD EDLVV+HGD CPFDQRLASRLPAAQARVEAGRVDADDFDDERLGQSPQQVFAQLCSTRPDHEDLVVSHGD | 193 |
| Query 191 | ACLPNIMVENGRFSGFIDCGRLGVADRYQDIALATRDIAEELG-GEWADRFLVLYGIAAP | 249 |
| Sbjct 194 | ACLPN+MV GRFSG IDCGRLGVADRYQD+ALA R + G W YG A ACLPNLMVSEGRFSGLIDCGRLGVADRYQDLALAARSLVHNFGDTRWVTPLFQRYGAVA- | 252 |
| Query 250  Sbjct 253 | DSQRIAFYRLLDEFF 264 D +R+AFYRLLDEFF DERRLAFYRLLDEFF 267 |  |

>CAB3814286.1 Aminoglycoside 3'-phosphotransferase [Achromobacter animicus] Length=266

Score = 249 bits (635), Expect = 6e-79, Method: Compositional matrix adjust.

| Identities | | = 127/264 (48%), Positives = 165/264 (63%), Gaps = 1/264 (0%) |  |
| --- | --- | --- | --- |
| Query 1  Sbjct 4 | | MIEQDGLHAGSPAAWVERLFGYDWAQQTIGCSDAAVFRLSAQGRPVLFVKTDLSGALNEL M Q +H P W RL G QQ+IG S A V+R+ + +P ++K++ +G L EL MHAQYSVHDSLPLQWQPRLAGARLEQQSIGESRADVYRIRSANQPDRYLKSEHTGTLPEL | 60  63 |
| Query 61  Sbjct 64 | | QDEAARLSWLATTGVPCAAVLDVVTEAGRDWLLLGEVPGQDLLSSHLAPAEKVSIMADAM DE RL W+A G+PC V+D R WLL+ V GQDL S+ L PA+ ++IMADA+ PDEIERLQWMAAHGLPCPTVIDAAATPTRHWLLMSAVAGQDLASAGLPPAQVIAIMADAL | 120  123 |
| Query 121  Sbjct 124 | | RRLHTLDPATCPFDHQAKHRIERARTRMEAGLVDQDDLDEEHQGLAPAELFARLKARMPD RLH + CPFDH + RI AR ++AGLVD+ D D+E G + FA + A P HRLHCIPIDQCPFDHTLERRIAEARRALDAGLVDESDFDDERIGRTAEDAFAEVLATRPT | 180  183 |
| Query 181  Sbjct 184 | | GEDLVVTHGDACLPNIMVENGRFSGFIDCGRLGVADRYQDIALATRDIAEELGGEWADRF DLVV HGDACLPN M + F+G+IDCGRLGV+DR+QD+ALA R I LG EW + F THDLVVAHGDACLPNFMADGDVFTGYIDCGRLGVSDRHQDLALAARSIERNLGREWIEPF | 240  243 |
| Query 241 | | LVLYGIAAPDSQRIAFYRLLDEFF 264 YG+ PD +R+AFY LLDEFF |  |
| Sbjct | 244 | FRSYGV-EPDDRRLAFYCLLDEFF 266 | |

>WP_039621131.1 aminoglycoside 3'-phosphotransferase [Rhizobium etli]

AJC80347.1 aminoglycoside 3'-phosphotransferase protein [Rhizobium etli bv. phaseoli str. IE4803]

Length=261

Score = 249 bits (635), Expect = 6e-79, Method: Compositional matrix adjust.

| Identities | = 127/254 (50%), Positives = 172/254 (68%), Gaps = 1/254 (0%) |  |
| --- | --- | --- |
| Query 12 | PAAWVERLFGYDWAQQTIGCSDAAVFRLSAQGRPVLFVKTDLSGALNELQDEAARLSWLA | 71 |
| Sbjct 8 | PAA ERL GY + + +G S A+VFRL +G P L++K + +G EL DEAARLSWL PAALGERLSGYRFERDALGRSAASVFRLEGEGLPALYLKVEAAGPFGELADEAARLSWLK | 67 |
| Query 72 | TTGVPCAAVLDVVTEAGRDWLLLGEVPGQDLLS-SHLAPAEKVSIMADAMRRLHTLDPAT | 130 |
| Sbjct 68 | G+PC V+ ++ R+WLL+G +PG DL S S L P +V ++A A+ LH L A+ AAGLPCPDVIARESDGKRNWLLIGALPGTDLASASALTPLTRVELLAAALSALHDLPIAS | 127 |
| Query 131 | CPFDHQAKHRIERARTRMEAGLVDQDDLDEEHQGLAPAELFARLKARMPDGEDLVVTHGD CPFDH+ ++R+ A+ RM+AG+VD++D D G LFA L++R P EDLVVTHGD | 190 |

| Sbjct | 128 | CPFDHRLENRLSVAKARMQAGIVDEEDFDAARLGQTAEALFAELESRRPGNEDLVVTHGD | 187 |
| --- | --- | --- | --- |
| Query | 191 | ACLPNIMVENGRFSGFIDCGRLGVADRYQDIALATRDIAEELGGEWADRFLVLYGIAAPD | 250 |
| Sbjct | 188 | ACLPN + +G+FSG+IDC RLGVADR+QD+ALA R IA G FL YG+ APD ACLPNFVASDGQFSGYIDCSRLGVADRHQDLALACRSIASNFGVALIQPFLDRYGLPAPD | 247 |
| Query | 251 | SQRIAFYRLLDEFF 264  +++ +++LLDEFF |  |
| Sbjct | 248 | PEKLHYFQLLDEFF 261 | |

>WP_174718601.1 APH(3') family aminoglycoside O-phosphotransferase [Rhizobium sp. 11515TR]

Length=266

Score = 249 bits (635), Expect = 6e-79, Method: Compositional matrix adjust.

| Identities | = 126/247 (51%), Positives = 164/247 (66%), Gaps = 1/247 (0%) |  |
| --- | --- | --- |
| Query 19 | LFGYDWAQQTIGCSDAAVFRLSAQGRPVLFVKTDLSGALNELQDEAARLSWLATTGVPCA | 78 |
| Sbjct 20 | L GY+W Q +G S A VFRL A GR +++KT+L+ AL EL E ARL WLA+ G+ C LAGYEWRQDGLGRSSAHVFRLEADGRAPVYLKTELADALGELAGEVARLRWLASHGLACP | 79 |
| Query 79 | AVLDVVTEAGRDWLLLGEVPGQDLLSSH-LAPAEKVSIMADAMRRLHTLDPATCPFDHQA | 137 |
| Sbjct 80 | V+ T+ R+WLL+ +PG DL+S+ + P E+V ++ADA+RRLH +D A CPFDH+ EVIAYETDGEREWLLMSALPGSDLVSAERVQPLERVQLLADALRRLHAVDIAACPFDHRL | 139 |
| Query 138 | KHRIERARTRMEAGLVDQDDLDEEHQGLAPAELFARLKARMPDGEDLVVTHGDACLPNIM | 197 |
| Sbjct 140 | + RI A+ + AG VD+DD DE G +LF L P EDLVVTHGDACLPN M ESRIGTAKAHLLAGRVDEDDFDETRLGRTAEDLFRELAETRPPKEDLVVTHGDACLPNFM | 199 |
| Query 198 | VENGRFSGFIDCGRLGVADRYQDIALATRDIAEELGGEWADRFLVLYGIAAPDSQRIAFY | 257 |
| Sbjct 200 | + +FSG+IDCGRLGVADR+QDIALA I G E FL YG P ++++A+Y ADGPKFSGYIDCGRLGVADRHQDIALACGSIEHNFGEELVAEFLKAYGDFEPQAEKMAYY | 259 |
| Query 258  Sbjct 260 | RLLDEFF 264  +LLDEFF  QLLDEFF 266 |  |

>WP_180845672.1 MULTISPECIES: APH(3')-II family aminoglycoside O-phosphotransferase [Stenotrophomonas]

MBA0259807.1 APH(3')-II family aminoglycoside O-phosphotransferase [Stenotrophomonas maltophilia]

MBB1135779.1 APH(3')-II family aminoglycoside O-phosphotransferase [Stenotrophomonas sp. I18B00994]

Length=267

Score = 249 bits (635), Expect = 6e-79, Method: Compositional matrix adjust.

| Identities | = 134/255 (53%), Positives = 168/255 (66%), Gaps = 3/255 (1%) |  |
| --- | --- | --- |
| Query 12 | PAAWVERLFGYDWAQQTIGCSDAAVFRLSAQGRPVLFVKTDLSGALNELQDEAARLSWLA | 71 |
| Sbjct 14 | P AW E L +Q+IG S A V R+ +G+ FVK+++ A +EL DE ARL WL PWAWQETLADARIERQSIGVSRADVARVQRRGQADAFVKSEVIDAFSELGDEIARLRWLQ | 73 |
| Query 72 | TTGVPCAAVLDVVTEAGRDWLLLGEVPGQDLLSS-HLAPAEKVSIMADAMRRLHTLDPAT | 130 |
| Sbjct 74 | G P V+ EAGR WLL+ +PG+DL SS LAP + V ++ADA+R LH L A EQGQPVPTVIATAEEAGRRWLLMSALPGRDLASSPELAPQQLVEMLADALRGLHALPLAA | 133 |
| Query 131 | CPFDHQAKHRIERARTRMEAGLVDQDDLDEEHQGLAPAELFARLKARMPDGEDLVVTHGD | 190 |
| Sbjct 134 | CPFD + R++ A+ R+EAGLVD DD D+E G +P ++FA L + PD EDLVV+HGD CPFDQRLASRLQAAQARVEAGLVDADDFDDERLGQSPQQVFAELCSTRPDHEDLVVSHGD | 193 |
| Query 191 | ACLPNIMVENGRFSGFIDCGRLGVADRYQDIALATRDIAEELG-GEWADRFLVLYGIAAP | 249 |
| Sbjct 194 | ACLPN+MV GRFSGFIDCGRLGVADRYQD+ALA R + G W YG+ A ACLPNLMVVEGRFSGFIDCGRLGVADRYQDLALAARSLVHNFGDTRWVAPLFQRYGMVA- | 252 |
| Query 250  Sbjct 253 | DSQRIAFYRLLDEFF 264 D +R+AFYRLLDEFF DERRLAFYRLLDEFF 267 |  |

>WP_088436185.1 APH(3')-II family aminoglycoside O-phosphotransferase [Stenotrophomonas

maltophilia]

AYZ69937.1 APH(3')-II family aminoglycoside O-phosphotransferase [Stenotrophomonas maltophilia]

OWQ64102.1 APH(3') family aminoglycoside O-phosphotransferase [Stenotrophomonas maltophilia]

GFF06301.1 aminoglycoside O-phosphotransferase APH(3')-IIb [Stenotrophomonas maltophilia]

Length=267

Score = 249 bits (635), Expect = 7e-79, Method: Compositional matrix adjust.

| Identities | = 134/255 (53%), Positives = 166/255 (65%), Gaps = 3/255 (1%) |  |
| --- | --- | --- |
| Query 12 | PAAWVERLFGYDWAQQTIGCSDAAVFRLSAQGRPVLFVKTDLSGALNELQDEAARLSWLA | 71 |
| Sbjct 14 | P AW E L +Q+IG S A V R+ G+P FVK+++ A +EL DE ARL WL PWAWQEALADARIERQSIGVSRADVARVQRPGQPDAFVKSEVIDAFSELGDEIARLRWLQ | 73 |
| Query 72 | TTGVPCAAVLDVVTEAGRDWLLLGEVPGQDLLSS-HLAPAEKVSIMADAMRRLHTLDPAT | 130 |
| Sbjct 74 | G P V+ EAGR WLL+ +PG+DL SS LAP + V ++ADA+R LH L A EQGQPAPTVIATAEEAGRRWLLMSALPGRDLASSPELAPQQLVELLADALRGLHALPLAA | 133 |
| Query 131 | CPFDHQAKHRIERARTRMEAGLVDQDDLDEEHQGLAPAELFARLKARMPDGEDLVVTHGD | 190 |
| Sbjct 134 | CPFD + R++ A+ R+EAGLVD DD D+E G +P ++FA L + PD EDLVV+HGD CPFDQRLASRLQAAQARVEAGLVDADDFDDERLGQSPQQVFAELCSTRPDHEDLVVSHGD | 193 |
| Query 191 | ACLPNIMVENGRFSGFIDCGRLGVADRYQDIALATRDIAEELG-GEWADRFLVLYGIAAP | 249 |
| Sbjct 194 | ACLPN+MV GRFSGFIDCGRLGVADRYQD+AL R + G W YG A ACLPNLMVVEGRFSGFIDCGRLGVADRYQDLALGARSLVHNFGDTRWVAPLFQRYGAVA- | 252 |
| Query 250  Sbjct 253 | DSQRIAFYRLLDEFF 264 D +R+AFYRLLDEFF DERRLAFYRLLDEFF 267 |  |

>WP_183812986.1 APH(3') family aminoglycoside O-phosphotransferase [Rhizobium sp. BK386]

MBB3508665.1 aminoglycoside 3'-phosphotransferase-2 [Rhizobium sp. BK386] Length=266

Score = 249 bits (635), Expect = 7e-79, Method: Compositional matrix adjust.

| Identities | = 125/247 (51%), Positives = 164/247 (66%), Gaps = 1/247 (0%) |  |
| --- | --- | --- |
| Query 19 | LFGYDWAQQTIGCSDAAVFRLSAQGRPVLFVKTDLSGALNELQDEAARLSWLATTGVPCA | 78 |
| Sbjct 20 | L GY+W Q +G S A VFRL A GR +++KT+++ L EL E ARL WLA+ G C LAGYEWRQDALGRSSAHVFRLEADGRAPVYLKTEVTDPLGELAGEVARLRWLASQGFACP | 79 |
| Query 79 | AVLDVVTEAGRDWLLLGEVPGQDLLSSH-LAPAEKVSIMADAMRRLHTLDPATCPFDHQA | 137 |
| Sbjct 80 | ++ + R+WLL+ +PG D +S+ L P E+V + A A+RRLH++D A CPFDH+ DIIAHEKDGEREWLLMSALPGSDFVSAEWLQPLERVRVFAGALRRLHSVDIAACPFDHRL | 139 |
| Query 138 | KHRIERARTRMEAGLVDQDDLDEEHQGLAPAELFARLKARMPDGEDLVVTHGDACLPNIM | 197 |
| Sbjct 140 | + RI AR + AG VD+DD DE G + A+LF L+A P EDLVVTHGDACLPN + ESRIGTARAHLLAGRVDEDDFDEARLGTSAADLFRELQATRPPTEDLVVTHGDACLPNFV | 199 |
| Query 198 | VENGRFSGFIDCGRLGVADRYQDIALATRDIAEELGGEWADRFLVLYGIAAPDSQRIAFY | 257 |
| Sbjct 200 | V+ RFSG+IDCGRLGVADR+QDIALA + G E FL YG P ++++A+Y VDGDRFSGYIDCGRLGVADRHQDIALACGSVEHNFGEELVGEFLKAYGDFEPQAEKMAYY | 259 |
| Query 258  Sbjct 260 | RLLDEFF 264 RLLDEFF RLLDEFF 266 |  |

>WP_037194572.1 MULTISPECIES: aminoglycoside 3'-phosphotransferase [Rhizobium] NKJ38709.1 aminoglycoside 3'-phosphotransferase-2 [Rhizobium sp. SG570] TWF18248.1 aminoglycoside 3'-phosphotransferase-2 [Rhizobium lusitanum]

Length=266

Score = 249 bits (635), Expect = 8e-79, Method: Compositional matrix adjust. Identities = 130/254 (51%), Positives = 167/254 (66%), Gaps = 1/254 (0%)

Query 12	PAAWVERLFGYDWAQQTIGCSDAAVFRLSAQGRPVLFVKTDLSGALNELQDEAARLSWLA 71 PA	+ L GYDW	+G S A VFRL A GR +++KT++	L EL E +RL WLA

| Sbjct | 13 | PARLRDLLAGYDWRPAELGRSPAHVFRLEAVGRAPIYLKTEIIHPLGELAGEVSRLRWLA | 72 |
| --- | --- | --- | --- |
| Query | 72 | TTGVPCAAVLDVVTEAGRDWLLLGEVPGQDLLSS-HLAPAEKVSIMADAMRRLHTLDPAT | 130 |
| Sbjct | 73 | + G+ C V+ T+ GR+WLL+ +PG DL+S+ L P E+V I+A A+RRLH LD AT SKGLACPEVIAHETDGGREWLLMSALPGTDLVSNDRLPPIERVRILAAALRRLHGLDIAT | 132 |
| Query | 131 | CPFDHQAKHRIERARTRMEAGLVDQDDLDEEHQGLAPAELFARLKARMPDGEDLVVTHGD | 190 |
| Sbjct | 133 | CPFDH+ ++RI A+ R+ AG VD+DD D+ G + +LF L+ R P EDLVVTHGD CPFDHRLENRIADAKARLLAGCVDEDDFDDARLGRSAQDLFRELEERRPKTEDLVVTHGD | 192 |
| Query | 191 | ACLPNIMVENGRFSGFIDCGRLGVADRYQDIALATRDIAEELGGEWADRFLVLYGIAAPD | 250 |
| Sbjct | 193 | ACLPN + G SG+IDCGRLGVADR+QDIALA IA G + FL YG ACLPNFVAHEGSISGYIDCGRLGVADRHQDIALACGSIARNFGEDLVADFLKAYGNFEMH | 252 |
| Query  Sbjct | 251  253 | SQRIAFYRLLDEFF 264 S++ A+YRLLDEFF SEKAAYYRLLDEFF 266 |  |

>WP_132653716.1 MULTISPECIES: aminoglycoside 3'-phosphotransferase [unclassified Rhizobium]

MBB3590148.1 aminoglycoside 3'-phosphotransferase-2 [Rhizobium sp. BK529] TCS04844.1 aminoglycoside 3'-phosphotransferase-2 [Rhizobium sp. BK418]

Length=265

Score = 249 bits (635), Expect = 8e-79, Method: Compositional matrix adjust.

| Identities | | = 126/254 (50%), Positives = 164/254 (65%), Gaps = 1/254 (0%) |  |
| --- | --- | --- | --- |
| Query 12  Sbjct 12 | | PAAWVERLFGYDWAQQTIGCSDAAVFRLSAQGRPVLFVKTDLSGALNELQDEAARLSWLA P + L GY W + +G S A VFRL +GRP L++K + +G EL DEA RL WL PVSLRSHLGGYQWRRDALGRSSACVFRLEGEGRPGLYLKGEEAGPFCELADEAERLVWLR | 71  71 |
| Query 72  Sbjct 72 | | TTGVPCAAVLDVVTEAGRDWLLLGEVPGQDLLSSH-LAPAEKVSIMADAMRRLHTLDPAT  + G+ C V+ ++ R +LL+ +PG DL+++ L P E+V I+A A+ LH LD SQGLDCPEVIAYESDGERSFLLMTALPGADLVTAAVLLPEERVEILASALSTLHNLDVNA | 130  131 |
| Query 131  Sbjct 132 | | CPFDHQAKHRIERARTRMEAGLVDQDDLDEEHQGLAPAELFARLKARMPDGEDLVVTHGD CPFDH+ ++R+ A RM AGLVD+ D DE+ G + LF L+ PD E+LVV HGD CPFDHRLENRLLTAEARMRAGLVDETDFDEKRLGKSAFFLFGELQRLRPDSENLVVAHGD | 190  191 |
| Query 191  Sbjct 192 | | ACLPNIMVENGRFSGFIDCGRLGVADRYQDIALATRDIAEELGGEWADRFLVLYGIAAPD ACLPN M GRF+G+IDC RLGVADRYQDIALA R IA GGE+ FL YG++ D ACLPNFMAAGGRFAGYIDCCRLGVADRYQDIALACRSIAHNFGGEFVRLFLNAYGLSIID | 250  251 |
| Query 251 | | SQRIAFYRLLDEFF 264  +RI FY+LLDEFF |  |
| Sbjct | 252 | PERIEFYQLLDEFF 265 | |

>WP_168842434.1 MULTISPECIES: APH(3') family aminoglycoside O-phosphotransferase [unclassified Rhizobium]

NLR87960.1 aminoglycoside 3'-phosphotransferase [Rhizobium sp. P28RR-XV] NLS19535.1 aminoglycoside 3'-phosphotransferase [Rhizobium sp. P40RR-XXII]

Length=266

Score = 248 bits (634), Expect = 8e-79, Method: Compositional matrix adjust.

| Identities | = 127/259 (49%), Positives = 169/259 (65%), Gaps = 1/259 (0%) |  |
| --- | --- | --- |
| Query 7 | LHAGSPAAWVERLFGYDWAQQTIGCSDAAVFRLSAQGRPVLFVKTDLSGALNELQDEAAR | 66 |
| Sbjct 8 | + A PA L GY+W Q +G S A VFRL A GR +++KT+L+ L EL E AR MEASIPARLRGLLAGYEWRQDVLGRSSAHVFRLEADGRAPVYLKTELADPLGELTGEVAR | 67 |
| Query 67 | LSWLATTGVPCAAVLDVVTEAGRDWLLLGEVPGQDLLSSH-LAPAEKVSIMADAMRRLHT | 125 |
| Sbjct 68 | L WLA+ G+ C V+ + R+WLL+ + G DL+S+ L P E+V ++ADA+RRLH LRWLASQGLTCPDVIAHEKDGEREWLLMSALSGNDLVSAERLQPLERVQLLADALRRLHA | 127 |
| Query 126 | LDPATCPFDHQAKHRIERARTRMEAGLVDQDDLDEEHQGLAPAELFARLKARMPDGEDLV | 185 |
| Sbjct 128 | +D A CPFD++ ++RI AR R+ AG VD+DD DE G + +LF L+AR P ED V VDIAACPFDNRLENRIGAARARLLAGHVDEDDFDETRLGRSAEDLFRELQARRPSTEDFV | 187 |
| Query 186 | VTHGDACLPNIMVENGRFSGFIDCGRLGVADRYQDIALATRDIAEELGGEWADRFLVLYG VTHGDACLPN +V+ +FSG+IDC RLG+ADR+QDIALA I G FL YG | 245 |

| Sbjct | 188 | VTHGDACLPNFIVDGDKFSGYIDCCRLGIADRHQDIALACGSIERNFGERLVSEFLKAYG 247 |
| --- | --- | --- |
| Query | 246 | IAAPDSQRIAFYRLLDEFF 264 |
| Sbjct | 248 | P ++++A+YRLLDEFF  NFEPKAEKMAYYRLLDEFF 266 |

>WP_049449629.1 APH(3')-II family aminoglycoside O-phosphotransferase [Stenotrophomonas maltophilia]

VUN96737.1 Aminoglycoside 3'-phosphotransferase [Stenotrophomonas maltophilia] VUP83865.1 Aminoglycoside 3'-phosphotransferase [Stenotrophomonas maltophilia]

Length=267

Score = 248 bits (634), Expect = 9e-79, Method: Compositional matrix adjust.

| Identities | = 133/255 (52%), Positives = 166/255 (65%), Gaps = 3/255 (1%) |  |
| --- | --- | --- |
| Query 12 | PAAWVERLFGYDWAQQTIGCSDAAVFRLSAQGRPVLFVKTDLSGALNELQDEAARLSWLA | 71 |
| Sbjct 14 | P AW E L +Q+IG S A V R+ G+P FVK+++ A +EL DE RL WL PRAWQEALADARIERQSIGVSRADVARVQQPGQPDAFVKSEVIDAFSELGDEITRLRWLQ | 73 |
| Query 72 | TTGVPCAAVLDVVTEAGRDWLLLGEVPGQDLLSS-HLAPAEKVSIMADAMRRLHTLDPAT | 130 |
| Sbjct 74 | G+P V+ EAGR WLL+ +PG+DL SS LAP + V ++ADA+R LH L A AQGLPAPTVIATAEEAGRRWLLMSALPGRDLASSPELAPQQLVELLADALRGLHALPVAA | 133 |
| Query 131 | CPFDHQAKHRIERARTRMEAGLVDQDDLDEEHQGLAPAELFARLKARMPDGEDLVVTHGD | 190 |
| Sbjct 134 | CPFD + R++ A+ R+EAGLVD DD D+E G + ++FA L + PD EDLVV+HGD CPFDQRLASRLQAAQARVEAGLVDADDFDDERLGQSAQQVFAELCSTRPDHEDLVVSHGD | 193 |
| Query 191 | ACLPNIMVENGRFSGFIDCGRLGVADRYQDIALATRDIAEELG-GEWADRFLVLYGIAAP | 249 |
| Sbjct 194 | ACLPN+MV GRFSGFIDCGRLGVADRYQD+ALA R + G W YG A ACLPNLMVAEGRFSGFIDCGRLGVADRYQDLALAARSLVHNFGDTRWVAPLFQRYGAVA- | 252 |
| Query 250  Sbjct 253 | DSQRIAFYRLLDEFF 264 D +R+AFYRLLDEFF DERRLAFYRLLDEFF 267 |  |

>WP_112411627.1 aminoglycoside 3'-phosphotransferase [Rhizobium sp. BK312] MBB3423580.1 aminoglycoside 3'-phosphotransferase-2 [Rhizobium sp. BK312]

Length=266

Score = 248 bits (634), Expect = 1e-78, Method: Compositional matrix adjust.

| Identities | | = 126/254 (50%), Positives = 168/254 (66%), Gaps = 1/254 (0%) |  |
| --- | --- | --- | --- |
| Query 12  Sbjct 13 | | PAAWVERLFGYDWAQQTIGCSDAAVFRLSAQGRPVLFVKTDLSGALNELQDEAARLSWLA P + L GY+W Q +G S A VFRL A R +++KT+L+ L EL E ARL WLA PVRLRDLLAGYEWRQDGLGRSSAHVFRLEADRRAPVYLKTELADPLGELAGEVARLRWLA | 71  72 |
| Query 72  Sbjct 73 | | TTGVPCAAVLDVVTEAGRDWLLLGEVPGQDLLSSH-LAPAEKVSIMADAMRRLHTLDPAT  + G C ++ T+ R+WLL+G +PG DL+S+ L P E+V ++ADA+R+LH +D A SQGFACPEIIAHETDGEREWLLMGALPGSDLVSAEWLQPLERVRLLADALRQLHAIDIAA | 130  132 |
| Query 131  Sbjct 133 | | CPFDHQAKHRIERARTRMEAGLVDQDDLDEEHQGLAPAELFARLKARMPDGEDLVVTHGD CPFDH+ ++RI A+ + AG VD+DD DE G + +LF L+A+ P EDLVVTHGD CPFDHRLENRIGTAKAHLLAGRVDEDDFDEARLGRSAEDLFRELQAKRPSMEDLVVTHGD | 190  192 |
| Query 191  Sbjct 193 | | ACLPNIMVENGRFSGFIDCGRLGVADRYQDIALATRDIAEELGGEWADRFLVLYGIAAPD ACLPN M + +FSG+IDCGRLGVADR+QDIALA I G E FL YG P ACLPNFMADGPKFSGYIDCGRLGVADRHQDIALACGSIEHNFGEELVAEFLKAYGDFQPQ | 250  252 |
| Query 251 | | SQRIAFYRLLDEFF 264  +++A+Y+LLDEFF |  |
| Sbjct | 253 | EEKMAYYQLLDEFF 266 | |

>WP_069612848.1 aminoglycoside 3'-phosphotransferase [Rhizobium sp. YK2]

OEC98466.1 APH(3') family aminoglycoside O-phosphotransferase [Rhizobium sp. YK2]

Length=266

Score = 248 bits (634), Expect = 1e-78, Method: Compositional matrix adjust. Identities = 125/247 (51%), Positives = 166/247 (67%), Gaps = 1/247 (0%)

| Query | 19 | LFGYDWAQQTIGCSDAAVFRLSAQGRPVLFVKTDLSGALNELQDEAARLSWLATTGVPCA | 78 |
| --- | --- | --- | --- |
| Sbjct | 20 | L GY+W Q +G S A VFRL A G +++KT+L+ L EL E ARL WLA+ G+ C LAGYEWRQDVLGRSSAHVFRLEADGCVPVYLKTELADPLGELAGEVARLRWLASQGLACP | 79 |
| Query | 79 | AVLDVVTEAGRDWLLLGEVPGQDLLSSH-LAPAEKVSIMADAMRRLHTLDPATCPFDHQA | 137 |
| Sbjct | 80 | ++ + R+WLL+ +PG D +S+ L P E+V + A A+RRLH++D A CPFDH+ DIIAHEKDGEREWLLMSALPGSDFVSAEWLQPLERVRLFAGALRRLHSVDIAACPFDHRL | 139 |
| Query | 138 | KHRIERARTRMEAGLVDQDDLDEEHQGLAPAELFARLKARMPDGEDLVVTHGDACLPNIM | 197 |
| Sbjct | 140 | ++RI +AR + AG VD+DD DE G + A+LF L+A P EDLVVTHGDACLPN + ENRIGKARAHLLAGRVDEDDFDEARLGRSAADLFCELQATRPSTEDLVVTHGDACLPNFV | 199 |
| Query | 198 | VENGRFSGFIDCGRLGVADRYQDIALATRDIAEELGGEWADRFLVLYGIAAPDSQRIAFY | 257 |
| Sbjct | 200 | V+ RFSG+IDCGRLGVADR+QDIALA + G E FL YG P ++++A+Y VDGDRFSGYIDCGRLGVADRHQDIALACGSVEHNFGQELVGEFLKAYGDFEPQAEKMAYY | 259 |
| Query  Sbjct | 258  260 | RLLDEFF 264 RLLDEFF RLLDEFF 266 |  |

>WP_154833201.1 aminoglycoside 3'-phosphotransferase [Delftia tsuruhatensis] OJX17202.1 APH(3') family aminoglycoside O-phosphotransferase [Delftia sp.

67-8]

QFS63173.1 APH(3') family aminoglycoside O-phosphotransferase [Delftia tsuruhatensis] Length=269

Score = 248 bits (634), Expect = 1e-78, Method: Compositional matrix adjust.

| Identities | | = 130/254 (51%), Positives = 164/254 (65%), Gaps = 2/254 (1%) |  |
| --- | --- | --- | --- |
| Query 12  Sbjct 17 | | PAAWVERLFGYDWAQQTIGCSDAAVFRLSAQGRPVLFVKTDLSGALNELQDEAARLSWLA PAAW R +Q IG S A VFRL LFVK++L+G L+EL E ARL W+ PAAWQARFPVTRAERQAIGESRADVFRLRPPEGGDLFVKSELAGPLSELPQEIARLRWMR | 71  76 |
| Query 72 | | TTGVPCAAVLDVVTEAGRDWLLLGEVPGQDLLS-SHLAPAEKVSIMADAMRRLHTLDPAT G+PC VL+ TE GR WLL+ V G+D+ S + L PA+ V I+A A+R LH L | 130 |
| Sbjct | 77 | QMGLPCPEVLEAATENGRHWLLMTAVAGRDMASATDLTPAQAVQIIAQALRHLHALPWQE | 136 |
| Query | 131 | CPFDHQAKHRIERARTRMEAGLVDQDDLDEEHQGLAPAELFARLKARMPDGEDLVVTHGD | 190 |
| Sbjct | 137 | CPFDH+ R+E A+ +++AG VD+ D D+E G A++ A L MP D VV HGD CPFDHRPAQRVEHAQAQVQAGRVDESDFDDERLGRTAADVLAELLEHMPQEVDAVVAHGD | 196 |
| Query | 191 | ACLPNIMVENGRFSGFIDCGRLGVADRYQDIALATRDIAEELGGEWADRFLVLYGIAAPD | 250 |
| Sbjct | 197 | ACLPN++V+ FSGFIDCGRLGV+DRYQD+ALA+R I LG +WA F YG+ PD ACLPNLIVDGAAFSGFIDCGRLGVSDRYQDLALASRSITRNLGAQWAAPFFEAYGV-VPD | 255 |
| Query  Sbjct | 251  256 | SQRIAFYRLLDEFF 264  +QRI FY LLDEFF  AQRIRFYNLLDEFF 269 |  |

>WP_053462776.1 APH(3')-II family aminoglycoside O-phosphotransferase [Stenotrophomonas maltophilia]

KOO76724.1 aminoglycoside phosphotransferase [Stenotrophomonas maltophilia] Length=267

Score = 248 bits (634), Expect = 1e-78, Method: Compositional matrix adjust.

| Identities | = 132/255 (52%), Positives = 165/255 (65%), Gaps = 3/255 (1%) |  |
| --- | --- | --- |
| Query 12 | PAAWVERLFGYDWAQQTIGCSDAAVFRLSAQGRPVLFVKTDLSGALNELQDEAARLSWLA | 71 |
| Sbjct 14 | P AW E L +Q+IG S A V R+ G+ F+K+++ A +EL DE ARL WL PHAWQEALADARIERQSIGVSRADVARVHRPGQADAFLKSEVIDAFSELGDEIARLRWLQ | 73 |
| Query 72 | TTGVPCAAVLDVVTEAGRDWLLLGEVPGQDLLSS-HLAPAEKVSIMADAMRRLHTLDPAT | 130 |
| Sbjct 74 | G P V+ E GR WLL+ +PG DL SS LAP +V ++ADA+R LH L AT AQGQPAPTVIATAEEEGRRWLLMSALPGHDLASSPALAPQHRVQLLADALRGLHALPVAT | 133 |
| Query 131 | CPFDHQAKHRIERARTRMEAGLVDQDDLDEEHQGLAPAELFARLKARMPDGEDLVVTHGD | 190 |
| Sbjct 134 | CPFD + R++ A+ R+EAG VD DD D+E G + ++FA L+A PD EDLVV+HGD  CPFDQRLASRLQAAQARVEAGRVDADDFDDERLGQSAQQVFAELRATRPDHEDLVVSHGD | 193 |

| Query | 191 | ACLPNIMVENGRFSGFIDCGRLGVADRYQDIALATRDIAEELG-GEWADRFLVLYGIAAP 249 |
| --- | --- | --- |
| Sbjct | 194 | ACLPN++ GRFSGFIDCGRLGVADRYQD+ALA R + G W R YGI P ACLPNLLAVEGRFSGFIDCGRLGVADRYQDLALAARSLVHNFGDTRWVARLFRSYGI-VP 252 |
| Query | 250 | DSQRIAFYRLLDEFF 264 |
| Sbjct | 253 | D +R+AFYRLLDEFF  DERRLAFYRLLDEFF 267 |

>WP_122606987.1 aminoglycoside 3'-phosphotransferase [Pseudomonas viridiflava] Length=256

Score = 248 bits (632), Expect = 1e-78, Method: Compositional matrix adjust.

| Identities | | = 127/254 (50%), Positives = 170/254 (67%), Gaps = 2/254 (1%) |  |
| --- | --- | --- | --- |
| Query 12  Sbjct 4 | | PAAWVERLFGYDWAQQTIGCSDAAVFRLSAQGRPVLFVKTDLSGALNELQDEAARLSWLA PA+W R D QQTIG S A V+R++ +G LFVKT+ EL E RL W+A PASWRSRFAHADIEQQTIGESRADVWRVTPKGSASLFVKTEPVMTWGELPGEVQRLRWMA | 71  63 |
| Query 72  Sbjct 64 | | TTGVPCAAVLDVVTEAGRDWLLLGEVPGQDLLSS-HLAPAEKVSIMADAMRRLHTLDPAT T +P VLD T A ++WLL+ VPG+DL SS HL+P + V++ A+A+R LH + + TQELPAPRVLDTTTAAEQNWLLMTAVPGRDLASSAHLSPQQIVTLTAEALRALHAVPIES | 130  123 |
| Query 131  Sbjct 124 | | CPFDHQAKHRIERARTRMEAGLVDQDDLDEEHQGLAPAELFARLKARMPDGEDLVVTHGD CPFDH+ + +I+ AR + AGL+D+ D D+ G + +F ++ A P EDLVVTHGD CPFDHRLEAKIKLARGHLLAGLIDEQDFDDSRLGQSAEAVFQQMLAARPAHEDLVVTHGD | 190  183 |
| Query 191  Sbjct 184 | | ACLPNIMVENGRFSGFIDCGRLGVADRYQDIALATRDIAEELGGEWADRFLVLYGIAAPD A LPN++ ++GRFSGF+DCGRLGVADRYQD+ALA I + LG +W F +YG+ PD AYLPNLLADDGRFSGFVDCGRLGVADRYQDLALAAHSITDNLGAQWLPVFFEVYGV-EPD | 250  242 |
| Query 251 | | SQRIAFYRLLDEFF 264 QRIAFY+LLDEFF |  |
| Sbjct | 243 | LQRIAFYQLLDEFF 256 | |

>WP_183013532.1 APH(3') family aminoglycoside O-phosphotransferase [Achromobacter sp. UMC46]

MBB1594303.1 APH(3') family aminoglycoside O-phosphotransferase [Achromobacter sp. UMC46]

Length=266

Score = 248 bits (633), Expect = 1e-78, Method: Compositional matrix adjust.

| Identities | = 127/253 (50%), Positives = 166/253 (66%), Gaps = 1/253 (0%) |  |
| --- | --- | --- |
| Query 12 | PAAWVERLFGYDWAQQTIGCSDAAVFRLSAQGRPVLFVKTDLSGALNELQDEAARLSWLA | 71 |
| Sbjct 15 | PA W QQ+IG S A VFR+ RP LF+K++L+G ++EL DE RL WLA PAKWRGDFAHASVEQQSIGESRADVFRVRRPNRPDLFLKSELAGGVSELPDEIDRLRWLA | 74 |
| Query 72 | TTGVPCAAVLDVVTEAGRDWLLLGEVPGQDLLSSHLAPAEKVSIMADAMRRLHTLDPATC | 131 |
| Sbjct 75 | G P VLD E R WLL+ + G+DL S+ LAPA+ + ++A A+R LH L TC QVGQPAPHVLDTAMEGDRHWLLMTAIRGRDLASAALAPAQIIGVLATALRNLHHLPIETC | 134 |
| Query 132 | PFDHQAKHRIERARTRMEAGLVDQDDLDEEHQGLAPAELFARLKARMPDGEDLVVTHGDA | 191 |
| Sbjct 135 | PFDH+ + RI +A+ ++AGLVD+ D D+E G + +++ A L + P D VV HGDA PFDHRLEARIAQAKRHVDAGLVDEADFDDERLGQSASQVLAELLSSCPTARDPVVAHGDA | 194 |
| Query 192 | CLPNIMVENGRFSGFIDCGRLGVADRYQDIALATRDIAEELGGEWADRFLVLYGIAAPDS | 251 |
| Sbjct 195 | CLPN MV+ +F+GFIDCGRLG+ADR+QD+ALA R IA +G +W F YGI APD+ CLPNFMVDGHQFTGFIDCGRLGIADRFQDLALAARSIARNVGEQWVPPFFRAYGI-APDA | 253 |
| Query 252  Sbjct 254 | QRIAFYRLLDEFF 264 QRI FY LLDEFF QRIRFYCLLDEFF 266 |  |

>WP_196710007.1 APH(3')-II family aminoglycoside O-phosphotransferase [Pseudomonas aeruginosa]

MBG4747171.1 APH(3')-II family aminoglycoside O-phosphotransferase [Pseudomonas aeruginosa]

Length=268

Score = 248 bits (633), Expect = 1e-78, Method: Compositional matrix adjust.

| Identities | | = 136/258 (53%), Positives = 168/258 (65%), Gaps = 5/258 (2%) |  |
| --- | --- | --- | --- |
| Query 11  Sbjct 12 | | SPAAWVERLFGYDWAQQTIGCSDAAVFRLSAQGRPVLFVKTDLSGALNELQDEAARLSWL  +P+ W + L GY W Q GCS A V RL A RP LFVK ++ A EL E ARL WL APSTWADYLAGYRWRGQGEGCSAATVHRLEAARRPTLFVKQEVLSAHAELPAEIARLRWL | 70  71 |
| Query 71  Sbjct 72 | | ATTGVPCAAVLDVVTEAGRDWLLLGEVPGQDLLS SHLAPAEKVSIMADAMRRLHTL  G+ C VL+ GR WLL+ VPG L + L P V ++A A+RRLH L HGAGIDCPQVLNETQSDGRQWLLMSAVPGDTLSALAQRGELEPERLVRLVAAALRRLHDL | 126  131 |
| Query 127  Sbjct 132 | | DPATCPFDHQAKHRIERARTRMEAGLVDQDDLDEEHQGLAPAELFARLKARMPDGEDLVV DPA CPFDH+ + R++ R R+EAGLVD+ D D++H+G + EL+ L R P EDLVV DPAACPFDHRLERRLDTVRQRVEAGLVDEADFDDDHRGRSATELYRLLLDRRPAVEDLVV | 186  191 |
| Query 187  Sbjct 192 | | THGDACLPNIMVENGRFSGFIDCGRLGVADRYQDIALATRDIAEELGGEWADRFLVLYGI HGDACLPN++ E RFSGFIDCGRLGVADR+QD+ALA RDI ELG A+ FLV YG AHGDACLPNLLAEGRRFSGFIDCGRLGVADRHQDLALAARDIEAELGAAGAEAFLVEYG- | 246  250 |
| Query | 247 AAPDSQRIAFYRLLDEFF 264 | | |
|  |  | D +R+A++RLLDEFF |  |
| Sbjct | 251 | GDIDGERLAYFRLLDEFF 268 | |

>WP_092577247.1 aminoglycoside 3'-phosphotransferase [Rhizobium lusitanum] NTJ07684.1 aminoglycoside 3'-phosphotransferase [Rhizobium lusitanum] SCB51935.1 aminoglycoside 3'-phosphotransferase-2 [Rhizobium lusitanum]

Length=266

Score = 248 bits (633), Expect = 1e-78, Method: Compositional matrix adjust.

| Identities | = 130/254 (51%), Positives = 166/254 (65%), Gaps = 1/254 (0%) |  |
| --- | --- | --- |
| Query 12 | PAAWVERLFGYDWAQQTIGCSDAAVFRLSAQGRPVLFVKTDLSGALNELQDEAARLSWLA | 71 |
| Sbjct 13 | PA + L GYDW +G S A VFRL A GR +++KT++ +L EL E +RL WLA PARLRDLLAGYDWRPAELGRSPAHVFRLEAVGRAPIYLKTEIIHSLGELAGEVSRLRWLA | 72 |
| Query 72 | TTGVPCAAVLDVVTEAGRDWLLLGEVPGQDLLSS-HLAPAEKVSIMADAMRRLHTLDPAT | 130 |
| Sbjct 73 | + G+ C V+ T GR+WLL+ +PG DL+S L P E+V I+A A+RRLH LD AT SKGLACPEVIAHETYGGREWLLMNALPGTDLVSDDRLPPIERVRILAAALRRLHGLDIAT | 132 |
| Query 131 | CPFDHQAKHRIERARTRMEAGLVDQDDLDEEHQGLAPAELFARLKARMPDGEDLVVTHGD | 190 |
| Sbjct 133 | CPFDH+ ++RI A+ R+ AG VD+DD D+ G + +LF L+ R P EDLVVTHGD CPFDHRLENRIADAKARLLAGCVDEDDFDDARLGRSAQDLFRELEERRPKTEDLVVTHGD | 192 |
| Query 191 | ACLPNIMVENGRFSGFIDCGRLGVADRYQDIALATRDIAEELGGEWADRFLVLYGIAAPD | 250 |
| Sbjct 193 | ACLPN + G SG+IDCGRLGVADR+QDIALA IA G + FL YG ACLPNFVAHEGSLSGYIDCGRLGVADRHQDIALACGSIARNFGEDLVADFLKAYGNFEMH | 252 |
| Query 251  Sbjct 253 | SQRIAFYRLLDEFF 264 S++ A+YRLLDEFF SEKAAYYRLLDEFF 266 |  |

>WP_170251222.1 APH(3') family aminoglycoside O-phosphotransferase [Rhizobium sp. SEMIA 4085]

Length=260

Score = 248 bits (633), Expect = 1e-78, Method: Compositional matrix adjust.

| Identities | = 122/244 (50%), Positives = 160/244 (66%), Gaps = 1/244 (0%) |  |
| --- | --- | --- |
| Query 22 | YDWAQQTIGCSDAAVFRLSAQGRPVLFVKTDLSGALNELQDEAARLSWLATTGVPCAAVL | 81 |
| Sbjct 17 | ++W + +GCS A VFRL+ GR F+K +++G EL EA RL WLA G+ C +V+ HEWKRDDLGCSSADVFRLAEAGRARYFLKGEIAGPFAELPLEAERLLWLAEQGLQCPSVV | 76 |
| Query 82 | DVVTEAGRDWLLLGEVPGQDLLSSHLAP-AEKVSIMADAMRRLHTLDPATCPFDHQAKHR | 140 |
| Sbjct 77 | + + GR+WL L + G DL SS L +++ I+A A+RRLH LDP TCPFDH+ R ALESHEGRNWLRLSALSGSDLASSKLTTDRQRIEILAAALRRLHELDPETCPFDHRLTAR | 136 |
| Query 141 | IERARTRMEAGLVDQDDLDEEHQGLAPAELFARLKARMPDGEDLVVTHGDACLPNIMVEN | 200 |
| Sbjct 137 | + A+ RMEAG VD+ D DEE Q A LFA L+A PD D VV HGDACLPN +  LRLAKARMEAGAVDEGDFDEERQNATAASLFAYLEAHQPDVIDPVVAHGDACLPNFLTHE | 196 |

| Query | 201 | GRFSGFIDCGRLGVADRYQDIALATRDIAEELGGEWADRFLVLYGIAAPDSQRIAFYRLL 260 |
| --- | --- | --- |
| Sbjct | 197 | G+F G++DCGRLGVADRYQDIALA R I +G EW FL YG++ + ++A+Y +L GQFGGYVDCGRLGVADRYQDIALALRSIRYNVGEEWVRHFLDCYGLSGINDAKVAYYMVL 256 |
| Query | 261 | DEFF 264 |
| Sbjct | 257 | DEFF  DEFF 260 |

>VUR21701.1 Aminoglycoside 3'-phosphotransferase [Stenotrophomonas maltophilia] Length=267

Score = 248 bits (633), Expect = 1e-78, Method: Compositional matrix adjust.

| Identities | = 133/255 (52%), Positives = 166/255 (65%), Gaps = 3/255 (1%) |  |
| --- | --- | --- |
| Query 12 | PAAWVERLFGYDWAQQTIGCSDAAVFRLSAQGRPVLFVKTDLSGALNELQDEAARLSWLA | 71 |
| Sbjct 14 | P AW E L +Q+IG S A V R+ G+ F+K+++ A +EL DE ARL WL PCAWQEALADARIERQSIGVSRADVARVHRPGQADAFLKSEVIDAFSELGDEIARLRWLQ | 73 |
| Query 72 | TTGVPCAAVLDVVTEAGRDWLLLGEVPGQDLLSS-HLAPAEKVSIMADAMRRLHTLDPAT | 130 |
| Sbjct 74 | G P V+ EAGR WLL+ +PG+DL SS LAP + V ++ADA+R LH L A AQGQPVPTVIATAEEAGRRWLLMSALPGRDLASSPELAPQQLVELLADALRGLHALPVAA | 133 |
| Query 131 | CPFDHQAKHRIERARTRMEAGLVDQDDLDEEHQGLAPAELFARLKARMPDGEDLVVTHGD | 190 |
| Sbjct 134 | CPFD + R++ A+ R+EAGLVD DD D+E G +P ++FA L + PD EDLVV+HGD CPFDQRLASRVQVAQARVEAGLVDADDFDDERMGQSPQQVFAELCSTRPDHEDLVVSHGD | 193 |
| Query 191 | ACLPNIMVENGRFSGFIDCGRLGVADRYQDIALATRDIAEELG-GEWADRFLVLYGIAAP | 249 |
| Sbjct 194 | ACLPN+MV GRFSGFIDCGRLGVADRYQD+ALA R + G W YG A ACLPNLMVVEGRFSGFIDCGRLGVADRYQDLALAARSLVHNFGDTRWVAPLFQRYGAVA- | 252 |
| Query 250  Sbjct 253 | DSQRIAFYRLLDEFF 264 D +R+AFYRLLDEFF DERRLAFYRLLDEFF 267 |  |

>WP_142807817.1 APH(3')-II family aminoglycoside O-phosphotransferase [Stenotrophomonas maltophilia]

Length=267

Score = 248 bits (633), Expect = 1e-78, Method: Compositional matrix adjust.

| Identities | = 134/255 (53%), Positives = 166/255 (65%), Gaps = 3/255 (1%) |  |
| --- | --- | --- |
| Query 12 | PAAWVERLFGYDWAQQTIGCSDAAVFRLSAQGRPVLFVKTDLSGALNELQDEAARLSWLA | 71 |
| Sbjct 14 | P AW E L +Q+IG S A V R+ G+ FVK+++ A +EL DE ARL WL PWAWQETLADARIERQSIGVSRADVARVQRPGQADAFVKSEVIDAFSELGDEIARLRWLQ | 73 |
| Query 72 | TTGVPCAAVLDVVTEAGRDWLLLGEVPGQDLLSS-HLAPAEKVSIMADAMRRLHTLDPAT | 130 |
| Sbjct 74 | G P V+ EAGR WLL+ +PG+DL SS LAP E V ++ADA+R LH L A AQGQPAPTVIATAEEAGRRWLLMSALPGRDLASSPELAPQELVELLADALRGLHALPVAA | 133 |
| Query 131 | CPFDHQAKHRIERARTRMEAGLVDQDDLDEEHQGLAPAELFARLKARMPDGEDLVVTHGD | 190 |
| Sbjct 134 | CPFD + R++ A+ R+EAG+VD DD D+E G +P ++FA L + PD EDLVV+HGD CPFDQRLASRLQAAQARVEAGMVDADDFDDERLGQSPQQVFAELCSTRPDHEDLVVSHGD | 193 |
| Query 191 | ACLPNIMVENGRFSGFIDCGRLGVADRYQDIALATRDIAEELG-GEWADRFLVLYGIAAP | 249 |
| Sbjct 194 | ACLPN+MV GRFSGFIDCGRLGVADRYQD+ALA R + G W YG A ACLPNLMVVEGRFSGFIDCGRLGVADRYQDLALAARSLVHNFGDTRWVAPLFQRYGAVA- | 252 |
| Query 250  Sbjct 253 | DSQRIAFYRLLDEFF 264 D +R+AFYRLLDEFF DERRLAFYRLLDEFF 267 |  |

>WP_005409358.1 APH(3')-II family aminoglycoside O-phosphotransferase [Stenotrophomonas maltophilia]

EJP77263.1 hypothetical protein A1OC_02073 [Stenotrophomonas maltophilia Ab55555]

MBA0233036.1 APH(3')-II family aminoglycoside O-phosphotransferase [Stenotrophomonas maltophilia]

MBA0239125.1 APH(3')-II family aminoglycoside O-phosphotransferase [Stenotrophomonas maltophilia]

MBA0266994.1 APH(3')-II family aminoglycoside O-phosphotransferase [Stenotrophomonas maltophilia]

MBA0331250.1 APH(3')-II family aminoglycoside O-phosphotransferase [Stenotrophomonas maltophilia]

Length=267

Score = 248 bits (633), Expect = 1e-78, Method: Compositional matrix adjust.

| Identities | = 134/255 (53%), Positives = 167/255 (65%), Gaps = 3/255 (1%) |  |
| --- | --- | --- |
| Query 12 | PAAWVERLFGYDWAQQTIGCSDAAVFRLSAQGRPVLFVKTDLSGALNELQDEAARLSWLA | 71 |
| Sbjct 14 | P AW E L +Q+IG S A V R+ G+ FVK+++ A +EL DE ARL WL PWAWQETLADARIERQSIGVSRADVARVQRPGQADAFVKSEVIDAFSELGDEIARLRWLQ | 73 |
| Query 72 | TTGVPCAAVLDVVTEAGRDWLLLGEVPGQDLLSS-HLAPAEKVSIMADAMRRLHTLDPAT | 130 |
| Sbjct 74 | G P V+ EAGR WLL+ +PG+DL SS LAP + V ++ADA+R LH L A EQGQPVPTVIATAEEAGRRWLLMSALPGRDLASSPELAPQQLVEMLADALRGLHALPLAA | 133 |
| Query 131 | CPFDHQAKHRIERARTRMEAGLVDQDDLDEEHQGLAPAELFARLKARMPDGEDLVVTHGD | 190 |
| Sbjct 134 | CPFD + R++ A+ R+EAGLVD DD D+E G +P ++FA L + PD EDLVV+HGD CPFDQRLASRLQAAQARVEAGLVDADDFDDERLGQSPQQVFAELCSTRPDHEDLVVSHGD | 193 |
| Query 191 | ACLPNIMVENGRFSGFIDCGRLGVADRYQDIALATRDIAEELG-GEWADRFLVLYGIAAP | 249 |
| Sbjct 194 | ACLPN+MV GRFSGFIDCGRLGVADRYQD+ALA R + G W YG+ A ACLPNLMVVEGRFSGFIDCGRLGVADRYQDLALAARSLVHNFGDTRWVAPLFQRYGMVA- | 252 |
| Query 250  Sbjct 253 | DSQRIAFYRLLDEFF 264 D +R+AFYRLLDEFF DERRLAFYRLLDEFF 267 |  |

>WP_175887290.1 APH(3') family aminoglycoside O-phosphotransferase [Burkholderia contaminans]

Length=278

Score = 248 bits (634), Expect = 1e-78, Method: Compositional matrix adjust.

| Identities | | = 128/254 (50%), Positives = 165/254 (65%), Gaps = 2/254 (1%) |  |
| --- | --- | --- | --- |
| Query 12  Sbjct 26 | | PAAWVERLFGYDWAQQTIGCSDAAVFRLSAQGRPVLFVKTDLSGALNELQDEAARLSWLA PAAW +QT+G S A VFRL Q +FVK++ + +L+EL E ARL W+ PAAWHAYFLDGGMERQTVGESRADVFRLRRQSGAEVFVKSERADSLSELPQEIARLRWMR | 71  85 |
| Query 72 | | TTGVPCAAVLDVVTEAGRDWLLLGEVPGQDLLSS-HLAPAEKVSIMADAMRRLHTLDPAT G+PC VL+ TE GR WLL+ V G+DL S+ +LAPA+ V I+A A+R LH + | 130 |
| Sbjct | 86 | QVGLPCPQVLEATTENGRHWLLMTAVAGRDLASAANLAPAQIVQIIAQALRSLHAIPWER | 145 |
| Query | 131 | CPFDHQAKHRIERARTRMEAGLVDQDDLDEEHQGLAPAELFARLKARMPDGEDLVVTHGD | 190 |
| Sbjct | 146 | CPFDH++ R+ AR R+EA +VD+ + D E G A++FA L + PD D VV HGD CPFDHRSGQRVIDARARVEAQVVDEANFDNERLGRTAADVFAELLSSTPDTVDAVVVHGD | 205 |
| Query | 191 | ACLPNIMVENGRFSGFIDCGRLGVADRYQDIALATRDIAEELGGEWADRFLVLYGIAAPD | 250 |
| Sbjct | 206 | ACLPN+M + FSGFIDCGRLGV+DRYQD+ALATR I LG +W F +YG+ PD ACLPNLMADGSTFSGFIDCGRLGVSDRYQDLALATRSITRNLGQQWVAPFFDVYGV-VPD | 264 |
| Query | 251 | SQRIAFYRLLDEFF 264  +RI FY LLDEFF |  |
| Sbjct | 265 | MRRIRFYNLLDEFF 278 | |

>WP_019661354.1 APH(3')-II family aminoglycoside O-phosphotransferase [Stenotrophomonas maltophilia]

Length=267

Score = 248 bits (633), Expect = 1e-78, Method: Compositional matrix adjust. Identities = 133/255 (52%), Positives = 165/255 (65%), Gaps = 3/255 (1%)

| Query | 12 | PAAWVERLFGYDWAQQTIGCSDAAVFRLSAQGRPVLFVKTDLSGALNELQDEAARLSWLA | 71 |
| --- | --- | --- | --- |
| Sbjct | 14 | P AW E L +Q+IG S A V R+ G+P FVK+++ A +EL DE RL WL PRAWQEALGDARIERQSIGVSPADVARVQQPGQPDAFVKSEVIDAFSELGDEIMRLRWLQ | 73 |
| Query | 72 | TTGVPCAAVLDVVTEAGRDWLLLGEVPGQDLLSS-HLAPAEKVSIMADAMRRLHTLDPAT | 130 |
| Sbjct | 74 | G+P V+ EAGR WLL+ +PG+DL SS LAP + V ++ADA+RRLH L A  AQGLPAPTVIATAEEAGRRWLLMSALPGRDLASSPELAPQQLVELLADALRRLHVLPVAA | 133 |

| Query | 131 | CPFDHQAKHRIERARTRMEAGLVDQDDLDEEHQGLAPAELFARLKARMPDGEDLVVTHGD | 190 |
| --- | --- | --- | --- |
| Sbjct | 134 | CPFD + R++ A R+EAGLVD DD D+E G + ++FA L + PD EDLVV+HGD CPFDQRLASRLQAAAARVEAGLVDADDFDDERLGQSAQQVFAELCSTRPDHEDLVVSHGD | 193 |
| Query | 191 | ACLPNIMVENGRFSGFIDCGRLGVADRYQDIALATRDIAEELG-GEWADRFLVLYGIAAP | 249 |
| Sbjct | 194 | ACLPN+M GRFSGFIDCGRLGVADRYQD+ALA R + G W YG A ACLPNLMAVEGRFSGFIDCGRLGVADRYQDLALAARSLVHNFGDTRWVAPLFQRYGAVA- | 252 |
| Query  Sbjct | 250  253 | DSQRIAFYRLLDEFF 264 D +R+AFYRLLDEFF DERRLAFYRLLDEFF 267 |  |

>RQT26395.1 aminoglycoside 3'-phosphotransferase [Burkholderia contaminans] Length=256

Score = 248 bits (632), Expect = 1e-78, Method: Compositional matrix adjust.

| Identities | | = 128/254 (50%), Positives = 165/254 (65%), Gaps = 2/254 (1%) |  |
| --- | --- | --- | --- |
| Query 12  Sbjct 4 | | PAAWVERLFGYDWAQQTIGCSDAAVFRLSAQGRPVLFVKTDLSGALNELQDEAARLSWLA PAAW +QT+G S A VFRL Q +FVK++ + +L+EL E ARL W+ PAAWHAYFLDGGMERQTVGESRADVFRLRRQSGAEVFVKSERADSLSELPQEIARLRWMR | 71  63 |
| Query 72 | | TTGVPCAAVLDVVTEAGRDWLLLGEVPGQDLLSS-HLAPAEKVSIMADAMRRLHTLDPAT G+PC VL+ TE GR WLL+ V G+DL S+ +LAPA+ V I+A A+R LH + | 130 |
| Sbjct | 64 | QVGLPCPQVLEATTENGRHWLLMTAVAGRDLASAANLAPAQIVQIIAQALRSLHAIPWER | 123 |
| Query | 131 | CPFDHQAKHRIERARTRMEAGLVDQDDLDEEHQGLAPAELFARLKARMPDGEDLVVTHGD | 190 |
| Sbjct | 124 | CPFDH++ R+ AR R+EA +VD+ + D E G A++FA L + PD D VV HGD CPFDHRSGQRVIDARARVEAQVVDEANFDNERLGRTAADVFAELLSSTPDTVDAVVVHGD | 183 |
| Query | 191 | ACLPNIMVENGRFSGFIDCGRLGVADRYQDIALATRDIAEELGGEWADRFLVLYGIAAPD | 250 |
| Sbjct | 184 | ACLPN+M + FSGFIDCGRLGV+DRYQD+ALATR I LG +W F +YG+ PD ACLPNLMADGSTFSGFIDCGRLGVSDRYQDLALATRSITRNLGQQWVAPFFDVYGV-VPD | 242 |
| Query | 251 | SQRIAFYRLLDEFF 264  +RI FY LLDEFF |  |
| Sbjct | 243 | MRRIRFYNLLDEFF 256 | |

>WP_060186137.1 aminoglycoside 3'-phosphotransferase [Alcaligenes faecalis] KVX07000.1 aminoglycoside phosphotransferase [Alcaligenes faecalis]

Length=256

Score = 248 bits (632), Expect = 2e-78, Method: Compositional matrix adjust.

| Identities | = 130/254 (51%), Positives = 162/254 (64%), Gaps = 2/254 (1%) |  |
| --- | --- | --- |
| Query 12 | PAAWVERLFGYDWAQQTIGCSDAAVFRLSAQGRPVLFVKTDLSGALNELQDEAARLSWLA | 71 |
| Sbjct 4 | P AW RL +QTIG S A V+RL FVK + L EL DE RL W+ PLAWQTRLAAAKVQRQTIGESGADVWRLDWPDGSCQFVKAEDDLLLAELPDEIERLRWMH | 63 |
| Query 72 | TTGVPCAAVLDVVTEAGRDWLLLGEVPGQDLLSSH-LAPAEKVSIMADAMRRLHTLDPAT | 130 |
| Sbjct 64 | T G+PC VLD + GR WLL+ VPG+DL + L+P + V ++A +++ LH L + TQGLPCPEVLDTLLAQGRHWLLMSAVPGRDLACTEGLSPQQTVELLASSLQSLHRLPLES | 123 |
| Query 131 | CPFDHQAKHRIERARTRMEAGLVDQDDLDEEHQGLAPAELFARLKARMPDGEDLVVTHGD | 190 |
| Sbjct 124 | CPFDH+ +HR+ AR R +AGL+D++D D+E QG+ L L A P EDLVVTHGD CPFDHRLEHRLADARARAKAGLIDEEDFDDERQGMPVQSLLDELYASRPGQEDLVVTHGD | 183 |
| Query 191 | ACLPNIMVENGRFSGFIDCGRLGVADRYQDIALATRDIAEELGGEWADRFLVLYGIAAPD | 250 |
| Sbjct 184 | ACLPN MV G+FSGFIDCGRLGVADRYQD+AL R I LG EW F LYG+ D ACLPNFMVHQGQFSGFIDCGRLGVADRYQDLALTARSIERNLGKEWLAPFFALYGVEM-D | 242 |
| Query 251  Sbjct 243 | SQRIAFYRLLDEFF 264  ++RIAF+ LLDEFF  AERIAFFCLLDEFF 256 |  |

>WP_115635296.1 aminoglycoside 3'-phosphotransferase [Alcaligenes faecalis] SUU82505.1 Aminoglycoside 3'-phosphotransferase [Alcaligenes faecalis subsp.

faecalis]

Length=256

Score = 248 bits (632), Expect = 2e-78, Method: Compositional matrix adjust.

| Identities | = 131/254 (52%), Positives = 161/254 (63%), Gaps = 2/254 (1%) |  |
| --- | --- | --- |
| Query 12 | PAAWVERLFGYDWAQQTIGCSDAAVFRLSAQGRPVLFVKTDLSGALNELQDEAARLSWLA | 71 |
| Sbjct 4 | P AW RL +QTIG S A V+RL FVK + L EL DE RL W+ PLAWQTRLAAAKAQRQTIGESGADVWRLDWPDGSCQFVKAEDDLPLAELPDEIERLRWMH | 63 |
| Query 72 | TTGVPCAAVLDVVTEAGRDWLLLGEVPGQDLLSSH-LAPAEKVSIMADAMRRLHTLDPAT | 130 |
| Sbjct 64 | T G+PC VLD + GR WLL+ VPG+DL + L+P + V ++A +++ LH L TQGLPCPEVLDTLLAQGRHWLLMSAVPGRDLACTEGLSPQQTVELLASSLQGLHRLPLEA | 123 |
| Query 131 | CPFDHQAKHRIERARTRMEAGLVDQDDLDEEHQGLAPAELFARLKARMPDGEDLVVTHGD | 190 |
| Sbjct 124 | CPFDH+ +HR+ AR R EAGL+D++D D+E QG+ L L A P EDLVVTHGD CPFDHRLEHRLADARARAEAGLIDEEDFDDERQGMPVQNLLDELYASRPGQEDLVVTHGD | 183 |
| Query 191 | ACLPNIMVENGRFSGFIDCGRLGVADRYQDIALATRDIAEELGGEWADRFLVLYGIAAPD | 250 |
| Sbjct 184 | ACLPN MV G+FSGFIDCGRLGVADRYQD+AL R I LG EW F LYG+ D ACLPNFMVHQGQFSGFIDCGRLGVADRYQDLALTARSIERNLGKEWLAPFFALYGVEM-D | 242 |
| Query 251  Sbjct 243 | SQRIAFYRLLDEFF 264  ++RIAF+ LLDEFF  AERIAFFCLLDEFF 256 |  |

>WP_049449616.1 APH(3')-II family aminoglycoside O-phosphotransferase [Stenotrophomonas maltophilia]

Length=269

Score = 248 bits (633), Expect = 2e-78, Method: Compositional matrix adjust.

| Identities | | = 135/262 (52%), Positives = 168/262 (64%), Gaps = 3/262 (1%) |  |
| --- | --- | --- | --- |
| Query 5  Sbjct 9 | | DGLHAGSPAAWVERLFGYDWAQQTIGCSDAAVFRLSAQGRPVLFVKTDLSGALNELQDEA DGL P AW E L +Q+IG S A V R+ G+P FVK+++ A +EL DE DGLPLPLPWAWQEALADARVERQSIGVSRADVARVQRPGQPDAFVKSEVIDAFSELGDEI | 64  68 |
| Query 65  Sbjct 69 | | ARLSWLATTGVPCAAVLDVVTEAGRDWLLLGEVPGQDLLSS-HLAPAEKVSIMADAMRRL ARL WL G P V+ E GR WLL+ +PG+DL SS LAP + V ++ADA+R L ARLRWLQEQGQPAPTVIATAEEGGRRWLLMSALPGRDLASSPELAPQQLVELLADALRGL | 123  128 |
| Query 124  Sbjct 129 | | HTLDPATCPFDHQAKHRIERARTRMEAGLVDQDDLDEEHQGLAPAELFARLKARMPDGED H L A CPFD + R++ A+ R+EAGLVD DD D+E G +P ++FA L + PD ED HALPVAACPFDQRLHLRLQAAQARVEAGLVDADDFDDERLGQSPQQVFAELCSTRPDHED | 183  188 |
| Query 184  Sbjct 189 | | LVVTHGDACLPNIMVENGRFSGFIDCGRLGVADRYQDIALATRDIAEEL-GGEWADRFLV LVV+HGDACLPN+M GRFSGFIDCGRLGVADRYQD+ALA R + W LVVSHGDACLPNLMAVGGRFSGFIDCGRLGVADRYQDLALAARSLVYNFDNTRWVAPLFK | 242  248 |
| Query 243 | | LYGIAAPDSQRIAFYRLLDEFF 264 YG+ A D +R+AFYRLLDEFF |  |
| Sbjct | 249 | RYGMVA-DERRLAFYRLLDEFF 269 | |

>OCP18229.1 APH(3') family aminoglycoside O-phosphotransferase [Ensifer sp. LC384]

OCP27834.1 APH(3') family aminoglycoside O-phosphotransferase [Ensifer sp. LC54]

OCP38249.1 APH(3') family aminoglycoside O-phosphotransferase [Ensifer sp. LC163]

Length=263

Score = 248 bits (632), Expect = 2e-78, Method: Compositional matrix adjust.

| Identities | = 125/253 (49%), Positives = 164/253 (65%), Gaps = 0/253 (0%) |  |
| --- | --- | --- |
| Query 12 | PAAWVERLFGYDWAQQTIGCSDAAVFRLSAQGRPVLFVKTDLSGALNELQDEAARLSWLA | 71 |
| Sbjct 11 | P L GYDW + ++G S+++VF A+ RP L +K + +G +EL DEA RL WL PPGLAPLLKGYDWRKDSLGQSESSVFLFDAKDRPPLVLKVEETGPFDELVDEAERLEWLG | 70 |
| Query 72 | TTGVPCAAVLDVVTEAGRDWLLLGEVPGQDLLSSHLAPAEKVSIMADAMRRLHTLDPATC | 131 |
| Sbjct 71 | G+PC ++ A ++WLL+ V G DL S L +E++ ++A+A+RR+H LD C  RHGMPCPRIVAREVHARQNWLLMEAVDGVDLASGTLLASEQIVLIAEALRRMHALDVDAC | 130 |

| Query | 132 | PFDHQAKHRIERARTRMEAGLVDQDDLDEEHQGLAPAELFARLKARMPDGEDLVVTHGDA | 191 |
| --- | --- | --- | --- |
| Sbjct | 131 | PFDH+ RI A+ R +AG+VD+ D D E G ELF L+AR+P LVVTHGDA PFDHRIGARIAIAKLRADAGIVDETDFDAERLGKTAEELFVELQARIPREGKLVVTHGDA | 190 |
| Query | 192 | CLPNIMVENGRFSGFIDCGRLGVADRYQDIALATRDIAEELGGEWADRFLVLYGIAAPDS | 251 |
| Sbjct | 191 | CLPNI+V +GRF+GFIDC RLGVAD YQD+AL R IA LG EW FL LYGI D CLPNIIVRDGRFAGFIDCSRLGVADPYQDLALTCRSIAYNLGEEWVQPFLDLYGIRRADP | 250 |
| Query  Sbjct | 252  251 | QRIAFYRLLDEFF 264  +++ FY LLDEFF  EKLDFYCLLDEFF 263 |  |

>HBJ70292.1 APH(3') family aminoglycoside O-phosphotransferase [Alcaligenes faecalis]

Length=256

Score = 248 bits (632), Expect = 2e-78, Method: Compositional matrix adjust.

| Identities | = 131/254 (52%), Positives = 161/254 (63%), Gaps = 2/254 (1%) |  |
| --- | --- | --- |
| Query 12 | PAAWVERLFGYDWAQQTIGCSDAAVFRLSAQGRPVLFVKTDLSGALNELQDEAARLSWLA | 71 |
| Sbjct 4 | P AW RL +QTIG S A V+RL FVK + L EL DE RL W+ PLAWQTRLAAAKVQRQTIGESGADVWRLDWPDGSCQFVKAEDDLPLAELPDEIERLRWMH | 63 |
| Query 72 | TTGVPCAAVLDVVTEAGRDWLLLGEVPGQDLLSSH-LAPAEKVSIMADAMRRLHTLDPAT | 130 |
| Sbjct 64 | T G+PC VLD + GR WLL+ VPG+DL + L+P + V ++A +++ LH L TQGLPCPEVLDTLLAQGRHWLLMSAVPGRDLACTEGLSPQQTVELLASSLQGLHRLPLEA | 123 |
| Query 131 | CPFDHQAKHRIERARTRMEAGLVDQDDLDEEHQGLAPAELFARLKARMPDGEDLVVTHGD | 190 |
| Sbjct 124 | CPFDH+ +HR+ AR R EAGL+D++D D+E QG+ L L A P EDLVVTHGD CPFDHRLEHRLADARARAEAGLIDEEDFDDERQGMPVQSLLDELYASRPGQEDLVVTHGD | 183 |
| Query 191 | ACLPNIMVENGRFSGFIDCGRLGVADRYQDIALATRDIAEELGGEWADRFLVLYGIAAPD | 250 |
| Sbjct 184 | ACLPN MV G+FSGFIDCGRLGVADRYQD+AL R I LG EW F LYG+ D ACLPNFMVHQGQFSGFIDCGRLGVADRYQDLALTARSIERNLGKEWLAPFFALYGVEM-D | 242 |
| Query 251  Sbjct 243 | SQRIAFYRLLDEFF 264  ++RIAF+ LLDEFF  AERIAFFCLLDEFF 256 |  |

>NNH33211.1 aminoglycoside 3'-phosphotransferase [Rhizobium sp. SEMIA 4085] Length=268

Score = 248 bits (633), Expect = 2e-78, Method: Compositional matrix adjust.

| Identities | | = 123/247 (50%), Positives = 161/247 (65%), Gaps = 1/247 (0%) |  |
| --- | --- | --- | --- |
| Query 19  Sbjct 22 | | LFGYDWAQQTIGCSDAAVFRLSAQGRPVLFVKTDLSGALNELQDEAARLSWLATTGVPCA L ++W + +GCS A VFRL+ GR F+K +++G EL EA RL WLA G+ C LSQHEWKRDDLGCSSADVFRLAEAGRARYFLKGEIAGPFAELPLEAERLLWLAEQGLQCP | 78  81 |
| Query 79  Sbjct 82 | | AVLDVVTEAGRDWLLLGEVPGQDLLSSHLAP-AEKVSIMADAMRRLHTLDPATCPFDHQA  +V+ + + GR+WL L + G DL SS L +++ I+A A+RRLH LDP TCPFDH+ SVVALESHEGRNWLRLSALSGSDLASSKLTTDRQRIEILAAALRRLHELDPETCPFDHRL | 137  141 |
| Query 138  Sbjct 142 | | KHRIERARTRMEAGLVDQDDLDEEHQGLAPAELFARLKARMPDGEDLVVTHGDACLPNIM R+ A+ RMEAG VD+ D DEE Q A LFA L+A PD D VV HGDACLPN + TARLRLAKARMEAGAVDEGDFDEERQNATAASLFAYLEAHQPDVIDPVVAHGDACLPNFL | 197  201 |
| Query 198  Sbjct 202 | | VENGRFSGFIDCGRLGVADRYQDIALATRDIAEELGGEWADRFLVLYGIAAPDSQRIAFY G+F G++DCGRLGVADRYQDIALA R I +G EW FL YG++ + ++A+Y THEGQFGGYVDCGRLGVADRYQDIALALRSIRYNVGEEWVRHFLDCYGLSGINDAKVAYY | 257  261 |
| Query 258 | | RLLDEFF 264  +LDEFF |  |
| Sbjct | 262 | MVLDEFF 268 | |

>HCW19070.1 APH(3')-II family aminoglycoside O-phosphotransferase [Achromobacter sp.]

Length=266

Score = 248 bits (633), Expect = 2e-78, Method: Compositional matrix adjust.

| Identities | = 128/253 (51%), Positives = 166/253 (66%), Gaps = 1/253 (0%) |  |
| --- | --- | --- |
| Query 12 | PAAWVERLFGYDWAQQTIGCSDAAVFRLSAQGRPVLFVKTDLSGALNELQDEAARLSWLA | 71 |
| Sbjct 15 | PA W E L Q+IG S A VFR+ +P LF+K++L+GA +EL DE RL WL PAKWSEDLAHASLEPQSIGESRADVFRVRRPKQPDLFLKSELAGAESELPDEIDRLRWLQ | 74 |
| Query 72 | TTGVPCAAVLDVVTEAGRDWLLLGEVPGQDLLSSHLAPAEKVSIMADAMRRLHTLDPATC | 131 |
| Sbjct 75 | G P V+D E GR WLL+ + G DL S+ L PA+ + ++A A+R LH L A C QVGQPAPHVVDTAMEGGRHWLLMTALRGADLASAALPPAQVIGVLAAALRDLHRLPIAAC | 134 |
| Query 132 | PFDHQAKHRIERARTRMEAGLVDQDDLDEEHQGLAPAELFARLKARMPDGEDLVVTHGDA | 191 |
| Sbjct 135 | PFDH+ + RI +A+ + AGLVD+ D D+E G + ++ A L + P DLVVTHGDA PFDHRLEARIAQAQRHVHAGLVDEADFDDERLGQSANQVLAELLSSRPPAHDLVVTHGDA | 194 |
| Query 192 | CLPNIMVENGRFSGFIDCGRLGVADRYQDIALATRDIAEELGGEWADRFLVLYGIAAPDS | 251 |
| Sbjct 195 | CLPN+M++ +F+GFIDCGRLG+ADR+QD+ALA R IA +G EW F YGI APD+ CLPNLMIDGHQFTGFIDCGRLGIADRFQDLALAARSIARNVGKEWVAVFFREYGI-APDT | 253 |
| Query 252  Sbjct 254 | QRIAFYRLLDEFF 264 QRI FY LLDEFF QRINFYCLLDEFF 266 |  |

>WP_035268392.1 aminoglycoside 3'-phosphotransferase [Alcaligenes faecalis] KGP03524.1 aminoglycoside phosphotransferase [Alcaligenes faecalis]

Length=256

Score = 248 bits (632), Expect = 2e-78, Method: Compositional matrix adjust.

| Identities | = 131/254 (52%), Positives = 161/254 (63%), Gaps = 2/254 (1%) |  |
| --- | --- | --- |
| Query 12 | PAAWVERLFGYDWAQQTIGCSDAAVFRLSAQGRPVLFVKTDLSGALNELQDEAARLSWLA | 71 |
| Sbjct 4 | P AW RL +QTIG S A V+RL FVK + L EL DE RL W+ PLAWQTRLAAAKVQRQTIGESGADVWRLDWPDGSCQFVKAEDDLPLAELPDEIERLRWMH | 63 |
| Query 72 | TTGVPCAAVLDVVTEAGRDWLLLGEVPGQDLLSSH-LAPAEKVSIMADAMRRLHTLDPAT | 130 |
| Sbjct 64 | T G+PC VLD + GR WLL+ VPG+DL + L+P + V ++A +++ LH L TQGLPCPEVLDTLLAQGRHWLLMSAVPGRDLACTEDLSPQQTVELLASSLQGLHRLPLEA | 123 |
| Query 131 | CPFDHQAKHRIERARTRMEAGLVDQDDLDEEHQGLAPAELFARLKARMPDGEDLVVTHGD | 190 |
| Sbjct 124 | CPFDH+ +HR+ AR R EAGL+D++D D+E QG+ L L A P EDLVVTHGD CPFDHRLEHRLADARARAEAGLIDEEDFDDERQGMPVQNLLDELYASRPGQEDLVVTHGD | 183 |
| Query 191 | ACLPNIMVENGRFSGFIDCGRLGVADRYQDIALATRDIAEELGGEWADRFLVLYGIAAPD | 250 |
| Sbjct 184 | ACLPN MV G+FSGFIDCGRLGVADRYQD+AL R I LG EW F LYG+ D ACLPNFMVHQGQFSGFIDCGRLGVADRYQDLALTARSIERNLGKEWLAPFFALYGVEM-D | 242 |
| Query 251  Sbjct 243 | SQRIAFYRLLDEFF 264  ++RIAF+ LLDEFF  AERIAFFCLLDEFF 256 |  |

>WP_137430814.1 aminoglycoside 3'-phosphotransferase [Alcaligenes faecalis] QCP82034.1 aminoglycoside 3'-phosphotransferase [Alcaligenes faecalis]

Length=256

Score = 248 bits (632), Expect = 2e-78, Method: Compositional matrix adjust.

| Identities | = 130/254 (51%), Positives = 161/254 (63%), Gaps = 2/254 (1%) |  |
| --- | --- | --- |
| Query 12 | PAAWVERLFGYDWAQQTIGCSDAAVFRLSAQGRPVLFVKTDLSGALNELQDEAARLSWLA | 71 |
| Sbjct 4 | P AW RL +QTIG S A V+RL FVK + L EL DE RL W+ PLAWQTRLAAAKVQRQTIGESGADVWRLDWPDGSCQFVKAEDDLPLAELPDEIERLRWMH | 63 |
| Query 72 | TTGVPCAAVLDVVTEAGRDWLLLGEVPGQDLLSSH-LAPAEKVSIMADAMRRLHTLDPAT | 130 |
| Sbjct 64 | T G+PC VLD + R WLL+ VPG+DL + L+P + V ++A +++ LH L + TQGLPCPEVLDTLLAQSRHWLLMSAVPGRDLACTEGLSPQQTVELLASSLQSLHRLPLES | 123 |
| Query 131 | CPFDHQAKHRIERARTRMEAGLVDQDDLDEEHQGLAPAELFARLKARMPDGEDLVVTHGD | 190 |
| Sbjct 124 | CPFDH+ +HR+ AR R EAGL+D++D D+E QG+ L L A P EDLVVTHGD  CPFDHRLEHRLADARARAEAGLIDEEDFDDERQGMPVQNLLDELYASRPGQEDLVVTHGD | 183 |

| Query | 191 | ACLPNIMVENGRFSGFIDCGRLGVADRYQDIALATRDIAEELGGEWADRFLVLYGIAAPD 250 |
| --- | --- | --- |
| Sbjct | 184 | ACLPN MV G+FSGFIDCGRLGVADRYQD+AL R I LG EW F LYG+ D ACLPNFMVHQGQFSGFIDCGRLGVADRYQDLALTARSIERNLGKEWLAPFFALYGVEM-D 242 |
| Query | 251 | SQRIAFYRLLDEFF 264 |
| Sbjct | 243 | ++RIAF+ LLDEFF  AERIAFFCLLDEFF 256 |

>WP_191862845.1 APH(3')-II family aminoglycoside O-phosphotransferase [Stenotrophomonas sp. AS012628]

Length=267

Score = 248 bits (633), Expect = 2e-78, Method: Compositional matrix adjust.

| Identities | = 132/255 (52%), Positives = 167/255 (65%), Gaps = 3/255 (1%) |  |
| --- | --- | --- |
| Query 12 | PAAWVERLFGYDWAQQTIGCSDAAVFRLSAQGRPVLFVKTDLSGALNELQDEAARLSWLA | 71 |
| Sbjct 14 | P AW E L +Q+IG S A V R+ G+P F+K+++ A +EL DE ARL WL PWAWREALANARIERQSIGVSRADVARVQRPGQPDAFLKSEVIDAFSELGDEIARLRWLQ | 73 |
| Query 72 | TTGVPCAAVLDVVTEAGRDWLLLGEVPGQDLLSS-HLAPAEKVSIMADAMRRLHTLDPAT | 130 |
| Sbjct 74 | G+P V+ EAGR WLL+ +PG+DL SS LAP + V ++ADA+R LH L A AQGLPAPTVIATAEEAGRRWLLMSALPGRDLASSSELAPQQLVELLADALRGLHALPVAA | 133 |
| Query 131 | CPFDHQAKHRIERARTRMEAGLVDQDDLDEEHQGLAPAELFARLKARMPDGEDLVVTHGD | 190 |
| Sbjct 134 | CPFD + R++ A+ R+EAG VD DD D+E G +P ++FA L + P EDLVV+HGD CPFDQRLASRLQAAQVRVEAGRVDADDFDDERLGQSPQQVFAELCSTRPAHEDLVVSHGD | 193 |
| Query 191 | ACLPNIMVENGRFSGFIDCGRLGVADRYQDIALATRDIAEELG-GEWADRFLVLYGIAAP | 249 |
| Sbjct 194 | ACLPN+MV GRFSGFIDCGRLGVADRYQD+ALA R + G W YG+ A ACLPNLMVVEGRFSGFIDCGRLGVADRYQDLALAARSLVHNFGDTRWVAPLFRRYGVVA- | 252 |
| Query 250  Sbjct 253 | DSQRIAFYRLLDEFF 264 D +R+AFYRLLDEFF DERRLAFYRLLDEFF 267 |  |

>WP_065197025.1 APH(3')-II family aminoglycoside O-phosphotransferase [Stenotrophomonas maltophilia]

OBU64154.1 APH(3') family aminoglycoside O-phosphotransferase [Stenotrophomonas maltophilia]

Length=267

Score = 248 bits (633), Expect = 2e-78, Method: Compositional matrix adjust.

| Identities | = 133/255 (52%), Positives = 166/255 (65%), Gaps = 3/255 (1%) |  |
| --- | --- | --- |
| Query 12 | PAAWVERLFGYDWAQQTIGCSDAAVFRLSAQGRPVLFVKTDLSGALNELQDEAARLSWLA | 71 |
| Sbjct 14 | P AW + L +Q++G S A V R+ RP F+K++L A +EL DE ARL WL PLAWQKALADARIERQSMGVSRADVARVLRPCRPDAFLKSELIDAFSELGDEIARLRWLQ | 73 |
| Query 72 | TTGVPCAAVLDVVTEAGRDWLLLGEVPGQDLLSS-HLAPAEKVSIMADAMRRLHTLDPAT | 130 |
| Sbjct 74 | G P V+ EAGR WLL+ +PG+DL SS LAP ++V I+ADA+R LH L A AQGQPAPTVIATAEEAGRHWLLMSALPGRDLASSPELAPQQRVEILADALRGLHALPVAA | 133 |
| Query 131 | CPFDHQAKHRIERARTRMEAGLVDQDDLDEEHQGLAPAELFARLKARMPDGEDLVVTHGD | 190 |
| Sbjct 134 | CPFD + R+ A+ R+EAG VD DD D+E G +P ++FA+L + PD EDLVV+HGD CPFDQRLASRLPAAQARVEAGRVDADDFDDERLGQSPQQVFAQLCSTRPDHEDLVVSHGD | 193 |
| Query 191 | ACLPNIMVENGRFSGFIDCGRLGVADRYQDIALATRDIAEELG-GEWADRFLVLYGIAAP | 249 |
| Sbjct 194 | ACLPN+MV GRFSGFIDCGRLGVADRYQD+ALA R + G W YG A ACLPNLMVAEGRFSGFIDCGRLGVADRYQDLALAARSLVHNFGDTRWVTPLFQRYGAVA- | 252 |
| Query 250  Sbjct 253 | DSQRIAFYRLLDEFF 264 D +R+AFYRLLDEFF DERRLAFYRLLDEFF 267 |  |

>WP_099589766.1 APH(3')-II family aminoglycoside O-phosphotransferase [Stenotrophomonas maltophilia]

VUJ72588.1 Aminoglycoside 3'-phosphotransferase [Stenotrophomonas maltophilia] VUJ82816.1 Aminoglycoside 3'-phosphotransferase [Stenotrophomonas maltophilia] VUL49424.1 Aminoglycoside 3'-phosphotransferase [Stenotrophomonas maltophilia]

VUM90783.1 Aminoglycoside 3'-phosphotransferase [Stenotrophomonas maltophilia] VUQ05930.1 Aminoglycoside 3'-phosphotransferase [Stenotrophomonas maltophilia]

Length=267

Score = 248 bits (632), Expect = 2e-78, Method: Compositional matrix adjust.

| Identities | = 134/255 (53%), Positives = 165/255 (65%), Gaps = 3/255 (1%) |  |
| --- | --- | --- |
| Query 12 | PAAWVERLFGYDWAQQTIGCSDAAVFRLSAQGRPVLFVKTDLSGALNELQDEAARLSWLA | 71 |
| Sbjct 14 | P AW E L +Q+IG S A V R+ G+P FVK+++ A +EL DE ARL WL PWAWREALANARIERQSIGVSRADVARVHRPGQPDAFVKSEVIDAFSELGDEIARLRWLQ | 73 |
| Query 72 | TTGVPCAAVLDVVTEAGRDWLLLGEVPGQDLLSS-HLAPAEKVSIMADAMRRLHTLDPAT | 130 |
| Sbjct 74 | G P V+ EAGR WLL+ +PG DL SS LAP + V ++ADA+R LH L A AQGQPAPTVIATTEEAGRRWLLMSALPGHDLASSPELAPQQLVDLLADALRGLHALPVAA | 133 |
| Query 131 | CPFDHQAKHRIERARTRMEAGLVDQDDLDEEHQGLAPAELFARLKARMPDGEDLVVTHGD | 190 |
| Sbjct 134 | CPFD + R++ A+ R+EAGLVD DD D+E G + ++FA L + PD EDLVV+HGD CPFDQRLASRLQAAQARVEAGLVDADDFDDERLGQSAQQVFAELCSTRPDHEDLVVSHGD | 193 |
| Query 191 | ACLPNIMVENGRFSGFIDCGRLGVADRYQDIALATRDIAEELG-GEWADRFLVLYGIAAP | 249 |
| Sbjct 194 | ACLPN+MV GRFSGFIDCGRLGVADRYQD+ALA R + G W YG A ACLPNLMVAEGRFSGFIDCGRLGVADRYQDLALAARSLVHNFGDTRWVAPLFQRYGAVA- | 252 |
| Query 250  Sbjct 253 | DSQRIAFYRLLDEFF 264 D +R+AFYRLLDEFF DERRLAFYRLLDEFF 267 |  |

>WP_083054316.1 aminoglycoside 3'-phosphotransferase [Alcaligenes faecalis]

OQV31728.1 APH(3') family aminoglycoside O-phosphotransferase [Alcaligenes faecalis subsp. phenolicus]

Length=256

Score = 247 bits (631), Expect = 2e-78, Method: Compositional matrix adjust.

| Identities | = 131/254 (52%), Positives = 161/254 (63%), Gaps = 2/254 (1%) |  |
| --- | --- | --- |
| Query 12 | PAAWVERLFGYDWAQQTIGCSDAAVFRLSAQGRPVLFVKTDLSGALNELQDEAARLSWLA | 71 |
| Sbjct 4 | P AW RL +QTIG S A V+RL FVK + L EL DE RL W+ PLAWQTRLAAAKVQRQTIGESGADVWRLDWPDGSCQFVKAEDDLPLAELPDEIERLRWMH | 63 |
| Query 72 | TTGVPCAAVLDVVTEAGRDWLLLGEVPGQDLLSSH-LAPAEKVSIMADAMRRLHTLDPAT | 130 |
| Sbjct 64 | T G+PC VLD + GR WLL+ VPG+DL + L+P + V ++A +++ LH L TQGLPCPEVLDTLLAQGRHWLLMSAVPGRDLACTEGLSPQQTVELLASSLQGLHRLPLEA | 123 |
| Query 131 | CPFDHQAKHRIERARTRMEAGLVDQDDLDEEHQGLAPAELFARLKARMPDGEDLVVTHGD | 190 |
| Sbjct 124 | CPFDH+ +HR+ AR R EAGL+D++D D+E QG+ L L A P EDLVVTHGD CPFDHRLEHRLADARARAEAGLIDEEDFDDERQGMPVQNLLDELYASRPGQEDLVVTHGD | 183 |
| Query 191 | ACLPNIMVENGRFSGFIDCGRLGVADRYQDIALATRDIAEELGGEWADRFLVLYGIAAPD | 250 |
| Sbjct 184 | ACLPN MV G+FSGFIDCGRLGVADRYQD+AL R I LG EW F LYG+ D ACLPNFMVHQGQFSGFIDCGRLGVADRYQDLALTARSIERNLGKEWLAPFFALYGVEM-D | 242 |
| Query 251  Sbjct 243 | SQRIAFYRLLDEFF 264  ++RIAF+ LLDEFF  AERIAFFCLLDEFF 256 |  |

>PSD16950.1 APH(3')-II family aminoglycoside O-phosphotransferase [Stenotrophomonas maltophilia]

Length=267

Score = 248 bits (632), Expect = 2e-78, Method: Compositional matrix adjust.

| Identities | = 133/255 (52%), Positives = 166/255 (65%), Gaps = 3/255 (1%) |  |
| --- | --- | --- |
| Query 12 | PAAWVERLFGYDWAQQTIGCSDAAVFRLSAQGRPVLFVKTDLSGALNELQDEAARLSWLA | 71 |
| Sbjct 14 | P AW E L +Q+IG S A V R+ G+ F+K+++ A +EL DE ARL WL PCAWQEALADARIERQSIGVSRADVARVHRPGQADAFLKSEVIDAFSELGDEIARLRWLQ | 73 |
| Query 72 | TTGVPCAAVLDVVTEAGRDWLLLGEVPGQDLLSS-HLAPAEKVSIMADAMRRLHTLDPAT | 130 |
| Sbjct 74 | G P V+ EAGR WLL+ +PG+DL SS LAP + V ++ADA+R LH L A  AQGQPVPTVIATAEEAGRRWLLMSALPGRDLASSPELAPQQLVELLADALRGLHALPVAA | 133 |

| Query | 131 | CPFDHQAKHRIERARTRMEAGLVDQDDLDEEHQGLAPAELFARLKARMPDGEDLVVTHGD | 190 |
| --- | --- | --- | --- |
| Sbjct | 134 | CPFD + R++ A+ R+EAGLVD DD D+E G +P ++FA L + PD EDLVV+HGD CPFDQRLASRVQVAQARIEAGLVDADDFDDERLGQSPQQVFAELCSIQPDHEDLVVSHGD | 193 |
| Query | 191 | ACLPNIMVENGRFSGFIDCGRLGVADRYQDIALATRDIAEELG-GEWADRFLVLYGIAAP | 249 |
| Sbjct | 194 | ACLPN+MV GRFSGFIDCGRLGVADRYQD+ALA R + G W YG A ACLPNLMVAEGRFSGFIDCGRLGVADRYQDLALAARSLVHNFGDTRWVAPLFQRYGAVA- | 252 |
| Query  Sbjct | 250  253 | DSQRIAFYRLLDEFF 264 D +R+AFYRLLDEFF DERRLAFYRLLDEFF 267 |  |

>WP_083222448.1 MULTISPECIES: aminoglycoside 3'-phosphotransferase [unclassified Ensifer]

Length=282

Score = 248 bits (633), Expect = 2e-78, Method: Compositional matrix adjust.

| Identities | = 125/253 (49%), Positives = 164/253 (65%), Gaps = 0/253 (0%) |  |
| --- | --- | --- |
| Query 12 | PAAWVERLFGYDWAQQTIGCSDAAVFRLSAQGRPVLFVKTDLSGALNELQDEAARLSWLA | 71 |
| Sbjct 30 | P L GYDW + ++G S+++VF A+ RP L +K + +G +EL DEA RL WL PPGLAPLLKGYDWRKDSLGQSESSVFLFDAKDRPPLVLKVEETGPFDELVDEAERLEWLG | 89 |
| Query 72 | TTGVPCAAVLDVVTEAGRDWLLLGEVPGQDLLSSHLAPAEKVSIMADAMRRLHTLDPATC | 131 |
| Sbjct 90 | G+PC ++ A ++WLL+ V G DL S L +E++ ++A+A+RR+H LD C RHGMPCPRIVAREVHARQNWLLMEAVDGVDLASGTLLASEQIVLIAEALRRMHALDVDAC | 149 |
| Query 132 | PFDHQAKHRIERARTRMEAGLVDQDDLDEEHQGLAPAELFARLKARMPDGEDLVVTHGDA | 191 |
| Sbjct 150 | PFDH+ RI A+ R +AG+VD+ D D E G ELF L+AR+P LVVTHGDA PFDHRIGARIAIAKLRADAGIVDETDFDAERLGKTAEELFVELQARIPREGKLVVTHGDA | 209 |
| Query 192 | CLPNIMVENGRFSGFIDCGRLGVADRYQDIALATRDIAEELGGEWADRFLVLYGIAAPDS | 251 |
| Sbjct 210 | CLPNI+V +GRF+GFIDC RLGVAD YQD+AL R IA LG EW FL LYGI D CLPNIIVRDGRFAGFIDCSRLGVADPYQDLALTCRSIAYNLGEEWVQPFLDLYGIRRADP | 269 |
| Query 252  Sbjct 270 | QRIAFYRLLDEFF 264  +++ FY LLDEFF  EKLDFYCLLDEFF 282 |  |

>NED64886.1 APH(3')-II family aminoglycoside O-phosphotransferase [Streptomyces sp. SID10244]

Length=267

Score = 248 bits (632), Expect = 2e-78, Method: Compositional matrix adjust.

| Identities | = 133/255 (52%), Positives = 166/255 (65%), Gaps = 3/255 (1%) |  |
| --- | --- | --- |
| Query 12 | PAAWVERLFGYDWAQQTIGCSDAAVFRLSAQGRPVLFVKTDLSGALNELQDEAARLSWLA | 71 |
| Sbjct 14 | P AW E L +Q+IG S A V R+ G+ F+K+++ A +EL DE ARL WL PCAWQEALADARIERQSIGVSRADVARVHRPGQADAFLKSEVIDAFSELGDEIARLRWLQ | 73 |
| Query 72 | TTGVPCAAVLDVVTEAGRDWLLLGEVPGQDLLSS-HLAPAEKVSIMADAMRRLHTLDPAT | 130 |
| Sbjct 74 | G P V+ EAGR WLL+ +PG+DL SS LAP + V ++ADA+R LH L A AQGQPVPTVIATAEEAGRRWLLMSALPGRDLASSPELAPQQLVELLADALRGLHALPVAA | 133 |
| Query 131 | CPFDHQAKHRIERARTRMEAGLVDQDDLDEEHQGLAPAELFARLKARMPDGEDLVVTHGD | 190 |
| Sbjct 134 | CPFD + R++ A+ R+EAGLVD DD D+E G +P ++FA L + PD EDLVV+HGD CPFDQRLASRVQVAQARIEAGLVDADDFDDERLGQSPQQVFAELCSTRPDHEDLVVSHGD | 193 |
| Query 191 | ACLPNIMVENGRFSGFIDCGRLGVADRYQDIALATRDIAEELG-GEWADRFLVLYGIAAP | 249 |
| Sbjct 194 | ACLPN+MV GRFSGFIDCGRLGVADRYQD+ALA R + G W YG A ACLPNLMVVEGRFSGFIDCGRLGVADRYQDLALAARSLVHNFGDTRWVAPLFQRYGAVA- | 252 |
| Query 250  Sbjct 253 | DSQRIAFYRLLDEFF 264 D +R+AFYRLLDEFF DERRLAFYRLLDEFF 267 |  |

>VUN57646.1 Aminoglycoside 3'-phosphotransferase [Stenotrophomonas maltophilia] Length=267

Score = 247 bits (631), Expect = 2e-78, Method: Compositional matrix adjust.

| Identities | = 133/255 (52%), Positives = 166/255 (65%), Gaps = 3/255 (1%) |  |
| --- | --- | --- |
| Query 12 | PAAWVERLFGYDWAQQTIGCSDAAVFRLSAQGRPVLFVKTDLSGALNELQDEAARLSWLA | 71 |
| Sbjct 14 | P AW E L +Q+IG S A V R+ G+ F+K+++ A +EL DE ARL WL PCAWQEALADARIERQSIGVSRADVARVHRPGQADAFLKSEVIDAFSELGDEIARLRWLQ | 73 |
| Query 72 | TTGVPCAAVLDVVTEAGRDWLLLGEVPGQDLLSS-HLAPAEKVSIMADAMRRLHTLDPAT | 130 |
| Sbjct 74 | G P V+ EAGR WLL+ +PG+DL SS LAP + V ++ADA+R LH L A AQGQPVPTVIATAEEAGRRWLLMSALPGRDLASSPELAPQQLVELLADALRGLHALPVAA | 133 |
| Query 131 | CPFDHQAKHRIERARTRMEAGLVDQDDLDEEHQGLAPAELFARLKARMPDGEDLVVTHGD | 190 |
| Sbjct 134 | CPFD + R++ A+ R+EAGLVD DD D+E G +P ++FA L + PD EDLVV+HGD CPFDQRLASRVQVAQARVEAGLVDADDFDDERLGQSPQQVFAELCSTRPDHEDLVVSHGD | 193 |
| Query 191 | ACLPNIMVENGRFSGFIDCGRLGVADRYQDIALATRDIAEELG-GEWADRFLVLYGIAAP | 249 |
| Sbjct 194 | ACLPN+MV GRFSGFIDCGRLGVADRYQD+ALA R + G W YG A ACLPNLMVVEGRFSGFIDCGRLGVADRYQDLALAARSLVHNFGDTRWVAPLFQRYGAVA- | 252 |
| Query 250  Sbjct 253 | DSQRIAFYRLLDEFF 264 D +R+AFYRLLDEFF DERRLAFYRLLDEFF 267 |  |

>KRG41425.1 aminoglycoside phosphotransferase [[Pseudomonas] geniculata ATCC 19374 = JCM 13324]

Length=267

Score = 247 bits (631), Expect = 2e-78, Method: Compositional matrix adjust.

| Identities | = 133/255 (52%), Positives = 166/255 (65%), Gaps = 3/255 (1%) |  |
| --- | --- | --- |
| Query 12 | PAAWVERLFGYDWAQQTIGCSDAAVFRLSAQGRPVLFVKTDLSGALNELQDEAARLSWLA | 71 |
| Sbjct 14 | P AW E L +Q+IG S A V R+ G+P F+K+++ A +EL DE ARL WL PRAWQEALADARIERQSIGVSRADVARVQQPGQPDAFLKSEVIDAFSELGDEIARLRWLQ | 73 |
| Query 72 | TTGVPCAAVLDVVTEAGRDWLLLGEVPGQDLLSS-HLAPAEKVSIMADAMRRLHTLDPAT | 130 |
| Sbjct 74 | G+P V+ EAGR WLL+ +PG+DL SS LAP + V ++ADA+RRLH L A AQGLPAPTVIATAEEAGRRWLLMSALPGRDLASSPELAPQQLVELLADALRRLHVLPVAA | 133 |
| Query 131 | CPFDHQAKHRIERARTRMEAGLVDQDDLDEEHQGLAPAELFARLKARMPDGEDLVVTHGD | 190 |
| Sbjct 134 | CPFD + R++ A R+EAGLVD DD D+E G + ++FA L + PD EDLVV+HGD CPFDQRLASRLQAAAARVEAGLVDADDFDDERLGQSAQQVFAELCSTRPDHEDLVVSHGD | 193 |
| Query 191 | ACLPNIMVENGRFSGFIDCGRLGVADRYQDIALATRDIAEELG-GEWADRFLVLYGIAAP | 249 |
| Sbjct 194 | ACLPN+M GRFSGFIDCGRLGVADRYQD+ALA R + G W YG A ACLPNLMAVEGRFSGFIDCGRLGVADRYQDLALAARSLVHNFGDTRWVAPLFQRYG-ALV | 252 |
| Query 250  Sbjct 253 | DSQRIAFYRLLDEFF 264 D +R+AFYRLLDEFF DERRLAFYRLLDEFF 267 |  |

```
>WP_054171560.1 APH(3')-II family aminoglycoside O-phosphotransferase [Stenotrophomonas maltophilia]
KPG83561.1 aminoglycoside phosphotransferase [Stenotrophomonas maltophilia]
MBA0242780.1 APH(3')-II family aminoglycoside O-phosphotransferase [Stenotrophomonas maltophilia]
MBA0247358.1 APH(3')-II family aminoglycoside O-phosphotransferase [Stenotrophomonas maltophilia]
MBA0306325.1 APH(3')-II family aminoglycoside O-phosphotransferase [Stenotrophomonas maltophilia]
MBA0438937.1 APH(3')-II family aminoglycoside O-phosphotransferase [Stenotrophomonas maltophilia]
Length=267

Score = 247 bits (631), Expect = 3e-78, Method: Compositional matrix adjust. Identities = 134/267 (50%), Positives = 173/267 (65%), Gaps = 8/267 (3%)

Query 5	DGLHAGS-----PAAWVERLFGYDWAQQTIGCSDAAVFRLSAQGRPVLFVKTDLSGALNE 59 D +HAG+	P AW + L	+Q+IG S A V R+	G+	F+K+++ A +E
Sbjct 2	DAIHAGNHGMPLPRAWQQGLADARIERQSIGVSRADVARVHRPGQADAFLKSEVIDAFSE 61
```

| Query | 60 | LQDEAARLSWLATTGVPCAAVLDVVTEAGRDWLLLGEVPGQDLLSS-HLAPAEKVSIMAD | 118 |
| --- | --- | --- | --- |
| Sbjct | 62 | L DE ARL WL G+P V+ EAGR WLL+ +PG+DL SS LAP + V ++AD LGDEIARLRWLQAQGLPAPTVIATAEEAGRRWLLMSALPGRDLASSPELAPQQLVELLAD | 121 |
| Query | 119 | AMRRLHTLDPATCPFDHQAKHRIERARTRMEAGLVDQDDLDEEHQGLAPAELFARLKARM | 178 |
| Sbjct | 122 | A+R LH L A CPFD + R++ A+ R+EAGLVD DD D+E G + ++FA L + ALRGLHVLPVAACPFDQRLASRLQAAQARVEAGLVDADDFDDERLGQSAQQVFAELCSTR | 181 |
| Query | 179 | PDGEDLVVTHGDACLPNIMVENGRFSGFIDCGRLGVADRYQDIALATRDIAEELGGE-WA | 237 |
| Sbjct | 182 | PD EDLVV+HGDACLPN+M GRFSGFIDCGRLGVADRYQD+ALA R + G W PDHEDLVVSHGDACLPNLMAVEGRFSGFIDCGRLGVADRYQDLALAARSLVHNFGDTCWV | 241 |
| Query  Sbjct | 238  242 | DRFLVLYGIAAPDSQRIAFYRLLDEFF 264  + YG+ A D +R+AFYRLLDEFF  EPLFRRYGVVA-DERRLAFYRLLDEFF 267 |  |

>WP_057500939.1 APH(3')-II family aminoglycoside O-phosphotransferase [Stenotrophomonas maltophilia]

KRG53639.1 aminoglycoside phosphotransferase [Stenotrophomonas maltophilia]

NNH48950.1 APH(3')-II family aminoglycoside O-phosphotransferase [Stenotrophomonas maltophilia]

VEE51814.1 Aminoglycoside 3'-phosphotransferase [Stenotrophomonas maltophilia] Length=267

Score = 247 bits (631), Expect = 3e-78, Method: Compositional matrix adjust.

| Identities | | = 134/255 (53%), Positives = 164/255 (64%), Gaps = 3/255 (1%) |  |
| --- | --- | --- | --- |
| Query 12  Sbjct 14 | | PAAWVERLFGYDWAQQTIGCSDAAVFRLSAQGRPVLFVKTDLSGALNELQDEAARLSWLA P AW E L +Q+IG S A V R+ G+P FVK+++ A +EL DE ARL WL PWAWREALANARIERQSIGVSRADVARVHRPGQPDAFVKSEVIDAFSELGDEIARLRWLQ | 71  73 |
| Query 72 | | TTGVPCAAVLDVVTEAGRDWLLLGEVPGQDLLSS-HLAPAEKVSIMADAMRRLHTLDPAT G P V+ EAGR WLL+ +PG DL SS LAP E V ++ADA+R LH L | 130 |
| Sbjct | 74 | AQGQPAPTVIATTEEAGRRWLLMSALPGHDLASSPELAPQELVELLADALRGLHALPVVA | 133 |
| Query | 131 | CPFDHQAKHRIERARTRMEAGLVDQDDLDEEHQGLAPAELFARLKARMPDGEDLVVTHGD | 190 |
| Sbjct | 134 | CPFD + R++ A+ R+EAGLVD DD D+E G + ++FA L + PD EDLVV+HGD CPFDQRLASRLQAAQARVEAGLVDADDFDDERLGQSAQQVFAELCSTRPDHEDLVVSHGD | 193 |
| Query | 191 | ACLPNIMVENGRFSGFIDCGRLGVADRYQDIALATRDIAEELG-GEWADRFLVLYGIAAP | 249 |
| Sbjct | 194 | ACLPN+MV GRFSGFIDCGRLGVADRYQD+ALA R + G W YG A ACLPNLMVVEGRFSGFIDCGRLGVADRYQDLALAARSLVHNFGDTRWVAPLFQRYGAVA- | 252 |
| Query  Sbjct | 250  253 | DSQRIAFYRLLDEFF 264 D +R+AFYRLLDEFF DERRLAFYRLLDEFF 267 |  |

>WP_122477756.1 aminoglycoside 3'-phosphotransferase [Burkholderia contaminans]

TCW72299.1 APH(3') family aminoglycoside O-phosphotransferase [Burkholderia sp. SRS-25]

Length=278

Score = 248 bits (632), Expect = 3e-78, Method: Compositional matrix adjust.

| Identities | | = 128/254 (50%), Positives = 164/254 (65%), Gaps = 2/254 (1%) |  |
| --- | --- | --- | --- |
| Query 12  Sbjct 26 | | PAAWVERLFGYDWAQQTIGCSDAAVFRLSAQGRPVLFVKTDLSGALNELQDEAARLSWLA PAAW +QT+G S A VFRL Q +FVK++ + +L+EL E ARL W+ PAAWHAYFLDGGMERQTVGESRADVFRLRRQSGAEVFVKSERADSLSELPQEIARLRWMR | 71  85 |
| Query 72 | | TTGVPCAAVLDVVTEAGRDWLLLGEVPGQDLLSS-HLAPAEKVSIMADAMRRLHTLDPAT G+PC VL+ TE GR WLL+ V G+DL S+ +LAPA+ V I+A A+R LH + | 130 |
| Sbjct | 86 | QVGLPCPQVLEATTENGRHWLLMTAVAGRDLASAANLAPAQIVQIIAQALRSLHAIPWER | 145 |
| Query | 131 | CPFDHQAKHRIERARTRMEAGLVDQDDLDEEHQGLAPAELFARLKARMPDGEDLVVTHGD | 190 |
| Sbjct | 146 | CPFDH+ R+ AR R+EA +VD+ + D E G A++FA L + PD D VV HGD CPFDHRPGQRVIDARARVEAQVVDEANFDNERLGRTAADVFAELLSSTPDTVDAVVVHGD | 205 |
| Query | 191 | ACLPNIMVENGRFSGFIDCGRLGVADRYQDIALATRDIAEELGGEWADRFLVLYGIAAPD | 250 |
| Sbjct | 206 | ACLPN+M + FSGFIDCGRLGV+DRYQD+ALATR I LG +W F +YG+ PD  ACLPNLMADGSTFSGFIDCGRLGVSDRYQDLALATRSITRNLGQQWVAPFFDVYGV-VPD | 264 |

Query 251 SQRIAFYRLLDEFF 264

+RI FY LLDEFF Sbjct 265 MRRIRFYNLLDEFF 278

>WP_088451364.1 aminoglycoside 3'-phosphotransferase [Alcaligenes faecalis]

ASC90297.1 APH(3') family aminoglycoside O-phosphotransferase [Alcaligenes faecalis]

Length=256

Score = 247 bits (630), Expect = 3e-78, Method: Compositional matrix adjust.

| Identities | = 131/254 (52%), Positives = 161/254 (63%), Gaps = 2/254 (1%) |  |
| --- | --- | --- |
| Query 12 | PAAWVERLFGYDWAQQTIGCSDAAVFRLSAQGRPVLFVKTDLSGALNELQDEAARLSWLA | 71 |
| Sbjct 4 | PAAW RL +QTIG S A V+RL FVK + L EL DE RL W+ PAAWQTRLAAAKVQRQTIGESGADVWRLDWPDGSCQFVKAEDDLPLAELPDEIERLRWMH | 63 |
| Query 72 | TTGVPCAAVLDVVTEAGRDWLLLGEVPGQDLLSSH-LAPAEKVSIMADAMRRLHTLDPAT | 130 |
| Sbjct 64 | T G+PC VLD + GR WLL+ VPG+DL + L+P + V ++A +++ LH L TQGLPCPEVLDTLVADGRHWLLMSAVPGRDLACTEGLSPQQTVELLASSLQGLHRLPLEA | 123 |
| Query 131 | CPFDHQAKHRIERARTRMEAGLVDQDDLDEEHQGLAPAELFARLKARMPDGEDLVVTHGD | 190 |
| Sbjct 124 | CPFDH+ +HR+ A R EAGL+D++D D+E QG+ L L A P EDLVVTHGD CPFDHRLEHRLADASARAEAGLIDEEDFDDERQGMPVQNLLDELYASRPMQEDLVVTHGD | 183 |
| Query 191 | ACLPNIMVENGRFSGFIDCGRLGVADRYQDIALATRDIAEELGGEWADRFLVLYGIAAPD | 250 |
| Sbjct 184 | ACLPN MV G+FSGFIDCGRLGVADRYQD+AL R I LG EW F LYG+ D ACLPNFMVHQGQFSGFIDCGRLGVADRYQDLALTARSIERNLGKEWLAPFFALYGVEM-D | 242 |
| Query 251  Sbjct 243 | SQRIAFYRLLDEFF 264  ++RIAF+ LLDEFF  AERIAFFCLLDEFF 256 |  |

>WP_060380027.1 APH(3')-II family aminoglycoside O-phosphotransferase [Stenotrophomonas maltophilia]

KUP03389.1 aminoglycoside phosphotransferase [Stenotrophomonas maltophilia] KZC91721.1 aminoglycoside phosphotransferase [Stenotrophomonas maltophilia]

OBU70926.1 APH(3') family aminoglycoside O-phosphotransferase [Stenotrophomonas maltophilia]

Length=267

Score = 247 bits (631), Expect = 3e-78, Method: Compositional matrix adjust.

| Identities | = 134/255 (53%), Positives = 167/255 (65%), Gaps = 3/255 (1%) |  |
| --- | --- | --- |
| Query 12 | PAAWVERLFGYDWAQQTIGCSDAAVFRLSAQGRPVLFVKTDLSGALNELQDEAARLSWLA | 71 |
| Sbjct 14 | P AW E L +Q+IG S A V R+ G+ FVK+++ A +EL DE ARL WL PWAWQETLADARIERQSIGVSRADVARVQRPGQADAFVKSEVIDAFSELVDEIARLRWLQ | 73 |
| Query 72 | TTGVPCAAVLDVVTEAGRDWLLLGEVPGQDLLSS-HLAPAEKVSIMADAMRRLHTLDPAT | 130 |
| Sbjct 74 | G P V+ EAGR WLL+ +PG+DL SS LAP + V ++ADA+R LH L A EQGQPVPTVIATAEEAGRRWLLMSALPGRDLASSPELAPQQLVELLADALRGLHALPLAA | 133 |
| Query 131 | CPFDHQAKHRIERARTRMEAGLVDQDDLDEEHQGLAPAELFARLKARMPDGEDLVVTHGD | 190 |
| Sbjct 134 | CPFD + R++ A+ R+EAGLVD DD D+E G +P ++FA L + PD EDLVV+HGD CPFDQRLHLRLQAAQARVEAGLVDADDFDDERLGQSPQQVFAELCSTRPDHEDLVVSHGD | 193 |
| Query 191 | ACLPNIMVENGRFSGFIDCGRLGVADRYQDIALATRDIAEELG-GEWADRFLVLYGIAAP | 249 |
| Sbjct 194 | ACLPN+MV GRFSGFIDCGRLGVADRYQD+ALA R + G W YG+ A ACLPNLMVVEGRFSGFIDCGRLGVADRYQDLALAARSLVHNFGDTRWVAPLFQRYGMVA- | 252 |
| Query 250  Sbjct 253 | DSQRIAFYRLLDEFF 264 D +R+AFYRLLDEFF DERRLAFYRLLDEFF 267 |  |

>WP_180868435.1 APH(3')-II family aminoglycoside O-phosphotransferase [Stenotrophomonas maltophilia]

MBA0387867.1 APH(3')-II family aminoglycoside O-phosphotransferase [Stenotrophomonas maltophilia]

MBA0392084.1 APH(3')-II family aminoglycoside O-phosphotransferase [Stenotrophomonas

maltophilia]

MBA0464654.1 APH(3')-II family aminoglycoside O-phosphotransferase [Stenotrophomonas maltophilia]

MBA0473950.1 APH(3')-II family aminoglycoside O-phosphotransferase [Stenotrophomonas maltophilia]

Length=267

Score = 247 bits (631), Expect = 3e-78, Method: Compositional matrix adjust.

| Identities | = 133/255 (52%), Positives = 166/255 (65%), Gaps = 3/255 (1%) |  |
| --- | --- | --- |
| Query 12 | PAAWVERLFGYDWAQQTIGCSDAAVFRLSAQGRPVLFVKTDLSGALNELQDEAARLSWLA | 71 |
| Sbjct 14 | P AW + L +Q++G S A V R+ QGRP F+K++L A +EL DE ARL WL PLAWQKALADARIERQSMGVSRADVARVLRQGRPDAFLKSELIDAFSELGDEIARLRWLQ | 73 |
| Query 72 | TTGVPCAAVLDVVTEAGRDWLLLGEVPGQDLLSS-HLAPAEKVSIMADAMRRLHTLDPAT | 130 |
| Sbjct 74 | G P V+ EAGR WLL+ +PG+DL SS LAP ++V I+ADA+R LH L A AQGQPAPTVIATAEEAGRRWLLMSALPGRDLASSPELAPQQRVEILADALRGLHALPVAA | 133 |
| Query 131 | CPFDHQAKHRIERARTRMEAGLVDQDDLDEEHQGLAPAELFARLKARMPDGEDLVVTHGD | 190 |
| Sbjct 134 | CPFD + R+ A+ R+EAG VD DD D+E G + ++FA+L + PD EDLVV+HGD CPFDQRLASRLPAAQARVEAGRVDADDFDDERLGQSTQQVFAQLCSTRPDHEDLVVSHGD | 193 |
| Query 191 | ACLPNIMVENGRFSGFIDCGRLGVADRYQDIALATRDIAEEL-GGEWADRFLVLYGIAAP | 249 |
| Sbjct 194 | ACLPN+MV GRFSGFIDCGRLGVADRYQD+ALA R + W YG A ACLPNLMVAEGRFSGFIDCGRLGVADRYQDLALAARSLVHNFDDTRWVTPLFQRYGAVA- | 252 |
| Query 250  Sbjct 253 | DSQRIAFYRLLDEFF 264 D +R+AFYRLLDEFF DERRLAFYRLLDEFF 267 |  |

>WP_047853731.1 MULTISPECIES: aminoglycoside 3'-phosphotransferase [Burkholderia] AKM45648.1 aminoglycoside phosphotransferase [Burkholderia contaminans] AOL09381.1 aminoglycoside phosphotransferase [Burkholderia contaminans] RQT18997.1 aminoglycoside 3'-phosphotransferase [Burkholderia contaminans]

Length=256

Score = 247 bits (630), Expect = 3e-78, Method: Compositional matrix adjust.

| Identities | | = 128/254 (50%), Positives = 164/254 (65%), Gaps = 2/254 (1%) |  |
| --- | --- | --- | --- |
| Query 12  Sbjct 4 | | PAAWVERLFGYDWAQQTIGCSDAAVFRLSAQGRPVLFVKTDLSGALNELQDEAARLSWLA PAAW +QT+G S A VFRL Q +FVK++ + +L+EL E ARL W+ PAAWHAYFLDGGMERQTVGESRADVFRLRRQSGAEVFVKSERADSLSELPQEIARLRWMR | 71  63 |
| Query 72 | | TTGVPCAAVLDVVTEAGRDWLLLGEVPGQDLLSS-HLAPAEKVSIMADAMRRLHTLDPAT G+PC VL+ TE GR WLL+ V G+DL S+ +LAPA+ V I+A A+R LH + | 130 |
| Sbjct | 64 | QVGLPCPQVLEATTENGRHWLLMTAVAGRDLASAANLAPAQIVQIIAQALRSLHAIPWER | 123 |
| Query | 131 | CPFDHQAKHRIERARTRMEAGLVDQDDLDEEHQGLAPAELFARLKARMPDGEDLVVTHGD | 190 |
| Sbjct | 124 | CPFDH+ R+ AR R+EA +VD+ + D E G A++FA L + PD D VV HGD CPFDHRPGQRVIDARARVEAQVVDEANFDNERLGRTAADVFAELLSSTPDTVDAVVVHGD | 183 |
| Query | 191 | ACLPNIMVENGRFSGFIDCGRLGVADRYQDIALATRDIAEELGGEWADRFLVLYGIAAPD | 250 |
| Sbjct | 184 | ACLPN+M + FSGFIDCGRLGV+DRYQD+ALATR I LG +W F +YG+ PD ACLPNLMADGSTFSGFIDCGRLGVSDRYQDLALATRSITRNLGQQWVAPFFDVYGV-VPD | 242 |
| Query | 251 | SQRIAFYRLLDEFF 264  +RI FY LLDEFF |  |
| Sbjct | 243 | MRRIRFYNLLDEFF 256 | |

```
>WP_099529527.1 APH(3')-II family aminoglycoside O-phosphotransferase [Stenotrophomonas maltophilia]
Length=269

Score = 247 bits (631), Expect = 3e-78, Method: Compositional matrix adjust. Identities = 136/262 (52%), Positives = 168/262 (64%), Gaps = 3/262 (1%)

Query 5	DGLHAGSPAAWVERLFGYDWAQQTIGCSDAAVFRLSAQGRPVLFVKTDLSGALNELQDEA 64 DGL	P AW E L	+Q+IG S A V R+	G+	FVK+++ A +EL DE
Sbjct 9	DGLPLPLPWAWQEALADARVERQSIGVSRADVARVQRPGQADAFVKSEVIDAFSELGDEI 68
```

| Query | 65 | ARLSWLATTGVPCAAVLDVVTEAGRDWLLLGEVPGQDLLSS-HLAPAEKVSIMADAMRRL | 123 |
| --- | --- | --- | --- |
| Sbjct | 69 | ARL WL G P V+ EAGR WLL+ +PG+DL SS L P + V ++ADA+R L ARLRWLQEQGQPAPTVIATAEEAGRRWLLMSALPGRDLASSPELGPQQLVELLADALRGL | 128 |
| Query | 124 | HTLDPATCPFDHQAKHRIERARTRMEAGLVDQDDLDEEHQGLAPAELFARLKARMPDGED | 183 |
| Sbjct | 129 | H L A CPFD + R++ A+ R+EAGLVD DD D+E G +P ++FA L + PD ED HALPLAACPFDQRLHLRLQAAQARVEAGLVDADDFDDERLGQSPQQVFAELCSTRPDHED | 188 |
| Query | 184 | LVVTHGDACLPNIMVENGRFSGFIDCGRLGVADRYQDIALATRDIAEELG-GEWADRFLV | 242 |
| Sbjct | 189 | LVV+HGDACLPN+MV GRFSGFIDCGRLGVADRYQD+ALA R + G W LVVSHGDACLPNLMVVEGRFSGFIDCGRLGVADRYQDLALAARSLVHNFGDTRWVAPLFQ | 248 |
| Query | 243 | LYGIAAPDSQRIAFYRLLDEFF 264 YG A D +R+AFYRLLDEFF |  |
| Sbjct | 249 | RYGAVA-DERRLAFYRLLDEFF 269 | |

>WP_154348935.1 APH(3')-II family aminoglycoside O-phosphotransferase [Stenotrophomonas maltophilia]

MBA0252700.1 APH(3')-II family aminoglycoside O-phosphotransferase [Stenotrophomonas maltophilia]

MBA0320285.1 APH(3')-II family aminoglycoside O-phosphotransferase [Stenotrophomonas maltophilia]

QGL92666.1 APH(3')-II family aminoglycoside O-phosphotransferase [Stenotrophomonas maltophilia]

Length=267

Score = 247 bits (631), Expect = 3e-78, Method: Compositional matrix adjust.

| Identities | = 133/255 (52%), Positives = 165/255 (65%), Gaps = 3/255 (1%) |  |
| --- | --- | --- |
| Query 12 | PAAWVERLFGYDWAQQTIGCSDAAVFRLSAQGRPVLFVKTDLSGALNELQDEAARLSWLA | 71 |
| Sbjct 14 | P AW E L +Q+IG S A V R+ G+P FVK+++ A +EL DE RL WL PRAWQEALADARIERQSIGVSRADVARVQQPGQPDAFVKSEVIDAFSELGDEITRLRWLQ | 73 |
| Query 72 | TTGVPCAAVLDVVTEAGRDWLLLGEVPGQDLLSS-HLAPAEKVSIMADAMRRLHTLDPAT | 130 |
| Sbjct 74 | G+P V+ EAGR WLL+ +PG+DL SS LAP + V ++ADA+RRLH L A AQGLPAPTVIATAEEAGRRWLLMSALPGRDLASSPELAPQQLVELLADALRRLHVLPVAA | 133 |
| Query 131 | CPFDHQAKHRIERARTRMEAGLVDQDDLDEEHQGLAPAELFARLKARMPDGEDLVVTHGD | 190 |
| Sbjct 134 | CPFD + R++ A R+EAGLVD DD D+E G + ++FA L + PD EDLVV+HGD CPFDQRLASRLQAAAARVEAGLVDADDFDDERLGQSAQQVFAELCSTRPDHEDLVVSHGD | 193 |
| Query 191 | ACLPNIMVENGRFSGFIDCGRLGVADRYQDIALATRDIAEELG-GEWADRFLVLYGIAAP | 249 |
| Sbjct 194 | ACLPN+M GRFSGFIDCGRLGVADRYQD+ALA R + G W YG A ACLPNLMAVEGRFSGFIDCGRLGVADRYQDLALAARSLVHNFGDTRWVAPLFQRYG-ALV | 252 |
| Query 250  Sbjct 253 | DSQRIAFYRLLDEFF 264 D +R+AFYRLLDEFF DERRLAFYRLLDEFF 267 |  |

>WP_132667369.1 APH(3')-II family aminoglycoside O-phosphotransferase [Pseudomonas aeruginosa]

Length=268

Score = 247 bits (631), Expect = 3e-78, Method: Compositional matrix adjust.

| Identities | = 135/258 (52%), Positives = 168/258 (65%), Gaps = 5/258 (2%) |  |
| --- | --- | --- |
| Query 11 | SPAAWVERLFGYDWAQQTIGCSDAAVFRLSAQGRPVLFVKTDLSGALNELQDEAARLSWL | 70 |
| Sbjct 12 | +P+ W + L GY W Q GCS A V RL A+ RP LFVK ++ A EL E ARL WL APSTWADYLAGYRWRGQGEGCSAATVHRLEAERRPTLFVKQEVLSAHAELPAEIARLRWL | 71 |
| Query 71 | ATTGVPCAAVLDVVTEAGRDWLLLGEVPGQDLLS SHLAPAEKVSIMADAMRRLHTL | 126 |
| Sbjct 72 | G+ C VL+ GR W L+ VPG L + L P V ++A A+RRLH L HGAGIDCPQVLNETQSDGRQWXLMSAVPGDTLSALAQRGELEPERLVRLVAAALRRLHDL | 131 |
| Query 127 | DPATCPFDHQAKHRIERARTRMEAGLVDQDDLDEEHQGLAPAELFARLKARMPDGEDLVV | 186 |
| Sbjct 132 | DPA CPFDH+ + R++ R R+EAGLVD+ D D++H+G + EL+ L R P EDLVV DPAACPFDHRLERRLDTVRQRVEAGLVDEADFDDDHRGRSATELYRLLLDRRPAVEDLVV | 191 |
| Query 187 | THGDACLPNIMVENGRFSGFIDCGRLGVADRYQDIALATRDIAEELGGEWADRFLVLYGI HGDACLPN++ E RFSGFIDCGRLGVADR+QD+ALA RDI ELG A+ FLV YG | 246 |

| Sbjct | 192 | AHGDACLPNLLAEGRRFSGFIDCGRLGVADRHQDLALAARDIEAELGAAXAEAFLVEYG- 250 |
| --- | --- | --- |
| Query | 247 | AAPDSQRIAFYRLLDEFF 264 |
| Sbjct | 251 | D +R+A++RLLDEFF  GDIDGERLAYFRLLDEFF 268 |

>WP_124492982.1 aminoglycoside 3'-phosphotransferase [Burkholderia contaminans] RQT07900.1 aminoglycoside 3'-phosphotransferase [Burkholderia contaminans] VWD05686.1 aminoglycoside phosphotransferase APH(3') [Burkholderia contaminans]

Length=278

Score = 247 bits (631), Expect = 4e-78, Method: Compositional matrix adjust.

| Identities | | = 128/254 (50%), Positives = 164/254 (65%), Gaps = 2/254 (1%) |  |
| --- | --- | --- | --- |
| Query 12  Sbjct 26 | | PAAWVERLFGYDWAQQTIGCSDAAVFRLSAQGRPVLFVKTDLSGALNELQDEAARLSWLA PAAW +QT+G S A VFRL Q +FVK++ + +L+EL E ARL W+ PAAWHAYFLDGGMERQTVGESRADVFRLRRQSGAEVFVKSERADSLSELPQEIARLRWMR | 71  85 |
| Query 72 | | TTGVPCAAVLDVVTEAGRDWLLLGEVPGQDLLSS-HLAPAEKVSIMADAMRRLHTLDPAT G+PC VL+ TE GR WLL+ V G+DL S+ +LAPA+ V I+A A+R LH + | 130 |
| Sbjct | 86 | QVGLPCPQVLEATTENGRHWLLMTAVAGRDLASAANLAPAQIVQIIAQALRSLHAIPWER | 145 |
| Query | 131 | CPFDHQAKHRIERARTRMEAGLVDQDDLDEEHQGLAPAELFARLKARMPDGEDLVVTHGD | 190 |
| Sbjct | 146 | CPFDH+ R+ AR R+EA +VD+ + D E G A++FA L + PD D VV HGD CPFDHRPGQRVIDARARVEAQVVDEANFDNERLGRTAADVFAELLSSTPDTVDAVVVHGD | 205 |
| Query | 191 | ACLPNIMVENGRFSGFIDCGRLGVADRYQDIALATRDIAEELGGEWADRFLVLYGIAAPD | 250 |
| Sbjct | 206 | ACLPN+M + FSGFIDCGRLGV+DRYQD+ALATR I LG +W F +YG+ PD ACLPNLMADGSTFSGFIDCGRLGVSDRYQDLALATRSITRNLGQQWVAPFFDVYGV-VPD | 264 |
| Query | 251 | SQRIAFYRLLDEFF 264  +RI FY LLDEFF |  |
| Sbjct | 265 | MRRIRFYNLLDEFF 278 | |

>WP_126924258.1 aminoglycoside 3'-phosphotransferase [Rhizobium vallis] RUM21451.1 aminoglycoside 3'-phosphotransferase [Rhizobium vallis]

Length=261

Score = 247 bits (630), Expect = 4e-78, Method: Compositional matrix adjust.

| Identities | | = 131/254 (52%), Positives = 170/254 (67%), Gaps = 1/254 (0%) |  |
| --- | --- | --- | --- |
| Query 12  Sbjct 8 | | PAAWVERLFGYDWAQQTIGCSDAAVFRLSAQGRPVLFVKTDLSGALNELQDEAARLSWLA PA RL GY Q IG S A+VFRL +G P L++K + +G EL DEAARL WL PAELAARLSGYRMEQDAIGRSTASVFRLEGEGLPALYLKVEEAGPFGELADEAARLGWLK | 71  67 |
| Query 72  Sbjct 68 | | TTGVPCAAVLDVVTEAGRDWLLLGEVPGQDLLS-SHLAPAEKVSIMADAMRRLHTLDPAT G+PC V+ ++ R+WLL+G +PG +L + S L P +V ++A A+ LH L ++ AAGLPCPDVIARESDGERNWLLIGALPGTNLANASVLTPLARVELLAAALLDLHRLPISS | 130  127 |
| Query 131  Sbjct 128 | | CPFDHQAKHRIERARTRMEAGLVDQDDLDEEHQGLAPAELFARLKARMPDGEDLVVTHGD CPFDH+ + R+ A+ RMEAG+VD++D D G + AELFA L++ P GEDLVV+HGD CPFDHRLEKRLPVAKARMEAGIVDEEDFDAARLGKSAAELFAELESLRPGGEDLVVSHGD | 190  187 |
| Query 191  Sbjct 188 | | ACLPNIMVENGRFSGFIDCGRLGVADRYQDIALATRDIAEELGGEWADRFLVLYGIAAPD ACLPN + +GRFSG+IDC RLGVADRYQDIALA R IA G FL+ YG+ APD ACLPNFIASDGRFSGYIDCSRLGVADRYQDIALACRSIAHNFGEALVLPFLIRYGLPAPD | 250  247 |
| Query 251 | | SQRIAFYRLLDEFF 264 R+ +YRLLDEFF |  |
| Sbjct | 248 | QARLDYYRLLDEFF 261 | |

>WP_123532154.1 aminoglycoside 3'-phosphotransferase [Pseudomonas fluorescens]

RON69877.1 APH(3') family aminoglycoside O-phosphotransferase [Pseudomonas fluorescens]

Length=256

Score = 246 bits (629), Expect = 4e-78, Method: Compositional matrix adjust. Identities = 126/254 (50%), Positives = 165/254 (65%), Gaps = 2/254 (1%)

| Query | 12 | PAAWVERLFGYDWAQQTIGCSDAAVFRLSAQGRPVLFVKTDLSGALNELQDEAARLSWLA | 71 |
| --- | --- | --- | --- |
| Sbjct | 4 | P W +R QQTIG S A V R++ +G LFVK++ EL E RL W+A PEQWRKRFADALIEQQTIGESRADVLRITPEGSAPLFVKSEQVMIRGELPGEVQRLRWMA | 63 |
| Query | 72 | TTGVPCAAVLDVVTEAGRDWLLLGEVPGQDLLSS-HLAPAEKVSIMADAMRRLHTLDPAT | 130 |
| Sbjct | 64 | + +P VL+ TEA R+WLL+ +PG+DL SS HLAP + V++ A+A+R LH + + SQDLPAPRVLETATEADRNWLLMTAIPGRDLASSAHLAPQQIVTLAANALRSLHAVSIDS | 123 |
| Query | 131 | CPFDHQAKHRIERARTRMEAGLVDQDDLDEEHQGLAPAELFARLKARMPDGEDLVVTHGD | 190 |
| Sbjct | 124 | CPFDH+ +I AR + AGLVD+ D D+ G +F ++ A PD EDLVVTHGD CPFDHRLDQKIPLAREHVLAGLVDEQDFDDARIGCTAENVFQQILAERPDHEDLVVTHGD | 183 |
| Query | 191 | ACLPNIMVENGRFSGFIDCGRLGVADRYQDIALATRDIAEELGGEWADRFLVLYGIAAPD | 250 |
| Sbjct | 184 | ACLPN++ ENG+FSGF+DCGRLGVADRYQD+ALAT I LG +W F +YG+ PD ACLPNLLAENGQFSGFVDCGRLGVADRYQDLALATHSIGYNLGEQWVPMFFEVYGV-EPD | 242 |
| Query | 251 | SQRIAFYRLLDEFF 264  + IAFY+LLDE F |  |
| Sbjct | 243 | QRLIAFYQLLDELF 256 | |

>WP_111189369.1 APH(3')-II family aminoglycoside O-phosphotransferase [Stenotrophomonas maltophilia]

PZS94043.1 APH(3') family aminoglycoside O-phosphotransferase [Stenotrophomonas maltophilia]

VUQ48816.1 Aminoglycoside 3'-phosphotransferase [Stenotrophomonas maltophilia] Length=267

Score = 247 bits (630), Expect = 4e-78, Method: Compositional matrix adjust.

| Identities | = 134/255 (53%), Positives = 166/255 (65%), Gaps = 3/255 (1%) |  |
| --- | --- | --- |
| Query 12 | PAAWVERLFGYDWAQQTIGCSDAAVFRLSAQGRPVLFVKTDLSGALNELQDEAARLSWLA | 71 |
| Sbjct 14 | P AW E L +Q+IG S A V R+ G+ FVK+++ A +EL DE ARL WL PWAWQEALADARIERQSIGVSRADVARVQRPGQADAFVKSEVIDAFSELGDEIARLRWLQ | 73 |
| Query 72 | TTGVPCAAVLDVVTEAGRDWLLLGEVPGQDLLSS-HLAPAEKVSIMADAMRRLHTLDPAT | 130 |
| Sbjct 74 | G P V+ EAGR WLL+ +PG+DL SS LAP + V ++ADA+R LH L A EQGQPAPTVIATAEEAGRRWLLMSALPGRDLASSPELAPQQLVELLADALRGLHALPLAA | 133 |
| Query 131 | CPFDHQAKHRIERARTRMEAGLVDQDDLDEEHQGLAPAELFARLKARMPDGEDLVVTHGD | 190 |
| Sbjct 134 | CPFD + R++ A+ R+EAGLVD DD D+E G +P ++FA L + PD EDLVV+HGD CPFDQRLASRLQAAQARVEAGLVDADDFDDERLGQSPQQVFAELCSTRPDHEDLVVSHGD | 193 |
| Query 191 | ACLPNIMVENGRFSGFIDCGRLGVADRYQDIALATRDIAEELG-GEWADRFLVLYGIAAP | 249 |
| Sbjct 194 | ACLPN+MV GRFSGFIDCGRLGVADRYQD+ALA R + G W YG A ACLPNLMVVEGRFSGFIDCGRLGVADRYQDLALAARSLVHNFGDTRWVAPLFQRYGAVA- | 252 |
| Query 250  Sbjct 253 | DSQRIAFYRLLDEFF 264 D +R+AFYRLLDEFF DERRLAFYRLLDEFF 267 |  |

>WP_062606334.1 APH(3')-II family aminoglycoside O-phosphotransferase [Stenotrophomonas maltophilia]

SSM90248.1 aminoglycoside 3'-phosphotransferase aphA1 [Acinetobacter baumannii] KUJ01594.1 aminoglycoside phosphotransferase [Stenotrophomonas maltophilia] KYK41493.1 aminoglycoside phosphotransferase [Stenotrophomonas maltophilia]

MBA0230480.1 APH(3')-II family aminoglycoside O-phosphotransferase [Stenotrophomonas maltophilia]

MBA0294677.1 APH(3')-II family aminoglycoside O-phosphotransferase [Stenotrophomonas maltophilia]

Length=267

Score = 247 bits (630), Expect = 4e-78, Method: Compositional matrix adjust. Identities = 134/255 (53%), Positives = 167/255 (65%), Gaps = 3/255 (1%)

| Query | 12 | PAAWVERLFGYDWAQQTIGCSDAAVFRLSAQGRPVLFVKTDLSGALNELQDEAARLSWLA | 71 |
| --- | --- | --- | --- |
| Sbjct | 14 | P AW E L +Q+IG S A V R+ G+ FVK+++ A +EL DE ARL WL PWAWQETLADARIERQSIGVSRADVARVQRPGQADAFVKSEVIDAFSELGDEIARLRWLQ | 73 |
| Query | 72 | TTGVPCAAVLDVVTEAGRDWLLLGEVPGQDLLSS-HLAPAEKVSIMADAMRRLHTLDPAT G P V+ EAGR WLL+ +PG+DL SS LAP + V ++ADA+R LH L A | 130 |

| Sbjct | 74 | EQGQPVPTVIATAEEAGRRWLLMSALPGRDLASSPELAPQQLVELLADALRGLHALPLAA | 133 |
| --- | --- | --- | --- |
| Query | 131 | CPFDHQAKHRIERARTRMEAGLVDQDDLDEEHQGLAPAELFARLKARMPDGEDLVVTHGD | 190 |
| Sbjct | 134 | CPFD + R++ A+ R+EAGLVD DD D+E G +P ++FA L + PD EDLVV+HGD CPFDQRLHLRLQAAQARVEAGLVDADDFDDERLGQSPQQVFAELCSTRPDHEDLVVSHGD | 193 |
| Query | 191 | ACLPNIMVENGRFSGFIDCGRLGVADRYQDIALATRDIAEELG-GEWADRFLVLYGIAAP | 249 |
| Sbjct | 194 | ACLPN+MV GRFSGFIDCGRLGVADRYQD+ALA R + G W YG+ A ACLPNLMVVEGRFSGFIDCGRLGVADRYQDLALAARSLVHNFGDTRWVAPLFQRYGMVA- | 252 |
| Query  Sbjct | 250  253 | DSQRIAFYRLLDEFF 264 D +R+AFYRLLDEFF DERRLAFYRLLDEFF 267 |  |

>WP_057494509.1 APH(3')-II family aminoglycoside O-phosphotransferase [Stenotrophomonas pavanii]

KRG79879.1 aminoglycoside phosphotransferase [Stenotrophomonas pavanii] Length=267

Score = 247 bits (630), Expect = 4e-78, Method: Compositional matrix adjust.

| Identities | = 132/255 (52%), Positives = 163/255 (64%), Gaps = 3/255 (1%) |  |
| --- | --- | --- |
| Query 12 | PAAWVERLFGYDWAQQTIGCSDAAVFRLSAQGRPVLFVKTDLSGALNELQDEAARLSWLA | 71 |
| Sbjct 14 | P AW E L QQ+IG S A V R+ G+ F+K+++ A +EL DE ARL WL PRAWQEALADARIEQQSIGVSRADVARVHRPGQADAFLKSEVIDAFSELGDEIARLRWLQ | 73 |
| Query 72 | TTGVPCAAVLDVVTEAGRDWLLLGEVPGQDLLSS-HLAPAEKVSIMADAMRRLHTLDPAT | 130 |
| Sbjct 74 | G P V+ E GR WLL+ +PG DL SS LAP +V ++ADA+R LH L A AQGQPAPTVIATAEEEGRRWLLMSALPGHDLASSPALAPQHRVQLLADALRGLHALPVAA | 133 |
| Query 131 | CPFDHQAKHRIERARTRMEAGLVDQDDLDEEHQGLAPAELFARLKARMPDGEDLVVTHGD | 190 |
| Sbjct 134 | CPFD + RIE A+ R+EAG VD DD D+E G + ++FA L+A PD EDLVV+HGD CPFDQRLASRIEAAQARVEAGRVDADDFDDERLGQSAQQVFAELRATRPDHEDLVVSHGD | 193 |
| Query 191 | ACLPNIMVENGRFSGFIDCGRLGVADRYQDIALATRDIAEELG-GEWADRFLVLYGIAAP | 249 |
| Sbjct 194 | ACLPN++ GRFSGFIDCGRLGVADRYQD+ALA R + G W YG+ P ACLPNLLAVEGRFSGFIDCGRLGVADRYQDLALAARSLVHNFGDSRWVAPLFRSYGV-VP | 252 |
| Query 250  Sbjct 253 | DSQRIAFYRLLDEFF 264 D +R+AFYRLLDEFF DERRLAFYRLLDEFF 267 |  |

>ACI97686.1 Aminoglycoside 3'-phosphotransferase, putative [Rhodospirillum centenum SW]

Length=270

Score = 247 bits (630), Expect = 4e-78, Method: Compositional matrix adjust.

| Identities | | = 141/254 (56%), Positives = 169/254 (67%), Gaps = 2/254 (1%) |  |
| --- | --- | --- | --- |
| Query 12  Sbjct 18 | | PAAWVERLFGYDWAQQTIGCSDAAVFRLSAQGRPVLFVKTDLSGALNELQDEAARLSWLA PA W ERL GY W + + GCSDAAVFRL A GRPVL+VKT+ + L EL E ARL WLA PAGWRERLEGYAWTRPSGGCSDAAVFRLDAPGRPVLYVKTEPAEPLAELPGEIARLRWLA | 71  77 |
| Query 72  Sbjct 78 | | TTGVPCAAVLDVVTEAGRDWLLLGEVPGQDLLSS-HLAPAEKVSIMADAMRRLHTLDPAT G+PC AVL EAGR WLL+ +PG L + L +V++ A A+R LH LD A  GQGIPCPAVLQATEEAGRHWLLMSALPGHVLEEAPDLDTGRRVALYAGALRTLHALDIAA | 130  137 |
| Query 131  Sbjct 138 | | CPFDHQAKHRIERARTRMEAGLVDQDDLDEEHQGLAPAELFARLKARMPDGEDLVVTHGD CPFD + +I A R+ AGLVD++D DE QG + A+L A L+AR P EDLVVTHGD CPFDQRIDGQIALAAARVAAGLVDEEDFDEVRQGRSAADLLAELRARRPAREDLVVTHGD | 190  197 |
| Query 191  Sbjct 198 | | ACLPNIMVENGRFSGFIDCGRLGVADRYQDIALATRDIAEELGGEWADRFLVLYGIAAPD AC N+M + GRF+GFIDCGRLGVADR+QD+ALA R IA+ G W FL YG A D ACPDNLMTDGGRFAGFIDCGRLGVADRHQDLALACRSIADCWGEAWIAPFLAHYGRPA-D | 250  256 |
| Query | 251 SQRIAFYRLLDEFF 264 | | |
|  |  | R+ Y LLDEFF |  |
| Sbjct | 257 | PDRLDCYCLLDEFF 270 | |

>WP_171968861.1 APH(3')-II family aminoglycoside O-phosphotransferase [Stenotrophomonas maltophilia]

Length=267

Score = 247 bits (630), Expect = 4e-78, Method: Compositional matrix adjust.

| Identities | = 134/255 (53%), Positives = 166/255 (65%), Gaps = 3/255 (1%) |  |
| --- | --- | --- |
| Query 12 | PAAWVERLFGYDWAQQTIGCSDAAVFRLSAQGRPVLFVKTDLSGALNELQDEAARLSWLA | 71 |
| Sbjct 14 | P AW E L +Q+IG S A V R+ G+ FVK+++ A +EL DE ARL WL PWAWQEALADARVERQSIGVSRADVARVQRPGQADAFVKSEVIDAFSELGDEIARLRWLQ | 73 |
| Query 72 | TTGVPCAAVLDVVTEAGRDWLLLGEVPGQDLLSS-HLAPAEKVSIMADAMRRLHTLDPAT | 130 |
| Sbjct 74 | G P V+ EAGR WLL+ +PG+DL SS LAP + V ++ADA+R LH L A EQGQPAPTVIATAEEAGRRWLLMSALPGRDLASSPELAPQQLVELLADALRGLHALPLAA | 133 |
| Query 131 | CPFDHQAKHRIERARTRMEAGLVDQDDLDEEHQGLAPAELFARLKARMPDGEDLVVTHGD | 190 |
| Sbjct 134 | CPFD + R++ A+ R+EAGLVD DD D+E G +P ++FA L + PD EDLVV+HGD CPFDQRLASRLQAAQARVEAGLVDADDFDDERLGQSPQQVFAELCSTRPDHEDLVVSHGD | 193 |
| Query 191 | ACLPNIMVENGRFSGFIDCGRLGVADRYQDIALATRDIAEELG-GEWADRFLVLYGIAAP | 249 |
| Sbjct 194 | ACLPN+MV GRFSGFIDCGRLGVADRYQD+ALA R + G W YG A ACLPNLMVVEGRFSGFIDCGRLGVADRYQDLALAARSLVHNFGDTRWVAPLFQRYGAVA- | 252 |
| Query 250  Sbjct 253 | DSQRIAFYRLLDEFF 264 D +R+AFYRLLDEFF DERRLAFYRLLDEFF 267 |  |

>WP_109338033.1 APH(3')-II family aminoglycoside O-phosphotransferase [Stenotrophomonas maltophilia]

MBA0290951.1 APH(3')-II family aminoglycoside O-phosphotransferase [Stenotrophomonas maltophilia]

MBA0371985.1 APH(3')-II family aminoglycoside O-phosphotransferase [Stenotrophomonas maltophilia]

PWI00815.1 APH(3')-II family aminoglycoside O-phosphotransferase [Stenotrophomonas maltophilia]

QGL71710.1 APH(3')-II family aminoglycoside O-phosphotransferase [Stenotrophomonas maltophilia]

QNG69696.1 aminoglycoside O-phosphotransferase APH(3')-IIc [Stenotrophomonas maltophilia]

Length=267

Score = 247 bits (630), Expect = 4e-78, Method: Compositional matrix adjust.

| Identities | = 134/255 (53%), Positives = 166/255 (65%), Gaps = 3/255 (1%) |  |
| --- | --- | --- |
| Query 12 | PAAWVERLFGYDWAQQTIGCSDAAVFRLSAQGRPVLFVKTDLSGALNELQDEAARLSWLA | 71 |
| Sbjct 14 | P AW E L +Q+IG S A V R+ G+ FVK+++ A +EL DE ARL WL PWAWQETLADARIERQSIGVSRADVARVQRPGQADAFVKSEVIDAFSELGDEIARLRWLQ | 73 |
| Query 72 | TTGVPCAAVLDVVTEAGRDWLLLGEVPGQDLLSS-HLAPAEKVSIMADAMRRLHTLDPAT | 130 |
| Sbjct 74 | G P V+ EAGR WLL+ +PG+DL SS LAP + V ++ADA+R LH L A EQGQPVPTVIATAEEAGRRWLLMSALPGRDLASSPELAPQQLVEMLADALRGLHALPLAA | 133 |
| Query 131 | CPFDHQAKHRIERARTRMEAGLVDQDDLDEEHQGLAPAELFARLKARMPDGEDLVVTHGD | 190 |
| Sbjct 134 | CPFD + R++ A+ R+EAGLVD DD D+E G +P ++FA L + PD EDLVV+HGD CPFDQRLASRLQAAQARVEAGLVDADDFDDERLGQSPQQVFAELCSTRPDHEDLVVSHGD | 193 |
| Query 191 | ACLPNIMVENGRFSGFIDCGRLGVADRYQDIALATRDIAEELG-GEWADRFLVLYGIAAP | 249 |
| Sbjct 194 | ACLPN+MV GRFSGFIDCGRLGVADRYQD+ALA R + G W YG A ACLPNLMVVEGRFSGFIDCGRLGVADRYQDLALAARSLVHNFGDTRWVAPLFQRYGAVA- | 252 |
| Query 250  Sbjct 253 | DSQRIAFYRLLDEFF 264 D +R+AFYRLLDEFF DERRLAFYRLLDEFF 267 |  |

>WP_041785050.1 aminoglycoside 3'-phosphotransferase [Rhodospirillum centenum] MBB4269036.1 aminoglycoside 3'-phosphotransferase-2 [Rhodospirillum centenum]

Length=257

Score = 246 bits (629), Expect = 4e-78, Method: Compositional matrix adjust. Identities = 141/254 (56%), Positives = 169/254 (67%), Gaps = 2/254 (1%)

| Query | 12 | PAAWVERLFGYDWAQQTIGCSDAAVFRLSAQGRPVLFVKTDLSGALNELQDEAARLSWLA | 71 |
| --- | --- | --- | --- |
| Sbjct | 5 | PA W ERL GY W + + GCSDAAVFRL A GRPVL+VKT+ + L EL E ARL WLA PAGWRERLEGYAWTRPSGGCSDAAVFRLDAPGRPVLYVKTEPAEPLAELPGEIARLRWLA | 64 |
| Query | 72 | TTGVPCAAVLDVVTEAGRDWLLLGEVPGQDLLSS-HLAPAEKVSIMADAMRRLHTLDPAT | 130 |
| Sbjct | 65 | G+PC AVL EAGR WLL+ +PG L + L +V++ A A+R LH LD A GQGIPCPAVLQATEEAGRHWLLMSALPGHVLEEAPDLDTGRRVALYAGALRTLHALDIAA | 124 |
| Query | 131 | CPFDHQAKHRIERARTRMEAGLVDQDDLDEEHQGLAPAELFARLKARMPDGEDLVVTHGD | 190 |
| Sbjct | 125 | CPFD + +I A R+ AGLVD++D DE QG + A+L A L+AR P EDLVVTHGD CPFDQRIDGQIALAAARVAAGLVDEEDFDEVRQGRSAADLLAELRARRPAREDLVVTHGD | 184 |
| Query | 191 | ACLPNIMVENGRFSGFIDCGRLGVADRYQDIALATRDIAEELGGEWADRFLVLYGIAAPD | 250 |
| Sbjct | 185 | AC N+M + GRF+GFIDCGRLGVADR+QD+ALA R IA+ G W FL YG A D  ACPDNLMTDGGRFAGFIDCGRLGVADRHQDLALACRSIADCWGEAWIAPFLAHYGRPA-D | 243 |
| Query | 251 SQRIAFYRLLDEFF 264 | | |
|  |  | R+ Y LLDEFF |  |
| Sbjct | 244 | PDRLDCYCLLDEFF 257 | |

>WP_094001862.1 APH(3')-II family aminoglycoside O-phosphotransferase [Stenotrophomonas sp. CC120222-04]

SNT84043.1 aminoglycoside 3'-phosphotransferase-2 [Stenotrophomonas sp. CC120222-04]

Length=267

Score = 247 bits (630), Expect = 5e-78, Method: Compositional matrix adjust.

| Identities | = 132/255 (52%), Positives = 165/255 (65%), Gaps = 3/255 (1%) |  |
| --- | --- | --- |
| Query 12 | PAAWVERLFGYDWAQQTIGCSDAAVFRLSAQGRPVLFVKTDLSGALNELQDEAARLSWLA | 71 |
| Sbjct 14 | P W E L +Q+IG S A V R+ G+ F+K+++ A +EL DE ARL WL PLPWQEALADARIERQSIGVSRADVARVHRPGQADAFLKSEVIDAFSELGDEIARLRWLQ | 73 |
| Query 72 | TTGVPCAAVLDVVTEAGRDWLLLGEVPGQDLLSS-HLAPAEKVSIMADAMRRLHTLDPAT | 130 |
| Sbjct 74 | G P V+ EAGR WLL+ +PG+DL SS LAP + V ++ADA+R LH L A AQGQPAPTVIATAEEAGRRWLLMSALPGRDLASSPELAPQQLVELLADALRGLHALSVAA | 133 |
| Query 131 | CPFDHQAKHRIERARTRMEAGLVDQDDLDEEHQGLAPAELFARLKARMPDGEDLVVTHGD | 190 |
| Sbjct 134 | CPFD + R++ A+ R+EAGLVD DD D+E G +P ++FA L + PD EDLVV+HGD CPFDQRLASRVQAAQARVEAGLVDADDFDDERLGQSPQQVFAELCSTRPDHEDLVVSHGD | 193 |
| Query 191 | ACLPNIMVENGRFSGFIDCGRLGVADRYQDIALATRDIAEELG-GEWADRFLVLYGIAAP | 249 |
| Sbjct 194 | ACLPN+MV GRFSGFIDCGRLGVADRYQD+ALA R + G W YG A ACLPNLMVAEGRFSGFIDCGRLGVADRYQDLALAARSLVHNFGDTRWVAPLFQRYGAVA- | 252 |
| Query 250  Sbjct 253 | DSQRIAFYRLLDEFF 264 D +R+AFYRLLDEFF DERRLAFYRLLDEFF 267 |  |

>WP_105556691.1 APH(3')-II family aminoglycoside O-phosphotransferase [Achromobacter sp. MYb9]

PQZ69385.1 APH(3') family aminoglycoside O-phosphotransferase [Achromobacter sp. MYb9]

Length=266

Score = 247 bits (630), Expect = 5e-78, Method: Compositional matrix adjust.

| Identities | = 127/253 (50%), Positives = 165/253 (65%), Gaps = 1/253 (0%) |  |
| --- | --- | --- |
| Query 12 | PAAWVERLFGYDWAQQTIGCSDAAVFRLSAQGRPVLFVKTDLSGALNELQDEAARLSWLA | 71 |
| Sbjct 15 | PA W E Q+IG S A VFR+ +P LF+K++L+GA +EL DE RL WL PAKWREDFAHASLEPQSIGESRADVFRVRRPKQPDLFLKSELAGAESELPDEIDRLRWLQ | 74 |
| Query 72 | TTGVPCAAVLDVVTEAGRDWLLLGEVPGQDLLSSHLAPAEKVSIMADAMRRLHTLDPATC | 131 |
| Sbjct 75 | G P V+D E GR WLL+ + G DL S+ L PA+ + ++A A+R LH L A C QVGQPAPHVVDTAMEGGRHWLLMTALRGADLASAALPPAQVIGVLATALRDLHRLPIAAC | 134 |
| Query 132 | PFDHQAKHRIERARTRMEAGLVDQDDLDEEHQGLAPAELFARLKARMPDGEDLVVTHGDA | 191 |
| Sbjct 135 | PFDH+ + RI +A+ + AGLVD+ D D+E G + ++ A L + P DLVVTHGDA  PFDHRLEARIAQAQRHVHAGLVDEADFDDERLGQSANQVLAELLSSRPPAHDLVVTHGDA | 194 |

| Query | 192 | CLPNIMVENGRFSGFIDCGRLGVADRYQDIALATRDIAEELGGEWADRFLVLYGIAAPDS 251 |
| --- | --- | --- |
| Sbjct | 195 | CLPN+M++ +F+GFIDCGRLG+ADR+QD+ALA R IA +G EW F YGI APD+ CLPNLMIDGHQFTGFIDCGRLGIADRFQDLALAARSIARNVGKEWVAAFFREYGI-APDT 253 |
| Query | 252 | QRIAFYRLLDEFF 264 |
| Sbjct | 254 | QRI FY LLDEFF  QRINFYCLLDEFF 266 |

>WP_071305756.1 APH(3')-II family aminoglycoside O-phosphotransferase [Stenotrophomonas maltophilia]

Length=269

Score = 247 bits (630), Expect = 5e-78, Method: Compositional matrix adjust.

| Identities | | = 134/262 (51%), Positives = 168/262 (64%), Gaps = 3/262 (1%) |  |
| --- | --- | --- | --- |
| Query 5  Sbjct 9 | | DGLHAGSPAAWVERLFGYDWAQQTIGCSDAAVFRLSAQGRPVLFVKTDLSGALNELQDEA DGL P AW E L +Q+IG S A V R+ G+ F+K+++ A +EL DE DGLPLPLPWAWQEALADAHIERQSIGVSRADVARVHRPGQADAFLKSEVIDAFSELGDEI | 64  68 |
| Query 65  Sbjct 69 | | ARLSWLATTGVPCAAVLDVVTEAGRDWLLLGEVPGQDLLSS-HLAPAEKVSIMADAMRRL RL WL G+P V+ EAGR WLL+ +PG+DL SS LAP + V ++ADA+RRL TRLRWLQAQGLPAPTVIATAEEAGRRWLLMSALPGRDLASSPELAPQQLVELLADALRRL | 123  128 |
| Query 124  Sbjct 129 | | HTLDPATCPFDHQAKHRIERARTRMEAGLVDQDDLDEEHQGLAPAELFARLKARMPDGED H L A CPFD + R++ A R+EAGLVD DD D+E G + ++FA L + PD ED HVLPVAACPFDQRLASRLQAAAARVEAGLVDADDFDDERLGQSAQQVFAELCSTRPDHED | 183  188 |
| Query 184  Sbjct 189 | | LVVTHGDACLPNIMVENGRFSGFIDCGRLGVADRYQDIALATRDIAEELG-GEWADRFLV LVV+HGDACLPN+M GRFSGFIDCGRLGVADRYQD+ALA R + G W LVVSHGDACLPNLMAVEGRFSGFIDCGRLGVADRYQDLALAARSLVHNFGDTRWVAPLFQ | 242  248 |
| Query 243 | | LYGIAAPDSQRIAFYRLLDEFF 264 YG+ A D +R+AFYRLLDEFF |  |
| Sbjct | 249 | RYGVVA-DERRLAFYRLLDEFF 269 | |

>VUR53297.1 Aminoglycoside 3'-phosphotransferase [Stenotrophomonas maltophilia] Length=267

Score = 247 bits (630), Expect = 5e-78, Method: Compositional matrix adjust.

| Identities | = 134/255 (53%), Positives = 167/255 (65%), Gaps = 3/255 (1%) |  |
| --- | --- | --- |
| Query 12 | PAAWVERLFGYDWAQQTIGCSDAAVFRLSAQGRPVLFVKTDLSGALNELQDEAARLSWLA | 71 |
| Sbjct 14 | P AW E L +Q+IG S A V R+ G+ FVK+++ A +EL DE ARL WL PWAWQETLADARIERQSIGVSRADVGRVQRPGQADAFVKSEVIDAFSELGDEIARLRWLQ | 73 |
| Query 72 | TTGVPCAAVLDVVTEAGRDWLLLGEVPGQDLLSS-HLAPAEKVSIMADAMRRLHTLDPAT | 130 |
| Sbjct 74 | G P V+ EAGR WLL+ +PG+DL SS LAP + V ++ADA+R LH L A EQGQPVPTVIATAEEAGRRWLLMSALPGRDLASSPELAPQQLVELLADALRGLHALPLAA | 133 |
| Query 131 | CPFDHQAKHRIERARTRMEAGLVDQDDLDEEHQGLAPAELFARLKARMPDGEDLVVTHGD | 190 |
| Sbjct 134 | CPFD + R++ A+ R+EAGLVD DD D+E G +P ++FA L + PD EDLVV+HGD CPFDQRLHLRLQAAQARVEAGLVDADDFDDERLGQSPQQVFAELCSTRPDHEDLVVSHGD | 193 |
| Query 191 | ACLPNIMVENGRFSGFIDCGRLGVADRYQDIALATRDIAEELG-GEWADRFLVLYGIAAP | 249 |
| Sbjct 194 | ACLPN+MV GRFSGFIDCGRLGVADRYQD+ALA R + G W YG+ A ACLPNLMVVEGRFSGFIDCGRLGVADRYQDLALAARSLVHNFGDTRWVAPLFQRYGMVA- | 252 |
| Query 250  Sbjct 253 | DSQRIAFYRLLDEFF 264 D +R+AFYRLLDEFF DERRLAFYRLLDEFF 267 |  |

>WP_180884419.1 APH(3')-II family aminoglycoside O-phosphotransferase [Stenotrophomonas maltophilia]

MBA0224574.1 APH(3')-II family aminoglycoside O-phosphotransferase [Stenotrophomonas maltophilia]

MBA0365739.1 APH(3')-II family aminoglycoside O-phosphotransferase [Stenotrophomonas maltophilia]

MBA0405081.1 APH(3')-II family aminoglycoside O-phosphotransferase [Stenotrophomonas

maltophilia]

VUR80051.1 Aminoglycoside 3'-phosphotransferase [Stenotrophomonas maltophilia] Length=257

Score = 246 bits (629), Expect = 5e-78, Method: Compositional matrix adjust.

| Identities | = 133/255 (52%), Positives = 166/255 (65%), Gaps = 3/255 (1%) |  |
| --- | --- | --- |
| Query 12 | PAAWVERLFGYDWAQQTIGCSDAAVFRLSAQGRPVLFVKTDLSGALNELQDEAARLSWLA | 71 |
| Sbjct 4 | P AW + L +Q++G S A V R+ GR F+K++L A +EL DE ARL WL PLAWQKALADARIERQSMGVSRADVARVLRPGRLDAFLKSELIDAFSELGDEIARLRWLQ | 63 |
| Query 72 | TTGVPCAAVLDVVTEAGRDWLLLGEVPGQDLLSS-HLAPAEKVSIMADAMRRLHTLDPAT | 130 |
| Sbjct 64 | G P V+ EAGR WLL+ +PG+DL SS LAP ++V I+ADA+R LH L A AQGQPAPTVIATAEEAGRHWLLMSALPGRDLASSPELAPQQRVEILADALRGLHALPVAA | 123 |
| Query 131 | CPFDHQAKHRIERARTRMEAGLVDQDDLDEEHQGLAPAELFARLKARMPDGEDLVVTHGD | 190 |
| Sbjct 124 | CPFD + R+ A+ R+EAG VD DD D+E G +P ++FA+L + PD EDLVV+HGD CPFDQRLASRLPAAQARVEAGRVDADDFDDERLGQSPQQVFAQLCSTRPDHEDLVVSHGD | 183 |
| Query 191 | ACLPNIMVENGRFSGFIDCGRLGVADRYQDIALATRDIAEELG-GEWADRFLVLYGIAAP | 249 |
| Sbjct 184 | ACLPN+MV GRFSGFIDCGRLGVADRYQD+ALA R + G W YG A ACLPNLMVAEGRFSGFIDCGRLGVADRYQDLALAARSLVHNFGDTRWITPLFQRYGAVA- | 242 |
| Query 250  Sbjct 243 | DSQRIAFYRLLDEFF 264 D +R+AFYRLLDEFF DERRLAFYRLLDEFF 257 |  |

>WP_184703200.1 APH(3') family aminoglycoside O-phosphotransferase [Rhizobium lusitanum]

MBB6484371.1 aminoglycoside 3'-phosphotransferase-2 [Rhizobium lusitanum] Length=266

Score = 246 bits (629), Expect = 5e-78, Method: Compositional matrix adjust.

| Identities | = 121/254 (48%), Positives = 168/254 (66%), Gaps = 1/254 (0%) |  |
| --- | --- | --- |
| Query 12 | PAAWVERLFGYDWAQQTIGCSDAAVFRLSAQGRPVLFVKTDLSGALNELQDEAARLSWLA | 71 |
| Sbjct 13 | P + L GY+W Q +GCS A VFRL A+GR +++KT+L L EL E ARL WLA PPRLQDLLAGYEWRQDALGCSSAYVFRLEAEGRAPVYLKTELIDPLGELSGEVARLRWLA | 72 |
| Query 72 | TTGVPCAAVLDVVTEAGRDWLLLGEVPGQDLLSSH-LAPAEKVSIMADAMRRLHTLDPAT | 130 |
| Sbjct 73 | + C V+ + R+WLL+ +PG DL+S ++P E+V ++A+A+RRLH +D AT LRELACPQVIAHERDDEREWLLMSALPGADLVSEDGMSPTERVHLLANALRRLHAVDIAT | 132 |
| Query 131 | CPFDHQAKHRIERARTRMEAGLVDQDDLDEEHQGLAPAELFARLKARMPDGEDLVVTHGD | 190 |
| Sbjct 133 | CPFDH+ +++ A+ + AG VD++D D+E G + +LF L+ R P ED VVTHGD CPFDHRLDNKVAEAKAHLLAGRVDEEDFDDERLGRSARDLFRELEERRPATEDFVVTHGD | 192 |
| Query 191 | ACLPNIMVENGRFSGFIDCGRLGVADRYQDIALATRDIAEELGGEWADRFLVLYGIAAPD | 250 |
| Sbjct 193 | ACLPN M + FSG+IDCGRLG+ADR+QDIALA I G E +FL +YG P ACLPNFMADGDTFSGYIDCGRLGIADRHQDIALACVSIERNFGKELISQFLKVYGDFEPQ | 252 |
| Query 251  Sbjct 253 | SQRIAFYRLLDEFF 264  ++++A+Y+LLDEFF  AEKMAYYQLLDEFF 266 |  |

>WP_111150888.1 APH(3')-II family aminoglycoside O-phosphotransferase [Stenotrophomonas maltophilia]

PZS50262.1 APH(3') family aminoglycoside O-phosphotransferase [Stenotrophomonas maltophilia]

Length=267

Score = 246 bits (629), Expect = 5e-78, Method: Compositional matrix adjust. Identities = 134/255 (53%), Positives = 166/255 (65%), Gaps = 3/255 (1%)

| Query | 12 | PAAWVERLFGYDWAQQTIGCSDAAVFRLSAQGRPVLFVKTDLSGALNELQDEAARLSWLA | 71 |
| --- | --- | --- | --- |
| Sbjct | 14 | P AW E L +Q+IG S A V R+ G+ FVK+++ A +EL DE ARL WL PWAWQEALADARIERQSIGVSRADVARVQRPGQADAFVKSEVIDAFSELGDEIARLRWLQ | 73 |
| Query | 72 | TTGVPCAAVLDVVTEAGRDWLLLGEVPGQDLLSS-HLAPAEKVSIMADAMRRLHTLDPAT G P V+ EAGR WLL+ +PG+DL SS LAP + V ++ADA+R LH L A | 130 |

| Sbjct | 74 | EQGQPAPTVIATAEEAGRRWLLMSALPGRDLASSPELAPQQLVELLADALRGLHALPVAA | 133 |
| --- | --- | --- | --- |
| Query | 131 | CPFDHQAKHRIERARTRMEAGLVDQDDLDEEHQGLAPAELFARLKARMPDGEDLVVTHGD | 190 |
| Sbjct | 134 | CPFD + R++ A+ R+EAGLVD DD D+E G +P ++FA L + PD EDLVV+HGD CPFDQRLVSRLQAAQARVEAGLVDADDFDDERLGQSPQQVFAELCSTRPDHEDLVVSHGD | 193 |
| Query | 191 | ACLPNIMVENGRFSGFIDCGRLGVADRYQDIALATRDIAEELG-GEWADRFLVLYGIAAP | 249 |
| Sbjct | 194 | ACLPN+MV GRFSGFIDCGRLGVADRYQD+ALA R + G W YG A ACLPNLMVVEGRFSGFIDCGRLGVADRYQDLALAARSLVHNFGDTRWVAPLFQRYGAVA- | 252 |
| Query  Sbjct | 250  253 | DSQRIAFYRLLDEFF 264 D +R+AFYRLLDEFF DERRLAFYRLLDEFF 267 |  |

>WP_076738960.1 APH(3')-II family aminoglycoside O-phosphotransferase [Stenotrophomonas sp. MB339]

OMO42378.1 APH(3') family aminoglycoside O-phosphotransferase [Stenotrophomonas sp. MB339]

Length=267

Score = 246 bits (629), Expect = 5e-78, Method: Compositional matrix adjust.

| Identities | = 133/255 (52%), Positives = 167/255 (65%), Gaps = 3/255 (1%) |  |
| --- | --- | --- |
| Query 12 | PAAWVERLFGYDWAQQTIGCSDAAVFRLSAQGRPVLFVKTDLSGALNELQDEAARLSWLA | 71 |
| Sbjct 14 | P AW E L +Q+IG S A V R+ G+ FVK+++ A +EL DE ARL WL PWAWQETLADARIERQSIGVSRADVARVQRPGQADAFVKSEVIDAFSELGDEIARLRWLQ | 73 |
| Query 72 | TTGVPCAAVLDVVTEAGRDWLLLGEVPGQDLLSS-HLAPAEKVSIMADAMRRLHTLDPAT | 130 |
| Sbjct 74 | G P V+ EAGR WLL+ +PG+DL SS LAP + V ++ADA+R LH L A EQGQPVPTVIATAEEAGRRWLLMSALPGRDLASSPELAPQQLVEMLADALRGLHALPLAA | 133 |
| Query 131 | CPFDHQAKHRIERARTRMEAGLVDQDDLDEEHQGLAPAELFARLKARMPDGEDLVVTHGD | 190 |
| Sbjct 134 | CPFD + R++ A+ R+EAGLVD D+ D+E G +P ++FA L + PD EDLVV+HGD CPFDQRLASRLQAAQARVEAGLVDADEFDDERLGQSPQQVFAELCSTRPDHEDLVVSHGD | 193 |
| Query 191 | ACLPNIMVENGRFSGFIDCGRLGVADRYQDIALATRDIAEELG-GEWADRFLVLYGIAAP | 249 |
| Sbjct 194 | ACLPN+MV GRFSGFIDCGRLGVADRYQD+ALA R + G W YG+ A ACLPNLMVVEGRFSGFIDCGRLGVADRYQDLALAARSLVHNFGDTRWVAPLFQRYGMVA- | 252 |
| Query 250  Sbjct 253 | DSQRIAFYRLLDEFF 264 D +R+AFYRLLDEFF DERRLAFYRLLDEFF 267 |  |

>WP_124647922.1 aminoglycoside 3'-phosphotransferase [Burkholderia contaminans] RQT11565.1 aminoglycoside 3'-phosphotransferase [Burkholderia contaminans]

Length=278

Score = 247 bits (630), Expect = 5e-78, Method: Compositional matrix adjust.

| Identities | | = 128/254 (50%), Positives = 164/254 (65%), Gaps = 2/254 (1%) |  |
| --- | --- | --- | --- |
| Query 12  Sbjct 26 | | PAAWVERLFGYDWAQQTIGCSDAAVFRLSAQGRPVLFVKTDLSGALNELQDEAARLSWLA PAAW +QT+G S A VFRL Q +FVK++ + +L+EL E ARL W+ PAAWHAYFLDGGMERQTVGESRADVFRLRRQSGAEVFVKSERADSLSELPQEIARLRWMR | 71  85 |
| Query 72 | | TTGVPCAAVLDVVTEAGRDWLLLGEVPGQDLLSS-HLAPAEKVSIMADAMRRLHTLDPAT G+PC VL+ TE GR WLL+ V G+DL S+ +LAPA+ V I+A A+R LH + | 130 |
| Sbjct | 86 | QVGLPCPQVLEATTENGRHWLLMTAVAGRDLASAANLAPAQIVQIIARALRSLHAIPWER | 145 |
| Query | 131 | CPFDHQAKHRIERARTRMEAGLVDQDDLDEEHQGLAPAELFARLKARMPDGEDLVVTHGD | 190 |
| Sbjct | 146 | CPFDH+ R+ AR R+EA +VD+ + D E G A++FA L + PD D VV HGD CPFDHRPGQRVIDARARVEAQVVDEANFDNERLGRTAADVFAELLSSTPDTVDAVVVHGD | 205 |
| Query | 191 | ACLPNIMVENGRFSGFIDCGRLGVADRYQDIALATRDIAEELGGEWADRFLVLYGIAAPD | 250 |
| Sbjct | 206 | ACLPN+M + FSGFIDCGRLGV+DRYQD+ALATR I LG +W F +YG+ PD ACLPNLMADGSTFSGFIDCGRLGVSDRYQDLALATRSITRNLGQQWVAPFFDVYGV-VPD | 264 |
| Query | 251 | SQRIAFYRLLDEFF 264  +RI FY LLDEFF |  |
| Sbjct | 265 | MRRIRFYNLLDEFF 278 | |

>WP_134735669.1 APH(3')-II family aminoglycoside O-phosphotransferase [Stenotrophomonas maltophilia]

Length=267

Score = 246 bits (629), Expect = 5e-78, Method: Compositional matrix adjust.

| Identities | = 132/255 (52%), Positives = 164/255 (64%), Gaps = 3/255 (1%) |  |
| --- | --- | --- |
| Query 12 | PAAWVERLFGYDWAQQTIGCSDAAVFRLSAQGRPVLFVKTDLSGALNELQDEAARLSWLA | 71 |
| Sbjct 14 | P AW E L +Q+IG S A V R+ G+P FVK+++ +EL DE RL WL PRAWQEALGDARIERQSIGVSPADVARVQQPGQPDAFVKSEVIDVFSELGDEIMRLRWLQ | 73 |
| Query 72 | TTGVPCAAVLDVVTEAGRDWLLLGEVPGQDLLSS-HLAPAEKVSIMADAMRRLHTLDPAT | 130 |
| Sbjct 74 | G+P V+ EAGR WLL+ +PG+DL SS LAP + V ++ADA+RRLH L A AQGLPAPTVIATAEEAGRRWLLMSALPGRDLASSPELAPQQLVELLADALRRLHVLPVAA | 133 |
| Query 131 | CPFDHQAKHRIERARTRMEAGLVDQDDLDEEHQGLAPAELFARLKARMPDGEDLVVTHGD | 190 |
| Sbjct 134 | CPFD + R++ A R+EAGLVD DD D+E G + ++FA L + PD EDLVV+HGD CPFDQRLASRLQAAAARVEAGLVDADDFDDERLGQSAQQVFAELCSTRPDHEDLVVSHGD | 193 |
| Query 191 | ACLPNIMVENGRFSGFIDCGRLGVADRYQDIALATRDIAEELG-GEWADRFLVLYGIAAP | 249 |
| Sbjct 194 | ACLPN+M GRFSGFIDCGRLGVADRYQD+ALA R + G W YG A ACLPNLMAVEGRFSGFIDCGRLGVADRYQDLALAARSLVHNFGDTRWVAPLFQRYGAVA- | 252 |
| Query 250  Sbjct 253 | DSQRIAFYRLLDEFF 264 D +R+AFYRLLDEFF DERRLAFYRLLDEFF 267 |  |

>WP_097628160.1 MULTISPECIES: aminoglycoside 3'-phosphotransferase [Rhizobium] MBB3430398.1 aminoglycoside 3'-phosphotransferase-2 [Rhizobium sp. BK327]

PDS83082.1 APH(3') family aminoglycoside O-phosphotransferase [Rhizobium sp. L18]

TBY45448.1 aminoglycoside 3'-phosphotransferase [Rhizobium leguminosarum bv. viciae]

Length=261

Score = 246 bits (629), Expect = 5e-78, Method: Compositional matrix adjust.

| Identities | | = 133/254 (52%), Positives = 167/254 (66%), Gaps = 1/254 (0%) |  |
| --- | --- | --- | --- |
| Query 12  Sbjct 8 | | PAAWVERLFGYDWAQQTIGCSDAAVFRLSAQGRPVLFVKTDLSGALNELQDEAARLSWLA PAA ERL GY + + T+G S A+VFRL +G+PVL++K + +G EL DEAARL WL PAALAERLSGYRFERDTLGRSTASVFRLEGEGQPVLYLKVEAAGPFGELADEAARLDWLK | 71  67 |
| Query 72  Sbjct 68 | | TTGVPCAAVLDVVTEAGRDWLLLGEVPGQDLLS-SHLAPAEKVSIMADAMRRLHTLDPAT T G+PC V+ + GR+WLL+G +PG DL S S L P +V ++A A+ LH L + TAGLPCPEVIGRESHGGRNWLLIGALPGNDLASASALTPLARVELLAAALLELHRLPITS | 130  127 |
| Query 131  Sbjct 128 | | CPFDHQAKHRIERARTRMEAGLVDQDDLDEEHQGLAPAELFARLKARMPDGEDLVVTHGD CPFDH+ + R+ A+ RM+AG VD+ D DE G + LFA L+ R P EDLVVTHGD CPFDHRREKRMAVAKARMQAGSVDETDFDETRLGKSAETLFAELETRRPGREDLVVTHGD | 190  187 |
| Query 191  Sbjct 188 | | ACLPNIMVENGRFSGFIDCGRLGVADRYQDIALATRDIAEELGGEWADRFLVLYGIAAPD ACLPN + FSG+IDC RLGVADRYQDIALA R IA G FL YG+ A D ACLPNFIASEDGFSGYIDCSRLGVADRYQDIALACRSIASNFGEALVQPFLDRYGMPAAD | 250  247 |
| Query 251 | | SQRIAFYRLLDEFF 264 R+ +YRLLDEFF |  |
| Sbjct | 248 | PVRLDYYRLLDEFF 261 | |

```
>WP_121341917.1 APH(3')-II family aminoglycoside O-phosphotransferase, partial [Pseudomonas aeruginosa]
Length=251

Score = 246 bits (628), Expect = 6e-78, Method: Compositional matrix adjust. Identities = 128/239 (54%), Positives = 155/239 (65%), Gaps = 4/239 (2%)

Query 11	SPAAWVERLFGYDWAQQTIGCSDAAVFRLSAQGRPVLFVKTDLSGALNELQDEAARLSWL 70
+PA W + L GY W Q GCS A V RL A RP LFVK ++ A EL E ARL WL Sbjct 12	APATWADYLAGYRWRGQGEGCSAATVHRLEAARRPTLFVKQEVLSAHAELPAEIARLRWL 71
```

| Query | 71 | ATTGVPCAAVLDVVTEAGRDWLLLGEVPGQDLLS SHLAPAEKVSIMADAMRRLHTL | 126 |
| --- | --- | --- | --- |
| Sbjct | 72 | G+ C VL+ GR WLL+ VPG L + L P V ++A A+RRLH L HGAGIDCPQVLNETQSDGRQWLLMSAVPGDTLSALAQRGELEPERLVRLVAAALRRLHDL | 131 |
| Query | 127 | DPATCPFDHQAKHRIERARTRMEAGLVDQDDLDEEHQGLAPAELFARLKARMPDGEDLVV | 186 |
| Sbjct | 132 | DPA CPFDH+ + R++ R R+EAGLVD+ D D++H+G + EL+ L R P EDLVV  DPAACPFDHRLERRLDTVRQRVEAGLVDEADFDDDHRGRSATELYRLLLDRRPAVEDLVV | 191 |

Query 187 THGDACLPNIMVENGRFSGFIDCGRLGVADRYQDIALATRDIAEELGGEWADRFLVLYG 245 HGDACLPN++ E RFSGFIDCGRLGVADR+QD+ALA RDI ELG WA+ FLV YG

Sbjct 192 AHGDACLPNLLAEGRRFSGFIDCGRLGVADRHQDLALAARDIEAELGAAWAEAFLVEYG 250

>VUL73972.1 Aminoglycoside 3'-phosphotransferase [Stenotrophomonas maltophilia] Length=267

Score = 246 bits (629), Expect = 6e-78, Method: Compositional matrix adjust.

| Identities | = 134/267 (50%), Positives = 172/267 (64%), Gaps = 8/267 (3%) |  |
| --- | --- | --- |
| Query 5 | DGLHAGS PAAWVERLFGYDWAQQTIGCSDAAVFRLSAQGRPVLFVKTDLSGALNE | 59 |
| Sbjct 2 | D +HAG+ P AW + L +Q+IG S A V R+ G+ F+K+++ A +E DAIHAGNHGMPLPRAWQQGLADARIERQSIGVSRADVARVHRPGQADAFLKSEVIDAFSE | 61 |
| Query 60 | LQDEAARLSWLATTGVPCAAVLDVVTEAGRDWLLLGEVPGQDLLSS-HLAPAEKVSIMAD | 118 |
| Sbjct 62 | L DE ARL WL G+P V+ EAGR WLL+ +PG+DL SS LAP + V ++AD LGDEIARLRWLQAQGLPAPTVIATAEEAGRRWLLMSALPGRDLASSPELAPQQLVELLAD | 121 |
| Query 119 | AMRRLHTLDPATCPFDHQAKHRIERARTRMEAGLVDQDDLDEEHQGLAPAELFARLKARM | 178 |
| Sbjct 122 | A+R LH L A CPFD + R++ A+ R+EAGLVD DD D+E G +P ++FA L + ALRGLHVLPVAACPFDQRLASRLQAAQARVEAGLVDADDFDDERLGQSPQQVFAELCSTR | 181 |
| Query 179 | PDGEDLVVTHGDACLPNIMVENGRFSGFIDCGRLGVADRYQDIALATRDIAEELG-GEWA | 237 |
| Sbjct 182 | P EDLVV+HGDACLPN+M GRFSGFIDCGRLGVADRYQD+ALA R + G W PAHEDLVVSHGDACLPNLMAVEGRFSGFIDCGRLGVADRYQDLALAARSLVHNFGDTRWI | 241 |
| Query 238  Sbjct 242 | DRFLVLYGIAAPDSQRIAFYRLLDEFF 264 YG+ A D +R+AFYRLLDEFF  APLFRRYGVVA-DERRLAFYRLLDEFF 267 |  |

>MBG6471870.1 APH(3')-II family aminoglycoside O-phosphotransferase [Pseudomonas aeruginosa]

Length=268

Score = 246 bits (629), Expect = 6e-78, Method: Compositional matrix adjust.

| Identities | | = 134/258 (52%), Positives = 164/258 (64%), Gaps = 5/258 (2%) |  |
| --- | --- | --- | --- |
| Query 11  Sbjct 12 | | SPAAWVERLFGYDWAQQTIGCSDAAVFRLSAQGRPVLFVKTDLSGALNELQDEAARLSWL  +P+ W + L GY W Q GCS A V RL+A RP LFVK ++ A EL E ARL WL APSTWADYLAGYRWRGQGEGCSAATVPRLAAARRPTLFVKQEVLSAHAELPAEIARLRWL | 70  71 |
| Query 71  Sbjct 72 | | ATTGVPCAAVLDVVTEAGRDWLLLGEVPGQDLLS SHLAPAEKVSIMADAMRRLHTL  G+ C VL+ GR WLL+ VPG L + L P V ++A A+RRLH L HGAGIDCPQVLNEPQSDGRQWLLMSAVPGDTLSALAQRGALEPERLVRLVAAALRRLHDL | 126  131 |
| Query 127  Sbjct 132 | | DPATCPFDHQAKHRIERARTRMEAGLVDQDDLDEEHQGLAPAELFARLKARMPDGEDLVV DPA CPF + R + R R EAGLVD+ D D++H+G + EL+ L AR P EDLVV DPAACPFAPRLDRRRDTVRQRGEAGLVDEADFDDDHRGRSATELYRLLLARRPAVEDLVV | 186  191 |
| Query 187  Sbjct 192 | | THGDACLPNIMVENGRFSGFIDCGRLGVADRYQDIALATRDIAEELGGEWADRFLVLYGI HGDACLPN++ E RFSGFIDCGRLG ADR+QD+ALA RD ELG WA+ FLV YG AHGDACLPNLLAEGRRFSGFIDCGRLGGADRHQDLALAARDSEAELGAAWAEAFLVEYG- | 246  250 |
| Query | 247 AAPDSQRIAFYRLLDEFF 264 | | |
|  |  | D +R+A++RLLDEFF |  |
| Sbjct | 251 | GDIDGERLAYFRLLDEFF 268 | |

>VUP53063.1 Aminoglycoside 3'-phosphotransferase [Stenotrophomonas maltophilia] Length=282

Score = 247 bits (630), Expect = 6e-78, Method: Compositional matrix adjust.

| Identities | = 131/255 (51%), Positives = 164/255 (64%), Gaps = 3/255 (1%) |  |
| --- | --- | --- |
| Query 12 | PAAWVERLFGYDWAQQTIGCSDAAVFRLSAQGRPVLFVKTDLSGALNELQDEAARLSWLA | 71 |
| Sbjct 29 | P AW E L +Q+IG S A V R+ G+ F+K+++ A +EL DE ARL WL PHAWQEALADARIERQSIGVSRADVARVHRPGQADAFLKSEVIDAFSELGDEIARLRWLQ | 88 |
| Query 72 | TTGVPCAAVLDVVTEAGRDWLLLGEVPGQDLLSSH-LAPAEKVSIMADAMRRLHTLDPAT | 130 |
| Sbjct 89 | G P V+ E GR WLL+ +PG DL SS LAP +V ++AD +R LH L AT AQGQPAPTVIATAEEEGRRWLLMSALPGHDLASSPALAPQHRVQLLADTLRGLHALPVAT | 148 |
| Query 131 | CPFDHQAKHRIERARTRMEAGLVDQDDLDEEHQGLAPAELFARLKARMPDGEDLVVTHGD | 190 |
| Sbjct 149 | CPFD + R++ A+ R+EAG VD DD D+E G + ++FA L+A PD EDLVV+HGD CPFDQRLASRLQAAQARVEAGRVDADDFDDERLGQSAQQVFAELRATRPDHEDLVVSHGD | 208 |
| Query 191 | ACLPNIMVENGRFSGFIDCGRLGVADRYQDIALATRDIAEELG-GEWADRFLVLYGIAAP | 249 |
| Sbjct 209 | ACLPN++ GRFSGFIDCGRLGVADRYQD+ALA R + G W R YGI P ACLPNLLAVEGRFSGFIDCGRLGVADRYQDLALAARSLVHNFGDTRWVARLFRSYGI-VP | 267 |
| Query 250  Sbjct 268 | DSQRIAFYRLLDEFF 264 D +R+AFYRLLDEFF DERRLAFYRLLDEFF 282 |  |

>WP_099478979.1 APH(3')-II family aminoglycoside O-phosphotransferase [Stenotrophomonas maltophilia]

Length=267

Score = 246 bits (629), Expect = 6e-78, Method: Compositional matrix adjust.

| Identities | = 134/255 (53%), Positives = 166/255 (65%), Gaps = 3/255 (1%) |  |
| --- | --- | --- |
| Query 12 | PAAWVERLFGYDWAQQTIGCSDAAVFRLSAQGRPVLFVKTDLSGALNELQDEAARLSWLA | 71 |
| Sbjct 14 | P AW E L +Q+IG S A V R+ G+ FVK+++ A +EL DE ARL WL PWAWQEALADARIERQSIGVSRADVARVQRPGQADAFVKSEVIDAFSELGDEIARLRWLQ | 73 |
| Query 72 | TTGVPCAAVLDVVTEAGRDWLLLGEVPGQDLLSS-HLAPAEKVSIMADAMRRLHTLDPAT | 130 |
| Sbjct 74 | G P V+ EAGR WLL+ +PG+DL SS LAP + V ++ADA+R LH L A EQGQPAPTVIATAEEAGRRWLLMSALPGRDLASSPELAPQQLVELLADALRGLHALPVAA | 133 |
| Query 131 | CPFDHQAKHRIERARTRMEAGLVDQDDLDEEHQGLAPAELFARLKARMPDGEDLVVTHGD | 190 |
| Sbjct 134 | CPFD + R++ A+ R+EAGLVD DD D+E G +P ++FA L + PD EDLVV+HGD CPFDQRLVSRLQAAQARVEAGLVDADDFDDERLGQSPQQVFAELCSTRPDHEDLVVSHGD | 193 |
| Query 191 | ACLPNIMVENGRFSGFIDCGRLGVADRYQDIALATRDIAEELG-GEWADRFLVLYGIAAP | 249 |
| Sbjct 194 | ACLPN+MV GRFSGFIDCGRLGVADRYQD+ALA R + G W YG A ACLPNLMVVEGRFSGFIDCGRLGVADRYQDLALAARSLVHNFGDTRWVAPLFQRYGAVA- | 252 |
| Query 250  Sbjct 253 | DSQRIAFYRLLDEFF 264 D +R+AFYRLLDEFF DERRLAFYRLLDEFF 267 |  |

>WP_099552881.1 APH(3')-II family aminoglycoside O-phosphotransferase [Stenotrophomonas maltophilia]

Length=267

Score = 246 bits (629), Expect = 6e-78, Method: Compositional matrix adjust.

| Identities | = 133/255 (52%), Positives = 165/255 (65%), Gaps = 3/255 (1%) |  |
| --- | --- | --- |
| Query 12 | PAAWVERLFGYDWAQQTIGCSDAAVFRLSAQGRPVLFVKTDLSGALNELQDEAARLSWLA | 71 |
| Sbjct 14 | P AW E L +Q+IG S A V R+ G+ FVK+++ A +EL DE ARL WL PWAWQETLADARIERQSIGVSRADVARVQRPGQADAFVKSEVIDAFSELGDEIARLRWLQ | 73 |
| Query 72 | TTGVPCAAVLDVVTEAGRDWLLLGEVPGQDLLSS-HLAPAEKVSIMADAMRRLHTLDPAT | 130 |
| Sbjct 74 | G P V+ EAGR WLL+ +PG+DL SS LAP E V ++ADA+R LH L A AQGQPAPTVIATAEEAGRRWLLMSALPGRDLASSPELAPQELVELLADALRGLHALPVAA | 133 |
| Query 131 | CPFDHQAKHRIERARTRMEAGLVDQDDLDEEHQGLAPAELFARLKARMPDGEDLVVTHGD | 190 |
| Sbjct 134 | CPFD + R++ A+ R+EAG+VD DD D+E G +P ++FA L + PD EDLVV+HGD CPFDQRLASRLQAAQARVEAGMVDADDFDDERLGQSPQQVFAELCSTRPDHEDLVVSHGD | 193 |
| Query 191 | ACLPNIMVENGRFSGFIDCGRLGVADRYQDIALATRDIAEELG-GEWADRFLVLYGIAAP ACLPN+MV GRFSGFIDCGRLGVADRYQD+ LA R + G W YG A | 249 |

| Sbjct | 194 | ACLPNLMVVEGRFSGFIDCGRLGVADRYQDLELAARSLVHNFGDTRWVAPLFQRYGAVA- 252 |
| --- | --- | --- |
| Query | 250 | DSQRIAFYRLLDEFF 264 |
| Sbjct | 253 | D +R+AFYRLLDEFF  DERRLAFYRLLDEFF 267 |

>VUM25775.1 Aminoglycoside 3'-phosphotransferase [Stenotrophomonas maltophilia] VUN74570.1 Aminoglycoside 3'-phosphotransferase [Stenotrophomonas maltophilia]

Length=267

Score = 246 bits (628), Expect = 7e-78, Method: Compositional matrix adjust.

| Identities | = 133/255 (52%), Positives = 166/255 (65%), Gaps = 3/255 (1%) |  |
| --- | --- | --- |
| Query 12 | PAAWVERLFGYDWAQQTIGCSDAAVFRLSAQGRPVLFVKTDLSGALNELQDEAARLSWLA | 71 |
| Sbjct 14 | P AW E L +Q+IG S A V R+ G+ F+K+++ A +EL DE ARL WL PWAWQEALADARIERQSIGVSRADVARVHRPGQADAFLKSEVIDAFSELGDEIARLRWLQ | 73 |
| Query 72 | TTGVPCAAVLDVVTEAGRDWLLLGEVPGQDLLSS-HLAPAEKVSIMADAMRRLHTLDPAT | 130 |
| Sbjct 74 | G P V+ EAGR WLL+ +PG+DL SS LAP + V ++ADA+R LH L A AQGQPVPTVIATAEEAGRRWLLMSALPGRDLASSPELAPQQLVELLADALRGLHALPVAA | 133 |
| Query 131 | CPFDHQAKHRIERARTRMEAGLVDQDDLDEEHQGLAPAELFARLKARMPDGEDLVVTHGD | 190 |
| Sbjct 134 | CPFD + R++ A+ R+EAGLVD DD D+E G +P ++FA L + PD EDLVV+HGD CPFDQRLASRVQVAQARVEAGLVDADDFDDERLGQSPQQVFAELCSTRPDHEDLVVSHGD | 193 |
| Query 191 | ACLPNIMVENGRFSGFIDCGRLGVADRYQDIALATRDIAEELG-GEWADRFLVLYGIAAP | 249 |
| Sbjct 194 | ACLPN+MV GRFSGFIDCGRLGVADRYQD+ALA R + G W YG A ACLPNLMVVEGRFSGFIDCGRLGVADRYQDLALAARSLVHNFGDTRWVAPLFQRYGAVA- | 252 |
| Query 250  Sbjct 253 | DSQRIAFYRLLDEFF 264 D +R+AFYRLLDEFF DERRLAFYRLLDEFF 267 |  |

>WP_059034263.1 APH(3')-II family aminoglycoside O-phosphotransferase [Stenotrophomonas maltophilia]

CRD49684.1 Aminoglycoside 3'-phosphotransferase [Stenotrophomonas maltophilia] Length=267

Score = 246 bits (628), Expect = 8e-78, Method: Compositional matrix adjust.

| Identities | = 134/255 (53%), Positives = 166/255 (65%), Gaps = 3/255 (1%) |  |
| --- | --- | --- |
| Query 12 | PAAWVERLFGYDWAQQTIGCSDAAVFRLSAQGRPVLFVKTDLSGALNELQDEAARLSWLA | 71 |
| Sbjct 14 | P AW E L +Q+IG S A V R+ G+ FVK+++ A +EL DE ARL WL PWAWQEALADARIERQSIGVSRADVARVQRPGQADAFVKSEVIDAFSELGDEIARLRWLQ | 73 |
| Query 72 | TTGVPCAAVLDVVTEAGRDWLLLGEVPGQDLLSS-HLAPAEKVSIMADAMRRLHTLDPAT | 130 |
| Sbjct 74 | G P V+ EAGR WLL+ +PG+DL SS LAP + V ++ADA+R LH L A EQGQPAPTVIATAEEAGRRWLLMSALPGRDLASSPELAPQQLVELLADALRGLHALPVAA | 133 |
| Query 131 | CPFDHQAKHRIERARTRMEAGLVDQDDLDEEHQGLAPAELFARLKARMPDGEDLVVTHGD | 190 |
| Sbjct 134 | CPFD + R++ A+ R+EAGLVD DD D+E G +P ++FA L + PD EDLVV+HGD CPFDQRLVSRLQAAQARVEAGLVDADDFDDERLGQSPQQVFAELCSPRPDHEDLVVSHGD | 193 |
| Query 191 | ACLPNIMVENGRFSGFIDCGRLGVADRYQDIALATRDIAEELG-GEWADRFLVLYGIAAP | 249 |
| Sbjct 194 | ACLPN+MV GRFSGFIDCGRLGVADRYQD+ALA R + G W YG A ACLPNLMVVEGRFSGFIDCGRLGVADRYQDLALAARSLVHNFGDTRWVAPLFQRYGAVA- | 252 |
| Query 250  Sbjct 253 | DSQRIAFYRLLDEFF 264 D +R+AFYRLLDEFF DERRLAFYRLLDEFF 267 |  |

>WP_071228923.1 APH(3')-II family aminoglycoside O-phosphotransferase [Stenotrophomonas maltophilia]

OHY65063.1 APH(3') family aminoglycoside O-phosphotransferase [Stenotrophomonas maltophilia]

Length=267

Score = 246 bits (628), Expect = 8e-78, Method: Compositional matrix adjust. Identities = 132/255 (52%), Positives = 166/255 (65%), Gaps = 3/255 (1%)

| Query | 12 | PAAWVERLFGYDWAQQTIGCSDAAVFRLSAQGRPVLFVKTDLSGALNELQDEAARLSWLA | 71 |
| --- | --- | --- | --- |
| Sbjct | 14 | P AW + L +Q+IG S A V R+ G+ F+K+++ A +EL DE ARL WL PWAWQDALADARIERQSIGVSRADVARVHRPGQADAFLKSEVIDAFSELGDEIARLRWLQ | 73 |
| Query | 72 | TTGVPCAAVLDVVTEAGRDWLLLGEVPGQDLLSS-HLAPAEKVSIMADAMRRLHTLDPAT | 130 |
| Sbjct | 74 | G P V+ EAGR WLL+ +PG+DL SS LAP + V ++ADA+R LH L A AQGQPVPTVIATAEEAGRRWLLMSALPGRDLASSPELAPQQLVELLADALRELHALPVAA | 133 |
| Query | 131 | CPFDHQAKHRIERARTRMEAGLVDQDDLDEEHQGLAPAELFARLKARMPDGEDLVVTHGD | 190 |
| Sbjct | 134 | CPFD + R++ A+ R+EAGLVD DD D+E G +P ++FA L + PD EDLVV+HGD CPFDQRLASRVQVAQARIEAGLVDADDFDDERLGQSPQQVFAELCSTRPDHEDLVVSHGD | 193 |
| Query | 191 | ACLPNIMVENGRFSGFIDCGRLGVADRYQDIALATRDIAEELG-GEWADRFLVLYGIAAP | 249 |
| Sbjct | 194 | ACLPN+MV GRFSGFIDCGRLGVADRYQD+ALA R + G W YG A ACLPNLMVAEGRFSGFIDCGRLGVADRYQDLALAARSLVHNFGDTRWVAPLFQRYGAVA- | 252 |
| Query  Sbjct | 250  253 | DSQRIAFYRLLDEFF 264 D +R+AFYRLLDEFF DERRLAFYRLLDEFF 267 |  |

>WP_034024097.1 aminoglycoside 3'-phosphotransferase [Pseudomonas aeruginosa] KSF25789.1 aminoglycoside 3'-phosphotransferase [Pseudomonas aeruginosa] KSF65663.1 aminoglycoside 3'-phosphotransferase [Pseudomonas aeruginosa]

Length=256

Score = 246 bits (627), Expect = 8e-78, Method: Compositional matrix adjust.

| Identities | = 130/254 (51%), Positives = 160/254 (63%), Gaps = 2/254 (1%) |  |
| --- | --- | --- |
| Query 12 | PAAWVERLFGYDWAQQTIGCSDAAVFRLSAQGRPVLFVKTDLSGALNELQDEAARLSWLA | 71 |
| Sbjct 4 | P AW RL +QTIG S A V+RL FVK + L EL DE RL W+ PLAWQTRLAAAKVQRQTIGESGADVWRLDWPDGSCQFVKAEDDLPLAELPDEIERLRWMH | 63 |
| Query 72 | TTGVPCAAVLDVVTEAGRDWLLLGEVPGQDLLSSH-LAPAEKVSIMADAMRRLHTLDPAT | 130 |
| Sbjct 64 | T G+PC VLD + GR WLL+ VPG+DL + L+P + V ++A +++ LH L TQGLPCPEVLDTLLSQGRHWLLMSAVPGRDLACTEGLSPQQTVELLASSLQGLHRLPLEA | 123 |
| Query 131 | CPFDHQAKHRIERARTRMEAGLVDQDDLDEEHQGLAPAELFARLKARMPDGEDLVVTHGD | 190 |
| Sbjct 124 | CPFDH+ +HR+ A R EAGL+D++D D+E QG+ L L A P EDLVVTHGD CPFDHRLEHRLADASARAEAGLIDEEDFDDERQGMPVQNLLDELYASRPGQEDLVVTHGD | 183 |
| Query 191 | ACLPNIMVENGRFSGFIDCGRLGVADRYQDIALATRDIAEELGGEWADRFLVLYGIAAPD | 250 |
| Sbjct 184 | ACLPN MV G+FSGFIDCGRLGVADRYQD+AL R I LG EW F LYG+ D ACLPNFMVHQGQFSGFIDCGRLGVADRYQDLALTARSIERNLGKEWLAPFFALYGVEM-D | 242 |
| Query 251  Sbjct 243 | SQRIAFYRLLDEFF 264  ++RIAF+ LLDEFF  AERIAFFCLLDEFF 256 |  |

>VUK53317.1 Aminoglycoside 3'-phosphotransferase [Stenotrophomonas maltophilia] VUK59186.1 Aminoglycoside 3'-phosphotransferase [Stenotrophomonas maltophilia] VUK97873.1 Aminoglycoside 3'-phosphotransferase [Stenotrophomonas maltophilia] VUL24859.1 Aminoglycoside 3'-phosphotransferase [Stenotrophomonas maltophilia]

Length=267

Score = 246 bits (628), Expect = 8e-78, Method: Compositional matrix adjust.

| Identities | = 134/267 (50%), Positives = 172/267 (64%), Gaps = 8/267 (3%) |  |
| --- | --- | --- |
| Query 5 | DGLHAGS PAAWVERLFGYDWAQQTIGCSDAAVFRLSAQGRPVLFVKTDLSGALNE | 59 |
| Sbjct 2 | D +HAG+ P AW + L +Q+IG S A V R+ G+ F+K+++ A +E DAIHAGNHGMPLPRAWQQGLADARIERQSIGVSRADVARMHRPGQADAFLKSEVIDAFSE | 61 |
| Query 60 | LQDEAARLSWLATTGVPCAAVLDVVTEAGRDWLLLGEVPGQDLLSS-HLAPAEKVSIMAD | 118 |
| Sbjct 62 | L DE ARL WL G+P V+ EAGR WLL+ +PG+DL SS LAP + V ++AD LGDEIARLRWLQAQGLPAPTVIATAEEAGRRWLLMSALPGRDLASSPELAPQQLVELLAD | 121 |
| Query 119 | AMRRLHTLDPATCPFDHQAKHRIERARTRMEAGLVDQDDLDEEHQGLAPAELFARLKARM | 178 |
| Sbjct 122 | A+R LH L A CPFD + R++ A+ R+EAGLVD DD D+E G + ++FA L +  ALRGLHALPVAACPFDQRLASRLQAAQARVEAGLVDADDFDDERLGQSAQQVFAELCSTR | 181 |

| Query | 179 | PDGEDLVVTHGDACLPNIMVENGRFSGFIDCGRLGVADRYQDIALATRDIAEELGGE-WA 237 |
| --- | --- | --- |
| Sbjct | 182 | PD EDLVV+HGDACLPN+M GRFSGFIDCGRLGVADRYQD+ALA R + G W PDHEDLVVSHGDACLPNLMAVEGRFSGFIDCGRLGVADRYQDLALAARSLVHNFGDTCWV 241 |
| Query | 238 | DRFLVLYGIAAPDSQRIAFYRLLDEFF 264 |
| Sbjct | 242 | YG+ A D +R+AFYRLLDEFF  APLFRRYGVVA-DERRLAFYRLLDEFF 267 |

>VUJ63248.1 Aminoglycoside 3'-phosphotransferase [Stenotrophomonas maltophilia] VUK64649.1 Aminoglycoside 3'-phosphotransferase [Stenotrophomonas maltophilia] VUK68773.1 Aminoglycoside 3'-phosphotransferase [Stenotrophomonas maltophilia] VUL65820.1 Aminoglycoside 3'-phosphotransferase [Stenotrophomonas maltophilia] VUN94284.1 Aminoglycoside 3'-phosphotransferase [Stenotrophomonas maltophilia]

Length=267

Score = 246 bits (628), Expect = 8e-78, Method: Compositional matrix adjust.

| Identities | = 132/255 (52%), Positives = 165/255 (65%), Gaps = 3/255 (1%) |  |
| --- | --- | --- |
| Query 12 | PAAWVERLFGYDWAQQTIGCSDAAVFRLSAQGRPVLFVKTDLSGALNELQDEAARLSWLA | 71 |
| Sbjct 14 | P AW E L +Q+IG S A V R+ G+ F+K+++ A +EL DE ARL WL PCAWQEALADARIERQSIGVSRADVARVHRPGQADAFLKSEVIDAFSELGDEIARLRWLQ | 73 |
| Query 72 | TTGVPCAAVLDVVTEAGRDWLLLGEVPGQDLLSS-HLAPAEKVSIMADAMRRLHTLDPAT | 130 |
| Sbjct 74 | G P V+ EAGR WLL+ +PG+DL SS LAP + V ++ADA+R LH L A AQGQPVPTVIATAEEAGRRWLLMSALPGRDLASSPELAPQQLVELLADALRGLHALPVAA | 133 |
| Query 131 | CPFDHQAKHRIERARTRMEAGLVDQDDLDEEHQGLAPAELFARLKARMPDGEDLVVTHGD | 190 |
| Sbjct 134 | CPFD + R++ A+ R+EAGLVD DD D+E G +P ++FA L + PD EDLVV+HGD CPFDQRLASRVQVAQARIEAGLVDADDFDDERLGQSPQQVFAELCSTRPDHEDLVVSHGD | 193 |
| Query 191 | ACLPNIMVENGRFSGFIDCGRLGVADRYQDIALATRDIAEELG-GEWADRFLVLYGIAAP | 249 |
| Sbjct 194 | ACLPN+M GRFSGFIDCGRLGVADRYQD+ALA R + G W YG A ACLPNLMAVEGRFSGFIDCGRLGVADRYQDLALAARSLVHNFGDTRWVAPLFQRYGAVA- | 252 |
| Query 250  Sbjct 253 | DSQRIAFYRLLDEFF 264 D +R+AFYRLLDEFF DERRLAFYRLLDEFF 267 |  |

>WP_164117190.1 APH(3')-II family aminoglycoside O-phosphotransferase [Stenotrophomonas maltophilia]

Length=267

Score = 246 bits (628), Expect = 9e-78, Method: Compositional matrix adjust.

| Identities | = 132/255 (52%), Positives = 165/255 (65%), Gaps = 3/255 (1%) |  |
| --- | --- | --- |
| Query 12 | PAAWVERLFGYDWAQQTIGCSDAAVFRLSAQGRPVLFVKTDLSGALNELQDEAARLSWLA | 71 |
| Sbjct 14 | P AW E L G + +Q+IG S A V R+ G+ F+K+++ A +EL DE RL WL PQAWQEVLAGARFERQSIGVSRADVARVHRPGQADAFLKSEVIDAFSELGDEIERLRWLQ | 73 |
| Query 72 | TTGVPCAAVLDVVTEAGRDWLLLGEVPGQDLLSS-HLAPAEKVSIMADAMRRLHTLDPAT | 130 |
| Sbjct 74 | G P V+ EAGR WLL+ +PG DL SS LAP V ++ADA+R LH L AT AQGQPAPMVIATAEEAGRRWLLMSALPGHDLASSPALAPQHLVELLADALRGLHALPVAT | 133 |
| Query 131 | CPFDHQAKHRIERARTRMEAGLVDQDDLDEEHQGLAPAELFARLKARMPDGEDLVVTHGD | 190 |
| Sbjct 134 | CPFD + R+ A+ R+EAGLVD DD D+E G + ++FA L+A PD EDLVV HGD CPFDQRLASRLRAAQARVEAGLVDADDFDDERLGQSAQQVFAELRATRPDHEDLVVCHGD | 193 |
| Query 191 | ACLPNIMVENGRFSGFIDCGRLGVADRYQDIALATRDIAEELG-GEWADRFLVLYGIAAP | 249 |
| Sbjct 194 | ACLPN++ GRFSGFIDCGRLGVADRYQD+ALA R + + G W YG+ A ACLPNLLAAEGRFSGFIDCGRLGVADRYQDLALAARSLVDNFGDTRWVAPLFRRYGVVA- | 252 |
| Query 250  Sbjct 253 | DSQRIAFYRLLDEFF 264 D +R+AFYRLLDEFF DERRLAFYRLLDEFF 267 |  |

>WP_108270053.1 APH(3')-II family aminoglycoside O-phosphotransferase [Stenotrophomonas maltophilia]

Length=267

Score = 246 bits (628), Expect = 9e-78, Method: Compositional matrix adjust.

| Identities | = 134/255 (53%), Positives = 166/255 (65%), Gaps = 3/255 (1%) |  |
| --- | --- | --- |
| Query 12 | PAAWVERLFGYDWAQQTIGCSDAAVFRLSAQGRPVLFVKTDLSGALNELQDEAARLSWLA | 71 |
| Sbjct 14 | P AW E L +Q+IG S A V R+ G+ FVK+++ A +EL DE ARL WL PWAWQEALADARIERQSIGVSRADVARVQRPGQADAFVKSEVIDAFSELGDEIARLRWLQ | 73 |
| Query 72 | TTGVPCAAVLDVVTEAGRDWLLLGEVPGQDLLSS-HLAPAEKVSIMADAMRRLHTLDPAT | 130 |
| Sbjct 74 | G P V+ EAGR WLL+ +PG+DL SS LAP + V ++ADA+R LH L A EQGQPAPTVIATAEEAGRRWLLMSALPGRDLASSPELAPQQLVELLADALRGLHALPLAA | 133 |
| Query 131 | CPFDHQAKHRIERARTRMEAGLVDQDDLDEEHQGLAPAELFARLKARMPDGEDLVVTHGD | 190 |
| Sbjct 134 | CPFD + R++ A+ R+EAGLVD DD D+E G +P ++FA L + PD EDLVV+HGD CPFDQRLHLRLQAAQARVEAGLVDADDFDDERLGQSPQQVFAELCSTRPDHEDLVVSHGD | 193 |
| Query 191 | ACLPNIMVENGRFSGFIDCGRLGVADRYQDIALATRDIAEELG-GEWADRFLVLYGIAAP | 249 |
| Sbjct 194 | ACLPN+MV GRFSGFIDCGRLGVADRYQD+ALA R + G W YG A ACLPNLMVVEGRFSGFIDCGRLGVADRYQDLALAARSLVHNFGDTRWVAPLFQRYGAVA- | 252 |
| Query 250  Sbjct 253 | DSQRIAFYRLLDEFF 264 D +R+AFYRLLDEFF DERRLAFYRLLDEFF 267 |  |

>VUQ68611.1 Aminoglycoside 3'-phosphotransferase [Stenotrophomonas maltophilia] Length=267

Score = 246 bits (628), Expect = 9e-78, Method: Compositional matrix adjust.

| Identities | = 133/255 (52%), Positives = 166/255 (65%), Gaps = 3/255 (1%) |  |
| --- | --- | --- |
| Query 12 | PAAWVERLFGYDWAQQTIGCSDAAVFRLSAQGRPVLFVKTDLSGALNELQDEAARLSWLA | 71 |
| Sbjct 14 | P AW + L +Q+IG S A V R+ G+ F+K+++ A +EL DE ARL WL PWAWQDALADARIERQSIGVSRADVARVHRPGQADAFLKSEVIDAFSELGDEIARLRWLQ | 73 |
| Query 72 | TTGVPCAAVLDVVTEAGRDWLLLGEVPGQDLLSS-HLAPAEKVSIMADAMRRLHTLDPAT | 130 |
| Sbjct 74 | G P V+ EAGR WLL+ +PG+DL SS LAP + V ++ADA+R LH L A AQGQPVPTVIATAEEAGRRWLLMSALPGRDLASSPELAPQQLVELLADALRGLHALPVAA | 133 |
| Query 131 | CPFDHQAKHRIERARTRMEAGLVDQDDLDEEHQGLAPAELFARLKARMPDGEDLVVTHGD | 190 |
| Sbjct 134 | CPFD + R++ A+ R+EAGLVD DD D+E G +P +LFA L + PD EDLVV+HGD CPFDQRLASRVQVAQARIEAGLVDADDFDDERLGQSPQQLFAELCSTRPDHEDLVVSHGD | 193 |
| Query 191 | ACLPNIMVENGRFSGFIDCGRLGVADRYQDIALATRDIAEELG-GEWADRFLVLYGIAAP | 249 |
| Sbjct 194 | ACLPN+MV GRFSGFIDCGRLGVADRYQD+ALA R + G W YG A ACLPNLMVVEGRFSGFIDCGRLGVADRYQDLALAARSLVHNFGDTRWVAPLFQRYGAVA- | 252 |
| Query 250  Sbjct 253 | DSQRIAFYRLLDEFF 264 D +R+AFYRLLDEFF DERRLAFYRLLDEFF 267 |  |

>VUL89945.1 Aminoglycoside 3'-phosphotransferase [Stenotrophomonas maltophilia] VUN69988.1 Aminoglycoside 3'-phosphotransferase [Stenotrophomonas maltophilia]

Length=267

Score = 246 bits (628), Expect = 1e-77, Method: Compositional matrix adjust.

| Identities | = 132/255 (52%), Positives = 165/255 (65%), Gaps = 3/255 (1%) |  |
| --- | --- | --- |
| Query 12 | PAAWVERLFGYDWAQQTIGCSDAAVFRLSAQGRPVLFVKTDLSGALNELQDEAARLSWLA | 71 |
| Sbjct 14 | P AW E L +Q+IG S A V R+ G+ F+K+++ A +EL DE ARL WL PCAWQEALADARIERQSIGVSRADVARVHRPGQADAFLKSEVIDAFSELGDEIARLRWLQ | 73 |
| Query 72 | TTGVPCAAVLDVVTEAGRDWLLLGEVPGQDLLSS-HLAPAEKVSIMADAMRRLHTLDPAT | 130 |
| Sbjct 74 | G P V+ EAGR WLL+ +PG+DL SS LAP + V ++ADA+R LH L A AQGQPVPTVIATAEEAGRRWLLMSALPGRDLASSPELAPQQLVELLADALRGLHALPVAA | 133 |
| Query 131 | CPFDHQAKHRIERARTRMEAGLVDQDDLDEEHQGLAPAELFARLKARMPDGEDLVVTHGD | 190 |
| Sbjct 134 | CPFD + R++ A+ R+EAGLVD DD D+E G +P ++FA L + PD EDLVV+HGD CPFDQRLASRVQVAQARVEAGLVDADDFDDERLGQSPQQVFAELCSTRPDHEDLVVSHGD | 193 |
| Query 191 | ACLPNIMVENGRFSGFIDCGRLGVADRYQDIALATRDIAEELG-GEWADRFLVLYGIAAP ACLPN+M GRFSGFIDCGRLGVADRYQD+ALA R + G W YG A | 249 |

| Sbjct | 194 | ACLPNLMAVEGRFSGFIDCGRLGVADRYQDLALAARSLVHNFGDTRWVAPLFQRYGAVA- 252 |
| --- | --- | --- |
| Query | 250 | DSQRIAFYRLLDEFF 264 |
| Sbjct | 253 | D +R+AFYRLLDEFF  DERRLAFYRLLDEFF 267 |

>WP_103302299.1 aminoglycoside 3'-phosphotransferase [Pseudomonas sp. MPR-ANC1]

POA50397.1 APH(3') family aminoglycoside O-phosphotransferase [Pseudomonas sp. MPR-ANC1]

Length=256

Score = 245 bits (626), Expect = 1e-77, Method: Compositional matrix adjust.

| Identities | | = 127/254 (50%), Positives = 164/254 (65%), Gaps = 2/254 (1%) |  |
| --- | --- | --- | --- |
| Query 12  Sbjct 4 | | PAAWVERLFGYDWAQQTIGCSDAAVFRLSAQGRPVLFVKTDLSGALNELQDEAARLSWLA PA W R QQTIG S A V R++ G LFVK++ EL E RL WLA PAIWHPRFADAQIEQQTIGESRADVLRITPAGSAPLFVKSEQVMTWGELPGEIQRLRWLA | 71  63 |
| Query 72  Sbjct 64 | | TTGVPCAAVLDVVTEAGRDWLLLGEVPGQDLL-SSHLAPAEKVSIMADAMRRLHTLDPAT  +P VLD TE R+WLL+ VPG+DL S+HLAP + V++ A A+R LH + + EQDLPAPRVLDTTTENERNWLLMTAVPGRDLARSAHLAPEQIVTLAATALRSLHAVAIDS | 130  123 |
| Query 131  Sbjct 124 | | CPFDHQAKHRIERARTRMEAGLVDQDDLDEEHQGLAPAELFARLKARMPDGEDLVVTHGD CPFDH+ +I AR + AGL+D++D D+ G + ++F ++ A PD EDLVVTHGD CPFDHRLDAKIALAREHLLAGLIDEEDFDDSRLGRSAEQVFQQMLAARPDHEDLVVTHGD | 190  183 |
| Query 191  Sbjct 184 | | ACLPNIMVENGRFSGFIDCGRLGVADRYQDIALATRDIAEELGGEWADRFLVLYGIAAPD ACLPN++ +NG+FSGF+DCGRLGV+DRYQD+ALA I LG +W F YG+ PD ACLPNLLADNGKFSGFVDCGRLGVSDRYQDLALAANSITYNLGEQWVPVFFEAYGV-EPD | 250  242 |
| Query | 251 SQRIAFYRLLDEFF 264 | | |
|  |  | QRIAFY+LLDEFF |  |
| Sbjct | 243 | LQRIAFYQLLDEFF 256 | |

>WP_012479999.1 aminoglycoside O-phosphotransferase APH(3')-IIc [Stenotrophomonas maltophilia]

KKD57309.1 aminoglycoside phosphotransferase [Stenotrophomonas maltophilia]

PJL20210.1 aminoglycoside O-phosphotransferase APH(3')-IIc [Stenotrophomonas maltophilia]

PJL42828.1 aminoglycoside O-phosphotransferase APH(3')-IIc [Stenotrophomonas maltophilia]

PZS80388.1 APH(3') family aminoglycoside O-phosphotransferase [Stenotrophomonas maltophilia]

QEU34508.1 aminoglycoside O-phosphotransferase APH(3')-IIc [Stenotrophomonas maltophilia]

Length=267

Score = 246 bits (627), Expect = 1e-77, Method: Compositional matrix adjust.

| Identities | = 134/255 (53%), Positives = 166/255 (65%), Gaps = 3/255 (1%) |  |
| --- | --- | --- |
| Query 12 | PAAWVERLFGYDWAQQTIGCSDAAVFRLSAQGRPVLFVKTDLSGALNELQDEAARLSWLA | 71 |
| Sbjct 14 | P AW E L +Q+IG S A V R+ G+ FVK+++ A +EL DE ARL WL PWAWQEALADARVERQSIGVSRADVARVQRPGQADAFVKSEVIDAFSELGDEIARLRWLQ | 73 |
| Query 72 | TTGVPCAAVLDVVTEAGRDWLLLGEVPGQDLLSS-HLAPAEKVSIMADAMRRLHTLDPAT | 130 |
| Sbjct 74 | G P V+ EAGR WLL+ +PG+DL SS LAP + V ++ADA+R LH L A EQGQPAPTVIATAEEAGRRWLLMSALPGRDLASSPELAPQQLVELLADALRGLHALPLAA | 133 |
| Query 131 | CPFDHQAKHRIERARTRMEAGLVDQDDLDEEHQGLAPAELFARLKARMPDGEDLVVTHGD | 190 |
| Sbjct 134 | CPFD + R++ A+ R+EAGLVD DD D+E G +P ++FA L + PD EDLVV+HGD CPFDQRLHLRLQAAQARVEAGLVDADDFDDERLGQSPQQVFAELCSTRPDHEDLVVSHGD | 193 |
| Query 191 | ACLPNIMVENGRFSGFIDCGRLGVADRYQDIALATRDIAEELG-GEWADRFLVLYGIAAP | 249 |
| Sbjct 194 | ACLPN+MV GRFSGFIDCGRLGVADRYQD+ALA R + G W YG A ACLPNLMVVEGRFSGFIDCGRLGVADRYQDLALAARSLVHNFGDTRWVAPLFQRYGAVA- | 252 |
| Query 250  Sbjct 253 | DSQRIAFYRLLDEFF 264 D +R+AFYRLLDEFF DERRLAFYRLLDEFF 267 |  |

>WP_049298729.1 APH(3')-II family aminoglycoside O-phosphotransferase, partial [Pseudomonas aeruginosa]

Length=252

Score = 245 bits (626), Expect = 1e-77, Method: Compositional matrix adjust.

| Identities | = 127/239 (53%), Positives = 155/239 (65%), Gaps = 4/239 (2%) |  |
| --- | --- | --- |
| Query 11 | SPAAWVERLFGYDWAQQTIGCSDAAVFRLSAQGRPVLFVKTDLSGALNELQDEAARLSWL | 70 |
| Sbjct 12 | +P+ W + L GY W Q GCS A V RL A RP LFVK ++ A EL E ARL WL APSTWADYLAGYRWRGQGEGCSAATVHRLEAARRPTLFVKQEVLSAHAELPAEIARLRWL | 71 |
| Query 71 | ATTGVPCAAVLDVVTEAGRDWLLLGEVPGQDLLS SHLAPAEKVSIMADAMRRLHTL | 126 |
| Sbjct 72 | G+ C VL+ GR WLL+ VPG L + L P V ++A A+RRLH L HGAGIDCPQVLNETQSDGRQWLLMSAVPGDTLSALAQRGELEPERLVRLVAAALRRLHDL | 131 |
| Query 127 | DPATCPFDHQAKHRIERARTRMEAGLVDQDDLDEEHQGLAPAELFARLKARMPDGEDLVV | 186 |
| Sbjct 132 | DPA CPFDH+ + R++ R R+EAGLVD+ D D++H+G + EL+ L R P EDLVV  DPAACPFDHRLERRLDTVRQRVEAGLVDEADFDDDHRGRSATELYRLLLDRRPAVEDLVV | 191 |

Query 187 THGDACLPNIMVENGRFSGFIDCGRLGVADRYQDIALATRDIAEELGGEWADRFLVLYG 245 HGDACLPN++ E RFSGFIDCGRLGVADR+QD+ALA RDI ELG WA+ FLV YG

Sbjct 192 AHGDACLPNLLAEGRRFSGFIDCGRLGVADRHQDLALAARDIEAELGAAWAEAFLVEYG 250

>ECZ5199100.1 aminoglycoside 3'-phosphotransferase [Salmonella enterica subsp. enterica serovar Enteritidis]

Length=118

Score = 240 bits (613), Expect = 1e-77, Method: Compositional matrix adjust. Identities = 118/118 (100%), Positives = 118/118 (100%), Gaps = 0/118 (0%)

| Query | 147 | RMEAGLVDQDDLDEEHQGLAPAELFARLKARMPDGEDLVVTHGDACLPNIMVENGRFSGF 206 |
| --- | --- | --- |
| Sbjct | 1 | RMEAGLVDQDDLDEEHQGLAPAELFARLKARMPDGEDLVVTHGDACLPNIMVENGRFSGF RMEAGLVDQDDLDEEHQGLAPAELFARLKARMPDGEDLVVTHGDACLPNIMVENGRFSGF 60 |
| Query | 207 | IDCGRLGVADRYQDIALATRDIAEELGGEWADRFLVLYGIAAPDSQRIAFYRLLDEFF 264 |
| Sbjct | 61 | IDCGRLGVADRYQDIALATRDIAEELGGEWADRFLVLYGIAAPDSQRIAFYRLLDEFF  IDCGRLGVADRYQDIALATRDIAEELGGEWADRFLVLYGIAAPDSQRIAFYRLLDEFF 118 |

>WP_003561190.1 aminoglycoside 3'-phosphotransferase [Rhizobium leguminosarum]

RWY87355.1 APH(3') family aminoglycoside O-phosphotransferase [Rhizobium leguminosarum]

Length=261

Score = 246 bits (627), Expect = 1e-77, Method: Compositional matrix adjust.

| Identities | | = 131/254 (52%), Positives = 168/254 (66%), Gaps = 1/254 (0%) |  |
| --- | --- | --- | --- |
| Query 12  Sbjct 8 | | PAAWVERLFGYDWAQQTIGCSDAAVFRLSAQGRPVLFVKTDLSGALNELQDEAARLSWLA PAA RL GY Q IG S A+VFRL +G P L++K + + EL DEAARL WL PAALAARLSGYRMEQDAIGRSAASVFRLEGEGLPALYLKVEEAAPFGELADEAARLGWLK | 71  67 |
| Query 72  Sbjct 68 | | TTGVPCAAVLDVVTEAGRDWLLLGEVPGQDLLS-SHLAPAEKVSIMADAMRRLHTLDPAT G+PC V+ ++ R+WLL+G +PG +L S S L P +V ++A A+ LH L A+ AAGLPCPDVIARESDGERNWLLIGALPGTNLASASSLTPLARVELLAAALLDLHRLPIAS | 130  127 |
| Query 131  Sbjct 128 | | CPFDHQAKHRIERARTRMEAGLVDQDDLDEEHQGLAPAELFARLKARMPDGEDLVVTHGD CPFDH+ + R+ A RMEAG+VD++ D G + AELFA L++ P EDLVV+HGD CPFDHRREKRLPVAEARMEAGIVDEEYFDAARLGKSAAELFAELESLKPGSEDLVVSHGD | 190  187 |
| Query 191  Sbjct 188 | | ACLPNIMVENGRFSGFIDCGRLGVADRYQDIALATRDIAEELGGEWADRFLVLYGIAAPD ACLPN + +GRFSG+IDC RLGVADRYQDIALA R IA G FL+ YG+ APD ACLPNFIASDGRFSGYIDCSRLGVADRYQDIALACRSIASNFGEALVPPFLIRYGLPAPD | 250  247 |
| Query 251 | | SQRIAFYRLLDEFF 264  +R+ +YRLLDEFF |  |
| Sbjct | 248 | PERVDYYRLLDEFF 261 | |

>WP_112334363.1 aminoglycoside 3'-phosphotransferase [Rhizobium sp. NFR17] RAS07461.1 aminoglycoside 3'-phosphotransferase-2 [Rhizobium sp. NFR17]

Length=266

Score = 246 bits (627), Expect = 1e-77, Method: Compositional matrix adjust.

| Identities | | = 127/254 (50%), Positives = 163/254 (64%), Gaps = 1/254 (0%) |  |
| --- | --- | --- | --- |
| Query 12  Sbjct 13 | | PAAWVERLFGYDWAQQTIGCSDAAVFRLSAQGRPVLFVKTDLSGALNELQDEAARLSWLA P + L GY+W Q +G S A VFRL A R +++KT+L+ L EL E ARL WLA PVRLRDLLAGYEWRQDGLGRSSAHVFRLEADRRAPVYLKTELADPLGELAGEVARLRWLA | 71  72 |
| Query 72  Sbjct 73 | | TTGVPCAAVLDVVTEAGRDWLLLGEVPGQDLLSSH-LAPAEKVSIMADAMRRLHTLDPAT  + G C ++ T+ R+WLL+ +PG DL+S+ L P E+V ++ADA+RRLH +D A SQGFACPEIIAHETDGEREWLLMRALPGSDLVSAEWLQPLERVRLLADALRRLHAIDIAA | 130  132 |
| Query 131  Sbjct 133 | | CPFDHQAKHRIERARTRMEAGLVDQDDLDEEHQGLAPAELFARLKARMPDGEDLVVTHGD CPFDH+ ++RI AR + AG VD+DD DE G +LF L P EDLVVTHGD CPFDHRLENRIGAARAHLLAGRVDEDDFDESRLGRPAEDLFRELVETRPSAEDLVVTHGD | 190  192 |
| Query 191  Sbjct 193 | | ACLPNIMVENGRFSGFIDCGRLGVADRYQDIALATRDIAEELGGEWADRFLVLYGIAAPD ACLPN M + +FSG+IDCGRLGVADR+QDIALA I G E FL YG P ACLPNFMADGPKFSGYIDCGRLGVADRHQDIALACGSIEHNFGEELVAEFLKAYGDFQPQ | 250  252 |
| Query 251 | | SQRIAFYRLLDEFF 264  +++A+YRLLDEFF |  |
| Sbjct | 253 | EEKMAYYRLLDEFF 266 | |

>WP_126905890.1 MULTISPECIES: aminoglycoside 3'-phosphotransferase [Rhizobium] RUM17993.1 aminoglycoside 3'-phosphotransferase [Rhizobium phaseoli]

Length=261

Score = 245 bits (626), Expect = 1e-77, Method: Compositional matrix adjust.

| Identities | | = 125/254 (49%), Positives = 165/254 (65%), Gaps = 1/254 (0%) |  |
| --- | --- | --- | --- |
| Query 12  Sbjct 8 | | PAAWVERLFGYDWAQQTIGCSDAAVFRLSAQGRPVLFVKTDLSGALNELQDEAARLSWLA PAA ERL GY + + +G S A+VFRL +G P L++K + +G EL DEAARLSWL PAALGERLSGYRFERDALGRSAASVFRLQGEGLPALYLKVEAAGPFGELADEAARLSWLK | 71  67 |
| Query 72  Sbjct 68 | | TTGVPCAAVLDVVTEAGRDWLLLGEVPGQDLLS-SHLAPAEKVSIMADAMRRLHTLDPAT G+PC V+ ++ R+WLL+ +PG DL S S L P +V ++A A+ LH L + AVGLPCPDVIARESDGERNWLLINALPGTDLASASALTPLTRVELLAAALSDLHDLPIVS | 130  127 |
| Query 131  Sbjct 128 | | CPFDHQAKHRIERARTRMEAGLVDQDDLDEEHQGLAPAELFARLKARMPDGEDLVVTHGD CPFDH+ + R+ A RM+AG+VD++D D G + A LF L P E+LVVTHGD CPFDHRLESRLSVAEARMQAGIVDEEDFDAARLGKSAAALFDDLMRLRPGDEELVVTHGD | 190  187 |
| Query 191  Sbjct 188 | | ACLPNIMVENGRFSGFIDCGRLGVADRYQDIALATRDIAEELGGEWADRFLVLYGIAAPD ACLPN + NG+FSG+IDC RLGVADR+QDIALA R IA G FL YG+ APD ACLPNFVASNGQFSGYIDCSRLGVADRHQDIALACRSIASNFGEALIRPFLDRYGLTAPD | 250  247 |
| Query 251 | | SQRIAFYRLLDEFF 264  ++ +++LLDEFF |  |
| Sbjct | 248 | PAKLHYFQLLDEFF 261 | |

>WP_095190961.1 aminoglycoside 3'-phosphotransferase [Pseudomonas sp. Irchel 3E19]

Length=256

Score = 245 bits (626), Expect = 1e-77, Method: Compositional matrix adjust.

| Identities | = 125/254 (49%), Positives = 164/254 (65%), Gaps = 2/254 (1%) |  |
| --- | --- | --- |
| Query 12 | PAAWVERLFGYDWAQQTIGCSDAAVFRLSAQGRPVLFVKTDLSGALNELQDEAARLSWLA | 71 |
| Sbjct 4 | P W +R QQTIG S A V R++ +G LFVK++ EL E RL W+A PEQWRKRFADALIEQQTIGESRADVLRITPEGSVPLFVKSEQVMIWGELPGEVQRLRWMA | 63 |
| Query 72 | TTGVPCAAVLDVVTEAGRDWLLLGEVPGQDLLSS-HLAPAEKVSIMADAMRRLHTLDPAT | 130 |
| Sbjct 64 | + +P VL+ TEA R+WLL+ +PG+DL SS HLAP + V++ A+A+R LH + SQDLPAPRVLETATEADRNWLLMTAIPGRDLASSAHLAPQQIVTLAANALRSLHAVAINR | 123 |
| Query 131 | CPFDHQAKHRIERARTRMEAGLVDQDDLDEEHQGLAPAELFARLKARMPDGEDLVVTHGD | 190 |
| Sbjct 124 | CPFDH+ +I AR + AGL+D+ D D+ G +F ++ A PD EDLVVTHGD  CPFDHRLDQKIPLAREHVLAGLIDEQDFDDARIGCTAENVFQQILAERPDHEDLVVTHGD | 183 |

| Query | 191 | ACLPNIMVENGRFSGFIDCGRLGVADRYQDIALATRDIAEELGGEWADRFLVLYGIAAPD 250 |
| --- | --- | --- |
| Sbjct | 184 | ACLPN++ ENG+FSGF+DCGRLGVADRYQD+ALAT I LG +W F +YG+ PD ACLPNLLAENGQFSGFVDCGRLGVADRYQDLALATHSIGSNLGKQWVPMFFEVYGV-EPD 242 |
| Query | 251 | SQRIAFYRLLDEFF 264 |
| Sbjct | 243 | + IAFY+LLDE F  QRLIAFYQLLDELF 256 |

>WP_021447065.1 aminoglycoside 3'-phosphotransferase [Alcaligenes sp. EGD-AK7] ERI33370.1 hypothetical protein N879_10320 [Alcaligenes sp. EGD-AK7]

Length=256

Score = 245 bits (625), Expect = 1e-77, Method: Compositional matrix adjust.

| Identities | = 130/254 (51%), Positives = 160/254 (63%), Gaps = 2/254 (1%) |  |
| --- | --- | --- |
| Query 12 | PAAWVERLFGYDWAQQTIGCSDAAVFRLSAQGRPVLFVKTDLSGALNELQDEAARLSWLA | 71 |
| Sbjct 4 | P AW RL +QTIG S A V+RL FVK + L EL DE RL W+ PLAWQTRLAAAKVQRQTIGESGADVWRLDWPDGSCQFVKAEDDLPLAELPDEIERLRWMH | 63 |
| Query 72 | TTGVPCAAVLDVVTEAGRDWLLLGEVPGQDLLSSH-LAPAEKVSIMADAMRRLHTLDPAT | 130 |
| Sbjct 64 | T G+PC VLD + GR WLL+ VPG+DL + L+P + V ++A +++ LH L TQGLPCPEVLDTLLAQGRHWLLMSAVPGRDLACTEGLSPQQTVELLASSLQGLHRLPLEA | 123 |
| Query 131 | CPFDHQAKHRIERARTRMEAGLVDQDDLDEEHQGLAPAELFARLKARMPDGEDLVVTHGD | 190 |
| Sbjct 124 | CPFDH+ +HR+ A R EAGL+D++D D+E QG+ L L A P EDLVVTHGD CPFDHRLEHRLADASARAEAGLIDEEDFDDERQGMPVQNLLDELYASRPGQEDLVVTHGD | 183 |
| Query 191 | ACLPNIMVENGRFSGFIDCGRLGVADRYQDIALATRDIAEELGGEWADRFLVLYGIAAPD | 250 |
| Sbjct 184 | ACLPN MV G+FSGFIDCGRLGVADRYQD+AL R I LG EW F LYG+ D ACLPNFMVHQGQFSGFIDCGRLGVADRYQDLALTARSIERNLGKEWLAPFFALYGVEM-D | 242 |
| Query 251  Sbjct 243 | SQRIAFYRLLDEFF 264  ++RIAF+ LLDEFF  AERIAFFCLLDEFF 256 |  |

>MBG7135377.1 APH(3')-II family aminoglycoside O-phosphotransferase [Pseudomonas aeruginosa]

Length=268

Score = 246 bits (627), Expect = 1e-77, Method: Compositional matrix adjust.

| Identities | | = 136/258 (53%), Positives = 166/258 (64%), Gaps = 5/258 (2%) |  |
| --- | --- | --- | --- |
| Query 11  Sbjct 12 | | SPAAWVERLFGYDWAQQTIGCSDAAVFRLSAQGRPVLFVKTDLSGALNELQDEAARLSWL  +PA W + L GY W Q GCS A V RL A RP LFVK ++ A EL E ARL WL APATWADYLAGYRWRGQGEGCSAATVHRLEAARRPTLFVKQEVLSAHAELPAEIARLRWL | 70  71 |
| Query 71  Sbjct 72 | | ATTGVPCAAVLDVVTEAGRDWLLLGEVPGQDLLS SHLAPAEKVSIMADAMRRLHTL  G+ C VL+ GR WLL+ VPG L + L P V ++A A+RRLH L HGAGIDCPQVLNETQSDGRQWLLMSAVPGDTLSALAQRGELEPERLVRLVAAALRRLHDL | 126  131 |
| Query 127  Sbjct 132 | | DPATCPFDHQAKHRIERARTRMEAGLVDQDDLDEEHQGLAPAELFARLKARMPDGEDLVV DPA CPFDH+ R++ R R+EAGLVD+ D D++H+G + EL+ L R P EDLVV DPAACPFDHRLDRRLDTVRQRVEAGLVDEADFDDDHRGRSATELYRLLLDRRPAVEDLVV | 186  191 |
| Query 187  Sbjct 192 | | THGDACLPNIMVENGRFSGFIDCGRLGVADRYQDIALATRDIAEELGGEWADRFLVLYGI HGDACLPN++ E RFSGFIDCGRLGVADR+QD+ALA R I ELG A+ FLV YG AHGDACLPNLLAEGRRFSGFIDCGRLGVADRHQDLALAARAIEAELGAAGAEAFLVEYG- | 246  250 |
| Query | 247 AAPDSQRIAFYRLLDEFF 264 | | |
|  |  | D +R+A++RLLDEFF |  |
| Sbjct | 251 | GDIDGERLAYFRLLDEFF 268 | |

>WP_132717099.1 aminoglycoside 3'-phosphotransferase [Rhizobium sp. BK315] TCR97041.1 aminoglycoside 3'-phosphotransferase-2 [Rhizobium sp. BK315]

Length=266

Score = 245 bits (626), Expect = 1e-77, Method: Compositional matrix adjust. Identities = 127/259 (49%), Positives = 167/259 (64%), Gaps = 1/259 (0%)

| Query | 7 | LHAGSPAAWVERLFGYDWAQQTIGCSDAAVFRLSAQGRPVLFVKTDLSGALNELQDEAAR | 66 |
| --- | --- | --- | --- |
| Sbjct | 8 | + A P + L G++W Q +G S A VFRL GR +++KT+L G L EL E AR MEASLPVRLRDLLAGHEWRQDGLGRSSAHVFRLEVDGRAPVYLKTELVGPLGELAGEVAR | 67 |
| Query | 67 | LSWLATTGVPCAAVLDVVTEAGRDWLLLGEVPGQDLLSSH-LAPAEKVSIMADAMRRLHT | 125 |
| Sbjct | 68 | L WLA+ G C ++ T+ R+WLL+G +PG DL+S+ L P E+V ++ADA+R+LH LRWLASQGFACPDIIAHETDGEREWLLMGALPGSDLVSAEWLQPLERVLLLADALRQLHA | 127 |
| Query | 126 | LDPATCPFDHQAKHRIERARTRMEAGLVDQDDLDEEHQGLAPAELFARLKARMPDGEDLV | 185 |
| Sbjct | 128 | +D A CPFDH+ ++RI A+ + AG VD+DD DE G A +LF L P EDLV IDIAACPFDHRLENRIGTAKGHLLAGRVDEDDFDEARLGRAAKDLFRELVEMRPLAEDLV | 187 |
| Query | 186 | VTHGDACLPNIMVENGRFSGFIDCGRLGVADRYQDIALATRDIAEELGGEWADRFLVLYG | 245 |
| Sbjct | 188 | VTHGDACLPN M + +FSG+IDCGRLGVADR+QDIALA I G E FL YG VTHGDACLPNFMADGPKFSGYIDCGRLGVADRHQDIALACGSIEHNFGEELVAEFLKAYG | 247 |
| Query | 246 | IAAPDSQRIAFYRLLDEFF 264 P +++A+Y+LLDEFF |  |
| Sbjct | 248 | DFQPQEEKMAYYQLLDEFF 266 | |

>WP_187757379.1 APH(3')-II family aminoglycoside O-phosphotransferase [Stenotrophomonas maltophilia]

MBC9116632.1 APH(3')-II family aminoglycoside O-phosphotransferase [Stenotrophomonas maltophilia]

Length=267

Score = 245 bits (626), Expect = 1e-77, Method: Compositional matrix adjust.

| Identities | = 134/255 (53%), Positives = 165/255 (65%), Gaps = 3/255 (1%) |  |
| --- | --- | --- |
| Query 12 | PAAWVERLFGYDWAQQTIGCSDAAVFRLSAQGRPVLFVKTDLSGALNELQDEAARLSWLA | 71 |
| Sbjct 14 | P AW E L +Q+IG S A V R+ G+ FVK+++ A +EL DE ARL WL PWAWQEALADARIERQSIGVSRADVARVQRPGQADAFVKSEVIDAFSELGDEIARLRWLQ | 73 |
| Query 72 | TTGVPCAAVLDVVTEAGRDWLLLGEVPGQDLLSS-HLAPAEKVSIMADAMRRLHTLDPAT | 130 |
| Sbjct 74 | G P V+ EAGR WLL+ +PG+DL SS LAP + V +ADA+R LH L A EQGQPAPTVIATAEEAGRRWLLMSALPGRDLASSPELAPQQLVEPLADALRGLHALPLAA | 133 |
| Query 131 | CPFDHQAKHRIERARTRMEAGLVDQDDLDEEHQGLAPAELFARLKARMPDGEDLVVTHGD | 190 |
| Sbjct 134 | CPFD + R++ A+ R+EAGLVD DD D+E G +P ++FA L + PD EDLVV+HGD CPFDQRLASRLQAAQARVEAGLVDADDFDDERLGQSPQQVFAELCSTRPDHEDLVVSHGD | 193 |
| Query 191 | ACLPNIMVENGRFSGFIDCGRLGVADRYQDIALATRDIAEELG-GEWADRFLVLYGIAAP | 249 |
| Sbjct 194 | ACLPN+MV GRFSGFIDCGRLGVADRYQD+ALA R + G W YG A ACLPNLMVVEGRFSGFIDCGRLGVADRYQDLALAARSLVHNFGDTRWVAPLFQCYGAVA- | 252 |
| Query 250  Sbjct 253 | DSQRIAFYRLLDEFF 264 D +R+AFYRLLDEFF DERRLAFYRLLDEFF 267 |  |

>WP_155001344.1 aminoglycoside 3'-phosphotransferase [Pseudomonas fluorescens] VVP09829.1 Aminoglycoside 3'-phosphotransferase [Pseudomonas fluorescens]

Length=256

Score = 245 bits (625), Expect = 1e-77, Method: Compositional matrix adjust.

| Identities | = 125/254 (49%), Positives = 163/254 (64%), Gaps = 2/254 (1%) |  |
| --- | --- | --- |
| Query 12 | PAAWVERLFGYDWAQQTIGCSDAAVFRLSAQGRPVLFVKTDLSGALNELQDEAARLSWLA | 71 |
| Sbjct 4 | PA W + G +QTIG S A VFR+ LFVK++ GA +EL E RL W+ PAQWSKTFSGAPIERQTIGESGAEVFRIRCINGKHLFVKSEPIGAHSELPQEIERLRWMN | 63 |
| Query 72 | TTGVPCAAVLDVVTEAGRDWLLLGEVPGQDLLSSH-LAPAEKVSIMADAMRRLHTLDPAT | 130 |
| Sbjct 64 | +P VLDV TE R WLL+ VPGQDL S++ L+P + +S++A A+R LH + T RLDLPGPTVLDVTTENNRHWLLMNAVPGQDLASANDLSPPQVISVLAMALRTLHQVPIET | 123 |
| Query 131 | CPFDHQAKHRIERARTRMEAGLVDQDDLDEEHQGLAPAELFARLKARMPDGEDLVVTHGD | 190 |
| Sbjct 124 | CPFDH + RI AR+R+ AGLVD+ D D+E QG ++F ++ + +P+ DL VTHGD CPFDHSLEQRIAAARSRVSAGLVDEADFDDERQGRTAEDVFTQMLSMLPETYDLAVTHGD | 183 |
| Query 191 | ACLPNIMVENGRFSGFIDCGRLGVADRYQDIALATRDIAEELGGEWADRFLVLYGIAAPD | 250 |

| Sbjct | 184 | ACLPN MVE F+G+IDCGRLG++D YQD+ALA R I LG W F YG+ PD ACLPNFMVEGSSFTGYIDCGRLGISDIYQDLALAARSIERNLGPAWGAPFFRAYGV-EPD 242 |
| --- | --- | --- |
| Query | 251 | SQRIAFYRLLDEFF 264 |
| Sbjct | 243 | +RIAFY LLDEF+  QRRIAFYCLLDEFY 256 |

>WP_189719573.1 APH(3')-II family aminoglycoside O-phosphotransferase [Stenotrophomonas maltophilia]

QDY49605.1 APH(3')-II family aminoglycoside O-phosphotransferase [Stenotrophomonas maltophilia]

Length=267

Score = 245 bits (626), Expect = 2e-77, Method: Compositional matrix adjust.

| Identities | = 132/255 (52%), Positives = 165/255 (65%), Gaps = 3/255 (1%) |  |
| --- | --- | --- |
| Query 12 | PAAWVERLFGYDWAQQTIGCSDAAVFRLSAQGRPVLFVKTDLSGALNELQDEAARLSWLA | 71 |
| Sbjct 14 | P AW E L +Q+IG S A V R+ G+ F+K+++ A +EL DE ARL WL PWAWQEALADARIERQSIGVSRADVARVHRPGQADAFLKSEVIDAFSELGDEIARLRWLQ | 73 |
| Query 72 | TTGVPCAAVLDVVTEAGRDWLLLGEVPGQDLLSS-HLAPAEKVSIMADAMRRLHTLDPAT | 130 |
| Sbjct 74 | G P V+ EAGR WLL+ +PG+DL SS LAP + V ++ADA+R LH L A AQGQPVPTVIATAEEAGRRWLLMSALPGRDLASSPELAPQQLVELLADALRGLHALPVAA | 133 |
| Query 131 | CPFDHQAKHRIERARTRMEAGLVDQDDLDEEHQGLAPAELFARLKARMPDGEDLVVTHGD | 190 |
| Sbjct 134 | CPFD + R++ A+ R+EAGLVD DD D+E G +P ++FA L + PD EDLVV+HGD CPFDQRLASRVQVAQARIEAGLVDADDFDDERLGQSPQQVFAELCSTQPDHEDLVVSHGD | 193 |
| Query 191 | ACLPNIMVENGRFSGFIDCGRLGVADRYQDIALATRDIAEELG-GEWADRFLVLYGIAAP | 249 |
| Sbjct 194 | ACLPN+M GRFSGFIDCGRLGVADRYQD+ALA R + G W YG A ACLPNLMAVEGRFSGFIDCGRLGVADRYQDLALAARSLVHNFGDTRWVAPLFQRYGAVA- | 252 |
| Query 250  Sbjct 253 | DSQRIAFYRLLDEFF 264 D +R+AFYRLLDEFF DERRLAFYRLLDEFF 267 |  |

>WP_169448694.1 APH(3')-II family aminoglycoside O-phosphotransferase [Stenotrophomonas maltophilia]

QJP20280.1 APH(3')-II family aminoglycoside O-phosphotransferase [Stenotrophomonas maltophilia]

Length=267

Score = 245 bits (626), Expect = 2e-77, Method: Compositional matrix adjust.

| Identities | = 133/255 (52%), Positives = 165/255 (65%), Gaps = 3/255 (1%) |  |
| --- | --- | --- |
| Query 12 | PAAWVERLFGYDWAQQTIGCSDAAVFRLSAQGRPVLFVKTDLSGALNELQDEAARLSWLA | 71 |
| Sbjct 14 | P AW E L +Q+IG S A V R+ G+ FVK+++ A +EL DE ARL WL PWAWQEALADARIERQSIGVSRADVARVQRPGQADAFVKSEVIDAFSELGDEIARLRWLQ | 73 |
| Query 72 | TTGVPCAAVLDVVTEAGRDWLLLGEVPGQDLLSS-HLAPAEKVSIMADAMRRLHTLDPAT | 130 |
| Sbjct 74 | G P V+ EAGR WLL+ +PG+DL SS LAP + V ++ADA+R LH L A EQGQPAPTVIATAEEAGRRWLLMSALPGRDLASSPELAPQQLVELLADALRGLHALPLAA | 133 |
| Query 131 | CPFDHQAKHRIERARTRMEAGLVDQDDLDEEHQGLAPAELFARLKARMPDGEDLVVTHGD | 190 |
| Sbjct 134 | CPFD + R++ A+ R+EAGLVD DD D+E G +P ++FA L + PD EDLVV+HGD CPFDQRLASRLQAAQARVEAGLVDADDFDDERLGQSPQQVFAELCSTRPDHEDLVVSHGD | 193 |
| Query 191 | ACLPNIMVENGRFSGFIDCGRLGVADRYQDIALATRDIAEELG-GEWADRFLVLYGIAAP | 249 |
| Sbjct 194 | ACLPN+MV GRFSGFIDCGRLGVADRYQD+AL R + G W YG A ACLPNLMVVEGRFSGFIDCGRLGVADRYQDLALGARSLVHNFGDTRWVAPLFQRYGAVA- | 252 |
| Query 250  Sbjct 253 | DSQRIAFYRLLDEFF 264 D +R+AFYRLLDEFF DERRLAFYRLLDEFF 267 |  |

>WP_164079688.1 APH(3')-II family aminoglycoside O-phosphotransferase [Stenotrophomonas maltophilia]

Length=267

Score = 245 bits (626), Expect = 2e-77, Method: Compositional matrix adjust.

| Identities | = 131/255 (51%), Positives = 165/255 (65%), Gaps = 3/255 (1%) |  |
| --- | --- | --- |
| Query 12 | PAAWVERLFGYDWAQQTIGCSDAAVFRLSAQGRPVLFVKTDLSGALNELQDEAARLSWLA | 71 |
| Sbjct 14 | P AW E L +Q+IG S A V R+ G+ F+K+++ A +EL DE ARL WL PCAWQEALADARIERQSIGVSRADVARVHRPGQADAFLKSEVIDAFSELGDEIARLRWLQ | 73 |
| Query 72 | TTGVPCAAVLDVVTEAGRDWLLLGEVPGQDLLSS-HLAPAEKVSIMADAMRRLHTLDPAT | 130 |
| Sbjct 74 | G P V+ EAGR WLL+ +PG+DL SS LAP + V ++ADA+R LH L A AQGQPVPTVIATAEEAGRRWLLMSALPGRDLASSPELAPQQLVELLADALRGLHALPVAA | 133 |
| Query 131 | CPFDHQAKHRIERARTRMEAGLVDQDDLDEEHQGLAPAELFARLKARMPDGEDLVVTHGD | 190 |
| Sbjct 134 | CPFD + R++ A+ R+EAGLVD DD D+E G +P ++FA L + PD EDLVV+HGD CPFDQRLASRVQAAQARVEAGLVDADDFDDERLGQSPQQVFAELCSTRPDHEDLVVSHGD | 193 |
| Query 191 | ACLPNIMVENGRFSGFIDCGRLGVADRYQDIALATRDIAEEL-GGEWADRFLVLYGIAAP | 249 |
| Sbjct 194 | ACLPN+M GRFSGFIDCGRLGVADRYQD+ALA R + W YG+ A ACLPNLMAAEGRFSGFIDCGRLGVADRYQDLALAVRSLVYNFDNTRWVAPLFKRYGMVA- | 252 |
| Query 250  Sbjct 253 | DSQRIAFYRLLDEFF 264 D +R+AFYRLLDEFF DERRLAFYRLLDEFF 267 |  |

>WP_121503112.1 APH(3')-II family aminoglycoside O-phosphotransferase [Stenotrophomonas sp. CF319]

RKQ55713.1 aminoglycoside 3'-phosphotransferase-2 [Stenotrophomonas sp. CF319]

Length=267

Score = 245 bits (626), Expect = 2e-77, Method: Compositional matrix adjust.

| Identities | = 132/255 (52%), Positives = 165/255 (65%), Gaps = 3/255 (1%) |  |
| --- | --- | --- |
| Query 12 | PAAWVERLFGYDWAQQTIGCSDAAVFRLSAQGRPVLFVKTDLSGALNELQDEAARLSWLA | 71 |
| Sbjct 14 | P AW E L +Q+IG S A V R+ G+ F+K+++ A +EL DE ARL WL PCAWQEALADARIERQSIGVSRADVARVHRPGQADAFLKSEVIDAFSELGDEIARLRWLQ | 73 |
| Query 72 | TTGVPCAAVLDVVTEAGRDWLLLGEVPGQDLLSS-HLAPAEKVSIMADAMRRLHTLDPAT | 130 |
| Sbjct 74 | G P V+ EAGR WLL+ +PG+DL SS LAP + V ++ADA+R LH L A AQGQPVPTVIATAEEAGRRWLLMSALPGRDLASSPELAPQQLVELLADALRGLHALPVAA | 133 |
| Query 131 | CPFDHQAKHRIERARTRMEAGLVDQDDLDEEHQGLAPAELFARLKARMPDGEDLVVTHGD | 190 |
| Sbjct 134 | CPFD + R++ A+ R+EAGLVD DD D+E G +P ++FA L + PD EDLVV+HGD CPFDQRLASRVQVAQARIEAGLVDADDFDDERLGQSPQQVFAELCSTRPDHEDLVVSHGD | 193 |
| Query 191 | ACLPNIMVENGRFSGFIDCGRLGVADRYQDIALATRDIAEELG-GEWADRFLVLYGIAAP | 249 |
| Sbjct 194 | ACLPN+MV GRFSGFIDCGRLGVADRYQD+ALA R + G W YG A ACLPNLMVAEGRFSGFIDCGRLGVADRYQDLALAARSLVHNFGDTRWVAPLFQRYGAVA- | 252 |
| Query 250  Sbjct 253 | DSQRIAFYRLLDEFF 264 D +R+AFYR LDEFF DERRLAFYRWLDEFF 267 |  |

```
>WP_150826461.1 MULTISPECIES: APH(3')-II family aminoglycoside O-phosphotransferase [unclassified Mesorhizobium]
TGR55652.1 APH(3')-II family aminoglycoside O-phosphotransferase [bacterium M00.F.Ca.ET.199.01.1.1]
TGT08716.1 APH(3')-II family aminoglycoside O-phosphotransferase [bacterium M00.F.Ca.ET.177.01.1.1]
TGU15563.1 APH(3')-II family aminoglycoside O-phosphotransferase [bacterium M00.F.Ca.ET.163.01.1.1]
TGV59955.1 APH(3')-II family aminoglycoside O-phosphotransferase [bacterium M00.F.Ca.ET.141.01.1.1]
TGT66650.1 APH(3')-II family aminoglycoside O-phosphotransferase [Mesorhizobium sp. M00.F.Ca.ET.170.01.1.1]
Length=267

Score = 245 bits (626), Expect = 2e-77, Method: Compositional matrix adjust. Identities = 131/255 (51%), Positives = 163/255 (64%), Gaps = 3/255 (1%)

Query 12	PAAWVERLFGYDWAQQTIGCSDAAVFRLSAQGRPVLFVKTDLSGALNELQDEAARLSWLA 71

P AW E L	+Q+IG S A V R+	G+	F+K+++ A +EL DE ARL WL
```

| Sbjct | 14 | PHAWQEALADARIERQSIGVSRADVARVHRPGQADAFLKSEVIDAFSELGDEIARLRWLQ | 73 |
| --- | --- | --- | --- |
| Query | 72 | TTGVPCAAVLDVVTEAGRDWLLLGEVPGQDLLSS-HLAPAEKVSIMADAMRRLHTLDPAT | 130 |
| Sbjct | 74 | G P V+ E GR WLL+ +PG DL SS LAP +V ++ADA+R LH L A AQGQPAPTVIATAEEEGRRWLLMSALPGHDLASSPALAPQHRVQLLADALRGLHALPVAA | 133 |
| Query | 131 | CPFDHQAKHRIERARTRMEAGLVDQDDLDEEHQGLAPAELFARLKARMPDGEDLVVTHGD | 190 |
| Sbjct | 134 | CPFD + RIE A+ R+EAG VD DD D+E G + ++FA L+A PD EDLVV+HGD CPFDQRLASRIEAAQARVEAGRVDADDFDDERLGQSAQQVFAELRATRPDHEDLVVSHGD | 193 |
| Query | 191 | ACLPNIMVENGRFSGFIDCGRLGVADRYQDIALATRDIAEELG-GEWADRFLVLYGIAAP | 249 |
| Sbjct | 194 | ACLPN++ GRFSGFIDCGRLGVADRYQD+ALA R + G W YG+ P ACLPNLLAVEGRFSGFIDCGRLGVADRYQDLALAARSLVHNFGDSRWVAPLFRSYGV-VP | 252 |
| Query  Sbjct | 250  253 | DSQRIAFYRLLDEFF 264 D +R+AFYRLLDEFF DERRLAFYRLLDEFF 267 |  |

>WP_097047956.1 APH(3')-II family aminoglycoside O-phosphotransferase [Stenotrophomonas sp. CC120223-11]

SNY60287.1 aminoglycoside 3'-phosphotransferase-2 [Stenotrophomonas sp. CC120223-11]

Length=267

Score = 245 bits (626), Expect = 2e-77, Method: Compositional matrix adjust.

| Identities | = 131/255 (51%), Positives = 165/255 (65%), Gaps = 3/255 (1%) |  |
| --- | --- | --- |
| Query 12 | PAAWVERLFGYDWAQQTIGCSDAAVFRLSAQGRPVLFVKTDLSGALNELQDEAARLSWLA | 71 |
| Sbjct 14 | P W E L +Q+IG S A V R+ G+ F+K+++ A +EL DE ARL WL PLPWQEALADARIERQSIGVSRADVARVHRPGQADAFLKSEVIDAFSELGDEIARLRWLQ | 73 |
| Query 72 | TTGVPCAAVLDVVTEAGRDWLLLGEVPGQDLLSS-HLAPAEKVSIMADAMRRLHTLDPAT | 130 |
| Sbjct 74 | G P V+ EAGR WLL+ +PG+DL SS LAP + V ++ADA+R LH L A AQGQPAPTVIATAEEAGRRWLLMSALPGRDLASSPELAPQQLVELLADALRGLHALSVAA | 133 |
| Query 131 | CPFDHQAKHRIERARTRMEAGLVDQDDLDEEHQGLAPAELFARLKARMPDGEDLVVTHGD | 190 |
| Sbjct 134 | CPFD + R++ A+ R+EAGLVD DD D+E G +P ++FA L + PD EDLVV+HGD CPFDQRLASRVQAAQARVEAGLVDADDFDDERLGQSPQQVFAELCSTRPDHEDLVVSHGD | 193 |
| Query 191 | ACLPNIMVENGRFSGFIDCGRLGVADRYQDIALATRDIAEELG-GEWADRFLVLYGIAAP | 249 |
| Sbjct 194 | ACLPN+MV GRFSGFIDCGRLGVADRYQD+ALA R + G W YG A ACLPNLMVAEGRFSGFIDCGRLGVADRYQDLALAARSLVHNFGDTRWVVPLFQRYGAVA- | 252 |
| Query 250  Sbjct 253 | DSQRIAFYRLLDEFF 264  + +R+AFYRLLDEFF  NERRLAFYRLLDEFF 267 |  |

>WP_180844097.1 APH(3')-II family aminoglycoside O-phosphotransferase [Stenotrophomonas maltophilia]

MBA0296988.1 APH(3')-II family aminoglycoside O-phosphotransferase [Stenotrophomonas maltophilia]

Length=267

Score = 245 bits (626), Expect = 2e-77, Method: Compositional matrix adjust.

| Identities | = 132/255 (52%), Positives = 165/255 (65%), Gaps = 3/255 (1%) |  |
| --- | --- | --- |
| Query 12 | PAAWVERLFGYDWAQQTIGCSDAAVFRLSAQGRPVLFVKTDLSGALNELQDEAARLSWLA | 71 |
| Sbjct 14 | P AW E L +Q+IG S A V R+ G+ F+K+++ A +EL DE ARL WL PWAWQEALADARIERQSIGVSRADVARVHRPGQADAFLKSEVIDAFSELGDEIARLRWLQ | 73 |
| Query 72 | TTGVPCAAVLDVVTEAGRDWLLLGEVPGQDLLSS-HLAPAEKVSIMADAMRRLHTLDPAT | 130 |
| Sbjct 74 | G P V+ EAGR WLL+ +PG+DL SS LAP + V ++ADA+R LH L A AQGQPAPTVIATAEEAGRRWLLMSALPGRDLASSPELAPQQLVELLADALRGLHALPVAA | 133 |
| Query 131 | CPFDHQAKHRIERARTRMEAGLVDQDDLDEEHQGLAPAELFARLKARMPDGEDLVVTHGD | 190 |
| Sbjct 134 | CPFD + R++ A+ R+EAGLVD DD D+E G +P ++FA L + PD EDLVV+HGD CPFDQRLASRVQVAQARIEAGLVDADDFDDERLGQSPQQVFAELCSTQPDHEDLVVSHGD | 193 |
| Query 191 | ACLPNIMVENGRFSGFIDCGRLGVADRYQDIALATRDIAEELG-GEWADRFLVLYGIAAP | 249 |

ACLPN+M	GRFSGFIDCGRLGVADRYQD+ALA R +	G	W	YG A

| Sbjct | 194 | ACLPNLMAVEGRFSGFIDCGRLGVADRYQDLALAARSLVHNFGDTRWVAPLFQRYGAVA- 252 |
| --- | --- | --- |
| Query | 250 | DSQRIAFYRLLDEFF 264 |
| Sbjct | 253 | D +R+AFYRLLDEFF  DERRLAFYRLLDEFF 267 |

>WP_058982626.1 APH(3')-II family aminoglycoside O-phosphotransferase [Stenotrophomonas maltophilia]

CRD63719.1 Aminoglycoside 3'-phosphotransferase [Stenotrophomonas maltophilia] Length=269

Score = 245 bits (626), Expect = 2e-77, Method: Compositional matrix adjust.

| Identities | | = 135/262 (52%), Positives = 166/262 (63%), Gaps = 3/262 (1%) |  |
| --- | --- | --- | --- |
| Query 5  Sbjct 9 | | DGLHAGSPAAWVERLFGYDWAQQTIGCSDAAVFRLSAQGRPVLFVKTDLSGALNELQDEA DGL P AW E L +Q+IG S A V R+ G+P FVK+++ A +EL DE DGLPLPLPWAWQEALADARIERQSIGVSRAYVARVQRPGQPDAFVKSEVIDAFSELGDEI | 64  68 |
| Query 65  Sbjct 69 | | ARLSWLATTGVPCAAVLDVVTEAGRDWLLLGEVPGQDLLSS-HLAPAEKVSIMADAMRRL ARL WL G P V+ EAGR WLL+ +PG+DL SS LAP + V ++ADA+R L ARLRWLQEQGQPAPTVIATAEEAGRRWLLMSALPGRDLASSPELAPQQLVELLADALRGL | 123  128 |
| Query 124  Sbjct 129 | | HTLDPATCPFDHQAKHRIERARTRMEAGLVDQDDLDEEHQGLAPAELFARLKARMPDGED H L A CPFD + R++ A+ R+EA LVD D D+E G +P ++FA L + PD ED HALPVAACPFDQRLASRLQAAQARVEASLVDAHDFDDERLGQSPQQVFAELCSTRPDHED | 183  188 |
| Query 184  Sbjct 189 | | LVVTHGDACLPNIMVENGRFSGFIDCGRLGVADRYQDIALATRDIAEELG-GEWADRFLV LVV HGDACLPN+MV G FSGFIDCGRLGVADRYQD+ALA R + G W LVVCHGDACLPNLMVAEGCFSGFIDCGRLGVADRYQDLALAARSLVHNFGDTRWVAPLFQ | 242  248 |
| Query 243 | | LYGIAAPDSQRIAFYRLLDEFF 264 YG A D +R+AFYRLLDEFF |  |
| Sbjct | 249 | RYGAVA-DERRLAFYRLLDEFF 269 | |

>WP_176354535.1 APH(3') family aminoglycoside O-phosphotransferase [Ensifer sp. HO-A22]

NVD41004.1 aminoglycoside 3'-phosphotransferase [Ensifer sp. HO-A22] Length=365

Score = 248 bits (634), Expect = 2e-77, Method: Compositional matrix adjust.

| Identities | = 125/253 (49%), Positives = 163/253 (64%), Gaps = 0/253 (0%) |  |
| --- | --- | --- |
| Query 12 | PAAWVERLFGYDWAQQTIGCSDAAVFRLSAQGRPVLFVKTDLSGALNELQDEAARLSWLA | 71 |
| Sbjct 113 | P + L GY W + ++G S+++VF A+ RP L +K + SG +EL DEA RL WL PPDFAPLLDGYSWRKDSLGQSESSVFLFGAEARPPLVLKVEQSGPFDELGDEAERLEWLG | 172 |
| Query 72 | TTGVPCAAVLDVVTEAGRDWLLLGEVPGQDLLSSHLAPAEKVSIMADAMRRLHTLDPATC | 131 |
| Sbjct 173 | G+PC V+ A R+WLL+ V G DL SS LA +E+V ++A+A+RR+H LD TC KQGMPCPRVIARDVYARRNWLLMEAVDGVDLASSTLAASEQVVLLAEAVRRMHALDAETC | 232 |
| Query 132 | PFDHQAKHRIERARTRMEAGLVDQDDLDEEHQGLAPAELFARLKARMPDGEDLVVTHGDA | 191 |
| Sbjct 233 | PFDH+ R+ A+ R EA ++D D DE G ELF L+A++P LVVTHGDA PFDHRVGARVAAAKLRAEADVIDVTDFDEARLGKTAEELFVELQAKIPREGKLVVTHGDA | 292 |
| Query 192 | CLPNIMVENGRFSGFIDCGRLGVADRYQDIALATRDIAEELGGEWADRFLVLYGIAAPDS | 251 |
| Sbjct 293 | CLPN++ +GRF+GFIDC RLGVAD YQD+AL R I LG EW FL LYGI D CLPNVIARDGRFAGFIDCSRLGVADPYQDLALTCRSITYNLGKEWVQPFLDLYGIRRADP | 352 |
| Query 252  Sbjct 353 | QRIAFYRLLDEFF 264  +++ FY LLDEFF  EKLDFYCLLDEFF 365 |  |

>NIK80981.1 aminoglycoside 3'-phosphotransferase-2 [Rhizobium sp. BK463] Length=266

Score = 245 bits (626), Expect = 2e-77, Method: Compositional matrix adjust. Identities = 122/254 (48%), Positives = 169/254 (67%), Gaps = 1/254 (0%)

| Query | 12 | PAAWVERLFGYDWAQQTIGCSDAAVFRLSAQGRPVLFVKTDLSGALNELQDEAARLSWLA | 71 |
| --- | --- | --- | --- |
| Sbjct | 13 | P + L GY+W Q +G S A VFRL A G +++KT+L+ L EL E ARL WLA PVRLRDLLAGYEWRQDGLGRSSAHVFRLEADGLAPVYLKTELADPLGELAGEVARLRWLA | 72 |
| Query | 72 | TTGVPCAAVLDVVTEAGRDWLLLGEVPGQDLLSSH-LAPAEKVSIMADAMRRLHTLDPAT | 130 |
| Sbjct | 73 | + G+ C ++ T+ R+WLL+ +PG+D +S+ L P E+ ++ADA+R+LH +D A SQGLACPEIIAHETDGEREWLLMSALPGRDFVSAERLQPLERARLLADALRQLHAVDIAA | 132 |
| Query | 131 | CPFDHQAKHRIERARTRMEAGLVDQDDLDEEHQGLAPAELFARLKARMPDGEDLVVTHGD | 190 |
| Sbjct | 133 | CPFDH+ ++RI A+ R+ AG VD+DD DE G + +LF L+A+ P E+LVVTHGD CPFDHRLENRIAAAKARLLAGRVDEDDFDEARLGRSAEDLFHELQAKRPSTENLVVTHGD | 192 |
| Query | 191 | ACLPNIMVENGRFSGFIDCGRLGVADRYQDIALATRDIAEELGGEWADRFLVLYGIAAPD | 250 |
| Sbjct | 193 | ACLPN M + +F+G+IDCGRLGVADR+QDIALA I G E FL YG P ACLPNFMADGPKFTGYIDCGRLGVADRHQDIALACGSIEHNFGEELVAEFLKAYGDFEPQ | 252 |
| Query  Sbjct | 251  253 | SQRIAFYRLLDEFF 264  ++++A+Y+LLDEFF  AEKMAYYQLLDEFF 266 |  |

>WP_113230447.1 aminoglycoside 3'-phosphotransferase [Rhizobium sp. BK463] Length=259

Score = 245 bits (625), Expect = 2e-77, Method: Compositional matrix adjust.

| Identities | = 122/254 (48%), Positives = 169/254 (67%), Gaps = 1/254 (0%) |  |
| --- | --- | --- |
| Query 12 | PAAWVERLFGYDWAQQTIGCSDAAVFRLSAQGRPVLFVKTDLSGALNELQDEAARLSWLA | 71 |
| Sbjct 6 | P + L GY+W Q +G S A VFRL A G +++KT+L+ L EL E ARL WLA PVRLRDLLAGYEWRQDGLGRSSAHVFRLEADGLAPVYLKTELADPLGELAGEVARLRWLA | 65 |
| Query 72 | TTGVPCAAVLDVVTEAGRDWLLLGEVPGQDLLSSH-LAPAEKVSIMADAMRRLHTLDPAT | 130 |
| Sbjct 66 | + G+ C ++ T+ R+WLL+ +PG+D +S+ L P E+ ++ADA+R+LH +D A SQGLACPEIIAHETDGEREWLLMSALPGRDFVSAERLQPLERARLLADALRQLHAVDIAA | 125 |
| Query 131 | CPFDHQAKHRIERARTRMEAGLVDQDDLDEEHQGLAPAELFARLKARMPDGEDLVVTHGD | 190 |
| Sbjct 126 | CPFDH+ ++RI A+ R+ AG VD+DD DE G + +LF L+A+ P E+LVVTHGD CPFDHRLENRIAAAKARLLAGRVDEDDFDEARLGRSAEDLFHELQAKRPSTENLVVTHGD | 185 |
| Query 191 | ACLPNIMVENGRFSGFIDCGRLGVADRYQDIALATRDIAEELGGEWADRFLVLYGIAAPD | 250 |
| Sbjct 186 | ACLPN M + +F+G+IDCGRLGVADR+QDIALA I G E FL YG P ACLPNFMADGPKFTGYIDCGRLGVADRHQDIALACGSIEHNFGEELVAEFLKAYGDFEPQ | 245 |
| Query 251  Sbjct 246 | SQRIAFYRLLDEFF 264  ++++A+Y+LLDEFF  AEKMAYYQLLDEFF 259 |  |

>VUP93328.1 Aminoglycoside 3'-phosphotransferase [Stenotrophomonas maltophilia] Length=267

Score = 245 bits (626), Expect = 2e-77, Method: Compositional matrix adjust.

| Identities | = 132/255 (52%), Positives = 166/255 (65%), Gaps = 3/255 (1%) |  |
| --- | --- | --- |
| Query 12 | PAAWVERLFGYDWAQQTIGCSDAAVFRLSAQGRPVLFVKTDLSGALNELQDEAARLSWLA | 71 |
| Sbjct 14 | P AW + L +Q+IG S A V R+ G+ F+K+++ A +EL DE ARL WL PWAWQDALADARIERQSIGVSRADVARVHRPGQADAFLKSEVIDAFSELGDEIARLRWLQ | 73 |
| Query 72 | TTGVPCAAVLDVVTEAGRDWLLLGEVPGQDLLSS-HLAPAEKVSIMADAMRRLHTLDPAT | 130 |
| Sbjct 74 | G P V+ EAGR WLL+ +PG+DL SS LAP + V ++ADA+R LH L A AQGQPVPTVIATAEEAGRRWLLMSALPGRDLASSPELAPQQLVELLADALRGLHALPVAA | 133 |
| Query 131 | CPFDHQAKHRIERARTRMEAGLVDQDDLDEEHQGLAPAELFARLKARMPDGEDLVVTHGD | 190 |
| Sbjct 134 | CPFD + R++ A+ R+EAGLVD DD D+E G +P ++FA L + PD EDLVV+HGD CPFDQRLASRVQVAQARIEAGLVDADDFDDERLGQSPQQVFAELCSTRPDHEDLVVSHGD | 193 |
| Query 191 | ACLPNIMVENGRFSGFIDCGRLGVADRYQDIALATRDIAEELG-GEWADRFLVLYGIAAP | 249 |
| Sbjct 194 | ACLPN+MV GRFSGFIDCGRLGVADRYQD+ALA R + G W YG A ACLPNLMVAEGRFSGFIDCGRLGVADRYQDLALAARSLVHNFGDTRWVAPLFQRYGAVA- | 252 |
| Query 250 | DSQRIAFYRLLDEFF 264 D +R+AFYRLLDEFF |  |

Sbjct 253 DERRLAFYRLLDEFF 267

>WP_043035050.1 APH(3')-II family aminoglycoside O-phosphotransferase [Stenotrophomonas maltophilia]

ASE52028.1 APH(3')-II family aminoglycoside O-phosphotransferase [Stenotrophomonas maltophilia]

KIS38070.1 aminoglycoside 3'-phosphotransferase [Stenotrophomonas maltophilia WJ66]

VUQ97863.1 Aminoglycoside 3'-phosphotransferase [Stenotrophomonas maltophilia] Length=267

Score = 245 bits (625), Expect = 2e-77, Method: Compositional matrix adjust.

| Identities | = 133/255 (52%), Positives = 165/255 (65%), Gaps = 3/255 (1%) |  |
| --- | --- | --- |
| Query 12 | PAAWVERLFGYDWAQQTIGCSDAAVFRLSAQGRPVLFVKTDLSGALNELQDEAARLSWLA | 71 |
| Sbjct 14 | P AW E L +Q+IG S A V R+ G+ FVK+++ A +EL DE ARL WL PWAWQEALADARIERQSIGVSRADVARVQRPGQADAFVKSEVIDAFSELGDEIARLRWLQ | 73 |
| Query 72 | TTGVPCAAVLDVVTEAGRDWLLLGEVPGQDLLSS-HLAPAEKVSIMADAMRRLHTLDPAT | 130 |
| Sbjct 74 | G P V+ EAGR WLL+ +PG+DL SS L P + V ++ADA+R LH L A EQGQPAPTVIATAEEAGRRWLLMSALPGRDLASSPELGPQQLVELLADALRGLHALPLAA | 133 |
| Query 131 | CPFDHQAKHRIERARTRMEAGLVDQDDLDEEHQGLAPAELFARLKARMPDGEDLVVTHGD | 190 |
| Sbjct 134 | CPFD + R++ A+ R+EAGLVD DD D+E G +P ++FA L + PD EDLVV+HGD CPFDQRLASRLQAAQARVEAGLVDADDFDDERLGQSPQQVFAELCSTRPDHEDLVVSHGD | 193 |
| Query 191 | ACLPNIMVENGRFSGFIDCGRLGVADRYQDIALATRDIAEELG-GEWADRFLVLYGIAAP | 249 |
| Sbjct 194 | ACLPN+MV GRFSGFIDCGRLGVADRYQD+ALA R + G W YG A ACLPNLMVVEGRFSGFIDCGRLGVADRYQDLALAARSLVHNFGDTRWVAPLFQRYGAVA- | 252 |
| Query 250  Sbjct 253 | DSQRIAFYRLLDEFF 264 D +R+AFYRLLDEFF DERRLAFYRLLDEFF 267 |  |

>WP_182267680.1 APH(3')-II family aminoglycoside O-phosphotransferase [Stenotrophomonas maltophilia]

Length=267

Score = 245 bits (625), Expect = 2e-77, Method: Compositional matrix adjust.

| Identities | = 131/255 (51%), Positives = 165/255 (65%), Gaps = 3/255 (1%) |  |
| --- | --- | --- |
| Query 12 | PAAWVERLFGYDWAQQTIGCSDAAVFRLSAQGRPVLFVKTDLSGALNELQDEAARLSWLA | 71 |
| Sbjct 14 | P AW E L +Q+IG S A V R+ G+ F+K+++ A +EL DE ARL WL PCAWQEALADARIERQSIGVSRADVARVHRPGQADAFLKSEVIDAFSELGDEIARLRWLQ | 73 |
| Query 72 | TTGVPCAAVLDVVTEAGRDWLLLGEVPGQDLLSS-HLAPAEKVSIMADAMRRLHTLDPAT | 130 |
| Sbjct 74 | G P V+ EAGR WLL+ +PG+DL SS LAP + V ++ADA+R LH L A AQGQPVPTVIATAEEAGRRWLLMSALPGRDLASSPELAPQQLVELLADALRGLHALPVAA | 133 |
| Query 131 | CPFDHQAKHRIERARTRMEAGLVDQDDLDEEHQGLAPAELFARLKARMPDGEDLVVTHGD | 190 |
| Sbjct 134 | CPFD + R++ A+ R+EAGLVD DD D+E G +P ++FA L + PD EDLVV+HGD CPFDQRLASRVQAAQARVEAGLVDADDFDDERLGQSPQQVFAELCSTRPDHEDLVVSHGD | 193 |
| Query 191 | ACLPNIMVENGRFSGFIDCGRLGVADRYQDIALATRDIAEEL-GGEWADRFLVLYGIAAP | 249 |
| Sbjct 194 | ACLPN+M GRFSGFIDCGRLGVADRYQD+ALA R + W YG+ A ACLPNLMAVQGRFSGFIDCGRLGVADRYQDLALAVRSLVYNFDNTRWVAPLFKRYGMVA- | 252 |
| Query 250  Sbjct 253 | DSQRIAFYRLLDEFF 264 D +R+AFYRLLDEFF DERRLAFYRLLDEFF 267 |  |

```
>WP_153228883.1 aminoglycoside 3'-phosphotransferase [Alcaligenes faecalis] QFY76495.1 aminoglycoside 3'-phosphotransferase [Alcaligenes faecalis]
Length=256

Score = 244 bits (624), Expect = 2e-77, Method: Compositional matrix adjust. Identities = 128/254 (50%), Positives = 161/254 (63%), Gaps = 2/254 (1%)

Query 12	PAAWVERLFGYDWAQQTIGCSDAAVFRLSAQGRPVLFVKTDLSGALNELQDEAARLSWLA 71

PAAW RL	+QTIG S A V+RL	FVK +	L EL DE RL W+
```

| Sbjct | 4 | PAAWQTRLAAAKVQRQTIGESGADVWRLEWPDGSCQFVKAEDDLPLAELPDEIERLRWMH | 63 |
| --- | --- | --- | --- |
| Query | 72 | TTGVPCAAVLDVVTEAGRDWLLLGEVPGQDLLSSH-LAPAEKVSIMADAMRRLHTLDPAT | 130 |
| Sbjct | 64 | G+PC VLD + GR WLL+ VPG+DL + L+P + V ++A +++ LH L + AQGLPCPEVLDTLVADGRHWLLMSAVPGRDLACTEGLSPQQTVELLASSLQSLHRLPIES | 123 |
| Query | 131 | CPFDHQAKHRIERARTRMEAGLVDQDDLDEEHQGLAPAELFARLKARMPDGEDLVVTHGD | 190 |
| Sbjct | 124 | CPFDH+ +HR+ A R +AGL+D++D D+E QG+ L L A P EDLVVTHGD CPFDHRLEHRLADASARAKAGLIDEEDFDDERQGMPVQSLLDELYASRPQQEDLVVTHGD | 183 |
| Query | 191 | ACLPNIMVENGRFSGFIDCGRLGVADRYQDIALATRDIAEELGGEWADRFLVLYGIAAPD | 250 |
| Sbjct | 184 | ACLPN MV G+FSGFIDCGRLGVADR+QD+AL R I LG EW F LYG+ D ACLPNFMVHQGQFSGFIDCGRLGVADRFQDLALTARSIERNLGKEWLAPFFALYGVEM-D | 242 |
| Query  Sbjct | 251  243 | SQRIAFYRLLDEFF 264  ++RIAF+ LLDEFF  AERIAFFCLLDEFF 256 |  |

>WP_032129737.1 APH(3')-II family aminoglycoside O-phosphotransferase [Stenotrophomonas maltophilia]

KGM22581.1 aminoglycoside phosphotransferase [Stenotrophomonas maltophilia] Length=267

Score = 245 bits (625), Expect = 2e-77, Method: Compositional matrix adjust.

| Identities | = 131/255 (51%), Positives = 165/255 (65%), Gaps = 3/255 (1%) |  |
| --- | --- | --- |
| Query 12 | PAAWVERLFGYDWAQQTIGCSDAAVFRLSAQGRPVLFVKTDLSGALNELQDEAARLSWLA | 71 |
| Sbjct 14 | P AW E L +Q+IG S A V R+ G+ F+K+++ A +EL DE ARL WL PCAWQEALADARIERQSIGVSRADVARVHRPGQADAFLKSEVIDAFSELGDEIARLRWLQ | 73 |
| Query 72 | TTGVPCAAVLDVVTEAGRDWLLLGEVPGQDLLSS-HLAPAEKVSIMADAMRRLHTLDPAT | 130 |
| Sbjct 74 | G P V+ EAGR WLL+ +PG+DL SS LAP + V ++ADA+R LH L A AQGQPVPTVIATAEEAGRRWLLMSALPGRDLASSPELAPQQLVELLADALRGLHALPVAA | 133 |
| Query 131 | CPFDHQAKHRIERARTRMEAGLVDQDDLDEEHQGLAPAELFARLKARMPDGEDLVVTHGD | 190 |
| Sbjct 134 | CPFD + R++ A+ R+EAGLVD DD D+E G +P ++FA L + PD EDLVV+HGD CPFDQRLASRVQVAQARVEAGLVDADDFDDERLGQSPQQVFAELCSTRPDHEDLVVSHGD | 193 |
| Query 191 | ACLPNIMVENGRFSGFIDCGRLGVADRYQDIALATRDIAEEL-GGEWADRFLVLYGIAAP | 249 |
| Sbjct 194 | ACLPN+M GRFSGFIDCGRLGVADRYQD+ALA R + W YG+ A ACLPNLMAVQGRFSGFIDCGRLGVADRYQDLALAVRSLVYNFDNTRWVAPLFKRYGMVA- | 252 |
| Query 250  Sbjct 253 | DSQRIAFYRLLDEFF 264 D +R+AFYRLLDEFF DERRLAFYRLLDEFF 267 |  |

>WP_088430213.1 APH(3')-II family aminoglycoside O-phosphotransferase [Stenotrophomonas maltophilia]

OWQ71284.1 APH(3') family aminoglycoside O-phosphotransferase [Stenotrophomonas maltophilia]

QGL67573.1 APH(3')-II family aminoglycoside O-phosphotransferase [Stenotrophomonas maltophilia]

VUO99053.1 Aminoglycoside 3'-phosphotransferase [Stenotrophomonas maltophilia] VUP47332.1 Aminoglycoside 3'-phosphotransferase [Stenotrophomonas maltophilia] VUP49214.1 Aminoglycoside 3'-phosphotransferase [Stenotrophomonas maltophilia]

Length=267

Score = 245 bits (625), Expect = 2e-77, Method: Compositional matrix adjust.

| Identities | = 133/255 (52%), Positives = 165/255 (65%), Gaps = 3/255 (1%) |  |
| --- | --- | --- |
| Query 12 | PAAWVERLFGYDWAQQTIGCSDAAVFRLSAQGRPVLFVKTDLSGALNELQDEAARLSWLA | 71 |
| Sbjct 14 | P AW E L +Q+IG S A V R+ G+ FVK+++ A +EL DE ARL WL PWAWQEALADARIERQSIGVSRADVARVQRPGQADAFVKSEVIDAFSELGDEIARLRWLQ | 73 |
| Query 72 | TTGVPCAAVLDVVTEAGRDWLLLGEVPGQDLLSS-HLAPAEKVSIMADAMRRLHTLDPAT | 130 |
| Sbjct 74 | G P V+ EAGR WLL+ +PG+DL SS LAP + V ++ADA+R LH L A EQGQPAPTVIATAEEAGRRWLLMSALPGRDLASSPELAPQQLVELLADALRGLHALPVAA | 133 |
| Query 131 | CPFDHQAKHRIERARTRMEAGLVDQDDLDEEHQGLAPAELFARLKARMPDGEDLVVTHGD | 190 |

CPFD +	R++ A+ R+EAGLVD DD D+E G +P ++FA L + PD EDLVV+HGD

| Sbjct | 134 | CPFDQRLASRLQAAQARVEAGLVDADDFDDERLGQSPQQVFAELCSTRPDHEDLVVSHGD | 193 |
| --- | --- | --- | --- |
| Query | 191 | ACLPNIMVENGRFSGFIDCGRLGVADRYQDIALATRDIAEELG-GEWADRFLVLYGIAAP | 249 |
| Sbjct | 194 | ACLPN+M GRFSGFIDCGRLGVADRYQD+ALA R + G W YG A ACLPNLMAVEGRFSGFIDCGRLGVADRYQDLALAARSLVHNFGDTRWVAPLFQRYGAVA- | 252 |
| Query  Sbjct | 250  253 | DSQRIAFYRLLDEFF 264 D +R+AFYRLLDEFF DERRLAFYRLLDEFF 267 |  |

>MBD3740732.1 APH(3')-II family aminoglycoside O-phosphotransferase [Stenotrophomonas sp.]

Length=267

Score = 245 bits (625), Expect = 2e-77, Method: Compositional matrix adjust.

| Identities | = 130/255 (51%), Positives = 165/255 (65%), Gaps = 3/255 (1%) |  |
| --- | --- | --- |
| Query 12 | PAAWVERLFGYDWAQQTIGCSDAAVFRLSAQGRPVLFVKTDLSGALNELQDEAARLSWLA | 71 |
| Sbjct 14 | P AW + L +Q+IG S A V R+ G+ F+K+++ A +EL DE ARL WL PCAWQDALADARIERQSIGVSRADVARVHRPGQADAFLKSEVIDAFSELGDEIARLRWLQ | 73 |
| Query 72 | TTGVPCAAVLDVVTEAGRDWLLLGEVPGQDLLSS-HLAPAEKVSIMADAMRRLHTLDPAT | 130 |
| Sbjct 74 | G P V+ EAGR WLL+ +PG+DL SS LAP + V ++ADA+R LH L A AQGQPVPTVIATAEEAGRRWLLMSALPGRDLASSPELAPQQLVELLADALRGLHALSVAA | 133 |
| Query 131 | CPFDHQAKHRIERARTRMEAGLVDQDDLDEEHQGLAPAELFARLKARMPDGEDLVVTHGD | 190 |
| Sbjct 134 | CPFD + R++ A+ R+EAGLVD DD D+E G +P ++FA L + PD EDLVV+HGD CPFDQRLASRVQAAQARIEAGLVDADDFDDERLGQSPQQVFAELCSTQPDHEDLVVSHGD | 193 |
| Query 191 | ACLPNIMVENGRFSGFIDCGRLGVADRYQDIALATRDIAEEL-GGEWADRFLVLYGIAAP | 249 |
| Sbjct 194 | ACLPN+M GRFSGFIDCGRLGVADRYQD+ALA R + W YG+ A ACLPNLMAVQGRFSGFIDCGRLGVADRYQDLALAVRSLVYNFDNTRWVAPLFKRYGMVA- | 252 |
| Query 250  Sbjct 253 | DSQRIAFYRLLDEFF 264 D +R+AFYRLLDEFF DERRLAFYRLLDEFF 267 |  |

>WP_049436299.1 APH(3')-II family aminoglycoside O-phosphotransferase [Stenotrophomonas maltophilia]

VUJ69010.1 Aminoglycoside 3'-phosphotransferase [Stenotrophomonas maltophilia] Length=267

Score = 245 bits (625), Expect = 2e-77, Method: Compositional matrix adjust.

| Identities | = 134/267 (50%), Positives = 171/267 (64%), Gaps = 8/267 (3%) |  |
| --- | --- | --- |
| Query 5 | DGLHAGS PAAWVERLFGYDWAQQTIGCSDAAVFRLSAQGRPVLFVKTDLSGALNE | 59 |
| Sbjct 2 | D +HAG+ P AW + L +Q+IG S A V R+ G+ F+K+++ A +E DAIHAGNHGMPLPRAWQQGLADARIERQSIGVSRADVARVHRPGQADAFLKSEVIDAFSE | 61 |
| Query 60 | LQDEAARLSWLATTGVPCAAVLDVVTEAGRDWLLLGEVPGQDLLSS-HLAPAEKVSIMAD | 118 |
| Sbjct 62 | L DE ARL WL G P V+ EAGR WLL+ +PG+DL SS LAP + V ++AD LGDEIARLRWLQAQGQPAPTVIATAEEAGRRWLLMSALPGRDLASSSELAPQQLVELLAD | 121 |
| Query 119 | AMRRLHTLDPATCPFDHQAKHRIERARTRMEAGLVDQDDLDEEHQGLAPAELFARLKARM | 178 |
| Sbjct 122 | A+R LH L A CPFD + R++ A+ R+EAGLVD DD D+E G + ++FA L + ALRGLHVLPVAACPFDQRLASRLQAAQARVEAGLVDADDFDDERLGQSAQQVFAELCSTR | 181 |
| Query 179 | PDGEDLVVTHGDACLPNIMVENGRFSGFIDCGRLGVADRYQDIALATRDIAEELGGE-WA | 237 |
| Sbjct 182 | PD EDLVV+HGDACLPN+M GRFSGFIDCGRLGVADRYQD+ALA R + G W PDHEDLVVSHGDACLPNLMAVEGRFSGFIDCGRLGVADRYQDLALAARSLVHNFGDTCWV | 241 |
| Query 238  Sbjct 242 | DRFLVLYGIAAPDSQRIAFYRLLDEFF 264 YG+ A D +R+AFYRLLDEFF  APLFRRYGVVA-DERRLAFYRLLDEFF 267 |  |

>WP_134731417.1 APH(3')-II family aminoglycoside O-phosphotransferase [Stenotrophomonas maltophilia]

Length=267

Score = 245 bits (625), Expect = 2e-77, Method: Compositional matrix adjust.

| Identities | = 132/255 (52%), Positives = 164/255 (64%), Gaps = 3/255 (1%) |  |
| --- | --- | --- |
| Query 12 | PAAWVERLFGYDWAQQTIGCSDAAVFRLSAQGRPVLFVKTDLSGALNELQDEAARLSWLA | 71 |
| Sbjct 14 | P AW E L +Q+IG S A V R+ G+P FVK+++ A +EL DE L WL PRAWQEALADARIERQSIGVSRADVARVQQPGQPDAFVKSEVIDAFSELGDEITHLRWLQ | 73 |
| Query 72 | TTGVPCAAVLDVVTEAGRDWLLLGEVPGQDLLSS-HLAPAEKVSIMADAMRRLHTLDPAT | 130 |
| Sbjct 74 | G+P V+ EAGR WLL+ +PG+DL SS LAP + V ++ADA+RRLH L A AQGLPAPTVIATAEEAGRRWLLMSALPGRDLASSPELAPQQLVELLADALRRLHVLPVAA | 133 |
| Query 131 | CPFDHQAKHRIERARTRMEAGLVDQDDLDEEHQGLAPAELFARLKARMPDGEDLVVTHGD | 190 |
| Sbjct 134 | CPFD + R++ A R+EAGLVD DD D+E G + ++FA L + PD EDLVV+HGD CPFDQRLASRLQAAAARVEAGLVDADDFDDERLGQSAQQVFAELCSTRPDHEDLVVSHGD | 193 |
| Query 191 | ACLPNIMVENGRFSGFIDCGRLGVADRYQDIALATRDIAEELG-GEWADRFLVLYGIAAP | 249 |
| Sbjct 194 | ACLPN+M GRFSGFIDCGRLGVADRYQD+ALA R + G W YG A ACLPNLMAVEGRFSGFIDCGRLGVADRYQDLALAARSLVHNFGDTRWVAPLFQRYG-ALV | 252 |
| Query 250  Sbjct 253 | DSQRIAFYRLLDEFF 264 D +R+AFYRLLDEFF DERRLAFYRLLDEFF 267 |  |

>WP_049445834.1 APH(3')-II family aminoglycoside O-phosphotransferase [Stenotrophomonas maltophilia]

KMU63691.1 Aminoglycoside 3'-phosphotransferase [Stenotrophomonas maltophilia] Length=267

Score = 245 bits (625), Expect = 2e-77, Method: Compositional matrix adjust.

| Identities | = 131/255 (51%), Positives = 165/255 (65%), Gaps = 3/255 (1%) |  |
| --- | --- | --- |
| Query 12 | PAAWVERLFGYDWAQQTIGCSDAAVFRLSAQGRPVLFVKTDLSGALNELQDEAARLSWLA | 71 |
| Sbjct 14 | P AW E L +Q+IG S A V R+ G+ F+K+++ A +EL DE ARL WL PHAWQEALADARIERQSIGVSRADVARVHRPGQADAFLKSEVIDAFSELGDEIARLRWLQ | 73 |
| Query 72 | TTGVPCAAVLDVVTEAGRDWLLLGEVPGQDLLSS-HLAPAEKVSIMADAMRRLHTLDPAT | 130 |
| Sbjct 74 | G P V+ E GR WLL+ +PG DL SS LAP V ++ADA+R LH L A+ VQGQPAPTVIATAEEGGRRWLLMSALPGHDLASSPALAPQHLVELLADALRGLHALPVAS | 133 |
| Query 131 | CPFDHQAKHRIERARTRMEAGLVDQDDLDEEHQGLAPAELFARLKARMPDGEDLVVTHGD | 190 |
| Sbjct 134 | CPFD + R++ A+TR+EAGLVD DD D+E G + ++FA L+A PD EDLVV+HGD CPFDQRLASRLQAAQTRVEAGLVDADDFDDERLGQSAQQVFAELRATRPDHEDLVVSHGD | 193 |
| Query 191 | ACLPNIMVENGRFSGFIDCGRLGVADRYQDIALATRDIAEELG-GEWADRFLVLYGIAAP | 249 |
| Sbjct 194 | ACLPN++ GRFSGFIDCGRLGVADRYQD+ALA R + G W YG+ A ACLPNLLAAEGRFSGFIDCGRLGVADRYQDLALAARSLVHNFGDTRWVAPLFRRYGVVA- | 252 |
| Query 250  Sbjct 253 | DSQRIAFYRLLDEFF 264 D +R+AFYRLLDEFF DERRLAFYRLLDEFF 267 |  |

>WP_064695926.1 aminoglycoside 3'-phosphotransferase [Rhizobium aegyptiacum] Length=262

Score = 245 bits (625), Expect = 2e-77, Method: Compositional matrix adjust.

| Identities | = 131/265 (49%), Positives = 168/265 (63%), Gaps = 4/265 (2%) |  |
| --- | --- | --- |
| Query 1 | MIEQDGLHAGSPAAWVERLFGYDWAQQTIGCSDAAVFRLSAQGRPVLFVKTDLSGALNEL | 60 |
| Sbjct 1 | M L AG A RL GY + +G S A+VFRL +G P L++K + +G EL MTSSKTLPAGPFGA---RLSGYRMERDALGRSAASVFRLEGEGLPALYLKVEAAGPFGEL | 57 |
| Query 61 | QDEAARLSWLATTGVPCAAVLDVVTEAGRDWLLLGEVPGQDLLS-SHLAPAEKVSIMADA | 119 |
| Sbjct 58 | DEAARLSWL +G+PC V+ + R+WLL+ +PG DL S S L P +V ++A+A ADEAARLSWLKASGLPCPEVIGRENDGERNWLLISALPGTDLASASVLTPLARVELLAEA | 117 |
| Query 120 | MRRLHTLDPATCPFDHQAKHRIERARTRMEAGLVDQDDLDEEHQGLAPAELFARLKARMP | 179 |
| Sbjct 118 | + LH L +CPFDH+ R+ AR RM+AG+VD+ D DE G +P LFA L+ R P  LLALHRLPITSCPFDHRLAGRVPLARARMQAGIVDETDFDETRLGKSPEALFAELENRRP | 177 |

Query 180 DGEDLVVTHGDACLPNIMVENGRFSGFIDCGRLGVADRYQDIALATRDIAEELGGEWADR 239 ED+VVTHGDACLPN + +G+FSG+IDC RLGVADRYQDIALA R I	G

| Sbjct | 178 | GSEDIVVTHGDACLPNFVASDGQFSGYIDCSRLGVADRYQDIALACRSITYNFGEALVQP 237 |
| --- | --- | --- |
| Query | 240 | FLVLYGIAAPDSQRIAFYRLLDEFF 264 |
| Sbjct | 238 | FL YG+ APD R+ +Y+LLDEFF  FLDRYGLPAPDPARLDYYQLLDEFF 262 |

>WP_096763339.1 MULTISPECIES: aminoglycoside 3'-phosphotransferase [Rhizobium]

PCK79998.1 APH(3') family aminoglycoside O-phosphotransferase [Rhizobium sophoriradicis]

Length=261

Score = 245 bits (625), Expect = 2e-77, Method: Compositional matrix adjust.

| Identities | | = 127/254 (50%), Positives = 170/254 (67%), Gaps = 1/254 (0%) |  |
| --- | --- | --- | --- |
| Query 12  Sbjct 8 | | PAAWVERLFGYDWAQQTIGCSDAAVFRLSAQGRPVLFVKTDLSGALNELQDEAARLSWLA PAA ERL GY + + +G S A+VFRL +G P L++K + +G EL DEAARLSWL PAALGERLTGYRFERDALGRSAASVFRLEGEGLPALYLKVEAAGPFGELADEAARLSWLK | 71  67 |
| Query 72  Sbjct 68 | | TTGVPCAAVLDVVTEAGRDWLLLGEVPGQDLLS-SHLAPAEKVSIMADAMRRLHTLDPAT G+PC V+ ++ R+WLL+G +PG DL S S L P +V ++A A+ LH L A+ AAGLPCPDVIARESDGKRNWLLIGALPGTDLASASALTPLTRVELLAAALSALHDLPIAS | 130  127 |
| Query 131  Sbjct 128 | | CPFDHQAKHRIERARTRMEAGLVDQDDLDEEHQGLAPAELFARLKARMPDGEDLVVTHGD CPFDH+ + R+ A+ RM+AG+VD++D D G + A LFA L P EDLVVTHGD CPFDHRLEKRLSVAKARMQAGIVDEEDFDAARLGKSAAALFADLMRLRPGNEDLVVTHGD | 190  187 |
| Query 191  Sbjct 188 | | ACLPNIMVENGRFSGFIDCGRLGVADRYQDIALATRDIAEELGGEWADRFLVLYGIAAPD ACLPN + +G+FSG+IDC RLGVADR+QD+ALA R IA G FL YG+ APD ACLPNFVASDGQFSGYIDCSRLGVADRHQDLALACRSIASNFGVALIQPFLDRYGLPAPD | 250  247 |
| Query 251 | | SQRIAFYRLLDEFF 264  +++ +++LLDEFF |  |
| Sbjct | 248 | PEKLHYFQLLDEFF 261 | |

>WP_065723058.1 APH(3')-II family aminoglycoside O-phosphotransferase [Stenotrophomonas maltophilia]

OCK48182.1 APH(3') family aminoglycoside O-phosphotransferase [Stenotrophomonas maltophilia]

Length=267

Score = 245 bits (625), Expect = 2e-77, Method: Compositional matrix adjust.

| Identities | = 132/255 (52%), Positives = 164/255 (64%), Gaps = 3/255 (1%) |  |
| --- | --- | --- |
| Query 12 | PAAWVERLFGYDWAQQTIGCSDAAVFRLSAQGRPVLFVKTDLSGALNELQDEAARLSWLA | 71 |
| Sbjct 14 | P+AW E L G +Q+IG S A V R+ G+ F+K+++ A +EL DE ARL WL PSAWREALAGARIERQSIGVSRADVARVHRPGQADAFLKSEVIDAFSELGDEIARLRWLQ | 73 |
| Query 72 | TTGVPCAAVLDVVTEAGRDWLLLGEVPGQDLLSS-HLAPAEKVSIMADAMRRLHTLDPAT | 130 |
| Sbjct 74 | G P V+ +AGR WLL+ +PG+DL SS L P V ++ADA+R LH L A AQGQPVPTVIATAEDAGRRWLLMSALPGRDLASSPELPPQRLVEVLADALRGLHALPVAA | 133 |
| Query 131 | CPFDHQAKHRIERARTRMEAGLVDQDDLDEEHQGLAPAELFARLKARMPDGEDLVVTHGD | 190 |
| Sbjct 134 | CPFD + R++ A R+EAGLVD DD D+E G +P ++ A L A P EDLVV+HGD CPFDQRLASRVQAAVVRVEAGLVDADDFDDERLGQSPQQVVAELLASQPAHEDLVVSHGD | 193 |
| Query 191 | ACLPNIMVENGRFSGFIDCGRLGVADRYQDIALATRDIAEELG-GEWADRFLVLYGIAAP | 249 |
| Sbjct 194 | ACLPN+MV GRFSGFIDCGRLGVADRYQD+ALA R + G W L YG A ACLPNLMVAEGRFSGFIDCGRLGVADRYQDLALAARSLVHNFGDTRWVAPLLQRYGAVA- | 252 |
| Query 250  Sbjct 253 | DSQRIAFYRLLDEFF 264 D +R+AFYRLLDEFF DERRLAFYRLLDEFF 267 |  |

>WP_134958273.1 APH(3')-II family aminoglycoside O-phosphotransferase [Stenotrophomonas maltophilia]

PSD47441.1 APH(3')-II family aminoglycoside O-phosphotransferase [Stenotrophomonas maltophilia]

Length=267

Score = 245 bits (625), Expect = 2e-77, Method: Compositional matrix adjust.

| Identities | = 131/255 (51%), Positives = 165/255 (65%), Gaps = 3/255 (1%) |  |
| --- | --- | --- |
| Query 12 | PAAWVERLFGYDWAQQTIGCSDAAVFRLSAQGRPVLFVKTDLSGALNELQDEAARLSWLA | 71 |
| Sbjct 14 | P AW E L +Q+IG S A V R+ G+ F+K+++ A +EL DE RL WL PWAWQEALADAHIERQSIGVSRADVARVHRPGQTDAFLKSEVIDAFSELGDEITRLRWLQ | 73 |
| Query 72 | TTGVPCAAVLDVVTEAGRDWLLLGEVPGQDLLSS-HLAPAEKVSIMADAMRRLHTLDPAT | 130 |
| Sbjct 74 | G+P V+ EAGR WLL+ +PG+DL SS LAP + V ++ADA+RRLH L A AQGLPAPTVIATAEEAGRRWLLMSALPGRDLASSPELAPQQLVELLADALRRLHVLPVAA | 133 |
| Query 131 | CPFDHQAKHRIERARTRMEAGLVDQDDLDEEHQGLAPAELFARLKARMPDGEDLVVTHGD | 190 |
| Sbjct 134 | CPFD + R++ A R+EAGLVD DD D+E G + ++FA L + PD EDLVV+HGD CPFDQRLASRVQAAAARVEAGLVDADDFDDERLGQSAQQVFAELCSTRPDHEDLVVSHGD | 193 |
| Query 191 | ACLPNIMVENGRFSGFIDCGRLGVADRYQDIALATRDIAEELG-GEWADRFLVLYGIAAP | 249 |
| Sbjct 194 | ACLPN+M GRFSGFIDCGRLGVADRYQD+ALA R + G W YG+ A ACLPNLMAVEGRFSGFIDCGRLGVADRYQDLALAARSLVHNFGDTRWVAPLFQRYGVVA- | 252 |
| Query 250  Sbjct 253 | DSQRIAFYRLLDEFF 264 D +R+AFYRLLDEFF DERRLAFYRLLDEFF 267 |  |

>WP_164212623.1 APH(3')-II family aminoglycoside O-phosphotransferase [Stenotrophomonas maltophilia]

Length=267

Score = 245 bits (625), Expect = 2e-77, Method: Compositional matrix adjust.

| Identities | = 133/255 (52%), Positives = 166/255 (65%), Gaps = 3/255 (1%) |  |
| --- | --- | --- |
| Query 12 | PAAWVERLFGYDWAQQTIGCSDAAVFRLSAQGRPVLFVKTDLSGALNELQDEAARLSWLA | 71 |
| Sbjct 14 | P AW E L +Q+IG S A V R+ G+ FVK+++ A +EL DE ARL WL PWAWQETLADARIERQSIGVSRADVARVQRPGQADAFVKSEVIDAFSELGDEIARLRWLQ | 73 |
| Query 72 | TTGVPCAAVLDVVTEAGRDWLLLGEVPGQDLLSS-HLAPAEKVSIMADAMRRLHTLDPAT | 130 |
| Sbjct 74 | G P V+ EAGR WLL+ +PG+DL SS LAP + V ++ADA+R LH L A EQGQPVPTVIATAEEAGRRWLLMSALPGRDLASSPELAPQQLVEMLADALRGLHALPLAA | 133 |
| Query 131 | CPFDHQAKHRIERARTRMEAGLVDQDDLDEEHQGLAPAELFARLKARMPDGEDLVVTHGD | 190 |
| Sbjct 134 | CPFD + R++ A+ R+EAGLVD DD D+E G +P ++FA L + PD EDLVV+HGD CPFDQRLASRLQAAQARVEAGLVDADDFDDERLGQSPQQVFAELCSTRPDHEDLVVSHGD | 193 |
| Query 191 | ACLPNIMVENGRFSGFIDCGRLGVADRYQDIALATRDIAEELG-GEWADRFLVLYGIAAP | 249 |
| Sbjct 194 | ACLPN+MV GRFSGFIDCGRL VADRYQD+ALA R + G W YG+ A ACLPNLMVVEGRFSGFIDCGRLVVADRYQDLALAARSLVHNFGDTRWVAPLFQRYGMVA- | 252 |
| Query 250  Sbjct 253 | DSQRIAFYRLLDEFF 264 D +R+AFYRLLDEFF DERRLAFYRLLDEFF 267 |  |

>WP_186450295.1 aminoglycoside 3'-phosphotransferase [Rhizobium sp. ERR1071] TWB18074.1 aminoglycoside 3'-phosphotransferase-2 [Rhizobium sp. ERR1071]

Length=266

Score = 245 bits (625), Expect = 2e-77, Method: Compositional matrix adjust.

| Identities | = 124/254 (49%), Positives = 165/254 (65%), Gaps = 1/254 (0%) |  |
| --- | --- | --- |
| Query 12 | PAAWVERLFGYDWAQQTIGCSDAAVFRLSAQGRPVLFVKTDLSGALNELQDEAARLSWLA | 71 |
| Sbjct 13 | P + L GY+W Q +G S A VFRL A GR +++KT+++ L EL E RL WLA PMRLRDALAGYEWRQDALGRSSAHVFRLEADGRVPVYLKTEVTDTLGELAGEVVRLRWLA | 72 |
| Query 72 | TTGVPCAAVLDVVTEAGRDWLLLGEVPGQDLLSSH-LAPAEKVSIMADAMRRLHTLDPAT | 130 |
| Sbjct 73 | + G+ C ++ + R+WLL+ +P D +S+ L P E+V + A A+RRLH +D A SQGLACPDIIAHEKDGEREWLLMSALPDSDFVSAEWLQPLERVRLFAGALRRLHAIDIAA | 132 |
| Query 131 | CPFDHQAKHRIERARTRMEAGLVDQDDLDEEHQGLAPAELFARLKARMPDGEDLVVTHGD | 190 |
| Sbjct 133 | CPFDH+ ++RI AR + AG VD+DD DE G + A+LF L+A P EDLVVTHGD  CPFDHRLENRIAAARAHLLAGRVDEDDFDEARLGRSAADLFRELQATRPTTEDLVVTHGD | 192 |

| Query | 191 | ACLPNIMVENGRFSGFIDCGRLGVADRYQDIALATRDIAEELGGEWADRFLVLYGIAAPD 250 |
| --- | --- | --- |
| Sbjct | 193 | ACLPN +V+ RFSG+IDCGRLGVADR+QDIALA + G E FL YG P ACLPNFVVDGDRFSGYIDCGRLGVADRHQDIALACGSVEHNFGQELVGEFLKAYGDFEPQ 252 |
| Query | 251 | SQRIAFYRLLDEFF 264 |
| Sbjct | 253 | ++++A+YRLLDEFF  AEKMAYYRLLDEFF 266 |

>WP_033838442.1 aminoglycoside 3'-phosphotransferase [Mesorhizobium loti] Length=262

Score = 244 bits (624), Expect = 3e-77, Method: Compositional matrix adjust.

| Identities | = 126/248 (51%), Positives = 165/248 (67%), Gaps = 1/248 (0%) |  |
| --- | --- | --- |
| Query 18 | RLFGYDWAQQTIGCSDAAVFRLSAQGRPVLFVKTDLSGALNELQDEAARLSWLATTGVPC | 77 |
| Sbjct 15 | R+ GY W++QTIG S+A VFRL+ +P LF+K + +G E+ E ARL WLA G+ C RIGGYRWSRQTIGRSNAGVFRLTGDNKPSLFLKIEEAGLFAEVAAEEARLRWLAGQGIAC | 74 |
| Query 78 | AAVLDVVTEAGRDWLLLGEVPGQDLLSSHLA-PAEKVSIMADAMRRLHTLDPATCPFDHQ | 136 |
| Sbjct 75 | AA++ AGR WLL+ +PGQDL S+ A V+IMADA+R LH +D +CPFDH+ AAIIAFEHHAGRHWLLMTALPGQDLASTGATDAASSVAIMADALRELHAIDAGSCPFDHR | 134 |
| Query 137 | AKHRIERARTRMEAGLVDQDDLDEEHQGLAPAELFARLKARMPDGEDLVVTHGDACLPNI | 196 |
| Sbjct 135 | + RI A R+EAG VD+ D D+E QG+ AE A+L A P ED+VVTHGDAC+PN LQQRIAEAWARVEAGTVDESDFDDERQGMTAAEALAQLAALKPASEDIVVTHGDACMPNF | 194 |
| Query 197 | MVENGRFSGFIDCGRLGVADRYQDIALATRDIAEELGGEWADRFLVLYGIAAPDSQRIAF | 256 |
| Sbjct 195 | + + FSGF+DCGRLG+ADR+QD+ALA I +G W FL YG D R+++ VAASKSFSGFVDCGRLGLADRHQDLALACWSIRHNIGERWVQPFLDRYGFMGVDPSRLSY | 254 |
| Query 257  Sbjct 255 | YRLLDEFF 264 YRLLDEFF YRLLDEFF 262 |  |

>WP_184371171.1 APH(3') family aminoglycoside O-phosphotransferase [Rhizobium sp. BK680]

MBB3716679.1 aminoglycoside 3'-phosphotransferase-2 [Rhizobium sp. BK680] Length=266

Score = 245 bits (625), Expect = 3e-77, Method: Compositional matrix adjust.

| Identities | | = 124/254 (49%), Positives = 167/254 (66%), Gaps = 1/254 (0%) |  |
| --- | --- | --- | --- |
| Query 12  Sbjct 13 | | PAAWVERLFGYDWAQQTIGCSDAAVFRLSAQGRPVLFVKTDLSGALNELQDEAARLSWLA P + L GY+W Q +G S A VFRL A R +++KT+L+ L EL E ARL WLA PVRLRDLLAGYEWRQDGLGRSSAHVFRLEADRRAPVYLKTELADPLGELAGEVARLRWLA | 71  72 |
| Query 72  Sbjct 73 | | TTGVPCAAVLDVVTEAGRDWLLLGEVPGQDLLSSH-LAPAEKVSIMADAMRRLHTLDPAT  + G+ C ++ + R+WLL+ +PG DL+S+ L P E+V ++ADA+R+LH +D A SQGLACPDIIAHEKDGEREWLLMSALPGSDLVSAEWLQPLERVRLLADALRQLHAIDIAA | 130  132 |
| Query 131  Sbjct 133 | | CPFDHQAKHRIERARTRMEAGLVDQDDLDEEHQGLAPAELFARLKARMPDGEDLVVTHGD CPFDH+ ++RI A+ + AG VD+DD DE G + +LF L+A+ P EDLVVTHGD CPFDHRLENRIGTAKAHLLAGRVDEDDFDEARLGRSAEDLFRELQAKRPSMEDLVVTHGD | 190  192 |
| Query 191  Sbjct 193 | | ACLPNIMVENGRFSGFIDCGRLGVADRYQDIALATRDIAEELGGEWADRFLVLYGIAAPD ACLPN M + +FSG+IDCGRLGVADR+QDIALA I G E FL YG P ACLPNFMADGPKFSGYIDCGRLGVADRHQDIALACGSIEHNFGEELVAEFLKAYGDFQPK | 250  252 |
| Query 251 | | SQRIAFYRLLDEFF 264  +++A+Y+LLDEFF |  |
| Sbjct | 253 | EEKMAYYQLLDEFF 266 | |

>WP_125892918.1 APH(3')-II family aminoglycoside O-phosphotransferase [Stenotrophomonas maltophilia]

RRU88839.1 APH(3')-II family aminoglycoside O-phosphotransferase [Stenotrophomonas maltophilia]

Length=267

Score = 245 bits (625), Expect = 3e-77, Method: Compositional matrix adjust.

| Identities | = 132/255 (52%), Positives = 166/255 (65%), Gaps = 3/255 (1%) |  |
| --- | --- | --- |
| Query 12 | PAAWVERLFGYDWAQQTIGCSDAAVFRLSAQGRPVLFVKTDLSGALNELQDEAARLSWLA | 71 |
| Sbjct 14 | P AW + L +Q+IG S A V R+ G+ F+K+++ A +EL DE ARL WL PWAWQDALADARIERQSIGVSRADVARVHRPGQADAFLKSEVIDAFSELGDEIARLRWLQ | 73 |
| Query 72 | TTGVPCAAVLDVVTEAGRDWLLLGEVPGQDLLSS-HLAPAEKVSIMADAMRRLHTLDPAT | 130 |
| Sbjct 74 | G P V+ EAGR WLL+ +PG+DL SS LAP + V ++ADA+R LH L A AQGQPVPTVIATAEEAGRRWLLMSALPGRDLASSPELAPQQLVELLADALRGLHALPVAA | 133 |
| Query 131 | CPFDHQAKHRIERARTRMEAGLVDQDDLDEEHQGLAPAELFARLKARMPDGEDLVVTHGD | 190 |
| Sbjct 134 | CPFD + R++ A+ R+EAGLVD DD D+E G +P ++FA L + PD EDLVV+HGD CPFDQRLASRVQAAQARVEAGLVDADDFDDERLGQSPQQVFAELCSTRPDHEDLVVSHGD | 193 |
| Query 191 | ACLPNIMVENGRFSGFIDCGRLGVADRYQDIALATRDIAEELG-GEWADRFLVLYGIAAP | 249 |
| Sbjct 194 | ACLPN+MV GRFSGFIDCGRLGVADRYQD+ALA R + G W YG A ACLPNLMVVEGRFSGFIDCGRLGVADRYQDLALAARSLVHNFGDTRWVAPLFQRYGAVA- | 252 |
| Query 250  Sbjct 253 | DSQRIAFYRLLDEFF 264 D +R+AFYRLLDEFF DERRLAFYRLLDEFF 267 |  |

>WP_153451750.1 aminoglycoside 3'-phosphotransferase [Sinorhizobium fredii] AWI59060.1 hypothetical protein AB395_00003425 [Sinorhizobium fredii CCBAU

45436]

KSV87245.1 hypothetical protein N181_19110 [Sinorhizobium fredii USDA 205] GEC34504.1 aminoglycoside phosphotransferase APH(3') [Sinorhizobium fredii]

Length=247

Score = 244 bits (623), Expect = 3e-77, Method: Compositional matrix adjust.

| Identities | = 124/231 (54%), Positives = 154/231 (67%), Gaps = 0/231 (0%) |  |
| --- | --- | --- |
| Query 34 | AAVFRLSAQGRPVLFVKTDLSGALNELQDEAARLSWLATTGVPCAAVLDVVTEAGRDWLL | 93 |
| Sbjct 17 | +AVF L A GR L +K + +G EL DEAARL WLA G+ C V AGR WLL SAVFLLKADGRARLVLKHEEAGPFGELADEAARLGWLAGEGLLCPRVKAQEIHAGRHWLL | 76 |
| Query 94 | LGEVPGQDLLSSHLAPAEKVSIMADAMRRLHTLDPATCPFDHQAKHRIERARTRMEAGLV | 153 |
| Sbjct 77 | + V G DL SS APA +++++A+A+RRLH LDP CPFDH+ RI A+ R++AGLV MEAVEGVDLASSRQAPARRIAVLAEALRRLHRLDPTRCPFDHRLDSRIAAAKARLDAGLV | 136 |
| Query 154 | DQDDLDEEHQGLAPAELFARLKARMPDGEDLVVTHGDACLPNIMVENGRFSGFIDCGRLG | 213 |
| Sbjct 137 | D+ D DEE G LF ++AR P GE LVVTHGDACLPNI+ F+GFIDC R+G DETDFDEERLGRTATTLFGEIQARRPKGERLVVTHGDACLPNIIAREDGFAGFIDCSRIG | 196 |
| Query 214  Sbjct 197 | VADRYQDIALATRDIAEELGGEWADRFLVLYGIAAPDSQRIAFYRLLDEFF 264 VAD +QDIALA R IA LG +W FL LYG+ PD +++ +Y LLDEFF VADVHQDIALACRSIAYNLGQDWVRPFLELYGLPNPDPEKLIYYCLLDEFF 247 |  |

>WP_164158428.1 APH(3')-II family aminoglycoside O-phosphotransferase [Stenotrophomonas maltophilia]

Length=267

Score = 244 bits (624), Expect = 3e-77, Method: Compositional matrix adjust.

| Identities | = 133/255 (52%), Positives = 164/255 (64%), Gaps = 3/255 (1%) |  |
| --- | --- | --- |
| Query 12 | PAAWVERLFGYDWAQQTIGCSDAAVFRLSAQGRPVLFVKTDLSGALNELQDEAARLSWLA | 71 |
| Sbjct 14 | P AW E L +Q+IG S A V R+ G+P FVK+++ A +EL DE A L WL PWAWREALANARIERQSIGVSRADVARVHRPGQPDAFVKSEVIDAFSELGDEIALLRWLQ | 73 |
| Query 72 | TTGVPCAAVLDVVTEAGRDWLLLGEVPGQDLLSS-HLAPAEKVSIMADAMRRLHTLDPAT | 130 |
| Sbjct 74 | G P V+ EAGR WLL+ +PG DL SS LAP + V ++ADA+R LH L A AQGQPAPTVIATTEEAGRRWLLMSALPGHDLASSPELAPQQLVDLLADALRGLHALPVAA | 133 |
| Query 131 | CPFDHQAKHRIERARTRMEAGLVDQDDLDEEHQGLAPAELFARLKARMPDGEDLVVTHGD | 190 |
| Sbjct 134 | CPFD + R++ A+ R+EAGLVD DD D+E G + ++FA L + PD EDLVV+HGD CPFDQRLASRLQAAQARVEAGLVDADDFDDERLGQSAQQVFAELCSTRPDHEDLVVSHGD | 193 |
| Query 191 | ACLPNIMVENGRFSGFIDCGRLGVADRYQDIALATRDIAEELG-GEWADRFLVLYGIAAP ACLPN+MV GRFSGFIDCGRLGVADRYQD+ALA R + G W YG A | 249 |

| Sbjct | 194 | ACLPNLMVAEGRFSGFIDCGRLGVADRYQDLALAARSLVHNFGDTRWVAPLFQRYGAVA- 252 |
| --- | --- | --- |
| Query | 250 | DSQRIAFYRLLDEFF 264 |
| Sbjct | 253 | D +R+AFYRLLDEFF  DERRLAFYRLLDEFF 267 |

>WP_111174387.1 APH(3')-II family aminoglycoside O-phosphotransferase [Stenotrophomonas maltophilia]

PZS53537.1 APH(3') family aminoglycoside O-phosphotransferase [Stenotrophomonas maltophilia]

VUN60415.1 Aminoglycoside 3'-phosphotransferase [Stenotrophomonas maltophilia] VUN73882.1 Aminoglycoside 3'-phosphotransferase [Stenotrophomonas maltophilia]

Length=267

Score = 244 bits (624), Expect = 3e-77, Method: Compositional matrix adjust.

| Identities | = 132/255 (52%), Positives = 165/255 (65%), Gaps = 3/255 (1%) |  |
| --- | --- | --- |
| Query 12 | PAAWVERLFGYDWAQQTIGCSDAAVFRLSAQGRPVLFVKTDLSGALNELQDEAARLSWLA | 71 |
| Sbjct 14 | P AW E L +Q+IG S A V R+ G+ FVK+++ A +EL DE ARL WL PWAWQEALADARIERQSIGVSRADVARVQRPGQADAFVKSEVIDAFSELGDEIARLRWLQ | 73 |
| Query 72 | TTGVPCAAVLDVVTEAGRDWLLLGEVPGQDLLSS-HLAPAEKVSIMADAMRRLHTLDPAT | 130 |
| Sbjct 74 | G P V+ EAGR WLL+ +PG+DL SS L P + V ++ADA+R LH L A EQGQPAPTVIATAEEAGRRWLLMSALPGRDLASSPELGPQQLVELLADALRGLHALPLAA | 133 |
| Query 131 | CPFDHQAKHRIERARTRMEAGLVDQDDLDEEHQGLAPAELFARLKARMPDGEDLVVTHGD | 190 |
| Sbjct 134 | CPFD + R++ A+ R+EAGLVD DD D+E G +P ++FA L + PD EDLVV+HGD CPFDQRLASRLQAAQARVEAGLVDADDFDDERLGQSPQQVFAELCSTRPDHEDLVVSHGD | 193 |
| Query 191 | ACLPNIMVENGRFSGFIDCGRLGVADRYQDIALATRDIAEELG-GEWADRFLVLYGIAAP | 249 |
| Sbjct 194 | ACLPN+MV GRFSGF+DCGRLGVADRYQD+ALA R + G W YG A ACLPNLMVVEGRFSGFVDCGRLGVADRYQDLALAARSLVHNFGDTRWVAPLFQRYGAVA- | 252 |
| Query 250  Sbjct 253 | DSQRIAFYRLLDEFF 264 D +R+AFYRLLDEFF DERRLAFYRLLDEFF 267 |  |

>WP_135967552.1 APH(3')-II family aminoglycoside O-phosphotransferase [Stenotrophomonas maltophilia]

TGW17327.1 APH(3')-II family aminoglycoside O-phosphotransferase [Stenotrophomonas maltophilia]

VUP91458.1 Aminoglycoside 3'-phosphotransferase [Stenotrophomonas maltophilia] Length=267

Score = 244 bits (624), Expect = 3e-77, Method: Compositional matrix adjust.

| Identities | = 131/255 (51%), Positives = 165/255 (65%), Gaps = 3/255 (1%) |  |
| --- | --- | --- |
| Query 12 | PAAWVERLFGYDWAQQTIGCSDAAVFRLSAQGRPVLFVKTDLSGALNELQDEAARLSWLA | 71 |
| Sbjct 14 | P AW E L +Q+IG S A V R+ G+ F+K+++ A +EL DE ARL WL PHAWQEALADARIERQSIGVSRADVARVHRPGQADAFLKSEVIDAFSELGDEIARLRWLQ | 73 |
| Query 72 | TTGVPCAAVLDVVTEAGRDWLLLGEVPGQDLLSS-HLAPAEKVSIMADAMRRLHTLDPAT | 130 |
| Sbjct 74 | G P V+ EAGR WLL+ +PG DL SS LAP V ++ADA+R LH L A+ VQGQPAPTVMATAEEAGRRWLLMSALPGHDLASSPALAPQHLVELLADALRGLHALPVAS | 133 |
| Query 131 | CPFDHQAKHRIERARTRMEAGLVDQDDLDEEHQGLAPAELFARLKARMPDGEDLVVTHGD | 190 |
| Sbjct 134 | CPFD + R++ A+ R+EAGLVD DD D+E G + ++FA L+A PD EDLVV+HGD CPFDQRLASRLQAAQARVEAGLVDADDFDDERLGQSAQQVFAELRATRPDHEDLVVSHGD | 193 |
| Query 191 | ACLPNIMVENGRFSGFIDCGRLGVADRYQDIALATRDIAEELG-GEWADRFLVLYGIAAP | 249 |
| Sbjct 194 | ACLPN++ GRFSGFIDCGRLGVADRYQD+ALA R + G W YG+ A ACLPNLLAAEGRFSGFIDCGRLGVADRYQDLALAARSLVHNFGDTRWVAPLFRRYGVVA- | 252 |
| Query 250  Sbjct 253 | DSQRIAFYRLLDEFF 264 D +R+AFYRLLDEFF DERRLAFYRLLDEFF 267 |  |

>WP_004116317.1 aminoglycoside 3'-phosphotransferase [Rhizobium freirei] ENN87859.1 putative aminoglycoside 3'-phosphotransferase protein [Rhizobium

freirei PRF 81] Length=266

Score = 244 bits (624), Expect = 3e-77, Method: Compositional matrix adjust.

| Identities | = 122/254 (48%), Positives = 167/254 (66%), Gaps = 1/254 (0%) |  |
| --- | --- | --- |
| Query 12 | PAAWVERLFGYDWAQQTIGCSDAAVFRLSAQGRPVLFVKTDLSGALNELQDEAARLSWLA | 71 |
| Sbjct 13 | P+ + L GY+W Q +G S A VFRL A GR +++KT+L L+EL E ARL WLA PSRLRDMLVGYEWRQDALGRSSAHVFRLEADGRAPVYLKTELVHPLSELPGEVARLRWLA | 72 |
| Query 72 | TTGVPCAAVLDVVTEAGRDWLLLGEVPGQDLLSSH-LAPAEKVSIMADAMRRLHTLDPAT | 130 |
| Sbjct 73 | + G C V+ + R+WLL+ + G DL+S ++PAE+V ++A A+RRLH +D A SRGFACPQVIAHERDEEREWLLMSALRGADLVSEEGMSPAERVRLLAHALRRLHDVDIAC | 132 |
| Query 131 | CPFDHQAKHRIERARTRMEAGLVDQDDLDEEHQGLAPAELFARLKARMPDGEDLVVTHGD | 190 |
| Sbjct 133 | CPFDH+ +++ A+ + AG VD+DD D+E G + +LF L+ R P ED VVTHGD CPFDHRLDNKVAEAKAHLLAGRVDEDDFDDERLGRSAQDLFRELEERRPATEDFVVTHGD | 192 |
| Query 191 | ACLPNIMVENGRFSGFIDCGRLGVADRYQDIALATRDIAEELGGEWADRFLVLYGIAAPD | 250 |
| Sbjct 193 | ACLPN M E+G FSG+IDCGRLG+ADR+QDIALA I G + FL YG P ACLPNFMAEDGEFSGYIDCGRLGIADRHQDIALACGSIERNFGSDLLAEFLKAYGDFEPQ | 252 |
| Query 251  Sbjct 253 | SQRIAFYRLLDEFF 264  ++++A+Y+LLDEFF  AEKMAYYQLLDEFF 266 |  |

>WP_165710397.1 APH(3')-II family aminoglycoside O-phosphotransferase [Stenotrophomonas maltophilia]

Length=267

Score = 244 bits (624), Expect = 3e-77, Method: Compositional matrix adjust.

| Identities | = 133/255 (52%), Positives = 166/255 (65%), Gaps = 3/255 (1%) |  |
| --- | --- | --- |
| Query 12 | PAAWVERLFGYDWAQQTIGCSDAAVFRLSAQGRPVLFVKTDLSGALNELQDEAARLSWLA | 71 |
| Sbjct 14 | P AW E L +Q+IG S A V R+ G+ FVK+++ A +EL DE ARL WL PWAWQETLADARIERQSIGVSRADVARVQRPGQADAFVKSEVIDAFSELGDEIARLRWLQ | 73 |
| Query 72 | TTGVPCAAVLDVVTEAGRDWLLLGEVPGQDLLSS-HLAPAEKVSIMADAMRRLHTLDPAT | 130 |
| Sbjct 74 | G P V+ EAGR WLL+ +PG+DL SS LAP + V ++ADA+R LH L A EQGQPVPTVIATAEEAGRRWLLMSALPGRDLASSPELAPQQLVEMLADALRGLHALPVAA | 133 |
| Query 131 | CPFDHQAKHRIERARTRMEAGLVDQDDLDEEHQGLAPAELFARLKARMPDGEDLVVTHGD | 190 |
| Sbjct 134 | CPF + R++ A+ R+EAGLVD DD D+E G +P ++FA L + PD EDLVV+HGD CPFHQRLHLRLQAAQARVEAGLVDADDFDDERLGQSPQQVFAELCSTRPDHEDLVVSHGD | 193 |
| Query 191 | ACLPNIMVENGRFSGFIDCGRLGVADRYQDIALATRDIAEELG-GEWADRFLVLYGIAAP | 249 |
| Sbjct 194 | ACLPN+MV GRFSGFIDCGRLGVADRYQD+ALA R + G W YG+ A ACLPNLMVVEGRFSGFIDCGRLGVADRYQDLALAARSLVHNFGDTRWVAPLFQRYGMVA- | 252 |
| Query 250  Sbjct 253 | DSQRIAFYRLLDEFF 264 D +R+AFYRLLDEFF DERRLAFYRLLDEFF 267 |  |

>WP_085860095.1 MULTISPECIES: aminoglycoside 3'-phosphotransferase [unclassified Rhizobium]

ARO25028.1 aminoglycoside 3'-phosphotransferase protein [Rhizobium sp. TAL182]

PDS98298.1 APH(3') family aminoglycoside O-phosphotransferase [Rhizobium sp. S9]

Length=261

Score = 244 bits (623), Expect = 4e-77, Method: Compositional matrix adjust. Identities = 130/254 (51%), Positives = 167/254 (66%), Gaps = 1/254 (0%)

| Query | 12 | PAAWVERLFGYDWAQQTIGCSDAAVFRLSAQGRPVLFVKTDLSGALNELQDEAARLSWLA | 71 |
| --- | --- | --- | --- |
| Sbjct | 8 | PAA ERL GY + + +G S A+VFRL G P L++K + +G EL DEAARL+WL PAALGERLSGYRFERDALGRSAASVFRLEGDGLPALYLKVEAAGRFGELADEAARLAWLK | 67 |
| Query | 72 | TTGVPCAAVLDVVTEAGRDWLLLGEVPGQDLLS-SHLAPAEKVSIMADAMRRLHTLDPAT | 130 |
| Sbjct | 68 | +PC V+ ++ R+WLL+ +PG DL S S L P V ++A+A+ LH L A+  ACRLPCPEVIARESDGERNWLLVSALPGTDLASASALTPLACVELLAEALLDLHRLPIAS | 127 |

| Query | 131 | CPFDHQAKHRIERARTRMEAGLVDQDDLDEEHQGLAPAELFARLKARMPDGEDLVVTHGD | 190 |
| --- | --- | --- | --- |
| Sbjct | 128 | CPFDH+ + R+ A+ RMEAG+VD++D D G AELFA L++ P EDLVVTHGD CPFDHRLEKRLPVAKVRMEAGIVDEEDFDPARLGKRAAELFAALESMRPTNEDLVVTHGD | 187 |
| Query | 191 | ACLPNIMVENGRFSGFIDCGRLGVADRYQDIALATRDIAEELGGEWADRFLVLYGIAAPD | 250 |
| Sbjct | 188 | ACLPN + G+FSG+IDC RLGVADRYQDIALA R IA G FL YG+ APD ACLPNFVASGGQFSGYIDCSRLGVADRYQDIALACRSIAYNFGEALVQPFLDRYGLPAPD | 247 |
| Query | 251 | SQRIAFYRLLDEFF 264 R+ +Y+LLDEFF |  |
| Sbjct | 248 | PARLGYYQLLDEFF 261 | |

>WP_005413136.1 APH(3')-II family aminoglycoside O-phosphotransferase [Stenotrophomonas maltophilia]

EMF60378.1 Aminoglycoside 3'-phosphotransferase [Stenotrophomonas maltophilia EPM1]

KWV52383.1 aminoglycoside phosphotransferase [Stenotrophomonas maltophilia]

MBA0459514.1 APH(3')-II family aminoglycoside O-phosphotransferase [Stenotrophomonas maltophilia]

MBC8774748.1 APH(3')-II family aminoglycoside O-phosphotransferase [Stenotrophomonas maltophilia]

QJC75086.1 APH(3')-II family aminoglycoside O-phosphotransferase [Stenotrophomonas maltophilia]

Length=267

Score = 244 bits (624), Expect = 4e-77, Method: Compositional matrix adjust.

| Identities | = 133/255 (52%), Positives = 165/255 (65%), Gaps = 3/255 (1%) |  |
| --- | --- | --- |
| Query 12 | PAAWVERLFGYDWAQQTIGCSDAAVFRLSAQGRPVLFVKTDLSGALNELQDEAARLSWLA | 71 |
| Sbjct 14 | P AW E L +Q+IG S A V R+ G+ FVK+++ A +EL DE ARL WL PWAWQEALADARIERQSIGVSRADVARVQRPGQADAFVKSEVIDAFSELGDEIARLRWLQ | 73 |
| Query 72 | TTGVPCAAVLDVVTEAGRDWLLLGEVPGQDLLSS-HLAPAEKVSIMADAMRRLHTLDPAT | 130 |
| Sbjct 74 | G P V+ EAGR WLL+ +PG+DL SS LAP + V ++ADA+R LH L A EQGQPAPTVIATAEEAGRRWLLMSALPGRDLASSPELAPQQLVELLADALRGLHALPVAA | 133 |
| Query 131 | CPFDHQAKHRIERARTRMEAGLVDQDDLDEEHQGLAPAELFARLKARMPDGEDLVVTHGD | 190 |
| Sbjct 134 | CPFD + R++ A+ R+EAGLVD DD D+E G +P ++FA L + PD EDLVV+HGD CPFDQRLHLRLQAAQARVEAGLVDADDFDDERLGQSPQQVFAELCSTRPDHEDLVVSHGD | 193 |
| Query 191 | ACLPNIMVENGRFSGFIDCGRLGVADRYQDIALATRDIAEELG-GEWADRFLVLYGIAAP | 249 |
| Sbjct 194 | ACLPN+M GRFSGFIDCGRLGVADRYQD+ALA R + G W YG A ACLPNLMAVEGRFSGFIDCGRLGVADRYQDLALAARSLVHNFGDTRWVAPLFQRYGAVA- | 252 |
| Query 250  Sbjct 253 | DSQRIAFYRLLDEFF 264 D +R+AFYRLLDEFF DERRLAFYRLLDEFF 267 |  |

>WP_173002436.1 MULTISPECIES: aminoglycoside 3'-phosphotransferase [unclassified Rhizobium]

GES45778.1 aminoglycoside phosphotransferase APH(3') [Rhizobium sp. S-62] GES48141.1 aminoglycoside phosphotransferase APH(3') [Rhizobium sp. S-93]

Length=266

Score = 244 bits (624), Expect = 4e-77, Method: Compositional matrix adjust.

| Identities | = 124/254 (49%), Positives = 166/254 (65%), Gaps = 1/254 (0%) |  |
| --- | --- | --- |
| Query 12 | PAAWVERLFGYDWAQQTIGCSDAAVFRLSAQGRPVLFVKTDLSGALNELQDEAARLSWLA | 71 |
| Sbjct 13 | P + L GY+W Q +G S A VFRL A GR +++K++L L EL E ARL WLA PVRLRDLLAGYEWRQDALGRSSAHVFRLEADGRVPVYLKSELVDPLGELAGEVARLRWLA | 72 |
| Query 72 | TTGVPCAAVLDVVTEAGRDWLLLGEVPGQDLLSSH-LAPAEKVSIMADAMRRLHTLDPAT | 130 |
| Sbjct 73 | + G+ C ++ + R+WLL+ +PG D +++ L P E+V + A A+RRLH++D A SQGLACPDIIAHEKDGEREWLLMSALPGSDFVNAEWLQPLERVRLFAGALRRLHSVDIAA | 132 |
| Query 131 | CPFDHQAKHRIERARTRMEAGLVDQDDLDEEHQGLAPAELFARLKARMPDGEDLVVTHGD | 190 |
| Sbjct 133 | CPFDH+ + R+ AR + AG VD+DD DE G + A+LF L+A P EDLVVTHGD  CPFDHRLESRMGTARAHLLAGRVDEDDFDEARLGKSAADLFRELQATRPPTEDLVVTHGD | 192 |

| Query | 191 | ACLPNIMVENGRFSGFIDCGRLGVADRYQDIALATRDIAEELGGEWADRFLVLYGIAAPD 250 |
| --- | --- | --- |
| Sbjct | 193 | ACLPN +V+ RFSG+IDCGRLGVADR+QDIALA + G E FL YG P ACLPNFVVDGDRFSGYIDCGRLGVADRHQDIALACGSVEHNFGQELVGEFLKAYGDFEPQ 252 |
| Query | 251 | SQRIAFYRLLDEFF 264 |
| Sbjct | 253 | ++++A+YRLLDEFF  AEKMAYYRLLDEFF 266 |

>WP_164086031.1 APH(3')-II family aminoglycoside O-phosphotransferase [Stenotrophomonas maltophilia]

MBA0433155.1 APH(3')-II family aminoglycoside O-phosphotransferase [Stenotrophomonas maltophilia]

VUJ57101.1 Aminoglycoside 3'-phosphotransferase [Stenotrophomonas maltophilia] VUK19216.1 Aminoglycoside 3'-phosphotransferase [Stenotrophomonas maltophilia] VUN34325.1 Aminoglycoside 3'-phosphotransferase [Stenotrophomonas maltophilia] VUP94238.1 Aminoglycoside 3'-phosphotransferase [Stenotrophomonas maltophilia]

Length=267

Score = 244 bits (624), Expect = 4e-77, Method: Compositional matrix adjust.

| Identities | = 132/255 (52%), Positives = 164/255 (64%), Gaps = 3/255 (1%) |  |
| --- | --- | --- |
| Query 12 | PAAWVERLFGYDWAQQTIGCSDAAVFRLSAQGRPVLFVKTDLSGALNELQDEAARLSWLA | 71 |
| Sbjct 14 | P AW E L +Q+IG S A V R+ G+ F+K+++ A +EL DE ARL WL PHAWQEALADARIERQSIGVSRADVARVHRPGQADAFLKSEVIDAFSELGDEIARLRWLQ | 73 |
| Query 72 | TTGVPCAAVLDVVTEAGRDWLLLGEVPGQDLLSS-HLAPAEKVSIMADAMRRLHTLDPAT | 130 |
| Sbjct 74 | G P V+ EAGR WLL+ +PG DL SS LAP V ++ADA+R LH L AT VQGQPAPTVIATAEEAGRRWLLMSALPGHDLASSPALAPQHLVELLADALRGLHALPVAT | 133 |
| Query 131 | CPFDHQAKHRIERARTRMEAGLVDQDDLDEEHQGLAPAELFARLKARMPDGEDLVVTHGD | 190 |
| Sbjct 134 | CPFD + R++ A+ R+EAGLVD DD D+E G + ++FA L+A PD EDLVV HGD CPFDQRLASRLQAAQARVEAGLVDADDFDDERLGQSAQQVFAELRATRPDHEDLVVCHGD | 193 |
| Query 191 | ACLPNIMVENGRFSGFIDCGRLGVADRYQDIALATRDIAEELG-GEWADRFLVLYGIAAP | 249 |
| Sbjct 194 | ACLPN++ GRFSGFIDCGRLGVADRYQD+ALA R + G W YG+ A ACLPNLLAAEGRFSGFIDCGRLGVADRYQDLALAARSLVHNFGDTRWVAPLFRRYGVEA- | 252 |
| Query 250  Sbjct 253 | DSQRIAFYRLLDEFF 264 D +R+AFYRLLDEFF DERRLAFYRLLDEFF 267 |  |

>WP_099497621.1 APH(3')-II family aminoglycoside O-phosphotransferase [Stenotrophomonas maltophilia]

Length=267

Score = 244 bits (624), Expect = 4e-77, Method: Compositional matrix adjust.

| Identities | = 133/255 (52%), Positives = 165/255 (65%), Gaps = 3/255 (1%) |  |
| --- | --- | --- |
| Query 12 | PAAWVERLFGYDWAQQTIGCSDAAVFRLSAQGRPVLFVKTDLSGALNELQDEAARLSWLA | 71 |
| Sbjct 14 | P AW E +Q+IG S A V R+ G+ FVK+++ A +EL DE ARL WL PWAWQETSADARIERQSIGVSRADVARVQRPGQADAFVKSEVIDAFSELGDEIARLRWLQ | 73 |
| Query 72 | TTGVPCAAVLDVVTEAGRDWLLLGEVPGQDLLSS-HLAPAEKVSIMADAMRRLHTLDPAT | 130 |
| Sbjct 74 | G P V+ EAGR WLL+ +PG+DL SS LAP + V ++ADA+R LH L A EQGQPVPTVIATAEEAGRRWLLMSALPGRDLASSPELAPQQLVEMLADALRGLHALPLAA | 133 |
| Query 131 | CPFDHQAKHRIERARTRMEAGLVDQDDLDEEHQGLAPAELFARLKARMPDGEDLVVTHGD | 190 |
| Sbjct 134 | CPFD + R++ A+ R+EAGLVD DD D+E G +P ++FA L + PD EDLVV+HGD CPFDQRLASRLQAAQARVEAGLVDADDFDDERLGQSPQQVFAELCSTRPDHEDLVVSHGD | 193 |
| Query 191 | ACLPNIMVENGRFSGFIDCGRLGVADRYQDIALATRDIAEELG-GEWADRFLVLYGIAAP | 249 |
| Sbjct 194 | ACLPN+MV GRFSGFIDCGRLGVADRYQD+ALA R + G W YG A ACLPNLMVVEGRFSGFIDCGRLGVADRYQDLALAARSLVHNFGDTRWVAPLFQRYGAVA- | 252 |
| Query 250  Sbjct 253 | DSQRIAFYRLLDEFF 264 D +R+AFYRLLDEFF DERRLAFYRLLDEFF 267 |  |

>VUN21478.1 Aminoglycoside 3'-phosphotransferase [Stenotrophomonas maltophilia]

Length=267

Score = 244 bits (624), Expect = 4e-77, Method: Compositional matrix adjust.

| Identities | = 132/255 (52%), Positives = 165/255 (65%), Gaps = 3/255 (1%) |  |
| --- | --- | --- |
| Query 12 | PAAWVERLFGYDWAQQTIGCSDAAVFRLSAQGRPVLFVKTDLSGALNELQDEAARLSWLA | 71 |
| Sbjct 14 | P AW E L +Q+IG S A V R+ G+ F+K+++ A +EL DE ARL WL PWAWQEALADARIERQSIGVSRADVARVHRPGQADAFLKSEVIDAFSELGDEIARLRWLQ | 73 |
| Query 72 | TTGVPCAAVLDVVTEAGRDWLLLGEVPGQDLLSS-HLAPAEKVSIMADAMRRLHTLDPAT | 130 |
| Sbjct 74 | G P V+ EAGR WLL+ +PG+DL SS LAP + V ++ADA+R LH L A AQGQPVPTVIATAEEAGRRWLLMSALPGRDLASSPELAPQQLVELLADALRGLHALPVAA | 133 |
| Query 131 | CPFDHQAKHRIERARTRMEAGLVDQDDLDEEHQGLAPAELFARLKARMPDGEDLVVTHGD | 190 |
| Sbjct 134 | CPFD + R++ A+ R+EAGLVD DD D+E G +P ++FA L + PD EDLVV+HGD CPFDQRLASRVQVAQARVEAGLVDADDFDDERLGQSPQQVFAELCSTRPDYEDLVVSHGD | 193 |
| Query 191 | ACLPNIMVENGRFSGFIDCGRLGVADRYQDIALATRDIAEELG-GEWADRFLVLYGIAAP | 249 |
| Sbjct 194 | ACLPN+M GRFSGFIDCGRLGVADRYQD+ALA R + G W YG A ACLPNLMAVEGRFSGFIDCGRLGVADRYQDLALAARSLVHNFGDTRWVAPLFQRYGAVA- | 252 |
| Query 250  Sbjct 253 | DSQRIAFYRLLDEFF 264 D +R+AFYRLLDEFF DERRLAFYRLLDEFF 267 |  |

>WP_128688214.1 APH(3')-II family aminoglycoside O-phosphotransferase, partial [Pseudomonas aeruginosa]

RPP20461.1 APH(3') family aminoglycoside O-phosphotransferase, partial [Pseudomonas aeruginosa]

Length=250

Score = 244 bits (622), Expect = 4e-77, Method: Compositional matrix adjust.

| Identities | = 126/239 (53%), Positives = 154/239 (64%), Gaps = 4/239 (2%) |  |
| --- | --- | --- |
| Query 11 | SPAAWVERLFGYDWAQQTIGCSDAAVFRLSAQGRPVLFVKTDLSGALNELQDEAARLSWL | 70 |
| Sbjct 12 | +P+ W + L GY W Q GCS A V RL RP LFVK ++ A EL E ARL WL APSTWADYLAGYRWRGQGEGCSAATVHRLEVARRPTLFVKQEVLSAHAELPAEIARLRWL | 71 |
| Query 71 | ATTGVPCAAVLDVVTEAGRDWLLLGEVPGQDLLS SHLAPAEKVSIMADAMRRLHTL | 126 |
| Sbjct 72 | G+ C VL+ GR WLL+ VPG L + L P V ++A A+RRLH L HGAGIDCPQVLNETQSDGRQWLLMSAVPGDTLSALAQRGELEPERLVRLVAAALRRLHDL | 131 |
| Query 127 | DPATCPFDHQAKHRIERARTRMEAGLVDQDDLDEEHQGLAPAELFARLKARMPDGEDLVV | 186 |
| Sbjct 132 | DPA CPFDH+ + R++ R R+EAGLVD+ D D++H+G + EL+ L R P EDLVV  DPAACPFDHRLERRLDTVRQRVEAGLVDEADFDDDHRGRSATELYRLLLDRRPAVEDLVV | 191 |

Query 187 THGDACLPNIMVENGRFSGFIDCGRLGVADRYQDIALATRDIAEELGGEWADRFLVLYG 245 HGDACLPN++ E RFSGFIDCGRLGVADR+QD+ALA RDI ELG WA+ FLV YG

Sbjct 192 AHGDACLPNLLAEGRRFSGFIDCGRLGVADRHQDLALAARDIEAELGAAWAEAFLVEYG 250

>WP_050984095.1 aminoglycoside 3'-phosphotransferase [Sinorhizobium fredii]

MQW94328.1 APH(3') family aminoglycoside O-phosphotransferase [Sinorhizobium fredii]

MQX10545.1 APH(3') family aminoglycoside O-phosphotransferase [Sinorhizobium fredii]

Length=253

Score = 244 bits (622), Expect = 4e-77, Method: Compositional matrix adjust.

| Identities | = 124/231 (54%), Positives = 154/231 (67%), Gaps = 0/231 (0%) |  |
| --- | --- | --- |
| Query 34 | AAVFRLSAQGRPVLFVKTDLSGALNELQDEAARLSWLATTGVPCAAVLDVVTEAGRDWLL | 93 |
| Sbjct 23 | +AVF L A GR L +K + +G EL DEAARL WLA G+ C V AGR WLL SAVFLLKADGRARLVLKHEEAGPFGELADEAARLGWLAGEGLLCPRVKAQEIHAGRHWLL | 82 |
| Query 94 | LGEVPGQDLLSSHLAPAEKVSIMADAMRRLHTLDPATCPFDHQAKHRIERARTRMEAGLV | 153 |
| Sbjct 83 | + V G DL SS APA +++++A+A+RRLH LDP CPFDH+ RI A+ R++AGLV MEAVEGVDLASSRQAPARRIAVLAEALRRLHRLDPTRCPFDHRLDSRIAAAKARLDAGLV | 142 |
| Query 154 | DQDDLDEEHQGLAPAELFARLKARMPDGEDLVVTHGDACLPNIMVENGRFSGFIDCGRLG D+ D DEE G LF ++AR P GE LVVTHGDACLPNI+ F+GFIDC R+G | 213 |

| Sbjct | 143 | DETDFDEERLGRTATTLFGEIQARRPKGERLVVTHGDACLPNIIAREDGFAGFIDCSRIG 202 |
| --- | --- | --- |
| Query | 214 | VADRYQDIALATRDIAEELGGEWADRFLVLYGIAAPDSQRIAFYRLLDEFF 264 |
| Sbjct | 203 | VAD +QDIALA R IA LG +W FL LYG+ PD +++ +Y LLDEFF  VADVHQDIALACRSIAYNLGQDWVRPFLELYGLPNPDPEKLIYYCLLDEFF 253 |

>WP_053442897.1 APH(3')-II family aminoglycoside O-phosphotransferase [Stenotrophomonas maltophilia]

ALA86376.1 aminoglycoside phosphotransferase [Stenotrophomonas maltophilia] ALA90332.1 aminoglycoside phosphotransferase [Stenotrophomonas maltophilia]

QHE20952.1 APH(3')-II family aminoglycoside O-phosphotransferase [Stenotrophomonas maltophilia]

VUK66163.1 Aminoglycoside 3'-phosphotransferase [Stenotrophomonas maltophilia] VUQ48580.1 Aminoglycoside 3'-phosphotransferase [Stenotrophomonas maltophilia]

Length=267

Score = 244 bits (623), Expect = 5e-77, Method: Compositional matrix adjust.

| Identities | = 132/255 (52%), Positives = 166/255 (65%), Gaps = 3/255 (1%) |  |
| --- | --- | --- |
| Query 12 | PAAWVERLFGYDWAQQTIGCSDAAVFRLSAQGRPVLFVKTDLSGALNELQDEAARLSWLA | 71 |
| Sbjct 14 | P AW E L +Q+IG S A V R+ G+ F+K+++ A +EL DE ARL WL PRAWQEALADARIERQSIGVSRADVARVHRPGQADAFLKSEVIDAFSELGDEIARLRWLQ | 73 |
| Query 72 | TTGVPCAAVLDVVTEAGRDWLLLGEVPGQDLLSS-HLAPAEKVSIMADAMRRLHTLDPAT | 130 |
| Sbjct 74 | GVP V+ EAGR WLL+ +PG+DL SS LAP + V ++ADA+RRLH L A AQGVPAPTVIATAEEAGRRWLLMSALPGRDLASSPELAPQQLVELLADALRRLHVLPVAA | 133 |
| Query 131 | CPFDHQAKHRIERARTRMEAGLVDQDDLDEEHQGLAPAELFARLKARMPDGEDLVVTHGD | 190 |
| Sbjct 134 | CPFD + R++ A+ R+EAGLVD DD D+E G + ++FA L + P+ EDLVV+HGD CPFDQRLASRLQAAQARVEAGLVDADDFDDERLGQSAQQVFAELCSTRPEHEDLVVSHGD | 193 |
| Query 191 | ACLPNIMVENGRFSGFIDCGRLGVADRYQDIALATRDIAEELG-GEWADRFLVLYGIAAP | 249 |
| Sbjct 194 | ACLPN+M GRFSGFIDCGRLGVADRYQD+ALA R + G W YG A ACLPNLMAVEGRFSGFIDCGRLGVADRYQDLALAARSLVHNFGDTRWVAPLFQRYG-ALV | 252 |
| Query 250  Sbjct 253 | DSQRIAFYRLLDEFF 264 D +R+AFYRLLDEFF DERRLAFYRLLDEFF 267 |  |

>NYB77909.1 APH(3')-II family aminoglycoside O-phosphotransferase [Stenotrophomonas maltophilia]

Length=267

Score = 244 bits (623), Expect = 5e-77, Method: Compositional matrix adjust.

| Identities | = 132/255 (52%), Positives = 165/255 (65%), Gaps = 3/255 (1%) |  |
| --- | --- | --- |
| Query 12 | PAAWVERLFGYDWAQQTIGCSDAAVFRLSAQGRPVLFVKTDLSGALNELQDEAARLSWLA | 71 |
| Sbjct 14 | P AW E L +Q+IG S A V R+ G+ FVK+++ A +EL DE ARL WL PWAWQEALADARIERQSIGVSRADVARVQRPGQADAFVKSEVIDAFSELGDEIARLRWLQ | 73 |
| Query 72 | TTGVPCAAVLDVVTEAGRDWLLLGEVPGQDLLSS-HLAPAEKVSIMADAMRRLHTLDPAT | 130 |
| Sbjct 74 | G P V+ EAGR WLL+ +PG+DL SS LAP + V ++ADA+R LH L A EQGQPAPTVIATAEEAGRRWLLMSALPGRDLASSPELAPQQLVELLADALRGLHALPLAA | 133 |
| Query 131 | CPFDHQAKHRIERARTRMEAGLVDQDDLDEEHQGLAPAELFARLKARMPDGEDLVVTHGD | 190 |
| Sbjct 134 | CPFD + R++ A+ R+EAGLVD DD D+E G +P ++FA L + PD EDLVV+HGD CPFDQRLASRLQAAQARVEAGLVDADDFDDERLGQSPQQVFAELCSTRPDHEDLVVSHGD | 193 |
| Query 191 | ACLPNIMVENGRFSGFIDCGRLGVADRYQDIALATRDIAEELG-GEWADRFLVLYGIAAP | 249 |
| Sbjct 194 | ACLPN+MV GRFSGFI+CGRLGVADRYQD+ALA R + G W YG A ACLPNLMVVEGRFSGFINCGRLGVADRYQDLALAARSLVHNFGDTRWVAPLFQRYGAVA- | 252 |
| Query 250  Sbjct 253 | DSQRIAFYRLLDEFF 264  + R+AFYRLLDEFF  NEHRLAFYRLLDEFF 267 |  |

>WP_049421316.1 APH(3')-II family aminoglycoside O-phosphotransferase [Stenotrophomonas maltophilia]

Length=267

Score = 244 bits (623), Expect = 5e-77, Method: Compositional matrix adjust.

| Identities | = 131/255 (51%), Positives = 165/255 (65%), Gaps = 3/255 (1%) |  |
| --- | --- | --- |
| Query 12 | PAAWVERLFGYDWAQQTIGCSDAAVFRLSAQGRPVLFVKTDLSGALNELQDEAARLSWLA | 71 |
| Sbjct 14 | P AW E L +Q+IG S A V R+ G+ F+K+++ A +EL DE ARL WL PWAWQEALADARIERQSIGVSRADVARVHRPGQADAFLKSEVIDAFSELGDEIARLRWLQ | 73 |
| Query 72 | TTGVPCAAVLDVVTEAGRDWLLLGEVPGQDLLSS-HLAPAEKVSIMADAMRRLHTLDPAT | 130 |
| Sbjct 74 | G P V+ EAGR WLL+ +PG+DL SS LAP + V ++ADA+R LH L A AQGQPAPTVIATAEEAGRRWLLMSALPGRDLASSPELAPQQLVELLADALRGLHALPVAA | 133 |
| Query 131 | CPFDHQAKHRIERARTRMEAGLVDQDDLDEEHQGLAPAELFARLKARMPDGEDLVVTHGD | 190 |
| Sbjct 134 | CPFD + R++ A+ R+EAGLVD DD D+E G +P ++FA L + PD EDLVV+HGD CPFDQRLASRVQVAQARIEAGLVDADDFDDERLGQSPQQVFAELCSTQPDHEDLVVSHGD | 193 |
| Query 191 | ACLPNIMVENGRFSGFIDCGRLGVADRYQDIALATRDIAEEL-GGEWADRFLVLYGIAAP | 249 |
| Sbjct 194 | ACLPN+M GRFSGFIDCGRLGVADRYQD+ALA R + W YG+ A ACLPNLMAVEGRFSGFIDCGRLGVADRYQDLALAARSLVYNFDNTRWVAPLFKRYGMVA- | 252 |
| Query 250  Sbjct 253 | DSQRIAFYRLLDEFF 264 D +R+AFYRLLDEFF DERRLAFYRLLDEFF 267 |  |

>WP_154993524.1 aminoglycoside 3'-phosphotransferase [Pseudomonas fluorescens] VVO76895.1 Aminoglycoside 3'-phosphotransferase [Pseudomonas fluorescens]

Length=256

Score = 244 bits (622), Expect = 5e-77, Method: Compositional matrix adjust.

| Identities | | = 124/254 (49%), Positives = 161/254 (63%), Gaps = 2/254 (1%) |  |
| --- | --- | --- | --- |
| Query 12  Sbjct 4 | | PAAWVERLFGYDWAQQTIGCSDAAVFRLSAQGRPVLFVKTDLSGALNELQDEAARLSWLA PA W + G +QTIG S A FR+ LFVK++ GA +EL E RL W+ PARWSQTFSGAPIERQTIGESGAEAFRIRCINGKHLFVKSEPIGAHSELPQEIERLRWMN | 71  63 |
| Query 72  Sbjct 64 | | TTGVPCAAVLDVVTEAGRDWLLLGEVPGQDLLSSH-LAPAEKVSIMADAMRRLHTLDPAT  +P VLDV TE R WLL+ V GQDL S++ L+P + +S++A A+R LH + T RLDLPGPTVLDVTTENNRHWLLMNAVSGQDLASANDLSPPQVISVLAMALRTLHQVPIET | 130  123 |
| Query 131  Sbjct 124 | | CPFDHQAKHRIERARTRMEAGLVDQDDLDEEHQGLAPAELFARLKARMPDGEDLVVTHGD CPFDH + RI AR+R+ AGLVD+ D D+E QG ++F + + +P+ DL VTHGD CPFDHSLEQRIAAARSRVSAGLVDEADFDDERQGRTAEDVFTEMLSTLPETYDLAVTHGD | 190  183 |
| Query 191  Sbjct 184 | | ACLPNIMVENGRFSGFIDCGRLGVADRYQDIALATRDIAEELGGEWADRFLVLYGIAAPD ACLPN MVE F+G+IDCGRLG++D YQD+ALA R I LG W F +YG+ PD ACLPNFMVEGSSFTGYIDCGRLGISDIYQDLALAARSIERNLGPAWVAPFFSIYGV-EPD | 250  242 |
| Query | 251 SQRIAFYRLLDEFF 264 | | |
|  |  | QRIAFY LLDEF+ |  |
| Sbjct | 243 | QQRIAFYCLLDEFY 256 | |

>WP_088025762.1 APH(3')-II family aminoglycoside O-phosphotransferase [Stenotrophomonas maltophilia]

ARQ89721.1 APH(3') family aminoglycoside O-phosphotransferase [Stenotrophomonas maltophilia]

Length=267

Score = 244 bits (623), Expect = 5e-77, Method: Compositional matrix adjust.

| Identities | = 130/255 (51%), Positives = 164/255 (64%), Gaps = 3/255 (1%) |  |
| --- | --- | --- |
| Query 12 | PAAWVERLFGYDWAQQTIGCSDAAVFRLSAQGRPVLFVKTDLSGALNELQDEAARLSWLA | 71 |
| Sbjct 14 | P AW E L +Q+IG S A V R+ G+ F+K+++ +EL DE ARL WL PCAWQEALADARIERQSIGVSRADVARVHRPGQADAFLKSEVIDTFSELGDEIARLRWLQ | 73 |
| Query 72 | TTGVPCAAVLDVVTEAGRDWLLLGEVPGQDLLSS-HLAPAEKVSIMADAMRRLHTLDPAT | 130 |
| Sbjct 74 | G P V+ EAGR WLL+ +PG+DL SS LAP + V ++AD +R LH L A AQGQPVPTVIATAEEAGRRWLLMSALPGRDLASSPELAPQQLVELLADVLRGLHALPVAA | 133 |
| Query 131 | CPFDHQAKHRIERARTRMEAGLVDQDDLDEEHQGLAPAELFARLKARMPDGEDLVVTHGD CPFD + R++ A+ R+EAGL+D DD D+E G +P ++FA L + PD EDLVV+HGD | 190 |

| Sbjct | 134 | CPFDQRLASRVQAAQARVEAGLMDADDFDDERLGQSPQQVFAELCSTRPDHEDLVVSHGD | 193 |
| --- | --- | --- | --- |
| Query | 191 | ACLPNIMVENGRFSGFIDCGRLGVADRYQDIALATRDIAEELG-GEWADRFLVLYGIAAP | 249 |
| Sbjct | 194 | ACLPN+MV GRFSGFIDCGRLGVADRYQD+ALA R + G W YG A ACLPNLMVAEGRFSGFIDCGRLGVADRYQDLALAARSLVHNFGDTRWVAPLFQHYGAVA- | 252 |
| Query  Sbjct | 250  253 | DSQRIAFYRLLDEFF 264 D +R+AFYRLLDEFF DERRLAFYRLLDEFF 267 |  |

>WP_092711567.1 aminoglycoside 3'-phosphotransferase [Rhizobium multihospitium] SCB25660.1 aminoglycoside 3'-phosphotransferase-2 [Rhizobium multihospitium]

Length=266

Score = 244 bits (623), Expect = 5e-77, Method: Compositional matrix adjust.

| Identities | = 124/247 (50%), Positives = 164/247 (66%), Gaps = 1/247 (0%) |  |
| --- | --- | --- |
| Query 19 | LFGYDWAQQTIGCSDAAVFRLSAQGRPVLFVKTDLSGALNELQDEAARLSWLATTGVPCA | 78 |
| Sbjct 20 | L GY+W Q +G S A VFRL A GR +++KT+L L EL E ARL WLA G+ C LAGYEWRQDALGRSSAHVFRLEADGRAPVYLKTELVDPLGELAGEVARLRWLALQGLACP | 79 |
| Query 79 | AVLDVVTEAGRDWLLLGEVPGQDLLSSH-LAPAEKVSIMADAMRRLHTLDPATCPFDHQA | 137 |
| Sbjct 80 | V+ + R+WLL+ +PG DL+S+ L P ++V ++A A+RRLH++D A CPFDH+ EVIAHEKDGEREWLLMSALPGSDLVSAEWLQPFDRVRLLASALRRLHSVDIAACPFDHRL | 139 |
| Query 138 | KHRIERARTRMEAGLVDQDDLDEEHQGLAPAELFARLKARMPDGEDLVVTHGDACLPNIM | 197 |
| Sbjct 140 | ++RI AR RM AG VD+DD D+ G + +LF L+ EDLVVTHGDACLPN M ENRIAEARARMLAGRVDEDDFDDTRLGRSAEDLFRELEETRSSMEDLVVTHGDACLPNFM | 199 |
| Query 198 | VENGRFSGFIDCGRLGVADRYQDIALATRDIAEELGGEWADRFLVLYGIAAPDSQRIAFY | 257 |
| Sbjct 200 | + +FSG+IDCGRLGVADR+QDIALA I G E FL +YG P ++++A+Y ADGPKFSGYIDCGRLGVADRHQDIALACGSIEYNFGEELVAEFLKVYGDFEPQAEKMAYY | 259 |
| Query 258  Sbjct 260 | RLLDEFF 264  +LLDEFF  QLLDEFF 266 |  |

>WP_183912555.1 APH(3') family aminoglycoside O-phosphotransferase [Rhizobium lentis]

MBB4572248.1 aminoglycoside 3'-phosphotransferase-2 [Rhizobium lentis] MBB5548561.1 aminoglycoside 3'-phosphotransferase-2 [Rhizobium lentis] MBB5559091.1 aminoglycoside 3'-phosphotransferase-2 [Rhizobium lentis] MBB5565386.1 aminoglycoside 3'-phosphotransferase-2 [Rhizobium lentis]

Length=261

Score = 244 bits (622), Expect = 5e-77, Method: Compositional matrix adjust.

| Identities | | = 130/254 (51%), Positives = 165/254 (65%), Gaps = 1/254 (0%) |  |
| --- | --- | --- | --- |
| Query 12  Sbjct 8 | | PAAWVERLFGYDWAQQTIGCSDAAVFRLSAQGRPVLFVKTDLSGALNELQDEAARLSWLA PAA ERL GY + + +G S A+VFRL + P L++K + +G EL DEAARL WL PAALGERLSGYRFERDALGRSSASVFRLEGECLPPLYLKVEAAGPFGELADEAARLGWLK | 71  67 |
| Query 72  Sbjct 68 | | TTGVPCAAVLDVVTEAGRDWLLLGEVPGQDLLS-SHLAPAEKVSIMADAMRRLHTLDPAT G+PC V+ ++ R+WLL+G +PG DL S S L P +V ++A A+ LH L + AAGLPCPDVIARESDGERNWLLIGALPGTDLASASALTPLARVGLLAAALADLHHLPIVS | 130  127 |
| Query 131  Sbjct 128 | | CPFDHQAKHRIERARTRMEAGLVDQDDLDEEHQGLAPAELFARLKARMPDGEDLVVTHGD CPFDH+ + R+ AR RM+AG+VD+ D DE G LFA L+ R PD E LVVTHGD CPFDHRLEKRLPAARARMQAGIVDETDFDETRLGKGAETLFAELERRRPDREHLVVTHGD | 190  187 |
| Query 191  Sbjct 188 | | ACLPNIMVENGRFSGFIDCGRLGVADRYQDIALATRDIAEELGGEWADRFLVLYGIAAPD ACLPN + G+FSG+IDC RLGVADRYQDIALA R IA G FL YG+ APD ACLPNFVASGGQFSGYIDCSRLGVADRYQDIALACRSIAGNFGAALVQPFLDRYGLTAPD | 250  247 |
| Query 251 | | SQRIAFYRLLDEFF 264 R+ +Y+LLDEFF |  |
| Sbjct | 248 | PARLDYYQLLDEFF 261 | |

>WP_134619153.1 APH(3')-II family aminoglycoside O-phosphotransferase, partial

[Pseudomonas aeruginosa] Length=250

Score = 243 bits (621), Expect = 6e-77, Method: Compositional matrix adjust.

| Identities | = 127/239 (53%), Positives = 154/239 (64%), Gaps = 4/239 (2%) |  |
| --- | --- | --- |
| Query 11 | SPAAWVERLFGYDWAQQTIGCSDAAVFRLSAQGRPVLFVKTDLSGALNELQDEAARLSWL | 70 |
| Sbjct 12 | +PA W + L GY W Q GCS A V RL A RP LFVK ++ A EL E ARL WL APATWADYLAGYRWRGQGEGCSAATVHRLEAARRPTLFVKQEVLSAHAELPAEIARLRWL | 71 |
| Query 71 | ATTGVPCAAVLDVVTEAGRDWLLLGEVPGQDLLS SHLAPAEKVSIMADAMRRLHTL | 126 |
| Sbjct 72 | G+ C VL+ GR WLL+ VPG L + L P V ++A A+RRLH L HGAGIDCPQVLNETQSDGRQWLLMSAVPGDTLSALAQRGELEPERLVRLVAAALRRLHDL | 131 |
| Query 127 | DPATCPFDHQAKHRIERARTRMEAGLVDQDDLDEEHQGLAPAELFARLKARMPDGEDLVV | 186 |
| Sbjct 132 | DPA CPFDH+ + R++ R R+EAGLVD+ D D++H+G + EL+ L R P EDLVV  DPAACPFDHRLERRLDTVRQRVEAGLVDEADFDDDHRGRSATELYRLLLDRRPAVEDLVV | 191 |

Query 187 THGDACLPNIMVENGRFSGFIDCGRLGVADRYQDIALATRDIAEELGGEWADRFLVLYG 245 HGDACLPN++ E RFSGFIDCGRLGVADR+QD+ALA RDI ELG W + FLV YG

Sbjct 192 AHGDACLPNLLAEGRRFSGFIDCGRLGVADRHQDLALAARDIEAELGAAWDEAFLVGYG 250

>WP_096772015.1 aminoglycoside 3'-phosphotransferase [Rhizobium sophoriradicis]

PCK83646.1 APH(3') family aminoglycoside O-phosphotransferase [Rhizobium sophoriradicis]

Length=261

Score = 244 bits (622), Expect = 6e-77, Method: Compositional matrix adjust.

| Identities | | = 128/254 (50%), Positives = 167/254 (66%), Gaps = 1/254 (0%) |  |
| --- | --- | --- | --- |
| Query 12  Sbjct 8 | | PAAWVERLFGYDWAQQTIGCSDAAVFRLSAQGRPVLFVKTDLSGALNELQDEAARLSWLA PAA ERL G + + +G S A+VFRL +G P L++K + +G EL DEAARL WL PAALGERLSGCRFERDALGRSAASVFRLEGEGLPALYLKVEAAGPFGELADEAARLGWLK | 71  67 |
| Query 72  Sbjct 68 | | TTGVPCAAVLDVVTEAGRDWLLLGEVPGQDLLS-SHLAPAEKVSIMADAMRRLHTLDPAT G+PC V+ ++ R+WLL+G +PG DL S S L P +V ++A A+ LH L + AAGLPCPDVIARESDGERNWLLIGALPGTDLASASALTPLARVELLAAALADLHDLPITS | 130  127 |
| Query 131  Sbjct 128 | | CPFDHQAKHRIERARTRMEAGLVDQDDLDEEHQGLAPAELFARLKARMPDGEDLVVTHGD CPFDH+ + R+ A RM+AG+VD+ D DE G + LFA L+ R PD EDLVVTHGD CPFDHRLEKRLPAAGARMQAGIVDETDFDETRLGKSAETLFAELEGRRPDREDLVVTHGD | 190  187 |
| Query 191  Sbjct 188 | | ACLPNIMVENGRFSGFIDCGRLGVADRYQDIALATRDIAEELGGEWADRFLVLYGIAAPD ACLPN + G+FSG+IDC RLG+ADRYQD+ALA R IA+ G FL YG+ APD ACLPNFVASGGQFSGYIDCSRLGIADRYQDLALACRSIADNFGAALVQPFLDRYGLTAPD | 250  247 |
| Query 251 | | SQRIAFYRLLDEFF 264 R+ +Y+LLDEFF |  |
| Sbjct | 248 | PARLDYYQLLDEFF 261 | |

>WP_171952084.1 APH(3')-II family aminoglycoside O-phosphotransferase [Stenotrophomonas maltophilia]

Length=267

Score = 244 bits (622), Expect = 6e-77, Method: Compositional matrix adjust.

| Identities | = 132/255 (52%), Positives = 164/255 (64%), Gaps = 3/255 (1%) |  |
| --- | --- | --- |
| Query 12 | PAAWVERLFGYDWAQQTIGCSDAAVFRLSAQGRPVLFVKTDLSGALNELQDEAARLSWLA | 71 |
| Sbjct 14 | P AW E L +Q+IG S A V R+ G+ F+K+++ A +EL DE ARL WL PRAWQEALADAHIERQSIGVSRADVARVHLPGQADAFLKSEVIDAFSELGDEIARLRWLQ | 73 |
| Query 72 | TTGVPCAAVLDVVTEAGRDWLLLGEVPGQDLLSS-HLAPAEKVSIMADAMRRLHTLDPAT | 130 |
| Sbjct 74 | G+P V+ EAGR WLL+ +PG+DL SS LAP + V ++ADA+R LH L A AQGLPAPTVIATAEEAGRRWLLMSALPGRDLASSPELAPQQLVELLADALRGLHALPVAA | 133 |
| Query 131 | CPFDHQAKHRIERARTRMEAGLVDQDDLDEEHQGLAPAELFARLKARMPDGEDLVVTHGD | 190 |
| Sbjct 134 | CPFD + R+E A+ R+EA LVD DD D+E G +P ++FA L PD EDLVV+HGD CPFDQRLASRLEAAQARVEASLVDADDFDDERLGQSPQQVFAELCNTRPDHEDLVVSHGD | 193 |
| Query 191 | ACLPNIMVENGRFSGFIDCGRLGVADRYQDIALATRDIAEELG-GEWADRFLVLYGIAAP | 249 |

ACLPN+M	GRFSGFIDCGRLGVADRYQD+ALA R +	G	W	YG A

| Sbjct | 194 | ACLPNLMAVEGRFSGFIDCGRLGVADRYQDLALAARSLVHNFGDTRWVAPLFQRYGAVA- 252 |
| --- | --- | --- |
| Query | 250 | DSQRIAFYRLLDEFF 264 |
| Sbjct | 253 | D +R+AFYRLLDEFF  DERRLAFYRLLDEFF 267 |

>WP_164089385.1 APH(3')-II family aminoglycoside O-phosphotransferase [Stenotrophomonas maltophilia]

MBA0276492.1 APH(3')-II family aminoglycoside O-phosphotransferase [Stenotrophomonas maltophilia]

MBA0412757.1 APH(3')-II family aminoglycoside O-phosphotransferase [Stenotrophomonas maltophilia]

MBA0496176.1 APH(3')-II family aminoglycoside O-phosphotransferase [Stenotrophomonas maltophilia]

MBA0500832.1 APH(3')-II family aminoglycoside O-phosphotransferase [Stenotrophomonas maltophilia]

MBA0506217.1 APH(3')-II family aminoglycoside O-phosphotransferase [Stenotrophomonas maltophilia]

Length=267

Score = 244 bits (622), Expect = 6e-77, Method: Compositional matrix adjust.

| Identities | = 130/255 (51%), Positives = 165/255 (65%), Gaps = 3/255 (1%) |  |
| --- | --- | --- |
| Query 12 | PAAWVERLFGYDWAQQTIGCSDAAVFRLSAQGRPVLFVKTDLSGALNELQDEAARLSWLA | 71 |
| Sbjct 14 | P AW + L +Q+IG S A V R+ G+ F+K+++ A +EL DE RL WL PWAWQDALADARIERQSIGVSRADVARVRRPGQADAFLKSEVIDAFSELGDEIVRLRWLQ | 73 |
| Query 72 | TTGVPCAAVLDVVTEAGRDWLLLGEVPGQDLLSS-HLAPAEKVSIMADAMRRLHTLDPAT | 130 |
| Sbjct 74 | G P V+ EAGR WLL+ +PG DL SS LAP + V ++ADA+R LH L A AQGQPAPTVIATAEEAGRRWLLMSALPGHDLASSPELAPQQLVELLADALRGLHALPVAA | 133 |
| Query 131 | CPFDHQAKHRIERARTRMEAGLVDQDDLDEEHQGLAPAELFARLKARMPDGEDLVVTHGD | 190 |
| Sbjct 134 | CPFD + R++ A+ R+EAGLVD DD D+E G +P ++FA L + PD EDLVV+HGD CPFDQRLASRVQVAQARVEAGLVDVDDFDDERLGQSPQQVFAELCSTRPDHEDLVVSHGD | 193 |
| Query 191 | ACLPNIMVENGRFSGFIDCGRLGVADRYQDIALATRDIAEELG-GEWADRFLVLYGIAAP | 249 |
| Sbjct 194 | ACLPN+MV GRFSGFIDCGRLGVADRYQD+ALA R + G W YG+ A ACLPNLMVVEGRFSGFIDCGRLGVADRYQDLALAARSLVHNFGDTRWVAPLFQRYGVVA- | 252 |
| Query 250  Sbjct 253 | DSQRIAFYRLLDEFF 264 D +R+AFYR+LDEFF DERRLAFYRMLDEFF 267 |  |

>WP_099559461.1 APH(3')-II family aminoglycoside O-phosphotransferase [Stenotrophomonas maltophilia]

Length=269

Score = 244 bits (623), Expect = 6e-77, Method: Compositional matrix adjust.

| Identities | | = 134/262 (51%), Positives = 167/262 (64%), Gaps = 3/262 (1%) |  |
| --- | --- | --- | --- |
| Query 5  Sbjct 9 | | DGLHAGSPAAWVERLFGYDWAQQTIGCSDAAVFRLSAQGRPVLFVKTDLSGALNELQDEA DGL P AW E L +Q+IG S A V R+ G+ F+K+++ A +EL DE DGLPLPLPWAWQEALADAHIERQSIGVSRADVARVHRPGQADAFLKSEVIDAFSELGDEI | 64  68 |
| Query 65  Sbjct 69 | | ARLSWLATTGVPCAAVLDVVTEAGRDWLLLGEVPGQDLLSS-HLAPAEKVSIMADAMRRL RL WL G+P V+ EAGR WLL+ +PG+DL SS LAP + V ++ADA+RRL TRLRWLQAQGLPAPTVIATAEEAGRRWLLMSALPGRDLASSPELAPQQLVELLADALRRL | 123  128 |
| Query 124  Sbjct 129 | | HTLDPATCPFDHQAKHRIERARTRMEAGLVDQDDLDEEHQGLAPAELFARLKARMPDGED H L A CPFD + R++ A R+EAGLVD DD D+E G + ++FA L + PD ED HVLPVAACPFDQRLASRLQAAAARVEAGLVDADDFDDERLGQSAQQVFAELCSTRPDHED | 183  188 |
| Query 184  Sbjct 189 | | LVVTHGDACLPNIMVENGRFSGFIDCGRLGVADRYQDIALATRDIAEELG-GEWADRFLV LVV+HGDACLPN+M GRFSGFIDCGRLGVADRYQD+ALA R + G W LVVSHGDACLPNLMAVEGRFSGFIDCGRLGVADRYQDLALAARSLVHNFGDTRWVAPLFQ | 242  248 |
| Query 243 | | LYGIAAPDSQRIAFYRLLDEFF 264 YG A D +R+AFYRLLDEFF |  |
| Sbjct | 249 | RYG-ALVDERRLAFYRLLDEFF 269 | |

>WP_168269291.1 APH(3') family aminoglycoside O-phosphotransferase [Rhizobium leguminosarum]

NKK68654.1 APH(3') family aminoglycoside O-phosphotransferase [Rhizobium leguminosarum bv. viciae]

NKL06620.1 APH(3') family aminoglycoside O-phosphotransferase [Rhizobium leguminosarum bv. viciae]

NKL87454.1 APH(3') family aminoglycoside O-phosphotransferase [Rhizobium leguminosarum bv. viciae]

NKL92967.1 APH(3') family aminoglycoside O-phosphotransferase [Rhizobium leguminosarum bv. viciae]

NKM96287.1 APH(3') family aminoglycoside O-phosphotransferase [Rhizobium leguminosarum bv. viciae]

Length=265

Score = 244 bits (622), Expect = 6e-77, Method: Compositional matrix adjust.

| Identities | = 128/255 (50%), Positives = 167/255 (65%), Gaps = 1/255 (0%) |  |
| --- | --- | --- |
| Query 11 | SPAAWVERLFGYDWAQQTIGCSDAAVFRLSAQGRPVLFVKTDLSGALNELQDEAARLSWL | 70 |
| Sbjct 11 | S +A RL GY + + +G S A+VFRL A G P L++K + +GA EL DEAARL WL SASALDARLAGYRFERDALGRSAASVFRLEALGLPTLYLKVEEAGAFGELADEAARLCWL | 70 |
| Query 71 | ATTGVPCAAVLDVVTEAGRDWLLLGEVPGQDLLS-SHLAPAEKVSIMADAMRRLHTLDPA | 129 |
| Sbjct 71 | +G+PC ++ ++ R+ LL+ +PG DL S S L P +V ++A A+ LH L A KASGLPCPDIIAEDSDGVRNRLLINALPGSDLASASVLTPLARVEMLAAALHDLHRLPIA | 130 |
| Query 130 | TCPFDHQAKHRIERARTRMEAGLVDQDDLDEEHQGLAPAELFARLKARMPDGEDLVVTHG | 189 |
| Sbjct 131 | +CPFDH+ + RI A+ RM+AG+VD+ D DE G +P LF L++ PD +DLVVTHG SCPFDHRLEARIAAAKARMQAGIVDETDFDETRLGKSPEALFDDLESGRPDRQDLVVTHG | 190 |
| Query 190 | DACLPNIMVENGRFSGFIDCGRLGVADRYQDIALATRDIAEELGGEWADRFLVLYGIAAP | 249 |
| Sbjct 191 | DACLPN +V FSG+IDC RLGVADRYQDIALA R IA G FL YG+ A DACLPNFVVSEDGFSGYIDCSRLGVADRYQDIALACRSIAHNFGEALVQPFLDRYGMPAT | 250 |
| Query 250  Sbjct 251 | DSQRIAFYRLLDEFF 264 D R+A+Y+LLDEFF DPARLAYYQLLDEFF 265 |  |

>WP_180874612.1 APH(3')-II family aminoglycoside O-phosphotransferase [Stenotrophomonas maltophilia]

MBA0376087.1 APH(3')-II family aminoglycoside O-phosphotransferase [Stenotrophomonas maltophilia]

MBA0546234.1 APH(3')-II family aminoglycoside O-phosphotransferase [Stenotrophomonas maltophilia]

VUO17474.1 Aminoglycoside 3'-phosphotransferase [Stenotrophomonas maltophilia] Length=267

Score = 244 bits (622), Expect = 6e-77, Method: Compositional matrix adjust.

| Identities | = 133/255 (52%), Positives = 165/255 (65%), Gaps = 3/255 (1%) |  |
| --- | --- | --- |
| Query 12 | PAAWVERLFGYDWAQQTIGCSDAAVFRLSAQGRPVLFVKTDLSGALNELQDEAARLSWLA | 71 |
| Sbjct 14 | P AW E L +Q+IG S A V R+ G+ FVK+++ A +EL DE ARL WL PWAWQETLADARIERQSIGVSRADVARVQRPGQADAFVKSEVIDAFSELGDEIARLRWLQ | 73 |
| Query 72 | TTGVPCAAVLDVVTEAGRDWLLLGEVPGQDLLSS-HLAPAEKVSIMADAMRRLHTLDPAT | 130 |
| Sbjct 74 | G P V+ EA R WLL+ +PG+DL SS LAP + V ++ADA+R LH L A EQGQPVPTVIATAEEARRRWLLMSALPGRDLASSPELAPQQLVEMLADALRGLHALPLAA | 133 |
| Query 131 | CPFDHQAKHRIERARTRMEAGLVDQDDLDEEHQGLAPAELFARLKARMPDGEDLVVTHGD | 190 |
| Sbjct 134 | CPFD + R++ A+ R+EAGLVD DD D+E G +P ++FA L + PD EDLVV+HGD CPFDQRLASRLQAAQARVEAGLVDADDFDDERLGQSPQQVFAELCSTRPDHEDLVVSHGD | 193 |
| Query 191 | ACLPNIMVENGRFSGFIDCGRLGVADRYQDIALATRDIAEELG-GEWADRFLVLYGIAAP | 249 |
| Sbjct 194 | ACLPN+MV GRFSGFIDCGRLGVADRYQD+ALA R + G W YG A ACLPNLMVVEGRFSGFIDCGRLGVADRYQDLALAARSLVHNFGDTRWVAPLFQRYGAVA- | 252 |
| Query 250  Sbjct 253 | DSQRIAFYRLLDEFF 264 D +R+AFYRLLDEFF DERRLAFYRLLDEFF 267 |  |

>VUN14167.1 Aminoglycoside 3'-phosphotransferase [Stenotrophomonas maltophilia] VUN14704.1 Aminoglycoside 3'-phosphotransferase [Stenotrophomonas maltophilia] VUO94675.1 Aminoglycoside 3'-phosphotransferase [Stenotrophomonas maltophilia]

Length=282

Score = 244 bits (623), Expect = 6e-77, Method: Compositional matrix adjust.

| Identities | = 132/255 (52%), Positives = 164/255 (64%), Gaps = 3/255 (1%) |  |
| --- | --- | --- |
| Query 12 | PAAWVERLFGYDWAQQTIGCSDAAVFRLSAQGRPVLFVKTDLSGALNELQDEAARLSWLA | 71 |
| Sbjct 29 | P AW E L +Q+IG S A V R+ G+ F+K+++ A +EL DE ARL WL PHAWQEALADARIERQSIGVSRADVARVHRPGQADAFLKSEVIDAFSELGDEIARLRWLQ | 88 |
| Query 72 | TTGVPCAAVLDVVTEAGRDWLLLGEVPGQDLLSS-HLAPAEKVSIMADAMRRLHTLDPAT | 130 |
| Sbjct 89 | G P V+ EAGR WLL+ +PG DL SS LAP V ++ADA+R LH L AT VQGQPAPTVIATAEEAGRRWLLMSALPGHDLASSPALAPQHLVELLADALRGLHALPVAT | 148 |
| Query 131 | CPFDHQAKHRIERARTRMEAGLVDQDDLDEEHQGLAPAELFARLKARMPDGEDLVVTHGD | 190 |
| Sbjct 149 | CPFD + R++ A+ R+EAGLVD DD D+E G + ++FA L+A PD EDLVV HGD CPFDQRLASRLQAAQARVEAGLVDADDFDDERLGQSAQQVFAELRATRPDHEDLVVCHGD | 208 |
| Query 191 | ACLPNIMVENGRFSGFIDCGRLGVADRYQDIALATRDIAEELG-GEWADRFLVLYGIAAP | 249 |
| Sbjct 209 | ACLPN++ GRFSGFIDCGRLGVADRYQD+ALA R + G W YG+ A ACLPNLLAAEGRFSGFIDCGRLGVADRYQDLALAARSLVHNFGDTRWVAPLFRRYGVEA- | 267 |
| Query 250  Sbjct 268 | DSQRIAFYRLLDEFF 264 D +R+AFYRLLDEFF DERRLAFYRLLDEFF 282 |  |

>VUM57579.1 Aminoglycoside 3'-phosphotransferase [Stenotrophomonas maltophilia] Length=267

Score = 244 bits (622), Expect = 7e-77, Method: Compositional matrix adjust.

| Identities | = 131/255 (51%), Positives = 164/255 (64%), Gaps = 3/255 (1%) |  |
| --- | --- | --- |
| Query 12 | PAAWVERLFGYDWAQQTIGCSDAAVFRLSAQGRPVLFVKTDLSGALNELQDEAARLSWLA | 71 |
| Sbjct 14 | P AW E L +Q+IG S A V R+ G+ F+K+++ A +EL DE ARL WL PCAWQEALADARIERQSIGVSRADVARVHRPGQADAFLKSEVIDAFSELGDEIARLRWLQ | 73 |
| Query 72 | TTGVPCAAVLDVVTEAGRDWLLLGEVPGQDLLSS-HLAPAEKVSIMADAMRRLHTLDPAT | 130 |
| Sbjct 74 | G P V+ EAGR WLL+ +PG+DL SS LAP + V ++AD +R LH L A AQGQPVPTVIATAEEAGRRWLLMSALPGRDLASSPELAPQQLVELLADGLRGLHALPVAA | 133 |
| Query 131 | CPFDHQAKHRIERARTRMEAGLVDQDDLDEEHQGLAPAELFARLKARMPDGEDLVVTHGD | 190 |
| Sbjct 134 | CPFD + R++ A+ R+EAGLVD DD D+E G +P ++FA L + PD EDLVV+HGD CPFDQRLASRVQVAQARIEAGLVDADDFDDERLGQSPQQVFAELCSIQPDHEDLVVSHGD | 193 |
| Query 191 | ACLPNIMVENGRFSGFIDCGRLGVADRYQDIALATRDIAEELG-GEWADRFLVLYGIAAP | 249 |
| Sbjct 194 | ACLPN+M GRFSGFIDCGRLGVADRYQD+ALA R + G W YG A ACLPNLMAVEGRFSGFIDCGRLGVADRYQDLALAARSLVHNFGDTRWVAPLFQRYGAVA- | 252 |
| Query 250  Sbjct 253 | DSQRIAFYRLLDEFF 264 D +R+AFYRLLDEFF DERRLAFYRLLDEFF 267 |  |

>VUL71239.1 Aminoglycoside 3'-phosphotransferase [Stenotrophomonas maltophilia] Length=267

Score = 244 bits (622), Expect = 7e-77, Method: Compositional matrix adjust.

| Identities | = 132/255 (52%), Positives = 165/255 (65%), Gaps = 3/255 (1%) |  |
| --- | --- | --- |
| Query 12 | PAAWVERLFGYDWAQQTIGCSDAAVFRLSAQGRPVLFVKTDLSGALNELQDEAARLSWLA | 71 |
| Sbjct 14 | P AW + L +Q+IG S A V R+ G+ F+K++ A +EL DE ARL WL PWAWQDALADARIERQSIGVSRADVARVHRPGQTDAFLKSEAIDAFSELGDEIARLRWLQ | 73 |
| Query 72 | TTGVPCAAVLDVVTEAGRDWLLLGEVPGQDLLSS-HLAPAEKVSIMADAMRRLHTLDPAT | 130 |
| Sbjct 74 | G P V+ EAGR WLL+ +PG+DL SS LAP + V ++ADA+R LH L A AQGQPVPTVIATAEEAGRRWLLMSALPGRDLASSPELAPQQLVELLADALRGLHALPVAA | 133 |
| Query 131 | CPFDHQAKHRIERARTRMEAGLVDQDDLDEEHQGLAPAELFARLKARMPDGEDLVVTHGD | 190 |

CPFD +	R++ A+ R+EAGLVD DD D+E G +P ++FA L + PD EDLVV+HGD

| Sbjct | 134 | CPFDQRLASRVQVAQARIEAGLVDADDFDDERLGQSPQQVFAELCSTRPDHEDLVVSHGD | 193 |
| --- | --- | --- | --- |
| Query | 191 | ACLPNIMVENGRFSGFIDCGRLGVADRYQDIALATRDIAEELG-GEWADRFLVLYGIAAP | 249 |
| Sbjct | 194 | ACLPN+MV GRFSGFIDCGRLGVADRYQD+ALA R + G W YG A ACLPNLMVVEGRFSGFIDCGRLGVADRYQDLALAARSLVHNFGDTRWVAPLFQRYGAVA- | 252 |
| Query  Sbjct | 250  253 | DSQRIAFYRLLDEFF 264 D +R+AFYRLLDEFF DERRLAFYRLLDEFF 267 |  |

>WP_086059731.1 aminoglycoside 3'-phosphotransferase [Alcaligenes faecalis]

OSZ43450.1 APH(3') family aminoglycoside O-phosphotransferase [Alcaligenes faecalis]

OSZ48319.1 APH(3') family aminoglycoside O-phosphotransferase [Alcaligenes faecalis]

OSZ54939.1 APH(3') family aminoglycoside O-phosphotransferase [Alcaligenes faecalis]

Length=256

Score = 243 bits (621), Expect = 7e-77, Method: Compositional matrix adjust.

| Identities | = 128/254 (50%), Positives = 161/254 (63%), Gaps = 2/254 (1%) |  |
| --- | --- | --- |
| Query 12 | PAAWVERLFGYDWAQQTIGCSDAAVFRLSAQGRPVLFVKTDLSGALNELQDEAARLSWLA | 71 |
| Sbjct 4 | PAAW RL +QTIG S A V+RL FVK + L EL DE RL W+ PAAWQTRLAAAKVQRQTIGESGADVWRLDWPDGSCQFVKAEDDLPLAELPDEIERLRWMH | 63 |
| Query 72 | TTGVPCAAVLDVVTEAGRDWLLLGEVPGQDLLSSH-LAPAEKVSIMADAMRRLHTLDPAT | 130 |
| Sbjct 64 | G+PC VLD + GR WLL+ VPG+DL + L+P + V ++A +++ LH L + AQGLPCPEVLDTLVADGRHWLLMSAVPGRDLACTKGLSPQQTVELLASSLQSLHRLPLES | 123 |
| Query 131 | CPFDHQAKHRIERARTRMEAGLVDQDDLDEEHQGLAPAELFARLKARMPDGEDLVVTHGD | 190 |
| Sbjct 124 | CPFDH+ +HR+ A R +AGL+D++D D+E QG+ L L A P EDLVVTHGD CPFDHRLEHRLADASARAKAGLIDEEDFDDERQGMPVQSLLDELYASRPMQEDLVVTHGD | 183 |
| Query 191 | ACLPNIMVENGRFSGFIDCGRLGVADRYQDIALATRDIAEELGGEWADRFLVLYGIAAPD | 250 |
| Sbjct 184 | ACLPN MV G+FSGFIDCGRLGVADR+QD+AL R I LG EW F LYG+ D ACLPNFMVHQGQFSGFIDCGRLGVADRFQDLALTARSIERNLGKEWLAPFFALYGVEM-D | 242 |
| Query 251  Sbjct 243 | SQRIAFYRLLDEFF 264  ++RIAF+ LLDEFF  AERIAFFCLLDEFF 256 |  |

```
>VUK78760.1 Aminoglycoside 3'-phosphotransferase [Stenotrophomonas maltophilia] Length=117

Score = 238 bits (608), Expect = 7e-77, Method: Compositional matrix adjust. Identities = 117/117 (100%), Positives = 117/117 (100%), Gaps = 0/117 (0%)

Query 148 MEAGLVDQDDLDEEHQGLAPAELFARLKARMPDGEDLVVTHGDACLPNIMVENGRFSGFI 207 MEAGLVDQDDLDEEHQGLAPAELFARLKARMPDGEDLVVTHGDACLPNIMVENGRFSGFI
Sbjct 1	MEAGLVDQDDLDEEHQGLAPAELFARLKARMPDGEDLVVTHGDACLPNIMVENGRFSGFI 60

Query 208 DCGRLGVADRYQDIALATRDIAEELGGEWADRFLVLYGIAAPDSQRIAFYRLLDEFF 264 DCGRLGVADRYQDIALATRDIAEELGGEWADRFLVLYGIAAPDSQRIAFYRLLDEFF
Sbjct 61	DCGRLGVADRYQDIALATRDIAEELGGEWADRFLVLYGIAAPDSQRIAFYRLLDEFF 117

>WP_150796062.1 aminoglycoside 3'-phosphotransferase [Pseudomonas fluorescens] VVN65335.1 Aminoglycoside 3'-phosphotransferase [Pseudomonas fluorescens] VVP73131.1 Aminoglycoside 3'-phosphotransferase [Pseudomonas fluorescens]
Length=256

Score = 243 bits (621), Expect = 7e-77, Method: Compositional matrix adjust. Identities = 126/254 (50%), Positives = 164/254 (65%), Gaps = 2/254 (1%)

Query 12	PAAWVERLFGYDWAQQTIGCSDAAVFRLSAQGRPVLFVKTDLSGALNELQDEAARLSWLA 71 P W +L G	+Q IG S A VFR+ +	LF+K+++ G LNEL DE RL WL
Sbjct 4	PTQWGNQLSGAQIERQAIGESRADVFRIRSSNGQDLFLKSEVVGLLNELPDEIERLRWLG 63
```

| Query | 72 | TTGVPCAAVLDVVTEAGRDWLLLGEVPGQDLLS-SHLAPAEKVSIMADAMRRLHTLDPAT | 130 |
| --- | --- | --- | --- |
| Sbjct | 64 | +P +VLD TE WLL+ VPGQDL S S+L+ ++ +SI+A A+R LH + A QLELPGPSVLDATTENNHHWLLMSAVPGQDLASASNLSASQIISIVAMALRTLHQVPIAK | 123 |
| Query | 131 | CPFDHQAKHRIERARTRMEAGLVDQDDLDEEHQGLAPAELFARLKARMPDGEDLVVTHGD | 190 |
| Sbjct | 124 | CPF+ + RI AR R+ AGLVD+ D DE+ G ++FA L + +P DLVVTHGD CPFESLLEQRIATARDRVSAGLVDETDFDEQRLGRTAEDVFAELLSTLPRTHDLVVTHGD | 183 |
| Query | 191 | ACLPNIMVENGRFSGFIDCGRLGVADRYQDIALATRDIAEELGGEWADRFLVLYGIAAPD | 250 |
| Sbjct | 184 | ACLPN+M + +F+GFIDCGRLGV+DR+QD+ALA R I LGG+W F YG+ PD ACLPNLMADADQFTGFIDCGRLGVSDRFQDLALAARSIERNLGGQWVAPFFQRYGV-EPD | 242 |
| Query | 251 | SQRIAFYRLLDEFF 264 QR+ FY LLDEFF |  |
| Sbjct | 243 | DQRMVFYCLLDEFF 256 | |

>WP_126928024.1 APH(3')-II family aminoglycoside O-phosphotransferase [Stenotrophomonas maltophilia]

RTQ91233.1 APH(3')-II family aminoglycoside O-phosphotransferase [Stenotrophomonas maltophilia]

Length=267

Score = 244 bits (622), Expect = 7e-77, Method: Compositional matrix adjust.

| Identities | = 132/255 (52%), Positives = 164/255 (64%), Gaps = 3/255 (1%) |  |
| --- | --- | --- |
| Query 12 | PAAWVERLFGYDWAQQTIGCSDAAVFRLSAQGRPVLFVKTDLSGALNELQDEAARLSWLA | 71 |
| Sbjct 14 | P AW E L +Q+IG S A V R+ G+ FVK+++ A +EL DE ARL WL PWAWQEALADARIERQSIGVSRADVARVQRPGQADAFVKSEVIDAFSELGDEIARLRWLQ | 73 |
| Query 72 | TTGVPCAAVLDVVTEAGRDWLLLGEVPGQDLLSS-HLAPAEKVSIMADAMRRLHTLDPAT | 130 |
| Sbjct 74 | G P V+ EAGR WLL+ +PG+DL SS LAP E V ++ADA+R LH L A AQGQPAPTVIATAEEAGRRWLLMSALPGRDLASSPELAPQELVELLADALRGLHALPVAA | 133 |
| Query 131 | CPFDHQAKHRIERARTRMEAGLVDQDDLDEEHQGLAPAELFARLKARMPDGEDLVVTHGD | 190 |
| Sbjct 134 | CPFD + R++ A+ R+EAG VD DD D+E G +P ++FA L + PD EDLVV+HGD CPFDQRLASRLQAAQARVEAGRVDADDFDDERLGQSPQQVFAELCSTRPDHEDLVVSHGD | 193 |
| Query 191 | ACLPNIMVENGRFSGFIDCGRLGVADRYQDIALATRDIAEEL-GGEWADRFLVLYGIAAP | 249 |
| Sbjct 194 | ACLPN+M GRFSGFIDCGRLGVADRYQD+ALA R + W YG+ A ACLPNLMAVEGRFSGFIDCGRLGVADRYQDLALAARSLVYNFDNTRWVAPLFKRYGVVA- | 252 |
| Query 250  Sbjct 253 | DSQRIAFYRLLDEFF 264 D +R+AFYRLLDEFF DERRLAFYRLLDEFF 267 |  |

>VUJ56063.1 Aminoglycoside 3'-phosphotransferase [Stenotrophomonas maltophilia] Length=267

Score = 244 bits (622), Expect = 7e-77, Method: Compositional matrix adjust.

| Identities | = 131/255 (51%), Positives = 165/255 (65%), Gaps = 3/255 (1%) |  |
| --- | --- | --- |
| Query 12 | PAAWVERLFGYDWAQQTIGCSDAAVFRLSAQGRPVLFVKTDLSGALNELQDEAARLSWLA | 71 |
| Sbjct 14 | P AW + L +Q+IG S A V R+ G+ F+K+++ A +EL DE ARL WL PWAWQDALADARIERQSIGVSRADVARVHRPGQADAFLKSEVIDAFSELGDETARLRWLQ | 73 |
| Query 72 | TTGVPCAAVLDVVTEAGRDWLLLGEVPGQDLLSS-HLAPAEKVSIMADAMRRLHTLDPAT | 130 |
| Sbjct 74 | G P V+ EAGR WLL+ +PG+DL SS LAP + V ++ADA+R LH L A AQGQPVPTVIATAEEAGRRWLLMSALPGRDLASSPELAPQQLVELLADALRGLHALPVAA | 133 |
| Query 131 | CPFDHQAKHRIERARTRMEAGLVDQDDLDEEHQGLAPAELFARLKARMPDGEDLVVTHGD | 190 |
| Sbjct 134 | CPFD + R++ A+ R+EAGLVD DD D+E G +P ++FA L + PD EDLVV+HGD CPFDQRLASRVQVAQARIEAGLVDADDFDDERLGQSPQQVFAELCSIQPDHEDLVVSHGD | 193 |
| Query 191 | ACLPNIMVENGRFSGFIDCGRLGVADRYQDIALATRDIAEELG-GEWADRFLVLYGIAAP | 249 |
| Sbjct 194 | ACLPN+M GRFSGFIDCGRLGVADRYQD+ALA R + G W YG A ACLPNLMAVQGRFSGFIDCGRLGVADRYQDLALAARSLVHNFGDTRWVAPLFQRYGAVA- | 252 |
| Query 250  Sbjct 253 | DSQRIAFYRLLDEFF 264 D +R+AFYRLLDEFF DERRLAFYRLLDEFF 267 |  |

>WP_154263322.1 APH(3')-II family aminoglycoside O-phosphotransferase [Stenotrophomonas maltophilia]

QGM00980.1 APH(3')-II family aminoglycoside O-phosphotransferase [Stenotrophomonas maltophilia]

VUQ74904.1 Aminoglycoside 3'-phosphotransferase [Stenotrophomonas maltophilia] Length=267

Score = 244 bits (622), Expect = 8e-77, Method: Compositional matrix adjust.

| Identities | = 131/255 (51%), Positives = 164/255 (64%), Gaps = 3/255 (1%) |  |
| --- | --- | --- |
| Query 12 | PAAWVERLFGYDWAQQTIGCSDAAVFRLSAQGRPVLFVKTDLSGALNELQDEAARLSWLA | 71 |
| Sbjct 14 | P AW E L +Q+IG S A V R+ G+ F+K+++ A +EL DE A L WL PCAWQEALADARIERQSIGVSRADVARVRRPGQADAFLKSEVIDAFSELGDEIACLRWLQ | 73 |
| Query 72 | TTGVPCAAVLDVVTEAGRDWLLLGEVPGQDLLSS-HLAPAEKVSIMADAMRRLHTLDPAT | 130 |
| Sbjct 74 | G P V+ EAGR WLL+ +PG DL SS LAP V ++ADA+R LH L A AEGQPAPTVIATAEEAGRRWLLMSALPGHDLASSPELAPQRLVELLADALRGLHALPVAA | 133 |
| Query 131 | CPFDHQAKHRIERARTRMEAGLVDQDDLDEEHQGLAPAELFARLKARMPDGEDLVVTHGD | 190 |
| Sbjct 134 | CPFD + R++ A+ R+EAGLVD DD D+E G +P ++FA L + PD EDLVV+HGD CPFDQRLASRVQVAQARVEAGLVDADDFDDERLGQSPQQVFAELCSTRPDHEDLVVSHGD | 193 |
| Query 191 | ACLPNIMVENGRFSGFIDCGRLGVADRYQDIALATRDIAEELG-GEWADRFLVLYGIAAP | 249 |
| Sbjct 194 | ACLPN+MV GRFSGFIDCGRLGVADRYQD+ALA R + G W YG+ A ACLPNLMVVEGRFSGFIDCGRLGVADRYQDLALAARSLVHNFGDTRWVAPLFQRYGVVA- | 252 |
| Query 250  Sbjct 253 | DSQRIAFYRLLDEFF 264 D +R+AFYR+LDEFF DERRLAFYRMLDEFF 267 |  |

>WP_111103743.1 APH(3')-II family aminoglycoside O-phosphotransferase [Stenotrophomonas maltophilia]

PZT03999.1 APH(3') family aminoglycoside O-phosphotransferase [Stenotrophomonas maltophilia]

VUO78818.1 Aminoglycoside 3'-phosphotransferase [Stenotrophomonas maltophilia] Length=267

Score = 244 bits (622), Expect = 8e-77, Method: Compositional matrix adjust.

| Identities | = 132/255 (52%), Positives = 164/255 (64%), Gaps = 3/255 (1%) |  |
| --- | --- | --- |
| Query 12 | PAAWVERLFGYDWAQQTIGCSDAAVFRLSAQGRPVLFVKTDLSGALNELQDEAARLSWLA | 71 |
| Sbjct 14 | P AW E L +Q+IG S A V R+ G+ F+K+++ A +EL DE ARL WL PRAWQEALADAHIERQSIGVSRADVARVHRPGQADAFLKSEVIDAFSELGDEIARLRWLQ | 73 |
| Query 72 | TTGVPCAAVLDVVTEAGRDWLLLGEVPGQDLLSS-HLAPAEKVSIMADAMRRLHTLDPAT | 130 |
| Sbjct 74 | G P V+ EAGR WLL+ +PG+DL SS LAP + V ++ADA+R LH L A EQGQPAPTVIATAEEAGRRWLLMSALPGRDLASSPELAPQQLVELLADALRGLHALPVAA | 133 |
| Query 131 | CPFDHQAKHRIERARTRMEAGLVDQDDLDEEHQGLAPAELFARLKARMPDGEDLVVTHGD | 190 |
| Sbjct 134 | CPFD + R+E A+ R+EA LVD DD D+E G +P ++FA L + PD EDLV+ HGD CPFDQRLASRLEAAQARVEASLVDADDFDDERLGQSPQQVFAELCSTRPDHEDLVICHGD | 193 |
| Query 191 | ACLPNIMVENGRFSGFIDCGRLGVADRYQDIALATRDIAEELG-GEWADRFLVLYGIAAP | 249 |
| Sbjct 194 | ACLPN+MV GRFSGFIDCGRLGVADRYQD+ALA R + G W YG A ACLPNLMVAEGRFSGFIDCGRLGVADRYQDLALAARSLVHNFGDTRWVAPLFQRYGALA- | 252 |
| Query 250  Sbjct 253 | DSQRIAFYRLLDEFF 264 D +R+AFYRLLDEFF DERRLAFYRLLDEFF 267 |  |

>WP_119007869.1 APH(3')-II family aminoglycoside O-phosphotransferase [Stenotrophomonas sp. AG209]

RIA19219.1 aminoglycoside 3'-phosphotransferase-2 [Stenotrophomonas sp. AG209]

Length=267

Score = 243 bits (621), Expect = 8e-77, Method: Compositional matrix adjust. Identities = 130/255 (51%), Positives = 164/255 (64%), Gaps = 3/255 (1%)

| Query | 12 | PAAWVERLFGYDWAQQTIGCSDAAVFRLSAQGRPVLFVKTDLSGALNELQDEAARLSWLA | 71 |
| --- | --- | --- | --- |
| Sbjct | 14 | P AW E L +Q+IG S A V R+ G+ F+K+++ A +EL DE ARL WL PHAWQEALADARIERQSIGVSRADVARVHRPGQADAFLKSEVIDAFSELGDEIARLRWLQ | 73 |
| Query | 72 | TTGVPCAAVLDVVTEAGRDWLLLGEVPGQDLLSS-HLAPAEKVSIMADAMRRLHTLDPAT | 130 |
| Sbjct | 74 | G P V+ E GR WLL+ +PG DL SS LAP V ++ADA+R LH L A+ VQGQPAPTVIATAEEGGRRWLLMSALPGHDLASSPALAPQHLVELLADALRGLHALPVAS | 133 |
| Query | 131 | CPFDHQAKHRIERARTRMEAGLVDQDDLDEEHQGLAPAELFARLKARMPDGEDLVVTHGD | 190 |
| Sbjct | 134 | CPFD + R++ A+TR+EAGLVD DD D+E G + ++FA L+A PD EDLVV+HGD CPFDQRLASRLQAAQTRVEAGLVDADDFDDERLGQSAQQVFAELRATRPDHEDLVVSHGD | 193 |
| Query | 191 | ACLPNIMVENGRFSGFIDCGRLGVADRYQDIALATRDIAEELG-GEWADRFLVLYGIAAP | 249 |
| Sbjct | 194 | ACLPN++ GRFSGFIDCGRLGVADRYQD+ALA R + G W YG+ ACLPNLLAAEGRFSGFIDCGRLGVADRYQDLALAARSLVHNFGDTRWVAPLFRRYGVVG- | 252 |
| Query  Sbjct | 250  253 | DSQRIAFYRLLDEFF 264 D +R+AFYRLLDEFF DERRLAFYRLLDEFF 267 |  |

>WP_180864567.1 APH(3')-II family aminoglycoside O-phosphotransferase [Stenotrophomonas maltophilia]

MBA0445880.1 APH(3')-II family aminoglycoside O-phosphotransferase [Stenotrophomonas maltophilia]

Length=267

Score = 243 bits (621), Expect = 9e-77, Method: Compositional matrix adjust.

| Identities | | = 128/254 (50%), Positives = 164/254 (65%), Gaps = 1/254 (0%) |  |
| --- | --- | --- | --- |
| Query 12  Sbjct 14 | | PAAWVERLFGYDWAQQTIGCSDAAVFRLSAQGRPVLFVKTDLSGALNELQDEAARLSWLA P AW + L G +Q+IG S A V R+ G+ F+K+++ A +EL DE ARL WL PQAWQDALAGARVERQSIGVSRADVARVHRSGQADAFLKSEVIDAFSELGDEIARLRWLQ | 71  73 |
| Query 72  Sbjct 74 | | TTGVPCAAVLDVVTEAGRDWLLLGEVPGQDLLSSHLAPAEKVSI-MADAMRRLHTLDPAT G P V+ EAGR WLL+ +PG+DL SS A +V++ MADA+R LH + A  AQGQPAPTVIATAEEAGRRWLLMSALPGRDLASSPELAARQVAVLMADALRGLHAMPVAN | 130  133 |
| Query 131  Sbjct 134 | | CPFDHQAKHRIERARTRMEAGLVDQDDLDEEHQGLAPAELFARLKARMPDGEDLVVTHGD CPFD + R++ + R+EAGLVD DD D+E G +P ++FA L+A P EDLVV+HGD CPFDQRLASRLQAVQARVEAGLVDADDFDDERLGQSPQQIFAELRATQPAHEDLVVSHGD | 190  193 |
| Query 191  Sbjct 194 | | ACLPNIMVENGRFSGFIDCGRLGVADRYQDIALATRDIAEELGGEWADRFLVLYGIAAPD ACLPN+MV NGRF+GFIDCGRLGVADRYQD+ALA R + G L A D ACLPNLMVANGRFTGFIDCGRLGVADRYQDLALAARSLVHNFGETSCVATLFQRYGAVAD | 250  253 |
| Query 251 | | SQRIAFYRLLDEFF 264  +R+AFYRLLDEFF |  |
| Sbjct | 254 | ERRMAFYRLLDEFF 267 | |

>WP_034504136.1 aminoglycoside 3'-phosphotransferase [Agrobacterium rhizogenes] Length=266

Score = 243 bits (621), Expect = 9e-77, Method: Compositional matrix adjust.

| Identities | = 125/254 (49%), Positives = 165/254 (65%), Gaps = 1/254 (0%) |  |
| --- | --- | --- |
| Query 12 | PAAWVERLFGYDWAQQTIGCSDAAVFRLSAQGRPVLFVKTDLSGALNELQDEAARLSWLA | 71 |
| Sbjct 13 | PA + L GYDW Q T+G S A VFRL A G +++KT++ L+EL E +RL WLA PARLRDLLAGYDWKQDTLGRSPAHVFRLEADGYAPVYLKTEIVHPLSELAGEVSRLRWLA | 72 |
| Query 72 | TTGVPCAAVLDVVTEAGRDWLLLGEVPGQDLLSS-HLAPAEKVSIMADAMRRLHTLDPAT | 130 |
| Sbjct 73 | + G+ C V+ T+ R+WLL+ +PG D +S L P E+V ++A+A+R+LH +D A SQGLACPEVIAHETDGEREWLLMSALPGTDFVSDDRLPPVERVRMLAEALRQLHGIDIAA | 132 |
| Query 131 | CPFDHQAKHRIERARTRMEAGLVDQDDLDEEHQGLAPAELFARLKARMPDGEDLVVTHGD | 190 |
| Sbjct 133 | CPFDH+ + RI A+ R+ AG VD+ D DEE G + +LF L+ P EDLVVTHGD CPFDHRLETRIAAAKARLLAGCVDESDFDEERLGRSAQDLFRELEQHRPMAEDLVVTHGD | 192 |
| Query 191 | ACLPNIMVENGRFSGFIDCGRLGVADRYQDIALATRDIAEELGGEWADRFLVLYGIAAPD ACLPN +V G FSG+IDCGRLGVADR+QDIALA A G + FL YG | 250 |

| Sbjct | 193 | ACLPNFIVNGGSFSGYIDCGRLGVADRHQDIALACGSTARNFGEDLVADFLKAYGNFKMH 252 |
| --- | --- | --- |
| Query | 251 | SQRIAFYRLLDEFF 264 |
| Sbjct | 253 | +++A+YRLLDEFF  PEKMAYYRLLDEFF 266 |

>WP_049471268.1 MULTISPECIES: APH(3')-II family aminoglycoside O-phosphotransferase [Stenotrophomonas]

MBC9080322.1 APH(3')-II family aminoglycoside O-phosphotransferase [Stenotrophomonas maltophilia]

MBC9090737.1 APH(3')-II family aminoglycoside O-phosphotransferase [Stenotrophomonas maltophilia]

OWR29110.1 aminoglycoside 3'-phosphotransferase [Stenotrophomonas pavanii] GCE67669.1 kanamycin kinase [Stenotrophomonas sp. Y]

Length=267

Score = 243 bits (621), Expect = 1e-76, Method: Compositional matrix adjust.

| Identities | = 129/255 (51%), Positives = 163/255 (64%), Gaps = 3/255 (1%) |  |
| --- | --- | --- |
| Query 12 | PAAWVERLFGYDWAQQTIGCSDAAVFRLSAQGRPVLFVKTDLSGALNELQDEAARLSWLA | 71 |
| Sbjct 14 | P AW E L +Q+IG S A V R+ G+ F+K+++ A +EL DE ARL WL PHAWQEALADARIERQSIGVSRADVARVHRPGQADAFLKSEVIDAFSELGDEIARLRWLQ | 73 |
| Query 72 | TTGVPCAAVLDVVTEAGRDWLLLGEVPGQDLLSS-HLAPAEKVSIMADAMRRLHTLDPAT | 130 |
| Sbjct 74 | G P V+ E GR WLL+ +PG DL SS LAP +V ++ADA+R LH L A AQGQPAPTVIATAEEEGRRWLLMSALPGHDLASSPALAPQHRVQLLADALRGLHALPVAV | 133 |
| Query 131 | CPFDHQAKHRIERARTRMEAGLVDQDDLDEEHQGLAPAELFARLKARMPDGEDLVVTHGD | 190 |
| Sbjct 134 | CPFD + R++ A+ R+EAG VD DD D+E G + ++FA L+A PD EDLVV+HGD CPFDQRLASRLQAAQARVEAGRVDADDFDDERLGQSAQQVFAELRATRPDHEDLVVSHGD | 193 |
| Query 191 | ACLPNIMVENGRFSGFIDCGRLGVADRYQDIALATRDIAEELG-GEWADRFLVLYGIAAP | 249 |
| Sbjct 194 | ACLPN++ GRFSGFIDCGRLGVADRYQD+ALA R + G W YG+ P ACLPNLLAVEGRFSGFIDCGRLGVADRYQDLALAARSLVHNFGDTRWVASLFRSYGV-VP | 252 |
| Query 250  Sbjct 253 | DSQRIAFYRLLDEFF 264 D +R+AFYRLLDEFF DERRLAFYRLLDEFF 267 |  |

>WP_064678486.1 aminoglycoside 3'-phosphotransferase [Pseudomonas silesiensis]

ANJ56980.1 APH(3') family aminoglycoside O-phosphotransferase [Pseudomonas silesiensis]

Length=256

Score = 243 bits (620), Expect = 1e-76, Method: Compositional matrix adjust.

| Identities | | = 125/254 (49%), Positives = 162/254 (64%), Gaps = 2/254 (1%) |  |
| --- | --- | --- | --- |
| Query 12  Sbjct 4 | | PAAWVERLFGYDWAQQTIGCSDAAVFRLSAQGRPVLFVKTDLSGALNELQDEAARLSWLA PA W + G +QTIG S A VFR+ LFVK++ GA +EL E RL W+ PAQWSKTFSGAPIERQTIGESGAEVFRIRCINGKHLFVKSEPIGAHSELPQEIERLRWMN | 71  63 |
| Query 72  Sbjct 64 | | TTGVPCAAVLDVVTEAGRDWLLLGEVPGQDLLSSH-LAPAEKVSIMADAMRRLHTLDPAT  +P VLDV TE R WLL+ VPGQDL S++ L+ + +S++A A+R LH + T RLDLPGPTVLDVTTENNRHWLLMNAVPGQDLASANDLSAPQVISVLAMALRTLHQVPIET | 130  123 |
| Query 131  Sbjct 124 | | CPFDHQAKHRIERARTRMEAGLVDQDDLDEEHQGLAPAELFARLKARMPDGEDLVVTHGD CPFDH + RI AR+R+ AGLVD+ D D+E QG E+F ++ + +P+ DL VTHGD CPFDHSLEQRIAAARSRVSAGLVDEADFDDERQGRTAEEVFTQMLSMLPETYDLAVTHGD | 190  183 |
| Query 191  Sbjct 184 | | ACLPNIMVENGRFSGFIDCGRLGVADRYQDIALATRDIAEELGGEWADRFLVLYGIAAPD ACLPN MVE F+G+IDCGRLG++D YQD+ALA R I LG W F YG+ PD ACLPNFMVEGSSFTGYIDCGRLGISDIYQDLALAARSIERNLGPAWGAPFFRAYGV-EPD | 250  242 |
| Query | 251 SQRIAFYRLLDEFF 264 | | |
|  |  | +RIAFY LLDEF+ |  |
| Sbjct | 243 | QRRIAFYCLLDEFY 256 | |

>WP_134545924.1 APH(3')-II family aminoglycoside O-phosphotransferase, partial [Pseudomonas aeruginosa]

Length=236

Score = 242 bits (618), Expect = 1e-76, Method: Compositional matrix adjust.

| Identities | | = 130/238 (55%), Positives = 160/238 (67%), Gaps = 7/238 (3%) |  |
| --- | --- | --- | --- |
| Query 32  Sbjct 1 | | SDAAVFRLSAQGRPVLFVKTDLSGALNELQDEAARLSWLATTGVPCAAVLDVVTEAGRDW S A V RL A+ RP LFVK ++ A EL E ARL WL G+ C VL+ GR W SAATVHRLEAERRPTLFVKQEVLSAHAELPAEIARLRWLHGAGIDCPQVLNETQSDGRQW | 91  60 |
| Query 92  Sbjct 61 | | LLLGEVPGQDLLSS HLAPAEKVSIMADAMRRLHTLDPATCPFDHQAKHRIERART  LL+ VPG D LS+ L P V ++A A+RRLH LDPA CPFDH+ + R++ R LLMSAVPG-DTLSALAQRGELEPERLVRLVAAALRRLHDLDPAACPFDHRLERRLDTVRQ | 146  119 |
| Query 147  Sbjct 120 | | RMEAGLVDQDDLDEEHQGLAPAELFARLKARMPDGEDLVVTHGDACLPNIMVENGRFSGF R+EAGLVD+ D D++H+G + EL+ L R P EDLVV HGDACLPN++ E RFSGF RVEAGLVDEADFDDDHRGRSATELYRLLLDRRPAVEDLVVAHGDACLPNLLAEGRRFSGF | 206  179 |
| Query Sbjct | 207  180 | IDCGRLGVADRYQDIALATRDIAEELGGEWADRFLVLYGIAAPDSQRIAFYRLLDEFF 264 IDCGRLGVADR+QD+ALA RDI ELG WA+ FLV YG D +R+A++RLLDEFF IDCGRLGVADRHQDLALAARDIEAELGAAWAEAFLVEYG-GDIDGERLAYFRLLDEFF 236 | |

>VUQ41399.1 Aminoglycoside 3'-phosphotransferase [Stenotrophomonas maltophilia] Length=267

Score = 243 bits (621), Expect = 1e-76, Method: Compositional matrix adjust.

| Identities | = 131/255 (51%), Positives = 165/255 (65%), Gaps = 3/255 (1%) |  |
| --- | --- | --- |
| Query 12 | PAAWVERLFGYDWAQQTIGCSDAAVFRLSAQGRPVLFVKTDLSGALNELQDEAARLSWLA | 71 |
| Sbjct 14 | P AW + L +Q+IG S A V R+ G+ F+K+++ A +EL DE ARL WL PWAWQDALADARIERQSIGVSRADVARVHRPGQADAFLKSEVIDAFSELGDEIARLRWLQ | 73 |
| Query 72 | TTGVPCAAVLDVVTEAGRDWLLLGEVPGQDLLSS-HLAPAEKVSIMADAMRRLHTLDPAT | 130 |
| Sbjct 74 | G P V+ EAGR WLL+ +PG+DL SS LAP + V ++ADA+R LH L A AQGQPVPTVIATAEEAGRRWLLMSALPGRDLASSPELAPQQLVELLADALRGLHALPVAA | 133 |
| Query 131 | CPFDHQAKHRIERARTRMEAGLVDQDDLDEEHQGLAPAELFARLKARMPDGEDLVVTHGD | 190 |
| Sbjct 134 | CPFD + R++ A+ R+EAGLVD DD D+E G +P ++FA L + PD EDLVV+HGD CPFDQRLASRVQVAQARIEAGLVDADDFDDERLGQSPQQVFAELCSTRPDHEDLVVSHGD | 193 |
| Query 191 | ACLPNIMVENGRFSGFIDCGRLGVADRYQDIALATRDIAEELG-GEWADRFLVLYGIAAP | 249 |
| Sbjct 194 | ACLPN+M GRFSGFIDCGRLGVADRYQD+ALA R + G W YG A ACLPNLMAVQGRFSGFIDCGRLGVADRYQDLALAARSLVHNFGDTRWVAPLFQRYGAVA- | 252 |
| Query 250  Sbjct 253 | DSQRIAFYRLLDEFF 264 D +R+AFYRLLDEFF DERRLAFYRLLDEFF 267 |  |

>WP_125303934.1 aminoglycoside 3'-phosphotransferase [Alcaligenes faecalis] RSE62800.1 aminoglycoside 3'-phosphotransferase [Alcaligenes faecalis]

Length=256

Score = 243 bits (620), Expect = 1e-76, Method: Compositional matrix adjust.

| Identities | = 128/254 (50%), Positives = 160/254 (63%), Gaps = 2/254 (1%) |  |
| --- | --- | --- |
| Query 12 | PAAWVERLFGYDWAQQTIGCSDAAVFRLSAQGRPVLFVKTDLSGALNELQDEAARLSWLA | 71 |
| Sbjct 4 | PAAW RL +QTIG S A V+RL FVK + L EL DE RL W+ PAAWQTRLAAAKVQRQTIGESGADVWRLDWPDGSCQFVKAEDDLPLAELPDEIERLGWMH | 63 |
| Query 72 | TTGVPCAAVLDVVTEAGRDWLLLGEVPGQDLLSSH-LAPAEKVSIMADAMRRLHTLDPAT | 130 |
| Sbjct 64 | G+PC VLD + GR WLL+ VPG+DL + L+P + V ++A +++ LH L + AQGLPCPEVLDTLVADGRHWLLMSAVPGRDLACTEGLSPQQTVELLALSLQSLHRLPLES | 123 |
| Query 131 | CPFDHQAKHRIERARTRMEAGLVDQDDLDEEHQGLAPAELFARLKARMPDGEDLVVTHGD | 190 |
| Sbjct 124 | CPFDH+ +HR+ A R +AGL+D++D D+E G+ L L A P EDLVVTHGD CPFDHRLEHRLADASARAKAGLIDEEDFDDERHGMPVQSLLDELYASRPKQEDLVVTHGD | 183 |
| Query 191 | ACLPNIMVENGRFSGFIDCGRLGVADRYQDIALATRDIAEELGGEWADRFLVLYGIAAPD | 250 |
| Sbjct 184 | ACLPN MV GRFSGFIDCGRLGVADR+QD+AL R I LG EW F LYG+ D  ACLPNFMVHQGRFSGFIDCGRLGVADRFQDLALTARSIERNLGKEWLAPFFALYGVEM-D | 242 |

Query 251 SQRIAFYRLLDEFF 264

++RIAF+ LLDEFF Sbjct 243 AERIAFFCLLDEFF 256

>WP_154352443.1 APH(3')-II family aminoglycoside O-phosphotransferase [Stenotrophomonas maltophilia]

QGM05326.1 APH(3')-II family aminoglycoside O-phosphotransferase [Stenotrophomonas maltophilia]

Length=267

Score = 243 bits (620), Expect = 1e-76, Method: Compositional matrix adjust.

| Identities | = 131/255 (51%), Positives = 164/255 (64%), Gaps = 3/255 (1%) |  |
| --- | --- | --- |
| Query 12 | PAAWVERLFGYDWAQQTIGCSDAAVFRLSAQGRPVLFVKTDLSGALNELQDEAARLSWLA | 71 |
| Sbjct 14 | P AW E L +Q+IG S A V R+ G+ F+K+++ A +EL DE A L WL PCAWQEALADARIERQSIGVSRADVARVRRPGQADAFLKSEVIDAFSELGDEIACLRWLQ | 73 |
| Query 72 | TTGVPCAAVLDVVTEAGRDWLLLGEVPGQDLLSS-HLAPAEKVSIMADAMRRLHTLDPAT | 130 |
| Sbjct 74 | G P V+ EAGR WLL+ +PG DL SS LAP V ++ADA+R LH L A AEGQPAPTVIATAEEAGRRWLLMSALPGHDLASSPELAPQRLVELLADALRGLHALPVAA | 133 |
| Query 131 | CPFDHQAKHRIERARTRMEAGLVDQDDLDEEHQGLAPAELFARLKARMPDGEDLVVTHGD | 190 |
| Sbjct 134 | CPFD + R++ A+ R+EAGLVD DD D+E G +P ++FA L + PD EDLVV+HGD CPFDQRLASRVQVAQARVEAGLVDADDFDDERLGQSPQQVFAELCSTRPDHEDLVVSHGD | 193 |
| Query 191 | ACLPNIMVENGRFSGFIDCGRLGVADRYQDIALATRDIAEELG-GEWADRFLVLYGIAAP | 249 |
| Sbjct 194 | ACLPN+MV GRFSGFIDCGRLGVADRYQD+ALA R + G W YG+ A ACLPNLMVVEGRFSGFIDCGRLGVADRYQDLALAARSLVHNFGDTRWVAPLFQRYGMVA- | 252 |
| Query 250  Sbjct 253 | DSQRIAFYRLLDEFF 264 D +R+AFYR+LDEFF DERRLAFYRMLDEFF 267 |  |

>WP_043397129.1 APH(3')-II family aminoglycoside O-phosphotransferase [Stenotrophomonas maltophilia]

Length=267

Score = 243 bits (620), Expect = 1e-76, Method: Compositional matrix adjust.

| Identities | = 132/255 (52%), Positives = 165/255 (65%), Gaps = 3/255 (1%) |  |
| --- | --- | --- |
| Query 12 | PAAWVERLFGYDWAQQTIGCSDAAVFRLSAQGRPVLFVKTDLSGALNELQDEAARLSWLA | 71 |
| Sbjct 14 | P AW E L +Q+IG S A V R+ G+ F+K+++ A +EL DE RL WL PWAWQEALADAHIERQSIGVSRADVARVHRPGQTDAFLKSEVIDAFSELGDEITRLRWLQ | 73 |
| Query 72 | TTGVPCAAVLDVVTEAGRDWLLLGEVPGQDLLSS-HLAPAEKVSIMADAMRRLHTLDPAT | 130 |
| Sbjct 74 | G+P V+ EAGR WLL+ +PG+DL SS LAP + V ++ADA+R LH L T AQGLPAPTVIATAEEAGRRWLLMSALPGRDLASSPELAPQQLVELLADALRGLHALPVVT | 133 |
| Query 131 | CPFDHQAKHRIERARTRMEAGLVDQDDLDEEHQGLAPAELFARLKARMPDGEDLVVTHGD | 190 |
| Sbjct 134 | CPFDH+ R++ A R+EAGLVD DD D+E G +P ++FA L + PD EDLVV+HGD CPFDHRLDLRLQAAAARVEAGLVDADDFDDERLGQSPRQVFAELCSTRPDHEDLVVSHGD | 193 |
| Query 191 | ACLPNIMVENGRFSGFIDCGRLGVADRYQDIALATRDIAEELG-GEWADRFLVLYGIAAP | 249 |
| Sbjct 194 | ACLPN+M GRFSGFIDCGRLGVADRYQD+ALA R + G W YG A ACLPNLMAVEGRFSGFIDCGRLGVADRYQDLALAARSLVHNFGDTRWVAPLFQRYG-ALV | 252 |
| Query 250  Sbjct 253 | DSQRIAFYRLLDEFF 264 D +R+AFYRLLDEFF DERRLAFYRLLDEFF 267 |  |

```
>PKA72529.1 aminoglycoside 3'-phosphotransferase-2 [Pseudomonas baetica] Length=258

Score = 243 bits (619), Expect = 1e-76, Method: Compositional matrix adjust. Identities = 122/240 (51%), Positives = 160/240 (67%), Gaps = 2/240 (1%)

Query 26	QQTIGCSDAAVFRLSAQGRPVLFVKTDLSGALNELQDEAARLSWLATTGVPCAAVLDVVT 85 QQTIG S A V R++ +G	LFVK++	EL E RL W+A+ +P	VL+ T
Sbjct 20	QQTIGESRADVLRITPEGSVPLFVKSEQVMIWGELPGEVQRLRWMASQDLPAPRVLETAT 79
```

| Query | 86 | EAGRDWLLLGEVPGQDLLSS-HLAPAEKVSIMADAMRRLHTLDPATCPFDHQAKHRIERA | 144 |
| --- | --- | --- | --- |
| Sbjct | 80 | EA R+WLL+ +PG+DL SS HLAP + V++ A+A+R LH + CPFDH+ +I A EADRNWLLMTAIPGRDLASSAHLAPQQIVTLAANALRSLHAVAINRCPFDHRLDQKIPLA | 139 |
| Query | 145 | RTRMEAGLVDQDDLDEEHQGLAPAELFARLKARMPDGEDLVVTHGDACLPNIMVENGRFS | 204 |
| Sbjct | 140 | R + AGL+D+ D D+ G +F ++ A PD EDLVVTHGDACLPN++ ENG+FS REHVLAGLIDEQDFDDARIGCTAENVFQQILAERPDHEDLVVTHGDACLPNLLAENGQFS | 199 |
| Query | 205 | GFIDCGRLGVADRYQDIALATRDIAEELGGEWADRFLVLYGIAAPDSQRIAFYRLLDEFF | 264 |
| Sbjct | 200 | GF+DCGRLGVADRYQD+ALAT I LG +W F +YG+ PD + IAFY+LLDE F  GFVDCGRLGVADRYQDLALATHSIGSNLGKQWVPMFFEVYGV-EPDQRLIAFYQLLDELF | 258 |

>WP_188249774.1 APH(3')-II family aminoglycoside O-phosphotransferase [Stenotrophomonas maltophilia]

Length=267

Score = 243 bits (620), Expect = 1e-76, Method: Compositional matrix adjust.

| Identities | = 131/255 (51%), Positives = 164/255 (64%), Gaps = 3/255 (1%) |  |
| --- | --- | --- |
| Query 12 | PAAWVERLFGYDWAQQTIGCSDAAVFRLSAQGRPVLFVKTDLSGALNELQDEAARLSWLA | 71 |
| Sbjct 14 | P AW E L +Q+IG S A V R+ G+ F+K+++ A +EL DE AR+ WL PRAWQEALADAHIERQSIGVSRADVARVHRPGQADAFLKSEVIDAFSELGDEIARMRWLQ | 73 |
| Query 72 | TTGVPCAAVLDVVTEAGRDWLLLGEVPGQDLLSS-HLAPAEKVSIMADAMRRLHTLDPAT | 130 |
| Sbjct 74 | G P V+ EAGR WLL+ +PG+DL SS LAP + V ++ADA+R LH L A EQGQPAPTVIATAEEAGRRWLLMSALPGRDLASSPELAPQQLVELLADALRGLHALPVAA | 133 |
| Query 131 | CPFDHQAKHRIERARTRMEAGLVDQDDLDEEHQGLAPAELFARLKARMPDGEDLVVTHGD | 190 |
| Sbjct 134 | CPFD + R+E A+ R+EA LVD DD D+E G +P ++FA L + PD EDLV+ HGD CPFDQRLASRLEAAQARVEASLVDADDFDDERLGQSPQQVFAELCSTRPDHEDLVICHGD | 193 |
| Query 191 | ACLPNIMVENGRFSGFIDCGRLGVADRYQDIALATRDIAEELG-GEWADRFLVLYGIAAP | 249 |
| Sbjct 194 | ACLPN+MV GRFSGFIDCGRLGVADRYQD+ALA R + G W YG A ACLPNLMVAEGRFSGFIDCGRLGVADRYQDLALAARSLVHNFGDTRWVAPLFQRYGALA- | 252 |
| Query 250  Sbjct 253 | DSQRIAFYRLLDEFF 264 D +R+AFYRLLDEFF DERRLAFYRLLDEFF 267 |  |

>WP_149909139.1 APH(3')-II family aminoglycoside O-phosphotransferase [Stenotrophomonas maltophilia]

Length=267

Score = 243 bits (620), Expect = 1e-76, Method: Compositional matrix adjust.

| Identities | = 131/255 (51%), Positives = 164/255 (64%), Gaps = 3/255 (1%) |  |
| --- | --- | --- |
| Query 12 | PAAWVERLFGYDWAQQTIGCSDAAVFRLSAQGRPVLFVKTDLSGALNELQDEAARLSWLA | 71 |
| Sbjct 14 | P AW + L +Q+IG S A V R+ G+ F+K+++ A +EL DE ARL WL PWAWQDALADARIERQSIGVSRADVARVHRPGQADAFLKSEVIDAFSELGDEIARLRWLQ | 73 |
| Query 72 | TTGVPCAAVLDVVTEAGRDWLLLGEVPGQDLLSS-HLAPAEKVSIMADAMRRLHTLDPAT | 130 |
| Sbjct 74 | G P V+ EAGR WLL+ +PG DL SS LAP + V ++ADA+R LH L A AQGQPAPTVIATAEEAGRRWLLMSALPGHDLASSPELAPQQLVELLADALRGLHALPVAA | 133 |
| Query 131 | CPFDHQAKHRIERARTRMEAGLVDQDDLDEEHQGLAPAELFARLKARMPDGEDLVVTHGD | 190 |
| Sbjct 134 | CPFD + R++ A+ R+EAGLVD DD D+E G +P ++FA L + PD EDLVV+HGD CPFDQRLASRVQVAQARVEAGLVDADDFDDERLGQSPQQVFAELCSTQPDHEDLVVSHGD | 193 |
| Query 191 | ACLPNIMVENGRFSGFIDCGRLGVADRYQDIALATRDIAEELG-GEWADRFLVLYGIAAP | 249 |
| Sbjct 194 | ACLPN+M GRFSGFIDCGRLGVADRYQD+ALA R + G W YG A ACLPNLMAVEGRFSGFIDCGRLGVADRYQDLALAARSLVHNFGDTRWVAPLFQRYGAVA- | 252 |
| Query 250  Sbjct 253 | DSQRIAFYRLLDEFF 264 D +R+AFYRLLDEFF DERRLAFYRLLDEFF 267 |  |

>NHC52627.1 APH(3') family aminoglycoside O-phosphotransferase [Pseudomonas sp. AU8050]

Length=256

Score = 243 bits (619), Expect = 1e-76, Method: Compositional matrix adjust.

| Identities | | = 131/254 (52%), Positives = 160/254 (63%), Gaps = 2/254 (1%) |  |
| --- | --- | --- | --- |
| Query 12  Sbjct 4 | | PAAWVERLFGYDWAQQTIGCSDAAVFRLSAQGRPVLFVKTDLSGALNELQDEAARLSWLA P W E+ QQ IG S A VFRL G LF+K++L EL DE RL WL PQKWREQFPHALIEQQAIGESRADVFRLRQDGGTDLFLKSELLEEHGELADEIDRLRWLQ | 71  63 |
| Query 72  Sbjct 64 | | TTGVPCAAVLDVVTEAGRDWLLLGEVPGQDLLS-SHLAPAEKVSIMADAMRRLHTLDPAT G+P VLD VT A R WLL+ VPGQDL S S L+ + ++I+A A+R LH L A  QMGLPAPVVLDEVTAAHRHWLLMTTVPGQDLASASGLSAEQIINILATALRTLHQLPVAL | 130  123 |
| Query 131  Sbjct 124 | | CPFDHQAKHRIERARTRMEAGLVDQDDLDEEHQGLAPAELFARLKARMPDGEDLVVTHGD CPFDH + RI RA+ + AGLVD+ D D+E G + ++FA L A P DLVVTHGD CPFDHSLQLRIARAQQHVSAGLVDETDFDDERLGQSAEDVFAELVATQPKTHDLVVTHGD | 190  183 |
| Query 191  Sbjct 184 | | ACLPNIMVENGRFSGFIDCGRLGVADRYQDIALATRDIAEELGGEWADRFLVLYGIAAPD ACLPN M G FSGFIDCGRLGV+DR+QD+A+A R I +G +W + F LYG+ PD ACLPNFMAAEGCFSGFIDCGRLGVSDRFQDLAIAARSIERNVGQKWVNPFFELYGV-EPD | 250  242 |
| Query | 251 SQRIAFYRLLDEFF 264 | | |
|  |  | Q+I FY LLDEFF |  |
| Sbjct | 243 | EQKIRFYCLLDEFF 256 | |

>VUR95505.1 Aminoglycoside 3'-phosphotransferase [Stenotrophomonas maltophilia] Length=282

Score = 243 bits (621), Expect = 2e-76, Method: Compositional matrix adjust.

| Identities | = 129/255 (51%), Positives = 163/255 (64%), Gaps = 3/255 (1%) |  |
| --- | --- | --- |
| Query 12 | PAAWVERLFGYDWAQQTIGCSDAAVFRLSAQGRPVLFVKTDLSGALNELQDEAARLSWLA | 71 |
| Sbjct 29 | P AW E L +Q+IG S A V R+ G+ F+K+++ A +EL DE ARL WL PRAWQEALADARIERQSIGVSRADVARVHRPGQADAFLKSEVIDAFSELGDEIARLRWLQ | 88 |
| Query 72 | TTGVPCAAVLDVVTEAGRDWLLLGEVPGQDLLSS-HLAPAEKVSIMADAMRRLHTLDPAT | 130 |
| Sbjct 89 | G P V+ E GR WLL+ +PG DL SS LAP +V ++ADA+R LH L A AQGQPAPTVIATAEEEGRRWLLMSALPGHDLASSPALAPQHRVQLLADALRGLHALPVAA | 148 |
| Query 131 | CPFDHQAKHRIERARTRMEAGLVDQDDLDEEHQGLAPAELFARLKARMPDGEDLVVTHGD | 190 |
| Sbjct 149 | CPFD + R++ A+ R+EAG VD DD D+E G + ++FA L+A PD EDLVV+HGD CPFDQRLASRLQAAQARVEAGRVDADDFDDERLGQSAQQVFAELRATRPDHEDLVVSHGD | 208 |
| Query 191 | ACLPNIMVENGRFSGFIDCGRLGVADRYQDIALATRDIAEELG-GEWADRFLVLYGIAAP | 249 |
| Sbjct 209 | ACLPN++ GRFSGFIDCGRLGVADRYQD+ALA R + G W YG+ P ACLPNLLAVEGRFSGFIDCGRLGVADRYQDLALAARSLVHNFGDTRWVASLFRSYGV-VP | 267 |
| Query 250  Sbjct 268 | DSQRIAFYRLLDEFF 264 D +R+AFYRLLDEFF DERRLAFYRLLDEFF 282 |  |

>VUO61439.1 Aminoglycoside 3'-phosphotransferase [Stenotrophomonas maltophilia] Length=267

Score = 243 bits (620), Expect = 2e-76, Method: Compositional matrix adjust.

| Identities | = 131/255 (51%), Positives = 165/255 (65%), Gaps = 3/255 (1%) |  |
| --- | --- | --- |
| Query 12 | PAAWVERLFGYDWAQQTIGCSDAAVFRLSAQGRPVLFVKTDLSGALNELQDEAARLSWLA | 71 |
| Sbjct 14 | P AW + L +Q+IG S A V R+ G+ F+K+++ A +EL DE ARL WL PWAWQDALADARIERQSIGVSRADVARVHRPGQADAFLKSEVIDAFSELGDEIARLRWLQ | 73 |
| Query 72 | TTGVPCAAVLDVVTEAGRDWLLLGEVPGQDLLSS-HLAPAEKVSIMADAMRRLHTLDPAT | 130 |
| Sbjct 74 | G P V+ EAGR WLL+ +PG+DL SS LAP + V ++ADA+R LH L A AQGQPVPTVIATAEEAGRRWLLMSALPGRDLASSPELAPQQLVELLADALRGLHALPVAA | 133 |
| Query 131 | CPFDHQAKHRIERARTRMEAGLVDQDDLDEEHQGLAPAELFARLKARMPDGEDLVVTHGD | 190 |
| Sbjct 134 | CPFD + R++ A+ R+EAGLVD DD D+E G +P ++FA L + PD EDLVV+HGD CPFDQRLASRVQVAQARVEAGLVDADDFDDERLGQSPQQVFAELCSIQPDHEDLVVSHGD | 193 |
| Query 191 | ACLPNIMVENGRFSGFIDCGRLGVADRYQDIALATRDIAEELG-GEWADRFLVLYGIAAP | 249 |

ACLPN+M	GRFSGFIDCGRLGVADRYQD+ALA R +	G	W	YG A

| Sbjct | 194 | ACLPNLMAVEGRFSGFIDCGRLGVADRYQDLALAARSLVHNFGDTRWVAPLFQRYGAVA- 252 |
| --- | --- | --- |
| Query | 250 | DSQRIAFYRLLDEFF 264 |
| Sbjct | 253 | D +R+AFYRLLDEFF  DERRLAFYRLLDEFF 267 |

>WP_053249296.1 aminoglycoside 3'-phosphotransferase [Ensifer adhaerens] KOF18957.1 aminoglycoside phosphotransferase [Ensifer adhaerens]

Length=263

Score = 243 bits (619), Expect = 2e-76, Method: Compositional matrix adjust.

| Identities | = 124/253 (49%), Positives = 163/253 (64%), Gaps = 0/253 (0%) |  |
| --- | --- | --- |
| Query 12 | PAAWVERLFGYDWAQQTIGCSDAAVFRLSAQGRPVLFVKTDLSGALNELQDEAARLSWLA | 71 |
| Sbjct 11 | P A L GYDW + ++G S+++VF A+ RP L +K + +G NEL DEA R+ WLA PPALAPLLKGYDWRKDSLGQSESSVFLFHAEDRPPLVLKVEETGPFNELVDEAERIKWLA | 70 |
| Query 72 | TTGVPCAAVLDVVTEAGRDWLLLGEVPGQDLLSSHLAPAEKVSIMADAMRRLHTLDPATC | 131 |
| Sbjct 71 | G+P ++ A ++WLL+ V G DL SS L +++V ++A+A+RR+H LD C KQGMPSPRIIAHEAYAQQNWLLMEAVDGVDLASSTLPASDQVILIAEALRRMHALDVDAC | 130 |
| Query 132 | PFDHQAKHRIERARTRMEAGLVDQDDLDEEHQGLAPAELFARLKARMPDGEDLVVTHGDA | 191 |
| Sbjct 131 | PFDH+ RI A+ R EA L+D D D+ G ELF L+A +P LVVTHGDA PFDHRIGARIAIAKLRAEADLIDVTDFDDVRLGKTTEELFVELQASIPREGKLVVTHGDA | 190 |
| Query 192 | CLPNIMVENGRFSGFIDCGRLGVADRYQDIALATRDIAEELGGEWADRFLVLYGIAAPDS | 251 |
| Sbjct 191 | CLPNI+ +GRF+GFIDC RLGVAD YQD+AL+ R IA LG EW FL LYGI D CLPNIIARDGRFAGFIDCSRLGVADPYQDLALSCRSIAYNLGEEWVQPFLDLYGIRRADP | 250 |
| Query 252  Sbjct 251 | QRIAFYRLLDEFF 264  +++ FY LLDEFF  EKLDFYCLLDEFF 263 |  |

>WP_183709535.1 MULTISPECIES: APH(3') family aminoglycoside O-phosphotransferase [unclassified Rhizobium]

MBB3381754.1 aminoglycoside 3'-phosphotransferase-2 [Rhizobium sp. BK098] MBB3613456.1 aminoglycoside 3'-phosphotransferase-2 [Rhizobium sp. BK609] MBB3679114.1 aminoglycoside 3'-phosphotransferase-2 [Rhizobium sp. BK612]

Length=266

Score = 243 bits (619), Expect = 2e-76, Method: Compositional matrix adjust.

| Identities | | = 124/254 (49%), Positives = 167/254 (66%), Gaps = 1/254 (0%) |  |
| --- | --- | --- | --- |
| Query 12  Sbjct 13 | | PAAWVERLFGYDWAQQTIGCSDAAVFRLSAQGRPVLFVKTDLSGALNELQDEAARLSWLA P + L GY+W Q +G S A VFRL A R +++KT+L+ L EL E ARL WLA PVRLRDLLAGYEWRQDGLGRSSAHVFRLEADRRAPVYLKTELADPLGELAGEVARLRWLA | 71  72 |
| Query 72  Sbjct 73 | | TTGVPCAAVLDVVTEAGRDWLLLGEVPGQDLLSSH-LAPAEKVSIMADAMRRLHTLDPAT  + G+ C ++ T+ R+WLL+ + G DL+S+ L P E+V ++ADA+R+LH +D A SQGLACPEIIAHETDDEREWLLMSALHGSDLVSAEWLQPLERVRLLADALRQLHAIDIAA | 130  132 |
| Query 131  Sbjct 133 | | CPFDHQAKHRIERARTRMEAGLVDQDDLDEEHQGLAPAELFARLKARMPDGEDLVVTHGD CPFDH+ ++RI A+ + AG VD+DD DE G + +LF L+A+ P EDLVVTHGD CPFDHRLENRIGTAKAHLLAGRVDEDDFDEARLGRSAEDLFRELQAKRPSMEDLVVTHGD | 190  192 |
| Query 191  Sbjct 193 | | ACLPNIMVENGRFSGFIDCGRLGVADRYQDIALATRDIAEELGGEWADRFLVLYGIAAPD ACLPN M + +FSG+IDCGRLGVADR+QDIALA I G E FL YG P ACLPNFMADGPKFSGYIDCGRLGVADRHQDIALACGSIKHNFGEELVAEFLKAYGDFQPK | 250  252 |
| Query 251 | | SQRIAFYRLLDEFF 264  +++A+Y+LLDEFF |  |
| Sbjct | 253 | EEKMAYYQLLDEFF 266 | |

>WP_097602636.1 aminoglycoside 3'-phosphotransferase [Rhizobium phaseoli]

PDS31061.1 APH(3') family aminoglycoside O-phosphotransferase [Rhizobium phaseoli]

Length=261

Score = 243 bits (619), Expect = 2e-76, Method: Compositional matrix adjust.

| Identities | | = 124/254 (49%), Positives = 167/254 (66%), Gaps = 1/254 (0%) |  |
| --- | --- | --- | --- |
| Query 12  Sbjct 8 | | PAAWVERLFGYDWAQQTIGCSDAAVFRLSAQGRPVLFVKTDLSGALNELQDEAARLSWLA PAA +RL GY + + +G S A+VFRL +G P L++K + +G EL DEAARLSWL PAALGKRLSGYRFERDALGRSAASVFRLQGEGLPALYLKVEAAGPFGELADEAARLSWLK | 71  67 |
| Query 72  Sbjct 68 | | TTGVPCAAVLDVVTEAGRDWLLLGEVPGQDLLS-SHLAPAEKVSIMADAMRRLHTLDPAT G+PC V+ ++ +WLL+ +PG DL S S L P +V ++A A+ LH L A+ AVGLPCPDVIARESDGECNWLLINALPGADLASASALTPLARVELLAAALSDLHDLPIAS | 130  127 |
| Query 131  Sbjct 128 | | CPFDHQAKHRIERARTRMEAGLVDQDDLDEEHQGLAPAELFARLKARMPDGEDLVVTHGD CPFDH+ ++R+ A+ RM+AG+VD++D D G + A LF L P E+LVVTHGD CPFDHRLENRLPIAKARMQAGIVDEEDFDAARLGKSAAALFDDLMRLRPGDEELVVTHGD | 190  187 |
| Query 191  Sbjct 188 | | ACLPNIMVENGRFSGFIDCGRLGVADRYQDIALATRDIAEELGGEWADRFLVLYGIAAPD ACLPN + NG+FSG+IDC RLGVADR+QDIALA R IA G FL YG+ APD ACLPNFVASNGQFSGYIDCSRLGVADRHQDIALACRSIASNFGEALIRPFLDRYGLTAPD | 250  247 |
| Query 251 | | SQRIAFYRLLDEFF 264  ++ +++LLDEFF |  |
| Sbjct | 248 | PAKLDYFQLLDEFF 261 | |

>WP_196648334.1 APH(3')-II family aminoglycoside O-phosphotransferase [Pseudomonas aeruginosa]

MBG4769585.1 APH(3')-II family aminoglycoside O-phosphotransferase [Pseudomonas aeruginosa]

Length=268

Score = 243 bits (619), Expect = 2e-76, Method: Compositional matrix adjust.

| Identities | | = 136/258 (53%), Positives = 165/258 (64%), Gaps = 5/258 (2%) |  |
| --- | --- | --- | --- |
| Query 11  Sbjct 12 | | SPAAWVERLFGYDWAQQTIGCSDAAVFRLSAQGRPVLFVKTDLSGALNELQDEAARLSWL  +PA W + L GY W Q GCS A V RL A RP LFVK ++ A EL E ARL WL APATWADYLAGYRWRGQGEGCSAATVHRLEAARRPTLFVKQEVLSAHAELPAEIARLRWL | 70  71 |
| Query 71  Sbjct 72 | | ATTGVPCAAVLDVVTEAGRDWLLLGEVPGQDLLS SHLAPAEKVSIMADAMRRLHTL  G+ C VL+ GR WLL+ VPG L + L P V ++A A+RRLH L HGAGIDCPQVLNEPQRDGRQWLLMSAVPGDTLSALAQRGELEPERLVRLVAAALRRLHDL | 126  131 |
| Query 127  Sbjct 132 | | DPATCPFDHQAKHRIERARTRMEAGLVDQDDLDEEHQGLAPAELFARLKARMPDGEDLVV DPA CPFDH+ + R++ R R+EAGLVD+ D D++H+G + EL+ L R P EDLVV DPAACPFDHRLERRLDTVRQRVEAGLVDEADFDDDHRGRSATELYRLLLDRRPAVEDLVV | 186  191 |
| Query 187  Sbjct 192 | | THGDACLPNIMVENGRFSGFIDCGRLGVADRYQDIALATRDIAEELGGEWADRFLVLYGI HGDACLPN+ E RFSGF DCGRLGVADR+QD ALA RDI ELG A+ FLV YG AHGDACLPNLRAEGRRFSGFSDCGRLGVADRHQDRALAARDIEAELGAAGAEAFLVEYG- | 246  250 |
| Query | 247 AAPDSQRIAFYRLLDEFF 264 | | |
|  |  | D +R+A++RLLDEFF |  |
| Sbjct | 251 | GDIDGERLAYFRLLDEFF 268 | |

>WP_053504986.1 APH(3')-II family aminoglycoside O-phosphotransferase [Stenotrophomonas maltophilia]

KOQ79015.1 aminoglycoside phosphotransferase [Stenotrophomonas maltophilia] Length=267

Score = 243 bits (619), Expect = 2e-76, Method: Compositional matrix adjust.

| Identities | = 130/255 (51%), Positives = 163/255 (64%), Gaps = 3/255 (1%) |  |
| --- | --- | --- |
| Query 12 | PAAWVERLFGYDWAQQTIGCSDAAVFRLSAQGRPVLFVKTDLSGALNELQDEAARLSWLA | 71 |
| Sbjct 14 | P AW E L +Q+IG S A V R+ G+ F+K+++ A +EL DE ARL WL PHAWQEALADARIERQSIGVSRADVARVHRPGQADAFLKSEVIDAFSELGDEIARLRWLQ | 73 |
| Query 72 | TTGVPCAAVLDVVTEAGRDWLLLGEVPGQDLLSS-HLAPAEKVSIMADAMRRLHTLDPAT | 130 |
| Sbjct 74 | G P V+ E GR WLL+ +PG DL SS LAP +V ++ADA+R LH L AT AQGQPAPTVIATAEEEGRRWLLMSALPGHDLASSPALAPQHRVQLLADALRGLHALPVAT | 133 |
| Query 131 | CPFDHQAKHRIERARTRMEAGLVDQDDLDEEHQGLAPAELFARLKARMPDGEDLVVTHGD CPFD + R++ A+ R+EAG VD D D+E G + ++FA L+A PD EDLVV+HGD | 190 |

| Sbjct | 134 | CPFDQRLASRLQAAQARVEAGRVDADHFDDERLGQSAQQVFAELRATRPDHEDLVVSHGD | 193 |
| --- | --- | --- | --- |
| Query | 191 | ACLPNIMVENGRFSGFIDCGRLGVADRYQDIALATRDIAEELG-GEWADRFLVLYGIAAP | 249 |
| Sbjct | 194 | ACLPN++ GRFSGFIDCGRLGVADRYQD+ALA R + G W YGI P ACLPNLLAVEGRFSGFIDCGRLGVADRYQDLALAARSLVHNFGDTRWVAPLFRSYGI-VP | 252 |
| Query  Sbjct | 250  253 | DSQRIAFYRLLDEFF 264 D +R+AFYRLLDEFF DERRLAFYRLLDEFF 267 |  |

>WP_100848512.1 aminoglycoside 3'-phosphotransferase [Pseudomonas baetica]

PTC17484.1 APH(3') family aminoglycoside O-phosphotransferase [Pseudomonas baetica]

Length=256

Score = 242 bits (618), Expect = 2e-76, Method: Compositional matrix adjust.

| Identities | = 122/240 (51%), Positives = 160/240 (67%), Gaps = 2/240 (1%) |  |
| --- | --- | --- |
| Query 26 | QQTIGCSDAAVFRLSAQGRPVLFVKTDLSGALNELQDEAARLSWLATTGVPCAAVLDVVT | 85 |
| Sbjct 18 | QQTIG S A V R++ +G LFVK++ EL E RL W+A+ +P VL+ T QQTIGESRADVLRITPEGSVPLFVKSEQVMIWGELPGEVQRLRWMASQDLPAPRVLETAT | 77 |
| Query 86 | EAGRDWLLLGEVPGQDLLSS-HLAPAEKVSIMADAMRRLHTLDPATCPFDHQAKHRIERA | 144 |
| Sbjct 78 | EA R+WLL+ +PG+DL SS HLAP + V++ A+A+R LH + CPFDH+ +I A EADRNWLLMTAIPGRDLASSAHLAPQQIVTLAANALRSLHAVAINRCPFDHRLDQKIPLA | 137 |
| Query 145 | RTRMEAGLVDQDDLDEEHQGLAPAELFARLKARMPDGEDLVVTHGDACLPNIMVENGRFS | 204 |
| Sbjct 138 | R + AGL+D+ D D+ G +F ++ A PD EDLVVTHGDACLPN++ ENG+FS REHVLAGLIDEQDFDDARIGCTAENVFQQILAERPDHEDLVVTHGDACLPNLLAENGQFS | 197 |
| Query 205 | GFIDCGRLGVADRYQDIALATRDIAEELGGEWADRFLVLYGIAAPDSQRIAFYRLLDEFF | 264 |
| Sbjct 198 | GF+DCGRLGVADRYQD+ALAT I LG +W F +YG+ PD + IAFY+LLDE F  GFVDCGRLGVADRYQDLALATHSIGSNLGKQWVPMFFEVYGV-EPDQRLIAFYQLLDELF | 256 |

>VUR34649.1 Aminoglycoside 3'-phosphotransferase [Stenotrophomonas maltophilia] Length=267

Score = 243 bits (619), Expect = 2e-76, Method: Compositional matrix adjust.

| Identities | = 131/255 (51%), Positives = 165/255 (65%), Gaps = 3/255 (1%) |  |
| --- | --- | --- |
| Query 12 | PAAWVERLFGYDWAQQTIGCSDAAVFRLSAQGRPVLFVKTDLSGALNELQDEAARLSWLA | 71 |
| Sbjct 14 | P AW + L +Q+IG S A V R+ G+ F+K+++ A +EL DE ARL WL PWAWQDALADARIERQSIGVSRADVARVHRPGQADAFLKSEVIDAFSELGDEIARLRWLQ | 73 |
| Query 72 | TTGVPCAAVLDVVTEAGRDWLLLGEVPGQDLLSS-HLAPAEKVSIMADAMRRLHTLDPAT | 130 |
| Sbjct 74 | G P V+ EAGR WLL+ +PG+DL SS LAP + V ++ADA+R LH L A AQGQPVPTVIATAEEAGRRWLLMSALPGRDLASSPELAPQQLVELLADALRGLHALPVAA | 133 |
| Query 131 | CPFDHQAKHRIERARTRMEAGLVDQDDLDEEHQGLAPAELFARLKARMPDGEDLVVTHGD | 190 |
| Sbjct 134 | CPFD + R++ A+ R+EAGLVD DD D+E G +P ++FA L + PD EDLVV+HGD CPFDQRLASRVQVAQARVEAGLVDADDFDDERLGQSPQQVFAELCSTRPDHEDLVVSHGD | 193 |
| Query 191 | ACLPNIMVENGRFSGFIDCGRLGVADRYQDIALATRDIAEELG-GEWADRFLVLYGIAAP | 249 |
| Sbjct 194 | ACLPN+M GRFSGFIDCGRLGVADRYQD+ALA R + G W YG A ACLPNLMAVEGRFSGFIDCGRLGVADRYQDLALAARSLVHNFGDTRWVAPLFQRYGAVA- | 252 |
| Query 250  Sbjct 253 | DSQRIAFYRLLDEFF 264 D +R+AFYRLLDEFF DERRLAFYRLLDEFF 267 |  |

>WP_107232491.1 MULTISPECIES: APH(3')-II family aminoglycoside O-phosphotransferase [unclassified Stenotrophomonas]

PTA71014.1 APH(3')-II family aminoglycoside O-phosphotransferase [Stenotrophomonas sp. Nf1]

PTA82186.1 APH(3')-II family aminoglycoside O-phosphotransferase [Stenotrophomonas sp. Nf4]

Length=267

Score = 243 bits (619), Expect = 2e-76, Method: Compositional matrix adjust.

| Identities | = 130/255 (51%), Positives = 167/255 (65%), Gaps = 3/255 (1%) |  |
| --- | --- | --- |
| Query 12 | PAAWVERLFGYDWAQQTIGCSDAAVFRLSAQGRPVLFVKTDLSGALNELQDEAARLSWLA | 71 |
| Sbjct 14 | P AW E L G +Q+IG S A V R+ G+ F+K+++ A +EL DE ARL WL PQAWREMLAGARIERQSIGVSRADVARVYRPGQADAFLKSEVIDAFSELGDEIARLRWLQ | 73 |
| Query 72 | TTGVPCAAVLDVVTEAGRDWLLLGEVPGQDLLSS-HLAPAEKVSIMADAMRRLHTLDPAT | 130 |
| Sbjct 74 | G P V+ EAGR WLL+ +PG+DL SS L P + V+++ADA+R LH L A AQGQPAPTVIATAEEAGRRWLLMSALPGRDLASSPDLPPQQVVAVLADALRGLHALPVAA | 133 |
| Query 131 | CPFDHQAKHRIERARTRMEAGLVDQDDLDEEHQGLAPAELFARLKARMPDGEDLVVTHGD | 190 |
| Sbjct 134 | CPFD + R++ A+ R++AGLVD DD D+E G + ++FA L A P+ EDLVV+HGD CPFDQRLASRLQAAQARVKAGLVDADDFDDERLGQSAQQVFAALDATRPEHEDLVVSHGD | 193 |
| Query 191 | ACLPNIMVENGRFSGFIDCGRLGVADRYQDIALATRDIAEEL-GGEWADRFLVLYGIAAP | 249 |
| Sbjct 194 | ACLPN+MV +GRFSGFIDCGRLGVADRYQD+ALA R + W YG+ A ACLPNLMVADGRFSGFIDCGRLGVADRYQDLALAARSLVHNFDDTRWVAPLFQRYGVIA- | 252 |
| Query 250  Sbjct 253 | DSQRIAFYRLLDEFF 264 D +R+AFYRLLDEFF DERRLAFYRLLDEFF 267 |  |

>WP_151591718.1 aminoglycoside 3'-phosphotransferase [Ochrobactrum sp. Kaboul] KAB2696411.1 aminoglycoside 3'-phosphotransferase [Ochrobactrum sp. Kaboul]

Length=266

Score = 243 bits (619), Expect = 2e-76, Method: Compositional matrix adjust.

| Identities | = 125/255 (49%), Positives = 162/255 (64%), Gaps = 2/255 (1%) |  |
| --- | --- | --- |
| Query 12 | PAAWVERLFGYDWAQQTIGCSDAAVFRLSAQGRPVLFVKTDLSGALNELQDEAARLSWLA | 71 |
| Sbjct 12 | P A + RL GY Q +G S A V L L +K + + ++EL DE ARL WL+ PDAMLHRLAGYSSQQDELGRSTANVLLLDHDAHEPLVLKIEPASTVSELADERARLEWLS | 71 |
| Query 72 | TTGVPCAAVLDVVTEAGRDWLLLGEVPGQDLLSS--HLAPAEKVSIMADAMRRLHTLDPA | 129 |
| Sbjct 72 | G+PC V+ T R +LL+ + G DL SS L P V+I+A A++ LH++DPA AQGLPCPRVVVFETMDDRHFLLMTRLAGSDLASSVGTLTPDRIVAILATALKALHSVDPA | 131 |
| Query 130 | TCPFDHQAKHRIERARTRMEAGLVDQDDLDEEHQGLAPAELFARLKARMPDGEDLVVTHG | 189 |
| Sbjct 132 | CPFDH+ +HRIE AR R+EAG V++DD D+EH+G + +LF+ L P ED+VVTHG ACPFDHRLEHRIEHARKRVEAGAVNEDDFDDEHEGRSAEDLFSELCRLKPSQEDIVVTHG | 191 |
| Query 190 | DACLPNIMVENGRFSGFIDCGRLGVADRYQDIALATRDIAEELGGEWADRFLVLYGIAAP | 249 |
| Sbjct 192 | DACLPN M +G F+GFIDCGRLG+ADR+QD+ LA I LG EW FL LYG DACLPNFMASDGAFTGFIDCGRLGLADRHQDLGLACWSIRYNLGDEWVKPFLDLYGATTI | 251 |
| Query 250  Sbjct 252 | DSQRIAFYRLLDEFF 264 D +I +YRLLDEFF DDAKITYYRLLDEFF 266 |  |

>WP_075851030.1 aminoglycoside 3'-phosphotransferase [Rhizobium hainanense] SCB09485.1 aminoglycoside 3'-phosphotransferase-2 [Rhizobium hainanense]

Length=266

Score = 243 bits (619), Expect = 2e-76, Method: Compositional matrix adjust.

| Identities | = 124/254 (49%), Positives = 167/254 (66%), Gaps = 1/254 (0%) |  |
| --- | --- | --- |
| Query 12 | PAAWVERLFGYDWAQQTIGCSDAAVFRLSAQGRPVLFVKTDLSGALNELQDEAARLSWLA | 71 |
| Sbjct 13 | P + L GY+W Q +G S A VFRL A GR +++KT+ L EL E RL WLA PVRLRDALAGYEWRQDALGRSSAHVFRLEADGRAPVYLKTEPLDPLGELVGEVERLRWLA | 72 |
| Query 72 | TTGVPCAAVLDVVTEAGRDWLLLGEVPGQDLLSSH-LAPAEKVSIMADAMRRLHTLDPAT | 130 |
| Sbjct 73 | + G+ C V+ + R+W+L+ +PG DL+S+ L P ++V ++A A+RRLH +D A SQGLACPDVIAHERDGEREWVLMSALPGSDLVSAEWLQPLDRVRLLAGALRRLHAVDIAA | 132 |
| Query 131 | CPFDHQAKHRIERARTRMEAGLVDQDDLDEEHQGLAPAELFARLKARMPDGEDLVVTHGD | 190 |
| Sbjct 133 | CPFDH+ ++RI AR R+ AG VD+DD DE G + +LF L+A+ P ED VVTHGD CPFDHRLENRIGAARARLLAGRVDEDDFDETRLGRSAEDLFRELEAKRPSTEDFVVTHGD | 192 |
| Query 191 | ACLPNIMVENGRFSGFIDCGRLGVADRYQDIALATRDIAEELGGEWADRFLVLYGIAAPD ACLPN +V+ RFSG+IDCGRLG+ADR+QDIALA IA G E FL YG P | 250 |

| Sbjct | 193 | ACLPNFVVDGDRFSGYIDCGRLGIADRHQDIALACGSIAYNFGEELVAEFLKAYGDFEPK 252 |
| --- | --- | --- |
| Query | 251 | SQRIAFYRLLDEFF 264 |
| Sbjct | 253 | ++++A+Y+LLDEFF  AEKMAYYQLLDEFF 266 |

>WP_183870768.1 APH(3') family aminoglycoside O-phosphotransferase [Rhizobium sp. BK491]

MBB3566795.1 aminoglycoside 3'-phosphotransferase-2 [Rhizobium sp. BK491] Length=266

Score = 243 bits (619), Expect = 2e-76, Method: Compositional matrix adjust.

| Identities | | = 124/254 (49%), Positives = 167/254 (66%), Gaps = 1/254 (0%) |  |
| --- | --- | --- | --- |
| Query 12  Sbjct 13 | | PAAWVERLFGYDWAQQTIGCSDAAVFRLSAQGRPVLFVKTDLSGALNELQDEAARLSWLA P + L GY+W Q +G S A VFRL A R +++KT+L+ L EL E ARL WLA PVRLRDLLAGYEWRQDGLGRSSAHVFRLEADRRAPVYLKTELADPLGELAGEVARLRWLA | 71  72 |
| Query 72  Sbjct 73 | | TTGVPCAAVLDVVTEAGRDWLLLGEVPGQDLLSSH-LAPAEKVSIMADAMRRLHTLDPAT  + G+ C ++ T+ R+WLL+ + G DL+S+ L P E+V ++ADA+R+LH +D A SQGLACPEIIAHETDDEREWLLMSALHGSDLVSAEWLQPLERVRLLADALRQLHAIDIAA | 130  132 |
| Query 131  Sbjct 133 | | CPFDHQAKHRIERARTRMEAGLVDQDDLDEEHQGLAPAELFARLKARMPDGEDLVVTHGD CPFDH+ ++RI A+ + AG VD+DD DE G + +LF L+A+ P EDLVVTHGD CPFDHRLENRIGTAKAHLLAGRVDEDDFDEARLGRSAEDLFRELQAKRPSMEDLVVTHGD | 190  192 |
| Query 191  Sbjct 193 | | ACLPNIMVENGRFSGFIDCGRLGVADRYQDIALATRDIAEELGGEWADRFLVLYGIAAPD ACLPN M + +FSG+IDCGRLGVADR+QDIALA I G E FL YG P ACLPNFMADGPKFSGYIDCGRLGVADRHQDIALACGSIEHNFGEELVAEFLKAYGDFQPK | 250  252 |
| Query 251 | | SQRIAFYRLLDEFF 264  +++A+Y+LLDEFF |  |
| Sbjct | 253 | EEKMAYYQLLDEFF 266 | |

>WP_140026165.1 aminoglycoside 3'-phosphotransferase [Ochrobactrum sp. LCB8] TNV10733.1 aminoglycoside 3'-phosphotransferase [Ochrobactrum sp. LCB8]

Length=266

Score = 243 bits (619), Expect = 2e-76, Method: Compositional matrix adjust.

| Identities | = 126/255 (49%), Positives = 161/255 (63%), Gaps = 2/255 (1%) |  |
| --- | --- | --- |
| Query 12 | PAAWVERLFGYDWAQQTIGCSDAAVFRLSAQGRPVLFVKTDLSGALNELQDEAARLSWLA | 71 |
| Sbjct 12 | P A + RL GY Q +G S A V L L +K + + ++EL DE ARL WL+ PDALLHRLAGYSSQQDELGRSSANVLLLDHDAHEPLVLKIEPASTVSELADERARLEWLS | 71 |
| Query 72 | TTGVPCAAVLDVVTEAGRDWLLLGEVPGQDLLSS--HLAPAEKVSIMADAMRRLHTLDPA | 129 |
| Sbjct 72 | G+PC +V+ A R +LL+ + G DL SS L P VSI+A A++ LH++DPA AQGLPCPSVVAYERMADRHFLLMTRLAGSDLASSVGTLTPDRIVSILATALKALHSVDPA | 131 |
| Query 130 | TCPFDHQAKHRIERARTRMEAGLVDQDDLDEEHQGLAPAELFARLKARMPDGEDLVVTHG | 189 |
| Sbjct 132 | CPFDH+ +HRI+ AR R+EAG VD+DD D E +G +LF+ L P ED+VVTHG ACPFDHRLEHRIKDARKRVEAGAVDEDDFDTEREGRTAEDLFSELHRLKPSQEDVVVTHG | 191 |
| Query 190 | DACLPNIMVENGRFSGFIDCGRLGVADRYQDIALATRDIAEELGGEWADRFLVLYGIAAP | 249 |
| Sbjct 192 | DACLPN M +G F+GFIDCGRLG+ADR+QD+ LA I LG EW FL LY A DACLPNFMASDGTFTGFIDCGRLGLADRHQDLGLACWSIRYNLGNEWVKPFLDLYSAEAI | 251 |
| Query 250  Sbjct 252 | DSQRIAFYRLLDEFF 264 D +IA+YRLLDEFF DEAKIAYYRLLDEFF 266 |  |

>WP_100441621.1 APH(3')-II family aminoglycoside O-phosphotransferase [Stenotrophomonas maltophilia]

PJL26092.1 APH(3') family aminoglycoside O-phosphotransferase [Stenotrophomonas maltophilia]

Length=267

Score = 243 bits (619), Expect = 2e-76, Method: Compositional matrix adjust. Identities = 130/255 (51%), Positives = 163/255 (64%), Gaps = 3/255 (1%)

| Query | 12 | PAAWVERLFGYDWAQQTIGCSDAAVFRLSAQGRPVLFVKTDLSGALNELQDEAARLSWLA | 71 |
| --- | --- | --- | --- |
| Sbjct | 14 | P AW L +Q+IG S A V R+ G F+K+++ A +EL DE ARL WL PRAWQHALAAARIERQSIGVSRADVARVHRPGHADAFLKSEVINAFSELGDEIARLRWLL | 73 |
| Query | 72 | TTGVPCAAVLDVVTEAGRDWLLLGEVPGQDLLSS-HLAPAEKVSIMADAMRRLHTLDPAT | 130 |
| Sbjct | 74 | G P AV+ E GR WLL+ +PG+DL SS LAP V ++ADA+R LH L A AQGQPAPAVIATAEETGRRWLLMSALPGRDLASSPELAPQHLVELLADALRGLHALPVAA | 133 |
| Query | 131 | CPFDHQAKHRIERARTRMEAGLVDQDDLDEEHQGLAPAELFARLKARMPDGEDLVVTHGD | 190 |
| Sbjct | 134 | CPFD + R++ A+ R+EAGLVD DD D+E G + ++FA L+A P EDLVV+HGD CPFDQRLASRLQAAQARVEAGLVDADDFDDERLGQSAQQVFAELRATRPGHEDLVVSHGD | 193 |
| Query | 191 | ACLPNIMVENGRFSGFIDCGRLGVADRYQDIALATRDIAEELG-GEWADRFLVLYGIAAP | 249 |
| Sbjct | 194 | ACLPN++ GRFSGFIDCGRLGVADRYQD+ALA R + G W YG+AA ACLPNLLAAEGRFSGFIDCGRLGVADRYQDLALAARSLVHNFGDTRWVAPLFRRYGVAA- | 252 |
| Query  Sbjct | 250  253 | DSQRIAFYRLLDEFF 264 D +R+AFYRLLDEFF DERRLAFYRLLDEFF 267 |  |

>WP_184454282.1 APH(3') family aminoglycoside O-phosphotransferase [Rhizobium aethiopicum]

MBB4190974.1 aminoglycoside 3'-phosphotransferase-2 [Rhizobium aethiopicum] MBB4578163.1 aminoglycoside 3'-phosphotransferase-2 [Rhizobium aethiopicum]

Length=261

Score = 242 bits (618), Expect = 2e-76, Method: Compositional matrix adjust.

| Identities | | = 125/254 (49%), Positives = 162/254 (64%), Gaps = 1/254 (0%) |  |
| --- | --- | --- | --- |
| Query 12  Sbjct 8 | | PAAWVERLFGYDWAQQTIGCSDAAVFRLSAQGRPVLFVKTDLSGALNELQDEAARLSWLA P RL GY + +G S A+VFRL+ +G P L++K + +G EL DEAARL WL PGPLAARLAGYRMQRDALGRSAASVFRLAGEGLPALYLKVEAAGPFGELADEAARLDWLK | 71  67 |
| Query 72  Sbjct 68 | | TTGVPCAAVLDVVTEAGRDWLLLGEVPGQDLLS-SHLAPAEKVSIMADAMRRLHTLDPAT  +PC V+ ++ R+WLL+ +PG DL S S L P +V ++A A+ LH L A+ ACRLPCPEVIARESDGERNWLLVSALPGTDLASASALTPLARVELLAGALLDLHRLPIAS | 130  127 |
| Query 131  Sbjct 128 | | CPFDHQAKHRIERARTRMEAGLVDQDDLDEEHQGLAPAELFARLKARMPDGEDLVVTHGD CPFDH+ + R+ A+ MEAG VD++D D G AELFA L++ P EDLVVTHGD CPFDHRLEKRLPVAKAHMEAGTVDEEDFDAARLGKRAAELFAALESMRPTDEDLVVTHGD | 190  187 |
| Query 191  Sbjct 188 | | ACLPNIMVENGRFSGFIDCGRLGVADRYQDIALATRDIAEELGGEWADRFLVLYGIAAPD ACLPN + G+FSG+IDC RLGVADR+QDIALA R IA G FL YG+ APD ACLPNFVASGGQFSGYIDCSRLGVADRHQDIALACRSIAYNFGEALLQPFLDRYGLPAPD | 250  247 |
| Query 251 | | SQRIAFYRLLDEFF 264 R+ +Y+LLDEFF |  |
| Sbjct | 248 | PARLGYYQLLDEFF 261 | |

>WP_109089015.1 aminoglycoside 3'-phosphotransferase [Alcaligenes faecalis]

PWE15039.1 APH(3') family aminoglycoside O-phosphotransferase [Alcaligenes faecalis]

Length=256

Score = 242 bits (618), Expect = 2e-76, Method: Compositional matrix adjust.

| Identities | = 127/254 (50%), Positives = 160/254 (63%), Gaps = 2/254 (1%) |  |
| --- | --- | --- |
| Query 12 | PAAWVERLFGYDWAQQTIGCSDAAVFRLSAQGRPVLFVKTDLSGALNELQDEAARLSWLA | 71 |
| Sbjct 4 | PA W RL +QTIG S A V+RL FVK + L EL DE RL W+ PAVWQTRLAAAKVQRQTIGESGADVWRLDWPDGSCQFVKAEDDLLLAELPDEIERLRWMH | 63 |
| Query 72 | TTGVPCAAVLDVVTEAGRDWLLLGEVPGQDLLSSH-LAPAEKVSIMADAMRRLHTLDPAT | 130 |
| Sbjct 64 | G+PC VLD + GR WLL+ VPG+DL + L+P + V ++A +++ LH L + AQGLPCPEVLDTLVADGRHWLLMSAVPGRDLACTEGLSPKQTVELLASSLQSLHRLPLES | 123 |
| Query 131 | CPFDHQAKHRIERARTRMEAGLVDQDDLDEEHQGLAPAELFARLKARMPDGEDLVVTHGD | 190 |
| Sbjct 124 | CPFDH+ +HR+ A R +AGL+D++D D+E QG+ L L A P EDLVVTHGD  CPFDHRLEHRLADASARAKAGLIDEEDFDDERQGMPVQSLLDELYASRPMQEDLVVTHGD | 183 |

| Query | 191 | ACLPNIMVENGRFSGFIDCGRLGVADRYQDIALATRDIAEELGGEWADRFLVLYGIAAPD 250 |
| --- | --- | --- |
| Sbjct | 184 | ACLPN MV G+FSGFIDCGRLGVADR+QD+AL R I LG EW F LYG+ D ACLPNFMVHQGQFSGFIDCGRLGVADRFQDLALTARSIERNLGKEWLAPFFALYGVEM-D 242 |
| Query | 251 | SQRIAFYRLLDEFF 264 |
| Sbjct | 243 | ++RIAF+ LLDEFF  AERIAFFCLLDEFF 256 |

>WP_038690273.1 aminoglycoside 3'-phosphotransferase [Rhizobium sp. IE4771] AIC28296.1 aminoglycoside 3'-phosphotransferase protein [Rhizobium sp. IE4771]

Length=261

Score = 242 bits (618), Expect = 2e-76, Method: Compositional matrix adjust.

| Identities | = 126/254 (50%), Positives = 170/254 (67%), Gaps = 1/254 (0%) |  |
| --- | --- | --- |
| Query 12 | PAAWVERLFGYDWAQQTIGCSDAAVFRLSAQGRPVLFVKTDLSGALNELQDEAARLSWLA | 71 |
| Sbjct 8 | PAA ERL GY + + +G S A+VFRL G P L++K + +G EL DEAARL WL PAALGERLTGYRFERDALGRSAASVFRLEGGGLPALYLKVEAAGPFGELADEAARLGWLK | 67 |
| Query 72 | TTGVPCAAVLDVVTEAGRDWLLLGEVPGQDLLS-SHLAPAEKVSIMADAMRRLHTLDPAT | 130 |
| Sbjct 68 | G+PC V+ ++ R+WLL+ +PG DL S S L P +V ++A A+ LH L A+ AVGLPCPDVIARESDGKRNWLLIVALPGTDLASASALTPLTRVELLAAALSALHDLPIAS | 127 |
| Query 131 | CPFDHQAKHRIERARTRMEAGLVDQDDLDEEHQGLAPAELFARLKARMPDGEDLVVTHGD | 190 |
| Sbjct 128 | CPFDH+ ++R+ A+ RM+AG+VD++D D G LFA L++R P EDLVVTHGD CPFDHRLENRLSVAKARMQAGIVDEEDFDAARLGQTAEALFAELESRRPGNEDLVVTHGD | 187 |
| Query 191 | ACLPNIMVENGRFSGFIDCGRLGVADRYQDIALATRDIAEELGGEWADRFLVLYGIAAPD | 250 |
| Sbjct 188 | ACLPN + +G+FSG+IDC RLGVADR+QDIALA R IA G FL YG+ APD ACLPNFVACDGQFSGYIDCSRLGVADRHQDIALACRSIASNFGEALIQPFLDRYGLPAPD | 247 |
| Query 251  Sbjct 248 | SQRIAFYRLLDEFF 264  ++++ +++LLDEFF  AEKLDYFQLLDEFF 261 |  |

>WP_076015284.1 aminoglycoside 3'-phosphotransferase [Pseudomonas sp. ATCC PTA-122608] OLY75059.1 aminoglycoside phosphotransferase [Pseudomonas sp. ATCC PTA-122608]

Length=256

Score = 242 bits (618), Expect = 2e-76, Method: Compositional matrix adjust.

| Identities | | = 130/254 (51%), Positives = 157/254 (62%), Gaps = 2/254 (1%) |  |
| --- | --- | --- | --- |
| Query 12  Sbjct 4 | | PAAWVERLFGYDWAQQTIGCSDAAVFRLSAQGRPVLFVKTDLSGALNELQDEAARLSWLA P W ++ QTIG S A VFRL LF+K++ G L EL DE RL WL PKKWRDQFPNALIEPQTIGESGADVFRLRCGNGEDLFLKSEPIGPLAELPDEIKRLRWLQ | 71  63 |
| Query 72  Sbjct 64 | | TTGVPCAAVLDVVTEAGRDWLLLGEVPGQDLLS-SHLAPAEKVSIMADAMRRLHTLDPAT  + +P AVLDV+TE R WLL+ VPGQDL S S L+ + + ++A A+R LH + A QSNLPGPAVLDVLTENHRHWLLMSAVPGQDLASASDLSAPQVIRLVASALRSLHQVSIAQ | 130  123 |
| Query 131  Sbjct 124 | | CPFDHQAKHRIERARTRMEAGLVDQDDLDEEHQGLAPAELFARLKARMPDGEDLVVTHGD CPFDH +HRI A R+ AGLVD+ D D+E G ++FA L + P DLVVTHGD CPFDHSLEHRIAAAGNRVNAGLVDETDFDDERLGRTATDVFAELLSTRPAAHDLVVTHGD | 190  183 |
| Query 191  Sbjct 184 | | ACLPNIMVENGRFSGFIDCGRLGVADRYQDIALATRDIAEELGGEWADRFLVLYGIAAPD ACLPN M E FSGFIDCGRLG++DRYQD+ALA R I LG W F YG+ PD ACLPNFMAEGDHFSGFIDCGRLGISDRYQDLALAARSIERNLGAAWVAPFFEQYGV-EPD | 250  242 |
| Query | 251 SQRIAFYRLLDEFF 264 | | |
|  |  | QRIA Y LLDEFF |  |
| Sbjct | 243 | EQRIALYCLLDEFF 256 | |

>WP_154330475.1 APH(3')-II family aminoglycoside O-phosphotransferase [Stenotrophomonas maltophilia]

QGL96936.1 APH(3')-II family aminoglycoside O-phosphotransferase [Stenotrophomonas maltophilia]

Length=267

Score = 243 bits (619), Expect = 2e-76, Method: Compositional matrix adjust.

| Identities | = 129/255 (51%), Positives = 163/255 (64%), Gaps = 3/255 (1%) |  |
| --- | --- | --- |
| Query 12 | PAAWVERLFGYDWAQQTIGCSDAAVFRLSAQGRPVLFVKTDLSGALNELQDEAARLSWLA | 71 |
| Sbjct 14 | P AW E L +Q+IG S A V R+ G+ F+K+++ A +EL DE ARL WL PRAWQEALADARIERQSIGVSRADVARVHRPGQADAFLKSEVIDAFSELGDEIARLRWLQ | 73 |
| Query 72 | TTGVPCAAVLDVVTEAGRDWLLLGEVPGQDLLSS-HLAPAEKVSIMADAMRRLHTLDPAT | 130 |
| Sbjct 74 | G P V+ E GR WLL+ +PG DL SS LAP +V ++ADA+R LH L A AQGQPAPTVIATAEEEGRRWLLMSALPGHDLASSPALAPQHRVQLLADALRGLHALPVAA | 133 |
| Query 131 | CPFDHQAKHRIERARTRMEAGLVDQDDLDEEHQGLAPAELFARLKARMPDGEDLVVTHGD | 190 |
| Sbjct 134 | CPFD + R++ A+ R+EAG VD DD D+E G + ++FA L+A PD EDLVV+HGD CPFDQRLASRLQAAQARVEAGRVDADDFDDERLGQSAQQVFAELRATRPDHEDLVVSHGD | 193 |
| Query 191 | ACLPNIMVENGRFSGFIDCGRLGVADRYQDIALATRDIAEELG-GEWADRFLVLYGIAAP | 249 |
| Sbjct 194 | ACLPN++ GRFSGFIDCGRLGVADRYQD+ALA R + G W YG+ P ACLPNLLAVEGRFSGFIDCGRLGVADRYQDLALAARSLVHNFGDTRWVAPLFRSYGV-VP | 252 |
| Query 250  Sbjct 253 | DSQRIAFYRLLDEFF 264 D +R+AFYRLLDEFF DERRLAFYRLLDEFF 267 |  |

>WP_064238319.1 MULTISPECIES: APH(3')-II family aminoglycoside O-phosphotransferase [Stenotrophomonas]

KAA3600637.1 APH(3')-II family aminoglycoside O-phosphotransferase [Stenotrophomonas maltophilia]

KOO78765.1 aminoglycoside phosphotransferase [Stenotrophomonas maltophilia]

PNY71236.1 APH(3')-II family aminoglycoside O-phosphotransferase [Stenotrophomonas pavanii]

Length=267

Score = 243 bits (619), Expect = 2e-76, Method: Compositional matrix adjust.

| Identities | = 129/255 (51%), Positives = 163/255 (64%), Gaps = 3/255 (1%) |  |
| --- | --- | --- |
| Query 12 | PAAWVERLFGYDWAQQTIGCSDAAVFRLSAQGRPVLFVKTDLSGALNELQDEAARLSWLA | 71 |
| Sbjct 14 | P AW E L +Q+IG S A V R+ G+ F+K+++ A +EL DE ARL WL PHAWQEALADARIERQSIGVSRADVARVHRPGQADAFLKSEVIDAFSELGDEIARLRWLQ | 73 |
| Query 72 | TTGVPCAAVLDVVTEAGRDWLLLGEVPGQDLLSS-HLAPAEKVSIMADAMRRLHTLDPAT | 130 |
| Sbjct 74 | G P V+ E GR WLL+ +PG DL SS LAP +V ++ADA+R LH L A AQGQPAPTVIATAEEEGRRWLLMSALPGHDLASSPALAPQHRVQLLADALRGLHALPVAA | 133 |
| Query 131 | CPFDHQAKHRIERARTRMEAGLVDQDDLDEEHQGLAPAELFARLKARMPDGEDLVVTHGD | 190 |
| Sbjct 134 | CPFD + R++ A+ R+EAG VD DD D+E G + ++FA L+A PD EDLVV+HGD CPFDQRLASRLQAAQARVEAGRVDADDFDDERLGQSAQQVFAELRATRPDHEDLVVSHGD | 193 |
| Query 191 | ACLPNIMVENGRFSGFIDCGRLGVADRYQDIALATRDIAEELG-GEWADRFLVLYGIAAP | 249 |
| Sbjct 194 | ACLPN++ GRFSGFIDCGRLGVADRYQD+ALA R + G W YG+ P ACLPNLLAVEGRFSGFIDCGRLGVADRYQDLALAARSLVHNFGDTRWVAPLFRSYGV-VP | 252 |
| Query 250  Sbjct 253 | DSQRIAFYRLLDEFF 264 D +R+AFYRLLDEFF DERRLAFYRLLDEFF 267 |  |

>WP_135273319.1 APH(3')-II family aminoglycoside O-phosphotransferase [Stenotrophomonas maltophilia]

TFZ45624.1 APH(3')-II family aminoglycoside O-phosphotransferase [Stenotrophomonas maltophilia]

Length=267

Score = 242 bits (618), Expect = 2e-76, Method: Compositional matrix adjust.

| Identities | = 131/254 (52%), Positives = 162/254 (64%), Gaps = 1/254 (0%) |  |
| --- | --- | --- |
| Query 12 | PAAWVERLFGYDWAQQTIGCSDAAVFRLSAQGRPVLFVKTDLSGALNELQDEAARLSWLA | 71 |
| Sbjct 14 | P AW E L +Q+IG S A V R+ G+ FVK+++ A +EL DE ARL WL PWAWREALADARIERQSIGVSRADVARVQRLGQADAFVKSEVIDAFSELGDEIARLRWLQ | 73 |
| Query 72 | TTGVPCAAVLDVVTEAGRDWLLLGEVPGQDLLSS-HLAPAEKVSIMADAMRRLHTLDPAT | 130 |
| Sbjct 74 | G P V+ EAGR WLL+ +PG+DL SS L P ++V ++ADA+R LH L A  AQGQPAPTVIATTEEAGRRWLLMSALPGRDLASSPELVPQQQVELLADALRGLHALPVAA | 133 |

| Query | 131 | CPFDHQAKHRIERARTRMEAGLVDQDDLDEEHQGLAPAELFARLKARMPDGEDLVVTHGD | 190 |
| --- | --- | --- | --- |
| Sbjct | 134 | CPFD + R++ A+ R+EAG VD DD D+E G P ++FA L A PD EDLVV+HGD CPFDQRLASRLQAAQARVEAGRVDADDFDDERLGQTPQQVFAELCATQPDHEDLVVSHGD | 193 |
| Query | 191 | ACLPNIMVENGRFSGFIDCGRLGVADRYQDIALATRDIAEELGGEWADRFLVLYGIAAPD | 250 |
| Sbjct | 194 | ACLPN+MV GRFSGFIDCGRLGVADRYQD+ALA R + G L A D ACLPNLMVAEGRFSGFIDCGRLGVADRYQDLALAARSLVHNFGDTRGVMPLFRRYGAVAD | 253 |
| Query | 251 | SQRIAFYRLLDEFF 264  +R+AFYRLLDEFF |  |
| Sbjct | 254 | ERRLAFYRLLDEFF 267 | |

>WP_154976088.1 aminoglycoside 3'-phosphotransferase [Pseudomonas fluorescens] VVO47184.1 Aminoglycoside 3'-phosphotransferase [Pseudomonas fluorescens]

Length=256

Score = 242 bits (617), Expect = 2e-76, Method: Compositional matrix adjust.

| Identities | | = 124/254 (49%), Positives = 160/254 (63%), Gaps = 2/254 (1%) |  |
| --- | --- | --- | --- |
| Query 12  Sbjct 4 | | PAAWVERLFGYDWAQQTIGCSDAAVFRLSAQGRPVLFVKTDLSGALNELQDEAARLSWLA PA W + G +QTIG S A FR+ LFVK++ GA +EL E RL W+ PARWSQTFSGAPIERQTIGESGAEAFRIRCINGKHLFVKSEPIGAHSELPQEIERLRWMN | 71  63 |
| Query 72  Sbjct 64 | | TTGVPCAAVLDVVTEAGRDWLLLGEVPGQDLLSSH-LAPAEKVSIMADAMRRLHTLDPAT  +P VLDV TE R WLL+ V GQDL S++ L+P + +S++A A+R LH + T RLDLPGPTVLDVTTENNRHWLLMNAVSGQDLASANDLSPPQVISVLAMALRTLHQVPIET | 130  123 |
| Query 131  Sbjct 124 | | CPFDHQAKHRIERARTRMEAGLVDQDDLDEEHQGLAPAELFARLKARMPDGEDLVVTHGD CPFDH + RI AR+R+ AGLVD+ D D+E QG ++F + + +P+ DL VTHGD CPFDHSLEQRIAAARSRVSAGLVDEADFDDERQGRTAEDVFTEMLSTLPETYDLAVTHGD | 190  183 |
| Query 191  Sbjct 184 | | ACLPNIMVENGRFSGFIDCGRLGVADRYQDIALATRDIAEELGGEWADRFLVLYGIAAPD ACLPN MVE F+G+IDCGRLG++D YQD+ALA R I LG W F YG+ PD ACLPNFMVEGSSFTGYIDCGRLGISDIYQDLALAARSIERNLGPAWVAPFFRTYGV-EPD | 250  242 |
| Query | 251 SQRIAFYRLLDEFF 264 | | |
|  |  | QRIAFY LLDEF+ |  |
| Sbjct | 243 | QQRIAFYCLLDEFY 256 | |

>WP_074726005.1 APH(3')-II family aminoglycoside O-phosphotransferase [Stenotrophomonas pavanii]

Length=267

Score = 242 bits (618), Expect = 2e-76, Method: Compositional matrix adjust.

| Identities | = 129/255 (51%), Positives = 163/255 (64%), Gaps = 3/255 (1%) |  |
| --- | --- | --- |
| Query 12 | PAAWVERLFGYDWAQQTIGCSDAAVFRLSAQGRPVLFVKTDLSGALNELQDEAARLSWLA | 71 |
| Sbjct 14 | P AW E L +Q+IG S A V R+ G+ F+K+++ A +EL DE ARL WL PHAWQEALADARIERQSIGVSRADVARVHRPGQADAFLKSEVIDAFSELGDEIARLRWLQ | 73 |
| Query 72 | TTGVPCAAVLDVVTEAGRDWLLLGEVPGQDLLSS-HLAPAEKVSIMADAMRRLHTLDPAT | 130 |
| Sbjct 74 | G P V+ E GR WLL+ +PG DL SS LAP +V ++ADA+R LH L A AQGQPAPTVIATAEEEGRRWLLMSALPGHDLASSPALAPQYRVQLLADALRGLHALPVAA | 133 |
| Query 131 | CPFDHQAKHRIERARTRMEAGLVDQDDLDEEHQGLAPAELFARLKARMPDGEDLVVTHGD | 190 |
| Sbjct 134 | CPFD + R++ A+ R+EAG VD DD D+E G + ++FA L+A PD EDLVV+HGD CPFDQRLASRLQAAQARVEAGRVDADDFDDERLGQSAQQVFAELRATRPDHEDLVVSHGD | 193 |
| Query 191 | ACLPNIMVENGRFSGFIDCGRLGVADRYQDIALATRDIAEELG-GEWADRFLVLYGIAAP | 249 |
| Sbjct 194 | ACLPN++ GRFSGFIDCGRLGVADRYQD+ALA R + G W YG+ P ACLPNLLAVEGRFSGFIDCGRLGVADRYQDLALAARSLVHNFGDTRWVASLFRSYGV-VP | 252 |
| Query 250  Sbjct 253 | DSQRIAFYRLLDEFF 264 D +R+AFYRLLDEFF DERRLAFYRLLDEFF 267 |  |

>WP_049410842.1 APH(3')-II family aminoglycoside O-phosphotransferase [Stenotrophomonas maltophilia]

Length=267

Score = 242 bits (618), Expect = 3e-76, Method: Compositional matrix adjust.

| Identities | = 131/255 (51%), Positives = 165/255 (65%), Gaps = 3/255 (1%) |  |
| --- | --- | --- |
| Query 12 | PAAWVERLFGYDWAQQTIGCSDAAVFRLSAQGRPVLFVKTDLSGALNELQDEAARLSWLA | 71 |
| Sbjct 14 | P AW E L +Q+IG S A V R+ G+ F+K+++ A +EL DE RL WL PWAWQEALADAHIERQSIGVSRADVARVHRPGQTDAFLKSEVIDAFSELGDEITRLRWLQ | 73 |
| Query 72 | TTGVPCAAVLDVVTEAGRDWLLLGEVPGQDLLSS-HLAPAEKVSIMADAMRRLHTLDPAT | 130 |
| Sbjct 74 | G+P V+ EAGR WLL+ +PG+DL SS LAP + V ++ADA+RRLH L A AQGLPAPTVIATAEEAGRRWLLMSALPGRDLASSPELAPQQLVELLADALRRLHVLPVAA | 133 |
| Query 131 | CPFDHQAKHRIERARTRMEAGLVDQDDLDEEHQGLAPAELFARLKARMPDGEDLVVTHGD | 190 |
| Sbjct 134 | CPFD + R++ A+ R+EAGLVD DD D+E G + ++FA L + PD EDLVV+HGD CPFDQRLASRLQAAQARVEAGLVDADDFDDERLGQSAQQVFAELCSTRPDHEDLVVSHGD | 193 |
| Query 191 | ACLPNIMVENGRFSGFIDCGRLGVADRYQDIALATRDIAEELG-GEWADRFLVLYGIAAP | 249 |
| Sbjct 194 | ACLPN+M GRFSGFIDCGRLGVADRYQD+ALA R + G W YG A ACLPNLMAVEGRFSGFIDCGRLGVADRYQDLALAARSLVHNFGDTRWVAPLFQRYG-ALV | 252 |
| Query 250  Sbjct 253 | DSQRIAFYRLLDEFF 264 D +R+AFYRLLDEFF DERRLAFYRLLDEFF 267 |  |

>WP_180832258.1 APH(3')-II family aminoglycoside O-phosphotransferase [Stenotrophomonas maltophilia]

MBA0327312.1 APH(3')-II family aminoglycoside O-phosphotransferase [Stenotrophomonas maltophilia]

Length=267

Score = 242 bits (618), Expect = 3e-76, Method: Compositional matrix adjust.

| Identities | = 131/255 (51%), Positives = 164/255 (64%), Gaps = 3/255 (1%) |  |
| --- | --- | --- |
| Query 12 | PAAWVERLFGYDWAQQTIGCSDAAVFRLSAQGRPVLFVKTDLSGALNELQDEAARLSWLA | 71 |
| Sbjct 14 | P AW E L +Q+IG S A V R+ G+ F+K+++ A +EL DE ARL WL PRAWQEALADAHIERQSIGVSRADVARVHRPGQADAFLKSEVIDAFSELGDEIARLRWLQ | 73 |
| Query 72 | TTGVPCAAVLDVVTEAGRDWLLLGEVPGQDLLSS-HLAPAEKVSIMADAMRRLHTLDPAT | 130 |
| Sbjct 74 | G P V+ EAGR WLL+ +PG+DL SS LAP + V ++ADA+R LH L A EQGQPAPTVIATAEEAGRRWLLMSALPGRDLASSPELAPQQLVELLADALRGLHALPVAA | 133 |
| Query 131 | CPFDHQAKHRIERARTRMEAGLVDQDDLDEEHQGLAPAELFARLKARMPDGEDLVVTHGD | 190 |
| Sbjct 134 | CPFD + R+E A+ R+EA LVD +D D+E G +P ++FA L + PD EDLV+ HGD CPFDQRLASRLEAAQARVEASLVDANDFDDERLGQSPQQVFAELCSTRPDHEDLVICHGD | 193 |
| Query 191 | ACLPNIMVENGRFSGFIDCGRLGVADRYQDIALATRDIAEELG-GEWADRFLVLYGIAAP | 249 |
| Sbjct 194 | ACLPN+MV GRFSGFIDCGRLGVADRYQD+ALA R + G W YG A ACLPNLMVAEGRFSGFIDCGRLGVADRYQDLALAARSLVHNFGDTRWVAPLFQRYGALA- | 252 |
| Query 250  Sbjct 253 | DSQRIAFYRLLDEFF 264 D +R+AFYRLLDEFF DERRLAFYRLLDEFF 267 |  |

>WP_125437553.1 APH(3')-II family aminoglycoside O-phosphotransferase [Stenotrophomonas maltophilia]

RRU08379.1 APH(3')-II family aminoglycoside O-phosphotransferase [Stenotrophomonas maltophilia]

RRU12654.1 APH(3')-II family aminoglycoside O-phosphotransferase [Stenotrophomonas maltophilia]

RRU33351.1 APH(3')-II family aminoglycoside O-phosphotransferase [Stenotrophomonas maltophilia]

RRU84931.1 APH(3')-II family aminoglycoside O-phosphotransferase [Stenotrophomonas maltophilia]

RRU97325.1 APH(3')-II family aminoglycoside O-phosphotransferase [Stenotrophomonas maltophilia]

Length=267

Score = 242 bits (618), Expect = 3e-76, Method: Compositional matrix adjust. Identities = 132/255 (52%), Positives = 165/255 (65%), Gaps = 3/255 (1%)

| Query | 12 | PAAWVERLFGYDWAQQTIGCSDAAVFRLSAQGRPVLFVKTDLSGALNELQDEAARLSWLA | 71 |
| --- | --- | --- | --- |
| Sbjct | 14 | P AW E L +Q+IG S A V R+ G+ F+K+++ A +EL DE ARL WL PRAWQEALADARIERQSIGVSRADVARVHRPGQADAFLKSEVIDAFSELGDEIARLRWLQ | 73 |
| Query | 72 | TTGVPCAAVLDVVTEAGRDWLLLGEVPGQDLLSS-HLAPAEKVSIMADAMRRLHTLDPAT | 130 |
| Sbjct | 74 | G+P V+ EAGR WLL+ +PG+DL SS LAP + V ++ADA+R LH L T AQGLPAPTVIATAEEAGRRWLLMSALPGRDLASSPELAPQQLVELLADALRGLHALPVVT | 133 |
| Query | 131 | CPFDHQAKHRIERARTRMEAGLVDQDDLDEEHQGLAPAELFARLKARMPDGEDLVVTHGD | 190 |
| Sbjct | 134 | CPFDH+ R++ A R+EAGLVD DD D+E G + ++FA L + PD EDLVV+HGD CPFDHRLDLRLQAAAARVEAGLVDADDFDDERLGQSAQQVFAELCSTRPDHEDLVVSHGD | 193 |
| Query | 191 | ACLPNIMVENGRFSGFIDCGRLGVADRYQDIALATRDIAEELG-GEWADRFLVLYGIAAP | 249 |
| Sbjct | 194 | ACLPN+M GRFSGFIDCGRLGVADRYQD+ALA R + G W YG A ACLPNLMAVEGRFSGFIDCGRLGVADRYQDLALAARSLVHNFGDTRWVAPLFQRYG-ALV | 252 |
| Query  Sbjct | 250  253 | DSQRIAFYRLLDEFF 264 D +R+AFYRLLDEFF DERRLAFYRLLDEFF 267 |  |

>SDK55887.1 aminoglycoside 3'-phosphotransferase-2 [Stenotrophomonas pavanii] Length=282

Score = 243 bits (619), Expect = 3e-76, Method: Compositional matrix adjust.

| Identities | = 129/255 (51%), Positives = 163/255 (64%), Gaps = 3/255 (1%) |  |
| --- | --- | --- |
| Query 12 | PAAWVERLFGYDWAQQTIGCSDAAVFRLSAQGRPVLFVKTDLSGALNELQDEAARLSWLA | 71 |
| Sbjct 29 | P AW E L +Q+IG S A V R+ G+ F+K+++ A +EL DE ARL WL PHAWQEALADARIERQSIGVSRADVARVHRPGQADAFLKSEVIDAFSELGDEIARLRWLQ | 88 |
| Query 72 | TTGVPCAAVLDVVTEAGRDWLLLGEVPGQDLLSS-HLAPAEKVSIMADAMRRLHTLDPAT | 130 |
| Sbjct 89 | G P V+ E GR WLL+ +PG DL SS LAP +V ++ADA+R LH L A AQGQPAPTVIATAEEEGRRWLLMSALPGHDLASSPALAPQYRVQLLADALRGLHALPVAA | 148 |
| Query 131 | CPFDHQAKHRIERARTRMEAGLVDQDDLDEEHQGLAPAELFARLKARMPDGEDLVVTHGD | 190 |
| Sbjct 149 | CPFD + R++ A+ R+EAG VD DD D+E G + ++FA L+A PD EDLVV+HGD CPFDQRLASRLQAAQARVEAGRVDADDFDDERLGQSAQQVFAELRATRPDHEDLVVSHGD | 208 |
| Query 191 | ACLPNIMVENGRFSGFIDCGRLGVADRYQDIALATRDIAEELG-GEWADRFLVLYGIAAP | 249 |
| Sbjct 209 | ACLPN++ GRFSGFIDCGRLGVADRYQD+ALA R + G W YG+ P ACLPNLLAVEGRFSGFIDCGRLGVADRYQDLALAARSLVHNFGDTRWVASLFRSYGV-VP | 267 |
| Query 250  Sbjct 268 | DSQRIAFYRLLDEFF 264 D +R+AFYRLLDEFF DERRLAFYRLLDEFF 282 |  |

>KOE99731.1 aminoglycoside phosphotransferase [[Pseudomonas] geniculata N1] Length=267

Score = 242 bits (618), Expect = 3e-76, Method: Compositional matrix adjust.

| Identities | = 132/255 (52%), Positives = 165/255 (65%), Gaps = 3/255 (1%) |  |
| --- | --- | --- |
| Query 12 | PAAWVERLFGYDWAQQTIGCSDAAVFRLSAQGRPVLFVKTDLSGALNELQDEAARLSWLA | 71 |
| Sbjct 14 | P AW E L +Q+IG S A V R+ G+ F+K+++ A +EL DE ARL WL PRAWQEALADARIERQSIGVSRADVARVHRPGQADAFLKSEVIDAFSELGDEIARLRWLQ | 73 |
| Query 72 | TTGVPCAAVLDVVTEAGRDWLLLGEVPGQDLLSS-HLAPAEKVSIMADAMRRLHTLDPAT | 130 |
| Sbjct 74 | GVP V+ EAGR WLL+ +PG+DL SS LAP + V ++ADA+RRLH L A AQGVPAPTVIATAEEAGRRWLLMSALPGRDLASSPELAPQQLVELLADALRRLHVLPVAA | 133 |
| Query 131 | CPFDHQAKHRIERARTRMEAGLVDQDDLDEEHQGLAPAELFARLKARMPDGEDLVVTHGD | 190 |
| Sbjct 134 | CPFD + R++ A+ R+EAGLVD DD D+E G + ++FA L + PD EDLVV+HGD CPFDQRLASRLQAAQARVEAGLVDADDFDDERLGQSAQQVFAELCSTRPDHEDLVVSHGD | 193 |
| Query 191 | ACLPNIMVENGRFSGFIDCGRLGVADRYQDIALATRDIAEELG-GEWADRFLVLYGIAAP | 249 |
| Sbjct 194 | ACLPN+M GRFSGFIDCG LGVADRYQD+ALA R + G W YG A ACLPNLMAVEGRFSGFIDCGWLGVADRYQDLALAARSLVHNFGDTRWVAPLFQRYG-ALV | 252 |
| Query 250 | DSQRIAFYRLLDEFF 264 |  |

D +R+AFYRLLDEFF Sbjct 253 DERRLAFYRLLDEFF 267

>WP_180836928.1 APH(3')-II family aminoglycoside O-phosphotransferase [Stenotrophomonas maltophilia]

MBA0419953.1 APH(3')-II family aminoglycoside O-phosphotransferase [Stenotrophomonas maltophilia]

VUK53360.1 Aminoglycoside 3'-phosphotransferase [Stenotrophomonas maltophilia] Length=267

Score = 242 bits (618), Expect = 3e-76, Method: Compositional matrix adjust.

| Identities | = 130/255 (51%), Positives = 164/255 (64%), Gaps = 3/255 (1%) |  |
| --- | --- | --- |
| Query 12 | PAAWVERLFGYDWAQQTIGCSDAAVFRLSAQGRPVLFVKTDLSGALNELQDEAARLSWLA | 71 |
| Sbjct 14 | P AW + L +Q+IG S A V R+ G+ F+K+++ A +EL DE ARL WL PWAWQDALADARIERQSIGVSRADVARVHRPGQADAFLKSEVIDAFSELGDEIARLRWLQ | 73 |
| Query 72 | TTGVPCAAVLDVVTEAGRDWLLLGEVPGQDLLSS-HLAPAEKVSIMADAMRRLHTLDPAT | 130 |
| Sbjct 74 | G P V+ EAGR WLL+ +PG+DL SS LAP + V ++ADA+R LH L A AQGQPVPTVIATAEEAGRRWLLMSALPGRDLASSPELAPQQLVELLADALRGLHALPVAA | 133 |
| Query 131 | CPFDHQAKHRIERARTRMEAGLVDQDDLDEEHQGLAPAELFARLKARMPDGEDLVVTHGD | 190 |
| Sbjct 134 | CPFD + R++ A+ R+EAGLVD DD D+E G +P ++FA L + PD EDLVV+HGD CPFDQRLASRVQVAQARIEAGLVDADDFDDERLGQSPQQVFAELCSTRPDHEDLVVSHGD | 193 |
| Query 191 | ACLPNIMVENGRFSGFIDCGRLGVADRYQDIALATRDIAEELG-GEWADRFLVLYGIAAP | 249 |
| Sbjct 194 | ACLPN+MV GRFSGFIDCGRLGVADRYQD+ALA R + G W Y A ACLPNLMVAEGRFSGFIDCGRLGVADRYQDLALAARSVVHNFGDTRWVAPLFQRYSAVA- | 252 |
| Query 250  Sbjct 253 | DSQRIAFYRLLDEFF 264 D +R+ FYRLLDEFF DERRLVFYRLLDEFF 267 |  |

>WP_134808795.1 APH(3')-II family aminoglycoside O-phosphotransferase [Stenotrophomonas maltophilia]

KLO00290.1 aminoglycoside phosphotransferase [Stenotrophomonas maltophilia] Length=267

Score = 242 bits (618), Expect = 3e-76, Method: Compositional matrix adjust.

| Identities | = 129/255 (51%), Positives = 164/255 (64%), Gaps = 3/255 (1%) |  |
| --- | --- | --- |
| Query 12 | PAAWVERLFGYDWAQQTIGCSDAAVFRLSAQGRPVLFVKTDLSGALNELQDEAARLSWLA | 71 |
| Sbjct 14 | P AW E L +Q+IG S A V R+ G+ F+K+++ A +EL DE ARL WL PWAWRETLADARIERQSIGVSRADVARVHRPGQADAFLKSEVIDAFSELGDEIARLRWLQ | 73 |
| Query 72 | TTGVPCAAVLDVVTEAGRDWLLLGEVPGQDLLSS-HLAPAEKVSIMADAMRRLHTLDPAT | 130 |
| Sbjct 74 | G P V+ EAGR WLL+ +PG+DL SS L P V ++ADA+R LH + A AQGQPAPTVIATAEEAGRRWLLMSALPGRDLASSPELPPQRLVELLADALRGLHAIPVAA | 133 |
| Query 131 | CPFDHQAKHRIERARTRMEAGLVDQDDLDEEHQGLAPAELFARLKARMPDGEDLVVTHGD | 190 |
| Sbjct 134 | CPFD + R++ A+ R+EAGLVD DD D+E G + ++FA L+A PD EDLVV+HGD CPFDQRLASRLQAAQERVEAGLVDADDFDDERLGQSAQQVFAELRATRPDHEDLVVSHGD | 193 |
| Query 191 | ACLPNIMVENGRFSGFIDCGRLGVADRYQDIALATRDIAEELG-GEWADRFLVLYGIAAP | 249 |
| Sbjct 194 | ACLPN++ GRFSGFIDCGRLGVADRYQD+ALA R + G W YG+ P ACLPNLLAAEGRFSGFIDCGRLGVADRYQDLALAARSLVHNFGDTRWVAPLFRRYGV-VP | 252 |
| Query 250  Sbjct 253 | DSQRIAFYRLLDEFF 264 D +R+AFYRLLDEFF DERRLAFYRLLDEFF 267 |  |

>WP_021202874.1 APH(3')-II family aminoglycoside O-phosphotransferase [Stenotrophomonas maltophilia]

EQM83034.1 aminoglycoside 3'-phosphotransferase [Stenotrophomonas maltophilia MF89]

Length=267

Score = 242 bits (618), Expect = 3e-76, Method: Compositional matrix adjust. Identities = 131/255 (51%), Positives = 164/255 (64%), Gaps = 3/255 (1%)

| Query | 12 | PAAWVERLFGYDWAQQTIGCSDAAVFRLSAQGRPVLFVKTDLSGALNELQDEAARLSWLA | 71 |
| --- | --- | --- | --- |
| Sbjct | 14 | P AW E L +Q+IG S A V R+ G+ F+K+++ A +EL DE ARL WL PRAWQEALADAHIERQSIGVSRADVARVHRPGQADAFLKSEVIDAFSELGDEIARLRWLQ | 73 |
| Query | 72 | TTGVPCAAVLDVVTEAGRDWLLLGEVPGQDLLSS-HLAPAEKVSIMADAMRRLHTLDPAT | 130 |
| Sbjct | 74 | G P V+ EAGR WLL+ +PG+DL SS LAP + V ++ADA+RRLH L A EQGQPAPTVIATAEEAGRRWLLMSALPGRDLASSPELAPQQLVELLADALRRLHVLPVAA | 133 |
| Query | 131 | CPFDHQAKHRIERARTRMEAGLVDQDDLDEEHQGLAPAELFARLKARMPDGEDLVVTHGD | 190 |
| Sbjct | 134 | CPFD + R++ A R++AGLVD DD D+E G + ++FA L + PD EDLVV+HGD CPFDQRLASRLQAAAARVDAGLVDADDFDDERLGQSAQQVFAELCSTRPDHEDLVVSHGD | 193 |
| Query | 191 | ACLPNIMVENGRFSGFIDCGRLGVADRYQDIALATRDIAEELG-GEWADRFLVLYGIAAP | 249 |
| Sbjct | 194 | ACLPN+M GRFSGFIDCGRLGVADRYQD+ALA R + G W YG A ACLPNLMAVEGRFSGFIDCGRLGVADRYQDLALAARSLVHNFGDTRWVAPLFQRYGAVA- | 252 |
| Query  Sbjct | 250  253 | DSQRIAFYRLLDEFF 264 D +R+AFYRLLDEFF DERRLAFYRLLDEFF 267 |  |

>WP_092850984.1 aminoglycoside 3'-phosphotransferase [Rhizobium miluonense] Length=267

Score = 242 bits (618), Expect = 3e-76, Method: Compositional matrix adjust.

| Identities | = 121/247 (49%), Positives = 162/247 (66%), Gaps = 1/247 (0%) |  |
| --- | --- | --- |
| Query 19 | LFGYDWAQQTIGCSDAAVFRLSAQGRPVLFVKTDLSGALNELQDEAARLSWLATTGVPCA | 78 |
| Sbjct 21 | L GY+W Q +G S A VFRL A GR +++KT+L L EL E ARL WL + G C LAGYEWRQDALGRSSAHVFRLEADGRAPVYLKTELVDPLGELAGEVARLRWLNSQGFACP | 80 |
| Query 79 | AVLDVVTEAGRDWLLLGEVPGQDLLSSHL-APAEKVSIMADAMRRLHTLDPATCPFDHQA | 137 |
| Sbjct 81 | ++ + R+WLL+ +PG DL+S+ L +PAE+V ++A A+RRLH +D A CPFDH+ EIIAHERDDEREWLLMSALPGSDLVSAELISPAERVRLLAHALRRLHAIDIAACPFDHRL | 140 |
| Query 138 | KHRIERARTRMEAGLVDQDDLDEEHQGLAPAELFARLKARMPDGEDLVVTHGDACLPNIM | 197 |
| Sbjct 141 | +RI AR R+ AG VD+DD DE G + +LF L+ + P +DLVVTHGDACLPN + ANRIGEARARLLAGCVDEDDFDETRLGRSGQDLFGELEEKRPVTDDLVVTHGDACLPNFI | 200 |
| Query 198 | VENGRFSGFIDCGRLGVADRYQDIALATRDIAEELGGEWADRFLVLYGIAAPDSQRIAFY | 257 |
| Sbjct 201 | + FSG+IDCGRLG+ADR+QDIALA I G + FL YG P +++I +Y ADGAGFSGYIDCGRLGIADRHQDIALACSSIERNFGSDLLAEFLSAYGDFEPQAEKIGYY | 260 |
| Query 258  Sbjct 261 | RLLDEFF 264  +LLDEFF  QLLDEFF 267 |  |

>SCB32369.1 aminoglycoside 3'-phosphotransferase-2 [Rhizobium miluonense] Length=266

Score = 242 bits (618), Expect = 3e-76, Method: Compositional matrix adjust.

| Identities | | = 121/247 (49%), Positives = 162/247 (66%), Gaps = 1/247 (0%) |  |
| --- | --- | --- | --- |
| Query 19  Sbjct 20 | | LFGYDWAQQTIGCSDAAVFRLSAQGRPVLFVKTDLSGALNELQDEAARLSWLATTGVPCA L GY+W Q +G S A VFRL A GR +++KT+L L EL E ARL WL + G C LAGYEWRQDALGRSSAHVFRLEADGRAPVYLKTELVDPLGELAGEVARLRWLNSQGFACP | 78  79 |
| Query 79  Sbjct 80 | | AVLDVVTEAGRDWLLLGEVPGQDLLSSHL-APAEKVSIMADAMRRLHTLDPATCPFDHQA  ++ + R+WLL+ +PG DL+S+ L +PAE+V ++A A+RRLH +D A CPFDH+ EIIAHERDDEREWLLMSALPGSDLVSAELISPAERVRLLAHALRRLHAIDIAACPFDHRL | 137  139 |
| Query 138  Sbjct 140 | | KHRIERARTRMEAGLVDQDDLDEEHQGLAPAELFARLKARMPDGEDLVVTHGDACLPNIM  +RI AR R+ AG VD+DD DE G + +LF L+ + P +DLVVTHGDACLPN + ANRIGEARARLLAGCVDEDDFDETRLGRSGQDLFGELEEKRPVTDDLVVTHGDACLPNFI | 197  199 |
| Query 198  Sbjct 200 | | VENGRFSGFIDCGRLGVADRYQDIALATRDIAEELGGEWADRFLVLYGIAAPDSQRIAFY  + FSG+IDCGRLG+ADR+QDIALA I G + FL YG P +++I +Y  ADGAGFSGYIDCGRLGIADRHQDIALACSSIERNFGSDLLAEFLSAYGDFEPQAEKIGYY | 257  259 |
| Query | 258 RLLDEFF 264 | | |

+LLDEFF Sbjct 260 QLLDEFF 266

>WP_164077566.1 APH(3')-II family aminoglycoside O-phosphotransferase [Stenotrophomonas maltophilia]

VUP83698.1 Aminoglycoside 3'-phosphotransferase [Stenotrophomonas maltophilia] Length=267

Score = 242 bits (618), Expect = 3e-76, Method: Compositional matrix adjust.

| Identities | = 129/255 (51%), Positives = 165/255 (65%), Gaps = 3/255 (1%) |  |
| --- | --- | --- |
| Query 12 | PAAWVERLFGYDWAQQTIGCSDAAVFRLSAQGRPVLFVKTDLSGALNELQDEAARLSWLA | 71 |
| Sbjct 14 | P AW E L +Q+IG S A V R+ G+ F+K+++ A +EL DE ARL WL PWAWRETLADARIERQSIGVSRADVARVHRPGQADAFLKSEVIDAFSELGDEIARLRWLQ | 73 |
| Query 72 | TTGVPCAAVLDVVTEAGRDWLLLGEVPGQDLLSS-HLAPAEKVSIMADAMRRLHTLDPAT | 130 |
| Sbjct 74 | G P AV+ EAGR WLL+ +PG+DL SS L P V ++ADA+R LH + A GQGQPAPAVIATAEEAGRRWLLMSALPGRDLASSPELPPQRLVELLADALRGLHAIPVAA | 133 |
| Query 131 | CPFDHQAKHRIERARTRMEAGLVDQDDLDEEHQGLAPAELFARLKARMPDGEDLVVTHGD | 190 |
| Sbjct 134 | CPFD + R++ A+ R+EAGLVD DD D+E G + ++FA L+A P+ EDLVV+HGD CPFDQRLASRLQAAQARVEAGLVDADDFDDERLGQSAQQVFAELRATRPNHEDLVVSHGD | 193 |
| Query 191 | ACLPNIMVENGRFSGFIDCGRLGVADRYQDIALATRDIAEELG-GEWADRFLVLYGIAAP | 249 |
| Sbjct 194 | ACLPN++ GRFSGFIDCGRLGVADRYQD+ALA R + G W YG+ P ACLPNLLAAEGRFSGFIDCGRLGVADRYQDLALAARSLVHNFGDTRWVAPLFRRYGV-VP | 252 |
| Query 250  Sbjct 253 | DSQRIAFYRLLDEFF 264 D +R+AFYRLLDEFF DERRLAFYRLLDEFF 267 |  |

>WP_085737875.1 aminoglycoside 3'-phosphotransferase [Rhizobium sp. CIAT894] Length=258

Score = 242 bits (617), Expect = 3e-76, Method: Compositional matrix adjust.

| Identities | | = 128/254 (50%), Positives = 165/254 (65%), Gaps = 1/254 (0%) |  |
| --- | --- | --- | --- |
| Query 12  Sbjct 5 | | PAAWVERLFGYDWAQQTIGCSDAAVFRLSAQGRPVLFVKTDLSGALNELQDEAARLSWLA P A V+RL GY + +G S A+VFRL +G PVL++K + G EL DEAARL WL PGALVDRLSGYRMERDALGRSAASVFRLEGEGLPVLYLKVEAMGPFGELADEAARLDWLK | 71  64 |
| Query 72  Sbjct 65 | | TTGVPCAAVLDVVTEAGRDWLLLGEVPGQDLLS-SHLAPAEKVSIMADAMRRLHTLDPAT  +G+PC V+ + GR+ LL+ +PG DL S S L P +V ++A A+ LH L A+ ASGLPCPEVIAWESSGGRNRLLISALPGADLASASTLTPLMRVELLAAALLGLHRLPIAS | 130  124 |
| Query 131  Sbjct 125 | | CPFDHQAKHRIERARTRMEAGLVDQDDLDEEHQGLAPAELFARLKARMPDGEDLVVTHGD CPFDH+ ++RI A+ RM+AG+VD+ D DE G + LFA L+ R P EDLVVTHGD CPFDHRLENRIAAAKARMQAGIVDETDFDETRLGKSAETLFAELENRRPGREDLVVTHGD | 190  184 |
| Query 191  Sbjct 185 | | ACLPNIMVENGRFSGFIDCGRLGVADRYQDIALATRDIAEELGGEWADRFLVLYGIAAPD ACLPN + FSG+IDC RLGVADR+QDIALA R IA GG FL YG+ PD ACLPNFIASAEGFSGYIDCSRLGVADRHQDIALACRSIAHNFGGALVQPFLDRYGLTTPD | 250  244 |
| Query 251 | | SQRIAFYRLLDEFF 264 R+ +++LLDEFF |  |
| Sbjct | 245 | PARLHYFQLLDEFF 258 | |

>VUO84224.1 Aminoglycoside 3'-phosphotransferase [Stenotrophomonas maltophilia] VUQ00416.1 Aminoglycoside 3'-phosphotransferase [Stenotrophomonas maltophilia]

Length=282

Score = 243 bits (619), Expect = 3e-76, Method: Compositional matrix adjust.

| Identities | = 129/255 (51%), Positives = 163/255 (64%), Gaps = 3/255 (1%) |  |
| --- | --- | --- |
| Query 12 | PAAWVERLFGYDWAQQTIGCSDAAVFRLSAQGRPVLFVKTDLSGALNELQDEAARLSWLA | 71 |
| Sbjct 29 | P AW E L +Q+IG S A V R+ G+ F+K+++ A +EL DE ARL WL PHAWQEALADARIERQSIGVSRADVARVHRPGQADAFLKSEVIDAFSELGDEIARLRWLQ | 88 |
| Query 72 | TTGVPCAAVLDVVTEAGRDWLLLGEVPGQDLLSSH-LAPAEKVSIMADAMRRLHTLDPAT | 130 |

G P	V+	E GR WLL+ +PG DL SS LAP +V ++ADA+R LH L A

| Sbjct | 89 | AQGQPAPTVIATAEEEGRRWLLMSALPGHDLASSPALAPQHRVQLLADALRGLHALPVAA | 148 |
| --- | --- | --- | --- |
| Query | 131 | CPFDHQAKHRIERARTRMEAGLVDQDDLDEEHQGLAPAELFARLKARMPDGEDLVVTHGD | 190 |
| Sbjct | 149 | CPFD + R++ A+ R+EAG VD DD D+E G + ++FA L+A PD EDLVV+HGD CPFDQRLASRLQAAQARVEAGRVDADDFDDERLGQSAQQVFAELRATRPDHEDLVVSHGD | 208 |
| Query | 191 | ACLPNIMVENGRFSGFIDCGRLGVADRYQDIALATRDIAEELG-GEWADRFLVLYGIAAP | 249 |
| Sbjct | 209 | ACLPN++ GRFSGFIDCGRLGVADRYQD+ALA R + G W YG+ P ACLPNLLAVEGRFSGFIDCGRLGVADRYQDLALAARSLVHNFGDTRWVAPLFRSYGV-VP | 267 |
| Query  Sbjct | 250  268 | DSQRIAFYRLLDEFF 264 D +R+AFYRLLDEFF DERRLAFYRLLDEFF 282 |  |

>VUK31393.1 Aminoglycoside 3'-phosphotransferase [Stenotrophomonas maltophilia] VUN10611.1 Aminoglycoside 3'-phosphotransferase [Stenotrophomonas maltophilia] VUO11818.1 Aminoglycoside 3'-phosphotransferase [Stenotrophomonas maltophilia]

Length=267

Score = 242 bits (617), Expect = 3e-76, Method: Compositional matrix adjust.

| Identities | = 131/255 (51%), Positives = 162/255 (64%), Gaps = 3/255 (1%) |  |
| --- | --- | --- |
| Query 12 | PAAWVERLFGYDWAQQTIGCSDAAVFRLSAQGRPVLFVKTDLSGALNELQDEAARLSWLA | 71 |
| Sbjct 14 | P+AW E L G +Q+IG S A V R+ G+ F+K+++ +EL DE ARL WL PSAWREALAGARIERQSIGVSRADVARVHRPGQADAFLKSEVIDPFSELGDEIARLRWLQ | 73 |
| Query 72 | TTGVPCAAVLDVVTEAGRDWLLLGEVPGQDLLSS-HLAPAEKVSIMADAMRRLHTLDPAT | 130 |
| Sbjct 74 | G P V+ +AGR WLL+ +PG+DL SS L P V ++ADA+R LH L A AQGQPVPTVIATAEDAGRRWLLMSALPGRDLASSPELVPQRLVEVLADALRGLHALPVAA | 133 |
| Query 131 | CPFDHQAKHRIERARTRMEAGLVDQDDLDEEHQGLAPAELFARLKARMPDGEDLVVTHGD | 190 |
| Sbjct 134 | CPFD + R++ A R EAGLVD DD D+E G + ++ A L A P EDLVV+HGD CPFDQRLASRVQAAVARAEAGLVDADDFDDERLGQSAQQVLAELLASQPAHEDLVVSHGD | 193 |
| Query 191 | ACLPNIMVENGRFSGFIDCGRLGVADRYQDIALATRDIAEELGG-EWADRFLVLYGIAAP | 249 |
| Sbjct 194 | ACLPN+MV GRFSGFIDCGRLGVADRYQD+ALATR + G W L YG A ACLPNLMVAEGRFSGFIDCGRLGVADRYQDLALATRSLVHNFGNTRWVAPLLQRYGAVA- | 252 |
| Query 250  Sbjct 253 | DSQRIAFYRLLDEFF 264 D +R+AFYRLLDEFF DERRLAFYRLLDEFF 267 |  |

>VUO71045.1 Aminoglycoside 3'-phosphotransferase [Stenotrophomonas maltophilia] Length=282

Score = 243 bits (619), Expect = 3e-76, Method: Compositional matrix adjust.

| Identities | = 129/255 (51%), Positives = 163/255 (64%), Gaps = 3/255 (1%) |  |
| --- | --- | --- |
| Query 12 | PAAWVERLFGYDWAQQTIGCSDAAVFRLSAQGRPVLFVKTDLSGALNELQDEAARLSWLA | 71 |
| Sbjct 29 | P AW E L +Q+IG S A V R+ G+ F+K+++ A +EL DE ARL WL PHAWQEALADARIERQSIGVSRADVARVHRPGQADAFLKSEVIDAFSELGDEIARLRWLQ | 88 |
| Query 72 | TTGVPCAAVLDVVTEAGRDWLLLGEVPGQDLLSSH-LAPAEKVSIMADAMRRLHTLDPAT | 130 |
| Sbjct 89 | G P V+ E GR WLL+ +PG DL SS LAP +V ++ADA+R LH L A AQGQPAPTVIATAEEEGRRWLLMSALPGHDLASSPALAPQHRVQLLADALRGLHALPVAA | 148 |
| Query 131 | CPFDHQAKHRIERARTRMEAGLVDQDDLDEEHQGLAPAELFARLKARMPDGEDLVVTHGD | 190 |
| Sbjct 149 | CPFD + R++ A+ R+EAG VD DD D+E G + ++FA L+A PD EDLVV+HGD CPFDQRLASRLQAAQARVEAGRVDADDFDDERLGQSAQQVFAELRATRPDHEDLVVSHGD | 208 |
| Query 191 | ACLPNIMVENGRFSGFIDCGRLGVADRYQDIALATRDIAEELG-GEWADRFLVLYGIAAP | 249 |
| Sbjct 209 | ACLPN++ GRFSGFIDCGRLGVADRYQD+ALA R + G W YG+ P ACLPNLLAVEGRFSGFIDCGRLGVADRYQDLALAARSLVHNFGDTRWVAPLFRSYGV-VP | 267 |
| Query 250  Sbjct 268 | DSQRIAFYRLLDEFF 264 D +R+AFYRLLDEFF DERRLAFYRLLDEFF 282 |  |

>ARM89293.1 aminoglycoside 3'-phosphotransferase protein [Rhizobium sp. CIAT894] Length=262

Score = 242 bits (617), Expect = 3e-76, Method: Compositional matrix adjust.

| Identities | | = 128/254 (50%), Positives = 165/254 (65%), Gaps = 1/254 (0%) |  |
| --- | --- | --- | --- |
| Query 12  Sbjct 9 | | PAAWVERLFGYDWAQQTIGCSDAAVFRLSAQGRPVLFVKTDLSGALNELQDEAARLSWLA P A V+RL GY + +G S A+VFRL +G PVL++K + G EL DEAARL WL PGALVDRLSGYRMERDALGRSAASVFRLEGEGLPVLYLKVEAMGPFGELADEAARLDWLK | 71  68 |
| Query 72  Sbjct 69 | | TTGVPCAAVLDVVTEAGRDWLLLGEVPGQDLLS-SHLAPAEKVSIMADAMRRLHTLDPAT  +G+PC V+ + GR+ LL+ +PG DL S S L P +V ++A A+ LH L A+ ASGLPCPEVIAWESSGGRNRLLISALPGADLASASTLTPLMRVELLAAALLGLHRLPIAS | 130  128 |
| Query 131  Sbjct 129 | | CPFDHQAKHRIERARTRMEAGLVDQDDLDEEHQGLAPAELFARLKARMPDGEDLVVTHGD CPFDH+ ++RI A+ RM+AG+VD+ D DE G + LFA L+ R P EDLVVTHGD CPFDHRLENRIAAAKARMQAGIVDETDFDETRLGKSAETLFAELENRRPGREDLVVTHGD | 190  188 |
| Query 191  Sbjct 189 | | ACLPNIMVENGRFSGFIDCGRLGVADRYQDIALATRDIAEELGGEWADRFLVLYGIAAPD ACLPN + FSG+IDC RLGVADR+QDIALA R IA GG FL YG+ PD ACLPNFIASAEGFSGYIDCSRLGVADRHQDIALACRSIAHNFGGALVQPFLDRYGLTTPD | 250  248 |
| Query 251 | | SQRIAFYRLLDEFF 264 R+ +++LLDEFF |  |
| Sbjct | 249 | PARLHYFQLLDEFF 262 | |

>WP_111206790.1 MULTISPECIES: APH(3')-II family aminoglycoside O-phosphotransferase [Stenotrophomonas]

PZT18671.1 APH(3') family aminoglycoside O-phosphotransferase [Stenotrophomonas maltophilia]

QCB32228.1 APH(3')-II family aminoglycoside O-phosphotransferase [Stenotrophomonas sp. PAMC25021]

QGL84315.1 APH(3')-II family aminoglycoside O-phosphotransferase [Stenotrophomonas maltophilia]

VUR02294.1 Aminoglycoside 3'-phosphotransferase [Stenotrophomonas maltophilia] VUR78356.1 Aminoglycoside 3'-phosphotransferase [Stenotrophomonas maltophilia]

Length=267

Score = 242 bits (617), Expect = 3e-76, Method: Compositional matrix adjust.

| Identities | = 129/255 (51%), Positives = 164/255 (64%), Gaps = 3/255 (1%) |  |
| --- | --- | --- |
| Query 12 | PAAWVERLFGYDWAQQTIGCSDAAVFRLSAQGRPVLFVKTDLSGALNELQDEAARLSWLA | 71 |
| Sbjct 14 | P AW E L +Q+IG S A V R+ G+ F+K+++ A +EL DE ARL WL PWAWRETLADARIERQSIGVSRADVARVHRPGQADAFLKSEVIDAFSELGDEIARLRWLQ | 73 |
| Query 72 | TTGVPCAAVLDVVTEAGRDWLLLGEVPGQDLLSS-HLAPAEKVSIMADAMRRLHTLDPAT | 130 |
| Sbjct 74 | G P V+ EAGR WLL+ +PG+DL SS L P V ++ADA+R LH + A AQGQPAPTVIATAEEAGRRWLLMSALPGRDLASSPELPPQRLVELLADALRGLHAIPVAA | 133 |
| Query 131 | CPFDHQAKHRIERARTRMEAGLVDQDDLDEEHQGLAPAELFARLKARMPDGEDLVVTHGD | 190 |
| Sbjct 134 | CPFD + R++ A+ R+EAGLVD DD D+E G + ++FA L+A PD EDLVV+HGD CPFDQRLASRLQAAQERVEAGLVDADDFDDERLGQSAQQVFAELRATRPDHEDLVVSHGD | 193 |
| Query 191 | ACLPNIMVENGRFSGFIDCGRLGVADRYQDIALATRDIAEELG-GEWADRFLVLYGIAAP | 249 |
| Sbjct 194 | ACLPN++ GRFSGFIDCGRLGVADRYQD+ALA R + G W YG+ P ACLPNLLAAEGRFSGFIDCGRLGVADRYQDLALAARSLVHNFGDTRWVAPLFRRYGV-VP | 252 |
| Query 250  Sbjct 253 | DSQRIAFYRLLDEFF 264 D +R+AFYRLLDEFF DERRLAFYRLLDEFF 267 |  |

```
>WP_111687175.1 APH(3')-II family aminoglycoside O-phosphotransferase [Stenotrophomonas maltophilia]
SQG10296.1 Aminoglycoside 3'-phosphotransferase [Stenotrophomonas maltophilia] Length=267

Score = 242 bits (617), Expect = 3e-76, Method: Compositional matrix adjust. Identities = 133/255 (52%), Positives = 164/255 (64%), Gaps = 3/255 (1%)

Query 12	PAAWVERLFGYDWAQQTIGCSDAAVFRLSAQGRPVLFVKTDLSGALNELQDEAARLSWLA 71

P AW E L	+Q+IG S A V R+	G+	FVK+++ A +EL DE ARL WL
```

| Sbjct | 14 | PWAWQEALADARIERQSIGVSRADVARVQRPGQADAFVKSEVIDAFSELGDEIARLRWLQ | 73 |
| --- | --- | --- | --- |
| Query | 72 | TTGVPCAAVLDVVTEAGRDWLLLGEVPGQDLLSS-HLAPAEKVSIMADAMRRLHTLDPAT | 130 |
| Sbjct | 74 | G P V+ EAGR WLL+ +P +DL SS LAP + V ++ADA+R LH L A EQGQPAPTVIATAEEAGRRWLLMSALPDRDLASSPELAPQQLVELLADALRGLHALPLAA | 133 |
| Query | 131 | CPFDHQAKHRIERARTRMEAGLVDQDDLDEEHQGLAPAELFARLKARMPDGEDLVVTHGD | 190 |
| Sbjct | 134 | CPFD + R++ A+ R+EAGLVD DD D E G +P ++FA L + PD EDLVV+HGD CPFDQRLHLRLQAAQARVEAGLVDADDFDVERLGQSPQQVFAELCSTRPDHEDLVVSHGD | 193 |
| Query | 191 | ACLPNIMVENGRFSGFIDCGRLGVADRYQDIALATRDIAEELG-GEWADRFLVLYGIAAP | 249 |
| Sbjct | 194 | ACLPN+MV GRFSGFIDCGRLGVADRYQD+ALA R + G W YG A ACLPNLMVVEGRFSGFIDCGRLGVADRYQDLALAARSLVHNFGDTRWVAPLFQRYGAVA- | 252 |
| Query  Sbjct | 250  253 | DSQRIAFYRLLDEFF 264 D +R+AFYRLLDEFF DERRLAFYRLLDEFF 267 |  |

>WP_077469385.1 aminoglycoside 3'-phosphotransferase [Rhizobium sp. P44RR-XXIV] TIX88581.1 aminoglycoside 3'-phosphotransferase [Rhizobium sp. P44RR-XXIV]

Length=266

Score = 242 bits (617), Expect = 4e-76, Method: Compositional matrix adjust.

| Identities | | = 124/254 (49%), Positives = 164/254 (65%), Gaps = 1/254 (0%) |  |
| --- | --- | --- | --- |
| Query 12  Sbjct 13 | | PAAWVERLFGYDWAQQTIGCSDAAVFRLSAQGRPVLFVKTDLSGALNELQDEAARLSWLA P + L GY+W Q +G S A VFRL A GR +++KT+ L EL E ARL WL PVPLRDLLAGYEWRQDALGRSSAHVFRLEADGRAPVYLKTEPVDPLGELVGEVARLRWLR | 71  72 |
| Query 72  Sbjct 73 | | TTGVPCAAVLDVVTEAGRDWLLLGEVPGQDLLSSHL-APAEKVSIMADAMRRLHTLDPAT  + G+ C ++ + R+WLL+ +PG DL+S+ L +PAE+V ++ADA+RRLH +D A SQGLACPEIIAHAKDEEREWLLMSALPGSDLVSAELISPAERVRLLADALRRLHAIDIAA | 130  132 |
| Query 131  Sbjct 133 | | CPFDHQAKHRIERARTRMEAGLVDQDDLDEEHQGLAPAELFARLKARMPDGEDLVVTHGD CPFDH+ +RI A+ R+ AG VD+DD DE G + +LF L+ + P EDLVVTHGD CPFDHRLANRIGDAKARLVAGCVDEDDFDETRLGHSGHDLFGELEDKRPATEDLVVTHGD | 190  192 |
| Query 191  Sbjct 193 | | ACLPNIMVENGRFSGFIDCGRLGVADRYQDIALATRDIAEELGGEWADRFLVLYGIAAPD ACLPN M + FSG+IDCGRLG+ADRYQD+ALA I G A FL YG ACLPNFMADGAGFSGYIDCGRLGIADRYQDLALACGSIQYNFGAALAADFLNAYGHFELH | 250  252 |
| Query 251 | | SQRIAFYRLLDEFF 264  +++A+YRLLDEFF |  |
| Sbjct | 253 | PEKMAYYRLLDEFF 266 | |

>WP_063689378.1 aminoglycoside 3'-phosphotransferase [Alcaligenes faecalis] ARP52215.1 aminoglycoside phosphotransferase [Alcaligenes faecalis]

Length=256

Score = 241 bits (616), Expect = 4e-76, Method: Compositional matrix adjust.

| Identities | | = 128/254 (50%), Positives = 160/254 (63%), Gaps = 2/254 (1%) |  |
| --- | --- | --- | --- |
| Query 12  Sbjct 4 | | PAAWVERLFGYDWAQQTIGCSDAAVFRLSAQGRPVLFVKTDLSGALNELQDEAARLSWLA PAAW RL +QTIG S A V+RL FVK + L EL DE RL W+ PAAWQTRLAAAKVQRQTIGESGAEVWRLDWPDGSCQFVKAEDDLPLAELPDEIERLGWMH | 71  63 |
| Query 72  Sbjct 64 | | TTGVPCAAVLDVVTEAGRDWLLLGEVPGQDLLSSH-LAPAEKVSIMADAMRRLHTLDPAT G+PC VLD + GR WLL+ VPG DL + L+P + V ++A +++ LH L + AQGLPCPEVLDTLVADGRHWLLMSAVPGLDLACTEGLSPQQTVELLALSLQSLHRLPLES | 130  123 |
| Query 131  Sbjct 124 | | CPFDHQAKHRIERARTRMEAGLVDQDDLDEEHQGLAPAELFARLKARMPDGEDLVVTHGD CPFDH+ +HR+ A R +AGL+D++D D+E QG+ L L A P EDLVVTHGD CPFDHRLEHRLADASARAKAGLIDEEDFDDERQGMPVQSLLDELYASRPMQEDLVVTHGD | 190  183 |
| Query 191  Sbjct 184 | | ACLPNIMVENGRFSGFIDCGRLGVADRYQDIALATRDIAEELGGEWADRFLVLYGIAAPD ACLPN MV G+FSGFIDCGRLGVADR+QD+AL R I LG EW F LYG+ D ACLPNFMVHQGQFSGFIDCGRLGVADRFQDLALTARSIERNLGKEWLAPFFALYGVEM-D | 250  242 |
| Query | 251 SQRIAFYRLLDEFF 264 | | |

++RIAF+ LLDEFF Sbjct 243 AERIAFFCLLDEFF 256

>WP_075076796.1 APH(3')-II family aminoglycoside O-phosphotransferase [Stenotrophomonas maltophilia]

KZE46042.1 aminoglycoside phosphotransferase [Stenotrophomonas maltophilia]

TNX97769.1 APH(3')-II family aminoglycoside O-phosphotransferase [Stenotrophomonas maltophilia]

TPD75201.1 APH(3')-II family aminoglycoside O-phosphotransferase [Stenotrophomonas maltophilia]

TPD76783.1 APH(3')-II family aminoglycoside O-phosphotransferase [Stenotrophomonas maltophilia]

TPD78685.1 APH(3')-II family aminoglycoside O-phosphotransferase [Stenotrophomonas maltophilia]

Length=267

Score = 242 bits (617), Expect = 4e-76, Method: Compositional matrix adjust.

| Identities | = 129/255 (51%), Positives = 165/255 (65%), Gaps = 3/255 (1%) |  |
| --- | --- | --- |
| Query 12 | PAAWVERLFGYDWAQQTIGCSDAAVFRLSAQGRPVLFVKTDLSGALNELQDEAARLSWLA | 71 |
| Sbjct 14 | P AW E L +Q+IG S A V R+ G+ F+K+++ A +EL DE ARL WL PWAWRETLADARIERQSIGVSRADVARVHRPGQADAFLKSEVIDAFSELGDEIARLRWLQ | 73 |
| Query 72 | TTGVPCAAVLDVVTEAGRDWLLLGEVPGQDLLSS-HLAPAEKVSIMADAMRRLHTLDPAT | 130 |
| Sbjct 74 | G P AV+ EAGR WLL+ +PG+DL SS L P V ++ADA+R LH + A GQGQPAPAVIATAEEAGRRWLLMSALPGRDLASSPELPPQRLVELLADALRGLHAIPVAA | 133 |
| Query 131 | CPFDHQAKHRIERARTRMEAGLVDQDDLDEEHQGLAPAELFARLKARMPDGEDLVVTHGD | 190 |
| Sbjct 134 | CPFD + R++ A+ R+EAGLVD DD D+E G + ++FA L+A P+ EDLVV+HGD CPFDQRLASRLQAAQARVEAGLVDADDFDDERLGQSAQQVFAELRATRPNHEDLVVSHGD | 193 |
| Query 191 | ACLPNIMVENGRFSGFIDCGRLGVADRYQDIALATRDIAEELG-GEWADRFLVLYGIAAP | 249 |
| Sbjct 194 | ACLPN++ GRFSGFIDCGRLGVADRYQD+ALA R + G W YG+ P ACLPNLLAAEGRFSGFIDCGRLGVADRYQDLALAARSLVHNFGDTRWVAPLFRRYGV-VP | 252 |
| Query 250  Sbjct 253 | DSQRIAFYRLLDEFF 264 D +R+AFYRLLDEFF DERRLAFYRLLDEFF 267 |  |

>WP_128096343.1 aminoglycoside 3'-phosphotransferase [Stenotrophomonas sp. HMWF003]

PTT63046.1 APH(3')-II family aminoglycoside O-phosphotransferase [Stenotrophomonas sp. HMWF003]

Length=270

Score = 242 bits (617), Expect = 4e-76, Method: Compositional matrix adjust.

| Identities | | = 135/266 (51%), Positives = 165/266 (62%), Gaps = 3/266 (1%) |  |
| --- | --- | --- | --- |
| Query 1  Sbjct 6 | | MIEQDGLHAGSPAAWVERLFGYDWAQQTIGCSDAAVFRLSAQGRPVLFVKTDLSG-ALNE  ++ D + P W ERL G Q IG S A V+R+ G +F+K + + E ILPHDTGNVPFPNLWRERLAGCVIEAQAIGASRADVYRVQGGGGRDVFIKREPADDPFGE | 59  65 |
| Query 60  Sbjct 66 | | LQDEAARLSWLATTGVPCAAVLDVVTEAGRDWLLLGEVPGQDLLSS-HLAPAEKVSIMAD L DE ARL WL T G+ A VLD +A WLL+ VPG+DL SS LAP + V +MAD LHDEVARLQWLQTQGMEAAQVLDTHDDANGRWLLMRAVPGRDLASSPDLAPLQVVHLMAD | 118  125 |
| Query 119  Sbjct 126 | | AMRRLHTLDPATCPFDHQAKHRIERARTRMEAGLVDQDDLDEEHQGLAPAELFARLKARM A++ LH L CPFDH+ HR+ AR RMEAG VD +D DE G ++FA L A + ALQALHRLPTDRCPFDHRLPHRLAAARARMEAGAVDAEDFDEARAGQTVQQVFAALLASV | 178  185 |
| Query 179  Sbjct 186 | | PDGEDLVVTHGDACLPNIMVENGRFSGFIDCGRLGVADRYQDIALATRDIAEELGGEWAD P DLVVTHGDACLPN+M GRF+GFIDCGRLGVADR+QD+ALA R IA LGG+W PAQVDLVVTHGDACLPNLMAAQGRFTGFIDCGRLGVADRHQDLALAARSIASNLGGQWVA | 238  245 |
| Query 239 | | RFLVLYGIAAPDSQRIAFYRLLDEFF 264 F YG PD+ +AFY LLDEFF |  |
| Sbjct | 246 | PFFARYGT-VPDTALLAFYCLLDEFF 270 | |

>WP_064711498.1 aminoglycoside 3'-phosphotransferase [Rhizobium bangladeshense] Length=262

Score = 241 bits (616), Expect = 4e-76, Method: Compositional matrix adjust.

| Identities | | = 128/254 (50%), Positives = 165/254 (65%), Gaps = 1/254 (0%) |  |
| --- | --- | --- | --- |
| Query 12  Sbjct 9 | | PAAWVERLFGYDWAQQTIGCSDAAVFRLSAQGRPVLFVKTDLSGALNELQDEAARLSWLA P E L GY + +G S A+VFRL +G P L++K + +G+ EL DEA RL WL PGRLAEHLSGYRMERDLLGRSAASVFRLEGEGLPALYLKVEEAGSFGELGDEAERLGWLK | 71  68 |
| Query 72  Sbjct 69 | | TTGVPCAAVLDVVTEAGRDWLLLGEVPGQDLLSSH-LAPAEKVSIMADAMRRLHTLDPAT  +GVPC + ++ R+WLL+ +PG DL S+ L P +V ++A A+ LH L A+ ASGVPCPESIARESDGERNWLLISALPGSDLASAAALTPLARVELLAAALADLHRLPIAS | 130  128 |
| Query 131  Sbjct 129 | | CPFDHQAKHRIERARTRMEAGLVDQDDLDEEHQGLAPAELFARLKARMPDGEDLVVTHGD CPFDH+ + R+ A+ RMEA +VD++D D G ELFA L+ PDGEDLVVTHGD CPFDHRLEQRLPVAKARMEAEIVDEEDFDAARLGKRAGELFAELERLKPDGEDLVVTHGD | 190  188 |
| Query 191  Sbjct 189 | | ACLPNIMVENGRFSGFIDCGRLGVADRYQDIALATRDIAEELGGEWADRFLVLYGIAAPD ACLPN +V +GRFSG+IDC RLGVADR+QDIALA R IA G FL YG+ APD ACLPNFIVSDGRFSGYIDCSRLGVADRHQDIALACRSIASNFGEALVQPFLDRYGMPAPD | 250  248 |
| Query 251 | | SQRIAFYRLLDEFF 264 R+ +YRLLDEFF |  |
| Sbjct | 249 | PARLHYYRLLDEFF 262 | |

>WP_124379363.1 aminoglycoside 3'-phosphotransferase [Pseudomonas synxantha] AZE57337.1 Aminoglycoside 3'-phosphotransferase [Pseudomonas synxantha]

Length=256

Score = 241 bits (616), Expect = 4e-76, Method: Compositional matrix adjust.

| Identities | | = 132/254 (52%), Positives = 160/254 (63%), Gaps = 2/254 (1%) |  |
| --- | --- | --- | --- |
| Query 12  Sbjct 4 | | PAAWVERLFGYDWAQQTIGCSDAAVFRLSAQGRPVLFVKTDLSGALNELQDEAARLSWLA P W E+ QQ IG S A VFRL G LF+K++ EL DE RL WL PQKWREQFPHALIEQQAIGESRADVFRLIQDGGTDLFLKSEPLEEHGELADEIDRLRWLQ | 71  63 |
| Query 72  Sbjct 64 | | TTGVPCAAVLDVVTEAGRDWLLLGEVPGQDLLSSHLAPA-EKVSIMADAMRRLHTLDPAT G+P AVLD VTEA R WLL+ VPG+DL+S++ PA + + I+A A+R LH L A  HMGLPAPAVLDEVTEAHRHWLLMTAVPGRDLVSTNELPAGQIIHILATALRALHQLPVAL | 130  123 |
| Query 131  Sbjct 124 | | CPFDHQAKHRIERARTRMEAGLVDQDDLDEEHQGLAPAELFARLKARMPDGEDLVVTHGD CPFDH + RI RA+ + AGLVD+ D D+E G + ++FA L A P DLVVTHGD CPFDHSLELRIARAQQHVSAGLVDETDFDDERLGQSAEDVFAELVATQPKTHDLVVTHGD | 190  183 |
| Query 191  Sbjct 184 | | ACLPNIMVENGRFSGFIDCGRLGVADRYQDIALATRDIAEELGGEWADRFLVLYGIAAPD ACLPN M G FSGFIDCGRLGV+DR+QD+ALA R I LG W F +YG+ PD ACLPNFMAAEGCFSGFIDCGRLGVSDRFQDLALAARSIESNLGQAWVKPFFEVYGV-EPD | 250  242 |
| Query | 251 SQRIAFYRLLDEFF 264 | | |
|  |  | QR+ FY LLDEFF |  |
| Sbjct | 243 | EQRMKFYCLLDEFF 256 | |

>VUK70898.1 Aminoglycoside 3'-phosphotransferase [Stenotrophomonas maltophilia] Length=267

Score = 242 bits (617), Expect = 4e-76, Method: Compositional matrix adjust.

| Identities | = 131/255 (51%), Positives = 164/255 (64%), Gaps = 3/255 (1%) |  |
| --- | --- | --- |
| Query 12 | PAAWVERLFGYDWAQQTIGCSDAAVFRLSAQGRPVLFVKTDLSGALNELQDEAARLSWLA | 71 |
| Sbjct 14 | P AW E L +Q+IG S A V R+ G+ F+K+++ A +EL DE ARL WL PRAWQEALADAHIERQSIGVSRADVARVHRPGQADAFLKSEVIDAFSELGDEIARLRWLQ | 73 |
| Query 72 | TTGVPCAAVLDVVTEAGRDWLLLGEVPGQDLLSS-HLAPAEKVSIMADAMRRLHTLDPAT | 130 |
| Sbjct 74 | G P V+ EAGR WLL+ +PG+DL SS LAP + V ++ADA+R LH L A EQGQPAPTVIATAEEAGRRWLLMSALPGRDLASSPELAPQQLVELLADALRGLHALPVAA | 133 |
| Query 131 | CPFDHQAKHRIERARTRMEAGLVDQDDLDEEHQGLAPAELFARLKARMPDGEDLVVTHGD | 190 |
| Sbjct 134 | CPFD + R+E A+ R+EA LVD DD D+E G +P ++FA L + PD EDLV+ HGD CPFDQRLASRLEAAQARVEASLVDADDFDDERLGQSPQQVFAELCSTRPDHEDLVICHGD | 193 |
| Query 191 | ACLPNIMVENGRFSGFIDCGRLGVADRYQDIALATRDIAEELG-GEWADRFLVLYGIAAP | 249 |

ACLPN+MV GRFSGFIDCGRLG+ADRYQD+ALA R +	G	W	YG A

| Sbjct | 194 | ACLPNLMVAEGRFSGFIDCGRLGLADRYQDLALAARSLVHNFGDTRWVAPLFQRYG-ALV 252 |
| --- | --- | --- |
| Query | 250 | DSQRIAFYRLLDEFF 264 |
| Sbjct | 253 | D +R+AFYRLLDEFF  DERRLAFYRLLDEFF 267 |

>WP_092748620.1 aminoglycoside 3'-phosphotransferase [Rhizobium aethiopicum] SCB57261.1 aminoglycoside 3'-phosphotransferase-2 [Rhizobium aethiopicum]

Length=262

Score = 241 bits (616), Expect = 4e-76, Method: Compositional matrix adjust.

| Identities | | = 125/248 (50%), Positives = 162/248 (65%), Gaps = 1/248 (0%) |  |
| --- | --- | --- | --- |
| Query 18  Sbjct 15 | | RLFGYDWAQQTIGCSDAAVFRLSAQGRPVLFVKTDLSGALNELQDEAARLSWLATTGVPC RL GY + +G S A+VFRL +G P L++K + +G EL DEAARL+WL +G+PC RLSGYRMERDALGRSAASVFRLEGEGLPALYLKVEAAGPFGELADEAARLAWLKASGLPC | 77  74 |
| Query 78  Sbjct 75 | | AAVLDVVTEAGRDWLLLGEVPGQDLLS-SHLAPAEKVSIMADAMRRLHTLDPATCPFDHQ V+ + R+WLL+ +PG DL S S L P +V ++A+A+ LH L +CPFDH+ PEVIGRENDGERNWLLISALPGTDLASASALTPLARVELLAEALLALHRLPITSCPFDHR | 136  134 |
| Query 137 | | AKHRIERARTRMEAGLVDQDDLDEEHQGLAPAELFARLKARMPDGEDLVVTHGDACLPNI R+ AR RM+AG+VD D DE G +P LFA L+ R P ED+VVTHGDACLPN | 196 |
| Sbjct | 135 | LAGRVPLARARMQAGIVDGTDFDETRLGKSPEALFAELENRRPCSEDIVVTHGDACLPNF | 194 |
| Query | 197 | MVENGRFSGFIDCGRLGVADRYQDIALATRDIAEELGGEWADRFLVLYGIAAPDSQRIAF | 256 |
| Sbjct | 195 | + +G+FSG+IDC RLGVADRYQDIALA R I G FL YG+ APD R+ + VASDGQFSGYIDCSRLGVADRYQDIALACRSITYNFGEALVQPFLDRYGLPAPDPARLDY | 254 |
| Query  Sbjct | 257  255 | YRLLDEFF 264 Y+LLDEFF YQLLDEFF 262 |  |

>WP_168329919.1 APH(3') family aminoglycoside O-phosphotransferase [Rhizobium leguminosarum]

NKM07317.1 APH(3') family aminoglycoside O-phosphotransferase [Rhizobium leguminosarum bv. viciae]

Length=265

Score = 242 bits (617), Expect = 4e-76, Method: Compositional matrix adjust.

| Identities | = 126/255 (49%), Positives = 163/255 (64%), Gaps = 1/255 (0%) |  |
| --- | --- | --- |
| Query 11 | SPAAWVERLFGYDWAQQTIGCSDAAVFRLSAQGRPVLFVKTDLSGALNELQDEAARLSWL | 70 |
| Sbjct 11 | S A RL GY + + +G S A+VFRL A G P L++K + +G EL DEAARL WL SAGALDARLAGYRFERDALGRSAASVFRLEAHGLPTLYLKVEEAGVFGELADEAARLRWL | 70 |
| Query 71 | ATTGVPCAAVLDVVTEAGRDWLLLGEVPGQDLLS-SHLAPAEKVSIMADAMRRLHTLDPA | 129 |
| Sbjct 71 | +G+PC V+ ++ R+ LL+ +PG DL S S L P +V ++A A+ LH L A KASGLPCPDVIAEDSDGARNRLLISALPGSDLASASVLTPLARVELLAAALHDLHRLPIA | 130 |
| Query 130 | TCPFDHQAKHRIERARTRMEAGLVDQDDLDEEHQGLAPAELFARLKARMPDGEDLVVTHG | 189 |
| Sbjct 131 | +CPFDH+ + RI A+ RM+AG+VD+ D DE G + LFA L++ P EDLVVTHG SCPFDHRLEARIAAAKARMQAGIVDETDFDERRLGKSAEALFAELESGKPSREDLVVTHG | 190 |
| Query 190 | DACLPNIMVENGRFSGFIDCGRLGVADRYQDIALATRDIAEELGGEWADRFLVLYGIAAP | 249 |
| Sbjct 191 | DACLPN + FSG+IDC RLGVADRYQDIALA R IA G FL YG++A DACLPNFIASEDGFSGYIDCSRLGVADRYQDIALACRSIAHNFGEALVQSFLDRYGMSAT | 250 |
| Query 250  Sbjct 251 | DSQRIAFYRLLDEFF 264 D R+ +Y+LLDEFF DPARLDYYQLLDEFF 265 |  |

>WP_166618453.1 APH(3') family aminoglycoside O-phosphotransferase [Pseudomonas sp. ODNR1LW]

NBB57840.1 APH(3') family aminoglycoside O-phosphotransferase [Pseudomonas sp. ODNR1LW]

Length=256

Score = 241 bits (616), Expect = 4e-76, Method: Compositional matrix adjust.

| Identities | | = 126/254 (50%), Positives = 163/254 (64%), Gaps = 2/254 (1%) |  |
| --- | --- | --- | --- |
| Query 12  Sbjct 4 | | PAAWVERLFGYDWAQQTIGCSDAAVFRLSAQGRPVLFVKTDLSGALNELQDEAARLSWLA PA W + +QTIG S A V R+ LF+K++ GA +EL E RL W+ PAQWAQTFSAAPIERQTIGESGAEVSRICCINGEHLFLKSEPIGAHSELPQEIERLRWMN | 71  63 |
| Query 72  Sbjct 64 | | TTGVPCAAVLDVVTEAGRDWLLLGEVPGQDLLSSH-LAPAEKVSIMADAMRRLHTLDPAT  +P VLDV TE R WLL+ VPG DL S++ L+ + +SI+A A+R LH + T RLDLPGPTVLDVTTENDRHWLLMSAVPGHDLASANDLSAPQVISILAMALRTLHQVSIET | 130  123 |
| Query 131  Sbjct 124 | | CPFDHQAKHRIERARTRMEAGLVDQDDLDEEHQGLAPAELFARLKARMPDGEDLVVTHGD CPFDH + RIE AR+R+ AGLVD+ D D+E +G ++FA L + +P+ DL VTHGD CPFDHSPELRIETARSRVSAGLVDEADFDDERKGRTAEDVFAELLSTLPETCDLAVTHGD | 190  183 |
| Query 191  Sbjct 184 | | ACLPNIMVENGRFSGFIDCGRLGVADRYQDIALATRDIAEELGGEWADRFLVLYGIAAPD ACLPN MVE GRF+G+IDCGRLG++D YQD+ALA R IA LG W F YG+ PD ACLPNFMVEGGRFTGYIDCGRLGISDIYQDLALAARSIARNLGQAWVAPFFRAYGV-EPD | 250  242 |
| Query | 251 SQRIAFYRLLDEFF 264 | | |
|  |  | +RIAFY LLDEF+ |  |
| Sbjct | 243 | QRRIAFYCLLDEFY 256 | |

>WP_019338462.1 APH(3')-II family aminoglycoside O-phosphotransferase [Stenotrophomonas maltophilia]

VUP82802.1 Aminoglycoside 3'-phosphotransferase [Stenotrophomonas maltophilia] Length=267

Score = 242 bits (617), Expect = 4e-76, Method: Compositional matrix adjust.

| Identities | = 130/255 (51%), Positives = 165/255 (65%), Gaps = 3/255 (1%) |  |
| --- | --- | --- |
| Query 12 | PAAWVERLFGYDWAQQTIGCSDAAVFRLSAQGRPVLFVKTDLSGALNELQDEAARLSWLA | 71 |
| Sbjct 14 | P AW E L +Q+IG S A V R+ G+ F+K+++ A +EL DE RL WL PRAWQEALADARIERQSIGVSRADVARVHRPGQTDAFLKSEVIDAFSELGDEITRLRWLQ | 73 |
| Query 72 | TTGVPCAAVLDVVTEAGRDWLLLGEVPGQDLLSS-HLAPAEKVSIMADAMRRLHTLDPAT | 130 |
| Sbjct 74 | G+P V+ EAGR WLL+ +PG+DL SS LAP + V ++ADA+RRLH L A AQGLPAPTVIATAEEAGRRWLLMSALPGRDLASSPELAPQQLVELLADALRRLHVLPVAA | 133 |
| Query 131 | CPFDHQAKHRIERARTRMEAGLVDQDDLDEEHQGLAPAELFARLKARMPDGEDLVVTHGD | 190 |
| Sbjct 134 | CPFD + R++ A+ R+EAGLVD DD D+E G + ++FA L + P+ EDLVV+HGD CPFDQRLASRLQAAQARVEAGLVDADDFDDERLGQSAQQVFAELCSTRPEHEDLVVSHGD | 193 |
| Query 191 | ACLPNIMVENGRFSGFIDCGRLGVADRYQDIALATRDIAEELG-GEWADRFLVLYGIAAP | 249 |
| Sbjct 194 | ACLPN+M GRFSGFIDCGRLGVADRYQD+ALA R + G W YG A ACLPNLMAVEGRFSGFIDCGRLGVADRYQDLALAARSLVHNFGDTRWVAPLFQRYG-ALV | 252 |
| Query 250  Sbjct 253 | DSQRIAFYRLLDEFF 264 D +R+AFYRLLDEFF DERRLAFYRLLDEFF 267 |  |

>WP_150787052.1 aminoglycoside 3'-phosphotransferase [Pseudomonas fluorescens] Length=256

Score = 241 bits (616), Expect = 4e-76, Method: Compositional matrix adjust.

| Identities | = 125/254 (49%), Positives = 161/254 (63%), Gaps = 2/254 (1%) |  |
| --- | --- | --- |
| Query 12 | PAAWVERLFGYDWAQQTIGCSDAAVFRLSAQGRPVLFVKTDLSGALNELQDEAARLSWLA | 71 |
| Sbjct 4 | P W + G +QTIG S A V R+ LFVK++ GA EL +E RL W+ PTQWSQTFSGAPIERQTIGESGAEVSRIRCINGEHLFVKSEPIGAHGELPEEIERLRWMN | 63 |
| Query 72 | TTGVPCAAVLDVVTEAGRDWLLLGEVPGQDLLSSH-LAPAEKVSIMADAMRRLHTLDPAT | 130 |
| Sbjct 64 | G+P VLDV TE R WLL+ V GQDL S++ L+P + +S++A A+R LH + T RLGLPGPTVLDVTTENNRHWLLMSAVLGQDLASANDLSPNQVISVLAMALRTLHQVPIET | 123 |
| Query 131 | CPFDHQAKHRIERARTRMEAGLVDQDDLDEEHQGLAPAELFARLKARMPDGEDLVVTHGD | 190 |
| Sbjct 124 | CPFDH + RI AR+R+ AGLVD+ D D+E QG A ++F + + +P+ DL VTHGD CPFDHSLEQRIAAARSRVNAGLVDEADFDDERQGRAAEDVFTEMLSTLPEIYDLAVTHGD | 183 |
| Query 191 | ACLPNIMVENGRFSGFIDCGRLGVADRYQDIALATRDIAEELGGEWADRFLVLYGIAAPD | 250 |

| Sbjct | 184 | ACLPN MVE F+G+IDCGRLG++D YQD+ALA R I LG W F YG+ PD ACLPNFMVEGSSFTGYIDCGRLGISDIYQDLALAARSIERNLGPAWVAPFFRAYGV-EPD 242 |
| --- | --- | --- |
| Query | 251 | SQRIAFYRLLDEFF 264 |
| Sbjct | 243 | QRIAFY LLDEF+  QQRIAFYCLLDEFY 256 |

>WP_033834473.1 APH(3')-II family aminoglycoside O-phosphotransferase [Stenotrophomonas maltophilia]

AVH90373.1 APH(3')-II family aminoglycoside O-phosphotransferase [Stenotrophomonas maltophilia]

KOO74651.1 aminoglycoside phosphotransferase [Stenotrophomonas maltophilia]

MBA0316177.1 APH(3')-II family aminoglycoside O-phosphotransferase [Stenotrophomonas maltophilia]

OWQ59872.1 aminoglycoside phosphotransferase APH(3') [Stenotrophomonas maltophilia]

PZS58660.1 APH(3') family aminoglycoside O-phosphotransferase [Stenotrophomonas maltophilia]

Length=267

Score = 241 bits (616), Expect = 5e-76, Method: Compositional matrix adjust.

| Identities | | = 131/261 (50%), Positives = 165/261 (63%), Gaps = 3/261 (1%) |  |
| --- | --- | --- | --- |
| Query 5  Sbjct 9 | | DGLHAGSPAAWVERLFGYDWAQQTIGCSDAAVFRLSAQGRPVLFVKTDLSGALNELQDEA DGL P AW E L +Q+IG S A V R+ G+ F+K+++ A +EL DE DGLRL--PRAWQEALADAHIERQSIGVSRADVARVHRPGQTDAFLKSEVIDAFSELGDEI | 64  66 |
| Query 65  Sbjct 67 | | ARLSWLATTGVPCAAVLDVVTEAGRDWLLLGEVPGQDLLSS-HLAPAEKVSIMADAMRRL ARL WL G V+ E GR WLL+ +PG+DL SS LAP ++ADA+R L ARLRWLQAQGQSAPTVIATTEEGGRRWLLMSALPGRDLASSPELAPRRVAELLADALRGL | 123  126 |
| Query 124  Sbjct 127 | | HTLDPATCPFDHQAKHRIERARTRMEAGLVDQDDLDEEHQGLAPAELFARLKARMPDGED H + A CPFD Q R++ A+ R+EAGLVD DD D+E G +P ++FA L+A P ED HAVPVANCPFDQQLASRLQAAQARVEAGLVDADDFDDERLGQSPQQVFAELRATRPAHED | 183  186 |
| Query 184  Sbjct 187 | | LVVTHGDACLPNIMVENGRFSGFIDCGRLGVADRYQDIALATRDIAEELGGEWADRFLVL LVV+HGDACLPN+MV +GRF+GFIDCGRLGVADRYQD+ALA R + G L LVVSHGDACLPNLMVTDGRFTGFIDCGRLGVADRYQDLALAARSLVHNFGESRCVATLFQ | 243  246 |
| Query 244 | | YGIAAPDSQRIAFYRLLDEFF 264 A PD +R+AFYRLLDEFF |  |
| Sbjct | 247 | RYGAVPDERRLAFYRLLDEFF 267 | |

>WP_056789071.1 MULTISPECIES: aminoglycoside 3'-phosphotransferase [Pseudomonas] KAA6192555.1 aminoglycoside 3'-phosphotransferase [Pseudomonas lactis] KRC94411.1 aminoglycoside phosphotransferase [Pseudomonas sp. Root9]

KWV73713.1 Aminoglycoside 3'-phosphotransferase [Pseudomonas fluorescens] Length=256

Score = 241 bits (615), Expect = 5e-76, Method: Compositional matrix adjust.

| Identities | | = 133/254 (52%), Positives = 159/254 (63%), Gaps = 2/254 (1%) |  |
| --- | --- | --- | --- |
| Query 12  Sbjct 4 | | PAAWVERLFGYDWAQQTIGCSDAAVFRLSAQGRPVLFVKTDLSGALNELQDEAARLSWLA P W E+ QQ IG S A VFRL G LFVK++ EL DE RL WL PQKWREQFPHALIEQQAIGESRADVFRLRQDGGTDLFVKSEPLEEHGELADEIDRLRWLQ | 71  63 |
| Query 72  Sbjct 64 | | TTGVPCAAVLDVVTEAGRDWLLLGEVPGQDLLSSHLAPA-EKVSIMADAMRRLHTLDPAT G+P AVLD VTEA WLL+ VPG+DL S++ PA + + I+A A+R LH L A  HMGLPAPAVLDEVTEAHHHWLLMTAVPGRDLASTNELPAGQIIHILATALRALHQLPVAL | 130  123 |
| Query 131  Sbjct 124 | | CPFDHQAKHRIERARTRMEAGLVDQDDLDEEHQGLAPAELFARLKARMPDGEDLVVTHGD CPFDH + RI RA+ + AGLVD+ D D+E G + ++FA L A P DLVVTHGD CPFDHSLELRIARAQQHVIAGLVDETDFDDERLGQSAEDVFAELVATQPKTHDLVVTHGD | 190  183 |
| Query 191  Sbjct 184 | | ACLPNIMVENGRFSGFIDCGRLGVADRYQDIALATRDIAEELGGEWADRFLVLYGIAAPD ACLPN M G FSGFIDCGRLGV+DR+QD+ALA R I LG EW F +YG+ PD ACLPNFMAAEGCFSGFIDCGRLGVSDRFQDLALAARSIESNLGQEWVKPFFEVYGV-EPD | 250  242 |
| Query | 251 SQRIAFYRLLDEFF 264 | | |
|  |  | QR+ FY LLDEFF |  |
| Sbjct | 243 | EQRMKFYCLLDEFF 256 | |

>WP_049462051.1 APH(3')-II family aminoglycoside O-phosphotransferase [Stenotrophomonas maltophilia]

MBA0353502.1 APH(3')-II family aminoglycoside O-phosphotransferase [Stenotrophomonas maltophilia]

PJL51427.1 aminoglycoside 3'-phosphotransferase [Stenotrophomonas maltophilia] VUP11198.1 Aminoglycoside 3'-phosphotransferase [Stenotrophomonas maltophilia]

Length=267

Score = 241 bits (616), Expect = 5e-76, Method: Compositional matrix adjust.

| Identities | | = 131/255 (51%), Positives = 165/255 (65%), Gaps = 3/255 (1%) |  |
| --- | --- | --- | --- |
| Query 12  Sbjct 14 | | PAAWVERLFGYDWAQQTIGCSDAAVFRLSAQGRPVLFVKTDLSGALNELQDEAARLSWLA P AW + L +Q+IG S A V R+ G+ F+K+++ A +EL DE ARL WL PWAWQDALADARIERQSIGVSRADVARVHRPGQADAFLKSEVIDAFSELGDEIARLRWLQ | 71  73 |
| Query 72 | | TTGVPCAAVLDVVTEAGRDWLLLGEVPGQDLLSS-HLAPAEKVSIMADAMRRLHTLDPAT P V+ EAGR WLL+ +PG+DL SS LAP + V ++ADA+R LH L A | 130 |
| Sbjct | 74 | AQEQPVPTVIATAEEAGRRWLLMSALPGRDLASSPELAPQQLVELLADALRGLHALPVAA | 133 |
| Query | 131 | CPFDHQAKHRIERARTRMEAGLVDQDDLDEEHQGLAPAELFARLKARMPDGEDLVVTHGD | 190 |
| Sbjct | 134 | CPFD + R++ A+ R+EAGLVD DD D+E G +P ++FA L + PD EDLVV+HGD CPFDQRLASRVQVAQARVEAGLVDADDFDDERLGQSPQQVFAELCSTRPDHEDLVVSHGD | 193 |
| Query | 191 | ACLPNIMVENGRFSGFIDCGRLGVADRYQDIALATRDIAEELG-GEWADRFLVLYGIAAP | 249 |
| Sbjct | 194 | ACLPN+MV GRFSGFIDCGRLGVADRYQD+ALA R + G W YG A ACLPNLMVVEGRFSGFIDCGRLGVADRYQDLALAARSLVHNFGDTRWVAPLFQRYGAVA- | 252 |
| Query  Sbjct | 250  253 | DSQRIAFYRLLDEFF 264 D +R+AFYRLLDEFF DERRLAFYRLLDEFF 267 |  |

>WP_183740996.1 APH(3') family aminoglycoside O-phosphotransferase [Rhizobium sp. BK196]

MBB3309142.1 aminoglycoside 3'-phosphotransferase-2 [Rhizobium sp. BK196] Length=265

Score = 241 bits (616), Expect = 5e-76, Method: Compositional matrix adjust.

| Identities | | = 122/254 (48%), Positives = 158/254 (62%), Gaps = 1/254 (0%) |  |
| --- | --- | --- | --- |
| Query 12  Sbjct 12 | | PAAWVERLFGYDWAQQTIGCSDAAVFRLSAQGRPVLFVKTDLSGALNELQDEAARLSWLA P + L GY+W + +G S A VFRL + RP L++K + G EL DEA RL WL PVSLRSHLGGYEWRRDALGRSSACVFRLEGEKRPGLYLKVEEEGPFCELADEAERLVWLR | 71  71 |
| Query 72  Sbjct 72 | | TTGVPCAAVLDVVTEAGRDWLLLGEVPGQDLLSSH-LAPAEKVSIMADAMRRLHTLDPAT  + G+ C V+ ++ R +LL+ +PG DL+++ L P E+V I+A A+ LH LD SRGLDCPEVIAHESDGERSFLLMTALPGADLVTAAALLPEERVEILASALSTLHNLDIGV | 130  131 |
| Query 131  Sbjct 132 | | CPFDHQAKHRIERARTRMEAGLVDQDDLDEEHQGLAPAELFARLKARMPDGEDLVVTHGD CPFDH+ R+ A RM AG+VD+ D DEE G + LF L+ P E+LVV HGD CPFDHRLDKRLAAAEIRMRAGVVDETDFDEERLGKSAFFLFGELQRLRPQSENLVVAHGD | 190  191 |
| Query 191  Sbjct 192 | | ACLPNIMVENGRFSGFIDCGRLGVADRYQDIALATRDIAEELGGEWADRFLVLYGIAAPD ACLPN M NGRF+G+IDC RLGVADRYQDIALA I G E+ FL YG++ D ACLPNFMAANGRFAGYIDCSRLGVADRYQDIALACHSITYNFGKEFVRLFLNAYGLSIID | 250  251 |
| Query 251 | | SQRIAFYRLLDEFF 264  +RI +YRLLDEFF |  |
| Sbjct | 252 | PKRIEYYRLLDEFF 265 | |

>WP_125428073.1 APH(3')-II family aminoglycoside O-phosphotransferase [Stenotrophomonas maltophilia]

RRU74100.1 APH(3')-II family aminoglycoside O-phosphotransferase [Stenotrophomonas maltophilia]

Length=267

Score = 241 bits (616), Expect = 5e-76, Method: Compositional matrix adjust. Identities = 130/255 (51%), Positives = 163/255 (64%), Gaps = 3/255 (1%)

| Query | 12 | PAAWVERLFGYDWAQQTIGCSDAAVFRLSAQGRPVLFVKTDLSGALNELQDEAARLSWLA | 71 |
| --- | --- | --- | --- |
| Sbjct | 14 | P AW E L +Q+IG S A V R+ G+ F+K+++ A +EL DE ARL WL PQAWQEMLTDAYIERQSIGVSRADVARIHRSGQADAFLKSEVIDAFSELGDEIARLRWLQ | 73 |
| Query | 72 | TTGVPCAAVLDVVTEAGRDWLLLGEVPGQDLLSS-HLAPAEKVSIMADAMRRLHTLDPAT | 130 |
| Sbjct | 74 | G P V+ EAGR WLL+ +PG+DL SS LAP + V ++ADA+R LH L A AQGQPVPTVIVTAEEAGRRWLLMSALPGRDLASSPELAPQQLVELLADALRGLHALPVAA | 133 |
| Query | 131 | CPFDHQAKHRIERARTRMEAGLVDQDDLDEEHQGLAPAELFARLKARMPDGEDLVVTHGD | 190 |
| Sbjct | 134 | CPFD + R++ A+ R+E GLVD DD D+E G +P ++FA L + PD ED VV+HGD CPFDQRLASRVQAAQARVEGGLVDADDFDDERLGQSPQQVFAELCSTRPDHEDPVVSHGD | 193 |
| Query | 191 | ACLPNIMVENGRFSGFIDCGRLGVADRYQDIALATRDIAEELG-GEWADRFLVLYGIAAP | 249 |
| Sbjct | 194 | ACLPN+M GRFSGFIDCGRLGVADRYQD+ALA R + G W YG A ACLPNLMAVQGRFSGFIDCGRLGVADRYQDLALAARSLVHNFGDTRWVAPLFQRYGEVA- | 252 |
| Query  Sbjct | 250  253 | DSQRIAFYRLLDEFF 264 D +R+AFYRLLDEFF DERRLAFYRLLDEFF 267 |  |

>VUN67894.1 Aminoglycoside 3'-phosphotransferase [Stenotrophomonas maltophilia] Length=267

Score = 241 bits (616), Expect = 5e-76, Method: Compositional matrix adjust.

| Identities | = 130/255 (51%), Positives = 165/255 (65%), Gaps = 3/255 (1%) |  |
| --- | --- | --- |
| Query 12 | PAAWVERLFGYDWAQQTIGCSDAAVFRLSAQGRPVLFVKTDLSGALNELQDEAARLSWLA | 71 |
| Sbjct 14 | P AW + L +Q+IG S A V R+ G+ F+K+++ A +EL DE ARL WL PWAWQDALADARIERQSIGVSRADVARVHRPGQADAFLKSEVIDAFSELGDEIARLRWLQ | 73 |
| Query 72 | TTGVPCAAVLDVVTEAGRDWLLLGEVPGQDLLSS-HLAPAEKVSIMADAMRRLHTLDPAT | 130 |
| Sbjct 74 | G P V+ EAGR WLL+ +PG+DL SS LAP + V ++ADA+R LH L A AQGQPVPTVIATAEEAGRRWLLMSALPGRDLASSPELAPQQLVELLADALRGLHALPVAA | 133 |
| Query 131 | CPFDHQAKHRIERARTRMEAGLVDQDDLDEEHQGLAPAELFARLKARMPDGEDLVVTHGD | 190 |
| Sbjct 134 | CPFD + R++ A+ R+EAGLVD DD D+E G +P ++FA L + PD EDLVV+HGD CPFDQRLASRVQVAQARVEAGLVDADDFDDERLGQSPQQVFAELCSIQPDHEDLVVSHGD | 193 |
| Query 191 | ACLPNIMVENGRFSGFIDCGRLGVADRYQDIALATRDIAEEL-GGEWADRFLVLYGIAAP | 249 |
| Sbjct 194 | ACLPN+M GRFSGFIDCGRLGVADRYQD+ALA R + W YG+ A ACLPNLMAVQGRFSGFIDCGRLGVADRYQDLALAARSLVYNFDNTRWVAPLFKRYGMVA- | 252 |
| Query 250  Sbjct 253 | DSQRIAFYRLLDEFF 264 D +R+AFYRLLDEFF DERRLAFYRLLDEFF 267 |  |

>WP_113750355.1 aminoglycoside 3'-phosphotransferase [Bordetella trematum] SPU49641.1 Aminoglycoside 3'-phosphotransferase [Bordetella trematum] VDH07394.1 Aminoglycoside 3'-phosphotransferase [Bordetella trematum]

Length=256

Score = 241 bits (615), Expect = 6e-76, Method: Compositional matrix adjust.

| Identities | = 127/240 (53%), Positives = 152/240 (63%), Gaps = 2/240 (1%) |  |
| --- | --- | --- |
| Query 26 | QQTIGCSDAAVFRLSAQGRPVLFVKTDLSGALNELQDEAARLSWLATTGVPCAAVLDVVT | 85 |
| Sbjct 18 | +QTIG SDAAV+RL+ F+K + + L EL E ARL W+ G+PC VL + RQTIGESDAAVWRLTDADGACRFLKAETAHPLAELPAEVARLRWMRVQGLPCPEVLQTLQ | 77 |
| Query 86 | EAGRDWLLLGEVPGQDLLSS-HLAPAEKVSIMADAMRRLHTLDPATCPFDHQAKHRIERA | 144 |
| Sbjct 78 | GR WLL+ VPG+DL SS L+P V+++A A++RLH PA CPFDH+ R+ A ADGRHWLLMSAVPGRDLASSPELSPQRTVALLAQALQRLHQTPPAACPFDHRLARRVAEA | 137 |
| Query 145 | RTRMEAGLVDQDDLDEEHQGLAPAELFARLKARMPDGEDLVVTHGDACLPNIMVENGRFS | 204 |
| Sbjct 138 | R +AGLVD + D+E QG A L L A P EDLVVTHGDACLPN M GRFS SRRAQAGLVDVGNFDQERQGQAVEALLRALVASQPPREDLVVTHGDACLPNFMAAEGRFS | 197 |
| Query 205 | GFIDCGRLGVADRYQDIALATRDIAEELGGEWADRFLVLYGIAAPDSQRIAFYRLLDEFF | 264 |
| Sbjct 198 | GFIDC RLGVADR+QD+AL R I LG EW F YGI PD R+AFY LLDEFF  GFIDCARLGVADRHQDLALTARSIERNLGAEWLTPFFTCYGI-EPDPARLAFYCLLDEFF | 256 |

>WP_046430302.1 MULTISPECIES: APH(3')-II family aminoglycoside O-phosphotransferase [Stenotrophomonas]

KKF88152.1 aminoglycoside phosphotransferase [Stenotrophomonas maltophilia]

MBA0256377.1 APH(3')-II family aminoglycoside O-phosphotransferase [Stenotrophomonas maltophilia]

MBA0380608.1 APH(3')-II family aminoglycoside O-phosphotransferase [Stenotrophomonas maltophilia]

MBA0409093.1 APH(3')-II family aminoglycoside O-phosphotransferase [Stenotrophomonas maltophilia]

MBA0426831.1 APH(3')-II family aminoglycoside O-phosphotransferase [Stenotrophomonas maltophilia]

Length=267

Score = 241 bits (616), Expect = 6e-76, Method: Compositional matrix adjust.

| Identities | = 129/255 (51%), Positives = 163/255 (64%), Gaps = 3/255 (1%) |  |
| --- | --- | --- |
| Query 12 | PAAWVERLFGYDWAQQTIGCSDAAVFRLSAQGRPVLFVKTDLSGALNELQDEAARLSWLA | 71 |
| Sbjct 14 | P AW E L +Q+IG S A V R+ G+ F+K+++ A +EL DE ARL WL PWAWRETLADARIERQSIGVSRADVARVHRPGQADAFLKSEVIDAFSELGDEIARLRWLQ | 73 |
| Query 72 | TTGVPCAAVLDVVTEAGRDWLLLGEVPGQDLLSS-HLAPAEKVSIMADAMRRLHTLDPAT | 130 |
| Sbjct 74 | G P V EAGR WLL+ +PG+DL SS L P V ++ADA+R LH + A AQGQPAPTVTATAEEAGRRWLLMSALPGRDLASSPELPPQRLVELLADALRGLHAIPVAA | 133 |
| Query 131 | CPFDHQAKHRIERARTRMEAGLVDQDDLDEEHQGLAPAELFARLKARMPDGEDLVVTHGD | 190 |
| Sbjct 134 | CPFD + R++ A+ R+EAGLVD DD D+E G + ++FA L+A PD EDLVV+HGD CPFDQRLASRLQAAQARVEAGLVDADDFDDERLGQSAQQVFAELRATRPDHEDLVVSHGD | 193 |
| Query 191 | ACLPNIMVENGRFSGFIDCGRLGVADRYQDIALATRDIAEELG-GEWADRFLVLYGIAAP | 249 |
| Sbjct 194 | ACLPN++ GRFSGFIDCGRLGVADRYQD+ALA R + G W YG+ P ACLPNLLAAEGRFSGFIDCGRLGVADRYQDLALAARSLVHNFGDTRWVAPLFRRYGV-VP | 252 |
| Query 250  Sbjct 253 | DSQRIAFYRLLDEFF 264 D +R+AFYRLLDEFF DERRLAFYRLLDEFF 267 |  |

>VUP55126.1 Aminoglycoside 3'-phosphotransferase [Stenotrophomonas maltophilia] VUR17770.1 Aminoglycoside 3'-phosphotransferase [Stenotrophomonas maltophilia]

Length=267

Score = 241 bits (616), Expect = 6e-76, Method: Compositional matrix adjust.

| Identities | = 131/255 (51%), Positives = 163/255 (64%), Gaps = 3/255 (1%) |  |
| --- | --- | --- |
| Query 12 | PAAWVERLFGYDWAQQTIGCSDAAVFRLSAQGRPVLFVKTDLSGALNELQDEAARLSWLA | 71 |
| Sbjct 14 | P AW E L +Q+IG S A V R+ G+ F+K+++ A +EL DE ARL WL PRAWQEALADAHIERQSIGVSRADVARVHRPGQADAFLKSEVIDAFSELGDEIARLRWLQ | 73 |
| Query 72 | TTGVPCAAVLDVVTEAGRDWLLLGEVPGQDLLSS-HLAPAEKVSIMADAMRRLHTLDPAT | 130 |
| Sbjct 74 | G P V+ EAGR WLL+ +PG+DL SS LAP + V ++ADA+R LH L A EQGQPAPTVIATAEEAGRRWLLMSALPGRDLASSPELAPQQLVELLADALRGLHALPVAA | 133 |
| Query 131 | CPFDHQAKHRIERARTRMEAGLVDQDDLDEEHQGLAPAELFARLKARMPDGEDLVVTHGD | 190 |
| Sbjct 134 | CPFD + R+E + R+EA LVD DD D+E G +P ++FA L + PD EDLVV+HGD CPFDQRLASRLEAVQARVEASLVDADDFDDERLGQSPQQVFAELCSTRPDHEDLVVSHGD | 193 |
| Query 191 | ACLPNIMVENGRFSGFIDCGRLGVADRYQDIALATRDIAEELG-GEWADRFLVLYGIAAP | 249 |
| Sbjct 194 | ACLPN+M GRFSGFIDCGRLGVADRYQD+ALA R + G W YG A ACLPNLMAVEGRFSGFIDCGRLGVADRYQDLALAARSLVHNFGDTRWVAPLFQRYG-AVV | 252 |
| Query 250  Sbjct 253 | DSQRIAFYRLLDEFF 264 D +R+AFYRLLDEFF DERRLAFYRLLDEFF 267 |  |

>WP_088673147.1 aminoglycoside 3'-phosphotransferase [Rhizobium sp. R339] OWV76792.1 aminoglycoside phosphotransferase [Rhizobium sp. R339]

Length=262

Score = 241 bits (615), Expect = 6e-76, Method: Compositional matrix adjust. Identities = 121/254 (48%), Positives = 163/254 (64%), Gaps = 1/254 (0%)

| Query | 12 | PAAWVERLFGYDWAQQTIGCSDAAVFRLSAQGRPVLFVKTDLSGALNELQDEAARLSWLA | 71 |
| --- | --- | --- | --- |
| Sbjct | 9 | P L GY + +G S A+VFRL +G P L++K + +G EL DEAARL WL PGRLAAHLTGYRMERDLLGRSAASVFRLEGEGLPALYLKVEAAGPFGELADEAARLGWLK | 68 |
| Query | 72 | TTGVPCAAVLDVVTEAGRDWLLLGEVPGQDLLSSH-LAPAEKVSIMADAMRRLHTLDPAT | 130 |
| Sbjct | 69 | +G+ C V+ ++ R+WLL+ +PG DL S+ L P +V ++A+A+ LH L A+ ASGLACPDVIARESDGERNWLLISALPGSDLASAAALTPLARVELLAEALADLHRLPIAS | 128 |
| Query | 131 | CPFDHQAKHRIERARTRMEAGLVDQDDLDEEHQGLAPAELFARLKARMPDGEDLVVTHGD | 190 |
| Sbjct | 129 | CPFDH+ + R+ A+ RMEAG+VD++D D G + ELFA L++ P EDLVVTHGD CPFDHRPEKRLPVAKARMEAGIVDEEDFDAARLGKSAGELFAALESMRPGDEDLVVTHGD | 188 |
| Query | 191 | ACLPNIMVENGRFSGFIDCGRLGVADRYQDIALATRDIAEELGGEWADRFLVLYGIAAPD | 250 |
| Sbjct | 189 | ACLPN + + RFSG+IDC RLG+ADR+QDIALA R I G FL YG+ APD ACLPNFIASDARFSGYIDCSRLGIADRHQDIALACRSITYNFGEALVQPFLDRYGMPAPD | 248 |
| Query | 251 | SQRIAFYRLLDEFF 264 R+ +Y+LLDEFF |  |
| Sbjct | 249 | PARLDYYQLLDEFF 262 | |

>WP_106467838.1 APH(3')-II family aminoglycoside O-phosphotransferase [Stenotrophomonas maltophilia]

AVO30134.1 APH(3')-II family aminoglycoside O-phosphotransferase [Stenotrophomonas maltophilia]

Length=267

Score = 241 bits (616), Expect = 6e-76, Method: Compositional matrix adjust.

| Identities | = 129/255 (51%), Positives = 164/255 (64%), Gaps = 3/255 (1%) |  |
| --- | --- | --- |
| Query 12 | PAAWVERLFGYDWAQQTIGCSDAAVFRLSAQGRPVLFVKTDLSGALNELQDEAARLSWLA | 71 |
| Sbjct 14 | P AW + L +Q+IG S A V R+ G+ F+K+++ A +EL DE RL WL PWAWQDALADARIERQSIGVSRADVARVRRPGQADAFLKSEVIDAFSELGDEIVRLRWLQ | 73 |
| Query 72 | TTGVPCAAVLDVVTEAGRDWLLLGEVPGQDLLSS-HLAPAEKVSIMADAMRRLHTLDPAT | 130 |
| Sbjct 74 | G P V+ EAGR WLL+ +PG DL SS LAP + V ++ADA+R LH L A AQGQPAPTVIATAEEAGRRWLLMSALPGHDLASSPELAPQQLVELLADALRGLHALPVAA | 133 |
| Query 131 | CPFDHQAKHRIERARTRMEAGLVDQDDLDEEHQGLAPAELFARLKARMPDGEDLVVTHGD | 190 |
| Sbjct 134 | CPF + R++ A+ R+EAGLVD DD D+E G +P ++FA L + PD EDLVV+HGD CPFGQRLASRVQVAQARVEAGLVDVDDFDDERLGQSPQQVFAELCSTRPDHEDLVVSHGD | 193 |
| Query 191 | ACLPNIMVENGRFSGFIDCGRLGVADRYQDIALATRDIAEELG-GEWADRFLVLYGIAAP | 249 |
| Sbjct 194 | ACLPN+MV GRFSGFIDCGRLGVADRYQD+ALA R + G W YG+ A ACLPNLMVVEGRFSGFIDCGRLGVADRYQDLALAARSLVHNFGDTRWVAPLFQRYGVVA- | 252 |
| Query 250  Sbjct 253 | DSQRIAFYRLLDEFF 264 D +R+AFYR+LDEFF DERRLAFYRMLDEFF 267 |  |

>EBO3241016.1 aminoglycoside 3'-phosphotransferase [Salmonella enterica subsp. enterica serovar Heidelberg]

EBO3241019.1 aminoglycoside 3'-phosphotransferase [Salmonella enterica subsp. enterica serovar Heidelberg]

EBO3241022.1 aminoglycoside 3'-phosphotransferase [Salmonella enterica subsp. enterica serovar Heidelberg]

Length=116

Score = 236 bits (601), Expect = 6e-76, Method: Compositional matrix adjust. Identities = 116/116 (100%), Positives = 116/116 (100%), Gaps = 0/116 (0%)

| Query | 149 | EAGLVDQDDLDEEHQGLAPAELFARLKARMPDGEDLVVTHGDACLPNIMVENGRFSGFID 208 |
| --- | --- | --- |
| Sbjct | 1 | EAGLVDQDDLDEEHQGLAPAELFARLKARMPDGEDLVVTHGDACLPNIMVENGRFSGFID EAGLVDQDDLDEEHQGLAPAELFARLKARMPDGEDLVVTHGDACLPNIMVENGRFSGFID 60 |
| Query | 209 | CGRLGVADRYQDIALATRDIAEELGGEWADRFLVLYGIAAPDSQRIAFYRLLDEFF 264 |
| Sbjct | 61 | CGRLGVADRYQDIALATRDIAEELGGEWADRFLVLYGIAAPDSQRIAFYRLLDEFF  CGRLGVADRYQDIALATRDIAEELGGEWADRFLVLYGIAAPDSQRIAFYRLLDEFF 116 |

>HBP04691.1 APH(3')-II family aminoglycoside O-phosphotransferase [Stenotrophomonas sp.]

Length=247

Score = 241 bits (614), Expect = 6e-76, Method: Compositional matrix adjust.

| Identities | = 127/241 (53%), Positives = 160/241 (66%), Gaps = 3/241 (1%) |  |
| --- | --- | --- |
| Query 26 | QQTIGCSDAAVFRLSAQGRPVLFVKTDLSGALNELQDEAARLSWLATTGVPCAAVLDVVT | 85 |
| Sbjct 8 | +Q+IG S A V R+ G+ F+K+++ A +EL DE ARL WL G P V+ RQSIGVSRADVARVHRPGQADAFLKSEVIDAFSELGDEIARLRWLQAQGQPVPTVIATAE | 67 |
| Query 86 | EAGRDWLLLGEVPGQDLLSS-HLAPAEKVSIMADAMRRLHTLDPATCPFDHQAKHRIERA | 144 |
| Sbjct 68 | EAGR WLL+ +PG+DL SS LAP + V ++ADA+R LH L A CPFD + R++ A EAGRRWLLMSALPGRDLASSPELAPQQLVELLADALRGLHALPVAACPFDQRLASRVQVA | 127 |
| Query 145 | RTRMEAGLVDQDDLDEEHQGLAPAELFARLKARMPDGEDLVVTHGDACLPNIMVENGRFS | 204 |
| Sbjct 128 | + R+EAGLVD DD D+E G +P ++FA L + PD EDLVV+HGDACLPN+M GRFS QARIEAGLVDADDFDDERLGQSPQQVFAELCSTQPDHEDLVVSHGDACLPNLMAVEGRFS | 187 |
| Query 205 | GFIDCGRLGVADRYQDIALATRDIAEELG-GEWADRFLVLYGIAAPDSQRIAFYRLLDEF | 263 |
| Sbjct 188 | GFIDCGRLGVADRYQD+ALA R + G W YG A D +R+AFYRLLDEF GFIDCGRLGVADRYQDLALAARSLVHNFGDTRWVAPLFQRYGAVA-DERRLAFYRLLDEF | 246 |
| Query 264  Sbjct 247 | F 264 F  F 247 |  |

>WP_164688020.1 APH(3')-II family aminoglycoside O-phosphotransferase [Stenotrophomonas pavanii]

NGM53759.1 APH(3')-II family aminoglycoside O-phosphotransferase [Stenotrophomonas pavanii]

Length=267

Score = 241 bits (616), Expect = 6e-76, Method: Compositional matrix adjust.

| Identities | = 129/255 (51%), Positives = 163/255 (64%), Gaps = 3/255 (1%) |  |
| --- | --- | --- |
| Query 12 | PAAWVERLFGYDWAQQTIGCSDAAVFRLSAQGRPVLFVKTDLSGALNELQDEAARLSWLA | 71 |
| Sbjct 14 | P AW E L +Q+IG S A V R+ G+ F+K+++ A +EL DE ARL WL PHAWQEALADARIERQSIGVSRADVARVHRPGQADAFLKSEVIDAFSELGDEIARLRWLQ | 73 |
| Query 72 | TTGVPCAAVLDVVTEAGRDWLLLGEVPGQDLLSS-HLAPAEKVSIMADAMRRLHTLDPAT | 130 |
| Sbjct 74 | G P V+ E GR WLL+ +PG DL SS LAP +V ++ADA+R LH L A AQGQPAPTVIATAEEEGRRWLLMSALPGHDLASSPALAPQHRVQLLADALRGLHALPVAV | 133 |
| Query 131 | CPFDHQAKHRIERARTRMEAGLVDQDDLDEEHQGLAPAELFARLKARMPDGEDLVVTHGD | 190 |
| Sbjct 134 | CPFD + R++ A+ R+EAG VD DD D+E G + ++FA L+A PD EDLVV+HGD CPFDQRLASRLQAAQARVEAGRVDADDFDDECLGQSAQQVFAELRATRPDHEDLVVSHGD | 193 |
| Query 191 | ACLPNIMVENGRFSGFIDCGRLGVADRYQDIALATRDIAEELG-GEWADRFLVLYGIAAP | 249 |
| Sbjct 194 | ACLPN++ GRFSGFIDCGRLGVADRYQD+ALA R + G W YG+ P ACLPNLLAVEGRFSGFIDCGRLGVADRYQDLALAARSLVHNFGDTRWVAPLFRSYGV-VP | 252 |
| Query 250  Sbjct 253 | DSQRIAFYRLLDEFF 264 D +R+AFYRLLDEFF DERRLAFYRLLDEFF 267 |  |

>WP_049403183.1 APH(3')-II family aminoglycoside O-phosphotransferase [Stenotrophomonas maltophilia]

KUJ04442.1 aminoglycoside phosphotransferase [Stenotrophomonas maltophilia] MBB5530903.1 aminoglycoside 3'-phosphotransferase-2 [Stenotrophomonas maltophilia]

NUH61355.1 APH(3')-II family aminoglycoside O-phosphotransferase [Stenotrophomonas maltophilia]

PZS49284.1 APH(3') family aminoglycoside O-phosphotransferase [Stenotrophomonas maltophilia]

Length=267

Score = 241 bits (615), Expect = 6e-76, Method: Compositional matrix adjust. Identities = 131/261 (50%), Positives = 165/261 (63%), Gaps = 3/261 (1%)

Query 5	DGLHAGSPAAWVERLFGYDWAQQTIGCSDAAVFRLSAQGRPVLFVKTDLSGALNELQDEA 64

| Sbjct | 9 | DGL P AW E L +Q+IG S A V R+ G+ F+K+++ A +EL DE DGLRL--PRAWQEALADAHIERQSIGVSRADVARVHRPGQTDAFLKSEVIDAFSELGDEI | 66 |
| --- | --- | --- | --- |
| Query | 65 | ARLSWLATTGVPCAAVLDVVTEAGRDWLLLGEVPGQDLLSS-HLAPAEKVSIMADAMRRL | 123 |
| Sbjct | 67 | ARL WL G V+ E GR WLL+ +PG+DL SS LAP ++ADA+R L ARLRWLQAQGQSAPTVIATTEEGGRRWLLMSALPGRDLASSPELAPRRVAELLADALRGL | 126 |
| Query | 124 | HTLDPATCPFDHQAKHRIERARTRMEAGLVDQDDLDEEHQGLAPAELFARLKARMPDGED | 183 |
| Sbjct | 127 | H + A CPFD Q R++ A+ R+EAGLVD DD D+E G +P ++FA L+A P ED HAVPVANCPFDQQLASRLQAAQARVEAGLVDADDFDDERLGQSPQQVFAELRATRPAHED | 186 |
| Query | 184 | LVVTHGDACLPNIMVENGRFSGFIDCGRLGVADRYQDIALATRDIAEELGGEWADRFLVL | 243 |
| Sbjct | 187 | LVV+HGDACLPN+MV +GRF+GFIDCGRLGVADRYQD+ALA R + G L LVVSHGDACLPNLMVADGRFTGFIDCGRLGVADRYQDLALAARSLVHNFGESRCVATLFQ | 246 |
| Query | 244 | YGIAAPDSQRIAFYRLLDEFF 264 A PD +R+AFYRLLDEFF |  |
| Sbjct | 247 | RYGAVPDERRLAFYRLLDEFF 267 | |

>WP_132809976.1 APH(3')-II family aminoglycoside O-phosphotransferase [Stenotrophomonas sp. ASS1]

QBL40661.1 APH(3')-II family aminoglycoside O-phosphotransferase [Stenotrophomonas sp. ASS1]

Length=267

Score = 241 bits (615), Expect = 7e-76, Method: Compositional matrix adjust.

| Identities | = 130/255 (51%), Positives = 163/255 (64%), Gaps = 3/255 (1%) |  |
| --- | --- | --- |
| Query 12 | PAAWVERLFGYDWAQQTIGCSDAAVFRLSAQGRPVLFVKTDLSGALNELQDEAARLSWLA | 71 |
| Sbjct 14 | P AW E L +Q+IG S A V R+ G+ F+K+++ A +EL DE ARL WL PCAWQEALADARIERQSIGVSRADVARVHRPGQADAFLKSEVIDAFSELGDEIARLRWLQ | 73 |
| Query 72 | TTGVPCAAVLDVVTEAGRDWLLLGEVPGQDLLSS-HLAPAEKVSIMADAMRRLHTLDPAT | 130 |
| Sbjct 74 | G V+ EAGR WLL+ +PG+DL SS LAP + V ++AD +R LH L A AQGQSVPTVIATAEEAGRRWLLMSALPGRDLASSPELAPQQLVELLADGLRGLHALPVAA | 133 |
| Query 131 | CPFDHQAKHRIERARTRMEAGLVDQDDLDEEHQGLAPAELFARLKARMPDGEDLVVTHGD | 190 |
| Sbjct 134 | CPFD + R++ A+ R+EAGLVD DD D+E G +P ++FA L + PD EDLVV+HGD CPFDQRLASRVQVAQARVEAGLVDADDFDDERLGQSPQQVFAELCSTQPDHEDLVVSHGD | 193 |
| Query 191 | ACLPNIMVENGRFSGFIDCGRLGVADRYQDIALATRDIAEELG-GEWADRFLVLYGIAAP | 249 |
| Sbjct 194 | ACLPN+M GRFSGFIDCGRLGVADRYQD+ALA R + G W YG A ACLPNLMAVEGRFSGFIDCGRLGVADRYQDLALAARSLVHNFGDTRWVAPLFQRYGAVA- | 252 |
| Query 250  Sbjct 253 | DSQRIAFYRLLDEFF 264 D +R+AFYRLLDEFF DERRLAFYRLLDEFF 267 |  |

>WP_100448432.1 MULTISPECIES: APH(3')-II family aminoglycoside O-phosphotransferase [Stenotrophomonas]

PJL67508.1 APH(3') family aminoglycoside O-phosphotransferase [Stenotrophomonas maltophilia]

Length=267

Score = 241 bits (615), Expect = 7e-76, Method: Compositional matrix adjust.

| Identities | = 131/255 (51%), Positives = 164/255 (64%), Gaps = 3/255 (1%) |  |
| --- | --- | --- |
| Query 12 | PAAWVERLFGYDWAQQTIGCSDAAVFRLSAQGRPVLFVKTDLSGALNELQDEAARLSWLA | 71 |
| Sbjct 14 | P AW E L +Q+IG S A V R+ G+ F+K+++ A +EL DE RL WL PWAWQEALADAHIERQSIGVSRADVARVHRPGQTDAFLKSEVIDAFSELGDEITRLRWLQ | 73 |
| Query 72 | TTGVPCAAVLDVVTEAGRDWLLLGEVPGQDLLSS-HLAPAEKVSIMADAMRRLHTLDPAT | 130 |
| Sbjct 74 | G+P V+ EAGR WLL+ +PG+DL SS LAP + V ++ADA+RRLH L A AQGLPAPTVIATAEEAGRRWLLMSALPGRDLASSPELAPQQLVELLADALRRLHVLPVAA | 133 |
| Query 131 | CPFDHQAKHRIERARTRMEAGLVDQDDLDEEHQGLAPAELFARLKARMPDGEDLVVTHGD | 190 |
| Sbjct 134 | CPFD + R++ A R+EAGLVD DD D+E G + ++FA L + PD EDLVV+HGD CPFDQRLASRLQAAAARVEAGLVDADDFDDERLGQSAQQVFAELCSTRPDHEDLVVSHGD | 193 |
| Query 191 | ACLPNIMVENGRFSGFIDCGRLGVADRYQDIALATRDIAEELG-GEWADRFLVLYGIAAP | 249 |

ACLPN+M	GRFSGFIDCGRLGVADRYQD+ALA R +	G	W	YG A

| Sbjct | 194 | ACLPNLMAVEGRFSGFIDCGRLGVADRYQDLALAARSLVHNFGDTRWVAPLFQRYG-ALV 252 |
| --- | --- | --- |
| Query | 250 | DSQRIAFYRLLDEFF 264 |
| Sbjct | 253 | D +R+AFYRLLDEFF  DERRLAFYRLLDEFF 267 |

>WP_109369674.1 aminoglycoside 3'-phosphotransferase [Ochrobactrum soli] RLL72699.1 aminoglycoside 3'-phosphotransferase [Ochrobactrum soli] RRD22276.1 aminoglycoside 3'-phosphotransferase [Brucellaceae bacterium

VT-16-1752]

SPL66201.1 Aminoglycoside phosphotransferase [Ochrobactrum soli] Length=266

Score = 241 bits (615), Expect = 7e-76, Method: Compositional matrix adjust.

| Identities | = 125/255 (49%), Positives = 161/255 (63%), Gaps = 2/255 (1%) |  |
| --- | --- | --- |
| Query 12 | PAAWVERLFGYDWAQQTIGCSDAAVFRLSAQGRPVLFVKTDLSGALNELQDEAARLSWLA | 71 |
| Sbjct 12 | P A + RL GY Q +G S A V L L +K + + ++EL DE ARL WL+ PDALLHRLTGYTSQQDELGRSSANVLLLDHDAHEPLVLKIEPASTVSELADERARLEWLS | 71 |
| Query 72 | TTGVPCAAVLDVVTEAGRDWLLLGEVPGQDLLSS--HLAPAEKVSIMADAMRRLHTLDPA | 129 |
| Sbjct 72 | G+PC +V+ A R +LL+ + G +L SS L P VSI+A A++ LH++DPA AQGLPCPSVVAYERMADRHFLLMTRLAGSNLASSVGTLTPDRIVSILATALKALHSVDPA | 131 |
| Query 130 | TCPFDHQAKHRIERARTRMEAGLVDQDDLDEEHQGLAPAELFARLKARMPDGEDLVVTHG | 189 |
| Sbjct 132 | CPFDH+ +HRI+ AR R+EAG VD+DD D E +G +LF+ L P ED+VVTHG ACPFDHRLEHRIKDARKRVEAGAVDEDDFDTEREGRTAEDLFSELHRLKPSQEDVVVTHG | 191 |
| Query 190 | DACLPNIMVENGRFSGFIDCGRLGVADRYQDIALATRDIAEELGGEWADRFLVLYGIAAP | 249 |
| Sbjct 192 | DACLPN M +G F+GFIDCGRLG+ADR+QD+ LA I LG EW FL LY A DACLPNFMASDGTFTGFIDCGRLGLADRHQDLGLACWSIRYNLGNEWVKPFLDLYSAEAI | 251 |
| Query 250  Sbjct 252 | DSQRIAFYRLLDEFF 264 D +IA+YRLLDEFF DEAKIAYYRLLDEFF 266 |  |

>WP_171962387.1 APH(3') family aminoglycoside O-phosphotransferase [Bordetella trematum]

Length=256

Score = 241 bits (614), Expect = 7e-76, Method: Compositional matrix adjust.

| Identities | = 127/240 (53%), Positives = 152/240 (63%), Gaps = 2/240 (1%) |  |
| --- | --- | --- |
| Query 26 | QQTIGCSDAAVFRLSAQGRPVLFVKTDLSGALNELQDEAARLSWLATTGVPCAAVLDVVT | 85 |
| Sbjct 18 | +QTIG SDAAV+RL+ F+K + + L EL E ARL W+ G+PC VL + RQTIGESDAAVWRLTDADGACRFLKAETAHPLAELPAEVARLRWMRVQGLPCPEVLQTLQ | 77 |
| Query 86 | EAGRDWLLLGEVPGQDLLSS-HLAPAEKVSIMADAMRRLHTLDPATCPFDHQAKHRIERA | 144 |
| Sbjct 78 | GR WLL+ VPG+DL SS L+P V+++A A++RLH PA CPFDH+ R+ A ADGRHWLLMSAVPGRDLASSPELSPQRTVALLAQALQRLHQTPPAACPFDHRLARRVAEA | 137 |
| Query 145 | RTRMEAGLVDQDDLDEEHQGLAPAELFARLKARMPDGEDLVVTHGDACLPNIMVENGRFS | 204 |
| Sbjct 138 | R +AGLVD + D+E QG A L L A P EDLVVTHGDACLPN M GRFS SRRAQAGLVDVGNFDQERQGQAVEALLRALVASQPPREDLVVTHGDACLPNFMAAEGRFS | 197 |
| Query 205 | GFIDCGRLGVADRYQDIALATRDIAEELGGEWADRFLVLYGIAAPDSQRIAFYRLLDEFF | 264 |
| Sbjct 198 | GFIDC RLGVADR+QD+AL R I LG EW F YGI PD R+AFY LLDEFF  GFIDCARLGVADRHQDLALTARSIERNLGAEWLTPFFTCYGI-EPDPARLAFYCLLDEFF | 256 |

>WP_164241085.1 APH(3')-II family aminoglycoside O-phosphotransferase [Stenotrophomonas maltophilia]

Length=267

Score = 241 bits (615), Expect = 7e-76, Method: Compositional matrix adjust. Identities = 129/255 (51%), Positives = 165/255 (65%), Gaps = 3/255 (1%)

Query 12	PAAWVERLFGYDWAQQTIGCSDAAVFRLSAQGRPVLFVKTDLSGALNELQDEAARLSWLA 71 P W E L G + +Q+IG S A V R+	G+	F+K+++ A +EL DE ARL WL

| Sbjct | 14 | PQTWQEVLAGARFERQSIGVSRADVARVHRPGQADAFLKSEVIDAFSELGDEIARLRWLQ | 73 |
| --- | --- | --- | --- |
| Query | 72 | TTGVPCAAVLDVVTEAGRDWLLLGEVPGQDLLSS-HLAPAEKVSIMADAMRRLHTLDPAT | 130 |
| Sbjct | 74 | G P V+ EAGR WLL+ +PG+DL +S L P + V+++ADA+R LH L AT AQGQPAPTVMATAEEAGRRWLLMSALPGRDLATSADLPPQQVVAVLADALRGLHALPVAT | 133 |
| Query | 131 | CPFDHQAKHRIERARTRMEAGLVDQDDLDEEHQGLAPAELFARLKARMPDGEDLVVTHGD | 190 |
| Sbjct | 134 | CPFD + R+ A+ R+EAG+VD DD D+E G + ++F L A P ED VV HGD CPFDQRLASRLHAAQARVEAGVVDADDFDDERLGQSAQQVFVELCASRPAHEDRVVCHGD | 193 |
| Query | 191 | ACLPNIMVENGRFSGFIDCGRLGVADRYQDIALATRDIAEELG-GEWADRFLVLYGIAAP | 249 |
| Sbjct | 194 | ACLPN+MV +GRFSGFIDCGRLGVADRYQD+ALA R +A G W YG+ A ACLPNLMVADGRFSGFIDCGRLGVADRYQDLALAARSLAHNFGDTRWVAPLFQRYGVIA- | 252 |
| Query  Sbjct | 250  253 | DSQRIAFYRLLDEFF 264 D +R+AFYRLLDEFF DERRLAFYRLLDEFF 267 |  |

>WP_049483201.1 APH(3')-II family aminoglycoside O-phosphotransferase [Stenotrophomonas maltophilia]

Length=271

Score = 241 bits (615), Expect = 7e-76, Method: Compositional matrix adjust.

| Identities | = 130/253 (51%), Positives = 164/253 (65%), Gaps = 3/253 (1%) |  |
| --- | --- | --- |
| Query 14 | AWVERLFGYDWAQQTIGCSDAAVFRLSAQGRPVLFVKTDLSGALNELQDEAARLSWLATT | 73 |
| Sbjct 20 | AW E L +Q+IG S A V R+ G+ F+K+++ A +EL DE RL WL AWQEALADAHIERQSIGVSRADVARVHRPGQTDAFLKSEVIDAFSELGDEITRLRWLHAQ | 79 |
| Query 74 | GVPCAAVLDVVTEAGRDWLLLGEVPGQDLLSS-HLAPAEKVSIMADAMRRLHTLDPATCP | 132 |
| Sbjct 80 | G+P V+ EAGR WLL+ +PG+DL SS LAP + V ++ADA+R LH L TCP GLPAPTVIATAEEAGRRWLLMSALPGRDLASSPELAPQQLVELLADALRGLHALPVVTCP | 139 |
| Query 133 | FDHQAKHRIERARTRMEAGLVDQDDLDEEHQGLAPAELFARLKARMPDGEDLVVTHGDAC | 192 |
| Sbjct 140 | FDH+ R++ A R+EAGLVD DD D+E G + ++FA L + PD EDLVV+HGDAC FDHRLDLRLQAAAARVEAGLVDADDFDDERLGQSAQQVFAELCSTRPDHEDLVVSHGDAC | 199 |
| Query 193 | LPNIMVENGRFSGFIDCGRLGVADRYQDIALATRDIAEELG-GEWADRFLVLYGIAAPDS | 251 |
| Sbjct 200 | LPN+M GRFSGFIDCGRLGVADRYQD+ALA R + G W YG+ A D LPNLMAVEGRFSGFIDCGRLGVADRYQDLALAARSLVHNFGDTRWVAPLFQRYGVVA-DE | 258 |
| Query 252  Sbjct 259 | QRIAFYRLLDEFF 264  +R+AFYRLLDEFF  RRLAFYRLLDEFF 271 |  |

>WP_049398550.1 APH(3')-II family aminoglycoside O-phosphotransferase [Stenotrophomonas maltophilia]

VUO08439.1 Aminoglycoside 3'-phosphotransferase [Stenotrophomonas maltophilia] Length=267

Score = 241 bits (615), Expect = 7e-76, Method: Compositional matrix adjust.

| Identities | = 130/255 (51%), Positives = 165/255 (65%), Gaps = 3/255 (1%) |  |
| --- | --- | --- |
| Query 12 | PAAWVERLFGYDWAQQTIGCSDAAVFRLSAQGRPVLFVKTDLSGALNELQDEAARLSWLA | 71 |
| Sbjct 14 | P AW + L +Q+IG S A V R+ G+ F+K+++ A +EL DE ARL WL PCAWQDALADARIERQSIGVSRADVARVHRPGQADAFLKSEVIDAFSELGDEIARLRWLQ | 73 |
| Query 72 | TTGVPCAAVLDVVTEAGRDWLLLGEVPGQDLLSS-HLAPAEKVSIMADAMRRLHTLDPAT | 130 |
| Sbjct 74 | G P V+ EAGR WLL+ +PG+DL SS LAP + V ++ADA+R LH L A AQGQPVPTVIATAEEAGRRWLLMSALPGRDLASSPELAPQQLVELLADALRGLHALPVAA | 133 |
| Query 131 | CPFDHQAKHRIERARTRMEAGLVDQDDLDEEHQGLAPAELFARLKARMPDGEDLVVTHGD | 190 |
| Sbjct 134 | CPFD + R++ A+ R+EAGLVD DD D+E G +P ++FA L + PD EDLVV+HGD CPFDQRLASRVQVAQARIEAGLVDADDFDDERLGQSPQQVFAELCSTRPDHEDLVVSHGD | 193 |
| Query 191 | ACLPNIMVENGRFSGFIDCGRLGVADRYQDIALATRDIAEEL-GGEWADRFLVLYGIAAP | 249 |
| Sbjct 194 | ACLPN+MV GRFSGFIDCGRLGVAD YQD+ALA R + W YG+ A ACLPNLMVVEGRFSGFIDCGRLGVADCYQDLALAVRSLVYNFDNTRWVAPLFKRYGMVA- | 252 |
| Query 250 | DSQRIAFYRLLDEFF 264 |  |

D +R+AFYRLLDEFF Sbjct 253 DERRLAFYRLLDEFF 267

>VUO98825.1 Aminoglycoside 3'-phosphotransferase [Stenotrophomonas maltophilia] Length=269

Score = 241 bits (615), Expect = 8e-76, Method: Compositional matrix adjust.

| Identities | = 129/255 (51%), Positives = 165/255 (65%), Gaps = 3/255 (1%) |  |
| --- | --- | --- |
| Query 12 | PAAWVERLFGYDWAQQTIGCSDAAVFRLSAQGRPVLFVKTDLSGALNELQDEAARLSWLA | 71 |
| Sbjct 16 | P W E L G + +Q+IG S A V R+ G+ F+K+++ A +EL DE ARL WL PQTWQEVLAGARFERQSIGVSRADVARVHRPGQADAFLKSEVIDAFSELGDEIARLRWLQ | 75 |
| Query 72 | TTGVPCAAVLDVVTEAGRDWLLLGEVPGQDLLSS-HLAPAEKVSIMADAMRRLHTLDPAT | 130 |
| Sbjct 76 | G P V+ EAGR WLL+ +PG+DL +S L P + V+++ADA+R LH L AT AQGQPAPTVMATAEEAGRRWLLMSALPGRDLATSADLPPQQVVAVLADALRGLHALPVAT | 135 |
| Query 131 | CPFDHQAKHRIERARTRMEAGLVDQDDLDEEHQGLAPAELFARLKARMPDGEDLVVTHGD | 190 |
| Sbjct 136 | CPFD + R+ A+ R+EAG+VD DD D+E G + ++F L A P ED VV HGD CPFDQRLASRLHAAQARVEAGVVDADDFDDERLGQSAQQVFVELCASRPAHEDRVVCHGD | 195 |
| Query 191 | ACLPNIMVENGRFSGFIDCGRLGVADRYQDIALATRDIAEELG-GEWADRFLVLYGIAAP | 249 |
| Sbjct 196 | ACLPN+MV +GRFSGFIDCGRLGVADRYQD+ALA R +A G W YG+ A ACLPNLMVADGRFSGFIDCGRLGVADRYQDLALAARSLAHNFGDTRWVAPLFQRYGVIA- | 254 |
| Query 250  Sbjct 255 | DSQRIAFYRLLDEFF 264 D +R+AFYRLLDEFF DERRLAFYRLLDEFF 269 |  |

>WP_136638745.1 APH(3')-II family aminoglycoside O-phosphotransferase [Stenotrophomonas maltophilia]

TIE18764.1 APH(3')-II family aminoglycoside O-phosphotransferase [Stenotrophomonas maltophilia]

TIE64844.1 APH(3')-II family aminoglycoside O-phosphotransferase [Stenotrophomonas maltophilia]

Length=267

Score = 241 bits (615), Expect = 8e-76, Method: Compositional matrix adjust.

| Identities | = 131/255 (51%), Positives = 163/255 (64%), Gaps = 3/255 (1%) |  |
| --- | --- | --- |
| Query 12 | PAAWVERLFGYDWAQQTIGCSDAAVFRLSAQGRPVLFVKTDLSGALNELQDEAARLSWLA | 71 |
| Sbjct 14 | P+AW E L G +Q+IG S A V R+ G+ F+K+++ A +EL E ARL WL PSAWREALAGARIERQSIGVSRADVARVHRPGQADAFLKSEVIDAFSELGHEIARLRWLQ | 73 |
| Query 72 | TTGVPCAAVLDVVTEAGRDWLLLGEVPGQDLLSS-HLAPAEKVSIMADAMRRLHTLDPAT | 130 |
| Sbjct 74 | G P V+ +AGR WLL+ +PG+DL SS LAP + V ++ADA+R LH L A AQGQPAPTVIATAEDAGRRWLLMSALPGRDLASSPELAPQQLVEVLADALRGLHALPVAA | 133 |
| Query 131 | CPFDHQAKHRIERARTRMEAGLVDQDDLDEEHQGLAPAELFARLKARMPDGEDLVVTHGD | 190 |
| Sbjct 134 | CPFD + R++ A R+EAGLVD DD D+E G + ++ A L A P EDLVV+HGD CPFDQRLASRVQAAVARVEAGLVDADDFDDERLGQSAQQVVAELLASQPAHEDLVVSHGD | 193 |
| Query 191 | ACLPNIMVENGRFSGFIDCGRLGVADRYQDIALATRDIAEELG-GEWADRFLVLYGIAAP | 249 |
| Sbjct 194 | ACLPN+MV GRFSGFIDCGRLGVADRYQD+ALA R I G W YG A ACLPNLMVAEGRFSGFIDCGRLGVADRYQDLALAARSIVHNFGDTRWVAPLFQRYGAVA- | 252 |
| Query 250  Sbjct 253 | DSQRIAFYRLLDEFF 264 D +R+AFYRLLDEFF DERRLAFYRLLDEFF 267 |  |

>WP_014646913.1 APH(3')-II family aminoglycoside O-phosphotransferase [Stenotrophomonas maltophilia]

AWB78161.1 APH(3')-II family aminoglycoside O-phosphotransferase [Stenotrophomonas maltophilia]

KDE89606.1 aminoglycoside phosphotransferase [Stenotrophomonas maltophilia M30]

KOO76563.1 aminoglycoside phosphotransferase [Stenotrophomonas maltophilia]

MBA0455575.1 APH(3')-II family aminoglycoside O-phosphotransferase [Stenotrophomonas maltophilia]

OWQ66535.1 aminoglycoside 3'-phosphotransferase [Stenotrophomonas maltophilia] Length=267

Score = 241 bits (615), Expect = 8e-76, Method: Compositional matrix adjust.

| Identities | = 128/255 (50%), Positives = 164/255 (64%), Gaps = 3/255 (1%) |  |
| --- | --- | --- |
| Query 12 | PAAWVERLFGYDWAQQTIGCSDAAVFRLSAQGRPVLFVKTDLSGALNELQDEAARLSWLA | 71 |
| Sbjct 14 | P AW E L +Q+IG S A V R+ G+ F+K+++ A +EL DE ARL WL PWAWRETLADARIERQSIGVSRADVARVHRPGQADAFLKSEVIDAFSELGDEIARLRWLQ | 73 |
| Query 72 | TTGVPCAAVLDVVTEAGRDWLLLGEVPGQDLLSS-HLAPAEKVSIMADAMRRLHTLDPAT | 130 |
| Sbjct 74 | G P V+ EAGR WLL+ +PG+DL SS L P V ++ADA+R LH + A AQGQPAPTVIATAEEAGRRWLLMSALPGRDLASSPELPPQRLVELLADALRGLHAIPVAA | 133 |
| Query 131 | CPFDHQAKHRIERARTRMEAGLVDQDDLDEEHQGLAPAELFARLKARMPDGEDLVVTHGD | 190 |
| Sbjct 134 | CPFD + R++ A+ R+EAGLVD DD D+E G + ++FA L+A P+ EDLVV+HGD CPFDQRLASRLQAAQARVEAGLVDADDFDDERLGQSAQQVFAELRATRPNHEDLVVSHGD | 193 |
| Query 191 | ACLPNIMVENGRFSGFIDCGRLGVADRYQDIALATRDIAEELG-GEWADRFLVLYGIAAP | 249 |
| Sbjct 194 | ACLPN++ GRFSGFIDCGRLGVADRYQD+ALA R + G W YG+ P ACLPNLLAAEGRFSGFIDCGRLGVADRYQDLALAARSLVHNFGDTRWVAPLFRRYGV-VP | 252 |
| Query 250  Sbjct 253 | DSQRIAFYRLLDEFF 264 D +R+AFYRLLDEFF DERRLAFYRLLDEFF 267 |  |

>WP_124418410.1 aminoglycoside 3'-phosphotransferase [Pseudomonas sp. LBUM920] AZF65900.1 Aminoglycoside 3'-phosphotransferase [Pseudomonas sp. LBUM920]

Length=256

Score = 241 bits (614), Expect = 9e-76, Method: Compositional matrix adjust.

| Identities | | = 126/254 (50%), Positives = 158/254 (62%), Gaps = 2/254 (1%) |  |
| --- | --- | --- | --- |
| Query 12  Sbjct 4 | | PAAWVERLFGYDWAQQTIGCSDAAVFRLSAQGRPVLFVKTDLSGALNELQDEAARLSWLA P W + QQ IG S A VFRL GR LF+K + A +EL DE RL WL PDKWRTQFAQALIEQQAIGESRADVFRLRQVGRKDLFLKAEPLVAFSELADEIERLRWLQ | 71  63 |
| Query 72 | | TTGVPCAAVLDVVTEAGRDWLLLGEVPGQDLLSSHLAPA-EKVSIMADAMRRLHTLDPAT  +P VLD + + WLL+ VPGQDL S++ PA + V I+A A+R LH + A | 130 |
| Sbjct | 64 | QVNLPAPVVLDELIDTHHHWLLMTAVPGQDLASANTLPAAQTVDILATALRTLHQVPIAQ | 123 |
| Query | 131 | CPFDHQAKHRIERARTRMEAGLVDQDDLDEEHQGLAPAELFARLKARMPDGEDLVVTHGD | 190 |
| Sbjct | 124 | CPFDH + RI AR + AGL+D+ D D+E G + ++FA L + P+ DLVVTHGD CPFDHALQQRIALARAHVNAGLIDETDFDDERLGRSLEDVFAELLSTQPNTHDLVVTHGD | 183 |
| Query | 191 | ACLPNIMVENGRFSGFIDCGRLGVADRYQDIALATRDIAEELGGEWADRFLVLYGIAAPD | 250 |
| Sbjct | 184 | ACLPN M +GRFSGFIDCGRLG++DRYQD+ALA R I +G +W F LYG+ PD ACLPNFMAADGRFSGFIDCGRLGISDRYQDLALAARSIERNVGPKWVKPFFELYGV-EPD | 242 |
| Query | 251 | SQRIAFYRLLDEFF 264 QR+ FY LLDEFF |  |
| Sbjct | 243 | EQRMKFYCLLDEFF 256 | |

>WP_089042367.1 aminoglycoside 3'-phosphotransferase [Pseudomonas fluorescens] MBC6622454.1 aminoglycoside 3'-phosphotransferase [Pseudomonas sp.]

Length=256

Score = 241 bits (614), Expect = 9e-76, Method: Compositional matrix adjust. Identities = 132/254 (52%), Positives = 158/254 (62%), Gaps = 2/254 (1%)

| Query | 12 | PAAWVERLFGYDWAQQTIGCSDAAVFRLSAQGRPVLFVKTDLSGALNELQDEAARLSWLA | 71 |
| --- | --- | --- | --- |
| Sbjct | 4 | P W E+ QQ IG S A VFRL G LF+K++L +EL DE RL WL PHKWREQFPHALIEQQAIGESRADVFRLRHDGGTDLFLKSELLEEHSELADEIDRLRWLQ | 63 |
| Query | 72 | TTGVPCAAVLDVVTEAGRDWLLLGEVPGQDLLS-SHLAPAEKVSIMADAMRRLHTLDPAT | 130 |
| Sbjct | 64 | G+P VLD VT A R WLL+ VPGQDL S S L+ + ++I+A A+R LH L A QMGLPAPVVLDEVTAAHRHWLLMTTVPGQDLASASELSAEQIINILATALRTLHQLPVAL | 123 |
| Query | 131 | CPFDHQAKHRIERARTRMEAGLVDQDDLDEEHQGLAPAELFARLKARMPDGEDLVVTHGD CPFDH + RI RA+ + AGLVD+ D D+E G + ++FA L A P DLVVTHGD | 190 |

| Sbjct | 124 | CPFDHSLQLRIARAQQHVSAGLVDETDFDDERLGQSAEDVFAELVATQPKTHDLVVTHGD | 183 |
| --- | --- | --- | --- |
| Query | 191 | ACLPNIMVENGRFSGFIDCGRLGVADRYQDIALATRDIAEELGGEWADRFLVLYGIAAPD | 250 |
| Sbjct | 184 | ACLPN M G FSGFIDCGRLGV+DR+QD+ALA R I LG W F YG+ PD ACLPNFMAAEGCFSGFIDCGRLGVSDRFQDLALAARSIESNLGLAWVRPFFEAYGV-EPD | 242 |
| Query | 251 | SQRIAFYRLLDEFF 264 QR+ FY LLDEFF |  |
| Sbjct | 243 | EQRMKFYCLLDEFF 256 | |

>WP_142234634.1 APH(3')-II family aminoglycoside O-phosphotransferase [Stenotrophomonas maltophilia]

TQM07960.1 aminoglycoside 3'-phosphotransferase-2 [Stenotrophomonas maltophilia] Length=267

Score = 241 bits (615), Expect = 9e-76, Method: Compositional matrix adjust.

| Identities | = 128/255 (50%), Positives = 164/255 (64%), Gaps = 3/255 (1%) |  |
| --- | --- | --- |
| Query 12 | PAAWVERLFGYDWAQQTIGCSDAAVFRLSAQGRPVLFVKTDLSGALNELQDEAARLSWLA | 71 |
| Sbjct 14 | P AW E L +Q+IG S A V R+ G+ F+K+++ A +EL DE ARL WL PWAWRETLADARIERQSIGVSRADVARVHRPGQADAFLKSEVIDAFSELGDEIARLRWLQ | 73 |
| Query 72 | TTGVPCAAVLDVVTEAGRDWLLLGEVPGQDLLSS-HLAPAEKVSIMADAMRRLHTLDPAT | 130 |
| Sbjct 74 | G P V+ EAGR WLL+ +PG+DL SS L P V ++ADA+R LH + A AQGQPAPTVIATAEEAGRRWLLMSALPGRDLASSPELPPQRLVELLADALRGLHAIPVAA | 133 |
| Query 131 | CPFDHQAKHRIERARTRMEAGLVDQDDLDEEHQGLAPAELFARLKARMPDGEDLVVTHGD | 190 |
| Sbjct 134 | CPFD + R++ A+ R+EAGLVD DD D+E G + ++FA L+A P+ EDLVV+HGD CPFDQRLASRLQAAQARVEAGLVDADDFDDERLGQSAQQVFAELRATRPNHEDLVVSHGD | 193 |
| Query 191 | ACLPNIMVENGRFSGFIDCGRLGVADRYQDIALATRDIAEELG-GEWADRFLVLYGIAAP | 249 |
| Sbjct 194 | ACLPN++ GRFSGFIDCGRLGVADRYQD+ALA R + G W YG+ P ACLPNLLAAEGRFSGFIDCGRLGVADRYQDLALAARSLVHNFGDTRWVAPLFRRYGV-VP | 252 |
| Query 250  Sbjct 253 | DSQRIAFYRLLDEFF 264 D +R+AFYRLLDEFF DERRLAFYRLLDEFF 267 |  |

>WP_101765352.1 APH(3')-II family aminoglycoside O-phosphotransferase [Stenotrophomonas maltophilia]

AUI07370.1 APH(3')-II family aminoglycoside O-phosphotransferase [Stenotrophomonas maltophilia]

Length=267

Score = 241 bits (614), Expect = 9e-76, Method: Compositional matrix adjust.

| Identities | = 129/255 (51%), Positives = 164/255 (64%), Gaps = 3/255 (1%) |  |
| --- | --- | --- |
| Query 12 | PAAWVERLFGYDWAQQTIGCSDAAVFRLSAQGRPVLFVKTDLSGALNELQDEAARLSWLA | 71 |
| Sbjct 14 | P AW E L +Q+IG S A V R+ G+ F+K+++ +EL DE ARL WL PHAWQEALADARIERQSIGVSRADVARVHRPGQADAFLKSEVIDTFSELGDEIARLRWLQ | 73 |
| Query 72 | TTGVPCAAVLDVVTEAGRDWLLLGEVPGQDLLSS-HLAPAEKVSIMADAMRRLHTLDPAT | 130 |
| Sbjct 74 | G P V+ EAGR WLL+ +PG DL SS LAP V ++A A+R LH L A+ AHGQPAPTVIATAEEAGRRWLLMSALPGHDLASSLALAPQHLVELLAGALRGLHALPVAS | 133 |
| Query 131 | CPFDHQAKHRIERARTRMEAGLVDQDDLDEEHQGLAPAELFARLKARMPDGEDLVVTHGD | 190 |
| Sbjct 134 | CPFD + R++ A+ R+EAGLVD DD D+E G + ++FA L+A PD EDLVV+HGD CPFDQRLASRLQAAQARVEAGLVDADDFDDERLGQSSQQVFAELRATRPDHEDLVVSHGD | 193 |
| Query 191 | ACLPNIMVENGRFSGFIDCGRLGVADRYQDIALATRDIAEELG-GEWADRFLVLYGIAAP | 249 |
| Sbjct 194 | ACLPN++ GRFSGFIDCGRLGVADRYQD+ALA R + G W YG+ A ACLPNLLAAEGRFSGFIDCGRLGVADRYQDLALAARSLVHNFGDTRWVAPLFRCYGVVA- | 252 |
| Query 250  Sbjct 253 | DSQRIAFYRLLDEFF 264 D++R+AFYRLLDEFF DARRLAFYRLLDEFF 267 |  |

>WP_037140469.1 MULTISPECIES: aminoglycoside 3'-phosphotransferase [Rhizobium] KEC73956.1 aminoglycoside 3'-phosphotransferase [Rhizobium leguminosarum

bv. phaseoli CCGM1]

PWI53807.1 aminoglycoside phosphotransferase APH(3') [Rhizobium phaseoli] Length=261

Score = 241 bits (614), Expect = 1e-75, Method: Compositional matrix adjust.

| Identities | | = 129/254 (51%), Positives = 169/254 (67%), Gaps = 1/254 (0%) |  |
| --- | --- | --- | --- |
| Query 12  Sbjct 8 | | PAAWVERLFGYDWAQQTIGCSDAAVFRLSAQGRPVLFVKTDLSGALNELQDEAARLSWLA PAA ERL GY + + +G S A+VFRL +G P L++K + +G EL DEAARLSWL PAALGERLSGYRFERDALGRSAASVFRLQGEGLPALYLKVEAAGPFGELADEAARLSWLK | 71  67 |
| Query 72  Sbjct 68 | | TTGVPCAAVLDVVTEAGRDWLLLGEVPGQDLLS-SHLAPAEKVSIMADAMRRLHTLDPAT G+PC V+ ++ R+WLL+G +PG DL S S L P +V ++A A+ LH L A+ AVGLPCPEVIARESDGERNWLLIGALPGTDLASASALTPLTRVELLAAALADLHDLPIAS | 130  127 |
| Query 131  Sbjct 128 | | CPFDHQAKHRIERARTRMEAGLVDQDDLDEEHQGLAPAELFARLKARMPDGEDLVVTHGD CPFDH+ + R+ A+ RM+AG+VD++D D G + A LF L P EDLVVTHGD CPFDHRLESRLPTAKARMQAGIVDEEDFDAARLGKSAAALFDDLMRLRPGDEDLVVTHGD | 190  187 |
| Query 191  Sbjct 188 | | ACLPNIMVENGRFSGFIDCGRLGVADRYQDIALATRDIAEELGGEWADRFLVLYGIAAPD ACLPN + NG+FSG+IDCGRLGVADR+QDIALA R IA G FL YG+ APD ACLPNFVASNGQFSGYIDCGRLGVADRHQDIALACRSIAYNFGEALIRPFLDRYGLTAPD | 250  247 |
| Query 251 | | SQRIAFYRLLDEFF 264  ++ +++LLDEFF |  |
| Sbjct | 248 | PAKLHYFQLLDEFF 261 | |

>RTY17322.1 APH(3')-II family aminoglycoside O-phosphotransferase [[Pseudomonas] geniculata]

Length=271

Score = 241 bits (614), Expect = 1e-75, Method: Compositional matrix adjust.

| Identities | = 130/253 (51%), Positives = 164/253 (65%), Gaps = 3/253 (1%) |  |
| --- | --- | --- |
| Query 14 | AWVERLFGYDWAQQTIGCSDAAVFRLSAQGRPVLFVKTDLSGALNELQDEAARLSWLATT | 73 |
| Sbjct 20 | AW E L +Q+IG S A V R+ G+ F+K+++ A +EL DE RL WL AWQEALADAHIERQSIGVSRADVARVHRPGQTDAFLKSEVIDAFSELGDEITRLRWLQAQ | 79 |
| Query 74 | GVPCAAVLDVVTEAGRDWLLLGEVPGQDLLSS-HLAPAEKVSIMADAMRRLHTLDPATCP | 132 |
| Sbjct 80 | G+P V+ EAGR WLL+ +PG+DL SS LAP + V ++ADA+RRLH L A CP GLPAPTVIATAEEAGRRWLLMSALPGRDLASSPELAPQQLVELLADALRRLHVLPVAACP | 139 |
| Query 133 | FDHQAKHRIERARTRMEAGLVDQDDLDEEHQGLAPAELFARLKARMPDGEDLVVTHGDAC | 192 |
| Sbjct 140 | FD + R++ A+ R+EAGLVD DD D+E G + ++FA L + PD EDLVV+HGDAC FDQRLASRLQAAQARVEAGLVDADDFDDERLGQSAQQVFAELCSTRPDHEDLVVSHGDAC | 199 |
| Query 193 | LPNIMVENGRFSGFIDCGRLGVADRYQDIALATRDIAEELG-GEWADRFLVLYGIAAPDS | 251 |
| Sbjct 200 | LPN+M GRFSGFIDCGRLGVADRYQD+ALA R + G W YG A D LPNLMAVEGRFSGFIDCGRLGVADRYQDLALAARSLVHNFGDTRWVAPLFQRYG-ALVDE | 258 |
| Query 252  Sbjct 259 | QRIAFYRLLDEFF 264  +R+AFYRLLDEFF  RRLAFYRLLDEFF 271 |  |

>WP_038368729.1 aminoglycoside 3'-phosphotransferase [Pseudomonas chlororaphis] KHA70465.1 aminoglycoside phosphotransferase [Pseudomonas chlororaphis]

Length=256

Score = 240 bits (613), Expect = 1e-75, Method: Compositional matrix adjust.

| Identities | = 125/254 (49%), Positives = 163/254 (64%), Gaps = 2/254 (1%) |  |
| --- | --- | --- |
| Query 12 | PAAWVERLFGYDWAQQTIGCSDAAVFRLSAQGRPVLFVKTDLSGALNELQDEAARLSWLA | 71 |
| Sbjct 4 | PAAW + QQTIG S A V+R++ G LFVK + EL E RL W+A PAAWRSQFAAAAIEQQTIGESRADVWRVTPAGAASLFVKAEQVMTWGELPGEVQRLRWMA | 63 |
| Query 72 | TTGVPCAAVLDVVTEAGRDWLLLGEVPGQDLLSS-HLAPAEKVSIMADAMRRLHTLDPAT | 130 |
| Sbjct 64 | T +P VLD + EA +WLL+ V G+DL SS HL + V++ A A+R LH + TQDLPAPRVLDTLVEADHNWLLMTAVAGRDLASSAHLTSQQIVTLAAQALRTLHAVAIDR | 123 |
| Query 131 | CPFDHQAKHRIERARTRMEAGLVDQDDLDEEHQGLAPAELFARLKARMPDGEDLVVTHGD | 190 |

| Sbjct | 124 | CPFDH + +I RAR + AGL+D++D D+ G + +F ++ A P EDLVVTHGD CPFDHCLEQKITRAREHLLAGLIDEEDFDDSRLGQSAEAVFQQMLAARPTHEDLVVTHGD | 183 |
| --- | --- | --- | --- |
| Query | 191 | ACLPNIMVENGRFSGFIDCGRLGVADRYQDIALATRDIAEELGGEWADRFLVLYGIAAPD | 250 |
| Sbjct | 184 | ACLPN++ ++GRFSGF+DCGRLGVADRYQD+ALA I + LG +W F +YG+ PD ACLPNLLADDGRFSGFVDCGRLGVADRYQDLALAAHSITDNLGAQWLPLFFEIYGV-EPD | 242 |
| Query | 251 | SQRIAFYRLLDEFF 264 QRIAFY+LLDEFF |  |
| Sbjct | 243 | LQRIAFYQLLDEFF 256 | |

>WP_102357651.1 aminoglycoside 3'-phosphotransferase [Pseudomonas sp. AD21] Length=256

Score = 240 bits (613), Expect = 1e-75, Method: Compositional matrix adjust.

| Identities | | = 126/254 (50%), Positives = 160/254 (63%), Gaps = 2/254 (1%) |  |
| --- | --- | --- | --- |
| Query 12  Sbjct 4 | | PAAWVERLFGYDWAQQTIGCSDAAVFRLSAQGRPVLFVKTDLSGALNELQDEAARLSWLA P W R QQTIG S A VFRL+ G LFVK++ EL E RL WLA PQTWRSRFADSACEQQTIGESRADVFRLTPAGSAPLFVKSEPRITFAELPGEVQRLRWLA | 71  63 |
| Query 72  Sbjct 64 | | TTGVPCAAVLDVVTEAGRDWLLLGEVPGQDLLSSH-LAPAEKVSIMADAMRRLHTLDPAT  +P A VLD E R WLL+ VPG+DL SS L P + V++ A+A+R LH LD EQDLPVARVLDSTEEHDRYWLLMSAVPGRDLASSERLTPQQIVTLAANALRTLHALDITQ | 130  123 |
| Query 131  Sbjct 124 | | CPFDHQAKHRIERARTRMEAGLVDQDDLDEEHQGLAPAELFARLKARMPDGEDLVVTHGD CPFDH+ +I AR + AGL+D++D D+ G + +F ++ A P EDLVVTHGD CPFDHRLDPKIALAREHLLAGLIDEEDFDDSRLGHSAEAVFQQMLAARPVSEDLVVTHGD | 190  183 |
| Query 191  Sbjct 184 | | ACLPNIMVENGRFSGFIDCGRLGVADRYQDIALATRDIAEELGGEWADRFLVLYGIAAPD ACLPN++ ++GRFSGF+DCGRLGVADR+QD+ALA I + LG +W F YG+ PD ACLPNLLADHGRFSGFVDCGRLGVADRFQDLALAAHSITDNLGEQWVPAFFEAYGV-QPD | 250  242 |
| Query | 251 SQRIAFYRLLDEFF 264 | | |
|  |  | Q+I FY+LLDEFF |  |
| Sbjct | 243 | PQKIDFYQLLDEFF 256 | |

>WP_178124262.1 APH(3') family aminoglycoside O-phosphotransferase [Pseudomonas sp. AN3A02]

Length=256

Score = 240 bits (613), Expect = 1e-75, Method: Compositional matrix adjust.

| Identities | | = 129/254 (51%), Positives = 157/254 (62%), Gaps = 2/254 (1%) |  |
| --- | --- | --- | --- |
| Query 12  Sbjct 4 | | PAAWVERLFGYDWAQQTIGCSDAAVFRLSAQGRPVLFVKTDLSGALNELQDEAARLSWLA P W ++ QTIG S A VFRL LF+K++ G EL DE RL WL PKKWRDQFPNALIEPQTIGESGADVFRLLRDNGEDLFLKSEPIGPHAELPDEIKRLRWLQ | 71  63 |
| Query 72  Sbjct 64 | | TTGVPCAAVLDVVTEAGRDWLLLGEVPGQDLL-SSHLAPAEKVSIMADAMRRLHTLDPAT  + +P AVLDV+TE R WLL+ VPGQDL +S L+ + + ++A A+R LH + A QSNLPGPAVLDVLTENHRHWLLMSAVPGQDLAGASDLSAPQVIHLVASALRSLHQVSIAQ | 130  123 |
| Query 131  Sbjct 124 | | CPFDHQAKHRIERARTRMEAGLVDQDDLDEEHQGLAPAELFARLKARMPDGEDLVVTHGD CPFDH +HRI A R+ AGLVD+ D D+E G ++FA L + P DLVVTHGD CPFDHSLEHRIAAAGNRVNAGLVDETDFDDERLGRTATDVFAELLSTRPAAHDLVVTHGD | 190  183 |
| Query 191  Sbjct 184 | | ACLPNIMVENGRFSGFIDCGRLGVADRYQDIALATRDIAEELGGEWADRFLVLYGIAAPD ACLPN M E FSGFIDCGRLG++DRYQD+ALA R I LG W F YG+ PD ACLPNFMAEGDHFSGFIDCGRLGISDRYQDLALAARSIERNLGAAWVAPFFEQYGV-EPD | 250  242 |
| Query | 251 SQRIAFYRLLDEFF 264 | | |
|  |  | QRIAFY LLDEFF |  |
| Sbjct | 243 | EQRIAFYCLLDEFF 256 | |

>WP_177048671.1 APH(3') family aminoglycoside O-phosphotransferase [Pseudomonas sp. F8002]

NWB57349.1 aminoglycoside 3'-phosphotransferase [Pseudomonas sp. F8002] Length=256

Score = 240 bits (613), Expect = 1e-75, Method: Compositional matrix adjust.

| Identities | | = 132/254 (52%), Positives = 159/254 (63%), Gaps = 2/254 (1%) |  |
| --- | --- | --- | --- |
| Query 12  Sbjct 4 | | PAAWVERLFGYDWAQQTIGCSDAAVFRLSAQGRPVLFVKTDLSGALNELQDEAARLSWLA P W ++ QTIG S A VFRL LF+K++ G L EL DE RL WL PKKWRDQFPDALIEPQTIGESGADVFRLRCGNGEDLFLKSEPIGPLAELPDEIERLRWLR | 71  63 |
| Query 72  Sbjct 64 | | TTGVPCAAVLDVVTEAGRDWLLLGEVPGQDLLS-SHLAPAEKVSIMADAMRRLHTLDPAT  + +P AVLDV+TE R WLL+ VPGQDL S S L+ + + ++A A+R LH + A QSNLPGPAVLDVLTEHYRHWLLMSAVPGQDLASASDLSAPQVIRLVASALRSLHQVPIAQ | 130  123 |
| Query 131  Sbjct 124 | | CPFDHQAKHRIERARTRMEAGLVDQDDLDEEHQGLAPAELFARLKARMPDGEDLVVTHGD CPFDH +HRI A R+ AGLVD+ D D++ G A ++FA L + P DLVVTHGD CPFDHSLEHRIAAAGNRVNAGLVDETDFDDQRLGRAATDVFAELLSTRPAAHDLVVTHGD | 190  183 |
| Query 191  Sbjct 184 | | ACLPNIMVENGRFSGFIDCGRLGVADRYQDIALATRDIAEELGGEWADRFLVLYGIAAPD ACLPN M E FSGFIDCG LGV+DRYQD+ALA R I LG W F YG+ APD ACLPNFMAEGDHFSGFIDCGGLGVSDRYQDLALAARSIERNLGVAWVAPFFEQYGV-APD | 250  242 |
| Query | 251 SQRIAFYRLLDEFF 264 | | |
|  |  | QRIAFY LLDEFF |  |
| Sbjct | 243 | QQRIAFYCLLDEFF 256 | |

>WP_133634335.1 aminoglycoside 3'-phosphotransferase [Pseudomonas sp. OV184] TDQ69068.1 aminoglycoside 3'-phosphotransferase-2 [Pseudomonas sp. OV184]

Length=256

Score = 240 bits (613), Expect = 1e-75, Method: Compositional matrix adjust.

| Identities | | = 131/254 (52%), Positives = 157/254 (62%), Gaps = 2/254 (1%) |  |
| --- | --- | --- | --- |
| Query 12  Sbjct 4 | | PAAWVERLFGYDWAQQTIGCSDAAVFRLSAQGRPVLFVKTDLSGALNELQDEAARLSWLA P W E+ QQ IG S A VFRL G LF+K++L +EL DE RL WL PHKWREQFPHAQIEQQAIGESRADVFRLRQDGGTDLFLKSELLEEHSELADEIDRLRWLQ | 71  63 |
| Query 72  Sbjct 64 | | TTGVPCAAVLDVVTEAGRDWLLLGEVPGQDLLS-SHLAPAEKVSIMADAMRRLHTLDPAT G+P VLD VT A WLL+ VPGQDL S S L+ + ++I+A A+R LH L A  QMGLPAPVVLDEVTAAHSHWLLMTTVPGQDLASASELSAEQIINILATALRALHQLPVAL | 130  123 |
| Query 131  Sbjct 124 | | CPFDHQAKHRIERARTRMEAGLVDQDDLDEEHQGLAPAELFARLKARMPDGEDLVVTHGD CPFDH + RI RA + AGLVD+ D D+E G + ++FA L A P DLVVTHGD CPFDHSLQLRIARAHQHVSAGLVDETDFDDERLGQSAEDVFAELVATQPKTHDLVVTHGD | 190  183 |
| Query 191  Sbjct 184 | | ACLPNIMVENGRFSGFIDCGRLGVADRYQDIALATRDIAEELGGEWADRFLVLYGIAAPD ACLPN M G FSGFIDCGRLGV+DR+QD+ALA R I LG W F +YG+ PD ACLPNFMAAEGCFSGFIDCGRLGVSDRFQDLALAARSIQSNLGLAWVRPFFEVYGV-EPD | 250  242 |
| Query | 251 SQRIAFYRLLDEFF 264 | | |
|  |  | QR+ FY LLDEFF |  |
| Sbjct | 243 | EQRMKFYCLLDEFF 256 | |

>PMQ09303.1 Aminoglycoside 3'-phosphotransferase [Pseudomonas sp. AD21] Length=258

Score = 240 bits (613), Expect = 1e-75, Method: Compositional matrix adjust.

| Identities | = 126/254 (50%), Positives = 160/254 (63%), Gaps = 2/254 (1%) |  |
| --- | --- | --- |
| Query 12 | PAAWVERLFGYDWAQQTIGCSDAAVFRLSAQGRPVLFVKTDLSGALNELQDEAARLSWLA | 71 |
| Sbjct 6 | P W R QQTIG S A VFRL+ G LFVK++ EL E RL WLA PQTWRSRFADSACEQQTIGESRADVFRLTPAGSAPLFVKSEPRITFAELPGEVQRLRWLA | 65 |
| Query 72 | TTGVPCAAVLDVVTEAGRDWLLLGEVPGQDLLSSH-LAPAEKVSIMADAMRRLHTLDPAT | 130 |
| Sbjct 66 | +P A VLD E R WLL+ VPG+DL SS L P + V++ A+A+R LH LD EQDLPVARVLDSTEEHDRYWLLMSAVPGRDLASSERLTPQQIVTLAANALRTLHALDITQ | 125 |
| Query 131 | CPFDHQAKHRIERARTRMEAGLVDQDDLDEEHQGLAPAELFARLKARMPDGEDLVVTHGD | 190 |
| Sbjct 126 | CPFDH+ +I AR + AGL+D++D D+ G + +F ++ A P EDLVVTHGD CPFDHRLDPKIALAREHLLAGLIDEEDFDDSRLGHSAEAVFQQMLAARPVSEDLVVTHGD | 185 |
| Query 191 | ACLPNIMVENGRFSGFIDCGRLGVADRYQDIALATRDIAEELGGEWADRFLVLYGIAAPD ACLPN++ ++GRFSGF+DCGRLGVADR+QD+ALA I + LG +W F YG+ PD | 250 |

| Sbjct | 186 | ACLPNLLADHGRFSGFVDCGRLGVADRFQDLALAAHSITDNLGEQWVPAFFEAYGV-QPD 244 |
| --- | --- | --- |
| Query | 251 | SQRIAFYRLLDEFF 264 |
| Sbjct | 245 | Q+I FY+LLDEFF  PQKIDFYQLLDEFF 258 |

>WP_169918582.1 APH(3') family aminoglycoside O-phosphotransferase [Pseudomonas sp. WS 5406]

NMX28535.1 aminoglycoside 3'-phosphotransferase [Pseudomonas sp. WS 5406] Length=256

Score = 240 bits (613), Expect = 1e-75, Method: Compositional matrix adjust.

| Identities | | = 132/254 (52%), Positives = 158/254 (62%), Gaps = 2/254 (1%) |  |
| --- | --- | --- | --- |
| Query 12  Sbjct 4 | | PAAWVERLFGYDWAQQTIGCSDAAVFRLSAQGRPVLFVKTDLSGALNELQDEAARLSWLA P W E+ QQ IG S A VFRL G LFVK++ L EL DE RL WL PQKWREQFPHALIEQQAIGESRADVFRLRQDGGTDLFVKSEPLEELGELADEIDRLRWLQ | 71  63 |
| Query 72  Sbjct 64 | | TTGVPCAAVLDVVTEAGRDWLLLGEVPGQDLLSSHLAPA-EKVSIMADAMRRLHTLDPAT G+P VLD V E R WLL+ VPG+DL S++ PA + + I+A A+R LH L A  HMGLPAPEVLDDVKEVHRHWLLMTAVPGRDLASTNELPAGQIIHILATALRALHQLPVAL | 130  123 |
| Query 131  Sbjct 124 | | CPFDHQAKHRIERARTRMEAGLVDQDDLDEEHQGLAPAELFARLKARMPDGEDLVVTHGD CPFDH + RI RA+ + AGLVD+ D D+E G + ++FA L A P DLVVTHGD CPFDHSLELRIARAQQHVIAGLVDETDFDDERLGQSAEDVFAELVATQPKTHDLVVTHGD | 190  183 |
| Query 191  Sbjct 184 | | ACLPNIMVENGRFSGFIDCGRLGVADRYQDIALATRDIAEELGGEWADRFLVLYGIAAPD ACLPN M G FSGFIDCGRLGV+DR+QD+ALA R I LG EW F +YG+ PD ACLPNFMAAEGCFSGFIDCGRLGVSDRFQDLALAARSIESNLGQEWVKPFFEVYGV-EPD | 250  242 |
| Query | 251 SQRIAFYRLLDEFF 264 | | |
|  |  | QR+ FY LLDEFF |  |
| Sbjct | 243 | EQRMKFYCLLDEFF 256 | |

>WP_179676762.1 APH(3') family aminoglycoside O-phosphotransferase [Pseudomonas sp. H1D6A]

NYK13562.1 aminoglycoside 3'-phosphotransferase-2 [Pseudomonas sp. H1D6A] Length=256

Score = 240 bits (613), Expect = 1e-75, Method: Compositional matrix adjust.

| Identities | = 124/240 (52%), Positives = 156/240 (65%), Gaps = 2/240 (1%) |  |
| --- | --- | --- |
| Query 26 | QQTIGCSDAAVFRLSAQGRPVLFVKTDLSGALNELQDEAARLSWLATTGVPCAAVLDVVT | 85 |
| Sbjct 18 | QQ IG S A VFRL GR LF+K + A +EL DE RL WL +P VLD + QQAIGESRADVFRLCQVGRKDLFLKAEPLVAFSELADEIERLRWLRQVNLPAPVVLDELI | 77 |
| Query 86 | EAGRDWLLLGEVPGQDLLSSH-LAPAEKVSIMADAMRRLHTLDPATCPFDHQAKHRIERA | 144 |
| Sbjct 78 | + WLL+ VPGQDL S++ L+ A+ V I+A A+R LH + A CPFDH + RI A DTHHHWLLMTAVPGQDLASANTLSAAQTVDILATALRTLHQVPIAQCPFDHALQQRIALA | 137 |
| Query 145 | RTRMEAGLVDQDDLDEEHQGLAPAELFARLKARMPDGEDLVVTHGDACLPNIMVENGRFS | 204 |
| Sbjct 138 | R + AGL+D+ D D+E G + ++FA L + P+ DLVVTHGDACLPN M +GRFS RAHVNAGLIDETDFDDERLGRSLEDVFAELLSNQPNTHDLVVTHGDACLPNFMAADGRFS | 197 |
| Query 205 | GFIDCGRLGVADRYQDIALATRDIAEELGGEWADRFLVLYGIAAPDSQRIAFYRLLDEFF | 264 |
| Sbjct 198 | GFIDCGRLG++DRYQD+ALA R I +G +W F LYG+ PD QR+ FY LLDEFF  GFIDCGRLGISDRYQDLALAARSIERNVGPKWVKPFFELYGV-EPDEQRMKFYCLLDEFF | 256 |

```
>WP_178122924.1 APH(3') family aminoglycoside O-phosphotransferase [Pseudomonas sp. AU8050]
Length=242

Score = 239 bits (611), Expect = 1e-75, Method: Compositional matrix adjust. Identities = 128/240 (53%), Positives = 156/240 (65%), Gaps = 2/240 (1%)

Query 26	QQTIGCSDAAVFRLSAQGRPVLFVKTDLSGALNELQDEAARLSWLATTGVPCAAVLDVVT 85 QQ IG S A VFRL	G	LF+K++L	EL DE RL WL	G+P	VLD VT
Sbjct 4	QQAIGESRADVFRLRQDGGTDLFLKSELLEEHGELADEIDRLRWLQQMGLPAPVVLDEVT 63
```

| Query | 86 | EAGRDWLLLGEVPGQDLLS-SHLAPAEKVSIMADAMRRLHTLDPATCPFDHQAKHRIERA | 144 |
| --- | --- | --- | --- |
| Sbjct | 64 | A R WLL+ VPGQDL S S L+ + ++I+A A+R LH L A CPFDH + RI RA AAHRHWLLMTTVPGQDLASASGLSAEQIINILATALRTLHQLPVALCPFDHSLQLRIARA | 123 |
| Query | 145 | RTRMEAGLVDQDDLDEEHQGLAPAELFARLKARMPDGEDLVVTHGDACLPNIMVENGRFS | 204 |
| Sbjct | 124 | + + AGLVD+ D D+E G + ++FA L A P DLVVTHGDACLPN M G FS QQHVSAGLVDETDFDDERLGQSAEDVFAELVATQPKTHDLVVTHGDACLPNFMAAEGCFS | 183 |
| Query | 205 | GFIDCGRLGVADRYQDIALATRDIAEELGGEWADRFLVLYGIAAPDSQRIAFYRLLDEFF | 264 |
| Sbjct | 184 | GFIDCGRLGV+DR+QD+A+A R I +G +W + F LYG+ PD Q+I FY LLDEFF  GFIDCGRLGVSDRFQDLAIAARSIERNVGQKWVNPFFELYGV-EPDEQKIRFYCLLDEFF | 242 |

>WP_076813318.1 aminoglycoside 3'-phosphotransferase [Achromobacter insolitus]

APX74330.1 APH(3') family aminoglycoside O-phosphotransferase [Achromobacter insolitus]

OWT61063.1 APH(3') family aminoglycoside O-phosphotransferase [Achromobacter insolitus]

CAB3690329.1 Aminoglycoside 3'-phosphotransferase [Achromobacter insolitus] VEG68620.1 Aminoglycoside 3'-phosphotransferase [Achromobacter insolitus]

Length=257

Score = 240 bits (613), Expect = 1e-75, Method: Compositional matrix adjust.

| Identities | | = 130/257 (51%), Positives = 161/257 (63%), Gaps = 3/257 (1%) |  |
| --- | --- | --- | --- |
| Query 10  Sbjct 2 | | GSPAAWVERLFGYDWAQQTIGCSDAAVFRLSAQGRPVLFVKTDLSGALNELQDEAARLSW  +PA W +R +Q IG S A VFR+ LF+K++ G LNEL DE ARL W NTPAKWRDRYTDATIERQNIGESGADVFRIRPGKGQDLFLKSEPVGMLNELPDEIARLRW | 69  61 |
| Query 70  Sbjct 62 | | LATTGVPCAAVLDVVTEAGRDWLLLGEVPGQDLLS-SHLAPAEKVSIMADAMRRLHTLDP L G+P +VLD TE R WLL+ +PG DL S + LAP + V IMA A+RRLH LNQHGLPAPSVLDTATENHRHWLLMTALPGHDLASATDLAPEQIVGIMAAALRRLHQTPV | 128  121 |
| Query 129  Sbjct 122 | | ATCPFDHQAKHRIERARTRMEAGLVDQDDLDEEHQGLAPAELFARLKARMPDG-EDLVVT A CPFDH+ RI A+ R AGLVD+ + DE+ QG +L L + P G +DLVVT AECPFDHRLDARIAAAQDRARAGLVDETNFDEQRQGQTAMDLLQELLSSRPMGADDLVVT | 187  181 |
| Query 188  Sbjct 182 | | HGDACLPNIMVENGRFSGFIDCGRLGVADRYQDIALATRDIAEELGGEWADRFLVLYGIA HGDACLPN M + G F+GF+DCGRLG++DR+QD+ALA R I LG W F YG+ HGDACLPNFMADAGEFTGFLDCGRLGISDRFQDLALAARSIERNLGAAWVAPFFREYGV- | 247  240 |
| Query 248 | | APDSQRIAFYRLLDEFF 264 PD +RIAFY LLDEFF |  |
| Sbjct | 241 | LPDERRIAFYCLLDEFF 257 | |

>WP_181461225.1 APH(3') family aminoglycoside O-phosphotransferase [Cupriavidus alkaliphilus]

Length=257

Score = 240 bits (613), Expect = 1e-75, Method: Compositional matrix adjust.

| Identities | = 125/255 (49%), Positives = 163/255 (64%), Gaps = 3/255 (1%) |  |
| --- | --- | --- |
[truncated: 5,173,948 more chars]
