# Supplementary material for: Safety Assessment of the CP4 EPSPS and NPTII Proteins in Eucalyptus
Source: GM Crops Food. 2023 Jun 19;14(1):1–14. doi: 10.1080/21645698.2023.2222436 (PMC10281460; doi:10.1080/21645698.2023.2222436)
Supplement: Supplemental Material [file KGMC_A_2222436_SM4294.zip › TableS06.docx]

BLASTP output of NPTII protein sequence against the UniProtKB protein database

BLASTP 2.9.0+

Reference: Stephen F. Altschul, Thomas L. Madden, Alejandro A. Schaffer, Jinghui Zhang, Zheng Zhang, Webb Miller, and David J. Lipman (1997), "Gapped BLAST and PSI-BLAST: a new generation of protein database search programs", Nucleic Acids Res. 25:3389-3402.

Reference for composition-based statistics: Alejandro A. Schaffer,

L. Aravind, Thomas L. Madden, Sergei Shavirin, John L. Spouge, Yuri

I. Wolf, Eugene V. Koonin, and Stephen F. Altschul (2001), "Improving the accuracy of PSI-BLAST protein database searches with composition-based statistics and other refinements", Nucleic Acids Res. 29:2994-3005.

Database: uniprotkb_refprotswissprot

57,391,823 sequences; 21,838,191,652 total letters

| Query= NPTII |  | |
| --- | --- | --- |
| Length=264 | Score | E |
| Sequences producing significant alignments: | (Bits) | Value |
| SP:P00552 KKA2_KLEPN Aminoglycoside 3'-phosphotransferase OS=Klebs... | 535 | 0.0 |
| TR:A0A149VWA2 A0A149VWA2_9PROT Aminoglycoside 3'-phosphotransferas... | 533 | 0.0 |
| TR:A0A2P7P4I2 A0A2P7P4I2_9GAMM Aminoglycoside 3'-phosphotransferas... | 533 | 0.0 |
| TR:A0A2U1C3P1 A0A2U1C3P1_9BURK Aminoglycoside 3'-phosphotransferas... | 484 | 2e-172 |
| TR:I0V5U7 I0V5U7_9PSEU Aminoglycoside phosphotransferase OS=Saccha... | 354 | 3e-121 |
| TR:A0A0Q9I9B9 A0A0Q9I9B9_9BRAD Aminoglycoside 3'-phosphotransferas... | 348 | 9e-119 |
| TR:W1SA25 W1SA25_9BACI Aminoglycoside phosphotransferase (Fragment... | 330 | 3e-113 |
| TR:A0A098U4E4 A0A098U4E4_9BURK Aminoglycoside 3'-phosphotransferas... | 332 | 1e-112 |
| TR:A0A160DZL2 A0A160DZL2_9GAMM Aminoglycoside 3'-phosphotransferas... | 313 | 5e-105 |
| TR:A0A542LUX5 A0A542LUX5_9BURK Aminoglycoside 3'-phosphotransferas... | 298 | 9e-99 |
| TR:A0A2S2CTZ1 A0A2S2CTZ1_9PROT Aminoglycoside 3'-phosphotransferas... | 293 | 4e-97 |
| TR:A0A2R4CEQ3 A0A2R4CEQ3_9BURK Aminoglycoside 3'-phosphotransferas... | 291 | 2e-96 |
| TR:A0A318THA2 A0A318THA2_9BRAD Aminoglycoside 3'-phosphotransferas... | 290 | 1e-95 |
| TR:A0A0Q8K3X2 A0A0Q8K3X2_9RHIZ Aminoglycoside 3'-phosphotransferas... | 281 | 2e-92 |
| TR:A0A0M3BDK3 A0A0M3BDK3_9RHIZ Aminoglycoside 3'-phosphotransferas... | 281 | 2e-92 |
| TR:A0A4S1ECS7 A0A4S1ECS7_9BURK Aminoglycoside 3'-phosphotransferas... | 280 | 5e-92 |
| TR:A0A0K2DCR3 A0A0K2DCR3_9RHIZ Aminoglycoside 3'-phosphotransferas... | 279 | 1e-91 |
| TR:A0A0Q6M9M4 A0A0Q6M9M4_9RHIZ Aminoglycoside 3'-phosphotransferas... | 276 | 2e-90 |
| TR:A0A5C8PGD3 A0A5C8PGD3_9PROT Aminoglycoside 3'-phosphotransferas... | 273 | 3e-89 |
| TR:A0A1C2ECT9 A0A1C2ECT9_9RHIZ Aminoglycoside 3'-phosphotransferas... | 270 | 6e-88 |
| TR:Q9HWR2 Q9HWR2_PSEAE Aminoglycoside 3'-phosphotransferase OS=Pse... | 269 | 2e-87 |
| TR:W3RM28 W3RM28_9BRAD Aminoglycoside 3'-phosphotransferase OS=Afi... | 267 | 5e-87 |
| TR:A0A2S9JF04 A0A2S9JF04_9RHIZ Aminoglycoside 3'-phosphotransferas... | 267 | 7e-87 |
| TR:A0A024KAC1 A0A024KAC1_9RHIZ Aminoglycoside 3'-phosphotransferas... | 267 | 9e-87 |
| TR:Q92LN7 Q92LN7_RHIME Aminoglycoside 3'-phosphotransferase OS=Rhi... | 266 | 1e-86 |
| TR:B6IQF0 B6IQF0_RHOCS Aminoglycoside 3'-phosphotransferase OS=Rho... | 265 | 4e-86 |
| TR:A0A1I5HYN7 A0A1I5HYN7_9BURK Aminoglycoside 3'-phosphotransferas... | 261 | 2e-84 |
| TR:A0A1H4UEE9 A0A1H4UEE9_9RHIZ Aminoglycoside 3'-phosphotransferas... | 254 | 1e-81 |
| TR:A0A085FM66 A0A085FM66_9RHIZ Aminoglycoside 3'-phosphotransferas... | 254 | 1e-81 |
| TR:A0A245Z5G3 A0A245Z5G3_9RHIZ Aminoglycoside 3'-phosphotransferas... | 252 | 5e-81 |
| TR:A0A0B4X2U9 A0A0B4X2U9_9RHIZ Aminoglycoside 3'-phosphotransferas... | 252 | 6e-81 |
| TR:J2VNI4 J2VNI4_9RHIZ Aminoglycoside 3'-phosphotransferase OS=Phy... | 252 | 7e-81 |
| TR:B2FNW7 B2FNW7_STRMK Aminoglycoside 3'-phosphotransferase OS=Ste... | 251 | 1e-80 |
| TR:A0A0A1PYP1 A0A0A1PYP1_9BACT Aminoglycoside 3'-phosphotransferas... | 272 | 3e-80 |
| TR:U1YD28 U1YD28_9BURK Aminoglycoside 3'-phosphotransferase OS=Alc... | 248 | 1e-79 |
| TR:A0A327XY80 A0A327XY80_RHIET Aminoglycoside 3'-phosphotransferas... | 240 | 3e-76 |
| TR:A0A157LWL8 A0A157LWL8_9BORD Aminoglycoside 3'-phosphotransferas... | 237 | 3e-75 |
| TR:A0A0D1NZF2 A0A0D1NZF2_BRAEL Aminoglycoside 3'-phosphotransferas... | 237 | 5e-75 |

| TR:B5ZXE0 B5ZXE0_RHILW Aminoglycoside 3'-phosphotransferase OS=Rhi... | 235 | 2e-74 |
| --- | --- | --- |
| TR:A6WYI3 A6WYI3_OCHA4 Aminoglycoside 3'-phosphotransferase OS=Och... | 233 | 1e-73 |
| TR:A0A135I064 A0A135I064_9RHIZ Aminoglycoside 3'-phosphotransferas... | 233 | 3e-73 |
| TR:A0A127CNG5 A0A127CNG5_9RHIZ Aminoglycoside 3'-phosphotransferas... | 232 | 7e-73 |
| TR:B9J736 B9J736_AGRRK Aminoglycoside 3'-phosphotransferase OS=Agr... | 231 | 8e-73 |
| TR:A0A0Q7EA18 A0A0Q7EA18_9RHIZ Aminoglycoside 3'-phosphotransferas... | 228 | 1e-71 |
| TR:A0A0U3QBG6 A0A0U3QBG6_9RHOB Aminoglycoside 3'-phosphotransferas... | 228 | 1e-71 |
| TR:J2KXC7 J2KXC7_9RHIZ Aminoglycoside 3'-phosphotransferase OS=Rhi... | 226 | 7e-71 |
| TR:A0A1N6GVZ6 A0A1N6GVZ6_9BURK Aminoglycoside 3'-phosphotransferas... | 226 | 1e-70 |
| TR:U4VD20 U4VD20_9RHIZ Aminoglycoside 3'-phosphotransferase OS=Och... | 219 | 4e-68 |
| TR:A0A3S4CF94 A0A3S4CF94_9RHIZ Aminoglycoside 3'-phosphotransferas... | 201 | 5e-61 |
| TR:A0A1I7NDQ9 A0A1I7NDQ9_9RHIZ Aminoglycoside 3'-phosphotransferas... | 198 | 8e-60 |
| TR:A0A2L1SA27 A0A2L1SA27_9RHIZ Aminoglycoside 3'-phosphotransferas... | 197 | 2e-59 |
| TR:A0A0Q8ISX4 A0A0Q8ISX4_9RHIZ Aminoglycoside 3'-phosphotransferas... | 196 | 3e-59 |
| TR:A0A0F5FLC5 A0A0F5FLC5_9RHIZ Aminoglycoside 3'-phosphotransferas... | 195 | 1e-58 |
| TR:A0A4P6JTH1 A0A4P6JTH1_9CHLR Aminoglycoside 3'-phosphotransferas... | 192 | 2e-57 |
| TR:A0A1H9FPI4 A0A1H9FPI4_9RHIZ Aminoglycoside 3'-phosphotransferas... | 189 | 4e-56 |
| TR:A0A4Y8LPN5 A0A4Y8LPN5_9BACL Aminoglycoside 3'-phosphotransferas... | 188 | 5e-56 |
| TR:A0A0F5L6W5 A0A0F5L6W5_9RHIZ Aminoglycoside 3'-phosphotransferas... | 187 | 1e-55 |
| TR:A0A554A1N3 A0A554A1N3_9BACI Aminoglycoside 3'-phosphotransferas... | 187 | 1e-55 |
| TR:K2N3F1 K2N3F1_9RHIZ Aminoglycoside 3'-phosphotransferase OS=Nit... | 184 | 2e-54 |
| TR:K9DXJ0 K9DXJ0_9BURK Aminoglycoside 3'-phosphotransferase OS=Mas... | 183 | 6e-54 |
| TR:A0A3A4AU57 A0A3A4AU57_9ACTN Aminoglycoside 3'-phosphotransferas... | 182 | 1e-53 |
| TR:A0A286CWL5 A0A286CWL5_9GAMM Aminoglycoside 3'-phosphotransferas... | 182 | 2e-53 |
| TR:A0A399F177 A0A399F177_9DEIN Aminoglycoside 3'-phosphotransferas... | 182 | 2e-53 |
| TR:A0A4U0FC53 A0A4U0FC53_9BACL Aminoglycoside 3'-phosphotransferas... | 180 | 7e-53 |
| TR:A0A0Q7JME4 A0A0Q7JME4_9BACL APH domain-containing protein OS=Pa... | 180 | 8e-53 |
| TR:A0A1L8ZNE0 A0A1L8ZNE0_9BACI APH domain-containing protein OS=Ba... | 178 | 4e-52 |
| TR:D7BCJ0 D7BCJ0_MEISD Aminoglycoside phosphotransferase OS=Meioth... | 177 | 1e-51 |
| TR:A0A1M5T3I1 A0A1M5T3I1_9BURK Aminoglycoside 3'-phosphotransferas... | 176 | 5e-51 |
| TR:A0A368VJC8 A0A368VJC8_9BACL Kanamycin kinase/aminoglycoside 3'-... | 174 | 1e-50 |
| TR:A0A285QF39 A0A285QF39_9SPHN Aminoglycoside 3'-phosphotransferas... | 174 | 3e-50 |
| TR:A0A1I6M7R6 A0A1I6M7R6_9SPHN Aminoglycoside 3'-phosphotransferas... | 173 | 5e-50 |
| TR:A0A177ZKY0 A0A177ZKY0_9BACI APH domain-containing protein OS=Ba... | 172 | 7e-50 |
| TR:A0A0B1Q485 A0A0B1Q485_9RHIZ Aminoglycoside 3'-phosphotransferas... | 172 | 1e-49 |
| TR:A0A179T5W8 A0A179T5W8_9BACI APH domain-containing protein OS=Me... | 171 | 2e-49 |
| TR:A0A4R2PAM0 A0A4R2PAM0_9SPHN Aminoglycoside 3'-phosphotransferas... | 171 | 3e-49 |
| TR:A9WI26 A9WI26_CHLAA Aminoglycoside phosphotransferase OS=Chloro... | 171 | 3e-49 |
| TR:A0A0Q4LIY2 A0A0Q4LIY2_9SPHN Aminoglycoside 3'-phosphotransferas... | 170 | 7e-49 |
| TR:A0A1I4UN46 A0A1I4UN46_9RHIZ Aminoglycoside 3'-phosphotransferas... | 170 | 8e-49 |
| TR:A0A1T4PRE7 A0A1T4PRE7_9ACTN Kanamycin kinase OS=Marinactinospor... | 170 | 1e-48 |
| TR:I2GER6 I2GER6_9BACT Aminoglycoside 3'-phosphotransferase OS=Fib... | 170 | 2e-48 |
| TR:A0A4Q1JS98 A0A4Q1JS98_9GAMM Aminoglycoside 3'-phosphotransferas... | 169 | 2e-48 |
| TR:A0A1H3NWN2 A0A1H3NWN2_9BACI Kanamycin kinase/aminoglycoside 3'-... | 168 | 4e-48 |
| TR:A0A318S577 A0A318S577_9DEIO Kanamycin kinase/aminoglycoside 3'-... | 168 | 4e-48 |
| TR:A0A1H0AE42 A0A1H0AE42_9PSEU Aminoglycoside 3'-phosphotransferas... | 167 | 1e-47 |
| TR:Q1IW34 Q1IW34_DEIGD Aminoglycoside phosphotransferase OS=Deinoc... | 167 | 1e-47 |
| TR:A0A512HAF7 A0A512HAF7_9PROT Aminoglycoside 3'-phosphotransferas... | 167 | 1e-47 |
| TR:A0A1G5KL09 A0A1G5KL09_9RHIZ Aminoglycoside 3'-phosphotransferas... | 166 | 1e-47 |
| TR:A0A2A4ICH6 A0A2A4ICH6_9SPHN Aminoglycoside 3'-phosphotransferas... | 167 | 1e-47 |
| TR:A0A2T0TND8 A0A2T0TND8_9BACT Aminoglycoside 3'-phosphotransferas... | 166 | 2e-47 |
| TR:A0A1C1A760 A0A1C1A760_9BACL APH domain-containing protein OS=Pa... | 166 | 3e-47 |
| TR:A0A096DMQ9 A0A096DMQ9_9CLOT APH domain-containing protein OS=Ca... | 164 | 1e-46 |
| TR:A0A3S0BKR3 A0A3S0BKR3_9BACL Aminoglycoside 3'-phosphotransferas... | 164 | 3e-46 |
| TR:A0A2R7JWN4 A0A2R7JWN4_9SPHN Aminoglycoside 3'-phosphotransferas... | 163 | 4e-46 |
| TR:A0A221ST19 A0A221ST19_9DEIO Aminoglycoside phosphotransferase A... | 162 | 7e-46 |
| TR:A0A150WR92 A0A150WR92_BDEBC Aminoglycoside 3'-phosphotransferas... | 161 | 2e-45 |
| TR:A0A1U7NWU8 A0A1U7NWU8_9DEIO Aminoglycoside 3'-phosphotransferas... | 161 | 3e-45 |
| TR:C1CZE3 C1CZE3_DEIDV Putative kanamycin kinase (Aminoglycoside 3... | 160 | 3e-45 |
| TR:E8U360 E8U360_DEIML Aminoglycoside phosphotransferase OS=Deinoc... | 161 | 8e-45 |
| TR:A0A0G2Z9P4 A0A0G2Z9P4_9BACT APH domain-containing protein OS=Ko... | 159 | 8e-45 |
| TR:H8GUT9 H8GUT9_DEIGI Aminoglycoside 3`-phosphotransferase OS=Dei... | 159 | 9e-45 |
| TR:Q9RY84 Q9RY84_DEIRA Aminoglycoside 3`-phosphotransferase OS=Dei... | 159 | 1e-44 |
| TR:A0A1I6IW28 A0A1I6IW28_9BURK Aminoglycoside 3'-phosphotransferas... | 159 | 1e-44 |
| TR:A0A0K9XNI0 A0A0K9XNI0_9ACTN Aminoglycoside phosphotransferase O... | 159 | 1e-44 |
| TR:A0A2K3UW40 A0A2K3UW40_9DEIO Aminoglycoside 3'-phosphotransferas... | 157 | 7e-44 |
| TR:A0A1I4WU92 A0A1I4WU92_9PSEU Kanamycin kinase OS=Saccharopolyspo... | 157 | 8e-44 |
| TR:A0A254N847 A0A254N847_9BURK Aminoglycoside 3'-phosphotransferas... | 157 | 8e-44 |
| TR:A0A553ZT10 A0A553ZT10_9BACI APH(3')-I family aminoglycoside O-p... | 157 | 1e-43 |
| TR:A0A1G6UA45 A0A1G6UA45_9PSEU Kanamycin kinase OS=Alloactinosynne... | 157 | 1e-43 |
| TR:A0A2K2F1D5 A0A2K2F1D5_9FIRM APH domain-containing protein OS=Ps... | 157 | 1e-43 |
| TR:A0A0F7N198 A0A0F7N198_9ACTN APH domain-containing protein OS=St... | 157 | 1e-43 |

| TR:A0A3D9KN32 A0A3D9KN32_9BURK Aminoglycoside 3'-phosphotransferas... | 156 | 2e-43 |
| --- | --- | --- |
| TR:A0A5E7YL26 A0A5E7YL26_9SPHN Aminoglycoside 3'-phosphotransferas... | 156 | 2e-43 |
| TR:B1N624 B1N624_ENTHI Aminoglycoside 3'-phosphotransferase, putat... | 155 | 2e-43 |
| TR:A0A1G7PFM6 A0A1G7PFM6_9SPHN Aminoglycoside 3'-phosphotransferas... | 156 | 3e-43 |
| TR:A0A3N7BPW6 A0A3N7BPW6_9BURK Aminoglycoside 3'-phosphotransferas... | 157 | 3e-43 |
| TR:A0A2P5GGN3 A0A2P5GGN3_9ENTR Aminoglycoside 3'-phosphotransferas... | 156 | 3e-43 |
| TR:B1VIB1 B1VIB1_CORU7 Aminoglycoside 3'-phosphotransferase OS=Cor... | 156 | 3e-43 |
| TR:A0A2S2FBP8 A0A2S2FBP8_9GAMM Aminoglycoside 3'-phosphotransferas... | 156 | 3e-43 |
| TR:M4R5K9 M4R5K9_BIBTR Aminoglycoside 3'-phosphotransferase OS=Bib... | 156 | 3e-43 |
| TR:A6TJ01 A6TJ01_KLEP7 Aminoglycoside 3'-phosphotransferase OS=Kle... | 156 | 3e-43 |
| TR:A0A345WUY0 A0A345WUY0_9SPHN Aminoglycoside 3'-phosphotransferas... | 156 | 3e-43 |
| TR:D6CJF7 D6CJF7_CORRG Aminoglycoside phosphotransferase OS=Coryne... | 156 | 3e-43 |
| SP:P00551 KKA1_ECOLX Aminoglycoside 3'-phosphotransferase OS=Esche... | 155 | 4e-43 |
| TR:A3UKS0 A3UKS0_9RHOB Aminoglycoside 3'-phosphotransferase OS=Oce... | 155 | 4e-43 |
| TR:A0A0M0HV15 A0A0M0HV15_9VIBR Aminoglycoside 3'-phosphotransferas... | 155 | 4e-43 |
| TR:A0A2S4XH80 A0A2S4XH80_9ACTN Aminoglycoside O-phosphotransferase... | 155 | 4e-43 |
| TR:A0A2S4Y376 A0A2S4Y376_9ACTN Aminoglycoside O-phosphotransferase... | 155 | 4e-43 |
| TR:A0A1T3P638 A0A1T3P638_9ACTN APH domain-containing protein OS=Em... | 155 | 5e-43 |
| TR:A0A1H6UK15 A0A1H6UK15_9DEIO Kanamycin kinase OS=Deinococcus ret... | 155 | 6e-43 |
| TR:F0RMC2 F0RMC2_DEIPM Aminoglycoside phosphotransferase OS=Deinoc... | 155 | 6e-43 |
| TR:A0A345WKK1 A0A345WKK1_9SPHN Aminoglycoside 3'-phosphotransferas... | 153 | 2e-42 |
| TR:A0A1W1V550 A0A1W1V550_9DEIO Kanamycin kinase OS=Deinococcus hop... | 153 | 3e-42 |
| TR:D3A171 D3A171_NEIMU Aminoglycoside 3'-phosphotransferase OS=Nei... | 152 | 4e-42 |
| TR:A0A1I2N3L4 A0A1I2N3L4_9BACL Kanamycin kinase OS=Planifilum fulg... | 152 | 4e-42 |
| TR:A0A1A9HRV5 A0A1A9HRV5_9BURK Aminoglycoside 3'-phosphotransferas... | 152 | 8e-42 |
| SP:P00555 KKA5_STRFR Aminoglycoside 3'-phosphotransferase OS=Strep... | 152 | 8e-42 |
| TR:A0A2T6BGV9 A0A2T6BGV9_9BACL Kanamycin kinase/aminoglycoside 3'-... | 152 | 8e-42 |
| TR:A0A1G5NZI3 A0A1G5NZI3_AFIMA Aminoglycoside 3'-phosphotransferas... | 152 | 1e-41 |
| TR:L0A5A4 L0A5A4_DEIPD Aminoglycoside phosphotransferase OS=Deinoc... | 151 | 1e-41 |
| TR:A0A561Q8L3 A0A561Q8L3_9BACL Kanamycin kinase/aminoglycoside 3'-... | 151 | 2e-41 |
| TR:A0A0W1QFZ6 A0A0W1QFZ6_9SPHN Aminoglycoside 3'-phosphotransferas... | 150 | 3e-41 |
| TR:A0A2I9CZQ6 A0A2I9CZQ6_9DEIO Aminoglycoside phosphotransferase O... | 150 | 3e-41 |
| SP:Q03447 KKA1_SALTM Aminoglycoside 3'-phosphotransferase OS=Salmo... | 150 | 3e-41 |
| TR:A0A3P7EH96 A0A3P7EH96_WUCBA APH domain-containing protein OS=Wu... | 155 | 4e-41 |
| TR:A0A0Q5QUZ1 A0A0Q5QUZ1_9SPHN Aminoglycoside 3'-phosphotransferas... | 150 | 5e-41 |
| TR:A0A418VBY7 A0A418VBY7_9DEIO Aminoglycoside 3'-phosphotransferas... | 149 | 7e-41 |
| TR:A0A0F7JMK4 A0A0F7JMK4_9DEIO Amino acid ABC transporter ATP-bind... | 149 | 1e-40 |
| TR:A0A1B2HPD3 A0A1B2HPD3_9PSEU APH domain-containing protein OS=Le... | 147 | 2e-40 |
| TR:U5W890 U5W890_9ACTN Aminoglycoside phosphotransferase OS=Actino... | 147 | 4e-40 |
| SP:P14509 KKA8_ECOLX Aminoglycoside 3'-phosphotransferase OS=Esche... | 148 | 4e-40 |
| TR:A0A172T670 A0A172T670_9DEIO Amino acid ABC transporter ATP-bind... | 147 | 5e-40 |
| TR:A0A1D8G9Y6 A0A1D8G9Y6_9ACTN Aminoglycoside 3'-phosphotransferas... | 155 | 6e-40 |
| TR:A0A5Q2RK27 A0A5Q2RK27_9ACTN APH(3') family aminoglycoside O-pho... | 147 | 9e-40 |
| TR:A0A3S8WKF5 A0A3S8WKF5_9ACTN Aminoglycoside O-phosphotransferase... | 147 | 1e-39 |
| TR:A0A5C8ZI13 A0A5C8ZI13_9ACTN Aminoglycoside 3'-phosphotransferas... | 145 | 1e-39 |
| TR:A0A418KVH4 A0A418KVH4_9ACTN Aminoglycoside 3'-phosphotransferas... | 145 | 1e-39 |
| TR:Q2MG02 Q2MG02_MICOL Kanamycin kinase OS=Micromonospora olivaste... | 146 | 2e-39 |
| TR:A0A563EHQ2 A0A563EHQ2_9PSEU Aminoglycoside 3'-phosphotransferas... | 145 | 3e-39 |
| TR:A0A0H5SK09 A0A0H5SK09_HERHM APH domain-containing protein OS=He... | 142 | 6e-39 |
| TR:A0A495X415 A0A495X415_9PSEU Kanamycin kinase/aminoglycoside 3'-... | 143 | 8e-39 |
| TR:A0A1H8FIN2 A0A1H8FIN2_9BACL Kanamycin kinase/aminoglycoside 3'-... | 143 | 2e-38 |
| TR:A0A561BZQ8 A0A561BZQ8_9ACTN Streptomycin 3'-kinase OS=Kribbella... | 143 | 2e-38 |
| TR:A0A4R4KXX2 A0A4R4KXX2_9ACTN Aminoglycoside 3'-phosphotransferas... | 142 | 2e-38 |
| TR:D2PKR4 D2PKR4_KRIFD Antimicrobial 3''-kinase OS=Kribbella flavi... | 142 | 3e-38 |
| TR:A0A2Z3JG07 A0A2Z3JG07_9DEIO Aminoglycoside 3'-phosphotransferas... | 143 | 3e-38 |
| TR:F5LER1 F5LER1_9BACL Kanamycin kinase OS=Paenibacillus sp. HGF7 ... | 142 | 5e-38 |
| TR:A0A221VYY1 A0A221VYY1_9PSEU Aminoglycoside 3'-phosphotransferas... | 141 | 1e-37 |
| TR:A0A0N0KCZ2 A0A0N0KCZ2_9SPHN Aminoglycoside 3'-phosphotransferas... | 141 | 2e-37 |
| TR:A0A2S6GIH1 A0A2S6GIH1_9PSEU Kanamycin kinase OS=Actinokineospor... | 140 | 2e-37 |
| TR:A0A164NZ73 A0A164NZ73_9NOCA Aminoglycoside phosphotransferase O... | 140 | 2e-37 |
| SP:P13250 KKA9_STRRI Aminoglycoside 3'-phosphotransferase OS=Strep... | 140 | 4e-37 |
| TR:W1SCY9 W1SCY9_9BACI Aminoglycoside phosphotransferase (Fragment... | 133 | 7e-37 |
| TR:A0A366DPY4 A0A366DPY4_9RHIZ Aminoglycoside 3'-phosphotransferas... | 139 | 7e-37 |
| TR:A0A2A2SDY7 A0A2A2SDY7_9SPHN Aminoglycoside 3'-phosphotransferas... | 139 | 8e-37 |
| TR:A0A4R4X9J2 A0A4R4X9J2_9ACTN Aminoglycoside 3'-phosphotransferas... | 138 | 1e-36 |
| TR:A0A2W2ERM5 A0A2W2ERM5_9ACTN Aminoglycoside 3'-phosphotransferas... | 138 | 2e-36 |
| TR:A0A1I5S7I3 A0A1I5S7I3_9SPHN Aminoglycoside 3'-phosphotransferas... | 135 | 3e-36 |
| TR:A0A1M4M9S6 A0A1M4M9S6_9FIRM Aminoglycoside 3'-phosphotransferas... | 137 | 7e-36 |
| SP:P00553 KKA4_BACCI Aminoglycoside 3'-phosphotransferase OS=Bacil... | 135 | 3e-35 |
| TR:A0A4Q7JFT4 A0A4Q7JFT4_9PSEU Aminoglycoside 3'-phosphotransferas... | 134 | 4e-35 |
| TR:A0A542DRV8 A0A542DRV8_9PSEU Kanamycin kinase OS=Amycolatopsis c... | 134 | 6e-35 |
| TR:A0A4R6PRW4 A0A4R6PRW4_9NOCA Kanamycin kinase OS=Nocardia ignora... | 137 | 7e-35 |

| TR:A0A365HB92 A0A365HB92_9ACTN Aminoglycoside 3'-phosphotransferas... | 134 | 1e-34 |
| --- | --- | --- |
| TR:A0A285LYT6 A0A285LYT6_9NOCA Kanamycin kinase OS=Nocardia amikac... | 137 | 1e-34 |
| TR:A0A1H9KTU8 A0A1H9KTU8_9PSEU Kanamycin kinase OS=Actinokineospor... | 133 | 1e-34 |
| TR:A0A370I256 A0A370I256_9NOCA Kanamycin kinase/aminoglycoside 3'-... | 132 | 2e-34 |
| TR:A0A4R5DQN1 A0A4R5DQN1_9ACTN Aminoglycoside 3'-phosphotransferas... | 131 | 3e-34 |
| TR:A0A1I2L6P9 A0A1I2L6P9_9ACTN Kanamycin kinase OS=Actinoplanes ph... | 131 | 5e-34 |
| TR:A0A366E427 A0A366E427_9NOCA Kanamycin kinase OS=Nocardia puris ... | 135 | 5e-34 |
| TR:A0A4R6KF00 A0A4R6KF00_9ACTN Streptomycin 3'-kinase OS=Kribbella... | 132 | 7e-34 |
| TR:A0A0M2VJQ1 A0A0M2VJQ1_9BACL Aminoglycoside phosphotransferase (... | 130 | 8e-34 |
| TR:T0MZ20 T0MZ20_9CLOT APH domain-containing protein OS=Clostridiu... | 131 | 1e-33 |
| TR:U5ED73 U5ED73_NOCAS Putative aminoglycoside 3'-O-phosphotransfe... | 132 | 5e-33 |
| TR:D6K107 D6K107_9ACTN Kanamycin kinase OS=Streptomyces sp. e14 OX... | 128 | 7e-33 |
| TR:A0A265QAG8 A0A265QAG8_9FIRM APH domain-containing protein OS=Ti... | 128 | 1e-32 |
| TR:A0A5C8JX64 A0A5C8JX64_9ACTN Aminoglycoside 3'-phosphotransferas... | 127 | 1e-32 |
| TR:A0A1J0VV95 A0A1J0VV95_9NOCA Aminoglycoside phosphotransferase A... | 131 | 1e-32 |
| TR:A0A5M3WX45 A0A5M3WX45_9ACTN Aminoglycoside O-phosphotransferase... | 127 | 1e-32 |
| TR:A0A1C4WLH3 A0A1C4WLH3_MICEC Kanamycin kinase OS=Micromonospora ... | 128 | 2e-32 |
| SP:P18150 APHE_STRGR Streptomycin 3''-kinase OS=Streptomyces grise... | 127 | 5e-32 |
| TR:A0A239HVP9 A0A239HVP9_9ACTN Kanamycin kinase/aminoglycoside 3'-... | 123 | 8e-32 |
| TR:S3AU80 S3AU80_9ACTN APH domain-containing protein OS=Streptomyc... | 126 | 1e-31 |
| TR:A0A4R7TFA0 A0A4R7TFA0_9ACTN Streptomycin 3'-kinase OS=Kribbella... | 125 | 1e-31 |
| TR:A0A1H7N6K2 A0A1H7N6K2_9ACTN Kanamycin kinase OS=Nonomuraea pusi... | 125 | 1e-31 |
| TR:K0F8X7 K0F8X7_9NOCA Aminoglycoside 3'-O-phosphotransferase OS=N... | 129 | 2e-31 |
| TR:A0A1V9K319 A0A1V9K319_9ACTN Aminoglycoside phosphotransferase A... | 125 | 3e-31 |
| TR:A0A6B2VAB2 A0A6B2VAB2_9ACTN Aminoglycoside 3'-phosphotransferas... | 124 | 4e-31 |
| TR:Q2J7L9 Q2J7L9_FRACC Aminoglycoside phosphotransferase OS=Franki... | 124 | 4e-31 |
| TR:A0A562VDH2 A0A562VDH2_9ACTN Kanamycin kinase OS=Stackebrandtia ... | 124 | 4e-31 |
| TR:H6R3X4 H6R3X4_NOCCG Putative aminoglycoside 3'-O-phosphotransfe... | 124 | 4e-31 |
| TR:A0A1Q4WCP7 A0A1Q4WCP7_9ACTN Aminoglycoside phosphotransferase O... | 124 | 6e-31 |
| TR:A0A1T4U3M6 A0A1T4U3M6_9BACT Kanamycin kinase OS=Chitinophaga ei... | 123 | 8e-31 |
| TR:A0A370I4X3 A0A370I4X3_9NOCA Streptomycin 3'-kinase OS=Nocardia ... | 124 | 8e-31 |
| TR:D2AUZ0 D2AUZ0_STRRD 3''-kinase OS=Streptosporangium roseum (str... | 123 | 1e-30 |
| TR:A0A5Q4TG46 A0A5Q4TG46_9ACTN APH(3'') family aminoglycoside O-ph... | 123 | 1e-30 |
| TR:A0A212TNI3 A0A212TNI3_9ACTN Streptomycin 3-kinase OS=Streptomyc... | 122 | 2e-30 |
| TR:A0A4U3CFI9 A0A4U3CFI9_9ACTN Aminoglycoside phosphotransferase A... | 122 | 2e-30 |
| TR:A0A1H5XY97 A0A1H5XY97_9ACTN Kanamycin kinase/aminoglycoside 3'-... | 121 | 3e-30 |
| TR:A0A2H5AUU2 A0A2H5AUU2_9ACTN Aminoglycoside phosphotransferase O... | 122 | 3e-30 |
| TR:A0A0Q7DQ20 A0A0Q7DQ20_9CAUL Aminoglycoside 3'-phosphotransferas... | 122 | 4e-30 |
| TR:A0A4R2JUF4 A0A4R2JUF4_9PSEU Streptomycin 3'-kinase OS=Actinocri... | 122 | 5e-30 |
| TR:A0A364K3T9 A0A364K3T9_9BACL Aminoglycoside 3'-phosphotransferas... | 121 | 6e-30 |
| TR:A0A2U9TCS3 A0A2U9TCS3_9GAMM Aminoglycoside 3'-phosphotransferas... | 118 | 6e-30 |
| TR:A0A2G5IDD5 A0A2G5IDD5_9ACTN APH domain-containing protein OS=St... | 121 | 7e-30 |
| TR:J7L841 J7L841_NOCAA Phosphotransferase enzyme family protein OS... | 121 | 7e-30 |
| TR:A0A132PMX6 A0A132PMX6_9MYCO 3'-kinase OS=Mycolicibacterium woli... | 121 | 8e-30 |
| TR:A0A1H0YKS3 A0A1H0YKS3_9ACTN Kanamycin kinase OS=Quadrisphaera s... | 121 | 8e-30 |
| TR:A0A0T0PUX4 A0A0T0PUX4_9SPHN Aminoglycoside 3'-phosphotransferas... | 120 | 8e-30 |
| TR:R4Z516 R4Z516_9ACTN Putative Kanamycin kinase OS=Candidatus Mic... | 120 | 1e-29 |
| TR:D2B8L9 D2B8L9_STRRD Kanamycin kinase OS=Streptosporangium roseu... | 120 | 1e-29 |
| TR:A0A0N9IEL0 A0A0N9IEL0_9PSEU 3'-kinase OS=Kibdelosporangium phyt... | 120 | 1e-29 |
| TR:A0A326U2D3 A0A326U2D3_9CHLR Kanamycin kinase/aminoglycoside 3'-... | 120 | 1e-29 |
| TR:A0A074LNS5 A0A074LNS5_9BACL APH domain-containing protein OS=Tu... | 120 | 2e-29 |
| TR:A0A1H6ESS3 A0A1H6ESS3_9ACTN Kanamycin kinase OS=Nonomuraea sola... | 120 | 2e-29 |
| TR:A0A1M5ZB99 A0A1M5ZB99_9FIRM Kanamycin kinase OS=Sporanaerobacte... | 119 | 3e-29 |
| TR:A0A318LPV1 A0A318LPV1_9PSEU APH(3'') family aminoglycoside O-ph... | 119 | 5e-29 |
| TR:A0A371PQL4 A0A371PQL4_9ACTN APH(3'') family aminoglycoside O-ph... | 119 | 5e-29 |
| TR:A0A511MYS9 A0A511MYS9_9DEIO Aminoglycoside 3'-phosphotransferas... | 118 | 5e-29 |
| TR:Q5YSZ5 Q5YSZ5_NOCFA Aminoglycoside 3'-O-phosphotransferase OS=N... | 119 | 5e-29 |
| TR:A0A212TEC1 A0A212TEC1_9ACTN Kanamycin kinase OS=Streptomyces sp... | 119 | 6e-29 |
| TR:A0A3N4SJR5 A0A3N4SJR5_9ACTN Kanamycin kinase OS=Streptomyces sp... | 119 | 6e-29 |
| TR:A0A2M8WS54 A0A2M8WS54_9MICO Kanamycin kinase OS=Luteimicrobium ... | 119 | 6e-29 |
| TR:A0A0F7N6E9 A0A0F7N6E9_9ACTN 3'-kinase OS=Streptomyces sp. CNQ-5... | 118 | 7e-29 |
| TR:A0A0A0B8D7 A0A0A0B8D7_9CELL Aminoglycoside phosphotransferase O... | 118 | 7e-29 |
| TR:A0A2A3I6G9 A0A2A3I6G9_9ACTN Kanamycin kinase OS=Streptomyces sp... | 118 | 8e-29 |
| TR:D3Q100 D3Q100_STANL 3''-kinase OS=Stackebrandtia nassauensis (s... | 118 | 9e-29 |
| TR:A0A366DUC7 A0A366DUC7_9NOCA Streptomycin 3'-kinase OS=Nocardia ... | 118 | 1e-28 |
| TR:A0A1H3RPA8 A0A1H3RPA8_9FIRM Kanamycin kinase OS=Proteiniborus e... | 117 | 1e-28 |
| TR:A0A4R0H3X8 A0A4R0H3X8_9ACTN Aminoglycoside 3'-phosphotransferas... | 117 | 2e-28 |
| TR:A0A0C5G322 A0A0C5G322_9ACTN 3'-kinase OS=Streptomyces cyaneogri... | 117 | 2e-28 |
| TR:A0A366ZFP9 A0A366ZFP9_9ACTN Aminoglycoside 3'-phosphotransferas... | 117 | 3e-28 |
| TR:A0A1N5H2U7 A0A1N5H2U7_9MYCO Probable streptomycin phosphotransf... | 116 | 4e-28 |
| TR:A0A4R2Q9K5 A0A4R2Q9K5_9PSEU Streptomycin 3'-kinase OS=Tamaricih... | 116 | 5e-28 |
| TR:A0A1Q4Y452 A0A1Q4Y452_9ACTN Aminoglycoside phosphotransferase A... | 115 | 7e-28 |

| TR:A0A2K4ZFP6 | A0A2K4ZFP6_9FIRM | Aminoglycoside 3'-phosphotransferas... | 115 | 8e-28 |
| --- | --- | --- | --- | --- |
| TR:A0A4R4YI30 | A0A4R4YI30_9ACTN | Aminoglycoside 3'-phosphotransferas... | 115 | 1e-27 |
| TR:A0A2W2E1A5 | A0A2W2E1A5_9ACTN | APH(3'') family aminoglycoside O-ph... | 115 | 1e-27 |
| TR:A0A1G6Y9F8 | A0A1G6Y9F8_9PSEU | Streptomycin 3'-kinase OS=Prauserel... | 115 | 2e-27 |
| TR:A0A1Y0CCJ5 | A0A1Y0CCJ5_9MYCO | Aminoglycoside phosphotransferase A... | 115 | 2e-27 |
| TR:A0A3N6DWL8 | A0A3N6DWL8_9ACTN | Aminoglycoside 3'-phosphotransferas... | 115 | 2e-27 |
| TR:A0A2T0N1P6 | A0A2T0N1P6_9ACTN | Kanamycin kinase OS=Nonomuraea fusc... | 114 | 3e-27 |
| TR:A0A4U3MHU9 | A0A4U3MHU9_9ACTN | Aminoglycoside 3'-phosphotransferas... | 114 | 3e-27 |
| TR:A0A164NGT2 | A0A164NGT2_9NOCA | APH domain-containing protein OS=No... | 113 | 3e-27 |
| TR:A0A4R8R5I2 | A0A4R8R5I2_9MYCO | Aminoglycoside 3'-phosphotransferas... | 114 | 4e-27 |
| TR:D9WPQ8 D9WPQ8_9ACTN Streptomycin 3''-kinase OS=Streptomyces him... | | | 114 | 5e-27 |
| TR:D1ALY5 D1ALY5_SEBTE Aminoglycoside phosphotransferase OS=Sebald... | | | 113 | 8e-27 |
| TR:A0A1X7LMZ4 A0A1X7LMZ4_9BURK Phosphotransferase enzyme family pr... | | | 108 | 9e-27 |
| TR:A0A1W6YZ55 A0A1W6YZ55_9BORD Aminoglycoside 3'-phosphotransferas... | | | 113 | 9e-27 |
| TR:D6CJG0 D6CJG0_CORRG Streptomycin phosphotransferase A OS=Coryne... | | | 113 | 9e-27 |
| TR:F3NKG7 F3NKG7_9ACTN Streptomycin 6-phosphotransferase OS=Strept... | | | 112 | 9e-27 |
| TR:A0A0N9XXU2 A0A0N9XXU2_MYCFO Aminoglycoside 3'-phosphotransferas... | | | 112 | 1e-26 |
| TR:A0A1T4PEC8 A0A1T4PEC8_9ACTN Streptomycin 3-kinase OS=Marinactin... | | | 113 | 1e-26 |
| TR:A0A1Q5GL35 A0A1Q5GL35_9ACTN 3'-kinase OS=Streptomyces sp. CB015... | | | 112 | 1e-26 |
| TR:A0A1M7YNK5 A0A1M7YNK5_9FIRM Kanamycin kinase OS=Anaerocolumna x... | | | 112 | 2e-26 |
| SP:P0A3Y5 KKA3_ENTFL Aminoglycoside 3'-phosphotransferase OS=Enter... | | | 112 | 2e-26 |
| TR:E8LFG4 E8LFG4_9FIRM Kanamycin kinase OS=Phascolarctobacterium s... | | | 112 | 2e-26 |
| TR:G2SZV9 G2SZV9_ROSHA Aminoglycoside 3'-phosphotransferase OS=Ros... | | | 112 | 2e-26 |
| TR:A0A1C0BVA1 A0A1C0BVA1_9FIRM APH(3') family aminoglycoside O-pho... | | | 112 | 2e-26 |
| TR:Q5HS59 Q5HS59_STAEQ Aminoglycoside 3'-phosphotransferase OS=Sta... | | | 112 | 2e-26 |
| TR:A0A3N0HWH4 A0A3N0HWH4_9FIRM Aminoglycoside O-phosphotransferase... | | | 112 | 2e-26 |
| TR:A0A3E2T6Z6 A0A3E2T6Z6_9FIRM Aminoglycoside O-phosphotransferase... | | | 112 | 2e-26 |
| TR:A0A143X0T6 A0A143X0T6_9ACTN Aminoglycoside 3'-phosphotransferas... | | | 112 | 2e-26 |
| TR:A0A1Y4TFF5 A0A1Y4TFF5_9FIRM Aminoglycoside O-phosphotransferase... | | | 112 | 2e-26 |
| TR:D2JEU4 D2JEU4_ENTFC APH(3') family aminoglycoside O-phosphotran... | | | 112 | 2e-26 |
| TR:T0V477 T0V477_9ENTE APH domain-containing protein OS=Enterococc... | | | 112 | 2e-26 |
| TR:E4ZFP6 E4ZFP6_9CAUD Aminoglycoside phosphotransferase type III ... | | | 112 | 2e-26 |
| TR:S5M7R5 S5M7R5_9CAUD Aminoglycoside 3'-phosphotransferase OS=Esc... | | | 112 | 2e-26 |
| TR:V2TI87 V2TI87_9GAMM Aminoglycoside 3'-phosphotransferase OS=Aci... | | | 112 | 2e-26 |
| TR:D5UBJ1 D5UBJ1_CELFN Aminoglycoside phosphotransferase OS=Cellul... | | | 112 | 2e-26 |
| TR:A0A077LEK5 A0A077LEK5_9PSED Aminoglycoside 3'-phosphotransferas... | | | 112 | 2e-26 |
| TR:A0A0V8T6W1 A0A0V8T6W1_9CELL Aminoglycoside phosphotransferase O... | | | 112 | 2e-26 |
| TR:R5B6I8 R5B6I8_9CLOT Aminoglycoside phosphotransferase OS=Clostr... | | | 112 | 2e-26 |
| TR:A0A1I3Y9R7 A0A1I3Y9R7_9CELL Kanamycin kinase OS=Cellulomonas sp... | | | 112 | 2e-26 |
| TR:D9VQD2 D9VQD2_9ACTN Streptomycin 6-phosphotransferase OS=Strept... | | | 112 | 3e-26 |
| TR:A0A1M6IJ84 A0A1M6IJ84_9ACTN Kanamycin kinase/aminoglycoside 3'-... | | | 111 | 3e-26 |
| TR:A0A416ESZ4 A0A416ESZ4_9FIRM Aminoglycoside 3'-phosphotransferas... | | | 111 | 4e-26 |
| TR:A0A6G4U730 A0A6G4U730_9ACTN APH(3'') family aminoglycoside O-ph... | | | 111 | 4e-26 |
| TR:A0A1B6AUD8 A0A1B6AUD8_9ACTN Kinase OS=Streptomyces sp. NBRC 110... | | | 111 | 5e-26 |
| TR:A0A6B3HQZ8 A0A6B3HQZ8_9ACTN APH(3'') family aminoglycoside O-ph... | | | 111 | 6e-26 |
| TR:A0A0C1X3I1 A0A0C1X3I1_9ACTN 3'-kinase OS=Streptomyces sp. AcH 5... | | | 111 | 6e-26 |
| TR:A0A4P6VWB3 A0A4P6VWB3_9GAMM Aminoglycoside 3'-phosphotransferas... | | | 110 | 7e-26 |
| TR:A0A2P5GGM4 A0A2P5GGM4_9ENTR Aminoglycoside 3'-phosphotransferas... | | | 110 | 7e-26 |
| TR:B1VIB4 B1VIB4_CORU7 Aminoglycoside-3''-phosphotransferase OS=Co... | | | 110 | 7e-26 |
| TR:A0A0H3GZY9 A0A0H3GZY9_KLEPH Aminoglycoside 3'-phosphotransferas... | | | 110 | 7e-26 |
| TR:J5KDL8 J5KDL8_PASMD Aminoglycoside 3'-phosphotransferase OS=Pas... | | | 110 | 7e-26 |
| TR:A0A0M2R0H0 A0A0M2R0H0_9PROT Aminoglycoside 3'-phosphotransferas... | | | 110 | 7e-26 |
| TR:A0A2S2FBY2 A0A2S2FBY2_9GAMM Aminoglycoside 3'-phosphotransferas... | | | 110 | 7e-26 |
| TR:A0A0R0BRS8 A0A0R0BRS8_9GAMM Aminoglycoside 3'-phosphotransferas... | | | 110 | 7e-26 |
| TR:A0A501WBA3 A0A501WBA3_9GAMM Aminoglycoside 3'-phosphotransferas... | | | 110 | 7e-26 |
| TR:A0A0M0HMR3 A0A0M0HMR3_9VIBR Aminoglycoside 3'-phosphotransferas... | | | 110 | 7e-26 |
| TR:A0A345WV32 A0A345WV32_9SPHN Aminoglycoside 3'-phosphotransferas... | | | 110 | 7e-26 |
| TR:A0A1Q2CIK9 A0A1Q2CIK9_9ACTN Aminoglycoside O-phosphotransferase... | | | 110 | 7e-26 |
| TR:A0A376BDS9 A0A376BDS9_9PAST Aminoglycoside 3'-phosphotransferas... | | | 110 | 7e-26 |
| TR:A6TIY6 A6TIY6_KLEP7 Aminoglycoside 3'-phosphotransferase OS=Kle... | | | 110 | 8e-26 |
| TR:A0A5C8PSK5 A0A5C8PSK5_9PROT Aminoglycoside 3'-phosphotransferas... | | | 110 | 9e-26 |
| TR:A0A2N3UL89 A0A2N3UL89_9ACTN Kanamycin kinase OS=Streptomyces sp... | | | 109 | 1e-25 |
| TR:A0A5C4T381 A0A5C4T381_9BACL Aminoglycoside 3'-phosphotransferas... | | | 110 | 1e-25 |
| TR:A0A512PFI8 A0A512PFI8_9CELL Aminoglycoside phosphotransferase A... | | | 110 | 1e-25 |
| TR:A0A239AVA4 A0A239AVA4_9ACTN Kanamycin kinase OS=Streptomyces gl... | | | 109 | 1e-25 |
| TR:A0A3G9JN98 A0A3G9JN98_9FIRM Aminoglycoside O-phosphotransferase... | | | 110 | 1e-25 |
| TR:W1DTX6 W1DTX6_KLEPN Aminoglycoside 3'-phosphotransferase OS=Kle... | | | 110 | 1e-25 |
| TR:A0A1Q9S1S5 A0A1Q9S1S5_9PSEU APH domain-containing protein OS=Ps... | | | 109 | 1e-25 |
| TR:D7BWG8 D7BWG8_STRBB Aminoglycoside phosphotransferase OS=Strept... | | | 110 | 2e-25 |
| TR:A0A5P9QDZ8 A0A5P9QDZ8_9MICO Kanamycin kinase OS=Luteimicrobium ... | | | 109 | 2e-25 |
| TR:A0A085LEB0 A0A085LEB0_9MICO Aminoglycoside phosphotransferase O... | | | 109 | 2e-25 |
| TR:A0A561UGU6 A0A561UGU6_9ACTN Kanamycin kinase OS=Kitasatospora v... | | | 109 | 2e-25 |

| TR:A0A1I3WQ17 A0A1I3WQ17_9PSEU Kanamycin kinase OS=Amycolatopsis s... | 108 | 2e-25 |
| --- | --- | --- |
| TR:L9PQ85 L9PQ85_9BURK Aminoglycoside 3'-phosphotransferase OS=Jan... | 108 | 3e-25 |
| TR:A0A285KQG3 A0A285KQG3_9NOCA Streptomycin 3-kinase OS=Nocardia a... | 108 | 5e-25 |
| TR:A0A1Y3WDH4 A0A1Y3WDH4_9FIRM APH domain-containing protein OS=Dr... | 108 | 6e-25 |
| TR:A0A431TTB2 A0A431TTB2_9BURK Aminoglycoside 3'-phosphotransferas... | 108 | 6e-25 |
| TR:A0A6G4VA64 A0A6G4VA64_9ACTN Aminoglycoside 3'-phosphotransferas... | 107 | 7e-25 |
| TR:V6JW13 V6JW13_STRNV APH domain-containing protein OS=Streptomyc... | 108 | 7e-25 |
| TR:A0A1H3THL2 A0A1H3THL2_9PSEU Streptomycin 3-kinase OS=Saccharopo... | 108 | 7e-25 |
| TR:A0A076NGZ5 A0A076NGZ5_9CORY Aminoglycoside phosphotransferase O... | 107 | 7e-25 |
| TR:M4R2W5 M4R2W5_BIBTR Aminoglycoside 3'-phosphotransferase OS=Bib... | 107 | 7e-25 |
| TR:A0A656YEK5 A0A656YEK5_SALNE Aminoglycoside 3'-phosphotransferas... | 107 | 7e-25 |
| TR:A0A4Y3R330 A0A4Y3R330_STRCI APH(3'') family aminoglycoside O-ph... | 107 | 7e-25 |
| TR:A0A5J6F9N2 A0A5J6F9N2_9ACTN APH(3'') family aminoglycoside O-ph... | 108 | 8e-25 |
| TR:A0A505D6T9 A0A505D6T9_9ACTN Aminoglycoside 3'-phosphotransferas... | 107 | 8e-25 |
| TR:A0A1H0PNC9 A0A1H0PNC9_9BACL Phosphotransferase enzyme family pr... | 103 | 8e-25 |
| TR:A0A239CX75 A0A239CX75_9ACTN Kanamycin kinase OS=Streptosporangi... | 107 | 1e-24 |
| TR:A0A2M9CPF0 A0A2M9CPF0_9CELL Kanamycin kinase OS=Sediminihabitan... | 108 | 1e-24 |
| TR:A0A329QTP9 A0A329QTP9_9ACTN Aminoglycoside 3'-phosphotransferas... | 107 | 1e-24 |
| TR:A0A2N3Y225 A0A2N3Y225_SACSN Streptomycin 3'-kinase OS=Saccharop... | 109 | 1e-24 |
| TR:A0A0M2LRK4 A0A0M2LRK4_9MICO Aminoglycoside phosphotransferase O... | 107 | 1e-24 |
| TR:A0A3N6F707 A0A3N6F707_9ACTN Aminoglycoside 3'-phosphotransferas... | 107 | 1e-24 |
| TR:A0A2P8DSU4 A0A2P8DSU4_9ACTN Streptomycin 3'-kinase OS=Murinocar... | 107 | 2e-24 |
| TR:A0A1X7E6V0 A0A1X7E6V0_CELCE Kanamycin kinase OS=Cellulosimicrob... | 107 | 2e-24 |
| TR:A0A164AWT6 A0A164AWT6_9MICO Aminoglycoside 3'-phosphotransferas... | 106 | 2e-24 |
| TR:D6AA68 D6AA68_STRV1 Aminoglycoside phosphotransferase OS=Strept... | 107 | 2e-24 |
| TR:A0A2N0FIZ2 A0A2N0FIZ2_9ACTN Kanamycin kinase OS=Streptomyces sp... | 106 | 3e-24 |
| TR:A0A2V1HU73 A0A2V1HU73_9MICO Aminoglycoside phosphotransferase A... | 106 | 3e-24 |
| TR:A0A0B1Q1B6 A0A0B1Q1B6_9RHIZ Aminoglycoside 3'-phosphotransferas... | 106 | 3e-24 |
| TR:A0A1Q9S8V5 A0A1Q9S8V5_9PSEU Aminoglycoside phosphotransferase A... | 106 | 3e-24 |
| TR:A0A1V2QTN1 A0A1V2QTN1_9PSEU Aminoglycoside phosphotransferase A... | 106 | 3e-24 |
| TR:A0A498B1H3 A0A498B1H3_9ACTN Kanamycin kinase OS=Streptomyces sp... | 105 | 3e-24 |
| TR:A0A1W2B0V5 A0A1W2B0V5_9FIRM Kanamycin kinase OS=Clostridiales b... | 106 | 4e-24 |
| TR:C6WFK3 C6WFK3_ACTMD Aminoglycoside phosphotransferase OS=Actino... | 105 | 4e-24 |
| TR:D7CCJ8 D7CCJ8_STRBB Phosphotransferase OS=Streptomyces bingchen... | 104 | 5e-24 |
| TR:A0A4R5U9I1 A0A4R5U9I1_9RHIZ Aminoglycoside 3'-phosphotransferas... | 105 | 6e-24 |
| TR:A0A2A9DRI3 A0A2A9DRI3_9MICO Kanamycin kinase OS=Microbacterium ... | 105 | 7e-24 |
| TR:A0A0F0KY17 A0A0F0KY17_9MICO Aminoglycoside 3'-phosphotransferas... | 104 | 8e-24 |
| TR:A0A0J1GBY8 A0A0J1GBY8_9FIRM APH domain-containing protein OS=Ro... | 105 | 9e-24 |
| TR:E0RY10 E0RY10_BUTPB Aminoglycoside phosphotransferase OS=Butyri... | 105 | 1e-23 |
| TR:A0A3N2D192 A0A3N2D192_9MICO Kanamycin kinase/streptomycin 3'-ki... | 104 | 1e-23 |
| TR:A0A6G4X282 A0A6G4X282_9ACTN APH(3'') family aminoglycoside O-ph... | 104 | 1e-23 |
| TR:C7QIF6 C7QIF6_CATAD Aminoglycoside phosphotransferase OS=Catenu... | 104 | 1e-23 |
| TR:A0A086YC10 A0A086YC10_9RHOB Aminoglycoside 3'-phosphotransferas... | 104 | 1e-23 |
| TR:A0A4R2JF33 A0A4R2JF33_9PSEU Kanamycin kinase OS=Actinocrispum w... | 104 | 1e-23 |
| TR:A0A4V1M245 A0A4V1M245_9STAP Aminoglycoside 3'-phosphotransferas... | 104 | 2e-23 |
| TR:A0A543G9W1 A0A543G9W1_9PSEU Kanamycin kinase OS=Pseudonocardia ... | 104 | 2e-23 |
| TR:A0A2V4NRL8 A0A2V4NRL8_9ACTN Aminoglycoside phosphotransferase A... | 104 | 2e-23 |
| TR:A0A3G6ZKC8 A0A3G6ZKC8_9MICO Aminoglycoside 3'-phosphotransferas... | 103 | 2e-23 |
| TR:A0A2A3YN51 A0A2A3YN51_9MICO APH(3'') family aminoglycoside O-ph... | 104 | 2e-23 |
| TR:A0A1M7CS78 A0A1M7CS78_9ACTN Kanamycin kinase OS=Streptomyces pa... | 103 | 2e-23 |
| TR:A0A0N0MZD3 A0A0N0MZD3_9ACTN Kanamycin kinase OS=Actinobacteria ... | 103 | 2e-23 |
| TR:A0A1J4N7A0 A0A1J4N7A0_9ACTN APH(3'') family aminoglycoside O-ph... | 103 | 4e-23 |
| SP:P0A3Y6 KKA3_STAAU Aminoglycoside 3'-phosphotransferase OS=Staph... | 103 | 5e-23 |
| TR:A0A365YVW7 A0A365YVW7_9ACTN APH(3'') family aminoglycoside O-ph... | 102 | 6e-23 |
| TR:A0A5B8M1N4 A0A5B8M1N4_9MICO Aminoglycoside 3'-phosphotransferas... | 103 | 6e-23 |
| TR:A0A1I1AXG3 A0A1I1AXG3_9CELL Kanamycin kinase OS=Cellulomonas ma... | 103 | 6e-23 |
| TR:D3D745 D3D745_9ACTN Aminoglycoside phosphotransferase OS=Franki... | 102 | 6e-23 |
| TR:A0A1I0MM21 A0A1I0MM21_9FIRM Kanamycin kinase/aminoglycoside 3'-... | 102 | 7e-23 |
| TR:A0A5R9EIG4 A0A5R9EIG4_9LACT Aminoglycoside 3'-phosphotransferas... | 102 | 8e-23 |
| TR:A0A263DYL7 A0A263DYL7_9PSEU Aminoglycoside O-phosphotransferase... | 102 | 8e-23 |
| TR:A0A1C7IAD6 A0A1C7IAD6_9FIRM APH domain-containing protein OS=Bl... | 102 | 9e-23 |
| TR:A0A066YSW7 A0A066YSW7_9ACTN Putative aminoglycoside 3'-phosphot... | 102 | 1e-22 |
| TR:A0A0D0PX29 A0A0D0PX29_KITGR Aminoglycoside phosphotransferase O... | 102 | 1e-22 |
| TR:A0A143WVH6 A0A143WVH6_9FIRM Aminoglycoside 3'-phosphotransferas... | 102 | 1e-22 |
| TR:A6W8Z6 A6W8Z6_KINRD Aminoglycoside phosphotransferase OS=Kineoc... | 101 | 2e-22 |
| TR:A0A143XDA0 A0A143XDA0_9FIRM Aminoglycoside 3'-phosphotransferas... | 101 | 2e-22 |
| TR:A0A1C6Q6K7 A0A1C6Q6K7_9ACTN Streptomycin 3-kinase OS=Streptomyc... | 101 | 2e-22 |
| TR:A0A0S4Q2S2 A0A0S4Q2S2_BLAVI Aminoglycoside 3'-phosphotransferas... | 99.4 | 3e-22 |
| TR:A0A4Y9FV78 A0A4Y9FV78_9MICO Aminoglycoside 3'-phosphotransferas... | 100 | 3e-22 |
| TR:A0A4R1D9F5 A0A4R1D9F5_9ACTN Aminoglycoside 3'-phosphotransferas... | 100 | 3e-22 |
| TR:A0A0M2HEX5 A0A0M2HEX5_9MICO Aminoglycoside 3'-phosphotransferas... | 100 | 3e-22 |
| TR:A0A1H7IXE6 A0A1H7IXE6_9ACTN Kanamycin kinase OS=Nonomuraea pusi... | 101 | 3e-22 |

| TR:A0A4Z1E7M6 A0A4Z1E7M6_9MICO Aminoglycoside phosphotransferase O... | 100 | 4e-22 |
| --- | --- | --- |
| TR:A0A4P7SLX7 A0A4P7SLX7_9CELL Aminoglycoside 3'-phosphotransferas... | 100 | 4e-22 |
| TR:A0A4U3MI75 A0A4U3MI75_9ACTN Aminoglycoside 3'-phosphotransferas... | 100 | 5e-22 |
| TR:A0A0Q5CBG7 A0A0Q5CBG7_9MICO Aminoglycoside phosphotransferase O... | 99.4 | 8e-22 |
| TR:A0A0Q6WPS9 A0A0Q6WPS9_9BURK Aminoglycoside 3'-phosphotransferas... | 99.8 | 9e-22 |
| TR:A0A543EQ83 A0A543EQ83_9MICO Kanamycin kinase OS=Microbacterium ... | 99.4 | 9e-22 |
| TR:A0A1C4LUU9 A0A1C4LUU9_9ACTN Kanamycin kinase OS=Streptomyces sp... | 99.8 | 9e-22 |
| TR:A0A2T0RA65 A0A2T0RA65_9ACTN Kanamycin kinase OS=Kineococcus rhi... | 100 | 9e-22 |
| TR:V2JBJ8 V2JBJ8_9BURK Aminoglycoside 3'-phosphotransferase OS=Cup... | 99.8 | 1e-21 |
| TR:A0A2W1Y1Y5 A0A2W1Y1Y5_9MICO Aminoglycoside 3'-phosphotransferas... | 99.4 | 1e-21 |
| TR:A0A0W7WBL7 A0A0W7WBL7_9MICO Aminoglycoside phosphotransferase O... | 99.8 | 1e-21 |
| TR:A0A4R6RZT0 A0A4R6RZT0_9MICO Kanamycin kinase OS=Leucobacter lut... | 99.8 | 1e-21 |
| TR:A0A511J994 A0A511J994_9CELL Putative phosphotransferase OS=Cell... | 98.6 | 1e-21 |
| TR:A0A2H3KMG1 A0A2H3KMG1_9CHLR APH(3') family aminoglycoside O-pho... | 99.4 | 1e-21 |
| TR:A0A1H1RG07 A0A1H1RG07_9CELL Kanamycin kinase OS=Paraoerskovia m... | 99.4 | 1e-21 |
| TR:A0A4Y9PX58 A0A4Y9PX58_9MICO Aminoglycoside 3'-phosphotransferas... | 99.0 | 2e-21 |
| TR:A0A126Z700 A0A126Z700_9MICO Aminoglycoside phosphotransferase O... | 98.6 | 2e-21 |
| TR:I0V1B4 I0V1B4_9PSEU Aminoglycoside phosphotransferase OS=Saccha... | 99.0 | 2e-21 |
| TR:D2B2K4 D2B2K4_STRRD Aminoglycoside 3'-phosphotransferase OS=Str... | 99.0 | 2e-21 |
| TR:A0A519GZG7 A0A519GZG7_9MICO Aminoglycoside 3'-phosphotransferas... | 98.2 | 2e-21 |
| TR:A0A1G7VTB2 A0A1G7VTB2_9MICO Kanamycin kinase OS=Microbacterium ... | 98.2 | 3e-21 |
| TR:X7YE21 X7YE21_MYCKA Phosphotransferase enzyme family protein OS... | 97.8 | 3e-21 |
| TR:A0A0Q5RH54 A0A0Q5RH54_9MICO Aminoglycoside phosphotransferase O... | 98.2 | 4e-21 |
| TR:A0A2S9QHZ7 A0A2S9QHZ7_9RHIZ Aminoglycoside 3'-phosphotransferas... | 98.2 | 5e-21 |
| TR:A0A1T5M0Z6 A0A1T5M0Z6_9CLOT Aminoglycoside phosphotransferase O... | 93.2 | 5e-21 |
| TR:A0A3N1HSX5 A0A3N1HSX5_9ACTN Kanamycin kinase OS=Pseudokineococc... | 97.8 | 5e-21 |
| TR:A0A371NNW3 A0A371NNW3_9MICO Aminoglycoside 3'-phosphotransferas... | 97.4 | 5e-21 |
| TR:A0A4R7VUS2 A0A4R7VUS2_9PSEU Streptomycin 3'-kinase OS=Actinophy... | 97.4 | 6e-21 |
| TR:A0A4R2CRE3 A0A4R2CRE3_9ACTN Kanamycin kinase/aminoglycoside 3'-... | 96.3 | 7e-21 |
| TR:R9N4E7 R9N4E7_9FIRM APH domain-containing protein OS=Lachnospir... | 97.4 | 8e-21 |
| TR:A0A553JZ49 A0A553JZ49_9ACTN Aminoglycoside 3'-phosphotransferas... | 97.4 | 8e-21 |
| TR:A0A2R5HEQ5 A0A2R5HEQ5_9MYCO Aminoglycoside phosphotransferase A... | 97.1 | 8e-21 |
| TR:A0A4P6EN66 A0A4P6EN66_9MICO Aminoglycoside 3'-phosphotransferas... | 97.1 | 9e-21 |
| TR:A0A031FWM9 A0A031FWM9_9MICO Aminoglycoside phosphotransferase O... | 96.7 | 9e-21 |
| TR:A0A562V9F0 A0A562V9F0_9ACTN Kanamycin kinase/aminoglycoside 3'-... | 97.1 | 1e-20 |
| TR:A0A399JCC3 A0A399JCC3_9MICC Aminoglycoside 3'-phosphotransferas... | 96.7 | 1e-20 |
| TR:A0A1S1NN44 A0A1S1NN44_9MYCO Aminoglycoside phosphotransferase A... | 96.3 | 1e-20 |
| TR:A0A498R2I3 A0A498R2I3_9MYCO Aminoglycoside 3'-phosphotransferas... | 96.3 | 1e-20 |
| TR:A0A6I6E2E6 A0A6I6E2E6_9MICO Aminoglycoside 3'-phosphotransferas... | 95.9 | 2e-20 |
| TR:A0A1P8U4Z7 A0A1P8U4Z7_9MICO Aminoglycoside phosphotransferase A... | 95.9 | 2e-20 |
| TR:A0A0P0EIW2 A0A0P0EIW2_9MICO APH domain-containing protein OS=Mi... | 95.9 | 2e-20 |
| TR:A0A0B2ADP6 A0A0B2ADP6_9MICO Aminoglycoside phosphotransferase O... | 95.5 | 3e-20 |
| TR:A0A222XAW6 A0A222XAW6_9CELL Aminoglycoside phosphotransferase A... | 95.5 | 3e-20 |
| TR:A0A542UQB3 A0A542UQB3_9CELL Kanamycin kinase OS=Cellulomonas sp... | 95.9 | 3e-20 |
| TR:A0A0Q4HAG9 A0A0Q4HAG9_9MICO APH domain-containing protein OS=Ag... | 95.5 | 3e-20 |
| TR:A0A0D0IMT7 A0A0D0IMT7_9MICO Aminoglycoside phosphotransferase O... | 95.9 | 3e-20 |
| TR:U2X9J7 U2X9J7_9MICO Aminoglycoside phosphotransferase OS=Microb... | 92.8 | 4e-20 |
| TR:A0A1I6L1U7 A0A1I6L1U7_9MICO Kanamycin kinase OS=Agrococcus bald... | 95.1 | 4e-20 |
| TR:E9SBN1 E9SBN1_RUMAL Putative kanamycin kinase OS=Ruminococcus a... | 95.1 | 5e-20 |
| TR:A0A0Q5QY89 A0A0Q5QY89_9NOCA Aminoglycoside phosphotransferase O... | 94.7 | 5e-20 |
| TR:A0A1I6MCT1 A0A1I6MCT1_9MICO Kanamycin kinase OS=Microbacterium ... | 94.4 | 7e-20 |
| TR:B2HML5 B2HML5_MYCMM Phosphotransferase OS=Mycobacterium marinum... | 94.4 | 7e-20 |
| TR:A0A1G8AJS0 A0A1G8AJS0_9MICO Kanamycin kinase OS=Agrococcus jeju... | 94.4 | 7e-20 |
| TR:A0A2U1ZXR4 A0A2U1ZXR4_9MICO Aminoglycoside phosphotransferase A... | 94.4 | 8e-20 |
| TR:A0A2N9JHG9 A0A2N9JHG9_9ACTN APH domain-containing protein OS=Mi... | 94.4 | 9e-20 |
| TR:A0A1G8EFL0 A0A1G8EFL0_9PSEU Kanamycin kinase OS=Pseudonocardia ... | 94.4 | 9e-20 |
| TR:A0A4R8QW26 A0A4R8QW26_9MYCO Aminoglycoside 3'-phosphotransferas... | 94.0 | 9e-20 |
| TR:D7B4D7 D7B4D7_NOCDD Aminoglycoside phosphotransferase OS=Nocard... | 95.1 | 1e-19 |
| TR:A0A0F2C8M1 A0A0F2C8M1_9MICO Aminoglycoside 3'-phosphotransferas... | 94.0 | 1e-19 |
| TR:A0A0N8GPB1 A0A0N8GPB1_9CHLR APH domain-containing protein OS=Le... | 94.4 | 1e-19 |
| TR:A0A1H9DI46 A0A1H9DI46_9SPIO Kanamycin kinase OS=Treponema bryan... | 94.4 | 1e-19 |
| TR:A0A0I9YP49 A0A0I9YP49_9MYCO Aminoglycoside phosphotransferase O... | 94.0 | 1e-19 |
| TR:A0A0C1U987 A0A0C1U987_9CLOT Phosphotransferase enzyme family pr... | 93.6 | 2e-19 |
| TR:A0A372PK99 A0A372PK99_9ACTN Aminoglycoside 3'-phosphotransferas... | 93.6 | 2e-19 |
| TR:A0A3N2JJJ7 A0A3N2JJJ7_9CELL Kanamycin kinase OS=Cellulomonas sp... | 94.0 | 2e-19 |
| TR:A0A0M2H7Z7 A0A0M2H7Z7_9MICO Aminoglycoside 3'-phosphotransferas... | 93.2 | 2e-19 |
| TR:A0A543BHZ6 A0A543BHZ6_9MICO Kanamycin kinase OS=Microbacterium ... | 93.2 | 2e-19 |
| TR:A0A249SRT6 A0A249SRT6_9BACT APH(3'') family aminoglycoside O-ph... | 93.6 | 2e-19 |
| TR:A0A1X1XNT1 A0A1X1XNT1_9MYCO Aminoglycoside phosphotransferase O... | 93.2 | 2e-19 |
| TR:A0A1R4HLD7 A0A1R4HLD7_9MICO Aminoglycoside phosphotransferase O... | 93.6 | 2e-19 |
| TR:A0A4R4XDR7 A0A4R4XDR7_9ACTN Aminoglycoside phosphotransferase O... | 92.0 | 3e-19 |
| TR:E8U5N2 E8U5N2_DEIML Kanamycin kinase OS=Deinococcus maricopensi... | 92.8 | 3e-19 |

TR:A0A4V3BVJ1 A0A4V3BVJ1_9MICO Kanamycin kinase OS=Microbacterium ... 92.4 4e-19 TR:A0A0Q9CLW6 A0A0Q9CLW6_9CELL Aminoglycoside phosphotransferase O... 93.2 4e-19 TR:A0A2U3P7J0 A0A2U3P7J0_9MYCO Aminoglycoside phosphotransferase (... 92.4 4e-19 TR:A0A1A3HJW7 A0A1A3HJW7_9MYCO Aminoglycoside phosphotransferase A... 92.0 5e-19 TR:A0A6H9WSB1 A0A6H9WSB1_9MICO Aminoglycoside 3'-phosphotransferas... 92.4 6e-19 TR:A0A1Y2FFT2 A0A1Y2FFT2_9FUNG Aminoglycoside phosphotransferase O... 92.0 7e-19 TR:A0A3R7HL97 A0A3R7HL97_9MICO Kanamycin kinase OS=Microbacterium ... 91.7 7e-19 TR:A0A1R4GJT3 A0A1R4GJT3_9MICO Aminoglycoside phosphotransferase O... 91.3 7e-19 TR:A0A1H0KHB2 A0A1H0KHB2_9ACTN Kanamycin kinase OS=Nakamurella pan... 92.0 7e-19 TR:A0A1R3X780 A0A1R3X780_9MICO Kanamycin kinase OS=Microbacterium ... 92.0 8e-19 TR:A0A5M3W3W7 A0A5M3W3W7_9ACTN Aminoglycoside phosphotransferase A... 91.7 8e-19 TR:A0A4R6JJQ2 A0A4R6JJQ2_9ACTN Kanamycin kinase/aminoglycoside 3'-... 90.5 1e-18 TR:A0A3B0B364 A0A3B0B364_9MICO Aminoglycoside 3'-phosphotransferas... 91.3 1e-18 TR:A0A0M2HM17 A0A0M2HM17_9MICO Aminoglycoside 3'-phosphotransferas... 91.3 1e-18

| TR:E4MZK3 E4MZK3_KITSK Putative aminoglycoside 3'-phosphotransfera... | 94.7 | 1e-18 |
| --- | --- | --- |
| TR:A0A397LR33 A0A397LR33_9MICO Kanamycin kinase OS=Promicromonospo... | 92.0 | 1e-18 |
| TR:A0A3N2G446 A0A3N2G446_9CELL Kanamycin kinase OS=Cellulomonas sp... | 90.9 | 1e-18 |
| TR:A0A0Q8LMY9 A0A0Q8LMY9_9MICO Aminoglycoside phosphotransferase O... | 90.9 | 1e-18 |
| TR:D2Q469 D2Q469_KRIFD Aminoglycoside phosphotransferase OS=Kribbe... | 90.1 | 1e-18 |
| TR:A0A4R7T9T0 A0A4R7T9T0_9ACTN Kanamycin kinase/aminoglycoside 3'-... | 90.1 | 1e-18 |
| TR:A0A5C4SX21 A0A5C4SX21_9BACL Aminoglycoside 3'-phosphotransferas... | 91.3 | 2e-18 |
| TR:A0A0Q5B127 A0A0Q5B127_9MICO Uncharacterized protein OS=Rathayib... | 89.7 | 2e-18 |
| TR:A0A4Q2UGS5 A0A4Q2UGS5_9BACT Aminoglycoside 3'-phosphotransferas... | 90.5 | 3e-18 |
| TR:A0A2S8WU98 A0A2S8WU98_9MICC Aminoglycoside phosphotransferase A... | 90.5 | 3e-18 |
| TR:F8A0B8 F8A0B8_CELGA Aminoglycoside phosphotransferase OS=Cellul... | 89.7 | 4e-18 |
| TR:A0A397LNG7 A0A397LNG7_9MICO Kanamycin kinase OS=Promicromonospo... | 90.1 | 4e-18 |
| TR:A0A1S1RKG9 A0A1S1RKG9_9ACTN Aminoglycoside phosphotransferase A... | 89.7 | 5e-18 |
| TR:A0A132PFY8 A0A132PFY8_9MYCO Aminoglycoside phosphotransferase O... | 89.4 | 5e-18 |
| TR:D5UXJ9 D5UXJ9_TSUPD Aminoglycoside phosphotransferase OS=Tsukam... | 89.7 | 5e-18 |
| TR:A0A511A8P8 A0A511A8P8_9MICO Aminoglycoside phosphotransferase A... | 89.4 | 6e-18 |
| TR:A0A1M6IMD1A0A1M6IMD1_9ACTN Kanamycin kinase OS=Tessaracoccus b... | 89.0 | 7e-18 |
| TR:A0A511ACM5A0A511ACM5_9MICO Putative phosphotransferase OS=Micr... | 89.0 | 7e-18 |
| TR:A0A0Q4FD09A0A0Q4FD09_9MICO Aminoglycoside phosphotransferase O... | 89.0 | 8e-18 |
| TR:A0A2I8DT11A0A2I8DT11_9BURK Aminoglycoside 3'-phosphotransferas... | 89.0 | 9e-18 |
| TR:A0A1X7NPX6A0A1X7NPX6_9MICO Kanamycin kinase OS=Rathayibacter o... | 88.6 | 1e-17 |
| TR:A0A1X2BR16A0A1X2BR16_9MYCO Aminoglycoside phosphotransferase O... | 88.6 | 1e-17 |
| TR:A0A5C8IEL5A0A5C8IEL5_9MICO Aminoglycoside 3'-phosphotransferas... | 88.2 | 1e-17 |
| TR:A0A516Q1Y0A0A516Q1Y0_9ACTN Aminoglycoside 3'-phosphotransferas... | 88.6 | 1e-17 |
| TR:A0A4P6EAP7A0A4P6EAP7_9MICO Aminoglycoside 3'-phosphotransferas... | 88.2 | 1e-17 |
| TR:A0A6I4P2F1A0A6I4P2F1_9MICO Phosphotransferase OS=Agromyces sp.... | 88.6 | 1e-17 |
| TR:A0A402AWP2A0A402AWP2_9CHLR APH domain-containing protein OS=Di... | 89.4 | 1e-17 |
| TR:M7N6T2M7N6T2_9MICC Aminoglycoside 3'-phosphotransferase OS=Pae... | 89.0 | 2e-17 |
| TR:R7WKW0R7WKW0_9NOCA APH domain-containing protein OS=Rhodococcu... | 87.8 | 2e-17 |
| TR:A0A0F0KM74A0A0F0KM74_9MICO Aminoglycoside 3'-phosphotransferas... | 87.8 | 2e-17 |
| TR:A0A3N1QT22A0A3N1QT22_9MICO Kanamycin kinase OS=Frondihabitans ... | 87.8 | 2e-17 |
| TR:A0A0M8MNW2A0A0M8MNW2_9MICO Aminoglycoside phosphotransferase O... | 87.4 | 3e-17 |
| TR:A0A1T5KQ69A0A1T5KQ69_9MICO Kanamycin kinase OS=Krasilnikoviell... | 87.4 | 3e-17 |
| TR:A0A1Q2CDL5A0A1Q2CDL5_9ACTN Kanamycin kinase OS=Tessaracoccus f... | 87.0 | 3e-17 |
| TR:A0A239J6B1A0A239J6B1_9MICO Kanamycin kinase OS=Micrococcales b... | 87.4 | 4e-17 |
| TR:A0A3N2BGP0A0A3N2BGP0_9MICO Kanamycin kinase OS=Bogoriella case... | 87.0 | 4e-17 |
| TR:A0A2M9BZZ8A0A2M9BZZ8_9MICO Kanamycin kinase OS=Compostimonas s... | 87.0 | 4e-17 |
| TR:A0A0E4CKY0A0A0E4CKY0_9MYCO Phosphotransferase OS=Mycobacterium... | 87.0 | 5e-17 |
| TR:A0A2W1TZZ4A0A2W1TZZ4_9MICO Aminoglycoside 3'-phosphotransferas... | 87.0 | 5e-17 |
| TR:A0A1N7FXR5A0A1N7FXR5_9ACTN Kanamycin kinase OS=Microbispora ro... | 86.7 | 6e-17 |
| TR:Q92N46Q92N46_RHIME Aminoglycoside 3'-phosphotransferase OS=Rhi... | 87.4 | 7e-17 |
| TR:A0A143WVJ8A0A143WVJ8_9FIRM Aminoglycoside 3'-phosphotransferas... | 86.3 | 1e-16 |
| TR:U1LKW4U1LKW4_9MICO Uncharacterized protein OS=Agrococcus pavil... | 85.5 | 1e-16 |
| TR:A0A4Q7PSR2A0A4Q7PSR2_9MICO Kanamycin kinase OS=Leifsonia sp. B... | 85.5 | 1e-16 |
| TR:A0A2T0Y0D2A0A2T0Y0D2_9MICO Kanamycin kinase OS=Isoptericola sp... | 85.5 | 1e-16 |
| TR:A0A6B2TZ19A0A6B2TZ19_9ACTN Phosphotransferase OS=Streptomyces ... | 84.3 | 2e-16 |
| TR:A8AVX1A8AVX1_STRGC 3''-aminoglycoside phosphotransferase-like ... | 85.5 | 2e-16 |
| TR:O07806O07806_MYCTU Possible phosphotransferase OS=Mycobacteriu... | 85.1 | 2e-16 |
| TR:A0A5J5L0M7A0A5J5L0M7_9MICC Aminoglycoside 3'-phosphotransferas... | 85.1 | 2e-16 |
| TR:A0A1G9LPD3A0A1G9LPD3_9ACTN Kanamycin kinase OS=Tessaracoccus o... | 84.7 | 2e-16 |
| TR:A0A1Y1XFQ1A0A1Y1XFQ1_9FUNG Aminoglycoside phosphotransferase O... | 85.1 | 3e-16 |
| TR:A0A4R0H4Z2A0A4R0H4Z2_9ACTN Aminoglycoside phosphotransferase O... | 84.0 | 3e-16 |
| TR:A0A1Q2CKW0A0A1Q2CKW0_9ACTN APH domain-containing protein OS=Te... | 84.3 | 3e-16 |
| TR:A0A1R4EVG4A0A1R4EVG4_9ACTO Aminoglycoside phosphotransferase O... | 84.7 | 3e-16 |
| TR:A0A498BT68A0A498BT68_9MICO Kanamycin kinase OS=Microbacterium ... | 84.3 | 4e-16 |
| TR:Q74I78Q74I78_LACJO APH domain-containing protein OS=Lactobacil... | 84.7 | 4e-16 |
| TR:A0A087DDT3A0A087DDT3_9BIFI Aminoglycoside phosphotransferase O... | 84.7 | 4e-16 |
| TR:A0A495ILW0A0A495ILW0_9MICO Kanamycin kinase OS=Frondihabitans ... | 83.6 | 7e-16 |

| TR:A0A3N4YLW5A0A3N4YLW5_9MICO Kanamycin kinase OS=Myceligenerans ... | 83.6 | 1e-15 |
| --- | --- | --- |
| TR:A0A4Y9FI64A0A4Y9FI64_9STRE Aminoglycoside 3'-phosphotransferas... | 83.2 | 1e-15 |
| TR:A0A143ZVT6A0A143ZVT6_9FIRM Aminoglycoside 3'-phosphotransferas... | 82.8 | 1e-15 |
| TR:R6BBM7R6BBM7_9CLOT ABC transporter related OS=Clostridium sp. ... | 85.5 | 2e-15 |
| TR:A0A1H1Z644A0A1H1Z644_9ACTN Kanamycin kinase OS=Microlunatus so... | 82.8 | 2e-15 |
| TR:A0A0T2L0U4A0A0T2L0U4_9MICO Aminoglycoside phosphotransferase O... | 82.4 | 2e-15 |
| TR:A0A2G5PEN7A0A2G5PEN7_9MYCO Aminoglycoside 3'-phosphotransferas... | 82.4 | 2e-15 |
| TR:A0A506XP55A0A506XP55_9MICO Phosphotransferase OS=Schumannella ... | 82.4 | 2e-15 |
| TR:A0A1I4T9U3A0A1I4T9U3_9BURK Aminoglycoside 3'-phosphotransferas... | 82.4 | 2e-15 |
| TR:D3QBL2D3QBL2_STANL Aminoglycoside phosphotransferase OS=Stacke... | 84.3 | 3e-15 |
| TR:A0A0U2WEG0A0A0U2WEG0_9BACL Aminoglycoside phosphotransferase O... | 82.0 | 3e-15 |
| TR:A0A1I7MG49A0A1I7MG49_9MICC Kanamycin kinase OS=Micrococcus ter... | 81.6 | 3e-15 |
| TR:A0A1X1QXF9A0A1X1QXF9_MYCFA APH domain-containing protein OS=My... | 81.6 | 3e-15 |
| TR:A0A1C0AID8A0A1C0AID8_9ACTN Uncharacterized protein OS=Tessarac... | 81.3 | 4e-15 |
| TR:A0A3D9UAT8A0A3D9UAT8_9GAMM Aminoglycoside 3'-phosphotransferas... | 81.3 | 6e-15 |
| TR:A0A2V3TQ51A0A2V3TQ51_9RHIZ Streptomycin 3'-kinase OS=Chelatoco... | 78.2 | 8e-15 |
| TR:T0JA54T0JA54_9SPHN APH domain-containing protein OS=Sphingobiu... | 78.2 | 8e-15 |
| TR:A0A0A1VE77A0A0A1VE77_9BURK Aminoglycoside phosphotransferase O... | 78.2 | 8e-15 |
| TR:A0A6B8VML3A0A6B8VML3_9CORY Aminoglycoside 3'-phosphotransferas... | 78.2 | 8e-15 |
| TR:A0A376BEX9A0A376BEX9_9PAST Streptomycin phosphotransferase Str... | 78.2 | 8e-15 |
| TR:A0A376C3I3A0A376C3I3_CITKO Aminoglycoside 3'-phosphotransferas... | 78.2 | 8e-15 |
| TR:A0A561BSL7A0A561BSL7_9ACTN Kanamycin kinase OS=Kribbella amoye... | 80.1 | 8e-15 |
| TR:A0A4Q8AH28A0A4Q8AH28_9MICC Kanamycin kinase OS=Zhihengliuella ... | 80.9 | 9e-15 |
| TR:A0A3P1VF18A0A3P1VF18_9STRE Aminoglycoside 3'-phosphotransferas... | 80.9 | 9e-15 |
| TR:K7S2Y9K7S2Y9_ACIA4 Aminoglycoside 3'-phosphotransferase OS=Aci... | 80.5 | 1e-14 |
| TR:A0A3G9JG38A0A3G9JG38_9BACL Aminoglycoside phosphotransferase A... | 80.9 | 1e-14 |
| TR:A0A3N4VI18A0A3N4VI18_9GAMM Aminoglycoside 3'-phosphotransferas... | 80.5 | 1e-14 |
| TR:F4H2N9F4H2N9_CELFA Aminoglycoside phosphotransferase OS=Cellul... | 80.5 | 1e-14 |
| TR:A0A318J1Z2A0A318J1Z2_9BURK Aminoglycoside 3'-phosphotransferas... | 80.5 | 2e-14 |
| TR:A0A0D1LE82A0A0D1LE82_9MYCO Aminoglycoside phosphotransferase O... | 79.3 | 2e-14 |
| TR:A0A2K4IX07A0A2K4IX07_9PSED Aminoglycoside 3'-phosphotransferas... | 79.7 | 2e-14 |
| TR:A0A5C8HR86A0A5C8HR86_9MICO Aminoglycoside 3'-phosphotransferas... | 79.0 | 4e-14 |
| TR:A0A4R4ZMC9A0A4R4ZMC9_9ACTN Aminoglycoside phosphotransferase O... | 78.2 | 4e-14 |
| TR:K9B0Y8K9B0Y8_9MICO 3''-kinase OS=Brevibacterium casei S18 OX=1... | 77.8 | 4e-14 |
| TR:A0A2A9E2H8A0A2A9E2H8_9MICO Kanamycin kinase OS=Sanguibacter an... | 79.0 | 4e-14 |
| TR:A0A255FX41A0A255FX41_9ACTN Aminoglycoside phosphotransferase A... | 78.6 | 5e-14 |
| TR:I3WKI2I3WKI2_BIFBI Putative aminoglycoside 3'-phospho transfer... | 79.0 | 5e-14 |
| TR:A0A087A046A0A087A046_9BIFI Aminoglycoside transferase OS=Bifid... | 79.0 | 5e-14 |
| TR:A0A1Q2CUC6A0A1Q2CUC6_9ACTN APH domain-containing protein OS=Te... | 78.2 | 6e-14 |
| TR:A0A420XVK2A0A420XVK2_9ACTN Kanamycin kinase OS=Motilibacter pe... | 78.2 | 7e-14 |
| TR:A0A261GCG4A0A261GCG4_9BIFI Aminoglycoside phosphotransferase A... | 78.6 | 7e-14 |
| TR:A0A367Y1N8A0A367Y1N8_9MICO Aminoglycoside 3'-phosphotransferas... | 78.2 | 7e-14 |
| TR:A0A2P8H182A0A2P8H182_9MICO Kanamycin kinase OS=Labedella gwakj... | 78.2 | 7e-14 |
| TR:I4BQY4I4BQY4_MYCCN Aminoglycoside phosphotransferase OS=Mycoba... | 77.8 | 8e-14 |
| TR:A0A4P6FDL8A0A4P6FDL8_9MICO Aminoglycoside 3'-phosphotransferas... | 78.2 | 8e-14 |
| TR:A0A261FXL1A0A261FXL1_9BIFI Aminoglycoside phosphotransferase A... | 78.2 | 9e-14 |
| TR:L7KMM7L7KMM7_9ACTN APH domain-containing protein OS=Gordonia a... | 77.8 | 9e-14 |
| TR:E1M643E1M643_9STRE Probable aminoglycoside 3'-phosphotransfera... | 77.4 | 1e-13 |
| TR:A0A1T2XFY3A0A1T2XFY3_9BACL APH domain-containing protein OS=Pa... | 77.4 | 2e-13 |
| TR:A0A061A9T2A0A061A9T2_9MOLU Similar to aminoglycoside 3-phospho... | 77.0 | 2e-13 |
| TR:S3N4B0S3N4B0_9GAMM Aminoglycoside 3'-phosphotransferase OS=Aci... | 77.0 | 2e-13 |
| TR:A0A1B8UWG8A0A1B8UWG8_9BACL APH domain-containing protein OS=Pa... | 77.0 | 3e-13 |
| TR:A0A1X3P9Y9A0A1X3P9Y9_9MICC APH domain-containing protein OS=Ne... | 77.0 | 3e-13 |
| TR:A0A430F6Q0A0A430F6Q0_9BIFI Aminoglycoside phosphotransferase A... | 76.6 | 3e-13 |
| TR:A0A077PK36A0A077PK36_XENBV Aminoglycoside 3'-phosphotransferas... | 76.3 | 4e-13 |
| TR:A0A0E4CT85A0A0E4CT85_9STRE 3'-aminoglycoside phosphotransferas... | 76.3 | 4e-13 |
| TR:R7ICL2R7ICL2_9FIRM Putative kanamycin kinase OS=Faecalibacteri... | 76.3 | 4e-13 |
| TR:E1VSE1E1VSE1_GLUAR Putative phosphotransferase OS=Glutamicibac... | 75.9 | 5e-13 |
| TR:A0A1H1TMJ1A0A1H1TMJ1_9ACTN Kanamycin kinase OS=Actinopolymorph... | 75.5 | 5e-13 |
| TR:W9B1C5W9B1C5_9MYCO Streptomycin phosphotransferase OS=Mycolici... | 73.2 | 6e-13 |
| TR:R5A6E5R5A6E5_9CLOT ABC transporter ATP-binding protein OS=Clos... | 77.8 | 6e-13 |
| TR:A3CPV7A3CPV7_STRSV Aminoglycoside phosphotransferase, putative... | 75.5 | 8e-13 |
| TR:A0A1X0D9W6A0A1X0D9W6_9MYCO APH domain-containing protein OS=My... | 75.1 | 9e-13 |
| TR:A0A085HJL1A0A085HJL1_9GAMM Aminoglycoside 3-phosphotransferase... | 72.0 | 9e-13 |
| TR:A0A143XCL0A0A143XCL0_9ACTN Aminoglycoside 3'-phosphotransferas... | 75.1 | 1e-12 |
| TR:A0A496PJQ8A0A496PJQ8_9MICC Aminoglycoside 3'-phosphotransferas... | 75.1 | 1e-12 |
| TR:B2GFM3B2GFM3_KOCRD APH domain-containing protein OS=Kocuria rh... | 74.3 | 1e-12 |
| TR:A0A1A3BRM6A0A1A3BRM6_9MYCO Aminoglycoside phosphotransferase A... | 73.9 | 2e-12 |
| TR:H3SH47H3SH47_9BACL Aminoglycoside 3'-phosphotransferase OS=Pae... | 74.7 | 2e-12 |
| TR:A0A1R4GFE7A0A1R4GFE7_9MICO Aminoglycoside phosphotransferase O... | 73.6 | 2e-12 |
| TR:A0A559K373A0A559K373_9BACL Aminoglycoside 3'-phosphotransferas... | 73.9 | 2e-12 |
| TR:A0A3E0I5S8A0A3E0I5S8_9PSEU Kanamycin kinase OS=Kutzneria burir... | 72.4 | 3e-12 |

| TR:Q5FJE9Q5FJE9_LACAC Kanamycin kinase OS=Lactobacillus acidophil... | 73.9 | 3e-12 |
| --- | --- | --- |
| TR:R9MHL6R9MHL6_9FIRM APH domain-containing protein OS=Lachnospir... | 74.3 | 3e-12 |
| TR:A0A0W8IGL2A0A0W8IGL2_9MICC APH domain-containing protein OS=Ne... | 73.9 | 3e-12 |
| TR:X8EWZ9X8EWZ9_MYCUL Phosphotransferase enzyme family protein OS... | 70.1 | 4e-12 |
| TR:A0A1R1IKH5A0A1R1IKH5_9ACTN APH domain-containing protein OS=Te... | 72.8 | 5e-12 |
| TR:A0A6I1GCI4A0A6I1GCI4_9BIFI Aminoglycoside phosphotransferase A... | 73.2 | 6e-12 |
| TR:H3SLV1H3SLV1_9BACL Aminoglycoside 3'-phosphotransferase OS=Pae... | 72.8 | 6e-12 |
| TR:A0A2V5JWE1A0A2V5JWE1_9BACL Aminoglycoside 3'-phosphotransferas... | 72.4 | 8e-12 |
| SP:P14508KKA7_CAMJU Aminoglycoside 3'-phosphotransferase OS=Campy... | 72.4 | 9e-12 |
| TR:A0A4Q2LP52A0A4Q2LP52_9BACL Aminoglycoside 3'-phosphotransferas... | 71.6 | 2e-11 |
| TR:A0A2A9CT09A0A2A9CT09_9ACTN Kanamycin kinase OS=Propionicimonas... | 71.6 | 2e-11 |
| TR:A0A261UAX2A0A261UAX2_9BORD Aminoglycoside 3'-phosphotransferas... | 71.2 | 2e-11 |
| TR:A0A087BM59A0A087BM59_9BIFI Aminoglycoside phosphotransferase O... | 72.0 | 3e-11 |
| TR:A0A3L9DLW9A0A3L9DLW9_9STRE Aminoglycoside 3'-phosphotransferas... | 71.2 | 3e-11 |
| TR:W7SQN8W7SQN8_9PSEU Mucin-7 OS=Kutzneria sp. 744 OX=345341 GN=K... | 69.7 | 3e-11 |
| TR:A0A6I5VUW7A0A6I5VUW7_9ACTN Phosphotransferase OS=Microlunatus ... | 70.5 | 4e-11 |
| TR:A0A0T1WEX7A0A0T1WEX7_9MYCO Aminoglycoside phosphotransferase O... | 70.1 | 4e-11 |
| TR:A0A089IMG6A0A089IMG6_9BACL APH domain-containing protein OS=Pa... | 70.5 | 5e-11 |
| TR:W0ZAR3W0ZAR3_9MICO Aminoglycoside phosphotransferase OS=Microb... | 70.1 | 5e-11 |
| TR:A0A0J6VHZ2A0A0J6VHZ2_MYCCU Aminoglycoside 3'-phosphotransferas... | 66.2 | 6e-11 |
| TR:A0A380KYX1A0A380KYX1_9STRE 3'-aminoglycoside phosphotransferas... | 68.9 | 8e-11 |
| TR:D3E898D3E898_GEOS4 Kanamycin kinase OS=Geobacillus sp. (strain... | 69.7 | 9e-11 |
| TR:I7LEC4I7LEC4_9LACO Kanamycin kinase OS=Lactobacillus pasteurii... | 69.7 | 9e-11 |
| TR:A0A4Q2LL01A0A4Q2LL01_9BACL APH domain-containing protein OS=Pa... | 69.3 | 1e-10 |
| TR:A0A0Q5VPB9A0A0Q5VPB9_9ACTN APH domain-containing protein OS=Ge... | 68.6 | 2e-10 |
| TR:U2EHS1U2EHS1_9FIRM APH domain-containing protein OS=Clostridia... | 67.4 | 3e-10 |
| TR:A0A255EHV5A0A255EHV5_9ACTN Aminoglycoside phosphotransferase A... | 68.2 | 3e-10 |
| TR:A0A1R4IYH0A0A1R4IYH0_9ACTN Aminoglycoside phosphotransferase O... | 63.5 | 6e-10 |
| TR:A0A3P1WTF6A0A3P1WTF6_9ACTN Aminoglycoside 3'-phosphotransferas... | 67.0 | 6e-10 |
| TR:Q091V1Q091V1_STIAD Adenylate kinase OS=Stigmatella aurantiaca ... | 68.9 | 6e-10 |
| TR:A0A5A7WYA1A0A5A7WYA1_9MYCO Aminoglycoside 3'-phosphotransferas... | 66.6 | 7e-10 |
| TR:A0A3S4E288A0A3S4E288_9MICO Aminoglycoside 3'-phosphotransferas... | 66.6 | 1e-09 |
| TR:A0A0D6SWE9A0A0D6SWE9_9PSED Aminoglycoside 3'-phosphotransferas... | 66.2 | 2e-09 |
| TR:F9PXB1F9PXB1_9STRE Phosphotransferase enzyme family protein OS... | 66.2 | 2e-09 |
| TR:A0A1W9EZW0A0A1W9EZW0_9GAMM Aminoglycoside phosphotransferase (... | 62.0 | 4e-09 |
| SP:P09885KKA6_ACIBA Aminoglycoside 3'-phosphotransferase OS=Acine... | 65.1 | 4e-09 |
| TR:A0A2S2F8N6A0A2S2F8N6_9GAMM Aminoglycoside 3'-phosphotransferas... | 65.1 | 4e-09 |
| TR:A0A1I4QCZ7A0A1I4QCZ7_9BACL Kanamycin kinase OS=Paenibacillus s... | 64.7 | 4e-09 |
| TR:T0S7I7T0S7I7_LACLC Aminoglycoside 3'-phosphotransferase OS=Lac... | 64.7 | 6e-09 |
| TR:A0A139P0N2A0A139P0N2_9STRE Putative aminoglycoside 3'-phosphot... | 63.2 | 2e-08 |
| TR:A0A1Y3NNY6A0A1Y3NNY6_PIRSE APH domain-containing protein OS=Pi... | 62.4 | 3e-08 |
| TR:A0A4R0QNW0A0A4R0QNW0_9STRE Aminoglycoside 3'-phosphotransferas... | 62.0 | 5e-08 |
| TR:B0N2T1B0N2T1_9FIRM Phosphotransferase enzyme family OS=Erysipe... | 61.2 | 8e-08 |
| TR:A0A1H3E0P0A0A1H3E0P0_9FIRM Phosphotransferase enzyme family pr... | 56.6 | 2e-07 |
| TR:D7CA47D7CA47_STRBB Phosphotransferase OS=Streptomyces bingchen... | 58.5 | 2e-07 |
| TR:A0A1G7E8N1A0A1G7E8N1_9ACTN Kanamycin kinase OS=Auraticoccus mo... | 60.1 | 2e-07 |
| SP:Q02149KKIH_LACLA Probable aminoglycoside 3'-phosphotransferase... | 59.7 | 3e-07 |
| TR:A0A397RZZ5A0A397RZZ5_9MOLU Kanamycin kinase/aminoglycoside 3'-... | 58.9 | 6e-07 |
| TR:M7NGN9M7NGN9_9BACL Aminoglycoside 3'-phosphotransferase OS=Bha... | 58.2 | 1e-06 |
| TR:A0A229UZF4A0A229UZF4_9BACL Aminoglycoside phosphotransferase O... | 57.8 | 1e-06 |
| TR:A0A3G9IW69A0A3G9IW69_9BACL Putative aminoglycoside 3'-phosphot... | 57.8 | 1e-06 |
| TR:A0A4R5KJH0A0A4R5KJH0_9BACL Aminoglycoside 3'-phosphotransferas... | 57.4 | 2e-06 |
| TR:A0A5S5CJF0A0A5S5CJF0_9BACL Kanamycin kinase OS=Paenibacillus m... | 57.0 | 2e-06 |
| TR:G5K1G0G5K1G0_9STRE Phosphotransferase enzyme family protein OS... | 57.0 | 3e-06 |
| TR:A0A089IMY8A0A089IMY8_9BACL Cyclic nucleotide-binding domain-co... | 54.3 | 3e-06 |
| TR:A0A261G110A0A261G110_9BIFI Aminoglycoside phosphotransferase O... | 56.6 | 4e-06 |
| TR:C5C0L7C5C0L7_BEUC1 Aminoglycoside phosphotransferase OS=Beuten... | 55.8 | 1e-05 |
| TR:A0A117V3D2A0A117V3D2_9SPHN Tyrosine protein kinase OS=Novosphi... | 55.8 | 1e-05 |
| TR:A0A1T5M197A0A1T5M197_9CLOT Kanamycin kinase/aminoglycoside 3'-... | 52.0 | 2e-05 |
| TR:A0A0C2RMP2A0A0C2RMP2_9BACL Amino acid transporter OS=Jeotgalib... | 54.3 | 2e-05 |
| TR:W9XP08W9XP08_9EURO APH domain-containing protein OS=Capronia c... | 54.7 | 3e-05 |
| TR:W7YSI2W7YSI2_9BACI APH domain-containing protein OS=Bacillus s... | 53.5 | 4e-05 |
| TR:A0A4R5XJ42A0A4R5XJ42_9BACL Aminoglycoside phosphotransferase f... | 53.5 | 4e-05 |
| TR:A0A093YDZ5A0A093YDZ5_9PEZI APH domain-containing protein OS=Ps... | 54.3 | 4e-05 |
| TR:A0A4V1R0D7A0A4V1R0D7_9BACL Aminoglycoside phosphotransferase f... | 53.5 | 4e-05 |
| TR:A0A0D9P141A0A0D9P141_METAN APH domain-containing protein OS=Me... | 53.9 | 4e-05 |
| TR:A0A402CZI9A0A402CZI9_9BACT APH domain-containing protein OS=Ca... | 51.6 | 1e-04 |
| TR:L8H2T2L8H2T2_ACACA Phosphotransferase enzyme family protein OS... | 52.4 | 1e-04 |
| TR:A0A4V3F714A0A4V3F714_9ACTN Putative kinase, aminoglycoside pho... | 52.0 | 2e-04 |
| TR:A0A4R6BS11A0A4R6BS11_9STAP Uncharacterized protein OS=Macrococ... | 48.1 | 2e-04 |
| TR:A0A094A129A0A094A129_9PEZI APH domain-containing protein OS=Ps... | 52.0 | 2e-04 |
| TR:M1W5L6M1W5L6_CLAP2 APH domain-containing protein OS=Claviceps ... | 52.0 | 2e-04 |

| TR:M1W247M1W247_CLAP2 APH domain-containing protein OS=Claviceps ... | 52.0 | 2e-04 |
| --- | --- | --- |
| TR:A0A6A9UZI0A0A6A9UZI0_9ACTN Phosphotransferase OS=Auraticoccus ... | 51.2 | 2e-04 |
| TR:Q2GBP4Q2GBP4_NOVAD Aminoglycoside phosphotransferase OS=Novosp... | 51.6 | 3e-04 |
| TR:M1WFZ8M1WFZ8_CLAP2 APH domain-containing protein OS=Claviceps ... | 51.6 | 3e-04 |
| TR:A0A561TSL2A0A561TSL2_9ACTN Aminoglycoside phosphotransferase (... | 50.8 | 4e-04 |
| TR:M1W4L6M1W4L6_CLAP2 APH domain-containing protein OS=Claviceps ... | 51.2 | 4e-04 |
| TR:A0A093X7R1A0A093X7R1_9PEZI APH domain-containing protein OS=Ps... | 51.2 | 4e-04 |
| TR:A0A069DJ88A0A069DJ88_9BACL APH domain-containing protein OS=Pa... | 50.4 | 4e-04 |
| TR:A0A1H0JY26A0A1H0JY26_9BACI Phosphotransferase enzyme family pr... | 50.4 | 4e-04 |
| TR:A0A0L0N1K5A0A0L0N1K5_TOLOC APH domain-containing protein OS=To... | 50.8 | 5e-04 |
| TR:A0A1M7EAK1A0A1M7EAK1_9STAP Aminoglycoside phosphotransferase O... | 49.7 | 6e-04 |
| TR:A0A0N1BWN5A0A0N1BWN5_9SPHN Tyrosine protein kinase OS=Novosphi... | 50.4 | 7e-04 |
| TR:A0A1I4R443A0A1I4R443_9BACL Aminoglycoside phosphotransferase O... | 49.7 | 8e-04 |

```
>SP:P00552 KKA2_KLEPN Aminoglycoside 3'-phosphotransferase OS=Klebsiella pneumoniae OX=573 GN=neo PE=1 SV=1
Length=264

Score = 535 bits (1378), Expect = 0.0
Identities = 264/264 (100%), Positives = 264/264 (100%), Gaps = 0/264 (0%)

Query 1	MIEQDGLHAGSPAAWVERLFGYDWAQQTIGCSDAAVFRLSAQGRPVLFVKTDLSGALNEL 60 MIEQDGLHAGSPAAWVERLFGYDWAQQTIGCSDAAVFRLSAQGRPVLFVKTDLSGALNEL
Sbjct 1	MIEQDGLHAGSPAAWVERLFGYDWAQQTIGCSDAAVFRLSAQGRPVLFVKTDLSGALNEL 60

Query 61	QDEAARLSWLATTGVPCAAVLDVVTEAGRDWLLLGEVPGQDLLSSHLAPAEKVSIMADAM 120 QDEAARLSWLATTGVPCAAVLDVVTEAGRDWLLLGEVPGQDLLSSHLAPAEKVSIMADAM
Sbjct 61	QDEAARLSWLATTGVPCAAVLDVVTEAGRDWLLLGEVPGQDLLSSHLAPAEKVSIMADAM 120

Query 121 RRLHTLDPATCPFDHQAKHRIERARTRMEAGLVDQDDLDEEHQGLAPAELFARLKARMPD 180 RRLHTLDPATCPFDHQAKHRIERARTRMEAGLVDQDDLDEEHQGLAPAELFARLKARMPD
Sbjct 121 RRLHTLDPATCPFDHQAKHRIERARTRMEAGLVDQDDLDEEHQGLAPAELFARLKARMPD 180

Query 181 GEDLVVTHGDACLPNIMVENGRFSGFIDCGRLGVADRYQDIALATRDIAEELGGEWADRF 240 GEDLVVTHGDACLPNIMVENGRFSGFIDCGRLGVADRYQDIALATRDIAEELGGEWADRF
Sbjct 181 GEDLVVTHGDACLPNIMVENGRFSGFIDCGRLGVADRYQDIALATRDIAEELGGEWADRF 240

Query 241 LVLYGIAAPDSQRIAFYRLLDEFF 264 LVLYGIAAPDSQRIAFYRLLDEFF
Sbjct 241 LVLYGIAAPDSQRIAFYRLLDEFF 264

>TR:A0A149VWA2 A0A149VWA2_9PROT Aminoglycoside 3'-phosphotransferase OS=Ferrovum sp. Z-31 OX=1789004 GN=neo PE=3 SV=1
Length=264

Score = 533 bits (1372), Expect = 0.0
```

| Identities | = 263/264 (99%), Positives = 263/264 (99%), Gaps = 0/264 (0%) |  |
| --- | --- | --- |
| Query 1 | MIEQDGLHAGSPAAWVERLFGYDWAQQTIGCSDAAVFRLSAQGRPVLFVKTDLSGALNEL | 60 |
| Sbjct 1 | MIEQDGLHAGSPAAWVERLFGYDWAQQTIGCSDAAVFRLSAQGRPVLFVKTDLSGALNEL MIEQDGLHAGSPAAWVERLFGYDWAQQTIGCSDAAVFRLSAQGRPVLFVKTDLSGALNEL | 60 |
| Query 61 | QDEAARLSWLATTGVPCAAVLDVVTEAGRDWLLLGEVPGQDLLSSHLAPAEKVSIMADAM | 120 |
| Sbjct 61 | QDEAARLSWLATTGVPCAAVLDVVTEAGRDWLLLGEVPGQDLLSSHLAPAEKVSIMADAM QDEAARLSWLATTGVPCAAVLDVVTEAGRDWLLLGEVPGQDLLSSHLAPAEKVSIMADAM | 120 |
| Query 121 | RRLHTLDPATCPFDHQAKHRIERARTRMEAGLVDQDDLDEEHQGLAPAELFARLKARMPD | 180 |
| Sbjct 121 | RRLHTLDPATCPFDHQAKHRIERARTRMEAGLVDQDDLDEEHQGLAPAELFARLKA MPD RRLHTLDPATCPFDHQAKHRIERARTRMEAGLVDQDDLDEEHQGLAPAELFARLKASMPD | 180 |
| Query 181 | GEDLVVTHGDACLPNIMVENGRFSGFIDCGRLGVADRYQDIALATRDIAEELGGEWADRF | 240 |
| Sbjct 181 | GEDLVVTHGDACLPNIMVENGRFSGFIDCGRLGVADRYQDIALATRDIAEELGGEWADRF GEDLVVTHGDACLPNIMVENGRFSGFIDCGRLGVADRYQDIALATRDIAEELGGEWADRF | 240 |
| Query 241  Sbjct 241 | LVLYGIAAPDSQRIAFYRLLDEFF 264 LVLYGIAAPDSQRIAFYRLLDEFF LVLYGIAAPDSQRIAFYRLLDEFF 264 |  |

>TR:A0A2P7P4I2 A0A2P7P4I2_9GAMM Aminoglycoside 3'-phosphotransferase OS=Halomonas

sp. ND22Bw OX=2054178 GN=aph(3')-II PE=3 SV=1

Length=264

Score = 533 bits (1372), Expect = 0.0

| Identities | = 263/264 (99%), Positives = 263/264 (99%), Gaps = 0/264 (0%) |  |
| --- | --- | --- |
| Query 1 | MIEQDGLHAGSPAAWVERLFGYDWAQQTIGCSDAAVFRLSAQGRPVLFVKTDLSGALNEL | 60 |
| Sbjct 1 | MIEQDGLHAGSPAAWVERLFGYDWAQQTIGCSDAAVFRLSAQGRPVLFVKTDLSGALNEL MIEQDGLHAGSPAAWVERLFGYDWAQQTIGCSDAAVFRLSAQGRPVLFVKTDLSGALNEL | 60 |
| Query 61 | QDEAARLSWLATTGVPCAAVLDVVTEAGRDWLLLGEVPGQDLLSSHLAPAEKVSIMADAM | 120 |
| Sbjct 61 | QDEAARLSWLATTGVPCAAVLDVVTEAGRDWLLLGEVPGQDLLSSHLAPAEKVSIMADAM QDEAARLSWLATTGVPCAAVLDVVTEAGRDWLLLGEVPGQDLLSSHLAPAEKVSIMADAM | 120 |
| Query 121 | RRLHTLDPATCPFDHQAKHRIERARTRMEAGLVDQDDLDEEHQGLAPAELFARLKARMPD | 180 |
| Sbjct 121 | RRLHTLDPATCPFDHQAKHRIERARTRMEAGLVDQDDLDEEHQGLAPAELFARLKA MPD RRLHTLDPATCPFDHQAKHRIERARTRMEAGLVDQDDLDEEHQGLAPAELFARLKASMPD | 180 |
| Query 181 | GEDLVVTHGDACLPNIMVENGRFSGFIDCGRLGVADRYQDIALATRDIAEELGGEWADRF | 240 |
| Sbjct 181 | GEDLVVTHGDACLPNIMVENGRFSGFIDCGRLGVADRYQDIALATRDIAEELGGEWADRF GEDLVVTHGDACLPNIMVENGRFSGFIDCGRLGVADRYQDIALATRDIAEELGGEWADRF | 240 |
| Query 241  Sbjct 241 | LVLYGIAAPDSQRIAFYRLLDEFF 264 LVLYGIAAPDSQRIAFYRLLDEFF LVLYGIAAPDSQRIAFYRLLDEFF 264 |  |

>TR:A0A2U1C3P1 A0A2U1C3P1_9BURK Aminoglycoside 3'-phosphotransferase OS=Simplicispira sp. 125 OX=2135643 GN=C8D04_0693 PE=3 SV=1

Length=260

Score = 484 bits (1245), Expect = 2e-172

| Identities | = 241/264 (91%), Positives = 245/264 (93%), Gaps = 4/264 (2%) |  |
| --- | --- | --- |
| Query 1 | MIEQDGLHAGSPAAWVERLFGYDWAQQTIGCSDAAVFRLSAQGRPVLFVKTDLSGALNEL | 60 |
| Sbjct 1 | MIEQDGLHAG PAAWVERL YDWA Q IGCS AAVFRLSAQGRPVLFVKTDLSGALNEL MIEQDGLHAGPPAAWVERLSSYDWAHQAIGCSHAAVFRLSAQGRPVLFVKTDLSGALNEL | 60 |
| Query 61 | QDEAARLSWLATTGVPCAAVLDVVTEAGRDWLLLGEVPGQDLLSSHLAPAEKVSIMADAM | 120 |
| Sbjct 61 | QDEAARLSWLATTGVPCA VLDVVTEAG DWLLLGEVPGQDLLSSHLAPAEKVS+MADA+ QDEAARLSWLATTGVPCATVLDVVTEAGTDWLLLGEVPGQDLLSSHLAPAEKVSLMADAL | 120 |
| Query 121 | RRLHTLDPATCPFDHQAKHRIERARTRMEAGLVDQDDLDEEHQGLAPAELFARLKARMPD | 180 |
| Sbjct 121 | RRLH LDPA CPFDHQAKHR+ERAR RMEAGLVDQDDLDE HQGLAPAELFARLKAR+PD RRLHALDPAACPFDHQAKHRVERARARMEAGLVDQDDLDEAHQGLAPAELFARLKARVPD | 180 |
| Query 181 | GEDLVVTHGDACLPNIMVENGRFSGFIDCGRLGVADRYQDIALATRDIAEELGGEWADRF | 240 |
| Sbjct 181 | GEDLV DACLPNIMVENGRFSGFIDCGRLGVADRYQDIALATRDIAEELGGEWADRF GEDLV DACLPNIMVENGRFSGFIDCGRLGVADRYQDIALATRDIAEELGGEWADRF | 236 |
| Query 241  Sbjct 237 | LVLYGIAAPDSQRIAFYRLLDEFF 264 LV YGI A DSQRIAFYRLLDEFF LVRYGIGASDSQRIAFYRLLDEFF 260 |  |

>TR:I0V5U7 I0V5U7_9PSEU Aminoglycoside phosphotransferase OS=Saccharomonospora xinjiangensis XJ-54 OX=882086 GN=SacxiDRAFT_3295 PE=3

SV=1

Length=263

Score = 354 bits (909), Expect = 3e-121

Identities = 170/253 (67%), Positives = 203/253 (80%), Gaps = 0/253 (0%)

| Query | 12 | PAAWVERLFGYDWAQQTIGCSDAAVFRLSAQGRPVLFVKTDLSGALNELQDEAARLSWLA | 71 |
| --- | --- | --- | --- |
| Sbjct | 11 | P AW ERL Y+W QTIGCS+AAV+RL ++G+P LF+KT+ +G L+EL EAARL WL+ PKAWKERLTSYEWVLQTIGCSEAAVYRLDSEGKPSLFIKTEPAGPLSELPGEAARLRWLS | 70 |
| Query | 72 | TTGVPCAAVLDVVTEAGRDWLLLGEVPGQDLLSSHLAPAEKVSIMADAMRRLHTLDPATC | 131 |
| Sbjct | 71 | T VPCA VLD V EAG+ WLLL VPG+DLLS+ + PA KV+I+A A+ LH LDP TC MTEVPCADVLDEVREAGQHWLLLSAVPGRDLLSASIDPASKVTILAQALHHLHRLDPTTC | 130 |
| Query | 132 | PFDHQAKHRIERARTRMEAGLVDQDDLDEEHQGLAPAELFARLKARMPDGEDLVVTHGDA | 191 |

PFDH+A+HR+E+AR R++AGLVDQDDLDEE+Q +	+LF L+AR P EDLVVTHGDA

| Sbjct | 131 | PFDHRAEHRVEQARARLDAGLVDQDDLDEENQNVELDDLFTELRARKPRREDLVVTHGDA | 190 |
| --- | --- | --- | --- |
| Query | 192 | CLPNIMVENGRFSGFIDCGRLGVADRYQDIALATRDIAEELGGEWADRFLVLYGIAAPDS | 251 |
| Sbjct | 191 | CLPNIMVE GRFSGFIDCGRLG+ADRYQD+ALATRDIAEELG +W FL YGI D CLPNIMVEKGRFSGFIDCGRLGLADRYQDVALATRDIAEELGEKWVPLFLDQYGIPELDG | 250 |
| Query  Sbjct | 252  251 | QRIAFYRLLDEFF 264  +RIAFYRLLDEF+  ERIAFYRLLDEFY 263 |  |

>TR:A0A0Q9I9B9 A0A0Q9I9B9_9BRAD Aminoglycoside 3'-phosphotransferase OS=Bosea sp. Root483D1 OX=1736544 GN=ASE66_24560 PE=3 SV=1

Length=257

Score = 348 bits (892), Expect = 9e-119

| Identities | | = 166/254 (65%), Positives = 200/254 (79%), Gaps = 0/254 (0%) |  |
| --- | --- | --- | --- |
| Query 11  Sbjct 4 | | SPAAWVERLFGYDWAQQTIGCSDAAVFRLSAQGRPVLFVKTDLSGALNELQDEAARLSWL  +P AW R+ Y W QQT+GCSDA VFRL G+P LFVKT+ + ++E+ DE RL WL APEAWKGRIAEYSWVQQTLGCSDAGVFRLVGNGKPPLFVKTEPASPMSEVPDEGNRLRWL | 70  63 |
| Query 71  Sbjct 64 | | ATTGVPCAAVLDVVTEAGRDWLLLGEVPGQDLLSSHLAPAEKVSIMADAMRRLHTLDPAT A+ G+P A V+D +EAGR WLLL VPG DLLS+ L P +K+ ++ADA+R LH LD A+ ASAGIPGAQVIDEASEAGRHWLLLSAVPGLDLLSASLEPLKKMEVIADALRLLHRLDTAS | 130  123 |
| Query 131  Sbjct 124 | | CPFDHQAKHRIERARTRMEAGLVDQDDLDEEHQGLAPAELFARLKARMPDGEDLVVTHGD CPFDH+A+HRI+RA+ RM AGLVD+DDLDEEHQGL P +LFARLKAR P EDLVVTHGD CPFDHRAEHRIQRAQARMLAGLVDEDDLDEEHQGLLPIDLFARLKARQPTHEDLVVTHGD | 190  183 |
| Query 191  Sbjct 184 | | ACLPNIMVENGRFSGFIDCGRLGVADRYQDIALATRDIAEELGGEWADRFLVLYGIAAPD ACLPNIMV+ G+FSGFIDCGRLGVADRYQD+AL TRDIAEELG ++ FL YG+A D ACLPNIMVDGGKFSGFIDCGRLGVADRYQDLALTTRDIAEELGDDFVKPFLERYGVAHLD | 250  243 |
| Query 251 | | SQRIAFYRLLDEFF 264 R+AFYRLLDEF+ |  |
| Sbjct | 244 | PDRVAFYRLLDEFY 257 | |

>TR:W1SA25 W1SA25_9BACI Aminoglycoside phosphotransferase (Fragment) OS=Bacillus vireti LMG 21834 OX=1131730 GN=BAVI_25314 PE=3 SV=1

Length=164

Score = 330 bits (846), Expect = 3e-113

| Identities | = 162/164 (99%), Positives = 162/164 (99%), Gaps = 0/164 (0%) |  |
| --- | --- | --- |
| Query 58 | NELQDEAARLSWLATTGVPCAAVLDVVTEAGRDWLLLGEVPGQDLLSSHLAPAEKVSIMA | 117 |
| Sbjct 1 | NELQDEAARLSWLATTGVPCAAVLDVVTEAGRDWLLLGEVPGQDLLSSHLAPAEKVSIMA NELQDEAARLSWLATTGVPCAAVLDVVTEAGRDWLLLGEVPGQDLLSSHLAPAEKVSIMA | 60 |
| Query 118 | DAMRRLHTLDPATCPFDHQAKHRIERARTRMEAGLVDQDDLDEEHQGLAPAELFARLKAR | 177 |
| Sbjct 61 | DAMRRLHTLDPATCPFDHQAKHRIERARTRMEAGLVDQDDLDEEHQGLAPAELFARLKA DAMRRLHTLDPATCPFDHQAKHRIERARTRMEAGLVDQDDLDEEHQGLAPAELFARLKAS | 120 |
| Query 178  Sbjct 121 | MPDGEDLVVTHGDACLPNIMVENGRFSGFIDCGRLGVADRYQDI 221 MPDGEDLVVTHGDACLPNIMVENGRFSGFIDCGRLGVADRYQ I MPDGEDLVVTHGDACLPNIMVENGRFSGFIDCGRLGVADRYQGI 164 |  |

>TR:A0A098U4E4 A0A098U4E4_9BURK Aminoglycoside 3'-phosphotransferase OS=Massilia sp. JS1662 OX=1519190 GN=IA69_26515 PE=3 SV=1

Length=255

Score = 332 bits (852), Expect = 1e-112

Identities = 164/254 (65%), Positives = 193/254 (76%), Gaps = 1/254 (0%)

| Query | 11 | SPAAWVERLFGYDWAQQTIGCSDAAVFRLSAQGRPVLFVKTDLSGALNELQDEAARLSWL | 70 |
| --- | --- | --- | --- |
|  |  | +P AW RL GY W +Q IGCS+A VFRL A RP+LF K + +G L EL+DE RL WL |  |
| Sbjct | 3 | TPTAWKTRLAGYRWTRQDIGCSEADVFRLDAPDRPILFAKFEPAGPLAELRDEGERLRWL | 62 |
| Query | 71 | ATTGVPCAAVLDVVTEAGRDWLLLGEVPGQDLLSSHLAPAEKVSIMADAMRRLHTLDPAT | 130 |

| Sbjct | 63 | A TG+PCA VLD TE G DWLLLG VPG+DLL++ L PA V +A A+R LH LDPAT AGTGIPCAPVLDTATEDGHDWLLLGAVPGRDLLAAGLEPAVAVERLAAALRALHALDPAT | 122 |
| --- | --- | --- | --- |
| Query | 131 | CPFDHQAKHRIERARTRMEAGLVDQDDLDEEHQGLAPAELFARLKARMPDGEDLVVTHGD | 190 |
| Sbjct | 123 | CPFDH+A RIERAR RMEAGLVD+DDLD+EHQG+APA LF RL A+ P EDLVVTHGD CPFDHRAARRIERARARMEAGLVDEDDLDDEHQGMAPAALFDRLAAQAPRSEDLVVTHGD | 182 |
| Query | 191 | ACLPNIMVENGRFSGFIDCGRLGVADRYQDIALATRDIAEELGGEWADRFLVLYGIAAPD | 250 |
| Sbjct | 183 | ACLPN++V+ +G+IDCGRLGVADR+QD+ALATRDIA+ELG W FL YG D ACLPNLIVDAAGRTGWIDCGRLGVADRWQDLALATRDIADELGEAWVVPFLAAYGTTF-D | 241 |
| Query  Sbjct | 251  242 | SQRIAFYRLLDEFF 264  + + +FYRLLDEFF  AAKSSFYRLLDEFF 255 |  |

>TR:A0A160DZL2 A0A160DZL2_9GAMM Aminoglycoside 3'-phosphotransferase OS=Dokdonella koreensis DS-123 OX=1300342 GN=I596_3655 PE=3 SV=1

Length=263

Score = 313 bits (802), Expect = 5e-105

| Identities | | = 159/253 (63%), Positives = 178/253 (70%), Gaps = 1/253 (0%) |  |
| --- | --- | --- | --- |
| Query 12  Sbjct 12 | | PAAWVERLFGYDWAQQTIGCSDAAVFRLSAQGRPVLFVKTDLSGALNELQDEAARLSWLA PA W E L GY QQTIGCSDAAVFR A GRP LF+KT+++ EL+DEA RL WLA PARWRETLEGYAVEQQTIGCSDAAVFRGVAPGRPTLFIKTEVADRFAELRDEALRLRWLA | 71  71 |
| Query 72  Sbjct 72 | | TTGVPCAAVLDVVTEAGRDWLLLGEVPGQDLLSSHLAPAEKVSIMADAMRRLHTLDPATC TTG PCA VLD V RDWLLL VPG+DL SS L P V I ADA+R LH +DPATC TTGRPCAGVLDQVLGPERDWLLLSAVPGRDLASSALGPETSVRIAADALRDLHRIDPATC | 131  131 |
| Query 132  Sbjct 132 | | PFDHQAKHRIERARTRMEAGLVDQDDLDEEHQGLAPAELFARLKARMPDGEDLVVTHGDA PFDH RI A R+EAG VD DD DE H+G +P L ARL+A P ED VV HGDA PFDHAVDRRIALAAARLEAGRVDADDFDEVHRGASPTALLARLRASRPAVEDRVVAHGDA | 191  191 |
| Query 192  Sbjct 192 | | CLPNIMVENGRFSGFIDCGRLGVADRYQDIALATRDIAEELGGEWADRFLVLYGIAAPDS CL N++ + GRF+GFIDCGRLGVADRYQD+ALA RDIAE G W FL YG+ PD CLSNLVADKGRFAGFIDCGRLGVADRYQDLALAARDIAETFGEAWVGPFLARYGV-TPDP | 251  250 |
| Query 252 | | QRIAFYRLLDEFF 264 R+AFYRLLDEFF |  |
| Sbjct | 251 | GRLAFYRLLDEFF 263 | |

>TR:A0A542LUX5 A0A542LUX5_9BURK Aminoglycoside 3'-phosphotransferase OS=Herbaspirillum sp. SJZ107 OX=2572881 GN=FBX97_3873 PE=3 SV=1

Length=274

Score = 298 bits (762), Expect = 9e-99

| Identities | = 154/256 (60%), Positives = 185/256 (72%), Gaps = 4/256 (2%) |  |
| --- | --- | --- |
| Query 12 | PAAWVERLFGYDWAQQTIGCSDAAVFRLSAQGRPVLFVKTDLSGALNELQDEAARLSWLA | 71 |
| Sbjct 20 | PA W RL GY W + GCS A+VFRL G+P L++KT+ +G L EL EA RL WLA PAGWRARLQGYRWTEALEGCSAASVFRLDTPGQPSLYLKTEPAGLLAELPGEAERLRWLA | 79 |
| Query 72 | TTGVPCAAVLDVVTEAGRDWLLLGEVPGQDLLSSHLAPAEKVSIMADAMRRLHTLDPATC | 131 |
| Sbjct 80 | G+PCA VL EAGR W+LLGE+ G++L + PA KV +MA A+R LH LD A C AAGIPCAPVLGWEQEAGRYWMLLGELRGRNLEDAAFDPACKVRLMAAALRSLHALDVAGC | 139 |
| Query 132 | PFDHQAKHRIERARTRMEAGLVDQDDLDEEHQGLAPAELFARLKARMPDGEDLVVTHGDA | 191 |
| Sbjct 140 | PFDH+A RI A R+ AGLVD+DDLD+ H+GLAPA+LFARL+A+ P EDLVVTHGDA PFDHRAALRIGHAEARLRAGLVDEDDLDDAHRGLAPADLFARLQAQAPAREDLVVTHGDA | 199 |
| Query 192 | CLPNIMV---ENGRFSGFIDCGRLGVADRYQDIALATRDIAEELGGEWADRFLVLYGIAA | 248 |
| Sbjct 200 | CLPN++V ++ R SG+IDCGRLGVADR+QD+ALATRDI E LG EW FL YG A CLPNLIVQHEQHERISGWIDCGRLGVADRWQDLALATRDIGEVLGPEWVAPFLREYG-AQ | 258 |
| Query 249  Sbjct 259 | PDSQRIAFYRLLDEFF 264 PD R +FYRLLDEFF PDPARTSFYRLLDEFF 274 |  |

>TR:A0A2S2CTZ1 A0A2S2CTZ1_9PROT Aminoglycoside 3'-phosphotransferase OS=Azospirillum thermophilum OX=2202148 GN=DEW08_09705 PE=3 SV=1

Length=268

Score = 293 bits (751), Expect = 4e-97

| Identities | | = 152/254 (60%), Positives = 176/254 (69%), Gaps = 2/254 (1%) |  |
| --- | --- | --- | --- |
| Query 12  Sbjct 16 | | PAAWVERLFGYDWAQQTIGCSDAAVFRLSAQGRPVLFVKTDLSGALNELQDEAARLSWLA P W ERL GY W +QTIGCS AAVFRL A RP LFVKT+ +G L EL+DE AR+ WL PVGWAERLAGYRWTRQTIGCSGAAVFRLEATDRPPLFVKTEPAGPLAELRDETARMRWLE | 71  75 |
| Query 72  Sbjct 76 | | TTGVPCAAVLDVVTEAGRDWLLLGEVPGQDLLSSHLA-PAEKVSIMADAMRRLHTLDPAT T GVP V+D A RDWLL+ V G DL S PA V + A+A+R LH +D A TVGVPAPRVIDAAATADRDWLLMTAVAGHDLASEQRPEPARIVRLAAEALRSLHRIDRAA | 130  135 |
| Query 131  Sbjct 136 | | CPFDHQAKHRIERARTRMEAGLVDQDDLDEEHQGLAPAELFARLKARMPDGEDLVVTHGD CPFDH A RI AR RMEAGLV+++D + + G ELFA L A P EDLVVTHGD CPFDHSAGVRIAHARARMEAGLVEEEDYEGKEDGRTVGELFALLVAGRPRTEDLVVTHGD | 190  195 |
| Query 191  Sbjct 196 | | ACLPNIMVENGRFSGFIDCGRLGVADRYQDIALATRDIAEELGGEWADRFLVLYGIAAPD ACLPN+M E+G F+GFID GRLG+ADR+QD+ALATRDIAEELG W FL YGIAA D ACLPNLMAEDGAFTGFIDVGRLGIADRHQDLALATRDIAEELGEAWIAPFLDRYGIAA-D | 250  254 |
| Query 251 | | SQRIAFYRLLDEFF 264  ++ FYRLLDEFF |  |
| Sbjct | 255 | PAKMRFYRLLDEFF 268 | |

>TR:A0A2R4CEQ3 A0A2R4CEQ3_9BURK Aminoglycoside 3'-phosphotransferase OS=Massilia armeniaca OX=2072590 GN=C9I28_21630 PE=3 SV=1

Length=264

Score = 291 bits (746), Expect = 2e-96

| Identities | | = 153/265 (58%), Positives = 182/265 (69%), Gaps = 7/265 (3%) |  |
| --- | --- | --- | --- |
| Query 5  Sbjct 2 | | DGLHAGSPAAWVERLFGYDWAQQTIGCSDAAVFRL--SAQGRPVLFVKTDLSGALNELQD D L P W ERL GY Q+ GCSDAAV R +A G P F+KT +G L ELQD DELDTTLPPTWRERLQGYAIVPQSGGCSDAAVLRAIPAAGGAPTFFIKTAAAGPLAELQD | 62  61 |
| Query 63  Sbjct 62 | | EAARLSWLATTGVPCAAVLDVVTEAGRDWLLLGEVPGQDLLSSHLAPAEKVSIMADAMRR EAARL WLA +G+ CA V+D T G+DWLLLG VPG +L +++ PA V++MA A+R EAARLRWLAQSGIACAPVIDTATTPGQDWLLLGAVPGTNL--TNVDPARAVAVMATALRT | 122  119 |
| Query 123  Sbjct 120 | | LHTLDPATCPFDHQAKHRIERARTRMEAGLVDQDDLDEEHQGLAPAELFARLKARMPDGE LH LD CPFDH+A RI A R++AGLVD DD DE++ G AP EL A L R P E LHALDVRECPFDHRADVRIGHALARLDAGLVDADDFDEDNAGCAPGELAALLAERKPLHE | 182  179 |
| Query 183  Sbjct 180 | | DLVVTHGDACLPNIMVENGRFSGFIDCGRLGVADRYQDIALATRDIAEELGGEWADRFLV DLVVTHGDACLPN++ +G F+GFIDCGRLGVADR+QD+ALA RDI EELG +W + FL DLVVTHGDACLPNVLAADGTFAGFIDCGRLGVADRHQDLALAVRDIGEELGEQWVEPFLA | 242  239 |
| Query 243 | | LYGIAAP---DSQRIAFYRLLDEFF 264 Y AP D+ R AFYRLLDEFF |  |
| Sbjct | 240 | QYFAPAPIAFDAGRAAFYRLLDEFF 264 | |

>TR:A0A318THA2 A0A318THA2_9BRAD Aminoglycoside 3'-phosphotransferase OS=Rhodopseudomonas faecalis OX=99655 GN=BJ122_12644 PE=3 SV=1

Length=281

Score = 290 bits (742), Expect = 1e-95

Identities = 149/253 (59%), Positives = 178/253 (70%), Gaps = 1/253 (0%)

| Query | 12 | PAAWVERLFGYDWAQQTIGCSDAAVFRLSAQGRPVLFVKTDLSGALNELQDEAARLSWLA | 71 |
| --- | --- | --- | --- |
| Sbjct | 30 | PAAW R+ G DW + T GCS+AAVFRL+A RP L+VKT S EL EAARL WL+ PAAWHCRIDGLDWTRATTGCSEAAVFRLAAPDRPTLYVKTAPSQPCGELAGEAARLRWLS | 89 |
| Query | 72 | TTGVPCAAVLDVVTEAGRDWLLLGEVPGQDLLSSHLAPAEKVSIMADAMRRLHTLDPATC | 131 |
| Sbjct | 90 | +TG+PCA VL RDWLLL VPG+DL ++ L+P E V++ A A+R+LH LD +C STGLPCAQVLASTRTNDRDWLLLSAVPGRDLSAAELSPRELVTLFAGALRQLHALDAGSC | 149 |
| Query | 132 | PFDHQAKHRIERARTRMEAGLVDQDDLDEEHQGLAPAELFARLKARMPDGEDLVVTHGDA | 191 |

| Sbjct | 150 | PFDH A RI AR R+ AGLVD +D D+E+ G+ PAEL +L A+ P VVTHGDA PFDHSADQRIALARERLAAGLVDPEDFDQENVGVPPAELLQQLVAQQPAVTAPVVTHGDA | 209 |
| --- | --- | --- | --- |
| Query | 192 | CLPNIMVENGRFSGFIDCGRLGVADRYQDIALATRDIAEELGGEWADRFLVLYGIAAPDS | 251 |
| Sbjct | 210 | CLPN+M E GRF+GFIDCGRLGVADR+QD+ALATRDIA ELG W FL YG D CLPNLMAEAGRFTGFIDCGRLGVADRHQDLALATRDIAAELGEGWVGPFLNSYG-QPLDW | 268 |
| Query | 252 | QRIAFYRLLDEFF 264  + AFYRLLDEFF |  |
| Sbjct | 269 | NKTAFYRLLDEFF 281 | |

>TR:A0A0Q8K3X2 A0A0Q8K3X2_9RHIZ Aminoglycoside 3'-phosphotransferase OS=Mesorhizobium sp. Root172 OX=1736481 GN=ASE05_25450 PE=3 SV=1

Length=262

Score = 281 bits (719), Expect = 2e-92

| Identities | | = 139/253 (55%), Positives = 174/253 (69%), Gaps = 0/253 (0%) |  |
| --- | --- | --- | --- |
| Query 12  Sbjct 10 | | PAAWVERLFGYDWAQQTIGCSDAAVFRLSAQGRPVLFVKTDLSGALNELQDEAARLSWLA P RL GY W +QTIG S A VFRL A +P LF+K + +G EL DEAARL WLA PRELKNRLSGYRWHRQTIGRSQAGVFRLVADDKPALFLKCERNGPFAELADEAARLRWLA | 71  69 |
| Query 72  Sbjct 70 | | TTGVPCAAVLDVVTEAGRDWLLLGEVPGQDLLSSHLAPAEKVSIMADAMRRLHTLDPATC G+ C V+ + + AG DWLL+ V G+DL S+ + PA+ + IMA A+R LH LD +C GQGIACPDVIAMESHAGHDWLLMSAVAGEDLASAPIDPADVIEIMASALRDLHALDIGSC | 131  129 |
| Query 132  Sbjct 130 | | PFDHQAKHRIERARTRMEAGLVDQDDLDEEHQGLAPAELFARLKARMPDGEDLVVTHGDA PFDH+ RI AR+RMEAG+VD+DD D+E QG +PA+ FA L+A P EDLVVTHGDA PFDHRLFRRIAAARSRMEAGVVDEDDFDDERQGWSPAKAFAELEALRPASEDLVVTHGDA | 191  189 |
| Query 192  Sbjct 190 | | CLPNIMVENGRFSGFIDCGRLGVADRYQDIALATRDIAEELGGEWADRFLVLYGIAAPDS CLPN+M GRFSGFIDCGRLGVADR+QD+ALA I +G W + FL YG + CLPNVMAARGRFSGFIDCGRLGVADRHQDLALACWSIRYNIGETWIEPFLERYGPPQAEP | 251  249 |
| Query 252 | | QRIAFYRLLDEFF 264  ++++YRLLDEFF |  |
| Sbjct | 250 | AKLSWYRLLDEFF 262 | |

>TR:A0A0M3BDK3 A0A0M3BDK3_9RHIZ Aminoglycoside 3'-phosphotransferase OS=Rhizobium sp. LC145 OX=1120688 GN=YH62_16080 PE=3 SV=1

Length=264

Score = 281 bits (719), Expect = 2e-92

| Identities | | = 138/254 (54%), Positives = 179/254 (70%), Gaps = 1/254 (0%) |  |
| --- | --- | --- | --- |
| Query 12  Sbjct 11 | | PAAWVERLFGYDWAQQTIGCSDAAVFRLSAQGRPVLFVKTDLSGALNELQDEAARLSWLA PA RL GYDW Q +G S A+VFRL A RP LF+K + +G EL DEA RL WLA PANLQARLAGYDWQQNMLGRSSASVFRLEAADRPTLFLKHEPAGPFTELPDEALRLRWLA | 71  70 |
| Query 72  Sbjct 71 | | TTGVPCAAVLDVVTEAGRDWLLLGEVPGQDLLSSH-LAPAEKVSIMADAMRRLHTLDPAT G+PCAAV+ +A R+WLLL V G+DL S+ ++ A++V+ +ADA+RRLH+LD AT AQGIPCAAVVAEAQDAERNWLLLTAVEGEDLASADGISAADRVTFLADALRRLHSLDIAT | 130  130 |
| Query 131  Sbjct 131 | | CPFDHQAKHRIERARTRMEAGLVDQDDLDEEHQGLAPAELFARLKARMPDGEDLVVTHGD CPFDH+ ++R+ R RM+AG+VD+ D D+E G + EL+ L+ P EDLVVTHGD CPFDHRLENRLPHVRARMQAGVVDESDFDDERIGRSSEELYRELETLKPANEDLVVTHGD | 190  190 |
| Query 191  Sbjct 191 | | ACLPNIMVENGRFSGFIDCGRLGVADRYQDIALATRDIAEELGGEWADRFLVLYGIAAPD ACLPNIM + F+GFIDC RLG+ADR+QDIAL+ R I LG EW FL LYG+ +PD ACLPNIMADPRGFTGFIDCARLGLADRHQDIALSCRSIRYNLGQEWVQPFLDLYGLPSPD | 250  250 |
| Query | 251 SQRIAFYRLLDEFF 264 | | |
|  |  | ++++YRLLDEFF |  |
| Sbjct | 251 | PAKLSYYRLLDEFF 264 | |

>TR:A0A4S1ECS7 A0A4S1ECS7_9BURK Aminoglycoside 3'-phosphotransferase OS=Alcaligenaceae bacterium 429 OX=2562948 GN=E4695_06975 PE=3 SV=1

Length=267

Score = 280 bits (717), Expect = 5e-92

| Identities | = 138/253 (55%), Positives = 174/253 (69%), Gaps = 0/253 (0%) |  |
| --- | --- | --- |
| Query 12 | PAAWVERLFGYDWAQQTIGCSDAAVFRLSAQGRPVLFVKTDLSGALNELQDEAARLSWLA | 71 |
| Sbjct 15 | PAAW + Y W QT GCSDAAV+RL A G +F+KT+ A EL DE RL WLA PAAWQSQFAAYSWTAQTEGCSDAAVYRLEAPGHDTVFIKTERMHANAELTDEEIRLRWLA | 74 |
| Query 72 | TTGVPCAAVLDVVTEAGRDWLLLGEVPGQDLLSSHLAPAEKVSIMADAMRRLHTLDPATC | 131 |
| Sbjct 75 | GV C V+D + R WLLL V G+DL S+ L EKV +MA A+R LH L A C AQGVSCPIVIDAHYQPERGWLLLSAVAGKDLYSASLPAHEKVKMMALALRDLHQLGVAHC | 134 |
| Query 132 | PFDHQAKHRIERARTRMEAGLVDQDDLDEEHQGLAPAELFARLKARMPDGEDLVVTHGDA | 191 |
| Sbjct 135 | PFDH A R+ A RM+AGL++ DD+D+E+QGL+ +LF +L A P ED V+THGDA PFDHNASQRVAHANARMQAGLIELDDVDDENQGLSVEQLFQKLLATQPLQEDKVLTHGDA | 194 |
| Query 192 | CLPNIMVENGRFSGFIDCGRLGVADRYQDIALATRDIAEELGGEWADRFLVLYGIAAPDS | 251 |
| Sbjct 195 | CLPN M E+G +G+IDCGRLGVADRYQD+ALA RDI+++LG E+ FL YG+A D+ CLPNFMAEDGVLTGYIDCGRLGVADRYQDLALAIRDISDDLGAEYVPLFLQHYGLAQLDT | 254 |
| Query 252  Sbjct 255 | QRIAFYRLLDEFF 264  +++AFY+LLDEFF  EKVAFYKLLDEFF 267 |  |

>TR:A0A0K2DCR3 A0A0K2DCR3_9RHIZ Aminoglycoside 3'-phosphotransferase OS=Chelatococcus sp. CO-6 OX=1702325 GN=AL346_10300 PE=3 SV=1

Length=258

Score = 279 bits (714), Expect = 1e-91

| Identities | | = 148/254 (58%), Positives = 174/254 (69%), Gaps = 2/254 (1%) |  |
| --- | --- | --- | --- |
| Query 12  Sbjct 6 | | PAAWVERLFGYDWAQQTIGCSDAAVFRLSAQGRPVLFVKTDLSGALNELQDEAARLSWLA P AW L Y QTIGCS+AAVFRL A+GRP LFVK + G L EL DE ARL WLA PRAWAADLSDYACRPQTIGCSEAAVFRLEAKGRPTLFVKAEPVGPLCELPDEIARLRWLA | 71  65 |
| Query 72  Sbjct 66 | | TTGVPCAAVLDVVTEAGRDWLLLGEVPGQDLLSS-HLAPAEKVSIMADAMRRLHTLDPAT V C VL EA R W+L+ V G+DL SS LAP V+I+A+A+R LH LD GQDVACPRVLAEAEEAQRHWVLMSAVDGRDLASSPDLAPERIVAIVAEALRGLHALDAHA | 130  125 |
| Query 131  Sbjct 126 | | CPFDHQAKHRIERARTRMEAGLVDQDDLDEEHQGLAPAELFARLKARMPDGEDLVVTHGD CPFDH+ + RI AR RMEAGLVD+ D DEE G + A+LF L A DGEDLVVTHGD CPFDHRLEGRIALARARMEAGLVDEADFDEERLGRSAADLFEDLLAGRLDGEDLVVTHGD | 190  185 |
| Query 191  Sbjct 186 | | ACLPNIMVENGRFSGFIDCGRLGVADRYQDIALATRDIAEELGGEWADRFLVLYGIAAPD ACLPN++ + GRF+GF+DCGRLGVADR+QD+ALA+ I LG +W + FL YG A D ACLPNLLAQAGRFTGFVDCGRLGVADRHQDLALASWSIRHNLGDDWVEPFLRCYG-GAVD | 250  244 |
| Query | 251 SQRIAFYRLLDEFF 264 | | |
|  |  | R+AFYRLLDEFF |  |
| Sbjct | 245 | PDRLAFYRLLDEFF 258 | |

>TR:A0A0Q6M9M4 A0A0Q6M9M4_9RHIZ Aminoglycoside 3'-phosphotransferase OS=Aminobacter sp. DSM 101952 OX=2735891 GN=ASC75_03245 PE=3 SV=1

Length=264

Score = 276 bits (706), Expect = 2e-90

| Identities | = 136/264 (52%), Positives = 172/264 (65%), Gaps = 0/264 (0%) |  |
| --- | --- | --- |
| Query 1 | MIEQDGLHAGSPAAWVERLFGYDWAQQTIGCSDAAVFRLSAQGRPVLFVKTDLSGALNEL | 60 |
| Sbjct 1 | M + + PA+ E L GYDW +QTIG S A VFRL A G+P LF+KT+ +G EL MTQHPAIEIDLPASLREALAGYDWQRQTIGASGAGVFRLEASGKPTLFLKTEETGQFAEL | 60 |
| Query 61 | QDEAARLSWLATTGVPCAAVLDVVTEAGRDWLLLGEVPGQDLLSSHLAPAEKVSIMADAM | 120 |
| Sbjct 61 | DE ARL WL + G+PC AVL G +WLL+ V G DL++ AP ++IMADA+ PDEVARLRWLDSQGIPCPAVLAFEAHGGHNWLLMSAVAGHDLVTQEAAPDRAIAIMADAL | 120 |
| Query 121 | RRLHTLDPATCPFDHQAKHRIERARTRMEAGLVDQDDLDEEHQGLAPAELFARLKARMPD | 180 |
| Sbjct 121 | RRLH LD A CPFDH RI A+ R +AG VD+DD D+E G + ++F L AR P RRLHALDAAACPFDHGLGPRIALAKARADAGEVDEDDFDDERLGKSAQQVFEELLARRPA | 180 |
| Query 181 | GEDLVVTHGDACLPNIMVENGRFSGFIDCGRLGVADRYQDIALATRDIAEELGGEWADRF | 240 |

E LVVTHGDA +PN M + GRF+GFIDC RLGVAD +QD+ALA	I	LG EW	F

| Sbjct | 181 | SETLVVTHGDASMPNFMADGGRFTGFIDCSRLGVADLHQDLALACWSIHLNLGEEWIAPF 240 |
| --- | --- | --- |
| Query | 241 | LVLYGIAAPDSQRIAFYRLLDEFF 264 |
| Sbjct | 241 | L LYG+ D+ ++++Y+LLDEFF  LALYGLPQADTAKLSYYQLLDEFF 264 |

>TR:A0A5C8PGD3 A0A5C8PGD3_9PROT Aminoglycoside 3'-phosphotransferase OS=Enhydrobacter sp. CC-CFT640 OX=2586908 GN=aph(3')-II PE=3 SV=1

Length=263

Score = 273 bits (698), Expect = 3e-89

| Identities | = 142/255 (56%), Positives = 174/255 (68%), Gaps = 2/255 (1%) |  |
| --- | --- | --- |
| Query 11 | SPAAWVERLFGYDWAQQTIGCSDAAVFRLSAQGRPVLFVKTDLSGALNELQDEAARLSWL | 70 |
| Sbjct 10 | +P AW L GY W +QTIG SDA VFRL A+GRP LF KT+ +G +EL EAARL WL TPRAWQSDLQGYAWHRQTIGHSDALVFRLEAEGRPTLFAKTEPAGPFSELPGEAARLRWL | 69 |
| Query 71 | ATTGVPCAAVLDVVTEAGRDWLLLGEVPGQDLLSS-HLAPAEKVSIMADAMRRLHTLDPA | 129 |
| Sbjct 70 | A+ V A VL + GR WLL VPG+DL SS L P+ V++ A A+R LH +D A ASRHVAAARVLAEALDEGRSWLLTTAVPGRDLASSAQLEPSRIVALAAGALRDLHRIDIA | 129 |
| Query 130 | TCPFDHQAKHRIERARTRMEAGLVDQDDLDEEHQGLAPAELFARLKARMPDGEDLVVTHG | 189 |
| Sbjct 130 | TCPFDH+ + RI +AR R+ AG+VD+ D D+E G ++L L AR P GEDL VTHG TCPFDHRLQQRIAQARARVAAGVVDESDFDDERLGRTASDLVEELTARRPQGEDLAVTHG | 189 |
| Query 190 | DACLPNIMVENGRFSGFIDCGRLGVADRYQDIALATRDIAEELGGEWADRFLVLYGIAAP | 249 |
| Sbjct 190 | DACLPN++V+ G F+GFIDCGRLGVADR+QD+ALA I LG EW + FL YG A DACLPNLLVDAGLFTGFIDCGRLGVADRHQDLALACWSIRYNLGEEWTEPFLRHYG-GAI | 248 |
| Query 250  Sbjct 249 | DSQRIAFYRLLDEFF 264 D ++ FYRLLDEFF DRGKLDFYRLLDEFF 263 |  |

>TR:A0A1C2ECT9 A0A1C2ECT9_9RHIZ Aminoglycoside 3'-phosphotransferase OS=Mesorhizobium hungaricum OX=1566387 GN=QV13_01800 PE=3 SV=1

Length=265

Score = 270 bits (690), Expect = 6e-88

| Identities | = 139/254 (55%), Positives = 169/254 (67%), Gaps = 1/254 (0%) |  |
| --- | --- | --- |
| Query 12 | PAAWVERLFGYDWAQQTIGCSDAAVFRLSAQGRPVLFVKTDLSGALNELQDEAARLSWLA | 71 |
| Sbjct 12 | P A + GY W + T+G SDA VFRL A RP LF+K + SG EL E RL WLA PPAIRAMIAGYAWDRDTLGHSDAGVFRLEATDRPRLFLKVEESGGHAELPAEVERLRWLA | 71 |
| Query 72 | TTGVPCAAVLDVVTEAGRDWLLLGEVPGQDLLSSHLAPAEK-VSIMADAMRRLHTLDPAT | 130 |
| Sbjct 72 | GV C VL T AGRDWLL+ +PG+DL+S+ AEK +++MA A+R LH LD + GQGVACPEVLAFETHAGRDWLLMSAMPGRDLVSAGPLSAEKAIAVMAGALRNLHALDIRS | 131 |
| Query 131 | CPFDHQAKHRIERARTRMEAGLVDQDDLDEEHQGLAPAELFARLKARMPDGEDLVVTHGD | 190 |
| Sbjct 132 | CPFDH+ +HRI AR +EAGLVD++D D+E G ++F + A P EDLVVTHGD CPFDHRLEHRIADARAHLEAGLVDEEDFDDERMGRTADDVFGDVIALRPSSEDLVVTHGD | 191 |
| Query 191 | ACLPNIMVENGRFSGFIDCGRLGVADRYQDIALATRDIAEELGGEWADRFLVLYGIAAPD | 250 |
| Sbjct 192 | ACLPN MV+ RFSGFIDCGRLGVADR QD+ALA I LG +W D FL YG D ACLPNFMVDGDRFSGFIDCGRLGVADRCQDLALACWSIHHNLGPQWVDPFLAHYGAPDID | 251 |
| Query 251  Sbjct 252 | SQRIAFYRLLDEFF 264 S ++A+YRLLDEFF SGKLAYYRLLDEFF 265 |  |

>TR:Q9HWR2 Q9HWR2_PSEAE Aminoglycoside 3'-phosphotransferase OS=Pseudomonas aeruginosa (strain ATCC 15692 / DSM 22644 / CIP 104116 /

JCM 14847 / LMG 12228 / 1C / PRS 101 / PAO1) OX=208964 GN=aph PE=3 SV=1

Length=268

Score = 269 bits (687), Expect = 2e-87

Identities = 137/258 (53%), Positives = 169/258 (66%), Gaps = 5/258 (2%)

| Query | 11 | SPAAWVERLFGYDWAQQTIGCSDAAVFRLSAQGRPVLFVKTDLSGALNELQDEAARLSWL | 70 |
| --- | --- | --- | --- |
| Sbjct | 12 | +P+ W + L GY W Q GCS A V RL A RP LFVK ++ A EL E ARL WL APSTWADYLAGYRWRGQGEGCSAATVHRLEAARRPTLFVKQEVLSAHAELPAEIARLRWL | 71 |
| Query | 71 | ATTGVPCAAVLDVVTEAGRDWLLLGEVPGQDLLS SHLAPAEKVSIMADAMRRLHTL | 126 |
| Sbjct | 72 | G+ C VL+ GR WLL+ VPG L + L P V ++A A+RRLH L HGAGIDCPQVLNETQSDGRQWLLMSAVPGDTLSALAQRGELEPERLVRLVAAALRRLHDL | 131 |
| Query | 127 | DPATCPFDHQAKHRIERARTRMEAGLVDQDDLDEEHQGLAPAELFARLKARMPDGEDLVV | 186 |
| Sbjct | 132 | DPA CPFDH+ + R++ R R+EAGLVD+ D D++H+G + EL+ L R P EDLVV DPAACPFDHRLERRLDTVRQRVEAGLVDEADFDDDHRGRSATELYRLLLDRRPAVEDLVV | 191 |
| Query | 187 | THGDACLPNIMVENGRFSGFIDCGRLGVADRYQDIALATRDIAEELGGEWADRFLVLYGI | 246 |
| Sbjct | 192 | HGDACLPN++ E RFSGFIDCGRLGVADR+QD+ALA RDI ELG WA+ FLV YG  AHGDACLPNLLAEGRRFSGFIDCGRLGVADRHQDLALAARDIEAELGAAWAEAFLVEYG- | 250 |
| Query | 247 AAPDSQRIAFYRLLDEFF 264 | | |
|  |  | D +R+A++RLLDEFF |  |
| Sbjct | 251 | GDIDGERLAYFRLLDEFF 268 | |

>TR:W3RM28 W3RM28_9BRAD Aminoglycoside 3'-phosphotransferase OS=Afipia sp. P52-10 OX=1429916 GN=X566_06580 PE=3 SV=1

Length=258

Score = 267 bits (683), Expect = 5e-87

| Identities | | = 133/254 (52%), Positives = 167/254 (66%), Gaps = 2/254 (1%) |  |
| --- | --- | --- | --- |
| Query 12  Sbjct 6 | | PAAWVERLFGYDWAQQTIGCSDAAVFRLSAQGRPVLFVKTDLSGALNELQDEAARLSWLA P W + L Y W QQTIG S AAVFRL A G+P LFVKT+ G N L EA RL WL PPTWTQELAHYAWRQQTIGRSGAAVFRLEANGQPTLFVKTEQRGGFNTLPAEAERLRWLG | 71  65 |
| Query 72  Sbjct 66 | | TTGVPCAAVLDVVTEAGRDWLLLGEVPGQDLLSS-HLAPAEKVSIMADAMRRLHTLDPAT TG+PC A+L + R+WLL +PG+DL SS HLAP + V+I ADA+R LH L P+ ETGIPCPAILKETRDGRRNWLLTTAIPGRDLASSPHLAPEQIVAIAADALRDLHRLAPSG | 130  125 |
| Query 131  Sbjct 126 | | CPFDHQAKHRIERARTRMEAGLVDQDDLDEEHQGLAPAELFARLKARMPDGEDLVVTHGD CPFDH+ + + A R +AGLVD+ D DE G + +LF L R PD EDLVV HGD CPFDHRLERALAEAEARTQAGLVDETDFDEPRLGRSAHDLFEELLRRRPDREDLVVAHGD | 190  185 |
| Query 191  Sbjct 186 | | ACLPNIMVENGRFSGFIDCGRLGVADRYQDIALATRDIAEELGGEWADRFLVLYGIAAPD LPN++ E GRFSGF+DC RLGVAD+++D+A+A+ + LG W FL YG A PD PSLPNLLAEAGRFSGFVDCARLGVADQHRDLAIASSSVRYNLGEAWVAPFLTRYG-AKPD | 250  244 |
| Query 251 | | SQRIAFYRLLDEFF 264  +R+ +Y+LLDEFF |  |
| Sbjct | 245 | PERMDYYQLLDEFF 258 | |

>TR:A0A2S9JF04 A0A2S9JF04_9RHIZ Aminoglycoside 3'-phosphotransferase OS=Phyllobacterium myrsinacearum OX=28101 GN=C5750_16760 PE=3 SV=1

Length=257

Score = 267 bits (682), Expect = 7e-87

| Identities | = 132/245 (54%), Positives = 164/245 (67%), Gaps = 1/245 (0%) |  |
| --- | --- | --- |
| Query 21 | GYDWAQQTIGCSDAAVFRLSAQGRPVLFVKTDLSGALNELQDEAARLSWLATTGVPCAAV | 80 |
| Sbjct 13 | GY W++ +GCSDA VF L +G P LF+K + EL DEAARL WL++ + C V GYSWSRDALGCSDAQVFMLQGEGLPQLFIKVEAVSPYGELPDEAARLRWLSSRHMLCPDV | 72 |
| Query 81 | LDVVTEAGRDWLLLGEVPGQDLLSSHLAPAE-KVSIMADAMRRLHTLDPATCPFDHQAKH | 139 |
| Sbjct 73 | L A R WLL+ VPG+DL S+ E ++ I+A A+R+LH LDPATCPFDH+ LFEGAHASRFWLLMSGVPGKDLASADSLSIETRIRILAGALRQLHALDPATCPFDHRLDM | 132 |
| Query 140 | RIERARTRMEAGLVDQDDLDEEHQGLAPAELFARLKARMPDGEDLVVTHGDACLPNIMVE | 199 |
| Sbjct 133 | RIE AR RM+AGLVD+ D D + G +ELF+RL A P D+VVTHGD CLPN M E RIEAARARMQAGLVDETDFDSDMLGKTASELFSRLVAERPGTADVVVTHGDGCLPNFMAE | 192 |
| Query 200 | NGRFSGFIDCGRLGVADRYQDIALATRDIAEELGGEWADRFLVLYGIAAPDSQRIAFYRL | 259 |
| Sbjct 193 | NG F+G+IDC RLG+ADRYQDIALA R IA+ G EW FL YG+ D ++A+YRL  NGIFTGYIDCARLGLADRYQDIALACRSIADNFGEEWVRPFLDCYGLVQADPAKLAYYRL | 252 |

Query 260 LDEFF 264

LDEFF Sbjct 253 LDEFF 257

>TR:A0A024KAC1 A0A024KAC1_9RHIZ Aminoglycoside 3'-phosphotransferase OS=Devosia sp. DBB001 OX=1380412 PE=3 SV=1

Length=264

Score = 267 bits (682), Expect = 9e-87

| Identities | = 139/259 (54%), Positives = 167/259 (64%), Gaps = 3/259 (1%) |  |
| --- | --- | --- |
| Query 8 | HAGS-PAAWVERLFGYDWAQQTIGCSDAAVFRLSAQGRPVLFVKTDLSGALNELQDEAAR | 66 |
| Sbjct 7 | HA + P W + L GY+W QTIG S A VFRL+A P LFVKT+ +EL+ EA R HASNLPPGWPD-LSGYEWVPQTIGQSGAMVFRLTAPQGPALFVKTESVSPHSELEGEAHR | 65 |
| Query 67 | LSWLATTGVPCAAVLDVVTEAGRDWLLLGEVPGQDLLSS-HLAPAEKVSIMADAMRRLHT | 125 |
| Sbjct 66 | L WLA P VL GR WLL+ V G DL SS L+P+ + I A ++R LH LEWLAGVDQPAPRVLGTAQHLGRQWLLMTGVDGLDLASSPDLSPSRIIEICATSLRALHA | 125 |
| Query 126 | LDPATCPFDHQAKHRIERARTRMEAGLVDQDDLDEEHQGLAPAELFARLKARMPDGEDLV | 185 |
| Sbjct 126 | +D A+CPFDH+ + AR R++AGLVD+ D D+ H G+ L+ARL P+ EDLV IDVASCPFDHRLDTTLATARERLDAGLVDETDFDDAHLGMPARTLYARLLTERPEAEDLV | 185 |
| Query 186 | VTHGDACLPNIMVENGRFSGFIDCGRLGVADRYQDIALATRDIAEELGGEWADRFLVLYG | 245 |
| Sbjct 186 | VTHGDACLPN M FSGFIDCGRLGVADRYQD+ALATR IA LG WA+ FL YG VTHGDACLPNFMASGREFSGFIDCGRLGVADRYQDLALATRSIAFNLGQVWAEPFLTAYG | 245 |
| Query 246  Sbjct 246 | IAAPDSQRIAFYRLLDEFF 264  + PD RI +YRLLDEFF  VGQPDRARIGYYRLLDEFF 264 |  |

>TR:Q92LN7 Q92LN7_RHIME Aminoglycoside 3'-phosphotransferase OS=Rhizobium meliloti (strain 1021) OX=266834 GN=SMc03094 PE=3 SV=1

Length=261

Score = 266 bits (681), Expect = 1e-86

| Identities | = 138/253 (55%), Positives = 163/253 (64%), Gaps = 0/253 (0%) |  |
| --- | --- | --- |
| Query 12 | PAAWVERLFGYDWAQQTIGCSDAAVFRLSAQGRPVLFVKTDLSGALNELQDEAARLSWLA | 71 |
| Sbjct 9 | P A+ L GY + + +G S + +F L AQ RP L +K + SG EL EAARL WLA PPAFRGLLSGYGFERDALGQSASTIFLLKAQSRPFLILKHEPSGPFTELTGEAARLHWLA | 68 |
| Query 72 | TTGVPCAAVLDVVTEAGRDWLLLGEVPGQDLLSSHLAPAEKVSIMADAMRRLHTLDPATC | 131 |
| Sbjct 69 | +PC VL AG WLLL G DL S L AE+++I+A+A+RRLH LDPA C AQDMPCPRVLGHDDHAGETWLLLEAAEGMDLASCRLPHAERIAILAEALRRLHRLDPAAC | 128 |
| Query 132 | PFDHQAKHRIERARTRMEAGLVDQDDLDEEHQGLAPAELFARLKARMPDGEDLVVTHGDA | 191 |
| Sbjct 129 | PFD + I A+ RMEAGLVD+ D DEE QG ELF L AR P E LVVTHGDA PFDRRLTASIAAAKARMEAGLVDESDFDEERQGRQTRELFDELLARRPKEEQLVVTHGDA | 188 |
| Query 192 | CLPNIMVENGRFSGFIDCGRLGVADRYQDIALATRDIAEELGGEWADRFLVLYGIAAPDS | 251 |
| Sbjct 189 | CLPN + +FSGFIDCGRLGVAD YQD+ALA I LG EW FL YG+AAPD CLPNFIAAGNQFSGFIDCGRLGVADIYQDLALACWSITHNLGEEWVAPFLDRYGLAAPDP | 248 |
| Query 252  Sbjct 249 | QRIAFYRLLDEFF 264  +++AFYRLLDEFF  EKLAFYRLLDEFF 261 |  |

>TR:B6IQF0 B6IQF0_RHOCS Aminoglycoside 3'-phosphotransferase OS=Rhodospirillum centenum (strain ATCC 51521 / SW) OX=414684 GN=butA PE=3

SV=1

Length=270

Score = 265 bits (678), Expect = 4e-86

Identities = 141/254 (56%), Positives = 169/254 (67%), Gaps = 2/254 (1%)

Query 12	PAAWVERLFGYDWAQQTIGCSDAAVFRLSAQGRPVLFVKTDLSGALNELQDEAARLSWLA 71 PA W ERL GY W + + GCSDAAVFRL A GRPVL+VKT+ + L EL E ARL WLA

| Sbjct | 18 | PAGWRERLEGYAWTRPSGGCSDAAVFRLDAPGRPVLYVKTEPAEPLAELPGEIARLRWLA | 77 |
| --- | --- | --- | --- |
| Query | 72 | TTGVPCAAVLDVVTEAGRDWLLLGEVPGQDLLSS-HLAPAEKVSIMADAMRRLHTLDPAT | 130 |
| Sbjct | 78 | G+PC AVL EAGR WLL+ +PG L + L +V++ A A+R LH LD A GQGIPCPAVLQATEEAGRHWLLMSALPGHVLEEAPDLDTGRRVALYAGALRTLHALDIAA | 137 |
| Query | 131 | CPFDHQAKHRIERARTRMEAGLVDQDDLDEEHQGLAPAELFARLKARMPDGEDLVVTHGD | 190 |
| Sbjct | 138 | CPFD + +I A R+ AGLVD++D DE QG + A+L A L+AR P EDLVVTHGD CPFDQRIDGQIALAAARVAAGLVDEEDFDEVRQGRSAADLLAELRARRPAREDLVVTHGD | 197 |
| Query | 191 | ACLPNIMVENGRFSGFIDCGRLGVADRYQDIALATRDIAEELGGEWADRFLVLYGIAAPD | 250 |
| Sbjct | 198 | AC N+M + GRF+GFIDCGRLGVADR+QD+ALA R IA+ G W FL YG A D  ACPDNLMTDGGRFAGFIDCGRLGVADRHQDLALACRSIADCWGEAWIAPFLAHYGRPA-D | 256 |
| Query | 251 SQRIAFYRLLDEFF 264 | | |
|  |  | R+ Y LLDEFF |  |
| Sbjct | 257 | PDRLDCYCLLDEFF 270 | |

>TR:A0A1I5HYN7 A0A1I5HYN7_9BURK Aminoglycoside 3'-phosphotransferase OS=Variovorax sp. PDC80 OX=1882827 GN=SAMN05443579_103638 PE=3 SV=1

Length=259

Score = 261 bits (666), Expect = 2e-84

| Identities | = 139/253 (55%), Positives = 164/253 (65%), Gaps = 1/253 (0%) |  |
| --- | --- | --- |
| Query 12 | PAAWVERLFGYDWAQQTIGCSDAAVFRLSAQGRPVLFVKTDLSGALNELQDEAARLSWLA | 71 |
| Sbjct 8 | P AW RL W +QTIG S A VFR+ A +LF+K++ L EL E ARL+WLA PHAWRARLADAHWRRQTIGESGADVFRIEAAHGELLFLKSEPVAPLAELPGEIARLAWLA | 67 |
| Query 72 | TTGVPCAAVLDVVTEAGRDWLLLGEVPGQDLLSSHLAPAEKVSIMADAMRRLHTLDPATC | 131 |
| Sbjct 68 | +P AVLD E R WLL+ VPG+DL S PAE V MADA+R LH + ATC RQDLPAPAVLDQALEHDRHWLLMSAVPGRDLASGPFGPAEIVVHMADALRLLHRVPVATC | 127 |
| Query 132 | PFDHQAKHRIERARTRMEAGLVDQDDLDEEHQGLAPAELFARLKARMPDGEDLVVTHGDA | 191 |
| Sbjct 128 | PFDH+ +RIE A R AGLVD+ D D+E G +LF RL+A P EDLVVTHGDA PFDHRLAYRIEDAHARAVAGLVDESDFDDERAGWTALDLFERLRATRPASEDLVVTHGDA | 187 |
| Query 192 | CLPNIMVENGRFSGFIDCGRLGVADRYQDIALATRDIAEELGGEWADRFLVLYGIAAPDS | 251 |
| Sbjct 188 | CLPN+M F+GFIDCGR+GVADR+QD+ALA R I LG W F YGI APD+ CLPNLMATADGFTGFIDCGRIGVADRWQDLALAARSIGFNLGEGWTAPFFERYGI-APDA | 246 |
| Query 252  Sbjct 247 | QRIAFYRLLDEFF 264  +RIAFY LLDEFF  ERIAFYCLLDEFF 259 |  |

>TR:A0A1H4UEE9 A0A1H4UEE9_9RHIZ Aminoglycoside 3'-phosphotransferase OS=Beijerinckia sp. 28-YEA-48 OX=1882748 GN=SAMN05443249_3242 PE=3

SV=1

Length=266

Score = 254 bits (649), Expect = 1e-81

| Identities | | = 135/254 (53%), Positives = 164/254 (65%), Gaps = 2/254 (1%) |  |
| --- | --- | --- | --- |
| Query 12  Sbjct 14 | | PAAWVERLFGYDWAQQTIGCSDAAVFRLSAQGRPVLFVKTDLSGALNELQDEAARLSWLA P W + L GY W QTIG S A VFRL A RP FVK + + A EL EAARL WLA PPHWRDDLAGYAWTTQTIGRSSATVFRLQADKRPSFFVKIEPADASGELLAEAARLRWLA | 71  73 |
| Query 72  Sbjct 74 | | TTGVPCAAVLDVVTEAGRDWLLLGEVPGQDLLSS-HLAPAEKVSIMADAMRRLHTLDPAT  +G+ C V + ++ R+WLL +PG+DL SS HLA + V+I ADA+R LH LDP+ ASGIACPQVENTSSDGQRNWLLTRALPGRDLASSPHLAAEQIVTIAADALRDLHRLDPSP | 130  133 |
| Query 131  Sbjct 134 | | CPFDHQAKHRIERARTRMEAGLVDQDDLDEEHQGLAPAELFARLKARMPDGEDLVVTHGD CPFD + RI A RM AGLVD+ D D+E G +FA L R P EDLVVTHGD CPFDQRLDSRIAAAEARMRAGLVDESDFDDERLGQPAHAVFADLLRRRPAQEDLVVTHGD | 190  193 |
| Query 191  Sbjct 194 | | ACLPNIMVENGRFSGFIDCGRLGVADRYQDIALATRDIAEELGGEWADRFLVLYGIAAPD ACLPN+M + RFSGF+DC RLGVADR+QD+ALA+ I LG W FL YG+ A D ACLPNLMADGERFSGFVDCARLGVADRHQDLALASWSIRYNLGQAWIAPFLQRYGLRA-D | 250  252 |
| Query | 251 SQRIAFYRLLDEFF 264 | | |

+ + FYRLLD+FF Sbjct 253 PELLDFYRLLDDFF 266

>TR:A0A085FM66 A0A085FM66_9RHIZ Aminoglycoside 3'-phosphotransferase OS=Devosia sp. LC5 OX=1502724 GN=neo PE=3 SV=1

Length=257

Score = 254 bits (648), Expect = 1e-81

| Identities | = 121/253 (48%), Positives = 165/253 (65%), Gaps = 2/253 (1%) |  |
| --- | --- | --- |
| Query 12 | PAAWVERLFGYDWAQQTIGCSDAAVFRLSAQGRPVLFVKTDLSGALNELQDEAARLSWLA | 71 |
| Sbjct 7 | P +W + W +QTIG S+A+V+RL+ LFVK++ L EL E ARL W+A PPSWRTKYADAVWERQTIGESNASVYRLTPARAGPLFVKSEQHAPLAELPGEIARLDWMA | 66 |
| Query 72 | TTGVPCAAVLDVVTEAGRDWLLLGEVPGQDLLSSHLAPAEKVSIMADAMRRLHTLDPATC | 131 |
| Sbjct 67 | G+PC ++DV A WLL+ VPG DL S +L+P V + +A++ LH LD A C QHGIPCPRIIDVADHADAHWLLMSAVPGHDLASGNLSPDRLVHLYVEALQPLHALDIADC | 126 |
| Query 132 | PFDHQAKHRIERARTRMEAGLVDQDDLDEEHQGLAPAELFARLKARMPDGEDLVVTHGDA | 191 |
| Sbjct 127 | PFD + + ++ RA R+ AGL+D+DD D++ G A++F + PD EDLVV HGDA PFDQRIETQLSRAAIRVAAGLIDEDDFDDDRTGWTAAQVFGAARQAAPDNEDLVVCHGDA | 186 |
| Query 192 | CLPNIMVENGRFSGFIDCGRLGVADRYQDIALATRDIAEELGGEWADRFLVLYGIAAPDS | 251 |
| Sbjct 187 | CLPN++ N +F+GFIDCGRLG+ADRYQD LA R +A+ L WAD FL YGIA PD CLPNLLARNSQFTGFIDCGRLGIADRYQD--LAHRSLADNLDASWADAFLTAYGIARPDR | 244 |
| Query 252  Sbjct 245 | QRIAFYRLLDEFF 264  +R+A++R+LDE F  ERMAYFRMLDELF 257 |  |

>TR:A0A245Z5G3 A0A245Z5G3_9RHIZ Aminoglycoside 3'-phosphotransferase OS=Rhizobium yanglingense OX=61013 GN=AJ87_25300 PE=3 SV=1

Length=262

Score = 252 bits (644), Expect = 5e-81

| Identities | = 124/244 (51%), Positives = 159/244 (65%), Gaps = 1/244 (0%) |  |
| --- | --- | --- |
| Query 22 | YDWAQQTIGCSDAAVFRLSAQGRPVLFVKTDLSGALNELQDEAARLSWLATTGVPCAAVL | 81 |
| Sbjct 19 | ++W Q +GCS A VFRL+ GR F+K +++G EL EA RL WLA G+ C +V+ HEWKQDDLGCSSADVFRLAEGGRTRFFLKGEIAGPFAELPLEAERLLWLAEQGLQCPSVV | 78 |
| Query 82 | DVVTEAGRDWLLLGEVPGQDLLSSHLAP-AEKVSIMADAMRRLHTLDPATCPFDHQAKHR | 140 |
| Sbjct 79 | + + GR+WLLL + G DL S L +++ I+A A+RRLH LDP TCPFDH+ R ALESHEGRNWLLLSALAGSDLASCKLTTDRQRIEILAAALRRLHELDPETCPFDHRLTAR | 138 |
| Query 141 | IERARTRMEAGLVDQDDLDEEHQGLAPAELFARLKARMPDGEDLVVTHGDACLPNIMVEN | 200 |
| Sbjct 139 | + A+ RMEAG VD+ D DEE Q A LFA L A PD D VV HGDACLPN M LRLAKARMEAGAVDESDFDEERQNATAASLFAYLDANQPDVTDPVVAHGDACLPNFMAHE | 198 |
| Query 201 | GRFSGFIDCGRLGVADRYQDIALATRDIAEELGGEWADRFLVLYGIAAPDSQRIAFYRLL | 260 |
| Sbjct 199 | G+FSG++DCGRLGVADRYQDIALA R I +G W FL YG++ + ++A+Y +L GQFSGYVDCGRLGVADRYQDIALALRSIRYNVGEAWVRHFLDCYGLSGINDAKVAYYMVL | 258 |
| Query 261  Sbjct 259 | DEFF 264 DEFF  DEFF 262 |  |

```
>TR:A0A0B4X2U9 A0A0B4X2U9_9RHIZ Aminoglycoside 3'-phosphotransferase OS=Rhizobium gallicum bv. gallicum R602 OX=1418105 GN=RGR602_CH01541
PE=3 SV=1
Length=260

Score = 252 bits (643), Expect = 6e-81
Identities = 123/244 (50%), Positives = 161/244 (66%), Gaps = 1/244 (0%)

Query 22	YDWAQQTIGCSDAAVFRLSAQGRPVLFVKTDLSGALNELQDEAARLSWLATTGVPCAAVL 81
++W + +GCS A VFRL+ GR	F+K +++G	EL EA RL WLA G+ C +V+ Sbjct 17	HEWKRDDLGCSSADVFRLAEAGRARYFLKGEIAGPFAELPLEAERLLWLAEQGLQCPSVV 76
```

| Query | 82 | DVVTEAGRDWLLLGEVPGQDLLSSHLAP-AEKVSIMADAMRRLHTLDPATCPFDHQAKHR | 140 |
| --- | --- | --- | --- |
| Sbjct | 77 | + + GR+WLLL + G DL SS L +++ I+A A+RRLH LDP TCPFDH+ R ALESHEGRNWLLLSALSGSDLASSKLTTDRQRIEILAAALRRLHELDPETCPFDHRLTAR | 136 |
| Query | 141 | IERARTRMEAGLVDQDDLDEEHQGLAPAELFARLKARMPDGEDLVVTHGDACLPNIMVEN | 200 |
| Sbjct | 137 | + A+ RMEAG VD+ D DEE Q A LFA L+A PD D VV HGDACLPN + LRLAKARMEAGAVDEGDFDEERQNATAASLFAYLEAHQPDVIDPVVAHGDACLPNFLTHE | 196 |
| Query | 201 | GRFSGFIDCGRLGVADRYQDIALATRDIAEELGGEWADRFLVLYGIAAPDSQRIAFYRLL | 260 |
| Sbjct | 197 | G+F G++DCGRLGVADRYQDIALA R I +G EW FL YG++ + ++A+Y +L GQFGGYVDCGRLGVADRYQDIALALRSIRYNVGEEWVRHFLDCYGLSGINDAKVAYYMVL | 256 |
| Query  Sbjct | 261  257 | DEFF 264 DEFF  DEFF 260 |  |

>TR:J2VNI4 J2VNI4_9RHIZ Aminoglycoside 3'-phosphotransferase OS=Phyllobacterium sp. YR531 OX=1144343 GN=PMI41_03333 PE=3 SV=1

Length=262

Score = 252 bits (643), Expect = 7e-81

| Identities | = 134/254 (53%), Positives = 162/254 (64%), Gaps = 4/254 (2%) |  |
| --- | --- | --- |
| Query 12 | PAAWVERLFGYDWAQQTIGCSDAAVFRLSAQGRPVLFVKTDLSGALNELQDEAARLSWLA | 71 |
| Sbjct 12 | P W RL Y W QTIG S A VFRL A G P LFVK++++G EL DE +RL WLA PPDWRGRLAHYGWTAQTIGESGALVFRLEASGLPTLFVKSEIAGEFGELPDEISRLRWLA | 71 |
| Query 72 | TTGVPCAAVLDVVTEAGRDWLLLGEVPGQDLLSSHLAPAEK-VSIMADAMRRLHTLDPAT | 130 |
| Sbjct 72 | G+ VLD + GR WLL+ +PG+DL S PAE V I A +++ LH+LD A GQGIAAPKVLDQTEDNGRIWLLMSALPGRDLASDKEIPAENIVRIAAQSLKDLHSLDIAH | 131 |
| Query 131 | CPFDHQAKHRIERARTRMEAGLVDQDDLDEEHQGLAPAELFARLKARMPDGEDLVVTHGD | 190 |
| Sbjct 132 | CPF+H +RI A RM A VD+ D DE G AELF RL DGEDLVVTHGD CPFNHTLSNRIPVAHARMIANQVDESDFDEV--GPTAAELFERLLKLRSDGEDLVVTHGD | 189 |
| Query 191 | ACLPNIMVENGRFSGFIDCGRLGVADRYQDIALATRDIAEELGGEWADRFLVLYGIAAPD | 250 |
| Sbjct 190 | ACLPNI+ + F+GF+DC RLG+ADRYQD+ALA+R I LG EW + F YG+ D ACLPNILSDGSVFTGFVDCARLGIADRYQDLALASRSIEHNLGVEWVEPFFRSYGVDM-D | 248 |
| Query 251  Sbjct 249 | SQRIAFYRLLDEFF 264 S R+ +YRLLDEFF SSRVEYYRLLDEFF 262 |  |

>TR:B2FNW7 B2FNW7_STRMK Aminoglycoside 3'-phosphotransferase OS=Stenotrophomonas maltophilia (strain K279a) OX=522373 GN=Smlt2120 PE=3

SV=1

Length=267

Score = 251 bits (642), Expect = 1e-80

| Identities | = 134/255 (53%), Positives = 166/255 (65%), Gaps = 3/255 (1%) |  |
| --- | --- | --- |
| Query 12 | PAAWVERLFGYDWAQQTIGCSDAAVFRLSAQGRPVLFVKTDLSGALNELQDEAARLSWLA | 71 |
| Sbjct 14 | P AW E L +Q+IG S A V R+ G+ FVK+++ A +EL DE ARL WL PWAWQEALADARVERQSIGVSRADVARVQRPGQADAFVKSEVIDAFSELGDEIARLRWLQ | 73 |
| Query 72 | TTGVPCAAVLDVVTEAGRDWLLLGEVPGQDLLSS-HLAPAEKVSIMADAMRRLHTLDPAT | 130 |
| Sbjct 74 | G P V+ EAGR WLL+ +PG+DL SS LAP + V ++ADA+R LH L A EQGQPAPTVIATAEEAGRRWLLMSALPGRDLASSPELAPQQLVELLADALRGLHALPLAA | 133 |
| Query 131 | CPFDHQAKHRIERARTRMEAGLVDQDDLDEEHQGLAPAELFARLKARMPDGEDLVVTHGD | 190 |
| Sbjct 134 | CPFD + R++ A+ R+EAGLVD DD D+E G +P ++FA L + PD EDLVV+HGD CPFDQRLHLRLQAAQARVEAGLVDADDFDDERLGQSPQQVFAELCSTRPDHEDLVVSHGD | 193 |
| Query 191 | ACLPNIMVENGRFSGFIDCGRLGVADRYQDIALATRDIAEELGG-EWADRFLVLYGIAAP | 249 |
| Sbjct 194 | ACLPN+MV GRFSGFIDCGRLGVADRYQD+ALA R + G W YG A ACLPNLMVVEGRFSGFIDCGRLGVADRYQDLALAARSLVHNFGDTRWVAPLFQRYG-AVA | 252 |
| Query 250  Sbjct 253 | DSQRIAFYRLLDEFF 264 D +R+AFYRLLDEFF DERRLAFYRLLDEFF 267 |  |

>TR:A0A0A1PYP1 A0A0A1PYP1_9BACT Aminoglycoside 3'-phosphotransferase OS=bacterium YEK0313 OX=1522316 GN=neo PE=3 SV=1

Length=1714

Score = 272 bits (695), Expect = 3e-80

Identities = 139/265 (52%), Positives = 168/265 (63%), Gaps = 1/265 (0%)

| Query | 1 | MIEQDGLHAGSPAAWVERLFGYDWAQQTIGCSDAAVFRLSAQGRPVLFVKTDLSGALNEL | 60 |
| --- | --- | --- | --- |
| Sbjct | 1450 | M + D P WV R G W +Q+IG SDA VFRL G L+VKT+ L+EL MPDIDATPPAPPPTWVRRFGGGRWTEQSIGRSDAHVFRLDEAGGSPLYVKTERIHPLSEL | 1509 |
| Query | 61 | QDEAARLSWLATTGVPCAAVLDVVTEAGRDWLLLGEVPGQDLLSSH-LAPAEKVSIMADA | 119 |
| Sbjct | 1510 | E ARL WLA +PC VLD G WL + +PG+DL S+ +AP + ++ A+A AGEIARLGWLAGQDIPCPRVLDTAEHDGWLWLAMSAIPGRDLASAEDMAPERRAAVAAEA | 1569 |
| Query | 120 | MRRLHTLDPATCPFDHQAKHRIERARTRMEAGLVDQDDLDEEHQGLAPAELFARLKARMP | 179 |
| Sbjct | 1570 | +RRLH LDPA CPFDH+ +RI ARTR+EAGLVD D DEE GL+ AE+F RL A P LRRLHALDPADCPFDHRLDNRIAAARTRVEAGLVDDSDFDEERLGLSAAEIFPRLAAERP | 1629 |
| Query | 180 | DGEDLVVTHGDACLPNIMVENGRFSGFIDCGRLGVADRYQDIALATRDIAEELGGEWADR | 239 |
| Sbjct | 1630 | GE LVVTHGDACLPN+M FSGF+DCGRLG+ D YQD+ALA I LG W TGETLVVTHGDACLPNLMATAEGFSGFVDCGRLGLVDSYQDLALARWSIIYNLGEAWVAP | 1689 |
| Query  Sbjct | 240  1690 | FLVLYGIAAPDSQRIAFYRLLDEFF 264 F YG+A PD R+A+Y LLDEFF FFRHYGLAEPDRDRLAYYCLLDEFF 1714 |  |

>TR:U1YD28 U1YD28_9BURK Aminoglycoside 3'-phosphotransferase OS=Alcaligenes sp. EGD-AK7 OX=1386079 GN=N879_10320 PE=3 SV=1

Length=256

Score = 248 bits (634), Expect = 1e-79

| Identities | = 130/254 (51%), Positives = 160/254 (63%), Gaps = 2/254 (1%) |  |
| --- | --- | --- |
| Query 12 | PAAWVERLFGYDWAQQTIGCSDAAVFRLSAQGRPVLFVKTDLSGALNELQDEAARLSWLA | 71 |
| Sbjct 4 | P AW RL +QTIG S A V+RL FVK + L EL DE RL W+ PLAWQTRLAAAKVQRQTIGESGADVWRLDWPDGSCQFVKAEDDLPLAELPDEIERLRWMH | 63 |
| Query 72 | TTGVPCAAVLDVVTEAGRDWLLLGEVPGQDLLSSH-LAPAEKVSIMADAMRRLHTLDPAT | 130 |
| Sbjct 64 | T G+PC VLD + GR WLL+ VPG+DL + L+P + V ++A +++ LH L TQGLPCPEVLDTLLAQGRHWLLMSAVPGRDLACTEGLSPQQTVELLASSLQGLHRLPLEA | 123 |
| Query 131 | CPFDHQAKHRIERARTRMEAGLVDQDDLDEEHQGLAPAELFARLKARMPDGEDLVVTHGD | 190 |
| Sbjct 124 | CPFDH+ +HR+ A R EAGL+D++D D+E QG+ L L A P EDLVVTHGD CPFDHRLEHRLADASARAEAGLIDEEDFDDERQGMPVQNLLDELYASRPGQEDLVVTHGD | 183 |
| Query 191 | ACLPNIMVENGRFSGFIDCGRLGVADRYQDIALATRDIAEELGGEWADRFLVLYGIAAPD | 250 |
| Sbjct 184 | ACLPN MV G+FSGFIDCGRLGVADRYQD+AL R I LG EW F LYG+ D ACLPNFMVHQGQFSGFIDCGRLGVADRYQDLALTARSIERNLGKEWLAPFFALYGVEM-D | 242 |
| Query 251  Sbjct 243 | SQRIAFYRLLDEFF 264  ++RIAF+ LLDEFF  AERIAFFCLLDEFF 256 |  |

>TR:A0A327XY80 A0A327XY80_RHIET Aminoglycoside 3'-phosphotransferase OS=Rhizobium etli OX=29449 GN=BCL34_104333 PE=3 SV=1

Length=257

Score = 240 bits (612), Expect = 3e-76

Identities = 124/255 (49%), Positives = 165/255 (65%), Gaps = 3/255 (1%)

| Query | 12 | PAAWVERLFGYDWAQQTIGCSDAAVFRLSAQGRPVLFVKTDLSGALNELQDEAARLSWLA | 71 |
| --- | --- | --- | --- |
| Sbjct | 4 | P A + GY W + +G S + VFRL +G P L++K + A EL DEAARL WL+ PVALDSFIAGYSWDRDPVGRSPSEVFRLEREGAPALYLKREAQSAYGELADEAARLRWLS | 63 |
| Query | 72 | TTGVPCAAVLDVVTEAGRDWLLLGEVPGQDLLSSHLAPAE-KVSIMADAMRRLHTLDPAT | 130 |
| Sbjct | 64 | G+PC ++ T+ G DWLL+ +PG+DL S AE +V ++A A+R LH+LD A+  GQGLPCPEIIASTTDDGYDWLLMSALPGRDLASDSAVDAEARVVMLASALRALHSLDIAS | 123 |

| Query | 131 | CPFDHQAKHRIERARTRMEAGLVDQDDLDEEHQGLAPAELFARLKARMPDGE-DLVVTHG | 189 |
| --- | --- | --- | --- |
| Sbjct | 124 | CPFDH+ R+ A RM AGLVD++D DE G + A+L + L +MP GE DLVVTHG CPFDHRWSKRLAVAERRMRAGLVDEEDFDETRLGRSAADLHSEL-CQMPVGEEDLVVTHG | 182 |
| Query | 190 | DACLPNIMVENGRFSGFIDCGRLGVADRYQDIALATRDIAEELGGEWADRFLVLYGIAAP | 249 |
| Sbjct | 183 | DACLPN + F+G+IDCGRLGVADRYQDIALA R IA G + + F YGI + DACLPNFVASGDAFAGYIDCGRLGVADRYQDIALACRSIAANFGEAFVEPFCRHYGIVSL | 242 |
| Query  Sbjct | 250  243 | DSQRIAFYRLLDEFF 264 D+ ++ Y++LDEFF DAVKMLRYQMLDEFF 257 |  |

>TR:A0A157LWL8 A0A157LWL8_9BORD Aminoglycoside 3'-phosphotransferase OS=Bordetella trematum OX=123899 GN=neo PE=3 SV=1

Length=256

Score = 237 bits (605), Expect = 3e-75

| Identities | = 126/240 (53%), Positives = 150/240 (63%), Gaps = 2/240 (1%) |  |
| --- | --- | --- |
| Query 26 | QQTIGCSDAAVFRLSAQGRPVLFVKTDLSGALNELQDEAARLSWLATTGVPCAAVLDVVT | 85 |
| Sbjct 18 | +QTIG SDA V+RL+ F+K + + L EL E ARL W+ G+PC VL + RQTIGESDATVWRLTDADGACRFLKAEAAHPLAELPAEVARLRWMRAQGLPCPEVLQTLQ | 77 |
| Query 86 | EAGRDWLLLGEVPGQDLLSS-HLAPAEKVSIMADAMRRLHTLDPATCPFDHQAKHRIERA | 144 |
| Sbjct 78 | GR WLL+ VPG+DL S+ L+P V+++A A++RLH PA CPFDH+ R+ A ADGRHWLLMSAVPGRDLASTPDLSPQRTVALLAHALQRLHQTPPAACPFDHRLALRVAEA | 137 |
| Query 145 | RTRMEAGLVDQDDLDEEHQGLAPAELFARLKARMPDGEDLVVTHGDACLPNIMVENGRFS | 204 |
| Sbjct 138 | R AGLVD D+ D QG A L L A P EDLVVTHGDACLPN MV GRFS SRRARAGLVDVDNFDPARQGQAVEALLRALVASQPPREDLVVTHGDACLPNFMVAEGRFS | 197 |
| Query 205 | GFIDCGRLGVADRYQDIALATRDIAEELGGEWADRFLVLYGIAAPDSQRIAFYRLLDEFF | 264 |
| Sbjct 198 | GFIDC RLGVADR+QD+AL R I LG EW F YGI PD R+AFY LLDEFF  GFIDCARLGVADRHQDLALTARSIERNLGPEWLTPFFTCYGI-EPDPARLAFYCLLDEFF | 256 |

>TR:A0A0D1NZF2 A0A0D1NZF2_BRAEL Aminoglycoside 3'-phosphotransferase OS=Bradyrhizobium elkanii OX=29448 GN=QU41_08680 PE=3 SV=1

Length=256

Score = 237 bits (604), Expect = 5e-75

| Identities | | = 128/254 (50%), Positives = 158/254 (62%), Gaps = 2/254 (1%) |  |
| --- | --- | --- | --- |
| Query 12  Sbjct 4 | | PAAWVERLFGYDWAQQTIGCSDAAVFRLSAQGRPVLFVKTDLSGALNELQDEAARLSWLA P W+ + + TIG S AAVFR+ LF+K++ EL E RL WLA PMQWLGEIADGHIERLTIGESGAAVFRIRRAYGGDLFIKSEPVDEFAELPGEIDRLRWLA | 71  63 |
| Query 72  Sbjct 64 | | TTGVPCAAVLDVVTEAGRDWLLLGEVPGQDLLS-SHLAPAEKVSIMADAMRRLHTLDPAT  + +P AVLDVV E R WLL+ E+ G DL + S L AE V + A A+R LH A SRDLPAPAVLDVVVEHQRHWLLMSEIAGHDLATASDLPAAETVMLAAAALRTLHQTPIAA | 130  123 |
| Query 131  Sbjct 124 | | CPFDHQAKHRIERARTRMEAGLVDQDDLDEEHQGLAPAELFARLKARMPDGEDLVVTHGD CPFD + R+E R+ AG +D+ D DEE G +L A L A P EDLVVTHGD CPFDQRLDQRLEAVGRRVAAGRIDEADFDEERLGRTTTDLLAELLATRPVSEDLVVTHGD | 190  183 |
| Query 191  Sbjct 184 | | ACLPNIMVENGRFSGFIDCGRLGVADRYQDIALATRDIAEELGGEWADRFLVLYGIAAPD ACLPN++ + GRF+GFIDCGRLG+ADR+QD+ALA R IA LG EW + F YGI APD ACLPNLLADGGRFTGFIDCGRLGIADRHQDLALAARSIASNLGVEWTEPFFRQYGI-APD | 250  242 |
| Query 251 | | SQRIAFYRLLDEFF 264  +R+AFYRLLDEFF |  |
| Sbjct | 243 | ERRLAFYRLLDEFF 256 | |

>TR:B5ZXE0 B5ZXE0_RHILW Aminoglycoside 3'-phosphotransferase OS=Rhizobium leguminosarum bv. trifolii (strain WSM2304) OX=395492 GN=Rleg2_2693

PE=3 SV=1

Length=263

Score = 235 bits (600), Expect = 2e-74

| Identities | = 122/248 (49%), Positives = 161/248 (65%), Gaps = 1/248 (0%) |  |
| --- | --- | --- |
| Query 18 | RLFGYDWAQQTIGCSDAAVFRLSAQGRPVLFVKTDLSGALNELQDEAARLSWLATTGVPC | 77 |
| Sbjct 16 | RL GY + + +G S A VFRL A G P L++K + +GA EL DEAARL WL +G+PC RLAGYRFERDALGRSAANVFRLEAGGLPTLYLKVEEAGAFGELADEAARLDWLKASGLPC | 75 |
| Query 78 | AAVLDVVTEAGRDWLLLGEVPGQDLLS-SHLAPAEKVSIMADAMRRLHTLDPATCPFDHQ | 136 |
| Sbjct 76 | V+ ++ GR+ LL+ +PG DL S S L P +V ++A A+ LH L A+CPFDH+ PEVIARESDGGRNRLLISALPGADLASASTLTPLLRVELLAAALLELHRLPIASCPFDHR | 135 |
| Query 137 | AKHRIERARTRMEAGLVDQDDLDEEHQGLAPAELFARLKARMPDGEDLVVTHGDACLPNI | 196 |
| Sbjct 136 | + R+ A+ RM+AG++D+ D DE G + LFA L++R P EDLVVTHGDACLPN LEKRMAVAKARMQAGIIDETDFDETRLGKSAEALFAELESRRPGREDLVVTHGDACLPNF | 195 |
| Query 197 | MVENGRFSGFIDCGRLGVADRYQDIALATRDIAEELGGEWADRFLVLYGIAAPDSQRIAF | 256 |
| Sbjct 196 | + FSG+IDC RLGVADRYQDIALA R I G FL YG+ A D ++ + IACEDGFSGYIDCSRLGVADRYQDIALACRSITYNFGEALVQPFLDRYGLTALDPAKLDY | 255 |
| Query 257  Sbjct 256 | YRLLDEFF 264  ++LLDEFF  FQLLDEFF 263 |  |

>TR:A6WYI3 A6WYI3_OCHA4 Aminoglycoside 3'-phosphotransferase OS=Ochrobactrum anthropi (strain ATCC 49188 / DSM 6882 / JCM 21032 / NBRC

15819 / NCTC 12168) OX=439375 GN=Oant_1320 PE=3 SV=1

Length=266

Score = 233 bits (595), Expect = 1e-73

| Identities | = 126/255 (49%), Positives = 159/255 (62%), Gaps = 2/255 (1%) |  |
| --- | --- | --- |
| Query 12 | PAAWVERLFGYDWAQQTIGCSDAAVFRLSAQGRPVLFVKTDLSGALNELQDEAARLSWLA | 71 |
| Sbjct 12 | P A + RL GY Q +G S A V L L +K + + ++EL E ARL WLA PDALLHRLAGYSSQQDELGRSTANVLLLDHDEHEPLVLKIEPASTVSELAGEGARLEWLA | 71 |
| Query 72 | TTGVPCAAVLDVVTEAGRDWLLLGEVPGQDLLSS-HLAPAEK-VSIMADAMRRLHTLDPA | 129 |
| Sbjct 72 | G+PC V+ T+A +LL+ + G DL SS PA+ VSI+A A++ LH++DPA RQGLPCPKVVAYETQADHHFLLMTRLAGSDLASSVGTLPADSIVSILATALKALHSVDPA | 131 |
| Query 130 | TCPFDHQAKHRIERARTRMEAGLVDQDDLDEEHQGLAPAELFARLKARMPDGEDLVVTHG | 189 |
| Sbjct 132 | CPFDH+ RIE AR R+EAG VD+DD D+E +G + +LF L P ED+VVTHG ACPFDHRLDRRIEDARKRVEAGAVDEDDFDDEREGRSAEDLFTELCRLKPSQEDVVVTHG | 191 |
| Query 190 | DACLPNIMVENGRFSGFIDCGRLGVADRYQDIALATRDIAEELGGEWADRFLVLYGIAAP | 249 |
| Sbjct 192 | DACLPN M F+GFIDCGRLG ADR+QD+ALA I LG EW FL +YG A DACLPNFMASECAFTGFIDCGRLGRADRHQDLALACWSIRYNLGDEWVQPFLDIYGGPAI | 251 |
| Query 250  Sbjct 252 | DSQRIAFYRLLDEFF 264 D +IA+YRLLDEFF DETKIAYYRLLDEFF 266 |  |

>TR:A0A135I064 A0A135I064_9RHIZ Aminoglycoside 3'-phosphotransferase OS=Paramesorhizobium deserti OX=1494590 GN=ATN84_03470 PE=3 SV=1

Length=266

Score = 233 bits (593), Expect = 3e-73

| Identities | = 124/255 (49%), Positives = 156/255 (61%), Gaps = 2/255 (1%) |  |
| --- | --- | --- |
| Query 12 | PAAWVERLFGYDWAQQTIGCSDAAVFRLSAQGRPVLFVKTDLSGALNELQDEAARLSWLA | 71 |
| Sbjct 12 | P + RL GY + +G S A + L + L +K + +G ++EL EAARL WL PWSLQNRLAGYRSRKDELGRSSANILLLEHETEQPLVLKIEPAGPVSELAGEAARLEWLG | 71 |
| Query 72 | TTGVPCAAVLDVVTEAGRDWLLLGEVPGQDLLSS--HLAPAEKVSIMADAMRRLHTLDPA | 129 |
| Sbjct 72 | + + C V +E R LL+ + G DL SS L PA V I+ADA++ +H +DPA SQRLACPRVPAFESEPERSLLLMTRLAGADLASSVGTLQPALIVQILADALKAMHAIDPA | 131 |
| Query 130 | TCPFDHQAKHRIERARTRMEAGLVDQDDLDEEHQGLAPAELFARLKARMPDGEDLVVTHG | 189 |
| Sbjct 132 | CPFDH+ RIE AR RMEAG VD++D D+E G LF L P GEDLVVTHG ACPFDHRLDIRIEDARARMEAGEVDEEDFDDERSGRTAESLFEELHRLRPAGEDLVVTHG | 191 |
| Query 190 | DACLPNIMVENGRFSGFIDCGRLGVADRYQDIALATRDIAEELGGEWADRFLVLYGIAAP | 249 |

DACLPN M + GRF+GFIDCGRLG+ADR+QD+ALA	I	LG EW	FL YG

| Sbjct | 192 | DACLPNFMADEGRFTGFIDCGRLGLADRHQDLALACWSIIHNLGQEWVQPFLAAYGGPPV 251 |
| --- | --- | --- |
| Query | 250 | DSQRIAFYRLLDEFF 264 |
| Sbjct | 252 | D ++A+YRLLDEFF  DESKLAYYRLLDEFF 266 |

>TR:A0A127CNG5 A0A127CNG5_9RHIZ Aminoglycoside 3'-phosphotransferase OS=Martelella sp. AD-3 OX=686597 GN=AZF01_20235 PE=3 SV=1

Length=272

Score = 232 bits (591), Expect = 7e-73

| Identities | = 121/254 (48%), Positives = 154/254 (61%), Gaps = 1/254 (0%) |  |
| --- | --- | --- |
| Query 12 | PAAWVERLFGYDWAQQTIGCSDAAVFRLSAQGRPVLFVKTDLSGALNELQDEAARLSWLA | 71 |
| Sbjct 19 | PA RL GYDW IGCS A VF L A GRP + +K++++GA +EL E RLSWL PAGLARRLAGYDWEGVDIGCSSADVFLLRAAGRPNVVLKSEIAGAFSELPAEVERLSWLR | 78 |
| Query 72 | TTGVPCAAVLDVVTEAGRDWLLLGEVPGQDLLSSHLAPA-EKVSIMADAMRRLHTLDPAT | 130 |
| Sbjct 79 | G+ + T GR +LL+ + G DL + P +V A A+R LH A REGLASPTPIVFETGGGRHFLLMSALSGSDLARADDIPVLARVEFAASALRTLHAQPVAG | 138 |
| Query 131 | CPFDHQAKHRIERARTRMEAGLVDQDDLDEEHQGLAPAELFARLKARMPDGEDLVVTHGD | 190 |
| Sbjct 139 | CPFDH+ ++R+ A RM AGLVD+DD D++++ ELFA L A P D+VVTHGD CPFDHRLENRLRLAEARMRAGLVDEDDFDDDNRDAGAQELFAWLTANRPQETDIVVTHGD | 198 |
| Query 191 | ACLPNIMVENGRFSGFIDCGRLGVADRYQDIALATRDIAEELGGEWADRFLVLYGIAAPD | 250 |
| Sbjct 199 | A +PN M FSG+IDCGRLGVADRYQDIA+A I G E FL YG++APD ASMPNFMATAEGFSGYIDCGRLGVADRYQDIAIAVSSIQSNFGAEAVAGFLKSYGLSAPD | 258 |
| Query 251  Sbjct 259 | SQRIAFYRLLDEFF 264  + R+ +YR LDEFF  AARMRYYRTLDEFF 272 |  |

>TR:B9J736 B9J736_AGRRK Aminoglycoside 3'-phosphotransferase OS=Agrobacterium radiobacter (strain K84 / ATCC BAA-868) OX=311403 GN=Arad_3116

PE=3 SV=1

Length=267

Score = 231 bits (590), Expect = 8e-73

| Identities | | = 124/256 (48%), Positives = 162/256 (63%), Gaps = 5/256 (2%) |  |
| --- | --- | --- | --- |
| Query 12  Sbjct 14 | | PAAWVERLFGYDWAQQTIGCSDAAVFRLSAQGRPVLFVKTDLSGALNELQDEAARLSWLA PA + L GY WA +G S A+VFRL A P L++K++++ + +EL E ARL WL PARLRDLLSGYSWAADELGRSSASVFRLEADSLPPLYLKSEINSSFSELAGEVARLRWLG | 71  73 |
| Query 72  Sbjct 74 | | TTGVPCAAVLDVVTEAG--RDWLLLGEVPGQDLLSSHL-APAEKVSIMADAMRRLHTLDP G+ C AV V EAG R+WLL+ + G DL S+ L +P E+V + A+A+R LH + AQGLVCPAV--VAHEAGGEREWLLMNALDGADLASASLISPLERVRMFAEALRDLHRIRI | 128  131 |
| Query 129  Sbjct 132 | | ATCPFDHQAKHRIERARTRMEAGLVDQDDLDEEHQGLAPAELFARLKARMPDGEDLVVTH CPFDH++ RI A+ RM AG VD+ D DE G + LF L+ R P EDLVVTH DACPFDHRSDARIAAAKARMLAGRVDETDFDEARLGRSAESLFRELEERRPAAEDLVVTH | 188  191 |
| Query 189  Sbjct 192 | | GDACLPNIMVENGRFSGFIDCGRLGVADRYQDIALATRDIAEELGGEWADRFLVLYGIAA GDACLPN + + RF+G+IDC RLGVADRYQDIALA I G FL YGI+ GDACLPNFIAADHRFAGYIDCCRLGVADRYQDIALACGSIVYNFGEALVASFLETYGISE | 248  251 |
| Query 249 | | PDSQRIAFYRLLDEFF 264 D++++ +YRLLDEFF |  |
| Sbjct | 252 | VDAEKMDYYRLLDEFF 267 | |

>TR:A0A0Q7EA18 A0A0Q7EA18_9RHIZ Aminoglycoside 3'-phosphotransferase OS=Devosia sp. Root413D1 OX=1736531 GN=ASC89_06525 PE=3 SV=1

Length=251

Score = 228 bits (581), Expect = 1e-71

Identities = 126/258 (49%), Positives = 156/258 (60%), Gaps = 18/258 (7%)

| Query | 12 | PAAWVERLFGYDWAQQTIGCSDAAVFRLSAQGRPVLFVKTDLSGALNELQDEAARLSWLA | 71 |
| --- | --- | --- | --- |
| Sbjct | 7 | P WV + D QTIG S A V+R A F+K++ L EL E RL WL PPGWVRNI---DLVPQTIGESGAWVYRAGAAH FIKSEPISPLAELPGEIERLRWLH | 59 |
| Query | 72 | TTGVPCAAVLDVVTEAGRDWLLLGEVPGQDLLS SHLAPAEKVSIMADAMRRLHTL | 126 |
| Sbjct | 60 | TG+PC AV+D GR WLL+ VPG DL S S + + ++A+A+R+LH L DTGLPCPAVIDAADHGGRHWLLMTAVPGHDLASTPGVTSEVTAETAIRVVAEALRQLHGL | 119 |
| Query | 127 | DPATCPFDHQAKHRIERARTRMEAGLVDQDDLDEEHQGLAPAELFARLKARMPDGEDLVV | 186 |
| Sbjct | 120 | DPA+CPFDH+A R+ A R AGL D +D D+ PA+ +ARL A +P EDLVV DPASCPFDHRASIRVAAASARYHAGLYDGNDPDQ GPAD-YARLIAGVPTAEDLVV | 173 |
| Query | 187 | THGDACLPNIMVENGRFSGFIDCGRLGVADRYQDIALATRDIAEELGGEWADRFLVLYGI | 246 |
| Sbjct | 174 | THGDAC PN M E+G F+GFIDCGRLGVADRYQD+ALA R + G FL YGI THGDACFPNFMAEDGHFTGFIDCGRLGVADRYQDLALACRSLDWNYGAASIPAFLAAYGI | 233 |
| Query  Sbjct | 247  234 | AAPDSQRIAFYRLLDEFF 264 A PD ++ +Y LLDEFF AEPDRAKLEWYTLLDEFF 251 |  |

>TR:A0A0U3QBG6 A0A0U3QBG6_9RHOB Aminoglycoside 3'-phosphotransferase OS=Pannonibacter phragmitetus OX=121719 GN=APZ00_03710 PE=3 SV=1

Length=251

Score = 228 bits (581), Expect = 1e-71

| Identities | = 120/249 (48%), Positives = 155/249 (62%), Gaps = 2/249 (1%) |  |
| --- | --- | --- |
| Query 17 | ERLFGYDWAQQTIGCSDAAVFRLSAQGRPVLFVKTDLSGALNELQDEAARLSWLATTGVP | 76 |
| Sbjct 4 | ERL GY + G S A VFR++ G P LF+K E++ EA+ L WL + G+ ERLKGYSGTAISTGRSSANVFRMAGSGVPALFLKHGFGEGAAEIEAEASCLRWLKSEGIA | 63 |
| Query 77 | CAAVLDVVTEAGRDWLLLGEVPGQDLLSS-HLAPAEKVSIMADAMRRLHTLDPATCPFDH | 135 |
| Sbjct 64 | CA V++ VT G WLL EV G+D+ +S LAP E V+I A+ +RRLH L+P+ CPFD CAEVIEQVTTDGSVWLLSSEVTGRDVATSMQLAPNEVVAIAAEGLRRLHALNPSKCPFDQ | 123 |
| Query 136 | QAKHRIERARTRMEAGLVDQDDLDEEHQGLAPAELFARLKARMPDGEDLVVTHGDACLPN | 195 |
| Sbjct 124 | + RI+ AR R+E LVD+ D D E G +F L A P EDLVVTHGDA N RLDRRIDLARERVEMNLVDETDFDAERMGRTARSVFEDLIAHRPRAEDLVVTHGDATFEN | 183 |
| Query 196 | IMVENGRFSGFIDCGRLGVADRYQDIALATRDIAEELGGEWADRFLVLYGIAAPDSQRIA | 255 |
| Sbjct 184 | +M +GRFSGF+DCGRLGVADRY+D+AL R I + LG +W F +YG D RI LMQCDGRFSGFVDCGRLGVADRYRDLALIARSIEDRLGRQWVQTFFDIYG-CHYDETRIR | 242 |
| Query 256  Sbjct 243 | FYRLLDEFF 264  +Y++LDEFF  YYQILDEFF 251 |  |

>TR:J2KXC7 J2KXC7_9RHIZ Aminoglycoside 3'-phosphotransferase OS=Rhizobium sp. CF142 OX=1144314 GN=PMI11_05783 PE=3 SV=1

Length=265

Score = 226 bits (577), Expect = 7e-71

| Identities | = 114/254 (45%), Positives = 161/254 (63%), Gaps = 1/254 (0%) |  |
| --- | --- | --- |
| Query 12 | PAAWVERLFGYDWAQQTIGCSDAAVFRLSAQGRPVLFVKTDLSGALNELQDEAARLSWLA | 71 |
| Sbjct 12 | P + L GY+W + +G S A VFRL + RP L++K + +G EL +EA RL WL PVSLRNHLGGYEWRRDALGRSSACVFRLEGERRPGLYLKIEDAGPFCELAEEAERLVWLR | 71 |
| Query 72 | TTGVPCAAVLDVVTEAGRDWLLLGEVPGQDLLSSH-LAPAEKVSIMADAMRRLHTLDPAT | 130 |
| Sbjct 72 | + G+ C V+ ++ R++LL+ +PG DL+++ L P E++ I+A A+ LH+LD SQGLDCPEVIAHESDGERNFLLMTALPGVDLVTAATLLPEERIEILASALSALHSLDINA | 131 |
| Query 131 | CPFDHQAKHRIERARTRMEAGLVDQDDLDEEHQGLAPAELFARLKARMPDGEDLVVTHGD | 190 |
| Sbjct 132 | CPFDH+ + R+ A RM AG+VD+ D DEE G + LF L+ P+ E+LV+THGD CPFDHRLEKRLAAAAARMRAGVVDETDFDEERLGKSAFFLFGELQRLRPESENLVITHGD | 191 |
| Query 191 | ACLPNIMVENGRFSGFIDCGRLGVADRYQDIALATRDIAEELGGEWADRFLVLYGIAAPD | 250 |
| Sbjct 192 | ACLPN M RF+G+IDC RLGVADRYQD+ALA I G E+ FL YG++ D  ACLPNFMAAGDRFAGYIDCCRLGVADRYQDVALACHSIGYNFGEEFVRLFLNAYGLSIID | 251 |

Query 251 SQRIAFYRLLDEFF 264

++ +Y+LLDEFF Sbjct 252 PVKMDYYQLLDEFF 265

>TR:A0A1N6GVZ6 A0A1N6GVZ6_9BURK Aminoglycoside 3'-phosphotransferase OS=Paraburkholderia phenazinium OX=60549 GN=SAMN05444165_0998 PE=3

SV=1

Length=256

Score = 226 bits (575), Expect = 1e-70

| Identities | | = 119/254 (47%), Positives = 156/254 (61%), Gaps = 2/254 (1%) |  |
| --- | --- | --- | --- |
| Query 12  Sbjct 4 | | PAAWVERLFGYDWAQQTIGCSDAAVFRLSAQGRPVLFVKTDLSGALNELQDEAARLSWLA P W ++ G +Q IG S A V R+ + LF+K++ +G +EL E RL W+ PTRWRKKFAGARVERQVIGESGAEVLRIRCRSGEYLFLKSEPTGLPSELPGEVERLRWMK | 71  63 |
| Query 72 | | TTGVPCAAVLDVVTEAGRDWLLLGEVPGQDLLSSH-LAPAEKVSIMADAMRRLHTLDPAT  +P VLD E R WLL+ VPG DL S+ L+P++ V I+A A+R LH + A | 130 |
| Sbjct | 64 | RLDLPAPTVLDDAVEDNRHWLLMTAVPGFDLASAKDLSPSQVVGILAMALRSLHQVPIAE | 123 |
| Query | 131 | CPFDHQAKHRIERARTRMEAGLVDQDDLDEEHQGLAPAELFARLKARMPDGEDLVVTHGD | 190 |
| Sbjct | 124 | CPF+H+ + RI A R++AGLVD+ D D E +G + A L + P+ DLVVTHGD CPFNHRIEERIAAASNRVDAGLVDEADFDTERRGRTATDALAELLSTRPETYDLVVTHGD | 183 |
| Query | 191 | ACLPNIMVENGRFSGFIDCGRLGVADRYQDIALATRDIAEELGGEWADRFLVLYGIAAPD | 250 |
| Sbjct | 184 | ACLPN M E F+GFIDCGRLGV+DRYQD+AL R IA LG W + F YG+ PD ACLPNFMAEGSGFTGFIDCGRLGVSDRYQDLALTARSIARNLGQAWVEPFFRDYGV-EPD | 242 |
| Query | 251 | SQRIAFYRLLDEFF 264  +R+AFY LLDEFF |  |
| Sbjct | 243 | HRRMAFYCLLDEFF 256 | |

>TR:U4VD20 U4VD20_9RHIZ Aminoglycoside 3'-phosphotransferase OS=Ochrobactrum intermedium 229E OX=1337887 GN=Q644_14020 PE=3 SV=1

Length=267

Score = 219 bits (559), Expect = 4e-68

| Identities | | = 119/256 (46%), Positives = 155/256 (61%), Gaps = 3/256 (1%) |  |
| --- | --- | --- | --- |
| Query 12  Sbjct 12 | | PAAWVERLFGYDWAQQTIGCSDAAVFRLSAQGRPVLFVKTDLSGALNELQDEAARLSWLA P A ++L Y Q +G S A V L + L +K + + EL DEAARL WL PQALRQQLRDYRSRQDELGRSTANVHLLDHPEQEPLVLKVEPQSPVGELADEAARLRWLR | 71  71 |
| Query 72  Sbjct 72 | | TTGVPCAAVLDVVTEAGRDWLLLGEVPGQDLLSS--HLAPAEKVSIMADAMRRLHTLDPA  + C VL +LL+ +PGQDL S+ LAP V I+A A++ +H +DPA QQNLACPEVLAFEIAPEHSFLLMTRLPGQDLASAVGTLAPERIVDILASALKSMHAIDPA | 129  131 |
| Query 130  Sbjct 132 | | TCPFDHQAKHRIERARTRMEAGLVDQDDL-DEEHQGLAPAELFARLKARMPDGEDLVVTH  +CPFDH+ + RIE AR R+EAG VD++D DEE +G P +F L P ED+VVTH SCPFDHRLEFRIEDARKRVEAGAVDEEDFXDEEREGRTPQGVFEELLRLKPQSEDIVVTH | 188  191 |
| Query 189  Sbjct 192 | | GDACLPNIMVENGRFSGFIDCGRLGVADRYQDIALATRDIAEELGGEWADRFLVLYGIAA GDACLPN + + F+GFIDCGRLG+ADR+QD+ALA I LG EW FL YG A GDACLPNFIADKDAFTGFIDCGRLGLADRHQDLALACWSIRYNLGEEWVAPFLATYGGPA | 248  251 |
| Query 249 | | PDSQRIAFYRLLDEFF 264  + +I++YRLLDEFF |  |
| Sbjct | 252 | IEEAKISYYRLLDEFF 267 | |

```
>TR:A0A3S4CF94 A0A3S4CF94_9RHIZ Aminoglycoside 3'-phosphotransferase OS=Devosia sp. M6-77 OX=2490941 GN=neo PE=3 SV=1
Length=258

Score = 201 bits (511), Expect = 5e-61
Identities = 107/242 (44%), Positives = 140/242 (58%), Gaps = 1/242 (0%)

Query 23	DWAQQTIGCSDAAVFRLSAQGRPVLFVKTDLSGALNELQDEAARLSWLATTGVPCAAVLD 82 DWA+ IG SD++V+R++	+F+K +	L EL E RL+WL	G	V+D
Sbjct 18	DWAEINIGQSDSSVWRITLGDGNAVFLKAEPRHELAELPGEIERLNWLTKMGFKAPRVVD 77
```

| Query | 83 | VVTEAGRDWLLLGEVPGQDLLSSHLAPAEKVSIMADAMRRLHTLDPATCPFDHQAKHRIE | 142 |
| --- | --- | --- | --- |
| Sbjct | 78 | A R WLL+ VPGQDL PA+ V A ++R+H LDP CPFDH R+ AEQSATRTWLLMTAVPGQDLTHYTDRPADFVRAYAQGLKRMHALDPTHCPFDHGIDARLA | 137 |
| Query | 143 | RARTRMEAGLVDQDDLDEEHQGLAPAELFARLKARMPDGEDLVVTHGDACLPNIMVENGR | 202 |
| Sbjct | 138 | R++AGLVD+DD D + +G E+ L A PD ++VTHGDA PNI+ E+GR EGAARLQAGLVDEDDFDSDRKGWTGLEVLDWLHANRPDTGPMIVTHGDASTPNILAEDGR | 197 |
| Query | 203 | FSGFIDCGRLGVADRYQDIALATRDIAEELGGEWADRFLVLYGIAAPDSQRIAFYRLLDE | 262 |
| Sbjct | 198 | FSG IDCGR+G AD +QD+A+A R IA LG + FL YG A D + FY LDE  FSGMIDCGRVGRADVWQDLAIACRSIAYNLGRDHVTAFLKAYG-AEWDEAKYTFYCTLDE | 256 |
| Query | 263 FF 264 | | |
|  |  | F |  |
| Sbjct | 257 | LF 258 | |

>TR:A0A1I7NDQ9 A0A1I7NDQ9_9RHIZ Aminoglycoside 3'-phosphotransferase OS=Devosia crocina OX=429728 GN=SAMN05216456_1766 PE=3 SV=1

Length=257

Score = 198 bits (503), Expect = 8e-60

| Identities | | = 108/246 (44%), Positives = 143/246 (58%), Gaps = 2/246 (1%) |  |
| --- | --- | --- | --- |
| Query 19  Sbjct 14 | | LFGYDWAQQTIGCSDAAVFRLSAQGRPVLFVKTDLSGALNELQDEAARLSWLATTGVPCA L ++W IG S AAV+R+ G+ L++K + + L E+ E RL +LA G P LDSHEWLPVAIGRSRAAVWRIGI-GKTALYLKAEPNHELAEMPRELLRLDYLAGMGFPAP | 78  72 |
| Query 79  Sbjct 73 | | AVLDVVTEAGRDWLLLGEVPGQDLLSSHLAPAEKVSIMADAMRRLHTLDPATCPFDHQAK V+D V WLL+ EV GQDL + + ++A+ +RRLH LDP CPFDH  RVVDHVQADDFSWLLMTEVKGQDLTRTLERRVDVARMLAEGLRRLHALDPVNCPFDHALD | 138  132 |
| Query 139  Sbjct 133 | | HRIERARTRMEAGLVDQDDLDEEHQGLAPAELFARLKARMPDGEDLVVTHGDACLPNIMV R+ A + AGLVD+ D D++ QG +F L A P E+ VVTHGDACLPN+M QRLADAERNLAAGLVDESDFDDDRQGWTGQAVFDWLVANRPAEEERVVTHGDACLPNVMS | 198  192 |
| Query 199  Sbjct 193 | | ENGRFSGFIDCGRLGVADRYQDIALATRDIAEELGGEWADRFLVLYGIAAPDSQRIAFYR  +GRFSG +DCGRLGVADR+QD+ALA R + G E FL YG D++R +Y  VDGRFSGVVDCGRLGVADRWQDLALACRSLQSNGGTEVVGEFLSAYGTEF-DAERYTYYC | 258  251 |
| Query | 259 LLDEFF 264 | | |
|  |  | LDE F |  |
| Sbjct | 252 | TLDEMF 257 | |

>TR:A0A2L1SA27 A0A2L1SA27_9RHIZ Aminoglycoside 3'-phosphotransferase OS=Devosia sp. I507 OX=2083786 GN=C4375_17355 PE=3 SV=1

Length=258

Score = 197 bits (501), Expect = 2e-59

| Identities | = 105/241 (44%), Positives = 134/241 (56%), Gaps = 1/241 (0%) |  |
| --- | --- | --- |
| Query 24 | WAQQTIGCSDAAVFRLSAQGRPVLFVKTDLSGALNELQDEAARLSWLATTGVPCAAVLDV | 83 |
| Sbjct 19 | W Q TIG S V+R++ +F+K + L EL E RL+WL G V+D WTQITIGRSTCDVWRIALGDGNAVFLKAETVHPLGELPGEIERLNWLTRMGFKAPRVVDA | 78 |
| Query 84 | VTEAGRDWLLLGEVPGQDLLSSHLAPAEKVSIMADAMRRLHTLDPATCPFDHQAKHRIER | 143 |
| Sbjct 79 | + R WLL+ VPG DL P V A A++RLH LDP TCPFDH R+ EQGSNRLWLLMSAVPGADLTHYDKEPEIVVRAYAQALKRLHALDPTTCPFDHSIDARLTL | 138 |
| Query 144 | ARTRMEAGLVDQDDLDEEHQGLAPAELFARLKARMPDGEDLVVTHGDACLPNIMVENGRF | 203 |
| Sbjct 139 | A R+ AGLVD+ D D E G ++ L A +P+ D +VTHGDAC PNI+ +GRF AEQRLAAGLVDETDFDAERLGWTGVQVLEWLHANLPETGDTIVTHGDACTPNILAVDGRF | 198 |
| Query 204 | SGFIDCGRLGVADRYQDIALATRDIAEELGGEWADRFLVLYGIAAPDSQRIAFYRLLDEF | 263 |
| Sbjct 199 | SG +DCGRLG AD +QD+A+A R I + +G E FL YG A D + FY LDE SGIVDCGRLGTADVWQDLAIACRSIRDNIGKEHIAPFLAAYG-AVWDEAKFRFYCTLDEM | 257 |
| Query 264  Sbjct 258 | F 264 F  F 258 |  |

>TR:A0A0Q8ISX4 A0A0Q8ISX4_9RHIZ Aminoglycoside 3'-phosphotransferase OS=Devosia sp. Root685 OX=1736587 GN=ASD83_03955 PE=3 SV=1

Length=257

Score = 196 bits (499), Expect = 3e-59

| Identities | | = 103/242 (43%), Positives = 143/242 (59%), Gaps = 2/242 (1%) |  |
| --- | --- | --- | --- |
| Query 23  Sbjct 18 | | DWAQQTIGCSDAAVFRLSAQGRPVLFVKTDLSGALNELQDEAARLSWLATTGVPCAAVLD  +W TIG S A+V+R+ A G LF+K + + L+E+ DE RL +LA G P V+D EWEPITIGESKASVWRI-AMGESALFLKAEPAHDLSEMADELLRLDYLAEMGFPAPRVVD | 82  76 |
| Query 83  Sbjct 77 | | VVTEAGRDWLLLGEVPGQDLLSSHLAPAEKVSIMADAMRRLHTLDPATCPFDHQAKHRIE V +WLL+ V G+DL P + ++AD ++RLH LDPA CPFDH R+ HVIGDDFNWLLMTAVKGRDLTHLGDEPQVLIQVLADGLKRLHALDPANCPFDHSLARRLA | 142  136 |
| Query 143  Sbjct 137 | | RARTRMEAGLVDQDDLDEEHQGLAPAELFARLKARMPDGEDLVVTHGDACLPNIMVENGR A + AG +D+ D D+E G ++F L + P+ VVTHGDACLPN + + R DAEANVLAGRIDETDFDDERNGWTGQQVFDWLASNRPEETSHVVTHGDACLPNFLADGNR | 202  196 |
| Query 203  Sbjct 197 | | FSGFIDCGRLGVADRYQDIALATRDIAEELGGEWADRFLVLYGIAAPDSQRIAFYRLLDE F G +DC RLGVADR+QD+ALA R + G ++ + FL YG A D++R AFY +DE FGGVVDCARLGVADRWQDLALACRSLEFNGGKDFVEEFLAAYG-AEFDAERYAFYCAMDE | 262  255 |
| Query | 263 FF 264 | | |
|  |  | F |  |
| Sbjct | 256 | LF 257 | |

>TR:A0A0F5FLC5 A0A0F5FLC5_9RHIZ Aminoglycoside 3'-phosphotransferase OS=Devosia chinhatensis OX=429727 GN=VE26_07165 PE=3 SV=1

Length=248

Score = 195 bits (495), Expect = 1e-58

| Identities | | = 102/242 (42%), Positives = 138/242 (57%), Gaps = 1/242 (0%) |  |
| --- | --- | --- | --- |
| Query 23  Sbjct 8 | | DWAQQTIGCSDAAVFRLSAQGRPVLFVKTDLSGALNELQDEAARLSWLATTGVPCAAVLD  +W + T+G S +V+R++ +F+K + L EL E RL+WL G V+D NWREITLGKSGCSVWRIALNDGNAVFLKAEPIHPLAELPGEIERLNWLTRMGFKSPRVID | 82  67 |
| Query 83  Sbjct 68 | | VVTEAGRDWLLLGEVPGQDLLSSHLAPAEKVSIMADAMRRLHTLDPATCPFDHQAKHRIE R WLL+ VPG+DL P V A ++R+H LDPA CPFDH +R+E TEQTQDRVWLLMSAVPGEDLTRYTARPDVFVRAYAQGLKRIHALDPAQCPFDHHPDNRLE | 142  127 |
| Query 143  Sbjct 128 | | RARTRMEAGLVDQDDLDEEHQGLAPAELFARLKARMPDGEDLVVTHGDACLPNIMVENGR AR R+EAGLVD+ D D EH+G + +++ L A P VVTHGDA PNI+ ++GR EARQRLEAGLVDEGDFDVEHEGWSAQQVYDWLFANRPSIGTRVVTHGDASTPNILAQDGR | 202  187 |
| Query 203  Sbjct 188 | | FSGFIDCGRLGVADRYQDIALATRDIAEELGGEWADRFLVLYGIAAPDSQRIAFYRLLDE FSG +DCGR+GVAD +QD+ +A R I +G E FL YG D + FY LDE FSGLVDCGRVGVADVWQDLTIACRSIRYNVGEEHVATFLAAYG-TEWDEAKYRFYNTLDE | 262  246 |
| Query | 263 FF 264 | | |
|  |  | F |  |
| Sbjct | 247 | LF 248 | |

>TR:A0A4P6JTH1 A0A4P6JTH1_9CHLR Aminoglycoside 3'-phosphotransferase OS=Ktedonosporobacter rubrisoli OX=2509675 GN=EPA93_24010 PE=3 SV=1

Length=258

Score = 192 bits (488), Expect = 2e-57

Identities = 114/265 (43%), Positives = 157/265 (59%), Gaps = 15/265 (6%)

| Query | 7 | LHAGSPAAWVERL-FGYDWAQQTIGCSDAAVFRLSAQGRPVLFVKTDLSGALNELQDEAA | 65 |
| --- | --- | --- | --- |
| Sbjct | 2 | L AG P A L G W TIGCS A+VFR+ + ++K GA + L +E LLAGLPPALRALLESGASWQPITIGCSGASVFRIGDR YLKIAPHGAADSLCEEEE | 56 |
| Query | 66 | RLSWLATTGVPCAAVLDVVTEAGRDWLLLGEVPG QDLLSSHLAPAEKVSIMADAMR | 121 |
| Sbjct | 57 | RL WL +P V +A R++LL+ EV G +++ + + V +MA +  RLQWLYGR-LPVPEVYYYGRDAEREYLLMSEVVGTMACEEVFKTDME--RLVVLMAQGLH | 113 |

| Query | 122 | RLHTLDPATCPFDHQAKHRIERARTRMEAGLVDQDDLDEEHQGLAPAELFARLKARMPDG | 181 |
| --- | --- | --- | --- |
| Sbjct | 114 | +H ++ A CPFD + R+E AR RM AG VD++D DEE +GL +L+A+L P MVHAVEIADCPFDQRLSIRLEEARRRMLAGQVDEEDFDEERRGLGVRQLYAQLLQARPAS | 173 |
| Query | 182 | EDLVVTHGDACLPNIMV--ENGRFSGFIDCGRLGVADRYQDIALATRDIAEELGGEWADR | 239 |
| Sbjct | 174 | EDLV THGD CLPNI++ E R +GFID GR G+ADRYQDIALA R +A LG EW + EDLVFTHGDFCLPNILIDREEMRVNGFIDLGRCGIADRYQDIALACRSLAFNLGPEWIPQ | 233 |
| Query  Sbjct | 240  234 | FLVLYGIAAPDSQRIAFYRLLDEFF 264 FL YG+++ + ++ FY+LLDEFF FLDAYGLSSVEQDKVHFYQLLDEFF 258 |  |

>TR:A0A1H9FPI4 A0A1H9FPI4_9RHIZ Aminoglycoside 3'-phosphotransferase OS=Devosia sp. YR412 OX=1881030 GN=SAMN05428969_2913 PE=3 SV=1

Length=286

Score = 189 bits (481), Expect = 4e-56

| Identities | = 105/237 (44%), Positives = 137/237 (58%), Gaps = 2/237 (1%) |  |
| --- | --- | --- |
| Query 28 | TIGCSDAAVFRLSAQGRPVLFVKTDLSGALNELQDEAARLSWLATTGVPCAAVLDVVTEA | 87 |
| Sbjct 52 | T+G SDA V+R+ +G+ LF+K + L+EL E RL WL T VP A V D +A TLGRSDAMVWRIEDEGQ-ALFLKAEAIHPLSELPGETRRLRWLEKTPVPAAKVRDSFADA | 110 |
| Query 88 | GRDWLLLGEVPGQDLLSSHLAPAEKVSIMADAMRRLHTLDPATCPFDHQAKHRIERARTR | 147 |
| Sbjct 111 | G +WLL+ +PG DL P + I+A +R +H LDP TCPFDH+ ++ GFNWLLMTALPGTDLTHLVDRPEDLRKILATGLRAMHALDPVTCPFDHRLDAKLSSGSAN | 170 |
| Query 148 | MEAGLVDQDDLDEEHQGLAPAELFARLKARMPDGEDLVVTHGDACLPNIMVENGRFSGFI | 207 |
| Sbjct 171 | ++AG VD+ D D+ G ++ L P EDLVVTHGDA LPNIM +GRF+G I VKAGRVDESDFDDARDGWTAHQVLDWLLDNRPPAEDLVVTHGDASLPNIMASDGRFAGVI | 230 |
| Query 208  Sbjct 231 | DCGRLGVADRYQDIALATRDIAEELGGEWADRFLVLYGIAAPDSQRIAFYRLLDEFF 264 D GRLGVADR+QD+ALA R + G E FL YG A + R +Y LDE F DVGRLGVADRWQDLALACRSLIYNCGPEHVAPFLSAYG-ADWNEARYHYYCTLDELF 286 |  |

>TR:A0A4Y8LPN5 A0A4Y8LPN5_9BACL Aminoglycoside 3'-phosphotransferase OS=Cohnella luojiensis OX=652876 GN=E2980_23155 PE=3 SV=1

Length=260

Score = 188 bits (478), Expect = 5e-56

| Identities | | = 110/246 (45%), Positives = 140/246 (57%), Gaps = 7/246 (3%) |  |
| --- | --- | --- | --- |
| Query 24  Sbjct 17 | | WAQQTIGCSDAAVFRLSAQGRPV-LFVKTDLSGALNELQDEAARLSWLATTGVPCAAVLD W TIGCS A FRL G V +++K LQ E RL WL +P ++D WTPITIGCSVAQTFRLQLPGHGVGMYLKMVDRDTRRSLQPEYERLVWLQGK-LPVPNIID | 82  75 |
| Query 83 | | VVTEAGRDWLLLGEVPGQ---DLLSSHLAPAEKVSIMADAMRRLHTLDPATCPFDHQAKH VTE R++LL E+PG D SS+ P V +A A+RR+H L CPF+ | 139 |
| Sbjct | 76 | FVTEDNREYLLTVELPGTHAADQSSSNSLPGI-VQELAQALRRIHALPIDDCPFEKNLTI | 134 |
| Query | 140 | RIERARTRMEAGLVDQDDLDEEHQGLAPAELFARLKARMPDGEDLVVTHGDACLPNIMV- | 198 |
| Sbjct | 135 | I AR ME GLVD +D D + G + L+ L P EDLV THGD CLPNI++ HIAEARQNMELGLVDVEDFDRKRSGRSSDSLYRELLITRPQNEDLVFTHGDYCLPNILLC | 194 |
| Query | 199 | ENGRFSGFIDCGRLGVADRYQDIALATRDIAEELGGEWADRFLVLYGIAAPDSQRIAFYR | 258 |
| Sbjct | 195 | E+GR SG+ID G G+ADRYQD+ALA R + G WA+ FL YG+ PD ++I FY+ EDGRLSGYIDWGNAGIADRYQDLALAHRSLIYNFGMTWAELFLHEYGLERPDREKIEFYQ | 254 |
| Query  Sbjct | 259  255 | LLDEFF 264 LLDEFF LLDEFF 260 |  |

>TR:A0A0F5L6W5 A0A0F5L6W5_9RHIZ Aminoglycoside 3'-phosphotransferase OS=Devosia soli OX=361041 GN=VW35_12425 PE=3 SV=1

Length=251

Score = 187 bits (475), Expect = 1e-55

Identities = 99/242 (41%), Positives = 139/242 (57%), Gaps = 2/242 (1%)

| Query | 23 | DWAQQTIGCSDAAVFRLSAQGRPVLFVKTDLSGALNELQDEAARLSWLATTGVPCAAVLD | 82 |
| --- | --- | --- | --- |
| Sbjct | 12 | +W TIG S A+V+R+ LF+K D + L+EL E RL +LA G P ++D EWHATTIGKSGASVWRIP-MAENALFLKADPAHELSELSAEVLRLDYLAEMGFPAPRIVD | 70 |
| Query | 83 | VVTEAGRDWLLLGEVPGQDLLSSHLAPAEKVSIMADAMRRLHTLDPATCPFDHQAKHRIE | 142 |
| Sbjct | 71 | V G +WLL+ EV G+DL P + ++AD ++RLH LDPA+CPFDH R+ YVDGEGFNWLLMTEVKGKDLTHLVDEPQVLIQVLADGLKRLHALDPASCPFDHSLDRRLS | 130 |
| Query | 143 | RARTRMEAGLVDQDDLDEEHQGLAPAELFARLKARMPDGEDLVVTHGDACLPNIMVENGR | 202 |
| Sbjct | 131 | + + G VD+ D D+ H+G +++ L A+ P D VVTHGDA LPN + + R DGKANILGGRVDETDFDDRHEGWTAQQVYDWLVAQRPAETDRVVTHGDASLPNFLAGDSR | 190 |
| Query | 203 | FSGFIDCGRLGVADRYQDIALATRDIAEELGGEWADRFLVLYGIAAPDSQRIAFYRLLDE | 262 |
| Sbjct | 191 | FSG +DC RLGVADR+QD+AL + G + FL YG+ D++R +Y LDE  FSGIVDCARLGVADRWQDLALTCWSLEFNGGMDMVHAFLDAYGVRF-DAERYQYYCALDE | 249 |
| Query | 263 FF 264 | | |
|  |  | F |  |
| Sbjct | 250 | LF 251 | |

>TR:A0A554A1N3 A0A554A1N3_9BACI Aminoglycoside 3'-phosphotransferase OS=Bacillus sp. P16(2019) OX=2597270 GN=FN960_03505 PE=3 SV=1

Length=256

Score = 187 bits (475), Expect = 1e-55

| Identities | | = 105/257 (41%), Positives = 148/257 (58%), Gaps = 8/257 (3%) |  |
| --- | --- | --- | --- |
| Query 12  Sbjct 4 | | PAAWVERLFGYDWAQQTIGCSDAAVFRLSAQGRPVLFVKT-DLSGALNELQDEAARLSWL P + E + GY W Q TIG S + V++L +G LF+KT D L DE +L WL PISIEELIQGYSWEQNTIGLSSSNVYQL--RGERTLFLKTQDRKHPFESLYDEKQKLEWL | 70  61 |
| Query 71  Sbjct 62 | | ATTGVPCAAVLDVVTEAGRDWLLLGEVPGQDLLSSHLAPAEKVSIM---ADAMRRLHTLD A +P VL ++LL+ +PG D S P + +I+ AD +++LH +D ADK-LPVPNVLFYEQAGQTEYLLMTALPGLDA-SRITEPTQLPTIIQAYADGLKQLHQID | 127  119 |
| Query 128  Sbjct 120 | | PATCPFDHQAKHRIERARTRMEAGLVDQDDLDEEHQGLAPAELFARLKARMPDGEDLVVT CPF+ Q ++++A ++AG+VD+DD D+ G + EL LK P EDLV T IWDCPFNEQNHIKVKKAEEHVKAGIVDEDDFDQIRLGKSSEELLEELKMFPPFEEDLVFT | 187  179 |
| Query 188  Sbjct 180 | | HGDACLPNIMVENGRFSGFIDCGRLGVADRYQDIALATRDIAEELGGEWADRFLVLYGIA HGD CLPNI++++G GFID GR G+ADRYQDIALA R I G EW F+ YG+ HGDYCLPNIIIKDGACQGFIDLGRAGIADRYQDIALAVRSITHNFGSEWVSTFVTHYGLT | 247  239 |
| Query 248 | | APDSQRIAFYRLLDEFF 264  + ++ +Y+LLDEFF |  |
| Sbjct | 240 | EFEQNKVFYYQLLDEFF 256 | |

>TR:K2N3F1 K2N3F1_9RHIZ Aminoglycoside 3'-phosphotransferase OS=Nitratireductor pacificus pht-3B OX=391937 GN=NA2_11360 PE=3 SV=1

Length=270

Score = 184 bits (468), Expect = 2e-54

| Identities | = 104/261 (40%), Positives = 153/261 (59%), Gaps = 7/261 (3%) |  |
| --- | --- | --- |
| Query 9 | AGSPAAWVERLFGYDWAQQTIGCSDAAVFRLSAQ-GRPVLFVKTDLSGALNELQDEAARL | 67 |
| Sbjct 12 | A PA E L GY WA+ TIG + V+RL + G P LF+K + N+L DE RL APMPAGLSEELTGYAWARNTIGQAGGTVYRLHGKPGAPDLFLKHGRNTVANDLVDEMVRL | 71 |
| Query 68 | SWLATTGVPCAAVLDVVTEAGRDWLLLGEVPGQDLLSSHLA-PAEKVSI---MADAMRRL | 123 |
| Sbjct 72 | WLA VP V V+ + WLL+ +PG+ + A PA +V+I +A+ +R L RWLADH-VPVPIVTHFVSTSDEAWLLMTALPGETAYQAIEAHPANRVAIVDALAEFLRTL | 130 |
| Query 124 | HTLDPATCPFDHQAKHRIERARTRMEAGLVDQDDLDEEHQGLAPAELFARLKARMPDGED | 183 |
| Sbjct 131 | HT+ ++CPF HR+ AR R++AGLVD+DD D++ G +++ ++ +P D HTIPISSCPFTSNHAHRLGLARKRIDAGLVDEDDFDDDRDGWTAEQVWQEMQKNLPLAPD | 190 |
| Query 184 | LVVTHGDACLPNIMVENGRFSGFIDCGRLGVADRYQDIALATRDIAEELGGEWADRFLVL | 243 |
| Sbjct 191 | VVTHGD L N+++ +GR +G +D GR+G+ADRYQD+A+ + E G +RFL  PVVTHGDFSLDNLLILDGRVTGCVDAGRVGIADRYQDLAILWNGLG-EFGEPLQERFLTR | 249 |

Query 244 YGIAAPDSQRIAFYRLLDEFF 264

YGI+ D ++ F+ LLDE F Sbjct 250 YGISEMDRGKLLFHLLLDELF 270

>TR:K9DXJ0 K9DXJ0_9BURK Aminoglycoside 3'-phosphotransferase OS=Massilia timonae CCUG 45783 OX=883126 GN=HMPREF9710_02755 PE=3 SV=1

Length=268

Score = 183 bits (465), Expect = 6e-54

| Identities | = 103/258 (40%), Positives = 151/258 (59%), Gaps = 7/258 (3%) |  |
| --- | --- | --- |
| Query 12 | PAAWVERLFGYDWAQQTIGCSDAAVFRLSAQ-GRPVLFVKTDLSGALNELQDEAARLSWL | 70 |
| Sbjct 13 | P + L GY WA+ +G S AV+RL + G P L++K + + DE RL WL PESMSAALAGYAWARDRVGESGGAVYRLHGKPGAPDLYLKHGRHAVADAIGDEMTRLRWL | 72 |
| Query 71 | ATTGVPCAAVLDVVTEAGRDWLLLGEVPGQDLLSSHLAPAEKVSIMADAM RRLHTL | 126 |
| Sbjct 73 | A +P AV+ A WLL+ VPG+ A + I+ DA+ +RLH + AGR-MPVPAVVHFDRSADEAWLLMTAVPGRTAYQLLQADPAQHDIVVDALARFLQRLHAI | 131 |
| Query 127 | DPATCPFDHQAKHRIERARTRMEAGLVDQDDLDEEHQGLAPAELFARLKARMPDGEDLVV | 186 |
| Sbjct 132 | + CPF+ +R+ AR+R+EAGLVD+DD DEE G +++ ++A +P D VV PTSACPFNSDHDYRLGLARSRIEAGLVDEDDFDEERAGWTARQVWDAMQALLPLAPDPVV | 191 |
| Query 187 | THGDACLPNIMVENGRFSGFIDCGRLGVADRYQDIALATRDIAEELGGEWADRFLVLYGI | 246 |
| Sbjct 192 | THGD L N+++ +G+ +G ID GR+G+ADRYQD+A+A + E GG +RFL YGI THGDYSLDNLLIVDGQVAGCIDAGRVGIADRYQDLAIAWNCLG-EFGGSLQERFLRQYGI | 250 |
| Query 247  Sbjct 251 | AAPDSQRIAFYRLLDEFF 264 AAPD +++ F+ +LDE F AAPDRRKLQFHLMLDELF 268 |  |

>TR:A0A3A4AU57 A0A3A4AU57_9ACTN Aminoglycoside 3'-phosphotransferase OS=Streptosporangiaceae bacterium YIM 75507 OX=2339229 GN=D5H75_09495

PE=3 SV=1

Length=264

Score = 182 bits (463), Expect = 1e-53

| Identities | = 115/260 (44%), Positives = 144/260 (55%), Gaps = 18/260 (7%) |  |
| --- | --- | --- |
| Query 18 | RLFGYDWAQQTIGCSDAAVFRLSAQGRPVLFVKTD--LSGALNELQDEAARLSWLATTGV | 75 |
| Sbjct 10 | R G++W G S A V+RL+ G P FVK D A L+ EA RL+WL +GV RYSGHEWEPVEAGRSGAGVWRLT--GTPSYFVKADGNRDHAGLSLRGEAERLAWLRASGV | 67 |
| Query 76 | PCAAVLDVVTEAGRDWLLLGEVPGQDLLSSHLAPAEKVSIMADAM RRLHTLDPATC | 131 |
| Sbjct 68 | P V++ + WL+ VPG+ P E+ + DA+ R LHT+ TC PVPEVVEQDADDDVAWLVTTVVPGRTAADDW--PRERHGALVDALAGFTRDLHTIPAGTC | 125 |
| Query 132 | PFDHQAKHRIERARTRMEAGLVDQDDLDEEHQGLAPAELFARLKARMPDGEDLVVTHGDA | 191 |
| Sbjct 126 | PFD + AR EAGLVD DDLD+E G + L A L A P EDLVV HGD PFDRTLAVTVPEAREIAEAGLVDLDDLDDERAGWSADRLVAELAATTPASEDLVVCHGDL | 185 |
| Query 192 | CLPNIMV--ENGRFSGFIDCGRLGVADRYQDIALATRDIAE ELGGEWADRFLVLY | 244 |
| Sbjct 186 | CLPN+++ E R +G +D GR GVADR QD+AL TR + + G ADRFL Y CLPNVLLDPETLRVTGVVDVGRAGVADRCQDLALMTRSLTSGELNPQYGPACADRFLARY | 245 |
| Query 245  Sbjct 246 | GIAAPDSQRIAFYRLLDEFF 264 G+ APD +IAFYRLLDEFF  GV-APDPSKIAFYRLLDEFF 264 |  |

```
>TR:A0A286CWL5 A0A286CWL5_9GAMM Aminoglycoside 3'-phosphotransferase OS=Pseudoxanthomonas wuyuanensis OX=1073196 GN=SAMN06296416_101306
PE=3 SV=1
Length=266

Score = 182 bits (461), Expect = 2e-53
Identities = 98/237 (41%), Positives = 141/237 (59%), Gaps = 2/237 (1%) Query 30	GCSDAAVFRLSAQGRPVLFVKTDLSGALNELQDEAARLSWL-ATTGVPCAAVLDVVTEAG 88

G S A V R+	G + +VK	EL++E R+ WL A T +P	V+	+ +
```

| Sbjct | 30 | GQSGARVIRVLMPGGDIAYVKYASGAFAEELREEHERIGWLTAQTRIPVPEVISYASTSN | 89 |
| --- | --- | --- | --- |
| Query | 89 | RDWLLLGEVPGQDLLSSHLA-PAEKVSIMADAMRRLHTLDPATCPFDHQAKHRIERARTR | 147 |
| Sbjct | 90 | LL + G++ + A P V+ MA A+R LH + P CPFD + R+++A R ATILLTKALVGRNAADAASADPTTIVAEMARALRELHAIAPEDCPFDERLHLRLKQALAR | 149 |
| Query | 148 | MEAGLVDQDDLDEEHQGLAPAELFARLKARMPDGEDLVVTHGDACLPNIMVENGRFSGFI | 207 |
| Sbjct | 150 | MEAGLVD++D D+ QG+ +++ +L +R+P E LVVTHGDAC N + F GFI MEAGLVDEEDFDQARQGMRVRDVYEQLLSRIPAAEQLVVTHGDACPENFIFRGDAFVGFI | 209 |
| Query  Sbjct | 208  210 | DCGRLGVADRYQDIALATRDIAEELGGEWADRFLVLYGIAAPDSQRIAFYRLLDEFF 264 DCGR+G+AD+YQD+ALA+R+I G E AD+F + YG P +I +YR+LDEFF DCGRVGLADKYQDLALASRNIDALFGPELADQFFIEYGEPNPSPVKIEYYRILDEFF 266 |  |

>TR:A0A399F177 A0A399F177_9DEIN Aminoglycoside 3'-phosphotransferase OS=Calidithermus roseus OX=1644118 GN=neo PE=3 SV=1

Length=267

Score = 182 bits (461), Expect = 2e-53

| Identities | | = 109/265 (41%), Positives = 150/265 (57%), Gaps = 15/265 (6%) |  |
| --- | --- | --- | --- |
| Query 12  Sbjct 6 | | PAAWVERLFGYDWAQQTIGCSDAAVFRLSAQGRPVLFVK TDLSGALNELQDEAARL  P A RL GY ++G S A V+RL G L +K +D L EA ++ PEAIARRLEGYQAEPISMGMSGAGVYRLRQPGATGLILKVQPQSDDPDPGFRLASEAEKM | 67  65 |
| Query 68  Sbjct 66 | | SWLATTGVPCAAVLDVVTEAGRDWLLLGEVPGQDLLSSHLAPAEK VSIMADAMRRL  WL+ VP VL+ + G +++L+ EV G+ + P+E+ V +A+A++RL RWLSAW-VPVPQVLEFCQQDGSEYMLMSEVAGRS--GAEDWPSEERARVVVAIAEALKRL | 123  122 |
| Query 124  Sbjct 123 | | HTLDPATCPFDHQAKHRIERARTRMEAGLVDQDDLDEEHQGLAPAELFARLKARMPDGED H++ CPFD + + +I +AR R E GLV+ +D DE QG + EL L P ED HSIPVEQCPFDQRLETKIAQARRRTELGLVNLEDFDERWQGKSAEELLEVLLETRPADED | 183  182 |
| Query 184 | | LVVTHGDACLPNIMVENGRFSGFIDCGRLGVADRYQDIALATRDIAEEL GGEWADR  VV HGD CLPN+++E G+ SG +D GR G+ADRYQD+AL TR + +L G W | 239 |
| Sbjct | 183 | WVVCHGDYCLPNVLLEQGKLSGLVDLGRAGIADRYQDLALMTRSLTSDLNPQFGEGWDRV | 242 |
| Query  Sbjct | 240  243 | FLVLYGIAAPDSQRIAFYRLLDEFF 264 FL YGI PD +RI FYRLLDEFF FLEAYGIPQPDWERIEFYRLLDEFF 267 |  |

>TR:A0A4U0FC53 A0A4U0FC53_9BACL Aminoglycoside 3'-phosphotransferase OS=Cohnella sp. CAU 1483 OX=2564100 GN=E5161_11360 PE=3 SV=1

Length=259

Score = 180 bits (457), Expect = 7e-53

| Identities | = 101/248 (41%), Positives = 145/248 (58%), Gaps = 8/248 (3%) |  |
| --- | --- | --- |
| Query 21 | GYDWAQQTIGCSDAAVFRLS-AQGRPVLFVKTDLSGALNELQDEAARLSWLATTGVPCAA | 79 |
| Sbjct 16 | G +W + TIG S+A F L+ A G ++K G++ L+ E R+ WL +P GLEWKRITIGYSEANTFLLTGAAGNR--YLKVLPVGSVETLRQEKERMEWLQGK-LPVPE | 72 |
| Query 80 | VLDVVTEAGRDWLLLGEVPGQDL---LSSHLAPAEKVSIMADAMRRLHTLDPATCPFDHQ | 136 |
| Sbjct 73 | +L + ++LL+ E+PG D L + P + + +A +R +H +D CPFD + ILYYEDDGSNEYLLMSEIPGLDASHRLYESMLP-QLMRELAAGLRAIHEVDIRNCPFDQR | 131 |
| Query 137 | AKHRIERARTRMEAGLVDQDDLDEEHQGLAPAELFARLKARMPDGEDLVVTHGDACLPNI | 196 |
| Sbjct 132 | + +I A R+E LVD+DD DE +G +LF L P EDLV THGD CLPN+ LERKIGEAGKRVENRLVDEDDFDEIREGCTAEQLFQELIREKPADEDLVFTHGDYCLPNV | 191 |
| Query 197 | MVENGRFSGFIDCGRLGVADRYQDIALATRDIAEELGGEWADRFLVLYGIAAPDSQRIAF | 256 |
| Sbjct 192 | ++++GR SGFID GR GVADRYQDIALA R I G E+ FL +YGI D ++++ ILQDGRLSGFIDWGRAGVADRYQDIALAVRSIRYNFGEEYVATFLDVYGITQLDESKVSY | 251 |
| Query 257  Sbjct 252 | YRLLDEFF 264 Y+L+DEFF YQLMDEFF 259 |  |

>TR:A0A0Q7JME4 A0A0Q7JME4_9BACL APH domain-containing protein OS=Paenibacillus sp. Root444D2 OX=1736538 GN=ASD40_33570 PE=3 SV=1

Length=260

Score = 180 bits (457), Expect = 8e-53

| Identities | = 105/247 (43%), Positives = 139/247 (56%), Gaps = 7/247 (3%) |  |
| --- | --- | --- |
| Query 23 | DWAQQTIGCSDAAVFRLSAQGRPV-LFVKTDLSGALNELQDEAARLSWLATTGVPCAAVL | 81 |
| Sbjct 16 | +W TIGCS A FRL + +++K L+ E RL WL +P ++ EWTPITIGCSVAQTFRLQMPDHGMGMYLKMVDKDTRRSLKQEFERLLWLQGK-LPVPNII | 74 |
| Query 82 | DVVTEAGRDWLLLGEVPGQ---DLLSSHLAPAEKVSIMADAMRRLHTLDPATCPFDHQAK | 138 |
| Sbjct 75 | D +TE R++LL E+PG D +S P V +A A+RR+H L CPFD DYITEDNREYLLTVELPGTHAADQSNSDRLPGI-VRELALALRRIHDLPIDDCPFDKSLA | 133 |
| Query 139 | HRIERARTRMEAGLVDQDDLDEEHQGLAPAELFARLKARMPDGEDLVVTHGDACLPNIMV | 198 |
| Sbjct 134 | I A+ +E GLVD +D D + G + LF L P EDLV THGD CLPNI++ VHINEAKQNIELGLVDVEDFDHKRSGRSSDSLFHELLITRPPNEDLVFTHGDYCLPNILL | 193 |
| Query 199 | -ENGRFSGFIDCGRLGVADRYQDIALATRDIAEELGGEWADRFLVLYGIAAPDSQRIAFY | 257 |
| Sbjct 194 | E+GR SG+ID G G+ADRYQD+ALA R + G WA+ FL YG+ PD ++I FY YEDGRLSGYIDWGNAGIADRYQDLALAHRSLIHNFGMTWAELFLHEYGLERPDREKIEFY | 253 |
| Query 258  Sbjct 254 | RLLDEFF 264  +LLDEFF  QLLDEFF 260 |  |

>TR:A0A1L8ZNE0 A0A1L8ZNE0_9BACI APH domain-containing protein OS=Bacillus obstructivus OX=1914540 GN=BLX88_08710 PE=3 SV=1

Length=258

Score = 178 bits (452), Expect = 4e-52

| Identities | = 97/249 (39%), Positives = 142/249 (57%), Gaps = 5/249 (2%) |  |
| --- | --- | --- |
| Query 19 | LFGYDWAQQTIGCSDAAVFRLSAQGRPVLFVKTDLSGALNELQDEAARLSWLATTGVPCA | 78 |
| Sbjct 12 | L G W + TIGCS++ FR+ F+K A +L +E RL WL +P LKGCSWEEITIGCSESNTFRVKGDNYHQ-FLKIQSINAKEKLLNEKERLEWLQGK-LPVP | 69 |
| Query 79 | AVLDVVTEAGRDWLLLGEVPGQDL---LSSHLAPAEKVSIMADAMRRLHTLDPATCPFDH | 135 |
| Sbjct 70 | VL + ++LL+ E+ G+D + + + + +A ++ +H++ CPFD KVLYYEQDDINEYLLISEIAGKDASVEIDKTTSVPDLMKQLALGLKMIHSIKIDQCPFDQ | 129 |
| Query 136 | QAKHRIERARTRMEAGLVDQDDLDEEHQGLAPAELFARLKARMPDGEDLVVTHGDACLPN | 195 |
| Sbjct 130 | + ++E AR R+E GLV++DD DEE +G+ LF LK +P EDLV HGD CLPN TVQKKLEEARLRVEKGLVNEDDFDEERKGIPSDVLFQHLKDSVPKDEDLVFAHGDYCLPN | 189 |
| Query 196 | IMVENGRFSGFIDCGRLGVADRYQDIALATRDIAEELGGEWADRFLVLYGIAAPDSQRIA | 255 |
| Sbjct 190 | I+++NG+ +GFID GR G++DRYQD+ALA R I G E F YG+ D +I IILQNGKINGFIDMGRAGISDRYQDLALAVRSITYNFGKEMLPYFFNEYGMQRYDKAKIN | 249 |
| Query 256  Sbjct 250 | FYRLLDEFF 264 FY+L+DEFF FYQLMDEFF 258 |  |

>TR:D7BCJ0 D7BCJ0_MEISD Aminoglycoside phosphotransferase OS=Meiothermus silvanus (strain ATCC 700542 / DSM 9946 / VI-R2) OX=526227 GN=Mesil_0966 PE=3 SV=1

Length=270

Score = 177 bits (450), Expect = 1e-51

Identities = 108/263 (41%), Positives = 149/263 (57%), Gaps = 13/263 (5%)

| Query | 12 | PAAWVERLFGYDWAQQTIGCSDAAVFRLSAQGRPVLFVKTDLSGALNE LQDEAARL | 67 |
| --- | --- | --- | --- |
| Sbjct | 9 | P A L GY+ T+G S A V+R G+P L +K + L EA ++ PQAVSRWLQGYEAEPVTVGMSGAGVYRWRKAGKPGLILKIRPDSHDPDPGFRLAPEAEKM | 68 |
| Query | 68 | SWLATTGVPCAAVLDVVTEAGRDWLLLGEVPGQDLLSSHLAPAEK---VSIMADAMRRLH | 124 |
| Sbjct | 69 | WL+ VP VL+ + G ++LL+ E+ G+ + + P E+ V +A+A+R+LH  RWLSAW-VPGPEVLEYSQQDGCEYLLMTEIVGRSG-AENWPPEERPRVVVAIAEALRKLH | 126 |

| Query | 125 | TLDPATCPFDHQAKHRIERARTRMEAGLVDQDDLDEEHQGLAPAELFARLKARMPDGEDL | 184 |
| --- | --- | --- | --- |
| Sbjct | 127 | ++ CPFD + + ++ +AR R E GLVD D DE QG + E L P EDL SIPLERCPFDQRLEAKLAQARQRTELGLVDLADFDERWQGKSAEEPLEVLLQTRPTDEDL | 186 |
| Query | 185 | VVTHGDACLPNIMVENGRFSGFIDCGRLGVADRYQDIALATRDIAEEL GGEWADRF | 240 |
| Sbjct | 187 | VV HGD CLPN++++ G+ SGF+D GRLGVADRYQD+AL TR + +L G W F VVCHGDYCLPNVLLQEGKLSGFVDLGRLGVADRYQDLALMTRSLTSDLNPQFGEGWDRVF | 246 |
| Query  Sbjct | 241  247 | LVLYGIAAPDSQRIAFYRLLDEF 263 L YGI PD R+ FYRLLDEF LEAYGIREPDWTRLEFYRLLDEF 269 |  |

>TR:A0A1M5T3I1 A0A1M5T3I1_9BURK Aminoglycoside 3'-phosphotransferase OS=Massilia sp. CF038 OX=1881045 GN=SAMN05428948_4097 PE=3 SV=1

Length=270

Score = 176 bits (446), Expect = 5e-51

| Identities | | = 104/258 (40%), Positives = 145/258 (56%), Gaps = 7/258 (3%) |  |
| --- | --- | --- | --- |
| Query 12  Sbjct 15 | | PAAWVERLFGYDWAQQTIGCSDAAVFRLSAQG-RPVLFVKTDLSGALNELQDEAARLSWL PA + GY WA+ T+G S V+RL + P LF+K +++ E ARL WL PANIASLVDGYAWARDTVGESGGTVYRLHGKSDAPDLFLKHGQGVFADDVIGEMARLRWL | 70  74 |
| Query 71  Sbjct 75 | | ATTGVPCAAVLDVVTEAGRDWLLLGEVPGQ---DLLSSHLAPAEKV-SIMADAMRRLHTL A A V V T G WLL+ +PG+ L+S V +AD +RRLH + AGHIAVPAIVQFVATPDGA-WLLMSAMPGKTAYQLMSDQAGSGNAVVDALADFLRRLHAI | 126  133 |
| Query 127  Sbjct 134 | | DPATCPFDHQAKHRIERARTRMEAGLVDQDDLDEEHQGLAPAELFARLKARMPDGEDLVV A CPF HR+ AR R++AGLVD DD D+E G + +++ ++ +P DLVV PVAACPFTSAHAHRLHLARARIDAGLVDADDFDDERAGWSAEQVWDAMQDLLPLAPDLVV | 186  193 |
| Query 187  Sbjct 194 | | THGDACLPNIMVENGRFSGFIDCGRLGVADRYQDIALATRDIAEELGGEWADRFLVLYGI THGD L N+ + +G+ SG ID GR+G+ADRYQD+A+A ++ E G RFL YG+ THGDYSLDNVFIVDGKVSGCIDAGRVGIADRYQDLAIAWNCLS-EFGPALQKRFLQQYGV | 246  252 |
| Query | 247 AAPDSQRIAFYRLLDEFF 264 | | |
|  |  | PD R+AF+ +LDE F |  |
| Sbjct | 253 | TQPDQARLAFHLMLDELF 270 | |

>TR:A0A368VJC8 A0A368VJC8_9BACL Kanamycin kinase/aminoglycoside 3'-phosphotransferase-2 OS=Paenibacillus prosopidis OX=630520 GN=DFP97_12331

PE=3 SV=1

Length=259

Score = 174 bits (442), Expect = 1e-50

| Identities | = 104/247 (42%), Positives = 136/247 (55%), Gaps = 6/247 (2%) |  |
| --- | --- | --- |
| Query 21 | GYDWAQQTIGCSDAAVFRLS-AQGRPVLFVKTDLSGALNELQDEAARLSWLATTGVPCAA | 79 |
| Sbjct 16 | G +W + TIG S++ +RL+ A G F+K A+ L+ E R+ WL +P GCEWNRVTIGLSESRTYRLTGASGN--YFIKIHSVDAVESLRAEKERMEWLQGK-LPVPE | 72 |
| Query 80 | VLDVVTEAGRDWLLLGEVPGQDLL--SSHLAPAEKVSIMADAMRRLHTLDPATCPFDHQA | 137 |
| Sbjct 73 | VL +A ++LLL EV G D S + + +A +R +H + CPFD + VLFYEKDASNEYLLLSEVAGLDASDKSYETMLPQLIRQLAVGLRAVHDVRIDDCPFDRRL | 132 |
| Query 138 | KHRIERARTRMEAGLVDQDDLDEEHQGLAPAELFARLKARMPDGEDLVVTHGDACLPNIM | 197 |
| Sbjct 133 | I A R E GLVD++D D G LF L R P EDLV THGD CLPNI+ DTVIAEAGRRAEHGLVDEEDFDSARNGAKAEALFQELLERRPAAEDLVFTHGDYCLPNII | 192 |
| Query 198 | VENGRFSGFIDCGRLGVADRYQDIALATRDIAEELGGEWADRFLVLYGIAAPDSQRIAFY | 257 |
| Sbjct 193 | ++NG SGFID GR G+ADRYQD+ALA R I G E FL YGI PD +I++Y LQNGEISGFIDWGRGGIADRYQDLALAVRSIIYNFGEEHVAAFLEEYGIREPDHAKISYY | 252 |
| Query 258  Sbjct 253 | RLLDEFF 264  +LLDEFF  QLLDEFF 259 |  |

>TR:A0A285QF39 A0A285QF39_9SPHN Aminoglycoside 3'-phosphotransferase OS=Sphingomonas guangdongensis OX=1141890 GN=SAMN06297144_0891 PE=3

SV=1

Length=270

Score = 174 bits (441), Expect = 3e-50

| Identities | | = 105/267 (39%), Positives = 147/267 (55%), Gaps = 7/267 (3%) |  |
| --- | --- | --- | --- |
| Query 3  Sbjct 6 | | EQDGLHAGSPAAWVERLFGYDWAQQTIGCSDAAVFRLSAQ-GRPVLFVKTDLSGALNELQ E+ A P W + L GY WA+ +G S AAV+RL + G P L++K +++ EEASAPARLPGGWADALGGYAWARDRVGESGAAVYRLHGRAGAPELYLKHGAGPTADDVV | 61  65 |
| Query 62  Sbjct 66 | | DEAARLSWLATTGVPCAAVLDVVTEAGRDWLLLGEVPG QDLLSSHLAPAEKVSIMA  DEA RL WLA +P AV+ T A WLL +PG Q L + A V +A DEAVRLRWLAAH-LPAPAVVRFATSADEAWLLTTALPGRTAYQLLETGGGEAAAVVDALA | 117  124 |
| Query 118  Sbjct 125 | | DAMRRLHTLDPATCPFDHQAKHRIERARTRMEAGLVDQDDLDEEHQGLAPAELFARLKAR D + RLH + C F R+ AR R+ AGLVD+DD DEE +G + +++ L DLLVRLHAVPVHACTFISDHARRLLLARARINAGLVDEDDFDEEREGWSAEQVWDALDEL | 177  184 |
| Query 178  Sbjct 185 | | MPDGEDLVVTHGDACLPNIMVENGRFSGFIDCGRLGVADRYQDIALATRDIAEELGGEWA  +P DLVVTHGD L NI++++ +G ID GRLG+ADRYQD+A+ + E G + LPLVPDLVVTHGDYSLDNILLQDEAVTGCIDVGRLGIADRYQDLAVMWNCLG-EFGSDLQ | 237  243 |
| Query 238 | | DRFLVLYGIAAPDSQRIAFYRLLDEFF 264 RFL YGI + +++ F+ LLDE F |  |
| Sbjct | 244 | QRFLARYGIDRLNERKLRFHLLLDELF 270 | |

>TR:A0A1I6M7R6 A0A1I6M7R6_9SPHN Aminoglycoside 3'-phosphotransferase OS=Sphingomonas jatrophae OX=1166337 GN=SAMN05192580_3620 PE=3 SV=1

Length=270

Score = 173 bits (439), Expect = 5e-50

| Identities | = 103/260 (40%), Positives = 149/260 (57%), Gaps = 11/260 (4%) |  |
| --- | --- | --- |
| Query 12 | PAAWVERLFGYDWAQQTIGCSDAAVFRLSAQ-GRPVLFVKTDLSGALNELQDEAARLSWL | 70 |
| Sbjct 15 | PA+ + GY+WA+ ++G S AV+RL + G P LF+K +++ DE RL WL PASLSPAVLGYEWARDSVGESGGAVYRLHGRSGVPDLFLKHGRGSVASDITDEMVRLRWL | 74 |
| Query 71 | ATTGVPCAAVLDVVTEAGRDWLLLGEVPGQ---DLLSSHLAPAEKVSI---MADAMRRLH | 124 |
| Sbjct 75 | A VP AV V WLL+ +PGQ LL +H P +V I +A +R LH AKH-VPAPAVEGFVATQDEAWLLMKALPGQTAYQLLEAH--PDARVGIVDALAAFLRGLH | 131 |
| Query 125 | TLDPATCPFDHQAKHRIERARTRMEAGLVDQDDLDEEHQGLAPAELFARLKARMPDGEDL | 184 |
| Sbjct 132 | + + CPF +R+ +AR R++AGL+D+ D D+E +G +++ ++ +P D AIPLSACPFTSDHAYRLAQARERIDAGLIDEHDFDDEREGWTAEQVWDAMQRLLPMQFDP | 191 |
| Query 185 | VVTHGDACLPNIMVENGRFSGFIDCGRLGVADRYQDIALATRDIAEELGGEWADRFLVLY | 244 |
| Sbjct 192 | VVTHGD L NI++ G G ID GR+GVADRYQD+A+ RD+ + DR LV Y VVTHGDFSLDNILLLGGDVIGCIDAGRVGVADRYQDLAILWRDLG-QFDQALQDRLLVQY | 250 |
| Query 245  Sbjct 251 | GIAAPDSQRIAFYRLLDEFF 264 GIA D+ ++ F+ LLDE F GIAGADAGKLQFHLLLDELF 270 |  |

>TR:A0A177ZKY0 A0A177ZKY0_9BACI APH domain-containing protein OS=Bacillus galactosidilyticus OX=217031 GN=ABB05_16965 PE=3 SV=1

Length=259

Score = 172 bits (437), Expect = 7e-50

Identities = 96/256 (38%), Positives = 142/256 (55%), Gaps = 6/256 (2%)

| Query | 12 | PAAWVERLFGYDWAQQTIGCSDAAVFRLSAQGRPVLFVKTDLSGALNELQDEAARLSWL- | 70 |
| --- | --- | --- | --- |
| Sbjct | 7 | P + + GY W Q TIG S+A+ F L +G L++K A+ L+ E ++ WL PTRLEKMIDGYTWTQVTIGHSEASTFLLK-KGNHHLYLKIQAISAIETLRQEKEKMEWLH | 65 |
| Query | 71 | ATTGVPCAAVLDVVTEAGRDWLLLGEVPGQDLLS--SHLAPAEKVSIMADAMRRLHTLDP | 128 |
| Sbjct | 66 | VP VL +++LL+ E+ G D + +H + + ++A +R +H++ GKLSVP--GVLCYERNEEKEFLLMTEITGMDASNPFNHADLTQLIKLLAKGLREIHSISI | 123 |
| Query | 129 | ATCPFDHQAKHRIERARTRMEAGLVDQDDLDEEHQGLAPAELFARLKARMPDGEDLVVTH CPF I A+ R+E LVD++D DEE +G EL+ L + P E+LV TH | 188 |

| Sbjct | 124 | DRCPFQRNLDMTIREAQERVEKNLVDREDFDEERKGKDAEELYQELLEKHPLDEELVFTH | 183 |
| --- | --- | --- | --- |
| Query | 189 | GDACLPNIMVENGRFSGFIDCGRLGVADRYQDIALATRDIAEELGGEWADRFLVLYGIAA | 248 |
| Sbjct | 184 | GD CLPNI++ N +GFID GR G+ADRYQDIALA R I +G + FL YG+ GDYCLPNIILHNKSINGFIDLGRAGIADRYQDIALAVRSITYNVGEQHIPLFLTEYGLKE | 243 |
| Query | 249 | PDSQRIAFYRLLDEFF 264  + ++ +Y+LLDEFF |  |
| Sbjct | 244 | LNQNKLRYYQLLDEFF 259 | |

>TR:A0A0B1Q485 A0A0B1Q485_9RHIZ Aminoglycoside 3'-phosphotransferase OS=Aureimonas altamirensis OX=370622 GN=LA66_00505 PE=3 SV=1

Length=271

Score = 172 bits (437), Expect = 1e-49

| Identities | = 98/259 (38%), Positives = 147/259 (57%), Gaps = 9/259 (3%) |  |
| --- | --- | --- |
| Query 12 | PAAWVERLFGYDWAQQTIGCSDAAVFRLSAQ-GRPVLFVKTDLSGALNELQDEAARLSWL | 70 |
| Sbjct 16 | P+ + GY WA+ TIG + AV+RL + G P LF+K +++ DE RL WL PSNMSVEVHGYKWARDTIGQAGGAVYRLYGRVGAPDLFLKHGRDEVADDVTDEMVRLRWL | 75 |
| Query 71 | AT-TGVPCAAVLDVVTEAGRDWLLLGEVPGQDLLSSHLAPAEKVSIMADA MRRLHT | 125 |
| Sbjct 76 | A T VP +V + V WLL+ +PG+ + A+ I+ DA +RR+H AGYTSVP--SVQNFVRTKDEAWLLMTAIPGKTVYQVLEENADARFIVVDAIAAFLRRVHA | 133 |
| Query 126 | LDPATCPFDHQAKHRIERARTRMEAGLVDQDDLDEEHQGLAPAELFARLKARMPDGEDLV | 185 |
| Sbjct 134 | + CPF+ +R+ RAR R+++GLVD DD D E +GL P +++ ++ +P D V IPVCECPFNSDYTYRLSRARARIDSGLVDADDFDTEREGLTPEQVWEEMQHLLPFPADPV | 193 |
| Query 186 | VTHGDACLPNIMVENGRFSGFIDCGRLGVADRYQDIALATRDIAEELGGEWADRFLVLYG | 245 |
| Sbjct 194 | VTHGD N+++ G G ID GR+G+ADRYQD+A+ + EE G +RF YG VTHGDFSPDNLLMNEGEILGCIDVGRVGIADRYQDLAILWNRL-EEFGSPLQERFFQRYG | 252 |
| Query 246  Sbjct 253 | IAAPDSQRIAFYRLLDEFF 264  +A D +++ F+ +LDEFF  VADVDRRKLQFHLMLDEFF 271 |  |

>TR:A0A179T5W8 A0A179T5W8_9BACI APH domain-containing protein OS=Metabacillus litoralis OX=152268 GN=A6K24_02630 PE=3 SV=1

Length=256

Score = 171 bits (434), Expect = 2e-49

| Identities | = 97/255 (38%), Positives = 142/255 (56%), Gaps = 5/255 (2%) |  |
| --- | --- | --- |
| Query 12 | PAAWVERLFGYDWAQQTIGCSDAAVFRLSAQGRPVLFVKTDLSGALNELQDEAARLSWLA | 71 |
| Sbjct 5 | P + + GY W Q TIG S++ F L ++K + LQDE RL WL PMTLTKLVNGYTWEQITIGLSESKTFLLKTNNHNQ-YLKIQSIKTIENLQDEKNRLEWLQ | 63 |
| Query 72 | TTGVPCAAVLDVVTEAGRDWLLLGEVPGQDLLSSHLAP--AEKVSIMADAMRRLHTLDPA | 129 |
| Sbjct 64 | +P VL + ++LLL E+ G+D + + + ++A ++ +H + GK-LPVPKVLHYEKDEENEYLLLSEIKGKDASNQFYENNLPQLMKLLAIGVKMMHGISIK | 122 |
| Query 130 | TCPFDHQAKHRIERARTRMEAGLVDQDDLDEEHQGLAPAELFARLKARMPDGEDLVVTHG | 189 |
| Sbjct 123 | CPF+ + + ++E AR R++ GLVD++D DE+ G +LF L + P E+LV THG DCPFNEKLEKKVEEARRRVKNGLVDEEDFDEKRSGKTAKQLFQDLIIKKPVNEELVFTHG | 182 |
| Query 190 | DACLPNIMVENGRFSGFIDCGRLGVADRYQDIALATRDIAEELGGEWADRFLVLYGIAAP | 249 |
| Sbjct 183 | D CLPNI+++N + SGFID GR GVADR+QD+ALA R I G E FL YG A DYCLPNIIMDNDKVSGFIDMGRAGVADRHQDLALAIRSIRYNFGEEMVPFFLNEYGYEA- | 241 |
| Query 250  Sbjct 242 | DSQRIAFYRLLDEFF 264 D +I +Y+LLDEF+ DETKIEYYQLLDEFY 256 |  |

>TR:A0A4R2PAM0 A0A4R2PAM0_9SPHN Aminoglycoside 3'-phosphotransferase OS=Sphingomonas sp. BK235 OX=2512131 GN=EV292_11083 PE=3 SV=1

Length=270

Score = 171 bits (434), Expect = 3e-49

| Identities | | = 100/258 (39%), Positives = 141/258 (55%), Gaps = 7/258 (3%) |  |
| --- | --- | --- | --- |
| Query 12  Sbjct 15 | | PAAWVERLFGYDWAQQTIGCSDAAVFRLSAQ-GRPVLFVKTDLSGALNELQDEAARLSWL P A L GY WA+ IG S AV+RL + G P LF+K ++ DE RL WL PPAMSSLLRGYAWARDRIGESGGAVYRLHGRAGAPDLFLKHGRGPVAGDVADEMVRLRWL | 70  74 |
| Query 71  Sbjct 75 | | ATTGVPCAAVLDVVTEAGRDWLLLGEVPGQDLLSSHLAPAEKVSIMADAM RRLHTL  A +P AV V + WLL+ +PGQ A + S + DA+ RR H + ARH-IPVPAVQRFVLDPDEAWLLMTALPGQTAYQLLEAGSHHHSAVVDALASLARRFHAI | 126  133 |
| Query 127  Sbjct 134 | | DPATCPFDHQAKHRIERARTRMEAGLVDQDDLDEEHQGLAPAELFARLKARMPDGEDLVV CPF HR+ +AR R++AG V++DD D+E +G +++ ++ +P DLVV PLGACPFTSDHAHRLTQARARIDAGWVEEDDFDDEREGWTSEQVWEAMQRLLPISPDLVV | 186  193 |
| Query 187  Sbjct 194 | | THGDACLPNIMVENGRFSGFIDCGRLGVADRYQDIALATRDIAEELGGEWADRFLVLYGI THGD L NI++ +G G ID GR G+ADRYQD+A+ ++ E + RFL YGI THGDLSLDNILLRDGEVVGCIDVGRTGIADRYQDLAIVWSNL-REFDPCLSRRFLERYGI | 246  252 |
| Query | 247 AAPDSQRIAFYRLLDEFF 264 | | |
|  |  | PD +++ F+ LLDE F |  |
| Sbjct | 253 | DHPDERKLQFHLLLDEMF 270 | |

>TR:A9WI26 A9WI26_CHLAA Aminoglycoside phosphotransferase OS=Chloroflexus aurantiacus (strain ATCC 29366 / DSM 635 / J-10-fl) OX=324602 GN=Caur_2511 PE=3 SV=1

Length=260

Score = 171 bits (433), Expect = 3e-49

| Identities | | = 103/256 (40%), Positives = 141/256 (55%), Gaps = 5/256 (2%) |  |
| --- | --- | --- | --- |
| Query 12  Sbjct 7 | | PAAWVERLFGYDWAQQTIGCSDAAVFRLSAQGRPVLFVKTDLSGALNELQDEAARLSWLA P A L G T GC+ A V+R+ GR +++K L EA L WL PVAVQRLLQGSSVELMTDGCTAAQVYRIEQDGR-CMYLKMQSLADTPSLHVEARILDWLQ | 71  65 |
| Query 72  Sbjct 66 | | TTGVPCAAVLDVVTEAGRDWLLLGEVPGQDLLSSH--LAPAEKVSIMADAMRRLHTLDPA  +P VL V + ++L+ EV GQ+ + + L V +MA +R +H LDPA GK-LPVPEVLVYVADDRYEYLVSTEVAGQNGVRAMATLRAEALVELMASGLRMIHALDPA | 129  124 |
| Query 130  Sbjct 125 | | TCPFDHQAKHRIERARTRMEAGLVDQDDLDEEHQGLAPAE-LFARLKARMPDGEDLVVTH TCPFD + R+ +AR + GLVD+ D D E G A+ + A L+ P +DLV TH TCPFDAGLELRLMQARANVLNGLVDETDFDPERLGSTTAQDILAALERERPATDDLVFTH | 188  184 |
| Query 189  Sbjct 185 | | GDACLPNIMVENGRFSGFIDCGRLGVADRYQDIALATRDIAEELGGEWADRFLVLYGIAA GD CLPNI+V +G GFID GR GVADRY D+A+A+R I LG ++ F YG+ GDYCLPNIIVRDGMIGGFIDWGRAGVADRYNDLAIASRSIRYNLGPQYEQYFFACYGLEQ | 248  244 |
| Query 249 | | PDSQRIAFYRLLDEFF 264 D+ +IA+YR+LDE F |  |
| Sbjct | 245 | VDAGKIAYYRMLDELF 260 | |

>TR:A0A0Q4LIY2 A0A0Q4LIY2_9SPHN Aminoglycoside 3'-phosphotransferase OS=Sphingomonas sp. Leaf42 OX=1736219 GN=ASE97_04905 PE=3 SV=1

Length=266

Score = 170 bits (431), Expect = 7e-49

| Identities | = 99/256 (39%), Positives = 140/256 (55%), Gaps = 5/256 (2%) |  |
| --- | --- | --- |
| Query 12 | PAAWVERLFGYDWAQQTIGCSDAAVFRLSAQ-GRPVLFVKTDLSGALNELQDEAARLSWL | 70 |
| Sbjct 13 | P E + GYDWA+ +G S AV+RL + G LF+K +++ DE RL WL PPGIAEAVAGYDWARDLVGESGGAVYRLHGRPGAADLFLKHGTGPVADDIADEMIRLRWL | 72 |
| Query 71 | ATTGVPCAAVLDVVTEAGRDWLLLGEVPGQDLLS--SHLAPAEKVSIMADAMRRLHTLDP | 128 |
| Sbjct 73 | A +P AV+ V WLL +PG+ S A V + +RRLH + AGR-MPVPAVVRFVGADDAAWLLTTALPGETAYQALSRRPDAALVDALVAFLRRLHAIPV | 131 |
| Query 129 | ATCPFDHQAKHRIERARTRMEAGLVDQDDLDEEHQGLAPAELFARLKARMPDGEDLVVTH | 188 |
| Sbjct 132 | A+CPF + R+ AR R++AG VD++D D+E G + +++A ++A +P D VVTH ASCPFASDHQRRMAAARARIDAGQVDEEDFDDERLGWSAGQVWAAMRALLPLDPDAVVTH | 191 |
| Query 189 | GDACLPNIMVENGRFSGFIDCGRLGVADRYQDIALATRDIAEELGGEWADRFLVLYGIAA | 248 |

GD L N++V+ G	G ID R GVADRYQD+A+	+ E G	+R	YGIA

| Sbjct | 192 | GDFSLDNLLVQGGAVVGCIDVARAGVADRYQDLAILFNCLG-EFGPGLQERMFAAYGIAE 250 |
| --- | --- | --- |
| Query | 249 | PDSQRIAFYRLLDEFF 264 |
| Sbjct | 251 | PD+ R+ F+ LLDE F  PDTDRLTFHLLLDELF 266 |

>TR:A0A1I4UN46 A0A1I4UN46_9RHIZ Aminoglycoside 3'-phosphotransferase OS=Pleomorphomonas diazotrophica OX=1166257 GN=aph(3')-I PE=3 SV=1

Length=269

Score = 170 bits (431), Expect = 8e-49

| Identities | = 103/267 (39%), Positives = 147/267 (55%), Gaps = 7/267 (3%) |  |
| --- | --- | --- |
| Query 3 | EQDGLHAGSPAAWVERLFGYDWAQQTIGCSDAAVFRLSAQ-GRPVLFVKTDLSGALNELQ | 61 |
| Sbjct 5 | ++D P + + GY WA+ TIG S AVFRL + G P L++K + + DRDDARPTVPPSLAAAVAGYAWARDTIGASGGAVFRLHGKAGAPNLYLKHGRDAVADAIT | 64 |
| Query 62 | DEAARLSWLATTGVPCAAVLDVVTEAGRDWLLLGEVPG QDLLSSHLAPAEKVSIMA | 117 |
| Sbjct 65 | +EAARL+WLA +P AV+ +LL+ +PG Q L++ A + + +A EEAARLNWLAAH-LPVPAVVKFRRTPHEAFLLMTALPGRTAYQVLVAEPDARPDVIDALA | 123 |
| Query 118 | DAMRRLHTLDPATCPFDHQAKHRIERARTRMEAGLVDQDDLDEEHQGLAPAELFARLKAR | 177 |
| Sbjct 124 | +RRLH + + CPF R+ AR R++AGLVD D DEE QG +++ L+A AFLRRLHAIPVSDCPFVAGHAQRLAVARARIDAGLVDVGDFDEERQGWTAEDVWDGLQAL | 183 |
| Query 178 | MPDGEDLVVTHGDACLPNIMVENGRFSGFIDCGRLGVADRYQDIALATRDIAEELGGEWA | 237 |
| Sbjct 184 | +P D VVTHGD L N++VE G G ID GR G+ADRYQD+A+ + E G LPLAADPVVTHGDFSLDNLIVEGGEVVGCIDVGRAGIADRYQDLAILW-NCLHEFGPALQ | 242 |
| Query 238  Sbjct 243 | DRFLVLYGIAAPDSQRIAFYRLLDEFF 264 DR YGIA PD +++ F+ +LDE F DRLFQSYGIARPDRRKLRFHLMLDELF 269 |  |

>TR:A0A1T4PRE7 A0A1T4PRE7_9ACTN Kanamycin kinase OS=Marinactinospora thermotolerans DSM 45154 OX=1122192 GN=SAMN02745673_01950 PE=3 SV=1

Length=282

Score = 170 bits (431), Expect = 1e-48

| Identities | | = 108/263 (41%), Positives = 143/263 (54%), Gaps = 17/263 (6%) |  |
| --- | --- | --- | --- |
| Query 12  Sbjct 27 | | PAAWVERLFGYDWAQQTIGCSDAAVFRLSAQGRPVLFVKTDLSGALNELQDEAARLSWLA PA R ++W + G S A V+RLS GR L+VK +EL EA R +WL PAPLRRRYRRHEWRRVRTGASGAGVWRLS--GRTSLYVKL---ADRDELTAEADRCAWLG | 71  81 |
| Query 72  Sbjct 82 | | TTGVPCAAVLDVVTEAGRDWLLLGEVPGQDLLSS HLAPAEKVSIMADAMRRLHTLD  G+ V+D+ + R WL+ EVPG+ L H AP V+ +A R LH L RAGIGAPEVVDLDDDGERAWLVTTEVPGRPLSGEWPAHHRAPI--VAALARFTRHLHDLP | 127  139 |
| Query 128  Sbjct 140 | | PATCPFDHQAKHRIERARTRMEAGLVDQDDLDEEHQGLAPAELFARLKARMPDGEDLVVT A CPFD + + AR R+EAGLVD D D E +G EL L+ P+GED V+ VADCPFDRRLSITLPLARQRVEAGLVDAADFDAERRGRTAGELLPLLEEGAPEGEDAVIC | 187  199 |
| Query 188  Sbjct 200 | | HGDACLPNIMVE--NGRFSGFIDCGRLGVADRYQDIALATRDIAEELGGEWAD RFL  HGD L N++V+ +SG +D GRLGVADR+QD+ALA R++A + G + FL HGDLTLDNVLVDPVTLDWSGVVDVGRLGVADRHQDLALACRELAADAAGRFGPPFVRAFL | 241  259 |
| Query 242 | | VLYGIAAPDSQRIAFYRLLDEFF 264 YG D R+ +YRLLDEFF |  |
| Sbjct | 260 | EHYGSHLVDPDRLDYYRLLDEFF 282 | |

>TR:I2GER6 I2GER6_9BACT Aminoglycoside 3'-phosphotransferase OS=Fibrisoma limi BUZ 3 OX=1185876 GN=BN8_01391 PE=3 SV=1

Length=280

Score = 170 bits (430), Expect = 2e-48

Identities = 96/242 (40%), Positives = 137/242 (57%), Gaps = 6/242 (2%) Query 24	WAQQTIGCSDAAVFRLSAQGRPVLFVKTDLSGALNELQDEAARLSWLATTGVPCAAVLDV 83

| Sbjct | 40 | W T+G S A FRL +G ++K + L+ EA L WL +P V+ WQADTLGMSSAQTFRL--EGPESFYLKINPVDPWGGLKTEADALVWLRAY-LPAPEVVYY | 96 |
| --- | --- | --- | --- |
| Query | 84 | VTEAGRDWLLLGEVPGQDLLSSHLA--PAEKVSIMADAMRRLHTLDPATCPFDHQAKHRI | 141 |
| Sbjct | 97 | G D+LL+ E+ G + P +A +M LH+L+PA+CPFD + ++ ERLTGIDYLLIRELVGSPASADEWKTNPKRLAETLAVSMLALHSLEPASCPFDQRTDTKL | 156 |
| Query | 142 | ERARTRMEAGLVDQDDLDEEHQGLAPAELFARLKARMPDGEDLVVTHGDACLPNIMVENG | 201 |
| Sbjct | 157 | + ++ GLVD DD D+E+Q P + +L A P +DLVVTHGD CLPN+++ + REVTSLVQRGLVDADDFDDENQARTPEHILEQLYAEQPADDDLVVTHGDFCLPNLILTDW | 216 |
| Query | 202 | RFSGFIDCGRLGVADRYQDIALATRDIAEELGGEWADR-FLVLYGIAAPDSQRIAFYRLL | 260 |
| Sbjct | 217 | R SGFID G LGV DRYQD+AL RD+A+ELG D FL YG+ A D+ ++ ++RLL RLSGFIDVGGLGVGDRYQDLALCLRDLADELGTNRYDACFLSAYGLRAVDTDKLRYFRLL | 276 |
| Query  Sbjct | 261  277 | DE 262 DE  DE 278 |  |

>TR:A0A4Q1JS98 A0A4Q1JS98_9GAMM Aminoglycoside 3'-phosphotransferase OS=Pseudoxanthomonas composti OX=2137479 GN=aph(3')-I PE=3 SV=1

Length=271

Score = 169 bits (428), Expect = 2e-48

| Identities | = 102/258 (40%), Positives = 143/258 (55%), Gaps = 7/258 (3%) |  |
| --- | --- | --- |
| Query 12 | PAAWVERLFGYDWAQQTIGCSDAAVFRLSAQ-GRPVLFVKTDLSGALNELQDEAARLSWL | 70 |
| Sbjct 16 | PA + GY WA+ +G S AV RL + G P LF+K ++ DE RL WL PATLATLVEGYAWARDKVGESGGAVHRLHGKRGAPDLFLKHGRGAVAGDITDEMVRLRWL | 75 |
| Query 71 | ATTGVPCAAVLDVVTEAGRDWLLLGEVPGQ---DLLSSHL-APAEKVSIMADAMRRLHTL | 126 |
| Sbjct 76 | A VP V + WLL+ VPGQ LL +H A AE V +A ++RLH + AGH-VPVPEVQAFLATQDEAWLLMTAVPGQTAYQLLEAHPEARAEIVDALAGFLQRLHAI | 134 |
| Query 127 | DPATCPFDHQAKHRIERARTRMEAGLVDQDDLDEEHQGLAPAELFARLKARMPDGEDLVV | 186 |
| Sbjct 135 | CPF +R+ RAR R++AGLVD+DD D E +G A+++ ++ +P D VV PVGDCPFTSDHAYRLARARERIDAGLVDEDDFDTEREGWTAAQVWEAMQKLLPLPPDPVV | 194 |
| Query 187 | THGDACLPNIMVENGRFSGFIDCGRLGVADRYQDIALATRDIAEELGGEWADRFLVLYGI | 246 |
| Sbjct 195 | THGD L NI++ +G+ +G ID GR+GVADRYQD+A+ + E RFL YG THGDYSLDNILMIDGQVTGCIDAGRVGVADRYQDLAILWNCLG-EFDASLQTRFLERYGS | 253 |
| Query 247  Sbjct 254 | AAPDSQRIAFYRLLDEFF 264 A + +++ F+ +LDE F AVVNERKLQFHLMLDEMF 271 |  |

>TR:A0A1H3NWN2 A0A1H3NWN2_9BACI Kanamycin kinase/aminoglycoside 3'-phosphotransferase-2 OS=Bacillus caseinilyticus OX=1503961 GN=SAMN05421736_104212

PE=3 SV=1

Length=265

Score = 168 bits (426), Expect = 4e-48

| Identities | = 95/245 (39%), Positives = 139/245 (57%), Gaps = 5/245 (2%) |  |
| --- | --- | --- |
| Query 24 | WAQQTIGCSDAAVFRL-SAQGRPVLFVKTDLSGALNELQDEAARLSWLATTGVPCAAVLD | 82 |
| Sbjct 22 | +++ T GCS++AVF + + ++K A L DEAA L WL +P V FSENTTGCSNSAVFHMQNVHQYGSAYLKVKQYDAAETLADEAAVLDWLKEK-IPVPEVYY | 80 |
| Query 83 | VVTEAGRDWLLLGEVPGQDLLSS--HLAPAEKVSIMADAMRRLHTLDPATCPFDHQAKHR | 140 |
| Sbjct 81 | +++LL+ E+ G D S+ +P V A+ ++ +H ++ CPF + + YKHYQEKEYLLMSEIKGFDCSSAVHKNSPENMVKAFAEGLKAIHNIEITNCPFKRTLEKK | 140 |
| Query 141 | IERARTRMEAGLVDQDDLDEEHQGLAPAELFARLKARMPDGEDLVVTHGDACLPNIMVEN | 200 |
| Sbjct 141 | I++AR R+E GLVD+ + +EE+ G +LF L ++ D EDLV THGD CLPN++++ IDKARYRVENGLVDEANFEEENWGKKAVDLFETLLSKKTDDEDLVFTHGDYCLPNVIIDG | 200 |
| Query 201 | GRFSGFIDCGRLGVADRYQDIALATRDIAEELGGE-WADRFLVLYGIAAPDSQRIAFYRL | 259 |
| Sbjct 201 | + SG ID GR GVADRYQDIALA R + L E W D F YG+ + +I FY L  NKLSGLIDLGRGGVADRYQDIALAVRSLKHNLQSEKWVDLFFDHYGMKDVNDAKIDFYIL | 260 |

Query 260 LDEFF 264

LDEFF Sbjct 261 LDEFF 265

>TR:A0A318S577 A0A318S577_9DEIO Kanamycin kinase/aminoglycoside 3'-phosphotransferase-2 OS=Deinococcus yavapaiensis KR-236 OX=694435 GN=DES52_1264

PE=3 SV=1

Length=255

Score = 168 bits (425), Expect = 4e-48

| Identities | = 94/243 (39%), Positives = 135/243 (56%), Gaps = 7/243 (3%) |  |
| --- | --- | --- |
| Query 24 | WAQQTIGCSDAAVFRLSAQGRPVLFVKTDLSGALNELQDEAARLSWLATTGVPCAAVLDV | 83 |
| Sbjct 18 | W + T+G S + V+R + R VL VK + L E +L WL VP V+ WEEVTVGMSSSRVYRST---RHVLKVKRTTE-PFDTLFAEKEKLRWLEGR-VPAPKVVAY | 72 |
| Query 84 | VTEAGRDWLLLGEVPGQDLL--SSHLAPAEKVSIMADAMRRLHTLDPATCPFDHQAKHRI | 141 |
| Sbjct 73 | + +A ++L + +PG D+ + L P ++A A+R LH L CPFD + ++ LKDATGEYLAVTRLPGVDMSHEDARLHPRRNAELLARALRELHALPIQDCPFDASLRTKL | 132 |
| Query 142 | ERARTRMEAGLVDQDDLDEEHQGLAPAELFARLKARMPDGEDLVVTHGDACLPNIMVENG | 201 |
| Sbjct 133 | AR R+ AG VD+ D D+E QG ++ L P EDLVVTHGD CLPN++V REARERVSAGFVDEQDFDDERQGRRAEDVLRELLGTRPSREDLVVTHGDPCLPNVIVSGE | 192 |
| Query 202 | RFSGFIDCGRLGVADRYQDIALATRDIAEELGGEWADRFLVLYGIAAPDSQRIAFYRLLD | 261 |
| Sbjct 193 | GF+D GR G+ADR+ D+ALA R ++ G E+A+ FL YG D+ ++A+YRLLD FVEGFVDVGRAGIADRHADLALAARSLSHNYGPEFAELFLDAYGRFLVDASKLAYYRLLD | 252 |
| Query 262  Sbjct 253 | EFF 264 E F  ELF 255 |  |

>TR:A0A1H0AE42 A0A1H0AE42_9PSEU Aminoglycoside 3'-phosphotransferase-2 OS=Allokutzneria albata OX=211114 GN=SAMN04489726_6027 PE=3 SV=1

Length=250

Score = 167 bits (422), Expect = 1e-47

| Identities | = 102/249 (41%), Positives = 137/249 (55%), Gaps = 15/249 (6%) |  |
| --- | --- | --- |
| Query 22 | YDWAQQTIGCSDAAVFRLSAQGRPVLFVKTDLSGALNELQDEAARLSWLATTGVPCAAVL | 81 |
| Sbjct 11 | + W Q TIG S A V+RLS G F+K+ G EL +EA RL WL G+P V+ HTWEQVTIGRSGAGVWRLS--GPTDHFLKSVDGGT--ELFEEAERLRWLRAQGLPAPEVV | 66 |
| Query 82 | DVVTEAGRDWLLLGEVPGQDLLS SHLAPAEKVSIMADAMRRLHTLDPATCPFDHQA | 137 |
| Sbjct 67 | ++ + W++ VPG+ L L A +++ +A+ R LH L A CPFD ELGGDDRASWMVTTAVPGKSLAELGGDEMLWTAGQITALAEIARALHALPVADCPFDRTL | 126 |
| Query 138 | KHRIERARTRMEAGLVDQDDLDEEHQGLAPAELFARLKARMPDGEDLVVTHGDACLPNIM | 197 |
| Sbjct 127 | + +AR+ EAGLVD+ D D+ G +L ++A PD EDLVV HGD C PN++ DVVVPQARSNAEAGLVDETDFDQHRLGRTALDLIKEVEATRPDREDLVVCHGDLCTPNVL | 186 |
| Query 198 | VENGRF--SGFIDCGRLGVADRYQDIALATRDIAEELGGEWADRFLVLYGIAAPDSQRIA | 255 |
| Sbjct 187 | ++ +G ID GRLG+ADRY D+AL R LG A F YG PD +R+ LDPDTLTVTGLIDVGRLGIADRYNDLALTAR SLGPANAGMFFEAYG-EVPDPERLE | 241 |
| Query 256  Sbjct 242 | FYRLLDEFF 264 FYRLLDEFF FYRLLDEFF 250 |  |

>TR:Q1IW34 Q1IW34_DEIGD Aminoglycoside phosphotransferase OS=Deinococcus geothermalis (strain DSM 11300 / AG-3a) OX=319795 GN=Dgeo_2256

PE=3 SV=1

Length=259

Score = 167 bits (422), Expect = 1e-47

Identities = 100/248 (40%), Positives = 134/248 (54%), Gaps = 16/248 (6%)

Query 24	WAQQTIGCSDAAVFR-----LSAQGRPVLFVKTDLSGALNELQDEAARLSWLATTGVPCA 78 W	T GCS A V+R	+ QGR L V T	L E RL WL	VP

| Sbjct | 21 | WEPVTEGCSGAGVWRSQNYVVKVQGRGGLPVST LLQERERLRWLRGR-VPVP | 71 |
| --- | --- | --- | --- |
| Query | 79 | AVLDVVTEAGRDWLLLGEVPGQDLL--SSHLAPAEKVSIMADAMRRLHTLDPATCPFDHQ | 136 |
| Sbjct | 72 | V+ ++L L +PG + + L P ++A A+R LH L CPF+ VVVGYEVTPTHEYLALTRLPGIPMSHPDATLHPERLTDLLARALRELHALPIRECPFNLS | 131 |
| Query | 137 | AKHRIERARTRMEAGLVDQDDLDEEHQGLAPAELFARLKARMPDGEDLVVTHGDACLPNI | 196 |
| Sbjct | 132 | + AR R+ AG+VD+ D D E QG A++ A L P+ ED+VVTHGDACLPN+ LPVTLRLARERVRAGVVDEADFDAERQGWRAADVLAELLRTRPEHEDIVVTHGDACLPNV | 191 |
| Query | 197 | MVENGRFSGFIDCGRLGVADRYQDIALATRDIAEELGGEWADRFLVLYGIAAPDSQRIAF | 256 |
| Sbjct | 192 | ++ GF+D GR G+ADR+ D+ALA R + LG E+A+RFL LYG A D ++A+ ILNGEYVEGFVDVGRAGLADRHADLALAWRSLTRNLGPEYAERFLDLYGRALVDQGKLAY | 251 |
| Query  Sbjct | 257  252 | YRLLDEFF 264 Y+LLDE F YQLLDELF 259 |  |

>TR:A0A512HAF7 A0A512HAF7_9PROT Aminoglycoside 3'-phosphotransferase OS=Pararhodospirillum oryzae OX=478448 GN=ROR02_25560 PE=3 SV=1

Length=272

Score = 167 bits (423), Expect = 1e-47

| Identities | = 101/258 (39%), Positives = 142/258 (55%), Gaps = 7/258 (3%) |  |
| --- | --- | --- |
| Query 12 | PAAWVERLFGYDWAQQTIGCSDAAVFRLSAQ-GRPVLFVKTDLSGALNELQDEAARLSWL | 70 |
| Sbjct 17 | PA+ + GYDWA+ +G S AV+RL + G P LF+K ++ DE RL WL PASLAADVEGYDWARDGVGESGGAVYRLHGKPGHPDLFLKQGEGTCATDVTDEMVRLMWL | 76 |
| Query 71 | ATTGVPCAAVLDVVTEAGRDWLLLGEVPGQDLLSSHLA-PAEKVSI---MADAMRRLHTL | 126 |
| Sbjct 77 | A +P AV V WLL+ +PG + A PAE+ +I +AD +RR H + AAF-LPVPAVRGFVRTPDAAWLLMTAIPGLTAWQALEAFPAERPAIVDALADFLRRFHAI | 135 |
| Query 127 | DPATCPFDHQAKHRIERARTRMEAGLVDQDDLDEEHQGLAPAELFARLKARMPDGEDLVV | 186 |
| Sbjct 136 | + CPF R+ +AR R++AGLVD +D DEE QG +++ + A +P D VV PVSDCPFTSNHACRLAQARRRLDAGLVDVEDFDEERQGWNAEQVWDAMTALLPLAPDSVV | 195 |
| Query 187 | THGDACLPNIMVENGRFSGFIDCGRLGVADRYQDIALATRDIAEELGGEWADRFLVLYGI | 246 |
| Sbjct 196 | THGD L N++ NG +G ID GR GVADRYQD+A+ + + R Y THGDFSLDNLLWHNGTITGCIDVGRAGVADRYQDLAILWNCLC-DFEAALQKRLFHRYAT | 254 |
| Query 247  Sbjct 255 | AAPDSQRIAFYRLLDEFF 264 AAPD +++ F+ +LDEFF AAPDWRKLNFHLMLDEFF 272 |  |

>TR:A0A1G5KL09 A0A1G5KL09_9RHIZ Aminoglycoside 3'-phosphotransferase OS=Microvirga guangxiensis OX=549386 GN=SAMN02927923_03403 PE=3 SV=1

Length=250

Score = 166 bits (421), Expect = 1e-47

| Identities | = 97/251 (39%), Positives = 138/251 (55%), Gaps = 7/251 (3%) |  |
| --- | --- | --- |
| Query 19 | LFGYDWAQQTIGCSDAAVFRLSAQGRPV-LFVKTDLSGALNELQDEAARLSWLATTGVPC | 77 |
| Sbjct 2 | L Y+WA+ T+G S AV+RL ++ L++K +E+ DE RL WL +P LTDYEWARNTVGQSGGAVYRLHSKADAAGLYLKYGEDAVADEITDEMVRLGWLKGY-IPV | 60 |
| Query 78 | AAVLDVVTEAGRDWLLLGEVPG QDLLSSHLAPAEKVSIMADAMRRLHTLDPATCPF | 133 |
| Sbjct 61 | A+ V + WLL+ +PG Q L S+ A V +A +RRLHT+ + CPF PAIKHFVRTHDQAWLLMTAMPGRTAYQILESNPDAGLAVVDALAAFLRRLHTIPISECPF | 120 |
| Query 134 | DHQAKHRIERARTRMEAGLVDQDDLDEEHQGLAPAELFARLKARMPDGEDLVVTHGDACL | 193 |
| Sbjct 121 | + R+ RAR R++ G VD DD DEE +G +++ + A +P D VVTHGD L NSDHTFRLHRARIRIDEGWVDADDFDEEREGWTAEQVWEAMSALLPLAPDPVVTHGDFSL | 180 |
| Query 194 | PNIMVENGRFSGFIDCGRLGVADRYQDIALATRDIAEELGGEWADRFLVLYGIAAPDSQR | 253 |
| Sbjct 181 | N+++ G G ID GR+GVADRYQD+A+ + E G +R YGIA PD + DNLLIHEGEVVGCIDVGRVGVADRYQDLAILWNCLG-EFGRSLQERLFHQYGIAEPDQHK | 239 |
| Query 254 | IAFYRLLDEFF 264  + F+ LLDE F |  |

Sbjct 240 LQFHLLLDELF 250

>TR:A0A2A4ICH6 A0A2A4ICH6_9SPHN Aminoglycoside 3'-phosphotransferase OS=Sphingomonas adhaesiva OX=28212 GN=COA07_00575 PE=3 SV=1

Length=263

Score = 167 bits (422), Expect = 1e-47

| Identities | = 108/260 (42%), Positives = 143/260 (55%), Gaps = 10/260 (4%) |  |
| --- | --- | --- |
| Query 12 | PAAWVERLFGYDWAQQTIGCSDAAVFRLSAQGRPVLFVKTDLSGALNELQDEAARLSWLA | 71 |
| Sbjct 7 | PA+W RL GY+W + G S + RL+ G P L++K + DEA RL WL PASWDARLAGYEWHRDEAGESGGVIHRLTRDGAPSLYLKYGTGAVAEAVVDEAVRLRWL- | 65 |
| Query 72 | TTGVPCAAVLDVVTEAGRDWLLLGEVPGQ--DLLSSHLAPAEKVSIMADA--MRRLHTLD | 127 |
| Sbjct 66 | GVP A + V E WLL V G+ D H V I A A ++RLH L QGGVPVARPVAFVDEGDAAWLLTAAVAGRTGDAWLEHDPAMLPVIIAAFAGFLKRLHALP | 125 |
| Query 128 | PATCPFDHQAKHRIERARTRMEAGLVDQDDLDEEHQGLAPAELFARLKARM-PDGEDLVV | 186 |
| Sbjct 126 | CPF+ A R+ AR R+ AG VD DD D++H G A + A +A PD VV  VDDCPFEAGAALRLAAARRRVAAGEVDVDDFDDDHLGWDAARMLAHTEALYDPDLHRRVV | 185 |
| Query 187 | THGDACLPNIMV-ENGRFSGFIDCGRLGVADRYQDIALATRDIAE-ELGGEWADRFLVLY | 244 |
| Sbjct 186 | THGD L N+++ E+G+ +G +D GR+GVAD YQDIA+ ++AE + E A FL THGDFSLGNLLMDEHGQVTGCLDVGRMGVADPYQDIAILCANLAEIDPAAERA--FLAAI | 243 |
| Query 245  Sbjct 244 | GIAAPDSQRIAFYRLLDEFF 264 G+A+PD QR+AF+R LDE F GVASPDRQRMAFHRCLDELF 263 |  |

>TR:A0A2T0TND8 A0A2T0TND8_9BACT Aminoglycoside 3'-phosphotransferase-2 OS=Spirosoma oryzae OX=1469603 GN=CLV58_101300 PE=3 SV=1

Length=259

Score = 166 bits (421), Expect = 2e-47

| Identities | | = 97/254 (38%), Positives = 138/254 (54%), Gaps = 6/254 (2%) |  |
| --- | --- | --- | --- |
| Query 12  Sbjct 7 | | PAAWVERLFGYDWAQQTIGCSDAAVFRLSAQGRPVLFVKTDLSGALNELQDEAARLSWLA PA L DW ++G S A ++ GR ++K + L+DEA L WL PATLQRLLPPTDWHPDSLGMSSAQTIFIN--GREPFYLKVNPVDPWGGLRDEADALVWLR | 71  64 |
| Query 72  Sbjct 65 | | TTGVPCAAVLDVVTEAGRDWLLLGEVPGQDLLSSHLA--PAEKVSIMADAMRRLHTLDPA T +P V+ G D+LL+ +PG P V +A+++R LH L+PA TY-LPAPEVVHYERTDGVDYLLMRALPGLPASDERWQADPERLVRRLAESLRMLHDLNPA | 129  123 |
| Query 130  Sbjct 124 | | TCPFDHQAKHRIERARTRMEAGLVDQDDLDEEHQGLAPAELFARLKARMPDGEDLVVTHG TCPFD + +I + LVD DD E +QG +P ++ A+L+ P +LVVTHG TCPFDQRNDVKISAVSVNVLHNLVDTDDFSEANQGRSPQDMLAQLRMEQPTQRELVVTHG | 189  183 |
| Query 190  Sbjct 184 | | DACLPNIMVENGRFSGFIDCGRLGVADRYQDIALATRDIAEELGGE-WADRFLVLYGIAA D CLPNI+++N SGFID GRLGV D YQD+AL TR + G + + FL YG+ A DFCLPNIILDNWSLSGFIDVGRLGVGDPYQDLALCTRSLIHNFGTDRFGALFLATYGLTA | 248  243 |
| Query 249 | | PDSQRIAFYRLLDE 262 D++R+ ++ LLDE |  |
| Sbjct | 244 | VDTERMRYFTLLDE 257 | |

>TR:A0A1C1A760 A0A1C1A760_9BACL APH domain-containing protein OS=Paenibacillus pectinilyticus OX=512399 GN=A8709_02955 PE=3 SV=1

Length=259

Score = 166 bits (420), Expect = 3e-47

Identities = 94/246 (38%), Positives = 137/246 (56%), Gaps = 4/246 (2%)

| Query | 21 | GYDWAQQTIGCSDAAVFRLSAQGRPVLFVKTDLSGALNELQDEAARLSWLATTGVPCAAV | 80 |
| --- | --- | --- | --- |
| Sbjct | 16 | GY W + TIG S++ + L L++K A+ L +E RL WL +P V GYTWRRNTIGHSESKTYLLIGSSCN-LYLKIQSLAAVESLLNEKERLEWLQGK-LPVPEV | 73 |
| Query | 81 | LDVVTEAGRDWLLLGEVPGQDLL--SSHLAPAEKVSIMADAMRRLHTLDPATCPFDHQAK  + + ++LL+ E+ G + S + + + +A +R +H + A CPF+ + | 138 |

| Sbjct | 74 | VYYGQDDSNEYLLITEIEGSNASEKSFEIILPQLMKQLAYGLRAVHDIHVAGCPFNQRLD | 133 |
| --- | --- | --- | --- |
| Query | 139 | HRIERARTRMEAGLVDQDDLDEEHQGLAPAELFARLKARMPDGEDLVVTHGDACLPNIMV | 198 |
| Sbjct | 134 | +I+ A+ R+E LVD+++ D QGL +LF L ++ P EDLV THGD CLPNI++ SKIKEAKKRVEDNLVDEENFDPHRQGLKAKDLFHELLSKKPVKEDLVFTHGDFCLPNIII | 193 |
| Query | 199 | ENGRFSGFIDCGRLGVADRYQDIALATRDIAEELGGEWADRFLVLYGIAAPDSQRIAFYR | 258 |
| Sbjct | 194 | NG+ GFID GR GVADRYQDIALA R I G E FL YG+ D + +Y+ NNGKVHGFIDWGRAGVADRYQDIALAIRSIKYNFGDEPIHAFLEHYGLTELDETKALYYQ | 253 |
| Query  Sbjct | 259  254 | LLDEFF 264 L+DEFF LMDEFF 259 |  |

>TR:A0A096DMQ9 A0A096DMQ9_9CLOT APH domain-containing protein OS=Caloranaerobacter azorensis H53214 OX=1156417 GN=Y919_05425 PE=3 SV=1

Length=265

Score = 164 bits (416), Expect = 1e-46

| Identities | = 93/248 (38%), Positives = 138/248 (56%), Gaps = 5/248 (2%) |  |
| --- | --- | --- |
| Query 21 | GYDWAQQTIGCSDAAVFRLSAQGRPV-LFVKTDLSGALNELQDEAARLSWLATTGVPCAA | 79 |
| Sbjct 19 | G W + TIG SDA ++ L ++K L +L L E + WL +P GSKWIKDTIGRSDAHIYYLKGISNDNNAYLKVVLQNSLLTLLPEKRVIEWLQGK-LPVPK | 77 |
| Query 80 | VLDVVTEAGRDWLLLGEVPGQDLLSS--HLAPAEKVSIMADAMRRLHTLDPATCPFDHQA | 137 |
| Sbjct 78 | +L ++LL+ E+ G+D S P + I+A ++R +HT+ CPFD + ILYYGIHKDIEYLLISEIEGEDASSKVFKTEPHNLIRILARSLRMIHTIPIENCPFDQKL | 137 |
| Query 138 | KHRIERARTRMEAGLVDQDDLDEEHQGLAPAELFARLKARMPDGEDLVVTHGDACLPNIM | 197 |
| Sbjct 138 | +++ A+ R+E GLVD++D + + G ++ + P EDLV THGD CLPNI+ DTKLKMAKIRVEKGLVDENDFEPAYLGKTAKYIYQEVFKIRPKDEDLVFTHGDYCLPNII | 197 |
| Query 198 | VENGRFSGFIDCGRLGVADRYQDIALATRDIAEELGG-EWADRFLVLYGIAAPDSQRIAF | 256 |
| Sbjct 198 | +++G+ SGFID GR GVADRYQD+ALA R I LG +W+D FL YG+ D ++ + IKDGKLSGFIDLGRAGVADRYQDLALAVRSIEYNLGSKKWSDMFLKEYGLDNIDVSKLKY | 257 |
| Query 257  Sbjct 258 | YRLLDEFF 264 Y LLDE F YILLDELF 265 |  |

>TR:A0A3S0BKR3 A0A3S0BKR3_9BACL Aminoglycoside 3'-phosphotransferase OS=Paenibacillus whitsoniae OX=2496558 GN=EJQ19_14745 PE=3 SV=1

Length=267

Score = 164 bits (414), Expect = 3e-46

| Identities | | = 99/259 (38%), Positives = 142/259 (55%), Gaps = 11/259 (4%) |  |
| --- | --- | --- | --- |
| Query 12  Sbjct 14 | | PAAWVERLFGYDWAQQTIGCSDAAVFRL-SAQGRPVLFVKTDLSGALNELQDEAARLSWL PA L G W ++TIG S + +++ A GR F+K A+ L +E RL WL PAPLESLLAGCVWRRETIGHSASQTYQVCGAAGR--YFLKIQSLQAVESLVEEKERLLWL | 70  71 |
| Query 71  Sbjct 72 | | ATTGVPCAAVLDVVTEAGRDWLLLGEVPG QDLLSSHLAPAEKVSIMADAMRRLHTL  +P V+ T+ ++LL+ E+PG DL L E + +A ++++H + KGR-LPVPEVVYYGTDEAHEFLLVTELPGVNASDDLWKDQLP--ELLRALARGLKQIHQV | 126  128 |
| Query 127 | | DPATCPFDHQAKHRIERARTRMEAGLVDQDDLDEEHQGLAPAELFARLKARMPDGEDLVV CPFD + ++E A R+ GLVD++D DE GL E+ +L +P EDLV | 186 |
| Sbjct | 129 | PIDDCPFDQRLAVKMEEAMLRVRQGLVDEEDFDESRLGLRAEEVLEQLLGSVPAEEDLVF | 188 |
| Query | 187 | THGDACLPN-IMVENGRFSGFIDCGRLGVADRYQDIALATRDIAEELGGEWADRFLVLYG | 245 |
| Sbjct | 189 | THGD C+PN IM+ G SGFID GR G+ADRYQD+ALA R I G F+ YG THGDYCMPNIIMLSEGELSGFIDVGRAGIADRYQDLALAIRSIRYNFGDAHVQTFVEAYG | 248 |
| Query  Sbjct | 246  249 | IAAPDSQRIAFYRLLDEFF 264  + D +++FY+L+DEFF  LDKLDEAKVSFYQLMDEFF 267 |  |

>TR:A0A2R7JWN4 A0A2R7JWN4_9SPHN Aminoglycoside 3'-phosphotransferase OS=Sphingomonas

sp. HMWF008 OX=2056845 GN=aph(3')-I PE=3 SV=1

Length=274

Score = 163 bits (413), Expect = 4e-46

| Identities | | = 96/262 (37%), Positives = 145/262 (55%), Gaps = 14/262 (5%) |  |
| --- | --- | --- | --- |
| Query 12  Sbjct 18 | | PAAWVERLFGYDWAQQTIGCSDAAVFRLSAQ-GRPVLFVKTDLSGALNELQDEAARLSWL PA E++ GY W++ +G S V+RL + G P LF+K G +++ DE RL WL PACMAEQINGYQWSRDLVGESGGVVYRLHDKAGAPDLFLKHGRDGVADDITDEMVRLRWL | 70  77 |
| Query 71  Sbjct 78 | | ATTGVPCAAVLDVVTEAGRDWLLLGEVPGQDL--LSSHLAPAEKVSI---MADAMRRLHT A +P V+ V WLL+ +PG+ L ++++I +A +RRLH AGH-LPVPEVVHFVRAPDEAWLLMTALPGRTAFQLLDDAGEDDRIAIVDALAGFLRRLHA | 125  136 |
| Query 126  Sbjct 137 | | LDPATCPFDHQAKHRIERARTRMEAGLVDQDDLDEEHQGLAPAELFARLKARMPDGEDLV  + + CPF +R+ RAR R++AGLV++DD D E +G +++ L+A++P D V IPLSDCPFASDHAYRLARARERIDAGLVEEDDFDAEREGWTAEQVWDALQAKLPLAPDPV | 185  196 |
| Query 186  Sbjct 197 | | VTHGDACLPNIMVENGRFSGFIDCGRLGVADRYQDIALATRDIAE---ELGGEWADRFLV VTHGD L NI++ +G G ID GR+G+ADRYQD+A+ + E L G DR VTHGDFSLDNILLRDGAVIGCIDAGRVGIADRYQDLAILWNCLGEFDPRLQGRLFDR--- | 242  253 |
| Query 243 | | LYGIAAPDSQRIAFYRLLDEFF 264 YG D ++ F+ +LDE F |  |
| Sbjct | 254 | -YGTTGVDRAKLHFHLMLDELF 274 | |

>TR:A0A221ST19 A0A221ST19_9DEIO Aminoglycoside phosphotransferase APH(3') OS=Deinococcus ficus OX=317577 GN=DFI_01070 PE=3 SV=1

Length=263

Score = 162 bits (411), Expect = 7e-46

| Identities | = 99/243 (41%), Positives = 132/243 (54%), Gaps = 6/243 (2%) |  |
| --- | --- | --- |
| Query 24 | WAQQTIGCSDAAVFRLSAQGRPVLFVKTDLSGALNELQDEAARLSWLATTGVPCAAVLDV | 83 |
| Sbjct 25 | W + T+G S A V+R R V+ ++ +++ L E RL WLA VP V+ WERVTVGESGAGVWRSQ---RFVVKLQPRTGLSVSSLLQERERLRWLAGR-VPVPQVVAY | 80 |
| Query 84 | VTEAGRDWLLLGEVPGQDLLS--SHLAPAEKVSIMADAMRRLHTLDPATCPFDHQAKHRI | 141 |
| Sbjct 81 | +AG+++L + +PG + + L P VS++A A+R LH L CPF R+ EQDAGQEFLAMTRLPGIPMSDDDARLHPERMVSLLARALRELHALPIRDCPFRETLDVRL | 140 |
| Query 142 | ERARTRMEAGLVDQDDLDEEHQGLAPAELFARLKARMPDGEDLVVTHGDACLPNIMVENG | 201 |
| Sbjct 141 | AR + G+VD DD DEE G P +F L P EDLVVTHGDACLPN +V HAARENVVRGVVDADDFDEERLGRTPESVFNELVRTRPVHEDLVVTHGDACLPNFIVNGE | 200 |
| Query 202 | RFSGFIDCGRLGVADRYQDIALATRDIAEELGGEWADRFLVLYGIAAPDSQRIAFYRLLD | 261 |
| Sbjct 201 | G ID GR G+ADR+ D+ALA R LG + A+R L YG A D +IA+Y LLD FVEGLIDVGRAGIADRHMDLALAWRSTRSNLGPDHAERLLDEYGRALVDEVKIAYYLLLD | 260 |
| Query 262  Sbjct 261 | EFF 264 E F  ELF 263 |  |

>TR:A0A150WR92 A0A150WR92_BDEBC Aminoglycoside 3'-phosphotransferase OS=Bdellovibrio bacteriovorus OX=959 GN=AZI86_07600 PE=3 SV=1

Length=261

Score = 161 bits (408), Expect = 2e-45

Identities = 94/244 (39%), Positives = 135/244 (55%), Gaps = 4/244 (2%)

| Query | 24 | WAQQTIGCSDAAVFRLSAQGRPVLFVKTDLSGALNELQDEAARLSWLATTGVPCAAVLDV | 83 |
| --- | --- | --- | --- |
| Sbjct | 19 | W Q +G S + F+++ + F+KT + + N E A WL + A VL WEQVNVGESGDSCFKVTLKNGNKAFLKTHANNSDNYFTHEIAAALWL-NQHISAAEVLAD | 77 |
| Query | 84 | VTEAGRDWLLLGEVPGQDLLS--SHLAPAEKVSIMADAMRRLHTLDPATCPFDHQAKHRI | 141 |
| Sbjct | 78 | ++ +LL + G+DL + S L+ E V+I A AMR+LH + A CP D + + +I GSQGPFSYLLTSALQGKDLSAWVSELSNLEIVNICAIAMRQLHMIPYANCPLDQKLQVKI | 137 |
| Query | 142 | ERARTRMEAGLVDQDDLDEEHQGLAPAELFARLKARMPDGEDLVVTHGDACLPNIMVENG A+ R+ GLVD +D D+ +G + L L +P EDLVVTHGD LPN++ E | 201 |

| Sbjct | 138 | PWAKQRLHDGLVDLEDFDDSRKGWSLERLLVELDKNIPKSEDLVVTHGDFSLPNLIAERS | 197 |
| --- | --- | --- | --- |
| Query | 202 | RFSGFIDCGRLGVADRYQDIALATRDIAEELGGEWADRFLVLYG-IAAPDSQRIAFYRLL | 260 |
| Sbjct | 198 | FSGFID GR GVADR+QD+A+ + LG + FL YG I D +++ FY+LL SFSGFIDLGRAGVADRHQDLAICFNSLERNLGDSFQTEFLESYGLITKLDEEKLEFYKLL | 257 |
| Query  Sbjct | 261  258 | DEFF 264 DEFF  DEFF 261 |  |

>TR:A0A1U7NWU8 A0A1U7NWU8_9DEIO Aminoglycoside 3'-phosphotransferase OS=Deinococcus marmoris OX=249408 GN=BOO71_0008875 PE=3 SV=1

Length=263

Score = 161 bits (407), Expect = 3e-45

| Identities | | = 98/248 (40%), Positives = 129/248 (52%), Gaps = 15/248 (6%) |  |
| --- | --- | --- | --- |
| Query 24  Sbjct 24 | | WAQQTIGCSDAAVFRLSAQGRPVLFVKTDLSGALN---ELQDEAARLSWLATTGVPCAAV W + +G S A V++ +K G + LQ E RL WLA VP V WERVNVGESGAGVWKSQK FVIKVQARGGVQFATSLQQERERLRWLAGR-VPVPRV | 80  77 |
| Query 81  Sbjct 78 | | LDVVTEAGRDWLLLGEVPGQDLLSSH LAPAEKVSIMADAMRRLHTLDPATCPFDHQ  + E G ++L L VPG D +SH P V+++A A+R LH L CPF+ VGYEVEDGLEYLALTRVPGLD--ASHPDALFHPDRLVNLLARALRELHALPLRECPFNMS | 136  135 |
| Query 137 | | AKHRIERARTRMEAGLVDQDDLDEEHQGLAPAELFARLKARMPDGEDLVVTHGDACLPNI  + R R+ AG+VD+ D D E +G +F L P+ EDLVVTHGDACLPN | 196 |
| Sbjct | 136 | LSVTLPHVRERVAAGVVDESDFDGEREGRTAISVFNELARTRPEKEDLVVTHGDACLPNF | 195 |
| Query | 197 | MVENGRFSGFIDCGRLGVADRYQDIALATRDIAEELGGEWADRFLVLYGIAAPDSQRIAF | 256 |
| Sbjct | 196 | +V G ID GR G+ADR+ D+AL R + L E A+ FL LYG D Q++A+ IVNGEFIEGLIDVGRAGIADRHADLALTHRSLIRNLSPEAAEEFLDLYGREFVDPQKLAY | 255 |
| Query  Sbjct | 257  256 | YRLLDEFF 264 YRLLDE F YRLLDELF 263 |  |

>TR:C1CZE3 C1CZE3_DEIDV Putative kanamycin kinase (Aminoglycoside 3-phosphotransferase) (Neomycin-kanamycin phosphotransferase) OS=Deinococcus

deserti (strain VCD115 / DSM 17065 / LMG 22923) OX=546414 GN=Deide_21650 PE=3 SV=2

Length=260

Score = 160 bits (406), Expect = 3e-45

| Identities | = 95/243 (39%), Positives = 135/243 (56%), Gaps = 6/243 (2%) |  |
| --- | --- | --- |
| Query 24 | WAQQTIGCSDAAVFRLSAQGRPVLFVKTDLSGALNELQDEAARLSWLATTGVPCAAVLDV | 83 |
| Sbjct 22 | W + ++G S A V+R + R V+ V+T + ++ LQ E RL + A VP V+ WERVSLGESGAGVWRST---RYVVKVQTRSAAPVSTLQQERERLRFFAGR-VPVPRVIGY | 77 |
| Query 84 | VTEAGRDWLLLGEVPGQDLLS--SHLAPAEKVSIMADAMRRLHTLDPATCPFDHQAKHRI | 141 |
| Sbjct 78 | T ++L + VPG + + L P V+++A A+R LH L CPF+ + + ETHGDTEYLAMTRVPGIPMSDPDATLHPERVVTLLARALRELHALPLRDCPFNMTLAYTL | 137 |
| Query 142 | ERARTRMEAGLVDQDDLDEEHQGLAPAELFARLKARMPDGEDLVVTHGDACLPNIMVENG | 201 |
| Sbjct 138 | AR R+EAG+VD+ D D QG + +F L P+ EDLVVTHGDACLPN ++ HVARERVEAGVVDEADFDSHRQGRSAVSVFNELVRCRPETEDLVVTHGDACLPNFIISGE | 197 |
| Query 202 | RFSGFIDCGRLGVADRYQDIALATRDIAEELGGEWADRFLVLYGIAAPDSQRIAFYRLLD | 261 |
| Sbjct 198 | G +D GR G+ADR+ D+ALA R + LG +A FL LYG A D R+ +Y LLD FVEGLVDVGRAGLADRHADLALAWRSVQYNLGETYAGMFLDLYGPALVDQSRLDYYALLD | 257 |
| Query 262  Sbjct 258 | EFF 264 E F  ELF 260 |  |

>TR:E8U360 E8U360_DEIML Aminoglycoside phosphotransferase OS=Deinococcus maricopensis (strain DSM 21211 / LMG 22137 / NRRL B-23946 /

LB-34) OX=709986 GN=Deima_0344 PE=3 SV=1

Length=304

Score = 161 bits (407), Expect = 8e-45

| Identities | = 102/260 (39%), Positives = 140/260 (54%), Gaps = 18/260 (7%) |  |
| --- | --- | --- |
| Query 12 | PAAWVERLFGYDWAQQTIGCSDAAVFRLSAQGRPVLFVKTDLSGALNELQDEAARLSWLA | 71 |
| Sbjct 56 | PA+ L G W T+G SDA V+R S +K SG + L+ E RL + A PASLRAMLSGERWEPVTVGESDADVYRSSR FVLKRQRSGDFDTLRGERERLGYFA | 110 |
| Query 72 | TTGVPCAAVLDVVT---EAGRDWLLLGEVPGQDLLSSHLA PAEKVSIMADAMRRLH | 124 |
| Sbjct 111 | V DVV E ++L++ +PG D+ SH A P ++A A+R +H GR VRVPDVVAYHVEDDVEYLVVERLPGTDM--SHPALQRDPRRAAELLARALREVH | 164 |
| Query 125 | TLDPATCPFDHQAKHRIERARTRMEAGLVDQDDLDEEHQGLAPAELFARLKARMPDGEDL | 184 |
| Sbjct 165 | L CPFD + + R+ AR R+ GLVD+DD D+E +G +L ++ P EDL ALPVHDCPFDRRLRVRLADARERVRRGLVDEDDFDDERRGRRATDLLNDVEQSCPADEDL | 224 |
| Query 185 | VVTHGDACLPNIMVENGRFSGFIDCGRLGVADRYQDIALATRDIAEELGGEWADRFLVLY | 244 |
| Sbjct 225 | VVTHGDA + N+MV +G +G ID GR GVADR+ D+ALA +A + G FL Y VVTHGDAYVHNVMVADGELAGLIDVGRAGVADRHMDLALAAGSLASDYGEGVDQVFLEAY | 284 |
| Query 245  Sbjct 285 | GIAAPDSQRIAFYRLLDEFF 264 G D +++ FYRLLDEFF GREHVDEEKLRFYRLLDEFF 304 |  |

>TR:A0A0G2Z9P4 A0A0G2Z9P4_9BACT APH domain-containing protein OS=Kosmotoga pacifica OX=1330330 GN=IX53_08290 PE=3 SV=1

Length=243

Score = 159 bits (402), Expect = 8e-45

| Identities | = 86/235 (37%), Positives = 134/235 (57%), Gaps = 3/235 (1%) |  |
| --- | --- | --- |
| Query 30 | GCSDAAVFRLSAQGRPVLFVKTDLSGALNELQDEAARLSWLATTGVPCAAVLDVVTEAGR | 89 |
| Sbjct 12 | G S V++L G V ++K + L+ E + WL +P VLDV T GLSGDTVYKLKRNGN-VFYLKISSPDRKDLLEREIRNIEWLGNY-LPVPKVLDVGTAESN | 69 |
| Query 90 | DWLLLGEVPGQDLLSSHLAPAEKVSIMADAMRRLHTLDPATCPFDHQAKHRIERARTRME | 149 |
| Sbjct 70 | ++L+ E+PG L S P + +SIMAD ++ H++D CP + + + ++ A ++ MYVLMSEIPGIPLHLSDEKPQKLISIMADGLKLFHSVDIPNCPLNQRLEVKLAAAFDNIK | 129 |
| Query 150 | AGLVDQDDLDEEHQGLAPAELFARLKARMPDGEDLVVTHGDACLPNIMVENGRFSGFIDC | 209 |
| Sbjct 130 | G V+ D +E ++ P +++ L + PD EDLV+ HGD CLPNI+V G+ SGFID KGRVNTDFFEERNRNRLPEQIYEELLKQKPD-EDLVLCHGDYCLPNILVYKGKLSGFIDL | 188 |
| Query 210  Sbjct 189 | GRLGVADRYQDIALATRDIAEELGGEWADRFLVLYGIAAPDSQRIAFYRLLDEFF 264 G GV+DRYQD+A+ R IA +G ++ +FL YG+ PD +++ +YR LDE F GLSGVSDRYQDLAIGARSIAYNIGEKYIQQFLEEYGLENPDERKMRYYRDLDELF 243 |  |

>TR:H8GUT9 H8GUT9_DEIGI Aminoglycoside 3`-phosphotransferase OS=Deinococcus gobiensis (strain DSM 21396 / JCM 16679 / CGMCC 1.7299 /

I-0) OX=745776 GN=DGo_CA0345 PE=3 SV=1

Length=260

Score = 159 bits (403), Expect = 9e-45

| Identities | = 97/243 (40%), Positives = 132/243 (54%), Gaps = 6/243 (2%) |  |
| --- | --- | --- |
| Query 24 | WAQQTIGCSDAAVFRLSAQGRPVLFVKTDLSGALNELQDEAARLSWLATTGVPCAAVLDV | 83 |
| Sbjct 22 | W + T+G S A V+R S R V+ V++ ++ L E RL +LA VP AV+ + WERVTLGHSGAGVWRSS---RHVVKVQSRGGLPVSTLPQERERLRYLAGR-VPVPAVVGL | 77 |
| Query 84 | VTEAGRDWLLLGEVPGQDLLSSHLA--PAEKVSIMADAMRRLHTLDPATCPFDHQAKHRI | 141 |
| Sbjct 78 | +E D+L + +PG + A PA ++A A+R LH L CPF + ESEGDFDYLAMTRLPGIPMSDPDAALHPARMAGLLARALRELHALPLRDCPFTMTLAVTL | 137 |
| Query 142 | ERARTRMEAGLVDQDDLDEEHQGLAPAELFARLKARMPDGEDLVVTHGDACLPNIMVENG | 201 |
| Sbjct 138 | AR R+ AG+VD+ D D G +F L P EDLVVTHGD CLPN++V PLARERVAAGVVDETDFDVSRSGRTATSVFNELARTRPGQEDLVVTHGDPCLPNLLVAGE | 197 |
| Query 202 | RFSGFIDCGRLGVADRYQDIALATRDIAEELGGEWADRFLVLYGIAAPDSQRIAFYRLLD GF+D GR G+ADR+ D+ALA R A LG E A+ FL YG A D ++A+Y+LLD | 261 |

| Sbjct | 198 | YVEGFVDVGRAGIADRHADLALAYRSAARNLGPEHAEAFLDQYGRALVDPHKLAYYQLLD 257 |
| --- | --- | --- |
| Query | 262 | EFF 264 |
| Sbjct | 258 | E F  ELF 260 |

>TR:Q9RY84 Q9RY84_DEIRA Aminoglycoside 3`-phosphotransferase OS=Deinococcus radiodurans (strain ATCC 13939 / DSM 20539 / JCM 16871 /

LMG 4051 / NBRC 15346 / NCIMB 9279 / R1 / VKM B-1422) OX=243230 GN=DR_0066 PE=3 SV=1

Length=263

Score = 159 bits (403), Expect = 1e-44

| Identities | | = 96/245 (39%), Positives = 137/245 (56%), Gaps = 10/245 (4%) |  |
| --- | --- | --- | --- |
| Query 24  Sbjct 25 | | WAQQTIGCSDAAVFRLSAQGRPVLFVKTDLSGALNELQDEAARLSWLATTGVPCAAVLDV W + T G S A V+R + R V+ V++ + L E RL W A +P V+ WERVTEGESGAGVWRST---RFVVKVQSRTLYPASTLLQERERLRWFAGR-LPVPQVVAY | 83  80 |
| Query 84 | | VTEAGRDWLLLGEVPGQDLLSSH LAPAEKVSIMADAMRRLHTLDPATCPFDHQAKH  + +++L + +PG + SH L P V+++A A+R LH L CPF+ | 139 |
| Sbjct | 81 | EDDGEQEYLAMTRLPG--IAMSHPDAALHPERMVNLLARALRELHALPVRDCPFNMSLGV | 138 |
| Query | 140 | RIERARTRMEAGLVDQDDLDEEHQGLAPAELFARLKARMPDGEDLVVTHGDACLPNIMVE | 199 |
| Sbjct | 139 | R++ AR R+ AG+VD+ D DEE QG + ++F +L P EDLVVTHGDACLPN++V+ RLKLARERVAAGVVDESDFDEERQGQSAVQVFNQLARTRPAEEDLVVTHGDACLPNVIVQ | 198 |
| Query | 200 | NGRFSGFIDCGRLGVADRYQDIALATRDIAEELGGEWADRFLVLYGIAAPDSQRIAFYRL | 259 |
| Sbjct | 199 | G ID GR G+ADR+ D+ALA R LG +A L LYG D ++A+Y+L GEYVEGLIDLGRAGIADRHMDLALAWRSTRRNLGATYAGMLLDLYGRELVDEGKLAYYQL | 258 |
| Query  Sbjct | 260  259 | LDEFF 264 LDE F LDELF 263 |  |

>TR:A0A1I6IW28 A0A1I6IW28_9BURK Aminoglycoside 3'-phosphotransferase OS=Mitsuaria sp. PDC51 OX=1881035 GN=SAMN05428960_0278 PE=3 SV=1

Length=276

Score = 159 bits (403), Expect = 1e-44

| Identities | = 96/258 (37%), Positives = 139/258 (54%), Gaps = 7/258 (3%) |  |
| --- | --- | --- |
| Query 12 | PAAWVERLFGYDWAQQTIGCSDAAVFRLSAQGRPVLFVKTDLSGALNELQDEAARLSWLA | 71 |
| Sbjct 21 | P + L G +W + +G + A V RL +G P L++K A + DE ARL WL+ PPSLAGALAGREWHRNLVGEAGALVHRLRREGSPDLYLKHGDGEAATAIVDEFARLRWLS | 80 |
| Query 72 | TTGVPCAAVLDVVTEAGRDWLLLGEVPGQDLLS-SHLAPAEKVSIMAD---AMRRLHTLD | 127 |
| Sbjct 81 | P AV G WLL +PG+ AP +I+A +RRLH L GRW-PVPAVAHFEATGGTAWLLTQALPGRTAYEWLEAAPDRAPAIVASIGRCLRRLHALP | 139 |
| Query 128 | PATCPFDHQAKHRIERARTRMEAGLVDQDDLDEEHQGLAPAELFARLKARMPDGEDLVVT | 187 |
| Sbjct 140 | TCPF + +++ AR R+EAGL+D DD D+ QG P +++ L +P ED VV+ VETCPFSASHRLQLDLARQRLEAGLIDADDFDDARQGWTPEQVWRALGELLPLEEDRVVS | 199 |
| Query 188 | HGDACLPNIMVE-NGRFSGFIDCGRLGVADRYQDIALATRDIAEELGGEWADRFLVLYGI | 246 |
| Sbjct 200 | HGD L N+++ G +G ID GR G+ADRY+D+A+ + +E G + YGI HGDFSLDNLLMSAEGEVAGVIDVGRAGIADRYRDLAILA-NCLDEFGPSLGEVLFSAYGI | 258 |
| Query 247  Sbjct 259 | AAPDSQRIAFYRLLDEFF 264 AAPD++++ F+ LLDE F AAPDARKLEFHLLLDECF 276 |  |

>TR:A0A0K9XNI0 A0A0K9XNI0_9ACTN Aminoglycoside phosphotransferase OS=Streptomyces caatingaensis OX=1678637 GN=AC230_04535 PE=3 SV=1

Length=264

Score = 159 bits (402), Expect = 1e-44

Identities = 102/255 (40%), Positives = 137/255 (54%), Gaps = 14/255 (5%)

| Query | 22 | YDWAQQTIGCSDAAVFRLSAQGRPVLFVKTD---LSGALNELQDEAARLSWLATTGVPCA | 78 |
| --- | --- | --- | --- |
| Sbjct | 12 | ++W T G S A V RL+ +P L+ K L + +L EA RL WLA G+P HEWNAVTDGDSGAFVHRLTGP-QPELYAKIAPRALDNSAFDLAGEADRLDWLARHGIPVP | 70 |
| Query | 79 | AVLDVVTEAGRDWLLLGEVPGQDLLSS---HLAPAEKVSIMADAMRRLHTLDPATCPFDH | 135 |
| Sbjct | 71 | +++ + W++ VPG H PA MA R LH L A CPFD RIVERGGDDTSVWIVTEAVPGVSAAEEWPEHQRPAV-AEAMARIARTLHDLPVAECPFDR | 129 |
| Query | 136 | QAKHRIERARTRMEAGLVDQDDLDEEHQGLAPAELFARLKARMPDGEDLVVTHGDACLPN | 195 |
| Sbjct | 130 | ++ AR + AGLVD +DLDE +G + A+L A L P+ ED+VV HGD C N SLAVAMDEARHNVRAGLVDLEDLDEARRGWSGAQLLAELDRTRPETEDIVVCHGDLCPDN | 189 |
| Query | 196 | IMV--ENGRFSGFIDCGRLGVADRYQDIALATRDIAEE LGGEWADRFLVLYGIAAP | 249 |
| Sbjct | 190 | +++ E +G ID GRLG+ADR+ D+ALATR++ E G E+A FL YG VVLDPETCEVTGVIDVGRLGLADRHADLALATRELEIEEDPWFGPEYARGFLTEYGGPGV | 249 |
| Query  Sbjct | 250  250 | DSQRIAFYRLLDEFF 264 D ++AFYRLLDEFF DKDKLAFYRLLDEFF 264 |  |

>TR:A0A2K3UW40 A0A2K3UW40_9DEIO Aminoglycoside 3'-phosphotransferase OS=Deinococcus koreensis OX=2054903 GN=CVO96_04640 PE=3 SV=1

Length=259

Score = 157 bits (397), Expect = 7e-44

| Identities | = 93/243 (38%), Positives = 129/243 (53%), Gaps = 6/243 (2%) |  |
| --- | --- | --- |
| Query 24 | WAQQTIGCSDAAVFRLSAQGRPVLFVKTDLSGALNELQDEAARLSWLATTGVPCAAVLDV | 83 |
| Sbjct 21 | W + T+G S A V+R + + V+ V+ ++ L E RL W A +P V+ WERVTLGKSGAGVWRST---KYVVKVQGRGGSPVSTLLQERERLRWFAGR-LPAPQVVGY | 76 |
| Query 84 | VTEAGRDWLLLGEVPGQDLLS--SHLAPAEKVSIMADAMRRLHTLDPATCPFDHQAKHRI | 141 |
| Sbjct 77 | G ++L + VPG + + L P V ++A A+R LH L CPF + EVTTGHEYLAMTRVPGIPMSDPDATLHPQRVVDLLARALRELHALPIRDCPFRMTLDVTL | 136 |
| Query 142 | ERARTRMEAGLVDQDDLDEEHQGLAPAELFARLKARMPDGEDLVVTHGDACLPNIMVENG | 201 |
| Sbjct 137 | AR ++EAG VD+ D DEE +G +F L PD EDLVVTHGD CLPN++V RLAREQVEAGTVDEADFDEERRGRTAVSVFNELARTRPDAEDLVVTHGDPCLPNLIVSGE | 196 |
| Query 202 | RFSGFIDCGRLGVADRYQDIALATRDIAEELGGEWADRFLVLYGIAAPDSQRIAFYRLLD | 261 |
| Sbjct 197 | G ID GR G+ADR+ D+ALA R + + L WA+R L LYG D +I ++ LLD HVEGLIDLGRAGIADRHADLALAWRSVRDILDEGWAERLLDLYGRDLVDRGKIGYFMLLD | 256 |
| Query 262  Sbjct 257 | EFF 264 E F  ELF 259 |  |

>TR:A0A1I4WU92 A0A1I4WU92_9PSEU Kanamycin kinase OS=Saccharopolyspora antimicrobica OX=455193 GN=SAMN05421805_10377 PE=3 SV=1

Length=262

Score = 157 bits (397), Expect = 8e-44

| Identities | = 104/255 (41%), Positives = 142/255 (56%), Gaps = 23/255 (9%) |  |
| --- | --- | --- |
| Query 25 | AQQTIGCSDAAVFRLSAQGRPVLFVKTDLSGALNELQ DEAARLSWLATTGVPCAAV | 80 |
| Sbjct 14 | AQ++ G A++R+ +GR +VKT ++L+ +EA RL WLA G+P V AQRSYG---TAIYRV--EGRRTYYVKTTPPRDPDDLRFHPGNEAERLGWLAGRGIPVPEV | 68 |
| Query 81 | LDVVTEAGRDWLLLGEVPGQDLLSSHLAPAEKVSIM---ADAMRRLHTLDPATCPFDHQA | 137 |
| Sbjct 69 | +++ WL+ VPG+ + AP E ++ AD R LH L CPFD VELGESEDLQWLVTTMVPGRPVAGGW-APHELPRVLESVADLARALHELPAGECPFDRGL | 127 |
| Query 138 | KHRIERARTRMEAGLVDQDDLDEEHQGLAPAELFARLKARMPDGE-DLVVTHGDACLPNI | 196 |
| Sbjct 128 | + + ART +E G VD DDLD EH G + L A+L A P E D+VV HGD CL N+ ESSLAGARTAVERGTVDLDDLDPEHDGWSGDRLLAKLDATPPPPEVDVVVCHGDLCLDNV | 187 |
| Query 197 | MV--ENGRFSGFIDCGRLGVADRYQDIALATRDIAEELGGEW ADRFLVLYGIAA | 248 |
| Sbjct 188 | +V E +G ID GRLGVADR+ D+A+ RD+ EE EW A+ FL YG+A  LVDPEGKSVTGVIDVGRLGVADRWLDLAIVLRDLGEET-REWGYDPDCAEVFLRRYGLAE | 246 |

Query 249 PDSQRIAFYRLLDEF 263

D +R +FYRL+DEF Sbjct 247 IDERRFSFYRLVDEF 261

>TR:A0A254N847 A0A254N847_9BURK Aminoglycoside 3'-phosphotransferase OS=Pelomonas puraquae OX=431059 GN=CDO81_10685 PE=3 SV=1

Length=276

Score = 157 bits (398), Expect = 8e-44

| Identities | | = 90/246 (37%), Positives = 133/246 (54%), Gaps = 7/246 (3%) |  |
| --- | --- | --- | --- |
| Query 24  Sbjct 33 | | WAQQTIGCSDAAVFRLSAQGRPVLFVKTDLSGALNELQDEAARLSWLATTGVPCAAVLDV W + +G + AAV+R++ GR L++K + DE RL WL +P A +L WHRDRVGEAGAAVYRVARPGRADLYLKHGRGAVAEAVVDEMVRLRWLQGH-LPVARLLHF | 83  91 |
| Query 84 | | VTEAGRDWLLLGEVPG QDLLSSHLAPAEKVSIMADAMRRLHTLDPATCPFDHQAKH  A WLL +PG Q L + + V+ +A + +LH + +CPF | 139 |
| Sbjct | 92 | EVAASEAWLLTHALPGRTAYQWLCDAPAHMPQIVTTLARTLAQLHRVPVPSCPFQAPLDQ | 151 |
| Query | 140 | RIERARTRMEAGLVDQDDLDEEHQGLAPAELFARLKARMPDGEDLVVTHGDACLPNIMVE | 199 |
| Sbjct | 152 | R+ AR R++AGL+D DD D+ QG PA+++ L+ P G D VV HGD L N++++ RLAHARQRLDAGLIDPDDFDDARQGWTPAQVWRELQRLRPTGHDAVVCHGDYSLDNLLLD | 211 |
| Query | 200 | -NGRFSGFIDCGRLGVADRYQDIALATRDIAEELGGEWADRFLVLYGIAAPDSQRIAFYR | 258 |
| Sbjct | 212 | GR +G ID GRLGVADR+QD+A+ + E G + YG+A PD++R+ FY AQGRLTGLIDLGRLGVADRFQDLAILWNSLG-EFGTDAQRHLFSAYGLAPPDARRLDFYL | 270 |
| Query | 259 | LLDEFF 264 LDE F |  |
| Sbjct | 271 | CLDECF 276 | |

>TR:A0A553ZT10 A0A553ZT10_9BACI APH(3')-I family aminoglycoside O-phosphotransferase OS=Bacillus sp. P16(2019) OX=2597270 GN=aph(3')-I PE=3

SV=1

Length=271

Score = 157 bits (397), Expect = 1e-43

| Identities | = 89/251 (35%), Positives = 134/251 (53%), Gaps = 7/251 (3%) |  |
| --- | --- | --- |
| Query 19 | LFGYDWAQQTIGCSDAAVFRLSAQ-GRPVLFVKTDLSGALNELQDEAARLSWLATTGVPC | 77 |
| Sbjct 23 | L+GY WA+ +G S A ++RL + P LF+K N++ DE RL+WL T +P LYGYKWARDNVGQSGATIYRLYGKPDAPELFLKHGKGSVANDVTDEMVRLNWL-TEFMPL | 81 |
| Query 78 | AAVLDVVTEAGRDWLLLGEVPGQ---DLLSSHLAPAEK-VSIMADAMRRLHTLDPATCPF | 133 |
| Sbjct 82 | + V+ WLL +PG+ +L + E V +A +RRLH++ CPF PTIKHVIRTPEDAWLLTTAIPGKTAFQVLEEYPDSGENIVDALAVFLRRLHSIPVCNCPF | 141 |
| Query 134 | DHQAKHRIERARTRMEAGLVDQDDLDEEHQGLAPAELFARLKARMPDGEDLVVTHGDACL | 193 |
| Sbjct 142 | + R+ +A++RM GLVD D D+E G +++ + +P D VVTHGD L NSDRVFRLAQAQSRMNNGLVDASDFDDERNGWPVEQVWKEMHKLLPFSPDSVVTHGDFSL | 201 |
| Query 194 | PNIMVENGRFSGFIDCGRLGVADRYQDIALATRDIAEELGGEWADRFLVLYGIAAPDSQR | 253 |
| Sbjct 202 | N++ + G+ G ID GR+G+ADRYQD+A+ + E R YGI PD + DNLIFDEGKLIGCIDVGRVGIADRYQDLAILWNCLG-EFSPSLQKRLFQKYGIDNPDMNK | 260 |
| Query 254  Sbjct 261 | IAFYRLLDEFF 264  + F+ +LDEFF  LQFHLMLDEFF 271 |  |

```
>TR:A0A1G6UA45 A0A1G6UA45_9PSEU Kanamycin kinase OS=Alloactinosynnema iranicum OX=1271860 GN=SAMN05216174_110177 PE=3 SV=1
Length=276

Score = 157 bits (397), Expect = 1e-43
Identities = 101/250 (40%), Positives = 137/250 (55%), Gaps = 13/250 (5%)

Query 23	DWAQQTIGCSDAAVFRLSAQGRPVLFVKTDLSGALNEL----QDEAARLSWLATTGVPCA 78 DW +	AA+ R+ +G	+VKT +	++L	++EA RLSWLA G P
Sbjct 30	DWHRVATRSYGAAIHRVQCRG--TYYVKTMSTVDRDDLRLHPENEAERLSWLAGYGFPVP 87
```

| Query | 79 | AVLDVVTEAGRDWLLLGEVPGQDLLSSHLAPAEKVSIM---ADAMRRLHTLDPATCPFDH | 135 |
| --- | --- | --- | --- |
| Sbjct | 88 | V++V G WL+ VPG S P E+V ++ AD LH++ PA CPFD EVVEVGAAEGLSWLVTTAVPGVPA-SGPWRPDERVRVLDAVADLTAALHSIPPAECPFDR | 146 |
| Query | 136 | QAKHRIERARTRMEAGLVDQDDLDEEHQGLAPAELFARLKARMPDGEDLVVTHGDACLPN | 195 |
| Sbjct | 147 | + R R +E G VD DDLDE H G + +L A L + ED+VV HGD L N TLAVTLPRCRVAVELGTVDLDDLDERHAGWSGQDLLAELDSLPAPREDVVVCHGDLGLDN | 206 |
| Query | 196 | IMVE--NGRFSGFIDCGRLGVADRYQDIALATRDIAEELGGEWADRFLVLYGIAAPDSQR | 253 |
| Sbjct | 207 | I+V+ + SG +D GRLG ADR+ D+A+A RDI EEL A FL YG++ D +R ILVDPRSLAVSGVLDAGRLGRADRWLDLAIAVRDITEELRVP-ATGFLKRYGVSTVDERR | 265 |
| Query | 254 | IAFYRLLDEF 263  +YRL+DEF |  |
| Sbjct | 266 | YFYYRLMDEF 275 | |

>TR:A0A2K2F1D5 A0A2K2F1D5_9FIRM APH domain-containing protein OS=Pseudoclostridium thermosuccinogenes OX=84032 GN=CDQ84_05660 PE=3 SV=1

Length=265

Score = 157 bits (396), Expect = 1e-43

| Identities | = 93/243 (38%), Positives = 130/243 (53%), Gaps = 3/243 (1%) |  |
| --- | --- | --- |
| Query 24 | WAQQTIGCSDAAVFRLSAQGRPVLFVKTDLSGALNELQDEAARLSWLATTGVPCAAVLDV | 83 |
| Sbjct 24 | W Q TIG S A + LS + ++K ++ E L WL +P VL WKQVTIGESGAYTYMLSDESGQNRYLKIMPRKLNLSMEREVDLLKWLEGK-LPVPKVLLF | 82 |
| Query 84 | VTEAGRDWLLLGEVPG-QDLLSSHLAPAEK-VSIMADAMRRLHTLDPATCPFDHQAKHRI | 141 |
| Sbjct 83 | + ++ ++ L+ EV G S + K V ++A +R +H++D CPFD + ++ IHDSEYEYFLMTEVKGIHSCAPSFESDLPKLVRLLAKGLRMIHSVDIKDCPFDQRLSIKL | 142 |
| Query 142 | ERARTRMEAGLVDQDDLDEEHQGLAPAELFARLKARMPDGEDLVVTHGDACLPNIMVENG | 201 |
| Sbjct 143 | + A R GLVD+DD D+ +G +LF L A P EDLV THGD CLPNI++ NG KEAEYRTVNGLVDEDDFDDIRKGRKAIDLFQELIATKPAEEDLVFTHGDYCLPNIIINNG | 202 |
| Query 202 | RFSGFIDCGRLGVADRYQDIALATRDIAEELGGEWADRFLVLYGIAAPDSQRIAFYRLLD | 261 |
| Sbjct 203 | GFID GR G+ADRYQDIAL R +A G +W YG+ D +I +Y+LLD DIGGFIDWGRGGIADRYQDIALVARSLAYNFGEKWIPLLFKEYGMDKIDYSKIEYYKLLD | 262 |
| Query 262  Sbjct 263 | EFF 264 EFF  EFF 265 |  |

>TR:A0A0F7N198 A0A0F7N198_9ACTN APH domain-containing protein OS=Streptomyces sp. CNQ-509 OX=444103 GN=AA958_02260 PE=3 SV=1

Length=271

Score = 157 bits (396), Expect = 1e-43

| Identities | = 109/266 (41%), Positives = 133/266 (50%), Gaps = 21/266 (8%) |  |
| --- | --- | --- |
| Query 18 | RLFGYDWAQQTIGCSDAAVFRLSAQGRPVLFVKTDLSG ALNELQDEAARLSWLAT | 72 |
| Sbjct 8 | R YDW T G S A V+RL+ G LFVKT +G +L+ EAA WL RYASYDWQPVTDGMSGAGVWRLTGPGE--LFVKTAAAGDGPAEPGADLRHEAACTRWLRA | 65 |
| Query 73 | TGVPCAAVLDVVTEAGRDWLLLGEVPGQDLLSSHLAP--AEKVSIMADAMRRLHTLDPAT | 130 |
| Sbjct 66 | G+P VLD G WL+ PG+ L A A V +A RRLH L A QGLPAPEVLDAGAADGTTWLVTRARPGRSLAEPWPADLRAGAVEALARFTRRLHELPVAD | 125 |
| Query 131 | CPFDHQAKHRIERARTRMEAGLVDQDDLDEEHQGLAPAELFARLKARMPDGEDLVVTHGD | 190 |
| Sbjct 126 | CPF + A + AGLVD++D D+ +G + A LFA L+ P EDLVV HGD CPFVRNLSVTVPLAGAAVAAGLVDEEDFDDGRRGRSAAGLFAELERTRPAVEDLVVCHGD | 185 |
| Query 191 | ACLPNIMVENG--RFSGFIDCGRLGVADRYQDIALATRDIAEE LGGEWADRFLVL | 243 |
| Sbjct 186 | C PN++V+ SG ID GRLGVADR+ D+ALA R ++E G A FL LCAPNVLVDPATLEVSGVIDLGRLGVADRHLDLALAVRSFSDEDLNPQHGPAAAGLFLRT | 245 |
| Query 244  Sbjct 246 | Y-----GIAAPDSQRIAFYRLLDEFF 264 Y G D R FY LLDEFF YRAAGEGDVRVDPARTEFYTLLDEFF 271 |  |

>TR:A0A3D9KN32 A0A3D9KN32_9BURK Aminoglycoside 3'-phosphotransferase OS=Duganella sp. GV023 OX=2135490 GN=C7506_102102 PE=3 SV=1

Length=270

Score = 156 bits (395), Expect = 2e-43

| Identities | = 93/259 (36%), Positives = 136/259 (53%), Gaps = 7/259 (3%) |  |
| --- | --- | --- |
| Query 11 | SPAAWVERLFGYDWAQQTIGCSDAAVFRL-SAQGRPVLFVKTDLSGALNELQDEAARLSW | 69 |
| Sbjct 14 | +P L GY+W T+G S V+RL S +G L++K +E+ E RLSW APLEIAAMLTGYEWLCNTVGESGGTVYRLRSKEGASDLYLKYGRDAVADEISTEMERLSW | 73 |
| Query 70 | LATTGVPCAAVLDVVTEAGRDWLLLGEVPGQDLLSSHLAPAEKVSIMADA MRRLHT | 125 |
| Sbjct 74 | LA + AV+ WLL+ VPG A + + DA +R+LH LARY-MTVPAVVHFTRTPDEAWLLMTAVPGTTAYQVLQAHPDYQLALVDAIARFLRQLHA | 132 |
| Query 126 | LDPATCPFDHQAKHRIERARTRMEAGLVDQDDLDEEHQGLAPAELFARLKARMPDGEDLV | 185 |
| Sbjct 133 | + CPF+ +R+ AR R++AGLVD+ D DEE G +++ ++ +P D V IPVNECPFNSGHAYRLSCARARIDAGLVDEGDFDEERDGWTAEQVWEAMQRLLPLTPDPV | 192 |
| Query 186 | VTHGDACLPNIMVENGRFSGFIDCGRLGVADRYQDIALATRDIAEELGGEWADRFLVLYG | 245 |
| Sbjct 193 | VTHGD L N+++++G G ID GR+G+ADRYQD+A+ + E +RFL YG VTHGDFSLDNLLIKDGEVIGCIDTGRVGIADRYQDLAIVLNCLT-EFDAALQERFLKQYG | 251 |
| Query 246  Sbjct 252 | IAAPDSQRIAFYRLLDEFF 264 I PD ++ F+ LLDE F IHEPDHDKLQFHLLLDELF 270 |  |

>TR:A0A5E7YL26 A0A5E7YL26_9SPHN Aminoglycoside 3'-phosphotransferase OS=Sphingomonas sp. EC-HK361 OX=2038397 GN=aphA PE=3 SV=1

Length=259

Score = 156 bits (394), Expect = 2e-43

| Identities | | = 96/257 (37%), Positives = 138/257 (54%), Gaps = 7/257 (3%) |  |
| --- | --- | --- | --- |
| Query 12  Sbjct 6 | | PAAWVERLFGYDWAQQTIGCSDAAVFRLSAQGRPVLFVKTDLSGALNELQDEAARLSWLA P + + + GY WA++ G S VFRL A+ P L++K ++ DEA RL+WLA PPSLADTIDGYRWARERSGESGGTVFRLDAEALPSLYLKHGKGRVAADIFDEATRLTWLA | 71  65 |
| Query 72  Sbjct 66 | | TTGVPCAAVLDVVTEAGRDWLLLGEVPGQD---LLSSHLAPAEKVSIMADA-MRRLHTLD  +P V V WLL +PGQ LL+ A + A A +RRLH + DV-LPVPEVRHFVATPDAAWLLTTAIPGQTADALLADDAANLPTIIDGAAAFLRRLHAIP | 127  124 |
| Query 128  Sbjct 125 | | PATCPFDHQAKHRIERARTRMEAGLVDQDDLDEEHQGLAPAELFARLKARMPDGEDLVVT A CPF+ HR+ AR ++AGLVD++D D +H G + +++ L A P + VT AANCPFEAGHAHRLAAARRNIDAGLVDEEDFDSDHDGWSAEDVWDELVALQPFAAERSVT | 187  184 |
| Query 188  Sbjct 185 | | HGDACLPNIMVENGRFSGFIDCGRLGVADRYQDIALATRDIAEELGGEWADRFLVLYGIA HGD L N++ +G G ID GR G+AD YQDIA+ +++A E G + F+ GI HGDYSLGNLLFADGAVVGCIDVGRAGLADPYQDIAIFWQNLA-EFGEGAQNAFVAALGIE | 247  243 |
| Query 248 | | APDSQRIAFYRLLDEFF 264 D +R+ F+R LDE F |  |
| Sbjct | 244 | L-DERRLRFHRCLDELF 259 | |

>TR:B1N624 B1N624_ENTHI Aminoglycoside 3'-phosphotransferase, putative OS=Entamoeba histolytica OX=5759 GN=EHI_117050 PE=3 SV=1

Length=253

Score = 155 bits (393), Expect = 2e-43

Identities = 88/251 (35%), Positives = 133/251 (53%), Gaps = 7/251 (3%)

| Query | 19 | LFGYDWAQQTIGCSDAAVFRLSAQ-GRPVLFVKTDLSGALNELQDEAARLSWLATTGVPC | 77 |
| --- | --- | --- | --- |
| Sbjct | 5 | L+GY WA+ +G S A ++RL + P LF+K N++ DE RL+WL T +P LYGYKWARDNVGQSGATIYRLYGKPDAPELFLKHGKGSVANDVTDEMVRLNWL-TEFMPL | 63 |
| Query | 78 | AAVLDVVTEAGRDWLLLGEVPGQ---DLLSSHLAPAEK-VSIMADAMRRLHTLDPATCPF | 133 |
| Sbjct | 64 | + + WLL +PG+ +L + E V +A +RRLH++ CPF  PTIKHFIRTPDDAWLLTTAIPGKTAFQVLEEYPDSGENIVDALAVFLRRLHSIPVCNCPF | 123 |

| Query | 134 | DHQAKHRIERARTRMEAGLVDQDDLDEEHQGLAPAELFARLKARMPDGEDLVVTHGDACL | 193 |
| --- | --- | --- | --- |
| Sbjct | 124 | + R+ +A++RM GLVD D D+E G +++ + +P D VVTHGD L NSDRVFRLAQAQSRMNNGLVDASDFDDERNGWPVEQVWKEMHKLLPFSPDSVVTHGDFSL | 183 |
| Query | 194 | PNIMVENGRFSGFIDCGRLGVADRYQDIALATRDIAEELGGEWADRFLVLYGIAAPDSQR | 253 |
| Sbjct | 184 | N++ + G+ G ID GR+G+ADRYQD+A+ + E R YGI PD + DNLIFDEGKLIGCIDVGRVGIADRYQDLAILWNCLG-EFSPSLQKRLFQKYGIDNPDMNK | 242 |
| Query  Sbjct | 254  243 | IAFYRLLDEFF 264  + F+ +LDEFF  LQFHLMLDEFF 253 |  |

>TR:A0A1G7PFM6 A0A1G7PFM6_9SPHN Aminoglycoside 3'-phosphotransferase OS=Sphingomonas carotinifaciens OX=1166323 GN=aph(3')-I PE=3 SV=1

Length=270

Score = 156 bits (394), Expect = 3e-43

| Identities | = 91/249 (37%), Positives = 136/249 (55%), Gaps = 7/249 (3%) |  |
| --- | --- | --- |
| Query 21 | GYDWAQQTIGCSDAAVFRLSA-QGRPVLFVKTDLSGALNELQDEAARLSWLATTGVPCAA | 79 |
| Sbjct 24 | GY W IG S V+RL Q P LF+K +++ DE RL WL+ + GYRWMHNMIGESGGGVYRLHGRQDAPDLFLKHGRGMVADDIADEMVRLRWLSDH-IAVPT | 82 |
| Query 80 | VLDVVTEAGRDWLLLGEVPGQDLLSSHLA-PAEKVSI---MADAMRRLHTLDPATCPFDH | 135 |
| Sbjct 83 | + V WLL+ +PGQ A P +++++ +A +R+LH + TCPF IRRFVHTGDEAWLLMTAIPGQTAYQHLTARPHDRLAVVDALATFLRQLHAIPVDTCPFIS | 142 |
| Query 136 | QAKHRIERARTRMEAGLVDQDDLDEEHQGLAPAELFARLKARMPDGEDLVVTHGDACLPN | 195 |
| Sbjct 143 | HR+ +AR R++ GLV +D+ DEE +G E++ ++ MP D VVTHGD L N DHHHRLMQARARIDQGLVAEDEFDEEREGWLAEEVWQAMQDLMPFTPDRVVTHGDFSLDN | 202 |
| Query 196 | IMVENGRFSGFIDCGRLGVADRYQDIALATRDIAEELGGEWADRFLVLYGIAAPDSQRIA | 255 |
| Sbjct 203 | I++E+G+ G ID GR+G+ADRYQDIA+ + E FL YG+A D++++ ILLEDGQVVGLIDAGRIGIADRYQDIAILWNCLG-EFDDALRSHFLRRYGVADVDNRKLQ | 261 |
| Query 256  Sbjct 262 | FYRLLDEFF 264 F+ +LDE F FHLMLDEMF 270 |  |

>TR:A0A3N7BPW6 A0A3N7BPW6_9BURK Aminoglycoside 3'-phosphotransferase OS=Paucibacter sp. KBW04 OX=2153361 GN=DBR47_22125 PE=3 SV=1

Length=310

Score = 157 bits (397), Expect = 3e-43

| Identities | = 99/260 (38%), Positives = 140/260 (54%), Gaps = 9/260 (3%) |  |
| --- | --- | --- |
| Query 12 | PAAWVERLFGYDWAQQTIGCSDAAVFRLS--AQGRPVLFVKTDLSGALNELQDEAARLSW | 69 |
| Sbjct 53 | P + +RL + W + +G + AAV+RL A + L++K L DEAARL W PPSLAQRLRHWQWRRNRVGQAGAAVYRLHKPAPNQQDLYLKQGRGEVAEALFDEAARLRW | 112 |
| Query 70 | LATTGVPCAAVLDVVTEAGRDWLLLGEVPGQDLLSSHLAPAEKVSIMADAM RRLHT | 125 |
| Sbjct 113 | L +P +L + WLL VPG+ E+ + +A A+ RLH LGQA-LPTNQLLGFEWDGEEAWLLSAAVPGRSAYECLRQRPEQAAEIAQALGAFLARLHA | 171 |
| Query 126 | LDPATCPFDHQAKHRIERARTRMEAGLVDQDDLDEEHQGLAPAELFARLKARMPDGEDLV | 185 |
| Sbjct 172 | L C F+ Q R+ +AR R+EAGLVD D D + G +++ +L+A +P +LV LPIEACAFNAQLPIRLAQARRRLEAGLVDAQDFDADRAGQTAEQVWLQLQALLPMQTELV | 231 |
| Query 186 | VTHGDACLPNIMV-ENGRFSGFIDCGRLGVADRYQDIALATRDIAEELGGEWADRFLVLY | 244 |
| Sbjct 232 | +THGD L NI++ E G SG ID GRLGVADRYQD+A+A + +E G F+ Y LTHGDYSLDNILLDEAGEVSGVIDLGRLGVADRYQDLAIAW-NCLQEFGSAAQQAFVSAY | 290 |
| Query 245  Sbjct 291 | GIAAPDSQRIAFYRLLDEFF 264 GI +PD +IA + LDEFF GIPSPDPHKIALHLCLDEFF 310 |  |

>TR:A0A2P5GGN3 A0A2P5GGN3_9ENTR Aminoglycoside 3'-phosphotransferase OS=Superficieibacter electus OX=2022662 GN=aph(3')-I PE=3 SV=1

Length=271

Score = 156 bits (394), Expect = 3e-43

| Identities | = 88/251 (35%), Positives = 133/251 (53%), Gaps = 7/251 (3%) |  |
| --- | --- | --- |
| Query 19 | LFGYDWAQQTIGCSDAAVFRLSAQ-GRPVLFVKTDLSGALNELQDEAARLSWLATTGVPC | 77 |
| Sbjct 23 | L+GY WA+ +G S A ++RL + P LF+K N++ DE RL+WL T +P LYGYRWARDNVGQSGATIYRLYGKPDAPELFLKHGKGSVANDVTDEMVRLNWL-TAFMPL | 81 |
| Query 78 | AAVLDVVTEAGRDWLLLGEVPGQ---DLLSSHLAPAEK-VSIMADAMRRLHTLDPATCPF | 133 |
| Sbjct 82 | + + WLL +PG+ +L + E V +A +RRLH++ CPF PTIKHFIRTPDDAWLLTTAIPGKTAFQVLEEYPDSGENIVDALAAFLRRLHSIPVCNCPF | 141 |
| Query 134 | DHQAKHRIERARTRMEAGLVDQDDLDEEHQGLAPAELFARLKARMPDGEDLVVTHGDACL | 193 |
| Sbjct 142 | + R+ +A++RM GLVD D D+E G +++ + +P D VVTHGD L NSDRVFRLAQAQSRMNNGLVDASDFDDERNGWPVEQVWKEMHKLLPFSPDSVVTHGDFSL | 201 |
| Query 194 | PNIMVENGRFSGFIDCGRLGVADRYQDIALATRDIAEELGGEWADRFLVLYGIAAPDSQR | 253 |
| Sbjct 202 | N++ + G+ G ID GR+G+ADRYQD+A+ + E R YGI PD + DNLIFDEGKLIGCIDVGRVGIADRYQDLAILWNCLG-EFSPSLQKRLFQKYGIDNPDMNK | 260 |
| Query 254  Sbjct 261 | IAFYRLLDEFF 264  + F+ +LDEFF  LQFHLMLDEFF 271 |  |

>TR:B1VIB1 B1VIB1_CORU7 Aminoglycoside 3'-phosphotransferase OS=Corynebacterium urealyticum (strain ATCC 43042 / DSM 7109) OX=504474

GN=aph(3')-Ia PE=3 SV=1

Length=271

Score = 156 bits (394), Expect = 3e-43

| Identities | = 88/251 (35%), Positives = 133/251 (53%), Gaps = 7/251 (3%) |  |
| --- | --- | --- |
| Query 19 | LFGYDWAQQTIGCSDAAVFRLSAQ-GRPVLFVKTDLSGALNELQDEAARLSWLATTGVPC | 77 |
| Sbjct 23 | L+GY WA+ +G S A ++RL + P LF+K N++ DE RL+WL T +P LYGYRWARDNVGQSGATIYRLYGKPNAPELFLKHGKGSVANDVTDEMVRLNWL-TAFMPL | 81 |
| Query 78 | AAVLDVVTEAGRDWLLLGEVPGQ---DLLSSHLAPAEK-VSIMADAMRRLHTLDPATCPF | 133 |
| Sbjct 82 | + + WLL +PG+ +L + E V +A +RRLH++ CPF PTIKHFIRTPDDAWLLTTAIPGKTAFQVLEEYPDSGENIVDALAVFLRRLHSIPVCNCPF | 141 |
| Query 134 | DHQAKHRIERARTRMEAGLVDQDDLDEEHQGLAPAELFARLKARMPDGEDLVVTHGDACL | 193 |
| Sbjct 142 | + R+ +A++RM GLVD D D+E G +++ + +P D VVTHGD L NSDRVFRLAQAQSRMNNGLVDASDFDDERNGWPVEQVWKEMHKLLPFSPDSVVTHGDFSL | 201 |
| Query 194 | PNIMVENGRFSGFIDCGRLGVADRYQDIALATRDIAEELGGEWADRFLVLYGIAAPDSQR | 253 |
| Sbjct 202 | N++ + G+ G ID GR+G+ADRYQD+A+ + E R YGI PD + DNLIFDEGKLIGCIDVGRVGIADRYQDLAILWNCLG-EFSPSLQKRLFQKYGIDNPDMNK | 260 |
| Query 254  Sbjct 261 | IAFYRLLDEFF 264  + F+ +LDEFF  LQFHLMLDEFF 271 |  |

>TR:A0A2S2FBP8 A0A2S2FBP8_9GAMM Aminoglycoside 3'-phosphotransferase OS=Acinetobacter defluvii OX=1871111 GN=DJ533_07430 PE=3 SV=1

Length=271

Score = 156 bits (394), Expect = 3e-43

Identities = 88/251 (35%), Positives = 133/251 (53%), Gaps = 7/251 (3%)

| Query | 19 | LFGYDWAQQTIGCSDAAVFRLSAQ-GRPVLFVKTDLSGALNELQDEAARLSWLATTGVPC | 77 |
| --- | --- | --- | --- |
| Sbjct | 23 | L+GY WA+ +G S A ++RL + P LF+K N++ DE RL+WL T +P LYGYRWARDNVGQSGATIYRLYGKPNAPELFLKHGKGSVANDVTDEMVRLNWL-TAFMPL | 81 |
| Query | 78 | AAVLDVVTEAGRDWLLLGEVPGQ---DLLSSHLAPAEK-VSIMADAMRRLHTLDPATCPF | 133 |
| Sbjct | 82 | + + WLL +PG+ +L + E V +A +RRLH++ CPF PTIKHFIRTPDDAWLLTTAIPGKTAFQVLEEYPDSGENIVDALAVFLRRLHSIPVCNCPF | 141 |
| Query | 134 | DHQAKHRIERARTRMEAGLVDQDDLDEEHQGLAPAELFARLKARMPDGEDLVVTHGDACL  + R+ +A++RM GLVD D D+E G +++ + +P D VVTHGD L | 193 |

| Sbjct | 142 | NSDRVFRLAQAQSRMNNGLVDASDFDDERNGWPVEQVWKEMHKLLPFSPDSVVTHGDFSL | 201 |
| --- | --- | --- | --- |
| Query | 194 | PNIMVENGRFSGFIDCGRLGVADRYQDIALATRDIAEELGGEWADRFLVLYGIAAPDSQR | 253 |
| Sbjct | 202 | N++ + G+ G ID GR+G+ADRYQD+A+ + E R YGI PD + DNLIFDEGKLIGCIDVGRVGIADRYQDLAILWNCLG-EFSPSLQKRLFQKYGIDNPDMNK | 260 |
| Query  Sbjct | 254  261 | IAFYRLLDEFF 264  + F+ +LDEFF  LQFHLMLDEFF 271 |  |

>TR:M4R5K9 M4R5K9_BIBTR Aminoglycoside 3'-phosphotransferase OS=Bibersteinia trehalosi USDA-ARS-USMARC-192 OX=1171377 GN=WQG_1070 PE=3

SV=1

Length=271

Score = 156 bits (394), Expect = 3e-43

| Identities | = 88/251 (35%), Positives = 134/251 (53%), Gaps = 7/251 (3%) |  |
| --- | --- | --- |
| Query 19 | LFGYDWAQQTIGCSDAAVFRLSAQ-GRPVLFVKTDLSGALNELQDEAARLSWLATTGVPC | 77 |
| Sbjct 23 | L+GY WA+ +G S A ++RL + P LF+K N++ DE RL+WL T +P LYGYKWARDNVGQSGATIYRLYGKPDAPELFLKHGKGSVANDVTDEMVRLNWL-TAFMPL | 81 |
| Query 78 | AAVLDVVTEAGRDWLLLGEVPGQ---DLLSSHLAPAEK-VSIMADAMRRLHTLDPATCPF | 133 |
| Sbjct 82 | + + WLL +PG+ +L + E V +A +RRLH++ + CPF PTIKHFIRTPDDAWLLTTALPGKTAFQVLEEYPDSGENIVDALAAFLRRLHSIPVSNCPF | 141 |
| Query 134 | DHQAKHRIERARTRMEAGLVDQDDLDEEHQGLAPAELFARLKARMPDGEDLVVTHGDACL | 193 |
| Sbjct 142 | + R+ +A++RM GLVD D D+E G +++ + +P D VVTHGD L NSDRVFRLAQAQSRMNNGLVDASDFDDERNGWPVEQVWKEMHKLLPFSPDSVVTHGDFSL | 201 |
| Query 194 | PNIMVENGRFSGFIDCGRLGVADRYQDIALATRDIAEELGGEWADRFLVLYGIAAPDSQR | 253 |
| Sbjct 202 | N++ + G+ G ID GR+G+ADRYQD+A+ + E R YGI PD + DNLIFDEGKLIGCIDVGRVGIADRYQDLAILWNCLG-EFSPSLQKRLFQKYGIDNPDMNK | 260 |
| Query 254  Sbjct 261 | IAFYRLLDEFF 264  + F+ +LDEFF  LQFHLMLDEFF 271 |  |

>TR:A6TJ01 A6TJ01_KLEP7 Aminoglycoside 3'-phosphotransferase OS=Klebsiella pneumoniae subsp. pneumoniae (strain ATCC 700721 / MGH 78578)

OX=272620 GN=aphA PE=3 SV=1

Length=271

Score = 156 bits (394), Expect = 3e-43

| Identities | = 88/251 (35%), Positives = 133/251 (53%), Gaps = 7/251 (3%) |  |
| --- | --- | --- |
| Query 19 | LFGYDWAQQTIGCSDAAVFRLSAQ-GRPVLFVKTDLSGALNELQDEAARLSWLATTGVPC | 77 |
| Sbjct 23 | L+GY WA+ +G S A ++RL + P LF+K N++ DE RL+WL T +P LYGYRWARDNVGQSGATIYRLYGKPNAPELFLKHGKGSVANDVTDEMVRLNWL-TAFMPL | 81 |
| Query 78 | AAVLDVVTEAGRDWLLLGEVPGQ---DLLSSHLAPAEK-VSIMADAMRRLHTLDPATCPF | 133 |
| Sbjct 82 | + + WLL +PG+ +L + E V +A +RRLH++ CPF PTIKHFIRTPDDAWLLTTAIPGKTAFQVLEEYPDSGENIVDALAVFLRRLHSIPVCNCPF | 141 |
| Query 134 | DHQAKHRIERARTRMEAGLVDQDDLDEEHQGLAPAELFARLKARMPDGEDLVVTHGDACL | 193 |
| Sbjct 142 | + R+ +A++RM GLVD D D+E G +++ + +P D VVTHGD L NSDRVFRLAQAQSRMNNGLVDASDFDDERNGWPVEQVWKEMHKLLPFSPDSVVTHGDFSL | 201 |
| Query 194 | PNIMVENGRFSGFIDCGRLGVADRYQDIALATRDIAEELGGEWADRFLVLYGIAAPDSQR | 253 |
| Sbjct 202 | N++ + G+ G ID GR+G+ADRYQD+A+ + E R YGI PD + DNLIFDEGKLIGCIDVGRVGIADRYQDLAILWNCLG-EFSPSLQKRLFQKYGIDNPDMNK | 260 |
| Query 254  Sbjct 261 | IAFYRLLDEFF 264  + F+ +LDEFF  LQFHLMLDEFF 271 |  |

>TR:A0A345WUY0 A0A345WUY0_9SPHN Aminoglycoside 3'-phosphotransferase OS=Sphingomonas sp. FARSPH OX=2219696 GN=aph(3')-I PE=3 SV=1

Length=271

Score = 156 bits (394), Expect = 3e-43

| Identities | = 88/251 (35%), Positives = 133/251 (53%), Gaps = 7/251 (3%) |  |
| --- | --- | --- |
| Query 19 | LFGYDWAQQTIGCSDAAVFRLSAQ-GRPVLFVKTDLSGALNELQDEAARLSWLATTGVPC | 77 |
| Sbjct 23 | L+GY WA+ +G S A ++RL + P LF+K N++ DE RL+WL T +P LYGYRWARDNVGQSGATIYRLYGKPNAPELFLKHGKGSVANDVTDEMVRLNWL-TAFMPL | 81 |
| Query 78 | AAVLDVVTEAGRDWLLLGEVPGQ---DLLSSHLAPAEK-VSIMADAMRRLHTLDPATCPF | 133 |
| Sbjct 82 | + + WLL +PG+ +L + E V +A +RRLH++ CPF PTIKHFIRTPDDAWLLTTAIPGKTAFQVLEEYPDSGENIVDALAVFLRRLHSIPVCNCPF | 141 |
| Query 134 | DHQAKHRIERARTRMEAGLVDQDDLDEEHQGLAPAELFARLKARMPDGEDLVVTHGDACL | 193 |
| Sbjct 142 | + R+ +A++RM GLVD D D+E G +++ + +P D VVTHGD L NSDRVFRLAQAQSRMNNGLVDASDFDDERNGWPVEQVWKEMHKLLPFSPDSVVTHGDFSL | 201 |
| Query 194 | PNIMVENGRFSGFIDCGRLGVADRYQDIALATRDIAEELGGEWADRFLVLYGIAAPDSQR | 253 |
| Sbjct 202 | N++ + G+ G ID GR+G+ADRYQD+A+ + E R YGI PD + DNLIFDEGKLIGCIDVGRVGIADRYQDLAILWNCLG-EFSPSLQKRLFQKYGIDNPDMNK | 260 |
| Query 254  Sbjct 261 | IAFYRLLDEFF 264  + F+ +LDEFF  LQFHLMLDEFF 271 |  |

>TR:D6CJF7 D6CJF7_CORRG Aminoglycoside phosphotransferase OS=Corynebacterium resistens (strain DSM 45100 / JCM 12819 / GTC 2026 / SICGH

158) OX=662755 GN=aphA1-IAB PE=3 SV=1 Length=271

Score = 156 bits (394), Expect = 3e-43

| Identities | = 88/251 (35%), Positives = 133/251 (53%), Gaps = 7/251 (3%) |  |
| --- | --- | --- |
| Query 19 | LFGYDWAQQTIGCSDAAVFRLSAQ-GRPVLFVKTDLSGALNELQDEAARLSWLATTGVPC | 77 |
| Sbjct 23 | L+GY WA+ +G S A ++RL + P LF+K N++ DE RL+WL T +P LYGYRWARDNVGQSGATIYRLYGKPNAPELFLKHGKGSVANDVTDEMVRLNWL-TAFMPL | 81 |
| Query 78 | AAVLDVVTEAGRDWLLLGEVPGQ---DLLSSHLAPAEK-VSIMADAMRRLHTLDPATCPF | 133 |
| Sbjct 82 | + + WLL +PG+ +L + E V +A +RRLH++ CPF PTIKHFIRTPDDAWLLTTAIPGKTAFQVLEEYPDSGENIVDALAVFLRRLHSIPVCNCPF | 141 |
| Query 134 | DHQAKHRIERARTRMEAGLVDQDDLDEEHQGLAPAELFARLKARMPDGEDLVVTHGDACL | 193 |
| Sbjct 142 | + R+ +A++RM GLVD D D+E G +++ + +P D VVTHGD L NSDRVFRLAQAQSRMNNGLVDASDFDDERNGWPVEQVWKEMHKLLPFSPDSVVTHGDFSL | 201 |
| Query 194 | PNIMVENGRFSGFIDCGRLGVADRYQDIALATRDIAEELGGEWADRFLVLYGIAAPDSQR | 253 |
| Sbjct 202 | N++ + G+ G ID GR+G+ADRYQD+A+ + E R YGI PD + DNLIFDEGKLIGCIDVGRVGIADRYQDLAILWNCLG-EFSPSLQKRLFQKYGIDNPDMNK | 260 |
| Query 254  Sbjct 261 | IAFYRLLDEFF 264  + F+ +LDEFF  LQFHLMLDEFF 271 |  |

>SP:P00551 KKA1_ECOLX Aminoglycoside 3'-phosphotransferase OS=Escherichia coli OX=562 GN=aphA1 PE=3 SV=2

Length=271

Score = 155 bits (393), Expect = 4e-43

| Identities | = 88/251 (35%), Positives = 133/251 (53%), Gaps = 7/251 (3%) |  |
| --- | --- | --- |
| Query 19 | LFGYDWAQQTIGCSDAAVFRLSAQ-GRPVLFVKTDLSGALNELQDEAARLSWLATTGVPC | 77 |
| Sbjct 23 | L+GY WA+ +G S A ++RL + P LF+K N++ DE RL+WL T +P LYGYKWARDNVGQSGATIYRLYGKPDAPELFLKHGKGSVANDVTDEMVRLNWL-TEFMPL | 81 |
| Query 78 | AAVLDVVTEAGRDWLLLGEVPGQ---DLLSSHLAPAEK-VSIMADAMRRLHTLDPATCPF | 133 |
| Sbjct 82 | + + WLL +PG+ +L + E V +A +RRLH++ CPF PTIKHFIRTPDDAWLLTTAIPGKTAFQVLEEYPDSGENIVDALAVFLRRLHSIPVCNCPF | 141 |
| Query 134 | DHQAKHRIERARTRMEAGLVDQDDLDEEHQGLAPAELFARLKARMPDGEDLVVTHGDACL | 193 |
| Sbjct 142 | + R+ +A++RM GLVD D D+E G +++ + +P D VVTHGD L  NSDRVFRLAQAQSRMNNGLVDASDFDDERNGWPVEQVWKEMHKLLPFSPDSVVTHGDFSL | 201 |

| Query | 194 | PNIMVENGRFSGFIDCGRLGVADRYQDIALATRDIAEELGGEWADRFLVLYGIAAPDSQR 253 |
| --- | --- | --- |
| Sbjct | 202 | N++ + G+ G ID GR+G+ADRYQD+A+ + E R YGI PD + DNLIFDEGKLIGCIDVGRVGIADRYQDLAILWNCLG-EFSPSLQKRLFQKYGIDNPDMNK 260 |
| Query | 254 | IAFYRLLDEFF 264 |
| Sbjct | 261 | + F+ +LDEFF  LQFHLMLDEFF 271 |

>TR:A3UKS0 A3UKS0_9RHOB Aminoglycoside 3'-phosphotransferase OS=Oceanicaulis sp. HTCC2633 OX=314254 GN=OA2633_15241 PE=3 SV=1

Length=271

Score = 155 bits (393), Expect = 4e-43

| Identities | = 88/251 (35%), Positives = 133/251 (53%), Gaps = 7/251 (3%) |  |
| --- | --- | --- |
| Query 19 | LFGYDWAQQTIGCSDAAVFRLSAQ-GRPVLFVKTDLSGALNELQDEAARLSWLATTGVPC | 77 |
| Sbjct 23 | L+GY WA+ +G S A ++RL + P LF+K N++ DE RL+WL T +P LYGYKWARDNVGQSGATIYRLYGKPDAPELFLKHGKGSVANDVTDEMVRLNWL-TEFMPL | 81 |
| Query 78 | AAVLDVVTEAGRDWLLLGEVPGQ---DLLSSHLAPAEK-VSIMADAMRRLHTLDPATCPF | 133 |
| Sbjct 82 | + + WLL +PG+ +L + E V +A +RRLH++ CPF PTIKHFIRTPDDAWLLTTAIPGKTAFQVLEEYPDSGENIVDALAVFLRRLHSIPVCNCPF | 141 |
| Query 134 | DHQAKHRIERARTRMEAGLVDQDDLDEEHQGLAPAELFARLKARMPDGEDLVVTHGDACL | 193 |
| Sbjct 142 | + R+ +A++RM GLVD D D+E G +++ + +P D VVTHGD L NSDRVFRLAQAQSRMNNGLVDASDFDDERNGWPVEQVWKEMHKLLPFSPDSVVTHGDFSL | 201 |
| Query 194 | PNIMVENGRFSGFIDCGRLGVADRYQDIALATRDIAEELGGEWADRFLVLYGIAAPDSQR | 253 |
| Sbjct 202 | N++ + G+ G ID GR+G+ADRYQD+A+ + E R YGI PD + DNLIFDEGKLIGCIDVGRVGIADRYQDLAILWNCLG-EFSPSLQKRLFQKYGIDNPDMNK | 260 |
| Query 254  Sbjct 261 | IAFYRLLDEFF 264  + F+ +LDEFF  LQFHLMLDEFF 271 |  |

>TR:A0A0M0HV15 A0A0M0HV15_9VIBR Aminoglycoside 3'-phosphotransferase OS=Vibrio hepatarius OX=171383 GN=AKJ31_20710 PE=3 SV=1

Length=271

Score = 155 bits (393), Expect = 4e-43

| Identities | = 88/251 (35%), Positives = 133/251 (53%), Gaps = 7/251 (3%) |  |
| --- | --- | --- |
| Query 19 | LFGYDWAQQTIGCSDAAVFRLSAQ-GRPVLFVKTDLSGALNELQDEAARLSWLATTGVPC | 77 |
| Sbjct 23 | L+GY WA+ +G S A ++RL + P LF+K N++ DE RL+WL T +P LYGYKWARDNVGQSGATIYRLYGKPDAPELFLKHGKGSVANDVTDEMVRLNWL-TEFMPL | 81 |
| Query 78 | AAVLDVVTEAGRDWLLLGEVPGQ---DLLSSHLAPAEK-VSIMADAMRRLHTLDPATCPF | 133 |
| Sbjct 82 | + + WLL +PG+ +L + E V +A +RRLH++ CPF PTIKHFIRTPDDAWLLTTAIPGKTAFQVLEEYPDSGENIVDALAVFLRRLHSIPVCNCPF | 141 |
| Query 134 | DHQAKHRIERARTRMEAGLVDQDDLDEEHQGLAPAELFARLKARMPDGEDLVVTHGDACL | 193 |
| Sbjct 142 | + R+ +A++RM GLVD D D+E G +++ + +P D VVTHGD L NSDRVFRLAQAQSRMNNGLVDASDFDDERNGWPVEQVWKEMHKLLPFSPDSVVTHGDFSL | 201 |
| Query 194 | PNIMVENGRFSGFIDCGRLGVADRYQDIALATRDIAEELGGEWADRFLVLYGIAAPDSQR | 253 |
| Sbjct 202 | N++ + G+ G ID GR+G+ADRYQD+A+ + E R YGI PD + DNLIFDEGKLIGCIDVGRVGIADRYQDLAILWNCLG-EFSPSLQKRLFQKYGIDNPDMNK | 260 |
| Query 254  Sbjct 261 | IAFYRLLDEFF 264  + F+ +LDEFF  LQFHLMLDEFF 271 |  |

>TR:A0A2S4XH80 A0A2S4XH80_9ACTN Aminoglycoside O-phosphotransferase APH(3')-Ia OS=Streptomyces sp. Ru73 OX=2080748 GN=aph(3')-I PE=3 SV=1

Length=271

Score = 155 bits (393), Expect = 4e-43

Identities = 88/251 (35%), Positives = 133/251 (53%), Gaps = 7/251 (3%)

| Query | 19 | LFGYDWAQQTIGCSDAAVFRLSAQ-GRPVLFVKTDLSGALNELQDEAARLSWLATTGVPC | 77 |
| --- | --- | --- | --- |
| Sbjct | 23 | L+GY WA+ +G S A ++RL + P LF+K N++ DE RL+WL T +P LYGYKWARDNVGQSGATIYRLYGKPDAPELFLKHGKGSVANDVTDEMVRLNWL-TEFMPL | 81 |
| Query | 78 | AAVLDVVTEAGRDWLLLGEVPGQ---DLLSSHLAPAEK-VSIMADAMRRLHTLDPATCPF | 133 |
| Sbjct | 82 | + + WLL +PG+ +L + E V +A +RRLH++ CPF PTIKHFIRTPDDAWLLTTAIPGKTAFQVLEEYPDSGENIVDALAVFLRRLHSIPVCNCPF | 141 |
| Query | 134 | DHQAKHRIERARTRMEAGLVDQDDLDEEHQGLAPAELFARLKARMPDGEDLVVTHGDACL | 193 |
| Sbjct | 142 | + R+ +A++RM GLVD D D+E G +++ + +P D VVTHGD L NSDRVFRLAQAQSRMNNGLVDASDFDDERNGWPVEQVWKEMHKLLPFSPDSVVTHGDFSL | 201 |
| Query | 194 | PNIMVENGRFSGFIDCGRLGVADRYQDIALATRDIAEELGGEWADRFLVLYGIAAPDSQR | 253 |
| Sbjct | 202 | N++ + G+ G ID GR+G+ADRYQD+A+ + E R YGI PD + DNLIFDEGKLIGCIDVGRVGIADRYQDLAILWNCLG-EFSPSLQKRLFQKYGIDNPDMNK | 260 |
| Query  Sbjct | 254  261 | IAFYRLLDEFF 264  + F+ +LDEFF  LQFHLMLDEFF 271 |  |

>TR:A0A2S4Y376 A0A2S4Y376_9ACTN Aminoglycoside O-phosphotransferase APH(3')-Ia OS=Streptomyces sp. Ru71 OX=2080746 GN=aph(3')-I PE=3 SV=1

Length=271

Score = 155 bits (393), Expect = 4e-43

| Identities | = 88/251 (35%), Positives = 133/251 (53%), Gaps = 7/251 (3%) |  |
| --- | --- | --- |
| Query 19 | LFGYDWAQQTIGCSDAAVFRLSAQ-GRPVLFVKTDLSGALNELQDEAARLSWLATTGVPC | 77 |
| Sbjct 23 | L+GY WA+ +G S A ++RL + P LF+K N++ DE RL+WL T +P LYGYKWARDNVGQSGATIYRLYGKPDAPELFLKHGKGSVANDVTDEMVRLNWL-TEFMPL | 81 |
| Query 78 | AAVLDVVTEAGRDWLLLGEVPGQ---DLLSSHLAPAEK-VSIMADAMRRLHTLDPATCPF | 133 |
| Sbjct 82 | + + WLL +PG+ +L + E V +A +RRLH++ CPF PTIKHFIRTPDDAWLLTTAIPGKTAFQVLEEYPDSGENIVDALAVFLRRLHSIPVCNCPF | 141 |
| Query 134 | DHQAKHRIERARTRMEAGLVDQDDLDEEHQGLAPAELFARLKARMPDGEDLVVTHGDACL | 193 |
| Sbjct 142 | + R+ +A++RM GLVD D D+E G +++ + +P D VVTHGD L NSDRVFRLAQAQSRMNNGLVDASDFDDERNGWPVEQVWKEMHKLLPFSPDSVVTHGDFSL | 201 |
| Query 194 | PNIMVENGRFSGFIDCGRLGVADRYQDIALATRDIAEELGGEWADRFLVLYGIAAPDSQR | 253 |
| Sbjct 202 | N++ + G+ G ID GR+G+ADRYQD+A+ + E R YGI PD + DNLIFDEGKLIGCIDVGRVGIADRYQDLAILWNCLG-EFSPSLQKRLFQKYGIDNPDMNK | 260 |
| Query 254  Sbjct 261 | IAFYRLLDEFF 264  + F+ +LDEFF  LQFHLMLDEFF 271 |  |

```
>TR:A0A1T3P638 A0A1T3P638_9ACTN APH domain-containing protein OS=Embleya scabrispora OX=159449 GN=B4N89_29590 PE=3 SV=1
Length=267

Score = 155 bits (392), Expect = 5e-43
Identities = 110/266 (41%), Positives = 138/266 (52%), Gaps = 27/266 (10%)

Query 18	RLFGYDWAQQTIGCSDAAVFRLSAQGRPVLFVKTDLSGALNELQD-------EAARLSWL 70 R	Y	T G S A V+RL +G	L++K	G NE D	EAAR W
Sbjct 8	RFAAYTREPVTDGMSGAGVYRLRGRGGEELYLKI---GTPNEHPDSGFHISAEAARGIWA 64

Query 71	ATTGVPCAAVLDVVTEAGRDWLLLGEVPGQDLLSSHLAPAEK----VSIMADAMRRLHTL 126 A G+P A +LD	+	WLL	V G+	++	PAE+	V +AD R LH L
Sbjct 65	AKQGLPVAEILDHEVDDAGGWLLTRAVAGRS--AADRWPAERRDAVVDALADLARDLHRL 122

Query 127 DPATCPFDHQAKHRIERARTRMEAGLVDQDDLDEEHQGLAPAELFARLKARMPDGEDLVV 186 A CPFD	+ A	+ AGLVD DDLDE+ G	AEL A L A +P EDLVV
Sbjct 123 PVADCPFDRTLAVTVPAAEQAIAAGLVDLDDLDEDRAGHTGAELLAELLATVPPTEDLVV 182

Query 187 THGDACLPNIMV--ENGRFSGFIDCGRLGVADRYQDIALATRDIAEELGGEW----ADRF 240 HGD C PN+++ + R +G ID GRLG DR+ D AL TR + ++ G++	A RF
Sbjct 183 GHGDYCPPNVLLDPDTARVTGLIDLGRLGRTDRHADPALLTRSLLDDDHGQYSAEHAARF 242

Query 241 LVLY---GIAAPDSQRIAFYRLLDEF 263 L Y	AP QR+ FYRLLDEF
Sbjct 243 LARYDPEDTLAP--QRLRFYRLLDEF 266

>TR:A0A1H6UK15 A0A1H6UK15_9DEIO Kanamycin kinase OS=Deinococcus reticulitermitis OX=856736 GN=SAMN04488058_102236 PE=3 SV=1
Length=260

Score = 155 bits (391), Expect = 6e-43
```

| Identities | = 95/243 (39%), Positives = 133/243 (55%), Gaps = 6/243 (2%) |  |
| --- | --- | --- |
| Query 24 | WAQQTIGCSDAAVFRLSAQGRPVLFVKTDLSGALNELQDEAARLSWLATTGVPCAAVLDV | 83 |
| Sbjct 22 | W + T+G S A V+R + R V+ V+ + + L E RL + A VP V+ WERVTLGESGAGVWRST---RFVVKVQPREAFPASTLLQERERLRFFAGR-VPVPRVVGY | 77 |
| Query 84 | VTEAGRDWLLLGEVPGQDLLSSHLA--PAEKVSIMADAMRRLHTLDPATCPFDHQAKHRI | 141 |
| Sbjct 78 | + R+ L L VPG + A P V ++A A+R LH L CPF+ ++ EGNSEREVLALTRVPGIPMSDPDAALHPERMVGLLARALRELHALPARECPFNMSLGIKL | 137 |
| Query 142 | ERARTRMEAGLVDQDDLDEEHQGLAPAELFARLKARMPDGEDLVVTHGDACLPNIMVENG | 201 |
| Sbjct 138 | AR R+ AG+VD+ D D+E QG + ++F L R P EDLVVTHGDACLPN +V HLARERVAAGVVDEADFDDERQGQSAVQVFNALARRRPVTEDLVVTHGDACLPNFIVGGE | 197 |
| Query 202 | RFSGFIDCGRLGVADRYQDIALATRDIAEELGGEWADRFLVLYGIAAPDSQRIAFYRLLD | 261 |
| Sbjct 198 | G ID GR G+ADR+ D+ALA R + LG +A FL LYG D +++ +Y++LD YVEGLIDLGRAGLADRHTDLALARRSVRHNLGERYAGMFLDLYGRDLVDEEKLEYYQMLD | 257 |
| Query 262  Sbjct 258 | EFF 264 E F  ELF 260 |  |

>TR:F0RMC2 F0RMC2_DEIPM Aminoglycoside phosphotransferase OS=Deinococcus proteolyticus (strain ATCC 35074 / DSM 20540 / JCM 6276 / NBRC

101906 / NCIMB 13154 / VKM Ac-1939 / CCM 2703 / MRP) OX=693977 GN=Deipr_1927 PE=3 SV=1

Length=260

Score = 155 bits (391), Expect = 6e-43

| Identities | = 92/241 (38%), Positives = 125/241 (52%), Gaps = 6/241 (2%) |  |
| --- | --- | --- |
| Query 24 | WAQQTIGCSDAAVFRLSAQGRPVLFVKTDLSGALNELQDEAARLSWLATTGVPCAAVLDV | 83 |
| Sbjct 22 | W T+G S A V+R + R VL ++ AL L DE RL WL VP VL WEPVTLGESGAEVWRST---RHVLKIQPHRHPALPSLADERERLRWLRGR-VPVPGVLGY | 77 |
| Query 84 | VTEAGRDWLLLGEVPGQDLL--SSHLAPAEKVSIMADAMRRLHTLDPATCPFDHQAKHRI | 141 |
| Sbjct 78 | ++ ++L VPG + + L ++A A+R LH+L CPF + FADSTHEYLATERVPGIPMSHPDATLHAERMAGLLARALRGLHSLPARDCPFTMTLPVSL | 137 |
| Query 142 | ERARTRMEAGLVDQDDLDEEHQGLAPAELFARLKARMPDGEDLVVTHGDACLPNIMVENG | 201 |
| Sbjct 138 | AR R+EAGLVD+ D + QG + ++ L P ED+VVTHGDACLPN +V+ RLARERVEAGLVDESGFDPQRQGQSAVDVLVELVHHRPQTEDVVVTHGDACLPNFIVQGE | 197 |
| Query 202 | RFSGFIDCGRLGVADRYQDIALATRDIAEELGGEWADRFLVLYGIAAPDSQRIAFYRLLD | 261 |
| Sbjct 198 | G +D GR G+ADR+ D+ALA R + LG +W D FL YG D R+ +Y LD YVEGLVDVGRAGLADRHADLALAARSVRRNLGVKWVDPFLDTYGRELVDESRLDYYAALD | 257 |
| Query 262  Sbjct 258 | E 262 E  E 258 |  |

```
>TR:A0A345WKK1 A0A345WKK1_9SPHN Aminoglycoside 3'-phosphotransferase OS=Sphingomonas sp. FARSPH OX=2219696 GN=DM480_00550 PE=3 SV=1
Length=259

Score = 153 bits (387), Expect = 2e-42
Identities = 99/242 (41%), Positives = 127/242 (52%), Gaps = 11/242 (5%) Query 30	GCSDAAVFRLSAQGRPVLFVKTDLSGALNELQDEAARLSWLATTGVPCAAVLDVVTEAGR 89

G S	+FRL+	V F+K	++ DEA RL WLA	+PCA L
```

| Sbjct | 22 | GESGGNIFRLTDTDGRVRFLKCGTGAVAEDIADEAHRLFWLAGR-MPCAEPLHFTRSVDG | 80 |
| --- | --- | --- | --- |
| Query | 90 | DWLLLGEVPGQDLLS SHLAPAEKVSIMADAMRRLHTLDPATCPFDHQAKHRIER | 143 |
| Sbjct | 81 | WLL V G+ + + +HL + MA +RRLH L A CPFD A R+ AWLLTDAVAGRTVDAWIEDDRAHLPMV--IDAMAAFLRRLHALPVADCPFDAGADVRMAH | 138 |
| Query | 144 | ARTRMEAGLVDQDDLDEEHQGLAPAELFARLKARMPDGEDLVVTHGDACLPNIMV-ENGR | 202 |
| Sbjct | 139 | AR ++A +VD DD D +H G +L+ RL A VTHGD L N+M+ E GR ARRLIDADMVDTDDFDSDHDGWIAEQLWDRLVALRRCVRPCAVTHGDYSLGNVMIDEGGR | 198 |
| Query | 203 | FSGFIDCGRLGVADRYQDIALATRDIAEELGGEWADRFLVLYGIAAPDSQRIAFYRLLDE | 262 |
| Sbjct | 199 | G ID GRLGVAD YQDIA+ +++ E G WA RFL YG+ D QR+ F+R LDE  VMGCIDVGRLGVADPYQDIAILWQNL-REFGEGWAARFLDAYGLEVVDWQRLEFHRCLDE | 257 |
| Query | 263 FF 264 | | |
|  |  | F |  |
| Sbjct | 258 | LF 259 | |

>TR:A0A1W1V550 A0A1W1V550_9DEIO Kanamycin kinase OS=Deinococcus hopiensis KR-140 OX=695939 GN=SAMN00790413_00065 PE=3 SV=1

Length=256

Score = 153 bits (386), Expect = 3e-42

| Identities | = 91/243 (37%), Positives = 130/243 (53%), Gaps = 6/243 (2%) |  |
| --- | --- | --- |
| Query 24 | WAQQTIGCSDAAVFRLSAQGRPVLFVKTDLSGALNELQDEAARLSWLATTGVPCAAVLDV | 83 |
| Sbjct 18 | W T G S A V+R R V+ V+ + + L E RL WLA VP V+ WEPVTGGKSGAGVWRAQ---RYVVKVQERGGVSASTLLQERERLRWLAGR-VPVPQVVGH | 73 |
| Query 84 | VTEAGRDWLLLGEVPG--QDLLSSHLAPAEKVSIMADAMRRLHTLDPATCPFDHQAKHRI | 141 |
| Sbjct 74 | A ++L + +PG D + L P ++A A+R LH + CPF+ + EVTATHEYLAMTRLPGVPMDHPDALLHPERITDLLARALRELHAVPVRECPFNVTLPVAL | 133 |
| Query 142 | ERARTRMEAGLVDQDDLDEEHQGLAPAELFARLKARMPDGEDLVVTHGDACLPNIMVENG | 201 |
| Sbjct 134 | AR +++AG VD+ D D+E +G AE++ L P+ ED+VVTHGDA LPN ++ RLAREKVQAGAVDESDFDDERRGWTAAEVWNELVRTRPEREDVVVTHGDAWLPNFILNGE | 193 |
| Query 202 | RFSGFIDCGRLGVADRYQDIALATRDIAEELGGEWADRFLVLYGIAAPDSQRIAFYRLLD | 261 |
| Sbjct 194 | G +D GR G+ADR+ D+AL + LG +WA+RFL LYG D ++A+YRLLD YVEGLVDVGRAGLADRHADLALTHHSLTHSLGAQWAERFLDLYGRTLVDEGKLAYYRLLD | 253 |
| Query 262  Sbjct 254 | EFF 264 E F  ELF 256 |  |

>TR:D3A171 D3A171_NEIMU Aminoglycoside 3'-phosphotransferase OS=Neisseria mucosa ATCC 25996 OX=546266 GN=NEIMUCOT_06662 PE=3 SV=1

Length=253

Score = 152 bits (385), Expect = 4e-42

| Identities | = 87/251 (35%), Positives = 132/251 (53%), Gaps = 7/251 (3%) |  |
| --- | --- | --- |
| Query 19 | LFGYDWAQQTIGCSDAAVFRLSAQ-GRPVLFVKTDLSGALNELQDEAARLSWLATTGVPC | 77 |
| Sbjct 5 | L+GY WA+ +G S A ++RL + P LF+K N++ DE RL+WL T +P LYGYKWARDNVGQSGATIYRLYGKPDAPELFLKHGKGSVANDVTDEMVRLNWL-TEFMPL | 63 |
| Query 78 | AAVLDVVTEAGRDWLLLGEVPGQ---DLLSSHLAPAEK-VSIMADAMRRLHTLDPATCPF | 133 |
| Sbjct 64 | + + WLL +PG+ +L + E V +A +RRLH++ CPF PTIKHFIRTPDDAWLLTTAIPGKTAFQVLEEYPDSGENIVDALAVFLRRLHSIPVCNCPF | 123 |
| Query 134 | DHQAKHRIERARTRMEAGLVDQDDLDEEHQGLAPAELFARLKARMPDGEDLVVTHGDACL | 193 |
| Sbjct 124 | + R+ +A++RM GLV D D+E G +++ + +P D VVTHGD L NGDRVFRLAQAQSRMNNGLVGASDFDDERNGWPVEQVWKEMHKLLPFSPDSVVTHGDFSL | 183 |
| Query 194 | PNIMVENGRFSGFIDCGRLGVADRYQDIALATRDIAEELGGEWADRFLVLYGIAAPDSQR | 253 |
| Sbjct 184 | N++ + G+ G ID GR+G+ADRYQD+A+ + E R YGI PD + DNLIFDEGKLIGCIDVGRVGIADRYQDLAILWNCLG-EFSPSLQKRLFQKYGIDNPDMNK | 242 |
| Query 254 | IAFYRLLDEFF 264 |  |

+ F+ +LDEFF Sbjct 243 LQFHLMLDEFF 253

>TR:A0A1I2N3L4 A0A1I2N3L4_9BACL Kanamycin kinase OS=Planifilum fulgidum OX=201973 GN=SAMN04488025_11128 PE=3 SV=1

Length=259

Score = 152 bits (385), Expect = 4e-42

| Identities | = 87/247 (35%), Positives = 136/247 (55%), Gaps = 5/247 (2%) |  |
| --- | --- | --- |
| Query 21 | GYDWAQQTIGCSDAAVFRLSAQGRPVLFVKTDLSG---ALNELQDEAARLSWLATTGVPC | 77 |
| Sbjct 15 | G +W + +G S V+RL++ R ++K G A L+ E+ L+WL +P GMNWEKNEVGKSGDEVYRLTSGARN-FYLKVKRIGKEAAAETLRRESEVLNWLKGR-LPV | 72 |
| Query 78 | AAVLDVVTEAGRDWLLLGEVPGQDLLSSHLAPAEKVSIMADAMRRLHTLDPATCPFDHQA | 137 |
| Sbjct 73 | + + +++LL+ EVPG + + + V +A+ +RR+H+L +CPF + PEAVCYHQDRSKEYLLITEVPGFAAMETGWDKKKVVCRLAEGLRRIHSLPIDSCPFVYPT | 132 |
| Query 138 | KHRIERARTRMEAGLVDQDDLDEEHQGLAPAELFARLKARMPDGEDLVVTHGDACLPNIM | 197 |
| Sbjct 133 | I A R+ AGLVD++D DE +G EL+ L P +DLV THGD CLPN++ GALIRLAAERIGAGLVDEEDFDECRKGKRAEELYEELVRSRPARDDLVFTHGDYCLPNVI | 192 |
| Query 198 | VENGRFSGFIDCGRLGVADRYQDIALATRDIAEELGGEWADRFLVLYGIAAPDSQRIAFY | 257 |
| Sbjct 193 | + + GFID GR GVADRY+D+ +A R I LG + F YG+ D +++ F+ LSDSGIGGFIDWGRGGVADRYRDLGIAARSITHNLGEGYVPYFFGFYGLKRVDFKKVKFF | 252 |
| Query 258  Sbjct 253 | RLLDEFF 264  +L+DEFF  QLMDEFF 259 |  |

>TR:A0A1A9HRV5 A0A1A9HRV5_9BURK Aminoglycoside 3'-phosphotransferase OS=Mitsuaria sp. 7 OX=1658665 GN=ABE85_18090 PE=3 SV=1

Length=253

Score = 152 bits (383), Expect = 8e-42

| Identities | = 98/252 (39%), Positives = 133/252 (53%), Gaps = 7/252 (3%) |  |
| --- | --- | --- |
| Query 18 | RLFGYDWAQQTIGCSDAAVFRLSAQGRPVLFVKTDLSGALNELQDEAARLSWLATTGVPC | 77 |
| Sbjct 4 | RL GYDW + +G + A V RL P L++K A + DE ARL W A P RLTGYDWHRNLVGEAGARVHRLHRADAPTLYLKHGDGNAAIAIVDEFARLQWFAERW-PV | 62 |
| Query 78 | AAVLDVVTEAGRDWLLLGEVPGQ---DLLSSHLAPAEK-VSIMADAMRRLHTLDPATCPF | 133 |
| Sbjct 63 | V WLL +PG+ + L A AE V+ + +R LH L TCPF PRVEHFEWAGDTAWLLTLALPGRTAYEWLEDDPARAEAIVTSLGRCLRDLHKLPVNTCPF | 122 |
| Query 134 | DHQAKHRIERARTRMEAGLVDQDDLDEEHQGLAPAELFARLKARMPDGEDLVVTHGDACL | 193 |
| Sbjct 123 | + + +++ AR R+ AGL+D DD DE QG P +++ L A +P ED VV+HGD L NANHRLQLDLARERLAAGLIDADDFDEARQGWTPQQVWDALVAMLPLAEDPVVSHGDFSL | 182 |
| Query 194 | PN-IMVENGRFSGFIDCGRLGVADRYQDIALATRDIAEELGGEWADRFLVLYGIAAPDSQ | 252 |
| Sbjct 183 | N +M E+G G ID GR GVADR +D+A+ + +E G D YGIA PD + DNLLMSEDGEVVGVIDLGRAGVADRSRDLAILA-NCLDEFGPTLRDTLFSAYGIAQPDLR | 241 |
| Query 253  Sbjct 242 | RIAFYRLLDEFF 264  ++ F+ LLDE F  KLEFHLLLDECF 253 |  |

>SP:P00555 KKA5_STRFR Aminoglycoside 3'-phosphotransferase OS=Streptomyces fradiae OX=1906 GN=aph PE=3 SV=1

Length=268

Score = 152 bits (384), Expect = 8e-42

Identities = 100/263 (38%), Positives = 137/263 (52%), Gaps = 27/263 (10%)

| Query | 22 | YDWAQQTIGCSDAAVFRLSA--QGRPVLFVKTDLSGALN---ELQDEAARLSWLATTGVP | 76 |
| --- | --- | --- | --- |
|  |  | ++W G S A V++L+ + +P L+ K N +L EA RL WL G+P |  |
| Sbjct | 13 | HEWHAVNEGDSGAFVYQLTGGPEPQPELYAKIAPRAPENSAFDLSGEADRLEWLHRHGIP | 72 |
| Query | 77 | CAAVLDVVTEAGRDWLLLGEVPGQDLLSSHLAPAEK VSIMADAMRRLHTLD | 127 |

| Sbjct | 73 | V++ + WL+ VPG +A AE+ V MA+ R LH L VPRVVERGADDTAAWLVTEAVPG VAAAEEWPEHQRFAVVEAMAELARALHELP | 125 |
| --- | --- | --- | --- |
| Query | 128 | PATCPFDHQAKHRIERARTRMEAGLVDQDDLDEEHQGLAPAELFARLKARMPDGEDLVVT | 187 |
| Sbjct | 126 | CP D + + AR + GLVD DDL EE G +L A L P+ EDLVV VEDCPSDRRLDAAVAEARRNVAEGLVDLDDLQEERAGWTGDQLLAELDRTRPEKEDLVVC | 185 |
| Query | 188 | HGDACLPNIMVENG--RFSGFIDCGRLGVADRYQDIALATRDIAEE LGGEWADRFL | 241 |
| Sbjct | 186 | HGD C N++++ G R +G ID GRLGVADR+ DIALA R++ + G +A+RFL HGDLCPNNVLLDPGTCRVTGVIDVGRLGVADRHADIALAARELEIDEDPWFGPAYAERFL | 245 |
| Query | 242 | VLYGIAAPDSQRIAFYRLLDEFF 264 YG D +++AFY+LLDEFF |  |
| Sbjct | 246 | ERYGAHRVDKEKLAFYQLLDEFF 268 | |

>TR:A0A2T6BGV9 A0A2T6BGV9_9BACL Kanamycin kinase/aminoglycoside 3'-phosphotransferase-2 OS=Melghirimyces profundicolus OX=1242148 GN=C8P63_11915

PE=3 SV=1

Length=256

Score = 152 bits (383), Expect = 8e-42

| Identities | = 88/245 (36%), Positives = 126/245 (51%), Gaps = 3/245 (1%) |  |
| --- | --- | --- |
| Query 21 | GYDWAQQTIGCSDAAVFRLSAQGRPVLFVKT-DLSGALNELQDEAARLSWLATTGVPCAA | 79 |
| Sbjct 14 | G +W ++++G S A VFRL +GR V ++K L L E L WL +P GMEWRRESVGKSGAMVFRLE-KGRQVQYLKVRPRDNRLEPLSREREVLRWLERR-LPVPE | 71 |
| Query 80 | VLDVVTEAGRDWLLLGEVPGQDLLSSHLAPAEKVSIMADAMRRLHTLDPATCPFDHQAKH | 139 |
| Sbjct 72 | VLD ++LL+ +PG+ + + + V + + +R +H L P CP+ VLDYEETRSAEFLLMSGIPGRMPMDLRVDKSRIVRWLGEGLRYIHELPPDPCPWSRPNDL | 131 |
| Query 140 | RIERARTRMEAGLVDQDDLDEEHQGLAPAELFARLKARMPDGEDLVVTHGDACLPNIMVE | 199 |
| Sbjct 132 | ++ A R+E G VD+ D D +G EL L P ED V THGD L NI++ LVKVAAARVEKGWVDESDFDPHRRGRTAEELLDELLRTCPREEDPVFTHGDYTLSNILMT | 191 |
| Query 200 | NGRFSGFIDCGRLGVADRYQDIALATRDIAEELGGEWADRFLVLYGIAAPDSQRIAFYRL | 259 |
| Sbjct 192 | SG ID GR VADRY D+A+ +R +A LG EW F YG+ + +++ FYRL ENGISGLIDWGRAAVADRYTDLAIGSRSLASHLGSEWVPFFFKAYGLNGVNWEKLRFYRL | 251 |
| Query 260  Sbjct 252 | LDEFF 264 LDEFF LDEFF 256 |  |

>TR:A0A1G5NZI3 A0A1G5NZI3_AFIMA Aminoglycoside 3'-phosphotransferase OS=Afifella marina DSM 2698 OX=1120955 GN=SAMN03080610_02899 PE=3 SV=1

Length=271

Score = 152 bits (383), Expect = 1e-41

| Identities | = 88/251 (35%), Positives = 133/251 (53%), Gaps = 7/251 (3%) |  |
| --- | --- | --- |
| Query 19 | LFGYDWAQQTIGCSDAAVFRLSAQ-GRPVLFVKTDLSGALNELQDEAARLSWLATTGVPC | 77 |
| Sbjct 23 | L GY W + +G S +VFRL + P F+K ++L DE RL WLA + LAGYRWMRDRVGQSGGSVFRLHGKLNGPDAFLKHGQDALADDLSDEMVRLRWLAGY-ISV | 81 |
| Query 78 | AAVLDVVTEAGRDWLLLGEVPG QDLLSSHLAPAEKVSIMADAMRRLHTLDPATCPF | 133 |
| Sbjct 82 | A++ + WL+ +PG Q L + A V +A +RRLH + CPF PALISFTRTSDEAWLVTTAIPGKTAYQVLEADPAAGLAIVDALAVFLRRLHAIPVCACPF | 141 |
| Query 134 | DHQAKHRIERARTRMEAGLVDQDDLDEEHQGLAPAELFARLKARMPDGEDLVVTHGDACL | 193 |
| Sbjct 142 | + + RAR R++AGLVD +D D+E +G +++ + +P D+VVTHGD L TSDHVYSLARARERLDAGLVDVEDFDDERKGWTAEQVWEEMHRLLPFAPDVVVTHGDFSL | 201 |
| Query 194 | PNIMVENGRFSGFIDCGRLGVADRYQDIALATRDIAEELGGEWADRFLVLYGIAAPDSQR | 253 |
| Sbjct 202 | N+ + +G +G ID G+ G+ADRYQD+A+ + + +RFL YG+ D QR DNLSILDGEVTGCIDVGKAGIADRYQDLAIMWNCLG-DFDRSLQERFLRQYGVVDVDWQR | 260 |
| Query 254  Sbjct 261 | IAFYRLLDEFF 264  + F+ +LDEFF  LQFHLMLDEFF 271 |  |

>TR:L0A5A4 L0A5A4_DEIPD Aminoglycoside phosphotransferase OS=Deinococcus peraridilitoris (strain DSM 19664 / LMG 22246 / CIP 109416

/ KR-200) OX=937777 GN=Deipe_2720 PE=3 SV=1

Length=263

Score = 151 bits (382), Expect = 1e-41

| Identities | = 91/243 (37%), Positives = 126/243 (52%), Gaps = 8/243 (3%) |  |
| --- | --- | --- |
| Query 24 | WAQQTIGCSDAAVFRLSAQGRPVLFVKTDLSGALNELQDEAARLSWLATTGVPCAAVLDV | 83 |
| Sbjct 27 | W + T+G S A V R S +K L E RL WLA+ +P V+ WEEVTVGESGARVLRSSR FMLKIQPKRIHGTLFAEKERLRWLASR-IPVPRVVAY | 80 |
| Query 84 | VTEAGRDWLLLGEVPGQDLL--SSHLAPAEKVSIMADAMRRLHTLDPATCPFDHQAKHRI | 141 |
| Sbjct 81 | E+G ++L + G D+ + L ++A A+R LH L CPFD + + HQESGTEYLATERLRGIDMSHPDAALHARRNTELLARALRELHALPVRDCPFDMSLRVML | 140 |
| Query 142 | ERARTRMEAGLVDQDDLDEEHQGLAPAELFARLKARMPDGEDLVVTHGDACLPNIMVENG | 201 |
| Sbjct 141 | AR R+ GLVD+ D D E G ++ + PD EDLV+THGDACLPN++V AEARERVGLGLVDEADFDGERAGHRARDVLREVVRTRPDREDLVLTHGDACLPNVIVAGE | 200 |
| Query 202 | RFSGFIDCGRLGVADRYQDIALATRDIAEELGGEWADRFLVLYGIAAPDSQRIAFYRLLD | 261 |
| Sbjct 201 | +GF+D GR G+ADR+ D+ALA R + G E+A FL YG A D ++ FY+LLD FVAGFVDVGRAGIADRHADLALAVRSLRRNYGEEFALLFLDTYGRALVDEDKLQFYQLLD | 260 |
| Query 262  Sbjct 261 | EFF 264 E F  ELF 263 |  |

>TR:A0A561Q8L3 A0A561Q8L3_9BACL Kanamycin kinase/aminoglycoside 3'-phosphotransferase-2 OS=Paenibacillus sp. 597 OX=2587047 GN=FHW43_10330

PE=3 SV=1

Length=259

Score = 151 bits (381), Expect = 2e-41

| Identities | | = 91/256 (36%), Positives = 132/256 (52%), Gaps = 6/256 (2%) |  |
| --- | --- | --- | --- |
| Query 12  Sbjct 7 | | PAAWVERLFGYDWAQQTIGCSDAAVFRLSAQGRPVLFVKTDLSGALNELQDEAARLSWLA PA + GY+W + T+G S + + L+ L++K A L E RL WL PAELSRLVNGYEWRRITLGLSPSHTYLLTGASHN-LYIKIQSITAAESLHREKERLLWLQ | 71  65 |
| Query 72  Sbjct 66 | | TTGVPCAAVLDVVTEAGRDWLLLGEVPG---QDLLSSHLAPAEKVSIMADAMRRLHTLDP  + V+ + ++LL+ E+ G D + P + +A +R +H + GK-LTVPEVVYFGQDDANEFLLMTEIEGINASDKSYESMLPT-LMQQLAIGLRIVHGVPV | 128  123 |
| Query 129  Sbjct 124 | | ATCPFDHQAKHRIERARTRMEAGLVDQDDLDEEHQGLAPAELFARLKARMPDGEDLVVTH CPFD +I AR R+E LVD++D D + +G +L L P EDLV TH EGCPFDQTLNAKIVEARRRVEQQLVDEEDFDRQREGRTAEDLAEELLQLKPAAEDLVFTH | 188  183 |
| Query 189  Sbjct 184 | | GDACLPNIMVENGRFSGFIDCGRLGVADRYQDIALATRDIAEELGGEWADRFLVLYGIAA GD CLPNI++++G+ GFID GR G+ADRYQDIALA R I G F YG+ A GDYCLPNIILKDGKVHGFIDWGRAGIADRYQDIALAIRSIRYNFGQGHVQTFFDAYGLTA | 248  243 |
| Query 249 | | PDSQRIAFYRLLDEFF 264 D ++ +Y+L+DEFF |  |
| Sbjct | 244 | LDEAKVHYYQLMDEFF 259 | |

>TR:A0A0W1QFZ6 A0A0W1QFZ6_9SPHN Aminoglycoside 3'-phosphotransferase OS=Sphingomonas sp. WG OX=1592629 GN=ATB93_17630 PE=3 SV=1

Length=264

Score = 150 bits (380), Expect = 3e-41

Identities = 93/248 (38%), Positives = 136/248 (55%), Gaps = 7/248 (3%)

| Query | 21 | GYDWAQQTIGCSDAAVFRLSAQGRPVLFVKTDLSGALNELQDEAARLSWLATTGVPCAAV | 80 |
| --- | --- | --- | --- |
| Sbjct | 20 | G W + +G S AV+RL+ G L++K + +L DEA RL WLA +P A + GRRWFRDRVGESGCAVYRLTEDGSDDLYLKVAHGPVVTDLVDEAVRLRWLAPY-LPVAPL | 78 |
| Query | 81 | LDVVTEAGRDWLLLGEVPGQ---DLLSSHLAPAEKVSI-MADAMRRLHTLDPATCPFDHQ V E+ WLL G +PG+ LL + A ++ +A +RRLH L CPF+ | 136 |

| Sbjct | 79 | HGFVLESDSAWLLTGRLPGRTAYQLLERDPSAAPVLAASLARFLRRLHALPADRCPFNAD | 138 |
| --- | --- | --- | --- |
| Query | 137 | AKHRIERARTRMEAGLVDQDDLDEEHQGLAPAELFARLKARMPDGEDLVVTHGDACLPNI | 196 |
| Sbjct | 139 | + R+ AR R++AGLVD+ D D+ G++ +++ + A +P D VVTHGD L N+ HRLRLAAARDRIDAGLVDESDFDDARIGMSAEQVWDAMMAALPPAFDRVVTHGDISLDNL | 198 |
| Query | 197 | MVENGRFSGFIDCGRLGVADRYQDIALATRDIAEELGGEWADRFLVLYGIAAPDSQRIAF | 256 |
| Sbjct | 199 | +V G G ID GR+GVAD +QD+A+ + +E G A L YG+A D R+ F LVVEGEVVGCIDLGRVGVADPWQDLAILWNSL-QEFGDGAAQAMLDAYGVAL-DGARLDF | 256 |
| Query  Sbjct | 257  257 | YRLLDEFF 264  + LDE F  HLRLDELF 264 |  |

>TR:A0A2I9CZQ6 A0A2I9CZQ6_9DEIO Aminoglycoside phosphotransferase OS=Deinococcus aerius OX=200253 GN=DAERI_160111 PE=3 SV=1

Length=256

Score = 150 bits (379), Expect = 3e-41

| Identities | = 91/243 (37%), Positives = 128/243 (53%), Gaps = 6/243 (2%) |  |
| --- | --- | --- |
| Query 24 | WAQQTIGCSDAAVFRLSAQGRPVLFVKTDLSGALNELQDEAARLSWLATTGVPCAAVLDV | 83 |
| Sbjct 18 | W T G S A V+R R V+ V+ ++ L E RL W VP AV+ WESVTHGESGAGVWRSQ---RFVVKVQERGGSPVSTLLQERERLRWFRGR-VPVPAVVGY | 73 |
| Query 84 | VTEAGRDWLLLGEVPGQDLLSSH--LAPAEKVSIMADAMRRLHTLDPATCPFDHQAKHRI | 141 |
| Sbjct 74 | ++L + +PG + L P ++A A+R LH L CPF+ + EVTDTHEYLAMTRLPGIPMSDPDALLHPGRVTDLLARALRELHALPVRECPFNMTLPVTL | 133 |
| Query 142 | ERARTRMEAGLVDQDDLDEEHQGLAPAELFARLKARMPDGEDLVVTHGDACLPNIMVENG | 201 |
| Sbjct 134 | AR ++EAG VD+ D D+E QG + A+++ L P ED+VVTHGD CLPN+++ RLAREQVEAGRVDEADFDDERQGWSAAQVWKELVRTRPTQEDVVVTHGDPCLPNLILNGA | 193 |
| Query 202 | RFSGFIDCGRLGVADRYQDIALATRDIAEELGGEWADRFLVLYGIAAPDSQRIAFYRLLD | 261 |
| Sbjct 194 | GFID GR G+ADR+ D+AL R + + E A+RFL LYG D ++A+YRLLD YVEGFIDVGRAGLADRHADLALTYRSLIRNIDQEHAERFLDLYGREFVDGGKLAYYRLLD | 253 |
| Query 262  Sbjct 254 | EFF 264 E F  ELF 256 |  |

>SP:Q03447 KKA1_SALTM Aminoglycoside 3'-phosphotransferase OS=Salmonella typhimurium OX=90371 PE=3 SV=1

Length=271

Score = 150 bits (380), Expect = 3e-41

| Identities | = 87/251 (35%), Positives = 131/251 (52%), Gaps = 7/251 (3%) |  |
| --- | --- | --- |
| Query 19 | LFGYDWAQQTIGCSDAAVFRLSAQ-GRPVLFVKTDLSGALNELQDEAARLSWLATTGVPC | 77 |
| Sbjct 23 | L+GY WA+ +G S A + RL + P LF+K N++ DE RL+W+ T +P LYGYKWARDNVGQSGATIDRLYGKPDAPELFLKHGKGSVANDVTDEMVRLNWM-TEFMPL | 81 |
| Query 78 | AAVLDVVTEAGRDWLLLGEVPGQ---DLLSSHLAPAEK-VSIMADAMRRLHTLDPATCPF | 133 |
| Sbjct 82 | + + WLL +PG+ +L + E V +A +RRLH++ CPF PTIKHFIRTPDDAWLLTTAIPGKTAFQVLEEYPDSGENIVDALAVFLRRLHSIPVCNCPF | 141 |
| Query 134 | DHQAKHRIERARTRMEAGLVDQDDLDEEHQGLAPAELFARLKARMPDGEDLVVTHGDACL | 193 |
| Sbjct 142 | + R+ +A++RM GLVD D D+E G +++ + +P D VVTHGD L NSDRVFRLVQAQSRMNNGLVDASDFDDERNGWPVEQVWKEMHKLLPFSPDSVVTHGDFSL | 201 |
| Query 194 | PNIMVENGRFSGFIDCGRLGVADRYQDIALATRDIAEELGGEWADRFLVLYGIAAPDSQR | 253 |
| Sbjct 202 | N++ + G+ G ID GR+G+ADRYQD+A+ + E R YGI PD + DNLIFDEGKLIGCIDVGRVGIADRYQDLAILWNCLG-EFSPSLQKRLFQKYGIDNPDMNK | 260 |
| Query 254  Sbjct 261 | IAFYRLLDEFF 264  + F +LDEFF  LQFTLMLDEFF 271 |  |

>TR:A0A3P7EH96 A0A3P7EH96_WUCBA APH domain-containing protein OS=Wuchereria

bancrofti OX=6293 GN=WBA_LOCUS12467 PE=3 SV=1

Length=484

Score = 155 bits (393), Expect = 4e-41

| Identities | = 88/251 (35%), Positives = 133/251 (53%), Gaps = 7/251 (3%) |  |
| --- | --- | --- |
| Query 19 | LFGYDWAQQTIGCSDAAVFRLSAQ-GRPVLFVKTDLSGALNELQDEAARLSWLATTGVPC | 77 |
| Sbjct 236 | L+GY WA+ +G S A ++RL + P LF+K N++ DE RL+WL T +P LYGYKWARDNVGQSGATIYRLYGKPDAPELFLKHGKGSVANDVTDEMVRLNWL-TEFMPL | 294 |
| Query 78 | AAVLDVVTEAGRDWLLLGEVPGQ---DLLSSHLAPAEK-VSIMADAMRRLHTLDPATCPF | 133 |
| Sbjct 295 | + + WLL +PG+ +L + E V +A +RRLH++ CPF PTIKHFIRTPDDAWLLTTAIPGKTAFQVLEEYPDSGENIVDALAVFLRRLHSIPVCNCPF | 354 |
| Query 134 | DHQAKHRIERARTRMEAGLVDQDDLDEEHQGLAPAELFARLKARMPDGEDLVVTHGDACL | 193 |
| Sbjct 355 | + R+ +A++RM GLVD D D+E G +++ + +P D VVTHGD L NSDRVFRLAQAQSRMNNGLVDASDFDDERNGWPVEQVWKEMHKLLPFSPDSVVTHGDFSL | 414 |
| Query 194 | PNIMVENGRFSGFIDCGRLGVADRYQDIALATRDIAEELGGEWADRFLVLYGIAAPDSQR | 253 |
| Sbjct 415 | N++ + G+ G ID GR+G+ADRYQD+A+ + E R YGI PD + DNLIFDEGKLIGCIDVGRVGIADRYQDLAILWNCLG-EFSPSLQKRLFQKYGIDNPDMNK | 473 |
| Query 254  Sbjct 474 | IAFYRLLDEFF 264  + F+ +LDEFF  LQFHLMLDEFF 484 |  |

>TR:A0A0Q5QUZ1 A0A0Q5QUZ1_9SPHN Aminoglycoside 3'-phosphotransferase OS=Sphingomonas sp. Leaf357 OX=1736350 GN=ASG11_02275 PE=3 SV=1

Length=271

Score = 150 bits (379), Expect = 5e-41

| Identities | = 94/259 (36%), Positives = 138/259 (53%), Gaps = 7/259 (3%) |  |
| --- | --- | --- |
| Query 11 | SPAAWVERLFGYDWAQQTIGCSDAAVFRLSAQG-RPVLFVKTDLSGALNELQDEAARLSW | 69 |
| Sbjct 15 | +P + L GYDW++ +G S AV+RL G P LF+K + ++ ++ E RL W TPTVLTDLLDGYDWSRDMVGESGDAVYRLRKTGVAPDLFLKRAPAISVRDVAGEMVRLQW | 74 |
| Query 70 | LATTGVPCAAVLDVVTEAGRDWLLLGEVPGQDLLS-SHLAPAEKVSI---MADAMRRLHT | 125 |
| Sbjct 75 | L + AV WLL+ +PG+ P ++ +I +A +R LH LRDH-IAVPAVRAFFATPDEAWLLITALPGKTAYQWLESCPDQRPAIVDRLARLLRCLHA | 133 |
| Query 126 | LDPATCPFDHQAKHRIERARTRMEAGLVDQDDLDEEHQGLAPAELFARLKARMPDGEDLV | 185 |
| Sbjct 134 | + A+CPF+ + R+ AR R++ GLVD DD D E G + ++ + +P D V IPVASCPFNSDHQLRLGEARWRIDRGLVDVDDFDVERAGWSAEAVWVAMTEVLPMAPDPV | 193 |
| Query 186 | VTHGDACLPNIMVENGRFSGFIDCGRLGVADRYQDIALATRDIAEELGGEWADRFLVLYG | 245 |
| Sbjct 194 | VTHGD L NI++ +G +G ID GR GVADRYQD+A+ +A E G R YG VTHGDFSLDNILLADGAEAGCIDVGRAGVADRYQDLAILWHGLA-EFGTALQRRLFDSYG | 252 |
| Query 246  Sbjct 253 | IAAPDSQRIAFYRLLDEFF 264 IA PD ++I F+ +LDE F IAEPDERKIRFHLMLDELF 271 |  |

>TR:A0A418VBY7 A0A418VBY7_9DEIO Aminoglycoside 3'-phosphotransferase OS=Deinococcus sp. K2S05-167 OX=2320857 GN=D3875_10380 PE=3 SV=1

Length=257

Score = 149 bits (377), Expect = 7e-41

Identities = 89/243 (37%), Positives = 126/243 (52%), Gaps = 6/243 (2%)

| Query | 24 | WAQQTIGCSDAAVFRLSAQGRPVLFVKTDLSGALNELQDEAARLSWLATTGVPCAAVLDV | 83 |
| --- | --- | --- | --- |
| Sbjct | 19 | W + T G S A V+R + R ++ ++ LQ E RL WL +P V+ WEEITTGKSGAQVWRST---RHIVKIQRHQGAVGVSLQQERGRLRWLQGR-LPAPQVVAY | 74 |
| Query | 84 | VTEAGRDWLLLGEVPGQDLL--SSHLAPAEKVSIMADAMRRLHTLDPATCPFDHQAKHRI | 141 |
| Sbjct | 75 | + ++L + +PG DL + L P V+++A A+R LH L CPF+ + DSTDTHEYLAMTRLPGIDLSHPDARLHPERVVNLLARALRELHALPLRDCPFNMSLNVAL | 134 |
| Query | 142 | ERARTRMEAGLVDQDDLDEEHQGLAPAELFARLKARMPDGEDLVVTHGDACLPNIMVENG AR R++AGL+D+ D DE QG +F L P EDLVVTHGD CLPN +V | 201 |

| Sbjct | 135 | HLARERVQAGLIDESDFDEARQGRTATSVFNELVKTRPVWEDLVVTHGDPCLPNFIVNGK | 194 |
| --- | --- | --- | --- |
| Query | 202 | RFSGFIDCGRLGVADRYQDIALATRDIAEELGGEWADRFLVLYGIAAPDSQRIAFYRLLD | 261 |
| Sbjct | 195 | G ID GRLG+ADR+ D+ LA R L E+A++ L YG D ++ +Y LLD LLEGLIDVGRLGIADRHADLGLAYRSTRHNLSVEYAEQLLDHYGREKVDMDKVRYYMLLD | 254 |
| Query  Sbjct | 262  255 | EFF 264 E +  ELW 257 |  |

>TR:A0A0F7JMK4 A0A0F7JMK4_9DEIO Amino acid ABC transporter ATP-binding protein OS=Deinococcus soli Cha et al. 2016 OX=1309411 GN=SY84_07575

PE=3 SV=1

Length=260

Score = 149 bits (375), Expect = 1e-40

| Identities | = 98/262 (37%), Positives = 136/262 (52%), Gaps = 14/262 (5%) |  |
| --- | --- | --- |
| Query 5 | DGLHAGSPAAWVERLFGYDWAQQTIGCSDAAVFRLSAQGRPVLFVKTDLSGALNELQDEA | 64 |
| Sbjct 11 | D L PAA ER+ G G S A V+R + R VL ++ + A + LQ E DALKRVLPAARWERVSG--------GQSGAQVWRST---RYVLKMQPRIPHATSTLQQER | 59 |
| Query 65 | ARLSWLATTGVPCAAVLDVVTEAGRDWLLLGEVPGQDLL--SSHLAPAEKVSIMADAMRR | 122 |
| Sbjct 60 | RL WL +P V+ E ++ L + + G + + L P V+++A A+R ERLRWLQGR-IPAPQVVAFEAEGDQEVLAMTRLTGIPMSHPDARLHPERVVNLLARALRE | 118 |
| Query 123 | LHTLDPATCPFDHQAKHRIERARTRMEAGLVDQDDLDEEHQGLAPAELFARLKARMPDGE | 182 |
| Sbjct 119 | LH L CPF + AR R++AGL+D D D++ G + +F L P + LHALPIRDCPFRQTLDVTLPLARERVQAGLIDGSDFDDDRAGRSATSVFNELARTRPTQD | 178 |
| Query 183 | DLVVTHGDACLPNIMVENGRFSGFIDCGRLGVADRYQDIALATRDIAEELGGEWADRFLV | 242 |
| Sbjct 179 | DLVV HGDA L NI++ G ID GRLG+ADR+ D+ALA R + ELG +A FL DLVVAHGDATLDNIILNGEYVEGLIDVGRLGIADRHADLALAHRSVRGELGERYAGMFLD | 238 |
| Query 243  Sbjct 239 | LYGIAAPDSQRIAFYRLLDEFF 264 LYG A D ++A+YRL DE F LYGRALVDDTKLAYYRLHDELF 260 |  |

>TR:A0A1B2HPD3 A0A1B2HPD3_9PSEU APH domain-containing protein OS=Lentzea guizhouensis OX=1586287 GN=BBK82_29490 PE=3 SV=1

Length=233

Score = 147 bits (372), Expect = 2e-40

| Identities | | = 88/204 (43%), Positives = 116/204 (57%), Gaps = 14/204 (7%) |  |
| --- | --- | --- | --- |
| Query 62  Sbjct 43 | | DEAARLSWLATT-GVPCAAVLDVVTEAGRDWLLLGEVPGQDLLSSHLAPAEKVSIMADAM DE ARL WL + VP + WL+L +V L + + P +++ + DEHARLRWLGSRFEVPAVLAFE DGWLVLQDVGAPSLAA--VEPVRAGTVLGTVL | 120  94 |
| Query 121  Sbjct 95 | | RRLHTLDPATCPFDHQAKHRIERARTRMEAGLVDQDDLDEEHQGLAPAELFARLKARMPD R LH++ CPFD + +E+AR +EAGLVD DD D+EH G +P ELF +L A P RELHSVPWQECPFDSRVAVAVEKARHNVEAGLVDPDDFDDEHLGTSPEELFEKLVAAAPA | 180  154 |
| Query 181  Sbjct 155 | | GEDLVVTHGDACLPNIMVENGRFSGFIDCGRLGVADRYQDIALATRDIAEELGGEWADRF EDLVV HGD C PN++V + ID GRLGVADR+ D+AL R ELG E A F DEDLVVVHGDFCPPNVLVRPDGSTVVIDVGRLGVADRHHDLALVHR ELGEEAAAAF | 240  210 |
| Query 241 | | LVLYGIAAPDSQRIAFYRLLDEFF 264 YG+ PD ++ +YRLLDEFF |  |
| Sbjct | 211 | DRAYGL-VPDPAKLYWYRLLDEFF 233 | |

```
>TR:U5W890 U5W890_9ACTN Aminoglycoside phosphotransferase OS=Actinoplanes friuliensis DSM 7358 OX=1246995 GN=AFR_29645 PE=3 SV=1
Length=231

Score = 147 bits (370), Expect = 4e-40
Identities = 109/248 (44%), Positives = 133/248 (54%), Gaps = 25/248 (10%) Query 24	WAQQTIGCSDAAVFRLSAQGRPVLFVKTDLSGALNELQDEAARLSWLATTGVPCAAVLDV 83

W	T G S A V R	R ++ KT +	++L EAARL+WL	G+P A VL
```

| Sbjct | 2 | WEIVTTGQSGATVSR RDGVYRKTS-NDPRDDLPGEAARLTWLRAHGIPAAQVLS- | 54 |
| --- | --- | --- | --- |
| Query | 84 | VTEAGRDWLLLGEVPGQDLLSSHLAPAEKVSIMADAMRRLHTLDPATCPFDHQAKHRIER | 143 |
| Sbjct | 55 | + L EVPG L + V +AD R LH L A CPFD + I  ---SAPGLLETAEVPG---LPASDYRHGVVDALADFTRVLHALPIADCPFDRRLAVTIPA | 108 |
| Query | 144 | ARTRMEAGLVDQDDLDEEHQGLAPAELFARLKARMPDGEDLVVTHGDACLPNIMV--ENG | 201 |
| Sbjct | 109 | A A VD DDLD+E G EL A+L A P EDLVV HGD CLPN+++ E AL AADVDLDDLDDERAGWTRDELVAQLHATRPVQEDLVVCHGDLCLPNVVLDREGS | 164 |
| Query | 202 | RFSGFIDCGRLGVADRYQDIALATRDIAE ELGGEW-ADRFLVLYGIAAPDSQRIAF | 256 |
| Sbjct | 165 | + +G ID GRLGVADR+ D+AL TR +++ G W ADRFL YGI APD + F QVTGLIDVGRLGVADRWTDLALMTRSLSDPGLNPGYGPWAADRFLTRYGI-APDPAKNDF | 223 |
| Query  Sbjct | 257  224 | YRLLDEFF 264 YRLLDEFF YRLLDEFF 231 |  |

>SP:P14509 KKA8_ECOLX Aminoglycoside 3'-phosphotransferase OS=Escherichia coli OX=562 GN=aphA PE=3 SV=1

Length=271

Score = 148 bits (373), Expect = 4e-40

| Identities | = 93/261 (36%), Positives = 133/261 (51%), Gaps = 7/261 (3%) |  |
| --- | --- | --- |
| Query 9 | AGSPAAWVERLFGYDWAQQTIGCSDAAVFRL-SAQGRPVLFVKTDLSGALNELQDEAARL | 67 |
| Sbjct 13 | A P + + GY WA+ +G S AV+RL S G LF+K +++ DE RL AAVPESMAAHVMGYKWARDKVGQSGCAVYRLHSKSGGSDLFLKHGKDAFADDVTDEMVRL | 72 |
| Query 68 | SWLATTGVPCAAVLDVVTEAGRDWLLLGEVPG QDLLSSHLAPAEKVSIMADAMRRL | 123 |
| Sbjct 73 | WLA + +V+ V + WLL + G Q L S A V +A MRRL RWLAGH-ISVPSVVSFVRTPNQAWLLTTAIHGKTAYQVLKSDFGARLVVVDALAAFMRRL | 131 |
| Query 124 | HTLDPATCPFDHQAKHRIERARTRMEAGLVDQDDLDEEHQGLAPAELFARLKARMPDGED | 183 |
| Sbjct 132 | H + + C H R +EAG+VD DD D+E +G +++ + +P D HAIPVSECSVQQWTTHAGLPERGSIEAGVVDVDDFDKEREGWTAEQVWEAMHRLLPLAPD | 191 |
| Query 184 | LVVTHGDACLPNIMVENGRFSGFIDCGRLGVADRYQDIALATRDIAEELGGEWADRFLVL | 243 |
| Sbjct 192 | VVTHGD L N+++ G+ G ID GR G+ADRYQD+A+ + EE +R + PVVTHGDFSLDNLLIVEGKVVGCIDVGRAGIADRYQDLAVLW-NCLEEFEPSLQERLVAQ | 250 |
| Query 244  Sbjct 251 | YGIAAPDSQRIAFYRLLDEFF 264 YGIA PD +++ F+ LLDE F YGIADPDRRKLQFHLLLDELF 271 |  |

>TR:A0A172T670 A0A172T670_9DEIO Amino acid ABC transporter ATP-binding protein OS=Deinococcus puniceus OX=1182568 GN=SU48_00375 PE=3 SV=1

Length=258

Score = 147 bits (371), Expect = 5e-40

| Identities | = 88/243 (36%), Positives = 128/243 (53%), Gaps = 6/243 (2%) |  |
| --- | --- | --- |
| Query 24 | WAQQTIGCSDAAVFRLSAQGRPVLFVKTDLSGALNELQDEAARLSWLATTGVPCAAVLDV | 83 |
| Sbjct 20 | W T G S A V++ + V+ V+ ++ LQ E RL WLA VP A++ WEAITEGQSGAGVWKSQ---KYVMKVQERGGHPVSTLQQERERLRWLAER-VPVPALIGF | 75 |
| Query 84 | VTEAGRDWLLLGEVPGQDLLSSH--LAPAEKVSIMADAMRRLHTLDPATCPFDHQAKHRI | 141 |
| Sbjct 76 | + ++L + +PG + + L P V ++A A+R+LH L CPF + EVSSAHEFLAMSRLPGIPMSHADALLHPERVVGLLARALRQLHALPVRDCPFSMILPVTL | 135 |
| Query 142 | ERARTRMEAGLVDQDDLDEEHQGLAPAELFARLKARMPDGEDLVVTHGDACLPNIMVENG | 201 |
| Sbjct 136 | AR ++EAG VD+ D DEE G + +F L P ED+VVTHGD CLPN+++ RLAREQVEAGAVDETDFDEERLGRSAVSIFNELARTRPPAEDIVVTHGDPCLPNLILNGE | 195 |
| Query 202 | RFSGFIDCGRLGVADRYQDIALATRDIAEELGGEWADRFLVLYGIAAPDSQRIAFYRLLD | 261 |
| Sbjct 196 | GF+D GR G+ADR+ D+ALA R + L A+ FL YG D +++ +Y LLD WVEGFVDVGRAGLADRHADLALAHRSLKFNLNAAHAEAFLDAYGREWVDPEKLEYYCLLD | 255 |
| Query 262 | EFF 264 |  |

```
E F Sbjct 256 ELF 258

>TR:A0A1D8G9Y6 A0A1D8G9Y6_9ACTN Aminoglycoside 3'-phosphotransferase OS=Streptomyces rubrolavendulae OX=285473 GN=neo PE=3 SV=1
Length=703

Score = 155 bits (392), Expect = 6e-40
Identities = 101/263 (38%), Positives = 138/263 (52%), Gaps = 27/263 (10%)

Query 22	YDWAQQTIGCSDAAVFRLSA--QGRPVLFVKTDLSGALN---ELQDEAARLSWLATTGVP 76
++W	G S A V++L+	+ +P L+ K	N	+L EA RL WL	G+P Sbjct 448 HEWHAVNEGDSGAFVYQLTGGPEPQPELYAKIAPRAPENSAFDLSGEADRLEWLHRHGIP 507

Query 77	CAAVLDVVTEAGRDWLLLGEVPGQDLLSSHLAPAEK---------VSIMADAMRRLHTLD 127 V++	+	WL+	VPG	+A AE+	V MA+ R LH L
Sbjct 508 VPRVVERGADDTAAWLVTEAVPG-------VAAAEEWPEHQRFAVVEAMAELARALHELP 560

Query 128 PATCPFDHQAKHRIERARTRMEAGLVDQDDLDEEHQGLAPAELFARLKARMPDGEDLVVT 187
```

| Sbjct | 561 | CPFD + + AR + GLVD DDL EE G +L A L P+ EDLVV VEDCPFDRRLDAAVAEARRNVAEGLVDLDDLQEERAGWTGDQLLAELDRTRPEKEDLVVC | 620 |
| --- | --- | --- | --- |
| Query | 188 | HGDACLPNIMVENG--RFSGFIDCGRLGVADRYQDIALATRDIAEE LGGEWADRFL | 241 |
| Sbjct | 621 | HGD C N++++ G R +G ID GRLGVADR+ DIALA R++ + G +A+RFL HGDLCPNNVLLDPGTCRVTGVIDVGRLGVADRHADIALAARELEIDEDPWFGPAYAERFL | 680 |
| Query | 242 | VLYGIAAPDSQRIAFYRLLDEFF 264 YG D +++AFY+LLDEFF |  |
| Sbjct | 681 | ERYGAHRVDKEKLAFYQLLDEFF 703 | |

>TR:A0A5Q2RK27 A0A5Q2RK27_9ACTN APH(3') family aminoglycoside O-phosphotransferase OS=Iamiaceae bacterium SCSIO 58843 OX=2664374 GN=aph(3')

PE=3 SV=1

Length=265

Score = 147 bits (370), Expect = 9e-40

| Identities | | = 93/258 (36%), Positives = 126/258 (49%), Gaps = 6/258 (2%) |  |
| --- | --- | --- | --- |
| Query 12  Sbjct 9 | | PAAWVERLFGYDWAQQTIGCSDAAVFRLSAQGRPVLFVK-TDLSGALNELQDEAARLSWL P++ L G A +G S + V R G LFVK D L E ARL WL PSSLAALLDGASLAPVNVGRSGSVVARAVRDGHADLFVKLVDEGSGAPVLGPEIARLQWL | 70  68 |
| Query 71  Sbjct 69 | | ATTGVPCAAVLDVVTEAGRDWLLLGEVPGQDLLSSHLA--PAEKVSIMADAMRRLHTLDP P ++D G +WL+ + G D S L P + A+R LH + P  QGRW-PVPELVDHGRADGAEWLVTVALDGVDATRSPLGADPEVLARALGTALRHLHDVGP | 128  127 |
| Query 129  Sbjct 128 | | AT-CPFDHQAKHRIERARTRMEAGLVDQDDLDEEHQGLAPAELFARLKARMP-DGEDLVV CPFD + + AR R+ AGLV+ D HQGL+ EL + R P D DLV+ VDGCPFDGRTAALVAHARARVAAGLVEPSDFQPIHQGLSAEELLDHVVDRQPEDPPDLVL | 186  187 |
| Query 187  Sbjct 188 | | THGDACLPNIMVENGRFSGFIDCGRLGVADRYQDIALATRDIAEELGGEWADRFLVLYGI THGD CLPN+++ RF G +DCG +GV DRY+D+ + R + + LG F YGI THGDFCLPNVVLAGDRFVGVLDCGLVGVGDRYRDLGIGARSVVQNLGPGAVGPFFDAYGI | 246  247 |
| Query 247 | | AAPDSQRIAFYRLLDEFF 264 PD R+ + ++DE F |  |
| Sbjct | 248 | EWPDLARVDAFVMIDELF 265 | |

```
>TR:A0A3S8WKF5 A0A3S8WKF5_9ACTN Aminoglycoside O-phosphotransferase APH(3')-Va OS=Streptomyces sp. WAC 01529 OX=2203205 GN=DMA15_01120 PE=3
SV=1
Length=274

Score = 147 bits (370), Expect = 1e-39
Identities = 103/274 (38%), Positives = 136/274 (50%), Gaps = 22/274 (8%)

Query 11	SPAAWVERLFGYDWAQQTIGCSDAAVFRLSAQGR--PVLFVK---TDLSGALNELQDEAA 65 S A	R	Y W	G S A V RL+ G	P L+VK	G+ +L EA
Sbjct 3	SARALRRRYAAYRWTPARNGASGAEVHRLTHPGGAGPELYVKIAPAAYEGSSLDLSGEAD 62
```

| Query | 66 | RLSWLATTGVPCAAVLDVVTEAGRDWLLLGEVPGQDLLSSHLAPAEK VSIMADAMR | 121 |
| --- | --- | --- | --- |
| Sbjct | 63 | RL+WL G+P +++ T+ WL+ +PG+ ++ P ++ V +AD R RLTWLGGRGIPVPRLVERGTDGEATWLVTEALPGRP--AAERWPEDRRFAVVEALADITR | 120 |
| Query | 122 | RLHTLDPATCPFDHQAKHRIERARTRMEAGLVDQDDLDEEHQGLAPAELFARLKARMPDG | 181 |
| Sbjct | 121 | LH L A CPFD +A ++ GLVD DDLDEE +G + L L P ALHELPSADCPFDRSLAVTQAQAVRNVQEGLVDLDDLDEEREGWSGRRLLDELDRTRPAA | 180 |
| Query | 182 | EDLVVTHGDACLPNIMV--ENGRFSGFIDCGRLGVADRYQDIALATRDIAEE LGGE | 235 |
| Sbjct | 181 | EDLVVTHGD N+++ E R +G ID RLG ADR+ D+A+A R++ E G EDLVVTHGDLTSENVLIDPETCRVTGVIDVIRLGRADRHTDLAIALRELESEEDPWFGPV | 240 |
| Query | 236 | WADRFLVLY-----GIAAPDSQRIAFYRLLDEFF 264 A FL Y G D R+AFYRLLDEFF |  |
| Sbjct | 241 | HAAHFLERYLQGAAGKVVVDEDRLAFYRLLDEFF 274 | |

>TR:A0A5C8ZI13 A0A5C8ZI13_9ACTN Aminoglycoside 3'-phosphotransferase OS=Quadrisphaera sp. DD2A OX=2593304 GN=FMM08_05305 PE=3 SV=1

Length=228

Score = 145 bits (366), Expect = 1e-39

| Identities | | = 86/209 (41%), Positives = 125/209 (60%), Gaps = 10/209 (5%) |  |
| --- | --- | --- | --- |
| Query 60  Sbjct 26 | | LQDEAARLSWLATTGVPCAAVLDVVTEAGRDWLLLGEVPGQDLLSSHLAPAEK---VSIM L EAARL+WL+ G+PC V+ + +A L+ VPG+ L + L PA + ++ + LAAEAARLTWLSAQGLPCPRVVSLSDDA LVTTTVPGRPLTA--LPPAAQPRAITAL | 116  79 |
| Query 117  Sbjct 80 | | ADAMRRLHTLDPATCPFDHQAKHRIERARTRMEAGLVDQDDLDEEHQGLAPAELFARLKA A+ +R LH+L A+CPFD + + AR+R GLVD D +G + L A L A AEVLRALHSLPVASCPFDARLRTTTAAARSRAALGLVDLTAPDGARRGWSVPLLLAELAA | 176  139 |
| Query 177  Sbjct 140 | | RMPDGEDLVVTHGDACLPNIMV-ENGRFSGFIDCGRLGVADRYQDIALATRDIAEELGGE  +P E+LVV HGD LPN++ ++G +G +D RLGVADR +D+A+A R + LGG  TLPTQEELVVCHGDPGLPNVLFDDDGEVTGLLDVERLGVADRCRDLAIAARSTRDRLGGT | 235  199 |
| Query | 236 WADRFLVLYGIAAPDSQRIAFYRLLDEFF 264 | | |
|  |  | A++ L YG + D+ + FYRLLDEFF |  |
| Sbjct | 200 | RAEQLLEHYGASDRDALVLPFYRLLDEFF 228 | |

>TR:A0A418KVH4 A0A418KVH4_9ACTN Aminoglycoside 3'-phosphotransferase OS=Jiangella sp. NEAU-YY265 OX=2293569 GN=DY240_05855 PE=3 SV=1

Length=231

Score = 145 bits (366), Expect = 1e-39

| Identities | = 101/247 (41%), Positives = 130/247 (53%), Gaps = 23/247 (9%) |  |
| --- | --- | --- |
| Query 23 | DWAQQTIGCSDAAVFRLSAQGRPVLFVKTDLSGALNELQDEAARLSWLATTGVPCAAVLD | 82 |
| Sbjct 3 | D Q T G S A V R + R A ++L EA RL WL G+P A VL DPEQVTTGRSGATVVRQAGAYRK ESATDDLLGEAGRLMWLRAHGIPAAEVL- | 53 |
| Query 83 | VVTEAGRDWLLLGEVPGQDLLSSHLAPAEK---VSIMADAMRRLHTLDPATCPFDHQAKH | 139 |
| Sbjct 54 | E G L+ EVPG+ PA + V +A+ R LH L A CPFD +  ---ECGPGLLVTAEVPGRPA-DDEWPPAARDRVVDALAELTRALHALPVADCPFDRRLDV | 109 |
| Query 140 | RIERARTRMEAGLVDQDDLDEEHQGLAPAELFARLKARMPDGEDLVVTHGDACLPNIMVE | 199 |
| Sbjct 110 | + A A VD DDLD+E G + L A L A P EDLVV HGD N++++ TVPAAL AADVDLDDLDDERAGWSRDRLVAELLATRPADEDLVVCHGDLTTQNVLID | 165 |
| Query 200 | NGRF--SGFIDCGRLGVADRYQDIALATRDIAEELGGEWADRFLVLYGIAAPDSQRIAFY | 257 |
| Sbjct 166 | SG ID GRLGV+DR+QD+A+ TR++A++LG + A R+L G+ PD + AFY PATLTVSGVIDAGRLGVSDRWQDLAVMTRELADDLGEDAAARYLRGCGV-EPDPAKQAFY | 224 |
| Query 258  Sbjct 225 | RLLDEFF 264 RLLDEFF RLLDEFF 231 |  |

>TR:Q2MG02 Q2MG02_MICOL Kanamycin kinase OS=Micromonospora olivasterospora OX=1880 GN=forP PE=3 SV=1

Length=274

Score = 146 bits (368), Expect = 2e-39

| Identities | = 95/236 (40%), Positives = 126/236 (53%), Gaps = 18/236 (8%) |  |
| --- | --- | --- |
| Query 44 | RPVLFVKTDLSGALNELQ DEAARLSWLATTGVPCAAVLDVVTEAGRDWLLLGEVPG | 99 |
| Sbjct 40 | R +VKT N+L+ EA RL WLA G P A V+DV + WL+ V G RHAFYVKTTPPRRDNDLRFHPGSEAERLQWLAARGFPVAEVVDVGGDDELMWLVTTAVEG | 99 |
| Query 100 | QDLL---SSHLAPAEKVSIMADAMRRLHTLDPATCPFDHQAKHRIERARTRMEAGLVDQD | 156 |
| Sbjct 100 | + ++H P + ++AD +R LH L CPFD + AR E G +D D RSAAGPWATHERPVV-LDVVADVVRALHELPLTDCPFDRTLAVTLPLARLAAEMGWIDLD | 158 |
| Query 157 | DLDEEHQGLAPAELFARLKARMPDG-EDLVVTHGDACLPNIMV--ENGRFSGFIDCGRLG | 213 |
| Sbjct 159 | DLD +H+G + +L L A P EDLVV HGD CL N +V + +G +D GRLG DLDPQHRGWSARQLLDELTATPPPAVEDLVVCHGDPCLDNFLVAPDTLALTGILDVGRLG | 218 |
| Query 214  Sbjct 219 | VADRYQDIALATRDIAEELGGEW------ADRFLVLYGIAAPDSQRIAFYRLLDEF 263 ADR++D+A+A RD+ E+ E A RFL YG A PD+ + FYRLLDEF  AADRWKDLAIAVRDVTEQCADEGDEGQAAAARFLARYG-ARPDAGKDRFYRLLDEF 273 |  |

>TR:A0A563EHQ2 A0A563EHQ2_9PSEU Aminoglycoside 3'-phosphotransferase OS=Lentzea sp. FXJ1.1311 OX=2591470 GN=FKR81_37175 PE=3 SV=1

Length=255

Score = 145 bits (366), Expect = 3e-39

| Identities | = 104/248 (42%), Positives = 132/248 (53%), Gaps = 23/248 (9%) |  |
| --- | --- | --- |
| Query 24 | WAQQTIGCSDAAVFRLSAQGRPVLFVKTDLSGALNELQDEAARLSWLATTGVPCAAVLDV | 83 |
| Sbjct 22 | W T G S A V R R ++D G +L E RL+WL G+P A VL WELVTGGMSGARVSRRDGVYRK ESDDPGI--DLAGEGERLTWLREHGIPAAEVL-- | 73 |
| Query 84 | VTEAGRDWLLLGEVPGQDLLSSHLAPAEKVSI--MADAMRRLHTLDPATCPFDHQAKHRI | 141 |
| Sbjct 74 | E L+ EVPG+ S A + + I +AD R LH L CPFD +  --ECRPGLLVTAEVPGRSAASPWPAESRPLIIDALADLARALHALPVDDCPFDRTLAVTV | 131 |
| Query 142 | ERARTRMEAGLVDQDDLDEEHQGLAPAELFARLKARMPDGEDLVVTHGDACLPNIMVE-- | 199 |
| Sbjct 132 | A +E+ VD DDLD+E G + +L A L A P EDLVV HGD CLPN++ + PAA---LESD-VDLDDLDDERLGWSREDLVAELLATRPPDEDLVVCHGDLCLPNVVFDPT | 187 |
| Query 200 | NGRFSGFIDCGRLGVADRYQDIALATRDIAEELGGEW ADRFLVLYGIAAPDSQRIA | 255 |
| Sbjct 188 | R SG +D G LGVADR+ D+A+ATR +A EL ++ ADRFL YGI APD + THRVSGVLDAGLLGVADRWADLAIATRSVAGELNPQYRPEHADRFLARYGI-APDPVKTT | 246 |
| Query 256  Sbjct 247 | FYRLLDEF 263 FYRLL+EF FYRLLEEF 254 |  |

>TR:A0A0H5SK09 A0A0H5SK09_HERHM APH domain-containing protein OS=Herbinix hemicellulosilytica OX=1564487 GN=HHT355_2249 PE=3 SV=1

Length=173

Score = 142 bits (357), Expect = 6e-39

Identities = 67/152 (44%), Positives = 93/152 (61%), Gaps = 0/152 (0%)

| Query | 113 | VSIMADAMRRLHTLDPATCPFDHQAKHRIERARTRMEAGLVDQDDLDEEHQGLAPAELFA | 172 |
| --- | --- | --- | --- |
| Sbjct | 22 | V ++A+ +R +H++D CPFD + + +++ A R++ GLVD+D+ D QG+ E+ VRLLAEGLRMIHSIDIKECPFDCRLEIKLKEAEYRVKNGLVDEDNFDHIRQGMKDVEVLE | 81 |
| Query | 173 | RLKARMPDGEDLVVTHGDACLPNIMVENGRFSGFIDCGRLGVADRYQDIALATRDIAEEL | 232 |
| Sbjct | 82 | L P EDLV THGD CLPNI + NG+ SGFID GR GVADRYQD+ALA R ELYKLKPADEDLVFTHGDYCLPNIFINNGKLSGFIDWGRGGVADRYQDLALAARSFEHNF | 141 |
| Query  Sbjct | 233  142 | GGEWADRFLVLYGIAAPDSQRIAFYRLLDEFF 264  +W YGI D ++ +Y+LLDE F  DAKWVPLLFKEYGIENIDYSKVEYYKLLDELF 173 |  |

>TR:A0A495X415 A0A495X415_9PSEU Kanamycin kinase/aminoglycoside 3'-phosphotransferase-2 OS=Saccharothrix variisporea OX=543527 GN=DFJ66_2194

PE=3 SV=1

Length=231

Score = 143 bits (361), Expect = 8e-39

| Identities | = 102/247 (41%), Positives = 127/247 (51%), Gaps = 23/247 (9%) |  |
| --- | --- | --- |
| Query 24 | WAQQTIGCSDAAVFRLSAQGRPVLFVKTDLSGALNELQDEAARLSWLATTGVPCAAVLDV | 83 |
| Sbjct 2 | W + TIG S AAV R R ++ KT + +L E RL WL G+P A VL+ WERVTIGQSGAAVSR-----RAGVYRKTS---DVEDLVGEGERLVWLRVQGIPAAEVLEC | 53 |
| Query 84 | VTEAGRDWLLLGEVPGQDLLSSHLAPAEKVSIMADAMRRLHTLDPATCPFDHQAKHRIER | 143 |
| Sbjct 54 | E G L+ EVPG+ P V +AD R LH L A CPFD +  --EPGL--LVTAEVPGRSAAEPWPDPDRIVDSLADFTRALHALPVADCPFDRSLAVTVPE | 109 |
| Query 144 | ARTRMEAGLVDQDDLDEEHQGLAPAELFARLKARMPDGEDLVVTHGDACLPNIMV--ENG | 201 |
| Sbjct 110 | A A D DDLD+E G L A L A P EDLVV HGD +PN++ E G AL AADPDLDDLDDERAGWTRDRLVAELLATRPADEDLVVCHGDLSVPNVLFDPETG | 165 |
| Query 202 | RFSGFIDCGRLGVADRYQDIALATRDIAEE LGGEWADRFLVLYGIAAPDSQRIAFY | 257 |
| Sbjct 166 | +G +D GRLGVADR+ D+A+ATR + +E G + A R+L G PD + AFY DVTGVVDAGRLGVADRWLDLAIATRSLVDERNSQYGPDAAARYLERCG-TEPDPAKTAFY | 224 |
| Query 258  Sbjct 225 | RLLDEFF 264 RLLDEFF RLLDEFF 231 |  |

>TR:A0A1H8FIN2 A0A1H8FIN2_9BACL Kanamycin kinase/aminoglycoside 3'-phosphotransferase-2 OS=Lihuaxuella thermophila OX=1173111 GN=SAMN05444955_108213

PE=3 SV=1

Length=257

Score = 143 bits (361), Expect = 2e-38

| Identities  Query 23 | | = 86/246 (35%), Positives = 139/246 (57%), Gaps = 9/246 (4%)  DWAQQTIGCSDAAVFRLSAQGRPV-LFVKTDLSGALNELQDEAARLSWLATTGVPCAAVL | 81 |
| --- | --- | --- | --- |
| Sbjct 17 | | +W + T+G S V++L+++ + L +K++ L +L+ + L+WL +P V+ NWRKDTVGRSGDEVYQLTSKTQHYYLKIKSEPGTELLKLESQV--LNWLKGK-LPVPEVV | 73 |
| Query 82 | | DVVTEAGRDWLLLGEVPGQDLLSSHLAPAEKVSI---MADAMRRLHTLDPATCPFDHQAK  ++ +++LL+ EVPG L++S +K + +A+ +R +H+L TCPF + A | 138 |
| Sbjct | 74 | CYHNDSFKEYLLITEVPG--LMASDPGWQDKKKVVRSLAEGLRWIHSLPIETCPFSYPAG | 131 |
| Query | 139 | HRIERARTRMEAGLVDQDDLDEEHQGLAPAELFARLKARMPDGEDLVVTHGDACLPNIMV | 198 |
| Sbjct | 132 | ++ A R+ G+VD+DD D G AEL+ L P D V THGD CLPN+++ LLVQAAGERVRLGMVDEDDFDPCRMGKRAAELYEELVRSEPVYNDEVFTHGDYCLPNVIL | 191 |
| Query | 199 | ENGRFSGFIDCGRLGVADRYQDIALATRDIAEELGGEWADRFLVLYGIAAPDSQRIAFYR | 258 |
| Sbjct | 192 | SGFID R G ADRY+D+ +A R IA+ G + F Y + A D ++I F++ SPSGVSGFIDWARGGAADRYRDLGIAARSIADNFGDTYIPYFFEAYELKAIDEEKIKFFQ | 251 |
| Query  Sbjct | 259  252 | LLDEFF 264 LLDEFF LLDEFF 257 |  |

>TR:A0A561BZQ8 A0A561BZQ8_9ACTN Streptomycin 3'-kinase OS=Kribbella amoyensis OX=996641 GN=FB561_5537 PE=3 SV=1

Length=259

Score = 143 bits (361), Expect = 2e-38

Identities = 104/249 (42%), Positives = 139/249 (56%), Gaps = 18/249 (7%)

| Query | 23 | DWAQQTIGCSDAAVFRLSAQGRPVLFVK-TDLSGALNELQDEAARLSWLATTGVPCAAVL | 81 |
| --- | --- | --- | --- |
| Sbjct | 14 | +W IG SDA V+R A G + K DL+G + EL+DE R+ WLA TG+P A V EWEPVEIGESDADVYR-RADGAE--YAKCADLAG-VGELRDERDRIVWLAGTGLPGATVA | 69 |
| Query | 82 | D-VVTEAGRDWLLLGEVPG---QDLLSSHLAPAEKVSIMADAMRRLHTLDPATCPFDHQA | 137 |
| Sbjct | 70 | D + T+AG L+ VPG DL +S A + +A +R LH LDPA CPF DWIETDAGA-CLVTTAVPGVAAADLPASAYDRATEQ--LAGVLRALHDLDPADCPFTRPL | 126 |
| Query | 138 | KHRIERARTRMEAGLVDQDDLDEEHQGLAPAELFARLKA---RMPDGEDLVVTHGDACLP | 194 |

+ +A	+ G V+ D L++E +	P +L AR++A	RM + DLVV HGDACLP

| Sbjct | 127 | TDVVRQAEDVVRRGAVNPDFLNDEWRAERPEDLLARIQADLPRMRESADLVVCHGDACLP | 186 |
| --- | --- | --- | --- |
| Query | 195 | NIMVENGRF--SGFIDCGRLGVADRYQDIALATRDIAEELGGEWADRFLVLYGIAAPDSQ | 252 |
| Sbjct | 187 | N+ V+ +G ID GRLG ADRY D+AL T + +E + A FL YG D NLFVDPETLAPTGLIDLGRLGTADRYTDLALVTTQLRDEWNADPAP-FLKAYGHPDADPA | 245 |
| Query  Sbjct | 253  246 | RIAFYRLLD 261  ++AFY LLD  KLAFYLLLD 254 |  |

```
>TR:A0A4R4KXX2 A0A4R4KXX2_9ACTN Aminoglycoside 3'-phosphotransferase OS=Micromonospora sp. KC723 OX=2530381 GN=E1165_15965 PE=3 SV=1
Length=235

Score = 142 bits (359), Expect = 2e-38
Identities = 104/249 (42%), Positives = 130/249 (52%), Gaps = 25/249 (10%)

Query 24	WAQQTIGCSDAAVFRLSAQGRPVLFVKTDLSGALNELQDEAARLSWLATTGVPCAAVLDV 83 W + T G S A V R +	R	+ +	+L E ARL WL	GVP	VLD
Sbjct 3	WERVTAGRSGARVSRRNGVYRK------ESTDPRIDLAGEGARLEWLRRQGVPAPEVLDC 56

Query 84	VTEAGRDWLLL-GEVPGQDLLSSHLAP--AEKVSIMADAMRRLHTLDPATCPFDHQAKHR 140 R LL+ EVPG+	A V +A+ R LH L A CPFD +
Sbjct 57	-----RPGLLVTAEVPGRSAADPWPVESRARVVDALAELTRALHALPIADCPFDRRLAVT 111

Query 141 IERARTRMEAGLVDQDDLDEEHQGLAPAELFARLKARMPDGEDLVVTHGDACLPNIMV-- 198
+ A	M A VD	EHQG + AEL A L A P EDLVV HGD C+PN+++
Sbjct 112 VPEA---MSAD-VDLAGPSAEHQGRSRAELVAELLATRPRHEDLVVCHGDLCVPNVLLDP 167

Query 199 ENGRFSGFIDCGRLGVADRYQDIALATRDIAEELG---GEW-ADRFLVLYGIAAPDSQRI 254
+ + SG ID GRLGVADR+ D+A+ATR + +L	G W ADR+L YG+ PD +I Sbjct 168 DTCKVSGVIDAGRLGVADRWVDLAIATRSLTIDLNPQYGPWAADRYLARYGV-RPDQSKI 226

Query 255 AFYRLLDEF 263
FYRLLDEF Sbjct 227 RFYRLLDEF 235

>TR:D2PKR4 D2PKR4_KRIFD Antimicrobial 3''-kinase OS=Kribbella flavida (strain DSM 17836 / JCM 10339 / NBRC 14399) OX=479435 GN=Kfla_3320
PE=3 SV=1
Length=254

Score = 142 bits (359), Expect = 3e-38
```

| Identities | | = 95/251 (38%), Positives = 134/251 (53%), Gaps = 24/251 (10%) |  |
| --- | --- | --- | --- |
| Query 24  Sbjct 10 | | WAQQTIGCSDAAVFRLSAQGRPVLFVKTDLSGALNELQDEAARLSWLATTGVPCAAVLDV W G SD VFR +G ++ K + EL+ E R+ WL+ TG+P A V D WEPAGSGESDTEVFR---RGDGAVYAKCAALRGIEELRAERDRVVWLSGTGIPGAEVADW | 83  66 |
| Query 84  Sbjct 67 | | VTEAGRDWLLLGEVPGQDLLSSHLAPAEK---VSIMADAMRRLHTLDPATCPFDHQAKHR  + LL VPG + + L P+ + V ++A+A+R LH L A CPF IETDDGACLLTTAVPG--IAAVDLPPSSRLKAVEVLAEALRGLHEL--ADCPFTRPLAEV | 140  122 |
| Query 141  Sbjct 123 | | IERARTRMEAGLVDQDDLDEEHQGLAPAELFARLKARMPDGE DLVVTHGDACLPN  + +A + G V+ D L +E + P +L A+++A P + DLVV HGD CLPN VAQATDVVRRGAVNPDFLTDEWRRERPEDLLAQIQADRPYADAQAAHDLVVCHGDPCLPN | 195  182 |
| Query 196  Sbjct 183 | | IMVENG--RFSGFIDCGRLGVADRYQDIALATRDIAEELGGEW---ADRFLVLYGIAAPD  ++++ G +GFID GRLG+ADRY D+AL T +LG EW F YG+ APD VLLDPGTLEITGFIDVGRLGLADRYADLALTT AQLGDEWDLDPASFHAAYGLDAPD | 250  238 |
| Query 251 | | SQRIAFYRLLD 261  +R+ FYRLLD |  |
| Sbjct | 239 | QRRLVFYRLLD 249 | |

>TR:A0A2Z3JG07 A0A2Z3JG07_9DEIO Aminoglycoside 3'-phosphotransferase OS=Deinococcus irradiatisoli OX=2202254 GN=DKM44_13465 PE=3 SV=1

Length=282

Score = 143 bits (361), Expect = 3e-38

| Identities | | = 82/213 (38%), Positives = 115/213 (54%), Gaps = 4/213 (2%) |  |
| --- | --- | --- | --- |
| Query 54  Sbjct 72 | | SGALNELQDEAARLSWLATTGVPCAAVLDVVTEAGRDWLLLGEVPGQDLL--SSHLAPAE  +G L L+ E R WLA +P A V+ ++A R++L +PG + + L AGQLESLRAEQLRWRWLAGR-LPAARVVGYASDAHREYLATTRLPGLPMHHPDALLHARR | 111  130 |
| Query 112  Sbjct 131 | | KVSIMADAMRRLHTLDPATCPFDHQAKHRIERARTRMEAGLVDQDDLDEEHQGLAPAELF  ++A A+R LH L CPF R+ +AR R+ AG VD + E + L P +L NADLLARALRELHALPIRDCPFSMSLAVRLRQARERVLAGAVDPQEFSEARRHLTPEQLL | 171  190 |
| Query 172  Sbjct 191 | | ARLKARMPDGEDLVVTHGDACLPNIMVENGRFSGFIDCGRLGVADRYQDIALATRDIAEE L R EDL VTHGDACLPN++V GF+D GRLG+ADR+ D+ALA R +  GWL-VRNRQNEDLAVTHGDACLPNVLVSGEYVEGFVDVGRLGLADRHTDLALACRSLRRN | 231  249 |
| Query 232 | | LGGEWADRFLVLYGIAAPDSQRIAFYRLLDEFF 264 G E A++FL YG D ++ +Y+ LDE F |  |
| Sbjct | 250 | CGDEMAEQFLDTYGRERVDPAKLEYYQTLDELF 282 | |

>TR:F5LER1 F5LER1_9BACL Kanamycin kinase OS=Paenibacillus sp. HGF7 OX=944559 GN=butA PE=3 SV=1

Length=262

Score = 142 bits (358), Expect = 5e-38

| Identities | = 91/259 (35%), Positives = 133/259 (51%), Gaps = 13/259 (5%) |  |
| --- | --- | --- |
| Query 15 | WVERLFGY DWAQQTIGCSDAAVFRLSAQGRPVLFVKTDLSGALNELQDEAARLSWL | 70 |
| Sbjct 8 | W E L G + IG S V+ + F+K S L+ E L+WL WPEELLGLLGQTELTVNKIGYSGDHVYHVKEYRGAPAFLKIAPSVWWRTLRPEIEALAWL | 67 |
| Query 71 | ATTGVPCAAVLDVVTEAGRDWLLLGEVPGQDLLSSH LAPAEKVSIMADAMRRLHTL | 126 |
| Sbjct 68 | +P +L G D+LL+ + G+D SH P V + A+++RR+H+L DGK-LPVPKILYTAEHGGMDYLLMEALAGKD--GSHETIQAKPEFFVKLYAESLRRIHSL | 124 |
| Query 127 | DPATCPFDHQAKHRIERARTRMEAGLVDQDDLDEEHQGLAPAELFARLKARMPDGEDLVV | 186 |
| Sbjct 125 | D CP + + ++ A+ ++ LVD D+ EE+ P EL+ L A P EDLV DIRECPLSNGLEKKLRDAKRIVDESLVDPTDIKEEYD-CTPEELYRLLLASKPVTEDLVF | 183 |
| Query 187 | THGDACLPNIMVENGRFSGFIDCGRLGVADRYQDIALATRDIAEELGGE-WADRFLVLYG | 245 |
| Sbjct 184 | HGD C PN++V+ + +GFID GR GVADRYQDI+LA R + + G + + FL LYG AHGDYCAPNLIVDGEKLTGFIDLGRAGVADRYQDISLAIRSLRHDYGDDRYKALFLELYG | 243 |
| Query 246  Sbjct 244 | IAAPDSQRIAFYRLLDEFF 264  + D ++ +Y LDEFF  LDGLDEDKVKYYIRLDEFF 262 |  |

>TR:A0A221VYY1 A0A221VYY1_9PSEU Aminoglycoside 3'-phosphotransferase OS=Actinoalloteichus hoggarensis OX=1470176 GN=neo PE=3 SV=1

Length=272

Score = 141 bits (356), Expect = 1e-37

| Identities | = 101/259 (39%), Positives = 134/259 (52%), Gaps = 23/259 (9%) |  |
| --- | --- | --- |
| Query 23 | DWAQQTIGCSDAAVFRLSAQGRPVLFVKT DLSGALNELQDEAARLSWLATTGVPCA | 78 |
| Sbjct 20 | +W + VFR+ +GR +VKT D + EA RL WL G P EWVRVADRSYGTEVFRV--EGRHTYYVKTTPPRDPDDPRFHPEGEARRLDWLRGRGFPVP | 77 |
| Query 79 | AVLDVVTEAGRDWLLLGEVPGQDLLSSHLAPAEKVSIM ADAMRRLHTLDPATCPFD | 134 |
| Sbjct 78 | V+DV + WL+ VPG L ++ PA + S + A+ + LH+ CPFD EVVDVGADDSSAWLVTTAVPG--LPAAARWPARQRSRVLAGVAELVGELHSSPVEDCPFD | 135 |
| Query 135 | HQAKHRIERARTRMEAGLVDQDDLDEEHQGLAPAELFARLKARMPDGE-DLVVTHGDACL | 193 |
| Sbjct 136 | +H + A + LVD DLDE H G A L A L++ P E DLVV HGD CL GGLRHTLAWAERATRSELVDVADLDEGHTGWTAARLLAELRSAAPPPEDDLVVCHGDPCL | 195 |
| Query 194 | PNIMVENG--RFSGFIDCGRLGVADRYQDIALATRDIAEELGGEWAD RFLVLYG | 245 |
| Sbjct 196 | N++V+ R +G ID GRLG ADR++D+A+ R++ E G WAD RFL +YG  DNVLVDPATLRPTGLIDVGRLGTADRWRDLAVLVRNLQGENDG-WADGADHASRFLAVYG | 254 |

Query 246 IAAPDSQRIAFYRLLDEFF 264

A D R A+YRLLDEFF Sbjct 255 -ADHDEYRAAYYRLLDEFF 272

>TR:A0A0N0KCZ2 A0A0N0KCZ2_9SPHN Aminoglycoside 3'-phosphotransferase OS=Novosphingobium sp. AAP93 OX=1523427 GN=IP83_00800 PE=3 SV=1

Length=280

Score = 141 bits (355), Expect = 2e-37

| Identities | | = 92/246 (37%), Positives = 125/246 (51%), Gaps = 7/246 (3%) |  |
| --- | --- | --- | --- |
| Query 24  Sbjct 37 | | WAQQTIGCSDAAVFRL-SAQGRPVLFVKTDLSGALNELQDEAARLSWLATTGVPCAAVLD W ++G S AAV+R+ +G P ++K +++EAARL WLA V A ++ WTLSSVGQSGAAVWRVRDPRGGPDHYLKHGAGDVAAAVREEAARLRWLAGR-VAVAELIA | 82  95 |
| Query 83 | | VVTEAGRDWLLLGEVPGQD LLSSHLAPAEKVSIMADAMRRLHTLDPATCPFDHQAK  EA WLL +PG+ L + + V +A + H L P CPFD A | 138 |
| Sbjct | 96 | FAEEADSAWLLTAALPGRTAWEVLDAGDVDAGAVVDAIAACLHEWHALAPQDCPFDASAT | 155 |
| Query | 139 | HRIERARTRMEAGLVDQDDLDEEHQGLAPAELFARLKARMPDGEDLVVTHGDACLPNIMV | 198 |
| Sbjct | 156 | R+ AR R++AGLVD DD D E G + +++ L A VVTHGD L NI++ QRLSAARARIDAGLVDVDDFDPERAGWSADQVWDALAALPLPSAPPVVTHGDFSLDNILI | 215 |
| Query | 199 | ENGRFSGFIDCGRLGVADRYQDIALATRDIAEELGGEWADRFLVLYGIAAPDSQRIAFYR | 258 |
| Sbjct | 216 | G +G ID GR G+ADRYQDIA+ + E G R L G + D RI F+ AEGVVTGCIDVGRAGLADRYQDIAILWNCLG-EFGPHLQARMLAALGEGSGDRARIDFHL | 274 |
| Query  Sbjct | 259  275 | LLDEFF 264 LLDE F LLDELF 280 |  |

>TR:A0A2S6GIH1 A0A2S6GIH1_9PSEU Kanamycin kinase OS=Actinokineospora auranticolor OX=155976 GN=CLV40_11650 PE=3 SV=1

Length=269

Score = 140 bits (354), Expect = 2e-37

| Identities | | = 93/252 (37%), Positives = 129/252 (51%), Gaps = 17/252 (7%) |  |
| --- | --- | --- | --- |
| Query 24  Sbjct 22 | | WAQQTIGCSDAAVFRLSAQGRPVLFVKTDLSGALNELQ DEAARLSWLATTGVPCAA  W V R+ P L+VK S + + EAA L WL G P A WEHVATRSYGTTVHRVVPDSGPALYVKATTSADETDPRFHPVAEAAALRWLRERGFPVAR | 79  81 |
| Query 80  Sbjct 82 | | VLDVVTEAGRDWLLLGEVPGQDLLSSHLAPAEKVSIMADAMR RLHTLDPATCPFDH  V++V + WL+ E+PG + +S A++V + DA+ LH L A CPFD VVEVGRDDEWSWLVTEELPG--VPASGPWRADQVDAVLDAVAGVAAALHRLPVARCPFDR | 135  139 |
| Query 136  Sbjct 140 | | QAKHRIERARTRMEAGLVDQDDLDEEHQGLAPAELFARLKARMPDGEDLVVTHGDACLPN  + + R +E G VD DDLDE H+G A+L A L A EDLVV HGD L N RLAVTLPACRRAVELGTVDLDDLDERHEGWTGAQLLAELAATPVPPEDLVVCHGDLGLDN | 195  199 |
| Query 196  Sbjct 200 | | IMVENGRF--SGFIDCGRLGVADRYQDIALATRDIAEELGGEWAD--RFLVLYGIAAPDS  ++++ +G +D GRLGVADR+ D+++ RD+AE W D F YG++ DS VLLDPDTLAPTGLLDAGRLGVADRWTDLSILLRDLAE---AGWCDPAGFTARYGLSDVDS | 251  256 |
| Query 252 | | QRIAFYRLLDEF 263  + +FYRL+DEF |  |
| Sbjct | 257 | AKDSFYRLMDEF 268 | |

```
>TR:A0A164NZ73 A0A164NZ73_9NOCA Aminoglycoside phosphotransferase OS=Nocardia terpenica OX=455432 GN=AWN90_23115 PE=3 SV=1
Length=244

Score = 140 bits (352), Expect = 2e-37
Identities = 81/202 (40%), Positives = 111/202 (55%), Gaps = 10/202 (5%)

Query 63	EAARLSWLATTGVPCAAVLDVVTEAGRDWLLLGEVPGQDLLSSHLAPAEKVSIMADAMRR 122 E RL+WLA G+P	V+	RD L+L +V	L S A	+ +	+RR
Sbjct 53	ERPRLNWLAGKGIPVPPVVAFE----RDVLILADVGAPSL--SAAADESVGATLGTVLRR 106
```

| Query | 123 | LHTLDPATCPFDHQAKHRIERARTRMEAGLVDQDDLDEEHQGLAPAELFARLKARMPDGE | 182 |
| --- | --- | --- | --- |
| Sbjct | 107 | LH + A CPFD + +ERAR +E GLVD +D+D +H GL P ++ RL P+ LHAIPIAECPFDGRLDVVLERARHLVEQGLVDPEDMDNDHYGLTPEQVLDRLYVERPEET | 166 |
| Query | 183 | DLVVTHGDACLPNIMVENGRFSGFIDCGRLGVADRYQDIALATRDIAEELGGEWADRFLV | 242 |
| Sbjct | 167 | DLVV HGD N++ R +D G LG+ADRY+D+ALA RD+ + G + F DLVVAHGDYTSGNVL RGGIVLDVGALGIADRYRDLALARRDLLDHHGLGQVEEFFA | 222 |
| Query | 243 | LYGIAAPDSQRIAFYRLLDEFF 264 YG+ PD R+ +YRLLDE F |  |
| Sbjct | 223 | AYGLVDPDPVRLDYYRLLDELF 244 | |

>SP:P13250 KKA9_STRRI Aminoglycoside 3'-phosphotransferase OS=Streptomyces ribosidificus OX=80859 GN=rph PE=3 SV=1

Length=263

Score = 140 bits (352), Expect = 4e-37

| Identities | | = 99/257 (39%), Positives = 137/257 (53%), Gaps = 19/257 (7%) |  |
| --- | --- | --- | --- |
| Query 22  Sbjct 12 | | YDWAQQTIGCSDAAVFRLSAQGRPVLFVK TDLSGALNELQDEAARLSWLATTGVPC  + W G S A V+RL+ G P L+ K T + A + L EA RL WLA G+ HTWHLVNEGDSGAFVYRLTGHG-PELYAKIAPRTPENSAFH-LDGEADRLDWLARHGISV | 77  69 |
| Query 78  Sbjct 70 | | AAVLDVVTEAGRDWLLLGEVPGQDLLSSHLAPAEKVSIMADAM RRLHTLDPATCPF  V++ + WL+ VPG +S P ++ + + DA+ R LH L + CPF PRVVERGADDTTAWLVTEAVPGA--AASEEWPEDERAAVVDAIAEMARTLHELPVSECPF | 133  127 |
| Query 134  Sbjct 128 | | DHQAKHRIERARTRMEAGLVDQDDLDEEHQGLAPAELFARLKARMPDGEDLVVTHGDACL D + E AR + GLVD DDL EE G +L A L P+ EDLVV HGD C DRRLDVTGE-ARHNVREGLVDLDDLQEEPAGWTGDQLLAELDLTRPEKEDLVVCHGDLCP | 193  186 |
| Query 194  Sbjct 187 | | PNIMV--ENGRFSGFIDCGRLGVADRYQDIALATRDIAEE LGGEWADRFLVLYGIA  N+++ E R +G ID GRL +A + D+ALA R++A + G +A+RFL YG  NNVLLDPETHRITGLIDVGRLRLATCHADLALAARELAIDEDPWFGPAYAERFLERYGAH | 247  246 |
| Query | 248 APDSQRIAFYRLLDEFF 264 | | |
|  |  | D +++AFY+LLDEFF |  |
| Sbjct | 247 | HVDQEKMAFYQLLDEFF 263 | |

>TR:W1SCY9 W1SCY9_9BACI Aminoglycoside phosphotransferase (Fragment) OS=Bacillus vireti LMG 21834 OX=1131730 GN=BAVI_25324 PE=4 SV=1

Length=64

Score = 133 bits (334), Expect = 7e-37

Identities = 64/64 (100%), Positives = 64/64 (100%), Gaps = 0/64 (0%)

| Query | 1 | MIEQDGLHAGSPAAWVERLFGYDWAQQTIGCSDAAVFRLSAQGRPVLFVKTDLSGALNEL 60 |
| --- | --- | --- |
| Sbjct | 1 | MIEQDGLHAGSPAAWVERLFGYDWAQQTIGCSDAAVFRLSAQGRPVLFVKTDLSGALNEL MIEQDGLHAGSPAAWVERLFGYDWAQQTIGCSDAAVFRLSAQGRPVLFVKTDLSGALNEL 60 |
| Query | 61 | QDEA 64 |
| Sbjct | 61 | QDEA  QDEA 64 |

>TR:A0A366DPY4 A0A366DPY4_9RHIZ Aminoglycoside 3'-phosphotransferase OS=Pseudochrobactrum asaccharolyticum OX=354351 GN=DFR47_10735 PE=3

SV=1

Length=270

Score = 139 bits (351), Expect = 7e-37

Identities = 82/244 (34%), Positives = 128/244 (52%), Gaps = 7/244 (3%)

| Query | 26 | QQTIGCSDAAVFRL-SAQGRPVLFVKTDLSGALNELQDEAARLSWLATTGVPCAAVLDVV | 84 |
| --- | --- | --- | --- |
| Sbjct | 29 | + +G S V+RL G+P L++K ++ DE RL+WL + V+ RNVVGMSGGTVYRLCDTAGKPDLYLKQGGGSVATDITDEMVRLNWLEQY-ISVPQVIAFT | 87 |
| Query | 85 | TEAGRDWLLLGEVPG QDLLSSHLAPAEKVSIMADAMRRLHTLDPATCPFDHQAKHR | 140 |
| Sbjct | 88 | ++ + WLL+ + G Q L A + V +A +R++HT+ +CPF+ R  AQSDQAWLLMSALTGRTAYQRLTEEPEARPQIVDALAGFLRQIHTIPAQSCPFNSDHHLR | 147 |

| Query | 141 | IERARTRMEAGLVDQDDLDEEHQGLAPAELFARLKARMPDGEDLVVTHGDACLPNIMVEN | 200 |
| --- | --- | --- | --- |
| Sbjct | 148 | + RA R++AGLVD DD ++G +++ + A +P VVTHGD L NI+++ LARAHQRLQAGLVDTDDFGTLYEGQTAQQVWDDITALLPLETKTVVTHGDFSLDNILMDG | 207 |
| Query | 201 | GRFSGFIDCGRLGVADRYQDIALATRDIAEELGGEWADRFLVLYGIAAPDSQRIAFYRLL | 260 |
| Sbjct | 208 | +G ID GR+G+AD YQD+A+ D E DR YGI APD +++ F+ L NTVTGCIDTGRVGLADPYQDLAILW-DCLGEFSPALQDRLFPAYGITAPDQKKLRFHLGL | 266 |
| Query  Sbjct | 261  267 | DEFF 264 DEFF  DEFF 270 |  |

>TR:A0A2A2SDY7 A0A2A2SDY7_9SPHN Aminoglycoside 3'-phosphotransferase OS=Sphingomonas lenta OX=1141887 GN=CKY28_13260 PE=3 SV=1

Length=251

Score = 139 bits (349), Expect = 8e-37

| Identities | = 90/248 (36%), Positives = 127/248 (51%), Gaps = 7/248 (3%) |  |
| --- | --- | --- |
| Query 21 | GYDWAQQTIGCSDAAVFRLSAQGRPVLFVKTDLSGALNELQDEAARLSWLATTGVPCAAV | 80 |
| Sbjct 7 | G W + G S V RL+ GR L++K + +E ARL WLA +P V GRRWTRDLDGESGGDVHRLNEPGRAPLYLKHGTGDVAVAITEEMARLRWLAGR-IPVPEV | 65 |
| Query 81 | LDVVTEAGRDWLLLGEVPG QDLLSSHLAPAEKVSIMADAMRRLHTLDPATCPFDHQ | 136 |
| Sbjct 66 | + V WLL +PG Q L++ E V+ +A +R +H L CPF+ Q VRFVCTPEEAWLLSTAMPGLTAYQRLVARPERRRETVAALAAFLRSIHELPVDECPFNAQ | 125 |
| Query 137 | AKHRIERARTRMEAGLVDQDDLDEEHQGLAPAELFARLKARMPDGEDLVVTHGDACLPNI | 196 |
| Sbjct 126 | A R+ AR R+E G D D E P ++ L A +P + VVTHGD + N+ APLRMAGARRRIETGWTPPPD-DPERDAWNPEPVWRELTALLPLSFERVVTHGDFSVGNV | 184 |
| Query 197 | MVENGRFSGFIDCGRLGVADRYQDIALATRDIAEELGGEWADRFLVLYGIAAPDSQRIAF | 256 |
| Sbjct 185 | ++E+GR +G ID GR+GVAD YQD+A+ ++ E G + YGIA PD +R+ F LIEDGRVTGCIDVGRVGVADPYQDLAILWDNLG-EFGEDLQRELFRAYGIAEPDQRRLRF | 243 |
| Query 257  Sbjct 244 | YRLLDEFF 264  + LLDE F  HLLLDELF 251 |  |

>TR:A0A4R4X9J2 A0A4R4X9J2_9ACTN Aminoglycoside 3'-phosphotransferase OS=Kribbella sp. 16K104 OX=2530375 GN=E1218_11150 PE=3 SV=1

Length=246

Score = 138 bits (348), Expect = 1e-36

| Identities | = 93/249 (37%), Positives = 129/249 (52%), Gaps = 18/249 (7%) |  |
| --- | --- | --- |
| Query 21 | GYDWAQQTIGCSDAAVFRLSAQGRPVLFVKTDLSGALNELQDEAARLSWLATTGVPCAAV | 80 |
| Sbjct 3 | G WA +G SD +V+R R +F K + + +L+ E R WLA TG+P A V GDGWAAVAVGDSDTSVYR RDGVFAKCCSTDGVRDLRGERDRAEWLAGTGLPAATV | 57 |
| Query 81 | LDVVTEAGRDWLLLGEVPGQDLLSSHLAPAEKVSIMAD---AMRRLHTLDPATCPFDHQA | 137 |
| Sbjct 58 | +D + + L+ VPG + + L PA M +R LH+L CPFD VDWLESSDGACLITSAVPG--VAGTDLPPAVHTRAMQSLGRTLRDLHSL--PDCPFDRPL | 113 |
| Query 138 | KHRIERARTRMEAGLVDQDDLDEEHQGLAPAELFARLKARMP---DGEDLVVTHGDACLP | 194 |
| Sbjct 114 | I A + G V+ L +E + L PAEL A++ A P G +L V HGDACLP ASVIGTAEDVVRRGAVNPQFLTDEWRLLEPAELLAQVVAERPYVETGAELAVCHGDACLP | 173 |
| Query 195 | NIMV--ENGRFSGFIDCGRLGVADRYQDIALATRDIAEELGGEWADRFLVLYGIAAPDSQ | 252 |
| Sbjct 174 | N+ E+ +G ID GRLG+AD Y D+AL T + +E + + FL YG+ PD + NVFFDPESLAVTGLIDVGRLGIADPYSDLALITIQLHDEWSAD-PEPFLRAYGVPEPDRR | 232 |
| Query 253  Sbjct 233 | RIAFYRLLD 261 R+ FYRLLD RLEFYRLLD 241 |  |

>TR:A0A2W2ERM5 A0A2W2ERM5_9ACTN Aminoglycoside 3'-phosphotransferase OS=Desertiactinospora gelatinilytica OX=2666298 GN=C1I98_33685 PE=3

SV=1

Length=256

Score = 138 bits (347), Expect = 2e-36

| Identities | | = 91/241 (38%), Positives = 126/241 (52%), Gaps = 14/241 (6%) |  |
| --- | --- | --- | --- |
| Query 24  Sbjct 30 | | WAQQTIGCSDAAVFRLSAQGRPVLFVKTDLSGALNELQDEAARLSWLATTGVPCAAVLDV W+ Q G S + A G V +VK G + + E RL WL AAV + WSDQHEGMSGQVMRVTGAAG--VFYVK---QGPIAVAEHE--RLRWLKRW AAVPGI | 83  78 |
| Query 84  Sbjct 79 | | VTEAGRDWLLLGEVPGQDLLSSHLAPAEKVSIMADAMRRLHTLDPATCPFDHQAKHRIER V G+ L+L +V L ++ AP E +++ +R LH ++ CPFD + R+ R VAFDGQS-LVLADVGAPSLETA--APPEIGTVLGRVLRGLHAIEVGECPFDERLDVRLAR | 143  135 |
| Query 144  Sbjct 136 | | ARTRMEAGLVDQDDLDEEHQGLAPAELFARLKARMPDGEDLVVTHGDACLPNIMVENGRF A TR+ GLVD DD DE+H G +P E+ RL A P ED VV HGD N++V AGTRVRGGLVDADDFDEDHAGCSPQEVLDRLIAERPAEEDPVVAHGDYTPGNVLVPPVGD | 203  195 |
| Query 204 | | SGFIDCGRLGVADRYQDIALATRDIAEELGGEWADRFLVLYGIAAPDSQRIAFYRLLDEF ID G LGVADRY D+A+A RD+ + G D F YG+ D +++ ++RLLDE | 263 |
| Sbjct | 196 | PVLIDVGGLGVADRYLDLAIAVRDLRADFGPPAVDDFFAAYGLDVLDERKLGYFRLLDEM | 255 |
| Query  Sbjct | 264  256 | F 264 F  F 256 |  |

>TR:A0A1I5S7I3 A0A1I5S7I3_9SPHN Aminoglycoside 3'-phosphotransferase OS=Sphingomonas rubra OX=634430 GN=SAMN04488241_10522 PE=3 SV=1

Length=186

Score = 135 bits (340), Expect = 3e-36

| Identities | = 76/185 (41%), Positives = 103/185 (56%), Gaps = 7/185 (4%) |  |
| --- | --- | --- |
| Query 86 | EAGRDWLLLGEVPGQDLL SSHLAPAEKVSIMADAMRRLHTLDPATCPFDHQAKH | 139 |
| Sbjct 3 | +A WLL+ + GQ +S +A E V +A + LH L A CP D A DADGAWLLMSALAGQTAYQCLADATSAVARGEVVDALARDLSALHALPAAECPLDSGAAV | 62 |
| Query 140 | RIERARTRMEAGLVDQDDLDEEHQGLAPAELFARLKARMPDGEDLVVTHGDACLPNIMVE | 199 |
| Sbjct 63 | R+ +AR R++AGLVD+DD E G +++ L P +D VVTHGD L NI++ RLVQARARIDAGLVDEDDFGEARAGWDAEQVWDELTRLRPRDDDRVVTHGDYSLDNILMA | 122 |
| Query 200 | NGRFSGFIDCGRLGVADRYQDIALATRDIAEELGGEWADRFLVLYGIAAPDSQRIAFYRL | 259 |
| Sbjct 123 | NGR G ID GR+G+ADRYQD+A+ D +E + DR YGIA PD+ R+ F+ NGRVVGLIDLGRVGMADRYQDLAILA-DCLDEFDDDLRDRLFATYGIAEPDTDRLDFHLC | 181 |
| Query 260  Sbjct 182 | LDEFF 264 LDE F LDEMF 186 |  |

>TR:A0A1M4M9S6 A0A1M4M9S6_9FIRM Aminoglycoside 3'-phosphotransferase OS=Proteiniborus sp. DW1 OX=1889883 GN=aphA PE=3 SV=1

Length=268

Score = 137 bits (344), Expect = 7e-36

| Identities | = 90/262 (34%), Positives = 129/262 (49%), Gaps = 6/262 (2%) |  |
| --- | --- | --- |
| Query 5 | DGLHAGSPAAWVERLFGYDWAQQTIGCSDAAVFRLSAQGRPVLFVKTDLSGALNELQDEA | 64 |
| Sbjct 11 | + L+ P E L G + IGCS+A V++ ++K S EL+ E NNLNTSYPKDLNELLAGMSCEENNIGCSNAGVYKYY-NDNVSYYLKIQPSS--QELEKEY | 67 |
| Query 65 | ARLSWLATTGVPCAAVLDVVTEAGRDWLLLGEVPGQDLLSSHLA--PAEKVSIMADAMRR | 122 |
| Sbjct 68 | + WL +P L + G D+LL+ EV G+ L S +L P E V ++AD ++ KIIDWLRDK-LPVPKALYFNSYNGLDYLLMTEVDGEMLCSDNLLSKPEETVKLLADGIKM | 126 |
| Query 123 | LHTLDPATCPFDHQAKHRIERARTRMEAGLVDQDDLDEEHQGLAPAELFARLKARMPDGE | 182 |
| Sbjct 127 | L ++ CPFD+ + +++ A +E VD D +E + P EL LK P LSSIPIDDCPFDNSLEIKLKDALYNIENNFVDMSDWEENNNFRTPEELLNYLKNNKPKAH | 186 |
| Query 183 | DLVVTHGDACLPNIMVENGRFSGFIDCGRLGVADRYQDIALATRDIAEELGGEWADRFLV V THGD CLPNI ++ + SGFID GR G+AD YQDIAL R + G + L | 242 |

| Sbjct | 187 | TPVFTHGDYCLPNIFGKDNKISGFIDLGRAGIADIYQDIALCVRSLQHNFGTDEYTELLF 246 |
| --- | --- | --- |
| Query | 243 | LYGIAAPDSQRIAFYRLLDEFF 264 |
| Sbjct | 247 | + D +RI +Y LLDE F  KHLDMEADWERINYYILLDELF 268 |

>SP:P00553 KKA4_BACCI Aminoglycoside 3'-phosphotransferase OS=Bacillus circulans OX=1397 GN=aphA4 PE=1 SV=1

Length=262

Score = 135 bits (339), Expect = 3e-35

| Identities | | = 86/256 (34%), Positives = 128/256 (50%), Gaps = 5/256 (2%) |  |
| --- | --- | --- | --- |
| Query 12  Sbjct 9 | | PAAWVERLFGYDWAQQTIGCSDAAVFRLSAQGRPVLFVKTDLSGALNELQDEAARLSWLA P +E L + IG S V+ + F+K S L+ E L+WL PEELLELLGQTELTVNKIGYSGDHVYHVKEYRGTPAFLKIAPSVWWRTLRPEIEALAWLD | 71  68 |
| Query 72  Sbjct 69 | | TTGVPCAAVLDVVTEAGRDWLLLGEVPGQDLLSSHLAPAEK--VSIMADAMRRLHTLDPA  +P +L G D+LL+ + G+D + K V + A+ +R +H LD GK-LPVPKILYTAEHGGMDYLLMEALGGKDGSHETIQAKRKLFVKLYAEGLRSVHGLDIR | 129  127 |
| Query 130  Sbjct 128 | | TCPFDHQAKHRIERARTRMEAGLVDQDDLDEEHQGLAPAELFARLKARMPDGEDLVVTHG CP + + ++ A+ ++ LVD D+ EE+ P EL+ L P EDLV HG ECPLSNGLEKKLRDAKRIVDESLVDPADIKEEYD-CTPEELYGLLLESKPVTEDLVFAHG | 189  186 |
| Query 190  Sbjct 187 | | DACLPNIMVENGRFSGFIDCGRLGVADRYQDIALATRDIAEELGGE-WADRFLVLYGIAA D C PN++++ + SGFID GR GVADRYQDI+LA R + + G + + FL LYG+ DYCAPNLIIDGEKLSGFIDLGRAGVADRYQDISLAIRSLRHDYGDDRYKALFLELYGLDG | 248  246 |
| Query 249 | | PDSQRIAFYRLLDEFF 264 D ++ +Y LDEFF |  |
| Sbjct | 247 | LDEDKVRYYIRLDEFF 262 | |

>TR:A0A4Q7JFT4 A0A4Q7JFT4_9PSEU Aminoglycoside 3'-phosphotransferase OS=Amycolatopsis suaedae OX=2510978 GN=EWH70_01480 PE=3 SV=1

Length=240

Score = 134 bits (337), Expect = 4e-35

| Identities | | = 97/244 (40%), Positives = 124/244 (51%), Gaps = 19/244 (8%) |  |
| --- | --- | --- | --- |
| Query 21  Sbjct 14 | | GYDWAQQTIGCSDAAVFRLSAQGRPVLFVKTDLSGALNELQDEAARLSWLATTGVPCAAV G +W+ G S A G FVK A E RL WL T VP V GAEWSADHEGQSGGVWKTTGAAG--TFFVKHGEDAAR-----EHDRLRWL-TGRVP---V | 80  62 |
| Query 81 | | LDVVTEAGRDWLLLGEVPGQDLLSSHLAPAEKVSIMADAMRRLHTLDPATCPFDHQAKHR  ++ G DWL+L + + + PA+ + M +RRLH L A CPFD + | 140 |
| Sbjct | 63 | PEIAAFTG-DWLVLADARAVSVATE--PPADPGAAMGAILRRLHLLPVARCPFDARTPVT | 119 |
| Query | 141 | IERARTRMEAGLVDQDDLDEEHQGLAPAELFARLKARMPDGEDLVVTHGDACLPNIMVEN | 200 |
| Sbjct | 120 | + +AR + AGLVD DD D++H G P EL RL A P EDLVV+HGD N+++ VGQARHNVAAGLVDADDFDDDHAGSTPGELLDRLVALCPADEDLVVSHGDFTAENVLLRP | 179 |
| Query | 201 | GRFSGFIDCGRLGVADRYQDIALATRDIAEELGGEWADRFLVLYG-IAAPDSQRIAFYRL | 259 |
| Sbjct | 180 | + D RLGVADR++DIALA R L G+ A FL YG I PD R+ +YRL DGSALLTDVSRLGVADRHRDIALACRG LTGDQAGSFLAAYGLIDRPDPARLYWYRL | 235 |
| Query  Sbjct | 260  236 | LDEF 263 LDEF  LDEF 239 |  |

```
>TR:A0A542DRV8 A0A542DRV8_9PSEU Kanamycin kinase OS=Amycolatopsis cihanbeyliensis OX=1128664 GN=FB471_5573 PE=3 SV=1
Length=271

Score = 134 bits (338), Expect = 6e-35
Identities = 92/260 (35%), Positives = 129/260 (50%), Gaps = 18/260 (7%)

Query 19	LFGYDWAQQTIGCSDAAVFRLSAQGRPVLFVKT----DLSGALNELQDEAARLSWLATTG 74
+ G +W	++ R+	G	+VKT	D +	++EAARL WLA G
```

| Sbjct | 14 | VLGGEWEHIATRSYGTSIHRVRGAG--TYYVKTSPIVDAADLRFHPENEAARLRWLARMG | 71 |
| --- | --- | --- | --- |
| Query | 75 | VPCAAVLDVVTEAGRDWLLLGEVPGQDLLSSHLAPAEK---VSIMADAMRRLHTLDPATC | 131 |
| Sbjct | 72 | P V++V A R WL+ V G + PAE+ V +A+ LH L C FPVPEVVEVGGTAERSWLVTTAVAGVPAAGPWI-PAERARVVGAVAELAAALHALPAREC | 130 |
| Query | 132 | PFDHQAKHRIERARTRMEAGLVDQDDLDEEHQGLAPAELFARLKAR-MPDGEDLVVTHGD | 190 |
| Sbjct | 131 | PFD + R RT + G VD DD+++ +G + L A L A +P +D+VV HGD PFDRTLAVTLPRCRTAVRLGTVDLDDIEDHRRGWSAERLLAELDATPVPPEDDIVVCHGD | 190 |
| Query | 191 | ACLPNIMVENGRFS--GFIDCGRLGVADRYQDIALATRDIAEE LGGEWADRFLVL | 243 |
| Sbjct | 191 | CL NI+++ S G +D GRLG ADR+ D+++A RDI EE G A F LCLDNILLDPETLSVTGILDTGRLGRADRWLDLSVALRDIGEERREWGFGPAHAALFRER | 250 |
| Query  Sbjct | 244  251 | YGIAAPDSQRIAFYRLLDEF 263 YG+ D + +YRLLDEF YGLPVLDHAKRDYYRLLDEF 270 |  |

>TR:A0A4R6PRW4 A0A4R6PRW4_9NOCA Kanamycin kinase OS=Nocardia ignorata OX=145285 GN=DFR75_101678 PE=3 SV=1

Length=408

Score = 137 bits (346), Expect = 7e-35

| Identities | | = 83/202 (41%), Positives = 105/202 (52%), Gaps = 8/202 (4%) |  |
| --- | --- | --- | --- |
| Query 63  Sbjct 215 | | EAARLSWLATTGVPCAAVLDVVTEAGRDWLLLGEVPGQDLLSSHLAPAEKVSIMADAMRR E RL WL G+ AV +V A D L+L +V G + S PA +++ +R EHERLIWLRDKGI---AVPEVAAFA-EDVLVLADVGGGSVSSQAPDPAAAGTLLGSRLRA | 122  270 |
| Query 123  Sbjct 271 | | LHTLDPATCPFDHQAKHRIERARTRMEAGLVDQDDLDEEHQGLAPAELFARLKARMPDGE LH L A CPFD + RA + G VD DD D +H+ PAEL A L R P E LHDLPAADCPFDAGLDVTLARAHRNVVEGFVDADDFDPDHRDRTPAELLAELVRRRPASE | 182  330 |
| Query 183  Sbjct 331 | | DLVVTHGDACLPNIMVENGRFSGFIDCGRLGVADRYQDIALATRDIAEELGGEWADRFLV DLVV HGD N++ ID LG ADRY+DIALA RD+ E G E F DLVVAHGDFTPGNVLAG GVLIDVAELGRADRYRDIALAERDLGENFGAEAVTAFRA | 242  386 |
| Query 243 | | LYGIAAPDSQRIAFYRLLDEFF 264 YG+ PD+ ++ +YRLLDE F |  |
| Sbjct | 387 | AYGLTEPDADKLDYYRLLDELF 408 | |

>TR:A0A365HB92 A0A365HB92_9ACTN Aminoglycoside 3'-phosphotransferase OS=Actinomadura sp. LHW63021 OX=2231787 GN=DPM19_04650 PE=3 SV=1

Length=277

Score = 134 bits (337), Expect = 1e-34

| Identities | = 83/217 (38%), Positives = 117/217 (54%), Gaps = 6/217 (3%) |  |
| --- | --- | --- |
| Query 48 | FVKTDLSGALNELQDEAARLSWLATTGVPCAAVLDVVTEAGRDWLLLGEVPGQDLLSSHL | 107 |
| Sbjct 58 | FVK +G + EL EAARL W A P VL++ + A DWL+ +PG++ ++ FVKWAPAGGVLELAAEAARLRW-ARAFTPVPEVLELGSGADGDWLVTAGLPGENAVAERW | 116 |
| Query 108 | A--PAEKVSIMADAMRRLHTLDP-ATCPFDHQAKHRIERARTRMEAGLVDQDDLDEEHQG | 164 |
| Sbjct 117 | PA V+ + +R H P A CPF A R+ AR R AGL+D +H+ LADPATAVAAIGAGLRAFHDALPVAECPFTWSAGERLADARRRASAGLIDPRGWHADHRH | 176 |
| Query 165 | LAPAELFARLKARMPDGEDLVVTHGDACLPNIMVE-NGRFSGFIDCGRLGVADRYQDIAL | 223 |
| Sbjct 177 | LA + + A P + LVV HGD C PN +++ +GR+SG +D G LGVADR+ D+A+ LAGVDEALAVLADAPPPDALVVCHGDTCAPNTLLDGDGRWSGHVDLGTLGVADRWADLAV | 236 |
| Query 224  Sbjct 237 | ATRDIAEELGGEWADRFLVLYGIAAPDSQRIAFYRLL 260 AT G W + L YG+ PD +R +YRLL ATWSTGWNYGPGWEEPLLAAYGV-EPDPERTRYYRLL 272 |  |

>TR:A0A285LYT6 A0A285LYT6_9NOCA Kanamycin kinase OS=Nocardia amikacinitolerans OX=756689 GN=SAMN04244553_5765 PE=3 SV=1

Length=412

Score = 137 bits (345), Expect = 1e-34

| Identities | | = 83/205 (40%), Positives = 109/205 (53%), Gaps = 11/205 (5%) |  |
| --- | --- | --- | --- |
| Query 63  Sbjct 216 | | EAARLSWLATTGVPCAAVLDVVTEAGRDWLLLGEVPGQDLLSSHLAPAEKVSI---MADA E RL WL G+ + V D L+L + L + SI M + ERDRLDWLRARGIR VPEVAAFAEDVLVLADAGAPSLAAREGDGGTSPSIGTVMGEL | 119  271 |
| Query 120  Sbjct 272 | | MRRLHTLDPATCPFDHQAKHRIERARTRMEAGLVDQDDLDEEHQGLAPAELFARLKARMP  +RRLH L A CPFD + + +AR + GLVD DD D+++ L P ++ RL A P LRRLHALPVAECPFDGRLDTVLAQARRHVLEGLVDPDDFDDDNSDLNPEQVLERLLAERP | 179  331 |
| Query 180 | | DGEDLVVTHGDACLPNIMVENGRFSGFIDCGRLGVADRYQDIALATRDIAEELGGEWADR EDLVV HGD PN++ ENG +D G LGVADRY+D+ALA RD+ E+ G | 239 |
| Sbjct | 332 | QTEDLVVAHGDFTPPNVL-ENGI---LLDVGGLGVADRYRDLALAERDLREDFGDHEVRA | 387 |
| Query  Sbjct | 240  388 | FLVLYGIAAPDSQRIAFYRLLDEFF 264 F YG+ APD R+ +YRLLDE F FFAAYGLDAPDRTRLDYYRLLDELF 412 |  |

>TR:A0A1H9KTU8 A0A1H9KTU8_9PSEU Kanamycin kinase OS=Actinokineospora terrae OX=155974 GN=SAMN04487818_101322 PE=3 SV=1

Length=245

Score = 133 bits (334), Expect = 1e-34

| Identities | = 85/231 (37%), Positives = 126/231 (55%), Gaps = 22/231 (10%) |  |
| --- | --- | --- |
| Query 42 | QGRPVLFVKTDLSGALNELQ DEAARLSWLATTGVPCAAVLDVVTEAGRDWLLLGEV | 97 |
| Sbjct 29 | +GR L+VKT ++ + +EAARL+WL G P V++V + W+++ EV EGRSTLYVKTTPPRRADDFRFNPSNEAARLAWLQERGFPVPEVIEVGASDEQSWMVMTEV | 88 |
| Query 98 | PGQDLLSSHLAP--AEKVSIMADAMRRLHTLDPATCPFDHQAKHRIERARTRMEAGLVDQ | 155 |
| Sbjct 89 | PG + + AP A V+ +A+ R LH L CPFD + AR + +G+VD PG---IPADTAPDRARAVATVAEFTRSLHAL--RDCPFDGSLTVTTDWARRAVASGVVDL | 143 |
| Query 156 | DDLDEEHQGLAPAELFARLKARMPDGEDLVVTHGDACLPNIMVENGRF--SGFIDCGRLG | 213 |
| Sbjct 144 | DDLD EH G + L +L EDLVV HGD L N++++ + +G ID GRLG DDLDPEHDGWSGQRLLEKLSGLPVPPEDLVVCHGDLGLDNVLIDPSTYELTGVIDVGRLG | 203 |
| Query 214  Sbjct 204 | VADRYQDIALATRDIAEELGGEWADRFLVLYGIAAPDSQRIAFYRLLDEFF 264  +AD ++D+A+ RD + L +YG+ D++R +YRLLDEFF  MADPWRDLAVLVRDAGRSV--------LEVYGVGF-DAERERYYRLLDEFF 245 |  |

>TR:A0A370I256 A0A370I256_9NOCA Kanamycin kinase/aminoglycoside 3'-phosphotransferase-2 OS=Nocardia pseudobrasiliensis OX=45979 GN=DFR76_107205

PE=3 SV=1

Length=243

Score = 132 bits (332), Expect = 2e-34

| Identities | = 80/201 (40%), Positives = 117/201 (58%), Gaps = 16/201 (8%) |  |
| --- | --- | --- |
| Query 66 | RLSWLATTG--VPCAAVLDVVTEAGRDWLLLGEVPGQDLLSSHLAPAEKVSIMADAMRRL | 123 |
| Sbjct 57 | RL+WL G VP A+ + D +L+ E G L+ H AP +++ +RRL RLTWLGERGIAVPAIAIFE-------DDVLVLEDAGVPSLADHPAPG---ALLGRVLRRL | 106 |
| Query 124 | HTLDPATCPFDHQAKHRIERARTRMEAGLVDQDDLDEEHQGLAPAELFARLKARMPDGED | 183 |
| Sbjct 107 | H + + CPFD + +E+AR ++ GLVD +D D+++ G P ++ +L A+ P ED HEIPVSACPFDGRLDVVLEQARRQVVDGLVDAEDFDDDNIGRTPEQVLEQLLAQRPAIED | 166 |
| Query 184 | LVVTHGDACLPNIMVENGRFSGFIDCGRLGVADRYQDIALATRDIAEELGGEWADRFLVL | 243 |
| Sbjct 167 | LVV HGD P+ ++E G ID GRLG+ADRY+D+ALA RD+ ++ G D F LVVAHGD-YTPSNVLEGGI---VIDTGRLGIADRYRDLALARRDLLDDFGQAEFDAFRAA | 222 |
| Query 244  Sbjct 223 | YGIAAPDSQRIAFYRLLDEFF 264 YG+A D R+ +YRLLDE F YGLAELDPIRLEYYRLLDELF 243 |  |

>TR:A0A4R5DQN1 A0A4R5DQN1_9ACTN Aminoglycoside 3'-phosphotransferase OS=Jiangella asiatica OX=2530372 GN=E1269_01420 PE=3 SV=1

Length=231

Score = 131 bits (330), Expect = 3e-34

| Identities | | = 99/250 (40%), Positives = 128/250 (51%), Gaps = 29/250 (12%) |  |
| --- | --- | --- | --- |
| Query 24  Sbjct 2 | | WAQQTIGCSDAAVFRLSAQGRPVLFVKTDLSGALNELQDEAARLSWLATTGVPCAAVLDV W + + G S A V R R ++D G ++L E RL WL G+P A VL+ WERVSAGQSGAEVSRRDGVFRK RSDHPG--DDLVGEGERLRWLRRHGIPAAEVLEC | 83  55 |
| Query 84  Sbjct 56 | | VTEAGRDWLLLGEVPGQDLLSSHLAPAEK VSIMADAMRRLHTLDPATCPFDHQAKH  GR L+ +VPG+ + P + V +AD R LH + A CPFD  --RPGR--LVTAQVPGRS--ADQPWPDDDLPRVVDALADLTRALHAIPVAECPFDR---- | 139  105 |
| Query 140  Sbjct 106 | | RIERARTRMEAGLV---DQDDLDEEHQGLAPAELFARLKARMPDGEDLVVTHGDACLPNI R T + A L D DDLD+E G +L A L A PDGEDL V HGD L N+  ---RLATTVPAALSADPDLDDLDDERAGWTRDQLVAELLATRPDGEDLAVCHGDLDLLNV | 196  162 |
| Query 197  Sbjct 163 | | MVE--NGRFSGFIDCGRLGVADRYQDIALATRDIAEELGGEWADRFLVLYGIAAPDSQRI  +++ +G ID GRLGVADR+ D+A+ TR + G E RFL YG+AA D + LLDPTTCTVTGVIDAGRLGVADRWLDLAVVTRSLEGNHGAESGRRFLERYGVAA-DPAKQ | 254  221 |
| Query 255 | | AFYRLLDEFF 264 FYRLLDEFF |  |
| Sbjct | 222 | DFYRLLDEFF 231 | |

>TR:A0A1I2L6P9 A0A1I2L6P9_9ACTN Kanamycin kinase OS=Actinoplanes philippinensis OX=35752 GN=SAMN05421541_12017 PE=3 SV=1

Length=231

Score = 131 bits (329), Expect = 5e-34

| Identities | = 86/207 (42%), Positives = 112/207 (54%), Gaps = 15/207 (7%) |  |
| --- | --- | --- |
| Query 63 | EAARLSWLATTGVPCAAVLDVVTEAGRDWLLL-GEVPGQDLLSSHLAPAEK--VSIMADA | 119 |
| Sbjct 35 | E RL WL G+P A VLD R LL+ VPG+ + A A + + +A+ EGNRLLWLRRHGIPAAEVLDC RPGLLVTAAVPGRSAAAPWPAEARERIIDALAEL | 89 |
| Query 120 | MRRLHTLDPATCPFDHQAKHRIERARTRMEAGLVDQDDLDEEHQGLAPAELFARLKARMP | 179 |
| Sbjct 90 | LH L A CP+D + I A A +D +LD E +G + L ARL + P TVALHALPVAGCPYDRRLAVTIPEAL AADIDLGNLDAERRGWSRDRLIARLLSTRP | 145 |
| Query 180 | DGEDLVVTHGDACLPNIMVE--NGRFSGFIDCGRLGVADRYQDIALATRDIAEELGGEWA | 237 |
| Sbjct 146 | EDLVV HGD LPNI+++ R SG ID GRLG ADR+ D+A+ TR + G A QHEDLVVCHGDLSLPNILLDPVTDRVSGVIDAGRLGAADRWADLAIITRSMTSRFGSAAA | 205 |
| Query 238  Sbjct 206 | DRFLVLYGIAAPDSQRIAFYRLLDEFF 264 DR+L YG+ P+ Q+ FYRLLDEFF DRYLDRYGV-DPNPQKTDFYRLLDEFF 231 |  |

>TR:A0A366E427 A0A366E427_9NOCA Kanamycin kinase OS=Nocardia puris OX=208602 GN=DFR74_101564 PE=3 SV=1

Length=407

Score = 135 bits (340), Expect = 5e-34

| Identities | | = 80/203 (39%), Positives = 108/203 (53%), Gaps = 12/203 (6%) |  |
| --- | --- | --- | --- |
| Query 63  Sbjct 216 | | EAARLSWLATTGVPCAAVLDVVTEAGRDWLLLGEVPGQDLLSSHLAPAEKV-SIMADAMR E RL WL G+P + V D L+L + L +AP + + M + +R EHDRLIWLRGRGIP----VPEVAVFAEDVLVLADAGAPSLA---VAPGDSPGAAMGEVLR | 121  268 |
| Query 122  Sbjct 269 | | RLHTLDPATCPFDHQAKHRIERARTRMEAGLVDQDDLDEEHQGLAPAELFARLKARMPDG RLH L + CPFD + + AR ++ GLVD D D ++ L P E+ ARL A P RLHALPVSECPFDGRLDVTLNLARRQVVEGLVDPADFDADNAALTPEEILARLVAERPAT | 181  328 |
| Query 182  Sbjct 329 | | EDLVVTHGDACLPNIMVENGRFSGFIDCGRLGVADRYQDIALATRDIAEELGGEWADRFL EDLVV HGD PN++ +D G LG+ADRY+D+ALA RD+ E+ G D F EDLVVAHGDFTPPNVLAG GTLLDVGALGLADRYRDLALAERDLREDFGPAEVDAFF | 241  384 |
| Query 242 | | VLYGIAAPDSQRIAFYRLLDEFF 264 YG+ PD R+ +YRLLDE F |  |
| Sbjct | 385 | AAYGLTDPDQVRLDYYRLLDEVF 407 | |

>TR:A0A4R6KF00 A0A4R6KF00_9ACTN Streptomycin 3'-kinase OS=Kribbella sp. VKM Ac-2527 OX=2512215 GN=EV643_108114 PE=3 SV=1

Length=273

Score = 132 bits (331), Expect = 7e-34

| Identities | = 92/250 (37%), Positives = 130/250 (52%), Gaps = 17/250 (7%) |  |
| --- | --- | --- |
| Query 23 | DWAQQTIGCSDAAVFRLSAQGRPVLFVKTDLSGALNELQDEAARLSWLATTGVPCAAVLD | 82 |
| Sbjct 25 | +W T G SD V+R +G + K + ++ L+ E R++WLA TG+P A VL+ EWVPVTSGESDTKVYR---RGDGAAYAKVAPASGIDALRGERDRVAWLAGTGIPGAEVLE | 81 |
| Query 83 | VVTEA-GRDWLLLGEVPGQDLLSSHLAPAEK---VSIMADAMRRLHTLDPATCPFDHQAK | 138 |
| Sbjct 82 | GR L+ V G + + L P + V +A +RRLH LD A CPF WSESGDGRACLVTSAVAG--VAAVELPPDARPKAVERLAGMLRRLHDLDVAACPFVRPLG | 139 |
| Query 139 | HRIERARTRMEAGLVDQDDLDEEHQGLAPAELFARLKARMPDGE DLVVTHGDACL | 193 |
| Sbjct 140 | +A + G V+ L +E + P +L ARL++ +P E DLVV HGDACL SVFGQAEDVVRRGAVNPQFLRDEWRRERPEDLLARLESELPYAEERAAGDLVVCHGDACL | 199 |
| Query 194 | PNIMV--ENGRFSGFIDCGRLGVADRYQDIALATRDIAEELGGEWADRFLVLYGIAAPDS | 251 |
| Sbjct 200 | PN + ++ +G ID GRLGVADRY D+AL + +E + A FL YG+ PD PNFLFDPDSLEITGLIDLGRLGVADRYADLALTAVQLVDEWDLDPAG-FLTAYGLPDPDR | 258 |
| Query 252  Sbjct 259 | QRIAFYRLLD 261  +R+ Y LLD  RRLDVYMLLD 268 |  |

>TR:A0A0M2VJQ1 A0A0M2VJQ1_9BACL Aminoglycoside phosphotransferase (Fragment) OS=Paenibacillus sp. DMB20 OX=1642570 GN=XI25_27810 PE=3 SV=1

Length=226

Score = 130 bits (327), Expect = 8e-34

| Identities | | = 72/205 (35%), Positives = 110/205 (54%), Gaps = 6/205 (3%) |  |
| --- | --- | --- | --- |
| Query 19  Sbjct 23 | | LFGYDWAQQTIGCSDAAVFRLSAQ-GRPVLFVKTDLSGALNELQDEAARLSWLATTGVPC L+GY WA+ +G S A ++RL + P LF+K N++ DE RL+WL T +P LYGYKWARDNVGQSGATIYRLYGKPDAPELFLKHGKGSVANDVTDEMVRLNWL-TEFMPL | 77  81 |
| Query 78  Sbjct 82 | | AAVLDVVTEAGRDWLLLGEVPGQ---DLLSSHLAPAEK-VSIMADAMRRLHTLDPATCPF  + + WLL +PG+ +L + E V +A +RRLH++ CPF PTIKHFIRTPDDAWLLTTAIPGKTAFQVLEEYPDSGENIVDALAVFLRRLHSIPVCNCPF | 133  141 |
| Query 134  Sbjct 142 | | DHQAKHRIERARTRMEAGLVDQDDLDEEHQGLAPAELFARLKARMPDGEDLVVTHGDACL  + R+ +A++RM GLVD D D+E G +++ + +P D VVTHGD L NSDRVFRLAQAQSRMNNGLVDASDFDDERNGWPVEQVWKEMHKLLPFSPDSVVTHGDFSL | 193  201 |
| Query 194 | | PNIMVENGRFSGFIDCGRLGVADRY 218 N++ + G+ G ID GR+G+ADRY |  |
| Sbjct | 202 | DNLIFDEGKLIGCIDVGRVGIADRY 226 | |

>TR:T0MZ20 T0MZ20_9CLOT APH domain-containing protein OS=Clostridium sp. BL8 OX=1354301 GN=M918_06875 PE=3 SV=1

Length=265

Score = 131 bits (329), Expect = 1e-33

| Identities | = 82/264 (31%), Positives = 132/264 (50%), Gaps = 6/264 (2%) |  |
| --- | --- | --- |
| Query 5 | DGLHAGSPAAWVERLFGYDWAQQTIGCSDAAVFR-LSAQGRPVLFVKTDLSGALNELQDE | 63 |
| Sbjct 4 | D L P E L G + + +GCSD V++ + G+ ++K S E DELICSFPKELKELLEGCRYEENKVGCSDTMVYKVIGKDGKDNRYLKVS-SNKFENFNYE | 62 |
| Query 64 | AARLSWLATTGVPCAAVLDVVTEAGRDWLLLGEVPGQD--LLSSHLAPAEKVSIMADAMR | 121 |
| Sbjct 63 | L+W+ +P VL ++ L+ E+ G+D ++ + + +A +R VDLLNWI-NGKLPVPRVLYHGHSGEYEFFLMSEICGKDSSVIDENTDKVKVAKALARGLR | 121 |
| Query 122 | RLHTLDPATCPFDHQAKHRIERARTRMEAGLVDQDDLDEEHQGLAPAELFARLKARMPDG | 181 |
| Sbjct 122 | +H++ C FD + +++ A +E LVD++D+ + P ++ L+ P+ VIHSVPIKHCDFDQSLETKLKIAEYNVENHLVDEEDIKSSDPNITPEKILKELQYNRPNS | 181 |
| Query 182 | EDLVVTHGDACLPNIMVENG-RFSGFIDCGRLGVADRYQDIALATRDIAEELGGEWADRF | 240 |

ED V+THGD CLPNI+++	SGFID GR GV+DR+QDI + R I + LG E	F

| Sbjct | 182 | EDSVLTHGDYCLPNIILDKDYNISGFIDWGRGGVSDRHQDIGIGCRSIKKNLGEEMVPVF | 241 |
| --- | --- | --- | --- |
| Query  Sbjct | 241  242 | LVLYGIAAPDSQRIAFYRLLDEFF 264 L YG+ D +I +Y LLDE F LEEYGLDKIDYSKIDYYILLDELF 265 |  |

>TR:U5ED73 U5ED73_NOCAS Putative aminoglycoside 3'-O-phosphotransferase OS=Nocardia asteroides NBRC 15531 OX=1110697 GN=NCAST_24_00090

PE=3 SV=1

Length=409

Score = 132 bits (333), Expect = 5e-33

| Identities | | = 79/203 (39%), Positives = 106/203 (52%), Gaps = 9/203 (4%) |  |
| --- | --- | --- | --- |
| Query 63  Sbjct 215 | | EAARLSWLATTGVPCAAVLDVVTEAGRDWLLLGEVPGQDLLSSHLAPAEKVSI-MADAMR E RL+WL + +P + VV D L+L + L A A V M +R EHDRLNWLRGSAIP VPVVAAFAEDVLVLADADAAALAERTTAEAAAVGERMGQTLR | 121  270 |
| Query 122  Sbjct 271 | | RLHTLDPATCPFDHQAKHRIERARTRMEAGLVDQDDLDEEHQGLAPAELFARLKARMPDG LH L CPFD + + +AR + LVD D D +H+ L PA + RL+ PD ALHALPVTGCPFDGRLDVTLAQARRNVVEELVDAADFDPDHRDLTPAAILERLRTERPDR | 181  330 |
| Query 182  Sbjct 331 | | EDLVVTHGDACLPNIMVENGRFSGFIDCGRLGVADRYQDIALATRDIAEELGGEWADRFL ED VV HGD N++ +NG +D G LG ADRY+D+ALA RD+AE+ G F EDTVVAHGDFTPGNVL-DNGVL---LDVGALGTADRYRDLALAERDLAEDFGPAAVTAFF | 241  386 |
| Query 242 | | VLYGIAAPDSQRIAFYRLLDEFF 264 YG+ APD ++ +YRLLDE F |  |
| Sbjct | 387 | TAYGLTAPDRAKLDYYRLLDELF 409 | |

>TR:D6K107 D6K107_9ACTN Kanamycin kinase OS=Streptomyces sp. e14 OX=645465 GN=SSTG_01440 PE=3 SV=1

Length=243

Score = 128 bits (322), Expect = 7e-33

| Identities | | = 84/220 (38%), Positives = 116/220 (53%), Gaps = 8/220 (4%) |  |
| --- | --- | --- | --- |
| Query 48  Sbjct 24 | | FVKTDLSGALNELQDEAARLSWLAT-TGVPCAAVLDVVTEAGRDWLLLGEVPGQDLLSSH FVK G+ +L E ARL W A T VP VL +A W++ +PG+ + H FVKWTPHGSRIDLSAEVARLRWAAGFTTVP--RVLGEGADASGSWIVTAGLPGRSAVDDH | 106  81 |
| Query 107  Sbjct 82 | | LA--PAEKVSIMADAMRRLHTLDP-ATCPFDHQAKHRIERARTRMEAGLVDQDDLDEEHQ PA V + +R LH P A CPFD A+ R+ R R AG +D D DE+ + SKRDPATAVRAVGAGLRALHDALPVADCPFDWSAERRLAAVRARAAAGRIDPADWDEDVR | 163  141 |
| Query 164  Sbjct 142 | | GLAPAELFARLKARMPDGEDLVVTHGDACLPNIMV-ENGRFSGFIDCGRLGVADRYQDIA  + + + +P ++LVV HGDAC PN +V ++G +G +D G LGVADR+ D+A  HHGTVQRALAVLSDIPPVDELVVCHGDACAPNTLVGDDGACTGHVDLGSLGVADRWADLA | 222  201 |
| Query | 223 LATRDIAEELGGEWADRFLVLYGIAAPDSQRIAFYRLLDE 262 | | |
|  |  | +AT G W L YG+ A D +RIA+YRLL E |  |
| Sbjct | 202 | VATWSTRWNYGPGWEKPLLDAYGVTA-DRERIAYYRLLWE 240 | |

>TR:A0A265QAG8 A0A265QAG8_9FIRM APH domain-containing protein OS=Tissierella sp. P1 OX=1280483 GN=CIW83_05225 PE=3 SV=1

Length=257

Score = 128 bits (322), Expect = 1e-32

Identities = 82/239 (34%), Positives = 122/239 (51%), Gaps = 8/239 (3%)

| Query | 29 | IGCSDAAVFRLSAQGRPVLFVKTDLSGALNELQDEAARLSWLATTGVPCAAVLDVVTEAG | 88 |
| --- | --- | --- | --- |
| Sbjct | 24 | IG S A V+R L + NEL+ E + WL +P + + G IGRSTAGVYRYY---NDKLSYYLKVQPTFNELEKEHEIMEWLQHK-LPVPRKIYFKSYGG | 79 |
| Query | 89 | RDWLLLGEVPGQDLLSSHLA--PAEKVSIMADAMRRLHTLDPATCPFDHQAKHRIERART | 146 |
| Sbjct | 80 | D+LL+ E+ G+ + S + P E V ++AD ++ L ++ CPFD+ + +++ A  FDYLLMTEIEGEMICSDYFLNNPEEAVRLLADGIKMLQSISIEDCPFDNSLETKLKEALY | 139 |

| Query | 147 | RMEAGLVDQDDLDEEHQGLAPAELFARLKARMPDGEDLVVTHGDACLPNIMVENGRFSGF 206 |
| --- | --- | --- |
| Sbjct | 140 | +E LVD D +E ++ P EL L+ P THGD CLPNI ++ + SGF NIENNLVDISDWEENNRFDTPMELLEYLRNNQPKEFTPAFTHGDYCLPNIFSKDNKISGF 199 |
| Query | 207 | IDCGRLGVADRYQDIALATRDIAEELG-GEWADRFLVLYGIAAPDSQRIAFYRLLDEFF 264 |
| Sbjct | 200 | ID GR G+AD YQDIAL R + G ++ D F GI PD +++ +Y LLDE F  IDLGRSGIADIYQDIALCVRSLKHNFGTDDYIDLFFKHLGI-QPDWEKVEYYILLDELF 257 |

>TR:A0A5C8JX64 A0A5C8JX64_9ACTN Aminoglycoside 3'-phosphotransferase OS=Nonomuraea sp. C10 OX=2600577 GN=FR742_22860 PE=3 SV=1

Length=240

Score = 127 bits (320), Expect = 1e-32

| Identities | | = 82/200 (41%), Positives = 107/200 (54%), Gaps = 11/200 (6%) |  |
| --- | --- | --- | --- |
| Query 63  Sbjct 50 | | EAARLSWLATTGVPCAAVLDVVTEAGRDWLLLGEVPGQDLLSSHLAPAEKVSIMADAMRR E RL WL A+V DVV G D L+L E + L H P + +IM A+R EHERLRWLKRW ASVPDVVAFEG-DVLVLAEAGWRSL--DHRPPPDAGTIMGRALRE | 122  102 |
| Query 123  Sbjct 103 | | LHTLDPATCPFDHQAKHRIERARTRMEAGLVDQDDLDEEHQGLAPAELFARLKARMPDGE LH + A CPFD + ++ RA + AGLVD +H GL P +++ RL A P GE LHEIPVAECPFDERLDVKLSRAAGHVRAGLVDPARFHGDHAGLTPEQVYDRLLAERPAGE | 182  162 |
| Query 183  Sbjct 163 | | DLVVTHGDACLPNIMVENGRFSGFIDCGRLGVADRYQDIALATRDIAEELGGEWADRFLV DLVVTHGD N++ + +D G LGVADR+ D+A+A R EL G A L DLVVTHGDFTPANVLASGSGEAILVDVGELGVADRHVDLAVALR ELDGRAAADLLA | 242  218 |
| Query 243 | | LYGIAAPDSQRIAFYRLLDE 262 YG A D R+++YRLL E |  |
| Sbjct | 219 | AYGPAEVDEARLSYYRLLAE 238 | |

>TR:A0A1J0VV95 A0A1J0VV95_9NOCA Aminoglycoside phosphotransferase APH(3') OS=Nocardia mangyaensis OX=2213200 GN=BOX37_20865 PE=4 SV=1

Length=407

Score = 131 bits (330), Expect = 1e-32

| Identities | | = 83/202 (41%), Positives = 113/202 (56%), Gaps = 9/202 (4%) |  |
| --- | --- | --- | --- |
| Query 63  Sbjct 215 | | EAARLSWLATTGVPCAAVLDVVTEAGRDWLLLGEVPGQDLLSSHLAPAEKVSIMADAMRR E RL+WL GV AV +V A D L+L +V G L + A A ++M +RR EYERLTWLRDHGV---AVPEVAAFA-EDVLVLADVDGGSLTGAGDAAAVG-TLMGRVLRR | 122  269 |
| Query 123  Sbjct 270 | | LHTLDPATCPFDHQAKHRIERARTRMEAGLVDQDDLDEEHQGLAPAELFARLKARMPDGE  +H L CPFD + RAR + G VD D D++H+ L+PAE+ ARL+ R P E VHDLPVDRCPFDSGLDVSLARARRAVVEGFVDPADFDDDHRDLSPAEVLARLRDRRPAEE | 182  329 |
| Query 183  Sbjct 330 | | DLVVTHGDACLPNIMVENGRFSGFIDCGRLGVADRYQDIALATRDIAEELGGEWADRFLV D+VVTHGD N++ ID LG DRY+D+ALA RD+AE+ G + F DVVVTHGDFTPGNVLAG GVLIDVAVLGRGDRYRDLALAHRDLAEDFGADAVAAFGA | 242  385 |
| Query 243 | | LYGIAAPDSQRIAFYRLLDEFF 264 YG++ D+ R+ +YRLLDE F |  |
| Sbjct | 386 | AYGLSDLDADRLYYYRLLDELF 407 | |

>TR:A0A5M3WX45 A0A5M3WX45_9ACTN Aminoglycoside O-phosphotransferase APH(3')-IIb OS=Acrocarpospora macrocephala OX=150177 GN=aph PE=3 SV=1

Length=244

Score = 127 bits (320), Expect = 1e-32

Identities = 92/243 (38%), Positives = 128/243 (53%), Gaps = 21/243 (9%)

| Query | 24 | WAQQTIGCSDAAVFRLSAQGRPVLFVKTD-LSGALNELQDEAARLSWL-ATTGVP-CAAV | 80 |
| --- | --- | --- | --- |
| Sbjct | 17 | W+ G S A+ +S G V +VK L+GA E RL WL +P A WSADHEGASGTALRVVS--GSKVFYVKHGPLAGA EHLRLGWLHGRVSIPEVVAF | 68 |
| Query | 81 | LDVVTEAGRDWLLLGEVPGQDLLSSHLAPAEKVSIMADAMRRLHTLDPATCPFDHQAKHR | 140 |
| Sbjct | 69 | D V L+L +V L ++ APA+ +++ +R LH L A CPFD +  EDPV LVLADVGAPSLEAA--APADVGAVLGRTLRMLHELPVAECPFDGRLSAV | 119 |

| Query | 141 | IERARTRMEAGLVDQDDLDEEHQGLAPAELFARLKARMPDGEDLVVTHGDACLPNIMVEN | 200 |
| --- | --- | --- | --- |
| Sbjct | 120 | +ERA + GLVD DD D +H GL P ++ RL A P EDLVV HGD L N+++ LERAGANVREGLVDPDDFDSDHLGLTPEVIYERLLAARPGVEDLVVAHGDYTLSNVLLPV | 179 |
| Query | 201 | GRFSGFIDCGRLGVADRYQDIALATRDIAEELGGEWADRFLVLYGIA-APDSQRIAFYRL | 259 |
| Sbjct | 180 | ID LGVADRY+D+A+A RD++E+ G + FL YG+ + +R+ +YRL SGEPVVIDVPALGVADRYRDLAIAHRDLSEDHGPAAWEGFLSAYGLVDHVEVERLYYYRL | 239 |
| Query  Sbjct | 260  240 | LDE 262 LDE  LDE 242 |  |

>TR:A0A1C4WLH3 A0A1C4WLH3_MICEC Kanamycin kinase OS=Micromonospora echinospora OX=1877 GN=GA0070618_2310 PE=3 SV=1

Length=268

Score = 128 bits (321), Expect = 2e-32

| Identities | | = 77/211 (36%), Positives = 116/211 (55%), Gaps = 12/211 (6%) |  |
| --- | --- | --- | --- |
| Query 63  Sbjct 59 | | EAARLSWLATTGVPCAAVLDVVTEAGRDWLLLGEVPGQDLLSSHLAPAEK---VSIMADA EA RL WLA G+P V+ + W++ +PG+ + H P E+ + ++AD EAERLRWLAAQGLPVPEVVALDANDELAWVVTRALPGRPA-ARHWKPEERWRVIDVVADV | 119  117 |
| Query 120 | | MRRLHTLDPATCPFDHQAKHRIERARTRMEAGLVDQDDLDEEHQGLAPAELFARL-KARM R LH L A CPF+ + I +A + M G +D DD+D H+G +L+ L K | 178 |
| Sbjct | 118 | ARTLHALPVAECPFERRLADLIHQASSSMALGALDLDDVDPSHEGWTGQQLWDELSKMTP | 177 |
| Query | 179 | PDGEDLVVTHGDACLPNIMV--ENGRFSGFIDCGRLGVADRYQDIALATRDIAEE L | 232 |
| Sbjct | 178 | P+ +DLVV HGD CL N++V E +G +D R GV+DR+ D+ALA +I ++ PEEDDLVVCHGDFCLDNVLVDPETLTLAGILDVDRAGVSDRWMDLALALYNIGQDDVWGY | 237 |
| Query  Sbjct | 233  238 | GGEWADRFLVLYGIAAPDSQRIAFYRLLDEF 263 G A+ FL YGI+ D ++ + +LLDEF GPPHAEHFLRRYGISV-DQHKLTYIQLLDEF 267 |  |

```
>SP:P18150 APHE_STRGR Streptomycin 3''-kinase OS=Streptomyces griseus OX=1911 GN=aphE PE=3 SV=1
Length=272

Score = 127 bits (318), Expect = 5e-32
Identities = 106/278 (38%), Positives = 136/278 (49%), Gaps = 38/278 (14%)

Query 8	HAGSPAAWVERLFGY--DWAQQTIGCSDAAVFRLSAQGRPVLFVKT-DLSGALNELQDEA 64 H G P A	LFG	DW	T G S A+VFR +	R	V	D +G	L+ E
Sbjct 4	HPG-PGAVTPELFGVGGDWLAVTAGESGASVFRAADATRYAKCVPAADAAG----LEAER 58
Query 65	ARLSWLATTGVPCAAVLDVVTEAGRDWLLLGEVPGQDLLSSHLAPAEKVSI	115
R++WL+ GVP	VLD	L+	VPG	PA++V
Sbjct 59	DRIAWLSGQGVPGPRVLDWYAGDAGACLVTRAVPG--------VPADRVGADDLRTAWGA	110
Query 116 MADAMRRLHTLDPATCPFDHQAKHRIERARTRMEAGLVDQDDLDEEHQGLAPAELFARL- 174
+ADA+RRLH + A+CPF	++ AR + G V + L E + + PAEL ARL Sbjct 111 VADAVRRLHEVPVASCPFRRGLDSVVDAARDVVARGAVHPEFLPVEQRLVPPAELLARLT	170
Query 175 ----KARMPDGEDLVVTHGDACLPNIMV--ENGRFSGFIDCGRLGVADRYQDIAL----A	224
+ R + D VV HGD CLPNI++ E	SGFID GRLG ADR+ D+AL	A Sbjct 171 GELARRRDQEAADTVVCHGDLCLPNIVLHPETLEVSGFIDLGRLGAADRHADLALLLANA	230
Query 225 TRDIAEELGGEWAD-RFLVLYGIAAPDSQRIAFYRLLD	261
+E	+AD F	YGI APD +R+ FY LD Sbjct 231 RETWVDEERARFADAAFAERYGI-APDPERLRFYLHLD	267

>TR:A0A239HVP9 A0A239HVP9_9ACTN Kanamycin kinase/aminoglycoside 3'-phosphotransferase-2 OS=Asanoa hainanensis OX=560556 GN=SAMN05421812_10267
PE=4 SV=1
Length=155

Score = 123 bits (308), Expect = 8e-32
Identities = 68/144 (47%), Positives = 83/144 (58%), Gaps = 2/144 (1%)
```

| Query | 123 | LHTLDPATCPFDHQAKHRIERARTRMEAGLVDQDDLDEEHQGLAPAELFARLKARMP-DG | 181 |
| --- | --- | --- | --- |
| Sbjct | 12 | LH L ATCPFD + I A VD D LD G + L A L A P D LHQLPTATCPFDRRLSTAIREAEAAARTKEVDLDSLDTTRAGWSIDRLLAELHATRPQDP | 71 |
| Query | 182 | EDLVVTHGDACLPNIM-VENGRFSGFIDCGRLGVADRYQDIALATRDIAEELGGEWADRF | 240 |
| Sbjct | 72 | EDLVV HGD CLPNI+ ++GR +G +D RLGVADR+ D+A+ATR++ A EDLVVGHGDPCLPNILCTDDGRPTGVVDVSRLGVADRHNDLAIATRNLTANWSKTEATTL | 131 |
| Query  Sbjct | 241  132 | LVLYGIAAPDSQRIAFYRLLDEFF 264 L YG+ PD ++AFYRLLDEFF LTAYGLENPDPAKLAFYRLLDEFF 155 |  |

>TR:S3AU80 S3AU80_9ACTN APH domain-containing protein OS=Streptomyces sp. HPH0547 OX=1203592 GN=HMPREF1486_04670 PE=3 SV=1

Length=294

Score = 126 bits (317), Expect = 1e-31

| Identities | = 83/217 (38%), Positives = 110/217 (51%), Gaps = 6/217 (3%) |  |
| --- | --- | --- |
| Query 48 | FVKTDLSGALNELQDEAARLSWLATTGVPCAAVLDVVTEAGRDWLLLGEVPGQDLLSSHL | 107 |
| Sbjct 75 | FVK G+ +L EA RL W + +AG WL +PG+ + +H FVKWAPHGSGLDLAAEAVRLRWAGRWATVPRVITHGTDDAG-SWLATHGLPGRSAVDAHW | 133 |
| Query 108 | A--PAEKVSIMADAMRRLHTLDP-ATCPFDHQAKHRIERARTRMEAGLVDQDDLDEEHQG | 164 |
| Sbjct 134 | P V + +R LH P A CPFD A R+ R R AG +D L E+ LRDPGTAVRAIGAGLRALHDALPVADCPFDWSAGSRLAAVRERAAAGRIDPAGLHEDLHH | 193 |
| Query 165 | LAPAELFARLKARMPDGEDLVVTHGDACLPNIMV-ENGRFSGFIDCGRLGVADRYQDIAL | 223 |
| Sbjct 194 | AE A PD + LVV HGDAC PN +V ++G ++G +D G LGVADR+ D+A+ FGTAEQALAALADPPDVDILVVCHGDACAPNTLVGDDGTWTGHVDLGALGVADRWADLAV | 253 |
| Query 224  Sbjct 254 | ATRDIAEELGGEWADRFLVLYGIAAPDSQRIAFYRLL 260 AT G W + L YG AAPD++RIA+YRLL ATWSTRWNYGPGWEEMLLDAYG-AAPDAERIAYYRLL 289 |  |

>TR:A0A4R7TFA0 A0A4R7TFA0_9ACTN Streptomycin 3'-kinase OS=Kribbella sp. VKM Ac-2575 OX=2512212 GN=EV138_3670 PE=3 SV=1

Length=254

Score = 125 bits (314), Expect = 1e-31

| Identities | = 87/244 (36%), Positives = 123/244 (50%), Gaps = 14/244 (6%) |  |
| --- | --- | --- |
| Query 24 | WAQQTIGCSDAAVFRLSAQGRPVLFVKTDLSGALNELQDEAARLSWLATTGVPCAAVLDV | 83 |
| Sbjct 9 | W G S VFR R ++ K L + EL+ E R+ WLA TG+P A V+D WEPVVGGESATEVFR RGGVYAKRCLVDGVAELRGERDRVEWLAGTGIPGAKVVDW | 63 |
| Query 84 | VTEAGRDWLLLGEVPGQDLLSSHLAPAEK-VSIMADAMRRLHTLDPATCPFDHQAKHRIE | 142 |
| Sbjct 64 | + + L+ VPG + P+ + V +A ++ LH + CPF+ + + IESSEGAVLMTTAVPGVG--GGDVPPSRRAVESLAAMVKALHEVPLVDCPFERRVDDVVR | 121 |
| Query 143 | RARTRMEAGLVDQDDLDEEHQGLAPAELFARLKARMPDGE---DLVVTHGDACLPNIMV- | 198 |
| Sbjct 122 | +A + G V+ D L +E + + L A L + + DLVV HGDACLPN + QAADVVRRGAVEPDFLTDEWRLVPSERLLAGLYGELDEVRAKADLVVCHGDACLPNFLFD | 181 |
| Query 199 | -ENGRFSGFIDCGRLGVADRYQDIALATRDIAEELGGEWADRFLVLYGIAAPDSQRIAFY | 257 |
| Sbjct 182 | E +G ID GRLG+ADRY D+AL I +E + A FL YG+ PD QR+ FY PETLECTGMIDVGRLGIADRYADLALLKAQIEDEWAVD-ATGFLKSYGLVEPDGQRLRFY | 240 |
| Query 258  Sbjct 241 | RLLD 261 RLLD  RLLD 244 |  |

>TR:A0A1H7N6K2 A0A1H7N6K2_9ACTN Kanamycin kinase OS=Nonomuraea pusilla OX=46177 GN=SAMN05660976_01931 PE=3 SV=1

Length=241

Score = 125 bits (313), Expect = 1e-31

Identities = 84/206 (41%), Positives = 111/206 (54%), Gaps = 17/206 (8%)

| Query | 63 | EAARLSWLATTGVPCAAVLDVVTEAGRDWLLLGEVPGQDLLSSHLAPAEKVSIMADAMRR | 122 |
| --- | --- | --- | --- |
| Sbjct | 49 | EA RL WL AAV DVV A D L+L + G L A ++M A+R EAERLLWLKRW AAVPDVVA-AEDDVLVLADA-GLPSLEGREADPVAGAVMGRALRA | 102 |
| Query | 123 | LHTLDPATCPFDHQAKHRIERARTRMEAGLVDQDDLDEEHQGLAPAELFARLKARMPDGE | 182 |
| Sbjct | 103 | LH + A CPFD + ++E+AR +E+GLVD D D++H P ++ RL P E LHAIPVAECPFDERLDVKLEQARVAVESGLVDPGDFDDDH---TPEHVYERLLRERPPIE | 159 |
| Query | 183 | DLVVTHGD ACLPNIMVENGRFSGFIDCGRLGVADRYQDIALATRDIAEELGGEWAD | 238 |
| Sbjct | 160 | DLVV HGD L +++ ++ ID GRLGV DR+ D+A+A R EL G DLVVAHGDFTPANVLADVLADDAGGPVLIDAGRLGVCDRHVDLAIALR ELEGPAVA | 215 |
| Query | 239 | RFLVLYGIAAPDSQRIAFYRLLDEFF 264 FL YG APD +R+ +YRLLDE F |  |
| Sbjct | 216 | SFLEAYGPPAPDPRRVEYYRLLDELF 241 | |

>TR:K0F8X7 K0F8X7_9NOCA Aminoglycoside 3'-O-phosphotransferase OS=Nocardia brasiliensis ATCC 700358 OX=1133849 GN=O3I_030060 PE=3 SV=1

Length=415

Score = 129 bits (323), Expect = 2e-31

| Identities | = 78/205 (38%), Positives = 111/205 (54%), Gaps = 11/205 (5%) |  |
| --- | --- | --- |
| Query 63 | EAARLSWLATTGVPCAAVLDVVTEAGRDWLLLGE--VPGQDLLSSHLAPAEKV-SIMADA | 119 |
| Sbjct 219 | E RL+WL G+ L +T D L+L + VP + A+ V +IM EQQRLNWLGERGIR LPEITVYAEDVLVLADAGVPSLAVRGESGTGADSVGTIMGTL | 274 |
| Query 120 | MRRLHTLDPATCPFDHQAKHRIERARTRMEAGLVDQDDLDEEHQGLAPAELFARLKARMP | 179 |
| Sbjct 275 | +R LH + A CPFD + + +AR ++ GLVD DD D+++Q +P ++ RL A P LRTLHGIPIAECPFDGRLDLVLAQARRQVIEGLVDADDFDDDNQDSSPEQVLERLLAERP | 334 |
| Query 180 | DGEDLVVTHGDACLPNIMVENGRFSGFIDCGRLGVADRYQDIALATRDIAEELGGEWADR | 239 |
| Sbjct 335 | DLVV HGD N++ + +D G LGVADRY+D+ALA RD+ ++ G AEPDLVVAHGDFTPANVL EGAILLDVGALGVADRYRDLALAVRDLRDDFGAAEVTA | 390 |
| Query 240  Sbjct 391 | FLVLYGIAAPDSQRIAFYRLLDEFF 264 F YG+A PD R+ +YRLLDE F FFAAYGLAEPDPVRLEYYRLLDELF 415 |  |

>TR:A0A1V9K319 A0A1V9K319_9ACTN Aminoglycoside phosphotransferase APH(3') OS=Streptomyces sp. M41(2017) OX=1955065 GN=B0675_27625 PE=3 SV=1

Length=267

Score = 125 bits (313), Expect = 3e-31

| Identities | | = 82/220 (37%), Positives = 115/220 (52%), Gaps = 8/220 (4%) |  |
| --- | --- | --- | --- |
| Query 48  Sbjct 48 | | FVKTDLSGALNELQDEAARLSWLAT-TGVPCAAVLDVVTEAGRDWLLLGEVPGQDLLSSH FVK +G+ + EAARL W T VP VLD + W++ +PG+ + H FVKWTPTGSGIDPAAEAARLRWAVRYTTVP--RVLDEGADGTGTWIVTRGLPGRMAVDDH | 106  105 |
| Query 107  Sbjct 106 | | LA--PAEKVSIMADAMRRLHTLDP-ATCPFDHQAKHRIERARTRMEAGLVDQDDLDEEHQ P V + +R LH P A CPFD A R++ AR+R AG + D +H+ WKRDPGTAVRAIGAGLRALHDALPVADCPFDWSAGRRLQSARSRAAAGRIHPTDWHHDHR | 163  165 |
| Query 164  Sbjct 166 | | GLAPAELFARLKARMPDGEDLVVTHGDACLPNIMV-ENGRFSGFIDCGRLGVADRYQDIA  + E + A +P + LVV HGDAC PN +V ++G ++G +D G LGVADR+ D+A  HIGTVERALDVLAAIPPVDRLVVCHGDACAPNTLVGDDGTWTGHVDLGTLGVADRWADLA | 222  225 |
| Query | 223 LATRDIAEELGGEWADRFLVLYGIAAPDSQRIAFYRLLDE 262 | | |
|  |  | +AT G W + L YG+ PD +RI YRLL E |  |
| Sbjct | 226 | VATWSTRWNYGPGWEEPLLEAYGV-EPDRERIRHYRLLRE 264 | |

>TR:A0A6B2VAB2 A0A6B2VAB2_9ACTN Aminoglycoside 3'-phosphotransferase OS=Streptomyces sp. SID10815 OX=2706027 GN=G3I35_27860 PE=3 SV=1

Length=267

Score = 124 bits (312), Expect = 4e-31

Identities = 82/224 (37%), Positives = 115/224 (51%), Gaps = 6/224 (3%)

| Query | 43 | GRPVLFVKTDLSGALNELQDEAARLSWLATTGVPCAAVLDVVTEAGRDWLLLGEVPGQDL | 102 |
| --- | --- | --- | --- |
| Sbjct | 43 | G+ FVK + +L E ARL W A V + + EAG W++ +PG+ GKTRQFVKWTPIDSGIDLAAEVARLRWAARFTVVPRVLGEGADEAG-SWIITRGLPGRMA | 101 |
| Query | 103 | LSSHLA--PAEKVSIMADAMRRLHTLDP-ATCPFDHQAKHRIERARTRMEAGLVDQDDLD | 159 |
| Sbjct | 102 | + H P V + +R LH P A CPFD A+ R+ER +R+ AG D VDDHWKRDPGTAVRAIGSGLRALHNALPVADCPFDWSAERRLERVWSRVAAGPTGPADWP | 161 |
| Query | 160 | EEHQGLAPAELFARLKARMPDGEDLVVTHGDACLPNIMV-ENGRFSGFIDCGRLGVADRY | 218 |
| Sbjct | 162 | E+ Q L E + +P + LVV HGDAC PN +V ++G +SG +D G LGVADR+ EDFQHLGTVEHALGILTDIPPVDRLVVCHGDACAPNTLVGDDGTWSGHVDLGTLGVADRW | 221 |
| Query  Sbjct | 219  222 | QDIALATRDIAEELGGEWADRFLVLYGIAAPDSQRIAFYRLLDE 262 D+A+AT G W + L YG+ PD +R+ +YRLL E  ADLAVATWSTRWNYGPGWEEPLLEAYGV-EPDPERVMYYRLLWE 264 |  |

>TR:Q2J7L9 Q2J7L9_FRACC Aminoglycoside phosphotransferase OS=Frankia casuarinae (strain DSM 45818 / CECT 9043 / CcI3) OX=106370 GN=Francci3_3368

PE=3 SV=1

Length=268

Score = 124 bits (312), Expect = 4e-31

| Identities | = 83/217 (38%), Positives = 116/217 (53%), Gaps = 15/217 (7%) |  |
| --- | --- | --- |
| Query 59 | ELQDEAARLSWLATTGVPCAAVLDVVTEAGRDWLLLGEVPGQDLLSSHLAPAEKVSIMAD | 118 |
| Sbjct 54 | + + EA RLSWLA G+P V+DV + WL+ EVPG + ++ PA+++ + D QAEAEAERLSWLARQGIPVPEVVDVGADETHMWLVTREVPG--VPAAGPWPADRLDRVID | 111 |
| Query 119 | AMRR LHTLDPATCPFDHQAKHRIERARTRMEAGLVDQDDLDEEHQGLAPAELFARL | 174 |
| Sbjct 112 | A+ LH L A CPFD + + AR + G VD DL+ H+G + +L L AVAELVLALHGLPAARCPFDRTLRITLPAARHAVRTGTVDLADLEPRHRGWSAQDLQREL | 171 |
| Query 175 | KAR-MPDGEDLVVTHGDACLPNIMVENGRF--SGFIDCGRLGVADRYQDIALATRDIAEE | 231 |
| Sbjct 172 | A P+ E LVV HGD CL N++++ + +D GR GVAD + D+A+A R+I EE DATPAPEQEQLVVCHGDLCLDNVLLDPDTLAPTALLDVGRCGVADVWLDLAVAVRNIGEE | 231 |
| Query 232  Sbjct 232 | -----LGGEWADRFLVLYGIAAPDSQRIAFYRLLDEF 263 G A R L G PD R +YRLLDEF  CTRWGYGPGHAARLLARCG-RQPDPVREHYYRLLDEF 267 |  |

>TR:A0A562VDH2 A0A562VDH2_9ACTN Kanamycin kinase OS=Stackebrandtia albiflava OX=406432 GN=LX16_1652 PE=3 SV=1

Length=268

Score = 124 bits (312), Expect = 4e-31

| Identities | | = 96/255 (38%), Positives = 125/255 (49%), Gaps = 19/255 (7%) |  |
| --- | --- | --- | --- |
| Query 22  Sbjct 19 | | YDWAQQTIGCSDAAVFRLSAQGRPVLFVKTDLSGALNE LQDEAARLSWLATTGVPC  ++W + A V RL G P L VKT A + L EAA +WLA GV C HEWTP--LAARTAHVHRL--HGDPALVVKTAPRPAGPDPGLDLATEAATCAWLADQGVRC | 77  74 |
| Query 78  Sbjct 75 | | AAVLDVVTEAGRDWLLLGEVPG QDLLSSHLAPAEKVSIMADAMRRLHTLDPATCPF  +D T +L++ PG DL + PA V +A A RLH L C F PTPVDTGTLDDHQYLVMTAAPGIPLDGDLPADLREPA--VDAVARATARLHRLPVTACGF | 133  132 |
| Query 134  Sbjct 133 | | DHQAKHRIERARTRMEAGLVDQDDLDEEHQGLAPAELFARL--KARMPDGEDLVVTHGDA D + + I A+ G VD DDLD +G L L +AR+ E++ V HGD DRRLRVTIPMAQESAGLGEVDLDDLDPPRRGWDVTRLTTELLTQARLIGREEVAVCHGDL | 191  192 |
| Query 192  Sbjct 193 | | CLPNIMVENGRFS-GFIDCGRLGVADRYQDIALATRDIAEE--LGGEWADRFLVLYGIAA N++++ + F+D RLG+ADRY D+AL TRD+ G ADRFL YG A  TPGNVLIDPDDLTVAFVDTARLGLADRYSDLALFTRDLTGTPGFGPAAADRFLHHYGEAP | 248  252 |
| Query 249 | | PDSQRIAFYRLLDEF 263 D QR+ FYRLLDEF |  |
| Sbjct | 253 | VDPQRLEFYRLLDEF 267 | |

>TR:H6R3X4 H6R3X4_NOCCG Putative aminoglycoside 3'-O-phosphotransferase

OS=Nocardia cyriacigeorgica (strain GUH-2) OX=1127134 GN=NOCYR_3725 PE=3 SV=1

Length=243

Score = 124 bits (310), Expect = 4e-31

| Identities | | = 80/204 (39%), Positives = 111/204 (54%), Gaps = 16/204 (8%) |  |
| --- | --- | --- | --- |
| Query 63  Sbjct 54 | | EAARLSWLA--TTGVPCAAVLDVVTEAGRDWLLLGEVPGQDLLSSHLAPAEKVSIMADAM E RL WLA VP A D D L+L ++ G L+ A ++M + + EYERLRWLAGRDISVPEVAAFD EDVLVLADM-GVPSLARRRGRAG--AVMGEVL | 120  104 |
| Query 121  Sbjct 105 | | RRLHTLDPATCPFDHQAKHRIERARTRMEAGLVDQDDLDEEHQGLAPAELFARLKARMPD R LH + A CPFD + + + +AR R+ G VD DD D+++ L P ++ RL P+ RGLHGVPVAGCPFDGRLEVMLPKARRRVLEGSVDADDFDDDNP-LTPEQVLRRLFDEQPE | 180  163 |
| Query 181  Sbjct 164 | | GEDLVVTHGDACLPNIMVENGRFSGFIDCGRLGVADRYQDIALATRDIAEELGGEWADRF EDLVV HGD N++ E G ID G LGVADRY+D+ALA RD+ E+ G F DEDLVVAHGDFTPANVL-EGGIL---IDVGALGVADRYRDLALAARDLREDFGAGEVGEF | 240  219 |
| Query 241 | | LVLYGIAAPDSQRIAFYRLLDEFF 264 YG+ PD +++ +YRLLDE F |  |
| Sbjct | 220 | FTAYGLGEPDQRKLDYYRLLDELF 243 | |

>TR:A0A1Q4WCP7 A0A1Q4WCP7_9ACTN Aminoglycoside phosphotransferase OS=Streptomyces sp. CB02056 OX=1703924 GN=AMK13_15030 PE=3 SV=1

Length=271

Score = 124 bits (311), Expect = 6e-31

| Identities | | = 80/208 (38%), Positives = 107/208 (51%), Gaps = 6/208 (3%) |  |
| --- | --- | --- | --- |
| Query 59  Sbjct 63 | | ELQDEAARLSWLATTGVPCAAVLDVVTEAGRDWLLLGEVPGQDLLSSHLA--PAEKVSIM  +L EA RL W A V + + E G WL+ +PG+ + H PA V + DLAAEAVRLRWAARFAVVPPVIGEGADETG-SWLVTQGLPGRSAVDEHWKRDPATAVRAI | 116  121 |
| Query 117 | | ADAMRRLHTLDP-ATCPFDHQAKHRIERARTRMEAGLVDQDDLDEEHQGLAPAELFARLK  +R LH P A CPFD A+ R+ AR + EAG D D EE + E | 175 |
| Sbjct | 122 | GAGLRALHDALPVADCPFDWSAETRLTNARAKAEAGRTDPADWPEELRHHGTVERALATL | 181 |
| Query | 176 | ARMPDGEDLVVTHGDACLPNIMV-ENGRFSGFIDCGRLGVADRYQDIALATRDIAEELGG | 234 |
| Sbjct | 182 | A +P + LVV HGDAC PN ++ ++G +G +D G LG+ADR+ D+A+AT G ADIPPIDHLVVCHGDACAPNTLIGDDGTHTGHVDLGTLGLADRWADLAIATWSTQWNYGP | 241 |
| Query | 235 | EWADRFLVLYGIAAPDSQRIAFYRLLDE 262 W L YGI APD +R A+YRLL E |  |
| Sbjct | 242 | GWEQPLLDAYGI-APDPERTAYYRLLWE 268 | |

>TR:A0A1T4U3M6 A0A1T4U3M6_9BACT Kanamycin kinase OS=Chitinophaga eiseniae OX=634771 GN=SAMN04488128_108175 PE=3 SV=1

Length=255

Score = 123 bits (309), Expect = 8e-31

| Identities | = 87/240 (36%), Positives = 119/240 (50%), Gaps = 8/240 (3%) |  |
| --- | --- | --- |
| Query 28 | TIGCSDAAVFRLSAQGRPVLFVKTDLSGALNELQDEAARLSWLATTGVPCAAVLDVVTEA | 87 |
| Sbjct 21 | TIG S AAV+R ++ V ++KT SG L+ + L+WL +P + T TIGYSAAAVYRYTS-AHSVFYLKTQASGQGLRLEYDV--LTWLQDK-LPVPVIQYFGTHD | 76 |
| Query 88 | GRDWLLLGEVPGQDLLSSHLA--PAEKVSIMADAMRRLHTLDPATCPFDHQAKHRIERAR | 145 |
| Sbjct 77 | G D+LL V G L + H P V ++AD ++ L L CPFD+ ++ A GIDYLLTTAVDGDMLCAGHYLDNPELTVKLLADGIQLLSALPIHDCPFDNSLDKKLAAAA | 136 |
| Query 146 | TRMEAGLVDQDDLDEEHQGLAPAELFARLKARMPDGEDLVVTHGDACLPNIMVENGRFSG | 205 |
| Sbjct 137 | ++ GLVD D + + P L L A P V THGD CLPNI + G+ SG YNVQHGLVDTSDWEGHNHFPTPQALLDHLIANKPAHYTPVFTHGDYCLPNIFGKQGQVSG | 196 |
| Query 206 | FIDCGRLGVADRYQDIALATRDIAEELG-GEWADRFLVLYGIAAPDSQRIAFYRLLDEFF | 264 |
| Sbjct 197 | FID G GVAD YQDIAL R + G ++A G D +R+A+Y +LDE F  FIDIGGAGVADIYQDIALCVRSLKHNFGTDDYAAGLFERLG-RERDEERVAYYVMLDELF | 255 |

>TR:A0A370I4X3 A0A370I4X3_9NOCA Streptomycin 3'-kinase OS=Nocardia pseudobrasiliensis OX=45979 GN=DFR76_10650 PE=3 SV=1

Length=273

Score = 124 bits (310), Expect = 8e-31

| Identities | | = 95/264 (36%), Positives = 130/264 (49%), Gaps = 24/264 (9%) |  |
| --- | --- | --- | --- |
| Query 15  Sbjct 12 | | WVERLFGYD-WAQQTIGCSDAAVFRLSAQGRPVLFVKTDLSGALNELQDEAARLSWLATT WV RL D W G S AAVFR R V+ D +G EL+DE R+ WLA+ WVGRLLARDGWEVVGDGESGAAVFRSPDGARYAKCVRPDRAG---ELEDERDRVEWLASQ | 73  68 |
| Query 74  Sbjct 69 | | GVPCAAVLDVVTEAGRDWLLLGEVPGQDLLSSHLAPAEKVSI MADAMRRLHTLDPA  G+P A+LD + L+ V G + ++L AE++ + A+RRLH L GIPGPAMLDWIDNDAGACLITSAVEG MPANLVSAEELGTAWESIGRAVRRLHALPVR | 129  125 |
| Query 130  Sbjct 126 | | TCPFDHQAKHRIERARTRMEAGLVDQDDLDEEHQGLAPAELFARLKARMP DGEDL  CPF + RAR + VD D L +EH+ L P L R+ +++ + + DCPFGRGLAEMVGRARDVVGRNAVDPDFLSDEHRRLVPEALLERVVSQVDRWLPREDAEA | 184  185 |
| Query 185  Sbjct 186 | | VVTHGDACLPNIMVE--NGRFSGFIDCGRLGVADRYQDIALATRDIAEELGGEWADR--- VV HGD CLPNI+++ +GFID GRLG AD Y D AL + E E R VVCHGDLCLPNIVLDPRTLEVTGFIDLGRLGRADPYTDFALLLANSRETWPDEQRARAAD | 239  245 |
| Query 240 | | --FLVLYGIAAPDSQRIAFYRLLD 261 F +YGI D++R+ FY LD |  |
| Sbjct | 246 | EAFARVYGIDL-DAERLRFYLDLD 268 | |

>TR:D2AUZ0 D2AUZ0_STRRD 3''-kinase OS=Streptosporangium roseum (strain ATCC 12428 / DSM 43021 / JCM 3005 / NI 9100) OX=479432 GN=Sros_3941

PE=3 SV=1

Length=273

Score = 123 bits (309), Expect = 1e-30

| Identities | = 103/277 (37%), Positives = 136/277 (49%), Gaps = 25/277 (9%) |  |
| --- | --- | --- |
| Query 1 | MIEQDGLHAGSPAAWVERLFGYDWAQQTIGCSDAAVFRLSAQGRPVLFVKTDLSGALNEL | 60 |
| Sbjct 1 | M + G A SP + + G DW T G S AAVFR + R + K + +L MSDHSGPQAISPV--LLSVGGGDWLPVTAGESGAAVFRNADATR---YAKCVPAADAADL | 55 |
| Query 61 | QDEAARLSWLATTGVPCAAVLDVVTEAGRDWLLLGEVPGQDLLSSHLAPAEKVSI M | 116 |
| Sbjct 56 | + E R++WL GVP VLD + L+ V G +S+ PAEK+ + + KAERDRVAWLNDQGVPGPRVLDWHSGDVGACLMTSAVSG---ISADQVPAEKLRVAWERI | 112 |
| Query 117 | ADAMRRLHTLDPATCPFDHQAKHRIERARTRMEAGLVDQDDLDEEHQGLAPAELFARLKA | 176 |
| Sbjct 113 | ADA+RRLH + CPF + AR + V+ + L E Q P EL RL ADAVRRLHEVPVPQCPFRRDLDAMVAVARGVVARDAVNPEFLPVEQQHTPPGELLDRLTR | 172 |
| Query 177 | RMP DGEDLVVTHGDACLPNIMVENGRF--SGFIDCGRLGVADRYQDIALATRDIA | 229 |
| Sbjct 173 | ++P + D VV HGD CLPNI+++ SGFID GRLG ADRY D+AL + QVPRRREQEAADTVVCHGDLCLPNIILDPRTLDVSGFIDLGRLGQADRYADLALLLANAR | 232 |
| Query 230  Sbjct 233 | E----ELGGEWAD-RFLVLYGIAAPDSQRIAFYRLLD 261 E E G AD F YGIA D +R+ FYR LD ETWKDEERGRAADMAFAERYGIAL-DHERLRFYRHLD 268 |  |

>TR:A0A5Q4TG46 A0A5Q4TG46_9ACTN APH(3'') family aminoglycoside O-phosphotransferase OS=Streptomyces sp. t39 OX=1828156 GN=aph(3'') PE=3

SV=1

Length=268

Score = 123 bits (308), Expect = 1e-30

Identities = 96/255 (38%), Positives = 125/255 (49%), Gaps = 23/255 (9%)

| Query | 23 | DWAQQTIGCSDAAVFRLSAQGRPVLFVKTDLSGALNELQDEAARLSWLATTGVPCAAVLD | 82 |
| --- | --- | --- | --- |
| Sbjct | 16 | DW T G S AAVFR + R V + AL +D R+ WLA VP VLD DWLPVTGGESGAAVFRSADATRYAKCVPVADAAALGAERD---RIDWLAVQDVPGPQVLD | 72 |
| Query | 83 | VVTEAGRDWLLLGEVPGQDLLSSHLAPAEKV SIMADAMRRLHTLDPATCPFDHQAK | 138 |
| Sbjct | 73 | + L+ VPG + + PA+ + + +A+RRLH + TCPFD  WYSGDAGACLVTSAVPG---VGADRLPADDLRAAWGAIGEAVRRLHAVPVETCPFDRDLD | 129 |

| Query | 139 | HRIERARTRMEAGLVDQDDLDEEHQGLAPAELFARLKARMP DGEDLVVTHGDACL | 193 |
| --- | --- | --- | --- |
| Sbjct | 130 | + AR + G V + L EE Q P L AR+ A++P + D VV HGD CL SMVAAARDAVARGAVRPEFLPEEQQHTPPGALLARVVAQVPRRREQEAADTVVCHGDLCL | 189 |
| Query | 194 | PNIMVE--NGRFSGFIDCGRLGVADRYQDIAL ATRDIAEELGGEWAD-RFLVLYGI | 246 |
| Sbjct | 190 | PNI+++ SGFID GRLG ADR+ D+AL A A+E AD F YG+ PNIVLDPRTLEVSGFIDLGRLGRADRHADVALLLATARETWADEERARAADAAFAERYGL | 249 |
| Query | 247 | AAPDSQRIAFYRLLD 261 APD R+ FY LD |  |
| Sbjct | 250 | -APDPGRLRFYLHLD 263 | |

>TR:A0A212TNI3 A0A212TNI3_9ACTN Streptomycin 3-kinase OS=Streptomyces sp. 2114.4 OX=1938836 GN=SAMN06272741_2033 PE=3 SV=1

Length=266

Score = 122 bits (307), Expect = 2e-30

| Identities | | = 97/254 (38%), Positives = 130/254 (51%), Gaps = 21/254 (8%) |  |
| --- | --- | --- | --- |
| Query 23  Sbjct 14 | | DWAQQTIGCSDAAVFRLSAQGRPVLFVKTDLSGALNELQDEAARLSWLATTGVPCAAVLD DW T G SDAAVFR SA G + K ++ +L+ E R++WL+ GVP VLD DWLPVTAGESDAAVFR-SADG--TRYAKCVVAADAADLKAERDRVAWLSDQGVPGPRVLD | 82  70 |
| Query 83 | | VVTEAGRDWLLLGEVPGQDLLSSHLAPAEKVSI---MADAMRRLHTLDPATCPFDHQAKH L+ G V G + + ++PA+ + +ADA+RRLH + CPF | 139 |
| Sbjct | 71 | WHCGDAGACLVTGAVSG--IPADQVSPADLRASWERIADAVRRLHEVPVQQCPFRRDLDK | 128 |
| Query | 140 | RIERARTRMEAGLVDQDDLDEEHQGLAPAELFARL KARMPDGEDLVVTHGDACLP | 194 |
| Sbjct | 129 | + AR + G V+ + L E Q +EL ARL + + + D VV HGD CLP MVAVARDVVTRGAVNPEFLPVEQQHTPASELLARLVPQVARRQGQEAADTVVCHGDLCLP | 188 |
| Query | 195 | NIMVENGRF--SGFIDCGRLGVADRYQDIALATRDIAE ELGGEWAD-RFLVLYGIA | 247 |
| Sbjct | 189 | NI+++ SGFID GRLG+ADRY D+AL + E E G AD F YGIA NIVLDPQTLDVSGFIDLGRLGLADRYADLALLLTNARETWPDEEGARAADAAFAERYGIA | 248 |
| Query | 248 | APDSQRIAFYRLLD 261 D R+ FY LD |  |
| Sbjct | 249 | I-DQDRLRFYLHLD 261 | |

>TR:A0A4U3CFI9 A0A4U3CFI9_9ACTN Aminoglycoside phosphotransferase APH(3') OS=Blastococcus sp. CCUG 61487 OX=1840703 GN=A6V29_08020 PE=3

SV=1

Length=267

Score = 122 bits (307), Expect = 2e-30

| Identities | = 88/217 (41%), Positives = 114/217 (53%), Gaps = 9/217 (4%) |  |
| --- | --- | --- |
| Query 48 | FVKTDLSGALNELQDEAARLSWLATTGVPCAAVLDVVTEAGRDWLLLGEVPGQDLLSS-H | 106 |
| Sbjct 51 | FVK +G+ L+DE RL W A P VLD + G WLL +PG+ + FVKWAPAGSGVRLEDEVERLRWAARFH-PVPPVLDHGADDGGTWLLTRGLPGRSAVDPVW | 109 |
| Query 107 | LA-PAEKVSIMADAMRRLHTLDPAT-CPFDHQAKHRIERARTRMEAGLVDQDDLDEEHQG | 164 |
| Sbjct 110 | LA P V + +R LH P CPF A R+ A M G H G LADPRRAVRGVGAGLRALHDALPVEGCPFGWTAATRVSEALDAM--GTWRPARWHAVHAG | 167 |
| Query 165 | LAPAELFARLKARMPDGEDLVVTHGDACLPN-IMVENGRFSGFIDCGRLGVADRYQDIAL | 223 |
| Sbjct 168 | LAPA++ ARL A +PD + LVV HGDAC PN ++ ++G +SG +D G LGVADR+ D+A+ LAPADVRARL-ADVPDVDRLVVCHGDACAPNTLLTDDGEWSGHVDLGSLGVADRWADLAI | 226 |
| Query 224  Sbjct 227 | ATRDIAEELGGEWADRFLVLYGIAAPDSQRIAFYRLL 260 AT G W L YG+ PD++R AFYRLL ATWSTEWNFGPGWEPHLLDAYGV-EPDAERTAFYRLL 262 |  |

>TR:A0A1H5XY97 A0A1H5XY97_9ACTN Kanamycin kinase/aminoglycoside 3'-phosphotransferase-2 OS=Nonomuraea solani OX=1144553 GN=SAMN05444920_10271

PE=3 SV=1

Length=239

Score = 121 bits (304), Expect = 3e-30

| Identities | = 89/243 (37%), Positives = 122/243 (50%), Gaps = 20/243 (8%) |  |
| --- | --- | --- |
| Query 21 | GYDWAQQTIGCSDAAVFRLSAQGRPVLFVKTDLSGALNELQDEAARLSWLATTGVPCAAV | 80 |
| Sbjct 14 | G W+ G S A + G V +VK G L + E RL WL VP V GATWSTDHEGASGTA--QRVTSGSTVFYVK---QGPL--ARAEHLRLRWLRGR-VPVPEV | 65 |
| Query 81 | LDVVTEAGRDWLLLGEVPGQDLLSSHLAPAEKVSIMADAMRRLHTLDPATCPFDHQAKHR | 140 |
| Sbjct 66 | + L+L +V L APA +++ +R+LH L A CPFD + VAYEDPV----LVLADVAAPSL---EAAPAGMGAVLGRTLRKLHALPVAECPFDGRLPAV | 118 |
| Query 141 | IERARTRMEAGLVDQDDLDEEHQGLAPAELFARLKARMPDGEDLVVTHGDACLPNIMVEN | 200 |
| Sbjct 119 | +ERA + GLVD DD D++H GL+P + RL A P EDLVV HGD N++V LERAAANVRDGLVDPDDFDDDHLGLSPETICDRLVATGPREEDLVVAHGDYTPSNVLVPA | 178 |
| Query 201 | GRFSGFIDCGRLGVADRYQDIALATRDIAEELGGEWADRFLVLYGIA-APDSQRIAFYRL | 259 |
| Sbjct 179 | ID LGVADRY+D+A+ R+++E W + FL YG+ D +R+++YRL SGEPVVIDVPALGVADRYRDLAIVRRELSE---AAW-EEFLAAYGLVDRIDEERLSYYRL | 234 |
| Query 260  Sbjct 235 | LDE 262 LDE  LDE 237 |  |

>TR:A0A2H5AUU2 A0A2H5AUU2_9ACTN Aminoglycoside phosphotransferase OS=Kitasatospora sp. MMS16-BH015 OX=2018025 GN=CFP65_1000 PE=3 SV=1

Length=274

Score = 122 bits (306), Expect = 3e-30

| Identities | | = 99/280 (35%), Positives = 136/280 (49%), Gaps = 40/280 (14%) |  |
| --- | --- | --- | --- |
| Query 6  Sbjct 8 | | GLHAGSPAAWVERLFGYDWAQQTIGCSDAAVFRLSAQGRPVLFVKTDLSGALNELQDEAA G+ G A R Y W T G S AAV+RL A+ L++K A EA GVRVGVRAVLGGRYDRYAWEPVTAGMSGAAVWRLGAE----LYLKVGPGVAA EAE | 65  58 |
| Query 66  Sbjct 59 | | RLSWLATTGVPCAAVLDV-VTEAGRDWLLLGEVPGQDLLSSHLAP--AEKVSIMADAMRR R +WLA G+P V++ + E +W+L + G+ A A V +AD R RCAWLAGYGLPAPEVVEHGIDEEHGEWVLSAALAGRSAAEPWPAGQRAGVVDALADLARA | 122  118 |
| Query 123  Sbjct 119 | | LHTLDPATCPFDHQAKHRIERARTRMEAGLVDQDDLDEEHQGLAPAELFARLKARMP--- LH L CPFD + AR + G + + +++ PA L ARL+A P LHGLPVGECPFDRSLAVTLPLARAAVADGSAEAEGVED PAGLLARLEAGWPAEG | 179  172 |
| Query 180  Sbjct 173 | | ------DG EDLVVTHGDACLPNIMVE--NGRFSGFIDCGRLGVADRYQDIALATR  +G EDL V HGD CLPN++++ + R +G ID GRLGVADR D+ L TR  GPVARHEGLRVLEEDLAVCHGDFCLPNVLLDPVSLRVTGVIDLGRLGVADRAADLGLMTR | 226  232 |
| Query | 227 DIAE-----ELGGEWADRFLVLYGIAAPDSQRIAFYRLLD 261 | | |
|  |  | +A+ + G A RFL YG A + QR+A+YR LD |  |
| Sbjct | 233 | SLADGRLNPQYGAAGAARFLDRYGGAVAE-QRLAYYRELD 271 | |

>TR:A0A0Q7DQ20 A0A0Q7DQ20_9CAUL Aminoglycoside 3'-phosphotransferase OS=Phenylobacterium sp. Root1277 OX=1736442 GN=ASC73_02595 PE=3 SV=1

Length=266

Score = 122 bits (305), Expect = 4e-30

| Identities | = 85/225 (38%), Positives = 111/225 (49%), Gaps = 11/225 (5%) |  |
| --- | --- | --- |
| Query 46 | VLFVKTDLSGALNELQDEAARLSWLATTGVPCAAVLDVVTEAGRDWLLLGEVPGQDLLSS | 105 |
| Sbjct 47 | V F D G+ L E + WL V AA L G L+ + GQ + S VKFAPGDHPGSAETLAREGEVVRWLGGR-VRVAATLWSGAFDGGFCLISERLHGQAV--S | 103 |
| Query 106 | HLAP---AEKVSIMADAMRRLHTLDPATCPFDHQAKHRIERARTRMEAGLVDQDDLDEEH | 162 |
| Sbjct 104 | H+AP A+ ++ D + RLH LD CPFD + A R+ GLVD++D D E HVAPHDAADALAATVDLLARLHALDAEGCPFDMSLSAKFALAERRVADGLVDEEDFDAER | 163 |
| Query 163 | QGLAPAELFARLKARMPDGEDLVVTHGDACLPNIMVENGRFSGFIDCGRLGVADRYQDIA | 222 |
| Sbjct 164 | G + R A P E LV+THGDA LPN + GR G +D GR G+ADRYQD+A LGWTARQALDRAIAARPAAERLVLTHGDASLPNFIWSPGRPVGMVDLGRFGLADRYQDLA | 223 |
| Query 223 | LATRDIAEELGGEWADRFLVL---YGIAAPDSQRIAFYRLLDEFF 264 |  |

L R + +	D	+L	Y ++A D	AFYRL+DEFF Sbjct 224 LFLR--SAKFNHPHLDAAAILRERYPLSAVDEAACAFYRLMDEFF 266

>TR:A0A4R2JUF4 A0A4R2JUF4_9PSEU Streptomycin 3'-kinase OS=Actinocrispum wychmicini OX=1213861 GN=EV192_102966 PE=3 SV=1

Length=277

Score = 122 bits (305), Expect = 5e-30

| Identities | | = 90/254 (35%), Positives = 125/254 (49%), Gaps = 17/254 (7%) |  |
| --- | --- | --- | --- |
| Query 21  Sbjct 23 | | GYDWAQQTIGCSDAAVFRLSAQGRPVLFVKTDLSGALNELQDEAARLSWLATTGVPCAAV G +W T G S AAVFRL +F K ++EL+ E R+ WLA T VP ++ GREWIPVTAGESRAAVFRLDDGS---VFAKCVAEDGIDELRGERDRVQWLADTPVPGPSI | 80  79 |
| Query 81  Sbjct 80 | | LDVVTEAGRDWLLLGEVPG--QDLLSSHLAPAEKVSIMADAMRRLHTLDPATCPFDHQAK  ++ + L+ VPG D LSS SI+ +RR+H L A CPF+ + VEWSSSRRGACLVTTAVPGLPADELSSSALLRAWPSIVR-MLRRMHELPTAECPFERRVS | 138  138 |
| Query 139  Sbjct 139 | | HRIERARTRMEAGLVDQDDLDEEHQGLAPAELFARLKA RMPDGEDLVVTHGDACL  A + V+ D L + +G+ L +L+ R + +DLVV HGDACL SMFRIAEDVVRRDAVNPDFLSADQRGVPSTTLLEQLRVELDERRAQEADDLVVCHGDACL | 193  198 |
| Query 194  Sbjct 199 | | PNIMVENGRFS--GFIDCGRLGVADRYQDIALATRDIAEELGGEW---ADRFL-VLYGIA PN +++ S G ID GRLG+ADR+ D+AL + E G+ ADR L YG PNFLIDTDTLSCTGLIDLGRLGIADRHADLALLQANARETWVGDHEARADRVLGTTYGAD | 247  258 |
| Query 248 | | APDSQRIAFYRLLD 261 DS+R FY LD |  |
| Sbjct | 259 | RIDSERSRFYLHLD 272 | |

>TR:A0A364K3T9 A0A364K3T9_9BACL Aminoglycoside 3'-phosphotransferase OS=Thermoflavimicrobium sp. FBKL4.011 OX=2137476 GN=DL897_10120 PE=3

SV=1

Length=273

Score = 121 bits (304), Expect = 6e-30

| Identities | = 85/244 (35%), Positives = 115/244 (47%), Gaps = 4/244 (2%) |  |
| --- | --- | --- |
| Query 24 | WAQQTIGCSDAAVFRLSAQGRPVLFVKTDLSGALNELQDEAARLSWLATTGVPCAAVLDV | 83 |
| Sbjct 31 | W IG S+A V+++ + + + + L+ E L WL +P + WLPNKIGRSNACVYQIISDQQRWYLKIQPYAPFESSLKKEKEILDWLQNK-LPVPRAIYY | 89 |
| Query 84 | VTEAGRDWLLLGEVPGQDLLSSHLA-PAEKVSI-MADAMRRLHTLDPATCPFDHQAKHRI | 141 |
| Sbjct 90 | A ++LLL EVPG AE V I +A ++ +H++ A CPF I KQFATNEFLLLSEVPGWMACDEKWRNQAEHVVIWLAKGLKMIHSIPIANCPFQSDPDKLI | 149 |
| Query 142 | ERARTRMEAGLVDQDDLDEEHQGLAPAELFARLKARMPDGEDLVVTHGDACLPNIMVENG | 201 |
| Sbjct 150 | + A RM G VD++D D G ELF L P D V HGD CLPNI++ QEAYLRMIQGYVDENDFDTARLGKTATELFDLLIQTKPTTYDPVWGHGDYCLPNILISEE | 209 |
| Query 202 | RFSGFIDCGRLGVADRYQDIALATRDIAEELG-GEWADRFLVLYGIAAPDSQRIAFYRLL | 260 |
| Sbjct 210 | GFID R GV DRY D+ALA R I LG E+ F Y + + +I FY+LL GIRGFIDWSRGGVTDRYFDLALAVRSITYNLGKKEYVPLFFQAYELENINQSKIEFYQLL | 269 |
| Query 261  Sbjct 270 | DEFF 264 DEFF  DEFF 273 |  |

```
>TR:A0A2U9TCS3 A0A2U9TCS3_9GAMM Aminoglycoside 3'-phosphotransferase OS=Lysobacter maris OX=1605891 GN=C9I47_1673 PE=3 SV=1
Length=167

Score = 118 bits (296), Expect = 6e-30
Identities = 63/152 (41%), Positives = 89/152 (59%), Gaps = 1/152 (1%)

Query 113 VSIMADAMRRLHTLDPATCPFDHQAKHRIERARTRMEAGLVDQDDLDEEHQGLAPAELFA 172 V +A +RRLH + A CPFD	+R+ +AR R++ G VD DD D+E +G	+++
Sbjct 17	VDAIAAFLRRLHAIPVAECPFDSDRAYRLIQARKRIDTGRVDVDDFDDEREGWTVEQVWK 76

Query 173 RLKARMPDGEDLVVTHGDACLPNIMVENGRFSGFIDCGRLGVADRYQDIALATRDIAEEL 232
+	P	D VVTHGD L N+++ G +G ID GRLGVADRYQD+A+	+
```

| Sbjct | 77 | TMLDLPPFEPDPVVTHGDFTLDNLLIREGEVTGCIDTGRLGVADRYQDLAILWNGLG-HF 135 |
| --- | --- | --- |
| Query | 233 | GGEWADRFLVLYGIAAPDSQRIAFYRLLDEFF 264 |
| Sbjct | 136 | G RFL YGIA + ++ F+ +LDEFF  GPLLQARFLQQYGIAEMNWDKLRFHLMLDEFF 167 |

>TR:A0A2G5IDD5 A0A2G5IDD5_9ACTN APH domain-containing protein OS=Streptomyces sp. HG99 OX=1958787 GN=B1C81_37685 PE=3 SV=1

Length=279

Score = 121 bits (304), Expect = 7e-30

| Identities | = 87/248 (35%), Positives = 127/248 (51%), Gaps = 15/248 (6%) |  |
| --- | --- | --- |
| Query 30 | GCSDAAVFR-LSAQGRPVLFVKTDLSGALN---ELQDEAARLSWLATTGVPCAAVLDVVT | 85 |
| Sbjct 24 | G S A V + L AQ R + FVK + EL+ EA RL WL GV VL + + GLSGARVIKVLDAQDRVLAFVKAASPTTPHLEEELRGEAERLIWLTGQGVRVPEVLGLTS | 83 |
| Query 86 | EAGRDWLLLGEVPGQDLLSSHLAPAEK---VSIMADAMRRLHTLDPATCPFDHQAKHRIE | 142 |
| Sbjct 84 | E G WL + G S H + E+ V+ +A+ +R LH+LDP +CPFD + EDGT-WLATRPLKGAPA-SDHFSSDERFAVVTAVAEGLRTLHSLDPLSCPFDRSLPSALA | 141 |
| Query 143 | RARTRMEAGLVDQDDLDEEHQGLAPAELFARLKARMPDGE--DLVVTHGDACLPNIMVEN | 200 |
| Sbjct 142 | A R GLVD + + A+ L+ + +LVV+HGD CLPN+M+ VAEKRTHEGLVDFSWRLAGKKDVTTADALRDLRQAISATVLGELVVSHGDFCLPNVMILP | 201 |
| Query 201 | GRFSGFIDCGRLGVADRYQDIALATRDIAEELGGEWAD RFLVLYGIAAPDSQRIAF | 256 |
| Sbjct 202 | +GF+D GR GVADR+ D+A R + + ++A+ FL +YG A +S+R+ HGDAGFVDVGRSGVADRHSDVADILRSLRSHMNPQYAEPHAQHFLDVYGRAGIESERLRL | 261 |
| Query 257  Sbjct 262 | YRLLDEFF 264  + L+EFF  HDKLEEFF 269 |  |

>TR:J7L841 J7L841_NOCAA Phosphotransferase enzyme family protein OS=Nocardiopsis alba (strain ATCC BAA-2165 / BE74) OX=1205910 GN=B005_4376

PE=3 SV=1

Length=280

Score = 121 bits (304), Expect = 7e-30

| Identities | = 81/248 (33%), Positives = 125/248 (50%), Gaps = 12/248 (5%) |  |
| --- | --- | --- |
| Query 29 | IGCSDAAVFRL-SAQGRPVLFVKT---DLSGALNELQDEAARLSWLATTGVPCAAVLDVV | 84 |
| Sbjct 22 | +G S A V R+ AQG PV VK + ++E + E ARL+WL GVP VL LGLSGARVLRMVDAQGVPVAVVKVVDHRMGHLVSEFEAETARLAWLRRLGVPVPEVLGSG | 81 |
| Query 85 | TEAGRDWLLLGEVPGQDLLSSHLAPAEK--VSIMADAMRRLHTLDPATCPFDHQAKHRIE | 142 |
| Sbjct 82 | + G WL + +PG+ A + V ++A A R LH PFD + VDEGESWLAMRALPGRPASDPWPAGEQDRVVDLLAHAARELHAASWEDTPFDRSLSSVLV | 141 |
| Query 143 | RARTRMEAGLVDQDDLDEEHQGLAPAELFARLKARMPD-GEDL-VVTHGDACLPNIMVEN | 200 |
| Sbjct 142 | R R+ +GLVD+ + +G A + L++ + G+ + VV+HGD CLPN++++ EVRERVASGLVDRSWVIAGKEGPPAAVVLEELESLAGELGDGIPVVSHGDFCLPNVLIDE | 201 |
| Query 201 | GRFSGFIDCGRLGVADRYQDIALATRDIAEELGGEW ADRFLVLYGIAAPDSQRIAF | 256 |
| Sbjct 202 | +G +D GR G+AD + D+A TR + + ++ A RFL YG D +R+ TGTAGLVDVGRAGLADPHSDLADMTRSLRSHMNPQYGEVSAHRFLDAYGRDGIDPERLRL | 261 |
| Query 257  Sbjct 262 | YRLLDEFF 264  + LL+ FF  HDLLESFF 269 |  |

>TR:A0A132PMX6 A0A132PMX6_9MYCO 3'-kinase OS=Mycolicibacterium wolinskyi OX=59750 GN=AFM11_12930 PE=3 SV=1

Length=266

Score = 121 bits (303), Expect = 8e-30

Identities = 90/250 (36%), Positives = 128/250 (51%), Gaps = 14/250 (6%)

| Query | 22 | YDWAQQTIGCSDAAVFRLSAQGRPVLFVKTDLSGALNELQDEAARLSWLATTGVPCAAVL | 81 |
| --- | --- | --- | --- |
| Sbjct | 2 | +DW T G S A VFR R + K + + +L E R+SW G+P AV+ FDWLPVTHGESGAGVFRSPDGTR---YAKMVAAAEVADLAAERDRVSWANEHGIPAPAVI | 58 |
| Query | 82 | DV-VTEAGRDWLLLGEVPG--QDLLSSHLAPAEKVSIMADAMRRLHTLDPATCPFDHQAK | 138 |
| Sbjct | 59 | D VT+ G +L+ V G D L+ A SI+A A+R LH + CP+ + DWGVTDGGGAYLVTTAVAGVPADRLTEAGLRAAWPSIVA-AVRALHDVPAHACPYRRELD | 117 |
| Query | 139 | HRIERARTRMEAGLVDQDDLDEEHQGLAPAELFARLKA RMPDGEDLVVTHGDACL | 193 |
| Sbjct | 118 | + RAR + A V+ D L +E + + EL AR++ R + DLVV HGD CL SMLARARRVVAADAVNPDFLPDEDRAVPATELLARVERDAAVRRTQEAHDLVVCHGDLCL | 177 |
| Query | 194 | PNIMVENGRFSGFIDCGRLGVADRYQDIALATRDIAEELGGEWADRFLVLYG-IAAP-DS | 251 |
| Sbjct | 178 | PNI+V++ +GFID GRLG ADR+ D+AL + A+ G A+ +L AP D PNILVDDAAVAGFIDLGRLGRADRHADLALLLANTADTFPGFAAEAAALLAAEYPAPIDD | 237 |
| Query | 252 | QRIAFYRLLD 261 R+ +Y LD |  |
| Sbjct | 238 | DRLTYYLDLD 247 | |

>TR:A0A1H0YKS3 A0A1H0YKS3_9ACTN Kanamycin kinase OS=Quadrisphaera sp. DSM 44207 OX=1881057 GN=SAMN05428996_0836 PE=3 SV=1

Length=267

Score = 121 bits (303), Expect = 8e-30

| Identities | = 85/218 (39%), Positives = 110/218 (50%), Gaps = 9/218 (4%) |  |
| --- | --- | --- |
| Query 48 | FVKTDLSGALNELQDEAARLSWLATTGVPCAAVLDVVTEAGRDWLLLGEVPGQDLLSSHL | 107 |
| Sbjct 47 | F K +G +L EA RL+W A P VL +A WLL +PG+ FAKWAPAGGPVDLAREAVRLAW-AVAWTPVPRVLGSGADAHGSWLLTAGLPGRSAADPRW | 105 |
| Query 108 | A--PAEKVSIMADAMRRLHTLDPAT-CPFDHQAKHRIERARTRMEAGLVDQDDLDEEHQG | 164 |
| Sbjct 106 | A PA V + A+R LH P CPF R+ AR R LVD EH+ AGDPAVAVPAVGAALRALHDALPVPGCPFSWSVADRMADARRRSSLRLVDPAQWQAEHRS | 165 |
| Query 165 | LA-PAELFARLKARMPDGEDLVVTHGDACLPNIMV-ENGRFSGFIDCGRLGVADRYQDIA | 222 |
| Sbjct 166 | L PA L L A +PD + LVV HGDA +PN ++ E+GR++G +D G LG ADR+ D+A LTVPAAL--ALAADVPDVDRLVVCHGDAAVPNTLLDEHGRWTGHVDLGLLGTADRWADLA | 223 |
| Query 223  Sbjct 224 | LATRDIAEELGGEWADRFLVLYGIAAPDSQRIAFYRLL 260  +AT G W +R L YG+ PD R +YRLL  VATWSSRWTHGPGWEERLLEAYGV-EPDPDRTRYYRLL 260 |  |

>TR:A0A0T0PUX4 A0A0T0PUX4_9SPHN Aminoglycoside 3'-phosphotransferase OS=Sphingomonas sp. Leaf412 OX=1736370 GN=ASG29_12990 PE=3 SV=1

Length=255

Score = 120 bits (302), Expect = 8e-30

| Identities | | = 92/258 (36%), Positives = 120/258 (47%), Gaps = 11/258 (4%) |  |
| --- | --- | --- | --- |
| Query 12  Sbjct 4 | | PAAWVERLFGYDWAQQTIGCSDAAVFRLSAQGRPVLFVKTDLSGALNELQDEAARLSWLA PAA + + T G S A VFR LF+KT + DEAARL W + PAAIADAIGDRRLVAVTDGQSGATVFRAGED LFLKTGRGRVAGLVADEAARLRW-S | 71  58 |
| Query 72 | | TTGVPCAAVLDVVTEAGRDWLLLGEVPGQ---DLLSSHLAPAEKV-SIMADAMRRLHTLD  +P V+ V E R WLL + G D + + A A V +A + RLH L | 127 |
| Sbjct | 59 | DGRIPVPRVIAAVGEGDRAWLLTSALRGMSAGDHIKADRARATAVPGSLAAFLTRLHALP | 118 |
| Query | 128 | PATCPFDHQAKHRIERARTRMEAGLVDQDDLDEEHQGLAPAELFARLKARMPDGEDLVVT | 187 |
| Sbjct | 119 | CPFD + R R+ GLVD D D+EH + A + AR++ E VV VRDCPFDSSVASWLPVVRRRVADGLVDTGDFDDEHADWSAARVLARVEELACHAEGRVVV | 178 |
| Query | 188 | HGDACLPNIMV-ENGRFSGFIDCGRLGVADRYQDIALATRDIAEELGGEWADRFLVLYGI | 246 |
| Sbjct | 179 | HGD L N++V ++G +G ID G LGV D Y+DI + RDI G + D FL GI HGDFSLGNVIVGDDGDIAGCIDVGLLGVGDPYRDIFIGWRDIG-GFGSDAQDAFLAALGI | 237 |
| Query  Sbjct | 247  238 | AAPDSQRIAFYRLLDEFF 264 R +R LDE F  DRLAEPRRDLHRALDELF 255 |  |

>TR:R4Z516 R4Z516_9ACTN Putative Kanamycin kinase OS=Candidatus Microthrix parvicella RN1 OX=1229780 GN=BN381_80318 PE=3 SV=1

Length=242

Score = 120 bits (300), Expect = 1e-29

| Identities | | = 73/207 (35%), Positives = 109/207 (53%), Gaps = 5/207 (2%) |  |
| --- | --- | --- | --- |
| Query 62  Sbjct 35 | | DEAARLSWLATTGVPCAAVLDVVTEA-GRDWLLLGEVPGQDLL--SSHLAPAEKVSIMAD DE+ RL WLA G P V++ V G LL +PG D + P E V + A DESQRLEWLAMAGAPVPKVIEFVDHGDGTATLLTERIPGFDATRREARARPEELVDLFAK | 118  94 |
| Query 119  Sbjct 95 | | AMRRLH-TLDPATCPFDHQAKHRIERARTRMEAGLVDQDDLDEEHQGLAPAELFARLKAR A+R H L A CPF R+E A+ R++ GL+ ++LD+ ++ + L A L ALRVWHDNLSVADCPFRSDWATRLEVAKARLKRGLIASENLDDAYRRVGFDTLVASLDRT | 177  154 |
| Query 178 | | MPDG-EDLVVTHGDACLPNIMVENGRFSGFIDCGRLGVADRYQDIALATRDIAEELGGEW PD +DLVV HGD LPN+++ G F+G++D GR+GV R+ D+ +A IA +G | 236 |
| Sbjct | 155 | DPDDHDDLVVIHGDPSLPNLILGAGGFAGWVDVGRVGVGSRWCDLMIAATTIASNMGPML | 214 |
| Query | 237 | ADRFLVLYGIAAPDSQRIAFYRLLDEF 263 YG + D+ + Y+LL++F |  |
| Sbjct | 215 | VPTLFEAYGADSADTLAVDAYQLLEQF 241 | |

>TR:D2B8L9 D2B8L9_STRRD Kanamycin kinase OS=Streptosporangium roseum (strain ATCC 12428 / DSM 43021 / JCM 3005 / NI 9100) OX=479432

GN=Sros_5038 PE=3 SV=1

Length=244

Score = 120 bits (300), Expect = 1e-29

| Identities | = 87/240 (36%), Positives = 123/240 (51%), Gaps = 15/240 (6%) |  |
| --- | --- | --- |
| Query 24 | WAQQTIGCSDAAVFRLSAQGRPVLFVKTDLSGALNELQDEAARLSWLATTGVPCAAVLDV | 83 |
| Sbjct 17 | W+ G S A+ +S G V +VK G L + E RL WL VP V+ WSTDHEGASGTALRVIS--GSKVFYVK---HGPL--ARAEHLRLRWLHGR-VPIPEVVAF | 68 |
| Query 84 | VTEAGRDWLLLGEVPGQDLLSSHLAPAEKVSIMADAMRRLHTLDPATCPFDHQAKHRIER | 143 |
| Sbjct 69 | A L+L +V L ++ APA+ +++ +R LH L CPFD + +ER EDPA LVLADVGAPSLEAA--APADVGAVLGRTLRMLHELPITECPFDGRLPAVLER | 122 |
| Query 144 | ARTRMEAGLVDQDDLDEEHQGLAPAELFARLKARMPDGEDLVVTHGDACLPNIMVENGRF | 203 |
| Sbjct 123 | A + GLVD DD D +H GL P ++ RL P EDLVV HGD N+++ AGANVRDGLVDPDDFDTDHVGLTPEAIYERLLVTRPRVEDLVVAHGDYTPSNVLLPASGE | 182 |
| Query 204 | SGFIDCGRLGVADRYQDIALATRDIAEELGGEWADRFLVLYGIAAP-DSQRIAFYRLLDE | 262 |
| Sbjct 183 | ID LGVADRY+D+A+ RD++E+ G + FL YG+ + +R+ +YRLLDE  PVVIDVPALGVADRYRDLAIVHRDLSEDHGPAAWEGFLSAYGLVDHIEEERLYYYRLLDE | 242 |

>TR:A0A0N9IEL0 A0A0N9IEL0_9PSEU 3'-kinase OS=Kibdelosporangium phytohabitans OX=860235 GN=AOZ06_22725 PE=3 SV=1

Length=274

Score = 120 bits (302), Expect = 1e-29

| Identities | = 94/254 (37%), Positives = 123/254 (48%), Gaps = 23/254 (9%) |  |
| --- | --- | --- |
| Query 24 | WAQQTIGCSDAAVFRLSAQGRPVLFVKTDLSGALNELQDEAARLSWLATTGVPCAAVLDV | 83 |
| Sbjct 23 | W T G S A VFR + R + K + + L E R++WLATTGVP +VLD WEPVTNGESGAFVFRSADGSR---YAKCVPAADEDVLTQERDRVAWLATTGVPGPSVLDW | 79 |
| Query 84 | VTEAGRDWLLLGEVPGQDLLSSHLAPAEKVS IMADAMRRLHTLDPATCPFDHQAKH | 139 |
| Sbjct 80 | +T L+ V G +S+ AE++ + +A RRLH L CPF + ITGTAGACLVTSAVHG---VSAERVSAEELRQAWPSITEATRRLHALPARDCPFSRDLEK | 136 |
| Query 140 | RIERARTRMEAGLVDQDDLDEEHQGLAPAELFARLKARM PDGEDLVVTHGDACLP | 194 |
| Sbjct 137 | A+ +E G V+ D L E+ P EL RL A++ + D VV HGD CLP MFTLAQDVVERGAVNPDFLPEDQVDTPPGELLGRLAAQLDLRVEQEAGDTVVCHGDLCLP | 196 |
| Query 195 | NIMVENG--RFSGFIDCGRLGVADRYQDIALATRDIAEELGGEWA DRFLVLYGIA  NI+++ F+GFID GRLG ADRY DI L + E GE A + F YGI | 247 |

| Sbjct | 197 | NIVLDPATLTFAGFIDLGRLGKADRYADICLLLANSRETWDGEDASVAADEAFARDYGIT 256 |
| --- | --- | --- |
| Query | 248 | APDSQRIAFYRLLD 261 |
| Sbjct | 257 | D R FY LD  L-DHARQEFYLHLD 269 |

>TR:A0A326U2D3 A0A326U2D3_9CHLR Kanamycin kinase/aminoglycoside 3'-phosphotransferase-2 OS=Thermosporothrix hazakensis OX=644383 GN=EI42_04292

PE=3 SV=1

Length=265

Score = 120 bits (301), Expect = 1e-29

| Identities | = 87/243 (36%), Positives = 127/243 (52%), Gaps = 21/243 (9%) |  |
| --- | --- | --- |
| Query 29 | IGCSDAAVFRLSAQGRPVLFVKTDLSGALNELQDEAARLSWLATTGVPCAAVLDVVTEAG | 88 |
| Sbjct 31 | IGCS + VF++ ++K A +L E RL WL +P V + IGCSLSRVFQIGPY YLKMTPESAAIKLLAEKERLEWLQGR-LPVPQVHFYGQDER | 84 |
| Query 89 | RDWLLLGEVPGQ---DLLSSHLAPAEKVSIMADAMRRLHTLDPATCPFDHQAKHRIERAR | 145 |
| Sbjct 85 | ++LLL VPG+ D L PA V ++A+ +R++H + A CPFD + + R+ RAR FEYLLLSTVPGKLSCDRLFLSDLPA-LVRLLAEGLRQIHAIPIADCPFDMRLEQRLSRAR | 143 |
| Query 146 | TRMEAGLVDQDDLDEEHQGLAPAELFARLKARMPDGEDLVVTHGDACLPNIMVENGRFSG | 205 |
| Sbjct 144 | + + + + L+ RL P ED V+THGD CLPNI++++ R SG SYVYSNRIADAPSWAFRSAEEARALYERLAQTQPP-EDPVLTHGDYCLPNILIQSWRISG | 202 |
| Query 206 | FIDCGRLGVADRYQDIALATRDI AEELGGEWADRFLVLYGIAAPDSQRIAFYRL | 259 |
| Sbjct 203 | FID + GVADRY D+ALA R + AE L E F YGI++ D +++ +YRL FIDLAQAGVADRYFDLALACRSLLYNFPEAEPLLPE FFQTYGISSVDREKLDYYRL | 258 |
| Query 260  Sbjct 259 | LDE 262 LDE  LDE 261 |  |

>TR:A0A074LNS5 A0A074LNS5_9BACL APH domain-containing protein OS=Tumebacillus flagellatus OX=1157490 GN=EL26_13470 PE=3 SV=1

Length=253

Score = 120 bits (300), Expect = 2e-29

| Identities | | = 84/249 (34%), Positives = 132/249 (53%), Gaps = 19/249 (8%) |  |
| --- | --- | --- | --- |
| Query 24  Sbjct 14 | | WAQQTIGCSDAAVFRL-SAQGRPVLFVKTDLSGALNELQDEAARLSWLATTGVPCAAVLD W + T+GCS A + RL +G+P ++K A ++L EA R W A +P VLD WEELTVGCSGATLHRLIGLEGQPTRYLKMMSRLAGSDLAFEAERTRWAAQF-LPAPDVLD | 82  72 |
| Query 83  Sbjct 73 | | VVTEAGRDWLLLGEVPGQDLLSSHLA--PAEKVSIMADAMRRLH-TLDPATCPFDHQAKH E ++LL+ EVPG H A A+ V ++A +RR+H TL CP+D + FREEGVFEYLLMSEVPGVHAADEHFAGRAADIVPLLAAGLRRVHETLPVNLCPYDMRTDV | 139  132 |
| Query 140  Sbjct 133 | | RIERARTRMEAGLVDQDDLDEEHQGLAPAELFARLKARMPDGE-DLVVTHGDACLPNIMV  ++ A+ + +GL+++ + E AEL +++R E DLVV HGD PN+M+ LLQVAKINLHSGLMEEGIENPE AELQELIQSRPETTEQDLVVIHGDYSFPNVML | 198  186 |
| Query 199  Sbjct 187 | | E--NGRFSGFIDCGRLGVADRYQDIALATRDIAEELGGEWADRFLVLYGIAAP--DSQRI SG++D G LG ADRY D+A+A + + +G W FL Y AP D +++ TPAGDAVSGYVDWGALGTADRYLDLAIAVKSVVYNVGESWVPVFLKSY---APDVDLRKL | 254  243 |
| Query | 255 AFYRLLDEF 263 | | |
|  |  | ++R ++ F |  |
| Sbjct | 244 | EWFRRVNRF 252 | |

```
>TR:A0A1H6ESS3 A0A1H6ESS3_9ACTN Kanamycin kinase OS=Nonomuraea solani OX=1144553 GN=SAMN05444920_114281 PE=3 SV=1
Length=268

Score = 120 bits (300), Expect = 2e-29
Identities = 90/230 (39%), Positives = 123/230 (53%), Gaps = 11/230 (5%) Query 37	FRLSAQGRPVLFVKTDLSGALN-ELQDEAARLSWLATTGVPCAAVLDVVTEAGRDWLLLG 95

FRL	G V + K	+G	+L EA RL+W A	V	VLD T+	WL+
```

| Sbjct | 40 | FRLDDPGGGVRYAKWVAAGTPEIDLPAEAVRLAW-AQRWVKVPPVLDHGTDGDGAWLVTE | 98 |
| --- | --- | --- | --- |
| Query | 96 | EVPGQDLLSSHLA--PAEKVSIMADAMRRLHTLDPA-TCPFDHQAKHRIERARTRMEAGL | 152 |
| Sbjct | 99 | VPG +S PA + + +R LH PA CPFD + R+ RA R+ G AVPGTSAVSPRWVTEPATAAAGIGRGLRLLHDALPAGECPFDWSVERRLPRADERIADG- | 157 |
| Query | 153 | VDQDDLDEEHQGLAPAELFARLKARMPDGEDLVVTHGDACLPNIMV-ENGRFSGFIDCGR | 211 |
| Sbjct | 158 | DD EH+ L AE ARL A P + LVV HGDAC+PN ++ ++G F+ +D G EGPDDWSPEHRHLGVAEARARLGAPPPI-DRLVVCHGDACVPNTLLHDDGTFAAHVDLGS | 216 |
| Query  Sbjct | 212  217 | LGVADRYQDIALATRDIAEELGGEWADRFLVL-YGIAAPDSQRIAFYRLL 260 LGVADR+ D+A+A + ++ G + D FL YG+ PD +R+A+YRLL LGVADRWADLAVAAWSLDQDYGPAF-DAFLYKGYGV-EPDGERLAYYRLL 264 |  |

>TR:A0A1M5ZB99 A0A1M5ZB99_9FIRM Kanamycin kinase OS=Sporanaerobacter acetigenes DSM 13106 OX=1123281 GN=SAMN02745180_02905 PE=3 SV=1

Length=261

Score = 119 bits (299), Expect = 3e-29

| Identities | | = 81/262 (31%), Positives = 133/262 (51%), Gaps = 23/262 (9%) |  |
| --- | --- | --- | --- |
| Query 17  Sbjct 9 | | ERLFGYDWAQQTIGCSDAAVFRLSAQGRPVLFVKTDLSGALNELQDEAARLSWLATTGVP E + G ++ TIG S+A V VL ++ E +E +SW++ +P EIIGGIPYSIDTIGMSNAKVICFEDM---VLKIEKQQE ESDNEHIMMSWISDK-LP | 76  60 |
| Query 77  Sbjct 61 | | CAAVLDVVTEAGRDWLLLGEVPGQDLLSSHLA--PAEKVSIMADAMRRLHTLDPATCPFD VL G ++LL+ + G+ SS L P V ++AD ++ L +D + CPF+ VPRVLCFEKHNGINYLLMSRIKGKMSCSSELLENPMHLVKLLADGLKMLWGVDISKCPFN | 134  120 |
| Query 135  Sbjct 121 | | HQAKHRIERARTRMEAGLVDQDDLDEEHQGL APAELFARLKARMPDGEDLVVTHGD  + +++ +A R+ L D +D + G +P +L LK PD E+LV +HGD NSIDNKLRQAEFRVRNNLCDMEDAEPTTYGAEGFESPEKLLQWLKENKPD-EELVFSHGD | 190  179 |
| Query 191 | | ACLPNIMVENGRFSGFIDCGRLGVADRYQDIALATRDIAEELGGEWADRFLVLYGIA--- CLPNI++++ + +GFID GR G+AD+YQDIAL R + G++ + + + | 247 |
| Sbjct | 180 | YCLPNIIIKDDKINGFIDLGRSGIADKYQDIALCYRSLQHNYDGKYGGKVYEGFDASILF | 239 |
| Query  Sbjct | 248  240 | -----APDSQRIAFYRLLDEFF 264 PD +I ++ LLDE F  EELNIVPDWNKIKYFILLDELF 261 |  |

>TR:A0A318LPV1 A0A318LPV1_9PSEU APH(3'') family aminoglycoside O-phosphotransferase OS=Prauserella sp. YIM 121212 OX=1477506 GN=BA062_07525

PE=3 SV=1

Length=259

Score = 119 bits (297), Expect = 5e-29

| Identities | | = 92/255 (36%), Positives = 119/255 (47%), Gaps = 23/255 (9%) |  |
| --- | --- | --- | --- |
| Query 23  Sbjct 7 | | DWAQQTIGCSDAAVFRLSAQGRPVLFVKTDLSGALNELQDEAARLSWLATTGVPCAAVLD DW T G S A V R + R + K + EL E R+ WLA TGVP VLD DWNPVTTGESGATVLRDTTGSR---YAKHVPASGREELAAERDRVGWLAGTGVPGPRVLD | 82  63 |
| Query 83 | | VVTEAGRDWLLLGEVPGQDLLSSHLAPAEKVSI MADAMRRLHTLDPATCPFDHQAK  + L+ VPG + + P E ++ +AD +R LH+L CPF | 138 |
| Sbjct | 64 | WAEDHTGACLVTSAVPG---VPADTVPPESLATAWPSIADTVRHLHSLSTVDCPFRRDLA | 120 |
| Query | 139 | HRIERARTRMEAGLVDQDDLDEEHQGLAPAELFARLKARM PDGEDLVVTHGDACL | 193 |
| Sbjct | 121 | AR + V+ D L E Q PAEL ARL+ ++ + D VV HGD CL LMFPLARDVVARDAVEPDFLPVEQQDTPPAELLARLEPQLDRRLAEEPADTVVCHGDLCL | 180 |
| Query | 194 | PNIMVENG--RFSGFIDCGRLGVADRYQDIALATRDIAEELGGEWADR FLVLYGI | 246 |
| Sbjct | 181 | PNI+++ G SG ID GRLG+ADR+ D+AL + E E R F YG PNIVLDPGTLEVSGLIDLGRLGLADRHADLALLLANSRETWPDETRARAADAVFAERYG- | 239 |
| Query | 247 | AAPDSQRIAFYRLLD 261 PD R+ FY LD |  |
| Sbjct | 240 | RRPDPDRLRFYLHLD 254 | |

```
>TR:A0A371PQL4 A0A371PQL4_9ACTN APH(3'') family aminoglycoside O-phosphotransferase OS=Streptomyces sp. NEAU-D10 OX=2293571 GN=aph(3'')
PE=3 SV=1
Length=275

Score = 119 bits (298), Expect = 5e-29
Identities = 103/282 (37%), Positives = 130/282 (46%), Gaps = 33/282 (12%)

Query 1	MIEQDGLHAGSPAAWVERLFGYDWAQQTIGCSDAAVFRLSAQGRPVLFVKTDLSGALNEL 60 M E+ G	SP	E	+W	T G S AAVFR SA G	+ K	+	+L
Sbjct 1	MSERPGPQTVSPVLLGEGGGEGNWLPVTAGESGAAVFR-SADG--TRYAKCVAAADAADL 57
Query 61	QDEAARLSWLATTGVPCAAVLDVVTEAGRDWLLLGEVPGQDLLSSHLAPAEKVSI	115
+ E R++WL+ GVP	VLD T	L+ G V G	PA++VS
Sbjct 58	KAERDRVAWLSDQGVPGPRVLDWHTGVAGACLVTGAVSG--------VPADQVSAADLRA	109
Query 116 ----MADAMRRLHTLDPATCPFDHQAKHRIERARTRMEAGLVDQDDLDEEHQGLAPAELF	171
+ADA+R LH L	CPF	+ AR + G V+ + L E Q	AEL Sbjct 110 SWERIADAVRGLHELPVQQCPFRRDVDGMVAIARDVVTRGAVNPEFLPVEQQHTPAAELL	169
Query 172 ARL-----KARMPDGEDLVVTHGDACLPNIMVENGRF--SGFIDCGRLGVADRYQDIALA	224
ARL	+ R + D VV HGD CLPNI+++	SGFID GRLG+ADRY D+AL Sbjct 170 ARLVPQVARRRDQEAADTVVCHGDLCLPNIVLDPQTLDVSGFIDLGRLGLADRYADLALL	229
Query 225 TRDIAEELGGEWADR-----FLVLYGIAAPDSQRIAFYRLLD	261
E	E	R	F	YGI+ D R+ FY LD Sbjct 230 LTSARETWTDEERARAADAAFAEKYGISL-DHDRLRFYLHLD	270

>TR:A0A511MYS9 A0A511MYS9_9DEIO Aminoglycoside 3'-phosphotransferase OS=Deinococcus cellulosilyticus NBRC 106333 = KACC 11606 OX=1223518
GN=DC3_11360 PE=3 SV=1
Length=248

Score = 118 bits (296), Expect = 5e-29
```

| Identities | = 79/241 (33%), Positives = 112/241 (46%), Gaps = 13/241 (5%) |  |
| --- | --- | --- |
| Query 24 | WAQQTIGCSDAAVFRLSAQGRPVLFVKTDLSGALNELQDEAARLSWLATTGVPCAAVLDV | 83 |
| Sbjct 19 | W Q IG S A V+ F+K + L+ + L WL VP VL WQQNDIGRSQAMVYFTDRW FLKIQPQASPETLRQDLQALKWLQQH-VPVPEVLGH | 72 |
| Query 84 | VTEAGRDWLLLGEVPGQDLLSSHLAPAEK-VSIMADAMRRLHTLDPATCPFDHQAKHRIE | 142 |
| Sbjct 73 | + G+++L+ + G+DL EK V A +R H CP D R+ AEQDGQEFLITSRIQGRDLSLLAQEDREKAVMHFARGLRLWHGQPTQDCPLDRSLDFRLV | 132 |
| Query 143 | RARTRMEAGLVDQDDLDEEHQGLAPAELFARLKARMPDGEDLVVTHGDACLPNIMVENGR | 202 |
| Sbjct 133 | AR R+E G + + +E +P ++ L PD EDLV+THGD C PN++V+ R EARQRLEQGQIALEAFTDE SPEDVLNWLHNNKPD-EDLVLTHGDYCFPNVLVDQDR | 187 |
| Query 203 | FSGFIDCGRLGVADRYQDIALATRDIAEELGGEWADRFLVLYGIAAPDSQRIAFYRLLDE | 262 |
| Sbjct 188 | GF+D GR G++DRY D+ LA G W FL YG D +++ +Y LLD ICGFVDVGRAGISDRYTDLTLALWSTEYNCGSGWKHVFLQAYGHDL-DQEKLHYYSLLDR | 246 |
| Query 263  Sbjct 247 | F 263 F  F 247 |  |

>TR:Q5YSZ5 Q5YSZ5_NOCFA Aminoglycoside 3'-O-phosphotransferase OS=Nocardia farcinica (strain IFM 10152) OX=247156 GN=aph(3') PE=3 SV=1

Length=263

Score = 119 bits (297), Expect = 5e-29

Identities = 84/205 (41%), Positives = 107/205 (52%), Gaps = 14/205 (7%)

| Query | 63 | EAARLSWLATTGVPCAAVLDVVTEAGRDWLLLGEVPGQDLLSSHLAPAEKVSI---MADA | 119 |
| --- | --- | --- | --- |
| Sbjct | 70 | E RL WL G+ V +VV AG L+L + L + A E V I M EYERLEWLRHNGI---RVPEVVVCAG-GVLVLADAGVPSLATR--ADTEGVPIGATMGAL | 123 |
| Query | 120 | MRRLHTLDPATCPFDHQAKHRIERARTRMEAGLVDQDDLDEEHQGLAPAELFARLKARMP  +RRLH L A CPFD + + +AR + GLVD DD D+++ P + A L R P | 179 |

| Sbjct | 124 | LRRLHALPVAGCPFDGRLDGVLAQARRNVIEGLVDADDFDDDNADHTPESVLAELLDRRP | 183 |
| --- | --- | --- | --- |
| Query | 180 | DGEDLVVTHGDACLPNIMVENGRFSGFIDCGRLGVADRYQDIALATRDIAEELGGEWADR | 239 |
| Sbjct | 184 | DLVV HGD PN++ +G ID G LGVADRY+D+ALA RD+ E G PEADLVVAHGDYTPPNVL--DGDL--LIDVGALGVADRYRDLALAERDL-REYGPAEVTA | 238 |
| Query  Sbjct | 240  239 | FLVLYGIAAPDSQRIAFYRLLDEFF 264 F YG+ PD R+ +YRLLDE F FFDAYGLPEPDPARLEYYRLLDELF 263 |  |

>TR:A0A212TEC1 A0A212TEC1_9ACTN Kanamycin kinase OS=Streptomyces sp. 2114.4 OX=1938836 GN=SAMN06272741_1076 PE=3 SV=1

Length=267

Score = 119 bits (297), Expect = 6e-29

| Identities | | = 78/219 (36%), Positives = 111/219 (51%), Gaps = 6/219 (3%) |  |
| --- | --- | --- | --- |
| Query 48  Sbjct 48 | | FVKTDLSGALNELQDEAARLSWLATTGVPCAAVLDVVTEAGRDWLLLGEVPGQDLLSSHL FVK +G+ +L E ARL W A + VL ++ W++ +PG+ + FVKWTPAGSGIDLAAEVARLRWAARF-IVVPRVLGEGSDRTGSWIVTHGLPGRMAVDDRW | 107  106 |
| Query 108 | | A--PAEKVSIMADAMRRLHTLDP-ATCPFDHQAKHRIERARTRMEAGLVDQDDLDEEHQG P V + +R LH P A CPFD A+ R+E R+R AG +D D E+ Q | 164 |
| Sbjct | 107 | KRDPGTAVRAIGSGLRALHDALPVANCPFDWSAEARLEAVRSRAAAGRIDPADWPEDLQH | 166 |
| Query | 165 | LAPAELFARLKARMPDGEDLVVTHGDACLPNIMV-ENGRFSGFIDCGRLGVADRYQDIAL | 223 |
| Sbjct | 167 | E + +P + LVV HGDAC PN +V ++G +G +D G LGVADR+ D+A+  FGTVERALGVLTDIPPVDRLVVCHGDACAPNTLVGDDGTCTGHVDLGTLGVADRWADLAI | 226 |
| Query | 224 ATRDIAEELGGEWADRFLVLYGIAAPDSQRIAFYRLLDE 262 | | |
|  |  | AT G W + L YG+ PD +R +YRLL E |  |
| Sbjct | 227 | ATWSTQWNYGPGWEEPLLKAYGV-EPDPERTRYYRLLWE 264 | |

>TR:A0A3N4SJR5 A0A3N4SJR5_9ACTN Kanamycin kinase OS=Streptomyces sp. Ag109_O5-1 OX=1938851 GN=EDD90_6904 PE=3 SV=1

Length=267

Score = 119 bits (297), Expect = 6e-29

| Identities | | = 91/261 (35%), Positives = 125/261 (48%), Gaps = 24/261 (9%) |  |
| --- | --- | --- | --- |
| Query 7  Sbjct 19 | | LHAGSPA--AWVERLFGYDWAQQTIGCSDAAVFRLSAQGRPVLFVKTDLSGALNELQDEA L AG PA W L G + IG DA F VK +G+ +L E LAAGRPARAVWKNELGGLTFQ---IGLGDARQF VKWAPAGSGLDLAAEV | 64  64 |
| Query 65  Sbjct 65 | | ARLSWLAT-TGVPCAAVLDVVTEAGRDWLLLGEVPGQDLLSSHLA--PAEKVSIMADAMR ARL W A T VP VLD + W++ +PG+ H P V + +R ARLRWAARFTVVP--RVLDEGADDTGSWIVTRGLPGRMAADDHWKRDPGTAVRAIGAGLR | 121  122 |
| Query 122  Sbjct 123 | | RLH-TLDPATCPFDHQAKHRIERARTRMEAGLVDQDDLDEEHQGLAPAELFARLKARMPD LH +L A CPFD A+ R++ R+R A +D D + + E + A +P ALHDSLPVANCPFDWSAERRLQAVRSRAAAQRIDPADWHRDLRHFGTVERALDVLADIPS | 180  182 |
| Query 181  Sbjct 183 | | GEDLVVTHGDACLPNIMV-ENGRFSGFIDCGRLGVADRYQDIALATRDIAEELGGEWADR  + LVV HGDAC PN +V ++G +G +D G LGVADR+ D+A+AT G W + ADRLVVCHGDACAPNTLVGDDGTCTGHVDLGALGVADRWADLAVATWSTQWNYGPGWEET | 239  242 |
| Query 240 | | FLVLYGIAAPDSQRIAFYRLL 260 L Y + PD +RI +YRLL |  |
| Sbjct | 243 | LLDAYEV-DPDRERITYYRLL 262 | |

>TR:A0A2M8WS54 A0A2M8WS54_9MICO Kanamycin kinase OS=Luteimicrobium subarcticum OX=620910 GN=CLV34_1257 PE=3 SV=1

Length=268

Score = 119 bits (297), Expect = 6e-29

Identities = 87/230 (38%), Positives = 113/230 (49%), Gaps = 23/230 (10%)

Query 37	FRLSAQ-GRPVLFVKTDLSGALN-ELQDEAARLSWLATTGVPCAAVLDVVTEAGRDWLLL 94 FRL + G	+VK	GA + +L EA RL W A+	P	VLD	+A	WL+

| Sbjct | 52 | FRLGPESGASTRYVKWAPVGAADLDLAAEAVRLRW-ASPFTPVPPVLDAGEDADGSWLVT | 110 |
| --- | --- | --- | --- |
| Query | 95 | GEVPGQDLLSSHLA--PAEKVSIMADAMRRLHTLDPAT-CPFDHQAKHRIERARTRMEAG | 151 |
| Sbjct | 111 | VPG + P + V + + +R LH P CPF R+ R+ AAVPGASAVDPVWLDRPRDAVRAIGEGLRALHDALPVDGCPFGWGVDERLARSVA----- | 165 |
| Query | 152 | LVDQDDLDEEHQGLAPAELFARLKARMPDGEDLVVTHGDACLPN-IMVENGRFSGFIDCG | 210 |
| Sbjct | 166 | D H AE A L+AR PD + LVV HGDAC PN ++ ++GR+S +D G  -------DGVH---TDAERAAALRARAPDVDRLVVCHGDACAPNTLLTDDGRWSAHVDLG | 215 |
| Query | 211 RLGVADRYQDIALATRDIAEELGGEWADRFLVLYGIAAPDSQRIAFYRLL 260 | | |
|  |  | RLGVADR+ D+A+AT G W D L YG+ APD R AFYR L |  |
| Sbjct | 216 | RLGVADRWADLAVATWSTQWNYGPGWEDELLAAYGV-APDPDRTAFYREL 264 | |

>TR:A0A0F7N6E9 A0A0F7N6E9_9ACTN 3'-kinase OS=Streptomyces sp. CNQ-509 OX=444103 GN=AA958_20680 PE=3 SV=1

Length=262

Score = 118 bits (296), Expect = 7e-29

| Identities | | = 94/260 (36%), Positives = 125/260 (48%), Gaps = 33/260 (13%) |  |
| --- | --- | --- | --- |
| Query 23  Sbjct 10 | | DWAQQTIGCSDAAVFRLSAQGRPVLFVKTDLSGALNELQDEAARLSWLATTGVPCAAVLD  +W T G S AAVFR + R V +++ EL E R++WLAT GVP VLD EWTPVTTGESGAAVFRSTDGTRYARCVPAEVAA---ELTGERDRIAWLATQGVPGPRVLD | 82  66 |
| Query 83  Sbjct 67 | | VVTEAGRDWLLLGEVPGQDLLSSHLAPAEKVSI MADAMRRLHTLDPATCPF  ++ L+ G VPG PA+++S +ADA+R LH + A CPF FRSDDVGACLVTGAVPG VPADQLSAADLRACWKRIADAVRALHEVPIAECPF | 133  118 |
| Query 134  Sbjct 119 | | DHQAKHRIERARTRMEAGLVDQDDLDEEHQGLAPAELFARL KARMPDGEDLVVTH  I ART + G V+ L E Q +L ARL + R + D VV H HRDLDLMISVARTVVAVGTVNPAFLPVEQQTTPAPQLLARLEPQLARRRAQEAADTVVCH | 188  178 |
| Query 189  Sbjct 179 | | GDACLPNIMVENGRF--SGFIDCGRLGVADRYQDIALATRDIAEELGGEWADR FL  GD LPNI+++ +GF+D GRLG+ADR+ D+AL + E E R F GDLTLPNIILDPATLDVAGFVDLGRLGLADRHADLALLLANSRETWPDEAEARAQDAAFA | 241  238 |
| Query 242 | | VLYGIAAPDSQRIAFYRLLD 261 YG APD R+ FY LD |  |
| Sbjct | 239 | GHYG-TAPDPDRLRFYLHLD 257 | |

>TR:A0A0A0B8D7 A0A0A0B8D7_9CELL Aminoglycoside phosphotransferase OS=Cellulomonas cellasea DSM 20118 OX=1408250 GN=Q760_00925 PE=3 SV=1

Length=263

Score = 118 bits (296), Expect = 7e-29

| Identities | = 83/217 (38%), Positives = 114/217 (53%), Gaps = 10/217 (5%) |  |
| --- | --- | --- |
| Query 48 | FVKTDLSGALNELQDEAARLSWLATTGVPCAAVLDVVTEAGRDWLLLGEVPGQDLLSSHL | 107 |
| Sbjct 48 | FVK +GA +L EA RL+W A P VLD ++A WL+ + G + FVKWLPAGARADLSAEAVRLAWAAPF-TPVPRVLDAGSDADGSWLVTAALDGTSAVDPRW | 106 |
| Query 108 | APAEKVSIMA--DAMRRLHTLDPAT-CPFDHQAKHRIERARTRMEAGLVDQDDLDEEHQG | 164 |
| Sbjct 107 | +V++ A + +R LH P CPF + R+ RA T G D +H VARPEVAVRALGEGLRALHDRLPVEDCPFAWDVRDRLTRALT---GGRDDPATWHPDHAH | 163 |
| Query 165 | LAPAELFARLKARMPDGEDLVVTHGDACLPNIMVEN-GRFSGFIDCGRLGVADRYQDIAL | 223 |
| Sbjct 164 | L AE ARL A +P + LVV HGDAC+PN ++++ GRF+ +D G LGVADR+ D+A+ LTVAEATARL-ADVPTVDRLVVCHGDACVPNTLLDDDGRFAAHVDLGTLGVADRWADLAI | 222 |
| Query 224  Sbjct 223 | ATRDIAEELGGEWADRFLVLYGIAAPDSQRIAFYRLL 260 AT G D L YG+ APD +R A+YRLL ATWSTQWNYGPGLDDVLLDAYGV-APDPERTAYYRLL 258 |  |

>TR:A0A2A3I6G9 A0A2A3I6G9_9ACTN Kanamycin kinase OS=Streptomyces sp. TLI_235 OX=1938860 GN=BX265_3238 PE=3 SV=1

Length=251

Score = 118 bits (295), Expect = 8e-29

| Identities | = 82/221 (37%), Positives = 111/221 (50%), Gaps = 14/221 (6%) |  |
| --- | --- | --- |
| Query 48 | FVKTDLSGALNELQDEAARLSWLATTGVPCAAVLDVVTEAGRDWLLLGEVPGQDLLSSHL | 107 |
| Sbjct 32 | FVK +G+ L E RL W A P VL+ + G W++ +PG+ + FVKWLPAGSDGGLTAEVVRLRWAARY-TPVPRVLEEGADEGGSWIVTAGLPGRSAVDDRW | 90 |
| Query 108 | A--PAEKVSIMADAMRRLHTLDPAT-CPFDHQAKHRIERARTRMEAGLVDQDDLDEEHQG | 164 |
| Sbjct 91 | PA V + + +R LH P CPF A+ R+ R R AG +D D G LRDPATAVRAVGEGLRALHEALPVRDCPFAWSAEQRLAAVRARAAAGRIDPAD---RPAG | 147 |
| Query 165 | LAP AELFARLKARMPDGEDLVVTHGDACLPNIMV-ENGRFSGFIDCGRLGVADRYQ | 219 |
| Sbjct 148 | L P E A L A +P + VV HGDAC PN ++ ++GR+SG +D G LG ADR+ LPPFRTVEEALAAL-ADVPPVDGPVVCHGDACAPNTLLGKDGRYSGHVDLGALGTADRWA | 206 |
| Query 220  Sbjct 207 | DIALATRDIAEELGGEWADRFLVLYGIAAPDSQRIAFYRLL 260 D+A+AT G W + L YGI APD +R A+YRLL DLAVATWSTRWNYGPGWEEPMLAAYGI-APDRERTAYYRLL 246 |  |

```
>TR:D3Q100 D3Q100_STANL 3''-kinase OS=Stackebrandtia nassauensis (strain DSM 44728 / CIP 108903 / NRRL B-16338 / NBRC 102104 / LLR-40K-21) OX=446470 GN=Snas_4099 PE=3 SV=1
Length=273

Score = 118 bits (296), Expect = 9e-29
Identities = 104/270 (39%), Positives = 130/270 (48%), Gaps = 26/270 (10%)

Query 12	PAAWVER--LFGYD---WAQQTIGCSDAAVFRLSAQGRPVLFVKTDLSGALNELQDEAAR 66 P AW	L G D	W	T G S AAVFR SA G	F K	+	ELQDE R
Sbjct 5	PEAWTASSVLLGVDEGAWLPITTGQSGAAVFR-SADG--TRFAKCVPAADAAELQDERDR 61

Query 67	LSWLATTGVPCAAVLDVVT-EAGRDWLL--LGEVPGQDLLSSHLAPAEKVSIMADAMRRL 123
+WL	GVP	VLD + EAG	+	+ VP	+ S L A	+ADA+R+L Sbjct 62	TTWLNDQGVPGPRVLDWRSGEAGACLVTTAVSGVPADQVSSEDLRRAW--GSIADALRQL 119
Query 124 HTLDPATCPFDHQAKHRIERARTRMEAGLVDQDDLDEEHQGLAPAELFARLKARMP	179
H L + CPF	+ AR +	V+ + L E +G	AEL R+ A++P
Sbjct 120 HELPVSQCPFRWGLDSMVALARDVVARDAVNPEFLPVEQRGTPAAELLERIVAQVPQRRE	179
Query 180 -DGEDLVVTHGDACLPNIMV--ENGRFSGFIDCGRLGVADRYQDIALATRDIAEELGGEW	236
+ D VV HGD CLPNI++ E	+GFID GRLG+ADRY D+AL	+ E	E Sbjct 180 QEAADTVVCHGDLCLPNIILDPETLDVAGFIDLGRLGLADRYIDVALLLTNARENWTDEE	239
Query 237 ADR-----FLVLYGIAAPDSQRIAFYRLLD	261
R	F	YGI	D R+ FY LD Sbjct 240 QARTADVAFAEKYGIDL-DHDRLRFYLHLD	268

>TR:A0A366DUC7 A0A366DUC7_9NOCA Streptomycin 3'-kinase OS=Nocardia puris OX=208602 GN=DFR74_102113 PE=3 SV=1
Length=268

Score = 118 bits (295), Expect = 1e-28
```

| Identities | | = 95/253 (38%), Positives = 119/253 (47%), Gaps = 21/253 (8%) |  |
| --- | --- | --- | --- |
| Query 24  Sbjct 17 | | WAQQTIGCSDAAVFRLSAQGRPVLFVKTDLSGALNELQDEAARLSWLATTGVPCAAVLDV W G S A VFR + R + K + + L E RL W +PCA VLD WTPVASGESGAGVFRSADGSR---YAKCVVDQDVPALDGERERLVWAGAHDIPCAPVLDW | 83  73 |
| Query 84 | | VTEAGRDWLLLGEVPG---QDLLSSHLAPAEKVSIMADAMRRLHTLDPATCPFDHQAKHR AG L+ G VPG + L + L A + +ADA+RRLH A CPFD | 140 |
| Sbjct | 74 | HAGAGIACLVTGTVPGVTAETLSADELGRAW--ASIADAVRRLHETPTADCPFDRGVARM | 131 |
| Query | 141 | IERARTRMEAGLVDQDDLDEEHQGLAPAELFARLKARM PDGEDLVVTHGDACLPN | 195 |
| Sbjct | 132 | A+ + V + L EE G PA L ARL AR+ + + VV GD CLPN FALAQDVVARDAVHPEFLPEEQVGTPPAVLLARLAARIDARLAQERANAVVCQGDLCLPN | 191 |
| Query | 196 | IMVE--NGRFSGFIDCGRLGVADRYQDIALATRDIAE ELGGEWADR-FLVLYGIAA | 248 |
| Sbjct | 192 | I+V+ +GF+D GRLG AD Y DIAL E E ADR F YG+ IVVDPATDTVTGFLDLGRLGTADPYADIALLLASSRETWPDEPSAAKADRDFAHRYGLTL | 251 |
| Query | 249 | PDSQRIAFYRLLD 261 |  |

DS+R+ FY LD Sbjct 252 -DSERLRFYLHLD 263

>TR:A0A1H3RPA8 A0A1H3RPA8_9FIRM Kanamycin kinase OS=Proteiniborus ethanoligenes OX=415015 GN=SAMN05660462_02468 PE=3 SV=1

Length=261

Score = 117 bits (294), Expect = 1e-28

| Identities | | = 80/262 (31%), Positives = 133/262 (51%), Gaps = 23/262 (9%) |  |
| --- | --- | --- | --- |
| Query 17  Sbjct 9 | | ERLFGYDWAQQTIGCSDAAVFRLSAQGRPVLFVKTDLSGALNELQDEAARLSWLATTGVP E + G ++ TIG S+A V VL ++ E +E + W++ +P EIIGGIPYSIDTIGMSNAKVICFEDM---VLKIEKQQE ESDNEHIMMLWISDK-LP | 76  60 |
| Query 77  Sbjct 61 | | CAAVLDVVTEAGRDWLLLGEVPGQDLLSSHLA--PAEKVSIMADAMRRLHTLDPATCPFD VL G ++LL+ + G+ SS L P V ++AD ++ L ++D + CPF+ VPRVLCFEKHNGINYLLMSRIKGKMSCSSELLENPMHLVKLLADGLKMLWSVDISKCPFN | 134  120 |
| Query 135  Sbjct 121 | | HQAKHRIERARTRMEAGLVDQDDLDEEHQGL APAELFARLKARMPDGEDLVVTHGD  + +++ +A R+ L D +D + G +P +L LK PD E+LV +HGD NSIDNKLRQAEFRVRNNLCDMEDAEPTTYGAEGFESPEKLLQWLKENKPD-EELVFSHGD | 190  179 |
| Query 191 | | ACLPNIMVENGRFSGFIDCGRLGVADRYQDIALATRDIAEELGGEWADRFLVLYGIA--- CLPNI++++ + +GFID GR G+AD+YQDIAL R + G++ + + + | 247 |
| Sbjct | 180 | YCLPNIIIKDDKINGFIDLGRSGIADKYQDIALFYRSLQHNYDGKYGGKVYEGFDASILF | 239 |
| Query  Sbjct | 248  240 | -----APDSQRIAFYRLLDEFF 264 PD +I ++ LLDE F  EELNIVPDWNKIKYFILLDELF 261 |  |

>TR:A0A4R0H3X8 A0A4R0H3X8_9ACTN Aminoglycoside 3'-phosphotransferase OS=Kribbella soli OX=1124743 GN=E0H45_33035 PE=3 SV=1

Length=255

Score = 117 bits (293), Expect = 2e-28

| Identities | = 91/253 (36%), Positives = 123/253 (49%), Gaps = 24/253 (9%) |  |
| --- | --- | --- |
| Query 23 | DWAQQTIGCSDAAVFRLSAQGRPVLFVKTDLSGALNELQDEAARLSWLATTGVPCAAVLD | 82 |
| Sbjct 8 | DW IG SD +V+R R F K + +L E R+ WLA TG P A V+D DWEPVDIGESDTSVYR RGDTFAKCCDPSGVADLAAERDRIRWLAGTGFPGATVVD | 62 |
| Query 83 | VV------TEAGRDWLLLGEVPGQDLLSSHLAPAEKVSIMAD---AMRRLHTLDPATCPF | 133 |
| Sbjct 63 | + + +G L+ VPG + L PA M + +R LH+L CPF WLEPDGPGSSSGGACLVTSAVPG--VPGDTLPPASHDRAMRNLGATLRDLHSL--TDCPF | 118 |
| Query 134 | DHQAKHRIERARTRMEAGLVDQDDLDEEHQGLAPAELFARLKARMPDGEDL---VVTHGD | 190 |
| Sbjct 119 | + I A + G V+ L +E + L P EL ++ A P E + VV HGD ERPLADVIATATDVVRRGAVNPAFLTDEWRRLQPTELLDQVLAERPYIESVLEPVVCHGD | 178 |
| Query 191 | ACLPNIMV--ENGRFSGFIDCGRLGVADRYQDIALATRDIAEELGGEWADRFLVLYGIAA | 248 |
| Sbjct 179 | ACLPN+ E +G ID GRLG+ADRY D+AL T +E + A F YG+ ACLPNVFFDPETLEVTGLIDLGRLGIADRYSDLALTTIQFHDEWSADPAP-FHEAYGVPD | 237 |
| Query 249  Sbjct 238 | PDSQRIAFYRLLD 261 PD +R+ F+RLLD PDPRRLYFFRLLD 250 |  |

>TR:A0A0C5G322 A0A0C5G322_9ACTN 3'-kinase OS=Streptomyces cyaneogriseus subsp. noncyanogenus OX=477245 GN=TU94_25875 PE=3 SV=1

Length=274

Score = 117 bits (294), Expect = 2e-28

Identities = 98/263 (37%), Positives = 125/263 (48%), Gaps = 35/263 (13%)

| Query | 21 | GYDWAQQTIGCSDAAVFRLSAQGRPVLFVKT-DLSGALNELQDEAARLSWLATTGVPCAA | 79 |
| --- | --- | --- | --- |
|  |  | G DW T G S A VFR + R V D +G L+ E R+ WL+ GVP |  |
| Sbjct | 20 | GGDWLPVTAGESGAFVFRSADAARYAKCVPAADAAG LEAERDRVEWLSGRGVPGPR | 75 |
| Query | 80 | VLDVVTEAGRDWLLLGEVPGQDLLSSHLAPAEKVSI MADAMRRLHTLDPAT | 130 |

| Sbjct | 76 | VLD + L+ V G PA++VS + DA+RRLH + VLDWSSGDAGACLVTSAVSG VPADQVSAEELRLSWGRVTDAVRRLHEVPVPE | 127 |
| --- | --- | --- | --- |
| Query | 131 | CPFDHQAKHRIERARTRMEAGLVDQDDLDEEHQGLAPAELFARLKARMP DGEDLV | 185 |
| Sbjct | 128 | CPF + AR + G V+ + L E Q L PAEL ARL ++P + D V CPFRRGLDAMVALARDVVARGAVNPEFLPVEQQHLPPAELLARLTGQVPRRREQEVADTV | 187 |
| Query | 186 | VTHGDACLPNIMVENGRF--SGFIDCGRLGVADRYQDIALATRDIAEELGGE-WA D | 238 |
| Sbjct | 188 | V HGD CLPNI+++ SGFID GRLG+ADR+ D+AL + E E WA VCHGDLCLPNIILDPRTLDVSGFIDLGRLGLADRHADLALLLANARETWPDEQWARAADA | 247 |
| Query | 239 | RFLVLYGIAAPDSQRIAFYRLLD 261 F YGIA D R+ FY LD |  |
| Sbjct | 248 | AFAERYGIAV-DHDRLRFYLHLD 269 | |

>TR:A0A366ZFP9 A0A366ZFP9_9ACTN Aminoglycoside 3'-phosphotransferase OS=Blastococcus sp. TF02-9 OX=2250576 GN=DQ239_14215 PE=3 SV=1

Length=265

Score = 117 bits (292), Expect = 3e-28

| Identities | = 84/217 (39%), Positives = 111/217 (51%), Gaps = 11/217 (5%) |  |
| --- | --- | --- |
| Query 48 | FVKTDLSGALNELQDEAARLSWLATTGVPCAAVLDVVTEAGRDWLLLGEVPGQDLLSSH- | 106 |
| Sbjct 51 | FVK + EL E RL W A P VL+ + +WLL +PG+ + FVKWAPAAIGLELASEVERLRWAARF-TPVPVVLEHGGDDDGEWLLTAGLPGRSAVDPRW | 109 |
| Query 107 | -LAPAEKVSIMADAMRRLHTLDPAT-CPFDHQAKHRIERARTRMEAGLVDQDDLDEEHQG | 164 |
| Sbjct 110 | P V + +R LH P CPFD A R+ A R EA + ++ EH LAEPGPAVDAIGLGLRALHDALPVVGCPFDWSAGARV--AGVRPEA--MRRERFWPEHAA | 165 |
| Query 165 | LAPAELFARLKARMPDGEDLVVTHGDACLPNIMVEN-GRFSGFIDCGRLGVADRYQDIAL | 223 |
| Sbjct 166 | L+ E+ ARL A +PD + LVV HGDAC PN +VE+ G +SG +D G LGVADR+ D+A+ LSVPEVLARL-ADVPDVDRLVVCHGDACAPNTLVEDDGSWSGHVDLGALGVADRWADLAV | 224 |
| Query 224  Sbjct 225 | ATRDIAEELGGEWADRFLVLYGIAAPDSQRIAFYRLL 260 A G W R L YG+ PD R+A+YRLL AAWSTGWNYGPGWESRLLAAYGV-EPDPGRMAYYRLL 260 |  |

>TR:A0A1N5H2U7 A0A1N5H2U7_9MYCO Probable streptomycin phosphotransferase OS=Mycobacteroides abscessus subsp. abscessus OX=1185650 GN=neo

PE=3 SV=1

Length=259

Score = 116 bits (291), Expect = 4e-28

| Identities | | = 93/254 (37%), Positives = 123/254 (48%), Gaps = 26/254 (10%) |  |
| --- | --- | --- | --- |
| Query 24  Sbjct 11 | | WAQQTIGCSDAAVFRLSAQGRPVLFVKTDLSGALNELQDEAARLSWLATTGVPCAAVLDV W G S A V+R R DL+G E R+ WL+ +P +VL+ WEPIVTGESGACVYRSPDGSRYAKKGGADLAG ERDRIEWLSAQRIPGPSVLEW | 83  63 |
| Query 84 | | VTEAGRDWLLLGEVPG---QDLLSSHLAPAEKVSIMADAMRRLHTLDPATCPFDHQAKHR T + L+ VPG L S L A S +A A+R LH L A CPF | 140 |
| Sbjct | 64 | ATTSDGPLLITSAVPGVPADQLDSEDLGRAW--SGIAAAVRDLHALPTAGCPFSRGLAAM | 121 |
| Query | 141 | IERARTRMEAGLVDQDDLDEEHQGLAPAELFARLKARMP DGEDLVVTHGDACLPN | 195 |
| Sbjct | 122 | AR + V+ D L +E + EL R+ A +P + +DLVV HGD CLPN ATLARDVVARDAVNPDFLTDEDRERPATELLCRVVAELPRRLAQEPDDLVVCHGDLCLPN | 181 |
| Query | 196 | IMVENGRFS--GFIDCGRLGVADRYQDIALATRDIAEELGGE-WA DRFLVLYGIA | 247 |
| Sbjct | 182 | I++ G FS GFID GRLG+ADR+ D+AL + E G+ W +RFL YG A IVIAPGDFSVAGFIDLGRLGLADRHADLALLFANARETWPGDGWVTAAAHERFLNRYG-A | 240 |
| Query  Sbjct | 248  241 | APDSQRIAFYRLLD 261  + D +R+AFY LD  SADPERLAFYLRLD 254 |  |

>TR:A0A4R2Q9K5 A0A4R2Q9K5_9PSEU Streptomycin 3'-kinase OS=Tamaricihabitans halophyticus OX=1262583 GN=EV191_12259 PE=3 SV=1

Length=275

Score = 116 bits (291), Expect = 5e-28

| Identities | = 96/278 (35%), Positives = 129/278 (46%), Gaps = 37/278 (13%) |  |
| --- | --- | --- |
| Query 9 | AGSPAAWVERLFG YDWAQQTIGCSDAAVFRLSAQGRPVLFVKTDLSGALNELQDEA | 64 |
| Sbjct 5 | A SP E L G ++W + T G S A V R + R + K +G + L +E AESPVRAWEHLLGPAAPHEWTRVTNGESGADVLRSTDGAR---YAKCVPAGQQDLLAEER | 61 |
| Query 65 | ARLSWLATTGVPCAAVLDVVTEAGRDWLLLGEVPGQDLLSSHLAPAEKVSI--------- | 115 |
| Sbjct 62 | R++WL+ G+PC VLD +A LL V G PA+ VS DRVAWLSQRGIPCPTVLDWTADATGACLLTSAVQG IPADSVSATELWRAWPS | 113 |
| Query 116 | MADAMRRLHTLDPATCPFDHQAKHRIERARTRMEAGLVDQDDLDEEHQGLAPAELFARLK | 175 |
| Sbjct 114 | + +A+R LH L CPF + A + V+ + L E Q + +L ARL IIEAVRELHALPSHDCPFHRDLRRMFAVAEDVVARDAVNPEFLPVEQQQMPGRDLLARLA | 173 |
| Query 176 | ARMPDG-----EDLVVTHGDACLPNIMVENGR--FSGFIDCGRLGVADRYQDIALA---T | 225 |
| Sbjct 174 | A++ D D VV HGD CLPNI+++ F+GFID GRLG ADRY DIAL + AQLDDRLAEEPADTVVCHGDLCLPNIILDADTLSFAGFIDLGRLGRADRYADIALLLANS | 233 |
| Query 226  Sbjct 234 | RDI--AEELGGEWADRFLVLYGIAAPDSQRIAFYRLLD 261 R++ E L D F YG D R+ FY LD REVWSEENLARRADDAFAADYG-GTLDRDRLRFYLHLD 270 |  |

>TR:A0A1Q4Y452 A0A1Q4Y452_9ACTN Aminoglycoside phosphotransferase APH(3') OS=Streptomyces sp. CB03911 OX=1804758 GN=A6A07_24560 PE=3 SV=1

Length=258

Score = 115 bits (289), Expect = 7e-28

| Identities | | = 81/226 (36%), Positives = 111/226 (49%), Gaps = 8/226 (4%) |  |
| --- | --- | --- | --- |
| Query 42  Sbjct 33 | | QGRPVLFVKTDLSGALNELQDEAARLSWLAT-TGVPCAAVLDVVTEAGRDWLLLGEVPGQ  +G F+K +G+ +L E RL W A T VP VL ++A W++ +PG+ EGASRQFLKWTPAGSGIDLPAEVVRLRWAAAHTVVP--EVLTEGSDASGAWIVTAGLPGR | 100  90 |
| Query 101  Sbjct 91 | | DLLSSHLA--PAEKVSIMADAMRRLHTLDPAT-CPFDHQAKHRIERARTRMEAGLVDQDD  + H PA V + +R LH P CPFD A R+ R R AG +D D TAVDDHWKRDPATAVRAVGAGLRALHDRLPVEECPFDWSAGRRLAAVRARAAAGRIDPAD | 157  150 |
| Query 158  Sbjct 151 | | LDEEHQGLAPAELFARLKARMPDGEDLVVTHGDACLPNIMV-ENGRFSGFIDCGRLGVAD DE + L + + A P + LVV HGDAC PN +V ++G G +D G LG AD WDESLRPLGTVQRALDVLADPPPVDRLVVCHGDACAPNTLVGDDGTCVGHVDLGALGRAD | 216  210 |
| Query | 217 RYQDIALATRDIAEELGGEWADRFLVLYGIAAPDSQRIAFYRLLDE 262 | | |
|  |  | R+ D+A+AT G W L YG+ PD +R +YRLL E |  |
| Sbjct | 211 | RWADLAVATWSTEWNYGPGWEGALLDAYGV-EPDPERTMYYRLLWE 255 | |

>TR:A0A2K4ZFP6 A0A2K4ZFP6_9FIRM Aminoglycoside 3'-phosphotransferase OS=Acetatifactor muris OX=879566 GN=aphA PE=3 SV=1

Length=264

Score = 115 bits (289), Expect = 8e-28

| Identities | | = 75/233 (32%), Positives = 125/233 (54%), Gaps = 18/233 (8%) |  |
| --- | --- | --- | --- |
| Query 47  Sbjct 35 | | LFVKTDLSGALNELQDEAARLSWLATTGVPCAAVLDVVTEAGRDWLLLGEVPGQDLLSS-  + +K + + E +E + WL +P VL G+ WLL+ ++PG+ L MVLKVQDADRMGEAANEYQMMQWLRGR-LPVPEVLAWEERDGKAWLLMTKLPGKMLCDEM | 105  93 |
| Query 106  Sbjct 94 | | HLAPAEKVS-IMADAMRRLHTLDPATCPFDHQAKHRIERARTRMEAGLVDQDDLDEEHQG  ++ E +S I+A++++ L +D + CP + +++ AR +E GLVD D+++ E G YMKNPELLSGILAESLKMLWRVDVSDCPCANDLDRKLQMARYNVENGLVDMDNVEPETFG | 164  153 |
| Query 165  Sbjct 154 | | ----LAPAELFARLKARMPDGEDLVVTHGDACLPNIMVENGRFSGFIDCGRLGVADRYQD  +P L L+ P E++V++HGD CLPNI E G+ GFID G+ G AD++ D  ENGFQSPEHLLNWLEENRP-AEEIVLSHGDFCLPNIFAEKGKPGGFIDLGKAGTADKWLD | 220  212 |
| Query | 221 IALATRDIAEELGGEW---------ADRFLVLYGIAAPDSQRIAFYRLLDEFF 264 | | |
|  |  | IALA R I+ G++ A++ G+ PD +++ +Y LLDE F |  |
| Sbjct | 213 | IALAFRSISHNYSGKYGGRAYEGFRAEQLFEQLGL-EPDYEKMKYYILLDELF 264 | |

>TR:A0A4R4YI30 A0A4R4YI30_9ACTN Aminoglycoside 3'-phosphotransferase OS=Kribbella antibiotica OX=190195 GN=E1263_40575 PE=3 SV=1

Length=258

Score = 115 bits (288), Expect = 1e-27

| Identities | = 89/262 (34%), Positives = 128/262 (49%), Gaps = 19/262 (7%) |  |
| --- | --- | --- |
| Query 9 | AGSPAAWVERLFGYDWAQQTIGCSDAAVFRLSAQGRPVLFVKTDLSGALNELQDEAARLS | 68 |
| Sbjct 2 | A A +V G W +G S +V+R R +F K + L+ E R++ AADRAGFVVIPAGDGWEPIEVGESATSVYR RGAVFAKCCDVAGVPALEGERDRVA | 56 |
| Query 69 | WLATTGVPCAAVLDVVTEAGRDWLLLGE VPGQDLLSSHLAPAEKVSIMADAMRRLH | 124 |
| Sbjct 57 | WLA TG+P A V+D ++ + LL V G DL S A + + +R LH WLAGTGLPGATVVDWLSSSEDGACLLTSAVPGVAGIDLPSWGWLGA--METLGRTLRELH | 114 |
| Query 125 | TLDPATCPFDHQAKHRIERARTRMEAGLVDQDDLDEEHQGLAPAELFARLKARMPDGEDL | 184 |
| Sbjct 115 | ++ CPF+ + + A + G V+ D L +E + P L A++ A P + SVPD--CPFERPLESVVAAAEDVVRRGAVNPDFLTDEWRERTPESLLAQVVAESPYVASV | 172 |
| Query 185 | V---VTHGDACLPNIMVE--NGRFSGFIDCGRLGVADRYQDIALATRDIAEELGGEWADR | 239 |
| Sbjct 173 | V HGDACLPN+ + R +GFID GRLGVADRY D+AL T + + + AEPGVCHGDACLPNVFFDPLTLRVTGFIDVGRLGVADRYSDLALTTIQLYDVWSVD-PTP | 231 |
| Query 240  Sbjct 232 | FLVLYGIAAPDSQRIAFYRLLD 261 FL YG+ PD +R+ FYRLLD FLTSYGLTDPDRRRLEFYRLLD 253 |  |

>TR:A0A2W2E1A5 A0A2W2E1A5_9ACTN APH(3'') family aminoglycoside O-phosphotransferase OS=Nonomuraea sp. KC333 OX=2070368 GN=aph(3'') PE=3

SV=1

Length=273

Score = 115 bits (288), Expect = 1e-27

| Identities | | = 93/259 (36%), Positives = 124/259 (48%), Gaps = 33/259 (13%) |  |
| --- | --- | --- | --- |
| Query 23  Sbjct 23 | | DWAQQTIGCSDAAVFRLSAQGRPVLFVKTDLSGALNELQDEAARLSWLATTGVPCAAVLD DW+ T G S A V R P + K + EL+ E R++WL GVP VLD DWSPVTGGESGAVVLR GPDAYAKCVPASGAAELEGERDRVAWLVERGVPGPRVLD | 82  77 |
| Query 83 | | VVTEAGRDWLLLGEVPGQDLLSSHLAPAEKVSI MADAMRRLHTLDPATCPFDHQAK  ++ L+ V G + + PA ++ I +ADA+RRLH + CPF | 138 |
| Sbjct | 78 | WHSDDTGACLVTSAVAG---VPADQMPAAELRIAWEHIADAVRRLHEVPVGDCPFRRDLD | 134 |
| Query | 139 | HRIERARTRMEAGLVDQDDLDEEHQGLAPAELFARL---KARMPDGE--DLVVTHGDACL | 193 |
| Sbjct | 135 | + AR + G V+ + L E+ Q P+EL ARL AR D E DLV+ HGD CL TMVAVARDVVARGAVNPEFLPEDQQRTPPSELLARLVPQLARRRDQEALDLVLCHGDLCL | 194 |
| Query | 194 | PNIMVENGRF--SGFIDCGRLGVADRYQDIALATRDIAEELGGEWADR FLV | 242 |
| Sbjct | 195 | PNI+++ SGF+D GRLG ADRY D+AL + E W D F PNIILDPRTLDVSGFVDLGRLGPADRYADLALLLTNARE TWEDEERALAADLAFAE | 250 |
| Query | 243 | LYGIAAPDSQRIAFYRLLD 261 YG+ D R+ FY LD |  |
| Sbjct | 251 | RYGVTL-DHDRLRFYLHLD 268 | |

>TR:A0A1G6Y9F8 A0A1G6Y9F8_9PSEU Streptomycin 3'-kinase OS=Prauserella marina OX=530584 GN=DES30_10344 PE=3 SV=1

Length=293

Score = 115 bits (289), Expect = 2e-27

Identities = 88/259 (34%), Positives = 122/259 (47%), Gaps = 33/259 (13%)

| Query | 24 | WAQQTIGCSDAAVFRLSAQGRPVLFVKTDLSGALNELQDEAARLSWLATTGVPCAAVLDV | 83 |
| --- | --- | --- | --- |
| Sbjct | 42 | W T G S A VFR VK + AL + +D R++WL+ GVP +VLD WQPVTEGESGAEVFRAPGGTEYAKCVKREHVAALEQERD---RVAWLSRHGVPGPSVLDW | 98 |
| Query | 84 | VTEAGRDWLLLGEVPGQDLLSSHLAPAEKVS IMADAMRRLHTLDPATCPFD | 134 |
| Sbjct | 99 | + LL V G PA+K +A+A R LH L CPF  TADETGACLLTSAVAG VPADKADDGELRRAWPSIAEATRALHELPAGDCPFG | 150 |

| Query | 135 | HQAKHRIERARTRMEAGLVDQDDLDEEHQGLAPAELFARLKAR MPDGEDLVVTHG | 189 |
| --- | --- | --- | --- |
| Sbjct | 151 | + + A + G VD D L + + +AP A L+A+ + D+VV HG RELSAMLALADDVVGRGAVDPDFLQPDQRDVAPERQLASLRAQEAKRAAQEAADIVVCHG | 210 |
| Query | 190 | DACLPNIMV--ENGRFSGFIDCGRLGVADRYQDIALATRDIA EELGGEWADRFLV | 242 |
| Sbjct | 211 | D CLPNIM+ E RF+GF+D GRLG ADR+ D+AL + EE ++F DLCLPNIMLDPETMRFTGFVDLGRLGRADRHADLALVLANARETWPDEERARHADEQFAK | 270 |
| Query | 243 | LYGIAAPDSQRIAFYRLLD 261 YG+ D++R+ FY +LD |  |
| Sbjct | 271 | YYGLPL-DAERLRFYLMLD 288 | |

>TR:A0A1Y0CCJ5 A0A1Y0CCJ5_9MYCO Aminoglycoside phosphotransferase APH(3') OS=Mycobacterium dioxanotrophicus OX=482462 GN=BTO20_33610 PE=3

SV=1

Length=270

Score = 115 bits (287), Expect = 2e-27

| Identities | | = 86/229 (38%), Positives = 118/229 (52%), Gaps = 9/229 (4%) |  |
| --- | --- | --- | --- |
| Query 37  Sbjct 42 | | FRLSAQGRPVLFVKTDLSGALN-ELQDEAARLSWLATTGVPCAAVLDVVTEAGRDWLLLG FRL +VK +G +L EA RL W A V VLD T+ WLL FRLDRSDGVTSYVKWVAAGTPEIDLVGEAERLVW-AQARVAVPPVLDHGTDLDGAWLLTA | 95  100 |
| Query 96  Sbjct 101 | | EVPGQDLLSSHLA--PAEKVSIMADAMRRLHTLDPAT-CPFDHQAKHRIERARTRMEAGL VPG+ + +H A + + + +R LH P CPFD + R+ RA R+ AG AVPGRSAVDAHWAGRATDVAAGIGRGLRLLHDALPVDECPFDWGIERRLRRADERIAAG- | 152  159 |
| Query 153 | | VDQDDLDEEHQGLAPAELFARLKARMPDGEDLVVTHGDACLPNIMV-ENGRFSGFIDCGR  + EH+ L P E ARL A P + LVV HGDAC+PN ++ ++ F+ +D G | 211 |
| Sbjct | 160 | EGPANWFPEHRHLDPDEARARLDAP-PAVDRLVVCHGDACVPNTLLHDDATFAAHVDLGS | 218 |
| Query  Sbjct | 212  219 | LGVADRYQDIALATRDIAEELGGEWADRFLVLYGIAAPDSQRIAFYRLL 260 LG+ADR+ D+A+AT + + G + D YGI PD RIA+YRLL LGIADRWADLAVATWSLGWDFGPGFDDVLFDAYGI-DPDPHRIAYYRLL 266 |  |

>TR:A0A3N6DWL8 A0A3N6DWL8_9ACTN Aminoglycoside 3'-phosphotransferase OS=Streptomyces sp. ADI93-02 OX=1522757 GN=neo PE=3 SV=1

Length=273

Score = 115 bits (287), Expect = 2e-27

| Identities | = 97/280 (35%), Positives = 131/280 (47%), Gaps = 31/280 (11%) |  |
| --- | --- | --- |
| Query 1 | MIEQDGLHAGSPAAWVERLFGYD---WAQQTIGCSDAAVFRLSAQGRPVLFVKTDLSGAL | 57 |
| Sbjct 1 | M ++ G A SP L G D W T G S A VFR + R + K + MRDRRGTQAVSPV-----LLGVDDSGWVPVTAGESGATVFRSADATR---YAKCVPAADA | 52 |
| Query 58 | NELQDEAARLSWLATTGVPCAAVLDVVTEAGRDWLLLGEVPGQDLLSSHLAPAEKVSI-- | 115 |
| Sbjct 53 | +L+ E R++WL+ GVP VLD + L+ G V G + + PAE + + ADLKAERDRVAWLSGQGVPGPRVLDWQSGDAGACLVTGAVSG---VPADQVPAEDLRVAW | 109 |
| Query 116 | --MADAMRRLHTLDPATCPFDHQAKHRIERARTRMEAGLVDQDDLDEEHQGLAPAELFAR | 173 |
| Sbjct 110 | +ADA+RRLH + CPF + AR + V+ + L + QG EL R ERIADAVRRLHGTPVSRCPFRRGLDAMVAVARDVVARDAVNAEFLPVDQQGTPATELLGR | 169 |
| Query 174 | LKARMP DGEDLVVTHGDACLPNIMVENGRF--SGFIDCGRLGVADRYQDIALATR | 226 |
| Sbjct 170 | L ++P + D VV HGD CLPNI+++ SGF+D GRLG+ADRY D+AL LTRQIPQRREQEAADTVVCHGDLCLPNIILDPQTLDVSGFVDLGRLGLADRYADLALLLA | 229 |
| Query 227  Sbjct 230 | DIAEELGGEWADR-----FLVLYGIAAPDSQRIAFYRLLD 261 D E GE R F YGI R+ FY LD DARETWTGEEQARAADTAFAERYGIDL-GHDRLRFYLHLD 268 |  |

>TR:A0A2T0N1P6 A0A2T0N1P6_9ACTN Kanamycin kinase OS=Nonomuraea fuscirosea OX=1291556 GN=B0I32_10644 PE=3 SV=1

Length=272

Score = 114 bits (286), Expect = 3e-27

| Identities | | = 86/229 (38%), Positives = 118/229 (52%), Gaps = 9/229 (4%) |  |
| --- | --- | --- | --- |
| Query 37  Sbjct 44 | | FRLSAQGRPVLFVKTDLSGALN-ELQDEAARLSWLATTGVPCAAVLDVVTEAGRDWLLLG FRL G V + K +G +L EA RL+W A V VL +A WL+ FRLDGAGGEVRYAKWVAAGTPEIDLGAEAERLAW-AGRWVRVPPVLTHGADADGAWLVTA | 95  102 |
| Query 96  Sbjct 103 | | EVPGQDLLSSHLA--PAEKVSIMADAMRRLHTLDP-ATCPFDHQAKHRIERARTRMEAGL VPG + + A PA + + +R LH P A CPFD + R+ R R+ G AVPGVSAVDARWAGAPAVAATAIGRGLRILHDALPVAECPFDWSVERRLPRVAERIADG- | 152  161 |
| Query 153 | | VDQDDLDEEHQGLAPAELFARLKARMPDGEDLVVTHGDACLPN-IMVENGRFSGFIDCGR D EH+ L AE ARL+A P + LVV HGDAC+PN +++++G + +D G | 211 |
| Sbjct | 162 | EGPGDWAPEHRPLGLAEARARLEAP-PPVDRLVVCHGDACVPNTLLLDDGTVAAHVDLGS | 220 |
| Query  Sbjct | 212  221 | LGVADRYQDIALATRDIAEELGGEWADRFLVLYGIAAPDSQRIAFYRLL 260 LGVADR+ D+A+A E+ G YGI PD++RIA+YRLL LGVADRWADLAVAAWSAEEDYGPGHDAHVYAGYGI-TPDAERIAYYRLL 268 |  |

>TR:A0A4U3MHU9 A0A4U3MHU9_9ACTN Aminoglycoside 3'-phosphotransferase OS=Herbidospora galbida OX=2575442 GN=FDA94_17175 PE=3 SV=1

Length=242

Score = 114 bits (284), Expect = 3e-27

| Identities | = 90/245 (37%), Positives = 123/245 (50%), Gaps = 27/245 (11%) |  |
| --- | --- | --- |
| Query 24 | WAQQTIGCSDAAVFRLSAQGRPVLFVKTD-LSGALNELQDEAARLSWL-ATTGVPCAAVL | 81 |
| Sbjct 17 | W+ G S A+ S G V +VK L+GA E RL WL VP WSSDHEGASGTALRVTS--GEKVFYVKHGPLAGA EHLRLRWLRGRVAVPEVVAF | 68 |
| Query 82 | DVVTEAGRDWLLLGEVPGQDLLSSHLAPAEKVSIMADAMRRLHTLDPATCPFDHQAKHRI | 141 |
| Sbjct 69 | D L+L +V L ++ APA+ +++ +R LH L A CPFD + + DDPV LVLADVGAPSLEAA--APADVGAVLGRTLRLLHELPVAGCPFDGRLPVVL | 120 |
| Query 142 | ERARTRMEAGLVDQDDLDEEHQGLAPAELFARLKARMPDGEDLVVTHGDACLPNIMVENG | 201 |
| Sbjct 121 | +RA + GLVD DD D++H GL P + RL AR P EDLVV HGD N+++ DRAAANVCGGLVDPDDFDDDHLGLTPEAVLDRLLARRPREEDLVVAHGDVTPANVLLPAP | 180 |
| Query 202 | RFSGFIDCGRLGVADRYQDIALATRDIAEELGGE---WADRFLVLYGIA-APDSQRIAFY | 257 |
| Sbjct 181 | ID LGVADR++D+AL R EL G+ W + FL YG+A D + + +Y GEPVVIDVPALGVADRHRDLALVHR ELDGDRVAW-ESFLAAYGLADRIDEELLHYY | 235 |
| Query 258  Sbjct 236 | RLLDE 262 RL+DE RLVDE 240 |  |

>TR:A0A164NGT2 A0A164NGT2_9NOCA APH domain-containing protein OS=Nocardia terpenica OX=455432 GN=AWN90_24980 PE=3 SV=1

Length=238

Score = 113 bits (283), Expect = 3e-27

| Identities | = 87/240 (36%), Positives = 122/240 (51%), Gaps = 17/240 (7%) |  |
| --- | --- | --- |
| Query 38 | RLSAQGRPVLFVKT-DLSGALNELQDEAARLSWLATTGVPCAAVLDVVTEAGRDWLLLGE | 96 |
| Sbjct 3 | R S GR + KT AL EL+DE RL WL+T V VLD G ++ RRSPDGRS--YAKTASRPAALAELEDECDRLMWLSTMSVGAPRVLDWGECDGSATMVTSA | 60 |
| Query 97 | VPGQDLLSSHLAPAEK---VSIMADAMRRLHTLDPATCPFDHQAKHRIERARTRMEAGLV | 153 |
| Sbjct 61 | + G + +S ++ AE V + + LH + CPFD + + A + AGLV LHG--IPASAVSSAEARTAVQGIVAFLGSLHAIAVRECPFDRRLEVTTALAAANVTAGLV | 118 |
| Query 154 | DQDDLDEEHQGLAPAELFARL KARMPDGEDLVVTHGDACLPNIMVENGRF--SGF | 206 |
| Sbjct 119 | D+DD DEE +G +L RL +A + DL V HGD CLPN++++ +G DEDDFDEERRGDTADQLLIRLLADRGRAEALEHSDLAVCHGDFCLPNVLLDPDTLTVTGI | 178 |
| Query 207 | IDCGRLGVADRYQDIALATRDI-AEELG-GEWADRFLVLYGIAAPDSQRIAFYRLLDEFF | 264 |
| Sbjct 179 | +D GRLG+ADR+ D+AL TR + A +L G + + D RI +YRLLDEFF  LDVGRLGIADRHLDVALLTRSMSAIDLNPGYGPELSAWVREQTNADPWRIEYYRLLDEFF | 238 |

>TR:A0A4R8R5I2 A0A4R8R5I2_9MYCO Aminoglycoside 3'-phosphotransferase OS=Mycobacteroides

franklinii OX=948102 GN=neo_1 PE=3 SV=1 Length=258

Score = 114 bits (284), Expect = 4e-27

| Identities | = 90/254 (35%), Positives = 127/254 (50%), Gaps = 27/254 (11%) |  |
| --- | --- | --- |
| Query 24 | WAQQTIGCSDAAVFRLSAQGRPVLFVKTDLSGALNELQDEAARLSWLATTGVPCAAVLDV | 83 |
| Sbjct 11 | W T G S A V+R R + KT G + +L E R+ WL++ +P + L+ WEPVTTGESGARVYRSPDHSR---YAKT---GGI-DLAAERDRIEWLSSQQIPGPSGLEW | 63 |
| Query 84 | VTEAGRDWLLLGEVPGQDLLSSHLAPAEKVS IMADAMRRLHTLDPATCPFDHQAKH | 139 |
| Sbjct 64 | T G L+ V G +S+ A+ + +ADA+RRLH+L CPF STAPGGPVLITSAVEG---ISADQLDADDLDRAWPAIADAVRRLHSLPTTCCPFSRDLAA | 120 |
| Query 140 | RIERARTRMEAGLVDQDDLDEEHQGLAPAELFARLKARM PDGEDLVVTHGDACLP | 194 |
| Sbjct 121 | + AR + V+ D L +E +G+ +L +R+ A++ + DLVV HGD CLP TVALARDVVARRAVNPDFLSDEDRGIPTQQLLSRVTAQLAERLAQEPGDLVVCHGDLCLP | 180 |
| Query 195 | NIMVENGRFS--GFIDCGRLGVADRYQDIALATRDIAEELGGEW ADRFLVLYGIA | 247 |
| Sbjct 181 | NI++ FS GFID GRLGVADR+ D+AL + E E RFL YG NIVISPEDFSVAGFIDLGRLGVADRHADLALLFANSRETWTEETRAISAQARFLDAYGTP | 240 |
| Query 248  Sbjct 241 | APDSQRIAFYRLLD 261 A D +R+AFY LD  A-DPERLAFYLRLD 253 |  |

>TR:D9WPQ8 D9WPQ8_9ACTN Streptomycin 3''-kinase OS=Streptomyces himastatinicus ATCC 53653 OX=457427 GN=SSOG_03626 PE=3 SV=1

Length=270

Score = 114 bits (284), Expect = 5e-27

| Identities | = 98/263 (37%), Positives = 124/263 (47%), Gaps = 35/263 (13%) |  |
| --- | --- | --- |
| Query 21 | GYDWAQQTIGCSDAAVFRLSAQGRPVLFVKTDLSGALNELQDEAARLSWLATTGVPCAAV | 80 |
| Sbjct 16 | G W T G S AAVFR + R + K + + L+ E R+ WL GVP A V GGGWEPVTAGESGAAVFRSADGSR---YGKCVPAHQVAALEAERDRVEWLHAQGVPGARV | 72 |
| Query 81 | LDV-VTEAGRDWLLLGEVPGQDLLSSHLAPAEKVSI MADAMRRLHTLDPAT | 130 |
| Sbjct 73 | LD VT+AG L+ G V G PA++V +AD++R+LH L P LDWRVTDAG-GCLVTGAVAG VPADRVPAATLRAAWEPIADSVRQLHDLPPRA | 123 |
| Query 131 | CPFDHQAKHRIERARTRMEAGLVDQDDLDEEHQGLAPAELFARL KARM-PDGEDLV | 185 |
| Sbjct 124 | CPF AR + G V+ + L + QG P EL ARL +AR+ + + V CPFSRDLSQMFATAREVVARGAVNPEFLPVDQQGTPPEELLARLAPQIEARLAQEAAEAV | 183 |
| Query 186 | VTHGDACLPNIMVENGRF--SGFIDCGRLGVADRYQDIALATRDIAEELGGE WAD | 238 |
| Sbjct 184 | V HGD CLPNI+++ +GFID GRLG AD Y DIAL E E D VCHGDLCLPNIVLDPTTLTVAGFIDLGRLGRADPYADIALLLATARETWPDERQATAAED | 243 |
| Query 239  Sbjct 244 | RFLVLYGIAAPDSQRIAFYRLLD 261 RF YG D R FY LD RFAHRYGTVL-DRDRERFYLHLD 265 |  |

>TR:D1ALY5 D1ALY5_SEBTE Aminoglycoside phosphotransferase OS=Sebaldella termitidis (strain ATCC 33386 / NCTC 11300) OX=526218 GN=Sterm_0369 PE=3 SV=1

Length=258

Score = 113 bits (282), Expect = 8e-27

Identities = 72/237 (30%), Positives = 118/237 (50%), Gaps = 6/237 (3%)

| Query | 30 | GCSDAAVFRLSAQGRPVLFVKTDLSGALNELQDEAARLSWLATTGVPCAAVLDVVTEAGR | 89 |
| --- | --- | --- | --- |
| Sbjct | 26 | G S A VF++S+ + A EL++E + WL + +L E GESGAKVFKVSSDTASYYL---KIETAAGELEEEYQKTRWLQGK-LEVPDILYFGEENKN | 81 |
| Query | 90 | DWLLLGEVPGQDLLSS-HLA-PAEKVSIMADAMRRLHTLDPATCPFDHQAKHRIERARTR | 147 |
| Sbjct | 82 | ++LL E+ G+ L H+ P + ++A+ + +L +D +CPFD++ + ++E+A KYMLLTEINGRILCDKEHIRNPEAAIKLLAEGLVKLGGVDTDSCPFDNRLEKKLEKAAEN | 141 |
| Query | 148 | MEAGLVDQDDLDEEHQGLAPAELFARLKARMPDGEDLVVTHGDACLPNIMVENGRFSGFI | 207 |

+	V+ D +E +	P L	L	P	+++ THGD CLPNI+ E	+GFI

| Sbjct | 142 | VRLNRVNMTDWEETTKFSNPESLLNYLYKNRPPENEVIFTHGDYCLPNIIAELDEITGFI 201 |
| --- | --- | --- |
| Query | 208 | DCGRLGVADRYQDIALATRDIAEELGGEWADRFLVLYGIAAPDSQRIAFYRLLDEFF 264 |
| Sbjct | 202 | D GR G+AD +QDIAL R + G + L+ Y D +++ +Y LLDE F  DLGRAGIADIWQDIALCMRSMHRNFGTRKYEELLLEYLGRKQDKEKLEYYILLDEMF 258 |

>TR:A0A1X7LMZ4 A0A1X7LMZ4_9BURK Phosphotransferase enzyme family protein OS=Paraburkholderia susongensis OX=1515439 GN=SAMN06265784_10722

PE=4 SV=1

Length=119

Score = 108 bits (271), Expect = 9e-27

Identities = 52/116 (45%), Positives = 74/116 (64%), Gaps = 1/116 (1%)

| Query | 97 | VPGQDLLSSH-LAPAEKVSIMADAMRRLHTLDPATCPFDHQAKHRIERARTRMEAGLVDQ 155 |
| --- | --- | --- |
| Sbjct | 4 | VPG DL +++ L+ + V ++A A+R LH + A CPF+H+ + I A+ R+ GL+D+ VPGLDLANANDLSSPQIVGMLAMALRILHEVPIAVCPFEHRLEEHIAAAKNRVNVGLIDE 63 |
| Query | 156 | DDLDEEHQGLAPAELFARLKARMPDGEDLVVTHGDACLPNIMVENGRFSGFIDCGR 211 |
| Sbjct | 64 | D D E QG ++ A L + +P+ DLVV HGDACLPN M F+GFIDCGR  ADFDNERQGQTATDVLADLLSTLPETYDLVVIHGDACLPNFMANGSNFTGFIDCGR 119 |

>TR:A0A1W6YZ55 A0A1W6YZ55_9BORD Aminoglycoside 3'-phosphotransferase OS=Bordetella genomosp. 9 OX=1416803 GN=CAL13_09290 PE=3 SV=1

Length=266

Score = 113 bits (282), Expect = 9e-27

| Identities | | = 84/258 (33%), Positives = 124/258 (48%), Gaps = 30/258 (12%) |  |
| --- | --- | --- | --- |
| Query 24  Sbjct 14 | | WAQQTIGCSDAAVFRLSAQGRPVLFVKTDLSGALNELQDEAARLSWLATTGVPCAAVLDV WA G S V+R + + + K +L E RL+WL GV C ++D WAAIGSGESGDFVYR---RDDGLAYAKIATGARAADLSAERDRLNWLQGRGVACPEIVDW | 83  70 |
| Query 84  Sbjct 71 | | VTEAGRDWLLLGEVPG QDLLSSHLAPAEKVSIMADAMRRLHTLDPATCPFDH  E L++ +PG DLL + + A+++++ LHTL CPF+ RQEEDGACLIMTAIPGIPAAGLSGDDLLKAWPSMAQQLAV LHTLPAGQCPFER | 135  123 |
| Query 136  Sbjct 124 | | QAKHRIERARTRMEAGLVDQDDLDEEHQGLAPAELFARLKARMP DGEDLVVTHGD  + RA + G V++D L +E Q + EL R+K +P + D+ V HGD RLSGLYSRAADVVSRGAVNRDFLPDEDQHIPQPELLMRVKRELPLRLEQETADMAVCHGD | 190  183 |
| Query 191  Sbjct 184 | | ACLPNIMVENGRF--SGFIDCGRLGVADRYQDIALATRDIAEE-LGGEWADR-FLVLYG- AC+PN +++ R +G ID GRLG ADRY D+AL + E + A+R F VL+ ACMPNFVIDPHRLQCTGLIDLGRLGTADRYADLALMVANAEESWTEPDQAERAFSVLFDI | 245  243 |
| Query 246 | | --IAAPDSQRIAFYRLLD 261 I PD R+AFY LD |  |
| Sbjct | 244 | MRIETPDRDRLAFYLRLD 261 | |

>TR:D6CJG0 D6CJG0_CORRG Streptomycin phosphotransferase A OS=Corynebacterium resistens (strain DSM 45100 / JCM 12819 / GTC 2026 / SICGH

158) OX=662755 GN=strA PE=3 SV=1 Length=267

Score = 113 bits (282), Expect = 9e-27

Identities = 88/254 (35%), Positives = 121/254 (48%), Gaps = 20/254 (8%)

| Query | 23 | DWAQQTIGCSDAAVFRLSAQGRPVLFVKTDLSGALNELQDEAARLSWLATTGVPCAAVLD | 82 |
| --- | --- | --- | --- |
| Sbjct | 14 | DW G S VFR +G F KT + EL E RL WL GV C V++ DWLPVRGGESGDFVFR---RGDGHAFAKTAPASRRGELAGERDRLIWLKGRGVACPEVIN | 70 |
| Query | 83 | VVTEAGRDWLLLGEVPG---QDLLSSHLAPAEKVSIMADAMRRLHTLDPATCPFDHQAKH | 139 |
| Sbjct | 71 | E L++ +PG DL + L A M + +H+L CPF+ + WQEEQEGACLVITAIPGVPAADLSGADLLKAWPS--MGQQLGAVHSLSVDQCPFERRLSR | 128 |
| Query | 140 | RIERARTRMEAGLVDQDDLDEEHQGLAPAELFARLKARMP DGEDLVVTHGDACLP | 194 |
| Sbjct | 129 | RA + V+ D L +E + +L AR++ +P + D+VV HGD C+P  MFGRAVDVVSRNAVNPDFLPDEDKSTPQLDLLARVERELPVRLDQERTDMVVCHGDPCMP | 188 |

| Query | 195 | NIMVENGRF--SGFIDCGRLGVADRYQDIALATRDIAEELGG-EWADR-FLVLY---GIA 247 |
| --- | --- | --- |
| Sbjct | 189 | N MV+ +G ID GRLG ADRY D+AL + E + A+R F VL+ GI NFMVDPKTLQCTGLIDLGRLGTADRYADLALMIANAEENWAAPDEAERAFAVLFNVLGIE 248 |
| Query | 248 | APDSQRIAFYRLLD 261 |
| Sbjct | 249 | APD +R+AFY LD  APDRERLAFYLRLD 262 |

>TR:F3NKG7 F3NKG7_9ACTN Streptomycin 6-phosphotransferase OS=Streptomyces griseoaurantiacus M045 OX=996637 GN=SGM_3631 PE=3 SV=1

Length=252

Score = 112 bits (281), Expect = 9e-27

| Identities | | = 88/232 (38%), Positives = 111/232 (48%), Gaps = 30/232 (13%) |  |
| --- | --- | --- | --- |
| Query 37  Sbjct 39 | | FRLSAQGRP VLFVKTDLSGALNELQDEAARLSWLATTGVPCAAVLDVVTEAGRDWL  FRL GRP LFVK SG+ L EA RL W A P V + ++A WL FRLDGDGRPGGEDPLFVKWAPSGSALPLAAEAERLRWAAPY-TPVPEVREYGSDADGSWL | 92  97 |
| Query 93  Sbjct 98 | | LLGEVPGQDLLSSHLA--PAEKVSIMADAMRRLHTLDP-ATCPFDHQAKHRIERARTRME  +P Q ++ PA V + +R LH P A CPF HR+E AR ATRALPAQSAVAPRWKADPATAVRALGTGLRALHDRLPVAECPFTWSVAHRLEVAR---R | 149  154 |
| Query 150  Sbjct 155 | | AGLVDQDDLDEEHQGLAPAELFARLKARMPDGEDLVVTHGDACLPNIMV-ENGRFSGFID AG+V DL + PA+L LVV HGD C PN ++ E+G ++ +D AGIVVPADLPDP----PPADL LVVCHGDPCAPNTLLHEDGSWAAHVD | 208  197 |
| Query 209 | | CGRLGVADRYQDIALATRDIAEELGGEWADRFLVLYGIAAPDSQRIAFYRLL 260 G LGVADR+ D+A+ TR+I G W L YGI APD R FYR L |  |
| Sbjct | 198 | MGALGVADRWADLAVVTRNIDWNYGPGWERTLLDAYGI-APDPVRTDFYRRL 248 | |

>TR:A0A0N9XXU2 A0A0N9XXU2_MYCFO Aminoglycoside 3'-phosphotransferase OS=Mycolicibacterium fortuitum OX=1766 GN=XA26_51950 PE=3 SV=1

Length=254

Score = 112 bits (281), Expect = 1e-26

| Identities | | = 92/251 (37%), Positives = 127/251 (51%), Gaps = 16/251 (6%) |  |
| --- | --- | --- | --- |
| Query 23  Sbjct 3 | | DWAQQTIGCSDAAVFRLSAQGRPVLFVKTDLSGALNELQDEAARLSWLATTGVPCAAVLD  +W T G S A VFR +A G + K + A+ +L E R+SW GVP AV+D EWLPVTRGESGAGVFR-NADGSS--YAKVVDAAAVADLAAERDRVSWAHRHGVPGPAVID | 82  59 |
| Query 83 | | V-VTEAGRDWLLLGEVPG--QDLLSSHLAPAEKVSIMADAMRRLHTLDPATCPFDHQAKH VTE G L+ V G D LS A +I+ +A+R LH L CP+ | 139 |
| Sbjct | 60 | WRVTEDGGACLITSTVRGVAADRLSESALRAAWPAIV-EAVRTLHALPADGCPYRRDLDD | 118 |
| Query | 140 | RIERARTRMEAGLVDQDDLDEEHQGLAPAELFARLKA RMPDGEDLVVTHGDACLP | 194 |
| Sbjct | 119 | + RAR + AG V+ + L +E + + L R++ R + D VV HGD CLP MLARARAVVGAGAVNPEFLSDEDREVPAEALLDRVEREADLRRRQEAADWVVCHGDLCLP | 178 |
| Query | 195 | NIMVENGRFS--GFIDCGRLGVADRYQDIALATRDIAEELGG--EWADRFLVLYGIAAPD | 250 |
| Sbjct | 179 | NI+V+ R + GFID GRLG+ADR+ D+AL + A+ + G E A L A D NILVDPDRHTVEGFIDLGRLGLADRHADLALLLANTADAVPGFAEKATAGLAAGYPAQVD | 238 |
| Query | 251 | SQRIAFYRLLD 261  +R+ FY LD |  |
| Sbjct | 239 | PERLRFYLALD 249 | |

>TR:A0A1T4PEC8 A0A1T4PEC8_9ACTN Streptomycin 3-kinase OS=Marinactinospora thermotolerans DSM 45154 OX=1122192 GN=SAMN02745673_01772 PE=3

SV=1

Length=275

Score = 113 bits (282), Expect = 1e-26

Identities = 97/264 (37%), Positives = 125/264 (47%), Gaps = 20/264 (8%)

Query 12	PAAWVERLFGYDWAQQTIGCSDAAVFRLSAQGRPVLFVKTDLSGALNELQDEAARLSWLA 71 P W RL G W	T+G S AAVFR +	R	V+ D + +L	+D	R+ WL+

| Sbjct | 13 | PPPWA-RLPGDGWTAVTVGESGAAVFRSADGSRYAKCVRGDQAVSLEAERD---RVEWLS | 68 |
| --- | --- | --- | --- |
| Query | 72 | TTGVPCAAVLDVVTEAGRDWLLLGEVPG--QDLLSSHLAPAEKVSIMADAMRRLHTLDPA | 129 |
| Sbjct | 69 | VP VLD L+ V G D LS+ A +I DA+RRLH L P+ AQAVPGPRVLDWRATDEGACLVTSAVRGVGADTLSASRLRAAWGTI-TDAVRRLHDLPPS | 127 |
| Query | 130 | TCPFDHQAKHRIERARTRMEAGLVDQDDLDEEHQGLAPAELFARLKARM PDGEDL | 184 |
| Sbjct | 128 | CPF AR + G V+ + L E Q P L ARL+ ++ + ++ RCPFSRDLTRMFAAARDVVARGAVNPEFLPVEQQRTPPERLLARLEPQLGHRLEQEADES | 187 |
| Query | 185 | VVTHGDACLPNIMV--ENGRFSGFIDCGRLGVADRYQDIALATRDIAE ELGGEWAD | 238 |
| Sbjct | 188 | VV HGD CLPNI++ E +GFID GRLG AD Y DIAL E E E A+ VVCHGDLCLPNIILDPETLDVAGFIDLGRLGRADPYADIALLLATARERWDDERQAEAAE | 247 |
| Query  Sbjct | 239  248 | R-FLVLYGIAAPDSQRIAFYRLLD 261  + F YG+ D R FY LD  KDFADRYGLVL-DRDRERFYLHLD 270 |  |

>TR:A0A1Q5GL35 A0A1Q5GL35_9ACTN 3'-kinase OS=Streptomyces sp. CB01580 OX=1703933 GN=AMK22_20765 PE=3 SV=1

Length=273

Score = 112 bits (281), Expect = 1e-26

| Identities | = 92/258 (36%), Positives = 118/258 (46%), Gaps = 29/258 (11%) |  |
| --- | --- | --- |
| Query 23 | DWAQQTIGCSDAAVFRLSAQGRPVLFVKTDLSGALNELQDEAARLSWLATTGVPCAAVLD | 82 |
| Sbjct 21 | DW G S A VFR + GR + K +L E R++WL G+P VLD DWLPVDTGESGAGVFRSADGGR---YAKCVAVAEAADLGAERDRIAWLGDQGIPGPRVLD | 77 |
| Query 83 | VVTEAGRDWLLLGEVPGQDLLSSHLAPAEKVSI---MADAMRRLHTLDPATCPFDHQAKH | 139 |
| Sbjct 78 | + L+ VPG + + L+ AE + +ADA+R LH + CPF WRSGDAGACLVTSTVPG--VSADRLSAAELRAAWDRIADAVRALHAMPARRCPFRRDLDG | 135 |
| Query 140 | RIERARTRMEAGLVDQDDLDEEHQGLAPAELFARL KARMPDGEDLVVTHGDACLP | 194 |
| Sbjct 136 | I RAR + G V + L E Q AEL RL R + D VV HGD CLP MIARARDVVARGAVHPEFLPVEQQRTPAAELLDRLVPQTDLRREQEAADTVVCHGDLCLP | 195 |
| Query 195 | NIMVENGRF--SGFIDCGRLGVADRYQDIALATRDIAEELGGEWADR FLVL | 243 |
| Sbjct 196 | NI+++ SGFID GRLG+ADRY D++L + E WAD F NIVLDPRTLDVSGFIDLGRLGLADRYADLSLLLANARE TWADEEEARAADAAFAER | 251 |
| Query 244  Sbjct 252 | YGIAAPDSQRIAFYRLLD 261 YGI D R+ FY LD YGITL-DHDRLRFYLHLD 268 |  |

>TR:A0A1M7YNK5 A0A1M7YNK5_9FIRM Kanamycin kinase OS=Anaerocolumna xylanovorans DSM 12503 OX=1121345 GN=SAMN02745217_04525 PE=3 SV=1

Length=263

Score = 112 bits (280), Expect = 2e-26

| Identities | = 77/249 (31%), Positives = 123/249 (49%), Gaps = 32/249 (13%) |  |
| --- | --- | --- |
| Query 31 | CSDAAVFRLSAQGRPVLFVKTDLSGALNELQDEAARLSWLATTGVPCAAVLDVVTEAGRD | 90 |
| Sbjct 32 | C D V ++ QG E +E + WLA + +L E G + CFDDMVLKIEKQGE ESDNEHRIMFWLADK-LSVPKILCSYKEDGIN | 76 |
| Query 91 | WLLLGEVPGQDLLSSHLA--PAEKVSIMADAMRRLHTLDPATCPFDHQAKHRIERARTRM | 148 |
| Sbjct 77 | +LL+ + G+ S L P E V ++A ++ L ++D + CP + +++++ A R+ YLLMSRIMGEMSCSPKLLENPKELVRLLAKGLKMLWSVDISDCPHSNSIENKLKLAEFRV | 136 |
| Query 149 | EAGLVDQDDLDEEHQGL APAELFARLKARMPDGEDLVVTHGDACLPNIMVENGRFS | 204 |
| Sbjct 137 | ++ L +D + + G +PA+L LK PD E+LV +HGD CLPNI +++ + QSNLCGMEDAEPKTYGKKGFESPAKLLEWLKENKPD-EELVFSHGDYCLPNIFIKDNNVN | 195 |
| Query 205 | GFIDCGRLGVADRYQDIALATRDIAEELGGEWADRFLVLY GIAAPDSQRIA | 255 |
| Sbjct 196 | GFID GR GVAD+YQDIAL R + G + + + GI PD +I GFIDLGRSGVADKYQDIALCYRSLQHNYNGSYGGKIYEDFNPELLFNELGI-LPDWDKIK | 254 |
| Query 256 | FYRLLDEFF 264  +Y L+DE F |  |

Sbjct 255 YYILMDELF 263

>SP:P0A3Y5 KKA3_ENTFL Aminoglycoside 3'-phosphotransferase OS=Enterococcus faecalis OX=1351 GN=aphA PE=1 SV=1

Length=264

Score = 112 bits (280), Expect = 2e-26

| Identities | = 80/251 (32%), Positives = 124/251 (49%), Gaps = 11/251 (4%) |  |
| --- | --- | --- |
| Query 22 | YDWAQQTIGCSDAAVFRLSAQGRPVLFVKTD--LSGALNELQDEAARLSWLATTGVPCAA | 79 |
| Sbjct 17 | Y + T G S A V++L + + TD G +++ E + WL +P YRCVKDTEGMSPAKVYKLVGENENLYLKMTDSRYKGTTYDVEREKDMMLWLEGK-LPVPK | 75 |
| Query 80 | VLDVVTEAGRDWLLLGEVPG---QDLLSSHLAPAEKVSIMADAMRRLHTLDPATCPFDHQ | 136 |
| Sbjct 76 | VL G LL+ E G + +P + + + A+ +R H++D + CP+ + VLHFERHDGWSNLLMSEADGVLCSEEYEDEQSPEKIIELYAECIRLFHSIDISDCPYTNS | 135 |
| Query 137 | AKHRIERARTRMEAGLVDQD--DLDEEHQGLAPAELFARLKARMPDGEDLVVTHGDACLP | 194 |
| Sbjct 136 | R+ + L D D + +E+ P EL+ LK P+ E+LV +HGD LDSRLAELDYLLNNDLADVDCENWEEDTPFKDPRELYDFLKTEKPE-EELVFSHGDLGDS | 194 |
| Query 195 | NIMVENGRFSGFIDCGRLGVADRYQDIALATRDIAEELGGE-WADRFLVLYGIAAPDSQR | 253 |
| Sbjct 195 | NI V++G+ SGFID GR G AD++ DIA R I E++G E + + F L GI PD ++ NIFVKDGKVSGFIDLGRSGRADKWYDIAFCVRSIREDIGEEQYVELFFDLLGI-KPDWEK | 253 |
| Query 254  Sbjct 254 | IAFYRLLDEFF 264 I +Y LLDE F IKYYILLDELF 264 |  |

>TR:E8LFG4 E8LFG4_9FIRM Kanamycin kinase OS=Phascolarctobacterium succinatutens YIT 12067 OX=626939 GN=aphA PE=3 SV=1

Length=264

Score = 112 bits (280), Expect = 2e-26

| Identities | = 80/251 (32%), Positives = 124/251 (49%), Gaps = 11/251 (4%) |  |
| --- | --- | --- |
| Query 22 | YDWAQQTIGCSDAAVFRLSAQGRPVLFVKTD--LSGALNELQDEAARLSWLATTGVPCAA | 79 |
| Sbjct 17 | Y + T G S A V++L + + TD G +++ E + WL +P YRCVKDTEGMSPAKVYKLVGENENLYLKMTDSRYKGTTYDVEREKDMMLWLEGK-LPVPK | 75 |
| Query 80 | VLDVVTEAGRDWLLLGEVPG---QDLLSSHLAPAEKVSIMADAMRRLHTLDPATCPFDHQ | 136 |
| Sbjct 76 | VL G LL+ E G + +P + + + A+ +R H++D + CP+ + VLHFERHDGWSNLLMSEADGVLCSEEYEDEQSPEKIIELYAECIRLFHSIDISDCPYTNS | 135 |
| Query 137 | AKHRIERARTRMEAGLVDQD--DLDEEHQGLAPAELFARLKARMPDGEDLVVTHGDACLP | 194 |
| Sbjct 136 | R+ + L D D + +E+ P EL+ LK P+ E+LV +HGD LDSRLAELDYLLNNDLADVDCENWEEDTPFKDPRELYDFLKTEKPE-EELVFSHGDLGDS | 194 |
| Query 195 | NIMVENGRFSGFIDCGRLGVADRYQDIALATRDIAEELGGE-WADRFLVLYGIAAPDSQR | 253 |
| Sbjct 195 | NI V++G+ SGFID GR G AD++ DIA R I E++G E + + F L GI PD ++ NIFVKDGKVSGFIDLGRSGRADKWYDIAFCVRSIREDIGEEQYVELFFDLLGI-KPDWEK | 253 |
| Query 254  Sbjct 254 | IAFYRLLDEFF 264 I +Y LLDE F IKYYILLDELF 264 |  |

>TR:G2SZV9 G2SZV9_ROSHA Aminoglycoside 3'-phosphotransferase OS=Roseburia hominis (strain DSM 16839 / JCM 17582 / NCIMB 14029 / A2-183)

OX=585394 GN=RHOM_01605 PE=3 SV=1

Length=264

Score = 112 bits (280), Expect = 2e-26

Identities = 80/251 (32%), Positives = 124/251 (49%), Gaps = 11/251 (4%)

| Query | 22 | YDWAQQTIGCSDAAVFRLSAQGRPVLFVKTD--LSGALNELQDEAARLSWLATTGVPCAA | 79 |
| --- | --- | --- | --- |
|  |  | Y + T G S A V++L + + TD G +++ E + WL +P |  |
| Sbjct | 17 | YRCVKDTEGMSPAKVYKLVGENENLYLKMTDSRYKGTTYDVEREKDMMLWLEGK-LPVPK | 75 |
| Query | 80 | VLDVVTEAGRDWLLLGEVPG---QDLLSSHLAPAEKVSIMADAMRRLHTLDPATCPFDHQ | 136 |

VL	G	LL+ E G	+	+P + + + A+ +R H++D + CP+ +

| Sbjct | 76 | VLHFERHDGWSNLLMSEADGVLCSEEYEDEQSPEKIIELYAECIRLFHSIDISDCPYTNS | 135 |
| --- | --- | --- | --- |
| Query | 137 | AKHRIERARTRMEAGLVDQD--DLDEEHQGLAPAELFARLKARMPDGEDLVVTHGDACLP | 194 |
| Sbjct | 136 | R+ + L D D + +E+ P EL+ LK P+ E+LV +HGD LDSRLAELDYLLNNDLADVDCENWEEDTPFKDPRELYDFLKTEKPE-EELVFSHGDLGDS | 194 |
| Query | 195 | NIMVENGRFSGFIDCGRLGVADRYQDIALATRDIAEELGGE-WADRFLVLYGIAAPDSQR | 253 |
| Sbjct | 195 | NI V++G+ SGFID GR G AD++ DIA R I E++G E + + F L GI PD ++ NIFVKDGKVSGFIDLGRSGRADKWYDIAFCVRSIREDIGEEQYVELFFDLLGI-KPDWEK | 253 |
| Query  Sbjct | 254  254 | IAFYRLLDEFF 264 I +Y LLDE F IKYYILLDELF 264 |  |

>TR:A0A1C0BVA1 A0A1C0BVA1_9FIRM APH(3') family aminoglycoside O-phosphotransferase OS=Erysipelotrichaceae bacterium MTC7 OX=1768196 GN=A4S06_09810

PE=3 SV=1

Length=264

Score = 112 bits (280), Expect = 2e-26

| Identities | = 80/251 (32%), Positives = 124/251 (49%), Gaps = 11/251 (4%) |  |
| --- | --- | --- |
| Query 22 | YDWAQQTIGCSDAAVFRLSAQGRPVLFVKTD--LSGALNELQDEAARLSWLATTGVPCAA | 79 |
| Sbjct 17 | Y + T G S A V++L + + TD G +++ E + WL +P YRCVKDTEGMSPAKVYKLVGENENLYLKMTDSRYKGTTYDVEREKDMMLWLEGK-LPVPK | 75 |
| Query 80 | VLDVVTEAGRDWLLLGEVPG---QDLLSSHLAPAEKVSIMADAMRRLHTLDPATCPFDHQ | 136 |
| Sbjct 76 | VL G LL+ E G + +P + + + A+ +R H++D + CP+ + VLHFERHDGWSNLLMSEADGVLCSEEYEDEQSPEKIIELYAECIRLFHSIDISDCPYTNS | 135 |
| Query 137 | AKHRIERARTRMEAGLVDQD--DLDEEHQGLAPAELFARLKARMPDGEDLVVTHGDACLP | 194 |
| Sbjct 136 | R+ + L D D + +E+ P EL+ LK P+ E+LV +HGD LDSRLAELDYLLNNDLADVDCENWEEDTPFKDPRELYDFLKTEKPE-EELVFSHGDLGDS | 194 |
| Query 195 | NIMVENGRFSGFIDCGRLGVADRYQDIALATRDIAEELGGE-WADRFLVLYGIAAPDSQR | 253 |
| Sbjct 195 | NI V++G+ SGFID GR G AD++ DIA R I E++G E + + F L GI PD ++ NIFVKDGKVSGFIDLGRSGRADKWYDIAFCVRSIREDIGEEQYVELFFDLLGI-KPDWEK | 253 |
| Query 254  Sbjct 254 | IAFYRLLDEFF 264 I +Y LLDE F IKYYILLDELF 264 |  |

>TR:Q5HS59 Q5HS59_STAEQ Aminoglycoside 3'-phosphotransferase OS=Staphylococcus epidermidis (strain ATCC 35984 / RP62A) OX=176279 GN=aphA

PE=3 SV=1

Length=264

Score = 112 bits (280), Expect = 2e-26

| Identities | = 80/251 (32%), Positives = 124/251 (49%), Gaps = 11/251 (4%) |  |
| --- | --- | --- |
| Query 22 | YDWAQQTIGCSDAAVFRLSAQGRPVLFVKTD--LSGALNELQDEAARLSWLATTGVPCAA | 79 |
| Sbjct 17 | Y + T G S A V++L + + TD G +++ E + WL +P YRCVKDTEGMSPAKVYKLVGENENLYLKMTDSRYKGTTYDVEREKDMMLWLEGK-LPVPK | 75 |
| Query 80 | VLDVVTEAGRDWLLLGEVPG---QDLLSSHLAPAEKVSIMADAMRRLHTLDPATCPFDHQ | 136 |
| Sbjct 76 | VL G LL+ E G + +P + + + A+ +R H++D + CP+ + VLHFERHDGWSNLLMSEADGVLCSEEYEDEQSPEKIIELYAECIRLFHSIDISDCPYTNS | 135 |
| Query 137 | AKHRIERARTRMEAGLVDQD--DLDEEHQGLAPAELFARLKARMPDGEDLVVTHGDACLP | 194 |
| Sbjct 136 | R+ + L D D + +E+ P EL+ LK P+ E+LV +HGD LDSRLAELDYLLNNDLADVDCENWEEDTPFKDPRELYDFLKTEKPE-EELVFSHGDLGDS | 194 |
| Query 195 | NIMVENGRFSGFIDCGRLGVADRYQDIALATRDIAEELGGE-WADRFLVLYGIAAPDSQR | 253 |
| Sbjct 195 | NI V++G+ SGFID GR G AD++ DIA R I E++G E + + F L GI PD ++ NIFVKDGKVSGFIDLGRSGRADKWYDIAFCVRSIREDIGEEQYVELFFDLLGI-KPDWEK | 253 |
| Query 254  Sbjct 254 | IAFYRLLDEFF 264 I +Y LLDE F IKYYILLDELF 264 |  |

>TR:A0A3N0HWH4 A0A3N0HWH4_9FIRM Aminoglycoside O-phosphotransferase APH(3')-IIIa OS=Absicoccus porci OX=2486576 GN=aph(3')-IIIa PE=3 SV=1

Length=264

Score = 112 bits (280), Expect = 2e-26

| Identities | = 80/251 (32%), Positives = 124/251 (49%), Gaps = 11/251 (4%) |  |
| --- | --- | --- |
| Query 22 | YDWAQQTIGCSDAAVFRLSAQGRPVLFVKTD--LSGALNELQDEAARLSWLATTGVPCAA | 79 |
| Sbjct 17 | Y + T G S A V++L + + TD G +++ E + WL +P YRCVKDTEGMSPAKVYKLVGENENLYLKMTDSRYKGTTYDVEREKDMMLWLEGK-LPVPK | 75 |
| Query 80 | VLDVVTEAGRDWLLLGEVPG---QDLLSSHLAPAEKVSIMADAMRRLHTLDPATCPFDHQ | 136 |
| Sbjct 76 | VL G LL+ E G + +P + + + A+ +R H++D + CP+ + VLHFERHDGWSNLLMSEADGVLCSEEYEDEQSPEKIIELYAECIRLFHSIDISDCPYTNS | 135 |
| Query 137 | AKHRIERARTRMEAGLVDQD--DLDEEHQGLAPAELFARLKARMPDGEDLVVTHGDACLP | 194 |
| Sbjct 136 | R+ + L D D + +E+ P EL+ LK P+ E+LV +HGD LDSRLAELDYLLNNDLADVDCENWEEDTPFKDPRELYDFLKTEKPE-EELVFSHGDLGDS | 194 |
| Query 195 | NIMVENGRFSGFIDCGRLGVADRYQDIALATRDIAEELGGE-WADRFLVLYGIAAPDSQR | 253 |
| Sbjct 195 | NI V++G+ SGFID GR G AD++ DIA R I E++G E + + F L GI PD ++ NIFVKDGKVSGFIDLGRSGRADKWYDIAFCVRSIREDIGEEQYVELFFDLLGI-KPDWEK | 253 |
| Query 254  Sbjct 254 | IAFYRLLDEFF 264 I +Y LLDE F IKYYILLDELF 264 |  |

>TR:A0A3E2T6Z6 A0A3E2T6Z6_9FIRM Aminoglycoside O-phosphotransferase APH(3')-IIIa OS=Harryflintia acetispora OX=1849041 GN=aph(3')-IIIa PE=3

SV=1

Length=264

Score = 112 bits (280), Expect = 2e-26

| Identities | = 80/251 (32%), Positives = 124/251 (49%), Gaps = 11/251 (4%) |  |
| --- | --- | --- |
| Query 22 | YDWAQQTIGCSDAAVFRLSAQGRPVLFVKTD--LSGALNELQDEAARLSWLATTGVPCAA | 79 |
| Sbjct 17 | Y + T G S A V++L + + TD G +++ E + WL +P YRCVKDTEGMSPAKVYKLVGENENLYLKMTDSRYKGTTYDVEREKDMMLWLEGK-LPVPK | 75 |
| Query 80 | VLDVVTEAGRDWLLLGEVPG---QDLLSSHLAPAEKVSIMADAMRRLHTLDPATCPFDHQ | 136 |
| Sbjct 76 | VL G LL+ E G + +P + + + A+ +R H++D + CP+ + VLHFERHDGWSNLLMSEADGVLCSEEYEDEQSPEKIIELYAECIRLFHSIDISDCPYTNS | 135 |
| Query 137 | AKHRIERARTRMEAGLVDQD--DLDEEHQGLAPAELFARLKARMPDGEDLVVTHGDACLP | 194 |
| Sbjct 136 | R+ + L D D + +E+ P EL+ LK P+ E+LV +HGD LDSRLAELDYLLNNDLADVDCENWEEDTPFKDPRELYDFLKTEKPE-EELVFSHGDLGDS | 194 |
| Query 195 | NIMVENGRFSGFIDCGRLGVADRYQDIALATRDIAEELGGE-WADRFLVLYGIAAPDSQR | 253 |
| Sbjct 195 | NI V++G+ SGFID GR G AD++ DIA R I E++G E + + F L GI PD ++ NIFVKDGKVSGFIDLGRSGRADKWYDIAFCVRSIREDIGEEQYVELFFDLLGI-KPDWEK | 253 |
| Query 254  Sbjct 254 | IAFYRLLDEFF 264 I +Y LLDE F IKYYILLDELF 264 |  |

>TR:A0A143X0T6 A0A143X0T6_9ACTN Aminoglycoside 3'-phosphotransferase OS=Coriobacteriaceae bacterium CHKCI002 OX=1780377 GN=aphA PE=3 SV=1

Length=264

Score = 112 bits (280), Expect = 2e-26

Identities = 80/251 (32%), Positives = 124/251 (49%), Gaps = 11/251 (4%)

| Query | 22 | YDWAQQTIGCSDAAVFRLSAQGRPVLFVKTD--LSGALNELQDEAARLSWLATTGVPCAA | 79 |
| --- | --- | --- | --- |
| Sbjct | 17 | Y + T G S A V++L + + TD G +++ E + WL +P YRCVKDTEGMSPAKVYKLVGENENLYLKMTDSRYKGTTYDVEREKDMMLWLEGK-LPVPK | 75 |
| Query | 80 | VLDVVTEAGRDWLLLGEVPG---QDLLSSHLAPAEKVSIMADAMRRLHTLDPATCPFDHQ VL G LL+ E G + +P + + + A+ +R H++D + CP+ + | 136 |

| Sbjct | 76 | VLHFERHDGWSNLLMSEADGVLCSEEYEDEQSPEKIIELYAECIRLFHSIDISDCPYTNS | 135 |
| --- | --- | --- | --- |
| Query | 137 | AKHRIERARTRMEAGLVDQD--DLDEEHQGLAPAELFARLKARMPDGEDLVVTHGDACLP | 194 |
| Sbjct | 136 | R+ + L D D + +E+ P EL+ LK P+ E+LV +HGD LDSRLAELDYLLNNDLADVDCENWEEDTPFKDPRELYDFLKTEKPE-EELVFSHGDLGDS | 194 |
| Query | 195 | NIMVENGRFSGFIDCGRLGVADRYQDIALATRDIAEELGGE-WADRFLVLYGIAAPDSQR | 253 |
| Sbjct | 195 | NI V++G+ SGFID GR G AD++ DIA R I E++G E + + F L GI PD ++ NIFVKDGKVSGFIDLGRSGRADKWYDIAFCVRSIREDIGEEQYVELFFDLLGI-KPDWEK | 253 |
| Query  Sbjct | 254  254 | IAFYRLLDEFF 264 I +Y LLDE F IKYYILLDELF 264 |  |

>TR:A0A1Y4TFF5 A0A1Y4TFF5_9FIRM Aminoglycoside O-phosphotransferase APH(3')-IIIa OS=Faecalibacterium sp. An121 OX=1965550 GN=B5E66_00970

PE=3 SV=1

Length=264

Score = 112 bits (280), Expect = 2e-26

| Identities | = 80/251 (32%), Positives = 124/251 (49%), Gaps = 11/251 (4%) |  |
| --- | --- | --- |
| Query 22 | YDWAQQTIGCSDAAVFRLSAQGRPVLFVKTD--LSGALNELQDEAARLSWLATTGVPCAA | 79 |
| Sbjct 17 | Y + T G S A V++L + + TD G +++ E + WL +P YRCVKDTEGMSPAKVYKLVGENENLYLKMTDSRYKGTTYDVEREKDMMLWLEGK-LPVPK | 75 |
| Query 80 | VLDVVTEAGRDWLLLGEVPG---QDLLSSHLAPAEKVSIMADAMRRLHTLDPATCPFDHQ | 136 |
| Sbjct 76 | VL G LL+ E G + +P + + + A+ +R H++D + CP+ + VLHFERHDGWSNLLMSEADGVLCSEEYEDEQSPEKIIELYAECIRLFHSIDISDCPYTNS | 135 |
| Query 137 | AKHRIERARTRMEAGLVDQD--DLDEEHQGLAPAELFARLKARMPDGEDLVVTHGDACLP | 194 |
| Sbjct 136 | R+ + L D D + +E+ P EL+ LK P+ E+LV +HGD LDSRLAELDYLLNNDLADVDCENWEEDTPFKDPRELYDFLKTEKPE-EELVFSHGDLGDS | 194 |
| Query 195 | NIMVENGRFSGFIDCGRLGVADRYQDIALATRDIAEELGGE-WADRFLVLYGIAAPDSQR | 253 |
| Sbjct 195 | NI V++G+ SGFID GR G AD++ DIA R I E++G E + + F L GI PD ++ NIFVKDGKVSGFIDLGRSGRADKWYDIAFCVRSIREDIGEEQYVELFFDLLGI-KPDWEK | 253 |
| Query 254  Sbjct 254 | IAFYRLLDEFF 264 I +Y LLDE F IKYYILLDELF 264 |  |

>TR:D2JEU4 D2JEU4_ENTFC APH(3') family aminoglycoside O-phosphotransferase OS=Enterococcus faecium OX=1352 GN=aph(3')-IIIa PE=3 SV=1

Length=264

Score = 112 bits (280), Expect = 2e-26

| Identities | = 80/251 (32%), Positives = 124/251 (49%), Gaps = 11/251 (4%) |  |
| --- | --- | --- |
| Query 22 | YDWAQQTIGCSDAAVFRLSAQGRPVLFVKTD--LSGALNELQDEAARLSWLATTGVPCAA | 79 |
| Sbjct 17 | Y + T G S A V++L + + TD G +++ E + WL +P YRCVKDTEGMSPAKVYKLVGENENLYLKMTDSRYKGTTYDVEREKDMMLWLEGK-LPVPK | 75 |
| Query 80 | VLDVVTEAGRDWLLLGEVPG---QDLLSSHLAPAEKVSIMADAMRRLHTLDPATCPFDHQ | 136 |
| Sbjct 76 | VL G LL+ E G + +P + + + A+ +R H++D + CP+ + VLHFERHDGWSNLLMSEADGVLCSEEYEDEQSPEKIIELYAECIRLFHSIDISDCPYTNS | 135 |
| Query 137 | AKHRIERARTRMEAGLVDQD--DLDEEHQGLAPAELFARLKARMPDGEDLVVTHGDACLP | 194 |
| Sbjct 136 | R+ + L D D + +E+ P EL+ LK P+ E+LV +HGD LDSRLAELDYLLNNDLADVDCENWEEDTPFKDPRELYDFLKTEKPE-EELVFSHGDLGDS | 194 |
| Query 195 | NIMVENGRFSGFIDCGRLGVADRYQDIALATRDIAEELGGE-WADRFLVLYGIAAPDSQR | 253 |
| Sbjct 195 | NI V++G+ SGFID GR G AD++ DIA R I E++G E + + F L GI PD ++ NIFVKDGKVSGFIDLGRSGRADKWYDIAFCVRSIREDIGEEQYVELFFDLLGI-KPDWEK | 253 |
| Query 254  Sbjct 254 | IAFYRLLDEFF 264 I +Y LLDE F IKYYILLDELF 264 |  |

>TR:T0V477 T0V477_9ENTE APH domain-containing protein OS=Enterococcus sp. HSIEG1 OX=1316414 GN=HSIEG1_2674 PE=3 SV=1

Length=264

Score = 112 bits (280), Expect = 2e-26

| Identities | = 80/251 (32%), Positives = 124/251 (49%), Gaps = 11/251 (4%) |  |
| --- | --- | --- |
| Query 22 | YDWAQQTIGCSDAAVFRLSAQGRPVLFVKTD--LSGALNELQDEAARLSWLATTGVPCAA | 79 |
| Sbjct 17 | Y + T G S A V++L + + TD G +++ E + WL +P YRCVKDTEGMSPAKVYKLVGENENLYLKMTDSRYKGTTYDVEREKDMMLWLEGK-LPVPK | 75 |
| Query 80 | VLDVVTEAGRDWLLLGEVPG---QDLLSSHLAPAEKVSIMADAMRRLHTLDPATCPFDHQ | 136 |
| Sbjct 76 | VL G LL+ E G + +P + + + A+ +R H++D + CP+ + VLHFERHDGWSNLLMSEADGVLCSEEYEDEQSPEKIIELYAECIRLFHSIDISDCPYTNS | 135 |
| Query 137 | AKHRIERARTRMEAGLVDQD--DLDEEHQGLAPAELFARLKARMPDGEDLVVTHGDACLP | 194 |
| Sbjct 136 | R+ + L D D + +E+ P EL+ LK P+ E+LV +HGD LDSRLAELDYLLNNDLADVDCENWEEDTPFKDPRELYDFLKTEKPE-EELVFSHGDLGDS | 194 |
| Query 195 | NIMVENGRFSGFIDCGRLGVADRYQDIALATRDIAEELGGE-WADRFLVLYGIAAPDSQR | 253 |
| Sbjct 195 | NI V++G+ SGFID GR G AD++ DIA R I E++G E + + F L GI PD ++ NIFVKDGKVSGFIDLGRSGRADKWYDIAFCVRSIREDIGEEQYVELFFDLLGI-KPDWEK | 253 |
| Query 254  Sbjct 254 | IAFYRLLDEFF 264 I +Y LLDE F IKYYILLDELF 264 |  |

>TR:E4ZFP6 E4ZFP6_9CAUD Aminoglycoside phosphotransferase type III OS=Streptococcus phage phi-SsUD.1 OX=861448 GN=aphA-3 PE=3 SV=1

Length=264

Score = 112 bits (280), Expect = 2e-26

| Identities | = 80/251 (32%), Positives = 124/251 (49%), Gaps = 11/251 (4%) |  |
| --- | --- | --- |
| Query 22 | YDWAQQTIGCSDAAVFRLSAQGRPVLFVKTD--LSGALNELQDEAARLSWLATTGVPCAA | 79 |
| Sbjct 17 | Y + T G S A V++L + + TD G +++ E + WL +P YRCVKDTEGMSPAKVYKLVGENENLYLKMTDSRYKGTTYDVEREKDMMLWLEGK-LPVPK | 75 |
| Query 80 | VLDVVTEAGRDWLLLGEVPG---QDLLSSHLAPAEKVSIMADAMRRLHTLDPATCPFDHQ | 136 |
| Sbjct 76 | VL G LL+ E G + +P + + + A+ +R H++D + CP+ + VLHFERHDGWSNLLMSEADGVLCSEEYEDEQSPEKIIELYAECIRLFHSIDISDCPYTNS | 135 |
| Query 137 | AKHRIERARTRMEAGLVDQD--DLDEEHQGLAPAELFARLKARMPDGEDLVVTHGDACLP | 194 |
| Sbjct 136 | R+ + L D D + +E+ P EL+ LK P+ E+LV +HGD LDSRLAELDYLLNNDLADVDCENWEEDTPFKDPRELYDFLKTEKPE-EELVFSHGDLGDS | 194 |
| Query 195 | NIMVENGRFSGFIDCGRLGVADRYQDIALATRDIAEELGGE-WADRFLVLYGIAAPDSQR | 253 |
| Sbjct 195 | NI V++G+ SGFID GR G AD++ DIA R I E++G E + + F L GI PD ++ NIFVKDGKVSGFIDLGRSGRADKWYDIAFCVRSIREDIGEEQYVELFFDLLGI-KPDWEK | 253 |
| Query 254  Sbjct 254 | IAFYRLLDEFF 264 I +Y LLDE F IKYYILLDELF 264 |  |

>TR:S5M7R5 S5M7R5_9CAUD Aminoglycoside 3'-phosphotransferase OS=Escherichia phage 1720a-02 OX=1115653 PE=3 SV=1

Length=264

Score = 112 bits (280), Expect = 2e-26

Identities = 80/251 (32%), Positives = 124/251 (49%), Gaps = 11/251 (4%)

| Query | 22 | YDWAQQTIGCSDAAVFRLSAQGRPVLFVKTD--LSGALNELQDEAARLSWLATTGVPCAA | 79 |
| --- | --- | --- | --- |
| Sbjct | 17 | Y + T G S A V++L + + TD G +++ E + WL +P YRCVKDTEGMSPAKVYKLVGENENLYLKMTDSRYKGTTYDVEREKDMMLWLEGK-LPVPK | 75 |
| Query | 80 | VLDVVTEAGRDWLLLGEVPG---QDLLSSHLAPAEKVSIMADAMRRLHTLDPATCPFDHQ | 136 |
| Sbjct | 76 | VL G LL+ E G + +P + + + A+ +R H++D + CP+ + VLHFERHDGWSNLLMSEADGVLCSEEYEDEQSPEKIIELYAECIRLFHSIDISDCPYTNS | 135 |
| Query | 137 | AKHRIERARTRMEAGLVDQD--DLDEEHQGLAPAELFARLKARMPDGEDLVVTHGDACLP | 194 |

R+	+	L D D + +E+	P EL+ LK	P+ E+LV +HGD

| Sbjct | 136 | LDSRLAELDYLLNNDLADVDCENWEEDTPFKDPRELYDFLKTEKPE-EELVFSHGDLGDS | 194 |
| --- | --- | --- | --- |
| Query | 195 | NIMVENGRFSGFIDCGRLGVADRYQDIALATRDIAEELGGE-WADRFLVLYGIAAPDSQR | 253 |
| Sbjct | 195 | NI V++G+ SGFID GR G AD++ DIA R I E++G E + + F L GI PD ++ NIFVKDGKVSGFIDLGRSGRADKWYDIAFCVRSIREDIGEEQYVELFFDLLGI-KPDWEK | 253 |
| Query  Sbjct | 254  254 | IAFYRLLDEFF 264 I +Y LLDE F IKYYILLDELF 264 |  |

>TR:V2TI87 V2TI87_9GAMM Aminoglycoside 3'-phosphotransferase OS=Acinetobacter nectaris CIP 110549 OX=1392540 GN=P256_02317 PE=3 SV=1

Length=265

Score = 112 bits (280), Expect = 2e-26

| Identities | = 68/220 (31%), Positives = 112/220 (51%), Gaps = 16/220 (7%) |  |
| --- | --- | --- |
| Query 59 | ELQDEAARLSWLATTGVPCAAVLDVVTEAGRDWLLLGEVPGQDLLSSH--LAPAEKVSIM | 116 |
| Sbjct 47 | E Q E A L WL +P ++ ++L+ + GQ S +P + + I+ ESQHEYAMLEWLVDK-LPIPQIIGYAQNNENSYILMSRIQGQLACSDDWLTSPKQLMKIL | 105 |
| Query 117 | ADAMRRLHTLDPATCPFDHQAKHRIERARTRMEAGLVDQ---DDLDEEHQGLAPAE-LFA | 172 |
| Sbjct 106 | A+ ++ L + CP++ H++ +++ GL + +D + G AE L AEGLKALWEIPMLNCPYNLSLDHKLNIEEQKIQQGLYKKPTGEDSTYHNSGFNSAEDLLY | 165 |
| Query 173 | RLKARMPDGEDLVVTHGDACLPNIMVENGRFSGFIDCGRLGVADRYQDIALATRDIAEEL | 232 |
| Sbjct 166 | LK PD EDLV +HGD CLPNI + + +GFID G+ G+ADRY+DIAL R + + L WLKNNKPD-EDLVFSHGDLCLPNIFINQNKINGFIDLGQSGIADRYRDIALCYRSLKDNL | 224 |
| Query 233  Sbjct 225 | GGEWADRFLVLYGIA--------APDSQRIAFYRLLDEFF 264 G ++ + + ++ +PD +IA+Y LLDE +  SGVYSGKIYSNFNLSDFFDALDISPDYDKIAYYILLDELY 264 |  |

>TR:D5UBJ1 D5UBJ1_CELFN Aminoglycoside phosphotransferase OS=Cellulomonas flavigena (strain ATCC 482 / DSM 20109 / NCIB 8073 / NRS 134)

OX=446466 GN=Cfla_1184 PE=3 SV=1

Length=282

Score = 112 bits (281), Expect = 2e-26

| Identities | | = 78/206 (38%), Positives = 107/206 (52%), Gaps = 8/206 (4%) |  |
| --- | --- | --- | --- |
| Query 59  Sbjct 77 | | ELQDEAARLSWLATTGVPCAAVLDVVTEAGRDWLLLGEVPGQDLLSSH--LAPAEKVSIM  +L EA RL+W A P VLD+ + WL+ +P + + PAE + + DLPAEALRLTW-AGRFTPVPEVLDLGVDDEGSWLVTAAIPARSAVDPMWLARPAEAAAAV | 116  135 |
| Query 117  Sbjct 136 | | ADAMRRLHTLDPA-TCPFDHQAKHRIERARTRMEAGLVDQDDLDEEHQGLAPAELFARLK  +R LH PA CPF Q + R+ERA + AG D H L+ A+ ARL GAGLRALHDALPAGECPFAWQVEARVERALEHLHAG-DSPTGWDPVHHHLSAADARARL- | 175  193 |
| Query 176  Sbjct 194 | | ARMPDGEDLVVTHGDACLPNIMV-ENGRFSGFIDCGRLGVADRYQDIALATRDIAEELGG P + LVV H DAC PN +V ++GR++ +D GRLGVADR+ D+A+AT G  LDAPPVDRLVVCHADACAPNTLVHDDGRWAAHVDLGRLGVADRWADLAIATWSTTWNYGP | 234  253 |
| Query 235 | | EWADRFLVLYGIAAPDSQRIAFYRLL 260  + L YGI PD +R A+YRLL |  |
| Sbjct | 254 | GYERHVLDAYGI-EPDDERTAYYRLL 278 | |

```
>TR:A0A077LEK5 A0A077LEK5_9PSED Aminoglycoside 3'-phosphotransferase OS=Pseudomonas sp. StFLB209 OX=1028989 GN=PSCI_1740 PE=3 SV=1
Length=267

Score = 112 bits (280), Expect = 2e-26
Identities = 88/254 (35%), Positives = 121/254 (48%), Gaps = 20/254 (8%)

Query 23	DWAQQTIGCSDAAVFRLSAQGRPVLFVKTDLSGALNELQDEAARLSWLATTGVPCAAVLD 82 DW	G S	VFR	+G	F K	+	EL E RL WL	GV C V++
Sbjct 14	DWLPVRGGESGDFVFR---RGDGHAFAKIAPASRRGELAGERDRLIWLKGRGVACPEVIN 70
```

| Query | 83 | VVTEAGRDWLLLGEVPG---QDLLSSHLAPAEKVSIMADAMRRLHTLDPATCPFDHQAKH | 139 |
| --- | --- | --- | --- |
| Sbjct | 71 | E L++ +PG DL + L A M + +H+L CPF+ + WQEEQEGACLVITAIPGVPAADLSGADLLKAWPS--MGQQLGAVHSLSVDQCPFERRLSR | 128 |
| Query | 140 | RIERARTRMEAGLVDQDDLDEEHQGLAPAELFARLKARMP DGEDLVVTHGDACLP | 194 |
| Sbjct | 129 | RA + V+ D L +E + +L AR++ +P + D+VV HGD C+P MFGRAVDVVSRNAVNPDFLPDEDKSTPQLDLLARVERELPVRLDQERTDMVVCHGDPCMP | 188 |
| Query | 195 | NIMVENG--RFSGFIDCGRLGVADRYQDIALATRDIAEELGG-EWADR-FLVLY---GIA | 247 |
| Sbjct | 189 | N MV+ R +G ID GRLG ADRY D+AL + E + A+R F VL+ GI NFMVDPKTLRCTGLIDLGRLGTADRYADLALMIANAEENWAAPDEAERAFAVLFNVLGIE | 248 |
| Query  Sbjct | 248  249 | APDSQRIAFYRLLD 261 APD +R+AFY LD APDRERLAFYLRLD 262 |  |

>TR:A0A0V8T6W1 A0A0V8T6W1_9CELL Aminoglycoside phosphotransferase OS=Cellulomonas sp. B6 OX=1295626 GN=ATM99_11980 PE=3 SV=1

Length=272

Score = 112 bits (280), Expect = 2e-26

| Identities | | = 94/259 (36%), Positives = 134/259 (52%), Gaps = 23/259 (9%) |  |
| --- | --- | --- | --- |
| Query 12  Sbjct 23 | | PAAWVERLFGYD WAQQTIGCSDAAVFRLSAQGRPVLFVKTDLSGALNELQDEAAR  PAA VER+ G W Q G + FR+ A+ R V + ++G +L EAAR PAA-VERVAGGARLEPVWENQLGGLT----FRVGAE-RYVKWAPAGVTGL--DLAAEAAR | 66  74 |
| Query 67  Sbjct 75 | | LSWLAT-TGVPCAAVLDVVTEAGRDWLLLGEVPGQDLLSSHLA--PAEKVSIMADAMRRL L+W A T VP VL+ + WL+ + G+ + + PAE + + + +R L LAWAARWTTVP--EVLEQGADDEGAWLVTAALRGRSAVEAPWVDRPAEAAAAIGEGLRAL | 123  132 |
| Query 124  Sbjct 133 | | HTLDPA-TCPFDHQAKHRIERARTRMEAGLVDQDDLDEEHQGLAPAELFARLKARMPDGE H P CPF R+ RA ++AG EH+ L AE ARL+ +PD + HDALPVGDCPFSWSTAERVARALEHLDAGDTPAG-WSPEHRDLTVAEARARLRD-VPDVD | 182  190 |
| Query 183  Sbjct 191 | | DLVVTHGDACLPNIMV-ENGRFSGFIDCGRLGVADRYQDIALATRDIAEELGGEWADRFL LVV HGDAC PN ++ ++GR++ +D G LGVADR+ D+A+A G +A  LLVVCHGDACAPNTLIGDDGRWAAHVDLGSLGVADRWADLAVAAWSTEWNYGPGYAGTVY | 241  250 |
| Query 242 | | VLYGIAAPDSQRIAFYRLL 260 YG+ APD RIAF+RLL |  |
| Sbjct | 251 | DAYGL-APDPDRIAFHRLL 268 | |

>TR:R5B6I8 R5B6I8_9CLOT Aminoglycoside phosphotransferase OS=Clostridium sp. CAG:226 OX=1262781 GN=BN545_01268 PE=3 SV=1

Length=260

Score = 112 bits (279), Expect = 2e-26

| Identities  Query 75 | | = 68/204 (33%), Positives = 104/204 (51%), Gaps = 16/204 (8%)  VPCAAVLDVVTEAGRDWLLLGEVPGQDLLSSHLAP--AEKVSIMADAMRRLHTLDPATCP | 132 |
| --- | --- | --- | --- |
| Sbjct 59 | | +P +++V E G ++LL+ + G+ + A + ++ADA+ L ++ A CP LPAPRIIEVAEENGTNYLLMTRIRGKMACAKEYIDDAATLLPLLADAIHMLWNVNTADCP | 118 |
| Query 133  Sbjct 119 | | FDHQAKHRIERARTRMEAGLVDQDDLDEEHQG LAPAELFARLKARMPDGEDLVVTH  + + + ++ A+ +E GL DD G PAEL+ L A P E + +H YQNMLEAKLAFAKRNVEQGLCSTDDAQPGTYGPNGFADPAELYNWLIANKPT-EQRIFSH | 188  177 |
| Query 189  Sbjct 178 | | GDACLPNIMVENGRFSGFIDCGRLGVADRYQDIALATRDIAEELGGEW ADRF  GD CLPN+ ++NG+ SGFID G GV D YQDIAL R + G + AD GDLCLPNVFIDNGKISGFIDLGWAGVTDIYQDIALCYRSLVNNRDGRYGTTHPYFDADAL | 240  237 |
| Query 241 | | LVLYGIAAPDSQRIAFYRLLDEFF 264 L GI PD ++ +Y +LDE F |  |
| Sbjct | 238 | FNLLGI-EPDRDKLFYYTMLDELF 260 | |

>TR:A0A1I3Y9R7 A0A1I3Y9R7_9CELL Kanamycin kinase OS=Cellulomonas sp. KH9 OX=1855324 GN=SAMN05216467_2680 PE=3 SV=1

Length=278

Score = 112 bits (280), Expect = 2e-26

| Identities | = 81/236 (34%), Positives = 119/236 (50%), Gaps = 11/236 (5%) |  |
| --- | --- | --- |
| Query 29 | IGCSDAAVFRLSAQGRPVLFVKTDLSGALNELQDEAARLSWLATTGVPCAAVLDVVTEAG | 88 |
| Sbjct 46 | + + FR+ R V +V T +G +L EA RL+W A P VLD+ +A VNVAGGVTFRVG-HDRYVKWVPTAATGL--DLAAEAVRLTW-ARQFTPVPEVLDLGADAE | 101 |
| Query 89 | RDWLLLGEVPGQDLLSSHLA--PAEKVSIMADAMRRLHTLDPA-TCPFDHQAKHRIERAR | 145 |
| Sbjct 102 | WL+ + + + + PA+ + + +R LH P CPFD A+ R+E A GTWLVTAAIDARSAVVPPWSSRPAQAAAAIGAGLRALHDALPVGDCPFDWSARSRVEVAL | 161 |
| Query 146 | TRMEAGLVDQDDLDEEHQGLAPAELFARLKARMPDGEDLVVTHGDACLPNIMV-ENGRFS | 204 |
| Sbjct 162 | + AG EH+GL E ARL +P + LVV H DAC PN ++ ++GR++ EHLAAGDTPSG-WSPEHRGLTATEARARL-LDVPPVDRLVVCHADACAPNTLIGDDGRWA | 219 |
| Query 205  Sbjct 220 | GFIDCGRLGVADRYQDIALATRDIAEELGGEWADRFLVLYGIAAPDSQRIAFYRLL 260  +D GRLGVADR+ D+A+A G + YG+ PD +RIA+YRLL  AHVDLGRLGVADRWADLAVAAWSTGWNYGAGYDTTVYEAYGV-EPDDERIAYYRLL 274 |  |

>TR:D9VQD2 D9VQD2_9ACTN Streptomycin 6-phosphotransferase OS=Streptomyces sp. C OX=253839 GN=SSNG_05821 PE=3 SV=1

Length=269

Score = 112 bits (279), Expect = 3e-26

| Identities | = 95/267 (36%), Positives = 125/267 (47%), Gaps = 26/267 (10%) |  |
| --- | --- | --- |
| Query 6 | GLHAGSPAAWVERLFGYDWAQQTIGCSDAAVFRLSAQGRPVLFVKTDLSGALNELQDEAA | 65 |
| Sbjct 13 | GL SPA G DW T G S A VFR + GR + K +L E GLRVVSPALLG---IGGDWLPVTTGESGAFVFRSADAGR---YAKCVPVADAADLAAERD | 66 |
| Query 66 | RLSWLATTGVPCAAVLDVVTEAGRDWLLLGEVPGQDLLSSHLAPAEKVSI MADAMR | 121 |
| Sbjct 67 | R++WL+ VP AVLD L+ V G + + PAE + +ADA+R RIAWLSARAVPGQAVLDWYDIDTGACLVTSAVHG---VPADRLPAEDLRASWDRIADAVR | 123 |
| Query 122 | RLHTLDPATCPFDHQAKHRIERARTRMEAGLVDQDDLDEEHQGLAPAELFARLKARMP-- | 179 |
| Sbjct 124 | RLH + + CPF + AR + V+ D L +E Q P +L ARL ++P RLHEVPVSQCPFHRGLDTMVTLARDVVARDAVNPDFLPDEQQHAPPTDLLARLTRQVPRR | 183 |
| Query 180 | ---DGEDLVVTHGDACLPNIMVENGRF--SGFIDCGRLGVADRYQDIALATRDIAEELGG | 234 |
| Sbjct 184 | + D VV HGD CLPNI+++ SGFID GRLG ADR+ D+AL + E REQEAGDTVVCHGDLCLPNIVLDPQTLDVSGFIDLGRLGRADRHADLALLLANARETWPD | 243 |
| Query 235  Sbjct 244 | EWADR-----FLVLYGIAAPDSQRIAF 256 E R F YGIA D +R+ F EEQARAADAAFAERYGIAL-DQERLRF 269 |  |

>TR:A0A1M6IJ84 A0A1M6IJ84_9ACTN Kanamycin kinase/aminoglycoside 3'-phosphotransferase-2 OS=Nocardiopsis flavescens OX=758803 GN=SAMN05421803_105184

PE=3 SV=1

Length=265

Score = 111 bits (278), Expect = 3e-26

| Identities | = 86/247 (35%), Positives = 120/247 (49%), Gaps = 12/247 (5%) |  |
| --- | --- | --- |
| Query 29 | IGCSDAAVFRLSAQGRPVLFVKT---DLSGALNELQDEAARLSWLATTGVPCAAVLDVVT | 85 |
| Sbjct 17 | +G S AAV RL GRP K+ D G + EL RL LA T +P +LD VGLSGAAVARLYTGGRPAAVFKSAREDRPGQVAELVGTVRRLRLLARTPLPVPRLLDSGR | 76 |
| Query 86 | EAGRDWLLLGEVPGQDLLS-SHLAPAEKVSIMADAMRRLHTLDPATCPFDHQAKHRIERA | 144 |
| Sbjct 77 | G WL + PG+ H PA ++++A A+R LH + A P +E+A WEGAYWLTMEHRPGRGAHEPGHDRPA-LIALLARALRELHAVPTADLPLPMGPDVLLEQA | 135 |
| Query 145 | RTRMEAGLVDQDDLDEEHQ-GLAPAELFARLKARMPDG--EDLVVTHGDACLPNIMVENG | 201 |
| Sbjct 136 | R R +G VD+ E H+ G AE+ + L R+ V+ HGD CLPN++V+ RGRTASGAVDRGWRAEGHRTGTTAAEVLSGLAGRLARTPVSGPVLLHGDYCLPNVLVDGR | 195 |
| Query 202 | RFSGFIDCGRLGVADRYQDIALATRDIAEEL GGEWADRFLVLYGIAAPDSQRIAFY | 257 |
| Sbjct 196 | ID GR+GV DR+ D+A T + L G A+ FL YG D + + Y  GGWSLIDLGRVGVGDRHLDLADMTGSMLSGLNPSFGPRDAEAFLDAYGRDLVDPELLDLY | 255 |

Query 258 RLLDEFF 264

R+LD+FF Sbjct 256 RVLDDFF 262

>TR:A0A416ESZ4 A0A416ESZ4_9FIRM Aminoglycoside 3'-phosphotransferase OS=Lachnotalea sp. AF33-28 OX=2292046 GN=DWZ56_01335 PE=3 SV=1

Length=268

Score = 111 bits (278), Expect = 4e-26

| Identities | = 78/251 (31%), Positives = 125/251 (50%), Gaps = 24/251 (10%) |  |
| --- | --- | --- |
| Query 29 | IGCSDAAVFRLSAQGRPVLFVKTDLSGALNELQDEAARLSWLATTGVPCAAVLDVVTEAG | 88 |
| Sbjct 27 | +G SDA V VL + + ++NE + + WLA +P VL V + G VGMSDAQVICFEDM---VLKIGPEREESVNEFR MMEWLAGR-LPVPEVLSYVCQDG | 78 |
| Query 89 | RDWLLLGEVPGQDLLSSHLA--PAEKVSIMADAMRRLHTLDPATCPFDHQAKHRIERART | 146 |
| Sbjct 79 | R +LL+ + G + P V ++A+ +R L T+D + CP D++ +++ A RCYLLMSRIDGVMACDGQMLEQPKRLVKLLAEGLRMLWTVDISHCPSDNRLDRKLKCAEA | 138 |
| Query 147 | RMEAGLVDQDDLDEEHQGL APAELFARLKARMPDGEDLVVTHGDACLPNIMVENGR | 202 |
| Sbjct 139 | R+ GL +D + + G +P +L LK P ED V +HGD CLPN+ ++G+ RVLNGLCSIEDAEPDTYGPGGFESPKQLLRWLKEHKPV-EDPVFSHGDYCLPNVFFKDGK | 197 |
| Query 203 | FSGFIDCGRLGVADRYQDIALATRDIAEELGGEWADR-FLVLYGIAA PDSQR | 253 |
| Sbjct 198 | +GFID GR G+ RYQDIAL R + G + + + + ++A PD + VNGFIDLGRSGIGGRYQDIALCCRSLQHNTAGCYGGKVYDEAFDLSAFFEELQMKPDYDK | 257 |
| Query 254  Sbjct 258 | IAFYRLLDEFF 264  + +Y LLDE F  VKYYILLDELF 268 |  |

>TR:A0A6G4U730 A0A6G4U730_9ACTN APH(3'') family aminoglycoside O-phosphotransferase OS=Streptomyces sp. A7024 OX=1128680 GN=aph(3'') PE=3

SV=1

Length=273

Score = 111 bits (278), Expect = 4e-26

| Identities | = 96/266 (36%), Positives = 124/266 (47%), Gaps = 36/266 (14%) |  |
| --- | --- | --- |
| Query 20 | FGYD---WAQQTIGCSDAAVFRLSAQGRPVLFVKTDLSGALNELQDEAARLSWLATTGVP | 76 |
| Sbjct 15 | FG D W T G S AAVFR + R F K + + L E R++WL GVP FGVDDGGWVPVTSGESGAAVFRSADAAR---FAKCVPAVDVVGLAAERDRVAWLRDQGVP | 71 |
| Query 77 | CAAVLDVVTEAGRDWLLLGEVPGQDLLSSHLAPAEKVSI MADAMRRLHTLD | 127 |
| Sbjct 72 | + VLD + L+ V G PA+++S +ADA+RRLH + GSRVLDWHSGEAGACLVTSAVSG VPADQLSADDLRGAWGRIADAVRRLHEVP | 123 |
| Query 128 | PATCPFDHQAKHRIERARTRMEAGLVDQDDLDEEHQGLAPAELFARL KARMPDGE | 182 |
| Sbjct 124 | + CPF + AR + G V+ + L E Q EL RL + R + VSQCPFRWGLDSMVALARDVVARGAVNPEFLPVEQQHTPGGELLDRLTPQLARRREQEAA | 183 |
| Query 183 | DLVVTHGDACLPNIMVENGRF--SGFIDCGRLGVADRYQDIALATRDI AEELGGE | 235 |
| Sbjct 184 | D VV HGD CLPNI+++ G SGFID GRLG+ADRY D+AL + AEE G DTVVCHGDLCLPNIILDPGTLDVSGFIDLGRLGLADRYADLALLLTNARETWKAEEQGRA | 243 |
| Query 236  Sbjct 244 | WADRFLVLYGIAAPDSQRIAFYRLLD 261 F YGI D R+ FY LD  ADMAFAERYGIDL-DHDRLRFYLHLD 268 |  |

>TR:A0A1B6AUD8 A0A1B6AUD8_9ACTN Kinase OS=Streptomyces sp. NBRC 110611 OX=1621259 GN=SSP35_25_00240 PE=3 SV=1

Length=279

Score = 111 bits (278), Expect = 5e-26

Identities = 93/260 (36%), Positives = 123/260 (47%), Gaps = 33/260 (13%)

Query 23	DWAQQTIGCSDAAVFRLSAQGRPVLFVKTDLSGALNELQDEAARLSWLATTGVPCAAVLD 82 DW	T G S AAVFR SA G	+ K	+	+L+ E R++WL+ GVP	VL

| Sbjct | 27 | DWVPVTTGESGAAVFR-SADG--TRYAKCVAAAEAADLKAERDRVAWLSDQGVPGPRVLG | 83 |
| --- | --- | --- | --- |
| Query | 83 | VVTEAGRDWLLLGEVPGQDLLSSHLAPAEKVSI MADAMRRLHTLDPATCPF | 133 |
| Sbjct | 84 | + L+ V G PA++VS +ADA+RRLH + CPF WHSGNAGACLVTAAVAG VPADQVSAEDLRAAWDRIADAVRRLHEVPVRQCPF | 135 |
| Query | 134 | DHQAKHRIERARTRMEAGLVDQDDLDEEHQGLAPAELFARL KARMPDGEDLVVTH | 188 |
| Sbjct | 136 | I RAR + V+ + L E Q EL +RL + R + + VV H RRDVDEMIARARDVVARNAVNPEFLPVEQQHTPATELLSRLLPQLGRRREQEAAETVVCH | 195 |
| Query | 189 | GDACLPNIMVENGRF--SGFIDCGRLGVADRYQDIALATRDIAE ELGGEWAD-RFL | 241 |
| Sbjct | 196 | GD CLPNI+++ SGF+D GRLG+ADRY D+AL + E E + AD F GDLCLPNIVLDPRTLDVSGFLDLGRLGLADRYADLALLLTNARETWQDEEQAQTADTAFA | 255 |
| Query | 242 | VLYGIAAPDSQRIAFYRLLD 261 YGI D R+ FY LD |  |
| Sbjct | 256 | ERYGIPL-DHHRLRFYLHLD 274 | |

>TR:A0A6B3HQZ8 A0A6B3HQZ8_9ACTN APH(3'') family aminoglycoside O-phosphotransferase OS=Streptomyces sp. SID8455 OX=2706104 GN=aph(3'') PE=3

SV=1

Length=273

Score = 111 bits (277), Expect = 6e-26

| Identities | | = 98/285 (34%), Positives = 135/285 (47%), Gaps = 41/285 (14%) |  |
| --- | --- | --- | --- |
| Query 1  Sbjct 1 | | MIEQDGLHAGSPAAWVERLFGYD---WAQQTIGCSDAAVFRLSAQGRPVLFVKTDLSGAL M ++ G+ A SP L G D W T G S A VFR SA G + K + + MSDRCGVRAVSPV LLGVDDGGWLAVTAGESGAVVFR-SADG--TRYAKCVGAAGV | 57  52 |
| Query 58  Sbjct 53 | | NELQDEAARLSWLATTGVPCAAVLDVVTEAGRDWLLLGEVPGQDLLSSHLAPAEKVSI--  +L E R+ WL+ GVP VLD + G L+ V G PA++VS ADLVAERERVGWLSGRGVPGPRVLDWQSGDGGACLVTSAVCG VPADQVSAAD | 115  104 |
| Query 116 | | -------MADAMRRLHTLDPATCPFDHQAKHRIERARTRMEAGLVDQDDLDEEHQGLAPA  +ADA+RRLH + + CPF + + AR + V+++ L + Q + | 168 |
| Sbjct | 105 | LHVAWERIADAVRRLHEVPVSQCPFRRGLEAMVGVARDVVSRDAVNREFLPVDQQQTSTV | 164 |
| Query | 169 | ELFARLKARMP DGEDLVVTHGDACLPNIMVENGRF--SGFIDCGRLGVADRYQDI | 221 |
| Sbjct | 165 | EL RL ++P + D VV HGD CLPNI+++ SGF+D GRLG+ADR+ D+ ELLDRLARQVPRRREQEAADTVVCHGDLCLPNIVLDPQTLDVSGFVDLGRLGLADRHADL | 224 |
| Query  Sbjct | 222  225 | ALATRDIAEELGGEWADR-----FLVLYGIAAPDSQRIAFYRLLD 261 AL + E E R F YGIA D R+ FY LD ALLLANARETWVDEGQARAADMAFAERYGIAL-DHDRLRFYLGLD 268 |  |

>TR:A0A0C1X3I1 A0A0C1X3I1_9ACTN 3'-kinase OS=Streptomyces sp. AcH 505 OX=352211 GN=HY68_12905 PE=3 SV=1

Length=275

Score = 111 bits (277), Expect = 6e-26

| Identities | | = 92/266 (35%), Positives = 118/266 (44%), Gaps = 41/266 (15%) |  |
| --- | --- | --- | --- |
| Query 21  Sbjct 21 | | GYDWAQQTIGCSDAAVFRLSAQGRPVLFVKTDLSGALNELQDEAARLSWLATTGVPCAAV G W T G S A VFR R + K + + L+ E R++WL T GVPC V GGGWQPVTDGESGARVFRSPDGSR---YAKCVAAEHVASLEAERNRVAWLGTQGVPCPRV | 80  77 |
| Query 81  Sbjct 78 | | LDVVTEAGRDWLLLGEVPGQDLLSSHLAPAEKVSI MADAMRRLHTLDPATC  LD A LL V G PA++V +A A+R+LH L P C LDWRVNANGAALLTSTVAG VPADRVPASLLRAAWRPIAGAVRQLHDLPPEEC | 131  129 |
| Query 132  Sbjct 130 | | PFDHQAKHRIERARTRMEAGLVDQDDLDEEHQGLAPAELFARLKARM PDGEDLVV  PF AR + G V+ D L ++ + P EL ARL+ ++ + + VV PFTRDLTQMFAAARDVVARGAVNPDFLPQDQRQTPPDELLARLEPQVDGRLAQEAAETVV | 186  189 |
| Query 187 | | THGDACLPNIMV--ENGRFSGFIDCGRLGVADRYQDIALATRDIAEELGGEWAD------  HGD CLPNI++ E+ +GFID GRLG AD Y DIAL + G W D | 238 |
| Sbjct | 190 | CHGDLCLPNIILDPESMTVAGFIDLGRLGRADPYADIAL LLATAGETWTDGQQSTA | 245 |
| Query | 239 | ---RFLVLYGIAAPDSQRIAFYRLLD 261 |  |

F	Y I	D R FY LD Sbjct 246 ADETFAHRYDIVL-DRDRADFYLHLD 270

>TR:A0A4P6VWB3 A0A4P6VWB3_9GAMM Aminoglycoside 3'-phosphotransferase OS=Rheinheimera sp. D18 OX=2545632 GN=aph(3'')-Ib PE=3 SV=1

Length=267

Score = 110 bits (276), Expect = 7e-26

| Identities | = 87/254 (34%), Positives = 120/254 (47%), Gaps = 20/254 (8%) |  |
| --- | --- | --- |
| Query 23 | DWAQQTIGCSDAAVFRLSAQGRPVLFVKTDLSGALNELQDEAARLSWLATTGVPCAAVLD | 82 |
| Sbjct 14 | DW G S VFR +G F K + EL E RL WL GV C V++ DWLPVRGGESGDFVFR---RGDGHAFAKIAPASRRGELAGERDRLIWLKGRGVACPEVIN | 70 |
| Query 83 | VVTEAGRDWLLLGEVPG---QDLLSSHLAPAEKVSIMADAMRRLHTLDPATCPFDHQAKH | 139 |
| Sbjct 71 | E L++ +PG DL + L A M + +H+L CPF+ + WQEEQEGACLVITAIPGVPAADLSGADLLKAWPS--MGQQLGAVHSLSVDQCPFERRLSR | 128 |
| Query 140 | RIERARTRMEAGLVDQDDLDEEHQGLAPAELFARLKARMP DGEDLVVTHGDACLP | 194 |
| Sbjct 129 | RA + V+ D L +E + +L AR++ +P + D+VV HGD C+P MFGRAVDVVSRNAVNPDFLPDEDKSTPQLDLLARVERELPVRLDQERTDMVVCHGDPCMP | 188 |
| Query 195 | NIMVENGRF--SGFIDCGRLGVADRYQDIALATRDIAEELGG-EWADR-FLVLY---GIA | 247 |
| Sbjct 189 | N MV+ +G ID GRLG ADRY D+AL + E + A+R F VL+ GI NFMVDPKTLQCTGLIDLGRLGTADRYADLALMIANAEENWAAPDEAERAFAVLFNVLGIE | 248 |
| Query 248  Sbjct 249 | APDSQRIAFYRLLD 261 APD +R+AFY LD APDRERLAFYLRLD 262 |  |

>TR:A0A2P5GGM4 A0A2P5GGM4_9ENTR Aminoglycoside 3'-phosphotransferase OS=Superficieibacter electus OX=2022662 GN=CHU32_27605 PE=3 SV=1

Length=267

Score = 110 bits (276), Expect = 7e-26

| Identities | = 87/254 (34%), Positives = 120/254 (47%), Gaps = 20/254 (8%) |  |
| --- | --- | --- |
| Query 23 | DWAQQTIGCSDAAVFRLSAQGRPVLFVKTDLSGALNELQDEAARLSWLATTGVPCAAVLD | 82 |
| Sbjct 14 | DW G S VFR +G F K + EL E RL WL GV C V++ DWLPVRGGESGDFVFR---RGDGHAFAKIAPASRRGELAGERDRLIWLKGRGVACPEVIN | 70 |
| Query 83 | VVTEAGRDWLLLGEVPG---QDLLSSHLAPAEKVSIMADAMRRLHTLDPATCPFDHQAKH | 139 |
| Sbjct 71 | E L++ +PG DL + L A M + +H+L CPF+ + WQEEQEGACLVITAIPGVPAADLSGADLLKAWPS--MGQQLGAVHSLSVDQCPFERRLSR | 128 |
| Query 140 | RIERARTRMEAGLVDQDDLDEEHQGLAPAELFARLKARMP DGEDLVVTHGDACLP | 194 |
| Sbjct 129 | RA + V+ D L +E + +L AR++ +P + D+VV HGD C+P MFGRAVDVVSRNAVNPDFLPDEDKSTPQLDLLARVERELPVRLDQERTDMVVCHGDPCMP | 188 |
| Query 195 | NIMVENGRF--SGFIDCGRLGVADRYQDIALATRDIAEELGG-EWADR-FLVLY---GIA | 247 |
| Sbjct 189 | N MV+ +G ID GRLG ADRY D+AL + E + A+R F VL+ GI NFMVDPKTLQCTGLIDLGRLGTADRYADLALMIANAEENWAAPDEAERAFAVLFNVLGIE | 248 |
| Query 248  Sbjct 249 | APDSQRIAFYRLLD 261 APD +R+AFY LD APDRERLAFYLRLD 262 |  |

```
>TR:B1VIB4 B1VIB4_CORU7 Aminoglycoside-3''-phosphotransferase OS=Corynebacterium urealyticum (strain ATCC 43042 / DSM 7109) OX=504474
GN=strA PE=3 SV=1
Length=267

Score = 110 bits (276), Expect = 7e-26
Identities = 87/254 (34%), Positives = 120/254 (47%), Gaps = 20/254 (8%)

Query 23	DWAQQTIGCSDAAVFRLSAQGRPVLFVKTDLSGALNELQDEAARLSWLATTGVPCAAVLD 82 DW	G S	VFR	+G	F K	+	EL E RL WL	GV C V++
Sbjct 14	DWLPVRGGESGDFVFR---RGDGHAFAKIAPASRRGELAGERDRLIWLKGRGVACPEVIN 70
```

| Query | 83 | VVTEAGRDWLLLGEVPG---QDLLSSHLAPAEKVSIMADAMRRLHTLDPATCPFDHQAKH | 139 |
| --- | --- | --- | --- |
| Sbjct | 71 | E L++ +PG DL + L A M + +H+L CPF+ + WQEEQEGACLVITAIPGVPAADLSGADLLKAWPS--MGQQLGAVHSLSVDQCPFERRLSR | 128 |
| Query | 140 | RIERARTRMEAGLVDQDDLDEEHQGLAPAELFARLKARMP DGEDLVVTHGDACLP | 194 |
| Sbjct | 129 | RA + V+ D L +E + +L AR++ +P + D+VV HGD C+P MFGRAVDVVSRNAVNPDFLPDEDKSTPQLDLLARVERELPVRLDQERTDMVVCHGDPCMP | 188 |
| Query | 195 | NIMVENGRF--SGFIDCGRLGVADRYQDIALATRDIAEELGG-EWADR-FLVLY---GIA | 247 |
| Sbjct | 189 | N MV+ +G ID GRLG ADRY D+AL + E + A+R F VL+ GI NFMVDPKTLQCTGLIDLGRLGTADRYADLALMIANAEENWAAPDEAERAFAVLFNVLGIE | 248 |
| Query  Sbjct | 248  249 | APDSQRIAFYRLLD 261 APD +R+AFY LD APDRERLAFYLRLD 262 |  |

>TR:A0A0H3GZY9 A0A0H3GZY9_KLEPH Aminoglycoside 3'-phosphotransferase OS=Klebsiella pneumoniae subsp. pneumoniae (strain HS11286) OX=1125630

GN=KPHS_p300870 PE=3 SV=1

Length=267

Score = 110 bits (276), Expect = 7e-26

| Identities | = 87/254 (34%), Positives = 120/254 (47%), Gaps = 20/254 (8%) |  |
| --- | --- | --- |
| Query 23 | DWAQQTIGCSDAAVFRLSAQGRPVLFVKTDLSGALNELQDEAARLSWLATTGVPCAAVLD | 82 |
| Sbjct 14 | DW G S VFR +G F K + EL E RL WL GV C V++ DWLPVRGGESGDFVFR---RGDGHAFAKIAPASRRGELAGERDRLIWLKGRGVACPEVIN | 70 |
| Query 83 | VVTEAGRDWLLLGEVPG---QDLLSSHLAPAEKVSIMADAMRRLHTLDPATCPFDHQAKH | 139 |
| Sbjct 71 | E L++ +PG DL + L A M + +H+L CPF+ + WQEEQEGACLVITAIPGVPAADLSGADLLKAWPS--MGQQLGAVHSLSVDQCPFERRLSR | 128 |
| Query 140 | RIERARTRMEAGLVDQDDLDEEHQGLAPAELFARLKARMP DGEDLVVTHGDACLP | 194 |
| Sbjct 129 | RA + V+ D L +E + +L AR++ +P + D+VV HGD C+P MFGRAVDVVSRNAVNPDFLPDEDKSTPQLDLLARVERELPVRLDQERTDMVVCHGDPCMP | 188 |
| Query 195 | NIMVENGRF--SGFIDCGRLGVADRYQDIALATRDIAEELGG-EWADR-FLVLY---GIA | 247 |
| Sbjct 189 | N MV+ +G ID GRLG ADRY D+AL + E + A+R F VL+ GI NFMVDPKTLQCTGLIDLGRLGTADRYADLALMIANAEENWAAPDEAERAFAVLFNVLGIE | 248 |
| Query 248  Sbjct 249 | APDSQRIAFYRLLD 261 APD +R+AFY LD APDRERLAFYLRLD 262 |  |

>TR:J5KDL8 J5KDL8_PASMD Aminoglycoside 3'-phosphotransferase OS=Pasteurella multocida subsp. multocida str. Anand1_buffalo OX=1032868

GN=AAUPMB_19980 PE=3 SV=1

Length=267

Score = 110 bits (276), Expect = 7e-26

| Identities | = 87/254 (34%), Positives = 120/254 (47%), Gaps = 20/254 (8%) |  |
| --- | --- | --- |
| Query 23 | DWAQQTIGCSDAAVFRLSAQGRPVLFVKTDLSGALNELQDEAARLSWLATTGVPCAAVLD | 82 |
| Sbjct 14 | DW G S VFR +G F K + EL E RL WL GV C V++ DWLPVRGGESGDFVFR---RGDGHAFAKIAPASRRGELAGERDRLIWLKGRGVACPEVIN | 70 |
| Query 83 | VVTEAGRDWLLLGEVPG---QDLLSSHLAPAEKVSIMADAMRRLHTLDPATCPFDHQAKH | 139 |
| Sbjct 71 | E L++ +PG DL + L A M + +H+L CPF+ + WQEEQEGACLVITAIPGVPAADLSGADLLKAWPS--MGQQLGAVHSLSVDQCPFERRLSR | 128 |
| Query 140 | RIERARTRMEAGLVDQDDLDEEHQGLAPAELFARLKARMP DGEDLVVTHGDACLP | 194 |
| Sbjct 129 | RA + V+ D L +E + +L AR++ +P + D+VV HGD C+P MFGRAVDVVSRNAVNPDFLPDEDKSTPQLDLLARVERELPVRLDQERTDMVVCHGDPCMP | 188 |
| Query 195 | NIMVENGRF--SGFIDCGRLGVADRYQDIALATRDIAEELGG-EWADR-FLVLY---GIA | 247 |
| Sbjct 189 | N MV+ +G ID GRLG ADRY D+AL + E + A+R F VL+ GI NFMVDPKTLQCTGLIDLGRLGTADRYADLALMIANAEENWAAPDEAERAFAVLFNVLGIE | 248 |
| Query 248 | APDSQRIAFYRLLD 261 APD +R+AFY LD |  |

Sbjct 249 APDRERLAFYLRLD 262

>TR:A0A0M2R0H0 A0A0M2R0H0_9PROT Aminoglycoside 3'-phosphotransferase OS=Kiloniella litopenaei OX=1549748 GN=WH95_19995 PE=3 SV=1

Length=267

Score = 110 bits (276), Expect = 7e-26

| Identities | = 87/254 (34%), Positives = 120/254 (47%), Gaps = 20/254 (8%) |  |
| --- | --- | --- |
| Query 23 | DWAQQTIGCSDAAVFRLSAQGRPVLFVKTDLSGALNELQDEAARLSWLATTGVPCAAVLD | 82 |
| Sbjct 14 | DW G S VFR +G F K + EL E RL WL GV C V++ DWLPVRGGESGDFVFR---RGDGHAFAKIAPASRRGELAGERDRLIWLKGRGVACPEVIN | 70 |
| Query 83 | VVTEAGRDWLLLGEVPG---QDLLSSHLAPAEKVSIMADAMRRLHTLDPATCPFDHQAKH | 139 |
| Sbjct 71 | E L++ +PG DL + L A M + +H+L CPF+ + WQEEQEGACLVITAIPGVPAADLSGADLLKAWPS--MGQQLGAVHSLSVDQCPFERRLSR | 128 |
| Query 140 | RIERARTRMEAGLVDQDDLDEEHQGLAPAELFARLKARMP DGEDLVVTHGDACLP | 194 |
| Sbjct 129 | RA + V+ D L +E + +L AR++ +P + D+VV HGD C+P MFGRAVDVVSRNAVNPDFLPDEDKSTPQLDLLARVERELPVRLDQERTDMVVCHGDPCMP | 188 |
| Query 195 | NIMVENGRF--SGFIDCGRLGVADRYQDIALATRDIAEELGG-EWADR-FLVLY---GIA | 247 |
| Sbjct 189 | N MV+ +G ID GRLG ADRY D+AL + E + A+R F VL+ GI NFMVDPKTLQCTGLIDLGRLGTADRYADLALMIANAEENWAAPDEAERAFAVLFNVLGIE | 248 |
| Query 248  Sbjct 249 | APDSQRIAFYRLLD 261 APD +R+AFY LD APDRERLAFYLRLD 262 |  |

>TR:A0A2S2FBY2 A0A2S2FBY2_9GAMM Aminoglycoside 3'-phosphotransferase OS=Acinetobacter defluvii OX=1871111 GN=aph(3'')-Ib PE=3 SV=1

Length=267

Score = 110 bits (276), Expect = 7e-26

| Identities | = 87/254 (34%), Positives = 120/254 (47%), Gaps = 20/254 (8%) |  |
| --- | --- | --- |
| Query 23 | DWAQQTIGCSDAAVFRLSAQGRPVLFVKTDLSGALNELQDEAARLSWLATTGVPCAAVLD | 82 |
| Sbjct 14 | DW G S VFR +G F K + EL E RL WL GV C V++ DWLPVRGGESGDFVFR---RGDGHAFAKIAPASRRGELAGERDRLIWLKGRGVACPEVIN | 70 |
| Query 83 | VVTEAGRDWLLLGEVPG---QDLLSSHLAPAEKVSIMADAMRRLHTLDPATCPFDHQAKH | 139 |
| Sbjct 71 | E L++ +PG DL + L A M + +H+L CPF+ + WQEEQEGACLVITAIPGVPAADLSGADLLKAWPS--MGQQLGAVHSLSVDQCPFERRLSR | 128 |
| Query 140 | RIERARTRMEAGLVDQDDLDEEHQGLAPAELFARLKARMP DGEDLVVTHGDACLP | 194 |
| Sbjct 129 | RA + V+ D L +E + +L AR++ +P + D+VV HGD C+P MFGRAVDVVSRNAVNPDFLPDEDKSTPQLDLLARVERELPVRLDQERTDMVVCHGDPCMP | 188 |
| Query 195 | NIMVENGRF--SGFIDCGRLGVADRYQDIALATRDIAEELGG-EWADR-FLVLY---GIA | 247 |
| Sbjct 189 | N MV+ +G ID GRLG ADRY D+AL + E + A+R F VL+ GI NFMVDPKTLQCTGLIDLGRLGTADRYADLALMIANAEENWAAPDEAERAFAVLFNVLGIE | 248 |
| Query 248  Sbjct 249 | APDSQRIAFYRLLD 261 APD +R+AFY LD APDRERLAFYLRLD 262 |  |

>TR:A0A0R0BRS8 A0A0R0BRS8_9GAMM Aminoglycoside 3'-phosphotransferase OS=Stenotrophomonas koreensis OX=266128 GN=ABB25_04415 PE=3 SV=1

Length=267

Score = 110 bits (276), Expect = 7e-26

Identities = 87/254 (34%), Positives = 120/254 (47%), Gaps = 20/254 (8%)

| Query | 23 | DWAQQTIGCSDAAVFRLSAQGRPVLFVKTDLSGALNELQDEAARLSWLATTGVPCAAVLD | 82 |
| --- | --- | --- | --- |
| Sbjct | 14 | DW G S VFR +G F K + EL E RL WL GV C V++ DWLPVRGGESGDFVFR---RGDGHAFAKIAPASRRGELAGERDRLIWLKGRGVACPEVIN | 70 |
| Query | 83 | VVTEAGRDWLLLGEVPG---QDLLSSHLAPAEKVSIMADAMRRLHTLDPATCPFDHQAKH E L++ +PG DL + L A M + +H+L CPF+ + | 139 |

| Sbjct | 71 | WQEEQEGACLVITAIPGVPAADLSGADLLKAWPS--MGQQLGAVHSLSVDQCPFERRLSR | 128 |
| --- | --- | --- | --- |
| Query | 140 | RIERARTRMEAGLVDQDDLDEEHQGLAPAELFARLKARMP DGEDLVVTHGDACLP | 194 |
| Sbjct | 129 | RA + V+ D L +E + +L AR++ +P + D+VV HGD C+P MFGRAVDVVSRNAVNPDFLPDEDKSTPQLDLLARVERELPVRLDQERTDMVVCHGDPCMP | 188 |
| Query | 195 | NIMVENGRF--SGFIDCGRLGVADRYQDIALATRDIAEELGG-EWADR-FLVLY---GIA | 247 |
| Sbjct | 189 | N MV+ +G ID GRLG ADRY D+AL + E + A+R F VL+ GI NFMVDPKTLQCTGLIDLGRLGTADRYADLALMIANAEENWAAPDEAERAFAVLFNVLGIE | 248 |
| Query  Sbjct | 248  249 | APDSQRIAFYRLLD 261 APD +R+AFY LD APDRERLAFYLRLD 262 |  |

>TR:A0A501WBA3 A0A501WBA3_9GAMM Aminoglycoside 3'-phosphotransferase OS=Marinomonas sp. HB171799 OX=2589817 GN=aph(3'')-Ib PE=3 SV=1

Length=267

Score = 110 bits (276), Expect = 7e-26

| Identities | = 87/254 (34%), Positives = 120/254 (47%), Gaps = 20/254 (8%) |  |
| --- | --- | --- |
| Query 23 | DWAQQTIGCSDAAVFRLSAQGRPVLFVKTDLSGALNELQDEAARLSWLATTGVPCAAVLD | 82 |
| Sbjct 14 | DW G S VFR +G F K + EL E RL WL GV C V++ DWLPVRGGESGDFVFR---RGDGHAFAKIAPASRRGELAGERDRLIWLKGRGVACPEVIN | 70 |
| Query 83 | VVTEAGRDWLLLGEVPG---QDLLSSHLAPAEKVSIMADAMRRLHTLDPATCPFDHQAKH | 139 |
| Sbjct 71 | E L++ +PG DL + L A M + +H+L CPF+ + WQEEQEGACLVITAIPGVPAADLSGADLLKAWPS--MGQQLGAVHSLSVDQCPFERRLSR | 128 |
| Query 140 | RIERARTRMEAGLVDQDDLDEEHQGLAPAELFARLKARMP DGEDLVVTHGDACLP | 194 |
| Sbjct 129 | RA + V+ D L +E + +L AR++ +P + D+VV HGD C+P MFGRAVDVVSRNAVNPDFLPDEDKSTPQLDLLARVERELPVRLDQERTDMVVCHGDPCMP | 188 |
| Query 195 | NIMVENGRF--SGFIDCGRLGVADRYQDIALATRDIAEELGG-EWADR-FLVLY---GIA | 247 |
| Sbjct 189 | N MV+ +G ID GRLG ADRY D+AL + E + A+R F VL+ GI NFMVDPKTLQCTGLIDLGRLGTADRYADLALMIANAEENWAAPDEAERAFAVLFNVLGIE | 248 |
| Query 248  Sbjct 249 | APDSQRIAFYRLLD 261 APD +R+AFY LD APDRERLAFYLRLD 262 |  |

>TR:A0A0M0HMR3 A0A0M0HMR3_9VIBR Aminoglycoside 3'-phosphotransferase OS=Vibrio hepatarius OX=171383 GN=AKJ31_22200 PE=3 SV=1

Length=267

Score = 110 bits (276), Expect = 7e-26

| Identities | = 87/254 (34%), Positives = 120/254 (47%), Gaps = 20/254 (8%) |  |
| --- | --- | --- |
| Query 23 | DWAQQTIGCSDAAVFRLSAQGRPVLFVKTDLSGALNELQDEAARLSWLATTGVPCAAVLD | 82 |
| Sbjct 14 | DW G S VFR +G F K + EL E RL WL GV C V++ DWLPVRGGESGDFVFR---RGDGHAFAKIAPASRRGELAGERDRLIWLKGRGVACPEVIN | 70 |
| Query 83 | VVTEAGRDWLLLGEVPG---QDLLSSHLAPAEKVSIMADAMRRLHTLDPATCPFDHQAKH | 139 |
| Sbjct 71 | E L++ +PG DL + L A M + +H+L CPF+ + WQEEQEGACLVITAIPGVPAADLSGADLLKAWPS--MGQQLGAVHSLSVDQCPFERRLSR | 128 |
| Query 140 | RIERARTRMEAGLVDQDDLDEEHQGLAPAELFARLKARMP DGEDLVVTHGDACLP | 194 |
| Sbjct 129 | RA + V+ D L +E + +L AR++ +P + D+VV HGD C+P MFGRAVDVVSRNAVNPDFLPDEDKSTPQLDLLARVERELPVRLDQERTDMVVCHGDPCMP | 188 |
| Query 195 | NIMVENGRF--SGFIDCGRLGVADRYQDIALATRDIAEELGG-EWADR-FLVLY---GIA | 247 |
| Sbjct 189 | N MV+ +G ID GRLG ADRY D+AL + E + A+R F VL+ GI NFMVDPKTLQCTGLIDLGRLGTADRYADLALMIANAEENWAAPDEAERAFAVLFNVLGIE | 248 |
| Query 248  Sbjct 249 | APDSQRIAFYRLLD 261 APD +R+AFY LD APDRERLAFYLRLD 262 |  |

>TR:A0A345WV32 A0A345WV32_9SPHN Aminoglycoside 3'-phosphotransferase OS=Sphingomonas

sp. FARSPH OX=2219696 GN=DM480_17840 PE=3 SV=1

Length=267

Score = 110 bits (276), Expect = 7e-26

| Identities | = 87/254 (34%), Positives = 120/254 (47%), Gaps = 20/254 (8%) |  |
| --- | --- | --- |
| Query 23 | DWAQQTIGCSDAAVFRLSAQGRPVLFVKTDLSGALNELQDEAARLSWLATTGVPCAAVLD | 82 |
| Sbjct 14 | DW G S VFR +G F K + EL E RL WL GV C V++ DWLPVRGGESGDFVFR---RGDGHAFAKIAPASRRGELAGERDRLIWLKGRGVACPEVIN | 70 |
| Query 83 | VVTEAGRDWLLLGEVPG---QDLLSSHLAPAEKVSIMADAMRRLHTLDPATCPFDHQAKH | 139 |
| Sbjct 71 | E L++ +PG DL + L A M + +H+L CPF+ + WQEEQEGACLVITAIPGVPAADLSGADLLKAWPS--MGQQLGAVHSLSVDQCPFERRLSR | 128 |
| Query 140 | RIERARTRMEAGLVDQDDLDEEHQGLAPAELFARLKARMP DGEDLVVTHGDACLP | 194 |
| Sbjct 129 | RA + V+ D L +E + +L AR++ +P + D+VV HGD C+P MFGRAVDVVSRNAVNPDFLPDEDKSTPQLDLLARVERELPVRLDQERTDMVVCHGDPCMP | 188 |
| Query 195 | NIMVENGRF--SGFIDCGRLGVADRYQDIALATRDIAEELGG-EWADR-FLVLY---GIA | 247 |
| Sbjct 189 | N MV+ +G ID GRLG ADRY D+AL + E + A+R F VL+ GI NFMVDPKTLQCTGLIDLGRLGTADRYADLALMIANAEENWAAPDEAERAFAVLFNVLGIE | 248 |
| Query 248  Sbjct 249 | APDSQRIAFYRLLD 261 APD +R+AFY LD APDRERLAFYLRLD 262 |  |

>TR:A0A1Q2CIK9 A0A1Q2CIK9_9ACTN Aminoglycoside O-phosphotransferase APH(3'')-Ib OS=Tessaracoccus flavus OX=1610493 GN=RPIT_14600 PE=3 SV=1

Length=267

Score = 110 bits (276), Expect = 7e-26

| Identities | = 87/254 (34%), Positives = 120/254 (47%), Gaps = 20/254 (8%) |  |
| --- | --- | --- |
| Query 23 | DWAQQTIGCSDAAVFRLSAQGRPVLFVKTDLSGALNELQDEAARLSWLATTGVPCAAVLD | 82 |
| Sbjct 14 | DW G S VFR +G F K + EL E RL WL GV C V++ DWLPVRGGESGDFVFR---RGDGHAFAKIAPASRRGELAGERDRLIWLKGRGVACPEVIN | 70 |
| Query 83 | VVTEAGRDWLLLGEVPG---QDLLSSHLAPAEKVSIMADAMRRLHTLDPATCPFDHQAKH | 139 |
| Sbjct 71 | E L++ +PG DL + L A M + +H+L CPF+ + WQEEQEGACLVITAIPGVPAADLSGADLLKAWPS--MGQQLGAVHSLSVDQCPFERRLSR | 128 |
| Query 140 | RIERARTRMEAGLVDQDDLDEEHQGLAPAELFARLKARMP DGEDLVVTHGDACLP | 194 |
| Sbjct 129 | RA + V+ D L +E + +L AR++ +P + D+VV HGD C+P MFGRAVDVVSRNAVNPDFLPDEDKSTPQLDLLARVERELPVRLDQERTDMVVCHGDPCMP | 188 |
| Query 195 | NIMVENGRF--SGFIDCGRLGVADRYQDIALATRDIAEELGG-EWADR-FLVLY---GIA | 247 |
| Sbjct 189 | N MV+ +G ID GRLG ADRY D+AL + E + A+R F VL+ GI NFMVDPKTLQCTGLIDLGRLGTADRYADLALMIANAEENWAAPDEAERAFAVLFNVLGIE | 248 |
| Query 248  Sbjct 249 | APDSQRIAFYRLLD 261 APD +R+AFY LD APDRERLAFYLRLD 262 |  |

>TR:A0A376BDS9 A0A376BDS9_9PAST Aminoglycoside 3'-phosphotransferase OS=[Actinobacillus] rossii OX=123820 GN=strA_3 PE=3 SV=1

Length=267

Score = 110 bits (276), Expect = 7e-26

Identities = 87/254 (34%), Positives = 120/254 (47%), Gaps = 20/254 (8%)

| Query | 23 | DWAQQTIGCSDAAVFRLSAQGRPVLFVKTDLSGALNELQDEAARLSWLATTGVPCAAVLD | 82 |
| --- | --- | --- | --- |
| Sbjct | 14 | DW G S VFR +G F K + EL E RL WL GV C V++ DWLPVRGGESGDFVFR---RGDGHAFAKIAPASRRGELAGERDRLIWLKGRGVACPEVIN | 70 |
| Query | 83 | VVTEAGRDWLLLGEVPG---QDLLSSHLAPAEKVSIMADAMRRLHTLDPATCPFDHQAKH | 139 |
| Sbjct | 71 | E L++ +PG DL + L A M + +H+L CPF+ + WQEEQEGACLVITAIPGVPAADLSGADLLKAWPS--MGQQLGAVHSLSVDQCPFERRLSR | 128 |
| Query | 140 | RIERARTRMEAGLVDQDDLDEEHQGLAPAELFARLKARMP DGEDLVVTHGDACLP  RA + V+ D L +E + +L AR++ +P + D+VV HGD C+P | 194 |

| Sbjct | 129 | MFGRAVDVVSRNAVNPDFLPDEDKSTPQLDLLARVERELPVRLDQERTDMVVCHGDPCMP | 188 |
| --- | --- | --- | --- |
| Query | 195 | NIMVENGRF--SGFIDCGRLGVADRYQDIALATRDIAEELGG-EWADR-FLVLY---GIA | 247 |
| Sbjct | 189 | N MV+ +G ID GRLG ADRY D+AL + E + A+R F VL+ GI NFMVDPKTLQCTGLIDLGRLGTADRYADLALMIANAEENWAAPDEAERAFAVLFNVLGIE | 248 |
| Query  Sbjct | 248  249 | APDSQRIAFYRLLD 261 APD +R+AFY LD APDRERLAFYLRLD 262 |  |

>TR:A6TIY6 A6TIY6_KLEP7 Aminoglycoside 3'-phosphotransferase OS=Klebsiella pneumoniae subsp. pneumoniae (strain ATCC 700721 / MGH 78578)

OX=272620 GN=str PE=3 SV=1

Length=272

Score = 110 bits (276), Expect = 8e-26

| Identities | = 87/254 (34%), Positives = 120/254 (47%), Gaps = 20/254 (8%) |  |
| --- | --- | --- |
| Query 23 | DWAQQTIGCSDAAVFRLSAQGRPVLFVKTDLSGALNELQDEAARLSWLATTGVPCAAVLD | 82 |
| Sbjct 19 | DW G S VFR +G F K + EL E RL WL GV C V++ DWLPVRGGESGDFVFR---RGDGHAFAKIAPASRRGELAGERDRLIWLKGRGVACPEVIN | 75 |
| Query 83 | VVTEAGRDWLLLGEVPG---QDLLSSHLAPAEKVSIMADAMRRLHTLDPATCPFDHQAKH | 139 |
| Sbjct 76 | E L++ +PG DL + L A M + +H+L CPF+ + WQEEQEGACLVITAIPGVPAADLSGADLLKAWPS--MGQQLGAVHSLSVDQCPFERRLSR | 133 |
| Query 140 | RIERARTRMEAGLVDQDDLDEEHQGLAPAELFARLKARMP DGEDLVVTHGDACLP | 194 |
| Sbjct 134 | RA + V+ D L +E + +L AR++ +P + D+VV HGD C+P MFGRAVDVVSRNAVNPDFLPDEDKSTPQLDLLARVERELPVRLDQERTDMVVCHGDPCMP | 193 |
| Query 195 | NIMVENGRF--SGFIDCGRLGVADRYQDIALATRDIAEELGG-EWADR-FLVLY---GIA | 247 |
| Sbjct 194 | N MV+ +G ID GRLG ADRY D+AL + E + A+R F VL+ GI NFMVDPKTLQCTGLIDLGRLGTADRYADLALMIANAEENWAAPDEAERAFAVLFNVLGIE | 253 |
| Query 248  Sbjct 254 | APDSQRIAFYRLLD 261 APD +R+AFY LD APDRERLAFYLRLD 267 |  |

>TR:A0A5C8PSK5 A0A5C8PSK5_9PROT Aminoglycoside 3'-phosphotransferase OS=Enhydrobacter sp. CC-CFT640 OX=2586908 GN=aph(3'') PE=3 SV=1

Length=262

Score = 110 bits (275), Expect = 9e-26

| Identities | = 86/252 (34%), Positives = 121/252 (48%), Gaps = 19/252 (8%) |  |
| --- | --- | --- |
| Query 24 | WAQQTIGCSDAAVFRLSAQGRPVLFVKTDLSGALNELQDEAARLSWLATTGVPCAAVLDV | 83 |
| Sbjct 11 | W G S V+R S+ G + K + L E R++WLA G+ A VLD WIAVHTGESGDRVYR-SSDG--AAYAKLATGARMAALDGERRRVAWLAPCGLGTARVLDW | 67 |
| Query 84 | VTEAGRDWLLLGEVPGQDLLSSHLAPAEKVSI---MADAMRRLHTLDPATCPFDHQAKHR | 140 |
| Sbjct 68 | +T G L+ VPG + +S L A+ + +A ++ LH L A CPF + ITVHGTACLITSTVPG--VPASDLPAADLIRAWPSIAQRLKALHALPVADCPFARGLETM | 125 |
| Query 141 | IERARTRMEAGLVDQDDLDEEHQGLAPAELFARLKARMP DGEDLVVTHGDACLPN | 195 |
| Sbjct 126 | + RA + V+ D LD + + + L ARL+A++P + D VV HGDACLPN MARAADVVARDAVNPDFLDPQLRDIPAPALLARLRAQLPGRLAQEQRDRVVCHGDACLPN | 185 |
| Query 196 | IMVENG--RFSGFIDCGRLGVADRYQDIALATRDIAEEL GGEWADRFLVLYGIAAP | 249 |
| Sbjct 186 | +MV+ R G +D GRLG ADR+ D+AL + E A L AP LMVDPATLRCVGLVDLGRLGTADRHADLALLLANARETWTSPDQARAAHESLFAALDIAP | 245 |
| Query 250  Sbjct 246 | DSQRIAFYRLLD 261 D +R+AFY LD DPERLAFYLHLD 257 |  |

>TR:A0A2N3UL89 A0A2N3UL89_9ACTN Kanamycin kinase OS=Streptomyces sp. GP55 OX=1798190 GN=AXZ45_2882 PE=3 SV=1

Length=245

Score = 109 bits (273), Expect = 1e-25

| Identities | | = 80/220 (36%), Positives = 110/220 (50%), Gaps = 23/220 (10%) |  |
| --- | --- | --- | --- |
| Query 48  Sbjct 44 | | FVKTDLSGALNELQDEAARLSWLATTGVPCAAVLDVVTEAGRDWLLLGEVPGQDLLSSHL FVK G+ +L EA RL W V VL + G WL+ +PG ++ FVKWAPVGSGLDLGAEAVRLGWAGRFAV-VPRVLGEGADGGGSWLVTAGLPGTMAVADRW | 107  102 |
| Query 108 | | A--PAEKVSIMADAMRRLHT-LDPATCPFDHQAKHRIERARTRMEAGLVDQDDLDEEHQG PA V + + +RRLH L CPF+ A+ R+ RAR +G V+ | 164 |
| Sbjct | 103 | KQEPATAVRAIGEGLRRLHEELPVGDCPFEWSAEQRVARARGG--SGTVE---------- | 150 |
| Query | 165 | LAPAELFARLKARMPDGEDLVVTHGDACLPNIMV-ENGRFSGFIDCGRLGVADRYQDIAL | 223 |
| Sbjct | 151 | E A L A +P + LVV HGDAC PN ++ ++G +G +D G LGVADR+ D+A+  ----EALAAL-ADIPPVDRLVVCHGDACAPNTLIGDDGHCTGHVDLGALGVADRWADLAV | 205 |
| Query | 224 ATRDIAEELGGEWADRFLVLYGIAAPDSQRIAFYRLLDEF 263 | | |
|  |  | AT G W + L YG+ PD +RI +YRLL EF |  |
| Sbjct | 206 | ATWSTVWNYGPGWEEPLLEAYGV-EPDQERIRYYRLLWEF 244 | |

>TR:A0A5C4T381 A0A5C4T381_9BACL Aminoglycoside 3'-phosphotransferase OS=Paenibacillus hemerocallicola OX=1172614 GN=FE784_24725 PE=3 SV=1

Length=261

Score = 110 bits (274), Expect = 1e-25

| Identities | = 74/235 (31%), Positives = 117/235 (50%), Gaps = 7/235 (3%) |  |
| --- | --- | --- |
| Query 32 | SDAAVFRLSAQGRPVLFVKTDLSGALNELQDEAARLSWLATTGVPCAAVLDVVTEAGRDW | 91 |
| Sbjct 29 | S A F ++ R ++K +G L +L WL +P L+ ++ ++ SGAYTFHFRSKDRSC-YLKILPTGHREPLVQYRDKLVWLKGR-LPVPEALEYEADSEYEY | 86 |
| Query 92 | LLLGEVPGQDLLSSHLA--PAEKVSIMADAMRRLHTLDPATCPFDHQAKHRIERARTRME | 149 |
| Sbjct 87 | LL+ E+ G+D VS++A +R +H++D A CPFD+ +R+ +R+ LLMSEITGRDATDELFRRNAEHTVSLLAKGLRAIHSVDIAHCPFDYSVNNRLRLIESRLS | 146 |
| Query 150 | AGLVDQDDLDEEHQGLAPAELFARLKARMPDGEDL-VVTHGDACLPNIMVENGRFSGFID | 208 |
| Sbjct 147 | GLVD+ + EE G L RL++ M ++ V HGD +PNI+++N SGFID EGLVDRGAV-EERFGDTLERLHERLQSDMTGLQETPVFCHGDYSVPNIVIDNNEISGFID | 205 |
| Query 209  Sbjct 206 | CGRLGVADRYQDIALATRDIAEELGGEWADRFLVLYGIAAPDSQRIAFYRLLDEF 263 GV+D Y+D A A R I G + F YGI PD +R+ Y L++ F  IADAGVSDPYRDFAAAHRSIIRNFGERFIPLFYETYGI-DPDMRRLRLYDLIEHF 259 |  |

>TR:A0A512PFI8 A0A512PFI8_9CELL Aminoglycoside phosphotransferase APH(3') OS=Cellulomonas soli OX=931535 GN=CSO01_26680 PE=3 SV=1

Length=295

Score = 110 bits (276), Expect = 1e-25

| Identities | | = 93/264 (35%), Positives = 129/264 (49%), Gaps = 32/264 (12%) |  |
| --- | --- | --- | --- |
| Query 10  Sbjct 47 | | GSP--AAWVERLFGYDWAQQTIGCSDAAVFRLSAQGRPVLFVKTDLSGALNELQDEAARL G+P A W G+ W L GRP L++K + +L+ EA RL GAPLEAVWHNEAGGFTWRA LDPDGRPTLYLKWSPADG-PDLEAEAQRL | 67  93 |
| Query 68  Sbjct 94 | | SWLATTGVPCAAVLDVVTEAGRD WLLLGEVPGQDLLSSH--LAPAEKVSIMADAMR  +W+A VP V DVVT G D WL+ + ++ P + +R AWVAEQ-VPGVRVPDVVTH-GSDGSGAWLVTRAIDADPAVADRWRAEPRTAARAIGAGLR | 121  151 |
| Query 122  Sbjct 152 | | RLHTLDP-ATCPFDHQAKHRIERARTRMEAGLVDQDDLDEEHQGLAPAELFARLKARMPD RLH P A+CPF A+ R+ AR R+ AG EH+ L A L A +P+ RLHDAAPVASCPFVWSAEQRLAAARGRVAAG-EGPSTWHPEHRSLDATRALALL-ADVPE | 180  209 |
| Query 181 | | GEDLVVTHGDACLPNIMVEN-GRFSGFIDCGRLGVADRYQDIALATRDIAEELGGE---W D VV HGDAC PN +++ GR++G +D GRLGVADR+ D+A+AT + G + | 236 |
| Sbjct | 210 | -PDPVVCHGDACAPNTLIDAAGRWAGLVDLGRLGVADRWADLAVATWSLGWNFGTDVPGL | 268 |
| Query  Sbjct | 237  269 | ADRFLVLYGIAAPDSQRIAFYRLL 260 L YGIAA D ++ A+YRLL  EAELLDAYGIAA-DPRKSAYYRLL 291 |  |

>TR:A0A239AVA4 A0A239AVA4_9ACTN Kanamycin kinase OS=Streptomyces glauciniger OX=235986 GN=SAMN05216252_102189 PE=3 SV=1

Length=248

Score = 109 bits (273), Expect = 1e-25

| Identities | = 85/229 (37%), Positives = 112/229 (49%), Gaps = 29/229 (13%) |  |
| --- | --- | --- |
| Query 37 | FRLSAQGRPVLFVKTDLSGALNELQDEAARLSWLAT-TGVPCAAVLDVVTEAGRDWLLLG | 95 |
| Sbjct 40 | FR+ + G LF K +G+ +L EA RL W T VP +VL+ ++ WLL FRIGS-GEDRLFAKWAPAGSGPDLGAEAERLEWARRFTSVP--SVLEHGSDDDGSWLLTR | 96 |
| Query 96 | EVPGQDLLSSHLA--PAEKVSIMADAMRRLHTLDPAT-CPFDHQAKHRIERARTRMEAGL | 152 |
| Sbjct 97 | +PG+ +S P V + +R LH P CP+D R+ERAR AG GLPGESAVSPRWTADPGTAVRALGAGLRALHERLPVDGCPYDWSVGARLERAR---RAGK | 153 |
| Query 153 | VDQDDLDEEHQGLAPAELFARLKARMPDGEDLVVTHGDACLPNIMV-ENGRFSGFIDCGR | 211 |
| Sbjct 154 | +DL + P E LVV HGD C PN ++ E+G +SG +D G RVPEDLPQA PPVERLVVCHGDPCAPNTLLGEDGTWSGHVDMGA | 196 |
| Query 212  Sbjct 197 | LGVADRYQDIALATRDIAEELGGEWADRFLVLYGIAAPDSQRIAFYRLL 260 LGVADR+ D+A+AT G W D L YGI APD +R AFYR L LGVADRWADLAVATWSTEWNYGPGWEDALLDAYGI-APDPERTAFYRRL 244 |  |

>TR:A0A3G9JN98 A0A3G9JN98_9FIRM Aminoglycoside O-phosphotransferase APH(3')-IIIa OS=Intestinibaculum porci OX=2487118 GN=aphA PE=3 SV=1

Length=264

Score = 110 bits (274), Expect = 1e-25

| Identities | = 79/251 (31%), Positives = 123/251 (49%), Gaps = 11/251 (4%) |  |
| --- | --- | --- |
| Query 22 | YDWAQQTIGCSDAAVFRLSAQGRPVLFVKTD--LSGALNELQDEAARLSWLATTGVPCAA | 79 |
| Sbjct 17 | Y + T G S A V++L + + TD G +++ E + WL +P YRCVKDTEGMSPAKVYKLVGENENLYLKMTDSRYKGTTYDVEREKDMMLWLEGK-LPVPK | 75 |
| Query 80 | VLDVVTEAGRDWLLLGEVPG---QDLLSSHLAPAEKVSIMADAMRRLHTLDPATCPFDHQ | 136 |
| Sbjct 76 | VL G LL+ E G + +P + + + A+ +R H++D + CP+ + VLHFERHDGWSNLLMSEADGVLCSEEYEDEQSPEKIIELYAECIRLFHSIDISDCPYTNS | 135 |
| Query 137 | AKHRIERARTRMEAGLVDQD--DLDEEHQGLAPAELFARLKARMPDGEDLVVTHGDACLP | 194 |
| Sbjct 136 | R+ + L D D + +E+ P EL+ LK P+ E+LV +HGD LDSRLAELDYLLNNDLADVDCENWEEDTPFKDPRELYDFLKTEKPE-EELVFSHGDLGDS | 194 |
| Query 195 | NIMVENGRFSGFIDCGRLGVADRYQDIALATRDIAEELGGE-WADRFLVLYGIAAPDSQR | 253 |
| Sbjct 195 | NI V++G+ S FID GR G AD++ DIA R I E++G E + + F L GI PD ++ NIFVKDGKVSSFIDLGRSGRADKWYDIAFCVRSIREDIGEEQYVELFFDLLGI-KPDWEK | 253 |
| Query 254  Sbjct 254 | IAFYRLLDEFF 264 I +Y LLDE F IKYYILLDELF 264 |  |

>TR:W1DTX6 W1DTX6_KLEPN Aminoglycoside 3'-phosphotransferase OS=Klebsiella pneumoniae IS43 OX=1432552 PE=3 SV=1

Length=303

Score = 110 bits (276), Expect = 1e-25

| Identities | = 87/254 (34%), Positives = 120/254 (47%), Gaps = 20/254 (8%) |  |
| --- | --- | --- |
| Query 23 | DWAQQTIGCSDAAVFRLSAQGRPVLFVKTDLSGALNELQDEAARLSWLATTGVPCAAVLD | 82 |
| Sbjct 50 | DW G S VFR +G F K + EL E RL WL GV C V++ DWLPVRGGESGDFVFR---RGDGHAFAKIAPASRRGELAGERDRLIWLKGRGVACPEVIN | 106 |
| Query 83 | VVTEAGRDWLLLGEVPG---QDLLSSHLAPAEKVSIMADAMRRLHTLDPATCPFDHQAKH | 139 |
| Sbjct 107 | E L++ +PG DL + L A M + +H+L CPF+ + WQEEQEGACLVITAIPGVPAADLSGADLLKAWPS--MGQQLGAVHSLSVDQCPFERRLSR | 164 |
| Query 140 | RIERARTRMEAGLVDQDDLDEEHQGLAPAELFARLKARMP DGEDLVVTHGDACLP | 194 |
| Sbjct 165 | RA + V+ D L +E + +L AR++ +P + D+VV HGD C+P MFGRAVDVVSRNAVNPDFLPDEDKSTPQLDLLARVERELPVRLDQERTDMVVCHGDPCMP | 224 |
| Query 195 | NIMVENGRF--SGFIDCGRLGVADRYQDIALATRDIAEELGG-EWADR-FLVLY---GIA | 247 |

N MV+	+G ID GRLG ADRY D+AL	+ E	+ A+R F VL+	GI

| Sbjct | 225 | NFMVDPKTLQCTGLIDLGRLGTADRYADLALMIANAEENWAAPDEAERAFAVLFNVLGIE 284 |
| --- | --- | --- |
| Query | 248 | APDSQRIAFYRLLD 261 |
| Sbjct | 285 | APD +R+AFY LD  APDRERLAFYLRLD 298 |

>TR:A0A1Q9S1S5 A0A1Q9S1S5_9PSEU APH domain-containing protein OS=Pseudonocardia sp. CNS-004 OX=1904967 GN=BJF90_25310 PE=3 SV=1

Length=255

Score = 109 bits (273), Expect = 1e-25

| Identities | | = 89/253 (35%), Positives = 119/253 (47%), Gaps = 19/253 (8%) |  |
| --- | --- | --- | --- |
| Query 23  Sbjct 3 | | DWAQQTIGCSDAAVFRLSAQGRPVLFVKTDLSGALNELQDEAARLSWLATTGVPCAAVLD  +W + G S A VFR + L K + +L E R+ WL TG+P +V+D EWIPVSTGESRADVFR---RDDGTLIAKRVGPSGVADLLAECRRIEWLTGTGIPGPSVVD | 82  59 |
| Query 83  Sbjct 60 | | VVTEAGRDWLLLGEVPGQ---DLLSSHLAPAEKVSIMADAMRRLHTLDPATCPFDHQAKH T L++ VPG +L S+ L A + +R LH L A CPF+  WSTSDDGARLVMTAVPGVAAGELDSATLLRAWPA--LVGVLRLLHELPTADCPFERTLAT | 139  117 |
| Query 140  Sbjct 118 | | RIERARTRMEAGLVDQDDLDEEHQGLAPAELFARLKARMPDG EDLVVTHGDACLP  A V + L E+ QG AEL A L+A++ + DLVV HGDACLP MFRIAEDVARRDAVIPEFLAEDQQGRPHAELLADLRAQLDERLDQERGDLVVCHGDACLP | 194  177 |
| Query 195  Sbjct 178 | | NIMVENGRF--SGFIDCGRLGVADRYQDIALATRDIAEELGGE---WADRFL-VLYGIAA N MV+ G +G ID GRLG+ADR+ D+AL E G+ A+R L YG NFMVDPGTLACTGLIDLGRLGIADRHADLALLRASATESWAGDDGTHAERLLSERYGDDR | 248  237 |
| Query 249 | | PDSQRIAFYRLLD 261 D R+ FY LD |  |
| Sbjct | 238 | IDPGRLRFYHHLD 250 | |

>TR:D7BWG8 D7BWG8_STRBB Aminoglycoside phosphotransferase OS=Streptomyces bingchenggensis (strain BCW-1) OX=749414 GN=SBI_00203 PE=3

SV=1

Length=287

Score = 110 bits (274), Expect = 2e-25

| Identities | = 91/262 (35%), Positives = 122/262 (47%), Gaps = 33/262 (13%) |  |
| --- | --- | --- |
| Query 21 | GYDWAQQTIGCSDAAVFRLSAQGRPVLFVKTDLSGALNELQDEAARLSWLATTGVPCAAV | 80 |
| Sbjct 33 | G DW T G S A+VFR + R + K + +L+ E R++WL+ GVP V GGDWLSVTAGESGASVFRSADTTR---YAKCVPAADATDLEAERDRIAWLSGQGVPGPRV | 89 |
| Query 81 | LDVVTEAGRDWLLLGEVPGQDLLSSHLAPAEKVSI MADAMRRLHTLDPATC | 131 |
| Sbjct 90 | LD + L+ V G PA++VS +ADA+RRLH L + C LDWYSGDSGACLVTSAVSG IPADQVSAEDLRMSWERIADAVRRLHELPVSEC | 141 |
| Query 132 | PFDHQAKHRIERARTRMEAGLVDQDDLDEEHQGLAPAELFARLKARMP DGEDLVV | 186 |
| Sbjct 142 | PF + A + V+ + L E Q EL R+ ++P + D VV PFRRGLDAMVAVAGDVVARDAVNPEFLPVEQQHTPAVELLTRVIRQVPRRREQEVADTVV | 201 |
| Query 187 | THGDACLPNIMVENGRF--SGFIDCGRLGVADRYQDIALATRDIAEELGGEWADR----- | 239 |
| Sbjct 202 | HGD CLPNI+++ SGFID GRLG+ADRY D+AL + E E R CHGDLCLPNIILDPQTLDVSGFIDLGRLGLADRYADLALVLANARETWTDEERARAADVA | 261 |
| Query 240  Sbjct 262 | FLVLYGIAAPDSQRIAFYRLLD 261 F YGIA D R+ FY LD FAERYGIAL-DHDRLRFYLHLD 282 |  |

>TR:A0A5P9QDZ8 A0A5P9QDZ8_9MICO Kanamycin kinase OS=Luteimicrobium xylanilyticum OX=1133546 GN=aphA PE=3 SV=1

Length=272

Score = 109 bits (273), Expect = 2e-25

Identities = 83/231 (36%), Positives = 109/231 (47%), Gaps = 25/231 (11%)

| Query | 37 | FRLS--AQGRPVLFVKTDLSGALNELQDEAARLSWLATTGVPCAAVLDVVTEA-GRDWLL | 93 |
| --- | --- | --- | --- |
| Sbjct | 56 | FRL ++ R V +V A +L EA RL+W A P VLD T+ G WL+ FRLGEGSRARYVKWVPPTERAAELDLVAEAERLAWAAPF-TPVPRVLDAGTDDDGAAWLM | 114 |
| Query | 94 | LGEVPGQDLLSSHLA--PAEKVSIMADAMRRLHTLDP-ATCPFDHQAKHRIERARTRMEA | 150 |
| Sbjct | 115 | +PG+ +S P V + + +R LH P A CPFD R+ R R R A TAALPGESAVSDRWRADPRTAVRAVGEGLRALHDALPVAECPFDWGVGSRVARGRLRPGA | 174 |
| Query | 151 | GLVDQDDLDEEHQGLAPAELFARLKARMPDGEDLVVTHGDACLPNIMV-ENGRFSGFIDC | 209 |
| Sbjct | 175 | L L RL A P+ E LVV HGDAC PN ++ +GR+ +D DLA LLDRLAAEPPEPERLVVCHGDACAPNTLLGHDGRWLAHVDL | 218 |
| Query  Sbjct | 210  219 | GRLGVADRYQDIALATRDIAEELGGEWADRFLVLYGIAAPDSQRIAFYRLL 260 G LGVAD + D+A+A G W D L YG A D++R A+YR L GTLGVADAWADLAVAAWSTQWNYGPGWEDELLAAYGTAV-DTERTAYYRAL 268 |  |

>TR:A0A085LEB0 A0A085LEB0_9MICO Aminoglycoside phosphotransferase OS=Cellulosimicrobium sp. MM OX=1523621 GN=IU11_12340 PE=3 SV=1

Length=275

Score = 109 bits (273), Expect = 2e-25

| Identities | | = 83/233 (36%), Positives = 116/233 (50%), Gaps = 13/233 (6%) |  |
| --- | --- | --- | --- |
| Query 37  Sbjct 43 | | FRLSAQG RPVLFVKTDLSGALN-ELQDEAARLSWLATTGVPCAAVLDVVTEAGRDW  FRL +G RPV + K +G +L EA RL+W+ P VLD + W FRLDPRGGEAARPVRYAKWVPAGVPEIDLAGEAERLAWVGDR-TPVPRVLDQGADDDGSW | 91  101 |
| Query 92  Sbjct 102 | | LLLGEVPGQDLLSSHLA--PAEKVSIMADAMRRLHTLDP-ATCPFDHQAKHRIERARTRM L+ V G+ + + PA + +R LH P A+CPF R+ A R+ LVTQAVAGRSAVEARWTGDPAVAAVAIGRGLRALHEALPVASCPFTWSVAQRVANADDRL | 148  161 |
| Query 149  Sbjct 162 | | EAGLVDQDDLDEEHQGLAPAELFARLKARMPDGEDLVVTHGDACLPNIMV-ENGRFSGFI G D+ EH+ LA AE ARL P + LVV HGDAC+PN ++ ++G F+ + ARGH-GPDEWFPEHRALAVAEARARLDDA-PPVDRLVVCHGDACVPNTLLHDDGTFAAHV | 207  219 |
| Query | 208 DCGRLGVADRYQDIALATRDIAEELGGEWADRFLVLYGIAAPDSQRIAFYRLL 260 | | |
|  |  | D G LGVADR+ D+A+A G + YG+ PD +RIA+YRLL |  |
| Sbjct | 220 | DLGSLGVADRWADLAVAAWSTEWNYGPGYDGHVYAGYGV-DPDPERIAYYRLL 271 | |

>TR:A0A561UGU6 A0A561UGU6_9ACTN Kanamycin kinase OS=Kitasatospora viridis OX=281105 GN=FHX73_112394 PE=3 SV=1

Length=260

Score = 109 bits (272), Expect = 2e-25

| Identities | | = 83/218 (38%), Positives = 103/218 (47%), Gaps = 13/218 (6%) |  |
| --- | --- | --- | --- |
| Query 48  Sbjct 47 | | FVKTDLSGALNELQDEAARLSWLATTG-VPCAAVLDVVTEAGRDWLLLGEVPGQDLLSSH FVK SG+ +L EA RL W VP VL EA WLL +PG + FVKWAPSGSGLDLAAEAPRLGWAGRFARVP--RVLGQGAEAAGQWLLTAALPGGMAVLDE | 106  104 |
| Query 107  Sbjct 105 | | LA--PAEKVSIMADAMRRLHTLDPAT-CPFDHQAKHRIERARTRMEAGLVDQDDLDEEHQ PA V + +R LH P CPFD R+ RAR D EH+ WKREPATAVRAIGRGLRALHEALPVEDCPFDWSTGTRLARARRGSGPA DWHPEHR | 163  159 |
| Query 164  Sbjct 160 | | GLAPAELFARLKARMPDGEDLVVTHGDACLPNIMV-ENGRFSGFIDCGRLGVADRYQDIA L AE L A P + LVV HGDAC PN ++ +G F+G +D G LG+ADR+ D+A HLGTAERALALLADPPPVDRLVVCHGDACAPNTLIGPDGEFAGHVDLGALGLADRWADLA | 222  219 |
| Query | 223 LATRDIAEELGGEWADRFLVLYGIAAPDSQRIAFYRLL 260 | | |
|  |  | +AT G W L YG+ PD R A+YRLL |  |
| Sbjct | 220 | VATWSTNWNYGPGWELPLLAAYGV-EPDPVRTAYYRLL 256 | |

>TR:A0A1I3WQ17 A0A1I3WQ17_9PSEU Kanamycin kinase OS=Amycolatopsis sacchari OX=115433 GN=SAMN05421835_113179 PE=3 SV=1

Length=247

Score = 108 bits (271), Expect = 2e-25

Identities = 80/220 (36%), Positives = 109/220 (50%), Gaps = 28/220 (13%)

| Query | 48 | FVKTDLSGALNELQDEAARLSWLAT-TGVPCAAVLDVVTEAGRDWLLLGEVPGQDLLSSH | 106 |
| --- | --- | --- | --- |
| Sbjct | 48 | FVK +G+ +L EA RL W A T VP VL + W++ + G+ + H FVKWTPAGSGIDLAAEAVRLRWAAEFTTVP--HVLGEGADETGSWIVTHGLTGRSAVDDH | 105 |
| Query | 107 | LA--PAEKVSIMADAMRRLH-TLDPATCPFDHQAKHRIERARTRMEAGLVDQDDLDEEHQ | 163 |
| Sbjct | 106 | PA V + +R LH TL A CPFD A+ R+E ARTR A L WKRDPATAVRAIGAGLRALHDTLPVAGCPFDWSAEARLENARTRAAAHL----------- | 154 |
| Query | 164 | GLAPAELFARLKARMPDGEDLVVTHGDACLPNIMV-ENGRFSGFIDCGRLGVADRYQDIA | 222 |
| Sbjct | 155 | + A +P + LVV HGDAC PN ++ ++GR +G +D G LGV DR+ D+A  ---------DVLADIPPVDRLVVCHGDACAPNTLIGDDGRCTGHVDLGALGVGDRWADLA | 205 |
| Query | 223 LATRDIAEELGGEWADRFLVLYGIAAPDSQRIAFYRLLDE 262 | | |
|  |  | +AT G W + L YG+ PD R ++YRLL E |  |
| Sbjct | 206 | VATWSTQWNYGPGWEEPLLRAYGV-EPDPDRTSYYRLLWE 244 | |

>TR:L9PQ85 L9PQ85_9BURK Aminoglycoside 3'-phosphotransferase OS=Janthinobacterium sp. HH01 OX=1198452 GN=aphA2 PE=3 SV=1

Length=258

Score = 108 bits (271), Expect = 3e-25

| Identities | | = 83/242 (34%), Positives = 125/242 (52%), Gaps = 12/242 (5%) |  |
| --- | --- | --- | --- |
| Query 29  Sbjct 23 | | IGCSDAAVFRLSAQGRPVLFVKTD---LSGALNELQDEAARLSWLA-TTGVPCAAVLDVV IG S V R +G V F+KT + + EAA L WLA VP + IGESPCQVHRFR-RGDEVFFLKTSPLVYASTTYSVMREAAVLQWLAGRVNVPEVVLAAPA | 84  81 |
| Query 85  Sbjct 82 | | TEAGRDWLLLGEVPGQDLLSSHLAPAEKVSIMADAMRRLHTLDPATCPFDHQAKHRIERA  +A ++++ VPG L S + + +A+R+L ++ A CPFD A R+ DDA--EYMITRAVPGVPLSSLIAEDRPVLELFREALRQLQSVSVADCPFDSGAAVRLREL | 144  139 |
| Query 145  Sbjct 140 | | RTRMEAGLVDQDDLDEEHQGLA-PAELFARLKARMPDGEDLVVTHGDACLPNIMVENGRF  + GL+D++ E+ GL PA+L ARL+ +P+ EDLV +HGD C N+ V+ DYLVGKGLIDEEYDFEQWPGLVTPADLLARLRTTIPN-EDLVFSHGDLCDNNVFVDQRGE | 203  198 |
| Query 204  Sbjct 199 | | SGFIDCGRLGVADRYQDIALATRDIAEELGGEWADRFLVLYGIAAPDS-QRIAFYRLLDE FID GR G+ADR+ DIA R++ +++ A +F L G+A PD+ + F+ LDE LHFIDAGRGGLADRWLDIAFIHRNLRDDVSERSATQF--LEGLAIPDAPAKREFFEQLDE | 262  256 |
| Query 263 | | FF 264 F |  |
| Sbjct | 257 | LF 258 | |

>TR:A0A285KQG3 A0A285KQG3_9NOCA Streptomycin 3-kinase OS=Nocardia amikacinitolerans OX=756689 GN=SAMN04244553_0360 PE=3 SV=1

Length=269

Score = 108 bits (270), Expect = 5e-25

| Identities | | = 87/261 (33%), Positives = 125/261 (48%), Gaps = 35/261 (13%) |  |
| --- | --- | --- | --- |
| Query 23  Sbjct 17 | | DWAQQTIGCSDAAVFRLSAQGRPVLFVKTDLSGALNELQDEAARLSWLATTGVPCA-AVL  +W G S AAVFR + R + K ++ +L++E R++W + G+P VL EWEPVDGGESGAAVFRAADGSR---YAKCVAEDSIPDLREECDRVAWFSGGGIPGPHPVL | 81  73 |
| Query 82  Sbjct 74 | | DVVTEAGRDWLLLGEVPGQDLLSSHLAPAEKVSI MADAMRRLHTLDPATCP  W +G+ + H PA++VS +A+A+R LH + TCP  ---------WRQVGDAACLVTTAVHGVPADRVSAEELSTAWASIAEAVRTLHEIPTDTCP | 132  124 |
| Query 133  Sbjct 125 | | FDHQAKHRIERARTRMEAGLVDQDDLDEEHQGLAPAELFARLKARMP DGEDLVVT  F+ +A + G V+ + L EE L ARL+A++ + + VV FERDVAKMFAKAADVVARGAVNPEFLPEEQLNTPTRVLLARLRAQLDLRIAQEAAEYVVC | 187  184 |
| Query 188  Sbjct 185 | | HGDACLPNIMVE--NGRFSGFIDCGRLGVADRYQDIALATRDIAE ELGGEWAD-RF  HGD CLPNI+++ G F+GF+D GRLGVAD Y DIAL + E E AD RF HGDLCLPNIVLDPDTGSFAGFLDLGRLGVADPYVDIALLLANSRETWPDEHSARDADRRF | 240  244 |
| Query 241 | | LVLYGIAAPDSQRIAFYRLLD 261 YGI D+ R FY LD |  |
| Sbjct | 245 | AERYGIEL-DADRQRFYLHLD 264 | |

>TR:A0A1Y3WDH4 A0A1Y3WDH4_9FIRM APH domain-containing protein OS=Drancourtella sp. An57 OX=1965647 GN=B5G11_03080 PE=3 SV=1

Length=261

Score = 108 bits (269), Expect = 6e-25

| Identities  Query 59 | | = 71/222 (32%), Positives = 122/222 (55%), Gaps = 20/222 (9%)  ELQDEAARLSWLATTGVPCAAVLDVVTEAGRD-WLLLGEVPGQDLLSSHLA--PAEKVSI | 115 |
| --- | --- | --- | --- |
| Sbjct 44 | | E + E + WL +P +L V E+G++ +LL+ + G+ + P + + ETEQELCMMEWLQDR-LPVPGIL-VKEESGKEQFLLMSRLEGEMMCEETYMENPVLQTKV | 101 |
| Query 116  Sbjct 102 | | MADAMRRLHTLDPATCPFDHQAKHRIERARTRMEAGLVDQDDLDEEHQGLA PAELF  +A+ ++RL +D + CP D + H++ RA ++E G VD D+++ + G P EL LAEGLKRLWAVDLSGCPADCRLDHKLMRAAEQVEQGRVDLDNVEPDTFGTDGFRDPEELL | 171  161 |
| Query 172  Sbjct 162 | | ARLKARMPDGEDLVVTHGDACLPNIMVENGRFSGFIDCGRLGVADRYQDIALATRDIAEE L P+ E+ V++HGD CLPN+ + R +G++D GR G+AD++ DIAL R +++  DWLLGNRPE-EEAVLSHGDYCLPNVFGKGDRLTGYLDLGRAGIADKWCDIALCYRSLSQN | 231  220 |
| Query | 232 LGGEWADRF------LVLY---GIAAPDSQRIAFYRLLDEFF 264 | | |
|  |  | G + + L+L+ GI APD ++I +Y LLDE F |  |
| Sbjct | 221 | YSGRFHGKAYSGYDDLLLFKELGI-APDWEKIRYYILLDELF 261 | |

>TR:A0A431TTB2 A0A431TTB2_9BURK Aminoglycoside 3'-phosphotransferase OS=Variovorax gossypii OX=1679495 GN=aph(3'') PE=3 SV=1

Length=278

Score = 108 bits (270), Expect = 6e-25

| Identities | | = 87/258 (34%), Positives = 121/258 (47%), Gaps = 22/258 (9%) |  |
| --- | --- | --- | --- |
| Query 21  Sbjct 21 | | GYDWAQQTIGCSDAAVFRLSAQGRPVLFVKTDLSGA--LNELQDEAARLSWLATTGVPCA G +W +G S +V+R S V F K +SG+ + L E R WLA + A GIEWLPVNLGESGDSVYRRSDG---VAFAKVSVSGSDGVESLAGERLRTEWLAPFDLGSA | 78  77 |
| Query 79  Sbjct 78 | | AVLDVVTEAGRDWLLLGEVPGQDLLSSHLAPAEKVSI---MADAMRRLHTLDPATCPFDH VLD L++ VPG + +S L + + MA +R LH + A+CPF  KVLDWHASQEGACLVMSAVPG--VPASELPAGQLLQAWPSMAHRLRLLHEIPAASCPFAR | 135  135 |
| Query 136  Sbjct 136 | | QAKHRIERARTRMEAGLVDQDDLDEEHQGLAPAELFARLKARMP DGEDLVVTHGD  ERA + V+++ LD E Q + P L L+ ++P + D VV HGD ALSTMFERACDVVARNAVNKEFLDPEQQDIPPPLLLDALRVQLPLRLAQEARDRVVCHGD | 190  195 |
| Query 191  Sbjct 196 | | ACLPNIMVE--NGRFSGFIDCGRLGVADRYQDIALATRDIAEELGG---EWADR--FLVL AC+PN MV+ R +G ID GRLG ADRY D++L + + G E A R ACMPNFMVDPRTLRCTGLIDLGRLGTADRYVDLSLFLANARDSWRGPDDELAARKSLFDT | 243  255 |
| Query 244 | | YGIAAPDSQRIAFYRLLD 261 I PD R+ FY LD |  |
| Sbjct | 256 | LEIGEPDDGRLDFYLGLD 273 | |

>TR:A0A6G4VA64 A0A6G4VA64_9ACTN Aminoglycoside 3'-phosphotransferase OS=Streptomyces sp. HC44 OX=2711217 GN=G5C60_24680 PE=3 SV=1

Length=249

Score = 107 bits (268), Expect = 7e-25

| Identities | = 84/239 (35%), Positives = 109/239 (46%), Gaps = 30/239 (13%) |  |
| --- | --- | --- |
| Query 24 | WAQQTIGCSDAAVFRLSAQGRPVLFVKTDLSGALNELQDEAARLSWLATTGVPCAAVLDV | 83 |
| Sbjct 31 | W Q G + FR+ GR LF K +G+ +L EA RL W A P V D WLNQLGGLT FRIGGDGREPLFAKWAPAGSGLDLAAEAERLRW-AGHFTPVPRVRDH | 85 |
| Query 84 | VTEAGRDWLLLGEVPGQDLLSSHLAP--AEKVSIMADAMRRLHTLDP-ATCPFDHQAKHR | 140 |
| Sbjct 86 | + WL+ +PG+ +S A V + +R LH P A CPF HR GGDEDGTWLVTHGLPGESAVSPRWKADAATAVRALGAGLRALHEALPVADCPFSWSVDHR | 145 |
| Query 141 | IERARTRMEAGLVDQDDLDEEHQGLAPAELFARLKARMPDGEDLVVTHGDACLPN-IMVE | 199 |
| Sbjct 146 | +ERAR AG+ DL + P + LVV HGD C PN ++ E  LERAR---RAGMEIPADLPDP PPVDRLVVCHGDPCAPNTLLYE | 185 |

Query 200 NGRFSGFIDCGRLGVADRYQDIALATRDIAEELGGEWADRFLVLYGIAAPDSQRIAFYR 258

+G +SG +D G LG+ADR+ D+A+AT	G W D L YGI PD R FYR Sbjct 186 DGSWSGHVDMGALGLADRWADLAVATWSTEWNYGPGWDDTLLEGYGI-EPDPHRTDFYR 243

>TR:V6JW13 V6JW13_STRNV APH domain-containing protein OS=Streptomyces niveus NCIMB 11891 OX=1352941 GN=M877_27630 PE=3 SV=1

Length=266

Score = 108 bits (269), Expect = 7e-25

| Identities | | = 97/259 (37%), Positives = 115/259 (44%), Gaps = 33/259 (13%) |  |
| --- | --- | --- | --- |
| Query 24  Sbjct 15 | | WAQQTIGCSDAAVFRLSAQGRPVLFVKTDLSGALNELQDEAARLSWLATTGVPCAAVLDV W T G S+AAVFR R V D + N L++E R+ WL+ GVP VLD WEPVTDGESEAAVFRSEDGTRYAKCVPAD---SANSLEEERDRVDWLSGQGVPGPRVLDW | 83  71 |
| Query 84 | | VTEAGRDWLLLGEVPG QDLLSSHLAPAEKVSIMADAMRRLHTLDPATCPFDHQAKH  LL V G Q S LA E + ADA+R LH L CPF | 139 |
| Sbjct | 72 | RVTPDAACLLTSTVAGIPADQVTASQLLAAWEPI---ADAVRHLHDLPARRCPFSRDLSL | 128 |
| Query | 140 | RIERARTRMEAGLVDQDDLDEEHQGLAPAELFARL KARMPDGEDLVVTHGDACL | 193 |
| Sbjct | 129 | AR + G V+ + L E+ Q EL A L + DGE VV HGD CL MFATARDVVARGAVNPEFLPEDQQRTPADELLALLVPQLEQRLAQEDGES-VVCHGDLCL | 187 |
| Query | 194 | PNIMVENGRFS--GFIDCGRLGVADRYQDIALATRDIAEELGGEWADR FLV | 242 |
| Sbjct | 188 | PNI+++ S GFID GRLG AD Y DIAL + E WAD F PNIVLDPETLSVAGFIDLGRLGRADPYADIALLLANSRE TWADERRATAADEAFAG | 243 |
| Query | 243 | LYGIAAPDSQRIAFYRLLD 261 YGI D +R FY LD |  |
| Sbjct | 244 | RYGIEL-DREREMFYLHLD 261 | |

>TR:A0A1H3THL2 A0A1H3THL2_9PSEU Streptomycin 3-kinase OS=Saccharopolyspora shandongensis OX=418495 GN=SAMN05216215_108228 PE=3 SV=1

Length=266

Score = 108 bits (269), Expect = 7e-25

| Identities | | = 91/273 (33%), Positives = 124/273 (45%), Gaps = 30/273 (11%) |  |
| --- | --- | --- | --- |
| Query 5  Sbjct 3 | | DGLHAGSPAAWVERLFGYDWAQQTIGCSDAAVFRLSAQGRPVLFVKTDLSGALNELQDEA  + L A PA G++W G S A FR + R V D +G L + +D+A ESLRASLPA GHEWQPVPGGESGAGTFRSADGSRYAKCVAADQAGELEQERDKA | 64  55 |
| Query 65  Sbjct 56 | | ARLSWLATTGVPCAAVLDVVTEAGRDWLLLGEVPGQDLLSSHLAPAEKVS IMADAM  WL G+P VLD + L+ V G + + PAE ++ ++D +  ---EWLRANGIPGPRVLDWSADDAGACLVTSAVAG VPADAVPAETLARAWESISDVV | 120  109 |
| Query 121  Sbjct 110 | | RRLHTLDPATCPFDHQAKHRIERARTRMEAGLVDQDDLDEEHQGLAPAELFARLKARMP- RRLH L +CPF A+ + G V+ + L E Q P L A L ++P RRLHELPADSCPFSRDLSKMFAIAQDVVARGAVNPEFLPVEQQLEEPVALLADLAPQLPQ | 179  169 |
| Query 180  Sbjct 170 | | ----DGEDLVVTHGDACLPNIMVENGRF--SGFIDCGRLGVADRYQDIALATRDIAE---  + + VV HGD CLPNI+++ +GFID GRLG ADRY DIAL + E RLEQEAAETVVCHGDLCLPNIILDPDALEVAGFIDLGRLGTADRYADIALLLANSRETWP | 230  229 |
| Query 231 | | -ELGGEWADR-FLVLYGIAAPDSQRIAFYRLLD 261 E AD F YGI D+ R FY LD |  |
| Sbjct | 230 | DESSASVADETFARRYGIEL-DTDRQRFYLHLD 261 | |

```
>TR:A0A076NGZ5 A0A076NGZ5_9CORY Aminoglycoside phosphotransferase OS=Corynebacterium imitans OX=156978 GN=CIMIT_00695 PE=3 SV=1
Length=252

Score = 107 bits (268), Expect = 7e-25
Identities = 85/247 (34%), Positives = 118/247 (48%), Gaps = 20/247 (8%)

Query 30	GCSDAAVFRLSAQGRPVLFVKTDLSGALNELQDEAARLSWLATTGVPCAAVLDVVTEAGR 89 G S	VFR	+G	F K	+	EL E RL WL	GV C V++	E
Sbjct 6	GESGDFVFR---RGDGHAFAKIAPASRRGELAGERDRLIWLKGRGVACPEVINWQEEQEG 62
```

| Query | 90 | DWLLLGEVPG---QDLLSSHLAPAEKVSIMADAMRRLHTLDPATCPFDHQAKHRIERART | 146 |
| --- | --- | --- | --- |
| Sbjct | 63 | L++ +PG DL + L A M + +H+L CPF+ + RA ACLVITAIPGVPAADLSGADLLKAWPS--MGQQLGAVHSLSVDQCPFERRLSRMFGRAVD | 120 |
| Query | 147 | RMEAGLVDQDDLDEEHQGLAPAELFARLKARMP DGEDLVVTHGDACLPNIMVENG | 201 |
| Sbjct | 121 | + V+ D L +E + +L AR++ +P + D+VV HGD C+PN MV+ VVSRNAVNPDFLPDEDKSTPQLDLLARVERELPVRLDQERTDMVVCHGDPCMPNFMVDPK | 180 |
| Query | 202 | RF--SGFIDCGRLGVADRYQDIALATRDIAEELGG-EWADR-FLVLY---GIAAPDSQRI | 254 |
| Sbjct | 181 | +G ID GRLG ADRY D+AL + E + A+R F VL+ GI APD +R+ TLQCTGLIDLGRLGTADRYADLALMIANAEENWAAPDEAERAFAVLFNVLGIEAPDRERL | 240 |
| Query  Sbjct | 255  241 | AFYRLLD 261 AFY LD AFYLRLD 247 |  |

>TR:M4R2W5 M4R2W5_BIBTR Aminoglycoside 3'-phosphotransferase OS=Bibersteinia trehalosi USDA-ARS-USMARC-192 OX=1171377 GN=WQG_1050 PE=3

SV=1

Length=252

Score = 107 bits (268), Expect = 7e-25

| Identities | = 85/247 (34%), Positives = 118/247 (48%), Gaps = 20/247 (8%) |  |
| --- | --- | --- |
| Query 30 | GCSDAAVFRLSAQGRPVLFVKTDLSGALNELQDEAARLSWLATTGVPCAAVLDVVTEAGR | 89 |
| Sbjct 6 | G S VFR +G F K + EL E RL WL GV C V++ E GESGDFVFR---RGDGHAFAKIAPASRRGELAGERDRLIWLKGRGVACPEVINWQEEQEG | 62 |
| Query 90 | DWLLLGEVPG---QDLLSSHLAPAEKVSIMADAMRRLHTLDPATCPFDHQAKHRIERART | 146 |
| Sbjct 63 | L++ +PG DL + L A M + +H+L CPF+ + RA ACLVITAIPGVPAADLSGADLLKAWPS--MGQQLGAVHSLSVDQCPFERRLSRMFGRAVD | 120 |
| Query 147 | RMEAGLVDQDDLDEEHQGLAPAELFARLKARMP DGEDLVVTHGDACLPNIMVENG | 201 |
| Sbjct 121 | + V+ D L +E + +L AR++ +P + D+VV HGD C+PN MV+ VVSRNAVNPDFLPDEDKSTPQLDLLARVERELPVRLDQERTDMVVCHGDPCMPNFMVDPK | 180 |
| Query 202 | RF--SGFIDCGRLGVADRYQDIALATRDIAEELGG-EWADR-FLVLY---GIAAPDSQRI | 254 |
| Sbjct 181 | +G ID GRLG ADRY D+AL + E + A+R F VL+ GI APD +R+ TLQCTGLIDLGRLGTADRYADLALMIANAEENWAAPDEAERAFAVLFNVLGIEAPDRERL | 240 |
| Query 255  Sbjct 241 | AFYRLLD 261 AFY LD AFYLRLD 247 |  |

>TR:A0A656YEK5 A0A656YEK5_SALNE Aminoglycoside 3'-phosphotransferase OS=Salmonella enterica subsp. enterica serovar Newport str. S09097

OX=1243581 GN=LFZ31_22805 PE=3 SV=1

Length=252

Score = 107 bits (268), Expect = 7e-25

| Identities | = 85/247 (34%), Positives = 118/247 (48%), Gaps = 20/247 (8%) |  |
| --- | --- | --- |
| Query 30 | GCSDAAVFRLSAQGRPVLFVKTDLSGALNELQDEAARLSWLATTGVPCAAVLDVVTEAGR | 89 |
| Sbjct 6 | G S VFR +G F K + EL E RL WL GV C V++ E GESGDFVFR---RGDGHAFAKIAPASRRGELAGERDRLIWLKGRGVACPEVINWQEEQEG | 62 |
| Query 90 | DWLLLGEVPG---QDLLSSHLAPAEKVSIMADAMRRLHTLDPATCPFDHQAKHRIERART | 146 |
| Sbjct 63 | L++ +PG DL + L A M + +H+L CPF+ + RA ACLVITAIPGVPAADLSGADLLKAWPS--MGQQLGAVHSLSVDQCPFERRLSRMFGRAVD | 120 |
| Query 147 | RMEAGLVDQDDLDEEHQGLAPAELFARLKARMP DGEDLVVTHGDACLPNIMVENG | 201 |
| Sbjct 121 | + V+ D L +E + +L AR++ +P + D+VV HGD C+PN MV+ VVSRNAVNPDFLPDEDKSTPQLDLLARVERELPVRLDQERTDMVVCHGDPCMPNFMVDPK | 180 |
| Query 202 | RF--SGFIDCGRLGVADRYQDIALATRDIAEELGG-EWADR-FLVLY---GIAAPDSQRI | 254 |
| Sbjct 181 | +G ID GRLG ADRY D+AL + E + A+R F VL+ GI APD +R+ TLQCTGLIDLGRLGTADRYADLALMIANAEENWAAPDEAERAFAVLFNVLGIEAPDRERL | 240 |
| Query 255 | AFYRLLD 261 AFY LD |  |

Sbjct 241 AFYLRLD 247

>TR:A0A4Y3R330 A0A4Y3R330_STRCI APH(3'') family aminoglycoside O-phosphotransferase OS=Streptomyces cacaoi subsp. cacaoi OX=66857 GN=SCA03_38680

PE=3 SV=1

Length=253

Score = 107 bits (268), Expect = 7e-25

| Identities | = 75/207 (36%), Positives = 105/207 (51%), Gaps = 13/207 (6%) |  |
| --- | --- | --- |
| Query 21 | GYDWAQQTIGCSDAAVFRLSAQGRPVLFVKTDLSGALNELQDEAARLSWLATTGVPCAAV | 80 |
| Sbjct 9 | G +W G S AAVFR S V + S L +D R++WLA TG+P V GREWDAVAAGESGAAVFRRSDGAAYAKCVPPERSSGLGAERD---RVAWLAGTGIPGPEV | 65 |
| Query 81 | LDVVTEAGRDWLLLGEVPG--QDLLSSHLAPAEKVSIMADAMRRLHTLDPATCPFDHQAK | 138 |
| Sbjct 66 | L+ + L+ VPG D L+S A +++ +RR+H L P CPF+ + + LEWASREAGACLVTSTVPGVPADTLTSGALRAAWPALVR-LLRRIHELPPGDCPFERRLE | 124 |
| Query 139 | HRIERARTRMEAGLVDQDDLDEEHQGLAPAELFARLKARMP DGEDLVVTHGDACL | 193 |
| Sbjct 125 | + A + G V+ L EE +G+ A L A L+A +P + DL V HGDACL TMVRVAEDVVRRGAVEPAFLAEEQRGVPAATLLADLRAELPARLAQEEADLAVCHGDACL | 184 |
| Query 194  Sbjct 185 | PNIMV--ENGRFSGFIDCGRLGVADRY 218 PN +V + +G ID GRLGVADR+ PNFLVDPDTSLCTGVIDLGRLGVADRW 211 |  |

>TR:A0A5J6F9N2 A0A5J6F9N2_9ACTN APH(3'') family aminoglycoside O-phosphotransferase OS=Streptomyces nitrosporeus OX=28894 GN=aph(3'') PE=3

SV=1

Length=273

Score = 108 bits (269), Expect = 8e-25

| Identities | = 93/256 (36%), Positives = 122/256 (48%), Gaps = 25/256 (10%) |  |
| --- | --- | --- |
| Query 23 | DWAQQTIGCSDAAVFRLSAQGRPVLFVKT-DLSGALNELQDEAARLSWLATTGVPCAAVL | 81 |
| Sbjct 21 | DW+ T G S AAVFR + R V D +G L+ E R++WL GVP VL DWSPVTAGESGAAVFRSADATRYAKCVSAADAAG LEAERDRVAWLNDQGVPGPRVL | 76 |
| Query 82 | DVVTEAGRDWLLLGEVPGQDLLSSHLAPAEKV SIMADAMRRLHTLDPATCPFDHQA | 137 |
| Sbjct 77 | D + L+ V G + + PAE + +ADA+RRLH + CPF DWRSGDAGACLMTSAVLG---IPADQVPAEDLWAAWGPIADAVRRLHGIPMPRCPFRRDL | 133 |
| Query 138 | KHRIERARTRMEAGLVDQDDLDEEHQGLAPAELFARLKARMP DGEDLVVTHGDAC | 192 |
| Sbjct 134 | + AR + V+ + L E + EL RL ++P + D VV HGD C DAMVALARDVVARDAVNPEFLPVEQEHTPAPELLDRLTRQVPRRREQEAADTVVCHGDLC | 193 |
| Query 193 | LPNIMVENGRF--SGFIDCGRLGVADRYQDIALATRDIAE ELGGEWAD-RFLVLYG | 245 |
| Sbjct 194 | LPNI+++ SGFID GRLG+ADRY D+AL + E E G AD F YG LPNIILDPRTLDVSGFIDLGRLGLADRYADLALLLANARETWPDEERGRAADTAFAEGYG | 253 |
| Query 246  Sbjct 254 | IAAPDSQRIAFYRLLD 261 I D R+ FY LD  ITL-DHDRLRFYLHLD 268 |  |

>TR:A0A505D6T9 A0A505D6T9_9ACTN Aminoglycoside 3'-phosphotransferase OS=Streptomyces sporangiiformans OX=2315329 GN=FGD71_036175 PE=3 SV=1

Length=257

Score = 107 bits (268), Expect = 8e-25

Identities = 80/225 (36%), Positives = 105/225 (47%), Gaps = 26/225 (12%)

| Query | 37 | FRLSAQGRPVLFVKTDLSGALNELQDEAARLSWLATTGVPCAAVLDVVTEAGRDWLLLGE | 96 |
| --- | --- | --- | --- |
| Sbjct | 48 | FR+ G LF K + +L EA RL W A P V D + G WL+ E FRIGDDGEGRLFAKWSPPDSGLDLAAEAERLRW-AGRFTPVPRVRDHGADEGGTWLVTHE | 106 |
| Query | 97 | VPGQDLLSSHLA--PAEKVSIMADAMRRLHTLDPAT-CPFDHQAKHRIERARTRMEAGLV | 153 |
| Sbjct | 107 | +PG+ ++ PA V + +R LH P T CPF HR+E AR AG+  LPGESAVTPRWKQDPATAVRALGAGLRALHERLPVTDCPFSWTVDHRLEVAR---HAGVE | 163 |

| Query | 154 | DQDDLDEEHQGLAPAELFARLKARMPDGEDLVVTHGDACLPNIMV-ENGRFSGFIDCGRL 212 |
| --- | --- | --- |
| Sbjct | 164 | DL E P + LVV HGD C+PN ++ E+G +SG +D G L VPADLPEP-----------------PPIDRLVVCHGDPCVPNTLLHEDGSWSGHVDLGAL 206 |
| Query | 213 | GVADRYQDIALATRDIAEELGGEWADRFLVLYGIAAPDSQRIAFY 257 |
| Sbjct | 207 | GVADR+ D+A+AT G W D L YG APD +R +Y  GVADRWADLAVATWSTEMNYGPGWEDALLDAYG-TAPDPRRTDYY 250 |

>TR:A0A1H0PNC9 A0A1H0PNC9_9BACL Phosphotransferase enzyme family protein OS=Paenibacillus sp. yr247 OX=1761880 GN=SAMN04487897_1342 PE=4

SV=1

Length=100

Score = 103 bits (256), Expect = 8e-25

Identities = 48/89 (54%), Positives = 61/89 (69%), Gaps = 0/89 (0%)

| Query | 176 | ARMPDGEDLVVTHGDACLPNIMVENGRFSGFIDCGRLGVADRYQDIALATRDIAEELGGE 235 |
| --- | --- | --- |
| Sbjct | 12 | ++ P EDLV THGD CLPNI++ NG+ GFID GR GVADRYQD+ALA R I G E SKKPITEDLVFTHGDYCLPNIIINNGKVHGFIDWGRAGVADRYQDLALAIRSIKYNFGNE 71 |
| Query | 236 | WADRFLVLYGIAAPDSQRIAFYRLLDEFF 264 |
| Sbjct | 72 | + FL YG+ D ++ +Y+L+DEFF  YIYAFLDNYGLTELDESKVIYYQLMDEFF 100 |

>TR:A0A239CX75 A0A239CX75_9ACTN Kanamycin kinase OS=Streptosporangium subroseum OX=106412 GN=SAMN05216276_1006121 PE=3 SV=1

Length=270

Score = 107 bits (268), Expect = 1e-24

| Identities | | = 78/206 (38%), Positives = 104/206 (50%), Gaps = 8/206 (4%) |  |
| --- | --- | --- | --- |
| Query 59  Sbjct 65 | | ELQDEAARLSWLATTGVPCAAVLDVVTEAGRDWLLLGEVPGQDLLSSHLA--PAEKVSIM  +L EA RL W A V V++ T+A WL+ VPG+ + PA + + DLPGEAERLDW-ARRWVNVPRVIEHGTDAAAAWLVTAAVPGRSAVDPRWIADPATAAAAI | 116  123 |
| Query 117 | | ADAMRRLHTLDPAT-CPFDHQAKHRIERARTRMEAGLVDQDDLDEEHQGLAPAELFARLK  +R H P CPFD R+ RA R+ G DD EH+ L A+ ARL | 175 |
| Sbjct | 124 | GRGLRLFHDALPVDECPFDWSVGRRLARADERIADG-EGSDDRFPEHRHLELAKARARL- | 181 |
| Query | 176 | ARMPDGEDLVVTHGDACLPNIMV-ENGRFSGFIDCGRLGVADRYQDIALATRDIAEELGG | 234 |
| Sbjct | 182 | P + LVV HGDAC+PN ++ ++G FS +D G LGVADR+ D+A+A G GEPPSIDRLVVCHGDACVPNTLLHDDGTFSAHVDLGSLGVADRWADLAVAAWSTEWNYGP | 241 |
| Query  Sbjct | 235  242 | EWADRFLVLYGIAAPDSQRIAFYRLL 260  + YGI APD RIA+YRLL  GYDGVLYDAYGI-APDPGRIAYYRLL 266 |  |

>TR:A0A2M9CPF0 A0A2M9CPF0_9CELL Kanamycin kinase OS=Sediminihabitans luteus OX=1138585 GN=CLV28_1255 PE=3 SV=1

Length=291

Score = 108 bits (269), Expect = 1e-24

| Identities | | = 86/230 (37%), Positives = 115/230 (50%), Gaps = 27/230 (12%) |  |
| --- | --- | --- | --- |
| Query 52  Sbjct 64 | | DLSGALNELQDEAARLSWLAT-TGVP-------CAAVLDVVTEA GRDWLLL  DL+G +L DEA RL W T VP AV + A G WLL DLAGV--DLDDEAQRLVWAGRWTPVPRVVRHGRVEAVPGITLPAPAASLPGSDGGTWLLT | 94  121 |
| Query 95  Sbjct 122 | | GEVPGQDLLSSHLAPAEKVSIMA--DAMRRLHTLDPAT-CPFDHQAKHRIERARTRMEAG  +PG+ + + A V+ A +R LH P CP+ A R AR R++AG ETIPGRSAVEPRWSAAPDVAARAVGAGLRALHDALPLLDCPYSWAAPQRAANARDRIDAG | 151  181 |
| Query 152  Sbjct 182 | | LVDQDDLDEEHQGLAPAELFARLKARMPDGEDLVVTHGDACLPNIMV-ENGRFSGFIDCG D EH+ L PA++ ARL A +PD DLVV HGDAC PN ++ ++G ++ +D G  -DGPDSWHPEHRDLTPADVLARL-ADVPD-PDLVVCHGDACAPNTLLHDDGSWAAHVDLG | 210  238 |
| Query | 211 RLGVADRYQDIALATRDIAEELGGEWADRFLVLYGIAAPDSQRIAFYRLL 260 | | |
|  |  | LGVADR+ D+A+A G + YGI APD +IA+YRLL |  |
| Sbjct | 239 | ALGVADRWADLAVAAWSTEWNYGPGYEHLVYDAYGI-APDPAKIAYYRLL 287 | |

>TR:A0A329QTP9 A0A329QTP9_9ACTN Aminoglycoside 3'-phosphotransferase OS=Phytoactinopolyspora halophila OX=1981511 GN=DPM12_09170 PE=3 SV=1

Length=275

Score = 107 bits (268), Expect = 1e-24

| Identities | | = 74/207 (36%), Positives = 106/207 (51%), Gaps = 12/207 (6%) |  |
| --- | --- | --- | --- |
| Query 60  Sbjct 71 | | LQDEAARLSWLATTGVPCAAVLDVVTEAGRDWLLLGEVPGQDLLSSH--LAPAEKVSIMA L DEA RLSW A + V++ + WL+ VPGQ ++ + P + + LDDEARRLSW-AQRWITVPRVIEHGIDDDGSWLVTLPVPGQSAVAPQWIVDPTSAATAVG | 117  129 |
| Query 118  Sbjct 130 | | DAMRRLH-TLDPATCPFDHQAKHRIERARTRMEAG--LVDQDDLDEEHQGLAPAELFARL  +R +H TL CPFD + R+ RAR R+ G LVD+ D D+ P L A RGLRMVHDTLPVEQCPFDWSVEQRLARARERLSDGEQLVDRLDPDQHPDVSDPNALIAE- | 174  188 |
| Query 175  Sbjct 189 | | KARMPDGEDLVVTHGDACLPNIMV-ENGRFSGFIDCGRLGVADRYQDIALATRDIAEELG P + VV HGDAC PN ++ ++G F+ +D G LGVADR+ D+A+A G  ---PPPTDVAVVCHGDACAPNTLLHDDGTFAAHVDLGSLGVADRWADLAVAAWSTVWNYG | 233  245 |
| Query 234 | | GEWADRFLVLYGIAAPDSQRIAFYRLL 260  + + YG+ PD +RIAFYRL+ |  |
| Sbjct | 246 | PGYEEIVYDAYGV-DPDPERIAFYRLI 271 | |

>TR:A0A2N3Y225 A0A2N3Y225_SACSN Streptomycin 3'-kinase OS=Saccharopolyspora spinosa OX=60894 GN=A8926_4807 PE=3 SV=1

Length=359

Score = 109 bits (272), Expect = 1e-24

| Identities | | = 91/253 (36%), Positives = 118/253 (47%), Gaps = 19/253 (8%) |  |
| --- | --- | --- | --- |
| Query 23  Sbjct 107 | | DWAQQTIGCSDAAVFRLSAQGRPVLFVKTDLSGALNELQDEAARLSWLATTGVPCAAVLD  +W G S A+ FR S+ G F K G + ELQ E R WL VP VL EWQPVQGGESGASTFR-SSDGS--CFAKCVAIGQVAELQQERDRAEWLCANRVPGPTVLA | 82  163 |
| Query 83  Sbjct 164 | | VVTEAGRDWLLLGEVPG--QDLLSSHLAPAEKVSIMADAMRRLHTLDPATCPFDHQAKHR  + L+ + G D +++ + SI +DA+RRLH L ATCPF WHADDAGACLVTRVMAGVSADAVAAPILARAWESI-SDAVRRLHDLPTATCPFSRDLAQM | 140  222 |
| Query 141  Sbjct 223 | | IERARTRMEAGLVDQDDLDEEHQGLAPAELFARLKARM PDGEDLVVTHGDACLPN  A+ + G V+ D L E QG PA L +RL ++ + +LVV HGD CLPN FATAQDVVARGAVNPDFLPVEQQGTEPALLLSRLAPQLEQRIEQEAAELVVCHGDLCLPN | 195  282 |
| Query 196  Sbjct 283 | | IMVENGRF--SGFIDCGRLGVADRYQDIALATRDIAEELGGEWADR FLVLYGIAA  I+++ +GFID GRLG AD Y DI+L + E GE R F YGI IILDPDTLDVAGFIDLGRLGTADPYADISLLLANARETWPGEAEARVTDGAFARRYGIEL | 248  342 |
| Query 249 | | PDSQRIAFYRLLD 261 D R FY LD |  |
| Sbjct | 343 | -DEDRRRFYLHLD 354 | |

>TR:A0A0M2LRK4 A0A0M2LRK4_9MICO Aminoglycoside phosphotransferase OS=Leucobacter sp. Ag1 OX=1642040 GN=XM48_08330 PE=3 SV=1

Length=267

Score = 107 bits (267), Expect = 1e-24

| Identities | = 78/250 (31%), Positives = 115/250 (46%), Gaps = 22/250 (9%) |  |
| --- | --- | --- |
| Query 15 | WVERLFGYDWAQQTIGCSDAAVFRLSAQGRPVLFVKTDLSGALNELQDEAARLSWLATTG | 74 |
| Sbjct 32 | W L G + +T G + A G P L +L EAARL W A G WENELGGLTFRDRTAG----RYLKWVAAGTPEL DLDAEAARLRWAAAQG | 76 |
| Query 75 | VPCAAVLDVVTEAGRDWLLLGEVPGQDLLSSH--LAPAEKVSIMADAMRRLHT-LDPATC | 131 |
| Sbjct 77 | +V++ ++ WL+ + G ++ P + +R LH L+ + C ARVPSVVEAGSDRSGSWLVTAALRGDSAVAPRWIAEPGTAARAIGAGLRELHDRLNASAC | 136 |
| Query 132 | PFDHQAKHRIERARTRMEAGLVDQDDLDEEHQGLAPAELFARLKARMPDGEDLVVTHGDA | 191 |
| Sbjct 137 | PFD + R+E+A R+ G + D EH+ L+ + L+ P E VV HGDA  PFDWSIERRLEQAEERLSRG-IGPADWSPEHRALSIRQARELLRDAPPVDEP-VVCHGDA | 194 |

| Query | 192 | CLPNIMV-ENGRFSGFIDCGRLGVADRYQDIALATRDIAEELGGEWADRFLVLYGIAAPD 250 |
| --- | --- | --- |
| Sbjct | 195 | C PN ++ E+G F +D G LG ADR+ D+A+A+ G + YGI PD CAPNTLLDESGAFLAHVDLGALGTADRWADLAIASWSTVWNYGPGYEAAVYAGYGI-EPD 253 |
| Query | 251 | SQRIAFYRLL 260 |
| Sbjct | 254 | +RIA+YRLL  PERIAYYRLL 263 |

>TR:A0A3N6F707 A0A3N6F707_9ACTN Aminoglycoside 3'-phosphotransferase OS=Streptomyces sp. ADI96-02 OX=1522760 GN=neo PE=3 SV=1

Length=267

Score = 107 bits (267), Expect = 1e-24

| Identities | | = 95/260 (37%), Positives = 116/260 (45%), Gaps = 28/260 (11%) |  |
| --- | --- | --- | --- |
| Query 21  Sbjct 12 | | GYDWAQQTIGCSDAAVFRLSAQGRPVLFVKTDLSGALNELQDEAARLSWLATTGVPCAAV G DW T G S A V+R + R V S AL QDE R+ WL GVP V GGDWEPVTGGESGAGVYRSADGSRYAKCVPAARSAAL---QDERDRVEWLGAQGVPVPRV | 80  68 |
| Query 81  Sbjct 69 | | LDVVTEA-GRDWLLLGEVPG--QDLLSSHLAPAEKVSIMADAMRRLHTLDPATCPFDHQA LD A G L+ V G D +S+ A SI A+A+R LH L CPF LDWRVRAEGAACLVTSTVEGVGADRVSARTLGAAWESI-AEAVRHLHRLPVERCPFSRDL | 137  127 |
| Query 138  Sbjct 128 | | KHRIERARTRMEAGLVDQDDLDEEHQGLAPAELFARLKARM PDGEDLVVTHGDAC  + A + G VD D L ++ + EL AR+ + + + VV HGD C ERMFALAGDVVARGAVDPDFLPDDDRNTPGGELLARIAPEVGQRLAQEAAEAVVCHGDLC | 192  187 |
| Query 193  Sbjct 188 | | LPNIMVE--NGRFSGFIDCGRLGVADRYQDIALATRDIAEELGGEWADRFLVL-------  LPNI+++ SGFID GR G AD Y DIAL + E WAD L LPNIVLDPRTLEVSGFIDLGRAGRADPYADIALLLANSRE TWADEDAALAADEAFG | 243  243 |
| Query 244 | | --YGIAAPDSQRIAFYRLLD 261 YGIA D R FY LD |  |
| Sbjct | 244 | RRYGIAL-DRGRERFYLRLD 262 | |

>TR:A0A2P8DSU4 A0A2P8DSU4_9ACTN Streptomycin 3'-kinase OS=Murinocardiopsis flavida OX=645275 GN=CLV63_102392 PE=3 SV=1

Length=288

Score = 107 bits (268), Expect = 2e-24

| Identities | | = 94/261 (36%), Positives = 120/261 (46%), Gaps = 31/261 (12%) |  |
| --- | --- | --- | --- |
| Query 21  Sbjct 34 | | GYDWAQQTIGCSDAAVFRLSAQGRPVLFVKTDLSGALNELQDEAARLSWLATTGVPCAAV G W T G S A VFR SA G + K + +L E R++WLA GVP A V GGRWEPVTGGESGAHVFR-SADG--TRYAKCVPAADAADLAGERDRVAWLAAQGVPGARV | 80  90 |
| Query 81  Sbjct 91 | | LDVVTEAGRDWLLLGEVPGQDLLSSHLAPAEKVSI MADAMRRLHTLDPATCPFDHQ  LD L+ V G + + PA + +ADA+RRLH L CPF + LDWHATGADACLVTSAVVG VPADRLPAPALDTAWPRIADAVRRLHDLPVHECPFSYG | 136  147 |
| Query 137 | | AKHRIERARTRMEAGLVDQDDLDEEHQGLAPAELFARLKARMP DGEDLVVTHGDA  + AR + G V + L E Q P EL AR++ ++P + + VV HGD | 191 |
| Sbjct | 148 | LADMVATARDVVARGAVHPEFLPVEQQRTPPEELLARIEPQVPHRLAQETAESVVGHGDL | 207 |
| Query | 192 | CLPNIMVENG--RFSGFIDCGRLGVADRYQDIALATRDIAEELGGEWAD RF | 240 |
| Sbjct | 208 | CLPNI+++ +GFID GRLG AD Y D+AL E WAD F CLPNIILDPATLEVAGFIDLGRLGRADPYADLALLFATARE AWADDHRAAAAEGAF | 263 |
| Query | 241 | LVLYGIAAPDSQRIAFYRLLD 261 LYGI D R FY LD |  |
| Sbjct | 264 | ADLYGIDL-DRDRERFYLHLD 283 | |

>TR:A0A1X7E6V0 A0A1X7E6V0_CELCE Kanamycin kinase OS=Cellulosimicrobium cellulans J1 OX=903523 GN=SAMN02744115_02424 PE=3 SV=1

Length=276

Score = 107 bits (267), Expect = 2e-24

Identities = 75/206 (36%), Positives = 107/206 (52%), Gaps = 8/206 (4%)

| Query | 59 | ELQDEAARLSWLATTGVPCAAVLDVVTEAGRDWLLLGEVPGQDLLSSHLA--PAEKVSIM | 116 |
| --- | --- | --- | --- |
| Sbjct | 71 | +L EAARL+W+ P VLD ++A WL+ VPG+ + + PA + + DLAAEAARLAWVGDR-TPVPRVLDHGSDADGAWLVTQAVPGRSAVEARETGDPAGAATAI | 129 |
| Query | 117 | ADAMRRLHTLDP-ATCPFDHQAKHRIERARTRMEAGLVDQDDLDEEHQGLAPAELFARLK | 175 |
| Sbjct | 130 | +R LH P A+CPF R+ A R+ G EH+G+ AE ARL GRGLRALHEALPVASCPFTWSVADRVANADARLARG-DGPAQWFPEHRGIPVAEARARLD | 188 |
| Query | 176 | ARMPDGEDLVVTHGDACLPNIMV-ENGRFSGFIDCGRLGVADRYQDIALATRDIAEELGG | 234 |
| Sbjct | 189 | P + LVV HGDAC+PN ++ ++G F+ +D G LGVADR+ D+A+A G DA-PAVDRLVVCHGDACVPNTLLHDDGSFAAHVDLGSLGVADRWADLAVAAWSTECHYGP | 247 |
| Query  Sbjct | 235  248 | EWADRFLVLYGIAAPDSQRIAFYRLL 260  + YG+ PD +RIA+YRLL  GYDGHVYAGYGV-EPDPERIAYYRLL 272 |  |

>TR:A0A164AWT6 A0A164AWT6_9MICO Aminoglycoside 3'-phosphotransferase OS=Microbacterium sp. TNHR37B OX=1775956 GN=neo PE=3 SV=1

Length=245

Score = 106 bits (265), Expect = 2e-24

| Identities | | = 78/218 (36%), Positives = 105/218 (48%), Gaps = 28/218 (13%) |  |
| --- | --- | --- | --- |
| Query 48  Sbjct 47 | | FVKTDLSGALNELQDEAARLSWLAT-TGVPCAAVLDVVTEAGRDWLLLGEVPGQDLLSSH F+K A + L DEA RL W A T VP V + T+ +WL+ +PG + H FIKYGPLNAESSLADEAVRLGWAARYTTVP--TVRETGTDGRHEWLVTAALPGLSAVDPH | 106  104 |
| Query 107 | | LAPAEKVSIMA--DAMRRLHTLDPAT-CPFDHQAKHRIERARTRMEAGLVDQDDLDEEHQ  +V++ A + +R LH P CPFD R+ RA R G+V L E | 163 |
| Sbjct | 105 | WRAQPEVAVRAVGEGLRALHDALPVDDCPFDWSVASRLARAAER---GIVVDATLHEP-- | 159 |
| Query | 164 | GLAPAELFARLKARMPDGEDLVVTHGDACLPNIMV-ENGRFSGFIDCGRLGVADRYQDIA | 222 |
| Sbjct | 160 | P + LVV H DAC PN ++ E+GR+ G +D G LGVADR+ DI+  ---------------PTIDRLVVCHADACCPNTLLGEDGRWLGHVDLGVLGVADRWADIS | 204 |
| Query | 223 LATRDIAEELGGEWADRFLVLYGIAAPDSQRIAFYRLL 260 | | |
|  |  | +A G W D L YGI A D +R+A+YR L |  |
| Sbjct | 205 | VAAMSTGWNYGPGWEDALLDAYGIDA-DEERMAYYRSL 241 | |

>TR:D6AA68 D6AA68_STRV1 Aminoglycoside phosphotransferase OS=Streptomyces viridosporus (strain ATCC 14672 / DSM 40746 / JCM 4963 / KCTC

9882 / NRRL B-12104 / FH 1290) OX=566461 GN=SSFG_07398 PE=3 SV=1

Length=273

Score = 107 bits (266), Expect = 2e-24

| Identities | = 95/280 (34%), Positives = 128/280 (46%), Gaps = 31/280 (11%) |  |
| --- | --- | --- |
| Query 1 | MIEQDGLHAGSPAAWVERLFGYD---WAQQTIGCSDAAVFRLSAQGRPVLFVKTDLSGAL | 57 |
| Sbjct 1 | M ++ G SP L G D W T G S A VFR + R + K + MSDRRGTQTMSPV-----LLGVDDGGWLPVTAGESGATVFRSADATR---YAKCVPAADA | 52 |
| Query 58 | NELQDEAARLSWLATTGVPCAAVLDVVTEAGRDWLLLGEVPGQDLLSSHLAPAEKV---- | 113 |
| Sbjct 53 | +L+ E R +WL+ GVP VLD + L+ V G + + PAE++ ADLKAECDRAAWLSGRGVPGPRVLDWQSGDAGACLVTSAVSG---IPADQVPAEELRGAW | 109 |
| Query 114 | SIMADAMRRLHTLDPATCPFDHQAKHRIERARTRMEAGLVDQDDLDEEHQGLAPAELFAR | 173 |
| Sbjct 110 | +ADA+RRLH + CPF + AR + V+ + L + Q EL R ERIADAVRRLHEAPVSQCPFRRGLDAMVAVARDVVTRDAVNAEFLPVDQQQTPTVELLDR | 169 |
| Query 174 | LKARMP DGEDLVVTHGDACLPNIMVENGRF--SGFIDCGRLGVADRYQDIALATR | 226 |
| Sbjct 170 | L ++P + D VV HGD CLPNI+++ SGFID GRLG+ADRY D+AL LTRQVPRRREQEAVDTVVCHGDLCLPNIILDPQTLDVSGFIDLGRLGLADRYADLALLLA | 229 |
| Query 227  Sbjct 230 | DIAEELGGEWADR-----FLVLYGIAAPDSQRIAFYRLLD 261  + E E R F YGIA D R+ FY LD  NARETWPDEEQARAADMAFAERYGIAL-DHDRLRFYLHLD 268 |  |

>TR:A0A2N0FIZ2 A0A2N0FIZ2_9ACTN Kanamycin kinase OS=Streptomyces sp. 4121.5 OX=1938847 GN=BX258_3409 PE=3 SV=1

Length=264

Score = 106 bits (265), Expect = 3e-24

| Identities | | = 93/261 (36%), Positives = 116/261 (44%), Gaps = 26/261 (10%) |  |
| --- | --- | --- | --- |
| Query 7  Sbjct 19 | | LHAGSP--AAWVERLFGYDWAQQTIGCSDAAVFRLSAQGRPVLFVKTDLSGALNELQDEA L AG P A W L G FRL G FVK +G+ +L EA LAAGEPVVAVWRNELHGL TFRLG-DGPGRRFVKWAPAGSPADLAVEA | 64  64 |
| Query 65  Sbjct 65 | | ARLSWLAT-TGVPCAAVLDVVTEAGRDWLLLGEVPGQDLLSS--HLAPAEKVSIMADAMR RL W T VP L AG WLL +PG+ + PA V + +R ERLGWARRFTTVPEVLALGGGEAAGGRWLLTAGLPGRSAVDEVWKRDPATAVRAIGAGLR | 121  124 |
| Query 122  Sbjct 125 | | RLHTLDP-ATCPFDHQAKHRIERARTRMEAGLVDQDDLDEEHQGLAPAELFARLKARMPD H P A CP+D A R+ RA DL H G L + A P  AFHEALPVAGCPYDWSAPGRVARAAALRRDPADWHPDL--RHHGTVERAL--AVLADPPP | 180  180 |
| Query 181  Sbjct 181 | | GEDLVVTHGDACLPNIMV-ENGRFSGFIDCGRLGVADRYQDIALATRDIAEELGGEWADR  + VV HGDAC PN +V +GR SG +D G LG ADR+ D+A+AT G W VDVEVVCHGDACAPNTLVGADGRPSGHVDLGALGTADRWADLAVATWSTVWNYGPGWERP | 239  240 |
| Query 240 | | FLVLYGIAAPDSQRIAFYRLL 260 L YG+ APD++R+A+YRLL |  |
| Sbjct | 241 | LLEAYGV-APDAERLAYYRLL 260 | |

>TR:A0A2V1HU73 A0A2V1HU73_9MICO Aminoglycoside phosphotransferase APH(3') OS=Amnibacterium sp. M8JJ-5 OX=2173173 GN=DDQ50_06780 PE=3 SV=1

Length=250

Score = 106 bits (264), Expect = 3e-24

| Identities | = 82/233 (35%), Positives = 112/233 (48%), Gaps = 26/233 (11%) |  |
| --- | --- | --- |
| Query 33 | DAAVFRLSAQ-GRPVLFVKTDLSGALNELQDEAARLSWLATTGVPCAAVLDVVTEAGRDW | 91 |
| Sbjct 35 | D F ++ + G +FVK +G +L DEA RL W A + P VLD + W DGITFEIAGRAGLHHVFVKVAATGDDVDLADEARRLGW-ARSFTPVPEVLDSGRDDEFQW | 93 |
| Query 92 | LLLGEVPGQDLLSSHLA--PAEKVSIMADAMRRLHTLDPA-TCPFDHQAKHRIERARTRM | 148 |
| Sbjct 94 | L+ V G ++ PA V + + +R LH P CPFD + R+ RA + LVTRAVAGTSAVAERWIADPAPAVRAIGEGLRALHDRLPVEACPFDWSIESRVRRAAAK- | 152 |
| Query 149 | EAGLVDQDDLDEEHQGLAPAELFARLKARMPDGEDLVVTHGDACLPNIMV-ENGRFSGFI | 207 |
| Sbjct 153 | G+ E + L+A PD VV HGDAC PN ++ E+GRF+ +  ---------------GIHVPE--SLLEAPPPDDHP-VVCHGDACAPNTLLDEDGRFTAHV | 194 |
| Query 208  Sbjct 195 | DCGRLGVADRYQDIALATRDIAEELGGEWADRFLVLYGIAAPDSQRIAFYRLL 260 D G LGVADR+ D+A+A+ + G W D L YGI PD RI FYR L DLGSLGVADRWADLAVASWSLEWNYGPGWDDLMLDAYGI-EPDPARIHFYRRL 246 |  |

>TR:A0A0B1Q1B6 A0A0B1Q1B6_9RHIZ Aminoglycoside 3'-phosphotransferase OS=Aureimonas altamirensis OX=370622 GN=LA66_17590 PE=3 SV=1

Length=250

Score = 106 bits (264), Expect = 3e-24

| Identities | | = 75/218 (34%), Positives = 107/218 (49%), Gaps = 17/218 (8%) |  |
| --- | --- | --- | --- |
| Query 59  Sbjct 30 | | ELQDEAARLSWLATTGVPCAAVLDVVTEAGRDWLLLGEVPGQ---DLLSSHLAPAEKVSI  +L DE RL+WL G+ C V+D L + +PG DL + L A DLADERDRLTWLHGRGIACPEVIDWRATGKGACLTMTAIPGVAAVDLSGADLLKAWPS-- | 115  87 |
| Query 116  Sbjct 88 | | MADAMRRLHTLDPATCPFDHQAKHRIERARTRMEAGLVDQDDLDEEHQGLAPAELFARLK MA + LH+L CPF+ A + V+ D L +E + + EL AR++ MAQQLGVLHSLPVVQCPFERSLSRMFASAVDVVSRNAVNPDFLPDEDKNIPQQELLARVE | 175  147 |
| Query 176  Sbjct 148 | | ARMP DGEDLVVTHGDACLPNIMVE--NGRFSGFIDCGRLGVADRYQDIALATRDI  +P + D+VV HGD C+PN +V+ + + +G ID GRLG ADRY D+AL +  RELPVRLDQERADMVVCHGDPCMPNFIVDPRSLQCAGVIDLGRLGTADRYADLALMVANA | 228  207 |
| Query | 229 AEE--LGGEWADRFLVLY---GIAAPDSQRIAFYRLLD 261 | | |

E	E	F +L+	GI APD +R+AFY LD Sbjct 208 GESWTTPHEAERAFAILFDILGINAPDRERLAFYLRLD 245

>TR:A0A1Q9S8V5 A0A1Q9S8V5_9PSEU Aminoglycoside phosphotransferase APH(3') OS=Pseudonocardia sp. CNS-004 OX=1904967 GN=BJF90_17000 PE=3 SV=1

Length=269

Score = 106 bits (265), Expect = 3e-24

| Identities | = 93/263 (35%), Positives = 124/263 (47%), Gaps = 20/263 (8%) |  |
| --- | --- | --- |
| Query 8 | HAGSPAAWVERLFGYD WAQQTIGCSDAAVFRLSAQGRPVLFVKTDLSGALN-ELQ | 61 |
| Sbjct 13 | H PAA V L G D W Q G + FRL GR V +VK +G + + HVPVPAA-VAGLAGADRIVPVWRNQLGGLT FRLDDGGR-VRYVKWAATGTPELDFR | 66 |
| Query 62 | DEAARLSWLATTGVPCAAVLDVVTEAGRDWLLLGEVPGQDLLSSH--LAPAEKVSIMADA | 119 |
| Sbjct 67 | EA RL W A VP VL +A WL+ PG+ + PA + + AEAERLRW-ARPRVPVPEVLAQDDDADGAWLVTAAQPGRSAVDPRWIAEPATAATAIGRG | 125 |
| Query 120 | MRRLHTLDPAT-CPFDHQAKHRIERARTRMEAGLVDQDDLDEEHQGLAPAELFARLKARM | 178 |
| Sbjct 126 | +R LH P CPFD R +A R+ G EH+ L AE AR+ LRLLHDALPVDDCPFDWSIGRRTAQADRRLADG-DSPASWATEHRNLGVAEARARI-GHP | 183 |
| Query 179 | PDGEDLVVTHGDACLPNIMV-ENGRFSGFIDCGRLGVADRYQDIALATRDIAEELGGEWA | 237 |
| Sbjct 184 | P + LVV HGDAC PN ++ ++G F+ +D G+LGVADR+ D+A+A G + PPIDRLVVCHGDACAPNTLLHDDGTFAAHVDLGKLGVADRWADLAIAAWSTEWNYGPGYE | 243 |
| Query 238  Sbjct 244 | DRFLVLYGIAAPDSQRIAFYRLL 260 YGI APD +RIA+YRL+  GHVFEGYGI-APDPRRIAYYRLI 265 |  |

>TR:A0A1V2QTN1 A0A1V2QTN1_9PSEU Aminoglycoside phosphotransferase APH(3') OS=Saccharothrix sp. ALI-22-I OX=1933778 GN=ALI22I_08295 PE=3

SV=1

Length=270

Score = 106 bits (265), Expect = 3e-24

| Identities | | = 84/229 (37%), Positives = 115/229 (50%), Gaps = 10/229 (4%) |  |
| --- | --- | --- | --- |
| Query 37  Sbjct 43 | | FRLSAQGRPVLFVKTDLSGALN-ELQDEAARLSWLATTGVPCAAVLDVVTEAGRDWLLLG FRL G +VK +G +L+ EA RL+W A VLD +A WL+ FRLDV-GDGARYVKWVAAGTPEIDLRGEAERLAW-AQRWARVPPVLDHGIDAEGAWLITA | 95  100 |
| Query 96  Sbjct 101 | | EVPGQDLLSSH--LAPAEKVSIMADAMRRLHTLDPAT-CPFDHQAKHRIERARTRMEAGL VPG+ + P + + +R LH PA CPFD + R+ RA R+ G AVPGRSAVEPRWKAEPGIAAAAIGRGLRLLHDTLPADRCPFDWSVERRLVRADERIADG- | 152  159 |
| Query 153 | | VDQDDLDEEHQGLAPAELFARLKARMPDGEDLVVTHGDACLPNIMV-ENGRFSGFIDCGR D EH+ L E ARL P + LVV HGDAC PN ++ ++G F+ +D G | 211 |
| Sbjct | 160 | EGVADWSPEHRHLGLTEARARL-GEPPAIDGLVVCHGDACTPNTLLHDDGTFAAHVDLGS | 218 |
| Query  Sbjct | 212  219 | LGVADRYQDIALATRDIAEELGGEWADRFLVLYGIAAPDSQRIAFYRLL 260 LGVADR+ D+A+AT + + G + YGI PD +RIA+YRLL LGVADRWADLAVATWSLDWDYGPGFDGILYDAYGI-DPDPERIAYYRLL 266 |  |

>TR:A0A498B1H3 A0A498B1H3_9ACTN Kanamycin kinase OS=Streptomyces sp. 57 OX=2035243 GN=CLZ79_5788 PE=3 SV=1

Length=247

Score = 105 bits (263), Expect = 3e-24

Identities = 79/217 (36%), Positives = 102/217 (47%), Gaps = 26/217 (12%)

| Query | 48 | FVKTDLSGALNELQDEAARLSWLATTGVPCAAVLDVVTEAGRDWLLLGEVPGQDLLSSH- | 106 |
| --- | --- | --- | --- |
| Sbjct | 49 | F K +G+ +L EA RL W A P V + +A WL+ +PG+ +S FAKWAPAGSGLDLAAEAVRLRW-AVAHTPVPRVREHGADAEGAWLVTHGLPGESAVSDRW | 107 |
| Query | 107 | -LAPAEKVSIMADAMRRLHTLDP-ATCPFDHQAKHRIERARTRMEAGLVDQDDLDEEHQG | 164 |
| Sbjct | 108 | P V + +R LH P A CPFD R+ERAR A  KAEPRRAVRALGAGLRSLHERLPVAECPFDWSVGSRLERARRSGRA-------------- | 153 |

Query 165 LAPAELFARLKARMPDGEDLVVTHGDACLPNIMV-ENGRFSGFIDCGRLGVADRYQDIAL 223 PA+L	A P + LVV HGD C PN ++ E+G +S +D G LGVADR+ D+A+

| Sbjct | 154 | -LPADL-----AEPPPDDRLVVCHGDPCAPNTLLREDGSWSAHVDMGALGVADRWADLAV 207 |
| --- | --- | --- |
| Query | 224 | ATRDIAEELGGEWADRFLVLYGIAAPDSQRIAFYRLL 260 |
| Sbjct | 208 | AT + G W D L YGI APD R FYR L  ATGNTYWNYGPGWEDELLDAYGI-APDPLRTGFYRRL 243 |

>TR:A0A1W2B0V5 A0A1W2B0V5_9FIRM Kanamycin kinase OS=Clostridiales bacterium OX=1898207 GN=SAMN06297397_1833 PE=3 SV=1

Length=268

Score = 106 bits (264), Expect = 4e-24

| Identities | | = 70/209 (33%), Positives = 98/209 (47%), Gaps = 15/209 (7%) |  |
| --- | --- | --- | --- |
| Query 67  Sbjct 60 | | LSWLATTGVPCAAVLDVVTEAGRDWLLLGEVPGQDLLSSHLA--PAEKVSIMADAMRRLH L WLA VP V + G DWLL+ + G+ L + P + MA+A+ L LRWLAGK-VPVPQVAAHAVQDGMDWLLMTRMKGKVLCDPSVMEKPTLLLDCMAEALHALW | 124  118 |
| Query 125  Sbjct 119 | | TLDPATCPFDHQAKHRIERARTRMEAGLVDQDDLDEEHQGLA PAELFARLKARMPD  + CPF+ K + A + AG D D + E G P L LKA +P DIPVENCPFERTVKANLANAEATILAGRFDASDSEPETFGPGGFENPMALLEWLKANLPP | 180  178 |
| Query 181  Sbjct 179 | | GEDLVVTHGDACLPNIMVENGRFSGFIDCGRLGVADRYQDIALATRDIAEELGGEWADRF  +D +VTHGD CLPN+ + RF+GFID G GV DR+QD+AL R + G + +  -QDRMVTHGDFCLPNLFTDGKRFTGFIDVGNAGVGDRWQDLALGWRSLKHNCDGHYGKVY | 240  237 |
| Query | 241 -------LVLYGIAAPDSQRIAFYRLLDE 262 | | |
|  |  | L D +++ +Y LLDE |  |
| Sbjct | 238 | PGINPDDLFRAAGVEKDEEKLRYYILLDE 266 | |

>TR:C6WFK3 C6WFK3_ACTMD Aminoglycoside phosphotransferase OS=Actinosynnema mirum (strain ATCC 29888 / DSM 43827 / NBRC 14064 / IMRU

3971) OX=446462 GN=Amir_1990 PE=3 SV=1

Length=243

Score = 105 bits (262), Expect = 4e-24

| Identities | | = 85/250 (34%), Positives = 116/250 (46%), Gaps = 17/250 (7%) |  |
| --- | --- | --- | --- |
| Query 23  Sbjct 3 | | DWAQQTIGCSDAAVFRLSAQGRPVLFVKTDLSGALNELQDEAARLSWLATTGVPCAAVLD DW T G S A V R S GR D++ L E RL WLATT V V+D DWTPVTTGLSGARV-RRSPDGRHHRKTSADVTA LAAERDRLLWLATTPVGAPEVVD | 82  57 |
| Query 83 | | VVTEAGRDWLLLGEVPGQDLLSSHLAPAEKVSIMADAMRRLHTLDPATCPFDHQAKHRIE L P DL + + PA +++A + LH L CPFD + + | 142 |
| Sbjct | 58 | WSDTGALVTTTLPGTPASDLPPTAV-PAAIAALLA-FLDVLHALPD--CPFDRRLAITVP | 113 |
| Query | 143 | RARTRMEAGLVDQDDLDEEHQGLAPAELFARLKA RMPDGEDLVVTHGDACLPNIM | 197 |
| Sbjct | 114 | A + AGLVD+ D D G +L L+A + DL V HGD LPN++ EAAAHVAAGLVDESDFDAPRLGRTAPDLLDLLRAGHDRAAAHEERDLAVCHGDFSLPNVL | 173 |
| Query | 198 | VENGRFS--GFIDCGRLGVADRYQDIALATRDIAE-ELGGEWADRFLVLYGIAAPDSQRI | 254 |
| Sbjct | 174 | ++ S G +D GRLG+ADR+QD+AL R + +L + +A D R+ LDPDTLSVTGILDVGRLGLADRHQDLALLARSAGDPDLNPAYGATPAAHEVLARTDPWRV | 233 |
| Query | 255 | AFYRLLDEFF 264  +Y+LLDEFF |  |
| Sbjct | 234 | DYYQLLDEFF 243 | |

>TR:D7CCJ8 D7CCJ8_STRBB Phosphotransferase OS=Streptomyces bingchenggensis (strain BCW-1) OX=749414 GN=SBI_05609 PE=3 SV=1

Length=222

Score = 104 bits (260), Expect = 5e-24

Identities = 78/218 (36%), Positives = 105/218 (48%), Gaps = 26/218 (12%)

| Query 47 | LFVKTDLSGALNELQDEAARLSWLATTGVPCAAVLDVVTEAGRDWLLLGEVPGQDLLSSH | 106 |
| --- | --- | --- |
|  | LF K D G+ +L+ EA RL W A P VLD+ + W++ +PG+ +S |  |
| Sbjct 23 | LFAKWDPKGSGADLEVEATRLRW-ARRYTPVPQVLDLGADEDGTWMITRGLPGESAVSPR | 81 |

| Query | 107 | LA--PAEKVSIMADAMRRLHTLDPAT-CPFDHQAKHRIERARTRMEAGLVDQDDLDEEHQ | 163 |
| --- | --- | --- | --- |
| Sbjct | 82 | P V + A+R LH P CPF HR++ AR WKADPRTAVRALGTALRALHERLPVDDCPFSWSVDHRLDVARR----------------- | 124 |
| Query | 164 | GLAPAELFARLKARMPDGEDLVVTHGDACLPNIMVEN-GRFSGFIDCGRLGVADRYQDIA | 222 |
| Sbjct | 125 | A AE+ A L A P + LVV HGD C PN ++ + G +S +D G LG+ADR+ D+A  --AGAEVPADLPAPPPI-DHLVVCHGDPCAPNTLLNSDGTWSAHVDMGALGLADRWADLA | 181 |
| Query | 223 LATRDIAEELGGEWADRFLVLYGIAAPDSQRIAFYRLL 260 | | |
|  |  | +AT G W D L YGI +PD +RI FYR L |  |
| Sbjct | 182 | VATWSTEWNYGPGWDDTLLDGYGI-SPDPERIDFYRRL 218 | |

>TR:A0A4R5U9I1 A0A4R5U9I1_9RHIZ Aminoglycoside 3'-phosphotransferase OS=Rhizobium deserti OX=2547961 GN=aph(3'') PE=3 SV=1

Length=276

Score = 105 bits (263), Expect = 6e-24

| Identities | = 75/225 (33%), Positives = 112/225 (50%), Gaps = 27/225 (12%) |  |
| --- | --- | --- |
| Query 57 | LNELQDEAARLSWLATTGVPCAAVLD VVTEAGRDWLLLGEVPGQDLLSSHLA | 108 |
| Sbjct 54 | L+ L E RL+WL G+ C ++D +T + ++ G DLL + + LDGLVGERDRLAWLYGRGIACPEMIDWRETEEGACLTMTAIPGVPAADLSGADLLKAWPS | 113 |
| Query 109 | PAEKVSIMADAMRRLHTLDPATCPFDHQAKHRIERARTRMEAGLVDQDDLDEEHQGLAPA | 168 |
| Sbjct 114 | A+++ IM H+L A CPF+ + H A + V+ D L +E + TAQQLGIM HSLPVAKCPFERRLSHMFGCAVDVVSRNAVNPDFLPDEDKDKPQQ | 166 |
| Query 169 | ELFARLKARMP DGEDLVVTHGDACLPNIMV--ENGRFSGFIDCGRLGVADRYQDI | 221 |
| Sbjct 167 | EL AR++ +P + D+VV HGD C+PN MV ++ R +G ID GRLG ADRY D+ ELLARVEQELPLRLDQEQADMVVCHGDPCMPNFMVDPQSLRCTGVIDLGRLGNADRYADL | 226 |
| Query 222  Sbjct 227 | ALATRDIAEE--LGGEWADRFLVLYG---IAAPDSQRIAFYRLLD 261 AL + E E F +L+ I+APD +R+AFY LD ALMVANAGESWTTPNEAERAFAILFNVLEISAPDRERLAFYLRLD 271 |  |

>TR:A0A2A9DRI3 A0A2A9DRI3_9MICO Kanamycin kinase OS=Microbacterium agarici OX=630514 GN=ATJ78_0100 PE=3 SV=1

Length=246

Score = 105 bits (261), Expect = 7e-24

| Identities | = 73/222 (33%), Positives = 106/222 (48%), Gaps = 26/222 (12%) |  |
| --- | --- | --- |
| Query 41 | AQGRPVLFVKTDLSGALNELQDEAARLSWLATTGVPCAAVLDVVTEAGRDWLLLGEVPGQ | 100 |
| Sbjct 41 | A G ++K A + EA RL+W A +P V++ +A +WL+ + GQ AAGDKARYIKWGPHDAETSMAAEAERLTWAARY-IPVPRVIEQGQDARHEWLVTEALSGQ | 99 |
| Query 101 | DLLSSHLA--PAEKVSIMADAMRRLHTLDPA-TCPFDHQAKHRIERARTRMEAGLVDQDD | 157 |
| Sbjct 100 | ++ PA V+ + +R LH P CPFD + R A R G+ + SAVAPRWIADPAVAVAAIGRGLRSLHDALPVDVCPFDWGVESRFANAAGR---GIHLPEP | 156 |
| Query 158 | LDEEHQGLAPAELFARLKARMPDGEDLVVTHGDACLPNIMVEN-GRFSGFIDCGRLGVAD | 216 |
| Sbjct 157 | L E PD + LVV HGDAC PN ++++ GR SG +D G LGVAD LKEA PDADKLVVCHGDACCPNTLIDDVGRCSGHVDFGALGVAD | 199 |
| Query 217  Sbjct 200 | RYQDIALATRDIAEELGGEWADRFLVLYGIAAPDSQRIAFYR 258 R+ DIA+A+ G W D + YG+ PD +R+A+YR RWADIAVASMSTEWNYGPGWEDALIEAYGV-EPDRERLAYYR 240 |  |

```
>TR:A0A0F0KY17 A0A0F0KY17_9MICO Aminoglycoside 3'-phosphotransferase OS=Microbacterium foliorum OX=104336 GN=neo PE=3 SV=1
Length=241

Score = 104 bits (260), Expect = 8e-24
Identities = 73/217 (34%), Positives = 106/217 (49%), Gaps = 26/217 (12%)

Query 48	FVKTDLSGALNELQDEAARLSWLATTGVPCAAVLDVVTEAGRDWLLLGEVPGQDLLSSHL 107
++K	A	++DEA R+ W A	+	VLD	+A +WL+	+PG	+
Sbjct 43	YIKWGPHDAEANMRDEAERMRW-ARRWIEVPEVLDQGQDATHEWLVTAALPGVSAVDPRW 101
```

| Query | 108 | A--PAEKVSIMADAMRRLHTLDPAT-CPFDHQAKHRIERARTRMEAGLVDQDDLDEEHQG | 164 |
| --- | --- | --- | --- |
| Sbjct | 102 | A PA V + A+R LH P CP+D + RI A +E G+V DDL ADEPATAVRAVGRALRSLHDALPVERCPWDWSPQWRISNA---LERGVVVPDDLHTP--- | 155 |
| Query | 165 | LAPAELFARLKARMPDGEDLVVTHGDACLPNIMVEN-GRFSGFIDCGRLGVADRYQDIAL | 223 |
| Sbjct | 156 | P + LVV HGDAC+PN ++++ GR +D LGVADR+ D+A  --------------PSIDRLVVCHGDACMPNTLLDDEGRPLAHVDLAALGVADRWADLAA | 201 |
| Query  Sbjct | 224  202 | ATRDIAEELGGEWADRFLVLYGIAAPDSQRIAFYRLL 260 A+ +G WAD + YG+ APD +R+ +YR L ASMSTEWNVGPGWADALIEAYGV-APDRERLEYYRRL 237 |  |

>TR:A0A0J1GBY8 A0A0J1GBY8_9FIRM APH domain-containing protein OS=Robinsoniella sp. RHS OX=1504536 GN=RHS_1545 PE=3 SV=1

Length=261

Score = 105 bits (261), Expect = 9e-24

| Identities | | = 72/221 (33%), Positives = 108/221 (49%), Gaps = 18/221 (8%) |  |
| --- | --- | --- | --- |
| Query 59  Sbjct 44 | | ELQDEAARLSWLATTGVPCAAVLDVVTEAGRDWLLLGEVPGQDLLSSHLA--PAEKVSIM E +E LSWL+ +P +L ++LL+ V G+ S+ P ++ ESDNEHQMLSWLSGR-LPVPKLLCSEKSCSTNYLLMERVVGEMSCSTEFLEDPELLTELL | 116  102 |
| Query 117  Sbjct 103 | | ADAMRRLHTLDPATCPFDHQAKHRIERARTRMEAGLVDQDDLDEEHQG LAPAELFA  A+ +R L +D + CP+ + +++ A + L + + ++ G +PA+L AEGLRMLWNVDISACPYHNGIDNKLRLAEILVSNNLCEMEGVEPGTYGDNGFASPADLLT | 172  162 |
| Query 173 | | RLKARMPDGEDLVVTHGDACLPNIMVENGRFSGFIDCGRLGVADRYQDIALATRDIAEEL LK P E+LV +HGD CLPNI + + SGFID GR G+AD+YQDIAL R + | 232 |
| Sbjct | 163 | WLKDNRP-AENLVFSHGDYCLPNIFINQNKVSGFIDLGRSGIADKYQDIALCYRSLKHNF | 221 |
| Query  Sbjct | 233  222 | GGEW---------ADRFLVLYGIAAPDSQRIAFYRLLDEFF 264 G + AD GI PD RI +Y LLDE F  EGRYGGKIYEDFNADILFDKLGI-EPDWDRIQYYILLDELF 261 |  |

>TR:E0RY10 E0RY10_BUTPB Aminoglycoside phosphotransferase OS=Butyrivibrio proteoclasticus (strain ATCC 51982 / DSM 14932 / B316) OX=515622 GN=bpr_I2232 PE=3 SV=1

Length=267

Score = 105 bits (261), Expect = 1e-23

| Identities  Query 61 | = 68/219 (31%), Positives = 109/219 (50%), Gaps = 18/219 (8%)  QDEAARLSWLATTGVPCAAVLDVVTEAGRDWLLLGEVPGQDLLSSHLA--PAEKVSIMAD | 118 |
| --- | --- | --- |
| Sbjct 52 | +D + WL +P V+ + +LL+ +V G+ + P E V +++ KDAVEMMRWLEGK-IPAPQVIVFEEDDSYSYLLMTKVRGKVACDKYYLERPKELVPLLSK | 110 |
| Query 119 | AMRRLHTLDPATCPFDHQAKHRIERARTRMEAGLVDQDDLDEEHQG LAPAELFAR | 173 |
| Sbjct 111 | +++ L ++D CP +++A R++ GLVD D + + G P EL + SIKMLQSIDITDCPVVKNVDRELKKAAYRVKNGLVDISDAEPDTFGENGRFRDPEELLSW | 170 |
| Query 174 | LKARMPDGEDLVVTHGDACLPNIMVENGRFSGFIDCGRLGVADRYQDIALATRDIAEELG | 233 |
| Sbjct 171 | L+ P E V++HGD CLPNI++E G SGFID G G+AD+++DIA+ R + LQENRPPYEP-VLSHGDLCLPNILIEKGNISGFIDMGNCGIADKWEDIAILYRSLRHNFD | 229 |
| Query 234  Sbjct 230 | GEWA--------DRFLVLYGIAAPDSQRIAFYRLLDEFF 264 G + D F GI PD ++I +Y LLDE F GTYGKIYPGLEPDSFFEELGI-EPDREKIDYYILLDELF 267 |  |

>TR:A0A3N2D192 A0A3N2D192_9MICO Kanamycin kinase/streptomycin 3'-kinase OS=Salana multivorans OX=120377 GN=EDD28_2952 PE=3 SV=1

Length=243

Score = 104 bits (259), Expect = 1e-23

Identities = 94/253 (37%), Positives = 120/253 (47%), Gaps = 32/253 (13%)

Query 23	DWAQQTIGCSDAAVFRLSAQGRPVLFVKTDLSGALNELQDEAARLSWLATTGVPCAAVLD 82 DW + TIG S AAV R S GR	+VK+	L E RL+WLATT V	VLD

| Sbjct | 6 | DWDRVTIGHS-AAVVRRSPDGRS--YVKSATGVERTALAAEHDRLTWLATTPVGAPRVLD | 62 |
| --- | --- | --- | --- |
| Query | 83 | VVT---EAGRDWLL LGEVPGQDLLSSHLAPAEKVSIMADAMRRLHTLDPATCP | 132 |
| Sbjct | 63 | V EAG + L VPG + + A A V M + + LH L PA CP WVDGSDEAGAAVRVSHLVTCALPGVPGSSVPRTDAARA--VRGMVELLAALHALSPADCP | 120 |
| Query | 133 | FDHQAKHRIERARTRMEAGLVDQDDLDEEHQGLAPAELFARLKARMPDGEDLVVTHGDAC | 192 |
| Sbjct | 121 | FD + + AR V D A + R++A P DL V HGD C FDRRLAVTVPLARAAGSPPAVVVD APILDRVRAAEP--ADLAVCHGDLC | 167 |
| Query | 193 | LPNIMVENG--RFSGFIDCGRLGVADRYQDIALATRDIAE-ELGGEWAD-RFLVLYGIAA | 248 |
| Sbjct | 168 | LPN ++ G R +G +D GRLGVADR+ D+AL R +A+ L + R L LPNFHLDPGTLRPTGVLDVGRLGVADRHLDLALLHRSMADVRLNPAFGPARARELAEATG | 227 |
| Query | 249 | PDSQRIAFYRLLD 261 D +R+ FYR+LD |  |
| Sbjct | 228 | ADPERLTFYRVLD 240 | |

>TR:A0A6G4X282 A0A6G4X282_9ACTN APH(3'') family aminoglycoside O-phosphotransferase OS=Streptomyces sp. SB3404 OX=2711219 GN=aph(3'') PE=3

SV=1

Length=264

Score = 104 bits (260), Expect = 1e-23

| Identities | | = 92/259 (36%), Positives = 120/259 (46%), Gaps = 33/259 (13%) |  |
| --- | --- | --- | --- |
| Query 24  Sbjct 13 | | WAQQTIGCSDAAVFRLSAQGRPVLFVKTDLSGALNELQDEAARLSWLATTGVPCAAVLDV W T G S AAVFR SA G + K + L+ E R++WL GVP VL WRPVTAGESGAAVFR-SADG--ARYAKCVPAAEAAGLRAERDRVAWLDGQGVPGPRVLGW | 83  69 |
| Query 84  Sbjct 70 | | VTEAGRDWLLLGEVPGQDLLSSHLAPAEKVSI MADAMRRLHTLDPATCPFD  L+ V G PA++VS +ADA+R LH + A CPF SAGDAGACLVTSAVRG VPADQVSAADLWDAWEGIADAVRALHEVPGARCPFR | 134  121 |
| Query 135  Sbjct 122 | | HQAKHRIERARTRMEAGLVDQDDLDEEHQGLAPAELFARL KARMPDGEDLVVTHG  + AR ++ G V + L + Q AEL RL R + D VV HG RGLDQMVALARDVVDRGAVHPEFLPADQQHTPAAELLDRLVPQVALRREQEAGDAVVCHG | 189  181 |
| Query 190  Sbjct 182 | | DACLPNIMVE--NGRFSGFIDCGRLGVADRYQDIALATRDIAE ELGGEWAD-RFLV  D CLPNI+++ + SGF+D GRLG+ADR+ D+AL + E E G AD F DLCLPNIVLDPRTRQVSGFVDLGRLGLADRHADLALLLANARETWRDEEGARAADAAFAE | 242  241 |
| Query 243 | | LYGIAAPDSQRIAFYRLLD 261 YG+A D R+ FY LD |  |
| Sbjct | 242 | RYGLALDDG-RLRFYLHLD 259 | |

>TR:C7QIF6 C7QIF6_CATAD Aminoglycoside phosphotransferase OS=Catenulispora acidiphila (strain DSM 44928 / JCM 14897 / NBRC 102108 /

NRRL B-24433 / ID139908) OX=479433 GN=Caci_6179 PE=3 SV=1

Length=267

Score = 104 bits (260), Expect = 1e-23

| Identities | = 74/219 (34%), Positives = 112/219 (51%), Gaps = 9/219 (4%) |  |
| --- | --- | --- |
| Query 48 | FVKTDLSGALNELQDEAARLSWLATTGVPCAAVLDVVTEAGRDWLLLGEVPGQDLLSSHL | 107 |
| Sbjct 51 | + K +G+ +L EAAR+ W A V V++ A L+ + G+ ++ YAKWAPAGSYLDLAGEAARIRW-AGAFVTVPQVVEQAASAEGSLLVTAALEGEMAVTERW | 109 |
| Query 108 | A--PAEKVSIMADAMRRLHTLDPA-TCPFDHQAKHRIERARTRMEAGLVDQDDLDEEHQG | 164 |
| Sbjct 110 | PA V ++ +++R H P +CPF A+ R+ A R L D+ + + G KQDPATAVRVIGESLRAFHDALPVDSCPFSFSAELRVAEA-VRYAEELKDRT-MHPDFAG | 167 |
| Query 165 | LAPAELFARLKARMPDGEDLVVTHGDACLPNIMVE-NGRFSGFIDCGRLGVADRYQDIAL | 223 |
| Sbjct 168 | L + R+ A +P + LVV HGD C PN ++ +GRF+G +D G LGVADR+ D+A+ LTREQALKRV-ADIPPVDKLVVCHGDTCAPNTLLSADGRFAGHVDLGDLGVADRWSDLAI | 226 |
| Query 224  Sbjct 227 | ATRDIAEELGGEWADRFLVLYGIAAPDSQRIAFYRLLDE 262 AT G W + L YGI APD +R +YRLL E ATWSTTWNYGDGWQEALLEAYGI-APDPERTEYYRLLWE 264 |  |

>TR:A0A086YC10 A0A086YC10_9RHOB Aminoglycoside 3'-phosphotransferase OS=Haematobacter massiliensis OX=195105 GN=aph(3'') PE=3 SV=1

Length=267

Score = 104 bits (260), Expect = 1e-23

| Identities | | = 88/276 (32%), Positives = 122/276 (44%), Gaps = 41/276 (15%) |  |
| --- | --- | --- | --- |
| Query 5  Sbjct 9 | | DGLHAGSPAAWVERLFGYDWAQQTIGCSDAAVFRLSAQGRPVLFVKTDLSGALNELQDEA DGLH+ +W G S VFR + + T L EL +E DGLHS-------------NWLPARGGESGNTVFRRADEQAYAKIAPTALR---KELAEER | 64  52 |
| Query 65  Sbjct 53 | | ARLSWLATTGVPCAAVLDVVTEAGRDWLLLGEVPG---QDLLSSHLAPAEKVSIMADAMR RL WL + GV C V+ E L++ +PG DL S L E M D + DRLIWLCSRGVACPEVISWREEQEGACLVMTAIPGVPAADLSGSDLL--EAWPSMVDQLN | 121  110 |
| Query 122  Sbjct 111 | | RLHTLDPATCPFDHQAKHRIERARTRMEAGLVDQDDLDEEHQGLAPAELFARLKARM---  +H+L CPF+ + RA + V+ D L +E + EL AR++ + AVHSLSAEECPFERRLSEMFARAVDVVSRNAVNPDFLPDEDKLTPQPELLARIERELAVR | 178  170 |
| Query 179  Sbjct 171 | | --PDGEDLVVTHGDACLPNIMV--ENGRFSGFIDCGRLGVADRYQDIALATRDIAEELGG  + +L V HGD C+PN MV ++ + +G ID GRLG AD Y D+ L + AEE+  LEQESRELTVCHGDPCMPNFMVSTKSLQCTGLIDVGRLGKADLYADLTLMISN-AEEI-- | 234  227 |
| Query | 235 EWA---------DRFLVLYGIAAPDSQRIAFYRLLD 261 | | |
|  |  | WA D GI APD +R+AFY LD |  |
| Sbjct | 228 | -WASSDEAKCAFDILFARLGIDAPDRERLAFYLRLD 262 | |

>TR:A0A4R2JF33 A0A4R2JF33_9PSEU Kanamycin kinase OS=Actinocrispum wychmicini OX=1213861 GN=EV192_108157 PE=3 SV=1

Length=251

Score = 104 bits (259), Expect = 1e-23

| Identities | | = 74/206 (36%), Positives = 103/206 (50%), Gaps = 8/206 (4%) |  |
| --- | --- | --- | --- |
| Query 59  Sbjct 46 | | ELQDEAARLSWLATTGVPCAAVLDVVTEAGRDWLLLGEVPGQDLLSSH--LAPAEKVSIM  +L EA RL W A V+D T++ DWL+ VPG + P + + DLPGEAERLEW-ARRWARVPRVVDHGTDSAGDWLVTAAVPGTSAVDPRWKAEPVTASAAI | 116  104 |
| Query 117 | | ADAMRRLHTLDPAT-CPFDHQAKHRIERARTRMEAGLVDQDDLDEEHQGLAPAELFARLK  +R LH P CPFD + R+ RA R+ G D EH+ L AE R+ | 175 |
| Sbjct | 105 | GHGLRLLHDTVPVDDCPFDWSVERRLVRADERIAEG-GGPADWAPEHRHLELAEARQRIG | 163 |
| Query | 176 | ARMPDGEDLVVTHGDACLPNIMV-ENGRFSGFIDCGRLGVADRYQDIALATRDIAEELGG | 234 |
| Sbjct | 164 | A P + LVV HGD C+PN ++ ++G F+ +D G LGVADR+ D+A+A + G AP-PAIDRLVVCHGDTCVPNTLIHDDGTFAAHVDLGSLGVADRWADLAVAAWSLGWNYGP | 222 |
| Query  Sbjct | 235  223 | EWADRFLVLYGIAAPDSQRIAFYRLL 260  + YGI PD +RIA+YRLL  GFDGILYDAYGI-EPDQERIAYYRLL 247 |  |

>TR:A0A4V1M245 A0A4V1M245_9STAP Aminoglycoside 3'-phosphotransferase OS=Macrococcus sp. DPC7161 OX=2507060 GN=ER639_04385 PE=3 SV=1

Length=255

Score = 104 bits (259), Expect = 2e-23

| Identities  Query 58 | = 66/217 (30%), Positives = 116/217 (53%), Gaps = 20/217 (9%)  NELQDEAARLSWLATTGVPCAAVLDVVTEAGRDWLLLGEVPGQDLLSSHLAPAEKVSIMA | 117 |
| --- | --- | --- |
| Sbjct 49 | +E ++E + WL +P +++ + + +LL+ + G+ L + + + SETENEIKLMKWL-NGRLPVPKIIEHCVKNDKSYLLMTRLEGEMLSKD GDSLELYT | 103 |
| Query 118 | DAMRRLHTLDPATCPFDHQAKHRIERARTRMEAGLVDQDDLDEEHQGLA PAELFAR | 173 |
| Sbjct 104 | A+ +L +D + CP D++ +H +++A +E GLVD +D + + G P EL + KAINKLKEIDFSDCPVDNRLQHYLKKAEYNVEKGLVDIEDANPDTYGPEGFKDPQELLSW | 163 |
| Query 174 | LKARMPDGEDLVVTHGDACLPNIMVENGRFSGFIDCGRLGVADRYQDIALATR D | 227 |
| Sbjct 164 | LK P+ E++ THGD C+PNI++++G SG+ID GR G+ D Y+DIAL R +  LKDNQPE-EEISYTHGDLCMPNILIKDGNISGYIDLGRGGLGDPYRDIALCYRSYLYNTE | 222 |

Query 228 IAEELGGEWADRFLVLYGIAAPDSQRIAFYRLLDEFF 264 I EE	E +	L G	D Q++ +Y L+DE +

Sbjct 223 INEE---ELKNELEHLLGFKL-DMQKVKYYILVDELY 255

>TR:A0A543G9W1 A0A543G9W1_9PSEU Kanamycin kinase OS=Pseudonocardia cypriaca OX=882449 GN=FB388_0213 PE=3 SV=1

Length=257

Score = 104 bits (259), Expect = 2e-23

| Identities | | = 80/229 (35%), Positives = 116/229 (51%), Gaps = 9/229 (4%) |  |
| --- | --- | --- | --- |
| Query 37  Sbjct 29 | | FRLSAQGRPVLFVKTDLSGALN-ELQDEAARLSWLATTGVPCAAVLDVVTEAGRDWLLLG FRL V +VK +G +L EA R++W A VP VL ++A WL+ FRLDDGNGRVRYVKWVAAGTPEIDLPGEAERIAW-AQGRVPVPRVLAQGSDADGSWLVTE | 95  87 |
| Query 96  Sbjct 88 | | EVPGQDLLSSH--LAPAEKVSIMADAMRRLH-TLDPATCPFDHQAKHRIERARTRMEAGL  +PG+ + PA + + +R LH L CPFD + R+ +A R+ G+ GLPGRSAVDPRWIAQPAVAAAAIGRGLRLLHDALAVDGCPFDWSVERRLAQADERIAGGM | 152  147 |
| Query 153 | | VDQDDLDEEHQGLAPAELFARLKARMPDGEDLVVTHGDACLPNIMV-ENGRFSGFIDCGR  + EH+ L AE A++ P + LVV HGDAC PN ++ ++G F+ +D G | 211 |
| Sbjct | 148 | -RPSEWAPEHRHLGLAEARAQI-GEQPPIDRLVVCHGDACAPNTLLHDDGTFAAHVDLGS | 205 |
| Query  Sbjct | 212  206 | LGVADRYQDIALATRDIAEELGGEWADRFLVLYGIAAPDSQRIAFYRLL 260 LGVADR+ D+A+A G + YGI APD +RIA+YRLL LGVADRWADLAVAAWSTEWNYGPGYDAFVYEGYGI-APDPRRIAYYRLL 253 |  |

>TR:A0A2V4NRL8 A0A2V4NRL8_9ACTN Aminoglycoside phosphotransferase APH(3') OS=Streptomyces tateyamensis OX=565073 GN=C7C46_08240 PE=3 SV=1

Length=257

Score = 104 bits (259), Expect = 2e-23

| Identities | = 73/217 (34%), Positives = 103/217 (47%), Gaps = 10/217 (5%) |  |
| --- | --- | --- |
| Query 48 | FVKTDLSGALNELQDEAARLSWLATTGVPCAAVLDVVTEAGRDWLLLGEVPGQDLLSSHL | 107 |
| Sbjct 43 | ++K +G+ +L EA RL W V + + G WL+ +PG + YLKWAPTGSGRDLGAEAVRLRWAGRFAVVPPVLAQGADQDG-SWLVTAALPGTMAVDDRW | 101 |
| Query 108 | A--PAEKVSIMADAMRRLHTLDP-ATCPFDHQAKHRIERARTRMEAGLVDQDDLDEEHQG | 164 |
| Sbjct 102 | PA V + + +R LH P A CPF+ A+ R+ A D + EH+ KREPATAVRAIGEGLRALHEALPVADCPFEWSAQQRLAVAAELR ADPAEWHAEHRH | 157 |
| Query 165 | LAPAELFARLKARMPDGEDLVVTHGDACLPNIMV-ENGRFSGFIDCGRLGVADRYQDIAL | 223 |
| Sbjct 158 | L E L A +P + VV HGD C PN ++ +GR+SG +D G LGVADR+ D+A+ LGTVERALELLAEVPPVDRAVVCHGDPCAPNTLLGPDGRWSGHVDLGALGVADRWADLAV | 217 |
| Query 224  Sbjct 218 | ATRDIAEELGGEWADRFLVLYGIAAPDSQRIAFYRLL 260 AT G W L YG+ PD R A+YRLL ATWSTGWNYGPGWERALLDAYGV-DPDPDRSAYYRLL 253 |  |

>TR:A0A3G6ZKC8 A0A3G6ZKC8_9MICO Aminoglycoside 3'-phosphotransferase OS=Microbacterium sp. ABRD_28 OX=2268461 GN=DT073_04635 PE=3 SV=1

Length=244

Score = 103 bits (258), Expect = 2e-23

| Identities | | = 75/219 (34%), Positives = 103/219 (47%), Gaps = 26/219 (12%) |  |
| --- | --- | --- | --- |
| Query 46  Sbjct 44 | | VLFVKTDLSGALNELQDEAARLSWLATTGVPCAAVLDVVTEAGRDWLLLGEVPGQDLLSS  + +VK A ++ EA RL W A P V+ + +WL+ +PG+ ++ IRYVKWGPRNAETTVEGEAERLVWAAAF-TPVPRVIARGGDDDEEWLVTEALPGESAVAP | 105  102 |
| Query 106 | | H--LAPAEKVSIMADAMRRLHTLDPAT-CPFDHQAKHRIERARTRMEAGLVDQDDLDEEH AP VS + +R LH P T CPF + R+ A R G+ + L E | 162 |
| Sbjct | 103 | RWIAAPGIAVSAIGVGLRSLHDRLPVTDCPFSWRVADRLAGAARR---GIRVPEPLREA- | 158 |
| Query | 163 | QGLAPAELFARLKARMPDGEDLVVTHGDACLPN-IMVENGRFSGFIDCGRLGVADRYQDI | 221 |
| Sbjct | 159 | PD EDLVV HGDAC PN ++ + G SG +D G LGVADR+ DI  ----------------PDEEDLVVCHGDACSPNTVLTDEGTVSGHVDLGALGVADRWADI | 202 |

Query 222 ALATRDIAEELGGEWADRFLVLYGIAAPDSQRIAFYRLL 260 A+A	+	G W + L YGI APD R+ +YR L

Sbjct 203 AVAAMSLEWNYGPGWTETLLEAYGI-APDRSRMTYYREL 240

>TR:A0A2A3YN51 A0A2A3YN51_9MICO APH(3'') family aminoglycoside O-phosphotransferase OS=Brachybacterium alimentarium OX=47845 GN=CIK66_02990

PE=3 SV=1

Length=260

Score = 104 bits (259), Expect = 2e-23

| Identities | = 78/211 (37%), Positives = 104/211 (49%), Gaps = 15/211 (7%) |  |
| --- | --- | --- |
| Query 23 | DWAQQTIGCSDAAVFRLSAQGRPVLFVKTDLSGALNELQDEAARLSWLATTGVPCAAVLD | 82 |
| Sbjct 10 | +W + G S A VFR + GR F K + EL++E R W A G+P A VLD EWHRIESGESAARVFRRT-DGRA--FAKLVPPSLVTELREERDRTEWAARAGIPSAPVLD | 66 |
| Query 83 | VVTEAGRDWLLLGEVPGQDLLSSHLAPAEKV---SIMADAMRRLHTLDPATCPFDHQAKH | 139 |
| Sbjct 67 | + L+ G VPG + + L P V ++ A RR+H L CPFD WTESSAGACLVTGAVPG--VPADGLDPERAVRAWPLILCAARRVHELASRDCPFDRGVDR | 124 |
| Query 140 | RIERARTRMEAGLVDQDDLDEEHQGLAPAELFARLKARMP DGEDLVVTHGDACLP | 194 |
| Sbjct 125 | I A + A V+ + L E Q + P L L +P + +DLVV HGD CLP MISLASAVVAARGVNHEFLRPEQQDVDPRLLLKGLLVDLPRRQEQEADDLVVCHGDLCLP | 184 |
| Query 195  Sbjct 185 | NIMVE--NGRFSGFIDCGRLGVADRYQDIAL 223 NI+V+ +GFID GRLG ADR+ D+AL NILVDPRTLEVTGFIDLGRLGRADRHVDLAL 215 |  |

>TR:A0A1M7CS78 A0A1M7CS78_9ACTN Kanamycin kinase OS=Streptomyces paucisporeus OX=310782 GN=SAMN05216499_105336 PE=3 SV=1

Length=248

Score = 103 bits (258), Expect = 2e-23

| Identities | | = 82/235 (35%), Positives = 112/235 (48%), Gaps = 31/235 (13%) |  |
| --- | --- | --- | --- |
| Query 32  Sbjct 34 | | SDAAVFRLSAQGRPVLFVKTDLSGALNELQDEAARLSWLATTGVPCAAVLDVVTE-AGR- S F+L QG LF K +G+ +L EAARL W A P V+D E AGR SGGVTFQLG-QGGGRLFAKWAPAGSGLDLGAEAARLRWAADF-TPVPRVVDHGLEGAGRG | 89  91 |
| Query 90  Sbjct 92 | | DWLLLGEVPGQDLLSSHLAPAEKVSIMA--DAMRRLHTLDPAT-CPFDHQAKHRIERART  +WL+ +PG +S+ K ++ A +R LH P CPF+ + R+ AR EWLVTVGLPGDSAVSARWRADPKTTVRALGKGLRALHDRLPVEDCPFEWSVERRVAEARA | 146  151 |
| Query 147  Sbjct 152 | | RMEAGLVDQDDLDEEHQGLAPAELFARLKARMPDGEDLVVTHGDACLPN-IMVENGRFSG E + P P + LVV HGDAC PN ++ E+G +S  E-----------GEPVPSMPP PPVDRLVVCHGDACSPNTLLAEDGSWSA | 205  189 |
| Query 206 | | FIDCGRLGVADRYQDIALATRDIAEELGGEWADRFLVLYGIAAPDSQRIAFYRLL 260  +D G LGVADR+ D+A+ + G W + + YGI APD +RIAFYR L |  |
| Sbjct | 190 | HVDLGALGVADRWADLAVGAWSVGVNYGSAWEEAYYDAYGI-APDPERIAFYRRL 243 | |

>TR:A0A0N0MZD3 A0A0N0MZD3_9ACTN Kanamycin kinase OS=Actinobacteria bacterium OK074 OX=1592327 GN=OK074_8789 PE=3 SV=1

Length=261

Score = 103 bits (258), Expect = 2e-23

Identities = 85/239 (36%), Positives = 111/239 (46%), Gaps = 40/239 (17%)

| Query | 37 | FRLSAQG---RPVLFVKTDLSGALNELQDEAARLSWLAT-TGVPCAAVLDVVTEAGRD-- | 90 |
| --- | --- | --- | --- |
| Sbjct | 48 | FR+ G R LF K +G +L EA RL W T VP V E GRD FRIDGDGVGDRETLFAKWVPAGTALDLAAEAERLRWARQFTPVPR VKEYGRDDD | 101 |
| Query | 91 | ---WLLLGEVPGQDLLSSH--LAPAEKVSIMADAMRRLHTLDP-ATCPFDHQAKHRIERA | 144 |
| Sbjct | 102 | WL+ +PG+ +S PA V + +R LH P A CPFD + R+ RA GAAWLVTYALPGESAVSPRWLAEPATAVRALGAGLRVLHEQLPVADCPFDWSVEERLARA | 161 |
| Query | 145 | RTRMEAGLVDQDDLDEEHQGLAPAELFARLKARMPDGEDLVVTHGDACLPNIMV-ENGRF | 203 |
| Sbjct | 162 | + GL PA P + LVV HGD C PN ++ ++G +  ARHSGRPVT----------GLPPA PPADRLVVCHGDPCAPNTLLHDDGSW | 201 |

Query 204 SGFIDCGRLGVADRYQDIALATRDIAEELGGEWADRFLVLYGIAAPDSQRIAFYRLLDE 262 SG +D G +GVADR+ D+A+A+ +	G W D L YGIA D +RI FYR L E

Sbjct 202 SGHVDLGTMGVADRWADLAVASWNTRMNYGPGWEDALLDAYGIAR-DPERIDFYRRLWE 259

>TR:A0A1J4N7A0 A0A1J4N7A0_9ACTN APH(3'') family aminoglycoside O-phosphotransferase OS=Nocardioides luteus OX=1844 GN=UG56_008305 PE=3 SV=1

Length=271

Score = 103 bits (257), Expect = 4e-23

| Identities | | = 87/254 (34%), Positives = 114/254 (45%), Gaps = 17/254 (7%) |  |
| --- | --- | --- | --- |
| Query 21  Sbjct 16 | | GYDWAQQTIGCSDAAVFRLSAQGRPVLFVKTDLSGALNELQDEAARLSWLATTGVPCAAV G W T G S A V + R V+ SG +L E R+ WL G+P A V GTSWEPVTAGESGAVVLHDRDRARYAKLVRAADSG---DLAGERDRIIWLGEAGLPAATV | 80  72 |
| Query 81  Sbjct 73 | | LDVVTEAGRDWLLLGEVPGQDLLSSHLAPAEKV-SIMADAMRRLHTLDPATCPFDHQAKH L L+ VPG A + +A R+LH L CPFD LAWRATDEGACLITTTVPGVPADQLDAAALRRAWPAIAAIARQLHALPAPDCPFDRGLGS | 139  132 |
| Query 140  Sbjct 133 | | RIERARTRMEAGLVDQDDLDEEHQGLAPAELFARLKARMPDGE DLVVTHGDACLP  + ART + V + L Q + PA + A+L+ ++P + + VV HGD CLP MMPLARTTVAEHRVQTEFLPVSLQQVPPARILAQLEDQLPQRQRQEETERVVCHGDLCLP | 194  192 |
| Query 195  Sbjct 193 | | NIMVENGRF--SGFIDCGRLGVADRYQDIALATRDIAE ELGGEWA-DRFLVLYGIA  NI+++ +G ID GRLGVAD Y DI+L + E EL A D F LYGI NILIDPDTMGVTGLIDLGRLGVADPYADISLLLANARETWPDELTARQADDEFSDLYGID | 247  252 |
| Query 248 | | APDSQRIAFYRLLD 261 D +R FY LLD |  |
| Sbjct | 253 | L-DQERQRFYLLLD 265 | |

>SP:P0A3Y6 KKA3_STAAU Aminoglycoside 3'-phosphotransferase OS=Staphylococcus aureus OX=1280 GN=aphA PE=3 SV=1

Length=263

Score = 103 bits (256), Expect = 5e-23

| Identities | | = 76/250 (30%), Positives = 120/250 (48%), Gaps = 10/250 (4%) |  |
| --- | --- | --- | --- |
| Query 22  Sbjct 17 | | YDWAQQTIGCSDAAVFRLSAQGRPVL-FVKTDLSGALNELQDEAARLSWLATTGVPCAAV Y + T G S A V++L L + G +++ E + WL +P V YRCVKDTEGMSPAKVYKLGENENLYLKMTDSRYKGTTYDVEREKDMMLWLEGK-LPVPKV | 80  75 |
| Query 81  Sbjct 76 | | LDVVTEAGRDWLLLGEVPG---QDLLSSHLAPAEKVSIMADAMRRLHTLDPATCPFDHQA  + G LL+ E G + +P + + + A+ +R H++D + CP+ + VHFERHDGWSNLLMSEADGVLCSEEYEDEQSPEKIIELYAECIRLFHSIDISDCPYTNSL | 137  135 |
| Query 138  Sbjct 136 | | KHRIERARTRMEAGLVDQD--DLDEEHQGLAPAELFARLKARMPDGEDLVVTHGDACLPN R+ + L D D + +E+ P EL+ LK P+ E+LV +HGD  DSRLAELDYLLNNDLADVDCENWEEDTPFKDPRELYDFLKTEKPE-EELVFSHGDLGDSK | 195  194 |
| Query 196  Sbjct 195 | | IMVENGRFSGFIDCGRLGVADRYQDIALATRDIAEELGGE-WADRFLVLYGIAAPDSQRI I V++G+ SGFID GR G AD++ DI R I E++G E + + F L GI PD ++I IFVKDGKVSGFIDLGRSGRADKWYDIPFCVRSIREDIGEEQYVELFFDLLGI-KPDWEKI | 254  253 |
| Query 255 | | AFYRLLDEFF 264  +Y LLDE F |  |
| Sbjct | 254 | KYYILLDELF 263 | |

```
>TR:A0A365YVW7 A0A365YVW7_9ACTN APH(3'') family aminoglycoside O-phosphotransferase OS=Streptomyces sp. PT12 OX=1510197 GN=aph(3'') PE=3
SV=1
Length=256

Score = 102 bits (255), Expect = 6e-23
Identities = 86/259 (33%), Positives = 113/259 (44%), Gaps = 31/259 (12%)

Query 23	DWAQQTIGCSDAAVFRLSAQGRPVLFVKTDLSGALNELQDEAARLSWLATTGVPCAAVLD 82
+W	G S A V R +	R	V + + AL	+D	R+ WL	G+P	VLD
```

| Sbjct | 4 | EWEPVAAGESGARVLRSADGSRYAKCVPAEATPALEAERD---RVGWLGAQGIPAPRVLD | 60 |
| --- | --- | --- | --- |
| Query | 83 | VVTEAGRDWLLLGEVPGQDLLSSHLAPAEKVSI MADAMRRLHTLDPATCPF | 133 |
| Sbjct | 61 | +A L+ V G PA++++ +ADA+RRLH L CPF WHADAAGARLVTSAVEG VPADRLTAPELWAAWEPIADAVRRLHELPVRRCPF | 112 |
| Query | 134 | DHQAKHRIERARTRMEAGLVDQDDLDEEHQGLAPAELFARLKARM PDGEDLVVTH | 188 |
| Sbjct | 113 | AR + G V+ D L EE + P L ARL ++ + + VV H GRGLAGMFALARDVVARGAVNPDFLPEEQRHTPPDALLARLAPQLERRLAQEAAEAVVCH | 172 |
| Query | 189 | GDACLPNIMV--ENGRFSGFIDCGRLGVADRYQDIALATRDIAEEL GGEWADRFLV | 242 |
| Sbjct | 173 | GD CLPNI++ E +GFID GRLG AD Y DIAL + E AD GDLCLPNIVLDPETREVAGFIDLGRLGRADPYADIALLLANARETWPDADRARAADEAFA | 232 |
| Query  Sbjct | 243  233 | LYGIAAPDSQRIAFYRLLD 261 A DS+R FY LD RRRGTALDSERQRFYLHLD 251 |  |

>TR:A0A5B8M1N4 A0A5B8M1N4_9MICO Aminoglycoside 3'-phosphotransferase OS=Humibacter sp. WJ7-1 OX=2599293 GN=FPZ11_03380 PE=3 SV=1

Length=274

Score = 103 bits (256), Expect = 6e-23

| Identities | | = 71/206 (34%), Positives = 103/206 (50%), Gaps = 22/206 (11%) |  |
| --- | --- | --- | --- |
| Query 59  Sbjct 83 | | ELQDEAARLSWLATTGVPCAAVLDVVTEAGRDWLLLGEVPGQDLLSSH--LAPAEKVSIM  +L+ E RL+W A V VLD ++A D L+ +PG++ S PA+ V + DLRAEVERLAWAAPY-VRVPRVLDFTSDADGDVLVTAAIPGENAASERWRAEPAQAVRAI | 116  141 |
| Query 117  Sbjct 142 | | ADAMRRLHTLDPAT-CPFDHQAKHRIERARTRMEAGLVDQDDLDEEHQGLAPAELFARLK A +R+LH P CPF R+ R+R ++G+ L AVGLRQLHDALPVPECPFSWSVDERMARSR NEGIRERILALDRL | 175  185 |
| Query 176  Sbjct 186 | | ARMPDGEDLVVTHGDACLPN-IMVENGRFSGFIDCGRLGVADRYQDIALATRDIAEELGG R+P + LVV H DAC+PN ++ ++G +G +D GRLGVADR+ DIA+ G PRVPAVDRLVVCHADACVPNTLLADDGSVAGHVDLGRLGVADRWADIAIGAWSTEWNYGP | 234  245 |
| Query 235 | | EWADRFLVLYGIAAPDSQRIAFYRLL 260  + D F YG+ A D +RIAFYR L |  |
| Sbjct | 246 | GYTDLFYQSYGVEA-DEERIAFYRAL 270 | |

>TR:A0A1I1AXG3 A0A1I1AXG3_9CELL Kanamycin kinase OS=Cellulomonas marina OX=988821 GN=SAMN05421867_12222 PE=3 SV=1

Length=292

Score = 103 bits (257), Expect = 6e-23

| Identities | = 80/220 (36%), Positives = 110/220 (50%), Gaps = 15/220 (7%) |  |
| --- | --- | --- |
| Query 48 | FVKTDLSGALNELQDEAARLSWLATTGVPCAAVLDVVT---EAGRDWLLLGEVPGQDLLS | 104 |
| Sbjct 77 | F+K G+ +L EAARL+W P AAV +V+ +A WL + G + FLKWAPHGSGLDLAAEAARLAWAR PHAAVPEVLARGEDADGTWLRTAALTGTSAVD | 132 |
| Query 105 | SHLA--PAEKVSIMADAMRRLHTLDP-ATCPFDHQAKHRIERARTRMEAGLVDQDDLDEE | 161 |
| Sbjct 133 | + PA + + +R LH P A CPF + R+ AR R+ AG E ARWRRDPARAAAGVGAGLRALHDALPVAGCPFTWSLEDRVAAARARLAAG-DGPAAWAPE | 191 |
| Query 162 | HQGLAPAELFARLKARMPDGEDLVVTHGDACLPNIMV-ENGRFSGFIDCGRLGVADRYQD | 220 |
| Sbjct 192 | H L PAE+ ARL P D VV GDAC PN ++ ++G +G +D GRLGVADR+ D HAALGPAEVLARLAD--PPPVDPVVCQGDACTPNALLGDDGAATGHVDLGRLGVADRWAD | 249 |
| Query 221  Sbjct 250 | IALATRDIAEELGGEWADRFLVLYGIAAPDSQRIAFYRLL 260  +A+A G + + YG+ PD +R AFYRLL  LAVAAWSFTWNHGPGYEHLVVAAYGV-TPDPRRNAFYRLL 288 |  |

>TR:D3D745 D3D745_9ACTN Aminoglycoside phosphotransferase OS=Frankia sp. EUN1f OX=102897 GN=FrEUN1fDRAFT_5617 PE=3 SV=1

Length=242

Score = 102 bits (254), Expect = 6e-23

| Identities | | = 77/219 (35%), Positives = 101/219 (46%), Gaps = 20/219 (9%) |  |
| --- | --- | --- | --- |
| Query 48  Sbjct 37 | | FVKTDLSGALNELQDEAARLSWLATTGVPCAAVLDVVTEAGRDWLLLGEVPGQDLLSS-- FVK +G L EA+RL W A P AVL + WL+ +PG++ + + FVKWAPAGTHLNLMAEASRLRWAAPF-TPVPAVLGEGRDGAGSWLITAGLPGRNAVHAIW | 105  95 |
| Query 106  Sbjct 96 | | HLAPAEKVSIMADAMRRLHTLDP-ATCPFDHQAKHRIERARTRMEAGLVDQDDLDEEHQG P V + +R H P A CPF R+ A R +H+  KAEPLRAVQAIGRGLRAFHEALPVAECPFSWSVADRLAEASARATTA QHR- | 164  145 |
| Query 165  Sbjct 146 | | LAPAELFARLKARMPDGEDLVVTHGDACLPNIMV-ENGRFSGFIDCGRLGVADRYQDIAL A L A P + LVV GDAC PN ++ E+GR+SG +D G LGVADR+ D+A+  ----RRLADLAADAPAVDVLVVCQGDACAPNTLLGEDGRWSGHVDLGALGVADRWADLAV | 223  201 |
| Query | 224 ATRDIAEELGGEWADRFLVLYGIAAPDSQRIAFYRLLDE 262 | | |
|  |  | AT G W L YG+ APD R AFYR L E |  |
| Sbjct | 202 | ATWSTEWNYGPGWEHVLLDAYGV-APDDDRTAFYRRLWE 239 | |

>TR:A0A1I0MM21 A0A1I0MM21_9FIRM Kanamycin kinase/aminoglycoside 3'-phosphotransferase-3 OS=Ruminococcaceae bacterium KH2T8 OX=1855400 GN=SAMN05216413_0484

PE=3 SV=1

Length=266

Score = 102 bits (255), Expect = 7e-23

| Identities  Query 61 | = 68/218 (31%), Positives = 107/218 (49%), Gaps = 16/218 (7%)  QDEAARLSWLATTGVPCAAVLDVVTEAGRDWLLLGEVPGQDLLSSHLA--PAEKVSIMAD | 118 |
| --- | --- | --- |
| Sbjct 51 | +D + WL +P V+ + +LL+ V G+ + P + ++A KDAVEMMRWLEGK-LPAPGVIAFTEDDIHCYLLMTRVKGKMPCDRYFMERPEVLIPLLAK | 109 |
| Query 119 | AMRRLHTLDPATCPFDHQAKHRIERARTRMEAGLVDQDDLDEEHQGLA PAELFAR | 173 |
| Sbjct 110 | +++ L ++DP CP +++A R+E GLVD D + + G P EL SIKMLWSIDPKDCPVVKDLDGELQKAEYRVEHGLVDISDAEPDTFGEGGRFKDPEELLTW | 169 |
| Query 174 | LKARMPDGEDLVVTHGDACLPNIMVENGRFSGFIDCGRLGVADRYQDIALATRDIAEELG | 233 |
| Sbjct 170 | LK P E V++HGD CLPNI++EN SGFID G G+AD+++DIA+ R + LKTNRPSYEP-VLSHGDLCLPNILIENSDISGFIDMGNCGIADKWEDIAMCYRSLRHNFD | 228 |
| Query 234  Sbjct 229 | GEWA------DRFLVLYGIA-APDSQRIAFYRLLDEFF 264 G + D L+ + PD ++I +Y LLDE F GTYGKFYQGIDPNLLFKELGIEPDWEKIDYYILLDELF 266 |  |

>TR:A0A5R9EIG4 A0A5R9EIG4_9LACT Aminoglycoside 3'-phosphotransferase OS=Facklamia tabacinasalis OX=87458 GN=FEZ33_02400 PE=3 SV=1

Length=270

Score = 102 bits (255), Expect = 8e-23

| Identities  Query 53 | = 67/232 (29%), Positives = 116/232 (50%), Gaps = 21/232 (9%)  LSGALNELQDEAARLSWLATTGVPCAAVLDVVTEAGRDWLLLGEVPGQDLLSSHL--APA | 110 |
| --- | --- | --- |
| Sbjct 40 | + + +E+++E L+W +P +L+ + G ++LL+ ++ G + + IESSSDEVKNEVLMLNWWNNYHLPVPEILENIQVEGINYLLMEKLKGDPSYEVIVRRSTE | 99 |
| Query 111 | EKVSIMADAMRRLHTLDPATCPFDHQAKHRIERARTRMEAGLVDQDDLDEEHQGL A | 166 |
| Sbjct 100 | + + ++AD+++ L +D CP + ++ A+ R+E LVD +D +E G + DLIELLADSLKLLWNVDITHCPSSQLLEDKLAAAKERVEKNLVDVNDAEEGTFGEDGFNS | 159 |
| Query 167 | PAELFARLKARMPDGEDLVVTHGDACLPNIMVENGRFSGFIDCGRLGVADRYQDIALATR | 226 |
| Sbjct 160 | P L+ L P+ E LV++HGD LPN++ +GFID G+ GV+D YQDIAL R PEALWDWLDNNRPEEEKLVLSHGDFSLPNLIASGSYLTGFIDLGQSGVSDIYQDIALCYR | 219 |
| Query 227  Sbjct 220 | DIAEELGGE--------------WADRFLVLYGIAAPDSQRIAFYRLLDEFF 264  + + L G+ + F GI PD +I +Y LLDE F  SLLDNLRGKFGVLRTYTEYEVENYGKLFFQKIGI-EPDWDKIKYYILLDELF 270 |  |

>TR:A0A263DYL7 A0A263DYL7_9PSEU Aminoglycoside O-phosphotransferase APH(3'')-Ia OS=Pseudonocardia sp. MH-G8 OX=1854588 GN=CFP66_04750 PE=3

SV=1

Length=255

Score = 102 bits (254), Expect = 8e-23

| Identities | | = 87/248 (35%), Positives = 114/248 (46%), Gaps = 15/248 (6%) |  |
| --- | --- | --- | --- |
| Query 23  Sbjct 10 | | DWAQQTIGCSDAAVFRLSAQGRPVLFVKTDLSGALNELQDEAARLSWLATTGVPCAAVLD DWA G S A V R + V D + AL +D R+ WL TGVP +VLD DWAPVPSGESGATVHRRTDGAAFAKCVDRDDAAALRGERD---RIHWLCGTGVPGPSVLD | 82  66 |
| Query 83 | | VVTEAGRDWLLLGEVPGQDL--LSSHLAPAEKVSIMADAMRRLHTLDPATCPFDHQAKHR L+ VPG L S A SI+ +R LH L A+CPF+ + | 140 |
| Sbjct | 67 | WSCSDLGACLVTSTVPGVPAGELPSGSLLAAWPSIVG-TLRGLHALPAASCPFERGLETM | 125 |
| Query | 141 | IERARTRMEAGLVDQDDLDEEHQGLAPAELFARLKARM PDGEDLVVTHGDACLPN | 195 |
| Sbjct | 126 | A + G V + L EE + + +L L+A + + DLVV HGDACLPN FATAEDVVARGAVHPEFLTEEQRDVPVTDLLTGLRAELGTRLAQEPGDLVVCHGDACLPN | 185 |
| Query | 196 | IMVENGRFS--GFIDCGRLGVADRYQDIALATRDIAEELGGEWADRFLVLYGIAAPDSQR | 253 |
| Sbjct | 186 | +V+ + G ID GRLG ADR+ D+AL + E G+ A R L G D R FLVDPDTLTCTGLIDLGRLGTADRHADLALLRANAGESWTGDDAARAEPLLG--GVDPAR | 243 |
| Query  Sbjct | 254  244 | IAFYRLLD 261  + FY LD  MRFYLHLD 251 |  |

>TR:A0A1C7IAD6 A0A1C7IAD6_9FIRM APH domain-containing protein OS=Blautia sp. YL58 OX=1796616 GN=A4V09_12765 PE=3 SV=2

Length=260

Score = 102 bits (254), Expect = 9e-23

| Identities  Query 6 | | = 80/266 (30%), Positives = 128/266 (48%), Gaps = 16/266 (6%)  GLHAGSPAAWVERLFGYDWAQQTIGCSDAAVFRLSAQGRPVLFVKTDLSGALNELQDEAA | 65 |
| --- | --- | --- | --- |
| Sbjct 4 | | G+ P + +++ D+++ TIG S + + S + +K D E ++E GMERKIPESIKKQIENKDYSRDTIGRSGSQILCFSD MVLKADRDR--EESRNEHE | 56 |
| Query 66  Sbjct 57 | | RLSWLATTGVPCAAVLDVVTEAGRDWLLLGEVPGQDLLSSHLA--PAEKVSIMADAMRRL LSWL +P ++ G +LL+ +V G L + P V ++A+ ++ L ILSWLQGR-LPVPKIICAEKMGGTRFLLMTKVKGAMLCDAQYLENPGLLVHLLAEGLKML | 123  115 |
| Query 124  Sbjct 116 | | HTLDPATCPFDHQAKHRIERARTRMEAGLV DQDDLDEEHQGLAPAELFARLKARMP  +D + CP ++++A + GL + DL + +P L L+ P RKVDISGCPAKRNLDFKLQQASVNVADGLELETGTEPDLYGTEEFHSPEGLLLWLREHKP | 179  175 |
| Query 180  Sbjct 176 | | DGEDLVVTHGDACLPNIMVENGRFSGFIDCGRLGVADRYQDIALATRDIAEELG-GEWAD E++V +HGD CLPNI EN SGFID GR G+AD +QDIAL R + +G E  R-EEMVFSHGDYCLPNIFAENEDISGFIDLGRAGIADFWQDIALCVRSLQYNIGKNEGCV | 238  234 |
| Query | 239 RFLVLYGIAAPDSQRIAFYRLLDEFF 264 | | |
|  |  | + L APD ++I +Y LLDE F |  |
| Sbjct | 235 | KQLFEELEIAPDWEKIRYYILLDELF 260 | |

>TR:A0A066YSW7 A0A066YSW7_9ACTN Putative aminoglycoside 3'-phosphotransferase OS=Kitasatospora cheerisanensis KCTC 2395 OX=1348663 GN=KCH_41130

PE=3 SV=1

Length=262

Score = 102 bits (253), Expect = 1e-22

| Identities | = 77/218 (35%), Positives = 105/218 (48%), Gaps = 12/218 (6%) |  |
| --- | --- | --- |
| Query 48 | FVKTDLSGALNELQDEAARLSWL-ATTGVPCAAVLDVVTEAGRDWLLLGEVPGQDLLSSH | 106 |
| Sbjct 48 | FVK +G+ +L EA RL W A T VP VL + W+L +PG+ + FVKWAPAGSGADLHAEAERLRWAGAFTTVP--EVLGTGGDETGRWMLTAGLPGRSAVDPE | 105 |
| Query 107 | L--APAEKVSIMADAMRRLHTLDP-ATCPFDHQAKHRIERARTRMEAGLVDQDDLDEEHQ | 163 |
| Sbjct 106 | L PA V + + +R LH P A CP+ A+ RI RA + D +H LLTTPAVAVRAIGEGLRALHEALPVAGCPYTWSAQERIGRAAAQRR DPATWHPDHH | 161 |
| Query 164 | GLAPAELFARLKARMPDGEDLVVTHGDACLPNIMV-ENGRFSGFIDCGRLGVADRYQDIA | 222 |
| Sbjct 162 | L + L A P + VV HGDAC PN +V +GR G +D G LG ADR+ D+A  HLGTVDRALALLADRPPVDVEVVCHGDACAPNTLVGADGRALGHVDLGALGTADRWADLA | 221 |

Query 223 LATRDIAEELGGEWADRFLVLYGIAAPDSQRIAFYRLL 260

+A+	G W	L YG+	D +R+A+YRLL Sbjct 222 VASWSTVWNYGPGWERPLLEAYGV-DEDRERLAYYRLL 258

>TR:A0A0D0PX29 A0A0D0PX29_KITGR Aminoglycoside phosphotransferase OS=Kitasatospora griseola OX=2064 GN=TR51_30515 PE=3 SV=1

Length=262

Score = 102 bits (253), Expect = 1e-22

| Identities | = 78/218 (36%), Positives = 108/218 (50%), Gaps = 12/218 (6%) |  |
| --- | --- | --- |
| Query 48 | FVKTDLSGALNELQDEAARLSWL-ATTGVPCAAVLDVVTEAGRDWLLLGEVPGQDLLSSH | 106 |
| Sbjct 48 | FVK +G+ +L EA RL W A T VP VL + + WLL +PG+ + FVKWAPAGSGADLDAEAERLRWARAFTTVP--EVLALGGDGTGRWLLTAGLPGRSAVDPE | 105 |
| Query 107 | LA--PAEKVSIMADAMRRLHTLDP-ATCPFDHQAKHRIERARTRMEAGLVDQDDLDEEHQ | 163 |
| Sbjct 106 | L PA V + + +R H P A CP+ + RI+RA DL H LTKTPAVAVRAIGEGLRAFHEALPVADCPYTWSLRDRIDRAVALRRDPADWHPDL--RHH | 163 |
| Query 164 | GLAPAELFARLKARMPDGEDLVVTHGDACLPNIMV-ENGRFSGFIDCGRLGVADRYQDIA | 222 |
| Sbjct 164 | G L + A P + VV HGD+C PN +V ++GR +G +D G LGVADR+ D+A GTVERAL--AILADPPPVDVEVVCHGDSCAPNTLVGDDGRPTGHVDLGTLGVADRWADLA | 221 |
| Query 223  Sbjct 222 | LATRDIAEELGGEWADRFLVLYGIAAPDSQRIAFYRLL 260  +A+ G W L YG+ PD +R+A+YRLL  VASWSTVWNYGPGWERPLLEAYGV-EPDEERLAYYRLL 258 |  |

>TR:A0A143WVH6 A0A143WVH6_9FIRM Aminoglycoside 3'-phosphotransferase OS=Clostridiales bacterium CHKCI006 OX=1780379 GN=aphA PE=3 SV=1

Length=263

Score = 102 bits (253), Expect = 1e-22

| Identities | | = 69/220 (31%), Positives = 111/220 (50%), Gaps = 16/220 (7%) |  |
| --- | --- | --- | --- |
| Query 59  Sbjct 46 | | ELQDEAARLSWLATTGVPCAAVLDVVTEAGRDWLLLGEVPGQDLLSS-HLAPAEK-VSIM EL +EA ++WL +P + V E G + L+ +PG+ L + ++ EK ++++ ELANEAQAIAWLQGR-LPVPEIRAFVIEDGWTYDLMTRLPGKMLCDTPYMEDEEKQLAVL | 116  104 |
| Query 117  Sbjct 105 | | ADAMRRLHTLDPATCPFDHQAKHRIERARTRMEAGLVDQDDLDEEHQG LAPAELFA  + L +D CP ++++AR + G VD ++ E G P +L TEVFDSLWAIDITDCPCQQILDIKLKQARENVLQGRVDLSLVEPETFGPDGFKDPMDLLC | 172  164 |
| Query 173  Sbjct 165 | | RLKARMPDGEDLVVTHGDACLPNIMVENGRFSGFIDCGRLGVADRYQDIALATRDIAEEL L+ P EDLV HGD CLPNI+ + + SG ID GR+GVADR+QD+AL R + +  YLETHRPT-EDLVFCHGDLCLPNILGHDQKLSGLIDLGRMGVADRWQDLALCYRSLRDNA | 232  223 |
| Query | 233 GGEWADRFLVLYGIA--------APDSQRIAFYRLLDEFF 264 | | |
|  |  | G + D+ + PD +++ +Y LLDE F |  |
| Sbjct | 224 | RGCFGDQVYANQAASKLFRRLGVVPDQEKLRYYLLLDELF 263 | |

>TR:A6W8Z6 A6W8Z6_KINRD Aminoglycoside phosphotransferase OS=Kineococcus radiotolerans (strain ATCC BAA-149 / DSM 14245 / SRS30216)

OX=266940 GN=Krad_1799 PE=3 SV=1

Length=256

Score = 101 bits (252), Expect = 2e-22

Identities = 84/256 (33%), Positives = 114/256 (45%), Gaps = 47/256 (18%)

| Query | 12 | PAAWVERLFGYDWAQQTIGCSDAAVFRLSAQGRPVLFVKTDLSGALNELQDEAARLSWLA | 71 |
| --- | --- | --- | --- |
| Sbjct | 40 | PAAWV G FRL +G +F K +G +L EA R W A PAAWVNAAGG-------------TTFRLEDRG---VFCKWAPAGVGLDLAAEADRSRW-A | 82 |
| Query | 72 | TTGVPCAAVLDVVTEAGRDWLLLGEVPGQDLLSS--HLAPAEKVSIMADAMRRLH-TLDP | 128 |
| Sbjct | 83 | + VL+ T +WL+ +PG+ ++ P V +A +RRLH TL VRWLAVPEVLEHGTCPEGEWLVTRGLPGRSAVAEDWRARPEAAVPALARGLRRLHDTLPV | 142 |
| Query | 129 | ATCPFDHQAKHRIERARTRMEAGLVDQDDLDEEHQGLAPAELFARLKARMPDGEDLVVTH A CPFD HR+ RA +E GL++ +D LVV H | 188 |

| Sbjct | 143 | AQCPFDWSVPHRLARAVGAVE-GLLEPPPVDR LVVCH | 178 |
| --- | --- | --- | --- |
| Query | 189 | GDACLPNIMVE--NGRFSGFIDCGRLGVADRYQDIALATRDIAEELGGEWADRFLVLYGI | 246 |
| Sbjct | 179 | GDAC PN ++ +G +D G LGVADR+ D+A+AT + G W +L YG+ GDACAPNTLLAEGSGEAVAHVDLGALGVADRWADLAVATLSLGWNYGPGWEGTYLAAYGV | 238 |
| Query | 247 | AAPDSQRIAFYRLLDE 262 PD R A+YR L E |  |
| Sbjct | 239 | -DPDPVRTAYYRALWE 253 | |

>TR:A0A143XDA0 A0A143XDA0_9FIRM Aminoglycoside 3'-phosphotransferase OS=Clostridiales bacterium CHKCI001 OX=1780378 GN=aphA PE=3 SV=1

Length=261

Score = 101 bits (252), Expect = 2e-22

| Identities | = 75/256 (29%), Positives = 130/256 (51%), Gaps = 25/256 (10%) |  |
| --- | --- | --- |
| Query 24 | WAQQTIGCSDAAVFRLSAQGRPVLFVKTDLSGALNELQDEAARLSWLATTGVPCAAVLDV | 83 |
| Sbjct 16 | + +IG SD++VF + +L V+ E ++E +SWL + VL YQSDSIGMSDSSVFIFHNK---ILKVQN CSKESENEYCMMSWLKGK-LQIPEVLAY | 67 |
| Query 84 | VTEAGRDWLLLGEVPGQDLLSSHLA--PAEKVSIMADAMRRLHTLDPATCPFDHQAKHRI | 141 |
| Sbjct 68 | + + +LL+ + G+ P +V ++A ++ L +D + CP D K+++ EVQNDKSYLLMSKCEGEMACDKKYMENPRMQVKLLATGIKNLWNIDISQCPSDWSLKNKL | 127 |
| Query 142 | ERARTRMEAGLVDQDDLDEEHQGLA PAELFARLKARMPDGEDLVVTHGDACLPNIM | 197 |
| Sbjct 128 | +AR +E LVD D+++ + G P L L P+ E+LV++HGD CLPNI AQARYNVENNLVDMDNIEPDTFGKNGFQNPQALLQWLYDNQPE-EELVLSHGDYCLPNIF | 186 |
| Query 198 | VENGRFSGFIDCGRLGVADRYQDIALATRDIAEELGGEWADRF------LVLY---GIAA | 248 |
| Sbjct 187 | ++ +G+ID G+ G+AD++ DIAL R ++ G + + L+L+ GI FKDNHITGYIDLGKTGIADKWCDIALCYRSLSHNYCGRYHGKAYSSLDDLLLFKELGI-K | 245 |
| Query 249  Sbjct 246 | PDSQRIAFYRLLDEFF 264 P+ ++I +Y LLDE F PNWEKIKYYLLLDELF 261 |  |

>TR:A0A1C6Q6K7 A0A1C6Q6K7_9ACTN Streptomycin 3-kinase OS=Streptomyces sp. SceaMP-e96 OX=1100824 GN=YWIDRAFT_07800 PE=3 SV=1

Length=273

Score = 101 bits (252), Expect = 2e-22

| Identities | | = 90/263 (34%), Positives = 113/263 (43%), Gaps = 40/263 (15%) |  |
| --- | --- | --- | --- |
| Query 24  Sbjct 21 | | WAQQTIGCSDAAVFRLSAQGRPVLFVKTDLSGALNELQDEAARLSWLATTGVPCAAVLDV W T G S+A+VFR R + K + + L+ E R+ WL GVP VLD WEPVTDGESEASVFRTEDGSR---YAKCVPAEGVASLEAERDRVDWLNRQGVPGPRVLDW | 83  77 |
| Query 84  Sbjct 78 | | VTEAGRDWLLLGEVPGQDLLSSHLAPAEKVSI MADAMRRLHTLDPATCPFD  LL V G PA++VS +A+A+R+LH L CPF RVGTAGAALLTSAVAG VPADRVSAPTLMTAWEPIAEAVRQLHALSAQQCPFS | 134  129 |
| Query 135  Sbjct 130 | | HQAKHRIERARTRMEAGLVDQDDLDEEHQGLAPAELFARLKARM PDGEDLVVTHG  AR + G V + L E Q P EL ARL ++ + + VV HG RDLTQMYAAARDVVARGAVKSEFLPVEQQQTPPDELLARLAPQLEHRLAQETAETVVCHG | 189  189 |
| Query 190  Sbjct 190 | | DACLPNIMV--ENGRFSGFIDCGRLGVADRYQDIALATRDIAEELGGEWAD---------  D CLPNI++ E +GFID GRLG AD Y DIAL E +W D DLCLPNIILDPEALTVAGFIDLGRLGRADPYADIALLLASARLE KWTDDQQADMAEE | 238  246 |
| Query 239 | | RFLVLYGIAAPDSQRIAFYRLLD 261 F YGI D R FY LD |  |
| Sbjct | 247 | TFADRYGITL-DRDRERFYLHLD 268 | |

>TR:A0A0S4Q2S2 A0A0S4Q2S2_BLAVI Aminoglycoside 3'-phosphotransferase OS=Blastochloris viridis OX=1079 GN=aphA PE=4 SV=1

Length=187

Score = 99.4 bits (246), Expect = 3e-22

| Identities | = 63/183 (34%), Positives = 92/183 (50%), Gaps = 3/183 (2%) |  |
| --- | --- | --- |
| Query 83 | VVTEAGRDWLLLGEVPGQDLLSSHLAPAEKVSIMADAMRRLHTLDPATCPFDHQAKHRIE | 142 |
| Sbjct 7 | V ++++ VPG+ L + A + DA+R L + CPFD + R+ VAASEDAEFMITRGVPGEPLAARIAAGRPVTELFRDALRLLQAVPVEGCPFDSRIAARLR | 66 |
| Query 143 | RARTRMEAGLVDQD-DLDEEHQGLAPAELFARLKARMPDGEDLVVTHGDACLPNIMVENG | 201 |
| Sbjct 67 | A + GL D D D+ P +L A L A PD EDLV THGD C N+ V++ EAEYLIAHGLCADDCDFDQWPGFAEPRDLLAHLDANRPD-EDLVFTHGDLCDNNVFVDDR | 125 |
| Query 202 | RFSGFIDCGRLGVADRYQDIALATRDIAEELGGEWADRFLVLYGIAAPDSQRIAFYRLLD | 261 |
| Sbjct 126 | FID GR G+ADR+ DIA R++ +++ + AD FL G A + ++ F+ LD DNLYFIDLGRGGLADRWSDIAFILRNLRDDVSEDAADNFLKAIG-TADNREKRTFFEQLD | 184 |
| Query 262  Sbjct 185 | EFF 264 E F  ELF 187 |  |

>TR:A0A4Y9FV78 A0A4Y9FV78_9MICO Aminoglycoside 3'-phosphotransferase OS=Microbacterium paludicola OX=300019 GN=E4U02_08620 PE=3 SV=1

Length=247

Score = 100 bits (250), Expect = 3e-22

| Identities | | = 75/206 (36%), Positives = 103/206 (50%), Gaps = 28/206 (14%) |  |
| --- | --- | --- | --- |
| Query 60  Sbjct 61 | | LQDEAARLSWLAT-TGVPCAAVLDVVTEAGRDWLLLGEVPGQDLLSSHLAPAEKVSI--M  + +EA RL W A T VP TEA +WL+ + G+ ++ H +V++ + MAEEAERLRWAAPFTPVPRVRAHGE-TEA-EEWLVTEALAGESAVAPHWVARPEVAVQAI | 116  118 |
| Query 117  Sbjct 119 | | ADAMRRLHTLDPAT-CPFDHQAKHRIERARTRMEAGLVDQDDLDEEHQGLAPAELFARLK A+ +R LH P CPFD R RA R G+V D+L + AEGLRALHDALPVDDCPFDWSVPARASRAAGR---GIVLPDELRDP-------------- | 175  161 |
| Query 176 | | ARMPDGEDLVVTHGDACLPNIMV-ENGRFSGFIDCGRLGVADRYQDIALATRDIAEELGG P + LVV HGDAC PN ++ E+GR++ +D G LGVADR+ DIA+AT + G | 234 |
| Sbjct | 162 | ---PPTDRLVVCHGDACAPNTLLGEDGRWAAHVDLGSLGVADRWADIAVATMSLEWNYGP | 218 |
| Query | 235 | EWADRFLVLYGIAAPDSQRIAFYRLL 260 W D FL YGI PD R +YR L |  |
| Sbjct | 219 | GWDDLFLEAYGI-DPDPLRTTYYRAL 243 | |

>TR:A0A4R1D9F5 A0A4R1D9F5_9ACTN Aminoglycoside 3'-phosphotransferase OS=Streptomyces sp. KM273126 OX=2545247 GN=E0500_37965 PE=3 SV=1

Length=247

Score = 100 bits (250), Expect = 3e-22

| Identities | = 80/226 (35%), Positives = 106/226 (47%), Gaps = 27/226 (12%) |  |
| --- | --- | --- |
| Query 37 | FRLSAQGRPVLFVKTDLSGALNELQDEAARLSWLATTGVPCAAVLDVVTEAGRDWLLLGE | 96 |
| Sbjct 39 | FR+ G +F K +G+ +L EA RL W A P V D WL+ E FRIG-DGEGRMFAKWAPAGSGLDLAAEAERLRW-AGGFTPVPRVRDQGASEDGAWLVTHE | 96 |
| Query 97 | VPGQDLLSSHLA--PAEKVSIMADAMRRLH-TLDPATCPFDHQAKHRIERARTRMEAGLV | 153 |
| Sbjct 97 | + G +S P V + +R+LH +L CPF + R+ RAR AG+ LRGDSAVSPQWKADPDTAVRALGVGLRQLHESLPVEDCPFSWSVERRLGRAR---RAGIE | 153 |
| Query 154 | DQDDLDEEHQGLAPAELFARLKARMPDGEDLVVTHGDACLPNIMV-ENGRFSGFIDCGRL | 212 |
| Sbjct 154 | DL E P + LVV HGDAC PN ++ ++G +SG +D G L VPADLPEA PPVDRLVVCHGDACAPNTLLGDDGSWSGHVDMGAL | 196 |
| Query 213  Sbjct 197 | GVADRYQDIALATRDIAEELGGEWADRFLVLYGIAAPDSQRIAFYR 258 GVADR+ D+A+AT G W D L YGI APD +RI FYR GVADRWADLAVATWSTQWNYGPGWEDLLLDAYGI-APDPERIEFYR 241 |  |

>TR:A0A0M2HEX5 A0A0M2HEX5_9MICO Aminoglycoside 3'-phosphotransferase OS=Microbacterium trichothecenolyticum OX=69370 GN=neo PE=3 SV=1

Length=241

Score = 100 bits (249), Expect = 3e-22

| Identities | = 74/218 (34%), Positives = 103/218 (47%), Gaps = 28/218 (13%) |  |
| --- | --- | --- |
| Query 48 | FVKTDLSGALNELQDEAARLSWLAT-TGVPCAAVLDVVTEAGRDWLLLGEVPGQDLLSSH | 106 |
| Sbjct 43 | ++K A EA RL W A T VP VL+ + +W++ +PG + YLKYGPRNAETSFAGEAERLRWAARYTAVP--RVLEAGGDDTHEWMVTAALPGLSAVDPR | 100 |
| Query 107 | LAP--AEKVSIMADAMRRLHTLDPA-TCPFDHQAKHRIERARTRMEAGLVDQDDLDEEHQ | 163 |
| Sbjct 101 | P A V + + +R LH P CPFD RI A E + WIPDAATAVRAVGEGLRALHDALPVGDCPFDWGVPGRIANA EGR | 144 |
| Query 164 | GLAPAELFARLKARMPDGEDLVVTHGDACLPNIMV-ENGRFSGFIDCGRLGVADRYQDIA | 222 |
| Sbjct 145 | G+ E F P + LVV HGDAC PN ++ ++GR+SG +D G LGVADR+ DIA GIRVPEAFHDA PPIDRLVVCHGDACCPNTLIGDDGRWSGHVDLGALGVADRWADIA | 200 |
| Query 223  Sbjct 201 | LATRDIAEELGGEWADRFLVLYGIAAPDSQRIAFYRLL 260  +A+ A G W D + YGI PD +R+++YR L  VASMSTAWNYGEGWEDALIEAYGI-EPDRERLSYYREL 237 |  |

>TR:A0A1H7IXE6 A0A1H7IXE6_9ACTN Kanamycin kinase OS=Nonomuraea pusilla OX=46177 GN=SAMN05660976_00950 PE=3 SV=1

Length=276

Score = 101 bits (251), Expect = 3e-22

| Identities | = 82/242 (34%), Positives = 118/242 (49%), Gaps = 14/242 (6%) |  |
| --- | --- | --- |
| Query 24 | WAQQTIGCSDAAVFRLSAQGRPVLFVKTDLSGALN-ELQDEAARLSWLATTGVPCAAVLD | 82 |
| Sbjct 40 | W Q G + FRL G V + K +G +L EA RL+W V VL+ WRNQLGGLT FRLEGPGG-VRYAKWVAAGTPELDLPGEAERLAWAGRWAV-VPRVLE | 93 |
| Query 83 | VVTEAGRDWLLLGEVPGQDLLSSHLA--PAEKVSIMADAMRRLHTLDP-ATCPFDHQAKH | 139 |
| Sbjct 94 | + +A WL+ E PG+ + PA + + +R LH P A CP+D + LGADAEGAWLVTAEAPGRSAVEPRWMADPATAAAGIGRGLRLLHDALPVAECPYDWSVER | 153 |
| Query 140 | RIERARTRMEAGLVDQDDLDEEHQGLAPAELFARLKARMPDGEDLVVTHGDACLPNIMV- | 198 |
| Sbjct 154 | R+ RA R+ G D EH+ L+ E ARL P + LVV HGDAC+PN ++ RLPRADERVAEG-EGPADWSPEHRHLSLEEARARL-GDPPPIDRLVVCHGDACVPNTLLH | 211 |
| Query 199 | ENGRFSGFIDCGRLGVADRYQDIALATRDIAEELGGEWADRFLVLYGIAAPDSQRIAFYR | 258 |
| Sbjct 212 | ++G + +D LGVADR+ D+A+A G + YG+ PD++RIA+YR DDGTPASHVDLDSLGVADRWADLAVAAWSTEWNYGPGYDTILYAAYGV-EPDAERIAYYR | 270 |
| Query 259  Sbjct 271 | LL 260 LL  LL 272 |  |

>TR:A0A4Z1E7M6 A0A4Z1E7M6_9MICO Aminoglycoside phosphotransferase OS=Serinibacter arcticus OX=1655435 GN=SERN_1565 PE=3 SV=1

Length=248

Score = 100 bits (249), Expect = 4e-22

| Identities | = 70/203 (34%), Positives = 99/203 (49%), Gaps = 26/203 (13%) |  |
| --- | --- | --- |
| Query 60 | LQDEAARLSWLATTGVPCAAVLDVVTEAGRDWLLLGEVPGQDLLSSHLA--PAEKVSIMA | 117 |
| Sbjct 62 | + DEA RL+W A +P VL+ ++A +WL+ +PG+ + P V + MADEADRLAWAAPF-IPVPEVLEHGSDATHEWLVTRALPGRSAVDPRWIADPETAVRAVG | 120 |
| Query 118 | DAMRRLHTLDPAT-CPFDHQAKHRIERARTRMEAGLVDQDDLDEEHQGLAPAELFARLKA | 176 |
| Sbjct 121 | +R LH P CPFD RI AR R G+V PA + QGLRALHDALPVDGCPFDWGVPARIANARRR---GVV------------VPATMH----- | 160 |
| Query 177 | RMPDGEDLVVTHGDACLPNIMVEN-GRFSGFIDCGRLGVADRYQDIALATRDIAEELGGE | 235 |
| Sbjct 161 | P + LVV HGDAC+PN ++++ G + G +D G LGVADR+ D+A+A G EAPPIDRLVVCHGDACVPNTLIDDAGAWVGHVDLGALGVADRWADLAVAAMSTEWNYGPG | 220 |
| Query 236  Sbjct 221 | WADRFLVLYGIAAPDSQRIAFYR 258 WAD + YG+ PD R A+YR WADALVAAYGV-EPDPVRAAYYR 242 |  |

>TR:A0A4P7SLX7 A0A4P7SLX7_9CELL Aminoglycoside 3'-phosphotransferase OS=Cellulomonas

shaoxiangyii OX=2566013 GN=E5225_12125 PE=3 SV=1

Length=268

Score = 100 bits (250), Expect = 4e-22

| Identities | | = 71/207 (34%), Positives = 105/207 (51%), Gaps = 10/207 (5%) |  |
| --- | --- | --- | --- |
| Query 59  Sbjct 63 | | ELQDEAARLSWLAT-TGVPCAAVLDVVTEAGRDWLLLGEVPGQDLLSSH--LAPAEKVSI  +L EA RL+W A T VP VL + WL+ + G+ + + P E + DLAAEAERLAWAAPFTSVP--RVLASGADDAGSWLVTAALDGRSAVDPYWLARPVEAATA | 115  120 |
| Query 116  Sbjct 121 | | MADAMRRLHTLDPA-TCPFDHQAKHRIERARTRMEAGLVDQDDLDEEHQGLAPAELFARL  + +R LH P +CP+ + R+ RA ++AG EH+ + AE RL IGRGLRALHDALPVGSCPYAWSVRDRLGRALENLDAGDTPAS-WAPEHRAMTAAEARYRL | 174  179 |
| Query 175  Sbjct 180 | | KARMPDGEDLVVTHGDACLPN-IMVENGRFSGFIDCGRLGVADRYQDIALATRDIAEELG PD + LVV H DAC PN ++ ++G +G +D GRLGVADR+ D+A+A + G  -TDPPDADVLVVCHADACAPNTLLADDGSVTGHVDLGRLGVADRWADLAVAAWSVDWNHG | 233  238 |
| Query | 234 GEWADRFLVLYGIAAPDSQRIAFYRLL 260 | | |
|  |  | + YG+ PD +RIA+YRLL |  |
| Sbjct | 239 | PGYDHHVYAGYGV-EPDPERIAYYRLL 264 | |

>TR:A0A4U3MI75 A0A4U3MI75_9ACTN Aminoglycoside 3'-phosphotransferase OS=Herbidospora galbida OX=2575442 GN=FDA94_10735 PE=3 SV=1

Length=249

Score = 100 bits (248), Expect = 5e-22

| Identities | = 94/252 (37%), Positives = 120/252 (48%), Gaps = 30/252 (12%) |  |
| --- | --- | --- |
| Query 23 | DWAQQTIGCSDAAVFRLSAQGRPVLFVKTDLSGALNELQDEAARLSWLATTGVPCAAVLD | 82 |
| Sbjct 10 | DW G S A VFR SA G + G EL E RL+WLA VP VLD DWTPVGHGESGARVFR-SADG ARYAKCGPAAELTAERDRLAWLAGHDVPGQRVLD | 63 |
| Query 83 | VVTEAGRDWLLLGEVPGQDLLSSHLAPAEKVSI---MADAMRRLHTLDPATCPFDHQAKH | 139 |
| Sbjct 64 | T R L+ V G + + ++P E +A+A+RRLH L CPF WRTAGDRACLVTSAVDG--VPAHQVSPGELERAWQPIAEAVRRLHGLPG--CPFSRDLDW | 119 |
| Query 140 | RIERARTRMEAGLVDQDDLDEEHQGLAPAELFARLK ARMPDGEDLVVTHGDACLP | 194 |
| Sbjct 120 | + RA + G V+ D L EE G P EL ARL+ R + D VV HGD LP MLARAEDVVARGAVNPDFLPEEQVGTPPPELLARLRPELDLRREQEARDGVVCHGDLTLP | 179 |
| Query 195 | NIMV--ENGRFSGFIDCGRLGVADRYQDIAL--ATRD-IAEELGGEWADRFLVLYGIAAP | 249 |
| Sbjct 180 | N+++ E+ +GF+D GRLG AD + D+ L AT D I L E L+G+ P NVILDPESLTVAGFVDVGRLGRADPHADLVLLFATADEIRPGLPREG LFGL-DP | 232 |
| Query 250  Sbjct 233 | DSQRIAFYRLLD 261 D R+ FY LD DPGRLRFYLHLD 244 |  |

>TR:A0A0Q5CBG7 A0A0Q5CBG7_9MICO Aminoglycoside phosphotransferase OS=Frondihabitans sp. Leaf304 OX=1736329 GN=ASF54_09190 PE=3 SV=1

Length=237

Score = 99.4 bits (246), Expect = 8e-22

| Identities | = 71/217 (33%), Positives = 102/217 (47%), Gaps = 24/217 (11%) |  |
| --- | --- | --- |
| Query 48 | FVKTDLSGALNELQDEAARLSWLATTGVPCAAVLDVVTEAGRDWLLLGEVPGQDLLSSH- | 106 |
| Sbjct 37 | F K G +L+ E RL W A + P VL + DWL+ +PG+ + + FAKWAPHGRGLDLRAEEERLRW-ARSFTPVPTVLASGRDPDADWLVTQGLPGRSAVDARW | 95 |
| Query 107 | -LAPAEKVSIMADAMRRLHTLDPAT-CPFDHQAKHRIERARTRMEAGLVDQDDLDEEHQG | 164 |
| Sbjct 96 | P V+ + +RRLH P CPFD + R+ R+ + + RAEPERAVAALGTGLRRLHDALPVDECPFDWSVETRLARS EKATTKDPA | 144 |
| Query 165 | LAPAELFARLKARMPDGEDLVVTHGDACLPNIMV-ENGRFSGFIDCGRLGVADRYQDIAL | 223 |
| Sbjct 145 | L P +P + LVV HGD C PN ++ ++ ++G +D LGVADR+ DIA+ LLPF GPVPSVDRLVVCHGDPCAPNTLIADDASWAGHVDLDALGVADRWADIAV | 197 |
| Query 224 | ATRDIAEELGGEWADRFLVLYGIAAPDSQRIAFYRLL 260 AT + G W D FL YG+ PD+QR AFYR L |  |

Sbjct 198 ATMSLGWNYGPGWDDVFLQAYGV-KPDAQRTAFYRSL 233

>TR:A0A0Q6WPS9 A0A0Q6WPS9_9BURK Aminoglycoside 3'-phosphotransferase OS=Massilia sp. Root351 OX=1736522 GN=ASD15_27525 PE=3 SV=1

Length=255

Score = 99.8 bits (247), Expect = 9e-22

| Identities | | = 75/222 (34%), Positives = 115/222 (52%), Gaps = 11/222 (5%) |  |
| --- | --- | --- | --- |
| Query 45  Sbjct 43 | | PVLFVKTDLSGALNELQDEAARLSWLA-TTGVPCAAVLDVVTEAGRDWLLLGEVPGQDLL P +F T S ++ EAA + WL+ VP + + + ++++ VPG L PAVFAPTTYS VRREAAIMRWLSGRLNVPEVVLAE--SSGSHEYMITRAVPGVPLS | 103  95 |
| Query 104  Sbjct 96 | | SSHLAPAEKVSIMADAMRRLHTLDPATCPFDHQAKHRIERARTRMEAGLVDQDDLDEEHQ  + +++ +A+R+L ++ A CPFD A+ R+ + GL+D+D E ARIDTGQPVMALFLEALRQLQSVPAAECPFDAGARLRLRELDYLLGKGLIDEDYDLEPWP | 163  155 |
| Query 164  Sbjct 156 | | GLA-PAELFARLKARMPDGEDLVVTHGDACLPNIMVENGRFSGFIDCGRLGVADRYQDIA GLA P +L ARL A +P EDLV HGD N++V+ FID GR G+ADR+ DIA GLATPQDLVARLHATVPQ-EDLVFAHGDLGDSNVLVDKRDQLYFIDFGRGGLADRWMDIA | 222  214 |
| Query 223 | | LATRDIAEELGGEWADRFLVLYGIAAPDSQRIAFYRLLDEFF 264 A R++ EE A + LV G+ ++R F+ LDE F |  |
| Sbjct | 215 | FAHRNLREEESEAAAAQLLVSLGMPDAGAKR-EFFEQLDELF 255 | |

>TR:A0A543EQ83 A0A543EQ83_9MICO Kanamycin kinase OS=Microbacterium kyungheense OX=1263636 GN=FB391_3133 PE=3 SV=1

Length=239

Score = 99.4 bits (246), Expect = 9e-22

| Identities | = 68/200 (34%), Positives = 97/200 (49%), Gaps = 24/200 (12%) |  |
| --- | --- | --- |
| Query 63 | EAARLSWLATTGVPCAAVLDVVTEAGRDWLLLGEVPGQDLLSSHLAPAEKVSIMADAMRR | 122 |
| Sbjct 58 | EA RL W A P VL+ + +W++ +PG + + A V + +R EADRLEW-AVAYTPVPRVLEHGGDDVHEWMVTASLPGISAVDPRMDAATAVREVGRGLRA | 116 |
| Query 123 | LHTLDP-ATCPFDHQAKHRIERARTRMEAGLVDQDDLDEEHQGLAPAELFARLKARMPDG | 181 |
| Sbjct 117 | LH P A CPF+ RIE A R G+ D L + P LHDALPVADCPFEWTVPRRIENAGIR---GIHLPDALRDA PPV | 156 |
| Query 182 | EDLVVTHGDACLPNIMV-ENGRFSGFIDCGRLGVADRYQDIALATRDIAEELGGEWADRF | 240 |
| Sbjct 157 | + LVV HGDAC+PN ++ ++GR+SG +D G LGV DR+ DIA+A+ G W D DRLVVCHGDACVPNTLIGDDGRWSGHVDLGALGVGDRWADIAVASMSTVWNYGEGWEDAL | 216 |
| Query 241  Sbjct 217 | LVLYGIAAPDSQRIAFYRLL 260  + YGI PD +R+A+Y+ L  IEAYGI-QPDRERLAYYQEL 235 |  |

>TR:A0A1C4LUU9 A0A1C4LUU9_9ACTN Kanamycin kinase OS=Streptomyces sp. DvalAA-14 OX=1839759 GN=GA0115240_12337 PE=3 SV=1

Length=257

Score = 99.8 bits (247), Expect = 9e-22

| Identities | = 78/218 (36%), Positives = 103/218 (47%), Gaps = 28/218 (13%) |  |
| --- | --- | --- |
| Query 48 | FVKTDLSGALNELQDEAARLSWLAT-TGVPCAAVLDVVTEAGRDWLLLGEVPGQDLLSSH | 106 |
| Sbjct 59 | F K +G+ +L EAARL+W T VP + G WL+ +PG +S FAKWAPAGSGLDLAAEAARLAWAGRFTTVPRVEARGA--DHGGAWLVTRGLPGDSAVSPR | 116 |
| Query 107 | LA--PAEKVSIMADAMRRLHTLDP-ATCPFDHQAKHRIERARTRMEAGLVDQDDLDEEHQ | 163 |
| Sbjct 117 | P + + +R LH P A CPF HR+ERAR AG+ WLADPRTAARALGEGLRALHDRLPVAGCPFSWSVGHRLERAR---RAGVE---------- | 163 |
| Query 164 | GLAPAELFARLKARMPDGEDLVVTHGDACLPNIMVE-NGRFSGFIDCGRLGVADRYQDIA | 222 |
| Sbjct 164 | PA+ P + LVV HGD C PN ++ +G +SG +D G LGVADR+ D+A  --LPAD PPPPPPVDRLVVCHGDPCAPNTLLRADGSWSGHVDLGALGVADRWADLA | 216 |
| Query 223 | LATRDIAEELGGEWADRFLVLYGIAAPDSQRIAFYRLL 260  +AT G W D L YG+ APD +R AFYR L |  |

Sbjct 217 VATWSTEWNYGPGWEDTLLDAYGV-APDPERTAFYRRL 253

>TR:A0A2T0RA65 A0A2T0RA65_9ACTN Kanamycin kinase OS=Kineococcus rhizosphaerae OX=559628 GN=CLV37_101293 PE=3 SV=1

Length=277

Score = 100 bits (248), Expect = 9e-22

| Identities | | = 84/254 (33%), Positives = 115/254 (45%), Gaps = 45/254 (18%) |  |
| --- | --- | --- | --- |
| Query 12  Sbjct 59 | | PAAWVERLFGYDWAQQTIGCSDAAVFRLSAQGRPVLFVKTDLSGALNELQDEAARLSWLA PAAWV G FR+ R +F K +G +L EA R +W A PAAWVNAAGG TTFRVQG-ARGAVFCKWAPAGTGLDLAAEAERAAWAA | 71  104 |
| Query 72 | | TTGVPCAAVLDVVTEAGRDWLLLGEVPGQDLLSSHLAPAEKVSIMA--DAMRRLHTLDPA VP VL+ +A WL+ +PG ++ A +V++ A A+R H P | 129 |
| Sbjct | 105 | AF-VPVPRVLEHGEDASGSWLVTRALPGASAVAPQWVTAPQVAVPALGRALRWWHDAVPV | 163 |
| Query | 130 | T-CPFDHQAKHRIERARTRMEAGLVDQDDLDEEHQGLAPAELFARLKARMPDGEDLVVTH | 188 |
| Sbjct | 164 | CPFD +R+ RA + D L E APA + LVV H GGCPFDWSVPYRLTRA-------VGDTTGLHE-----APAV DRLVVCH | 199 |
| Query | 189 | GDACLPNIMVENGRFS--GFIDCGRLGVADRYQDIALATRDIAEELGGEWADRFLVLYGI | 246 |
| Sbjct | 200 | GDAC PN ++ +G GF+D +GVADR+ D+A+AT + G W + FL YG+ GDACSPNTLLADGSGEPVGFVDLAAVGVADRWADLAVATVSLDWNYGEGWQETFLAAYGV | 259 |
| Query | 247 | AAPDSQRIAFYRLL 260 APD R A+YR L |  |
| Sbjct | 260 | -APDPVRTAYYRAL 272 | |

>TR:V2JBJ8 V2JBJ8_9BURK Aminoglycoside 3'-phosphotransferase OS=Cupriavidus sp. HPC(L) OX=1217418 GN=B551_0203615 PE=3 SV=1

Length=266

Score = 99.8 bits (247), Expect = 1e-21

| Identities | | = 86/259 (33%), Positives = 116/259 (45%), Gaps = 33/259 (13%) |  |
| --- | --- | --- | --- |
| Query 24  Sbjct 15 | | WAQQTIGCSDAAVFRLSAQGRPVLFVKTDLSGALNELQDEAARLSWLATTGVPCAAVLDV W ++G S V+R GR F K EL E RL+WL G+ C VLD WMPVSMGESGDLVYRRD-DGRA--FAKVVPESRSAELAGERDRLAWLEGQGIACPKVLDW | 83  71 |
| Query 84  Sbjct 72 | | VTEAGRDWLLL GEVPGQDLLSSHLAPAEKVSIMADAMRRLHTLDPATCPFD  EAG + L+ ++PG DLL E M + LH L CPF+  -REAGNEVCLVMTAVPGVPAADLPGTDLL EAWPSMVRELGALHALSSGQCPFE | 134  123 |
| Query 135  Sbjct 124 | | HQAKHRIERARTRMEAGLVDQDDLDEEHQGLAPAELFARLKARMP DGEDLVVTHG  A + V+ D L +E + EL AR++ +P + VV HG RSLSAMFAHAVDVVNRDAVNPDFLPDEDKRTPAPELLARVERELPVRLAQEATSTVVCHG | 189  183 |
| Query 190  Sbjct 184 | | DACLPNIMVENG--RFSGFIDCGRLGVADRYQDIALATRDIAE--ELGGEWADRFLVLY- D CLPN MV+ R +G ID GRLG ADRY D+AL + E + + F +L+ DPCLPNFMVDPHTLRCTGIIDLGRLGRADRYADLALMVANAEESWQTPQQAEQAFAMLFD | 244  243 |
| Query 245 | | --GIAAPDSQRIAFYRLLD 261 G+ A D +R+AFY LD |  |
| Sbjct | 244 | TLGLTA-DRERLAFYLRLD 261 | |

>TR:A0A2W1Y1Y5 A0A2W1Y1Y5_9MICO Aminoglycoside 3'-phosphotransferase OS=Curtobacterium sp. MCBD17_040 OX=2175674 GN=DEI94_17160 PE=3 SV=1

Length=253

Score = 99.4 bits (246), Expect = 1e-21

Identities = 76/232 (33%), Positives = 107/232 (46%), Gaps = 28/232 (12%)

| Query | 33 | DAAVFRLSAQGRPVLFVKTDLSGALNELQDEAARLSWLATTGVPCAAVLDVVTEAGRDWL | 92 |
| --- | --- | --- | --- |
| Sbjct | 35 | D FRL +G +VK G +L E ARLSW A + P V+D + WL DGRTFRLG-EGHAAEYVKWAPHGVRLDLLGEIARLSW-AGSYTPVPRVVDHGEDPDGRWL | 92 |
| Query | 93 | LLGEVPGQDLLSSHLAPAEKVSIMA--DAMRRLHTLDP-ATCPFDHQAKHRIERARTRME  +PG+ + + +++A +R LH P A CPF+ RI R+ | 149 |

| Sbjct | 93 | RTVALPGRSAVDPRFQADPRPAVIAAGHGLRALHDALPVADCPFEWSVTQRIARSGAD-- | 150 |
| --- | --- | --- | --- |
| Query | 150 | AGLVDQDDLDEEHQGLAPAELFARLKARMPDGEDLVVTHGDACLPNIMV-ENGRFSGFID | 208 |
| Sbjct | 151 | D+ L P P+ + +VV HGD C PN +V E+G++ G +D  ---------DDTVAALGPR PEPDRIVVCHGDPCTPNTIVGEDGQWVGHVD | 191 |
| Query | 209 | CGRLGVADRYQDIALATRDIAEELGGEWADRFLVLYGIAAPDSQRIAFYRLL 260 LG+ADR+ DIA+AT + G W FL YGI APD +R +YR L |  |
| Sbjct | 192 | LDTLGIADRWADIAVATMALGWNFGDGWESLFLDAYGI-APDPERTRWYRAL 242 | |

>TR:A0A0W7WBL7 A0A0W7WBL7_9MICO Aminoglycoside phosphotransferase OS=Leucobacter sp. G161 OX=663704 GN=AUL38_06665 PE=3 SV=1

Length=273

Score = 99.8 bits (247), Expect = 1e-21

| Identities | | = 76/231 (33%), Positives = 109/231 (47%), Gaps = 8/231 (3%) |  |
| --- | --- | --- | --- |
| Query 37  Sbjct 44 | | FRLSAQGRPVLFVKTDLSGALN-ELQDEAARLSWLATTGVPCAAVLDVVTEAGRDWLLLG FR + G FVK +G +L EAARL W G V++ T A WL+ FRDAGPGDGARFVKWVAAGTPEIDLAAEAARLRWAGQHGAVAPTVIEQGTNAHGSWLITA | 95  103 |
| Query 96  Sbjct 104 | | EVPGQDLLSSH--LAPAEKVSIMADAMRRLH-TLDPATCPFDHQAKHRIERARTRMEAGL  + G+ +S P + +R LH TL +CP+ R + R+ AG ALAGESAVSPRWIAEPETAARAIGAGLRLLHDTLPVVSCPYRWDVTTRAAQFEQRIRAG- | 152  162 |
| Query 153 | | VDQDDLDEEHQGLAPAELFARLKARMPDGEDLVVTHGDACLPNIMVE-NGRFSGFIDCGR D ++GL+ A + L+ P D VV HGD C PN ++ +G F+G +D G | 211 |
| Sbjct | 163 | AGPADWAPAYRGLSVARVREILR-ESPAVADPVVCHGDPCAPNTLIGGDGTFAGHVDLGD | 221 |
| Query  Sbjct | 212  222 | LGVADRYQDIALATRDIAEELGGEWADRFLVLYGIAAPDSQRIAFYRLLDE 262 LGV DR+ D+A+A G + + YG+ A D++RIAFYRLL E LGVGDRWADLAVAAWSTEWNYGPGFDEHVYEGYGVTA-DAERIAFYRLLWE 271 |  |

>TR:A0A4R6RZT0 A0A4R6RZT0_9MICO Kanamycin kinase OS=Leucobacter luti OX=340320 GN=EDF62_1892 PE=3 SV=1

Length=273

Score = 99.8 bits (247), Expect = 1e-21

| Identities | | = 71/206 (34%), Positives = 100/206 (49%), Gaps = 7/206 (3%) |  |
| --- | --- | --- | --- |
| Query 59  Sbjct 67 | | ELQDEAARLSWLATTGVPCAAVLDVVTEAGRDWLLLGEVPGQDLLSSH--LAPAEKVSIM  +L+ EA RL+W G VL + WL+ + G+ ++ P + DLEAEAERLAWADEHGALVPRVLASGADESGSWLITAALAGESAVAPRWIAEPVTAARAI | 116  126 |
| Query 117 | | ADAMRRLH-TLDPATCPFDHQAKHRIERARTRMEAGLVDQDDLDEEHQGLAPAELFARLK  +R LH TL A CP+ A RIE+ R+ G + D H+ L A+ ARL | 175 |
| Sbjct | 127 | GAGLRILHDTLPVAECPYSWSAAERIEQFEQRLRTGS-GPSEWDPAHRHLTLADARARL- | 184 |
| Query | 176 | ARMPDGEDLVVTHGDACLPNIMVE-NGRFSGFIDCGRLGVADRYQDIALATRDIAEELGG | 234 |
| Sbjct | 185 | A +PD VV HGDAC PN +++ GRF+ +D G LGVAD + D+A+A G ADIPDLAHPVVCHGDACAPNTLLDATGRFTAHVDLGTLGVADPWADLAIAAWSTEWNYGT | 244 |
| Query | 235 | EWADRFLVLYGIAAPDSQRIAFYRLL 260  + D YGI D RI++YRLL |  |
| Sbjct | 245 | GFEDLVYEGYGIER-DPARISYYRLL 269 | |

>TR:A0A511J994 A0A511J994_9CELL Putative phosphotransferase OS=Cellulomonas composti OX=266130 GN=CCO02nite_12180 PE=3 SV=1

Length=226

Score = 98.6 bits (244), Expect = 1e-21

Identities = 76/203 (37%), Positives = 95/203 (47%), Gaps = 26/203 (13%)

| Query | 62 | DEAARLSWLATTGVPCAAVLDVVTEAGRDWLLLGEVPGQDLLSSHLA--PAEKVSIMADA | 119 |
| --- | --- | --- | --- |
| Sbjct | 42 | DEA RL W A P V+ ++ WL+ VPG + A P V + DEARRLRW-AEGRTPVPRVVSTGSDENGAWLVTATVPGTSAVDPRWAADPDTAVRAIGAG | 100 |
| Query | 120 | MRRLHTLDP-ATCPFDHQAKHRIERARTRMEAGLVDQDDLDEEHQGLAPAELFARLKARM  +R LH P A CPFD RI RA R G+V PA L | 178 |

| Sbjct | 101 | LRALHDALPVAGCPFDWGVPARIARAAAR---GVV------------VPASL VEA | 140 |
| --- | --- | --- | --- |
| Query | 179 | PDGEDLVVTHGDACLPNIMV-ENGRFSGFIDCGRLGVADRYQDIALATRDIAEELGGEWA | 237 |
| Sbjct | 141 | P + LVV HGDAC PN +V ++GR++ +D G LGVADR+ D+A+AT G PPVDRLVVCHGDACAPNTLVGDDGRWTAHVDLGALGVADRWADLAIATWSTVWNYGPGHE | 200 |
| Query  Sbjct | 238  201 | DRFLVLYGIAAPDSQRIAFYRLL 260 D L YGI APD R A+YR L DALLDAYGI-APDDVRTAYYRAL 222 |  |

>TR:A0A2H3KMG1 A0A2H3KMG1_9CHLR APH(3') family aminoglycoside O-phosphotransferase OS=Candidatus Chloroploca asiatica OX=1506545 GN=A9Q02_13060

PE=3 SV=1

Length=265

Score = 99.4 bits (246), Expect = 1e-21

| Identities | | = 76/253 (30%), Positives = 119/253 (47%), Gaps = 14/253 (6%) |  |
| --- | --- | --- | --- |
| Query 22  Sbjct 17 | | YDWAQQTIGCSDAAVFRLSAQGRPVLFVKTD---LSGALNELQDEAARLSWLATTGVPCA Y + T G S A+V++L + L++K +G +++ E + WL +P YRFVPDTAGMSPASVYQLIGRNDH-LYLKVSPRQYNGTTYDVEREKDVMLWLQGQ-LPVP | 78  74 |
| Query 79  Sbjct 75 | | AVLDVVTEAGRDWLLLGEVPG---QDLLSSHLAPAEKVSIMADAMRRLHTLDPATCPFDH VL G +LL+ V G + H P V I +D ++R H + CPFD EVLHFEQYEGNKFLLMRAVDGVVGSEDYKHHRDPKRMVHIYSDGIKRFHAIRIEDCPFDS | 135  134 |
| Query 136  Sbjct 135 | | QAKHRIERARTRMEAGLVD--QDDLDEEHQGLAPAELFARLKARMPDGEDLVVTHGDACL  +R+ + L D ++ +++ P L+A L+ P+ E+LV +HGD SVDYRLAELAYFLRHNLADINTENWEDDTPFADPDALYAFLQTHKPN-EELVFSHGDFGD | 193  193 |
| Query 194  Sbjct 194 | | PNIMVENGRFSGFIDCGRLGVADRYQDIALATRDIAEELGGE--WADRFLVLYGIAAPDS NI + N R SG ID GR G AD++ DIA + I ++G + + D L I PD SNIFIRNDRISGLIDLGRSGKADKWYDIAFCVQSIQHDIGNDKAYVDLLFNLLEI-EPDW | 251  252 |
| Query | 252 QRIAFYRLLDEFF 264 | | |
|  |  | +I +Y LLDE F |  |
| Sbjct | 253 | AKIRYYILLDELF 265 | |

>TR:A0A1H1RG07 A0A1H1RG07_9CELL Kanamycin kinase OS=Paraoerskovia marina OX=545619 GN=SAMN04489860_1344 PE=3 SV=1

Length=266

Score = 99.4 bits (246), Expect = 1e-21

| Identities | | = 72/206 (35%), Positives = 105/206 (51%), Gaps = 17/206 (8%) |  |
| --- | --- | --- | --- |
| Query 63  Sbjct 66 | | EAARLSWLATTGVPCAAVLDVVTEAGR---DWLLLGEVPGQDLLSSHLA--PAEKVSIMA EA R+SW A P AV +VV +A W++ +PG ++ P + EADRISWAA PHVAVPEVVGQASSPEGSWMVTRTLPGSSAVAERWLADPEAAARAVG | 117  121 |
| Query 118  Sbjct 122 | | DAMRRLH-TLDPATCPFDHQAKHRIERARTRMEAGLVDQDDL-DEEHQGLAPAELFARLK  +R H +L CP + R+ ARTR++ G D L EH+G + E + L RGLRTFHDSLPVEACPTEWSIAARVANARTRVDRG--DSRALWAPEHRGYSIGEALSLLA | 175  179 |
| Query 176  Sbjct 180 | | ARMPDGEDLVVTHGDACLPNIMV-ENGRFSGFIDCGRLGVADRYQDIALATRDIAEELGG  + P DLVV HGDACLPN ++ ++G +G +D G LGVADR+ D+A+A G S--PPPLDLVVCHGDACLPNTLLHDDGALAGHVDLGTLGVADRWADLAVAAWSTGWNYGE | 234  237 |
| Query 235 | | EWADRFLVLYGIAAPDSQRIAFYRLL 260  + D YG+ APD +I++YRLL |  |
| Sbjct | 238 | GYEDALYAGYGL-APDPAKISYYRLL 262 | |

```
>TR:A0A4Y9PX58 A0A4Y9PX58_9MICO Aminoglycoside 3'-phosphotransferase OS=Microbacterium sp. dk485 OX=2560021 GN=E4V99_07920 PE=3 SV=1
Length=255

Score = 99.0 bits (245), Expect = 2e-21
Identities = 73/219 (33%), Positives = 102/219 (47%), Gaps = 26/219 (12%) Query 46	VLFVKTDLSGALNELQDEAARLSWLATTGVPCAAVLDVVTEAGRDWLLLGEVPGQDLLSS 105

V ++K	A	+ EA R+ W A+	P	VL V +	+WL+	+PG	+
```

| Sbjct | 55 | VRYIKYGPLNAETSMAAEAERMKW-ASAYTPVPRVLAVGADDAHEWLVTSGLPGLSAVDP | 113 |
| --- | --- | --- | --- |
| Query | 106 | HLA--PAEKVSIMADAMRRLHTLDPAT-CPFDHQAKHRIERARTRMEAGLVDQDDLDEEH | 162 |
| Sbjct | 114 | PA V + +R LH P CPFD R+ A R G++ RWTAEPATAVRAIGRGLRALHEALPVDECPFDWTVPTRVANAAGR---GII--------- | 161 |
| Query | 163 | QGLAPAELFARLKARMPDGEDLVVTHGDACLPNIMV-ENGRFSGFIDCGRLGVADRYQDI | 221 |
| Sbjct | 162 | PA L P + LVV HGDAC PN +V ++G +SG +D G LGVADR+ DI  ---LPAAL REPPPIDRLVVCHGDACAPNTLVGDDGAWSGHVDLGALGVADRWADI | 213 |
| Query  Sbjct | 222  214 | ALATRDIAEELGGEWADRFLVLYGIAAPDSQRIAFYRLL 260 A+A G W D + YGI +PD +R+A+Y+ L AVAAMSTEWNYGPGWHDALVEAYGI-SPDRERLAYYQAL 251 |  |

>TR:A0A126Z700 A0A126Z700_9MICO Aminoglycoside phosphotransferase OS=Frondihabitans sp. PAMC 28766 OX=1795630 GN=AX769_02465 PE=3 SV=1

Length=246

Score = 98.6 bits (244), Expect = 2e-21

| Identities | = 75/218 (34%), Positives = 101/218 (46%), Gaps = 29/218 (13%) |  |
| --- | --- | --- |
| Query 48 | FVKTDLSGALNELQDEAARLSW-LATTGVPCAAVLDVVTEAGRDWLLLGEVPGQDLLSSH | 106 |
| Sbjct 49 | F K G +L E ARL W +A T VP VL T DWL+ VPG+ + FAKWAPHGVGLDLAAEEARLRWAVAYTSVP--RVLASGTGPDADWLVTAGVPGRSAVDPR | 106 |
| Query 107 | --LAPAEKVSIMADAMRRLHTLDPA-TCPFDHQAKHRIERARTRMEAGLVDQDDLDEEHQ | 163 |
| Sbjct 107 | P V+ + +R H P CPFD + R+ R+ + + WRAEPVRAVAALGQGLRAFHDALPVEACPFDWSVEMRLARS EKARRQDP | 155 |
| Query 164 | GLAPAELFARLKARMPDGEDLVVTHGDACLPNIMV-ENGRFSGFIDCGRLGVADRYQDIA | 222 |
| Sbjct 156 | GL P +P + LVV GD C PN ++ ++G +SG +D LGVADR+ DIA GLPP VPSLDRLVVCQGDPCSPNTLIADDGAWSGHVDLDALGVADRWADIA | 205 |
| Query 223  Sbjct 206 | LATRDIAEELGGEWADRFLVLYGIAAPDSQRIAFYRLL 260  +AT + G W FL YGI PDS+R +FYR L  VATMSLEWNYGLGWDAAFLEAYGI-EPDSERTSFYRAL 242 |  |

>TR:I0V1B4 I0V1B4_9PSEU Aminoglycoside phosphotransferase OS=Saccharomonospora xinjiangensis XJ-54 OX=882086 GN=SacxiDRAFT_1674 PE=3

SV=1

Length=270

Score = 99.0 bits (245), Expect = 2e-21

| Identities | | = 80/229 (35%), Positives = 111/229 (48%), Gaps = 9/229 (4%) |  |
| --- | --- | --- | --- |
| Query 37  Sbjct 42 | | FRLSAQGRPVLFVKTDLSGALN-ELQDEAARLSWLATTGVPCAAVLDVVTEAGRDWLLLG FRL A+ +VK G +L EA RL+W A+ V VL + A WL+ FRLDARIGGSRYVKWVAHGTPEIDLAAEADRLAW-ASRWVRVPRVLGQGSNAEGSWLVTA | 95  100 |
| Query 96  Sbjct 101 | | EVPGQDLLSSHLA--PAEKVSIMADAMRRLHTLDP-ATCPFDHQAKHRIERARTRMEAGL VPG+ + + PA + + +R LH P A CP+ R+ RA R+ G PVPGRSAVDTRWLADPATAATAIGHGLRVLHDALPVAECPYTWSVADRLARADERIADG- | 152  159 |
| Query 153 | | VDQDDLDEEHQGLAPAELFARLKARMPDGEDLVVTHGDACLPNIMV-ENGRFSGFIDCGR D EH+ L AE L P + LVV HGDAC PN ++ ++G F+ +D G | 211 |
| Sbjct | 160 | EGPGDWFPEHRHLTVAEARGLLDDA-PPVDRLVVCHGDACAPNTLLHDDGTFAAHVDLGS | 218 |
| Query  Sbjct | 212  219 | LGVADRYQDIALATRDIAEELGGEWADRFLVLYGIAAPDSQRIAFYRLL 260 LGVADR+ D+A+A G + YG+ PD +RIA+YRLL LGVADRWADLAVAAWSTEWNHGPGYDHLVYAAYGV-EPDPERIAYYRLL 266 |  |

>TR:D2B2K4 D2B2K4_STRRD Aminoglycoside 3'-phosphotransferase OS=Streptosporangium roseum (strain ATCC 12428 / DSM 43021 / JCM 3005 /

NI 9100) OX=479432 GN=Sros_0454 PE=3 SV=1

Length=270

Score = 99.0 bits (245), Expect = 2e-21

Identities = 72/206 (35%), Positives = 104/206 (50%), Gaps = 8/206 (4%)

| Query | 59 | ELQDEAARLSWLATTGVPCAAVLDVVTEAGRDWLLLGEVPGQDLLSSHLA--PAEKVSIM | 116 |
| --- | --- | --- | --- |
| Sbjct | 65 | +L EA RL+W A V++ T+A WL+ VPG+ + PA + + DLPAEAERLAW-AQRWATVPRVIEHGTDADGAWLVTEAVPGRSAVDPRWIADPATAAAAI | 123 |
| Query | 117 | ADAMRRLHTLDPAT-CPFDHQAKHRIERARTRMEAGLVDQDDLDEEHQGLAPAELFARLK | 175 |
| Sbjct | 124 | +R LH + P CPFD R+ RA R+ G + EH+ L A+ AR+ GRGLRLLHDVLPVEQCPFDWSIDRRLARADERIADG-EEPAGWSPEHRHLDLAKARARI- | 181 |
| Query | 176 | ARMPDGEDLVVTHGDACLPNIMV-ENGRFSGFIDCGRLGVADRYQDIALATRDIAEELGG | 234 |
| Sbjct | 182 | P + LVV HGDAC+PN ++ ++G F+ +D G LGVADR+ D+A+ G GEPPAIDRLVVCHGDACVPNTLLHDDGTFAAHVDLGSLGVADRWADLAVTAWSTEWNYGP | 241 |
| Query  Sbjct | 235  242 | EWADRFLVLYGIAAPDSQRIAFYRLL 260  + YGI PD +RIA+YRLL  GYDGILYDAYGI-TPDPERIAYYRLL 266 |  |

>TR:A0A519GZG7 A0A519GZG7_9MICO Aminoglycoside 3'-phosphotransferase OS=Microbacterium sp. OX=51671 GN=EOO67_06710 PE=3 SV=1

Length=241

Score = 98.2 bits (243), Expect = 2e-21

| Identities | | = 72/216 (33%), Positives = 99/216 (46%), Gaps = 28/216 (13%) |  |
| --- | --- | --- | --- |
| Query 48  Sbjct 43 | | FVKTDLSGALNELQDEAARLSWLATTGVPCAAVLDVVTEAGRDWLLLGEVPGQDLLSSHL FVK A ++ EA RL W A + VLD +A +WL+ +PG + FVKHGPRNAETSMRAEAERLRW-AGLRIRVPEVLDTGEDADTEWLVTRAIPGLSAVDPRW | 107  101 |
| Query 108 | | A--PAEKVSIMADAMRRLHTLDPAT-CPFDHQAKHRIERARTRMEAGLVDQDDLDEEHQG PA V + + +R LH P CPFD R+ A R G+ + L | 164 |
| Sbjct | 102 | LTDPATAVRAVGEGLRALHDALPVDDCPFDWTVPRRLANAAAR---GITVPESL------ | 152 |
| Query | 165 | LAPAELFARLKARMPDGED-LVVTHGDACLPNIMV-ENGRFSGFIDCGRLGVADRYQDIA | 222 |
| Sbjct | 153 | R+P D LVV HGDAC PN ++ E+GR+ +D G LGV DR+ DIA  ------------RIPPPVDRLVVCHGDACCPNTLLGEDGRWLAHVDLGALGVGDRWADIA | 200 |
| Query | 223 LATRDIAEELGGEWADRFLVLYGIAAPDSQRIAFYR 258 | | |
|  |  | +A G W D + YG+ PD +R+A+YR |  |
| Sbjct | 201 | VAAMSTEWNYGPGWEDALVDAYGV-TPDRERMAYYR 235 | |

>TR:A0A1G7VTB2 A0A1G7VTB2_9MICO Kanamycin kinase OS=Microbacterium pygmaeum OX=370764 GN=SAMN04489810_0818 PE=3 SV=1

Length=244

Score = 98.2 bits (243), Expect = 3e-21

| Identities | = 71/220 (32%), Positives = 101/220 (46%), Gaps = 32/220 (15%) |  |
| --- | --- | --- |
| Query 48 | FVKTDLSGALNELQDEAARLSWLATTGVPCAAVLDVVTEAGRD---WLLLGEVPGQDLLS | 104 |
| Sbjct 46 | ++K G + L DE RL W P V +V+ G D WL+ +PGQ ++ YIKWAPRGDESSLADEVTRLRWAG PFLRVPEVIEYGGDDTHEWLVTAALPGQSAVA | 101 |
| Query 105 | SHLAP--AEKVSIMADAMRRLHTLDPAT-CPFDHQAKHRIERARTRMEAGLVDQDDLDEE | 161 |
| Sbjct 102 | H A V + + +R +H P CPF+ RIE A R G+ D L E PHWVADAATAVRAVGEGLRAMHDRLPVLDCPFEWSVASRIENAADR---GIRVPDTLREP | 158 |
| Query 162 | HQGLAPAELFARLKARMPDGEDLVVTHGDACLPNIMV-ENGRFSGFIDCGRLGVADRYQD | 220 |
| Sbjct 159 | P + LVV HGDAC PN ++ ++GR+ +D G+LG DR+ D  -----------------PPIDRLVVCHGDACCPNTLIGDDGRWLAHVDLGQLGTGDRWAD | 201 |
| Query 221  Sbjct 202 | IALATRDIAEELGGEWADRFLVLYGIAAPDSQRIAFYRLL 260 IA+A+ G W D + YG+ PD R+A+YR L IAVASMSTEWNYGPGWEDALIEAYGV-EPDRPRLAYYREL 240 |  |

>TR:X7YE21 X7YE21_MYCKA Phosphotransferase enzyme family protein OS=Mycobacterium kansasii 824 OX=1299328 GN=I547_2784 PE=3 SV=1

Length=235

Score = 97.8 bits (242), Expect = 3e-21

Identities = 74/218 (34%), Positives = 100/218 (46%), Gaps = 31/218 (14%)

| Query | 48 | FVKTDLSGALNELQDEAARLSWLATTGVPCAAVLDVVTEAGRDWLLLGEVPGQDLLSSH- | 106 |
| --- | --- | --- | --- |
| Sbjct | 35 | FVK G + + DEA RL W A P +VL + R WL G +PG + FVKVAGVGTV-DFADEARRLRWAAQY-TPVPSVLGFGLDGDRAWLRTGGLPGVSAVHPRW | 92 |
| Query | 107 | -LAPAEKVSIMADAMRRLH-TLDPATCPFDHQAKHRIERARTRMEAGLVDQDDLDEEHQG | 164 |
| Sbjct | 93 | AP V +A+ +R LH TL ++CPFD A R+ R A L D +D LAAPDVAVRAIAEGLRTLHDTLPVSSCPFDWSAAGRLARLSPAWRAKLGDPPPVDR---- | 148 |
| Query | 165 | LAPAELFARLKARMPDGEDLVVTHGDACLPNIMV-ENGRFSGFIDCGRLGVADRYQDIAL | 223 |
| Sbjct | 149 | +VV HGDAC PN ++ E+GR G +D +GVADR+ D+A+  -------------------VVVCHGDACSPNTLIDEHGRCCGHVDVDAMGVADRWADLAV | 189 |
| Query  Sbjct | 224  190 | ATRDIAEELGGE-WADRFLVLYGIAAPDSQRIAFYRLL 260 AT + G W F YG+ PD R+ +YR L ATLSLGWNYPGRGWDTMFFEAYGV-EPDPPRLDYYRRL 226 |  |

>TR:A0A0Q5RH54 A0A0Q5RH54_9MICO Aminoglycoside phosphotransferase OS=Curtobacterium sp. Leaf183 OX=1736291 GN=ASG04_10065 PE=3 SV=1

Length=261

Score = 98.2 bits (243), Expect = 4e-21

| Identities | | = 76/202 (38%), Positives = 101/202 (50%), Gaps = 27/202 (13%) |  |
| --- | --- | --- | --- |
| Query 63  Sbjct 71 | | EAARLSWLATTGVPCAAVLDVVTEAGRDWLLLGEVPGQDLLSSHLA--PAEKVSIMADAM EA RL W A + VLD + WL VPG + P V +A+ + EAERLRW-AGRWLRVPEVLDHGADDDGAWLRTRAVPGWSAVDPRWRDEPRTAVVAIAEGL | 120  129 |
| Query 121  Sbjct 130 | | RRLHTLDP-ATCPFDHQAKHRIERARTRMEAGLVDQDDLDEEHQGLAPAELFARLKARMP R +H P A CPFD A R++R+R AG+ D+D L PA P RAMHDALPVAECPFDWSAAMRLDRSRA---AGV----DVDR----LGPA P | 179  168 |
| Query 180  Sbjct 169 | | DGEDLVVTHGDACLPNIMV-ENGRFSGFIDCGRLGVADRYQDIALATRDIAEELGGEWAD D + LVV HGDAC PN ++ ++GR+ G +D G LGVADR+ DIA+AT + G W D DPDVLVVCHGDACSPNTLLGDDGRWIGHVDLGSLGVADRWADIAVATMALGWNYGPGWDD | 238  228 |
| Query 239 | | RFLVLYGIAAPDSQRIAFYRLL 260 F YG+ D +R A+YR L |  |
| Sbjct | 229 | VFHGAYGVMV-DRERTAWYRAL 249 | |

>TR:A0A2S9QHZ7 A0A2S9QHZ7_9RHIZ Aminoglycoside 3'-phosphotransferase OS=Labrys okinawensis OX=346911 GN=aph(3'') PE=3 SV=1

Length=272

Score = 98.2 bits (243), Expect = 5e-21

| Identities | = 87/257 (34%), Positives = 116/257 (45%), Gaps = 22/257 (9%) |  |
| --- | --- | --- |
| Query 21 | GYDWAQQTIGCSDAAVFRLSAQGRPVLFVKTDLSGALNELQDEAARLSWLATTGVPCAAV | 80 |
| Sbjct 17 | G +W + G S +V+R S G + D GAL L+ E + WL+ + + GKEWLAVSHGESGDSVYRRS-DGAAFAKISQD-RGAL-ALEGERRPIEWLSRFDLGSPQM | 73 |
| Query 81 | LDVVTEAGRDWLLLGEVPG---QDLLSSHLAPAEKVSIMADAMRRLHTLDPATCPFDHQA | 137 |
| Sbjct 74 | LD L+ VPG DL L A +A + LH L CPF+ LDWRIGEEGACLITSAVPGVPASDLDGRQLLQAWPS--IAARIGALHALAAEDCPFERGL | 131 |
| Query 138 | KHRIERARTRMEAGLVDQDDLDEEHQGLAPAELFARLKARMP DGEDLVVTHGDAC | 192 |
| Sbjct 132 | + RA + G V+ D L E Q PA+L L+A + D VV HGDAC STMLARAADVVSRGAVNPDFLRPEQQETDPADLLLALEAEREKRLAEEAGDRVVCHGDAC | 191 |
| Query 193 | LPNIMVENG--RFSGFIDCGRLGVADRYQDIALATRDIAEELGG EWADRFLVLY | 244 |
| Sbjct 192 | +PN MV+ R +G ID GRLG ADRY D+AL + E G +A F VL MPNFMVDPATLRCTGMIDLGRLGTADRYVDLALMLANAGESWSGPDQAQAAFASLFEVL- | 250 |
| Query 245  Sbjct 251 | GIAAPDSQRIAFYRLLD 261 G+ PD +R+ FY LD GLERPDRERLDFYLSLD 267 |  |

>TR:A0A1T5M0Z6 A0A1T5M0Z6_9CLOT Aminoglycoside phosphotransferase OS=Maledivibacter halophilus OX=36842 GN=SAMN02194393_03673 PE=4 SV=1

Length=93

Score = 93.2 bits (230), Expect = 5e-21

Identities = 47/89 (53%), Positives = 55/89 (62%), Gaps = 1/89 (1%)

| Query | 177 | RMPDGEDLVVTHGDACLPNIMVENGRFSGFIDCGRLGVADRYQDIALATRDIAEELG-GE 235 |
| --- | --- | --- |
| Sbjct | 5 | + PD ED V THGD C+ NI++ + SGFID GR GV+DRYQDIALA R G + KKPDNEDFVFTHGDYCMANIILLGNKLSGFIDLGRAGVSDRYQDIALAVRSFEHNFGTDK 64 |
| Query | 236 | WADRFLVLYGIAAPDSQRIAFYRLLDEFF 264 |
| Sbjct | 65 | W D F YGI D +I FY LLDE F  WNDLFYKEYGIEDVDYSKIEFYILLDELF 93 |

>TR:A0A3N1HSX5 A0A3N1HSX5_9ACTN Kanamycin kinase OS=Pseudokineococcus lusitanus OX=763993 GN=EDC03_0191 PE=3 SV=1

Length=258

Score = 97.8 bits (242), Expect = 5e-21

| Identities | | = 75/221 (34%), Positives = 111/221 (50%), Gaps = 26/221 (12%) |  |
| --- | --- | --- | --- |
| Query 45  Sbjct 54 | | PVLFVK-TDLSGALNELQDEAARLSWLATTGVPCAAVLDVVTEAGRDWLLLGEVPGQDLL P VK T A ++ EA RL WLA V V+ V + G L+ +PG+ + PARHVKWTPHGAAALDIPAEAGRLRWLAGR-VVVPEVVAVEHDDGGTVLVTATLPGRSAV | 103  112 |
| Query 104 | | SSHLAPAEKVSIMA--DAMRRLHTLDPAT-CPFDHQAKHRIERARTRMEAGLVDQDDLDE A +V++ A +R H P CPF A+HR+ R R + | 160 |
| Sbjct | 113 | DPRWLAAPEVAVTAVGRGLRAFHDALPVEGCPFSWTAEHRLARLRPDV------------ | 160 |
| Query | 161 | EHQGLAPAELFARLKARMPDGEDLVVTHGDACLPNIMVEN-GRFSGFIDCGRLGVADRYQ | 219 |
| Sbjct | 161 | PAE+ ARL +PD + LVV HGDAC+PN ++++ GR + +D G LG+ADR+  ------PAEVRARL-GDVPDVDHLVVAHGDACMPNTLLDDDGRPAAHVDVGALGLADRWA | 213 |
| Query  Sbjct | 220  214 | DIALATRDIAEELGGEWADRFLVLYGIAAPDSQRIAFYRLL 260 D+A+A+ A +G + D L YG+ D+ R+A+YR L DLAVASWSTAWNVGPGYEDLLLEAYGVERDDA-RVAYYREL 253 |  |

>TR:A0A371NNW3 A0A371NNW3_9MICO Aminoglycoside 3'-phosphotransferase OS=Microbacterium sp. NEAU-LLE OX=2293570 GN=DY023_16105 PE=3 SV=1

Length=245

Score = 97.4 bits (241), Expect = 5e-21

| Identities | = 71/226 (31%), Positives = 104/226 (46%), Gaps = 26/226 (12%) |  |
| --- | --- | --- |
| Query 37 | FRLSAQGRPVLFVKTDLSGALNELQDEAARLSWLATTGVPCAAVLDVVTEAGRDWLLLGE | 96 |
| Sbjct 36 | FR + V F+K A L+DEA R++W A + VLD + +WL+ FRATPAAGDVFFIKWGPRNAETSLRDEAERMTW-AGRFIRVPRVLDQGDDDTHEWLVTAA | 94 |
| Query 97 | VPGQDLLSSHLA--PAEKVSIMADAMRRLHTLDPAT-CPFDHQAKHRIERARTRMEAGLV | 153 |
| Sbjct 95 | + G+ ++ PA V + + +R LH PA+ CP+D RI A R G+ IDGESAVAPRWVAEPAVAVRAVGEGLRMLHDRLPASECPWDWGVPSRIANAAGR---GIR | 151 |
| Query 154 | DQDDLDEEHQGLAPAELFARLKARMPDGEDLVVTHGDACLPNIMV-ENGRFSGFIDCGRL | 212 |
| Sbjct 152 | + L E P E LVV H DAC PN ++ ++GR +D L VPEHLREA PPVERLVVCHADACCPNTLIGDDGRVLAHVDLAAL | 194 |
| Query 213  Sbjct 195 | GVADRYQDIALATRDIAEELGGEWADRFLVLYGIAAPDSQRIAFYR 258 G ADR+ DIA+A+ G W D + YG+ PD +R+A+YR GTADRWADIAVASMSTEWNYGPGWEDALIEAYGV-EPDRERLAYYR 239 |  |

>TR:A0A4R7VUS2 A0A4R7VUS2_9PSEU Streptomycin 3'-kinase OS=Actinophytocola oryzae OX=502181 GN=CLV71_104201 PE=3 SV=1

Length=253

Score = 97.4 bits (241), Expect = 6e-21

Identities = 88/256 (34%), Positives = 111/256 (43%), Gaps = 32/256 (13%)

| Query 24 | WAQQTIGCSDAAVFRLSAQGRPVLFVKTDLSGALNELQDEAARLSWLATTGVPCAAVLDV | 83 |
| --- | --- | --- |
|  | W + T G S A V R S GR K+ + EL E R++WLATTGVP V+D |  |
| Sbjct 7 | WTRVTSGESGAEVHR-SPDGRQ--HAKSVPAAREAELAGERDRIAWLATTGVPGPTVVDW | 63 |

| Query | 84 | VTEAGRDWLLLGEVPGQDLLSSHLAPAEKVSIMADAMRR LHTLDPATCP | 132 |
| --- | --- | --- | --- |
| Sbjct | 64 | + G L+ V G PA+ VS A +RR LH L CP HVDEGGACLVTSTVEG VPADSVS--AGTLRRAWPAIVELVGALHGLPAGDCP | 113 |
| Query | 133 | FDHQAKHRIERARTRMEAGLVDQDDLDEEHQGLAPAELFARLKARM PDGEDLVVT | 187 |
| Sbjct | 114 | F AR + G V+ L E Q PAEL ARL A + + D VV FSRDLAAMWAVARDVVARGAVNAAFLPVEQQSTPPAELLARLSAELETRLAQEPADTVVC | 173 |
| Query | 188 | HGDACLPNIMV--ENGRFSGFIDCGRLGVADRYQDIALATRDIAEELGGEWADRFLVLYG | 245 |
| Sbjct | 174 | HGD CLPNI++ + +G ID GRLG AD + D+AL + E E ++ HGDLCLPNIILTPDGSAVAGLIDLGRLGRADSHADLALLLTNARETWTSE-SEATAADEA | 232 |
| Query  Sbjct | 246  233 | IAAPDSQRIAFYRLLD 261 D R FY LD  FTDIDDDRRRFYLHLD 248 |  |

>TR:A0A4R2CRE3 A0A4R2CRE3_9ACTN Kanamycin kinase/aminoglycoside 3'-phosphotransferase-2 OS=Kribbella sp. VKM Ac-2500 OX=2512214 GN=EV642_101112

PE=4 SV=1

Length=211

Score = 96.3 bits (238), Expect = 7e-21

| Identities | = 80/252 (32%), Positives = 109/252 (43%), Gaps = 65/252 (26%) |  |
| --- | --- | --- |
| Query 20 | FGYDWAQQT-IGCSDAAVFRLSAQGRPVLFVKTDLSGALNELQDEAARLSWLATTGVPCA | 78 |
| Sbjct 10 | F W + +G S AA+ RL +G L+ K A + + DEA RL+WL TG PC FNARWGEPIHLGLSGAALVRLG-KGAETLYHK AGSGVDDEADRLAWLGATGFPCP | 63 |
| Query 79 | AVLDVVTEAGRDWLLLGEVPGQDLLSSHLAPAEK--VSIMADAMRRLHTLDPATCPFDHQ | 136 |
| Sbjct 64 | ++D G W+L E+PG+D A ++ MAD +R LH L CPF+ RIVD RGNGWMLTRELPGRDASQPWSASDRPGVLAAMADGLRALHQL--TECPFESP | 117 |
| Query 137 | AKHRIERARTRMEAGLVDQDDLDEEHQGLAPAELFARLKARMPDGEDLVVTHGDACLPNI | 196 |
| Sbjct 118 | GE VTHGD C PN+ FP GERAAVTHGDYCAPNV | 135 |
| Query 197 | MVE--NGRFSGFIDCGRLGVADRYQDIALATRDIAEELGGEW ADRFLVLYGIAAP | 249 |
| Sbjct 136 | ++ FSG +D GRLG+ D Y D+AL + ++ EL ++ A RF YG A P FIDPVTLSFSGVLDIGRLGLGDPYLDVALMVKSLSGELNPQYGGPPAARRFAETYG-ADP | 194 |
| Query 250  Sbjct 195 | DSQRIAFYRLLD 261  + RIA Y LD  EDPRIAHYIDLD 206 |  |

>TR:R9N4E7 R9N4E7_9FIRM APH domain-containing protein OS=Lachnospiraceae bacterium 10-1 OX=1235800 GN=C819_00316 PE=3 SV=1

Length=264

Score = 97.4 bits (241), Expect = 8e-21

| Identities | = 75/253 (30%), Positives = 123/253 (49%), Gaps = 28/253 (11%) |  |
| --- | --- | --- |
| Query 30 | GCSDAAVFRLSAQGRPVLFVKTDLSGALNELQDEAARLSWLATTGVPCAAVLDVVTEAGR | 89 |
| Sbjct 22 | G S+A VF G VL V+ + A NE R+ VP + + GMSEANVFIF---GNKVLKVQENNEEAANEY RMLQYLQGKVPMPRIYAYEIADKK | 73 |
| Query 90 | DWLLLGEVPGQDLLSSHLA--PAEKVSIMADAMRRLHTLDPATCPFDHQAKHRIERARTR | 147 |
| Sbjct 74 | +LL+ + G+ S P ++ADA++RL +++ + CP D + +++ +A SYLLMSKCEGKMACSHEYMENPFSLCKLLADALKRLWSINISDCPSDQRLSNKLLQAEYN | 133 |
| Query 148 | MEAGLVDQDDLDEEHQG LAPAELFARLKARMPDGEDLVVTHGDACLPNIMVENGRF | 203 |
| Sbjct 134 | ++ G VD D ++ + G P++L L P+ E+LV++HGD CLPNI + + IKNGYVDLDHVEPDTFGENGFRDPSQLLEWLYENRPE-EELVMSHGDFCLPNIFGIHDKV | 192 |
| Query 204 | SGFIDCGRLGVADRYQDIALATRDIAEELGGEWADRFLVLY GIAAPDS | 251 |
| Sbjct 193 | +G+ID G+ G+AD++ DIAL R I G++ Y GI AP+ TGYIDLGKAGIADKWCDIALCCRSILHNYSGKYGGNEAYAYTDYDESLLFQELGI-APNW | 251 |
| Query 252 | QRIAFYRLLDEFF 264  ++I +Y LLDE F |  |

Sbjct 252 EKIRYYILLDELF 264

>TR:A0A553JZ49 A0A553JZ49_9ACTN Aminoglycoside 3'-phosphotransferase OS=Tessaracoccus rhinocerotis OX=1689449 GN=FOJ82_10610 PE=3 SV=1

Length=268

Score = 97.4 bits (241), Expect = 8e-21

| Identities | = 81/233 (35%), Positives = 107/233 (46%), Gaps = 43/233 (18%) |  |
| --- | --- | --- |
| Query 48 | FVKTDLSGALNELQDEAARLSWLATTGVPCAAVLDVVTEAGRD WLLLGEVPGQDLL | 103 |
| Sbjct 55 | F K + +G+ L DE RL WLA P V G D WL+ + G+D + FAKWNPAGSPVSLDDERLRLEWLARN--PPTVAFPVPLGHGSDVDGEWLVTRGMAGRDAV | 112 |
| Query 104 | S--SHLAPAEKVSIMADAMRRLH-TLDPATCPFDHQAKHRIERARTRMEAGLVDQDDLDE | 160 |
| Sbjct 113 | S +H P S M + +R LH +LDPA CPFD R+ A AG SGPAHEHPEVAASAMGEGLRLLHDSLDPADCPFDWGVPARLRAAAL---AG--------- | 160 |
| Query 161 | EHQGLAPAELFARLKARMPDGEDLVVTHGDACLPN-IMVENGRFSGFIDCGRLGVADRYQ | 219 |
| Sbjct 161 | G+ PA L R P VV HGD C+PN ++ +G +G +D G LGVADR+  ---GVVPAALH RPPSLARAVVCHGDPCVPNTLLTPDGALAGIVDLGALGVADRWA | 212 |
| Query 220  Sbjct 213 | DIALATRDIAEELGGEWADR------------FLVLYGIAAPDSQRIAFYRLL 260 D+A+AT +A G D L YGI APD +R+ +YR L DLAVATWSLAWNFEGASPDGPVTGTPSELELVLLDAYGI-APDLERMRYYRAL 264 |  |

>TR:A0A2R5HEQ5 A0A2R5HEQ5_9MYCO Aminoglycoside phosphotransferase APH(3') OS=Mycobacterium montefiorense OX=154654 GN=MmonteBS_52710 PE=3

SV=1

Length=251

Score = 97.1 bits (240), Expect = 8e-21

| Identities | | = 89/268 (33%), Positives = 114/268 (43%), Gaps = 48/268 (18%) |  |
| --- | --- | --- | --- |
| Query 5  Sbjct 15 | | DGLHAGSP--AAWVERLFGYDWAQQTIGCSDAAVFRLSAQGRPVLFVKTDLSGALNELQD DG G P A WV G FRL FVK + + DGFAGGRPVQAVWVNDEGG VTFRLGLASSDREFVKV-ANARGTDFAG | 62  60 |
| Query 63  Sbjct 61 | | EAARLSWLATTGVPCAAVLDVVTEAGRDWLLLGEVPGQDLLSSHLAPAEKVSIMA--DAM EA RL W A V VL +A R WL +PG + + A V++ A + EARRLRWAARY-VAVPQVLGFGVDAERAWLYTRGLPGLSAVHARWVAAPDVAVRAIGAGL | 120  119 |
| Query 121  Sbjct 120 | | RRLHT-LDPATCPFDHQAKHRIERARTRMEAGLVDQDDLDEEHQGLAPAELFARLKARMP R LH L TCPF+ A R+ L+PA ARL+ P RELHDGLPTHTCPFEWSATSRLA---------------------ALSPA---ARLRVGEP | 179  155 |
| Query 180  Sbjct 156 | | DGED-LVVTHGDACLPNIMVEN-GRFSGFIDCGRLGVADRYQDIALATRDIAEELGGE-W D LVV HGDAC PN ++++ GR G +D G LGVADR+ D+A+AT + G W PPVDRLVVCHGDACAPNTLIDDDGRCCGHVDLGELGVADRWADLAVATLSLGWNYPGRAW | 236  215 |
| Query | 237 ADRFLVLYGIAAPDSQRIAFYRLLDEFF 264 | | |
|  |  | F YG+ APD RI +YR L + F |  |
| Sbjct | 216 | EAEFFTAYGV-APDPARIDYYRRLWQAF 242 | |

>TR:A0A4P6EN66 A0A4P6EN66_9MICO Aminoglycoside 3'-phosphotransferase OS=Xylanimonas allomyrinae OX=2509459 GN=ET495_07705 PE=3 SV=1

Length=255

Score = 97.1 bits (240), Expect = 9e-21

Identities = 83/239 (35%), Positives = 113/239 (47%), Gaps = 40/239 (17%)

| Query | 37 | FRLSAQG---RPVLFVKTDLSGALNELQDEAARLSWLATTGVPCAAVL DVVTEA | 87 |
| --- | --- | --- | --- |
| Sbjct | 38 | FRL A G P FVK +G L EA RL W A P VL D + FRLDAVGGSATPTRFVKWSRTG LAAEADRLRW-AAPWTPVPRVLAHGTAPDDTSPG | 92 |
| Query | 88 | GR--DWLLLGEVPGQDLLSSH--LAPAEKVSIMADAMRRLHTLDP-ATCPFDHQAKHRIE | 142 |
| Sbjct | 93 | G ++L+ VPG+ + APA + + +R +H P A CPFD + R+ GEPAEYLVTAAVPGRSAVDPRWLAAPATAARALGEGLRAMHDALPVAACPFDWGVRERL- | 151 |
| Query | 143 | RARTRMEAGLVDQDDLDEEHQGLAPAELFARLKARMPDGEDLVVTHGDACLPNIMVEN-G | 201 |

AR	E G	E+ ARL+ +PD + LVV HGDAC PN ++++ G

| Sbjct | 152 | -ARLSPEVG----------------PEVRARLRD-VPDVDRLVVCHGDACAPNTLLDDDG 193 |
| --- | --- | --- |
| Query | 202 | RFSGFIDCGRLGVADRYQDIALATRDIAEELGGEWADRFLVLYGIAAPDSQRIAFYRLL 260 |
| Sbjct | 194 | R+ +D GRLG ADR+ D+A+A G + YG+ PD+ RIA+YRLL  RWCAHVDLGRLGAADRWADLAVAAMSTTWNYGPGFGHLVYEGYGV-VPDAARIAYYRLL 251 |

>TR:A0A031FWM9 A0A031FWM9_9MICO Aminoglycoside phosphotransferase OS=Microbacterium oleivorans OX=273677 GN=BW34_00766 PE=3 SV=1

Length=241

Score = 96.7 bits (239), Expect = 9e-21

| Identities | = 70/215 (33%), Positives = 101/215 (47%), Gaps = 26/215 (12%) |  |
| --- | --- | --- |
| Query 48 | FVKTDLSGALNELQDEAARLSWLATTGVPCAAVLDVVTEAGRDWLLLGEVPGQDLLSSHL | 107 |
| Sbjct 43 | F+K + DEA R+SW A + V++ + +WL+ + G + FIKVGPVHLETSMADEAERMSWAAPF-IRVPTVVEQGDDGDTEWLVTRSITGLSAVHPQW | 101 |
| Query 108 | A--PAEKVSIMADAMRRLHTLDP-ATCPFDHQAKHRIERARTRMEAGLVDQDDLDEEHQG | 164 |
| Sbjct 102 | P V + + +R LH P A+CPFD RI A R G++ + G VAQPETAVRAIGEGLRALHERLPVASCPFDWTVPSRISNAAER---GVIVPE G | 151 |
| Query 165 | LAPAELFARLKARMPDGEDLVVTHGDACLPNIMV-ENGRFSGFIDCGRLGVADRYQDIAL | 223 |
| Sbjct 152 | L A P + LVV HGDAC PN +V E+GR+SG +D G+LG+ DR+ DIA+ LRAA PPIDVLVVCHGDACAPNTLVSEDGRWSGHVDMGQLGLGDRWGDIAV | 201 |
| Query 224  Sbjct 202 | ATRDIAEELGGEWADRFLVLYGIAAPDSQRIAFYR 258 A G W L YG+ PD +R+A+YR AAMSTGWNYGPGWGGLLLEAYGV-EPDEERLAYYR 235 |  |

>TR:A0A562V9F0 A0A562V9F0_9ACTN Kanamycin kinase/aminoglycoside 3'-phosphotransferase-2 OS=Stackebrandtia albiflava OX=406432 GN=LX16_0200

PE=3 SV=1

Length=260

Score = 97.1 bits (240), Expect = 1e-20

| Identities | = 83/256 (32%), Positives = 117/256 (46%), Gaps = 30/256 (12%) |  |
| --- | --- | --- |
| Query 22 | YDWAQQTIGCSDAAVFRLSAQGRPVLFVKT DLSGALNELQDEAARLSWLATTG | 74 |
| Sbjct 16 | +DW G S A V+RL+ G LFVK D G + EA RL WLA G HDWESIGDGYSGATVYRLT--GPSALFVKLAPWPVGPDTGG---DPTAEADRLRWLAANG | 70 |
| Query 75 | VPCAAVLDVVTEAGRDWLLLGEVPGQDLLSSHLAP--AEKVSIMADAMRRLHTLDPATCP | 132 |
| Sbjct 71 | VP D +L+ +PG+ P A V +AD LH+L CP VPAPEPWDAGVIGDTAFLVSAGLPGRSAAEPWPEPVRAAVVDAVADFAVWLHSLPVDGCP | 130 |
| Query 133 | FDHQAKHRIERARTRMEAGLVDQDDLDEEHQGLAPAELFARLKARMPDGEDLVVTHGDAC | 192 |
| Sbjct 131 | F + ++ + V +DD + A + A L A + +G + + HGD C FTRDVPVILAQS-----SEAVARDDSGDRR---AVLDRTATLAAGLTEGAE-ALCHGDFC | 181 |
| Query 193 | LPNIMVENGRFS--GFIDCGRLGVADRYQDIALATRDIAE ELGGEWADRFLVLYG | 245 |
| Sbjct 182 | LPN++++ + G +D GR G +D Y D+AL TR +A+ + G E+A RFL YG LPNVLLDPDSLAVTGVVDVGRFGRSDPYADLALVTRSLADHRLNPQYGPEYARRFLRRYG | 241 |
| Query 246  Sbjct 242 | IAAPDSQRIAFYRLLD 261 DS RI +R LD  ADPVDSDRIDLFRSLD 257 |  |

```
>TR:A0A399JCC3 A0A399JCC3_9MICC Aminoglycoside 3'-phosphotransferase OS=Galactobacter valiniphilus OX=2676122 GN=DWB68_11565 PE=3 SV=1
Length=246

Score = 96.7 bits (239), Expect = 1e-20
Identities = 72/230 (31%), Positives = 108/230 (47%), Gaps = 29/230 (13%)

Query 35	AVFRLSAQGRPVLFVKTDLSGALNELQDEAARLSWLATTGVPCAAVLDVVTEAGRDWLLL 94 AV	+ G	+++K	N + EAARLSW A	P	V+D ++ ++WL+
Sbjct 33	AVTVRAGDGPDAVYIKCGPLNDENSAEGEAARLSW-AGAHTPVPRVVDHGSDGKQEWLVT 91
```

| Query | 95 | GEVPGQDLLSSHL--APAEKVSIMADAMRRLHTLDP-ATCPFDHQAKHRIERARTRMEAG | 151 |
| --- | --- | --- | --- |
| Sbjct | 92 | +P ++ + AP V + +R LH P A C FD R+ AALPYRNAVDRRWISAPEPAVRALGAGLRALHEALPVAGCRFDWGTASRLG--------- | 142 |
| Query | 152 | LVDQDDLDEEHQGLAPAELFARLKARMPDGEDLVVTHGDACLPN-IMVENGRFSGFIDCG | 210 |
| Sbjct | 143 | D L PA P + LVV HGDAC PN ++ ++GR++ +D G  ----DLPPSGRAALGPA PSIDRLVVCHGDACAPNTLLTDDGRWAAHVDLG | 188 |
| Query | 211 | RLGVADRYQDIALATRDIAEELGGEWADRFLVLYGIAAPDSQRIAFYRLL 260 LGVADR+ D+A+A+ ++ G W F YGI APD++ +A+YR L |  |
| Sbjct | 189 | SLGVADRWADLAVASANLTLNYGPGWEATFFEAYGI-APDARLLAYYRAL 237 | |

>TR:A0A1S1NN44 A0A1S1NN44_9MYCO Aminoglycoside phosphotransferase APH(3') OS=Mycobacterium talmoniae OX=1858794 GN=BKN37_08910 PE=3 SV=1

Length=234

Score = 96.3 bits (238), Expect = 1e-20

| Identities | | = 86/258 (33%), Positives = 107/258 (41%), Gaps = 53/258 (21%) |  |
| --- | --- | --- | --- |
| Query 9  Sbjct 19 | | AGSP--AAWVERLFGYDWAQQTIGCSDAAVFRLSAQGRPVLFVKTDLSGALNELQDEAAR AG P A WV L G + +G DA F V DLS EA R AGRPVRAVWVNELGGVTFR---VGDRDAREF--------VKVSSADLSA EAER | 66  60 |
| Query 67  Sbjct 61 | | LSWLATTGVPCAAVLDVVTEAGRDWLLLGEVPGQDLLSSH--LAPAEKVSIMADAMRRLH L W A P VL V G WL +PG + P + V + +R LH LRW-AGRYRPVPPVLGV GDGWLHTAGLPGVSAVHPRWLARPRQAVRALGAGLRALH | 124  115 |
| Query 125  Sbjct 116 | | TLDPAT-CPFDHQAKHRIERARTRMEAGLVDQDDLDEEHQGLAPAELFARLKARMPDGED P CPFD R+ + AGL D P +  DGLPVDDCPFDWSVPARLAQLAPPRRAGLEDP PPIQQ | 183  152 |
| Query 184  Sbjct 153 | | LVVTHGDACLPNIMV-ENGRFSGFIDCGRLGVADRYQDIALATRDIAEELGGEWADRFLV LVV HGDAC PN ++ E+GR G +D G LGVADR+ D+A+AT + G+W D F LVVCHGDACAPNTLLDEDGRCCGHVDLGELGVADRWADLAVATLSLGWNYPGQWQDDFFT | 242  212 |
| Query 243 | | LYGIAAPDSQRIAFYRLL 260 YG+ PD RI +YR L |  |
| Sbjct | 213 | AYGV-EPDPVRIGYYRRL 229 | |

>TR:A0A498R2I3 A0A498R2I3_9MYCO Aminoglycoside 3'-phosphotransferase OS=Mycobacterium pseudokansasii OX=2341080 GN=neo PE=3 SV=1

Length=244

Score = 96.3 bits (238), Expect = 1e-20

| Identities | = 75/218 (34%), Positives = 100/218 (46%), Gaps = 31/218 (14%) |  |
| --- | --- | --- |
| Query 48 | FVKTDLSGALNELQDEAARLSWLATTGVPCAAVLDVVTEAGRDWLLLGEVPGQDLLSSHL | 107 |
| Sbjct 44 | FVK +G + + DEA RL W A P VL + R WL G +PG + FVKVAGAGTV-DFADEARRLHWAAQY-TPVPPVLGFGLDGDRAWLRTGGLPGVSAVHPRW | 101 |
| Query 108 | APAEKVSIMADA--MRRLH-TLDPATCPFDHQAKHRIERARTRMEAGLVDQDDLDEEHQG | 164 |
| Sbjct 102 | A +V++ A A +R LH TL +CPFD A R+ A L D +D LAAPEVAVCAIAVGLRTLHDTLPVPSCPFDWSATGRLATLAPARRAKLGDPPPVDR---- | 157 |
| Query 165 | LAPAELFARLKARMPDGEDLVVTHGDACLPNIMV-ENGRFSGFIDCGRLGVADRYQDIAL | 223 |
| Sbjct 158 | LVV HGDAC PN ++ E+G+ G +D LGVADR+ DIA+  -------------------LVVCHGDACAPNTLLDEHGQCCGHVDVDALGVADRWADIAV | 198 |
| Query 224  Sbjct 199 | ATRDIAEEL-GGEWADRFLVLYGIAAPDSQRIAFYRLL 260 AT + G +W F YG+ PD R+ +YR L ATLSLGWNYPGRDWDAEFFAAYGV-EPDPLRLDYYRRL 235 |  |

>TR:A0A6I6E2E6 A0A6I6E2E6_9MICO Aminoglycoside 3'-phosphotransferase OS=Microbacterium oryzae OX=743009 GN=D7D94_12160 PE=3 SV=1

Length=241

Score = 95.9 bits (237), Expect = 2e-20

Identities = 65/200 (33%), Positives = 97/200 (49%), Gaps = 26/200 (13%)

| Query | 63 | EAARLSWLATTGVPCAAVLDVVTEAGRDWLLLGEVPGQDLLSSHLAP--AEKVSIMADAM | 120 |
| --- | --- | --- | --- |
| Sbjct | 58 | E+ RL+W A V VL+ ++ +WL+ +PG+ + A V + + + ESERLAW-AAAWVRVPEVLEQGSDDTHEWLVTRALPGESAAAPRWTADAATAVRAVGEGL | 116 |
| Query | 121 | RRLHTLDPA-TCPFDHQAKHRIERARTRMEAGLVDQDDLDEEHQGLAPAELFARLKARMP | 179 |
| Sbjct | 117 | R LH P CP+ Q R+ AR R G+A E + P RALHDALPVEACPWSWQVPDRVANARRR----------------GVAVPEALRQ GP | 156 |
| Query | 180 | DGEDLVVTHGDACLPN-IMVENGRFSGFIDCGRLGVADRYQDIALATRDIAEELGGEWAD | 238 |
| Sbjct | 157 | + LVV HGD C PN ++ ++GR++G +D G LGVADR+ DIA+A G WAD SVDRLVVCHGDPCCPNTLLADDGRWTGHVDLGALGVADRWADIAVAAMSTEWNYGPGWAD | 216 |
| Query | 239 | RFLVLYGIAAPDSQRIAFYR 258  + YG+ PD +R+A+YR |  |
| Sbjct | 217 | ALVAAYGV-EPDRERMAYYR 235 | |

>TR:A0A1P8U4Z7 A0A1P8U4Z7_9MICO Aminoglycoside phosphotransferase APH(3') OS=Microbacterium aurum OX=36805 GN=BOH66_01830 PE=3 SV=1

Length=242

Score = 95.9 bits (237), Expect = 2e-20

| Identities | | = 69/220 (31%), Positives = 102/220 (46%), Gaps = 35/220 (16%) |  |
| --- | --- | --- | --- |
| Query 48  Sbjct 43 | | FVKTDLSGALNELQDEAARLSWL-ATTGVPCAAVLDVVTEAGRDWLLLGEVPGQDLLSSH  ++K A ++ EA R+ W A T VP VL+ + +WL+ + G+ + H YIKYGPQQAETSMRAEAERMEWAHAFTRVP--EVLESGEDDAHEWLVTRAIDGRSAVDPH | 106  100 |
| Query 107 | | LA---PAEKVSIMADAMRRLHTLDP-ATCPFDHQAKHRIERARTRMEAGLVDQDDLDEEH PA V + + +R LH P A CPFD + R+ A R | 162 |
| Sbjct | 101 | WLADDPATAVRAVGEGLRALHEALPVADCPFDWSVRSRVSHAAAR--------------- | 145 |
| Query | 163 | QGLAPAELFARLKARMPDG---EDLVVTHGDACLPNIMV-ENGRFSGFIDCGRLGVADRY | 218 |
| Sbjct | 146 | G+ RL + +G + LVV HGDAC PN ++ ++G + +D G LGV DR+  -GI RLPVALQEGPPVDQLVVCHGDACCPNTLLGDDGGWLAHVDLGALGVGDRW | 197 |
| Query  Sbjct | 219  198 | QDIALATRDIAEELGGEWADRFLVLYGIAAPDSQRIAFYR 258 DIA+A G W D + YG+ APD R+A+YR  ADIAVAAMSTEWNYGPGWEDALIAAYGV-APDRDRLAYYR 236 |  |

>TR:A0A0P0EIW2 A0A0P0EIW2_9MICO APH domain-containing protein OS=Microbacterium sp. No. 7 OX=1714373 GN=AOA12_18300 PE=3 SV=1

Length=253

Score = 95.9 bits (237), Expect = 2e-20

| Identities | | = 73/217 (34%), Positives = 103/217 (47%), Gaps = 29/217 (13%) |  |
| --- | --- | --- | --- |
| Query 47  Sbjct 59 | | LFVKTDLSGALNELQDEAARLSWLATTGVPCAAVLDVVTEAGRDWLLLGEVPGQDLLSSH L+ K + + L DEA RL W+A P AV+D++T+AG + LL +PG+ +S LYAKWNPVASGESLGDEAERLRWIAGRH-PAPAVVDLMTDAGEEVLLTRALPGESAVSER | 106  117 |
| Query 107 | | LA--PAEKVSIMADAMRRLHTLDPATCPFDHQAKHRIERARTRMEAGLVDQDDLDEEHQG A P + + +RRLH + CPFD HR+ A + AG V+ | 164 |
| Sbjct | 118 | WARDPDAALRALGTGLRRLHDVPLDGCPFDGGVAHRLRAAH--VPAGTVE---------- | 165 |
| Query | 165 | LAPAELFARLKARMPDGEDLVVTHGDACLPN-IMVENGRFSGFIDCGRLGVADRYQDIAL | 223 |
| Sbjct | 166 | PA P + LV+ GD C PN ++ +G F +D RLG ADR+ D+A+  --PA PPIDRLVLCQGDPCAPNTLLAGDGSFLAHVDLARLGAADRWADLAV | 213 |
| Query | 224 | ATRDIAEELGGEWADRFLVLYGIAAPDSQRIAFYRLL 260 T + G F YG A PD RIA+YR L |  |
| Sbjct | 214 | MTMSLEWNYRGYDEAVFWEAYG-AEPDPIRIAYYRRL 249 | |

>TR:A0A0B2ADP6 A0A0B2ADP6_9MICO Aminoglycoside phosphotransferase OS=Microbacterium mangrovi OX=1348253 GN=LK09_00280 PE=3 SV=1

Length=241

Score = 95.5 bits (236), Expect = 3e-20

Identities = 71/218 (33%), Positives = 100/218 (46%), Gaps = 28/218 (13%)

| Query | 48 | FVKTDLSGALNELQDEAARLSWLATTGVPCAAVLDVVTEAGRDWLLLGEVPGQDLLSSHL | 107 |
| --- | --- | --- | --- |
| Sbjct | 43 | +VK + DE ARL+W A P V+D +A +WL+ + G+ ++ YVKHGPRSPETSIADEVARLAW-AGRFTPVPRVIDSGRDATDEWLVTAAIEGESAVAPRW | 101 |
| Query | 108 | AP--AEKVSIMADAMRRLHTLDPAT-CPFDHQAKHRIERARTRMEAGLVDQDDLDEEHQG | 164 |
| Sbjct | 102 | A V + + +R LH P CPFD RI A TR G IADAATAVRAVGEGLRALHEALPVDDCPFDWSVPTRIANAATR G | 145 |
| Query | 165 | L-APAELFARLKARMPDGEDLVVTHGDACLPNIMVEN-GRFSGFIDCGRLGVADRYQDIA | 222 |
| Sbjct | 146 | + PA L P + VV HGDAC PN +++ G ++ +D G LGVADR+ DIA IRVPASL QDAPPVDVAVVCHGDACCPNTLLDAAGAWTAHVDLGALGVADRWADIA | 200 |
| Query  Sbjct | 223  201 | LATRDIAEELGGEWADRFLVLYGIAAPDSQRIAFYRLL 260  +A+ G W D + YG+ PD +RIA+YR L  VASMSTEWNYGPGWEDALIAAYGV-DPDRERIAYYRAL 237 |  |

>TR:A0A222XAW6 A0A222XAW6_9CELL Aminoglycoside phosphotransferase APH(3') OS=Cellulomonas sp. PSBB021 OX=2003551 GN=CBP52_08815 PE=3 SV=1

Length=251

Score = 95.5 bits (236), Expect = 3e-20

| Identities | = 74/221 (33%), Positives = 105/221 (48%), Gaps = 34/221 (15%) |  |
| --- | --- | --- |
| Query 47 | LFVKTDLSGALNELQDEAARLSWLATTGVPCAAVLDVVTEAGRD---WLLLGEVPGQDLL | 103 |
| Sbjct 49 | +FVK +G +L EA RL W + V +VVT+ D WL+ VPG+ + VFVKWAPAGTPFDLAAEAERLVWAGRF SPVPEVVTQGADDEGAWLVTRAVPGRSAV | 104 |
| Query 104 | SSH--LAPAEKVSIMADAMRRLHTLDPAT-CPFDHQAKHRIERARTRMEAGLVDQDDLDE | 160 |
| Sbjct 105 | P + V + +R LH P CPF + R+ R LD+ DPLWLARPLDAVRAVGAGLRALHDALPVVGCPFSWSVEDRLGR LDD | 150 |
| Query 161 | EHQGLAPAELFARLKARMPDGEDLVVTHGDACLPNIMV-ENGRFSGFIDCGRLGVADRYQ | 219 |
| Sbjct 151 | + A AE P + LVV HGDAC+PN ++ ++G + +D GRLGVADR+ RDRAAALAEA PPVDRLVVCHGDACVPNTLLSDDGAWLAHVDLGRLGVADRWA | 202 |
| Query 220  Sbjct 203 | DIALATRDIAEELGGEWADRFLVLYGIAAPDSQRIAFYRLL 260 D+A+AT G + D L YG+ APD +R A+YR L DLAIATWSTVWNYGPGYEDALLDAYGV-APDLERTAYYRAL 242 |  |

>TR:A0A542UQB3 A0A542UQB3_9CELL Kanamycin kinase OS=Cellulomonas sp. GX59 OX=1042957 GN=FBY24_0211 PE=3 SV=1

Length=269

Score = 95.9 bits (237), Expect = 3e-20

| Identities | = 83/246 (34%), Positives = 114/246 (46%), Gaps = 14/246 (6%) |  |
| --- | --- | --- |
| Query 21 | GYDWAQQTIGCSDAAVFRLSAQGRPVLFVKTDLSGALNELQDEAARLSWL-ATTGVPCAA | 79 |
| Sbjct 28 | G D + FRL A R V +V D A +L EAARL+W T VP GTDPVAVWVNARGGLTFRLDAH-RFVKWVPAD--AAELDLAAEAARLTWSRGWTSVP--E | 82 |
| Query 80 | VLDVVTEAGRDWLLLGEVPGQDLLSS---HLAPAEKVSIMADAMRRLHTLDPAT-CPFDH | 135 |
| Sbjct 83 | VLD+ + WL+ +PG+ + P E + + +R LH P CP+ VLDLGRDGEGSWLVTAALPGRSAVDPAWRDSRPVEAATAVGRGLRALHDALPVDLCPWAW | 142 |
| Query 136 | QAKHRIERARTRMEAGLVDQDDLDEEHQGLAPAELFARLKARMPDGEDLVVTHGDACLPN | 195 |
| Sbjct 143 | R RA ++AG EHQ L+ + ARL A P + LVV GD C PN SVHERTRRAAEHLDAG-DSPTSWSAEHQHLSADDARARLAAP-PAPDVLVVCQGDPCAPN | 200 |
| Query 196 | IMVEN-GRFSGFIDCGRLGVADRYQDIALATRDIAEELGGEWADRFLVLYGIAAPDSQRI | 254 |
| Sbjct 201 | ++++ G +G +D G LGVADR+ D+A+A G + YG+ PD RI TLLDDDGTVTGHVDLGTLGVADRWADLAVAGWSANWNYGPGHDEHVYAGYGV-DPDPVRI | 259 |
| Query 255  Sbjct 260 | AFYRLL 260 A+YRLL AYYRLL 265 |  |

>TR:A0A0Q4HAG9 A0A0Q4HAG9_9MICO APH domain-containing protein OS=Agromyces sp. Leaf222 OX=1735688 GN=ASE68_08455 PE=3 SV=1

Length=252

Score = 95.5 bits (236), Expect = 3e-20

| Identities | | = 73/217 (34%), Positives = 99/217 (46%), Gaps = 26/217 (12%) |  |
| --- | --- | --- | --- |
| Query 48  Sbjct 54 | | FVKTDLSGALNELQDEAARLSWLATTGVPCAAVLDVVTEAGRDWLLLGEVPGQDLLSSHL FVK + +G L DE RLSWL V E G +WL + +S+ FVKWNPNGTGESLADEERRLSWLRGRIAAPRVVASGHDERG-EWLATEAIDAASAVSAAW | 107  112 |
| Query 108  Sbjct 113 | | APAEKVSI--MADAMRRLH-TLDPATCPFDHQAKHRIERARTRMEAGLVDQDDLDEEHQG  + V+ M +RRLH +DP CPFD RI A E+ SARADVAARAMGTGLRRLHDAVDPDECPFDWSVAARIAVA EDSWL | 164  157 |
| Query 165  Sbjct 158 | | LAPAELFARLKARMPDGEDLVVTHGDACLPNIMVE-NGRFSGFIDCGRLGVADRYQDIAL  + P L P + LVV HGD C PN +++ G F+G +D RLGVADR+ D+A+ VVPPHL RDAPPIDRLVVCHGDPCAPNTLIDVRGAFAGIVDVARLGVADRWADLAV | 223  212 |
| Query 224 | | ATRDIAEELGGEWADRFLVLYGIAAPDSQRIAFYRLL 260  + + GG F YGI PD++RIA+YR L |  |
| Sbjct | 213 | GSWSLEWNFGGGSEPAFFEAYGI-EPDAERIAYYRAL 248 | |

>TR:A0A0D0IMT7 A0A0D0IMT7_9MICO Aminoglycoside phosphotransferase OS=Leucobacter komagatae OX=55969 GN=SD72_09380 PE=3 SV=1

Length=274

Score = 95.9 bits (237), Expect = 3e-20

| Identities | | = 79/250 (32%), Positives = 115/250 (46%), Gaps = 18/250 (7%) |  |
| --- | --- | --- | --- |
| Query 15  Sbjct 35 | | WVERLFGYDWAQQTIGCSDAAVFRLSAQGRPVLFVKTDLSGALNELQDEAARLSWLATTG WV L G + + A+ + A G P L +L EA RL+W G WVNGLGGLTFRDGGADGAAASYIKWVAAGTPEL DLAAEAERLAWARRHG | 74  83 |
| Query 75  Sbjct 84 | | VPCAAVLDVVTEAGRDWLLLGEVPGQDLLSSH--LAPAEKVSIMADAMRRLHTLDP-ATC VL +A WL+ + G+ ++ PA + +R LH L P A C AAVPEVLAHGADASGSWLITRALSGESAVAPTWIAEPATAARAIGAGLRALHELLPVAEC | 131  143 |
| Query 132  Sbjct 144 | | PFDHQAKHRIERARTRMEAGLVDQDDLDEEHQGLAPAELFARLKARMPDGEDLVVTHGDA PF+ RI R R+ +G D ++++ L+ A A L P + LVV HGDA PFEWAIPARIARFEERVASG-ESPADWSDDYRELSVATARALLHDP-PAVDRLVVCHGDA | 191  201 |
| Query 192  Sbjct 202 | | CLPNIMV-ENGRFSGFIDCGRLGVADRYQDIALATRDIAEELGGEWADRFLVLYGIAAPD C PN ++ +G F+ +D G LG+ADR+ D+A+A I G + YGI PD CAPNTLLGADGEFAAHVDLGSLGLADRWADLAVAAWSIDWNHGPGFDHLVYEGYGI-EPD | 250  260 |
| Query 251 | | SQRIAFYRLL 260  +RI++YRLL |  |
| Sbjct | 261 | QERISYYRLL 270 | |

>TR:U2X9J7 U2X9J7_9MICO Aminoglycoside phosphotransferase OS=Microbacterium sp. TS-1 OX=1344956 GN=MTS1_01447 PE=4 SV=1

Length=155

Score = 92.8 bits (229), Expect = 4e-20

Identities = 60/154 (39%), Positives = 79/154 (51%), Gaps = 23/154 (15%)

| Query | 109 | PAEKVSIMADAMRRLHTLDPAT-CPFDHQAKHRIERARTRMEAGLVDQDDLDEEHQGLAP | 167 |
| --- | --- | --- | --- |
| Sbjct | 19 | PA V + + +R LH PA CPFD +HR+ A R LD P PATAVRAVGEGLRALHDALPAAGCPFDWGVEHRLRTAAAR---------GLD VP | 63 |
| Query | 168 | AELFARLKARMPDGEDLVVTHGDACLPNIMVEN-GRFSGFIDCGRLGVADRYQDIALATR | 226 |
| Sbjct | 64 | A L A P+ + LVV HGDAC PN +V++ GR+ +D G LGVADR+ DIA+A ASLHAA PEPDLLVVCHGDACCPNSLVDDDGRWLAHVDLGALGVADRWADIAVAAM | 118 |
| Query  Sbjct | 227  119 | DIAEELGGEWADRFLVLYGIAAPDSQRIAFYRLL 260 G W D + YG+ PD +R+A+YR L  STEWNYGPGWEDALIAAYGV-TPDPERLAYYRSL 151 |  |

>TR:A0A1I6L1U7 A0A1I6L1U7_9MICO Kanamycin kinase OS=Agrococcus baldri OX=153730 GN=SAMN04487783_0067 PE=3 SV=1

Length=244

Score = 95.1 bits (235), Expect = 4e-20

| Identities | = 66/217 (30%), Positives = 100/217 (46%), Gaps = 33/217 (15%) |  |
| --- | --- | --- |
| Query 47 | LFVKTDLSGALNELQDEAARLSWLATTG-VPCAAVLDVVTEAGRDWLLLGEVPGQDLLSS | 105 |
| Sbjct 50 | ++ K +G+ L DEA R+ WLA G +P VLD ++A + L+ + ++S+ VYAKWSPAGSGESLADEAERMRWLAQAGPLPVPRVLDQASDAAGETLVTAAIDAASIVSA | 109 |
| Query 106 | H--LAPAEKVSIMADAMRRLHTLDPATCPFDHQAKHRIERARTRMEAGLVDQDDLDEEHQ | 163 |
| Sbjct 110 | P + + + +R LH L A CPF A LD AGLRDPERAAAALGEGLRCLHALPVADCPFP APDWTASTLDSAAD | 154 |
| Query 164 | GLAPAELFARLKARMPDGEDLVVTHGDACLPNIMVENGRFSGFIDCGRLGVADRYQDIAL | 223 |
| Sbjct 155 | G P +DLVV HGD C PN ++ +GRF+G +D GR+GV DR+ D+A+ GAGP DDLVVCHGDPCAPNTLLASGRFAGLVDLGRVGVGDRWSDLAI | 200 |
| Query 224  Sbjct 201 | ATRDIAEELGGEWADRFLVLYGIAAPDSQRIAFYRLL 260 A+ + + F YG+ PD+ RIA +R L ASWSLEWNGLADAEPAFWRAYGV-VPDAARIAGWRAL 236 |  |

>TR:E9SBN1 E9SBN1_RUMAL Putative kanamycin kinase OS=Ruminococcus albus 8 OX=246199 GN=CUS_5510 PE=3 SV=1

Length=256

Score = 95.1 bits (235), Expect = 5e-20

| Identities | = 64/212 (30%), Positives = 104/212 (49%), Gaps = 14/212 (7%) |  |
| --- | --- | --- |
| Query 62 | DEAARLSWLATTGVPCAAVLDVVTEAGRDWLLLGEVPGQDLLSSHL--APAEKVSIMADA | 119 |
| Sbjct 50 | +E L WL +P VL A + +LL+ ++ G+ AP + ++ADA EEVKMLKWLDGR-MPVPKVLYHGVSANKSYLLMTKIKGRMACDPIYLTAPELLIPLLADA | 108 |
| Query 120 | MRRLHTLDPATCPFDHQAKHRIERARTRMEAGLVDQDDLDEEHQGLAPAELFARLKARMP | 179 |
| Sbjct 109 | ++RL + D TCP + R++ +++ + +P ELF L P LKRLWSADITTCPRKRTLDVELSEIEPRLD--ILETTQFMRKEGFSSPHELFRWLIENRP | 166 |
| Query 180 | DGEDLVVTHGDACLPNIMVENGRFSGFIDCGRLGVADRYQDIALATRDIAEELGGEWA-- | 237 |
| Sbjct 167 | E+ V++HGD CLPNI + FSG+ID G G+AD+++DI+L R + + G +  Q-EEYVLSHGDFCLPNIFLSGESFSGYIDLGDAGIADKWRDISLCYRSLKHNVDGTYGRY | 225 |
| Query 238  Sbjct 226 | -----DRFLVLYGIAAPDSQRIAFYRLLDEFF 264 DR + GI D +++ +Y LLDE F EGVDPDRLFIELGI-KKDEKKLRYYLLLDELF 256 |  |

>TR:A0A0Q5QY89 A0A0Q5QY89_9NOCA Aminoglycoside phosphotransferase OS=Williamsia sp. Leaf354 OX=1736349 GN=ASG12_09470 PE=3 SV=1

Length=244

Score = 94.7 bits (234), Expect = 5e-20

| Identities | | = 67/203 (33%), Positives = 100/203 (49%), Gaps = 26/203 (13%) |  |
| --- | --- | --- | --- |
| Query 62  Sbjct 60 | | DEAARLSWLATTGVPCAAVLDVVTEAGRDWLLLGEVPGQDLLSSHL--APAEKVSIMADA DE A+L+W A V VLD + A +W++ + G+ ++ +P VS + DEVAKLAW-ARAFVVVPRVLDHGSTADAEWIVTEALDGRSAVAPEWIGSPEVAVSAVGRG | 119  118 |
| Query 120  Sbjct 119 | | MRRLHTLDP-ATCPFDHQAKHRIERARTRMEAGLVDQDDLDEEHQGLAPAELFARLKARM  +R++H P A C + K R+E+AR R GL +DLD+ LRQMHDALPVAQCGWAWGVKERLEQARAR---GLTLPEDLDDP----------------- | 178  158 |
| Query 179  Sbjct 159 | | PDGEDLVVTHGDACLPN-IMVENGRFSGFIDCGRLGVADRYQDIALATRDIAEELGGEWA P + LVV HGDAC PN I+ +G +G +D G LG ADR+ D+A+A G W PSVDRLVVCHGDACAPNSILASDGTPAGHVDLGSLGTADRWADLAVAAASTEWNYGPGWR | 237  218 |
| Query 238 | | DRFLVLYGIAAPDSQRIAFYRLL 260 R + YG+ PD +RI++Y L |  |
| Sbjct | 219 | HRLIEAYGL-EPDERRISYYERL 240 | |

>TR:A0A1I6MCT1 A0A1I6MCT1_9MICO Kanamycin kinase OS=Microbacterium sp. cf046 OX=1761803 GN=SAMN04487846_2680 PE=3 SV=1

Length=241

Score = 94.4 bits (233), Expect = 7e-20

| Identities | = 73/226 (32%), Positives = 103/226 (46%), Gaps = 31/226 (14%) |  |
| --- | --- | --- |
| Query 40 | SAQGRPVLFVKTDLSGALNELQDEAARLSWLATTG-VPCAAVLDVVTEAGRDWLLLGEVP | 98 |
| Sbjct 38 | +A GR F+K A EA RL+W T G P VL+V +A +WL+ TADGR---FIKYGPCNAETSFSAEAERLAW--TVGFTPVPRVLEVGADATHEWLVTTAAR | 92 |
| Query 99 | GQDLLSSHLA--PAEKVSIMADAMRRLHTLDPA-TCPFDHQAKHRIERARTRMEAGLVDQ | 155 |
| Sbjct 93 | G+ ++ PA V + +R LH PA CPF+ R+ A R G+ GESAVAPRWVAEPATAVRAVGAGLRALHEALPAQACPFEWSVPSRLANAAGR---GIRIP | 149 |
| Query 156 | DDLDEEHQGLAPAELFARLKARMPDGEDLVVTHGDACLPNIMV-ENGRFSGFIDCGRLGV | 214 |
| Sbjct 150 | L E P + LVV HGDAC PN ++ ++GR+S +D G LGV GALREP PPVDRLVVCHGDACCPNTLIGDDGRWSAHVDLGALGV | 192 |
| Query 215  Sbjct 193 | ADRYQDIALATRDIAEELGGEWADRFLVLYGIAAPDSQRIAFYRLL 260 DR+ DIA+A G W + + YG+ PD +R+ +YR L  GDRWADIAVAAMSTEWNYGPGWEELLIEAYGV-EPDGERLEYYREL 237 |  |

>TR:B2HML5 B2HML5_MYCMM Phosphotransferase OS=Mycobacterium marinum (strain ATCC BAA-535 / M) OX=216594 GN=MMAR_5380 PE=3 SV=1

Length=244

Score = 94.4 bits (233), Expect = 7e-20

| Identities | = 72/218 (33%), Positives = 98/218 (45%), Gaps = 31/218 (14%) |  |
| --- | --- | --- |
| Query 48 | FVKTDLSGALNELQDEAARLSWLATTGVPCAAVLDVVTEAGRDWLLLGEVPGQDLLSSHL | 107 |
| Sbjct 44 | F+K +G + EA RL W A+ + AVLD E WL +PG + FIKVARTGVF-DFTAEAQRLRW-ASAHLTVPAVLDFGVEQDWAWLRTAGLPGVSAVHPRW | 101 |
| Query 108 | APAEKVSIMADA--MRRLHTLDPA-TCPFDHQAKHRIERARTRMEAGLVDQDDLDEEHQG | 164 |
| Sbjct 102 | A V++ A +R LH P CPFD A+ R+ R A + +D VAAPDVAVRAIGVGLRTLHERLPVRACPFDWSAQSRLARLTAAGRANVGAPPPIDR---- | 157 |
| Query 165 | LAPAELFARLKARMPDGEDLVVTHGDACLPNIMV-ENGRFSGFIDCGRLGVADRYQDIAL | 223 |
| Sbjct 158 | LVV HGDAC PN ++ E+G G +D G LGVADR+ D+A+  -------------------LVVCHGDACAPNTLIAEDGNCCGHVDLGTLGVADRWADLAV | 198 |
| Query 224  Sbjct 199 | ATRDIAEELGGE-WADRFLVLYGIAAPDSQRIAFYRLL 260 AT + G W +F YG+ APD +RI +YR L ATLSLQWNFPGHGWEAKFFAAYGV-APDPRRIDYYRRL 235 |  |

>TR:A0A1G8AJS0 A0A1G8AJS0_9MICO Kanamycin kinase OS=Agrococcus jejuensis OX=399736 GN=SAMN04489720_0414 PE=3 SV=1

Length=245

Score = 94.4 bits (233), Expect = 7e-20

| Identities | | = 67/204 (33%), Positives = 96/204 (47%), Gaps = 25/204 (12%) |  |
| --- | --- | --- | --- |
| Query 60  Sbjct 59 | | LQDEAARLSWLATTGVPCAAVLDVVTEAGRDWLLLGEVPGQDLLSSHLA--PAEKVSIMA L EA R+ WLAT P V + V + L+ +PG +++ P V +A LAGEAERMRWLATRH-PAPLVREHVVVDDGELLVTEALPGDGAVANRWLTDPETAVRAIA | 117  117 |
| Query 118  Sbjct 118 | | DAMRRLHTLDPATCPFDHQAKHRIERARTRMEAGLVDQDDLDEEHQGLAPAELFARLKAR  + +RRLH L CP+ RI AR H + P L EGLRRLHALPTDDCPWTWLPADRISSARA---------------HGSIVPTPL EH | 177  157 |
| Query 178  Sbjct 158 | | MPDGEDLVVTHGDACLPNIMVEN-GRFSGFIDCGRLGVADRYQDIALATRDIAEELGGEW P + LVV HGDAC PN ++++ G F +D GRLG+ADR+ D+A+AT + G + APSIDHLVVGHGDACAPNTLLDDAGAFLATVDVGRLGLADRWSDLAVATMSLEWNYGPGF | 236  217 |
| Query | 237 ADRFLVLYGIAAPDSQRIAFYRLL 260 | | |
|  |  | F YG+ PD +R+A+YR L |  |
| Sbjct | 218 | EPAFWQAYGM-EPDVERVAYYRAL 240 | |

>TR:A0A2U1ZXR4 A0A2U1ZXR4_9MICO Aminoglycoside phosphotransferase APH(3') OS=Miniimonas sp. PCH200 OX=2135789 GN=C8046_14715 PE=3 SV=1

Length=249

Score = 94.4 bits (233), Expect = 8e-20

| Identities | | = 69/218 (32%), Positives = 103/218 (47%), Gaps = 28/218 (13%) |  |
| --- | --- | --- | --- |
| Query 45  Sbjct 48 | | PVLFVKTDLSGALNELQDEAARLSWLAT-TGVPCAAVLDVVTEAGRDWLLLGEVPGQDLL P ++K A + EA RL W T VP VL+ + +WL+ V G+ + PTRYLKWGPWNAETSMAAEAERLVWAGRYTRVP--EVLEHGADGEHEWLVTAAVAGRSAV | 103  105 |
| Query 104  Sbjct 106 | | SSHLA--PAEKVSIMADAMRRLHTLDP-ATCPFDHQAKHRIERARTRMEAGLVDQDDLDE PA V ++ + +R LH P A CPFD R+ A ++ G+ ++L E DPRWLADPATAVRVVGEGLRALHDALPVADCPFDWGVHARVANA---LQRGIEVPEELLE | 160  162 |
| Query 161 | | EHQGLAPAELFARLKARMPDGEDLVVTHGDACLPNIMV-ENGRFSGFIDCGRLGVADRYQ  P + LVV HGDAC+PN +V ++G ++G +D G LGVADR+ | 219 |
| Sbjct | 163 | P PSIDRLVVCHGDACVPNTLVHDDGAWAGHVDLGALGVADRWA | 205 |
| Query  Sbjct | 220  206 | DIALATRDIAEELGGEWADRFLVLYGIAAPDSQRIAFY 257 D+A+A G W+D + YG+ PD R A+Y DLAVAAMSTGWNYGPGWSDALIAAYGV-EPDPLRQAYY 242 |  |

>TR:A0A2N9JHG9 A0A2N9JHG9_9ACTN APH domain-containing protein OS=Micropruina glycogenica OX=75385 GN=MPLG2_2485 PE=3 SV=1

Length=253

Score = 94.4 bits (233), Expect = 9e-20

| Identities | | = 71/222 (32%), Positives = 100/222 (45%), Gaps = 31/222 (14%) |  |
| --- | --- | --- | --- |
| Query 43  Sbjct 45 | | GRPVLFVKTDLSGALNELQDEAARLSWLATTGVPCAAVLDVVTEAGRDWLLLGEVPGQDL G ++K G + E RL+W+A VP VL + WLL +PG+ GAGAAYLKAQRPGVDWQPDAERLRLAWVAPF-VPAPRVLAHGVQGDEHWLLTAGLPGRSA | 102  103 |
| Query 103  Sbjct 104 | | LSSHLA--PAEKVSIMADAMRRLHTLDPAT-CPFDHQAKHRIERARTRMEAGLVDQDDLD  ++ PA V + A+RR H P CPFD +R+ VALPWRDRPAVTVPQLGRALRRFHDTVPVNGCPFDWSVTYRVR----------------- | 159  146 |
| Query 160  Sbjct 147 | | EEHQGLAPAELFARLKARMPDGEDLVVTHGDACLPN-IMVENGRFSGFIDCGRLGVADRY H L E F RL + D VV HGDAC PN ++ ++G FSG++D G LGVADR+  --HYSLN--EAFVRLAPPL DAVVCHGDACNPNFLLTDDGAFSGYVDLGGLGVADRW | 218  198 |
| Query | 219 QDIALATRDIAEELGGEWADRFLVLYGIAAPDSQRIAFYRLL 260 | | |
|  |  | D+A A + G W FL YG+ D+ +++FY L |  |
| Sbjct | 199 | ADLAPALLSLGWNFGPGWQPTFLDAYGVTLDDA-KLSFYTAL 239 | |

>TR:A0A1G8EFL0 A0A1G8EFL0_9PSEU Kanamycin kinase OS=Pseudonocardia oroxyli OX=366584 GN=SAMN05216377_13514 PE=3 SV=1

Length=254

Score = 94.4 bits (233), Expect = 9e-20

| Identities | = 72/206 (35%), Positives = 101/206 (49%), Gaps = 18/206 (9%) |  |
| --- | --- | --- |
| Query 59 | ELQDEAARLSWLATTGVPCAAVLDVVTEAGRDWLLLGEVPGQDLLSSH--LAPAEKVSIM | 116 |
| Sbjct 59 | +L EA RL+W A V +L+ ++ WL+ +PG++ + + P V + DLAPEAERLAWAAPF-VTVPTLLEHGADSDGSWLVTAPLPGRNAVENRWLAEPERAVEAV | 117 |
| Query 117 | ADAMRRLHTLDP-ATCPFDHQAKHRIERARTRMEAGLVDQDDLDEEHQGLAPAELFARLK | 175 |
| Sbjct 118 | +R LH P A CPF R RAR + + D GL E R+ GAGLRALHDALPVAECPFSWDVATR--RARIQ NPDPHRFGLTLDEAL-RIL | 165 |
| Query 176 | ARMPDGEDLVVTHGDACLPNIMVEN-GRFSGFIDCGRLGVADRYQDIALATRDIAEELGG | 234 |
| Sbjct 166 | P+ + LVV HGDAC+PN ++++ GR G +D G LGVADR+ D+A AT G DDPPEADRLVVCHGDACVPNTLLDDDGRCCGHVDLGSLGVADRWADLAAATWSTIWNYGP | 225 |
| Query 235  Sbjct 226 | EWADRFLVLYGIAAPDSQRIAFYRLL 260 R L YG+ APD +R FYRLL  GHEHRLLDAYGV-APDPERQFFYRLL 250 |  |

>TR:A0A4R8QW26 A0A4R8QW26_9MYCO Aminoglycoside 3'-phosphotransferase OS=Mycobacteroides franklinii OX=948102 GN=neo_2 PE=3 SV=1

Length=240

Score = 94.0 bits (232), Expect = 9e-20

| Identities | = 74/228 (32%), Positives = 103/228 (45%), Gaps = 32/228 (14%) |  |
| --- | --- | --- |
| Query 37 | FRLSAQGRPVLFVKTDLSGALNELQDEAARLSWLATTGVPCAAVLDVVTEAGRDWLLLGE | 96 |
| Sbjct 36 | F+L A GR FVK +L+ EA +L+W A AVL V T+ WL FQLGA-GRGREFVKVSAPHPAIDLRAEAEKLTW-AGRYATVPAVLAVNTDGPLHWLHTAG | 93 |
| Query 97 | VPGQDLLSSHLA--PAEKVSIMADAMRRLHTLDPA-TCPFDHQAKHRIERARTRMEAGLV | 153 |
| Sbjct 94 | +PG + P V + +R LH L P CP+ RI R + IPGDSAVVPRWIDDPEPAVRAIGSGLRALHDLLPVGDCPYSWSVADRIAR VA | 145 |
| Query 154 | DQDDLDEEHQGLAPAELFARLKARMPDGEDLVVTHGDACLPNIMVEN-GRFSGFIDCGRL | 212 |
| Sbjct 146 | + D L E P + LVV HGDAC PN ++++ G + G +D G L NPDGLSEH PPIDHLVVCHGDACAPNTLLDDDGNWCGHVDFGEL | 188 |
| Query 213  Sbjct 189 | GVADRYQDIALATRDIAEELGGEWADRFLVLYGIAAPDSQRIAFYRLL 260 GVADR+ D+A+A+ + GG W F YG+ A D R+++YR L GVADRWADLAVASWSLEWNYGGGWDATFFEAYGVPA-DPDRMSYYRRL 235 |  |

>TR:D7B4D7 D7B4D7_NOCDD Aminoglycoside phosphotransferase OS=Nocardiopsis dassonvillei (strain ATCC 23218 / DSM 43111 / CIP 107115 /

JCM 7437 / KCTC 9190 / NBRC 14626 / NCTC 10488 / NRRL B-5397

/ IMRU 509) OX=446468 GN=Ndas_3531 PE=3 SV=1

Length=300

Score = 95.1 bits (235), Expect = 1e-19

| Identities | = 81/281 (29%), Positives = 119/281 (42%), Gaps = 45/281 (16%) |  |
| --- | --- | --- |
| Query 29 | IGCSDAAVFRL-SAQGRPVLFVKT---DLSGALNELQDEAARLSWLATTGVPCAAVLDVV | 84 |
| Sbjct 17 | +G S A V RL A+ RP +K+ D + EL + RL WLA G+P VLD VGLSGARVARLFDARDRPFAVLKSVDADRPDLVAELLGQERRLRWLAGRGLPVPHVLDSG | 76 |
| Query 85 | TEAGRDWLLLGEVPGQDLLSSHLAP--AEKVSIMADAMRRLHTLDPATCPFDHQAKHRIE | 142 |
| Sbjct 77 | AG WL + + G+ A + V ++A A R LH A CPFD ++E AWAGLRWLTMSHLDGRAAHEPWPAGDRSRVVDLLARAARALHRAPAAGCPFDMTLPAQLE | 136 |
| Query 143 | RARTRMEAGLVDQDDLDEEHQGLAPAELFARLK--ARMPDGEDLVVTHGDACLPNIMVEN | 200 |
| Sbjct 137 | RAR R+ +GLV++ +G AE+ + L AR + HGD CLPN++++ RARERVASGLVERSWSAAGREGPPAAEVLSELAGLARALGPGRTALVHGDYCLPNVLLDT | 196 |
| Query 201 | GRFSG FIDCGRLGVADRYQDIALATRD | 227 |
| Sbjct 197 | G G +D GR G+ D + D+A  GAVRGVAARTHRSAGGVPGPARPDGPPPAGDGRADGWALVDLGRAGLGDPHADLADMVGS | 256 |
| Query 228  Sbjct 257 | IAE----ELGGEWADRFLVLYGIAAPDSQRIAFYRLLDEFF 264  + + G A+RFL YG D +R+ ++ FF  LLSPMNPQFGPSDAERFLDAYGREDVDPERLRLCAGVNSFF 297 |  |

>TR:A0A0F2C8M1 A0A0F2C8M1_9MICO Aminoglycoside 3'-phosphotransferase OS=Microbacterium sp. SA39 OX=1263625 GN=neo PE=3 SV=1

Length=244

Score = 94.0 bits (232), Expect = 1e-19

| Identities | = 70/227 (31%), Positives = 105/227 (46%), Gaps = 29/227 (13%) |  |
| --- | --- | --- |
| Query 37 | FRLSAQGRPVLFVKTDLSGALNELQDEAARLSWLATTGVPCAAVLDVVTEAGRDWLLLGE | 96 |
| Sbjct 36 | FR + G P F+K LQDEA R++W A+ + VL+ + +WL+ FRATGAGDP-FFIKWGPRNLEFTLQDEAERMAW-ASRWITVPTVLEQGQDDTHEWLVTAA | 93 |
| Query 97 | VPGQDLLSSHLA--PAEKVSIMADAMRRLH-TLDPATCPFDHQAKHRIERARTRMEAGLV | 153 |
| Sbjct 94 | + G + PA V + +A+R +H TL CP++ RI A IEGLSAVDPRWVAEPATAVRAVGEALRAMHDTLPVDECPWEWSVASRIANA--------- | 144 |
| Query 154 | DQDDLDEEHQGL-APAELFARLKARMPDGEDLVVTHGDACLPNIMV-ENGRFSGFIDCGR | 211 |
| Sbjct 145 | E +G+ P EL P + L V+HGDAC PN ++ +GR+ +D  -------ERRGIRVPEELHTA PPIDRLAVSHGDACCPNTLIGADGRWVAHVDLSL | 192 |
| Query 212 | LGVADRYQDIALATRDIAEELGGEWADRFLVLYGIAAPDSQRIAFYR 258 LG ADR+ D+A+A+ G W D + YGI PD +R+A+YR |  |

Sbjct 193 LGTADRWADLAVASMSTEWNYGPGWEDALIEAYGI-EPDRERLAYYR 238

>TR:A0A0N8GPB1 A0A0N8GPB1_9CHLR APH domain-containing protein OS=Levilinea saccharolytica OX=229921 GN=ADN01_12010 PE=3 SV=1

Length=265

Score = 94.4 bits (233), Expect = 1e-19

| Identities | | = 83/256 (32%), Positives = 116/256 (45%), Gaps = 14/256 (5%) |  |
| --- | --- | --- | --- |
| Query 19  Sbjct 14 | | LFGYDWAQQTIGCSDAAVFRLSAQGRPVLFVKTD---LSGALNELQDEAARLSWLATTGV L GY + G S A V+ L+ + +P L++KT G +++ E L WL + LRGYQRTPDSRGQSPARVYALTGR-QPTLYLKTSDRRCQGTSYDVEREKDLLLWLQDR-L | 75  71 |
| Query 76  Sbjct 72 | | PCAAVLDVVTEAGRDWLLLGEVPGQDLLSSHLA---PAEKVSIMADAMRRLHTLDPATCP P VL AG +LL+ G + P A+A++ L ++ A CP PVPEVLHYEEYAGVRFLLMSAAEGTAVCEDQRQQPDPERLARHYAEAVQLLRSVSIADCP | 132  131 |
| Query 133  Sbjct 132 | | FDHQAKHRIERARTRMEAGLVDQDDLD-EEHQGLAPAE-LFARLKARMPDGEDLVVTHGD  + + R++ + L D + D E A AE L A L+ P E+ THGD YPYGLDQRLQELEVLLARQLTDVERTDWEADTPFADAESLAAHLQTHRPP-EEWGFTHGD | 190  190 |
| Query 191 | | ACLPNIMVENGRFSGFIDCGRLGVADRYQDIALATRDIAEELGG--EWADRFLVLYGIAA N++V GR SGFID GR GVAD + DIAL R + GG E D F I | 248 |
| Sbjct | 191 | LGRENLLVRGGRISGFIDVGRSGVADPWLDIALCVRSLQHTCGGRREPLDLFFKWLEI-E | 249 |
| Query  Sbjct | 249  250 | PDSQRIAFYRLLDEFF 264 PD +I +Y LDE F PDWDKIRYYIWLDELF 265 |  |

>TR:A0A1H9DI46 A0A1H9DI46_9SPIO Kanamycin kinase OS=Treponema bryantii OX=163 GN=SAMN04487977_102586 PE=3 SV=1

Length=267

Score = 94.4 bits (233), Expect = 1e-19

| Identities | = 66/205 (32%), Positives = 97/205 (47%), Gaps = 17/205 (8%) |  |
| --- | --- | --- |
| Query 75 | VPCAAVLDVVTEAGRDWLLLGEVPGQDLLSSHLA--PAEKVSIMADAMRRLHTLDPATCP | 132 |
| Sbjct 65 | VP + AG +LL+ V G+ + P E ++A A++ L ++D + CP VPKVIAYEKDEAAGLQFLLMSRVTGKMSCDDYYMSRPKELCKLLAQALKLLWSVDISDCP | 124 |
| Query 133 | FDHQAKHRIERARTRMEAGLVDQDDLDEEHQGLA PAELFARLKARMPDGEDLVVTH | 188 |
| Sbjct 125 | ++ AR R+E L+D D + G P L L+ P+ E V +H RCITLDDELKEARFRVENNLIDVSDAELTTFGPDGFENPMALLEWLENNRPEREP-VFSH | 183 |
| Query 189 | GDACLPNIMVENGRFSGFIDCGRLGVADRYQDIALATRDIAEELGGEWADR--------- | 239 |
| Sbjct 184 | GD CLPNI++E+G+ SG ID G GV D++ DIAL R + G W + GDFCLPNILIEDGKLSGLIDLGDTGVNDKWLDIALCYRSLKHNADGTWGKKIYQDFNPEL | 243 |
| Query 240  Sbjct 244 | FLVLYGIAAPDSQRIAFYRLLDEFF 264 F G+ PD +I +Y LLDE F FFEALGV-EPDWDKINYYILLDELF 267 |  |

>TR:A0A0I9YP49 A0A0I9YP49_9MYCO Aminoglycoside phosphotransferase OS=Mycobacterium haemophilum OX=29311 GN=ABH38_12965 PE=3 SV=1

Length=251

Score = 94.0 bits (232), Expect = 1e-19

Identities = 75/229 (33%), Positives = 102/229 (45%), Gaps = 23/229 (10%)

| Query | 37 | FRLSAQGRPVLFVKTDLSGALNELQDEAARLSWLATTGVPCAAVLDVVTEAGRDWLLLGE | 96 |
| --- | --- | --- | --- |
| Sbjct | 36 | FR+++ F+K +GA+ + DEA RL W A V VL V + WL FRVNSTSCDSEFIKVAEAGAV-DFADEARRLRWAARY-VTAPRVLGVGRDGDWAWLRTRA | 93 |
| Query | 97 | VPGQDLLSSHLAPAEKVSIMA--DAMRRLHTLDPAT-CPFDHQAKHRIERARTRMEAGLV | 153 |
| Sbjct | 94 | +PG+ + A V++ A +RRLH P CPFD R+ LPGRSAVHPRWVAAPDVAVRAIGAGLRRLHDQLPVQLCPFDWSVASRLATLSPA------ | 147 |
| Query | 154 | DQDDLDEEHQGLAPAELFARLKARMPDGEDLVVTHGDACLPNIMVEN-GRFSGFIDCGRL G P P + LVV HGDAC PN ++++ GR G +D G L | 212 |

| Sbjct | 148 | ---------SGRYPQPAARAGLGEPPPVDQLVVCHGDACSPNTLIDDDGRCCGHVDFGSL 198 |
| --- | --- | --- |
| Query | 213 | GVADRYQDIALATRDIAEELGGE-WADRFLVLYGIAAPDSQRIAFYRLL 260 |
| Sbjct | 199 | GVADR+ D+A+AT ++ G W F YGI PD RI +YR L  GVADRWADLAVATLSLSWNYPGRVWDAEFFAAYGI-EPDPPRIDYYRRL 246 |

>TR:A0A0C1U987 A0A0C1U987_9CLOT Phosphotransferase enzyme family protein OS=Clostridium argentinense CDC 2741 OX=1418104 GN=U732_4019 PE=3

SV=1

Length=252

Score = 93.6 bits (231), Expect = 2e-19

| Identities | | = 64/215 (30%), Positives = 111/215 (52%), Gaps = 11/215 (5%) |  |
| --- | --- | --- | --- |
| Query 57  Sbjct 42 | | LNELQDEAAR LSWLATTGVPCAAVLDVVTEAGRDWLLLGEVPGQDLLSS--HLAPA  + ++ DE R WL +P ++ V E ++L+ G+ L P IEKINDEVIREYEMYQWLIGK-LPVPKIVYRVVEDNVSYMLIEAARGKMLEDDTYRSRPE | 110  100 |
| Query 111  Sbjct 101 | | EKVSIMADAMRRLHTLDPATCPFDHQAKHRIERARTRMEAGLVDQDDLDEEHQGL-APAE  + V++ A ++ L ++D CPFD + ++E+AR R+E+ L + D + +GL P + KLVALAAKGIKLLQSVDVHECPFDSRIDGKLEKARKRIESDLAGRIDKNVYTEGLYTPED | 169  160 |
| Query 170  Sbjct 161 | | LFARLKARMPDGEDLVVTHGDACLPNIMVENGRFSGFIDCGRLGVADRYQDIALATRDIA  ++ L P+ E+L+ +HGD C N + FID GR GV+D YQDIAL R++ VYRYLIENKPE-EELIFSHGDYCFNNYFTNGTEITDFIDMGRAGVSDFYQDIALCVRELM | 229  219 |
| Query 230 | | EELGGEWADRFLVLYGIAAPDSQRIAFYRLLDEFF 264  + ++ + F+ GI + + ++I +Y LLDE F |  |
| Sbjct | 220 | -DFEPKYTEMFIKELGIES-NWEKIKYYILLDELF 252 | |

>TR:A0A372PK99 A0A372PK99_9ACTN Aminoglycoside 3'-phosphotransferase OS=Microbispora triticiradicis OX=2200763 GN=DI270_000735 PE=3 SV=1

Length=257

Score = 93.6 bits (231), Expect = 2e-19

| Identities | | = 72/207 (35%), Positives = 99/207 (48%), Gaps = 26/207 (13%) |  |
| --- | --- | --- | --- |
| Query 59  Sbjct 68 | | ELQDEAARLSWLAT-TGVPCAAVLDVVTEAGRDWLLLGEVPGQDLLSSHLA--PAEKVSI  +L EA RL+W T VP VL ++A WL+ +PG+ + P + DLPREAERLTWARQWTTVP--RVLAYGSDADGAWLVTAALPGRSAVDPRWTADPRTAAAA | 115  125 |
| Query 116  Sbjct 126 | | MADAMRRLHTLDP-ATCPFDHQAKHRIERARTRMEAGLVDQDDLDEEHQGLAPAELFARL  + +R LH P A CP+D +HR+ RA R L AE RL IGRGLRLLHDALPVARCPYDWGVEHRLRRAGER LGRAEARRRL | 174  168 |
| Query 175  Sbjct 169 | | KARMPDGEDLVVTHGDACLPNIMV-ENGRFSGFIDCGRLGVADRYQDIALATRDIAEELG R P + LVV HGDAC PN ++ ++G +G +D G LGVADR+ D+A+A G  -GRPPAPDRLVVCHGDACAPNTLLHDDGTVAGHVDLGSLGVADRWADLAVAAWSTEWNYG | 233  227 |
| Query 234 | | GEWADRFLVLYGIAAPDSQRIAFYRLL 260  YG+ PD +RIA+YRLL |  |
| Sbjct | 228 | PGHDGEVYEAYGV-VPDPERIAYYRLL 253 | |

>TR:A0A3N2JJJ7 A0A3N2JJJ7_9CELL Kanamycin kinase OS=Cellulomonas sp. PhB143 OX=2485186 GN=EDF32_2387 PE=3 SV=1

Length=277

Score = 94.0 bits (232), Expect = 2e-19

Identities = 71/214 (33%), Positives = 102/214 (48%), Gaps = 16/214 (7%)

| Query | 59 | ELQDEAARLSWLATTGVPCAAVL DVVTEAGRD--WLLLGEVPGQDLLSSHLA-- | 108 |
| --- | --- | --- | --- |
| Sbjct | 64 | +L EA R++W V V + E R WL+ +PG ++ DLVAEAERMAWARAYAVVPTVVGHGRAEDEAAPEDARQGTWLVTRTLPGASAVAPRWLAD | 123 |
| Query | 109 | PAEKVSIMADAMRRLHTLDPA-TCPFDHQAKHRIERARTRMEAGLVDQDDLDEEHQGLAP | 167 |
| Sbjct | 124 | P + +R LH P CPF A+ R+ AR R +AG + +H+ + PETAARAIGAGLRTLHDALPVGECPFSWSARERLADARDRADAG-DGPESWSPQHRTVPV | 182 |
| Query | 168 | AELFARLKARMPDGEDLVVTHGDACLPNIMVEN-GRFSGFIDCGRLGVADRYQDIALATR | 226 |

AE ARL	P	DLVV HGDAC PN ++++ G +G +D G LGVADR+ D+A+A

| Sbjct | 183 | AEALARLAD--PPAPDLVVCHGDACAPNTLLDDDGAPAGHVDLGSLGVADRWADLAVAAW 240 |
| --- | --- | --- |
| Query | 227 | DIAEELGGEWADRFLVLYGIAAPDSQRIAFYRLL 260 |
| Sbjct | 241 | G + YGI PD+++IA+YRLL  STEWNYGPGYDAPLYEAYGI-EPDAEKIAYYRLL 273 |

>TR:A0A0M2H7Z7 A0A0M2H7Z7_9MICO Aminoglycoside 3'-phosphotransferase OS=Microbacterium ketosireducens OX=92835 GN=neo PE=3 SV=1

Length=241

Score = 93.2 bits (230), Expect = 2e-19

| Identities | | = 66/202 (33%), Positives = 94/202 (47%), Gaps = 26/202 (13%) |  |
| --- | --- | --- | --- |
| Query 63  Sbjct 58 | | EAARLSWLATTGVPCAAVLDVVTEAGRDWLLLGEVPGQDLLSSHLA--PAEKVSIMADAM EA R++W A + VL+V + +W++ +PG + PA V + + EAERMAW-AGAFIRVPQVLEVGGDETHEWMVTTALPGLSAVDPRWIADPATAVRAVGAGL | 120  116 |
| Query 121  Sbjct 117 | | RRLHTLDPAT-CPFDHQAKHRIERARTRMEAGLVDQDDLDEEHQGLAPAELFARLKARMP R LH P CPFD R+ A R G+ P E P RALHDALPVVDCPFDWGVPARLANAAGR----------------GIRPPEA GDDAP | 179  156 |
| Query 180  Sbjct 157 | | DGEDLVVTHGDACLPNIMV-ENGRFSGFIDCGRLGVADRYQDIALATRDIAEELGGEWAD  ++LVV HGDAC PN ++ ++G + G +D G LGVADR+ DIA+A+ G W D PVDELVVCHGDACCPNTLIGDDGEWCGHVDLGALGVADRWADIAVASMSTEWNYGPGWED | 238  216 |
| Query 239 | | RFLVLYGIAAPDSQRIAFYRLL 260  + YGI PD +R+ FYR L |  |
| Sbjct | 217 | ALIEAYGI-EPDRERLTFYREL 237 | |

>TR:A0A543BHZ6 A0A543BHZ6_9MICO Kanamycin kinase OS=Microbacterium saperdae OX=69368 GN=FB560_0059 PE=3 SV=1

Length=241

Score = 93.2 bits (230), Expect = 2e-19

| Identities | | = 71/222 (32%), Positives = 106/222 (48%), Gaps = 29/222 (13%) |  |
| --- | --- | --- | --- |
| Query 43  Sbjct 41 | | GRPVLFVKTDLSGALNELQDEAARLSWLATTGVPCAAVLDVVTEAGRDWLLLGEVPGQDL GR + + DL +L +DEA R+ W A + VL + +WL+ + + GRYIKWGPRDLEASL---RDEAERMRW-ARHWITAPEVLAQGQDDTHEWLVTVALDARSA | 102  96 |
| Query 103  Sbjct 97 | | LSSHLAPAEKVSIMA--DAMRRLHTLDPAT-CPFDHQAKHRIERARTRMEAGLVDQDDLD  + ++S+ A + +R LH P CP++ R+ A R D+D VDPRWVEEPELSVRAVGEGLRTLHDALPVDDCPWEWSVAERLANAAAR DVD | 159  147 |
| Query 160  Sbjct 148 | | EEHQGLAPAELFARLKARMPDGEDLVVTHGDACLPNIMVEN-GRFSGFIDCGRLGVADRY PAEL P + LVV HGDAC+PN ++++ GR GF+D LG ADR+  ------VPAEL RDAPSIDRLVVCHGDACMPNTLLDDAGRPVGFVDLAALGTADRW | 218  196 |
| Query 219 | | QDIALATRDIAEELGGEWADRFLVLYGIAAPDSQRIAFYRLL 260 DIA+A+ A G W D + YGI PD +R+A+YR L |  |
| Sbjct | 197 | ADIAVASMSTAWNFGPGWEDTLIEAYGI-EPDRERLAYYRRL 237 | |

>TR:A0A249SRT6 A0A249SRT6_9BACT APH(3'') family aminoglycoside O-phosphotransferase OS=Chitinophaga sp. MD30 OX=2033437 GN=CK934_04955 PE=3

SV=1

Length=267

Score = 93.6 bits (231), Expect = 2e-19

Identities = 75/222 (34%), Positives = 100/222 (45%), Gaps = 27/222 (12%)

| Query | 60 | LQDEAARLSWLATTGVPCAAVLDVVTEAGRDWLLLGEVPGQDLLSSHLAPAEKVSIMADA | 119 |
| --- | --- | --- | --- |
| Sbjct | 48 | L E RL WLA +P VLD + A LL+ +PG + + AE++SI + LAAEKDRLLWLADKDLPTPKVLDWIETATGSCLLISPIPG---IPASKLTAEQLSIAWTS | 104 |
| Query | 120 | MRR LHTLDPATCPFDHQAKHRIERARTRMEAGLVDQDDLDEEHQGLAPAELFARLK | 175 |
| Sbjct | 105 | + R LH L CPFD + +AR + G V+ D L E + A L A L+  ITRQLQALHQLPVNNCPFDRRLSLMFAQARDVVARGAVNPDFLRPEQHHIPAATLLANLE | 164 |

| Query | 176 | ARM-----PDGEDLVVTHGDACLPNIMVE--NGRFSGFIDCGRLGVADRYQDIALATRDI 228 |
| --- | --- | --- |
| Sbjct | 165 | + + + VV HGDACLPN MV+ + +G ID GRLG AD Y D L + MSLQERLAQESAEQVVCHGDACLPNFMVDPVTLQCTGMIDLGRLGKADPYVDYTLLLANS 224 |
| Query | 229 | AEELGGEWAD---------RFLVLYGIAAPDSQRIAFYRLLD 261 |
| Sbjct | 225 | E W R L I PD+ R+ FY LD  EE----TWPQADQQQAAYIRLFQLLQIDHPDTSRLDFYLQLD 262 |

>TR:A0A1X1XNT1 A0A1X1XNT1_9MYCO Aminoglycoside phosphotransferase OS=Mycobacterium lacus OX=169765 GN=AWC15_08580 PE=3 SV=1

Length=249

Score = 93.2 bits (230), Expect = 2e-19

| Identities | | = 83/262 (32%), Positives = 108/262 (41%), Gaps = 48/262 (18%) |  |
| --- | --- | --- | --- |
| Query 9  Sbjct 19 | | AGSP--AAWVERLFGYDWAQQTIGCSDAAVFRLSAQGRPVLFVKTDLSGALN---ELQDE AG P A WV L A +R+ A F+K GA + + DE AGRPVRAVWVNEL GAVTYRVGAATSNSEFIKVGKCGATDFAADFADE | 63  65 |
| Query 64  Sbjct 66 | | AARLSWLATTGVPCAAVLDVVTEAGRDWLLLGEVPGQDLLSSH--LAPAEKVSIMADAMR A RL W A V VL + WL +PG + +P V +A +R ARRLRWAAPY-VAAPPVLGAGLDGDWAWLRTAGLPGVSAVHPRWQASPDAAVRAIAAGLR | 121  124 |
| Query 122  Sbjct 125 | | RLH-TLDPATCPFDHQAKHRIERARTRMEAGLVDQDDLDEEHQGLAPAELFARLKARMPD LH +L ++CPFD A R+ R A L + P TLHDSLPVSSCPFDWSAASRLARVAPARRASLGEP PP | 180  161 |
| Query 181  Sbjct 162 | | GEDLVVTHGDACLPNIMVEN-GRFSGFIDCGRLGVADRYQDIALATRDIAEELGGEWAD- E L V HGDAC PN ++ N GR G +D G LGVADR+ D+A+AT + G D VEKLAVCHGDACSPNTLIGNDGRCCGHVDFGDLGVADRWADLAVATLSLRWNYPGRGRDA | 238  221 |
| Query | 239 RFLVLYGIAAPDSQRIAFYRLL 260 | | |
|  |  | F YG+ PD RI +YR L |  |
| Sbjct | 222 | EFFAAYGV-EPDLARIEYYRRL 242 | |

>TR:A0A1R4HLD7 A0A1R4HLD7_9MICO Aminoglycoside phosphotransferase OS=Leucobacter sp. 7(1) OX=1255613 GN=FM113_02205 PE=3 SV=1

Length=274

Score = 93.6 bits (231), Expect = 2e-19

| Identities | = 78/252 (31%), Positives = 114/252 (45%), Gaps = 18/252 (7%) |  |
| --- | --- | --- |
| Query 13 | AAWVERLFGYDWAQQTIGCSDAAVFRLSAQGRPVLFVKTDLSGALNELQDEAARLSWLAT | 72 |
| Sbjct 33 | + W+ L G + ++A + +A G P + +L EA RL+W A SVWINGLGGVTFRDPGPAGTEARFVKWTAAGTPEI DLAQEAHRLAWAAA | 81 |
| Query 73 | TGVPCAAVLDVVTEAGRDWLLLGEVPGQDLLSSH--LAPAEKVSIMADAMRRLHTLDPAT | 130 |
| Sbjct 82 | G VLD ++A WL+ + G+ +S P + +R LH P + HGAAVPRVLDHGSDAAGSWLVTAALNGESAVSPRWIAEPETAARAIGVGLRVLHEQLPVS | 141 |
| Query 131 | -CPFDHQAKHRIERARTRMEAGLVDQDDLDEEHQGLAPAELFARLKARMPDGEDLVVTHG | 189 |
| Sbjct 142 | P + RI + R+ AG E++ L+ AE ARL A P + VV HG EAPAAWSIERRIAQFEDRVRAG-DGPAHWSAEYRQLSVAEARARL-ADAPPLDGPVVCHG | 199 |
| Query 190 | DACLPNIMVEN-GRFSGFIDCGRLGVADRYQDIALATRDIAEELGGEWADRFLVLYGIAA | 248 |
| Sbjct 200 | DAC PN +++ GRF +D LGVADR+ D+A+A G + Y I A DACAPNTLLDGAGRFLAHVDLQELGVADRWADLAVAAWSTEWNYGAGFDHLVYEGYEI-A | 258 |
| Query 249  Sbjct 259 | PDSQRIAFYRLL 260 PD +RIA+YRLL PDPERIAYYRLL 270 |  |

>TR:A0A4R4XDR7 A0A4R4XDR7_9ACTN Aminoglycoside phosphotransferase OS=Kribbella sp. 16K104 OX=2530375 GN=E1218_05890 PE=4 SV=1

Length=211

Score = 92.0 bits (227), Expect = 3e-19

Identities = 82/257 (32%), Positives = 110/257 (43%), Gaps = 62/257 (24%)

| Query | 14 | AWVERLFGYDWAQQTIGCSDAAVFRLSAQGRPVLFVKTDLSGALNELQDEAARLSWLATT | 73 |
| --- | --- | --- | --- |
| Sbjct | 5 | A V+R + +G S A V RL + LF K + DEA RL+WL +T ALVDRFGAHSAEPIHLGMSAATVVRLDRRSER-LFYKVGPG VDDEADRLAWLGST | 58 |
| Query | 74 | GVPCAAVLDVVTEAGRDWLLLGEVPGQDLLSSHLAPAEKVSIMADAMRRLHTLDPAT-CP | 132 |
| Sbjct | 59 | G PC ++D G +WLL E+PG+D S A++ +++A L LD T CP GFPCPQIVD RGNNWLLTTELPGRDT-SQPWPAADRPAVLAAMADGLRALDELTDCP | 113 |
| Query | 133 | FDHQAKHRIERARTRMEAGLVDQDDLDEEHQGLAPAELFARLKARMPDGEDLVVTHGDAC | 192 |
| Sbjct | 114 | F G+ VTHGD C  FGSPFP GDGAAVTHGDYC | 131 |
| Query | 193 | LPNIMV--ENGRFSGFIDCGRLGVADRYQDIALATRDIAEEL GGE-WADRFLVLYG | 245 |
| Sbjct | 132 | PN+ V E RF+G +D RLG DR D+AL + ++ L GGE A RF+ YG  CPNVFVDPETLRFTGVLDVSRLGTGDRCVDLALMYKSLSGTLNPQYGGEPAARRFVEAYG | 191 |
| Query | 246 IAAPDSQRIAFYRLLDE 262 | | |
|  |  | A PD RI +Y LD+ |  |
| Sbjct | 192 | -ADPDDSRITYYIDLDD 207 | |

>TR:E8U5N2 E8U5N2_DEIML Kanamycin kinase OS=Deinococcus maricopensis (strain DSM 21211 / LMG 22137 / NRRL B-23946 / LB-34) OX=709986

GN=Deima_0715 PE=3 SV=1

Length=249

Score = 92.8 bits (229), Expect = 3e-19

| Identities | = 67/218 (31%), Positives = 95/218 (44%), Gaps = 18/218 (8%) |  |
| --- | --- | --- |
| Query 49 | VKTDLSGALNELQDEAARLSWLATTGVPCAAVLDVVTEAGRDWLLLGEVPGQDLL--SSH | 106 |
| Sbjct 42 | +K G + L E RL WLA VP + +A +L L +PG + + IKVHPRGPSHPLFSEKERLRWLAPR-VPVPPLAGYAEDADHAYLALARLPGMPMNHPDAR | 100 |
| Query 107 | LAPAEKVSIMADAMRRLHTLDPATCPFDHQAKHRIERARTRMEAGLVDQDDLDEEHQGLA | 166 |
| Sbjct 101 | L ++A A+ LH L CPF H R+ RTR+ A G LHARRNADLLARALAELHALPIRDCPFTHTLTERLRDLRTRLTAS GEI | 148 |
| Query 167 | P---AELFARLKARMPDGEDLVVTHGDACLPNIMVENGRFSGFIDCGRLGVADRYQDIAL | 223 |
| Sbjct 149 | P AE F L + P EDLVVTHG A L +++V GR G+ADR+ D+A PTPVAERFNALVRQRPHDEDLVVTHGHATLEHVLVNGEYVEALTGVGRAGLADRHVDLAA | 208 |
| Query 224  Sbjct 209 | ATRDIAEELGGEWADRFLVLYGIAAPDSQRIAFYRLLD 261 A + ++ + A FL YG A D ++ +Y L+ AHTSLTQDYDPDAAAHFLDTYGRARIDLHKLDYYARLN 246 |  |

>TR:A0A4V3BVJ1 A0A4V3BVJ1_9MICO Kanamycin kinase OS=Microbacterium sp. BK668 OX=2512118 GN=EV279_0857 PE=3 SV=1

Length=241

Score = 92.4 bits (228), Expect = 4e-19

| Identities | | = 68/203 (33%), Positives = 97/203 (48%), Gaps = 28/203 (14%) |  |
| --- | --- | --- | --- |
| Query 63  Sbjct 58 | | EAARLSWLAT-TGVPCAAVLDVVTEAGRDWLLLGEVPGQDLLSSH--LAPAEKVSIMADA EA RL W T VP V++ + +W++ VPG+ ++ PA V + + EAERLDWAGRYTDVP--RVVEFGGDETHEWMVTETVPGESAVAPRWIAEPATAVRAIGEG | 119  115 |
| Query 120  Sbjct 116 | | MRRLHTLDP-ATCPFDHQAKHRIERARTRMEAGLVDQDDLDEEHQGLAPAELFARLKARM  +R LH P A CPF+ A RI +A R G+ D L E LRALHDALPVADCPFERSAASRIAKAAVR---GIRLPDRLREP----------------- | 178  155 |
| Query 179  Sbjct 156 | | PDGEDLVVTHGDACLPNIMVE-NGRFSGFIDCGRLGVADRYQDIALATRDIAEELGGEWA P + LVV HGDAC PN +++ GR++ +D G LGV DR+ D+A+A+ G W PAVDALVVCHGDACSPNTLLDARGRWTAHVDLGALGVGDRWADLAVASMSTEWNYGPGWD | 237  215 |
| Query 238 | | DRFLVLYGIAAPDSQRIAFYRLL 260 L YGI PD++R +YR L |  |
| Sbjct | 216 | GALLAAYGI-QPDAERTDYYREL 237 | |

>TR:A0A0Q9CLW6 A0A0Q9CLW6_9CELL Aminoglycoside phosphotransferase OS=Oerskovia sp. Root918 OX=1736607 GN=ASE27_19325 PE=3 SV=1

Length=281

Score = 93.2 bits (230), Expect = 4e-19

| Identities | = 73/207 (35%), Positives = 99/207 (48%), Gaps = 10/207 (5%) |  |
| --- | --- | --- |
| Query 59 | ELQDEAARLSWLAT-TGVPCAAVLDVVTEAGRDWLLLGEVPGQDLLSSHLA--PAEKVSI | 115 |
| Sbjct 76 | +L EA RL+W + T VP VLD + WL+ + + PA + DLLAEAERLAWASQHTAVP--HVLDAGQDELGTWLVTEAIDAASAVDPRWVAEPATAAAA | 133 |
| Query 116 | MADAMRRLHTLDPAT-CPFDHQAKHRIERARTRMEAGLVDQDDLDEEHQGLAPAELFARL | 174 |
| Sbjct 134 | + + +R LH P CPF + R+ RA R+ G + EH L E ARL IGNGLRVLHDALPVEGCPFTWSVEERVARADERIADG-EGPTEWFPEHAALTVTEARARL | 192 |
| Query 175 | KARMPDGEDLVVTHGDACLPNIMVE-NGRFSGFIDCGRLGVADRYQDIALATRDIAEELG | 233 |
| Sbjct 193 | P + LVV HGDAC PN +V +G F+ +D G LGVADR+ D+A+A G DLA-PPVDRLVVCHGDACAPNTLVHTDGSFAAHVDLGSLGVADRWADLAVAAWSTEWNYG | 251 |
| Query 234  Sbjct 252 | GEWADRFLVLYGIAAPDSQRIAFYRLL 260  + YGI PD +RIA+YRLL  PGYDRIVYDAYGI-EPDEERIAYYRLL 277 |  |

>TR:A0A2U3P7J0 A0A2U3P7J0_9MYCO Aminoglycoside phosphotransferase (Fragment) OS=Mycobacterium numidiamassiliense OX=1841861 GN=MNAB215_1925

PE=3 SV=1

Length=251

Score = 92.4 bits (228), Expect = 4e-19

| Identities | = 76/230 (33%), Positives = 105/230 (46%), Gaps = 33/230 (14%) |  |
| --- | --- | --- |
| Query 37 | FRLSAQGRPVLFVKTDLSGALNELQDEAARLSWLAT-TGVPCAAVLDVVTEAGRDWLLLG | 95 |
| Sbjct 36 | FRL + G FVK ++ A + EA RL W A VP V R WL G FRLGS-GISAEFVK--VADATVDFAAEARRLRWAAPHLTVPPVLGFGVDEAGDRAWLHTG | 92 |
| Query 96 | EVPGQDLLSSHLAPAEKVSIMA--DAMRRLHTLDPA-TCPFDHQAKHRIERARTRMEAGL | 152 |
| Sbjct 93 | +PG + A +V++ A +R LH P +CPFD A +R++R ++ A L ALPGLSAVHPRWLAAPEVAVRAVGAGLRVLHDRLPVWSCPFDWSATNRLDRLSSKGRAQL | 152 |
| Query 153 | VDQDDLDEEHQGLAPAELFARLKARMPDGEDLVVTHGDACLPNIMVEN-GRFSGFIDCGR | 211 |
| Sbjct 153 | + +D LVV HGDAC PN ++++ GR G +D G GEPPPIDR LVVCHGDACSPNTLIDDDGRCCGHVDFGE | 189 |
| Query 212  Sbjct 190 | LGVADRYQDIALATRDIAEEL-GGEWADRFLVLYGIAAPDSQRIAFYRLL 260 LGVADR+ D+A+AT + G W YG+ PD RI +YR L LGVADRWADLAVATLSLGWNYPGRSWEAELFTAYGV-RPDPVRIDYYRRL 238 |  |

>TR:A0A1A3HJW7 A0A1A3HJW7_9MYCO Aminoglycoside phosphotransferase APH(3') OS=Mycobacterium sp. 1423905.2 OX=1856859 GN=A9W95_05690 PE=3

SV=1

Length=240

Score = 92.0 bits (227), Expect = 5e-19

| Identities | = 74/218 (34%), Positives = 96/218 (44%), Gaps = 31/218 (14%) |  |
| --- | --- | --- |
| Query 48 | FVKTDLSGALNELQDEAARLSWLATTGVPCAAVLDVVTEAGRDWLLLGEVPGQDLLSSHL | 107 |
| Sbjct 44 | FVK A + +EA RL W A V VL V G WL +PG+ + H FVKV-ADAATVDFTEEARRLRWAAAY-VTVPEVLGVGAGDGWAWLRTRGLPGRSAVHPHW | 101 |
| Query 108 | APAEKVSIMADA--MRRLHTLDPA-TCPFDHQAKHRIERARTRMEAGLVDQDDLDEEHQG | 164 |
| Sbjct 102 | A V++ A +R LH P +CPFD A R+ A L D +D LAAPHVAVRAIGVGLRALHDRLPVLSCPFDWSAPSRLAGLSPTARAELGDPPPVDR---- | 157 |
| Query 165 | LAPAELFARLKARMPDGEDLVVTHGDACLPNIMV-ENGRFSGFIDCGRLGVADRYQDIAL | 223 |
| Sbjct 158 | LVV HGDAC PN ++ +G SG +D G LGVADR+ D+A+  -------------------LVVCHGDACSPNTLIGPDGNCSGHVDMGSLGVADRWADLAI | 198 |
| Query 224  Sbjct 199 | ATRDIAEEL-GGEWADRFLVLYGIAAPDSQRIAFYRLL 260 AT + G +W F YG A PD R+ +YR L ATLSLQWNYPGRQWEAEFFAAYG-ARPDPARVDYYRRL 235 |  |

>TR:A0A6H9WSB1 A0A6H9WSB1_9MICO Aminoglycoside 3'-phosphotransferase OS=Pseudoclavibacter endophyticus OX=1778590 GN=F8O04_04975 PE=3 SV=1

Length=265

Score = 92.4 bits (228), Expect = 6e-19

| Identities | | = 72/221 (33%), Positives = 102/221 (46%), Gaps = 39/221 (18%) |  |
| --- | --- | --- | --- |
| Query 48  Sbjct 69 | | FVKTDLSGALNELQDEAARLSWL-ATTGVPCAAVLDVVTEAGRDWLLLGEVPGQDLLSSH  +VK + G+ L DE RL+WL VP A + ++ L+ +PG+ +S YVKWNPQGSGESLADEVDRLNWLEGRHAVPVVAFF--TADRAQEVLVTRALPGESAVSRR | 106  126 |
| Query 107 | | LA--PAEKVSIMADAMRRLHTLDPATCPFDHQAKHRIERARTRMEAGLVDQDDLDEEHQG PA+ + + +RRLH L P CPFD +HR+ | 164 |
| Sbjct | 127 | WRREPADAMRALGVGLRRLHELPPDECPFDWGVQHRLRSVG------------------- | 167 |
| Query | 165 | LAPAELFARLKARMPDGEDLVVTHGDACLPNIMV-ENGRFSGFIDCGRLGVADRYQDIAL | 223 |
| Sbjct | 168 | AP E P + LVV HGD C PN ++ ++GRF +D RLG ADR+ D+A+  -APRESL GEAPPVDRLVVCHGDPCAPNTLLGDDGRFLAHVDLARLGTADRWADLAV | 222 |
| Query  Sbjct | 224  223 | ATRDIAEELGGEWADR----FLVLYGIAAPDSQRIAFYRLL 260  ++ LG +AD F YGI PD RIA+YR L  ----MSMSLGWNYADHDEAVFWQAYGI-DPDPVRIAYYRRL 258 |  |

>TR:A0A1Y2FFT2 A0A1Y2FFT2_9FUNG Aminoglycoside phosphotransferase OS=Neocallimastix californiae OX=1754190 GN=LY90DRAFT_647833 PE=3 SV=1

Length=258

Score = 92.0 bits (227), Expect = 7e-19

| Identities | = 63/246 (26%), Positives = 123/246 (50%), Gaps = 20/246 (8%) |  |
| --- | --- | --- |
| Query 29 | IGCSDAAVFRLSAQGRPVLFVKTDLSGALNELQDEAARLSWLATTGVPCAAVLDVVTEAG | 88 |
| Sbjct 23 | IG SD+ V VL ++ D+ A ++ + WL +P V+ IGFSDSTVLIYDDM---VLKIENDVLKA KETVIMMKWLKGK-IPVPEVIYHEVSNN | 74 |
| Query 89 | RDWLLLGEVPGQDLLSSHLA--PAEKVSIMADAMRRLHTLDPATCPFDHQAKHRIERART | 146 |
| Sbjct 75 | + +LL+ + G+ P + ++I+++A++ L ++ + CP + +++ R KSYLLMSRITGKMSCDEFYLEQPEKLLAILSEALKSLWKINVSDCPRNKNIDTKLKEVRK | 134 |
| Query 147 | RMEAGLVDQDDLDEEHQGLAPAELFARLKARMPDGEDLVVTHGDACLPNIMVENGRFSGF | 206 |
| Sbjct 135 | R+E GLV+ ++ D+ + P EL L+ PD E V++HGD CLPNI++E+ + +GF RVENGLVNLEEFDKS-EFKNPEELLLWLEKNKPDDEP-VLSHGDFCLPNIILEDDKINGF | 192 |
| Query 207 | IDCGRLGVADRYQDIALATRDIAEELGGEWADRFLVLYGIAA PDSQRIAFYR | 258 |
| Sbjct 193 | ID G G++D++ DIA+ R + G + + + A P+ +++ ++ IDLGDSGISDKWYDIAICYRSLKNNFSGIYGGKVYPDFDPNALFKALNLKPNHEKLRYFL | 252 |
| Query 259  Sbjct 253 | LLDEFF 264 L++E F LMEELF 258 |  |

>TR:A0A3R7HL97 A0A3R7HL97_9MICO Kanamycin kinase OS=Microbacterium ginsengiterrae OX=546115 GN=C7323_0054 PE=3 SV=1

Length=241

Score = 91.7 bits (226), Expect = 7e-19

Identities = 73/218 (33%), Positives = 103/218 (47%), Gaps = 28/218 (13%)

| Query | 48 | FVKTDLSGALNELQDEAARLSWLAT-TGVPCAAVLDVVTEAGRDWLLLGEVPGQDLLS-S | 105 |
| --- | --- | --- | --- |
| Sbjct | 43 | ++K A ++DEA R+ W T VP VLD +A +WL+ VPG+ + S YIKWGPLDAEANMRDEAERMRWARRWTRVP--EVLDQGQDAEHEWLVTAAVPGRSAVDPS | 100 |
| Query | 106 | HLA-PAEKVSIMADAMRRLHTLDPAT-CPFDHQAKHRIERARTRMEAGLVDQDDLDEEHQ | 163 |
| Sbjct | 101 | +A P V + +R LH P CP+ A RI A R G+ DDL WIARPEATVRAVGLGLRMLHDALPVDECPWAWTAASRIANAGRR---GVRIPDDL----- | 152 |
| Query | 164 | GLAPAELFARLKARMPDGEDLVVTHGDACLPNIMV-ENGRFSGFIDCGRLGVADRYQDIA | 222 |
| Sbjct | 153 | P ++LVV HGDAC+PN ++ E GR +D LGV DR+ DIA  ------------RTAPPVDELVVCHGDACMPNTLLDEEGRPLAHVDLAVLGVGDRWADIA | 200 |
| Query | 223 LATRDIAEELGGEWADRFLVLYGIAAPDSQRIAFYRLL 260 | | |

+A+	+G W D + YG+ PD +R+ +YR L Sbjct 201 VASMSTLWNVGPGWEDALIEAYGV-EPDRERLEYYRAL 237

>TR:A0A1R4GJT3 A0A1R4GJT3_9MICO Aminoglycoside phosphotransferase OS=Agrococcus casei LMG 22410 OX=1255656 GN=CZ674_12530 PE=3 SV=1

Length=225

Score = 91.3 bits (225), Expect = 7e-19

| Identities | = 65/210 (31%), Positives = 96/210 (46%), Gaps = 44/210 (21%) |  |
| --- | --- | --- |
| Query 48 | FVKTDLSGALNELQDEAARLSWLATTGVPCAAVLDVVTEAGRDWLLLGEVPGQDLLSSHL | 107 |
| Sbjct 46 | +VK D + + L +E R+ WL P ++ E + L+ +PG ++ YVKCDPADSSESLGEEFMRMRWLDGR-FPAPQPIEFAAEDDGEVLVTAALPGAGAVTDEW | 104 |
| Query 108 | A--PAEKVSIMADAMRRLHTLDPATCPFDHQAKHRIERARTRMEAGLVDQDDLDEEHQGL | 165 |
| Sbjct 105 | P++ V +A +RRLHTLD A CPF M + DD LRRPSDAVRAIATGLRRLHTLDAADCPF--------------MSSAPASADD-------- | 142 |
| Query 166 | APAELFARLKARMPDGEDLVVTHGDACLPN-IMVENGRFSGFIDCGRLGVADRYQDIALA | 224 |
| Sbjct 143 | DLVV+HGDAC PN ++ +G F+G +D R+GVADR+ D+A+A  -----------------DLVVSHGDACAPNTVIAPDGSFAGIVDVARVGVADRWLDLAIA | 185 |
| Query 225  Sbjct 186 | TRDIAEELGGEWADRFLVLYGIAAPDSQRI 254  +R + G + F YGI APD+ RI  SRSLGWNYGQGFEPLFFDAYGI-APDAARI 214 |  |

>TR:A0A1H0KHB2 A0A1H0KHB2_9ACTN Kanamycin kinase OS=Nakamurella panacisegetis OX=1090615 GN=SAMN04515671_1309 PE=3 SV=1

Length=262

Score = 92.0 bits (227), Expect = 7e-19

| Identities | | = 79/220 (36%), Positives = 106/220 (48%), Gaps = 17/220 (8%) |  |
| --- | --- | --- | --- |
| Query 48  Sbjct 48 | | FVKTDLSGALNELQDEAARLSWLAT-TGVPCAAVLDVVTEAGRDWLLLGEVPGQDLLSSH FVK G +L+ E RL W+A VP AVL+ ++ +LL +PG++ ++ FVKHSPPGG-PDLRREVQRLRWVAPYASVP--AVLESGSDEHGQFLLTAGIPGENAVTER | 106  104 |
| Query 107 | | LA--PAEKVSIMADAMRRLHTLDPAT-CPFDHQAKHRIERARTRMEAGLVDQDDLDEE-- PA V + +RRLH P CPFD A R RT +E G+ QD | 161 |
| Sbjct | 105 | WRADPAAAVRAIGSGLRRLHDAAPVEDCPFDWSADTR RTVIE-GMPRQDPSRWHPI | 159 |
| Query | 162 | HQGLAPAELFARLKARMPDGEDLVVTHGDACLPNIMV-ENGRFSGFIDCGRLGVADRYQD | 220 |
| Sbjct | 160 | H L L+ P + VV HGD+C PN ++ E G + +D G LGVADR+ D HAHLDHDRALDILR-NPPPVDKAVVCHGDSCAPNTLLDEAGNYRALVDLGALGVADRWAD | 218 |
| Query  Sbjct | 221  219 | IALATRDIAEELGGEWADRFLVLYGIAAPDSQRIAFYRLL 260  +A+AT G W L YGI PD R A+YRLL  LAVATWSTNWNYGQGWEPALLDAYGI-DPDPDRTAYYRLL 257 |  |

>TR:A0A1R3X780 A0A1R3X780_9MICO Kanamycin kinase OS=Microbacterium sp. RU33B OX=1907390 GN=SAMN05880545_2581 PE=3 SV=1

Length=265

Score = 92.0 bits (227), Expect = 8e-19

Identities = 69/217 (32%), Positives = 98/217 (45%), Gaps = 26/217 (12%)

| Query | 48 | FVKTDLSGALNELQDEAARLSWLATTGVPCAAVLDVVTEAGRDWLLLGEVPGQDLLSSH- | 106 |
| --- | --- | --- | --- |
| Sbjct | 67 | F+K A + + EA RL W A + +L+ + +WL+ +PG + FLKFGPRNAESTMAGEAERLRW-AGGWLTVPRILEAGADDTHEWLVTAALPGLSAVDPRW | 125 |
| Query | 107 | -LAPAEKVSIMADAMRRLHTLDPAT-CPFDHQAKHRIERARTRMEAGLVDQDDLDEEHQG | 164 |
| Sbjct | 126 | APA V + + +R LH P C FD + RI A R G+ DDL IAAPAVAVRAVGEGLRALHDALPVEDCAFDWSVERRIANAAGR---GIRVPDDL------ | 176 |
| Query | 165 | LAPAELFARLKARMPDGEDLVVTHGDACLPNIMV-ENGRFSGFIDCGRLGVADRYQDIAL | 223 |
| Sbjct | 177 | P + LVV H DAC PN ++ ++GR+ +D G LG DR+ DIA+  -----------RTAPPIDRLVVCHADACCPNTLLGDDGRWLAHVDLGLLGTGDRWGDIAV | 225 |
| Query | 224 ATRDIAEELGGEWADRFLVLYGIAAPDSQRIAFYRLL 260 | | |

A	G W D L YG+ APD +R+A+YR L Sbjct 226 AAMSTEWNYGPGWEDALLEAYGV-APDHERLAYYREL 261

>TR:A0A5M3W3W7 A0A5M3W3W7_9ACTN Aminoglycoside phosphotransferase APH(3') OS=Acrocarpospora corrugata OX=35763 GN=Acor_33620 PE=3 SV=1

Length=251

Score = 91.7 bits (226), Expect = 8e-19

| Identities | = 80/248 (32%), Positives = 113/248 (46%), Gaps = 27/248 (11%) |  |
| --- | --- | --- |
| Query 24 | WAQQTIGCSDAAVFRLSAQGRPVLFVKTDLSGALN-ELQDEAARLSWLATTGVPCAAVLD | 82 |
| Sbjct 20 | WA + G + FR+ +G PV +VK SG +L E RLSW A V V++ WANEVGGLT FRI--EGEPVRYVKWVASGTPEIDLYSEMLRLSW-ARRWVAVPPVIE | 72 |
| Query 83 | VVTEAGRDWLLLGEVPGQDLLSSHLA--PAEKVSIMADAMRRLHTLDPAT-CPFDHQAKH | 139 |
| Sbjct 73 | + +WL+ VPG + P + +R H P CPFD HGADDEGEWLVTAAVPGLSAVDPRRVTDPVTAAVAIGRGLRVFHDALPVDECPFDWGVGS | 132 |
| Query 140 | RIERARTRMEAGLVDQDDLDEEHQGLAPAEL-FARLKARMPDG---EDLVVTHGDACLPN | 195 |
| Sbjct 133 | R+ A R E G +P L A ++A + D + LVV HGDAC+PN RLANAAERAEGA EAGGSPERLRLAEVRAGIGDAPAIDRLVVCHGDACVPN | 182 |
| Query 196 | IMVE-NGRFSGFIDCGRLGVADRYQDIALATRDIAEELGGEWADRFLVLYGIAAPDSQRI | 254 |
| Sbjct 183 | + +G F+ +D G LG ADR+ D+A+A + + YGI APD++RI TLSHADGTFAAHVDLGSLGRADRWADLAVAAWSMDWNFSPGYDGLVYEAYGI-APDAERI | 241 |
| Query 255  Sbjct 242 | AFYRLLDE 262 A+YRLL E AYYRLLWE 249 |  |

>TR:A0A4R6JJQ2 A0A4R6JJQ2_9ACTN Kanamycin kinase/aminoglycoside 3'-phosphotransferase-2 OS=Kribbella sp. VKM Ac-2527 OX=2512215 GN=EV643_120152

PE=4 SV=1

Length=209

Score = 90.5 bits (223), Expect = 1e-18

| Identities | | = 80/255 (31%), Positives = 108/255 (42%), Gaps = 64/255 (25%) |  |
| --- | --- | --- | --- |
| Query 16  Sbjct 7 | | VERLFGYDWAQQTIGCSDAAVFRLSAQGRPVLFVKTDLSGALNELQDEAARLSWLATTGV V+R +D +G S A V RL V + A + DEA RL WL TG VDRFGAHDAEPIHVGFSGATVIRLHRATERVFYK AGEGVADEADRLEWLGGTGF | 75  60 |
| Query 76  Sbjct 61 | | PCAAVLDVVTEAGRDWLLLGEVPGQDLLSSHLAPAEKVSI---MADAMRRLHTLDPATCP PC +LD G DW+L E+PG+D S + A++ ++ MA+ +R+L L A CP PCPRLLD RGDDWMLTAELPGRD-ASQPWSAADRPAVLTAMAEGLRQLDAL--AACP | 132  113 |
| Query 133  Sbjct 114 | | FDHQAKHRIERARTRMEAGLVDQDDLDEEHQGLAPAELFARLKARMPDGEDLVVTHGDAC F PA+ VTHGD C  FPSPFP---------------------------GPAD AVTHGDYC | 192  131 |
| Query 193 | | LPNIMVENG--RFSGFIDCGRLGVADRYQDIALATRDIAEEL GGEWADRFLVLYGI  PN+ ++ RF+G +D G LG DRY D+AL + +A L GG A R V | 246 |
| Sbjct | 132 | APNVFIDPATLRFTGVLDVGALGANDRYVDLALMYKSLAGNLNPQYGGPSAARSFVQQYG | 191 |
| Query  Sbjct | 247  192 | AAPDSQRIAFYRLLD 261 A P+ RI +Y LD ADPEDPRIKYYIDLD 206 |  |

```
>TR:A0A3B0B364 A0A3B0B364_9MICO Aminoglycoside 3'-phosphotransferase OS=Microbacterium sp. CGR2 OX=1805820 GN=D7252_03275 PE=3 SV=1
Length=244

Score = 91.3 bits (225), Expect = 1e-18
Identities = 73/227 (32%), Positives = 104/227 (46%), Gaps = 29/227 (13%)

Query 37	FRLSAQGRPVLFVKTDLSGALNELQDEAARLSWLATTGVPCAAVLDVVTEAGRDWLLLGE 96 FR +	P ++K	A + L+DEA RL+W A+ +	VLD	+	+WL+
Sbjct 36	FRATGADEP-FYIKWGPRNAESTLEDEAERLTW-ASRWITVPTVLDHGHDDTHEWLVTAA 93
```

| Query | 97 | VPGQDLLSSHLA--PAEKVSIMADAMRRLH-TLDPATCPFDHQAKHRIERARTRMEAGLV | 153 |
| --- | --- | --- | --- |
| Sbjct | 94 | V G + P V + +R LH +L A CP+D RI A VEGLSAVDPVWVAQPETAVRAVGAGLRVLHDSLPVAVCPWDWSVAWRIANA--------- | 144 |
| Query | 154 | DQDDLDEEHQGLA-PAELFARLKARMPDGEDLVVTHGDACLPNIMVE-NGRFSGFIDCGR | 211 |
| Sbjct | 145 | E +G+ PA L P + LVV+HGDAC PN +++ GR +D  -------EERGIEIPAALHTP PPIDRLVVSHGDACCPNTLLDAEGRPLAHVDLAL | 192 |
| Query  Sbjct | 212  193 | LGVADRYQDIALATRDIAEELGGEWADRFLVLYGIAAPDSQRIAFYR 258 LGVADR+ DIA+A G W D + YG+ PD +R+A+YR LGVADRWADIAVAAMSTEWNYGPGWEDTLIEAYGV-EPDRERLAYYR 238 |  |

>TR:A0A0M2HM17 A0A0M2HM17_9MICO Aminoglycoside 3'-phosphotransferase OS=Microbacterium hydrocarbonoxydans OX=273678 GN=neo PE=3 SV=1

Length=244

Score = 91.3 bits (225), Expect = 1e-18

| Identities  Query 48 | = 66/217 (30%), Positives = 100/217 (46%), Gaps = 26/217 (12%)  FVKTDLSGALNELQDEAARLSWLATTGVPCAAVLDVVTEAGRDWLLLGEVPGQDLLSSHL | 107 |
| --- | --- | --- |
| Sbjct 46 | ++K A ++DEA R+ W A+ + VL+ ++ ++WL+ + G+ YIKWGPHDAEANMRDEAERMRW-ASRWITVPDVLEQGQDSEQEWLVTIALDGRSAADPRW | 104 |
| Query 108 | A--PAEKVSIMADAMRRLHTLDPAT-CPFDHQAKHRIERARTRMEAGLVDQDDLDEEHQG | 164 |
| Sbjct 105 | P V + + +R LH P CP++ RI RA E REDPERAVRAVGEGLRMLHDKLPVEECPWEWSIDSRIARAA ERGT | 149 |
| Query 165 | LAPAELFARLKARMPDGEDLVVTHGDACLPN-IMVENGRFSGFIDCGRLGVADRYQDIAL | 223 |
| Sbjct 150 | PA+L P + LVV HGDACLPN ++ ++G +G +D LG ADR+ DIA+ FVPADL REAPSIDRLVVCHGDACLPNTLLADDGAVAGHVDLAALGTADRWADIAV | 204 |
| Query 224  Sbjct 205 | ATRDIAEELGGEWADRFLVLYGIAAPDSQRIAFYRLL 260 A+ LG W D + YG+ PD R+ FYR L ASMSTIWNLGEGWEDTLIEAYGV-EPDRARLEFYRRL 240 |  |

>TR:E4MZK3 E4MZK3_KITSK Putative aminoglycoside 3'-phosphotransferase OS=Kitasatospora setae (strain ATCC 33774 / DSM 43861 / JCM 3304

/ KCC A-0304 / NBRC 14216 / KM-6054) OX=452652 GN=KSE_41510 PE=3 SV=1

Length=776

Score = 94.7 bits (234), Expect = 1e-18

| Identities | | = 84/254 (33%), Positives = 113/254 (44%), Gaps = 13/254 (5%) |  |
| --- | --- | --- | --- |
| Query 12  Sbjct 527 | | PAAWVERLFGYDWAQQTIGCSDAAVFRLSAQGRPVLFVKTDLSGALNELQDEAARLSWLA PAA VE G A FRL G F K +G+ +L EA R W PAAVVELAVGEPIAAVWRNELKGVTFRLG-DGPGRRFAKWAPAGSGVDLAVEAERTRWAR | 71  585 |
| Query 72  Sbjct 586 | | T-TGVPCAAVLDVVTEAGRDWLLLGEVPGQDLLSSHLA--PAEKVSIMADAMRRLHTLDP T VP VL + + WLL +PG+ + L P V + + +R LH P PFTAVP--EVLGLGGDEHGSWLLTAGLPGRSAVEPELRADPETAVRAIGEGLRALHEALP | 128  643 |
| Query 129  Sbjct 644 | | -ATCPFDHQAKHRIERARTRMEAGLVDQDDLDEEHQGLAPAELFARLKARMPDGEDLVVT A CP++ R+ RA L H G L + A P + VV VADCPYEWSTAERVARAAELRRPPEEWYPAL--RHHGTVERAL--AVLADPPPVDRAVVC | 187  699 |
| Query 188  Sbjct 700 | | HGDACLPNIMV-ENGRFSGFIDCGRLGVADRYQDIALATRDIAEELGGEWADRFLVLYGI HGD C PN +V ++GR SG +D G LGVADR+ D+A+A+ G W L YG+ HGDPCAPNTLVGDDGRPSGHVDLGALGVADRWADLAVASWSTVWNYGPGWELPLLAAYGV | 246  759 |
| Query 247 | | AAPDSQRIAFYRLL 260 PD++R+ +YRLL |  |
| Sbjct | 760 | -EPDAERLDYYRLL 772 | |

>TR:A0A397LR33 A0A397LR33_9MICO Kanamycin kinase OS=Promicromonospora thailandica OX=765201 GN=C7338_3323 PE=4 SV=1

Length=284

Score = 92.0 bits (227), Expect = 1e-18

| Identities | = 75/208 (36%), Positives = 102/208 (49%), Gaps = 24/208 (12%) |  |
| --- | --- | --- |
| Query 63 | EAARLSWLAT-TGVPCAAVLDVVTEAGRDWLLLGEVPGQ-DLLSSHLAP AEKVSI | 115 |
| Sbjct 76 | EA RL W + T VP VLD +A WLL V D+ +S + P AE+ +I EADRLLWAGSFTRVP--HVLDAGRDAHGSWLLTAAVEADGDVATSAVEPRWHAYAERAAI | 133 |
| Query 116 | -MADAMRRLHTLDPA-TCPFDHQAKHRIERARTRMEAGLVDQDDLDEEHQGLAPAELFAR | 173 |
| Sbjct 134 | + +RR H P TCPF + R+ T A L + + A + A AIGVGLRRFHDALPTDTCPFVRTPQMRVHALTTDARAPLAEAER ATISAA | 183 |
| Query 174 | LKARMPDGEDLVVTHGDACLPNIMVEN-GRFSGFIDCGRLGVADRYQDIALATRDIAEEL | 232 |
| Sbjct 184 | L P + LVV HGDA +PN +++ GRF+ +D G LGVADR+ DIA+ TR +  L-GDPPPVDRLVVCHGDATVPNTLLDQVGRFAAHVDVGDLGVADRWSDIAVTTRSVTRRY | 242 |
| Query 233  Sbjct 243 | GGEWADRFLVLYGIAAPDSQRIAFYRLL 260 G L YG+ APD +R A+YR L GPGLEPLVLAAYGV-APDRERTAYYRAL 269 |  |

>TR:A0A3N2G446 A0A3N2G446_9CELL Kanamycin kinase OS=Cellulomonas sp. PhB150 OX=2485188 GN=EDF34_1049 PE=3 SV=1

Length=236

Score = 90.9 bits (224), Expect = 1e-18

| Identities | | = 79/253 (31%), Positives = 112/253 (44%), Gaps = 39/253 (15%) |  |
| --- | --- | --- | --- |
| Query 12  Sbjct 15 | | PAAWVERLFGYDWAQQTIGCSDAAVFRLSAQGRPVLFVKTDLSGALNELQDEAARLSWLA PAA V RL D A + + ++A A R + + TDL+ EAARL W A PAA-VARLARGD-ALEPVWANEAGGLTFRAADRYLKWAPTDLA PEAARLGW-A | 71  64 |
| Query 72  Sbjct 65 | | TTGVPCAAVLDVVTEAGRDWLLLGEVPGQDLLSSHLA--PAEKVSIMADAMRRLHTLDP- P VL+ ++ WL+ +P + + PA + +R LH P  AAWTPVPEVLEHGSDDEGSWLVTRAIPARSAVDPRWVAEPAVAARAIGAGLRALHDALPV | 128  124 |
| Query 129  Sbjct 125 | | ATCPFDHQAKHRIERARTRMEAGLVDQDDLDEEHQGLAPAELFARLKARMPDGEDLVVTH A CPFD R+ RA + E L A P + LVV H AECPFDWGVASRVRRAGSSAE LVAEAPAVDVLVVCH | 188  160 |
| Query 189  Sbjct 161 | | GDACLPN-IMVENGRFSGFIDCGRLGVADRYQDIALATRDIAEELGGEWADRFLVLYGIA GDAC PN ++ ++G ++ +D G LG ADR+ D+A+A G +A YG+ GDACAPNTLLADDGTWAAHVDLGTLGTADRWADLAIAAWSTEWNYGPGFAPVVYEAYGV- | 247  219 |
| Query 248 | | APDSQRIAFYRLL 260 PD++RIAFYR L |  |
| Sbjct | 220 | DPDAERIAFYRRL 232 | |

>TR:A0A0Q8LMY9 A0A0Q8LMY9_9MICO Aminoglycoside phosphotransferase OS=Microbacterium sp. Root180 OX=1736483 GN=ASD93_11990 PE=3 SV=1

Length=241

Score = 90.9 bits (224), Expect = 1e-18

| Identities | = 68/217 (31%), Positives = 99/217 (46%), Gaps = 26/217 (12%) |  |
| --- | --- | --- |
| Query 48 | FVKTDLSGALNELQDEAARLSWLATTGVPCAAVLDVVTEAGRDWLLLGEVPGQDLLSSHL | 107 |
| Sbjct 43 | F+K A + L DE RL W A + VL+ + +WL+ +PG+ ++ FIKHAPHNAESSLADETVRLEW-AGRFLRVPHVLESGQDDTHEWLVTRALPGESAVAPRW | 101 |
| Query 108 | A--PAEKVSIMADAMRRLHTLDPAT-CPFDHQAKHRIERARTRMEAGLVDQDDLDEEHQG | 164 |
| Sbjct 102 | PA V + + +R LH P CPF+ RI A R G+ D L E VAQPAVAVRAVGEGLRALHEALPVEECPFEWSVPSRIANAAGR---GIRIPDALREP--- | 155 |
| Query 165 | LAPAELFARLKARMPDGEDLVVTHGDACLPNIMV-ENGRFSGFIDCGRLGVADRYQDIAL | 223 |
| Sbjct 156 | P + LVV H DAC PN ++ ++GR+S +D G LG DR+ DIA+  --------------PPIDRLVVCHADACCPNTLIGDDGRWSAHVDLGLLGTGDRWGDIAV | 201 |
| Query 224  Sbjct 202 | ATRDIAEELGGEWADRFLVLYGIAAPDSQRIAFYRLL 260 A+ G W D + YG+ PD R+++YR L ASMSTEWNYGPGWEDALIEAYGV-EPDRTRLSYYREL 237 |  |

>TR:D2Q469 D2Q469_KRIFD Aminoglycoside phosphotransferase OS=Kribbella flavida

(strain DSM 17836 / JCM 10339 / NBRC 14399) OX=479435 GN=Kfla_1280 PE=4 SV=1

Length=212

Score = 90.1 bits (222), Expect = 1e-18

| Identities | | = 80/246 (33%), Positives = 107/246 (43%), Gaps = 69/246 (28%) |  |
| --- | --- | --- | --- |
| Query 29  Sbjct 20 | | IGCSDAAVFRLSAQGRPVLFVKTDLSGALNELQDEAARLSWLATTGVPCAAVLDVVTEAG  +G S A V RL +G L+ K D + + +A RL WL+ TG PC VLD G VGHSGATVVRLR-RGAETLYYKEDPA-----VGADADRLVWLSGTGFPCPRVLD RG | 88  69 |
| Query 89  Sbjct 70 | | RDWLLLGEVPGQDLLSSHLAPAEK VSIMADAMRRLHTLDPATCPFDHQAKHRIERA  W+L E+PG+D +S PA ++ +A +R LH L P CPF DGWMLTSELPGRD--ASQPWPAADRPHVLAAIAAGLRELHALPP-DCPF----------- | 144  115 |
| Query 145 | | RTRMEAGLVDQDDLDEEHQGLAPAELFARLKARMP-DGEDLVVTHGDACLPNIMVENG--  R P G VVTHGD PN+ V+ | 201 |
| Sbjct | 116 | --------------------------------RSPFPGSAQVVTHGDYAAPNVFVDPDTL | 143 |
| Query | 202 | RFSGFIDCGRLGVADRYQDIALATRDIAEELGGEW ADRFLVLYGIAAPDSQRIAF | 256 |
| Sbjct | 144 | RF+G +D RLG+ DRY DIAL + ++ L ++ A F YG A PD RI + RFAGVLDISRLGLGDRYVDIALMVKSLSGRLNPQYGGPPAARTFATAYG-ADPDDPRIPY | 202 |
| Query  Sbjct | 257  203 | YRLLDE 262 Y LD+ YTALDD 208 |  |

>TR:A0A4R7T9T0 A0A4R7T9T0_9ACTN Kanamycin kinase/aminoglycoside 3'-phosphotransferase-2 OS=Kribbella sp. VKM Ac-2575 OX=2512212 GN=EV138_2257

PE=4 SV=1

Length=213

Score = 90.1 bits (222), Expect = 1e-18

| Identities | | = 80/243 (33%), Positives = 104/243 (43%), Gaps = 64/243 (26%) |  |
| --- | --- | --- | --- |
| Query 29  Sbjct 20 | | IGCSDAAVFRLSAQGRPVLFVKTDLSGALNELQDEAARLSWLATTGVPCAAVLDVVTEAG  +G S A V RL + L+ K A +EA RLSWLA T +PC VLD G LGWSGATVVRLE-RAAETLYYKAGAGVA-----EEAERLSWLAGTEIPCPRVLD RG | 88  69 |
| Query 89 | | RDWLLLGEVPGQDLLSSHLAPAEKVSIMA---DAMRRLHTLDPATCPFDHQAKHRIERAR DW+L ++ G+D + A AE+ +++A D +RRL L CPFD | 145 |
| Sbjct | 70 | DDWMLTTQLHGRDASQTWPA-AERPAVLAAVADGVRRLRGL--TECPFDSPFP------- | 119 |
| Query | 146 | TRMEAGLVDQDDLDEEHQGLAPAELFARLKARMPDGEDLVVTHGDACLPNIMV--ENGRF | 203 |
| Sbjct | 120 | G VVTHGD C PN+ + E RF  -----------------------------------GPREVVTHGDYCAPNVFIDPETLRF | 144 |
| Query | 204 | SGFIDCGRLGVADRYQDIALATRDIAEEL GGEWADRFLVLYGIAAPDSQRIAFYRL | 259 |
| Sbjct | 145 | SG +D RLG+ DRY D AL + ++ L GG A R V+ PD RI Y SGILDLSRLGLGDRYVDFALMHKSLSNGLNPQYGGPPAAREFVVLAGGDPDDPRIQHYTD | 204 |
| Query  Sbjct | 260  205 | LDE 262 LD+  LDD 207 |  |

>TR:A0A5C4SX21 A0A5C4SX21_9BACL Aminoglycoside 3'-phosphotransferase OS=Paenibacillus hemerocallicola OX=1172614 GN=FE784_40545 PE=3 SV=1

Length=271

Score = 91.3 bits (225), Expect = 2e-18

Identities = 55/217 (25%), Positives = 101/217 (47%), Gaps = 19/217 (9%)

| Query | 57 | LNELQDEAARLSWLATTGVPCAAVLDVVTEAGRDWLLLGEVPGQDLLSSH--LAPAEKVS | 114 |
| --- | --- | --- | --- |
| Sbjct | 53 | +N++Q EA +L GV C VL ++ RD+L+ +PG D +S + P INQMQTEALMTDYLYKYGV-CPRVLHYSSDDKRDYLITDRIPGSDAVSDEYLMQPERLTE | 111 |
| Query | 115 | IMADAMRRLHTLDPATCPFDHQAKHRIERARTRMEAGLVDQD DLDEEHQGLA | 166 |
| Sbjct | 112 | + A+++ +LH ++ + CP + + + RA G ++ ++ H+ +  VFAESLLKLHQVEYSDCPIINGLEEMVIRAEGNYREGRAEKGILRYLGYTSIESAHENMI | 171 |

| Query | 167 | PAELFARLKARMPDGEDLVVTHGDACLPNIMVENGRFSGFIDCGRLGVADRYQDIALATR 226 |
| --- | --- | --- |
| Sbjct | 172 | + ED VV HGD CLPN+++ + + +G++D G+ DR+ DI  NLYKYVN--------EDKVVIHGDHCLPNLILHDFKMNGYVDVAYGGIGDRHYDIFWGLW 223 |
| Query | 227 | DIAEELGGEWADRFLVLYGIAAPDSQRIAFYRLLDEF 263 |
| Sbjct | 224 | + L G+++ +F+ YG D R+ LL F  SLQHNLKGDYSQQFIQTYGNHLIDPDRLRLCGLLSVF 260 |

>TR:A0A0Q5B127 A0A0Q5B127_9MICO Uncharacterized protein OS=Rathayibacter sp. Leaf296 OX=1736327 GN=ASF46_09425 PE=3 SV=1

Length=219

Score = 89.7 bits (221), Expect = 2e-18

| Identities | = 79/257 (31%), Positives = 113/257 (44%), Gaps = 56/257 (22%) |  |
| --- | --- | --- |
| Query 14 | AWVERLFGYD WAQQTIGCSDAAVFRLSAQGRPVLFVKTDLSGALNELQDEAARLSW | 69 |
| Sbjct 4 | A RL G D W Q +D FR+ P ++K + +G +L+ E+ARL W AIARRLGGGDPELAWRNQ----ADGLTFRV PAGYLKWNPAGTGIDLRRESARLRW | 54 |
| Query 70 | LATTGVPCAAVLDVVTEAGRDWLLLGEVPGQDLLSSH--LAPAEKVSIMADAMRRLHTLD | 127 |
| Sbjct 55 | L P VLD E G WLL + G+ + P V +A +R LH + LEGRH-PSPRVLDEGDEDGGQWLLTAPIAGRSAVDPLWIARPGTAVRAIARNLRALHAVP | 113 |
| Query 128 | PATCPFDHQAKHRIERARTRMEAGLVDQDDLDEE---HQGLAPAELFARLKARMPDGEDL | 184 |
| Sbjct 114 | P D+L + H+G AP + E AGDLP-----------------------DELRRDSWFHRGAAP EPERP | 138 |
| Query 185 | VVTHGDACLPNIMV-ENGRFSGFIDCGRLGVADRYQDIALATRDIAEELGGEWADRFLVL | 243 |
| Sbjct 139 | V+ HGDAC PN ++ ++G +SG +D G LG+ DR+ D+A+AT + G + D L VLVHGDACAPNTLIGDDGAWSGTVDLGDLGLGDRWADLAVATMSLHWNYGDGFTDLLLEE | 198 |
| Query 244  Sbjct 199 | YGIAAPDSQRIAFYRLL 260 YGI APD +R +FYR L  YGI-APDEERTSFYRAL 214 |  |

>TR:A0A4Q2UGS5 A0A4Q2UGS5_9BACT Aminoglycoside 3'-phosphotransferase OS=Spirosoma sordidisoli OX=2502893 GN=EQG79_28890 PE=3 SV=1

Length=264

Score = 90.5 bits (223), Expect = 3e-18

| Identities | | = 79/244 (32%), Positives = 115/244 (47%), Gaps = 12/244 (5%) |  |
| --- | --- | --- | --- |
| Query 28  Sbjct 26 | | TIGCSDAAVFRLSAQGRPVLFVKTD---LSGALNELQDEAARLSWLA-TTGVPCAAVLDV T+G S V+R +G F+K S ++ EA L WL+ VP V+ V TVGESPCPVYRFK-RGNDSFFLKMCSRVYSPTTFSVRREADVLGWLSGQLNVP--EVVGV | 83  82 |
| Query 84 | | VTEAGRDWLLLGEVPGQDLLSSHLAPAEKVSIMADAMRRLHTLDPATCPFDHQAKHRIER  + ++++ VPGQ L A + + +A+ +L L TCPFD R++ | 143 |
| Sbjct | 83 | AESSEGEFMITRSVPGQPLYQRIDAQQPVLHLFQEALAQLQALPITTCPFDGGVAFRLKE | 142 |
| Query | 144 | ARTRMEAGLVDQD-DLDE--EHQGLAPAELFARLKARMPDGEDLVVTHGDACLPNIMVEN | 200 |
| Sbjct | 143 | + GL+ ++ DL + + + P E A L A P E+LV HGD N+ V+ LEYLLANGLLAEEYDLHQWPDVEANNPQEFVAHLFATQPT-EELVFAHGDLTDSNLFVDA | 201 |
| Query | 201 | GRFSGFIDCGRLGVADRYQDIALATRDIAEELGGEWADRFLVLYGIAAPDSQRIAFYRLL | 260 |
| Sbjct | 202 | FID GR GVADR+ D AL ++ EE+ AD FL G A +R+ FY L QDRLYFIDWGRGGVADRWMDRALVYHNLLEEVSPTQADAFLAELGEADNPRKRV-FYEQL | 260 |
| Query  Sbjct | 261  261 | DEFF 264 DE F  DELF 264 |  |

>TR:A0A2S8WU98 A0A2S8WU98_9MICC Aminoglycoside phosphotransferase APH(3') OS=Arthrobacter sp. MYb227 OX=1848601 GN=CQ018_07275 PE=3 SV=1

Length=271

Score = 90.5 bits (223), Expect = 3e-18

Identities = 67/230 (29%), Positives = 113/230 (49%), Gaps = 10/230 (4%)

| Query | 37 | FRLSAQ-GRPVLFVKTDLSGAL-NELQDEAARLSWLATTGVPCAAVLDVVTEAGRDWLLL | 94 |
| --- | --- | --- | --- |
| Sbjct | 42 | FR+ + G FVK ++G ++ + EA +++W+ + VL+V + WL+ FRIGDETGLATRFVKYAVAGTPESDFEREATKMAWVGKFSL-VPQVLEVGQDEAGSWLMT | 100 |
| Query | 95 | GEVPGQDLLSSHLAPAEKVSIMA--DAMRRLHTLDP-ATCPFDHQAKHRIERARTRMEAG | 151 |
| Sbjct | 101 | +PG + +++ A +R LH P A C F R++ R+ G RALPGSSAVDPRWIARPEIAARAIGAGLRTLHDALPVAGCEFSWSVSERLKGFEQRISTG | 160 |
| Query | 152 | LVDQDDLDEEHQGLAPAELFARLKARMPDGEDLVVTHGDACLPNIMVEN-GRFSGFIDCG | 210 |
| Sbjct | 161 | D +E+ L+ AE L ++ P+ + LVV HGDAC PN ++++ G F+G +D G  ETP-DSWSKEYAHLSIAEA-RELLSQPPEIQKLVVCHGDACAPNTLLDDEGNFAGHVDLG | 218 |
| Query | 211 RLGVADRYQDIALATRDIAEELGGEWADRFLVLYGIAAPDSQRIAFYRLL 260 | | |
|  |  | +GV DR+ D+A+A G + YG+ PD RIA+Y++L |  |
| Sbjct | 219 | EVGVGDRWADLAIAAWSTEWNYGPGYEGFVYQGYGV-VPDQVRIAYYKML 267 | |

>TR:F8A0B8 F8A0B8_CELGA Aminoglycoside phosphotransferase OS=Cellulomonas gilvus (strain ATCC 13127 / NRRL B-14078) OX=593907 GN=Celgi_0943

PE=3 SV=1

Length=240

Score = 89.7 bits (221), Expect = 4e-18

| Identities | | = 72/219 (33%), Positives = 102/219 (47%), Gaps = 30/219 (14%) |  |
| --- | --- | --- | --- |
| Query 47  Sbjct 43 | | LFVKTDLSGALNELQDEAARLSWLAT-TGVPCAAVLDVVTEAGRDWLLLGEVPGQDLLSS L+ K +G +L DEA RL+W+A T VP E G WL+ + G+ + LYAKWAPAGTEGDLADEAERLAWVARWTPVPHVVETGADDEGG--WLVTRAIEGRSAVHP | 105  100 |
| Query 106 | | HLAPAEKVSIMA--DAMRRLHTLDP-ATCPFDHQAKHRIERARTRMEAGLVDQDDLDEEH  +V++ A +R LH P A CP+ A+ R+ R R A + | 162 |
| Sbjct | 101 | QWVAHPEVAVRAVGAGLRALHDALPVAGCPWTWSARDRLARVADRAAAAALG-------- | 152 |
| Query | 163 | QGLAPAELFARLKARMPDGEDLVVTHGDACLPNIMV-ENGRFSGFIDCGRLGVADRYQDI | 221 |
| Sbjct | 153 | AP P + LVV HGDAC+PN ++ ++G + +D G LGVADR+ D+  ---AP PPVDRLVVCHGDACVPNTLLSDDGTWLAHVDLGSLGVADRWADL | 198 |
| Query  Sbjct | 222  199 | ALATRDIAEELGGEWADRFLVLYGIAAPDSQRIAFYRLL 260 A+AT G + L YGI APD R A+YR L AVATWSTVWNYGPGHEEALLDAYGI-APDPVRTAYYRTL 236 |  |

>TR:A0A397LNG7 A0A397LNG7_9MICO Kanamycin kinase OS=Promicromonospora thailandica OX=765201 GN=C7338_3321 PE=3 SV=1

Length=262

Score = 90.1 bits (222), Expect = 4e-18

| Identities | | = 72/210 (34%), Positives = 105/210 (50%), Gaps = 18/210 (9%) |  |
| --- | --- | --- | --- |
| Query 59  Sbjct 65 | | ELQDEAARLSWLAT-TGVPCAAVLDVVTEAGRDWLLLGEVPGQDLLSSH--LAPAEKVSI  +L DEAAR+ W T VP VL+V ++ DWL+ G + + + P DLVDEAARMRWAEPYTTVP--HVLEVGSDGEVDWLVTGALDARSAVDPRWLAEPETAARA | 115  122 |
| Query 116  Sbjct 123 | | MADAMRRLHTLDPAT-CPFDHQAKHRIERARTRMEAGLVDQDDLDEEHQGLAPAELFARL  + +R LH P CP+ + R+ A R G G + AE+ ARL IGAGLRALHDALPVDRCPWTWSIEDRMAVAEDRAARG HAADGWS-AEVRARL | 174  173 |
| Query 175  Sbjct 174 | | KARMPDGEDLVVTHGDACLPNIMV-ENGRFSGFIDCGRLGVADRYQDIALATRDIAEELG A PD + LVV HGDAC PN ++ ++G + G +D G LGVADR+ D+A+AT + G  -ADAPDHDRLVVCHGDACAPNTLLSDDGAWFGHVDLGALGVADRWADLAVATYSLGWNYG | 233  232 |
| Query | 234 GEWADRFLVLYGIAAPDSQRIAFYRLLDEF 263 | | |
|  |  | + YGI D +R+A+YR+L +F |  |
| Sbjct | 233 | PGYDHLVYEGYGI-DEDPERVAWYRMLWDF 261 | |

>TR:A0A1S1RKG9 A0A1S1RKG9_9ACTN Aminoglycoside phosphotransferase APH(3') OS=Frankia sp. Cc1.17 OX=573497 GN=CC117_00075 PE=3 SV=1

Length=254

Score = 89.7 bits (221), Expect = 5e-18

Identities = 69/217 (32%), Positives = 95/217 (44%), Gaps = 20/217 (9%)

| Query | 48 | FVKTDLSGALNELQDEAARLSWLATTGVPCAAVLDVVTEAGRDWLLLGEVPGQDLLSSHL | 107 |
| --- | --- | --- | --- |
| Sbjct | 49 | FVK +G+ +L EA RL W A P VL + WL+ +PG++ + FVKWAPAGSGLDLTAEADRLRWAAPF-TPVPTVLGEGRDGAGSWLVTAGLPGRNAVDERW | 107 |
| Query | 108 | A--PAEKVSIMADAMRRLHTLDP-ATCPFDHQAKHRIERARTRMEAGLVDQDDLDEEHQG | 164 |
| Sbjct | 108 | P V + +R H P A CPF R+ A E + KADPRRAVQAIGRGLRAFHDALPVAVCPFSWSVADRLADAAPWATT ERRR- | 157 |
| Query | 165 | LAPAELFARLKARMPDGEDLVVTHGDACLPN-IMVENGRFSGFIDCGRLGVADRYQDIAL | 223 |
| Sbjct | 158 | A L A P + LVV GDAC PN ++ ++G +SG +D G +GVADR+ D+A+  ----RCLAELAAGAPPVDVLVVCQGDACPPNTLLADDGTWSGHVDLGAMGVADRWADLAV | 213 |
| Query  Sbjct | 224  214 | ATRDIAEELGGEWADRFLVLYGIAAPDSQRIAFYRLL 260 AT + G W L YG+ PD R AFY L ATCSLGWNYGPGWEPALLDAYGV-VPDDDRTAFYNRL 249 |  |

>TR:A0A132PFY8 A0A132PFY8_9MYCO Aminoglycoside phosphotransferase OS=Mycolicibacterium wolinskyi OX=59750 GN=AFM11_26685 PE=3 SV=1

Length=242

Score = 89.4 bits (220), Expect = 5e-18

| Identities | = 65/217 (30%), Positives = 92/217 (42%), Gaps = 29/217 (13%) |  |
| --- | --- | --- |
| Query 48 | FVKTDLSGALNELQDEAARLSWLATTGVPCAAVLDVVTEAGRDWLLLGEVPGQDLLSSH- | 106 |
| Sbjct 46 | FVK +L EA +L W A + VLDV + +WL + G+ + FVKVAPPHPSVDLHREAIKLRW-ARQYLTVPPVLDVGRDGATEWLHTAGIAGRSAADPYW | 104 |
| Query 107 | -LAPAEKVSIMADAMRRLHTLDPAT-CPFDHQAKHRIERARTRMEAGLVDQDDLDEEHQG | 164 |
| Sbjct 105 | P V +A +R +H P CPF R+ L D +D IARPRLAVRAIATGLRAMHERAPVDRCPFSWSVPERLANIDDAARRRLPDAPPIDR---- | 160 |
| Query 165 | LAPAELFARLKARMPDGEDLVVTHGDACLPNIMVEN-GRFSGFIDCGRLGVADRYQDIAL | 223 |
| Sbjct 161 | LVV HGD+C PN ++++ GR+ G +D G LGVADR+ D+A+  -------------------LVVCHGDSCAPNTLLDDDGRWCGHVDLGELGVADRWADLAV | 201 |
| Query 224  Sbjct 202 | ATRDIAEELGGEWADRFLVLYGIAAPDSQRIAFYRLL 260 AT + G+W F YG+ PD RI +YR L ATLSLGWNYAGDWEAEFFGAYGV-TPDPVRIDYYRRL 237 |  |

>TR:D5UXJ9 D5UXJ9_TSUPD Aminoglycoside phosphotransferase OS=Tsukamurella paurometabola (strain ATCC 8368 / DSM 20162 / JCM 10117 /

NBRC 16120 / NCTC 13040) OX=521096 GN=Tpau_1464 PE=3 SV=1

Length=261

Score = 89.7 bits (221), Expect = 5e-18

| Identities | = 71/200 (36%), Positives = 90/200 (45%), Gaps = 29/200 (15%) |  |
| --- | --- | --- |
| Query 63 | EAARLSWLATTGVPCAAVLDVVTEAGRDWLLLGEVPGQDLLSSHLA--PAEKVSIMADAM | 120 |
| Sbjct 59 | EA RL W A V V+ + G +WL+ + G + A PA + + EAVRLRW-AQPHVTVPRVIARGSSDGAEWLVTAAIAGTSAIDPRWADSPAIAARAIGAGL | 117 |
| Query 121 | RRLH-TLDPATCPFDHQAKHRIERARTRMEAGLVDQDDLDEEHQGLAPAELFARLKARMP | 179 |
| Sbjct 118 | R LH TL CPFD ART GL PAE A P RHLHDTLPVHVCPFDWSVS-----ART VGLTPAERAAL--GPTP | 154 |
| Query 180 | DGEDLVVTHGDACLPNIMV-ENGRFSGFIDCGRLGVADRYQDIALATRDIAEELGGEWAD | 238 |
| Sbjct 155 | + LVV HGDAC PN +V E+GRF+G +D G LG ADR+ D+A+A + D PIDRLVVCHGDACAPNTLVGEDGRFAGHVDLGSLGTADRWADLAVAAMSLDWNFDQPRPD | 214 |
| Query 239  Sbjct 215 | RFLVLYGIAAPDSQRIAFYR 258 YG+ APD R+AFYR  VLFEAYGV-APDEARLAFYR 233 |  |

>TR:A0A511A8P8 A0A511A8P8_9MICO Aminoglycoside phosphotransferase APH(3') OS=Frigoribacterium faeni OX=145483 GN=FFA01_19620 PE=3 SV=1

Length=246

Score = 89.4 bits (220), Expect = 6e-18

| Identities | | = 70/217 (32%), Positives = 104/217 (48%), Gaps = 27/217 (12%) |  |
| --- | --- | --- | --- |
| Query 48  Sbjct 49 | | FVKTDLSGALNELQDEAARLSWLATTGVPCAAVLDVVTEAGRDWLLLGEVPGQDLLSSHL F K G+ +L E RL W A P VL++ ++ D+LL +PG+ + + FAKWAPHGSGLDLHAEELRLRWAAEF-TPVPRVLEIGSDDDGDYLLTAGLPGRSAVDARW | 107  107 |
| Query 108 | | APAEKVSIMADA--MRRLHTLDPAT-CPFDHQAKHRIERARTRMEAGLVDQDDLDEEHQG A + ++ A +R LH P CPF + RI + T A | 164 |
| Sbjct | 108 | VAAPEAAVRAAGRGLRLLHDALPVDECPFGWSVEERIATSATARTAP------------- | 154 |
| Query | 165 | LAPAELFARLKARMPDGEDLVVTHGDACLPNIMV-ENGRFSGFIDCGRLGVADRYQDIAL | 223 |
| Sbjct | 155 | RL PD + +VV HGDAC+PN +V ++G ++G +D LGVADR+ D+A+  -------PRLGDPPPD-DLVVVCHGDACVPNTLVHDDGTWAGHVDLDALGVADRWADLAV | 206 |
| Query  Sbjct | 224  207 | ATRDIAEELGGEWADRFLVLYGIAAPDSQRIAFYRLL 260 AT + G + +L YGIAA D R+A+YR L ATMSLEWNYGPGYERTYLDAYGIAA-DPVRLAYYRAL 242 |  |

| Lambda | K | H |
| --- | --- | --- |
| 0.322 | 0.138 | 0.423 |
| Gapped Lambda | K | H |
| 0.267 | 0.0410 | 0.140 |

Effective search space used: 1567986459507

Database: uniprotkb_refprotswissprot Posted date: Dec 02, 2020 12:29 PM

Number of letters in database: 21,838,191,652 Number of sequences in database: 57,391,823

Matrix: BLOSUM62

Gap Penalties: Existence: 11, Extension: 1 Neighboring words threshold: 11

Window for multiple hits: 40
