# Supplementary material for: Safety Assessment of the CP4 EPSPS and NPTII Proteins in Eucalyptus
Source: GM Crops Food. 2023 Jun 19;14(1):1–14. doi: 10.1080/21645698.2023.2222436 (PMC10281460; doi:10.1080/21645698.2023.2222436)
Supplement: Supplemental Material [file KGMC_A_2222436_SM4294.zip › TableS07.docx]

BLASTP output of the CP4-EPSPS protein against the NCBI nr protein database

BLASTP 2.11.0+

Reference: Stephen F. Altschul, Thomas L. Madden, Alejandro

A. Schaffer, Jinghui Zhang, Zheng Zhang, Webb Miller, and David J. Lipman (1997), "Gapped BLAST and PSI-BLAST: a new generation of protein database search programs", Nucleic Acids Res. 25:3389-3402.

Reference for compositional score matrix adjustment: Stephen

F. Altschul, John C. Wootton, E. Michael Gertz, Richa Agarwala, Aleksandr Morgulis, Alejandro A. Schaffer, and Yi-Kuo Yu (2005) "Protein database searches using compositionally adjusted substitution matrices", FEBS J. 272:5101-5109.

RID: X2SS55HW016

Database: All non-redundant GenBank CDS translations+PDB+SwissProt+PIR+PRF excluding environmental samples from WGS projects

329,899,723 sequences; 119,339,821,200 total letters

| Query= CP$-EPSPS |  | | |
| --- | --- | --- | --- |
| Length=455 |  |  |  |
| Sequences producing significant alignments: | Score (Bits) | E  Value | Max Ident |
| ARW80140.1 Ctp2 CP4 EPSPS [synthetic construct] [Zea mays] | 887 | 0.0 | 100% |
| ARW80139.1 CTP2 CP4-EPSPS [synthetic construct] [Zea mays] | 885 | 0.0 | 100% |
| AAL67577.1 CP4EPSPS [Glycine max] | 885 | 0.0 | 100% |
| AII71485.1 5-enolpyruvulshikimate-3-phosphate synthase [standa... | 882 | 0.0 | 100% |
| Q9R4E4.2 RecName: Full=3-phosphoshikimate 1-carboxyvinyltransf... | 881 | 0.0 | 100% |
| 2GGD_A CP4 EPSP synthase Ala100Gly liganded with S3P and Glyph... | 879 | 0.0 | 100% |
| AII80555.1 5-enol-pyruvylshikimate-3-phospate synthase [synthe... | 879 | 0.0 | 99% |
| ADZ55658.1 CP4EPSPS protein [synthetic construct] | 877 | 0.0 | 99% |
| AJW67920.1 CP4 EPSPS protein [synthetic construct] | 874 | 0.0 | 99% |
| AEK64772.1 wheat CP4EPSPS protein [synthetic construct] | 867 | 0.0 | 98% |
| 2PQB_A CP4 EPSPS liganded with (R)-difluoromethyl tetrahedral ... | 863 | 0.0 | 100% |
| 2PQD_A A100G CP4 EPSPS liganded with (R)-difluoromethyl tetrah... | 860 | 0.0 | 100% |
| WP_117368106.1 3-phosphoshikimate 1-carboxyvinyltransferase [S... | 854 | 0.0 | 98% |
| WP_160786970.1 3-phosphoshikimate 1-carboxyvinyltransferase [S... | 848 | 0.0 | 95% |
| WP_184145208.1 3-phosphoshikimate 1-carboxyvinyltransferase [S... | 847 | 0.0 | 95% |
| WP_134649646.1 MULTISPECIES: 3-phosphoshikimate 1-carboxyvinyl... | 847 | 0.0 | 95% |
| WP_160870076.1 3-phosphoshikimate 1-carboxyvinyltransferase [S... | 846 | 0.0 | 95% |
| WP_037405162.1 3-phosphoshikimate 1-carboxyvinyltransferase [S... | 844 | 0.0 | 96% |
| EYR84130.1 3-phosphoshikimate 1-carboxyvinyltransferase AroA [... | 843 | 0.0 | 96% |
| WP_050745479.1 MULTISPECIES: 3-phosphoshikimate 1-carboxyvinyl... | 843 | 0.0 | 96% |
| WP_130516341.1 3-phosphoshikimate 1-carboxyvinyltransferase [S... | 842 | 0.0 | 96% |
| WP_119257645.1 3-phosphoshikimate 1-carboxyvinyltransferase [S... | 841 | 0.0 | 95% |
| TCN38546.1 3-phosphoshikimate 1-carboxyvinyltransferase [Shine... | 841 | 0.0 | 95% |
| WP_133035983.1 3-phosphoshikimate 1-carboxyvinyltransferase [S... | 840 | 0.0 | 95% |
| WP_064328835.1 3-phosphoshikimate 1-carboxyvinyltransferase [S... | 838 | 0.0 | 95% |
| WP_069060003.1 3-phosphoshikimate 1-carboxyvinyltransferase [S... | 837 | 0.0 | 95% |
| WP_160858055.1 3-phosphoshikimate 1-carboxyvinyltransferase [S... | 836 | 0.0 | 95% |
| WP_176950611.1 3-phosphoshikimate 1-carboxyvinyltransferase [R... | 820 | 0.0 | 93% |
| WP_163902602.1 3-phosphoshikimate 1-carboxyvinyltransferase [R... | 820 | 0.0 | 92% |
| WP_099056063.1 3-phosphoshikimate 1-carboxyvinyltransferase [R... | 819 | 0.0 | 92% |
| WP_108001699.1 3-phosphoshikimate 1-carboxyvinyltransferase [M... | 818 | 0.0 | 92% |
| WP_183801805.1 3-phosphoshikimate 1-carboxyvinyltransferase [R... | 817 | 0.0 | 93% |
| PZU81186.1 3-phosphoshikimate 1-carboxyvinyltransferase [Shine... | 817 | 0.0 | 92% |
| WP_109458669.1 3-phosphoshikimate 1-carboxyvinyltransferase [R... | 815 | 0.0 | 91% |
| WP_097140341.1 3-phosphoshikimate 1-carboxyvinyltransferase [R... | 815 | 0.0 | 93% |

| OJT97432.1 3-phosphoshikimate 1-carboxyvinyltransferase [Rhizo... | 815 | 0.0 | 91% |
| --- | --- | --- | --- |
| WP_153442469.1 3-phosphoshikimate 1-carboxyvinyltransferase [S... | 812 | 0.0 | 91% |
| WP_071024014.1 3-phosphoshikimate 1-carboxyvinyltransferase [E... | 811 | 0.0 | 91% |
| WP_034853513.1 3-phosphoshikimate 1-carboxyvinyltransferase [E... | 811 | 0.0 | 91% |
| WP_066867978.1 3-phosphoshikimate 1-carboxyvinyltransferase [S... | 811 | 0.0 | 91% |
| WP_026613614.1 MULTISPECIES: 3-phosphoshikimate 1-carboxyvinyl... | 811 | 0.0 | 91% |
| WP_003534382.1 3-phosphoshikimate 1-carboxyvinyltransferase [S... | 810 | 0.0 | 91% |
| WP_180939806.1 3-phosphoshikimate 1-carboxyvinyltransferase [E... | 810 | 0.0 | 91% |
| WP_017271506.1 3-phosphoshikimate 1-carboxyvinyltransferase [S... | 810 | 0.0 | 91% |
| WP_153420160.1 3-phosphoshikimate 1-carboxyvinyltransferase [S... | 809 | 0.0 | 91% |
| WP_081157816.1 3-phosphoshikimate 1-carboxyvinyltransferase [E... | 808 | 0.0 | 91% |
| WP_136509256.1 3-phosphoshikimate 1-carboxyvinyltransferase [E... | 808 | 0.0 | 91% |
| WP_010968452.1 MULTISPECIES: 3-phosphoshikimate 1-carboxyvinyl... | 808 | 0.0 | 91% |
| WP_085031493.1 3-phosphoshikimate 1-carboxyvinyltransferase [E... | 808 | 0.0 | 91% |
| WP_085024845.1 3-phosphoshikimate 1-carboxyvinyltransferase [E... | 808 | 0.0 | 91% |
| WP_127661569.1 3-phosphoshikimate 1-carboxyvinyltransferase [S... | 808 | 0.0 | 90% |
| WP_018093872.1 MULTISPECIES: 3-phosphoshikimate 1-carboxyvinyl... | 807 | 0.0 | 91% |
| WP_127622322.1 3-phosphoshikimate 1-carboxyvinyltransferase [S... | 807 | 0.0 | 90% |
| WP_127631404.1 3-phosphoshikimate 1-carboxyvinyltransferase [S... | 807 | 0.0 | 90% |
| WP_192249075.1 3-phosphoshikimate 1-carboxyvinyltransferase [R... | 807 | 0.0 | 92% |
| WP_127510767.1 3-phosphoshikimate 1-carboxyvinyltransferase [S... | 806 | 0.0 | 90% |
| WP_100669854.1 3-phosphoshikimate 1-carboxyvinyltransferase [S... | 805 | 0.0 | 90% |
| WP_013844999.1 3-phosphoshikimate 1-carboxyvinyltransferase [S... | 805 | 0.0 | 90% |
| WP_127535276.1 3-phosphoshikimate 1-carboxyvinyltransferase [S... | 805 | 0.0 | 90% |
| WP_127597035.1 3-phosphoshikimate 1-carboxyvinyltransferase [S... | 804 | 0.0 | 90% |
| WP_037398737.1 3-phosphoshikimate 1-carboxyvinyltransferase [S... | 803 | 0.0 | 92% |
| WP_151611883.1 3-phosphoshikimate 1-carboxyvinyltransferase [E... | 803 | 0.0 | 90% |
| WP_037456937.1 3-phosphoshikimate 1-carboxyvinyltransferase [S... | 802 | 0.0 | 92% |
| WP_014330677.1 3-phosphoshikimate 1-carboxyvinyltransferase [S... | 801 | 0.0 | 92% |
| OJU68221.1 3-phosphoshikimate 1-carboxyvinyltransferase [Rhizo... | 801 | 0.0 | 89% |
| WP_037377818.1 MULTISPECIES: 3-phosphoshikimate 1-carboxyvinyl... | 800 | 0.0 | 91% |
| WP_012710050.1 3-phosphoshikimate 1-carboxyvinyltransferase [S... | 800 | 0.0 | 92% |
| WP_040959218.1 3-phosphoshikimate 1-carboxyvinyltransferase [S... | 800 | 0.0 | 91% |
| WP_069459855.1 3-phosphoshikimate 1-carboxyvinyltransferase [E... | 800 | 0.0 | 90% |
| WP_145161583.1 3-phosphoshikimate 1-carboxyvinyltransferase [S... | 799 | 0.0 | 90% |
| WP_026621852.1 3-phosphoshikimate 1-carboxyvinyltransferase [E... | 799 | 0.0 | 90% |
| WP_037432760.1 3-phosphoshikimate 1-carboxyvinyltransferase [S... | 799 | 0.0 | 92% |
| WP_018209105.1 3-phosphoshikimate 1-carboxyvinyltransferase [S... | 798 | 0.0 | 90% |
| HCL64536.1 TPA: 3-phosphoshikimate 1-carboxyvinyltransferase [... | 797 | 0.0 | 89% |
| WP_132072694.1 3-phosphoshikimate 1-carboxyvinyltransferase [S... | 797 | 0.0 | 91% |
| WP_018235235.1 3-phosphoshikimate 1-carboxyvinyltransferase [E... | 796 | 0.0 | 91% |
| WP_064241696.1 3-phosphoshikimate 1-carboxyvinyltransferase [E... | 796 | 0.0 | 91% |
| WP_012067654.1 MULTISPECIES: 3-phosphoshikimate 1-carboxyvinyl... | 796 | 0.0 | 90% |
| WP_037422330.1 3-phosphoshikimate 1-carboxyvinyltransferase [S... | 796 | 0.0 | 90% |
| WP_127617043.1 3-phosphoshikimate 1-carboxyvinyltransferase [S... | 795 | 0.0 | 90% |
| WP_018013258.1 3-phosphoshikimate 1-carboxyvinyltransferase [S... | 795 | 0.0 | 90% |
| WP_097519311.1 3-phosphoshikimate 1-carboxyvinyltransferase [S... | 794 | 0.0 | 91% |
| WP_104838674.1 3-phosphoshikimate 1-carboxyvinyltransferase [S... | 794 | 0.0 | 90% |
| WP_097537683.1 MULTISPECIES: 3-phosphoshikimate 1-carboxyvinyl... | 793 | 0.0 | 90% |
| WP_014766697.1 3-phosphoshikimate 1-carboxyvinyltransferase [S... | 793 | 0.0 | 90% |
| WP_192119985.1 unnamed protein product | 789 | 0.0 | 90% |
| AMM72836.1 5-enolypyruvylshikimate-3-phosphate synthase, parti... | 781 | 0.0 | 98% |
| WP_173511260.1 3-phosphoshikimate 1-carboxyvinyltransferase [E... | 780 | 0.0 | 91% |
| WP_168265554.1 3-phosphoshikimate 1-carboxyvinyltransferase [R... | 776 | 0.0 | 88% |
| WP_180194384.1 3-phosphoshikimate 1-carboxyvinyltransferase [R... | 776 | 0.0 | 87% |
| WP_105007830.1 3-phosphoshikimate 1-carboxyvinyltransferase [R... | 776 | 0.0 | 88% |
| WP_130653374.1 3-phosphoshikimate 1-carboxyvinyltransferase [R... | 774 | 0.0 | 87% |
| WP_065276731.1 3-phosphoshikimate 1-carboxyvinyltransferase [R... | 774 | 0.0 | 87% |
| WP_163878748.1 3-phosphoshikimate 1-carboxyvinyltransferase [R... | 774 | 0.0 | 88% |
| WP_017989914.1 3-phosphoshikimate 1-carboxyvinyltransferase [R... | 774 | 0.0 | 88% |
| WP_138388525.1 3-phosphoshikimate 1-carboxyvinyltransferase [R... | 774 | 0.0 | 87% |
| WP_168349154.1 3-phosphoshikimate 1-carboxyvinyltransferase [R... | 774 | 0.0 | 87% |
| WP_130759700.1 3-phosphoshikimate 1-carboxyvinyltransferase [R... | 774 | 0.0 | 87% |
| WP_131702060.1 3-phosphoshikimate 1-carboxyvinyltransferase [R... | 774 | 0.0 | 87% |
| WP_128400242.1 3-phosphoshikimate 1-carboxyvinyltransferase [R... | 773 | 0.0 | 87% |
| WP_130784542.1 3-phosphoshikimate 1-carboxyvinyltransferase [R... | 773 | 0.0 | 87% |
| WP_164046176.1 3-phosphoshikimate 1-carboxyvinyltransferase [R... | 773 | 0.0 | 87% |
| WP_131605380.1 3-phosphoshikimate 1-carboxyvinyltransferase [R... | 773 | 0.0 | 87% |
| WP_047617633.1 3-phosphoshikimate 1-carboxyvinyltransferase [R... | 772 | 0.0 | 87% |
| WP_003544289.1 3-phosphoshikimate 1-carboxyvinyltransferase [R... | 772 | 0.0 | 87% |
| WP_072640868.1 3-phosphoshikimate 1-carboxyvinyltransferase [R... | 772 | 0.0 | 87% |
| WP_130718486.1 3-phosphoshikimate 1-carboxyvinyltransferase [R... | 772 | 0.0 | 87% |

| WP_145638220.1 | 3-phosphoshikimate | 1-carboxyvinyltransferase | [N... | 772 | 0.0 | 88% |
| --- | --- | --- | --- | --- | --- | --- |
| WP_131631191.1 | 3-phosphoshikimate | 1-carboxyvinyltransferase | [R... | 772 | 0.0 | 87% |
| WP_112905641.1 | 3-phosphoshikimate | 1-carboxyvinyltransferase | [R... | 772 | 0.0 | 87% |
| WP_028002217.1 | 3-phosphoshikimate | 1-carboxyvinyltransferase | [S... | 771 | 0.0 | 89% |
| WP_131719773.1 | 3-phosphoshikimate | 1-carboxyvinyltransferase | [R... | 771 | 0.0 | 87% |
| WP_130712832.1 | 3-phosphoshikimate | 1-carboxyvinyltransferase | [R... | 771 | 0.0 | 86% |
| WP_017962713.1 | 3-phosphoshikimate | 1-carboxyvinyltransferase | [R... | 771 | 0.0 | 87% |
| WP_130809592.1 | 3-phosphoshikimate | 1-carboxyvinyltransferase | [R... | 771 | 0.0 | 87% |
| WP_065281752.1 | 3-phosphoshikimate | 1-carboxyvinyltransferase | [R... | 771 | 0.0 | 87% |
| WP_003555761.1 | 3-phosphoshikimate | 1-carboxyvinyltransferase | [R... | 771 | 0.0 | 86% |
| WP_164026695.1 | 3-phosphoshikimate | 1-carboxyvinyltransferase | [R... | 771 | 0.0 | 87% |
| WP_131665415.1 | 3-phosphoshikimate | 1-carboxyvinyltransferase | [R... | 770 | 0.0 | 87% |
| WP_151517755.1 | 3-phosphoshikimate | 1-carboxyvinyltransferase | [R... | 770 | 0.0 | 86% |
| WP_130872618.1 | 3-phosphoshikimate | 1-carboxyvinyltransferase | [R... | 770 | 0.0 | 87% |
| WP_029618989.1 | 3-phosphoshikimate | 1-carboxyvinyltransferase | [R... | 770 | 0.0 | 86% |
| WP_011649931.1 | 3-phosphoshikimate | 1-carboxyvinyltransferase | [R... | 770 | 0.0 | 87% |
| WP_033181070.1 | 3-phosphoshikimate | 1-carboxyvinyltransferase | [R... | 770 | 0.0 | 86% |
| WP_168277711.1 | 3-phosphoshikimate | 1-carboxyvinyltransferase | [R... | 770 | 0.0 | 87% |
| WP_130659400.1 | 3-phosphoshikimate | 1-carboxyvinyltransferase | [R... | 769 | 0.0 | 86% |
| WP_139675970.1 | 3-phosphoshikimate | 1-carboxyvinyltransferase | [R... | 769 | 0.0 | 86% |
| WP_018244244.1 | 3-phosphoshikimate | 1-carboxyvinyltransferase | [R... | 769 | 0.0 | 87% |
| WP_168331254.1 | 3-phosphoshikimate | 1-carboxyvinyltransferase | [R... | 769 | 0.0 | 86% |
| WP_168268235.1 | 3-phosphoshikimate | 1-carboxyvinyltransferase | [R... | 769 | 0.0 | 86% |
| WP_062941108.1 | 3-phosphoshikimate | 1-carboxyvinyltransferase | [R... | 769 | 0.0 | 87% |
| WP_130709147.1 | 3-phosphoshikimate | 1-carboxyvinyltransferase | [R... | 769 | 0.0 | 87% |
| WP_130711011.1 | 3-phosphoshikimate | 1-carboxyvinyltransferase | [R... | 769 | 0.0 | 86% |
| WP_131640109.1 | 3-phosphoshikimate | 1-carboxyvinyltransferase | [R... | 769 | 0.0 | 87% |
| WP_164070240.1 | 3-phosphoshikimate | 1-carboxyvinyltransferase | [R... | 769 | 0.0 | 86% |
| WP_168283252.1 | 3-phosphoshikimate | 1-carboxyvinyltransferase | [R... | 769 | 0.0 | 86% |
| WP_168290947.1 | 3-phosphoshikimate | 1-carboxyvinyltransferase | [R... | 769 | 0.0 | 87% |
| WP_138332068.1 | 3-phosphoshikimate | 1-carboxyvinyltransferase | [R... | 769 | 0.0 | 86% |
| WP_181293776.1 | 3-phosphoshikimate | 1-carboxyvinyltransferase | [R... | 769 | 0.0 | 86% |
| WP_094087019.1 | 3-phosphoshikimate | 1-carboxyvinyltransferase | [R... | 769 | 0.0 | 87% |
| WP_129422250.1 | 3-phosphoshikimate | 1-carboxyvinyltransferase | [R... | 768 | 0.0 | 86% |
| WP_193446494.1 | 3-phosphoshikimate | 1-carboxyvinyltransferase | [R... | 768 | 0.0 | 86% |
| WP_179038807.1 | 3-phosphoshikimate | 1-carboxyvinyltransferase | [R... | 768 | 0.0 | 86% |
| WP_180694191.1 | 3-phosphoshikimate | 1-carboxyvinyltransferase | [R... | 768 | 0.0 | 86% |
| WP_168336420.1 | 3-phosphoshikimate | 1-carboxyvinyltransferase | [R... | 768 | 0.0 | 86% |
| WP_152336385.1 | 3-phosphoshikimate | 1-carboxyvinyltransferase | [R... | 768 | 0.0 | 86% |
| WP_168323482.1 | 3-phosphoshikimate | 1-carboxyvinyltransferase | [R... | 768 | 0.0 | 86% |
| WP_012759631.1 | 3-phosphoshikimate | 1-carboxyvinyltransferase | [R... | 768 | 0.0 | 87% |
| WP_183906586.1 | 3-phosphoshikimate | 1-carboxyvinyltransferase | [R... | 768 | 0.0 | 86% |
| WP_152341413.1 | 3-phosphoshikimate | 1-carboxyvinyltransferase | [R... | 768 | 0.0 | 87% |
| WP_132576056.1 | 3-phosphoshikimate | 1-carboxyvinyltransferase | [R... | 768 | 0.0 | 86% |
| WP_063473421.1 | MULTISPECIES: 3-phosphoshikimate 1-carboxyvinyl... | | | 768 | 0.0 | 86% |
| WP_130678697.1 | 3-phosphoshikimate 1-carboxyvinyltransferase [R... | | | 767 | 0.0 | 86% |
| WP_052641111.1 | 3-phosphoshikimate 1-carboxyvinyltransferase [R... | | | 767 | 0.0 | 87% |
| WP_168345505.1 | 3-phosphoshikimate 1-carboxyvinyltransferase [R... | | | 767 | 0.0 | 86% |
| WP_131707667.1 | 3-phosphoshikimate 1-carboxyvinyltransferase [R... | | | 767 | 0.0 | 86% |
| WP_018448039.1 | MULTISPECIES: 3-phosphoshikimate 1-carboxyvinyl... | | | 767 | 0.0 | 86% |
| WP_171599944.1 | 3-phosphoshikimate 1-carboxyvinyltransferase [R... | | | 767 | 0.0 | 86% |
| WP_130703641.1 | 3-phosphoshikimate 1-carboxyvinyltransferase [R... | | | 767 | 0.0 | 86% |
| WP_027669080.1 | 3-phosphoshikimate 1-carboxyvinyltransferase [R... | | | 767 | 0.0 | 86% |
| WP_168287538.1 | 3-phosphoshikimate 1-carboxyvinyltransferase [R... | | | 767 | 0.0 | 86% |
| WP_018068678.1 | 3-phosphoshikimate 1-carboxyvinyltransferase [R... | | | 767 | 0.0 | 86% |
| WP_168312063.1 | MULTISPECIES: 3-phosphoshikimate 1-carboxyvinyl... | | | 766 | 0.0 | 86% |
| WP_130684197.1 | 3-phosphoshikimate 1-carboxyvinyltransferase [R... | | | 766 | 0.0 | 86% |
| WP_077977105.1 | 3-phosphoshikimate 1-carboxyvinyltransferase [R... | | | 766 | 0.0 | 86% |
| WP_028731871.1 | 3-phosphoshikimate 1-carboxyvinyltransferase [R... | | | 766 | 0.0 | 86% |
| WP_162115601.1 | 3-phosphoshikimate 1-carboxyvinyltransferase [R... | | | 766 | 0.0 | 86% |
| WP_027682251.1 | 3-phosphoshikimate 1-carboxyvinyltransferase [R... | | | 766 | 0.0 | 86% |
| WP_020052313.1 | 3-phosphoshikimate 1-carboxyvinyltransferase [R... | | | 766 | 0.0 | 86% |
| WP_164578062.1 | 3-phosphoshikimate 1-carboxyvinyltransferase [R... | | | 766 | 0.0 | 86% |
| WP_130765412.1 | 3-phosphoshikimate 1-carboxyvinyltransferase [R... | | | 766 | 0.0 | 86% |
| WP_029873774.1 | 3-phosphoshikimate 1-carboxyvinyltransferase [R... | | | 765 | 0.0 | 86% |
| WP_130670010.1 | 3-phosphoshikimate 1-carboxyvinyltransferase [R... | | | 765 | 0.0 | 86% |
| WP_130665327.1 | 3-phosphoshikimate 1-carboxyvinyltransferase [R... | | | 765 | 0.0 | 86% |
| WP_184499397.1 | 3-phosphoshikimate 1-carboxyvinyltransferase [R... | | | 765 | 0.0 | 86% |
| WP_130822769.1 | 3-phosphoshikimate 1-carboxyvinyltransferase [R... | | | 765 | 0.0 | 86% |
| WP_017995641.1 | 3-phosphoshikimate 1-carboxyvinyltransferase [R... | | | 765 | 0.0 | 86% |
| WP_168317284.1 | 3-phosphoshikimate 1-carboxyvinyltransferase [R... | | | 765 | 0.0 | 86% |
| WP_130676013.1 | 3-phosphoshikimate 1-carboxyvinyltransferase [R... | | | 765 | 0.0 | 86% |

| WP_164564939.1 | 3-phosphoshikimate 1-carboxyvinyltransferase [R... | 764 | 0.0 | 86% |
| --- | --- | --- | --- | --- |
| WP_130769267.1 | 3-phosphoshikimate 1-carboxyvinyltransferase [R... | 764 | 0.0 | 86% |
| WP_130826003.1 | 3-phosphoshikimate 1-carboxyvinyltransferase [R... | 764 | 0.0 | 85% |
| WP_183637345.1 | 3-phosphoshikimate 1-carboxyvinyltransferase [R... | 764 | 0.0 | 86% |
| WP_092995433.1 | 3-phosphoshikimate 1-carboxyvinyltransferase [R... | 764 | 0.0 | 85% |
| WP_047619821.1 | MULTISPECIES: 3-phosphoshikimate 1-carboxyvinyl... | 764 | 0.0 | 86% |
| WP_024323105.1 | 3-phosphoshikimate 1-carboxyvinyltransferase [R... | 764 | 0.0 | 86% |
| WP_064653653.1 | 3-phosphoshikimate 1-carboxyvinyltransferase [R... | 764 | 0.0 | 86% |
| WP_164055205.1 | 3-phosphoshikimate 1-carboxyvinyltransferase [R... | 764 | 0.0 | 86% |
| WP_130763894.1 | 3-phosphoshikimate 1-carboxyvinyltransferase [R... | 764 | 0.0 | 86% |
| WP_168334042.1 | 3-phosphoshikimate 1-carboxyvinyltransferase [R... | 764 | 0.0 | 86% |
| WP_037167849.1 | 3-phosphoshikimate 1-carboxyvinyltransferase [P... | 763 | 0.0 | 86% |
| WP_064250148.1 | 3-phosphoshikimate 1-carboxyvinyltransferase [R... | 763 | 0.0 | 85% |
| WP_128405682.1 | 3-phosphoshikimate 1-carboxyvinyltransferase [R... | 763 | 0.0 | 86% |
| WP_168304617.1 | 3-phosphoshikimate 1-carboxyvinyltransferase [R... | 763 | 0.0 | 86% |
| WP_168330041.1 | 3-phosphoshikimate 1-carboxyvinyltransferase [R... | 763 | 0.0 | 86% |
| WP_028743594.1 | 3-phosphoshikimate 1-carboxyvinyltransferase [R... | 763 | 0.0 | 86% |
| WP_164580775.1 | 3-phosphoshikimate 1-carboxyvinyltransferase [R... | 763 | 0.0 | 85% |
| WP_130786092.1 | 3-phosphoshikimate 1-carboxyvinyltransferase [R... | 763 | 0.0 | 86% |
| WP_018483104.1 | 3-phosphoshikimate 1-carboxyvinyltransferase [R... | 763 | 0.0 | 86% |
| WP_182209093.1 | 3-phosphoshikimate 1-carboxyvinyltransferase [R... | 763 | 0.0 | 86% |
| WP_142589029.1 | 3-phosphoshikimate 1-carboxyvinyltransferase [R... | 763 | 0.0 | 86% |
| WP_127431863.1 | 3-phosphoshikimate 1-carboxyvinyltransferase [R... | 763 | 0.0 | 85% |
| WP_183904688.1 | 3-phosphoshikimate 1-carboxyvinyltransferase [R... | 763 | 0.0 | 86% |
| WP_130697986.1 | 3-phosphoshikimate 1-carboxyvinyltransferase [R... | 763 | 0.0 | 86% |
| WP_077548496.1 | 3-phosphoshikimate 1-carboxyvinyltransferase [R... | 762 | 0.0 | 86% |
| WP_168574935.1 | 3-phosphoshikimate 1-carboxyvinyltransferase [R... | 762 | 0.0 | 86% |
| WP_133317022.1 | 3-phosphoshikimate 1-carboxyvinyltransferase [R... | 762 | 0.0 | 85% |
| WP_130656708.1 | 3-phosphoshikimate 1-carboxyvinyltransferase [R... | 762 | 0.0 | 86% |
| WP_018494545.1 | 3-phosphoshikimate 1-carboxyvinyltransferase [R... | 762 | 0.0 | 86% |
| WP_148146226.1 | 3-phosphoshikimate 1-carboxyvinyltransferase [R... | 761 | 0.0 | 85% |
| WP_097545160.1 | 3-phosphoshikimate 1-carboxyvinyltransferase [R... | 761 | 0.0 | 85% |
| WP_025396586.1 | 3-phosphoshikimate 1-carboxyvinyltransferase [R... | 761 | 0.0 | 86% |
| WP_185827687.1 | 3-phosphoshikimate 1-carboxyvinyltransferase [R... | 761 | 0.0 | 86% |
| WP_166478212.1 | 3-phosphoshikimate 1-carboxyvinyltransferase [R... | 761 | 0.0 | 86% |
| WP_170282642.1 | 3-phosphoshikimate 1-carboxyvinyltransferase [R... | 761 | 0.0 | 85% |
| WP_183367161.1 | 3-phosphoshikimate 1-carboxyvinyltransferase [G... | 761 | 0.0 | 86% |
| WP_130662575.1 | 3-phosphoshikimate 1-carboxyvinyltransferase [R... | 761 | 0.0 | 86% |
| WP_130745589.1 | 3-phosphoshikimate 1-carboxyvinyltransferase [R... | 761 | 0.0 | 86% |
| WP_168251672.1 | MULTISPECIES: 3-phosphoshikimate 1-carboxyvinyl... | 760 | 0.0 | 86% |
| WP_168282289.1 | 3-phosphoshikimate 1-carboxyvinyltransferase [R... | 760 | 0.0 | 85% |
| QND16088.1 3-phosphoshikimate 1-carboxyvinyltransferase [Rhizo... | | 760 | 0.0 | 85% |
| QND21961.1 3-phosphoshikimate 1-carboxyvinyltransferase [Rhizo... | | 760 | 0.0 | 85% |
| WP_164593118.1 3-phosphoshikimate 1-carboxyvinyltransferase [R... | | 760 | 0.0 | 86% |
| WP_017966433.1 3-phosphoshikimate 1-carboxyvinyltransferase [R... | | 759 | 0.0 | 85% |
| WP_130673329.1 3-phosphoshikimate 1-carboxyvinyltransferase [R... | | 759 | 0.0 | 86% |
| WP_170269375.1 3-phosphoshikimate 1-carboxyvinyltransferase [R... | | 758 | 0.0 | 85% |
| WP_183823914.1 3-phosphoshikimate 1-carboxyvinyltransferase [R... | | 758 | 0.0 | 85% |
| WP_142592023.1 3-phosphoshikimate 1-carboxyvinyltransferase [R... | | 758 | 0.0 | 84% |
| WP_130853502.1 3-phosphoshikimate 1-carboxyvinyltransferase [R... | | 757 | 0.0 | 85% |
| WP_171602083.1 3-phosphoshikimate 1-carboxyvinyltransferase [R... | | 756 | 0.0 | 85% |
| WP_183992976.1 3-phosphoshikimate 1-carboxyvinyltransferase [R... | | 756 | 0.0 | 84% |
| WP_131718266.1 3-phosphoshikimate 1-carboxyvinyltransferase [R... | | 755 | 0.0 | 85% |
| WP_170277187.1 3-phosphoshikimate 1-carboxyvinyltransferase [R... | | 755 | 0.0 | 85% |
| WP_192078030.1 unnamed protein product | | 753 | 0.0 | 85% |
| WP_170263476.1 3-phosphoshikimate 1-carboxyvinyltransferase [R... | | 753 | 0.0 | 85% |
| WP_092664007.1 3-phosphoshikimate 1-carboxyvinyltransferase [R... | | 752 | 0.0 | 85% |
| WP_174195395.1 3-phosphoshikimate 1-carboxyvinyltransferase [N... | | 752 | 0.0 | 85% |
| WP_062578552.1 3-phosphoshikimate 1-carboxyvinyltransferase [R... | | 750 | 0.0 | 85% |
| WP_183837182.1 3-phosphoshikimate 1-carboxyvinyltransferase [R... | | 750 | 0.0 | 85% |
| WP_168827834.1 3-phosphoshikimate 1-carboxyvinyltransferase [R... | | 749 | 0.0 | 85% |
| WP_126925121.1 3-phosphoshikimate 1-carboxyvinyltransferase [R... | | 749 | 0.0 | 85% |
| WP_184654285.1 3-phosphoshikimate 1-carboxyvinyltransferase [R... | | 749 | 0.0 | 85% |
| WP_170257465.1 3-phosphoshikimate 1-carboxyvinyltransferase [R... | | 749 | 0.0 | 84% |
| MBA4785050.1 3-phosphoshikimate 1-carboxyvinyltransferase [Rhi... | | 749 | 0.0 | 86% |
| WP_192087715.1 3-phosphoshikimate 1-carboxyvinyltransferase [R... | | 749 | 0.0 | 85% |
| WP_133282785.1 3-phosphoshikimate 1-carboxyvinyltransferase [P... | | 749 | 0.0 | 85% |
| WP_062454213.1 3-phosphoshikimate 1-carboxyvinyltransferase [R... | | 748 | 0.0 | 85% |
| WP_119942315.1 3-phosphoshikimate 1-carboxyvinyltransferase [N... | | 747 | 0.0 | 85% |
| WP_128443486.1 3-phosphoshikimate 1-carboxyvinyltransferase [R... | | 746 | 0.0 | 85% |
| MAZ85107.1 3-phosphoshikimate 1-carboxyvinyltransferase [Hoefl... | | 742 | 0.0 | 84% |
| WP_126828979.1 3-phosphoshikimate 1-carboxyvinyltransferase [R... | | 741 | 0.0 | 83% |

| WP_125846735.1 | 3-phosphoshikimate 1-carboxyvinyltransferase [R... | 741 | 0.0 | 84% |
| --- | --- | --- | --- | --- |
| WP_121981347.1 | MULTISPECIES: 3-phosphoshikimate 1-carboxyvinyl... | 738 | 0.0 | 83% |
| WP_094506051.1 | 3-phosphoshikimate 1-carboxyvinyltransferase [O... | 737 | 0.0 | 83% |
| WP_105518014.1 | MULTISPECIES: 3-phosphoshikimate 1-carboxyvinyl... | 737 | 0.0 | 82% |
| WP_064322336.1 | MULTISPECIES: 3-phosphoshikimate 1-carboxyvinyl... | 736 | 0.0 | 82% |
| WP_113485490.1 | 3-phosphoshikimate 1-carboxyvinyltransferase [O... | 736 | 0.0 | 82% |
| WP_100647755.1 | MULTISPECIES: 3-phosphoshikimate 1-carboxyvinyl... | 735 | 0.0 | 82% |
| WP_006465745.1 | MULTISPECIES: 3-phosphoshikimate 1-carboxyvinyl... | 735 | 0.0 | 82% |
| WP_134153901.1 | 3-phosphoshikimate 1-carboxyvinyltransferase [O... | 735 | 0.0 | 82% |
| WP_007872980.1 | MULTISPECIES: 3-phosphoshikimate 1-carboxyvinyl... | 735 | 0.0 | 82% |
| AVC52229.1 3-phosphoshikimate 1-carboxyvinyltransferase [Rhizo... | | 734 | 0.0 | 87% |
| WP_151645746.1 3-phosphoshikimate 1-carboxyvinyltransferase [O... | | 734 | 0.0 | 82% |
| WP_128092818.1 3-phosphoshikimate 1-carboxyvinyltransferase [O... | | 734 | 0.0 | 82% |
| WP_151688841.1 3-phosphoshikimate 1-carboxyvinyltransferase [O... | | 734 | 0.0 | 82% |
| WP_105734053.1 3-phosphoshikimate 1-carboxyvinyltransferase [P... | | 734 | 0.0 | 82% |
| WP_176036429.1 3-phosphoshikimate 1-carboxyvinyltransferase [O... | | 734 | 0.0 | 82% |
| WP_025090682.1 3-phosphoshikimate 1-carboxyvinyltransferase [B... | | 734 | 0.0 | 82% |
| WP_094541214.1 3-phosphoshikimate 1-carboxyvinyltransferase [O... | | 734 | 0.0 | 82% |
| WP_176073658.1 3-phosphoshikimate 1-carboxyvinyltransferase [B... | | 733 | 0.0 | 82% |
| WP_125300483.1 3-phosphoshikimate 1-carboxyvinyltransferase [O... | | 733 | 0.0 | 82% |
| WP_183515843.1 MULTISPECIES: 3-phosphoshikimate 1-carboxyvinyl... | | 733 | 0.0 | 82% |
| WP_021587681.1 MULTISPECIES: 3-phosphoshikimate 1-carboxyvinyl... | | 733 | 0.0 | 83% |
| WP_151660289.1 3-phosphoshikimate 1-carboxyvinyltransferase [B... | | 733 | 0.0 | 82% |
| ADG03677.1 5-enolpyruvylshikimate 3-phosphate synthase [Ochrob... | | 733 | 0.0 | 82% |
| WP_133704665.1 MULTISPECIES: 3-phosphoshikimate 1-carboxyvinyl... | | 733 | 0.0 | 83% |
| ANN25048.1 5-enolpyruvylshikimate-3-phosphate synthase [Brucel... | | 733 | 0.0 | 82% |
| WP_061344725.1 3-phosphoshikimate 1-carboxyvinyltransferase [O... | | 733 | 0.0 | 82% |
| WP_132226789.1 3-phosphoshikimate 1-carboxyvinyltransferase [O... | | 733 | 0.0 | 82% |
| WP_063619892.1 MULTISPECIES: 3-phosphoshikimate 1-carboxyvinyl... | | 732 | 0.0 | 82% |
| WP_151662889.1 3-phosphoshikimate 1-carboxyvinyltransferase [O... | | 732 | 0.0 | 82% |
| WP_094578647.1 3-phosphoshikimate 1-carboxyvinyltransferase [O... | | 732 | 0.0 | 82% |
| WP_094514590.1 MULTISPECIES: 3-phosphoshikimate 1-carboxyvinyl... | | 732 | 0.0 | 82% |
| WP_024898290.1 MULTISPECIES: 3-phosphoshikimate 1-carboxyvinyl... | | 732 | 0.0 | 82% |
| WP_106712475.1 3-phosphoshikimate 1-carboxyvinyltransferase [P... | | 732 | 0.0 | 82% |
| WP_104756599.1 3-phosphoshikimate 1-carboxyvinyltransferase [O... | | 732 | 0.0 | 82% |
| WP_071632039.1 3-phosphoshikimate 1-carboxyvinyltransferase [O... | | 732 | 0.0 | 82% |
| WP_151658442.1 3-phosphoshikimate 1-carboxyvinyltransferase [B... | | 731 | 0.0 | 83% |
| TDQ32008.1 3-phosphoshikimate 1-carboxyvinyltransferase [Phyll... | | 731 | 0.0 | 82% |
| WP_010657837.1 MULTISPECIES: 3-phosphoshikimate 1-carboxyvinyl... | | 731 | 0.0 | 82% |
| WP_105705525.1 3-phosphoshikimate 1-carboxyvinyltransferase [O... | | 731 | 0.0 | 82% |
| WP_187543617.1 3-phosphoshikimate 1-carboxyvinyltransferase [O... | | 731 | 0.0 | 81% |
| WP_011982268.1 3-phosphoshikimate 1-carboxyvinyltransferase [O... | | 731 | 0.0 | 82% |
| WP_196108699.1 MULTISPECIES: 3-phosphoshikimate 1-carboxyvinyl... | | 730 | 0.0 | 82% |
| AWH63006.1 5-enolpyruvylshikimate-3-phosphate (EPSP) synthase ... | | 730 | 0.0 | 83% |
| WP_139975008.1 3-phosphoshikimate 1-carboxyvinyltransferase [O... | | 730 | 0.0 | 81% |
| WP_100558340.1 MULTISPECIES: 3-phosphoshikimate 1-carboxyvinyl... | | 730 | 0.0 | 82% |
| MAY63235.1 3-phosphoshikimate 1-carboxyvinyltransferase [Rhizo... | | 730 | 0.0 | 82% |
| WP_140020666.1 3-phosphoshikimate 1-carboxyvinyltransferase [O... | | 729 | 0.0 | 82% |
| WP_151677450.1 3-phosphoshikimate 1-carboxyvinyltransferase [O... | | 729 | 0.0 | 82% |
| WP_174049715.1 3-phosphoshikimate 1-carboxyvinyltransferase [A... | | 729 | 0.0 | 83% |
| WP_109987137.1 3-phosphoshikimate 1-carboxyvinyltransferase [O... | | 729 | 0.0 | 82% |
| WP_114214379.1 3-phosphoshikimate 1-carboxyvinyltransferase [O... | | 729 | 0.0 | 82% |
| WP_091947273.1 MULTISPECIES: 3-phosphoshikimate 1-carboxyvinyl... | | 729 | 0.0 | 83% |
| WP_119044001.1 3-phosphoshikimate 1-carboxyvinyltransferase [O... | | 729 | 0.0 | 82% |
| WP_043062739.1 3-phosphoshikimate 1-carboxyvinyltransferase [O... | | 728 | 0.0 | 82% |
| WP_027230834.1 3-phosphoshikimate 1-carboxyvinyltransferase [P... | | 728 | 0.0 | 83% |
| WP_151557108.1 3-phosphoshikimate 1-carboxyvinyltransferase [O... | | 728 | 0.0 | 82% |
| WP_028754674.1 3-phosphoshikimate 1-carboxyvinyltransferase [R... | | 728 | 0.0 | 82% |
| WP_091879195.1 3-phosphoshikimate 1-carboxyvinyltransferase [P... | | 728 | 0.0 | 81% |
| WP_007690336.1 MULTISPECIES: 3-phosphoshikimate 1-carboxyvinyl... | | 728 | 0.0 | 83% |
| WP_029926097.1 MULTISPECIES: 3-phosphoshikimate 1-carboxyvinyl... | | 728 | 0.0 | 81% |
| WP_047466864.1 3-phosphoshikimate 1-carboxyvinyltransferase [A... | | 728 | 0.0 | 83% |
| WP_110032593.1 3-phosphoshikimate 1-carboxyvinyltransferase [H... | | 727 | 0.0 | 85% |
| WP_151614879.1 3-phosphoshikimate 1-carboxyvinyltransferase [O... | | 727 | 0.0 | 82% |
| WP_031352532.1 3-phosphoshikimate 1-carboxyvinyltransferase [B... | | 727 | 0.0 | 83% |
| WP_105540776.1 3-phosphoshikimate 1-carboxyvinyltransferase [O... | | 727 | 0.0 | 82% |
| WP_182519086.1 3-phosphoshikimate 1-carboxyvinyltransferase [O... | | 727 | 0.0 | 82% |
| OJY06829.1 3-phosphoshikimate 1-carboxyvinyltransferase [Rhizo... | | 726 | 0.0 | 83% |
| WP_171316908.1 3-phosphoshikimate 1-carboxyvinyltransferase [O... | | 726 | 0.0 | 81% |
| WP_174041607.1 3-phosphoshikimate 1-carboxyvinyltransferase [A... | | 726 | 0.0 | 82% |
| WP_109368078.1 3-phosphoshikimate 1-carboxyvinyltransferase [O... | | 726 | 0.0 | 81% |
| WP_174015292.1 3-phosphoshikimate 1-carboxyvinyltransferase [A... | | 726 | 0.0 | 82% |

| WP_174013348.1 3-phosphoshikimate 1-carboxyvinyltransferase [A... | 726 | 0.0 | 82% |
| --- | --- | --- | --- |
| WP_174017532.1 3-phosphoshikimate 1-carboxyvinyltransferase [A... | 726 | 0.0 | 82% |
| WP_091920605.1 3-phosphoshikimate 1-carboxyvinyltransferase [P... | 726 | 0.0 | 83% |
| WP_042476496.1 3-phosphoshikimate 1-carboxyvinyltransferase [A... | 726 | 0.0 | 82% |
| WP_153666806.1 3-phosphoshikimate 1-carboxyvinyltransferase [P... | 726 | 0.0 | 83% |
| WP_140024540.1 3-phosphoshikimate 1-carboxyvinyltransferase [O... | 725 | 0.0 | 81% |
| WP_151588586.1 3-phosphoshikimate 1-carboxyvinyltransferase [O... | 725 | 0.0 | 82% |
| WP_185917167.1 3-phosphoshikimate 1-carboxyvinyltransferase [O... | 725 | 0.0 | 81% |
| WP_174005577.1 3-phosphoshikimate 1-carboxyvinyltransferase [A... | 725 | 0.0 | 82% |
| NKC47702.1 3-phosphoshikimate 1-carboxyvinyltransferase [Ochro... | 725 | 0.0 | 82% |
| WP_174031631.1 3-phosphoshikimate 1-carboxyvinyltransferase [A... | 725 | 0.0 | 82% |
| WP_138783991.1 3-phosphoshikimate 1-carboxyvinyltransferase [O... | 724 | 0.0 | 81% |
| WP_169592424.1 3-phosphoshikimate 1-carboxyvinyltransferase [R... | 724 | 0.0 | 82% |
| WP_100000714.1 3-phosphoshikimate 1-carboxyvinyltransferase [P... | 724 | 0.0 | 82% |
| WP_174167749.1 3-phosphoshikimate 1-carboxyvinyltransferase [A... | 724 | 0.0 | 82% |
| WP_174038288.1 3-phosphoshikimate 1-carboxyvinyltransferase [A... | 724 | 0.0 | 82% |
| WP_095445804.1 MULTISPECIES: 3-phosphoshikimate 1-carboxyvinyl... | 724 | 0.0 | 81% |
| WP_065706665.1 3-phosphoshikimate 1-carboxyvinyltransferase [A... | 724 | 0.0 | 82% |
| WP_182514632.1 3-phosphoshikimate 1-carboxyvinyltransferase [O... | 723 | 0.0 | 81% |
| WP_182548377.1 3-phosphoshikimate 1-carboxyvinyltransferase [P... | 723 | 0.0 | 81% |
| WP_142889431.1 MULTISPECIES: 3-phosphoshikimate 1-carboxyvinyl... | 723 | 0.0 | 82% |
| WP_109704805.1 3-phosphoshikimate 1-carboxyvinyltransferase [F... | 723 | 0.0 | 81% |
| WP_047453994.1 3-phosphoshikimate 1-carboxyvinyltransferase [A... | 722 | 0.0 | 82% |
| WP_174059048.1 3-phosphoshikimate 1-carboxyvinyltransferase [A... | 722 | 0.0 | 82% |
| WP_182509161.1 3-phosphoshikimate 1-carboxyvinyltransferase [P... | 722 | 0.0 | 81% |
| WP_107106441.1 MULTISPECIES: 3-phosphoshikimate 1-carboxyvinyl... | 722 | 0.0 | 82% |
| WP_184364822.1 3-phosphoshikimate 1-carboxyvinyltransferase [R... | 721 | 0.0 | 82% |
| WP_111573814.1 3-phosphoshikimate 1-carboxyvinyltransferase [F... | 721 | 0.0 | 80% |
| WP_058326193.1 3-phosphoshikimate 1-carboxyvinyltransferase [S... | 720 | 0.0 | 82% |
| WP_008127565.1 3-phosphoshikimate 1-carboxyvinyltransferase [P... | 720 | 0.0 | 81% |
| WP_112529618.1 3-phosphoshikimate 1-carboxyvinyltransferase [P... | 719 | 0.0 | 80% |
| WP_183690442.1 3-phosphoshikimate 1-carboxyvinyltransferase [R... | 719 | 0.0 | 82% |
| WP_104821503.1 3-phosphoshikimate 1-carboxyvinyltransferase [R... | 718 | 0.0 | 82% |
| WP_095436112.1 3-phosphoshikimate 1-carboxyvinyltransferase [R... | 718 | 0.0 | 80% |
| WP_114429810.1 3-phosphoshikimate 1-carboxyvinyltransferase [P... | 717 | 0.0 | 80% |
| WP_188344663.1 unnamed protein product | 717 | 0.0 | 79% |
| WP_093234324.1 3-phosphoshikimate 1-carboxyvinyltransferase [S... | 717 | 0.0 | 82% |
| WP_106662274.1 3-phosphoshikimate 1-carboxyvinyltransferase [P... | 716 | 0.0 | 81% |
| WP_153370279.1 3-phosphoshikimate 1-carboxyvinyltransferase [R... | 715 | 0.0 | 81% |
| WP_113232730.1 3-phosphoshikimate 1-carboxyvinyltransferase [R... | 714 | 0.0 | 80% |
| WP_065372074.1 3-phosphoshikimate 1-carboxyvinyltransferase [E... | 714 | 0.0 | 81% |
| WP_053251884.1 3-phosphoshikimate 1-carboxyvinyltransferase [E... | 712 | 0.0 | 80% |
| WP_009364909.1 3-phosphoshikimate 1-carboxyvinyltransferase [B... | 712 | 0.0 | 82% |
| WP_183814360.1 3-phosphoshikimate 1-carboxyvinyltransferase [R... | 710 | 0.0 | 79% |
| WP_077962779.1 3-phosphoshikimate 1-carboxyvinyltransferase [E... | 709 | 0.0 | 81% |
| WP_187959664.1 3-phosphoshikimate 1-carboxyvinyltransferase [B... | 709 | 0.0 | 82% |
| WP_040448875.1 3-phosphoshikimate 1-carboxyvinyltransferase [H... | 709 | 0.0 | 81% |
| WP_140904801.1 3-phosphoshikimate 1-carboxyvinyltransferase [O... | 708 | 0.0 | 80% |
| WP_153527654.1 3-phosphoshikimate 1-carboxyvinyltransferase [B... | 708 | 0.0 | 82% |
| WP_076780315.1 MULTISPECIES: 3-phosphoshikimate 1-carboxyvinyl... | 708 | 0.0 | 82% |
| WP_104662475.1 3-phosphoshikimate 1-carboxyvinyltransferase [E... | 708 | 0.0 | 80% |
| WP_069714071.1 MULTISPECIES: 3-phosphoshikimate 1-carboxyvinyl... | 707 | 0.0 | 82% |
| MBC7281698.1 3-phosphoshikimate 1-carboxyvinyltransferase [Hoe... | 707 | 0.0 | 81% |
| WP_134885619.1 3-phosphoshikimate 1-carboxyvinyltransferase [E... | 707 | 0.0 | 80% |
| WP_183646676.1 3-phosphoshikimate 1-carboxyvinyltransferase [O... | 706 | 0.0 | 82% |
| WP_070996949.1 MULTISPECIES: 3-phosphoshikimate 1-carboxyvinyl... | 706 | 0.0 | 81% |
| EEH15273.1 3-phosphoshikimate 1-carboxyvinyltransferase [Bruce... | 705 | 0.0 | 81% |
| WP_008935122.1 MULTISPECIES: 3-phosphoshikimate 1-carboxyvinyl... | 705 | 0.0 | 82% |
| WP_004691209.1 MULTISPECIES: 3-phosphoshikimate 1-carboxyvinyl... | 705 | 0.0 | 81% |
| WP_068880270.1 3-phosphoshikimate 1-carboxyvinyltransferase [P... | 705 | 0.0 | 81% |
| KSV66437.1 3-phosphoshikimate 1-carboxyvinyltransferase [Sinor... | 705 | 0.0 | 79% |
| WP_092720367.1 3-phosphoshikimate 1-carboxyvinyltransferase [R... | 704 | 0.0 | 79% |
| WP_114439432.1 3-phosphoshikimate 1-carboxyvinyltransferase [P... | 704 | 0.0 | 81% |
| EEP63548.1 3-phosphoshikimate 1-carboxyvinyltransferase [Bruce... | 704 | 0.0 | 81% |
| WP_004685324.1 MULTISPECIES: 3-phosphoshikimate 1-carboxyvinyl... | 704 | 0.0 | 81% |
| CUW43595.1 3-phosphoshikimate 1-carboxyvinyltransferase [Bruce... | 704 | 0.0 | 82% |
| WP_192437361.1 3-phosphoshikimate 1-carboxyvinyltransferase [E... | 704 | 0.0 | 79% |
| ENR88457.1 3-phosphoshikimate 1-carboxyvinyltransferase [Bruce... | 704 | 0.0 | 81% |
| WP_147836101.1 3-phosphoshikimate 1-carboxyvinyltransferase [P... | 704 | 0.0 | 78% |
| WP_008503805.1 3-phosphoshikimate 1-carboxyvinyltransferase [B... | 704 | 0.0 | 81% |
| WP_060519521.1 MULTISPECIES: 3-phosphoshikimate 1-carboxyvinyl... | 703 | 0.0 | 79% |
| WP_069615366.1 3-phosphoshikimate 1-carboxyvinyltransferase [R... | 703 | 0.0 | 80% |

| WP_112770687.1 3-phosphoshikimate 1-carboxyvinyltransferase [E... | 703 | 0.0 | 79% |
| --- | --- | --- | --- |
| WP_012783809.1 3-phosphoshikimate 1-carboxyvinyltransferase [B... | 703 | 0.0 | 81% |
| WP_105985360.1 3-phosphoshikimate 1-carboxyvinyltransferase [B... | 703 | 0.0 | 81% |
| WP_076770992.1 MULTISPECIES: 3-phosphoshikimate 1-carboxyvinyl... | 703 | 0.0 | 81% |
| EEW80290.1 5-enolpyruvyl shikimate 3-phosphate synthase [Bruce... | 703 | 0.0 | 81% |
| OWZ94747.1 3-phosphoshikimate 1-carboxyvinyltransferase [Sinor... | 703 | 0.0 | 79% |
| WP_110818568.1 MULTISPECIES: 3-phosphoshikimate 1-carboxyvinyl... | 703 | 0.0 | 79% |
| WP_127889705.1 MULTISPECIES: 3-phosphoshikimate 1-carboxyvinyl... | 702 | 0.0 | 79% |
| WP_043611868.1 3-phosphoshikimate 1-carboxyvinyltransferase [E... | 702 | 0.0 | 79% |
| WP_002965272.1 MULTISPECIES: 3-phosphoshikimate 1-carboxyvinyl... | 702 | 0.0 | 81% |
| WP_008933634.1 3-phosphoshikimate 1-carboxyvinyltransferase [B... | 702 | 0.0 | 81% |
| WP_065874153.1 3-phosphoshikimate 1-carboxyvinyltransferase [B... | 702 | 0.0 | 81% |
| WP_090296221.1 MULTISPECIES: 3-phosphoshikimate 1-carboxyvinyl... | 702 | 0.0 | 79% |
| AAL53098.1 3-phosphoshikimate 1-carboxyvinyltransferase [Bruce... | 702 | 0.0 | 81% |
| WP_154718478.1 3-phosphoshikimate 1-carboxyvinyltransferase [B... | 702 | 0.0 | 81% |
| WP_106715944.1 3-phosphoshikimate 1-carboxyvinyltransferase [P... | 702 | 0.0 | 78% |
| ABQ60885.1 3-phosphoshikimate 1-carboxyvinyltransferase [Bruce... | 702 | 0.0 | 81% |
| WP_174164335.1 3-phosphoshikimate 1-carboxyvinyltransferase [A... | 701 | 0.0 | 80% |
| WP_058324529.1 MULTISPECIES: 3-phosphoshikimate 1-carboxyvinyl... | 701 | 0.0 | 82% |
| QOK51506.1 3-phosphoshikimate 1-carboxyvinyltransferase [Bruce... | 701 | 0.0 | 81% |
| WP_117346186.1 3-phosphoshikimate 1-carboxyvinyltransferase [B... | 701 | 0.0 | 81% |
| WP_006206089.1 3-phosphoshikimate 1-carboxyvinyltransferase [B... | 701 | 0.0 | 81% |
| WP_006014665.1 3-phosphoshikimate 1-carboxyvinyltransferase [B... | 701 | 0.0 | 81% |
| WP_002968066.1 3-phosphoshikimate 1-carboxyvinyltransferase [B... | 701 | 0.0 | 81% |
| WP_145324223.1 MULTISPECIES: 3-phosphoshikimate 1-carboxyvinyl... | 701 | 0.0 | 79% |
| WP_127664702.1 3-phosphoshikimate 1-carboxyvinyltransferase [S... | 700 | 0.0 | 92% |
| WP_034804733.1 MULTISPECIES: 3-phosphoshikimate 1-carboxyvinyl... | 700 | 0.0 | 79% |
| WP_037100378.1 3-phosphoshikimate 1-carboxyvinyltransferase [R... | 700 | 0.0 | 78% |
| WP_060580572.1 3-phosphoshikimate 1-carboxyvinyltransferase [E... | 700 | 0.0 | 79% |
| WP_004684607.1 MULTISPECIES: 3-phosphoshikimate 1-carboxyvinyl... | 700 | 0.0 | 81% |
| WP_011265281.1 3-phosphoshikimate 1-carboxyvinyltransferase [B... | 700 | 0.0 | 81% |
| WP_113397470.1 MULTISPECIES: 3-phosphoshikimate 1-carboxyvinyl... | 700 | 0.0 | 79% |
| WP_192338922.1 unnamed protein product | 700 | 0.0 | 79% |
| WP_154167325.1 3-phosphoshikimate 1-carboxyvinyltransferase [B... | 700 | 0.0 | 81% |
| WP_188823230.1 3-phosphoshikimate 1-carboxyvinyltransferase [O... | 699 | 0.0 | 80% |
| WP_007814132.1 3-phosphoshikimate 1-carboxyvinyltransferase [R... | 699 | 0.0 | 78% |
| WP_132653128.1 MULTISPECIES: 3-phosphoshikimate 1-carboxyvinyl... | 699 | 0.0 | 77% |
| WP_006104394.1 3-phosphoshikimate 1-carboxyvinyltransferase [B... | 699 | 0.0 | 81% |
| WP_110749612.1 3-phosphoshikimate 1-carboxyvinyltransferase [P... | 698 | 0.0 | 80% |
| WP_183431406.1 3-phosphoshikimate 1-carboxyvinyltransferase [M... | 698 | 0.0 | 80% |
| GGA89126.1 3-phosphoshikimate 1-carboxyvinyltransferase [Ochro... | 698 | 0.0 | 80% |
| WP_184221298.1 3-phosphoshikimate 1-carboxyvinyltransferase [P... | 698 | 0.0 | 79% |
| WP_183823742.1 3-phosphoshikimate 1-carboxyvinyltransferase [R... | 697 | 0.0 | 77% |
| WP_173515182.1 3-phosphoshikimate 1-carboxyvinyltransferase [E... | 697 | 0.0 | 80% |
| AAK27445.1 5-enolpyruvyl shikimate 3-phosphate synthase [Bruce... | 697 | 0.0 | 80% |
| NUS20768.1 3-phosphoshikimate 1-carboxyvinyltransferase [Mesor... | 696 | 0.0 | 79% |
| WP_152096677.1 3-phosphoshikimate 1-carboxyvinyltransferase [R... | 696 | 0.0 | 78% |
| WP_099303422.1 3-phosphoshikimate 1-carboxyvinyltransferase [Z... | 696 | 0.0 | 79% |
| WP_065797057.1 3-phosphoshikimate 1-carboxyvinyltransferase [E... | 696 | 0.0 | 79% |
| WP_105741050.1 3-phosphoshikimate 1-carboxyvinyltransferase [P... | 696 | 0.0 | 80% |
| WP_025425507.1 MULTISPECIES: 3-phosphoshikimate 1-carboxyvinyl... | 695 | 0.0 | 79% |
| WP_057247832.1 MULTISPECIES: 3-phosphoshikimate 1-carboxyvinyl... | 695 | 0.0 | 81% |
| WP_113943644.1 3-phosphoshikimate 1-carboxyvinyltransferase [P... | 694 | 0.0 | 78% |
| WP_120702574.1 3-phosphoshikimate 1-carboxyvinyltransferase [R... | 694 | 0.0 | 80% |
| WP_022710242.1 MULTISPECIES: 3-phosphoshikimate 1-carboxyvinyl... | 694 | 0.0 | 77% |
| WP_127861800.1 MULTISPECIES: 3-phosphoshikimate 1-carboxyvinyl... | 694 | 0.0 | 78% |
| WP_183801150.1 3-phosphoshikimate 1-carboxyvinyltransferase [R... | 694 | 0.0 | 78% |
| WP_168843475.1 MULTISPECIES: 3-phosphoshikimate 1-carboxyvinyl... | 694 | 0.0 | 77% |
| WP_151158754.1 3-phosphoshikimate 1-carboxyvinyltransferase [P... | 694 | 0.0 | 77% |
| RWE74684.1 3-phosphoshikimate 1-carboxyvinyltransferase [Mesor... | 694 | 0.0 | 79% |
| WP_149635011.1 3-phosphoshikimate 1-carboxyvinyltransferase [R... | 693 | 0.0 | 78% |
| WP_127424639.1 MULTISPECIES: 3-phosphoshikimate 1-carboxyvinyl... | 693 | 0.0 | 79% |
| WP_059187322.1 3-phosphoshikimate 1-carboxyvinyltransferase [M... | 693 | 0.0 | 78% |
| RWK01831.1 3-phosphoshikimate 1-carboxyvinyltransferase [Mesor... | 693 | 0.0 | 78% |
| WP_127866572.1 3-phosphoshikimate 1-carboxyvinyltransferase [M... | 693 | 0.0 | 79% |
| WP_095799189.1 3-phosphoshikimate 1-carboxyvinyltransferase [M... | 693 | 0.0 | 79% |
| RWK26142.1 3-phosphoshikimate 1-carboxyvinyltransferase [Mesor... | 693 | 0.0 | 78% |
| WP_127311342.1 MULTISPECIES: 3-phosphoshikimate 1-carboxyvinyl... | 693 | 0.0 | 78% |
| RWM20009.1 3-phosphoshikimate 1-carboxyvinyltransferase [Mesor... | 692 | 0.0 | 79% |
| WP_140866714.1 3-phosphoshikimate 1-carboxyvinyltransferase [M... | 692 | 0.0 | 78% |
| WP_140684744.1 MULTISPECIES: 3-phosphoshikimate 1-carboxyvinyl... | 692 | 0.0 | 78% |
| WP_183745483.1 3-phosphoshikimate 1-carboxyvinyltransferase [R... | 692 | 0.0 | 77% |

| WP_192250998.1 3-phosphoshikimate 1-carboxyvinyltransferase [M... | 692 | 0.0 | 79% |
| --- | --- | --- | --- |
| WP_127239465.1 MULTISPECIES: 3-phosphoshikimate 1-carboxyvinyl... | 692 | 0.0 | 79% |
| WP_169646177.1 3-phosphoshikimate 1-carboxyvinyltransferase [R... | 691 | 0.0 | 78% |
| WP_145646366.1 3-phosphoshikimate 1-carboxyvinyltransferase [R... | 691 | 0.0 | 78% |
| WP_112128512.1 3-phosphoshikimate 1-carboxyvinyltransferase [M... | 691 | 0.0 | 78% |
| RWF25187.1 3-phosphoshikimate 1-carboxyvinyltransferase [Mesor... | 691 | 0.0 | 79% |
| WP_140753761.1 3-phosphoshikimate 1-carboxyvinyltransferase [M... | 691 | 0.0 | 78% |
| RWA83849.1 3-phosphoshikimate 1-carboxyvinyltransferase [Mesor... | 691 | 0.0 | 78% |
| WP_135908800.1 3-phosphoshikimate 1-carboxyvinyltransferase [M... | 691 | 0.0 | 78% |
| RWM09552.1 3-phosphoshikimate 1-carboxyvinyltransferase [Mesor... | 691 | 0.0 | 79% |
| WP_127876384.1 3-phosphoshikimate 1-carboxyvinyltransferase [M... | 691 | 0.0 | 78% |
| WP_067003404.1 MULTISPECIES: 3-phosphoshikimate 1-carboxyvinyl... | 691 | 0.0 | 79% |
| WP_127256349.1 MULTISPECIES: 3-phosphoshikimate 1-carboxyvinyl... | 691 | 0.0 | 78% |
| RWG25457.1 3-phosphoshikimate 1-carboxyvinyltransferase [Mesor... | 691 | 0.0 | 79% |
| WP_040974942.1 3-phosphoshikimate 1-carboxyvinyltransferase [M... | 691 | 0.0 | 79% |
| WP_192728618.1 3-phosphoshikimate 1-carboxyvinyltransferase [R... | 691 | 0.0 | 77% |
| WP_135860344.1 MULTISPECIES: 3-phosphoshikimate 1-carboxyvinyl... | 691 | 0.0 | 79% |
| WP_127284415.1 3-phosphoshikimate 1-carboxyvinyltransferase [M... | 691 | 0.0 | 79% |
| RWC96807.1 3-phosphoshikimate 1-carboxyvinyltransferase [Mesor... | 690 | 0.0 | 78% |
| WP_095831201.1 3-phosphoshikimate 1-carboxyvinyltransferase [M... | 690 | 0.0 | 78% |
| WP_095782258.1 3-phosphoshikimate 1-carboxyvinyltransferase [M... | 690 | 0.0 | 78% |
| WP_067324710.1 3-phosphoshikimate 1-carboxyvinyltransferase [M... | 690 | 0.0 | 78% |
| WP_183727425.1 3-phosphoshikimate 1-carboxyvinyltransferase [R... | 690 | 0.0 | 77% |
| RWE17666.1 3-phosphoshikimate 1-carboxyvinyltransferase [Mesor... | 690 | 0.0 | 78% |
| PBB80549.1 3-phosphoshikimate 1-carboxyvinyltransferase [Mesor... | 690 | 0.0 | 79% |
| WP_126096578.1 3-phosphoshikimate 1-carboxyvinyltransferase [M... | 690 | 0.0 | 78% |
| WP_127391261.1 MULTISPECIES: 3-phosphoshikimate 1-carboxyvinyl... | 689 | 0.0 | 79% |
| WP_127247776.1 3-phosphoshikimate 1-carboxyvinyltransferase [M... | 689 | 0.0 | 78% |
| RWH72139.1 3-phosphoshikimate 1-carboxyvinyltransferase [Mesor... | 689 | 0.0 | 78% |
| WP_112340061.1 3-phosphoshikimate 1-carboxyvinyltransferase [R... | 689 | 0.0 | 78% |
| WP_057204358.1 3-phosphoshikimate 1-carboxyvinyltransferase [E... | 689 | 0.0 | 79% |
| RWD72893.1 3-phosphoshikimate 1-carboxyvinyltransferase [Mesor... | 689 | 0.0 | 78% |
| TIW94727.1 3-phosphoshikimate 1-carboxyvinyltransferase [Mesor... | 689 | 0.0 | 79% |
| CDX26639.1 3-phosphoshikimate 1-carboxyvinyltransferase [Mesor... | 689 | 0.0 | 79% |
| WP_126086286.1 3-phosphoshikimate 1-carboxyvinyltransferase [M... | 689 | 0.0 | 78% |
| WP_095806734.1 MULTISPECIES: 3-phosphoshikimate 1-carboxyvinyl... | 689 | 0.0 | 78% |
| CDX50135.1 3-phosphoshikimate 1-carboxyvinyltransferase [Mesor... | 689 | 0.0 | 78% |
| WP_149790164.1 3-phosphoshikimate 1-carboxyvinyltransferase [M... | 689 | 0.0 | 78% |
| WP_124999662.1 3-phosphoshikimate 1-carboxyvinyltransferase [M... | 689 | 0.0 | 78% |
| RWM41150.1 3-phosphoshikimate 1-carboxyvinyltransferase [Mesor... | 689 | 0.0 | 78% |
| TIT78119.1 3-phosphoshikimate 1-carboxyvinyltransferase [Mesor... | 689 | 0.0 | 78% |
| WP_127878736.1 MULTISPECIES: 3-phosphoshikimate 1-carboxyvinyl... | 689 | 0.0 | 78% |
| RWB78626.1 3-phosphoshikimate 1-carboxyvinyltransferase [Mesor... | 688 | 0.0 | 78% |
| WP_040994398.1 3-phosphoshikimate 1-carboxyvinyltransferase [M... | 688 | 0.0 | 78% |
| WP_127302779.1 MULTISPECIES: 3-phosphoshikimate 1-carboxyvinyl... | 688 | 0.0 | 78% |
| WP_071036801.1 3-phosphoshikimate 1-carboxyvinyltransferase [M... | 688 | 0.0 | 78% |
| WP_040583313.1 3-phosphoshikimate 1-carboxyvinyltransferase [M... | 688 | 0.0 | 78% |
| RWL45835.1 3-phosphoshikimate 1-carboxyvinyltransferase [Mesor... | 688 | 0.0 | 78% |
| RWC46869.1 3-phosphoshikimate 1-carboxyvinyltransferase [Mesor... | 687 | 0.0 | 78% |
| WP_127249697.1 MULTISPECIES: 3-phosphoshikimate 1-carboxyvinyl... | 687 | 0.0 | 78% |
| CDX12151.1 3-phosphoshikimate 1-carboxyvinyltransferase [Mesor... | 687 | 0.0 | 78% |
| RWM27419.1 3-phosphoshikimate 1-carboxyvinyltransferase [Mesor... | 687 | 0.0 | 78% |
| WP_065784613.1 MULTISPECIES: 3-phosphoshikimate 1-carboxyvinyl... | 687 | 0.0 | 79% |
| RWA68673.1 3-phosphoshikimate 1-carboxyvinyltransferase [Mesor... | 687 | 0.0 | 78% |
| WP_145718824.1 3-phosphoshikimate 1-carboxyvinyltransferase [M... | 687 | 0.0 | 78% |
| TIQ33697.1 3-phosphoshikimate 1-carboxyvinyltransferase [Mesor... | 687 | 0.0 | 78% |
| WP_071069874.1 3-phosphoshikimate 1-carboxyvinyltransferase [M... | 687 | 0.0 | 78% |
| CDX14990.1 3-phosphoshikimate 1-carboxyvinyltransferase [Mesor... | 687 | 0.0 | 78% |
| WP_128342427.1 3-phosphoshikimate 1-carboxyvinyltransferase [M... | 686 | 0.0 | 78% |
| WP_135920479.1 3-phosphoshikimate 1-carboxyvinyltransferase [M... | 686 | 0.0 | 78% |
| TIS77745.1 3-phosphoshikimate 1-carboxyvinyltransferase [Mesor... | 686 | 0.0 | 78% |
| WP_188256751.1 3-phosphoshikimate 1-carboxyvinyltransferase [P... | 686 | 0.0 | 79% |
| WP_140628796.1 3-phosphoshikimate 1-carboxyvinyltransferase [M... | 686 | 0.0 | 78% |
| WP_127229749.1 MULTISPECIES: 3-phosphoshikimate 1-carboxyvinyl... | 686 | 0.0 | 78% |
| WP_140782444.1 MULTISPECIES: 3-phosphoshikimate 1-carboxyvinyl... | 686 | 0.0 | 78% |
| RWD84160.1 3-phosphoshikimate 1-carboxyvinyltransferase [Mesor... | 686 | 0.0 | 78% |
| WP_183765354.1 MULTISPECIES: 3-phosphoshikimate 1-carboxyvinyl... | 686 | 0.0 | 77% |
| WP_126063101.1 3-phosphoshikimate 1-carboxyvinyltransferase [M... | 686 | 0.0 | 78% |
| RVD73587.1 3-phosphoshikimate 1-carboxyvinyltransferase [Mesor... | 686 | 0.0 | 78% |
| RWB57618.1 3-phosphoshikimate 1-carboxyvinyltransferase [Mesor... | 686 | 0.0 | 77% |
| RWL90264.1 3-phosphoshikimate 1-carboxyvinyltransferase [Mesor... | 686 | 0.0 | 78% |
| WP_192448167.1 3-phosphoshikimate 1-carboxyvinyltransferase [E... | 685 | 0.0 | 79% |

| WP_149759219.1 3-phosphoshikimate 1-carboxyvinyltransferase [M... | 685 | 0.0 | 78% |
| --- | --- | --- | --- |
| WP_095769357.1 3-phosphoshikimate 1-carboxyvinyltransferase [M... | 685 | 0.0 | 78% |
| RTM07576.1 3-phosphoshikimate 1-carboxyvinyltransferase [Rhizo... | 685 | 0.0 | 78% |
| TGP51891.1 3-phosphoshikimate 1-carboxyvinyltransferase [bacte... | 685 | 0.0 | 78% |
| WP_126090586.1 3-phosphoshikimate 1-carboxyvinyltransferase [M... | 685 | 0.0 | 77% |
| WP_127236872.1 3-phosphoshikimate 1-carboxyvinyltransferase [M... | 685 | 0.0 | 77% |
| WP_127328864.1 3-phosphoshikimate 1-carboxyvinyltransferase [M... | 685 | 0.0 | 77% |
| WP_073989321.1 3-phosphoshikimate 1-carboxyvinyltransferase [M... | 685 | 0.0 | 77% |
| WP_135933667.1 3-phosphoshikimate 1-carboxyvinyltransferase [M... | 685 | 0.0 | 78% |
| RWD50221.1 3-phosphoshikimate 1-carboxyvinyltransferase [Mesor... | 685 | 0.0 | 78% |
| RWF54871.1 3-phosphoshikimate 1-carboxyvinyltransferase [Mesor... | 685 | 0.0 | 78% |
| WP_006325315.1 3-phosphoshikimate 1-carboxyvinyltransferase [M... | 685 | 0.0 | 78% |
| WP_095810358.1 3-phosphoshikimate 1-carboxyvinyltransferase [M... | 685 | 0.0 | 78% |
| WP_140900106.1 3-phosphoshikimate 1-carboxyvinyltransferase [M... | 684 | 0.0 | 77% |
| RWO84248.1 3-phosphoshikimate 1-carboxyvinyltransferase [Mesor... | 684 | 0.0 | 77% |
| RWO81408.1 3-phosphoshikimate 1-carboxyvinyltransferase [Mesor... | 684 | 0.0 | 78% |
| RUU13362.1 3-phosphoshikimate 1-carboxyvinyltransferase [Mesor... | 684 | 0.0 | 77% |
| WP_127395335.1 3-phosphoshikimate 1-carboxyvinyltransferase [M... | 684 | 0.0 | 77% |
| WP_107649170.1 3-phosphoshikimate 1-carboxyvinyltransferase [M... | 684 | 0.0 | 77% |
| WP_140832121.1 3-phosphoshikimate 1-carboxyvinyltransferase [M... | 684 | 0.0 | 78% |
| TIR86484.1 3-phosphoshikimate 1-carboxyvinyltransferase [Mesor... | 684 | 0.0 | 78% |
| WP_151983503.1 3-phosphoshikimate 1-carboxyvinyltransferase [R... | 684 | 0.0 | 78% |
| RWE58108.1 3-phosphoshikimate 1-carboxyvinyltransferase [Mesor... | 684 | 0.0 | 78% |
| WP_123146362.1 3-phosphoshikimate 1-carboxyvinyltransferase [M... | 684 | 0.0 | 77% |
| CDX61544.1 3-phosphoshikimate 1-carboxyvinyltransferase [Mesor... | 684 | 0.0 | 77% |
| WP_095817868.1 3-phosphoshikimate 1-carboxyvinyltransferase [M... | 684 | 0.0 | 78% |
| TIN70018.1 3-phosphoshikimate 1-carboxyvinyltransferase [Mesor... | 684 | 0.0 | 77% |
| RWB99656.1 3-phosphoshikimate 1-carboxyvinyltransferase [Mesor... | 684 | 0.0 | 77% |
| TIV34104.1 3-phosphoshikimate 1-carboxyvinyltransferase [Mesor... | 684 | 0.0 | 77% |
| WP_140722107.1 3-phosphoshikimate 1-carboxyvinyltransferase [M... | 684 | 0.0 | 78% |
| RWO34190.1 3-phosphoshikimate 1-carboxyvinyltransferase [Mesor... | 684 | 0.0 | 78% |
| WP_140845573.1 3-phosphoshikimate 1-carboxyvinyltransferase [M... | 684 | 0.0 | 78% |
| WP_140687181.1 3-phosphoshikimate 1-carboxyvinyltransferase [M... | 683 | 0.0 | 78% |
| RWE69018.1 3-phosphoshikimate 1-carboxyvinyltransferase [Mesor... | 683 | 0.0 | 78% |
| TIM06126.1 3-phosphoshikimate 1-carboxyvinyltransferase [Mesor... | 683 | 0.0 | 77% |
| TIR30984.1 3-phosphoshikimate 1-carboxyvinyltransferase [Mesor... | 683 | 0.0 | 78% |
| TIM83740.1 3-phosphoshikimate 1-carboxyvinyltransferase [Mesor... | 683 | 0.0 | 78% |
| TKD39474.1 3-phosphoshikimate 1-carboxyvinyltransferase [Mesor... | 683 | 0.0 | 78% |
| WP_023796773.1 3-phosphoshikimate 1-carboxyvinyltransferase [M... | 683 | 0.0 | 78% |
| WP_140545274.1 3-phosphoshikimate 1-carboxyvinyltransferase [M... | 683 | 0.0 | 78% |
| WP_126056883.1 MULTISPECIES: 3-phosphoshikimate 1-carboxyvinyl... | 683 | 0.0 | 78% |
| RWO92749.1 3-phosphoshikimate 1-carboxyvinyltransferase [Mesor... | 683 | 0.0 | 78% |
| RUU39409.1 3-phosphoshikimate 1-carboxyvinyltransferase [Mesor... | 683 | 0.0 | 77% |
| WP_136618322.1 MULTISPECIES: 3-phosphoshikimate 1-carboxyvinyl... | 683 | 0.0 | 77% |
| RWN54766.1 3-phosphoshikimate 1-carboxyvinyltransferase [Mesor... | 682 | 0.0 | 77% |
| WP_192179333.1 3-phosphoshikimate 1-carboxyvinyltransferase [M... | 682 | 0.0 | 78% |
| TIP95793.1 3-phosphoshikimate 1-carboxyvinyltransferase [Mesor... | 682 | 0.0 | 77% |
| TIO06699.1 3-phosphoshikimate 1-carboxyvinyltransferase [Mesor... | 682 | 0.0 | 78% |
| RWB01057.1 3-phosphoshikimate 1-carboxyvinyltransferase [Mesor... | 682 | 0.0 | 78% |
| WP_128364101.1 3-phosphoshikimate 1-carboxyvinyltransferase [M... | 682 | 0.0 | 77% |
| OYR27757.1 3-phosphoshikimate 1-carboxyvinyltransferase [Ochro... | 682 | 0.0 | 83% |
| RWQ18689.1 3-phosphoshikimate 1-carboxyvinyltransferase [Mesor... | 682 | 0.0 | 77% |
| RWB27226.1 3-phosphoshikimate 1-carboxyvinyltransferase [Mesor... | 682 | 0.0 | 78% |
| RWN67225.1 3-phosphoshikimate 1-carboxyvinyltransferase [Mesor... | 682 | 0.0 | 77% |
| RWH78490.1 3-phosphoshikimate 1-carboxyvinyltransferase [Mesor... | 682 | 0.0 | 77% |
| RWO02711.1 3-phosphoshikimate 1-carboxyvinyltransferase [Mesor... | 682 | 0.0 | 77% |
| WP_023718021.1 3-phosphoshikimate 1-carboxyvinyltransferase [M... | 682 | 0.0 | 78% |
| RWL83793.1 3-phosphoshikimate 1-carboxyvinyltransferase [Mesor... | 682 | 0.0 | 77% |
| RWN61857.1 3-phosphoshikimate 1-carboxyvinyltransferase [Mesor... | 682 | 0.0 | 78% |
| WP_027151509.1 3-phosphoshikimate 1-carboxyvinyltransferase [M... | 682 | 0.0 | 78% |
| WP_127220727.1 3-phosphoshikimate 1-carboxyvinyltransferase [M... | 682 | 0.0 | 77% |
| WP_184394089.1 3-phosphoshikimate 1-carboxyvinyltransferase [R... | 682 | 0.0 | 76% |
| WP_126007218.1 3-phosphoshikimate 1-carboxyvinyltransferase [G... | 682 | 0.0 | 78% |
| TIX55651.1 3-phosphoshikimate 1-carboxyvinyltransferase [Mesor... | 682 | 0.0 | 78% |
| RWO74798.1 3-phosphoshikimate 1-carboxyvinyltransferase [Mesor... | 681 | 0.0 | 77% |
| RWN01664.1 3-phosphoshikimate 1-carboxyvinyltransferase [Mesor... | 681 | 0.0 | 78% |
| ERM02521.1 3-phosphoshikimate 1-carboxyvinyltransferase [Ochro... | 681 | 0.0 | 83% |
| TIS60377.1 3-phosphoshikimate 1-carboxyvinyltransferase [Mesor... | 681 | 0.0 | 77% |
| RWM21195.1 3-phosphoshikimate 1-carboxyvinyltransferase [Mesor... | 681 | 0.0 | 77% |
| RWQ24501.1 3-phosphoshikimate 1-carboxyvinyltransferase [Mesor... | 681 | 0.0 | 77% |
| CDX42125.1 3-phosphoshikimate 1-carboxyvinyltransferase [Mesor... | 681 | 0.0 | 77% |
| WP_091592754.1 3-phosphoshikimate 1-carboxyvinyltransferase [M... | 681 | 0.0 | 78% |

| RWM82345.1 3-phosphoshikimate 1-carboxyvinyltransferase [Mesor... | 681 | 0.0 | 77% |
| --- | --- | --- | --- |
| WP_127405880.1 MULTISPECIES: 3-phosphoshikimate 1-carboxyvinyl... | 681 | 0.0 | 78% |
| TIL28789.1 3-phosphoshikimate 1-carboxyvinyltransferase [Mesor... | 681 | 0.0 | 77% |
| RWK64962.1 3-phosphoshikimate 1-carboxyvinyltransferase [Mesor... | 681 | 0.0 | 77% |
| WP_095082926.1 3-phosphoshikimate 1-carboxyvinyltransferase [M... | 681 | 0.0 | 77% |
| TIL45112.1 3-phosphoshikimate 1-carboxyvinyltransferase [Mesor... | 681 | 0.0 | 77% |
| RWP32998.1 3-phosphoshikimate 1-carboxyvinyltransferase [Mesor... | 680 | 0.0 | 77% |
| WP_109665793.1 3-phosphoshikimate 1-carboxyvinyltransferase [M... | 680 | 0.0 | 77% |
| TIL95137.1 3-phosphoshikimate 1-carboxyvinyltransferase [Mesor... | 680 | 0.0 | 77% |
| WP_029347642.1 3-phosphoshikimate 1-carboxyvinyltransferase [M... | 680 | 0.0 | 77% |
| WP_095517360.1 3-phosphoshikimate 1-carboxyvinyltransferase [M... | 680 | 0.0 | 77% |
| WP_127853519.1 3-phosphoshikimate 1-carboxyvinyltransferase [M... | 680 | 0.0 | 77% |
| RWO22386.1 3-phosphoshikimate 1-carboxyvinyltransferase [Mesor... | 680 | 0.0 | 77% |
| WP_095493879.1 3-phosphoshikimate 1-carboxyvinyltransferase [M... | 680 | 0.0 | 77% |
| TJV72134.1 3-phosphoshikimate 1-carboxyvinyltransferase [Mesor... | 679 | 0.0 | 77% |
| WP_023676281.1 3-phosphoshikimate 1-carboxyvinyltransferase [M... | 679 | 0.0 | 78% |
| TIS36796.1 3-phosphoshikimate 1-carboxyvinyltransferase [Mesor... | 679 | 0.0 | 77% |
| WP_144702376.1 3-phosphoshikimate 1-carboxyvinyltransferase [A... | 679 | 0.0 | 79% |
| WP_035025239.1 3-phosphoshikimate 1-carboxyvinyltransferase [A... | 679 | 0.0 | 77% |
| WP_024847074.1 3-phosphoshikimate 1-carboxyvinyltransferase [A... | 679 | 0.0 | 79% |
| WP_162654266.1 3-phosphoshikimate 1-carboxyvinyltransferase [L... | 679 | 0.0 | 78% |
| RWA77644.1 3-phosphoshikimate 1-carboxyvinyltransferase [Mesor... | 679 | 0.0 | 78% |
| RWO60206.1 3-phosphoshikimate 1-carboxyvinyltransferase [Mesor... | 679 | 0.0 | 78% |
| TIP26977.1 3-phosphoshikimate 1-carboxyvinyltransferase [Mesor... | 679 | 0.0 | 77% |
| RWN38360.1 3-phosphoshikimate 1-carboxyvinyltransferase [Mesor... | 679 | 0.0 | 77% |
| TIR25212.1 3-phosphoshikimate 1-carboxyvinyltransferase [Mesor... | 678 | 0.0 | 78% |
| WP_129412169.1 3-phosphoshikimate 1-carboxyvinyltransferase [M... | 678 | 0.0 | 78% |
| WP_077375103.1 3-phosphoshikimate 1-carboxyvinyltransferase [M... | 678 | 0.0 | 77% |
| TIL70542.1 3-phosphoshikimate 1-carboxyvinyltransferase [Mesor... | 678 | 0.0 | 77% |
| WP_065006055.1 3-phosphoshikimate 1-carboxyvinyltransferase [M... | 678 | 0.0 | 77% |
| RWK53918.1 3-phosphoshikimate 1-carboxyvinyltransferase [Mesor... | 678 | 0.0 | 77% |
| TIP80571.1 3-phosphoshikimate 1-carboxyvinyltransferase [Mesor... | 678 | 0.0 | 77% |
| RWG78543.1 3-phosphoshikimate 1-carboxyvinyltransferase [Mesor... | 678 | 0.0 | 78% |
| RWK33405.1 3-phosphoshikimate 1-carboxyvinyltransferase [Mesor... | 677 | 0.0 | 77% |
| WP_095484342.1 MULTISPECIES: 3-phosphoshikimate 1-carboxyvinyl... | 677 | 0.0 | 77% |
| WP_126079852.1 3-phosphoshikimate 1-carboxyvinyltransferase [M... | 677 | 0.0 | 78% |
| TIP01447.1 3-phosphoshikimate 1-carboxyvinyltransferase [Mesor... | 677 | 0.0 | 77% |
| WP_144708598.1 3-phosphoshikimate 1-carboxyvinyltransferase [A... | 677 | 0.0 | 79% |
| TIT21188.1 3-phosphoshikimate 1-carboxyvinyltransferase [Mesor... | 677 | 0.0 | 77% |
| WP_008834206.1 3-phosphoshikimate 1-carboxyvinyltransferase [M... | 676 | 0.0 | 77% |
| WP_140497241.1 MULTISPECIES: 3-phosphoshikimate 1-carboxyvinyl... | 676 | 0.0 | 79% |
| WP_095200843.1 3-phosphoshikimate 1-carboxyvinyltransferase [M... | 676 | 0.0 | 78% |
| WP_184873211.1 3-phosphoshikimate 1-carboxyvinyltransferase [M... | 676 | 0.0 | 77% |
| WP_126103907.1 3-phosphoshikimate 1-carboxyvinyltransferase [M... | 676 | 0.0 | 77% |
| WP_140802303.1 3-phosphoshikimate 1-carboxyvinyltransferase [M... | 676 | 0.0 | 79% |
| WP_140506683.1 3-phosphoshikimate 1-carboxyvinyltransferase [M... | 676 | 0.0 | 78% |
| WP_140624910.1 3-phosphoshikimate 1-carboxyvinyltransferase [M... | 676 | 0.0 | 78% |
| WP_140657796.1 MULTISPECIES: 3-phosphoshikimate 1-carboxyvinyl... | 676 | 0.0 | 79% |
| WP_128242333.1 3-phosphoshikimate 1-carboxyvinyltransferase [M... | 675 | 0.0 | 77% |
| RWQ67813.1 3-phosphoshikimate 1-carboxyvinyltransferase [Mesor... | 675 | 0.0 | 77% |
| WP_140550305.1 3-phosphoshikimate 1-carboxyvinyltransferase [M... | 675 | 0.0 | 78% |
| WP_140815185.1 MULTISPECIES: 3-phosphoshikimate 1-carboxyvinyl... | 675 | 0.0 | 79% |
| TJX01309.1 3-phosphoshikimate 1-carboxyvinyltransferase [Mesor... | 674 | 0.0 | 77% |
| TIP47873.1 3-phosphoshikimate 1-carboxyvinyltransferase [Mesor... | 674 | 0.0 | 77% |
| RWI29033.1 3-phosphoshikimate 1-carboxyvinyltransferase [Mesor... | 674 | 0.0 | 77% |
| WP_140580572.1 MULTISPECIES: 3-phosphoshikimate 1-carboxyvinyl... | 674 | 0.0 | 78% |
| WP_140768585.1 3-phosphoshikimate 1-carboxyvinyltransferase [M... | 673 | 0.0 | 78% |
| WP_140643738.1 3-phosphoshikimate 1-carboxyvinyltransferase [M... | 673 | 0.0 | 78% |
| RWF32751.1 3-phosphoshikimate 1-carboxyvinyltransferase [Mesor... | 673 | 0.0 | 79% |
| WP_140559770.1 MULTISPECIES: 3-phosphoshikimate 1-carboxyvinyl... | 673 | 0.0 | 77% |
| WP_140649465.1 MULTISPECIES: 3-phosphoshikimate 1-carboxyvinyl... | 672 | 0.0 | 78% |
| WP_140512883.1 3-phosphoshikimate 1-carboxyvinyltransferase [M... | 672 | 0.0 | 78% |
| WP_096452931.1 3-phosphoshikimate 1-carboxyvinyltransferase [M... | 672 | 0.0 | 78% |
| WP_173418650.1 3-phosphoshikimate 1-carboxyvinyltransferase [E... | 672 | 0.0 | 76% |
| WP_054310093.1 3-phosphoshikimate 1-carboxyvinyltransferase [M... | 672 | 0.0 | 77% |
| WP_011582919.1 MULTISPECIES: 3-phosphoshikimate 1-carboxyvinyl... | 672 | 0.0 | 76% |
| WP_101939518.1 3-phosphoshikimate 1-carboxyvinyltransferase [M... | 672 | 0.0 | 78% |
| MAW88510.1 3-phosphoshikimate 1-carboxyvinyltransferase [Phyll... | 671 | 0.0 | 78% |
| WP_077499323.1 3-phosphoshikimate 1-carboxyvinyltransferase [S... | 671 | 0.0 | 76% |
| WP_112097278.1 3-phosphoshikimate 1-carboxyvinyltransferase [M... | 671 | 0.0 | 79% |
| WP_195164173.1 3-phosphoshikimate 1-carboxyvinyltransferase [M... | 671 | 0.0 | 76% |
| RWP91139.1 3-phosphoshikimate 1-carboxyvinyltransferase [Mesor... | 671 | 0.0 | 78% |

| WP_106726316.1 3-phosphoshikimate 1-carboxyvinyltransferase [M... | 671 | 0.0 | 77% |
| --- | --- | --- | --- |
| WP_140556906.1 MULTISPECIES: 3-phosphoshikimate 1-carboxyvinyl... | 671 | 0.0 | 78% |
| WP_056243951.1 3-phosphoshikimate 1-carboxyvinyltransferase [M... | 671 | 0.0 | 78% |
| WP_140564979.1 MULTISPECIES: 3-phosphoshikimate 1-carboxyvinyl... | 670 | 0.0 | 78% |
| WP_027166167.1 3-phosphoshikimate 1-carboxyvinyltransferase [M... | 670 | 0.0 | 78% |
| WP_140702313.1 3-phosphoshikimate 1-carboxyvinyltransferase [M... | 670 | 0.0 | 78% |
| OJX77951.1 3-phosphoshikimate 1-carboxyvinyltransferase [Mesor... | 670 | 0.0 | 79% |
| WP_106772857.1 3-phosphoshikimate 1-carboxyvinyltransferase [M... | 670 | 0.0 | 77% |
| WP_140738324.1 MULTISPECIES: 3-phosphoshikimate 1-carboxyvinyl... | 669 | 0.0 | 79% |
| WP_042645218.1 3-phosphoshikimate 1-carboxyvinyltransferase [M... | 669 | 0.0 | 78% |
| TIM22261.1 3-phosphoshikimate 1-carboxyvinyltransferase [Mesor... | 669 | 0.0 | 77% |
| RWN46227.1 3-phosphoshikimate 1-carboxyvinyltransferase [Mesor... | 669 | 0.0 | 78% |
| WP_140630142.1 MULTISPECIES: 3-phosphoshikimate 1-carboxyvinyl... | 669 | 0.0 | 78% |
| RWE46321.1 3-phosphoshikimate 1-carboxyvinyltransferase [Mesor... | 669 | 0.0 | 79% |
| WP_136657667.1 3-phosphoshikimate 1-carboxyvinyltransferase [N... | 669 | 0.0 | 78% |
| CDX40919.1 3-phosphoshikimate 1-carboxyvinyltransferase [Mesor... | 669 | 0.0 | 78% |
| TGQ65525.1 3-phosphoshikimate 1-carboxyvinyltransferase [bacte... | 669 | 0.0 | 77% |
| WP_131564968.1 3-phosphoshikimate 1-carboxyvinyltransferase [O... | 669 | 0.0 | 76% |
| WP_027040543.1 3-phosphoshikimate 1-carboxyvinyltransferase [M... | 669 | 0.0 | 78% |
| WP_027032687.1 3-phosphoshikimate 1-carboxyvinyltransferase [M... | 668 | 0.0 | 78% |
| WP_091580881.1 3-phosphoshikimate 1-carboxyvinyltransferase [M... | 667 | 0.0 | 78% |
| WP_023811518.1 3-phosphoshikimate 1-carboxyvinyltransferase [M... | 667 | 0.0 | 78% |
| WP_024924358.1 MULTISPECIES: 3-phosphoshikimate 1-carboxyvinyl... | 667 | 0.0 | 76% |
| WP_053998386.1 3-phosphoshikimate 1-carboxyvinyltransferase [A... | 666 | 0.0 | 75% |
| WP_144378739.1 3-phosphoshikimate 1-carboxyvinyltransferase [M... | 666 | 0.0 | 79% |
| WP_097576630.1 3-phosphoshikimate 1-carboxyvinyltransferase [M... | 666 | 0.0 | 78% |
| RWN17211.1 3-phosphoshikimate 1-carboxyvinyltransferase [Mesor... | 666 | 0.0 | 78% |
| WP_195176840.1 3-phosphoshikimate 1-carboxyvinyltransferase [M... | 666 | 0.0 | 76% |
| WP_115418752.1 3-phosphoshikimate 1-carboxyvinyltransferase [E... | 665 | 0.0 | 76% |
| WP_140650592.1 MULTISPECIES: 3-phosphoshikimate 1-carboxyvinyl... | 665 | 0.0 | 78% |
| MBA3446854.1 3-phosphoshikimate 1-carboxyvinyltransferase [Pse... | 665 | 0.0 | 77% |
| WP_189504106.1 3-phosphoshikimate 1-carboxyvinyltransferase [C... | 665 | 0.0 | 79% |
| WP_128302807.1 3-phosphoshikimate 1-carboxyvinyltransferase [M... | 665 | 0.0 | 78% |
| WP_127289806.1 3-phosphoshikimate 1-carboxyvinyltransferase [M... | 665 | 0.0 | 78% |
| WP_141836710.1 3-phosphoshikimate 1-carboxyvinyltransferase [M... | 665 | 0.0 | 75% |
| WP_023761479.1 3-phosphoshikimate 1-carboxyvinyltransferase [M... | 665 | 0.0 | 78% |
| RUV11441.1 3-phosphoshikimate 1-carboxyvinyltransferase [Mesor... | 665 | 0.0 | 77% |
| WP_120011925.1 3-phosphoshikimate 1-carboxyvinyltransferase [M... | 665 | 0.0 | 78% |
| WP_018688986.1 3-phosphoshikimate 1-carboxyvinyltransferase [A... | 665 | 0.0 | 74% |
| WP_127381465.1 3-phosphoshikimate 1-carboxyvinyltransferase [M... | 665 | 0.0 | 77% |
| WP_140644739.1 3-phosphoshikimate 1-carboxyvinyltransferase [M... | 664 | 0.0 | 78% |
| WP_026479127.1 3-phosphoshikimate 1-carboxyvinyltransferase [A... | 664 | 0.0 | 74% |
| RWN96981.1 3-phosphoshikimate 1-carboxyvinyltransferase [Mesor... | 664 | 0.0 | 77% |
| WP_183445833.1 3-phosphoshikimate 1-carboxyvinyltransferase [M... | 664 | 0.0 | 78% |
| WP_127808188.1 MULTISPECIES: 3-phosphoshikimate 1-carboxyvinyl... | 664 | 0.0 | 77% |
| WP_027061881.1 3-phosphoshikimate 1-carboxyvinyltransferase [M... | 664 | 0.0 | 77% |
| WP_139953887.1 3-phosphoshikimate 1-carboxyvinyltransferase [M... | 664 | 0.0 | 78% |
| WP_140606778.1 3-phosphoshikimate 1-carboxyvinyltransferase [M... | 664 | 0.0 | 75% |
| RWC31844.1 3-phosphoshikimate 1-carboxyvinyltransferase [Mesor... | 664 | 0.0 | 78% |
| WP_127272415.1 MULTISPECIES: 3-phosphoshikimate 1-carboxyvinyl... | 663 | 0.0 | 77% |
| WP_056589697.1 3-phosphoshikimate 1-carboxyvinyltransferase [M... | 663 | 0.0 | 78% |
| WP_127315841.1 3-phosphoshikimate 1-carboxyvinyltransferase [M... | 663 | 0.0 | 78% |
| WP_013528181.1 MULTISPECIES: 3-phosphoshikimate 1-carboxyvinyl... | 663 | 0.0 | 75% |
| WP_080918705.1 3-phosphoshikimate 1-carboxyvinyltransferase [P... | 663 | 0.0 | 77% |
| WP_140538724.1 3-phosphoshikimate 1-carboxyvinyltransferase [M... | 663 | 0.0 | 77% |
| WP_136357407.1 3-phosphoshikimate 1-carboxyvinyltransferase [M... | 663 | 0.0 | 78% |
| WP_057142573.1 3-phosphoshikimate 1-carboxyvinyltransferase [M... | 663 | 0.0 | 78% |
| WP_176351182.1 3-phosphoshikimate 1-carboxyvinyltransferase [E... | 663 | 0.0 | 76% |
| WP_027023824.1 3-phosphoshikimate 1-carboxyvinyltransferase [M... | 662 | 0.0 | 75% |
| RWC62810.1 3-phosphoshikimate 1-carboxyvinyltransferase [Mesor... | 662 | 0.0 | 77% |
| WP_036484870.1 3-phosphoshikimate 1-carboxyvinyltransferase [N... | 662 | 0.0 | 76% |
| WP_027039823.1 3-phosphoshikimate 1-carboxyvinyltransferase [M... | 662 | 0.0 | 77% |
| RWC42594.1 3-phosphoshikimate 1-carboxyvinyltransferase [Mesor... | 662 | 0.0 | 75% |
| RWO47749.1 3-phosphoshikimate 1-carboxyvinyltransferase [Mesor... | 662 | 0.0 | 77% |
| WP_127419381.1 3-phosphoshikimate 1-carboxyvinyltransferase [M... | 662 | 0.0 | 75% |
| WP_047147080.1 3-phosphoshikimate 1-carboxyvinyltransferase [A... | 662 | 0.0 | 77% |
| WP_127390000.1 3-phosphoshikimate 1-carboxyvinyltransferase [M... | 662 | 0.0 | 75% |
| WP_140764905.1 3-phosphoshikimate 1-carboxyvinyltransferase [M... | 662 | 0.0 | 75% |
| WP_008872511.1 3-phosphoshikimate 1-carboxyvinyltransferase [M... | 662 | 0.0 | 77% |
| RWF87413.1 3-phosphoshikimate 1-carboxyvinyltransferase [Mesor... | 662 | 0.0 | 77% |
| WP_091916395.1 3-phosphoshikimate 1-carboxyvinyltransferase [M... | 662 | 0.0 | 76% |
| WP_140695278.1 3-phosphoshikimate 1-carboxyvinyltransferase [M... | 662 | 0.0 | 75% |

| WP_035523551.1 3-phosphoshikimate 1-carboxyvinyltransferase [H... | 662 | 0.0 | 77% |
| --- | --- | --- | --- |
| WP_127320562.1 MULTISPECIES: 3-phosphoshikimate 1-carboxyvinyl... | 662 | 0.0 | 77% |
| WP_165025996.1 3-phosphoshikimate 1-carboxyvinyltransferase [M... | 662 | 0.0 | 78% |
| TIS06620.1 3-phosphoshikimate 1-carboxyvinyltransferase [Mesor... | 662 | 0.0 | 79% |
| KFB08301.1 3-phosphoshikimate 1-carboxyvinyltransferase [Nitra... | 662 | 0.0 | 75% |
| WP_119821199.1 3-phosphoshikimate 1-carboxyvinyltransferase [M... | 661 | 0.0 | 76% |
| WP_099867085.1 3-phosphoshikimate 1-carboxyvinyltransferase [P... | 661 | 0.0 | 74% |
| WP_071094554.1 3-phosphoshikimate 1-carboxyvinyltransferase [M... | 661 | 0.0 | 77% |
| WP_111544913.1 3-phosphoshikimate 1-carboxyvinyltransferase [M... | 661 | 0.0 | 78% |
| HHZ09513.1 TPA: 3-phosphoshikimate 1-carboxyvinyltransferase [... | 661 | 0.0 | 77% |
| WP_127256236.1 3-phosphoshikimate 1-carboxyvinyltransferase [M... | 661 | 0.0 | 77% |
| WP_127373576.1 3-phosphoshikimate 1-carboxyvinyltransferase [M... | 661 | 0.0 | 77% |
| WP_056113325.1 MULTISPECIES: 3-phosphoshikimate 1-carboxyvinyl... | 661 | 0.0 | 77% |
| ODT31455.1 3-phosphoshikimate 1-carboxyvinyltransferase [Kaist... | 661 | 0.0 | 77% |
| WP_128627844.1 3-phosphoshikimate 1-carboxyvinyltransferase [P... | 660 | 0.0 | 78% |
| WP_127413364.1 3-phosphoshikimate 1-carboxyvinyltransferase [M... | 660 | 0.0 | 77% |
| WP_128371227.1 3-phosphoshikimate 1-carboxyvinyltransferase [M... | 660 | 0.0 | 79% |
| WP_140745133.1 3-phosphoshikimate 1-carboxyvinyltransferase [M... | 660 | 0.0 | 75% |
| WP_126067394.1 3-phosphoshikimate 1-carboxyvinyltransferase [M... | 660 | 0.0 | 77% |
| WP_126037197.1 3-phosphoshikimate 1-carboxyvinyltransferase [M... | 660 | 0.0 | 77% |
| WP_137929092.1 3-phosphoshikimate 1-carboxyvinyltransferase [M... | 660 | 0.0 | 79% |
| EHH09928.1 3-phosphoshikimate 1-carboxyvinyltransferase [Mesor... | 659 | 0.0 | 79% |
| WP_065773842.1 MULTISPECIES: 3-phosphoshikimate 1-carboxyvinyl... | 659 | 0.0 | 75% |
| WP_019172734.1 3-phosphoshikimate 1-carboxyvinyltransferase [P... | 659 | 0.0 | 77% |
| WP_056099160.1 3-phosphoshikimate 1-carboxyvinyltransferase [M... | 659 | 0.0 | 76% |
| WP_115141210.1 3-phosphoshikimate 1-carboxyvinyltransferase [M... | 659 | 0.0 | 78% |
| WP_165119556.1 3-phosphoshikimate 1-carboxyvinyltransferase [M... | 659 | 0.0 | 76% |
| WP_027164217.1 MULTISPECIES: 3-phosphoshikimate 1-carboxyvinyl... | 659 | 0.0 | 75% |
| WP_140660049.1 3-phosphoshikimate 1-carboxyvinyltransferase [M... | 659 | 0.0 | 76% |
| WP_064992914.1 3-phosphoshikimate 1-carboxyvinyltransferase [M... | 659 | 0.0 | 75% |
| WP_135917326.1 MULTISPECIES: 3-phosphoshikimate 1-carboxyvinyl... | 659 | 0.0 | 78% |
| WP_140614977.1 MULTISPECIES: 3-phosphoshikimate 1-carboxyvinyl... | 659 | 0.0 | 76% |
| WP_135930572.1 MULTISPECIES: 3-phosphoshikimate 1-carboxyvinyl... | 658 | 0.0 | 78% |
| WP_127367007.1 3-phosphoshikimate 1-carboxyvinyltransferase [M... | 658 | 0.0 | 75% |
| TJV02541.1 3-phosphoshikimate 1-carboxyvinyltransferase [Mesor... | 657 | 0.0 | 78% |
| RUU13969.1 3-phosphoshikimate 1-carboxyvinyltransferase [Mesor... | 656 | 0.0 | 77% |
| WP_140777867.1 3-phosphoshikimate 1-carboxyvinyltransferase [M... | 656 | 0.0 | 77% |
| WP_109612751.1 3-phosphoshikimate 1-carboxyvinyltransferase [P... | 656 | 0.0 | 77% |
| WP_188162617.1 3-phosphoshikimate 1-carboxyvinyltransferase [T... | 656 | 0.0 | 76% |
| PZO80986.1 3-phosphoshikimate 1-carboxyvinyltransferase [Mesor... | 656 | 0.0 | 75% |
| WP_140709369.1 3-phosphoshikimate 1-carboxyvinyltransferase [M... | 656 | 0.0 | 78% |
| WP_121579041.1 3-phosphoshikimate 1-carboxyvinyltransferase [M... | 655 | 0.0 | 76% |
| WP_183886930.1 MULTISPECIES: 3-phosphoshikimate 1-carboxyvinyl... | 655 | 0.0 | 72% |
| WP_149905641.1 3-phosphoshikimate 1-carboxyvinyltransferase [M... | 655 | 0.0 | 78% |
| WP_023775876.1 3-phosphoshikimate 1-carboxyvinyltransferase [M... | 655 | 0.0 | 78% |
| WP_140821633.1 MULTISPECIES: 3-phosphoshikimate 1-carboxyvinyl... | 655 | 0.0 | 78% |
| WP_140714325.1 3-phosphoshikimate 1-carboxyvinyltransferase [M... | 655 | 0.0 | 76% |
| RWE36969.1 3-phosphoshikimate 1-carboxyvinyltransferase [Mesor... | 655 | 0.0 | 78% |
| WP_140736040.1 3-phosphoshikimate 1-carboxyvinyltransferase [M... | 655 | 0.0 | 78% |
| WP_013891534.1 3-phosphoshikimate 1-carboxyvinyltransferase [M... | 655 | 0.0 | 78% |
| WP_140723342.1 3-phosphoshikimate 1-carboxyvinyltransferase [M... | 655 | 0.0 | 78% |
| WP_116621819.1 MULTISPECIES: 3-phosphoshikimate 1-carboxyvinyl... | 655 | 0.0 | 76% |
| WP_113207245.1 3-phosphoshikimate 1-carboxyvinyltransferase [R... | 654 | 0.0 | 72% |
| WP_140570552.1 MULTISPECIES: 3-phosphoshikimate 1-carboxyvinyl... | 654 | 0.0 | 78% |
| WP_127872952.1 MULTISPECIES: 3-phosphoshikimate 1-carboxyvinyl... | 654 | 0.0 | 78% |
| WP_047559886.1 3-phosphoshikimate 1-carboxyvinyltransferase [M... | 654 | 0.0 | 76% |
| WP_015314257.1 3-phosphoshikimate 1-carboxyvinyltransferase [M... | 654 | 0.0 | 76% |
| WP_023767473.1 3-phosphoshikimate 1-carboxyvinyltransferase [M... | 654 | 0.0 | 78% |
| WP_106570401.1 3-phosphoshikimate 1-carboxyvinyltransferase [N... | 654 | 0.0 | 76% |
| WP_056569280.1 MULTISPECIES: 3-phosphoshikimate 1-carboxyvinyl... | 654 | 0.0 | 78% |
| WP_140812040.1 3-phosphoshikimate 1-carboxyvinyltransferase [M... | 654 | 0.0 | 78% |
| WP_038651639.1 3-phosphoshikimate 1-carboxyvinyltransferase [M... | 653 | 0.0 | 78% |
| WP_183847653.1 3-phosphoshikimate 1-carboxyvinyltransferase [R... | 653 | 0.0 | 72% |
| WP_140770091.1 3-phosphoshikimate 1-carboxyvinyltransferase [M... | 653 | 0.0 | 78% |
| WP_172347288.1 3-phosphoshikimate 1-carboxyvinyltransferase [M... | 653 | 0.0 | 78% |
| WP_069090822.1 3-phosphoshikimate 1-carboxyvinyltransferase [M... | 653 | 0.0 | 78% |
| WP_136091297.1 3-phosphoshikimate 1-carboxyvinyltransferase [M... | 652 | 0.0 | 78% |
| WP_064984411.1 MULTISPECIES: 3-phosphoshikimate 1-carboxyvinyl... | 652 | 0.0 | 78% |
| WP_023772435.1 3-phosphoshikimate 1-carboxyvinyltransferase [M... | 652 | 0.0 | 76% |
| TIT38549.1 3-phosphoshikimate 1-carboxyvinyltransferase [Mesor... | 652 | 0.0 | 78% |
| WP_027052745.1 3-phosphoshikimate 1-carboxyvinyltransferase [M... | 652 | 0.0 | 78% |
| WP_143976821.1 3-phosphoshikimate 1-carboxyvinyltransferase [M... | 652 | 0.0 | 78% |

| WP_023832571.1 3-phosphoshikimate 1-carboxyvinyltransferase [M... | 652 | 0.0 | 78% |
| --- | --- | --- | --- |
| WP_140716422.1 3-phosphoshikimate 1-carboxyvinyltransferase [M... | 652 | 0.0 | 75% |
| WP_132957664.1 3-phosphoshikimate 1-carboxyvinyltransferase [R... | 652 | 0.0 | 73% |
| WP_019862792.1 3-phosphoshikimate 1-carboxyvinyltransferase [M... | 652 | 0.0 | 78% |
| RUX00791.1 3-phosphoshikimate 1-carboxyvinyltransferase [Mesor... | 652 | 0.0 | 78% |
| WP_023792912.1 3-phosphoshikimate 1-carboxyvinyltransferase [M... | 651 | 0.0 | 76% |
| WP_172220501.1 3-phosphoshikimate 1-carboxyvinyltransferase [M... | 651 | 0.0 | 77% |
| WP_192691534.1 3-phosphoshikimate 1-carboxyvinyltransferase [M... | 651 | 0.0 | 76% |
| WP_135862389.1 MULTISPECIES: 3-phosphoshikimate 1-carboxyvinyl... | 651 | 0.0 | 78% |
| WP_135921636.1 MULTISPECIES: 3-phosphoshikimate 1-carboxyvinyl... | 651 | 0.0 | 78% |
| WP_140762762.1 3-phosphoshikimate 1-carboxyvinyltransferase [M... | 651 | 0.0 | 78% |
| WP_095792041.1 MULTISPECIES: 3-phosphoshikimate 1-carboxyvinyl... | 650 | 0.0 | 78% |
| WP_127275814.1 3-phosphoshikimate 1-carboxyvinyltransferase [M... | 650 | 0.0 | 78% |
| WP_023695918.1 MULTISPECIES: 3-phosphoshikimate 1-carboxyvinyl... | 650 | 0.0 | 77% |
| WP_140888393.1 3-phosphoshikimate 1-carboxyvinyltransferase [M... | 650 | 0.0 | 78% |
| PHR17808.1 3-phosphoshikimate 1-carboxyvinyltransferase [Hoefl... | 650 | 0.0 | 75% |
| WP_135933027.1 MULTISPECIES: 3-phosphoshikimate 1-carboxyvinyl... | 650 | 0.0 | 78% |
| TGV09609.1 3-phosphoshikimate 1-carboxyvinyltransferase [Mesor... | 650 | 0.0 | 78% |
| WP_032932707.1 MULTISPECIES: 3-phosphoshikimate 1-carboxyvinyl... | 650 | 0.0 | 78% |
| RWG50159.1 3-phosphoshikimate 1-carboxyvinyltransferase [Mesor... | 650 | 0.0 | 79% |
| WP_027142836.1 3-phosphoshikimate 1-carboxyvinyltransferase [M... | 650 | 0.0 | 75% |
| WP_023739675.1 MULTISPECIES: 3-phosphoshikimate 1-carboxyvinyl... | 650 | 0.0 | 77% |
| WP_023671314.1 MULTISPECIES: 3-phosphoshikimate 1-carboxyvinyl... | 650 | 0.0 | 77% |
| WP_095771614.1 MULTISPECIES: 3-phosphoshikimate 1-carboxyvinyl... | 650 | 0.0 | 77% |
| WP_183826574.1 3-phosphoshikimate 1-carboxyvinyltransferase [A... | 650 | 0.0 | 75% |
| WP_023822061.1 3-phosphoshikimate 1-carboxyvinyltransferase [M... | 649 | 0.0 | 77% |
| ODT04152.1 3-phosphoshikimate 1-carboxyvinyltransferase [Mesor... | 649 | 0.0 | 76% |
| WP_150830184.1 3-phosphoshikimate 1-carboxyvinyltransferase [M... | 649 | 0.0 | 76% |
| WP_023746535.1 3-phosphoshikimate 1-carboxyvinyltransferase [M... | 649 | 0.0 | 77% |
| WP_123168731.1 MULTISPECIES: 3-phosphoshikimate 1-carboxyvinyl... | 649 | 0.0 | 78% |
| WP_023703673.1 MULTISPECIES: 3-phosphoshikimate 1-carboxyvinyl... | 649 | 0.0 | 77% |
| WP_095789459.1 3-phosphoshikimate 1-carboxyvinyltransferase [M... | 649 | 0.0 | 78% |
| WP_140746102.1 MULTISPECIES: 3-phosphoshikimate 1-carboxyvinyl... | 649 | 0.0 | 75% |
| WP_192255136.1 unnamed protein product | 649 | 0.0 | 77% |
| WP_023826650.1 3-phosphoshikimate 1-carboxyvinyltransferase [M... | 649 | 0.0 | 77% |
| WP_023781693.1 3-phosphoshikimate 1-carboxyvinyltransferase [M... | 649 | 0.0 | 76% |
| WP_023815974.1 3-phosphoshikimate 1-carboxyvinyltransferase [M... | 649 | 0.0 | 77% |
| WP_064990344.1 MULTISPECIES: 3-phosphoshikimate 1-carboxyvinyl... | 649 | 0.0 | 77% |
| WP_023693131.1 MULTISPECIES: 3-phosphoshikimate 1-carboxyvinyl... | 649 | 0.0 | 77% |
| WP_172371117.1 3-phosphoshikimate 1-carboxyvinyltransferase [M... | 649 | 0.0 | 78% |
| RWE49186.1 3-phosphoshikimate 1-carboxyvinyltransferase [Mesor... | 649 | 0.0 | 78% |
| WP_142886122.1 MULTISPECIES: 3-phosphoshikimate 1-carboxyvinyl... | 649 | 0.0 | 78% |
| WP_192312676.1 3-phosphoshikimate 1-carboxyvinyltransferase [A... | 648 | 0.0 | 75% |
| WP_181056632.1 3-phosphoshikimate 1-carboxyvinyltransferase [M... | 648 | 0.0 | 77% |
| WP_028034550.1 3-phosphoshikimate 1-carboxyvinyltransferase [C... | 648 | 0.0 | 73% |
| WP_027049184.1 3-phosphoshikimate 1-carboxyvinyltransferase [M... | 648 | 0.0 | 77% |
| WP_184768513.1 3-phosphoshikimate 1-carboxyvinyltransferase [A... | 648 | 0.0 | 75% |
| WP_023683537.1 MULTISPECIES: 3-phosphoshikimate 1-carboxyvinyl... | 647 | 0.0 | 77% |
| WP_154385240.1 MULTISPECIES: 3-phosphoshikimate 1-carboxyvinyl... | 647 | 0.0 | 75% |
| WP_023829348.1 3-phosphoshikimate 1-carboxyvinyltransferase [M... | 647 | 0.0 | 77% |
| WP_023725957.1 3-phosphoshikimate 1-carboxyvinyltransferase [M... | 647 | 0.0 | 77% |
| WP_023732647.1 3-phosphoshikimate 1-carboxyvinyltransferase [M... | 647 | 0.0 | 77% |
| WP_067955592.1 3-phosphoshikimate 1-carboxyvinyltransferase [A... | 647 | 0.0 | 75% |
| WP_142876200.1 3-phosphoshikimate 1-carboxyvinyltransferase [M... | 647 | 0.0 | 77% |
| WP_142867910.1 MULTISPECIES: 3-phosphoshikimate 1-carboxyvinyl... | 647 | 0.0 | 77% |
| TIM79233.1 3-phosphoshikimate 1-carboxyvinyltransferase [Mesor... | 646 | 0.0 | 77% |
| WP_192566122.1 3-phosphoshikimate 1-carboxyvinyltransferase [A... | 646 | 0.0 | 76% |
| WP_163272264.1 3-phosphoshikimate 1-carboxyvinyltransferase [C... | 646 | 0.0 | 76% |
| WP_115729922.1 3-phosphoshikimate 1-carboxyvinyltransferase [A... | 646 | 0.0 | 76% |
| WP_184699920.1 3-phosphoshikimate 1-carboxyvinyltransferase [A... | 645 | 0.0 | 76% |
| WP_159589909.1 3-phosphoshikimate 1-carboxyvinyltransferase [C... | 645 | 0.0 | 76% |
| WP_023823897.1 3-phosphoshikimate 1-carboxyvinyltransferase [M... | 644 | 0.0 | 77% |
| WP_183263252.1 3-phosphoshikimate 1-carboxyvinyltransferase [A... | 644 | 0.0 | 76% |
| TIU23855.1 3-phosphoshikimate 1-carboxyvinyltransferase [Mesor... | 644 | 0.0 | 78% |
| WP_055982159.1 3-phosphoshikimate 1-carboxyvinyltransferase [A... | 643 | 0.0 | 76% |
| WP_018430635.1 3-phosphoshikimate 1-carboxyvinyltransferase [H... | 642 | 0.0 | 75% |
| WP_157016237.1 3-phosphoshikimate 1-carboxyvinyltransferase [M... | 642 | 0.0 | 75% |
| WP_156711645.1 3-phosphoshikimate 1-carboxyvinyltransferase [N... | 642 | 0.0 | 75% |
| WP_108606019.1 3-phosphoshikimate 1-carboxyvinyltransferase [A... | 642 | 0.0 | 76% |
| KAB2954812.1 3-phosphoshikimate 1-carboxyvinyltransferase [Rhi... | 641 | 0.0 | 76% |
| WP_091522196.1 3-phosphoshikimate 1-carboxyvinyltransferase [A... | 641 | 0.0 | 76% |
| AQS41064.1 3-phosphoshikimate 1-carboxyvinyltransferase [Candi... | 640 | 0.0 | 74% |

| WP_193178040.1 3-phosphoshikimate 1-carboxyvinyltransferase [O... | 640 | 0.0 | 75% |
| --- | --- | --- | --- |
| WP_109577920.1 3-phosphoshikimate 1-carboxyvinyltransferase [A... | 640 | 0.0 | 75% |
| WP_127312607.1 MULTISPECIES: 3-phosphoshikimate 1-carboxyvinyl... | 639 | 0.0 | 77% |
| OJU02442.1 3-phosphoshikimate 1-carboxyvinyltransferase [Rhizo... | 639 | 0.0 | 73% |
| WP_148915517.1 3-phosphoshikimate 1-carboxyvinyltransferase [M... | 639 | 0.0 | 76% |
| RWO95276.1 3-phosphoshikimate 1-carboxyvinyltransferase [Mesor... | 639 | 0.0 | 79% |
| WP_127269106.1 MULTISPECIES: 3-phosphoshikimate 1-carboxyvinyl... | 638 | 0.0 | 79% |
| WP_127520378.1 3-phosphoshikimate 1-carboxyvinyltransferase [M... | 638 | 0.0 | 76% |
| WP_182418150.1 3-phosphoshikimate 1-carboxyvinyltransferase [B... | 638 | 0.0 | 74% |
| WP_152010841.1 3-phosphoshikimate 1-carboxyvinyltransferase [H... | 638 | 0.0 | 76% |
| WP_097107526.1 3-phosphoshikimate 1-carboxyvinyltransferase [H... | 637 | 0.0 | 73% |
| WP_121646269.1 3-phosphoshikimate 1-carboxyvinyltransferase [N... | 637 | 0.0 | 72% |
| WP_127423505.1 3-phosphoshikimate 1-carboxyvinyltransferase [M... | 637 | 0.0 | 76% |
| WP_173188499.1 3-phosphoshikimate 1-carboxyvinyltransferase [M... | 636 | 0.0 | 77% |
| RCL02308.1 3-phosphoshikimate 1-carboxyvinyltransferase [Candi... | 636 | 0.0 | 72% |
| MBE7184227.1 3-phosphoshikimate 1-carboxyvinyltransferase [Met... | 635 | 0.0 | 74% |
| WP_094075850.1 3-phosphoshikimate 1-carboxyvinyltransferase [N... | 634 | 0.0 | 72% |
| AYE89274.1 5-enolpyruvylshikimate-3-phosphate synthase [Barton... | 634 | 0.0 | 74% |
| WP_183879971.1 3-phosphoshikimate 1-carboxyvinyltransferase [R... | 634 | 0.0 | 72% |
| WP_188848488.1 3-phosphoshikimate 1-carboxyvinyltransferase [A... | 634 | 0.0 | 74% |
| WP_127212524.1 3-phosphoshikimate 1-carboxyvinyltransferase [M... | 633 | 0.0 | 78% |
| WP_127265481.1 3-phosphoshikimate 1-carboxyvinyltransferase [M... | 632 | 0.0 | 78% |
| WP_114388945.1 3-phosphoshikimate 1-carboxyvinyltransferase [N... | 632 | 0.0 | 72% |
| WP_128369389.1 3-phosphoshikimate 1-carboxyvinyltransferase [M... | 632 | 0.0 | 77% |
| TIS98294.1 3-phosphoshikimate 1-carboxyvinyltransferase [Mesor... | 631 | 0.0 | 78% |
| WP_062224506.1 3-phosphoshikimate 1-carboxyvinyltransferase [A... | 630 | 0.0 | 73% |
| WP_077970042.1 3-phosphoshikimate 1-carboxyvinyltransferase [B... | 629 | 0.0 | 74% |
| WP_075869698.1 3-phosphoshikimate 1-carboxyvinyltransferase [B... | 629 | 0.0 | 74% |
| WP_075913613.1 3-phosphoshikimate 1-carboxyvinyltransferase [B... | 629 | 0.0 | 74% |
| WP_024587966.1 3-phosphoshikimate 1-carboxyvinyltransferase [A... | 628 | 0.0 | 76% |
| WP_127218887.1 3-phosphoshikimate 1-carboxyvinyltransferase [M... | 627 | 0.0 | 76% |
| WP_062230133.1 3-phosphoshikimate 1-carboxyvinyltransferase [A... | 627 | 0.0 | 72% |
| WP_078038922.1 3-phosphoshikimate 1-carboxyvinyltransferase [B... | 627 | 0.0 | 74% |
| WP_008038183.1 3-phosphoshikimate 1-carboxyvinyltransferase [B... | 626 | 0.0 | 74% |
| WP_058598851.1 3-phosphoshikimate 1-carboxyvinyltransferase [A... | 626 | 0.0 | 73% |
| RIK86562.1 3-phosphoshikimate 1-carboxyvinyltransferase [Rhizo... | 626 | 0.0 | 76% |
| WP_077990537.1 3-phosphoshikimate 1-carboxyvinyltransferase [B... | 626 | 0.0 | 73% |
| WP_127244068.1 MULTISPECIES: 3-phosphoshikimate 1-carboxyvinyl... | 625 | 0.0 | 76% |
| WP_188907550.1 unnamed protein product | 625 | 0.0 | 71% |
| WP_007008519.1 3-phosphoshikimate 1-carboxyvinyltransferase [N... | 625 | 0.0 | 74% |
| WP_025031120.1 3-phosphoshikimate 1-carboxyvinyltransferase [N... | 625 | 0.0 | 74% |
| WP_141167550.1 3-phosphoshikimate 1-carboxyvinyltransferase [P... | 625 | 0.0 | 73% |
| RUU65785.1 3-phosphoshikimate 1-carboxyvinyltransferase [Mesor... | 624 | 0.0 | 78% |
| WP_113156111.1 3-phosphoshikimate 1-carboxyvinyltransferase [N... | 624 | 0.0 | 74% |
| WP_144864065.1 3-phosphoshikimate 1-carboxyvinyltransferase [M... | 624 | 0.0 | 73% |
| WP_090329024.1 3-phosphoshikimate 1-carboxyvinyltransferase [N... | 624 | 0.0 | 74% |
| WP_065816634.1 3-phosphoshikimate 1-carboxyvinyltransferase [N... | 623 | 0.0 | 73% |
| WP_188719795.1 3-phosphoshikimate 1-carboxyvinyltransferase [N... | 622 | 0.0 | 72% |
| MAW86831.1 3-phosphoshikimate 1-carboxyvinyltransferase [Phyll... | 622 | 0.0 | 73% |
| WP_141066607.1 3-phosphoshikimate 1-carboxyvinyltransferase [B... | 621 | 0.0 | 82% |
| NGR07202.1 3-phosphoshikimate 1-carboxyvinyltransferase [bacte... | 621 | 0.0 | 71% |
| WP_146298775.1 3-phosphoshikimate 1-carboxyvinyltransferase [N... | 621 | 0.0 | 73% |
| WP_008596251.1 3-phosphoshikimate 1-carboxyvinyltransferase [N... | 620 | 0.0 | 73% |
| WP_173935033.1 3-phosphoshikimate 1-carboxyvinyltransferase [C... | 620 | 0.0 | 72% |
| GGD96629.1 3-phosphoshikimate 1-carboxyvinyltransferase [Aurei... | 620 | 0.0 | 72% |
| WP_055849546.1 3-phosphoshikimate 1-carboxyvinyltransferase [A... | 617 | 0.0 | 73% |
| WP_127324264.1 3-phosphoshikimate 1-carboxyvinyltransferase [M... | 617 | 0.0 | 79% |
| WP_150967936.1 3-phosphoshikimate 1-carboxyvinyltransferase [A... | 616 | 0.0 | 71% |
| WP_116684370.1 3-phosphoshikimate 1-carboxyvinyltransferase [F... | 616 | 0.0 | 72% |
| WP_127372465.1 3-phosphoshikimate 1-carboxyvinyltransferase [M... | 616 | 0.0 | 78% |
| WP_061933195.1 3-phosphoshikimate 1-carboxyvinyltransferase [A... | 615 | 0.0 | 73% |
| WP_062115027.1 3-phosphoshikimate 1-carboxyvinyltransferase [A... | 615 | 0.0 | 73% |
| WP_061973116.1 3-phosphoshikimate 1-carboxyvinyltransferase [A... | 615 | 0.0 | 73% |
| WP_090963268.1 3-phosphoshikimate 1-carboxyvinyltransferase [A... | 614 | 0.0 | 73% |
| WP_055891398.1 3-phosphoshikimate 1-carboxyvinyltransferase [A... | 613 | 0.0 | 73% |
| MAU95573.1 3-phosphoshikimate 1-carboxyvinyltransferase [Fulvi... | 610 | 0.0 | 72% |
| WP_127300022.1 3-phosphoshikimate 1-carboxyvinyltransferase [M... | 610 | 0.0 | 78% |
| WP_083591677.1 3-phosphoshikimate 1-carboxyvinyltransferase [A... | 609 | 0.0 | 72% |
| WP_183199165.1 3-phosphoshikimate 1-carboxyvinyltransferase [A... | 608 | 0.0 | 73% |
| WP_062015223.1 3-phosphoshikimate 1-carboxyvinyltransferase [A... | 608 | 0.0 | 72% |
| WP_119538726.1 3-phosphoshikimate 1-carboxyvinyltransferase [A... | 608 | 0.0 | 72% |
| HHV68647.1 TPA: 3-phosphoshikimate 1-carboxyvinyltransferase [... | 607 | 0.0 | 82% |

| MAS05025.1 3-phosphoshikimate 1-carboxyvinyltransferase [Ahren... | 607 | 0.0 | 69% |
| --- | --- | --- | --- |
| WP_062209085.1 3-phosphoshikimate 1-carboxyvinyltransferase [A... | 606 | 0.0 | 72% |
| WP_182083852.1 3-phosphoshikimate 1-carboxyvinyltransferase [A... | 605 | 0.0 | 73% |
| WP_163460489.1 3-phosphoshikimate 1-carboxyvinyltransferase [J... | 605 | 0.0 | 70% |
| WP_134761508.1 3-phosphoshikimate 1-carboxyvinyltransferase [J... | 604 | 0.0 | 71% |
| WP_127374796.1 3-phosphoshikimate 1-carboxyvinyltransferase [M... | 603 | 0.0 | 78% |
| WP_019994581.1 3-phosphoshikimate 1-carboxyvinyltransferase [A... | 602 | 0.0 | 72% |
| WP_169288210.1 3-phosphoshikimate 1-carboxyvinyltransferase [C... | 602 | 0.0 | 72% |
| RCL01181.1 3-phosphoshikimate 1-carboxyvinyltransferase [Candi... | 602 | 0.0 | 69% |
| RCL02747.1 3-phosphoshikimate 1-carboxyvinyltransferase [Candi... | 602 | 0.0 | 67% |
| WP_072602358.1 3-phosphoshikimate 1-carboxyvinyltransferase [M... | 601 | 0.0 | 71% |
| WP_090675031.1 3-phosphoshikimate 1-carboxyvinyltransferase [A... | 600 | 0.0 | 70% |
| WP_183210726.1 3-phosphoshikimate 1-carboxyvinyltransferase [A... | 600 | 0.0 | 72% |
| WP_060610358.1 3-phosphoshikimate 1-carboxyvinyltransferase [A... | 600 | 0.0 | 71% |
| WP_040488910.1 3-phosphoshikimate 1-carboxyvinyltransferase [F... | 599 | 0.0 | 71% |
| WP_187392436.1 3-phosphoshikimate 1-carboxyvinyltransferase [A... | 599 | 0.0 | 70% |
| WP_056692696.1 MULTISPECIES: 3-phosphoshikimate 1-carboxyvinyl... | 599 | 0.0 | 73% |
| MBB6044674.1 3-phosphoshikimate 1-carboxyvinyltransferase [Aur... | 598 | 0.0 | 70% |
| WP_024352250.1 3-phosphoshikimate 1-carboxyvinyltransferase [A... | 598 | 0.0 | 70% |
| WP_040326053.1 3-phosphoshikimate 1-carboxyvinyltransferase [A... | 598 | 0.0 | 70% |
| WP_009449224.1 3-phosphoshikimate 1-carboxyvinyltransferase [N... | 598 | 0.0 | 72% |
| SFQ24155.1 3-phosphoshikimate 1-carboxyvinyltransferase [Nitra... | 597 | 0.0 | 73% |
| WP_056508570.1 3-phosphoshikimate 1-carboxyvinyltransferase [A... | 597 | 0.0 | 73% |
| EAS48526.1 3-phosphoshikimate 1-carboxyvinyltransferase [Auran... | 597 | 0.0 | 70% |
| WP_048649207.1 3-phosphoshikimate 1-carboxyvinyltransferase [N... | 596 | 0.0 | 71% |
| WP_163045247.1 3-phosphoshikimate 1-carboxyvinyltransferase [A... | 596 | 0.0 | 71% |
| WP_185985412.1 3-phosphoshikimate 1-carboxyvinyltransferase [A... | 594 | 0.0 | 73% |
| WP_004856123.1 3-phosphoshikimate 1-carboxyvinyltransferase [B... | 593 | 0.0 | 69% |
| WP_052194983.1 3-phosphoshikimate 1-carboxyvinyltransferase [A... | 593 | 0.0 | 71% |
| WP_128369818.1 3-phosphoshikimate 1-carboxyvinyltransferase [M... | 593 | 0.0 | 78% |
| WP_015272530.1 3-phosphoshikimate 1-carboxyvinyltransferase [L... | 592 | 0.0 | 68% |
| WP_127398171.1 3-phosphoshikimate 1-carboxyvinyltransferase [M... | 592 | 0.0 | 75% |
| RWH33120.1 3-phosphoshikimate 1-carboxyvinyltransferase [Mesor... | 592 | 0.0 | 78% |
| WP_102959749.1 3-phosphoshikimate 1-carboxyvinyltransferase [M... | 592 | 0.0 | 70% |
| WP_074381135.1 3-phosphoshikimate 1-carboxyvinyltransferase [B... | 591 | 0.0 | 69% |
| WP_084409415.1 3-phosphoshikimate 1-carboxyvinyltransferase [F... | 591 | 0.0 | 72% |
| WP_100129788.1 3-phosphoshikimate 1-carboxyvinyltransferase [B... | 591 | 0.0 | 70% |
| WP_128360714.1 3-phosphoshikimate 1-carboxyvinyltransferase [M... | 590 | 0.0 | 75% |
| PZN49298.1 3-phosphoshikimate 1-carboxyvinyltransferase [Prote... | 589 | 0.0 | 76% |
| HDZ73356.1 TPA: 3-phosphoshikimate 1-carboxyvinyltransferase [... | 589 | 0.0 | 71% |
| KHJ53559.1 3-phosphoshikimate 1-carboxyvinyltransferase [Aurei... | 589 | 0.0 | 72% |
| WP_128301267.1 3-phosphoshikimate 1-carboxyvinyltransferase [M... | 588 | 0.0 | 75% |
| WP_192389097.1 3-phosphoshikimate 1-carboxyvinyltransferase [A... | 587 | 0.0 | 70% |
| RUV49141.1 3-phosphoshikimate 1-carboxyvinyltransferase [Mesor... | 587 | 0.0 | 77% |
| EAU42291.1 3-phosphoshikimate 1-carboxyvinyltransferase [Fulvi... | 586 | 0.0 | 72% |
| WP_006925766.1 3-phosphoshikimate 1-carboxyvinyltransferase [B... | 585 | 0.0 | 68% |
| WP_039759343.1 3-phosphoshikimate 1-carboxyvinyltransferase [B... | 585 | 0.0 | 68% |
| WP_006923770.1 3-phosphoshikimate 1-carboxyvinyltransferase [B... | 583 | 0.0 | 68% |
| WP_120100265.1 3-phosphoshikimate 1-carboxyvinyltransferase [B... | 583 | 0.0 | 69% |
| WP_127307647.1 3-phosphoshikimate 1-carboxyvinyltransferase [M... | 583 | 0.0 | 75% |
| BAT25294.1 3-phosphoshikimate 1-carboxyvinyltransferase [Aurei... | 583 | 0.0 | 72% |
| WP_116002625.1 3-phosphoshikimate 1-carboxyvinyltransferase [C... | 583 | 0.0 | 70% |
| WP_011178922.1 3-phosphoshikimate 1-carboxyvinyltransferase [B... | 583 | 0.0 | 68% |
| WP_005774413.1 3-phosphoshikimate 1-carboxyvinyltransferase [B... | 583 | 0.0 | 69% |
| WP_158869886.1 3-phosphoshikimate 1-carboxyvinyltransferase [J... | 582 | 0.0 | 70% |
| SKA08290.1 3-phosphoshikimate 1-carboxyvinyltransferase [Conso... | 582 | 0.0 | 70% |
| WP_004862282.1 3-phosphoshikimate 1-carboxyvinyltransferase [B... | 582 | 0.0 | 68% |
| WP_085463010.1 3-phosphoshikimate 1-carboxyvinyltransferase [M... | 582 | 0.0 | 70% |
| WP_014923635.1 3-phosphoshikimate 1-carboxyvinyltransferase [B... | 581 | 0.0 | 68% |
| WP_161510220.1 3-phosphoshikimate 1-carboxyvinyltransferase [B... | 580 | 0.0 | 68% |
| WP_142416210.1 3-phosphoshikimate 1-carboxyvinyltransferase [B... | 580 | 0.0 | 69% |
| WP_156850670.1 3-phosphoshikimate 1-carboxyvinyltransferase [B... | 579 | 0.0 | 67% |
| WP_175868407.1 3-phosphoshikimate 1-carboxyvinyltransferase [B... | 579 | 0.0 | 69% |
| WP_072342719.1 3-phosphoshikimate 1-carboxyvinyltransferase [D... | 578 | 0.0 | 68% |
| WP_127189083.1 3-phosphoshikimate 1-carboxyvinyltransferase [A... | 577 | 0.0 | 66% |
| SFZ84809.1 3-phosphoshikimate 1-carboxyvinyltransferase [Devos... | 577 | 0.0 | 68% |
| PCH50806.1 3-phosphoshikimate 1-carboxyvinyltransferase [Rhizo... | 577 | 0.0 | 68% |
| WP_012230402.1 3-phosphoshikimate 1-carboxyvinyltransferase [B... | 577 | 0.0 | 68% |
| WP_183228861.1 3-phosphoshikimate 1-carboxyvinyltransferase [B... | 576 | 0.0 | 69% |
| WP_004858272.1 3-phosphoshikimate 1-carboxyvinyltransferase [B... | 576 | 0.0 | 68% |
| MBE0691991.1 3-phosphoshikimate 1-carboxyvinyltransferase [Aqu... | 576 | 0.0 | 76% |
| WP_019223165.1 3-phosphoshikimate 1-carboxyvinyltransferase [B... | 576 | 0.0 | 68% |

| WP_019219167.1 3-phosphoshikimate 1-carboxyvinyltransferase [B... | 576 | 0.0 | 68% |
| --- | --- | --- | --- |
| WP_139412656.1 3-phosphoshikimate 1-carboxyvinyltransferase [B... | 576 | 0.0 | 68% |
| AQX20646.1 3-phosphoshikimate 1-carboxyvinyltransferase [Barto... | 575 | 0.0 | 67% |
| RVC44860.1 3-phosphoshikimate 1-carboxyvinyltransferase [Mesor... | 575 | 0.0 | 80% |
| WP_078691505.1 3-phosphoshikimate 1-carboxyvinyltransferase [B... | 574 | 0.0 | 67% |
| WP_006590051.1 3-phosphoshikimate 1-carboxyvinyltransferase [B... | 573 | 0.0 | 67% |
| WP_019220849.1 3-phosphoshikimate 1-carboxyvinyltransferase [B... | 573 | 0.0 | 67% |
| WP_078706184.1 3-phosphoshikimate 1-carboxyvinyltransferase [B... | 573 | 0.0 | 67% |
| WP_011180056.1 3-phosphoshikimate 1-carboxyvinyltransferase [B... | 572 | 0.0 | 67% |
| WP_038486723.1 3-phosphoshikimate 1-carboxyvinyltransferase [B... | 572 | 0.0 | 67% |
| WP_082252423.1 3-phosphoshikimate 1-carboxyvinyltransferase [B... | 572 | 0.0 | 67% |
| WP_005866359.1 3-phosphoshikimate 1-carboxyvinyltransferase [B... | 572 | 0.0 | 67% |
| WP_128310133.1 3-phosphoshikimate 1-carboxyvinyltransferase [M... | 572 | 0.0 | 77% |
| WP_100128177.1 3-phosphoshikimate 1-carboxyvinyltransferase [B... | 572 | 0.0 | 67% |
| WP_012754502.1 3-phosphoshikimate 1-carboxyvinyltransferase [B... | 572 | 0.0 | 67% |
| WP_007552343.1 3-phosphoshikimate 1-carboxyvinyltransferase [B... | 571 | 0.0 | 66% |
| VEJ44697.1 3-phosphoshikimate 1-carboxyvinyltransferase [Barto... | 570 | 0.0 | 69% |
| WP_010703246.1 3-phosphoshikimate 1-carboxyvinyltransferase [B... | 570 | 0.0 | 67% |
| WP_102829880.1 3-phosphoshikimate 1-carboxyvinyltransferase [B... | 570 | 0.0 | 67% |
| WP_062226669.1 3-phosphoshikimate 1-carboxyvinyltransferase [A... | 570 | 0.0 | 69% |
| MAZ19007.1 3-phosphoshikimate 1-carboxyvinyltransferase [Ahren... | 570 | 0.0 | 68% |
| WP_007476158.1 3-phosphoshikimate 1-carboxyvinyltransferase [B... | 570 | 0.0 | 66% |
| WP_078663990.1 3-phosphoshikimate 1-carboxyvinyltransferase [B... | 569 | 0.0 | 66% |
| WP_010700656.1 3-phosphoshikimate 1-carboxyvinyltransferase [B... | 569 | 0.0 | 66% |
| WP_114645333.1 3-phosphoshikimate 1-carboxyvinyltransferase [P... | 569 | 0.0 | 67% |
| WP_010704585.1 3-phosphoshikimate 1-carboxyvinyltransferase [B... | 569 | 0.0 | 69% |
| WP_126602292.1 3-phosphoshikimate 1-carboxyvinyltransferase [B... | 568 | 0.0 | 69% |
| WP_078689328.1 3-phosphoshikimate 1-carboxyvinyltransferase [B... | 568 | 0.0 | 67% |
| WP_034458243.1 3-phosphoshikimate 1-carboxyvinyltransferase [B... | 568 | 0.0 | 66% |
| TIV51588.1 3-phosphoshikimate 1-carboxyvinyltransferase [Mesor... | 568 | 0.0 | 79% |
| WP_007347335.1 3-phosphoshikimate 1-carboxyvinyltransferase [B... | 566 | 0.0 | 67% |
| WP_144755690.1 3-phosphoshikimate 1-carboxyvinyltransferase [B... | 565 | 0.0 | 67% |
| WP_182479990.1 3-phosphoshikimate 1-carboxyvinyltransferase [B... | 564 | 0.0 | 66% |
| WP_196260069.1 3-phosphoshikimate 1-carboxyvinyltransferase [P... | 563 | 0.0 | 65% |
| WP_047032803.1 3-phosphoshikimate 1-carboxyvinyltransferase [H... | 563 | 0.0 | 69% |
| WP_182016833.1 3-phosphoshikimate 1-carboxyvinyltransferase [C... | 563 | 0.0 | 64% |
| AKI02702.1 3-phosphoshikimate 1-carboxyvinyltransferase [Hoefl... | 563 | 0.0 | 69% |
| NRB29749.1 3-phosphoshikimate 1-carboxyvinyltransferase [Rhizo... | 563 | 0.0 | 65% |
| MBN15267.1 3-phosphoshikimate 1-carboxyvinyltransferase [Pelag... | 563 | 0.0 | 65% |
| WP_014132602.1 3-phosphoshikimate 1-carboxyvinyltransferase [P... | 562 | 0.0 | 65% |
| WP_010701907.1 3-phosphoshikimate 1-carboxyvinyltransferase [B... | 561 | 0.0 | 66% |
| MAN78562.1 3-phosphoshikimate 1-carboxyvinyltransferase [Rhizo... | 561 | 0.0 | 65% |
| WP_149301409.1 3-phosphoshikimate 1-carboxyvinyltransferase [A... | 560 | 0.0 | 69% |
| WP_116652356.1 3-phosphoshikimate 1-carboxyvinyltransferase [P... | 559 | 0.0 | 66% |
| WP_127071647.1 3-phosphoshikimate 1-carboxyvinyltransferase [P... | 559 | 0.0 | 65% |
| KJS20089.1 3-phosphoshikimate 1-carboxyvinyltransferase [Hoefl... | 558 | 0.0 | 65% |
| WP_120121591.1 3-phosphoshikimate 1-carboxyvinyltransferase [B... | 558 | 0.0 | 67% |
| WP_090591547.1 3-phosphoshikimate 1-carboxyvinyltransferase [P... | 558 | 0.0 | 66% |
| HCO55142.1 TPA: 3-phosphoshikimate 1-carboxyvinyltransferase [... | 558 | 0.0 | 65% |
| WP_183193578.1 3-phosphoshikimate 1-carboxyvinyltransferase [B... | 557 | 0.0 | 66% |
| WP_075216419.1 3-phosphoshikimate 1-carboxyvinyltransferase [M... | 557 | 0.0 | 67% |
| EYS88931.1 3-phosphoshikimate 1-carboxyvinyltransferase [Barto... | 556 | 0.0 | 64% |
| WP_005768078.1 3-phosphoshikimate 1-carboxyvinyltransferase [B... | 555 | 0.0 | 64% |
| WP_133573785.1 3-phosphoshikimate 1-carboxyvinyltransferase [M... | 555 | 0.0 | 66% |
| WP_066183134.1 3-phosphoshikimate 1-carboxyvinyltransferase [H... | 553 | 0.0 | 64% |
| WP_013544460.1 3-phosphoshikimate 1-carboxyvinyltransferase [B... | 552 | 0.0 | 63% |
| RUY21393.1 3-phosphoshikimate 1-carboxyvinyltransferase [Mesor... | 552 | 0.0 | 80% |
| WP_161678187.1 MULTISPECIES: 3-phosphoshikimate 1-carboxyvinyl... | 551 | 0.0 | 65% |
| WP_153770444.1 3-phosphoshikimate 1-carboxyvinyltransferase [L... | 551 | 0.0 | 62% |
| KEG15787.1 3-phosphoshikimate 1-carboxyvinyltransferase [Barto... | 551 | 0.0 | 64% |
| WP_141183782.1 3-phosphoshikimate 1-carboxyvinyltransferase [L... | 551 | 0.0 | 64% |
| EYS90892.1 3-phosphoshikimate 1-carboxyvinyltransferase [Barto... | 551 | 0.0 | 64% |
| WP_192110922.1 3-phosphoshikimate 1-carboxyvinyltransferase [L... | 550 | 0.0 | 62% |
| WP_189435807.1 3-phosphoshikimate 1-carboxyvinyltransferase [P... | 550 | 0.0 | 64% |
| WP_190292509.1 3-phosphoshikimate 1-carboxyvinyltransferase [L... | 549 | 0.0 | 63% |
| WP_035454831.1 3-phosphoshikimate 1-carboxyvinyltransferase [B... | 549 | 0.0 | 64% |
| TIX21068.1 3-phosphoshikimate 1-carboxyvinyltransferase [Mesor... | 549 | 0.0 | 78% |
| WP_042995233.1 3-phosphoshikimate 1-carboxyvinyltransferase [B... | 549 | 0.0 | 64% |
| WP_068005683.1 3-phosphoshikimate 1-carboxyvinyltransferase [P... | 548 | 0.0 | 64% |
| WP_192147899.1 3-phosphoshikimate 1-carboxyvinyltransferase [L... | 548 | 0.0 | 64% |
| WP_155144717.1 3-phosphoshikimate 1-carboxyvinyltransferase [R... | 547 | 0.0 | 63% |
| PTL86954.1 3-phosphoshikimate 1-carboxyvinyltransferase [Candi... | 547 | 0.0 | 63% |

| WP_013654899.1 3-phosphoshikimate 1-carboxyvinyltransferase [P... | 547 | 0.0 | 65% |
| --- | --- | --- | --- |
| SFJ99152.1 3-phosphoshikimate 1-carboxyvinyltransferase [Pseud... | 546 | 0.0 | 63% |
| WP_133949076.1 3-phosphoshikimate 1-carboxyvinyltransferase [L... | 546 | 0.0 | 63% |
| WP_054783073.1 3-phosphoshikimate 1-carboxyvinyltransferase [P... | 545 | 0.0 | 63% |
| WP_181707969.1 3-phosphoshikimate 1-carboxyvinyltransferase [C... | 545 | 0.0 | 66% |
| WP_041768853.1 3-phosphoshikimate 1-carboxyvinyltransferase [P... | 545 | 0.0 | 63% |
| SHL29410.1 3-phosphoshikimate 1-carboxyvinyltransferase [Labre... | 545 | 0.0 | 63% |
| WP_139250975.1 3-phosphoshikimate 1-carboxyvinyltransferase [L... | 545 | 0.0 | 63% |
| MBA4781949.1 3-phosphoshikimate 1-carboxyvinyltransferase [Rhi... | 545 | 0.0 | 67% |
| WP_063294970.1 3-phosphoshikimate 1-carboxyvinyltransferase [P... | 545 | 0.0 | 63% |
| WP_152503898.1 3-phosphoshikimate 1-carboxyvinyltransferase [L... | 545 | 0.0 | 63% |
| WP_075997740.1 3-phosphoshikimate 1-carboxyvinyltransferase [S... | 545 | 0.0 | 67% |
| WP_063299489.1 3-phosphoshikimate 1-carboxyvinyltransferase [P... | 545 | 0.0 | 63% |
| WP_181952052.1 3-phosphoshikimate 1-carboxyvinyltransferase [P... | 544 | 0.0 | 63% |
| RCL03496.1 3-phosphoshikimate 1-carboxyvinyltransferase [Candi... | 544 | 0.0 | 63% |
| TYC49519.1 3-phosphoshikimate 1-carboxyvinyltransferase [Rhodo... | 544 | 0.0 | 64% |
| WP_027835568.1 3-phosphoshikimate 1-carboxyvinyltransferase [M... | 544 | 0.0 | 65% |
| WP_117396645.1 3-phosphoshikimate 1-carboxyvinyltransferase [M... | 544 | 0.0 | 65% |
| OJJ12782.1 3-phosphoshikimate 1-carboxyvinyltransferase [Alpha... | 544 | 0.0 | 63% |
| WP_057462755.1 3-phosphoshikimate 1-carboxyvinyltransferase [P... | 544 | 0.0 | 63% |
| TIX39132.1 3-phosphoshikimate 1-carboxyvinyltransferase [Mesor... | 544 | 0.0 | 79% |
| WP_050473401.1 3-phosphoshikimate 1-carboxyvinyltransferase [P... | 543 | 0.0 | 66% |
| NVK35595.1 3-phosphoshikimate 1-carboxyvinyltransferase [Rhodo... | 543 | 0.0 | 63% |
| WP_134853052.1 3-phosphoshikimate 1-carboxyvinyltransferase [L... | 543 | 0.0 | 62% |
| WP_075701102.1 3-phosphoshikimate 1-carboxyvinyltransferase [P... | 543 | 0.0 | 64% |
| WP_009758751.1 3-phosphoshikimate 1-carboxyvinyltransferase [A... | 543 | 0.0 | 65% |
| WP_123193343.1 3-phosphoshikimate 1-carboxyvinyltransferase [P... | 543 | 0.0 | 66% |
| WP_074881946.1 3-phosphoshikimate 1-carboxyvinyltransferase [P... | 543 | 0.0 | 64% |
| TIT91887.1 3-phosphoshikimate 1-carboxyvinyltransferase [Mesor... | 542 | 0.0 | 79% |
| WP_055454499.1 3-phosphoshikimate 1-carboxyvinyltransferase [P... | 542 | 0.0 | 66% |
| AVX05912.1 3-phosphoshikimate 1-carboxyvinyltransferase [Marit... | 542 | 0.0 | 65% |
| WP_093517074.1 3-phosphoshikimate 1-carboxyvinyltransferase [P... | 541 | 0.0 | 64% |
| WP_119396577.1 3-phosphoshikimate 1-carboxyvinyltransferase [P... | 541 | 0.0 | 66% |
| WP_063286572.1 MULTISPECIES: 3-phosphoshikimate 1-carboxyvinyl... | 541 | 0.0 | 63% |
| WP_058898281.1 MULTISPECIES: 3-phosphoshikimate 1-carboxyvinyl... | 541 | 0.0 | 66% |
| WP_149893641.1 3-phosphoshikimate 1-carboxyvinyltransferase [R... | 541 | 0.0 | 64% |
| MTI17791.1 3-phosphoshikimate 1-carboxyvinyltransferase [Rhodo... | 541 | 0.0 | 64% |
| AEV35188.1 3-phosphoshikimate 1-carboxyvinyltransferase/5-enol... | 541 | 0.0 | 64% |
| WP_193757816.1 MULTISPECIES: 3-phosphoshikimate 1-carboxyvinyl... | 541 | 0.0 | 63% |
| WP_022998948.1 3-phosphoshikimate 1-carboxyvinyltransferase [L... | 541 | 0.0 | 63% |
| WP_055672935.1 3-phosphoshikimate 1-carboxyvinyltransferase [L... | 541 | 0.0 | 62% |
| PTW62741.1 3-phosphoshikimate 1-carboxyvinyltransferase [Breog... | 541 | 0.0 | 64% |
| WP_173013757.1 MULTISPECIES: 3-phosphoshikimate 1-carboxyvinyl... | 540 | 0.0 | 63% |
| WP_083416927.1 3-phosphoshikimate 1-carboxyvinyltransferase [P... | 540 | 0.0 | 64% |
| WP_106752717.1 3-phosphoshikimate 1-carboxyvinyltransferase [P... | 540 | 0.0 | 65% |
| WP_182163977.1 3-phosphoshikimate 1-carboxyvinyltransferase [S... | 540 | 0.0 | 63% |
| WP_116699292.1 3-phosphoshikimate 1-carboxyvinyltransferase [L... | 540 | 0.0 | 63% |
| WP_026355520.1 3-phosphoshikimate 1-carboxyvinyltransferase [P... | 540 | 0.0 | 66% |
| WP_108820002.1 3-phosphoshikimate 1-carboxyvinyltransferase [P... | 540 | 0.0 | 64% |
| WP_189640086.1 3-phosphoshikimate 1-carboxyvinyltransferase [L... | 540 | 0.0 | 63% |
| WP_082596748.1 3-phosphoshikimate 1-carboxyvinyltransferase [P... | 539 | 0.0 | 64% |
| WP_127145193.1 3-phosphoshikimate 1-carboxyvinyltransferase [P... | 539 | 0.0 | 66% |
| WP_068409180.1 3-phosphoshikimate 1-carboxyvinyltransferase [L... | 539 | 0.0 | 63% |
| WP_173017255.1 3-phosphoshikimate 1-carboxyvinyltransferase [L... | 539 | 0.0 | 63% |
| WP_094073541.1 3-phosphoshikimate 1-carboxyvinyltransferase [L... | 538 | 0.0 | 62% |
| WP_103220754.1 3-phosphoshikimate 1-carboxyvinyltransferase [L... | 538 | 0.0 | 63% |
| WP_114007697.1 3-phosphoshikimate 1-carboxyvinyltransferase [C... | 538 | 0.0 | 64% |
| WP_138148485.1 3-phosphoshikimate 1-carboxyvinyltransferase [C... | 538 | 0.0 | 64% |
| WP_055677096.1 3-phosphoshikimate 1-carboxyvinyltransferase [L... | 536 | 0.0 | 63% |
| WP_107989066.1 3-phosphoshikimate 1-carboxyvinyltransferase [B... | 536 | 0.0 | 64% |
| SEQ63393.1 3-phosphoshikimate 1-carboxyvinyltransferase [bacte... | 536 | 0.0 | 65% |
| EAV40333.1 3-phosphoshikimate 1-carboxyvinyltransferase [Stapp... | 536 | 0.0 | 63% |
| SDR23290.1 3-phosphoshikimate 1-carboxyvinyltransferase [Pseud... | 536 | 0.0 | 63% |
| WP_009466663.1 3-phosphoshikimate 1-carboxyvinyltransferase [R... | 536 | 0.0 | 62% |
| WP_172972065.1 3-phosphoshikimate 1-carboxyvinyltransferase [R... | 536 | 0.0 | 62% |
| WP_008548787.1 3-phosphoshikimate 1-carboxyvinyltransferase [P... | 536 | 0.0 | 64% |
| WP_140940853.1 3-phosphoshikimate 1-carboxyvinyltransferase [P... | 536 | 0.0 | 65% |
| WP_131721412.1 3-phosphoshikimate 1-carboxyvinyltransferase [R... | 535 | 0.0 | 86% |
| WP_155191076.1 3-phosphoshikimate 1-carboxyvinyltransferase [R... | 535 | 0.0 | 61% |
| WP_192913768.1 3-phosphoshikimate 1-carboxyvinyltransferase [R... | 535 | 0.0 | 62% |
| WP_181700821.1 3-phosphoshikimate 1-carboxyvinyltransferase [C... | 535 | 0.0 | 66% |
| WP_054358937.1 3-phosphoshikimate 1-carboxyvinyltransferase [P... | 535 | 0.0 | 64% |

| MBG6207457.1 3-phosphoshikimate 1-carboxyvinyltransferase [Lab... | 534 | 0.0 | 66% |
| --- | --- | --- | --- |
| NKB53260.1 3-phosphoshikimate 1-carboxyvinyltransferase [Rhizo... | 534 | 0.0 | 63% |
| WP_104701840.1 3-phosphoshikimate 1-carboxyvinyltransferase [L... | 534 | 0.0 | 66% |
| WP_035907724.1 MULTISPECIES: 3-phosphoshikimate 1-carboxyvinyl... | 534 | 0.0 | 64% |
| RKD50842.1 3-phosphoshikimate 1-carboxyvinyltransferase [Rhizo... | 534 | 0.0 | 79% |
| WP_078719469.1 3-phosphoshikimate 1-carboxyvinyltransferase [B... | 533 | 0.0 | 60% |
| MBC00633.1 3-phosphoshikimate 1-carboxyvinyltransferase [Rhodo... | 533 | 0.0 | 63% |
| MBG6199973.1 3-phosphoshikimate 1-carboxyvinyltransferase [Lab... | 532 | 0.0 | 64% |
| OLL45451.1 3-phosphoshikimate 1-carboxyvinyltransferase [Labre... | 532 | 0.0 | 64% |
| WP_055119777.1 MULTISPECIES: 3-phosphoshikimate 1-carboxyvinyl... | 532 | 0.0 | 65% |
| CTQ61348.1 3-phosphoshikimate 1-carboxyvinyltransferase [Labre... | 532 | 0.0 | 65% |
| WP_046137153.1 3-phosphoshikimate 1-carboxyvinyltransferase [D... | 532 | 0.0 | 63% |
| WP_062491989.1 3-phosphoshikimate 1-carboxyvinyltransferase [L... | 532 | 0.0 | 64% |
| TIW59152.1 3-phosphoshikimate 1-carboxyvinyltransferase [Mesor... | 531 | 0.0 | 79% |
| KEG21195.1 3-phosphoshikimate 1-carboxyvinyltransferase [Barto... | 531 | 0.0 | 64% |
| WP_131307514.1 3-phosphoshikimate 1-carboxyvinyltransferase [S... | 531 | 0.0 | 65% |
| WP_077293198.1 3-phosphoshikimate 1-carboxyvinyltransferase [L... | 531 | 0.0 | 64% |
| WP_093189134.1 3-phosphoshikimate 1-carboxyvinyltransferase [P... | 531 | 0.0 | 63% |
| WP_029064272.1 3-phosphoshikimate 1-carboxyvinyltransferase [L... | 531 | 0.0 | 65% |
| WP_168686690.1 3-phosphoshikimate 1-carboxyvinyltransferase [L... | 531 | 0.0 | 64% |
| WP_068085148.1 3-phosphoshikimate 1-carboxyvinyltransferase [P... | 530 | 0.0 | 63% |
| WP_041848941.1 3-phosphoshikimate 1-carboxyvinyltransferase [B... | 530 | 0.0 | 64% |
| WP_148582400.1 3-phosphoshikimate 1-carboxyvinyltransferase [S... | 530 | 0.0 | 64% |
| WP_186388761.1 3-phosphoshikimate 1-carboxyvinyltransferase [S... | 530 | 0.0 | 62% |
| WP_117411063.1 3-phosphoshikimate 1-carboxyvinyltransferase [B... | 529 | 0.0 | 65% |
| WP_160776977.1 3-phosphoshikimate 1-carboxyvinyltransferase [S... | 529 | 0.0 | 62% |
| WP_126540331.1 3-phosphoshikimate 1-carboxyvinyltransferase [O... | 529 | 0.0 | 66% |
| WP_068311491.1 3-phosphoshikimate 1-carboxyvinyltransferase [P... | 529 | 0.0 | 63% |
| WP_081455389.1 3-phosphoshikimate 1-carboxyvinyltransferase [L... | 528 | 0.0 | 64% |
| MAA99356.1 3-phosphoshikimate 1-carboxyvinyltransferase [Stapp... | 528 | 0.0 | 64% |
| TIU98435.1 3-phosphoshikimate 1-carboxyvinyltransferase [Mesor... | 528 | 0.0 | 80% |
| WP_088941674.1 3-phosphoshikimate 1-carboxyvinyltransferase [S... | 528 | 0.0 | 61% |
| WP_186399192.1 3-phosphoshikimate 1-carboxyvinyltransferase [S... | 528 | 0.0 | 62% |
| WP_186397172.1 3-phosphoshikimate 1-carboxyvinyltransferase [S... | 528 | 0.0 | 62% |
| TJW81642.1 3-phosphoshikimate 1-carboxyvinyltransferase [Mesor... | 527 | 0.0 | 79% |
| SFN90139.1 3-phosphoshikimate 1-carboxyvinyltransferase [Cohae... | 527 | 0.0 | 63% |
| WP_145343545.1 3-phosphoshikimate 1-carboxyvinyltransferase [R... | 527 | 0.0 | 62% |
| WP_097175920.1 3-phosphoshikimate 1-carboxyvinyltransferase [S... | 526 | 0.0 | 60% |
| WP_090070389.1 3-phosphoshikimate 1-carboxyvinyltransferase [C... | 526 | 0.0 | 63% |
| WP_181761654.1 3-phosphoshikimate 1-carboxyvinyltransferase [S... | 526 | 0.0 | 61% |
| WP_063309381.1 3-phosphoshikimate 1-carboxyvinyltransferase [P... | 525 | 0.0 | 62% |
| WP_111436645.1 3-phosphoshikimate 1-carboxyvinyltransferase [R... | 525 | 0.0 | 64% |
| WP_184443470.1 3-phosphoshikimate 1-carboxyvinyltransferase [R... | 525 | 0.0 | 64% |
| WP_088946121.1 3-phosphoshikimate 1-carboxyvinyltransferase [S... | 525 | 0.0 | 60% |
| WP_120270476.1 MULTISPECIES: 3-phosphoshikimate 1-carboxyvinyl... | 524 | 0.0 | 60% |
| MBA3520442.1 3-phosphoshikimate 1-carboxyvinyltransferase [Rhi... | 524 | 0.0 | 63% |
| RWF23126.1 3-phosphoshikimate 1-carboxyvinyltransferase [Mesor... | 524 | 0.0 | 78% |
| WP_158194679.1 3-phosphoshikimate 1-carboxyvinyltransferase [S... | 523 | 0.0 | 61% |
| WP_028480630.1 3-phosphoshikimate 1-carboxyvinyltransferase [N... | 523 | 0.0 | 61% |
| WP_099559114.1 3-phosphoshikimate 1-carboxyvinyltransferase [H... | 523 | 0.0 | 65% |
| WP_101289949.1 3-phosphoshikimate 1-carboxyvinyltransferase [P... | 522 | 0.0 | 63% |
| WP_134183084.1 3-phosphoshikimate 1-carboxyvinyltransferase [P... | 522 | 0.0 | 62% |
| TIX79296.1 3-phosphoshikimate 1-carboxyvinyltransferase [Mesor... | 521 | 0.0 | 78% |
| MQT12661.1 3-phosphoshikimate 1-carboxyvinyltransferase [Rhizo... | 522 | 1e-180 | 65% |
| WP_097154998.1 3-phosphoshikimate 1-carboxyvinyltransferase [C... | 521 | 2e-180 | 62% |
| PLX38031.1 3-phosphoshikimate 1-carboxyvinyltransferase [Rhizo... | 521 | 3e-180 | 64% |
| WP_102868110.1 3-phosphoshikimate 1-carboxyvinyltransferase [N... | 520 | 6e-180 | 61% |
| WP_166902362.1 MULTISPECIES: 3-phosphoshikimate 1-carboxyvinyl... | 520 | 6e-180 | 62% |
| WP_029057711.1 3-phosphoshikimate 1-carboxyvinyltransferase [S... | 519 | 2e-179 | 63% |
| WP_026791560.1 3-phosphoshikimate 1-carboxyvinyltransferase [P... | 519 | 2e-179 | 63% |
| NMD08018.1 3-phosphoshikimate 1-carboxyvinyltransferase [Phyll... | 518 | 2e-179 | 61% |
| WP_128370261.1 3-phosphoshikimate 1-carboxyvinyltransferase [M... | 514 | 4e-179 | 80% |
| WP_100081973.1 3-phosphoshikimate 1-carboxyvinyltransferase [P... | 518 | 4e-179 | 63% |
| WP_183857034.1 3-phosphoshikimate 1-carboxyvinyltransferase [P... | 518 | 4e-179 | 65% |
| TIW43317.1 3-phosphoshikimate 1-carboxyvinyltransferase [Mesor... | 513 | 6e-179 | 79% |
| TIX64227.1 3-phosphoshikimate 1-carboxyvinyltransferase [Mesor... | 513 | 7e-179 | 79% |
| MAU61588.1 3-phosphoshikimate 1-carboxyvinyltransferase [Parvi... | 517 | 1e-178 | 63% |
| SCM77779.1 3-phosphoshikimate 1-carboxyvinyltransferase [uncul... | 516 | 2e-178 | 63% |
| TIS01320.1 3-phosphoshikimate 1-carboxyvinyltransferase [Mesor... | 511 | 4e-178 | 80% |
| NRG16459.1 3-phosphoshikimate 1-carboxyvinyltransferase [Rhizo... | 515 | 5e-178 | 60% |
| HAC57397.1 TPA: 3-phosphoshikimate 1-carboxyvinyltransferase [... | 515 | 5e-178 | 63% |
| WP_096173304.1 3-phosphoshikimate 1-carboxyvinyltransferase [C... | 515 | 6e-178 | 62% |

| WP_119309197.1 3-phosphoshikimate 1-carboxyvinyltransferase [C... | 514 | 7e-178 | 63% |
| --- | --- | --- | --- |
| TIW31195.1 3-phosphoshikimate 1-carboxyvinyltransferase [Mesor... | 509 | 2e-177 | 77% |
| WP_101535147.1 3-phosphoshikimate 1-carboxyvinyltransferase [C... | 513 | 2e-177 | 62% |
| WP_090875868.1 3-phosphoshikimate 1-carboxyvinyltransferase [B... | 512 | 1e-176 | 62% |
| WP_192663925.1 3-phosphoshikimate 1-carboxyvinyltransferase [N... | 512 | 1e-176 | 62% |
| WP_093811412.1 3-phosphoshikimate 1-carboxyvinyltransferase [S... | 511 | 3e-176 | 63% |
| PCI17695.1 3-phosphoshikimate 1-carboxyvinyltransferase [Rhodo... | 511 | 4e-176 | 61% |
| WP_164851744.1 3-phosphoshikimate 1-carboxyvinyltransferase [S... | 504 | 4e-176 | 90% |
| WP_007556833.1 3-phosphoshikimate 1-carboxyvinyltransferase [C... | 509 | 1e-175 | 59% |
| WP_092813689.1 3-phosphoshikimate 1-carboxyvinyltransferase [A... | 508 | 5e-175 | 64% |
| KAB2876106.1 3-phosphoshikimate 1-carboxyvinyltransferase [Bau... | 504 | 7e-174 | 64% |
| TIN07126.1 3-phosphoshikimate 1-carboxyvinyltransferase [Mesor... | 500 | 8e-174 | 80% |
| WP_053240115.1 3-phosphoshikimate 1-carboxyvinyltransferase [P... | 503 | 3e-173 | 63% |
| WP_015397500.1 3-phosphoshikimate 1-carboxyvinyltransferase [B... | 503 | 4e-173 | 62% |
| WP_026380010.1 3-phosphoshikimate 1-carboxyvinyltransferase [A... | 503 | 4e-173 | 62% |
| WP_128290748.1 3-phosphoshikimate 1-carboxyvinyltransferase [A... | 502 | 8e-173 | 63% |
| WP_029042157.1 3-phosphoshikimate 1-carboxyvinyltransferase [C... | 501 | 3e-172 | 62% |
| WP_068091776.1 3-phosphoshikimate 1-carboxyvinyltransferase [N... | 500 | 3e-172 | 60% |
| MYZ48636.1 3-phosphoshikimate 1-carboxyvinyltransferase [Rhizo... | 501 | 4e-172 | 61% |
| RWF61461.1 3-phosphoshikimate 1-carboxyvinyltransferase [Mesor... | 495 | 4e-172 | 79% |
| MAM94433.1 3-phosphoshikimate 1-carboxyvinyltransferase [Parvi... | 500 | 4e-172 | 61% |
| WP_038360867.1 3-phosphoshikimate 1-carboxyvinyltransferase [B... | 500 | 6e-172 | 61% |
| MBC8035898.1 3-phosphoshikimate 1-carboxyvinyltransferase [Rhi... | 499 | 1e-171 | 61% |
| WP_196504929.1 3-phosphoshikimate 1-carboxyvinyltransferase [A... | 499 | 2e-171 | 58% |
| WP_175563699.1 3-phosphoshikimate 1-carboxyvinyltransferase [P... | 498 | 3e-171 | 62% |
| TMJ40172.1 3-phosphoshikimate 1-carboxyvinyltransferase [Alpha... | 498 | 4e-171 | 60% |
| PKQ01795.1 3-phosphoshikimate 1-carboxyvinyltransferase [Alpha... | 496 | 1e-170 | 61% |
| TDI57055.1 3-phosphoshikimate 1-carboxyvinyltransferase [Alpha... | 496 | 1e-170 | 61% |
| GGB47201.1 3-phosphoshikimate 1-carboxyvinyltransferase [Rosei... | 495 | 2e-170 | 63% |
| TMJ35260.1 3-phosphoshikimate 1-carboxyvinyltransferase [Alpha... | 496 | 2e-170 | 60% |
| WP_144341404.1 3-phosphoshikimate 1-carboxyvinyltransferase [S... | 496 | 2e-170 | 61% |
| MBC7101758.1 3-phosphoshikimate 1-carboxyvinyltransferase [Par... | 495 | 6e-170 | 60% |
| NOW46609.1 3-phosphoshikimate 1-carboxyvinyltransferase [Novos... | 494 | 7e-170 | 60% |
| TIX98404.1 3-phosphoshikimate 1-carboxyvinyltransferase [Mesor... | 489 | 1e-169 | 79% |
| TWI87178.1 3-phosphoshikimate 1-carboxyvinyltransferase [Rosei... | 493 | 1e-169 | 63% |
| WP_172341140.1 3-phosphoshikimate 1-carboxyvinyltransferase [N... | 494 | 4e-169 | 60% |
| WP_104219476.1 3-phosphoshikimate 1-carboxyvinyltransferase [K... | 493 | 6e-169 | 63% |
| PKP78875.1 3-phosphoshikimate 1-carboxyvinyltransferase [Alpha... | 492 | 6e-169 | 61% |
| TIY09784.1 3-phosphoshikimate 1-carboxyvinyltransferase [Mesor... | 487 | 9e-169 | 79% |
| WP_121880707.1 3-phosphoshikimate 1-carboxyvinyltransferase [T... | 491 | 1e-168 | 60% |
| QPC45228.1 3-phosphoshikimate 1-carboxyvinyltransferase [Alpha... | 491 | 1e-168 | 61% |
| WP_045448780.1 3-phosphoshikimate 1-carboxyvinyltransferase [T... | 491 | 1e-168 | 60% |
| TIN50024.1 3-phosphoshikimate 1-carboxyvinyltransferase [Mesor... | 486 | 2e-168 | 80% |
| TMJ66676.1 3-phosphoshikimate 1-carboxyvinyltransferase [Alpha... | 491 | 2e-168 | 60% |
| NKJ00509.1 3-phosphoshikimate 1-carboxyvinyltransferase [Novos... | 490 | 3e-168 | 60% |
| WP_096358373.1 3-phosphoshikimate 1-carboxyvinyltransferase [V... | 490 | 3e-168 | 60% |
| WP_111198970.1 3-phosphoshikimate 1-carboxyvinyltransferase [A... | 490 | 3e-168 | 60% |
| TMJ99398.1 3-phosphoshikimate 1-carboxyvinyltransferase [Alpha... | 490 | 4e-168 | 60% |
| MBA4210086.1 3-phosphoshikimate 1-carboxyvinyltransferase [Par... | 490 | 5e-168 | 61% |
| WP_183621601.1 3-phosphoshikimate 1-carboxyvinyltransferase [N... | 489 | 1e-167 | 59% |
| MBG54141.1 3-phosphoshikimate 1-carboxyvinyltransferase [Rhodo... | 488 | 2e-167 | 59% |
| WP_168604071.1 MULTISPECIES: 3-phosphoshikimate 1-carboxyvinyl... | 487 | 4e-167 | 61% |
| WP_165167713.1 3-phosphoshikimate 1-carboxyvinyltransferase [N... | 487 | 5e-167 | 59% |
| WP_104954407.1 3-phosphoshikimate 1-carboxyvinyltransferase [S... | 487 | 6e-167 | 61% |
| WP_026319199.1 3-phosphoshikimate 1-carboxyvinyltransferase [A... | 487 | 6e-167 | 60% |
| WP_152214261.1 3-phosphoshikimate 1-carboxyvinyltransferase [P... | 487 | 9e-167 | 59% |
| TMK01383.1 3-phosphoshikimate 1-carboxyvinyltransferase [Alpha... | 486 | 1e-166 | 60% |
| WP_011995065.1 3-phosphoshikimate 1-carboxyvinyltransferase [P... | 486 | 2e-166 | 60% |
| ODU82490.1 3-phosphoshikimate 1-carboxyvinyltransferase [Novos... | 486 | 2e-166 | 59% |
| TMJ44386.1 3-phosphoshikimate 1-carboxyvinyltransferase [Alpha... | 486 | 2e-166 | 60% |
| OLB69734.1 3-phosphoshikimate 1-carboxyvinyltransferase [Alpha... | 485 | 3e-166 | 59% |
| TIU18120.1 3-phosphoshikimate 1-carboxyvinyltransferase [Mesor... | 480 | 4e-166 | 79% |
| CDO38762.1 3-phosphoshikimate 1-carboxyvinyltransferase (5-eno... | 484 | 7e-166 | 59% |
| TMJ32682.1 3-phosphoshikimate 1-carboxyvinyltransferase [Alpha... | 485 | 7e-166 | 59% |
| WP_170014726.1 3-phosphoshikimate 1-carboxyvinyltransferase [S... | 484 | 1e-165 | 60% |
| PTM51451.1 3-phosphoshikimate 1-carboxyvinyltransferase [Phrea... | 483 | 2e-165 | 62% |
| WP_183939368.1 3-phosphoshikimate 1-carboxyvinyltransferase [S... | 483 | 2e-165 | 58% |
| OPB32490.1 3-phosphoshikimate 1-carboxyvinyltransferase [Barto... | 483 | 3e-165 | 56% |
| WP_054948330.1 3-phosphoshikimate 1-carboxyvinyltransferase [N... | 483 | 3e-165 | 59% |
| WP_053193435.1 3-phosphoshikimate 1-carboxyvinyltransferase [C... | 483 | 4e-165 | 61% |
| WP_078680986.1 3-phosphoshikimate 1-carboxyvinyltransferase [B... | 482 | 4e-165 | 56% |
| MSO75272.1 3-phosphoshikimate 1-carboxyvinyltransferase [Alpha... | 482 | 5e-165 | 60% |

| TMJ84064.1 3-phosphoshikimate 1-carboxyvinyltransferase [Alpha... | 482 | 6e-165 | 60% |
| --- | --- | --- | --- |
| WP_013168634.1 3-phosphoshikimate 1-carboxyvinyltransferase [S... | 482 | 8e-165 | 61% |
| WP_192639958.1 3-phosphoshikimate 1-carboxyvinyltransferase [S... | 482 | 8e-165 | 60% |
| WP_107718106.1 3-phosphoshikimate 1-carboxyvinyltransferase [C... | 481 | 1e-164 | 60% |
| WP_108179128.1 3-phosphoshikimate 1-carboxyvinyltransferase [P... | 483 | 1e-164 | 62% |
| WP_020186149.1 3-phosphoshikimate 1-carboxyvinyltransferase [M... | 481 | 1e-164 | 60% |
| WP_056800231.1 3-phosphoshikimate 1-carboxyvinyltransferase [B... | 480 | 2e-164 | 61% |
| WP_144068275.1 3-phosphoshikimate 1-carboxyvinyltransferase [F... | 481 | 2e-164 | 58% |
| TQS71685.1 3-phosphoshikimate 1-carboxyvinyltransferase [Rhodo... | 480 | 3e-164 | 58% |
| WP_100693893.1 3-phosphoshikimate 1-carboxyvinyltransferase [B... | 480 | 3e-164 | 55% |
| WP_183398893.1 3-phosphoshikimate 1-carboxyvinyltransferase [K... | 480 | 5e-164 | 61% |
| WP_108502252.1 3-phosphoshikimate 1-carboxyvinyltransferase [P... | 479 | 5e-164 | 57% |
| PZQ84716.1 3-phosphoshikimate 1-carboxyvinyltransferase [Stark... | 480 | 6e-164 | 60% |
| WP_143957356.1 3-phosphoshikimate 1-carboxyvinyltransferase [R... | 479 | 6e-164 | 59% |
| ADR52095.1 3-phosphoshikimate 1-carboxyvinyltransferase [Candi... | 481 | 7e-164 | 56% |
| WP_161717198.1 3-phosphoshikimate 1-carboxyvinyltransferase [N... | 481 | 8e-164 | 59% |
| WP_072296262.1 3-phosphoshikimate 1-carboxyvinyltransferase [P... | 479 | 8e-164 | 57% |
| WP_167095610.1 3-phosphoshikimate 1-carboxyvinyltransferase [P... | 478 | 1e-163 | 59% |
| WP_192646098.1 3-phosphoshikimate 1-carboxyvinyltransferase [S... | 479 | 1e-163 | 60% |
| WP_086087942.1 3-phosphoshikimate 1-carboxyvinyltransferase [P... | 479 | 1e-163 | 59% |
| SLK08477.1 3-phosphoshikimate 1-carboxyvinyltransferase [Novos... | 479 | 1e-163 | 59% |
| WP_050780907.1 3-phosphoshikimate 1-carboxyvinyltransferase [C... | 479 | 1e-163 | 56% |
| WP_039574803.1 3-phosphoshikimate 1-carboxyvinyltransferase [S... | 478 | 2e-163 | 59% |
| OYY14604.1 3-phosphoshikimate 1-carboxyvinyltransferase [Sphin... | 478 | 2e-163 | 57% |
| WP_174540220.1 3-phosphoshikimate 1-carboxyvinyltransferase [M... | 478 | 2e-163 | 58% |
| MBF0251916.1 3-phosphoshikimate 1-carboxyvinyltransferase [Alp... | 479 | 2e-163 | 60% |
| WP_037511468.1 3-phosphoshikimate 1-carboxyvinyltransferase [S... | 478 | 2e-163 | 59% |
| WP_079731575.1 3-phosphoshikimate 1-carboxyvinyltransferase [N... | 478 | 2e-163 | 59% |
| WP_111297935.1 3-phosphoshikimate 1-carboxyvinyltransferase [P... | 478 | 2e-163 | 57% |
| WP_103121979.1 3-phosphoshikimate 1-carboxyvinyltransferase [M... | 478 | 3e-163 | 61% |
| WP_183316588.1 3-phosphoshikimate 1-carboxyvinyltransferase [C... | 478 | 3e-163 | 61% |
| WP_133863462.1 3-phosphoshikimate 1-carboxyvinyltransferase [A... | 478 | 4e-163 | 61% |
| PCJ68127.1 3-phosphoshikimate 1-carboxyvinyltransferase [Rhodo... | 478 | 4e-163 | 59% |
| WP_062217123.1 3-phosphoshikimate 1-carboxyvinyltransferase [C... | 478 | 4e-163 | 58% |
| WP_012113130.1 3-phosphoshikimate 1-carboxyvinyltransferase [X... | 478 | 4e-163 | 61% |
| WP_075219354.1 3-phosphoshikimate 1-carboxyvinyltransferase [A... | 478 | 4e-163 | 59% |
| WP_058096637.1 3-phosphoshikimate 1-carboxyvinyltransferase [P... | 477 | 5e-163 | 57% |
| WP_138399597.1 3-phosphoshikimate 1-carboxyvinyltransferase [X... | 478 | 5e-163 | 61% |
| TIT43065.1 3-phosphoshikimate 1-carboxyvinyltransferase [Mesor... | 472 | 5e-163 | 80% |
| WP_035005505.1 3-phosphoshikimate 1-carboxyvinyltransferase [B... | 477 | 5e-163 | 55% |
| WP_078688202.1 MULTISPECIES: 3-phosphoshikimate 1-carboxyvinyl... | 477 | 6e-163 | 55% |
| RWF93176.1 3-phosphoshikimate 1-carboxyvinyltransferase [Mesor... | 471 | 6e-163 | 80% |
| WP_127710704.1 3-phosphoshikimate 1-carboxyvinyltransferase [N... | 477 | 6e-163 | 60% |
| WP_026868339.1 3-phosphoshikimate 1-carboxyvinyltransferase [H... | 476 | 9e-163 | 58% |
| RYG98830.1 3-phosphoshikimate 1-carboxyvinyltransferase [Alpha... | 469 | 1e-162 | 88% |
| WP_068013353.1 3-phosphoshikimate 1-carboxyvinyltransferase [R... | 476 | 1e-162 | 59% |
| WP_188520222.1 3-phosphoshikimate 1-carboxyvinyltransferase [A... | 476 | 1e-162 | 61% |
| WP_036637662.1 3-phosphoshikimate 1-carboxyvinyltransferase [P... | 476 | 1e-162 | 59% |
| WP_111559424.1 3-phosphoshikimate 1-carboxyvinyltransferase [P... | 476 | 1e-162 | 57% |
| ODT58996.1 3-phosphoshikimate 1-carboxyvinyltransferase [Parac... | 476 | 2e-162 | 57% |
| WP_159600889.1 3-phosphoshikimate 1-carboxyvinyltransferase [S... | 476 | 2e-162 | 61% |
| WP_009572058.1 3-phosphoshikimate 1-carboxyvinyltransferase [C... | 476 | 2e-162 | 60% |
| WP_160771430.1 3-phosphoshikimate 1-carboxyvinyltransferase [A... | 476 | 2e-162 | 59% |
| WP_156912096.1 3-phosphoshikimate 1-carboxyvinyltransferase [K... | 476 | 2e-162 | 58% |
| ADJ24126.1 3-phosphoshikimate 1-carboxyvinyltransferase [Hypho... | 476 | 2e-162 | 57% |
| WP_085378010.1 3-phosphoshikimate 1-carboxyvinyltransferase [P... | 475 | 2e-162 | 58% |
| WP_183507186.1 3-phosphoshikimate 1-carboxyvinyltransferase [M... | 476 | 2e-162 | 61% |
| WP_022724471.1 3-phosphoshikimate 1-carboxyvinyltransferase [R... | 475 | 2e-162 | 58% |
| CAA7619491.1 3-phosphoshikimate 1-carboxyvinyltransferase [Mag... | 475 | 3e-162 | 59% |
| WP_089219199.1 3-phosphoshikimate 1-carboxyvinyltransferase [S... | 476 | 3e-162 | 59% |
| WP_107966118.1 3-phosphoshikimate 1-carboxyvinyltransferase [S... | 475 | 3e-162 | 60% |
| PKP72306.1 3-phosphoshikimate 1-carboxyvinyltransferase [Alpha... | 475 | 3e-162 | 58% |
| WP_147163118.1 3-phosphoshikimate 1-carboxyvinyltransferase [P... | 475 | 3e-162 | 58% |
| MBD3747717.1 3-phosphoshikimate 1-carboxyvinyltransferase [Sph... | 475 | 4e-162 | 59% |
| PCJ83729.1 3-phosphoshikimate 1-carboxyvinyltransferase [Rhizo... | 474 | 4e-162 | 60% |
| OYU02279.1 3-phosphoshikimate 1-carboxyvinyltransferase [Sphin... | 474 | 4e-162 | 57% |
| MAB15079.1 3-phosphoshikimate 1-carboxyvinyltransferase [Parvi... | 474 | 4e-162 | 60% |
| WP_090610237.1 3-phosphoshikimate 1-carboxyvinyltransferase [P... | 474 | 5e-162 | 56% |
| WP_007792286.1 3-phosphoshikimate 1-carboxyvinyltransferase [S... | 474 | 5e-162 | 59% |
| WP_173976064.1 3-phosphoshikimate 1-carboxyvinyltransferase [M... | 474 | 5e-162 | 59% |
| WP_088443504.1 3-phosphoshikimate 1-carboxyvinyltransferase [S... | 474 | 5e-162 | 60% |
| WP_074642921.1 3-phosphoshikimate 1-carboxyvinyltransferase [C... | 474 | 5e-162 | 60% |

| WP_055460733.1 3-phosphoshikimate 1-carboxyvinyltransferase [C... | 474 | 5e-162 | 60% |
| --- | --- | --- | --- |
| WP_147047185.1 3-phosphoshikimate 1-carboxyvinyltransferase [M... | 474 | 5e-162 | 60% |
| WP_187140341.1 3-phosphoshikimate 1-carboxyvinyltransferase [S... | 474 | 6e-162 | 60% |
| WP_043234835.1 3-phosphoshikimate 1-carboxyvinyltransferase [B... | 474 | 6e-162 | 60% |
| OAN55141.1 3-phosphoshikimate 1-carboxyvinyltransferase [Magne... | 474 | 6e-162 | 58% |
| WP_188574582.1 3-phosphoshikimate 1-carboxyvinyltransferase [A... | 475 | 6e-162 | 60% |
| MBF0327127.1 3-phosphoshikimate 1-carboxyvinyltransferase [Alp... | 474 | 6e-162 | 59% |
| TCM21579.1 3-phosphoshikimate 1-carboxyvinyltransferase [Novos... | 474 | 7e-162 | 58% |
| WP_132385721.1 3-phosphoshikimate 1-carboxyvinyltransferase [N... | 474 | 7e-162 | 58% |
| WP_139939645.1 3-phosphoshikimate 1-carboxyvinyltransferase [E... | 474 | 8e-162 | 58% |
| WP_063189722.1 3-phosphoshikimate 1-carboxyvinyltransferase [C... | 474 | 8e-162 | 60% |
| PPD14822.1 3-phosphoshikimate 1-carboxyvinyltransferase [Methy... | 474 | 8e-162 | 59% |
| MAE91452.1 3-phosphoshikimate 1-carboxyvinyltransferase [Pelag... | 474 | 9e-162 | 59% |
| WP_188715487.1 3-phosphoshikimate 1-carboxyvinyltransferase [P... | 474 | 9e-162 | 57% |
| SNS47593.1 3-phosphoshikimate 1-carboxyvinyltransferase [Sphin... | 475 | 9e-162 | 59% |
| WP_029005438.1 3-phosphoshikimate 1-carboxyvinyltransferase [A... | 474 | 9e-162 | 61% |
| WP_058811852.1 3-phosphoshikimate 1-carboxyvinyltransferase [S... | 474 | 9e-162 | 59% |
| WP_083547751.1 3-phosphoshikimate 1-carboxyvinyltransferase [S... | 474 | 9e-162 | 59% |
| NDF11239.1 3-phosphoshikimate 1-carboxyvinyltransferase [Prote... | 474 | 1e-161 | 58% |
| KFL44973.1 3-phosphoshikimate 1-carboxyvinyltransferase [Sphin... | 474 | 1e-161 | 59% |
| MAJ63162.1 3-phosphoshikimate 1-carboxyvinyltransferase [Alpha... | 474 | 1e-161 | 58% |
| WP_136857659.1 3-phosphoshikimate 1-carboxyvinyltransferase [P... | 473 | 1e-161 | 56% |
| WP_069689234.1 3-phosphoshikimate 1-carboxyvinyltransferase [B... | 474 | 1e-161 | 60% |
| WP_132264198.1 3-phosphoshikimate 1-carboxyvinyltransferase [N... | 473 | 1e-161 | 59% |
| TVP72557.1 3-phosphoshikimate 1-carboxyvinyltransferase [Rhodo... | 474 | 1e-161 | 59% |
| MAN80221.1 3-phosphoshikimate 1-carboxyvinyltransferase [Magne... | 475 | 1e-161 | 59% |
| TCU61986.1 3-phosphoshikimate 1-carboxyvinyltransferase [Novos... | 473 | 1e-161 | 59% |
| WP_082910729.1 3-phosphoshikimate 1-carboxyvinyltransferase [M... | 473 | 2e-161 | 58% |
| WP_020181440.1 3-phosphoshikimate 1-carboxyvinyltransferase [M... | 473 | 2e-161 | 61% |
| MBB3770621.1 3-phosphoshikimate 1-carboxyvinyltransferase [Ang... | 474 | 2e-161 | 59% |
| PPD01903.1 3-phosphoshikimate 1-carboxyvinyltransferase [Hypho... | 473 | 2e-161 | 58% |
| OHD02948.1 3-phosphoshikimate 1-carboxyvinyltransferase [Sphin... | 473 | 2e-161 | 60% |
| WP_109896560.1 3-phosphoshikimate 1-carboxyvinyltransferase [M... | 473 | 2e-161 | 60% |
| WP_040590081.1 3-phosphoshikimate 1-carboxyvinyltransferase [S... | 473 | 2e-161 | 59% |
| WP_077148129.1 3-phosphoshikimate 1-carboxyvinyltransferase [S... | 473 | 2e-161 | 59% |
| WP_128778118.1 3-phosphoshikimate 1-carboxyvinyltransferase [H... | 473 | 3e-161 | 60% |
| WP_086457869.1 3-phosphoshikimate 1-carboxyvinyltransferase [S... | 473 | 3e-161 | 59% |
| WP_145149455.1 3-phosphoshikimate 1-carboxyvinyltransferase [S... | 472 | 3e-161 | 60% |
| WP_031341758.1 3-phosphoshikimate 1-carboxyvinyltransferase [N... | 473 | 3e-161 | 59% |
| TXH37697.1 3-phosphoshikimate 1-carboxyvinyltransferase [Rhodo... | 473 | 3e-161 | 60% |
| WP_152303718.1 3-phosphoshikimate 1-carboxyvinyltransferase [A... | 473 | 3e-161 | 61% |
| WP_150103388.1 3-phosphoshikimate 1-carboxyvinyltransferase [H... | 472 | 3e-161 | 59% |
| EQB17607.1 3-phosphoshikimate 1-carboxyvinyltransferase [Novos... | 473 | 3e-161 | 59% |
| WP_136693321.1 3-phosphoshikimate 1-carboxyvinyltransferase [A... | 472 | 4e-161 | 61% |
| ENY81248.1 3-phosphoshikimate 1-carboxyvinyltransferase [Sphin... | 473 | 4e-161 | 59% |
| WP_056432674.1 MULTISPECIES: 3-phosphoshikimate 1-carboxyvinyl... | 473 | 4e-161 | 61% |
| WP_149576954.1 3-phosphoshikimate 1-carboxyvinyltransferase [X... | 473 | 4e-161 | 61% |
| WP_076485713.1 MULTISPECIES: 3-phosphoshikimate 1-carboxyvinyl... | 472 | 4e-161 | 59% |
| WP_078673487.1 MULTISPECIES: 3-phosphoshikimate 1-carboxyvinyl... | 472 | 4e-161 | 54% |
| WP_081434058.1 3-phosphoshikimate 1-carboxyvinyltransferase [A... | 473 | 5e-161 | 60% |
| WP_192643498.1 3-phosphoshikimate 1-carboxyvinyltransferase [B... | 472 | 5e-161 | 60% |
| WP_066706265.1 3-phosphoshikimate 1-carboxyvinyltransferase [C... | 472 | 5e-161 | 58% |
| WP_066964777.1 3-phosphoshikimate 1-carboxyvinyltransferase [R... | 472 | 6e-161 | 58% |
| WP_131003514.1 3-phosphoshikimate 1-carboxyvinyltransferase [H... | 472 | 6e-161 | 60% |
| WP_163075125.1 3-phosphoshikimate 1-carboxyvinyltransferase [A... | 473 | 6e-161 | 57% |
| WP_089214970.1 3-phosphoshikimate 1-carboxyvinyltransferase [S... | 471 | 8e-161 | 59% |
| MBD3762339.1 3-phosphoshikimate 1-carboxyvinyltransferase [Rhi... | 471 | 8e-161 | 59% |
| WP_045999306.1 3-phosphoshikimate 1-carboxyvinyltransferase [P... | 471 | 8e-161 | 57% |
| WP_179495510.1 3-phosphoshikimate 1-carboxyvinyltransferase [S... | 471 | 9e-161 | 59% |
| BAF89929.1 3-phosphoshikimate 1-carboxyvinyltransferase [Azorh... | 472 | 9e-161 | 60% |
| SBV32465.1 3-phosphoshikimate 1-carboxyvinyltransferase [uncul... | 471 | 1e-160 | 59% |
| WP_022708032.1 3-phosphoshikimate 1-carboxyvinyltransferase [P... | 471 | 1e-160 | 56% |
| PZO90303.1 3-phosphoshikimate 1-carboxyvinyltransferase [Sphin... | 471 | 1e-160 | 57% |
| WP_056252388.1 3-phosphoshikimate 1-carboxyvinyltransferase [M... | 471 | 1e-160 | 60% |
| WP_183189235.1 3-phosphoshikimate 1-carboxyvinyltransferase [A... | 471 | 1e-160 | 60% |
| WP_058816608.1 3-phosphoshikimate 1-carboxyvinyltransferase [S... | 471 | 1e-160 | 57% |
| WP_129404373.1 3-phosphoshikimate 1-carboxyvinyltransferase [S... | 471 | 1e-160 | 59% |
| WP_108045868.1 3-phosphoshikimate 1-carboxyvinyltransferase [B... | 471 | 1e-160 | 60% |
| WP_106337259.1 3-phosphoshikimate 1-carboxyvinyltransferase [A... | 471 | 1e-160 | 60% |
| SMQ79048.1 3-phosphoshikimate 1-carboxyvinyltransferase [Sphin... | 472 | 1e-160 | 59% |
| AMU96088.1 3-phosphoshikimate 1-carboxyvinyltransferase [Sphin... | 471 | 1e-160 | 59% |
| WP_161723388.1 MULTISPECIES: 3-phosphoshikimate 1-carboxyvinyl... | 471 | 1e-160 | 61% |

| MBB6227577.1 3-phosphoshikimate 1-carboxyvinyltransferase [Pol... | 471 | 1e-160 | 59% |
| --- | --- | --- | --- |
| WP_182745551.1 3-phosphoshikimate 1-carboxyvinyltransferase [R... | 471 | 1e-160 | 58% |
| NNE25191.1 3-phosphoshikimate 1-carboxyvinyltransferase [Rhizo... | 471 | 2e-160 | 57% |
| WP_192215968.1 3-phosphoshikimate 1-carboxyvinyltransferase [M... | 471 | 2e-160 | 60% |
| WP_072245419.1 3-phosphoshikimate 1-carboxyvinyltransferase [R... | 471 | 2e-160 | 59% |
| NBB98505.1 3-phosphoshikimate 1-carboxyvinyltransferase [Alpha... | 471 | 2e-160 | 59% |
| TDW62968.1 3-phosphoshikimate 1-carboxyvinyltransferase [Novos... | 471 | 2e-160 | 59% |
| RTL66005.1 3-phosphoshikimate 1-carboxyvinyltransferase [Rhizo... | 471 | 2e-160 | 59% |
| AKM12419.1 5-enolpyruvylshikimate 3-phosphate synthase [Paraco... | 470 | 2e-160 | 57% |
| WP_038127376.1 3-phosphoshikimate 1-carboxyvinyltransferase [T... | 471 | 2e-160 | 58% |
| WP_112664350.1 3-phosphoshikimate 1-carboxyvinyltransferase [M... | 470 | 2e-160 | 60% |
| WP_167622184.1 3-phosphoshikimate 1-carboxyvinyltransferase [P... | 470 | 2e-160 | 57% |
| WP_023431417.1 3-phosphoshikimate 1-carboxyvinyltransferase [L... | 471 | 2e-160 | 61% |
| WP_184198405.1 3-phosphoshikimate 1-carboxyvinyltransferase [P... | 471 | 2e-160 | 59% |
| WP_134095455.1 3-phosphoshikimate 1-carboxyvinyltransferase [N... | 470 | 2e-160 | 59% |
| PZU92720.1 3-phosphoshikimate 1-carboxyvinyltransferase [Chela... | 471 | 2e-160 | 60% |
| WP_074555387.1 3-phosphoshikimate 1-carboxyvinyltransferase [R... | 470 | 2e-160 | 57% |
| AGK58596.1 3-phosphoshikimate 1-carboxyvinyltransferase [Hypho... | 470 | 2e-160 | 57% |
| WP_109598923.1 3-phosphoshikimate 1-carboxyvinyltransferase [M... | 470 | 3e-160 | 61% |
| WP_191307423.1 3-phosphoshikimate 1-carboxyvinyltransferase [P... | 470 | 3e-160 | 57% |
| WP_056158172.1 MULTISPECIES: 3-phosphoshikimate 1-carboxyvinyl... | 470 | 3e-160 | 60% |
| WP_147428177.1 3-phosphoshikimate 1-carboxyvinyltransferase [P... | 470 | 3e-160 | 57% |
| WP_183567256.1 3-phosphoshikimate 1-carboxyvinyltransferase [M... | 470 | 3e-160 | 60% |
| WP_172788605.1 3-phosphoshikimate 1-carboxyvinyltransferase [B... | 470 | 3e-160 | 57% |
| WP_168203054.1 MULTISPECIES: 3-phosphoshikimate 1-carboxyvinyl... | 470 | 3e-160 | 58% |
| NBU78266.1 3-phosphoshikimate 1-carboxyvinyltransferase [Sphin... | 470 | 3e-160 | 57% |
| WP_062765185.1 3-phosphoshikimate 1-carboxyvinyltransferase [S... | 470 | 3e-160 | 59% |
| WP_037553533.1 3-phosphoshikimate 1-carboxyvinyltransferase [S... | 470 | 3e-160 | 59% |
| WP_154765464.1 3-phosphoshikimate 1-carboxyvinyltransferase [P... | 470 | 4e-160 | 57% |
| WP_186401922.1 3-phosphoshikimate 1-carboxyvinyltransferase [S... | 469 | 4e-160 | 58% |
| WP_089344693.1 3-phosphoshikimate 1-carboxyvinyltransferase [P... | 469 | 4e-160 | 57% |
| WP_135074990.1 3-phosphoshikimate 1-carboxyvinyltransferase [T... | 469 | 4e-160 | 57% |
| WP_119062223.1 3-phosphoshikimate 1-carboxyvinyltransferase [D... | 469 | 4e-160 | 58% |
| WP_018042728.1 3-phosphoshikimate 1-carboxyvinyltransferase [M... | 470 | 4e-160 | 60% |
| WP_175369405.1 3-phosphoshikimate 1-carboxyvinyltransferase [T... | 470 | 4e-160 | 58% |
| WP_117353424.1 3-phosphoshikimate 1-carboxyvinyltransferase [B... | 470 | 4e-160 | 58% |
| WP_090061854.1 3-phosphoshikimate 1-carboxyvinyltransferase [C... | 470 | 4e-160 | 58% |
| WP_013066859.1 3-phosphoshikimate 1-carboxyvinyltransferase [R... | 470 | 4e-160 | 58% |
| WP_071797542.1 3-phosphoshikimate 1-carboxyvinyltransferase [N... | 469 | 4e-160 | 57% |
| WP_192335445.1 3-phosphoshikimate 1-carboxyvinyltransferase [P... | 469 | 4e-160 | 56% |
| OJW28520.1 3-phosphoshikimate 1-carboxyvinyltransferase [Sphin... | 469 | 5e-160 | 59% |
| WP_056282096.1 MULTISPECIES: 3-phosphoshikimate 1-carboxyvinyl... | 469 | 5e-160 | 60% |
| MBC7586546.1 3-phosphoshikimate 1-carboxyvinyltransferase [Tar... | 469 | 5e-160 | 58% |
| WP_194390596.1 3-phosphoshikimate 1-carboxyvinyltransferase [B... | 469 | 5e-160 | 58% |
| TIW15775.1 3-phosphoshikimate 1-carboxyvinyltransferase [Mesor... | 463 | 5e-160 | 80% |
| WP_147818283.1 3-phosphoshikimate 1-carboxyvinyltransferase [M... | 469 | 5e-160 | 60% |
| WP_193378929.1 3-phosphoshikimate 1-carboxyvinyltransferase [S... | 469 | 5e-160 | 59% |
| QDM19262.1 3-phosphoshikimate 1-carboxyvinyltransferase [Tardi... | 470 | 5e-160 | 58% |
| OQW84099.1 3-phosphoshikimate 1-carboxyvinyltransferase [Prote... | 469 | 6e-160 | 59% |
| WP_140041791.1 3-phosphoshikimate 1-carboxyvinyltransferase [S... | 469 | 6e-160 | 58% |
| WP_011500621.1 3-phosphoshikimate 1-carboxyvinyltransferase [R... | 469 | 6e-160 | 58% |
| WP_119765599.1 3-phosphoshikimate 1-carboxyvinyltransferase [S... | 469 | 6e-160 | 61% |
| WP_010392892.1 MULTISPECIES: 3-phosphoshikimate 1-carboxyvinyl... | 469 | 7e-160 | 57% |
| WP_131576442.1 3-phosphoshikimate 1-carboxyvinyltransferase [P... | 469 | 7e-160 | 56% |
| WP_023918327.1 3-phosphoshikimate 1-carboxyvinyltransferase [R... | 469 | 7e-160 | 58% |
| PBN45434.1 3-phosphoshikimate 1-carboxyvinyltransferase [Sphin... | 469 | 7e-160 | 59% |
| WP_080622089.1 3-phosphoshikimate 1-carboxyvinyltransferase [P... | 469 | 7e-160 | 57% |
| WP_056350492.1 3-phosphoshikimate 1-carboxyvinyltransferase [S... | 469 | 8e-160 | 59% |
| WP_139597572.1 3-phosphoshikimate 1-carboxyvinyltransferase [P... | 469 | 8e-160 | 56% |
| WP_133654681.1 3-phosphoshikimate 1-carboxyvinyltransferase [S... | 469 | 8e-160 | 58% |
| WP_091439612.1 3-phosphoshikimate 1-carboxyvinyltransferase [A... | 469 | 8e-160 | 60% |
| TMK12027.1 3-phosphoshikimate 1-carboxyvinyltransferase [Alpha... | 469 | 9e-160 | 60% |
| WP_167101194.1 3-phosphoshikimate 1-carboxyvinyltransferase [S... | 469 | 9e-160 | 59% |
| WP_120440791.1 3-phosphoshikimate 1-carboxyvinyltransferase [P... | 469 | 9e-160 | 57% |
| WP_018388684.1 3-phosphoshikimate 1-carboxyvinyltransferase [A... | 469 | 9e-160 | 60% |
| WP_046848545.1 3-phosphoshikimate 1-carboxyvinyltransferase [H... | 469 | 9e-160 | 57% |
| WP_058803770.1 3-phosphoshikimate 1-carboxyvinyltransferase [S... | 469 | 1e-159 | 58% |
| WP_127898378.1 MULTISPECIES: 3-phosphoshikimate 1-carboxyvinyl... | 469 | 1e-159 | 56% |
| WP_138010815.1 3-phosphoshikimate 1-carboxyvinyltransferase [C... | 469 | 1e-159 | 58% |
| WP_058536265.1 MULTISPECIES: 3-phosphoshikimate 1-carboxyvinyl... | 468 | 1e-159 | 59% |
| WP_108658574.1 3-phosphoshikimate 1-carboxyvinyltransferase [A... | 468 | 1e-159 | 61% |
| EXS70363.1 3-phosphoshikimate 1-carboxyvinyltransferase [Sphin... | 468 | 1e-159 | 58% |

| WP_023915210.1 3-phosphoshikimate 1-carboxyvinyltransferase [R... | 468 | 1e-159 | 58% |
| --- | --- | --- | --- |
| WP_188236860.1 3-phosphoshikimate 1-carboxyvinyltransferase [S... | 468 | 1e-159 | 60% |
| WP_126281041.1 3-phosphoshikimate 1-carboxyvinyltransferase [A... | 469 | 1e-159 | 60% |
| WP_028711956.1 MULTISPECIES: 3-phosphoshikimate 1-carboxyvinyl... | 468 | 1e-159 | 56% |
| WP_024843149.1 3-phosphoshikimate 1-carboxyvinyltransferase [P... | 468 | 1e-159 | 56% |
| WP_194405182.1 3-phosphoshikimate 1-carboxyvinyltransferase [B... | 468 | 1e-159 | 57% |
| WP_155038115.1 3-phosphoshikimate 1-carboxyvinyltransferase [P... | 468 | 1e-159 | 56% |
| WP_045980815.1 3-phosphoshikimate 1-carboxyvinyltransferase [P... | 468 | 1e-159 | 56% |
| EHJ60167.1 3-phosphoshikimate 1-carboxyvinyltransferase [Novos... | 468 | 1e-159 | 58% |
| TIM60369.1 3-phosphoshikimate 1-carboxyvinyltransferase [Mesor... | 463 | 1e-159 | 80% |
| WP_160353864.1 3-phosphoshikimate 1-carboxyvinyltransferase [S... | 469 | 1e-159 | 56% |
| WP_069878524.1 3-phosphoshikimate 1-carboxyvinyltransferase [B... | 468 | 2e-159 | 59% |
| WP_055886211.1 3-phosphoshikimate 1-carboxyvinyltransferase [M... | 468 | 2e-159 | 60% |
| WP_107815771.1 3-phosphoshikimate 1-carboxyvinyltransferase [C... | 468 | 2e-159 | 58% |
| WP_043151280.1 3-phosphoshikimate 1-carboxyvinyltransferase [S... | 468 | 2e-159 | 58% |
| WP_125231049.1 3-phosphoshikimate 1-carboxyvinyltransferase [S... | 468 | 2e-159 | 58% |
| WP_013832671.1 3-phosphoshikimate 1-carboxyvinyltransferase [N... | 468 | 2e-159 | 59% |
| WP_192667116.1 3-phosphoshikimate 1-carboxyvinyltransferase [S... | 468 | 2e-159 | 59% |
| MAO55009.1 3-phosphoshikimate 1-carboxyvinyltransferase [Rhodo... | 469 | 2e-159 | 58% |
| WP_055209994.1 3-phosphoshikimate 1-carboxyvinyltransferase [R... | 468 | 2e-159 | 57% |
| WP_034465126.1 3-phosphoshikimate 1-carboxyvinyltransferase [A... | 468 | 2e-159 | 56% |
| OUS12053.1 3-phosphoshikimate 1-carboxyvinyltransferase [Rhodo... | 468 | 2e-159 | 58% |
| NJM28810.1 3-phosphoshikimate 1-carboxyvinyltransferase [Rhizo... | 468 | 2e-159 | 56% |
| GAD48294.1 3-phosphoshikimate 1-carboxyvinyltransferase [Novos... | 468 | 2e-159 | 59% |
| WP_182553874.1 3-phosphoshikimate 1-carboxyvinyltransferase [M... | 468 | 2e-159 | 60% |
| WP_176454678.1 3-phosphoshikimate 1-carboxyvinyltransferase [T... | 468 | 2e-159 | 58% |
| WP_061880269.1 3-phosphoshikimate 1-carboxyvinyltransferase [B... | 468 | 2e-159 | 57% |
| WP_056144372.1 3-phosphoshikimate 1-carboxyvinyltransferase [M... | 468 | 2e-159 | 60% |
| WP_119886705.1 3-phosphoshikimate 1-carboxyvinyltransferase [P... | 468 | 3e-159 | 57% |
| NDE90534.1 3-phosphoshikimate 1-carboxyvinyltransferase [Alpha... | 468 | 3e-159 | 57% |
| OYW66679.1 3-phosphoshikimate 1-carboxyvinyltransferase [Bosea... | 468 | 3e-159 | 60% |
| WP_137753877.1 3-phosphoshikimate 1-carboxyvinyltransferase [S... | 468 | 3e-159 | 59% |
| WP_028718187.1 3-phosphoshikimate 1-carboxyvinyltransferase [P... | 468 | 3e-159 | 57% |
| AWZ02860.1 3-phosphoshikimate 1-carboxyvinyltransferase [Rhodo... | 468 | 3e-159 | 57% |
| WP_025032953.1 3-phosphoshikimate 1-carboxyvinyltransferase [B... | 468 | 3e-159 | 57% |
| WP_106690344.1 3-phosphoshikimate 1-carboxyvinyltransferase [M... | 468 | 3e-159 | 57% |
| MBC7136590.1 3-phosphoshikimate 1-carboxyvinyltransferase [Oce... | 468 | 3e-159 | 59% |
| WP_086464128.1 3-phosphoshikimate 1-carboxyvinyltransferase [O... | 468 | 3e-159 | 59% |
| WP_187791653.1 3-phosphoshikimate 1-carboxyvinyltransferase [P... | 467 | 3e-159 | 56% |
| WP_194952734.1 3-phosphoshikimate 1-carboxyvinyltransferase [S... | 467 | 3e-159 | 59% |
| PPD40785.1 3-phosphoshikimate 1-carboxyvinyltransferase [Methy... | 467 | 3e-159 | 59% |
| WP_090739919.1 3-phosphoshikimate 1-carboxyvinyltransferase [P... | 467 | 3e-159 | 55% |
| PKP88896.1 3-phosphoshikimate 1-carboxyvinyltransferase [Alpha... | 467 | 3e-159 | 59% |
| WP_148911705.1 3-phosphoshikimate 1-carboxyvinyltransferase [P... | 467 | 4e-159 | 56% |
| WP_156927670.1 3-phosphoshikimate 1-carboxyvinyltransferase [B... | 468 | 4e-159 | 57% |
| WP_126155260.1 3-phosphoshikimate 1-carboxyvinyltransferase [P... | 467 | 4e-159 | 57% |
| WP_020951194.1 3-phosphoshikimate 1-carboxyvinyltransferase [P... | 467 | 4e-159 | 56% |
| WP_130363940.1 3-phosphoshikimate 1-carboxyvinyltransferase [B... | 468 | 4e-159 | 57% |
| HCF16892.1 TPA: 3-phosphoshikimate 1-carboxyvinyltransferase [... | 467 | 4e-159 | 59% |
| KYG22913.1 3-phosphoshikimate 1-carboxyvinyltransferase [Brady... | 467 | 5e-159 | 57% |
| WP_183332690.1 3-phosphoshikimate 1-carboxyvinyltransferase [C... | 467 | 5e-159 | 59% |
| WP_184101109.1 3-phosphoshikimate 1-carboxyvinyltransferase [S... | 467 | 5e-159 | 59% |
| HBV55587.1 TPA: 3-phosphoshikimate 1-carboxyvinyltransferase [... | 467 | 5e-159 | 58% |
| WP_106000018.1 3-phosphoshikimate 1-carboxyvinyltransferase [S... | 467 | 5e-159 | 59% |
| WP_101251686.1 3-phosphoshikimate 1-carboxyvinyltransferase [T... | 467 | 5e-159 | 60% |
| WP_051112076.1 3-phosphoshikimate 1-carboxyvinyltransferase [H... | 466 | 6e-159 | 58% |
| WP_165406042.1 3-phosphoshikimate 1-carboxyvinyltransferase [B... | 467 | 6e-159 | 57% |
| WP_137101929.1 3-phosphoshikimate 1-carboxyvinyltransferase [P... | 466 | 6e-159 | 60% |
| WP_186467242.1 3-phosphoshikimate 1-carboxyvinyltransferase [B... | 467 | 6e-159 | 57% |
| WP_056380827.1 3-phosphoshikimate 1-carboxyvinyltransferase [S... | 466 | 6e-159 | 58% |
| WP_067617417.1 3-phosphoshikimate 1-carboxyvinyltransferase [N... | 467 | 6e-159 | 58% |
| TWB07090.1 3-phosphoshikimate 1-carboxyvinyltransferase [Brady... | 467 | 7e-159 | 57% |
| WP_099649112.1 3-phosphoshikimate 1-carboxyvinyltransferase [P... | 466 | 7e-159 | 57% |
| WP_011388048.1 3-phosphoshikimate 1-carboxyvinyltransferase [R... | 467 | 7e-159 | 59% |
| WP_069051599.1 3-phosphoshikimate 1-carboxyvinyltransferase [B... | 466 | 7e-159 | 57% |
| WP_038577061.1 3-phosphoshikimate 1-carboxyvinyltransferase [N... | 466 | 8e-159 | 58% |
| WP_027174258.1 3-phosphoshikimate 1-carboxyvinyltransferase [M... | 466 | 8e-159 | 60% |
| WP_129927008.1 3-phosphoshikimate 1-carboxyvinyltransferase [S... | 466 | 8e-159 | 59% |
| OHC96004.1 3-phosphoshikimate 1-carboxyvinyltransferase [Sphin... | 466 | 8e-159 | 59% |
| WP_134678815.1 3-phosphoshikimate 1-carboxyvinyltransferase [P... | 466 | 8e-159 | 57% |
| WP_126256966.1 3-phosphoshikimate 1-carboxyvinyltransferase [B... | 466 | 8e-159 | 57% |
| TMK33151.1 3-phosphoshikimate 1-carboxyvinyltransferase [Alpha... | 467 | 8e-159 | 59% |

| OQW51661.1 3-phosphoshikimate 1-carboxyvinyltransferase [Prote... | 466 | 8e-159 | 59% |
| --- | --- | --- | --- |
| WP_147219414.1 3-phosphoshikimate 1-carboxyvinyltransferase [N... | 466 | 9e-159 | 59% |
| WP_172980185.1 3-phosphoshikimate 1-carboxyvinyltransferase [T... | 466 | 9e-159 | 58% |
| WP_137827481.1 3-phosphoshikimate 1-carboxyvinyltransferase [M... | 466 | 9e-159 | 60% |
| WP_196271749.1 3-phosphoshikimate 1-carboxyvinyltransferase [M... | 466 | 9e-159 | 61% |
| WP_135170068.1 3-phosphoshikimate 1-carboxyvinyltransferase [B... | 466 | 1e-158 | 57% |
| WP_038972341.1 MULTISPECIES: 3-phosphoshikimate 1-carboxyvinyl... | 466 | 1e-158 | 57% |
| OYU14696.1 3-phosphoshikimate 1-carboxyvinyltransferase [Alpha... | 466 | 1e-158 | 57% |
| WP_114348627.1 3-phosphoshikimate 1-carboxyvinyltransferase [P... | 466 | 1e-158 | 57% |
| WP_042250393.1 3-phosphoshikimate 1-carboxyvinyltransferase [P... | 466 | 1e-158 | 56% |
| WP_056709693.1 3-phosphoshikimate 1-carboxyvinyltransferase [B... | 466 | 1e-158 | 59% |
| WP_170849631.1 3-phosphoshikimate 1-carboxyvinyltransferase [T... | 466 | 1e-158 | 58% |
| WP_091942277.1 3-phosphoshikimate 1-carboxyvinyltransferase [M... | 466 | 1e-158 | 60% |
| WP_171010043.1 3-phosphoshikimate 1-carboxyvinyltransferase [S... | 466 | 1e-158 | 58% |
| WP_061940042.1 3-phosphoshikimate 1-carboxyvinyltransferase [S... | 466 | 1e-158 | 58% |
| TMJ33177.1 3-phosphoshikimate 1-carboxyvinyltransferase [Alpha... | 466 | 1e-158 | 59% |
| WP_023921087.1 3-phosphoshikimate 1-carboxyvinyltransferase [R... | 466 | 1e-158 | 57% |
| AOF97576.1 3-phosphoshikimate 1-carboxyvinyltransferase [Sphin... | 466 | 1e-158 | 58% |
| WP_036740623.1 3-phosphoshikimate 1-carboxyvinyltransferase [P... | 466 | 1e-158 | 57% |
| WP_044662093.1 3-phosphoshikimate 1-carboxyvinyltransferase [S... | 466 | 1e-158 | 58% |
| WP_127690742.1 3-phosphoshikimate 1-carboxyvinyltransferase [S... | 466 | 1e-158 | 58% |
| MPT24229.1 3-phosphoshikimate 1-carboxyvinyltransferase [Stark... | 466 | 1e-158 | 60% |
| WP_122400172.1 3-phosphoshikimate 1-carboxyvinyltransferase [B... | 466 | 1e-158 | 57% |
| WP_131870515.1 MULTISPECIES: 3-phosphoshikimate 1-carboxyvinyl... | 466 | 1e-158 | 58% |
| KAA0077767.1 3-phosphoshikimate 1-carboxyvinyltransferase [Tar... | 466 | 1e-158 | 58% |
| TMJ52324.1 3-phosphoshikimate 1-carboxyvinyltransferase [Alpha... | 466 | 1e-158 | 59% |
| PZU50764.1 3-phosphoshikimate 1-carboxyvinyltransferase [Sphin... | 466 | 1e-158 | 59% |
| WP_071482003.1 MULTISPECIES: 3-phosphoshikimate 1-carboxyvinyl... | 466 | 1e-158 | 59% |
| WP_151645713.1 3-phosphoshikimate 1-carboxyvinyltransferase [B... | 466 | 1e-158 | 57% |
| WP_118052674.1 3-phosphoshikimate 1-carboxyvinyltransferase [A... | 465 | 1e-158 | 59% |
| TGV76805.1 3-phosphoshikimate 1-carboxyvinyltransferase [Mesor... | 459 | 2e-158 | 77% |
| WP_120498474.1 3-phosphoshikimate 1-carboxyvinyltransferase [K... | 465 | 2e-158 | 59% |
| WP_038950990.1 3-phosphoshikimate 1-carboxyvinyltransferase [B... | 466 | 2e-158 | 57% |
| TMJ92196.1 3-phosphoshikimate 1-carboxyvinyltransferase [Alpha... | 466 | 2e-158 | 57% |
| WP_088470955.1 3-phosphoshikimate 1-carboxyvinyltransferase [S... | 466 | 2e-158 | 58% |
| WP_095424882.1 3-phosphoshikimate 1-carboxyvinyltransferase [B... | 466 | 2e-158 | 57% |
| WP_076399591.1 3-phosphoshikimate 1-carboxyvinyltransferase [I... | 466 | 2e-158 | 58% |
| KUO57998.1 3-phosphoshikimate 1-carboxyvinyltransferase [Sphin... | 465 | 2e-158 | 59% |
| WP_155093638.1 3-phosphoshikimate 1-carboxyvinyltransferase [P... | 465 | 2e-158 | 57% |
| WP_008996815.1 3-phosphoshikimate 1-carboxyvinyltransferase [N... | 466 | 2e-158 | 59% |
| WP_170825577.1 MULTISPECIES: 3-phosphoshikimate 1-carboxyvinyl... | 465 | 2e-158 | 58% |
| GGN47341.1 3-phosphoshikimate 1-carboxyvinyltransferase [Novos... | 465 | 2e-158 | 58% |
| WP_121621319.1 3-phosphoshikimate 1-carboxyvinyltransferase [X... | 466 | 2e-158 | 60% |
| WP_135163605.1 3-phosphoshikimate 1-carboxyvinyltransferase [B... | 465 | 2e-158 | 57% |
| WP_154149591.1 3-phosphoshikimate 1-carboxyvinyltransferase [R... | 466 | 2e-158 | 58% |
| ORE95213.1 3-phosphoshikimate 1-carboxyvinyltransferase [Stapp... | 465 | 2e-158 | 59% |
| PZN92524.1 3-phosphoshikimate 1-carboxyvinyltransferase [Alpha... | 465 | 2e-158 | 61% |
| WP_014438739.1 3-phosphoshikimate 1-carboxyvinyltransferase [B... | 465 | 2e-158 | 58% |
| WP_103730090.1 3-phosphoshikimate 1-carboxyvinyltransferase [N... | 465 | 2e-158 | 58% |
| WP_085906538.1 3-phosphoshikimate 1-carboxyvinyltransferase [K... | 465 | 2e-158 | 59% |
| WP_008071416.1 3-phosphoshikimate 1-carboxyvinyltransferase [N... | 465 | 2e-158 | 59% |
| WP_023788739.1 3-phosphoshikimate 1-carboxyvinyltransferase [H... | 466 | 2e-158 | 59% |
| OHD04964.1 3-phosphoshikimate 1-carboxyvinyltransferase [Sphin... | 465 | 2e-158 | 59% |
| WP_061850547.1 3-phosphoshikimate 1-carboxyvinyltransferase [B... | 465 | 2e-158 | 57% |
| WP_028133603.1 3-phosphoshikimate 1-carboxyvinyltransferase [B... | 465 | 2e-158 | 57% |
| WP_148740990.1 3-phosphoshikimate 1-carboxyvinyltransferase [B... | 465 | 2e-158 | 57% |
| GGB94206.1 3-phosphoshikimate 1-carboxyvinyltransferase [Novos... | 465 | 2e-158 | 59% |
| WP_131833520.1 3-phosphoshikimate 1-carboxyvinyltransferase [A... | 465 | 3e-158 | 59% |
| WP_142849234.1 3-phosphoshikimate 1-carboxyvinyltransferase [T... | 465 | 3e-158 | 58% |
| WP_176482648.1 3-phosphoshikimate 1-carboxyvinyltransferase [S... | 464 | 3e-158 | 58% |
| WP_129142371.1 3-phosphoshikimate 1-carboxyvinyltransferase [B... | 465 | 3e-158 | 57% |
| WP_100178636.1 3-phosphoshikimate 1-carboxyvinyltransferase [B... | 465 | 3e-158 | 57% |
| OYU34857.1 3-phosphoshikimate 1-carboxyvinyltransferase [Novos... | 465 | 3e-158 | 58% |
| NBU13217.1 3-phosphoshikimate 1-carboxyvinyltransferase [Alpha... | 465 | 3e-158 | 62% |
| WP_100235220.1 3-phosphoshikimate 1-carboxyvinyltransferase [B... | 465 | 3e-158 | 57% |
| TMJ78338.1 3-phosphoshikimate 1-carboxyvinyltransferase [Alpha... | 465 | 3e-158 | 59% |
| WP_142637507.1 3-phosphoshikimate 1-carboxyvinyltransferase [S... | 465 | 3e-158 | 59% |
| WP_071470090.1 3-phosphoshikimate 1-carboxyvinyltransferase [R... | 465 | 3e-158 | 59% |
| NOZ65952.1 3-phosphoshikimate 1-carboxyvinyltransferase [Alpha... | 464 | 3e-158 | 57% |
| WP_128232588.1 3-phosphoshikimate 1-carboxyvinyltransferase [S... | 465 | 3e-158 | 59% |
| WP_167601783.1 3-phosphoshikimate 1-carboxyvinyltransferase [C... | 465 | 4e-158 | 57% |
| WP_104491853.1 3-phosphoshikimate 1-carboxyvinyltransferase [P... | 464 | 4e-158 | 56% |

| BBO09269.1 3-phosphoshikimate 1-carboxyvinyltransferase [Brady... | 465 | 4e-158 | 57% |
| --- | --- | --- | --- |
| WP_027576917.1 3-phosphoshikimate 1-carboxyvinyltransferase [B... | 464 | 4e-158 | 58% |
| WP_106750623.1 3-phosphoshikimate 1-carboxyvinyltransferase [P... | 464 | 4e-158 | 61% |
| MBB4208287.1 3-phosphoshikimate 1-carboxyvinyltransferase [Rho... | 466 | 4e-158 | 59% |
| WP_147076059.1 3-phosphoshikimate 1-carboxyvinyltransferase [M... | 464 | 4e-158 | 59% |
| NBS22827.1 3-phosphoshikimate 1-carboxyvinyltransferase [Alter... | 464 | 4e-158 | 59% |
| WP_097068104.1 3-phosphoshikimate 1-carboxyvinyltransferase [R... | 464 | 4e-158 | 57% |
| WP_067732857.1 3-phosphoshikimate 1-carboxyvinyltransferase [N... | 464 | 5e-158 | 58% |
| WP_092680573.1 3-phosphoshikimate 1-carboxyvinyltransferase [A... | 464 | 5e-158 | 59% |
| TCU70383.1 3-phosphoshikimate 1-carboxyvinyltransferase [Brady... | 465 | 5e-158 | 57% |
| WP_047762692.1 3-phosphoshikimate 1-carboxyvinyltransferase [K... | 464 | 5e-158 | 58% |
| WP_028180116.1 MULTISPECIES: 3-phosphoshikimate 1-carboxyvinyl... | 464 | 5e-158 | 57% |
| WP_188819204.1 3-phosphoshikimate 1-carboxyvinyltransferase [N... | 464 | 5e-158 | 58% |
| WP_148749071.1 3-phosphoshikimate 1-carboxyvinyltransferase [B... | 464 | 5e-158 | 57% |
| WP_072598188.1 3-phosphoshikimate 1-carboxyvinyltransferase [T... | 464 | 5e-158 | 59% |
| WP_175500306.1 3-phosphoshikimate 1-carboxyvinyltransferase [B... | 464 | 5e-158 | 58% |
| WP_028149795.1 3-phosphoshikimate 1-carboxyvinyltransferase [B... | 464 | 5e-158 | 58% |
| WP_067555060.1 3-phosphoshikimate 1-carboxyvinyltransferase [O... | 465 | 5e-158 | 58% |
| BCJ92131.1 3-phosphoshikimate 1-carboxyvinyltransferase [Rhizo... | 464 | 6e-158 | 58% |
| WP_188769252.1 3-phosphoshikimate 1-carboxyvinyltransferase [N... | 464 | 6e-158 | 60% |
| NVJ93233.1 3-phosphoshikimate 1-carboxyvinyltransferase [Methy... | 464 | 6e-158 | 56% |
| WP_157095642.1 3-phosphoshikimate 1-carboxyvinyltransferase [B... | 465 | 6e-158 | 57% |
| TAJ31234.1 3-phosphoshikimate 1-carboxyvinyltransferase [Bosea... | 464 | 6e-158 | 59% |
| WP_134496957.1 3-phosphoshikimate 1-carboxyvinyltransferase [M... | 464 | 6e-158 | 60% |
| NDB68237.1 3-phosphoshikimate 1-carboxyvinyltransferase [Methy... | 464 | 7e-158 | 57% |
| WP_183250082.1 3-phosphoshikimate 1-carboxyvinyltransferase [B... | 464 | 7e-158 | 57% |
| WP_123153430.1 3-phosphoshikimate 1-carboxyvinyltransferase [A... | 464 | 7e-158 | 58% |
| WP_179732574.1 MULTISPECIES: 3-phosphoshikimate 1-carboxyvinyl... | 464 | 7e-158 | 58% |
| WP_044436115.1 3-phosphoshikimate 1-carboxyvinyltransferase [S... | 464 | 7e-158 | 58% |
| WP_091962331.1 3-phosphoshikimate 1-carboxyvinyltransferase [B... | 464 | 7e-158 | 57% |
| WP_185678833.1 3-phosphoshikimate 1-carboxyvinyltransferase [N... | 464 | 7e-158 | 58% |
| NBQ38755.1 3-phosphoshikimate 1-carboxyvinyltransferase [Alpha... | 464 | 8e-158 | 60% |
| WP_040715081.1 3-phosphoshikimate 1-carboxyvinyltransferase [N... | 464 | 8e-158 | 60% |
| WP_156678099.1 3-phosphoshikimate 1-carboxyvinyltransferase [S... | 464 | 8e-158 | 58% |
| WP_036523275.1 3-phosphoshikimate 1-carboxyvinyltransferase [N... | 463 | 8e-158 | 58% |
| WP_136905498.1 3-phosphoshikimate 1-carboxyvinyltransferase [R... | 464 | 8e-158 | 57% |
| WP_165197711.1 3-phosphoshikimate 1-carboxyvinyltransferase [A... | 464 | 8e-158 | 59% |
| WP_173664377.1 MULTISPECIES: 3-phosphoshikimate 1-carboxyvinyl... | 463 | 8e-158 | 58% |
| WP_107750619.1 3-phosphoshikimate 1-carboxyvinyltransferase [P... | 464 | 8e-158 | 58% |
| WP_089177597.1 3-phosphoshikimate 1-carboxyvinyltransferase [B... | 464 | 9e-158 | 59% |
| MBS88029.1 3-phosphoshikimate 1-carboxyvinyltransferase [Sphin... | 464 | 9e-158 | 59% |
| MBD3788310.1 3-phosphoshikimate 1-carboxyvinyltransferase [Sph... | 464 | 9e-158 | 57% |
| WP_074965695.1 3-phosphoshikimate 1-carboxyvinyltransferase [P... | 464 | 9e-158 | 57% |
| MTJ84173.1 3-phosphoshikimate 1-carboxyvinyltransferase [Telma... | 464 | 9e-158 | 59% |
| WP_007678219.1 3-phosphoshikimate 1-carboxyvinyltransferase [N... | 464 | 9e-158 | 58% |
| WP_076440152.1 3-phosphoshikimate 1-carboxyvinyltransferase [B... | 464 | 9e-158 | 59% |
| WP_036040971.1 3-phosphoshikimate 1-carboxyvinyltransferase [B... | 464 | 1e-157 | 57% |
| RZM92641.1 3-phosphoshikimate 1-carboxyvinyltransferase [Brady... | 464 | 1e-157 | 57% |
| WP_128921182.1 3-phosphoshikimate 1-carboxyvinyltransferase [B... | 464 | 1e-157 | 57% |
| WP_152276924.1 3-phosphoshikimate 1-carboxyvinyltransferase [M... | 464 | 1e-157 | 60% |
| WP_124162402.1 3-phosphoshikimate 1-carboxyvinyltransferase [B... | 464 | 1e-157 | 57% |
| WP_142248635.1 3-phosphoshikimate 1-carboxyvinyltransferase [B... | 463 | 1e-157 | 57% |
| WP_008539993.1 3-phosphoshikimate 1-carboxyvinyltransferase [B... | 463 | 1e-157 | 57% |
| WP_036716752.1 3-phosphoshikimate 1-carboxyvinyltransferase [P... | 463 | 1e-157 | 58% |
| WP_011155632.1 3-phosphoshikimate 1-carboxyvinyltransferase [R... | 463 | 1e-157 | 59% |
| WP_069331566.1 3-phosphoshikimate 1-carboxyvinyltransferase [R... | 463 | 1e-157 | 59% |
| WP_036751442.1 3-phosphoshikimate 1-carboxyvinyltransferase [P... | 463 | 1e-157 | 57% |
| WP_027278945.1 3-phosphoshikimate 1-carboxyvinyltransferase [R... | 463 | 1e-157 | 58% |
| WP_145397259.1 3-phosphoshikimate 1-carboxyvinyltransferase [P... | 463 | 1e-157 | 57% |
| WP_069709105.1 3-phosphoshikimate 1-carboxyvinyltransferase [N... | 463 | 1e-157 | 58% |
| WP_165323827.1 3-phosphoshikimate 1-carboxyvinyltransferase [S... | 463 | 1e-157 | 58% |
| WP_047187715.1 3-phosphoshikimate 1-carboxyvinyltransferase [M... | 463 | 1e-157 | 60% |
| RDK94433.1 3-phosphoshikimate 1-carboxyvinyltransferase [Tardi... | 463 | 1e-157 | 58% |
| WP_011748513.1 3-phosphoshikimate 1-carboxyvinyltransferase [P... | 463 | 1e-157 | 56% |
| RTL53876.1 3-phosphoshikimate 1-carboxyvinyltransferase [Brady... | 463 | 1e-157 | 58% |
| WP_141935711.1 3-phosphoshikimate 1-carboxyvinyltransferase [B... | 463 | 1e-157 | 57% |
| WP_085405020.1 3-phosphoshikimate 1-carboxyvinyltransferase [B... | 463 | 1e-157 | 58% |
| WP_165826664.1 3-phosphoshikimate 1-carboxyvinyltransferase [T... | 463 | 1e-157 | 58% |
| PZQ54206.1 3-phosphoshikimate 1-carboxyvinyltransferase [Novos... | 463 | 1e-157 | 59% |
| WP_036009135.1 3-phosphoshikimate 1-carboxyvinyltransferase [B... | 463 | 1e-157 | 57% |
| WP_183259490.1 3-phosphoshikimate 1-carboxyvinyltransferase [B... | 463 | 1e-157 | 58% |
| WP_046506249.1 3-phosphoshikimate 1-carboxyvinyltransferase [K... | 463 | 1e-157 | 59% |

| GEK66894.1 3-phosphoshikimate 1-carboxyvinyltransferase [Parac... | 464 | 1e-157 | 56% |
| --- | --- | --- | --- |
| WP_097663331.1 3-phosphoshikimate 1-carboxyvinyltransferase [B... | 463 | 1e-157 | 57% |
| WP_025771913.1 MULTISPECIES: 3-phosphoshikimate 1-carboxyvinyl... | 463 | 1e-157 | 58% |
| WP_157285994.1 3-phosphoshikimate 1-carboxyvinyltransferase [B... | 463 | 2e-157 | 57% |
| WP_003600037.1 3-phosphoshikimate 1-carboxyvinyltransferase [M... | 463 | 2e-157 | 60% |
| NQZ13464.1 3-phosphoshikimate 1-carboxyvinyltransferase [Alpha... | 463 | 2e-157 | 57% |
| ODU23022.1 3-phosphoshikimate 1-carboxyvinyltransferase [Sphin... | 462 | 2e-157 | 59% |
| WP_067107501.1 3-phosphoshikimate 1-carboxyvinyltransferase [S... | 463 | 2e-157 | 59% |
| PNP98698.1 3-phosphoshikimate 1-carboxyvinyltransferase [Sphin... | 463 | 2e-157 | 58% |
| MPZ36830.1 3-phosphoshikimate 1-carboxyvinyltransferase [Rhizo... | 463 | 2e-157 | 58% |
| WP_086493449.1 3-phosphoshikimate 1-carboxyvinyltransferase [N... | 462 | 2e-157 | 59% |
| WP_192092274.1 3-phosphoshikimate 1-carboxyvinyltransferase [S... | 463 | 2e-157 | 58% |
| WP_063980174.1 MULTISPECIES: 3-phosphoshikimate 1-carboxyvinyl... | 463 | 2e-157 | 58% |
| WP_018000831.1 3-phosphoshikimate 1-carboxyvinyltransferase [P... | 463 | 2e-157 | 57% |
| WP_077029240.1 3-phosphoshikimate 1-carboxyvinyltransferase [S... | 463 | 2e-157 | 58% |
| WP_114828588.1 3-phosphoshikimate 1-carboxyvinyltransferase [B... | 463 | 2e-157 | 60% |
| WP_070158132.1 3-phosphoshikimate 1-carboxyvinyltransferase [S... | 463 | 2e-157 | 58% |
| WP_182915265.1 3-phosphoshikimate 1-carboxyvinyltransferase [R... | 463 | 2e-157 | 58% |
| WP_183238322.1 3-phosphoshikimate 1-carboxyvinyltransferase [B... | 463 | 2e-157 | 57% |
| WP_085907578.1 3-phosphoshikimate 1-carboxyvinyltransferase [K... | 462 | 2e-157 | 59% |
| NVK19191.1 3-phosphoshikimate 1-carboxyvinyltransferase [Methy... | 462 | 2e-157 | 58% |
| WP_121093978.1 3-phosphoshikimate 1-carboxyvinyltransferase [R... | 463 | 2e-157 | 58% |
| WP_100238519.1 3-phosphoshikimate 1-carboxyvinyltransferase [S... | 462 | 2e-157 | 57% |
| WP_037488078.1 3-phosphoshikimate 1-carboxyvinyltransferase [S... | 462 | 2e-157 | 59% |
| WP_135818293.1 3-phosphoshikimate 1-carboxyvinyltransferase [P... | 462 | 2e-157 | 56% |
| WP_161532846.1 3-phosphoshikimate 1-carboxyvinyltransferase [B... | 462 | 2e-157 | 57% |
| WP_128269602.1 3-phosphoshikimate 1-carboxyvinyltransferase [S... | 463 | 2e-157 | 58% |
| WP_093511513.1 3-phosphoshikimate 1-carboxyvinyltransferase [S... | 462 | 2e-157 | 57% |
| EZP84457.1 3-phosphoshikimate 1-carboxyvinyltransferase [Novos... | 462 | 2e-157 | 58% |
| WP_169573261.1 3-phosphoshikimate 1-carboxyvinyltransferase [S... | 462 | 2e-157 | 58% |
| WP_115031979.1 3-phosphoshikimate 1-carboxyvinyltransferase [T... | 463 | 2e-157 | 58% |
| WP_037235355.1 3-phosphoshikimate 1-carboxyvinyltransferase [R... | 463 | 2e-157 | 57% |
| WP_011339439.1 3-phosphoshikimate 1-carboxyvinyltransferase [R... | 462 | 2e-157 | 58% |
| WP_110781157.1 3-phosphoshikimate 1-carboxyvinyltransferase [R... | 462 | 2e-157 | 58% |
| MAS42375.1 3-phosphoshikimate 1-carboxyvinyltransferase [Rhodo... | 463 | 2e-157 | 58% |
| TXG84749.1 3-phosphoshikimate 1-carboxyvinyltransferase [Sphin... | 462 | 2e-157 | 57% |
| WP_015740219.1 3-phosphoshikimate 1-carboxyvinyltransferase [Z... | 463 | 2e-157 | 56% |
| WP_127075928.1 3-phosphoshikimate 1-carboxyvinyltransferase [R... | 463 | 3e-157 | 56% |
| WP_142084538.1 3-phosphoshikimate 1-carboxyvinyltransferase [R... | 462 | 3e-157 | 58% |
| WP_128237884.1 3-phosphoshikimate 1-carboxyvinyltransferase [S... | 462 | 3e-157 | 58% |
| WP_024022182.1 3-phosphoshikimate 1-carboxyvinyltransferase [S... | 462 | 3e-157 | 57% |
| WP_157216461.1 3-phosphoshikimate 1-carboxyvinyltransferase [S... | 462 | 3e-157 | 60% |
| MBA4091099.1 3-phosphoshikimate 1-carboxyvinyltransferase [Sph... | 462 | 3e-157 | 57% |
| WP_115754239.1 3-phosphoshikimate 1-carboxyvinyltransferase [P... | 462 | 3e-157 | 57% |
| WP_008944027.1 3-phosphoshikimate 1-carboxyvinyltransferase [O... | 462 | 3e-157 | 58% |
| MBA3940452.1 3-phosphoshikimate 1-carboxyvinyltransferase [Sph... | 463 | 3e-157 | 56% |
| WP_108512703.1 3-phosphoshikimate 1-carboxyvinyltransferase [B... | 462 | 3e-157 | 57% |
| WP_166939611.1 3-phosphoshikimate 1-carboxyvinyltransferase [S... | 462 | 3e-157 | 58% |
| WP_164604651.1 3-phosphoshikimate 1-carboxyvinyltransferase [R... | 462 | 3e-157 | 59% |
| WP_085902304.1 3-phosphoshikimate 1-carboxyvinyltransferase [K... | 462 | 4e-157 | 59% |
| WP_184512586.1 3-phosphoshikimate 1-carboxyvinyltransferase [T... | 462 | 4e-157 | 58% |
| RBP95644.1 3-phosphoshikimate 1-carboxyvinyltransferase [Rhodo... | 462 | 4e-157 | 58% |
| MAZ76262.1 3-phosphoshikimate 1-carboxyvinyltransferase [Micav... | 462 | 4e-157 | 58% |
| WP_129609299.1 3-phosphoshikimate 1-carboxyvinyltransferase [R... | 462 | 4e-157 | 60% |
| WP_094976065.1 3-phosphoshikimate 1-carboxyvinyltransferase [B... | 462 | 4e-157 | 57% |
| GFE72417.1 3-phosphoshikimate 1-carboxyvinyltransferase [Novos... | 462 | 4e-157 | 59% |
| WP_114534865.1 3-phosphoshikimate 1-carboxyvinyltransferase [P... | 462 | 4e-157 | 57% |
| WP_068299620.1 3-phosphoshikimate 1-carboxyvinyltransferase [P... | 462 | 4e-157 | 57% |
| WP_188663067.1 3-phosphoshikimate 1-carboxyvinyltransferase [T... | 462 | 4e-157 | 57% |
| KQO89096.1 3-phosphoshikimate 1-carboxyvinyltransferase [Methy... | 462 | 4e-157 | 60% |
| WP_017667316.1 3-phosphoshikimate 1-carboxyvinyltransferase [S... | 462 | 4e-157 | 60% |
| WP_175310701.1 3-phosphoshikimate 1-carboxyvinyltransferase [S... | 462 | 4e-157 | 57% |
| WP_014501016.1 3-phosphoshikimate 1-carboxyvinyltransferase [Z... | 462 | 4e-157 | 56% |
| PZN85980.1 3-phosphoshikimate 1-carboxyvinyltransferase [Prote... | 462 | 4e-157 | 60% |
| WP_183983039.1 3-phosphoshikimate 1-carboxyvinyltransferase [S... | 462 | 5e-157 | 58% |
| WP_072461151.1 3-phosphoshikimate 1-carboxyvinyltransferase [P... | 462 | 5e-157 | 57% |
| WP_018323432.1 3-phosphoshikimate 1-carboxyvinyltransferase [B... | 462 | 5e-157 | 57% |
| WP_046192749.1 3-phosphoshikimate 1-carboxyvinyltransferase [S... | 462 | 5e-157 | 57% |
| WP_159828200.1 3-phosphoshikimate 1-carboxyvinyltransferase [N... | 462 | 5e-157 | 59% |
| WP_125726367.1 3-phosphoshikimate 1-carboxyvinyltransferase [S... | 462 | 5e-157 | 58% |
| SFK53398.1 3-phosphoshikimate 1-carboxyvinyltransferase [Methy... | 464 | 5e-157 | 60% |
| WP_021223078.1 MULTISPECIES: 3-phosphoshikimate 1-carboxyvinyl... | 462 | 5e-157 | 57% |

| WP_111343798.1 3-phosphoshikimate 1-carboxyvinyltransferase [A... | 462 | 5e-157 | 60% |
| --- | --- | --- | --- |
| WP_166063843.1 3-phosphoshikimate 1-carboxyvinyltransferase [B... | 462 | 5e-157 | 58% |
| TAH68816.1 3-phosphoshikimate 1-carboxyvinyltransferase [Rhodo... | 462 | 5e-157 | 58% |
| WP_038072928.1 3-phosphoshikimate 1-carboxyvinyltransferase [T... | 462 | 5e-157 | 56% |
| WP_177234855.1 3-phosphoshikimate 1-carboxyvinyltransferase [B... | 462 | 5e-157 | 57% |
| WP_040307946.1 3-phosphoshikimate 1-carboxyvinyltransferase [A... | 462 | 5e-157 | 59% |
| WP_177243568.1 3-phosphoshikimate 1-carboxyvinyltransferase [B... | 462 | 5e-157 | 57% |
| OJY41141.1 3-phosphoshikimate 1-carboxyvinyltransferase [Rhizo... | 462 | 5e-157 | 58% |
| WP_076622116.1 MULTISPECIES: 3-phosphoshikimate 1-carboxyvinyl... | 462 | 5e-157 | 59% |
| WP_027561581.1 MULTISPECIES: 3-phosphoshikimate 1-carboxyvinyl... | 462 | 5e-157 | 56% |
| NNE87737.1 3-phosphoshikimate 1-carboxyvinyltransferase [Silic... | 462 | 6e-157 | 59% |
| WP_164637370.1 3-phosphoshikimate 1-carboxyvinyltransferase [R... | 462 | 6e-157 | 59% |
| WP_147160709.1 3-phosphoshikimate 1-carboxyvinyltransferase [N... | 462 | 6e-157 | 58% |
| WP_025550490.1 3-phosphoshikimate 1-carboxyvinyltransferase [S... | 462 | 6e-157 | 58% |
| WP_096430906.1 3-phosphoshikimate 1-carboxyvinyltransferase [T... | 462 | 6e-157 | 57% |
| WP_171899301.1 3-phosphoshikimate 1-carboxyvinyltransferase [S... | 461 | 6e-157 | 58% |
| WP_116223276.1 MULTISPECIES: 3-phosphoshikimate 1-carboxyvinyl... | 461 | 6e-157 | 57% |
| WP_110156976.1 3-phosphoshikimate 1-carboxyvinyltransferase [N... | 461 | 6e-157 | 59% |
| WP_152587178.1 3-phosphoshikimate 1-carboxyvinyltransferase [M... | 462 | 6e-157 | 62% |
| WP_012493896.1 3-phosphoshikimate 1-carboxyvinyltransferase [R... | 461 | 6e-157 | 59% |
| WP_066602939.1 3-phosphoshikimate 1-carboxyvinyltransferase [S... | 461 | 6e-157 | 58% |
| WP_160592653.1 3-phosphoshikimate 1-carboxyvinyltransferase [S... | 461 | 6e-157 | 59% |
| WP_057027883.1 3-phosphoshikimate 1-carboxyvinyltransferase [B... | 461 | 6e-157 | 57% |
| WP_171745366.1 3-phosphoshikimate 1-carboxyvinyltransferase [S... | 461 | 6e-157 | 59% |
| WP_060909562.1 3-phosphoshikimate 1-carboxyvinyltransferase [B... | 461 | 6e-157 | 57% |
| WP_002713808.1 3-phosphoshikimate 1-carboxyvinyltransferase [A... | 461 | 6e-157 | 57% |
| WP_058746242.1 3-phosphoshikimate 1-carboxyvinyltransferase [S... | 461 | 7e-157 | 57% |
| TMV92373.1 3-phosphoshikimate 1-carboxyvinyltransferase [Thioc... | 461 | 7e-157 | 56% |
| PZO65945.1 3-phosphoshikimate 1-carboxyvinyltransferase [Parac... | 461 | 7e-157 | 56% |
| WP_121534746.1 3-phosphoshikimate 1-carboxyvinyltransferase [S... | 461 | 7e-157 | 58% |
| WP_173676449.1 MULTISPECIES: 3-phosphoshikimate 1-carboxyvinyl... | 461 | 7e-157 | 58% |
| WP_101327728.1 3-phosphoshikimate 1-carboxyvinyltransferase [R... | 461 | 7e-157 | 58% |
| WP_046762774.1 3-phosphoshikimate 1-carboxyvinyltransferase [S... | 461 | 7e-157 | 57% |
| WP_092027486.1 3-phosphoshikimate 1-carboxyvinyltransferase [B... | 461 | 7e-157 | 57% |
| WP_119018854.1 3-phosphoshikimate 1-carboxyvinyltransferase [R... | 461 | 8e-157 | 59% |
| TQM93757.1 3-phosphoshikimate 1-carboxyvinyltransferase [Rosei... | 462 | 8e-157 | 58% |
| WP_038965151.1 3-phosphoshikimate 1-carboxyvinyltransferase [B... | 461 | 8e-157 | 57% |
| WP_011241530.1 3-phosphoshikimate 1-carboxyvinyltransferase [Z... | 461 | 9e-157 | 56% |
| WP_041670501.1 3-phosphoshikimate 1-carboxyvinyltransferase [R... | 461 | 9e-157 | 58% |
| WP_123690072.1 3-phosphoshikimate 1-carboxyvinyltransferase [S... | 461 | 9e-157 | 59% |
| WP_193377937.1 3-phosphoshikimate 1-carboxyvinyltransferase [S... | 461 | 9e-157 | 57% |
| WP_107353703.1 3-phosphoshikimate 1-carboxyvinyltransferase [R... | 461 | 9e-157 | 59% |
| EPR14491.1 3-phosphoshikimate 1-carboxyvinyltransferase [Sphin... | 461 | 9e-157 | 57% |
| WP_024338603.1 MULTISPECIES: 3-phosphoshikimate 1-carboxyvinyl... | 461 | 1e-156 | 56% |
| RKE51899.1 3-phosphoshikimate 1-carboxyvinyltransferase [Tardi... | 461 | 1e-156 | 58% |
| WP_011439544.1 3-phosphoshikimate 1-carboxyvinyltransferase [R... | 461 | 1e-156 | 57% |
| WP_014415453.1 3-phosphoshikimate 1-carboxyvinyltransferase [P... | 461 | 1e-156 | 59% |
| CCW15849.1 5-Enolpyruvylshikimate-3-phosphate synthase [Sphing... | 461 | 1e-156 | 59% |
| WP_164571889.1 3-phosphoshikimate 1-carboxyvinyltransferase [R... | 461 | 1e-156 | 59% |
| WP_023593619.1 3-phosphoshikimate 1-carboxyvinyltransferase [Z... | 461 | 1e-156 | 56% |
| WP_116092202.1 3-phosphoshikimate 1-carboxyvinyltransferase [S... | 461 | 1e-156 | 58% |
| WP_128150383.1 3-phosphoshikimate 1-carboxyvinyltransferase [S... | 461 | 1e-156 | 58% |
| WP_067184269.1 MULTISPECIES: 3-phosphoshikimate 1-carboxyvinyl... | 461 | 1e-156 | 58% |
| WP_013418594.1 3-phosphoshikimate 1-carboxyvinyltransferase [R... | 461 | 1e-156 | 57% |
| WP_056507322.1 3-phosphoshikimate 1-carboxyvinyltransferase [S... | 461 | 1e-156 | 58% |
| WP_119805555.1 3-phosphoshikimate 1-carboxyvinyltransferase [P... | 461 | 1e-156 | 57% |
| WP_118999439.1 3-phosphoshikimate 1-carboxyvinyltransferase [R... | 461 | 1e-156 | 58% |
| WP_177236840.1 MULTISPECIES: 3-phosphoshikimate 1-carboxyvinyl... | 461 | 1e-156 | 57% |
| WP_160986073.1 3-phosphoshikimate 1-carboxyvinyltransferase [N... | 461 | 1e-156 | 59% |
| WP_172451905.1 3-phosphoshikimate 1-carboxyvinyltransferase [S... | 461 | 1e-156 | 58% |
| WP_035966985.1 3-phosphoshikimate 1-carboxyvinyltransferase [B... | 461 | 1e-156 | 57% |
| WP_011661897.1 3-phosphoshikimate 1-carboxyvinyltransferase [R... | 461 | 1e-156 | 58% |
| WP_026002309.1 3-phosphoshikimate 1-carboxyvinyltransferase [S... | 461 | 1e-156 | 58% |
| WP_091973892.1 3-phosphoshikimate 1-carboxyvinyltransferase [M... | 461 | 1e-156 | 61% |
| WP_012561643.1 3-phosphoshikimate 1-carboxyvinyltransferase [O... | 461 | 1e-156 | 58% |
| BAI96985.1 3-phosphoshikimate 1-carboxyvinyltransferase [Sphin... | 461 | 1e-156 | 57% |
| WP_183952208.1 3-phosphoshikimate 1-carboxyvinyltransferase [S... | 461 | 1e-156 | 57% |
| WP_124255390.1 3-phosphoshikimate 1-carboxyvinyltransferase [R... | 461 | 1e-156 | 58% |
| WP_173948477.1 3-phosphoshikimate 1-carboxyvinyltransferase [M... | 461 | 1e-156 | 61% |
| MBC7578873.1 3-phosphoshikimate 1-carboxyvinyltransferase [Tar... | 461 | 1e-156 | 58% |
| WP_110786768.1 3-phosphoshikimate 1-carboxyvinyltransferase [R... | 461 | 1e-156 | 59% |
| WP_078528210.1 3-phosphoshikimate 1-carboxyvinyltransferase [T... | 461 | 1e-156 | 57% |

| WP_140481737.1 3-phosphoshikimate 1-carboxyvinyltransferase [B... | 461 | 1e-156 | 57% |
| --- | --- | --- | --- |
| TIU64215.1 3-phosphoshikimate 1-carboxyvinyltransferase [Mesor... | 455 | 1e-156 | 81% |
| WP_135312748.1 3-phosphoshikimate 1-carboxyvinyltransferase [P... | 461 | 1e-156 | 55% |
| WP_062563316.1 3-phosphoshikimate 1-carboxyvinyltransferase [P... | 461 | 1e-156 | 57% |
| WP_028172491.1 3-phosphoshikimate 1-carboxyvinyltransferase [B... | 461 | 1e-156 | 57% |
| SCA56118.1 3-phosphoshikimate 1-carboxyvinyltransferase [Candi... | 461 | 1e-156 | 56% |
| WP_018645074.1 3-phosphoshikimate 1-carboxyvinyltransferase [B... | 461 | 1e-156 | 56% |
| WP_164934836.1 3-phosphoshikimate 1-carboxyvinyltransferase [B... | 461 | 1e-156 | 57% |
| WP_167533290.1 3-phosphoshikimate 1-carboxyvinyltransferase [B... | 461 | 1e-156 | 57% |
| WP_110805938.1 3-phosphoshikimate 1-carboxyvinyltransferase [R... | 461 | 1e-156 | 57% |
| WP_115475859.1 3-phosphoshikimate 1-carboxyvinyltransferase [R... | 461 | 1e-156 | 58% |
| TWE53678.1 3-phosphoshikimate 1-carboxyvinyltransferase [Novos... | 460 | 1e-156 | 60% |
| EKS30100.1 3-phosphoshikimate 1-carboxyvinyltransferase [Afipi... | 461 | 1e-156 | 60% |
| WP_185665693.1 3-phosphoshikimate 1-carboxyvinyltransferase [N... | 460 | 1e-156 | 59% |
| WP_156947132.1 3-phosphoshikimate 1-carboxyvinyltransferase [B... | 461 | 1e-156 | 57% |
| WP_128182701.1 3-phosphoshikimate 1-carboxyvinyltransferase [S... | 461 | 2e-156 | 58% |
| WP_011842229.1 3-phosphoshikimate 1-carboxyvinyltransferase [R... | 461 | 2e-156 | 58% |
| WP_038065270.1 3-phosphoshikimate 1-carboxyvinyltransferase [T... | 461 | 2e-156 | 56% |
| WP_028155444.1 3-phosphoshikimate 1-carboxyvinyltransferase [B... | 461 | 2e-156 | 57% |
| WP_167520348.1 3-phosphoshikimate 1-carboxyvinyltransferase [B... | 461 | 2e-156 | 57% |
| WP_166811772.1 3-phosphoshikimate 1-carboxyvinyltransferase [B... | 461 | 2e-156 | 57% |
| WP_109952126.1 3-phosphoshikimate 1-carboxyvinyltransferase [M... | 461 | 2e-156 | 60% |
| WP_184105336.1 3-phosphoshikimate 1-carboxyvinyltransferase [S... | 460 | 2e-156 | 58% |
| WP_089257017.1 3-phosphoshikimate 1-carboxyvinyltransferase [R... | 461 | 2e-156 | 58% |
| WP_142884063.1 3-phosphoshikimate 1-carboxyvinyltransferase [R... | 461 | 2e-156 | 59% |
| WP_149765186.1 3-phosphoshikimate 1-carboxyvinyltransferase [P... | 460 | 2e-156 | 57% |
| WP_061781415.1 3-phosphoshikimate 1-carboxyvinyltransferase [S... | 460 | 2e-156 | 57% |
| WP_124257447.1 3-phosphoshikimate 1-carboxyvinyltransferase [R... | 461 | 2e-156 | 58% |
| WP_136174148.1 3-phosphoshikimate 1-carboxyvinyltransferase [S... | 460 | 2e-156 | 58% |
| MBA4754723.1 3-phosphoshikimate 1-carboxyvinyltransferase [Sph... | 460 | 2e-156 | 59% |
| WP_076715512.1 MULTISPECIES: 3-phosphoshikimate 1-carboxyvinyl... | 460 | 2e-156 | 57% |
| WP_034441284.1 3-phosphoshikimate 1-carboxyvinyltransferase [C... | 460 | 2e-156 | 56% |
| WP_104465331.1 3-phosphoshikimate 1-carboxyvinyltransferase [B... | 460 | 2e-156 | 57% |
| WP_129270366.1 3-phosphoshikimate 1-carboxyvinyltransferase [B... | 460 | 2e-156 | 57% |
| WP_055848761.1 3-phosphoshikimate 1-carboxyvinyltransferase [S... | 460 | 2e-156 | 59% |
| RYX99530.1 3-phosphoshikimate 1-carboxyvinyltransferase [Brady... | 461 | 2e-156 | 57% |
| WP_143842651.1 3-phosphoshikimate 1-carboxyvinyltransferase [B... | 460 | 2e-156 | 57% |
| WP_111384676.1 3-phosphoshikimate 1-carboxyvinyltransferase [R... | 460 | 2e-156 | 60% |
| TVP98400.1 3-phosphoshikimate 1-carboxyvinyltransferase [Rosei... | 461 | 2e-156 | 58% |
| OYU48958.1 3-phosphoshikimate 1-carboxyvinyltransferase [Rhizo... | 460 | 2e-156 | 58% |
| WP_103719139.1 3-phosphoshikimate 1-carboxyvinyltransferase [B... | 460 | 2e-156 | 59% |
| WP_078573277.1 3-phosphoshikimate 1-carboxyvinyltransferase [T... | 461 | 2e-156 | 57% |
| WP_097668266.1 3-phosphoshikimate 1-carboxyvinyltransferase [B... | 460 | 2e-156 | 57% |
| WP_019052008.1 3-phosphoshikimate 1-carboxyvinyltransferase [S... | 460 | 2e-156 | 59% |
| QHP67091.1 3-phosphoshikimate 1-carboxyvinyltransferase [Brady... | 461 | 2e-156 | 56% |
| WP_039155160.1 3-phosphoshikimate 1-carboxyvinyltransferase [B... | 460 | 2e-156 | 57% |
| WP_066717701.1 MULTISPECIES: 3-phosphoshikimate 1-carboxyvinyl... | 460 | 2e-156 | 60% |
| WP_167671936.1 3-phosphoshikimate 1-carboxyvinyltransferase [M... | 461 | 2e-156 | 60% |
| GHC92952.1 3-phosphoshikimate 1-carboxyvinyltransferase [Novos... | 460 | 2e-156 | 57% |
| WP_018631480.1 3-phosphoshikimate 1-carboxyvinyltransferase [N... | 460 | 2e-156 | 56% |
| WP_106948807.1 3-phosphoshikimate 1-carboxyvinyltransferase [B... | 460 | 2e-156 | 57% |
| WP_163639424.1 3-phosphoshikimate 1-carboxyvinyltransferase [M... | 460 | 2e-156 | 61% |
| TVR46436.1 3-phosphoshikimate 1-carboxyvinyltransferase [Rhodo... | 460 | 2e-156 | 57% |
| WP_061023493.1 3-phosphoshikimate 1-carboxyvinyltransferase [B... | 460 | 2e-156 | 57% |
| WP_024576709.1 MULTISPECIES: 3-phosphoshikimate 1-carboxyvinyl... | 460 | 2e-156 | 57% |
| WP_160477589.1 3-phosphoshikimate 1-carboxyvinyltransferase [R... | 460 | 2e-156 | 58% |
| EJN10251.1 3-phosphoshikimate 1-carboxyvinyltransferase [Brady... | 461 | 2e-156 | 57% |
| RJF76043.1 3-phosphoshikimate 1-carboxyvinyltransferase [Rhodo... | 461 | 2e-156 | 58% |
| WP_164939837.1 3-phosphoshikimate 1-carboxyvinyltransferase [B... | 460 | 2e-156 | 57% |
| WP_055786333.1 MULTISPECIES: 3-phosphoshikimate 1-carboxyvinyl... | 460 | 2e-156 | 57% |
| WP_101461168.1 3-phosphoshikimate 1-carboxyvinyltransferase [P... | 460 | 2e-156 | 56% |
| WP_159011191.1 3-phosphoshikimate 1-carboxyvinyltransferase [B... | 460 | 2e-156 | 57% |
| WP_166467646.1 3-phosphoshikimate 1-carboxyvinyltransferase [B... | 460 | 2e-156 | 57% |
| WP_112331048.1 3-phosphoshikimate 1-carboxyvinyltransferase [R... | 460 | 2e-156 | 58% |
| WP_028139471.1 MULTISPECIES: 3-phosphoshikimate 1-carboxyvinyl... | 460 | 2e-156 | 57% |
| PIW29394.1 3-phosphoshikimate 1-carboxyvinyltransferase [Rhodo... | 460 | 3e-156 | 61% |
| PCJ33379.1 3-phosphoshikimate 1-carboxyvinyltransferase [Alpha... | 460 | 3e-156 | 56% |
| THD45837.1 3-phosphoshikimate 1-carboxyvinyltransferase [Brady... | 460 | 3e-156 | 62% |
| WP_089388287.1 3-phosphoshikimate 1-carboxyvinyltransferase [P... | 460 | 3e-156 | 56% |
| WP_002724552.1 3-phosphoshikimate 1-carboxyvinyltransferase [R... | 460 | 3e-156 | 58% |
| WP_069186803.1 3-phosphoshikimate 1-carboxyvinyltransferase [C... | 460 | 3e-156 | 56% |
| WP_109145545.1 3-phosphoshikimate 1-carboxyvinyltransferase [B... | 460 | 3e-156 | 57% |

| WP_161352967.1 3-phosphoshikimate 1-carboxyvinyltransferase [M... | 460 | 3e-156 | 59% |
| --- | --- | --- | --- |
| WP_013499909.1 3-phosphoshikimate 1-carboxyvinyltransferase [R... | 460 | 3e-156 | 59% |
| WP_080134050.1 3-phosphoshikimate 1-carboxyvinyltransferase [B... | 460 | 3e-156 | 57% |
| WP_038932748.1 3-phosphoshikimate 1-carboxyvinyltransferase [B... | 460 | 3e-156 | 57% |
| WP_171712786.1 3-phosphoshikimate 1-carboxyvinyltransferase [B... | 460 | 3e-156 | 57% |
| WP_054121785.1 3-phosphoshikimate 1-carboxyvinyltransferase [N... | 460 | 3e-156 | 57% |
| WP_035704204.1 3-phosphoshikimate 1-carboxyvinyltransferase [B... | 460 | 3e-156 | 57% |
| OYW47458.1 3-phosphoshikimate 1-carboxyvinyltransferase [Novos... | 459 | 3e-156 | 58% |
| WP_128157279.1 3-phosphoshikimate 1-carboxyvinyltransferase [S... | 460 | 3e-156 | 58% |
| WP_014490917.1 MULTISPECIES: 3-phosphoshikimate 1-carboxyvinyl... | 460 | 3e-156 | 57% |
| WP_166476620.1 3-phosphoshikimate 1-carboxyvinyltransferase [B... | 460 | 3e-156 | 57% |
| WP_074121866.1 3-phosphoshikimate 1-carboxyvinyltransferase [B... | 460 | 3e-156 | 56% |
| WP_147709184.1 3-phosphoshikimate 1-carboxyvinyltransferase [M... | 460 | 3e-156 | 60% |
| WP_040878438.1 3-phosphoshikimate 1-carboxyvinyltransferase [K... | 459 | 3e-156 | 57% |
| KQC49413.1 3-phosphoshikimate 1-carboxyvinyltransferase [Candi... | 460 | 3e-156 | 56% |
| WP_026261194.1 3-phosphoshikimate 1-carboxyvinyltransferase [K... | 459 | 3e-156 | 59% |
| WP_072383854.1 3-phosphoshikimate 1-carboxyvinyltransferase [N... | 460 | 3e-156 | 57% |
| NMM44769.1 3-phosphoshikimate 1-carboxyvinyltransferase [Rhodo... | 460 | 4e-156 | 58% |
| RTL75191.1 3-phosphoshikimate 1-carboxyvinyltransferase [Brady... | 459 | 4e-156 | 59% |
| GGZ02336.1 3-phosphoshikimate 1-carboxyvinyltransferase [Novos... | 460 | 4e-156 | 57% |
| WP_191324240.1 3-phosphoshikimate 1-carboxyvinyltransferase [N... | 459 | 4e-156 | 57% |
| WP_056002888.1 3-phosphoshikimate 1-carboxyvinyltransferase [S... | 459 | 4e-156 | 57% |
| WP_107342334.1 3-phosphoshikimate 1-carboxyvinyltransferase [R... | 459 | 4e-156 | 58% |
| MBA4097574.1 3-phosphoshikimate 1-carboxyvinyltransferase [Rho... | 459 | 4e-156 | 58% |
| WP_119003735.1 3-phosphoshikimate 1-carboxyvinyltransferase [R... | 459 | 4e-156 | 58% |
| WP_160884154.1 3-phosphoshikimate 1-carboxyvinyltransferase [M... | 459 | 4e-156 | 60% |
| WP_154336730.1 3-phosphoshikimate 1-carboxyvinyltransferase [P... | 459 | 4e-156 | 58% |
| NCO03870.1 3-phosphoshikimate 1-carboxyvinyltransferase [Alpha... | 459 | 4e-156 | 58% |
| RTM08224.1 3-phosphoshikimate 1-carboxyvinyltransferase [Brady... | 459 | 4e-156 | 57% |
| WP_037447827.1 3-phosphoshikimate 1-carboxyvinyltransferase [S... | 459 | 4e-156 | 59% |
| HBM89721.1 TPA: 3-phosphoshikimate 1-carboxyvinyltransferase [... | 459 | 4e-156 | 57% |
| GHA04039.1 3-phosphoshikimate 1-carboxyvinyltransferase [Novos... | 459 | 4e-156 | 60% |
| NYJ39998.1 3-phosphoshikimate 1-carboxyvinyltransferase [Novos... | 459 | 4e-156 | 59% |
| WP_137459253.1 3-phosphoshikimate 1-carboxyvinyltransferase [R... | 459 | 4e-156 | 58% |
| WP_137388680.1 3-phosphoshikimate 1-carboxyvinyltransferase [R... | 459 | 4e-156 | 58% |
| WP_092172993.1 3-phosphoshikimate 1-carboxyvinyltransferase [B... | 459 | 4e-156 | 60% |
| SFJ74457.1 3-phosphoshikimate 1-carboxyvinyltransferase [Brady... | 460 | 5e-156 | 57% |
| WP_128265128.1 3-phosphoshikimate 1-carboxyvinyltransferase [B... | 459 | 5e-156 | 57% |
| WP_158670101.1 3-phosphoshikimate 1-carboxyvinyltransferase [B... | 459 | 5e-156 | 57% |
| WP_189542313.1 3-phosphoshikimate 1-carboxyvinyltransferase [N... | 459 | 5e-156 | 60% |
| WP_143199440.1 3-phosphoshikimate 1-carboxyvinyltransferase [B... | 459 | 5e-156 | 56% |
| WP_189620799.1 3-phosphoshikimate 1-carboxyvinyltransferase [N... | 459 | 5e-156 | 57% |
| WP_038954456.1 3-phosphoshikimate 1-carboxyvinyltransferase [B... | 459 | 5e-156 | 57% |
| WP_019199349.1 MULTISPECIES: 3-phosphoshikimate 1-carboxyvinyl... | 459 | 5e-156 | 58% |
| WP_120287730.1 3-phosphoshikimate 1-carboxyvinyltransferase [T... | 460 | 5e-156 | 58% |
| WP_047307655.1 3-phosphoshikimate 1-carboxyvinyltransferase [R... | 459 | 5e-156 | 59% |
| WP_104512393.1 3-phosphoshikimate 1-carboxyvinyltransferase [R... | 459 | 5e-156 | 59% |
| WP_100094011.1 3-phosphoshikimate 1-carboxyvinyltransferase [S... | 459 | 5e-156 | 56% |
| WP_079649747.1 3-phosphoshikimate 1-carboxyvinyltransferase [S... | 459 | 5e-156 | 58% |
| SEM97074.1 3-phosphoshikimate 1-carboxyvinyltransferase [Brady... | 460 | 5e-156 | 56% |
| WP_066133078.1 3-phosphoshikimate 1-carboxyvinyltransferase [H... | 459 | 5e-156 | 56% |
| SFJ69291.1 3-phosphoshikimate 1-carboxyvinyltransferase [Brady... | 460 | 5e-156 | 57% |
| WP_037521746.1 3-phosphoshikimate 1-carboxyvinyltransferase [S... | 459 | 5e-156 | 58% |
| WP_041041977.1 3-phosphoshikimate 1-carboxyvinyltransferase [M... | 459 | 5e-156 | 58% |
| WP_166347343.1 3-phosphoshikimate 1-carboxyvinyltransferase [B... | 459 | 5e-156 | 57% |
| WP_028639980.1 3-phosphoshikimate 1-carboxyvinyltransferase [N... | 459 | 5e-156 | 57% |
| WP_150943717.1 3-phosphoshikimate 1-carboxyvinyltransferase [M... | 459 | 6e-156 | 61% |
| WP_085837351.1 3-phosphoshikimate 1-carboxyvinyltransferase [A... | 459 | 6e-156 | 57% |
| PCJ39708.1 3-phosphoshikimate 1-carboxyvinyltransferase [Alpha... | 459 | 6e-156 | 57% |
| MBD3804231.1 3-phosphoshikimate 1-carboxyvinyltransferase [Thi... | 459 | 6e-156 | 56% |
| WP_082884937.1 3-phosphoshikimate 1-carboxyvinyltransferase [B... | 460 | 6e-156 | 57% |
| WP_121220856.1 3-phosphoshikimate 1-carboxyvinyltransferase [O... | 459 | 6e-156 | 58% |
| WP_141951281.1 3-phosphoshikimate 1-carboxyvinyltransferase [M... | 459 | 6e-156 | 60% |
| WP_192360918.1 3-phosphoshikimate 1-carboxyvinyltransferase [M... | 453 | 6e-156 | 78% |
| WP_022680183.1 3-phosphoshikimate 1-carboxyvinyltransferase [S... | 459 | 6e-156 | 58% |
| WP_088309665.1 3-phosphoshikimate 1-carboxyvinyltransferase [N... | 459 | 6e-156 | 58% |
| WP_095385982.1 3-phosphoshikimate 1-carboxyvinyltransferase [S... | 459 | 7e-156 | 59% |
| Q89WF2.1 RecName: Full=3-phosphoshikimate 1-carboxyvinyltransf... | 460 | 7e-156 | 57% |
| WP_068730322.1 3-phosphoshikimate 1-carboxyvinyltransferase [T... | 459 | 7e-156 | 58% |
| WP_185998822.1 3-phosphoshikimate 1-carboxyvinyltransferase [N... | 459 | 7e-156 | 59% |
| WP_114356825.1 MULTISPECIES: 3-phosphoshikimate 1-carboxyvinyl... | 459 | 7e-156 | 59% |
| WP_183708542.1 3-phosphoshikimate 1-carboxyvinyltransferase [B... | 459 | 7e-156 | 57% |

| WP_042468914.1 MULTISPECIES: 3-phosphoshikimate 1-carboxyvinyl... | 459 | 7e-156 | 57% |
| --- | --- | --- | --- |
| WP_136884046.1 3-phosphoshikimate 1-carboxyvinyltransferase [P... | 459 | 7e-156 | 55% |
| MSO69281.1 3-phosphoshikimate 1-carboxyvinyltransferase [Alpha... | 459 | 7e-156 | 58% |
| WP_120215890.1 3-phosphoshikimate 1-carboxyvinyltransferase [S... | 459 | 8e-156 | 57% |
| WP_071908895.1 3-phosphoshikimate 1-carboxyvinyltransferase [B... | 459 | 8e-156 | 57% |
| KAF0125377.1 aroA [Methylocystaceae bacterium] | 459 | 8e-156 | 60% |
| SEB87957.1 3-phosphoshikimate 1-carboxyvinyltransferase [Rhizo... | 459 | 8e-156 | 58% |
| APO49317.1 3-phosphoshikimate 1-carboxyvinyltransferase [Brady... | 459 | 8e-156 | 56% |
| WP_011541786.1 3-phosphoshikimate 1-carboxyvinyltransferase [S... | 459 | 8e-156 | 58% |
| WP_109799417.1 3-phosphoshikimate 1-carboxyvinyltransferase [N... | 458 | 8e-156 | 59% |
| WP_093019773.1 3-phosphoshikimate 1-carboxyvinyltransferase [S... | 459 | 8e-156 | 58% |
| WP_056439413.1 3-phosphoshikimate 1-carboxyvinyltransferase [S... | 459 | 9e-156 | 57% |
| WP_055759123.1 MULTISPECIES: 3-phosphoshikimate 1-carboxyvinyl... | 459 | 9e-156 | 57% |
| WP_128928669.1 3-phosphoshikimate 1-carboxyvinyltransferase [B... | 459 | 9e-156 | 56% |
| WP_114946022.1 3-phosphoshikimate 1-carboxyvinyltransferase [M... | 459 | 9e-156 | 62% |
| WP_120249756.1 MULTISPECIES: 3-phosphoshikimate 1-carboxyvinyl... | 459 | 9e-156 | 57% |
| WP_056093061.1 3-phosphoshikimate 1-carboxyvinyltransferase [M... | 459 | 9e-156 | 60% |
| WP_160589653.1 3-phosphoshikimate 1-carboxyvinyltransferase [A... | 459 | 9e-156 | 59% |
| NQV95360.1 3-phosphoshikimate 1-carboxyvinyltransferase [Sphin... | 459 | 1e-155 | 56% |
| WP_074076470.1 3-phosphoshikimate 1-carboxyvinyltransferase [B... | 459 | 1e-155 | 56% |
| WP_155479567.1 3-phosphoshikimate 1-carboxyvinyltransferase [R... | 458 | 1e-155 | 60% |
| WP_156255767.1 3-phosphoshikimate 1-carboxyvinyltransferase [S... | 459 | 1e-155 | 57% |
| WP_188513961.1 unnamed protein product | 459 | 1e-155 | 57% |
| RED38670.1 3-phosphoshikimate 1-carboxyvinyltransferase [Rhodo... | 459 | 1e-155 | 59% |
| OSZ63267.1 3-phosphoshikimate 1-carboxyvinyltransferase [Sphin... | 459 | 1e-155 | 58% |
| MBA4771465.1 3-phosphoshikimate 1-carboxyvinyltransferase [Sph... | 457 | 1e-155 | 59% |
| WP_036024496.1 3-phosphoshikimate 1-carboxyvinyltransferase [B... | 458 | 1e-155 | 57% |
| WP_149141005.1 3-phosphoshikimate 1-carboxyvinyltransferase [G... | 459 | 1e-155 | 58% |
| WP_188789175.1 3-phosphoshikimate 1-carboxyvinyltransferase [P... | 458 | 1e-155 | 58% |
| WP_116527343.1 3-phosphoshikimate 1-carboxyvinyltransferase [M... | 458 | 1e-155 | 61% |
| TCM39995.1 3-phosphoshikimate 1-carboxyvinyltransferase [Novos... | 458 | 1e-155 | 59% |
| SFM36026.1 3-phosphoshikimate 1-carboxyvinyltransferase [Brady... | 459 | 1e-155 | 57% |
| OYZ13825.1 3-phosphoshikimate 1-carboxyvinyltransferase [Sphin... | 459 | 1e-155 | 59% |
| PYG21306.1 3-phosphoshikimate 1-carboxyvinyltransferase [Novos... | 458 | 1e-155 | 58% |
| WP_012567657.1 3-phosphoshikimate 1-carboxyvinyltransferase [R... | 458 | 1e-155 | 58% |
| WP_009564253.1 3-phosphoshikimate 1-carboxyvinyltransferase [R... | 458 | 1e-155 | 58% |
| WP_107974610.1 3-phosphoshikimate 1-carboxyvinyltransferase [R... | 458 | 1e-155 | 58% |
| MVT77314.1 3-phosphoshikimate 1-carboxyvinyltransferase [Brady... | 459 | 1e-155 | 57% |
| WP_174827297.1 3-phosphoshikimate 1-carboxyvinyltransferase [S... | 458 | 1e-155 | 59% |
| WP_014849176.1 3-phosphoshikimate 1-carboxyvinyltransferase [Z... | 458 | 1e-155 | 56% |
| KAF0173816.1 3-phosphoshikimate 1-carboxyvinyltransferase [Rho... | 458 | 1e-155 | 58% |
| WP_194478696.1 3-phosphoshikimate 1-carboxyvinyltransferase [B... | 458 | 1e-155 | 57% |
| WP_164938352.1 3-phosphoshikimate 1-carboxyvinyltransferase [B... | 458 | 1e-155 | 57% |
| WP_132469116.1 3-phosphoshikimate 1-carboxyvinyltransferase [N... | 458 | 1e-155 | 59% |
| WP_184643703.1 3-phosphoshikimate 1-carboxyvinyltransferase [S... | 458 | 1e-155 | 61% |
| NBW76347.1 3-phosphoshikimate 1-carboxyvinyltransferase [Sphin... | 458 | 1e-155 | 57% |
| OYU89401.1 3-phosphoshikimate 1-carboxyvinyltransferase [Brady... | 459 | 1e-155 | 57% |
| NWH07108.1 3-phosphoshikimate 1-carboxyvinyltransferase [Alpha... | 458 | 1e-155 | 60% |
| WP_128957928.1 3-phosphoshikimate 1-carboxyvinyltransferase [B... | 458 | 1e-155 | 57% |
| TWB65260.1 3-phosphoshikimate 1-carboxyvinyltransferase [Brady... | 459 | 1e-155 | 56% |
| RZJ99137.1 3-phosphoshikimate 1-carboxyvinyltransferase [Novos... | 458 | 1e-155 | 57% |
| WP_135414581.1 3-phosphoshikimate 1-carboxyvinyltransferase [M... | 458 | 1e-155 | 59% |
| NDD08952.1 3-phosphoshikimate 1-carboxyvinyltransferase [Rhodo... | 458 | 1e-155 | 56% |
| WP_058734746.1 3-phosphoshikimate 1-carboxyvinyltransferase [S... | 458 | 1e-155 | 57% |
| WP_131196187.1 3-phosphoshikimate 1-carboxyvinyltransferase [L... | 459 | 1e-155 | 60% |
| WP_176400312.1 3-phosphoshikimate 1-carboxyvinyltransferase [S... | 457 | 1e-155 | 59% |
| WP_152716111.1 3-phosphoshikimate 1-carboxyvinyltransferase [M... | 458 | 1e-155 | 61% |
| SDR61634.1 3-phosphoshikimate 1-carboxyvinyltransferase [Rhizo... | 458 | 1e-155 | 58% |
| WP_120219046.1 3-phosphoshikimate 1-carboxyvinyltransferase [S... | 458 | 2e-155 | 60% |
| WP_054163311.1 3-phosphoshikimate 1-carboxyvinyltransferase [R... | 458 | 2e-155 | 57% |
| WP_054210400.1 3-phosphoshikimate 1-carboxyvinyltransferase [B... | 458 | 2e-155 | 60% |
| TWI07747.1 3-phosphoshikimate 1-carboxyvinyltransferase [Brady... | 459 | 2e-155 | 56% |
| WP_141920517.1 3-phosphoshikimate 1-carboxyvinyltransferase [Z... | 458 | 2e-155 | 56% |
| RXG92471.1 3-phosphoshikimate 1-carboxyvinyltransferase [Brady... | 459 | 2e-155 | 57% |
| WP_135297742.1 3-phosphoshikimate 1-carboxyvinyltransferase [M... | 458 | 2e-155 | 60% |
| WP_076527013.1 3-phosphoshikimate 1-carboxyvinyltransferase [P... | 458 | 2e-155 | 56% |
| WP_188099355.1 3-phosphoshikimate 1-carboxyvinyltransferase [B... | 458 | 2e-155 | 57% |
| WP_125657987.1 3-phosphoshikimate 1-carboxyvinyltransferase [B... | 458 | 2e-155 | 57% |
| WP_024275320.1 3-phosphoshikimate 1-carboxyvinyltransferase [H... | 458 | 2e-155 | 59% |
| WP_183943406.1 3-phosphoshikimate 1-carboxyvinyltransferase [S... | 458 | 2e-155 | 58% |
| QBR71008.1 3-phosphoshikimate 1-carboxyvinyltransferase [Beije... | 458 | 2e-155 | 57% |
| MBC7951896.1 3-phosphoshikimate 1-carboxyvinyltransferase [Rho... | 457 | 2e-155 | 57% |

| ERL97189.1 3-phosphoshikimate 1-carboxyvinyltransferase [Rhodo... | 458 | 2e-155 | 57% |
| --- | --- | --- | --- |
| WP_058752317.1 3-phosphoshikimate 1-carboxyvinyltransferase [S... | 457 | 2e-155 | 57% |
| WP_164940037.1 3-phosphoshikimate 1-carboxyvinyltransferase [B... | 457 | 2e-155 | 56% |
| WP_169545162.1 3-phosphoshikimate 1-carboxyvinyltransferase [N... | 458 | 2e-155 | 57% |
| WP_103171026.1 3-phosphoshikimate 1-carboxyvinyltransferase [P... | 457 | 2e-155 | 57% |
| ABQ68817.1 3-phosphoshikimate 1-carboxyvinyltransferase [Sphin... | 459 | 2e-155 | 58% |
| RTL77824.1 3-phosphoshikimate 1-carboxyvinyltransferase [Brady... | 457 | 2e-155 | 57% |
| WP_043869001.1 3-phosphoshikimate 1-carboxyvinyltransferase [C... | 457 | 2e-155 | 57% |
| WP_164157432.1 3-phosphoshikimate 1-carboxyvinyltransferase [S... | 457 | 2e-155 | 57% |
| OKO81637.1 3-phosphoshikimate 1-carboxyvinyltransferase [Brady... | 458 | 2e-155 | 56% |
| WP_055822568.1 MULTISPECIES: 3-phosphoshikimate 1-carboxyvinyl... | 457 | 2e-155 | 58% |
| WP_186378772.1 3-phosphoshikimate 1-carboxyvinyltransferase [S... | 457 | 2e-155 | 58% |
| WP_056384844.1 3-phosphoshikimate 1-carboxyvinyltransferase [M... | 457 | 2e-155 | 61% |
| WP_017182692.1 MULTISPECIES: 3-phosphoshikimate 1-carboxyvinyl... | 457 | 2e-155 | 58% |
| CEJ10785.1 3-phosphoshikimate 1-carboxyvinyltransferase [bacte... | 457 | 2e-155 | 61% |
| WP_056247558.1 3-phosphoshikimate 1-carboxyvinyltransferase [M... | 457 | 3e-155 | 60% |
| MBB3880099.1 3-phosphoshikimate 1-carboxyvinyltransferase [Sph... | 457 | 3e-155 | 57% |
| NBR32068.1 3-phosphoshikimate 1-carboxyvinyltransferase [Sphin... | 463 | 3e-155 | 58% |
| EQB16964.1 3-phosphoshikimate 1-carboxyvinyltransferase [Sphin... | 457 | 3e-155 | 58% |
| WP_184271602.1 MULTISPECIES: 3-phosphoshikimate 1-carboxyvinyl... | 457 | 3e-155 | 57% |
| WP_114770334.1 MULTISPECIES: 3-phosphoshikimate 1-carboxyvinyl... | 457 | 3e-155 | 61% |
| MBB3692541.1 3-phosphoshikimate 1-carboxyvinyltransferase [Sph... | 457 | 3e-155 | 58% |
| WP_066108696.1 3-phosphoshikimate 1-carboxyvinyltransferase [Y... | 457 | 3e-155 | 57% |
| MPT47399.1 3-phosphoshikimate 1-carboxyvinyltransferase [Sphin... | 457 | 3e-155 | 58% |
| WP_066859367.1 3-phosphoshikimate 1-carboxyvinyltransferase [S... | 457 | 3e-155 | 57% |
| RUO99821.1 3-phosphoshikimate 1-carboxyvinyltransferase [Hypho... | 457 | 3e-155 | 60% |
| WP_155064176.1 3-phosphoshikimate 1-carboxyvinyltransferase [P... | 457 | 3e-155 | 57% |
| WP_092863962.1 3-phosphoshikimate 1-carboxyvinyltransferase [A... | 457 | 3e-155 | 58% |
| WP_040603623.1 3-phosphoshikimate 1-carboxyvinyltransferase [S... | 457 | 3e-155 | 58% |
| WP_063681439.1 3-phosphoshikimate 1-carboxyvinyltransferase [B... | 457 | 3e-155 | 56% |
| WP_024281091.1 3-phosphoshikimate 1-carboxyvinyltransferase [X... | 457 | 3e-155 | 60% |
| WP_155186378.1 3-phosphoshikimate 1-carboxyvinyltransferase [S... | 457 | 3e-155 | 58% |
| SED29536.1 3-phosphoshikimate 1-carboxyvinyltransferase [Rhizo... | 457 | 3e-155 | 58% |
| WP_188762751.1 3-phosphoshikimate 1-carboxyvinyltransferase [P... | 457 | 3e-155 | 60% |
| ACM03623.1 3-phosphoshikimate 1-carboxyvinyltransferase [Rhodo... | 457 | 3e-155 | 58% |
| WP_176943385.1 3-phosphoshikimate 1-carboxyvinyltransferase [S... | 457 | 3e-155 | 57% |
| WP_148625632.1 3-phosphoshikimate 1-carboxyvinyltransferase [N... | 457 | 3e-155 | 56% |
| WP_174544420.1 3-phosphoshikimate 1-carboxyvinyltransferase [S... | 457 | 3e-155 | 59% |
| WP_156786860.1 3-phosphoshikimate 1-carboxyvinyltransferase [A... | 457 | 4e-155 | 58% |
| WP_094894659.1 3-phosphoshikimate 1-carboxyvinyltransferase [B... | 457 | 4e-155 | 57% |
| WP_172332108.1 3-phosphoshikimate 1-carboxyvinyltransferase [M... | 457 | 4e-155 | 56% |
| PSO10795.1 3-phosphoshikimate 1-carboxyvinyltransferase [Sphin... | 457 | 4e-155 | 58% |
| NOT70168.1 3-phosphoshikimate 1-carboxyvinyltransferase [Hypho... | 457 | 4e-155 | 57% |
| WP_060772232.1 3-phosphoshikimate 1-carboxyvinyltransferase [M... | 457 | 4e-155 | 60% |
| WP_115692721.1 3-phosphoshikimate 1-carboxyvinyltransferase [P... | 457 | 4e-155 | 56% |
| OJW63237.1 3-phosphoshikimate 1-carboxyvinyltransferase [Afipi... | 457 | 4e-155 | 59% |
| EFI53672.1 3-phosphoshikimate 1-carboxyvinyltransferase [Afipi... | 457 | 4e-155 | 58% |
| WP_016921782.1 3-phosphoshikimate 1-carboxyvinyltransferase [M... | 457 | 4e-155 | 59% |
| WP_150292349.1 3-phosphoshikimate 1-carboxyvinyltransferase [S... | 457 | 4e-155 | 57% |
| WP_079565740.1 3-phosphoshikimate 1-carboxyvinyltransferase [B... | 457 | 4e-155 | 56% |
| WP_177248091.1 3-phosphoshikimate 1-carboxyvinyltransferase [B... | 457 | 4e-155 | 57% |
| AEG48347.1 3-phosphoshikimate 1-carboxyvinyltransferase [Sphin... | 457 | 4e-155 | 58% |
| WP_128207678.1 3-phosphoshikimate 1-carboxyvinyltransferase [S... | 457 | 4e-155 | 58% |
| WP_056402580.1 MULTISPECIES: 3-phosphoshikimate 1-carboxyvinyl... | 457 | 4e-155 | 57% |
| OUS05793.1 3-phosphoshikimate 1-carboxyvinyltransferase [Rhodo... | 457 | 4e-155 | 57% |
| OJU29870.1 3-phosphoshikimate 1-carboxyvinyltransferase [Nitro... | 457 | 4e-155 | 58% |
| WP_159870974.1 3-phosphoshikimate 1-carboxyvinyltransferase [N... | 457 | 4e-155 | 58% |
| WP_160732658.1 3-phosphoshikimate 1-carboxyvinyltransferase [A... | 456 | 4e-155 | 61% |
| BBO01557.1 3-phosphoshikimate 1-carboxyvinyltransferase [Brady... | 457 | 4e-155 | 57% |
| KWV57498.1 3-phosphoshikimate 1-carboxyvinyltransferase [Brady... | 457 | 4e-155 | 57% |
| WP_184086940.1 3-phosphoshikimate 1-carboxyvinyltransferase [A... | 457 | 4e-155 | 57% |
| WP_069053328.1 3-phosphoshikimate 1-carboxyvinyltransferase [B... | 457 | 4e-155 | 61% |
| PZQ21121.1 3-phosphoshikimate 1-carboxyvinyltransferase [Sphin... | 457 | 4e-155 | 59% |
| WP_168818700.1 3-phosphoshikimate 1-carboxyvinyltransferase [S... | 457 | 4e-155 | 58% |
| WP_136636570.1 3-phosphoshikimate 1-carboxyvinyltransferase [P... | 457 | 4e-155 | 58% |
| WP_058717745.1 3-phosphoshikimate 1-carboxyvinyltransferase [S... | 457 | 4e-155 | 57% |
| WP_168021298.1 MULTISPECIES: 3-phosphoshikimate 1-carboxyvinyl... | 457 | 5e-155 | 56% |
| WP_066557501.1 MULTISPECIES: 3-phosphoshikimate 1-carboxyvinyl... | 457 | 5e-155 | 58% |
| HBK07259.1 TPA: 3-phosphoshikimate 1-carboxyvinyltransferase [... | 457 | 5e-155 | 58% |
| WP_164633094.1 3-phosphoshikimate 1-carboxyvinyltransferase [R... | 457 | 5e-155 | 59% |
| WP_006023248.1 3-phosphoshikimate 1-carboxyvinyltransferase [A... | 457 | 5e-155 | 57% |
| WP_109355742.1 3-phosphoshikimate 1-carboxyvinyltransferase [S... | 457 | 5e-155 | 55% |

| WP_099510768.1 3-phosphoshikimate 1-carboxyvinyltransferase [M... | 457 | 5e-155 | 61% |
| --- | --- | --- | --- |
| WP_093084863.1 3-phosphoshikimate 1-carboxyvinyltransferase [S... | 457 | 5e-155 | 57% |
| WP_019831829.1 3-phosphoshikimate 1-carboxyvinyltransferase [S... | 457 | 5e-155 | 58% |
| WP_019903188.1 3-phosphoshikimate 1-carboxyvinyltransferase [M... | 457 | 5e-155 | 60% |
| WP_125324647.1 3-phosphoshikimate 1-carboxyvinyltransferase [T... | 457 | 5e-155 | 57% |
| RMD47160.1 3-phosphoshikimate 1-carboxyvinyltransferase [Alpha... | 457 | 5e-155 | 56% |
| KAB2882011.1 3-phosphoshikimate 1-carboxyvinyltransferase [Pse... | 457 | 5e-155 | 59% |
| WP_184024628.1 3-phosphoshikimate 1-carboxyvinyltransferase [S... | 457 | 5e-155 | 58% |
| WP_056627550.1 3-phosphoshikimate 1-carboxyvinyltransferase [S... | 456 | 6e-155 | 57% |
| WP_120499794.1 3-phosphoshikimate 1-carboxyvinyltransferase [R... | 457 | 6e-155 | 57% |
| MBA4132392.1 3-phosphoshikimate 1-carboxyvinyltransferase [Hyp... | 456 | 6e-155 | 59% |
| WP_181051495.1 3-phosphoshikimate 1-carboxyvinyltransferase [M... | 457 | 6e-155 | 62% |
| WP_128948778.1 3-phosphoshikimate 1-carboxyvinyltransferase [B... | 456 | 6e-155 | 56% |
| WP_106944030.1 3-phosphoshikimate 1-carboxyvinyltransferase [B... | 456 | 6e-155 | 58% |
| WP_011444984.1 MULTISPECIES: 3-phosphoshikimate 1-carboxyvinyl... | 456 | 6e-155 | 58% |
| QOZ49994.1 3-phosphoshikimate 1-carboxyvinyltransferase [Brady... | 457 | 6e-155 | 56% |
| WP_091138879.1 3-phosphoshikimate 1-carboxyvinyltransferase [M... | 456 | 6e-155 | 60% |
| PHO02985.1 3-phosphoshikimate 1-carboxyvinyltransferase [Rhodo... | 457 | 6e-155 | 58% |
| WP_076969755.1 3-phosphoshikimate 1-carboxyvinyltransferase [C... | 457 | 6e-155 | 55% |
| WP_046828075.1 3-phosphoshikimate 1-carboxyvinyltransferase [A... | 456 | 6e-155 | 57% |
| WP_054107525.1 3-phosphoshikimate 1-carboxyvinyltransferase [N... | 456 | 6e-155 | 58% |
| WP_128253347.1 3-phosphoshikimate 1-carboxyvinyltransferase [F... | 456 | 6e-155 | 58% |
| WP_149779951.1 3-phosphoshikimate 1-carboxyvinyltransferase [R... | 456 | 7e-155 | 58% |
| WP_067201349.1 3-phosphoshikimate 1-carboxyvinyltransferase [S... | 456 | 7e-155 | 55% |
| WP_101751376.1 3-phosphoshikimate 1-carboxyvinyltransferase [P... | 456 | 7e-155 | 57% |
| WP_078550131.1 3-phosphoshikimate 1-carboxyvinyltransferase [T... | 456 | 7e-155 | 56% |
| WP_167303147.1 3-phosphoshikimate 1-carboxyvinyltransferase [S... | 456 | 7e-155 | 57% |
| WP_066697122.1 3-phosphoshikimate 1-carboxyvinyltransferase [S... | 456 | 7e-155 | 59% |
| WP_027315859.1 3-phosphoshikimate 1-carboxyvinyltransferase [M... | 456 | 7e-155 | 60% |
| WP_057862160.1 3-phosphoshikimate 1-carboxyvinyltransferase [B... | 456 | 7e-155 | 56% |
| VWX51724.1 3-phosphoshikimate 1-carboxyvinyltransferase [Novos... | 456 | 7e-155 | 58% |
| WP_013946177.1 3-phosphoshikimate 1-carboxyvinyltransferase [H... | 456 | 7e-155 | 58% |
| WP_038146924.1 3-phosphoshikimate 1-carboxyvinyltransferase [T... | 456 | 7e-155 | 56% |
| WP_062179822.1 3-phosphoshikimate 1-carboxyvinyltransferase [S... | 456 | 7e-155 | 57% |
| WP_174544792.1 3-phosphoshikimate 1-carboxyvinyltransferase [N... | 456 | 8e-155 | 57% |
| WP_168452540.1 3-phosphoshikimate 1-carboxyvinyltransferase [S... | 456 | 8e-155 | 57% |
| AQX29939.1 3-phosphoshikimate 1-carboxyvinyltransferase [Barto... | 454 | 8e-155 | 60% |
| WP_011508772.1 3-phosphoshikimate 1-carboxyvinyltransferase [N... | 456 | 8e-155 | 58% |
| WP_094475224.1 3-phosphoshikimate 1-carboxyvinyltransferase [S... | 456 | 9e-155 | 57% |
| WP_176392327.1 3-phosphoshikimate 1-carboxyvinyltransferase [S... | 456 | 9e-155 | 59% |
| WP_101339914.1 3-phosphoshikimate 1-carboxyvinyltransferase [R... | 456 | 9e-155 | 58% |
| MBC7520893.1 3-phosphoshikimate 1-carboxyvinyltransferase [San... | 456 | 9e-155 | 60% |
| WP_056459644.1 3-phosphoshikimate 1-carboxyvinyltransferase [S... | 456 | 9e-155 | 58% |
| WP_166929367.1 3-phosphoshikimate 1-carboxyvinyltransferase [R... | 456 | 1e-154 | 56% |
| WP_063701552.1 3-phosphoshikimate 1-carboxyvinyltransferase [B... | 456 | 1e-154 | 55% |
| WP_168069721.1 3-phosphoshikimate 1-carboxyvinyltransferase [H... | 456 | 1e-154 | 56% |
| OYY69256.1 3-phosphoshikimate 1-carboxyvinyltransferase [Sphin... | 456 | 1e-154 | 55% |
| WP_150424435.1 3-phosphoshikimate 1-carboxyvinyltransferase [S... | 456 | 1e-154 | 57% |
| WP_131114624.1 3-phosphoshikimate 1-carboxyvinyltransferase [L... | 456 | 1e-154 | 59% |
| WP_058309962.1 3-phosphoshikimate 1-carboxyvinyltransferase [S... | 456 | 1e-154 | 57% |
| WP_142661214.1 3-phosphoshikimate 1-carboxyvinyltransferase [P... | 456 | 1e-154 | 56% |
| KJC44733.1 3-phosphoshikimate 1-carboxyvinyltransferase [Brady... | 456 | 1e-154 | 57% |
| WP_098411111.1 3-phosphoshikimate 1-carboxyvinyltransferase [T... | 456 | 1e-154 | 56% |
| WP_167922936.1 3-phosphoshikimate 1-carboxyvinyltransferase [S... | 456 | 1e-154 | 59% |
| OYW15058.1 3-phosphoshikimate 1-carboxyvinyltransferase [Sphin... | 456 | 1e-154 | 57% |
| MBP08240.1 3-phosphoshikimate 1-carboxyvinyltransferase [Marin... | 456 | 1e-154 | 57% |
| WP_166328359.1 3-phosphoshikimate 1-carboxyvinyltransferase [P... | 456 | 1e-154 | 55% |
| WP_066923688.1 3-phosphoshikimate 1-carboxyvinyltransferase [M... | 456 | 1e-154 | 60% |
| TWI32453.1 3-phosphoshikimate 1-carboxyvinyltransferase [Brady... | 456 | 1e-154 | 57% |
| WP_035993851.1 3-phosphoshikimate 1-carboxyvinyltransferase [B... | 456 | 1e-154 | 58% |
| HAF40483.1 TPA: 3-phosphoshikimate 1-carboxyvinyltransferase [... | 456 | 1e-154 | 57% |
| MBE1383714.1 3-phosphoshikimate 1-carboxyvinyltransferase [Bra... | 456 | 1e-154 | 56% |
| PZR00618.1 3-phosphoshikimate 1-carboxyvinyltransferase [Rhodo... | 456 | 1e-154 | 57% |
| WP_029624491.1 3-phosphoshikimate 1-carboxyvinyltransferase [S... | 456 | 1e-154 | 58% |
| THD66159.1 3-phosphoshikimate 1-carboxyvinyltransferase [Brady... | 456 | 1e-154 | 57% |
| WP_054725991.1 3-phosphoshikimate 1-carboxyvinyltransferase [S... | 456 | 1e-154 | 60% |
| WP_114186486.1 3-phosphoshikimate 1-carboxyvinyltransferase [M... | 456 | 1e-154 | 60% |
| WP_157027449.1 MULTISPECIES: 3-phosphoshikimate 1-carboxyvinyl... | 456 | 1e-154 | 59% |
| WP_085804679.1 3-phosphoshikimate 1-carboxyvinyltransferase [R... | 456 | 1e-154 | 57% |
| WP_014746734.1 3-phosphoshikimate 1-carboxyvinyltransferase [T... | 456 | 1e-154 | 58% |
| WP_176592850.1 3-phosphoshikimate 1-carboxyvinyltransferase [S... | 455 | 1e-154 | 58% |
| WP_130989344.1 3-phosphoshikimate 1-carboxyvinyltransferase [P... | 456 | 1e-154 | 55% |

| WP_183393279.1 3-phosphoshikimate 1-carboxyvinyltransferase [H... | 456 | 1e-154 | 58% |
| --- | --- | --- | --- |
| MBB3980650.1 3-phosphoshikimate 1-carboxyvinyltransferase [Sph... | 456 | 1e-154 | 57% |
| WP_057752375.1 3-phosphoshikimate 1-carboxyvinyltransferase [B... | 456 | 1e-154 | 56% |
| WP_118133382.1 3-phosphoshikimate 1-carboxyvinyltransferase [O... | 456 | 1e-154 | 57% |
| WP_089961797.1 3-phosphoshikimate 1-carboxyvinyltransferase [S... | 456 | 1e-154 | 57% |
| QAU36328.1 3-phosphoshikimate 1-carboxyvinyltransferase [Brady... | 456 | 1e-154 | 57% |
| WP_193742857.1 3-phosphoshikimate 1-carboxyvinyltransferase [S... | 455 | 1e-154 | 59% |
| WP_194465208.1 3-phosphoshikimate 1-carboxyvinyltransferase [B... | 456 | 1e-154 | 57% |
| WP_133613445.1 3-phosphoshikimate 1-carboxyvinyltransferase [D... | 456 | 1e-154 | 57% |
| WP_012453990.1 3-phosphoshikimate 1-carboxyvinyltransferase [M... | 456 | 1e-154 | 60% |
| WP_013934613.1 3-phosphoshikimate 1-carboxyvinyltransferase [Z... | 456 | 2e-154 | 56% |
| MBA4308564.1 3-phosphoshikimate 1-carboxyvinyltransferase [Sph... | 456 | 2e-154 | 56% |
| WP_024509548.1 3-phosphoshikimate 1-carboxyvinyltransferase [B... | 455 | 2e-154 | 57% |
| WP_126974614.1 3-phosphoshikimate 1-carboxyvinyltransferase [D... | 456 | 2e-154 | 58% |
| WP_027550076.1 3-phosphoshikimate 1-carboxyvinyltransferase [B... | 455 | 2e-154 | 55% |
| PCI31853.1 3-phosphoshikimate 1-carboxyvinyltransferase [Alpha... | 455 | 2e-154 | 55% |
| MBC7906367.1 3-phosphoshikimate 1-carboxyvinyltransferase [Rho... | 455 | 2e-154 | 58% |
| OJV03854.1 3-phosphoshikimate 1-carboxyvinyltransferase [Nitro... | 455 | 2e-154 | 57% |
| EIG60151.1 3-phosphoshikimate 1-carboxyvinyltransferase [Brady... | 456 | 2e-154 | 58% |
| WP_137871666.1 3-phosphoshikimate 1-carboxyvinyltransferase [S... | 455 | 2e-154 | 57% |
| WP_174273873.1 3-phosphoshikimate 1-carboxyvinyltransferase [S... | 456 | 2e-154 | 58% |
| ARR54318.1 3-phosphoshikimate 1-carboxyvinyltransferase [Sphin... | 455 | 2e-154 | 58% |
| RXH00570.1 3-phosphoshikimate 1-carboxyvinyltransferase [Brady... | 456 | 2e-154 | 56% |
| WP_136626213.1 3-phosphoshikimate 1-carboxyvinyltransferase [B... | 456 | 2e-154 | 57% |
| WP_056300305.1 3-phosphoshikimate 1-carboxyvinyltransferase [A... | 455 | 2e-154 | 57% |
| WP_089843338.1 3-phosphoshikimate 1-carboxyvinyltransferase [S... | 455 | 2e-154 | 56% |
| PCJ73856.1 3-phosphoshikimate 1-carboxyvinyltransferase [Rhodo... | 455 | 2e-154 | 58% |
| WP_174269597.1 3-phosphoshikimate 1-carboxyvinyltransferase [S... | 455 | 2e-154 | 59% |
| WP_176277982.1 3-phosphoshikimate 1-carboxyvinyltransferase [P... | 455 | 2e-154 | 56% |
| WP_156186929.1 3-phosphoshikimate 1-carboxyvinyltransferase [A... | 455 | 2e-154 | 58% |
| WP_040885175.1 3-phosphoshikimate 1-carboxyvinyltransferase [M... | 455 | 2e-154 | 58% |
| MAQ39134.1 3-phosphoshikimate 1-carboxyvinyltransferase [Thioc... | 455 | 2e-154 | 56% |
| WP_108221145.1 MULTISPECIES: 3-phosphoshikimate 1-carboxyvinyl... | 455 | 2e-154 | 58% |
| WP_044416292.1 3-phosphoshikimate 1-carboxyvinyltransferase [R... | 455 | 2e-154 | 58% |
| WP_050524195.1 3-phosphoshikimate 1-carboxyvinyltransferase [P... | 455 | 2e-154 | 57% |
| PWB82868.1 3-phosphoshikimate 1-carboxyvinyltransferase [Methy... | 455 | 2e-154 | 59% |
| WP_127045416.1 3-phosphoshikimate 1-carboxyvinyltransferase [P... | 455 | 2e-154 | 57% |
| WP_129682945.1 3-phosphoshikimate 1-carboxyvinyltransferase [L... | 455 | 3e-154 | 58% |
| WP_095639345.1 3-phosphoshikimate 1-carboxyvinyltransferase [P... | 455 | 3e-154 | 57% |
| ABP72735.1 3-phosphoshikimate 1-carboxyvinyltransferase [Rhodo... | 455 | 3e-154 | 58% |
| WP_062115994.1 3-phosphoshikimate 1-carboxyvinyltransferase [S... | 455 | 3e-154 | 57% |
| WP_047168465.1 3-phosphoshikimate 1-carboxyvinyltransferase [S... | 455 | 3e-154 | 58% |
| WP_072396545.1 3-phosphoshikimate 1-carboxyvinyltransferase [H... | 455 | 3e-154 | 58% |
| WP_183611914.1 3-phosphoshikimate 1-carboxyvinyltransferase [N... | 455 | 3e-154 | 58% |
| NVO15882.1 3-phosphoshikimate 1-carboxyvinyltransferase [Rhodo... | 455 | 3e-154 | 58% |
| WP_057850014.1 3-phosphoshikimate 1-carboxyvinyltransferase [B... | 455 | 3e-154 | 56% |
| WP_054142557.1 3-phosphoshikimate 1-carboxyvinyltransferase [B... | 455 | 3e-154 | 60% |
| WP_057844875.1 3-phosphoshikimate 1-carboxyvinyltransferase [B... | 455 | 3e-154 | 56% |
| WP_073034096.1 3-phosphoshikimate 1-carboxyvinyltransferase [R... | 455 | 3e-154 | 58% |
| WP_103847055.1 3-phosphoshikimate 1-carboxyvinyltransferase [C... | 455 | 3e-154 | 55% |
| OHC59255.1 3-phosphoshikimate 1-carboxyvinyltransferase [Rhodo... | 455 | 3e-154 | 57% |
| WP_027514725.1 3-phosphoshikimate 1-carboxyvinyltransferase [B... | 455 | 3e-154 | 58% |
| WP_027528283.1 3-phosphoshikimate 1-carboxyvinyltransferase [B... | 455 | 3e-154 | 56% |
| MAU44203.1 3-phosphoshikimate 1-carboxyvinyltransferase [Yangi... | 455 | 3e-154 | 57% |
| WP_143776527.1 3-phosphoshikimate 1-carboxyvinyltransferase [S... | 455 | 3e-154 | 56% |
| WP_072705523.1 3-phosphoshikimate 1-carboxyvinyltransferase [D... | 455 | 3e-154 | 57% |
| WP_172627862.1 3-phosphoshikimate 1-carboxyvinyltransferase [B... | 454 | 3e-154 | 57% |
| WP_189633320.1 3-phosphoshikimate 1-carboxyvinyltransferase [G... | 455 | 3e-154 | 56% |
| WP_119900600.1 3-phosphoshikimate 1-carboxyvinyltransferase [P... | 454 | 3e-154 | 56% |
| WP_066766330.1 3-phosphoshikimate 1-carboxyvinyltransferase [S... | 454 | 3e-154 | 57% |
| WP_056498049.1 3-phosphoshikimate 1-carboxyvinyltransferase [S... | 454 | 3e-154 | 57% |
| WP_024920193.1 MULTISPECIES: 3-phosphoshikimate 1-carboxyvinyl... | 454 | 3e-154 | 57% |
| MBB3859020.1 3-phosphoshikimate 1-carboxyvinyltransferase [Nov... | 455 | 3e-154 | 58% |
| KAB7785623.1 3-phosphoshikimate 1-carboxyvinyltransferase [Met... | 454 | 3e-154 | 61% |
| MBD3732238.1 3-phosphoshikimate 1-carboxyvinyltransferase [Sph... | 454 | 3e-154 | 58% |
| WP_106267453.1 3-phosphoshikimate 1-carboxyvinyltransferase [D... | 454 | 4e-154 | 57% |
| WP_028096098.1 3-phosphoshikimate 1-carboxyvinyltransferase [D... | 455 | 4e-154 | 59% |
| WP_084278879.1 3-phosphoshikimate 1-carboxyvinyltransferase [N... | 454 | 4e-154 | 58% |
| WP_078520832.1 MULTISPECIES: 3-phosphoshikimate 1-carboxyvinyl... | 454 | 4e-154 | 56% |
| WP_134726517.1 3-phosphoshikimate 1-carboxyvinyltransferase [P... | 454 | 4e-154 | 58% |
| RJG53510.1 3-phosphoshikimate 1-carboxyvinyltransferase [Sphin... | 454 | 4e-154 | 57% |
| WP_076711832.1 3-phosphoshikimate 1-carboxyvinyltransferase [S... | 454 | 4e-154 | 57% |

| WP_059150824.1 3-phosphoshikimate 1-carboxyvinyltransferase [N... | 454 | 4e-154 | 58% |
| --- | --- | --- | --- |
| WP_092886573.1 3-phosphoshikimate 1-carboxyvinyltransferase [R... | 454 | 4e-154 | 58% |
| WP_085848874.1 3-phosphoshikimate 1-carboxyvinyltransferase [P... | 454 | 4e-154 | 57% |
| NNE56534.1 3-phosphoshikimate 1-carboxyvinyltransferase [Helle... | 454 | 4e-154 | 57% |
| WP_108037395.1 MULTISPECIES: 3-phosphoshikimate 1-carboxyvinyl... | 454 | 4e-154 | 60% |
| OJX68359.1 3-phosphoshikimate 1-carboxyvinyltransferase [Magne... | 454 | 4e-154 | 57% |
| WP_068765751.1 3-phosphoshikimate 1-carboxyvinyltransferase [P... | 454 | 4e-154 | 56% |
| WP_051610136.1 3-phosphoshikimate 1-carboxyvinyltransferase [T... | 454 | 4e-154 | 56% |
| WP_110873050.1 3-phosphoshikimate 1-carboxyvinyltransferase [N... | 457 | 4e-154 | 58% |
| WP_108258800.1 3-phosphoshikimate 1-carboxyvinyltransferase [M... | 454 | 4e-154 | 56% |
| WP_147097698.1 3-phosphoshikimate 1-carboxyvinyltransferase [P... | 454 | 4e-154 | 56% |
| MAM74127.1 3-phosphoshikimate 1-carboxyvinyltransferase [Tistr... | 454 | 4e-154 | 58% |
| WP_058454095.1 MULTISPECIES: 3-phosphoshikimate 1-carboxyvinyl... | 454 | 5e-154 | 58% |
| MAN56392.1 3-phosphoshikimate 1-carboxyvinyltransferase [Parac... | 454 | 5e-154 | 55% |
| MAU41384.1 3-phosphoshikimate 1-carboxyvinyltransferase [Kordi... | 454 | 5e-154 | 56% |
| WP_043977630.1 3-phosphoshikimate 1-carboxyvinyltransferase [N... | 454 | 5e-154 | 58% |
| WP_055761847.1 3-phosphoshikimate 1-carboxyvinyltransferase [S... | 454 | 5e-154 | 57% |
| KHS46569.1 3-phosphoshikimate 1-carboxyvinyltransferase [Novos... | 454 | 5e-154 | 57% |
| NMN10562.1 3-phosphoshikimate 1-carboxyvinyltransferase [Novos... | 454 | 5e-154 | 56% |
| WP_171793885.1 3-phosphoshikimate 1-carboxyvinyltransferase [N... | 454 | 5e-154 | 56% |
| WP_154490958.1 MULTISPECIES: 3-phosphoshikimate 1-carboxyvinyl... | 454 | 5e-154 | 57% |
| KAF0122149.1 3-phosphoshikimate 1-carboxyvinyltransferase [Xan... | 454 | 5e-154 | 61% |
| EEX14637.1 3-phosphoshikimate 1-carboxyvinyltransferase [Citre... | 454 | 5e-154 | 58% |
| MYM57162.1 3-phosphoshikimate 1-carboxyvinyltransferase [Rhodo... | 454 | 5e-154 | 57% |
| GGO54506.1 3-phosphoshikimate 1-carboxyvinyltransferase [Roseo... | 455 | 5e-154 | 58% |
| PZO77686.1 3-phosphoshikimate 1-carboxyvinyltransferase [Sphin... | 454 | 5e-154 | 57% |
| WP_181316793.1 MULTISPECIES: 3-phosphoshikimate 1-carboxyvinyl... | 454 | 6e-154 | 58% |
| WP_176597735.1 3-phosphoshikimate 1-carboxyvinyltransferase [S... | 454 | 6e-154 | 58% |
| WP_071166569.1 3-phosphoshikimate 1-carboxyvinyltransferase [R... | 454 | 6e-154 | 56% |
| WP_163682705.1 3-phosphoshikimate 1-carboxyvinyltransferase [M... | 454 | 6e-154 | 59% |
| WP_060979322.1 MULTISPECIES: 3-phosphoshikimate 1-carboxyvinyl... | 454 | 6e-154 | 57% |
| TDQ82138.1 3-phosphoshikimate 1-carboxyvinyltransferase [Dongi... | 455 | 6e-154 | 57% |
| WP_127114850.1 3-phosphoshikimate 1-carboxyvinyltransferase [T... | 454 | 6e-154 | 57% |
| EGI54479.1 LOW QUALITY PROTEIN: EPSP synthase family protein [... | 454 | 6e-154 | 57% |
| WP_155044234.1 MULTISPECIES: 3-phosphoshikimate 1-carboxyvinyl... | 454 | 6e-154 | 56% |
| WP_074206154.1 3-phosphoshikimate 1-carboxyvinyltransferase [P... | 454 | 7e-154 | 55% |
| MBQ66057.1 3-phosphoshikimate 1-carboxyvinyltransferase [Marin... | 454 | 7e-154 | 56% |
| SFN92536.1 3-phosphoshikimate 1-carboxyvinyltransferase [Brady... | 455 | 7e-154 | 57% |
| WP_056470369.1 3-phosphoshikimate 1-carboxyvinyltransferase [M... | 454 | 7e-154 | 60% |
| WP_164183709.1 MULTISPECIES: 3-phosphoshikimate 1-carboxyvinyl... | 454 | 7e-154 | 57% |
| WP_079606504.1 3-phosphoshikimate 1-carboxyvinyltransferase [B... | 454 | 7e-154 | 58% |
| WP_160599125.1 3-phosphoshikimate 1-carboxyvinyltransferase [A... | 453 | 7e-154 | 58% |
| SBW12154.1 3-phosphoshikimate 1-carboxyvinyltransferase [uncul... | 454 | 7e-154 | 56% |
| WP_058806845.1 MULTISPECIES: 3-phosphoshikimate 1-carboxyvinyl... | 454 | 7e-154 | 58% |
| WP_035973472.1 3-phosphoshikimate 1-carboxyvinyltransferase [B... | 454 | 7e-154 | 57% |
| MBF21962.1 3-phosphoshikimate 1-carboxyvinyltransferase [Marin... | 454 | 7e-154 | 54% |
| WP_056358983.1 3-phosphoshikimate 1-carboxyvinyltransferase [M... | 454 | 7e-154 | 60% |
| WP_194471978.1 3-phosphoshikimate 1-carboxyvinyltransferase [B... | 454 | 8e-154 | 56% |
| WP_131855366.1 3-phosphoshikimate 1-carboxyvinyltransferase [B... | 454 | 8e-154 | 60% |
| WP_156163070.1 3-phosphoshikimate 1-carboxyvinyltransferase [B... | 454 | 8e-154 | 57% |
| WP_184261473.1 3-phosphoshikimate 1-carboxyvinyltransferase [R... | 454 | 8e-154 | 58% |
| NVK58888.1 3-phosphoshikimate 1-carboxyvinyltransferase [Rhodo... | 454 | 8e-154 | 57% |
| WP_029555952.1 3-phosphoshikimate 1-carboxyvinyltransferase [X... | 454 | 8e-154 | 59% |
| WP_109533145.1 3-phosphoshikimate 1-carboxyvinyltransferase [P... | 454 | 8e-154 | 58% |
| WP_085869900.1 3-phosphoshikimate 1-carboxyvinyltransferase [P... | 454 | 9e-154 | 57% |
| WP_169124792.1 3-phosphoshikimate 1-carboxyvinyltransferase [X... | 454 | 9e-154 | 59% |
| WP_039334255.1 3-phosphoshikimate 1-carboxyvinyltransferase [N... | 453 | 9e-154 | 57% |
| HBT00362.1 TPA: 3-phosphoshikimate 1-carboxyvinyltransferase [... | 454 | 9e-154 | 56% |
| WP_157174768.1 3-phosphoshikimate 1-carboxyvinyltransferase [S... | 454 | 9e-154 | 59% |
| EDM69814.1 3-phosphoshikimate 1-carboxyvinyltransferase [Roseo... | 454 | 9e-154 | 57% |
| WP_136683606.1 3-phosphoshikimate 1-carboxyvinyltransferase [R... | 453 | 9e-154 | 58% |
| WP_063992184.1 3-phosphoshikimate 1-carboxyvinyltransferase [B... | 453 | 9e-154 | 57% |
| WP_037465648.1 3-phosphoshikimate 1-carboxyvinyltransferase [S... | 453 | 1e-153 | 58% |
| WP_097030851.1 3-phosphoshikimate 1-carboxyvinyltransferase [R... | 453 | 1e-153 | 57% |
| MBB5729172.1 3-phosphoshikimate 1-carboxyvinyltransferase [Sph... | 454 | 1e-153 | 59% |
| WP_138421726.1 3-phosphoshikimate 1-carboxyvinyltransferase [M... | 454 | 1e-153 | 57% |
| NOU02046.1 3-phosphoshikimate 1-carboxyvinyltransferase [Novos... | 453 | 1e-153 | 58% |
| WP_179045662.1 3-phosphoshikimate 1-carboxyvinyltransferase [S... | 453 | 1e-153 | 57% |
| WP_022727575.1 3-phosphoshikimate 1-carboxyvinyltransferase [F... | 453 | 1e-153 | 57% |
| WP_103094548.1 3-phosphoshikimate 1-carboxyvinyltransferase [N... | 453 | 1e-153 | 56% |
| WP_078601316.1 3-phosphoshikimate 1-carboxyvinyltransferase [T... | 453 | 1e-153 | 56% |
| WP_176945408.1 3-phosphoshikimate 1-carboxyvinyltransferase [B... | 453 | 1e-153 | 57% |

| WP_159982696.1 MULTISPECIES: 3-phosphoshikimate 1-carboxyvinyl... | 453 | 1e-153 | 59% |
| --- | --- | --- | --- |
| WP_193828438.1 3-phosphoshikimate 1-carboxyvinyltransferase [N... | 453 | 1e-153 | 57% |
| OYU19678.1 3-phosphoshikimate 1-carboxyvinyltransferase [Rhodo... | 453 | 1e-153 | 58% |
| WP_041208492.1 3-phosphoshikimate 1-carboxyvinyltransferase [S... | 453 | 1e-153 | 57% |
| HBM60736.1 TPA: 3-phosphoshikimate 1-carboxyvinyltransferase [... | 453 | 1e-153 | 56% |
| KJC61808.1 3-phosphoshikimate 1-carboxyvinyltransferase [Brady... | 453 | 1e-153 | 56% |
| EAQ10494.1 3-phosphoshikimate 1-carboxyvinyltransferase [Rhodo... | 453 | 1e-153 | 58% |
| WP_195801374.1 3-phosphoshikimate 1-carboxyvinyltransferase [B... | 453 | 1e-153 | 56% |
| WP_040650449.1 3-phosphoshikimate 1-carboxyvinyltransferase [R... | 453 | 1e-153 | 57% |
| WP_147027759.1 3-phosphoshikimate 1-carboxyvinyltransferase [M... | 453 | 1e-153 | 59% |
| WP_066711661.1 3-phosphoshikimate 1-carboxyvinyltransferase [S... | 453 | 1e-153 | 58% |
| WP_176502254.1 3-phosphoshikimate 1-carboxyvinyltransferase [N... | 453 | 1e-153 | 58% |
| WP_133358862.1 3-phosphoshikimate 1-carboxyvinyltransferase [R... | 453 | 1e-153 | 57% |
| WP_076745347.1 3-phosphoshikimate 1-carboxyvinyltransferase [S... | 453 | 1e-153 | 57% |
| WP_114393446.1 3-phosphoshikimate 1-carboxyvinyltransferase [O... | 453 | 1e-153 | 57% |
| WP_182792941.1 MULTISPECIES: 3-phosphoshikimate 1-carboxyvinyl... | 453 | 1e-153 | 56% |
| WP_093992625.1 3-phosphoshikimate 1-carboxyvinyltransferase [F... | 453 | 1e-153 | 57% |
| WP_068429388.1 3-phosphoshikimate 1-carboxyvinyltransferase [M... | 453 | 1e-153 | 58% |
| OYZ97343.1 3-phosphoshikimate 1-carboxyvinyltransferase [Novos... | 453 | 1e-153 | 57% |
| KRO90788.1 3-phosphoshikimate 1-carboxyvinyltransferase [Rhodo... | 453 | 1e-153 | 57% |
| MAB62456.1 3-phosphoshikimate 1-carboxyvinyltransferase [Marin... | 453 | 1e-153 | 54% |
| TMJ60203.1 3-phosphoshikimate 1-carboxyvinyltransferase [Alpha... | 453 | 1e-153 | 58% |
| WP_143793334.1 3-phosphoshikimate 1-carboxyvinyltransferase [P... | 453 | 1e-153 | 56% |
| WP_050629023.1 3-phosphoshikimate 1-carboxyvinyltransferase [B... | 453 | 1e-153 | 57% |
| WP_056369485.1 MULTISPECIES: 3-phosphoshikimate 1-carboxyvinyl... | 453 | 2e-153 | 58% |
| WP_099474354.1 3-phosphoshikimate 1-carboxyvinyltransferase [E... | 453 | 2e-153 | 56% |
| QOZ22285.1 3-phosphoshikimate 1-carboxyvinyltransferase [Brady... | 453 | 2e-153 | 57% |
| WP_011569982.1 3-phosphoshikimate 1-carboxyvinyltransferase [R... | 453 | 2e-153 | 57% |
| WP_079445220.1 3-phosphoshikimate 1-carboxyvinyltransferase [N... | 453 | 2e-153 | 57% |
| WP_027566168.1 3-phosphoshikimate 1-carboxyvinyltransferase [B... | 453 | 2e-153 | 57% |
| WP_194135518.1 3-phosphoshikimate 1-carboxyvinyltransferase [S... | 453 | 2e-153 | 57% |
| WP_154450739.1 3-phosphoshikimate 1-carboxyvinyltransferase [M... | 453 | 2e-153 | 60% |
| OAH07148.1 3-phosphoshikimate 1-carboxyvinyltransferase [Rhodo... | 453 | 2e-153 | 54% |
| WP_062770573.1 3-phosphoshikimate 1-carboxyvinyltransferase [T... | 453 | 2e-153 | 57% |
| NDH72606.1 3-phosphoshikimate 1-carboxyvinyltransferase [Rhodo... | 453 | 2e-153 | 56% |
| MAN11575.1 3-phosphoshikimate 1-carboxyvinyltransferase [Sphin... | 453 | 2e-153 | 56% |
| TMJ00164.1 3-phosphoshikimate 1-carboxyvinyltransferase [Alpha... | 452 | 2e-153 | 58% |
| WP_100928125.1 3-phosphoshikimate 1-carboxyvinyltransferase [S... | 453 | 2e-153 | 56% |
| WP_181744669.1 3-phosphoshikimate 1-carboxyvinyltransferase [P... | 452 | 2e-153 | 55% |
| WP_057837503.1 3-phosphoshikimate 1-carboxyvinyltransferase [B... | 452 | 2e-153 | 56% |
| MBC8338867.1 3-phosphoshikimate 1-carboxyvinyltransferase [Alp... | 453 | 2e-153 | 58% |
| WP_066012792.1 3-phosphoshikimate 1-carboxyvinyltransferase [T... | 453 | 2e-153 | 57% |
| WP_137679457.1 3-phosphoshikimate 1-carboxyvinyltransferase [E... | 453 | 2e-153 | 58% |
| WP_051134435.1 3-phosphoshikimate 1-carboxyvinyltransferase [M... | 453 | 2e-153 | 58% |
| WP_066690365.1 3-phosphoshikimate 1-carboxyvinyltransferase [S... | 452 | 2e-153 | 57% |
| WP_056262868.1 3-phosphoshikimate 1-carboxyvinyltransferase [M... | 452 | 2e-153 | 60% |
| WP_168903834.1 3-phosphoshikimate 1-carboxyvinyltransferase [N... | 452 | 2e-153 | 57% |
| WP_046347746.1 3-phosphoshikimate 1-carboxyvinyltransferase [S... | 452 | 2e-153 | 57% |
| WP_168457402.1 MULTISPECIES: 3-phosphoshikimate 1-carboxyvinyl... | 453 | 2e-153 | 59% |
| EKU76953.1 3-phosphoshikimate 1-carboxyvinyltransferase [Sphin... | 452 | 2e-153 | 57% |
| WP_078570977.1 MULTISPECIES: 3-phosphoshikimate 1-carboxyvinyl... | 452 | 2e-153 | 55% |
| OFW71470.1 3-phosphoshikimate 1-carboxyvinyltransferase [Alpha... | 452 | 2e-153 | 56% |
| PZU08275.1 3-phosphoshikimate 1-carboxyvinyltransferase [Sphin... | 452 | 2e-153 | 58% |
| WP_111422349.1 3-phosphoshikimate 1-carboxyvinyltransferase [R... | 452 | 2e-153 | 58% |
| WP_090734202.1 3-phosphoshikimate 1-carboxyvinyltransferase [P... | 452 | 2e-153 | 56% |
| WP_081911733.1 3-phosphoshikimate 1-carboxyvinyltransferase [S... | 452 | 2e-153 | 57% |
| WP_169049296.1 3-phosphoshikimate 1-carboxyvinyltransferase [N... | 452 | 2e-153 | 56% |
| WP_160603965.1 3-phosphoshikimate 1-carboxyvinyltransferase [A... | 452 | 2e-153 | 58% |
| WP_136462751.1 3-phosphoshikimate 1-carboxyvinyltransferase [A... | 452 | 3e-153 | 56% |
| MBC7506827.1 3-phosphoshikimate 1-carboxyvinyltransferase [San... | 452 | 3e-153 | 58% |
| WP_094410158.1 3-phosphoshikimate 1-carboxyvinyltransferase [E... | 452 | 3e-153 | 55% |
| NDD42179.1 3-phosphoshikimate 1-carboxyvinyltransferase [Rhodo... | 452 | 3e-153 | 57% |
| MTI11377.1 3-phosphoshikimate 1-carboxyvinyltransferase [Rhodo... | 452 | 3e-153 | 57% |
| WP_132858837.1 3-phosphoshikimate 1-carboxyvinyltransferase [S... | 452 | 3e-153 | 57% |
| WP_066486677.1 3-phosphoshikimate 1-carboxyvinyltransferase [S... | 452 | 3e-153 | 58% |
| WP_065746756.1 3-phosphoshikimate 1-carboxyvinyltransferase [B... | 452 | 3e-153 | 55% |
| WP_067908426.1 3-phosphoshikimate 1-carboxyvinyltransferase [N... | 453 | 3e-153 | 56% |
| PDH66632.1 3-phosphoshikimate 1-carboxyvinyltransferase [Sphin... | 452 | 3e-153 | 56% |
| KJC47427.1 3-phosphoshikimate 1-carboxyvinyltransferase [Brady... | 452 | 3e-153 | 56% |
| WP_125944836.1 3-phosphoshikimate 1-carboxyvinyltransferase [S... | 452 | 3e-153 | 56% |
| EHQ99609.1 3-phosphoshikimate 1-carboxyvinyltransferase [Brady... | 453 | 3e-153 | 57% |
| KQO78421.1 3-phosphoshikimate 1-carboxyvinyltransferase [Methy... | 452 | 3e-153 | 60% |

| WP_142898473.1 3-phosphoshikimate 1-carboxyvinyltransferase [D... | 452 | 3e-153 | 58% |
| --- | --- | --- | --- |
| QEX20292.1 3-phosphoshikimate 1-carboxyvinyltransferase [Hyper... | 452 | 3e-153 | 58% |
| WP_076071969.1 3-phosphoshikimate 1-carboxyvinyltransferase [S... | 452 | 3e-153 | 57% |
| WP_138166313.1 3-phosphoshikimate 1-carboxyvinyltransferase [M... | 452 | 3e-153 | 60% |
| WP_118073316.1 3-phosphoshikimate 1-carboxyvinyltransferase [N... | 452 | 3e-153 | 57% |
| WP_056215810.1 MULTISPECIES: 3-phosphoshikimate 1-carboxyvinyl... | 452 | 4e-153 | 60% |
| WP_033073497.1 3-phosphoshikimate 1-carboxyvinyltransferase [S... | 452 | 4e-153 | 58% |
| WP_067747944.1 3-phosphoshikimate 1-carboxyvinyltransferase [N... | 452 | 4e-153 | 58% |
| EZP72430.1 3-phosphoshikimate 1-carboxyvinyltransferase [Sphin... | 452 | 4e-153 | 56% |
| WP_196277199.1 3-phosphoshikimate 1-carboxyvinyltransferase [N... | 452 | 4e-153 | 57% |
| WP_183754693.1 3-phosphoshikimate 1-carboxyvinyltransferase [P... | 452 | 4e-153 | 59% |
| MAC72402.1 3-phosphoshikimate 1-carboxyvinyltransferase [Marin... | 452 | 4e-153 | 56% |
| WP_182568972.1 3-phosphoshikimate 1-carboxyvinyltransferase [M... | 452 | 4e-153 | 60% |
| WP_066616718.1 3-phosphoshikimate 1-carboxyvinyltransferase [B... | 452 | 4e-153 | 58% |
| WP_062347616.1 3-phosphoshikimate 1-carboxyvinyltransferase [N... | 452 | 4e-153 | 57% |
| WP_046411473.1 3-phosphoshikimate 1-carboxyvinyltransferase [S... | 452 | 4e-153 | 56% |
| WP_009763477.1 3-phosphoshikimate 1-carboxyvinyltransferase [M... | 452 | 4e-153 | 62% |
| WP_083077148.1 3-phosphoshikimate 1-carboxyvinyltransferase [T... | 452 | 4e-153 | 55% |
| NMW31655.1 3-phosphoshikimate 1-carboxyvinyltransferase [Alter... | 451 | 4e-153 | 59% |
| NCV28591.1 3-phosphoshikimate 1-carboxyvinyltransferase [Rhodo... | 452 | 4e-153 | 56% |
| HAM90363.1 TPA: 3-phosphoshikimate 1-carboxyvinyltransferase [... | 452 | 4e-153 | 57% |
| WP_054004522.1 3-phosphoshikimate 1-carboxyvinyltransferase [P... | 452 | 4e-153 | 56% |
| SMX42573.1 3-phosphoshikimate 1-carboxyvinyltransferase [Malip... | 452 | 4e-153 | 57% |
| WP_056493486.1 3-phosphoshikimate 1-carboxyvinyltransferase [M... | 452 | 4e-153 | 60% |
| REF86523.1 3-phosphoshikimate 1-carboxyvinyltransferase [Methy... | 452 | 4e-153 | 60% |
| TVS02724.1 3-phosphoshikimate 1-carboxyvinyltransferase [Rhodo... | 452 | 4e-153 | 56% |
| WP_092419284.1 3-phosphoshikimate 1-carboxyvinyltransferase [S... | 452 | 4e-153 | 57% |
| NDI03020.1 3-phosphoshikimate 1-carboxyvinyltransferase [Rhodo... | 451 | 5e-153 | 56% |
| WP_089134447.1 3-phosphoshikimate 1-carboxyvinyltransferase [S... | 452 | 5e-153 | 55% |
| WP_183617551.1 3-phosphoshikimate 1-carboxyvinyltransferase [N... | 451 | 5e-153 | 56% |
| WP_156179675.1 3-phosphoshikimate 1-carboxyvinyltransferase [B... | 452 | 5e-153 | 56% |
| WP_068337758.1 3-phosphoshikimate 1-carboxyvinyltransferase [R... | 452 | 5e-153 | 56% |
| WP_042490053.1 3-phosphoshikimate 1-carboxyvinyltransferase [S... | 451 | 5e-153 | 56% |
| WP_174525932.1 3-phosphoshikimate 1-carboxyvinyltransferase [S... | 451 | 5e-153 | 57% |
| WP_022675694.1 MULTISPECIES: 3-phosphoshikimate 1-carboxyvinyl... | 452 | 5e-153 | 57% |
| WP_145108701.1 3-phosphoshikimate 1-carboxyvinyltransferase [R... | 451 | 5e-153 | 57% |
| MSP45043.1 3-phosphoshikimate 1-carboxyvinyltransferase [Xanth... | 451 | 5e-153 | 57% |
| WP_091148278.1 3-phosphoshikimate 1-carboxyvinyltransferase [N... | 451 | 5e-153 | 58% |
| WP_136451457.1 MULTISPECIES: 3-phosphoshikimate 1-carboxyvinyl... | 451 | 5e-153 | 56% |
| TDS22069.1 3-phosphoshikimate 1-carboxyvinyltransferase [Novos... | 452 | 5e-153 | 57% |
| WP_009796518.1 3-phosphoshikimate 1-carboxyvinyltransferase [N... | 451 | 6e-153 | 57% |
| MBB3940921.1 3-phosphoshikimate 1-carboxyvinyltransferase [Nov... | 452 | 6e-153 | 56% |
| WP_188073207.1 unnamed protein product | 451 | 6e-153 | 57% |
| ABD85975.1 3-phosphoshikimate 1-carboxyvinyltransferase [Rhodo... | 452 | 6e-153 | 58% |
| WP_035842278.1 3-phosphoshikimate 1-carboxyvinyltransferase [A... | 451 | 6e-153 | 56% |
| WP_054132738.1 3-phosphoshikimate 1-carboxyvinyltransferase [N... | 451 | 6e-153 | 57% |
| WP_165047891.1 3-phosphoshikimate 1-carboxyvinyltransferase [R... | 451 | 6e-153 | 57% |
| OJU31482.1 3-phosphoshikimate 1-carboxyvinyltransferase [Alpha... | 451 | 6e-153 | 58% |
| WP_080507295.1 3-phosphoshikimate 1-carboxyvinyltransferase [R... | 452 | 6e-153 | 58% |
| KUO64585.1 3-phosphoshikimate 1-carboxyvinyltransferase [Alpha... | 451 | 6e-153 | 59% |
| WP_115837043.1 3-phosphoshikimate 1-carboxyvinyltransferase [M... | 452 | 6e-153 | 60% |
| WP_170362445.1 3-phosphoshikimate 1-carboxyvinyltransferase [R... | 451 | 6e-153 | 56% |
| SHK64294.1 3-phosphoshikimate 1-carboxyvinyltransferase [Brady... | 451 | 6e-153 | 57% |
| WP_056169540.1 3-phosphoshikimate 1-carboxyvinyltransferase [M... | 451 | 7e-153 | 60% |
| WP_078540686.1 3-phosphoshikimate 1-carboxyvinyltransferase [T... | 451 | 7e-153 | 55% |
| WP_107395010.1 3-phosphoshikimate 1-carboxyvinyltransferase [S... | 452 | 7e-153 | 59% |
| TNE41880.1 3-phosphoshikimate 1-carboxyvinyltransferase [Sphin... | 451 | 7e-153 | 58% |
| WP_136439598.1 3-phosphoshikimate 1-carboxyvinyltransferase [P... | 451 | 7e-153 | 57% |
| WP_156164542.1 3-phosphoshikimate 1-carboxyvinyltransferase [B... | 452 | 7e-153 | 56% |
| WP_136497210.1 3-phosphoshikimate 1-carboxyvinyltransferase [M... | 451 | 7e-153 | 58% |
| KRO89933.1 3-phosphoshikimate 1-carboxyvinyltransferase [Rhodo... | 451 | 7e-153 | 58% |
| HHW33878.1 TPA: 3-phosphoshikimate 1-carboxyvinyltransferase [... | 451 | 7e-153 | 56% |
| WP_085373703.1 3-phosphoshikimate 1-carboxyvinyltransferase [M... | 451 | 7e-153 | 58% |
| WP_028657711.1 3-phosphoshikimate 1-carboxyvinyltransferase [N... | 451 | 7e-153 | 57% |
| EJK84363.1 3-phosphoshikimate 1-carboxyvinyltransferase [Sphin... | 451 | 8e-153 | 57% |
| MAM60362.1 3-phosphoshikimate 1-carboxyvinyltransferase [Marit... | 451 | 8e-153 | 57% |
| KAF0229348.1 3-phosphoshikimate [Beijerinckiaceae bacterium] | 451 | 8e-153 | 60% |
| WP_056304674.1 3-phosphoshikimate 1-carboxyvinyltransferase [M... | 451 | 8e-153 | 60% |
| WP_160586930.1 3-phosphoshikimate 1-carboxyvinyltransferase [P... | 452 | 8e-153 | 56% |
| TMJ51321.1 3-phosphoshikimate 1-carboxyvinyltransferase [Alpha... | 451 | 8e-153 | 57% |
| WP_121630337.1 3-phosphoshikimate 1-carboxyvinyltransferase [T... | 451 | 8e-153 | 57% |
| EME69421.1 3-phosphoshikimate 1-carboxyvinyltransferase [Magne... | 451 | 8e-153 | 58% |

| WP_085394672.1 3-phosphoshikimate 1-carboxyvinyltransferase [B... | 451 | 8e-153 | 57% |
| --- | --- | --- | --- |
| WP_072386271.1 3-phosphoshikimate 1-carboxyvinyltransferase [H... | 451 | 8e-153 | 58% |
| WP_171795043.1 MULTISPECIES: 3-phosphoshikimate 1-carboxyvinyl... | 451 | 8e-153 | 57% |
| WP_008560786.1 3-phosphoshikimate 1-carboxyvinyltransferase [P... | 451 | 9e-153 | 56% |
| NOW80935.1 3-phosphoshikimate 1-carboxyvinyltransferase [Novos... | 451 | 9e-153 | 57% |
| WP_122114410.1 3-phosphoshikimate 1-carboxyvinyltransferase [P... | 451 | 9e-153 | 55% |
| WP_147125751.1 3-phosphoshikimate 1-carboxyvinyltransferase [S... | 451 | 9e-153 | 55% |
| WP_170414141.1 3-phosphoshikimate 1-carboxyvinyltransferase [R... | 451 | 9e-153 | 56% |
| WP_070933128.1 3-phosphoshikimate 1-carboxyvinyltransferase [S... | 451 | 9e-153 | 59% |
| MBB5310064.1 3-phosphoshikimate 1-carboxyvinyltransferase [Nov... | 451 | 9e-153 | 56% |
| NKB27920.1 3-phosphoshikimate 1-carboxyvinyltransferase [Rhodo... | 451 | 9e-153 | 56% |
| WP_172804619.1 3-phosphoshikimate 1-carboxyvinyltransferase [A... | 451 | 9e-153 | 56% |
| WP_147017874.1 3-phosphoshikimate 1-carboxyvinyltransferase [M... | 451 | 9e-153 | 60% |
| NCN84222.1 3-phosphoshikimate 1-carboxyvinyltransferase [Sphin... | 451 | 9e-153 | 56% |
| WP_088520877.1 3-phosphoshikimate 1-carboxyvinyltransferase [R... | 451 | 1e-152 | 60% |
| WP_036257937.1 3-phosphoshikimate 1-carboxyvinyltransferase [M... | 451 | 1e-152 | 57% |
| WP_179563327.1 MULTISPECIES: 3-phosphoshikimate 1-carboxyvinyl... | 451 | 1e-152 | 56% |
| WP_182913990.1 3-phosphoshikimate 1-carboxyvinyltransferase [S... | 451 | 1e-152 | 58% |
| WP_140980683.1 3-phosphoshikimate 1-carboxyvinyltransferase [B... | 451 | 1e-152 | 56% |
| WP_172626582.1 3-phosphoshikimate 1-carboxyvinyltransferase [B... | 451 | 1e-152 | 56% |
| WP_184148442.1 3-phosphoshikimate 1-carboxyvinyltransferase [A... | 451 | 1e-152 | 57% |
| WP_190843426.1 3-phosphoshikimate 1-carboxyvinyltransferase [M... | 451 | 1e-152 | 60% |
| WP_152426866.1 3-phosphoshikimate 1-carboxyvinyltransferase [M... | 451 | 1e-152 | 58% |
| MBB4612255.1 3-phosphoshikimate 1-carboxyvinyltransferase [Nov... | 451 | 1e-152 | 57% |
| WP_108782915.1 3-phosphoshikimate 1-carboxyvinyltransferase [P... | 451 | 1e-152 | 55% |
| WP_110297104.1 3-phosphoshikimate 1-carboxyvinyltransferase [B... | 451 | 1e-152 | 57% |
| WP_099911347.1 3-phosphoshikimate 1-carboxyvinyltransferase [P... | 451 | 1e-152 | 56% |
| WP_056274115.1 3-phosphoshikimate 1-carboxyvinyltransferase [M... | 451 | 1e-152 | 59% |
| SDG94630.1 3-phosphoshikimate 1-carboxyvinyltransferase [Brady... | 451 | 1e-152 | 56% |
| WP_115547455.1 3-phosphoshikimate 1-carboxyvinyltransferase [S... | 451 | 1e-152 | 57% |
| WP_096701725.1 3-phosphoshikimate 1-carboxyvinyltransferase [M... | 451 | 1e-152 | 58% |
| OYW56281.1 3-phosphoshikimate 1-carboxyvinyltransferase [Hypho... | 451 | 1e-152 | 58% |
| WP_076529554.1 3-phosphoshikimate 1-carboxyvinyltransferase [G... | 451 | 1e-152 | 57% |
| EAQ24222.1 3-phosphoshikimate 1-carboxyvinyltransferase [Roseo... | 450 | 1e-152 | 57% |
| OYX71356.1 3-phosphoshikimate 1-carboxyvinyltransferase [Rhizo... | 451 | 1e-152 | 58% |
| WP_190288192.1 3-phosphoshikimate 1-carboxyvinyltransferase [S... | 450 | 1e-152 | 57% |
| WP_184260833.1 3-phosphoshikimate 1-carboxyvinyltransferase [N... | 451 | 1e-152 | 56% |
| WP_065732304.1 3-phosphoshikimate 1-carboxyvinyltransferase [B... | 451 | 1e-152 | 56% |
| WP_018263257.1 3-phosphoshikimate 1-carboxyvinyltransferase [M... | 451 | 1e-152 | 60% |
| WP_076643906.1 3-phosphoshikimate 1-carboxyvinyltransferase [M... | 451 | 1e-152 | 60% |
| KJC42141.1 3-phosphoshikimate 1-carboxyvinyltransferase [Brady... | 451 | 1e-152 | 57% |
| WP_085362032.1 3-phosphoshikimate 1-carboxyvinyltransferase [B... | 451 | 1e-152 | 57% |
| WP_093254860.1 3-phosphoshikimate 1-carboxyvinyltransferase [R... | 451 | 1e-152 | 57% |
| WP_131673188.1 3-phosphoshikimate 1-carboxyvinyltransferase [P... | 450 | 1e-152 | 55% |
| WP_088332058.1 3-phosphoshikimate 1-carboxyvinyltransferase [S... | 451 | 1e-152 | 57% |
| NDH21727.1 3-phosphoshikimate 1-carboxyvinyltransferase [Rhodo... | 451 | 1e-152 | 56% |
| MBA4088378.1 3-phosphoshikimate 1-carboxyvinyltransferase [Nov... | 450 | 1e-152 | 57% |
| WP_012336181.1 3-phosphoshikimate 1-carboxyvinyltransferase [M... | 451 | 1e-152 | 60% |
| MAR18762.1 3-phosphoshikimate 1-carboxyvinyltransferase [Rhodo... | 450 | 1e-152 | 55% |
| Q2VZA8.1 RecName: Full=3-phosphoshikimate 1-carboxyvinyltransf... | 450 | 1e-152 | 58% |
| WP_158902488.1 3-phosphoshikimate 1-carboxyvinyltransferase [S... | 451 | 1e-152 | 56% |
| WP_015950858.1 3-phosphoshikimate 1-carboxyvinyltransferase [M... | 451 | 1e-152 | 60% |
| WP_168783604.1 3-phosphoshikimate 1-carboxyvinyltransferase [P... | 450 | 2e-152 | 55% |
| WP_041357747.1 3-phosphoshikimate 1-carboxyvinyltransferase [M... | 451 | 2e-152 | 60% |
| WP_184475564.1 3-phosphoshikimate 1-carboxyvinyltransferase [R... | 450 | 2e-152 | 58% |
| WP_161314375.1 3-phosphoshikimate 1-carboxyvinyltransferase [S... | 451 | 2e-152 | 56% |
| OYZ75763.1 3-phosphoshikimate 1-carboxyvinyltransferase [Rhizo... | 451 | 2e-152 | 59% |
| WP_043745553.1 3-phosphoshikimate 1-carboxyvinyltransferase [M... | 450 | 2e-152 | 58% |
| TIR66067.1 3-phosphoshikimate 1-carboxyvinyltransferase [Mesor... | 444 | 2e-152 | 78% |
| WP_109972058.1 3-phosphoshikimate 1-carboxyvinyltransferase [M... | 451 | 2e-152 | 58% |
| TKW67555.1 3-phosphoshikimate 1-carboxyvinyltransferase [Parac... | 450 | 2e-152 | 56% |
| WP_029993739.1 3-phosphoshikimate 1-carboxyvinyltransferase [S... | 451 | 2e-152 | 56% |
| WP_144768620.1 3-phosphoshikimate 1-carboxyvinyltransferase [M... | 451 | 2e-152 | 60% |
| ODU20812.1 3-phosphoshikimate 1-carboxyvinyltransferase [Sphin... | 450 | 2e-152 | 57% |
| WP_148773438.1 3-phosphoshikimate 1-carboxyvinyltransferase [B... | 450 | 2e-152 | 57% |
| MBK18234.1 3-phosphoshikimate 1-carboxyvinyltransferase [Rhodo... | 450 | 2e-152 | 58% |
| WP_056503686.1 MULTISPECIES: 3-phosphoshikimate 1-carboxyvinyl... | 451 | 2e-152 | 60% |
| WP_152578772.1 3-phosphoshikimate 1-carboxyvinyltransferase [P... | 450 | 2e-152 | 59% |
| WP_127110569.1 3-phosphoshikimate 1-carboxyvinyltransferase [P... | 450 | 2e-152 | 57% |
| WP_123792814.1 3-phosphoshikimate 1-carboxyvinyltransferase [P... | 450 | 2e-152 | 57% |
| MBB3927690.1 3-phosphoshikimate 1-carboxyvinyltransferase [Sph... | 451 | 2e-152 | 57% |
| MBB3360337.1 3-phosphoshikimate 1-carboxyvinyltransferase [Nov... | 450 | 2e-152 | 57% |

| PZP87082.1 3-phosphoshikimate 1-carboxyvinyltransferase [Azosp... | 451 | 2e-152 | 56% |
| --- | --- | --- | --- |
| WP_079540289.1 3-phosphoshikimate 1-carboxyvinyltransferase [B... | 451 | 2e-152 | 57% |
| WP_056186602.1 3-phosphoshikimate 1-carboxyvinyltransferase [M... | 450 | 2e-152 | 59% |
| WP_170142236.1 3-phosphoshikimate 1-carboxyvinyltransferase [P... | 451 | 2e-152 | 55% |
| WP_100865475.1 3-phosphoshikimate 1-carboxyvinyltransferase [N... | 450 | 2e-152 | 58% |
| WP_095731857.1 3-phosphoshikimate 1-carboxyvinyltransferase [B... | 451 | 2e-152 | 56% |
| WP_133719923.1 MULTISPECIES: 3-phosphoshikimate 1-carboxyvinyl... | 452 | 2e-152 | 57% |
| WP_103873080.1 3-phosphoshikimate 1-carboxyvinyltransferase [B... | 450 | 2e-152 | 58% |
| GAM07132.1 3-phosphoshikimate 1-carboxyvinyltransferase [Novos... | 450 | 2e-152 | 57% |
| WP_028347901.1 3-phosphoshikimate 1-carboxyvinyltransferase [B... | 450 | 2e-152 | 55% |
| WP_115516163.1 3-phosphoshikimate 1-carboxyvinyltransferase [P... | 450 | 2e-152 | 58% |
| WP_168212734.1 MULTISPECIES: 3-phosphoshikimate 1-carboxyvinyl... | 450 | 2e-152 | 56% |
| WP_160365802.1 3-phosphoshikimate 1-carboxyvinyltransferase [S... | 450 | 2e-152 | 58% |
| WP_171799630.1 MULTISPECIES: 3-phosphoshikimate 1-carboxyvinyl... | 450 | 2e-152 | 57% |
| HBM69480.1 TPA: 3-phosphoshikimate 1-carboxyvinyltransferase [... | 450 | 2e-152 | 55% |
| WP_068077886.1 3-phosphoshikimate 1-carboxyvinyltransferase [N... | 451 | 2e-152 | 56% |
| WP_056681877.1 3-phosphoshikimate 1-carboxyvinyltransferase [S... | 450 | 2e-152 | 57% |
| WP_103911067.1 3-phosphoshikimate 1-carboxyvinyltransferase [T... | 450 | 2e-152 | 57% |
| WP_181267399.1 3-phosphoshikimate 1-carboxyvinyltransferase [S... | 450 | 2e-152 | 56% |
| WP_036356501.1 3-phosphoshikimate 1-carboxyvinyltransferase [M... | 450 | 2e-152 | 60% |
| WP_171578847.1 3-phosphoshikimate 1-carboxyvinyltransferase [B... | 450 | 2e-152 | 55% |
| HBS38655.1 TPA: 3-phosphoshikimate 1-carboxyvinyltransferase [... | 450 | 2e-152 | 56% |
| WP_127657379.1 3-phosphoshikimate 1-carboxyvinyltransferase [P... | 450 | 2e-152 | 57% |
| WP_137776198.1 3-phosphoshikimate 1-carboxyvinyltransferase [P... | 450 | 2e-152 | 57% |
| MBF0268519.1 3-phosphoshikimate 1-carboxyvinyltransferase [Alp... | 451 | 2e-152 | 58% |
| WP_056479693.1 3-phosphoshikimate 1-carboxyvinyltransferase [M... | 450 | 2e-152 | 59% |
| WP_170465696.1 3-phosphoshikimate 1-carboxyvinyltransferase [R... | 450 | 2e-152 | 57% |
| WP_144903109.1 3-phosphoshikimate 1-carboxyvinyltransferase [N... | 450 | 2e-152 | 58% |
| WP_193184064.1 3-phosphoshikimate 1-carboxyvinyltransferase [N... | 449 | 3e-152 | 57% |
| WP_081816430.1 3-phosphoshikimate 1-carboxyvinyltransferase [F... | 450 | 3e-152 | 56% |
| MBE0553346.1 3-phosphoshikimate 1-carboxyvinyltransferase [Rho... | 450 | 3e-152 | 57% |
| PIL19703.1 3-phosphoshikimate 1-carboxyvinyltransferase [Punic... | 451 | 3e-152 | 56% |
| WP_147844045.1 3-phosphoshikimate 1-carboxyvinyltransferase [M... | 450 | 3e-152 | 59% |
| OYX61362.1 3-phosphoshikimate 1-carboxyvinyltransferase [Novos... | 449 | 3e-152 | 59% |
| WP_050672888.1 3-phosphoshikimate 1-carboxyvinyltransferase [P... | 450 | 3e-152 | 56% |
| PCJ06578.1 3-phosphoshikimate 1-carboxyvinyltransferase [Rhodo... | 450 | 3e-152 | 57% |
| WP_085825868.1 3-phosphoshikimate 1-carboxyvinyltransferase [R... | 450 | 3e-152 | 57% |
| WP_183953653.1 3-phosphoshikimate 1-carboxyvinyltransferase [S... | 449 | 3e-152 | 57% |
| WP_057817833.1 3-phosphoshikimate 1-carboxyvinyltransferase [R... | 450 | 3e-152 | 57% |
| WP_142583348.1 3-phosphoshikimate 1-carboxyvinyltransferase [M... | 450 | 3e-152 | 60% |
| WP_007256702.1 3-phosphoshikimate 1-carboxyvinyltransferase [O... | 450 | 3e-152 | 57% |
| HCS68476.1 TPA: 3-phosphoshikimate 1-carboxyvinyltransferase [... | 451 | 3e-152 | 59% |
| WP_185686443.1 3-phosphoshikimate 1-carboxyvinyltransferase [N... | 450 | 3e-152 | 57% |
| WP_093092409.1 3-phosphoshikimate 1-carboxyvinyltransferase [S... | 450 | 3e-152 | 56% |
| HAX91653.1 TPA: 3-phosphoshikimate 1-carboxyvinyltransferase [... | 449 | 3e-152 | 56% |
| WP_170011312.1 3-phosphoshikimate 1-carboxyvinyltransferase [A... | 451 | 3e-152 | 59% |
| WP_056197384.1 3-phosphoshikimate 1-carboxyvinyltransferase [M... | 450 | 3e-152 | 60% |
| ETA52836.1 3-phosphoshikimate 1-carboxyvinyltransferase [Rhodo... | 450 | 3e-152 | 56% |
| WP_147091619.1 3-phosphoshikimate 1-carboxyvinyltransferase [N... | 449 | 3e-152 | 57% |
| WP_090028094.1 3-phosphoshikimate 1-carboxyvinyltransferase [L... | 449 | 3e-152 | 57% |
| HBZ44504.1 TPA: 3-phosphoshikimate 1-carboxyvinyltransferase [... | 450 | 3e-152 | 57% |
| WP_107718291.1 MULTISPECIES: 3-phosphoshikimate 1-carboxyvinyl... | 450 | 3e-152 | 56% |
| WP_065752461.1 3-phosphoshikimate 1-carboxyvinyltransferase [B... | 449 | 3e-152 | 55% |
| WP_100385066.1 3-phosphoshikimate 1-carboxyvinyltransferase [A... | 449 | 3e-152 | 57% |
| WP_058636916.1 3-phosphoshikimate 1-carboxyvinyltransferase [A... | 445 | 3e-152 | 73% |
| WP_126014324.1 3-phosphoshikimate 1-carboxyvinyltransferase [S... | 449 | 4e-152 | 57% |
| OJY31706.1 3-phosphoshikimate 1-carboxyvinyltransferase [Rhodo... | 449 | 4e-152 | 57% |
| WP_148203900.1 3-phosphoshikimate 1-carboxyvinyltransferase [N... | 449 | 4e-152 | 57% |
| MXO59144.1 3-phosphoshikimate 1-carboxyvinyltransferase [Alter... | 449 | 4e-152 | 58% |
| WP_143273913.1 3-phosphoshikimate 1-carboxyvinyltransferase [B... | 450 | 4e-152 | 57% |
| WP_093032177.1 3-phosphoshikimate 1-carboxyvinyltransferase [R... | 449 | 4e-152 | 57% |
| WP_170378969.1 3-phosphoshikimate 1-carboxyvinyltransferase [R... | 449 | 4e-152 | 56% |
| NKX40817.1 3-phosphoshikimate 1-carboxyvinyltransferase [Rhodo... | 449 | 4e-152 | 56% |
| WP_064260760.1 3-phosphoshikimate 1-carboxyvinyltransferase [R... | 449 | 4e-152 | 57% |
| MBA2398882.1 3-phosphoshikimate 1-carboxyvinyltransferase [Bra... | 449 | 4e-152 | 56% |
| WP_008026908.1 3-phosphoshikimate 1-carboxyvinyltransferase [R... | 449 | 4e-152 | 57% |
| HBR62641.1 TPA: 3-phosphoshikimate 1-carboxyvinyltransferase [... | 449 | 4e-152 | 56% |
| NOW69054.1 3-phosphoshikimate 1-carboxyvinyltransferase [Novos... | 449 | 4e-152 | 56% |
| WP_011241800.1 3-phosphoshikimate 1-carboxyvinyltransferase [R... | 449 | 4e-152 | 56% |
| WP_090203480.1 3-phosphoshikimate 1-carboxyvinyltransferase [Y... | 449 | 4e-152 | 57% |
| ARS25733.1 3-phosphoshikimate 1-carboxyvinyltransferase [Sphin... | 449 | 4e-152 | 58% |
| WP_170506563.1 3-phosphoshikimate 1-carboxyvinyltransferase [R... | 449 | 4e-152 | 56% |

| WP_187336780.1 3-phosphoshikimate 1-carboxyvinyltransferase [N... | 449 | 5e-152 | 56% |
| --- | --- | --- | --- |
| WP_100415698.1 3-phosphoshikimate 1-carboxyvinyltransferase [B... | 449 | 5e-152 | 55% |
| WP_054133525.1 3-phosphoshikimate 1-carboxyvinyltransferase [B... | 449 | 5e-152 | 57% |
| RYI33451.1 3-phosphoshikimate 1-carboxyvinyltransferase [Aceto... | 449 | 5e-152 | 56% |
| NOX73069.1 3-phosphoshikimate 1-carboxyvinyltransferase [Alpha... | 449 | 5e-152 | 56% |
| WP_058264142.1 3-phosphoshikimate 1-carboxyvinyltransferase [T... | 449 | 5e-152 | 57% |
| BBK34984.1 3-phosphoshikimate 1-carboxyvinyltransferase [Stell... | 449 | 5e-152 | 57% |
| WP_112142999.1 3-phosphoshikimate 1-carboxyvinyltransferase [M... | 449 | 5e-152 | 57% |
| MAL00017.1 3-phosphoshikimate 1-carboxyvinyltransferase [Citro... | 449 | 5e-152 | 57% |
| KJS40667.1 3-phosphoshikimate 1-carboxyvinyltransferase [Roseo... | 449 | 5e-152 | 57% |
| WP_170385226.1 3-phosphoshikimate 1-carboxyvinyltransferase [R... | 449 | 5e-152 | 56% |
| WP_142494698.1 3-phosphoshikimate 1-carboxyvinyltransferase [L... | 449 | 5e-152 | 56% |
| WP_126003309.1 3-phosphoshikimate 1-carboxyvinyltransferase [S... | 449 | 5e-152 | 59% |
| WP_174320766.1 MULTISPECIES: 3-phosphoshikimate 1-carboxyvinyl... | 449 | 5e-152 | 59% |
| WP_155805337.1 3-phosphoshikimate 1-carboxyvinyltransferase [B... | 449 | 6e-152 | 56% |
| WP_084592265.1 3-phosphoshikimate 1-carboxyvinyltransferase [N... | 450 | 6e-152 | 57% |
| WP_151114541.1 3-phosphoshikimate 1-carboxyvinyltransferase [H... | 453 | 6e-152 | 58% |
| WP_127562346.1 3-phosphoshikimate 1-carboxyvinyltransferase [N... | 449 | 6e-152 | 57% |
| WP_119134817.1 3-phosphoshikimate 1-carboxyvinyltransferase [G... | 449 | 6e-152 | 57% |
| WP_185683937.1 3-phosphoshikimate 1-carboxyvinyltransferase [N... | 449 | 6e-152 | 57% |
| WP_088726476.1 3-phosphoshikimate 1-carboxyvinyltransferase [T... | 449 | 6e-152 | 56% |
| WP_171796639.1 3-phosphoshikimate 1-carboxyvinyltransferase [N... | 449 | 6e-152 | 56% |
| WP_192533666.1 3-phosphoshikimate 1-carboxyvinyltransferase [P... | 449 | 6e-152 | 55% |
| WP_132939492.1 3-phosphoshikimate 1-carboxyvinyltransferase [V... | 449 | 6e-152 | 58% |
| WP_103258808.1 3-phosphoshikimate 1-carboxyvinyltransferase [T... | 449 | 6e-152 | 56% |
| PZQ17237.1 3-phosphoshikimate 1-carboxyvinyltransferase [Stark... | 449 | 6e-152 | 58% |
| WP_048426810.1 3-phosphoshikimate 1-carboxyvinyltransferase [M... | 449 | 6e-152 | 58% |
| WP_066575156.1 3-phosphoshikimate 1-carboxyvinyltransferase [S... | 449 | 6e-152 | 58% |
| WP_096610591.1 3-phosphoshikimate 1-carboxyvinyltransferase [S... | 449 | 6e-152 | 58% |
| WP_074820885.1 MULTISPECIES: 3-phosphoshikimate 1-carboxyvinyl... | 449 | 6e-152 | 56% |
| WP_181195926.1 3-phosphoshikimate 1-carboxyvinyltransferase [R... | 449 | 6e-152 | 56% |
| WP_159793318.1 3-phosphoshikimate 1-carboxyvinyltransferase [A... | 449 | 7e-152 | 58% |
| WP_176936797.1 3-phosphoshikimate 1-carboxyvinyltransferase [B... | 449 | 7e-152 | 56% |
| WP_108854659.1 3-phosphoshikimate 1-carboxyvinyltransferase [D... | 449 | 7e-152 | 57% |
| Q3SW64.1 RecName: Full=3-phosphoshikimate 1-carboxyvinyltransf... | 449 | 7e-152 | 57% |
| WP_068489455.1 3-phosphoshikimate 1-carboxyvinyltransferase [M... | 449 | 7e-152 | 58% |
| WP_072818516.1 3-phosphoshikimate 1-carboxyvinyltransferase [B... | 449 | 7e-152 | 57% |
| WP_167770878.1 3-phosphoshikimate 1-carboxyvinyltransferase [B... | 449 | 7e-152 | 56% |
| WP_171669332.1 3-phosphoshikimate 1-carboxyvinyltransferase [R... | 449 | 7e-152 | 56% |
| TXI01591.1 3-phosphoshikimate 1-carboxyvinyltransferase [Pseud... | 449 | 7e-152 | 58% |
| HAU22386.1 TPA: 3-phosphoshikimate 1-carboxyvinyltransferase [... | 449 | 7e-152 | 57% |
| NVN88420.1 3-phosphoshikimate 1-carboxyvinyltransferase [Rhodo... | 449 | 7e-152 | 57% |
| MAO93715.1 3-phosphoshikimate 1-carboxyvinyltransferase [Rhodo... | 448 | 7e-152 | 58% |
| WP_069956808.1 3-phosphoshikimate 1-carboxyvinyltransferase [M... | 449 | 7e-152 | 55% |
| WP_170426783.1 3-phosphoshikimate 1-carboxyvinyltransferase [R... | 449 | 8e-152 | 56% |
| NJM49494.1 3-phosphoshikimate 1-carboxyvinyltransferase [Sphin... | 449 | 8e-152 | 57% |
| WP_058619794.1 3-phosphoshikimate 1-carboxyvinyltransferase [M... | 449 | 8e-152 | 58% |
| WP_194457496.1 3-phosphoshikimate 1-carboxyvinyltransferase [B... | 449 | 8e-152 | 56% |
| WP_167199045.1 3-phosphoshikimate 1-carboxyvinyltransferase [R... | 449 | 8e-152 | 58% |
| WP_119775321.1 3-phosphoshikimate 1-carboxyvinyltransferase [O... | 448 | 8e-152 | 59% |
| WP_173982348.1 3-phosphoshikimate 1-carboxyvinyltransferase [M... | 448 | 8e-152 | 57% |
| WP_141384293.1 3-phosphoshikimate 1-carboxyvinyltransferase [N... | 448 | 8e-152 | 57% |
| PZU57836.1 3-phosphoshikimate 1-carboxyvinyltransferase [Sphin... | 448 | 8e-152 | 58% |
| WP_099592256.1 3-phosphoshikimate 1-carboxyvinyltransferase [A... | 449 | 8e-152 | 56% |
| WP_156175557.1 3-phosphoshikimate 1-carboxyvinyltransferase [B... | 449 | 8e-152 | 56% |
| OJY12932.1 3-phosphoshikimate 1-carboxyvinyltransferase [Rhizo... | 449 | 8e-152 | 56% |
| MBE5073035.1 3-phosphoshikimate 1-carboxyvinyltransferase [Ery... | 448 | 9e-152 | 60% |
| WP_161851072.1 3-phosphoshikimate 1-carboxyvinyltransferase [B... | 448 | 9e-152 | 55% |
| WP_168856210.1 3-phosphoshikimate 1-carboxyvinyltransferase [B... | 448 | 9e-152 | 56% |
| WP_142114415.1 3-phosphoshikimate 1-carboxyvinyltransferase [B... | 448 | 9e-152 | 59% |
| WP_171947923.1 3-phosphoshikimate 1-carboxyvinyltransferase [B... | 448 | 9e-152 | 56% |
| WP_093570906.1 3-phosphoshikimate 1-carboxyvinyltransferase [M... | 449 | 9e-152 | 58% |
| KZY47210.1 3-phosphoshikimate 1-carboxyvinyltransferase [Roseo... | 448 | 9e-152 | 57% |
| WP_166059246.1 MULTISPECIES: 3-phosphoshikimate 1-carboxyvinyl... | 449 | 9e-152 | 59% |
| WP_015459818.1 3-phosphoshikimate 1-carboxyvinyltransferase [S... | 449 | 1e-151 | 58% |
| WP_183932066.1 3-phosphoshikimate 1-carboxyvinyltransferase [S... | 448 | 1e-151 | 58% |
| WP_149786658.1 3-phosphoshikimate 1-carboxyvinyltransferase [L... | 448 | 1e-151 | 57% |
| WP_026091177.1 3-phosphoshikimate 1-carboxyvinyltransferase [B... | 448 | 1e-151 | 57% |
| WP_183448456.1 3-phosphoshikimate 1-carboxyvinyltransferase [M... | 448 | 1e-151 | 60% |
| TMM08221.1 3-phosphoshikimate 1-carboxyvinyltransferase [Actin... | 448 | 1e-151 | 57% |
| PHR12084.1 3-phosphoshikimate 1-carboxyvinyltransferase [Sphin... | 448 | 1e-151 | 55% |
| WP_035743626.1 3-phosphoshikimate 1-carboxyvinyltransferase [H... | 448 | 1e-151 | 57% |

| WP_042508819.1 3-phosphoshikimate 1-carboxyvinyltransferase [M... | 449 | 1e-151 | 59% |
| --- | --- | --- | --- |
| GEC16652.1 3-phosphoshikimate 1-carboxyvinyltransferase [Nitro... | 448 | 1e-151 | 57% |
| WP_149587888.1 3-phosphoshikimate 1-carboxyvinyltransferase [R... | 448 | 1e-151 | 56% |
| SPH24203.1 3-phosphoshikimate 1-carboxyvinyltransferase [Deflu... | 449 | 1e-151 | 57% |
| WP_173977698.1 3-phosphoshikimate 1-carboxyvinyltransferase [M... | 448 | 1e-151 | 58% |
| NCT40125.1 3-phosphoshikimate 1-carboxyvinyltransferase [Alpha... | 448 | 1e-151 | 55% |
| WP_037507408.1 3-phosphoshikimate 1-carboxyvinyltransferase [S... | 448 | 1e-151 | 57% |
| WP_093063286.1 3-phosphoshikimate 1-carboxyvinyltransferase [S... | 448 | 1e-151 | 56% |
| WP_193378295.1 3-phosphoshikimate 1-carboxyvinyltransferase [S... | 448 | 1e-151 | 57% |
| WP_159758913.1 3-phosphoshikimate 1-carboxyvinyltransferase [S... | 448 | 1e-151 | 57% |
| WP_171238956.1 3-phosphoshikimate 1-carboxyvinyltransferase [R... | 448 | 1e-151 | 56% |
| WP_182297402.1 3-phosphoshikimate 1-carboxyvinyltransferase [S... | 448 | 1e-151 | 57% |
| WP_104071253.1 3-phosphoshikimate 1-carboxyvinyltransferase [A... | 448 | 1e-151 | 57% |
| WP_170351765.1 3-phosphoshikimate 1-carboxyvinyltransferase [R... | 448 | 1e-151 | 56% |
| WP_084302570.1 3-phosphoshikimate 1-carboxyvinyltransferase [B... | 449 | 1e-151 | 56% |
| WP_128486178.1 3-phosphoshikimate 1-carboxyvinyltransferase [G... | 448 | 1e-151 | 57% |
| WP_149125190.1 3-phosphoshikimate 1-carboxyvinyltransferase [R... | 448 | 1e-151 | 57% |
| WP_029585498.1 3-phosphoshikimate 1-carboxyvinyltransferase [B... | 448 | 1e-151 | 57% |
| WP_088724378.1 MULTISPECIES: 3-phosphoshikimate 1-carboxyvinyl... | 448 | 1e-151 | 56% |
| WP_196226522.1 3-phosphoshikimate 1-carboxyvinyltransferase [S... | 447 | 1e-151 | 58% |
| WP_136650373.1 3-phosphoshikimate 1-carboxyvinyltransferase [P... | 448 | 1e-151 | 56% |
| WP_193368962.1 3-phosphoshikimate 1-carboxyvinyltransferase [P... | 448 | 1e-151 | 57% |
| HAM47838.1 TPA: 3-phosphoshikimate 1-carboxyvinyltransferase [... | 448 | 1e-151 | 55% |
| WP_058318323.1 3-phosphoshikimate 1-carboxyvinyltransferase [T... | 448 | 1e-151 | 57% |
| WP_127744510.1 3-phosphoshikimate 1-carboxyvinyltransferase [S... | 448 | 1e-151 | 56% |
| MBB3017522.1 3-phosphoshikimate 1-carboxyvinyltransferase [Mic... | 449 | 1e-151 | 60% |
| OSI25886.1 3-phosphoshikimate 1-carboxyvinyltransferase [Brady... | 448 | 1e-151 | 57% |
| TCS61624.1 3-phosphoshikimate 1-carboxyvinyltransferase [Varun... | 449 | 1e-151 | 58% |
| WP_026299654.1 3-phosphoshikimate 1-carboxyvinyltransferase [E... | 448 | 1e-151 | 56% |
| WP_188408330.1 3-phosphoshikimate 1-carboxyvinyltransferase [A... | 448 | 1e-151 | 60% |
| WP_107663924.1 3-phosphoshikimate 1-carboxyvinyltransferase [C... | 448 | 1e-151 | 56% |
| MBE0452919.1 3-phosphoshikimate 1-carboxyvinyltransferase [Ros... | 448 | 1e-151 | 56% |
| WP_176534640.1 3-phosphoshikimate 1-carboxyvinyltransferase [B... | 448 | 1e-151 | 56% |
| WP_038378541.1 3-phosphoshikimate 1-carboxyvinyltransferase [B... | 448 | 1e-151 | 57% |
| WP_029548965.1 3-phosphoshikimate 1-carboxyvinyltransferase [S... | 449 | 1e-151 | 56% |
| WP_152824585.1 3-phosphoshikimate 1-carboxyvinyltransferase [F... | 448 | 1e-151 | 57% |
| WP_030540465.1 3-phosphoshikimate 1-carboxyvinyltransferase [S... | 447 | 1e-151 | 57% |
| WP_132250293.1 3-phosphoshikimate 1-carboxyvinyltransferase [M... | 448 | 1e-151 | 59% |
| WP_111359923.1 3-phosphoshikimate 1-carboxyvinyltransferase [R... | 448 | 1e-151 | 58% |
| WP_163153989.1 3-phosphoshikimate 1-carboxyvinyltransferase [B... | 448 | 1e-151 | 56% |
| WP_170761576.1 3-phosphoshikimate 1-carboxyvinyltransferase [R... | 448 | 2e-151 | 56% |
| WP_058243196.1 3-phosphoshikimate 1-carboxyvinyltransferase [T... | 448 | 2e-151 | 56% |
| GGE32578.1 3-phosphoshikimate 1-carboxyvinyltransferase [Agari... | 447 | 2e-151 | 60% |
| SEG42948.1 3-phosphoshikimate 1-carboxyvinyltransferase [Bosea... | 448 | 2e-151 | 58% |
| WP_075152059.1 3-phosphoshikimate 1-carboxyvinyltransferase [S... | 447 | 2e-151 | 59% |
| MBF6603708.1 3-phosphoshikimate 1-carboxyvinyltransferase [Sph... | 448 | 2e-151 | 57% |
| WP_155262625.1 MULTISPECIES: 3-phosphoshikimate 1-carboxyvinyl... | 447 | 2e-151 | 57% |
| WP_088722851.1 3-phosphoshikimate 1-carboxyvinyltransferase [T... | 448 | 2e-151 | 55% |
| WP_057020409.1 3-phosphoshikimate 1-carboxyvinyltransferase [B... | 447 | 2e-151 | 56% |
| WP_137111817.1 3-phosphoshikimate 1-carboxyvinyltransferase [R... | 448 | 2e-151 | 56% |
| RMH48607.1 3-phosphoshikimate 1-carboxyvinyltransferase [Alpha... | 447 | 2e-151 | 59% |
| WP_094021526.1 3-phosphoshikimate 1-carboxyvinyltransferase [M... | 447 | 2e-151 | 57% |
| PWG17977.1 3-phosphoshikimate 1-carboxyvinyltransferase [Rhodo... | 448 | 2e-151 | 56% |
| WP_069322074.1 3-phosphoshikimate 1-carboxyvinyltransferase [S... | 447 | 2e-151 | 59% |
| MTV17463.1 3-phosphoshikimate 1-carboxyvinyltransferase [Brady... | 448 | 2e-151 | 57% |
| WP_108855367.1 3-phosphoshikimate 1-carboxyvinyltransferase [A... | 448 | 2e-151 | 57% |
| WP_021082602.1 3-phosphoshikimate 1-carboxyvinyltransferase [B... | 447 | 2e-151 | 56% |
| WP_016843524.1 MULTISPECIES: 3-phosphoshikimate 1-carboxyvinyl... | 447 | 2e-151 | 57% |
| WP_054302421.1 3-phosphoshikimate 1-carboxyvinyltransferase [G... | 447 | 2e-151 | 57% |
| WP_166418870.1 3-phosphoshikimate 1-carboxyvinyltransferase [P... | 447 | 2e-151 | 56% |
| WP_169475971.1 MULTISPECIES: 3-phosphoshikimate 1-carboxyvinyl... | 447 | 2e-151 | 58% |
| WP_071672476.1 3-phosphoshikimate 1-carboxyvinyltransferase [N... | 448 | 2e-151 | 57% |
| HAL76273.1 TPA: 3-phosphoshikimate 1-carboxyvinyltransferase [... | 448 | 2e-151 | 55% |
| WP_166212774.1 MULTISPECIES: 3-phosphoshikimate 1-carboxyvinyl... | 447 | 2e-151 | 56% |
| ALE16712.1 5-Enolpyruvylshikimate-3-phosphate synthase [Altere... | 447 | 2e-151 | 59% |
| WP_069094364.1 3-phosphoshikimate 1-carboxyvinyltransferase [M... | 447 | 2e-151 | 56% |
| WP_085964750.1 3-phosphoshikimate 1-carboxyvinyltransferase [B... | 448 | 2e-151 | 57% |
| QDW35746.1 3-phosphoshikimate 1-carboxyvinyltransferase [Brady... | 448 | 2e-151 | 55% |
| WP_170553685.1 3-phosphoshikimate 1-carboxyvinyltransferase [R... | 447 | 2e-151 | 56% |
| NBE58291.1 3-phosphoshikimate 1-carboxyvinyltransferase [Rhodo... | 447 | 2e-151 | 57% |
| WP_037518460.1 3-phosphoshikimate 1-carboxyvinyltransferase [S... | 447 | 2e-151 | 58% |
| HAD26001.1 TPA: 3-phosphoshikimate 1-carboxyvinyltransferase [... | 448 | 2e-151 | 55% |

| WP_184016997.1 | 3-phosphoshikimate | 1-carboxyvinyltransferase | [S... | 447 | 2e-151 | 56% |
| --- | --- | --- | --- | --- | --- | --- |
| WP_114286497.1 | 3-phosphoshikimate | 1-carboxyvinyltransferase | [C... | 447 | 2e-151 | 56% |
| WP_134332679.1 | 3-phosphoshikimate | 1-carboxyvinyltransferase | [T... | 447 | 2e-151 | 55% |
| WP_142851981.1 | 3-phosphoshikimate | 1-carboxyvinyltransferase | [A... | 447 | 2e-151 | 57% |
| WP_137042732.1 | 3-phosphoshikimate | 1-carboxyvinyltransferase | [P... | 447 | 2e-151 | 58% |
| WP_160755001.1 | 3-phosphoshikimate | 1-carboxyvinyltransferase | [A... | 447 | 2e-151 | 58% |
| WP_138934624.1 | 3-phosphoshikimate | 1-carboxyvinyltransferase | [R... | 447 | 2e-151 | 57% |
| WP_120350583.1 | 3-phosphoshikimate | 1-carboxyvinyltransferase | [S... | 447 | 2e-151 | 57% |
| WP_142639441.1 | 3-phosphoshikimate | 1-carboxyvinyltransferase | [R... | 447 | 2e-151 | 56% |
| WP_092866131.1 | 3-phosphoshikimate | 1-carboxyvinyltransferase | [H... | 447 | 2e-151 | 58% |
| WP_183969440.1 | 3-phosphoshikimate | 1-carboxyvinyltransferase | [S... | 447 | 2e-151 | 56% |
| WP_170517717.1 | 3-phosphoshikimate | 1-carboxyvinyltransferase | [R... | 447 | 2e-151 | 56% |
| WP_073062467.1 | 3-phosphoshikimate | 1-carboxyvinyltransferase | [P... | 447 | 2e-151 | 56% |
| WP_028467654.1 | 3-phosphoshikimate | 1-carboxyvinyltransferase | [N... | 447 | 2e-151 | 57% |
| MAO89962.1 3-phosphoshikimate 1-carboxyvinyltransferase [Rhodo... | | | | 447 | 3e-151 | 58% |
| MAC02454.1 3-phosphoshikimate 1-carboxyvinyltransferase [Marin... | | | | 447 | 3e-151 | 56% |
| WP_085355181.1 3-phosphoshikimate 1-carboxyvinyltransferase [B... | | | | 447 | 3e-151 | 57% |
| WP_069279030.1 3-phosphoshikimate 1-carboxyvinyltransferase [B... | | | | 447 | 3e-151 | 57% |
| WP_075774957.1 MULTISPECIES: 3-phosphoshikimate 1-carboxyvinyl... | | | | 447 | 3e-151 | 55% |
| WP_066681625.1 3-phosphoshikimate 1-carboxyvinyltransferase [S... | | | | 447 | 3e-151 | 58% |
| WP_085409890.1 3-phosphoshikimate 1-carboxyvinyltransferase [B... | | | | 447 | 3e-151 | 57% |
| WP_044025178.1 3-phosphoshikimate 1-carboxyvinyltransferase [R... | | | | 447 | 3e-151 | 56% |
| WP_132907937.1 3-phosphoshikimate 1-carboxyvinyltransferase [S... | | | | 447 | 3e-151 | 58% |
| WP_171181435.1 MULTISPECIES: 3-phosphoshikimate 1-carboxyvinyl... | | | | 447 | 3e-151 | 56% |
| WP_139247098.1 3-phosphoshikimate 1-carboxyvinyltransferase [H... | | | | 447 | 3e-151 | 56% |
| WP_072746470.1 3-phosphoshikimate 1-carboxyvinyltransferase [O... | | | | 447 | 3e-151 | 56% |
| WP_025050799.1 3-phosphoshikimate 1-carboxyvinyltransferase [S... | | | | 447 | 3e-151 | 55% |
| WP_184504344.1 3-phosphoshikimate 1-carboxyvinyltransferase [S... | | | | 447 | 3e-151 | 57% |
| QJR01589.1 3-phosphoshikimate 1-carboxyvinyltransferase [Sphin... | | | | 447 | 3e-151 | 57% |
| WP_128515135.1 3-phosphoshikimate 1-carboxyvinyltransferase [R... | | | | 447 | 3e-151 | 57% |
| WP_076705555.1 3-phosphoshikimate 1-carboxyvinyltransferase [P... | | | | 447 | 3e-151 | 57% |
| WP_126618140.1 3-phosphoshikimate 1-carboxyvinyltransferase [A... | | | | 447 | 3e-151 | 58% |
| WP_143273208.1 MULTISPECIES: 3-phosphoshikimate 1-carboxyvinyl... | | | | 448 | 3e-151 | 57% |
| WP_073197375.1 3-phosphoshikimate 1-carboxyvinyltransferase [R... | | | | 447 | 3e-151 | 57% |
| WP_193183603.1 3-phosphoshikimate 1-carboxyvinyltransferase [M... | | | | 447 | 3e-151 | 56% |
| WP_114963430.1 3-phosphoshikimate 1-carboxyvinyltransferase [E... | | | | 447 | 3e-151 | 56% |
| WP_149076405.1 MULTISPECIES: 3-phosphoshikimate 1-carboxyvinyl... | | | | 447 | 3e-151 | 57% |
| WP_040604691.1 3-phosphoshikimate 1-carboxyvinyltransferase [S... | | | | 447 | 3e-151 | 56% |
| QOZ30384.1 3-phosphoshikimate 1-carboxyvinyltransferase [Brady... | | | | 447 | 3e-151 | 56% |
| WP_108434447.1 3-phosphoshikimate 1-carboxyvinyltransferase [G... | | | | 447 | 3e-151 | 57% |
| WP_171131600.1 MULTISPECIES: 3-phosphoshikimate 1-carboxyvinyl... | | | | 447 | 3e-151 | 57% |
| WP_171235361.1 3-phosphoshikimate 1-carboxyvinyltransferase [R... | | | | 447 | 3e-151 | 56% |
| THD46793.1 3-phosphoshikimate 1-carboxyvinyltransferase [Brady... | | | | 447 | 3e-151 | 57% |
| NBX66466.1 3-phosphoshikimate 1-carboxyvinyltransferase [Prote... | | | | 446 | 3e-151 | 56% |
| WP_164115732.1 3-phosphoshikimate 1-carboxyvinyltransferase [S... | | | | 447 | 3e-151 | 57% |
| MBF9025526.1 3-phosphoshikimate 1-carboxyvinyltransferase [Rho... | | | | 447 | 3e-151 | 56% |
| WP_037263828.1 3-phosphoshikimate 1-carboxyvinyltransferase [R... | | | | 447 | 4e-151 | 55% |
| WP_074272185.1 3-phosphoshikimate 1-carboxyvinyltransferase [B... | | | | 447 | 4e-151 | 56% |
| WP_054004088.1 3-phosphoshikimate 1-carboxyvinyltransferase [S... | | | | 447 | 4e-151 | 56% |
| WP_160600525.1 3-phosphoshikimate 1-carboxyvinyltransferase [A... | | | | 447 | 4e-151 | 58% |
| EJU13553.1 3-phosphoshikimate 1-carboxyvinyltransferase [Sphin... | | | | 447 | 4e-151 | 58% |
| WP_128089907.1 3-phosphoshikimate 1-carboxyvinyltransferase [M... | | | | 447 | 4e-151 | 60% |
| WP_046477896.1 MULTISPECIES: 3-phosphoshikimate 1-carboxyvinyl... | | | | 447 | 4e-151 | 57% |
| HCS22442.1 TPA: 3-phosphoshikimate 1-carboxyvinyltransferase [... | | | | 447 | 4e-151 | 56% |
| MBB3475570.1 3-phosphoshikimate 1-carboxyvinyltransferase [Sph... | | | | 446 | 4e-151 | 59% |
| VVB42963.1 3-phosphoshikimate 1-carboxyvinyltransferase [Beije... | | | | 447 | 4e-151 | 59% |
| KEO87448.1 3-phosphoshikimate 1-carboxyvinyltransferase [Eryth... | | | | 446 | 4e-151 | 57% |
| EEW60351.1 3-phosphoshikimate 1-carboxyvinyltransferase [Silic... | | | | 447 | 4e-151 | 56% |
| WP_065334340.1 3-phosphoshikimate 1-carboxyvinyltransferase [E... | | | | 447 | 4e-151 | 56% |
| PZR85444.1 3-phosphoshikimate 1-carboxyvinyltransferase [Rhizo... | | | | 447 | 4e-151 | 58% |
| SFV26215.1 3-phosphoshikimate 1-carboxyvinyltransferase [Hypho... | | | | 447 | 4e-151 | 58% |
| WP_165087556.1 3-phosphoshikimate 1-carboxyvinyltransferase [M... | | | | 447 | 4e-151 | 58% |
| WP_122129513.1 3-phosphoshikimate 1-carboxyvinyltransferase [S... | | | | 447 | 4e-151 | 57% |
| WP_037310887.1 3-phosphoshikimate 1-carboxyvinyltransferase [R... | | | | 447 | 4e-151 | 56% |
| ABY30523.1 3-phosphoshikimate 1-carboxyvinyltransferase [Methy... | | | | 447 | 4e-151 | 59% |
| TAK46702.1 3-phosphoshikimate 1-carboxyvinyltransferase [Xanth... | | | | 447 | 4e-151 | 56% |
| WP_191123724.1 3-phosphoshikimate 1-carboxyvinyltransferase [B... | | | | 447 | 4e-151 | 60% |
| EJW09515.1 5-Enolpyruvylshikimate-3-phosphate synthase [Rhodov... | | | | 447 | 4e-151 | 57% |
| WP_132245913.1 3-phosphoshikimate 1-carboxyvinyltransferase [P... | | | | 447 | 4e-151 | 56% |
| WP_127907399.1 3-phosphoshikimate 1-carboxyvinyltransferase [T... | | | | 447 | 4e-151 | 54% |
| WP_088216912.1 3-phosphoshikimate 1-carboxyvinyltransferase [H... | | | | 447 | 4e-151 | 57% |
| WP_117869790.1 3-phosphoshikimate 1-carboxyvinyltransferase [R... | | | | 447 | 4e-151 | 56% |

| WP_147764024.1 MULTISPECIES: 3-phosphoshikimate 1-carboxyvinyl... | 447 | 4e-151 | 59% |
| --- | --- | --- | --- |
| WP_056406030.1 3-phosphoshikimate 1-carboxyvinyltransferase [S... | 447 | 4e-151 | 57% |
| WP_171094339.1 MULTISPECIES: 3-phosphoshikimate 1-carboxyvinyl... | 447 | 4e-151 | 56% |
| WP_065329133.1 3-phosphoshikimate 1-carboxyvinyltransferase [E... | 447 | 5e-151 | 56% |
| WP_067627293.1 MULTISPECIES: 3-phosphoshikimate 1-carboxyvinyl... | 447 | 5e-151 | 56% |
| WP_108968363.1 3-phosphoshikimate 1-carboxyvinyltransferase [S... | 447 | 5e-151 | 56% |
| NCP18761.1 3-phosphoshikimate 1-carboxyvinyltransferase [Eryth... | 446 | 5e-151 | 60% |
| WP_028965401.1 3-phosphoshikimate 1-carboxyvinyltransferase [S... | 446 | 5e-151 | 57% |
| WP_040622225.1 3-phosphoshikimate 1-carboxyvinyltransferase [R... | 447 | 5e-151 | 57% |
| PIE10304.1 3-phosphoshikimate 1-carboxyvinyltransferase [Rhodo... | 447 | 5e-151 | 56% |
| WP_146689381.1 3-phosphoshikimate 1-carboxyvinyltransferase [B... | 446 | 5e-151 | 56% |
| WP_085555968.1 3-phosphoshikimate 1-carboxyvinyltransferase [A... | 446 | 5e-151 | 57% |
| WP_160735330.1 3-phosphoshikimate 1-carboxyvinyltransferase [A... | 446 | 5e-151 | 57% |
| NTU76582.1 3-phosphoshikimate 1-carboxyvinyltransferase [Alpha... | 446 | 5e-151 | 58% |
| WP_011537567.1 3-phosphoshikimate 1-carboxyvinyltransferase [R... | 447 | 5e-151 | 56% |
| WP_126994204.1 3-phosphoshikimate 1-carboxyvinyltransferase [A... | 446 | 5e-151 | 57% |
| WP_146363514.1 3-phosphoshikimate 1-carboxyvinyltransferase [L... | 446 | 6e-151 | 56% |
| WP_174861168.1 3-phosphoshikimate 1-carboxyvinyltransferase [S... | 446 | 6e-151 | 56% |
| WP_171100542.1 3-phosphoshikimate 1-carboxyvinyltransferase [R... | 447 | 6e-151 | 57% |
| MPZ55731.1 3-phosphoshikimate 1-carboxyvinyltransferase [Rhizo... | 446 | 6e-151 | 58% |
| PHP18121.1 3-phosphoshikimate 1-carboxyvinyltransferase [Sphin... | 446 | 6e-151 | 57% |
| NJS38798.1 3-phosphoshikimate 1-carboxyvinyltransferase [Rhodo... | 447 | 6e-151 | 57% |
| WP_173639765.1 3-phosphoshikimate 1-carboxyvinyltransferase [B... | 446 | 6e-151 | 56% |
| NLS69439.1 3-phosphoshikimate 1-carboxyvinyltransferase [Brady... | 447 | 6e-151 | 57% |
| OAO01084.1 3-phosphoshikimate 1-carboxyvinyltransferase [Sphin... | 446 | 6e-151 | 54% |
| WP_068363952.1 3-phosphoshikimate 1-carboxyvinyltransferase [J... | 446 | 6e-151 | 56% |
| NEU96987.1 3-phosphoshikimate 1-carboxyvinyltransferase [Brady... | 447 | 6e-151 | 56% |
| WP_074131069.1 3-phosphoshikimate 1-carboxyvinyltransferase [B... | 446 | 6e-151 | 55% |
| WP_096787866.1 3-phosphoshikimate 1-carboxyvinyltransferase [R... | 446 | 6e-151 | 57% |
| MBB4374930.1 3-phosphoshikimate 1-carboxyvinyltransferase [Bra... | 445 | 6e-151 | 58% |
| WP_171363664.1 3-phosphoshikimate 1-carboxyvinyltransferase [R... | 446 | 7e-151 | 56% |
| WP_170399154.1 3-phosphoshikimate 1-carboxyvinyltransferase [R... | 446 | 7e-151 | 56% |
| WP_171187027.1 MULTISPECIES: 3-phosphoshikimate 1-carboxyvinyl... | 446 | 7e-151 | 56% |
| BAR56881.1 3-phosphoshikimate 1-carboxyvinyltransferase [Brady... | 445 | 7e-151 | 58% |
| WP_106940427.1 3-phosphoshikimate 1-carboxyvinyltransferase [B... | 446 | 7e-151 | 55% |
| WP_136792682.1 3-phosphoshikimate 1-carboxyvinyltransferase [C... | 446 | 7e-151 | 56% |
| PKP71493.1 3-phosphoshikimate 1-carboxyvinyltransferase [Alpha... | 446 | 7e-151 | 57% |
| WP_083440110.1 3-phosphoshikimate 1-carboxyvinyltransferase [A... | 447 | 7e-151 | 59% |
| WP_171728099.1 3-phosphoshikimate 1-carboxyvinyltransferase [R... | 446 | 7e-151 | 56% |
| TNY31416.1 3-phosphoshikimate 1-carboxyvinyltransferase [Rhodo... | 446 | 8e-151 | 56% |
| WP_168556743.1 3-phosphoshikimate 1-carboxyvinyltransferase [N... | 446 | 8e-151 | 57% |
| WP_071691949.1 MULTISPECIES: 3-phosphoshikimate 1-carboxyvinyl... | 446 | 8e-151 | 56% |
| WP_156260725.1 3-phosphoshikimate 1-carboxyvinyltransferase [O... | 446 | 8e-151 | 56% |
| TIX56143.1 3-phosphoshikimate 1-carboxyvinyltransferase [Mesor... | 440 | 8e-151 | 77% |
| APZ55113.1 3-phosphoshikimate 1-carboxyvinyltransferase [Pelag... | 446 | 9e-151 | 57% |
| MBD1204083.1 3-phosphoshikimate 1-carboxyvinyltransferase [Rho... | 446 | 9e-151 | 57% |
| AEI92546.1 3-phosphoshikimate 1-carboxyvinyltransferase AroA [... | 447 | 9e-151 | 56% |
| WP_195820194.1 3-phosphoshikimate 1-carboxyvinyltransferase [R... | 446 | 9e-151 | 56% |
| WP_184012612.1 3-phosphoshikimate 1-carboxyvinyltransferase [R... | 446 | 9e-151 | 56% |
| WP_035713374.1 3-phosphoshikimate 1-carboxyvinyltransferase [H... | 446 | 9e-151 | 56% |
| WP_028341707.1 3-phosphoshikimate 1-carboxyvinyltransferase [B... | 446 | 9e-151 | 57% |
| WP_141340870.1 MULTISPECIES: 3-phosphoshikimate 1-carboxyvinyl... | 446 | 9e-151 | 57% |
| WP_081826140.1 3-phosphoshikimate 1-carboxyvinyltransferase [C... | 446 | 9e-151 | 56% |
| WP_084366947.1 3-phosphoshikimate 1-carboxyvinyltransferase [N... | 446 | 9e-151 | 56% |
| NKX37714.1 3-phosphoshikimate 1-carboxyvinyltransferase [Rhodo... | 446 | 9e-151 | 56% |
| NKB45766.1 3-phosphoshikimate 1-carboxyvinyltransferase [Alpha... | 446 | 1e-150 | 56% |
| EBA07679.1 3-phosphoshikimate 1-carboxyvinyltransferase [Sagit... | 446 | 1e-150 | 56% |
| RYD57205.1 3-phosphoshikimate 1-carboxyvinyltransferase [Sphin... | 446 | 1e-150 | 56% |
| WP_114275343.1 3-phosphoshikimate 1-carboxyvinyltransferase [S... | 446 | 1e-150 | 56% |
| WP_147753586.1 3-phosphoshikimate 1-carboxyvinyltransferase [M... | 446 | 1e-150 | 58% |
| WP_171209448.1 MULTISPECIES: 3-phosphoshikimate 1-carboxyvinyl... | 446 | 1e-150 | 57% |
| OYU37940.1 3-phosphoshikimate 1-carboxyvinyltransferase [Pseud... | 446 | 1e-150 | 56% |
| WP_058315452.1 3-phosphoshikimate 1-carboxyvinyltransferase [C... | 446 | 1e-150 | 56% |
| RUP08589.1 3-phosphoshikimate 1-carboxyvinyltransferase [Hypho... | 446 | 1e-150 | 57% |
| WP_183920183.1 3-phosphoshikimate 1-carboxyvinyltransferase [S... | 446 | 1e-150 | 58% |
| WP_088718518.1 3-phosphoshikimate 1-carboxyvinyltransferase [T... | 446 | 1e-150 | 55% |
| OQW61081.1 3-phosphoshikimate 1-carboxyvinyltransferase [Prote... | 446 | 1e-150 | 55% |
| NWG45570.1 3-phosphoshikimate 1-carboxyvinyltransferase [Alpha... | 446 | 1e-150 | 58% |
| WP_125993157.1 3-phosphoshikimate 1-carboxyvinyltransferase [S... | 446 | 1e-150 | 57% |
| TFV47420.1 3-phosphoshikimate 1-carboxyvinyltransferase [Brady... | 446 | 1e-150 | 55% |
| PKQ13599.1 3-phosphoshikimate 1-carboxyvinyltransferase [Alpha... | 446 | 1e-150 | 56% |
| PID36164.1 3-phosphoshikimate 1-carboxyvinyltransferase [Rhodo... | 446 | 1e-150 | 57% |

| WP_192736225.1 3-phosphoshikimate 1-carboxyvinyltransferase [B... | 446 | 1e-150 | 56% |
| --- | --- | --- | --- |
| WP_028093850.1 3-phosphoshikimate 1-carboxyvinyltransferase [P... | 446 | 1e-150 | 56% |
| WP_006613118.1 3-phosphoshikimate 1-carboxyvinyltransferase [B... | 446 | 1e-150 | 57% |
| WP_073251603.1 3-phosphoshikimate 1-carboxyvinyltransferase [S... | 446 | 1e-150 | 56% |
| WP_109907353.1 3-phosphoshikimate 1-carboxyvinyltransferase [Z... | 446 | 1e-150 | 58% |
| WP_136645022.1 3-phosphoshikimate 1-carboxyvinyltransferase [T... | 446 | 1e-150 | 56% |
| WP_189380952.1 3-phosphoshikimate 1-carboxyvinyltransferase [G... | 446 | 1e-150 | 57% |
| WP_039285032.1 3-phosphoshikimate 1-carboxyvinyltransferase [N... | 445 | 1e-150 | 58% |
| WP_166401415.1 3-phosphoshikimate 1-carboxyvinyltransferase [R... | 446 | 1e-150 | 56% |
| MBA4761315.1 3-phosphoshikimate 1-carboxyvinyltransferase [Sph... | 446 | 1e-150 | 59% |
| KGJ65176.1 putative 3-phosphoshikimate 1-carboxyvinyltransfera... | 444 | 1e-150 | 58% |
| MBA9069169.1 3-phosphoshikimate 1-carboxyvinyltransferase [Met... | 445 | 1e-150 | 61% |
| WP_102874823.1 3-phosphoshikimate 1-carboxyvinyltransferase [P... | 446 | 1e-150 | 56% |
| WP_065326226.1 3-phosphoshikimate 1-carboxyvinyltransferase [E... | 446 | 1e-150 | 56% |
| WP_101798649.1 3-phosphoshikimate 1-carboxyvinyltransferase [N... | 445 | 1e-150 | 58% |
| WP_119751807.1 3-phosphoshikimate 1-carboxyvinyltransferase [P... | 446 | 1e-150 | 54% |
| MAQ86161.1 3-phosphoshikimate 1-carboxyvinyltransferase [Marit... | 446 | 1e-150 | 57% |
| WP_038035161.1 3-phosphoshikimate 1-carboxyvinyltransferase [T... | 446 | 1e-150 | 56% |
| WP_051279868.1 3-phosphoshikimate 1-carboxyvinyltransferase [H... | 446 | 1e-150 | 54% |
| WP_050527880.1 3-phosphoshikimate 1-carboxyvinyltransferase [P... | 445 | 1e-150 | 57% |
| WP_170769913.1 3-phosphoshikimate 1-carboxyvinyltransferase [R... | 445 | 2e-150 | 56% |
| WP_171241061.1 3-phosphoshikimate 1-carboxyvinyltransferase [R... | 445 | 2e-150 | 56% |
| WP_149522171.1 3-phosphoshikimate 1-carboxyvinyltransferase [S... | 445 | 2e-150 | 55% |
| WP_177158681.1 3-phosphoshikimate 1-carboxyvinyltransferase [D... | 445 | 2e-150 | 57% |
| WP_024584328.1 MULTISPECIES: 3-phosphoshikimate 1-carboxyvinyl... | 445 | 2e-150 | 56% |
| WP_066798914.1 3-phosphoshikimate 1-carboxyvinyltransferase [S... | 445 | 2e-150 | 56% |
| WP_076863574.1 3-phosphoshikimate 1-carboxyvinyltransferase [B... | 445 | 2e-150 | 55% |
| SHE90971.1 3-phosphoshikimate 1-carboxyvinyltransferase [Acido... | 446 | 2e-150 | 55% |
| PWR03251.1 3-phosphoshikimate 1-carboxyvinyltransferase [Rhodo... | 445 | 2e-150 | 56% |
| WP_170326973.1 3-phosphoshikimate 1-carboxyvinyltransferase [R... | 445 | 2e-150 | 56% |
| WP_140047969.1 3-phosphoshikimate 1-carboxyvinyltransferase [S... | 445 | 2e-150 | 58% |
| KZZ21568.1 3-phosphoshikimate 1-carboxyvinyltransferase [Sulfi... | 445 | 2e-150 | 56% |
| WP_184003972.1 3-phosphoshikimate 1-carboxyvinyltransferase [S... | 445 | 2e-150 | 55% |
| WP_050383457.1 3-phosphoshikimate 1-carboxyvinyltransferase [B... | 445 | 2e-150 | 55% |
| CAA6604547.1 3-phosphoshikimate 1-carboxyvinyltransferase [Rho... | 446 | 2e-150 | 59% |
| WP_182115379.1 3-phosphoshikimate 1-carboxyvinyltransferase [S... | 445 | 2e-150 | 57% |
| WP_054587435.1 3-phosphoshikimate 1-carboxyvinyltransferase [S... | 445 | 2e-150 | 58% |
| WP_048879587.1 3-phosphoshikimate 1-carboxyvinyltransferase [A... | 445 | 2e-150 | 55% |
| WP_135504658.1 3-phosphoshikimate 1-carboxyvinyltransferase [R... | 445 | 2e-150 | 57% |
| WP_091857251.1 3-phosphoshikimate 1-carboxyvinyltransferase [B... | 445 | 2e-150 | 59% |
| ANI76726.1 3-phosphoshikimate 1-carboxyvinyltransferase [Sphin... | 445 | 2e-150 | 57% |
| WP_109329166.1 3-phosphoshikimate 1-carboxyvinyltransferase [A... | 445 | 2e-150 | 58% |
| WP_088367739.1 3-phosphoshikimate 1-carboxyvinyltransferase [S... | 445 | 2e-150 | 56% |
| WP_179512013.1 MULTISPECIES: 3-phosphoshikimate 1-carboxyvinyl... | 445 | 2e-150 | 56% |
| NBR53521.1 3-phosphoshikimate 1-carboxyvinyltransferase [Rhodo... | 445 | 2e-150 | 57% |
| WP_192708089.1 3-phosphoshikimate 1-carboxyvinyltransferase [M... | 445 | 2e-150 | 60% |
| CAA2137178.1 3-phosphoshikimate 1-carboxyvinyltransferase [Hyp... | 446 | 2e-150 | 58% |
| WP_183669926.1 3-phosphoshikimate 1-carboxyvinyltransferase [M... | 445 | 2e-150 | 60% |
| WP_096707348.1 3-phosphoshikimate 1-carboxyvinyltransferase [P... | 445 | 2e-150 | 56% |
| WP_072791061.1 3-phosphoshikimate 1-carboxyvinyltransferase [C... | 445 | 2e-150 | 57% |
| WP_115378911.1 3-phosphoshikimate 1-carboxyvinyltransferase [S... | 445 | 2e-150 | 58% |
| WP_170430099.1 3-phosphoshikimate 1-carboxyvinyltransferase [R... | 445 | 2e-150 | 56% |
| OUR78492.1 3-phosphoshikimate 1-carboxyvinyltransferase [Alpha... | 445 | 2e-150 | 58% |
| WP_170403936.1 3-phosphoshikimate 1-carboxyvinyltransferase [R... | 445 | 2e-150 | 56% |
| WP_152457170.1 3-phosphoshikimate 1-carboxyvinyltransferase [R... | 445 | 2e-150 | 56% |
| WP_179506561.1 MULTISPECIES: 3-phosphoshikimate 1-carboxyvinyl... | 444 | 2e-150 | 57% |
| WP_093928555.1 3-phosphoshikimate 1-carboxyvinyltransferase [S... | 445 | 2e-150 | 56% |
| NRB36278.1 3-phosphoshikimate 1-carboxyvinyltransferase [Rhodo... | 445 | 2e-150 | 57% |
| RTL15440.1 3-phosphoshikimate 1-carboxyvinyltransferase [Sphin... | 445 | 2e-150 | 57% |
| WP_080663988.1 3-phosphoshikimate 1-carboxyvinyltransferase [B... | 446 | 2e-150 | 56% |
| WP_113825142.1 3-phosphoshikimate 1-carboxyvinyltransferase [P... | 445 | 2e-150 | 56% |
| WP_050404281.1 3-phosphoshikimate 1-carboxyvinyltransferase [B... | 445 | 2e-150 | 55% |
| PZN17384.1 3-phosphoshikimate 1-carboxyvinyltransferase [Prote... | 444 | 2e-150 | 60% |
| OYX51009.1 3-phosphoshikimate 1-carboxyvinyltransferase [Sphin... | 445 | 2e-150 | 59% |
| WP_152535582.1 3-phosphoshikimate 1-carboxyvinyltransferase [B... | 446 | 2e-150 | 56% |
| MBB4257690.1 3-phosphoshikimate 1-carboxyvinyltransferase [Bra... | 444 | 2e-150 | 58% |
| MBB5719320.1 3-phosphoshikimate 1-carboxyvinyltransferase [Sta... | 445 | 2e-150 | 55% |
| WP_108940054.1 3-phosphoshikimate 1-carboxyvinyltransferase [M... | 445 | 3e-150 | 60% |
| WP_029010516.1 3-phosphoshikimate 1-carboxyvinyltransferase [A... | 445 | 3e-150 | 57% |
| NOT42123.1 3-phosphoshikimate 1-carboxyvinyltransferase [Alpha... | 445 | 3e-150 | 56% |
| WP_066523881.1 3-phosphoshikimate 1-carboxyvinyltransferase [S... | 445 | 3e-150 | 57% |
| WP_033925957.1 3-phosphoshikimate 1-carboxyvinyltransferase [S... | 445 | 3e-150 | 57% |

| WP_029082480.1 3-phosphoshikimate 1-carboxyvinyltransferase [B... | 444 | 3e-150 | 56% |
| --- | --- | --- | --- |
| MBG78836.1 3-phosphoshikimate 1-carboxyvinyltransferase [Alpha... | 445 | 3e-150 | 55% |
| WP_116460857.1 MULTISPECIES: 3-phosphoshikimate 1-carboxyvinyl... | 445 | 3e-150 | 58% |
| WP_058754670.1 3-phosphoshikimate 1-carboxyvinyltransferase [S... | 444 | 3e-150 | 57% |
| WP_110364904.1 3-phosphoshikimate 1-carboxyvinyltransferase [R... | 445 | 3e-150 | 56% |
| WP_170319032.1 3-phosphoshikimate 1-carboxyvinyltransferase [S... | 444 | 3e-150 | 57% |
| WP_163894500.1 3-phosphoshikimate 1-carboxyvinyltransferase [P... | 445 | 3e-150 | 55% |
| WP_196264403.1 3-phosphoshikimate 1-carboxyvinyltransferase [M... | 444 | 3e-150 | 60% |
| KAF0183921.1 3-phosphoshikimate 1-carboxyvinyltransferase [Hyp... | 444 | 3e-150 | 54% |
| WP_187429417.1 3-phosphoshikimate 1-carboxyvinyltransferase [R... | 444 | 3e-150 | 56% |
| RYY23537.1 3-phosphoshikimate 1-carboxyvinyltransferase [Sphin... | 444 | 3e-150 | 55% |
| WP_170572991.1 3-phosphoshikimate 1-carboxyvinyltransferase [R... | 444 | 3e-150 | 56% |
| PHS77794.1 3-phosphoshikimate 1-carboxyvinyltransferase [Rhodo... | 444 | 3e-150 | 55% |
| WP_024081958.1 3-phosphoshikimate 1-carboxyvinyltransferase [M... | 444 | 3e-150 | 57% |
| WP_171121779.1 MULTISPECIES: 3-phosphoshikimate 1-carboxyvinyl... | 444 | 3e-150 | 56% |
| AMK17839.1 3-phosphoshikimate 1-carboxyvinyltransferase [Sphin... | 443 | 4e-150 | 58% |
| WP_169863241.1 3-phosphoshikimate 1-carboxyvinyltransferase [S... | 444 | 4e-150 | 58% |
| MBB5557016.1 3-phosphoshikimate 1-carboxyvinyltransferase [Sph... | 444 | 4e-150 | 57% |
| WP_014879147.1 3-phosphoshikimate 1-carboxyvinyltransferase [P... | 444 | 4e-150 | 56% |
| MBB3346078.1 3-phosphoshikimate 1-carboxyvinyltransferase [Sph... | 444 | 4e-150 | 58% |
| WP_164189525.1 MULTISPECIES: 3-phosphoshikimate 1-carboxyvinyl... | 444 | 4e-150 | 56% |
| HCI20021.1 TPA: 3-phosphoshikimate 1-carboxyvinyltransferase [... | 444 | 4e-150 | 54% |
| MAE47522.1 3-phosphoshikimate 1-carboxyvinyltransferase [SAR11... | 444 | 4e-150 | 54% |
| WP_130579844.1 3-phosphoshikimate 1-carboxyvinyltransferase [B... | 445 | 4e-150 | 56% |
| WP_137195333.1 3-phosphoshikimate 1-carboxyvinyltransferase [P... | 444 | 4e-150 | 57% |
| WP_085824275.1 3-phosphoshikimate 1-carboxyvinyltransferase [R... | 444 | 4e-150 | 55% |
| WP_167637212.1 3-phosphoshikimate 1-carboxyvinyltransferase [M... | 444 | 4e-150 | 56% |
| MBF0169022.1 3-phosphoshikimate 1-carboxyvinyltransferase [Alp... | 444 | 4e-150 | 58% |
| WP_110762830.1 3-phosphoshikimate 1-carboxyvinyltransferase [N... | 444 | 4e-150 | 57% |
| WP_124963527.1 3-phosphoshikimate 1-carboxyvinyltransferase [F... | 444 | 4e-150 | 57% |
| WP_092778340.1 3-phosphoshikimate 1-carboxyvinyltransferase [J... | 444 | 4e-150 | 57% |
| WP_003615654.1 MULTISPECIES: 3-phosphoshikimate 1-carboxyvinyl... | 444 | 4e-150 | 57% |
| WP_031294933.1 3-phosphoshikimate 1-carboxyvinyltransferase [S... | 444 | 4e-150 | 58% |
| NBD28623.1 3-phosphoshikimate 1-carboxyvinyltransferase [Alpha... | 444 | 4e-150 | 56% |
| WP_163037782.1 3-phosphoshikimate 1-carboxyvinyltransferase [A... | 444 | 4e-150 | 57% |
| WP_056365363.1 MULTISPECIES: 3-phosphoshikimate 1-carboxyvinyl... | 444 | 4e-150 | 58% |
| WP_180284078.1 3-phosphoshikimate 1-carboxyvinyltransferase [A... | 444 | 4e-150 | 58% |
| WP_133489315.1 3-phosphoshikimate 1-carboxyvinyltransferase [A... | 444 | 4e-150 | 57% |
| WP_171205597.1 MULTISPECIES: 3-phosphoshikimate 1-carboxyvinyl... | 444 | 4e-150 | 56% |
| WP_095590506.1 3-phosphoshikimate 1-carboxyvinyltransferase [A... | 444 | 4e-150 | 56% |
| WP_115493064.1 3-phosphoshikimate 1-carboxyvinyltransferase [E... | 444 | 5e-150 | 56% |
| WP_180275748.1 3-phosphoshikimate 1-carboxyvinyltransferase [S... | 444 | 5e-150 | 58% |
| WP_108128945.1 3-phosphoshikimate 1-carboxyvinyltransferase [G... | 444 | 5e-150 | 57% |
| WP_091883846.1 3-phosphoshikimate 1-carboxyvinyltransferase [M... | 444 | 5e-150 | 59% |
| WP_171125797.1 MULTISPECIES: 3-phosphoshikimate 1-carboxyvinyl... | 444 | 5e-150 | 56% |
| WP_037458751.1 3-phosphoshikimate 1-carboxyvinyltransferase [S... | 444 | 5e-150 | 58% |
| WP_053553764.1 3-phosphoshikimate 1-carboxyvinyltransferase [S... | 444 | 5e-150 | 59% |
| WP_097928210.1 3-phosphoshikimate 1-carboxyvinyltransferase [M... | 444 | 5e-150 | 55% |
| MBA4035415.1 3-phosphoshikimate 1-carboxyvinyltransferase [Bra... | 444 | 5e-150 | 55% |
| WP_184067007.1 3-phosphoshikimate 1-carboxyvinyltransferase [S... | 444 | 5e-150 | 59% |
| WP_108886921.1 3-phosphoshikimate 1-carboxyvinyltransferase [P... | 444 | 5e-150 | 56% |
| WP_028287263.1 3-phosphoshikimate 1-carboxyvinyltransferase [P... | 444 | 5e-150 | 56% |
| WP_113890659.1 3-phosphoshikimate 1-carboxyvinyltransferase [R... | 444 | 5e-150 | 60% |
| WP_090709449.1 3-phosphoshikimate 1-carboxyvinyltransferase [B... | 444 | 5e-150 | 59% |
| WP_050426524.1 3-phosphoshikimate 1-carboxyvinyltransferase [B... | 444 | 5e-150 | 55% |
| WP_108788325.1 3-phosphoshikimate 1-carboxyvinyltransferase [R... | 444 | 5e-150 | 56% |
| WP_147859421.1 MULTISPECIES: 3-phosphoshikimate 1-carboxyvinyl... | 444 | 5e-150 | 59% |
| WP_185707424.1 3-phosphoshikimate 1-carboxyvinyltransferase [S... | 444 | 5e-150 | 57% |
| MBB4366479.1 3-phosphoshikimate 1-carboxyvinyltransferase [Bra... | 443 | 5e-150 | 58% |
| WP_171330064.1 3-phosphoshikimate 1-carboxyvinyltransferase [R... | 444 | 6e-150 | 56% |
| WP_191286133.1 3-phosphoshikimate 1-carboxyvinyltransferase [A... | 444 | 6e-150 | 57% |
| WP_173484988.1 MULTISPECIES: 3-phosphoshikimate 1-carboxyvinyl... | 444 | 6e-150 | 56% |
| SED00662.1 3-phosphoshikimate 1-carboxyvinyltransferase [Beije... | 445 | 6e-150 | 59% |
| WP_076619008.1 3-phosphoshikimate 1-carboxyvinyltransferase [P... | 444 | 6e-150 | 56% |
| PWL26442.1 3-phosphoshikimate 1-carboxyvinyltransferase [Alter... | 443 | 6e-150 | 59% |
| TAL78363.1 3-phosphoshikimate 1-carboxyvinyltransferase [Beije... | 444 | 6e-150 | 59% |
| WP_017485476.1 3-phosphoshikimate 1-carboxyvinyltransferase [M... | 444 | 6e-150 | 59% |
| WP_045837245.1 3-phosphoshikimate 1-carboxyvinyltransferase [H... | 444 | 6e-150 | 58% |
| MAZ02541.1 3-phosphoshikimate 1-carboxyvinyltransferase [Sneat... | 444 | 6e-150 | 56% |
| WP_170753645.1 3-phosphoshikimate 1-carboxyvinyltransferase [R... | 444 | 6e-150 | 56% |
| WP_135448927.1 3-phosphoshikimate 1-carboxyvinyltransferase [R... | 444 | 6e-150 | 56% |
| WP_136964539.1 3-phosphoshikimate 1-carboxyvinyltransferase [P... | 443 | 7e-150 | 61% |

| WP_169492123.1 3-phosphoshikimate 1-carboxyvinyltransferase [N... | 444 | 7e-150 | 60% |
| --- | --- | --- | --- |
| WP_166948356.1 3-phosphoshikimate 1-carboxyvinyltransferase [P... | 444 | 7e-150 | 59% |
| WP_050521463.1 3-phosphoshikimate 1-carboxyvinyltransferase [P... | 444 | 7e-150 | 57% |
| WP_039532104.1 3-phosphoshikimate 1-carboxyvinyltransferase [R... | 444 | 7e-150 | 56% |
| MBB4426971.1 3-phosphoshikimate 1-carboxyvinyltransferase [Bra... | 442 | 7e-150 | 58% |
| WP_093312157.1 3-phosphoshikimate 1-carboxyvinyltransferase [S... | 444 | 7e-150 | 57% |
| WP_026439441.1 3-phosphoshikimate 1-carboxyvinyltransferase [A... | 444 | 7e-150 | 55% |
| WP_055190210.1 3-phosphoshikimate 1-carboxyvinyltransferase [A... | 444 | 7e-150 | 56% |
| MSQ87255.1 3-phosphoshikimate 1-carboxyvinyltransferase [Alpha... | 444 | 7e-150 | 55% |
| WP_184034339.1 3-phosphoshikimate 1-carboxyvinyltransferase [S... | 444 | 8e-150 | 57% |
| WP_151974224.1 3-phosphoshikimate 1-carboxyvinyltransferase [E... | 445 | 8e-150 | 58% |
| WP_176855928.1 3-phosphoshikimate 1-carboxyvinyltransferase [D... | 444 | 8e-150 | 57% |
| WP_170492389.1 3-phosphoshikimate 1-carboxyvinyltransferase [R... | 444 | 8e-150 | 56% |
| WP_008493564.1 3-phosphoshikimate 1-carboxyvinyltransferase [A... | 443 | 8e-150 | 55% |
| WP_109073329.1 MULTISPECIES: 3-phosphoshikimate 1-carboxyvinyl... | 444 | 8e-150 | 57% |
| WP_058288670.1 3-phosphoshikimate 1-carboxyvinyltransferase [E... | 444 | 8e-150 | 56% |
| WP_119592338.1 3-phosphoshikimate 1-carboxyvinyltransferase [E... | 444 | 8e-150 | 57% |
| MBE0412461.1 3-phosphoshikimate 1-carboxyvinyltransferase [Yoo... | 444 | 8e-150 | 57% |
| WP_149131561.1 3-phosphoshikimate 1-carboxyvinyltransferase [B... | 443 | 8e-150 | 59% |
| WP_145072128.1 3-phosphoshikimate 1-carboxyvinyltransferase [S... | 443 | 8e-150 | 56% |
| WP_100250770.1 3-phosphoshikimate 1-carboxyvinyltransferase [M... | 444 | 8e-150 | 59% |
| WP_060847950.1 3-phosphoshikimate 1-carboxyvinyltransferase [M... | 444 | 8e-150 | 59% |
| WP_040603238.1 3-phosphoshikimate 1-carboxyvinyltransferase [S... | 443 | 8e-150 | 56% |
| WP_156928710.1 3-phosphoshikimate 1-carboxyvinyltransferase [B... | 444 | 8e-150 | 56% |
| WP_171726658.1 3-phosphoshikimate 1-carboxyvinyltransferase [R... | 443 | 9e-150 | 56% |
| WP_101922629.1 3-phosphoshikimate 1-carboxyvinyltransferase [T... | 443 | 9e-150 | 57% |
| WP_093416631.1 3-phosphoshikimate 1-carboxyvinyltransferase [T... | 443 | 9e-150 | 57% |
| HAE04204.1 TPA: 3-phosphoshikimate 1-carboxyvinyltransferase [... | 443 | 9e-150 | 57% |
| WP_024098368.1 3-phosphoshikimate 1-carboxyvinyltransferase [P... | 443 | 9e-150 | 56% |
| WP_132445770.1 3-phosphoshikimate 1-carboxyvinyltransferase [S... | 444 | 9e-150 | 56% |
| WP_167643501.1 3-phosphoshikimate 1-carboxyvinyltransferase [P... | 444 | 9e-150 | 56% |
| WP_129435064.1 3-phosphoshikimate 1-carboxyvinyltransferase [R... | 443 | 9e-150 | 56% |
| WP_102897633.1 3-phosphoshikimate 1-carboxyvinyltransferase [P... | 443 | 9e-150 | 56% |
| WP_050603103.1 3-phosphoshikimate 1-carboxyvinyltransferase [R... | 443 | 9e-150 | 56% |
| WP_171724475.1 3-phosphoshikimate 1-carboxyvinyltransferase [R... | 443 | 9e-150 | 56% |
| WP_102226125.1 3-phosphoshikimate 1-carboxyvinyltransferase [A... | 443 | 9e-150 | 56% |
| WP_056425802.1 3-phosphoshikimate 1-carboxyvinyltransferase [S... | 443 | 9e-150 | 57% |
| WP_170508953.1 3-phosphoshikimate 1-carboxyvinyltransferase [R... | 443 | 9e-150 | 56% |
| NBT30846.1 3-phosphoshikimate 1-carboxyvinyltransferase [Rhodo... | 443 | 9e-150 | 57% |
| WP_047093472.1 3-phosphoshikimate 1-carboxyvinyltransferase [E... | 443 | 9e-150 | 57% |
| WP_171636755.1 3-phosphoshikimate 1-carboxyvinyltransferase [R... | 443 | 1e-149 | 56% |
| WP_152143079.1 3-phosphoshikimate 1-carboxyvinyltransferase [A... | 443 | 1e-149 | 56% |
| TNE57399.1 3-phosphoshikimate 1-carboxyvinyltransferase [Alpha... | 444 | 1e-149 | 59% |
| WP_102857921.1 3-phosphoshikimate 1-carboxyvinyltransferase [P... | 443 | 1e-149 | 56% |
| HIF77722.1 TPA: 3-phosphoshikimate 1-carboxyvinyltransferase [... | 443 | 1e-149 | 56% |
| MVT65336.1 3-phosphoshikimate 1-carboxyvinyltransferase [Brady... | 444 | 1e-149 | 55% |
| MAL75566.1 3-phosphoshikimate 1-carboxyvinyltransferase [Rhodo... | 443 | 1e-149 | 57% |
| WP_165328108.1 3-phosphoshikimate 1-carboxyvinyltransferase [S... | 443 | 1e-149 | 57% |
| WP_047996595.1 3-phosphoshikimate 1-carboxyvinyltransferase [P... | 443 | 1e-149 | 56% |
| WP_096870586.1 3-phosphoshikimate 1-carboxyvinyltransferase [P... | 443 | 1e-149 | 56% |
| WP_068345567.1 3-phosphoshikimate 1-carboxyvinyltransferase [R... | 443 | 1e-149 | 56% |
| WP_126138179.1 3-phosphoshikimate 1-carboxyvinyltransferase [E... | 443 | 1e-149 | 56% |
| MBB4146939.1 3-phosphoshikimate 1-carboxyvinyltransferase [Sph... | 443 | 1e-149 | 56% |
| GBF28379.1 3-phosphoshikimate 1-carboxyvinyltransferase [bacte... | 445 | 1e-149 | 56% |
| WP_093009468.1 3-phosphoshikimate 1-carboxyvinyltransferase [R... | 443 | 1e-149 | 55% |
| WP_027248014.1 3-phosphoshikimate 1-carboxyvinyltransferase [P... | 443 | 1e-149 | 56% |
| WP_168136134.1 3-phosphoshikimate 1-carboxyvinyltransferase [S... | 443 | 1e-149 | 58% |
| WP_176272150.1 3-phosphoshikimate 1-carboxyvinyltransferase [A... | 444 | 1e-149 | 59% |
| WP_067469060.1 3-phosphoshikimate 1-carboxyvinyltransferase [E... | 442 | 1e-149 | 58% |
| WP_082922069.1 3-phosphoshikimate 1-carboxyvinyltransferase [C... | 444 | 1e-149 | 56% |
| GGH33899.1 3-phosphoshikimate 1-carboxyvinyltransferase [Alsob... | 442 | 1e-149 | 61% |
| WP_061971734.1 3-phosphoshikimate 1-carboxyvinyltransferase [B... | 443 | 1e-149 | 59% |
| WP_171210998.1 3-phosphoshikimate 1-carboxyvinyltransferase [R... | 443 | 1e-149 | 55% |
| WP_167529013.1 3-phosphoshikimate 1-carboxyvinyltransferase [B... | 443 | 1e-149 | 55% |
| WP_170454307.1 3-phosphoshikimate 1-carboxyvinyltransferase [R... | 443 | 1e-149 | 56% |
| WP_072628952.1 3-phosphoshikimate 1-carboxyvinyltransferase [P... | 443 | 1e-149 | 56% |
| WP_171616358.1 3-phosphoshikimate 1-carboxyvinyltransferase [R... | 443 | 1e-149 | 56% |
| MBU11464.1 3-phosphoshikimate 1-carboxyvinyltransferase [Rhodo... | 443 | 1e-149 | 54% |
| WP_161767322.1 3-phosphoshikimate 1-carboxyvinyltransferase [R... | 443 | 1e-149 | 57% |
| WP_065323400.1 3-phosphoshikimate 1-carboxyvinyltransferase [E... | 443 | 1e-149 | 55% |
| OJV10283.1 3-phosphoshikimate 1-carboxyvinyltransferase [Bosea... | 443 | 1e-149 | 59% |
| MBF0355634.1 3-phosphoshikimate 1-carboxyvinyltransferase [Alp... | 450 | 1e-149 | 57% |

| WP_072505610.1 3-phosphoshikimate 1-carboxyvinyltransferase [P... | 443 | 1e-149 | 56% |
| --- | --- | --- | --- |
| WP_102848613.1 MULTISPECIES: 3-phosphoshikimate 1-carboxyvinyl... | 443 | 1e-149 | 56% |
| AUJ62921.1 3-phosphoshikimate 1-carboxyvinyltransferase [Rhodo... | 443 | 1e-149 | 55% |
| WP_021246697.1 MULTISPECIES: 3-phosphoshikimate 1-carboxyvinyl... | 442 | 1e-149 | 57% |
| WP_150281445.1 3-phosphoshikimate 1-carboxyvinyltransferase [S... | 443 | 1e-149 | 56% |
| WP_102915539.1 3-phosphoshikimate 1-carboxyvinyltransferase [P... | 443 | 1e-149 | 56% |
| WP_085770653.1 3-phosphoshikimate 1-carboxyvinyltransferase [M... | 443 | 1e-149 | 58% |
| WP_150961204.1 3-phosphoshikimate 1-carboxyvinyltransferase [M... | 443 | 1e-149 | 61% |
| WP_018408126.1 3-phosphoshikimate 1-carboxyvinyltransferase [M... | 443 | 1e-149 | 59% |
| WP_046002570.1 3-phosphoshikimate 1-carboxyvinyltransferase [E... | 443 | 1e-149 | 55% |
| WP_085308479.1 3-phosphoshikimate 1-carboxyvinyltransferase [P... | 443 | 1e-149 | 55% |
| WP_038364847.1 3-phosphoshikimate 1-carboxyvinyltransferase [B... | 443 | 2e-149 | 59% |
| MBG04961.1 3-phosphoshikimate 1-carboxyvinyltransferase [Rhodo... | 442 | 2e-149 | 57% |
| OYX25435.1 3-phosphoshikimate 1-carboxyvinyltransferase [Rhodo... | 443 | 2e-149 | 56% |
| WP_116557788.1 3-phosphoshikimate 1-carboxyvinyltransferase [P... | 443 | 2e-149 | 56% |
| WP_090755598.1 3-phosphoshikimate 1-carboxyvinyltransferase [P... | 442 | 2e-149 | 57% |
| WP_107494960.1 3-phosphoshikimate 1-carboxyvinyltransferase [T... | 443 | 2e-149 | 55% |
| WP_168226274.1 3-phosphoshikimate 1-carboxyvinyltransferase [B... | 442 | 2e-149 | 55% |
| WP_014873582.1 3-phosphoshikimate 1-carboxyvinyltransferase [P... | 443 | 2e-149 | 56% |
| WP_151175405.1 3-phosphoshikimate 1-carboxyvinyltransferase [H... | 442 | 2e-149 | 57% |
| PPD29112.1 3-phosphoshikimate 1-carboxyvinyltransferase [Hypho... | 442 | 2e-149 | 60% |
| WP_008963160.1 3-phosphoshikimate 1-carboxyvinyltransferase [B... | 442 | 2e-149 | 57% |
| WP_135986755.1 3-phosphoshikimate 1-carboxyvinyltransferase [S... | 442 | 2e-149 | 56% |
| WP_170332913.1 3-phosphoshikimate 1-carboxyvinyltransferase [R... | 442 | 2e-149 | 56% |
| WP_067548038.1 MULTISPECIES: 3-phosphoshikimate 1-carboxyvinyl... | 442 | 2e-149 | 55% |
| WP_107323849.1 3-phosphoshikimate 1-carboxyvinyltransferase [R... | 442 | 2e-149 | 57% |
| EIE49217.1 3-phosphoshikimate 1-carboxyvinyltransferase [Citre... | 442 | 2e-149 | 56% |
| WP_180886073.1 3-phosphoshikimate 1-carboxyvinyltransferase [R... | 442 | 2e-149 | 55% |
| OJY00076.1 3-phosphoshikimate 1-carboxyvinyltransferase [Rhizo... | 442 | 2e-149 | 59% |
| WP_170371755.1 3-phosphoshikimate 1-carboxyvinyltransferase [R... | 442 | 2e-149 | 56% |
| WP_193173403.1 3-phosphoshikimate 1-carboxyvinyltransferase [N... | 442 | 2e-149 | 57% |
| WP_039192116.1 3-phosphoshikimate 1-carboxyvinyltransferase [L... | 442 | 2e-149 | 56% |
| QJE74958.1 3-phosphoshikimate 1-carboxyvinyltransferase [Rhodo... | 442 | 2e-149 | 58% |
| WP_153215954.1 3-phosphoshikimate 1-carboxyvinyltransferase [E... | 442 | 2e-149 | 56% |
| WP_133344444.1 3-phosphoshikimate 1-carboxyvinyltransferase [F... | 442 | 2e-149 | 55% |
| RZV31698.1 3-phosphoshikimate 1-carboxyvinyltransferase [Sphin... | 442 | 2e-149 | 56% |
| WP_172240428.1 3-phosphoshikimate 1-carboxyvinyltransferase [B... | 442 | 2e-149 | 56% |
| WP_109116903.1 3-phosphoshikimate 1-carboxyvinyltransferase [A... | 442 | 2e-149 | 57% |
| WP_171131573.1 3-phosphoshikimate 1-carboxyvinyltransferase [S... | 442 | 2e-149 | 58% |
| WP_101498433.1 3-phosphoshikimate 1-carboxyvinyltransferase [P... | 442 | 2e-149 | 57% |
| WP_128893537.1 3-phosphoshikimate 1-carboxyvinyltransferase [E... | 442 | 2e-149 | 58% |
| WP_067599167.1 3-phosphoshikimate 1-carboxyvinyltransferase [A... | 442 | 2e-149 | 59% |
| ESZ85726.1 3-phosphoshikimate 1-carboxyvinyltransferase [Blast... | 442 | 2e-149 | 58% |
| HHN67766.1 TPA: 3-phosphoshikimate 1-carboxyvinyltransferase [... | 442 | 2e-149 | 57% |
| WP_096485120.1 3-phosphoshikimate 1-carboxyvinyltransferase [M... | 442 | 2e-149 | 60% |
| WP_104509503.1 3-phosphoshikimate 1-carboxyvinyltransferase [R... | 442 | 2e-149 | 58% |
| WP_142861450.1 3-phosphoshikimate 1-carboxyvinyltransferase [M... | 443 | 2e-149 | 57% |
| KPQ08873.1 3-phosphoshikimate 1-carboxyvinyltransferase AroA [... | 442 | 2e-149 | 56% |
| WP_171641334.1 3-phosphoshikimate 1-carboxyvinyltransferase [R... | 442 | 2e-149 | 56% |
| WP_171117402.1 MULTISPECIES: 3-phosphoshikimate 1-carboxyvinyl... | 442 | 3e-149 | 56% |
| WP_150296814.1 3-phosphoshikimate 1-carboxyvinyltransferase [S... | 442 | 3e-149 | 56% |
| WP_170344918.1 3-phosphoshikimate 1-carboxyvinyltransferase [R... | 442 | 3e-149 | 56% |
| WP_091682004.1 MULTISPECIES: 3-phosphoshikimate 1-carboxyvinyl... | 442 | 3e-149 | 59% |
| QPD00258.1 3-phosphoshikimate 1-carboxyvinyltransferase [Eryth... | 442 | 3e-149 | 59% |
| WP_091925065.1 3-phosphoshikimate 1-carboxyvinyltransferase [M... | 442 | 3e-149 | 59% |
| WP_074787138.1 3-phosphoshikimate 1-carboxyvinyltransferase [R... | 442 | 3e-149 | 56% |
| WP_190787089.1 3-phosphoshikimate 1-carboxyvinyltransferase [E... | 442 | 3e-149 | 57% |
| WP_092835267.1 3-phosphoshikimate 1-carboxyvinyltransferase [R... | 442 | 3e-149 | 56% |
| WP_172132545.1 MULTISPECIES: 3-phosphoshikimate 1-carboxyvinyl... | 442 | 3e-149 | 57% |
| WP_188658247.1 unnamed protein product | 442 | 3e-149 | 56% |
| WP_019297669.1 MULTISPECIES: 3-phosphoshikimate 1-carboxyvinyl... | 442 | 3e-149 | 56% |
| WP_169566567.1 3-phosphoshikimate 1-carboxyvinyltransferase [S... | 442 | 3e-149 | 56% |
| WP_119830374.1 3-phosphoshikimate 1-carboxyvinyltransferase [A... | 442 | 3e-149 | 57% |
| WP_150996140.1 3-phosphoshikimate 1-carboxyvinyltransferase [M... | 442 | 3e-149 | 60% |
| GEP10908.1 3-phosphoshikimate 1-carboxyvinyltransferase [Methy... | 441 | 3e-149 | 61% |
| WP_105515309.1 3-phosphoshikimate 1-carboxyvinyltransferase [D... | 442 | 3e-149 | 58% |
| WP_093293933.1 3-phosphoshikimate 1-carboxyvinyltransferase [S... | 442 | 3e-149 | 57% |
| MAI44734.1 3-phosphoshikimate 1-carboxyvinyltransferase [Filom... | 442 | 3e-149 | 58% |
| KAB2914323.1 3-phosphoshikimate 1-carboxyvinyltransferase [Hyp... | 442 | 3e-149 | 59% |
| WP_183436244.1 3-phosphoshikimate 1-carboxyvinyltransferase [M... | 442 | 3e-149 | 60% |
| PYC46478.1 3-phosphoshikimate 1-carboxyvinyltransferase [Rhodo... | 442 | 3e-149 | 57% |
| WP_152462945.1 3-phosphoshikimate 1-carboxyvinyltransferase [R... | 442 | 3e-149 | 55% |

| BBK45021.1 3-phosphoshikimate 1-carboxyvinyltransferase [Stell... | 442 | 3e-149 | 57% |
| --- | --- | --- | --- |
| WP_174474144.1 3-phosphoshikimate 1-carboxyvinyltransferase [A... | 442 | 3e-149 | 57% |
| EDZ44360.1 3-phosphoshikimate 1-carboxyvinyltransferase [Rhodo... | 442 | 3e-149 | 56% |
| MBU96398.1 3-phosphoshikimate 1-carboxyvinyltransferase [Rhodo... | 442 | 3e-149 | 54% |
| WP_069298825.1 3-phosphoshikimate 1-carboxyvinyltransferase [N... | 442 | 3e-149 | 56% |
| WP_045364250.1 3-phosphoshikimate 1-carboxyvinyltransferase [M... | 441 | 4e-149 | 56% |
| WP_137788146.1 3-phosphoshikimate 1-carboxyvinyltransferase [S... | 442 | 4e-149 | 55% |
| MBB4379353.1 3-phosphoshikimate 1-carboxyvinyltransferase [Bra... | 441 | 4e-149 | 58% |
| WP_119677337.1 3-phosphoshikimate 1-carboxyvinyltransferase [I... | 442 | 4e-149 | 56% |
| NNC60335.1 3-phosphoshikimate 1-carboxyvinyltransferase [Eryth... | 442 | 4e-149 | 56% |
| PKP74916.1 3-phosphoshikimate 1-carboxyvinyltransferase [Alpha... | 442 | 4e-149 | 56% |
| KPF78446.1 3-phosphoshikimate 1-carboxyvinyltransferase [alpha... | 442 | 4e-149 | 58% |
| OJY61649.1 3-phosphoshikimate 1-carboxyvinyltransferase [Sphin... | 441 | 4e-149 | 57% |
| WP_164132173.1 3-phosphoshikimate 1-carboxyvinyltransferase [R... | 442 | 4e-149 | 55% |
| NCQ23889.1 3-phosphoshikimate 1-carboxyvinyltransferase [Roseo... | 442 | 4e-149 | 56% |
| WP_111549579.1 3-phosphoshikimate 1-carboxyvinyltransferase [S... | 442 | 4e-149 | 56% |
| WP_130403973.1 3-phosphoshikimate 1-carboxyvinyltransferase [T... | 442 | 4e-149 | 55% |
| TVQ53780.1 3-phosphoshikimate 1-carboxyvinyltransferase [Rhodo... | 441 | 4e-149 | 56% |
| WP_096342280.1 3-phosphoshikimate 1-carboxyvinyltransferase [S... | 442 | 4e-149 | 58% |
| WP_189639910.1 3-phosphoshikimate 1-carboxyvinyltransferase [A... | 441 | 5e-149 | 56% |
| NVO57107.1 3-phosphoshikimate 1-carboxyvinyltransferase [Rhodo... | 442 | 5e-149 | 55% |
| WP_067261419.1 MULTISPECIES: 3-phosphoshikimate 1-carboxyvinyl... | 442 | 5e-149 | 56% |
| WP_174211127.1 3-phosphoshikimate 1-carboxyvinyltransferase [T... | 441 | 5e-149 | 56% |
| WP_136704629.1 3-phosphoshikimate 1-carboxyvinyltransferase [A... | 441 | 5e-149 | 57% |
| RMD65225.1 3-phosphoshikimate 1-carboxyvinyltransferase [Alpha... | 442 | 5e-149 | 57% |
| WP_168844945.1 3-phosphoshikimate 1-carboxyvinyltransferase [S... | 441 | 5e-149 | 56% |
| WP_110353420.1 3-phosphoshikimate 1-carboxyvinyltransferase [M... | 442 | 5e-149 | 59% |
| WP_114583720.1 3-phosphoshikimate 1-carboxyvinyltransferase [F... | 441 | 5e-149 | 56% |
| WP_050662389.1 3-phosphoshikimate 1-carboxyvinyltransferase [R... | 441 | 5e-149 | 56% |
| NVJ94593.1 3-phosphoshikimate 1-carboxyvinyltransferase [Mariv... | 441 | 5e-149 | 55% |
| NEY90940.1 3-phosphoshikimate 1-carboxyvinyltransferase [Rhodo... | 441 | 5e-149 | 57% |
| WP_142022395.1 3-phosphoshikimate 1-carboxyvinyltransferase [B... | 441 | 5e-149 | 59% |
| WP_040171018.1 MULTISPECIES: 3-phosphoshikimate 1-carboxyvinyl... | 441 | 5e-149 | 56% |
| WP_108815373.1 3-phosphoshikimate 1-carboxyvinyltransferase [L... | 441 | 5e-149 | 57% |
| WP_166145899.1 3-phosphoshikimate 1-carboxyvinyltransferase [M... | 442 | 5e-149 | 57% |
| WP_089276671.1 3-phosphoshikimate 1-carboxyvinyltransferase [A... | 441 | 6e-149 | 58% |
| WP_011453833.1 3-phosphoshikimate 1-carboxyvinyltransferase [J... | 441 | 6e-149 | 55% |
| WP_066719237.1 3-phosphoshikimate 1-carboxyvinyltransferase [S... | 441 | 6e-149 | 56% |
| WP_121074898.1 3-phosphoshikimate 1-carboxyvinyltransferase [S... | 441 | 6e-149 | 57% |
| WP_172260445.1 3-phosphoshikimate 1-carboxyvinyltransferase [R... | 441 | 6e-149 | 55% |
| NQV21650.1 3-phosphoshikimate 1-carboxyvinyltransferase [Rhodo... | 441 | 6e-149 | 53% |
| WP_091428468.1 3-phosphoshikimate 1-carboxyvinyltransferase [A... | 441 | 6e-149 | 56% |
| WP_196409366.1 3-phosphoshikimate 1-carboxyvinyltransferase [M... | 442 | 6e-149 | 57% |
| WP_132806531.1 3-phosphoshikimate 1-carboxyvinyltransferase [T... | 442 | 6e-149 | 61% |
| WP_132366103.1 3-phosphoshikimate 1-carboxyvinyltransferase [M... | 441 | 6e-149 | 59% |
| WP_086051690.1 3-phosphoshikimate 1-carboxyvinyltransferase [P... | 441 | 6e-149 | 55% |
| WP_056532002.1 3-phosphoshikimate 1-carboxyvinyltransferase [M... | 441 | 6e-149 | 59% |
| WP_092499653.1 3-phosphoshikimate 1-carboxyvinyltransferase [A... | 441 | 6e-149 | 56% |
| WP_155983111.1 3-phosphoshikimate 1-carboxyvinyltransferase [O... | 441 | 6e-149 | 57% |
| WP_152491290.1 3-phosphoshikimate 1-carboxyvinyltransferase [R... | 441 | 6e-149 | 56% |
| WP_165055884.1 3-phosphoshikimate 1-carboxyvinyltransferase [M... | 441 | 6e-149 | 58% |
| WP_099248993.1 3-phosphoshikimate 1-carboxyvinyltransferase [T... | 441 | 6e-149 | 56% |
| WP_097671322.1 3-phosphoshikimate 1-carboxyvinyltransferase [B... | 441 | 7e-149 | 55% |
| WP_069443836.1 3-phosphoshikimate 1-carboxyvinyltransferase [M... | 441 | 7e-149 | 56% |
| WP_152463977.1 3-phosphoshikimate 1-carboxyvinyltransferase [S... | 441 | 7e-149 | 56% |
| AUC93838.1 3-phosphoshikimate 1-carboxyvinyltransferase [Brady... | 441 | 7e-149 | 55% |
| WP_159978971.1 3-phosphoshikimate 1-carboxyvinyltransferase [R... | 441 | 7e-149 | 56% |
| KUO53456.1 3-phosphoshikimate 1-carboxyvinyltransferase [Sphin... | 441 | 7e-149 | 58% |
| WP_109107513.1 3-phosphoshikimate 1-carboxyvinyltransferase [A... | 441 | 7e-149 | 57% |
| OYX66312.1 3-phosphoshikimate 1-carboxyvinyltransferase [Sphin... | 442 | 7e-149 | 57% |
| WP_115393464.1 3-phosphoshikimate 1-carboxyvinyltransferase [R... | 441 | 7e-149 | 56% |
| WP_172113572.1 3-phosphoshikimate 1-carboxyvinyltransferase [B... | 441 | 7e-149 | 57% |
| WP_091980132.1 3-phosphoshikimate 1-carboxyvinyltransferase [M... | 441 | 7e-149 | 59% |
| WP_068863322.1 3-phosphoshikimate 1-carboxyvinyltransferase [P... | 441 | 8e-149 | 57% |
| WP_090846930.1 3-phosphoshikimate 1-carboxyvinyltransferase [P... | 441 | 8e-149 | 54% |
| MBA3055076.1 3-phosphoshikimate 1-carboxyvinyltransferase [Sph... | 441 | 8e-149 | 56% |
| NQU62493.1 3-phosphoshikimate 1-carboxyvinyltransferase [Rhodo... | 441 | 8e-149 | 55% |
| PZQ51566.1 3-phosphoshikimate 1-carboxyvinyltransferase [Rhodo... | 441 | 8e-149 | 57% |
| WP_160738580.1 3-phosphoshikimate 1-carboxyvinyltransferase [A... | 441 | 8e-149 | 58% |
| MBC7131776.1 3-phosphoshikimate 1-carboxyvinyltransferase [Ros... | 441 | 8e-149 | 57% |
| WP_166300063.1 3-phosphoshikimate 1-carboxyvinyltransferase [B... | 441 | 8e-149 | 55% |
| WP_026008960.1 MULTISPECIES: 3-phosphoshikimate 1-carboxyvinyl... | 441 | 8e-149 | 57% |

| WP_174521937.1 3-phosphoshikimate 1-carboxyvinyltransferase [S... | 441 | 8e-149 | 57% |
| --- | --- | --- | --- |
| WP_012972917.1 3-phosphoshikimate 1-carboxyvinyltransferase [A... | 441 | 9e-149 | 57% |
| WP_061047972.1 3-phosphoshikimate 1-carboxyvinyltransferase [P... | 441 | 9e-149 | 56% |
| WP_161340568.1 3-phosphoshikimate 1-carboxyvinyltransferase [S... | 441 | 9e-149 | 56% |
| WP_121102525.1 3-phosphoshikimate 1-carboxyvinyltransferase [L... | 441 | 9e-149 | 55% |
| WP_058249380.1 3-phosphoshikimate 1-carboxyvinyltransferase [T... | 441 | 9e-149 | 56% |
| RYE32096.1 3-phosphoshikimate 1-carboxyvinyltransferase [Rhizo... | 441 | 9e-149 | 58% |
| WP_111734393.1 3-phosphoshikimate 1-carboxyvinyltransferase [R... | 441 | 9e-149 | 57% |
| HIQ18401.1 TPA: 3-phosphoshikimate 1-carboxyvinyltransferase [... | 441 | 9e-149 | 57% |
| WP_039016885.1 3-phosphoshikimate 1-carboxyvinyltransferase [H... | 441 | 9e-149 | 55% |
| NHO42099.1 3-phosphoshikimate 1-carboxyvinyltransferase [Aceto... | 441 | 9e-149 | 55% |
| WP_181335950.1 3-phosphoshikimate 1-carboxyvinyltransferase [H... | 441 | 9e-149 | 57% |
| PIR38993.1 3-phosphoshikimate 1-carboxyvinyltransferase [Alpha... | 441 | 1e-148 | 59% |
| WP_039171759.1 3-phosphoshikimate 1-carboxyvinyltransferase [L... | 441 | 1e-148 | 55% |
| WP_011942628.1 3-phosphoshikimate 1-carboxyvinyltransferase [B... | 441 | 1e-148 | 56% |
| OZA18671.1 3-phosphoshikimate 1-carboxyvinyltransferase [Novos... | 441 | 1e-148 | 57% |
| WP_074770335.1 3-phosphoshikimate 1-carboxyvinyltransferase [P... | 441 | 1e-148 | 57% |
| WP_092042708.1 3-phosphoshikimate 1-carboxyvinyltransferase [M... | 441 | 1e-148 | 59% |
| MBC7985472.1 3-phosphoshikimate 1-carboxyvinyltransferase [Sph... | 442 | 1e-148 | 56% |
| NQV44838.1 3-phosphoshikimate 1-carboxyvinyltransferase [Rhodo... | 441 | 1e-148 | 56% |
| HBH41298.1 TPA: 3-phosphoshikimate 1-carboxyvinyltransferase [... | 440 | 1e-148 | 55% |
| NNC52862.1 3-phosphoshikimate 1-carboxyvinyltransferase [Eryth... | 441 | 1e-148 | 59% |
| WP_040701792.1 3-phosphoshikimate 1-carboxyvinyltransferase [S... | 441 | 1e-148 | 55% |
| WP_184429785.1 3-phosphoshikimate 1-carboxyvinyltransferase [R... | 441 | 1e-148 | 59% |
| WP_152496467.1 3-phosphoshikimate 1-carboxyvinyltransferase [R... | 441 | 1e-148 | 56% |
| GCD54780.1 3-phosphoshikimate 1-carboxyvinyltransferase [Aceto... | 441 | 1e-148 | 56% |
| WP_027263104.1 3-phosphoshikimate 1-carboxyvinyltransferase [S... | 441 | 1e-148 | 55% |
| CCG42636.1 3-phosphoshikimate 1-carboxyvinyltransferase (5-eno... | 441 | 1e-148 | 57% |
| NNL18051.1 3-phosphoshikimate 1-carboxyvinyltransferase [Boseo... | 441 | 1e-148 | 55% |
| WP_089269114.1 3-phosphoshikimate 1-carboxyvinyltransferase [P... | 441 | 1e-148 | 56% |
| WP_109757594.1 3-phosphoshikimate 1-carboxyvinyltransferase [S... | 441 | 1e-148 | 56% |
| WP_170561110.1 3-phosphoshikimate 1-carboxyvinyltransferase [R... | 441 | 1e-148 | 55% |
| WP_029012256.1 3-phosphoshikimate 1-carboxyvinyltransferase [N... | 440 | 1e-148 | 57% |
| WP_072558570.1 3-phosphoshikimate 1-carboxyvinyltransferase [S... | 440 | 1e-148 | 57% |
| WP_056772308.1 3-phosphoshikimate 1-carboxyvinyltransferase [N... | 440 | 1e-148 | 57% |
| WP_051949144.1 3-phosphoshikimate 1-carboxyvinyltransferase [M... | 441 | 1e-148 | 57% |
| WP_108692777.1 3-phosphoshikimate 1-carboxyvinyltransferase [P... | 441 | 1e-148 | 57% |
| WP_141492073.1 3-phosphoshikimate 1-carboxyvinyltransferase [N... | 440 | 1e-148 | 57% |
| WP_165810447.1 3-phosphoshikimate 1-carboxyvinyltransferase [E... | 441 | 1e-148 | 56% |
| HHY01440.1 TPA: 3-phosphoshikimate 1-carboxyvinyltransferase [... | 440 | 1e-148 | 55% |
| WP_039893136.1 3-phosphoshikimate 1-carboxyvinyltransferase [M... | 440 | 1e-148 | 59% |
| EEE39199.1 3-phosphoshikimate 1-carboxyvinyltransferase [Rhodo... | 440 | 1e-148 | 56% |
| HIM25307.1 TPA: 3-phosphoshikimate 1-carboxyvinyltransferase [... | 440 | 1e-148 | 57% |
| OJU56345.1 3-phosphoshikimate 1-carboxyvinyltransferase [Alpha... | 440 | 1e-148 | 55% |
| WP_050928210.1 3-phosphoshikimate 1-carboxyvinyltransferase [A... | 440 | 1e-148 | 56% |
| NOX41252.1 3-phosphoshikimate 1-carboxyvinyltransferase [Alpha... | 440 | 2e-148 | 55% |
| WP_054526009.1 3-phosphoshikimate 1-carboxyvinyltransferase [C... | 440 | 2e-148 | 56% |
| WP_012778677.1 3-phosphoshikimate 1-carboxyvinyltransferase [C... | 440 | 2e-148 | 51% |
| WP_147940803.1 3-phosphoshikimate 1-carboxyvinyltransferase [M... | 440 | 2e-148 | 60% |
| WP_035362368.1 3-phosphoshikimate 1-carboxyvinyltransferase [A... | 440 | 2e-148 | 56% |
| WP_043920517.1 3-phosphoshikimate 1-carboxyvinyltransferase [J... | 440 | 2e-148 | 56% |
| MBC6440809.1 3-phosphoshikimate 1-carboxyvinyltransferase [Rho... | 440 | 2e-148 | 56% |
| NYS25185.1 3-phosphoshikimate 1-carboxyvinyltransferase [Rhodo... | 440 | 2e-148 | 57% |
| MBB97326.1 3-phosphoshikimate 1-carboxyvinyltransferase [Rhodo... | 441 | 2e-148 | 54% |
| WP_086646943.1 3-phosphoshikimate 1-carboxyvinyltransferase [A... | 440 | 2e-148 | 56% |
| WP_165196656.1 3-phosphoshikimate 1-carboxyvinyltransferase [P... | 440 | 2e-148 | 56% |
| WP_189411110.1 3-phosphoshikimate 1-carboxyvinyltransferase [G... | 440 | 2e-148 | 57% |
| WP_043744828.1 3-phosphoshikimate 1-carboxyvinyltransferase [P... | 440 | 2e-148 | 56% |
| WP_029936415.1 3-phosphoshikimate 1-carboxyvinyltransferase [S... | 440 | 2e-148 | 56% |
| WP_118864166.1 3-phosphoshikimate 1-carboxyvinyltransferase [S... | 440 | 2e-148 | 58% |
| TAN70319.1 3-phosphoshikimate 1-carboxyvinyltransferase [Magne... | 440 | 2e-148 | 57% |
| WP_084717857.1 3-phosphoshikimate 1-carboxyvinyltransferase [S... | 441 | 2e-148 | 58% |
| WP_040567413.1 3-phosphoshikimate 1-carboxyvinyltransferase [P... | 440 | 2e-148 | 57% |
| WP_045581665.1 3-phosphoshikimate 1-carboxyvinyltransferase [A... | 440 | 2e-148 | 57% |
| WP_149229452.1 3-phosphoshikimate 1-carboxyvinyltransferase [A... | 440 | 2e-148 | 57% |
| WP_147828560.1 3-phosphoshikimate 1-carboxyvinyltransferase [M... | 440 | 2e-148 | 58% |
| WP_051079627.1 3-phosphoshikimate 1-carboxyvinyltransferase [M... | 441 | 2e-148 | 57% |
| HIF09587.1 TPA: 3-phosphoshikimate 1-carboxyvinyltransferase [... | 440 | 2e-148 | 54% |
| HCM83774.1 TPA: 3-phosphoshikimate 1-carboxyvinyltransferase [... | 440 | 2e-148 | 53% |
| WP_188672861.1 3-phosphoshikimate 1-carboxyvinyltransferase [A... | 440 | 2e-148 | 57% |
| OUS20317.1 3-phosphoshikimate 1-carboxyvinyltransferase [Rhodo... | 440 | 2e-148 | 57% |
| WP_116469769.1 3-phosphoshikimate 1-carboxyvinyltransferase [S... | 440 | 2e-148 | 56% |

| WP_017500900.1 3-phosphoshikimate 1-carboxyvinyltransferase [S... | 441 | 2e-148 | 54% |
| --- | --- | --- | --- |
| MXU64437.1 3-phosphoshikimate 1-carboxyvinyltransferase [Rhodo... | 440 | 2e-148 | 56% |
| WP_058283224.1 3-phosphoshikimate 1-carboxyvinyltransferase [R... | 440 | 2e-148 | 55% |
| WP_019516806.1 3-phosphoshikimate 1-carboxyvinyltransferase [S... | 440 | 2e-148 | 56% |
| WP_169945730.1 3-phosphoshikimate 1-carboxyvinyltransferase [S... | 441 | 2e-148 | 54% |
| MBA4350602.1 3-phosphoshikimate 1-carboxyvinyltransferase [Rho... | 440 | 2e-148 | 57% |
| HCL49020.1 TPA: 3-phosphoshikimate 1-carboxyvinyltransferase [... | 440 | 2e-148 | 56% |
| WP_159805280.1 3-phosphoshikimate 1-carboxyvinyltransferase [L... | 440 | 2e-148 | 56% |
| TVR11815.1 3-phosphoshikimate 1-carboxyvinyltransferase [Salin... | 440 | 2e-148 | 58% |
| WP_045387367.1 3-phosphoshikimate 1-carboxyvinyltransferase [F... | 440 | 2e-148 | 55% |
| MTJ03155.1 3-phosphoshikimate 1-carboxyvinyltransferase [Sedim... | 440 | 2e-148 | 55% |
| MBN34756.1 3-phosphoshikimate 1-carboxyvinyltransferase [Rhodo... | 440 | 2e-148 | 56% |
| WP_140013029.1 3-phosphoshikimate 1-carboxyvinyltransferase [O... | 440 | 2e-148 | 56% |
| WP_036704129.1 3-phosphoshikimate 1-carboxyvinyltransferase [P... | 439 | 2e-148 | 56% |
| WP_194301343.1 3-phosphoshikimate 1-carboxyvinyltransferase [A... | 441 | 2e-148 | 55% |
| WP_091747646.1 3-phosphoshikimate 1-carboxyvinyltransferase [M... | 440 | 2e-148 | 59% |
| SCW84558.1 3-phosphoshikimate 1-carboxyvinyltransferase [Sphin... | 440 | 2e-148 | 56% |
| WP_171170927.1 3-phosphoshikimate 1-carboxyvinyltransferase [R... | 440 | 2e-148 | 55% |
| MBF9035412.1 3-phosphoshikimate 1-carboxyvinyltransferase [Rho... | 440 | 2e-148 | 55% |
| WP_012812282.1 3-phosphoshikimate 1-carboxyvinyltransferase [A... | 441 | 2e-148 | 56% |
| WP_145625511.1 3-phosphoshikimate 1-carboxyvinyltransferase [A... | 439 | 2e-148 | 57% |
| WP_112874274.1 3-phosphoshikimate 1-carboxyvinyltransferase [P... | 439 | 2e-148 | 56% |
| WP_046900901.1 3-phosphoshikimate 1-carboxyvinyltransferase [G... | 439 | 3e-148 | 56% |
| WP_085794490.1 3-phosphoshikimate 1-carboxyvinyltransferase [R... | 440 | 3e-148 | 56% |
| WP_020092866.1 3-phosphoshikimate 1-carboxyvinyltransferase [M... | 440 | 3e-148 | 59% |
| WP_169641350.1 3-phosphoshikimate 1-carboxyvinyltransferase [R... | 440 | 3e-148 | 55% |
| RCL07549.1 3-phosphoshikimate 1-carboxyvinyltransferase [Aceto... | 440 | 3e-148 | 56% |
| WP_087652130.1 3-phosphoshikimate 1-carboxyvinyltransferase [A... | 440 | 3e-148 | 55% |
| NLS25797.1 3-phosphoshikimate 1-carboxyvinyltransferase [Sphin... | 440 | 3e-148 | 56% |
| WP_161730396.1 3-phosphoshikimate 1-carboxyvinyltransferase [S... | 440 | 3e-148 | 55% |
| MBA84590.1 3-phosphoshikimate 1-carboxyvinyltransferase [Rhodo... | 439 | 3e-148 | 58% |
| WP_189585275.1 3-phosphoshikimate 1-carboxyvinyltransferase [L... | 439 | 3e-148 | 54% |
| WP_109154680.1 3-phosphoshikimate 1-carboxyvinyltransferase [A... | 439 | 3e-148 | 57% |
| WP_011923369.1 3-phosphoshikimate 1-carboxyvinyltransferase [B... | 439 | 3e-148 | 56% |
| GCD53928.1 3-phosphoshikimate 1-carboxyvinyltransferase [Aceto... | 439 | 3e-148 | 56% |
| WP_086449643.1 3-phosphoshikimate 1-carboxyvinyltransferase [M... | 439 | 3e-148 | 56% |
| WP_104018451.1 3-phosphoshikimate 1-carboxyvinyltransferase [R... | 439 | 3e-148 | 55% |
| WP_122161231.1 3-phosphoshikimate 1-carboxyvinyltransferase [M... | 439 | 3e-148 | 59% |
| WP_109448573.1 3-phosphoshikimate 1-carboxyvinyltransferase [A... | 439 | 3e-148 | 57% |
| WP_076532117.1 3-phosphoshikimate 1-carboxyvinyltransferase [R... | 439 | 3e-148 | 56% |
| WP_076365410.1 3-phosphoshikimate 1-carboxyvinyltransferase [R... | 439 | 3e-148 | 57% |
| WP_151886529.1 3-phosphoshikimate 1-carboxyvinyltransferase [E... | 439 | 3e-148 | 58% |
| WP_167689072.1 3-phosphoshikimate 1-carboxyvinyltransferase [P... | 439 | 3e-148 | 56% |
| HAG24373.1 TPA: 3-phosphoshikimate 1-carboxyvinyltransferase [... | 439 | 3e-148 | 55% |
| WP_185332770.1 3-phosphoshikimate 1-carboxyvinyltransferase [A... | 440 | 4e-148 | 56% |
| MBR69842.1 3-phosphoshikimate 1-carboxyvinyltransferase [SAR11... | 439 | 4e-148 | 55% |
| WP_026756484.1 3-phosphoshikimate 1-carboxyvinyltransferase [S... | 439 | 4e-148 | 56% |
| AKH41277.1 3-phosphoshikimate 1-carboxyvinyltransferase [Alter... | 439 | 4e-148 | 58% |
| WP_138841431.1 3-phosphoshikimate 1-carboxyvinyltransferase [R... | 439 | 4e-148 | 56% |
| WP_050734265.1 3-phosphoshikimate 1-carboxyvinyltransferase [M... | 439 | 4e-148 | 59% |
| WP_176496287.1 3-phosphoshikimate 1-carboxyvinyltransferase [S... | 439 | 4e-148 | 57% |
| PZO73256.1 3-phosphoshikimate 1-carboxyvinyltransferase [Sphin... | 439 | 4e-148 | 56% |
| WP_039180893.1 3-phosphoshikimate 1-carboxyvinyltransferase [L... | 439 | 4e-148 | 55% |
| WP_098738943.1 3-phosphoshikimate 1-carboxyvinyltransferase [A... | 439 | 4e-148 | 57% |
| MAE11963.1 3-phosphoshikimate 1-carboxyvinyltransferase [SAR11... | 439 | 4e-148 | 55% |
| WP_174192438.1 3-phosphoshikimate 1-carboxyvinyltransferase [S... | 439 | 4e-148 | 56% |
| WP_133560388.1 3-phosphoshikimate 1-carboxyvinyltransferase [B... | 439 | 4e-148 | 59% |
| WP_175485338.1 3-phosphoshikimate 1-carboxyvinyltransferase [S... | 439 | 4e-148 | 57% |
| WP_161344919.1 3-phosphoshikimate 1-carboxyvinyltransferase [F... | 439 | 4e-148 | 55% |
| WP_068244786.1 3-phosphoshikimate 1-carboxyvinyltransferase [T... | 439 | 4e-148 | 56% |
| WP_095351755.1 3-phosphoshikimate 1-carboxyvinyltransferase [A... | 439 | 4e-148 | 56% |
| WP_147909751.1 MULTISPECIES: 3-phosphoshikimate 1-carboxyvinyl... | 439 | 5e-148 | 60% |
| WP_085889232.1 3-phosphoshikimate 1-carboxyvinyltransferase [P... | 439 | 5e-148 | 55% |
| MBF9037678.1 3-phosphoshikimate 1-carboxyvinyltransferase [Rho... | 439 | 5e-148 | 57% |
| MBA4747889.1 3-phosphoshikimate 1-carboxyvinyltransferase [Sph... | 439 | 5e-148 | 58% |
| TPG42349.1 3-phosphoshikimate 1-carboxyvinyltransferase [Sphin... | 439 | 5e-148 | 57% |
| TNE38742.1 3-phosphoshikimate 1-carboxyvinyltransferase [Alpha... | 439 | 5e-148 | 59% |
| WP_049835242.1 3-phosphoshikimate 1-carboxyvinyltransferase [O... | 439 | 5e-148 | 56% |
| PXW53938.1 3-phosphoshikimate 1-carboxyvinyltransferase [Methy... | 441 | 5e-148 | 59% |
| WP_090219335.1 3-phosphoshikimate 1-carboxyvinyltransferase [E... | 439 | 5e-148 | 56% |
| WP_088632386.1 3-phosphoshikimate 1-carboxyvinyltransferase [P... | 439 | 5e-148 | 55% |
| MBB4393290.1 3-phosphoshikimate 1-carboxyvinyltransferase [Bra... | 438 | 5e-148 | 58% |

| WP_080456534.1 3-phosphoshikimate 1-carboxyvinyltransferase [R... | 439 | 5e-148 | 55% |
| --- | --- | --- | --- |
| WP_068309019.1 3-phosphoshikimate 1-carboxyvinyltransferase [K... | 439 | 5e-148 | 57% |
| WP_114077159.1 3-phosphoshikimate 1-carboxyvinyltransferase [P... | 439 | 5e-148 | 57% |
| RTL92066.1 3-phosphoshikimate 1-carboxyvinyltransferase [Brady... | 439 | 5e-148 | 56% |
| WP_093400354.1 3-phosphoshikimate 1-carboxyvinyltransferase [S... | 439 | 5e-148 | 56% |
| WP_015663268.1 3-phosphoshikimate 1-carboxyvinyltransferase [B... | 439 | 5e-148 | 56% |
| HCQ57853.1 TPA: 3-phosphoshikimate 1-carboxyvinyltransferase [... | 439 | 5e-148 | 56% |
| WP_084353411.1 3-phosphoshikimate 1-carboxyvinyltransferase [P... | 439 | 5e-148 | 56% |
| PPR73165.1 3-phosphoshikimate 1-carboxyvinyltransferase [Alpha... | 439 | 5e-148 | 57% |
| WP_094062925.1 3-phosphoshikimate 1-carboxyvinyltransferase [E... | 438 | 5e-148 | 58% |
| WP_126847342.1 3-phosphoshikimate 1-carboxyvinyltransferase [S... | 439 | 6e-148 | 57% |
| WP_096788603.1 3-phosphoshikimate 1-carboxyvinyltransferase [P... | 439 | 6e-148 | 56% |
| OAZ71958.1 3-phosphoshikimate 1-carboxyvinyltransferase [Aceto... | 439 | 6e-148 | 55% |
| WP_128104736.1 3-phosphoshikimate 1-carboxyvinyltransferase [A... | 439 | 6e-148 | 55% |
| WP_046904680.1 3-phosphoshikimate 1-carboxyvinyltransferase [A... | 438 | 6e-148 | 58% |
| RMF41239.1 3-phosphoshikimate 1-carboxyvinyltransferase [Alpha... | 439 | 6e-148 | 56% |
| OQW41335.1 3-phosphoshikimate 1-carboxyvinyltransferase [Prote... | 438 | 6e-148 | 58% |
| PHQ60505.1 3-phosphoshikimate 1-carboxyvinyltransferase [Sphin... | 437 | 6e-148 | 57% |
| WP_149164119.1 3-phosphoshikimate 1-carboxyvinyltransferase [A... | 439 | 6e-148 | 57% |
| HAN63203.1 TPA: 3-phosphoshikimate 1-carboxyvinyltransferase [... | 438 | 6e-148 | 55% |
| SFO37195.1 3-phosphoshikimate 1-carboxyvinyltransferase [Sphin... | 438 | 6e-148 | 56% |
| PKP90959.1 3-phosphoshikimate 1-carboxyvinyltransferase [Alpha... | 439 | 6e-148 | 56% |
| WP_192284189.1 3-phosphoshikimate 1-carboxyvinyltransferase [M... | 439 | 6e-148 | 60% |
| WP_135429695.1 3-phosphoshikimate 1-carboxyvinyltransferase [T... | 439 | 6e-148 | 56% |
| ARW47597.1 3-phosphoshikimate 1-carboxyvinyltransferase [Aceto... | 439 | 7e-148 | 55% |
| WP_124296303.1 3-phosphoshikimate 1-carboxyvinyltransferase [A... | 439 | 7e-148 | 56% |
| WP_184242367.1 3-phosphoshikimate 1-carboxyvinyltransferase [N... | 439 | 7e-148 | 56% |
| WP_194097998.1 3-phosphoshikimate 1-carboxyvinyltransferase [M... | 439 | 7e-148 | 55% |
| WP_173049052.1 3-phosphoshikimate 1-carboxyvinyltransferase [B... | 439 | 7e-148 | 58% |
| WP_106161918.1 3-phosphoshikimate 1-carboxyvinyltransferase [E... | 439 | 7e-148 | 56% |
| WP_065266123.1 3-phosphoshikimate 1-carboxyvinyltransferase [L... | 439 | 7e-148 | 55% |
| WP_138572364.1 3-phosphoshikimate 1-carboxyvinyltransferase [P... | 439 | 7e-148 | 56% |
| WP_043144981.1 3-phosphoshikimate 1-carboxyvinyltransferase [M... | 439 | 7e-148 | 57% |
| WP_003629416.1 3-phosphoshikimate 1-carboxyvinyltransferase [A... | 439 | 7e-148 | 56% |
| NNE51129.1 3-phosphoshikimate 1-carboxyvinyltransferase [Sulfi... | 439 | 7e-148 | 55% |
| WP_089420917.1 MULTISPECIES: 3-phosphoshikimate 1-carboxyvinyl... | 439 | 7e-148 | 55% |
| WP_113777478.1 3-phosphoshikimate 1-carboxyvinyltransferase [L... | 439 | 7e-148 | 56% |
| RGP40384.1 3-phosphoshikimate 1-carboxyvinyltransferase [Alter... | 437 | 7e-148 | 58% |
| WP_181577703.1 3-phosphoshikimate 1-carboxyvinyltransferase [C... | 439 | 7e-148 | 51% |
| WP_027236355.1 3-phosphoshikimate 1-carboxyvinyltransferase [L... | 439 | 8e-148 | 55% |
| WP_147899427.1 3-phosphoshikimate 1-carboxyvinyltransferase [M... | 439 | 8e-148 | 60% |
| WP_175483255.1 3-phosphoshikimate 1-carboxyvinyltransferase [S... | 438 | 8e-148 | 56% |
| WP_054530129.1 3-phosphoshikimate 1-carboxyvinyltransferase [E... | 438 | 8e-148 | 58% |
| WP_137703066.1 3-phosphoshikimate 1-carboxyvinyltransferase [M... | 439 | 8e-148 | 55% |
| WP_166193493.1 3-phosphoshikimate 1-carboxyvinyltransferase [M... | 438 | 8e-148 | 55% |
| WP_046022894.1 3-phosphoshikimate 1-carboxyvinyltransferase [M... | 438 | 8e-148 | 56% |
| GGD21030.1 3-phosphoshikimate 1-carboxyvinyltransferase [Pyruv... | 437 | 8e-148 | 57% |
| MBB5732795.1 3-phosphoshikimate 1-carboxyvinyltransferase [Alt... | 438 | 8e-148 | 58% |
| BBU62148.1 3-phosphoshikimate 1-carboxyvinyltransferase [Methy... | 438 | 8e-148 | 57% |
| WP_130226707.1 3-phosphoshikimate 1-carboxyvinyltransferase [B... | 439 | 8e-148 | 56% |
| WP_160671089.1 3-phosphoshikimate 1-carboxyvinyltransferase [A... | 437 | 8e-148 | 59% |
| WP_069207901.1 MULTISPECIES: 3-phosphoshikimate 1-carboxyvinyl... | 438 | 9e-148 | 57% |
| WP_122073111.1 3-phosphoshikimate 1-carboxyvinyltransferase [P... | 438 | 9e-148 | 55% |
| WP_161913528.1 3-phosphoshikimate 1-carboxyvinyltransferase [M... | 439 | 9e-148 | 57% |
| WP_160729615.1 3-phosphoshikimate 1-carboxyvinyltransferase [A... | 438 | 9e-148 | 59% |
| WP_196400429.1 3-phosphoshikimate 1-carboxyvinyltransferase [M... | 438 | 9e-148 | 58% |
| WP_111065442.1 3-phosphoshikimate 1-carboxyvinyltransferase [A... | 439 | 9e-148 | 57% |
| MAI50606.1 3-phosphoshikimate 1-carboxyvinyltransferase [Rhodo... | 438 | 9e-148 | 56% |
| WP_120504272.1 3-phosphoshikimate 1-carboxyvinyltransferase [S... | 438 | 9e-148 | 55% |
| MBB6457683.1 3-phosphoshikimate 1-carboxyvinyltransferase [Ace... | 438 | 9e-148 | 55% |
| WP_093360758.1 3-phosphoshikimate 1-carboxyvinyltransferase [T... | 438 | 9e-148 | 57% |
| MBT55699.1 3-phosphoshikimate 1-carboxyvinyltransferase [Mamel... | 438 | 9e-148 | 57% |
| WP_090519942.1 3-phosphoshikimate 1-carboxyvinyltransferase [P... | 438 | 1e-147 | 55% |
| MAO03685.1 3-phosphoshikimate 1-carboxyvinyltransferase [Citro... | 438 | 1e-147 | 57% |
| AKM06722.1 3-phosphoshikimate 1-carboxyvinyltransferase [Alter... | 438 | 1e-147 | 57% |
| WP_141986355.1 3-phosphoshikimate 1-carboxyvinyltransferase [S... | 438 | 1e-147 | 56% |
| PAK78654.1 3-phosphoshikimate 1-carboxyvinyltransferase [Aceto... | 438 | 1e-147 | 55% |
| CAA7613141.1 3-phosphoshikimate 1-carboxyvinyltransferase [Can... | 438 | 1e-147 | 57% |
| WP_066585247.1 3-phosphoshikimate 1-carboxyvinyltransferase [S... | 438 | 1e-147 | 56% |
| WP_055931434.1 3-phosphoshikimate 1-carboxyvinyltransferase [S... | 439 | 1e-147 | 55% |
| MBS46853.1 3-phosphoshikimate 1-carboxyvinyltransferase [Sphin... | 438 | 1e-147 | 58% |
| MRG71144.1 3-phosphoshikimate 1-carboxyvinyltransferase [Alpha... | 438 | 1e-147 | 56% |

| TPG18587.1 3-phosphoshikimate 1-carboxyvinyltransferase [Sphin... | 438 | 1e-147 | 57% |
| --- | --- | --- | --- |
| WP_195786711.1 unnamed protein product | 438 | 1e-147 | 56% |
| WP_043749908.1 3-phosphoshikimate 1-carboxyvinyltransferase [M... | 438 | 1e-147 | 60% |
| MBF9026379.1 3-phosphoshikimate 1-carboxyvinyltransferase [Rho... | 438 | 1e-147 | 57% |
| NNK16142.1 3-phosphoshikimate 1-carboxyvinyltransferase [Sulfi... | 438 | 1e-147 | 56% |
| WP_145845427.1 3-phosphoshikimate 1-carboxyvinyltransferase [S... | 438 | 1e-147 | 58% |
| MAH05601.1 3-phosphoshikimate 1-carboxyvinyltransferase [Alpha... | 439 | 1e-147 | 55% |
| WP_007564916.1 3-phosphoshikimate 1-carboxyvinyltransferase [M... | 438 | 1e-147 | 59% |
| WP_048854949.1 3-phosphoshikimate 1-carboxyvinyltransferase [A... | 438 | 1e-147 | 56% |
| EDQ03614.1 3-phosphoshikimate 1-carboxyvinyltransferase [Sulfi... | 438 | 1e-147 | 55% |
| WP_152475907.1 3-phosphoshikimate 1-carboxyvinyltransferase [R... | 438 | 1e-147 | 55% |
| WP_088671178.1 3-phosphoshikimate 1-carboxyvinyltransferase [M... | 438 | 1e-147 | 57% |
| TAD89014.1 3-phosphoshikimate 1-carboxyvinyltransferase [Alpha... | 438 | 1e-147 | 55% |
| WP_126000790.1 3-phosphoshikimate 1-carboxyvinyltransferase [S... | 438 | 1e-147 | 56% |
| WP_192145279.1 3-phosphoshikimate 1-carboxyvinyltransferase [S... | 438 | 1e-147 | 56% |
| WP_193715331.1 3-phosphoshikimate 1-carboxyvinyltransferase [S... | 438 | 1e-147 | 57% |
| OUI97351.1 3-phosphoshikimate 1-carboxyvinyltransferase [Aceto... | 437 | 1e-147 | 55% |
| WP_009026305.1 3-phosphoshikimate 1-carboxyvinyltransferase [B... | 438 | 1e-147 | 56% |
| WP_120299298.1 MULTISPECIES: 3-phosphoshikimate 1-carboxyvinyl... | 438 | 1e-147 | 56% |
| WP_125999769.1 3-phosphoshikimate 1-carboxyvinyltransferase [S... | 438 | 1e-147 | 55% |
| WP_184382686.1 3-phosphoshikimate 1-carboxyvinyltransferase [A... | 439 | 1e-147 | 55% |
| WP_141458818.1 3-phosphoshikimate 1-carboxyvinyltransferase [P... | 437 | 1e-147 | 58% |
| WP_081461454.1 MULTISPECIES: 3-phosphoshikimate 1-carboxyvinyl... | 438 | 1e-147 | 55% |
| HER04292.1 TPA: 3-phosphoshikimate 1-carboxyvinyltransferase [... | 438 | 1e-147 | 59% |
| WP_167384170.1 3-phosphoshikimate 1-carboxyvinyltransferase [A... | 438 | 1e-147 | 56% |
| WP_125972789.1 MULTISPECIES: 3-phosphoshikimate 1-carboxyvinyl... | 437 | 1e-147 | 56% |
| MAX15536.1 3-phosphoshikimate 1-carboxyvinyltransferase [Sphin... | 437 | 1e-147 | 58% |
| WP_196767385.1 3-phosphoshikimate 1-carboxyvinyltransferase [A... | 438 | 2e-147 | 55% |
| WP_054523853.1 MULTISPECIES: 3-phosphoshikimate 1-carboxyvinyl... | 437 | 2e-147 | 57% |
| WP_159366615.1 3-phosphoshikimate 1-carboxyvinyltransferase [S... | 438 | 2e-147 | 54% |
| WP_100164808.1 3-phosphoshikimate 1-carboxyvinyltransferase [P... | 438 | 2e-147 | 56% |
| WP_160256579.1 3-phosphoshikimate 1-carboxyvinyltransferase [A... | 438 | 2e-147 | 55% |
| WP_180537943.1 3-phosphoshikimate 1-carboxyvinyltransferase [A... | 438 | 2e-147 | 55% |
| WP_066650305.1 3-phosphoshikimate 1-carboxyvinyltransferase [S... | 437 | 2e-147 | 57% |
| MBO91998.1 3-phosphoshikimate 1-carboxyvinyltransferase [Rhodo... | 437 | 2e-147 | 55% |
| WP_108642156.1 3-phosphoshikimate 1-carboxyvinyltransferase [T... | 438 | 2e-147 | 58% |
| WP_093743008.1 3-phosphoshikimate 1-carboxyvinyltransferase [S... | 438 | 2e-147 | 56% |
| WP_149269897.1 3-phosphoshikimate 1-carboxyvinyltransferase [A... | 437 | 2e-147 | 57% |
| WP_133018746.1 3-phosphoshikimate 1-carboxyvinyltransferase [S... | 437 | 2e-147 | 56% |
| OUI98268.1 3-phosphoshikimate 1-carboxyvinyltransferase [Aceto... | 439 | 2e-147 | 56% |
| MAU52670.1 3-phosphoshikimate 1-carboxyvinyltransferase [Roseo... | 437 | 2e-147 | 56% |
| AKR49855.1 3-phosphoshikimate 1-carboxyvinyltransferase [Aceto... | 437 | 2e-147 | 55% |
| WP_136162370.1 3-phosphoshikimate 1-carboxyvinyltransferase [S... | 439 | 2e-147 | 57% |
| WP_171216597.1 3-phosphoshikimate 1-carboxyvinyltransferase [E... | 438 | 2e-147 | 58% |
| WP_099954816.1 3-phosphoshikimate 1-carboxyvinyltransferase [M... | 437 | 2e-147 | 58% |
| WP_173957506.1 3-phosphoshikimate 1-carboxyvinyltransferase [A... | 437 | 2e-147 | 55% |
| WP_080617069.1 3-phosphoshikimate 1-carboxyvinyltransferase [R... | 437 | 2e-147 | 57% |
| WP_075788152.1 3-phosphoshikimate 1-carboxyvinyltransferase [R... | 437 | 2e-147 | 57% |
| WP_034159984.1 3-phosphoshikimate 1-carboxyvinyltransferase [S... | 437 | 2e-147 | 55% |
| WP_156500327.1 3-phosphoshikimate 1-carboxyvinyltransferase [C... | 439 | 2e-147 | 58% |
| WP_115938038.1 3-phosphoshikimate 1-carboxyvinyltransferase [A... | 437 | 2e-147 | 57% |
| WP_048938431.1 MULTISPECIES: 3-phosphoshikimate 1-carboxyvinyl... | 438 | 2e-147 | 55% |
| WP_036736066.1 3-phosphoshikimate 1-carboxyvinyltransferase [P... | 437 | 2e-147 | 55% |
| WP_159644497.1 3-phosphoshikimate 1-carboxyvinyltransferase [S... | 437 | 2e-147 | 55% |
| WP_113910676.1 3-phosphoshikimate 1-carboxyvinyltransferase [P... | 437 | 2e-147 | 56% |
| RZM36658.1 3-phosphoshikimate 1-carboxyvinyltransferase [Sphin... | 437 | 2e-147 | 57% |
| WP_099274738.1 3-phosphoshikimate 1-carboxyvinyltransferase [L... | 437 | 2e-147 | 56% |
| WP_179290134.1 3-phosphoshikimate 1-carboxyvinyltransferase [A... | 438 | 2e-147 | 55% |
| CCQ72605.1 3-phosphoshikimate 1-carboxyvinyltransferase (5-eno... | 438 | 2e-147 | 56% |
| WP_048454019.1 3-phosphoshikimate 1-carboxyvinyltransferase [M... | 437 | 2e-147 | 58% |
| WP_018251081.1 3-phosphoshikimate 1-carboxyvinyltransferase [S... | 437 | 2e-147 | 59% |
| WP_037284315.1 3-phosphoshikimate 1-carboxyvinyltransferase [R... | 437 | 2e-147 | 55% |
| OYZ98867.1 3-phosphoshikimate 1-carboxyvinyltransferase [Rhizo... | 437 | 2e-147 | 59% |
| WP_148057151.1 3-phosphoshikimate 1-carboxyvinyltransferase [O... | 437 | 2e-147 | 57% |
| PCJ56671.1 3-phosphoshikimate 1-carboxyvinyltransferase [Rhodo... | 437 | 2e-147 | 54% |
| WP_058735457.1 3-phosphoshikimate 1-carboxyvinyltransferase [N... | 437 | 2e-147 | 55% |
| RKE53573.1 3-phosphoshikimate 1-carboxyvinyltransferase [Sphin... | 438 | 2e-147 | 56% |
| PLX34802.1 3-phosphoshikimate 1-carboxyvinyltransferase [Rhizo... | 437 | 2e-147 | 55% |
| EGE47587.1 3-phosphoshikimate 1-carboxyvinyltransferase [Aceto... | 439 | 3e-147 | 55% |
| NHO40038.1 3-phosphoshikimate 1-carboxyvinyltransferase [Aceto... | 437 | 3e-147 | 55% |
| MBC7153150.1 3-phosphoshikimate 1-carboxyvinyltransferase [Rho... | 437 | 3e-147 | 55% |
| WP_075782734.1 3-phosphoshikimate 1-carboxyvinyltransferase [R... | 437 | 3e-147 | 57% |

| WP_037246703.1 3-phosphoshikimate 1-carboxyvinyltransferase [R... | 437 | 3e-147 | 55% |
| --- | --- | --- | --- |
| WP_091863170.1 3-phosphoshikimate 1-carboxyvinyltransferase [M... | 437 | 3e-147 | 59% |
| WP_144299674.1 3-phosphoshikimate 1-carboxyvinyltransferase [E... | 437 | 3e-147 | 57% |
| WP_168853336.1 3-phosphoshikimate 1-carboxyvinyltransferase [N... | 437 | 3e-147 | 57% |
| WP_102844574.1 3-phosphoshikimate 1-carboxyvinyltransferase [M... | 437 | 3e-147 | 58% |
| WP_099624023.1 3-phosphoshikimate 1-carboxyvinyltransferase [C... | 437 | 3e-147 | 56% |
| WP_188478730.1 3-phosphoshikimate 1-carboxyvinyltransferase [P... | 437 | 3e-147 | 56% |
| WP_046862123.1 3-phosphoshikimate 1-carboxyvinyltransferase [M... | 437 | 3e-147 | 59% |
| WP_145681283.1 3-phosphoshikimate 1-carboxyvinyltransferase [A... | 437 | 3e-147 | 56% |
| PHY92731.1 3-phosphoshikimate 1-carboxyvinyltransferase [Aceto... | 438 | 3e-147 | 56% |
| WP_025049825.1 3-phosphoshikimate 1-carboxyvinyltransferase [S... | 437 | 3e-147 | 55% |
| WP_037908187.1 3-phosphoshikimate 1-carboxyvinyltransferase [S... | 437 | 3e-147 | 55% |
| WP_052249437.1 3-phosphoshikimate 1-carboxyvinyltransferase [T... | 437 | 3e-147 | 56% |
| WP_183472098.1 3-phosphoshikimate 1-carboxyvinyltransferase [L... | 437 | 3e-147 | 56% |
| WP_109051899.1 3-phosphoshikimate 1-carboxyvinyltransferase [A... | 437 | 3e-147 | 57% |
| WP_191249887.1 3-phosphoshikimate 1-carboxyvinyltransferase [K... | 437 | 3e-147 | 56% |
| WP_196332603.1 3-phosphoshikimate 1-carboxyvinyltransferase [A... | 437 | 3e-147 | 55% |
| WP_109085031.1 3-phosphoshikimate 1-carboxyvinyltransferase [A... | 437 | 3e-147 | 56% |
| WP_181559141.1 MULTISPECIES: 3-phosphoshikimate 1-carboxyvinyl... | 437 | 3e-147 | 56% |
| WP_109923270.1 3-phosphoshikimate 1-carboxyvinyltransferase [Z... | 437 | 3e-147 | 59% |
| TCP93985.1 3-phosphoshikimate 1-carboxyvinyltransferase [Sphin... | 438 | 3e-147 | 56% |
| WP_136186885.1 3-phosphoshikimate 1-carboxyvinyltransferase [S... | 437 | 3e-147 | 55% |
| WP_069440634.1 3-phosphoshikimate 1-carboxyvinyltransferase [M... | 437 | 3e-147 | 57% |
| WP_172430928.1 3-phosphoshikimate 1-carboxyvinyltransferase [M... | 437 | 3e-147 | 57% |
| MBF9023839.1 3-phosphoshikimate 1-carboxyvinyltransferase [Rho... | 437 | 3e-147 | 57% |
| WP_147111507.1 3-phosphoshikimate 1-carboxyvinyltransferase [T... | 437 | 3e-147 | 56% |
| WP_116130978.1 3-phosphoshikimate 1-carboxyvinyltransferase [T... | 437 | 3e-147 | 57% |
| HCW60321.1 TPA: 3-phosphoshikimate 1-carboxyvinyltransferase [... | 437 | 3e-147 | 58% |
| WP_161393345.1 3-phosphoshikimate 1-carboxyvinyltransferase [M... | 437 | 3e-147 | 59% |
| WP_088651674.1 3-phosphoshikimate 1-carboxyvinyltransferase [M... | 437 | 3e-147 | 54% |
| WP_164353296.1 3-phosphoshikimate 1-carboxyvinyltransferase [S... | 437 | 3e-147 | 55% |
| WP_108385833.1 3-phosphoshikimate 1-carboxyvinyltransferase [Y... | 437 | 3e-147 | 56% |
| WP_068352646.1 3-phosphoshikimate 1-carboxyvinyltransferase [P... | 436 | 3e-147 | 59% |
| WP_141462342.1 3-phosphoshikimate 1-carboxyvinyltransferase [S... | 437 | 3e-147 | 55% |
| WP_116599509.1 3-phosphoshikimate 1-carboxyvinyltransferase [P... | 437 | 4e-147 | 55% |
| WP_113791625.1 3-phosphoshikimate 1-carboxyvinyltransferase [R... | 437 | 4e-147 | 55% |
| WP_103334010.1 3-phosphoshikimate 1-carboxyvinyltransferase [T... | 437 | 4e-147 | 56% |
| WP_106608437.1 3-phosphoshikimate 1-carboxyvinyltransferase [S... | 437 | 4e-147 | 55% |
| WP_043133871.1 3-phosphoshikimate 1-carboxyvinyltransferase [P... | 436 | 4e-147 | 55% |
| MAP15927.1 3-phosphoshikimate 1-carboxyvinyltransferase [Sulfi... | 437 | 4e-147 | 55% |
| WP_196104438.1 unnamed protein product | 436 | 4e-147 | 55% |
| WP_182591121.1 3-phosphoshikimate 1-carboxyvinyltransferase [M... | 437 | 4e-147 | 60% |
| WP_039124393.1 3-phosphoshikimate 1-carboxyvinyltransferase [L... | 437 | 4e-147 | 56% |
| NNJ66855.1 3-phosphoshikimate 1-carboxyvinyltransferase [Boseo... | 437 | 4e-147 | 55% |
| ACL61849.1 3-phosphoshikimate 1-carboxyvinyltransferase [Methy... | 437 | 4e-147 | 60% |
| WP_088622314.1 3-phosphoshikimate 1-carboxyvinyltransferase [O... | 437 | 4e-147 | 56% |
| WP_114967208.1 3-phosphoshikimate 1-carboxyvinyltransferase [A... | 437 | 4e-147 | 57% |
| KPP89555.1 3-phosphoshikimate 1-carboxyvinyltransferase AroA [... | 437 | 4e-147 | 57% |
| WP_171176785.1 3-phosphoshikimate 1-carboxyvinyltransferase [R... | 437 | 4e-147 | 55% |
| WP_179697799.1 MULTISPECIES: 3-phosphoshikimate 1-carboxyvinyl... | 437 | 4e-147 | 60% |
| WP_101654312.1 3-phosphoshikimate 1-carboxyvinyltransferase [A... | 436 | 4e-147 | 56% |
| WP_074623481.1 3-phosphoshikimate 1-carboxyvinyltransferase [M... | 436 | 4e-147 | 57% |
| WP_141889418.1 3-phosphoshikimate 1-carboxyvinyltransferase [L... | 437 | 4e-147 | 56% |
| ANA14819.1 3-phosphoshikimate 1-carboxyvinyltransferase [Aceto... | 437 | 4e-147 | 55% |
| WP_025045629.1 3-phosphoshikimate 1-carboxyvinyltransferase [S... | 437 | 4e-147 | 54% |
| MAQ78359.1 3-phosphoshikimate 1-carboxyvinyltransferase [Rhodo... | 436 | 4e-147 | 55% |
| WP_058335610.1 3-phosphoshikimate 1-carboxyvinyltransferase [P... | 436 | 4e-147 | 55% |
| WP_108860960.1 3-phosphoshikimate 1-carboxyvinyltransferase [R... | 437 | 5e-147 | 55% |
| WP_153876083.1 3-phosphoshikimate 1-carboxyvinyltransferase [M... | 436 | 5e-147 | 60% |
| OYV42800.1 3-phosphoshikimate 1-carboxyvinyltransferase [Acido... | 436 | 5e-147 | 56% |
| WP_058241227.1 3-phosphoshikimate 1-carboxyvinyltransferase [S... | 436 | 5e-147 | 55% |
| CAA2107799.1 3-phosphoshikimate 1-carboxyvinyltransferase [Met... | 435 | 5e-147 | 61% |
| HEL41339.1 TPA: 3-phosphoshikimate 1-carboxyvinyltransferase [... | 436 | 5e-147 | 56% |
| WP_099211139.1 3-phosphoshikimate 1-carboxyvinyltransferase [G... | 436 | 5e-147 | 56% |
| WP_128454228.1 3-phosphoshikimate 1-carboxyvinyltransferase [S... | 436 | 5e-147 | 56% |
| PQO23204.1 3-phosphoshikimate 1-carboxyvinyltransferase [Rhodo... | 437 | 5e-147 | 57% |
| BBI21129.1 3-phosphoshikimate 1-carboxyvinyltransferase [Eryth... | 436 | 5e-147 | 58% |
| WP_095596634.1 3-phosphoshikimate 1-carboxyvinyltransferase [A... | 436 | 5e-147 | 56% |
| WP_127726898.1 3-phosphoshikimate 1-carboxyvinyltransferase [M... | 436 | 5e-147 | 59% |
| KXV39110.1 3-phosphoshikimate 1-carboxyvinyltransferase [Gluco... | 436 | 5e-147 | 55% |
| WP_067500669.1 3-phosphoshikimate 1-carboxyvinyltransferase [E... | 436 | 5e-147 | 57% |
| MAP43832.1 3-phosphoshikimate 1-carboxyvinyltransferase [Sphin... | 436 | 5e-147 | 58% |

| WP_058286291.1 3-phosphoshikimate 1-carboxyvinyltransferase [L... | 436 | 5e-147 | 56% |
| --- | --- | --- | --- |
| WP_076445877.1 3-phosphoshikimate 1-carboxyvinyltransferase [R... | 436 | 5e-147 | 55% |
| WP_141855940.1 3-phosphoshikimate 1-carboxyvinyltransferase [E... | 436 | 6e-147 | 57% |
| WP_066817327.1 3-phosphoshikimate 1-carboxyvinyltransferase [S... | 436 | 6e-147 | 55% |
| WP_152448889.1 MULTISPECIES: 3-phosphoshikimate 1-carboxyvinyl... | 436 | 6e-147 | 56% |
| WP_174149188.1 3-phosphoshikimate 1-carboxyvinyltransferase [L... | 436 | 6e-147 | 55% |
| TNF22812.1 3-phosphoshikimate 1-carboxyvinyltransferase [Rhodo... | 436 | 6e-147 | 56% |
| WP_112382311.1 3-phosphoshikimate 1-carboxyvinyltransferase [S... | 436 | 6e-147 | 57% |
| PIY72691.1 3-phosphoshikimate 1-carboxyvinyltransferase [Rhodo... | 436 | 6e-147 | 57% |
| WP_167648320.1 3-phosphoshikimate 1-carboxyvinyltransferase [M... | 436 | 6e-147 | 56% |
| NVK13392.1 3-phosphoshikimate 1-carboxyvinyltransferase [Rhodo... | 436 | 6e-147 | 55% |
| WP_132031658.1 3-phosphoshikimate 1-carboxyvinyltransferase [A... | 436 | 6e-147 | 58% |
| WP_093321952.1 3-phosphoshikimate 1-carboxyvinyltransferase [S... | 436 | 6e-147 | 57% |
| HGG06400.1 TPA: 3-phosphoshikimate 1-carboxyvinyltransferase [... | 436 | 6e-147 | 57% |
| WP_170334138.1 3-phosphoshikimate 1-carboxyvinyltransferase [R... | 436 | 6e-147 | 54% |
| WP_021692592.1 3-phosphoshikimate 1-carboxyvinyltransferase [L... | 436 | 7e-147 | 56% |
| WP_068109831.1 3-phosphoshikimate 1-carboxyvinyltransferase [P... | 436 | 7e-147 | 57% |
| MBF9021271.1 3-phosphoshikimate 1-carboxyvinyltransferase [Rho... | 436 | 7e-147 | 57% |
| PWB61509.1 3-phosphoshikimate 1-carboxyvinyltransferase [Brady... | 436 | 7e-147 | 56% |
| WP_191075241.1 3-phosphoshikimate 1-carboxyvinyltransferase [S... | 436 | 7e-147 | 54% |
| WP_130587335.1 3-phosphoshikimate 1-carboxyvinyltransferase [E... | 435 | 7e-147 | 58% |
| WP_150287806.1 3-phosphoshikimate 1-carboxyvinyltransferase [R... | 436 | 7e-147 | 58% |
| PIE11869.1 3-phosphoshikimate 1-carboxyvinyltransferase [Rhodo... | 436 | 7e-147 | 56% |
| WP_167222387.1 3-phosphoshikimate 1-carboxyvinyltransferase [P... | 436 | 7e-147 | 56% |
| HIJ92698.1 TPA: 3-phosphoshikimate 1-carboxyvinyltransferase [... | 436 | 7e-147 | 57% |
| WP_150115260.1 3-phosphoshikimate 1-carboxyvinyltransferase [A... | 435 | 8e-147 | 57% |
| WP_034958014.1 3-phosphoshikimate 1-carboxyvinyltransferase [E... | 436 | 8e-147 | 56% |
| WP_050528917.1 3-phosphoshikimate 1-carboxyvinyltransferase [P... | 436 | 8e-147 | 56% |
| WP_135513904.1 3-phosphoshikimate 1-carboxyvinyltransferase [T... | 436 | 8e-147 | 55% |
| WP_058273164.1 3-phosphoshikimate 1-carboxyvinyltransferase [R... | 436 | 8e-147 | 55% |
| WP_167085141.1 3-phosphoshikimate 1-carboxyvinyltransferase [R... | 436 | 8e-147 | 56% |
| WP_172793752.1 3-phosphoshikimate 1-carboxyvinyltransferase [A... | 436 | 8e-147 | 55% |
| WP_123176329.1 3-phosphoshikimate 1-carboxyvinyltransferase [M... | 436 | 8e-147 | 58% |
| WP_055730033.1 3-phosphoshikimate 1-carboxyvinyltransferase [B... | 436 | 8e-147 | 58% |
| WP_150177888.1 3-phosphoshikimate 1-carboxyvinyltransferase [A... | 436 | 8e-147 | 55% |
| WP_055082534.1 3-phosphoshikimate 1-carboxyvinyltransferase [J... | 436 | 8e-147 | 55% |
| MBA4788878.1 3-phosphoshikimate 1-carboxyvinyltransferase [Rhi... | 435 | 9e-147 | 58% |
| WP_093161972.1 3-phosphoshikimate 1-carboxyvinyltransferase [P... | 436 | 9e-147 | 56% |
| WP_010236686.1 MULTISPECIES: 3-phosphoshikimate 1-carboxyvinyl... | 436 | 9e-147 | 56% |
| RCL08967.1 3-phosphoshikimate 1-carboxyvinyltransferase [Aceto... | 436 | 9e-147 | 55% |
| HIM77589.1 TPA: 3-phosphoshikimate 1-carboxyvinyltransferase [... | 436 | 9e-147 | 57% |
| WP_074256592.1 3-phosphoshikimate 1-carboxyvinyltransferase [O... | 436 | 9e-147 | 56% |
| PKP65101.1 3-phosphoshikimate 1-carboxyvinyltransferase [Alpha... | 436 | 9e-147 | 57% |
| WP_184116785.1 3-phosphoshikimate 1-carboxyvinyltransferase [S... | 436 | 9e-147 | 57% |
| WP_048840890.1 3-phosphoshikimate 1-carboxyvinyltransferase [A... | 435 | 9e-147 | 55% |
| KIC50879.1 3-phosphoshikimate 1-carboxyvinyltransferase [Tatey... | 436 | 9e-147 | 56% |
| MBE1289248.1 3-phosphoshikimate 1-carboxyvinyltransferase [Rho... | 436 | 9e-147 | 57% |
| WP_185804454.1 3-phosphoshikimate 1-carboxyvinyltransferase [M... | 436 | 1e-146 | 55% |
| OYW58744.1 3-phosphoshikimate 1-carboxyvinyltransferase [Rhizo... | 436 | 1e-146 | 59% |
| WP_162104743.1 3-phosphoshikimate 1-carboxyvinyltransferase [R... | 436 | 1e-146 | 56% |
| OUI82465.1 3-phosphoshikimate 1-carboxyvinyltransferase [Aceto... | 435 | 1e-146 | 55% |
| WP_077511643.1 3-phosphoshikimate 1-carboxyvinyltransferase [S... | 436 | 1e-146 | 56% |
| RNJ63214.1 3-phosphoshikimate 1-carboxyvinyltransferase [Porph... | 436 | 1e-146 | 58% |
| WP_041926722.1 3-phosphoshikimate 1-carboxyvinyltransferase [M... | 436 | 1e-146 | 58% |
| EDZ47172.1 3-phosphoshikimate 1-carboxyvinyltransferase [Rhodo... | 436 | 1e-146 | 55% |
| WP_036710752.1 3-phosphoshikimate 1-carboxyvinyltransferase [P... | 436 | 1e-146 | 55% |
| MBA3880863.1 3-phosphoshikimate 1-carboxyvinyltransferase [Sph... | 436 | 1e-146 | 56% |
| WP_091299768.1 3-phosphoshikimate 1-carboxyvinyltransferase [G... | 436 | 1e-146 | 56% |
| WP_056486787.1 3-phosphoshikimate 1-carboxyvinyltransferase [S... | 436 | 1e-146 | 56% |
| OIQ40701.1 3-phosphoshikimate 1-carboxyvinyltransferase [Roseo... | 436 | 1e-146 | 55% |
| WP_179508992.1 3-phosphoshikimate 1-carboxyvinyltransferase [S... | 435 | 1e-146 | 57% |
| WP_082823094.1 3-phosphoshikimate 1-carboxyvinyltransferase [A... | 436 | 1e-146 | 55% |
| WP_135965201.1 3-phosphoshikimate 1-carboxyvinyltransferase [S... | 435 | 1e-146 | 55% |
| WP_082719703.1 3-phosphoshikimate 1-carboxyvinyltransferase [K... | 435 | 1e-146 | 55% |
| WP_038525883.1 3-phosphoshikimate 1-carboxyvinyltransferase [A... | 436 | 1e-146 | 57% |
| WP_008971956.1 3-phosphoshikimate 1-carboxyvinyltransferase [B... | 436 | 1e-146 | 57% |
| WP_107952120.1 3-phosphoshikimate 1-carboxyvinyltransferase [S... | 435 | 1e-146 | 56% |
| MAX00756.1 3-phosphoshikimate 1-carboxyvinyltransferase [Sphin... | 436 | 1e-146 | 57% |
| MBC7164770.1 3-phosphoshikimate 1-carboxyvinyltransferase [Ros... | 436 | 1e-146 | 55% |
| WP_114859073.1 3-phosphoshikimate 1-carboxyvinyltransferase [A... | 435 | 1e-146 | 57% |
| WP_194911389.1 3-phosphoshikimate 1-carboxyvinyltransferase [A... | 436 | 1e-146 | 57% |
| OYW49433.1 3-phosphoshikimate 1-carboxyvinyltransferase [Novos... | 435 | 1e-146 | 57% |

| WP_154738721.1 3-phosphoshikimate 1-carboxyvinyltransferase [H... | 437 | 1e-146 | 56% |
| --- | --- | --- | --- |
| WP_127765477.1 3-phosphoshikimate 1-carboxyvinyltransferase [H... | 436 | 1e-146 | 55% |
| MSO98377.1 3-phosphoshikimate 1-carboxyvinyltransferase [Rhodo... | 436 | 1e-146 | 54% |
| CBS85586.1 3-phosphoshikimate 1-carboxyvinyltransferase [Azosp... | 436 | 1e-146 | 57% |
| WP_043381639.1 MULTISPECIES: 3-phosphoshikimate 1-carboxyvinyl... | 436 | 1e-146 | 60% |
| WP_026606222.1 3-phosphoshikimate 1-carboxyvinyltransferase [M... | 435 | 1e-146 | 56% |
| BBC80447.1 3-phosphoshikimate 1-carboxyvinyltransferase [Aceto... | 436 | 1e-146 | 55% |
| WP_137899160.1 3-phosphoshikimate 1-carboxyvinyltransferase [S... | 435 | 1e-146 | 57% |
| WP_137115136.1 3-phosphoshikimate 1-carboxyvinyltransferase [A... | 435 | 1e-146 | 57% |
| WP_015501327.1 3-phosphoshikimate 1-carboxyvinyltransferase [O... | 436 | 1e-146 | 57% |
| OYY08385.1 3-phosphoshikimate 1-carboxyvinyltransferase [Rhizo... | 436 | 1e-146 | 59% |
| WP_137678163.1 3-phosphoshikimate 1-carboxyvinyltransferase [A... | 435 | 1e-146 | 58% |
| PZU18381.1 3-phosphoshikimate 1-carboxyvinyltransferase [Citro... | 435 | 1e-146 | 57% |
| WP_160753348.1 3-phosphoshikimate 1-carboxyvinyltransferase [P... | 436 | 1e-146 | 58% |
| WP_181014210.1 3-phosphoshikimate 1-carboxyvinyltransferase [A... | 436 | 1e-146 | 55% |
| WP_085090352.1 3-phosphoshikimate 1-carboxyvinyltransferase [A... | 435 | 1e-146 | 57% |
| NYD90508.1 3-phosphoshikimate 1-carboxyvinyltransferase [Sphin... | 435 | 1e-146 | 57% |
| WP_170357083.1 3-phosphoshikimate 1-carboxyvinyltransferase [R... | 435 | 1e-146 | 56% |
| CEF54039.1 3-phosphoshikimate 1-carboxyvinyltransferase [Aceto... | 437 | 1e-146 | 55% |
| WP_103011853.1 3-phosphoshikimate 1-carboxyvinyltransferase [R... | 436 | 1e-146 | 54% |
| WP_018980129.1 3-phosphoshikimate 1-carboxyvinyltransferase [S... | 435 | 2e-146 | 54% |
| WP_056612031.1 3-phosphoshikimate 1-carboxyvinyltransferase [S... | 435 | 2e-146 | 56% |
| WP_170527602.1 3-phosphoshikimate 1-carboxyvinyltransferase [R... | 435 | 2e-146 | 56% |
| WP_027245564.1 3-phosphoshikimate 1-carboxyvinyltransferase [L... | 435 | 2e-146 | 55% |
| WP_060526649.1 3-phosphoshikimate 1-carboxyvinyltransferase [S... | 435 | 2e-146 | 56% |
| MBE1292914.1 3-phosphoshikimate 1-carboxyvinyltransferase [Rho... | 435 | 2e-146 | 55% |
| WP_109027700.1 3-phosphoshikimate 1-carboxyvinyltransferase [M... | 435 | 2e-146 | 59% |
| WP_179193554.1 3-phosphoshikimate 1-carboxyvinyltransferase [A... | 435 | 2e-146 | 55% |
| WP_170364915.1 3-phosphoshikimate 1-carboxyvinyltransferase [R... | 435 | 2e-146 | 55% |
| WP_069311268.1 3-phosphoshikimate 1-carboxyvinyltransferase [P... | 435 | 2e-146 | 57% |
| WP_136339913.1 3-phosphoshikimate 1-carboxyvinyltransferase [Y... | 435 | 2e-146 | 57% |
| WP_109312559.1 3-phosphoshikimate 1-carboxyvinyltransferase [R... | 435 | 2e-146 | 55% |
| MAF97347.1 3-phosphoshikimate 1-carboxyvinyltransferase [Micav... | 435 | 2e-146 | 54% |
| MBE1283781.1 3-phosphoshikimate 1-carboxyvinyltransferase [Rho... | 435 | 2e-146 | 55% |
| MBF9056956.1 3-phosphoshikimate 1-carboxyvinyltransferase [Rho... | 435 | 2e-146 | 57% |
| WP_070323966.1 3-phosphoshikimate 1-carboxyvinyltransferase [A... | 435 | 2e-146 | 55% |
| WP_170538015.1 3-phosphoshikimate 1-carboxyvinyltransferase [R... | 435 | 2e-146 | 55% |
| WP_092692298.1 3-phosphoshikimate 1-carboxyvinyltransferase [R... | 435 | 2e-146 | 55% |
| WP_158815524.1 3-phosphoshikimate 1-carboxyvinyltransferase [M... | 435 | 2e-146 | 59% |
| WP_136189424.1 3-phosphoshikimate 1-carboxyvinyltransferase [S... | 435 | 2e-146 | 56% |
| WP_039147386.1 3-phosphoshikimate 1-carboxyvinyltransferase [L... | 435 | 2e-146 | 56% |
| WP_108894239.1 3-phosphoshikimate 1-carboxyvinyltransferase [P... | 435 | 2e-146 | 56% |
| MBD59834.1 3-phosphoshikimate 1-carboxyvinyltransferase [Citro... | 436 | 2e-146 | 56% |
| MBA4759673.1 3-phosphoshikimate 1-carboxyvinyltransferase [Sph... | 434 | 2e-146 | 59% |
| OJV31828.1 3-phosphoshikimate 1-carboxyvinyltransferase [Sphin... | 436 | 2e-146 | 52% |
| WP_085938464.1 3-phosphoshikimate 1-carboxyvinyltransferase [A... | 435 | 2e-146 | 57% |
| GAP25293.1 3-phosphoshikimate 1-carboxyvinyltransferase [Gluco... | 435 | 2e-146 | 55% |
| CAA2155560.1 3-phosphoshikimate 1-carboxyvinyltransferase [Met... | 435 | 2e-146 | 59% |
| OHC73788.1 3-phosphoshikimate 1-carboxyvinyltransferase [Rhodo... | 434 | 2e-146 | 55% |
| OAJ68022.1 3-phosphoshikimate 1-carboxyvinyltransferase [Gluco... | 435 | 2e-146 | 55% |
| WP_089179855.1 MULTISPECIES: 3-phosphoshikimate 1-carboxyvinyl... | 436 | 2e-146 | 55% |
| WP_068460679.1 3-phosphoshikimate 1-carboxyvinyltransferase [H... | 435 | 2e-146 | 58% |
| CAA2145433.1 3-phosphoshikimate 1-carboxyvinyltransferase [Met... | 434 | 2e-146 | 60% |
| WP_149681512.1 3-phosphoshikimate 1-carboxyvinyltransferase [S... | 435 | 2e-146 | 57% |
| WP_170451013.1 3-phosphoshikimate 1-carboxyvinyltransferase [R... | 435 | 2e-146 | 55% |
| WP_170978439.1 3-phosphoshikimate 1-carboxyvinyltransferase [S... | 435 | 2e-146 | 56% |
| KXV28553.1 3-phosphoshikimate 1-carboxyvinyltransferase [Gluco... | 435 | 2e-146 | 55% |
| WP_194257334.1 3-phosphoshikimate 1-carboxyvinyltransferase [G... | 434 | 2e-146 | 55% |
| WP_036561925.1 3-phosphoshikimate 1-carboxyvinyltransferase [O... | 435 | 2e-146 | 55% |
| WP_071970751.1 3-phosphoshikimate 1-carboxyvinyltransferase [S... | 435 | 2e-146 | 56% |
| WP_088346903.1 MULTISPECIES: 3-phosphoshikimate 1-carboxyvinyl... | 434 | 2e-146 | 58% |
| WP_170420221.1 3-phosphoshikimate 1-carboxyvinyltransferase [R... | 435 | 2e-146 | 55% |
| WP_149197833.1 3-phosphoshikimate 1-carboxyvinyltransferase [A... | 435 | 2e-146 | 57% |
| WP_093031308.1 3-phosphoshikimate 1-carboxyvinyltransferase [R... | 435 | 2e-146 | 57% |
| ARW10308.1 3-phosphoshikimate 1-carboxyvinyltransferase [Aceto... | 435 | 2e-146 | 55% |
| WP_019089004.1 MULTISPECIES: 3-phosphoshikimate 1-carboxyvinyl... | 435 | 2e-146 | 55% |
| WP_012589657.1 3-phosphoshikimate 1-carboxyvinyltransferase [M... | 434 | 2e-146 | 58% |
| WP_089887969.1 3-phosphoshikimate 1-carboxyvinyltransferase [L... | 435 | 2e-146 | 55% |
| KXV26573.1 3-phosphoshikimate 1-carboxyvinyltransferase [Gluco... | 434 | 2e-146 | 55% |
| EAT07607.1 3-phosphoshikimate 1-carboxyvinyltransferase [Sphin... | 434 | 2e-146 | 58% |
| QEW18648.1 3-phosphoshikimate 1-carboxyvinyltransferase [Rhodo... | 435 | 3e-146 | 53% |
| WP_044330315.1 3-phosphoshikimate 1-carboxyvinyltransferase [S... | 434 | 3e-146 | 56% |

| WP_170390863.1 3-phosphoshikimate 1-carboxyvinyltransferase [R... | 434 | 3e-146 | 56% |
| --- | --- | --- | --- |
| WP_159732769.1 3-phosphoshikimate 1-carboxyvinyltransferase [M... | 434 | 3e-146 | 58% |
| WP_121884400.1 3-phosphoshikimate 1-carboxyvinyltransferase [S... | 434 | 3e-146 | 56% |
| WP_093974129.1 3-phosphoshikimate 1-carboxyvinyltransferase [B... | 435 | 3e-146 | 55% |
| WP_187729453.1 3-phosphoshikimate 1-carboxyvinyltransferase [S... | 434 | 3e-146 | 56% |
| WP_170598855.1 3-phosphoshikimate 1-carboxyvinyltransferase [R... | 434 | 3e-146 | 55% |
| MAC79763.1 3-phosphoshikimate 1-carboxyvinyltransferase [Rhodo... | 435 | 3e-146 | 53% |
| WP_170441549.1 3-phosphoshikimate 1-carboxyvinyltransferase [R... | 434 | 3e-146 | 55% |
| WP_038662758.1 3-phosphoshikimate 1-carboxyvinyltransferase [S... | 434 | 3e-146 | 56% |
| QLQ19708.1 3-phosphoshikimate 1-carboxyvinyltransferase [Exigu... | 434 | 3e-146 | 57% |
| WP_093964596.1 3-phosphoshikimate 1-carboxyvinyltransferase [R... | 434 | 3e-146 | 55% |
| OUJ08060.1 3-phosphoshikimate 1-carboxyvinyltransferase [Gluco... | 434 | 3e-146 | 55% |
| WP_099899384.1 3-phosphoshikimate 1-carboxyvinyltransferase [M... | 434 | 3e-146 | 58% |
| WP_127090696.1 3-phosphoshikimate 1-carboxyvinyltransferase [A... | 435 | 3e-146 | 59% |
| WP_121047395.1 3-phosphoshikimate 1-carboxyvinyltransferase [S... | 434 | 3e-146 | 59% |
| ESW59728.1 3-phosphoshikimate 1-carboxyvinyltransferase [Rhodo... | 435 | 3e-146 | 57% |
| WP_086633805.1 3-phosphoshikimate 1-carboxyvinyltransferase [G... | 434 | 3e-146 | 55% |
| PCI54892.1 3-phosphoshikimate 1-carboxyvinyltransferase [Alpha... | 435 | 3e-146 | 55% |
| WP_160383437.1 3-phosphoshikimate 1-carboxyvinyltransferase [P... | 434 | 3e-146 | 56% |
| WP_064274152.1 3-phosphoshikimate 1-carboxyvinyltransferase [G... | 434 | 3e-146 | 55% |
| NKB18939.1 3-phosphoshikimate 1-carboxyvinyltransferase [Alpha... | 434 | 3e-146 | 56% |
| WP_160799196.1 MULTISPECIES: 3-phosphoshikimate 1-carboxyvinyl... | 434 | 3e-146 | 53% |
| WP_132605418.1 3-phosphoshikimate 1-carboxyvinyltransferase [R... | 434 | 3e-146 | 56% |
| OAG73308.1 3-phosphoshikimate 1-carboxyvinyltransferase [Gluco... | 434 | 3e-146 | 55% |
| WP_054459314.1 3-phosphoshikimate 1-carboxyvinyltransferase [P... | 434 | 3e-146 | 55% |
| WP_039966345.1 3-phosphoshikimate 1-carboxyvinyltransferase [R... | 434 | 3e-146 | 55% |
| WP_196391941.1 MULTISPECIES: 3-phosphoshikimate 1-carboxyvinyl... | 434 | 3e-146 | 58% |
| PZO70273.1 3-phosphoshikimate 1-carboxyvinyltransferase [Alter... | 434 | 3e-146 | 58% |
| WP_093450348.1 3-phosphoshikimate 1-carboxyvinyltransferase [P... | 434 | 3e-146 | 58% |
| WP_022704596.1 3-phosphoshikimate 1-carboxyvinyltransferase [P... | 434 | 3e-146 | 56% |
| WP_027442212.1 3-phosphoshikimate 1-carboxyvinyltransferase [P... | 434 | 3e-146 | 58% |
| WP_172491993.1 3-phosphoshikimate 1-carboxyvinyltransferase [G... | 434 | 3e-146 | 56% |
| KXV22785.1 3-phosphoshikimate 1-carboxyvinyltransferase [Gluco... | 434 | 3e-146 | 55% |
| WP_092618056.1 3-phosphoshikimate 1-carboxyvinyltransferase [R... | 434 | 3e-146 | 55% |
| AOL95094.1 3-phosphoshikimate 1-carboxyvinyltransferase [Porph... | 435 | 3e-146 | 57% |
| OIQ43729.1 3-phosphoshikimate 1-carboxyvinyltransferase [Roseo... | 434 | 3e-146 | 55% |
| WP_170585336.1 3-phosphoshikimate 1-carboxyvinyltransferase [R... | 434 | 4e-146 | 56% |
| WP_103763327.1 3-phosphoshikimate 1-carboxyvinyltransferase [R... | 434 | 4e-146 | 55% |
| WP_007283224.1 3-phosphoshikimate 1-carboxyvinyltransferase [G... | 434 | 4e-146 | 55% |
| WP_074735927.1 3-phosphoshikimate 1-carboxyvinyltransferase [R... | 434 | 4e-146 | 55% |
| WP_091715738.1 3-phosphoshikimate 1-carboxyvinyltransferase [M... | 434 | 4e-146 | 59% |
| WP_093733565.1 3-phosphoshikimate 1-carboxyvinyltransferase [S... | 434 | 4e-146 | 54% |
| WP_170339889.1 3-phosphoshikimate 1-carboxyvinyltransferase [R... | 434 | 4e-146 | 55% |
| WP_124738395.1 3-phosphoshikimate 1-carboxyvinyltransferase [M... | 434 | 4e-146 | 58% |
| WP_069436668.1 3-phosphoshikimate 1-carboxyvinyltransferase [M... | 434 | 4e-146 | 57% |
| WP_169555727.1 3-phosphoshikimate 1-carboxyvinyltransferase [S... | 434 | 4e-146 | 55% |
| WP_170404971.1 3-phosphoshikimate 1-carboxyvinyltransferase [R... | 434 | 4e-146 | 55% |
| MBD3678259.1 3-phosphoshikimate 1-carboxyvinyltransferase [Rho... | 434 | 4e-146 | 57% |
| WP_093002677.1 3-phosphoshikimate 1-carboxyvinyltransferase [S... | 434 | 4e-146 | 56% |
| KXV53174.1 3-phosphoshikimate 1-carboxyvinyltransferase [Gluco... | 434 | 4e-146 | 55% |
| WP_042463524.1 3-phosphoshikimate 1-carboxyvinyltransferase [R... | 434 | 4e-146 | 56% |
| WP_170434365.1 3-phosphoshikimate 1-carboxyvinyltransferase [R... | 434 | 4e-146 | 55% |
| WP_112312726.1 3-phosphoshikimate 1-carboxyvinyltransferase [P... | 434 | 4e-146 | 57% |
| WP_058279467.1 3-phosphoshikimate 1-carboxyvinyltransferase [R... | 434 | 4e-146 | 55% |
| WP_082905366.1 3-phosphoshikimate 1-carboxyvinyltransferase [G... | 434 | 4e-146 | 55% |
| MPZ09416.1 3-phosphoshikimate 1-carboxyvinyltransferase [Kilon... | 434 | 4e-146 | 56% |
| WP_170460254.1 3-phosphoshikimate 1-carboxyvinyltransferase [R... | 434 | 4e-146 | 55% |
| WP_085893793.1 3-phosphoshikimate 1-carboxyvinyltransferase [P... | 434 | 4e-146 | 56% |
| NQX74902.1 3-phosphoshikimate 1-carboxyvinyltransferase [Epiba... | 434 | 4e-146 | 54% |
| PTW49065.1 3-phosphoshikimate 1-carboxyvinyltransferase [Sphin... | 435 | 4e-146 | 56% |
| NRB00470.1 3-phosphoshikimate 1-carboxyvinyltransferase [Rhodo... | 434 | 4e-146 | 56% |
| WP_026598220.1 MULTISPECIES: 3-phosphoshikimate 1-carboxyvinyl... | 434 | 4e-146 | 58% |
| NBT22689.1 3-phosphoshikimate 1-carboxyvinyltransferase [Methy... | 434 | 4e-146 | 56% |
| WP_107846615.1 3-phosphoshikimate 1-carboxyvinyltransferase [L... | 434 | 4e-146 | 55% |
| OIQ27650.1 3-phosphoshikimate 1-carboxyvinyltransferase [Alpha... | 434 | 5e-146 | 55% |
| OUJ15496.1 3-phosphoshikimate 1-carboxyvinyltransferase [Aceto... | 434 | 5e-146 | 55% |
| WP_043767612.1 3-phosphoshikimate 1-carboxyvinyltransferase [R... | 434 | 5e-146 | 55% |
| WP_079639314.1 3-phosphoshikimate 1-carboxyvinyltransferase [S... | 434 | 5e-146 | 58% |
| WP_005978051.1 3-phosphoshikimate 1-carboxyvinyltransferase [R... | 434 | 5e-146 | 55% |
| WP_157096424.1 3-phosphoshikimate 1-carboxyvinyltransferase [M... | 434 | 5e-146 | 58% |
| WP_021131750.1 3-phosphoshikimate 1-carboxyvinyltransferase [P... | 434 | 5e-146 | 58% |
| WP_108643208.1 3-phosphoshikimate 1-carboxyvinyltransferase [R... | 434 | 5e-146 | 56% |

| GDX39375.1 3-phosphoshikimate 1-carboxyvinyltransferase [Methy... | 434 | 5e-146 | 56% |
| --- | --- | --- | --- |
| HDY94899.1 TPA: 3-phosphoshikimate 1-carboxyvinyltransferase [... | 434 | 5e-146 | 54% |
| WP_139020360.1 3-phosphoshikimate 1-carboxyvinyltransferase [C... | 434 | 5e-146 | 55% |
| MBO81964.1 3-phosphoshikimate 1-carboxyvinyltransferase [Citro... | 434 | 5e-146 | 55% |
| WP_185798797.1 3-phosphoshikimate 1-carboxyvinyltransferase [G... | 434 | 5e-146 | 58% |
| WP_108835397.1 3-phosphoshikimate 1-carboxyvinyltransferase [T... | 434 | 5e-146 | 56% |
| WP_169559011.1 3-phosphoshikimate 1-carboxyvinyltransferase [S... | 434 | 5e-146 | 55% |
| RYF93958.1 3-phosphoshikimate 1-carboxyvinyltransferase [Caulo... | 434 | 5e-146 | 57% |
| WP_149819735.1 3-phosphoshikimate 1-carboxyvinyltransferase [S... | 434 | 5e-146 | 59% |
| WP_170365349.1 3-phosphoshikimate 1-carboxyvinyltransferase [R... | 434 | 6e-146 | 55% |
| WP_138922795.1 3-phosphoshikimate 1-carboxyvinyltransferase [S... | 434 | 6e-146 | 54% |
| WP_060834119.1 3-phosphoshikimate 1-carboxyvinyltransferase [R... | 434 | 6e-146 | 56% |
| MAQ71652.1 3-phosphoshikimate 1-carboxyvinyltransferase [Alpha... | 435 | 6e-146 | 56% |
| ATI80911.1 3-phosphoshikimate 1-carboxyvinyltransferase [Sphin... | 434 | 6e-146 | 54% |
| OIQ35426.1 3-phosphoshikimate 1-carboxyvinyltransferase [Roseo... | 434 | 6e-146 | 55% |
| WP_005849419.1 3-phosphoshikimate 1-carboxyvinyltransferase [S... | 434 | 6e-146 | 54% |
| WP_170828736.1 3-phosphoshikimate 1-carboxyvinyltransferase [S... | 434 | 6e-146 | 56% |
| WP_075381439.1 3-phosphoshikimate 1-carboxyvinyltransferase [M... | 434 | 6e-146 | 59% |
| WP_072674421.1 3-phosphoshikimate 1-carboxyvinyltransferase [P... | 433 | 6e-146 | 56% |
| WP_168352858.1 3-phosphoshikimate 1-carboxyvinyltransferase [N... | 434 | 6e-146 | 56% |
| WP_069205551.1 3-phosphoshikimate 1-carboxyvinyltransferase [S... | 434 | 6e-146 | 58% |
| WP_087635614.1 3-phosphoshikimate 1-carboxyvinyltransferase [A... | 434 | 6e-146 | 55% |
| WP_146348105.1 3-phosphoshikimate 1-carboxyvinyltransferase [P... | 434 | 7e-146 | 55% |
| WP_037542284.1 3-phosphoshikimate 1-carboxyvinyltransferase [S... | 434 | 7e-146 | 58% |
| OAI23321.1 3-phosphoshikimate 1-carboxyvinyltransferase [Methy... | 434 | 7e-146 | 58% |
| WP_170340448.1 3-phosphoshikimate 1-carboxyvinyltransferase [R... | 434 | 7e-146 | 55% |
| WP_170369729.1 3-phosphoshikimate 1-carboxyvinyltransferase [R... | 434 | 7e-146 | 55% |
| WP_179193375.1 3-phosphoshikimate 1-carboxyvinyltransferase [A... | 434 | 7e-146 | 55% |
| WP_121904223.1 3-phosphoshikimate 1-carboxyvinyltransferase [S... | 433 | 7e-146 | 56% |
| GFE95028.1 3-phosphoshikimate 1-carboxyvinyltransferase [Gluco... | 433 | 7e-146 | 56% |
| WP_119584362.1 3-phosphoshikimate 1-carboxyvinyltransferase [E... | 433 | 7e-146 | 57% |
| WP_097144741.1 3-phosphoshikimate 1-carboxyvinyltransferase [P... | 433 | 7e-146 | 54% |
| WP_132001200.1 3-phosphoshikimate 1-carboxyvinyltransferase [C... | 434 | 8e-146 | 57% |
| WP_183177317.1 3-phosphoshikimate 1-carboxyvinyltransferase [A... | 433 | 8e-146 | 56% |
| MBC7137643.1 3-phosphoshikimate 1-carboxyvinyltransferase [Def... | 434 | 8e-146 | 55% |
| TCO15815.1 3-phosphoshikimate 1-carboxyvinyltransferase [Camel... | 434 | 8e-146 | 57% |
| KAF0223179.1 3-phosphoshikimate [Rhodospirillaceae bacterium] | 433 | 8e-146 | 56% |
| WP_082784552.1 3-phosphoshikimate 1-carboxyvinyltransferase [G... | 433 | 8e-146 | 55% |
| WP_160792920.1 3-phosphoshikimate 1-carboxyvinyltransferase [S... | 433 | 8e-146 | 53% |
| WP_037953845.1 MULTISPECIES: 3-phosphoshikimate 1-carboxyvinyl... | 433 | 8e-146 | 54% |
| WP_132897734.1 3-phosphoshikimate 1-carboxyvinyltransferase [S... | 433 | 8e-146 | 56% |
| BBC72421.1 3-phosphoshikimate 1-carboxyvinyltransferase [Alter... | 433 | 9e-146 | 57% |
| WP_160614155.1 3-phosphoshikimate 1-carboxyvinyltransferase [A... | 434 | 9e-146 | 58% |
| WP_092895957.1 3-phosphoshikimate 1-carboxyvinyltransferase [R... | 433 | 9e-146 | 57% |
| OJW67395.1 3-phosphoshikimate 1-carboxyvinyltransferase [Sphin... | 433 | 9e-146 | 58% |
| WP_023944710.1 3-phosphoshikimate 1-carboxyvinyltransferase [G... | 433 | 9e-146 | 55% |
| WP_085817236.1 3-phosphoshikimate 1-carboxyvinyltransferase [R... | 433 | 9e-146 | 56% |
| WP_151954771.1 3-phosphoshikimate 1-carboxyvinyltransferase [S... | 433 | 9e-146 | 56% |
| RMB37085.1 3-phosphoshikimate 1-carboxyvinyltransferase [Sphin... | 434 | 9e-146 | 56% |
| WP_108683720.1 3-phosphoshikimate 1-carboxyvinyltransferase [M... | 433 | 9e-146 | 56% |
| WP_107672370.1 3-phosphoshikimate 1-carboxyvinyltransferase [R... | 433 | 9e-146 | 56% |
| WP_048532978.1 3-phosphoshikimate 1-carboxyvinyltransferase [M... | 433 | 1e-145 | 56% |
| KXV32364.1 3-phosphoshikimate 1-carboxyvinyltransferase [Gluco... | 433 | 1e-145 | 55% |
| WP_027288128.1 3-phosphoshikimate 1-carboxyvinyltransferase [R... | 433 | 1e-145 | 56% |
| KDE21437.1 3-phosphoshikimate 1-carboxyvinyltransferase [Aceto... | 434 | 1e-145 | 55% |
| WP_094034620.1 3-phosphoshikimate 1-carboxyvinyltransferase [A... | 433 | 1e-145 | 57% |
| WP_009806221.1 3-phosphoshikimate 1-carboxyvinyltransferase [P... | 433 | 1e-145 | 56% |
| WP_137861527.1 MULTISPECIES: 3-phosphoshikimate 1-carboxyvinyl... | 433 | 1e-145 | 55% |
| WP_169543787.1 3-phosphoshikimate 1-carboxyvinyltransferase [S... | 433 | 1e-145 | 55% |
| WP_115380901.1 3-phosphoshikimate 1-carboxyvinyltransferase [J... | 433 | 1e-145 | 56% |
| WP_015072566.1 MULTISPECIES: 3-phosphoshikimate 1-carboxyvinyl... | 433 | 1e-145 | 55% |
| WP_037293809.1 3-phosphoshikimate 1-carboxyvinyltransferase [R... | 433 | 1e-145 | 57% |
| WP_173952520.1 3-phosphoshikimate 1-carboxyvinyltransferase [H... | 433 | 1e-145 | 58% |
| WP_108917551.1 3-phosphoshikimate 1-carboxyvinyltransferase [M... | 433 | 1e-145 | 58% |
| WP_188581245.1 3-phosphoshikimate 1-carboxyvinyltransferase [T... | 434 | 1e-145 | 55% |
| WP_025311847.1 3-phosphoshikimate 1-carboxyvinyltransferase [R... | 433 | 1e-145 | 57% |
| MAX77114.1 3-phosphoshikimate 1-carboxyvinyltransferase [Roseo... | 433 | 1e-145 | 54% |
| MAM41311.1 3-phosphoshikimate 1-carboxyvinyltransferase [Eryth... | 432 | 1e-145 | 58% |
| WP_082785480.1 3-phosphoshikimate 1-carboxyvinyltransferase [G... | 432 | 1e-145 | 55% |
| WP_049643220.1 3-phosphoshikimate 1-carboxyvinyltransferase [C... | 433 | 1e-145 | 56% |
| WP_082785274.1 3-phosphoshikimate 1-carboxyvinyltransferase [G... | 432 | 1e-145 | 55% |
| TMJ71999.1 3-phosphoshikimate 1-carboxyvinyltransferase [Alpha... | 433 | 1e-145 | 57% |

| ANQ40985.1 3-phosphoshikimate 1-carboxyvinyltransferase [Gluco... | 432 | 1e-145 | 55% |
| --- | --- | --- | --- |
| WP_017929699.1 3-phosphoshikimate 1-carboxyvinyltransferase [L... | 433 | 1e-145 | 55% |
| MBC7179703.1 3-phosphoshikimate 1-carboxyvinyltransferase [Ros... | 433 | 1e-145 | 58% |
| WP_022672661.1 3-phosphoshikimate 1-carboxyvinyltransferase [N... | 432 | 1e-145 | 56% |
| WP_034921279.1 3-phosphoshikimate 1-carboxyvinyltransferase [E... | 432 | 1e-145 | 59% |
| WP_093965717.1 3-phosphoshikimate 1-carboxyvinyltransferase [C... | 433 | 1e-145 | 55% |
| WP_018302820.1 3-phosphoshikimate 1-carboxyvinyltransferase [W... | 433 | 1e-145 | 56% |
| WP_085853231.1 3-phosphoshikimate 1-carboxyvinyltransferase [P... | 433 | 1e-145 | 56% |
| WP_090159153.1 3-phosphoshikimate 1-carboxyvinyltransferase [L... | 433 | 1e-145 | 55% |
| WP_193141672.1 3-phosphoshikimate 1-carboxyvinyltransferase [F... | 433 | 1e-145 | 55% |
| WP_039538653.1 3-phosphoshikimate 1-carboxyvinyltransferase [R... | 433 | 1e-145 | 55% |
| WP_012322503.1 3-phosphoshikimate 1-carboxyvinyltransferase [M... | 433 | 1e-145 | 59% |
| WP_010545512.1 3-phosphoshikimate 1-carboxyvinyltransferase [S... | 432 | 1e-145 | 56% |
| WP_119082673.1 3-phosphoshikimate 1-carboxyvinyltransferase [A... | 434 | 1e-145 | 57% |
| WP_174525668.1 3-phosphoshikimate 1-carboxyvinyltransferase [S... | 432 | 1e-145 | 57% |
| WP_168194141.1 3-phosphoshikimate 1-carboxyvinyltransferase [A... | 433 | 1e-145 | 56% |
| MSP83784.1 3-phosphoshikimate 1-carboxyvinyltransferase [Alpha... | 432 | 1e-145 | 57% |
| AVO38157.1 3-phosphoshikimate 1-carboxyvinyltransferase [Rhodo... | 433 | 2e-145 | 56% |
| WP_132831464.1 3-phosphoshikimate 1-carboxyvinyltransferase [S... | 433 | 2e-145 | 56% |
| WP_055951263.1 MULTISPECIES: 3-phosphoshikimate 1-carboxyvinyl... | 432 | 2e-145 | 60% |
| WP_063085979.1 MULTISPECIES: 3-phosphoshikimate 1-carboxyvinyl... | 432 | 2e-145 | 55% |
| WP_150042512.1 3-phosphoshikimate 1-carboxyvinyltransferase [R... | 433 | 2e-145 | 56% |
| WP_082778403.1 3-phosphoshikimate 1-carboxyvinyltransferase [G... | 432 | 2e-145 | 55% |
| WP_027239140.1 3-phosphoshikimate 1-carboxyvinyltransferase [P... | 432 | 2e-145 | 54% |
| EAQ47096.1 3-phosphoshikimate 1-carboxyvinyltransferase [Roseo... | 432 | 2e-145 | 55% |
| OYY04819.1 3-phosphoshikimate 1-carboxyvinyltransferase [Acido... | 432 | 2e-145 | 55% |
| WP_114884352.1 3-phosphoshikimate 1-carboxyvinyltransferase [S... | 432 | 2e-145 | 55% |
| WP_118150599.1 3-phosphoshikimate 1-carboxyvinyltransferase [T... | 432 | 2e-145 | 56% |
| WP_149775646.1 3-phosphoshikimate 1-carboxyvinyltransferase [R... | 432 | 2e-145 | 55% |
| WP_170444475.1 3-phosphoshikimate 1-carboxyvinyltransferase [R... | 432 | 2e-145 | 55% |
| WP_039161804.1 3-phosphoshikimate 1-carboxyvinyltransferase [L... | 432 | 2e-145 | 55% |
| NKW92043.1 3-phosphoshikimate 1-carboxyvinyltransferase [Rhodo... | 432 | 2e-145 | 55% |
| WP_149228174.1 3-phosphoshikimate 1-carboxyvinyltransferase [A... | 433 | 2e-145 | 57% |
| WP_055781338.1 3-phosphoshikimate 1-carboxyvinyltransferase [S... | 432 | 2e-145 | 55% |
| MBA3911426.1 3-phosphoshikimate 1-carboxyvinyltransferase [Rho... | 432 | 2e-145 | 57% |
| WP_066530080.1 MULTISPECIES: 3-phosphoshikimate 1-carboxyvinyl... | 432 | 2e-145 | 58% |
| WP_099181567.1 3-phosphoshikimate 1-carboxyvinyltransferase [G... | 432 | 2e-145 | 55% |
| WP_066765757.1 3-phosphoshikimate 1-carboxyvinyltransferase [C... | 432 | 2e-145 | 57% |
| WP_133496107.1 3-phosphoshikimate 1-carboxyvinyltransferase [S... | 432 | 2e-145 | 56% |
| SHN58072.1 3-phosphoshikimate 1-carboxyvinyltransferase [Porph... | 433 | 2e-145 | 56% |
| WP_085897098.1 3-phosphoshikimate 1-carboxyvinyltransferase [L... | 432 | 2e-145 | 56% |
| NBR01775.1 3-phosphoshikimate 1-carboxyvinyltransferase [Alpha... | 432 | 2e-145 | 55% |
| MBE0533188.1 3-phosphoshikimate 1-carboxyvinyltransferase [Rho... | 432 | 2e-145 | 57% |
| WP_109425491.1 3-phosphoshikimate 1-carboxyvinyltransferase [M... | 432 | 2e-145 | 56% |
| MBA3863829.1 3-phosphoshikimate 1-carboxyvinyltransferase [Ery... | 432 | 2e-145 | 57% |
| WP_120223755.1 3-phosphoshikimate 1-carboxyvinyltransferase [T... | 432 | 2e-145 | 55% |
| WP_074634656.1 3-phosphoshikimate 1-carboxyvinyltransferase [S... | 432 | 2e-145 | 54% |
| TCQ05133.1 3-phosphoshikimate 1-carboxyvinyltransferase [Sphin... | 433 | 2e-145 | 56% |
| WP_037944692.1 MULTISPECIES: 3-phosphoshikimate 1-carboxyvinyl... | 432 | 2e-145 | 54% |
| WP_108546678.1 3-phosphoshikimate 1-carboxyvinyltransferase [A... | 432 | 2e-145 | 57% |
| WP_085120838.1 3-phosphoshikimate 1-carboxyvinyltransferase [T... | 432 | 3e-145 | 58% |
| WP_090270318.1 3-phosphoshikimate 1-carboxyvinyltransferase [L... | 432 | 3e-145 | 57% |
| WP_148473665.1 3-phosphoshikimate 1-carboxyvinyltransferase [O... | 432 | 3e-145 | 56% |
| WP_153429992.1 3-phosphoshikimate 1-carboxyvinyltransferase [G... | 432 | 3e-145 | 55% |
| EAU51887.1 EPSP synthase, 3-phosphoshikimate 1-carboxyvinyltra... | 432 | 3e-145 | 55% |
| NKB55778.1 3-phosphoshikimate 1-carboxyvinyltransferase [Alpha... | 433 | 3e-145 | 57% |
| EBA12436.1 3-phosphoshikimate 1-carboxyvinyltransferase [Roseo... | 432 | 3e-145 | 57% |
| WP_185208163.1 3-phosphoshikimate 1-carboxyvinyltransferase [S... | 432 | 3e-145 | 54% |
| WP_064217081.1 3-phosphoshikimate 1-carboxyvinyltransferase [S... | 432 | 3e-145 | 54% |
| RMB54193.1 3-phosphoshikimate 1-carboxyvinyltransferase [Sphin... | 433 | 3e-145 | 56% |
| WP_035048521.1 3-phosphoshikimate 1-carboxyvinyltransferase [C... | 432 | 3e-145 | 58% |
| OJU61177.1 3-phosphoshikimate 1-carboxyvinyltransferase [Alter... | 432 | 3e-145 | 58% |
| PZQ61084.1 3-phosphoshikimate 1-carboxyvinyltransferase [Sphin... | 432 | 3e-145 | 57% |
| WP_140454499.1 3-phosphoshikimate 1-carboxyvinyltransferase [A... | 432 | 3e-145 | 56% |
| EDL50531.1 3-phosphoshikimate 1-carboxyvinyltransferase [Eryth... | 432 | 3e-145 | 59% |
| WP_144095447.1 3-phosphoshikimate 1-carboxyvinyltransferase [C... | 432 | 3e-145 | 58% |
| WP_114869114.1 3-phosphoshikimate 1-carboxyvinyltransferase [S... | 432 | 3e-145 | 55% |
| WP_188607287.1 3-phosphoshikimate 1-carboxyvinyltransferase [C... | 432 | 3e-145 | 59% |
| WP_175094385.1 3-phosphoshikimate 1-carboxyvinyltransferase [M... | 432 | 3e-145 | 58% |
| KTS08888.1 3-phosphoshikimate 1-carboxyvinyltransferase [Methy... | 432 | 3e-145 | 59% |
| WP_174139851.1 3-phosphoshikimate 1-carboxyvinyltransferase [S... | 432 | 3e-145 | 56% |
| WP_110814604.1 3-phosphoshikimate 1-carboxyvinyltransferase [P... | 432 | 3e-145 | 55% |

| WP_097064382.1 3-phosphoshikimate 1-carboxyvinyltransferase [S... | 432 | 3e-145 | 56% |
| --- | --- | --- | --- |
| WP_187502767.1 3-phosphoshikimate 1-carboxyvinyltransferase [S... | 432 | 3e-145 | 56% |
| WP_114100560.1 3-phosphoshikimate 1-carboxyvinyltransferase [T... | 432 | 3e-145 | 55% |
| WP_138014630.1 3-phosphoshikimate 1-carboxyvinyltransferase [P... | 432 | 3e-145 | 54% |
| WP_086639970.1 3-phosphoshikimate 1-carboxyvinyltransferase [A... | 432 | 4e-145 | 54% |
| WP_100197504.1 3-phosphoshikimate 1-carboxyvinyltransferase [P... | 432 | 4e-145 | 57% |
| NRB02615.1 3-phosphoshikimate 1-carboxyvinyltransferase [Rhodo... | 432 | 4e-145 | 56% |
| WP_092642759.1 3-phosphoshikimate 1-carboxyvinyltransferase [J... | 432 | 4e-145 | 57% |
| WP_151978092.1 MULTISPECIES: 3-phosphoshikimate 1-carboxyvinyl... | 432 | 4e-145 | 57% |
| WP_170416872.1 3-phosphoshikimate 1-carboxyvinyltransferase [R... | 432 | 4e-145 | 54% |
| WP_104006903.1 3-phosphoshikimate 1-carboxyvinyltransferase [J... | 432 | 4e-145 | 56% |
| GAO56953.1 5-Enolpyruvylshikimate-3-phosphate synthase [Novosp... | 432 | 4e-145 | 57% |
| WP_111363438.1 3-phosphoshikimate 1-carboxyvinyltransferase [A... | 432 | 4e-145 | 54% |
| MBC7831757.1 3-phosphoshikimate 1-carboxyvinyltransferase [Hyp... | 431 | 4e-145 | 56% |
| WP_161390614.1 3-phosphoshikimate 1-carboxyvinyltransferase [A... | 434 | 4e-145 | 59% |
| WP_082719838.1 3-phosphoshikimate 1-carboxyvinyltransferase [K... | 432 | 4e-145 | 56% |
| MAV62865.1 3-phosphoshikimate 1-carboxyvinyltransferase [Rhodo... | 432 | 4e-145 | 51% |
| ODS98719.1 3-phosphoshikimate 1-carboxyvinyltransferase [Eryth... | 431 | 4e-145 | 61% |
| KZB99114.1 3-phosphoshikimate 1-carboxyvinyltransferase [Methy... | 431 | 4e-145 | 59% |
| WP_167445741.1 3-phosphoshikimate 1-carboxyvinyltransferase [T... | 431 | 5e-145 | 56% |
| QDM40275.1 3-phosphoshikimate 1-carboxyvinyltransferase [Alter... | 431 | 5e-145 | 57% |
| MBC6438630.1 3-phosphoshikimate 1-carboxyvinyltransferase [Rho... | 431 | 5e-145 | 55% |
| WP_056037996.1 3-phosphoshikimate 1-carboxyvinyltransferase [L... | 431 | 5e-145 | 55% |
| WP_139077196.1 3-phosphoshikimate 1-carboxyvinyltransferase [R... | 431 | 5e-145 | 55% |
| WP_108504452.1 MULTISPECIES: 3-phosphoshikimate 1-carboxyvinyl... | 431 | 5e-145 | 58% |
| WP_134492340.1 3-phosphoshikimate 1-carboxyvinyltransferase [M... | 431 | 5e-145 | 57% |
| WP_056631662.1 3-phosphoshikimate 1-carboxyvinyltransferase [S... | 431 | 5e-145 | 57% |
| OAI44948.1 3-phosphoshikimate 1-carboxyvinyltransferase [Rhizo... | 431 | 5e-145 | 56% |
| WP_036173467.1 3-phosphoshikimate 1-carboxyvinyltransferase [P... | 431 | 5e-145 | 55% |
| WP_155148944.1 3-phosphoshikimate 1-carboxyvinyltransferase [R... | 431 | 5e-145 | 54% |
| MAM34595.1 3-phosphoshikimate 1-carboxyvinyltransferase [Micav... | 432 | 5e-145 | 55% |
| WP_109959674.1 3-phosphoshikimate 1-carboxyvinyltransferase [M... | 431 | 5e-145 | 58% |
| NNF91979.1 3-phosphoshikimate 1-carboxyvinyltransferase [Boseo... | 431 | 5e-145 | 54% |
| HBR25911.1 TPA: 3-phosphoshikimate 1-carboxyvinyltransferase [... | 431 | 5e-145 | 55% |
| WP_150446694.1 3-phosphoshikimate 1-carboxyvinyltransferase [H... | 431 | 5e-145 | 55% |
| WP_028030056.1 3-phosphoshikimate 1-carboxyvinyltransferase [G... | 431 | 5e-145 | 57% |
| WP_043075112.1 3-phosphoshikimate 1-carboxyvinyltransferase [M... | 431 | 6e-145 | 59% |
| WP_114511376.1 3-phosphoshikimate 1-carboxyvinyltransferase [T... | 431 | 6e-145 | 55% |
| WP_189674888.1 3-phosphoshikimate 1-carboxyvinyltransferase [S... | 431 | 6e-145 | 57% |
| WP_158046752.1 3-phosphoshikimate 1-carboxyvinyltransferase [S... | 431 | 6e-145 | 56% |
| WP_012177695.1 3-phosphoshikimate 1-carboxyvinyltransferase [D... | 431 | 6e-145 | 55% |
| WP_053621804.1 MULTISPECIES: 3-phosphoshikimate 1-carboxyvinyl... | 431 | 6e-145 | 59% |
| WP_043060544.1 3-phosphoshikimate 1-carboxyvinyltransferase [S... | 431 | 6e-145 | 58% |
| NND19462.1 3-phosphoshikimate 1-carboxyvinyltransferase [Silic... | 431 | 6e-145 | 55% |
| WP_037966240.1 3-phosphoshikimate 1-carboxyvinyltransferase [S... | 431 | 7e-145 | 54% |
| WP_089230903.1 3-phosphoshikimate 1-carboxyvinyltransferase [T... | 431 | 7e-145 | 55% |
| WP_149312702.1 3-phosphoshikimate 1-carboxyvinyltransferase [M... | 431 | 7e-145 | 60% |
| MAW45253.1 3-phosphoshikimate 1-carboxyvinyltransferase [Marin... | 431 | 7e-145 | 53% |
| WP_095011320.1 3-phosphoshikimate 1-carboxyvinyltransferase [A... | 431 | 7e-145 | 58% |
| MBF0852503.1 3-phosphoshikimate 1-carboxyvinyltransferase [Glu... | 431 | 7e-145 | 55% |
| WP_033922320.1 3-phosphoshikimate 1-carboxyvinyltransferase [S... | 431 | 7e-145 | 56% |
| WP_073328913.1 3-phosphoshikimate 1-carboxyvinyltransferase [W... | 431 | 7e-145 | 56% |
| MBE2274870.1 3-phosphoshikimate 1-carboxyvinyltransferase [Rho... | 431 | 7e-145 | 55% |
| WP_091786525.1 3-phosphoshikimate 1-carboxyvinyltransferase [M... | 431 | 7e-145 | 59% |
| PDH50557.1 3-phosphoshikimate 1-carboxyvinyltransferase [alpha... | 431 | 8e-145 | 51% |
| WP_107891678.1 3-phosphoshikimate 1-carboxyvinyltransferase [R... | 431 | 8e-145 | 57% |
| WP_194688708.1 3-phosphoshikimate 1-carboxyvinyltransferase [G... | 431 | 8e-145 | 55% |
| WP_170440513.1 3-phosphoshikimate 1-carboxyvinyltransferase [R... | 431 | 8e-145 | 55% |
| WP_085801622.1 3-phosphoshikimate 1-carboxyvinyltransferase [R... | 431 | 8e-145 | 55% |
| WP_146792937.1 3-phosphoshikimate 1-carboxyvinyltransferase [G... | 431 | 8e-145 | 55% |
| MAN01287.1 3-phosphoshikimate 1-carboxyvinyltransferase [Rhodo... | 431 | 8e-145 | 53% |
| VFU08095.1 3-phosphoshikimate 1-carboxyvinyltransferase [Methy... | 431 | 8e-145 | 57% |
| OUJ10345.1 3-phosphoshikimate 1-carboxyvinyltransferase [Aceto... | 431 | 9e-145 | 54% |
| WP_084573110.1 3-phosphoshikimate 1-carboxyvinyltransferase [E... | 431 | 9e-145 | 58% |
| WP_066810071.1 3-phosphoshikimate 1-carboxyvinyltransferase [D... | 431 | 9e-145 | 55% |
| KAB2883736.1 3-phosphoshikimate 1-carboxyvinyltransferase [Def... | 431 | 9e-145 | 57% |
| OED48958.1 3-phosphoshikimate 1-carboxyvinyltransferase [Rhodo... | 431 | 9e-145 | 55% |
| WP_024879515.1 3-phosphoshikimate 1-carboxyvinyltransferase [M... | 431 | 9e-145 | 57% |
| WP_125957268.1 3-phosphoshikimate 1-carboxyvinyltransferase [N... | 430 | 1e-144 | 58% |
| OYV37459.1 3-phosphoshikimate 1-carboxyvinyltransferase [Rhodo... | 431 | 1e-144 | 55% |
| WP_113289868.1 3-phosphoshikimate 1-carboxyvinyltransferase [R... | 431 | 1e-144 | 55% |
| MBO33872.1 3-phosphoshikimate 1-carboxyvinyltransferase [Rhodo... | 430 | 1e-144 | 55% |

| MBC8239301.1 3-phosphoshikimate 1-carboxyvinyltransferase [Alp... | 431 | 1e-144 | 55% |
| --- | --- | --- | --- |
| NQY61058.1 3-phosphoshikimate 1-carboxyvinyltransferase [Cogna... | 431 | 1e-144 | 55% |
| MBA4165452.1 3-phosphoshikimate 1-carboxyvinyltransferase [Ery... | 432 | 1e-144 | 56% |
| WP_024091304.1 3-phosphoshikimate 1-carboxyvinyltransferase [L... | 431 | 1e-144 | 55% |
| WP_068314942.1 MULTISPECIES: 3-phosphoshikimate 1-carboxyvinyl... | 431 | 1e-144 | 55% |
| WP_164614048.1 3-phosphoshikimate 1-carboxyvinyltransferase [R... | 431 | 1e-144 | 57% |
| WP_173214881.1 3-phosphoshikimate 1-carboxyvinyltransferase [E... | 430 | 1e-144 | 57% |
| NQV83232.1 3-phosphoshikimate 1-carboxyvinyltransferase [Rhodo... | 430 | 1e-144 | 57% |
| WP_155445304.1 3-phosphoshikimate 1-carboxyvinyltransferase [R... | 430 | 1e-144 | 59% |
| WP_146285064.1 3-phosphoshikimate 1-carboxyvinyltransferase [G... | 431 | 1e-144 | 57% |
| WP_035250709.1 3-phosphoshikimate 1-carboxyvinyltransferase [A... | 431 | 1e-144 | 55% |
| WP_074840257.1 3-phosphoshikimate 1-carboxyvinyltransferase [M... | 431 | 1e-144 | 56% |
| HGG64525.1 TPA: 3-phosphoshikimate 1-carboxyvinyltransferase [... | 431 | 1e-144 | 53% |
| NBX03364.1 3-phosphoshikimate 1-carboxyvinyltransferase [Alpha... | 438 | 1e-144 | 55% |
| WP_160761186.1 3-phosphoshikimate 1-carboxyvinyltransferase [E... | 430 | 1e-144 | 57% |
| WP_048435695.1 3-phosphoshikimate 1-carboxyvinyltransferase [M... | 430 | 1e-144 | 58% |
| EAP81736.1 3-phosphoshikimate 1-carboxyvinyltransferase [Sulfi... | 430 | 1e-144 | 55% |
| KAB2939779.1 3-phosphoshikimate 1-carboxyvinyltransferase [Hyp... | 430 | 1e-144 | 57% |
| GEB41920.1 3-phosphoshikimate 1-carboxyvinyltransferase [Gluco... | 431 | 1e-144 | 55% |
| WP_151718555.1 3-phosphoshikimate 1-carboxyvinyltransferase [G... | 430 | 1e-144 | 56% |
| WP_081507030.1 3-phosphoshikimate 1-carboxyvinyltransferase [R... | 430 | 1e-144 | 57% |
| WP_151991314.1 3-phosphoshikimate 1-carboxyvinyltransferase [S... | 430 | 1e-144 | 57% |
| WP_101301202.1 3-phosphoshikimate 1-carboxyvinyltransferase [T... | 430 | 1e-144 | 56% |
| PCI90222.1 3-phosphoshikimate 1-carboxyvinyltransferase [Ectot... | 430 | 1e-144 | 54% |
| WP_172438782.1 3-phosphoshikimate 1-carboxyvinyltransferase [S... | 430 | 1e-144 | 55% |
| WP_085615388.1 3-phosphoshikimate 1-carboxyvinyltransferase [T... | 430 | 2e-144 | 57% |
| WP_172292999.1 3-phosphoshikimate 1-carboxyvinyltransferase [P... | 430 | 2e-144 | 56% |
| WP_100369029.1 3-phosphoshikimate 1-carboxyvinyltransferase [Y... | 430 | 2e-144 | 57% |
| WP_159713347.1 3-phosphoshikimate 1-carboxyvinyltransferase [S... | 430 | 2e-144 | 54% |
| WP_140928182.1 3-phosphoshikimate 1-carboxyvinyltransferase [S... | 430 | 2e-144 | 57% |
| MBA4764473.1 3-phosphoshikimate 1-carboxyvinyltransferase [Ery... | 429 | 2e-144 | 58% |
| WP_184080284.1 3-phosphoshikimate 1-carboxyvinyltransferase [S... | 430 | 2e-144 | 57% |
| HCW65677.1 TPA: 3-phosphoshikimate 1-carboxyvinyltransferase [... | 430 | 2e-144 | 56% |
| WP_064788321.1 MULTISPECIES: 3-phosphoshikimate 1-carboxyvinyl... | 430 | 2e-144 | 55% |
| PYE84070.1 3-phosphoshikimate 1-carboxyvinyltransferase [Pseud... | 430 | 2e-144 | 55% |
| WP_085792317.1 3-phosphoshikimate 1-carboxyvinyltransferase [R... | 430 | 2e-144 | 54% |
| WP_085864733.1 3-phosphoshikimate 1-carboxyvinyltransferase [P... | 430 | 2e-144 | 58% |
| WP_141350800.1 3-phosphoshikimate 1-carboxyvinyltransferase [G... | 430 | 2e-144 | 55% |
| RZM35273.1 3-phosphoshikimate 1-carboxyvinyltransferase [Sphin... | 429 | 2e-144 | 57% |
| WP_058861837.1 3-phosphoshikimate 1-carboxyvinyltransferase [P... | 430 | 2e-144 | 56% |
| SMC13622.1 3-phosphoshikimate 1-carboxyvinyltransferase [Roseo... | 431 | 2e-144 | 55% |
| MBC7142437.1 3-phosphoshikimate 1-carboxyvinyltransferase [Rho... | 430 | 2e-144 | 58% |
| PTS74306.1 3-phosphoshikimate 1-carboxyvinyltransferase [Sphin... | 430 | 2e-144 | 56% |
| MBB6123970.1 3-phosphoshikimate 1-carboxyvinyltransferase [Sph... | 430 | 2e-144 | 57% |
| MBE71625.1 3-phosphoshikimate 1-carboxyvinyltransferase [Thala... | 430 | 2e-144 | 55% |
| WP_102784632.1 3-phosphoshikimate 1-carboxyvinyltransferase [T... | 430 | 2e-144 | 56% |
| OAN71784.1 3-phosphoshikimate 1-carboxyvinyltransferase [Rhodo... | 430 | 2e-144 | 56% |
| WP_011252175.1 3-phosphoshikimate 1-carboxyvinyltransferase [G... | 429 | 2e-144 | 55% |
| WP_140872715.1 3-phosphoshikimate 1-carboxyvinyltransferase [S... | 430 | 2e-144 | 56% |
| WP_191319867.1 3-phosphoshikimate 1-carboxyvinyltransferase [C... | 429 | 3e-144 | 57% |
| GEC59735.1 3-phosphoshikimate 1-carboxyvinyltransferase [Gluco... | 430 | 3e-144 | 55% |
| WP_184563712.1 3-phosphoshikimate 1-carboxyvinyltransferase [S... | 430 | 3e-144 | 54% |
| WP_031441627.1 MULTISPECIES: 3-phosphoshikimate 1-carboxyvinyl... | 429 | 3e-144 | 57% |
| NNC72920.1 3-phosphoshikimate 1-carboxyvinyltransferase [Sphin... | 429 | 3e-144 | 56% |
| OWU69247.1 3-phosphoshikimate 1-carboxyvinyltransferase [Roseo... | 429 | 3e-144 | 55% |
| WP_090113976.1 3-phosphoshikimate 1-carboxyvinyltransferase [L... | 429 | 3e-144 | 57% |
| WP_194259645.1 3-phosphoshikimate 1-carboxyvinyltransferase [G... | 429 | 3e-144 | 55% |
| RTL86647.1 3-phosphoshikimate 1-carboxyvinyltransferase [Rhizo... | 429 | 3e-144 | 58% |
| WP_067671070.1 3-phosphoshikimate 1-carboxyvinyltransferase [E... | 429 | 3e-144 | 58% |
| NBC95665.1 3-phosphoshikimate 1-carboxyvinyltransferase [Deino... | 429 | 3e-144 | 55% |
| WP_194921187.1 3-phosphoshikimate 1-carboxyvinyltransferase [N... | 429 | 3e-144 | 58% |
| WP_171558573.1 MULTISPECIES: 3-phosphoshikimate 1-carboxyvinyl... | 429 | 3e-144 | 55% |
| MBB3993373.1 3-phosphoshikimate 1-carboxyvinyltransferase [Sul... | 430 | 3e-144 | 54% |
| MAB86529.1 3-phosphoshikimate 1-carboxyvinyltransferase [Rhodo... | 429 | 3e-144 | 55% |
| MBA4325634.1 3-phosphoshikimate 1-carboxyvinyltransferase [Rho... | 429 | 3e-144 | 55% |
| WP_029651469.1 3-phosphoshikimate 1-carboxyvinyltransferase [M... | 429 | 3e-144 | 58% |
| WP_010142076.1 3-phosphoshikimate 1-carboxyvinyltransferase [O... | 429 | 3e-144 | 54% |
| WP_055918419.1 3-phosphoshikimate 1-carboxyvinyltransferase [A... | 429 | 3e-144 | 58% |
| WP_076626742.1 3-phosphoshikimate 1-carboxyvinyltransferase [T... | 429 | 3e-144 | 55% |
| WP_055984796.1 3-phosphoshikimate 1-carboxyvinyltransferase [S... | 429 | 3e-144 | 58% |
| MBF9047820.1 3-phosphoshikimate 1-carboxyvinyltransferase [Rho... | 429 | 3e-144 | 55% |
| WP_101067796.1 3-phosphoshikimate 1-carboxyvinyltransferase [R... | 429 | 3e-144 | 56% |

| NNE81054.1 3-phosphoshikimate 1-carboxyvinyltransferase [Silic... | 429 | 3e-144 | 56% |
| --- | --- | --- | --- |
| WP_090213485.1 3-phosphoshikimate 1-carboxyvinyltransferase [L... | 429 | 3e-144 | 55% |
| WP_184581637.1 3-phosphoshikimate 1-carboxyvinyltransferase [S... | 429 | 3e-144 | 54% |
| WP_160606788.1 3-phosphoshikimate 1-carboxyvinyltransferase [E... | 430 | 3e-144 | 57% |
| MBB4085109.1 3-phosphoshikimate 1-carboxyvinyltransferase [Sph... | 428 | 3e-144 | 57% |
| WP_097372745.1 3-phosphoshikimate 1-carboxyvinyltransferase [C... | 429 | 3e-144 | 55% |
| WP_148621152.1 3-phosphoshikimate 1-carboxyvinyltransferase [G... | 429 | 4e-144 | 55% |
| WP_192141460.1 3-phosphoshikimate 1-carboxyvinyltransferase [S... | 429 | 4e-144 | 56% |
| EBA02168.1 3-phosphoshikimate 1-carboxyvinyltransferase [Rhodo... | 429 | 4e-144 | 54% |
| NBS35373.1 3-phosphoshikimate 1-carboxyvinyltransferase [Methy... | 429 | 4e-144 | 55% |
| MAY34423.1 3-phosphoshikimate 1-carboxyvinyltransferase [Rhodo... | 429 | 4e-144 | 55% |
| NBT97344.1 3-phosphoshikimate 1-carboxyvinyltransferase [Methy... | 429 | 4e-144 | 55% |
| WP_085877642.1 3-phosphoshikimate 1-carboxyvinyltransferase [R... | 429 | 4e-144 | 56% |
| WP_062448344.1 3-phosphoshikimate 1-carboxyvinyltransferase [G... | 429 | 4e-144 | 55% |
| OYW44467.1 3-phosphoshikimate 1-carboxyvinyltransferase [Sphin... | 431 | 4e-144 | 59% |
| WP_022685844.1 3-phosphoshikimate 1-carboxyvinyltransferase [S... | 429 | 4e-144 | 56% |
| WP_072900511.1 3-phosphoshikimate 1-carboxyvinyltransferase [C... | 429 | 4e-144 | 56% |
| WP_062507947.1 3-phosphoshikimate 1-carboxyvinyltransferase [G... | 429 | 4e-144 | 55% |
| WP_149757147.1 3-phosphoshikimate 1-carboxyvinyltransferase [R... | 429 | 4e-144 | 55% |
| WP_108829045.1 3-phosphoshikimate 1-carboxyvinyltransferase [A... | 429 | 4e-144 | 54% |
| WP_062458126.1 3-phosphoshikimate 1-carboxyvinyltransferase [G... | 429 | 5e-144 | 55% |
| WP_058193397.1 3-phosphoshikimate 1-carboxyvinyltransferase [M... | 429 | 5e-144 | 59% |
| WP_136957461.1 3-phosphoshikimate 1-carboxyvinyltransferase [N... | 429 | 5e-144 | 58% |
| MAQ47118.1 3-phosphoshikimate 1-carboxyvinyltransferase [Confl... | 429 | 5e-144 | 55% |
| WP_189678478.1 3-phosphoshikimate 1-carboxyvinyltransferase [S... | 429 | 5e-144 | 57% |
| WP_107045323.1 3-phosphoshikimate 1-carboxyvinyltransferase [S... | 429 | 5e-144 | 57% |
| WP_166175383.1 3-phosphoshikimate 1-carboxyvinyltransferase [A... | 429 | 5e-144 | 58% |
| WP_048834855.1 3-phosphoshikimate 1-carboxyvinyltransferase [A... | 429 | 5e-144 | 55% |
| WP_133188644.1 3-phosphoshikimate 1-carboxyvinyltransferase [S... | 429 | 5e-144 | 57% |
| WP_184152418.1 MULTISPECIES: 3-phosphoshikimate 1-carboxyvinyl... | 429 | 5e-144 | 55% |
| NCF78350.1 3-phosphoshikimate 1-carboxyvinyltransferase [Alpha... | 428 | 6e-144 | 56% |
| WP_037297902.1 3-phosphoshikimate 1-carboxyvinyltransferase [R... | 429 | 6e-144 | 57% |
| NHO57587.1 3-phosphoshikimate 1-carboxyvinyltransferase [Aceto... | 429 | 6e-144 | 54% |
| RUP21917.1 3-phosphoshikimate 1-carboxyvinyltransferase [Methy... | 429 | 6e-144 | 59% |
| TDI59597.1 3-phosphoshikimate 1-carboxyvinyltransferase [Alpha... | 428 | 6e-144 | 56% |
| PIE08060.1 3-phosphoshikimate 1-carboxyvinyltransferase [Rhodo... | 429 | 6e-144 | 55% |
| WP_120325190.1 3-phosphoshikimate 1-carboxyvinyltransferase [A... | 428 | 6e-144 | 59% |
| MBF52834.1 3-phosphoshikimate 1-carboxyvinyltransferase [Confl... | 429 | 7e-144 | 55% |
| WP_133372776.1 3-phosphoshikimate 1-carboxyvinyltransferase [M... | 422 | 7e-144 | 81% |
| MAM26092.1 3-phosphoshikimate 1-carboxyvinyltransferase [Rhodo... | 429 | 7e-144 | 53% |
| WP_147853284.1 3-phosphoshikimate 1-carboxyvinyltransferase [M... | 427 | 7e-144 | 61% |
| WP_107930129.1 3-phosphoshikimate 1-carboxyvinyltransferase [S... | 428 | 8e-144 | 56% |
| WP_089882715.1 3-phosphoshikimate 1-carboxyvinyltransferase [C... | 428 | 8e-144 | 55% |
| WP_189369169.1 3-phosphoshikimate 1-carboxyvinyltransferase [T... | 428 | 8e-144 | 55% |
| GEB02545.1 3-phosphoshikimate 1-carboxyvinyltransferase [Gluco... | 429 | 8e-144 | 55% |
| WP_084582161.1 3-phosphoshikimate 1-carboxyvinyltransferase [S... | 428 | 8e-144 | 56% |
| WP_172366560.1 3-phosphoshikimate 1-carboxyvinyltransferase [X... | 428 | 9e-144 | 57% |
| WP_188447235.1 3-phosphoshikimate 1-carboxyvinyltransferase [S... | 428 | 9e-144 | 56% |
| WP_109565780.1 3-phosphoshikimate 1-carboxyvinyltransferase [J... | 428 | 9e-144 | 57% |
| WP_188644522.1 3-phosphoshikimate 1-carboxyvinyltransferase [A... | 428 | 9e-144 | 59% |
| WP_022734001.1 3-phosphoshikimate 1-carboxyvinyltransferase [T... | 428 | 9e-144 | 56% |
| WP_114686348.1 3-phosphoshikimate 1-carboxyvinyltransferase [S... | 428 | 9e-144 | 57% |
| WP_131625298.1 3-phosphoshikimate 1-carboxyvinyltransferase [F... | 429 | 9e-144 | 56% |
| WP_149468411.1 3-phosphoshikimate 1-carboxyvinyltransferase [R... | 428 | 9e-144 | 57% |
| WP_055879645.1 3-phosphoshikimate 1-carboxyvinyltransferase [S... | 428 | 1e-143 | 56% |
| WP_169333819.1 3-phosphoshikimate 1-carboxyvinyltransferase [K... | 427 | 1e-143 | 55% |
| WP_120634330.1 3-phosphoshikimate 1-carboxyvinyltransferase [R... | 428 | 1e-143 | 55% |
| WP_067598184.1 3-phosphoshikimate 1-carboxyvinyltransferase [E... | 428 | 1e-143 | 55% |
| OYV24549.1 3-phosphoshikimate 1-carboxyvinyltransferase [Rhodo... | 428 | 1e-143 | 54% |
| WP_137902333.1 3-phosphoshikimate 1-carboxyvinyltransferase [S... | 428 | 1e-143 | 56% |
| WP_194263843.1 MULTISPECIES: 3-phosphoshikimate 1-carboxyvinyl... | 428 | 1e-143 | 55% |
| MBV00557.1 3-phosphoshikimate 1-carboxyvinyltransferase [Rhodo... | 428 | 1e-143 | 54% |
| WP_040507313.1 3-phosphoshikimate 1-carboxyvinyltransferase [G... | 428 | 1e-143 | 54% |
| WP_134077707.1 3-phosphoshikimate 1-carboxyvinyltransferase [R... | 428 | 1e-143 | 56% |
| WP_085809119.1 3-phosphoshikimate 1-carboxyvinyltransferase [S... | 428 | 1e-143 | 54% |
| WP_132543645.1 3-phosphoshikimate 1-carboxyvinyltransferase [R... | 428 | 1e-143 | 56% |
| WP_047006444.1 3-phosphoshikimate 1-carboxyvinyltransferase [E... | 427 | 1e-143 | 56% |
| MBD3765883.1 3-phosphoshikimate 1-carboxyvinyltransferase [Rho... | 428 | 1e-143 | 57% |
| WP_192097560.1 3-phosphoshikimate 1-carboxyvinyltransferase [S... | 427 | 1e-143 | 56% |
| THD74525.1 3-phosphoshikimate 1-carboxyvinyltransferase [Pheny... | 428 | 1e-143 | 58% |
| WP_183937048.1 3-phosphoshikimate 1-carboxyvinyltransferase [S... | 427 | 1e-143 | 56% |
| WP_040820424.1 3-phosphoshikimate 1-carboxyvinyltransferase [T... | 427 | 1e-143 | 56% |

| WP_066806389.1 3-phosphoshikimate 1-carboxyvinyltransferase [S... | 427 | 1e-143 | 55% |
| --- | --- | --- | --- |
| WP_155860071.1 3-phosphoshikimate 1-carboxyvinyltransferase [K... | 427 | 1e-143 | 55% |
| WP_129369484.1 3-phosphoshikimate 1-carboxyvinyltransferase [L... | 427 | 1e-143 | 54% |
| HAB75768.1 TPA: 3-phosphoshikimate 1-carboxyvinyltransferase [... | 427 | 1e-143 | 55% |
| WP_166547568.1 3-phosphoshikimate 1-carboxyvinyltransferase [P... | 427 | 2e-143 | 57% |
| WP_010402713.1 3-phosphoshikimate 1-carboxyvinyltransferase [S... | 427 | 2e-143 | 56% |
| RMF10794.1 3-phosphoshikimate 1-carboxyvinyltransferase [Alpha... | 427 | 2e-143 | 57% |
| PZU05804.1 3-phosphoshikimate 1-carboxyvinyltransferase [Sphin... | 427 | 2e-143 | 57% |
| CCJ05474.1 3-phosphoshikimate 1-carboxyvinyltransferase, 5-eno... | 427 | 2e-143 | 58% |
| WP_138862746.1 3-phosphoshikimate 1-carboxyvinyltransferase [M... | 427 | 2e-143 | 56% |
| WP_093997477.1 3-phosphoshikimate 1-carboxyvinyltransferase [O... | 427 | 2e-143 | 57% |
| WP_174295147.1 3-phosphoshikimate 1-carboxyvinyltransferase [A... | 427 | 2e-143 | 56% |
| PCJ03197.1 3-phosphoshikimate 1-carboxyvinyltransferase [Alpha... | 428 | 2e-143 | 56% |
| WP_162471532.1 3-phosphoshikimate 1-carboxyvinyltransferase [B... | 427 | 2e-143 | 55% |
| MQA67144.1 3-phosphoshikimate 1-carboxyvinyltransferase [Alpha... | 427 | 2e-143 | 55% |
| KOX58951.1 3-phosphoshikimate 1-carboxyvinyltransferase [Strep... | 427 | 2e-143 | 60% |
| WP_174511407.1 3-phosphoshikimate 1-carboxyvinyltransferase [M... | 427 | 2e-143 | 57% |
| GAD59082.1 5-Enolpyruvylshikimate-3-phosphate synthase [Brevun... | 427 | 2e-143 | 55% |
| WP_176501359.1 3-phosphoshikimate 1-carboxyvinyltransferase [S... | 427 | 2e-143 | 55% |
| WP_130324334.1 3-phosphoshikimate 1-carboxyvinyltransferase [S... | 427 | 2e-143 | 56% |
| EHH68821.1 3-phosphoshikimate 1-carboxyvinyltransferase [Gluco... | 427 | 2e-143 | 54% |
| WP_133395736.1 3-phosphoshikimate 1-carboxyvinyltransferase [P... | 427 | 2e-143 | 54% |
| NQV99735.1 3-phosphoshikimate 1-carboxyvinyltransferase [Rhodo... | 427 | 2e-143 | 55% |
| WP_184055446.1 3-phosphoshikimate 1-carboxyvinyltransferase [S... | 427 | 2e-143 | 56% |
| WP_089898901.1 3-phosphoshikimate 1-carboxyvinyltransferase [L... | 427 | 2e-143 | 55% |
| NBZ88444.1 3-phosphoshikimate 1-carboxyvinyltransferase [Rhodo... | 427 | 2e-143 | 54% |
| WP_194301179.1 3-phosphoshikimate 1-carboxyvinyltransferase [A... | 428 | 2e-143 | 54% |
| WP_152434860.1 3-phosphoshikimate 1-carboxyvinyltransferase [E... | 427 | 2e-143 | 56% |
| RYD67987.1 3-phosphoshikimate 1-carboxyvinyltransferase [Sphin... | 427 | 2e-143 | 54% |
| WP_126172733.1 3-phosphoshikimate 1-carboxyvinyltransferase [A... | 427 | 2e-143 | 57% |
| HAJ90568.1 TPA: 3-phosphoshikimate 1-carboxyvinyltransferase [... | 427 | 2e-143 | 53% |
| WP_119838281.1 3-phosphoshikimate 1-carboxyvinyltransferase [P... | 427 | 2e-143 | 55% |
| WP_166119960.1 3-phosphoshikimate 1-carboxyvinyltransferase [A... | 427 | 3e-143 | 54% |
| WP_064780490.1 3-phosphoshikimate 1-carboxyvinyltransferase [T... | 427 | 3e-143 | 55% |
| WP_166746175.1 3-phosphoshikimate 1-carboxyvinyltransferase [S... | 427 | 3e-143 | 56% |
| WP_121896568.1 3-phosphoshikimate 1-carboxyvinyltransferase [R... | 427 | 3e-143 | 55% |
| MPY73276.1 3-phosphoshikimate 1-carboxyvinyltransferase [Alpha... | 427 | 3e-143 | 57% |
| WP_090199498.1 3-phosphoshikimate 1-carboxyvinyltransferase [Y... | 427 | 3e-143 | 57% |
| NCV17813.1 3-phosphoshikimate 1-carboxyvinyltransferase [Rhodo... | 427 | 3e-143 | 53% |
| WP_044050236.1 3-phosphoshikimate 1-carboxyvinyltransferase [P... | 427 | 3e-143 | 55% |
| EDM31728.1 3-phosphoshikimate 1-carboxyvinyltransferase [Roseo... | 426 | 3e-143 | 57% |
| WP_119399853.1 3-phosphoshikimate 1-carboxyvinyltransferase [P... | 427 | 3e-143 | 56% |
| HCQ65438.1 TPA: 3-phosphoshikimate 1-carboxyvinyltransferase [... | 427 | 3e-143 | 55% |
| WP_037253198.1 3-phosphoshikimate 1-carboxyvinyltransferase [R... | 427 | 3e-143 | 54% |
| WP_119035104.1 3-phosphoshikimate 1-carboxyvinyltransferase [H... | 427 | 3e-143 | 56% |
| KPP85307.1 3-phosphoshikimate 1-carboxyvinyltransferase [Rhodo... | 427 | 3e-143 | 54% |
| WP_159511311.1 3-phosphoshikimate 1-carboxyvinyltransferase [S... | 427 | 3e-143 | 56% |
| WP_056523956.1 3-phosphoshikimate 1-carboxyvinyltransferase [S... | 427 | 3e-143 | 58% |
| WP_166775955.1 3-phosphoshikimate 1-carboxyvinyltransferase [E... | 426 | 3e-143 | 58% |
| WP_073975254.1 3-phosphoshikimate 1-carboxyvinyltransferase [P... | 426 | 3e-143 | 57% |
| MBD0865283.1 3-phosphoshikimate 1-carboxyvinyltransferase [Rho... | 427 | 3e-143 | 54% |
| BCA64214.1 3-phosphoshikimate 1-carboxyvinyltransferase [Sphin... | 426 | 3e-143 | 56% |
| WP_062497715.1 3-phosphoshikimate 1-carboxyvinyltransferase [G... | 426 | 4e-143 | 55% |
| WP_184049332.1 3-phosphoshikimate 1-carboxyvinyltransferase [S... | 427 | 4e-143 | 55% |
| WP_111992752.1 3-phosphoshikimate 1-carboxyvinyltransferase [E... | 426 | 4e-143 | 56% |
| WP_184086608.1 3-phosphoshikimate 1-carboxyvinyltransferase [S... | 426 | 4e-143 | 57% |
| WP_109385207.1 3-phosphoshikimate 1-carboxyvinyltransferase [P... | 427 | 4e-143 | 57% |
| WP_067287207.1 3-phosphoshikimate 1-carboxyvinyltransferase [S... | 427 | 4e-143 | 56% |
| WP_086607795.1 3-phosphoshikimate 1-carboxyvinyltransferase [P... | 426 | 4e-143 | 58% |
| WP_109666872.1 3-phosphoshikimate 1-carboxyvinyltransferase [R... | 427 | 4e-143 | 55% |
| WP_076647712.1 3-phosphoshikimate 1-carboxyvinyltransferase [P... | 427 | 4e-143 | 53% |
| WP_184169441.1 3-phosphoshikimate 1-carboxyvinyltransferase [S... | 426 | 4e-143 | 55% |
| OYV51434.1 3-phosphoshikimate 1-carboxyvinyltransferase [Rhodo... | 426 | 4e-143 | 52% |
| MAH15595.1 3-phosphoshikimate 1-carboxyvinyltransferase [Sphin... | 426 | 4e-143 | 57% |
| WP_183530873.1 3-phosphoshikimate 1-carboxyvinyltransferase [L... | 427 | 4e-143 | 56% |
| WP_010438017.1 3-phosphoshikimate 1-carboxyvinyltransferase [R... | 427 | 4e-143 | 55% |
| WP_077931748.1 3-phosphoshikimate 1-carboxyvinyltransferase [A... | 426 | 4e-143 | 54% |
| WP_129631973.1 3-phosphoshikimate 1-carboxyvinyltransferase [T... | 426 | 4e-143 | 56% |
| WP_007088346.1 3-phosphoshikimate 1-carboxyvinyltransferase [T... | 426 | 4e-143 | 56% |
| WP_029625380.1 3-phosphoshikimate 1-carboxyvinyltransferase [S... | 426 | 4e-143 | 56% |
| WP_104942587.1 3-phosphoshikimate 1-carboxyvinyltransferase [P... | 426 | 4e-143 | 58% |
| WP_067927454.1 MULTISPECIES: 3-phosphoshikimate 1-carboxyvinyl... | 427 | 4e-143 | 53% |

| WP_047003166.1 3-phosphoshikimate 1-carboxyvinyltransferase [E... | 426 | 4e-143 | 57% |
| --- | --- | --- | --- |
| KWT70027.1 5-Enolpyruvylshikimate-3-phosphate synthase [Hyphom... | 426 | 4e-143 | 59% |
| WP_013639133.1 3-phosphoshikimate 1-carboxyvinyltransferase [A... | 426 | 4e-143 | 54% |
| VTZ48970.1 3-phosphoshikimate 1-carboxyvinyltransferase [Methy... | 427 | 4e-143 | 57% |
| WP_096053133.1 3-phosphoshikimate 1-carboxyvinyltransferase [C... | 426 | 5e-143 | 56% |
| MBC44730.1 3-phosphoshikimate 1-carboxyvinyltransferase [Thala... | 426 | 5e-143 | 56% |
| WP_107957234.1 3-phosphoshikimate 1-carboxyvinyltransferase [S... | 426 | 5e-143 | 56% |
| MBU74955.1 3-phosphoshikimate 1-carboxyvinyltransferase [Rhodo... | 426 | 5e-143 | 53% |
| WP_059398951.1 MULTISPECIES: 3-phosphoshikimate 1-carboxyvinyl... | 426 | 5e-143 | 57% |
| WP_185800497.1 3-phosphoshikimate 1-carboxyvinyltransferase [P... | 426 | 5e-143 | 54% |
| WP_158965602.1 3-phosphoshikimate 1-carboxyvinyltransferase [P... | 426 | 6e-143 | 56% |
| PQM61193.1 3-phosphoshikimate 1-carboxyvinyltransferase [Rhodo... | 426 | 6e-143 | 53% |
| NCO87585.1 3-phosphoshikimate 1-carboxyvinyltransferase [Rhodo... | 426 | 6e-143 | 55% |
| TDI59089.1 3-phosphoshikimate 1-carboxyvinyltransferase [Alpha... | 426 | 6e-143 | 55% |
| WP_100283944.1 3-phosphoshikimate 1-carboxyvinyltransferase [S... | 426 | 6e-143 | 56% |
| WP_139664869.1 3-phosphoshikimate 1-carboxyvinyltransferase [P... | 426 | 6e-143 | 57% |
| WP_114376909.1 3-phosphoshikimate 1-carboxyvinyltransferase [E... | 426 | 6e-143 | 56% |
| MAE51157.1 3-phosphoshikimate 1-carboxyvinyltransferase [Micav... | 426 | 7e-143 | 55% |
| WP_063688506.1 3-phosphoshikimate 1-carboxyvinyltransferase [S... | 426 | 7e-143 | 60% |
| WP_062957212.1 MULTISPECIES: 3-phosphoshikimate 1-carboxyvinyl... | 426 | 7e-143 | 56% |
| WP_174439122.1 3-phosphoshikimate 1-carboxyvinyltransferase [A... | 426 | 7e-143 | 57% |
| EET47754.1 3-phosphoshikimate 1-carboxyvinyltransferase [Thala... | 427 | 7e-143 | 56% |
| WP_183264946.1 3-phosphoshikimate 1-carboxyvinyltransferase [A... | 426 | 7e-143 | 53% |
| WP_179380180.1 3-phosphoshikimate 1-carboxyvinyltransferase [J... | 426 | 8e-143 | 55% |
| WP_142835334.1 3-phosphoshikimate 1-carboxyvinyltransferase [P... | 426 | 8e-143 | 56% |
| WP_158809877.1 3-phosphoshikimate 1-carboxyvinyltransferase [B... | 426 | 8e-143 | 57% |
| WP_121061404.1 3-phosphoshikimate 1-carboxyvinyltransferase [C... | 426 | 8e-143 | 54% |
| WP_147036527.1 3-phosphoshikimate 1-carboxyvinyltransferase [S... | 426 | 8e-143 | 57% |
| EBA18244.1 3-phosphoshikimate 1-carboxyvinyltransferase [Roseo... | 426 | 8e-143 | 54% |
| SKB95202.1 3-phosphoshikimate 1-carboxyvinyltransferase [Sphin... | 424 | 8e-143 | 59% |
| WP_056674330.1 MULTISPECIES: 3-phosphoshikimate 1-carboxyvinyl... | 426 | 9e-143 | 56% |
| HBS50587.1 TPA: 3-phosphoshikimate 1-carboxyvinyltransferase [... | 426 | 9e-143 | 56% |
| WP_073957103.1 3-phosphoshikimate 1-carboxyvinyltransferase [T... | 426 | 9e-143 | 56% |
| WP_056913064.1 3-phosphoshikimate 1-carboxyvinyltransferase [P... | 425 | 9e-143 | 56% |
| OYX05295.1 3-phosphoshikimate 1-carboxyvinyltransferase [Caulo... | 425 | 1e-142 | 56% |
| WP_179405816.1 3-phosphoshikimate 1-carboxyvinyltransferase [N... | 426 | 1e-142 | 56% |
| WP_194267606.1 3-phosphoshikimate 1-carboxyvinyltransferase [G... | 425 | 1e-142 | 55% |
| WP_098105277.1 3-phosphoshikimate 1-carboxyvinyltransferase [N... | 425 | 1e-142 | 57% |
| WP_123642441.1 3-phosphoshikimate 1-carboxyvinyltransferase [H... | 426 | 1e-142 | 56% |
| WP_124809078.1 3-phosphoshikimate 1-carboxyvinyltransferase [N... | 425 | 1e-142 | 57% |
| NIJ16535.1 3-phosphoshikimate 1-carboxyvinyltransferase [Sphin... | 424 | 1e-142 | 57% |
| RVD48397.1 3-phosphoshikimate 1-carboxyvinyltransferase [Mesor... | 419 | 1e-142 | 78% |
| WP_157706042.1 3-phosphoshikimate 1-carboxyvinyltransferase [R... | 426 | 1e-142 | 55% |
| WP_166581420.1 3-phosphoshikimate 1-carboxyvinyltransferase [R... | 425 | 1e-142 | 54% |
| WP_194261702.1 3-phosphoshikimate 1-carboxyvinyltransferase [G... | 425 | 1e-142 | 55% |
| WP_092810408.1 3-phosphoshikimate 1-carboxyvinyltransferase [R... | 425 | 1e-142 | 54% |
| PZR35630.1 3-phosphoshikimate 1-carboxyvinyltransferase [Caulo... | 425 | 1e-142 | 57% |
| WP_086612179.1 3-phosphoshikimate 1-carboxyvinyltransferase [A... | 425 | 1e-142 | 54% |
| WP_099537175.1 3-phosphoshikimate 1-carboxyvinyltransferase [C... | 425 | 1e-142 | 56% |
| WP_062065833.1 3-phosphoshikimate 1-carboxyvinyltransferase [S... | 425 | 1e-142 | 56% |
| WP_033070168.1 3-phosphoshikimate 1-carboxyvinyltransferase [T... | 425 | 1e-142 | 56% |
| QFT78152.1 3-phosphoshikimate 1-carboxyvinyltransferase [Eryth... | 426 | 1e-142 | 56% |
| OIN86916.1 3-phosphoshikimate 1-carboxyvinyltransferase [Alpha... | 426 | 1e-142 | 54% |
| WP_194254052.1 3-phosphoshikimate 1-carboxyvinyltransferase [G... | 425 | 1e-142 | 55% |
| MAF96227.1 3-phosphoshikimate 1-carboxyvinyltransferase [Rhodo... | 425 | 1e-142 | 56% |
| WP_101267633.1 3-phosphoshikimate 1-carboxyvinyltransferase [T... | 425 | 1e-142 | 57% |
| WP_179194573.1 3-phosphoshikimate 1-carboxyvinyltransferase [A... | 425 | 1e-142 | 54% |
| WP_146858861.1 3-phosphoshikimate 1-carboxyvinyltransferase [G... | 425 | 1e-142 | 55% |
| MAO00414.1 3-phosphoshikimate 1-carboxyvinyltransferase [Roseo... | 425 | 1e-142 | 58% |
| WP_135245861.1 3-phosphoshikimate 1-carboxyvinyltransferase [P... | 425 | 1e-142 | 57% |
| MBS40844.1 3-phosphoshikimate 1-carboxyvinyltransferase [Rhodo... | 425 | 1e-142 | 52% |
| THD35285.1 3-phosphoshikimate 1-carboxyvinyltransferase [Sphin... | 425 | 1e-142 | 55% |
| WP_010217257.1 3-phosphoshikimate 1-carboxyvinyltransferase [S... | 425 | 2e-142 | 55% |
| WP_114877485.1 3-phosphoshikimate 1-carboxyvinyltransferase [S... | 425 | 2e-142 | 55% |
| WP_044830110.1 3-phosphoshikimate 1-carboxyvinyltransferase [T... | 425 | 2e-142 | 55% |
| MBO23472.1 3-phosphoshikimate 1-carboxyvinyltransferase [Rhodo... | 425 | 2e-142 | 54% |
| RZO42281.1 3-phosphoshikimate 1-carboxyvinyltransferase [Rhodo... | 425 | 2e-142 | 54% |
| WP_062501880.1 3-phosphoshikimate 1-carboxyvinyltransferase [G... | 425 | 2e-142 | 55% |
| MBA4008320.1 3-phosphoshikimate 1-carboxyvinyltransferase [Ery... | 425 | 2e-142 | 58% |
| WP_056020627.1 3-phosphoshikimate 1-carboxyvinyltransferase [S... | 425 | 2e-142 | 56% |
| WP_061501381.1 3-phosphoshikimate 1-carboxyvinyltransferase [A... | 425 | 2e-142 | 54% |
| WP_041111183.1 3-phosphoshikimate 1-carboxyvinyltransferase [G... | 425 | 2e-142 | 54% |

| PTQ61011.1 3-phosphoshikimate 1-carboxyvinyltransferase [Sphin... | 426 | 2e-142 | 56% |
| --- | --- | --- | --- |
| WP_119508059.1 3-phosphoshikimate 1-carboxyvinyltransferase [A... | 425 | 2e-142 | 57% |
| WP_007424234.1 MULTISPECIES: 3-phosphoshikimate 1-carboxyvinyl... | 425 | 2e-142 | 54% |
| NCC03864.1 3-phosphoshikimate 1-carboxyvinyltransferase [Prote... | 425 | 2e-142 | 54% |
| WP_163846845.1 3-phosphoshikimate 1-carboxyvinyltransferase [P... | 425 | 2e-142 | 54% |
| WP_160746006.1 3-phosphoshikimate 1-carboxyvinyltransferase [A... | 424 | 2e-142 | 59% |
| EJW20938.1 hypothetical protein IMCC14465_07340 [alpha proteob... | 426 | 2e-142 | 52% |
| WP_101791979.1 3-phosphoshikimate 1-carboxyvinyltransferase [P... | 424 | 2e-142 | 57% |
| WP_037276771.1 3-phosphoshikimate 1-carboxyvinyltransferase [R... | 425 | 2e-142 | 58% |
| MAY87654.1 3-phosphoshikimate 1-carboxyvinyltransferase [Pseud... | 425 | 2e-142 | 55% |
| WP_063092148.1 MULTISPECIES: 3-phosphoshikimate 1-carboxyvinyl... | 425 | 2e-142 | 56% |
| WP_114094124.1 3-phosphoshikimate 1-carboxyvinyltransferase [T... | 425 | 2e-142 | 56% |
| TAL01789.1 3-phosphoshikimate 1-carboxyvinyltransferase [Rhodo... | 426 | 2e-142 | 56% |
| WP_070402017.1 3-phosphoshikimate 1-carboxyvinyltransferase [K... | 425 | 2e-142 | 54% |
| MBA4050306.1 3-phosphoshikimate 1-carboxyvinyltransferase [Ery... | 424 | 2e-142 | 58% |
| MBB53916.1 3-phosphoshikimate 1-carboxyvinyltransferase [Magne... | 425 | 2e-142 | 54% |
| WP_021102209.1 3-phosphoshikimate 1-carboxyvinyltransferase [L... | 425 | 2e-142 | 54% |
| WP_160894400.1 3-phosphoshikimate 1-carboxyvinyltransferase [P... | 425 | 2e-142 | 55% |
| WP_062031867.1 3-phosphoshikimate 1-carboxyvinyltransferase [G... | 424 | 2e-142 | 55% |
| GEK95285.1 3-phosphoshikimate 1-carboxyvinyltransferase [Gluco... | 425 | 2e-142 | 55% |
| WP_062109394.1 3-phosphoshikimate 1-carboxyvinyltransferase [G... | 424 | 2e-142 | 55% |
| WP_048845250.1 3-phosphoshikimate 1-carboxyvinyltransferase [A... | 425 | 2e-142 | 54% |
| WP_061505449.1 3-phosphoshikimate 1-carboxyvinyltransferase [A... | 425 | 2e-142 | 54% |
| WP_056053981.1 MULTISPECIES: 3-phosphoshikimate 1-carboxyvinyl... | 424 | 2e-142 | 57% |
| WP_124088256.1 3-phosphoshikimate 1-carboxyvinyltransferase [X... | 424 | 2e-142 | 56% |
| WP_091845457.1 3-phosphoshikimate 1-carboxyvinyltransferase [M... | 425 | 2e-142 | 55% |
| WP_052403767.1 3-phosphoshikimate 1-carboxyvinyltransferase [A... | 424 | 2e-142 | 54% |
| WP_077803771.1 3-phosphoshikimate 1-carboxyvinyltransferase [G... | 424 | 2e-142 | 55% |
| WP_092079150.1 3-phosphoshikimate 1-carboxyvinyltransferase [P... | 424 | 2e-142 | 54% |
| MBC04709.1 3-phosphoshikimate 1-carboxyvinyltransferase [Thala... | 424 | 3e-142 | 55% |
| WP_174838252.1 3-phosphoshikimate 1-carboxyvinyltransferase [S... | 424 | 3e-142 | 55% |
| WP_074221237.1 3-phosphoshikimate 1-carboxyvinyltransferase [R... | 424 | 3e-142 | 56% |
| WP_092062052.1 3-phosphoshikimate 1-carboxyvinyltransferase [P... | 424 | 3e-142 | 54% |
| NBC33554.1 3-phosphoshikimate 1-carboxyvinyltransferase [Alpha... | 424 | 3e-142 | 56% |
| TPV97665.1 3-phosphoshikimate 1-carboxyvinyltransferase [Beije... | 432 | 3e-142 | 57% |
| WP_144335037.1 3-phosphoshikimate 1-carboxyvinyltransferase [S... | 424 | 3e-142 | 56% |
| NNE50587.1 3-phosphoshikimate 1-carboxyvinyltransferase [Alter... | 424 | 3e-142 | 58% |
| MBH69141.1 3-phosphoshikimate 1-carboxyvinyltransferase [Rhodo... | 424 | 3e-142 | 54% |
| OAG77973.1 5-Enolpyruvylshikimate-3-phosphate synthase [Acetob... | 424 | 3e-142 | 54% |
| WP_154692872.1 3-phosphoshikimate 1-carboxyvinyltransferase [N... | 424 | 3e-142 | 57% |
| WP_021120531.1 3-phosphoshikimate 1-carboxyvinyltransferase [S... | 424 | 3e-142 | 54% |
| WP_043842950.1 3-phosphoshikimate 1-carboxyvinyltransferase [R... | 424 | 3e-142 | 54% |
| HBN31644.1 TPA: 3-phosphoshikimate 1-carboxyvinyltransferase [... | 424 | 3e-142 | 56% |
| WP_116489144.1 3-phosphoshikimate 1-carboxyvinyltransferase [C... | 424 | 3e-142 | 57% |
| PCI64366.1 3-phosphoshikimate 1-carboxyvinyltransferase [Kordi... | 424 | 3e-142 | 54% |
| WP_055686298.1 3-phosphoshikimate 1-carboxyvinyltransferase [L... | 424 | 3e-142 | 56% |
| OYV56270.1 3-phosphoshikimate 1-carboxyvinyltransferase [Acidi... | 424 | 3e-142 | 54% |
| WP_109806272.1 3-phosphoshikimate 1-carboxyvinyltransferase [S... | 424 | 3e-142 | 54% |
| WP_085642885.1 3-phosphoshikimate 1-carboxyvinyltransferase [T... | 424 | 3e-142 | 56% |
| WP_183994018.1 3-phosphoshikimate 1-carboxyvinyltransferase [S... | 424 | 3e-142 | 55% |
| WP_190261097.1 3-phosphoshikimate 1-carboxyvinyltransferase [D... | 424 | 3e-142 | 53% |
| WP_174502927.1 3-phosphoshikimate 1-carboxyvinyltransferase [A... | 424 | 4e-142 | 54% |
| WP_086653723.1 3-phosphoshikimate 1-carboxyvinyltransferase [A... | 424 | 4e-142 | 54% |
| WP_073128319.1 3-phosphoshikimate 1-carboxyvinyltransferase [M... | 424 | 4e-142 | 55% |
| WP_163958472.1 3-phosphoshikimate 1-carboxyvinyltransferase [S... | 424 | 4e-142 | 55% |
| WP_101720491.1 3-phosphoshikimate 1-carboxyvinyltransferase [C... | 424 | 4e-142 | 57% |
| WP_008888953.1 3-phosphoshikimate 1-carboxyvinyltransferase [T... | 424 | 4e-142 | 55% |
| WP_061508293.1 3-phosphoshikimate 1-carboxyvinyltransferase [A... | 424 | 4e-142 | 54% |
| WP_116082687.1 3-phosphoshikimate 1-carboxyvinyltransferase [T... | 424 | 4e-142 | 53% |
| MBE9475791.1 3-phosphoshikimate 1-carboxyvinyltransferase [Pro... | 424 | 4e-142 | 54% |
| WP_093219355.1 3-phosphoshikimate 1-carboxyvinyltransferase [S... | 424 | 4e-142 | 56% |
| MBB05530.1 3-phosphoshikimate 1-carboxyvinyltransferase [Pseud... | 424 | 4e-142 | 56% |
| NNM55748.1 3-phosphoshikimate 1-carboxyvinyltransferase [Acido... | 424 | 4e-142 | 54% |
| WP_138380864.1 3-phosphoshikimate 1-carboxyvinyltransferase [E... | 424 | 4e-142 | 57% |
| WP_114090363.1 3-phosphoshikimate 1-carboxyvinyltransferase [T... | 424 | 4e-142 | 56% |
| WP_063907352.1 3-phosphoshikimate 1-carboxyvinyltransferase [A... | 424 | 4e-142 | 54% |
| TIR10309.1 3-phosphoshikimate 1-carboxyvinyltransferase [Mesor... | 417 | 4e-142 | 77% |
| RZJ17566.1 3-phosphoshikimate 1-carboxyvinyltransferase [Brevu... | 423 | 4e-142 | 55% |
| ODU81016.1 3-phosphoshikimate 1-carboxyvinyltransferase [Novos... | 424 | 4e-142 | 57% |
| WP_167071663.1 3-phosphoshikimate 1-carboxyvinyltransferase [S... | 424 | 5e-142 | 53% |
| OYX44771.1 3-phosphoshikimate 1-carboxyvinyltransferase [Rhodo... | 424 | 5e-142 | 58% |
| WP_188527746.1 3-phosphoshikimate 1-carboxyvinyltransferase [M... | 424 | 5e-142 | 55% |

| WP_170004659.1 3-phosphoshikimate 1-carboxyvinyltransferase [P... | 424 | 5e-142 | 55% |
| --- | --- | --- | --- |
| OUJ05400.1 3-phosphoshikimate 1-carboxyvinyltransferase [Aceto... | 424 | 5e-142 | 54% |
| WP_165258723.1 3-phosphoshikimate 1-carboxyvinyltransferase [C... | 424 | 5e-142 | 57% |
| EQB03337.1 hypothetical protein L288_14840 [Sphingobium quisqu... | 422 | 5e-142 | 56% |
| WP_192070702.1 3-phosphoshikimate 1-carboxyvinyltransferase [S... | 424 | 5e-142 | 57% |
| WP_127611133.1 3-phosphoshikimate 1-carboxyvinyltransferase [C... | 424 | 5e-142 | 58% |
| MBE09859.1 3-phosphoshikimate 1-carboxyvinyltransferase [Rhodo... | 424 | 5e-142 | 55% |
| WP_130159472.1 3-phosphoshikimate 1-carboxyvinyltransferase [S... | 424 | 5e-142 | 57% |
| WP_036800775.1 3-phosphoshikimate 1-carboxyvinyltransferase [P... | 423 | 5e-142 | 57% |
| WP_035258004.1 3-phosphoshikimate 1-carboxyvinyltransferase [A... | 424 | 5e-142 | 54% |
| WP_048462773.1 3-phosphoshikimate 1-carboxyvinyltransferase [M... | 424 | 5e-142 | 58% |
| MAK57623.1 3-phosphoshikimate 1-carboxyvinyltransferase [Rhizo... | 424 | 5e-142 | 53% |
| WP_025884509.1 3-phosphoshikimate 1-carboxyvinyltransferase [A... | 424 | 6e-142 | 54% |
| PKP90070.1 3-phosphoshikimate 1-carboxyvinyltransferase [Alpha... | 423 | 6e-142 | 55% |
| WP_099444253.1 3-phosphoshikimate 1-carboxyvinyltransferase [C... | 423 | 6e-142 | 56% |
| WP_056476624.1 3-phosphoshikimate 1-carboxyvinyltransferase [S... | 423 | 6e-142 | 56% |
| WP_066624141.1 3-phosphoshikimate 1-carboxyvinyltransferase [B... | 423 | 6e-142 | 54% |
| WP_092764075.1 3-phosphoshikimate 1-carboxyvinyltransferase [J... | 423 | 6e-142 | 56% |
| MAT88571.1 3-phosphoshikimate 1-carboxyvinyltransferase [Aestu... | 424 | 6e-142 | 55% |
| PZP56436.1 3-phosphoshikimate 1-carboxyvinyltransferase [Micav... | 424 | 6e-142 | 53% |
| WP_160726982.1 3-phosphoshikimate 1-carboxyvinyltransferase [E... | 423 | 6e-142 | 58% |
| WP_062150482.1 3-phosphoshikimate 1-carboxyvinyltransferase [C... | 423 | 6e-142 | 57% |
| PPR77511.1 3-phosphoshikimate 1-carboxyvinyltransferase [Alpha... | 424 | 6e-142 | 52% |
| NCF49567.1 3-phosphoshikimate 1-carboxyvinyltransferase [Bacte... | 423 | 6e-142 | 53% |
| MBA15149.1 3-phosphoshikimate 1-carboxyvinyltransferase [Sphin... | 423 | 6e-142 | 54% |
| WP_140849150.1 3-phosphoshikimate 1-carboxyvinyltransferase [S... | 424 | 6e-142 | 56% |
| WP_061491495.1 3-phosphoshikimate 1-carboxyvinyltransferase [A... | 424 | 6e-142 | 54% |
| MBA4044749.1 3-phosphoshikimate 1-carboxyvinyltransferase [Ery... | 423 | 6e-142 | 57% |
| WP_066281930.1 3-phosphoshikimate 1-carboxyvinyltransferase [B... | 422 | 6e-142 | 57% |
| WP_092362387.1 3-phosphoshikimate 1-carboxyvinyltransferase [C... | 424 | 6e-142 | 55% |
| WP_062949763.1 MULTISPECIES: 3-phosphoshikimate 1-carboxyvinyl... | 423 | 6e-142 | 56% |
| KFL90541.1 5-Enolpyruvylshikimate-3-phosphate synthase [Acetob... | 425 | 7e-142 | 54% |
| WP_134828868.1 3-phosphoshikimate 1-carboxyvinyltransferase [L... | 423 | 7e-142 | 55% |
| WP_114109155.1 3-phosphoshikimate 1-carboxyvinyltransferase [T... | 423 | 7e-142 | 56% |
| WP_048839160.1 3-phosphoshikimate 1-carboxyvinyltransferase [A... | 424 | 7e-142 | 55% |
| WP_159964846.1 3-phosphoshikimate 1-carboxyvinyltransferase [P... | 424 | 8e-142 | 55% |
| WP_062141253.1 3-phosphoshikimate 1-carboxyvinyltransferase [A... | 423 | 8e-142 | 54% |
| PCJ97796.1 3-phosphoshikimate 1-carboxyvinyltransferase [Zetap... | 424 | 8e-142 | 56% |
| WP_089947468.1 3-phosphoshikimate 1-carboxyvinyltransferase [L... | 423 | 8e-142 | 54% |
| MBD19997.1 3-phosphoshikimate 1-carboxyvinyltransferase [Rhodo... | 423 | 8e-142 | 52% |
| WP_126176597.1 3-phosphoshikimate 1-carboxyvinyltransferase [A... | 423 | 8e-142 | 57% |
| WP_118944325.1 3-phosphoshikimate 1-carboxyvinyltransferase [P... | 423 | 8e-142 | 53% |
| THD54346.1 3-phosphoshikimate 1-carboxyvinyltransferase [Pheny... | 423 | 8e-142 | 57% |
| WP_086734794.1 3-phosphoshikimate 1-carboxyvinyltransferase [P... | 423 | 8e-142 | 57% |
| WP_149648266.1 3-phosphoshikimate 1-carboxyvinyltransferase [A... | 423 | 8e-142 | 57% |
| WP_106356514.1 3-phosphoshikimate 1-carboxyvinyltransferase [Y... | 423 | 8e-142 | 57% |
| WP_007164484.1 3-phosphoshikimate 1-carboxyvinyltransferase [E... | 422 | 9e-142 | 56% |
| GAN60992.1 3-phosphoshikimate 1-carboxyvinyltransferase [Aceto... | 423 | 9e-142 | 55% |
| WP_127749948.1 3-phosphoshikimate 1-carboxyvinyltransferase [P... | 423 | 9e-142 | 54% |
| WP_085527074.1 3-phosphoshikimate 1-carboxyvinyltransferase [M... | 423 | 1e-141 | 56% |
| WP_094301664.1 3-phosphoshikimate 1-carboxyvinyltransferase [A... | 423 | 1e-141 | 57% |
| MAH38624.1 3-phosphoshikimate 1-carboxyvinyltransferase [Marin... | 423 | 1e-141 | 52% |
| BAK66334.1 3-phosphoshikimate 1-carboxyvinyltransferase [Sphin... | 423 | 1e-141 | 55% |
| WP_137103578.1 3-phosphoshikimate 1-carboxyvinyltransferase [A... | 423 | 1e-141 | 57% |
| WP_037989161.1 3-phosphoshikimate 1-carboxyvinyltransferase [T... | 423 | 1e-141 | 56% |
| WP_128562857.1 3-phosphoshikimate 1-carboxyvinyltransferase [M... | 423 | 1e-141 | 60% |
| WP_037923926.1 3-phosphoshikimate 1-carboxyvinyltransferase [S... | 423 | 1e-141 | 55% |
| WP_056051419.1 3-phosphoshikimate 1-carboxyvinyltransferase [S... | 422 | 1e-141 | 56% |
| WP_109070685.1 3-phosphoshikimate 1-carboxyvinyltransferase [A... | 422 | 1e-141 | 57% |
| WP_042055236.1 3-phosphoshikimate 1-carboxyvinyltransferase [A... | 422 | 1e-141 | 53% |
| HDZ82487.1 TPA: 3-phosphoshikimate 1-carboxyvinyltransferase [... | 423 | 1e-141 | 54% |
| HBD91226.1 TPA: 3-phosphoshikimate 1-carboxyvinyltransferase [... | 422 | 1e-141 | 57% |
| WP_055681621.1 3-phosphoshikimate 1-carboxyvinyltransferase [J... | 422 | 1e-141 | 56% |
| WP_100261084.1 3-phosphoshikimate 1-carboxyvinyltransferase [E... | 422 | 1e-141 | 58% |
| WP_170604593.1 3-phosphoshikimate 1-carboxyvinyltransferase [R... | 422 | 1e-141 | 54% |
| NCX69814.1 3-phosphoshikimate 1-carboxyvinyltransferase [Rhodo... | 422 | 1e-141 | 52% |
| WP_086651444.1 3-phosphoshikimate 1-carboxyvinyltransferase [A... | 423 | 1e-141 | 55% |
| PDH61514.1 3-phosphoshikimate 1-carboxyvinyltransferase [Rhodo... | 422 | 1e-141 | 52% |
| OUJ01936.1 3-phosphoshikimate 1-carboxyvinyltransferase [Aceto... | 422 | 1e-141 | 55% |
| KQB97124.1 3-phosphoshikimate 1-carboxyvinyltransferase [Lokta... | 422 | 1e-141 | 56% |
| MAI12606.1 3-phosphoshikimate 1-carboxyvinyltransferase [Rhodo... | 422 | 1e-141 | 54% |
| WP_170473848.1 3-phosphoshikimate 1-carboxyvinyltransferase [R... | 422 | 2e-141 | 54% |

| MAI49833.1 3-phosphoshikimate 1-carboxyvinyltransferase [Rhodo... | 422 | 2e-141 | 52% |
| --- | --- | --- | --- |
| KAF0676247.1 3-phosphoshikimate 1-carboxyvinyltransferase [Pro... | 424 | 2e-141 | 55% |
| WP_129764201.1 3-phosphoshikimate 1-carboxyvinyltransferase [S... | 422 | 2e-141 | 54% |
| WP_014239235.1 MULTISPECIES: 3-phosphoshikimate 1-carboxyvinyl... | 422 | 2e-141 | 57% |
| WP_126401412.1 3-phosphoshikimate 1-carboxyvinyltransferase [B... | 422 | 2e-141 | 56% |
| WP_083931526.1 3-phosphoshikimate 1-carboxyvinyltransferase [M... | 421 | 2e-141 | 58% |
| WP_145674940.1 3-phosphoshikimate 1-carboxyvinyltransferase [A... | 422 | 2e-141 | 57% |
| WP_045996651.1 3-phosphoshikimate 1-carboxyvinyltransferase [L... | 422 | 2e-141 | 56% |
| OQW76860.1 3-phosphoshikimate 1-carboxyvinyltransferase [Prote... | 422 | 2e-141 | 57% |
| WP_114520173.1 3-phosphoshikimate 1-carboxyvinyltransferase [A... | 422 | 2e-141 | 57% |
| WP_137794442.1 3-phosphoshikimate 1-carboxyvinyltransferase [C... | 422 | 2e-141 | 55% |
| WP_058348970.1 3-phosphoshikimate 1-carboxyvinyltransferase [C... | 422 | 2e-141 | 57% |
| WP_064313179.1 3-phosphoshikimate 1-carboxyvinyltransferase [S... | 422 | 2e-141 | 56% |
| WP_180141723.1 3-phosphoshikimate 1-carboxyvinyltransferase [S... | 422 | 2e-141 | 54% |
| WP_057884396.1 3-phosphoshikimate 1-carboxyvinyltransferase [A... | 422 | 2e-141 | 56% |
| WP_148378743.1 3-phosphoshikimate 1-carboxyvinyltransferase [B... | 422 | 2e-141 | 55% |
| MAH84831.1 3-phosphoshikimate 1-carboxyvinyltransferase [Magne... | 422 | 2e-141 | 54% |
| WP_021777738.1 3-phosphoshikimate 1-carboxyvinyltransferase [C... | 422 | 2e-141 | 53% |
| WP_099286340.1 3-phosphoshikimate 1-carboxyvinyltransferase [G... | 422 | 2e-141 | 54% |
| WP_184715364.1 3-phosphoshikimate 1-carboxyvinyltransferase [C... | 422 | 2e-141 | 56% |
| WP_037534619.1 3-phosphoshikimate 1-carboxyvinyltransferase [S... | 422 | 2e-141 | 56% |
| WP_064224658.1 3-phosphoshikimate 1-carboxyvinyltransferase [S... | 422 | 2e-141 | 54% |
| MBG62671.1 3-phosphoshikimate 1-carboxyvinyltransferase [Roseo... | 422 | 2e-141 | 53% |
| WP_116838541.1 3-phosphoshikimate 1-carboxyvinyltransferase [S... | 422 | 2e-141 | 54% |
| WP_028793819.1 3-phosphoshikimate 1-carboxyvinyltransferase [T... | 422 | 2e-141 | 56% |
| NPD66905.1 3-phosphoshikimate 1-carboxyvinyltransferase [Aceto... | 422 | 2e-141 | 54% |
| NNE84732.1 3-phosphoshikimate 1-carboxyvinyltransferase [Alpha... | 422 | 2e-141 | 54% |
| WP_160616430.1 3-phosphoshikimate 1-carboxyvinyltransferase [A... | 422 | 2e-141 | 57% |
| WP_027257635.1 3-phosphoshikimate 1-carboxyvinyltransferase [L... | 422 | 2e-141 | 54% |
| WP_066843746.1 3-phosphoshikimate 1-carboxyvinyltransferase [C... | 421 | 2e-141 | 60% |
| WP_055805257.1 3-phosphoshikimate 1-carboxyvinyltransferase [B... | 422 | 3e-141 | 54% |
| WP_043643070.1 3-phosphoshikimate 1-carboxyvinyltransferase [C... | 422 | 3e-141 | 53% |
| WP_028958451.1 3-phosphoshikimate 1-carboxyvinyltransferase [S... | 422 | 3e-141 | 55% |
| WP_112863837.1 3-phosphoshikimate 1-carboxyvinyltransferase [B... | 421 | 3e-141 | 54% |
| WP_010164034.1 3-phosphoshikimate 1-carboxyvinyltransferase [S... | 422 | 3e-141 | 55% |
| WP_029603913.1 3-phosphoshikimate 1-carboxyvinyltransferase [K... | 422 | 3e-141 | 54% |
| WP_147080063.1 3-phosphoshikimate 1-carboxyvinyltransferase [S... | 422 | 3e-141 | 54% |
| WP_115978540.1 3-phosphoshikimate 1-carboxyvinyltransferase [R... | 422 | 3e-141 | 55% |
| WP_102154172.1 MULTISPECIES: 3-phosphoshikimate 1-carboxyvinyl... | 422 | 3e-141 | 57% |
| PZO02199.1 3-phosphoshikimate 1-carboxyvinyltransferase [Alpha... | 421 | 3e-141 | 55% |
| WP_137139362.1 3-phosphoshikimate 1-carboxyvinyltransferase [A... | 422 | 3e-141 | 57% |
| WP_066687922.1 3-phosphoshikimate 1-carboxyvinyltransferase [C... | 421 | 3e-141 | 56% |
| WP_146570203.1 3-phosphoshikimate 1-carboxyvinyltransferase [S... | 421 | 3e-141 | 55% |
| WP_056024747.1 MULTISPECIES: 3-phosphoshikimate 1-carboxyvinyl... | 421 | 3e-141 | 56% |
| MBS06296.1 3-phosphoshikimate 1-carboxyvinyltransferase [Marin... | 422 | 3e-141 | 52% |
| WP_086648286.1 3-phosphoshikimate 1-carboxyvinyltransferase [G... | 421 | 4e-141 | 55% |
| MBM61051.1 3-phosphoshikimate 1-carboxyvinyltransferase [Rhodo... | 421 | 4e-141 | 53% |
| PZU73779.1 3-phosphoshikimate 1-carboxyvinyltransferase [Brevu... | 421 | 4e-141 | 55% |
| WP_148276707.1 3-phosphoshikimate 1-carboxyvinyltransferase [S... | 423 | 4e-141 | 55% |
| WP_047403883.1 3-phosphoshikimate 1-carboxyvinyltransferase [C... | 421 | 4e-141 | 57% |
| VUX46825.1 3-phosphoshikimate 1-carboxyvinyltransferase [Candi... | 421 | 4e-141 | 54% |
| WP_153548573.1 3-phosphoshikimate 1-carboxyvinyltransferase [E... | 421 | 4e-141 | 54% |
| WP_121482245.1 3-phosphoshikimate 1-carboxyvinyltransferase [B... | 421 | 4e-141 | 55% |
| HBK08165.1 TPA: 3-phosphoshikimate 1-carboxyvinyltransferase [... | 421 | 4e-141 | 55% |
| RPF90843.1 3-phosphoshikimate 1-carboxyvinyltransferase [Rhodo... | 421 | 5e-141 | 51% |
| NQZ46008.1 3-phosphoshikimate 1-carboxyvinyltransferase [Eryth... | 421 | 5e-141 | 55% |
| WP_073145406.1 3-phosphoshikimate 1-carboxyvinyltransferase [L... | 421 | 5e-141 | 54% |
| WP_142788685.1 3-phosphoshikimate 1-carboxyvinyltransferase [E... | 421 | 5e-141 | 55% |
| QMU56943.1 3-phosphoshikimate 1-carboxyvinyltransferase [Boseo... | 421 | 5e-141 | 55% |
| WP_133365106.1 3-phosphoshikimate 1-carboxyvinyltransferase [Q... | 421 | 6e-141 | 56% |
| WP_124111248.1 3-phosphoshikimate 1-carboxyvinyltransferase [M... | 421 | 6e-141 | 55% |
| WP_165014292.1 3-phosphoshikimate 1-carboxyvinyltransferase [R... | 421 | 6e-141 | 57% |
| WP_132949983.1 3-phosphoshikimate 1-carboxyvinyltransferase [R... | 421 | 6e-141 | 56% |
| WP_139035705.1 3-phosphoshikimate 1-carboxyvinyltransferase [M... | 421 | 6e-141 | 59% |
| PCJ06265.1 3-phosphoshikimate 1-carboxyvinyltransferase [Rhodo... | 421 | 6e-141 | 54% |
| WP_165115380.1 3-phosphoshikimate 1-carboxyvinyltransferase [B... | 421 | 6e-141 | 54% |
| MBC6443640.1 3-phosphoshikimate 1-carboxyvinyltransferase [Rho... | 421 | 6e-141 | 53% |
| WP_099575295.1 3-phosphoshikimate 1-carboxyvinyltransferase [C... | 421 | 6e-141 | 56% |
| OFW88955.1 3-phosphoshikimate 1-carboxyvinyltransferase [Alpha... | 421 | 6e-141 | 54% |
| WP_056419987.1 3-phosphoshikimate 1-carboxyvinyltransferase [S... | 421 | 7e-141 | 56% |
| RCL81548.1 3-phosphoshikimate 1-carboxyvinyltransferase [Rhodo... | 421 | 7e-141 | 51% |
| WP_138466785.1 3-phosphoshikimate 1-carboxyvinyltransferase [P... | 421 | 7e-141 | 54% |

| WP_101246837.1 MULTISPECIES: 3-phosphoshikimate 1-carboxyvinyl... | 421 | 7e-141 | 55% |
| --- | --- | --- | --- |
| WP_055753320.1 3-phosphoshikimate 1-carboxyvinyltransferase [B... | 420 | 8e-141 | 54% |
| WP_121165320.1 3-phosphoshikimate 1-carboxyvinyltransferase [R... | 421 | 8e-141 | 53% |
| WP_188258921.1 3-phosphoshikimate 1-carboxyvinyltransferase [A... | 421 | 8e-141 | 56% |
| SUS08181.1 3-phosphoshikimate 1-carboxyvinyltransferase [uncul... | 421 | 8e-141 | 53% |
| SPU47285.1 3-phosphoshikimate 1-carboxyvinyltransferase [Brevu... | 421 | 8e-141 | 55% |
| HBU15069.1 TPA: 3-phosphoshikimate 1-carboxyvinyltransferase [... | 420 | 8e-141 | 56% |
| WP_109468800.1 3-phosphoshikimate 1-carboxyvinyltransferase [R... | 421 | 9e-141 | 57% |
| MBA4767299.1 3-phosphoshikimate 1-carboxyvinyltransferase [Por... | 420 | 9e-141 | 58% |
| WP_133030356.1 3-phosphoshikimate 1-carboxyvinyltransferase [S... | 420 | 1e-140 | 56% |
| WP_190275484.1 3-phosphoshikimate 1-carboxyvinyltransferase [C... | 420 | 1e-140 | 52% |
| NQV61747.1 3-phosphoshikimate 1-carboxyvinyltransferase [Alpha... | 420 | 1e-140 | 55% |
| WP_087121382.1 3-phosphoshikimate 1-carboxyvinyltransferase [B... | 420 | 1e-140 | 55% |
| WP_107961666.1 3-phosphoshikimate 1-carboxyvinyltransferase [S... | 420 | 1e-140 | 56% |
| WP_166573036.1 3-phosphoshikimate 1-carboxyvinyltransferase [C... | 420 | 1e-140 | 56% |
| WP_158921841.1 3-phosphoshikimate 1-carboxyvinyltransferase [A... | 420 | 1e-140 | 56% |
| OJY77421.1 3-phosphoshikimate 1-carboxyvinyltransferase [Rhodo... | 421 | 1e-140 | 53% |
| WP_010921418.1 3-phosphoshikimate 1-carboxyvinyltransferase [C... | 420 | 1e-140 | 56% |
| WP_093148425.1 3-phosphoshikimate 1-carboxyvinyltransferase [T... | 420 | 1e-140 | 56% |
| GGX51461.1 3-phosphoshikimate 1-carboxyvinyltransferase [Tatey... | 419 | 1e-140 | 56% |
| WP_081463090.1 3-phosphoshikimate 1-carboxyvinyltransferase [M... | 420 | 1e-140 | 55% |
| EGP09679.1 5-enolpyruvylshikimate-3-phosphate synthase [Bradyr... | 419 | 1e-140 | 58% |
| WP_145688674.1 MULTISPECIES: 3-phosphoshikimate 1-carboxyvinyl... | 420 | 1e-140 | 56% |
| PCH96059.1 3-phosphoshikimate 1-carboxyvinyltransferase [Rhodo... | 420 | 1e-140 | 55% |
| ADE39493.1 5-enolpyruvylshikimate-3-phosphate synthase [Candid... | 420 | 1e-140 | 52% |
| TAL34803.1 3-phosphoshikimate 1-carboxyvinyltransferase [Alpha... | 420 | 1e-140 | 55% |
| WP_054120107.1 3-phosphoshikimate 1-carboxyvinyltransferase [P... | 420 | 1e-140 | 57% |
| WP_120078348.1 3-phosphoshikimate 1-carboxyvinyltransferase [E... | 420 | 1e-140 | 57% |
| WP_187762671.1 3-phosphoshikimate 1-carboxyvinyltransferase [S... | 420 | 1e-140 | 55% |
| WP_174286832.1 3-phosphoshikimate 1-carboxyvinyltransferase [S... | 420 | 2e-140 | 56% |
| WP_167719596.1 3-phosphoshikimate 1-carboxyvinyltransferase [T... | 420 | 2e-140 | 55% |
| MBA3325987.1 3-phosphoshikimate 1-carboxyvinyltransferase [Rho... | 420 | 2e-140 | 56% |
| WP_090479739.1 3-phosphoshikimate 1-carboxyvinyltransferase [E... | 419 | 2e-140 | 57% |
| WP_076659194.1 3-phosphoshikimate 1-carboxyvinyltransferase [Y... | 420 | 2e-140 | 56% |
| WP_125159594.1 3-phosphoshikimate 1-carboxyvinyltransferase [C... | 419 | 2e-140 | 56% |
| WP_086617439.1 3-phosphoshikimate 1-carboxyvinyltransferase [P... | 419 | 2e-140 | 58% |
| OYW29162.1 3-phosphoshikimate 1-carboxyvinyltransferase [Caulo... | 419 | 2e-140 | 56% |
| WP_114096222.1 3-phosphoshikimate 1-carboxyvinyltransferase [T... | 420 | 2e-140 | 55% |
| WP_093923157.1 3-phosphoshikimate 1-carboxyvinyltransferase [S... | 420 | 2e-140 | 54% |
| MBA96987.1 3-phosphoshikimate 1-carboxyvinyltransferase [Roseo... | 420 | 2e-140 | 55% |
| SPU55624.1 3-phosphoshikimate 1-carboxyvinyltransferase [Brevu... | 421 | 2e-140 | 54% |
| WP_095998514.1 3-phosphoshikimate 1-carboxyvinyltransferase [S... | 419 | 2e-140 | 55% |
| WP_192964475.1 3-phosphoshikimate 1-carboxyvinyltransferase [P... | 419 | 2e-140 | 55% |
| KRA64603.1 3-phosphoshikimate 1-carboxyvinyltransferase [Caulo... | 419 | 2e-140 | 56% |
| WP_067538731.1 3-phosphoshikimate 1-carboxyvinyltransferase [A... | 419 | 2e-140 | 57% |
| WP_166620623.1 3-phosphoshikimate 1-carboxyvinyltransferase [P... | 419 | 2e-140 | 54% |
| WP_156811942.1 3-phosphoshikimate 1-carboxyvinyltransferase [R... | 420 | 2e-140 | 55% |
| RVT85760.1 3-phosphoshikimate 1-carboxyvinyltransferase [Rhodo... | 419 | 2e-140 | 54% |
| MBC8238933.1 3-phosphoshikimate 1-carboxyvinyltransferase [Alp... | 419 | 2e-140 | 54% |
| TCP71367.1 3-phosphoshikimate 1-carboxyvinyltransferase [Sphin... | 420 | 2e-140 | 56% |
| WP_134015767.1 3-phosphoshikimate 1-carboxyvinyltransferase [L... | 419 | 3e-140 | 54% |
| MBE1295833.1 3-phosphoshikimate 1-carboxyvinyltransferase [Rho... | 419 | 3e-140 | 55% |
| WP_045809451.1 MULTISPECIES: 3-phosphoshikimate 1-carboxyvinyl... | 419 | 3e-140 | 54% |
| AKQ43278.1 3-phosphoshikimate 1-carboxyvinyltransferase [Eryth... | 419 | 3e-140 | 56% |
| WP_088914551.1 3-phosphoshikimate 1-carboxyvinyltransferase [P... | 419 | 3e-140 | 57% |
| WP_106745720.1 3-phosphoshikimate 1-carboxyvinyltransferase [Y... | 419 | 3e-140 | 56% |
| PCI99841.1 3-phosphoshikimate 1-carboxyvinyltransferase [OCS11... | 419 | 3e-140 | 52% |
| MBC6412723.1 3-phosphoshikimate 1-carboxyvinyltransferase [Hyp... | 419 | 3e-140 | 54% |
| WP_127903458.1 3-phosphoshikimate 1-carboxyvinyltransferase [S... | 419 | 3e-140 | 56% |
| RZJ93701.1 3-phosphoshikimate 1-carboxyvinyltransferase [Brevu... | 419 | 3e-140 | 54% |
| HIM41753.1 TPA: 3-phosphoshikimate 1-carboxyvinyltransferase [... | 419 | 3e-140 | 55% |
| OYY65358.1 3-phosphoshikimate 1-carboxyvinyltransferase [Sphin... | 419 | 3e-140 | 55% |
| KPQ07631.1 3-phosphoshikimate 1-carboxyvinyltransferase AroA [... | 419 | 3e-140 | 56% |
| MBQ05115.1 3-phosphoshikimate 1-carboxyvinyltransferase [Roseo... | 419 | 3e-140 | 52% |
| WP_047264218.1 3-phosphoshikimate 1-carboxyvinyltransferase [C... | 419 | 3e-140 | 50% |
| PTQ65552.1 3-phosphoshikimate 1-carboxyvinyltransferase [Sphin... | 420 | 3e-140 | 56% |
| WP_188641555.1 3-phosphoshikimate 1-carboxyvinyltransferase [B... | 419 | 3e-140 | 57% |
| RZM10818.1 3-phosphoshikimate 1-carboxyvinyltransferase [Sphin... | 418 | 4e-140 | 58% |
| PZT94726.1 3-phosphoshikimate 1-carboxyvinyltransferase [Brevu... | 418 | 4e-140 | 55% |
| WP_132096231.1 3-phosphoshikimate 1-carboxyvinyltransferase [C... | 419 | 4e-140 | 55% |
| WP_013081129.1 3-phosphoshikimate 1-carboxyvinyltransferase [C... | 419 | 4e-140 | 56% |
| TNF59098.1 3-phosphoshikimate 1-carboxyvinyltransferase [Rhodo... | 419 | 4e-140 | 55% |

| HAJ85011.1 TPA: 3-phosphoshikimate 1-carboxyvinyltransferase [... | 418 | 4e-140 | 54% |
| --- | --- | --- | --- |
| MAP82075.1 3-phosphoshikimate 1-carboxyvinyltransferase [Marin... | 419 | 4e-140 | 52% |
| MBC6402499.1 3-phosphoshikimate 1-carboxyvinyltransferase [Hyp... | 419 | 4e-140 | 53% |
| WP_132738984.1 3-phosphoshikimate 1-carboxyvinyltransferase [S... | 419 | 4e-140 | 56% |
| WP_155142525.1 3-phosphoshikimate 1-carboxyvinyltransferase [P... | 419 | 4e-140 | 56% |
| PKP90858.1 3-phosphoshikimate 1-carboxyvinyltransferase [Alpha... | 418 | 5e-140 | 57% |
| WP_101711182.1 3-phosphoshikimate 1-carboxyvinyltransferase [C... | 418 | 5e-140 | 56% |
| WP_056757969.1 MULTISPECIES: 3-phosphoshikimate 1-carboxyvinyl... | 418 | 5e-140 | 55% |
| WP_056456663.1 3-phosphoshikimate 1-carboxyvinyltransferase [C... | 418 | 5e-140 | 56% |
| WP_109101210.1 3-phosphoshikimate 1-carboxyvinyltransferase [C... | 419 | 5e-140 | 56% |
| WP_160618147.1 3-phosphoshikimate 1-carboxyvinyltransferase [B... | 418 | 5e-140 | 51% |
| WP_168196907.1 3-phosphoshikimate 1-carboxyvinyltransferase [B... | 418 | 5e-140 | 55% |
| TVZ32703.1 3-phosphoshikimate 1-carboxyvinyltransferase [Rhodo... | 419 | 5e-140 | 55% |
| WP_008264235.1 MULTISPECIES: 3-phosphoshikimate 1-carboxyvinyl... | 418 | 6e-140 | 55% |
| WP_172267363.1 3-phosphoshikimate 1-carboxyvinyltransferase [C... | 418 | 6e-140 | 56% |
| WP_174452289.1 3-phosphoshikimate 1-carboxyvinyltransferase [A... | 418 | 6e-140 | 57% |
| WP_116569404.1 3-phosphoshikimate 1-carboxyvinyltransferase [C... | 418 | 6e-140 | 56% |
| WP_081531547.1 3-phosphoshikimate 1-carboxyvinyltransferase [R... | 419 | 6e-140 | 56% |
| MBE90041.1 3-phosphoshikimate 1-carboxyvinyltransferase [Rhodo... | 418 | 6e-140 | 53% |
| WP_160682476.1 3-phosphoshikimate 1-carboxyvinyltransferase [A... | 420 | 6e-140 | 56% |
| AGH96986.1 5-Enolpyruvylshikimate-3-phosphate synthase [Micavi... | 418 | 6e-140 | 54% |
| PZO88915.1 3-phosphoshikimate 1-carboxyvinyltransferase [Micav... | 419 | 6e-140 | 54% |
| RME64154.1 3-phosphoshikimate 1-carboxyvinyltransferase [Alpha... | 418 | 7e-140 | 55% |
| NCX54820.1 3-phosphoshikimate 1-carboxyvinyltransferase [Rhodo... | 418 | 7e-140 | 52% |
| EQB32171.1 3-phosphoshikimate 1-carboxyvinyltransferase [Sphin... | 417 | 8e-140 | 58% |
| WP_167298029.1 3-phosphoshikimate 1-carboxyvinyltransferase [S... | 418 | 8e-140 | 55% |
| WP_017665371.1 3-phosphoshikimate 1-carboxyvinyltransferase [P... | 418 | 8e-140 | 58% |
| WP_025824011.1 3-phosphoshikimate 1-carboxyvinyltransferase [A... | 418 | 8e-140 | 53% |
| RZP19260.1 3-phosphoshikimate 1-carboxyvinyltransferase [Eryth... | 419 | 8e-140 | 57% |
| WP_113257182.1 3-phosphoshikimate 1-carboxyvinyltransferase [R... | 418 | 8e-140 | 54% |
| WP_192974338.1 3-phosphoshikimate 1-carboxyvinyltransferase [S... | 418 | 8e-140 | 56% |
| MBF0130428.1 3-phosphoshikimate 1-carboxyvinyltransferase [Alp... | 418 | 8e-140 | 55% |
| WP_007425404.1 3-phosphoshikimate 1-carboxyvinyltransferase [O... | 418 | 9e-140 | 55% |
| WP_168622452.1 3-phosphoshikimate 1-carboxyvinyltransferase [R... | 418 | 9e-140 | 54% |
| OUS35445.1 3-phosphoshikimate 1-carboxyvinyltransferase [Rhodo... | 418 | 9e-140 | 54% |
| MBC7478111.1 3-phosphoshikimate 1-carboxyvinyltransferase [Pse... | 418 | 9e-140 | 55% |
| WP_146586322.1 3-phosphoshikimate 1-carboxyvinyltransferase [P... | 418 | 9e-140 | 54% |
| WP_147845347.1 3-phosphoshikimate 1-carboxyvinyltransferase [E... | 419 | 9e-140 | 57% |
| PIV75645.1 3-phosphoshikimate 1-carboxyvinyltransferase [Rhodo... | 418 | 1e-139 | 54% |
| MAW54711.1 3-phosphoshikimate 1-carboxyvinyltransferase [Rhodo... | 417 | 1e-139 | 54% |
| RFP85765.1 3-phosphoshikimate 1-carboxyvinyltransferase [Rhodo... | 418 | 1e-139 | 55% |
| MBA3836369.1 3-phosphoshikimate 1-carboxyvinyltransferase [Zym... | 418 | 1e-139 | 57% |
| WP_114292823.1 3-phosphoshikimate 1-carboxyvinyltransferase [S... | 418 | 1e-139 | 55% |
| NQZ72221.1 3-phosphoshikimate 1-carboxyvinyltransferase [Dinor... | 418 | 1e-139 | 54% |
| WP_077808064.1 3-phosphoshikimate 1-carboxyvinyltransferase [N... | 418 | 1e-139 | 53% |
| PPR62931.1 3-phosphoshikimate 1-carboxyvinyltransferase [Alpha... | 418 | 1e-139 | 55% |
| HBG98407.1 TPA: 3-phosphoshikimate 1-carboxyvinyltransferase [... | 418 | 1e-139 | 54% |
| WP_082863765.1 3-phosphoshikimate 1-carboxyvinyltransferase [E... | 419 | 1e-139 | 56% |
| MBD3835690.1 3-phosphoshikimate 1-carboxyvinyltransferase [Bre... | 417 | 1e-139 | 54% |
| MSO80931.1 3-phosphoshikimate 1-carboxyvinyltransferase [Alpha... | 418 | 1e-139 | 54% |
| OMG59346.1 3-phosphoshikimate 1-carboxyvinyltransferase [Brevu... | 417 | 1e-139 | 54% |
| HBC14747.1 TPA: 3-phosphoshikimate 1-carboxyvinyltransferase [... | 418 | 1e-139 | 57% |
| WP_120109960.1 3-phosphoshikimate 1-carboxyvinyltransferase [A... | 418 | 1e-139 | 56% |
| TCP98748.1 3-phosphoshikimate 1-carboxyvinyltransferase [Sphin... | 419 | 1e-139 | 56% |
| MBF0392817.1 3-phosphoshikimate 1-carboxyvinyltransferase [Alp... | 417 | 1e-139 | 56% |
| WP_111536074.1 3-phosphoshikimate 1-carboxyvinyltransferase [H... | 417 | 1e-139 | 55% |
| MBD82454.1 3-phosphoshikimate 1-carboxyvinyltransferase [Sulfi... | 417 | 1e-139 | 54% |
| QDH74461.1 3-phosphoshikimate 1-carboxyvinyltransferase [Brevu... | 417 | 1e-139 | 55% |
| MBE9556176.1 3-phosphoshikimate 1-carboxyvinyltransferase [Pro... | 417 | 1e-139 | 55% |
| WP_185885663.1 3-phosphoshikimate 1-carboxyvinyltransferase [C... | 417 | 1e-139 | 59% |
| NBP48938.1 3-phosphoshikimate 1-carboxyvinyltransferase [Alpha... | 417 | 2e-139 | 53% |
| HCA13343.1 TPA: 3-phosphoshikimate 1-carboxyvinyltransferase [... | 417 | 2e-139 | 53% |
| MAI22287.1 3-phosphoshikimate 1-carboxyvinyltransferase [Rhodo... | 417 | 2e-139 | 53% |
| WP_067787222.1 3-phosphoshikimate 1-carboxyvinyltransferase [A... | 417 | 2e-139 | 57% |
| MAA55911.1 3-phosphoshikimate 1-carboxyvinyltransferase [Rhodo... | 417 | 2e-139 | 53% |
| GGC21143.1 3-phosphoshikimate 1-carboxyvinyltransferase [Asaia... | 417 | 2e-139 | 54% |
| NCC20946.1 3-phosphoshikimate 1-carboxyvinyltransferase [Alpha... | 417 | 2e-139 | 55% |
| WP_188424889.1 3-phosphoshikimate 1-carboxyvinyltransferase [A... | 417 | 2e-139 | 54% |
| WP_112316208.1 3-phosphoshikimate 1-carboxyvinyltransferase [R... | 417 | 2e-139 | 56% |
| WP_148262713.1 3-phosphoshikimate 1-carboxyvinyltransferase [A... | 417 | 2e-139 | 54% |
| HBF61037.1 TPA: 3-phosphoshikimate 1-carboxyvinyltransferase [... | 417 | 2e-139 | 55% |
| WP_166131842.1 3-phosphoshikimate 1-carboxyvinyltransferase [B... | 417 | 2e-139 | 55% |

| WP_174086575.1 3-phosphoshikimate 1-carboxyvinyltransferase [B... | 417 | 2e-139 | 54% |
| --- | --- | --- | --- |
| PDH62400.1 3-phosphoshikimate 1-carboxyvinyltransferase [SAR11... | 417 | 2e-139 | 53% |
| MBC8020220.1 3-phosphoshikimate 1-carboxyvinyltransferase [Met... | 417 | 2e-139 | 55% |
| WP_146539359.1 3-phosphoshikimate 1-carboxyvinyltransferase [R... | 417 | 3e-139 | 56% |
| WP_025054384.1 3-phosphoshikimate 1-carboxyvinyltransferase [S... | 417 | 3e-139 | 54% |
| NOZ41861.1 3-phosphoshikimate 1-carboxyvinyltransferase [Alpha... | 416 | 3e-139 | 52% |
| OBX18425.1 3-phosphoshikimate 1-carboxyvinyltransferase [Eryth... | 417 | 3e-139 | 58% |
| WP_089929071.1 3-phosphoshikimate 1-carboxyvinyltransferase [C... | 416 | 3e-139 | 56% |
| OHC83051.1 3-phosphoshikimate 1-carboxyvinyltransferase [Rhodo... | 417 | 3e-139 | 53% |
| NBX06604.1 3-phosphoshikimate 1-carboxyvinyltransferase [Prote... | 417 | 3e-139 | 51% |
| WP_056060405.1 3-phosphoshikimate 1-carboxyvinyltransferase [S... | 416 | 3e-139 | 56% |
| WP_110377228.1 3-phosphoshikimate 1-carboxyvinyltransferase [C... | 417 | 3e-139 | 57% |
| WP_145916930.1 3-phosphoshikimate 1-carboxyvinyltransferase [E... | 417 | 3e-139 | 58% |
| WP_194745493.1 MULTISPECIES: 3-phosphoshikimate 1-carboxyvinyl... | 417 | 3e-139 | 54% |
| KXV61388.1 3-phosphoshikimate 1-carboxyvinyltransferase [Aceto... | 417 | 3e-139 | 54% |
| WP_034904860.1 3-phosphoshikimate 1-carboxyvinyltransferase [E... | 416 | 3e-139 | 56% |
| MBD3728399.1 3-phosphoshikimate 1-carboxyvinyltransferase [Sph... | 416 | 3e-139 | 57% |
| OJY52942.1 3-phosphoshikimate 1-carboxyvinyltransferase [Sphin... | 416 | 4e-139 | 55% |
| WP_115416978.1 3-phosphoshikimate 1-carboxyvinyltransferase [E... | 416 | 4e-139 | 58% |
| HCQ82933.1 TPA: 3-phosphoshikimate 1-carboxyvinyltransferase [... | 416 | 4e-139 | 55% |
| WP_110279904.1 3-phosphoshikimate 1-carboxyvinyltransferase [L... | 416 | 4e-139 | 55% |
| MAV72555.1 3-phosphoshikimate 1-carboxyvinyltransferase [Marin... | 416 | 4e-139 | 53% |
| WP_048846548.1 3-phosphoshikimate 1-carboxyvinyltransferase [T... | 416 | 4e-139 | 54% |
| WP_086640657.1 MULTISPECIES: 3-phosphoshikimate 1-carboxyvinyl... | 416 | 4e-139 | 53% |
| WP_150097206.1 3-phosphoshikimate 1-carboxyvinyltransferase [B... | 416 | 4e-139 | 56% |
| SPU42695.1 3-phosphoshikimate 1-carboxyvinyltransferase [Brevu... | 416 | 4e-139 | 54% |
| WP_035378874.1 3-phosphoshikimate 1-carboxyvinyltransferase [A... | 416 | 4e-139 | 54% |
| WP_156842108.1 3-phosphoshikimate 1-carboxyvinyltransferase [N... | 416 | 4e-139 | 55% |
| WP_192126858.1 3-phosphoshikimate 1-carboxyvinyltransferase [S... | 416 | 4e-139 | 57% |
| WP_141375876.1 3-phosphoshikimate 1-carboxyvinyltransferase [A... | 416 | 4e-139 | 54% |
| WP_072857758.1 3-phosphoshikimate 1-carboxyvinyltransferase [L... | 416 | 4e-139 | 56% |
| WP_125901226.1 3-phosphoshikimate 1-carboxyvinyltransferase [B... | 416 | 4e-139 | 55% |
| WP_035080289.1 3-phosphoshikimate 1-carboxyvinyltransferase [C... | 416 | 4e-139 | 56% |
| WP_058987672.1 MULTISPECIES: 3-phosphoshikimate 1-carboxyvinyl... | 416 | 4e-139 | 53% |
| WP_176266287.1 3-phosphoshikimate 1-carboxyvinyltransferase [A... | 416 | 4e-139 | 57% |
| KNH01133.1 3-phosphoshikimate 1-carboxyvinyltransferase [Eryth... | 416 | 4e-139 | 58% |
| MSP00136.1 3-phosphoshikimate 1-carboxyvinyltransferase [Aceto... | 416 | 4e-139 | 56% |
| MAI68692.1 3-phosphoshikimate 1-carboxyvinyltransferase [Rhodo... | 416 | 5e-139 | 55% |
| MBF9043511.1 3-phosphoshikimate 1-carboxyvinyltransferase [Rho... | 416 | 5e-139 | 54% |
| WP_110120433.1 MULTISPECIES: 3-phosphoshikimate 1-carboxyvinyl... | 416 | 5e-139 | 57% |
| WP_025059821.1 3-phosphoshikimate 1-carboxyvinyltransferase [S... | 416 | 5e-139 | 55% |
| MSO66396.1 3-phosphoshikimate 1-carboxyvinyltransferase [Pseud... | 416 | 5e-139 | 55% |
| MSO54945.1 3-phosphoshikimate 1-carboxyvinyltransferase [Rhodo... | 416 | 6e-139 | 56% |
| ODT99234.1 3-phosphoshikimate 1-carboxyvinyltransferase [Rhodo... | 416 | 6e-139 | 57% |
| NQW54267.1 3-phosphoshikimate 1-carboxyvinyltransferase [Rhodo... | 417 | 6e-139 | 56% |
| WP_192605201.1 3-phosphoshikimate 1-carboxyvinyltransferase [S... | 416 | 6e-139 | 56% |
| WP_083437397.1 3-phosphoshikimate 1-carboxyvinyltransferase [E... | 417 | 6e-139 | 58% |
| WP_164661833.1 3-phosphoshikimate 1-carboxyvinyltransferase [T... | 416 | 6e-139 | 56% |
| WP_193323458.1 MULTISPECIES: 3-phosphoshikimate 1-carboxyvinyl... | 417 | 6e-139 | 54% |
| MAX74339.1 3-phosphoshikimate 1-carboxyvinyltransferase [Nioel... | 416 | 7e-139 | 55% |
| NCP13542.1 3-phosphoshikimate 1-carboxyvinyltransferase [Sphin... | 415 | 7e-139 | 57% |
| AEP08498.1 3-phosphoshikimate 1-carboxyvinyltransferase [Micav... | 415 | 7e-139 | 55% |
| MBO42418.1 3-phosphoshikimate 1-carboxyvinyltransferase [Rhodo... | 416 | 7e-139 | 52% |
| WP_086659435.1 3-phosphoshikimate 1-carboxyvinyltransferase [A... | 416 | 8e-139 | 54% |
| MBF9028901.1 3-phosphoshikimate 1-carboxyvinyltransferase [Rho... | 416 | 8e-139 | 54% |
| WP_057792190.1 3-phosphoshikimate 1-carboxyvinyltransferase [R... | 416 | 8e-139 | 55% |
| HBV79308.1 TPA: 3-phosphoshikimate 1-carboxyvinyltransferase [... | 416 | 8e-139 | 52% |
| TIW71151.1 3-phosphoshikimate 1-carboxyvinyltransferase [Mesor... | 409 | 8e-139 | 79% |
| WP_087208371.1 3-phosphoshikimate 1-carboxyvinyltransferase [Y... | 416 | 8e-139 | 56% |
| MAK79532.1 3-phosphoshikimate 1-carboxyvinyltransferase [Marin... | 416 | 9e-139 | 52% |
| KZY93836.1 3-phosphoshikimate 1-carboxyvinyltransferase [Eryth... | 415 | 9e-139 | 57% |
| PXW54745.1 3-phosphoshikimate 1-carboxyvinyltransferase [Chela... | 416 | 9e-139 | 57% |
| WP_121023390.1 3-phosphoshikimate 1-carboxyvinyltransferase [L... | 416 | 9e-139 | 53% |
| CEF40908.1 cytidylate kinase [Acetobacter senegalensis] | 416 | 9e-139 | 53% |
| WP_114815901.1 3-phosphoshikimate 1-carboxyvinyltransferase [B... | 415 | 1e-138 | 55% |
| EDP65319.1 5-enolpyruvylshikimate-3-phosphate synthase [alpha ... | 415 | 1e-138 | 56% |
| WP_119511472.1 3-phosphoshikimate 1-carboxyvinyltransferase [A... | 416 | 1e-138 | 56% |
| WP_167954001.1 3-phosphoshikimate 1-carboxyvinyltransferase [S... | 415 | 1e-138 | 56% |
| WP_182040947.1 3-phosphoshikimate 1-carboxyvinyltransferase [B... | 415 | 1e-138 | 52% |
| WP_056438234.1 3-phosphoshikimate 1-carboxyvinyltransferase [C... | 415 | 1e-138 | 55% |
| HAW36675.1 TPA: 3-phosphoshikimate 1-carboxyvinyltransferase [... | 416 | 1e-138 | 58% |
| WP_184269660.1 3-phosphoshikimate 1-carboxyvinyltransferase [B... | 415 | 1e-138 | 53% |

| NRB16019.1 3-phosphoshikimate 1-carboxyvinyltransferase [Rhodo... | 415 | 1e-138 | 54% |
| --- | --- | --- | --- |
| MAJ94148.1 3-phosphoshikimate 1-carboxyvinyltransferase [Marin... | 415 | 1e-138 | 53% |
| OYV41314.1 3-phosphoshikimate 1-carboxyvinyltransferase [Rhodo... | 415 | 1e-138 | 53% |
| OJU54180.1 3-phosphoshikimate 1-carboxyvinyltransferase [Brevu... | 415 | 1e-138 | 54% |
| GBE44732.1 3-phosphoshikimate 1-carboxyvinyltransferase [bacte... | 415 | 1e-138 | 56% |
| WP_115366393.1 3-phosphoshikimate 1-carboxyvinyltransferase [A... | 414 | 1e-138 | 57% |
| RCL77452.1 3-phosphoshikimate 1-carboxyvinyltransferase [PS1 c... | 416 | 1e-138 | 52% |
| WP_008229026.1 3-phosphoshikimate 1-carboxyvinyltransferase [R... | 415 | 1e-138 | 54% |
| WP_048599346.1 3-phosphoshikimate 1-carboxyvinyltransferase [N... | 415 | 1e-138 | 54% |
| WP_192848368.1 3-phosphoshikimate 1-carboxyvinyltransferase [B... | 415 | 1e-138 | 53% |
| WP_082847510.1 MULTISPECIES: 3-phosphoshikimate 1-carboxyvinyl... | 416 | 1e-138 | 57% |
| WP_106161338.1 3-phosphoshikimate 1-carboxyvinyltransferase [H... | 415 | 1e-138 | 53% |
| WP_183414821.1 3-phosphoshikimate 1-carboxyvinyltransferase [L... | 414 | 2e-138 | 56% |
| MAJ20827.1 3-phosphoshikimate 1-carboxyvinyltransferase [Marin... | 415 | 2e-138 | 52% |
| MAC61793.1 3-phosphoshikimate 1-carboxyvinyltransferase [SAR11... | 414 | 2e-138 | 48% |
| WP_097281144.1 3-phosphoshikimate 1-carboxyvinyltransferase [C... | 415 | 2e-138 | 55% |
| OYW22275.1 3-phosphoshikimate 1-carboxyvinyltransferase [Sphin... | 415 | 2e-138 | 54% |
| MAG06420.1 3-phosphoshikimate 1-carboxyvinyltransferase [Sphin... | 416 | 2e-138 | 57% |
| MBG53102.1 3-phosphoshikimate 1-carboxyvinyltransferase [Rhodo... | 411 | 2e-138 | 62% |
| MAI18799.1 3-phosphoshikimate 1-carboxyvinyltransferase [Marin... | 415 | 2e-138 | 51% |
| WP_055038151.1 3-phosphoshikimate 1-carboxyvinyltransferase [B... | 414 | 2e-138 | 56% |
| WP_135193714.1 3-phosphoshikimate 1-carboxyvinyltransferase [B... | 414 | 2e-138 | 54% |
| WP_085587739.1 3-phosphoshikimate 1-carboxyvinyltransferase [T... | 414 | 2e-138 | 56% |
| RPG86915.1 3-phosphoshikimate 1-carboxyvinyltransferase [Candi... | 414 | 2e-138 | 53% |
| WP_193079363.1 3-phosphoshikimate 1-carboxyvinyltransferase [P... | 414 | 2e-138 | 56% |
| WP_012284197.1 3-phosphoshikimate 1-carboxyvinyltransferase [C... | 414 | 2e-138 | 55% |
| WP_154725801.1 3-phosphoshikimate 1-carboxyvinyltransferase [B... | 414 | 2e-138 | 54% |
| WP_152949456.1 3-phosphoshikimate 1-carboxyvinyltransferase [B... | 414 | 2e-138 | 54% |
| WP_043557972.1 MULTISPECIES: 3-phosphoshikimate 1-carboxyvinyl... | 414 | 2e-138 | 53% |
| WP_145912945.1 3-phosphoshikimate 1-carboxyvinyltransferase [A... | 414 | 2e-138 | 54% |
| WP_106204306.1 3-phosphoshikimate 1-carboxyvinyltransferase [P... | 414 | 2e-138 | 56% |
| MBL23462.1 3-phosphoshikimate 1-carboxyvinyltransferase [Rhodo... | 414 | 3e-138 | 54% |
| MBC7167432.1 3-phosphoshikimate 1-carboxyvinyltransferase [Phe... | 414 | 3e-138 | 56% |
| WP_108023778.1 3-phosphoshikimate 1-carboxyvinyltransferase [R... | 414 | 3e-138 | 56% |
| ANF53423.1 3-phosphoshikimate 1-carboxyvinyltransferase [Brevu... | 414 | 3e-138 | 55% |
| MBD3834762.1 3-phosphoshikimate 1-carboxyvinyltransferase [Bre... | 414 | 3e-138 | 55% |
| WP_159849092.1 3-phosphoshikimate 1-carboxyvinyltransferase [B... | 414 | 3e-138 | 54% |
| WP_028038989.1 3-phosphoshikimate 1-carboxyvinyltransferase [C... | 414 | 3e-138 | 55% |
| HBY43577.1 TPA: 3-phosphoshikimate 1-carboxyvinyltransferase [... | 413 | 3e-138 | 55% |
| WP_025063179.1 3-phosphoshikimate 1-carboxyvinyltransferase [S... | 414 | 3e-138 | 55% |
| WP_086002697.1 3-phosphoshikimate 1-carboxyvinyltransferase [R... | 415 | 3e-138 | 57% |
| WP_045543633.1 3-phosphoshikimate 1-carboxyvinyltransferase [A... | 414 | 3e-138 | 54% |
| MAC31911.1 3-phosphoshikimate 1-carboxyvinyltransferase [Eryth... | 415 | 3e-138 | 57% |
| VVC53596.1 3-phosphoshikimate 1-carboxyvinyltransferase [Beije... | 412 | 3e-138 | 59% |
| WP_149278686.1 3-phosphoshikimate 1-carboxyvinyltransferase [A... | 414 | 3e-138 | 55% |
| WP_183111615.1 3-phosphoshikimate 1-carboxyvinyltransferase [S... | 414 | 3e-138 | 55% |
| WP_066555098.1 3-phosphoshikimate 1-carboxyvinyltransferase [B... | 414 | 3e-138 | 54% |
| MBA4793633.1 3-phosphoshikimate 1-carboxyvinyltransferase [Phe... | 414 | 3e-138 | 56% |
| NCX86883.1 3-phosphoshikimate 1-carboxyvinyltransferase [Rhodo... | 414 | 3e-138 | 50% |
| WP_158742224.1 3-phosphoshikimate 1-carboxyvinyltransferase [A... | 414 | 3e-138 | 54% |
| TMJ63974.1 3-phosphoshikimate 1-carboxyvinyltransferase [Alpha... | 414 | 3e-138 | 55% |
| NQX79709.1 3-phosphoshikimate 1-carboxyvinyltransferase [Hypho... | 414 | 4e-138 | 52% |
| ONG51495.1 3-phosphoshikimate 1-carboxyvinyltransferase [Roseo... | 414 | 4e-138 | 54% |
| WP_136393216.1 3-phosphoshikimate 1-carboxyvinyltransferase [G... | 414 | 4e-138 | 56% |
| NCG10635.1 3-phosphoshikimate 1-carboxyvinyltransferase [Alpha... | 414 | 4e-138 | 52% |
| WP_178824397.1 3-phosphoshikimate 1-carboxyvinyltransferase [B... | 414 | 4e-138 | 54% |
| WP_154981120.1 3-phosphoshikimate 1-carboxyvinyltransferase [B... | 414 | 4e-138 | 53% |
| WP_056731511.1 3-phosphoshikimate 1-carboxyvinyltransferase [P... | 413 | 4e-138 | 56% |
| VTO11842.1 3-phosphoshikimate 1-carboxyvinyltransferase [Brevu... | 414 | 4e-138 | 54% |
| WP_093329869.1 3-phosphoshikimate 1-carboxyvinyltransferase [S... | 414 | 4e-138 | 56% |
| GAL99224.1 3-phosphoshikimate 1-carboxyvinyltransferase [Aceto... | 414 | 4e-138 | 54% |
| MBA2588442.1 3-phosphoshikimate 1-carboxyvinyltransferase [Alp... | 414 | 5e-138 | 53% |
| WP_093665478.1 3-phosphoshikimate 1-carboxyvinyltransferase [S... | 413 | 5e-138 | 56% |
| HIF60492.1 TPA: 3-phosphoshikimate 1-carboxyvinyltransferase [... | 413 | 5e-138 | 51% |
| WP_085584324.1 3-phosphoshikimate 1-carboxyvinyltransferase [T... | 414 | 5e-138 | 56% |
| WP_189987216.1 3-phosphoshikimate 1-carboxyvinyltransferase [T... | 413 | 5e-138 | 58% |
| RZO31290.1 3-phosphoshikimate 1-carboxyvinyltransferase [SAR11... | 413 | 5e-138 | 51% |
| WP_137720929.1 3-phosphoshikimate 1-carboxyvinyltransferase [B... | 413 | 6e-138 | 54% |
| MBF9058274.1 3-phosphoshikimate 1-carboxyvinyltransferase [Rho... | 413 | 6e-138 | 56% |
| MAK64615.1 3-phosphoshikimate 1-carboxyvinyltransferase [Maric... | 413 | 6e-138 | 57% |
| WP_035307012.1 3-phosphoshikimate 1-carboxyvinyltransferase [B... | 413 | 7e-138 | 54% |
| PDH60158.1 3-phosphoshikimate 1-carboxyvinyltransferase [SAR11... | 413 | 7e-138 | 53% |

| WP_083747595.1 3-phosphoshikimate 1-carboxyvinyltransferase [R... | 414 | 7e-138 | 54% |
| --- | --- | --- | --- |
| HCN32997.1 TPA: 3-phosphoshikimate 1-carboxyvinyltransferase [... | 414 | 7e-138 | 52% |
| QIQ85858.1 3-phosphoshikimate 1-carboxyvinyltransferase [Eryth... | 413 | 7e-138 | 55% |
| MAF49552.1 3-phosphoshikimate 1-carboxyvinyltransferase [Rhodo... | 413 | 8e-138 | 52% |
| PIU07144.1 3-phosphoshikimate 1-carboxyvinyltransferase [Methy... | 413 | 8e-138 | 59% |
| WP_090187866.1 3-phosphoshikimate 1-carboxyvinyltransferase [L... | 413 | 8e-138 | 54% |
| WP_091749195.1 3-phosphoshikimate 1-carboxyvinyltransferase [B... | 412 | 9e-138 | 53% |
| WP_042694357.1 3-phosphoshikimate 1-carboxyvinyltransferase [A... | 412 | 9e-138 | 57% |
| WP_148285153.1 3-phosphoshikimate 1-carboxyvinyltransferase [M... | 412 | 1e-137 | 54% |
| WP_065272330.1 MULTISPECIES: 3-phosphoshikimate 1-carboxyvinyl... | 413 | 1e-137 | 55% |
| WP_072548972.1 3-phosphoshikimate 1-carboxyvinyltransferase [G... | 413 | 1e-137 | 53% |
| WP_081562843.1 3-phosphoshikimate 1-carboxyvinyltransferase [P... | 412 | 1e-137 | 53% |
| WP_175578919.1 3-phosphoshikimate 1-carboxyvinyltransferase [C... | 413 | 1e-137 | 57% |
| WP_188158369.1 3-phosphoshikimate 1-carboxyvinyltransferase [A... | 412 | 1e-137 | 55% |
| WP_076980473.1 3-phosphoshikimate 1-carboxyvinyltransferase [B... | 413 | 1e-137 | 55% |
| TAJ86659.1 3-phosphoshikimate 1-carboxyvinyltransferase [Reyra... | 412 | 1e-137 | 56% |
| NNL72927.1 3-phosphoshikimate 1-carboxyvinyltransferase [Silic... | 412 | 1e-137 | 54% |
| TDR85513.1 3-phosphoshikimate 1-carboxyvinyltransferase [Enter... | 412 | 1e-137 | 58% |
| OQW74591.1 3-phosphoshikimate 1-carboxyvinyltransferase [Prote... | 412 | 1e-137 | 55% |
| WP_021096679.1 3-phosphoshikimate 1-carboxyvinyltransferase [R... | 412 | 1e-137 | 53% |
| KZX90514.1 3-phosphoshikimate 1-carboxyvinyltransferase [Eryth... | 412 | 1e-137 | 57% |
| HAB36738.1 TPA: 3-phosphoshikimate 1-carboxyvinyltransferase [... | 412 | 1e-137 | 52% |
| MBC6406854.1 3-phosphoshikimate 1-carboxyvinyltransferase [Rho... | 412 | 1e-137 | 54% |
| MBR46410.1 3-phosphoshikimate 1-carboxyvinyltransferase [Rhodo... | 413 | 2e-137 | 52% |
| HCV88019.1 TPA: 3-phosphoshikimate 1-carboxyvinyltransferase [... | 412 | 2e-137 | 53% |
| HIJ43621.1 TPA: 3-phosphoshikimate 1-carboxyvinyltransferase [... | 412 | 2e-137 | 53% |
| MBF0373312.1 3-phosphoshikimate 1-carboxyvinyltransferase [Alp... | 412 | 2e-137 | 56% |
| MBA4808950.1 3-phosphoshikimate 1-carboxyvinyltransferase [Bre... | 412 | 2e-137 | 54% |
| WP_176249683.1 3-phosphoshikimate 1-carboxyvinyltransferase [S... | 412 | 2e-137 | 54% |
| WP_133774670.1 3-phosphoshikimate 1-carboxyvinyltransferase [E... | 412 | 2e-137 | 58% |
| WP_136941958.1 3-phosphoshikimate 1-carboxyvinyltransferase [S... | 412 | 2e-137 | 56% |
| RYY37136.1 3-phosphoshikimate 1-carboxyvinyltransferase [Sphin... | 410 | 2e-137 | 57% |
| NOR31147.1 3-phosphoshikimate 1-carboxyvinyltransferase [Sulfi... | 412 | 2e-137 | 54% |
| WP_105562590.1 MULTISPECIES: 3-phosphoshikimate 1-carboxyvinyl... | 412 | 2e-137 | 54% |
| WP_048445242.1 3-phosphoshikimate 1-carboxyvinyltransferase [M... | 412 | 2e-137 | 59% |
| WP_072573148.1 3-phosphoshikimate 1-carboxyvinyltransferase [G... | 412 | 2e-137 | 54% |
| WP_187449503.1 3-phosphoshikimate 1-carboxyvinyltransferase [C... | 412 | 2e-137 | 57% |
| MAK76856.1 3-phosphoshikimate 1-carboxyvinyltransferase [Nisae... | 412 | 2e-137 | 54% |
| WP_135375418.1 3-phosphoshikimate 1-carboxyvinyltransferase [B... | 412 | 2e-137 | 53% |
| NBR12456.1 3-phosphoshikimate 1-carboxyvinyltransferase [Alpha... | 409 | 2e-137 | 63% |
| NNG05013.1 3-phosphoshikimate 1-carboxyvinyltransferase [Inqui... | 412 | 2e-137 | 55% |
| WP_153925612.1 3-phosphoshikimate 1-carboxyvinyltransferase [B... | 411 | 3e-137 | 53% |
| WP_112999750.1 3-phosphoshikimate 1-carboxyvinyltransferase [B... | 411 | 3e-137 | 54% |
| MBI36389.1 3-phosphoshikimate 1-carboxyvinyltransferase [Alpha... | 412 | 3e-137 | 52% |
| WP_089994970.1 3-phosphoshikimate 1-carboxyvinyltransferase [C... | 412 | 3e-137 | 57% |
| WP_025287347.1 3-phosphoshikimate 1-carboxyvinyltransferase [G... | 412 | 3e-137 | 53% |
| PZO05222.1 3-phosphoshikimate 1-carboxyvinyltransferase [Alpha... | 411 | 3e-137 | 54% |
| MAP33721.1 3-phosphoshikimate 1-carboxyvinyltransferase [Ricke... | 411 | 3e-137 | 50% |
| HEV42187.1 TPA: 3-phosphoshikimate 1-carboxyvinyltransferase [... | 411 | 3e-137 | 54% |
| MBF0333133.1 3-phosphoshikimate 1-carboxyvinyltransferase [Alp... | 412 | 3e-137 | 55% |
| WP_050796724.1 3-phosphoshikimate 1-carboxyvinyltransferase [B... | 411 | 3e-137 | 54% |
| MXQ08878.1 3-phosphoshikimate 1-carboxyvinyltransferase [Alpha... | 411 | 3e-137 | 55% |
| WP_082835577.1 MULTISPECIES: 3-phosphoshikimate 1-carboxyvinyl... | 412 | 3e-137 | 57% |
| WP_125404604.1 3-phosphoshikimate 1-carboxyvinyltransferase [R... | 411 | 4e-137 | 57% |
| WP_093915641.1 3-phosphoshikimate 1-carboxyvinyltransferase [S... | 411 | 4e-137 | 54% |
| WP_086436048.1 3-phosphoshikimate 1-carboxyvinyltransferase [A... | 412 | 4e-137 | 56% |

ALIGNMENTS

>ARW80140.1 Ctp2 CP4 EPSPS [synthetic construct] [Zea mays] QLF98898.1 CTP2-CP4-EPSPS [synthetic construct] [Triticum aestivum] QLF98899.1 CTP2-CP4-EPSPS [synthetic construct] [Triticum aestivum]

Length=531

Score = 887 bits (2293), Expect = 0.0, Method: Compositional matrix adjust. Identities = 455/455 (100%), Positives = 455/455 (100%), Gaps = 0/455 (0%)

| Query | 1 | MLHGASSRPATARKSSGLSGTVRIPGDKSISHRSFMFGGLASGETRITGLLEGEDVINTG | 60 |
| --- | --- | --- | --- |
| Sbjct | 77 | MLHGASSRPATARKSSGLSGTVRIPGDKSISHRSFMFGGLASGETRITGLLEGEDVINTG MLHGASSRPATARKSSGLSGTVRIPGDKSISHRSFMFGGLASGETRITGLLEGEDVINTG | 136 |
| Query | 61 | KAMQAMGARIRKEGDTWIIDGVGNGGLLAPEAPLDFGNAATGCRLTMGLVGVYDFDSTFI KAMQAMGARIRKEGDTWIIDGVGNGGLLAPEAPLDFGNAATGCRLTMGLVGVYDFDSTFI | 120 |

| Sbjct | 137 | KAMQAMGARIRKEGDTWIIDGVGNGGLLAPEAPLDFGNAATGCRLTMGLVGVYDFDSTFI | 196 |
| --- | --- | --- | --- |
| Query | 121 | GDASLTKRPMGRVLNPLREMGVQVKSEDGDRLPVTLRGPKTPTPITYRVPMASAQVKSAV | 180 |
| Sbjct | 197 | GDASLTKRPMGRVLNPLREMGVQVKSEDGDRLPVTLRGPKTPTPITYRVPMASAQVKSAV GDASLTKRPMGRVLNPLREMGVQVKSEDGDRLPVTLRGPKTPTPITYRVPMASAQVKSAV | 256 |
| Query | 181 | LLAGLNTPGITTVIEPIMTRDHTEKMLQGFGANLTVETDADGVRTIRLEGRGKLTGQVID | 240 |
| Sbjct | 257 | LLAGLNTPGITTVIEPIMTRDHTEKMLQGFGANLTVETDADGVRTIRLEGRGKLTGQVID LLAGLNTPGITTVIEPIMTRDHTEKMLQGFGANLTVETDADGVRTIRLEGRGKLTGQVID | 316 |
| Query | 241 | VPGDPSSTAFPLVAALLVPGSDVTILNVLMNPTRTGLILTLQEMGADIEVINPRLAGGED | 300 |
| Sbjct | 317 | VPGDPSSTAFPLVAALLVPGSDVTILNVLMNPTRTGLILTLQEMGADIEVINPRLAGGED VPGDPSSTAFPLVAALLVPGSDVTILNVLMNPTRTGLILTLQEMGADIEVINPRLAGGED | 376 |
| Query | 301 | VADLRVRSSTLKGVTVPEDRAPSMIDEYPILavaaafaegaTVMNGLEELRVKESDRLSA | 360 |
| Sbjct | 377 | VADLRVRSSTLKGVTVPEDRAPSMIDEYPILAVAAAFAEGATVMNGLEELRVKESDRLSA VADLRVRSSTLKGVTVPEDRAPSMIDEYPILAVAAAFAEGATVMNGLEELRVKESDRLSA | 436 |
| Query | 361 | VANGLKLNGVDCDEGETSLVVRGRPDGKGLGNASGAAVATHLDHRIAMSFLVMGLVSENP | 420 |
| Sbjct | 437 | VANGLKLNGVDCDEGETSLVVRGRPDGKGLGNASGAAVATHLDHRIAMSFLVMGLVSENP VANGLKLNGVDCDEGETSLVVRGRPDGKGLGNASGAAVATHLDHRIAMSFLVMGLVSENP | 496 |
| Query  Sbjct | 421  497 | VTVDDATMIATSFPEFMDLMAGLGAKIELSDTKAA 455 VTVDDATMIATSFPEFMDLMAGLGAKIELSDTKAA VTVDDATMIATSFPEFMDLMAGLGAKIELSDTKAA 531 |  |

>ARW80139.1 CTP2 CP4-EPSPS [synthetic construct] [Zea mays] Length=531

Score = 885 bits (2287), Expect = 0.0, Method: Compositional matrix adjust.

| Identities | = 454/455 (99%), Positives = 454/455 (99%), Gaps = 0/455 (0%) |  |
| --- | --- | --- |
| Query 1 | MLHGASSRPATARKSSGLSGTVRIPGDKSISHRSFMFGGLASGETRITGLLEGEDVINTG | 60 |
| Sbjct 77 | MLHGASSRPATARKSSGLSGTVRIPGDKSISHRSFMFGGLASGETRITGLLEGEDVINTG MLHGASSRPATARKSSGLSGTVRIPGDKSISHRSFMFGGLASGETRITGLLEGEDVINTG | 136 |
| Query 61 | KAMQAMGARIRKEGDTWIIDGVGNGGLLAPEAPLDFGNAATGCRLTMGLVGVYDFDSTFI | 120 |
| Sbjct 137 | KAMQAMGARIRKEGDTWIIDGVGNGGLLAPEAPLDFGNAATGCRLTMGLVGVYDFDSTFI KAMQAMGARIRKEGDTWIIDGVGNGGLLAPEAPLDFGNAATGCRLTMGLVGVYDFDSTFI | 196 |
| Query 121 | GDASLTKRPMGRVLNPLREMGVQVKSEDGDRLPVTLRGPKTPTPITYRVPMASAQVKSAV | 180 |
| Sbjct 197 | GDASLTKRPMGRVLNPLREMGVQVKSEDGDRLPVTLRGPKTPTPITYRVPMASAQVKSAV GDASLTKRPMGRVLNPLREMGVQVKSEDGDRLPVTLRGPKTPTPITYRVPMASAQVKSAV | 256 |
| Query 181 | LLAGLNTPGITTVIEPIMTRDHTEKMLQGFGANLTVETDADGVRTIRLEGRGKLTGQVID | 240 |
| Sbjct 257 | LLAGLNTPGITTVIEPIMTRDHTEKMLQGFGAN TVETDADGVRTIRLEGRGKLTGQVID LLAGLNTPGITTVIEPIMTRDHTEKMLQGFGANPTVETDADGVRTIRLEGRGKLTGQVID | 316 |
| Query 241 | VPGDPSSTAFPLVAALLVPGSDVTILNVLMNPTRTGLILTLQEMGADIEVINPRLAGGED | 300 |
| Sbjct 317 | VPGDPSSTAFPLVAALLVPGSDVTILNVLMNPTRTGLILTLQEMGADIEVINPRLAGGED VPGDPSSTAFPLVAALLVPGSDVTILNVLMNPTRTGLILTLQEMGADIEVINPRLAGGED | 376 |
| Query 301 | VADLRVRSSTLKGVTVPEDRAPSMIDEYPILavaaafaegaTVMNGLEELRVKESDRLSA | 360 |
| Sbjct 377 | VADLRVRSSTLKGVTVPEDRAPSMIDEYPILAVAAAFAEGATVMNGLEELRVKESDRLSA VADLRVRSSTLKGVTVPEDRAPSMIDEYPILAVAAAFAEGATVMNGLEELRVKESDRLSA | 436 |
| Query 361 | VANGLKLNGVDCDEGETSLVVRGRPDGKGLGNASGAAVATHLDHRIAMSFLVMGLVSENP | 420 |
| Sbjct 437 | VANGLKLNGVDCDEGETSLVVRGRPDGKGLGNASGAAVATHLDHRIAMSFLVMGLVSENP VANGLKLNGVDCDEGETSLVVRGRPDGKGLGNASGAAVATHLDHRIAMSFLVMGLVSENP | 496 |
| Query 421  Sbjct 497 | VTVDDATMIATSFPEFMDLMAGLGAKIELSDTKAA 455 VTVDDATMIATSFPEFMDLMAGLGAKIELSDTKAA VTVDDATMIATSFPEFMDLMAGLGAKIELSDTKAA 531 |  |

```
>AAL67577.1 CP4EPSPS [Glycine max]
AAO17037.1 CP4EPSPS protein [synthetic construct] AEP17821.1 CP4 EPSPS [Expression vector pMON108080] AEP17824.1 CP4 EPSPS [Expression vector pMON98939] AEP17826.1 CP4 EPSPS [Expression vector pMON93914] AEP17829.1 CP4 EPSPS [Expression vector pMON81312]

AEP17832.1 CP4 EPSPS [Expression vector pMON100407] AEP17834.1 CP4 EPSPS [Expression vector pMON74532] AEP17836.1 CP4 EPSPS [Expression vector pMON82053]
Length=455

Score = 885 bits (2286), Expect = 0.0, Method: Compositional matrix adjust. Identities = 455/455 (100%), Positives = 455/455 (100%), Gaps = 0/455 (0%)

Query 1	MLHGASSRPATARKSSGLSGTVRIPGDKSISHRSFMFGGLASGETRITGLLEGEDVINTG 60 MLHGASSRPATARKSSGLSGTVRIPGDKSISHRSFMFGGLASGETRITGLLEGEDVINTG
Sbjct 1	MLHGASSRPATARKSSGLSGTVRIPGDKSISHRSFMFGGLASGETRITGLLEGEDVINTG 60

Query 61	KAMQAMGARIRKEGDTWIIDGVGNGGLLAPEAPLDFGNAATGCRLTMGLVGVYDFDSTFI 120 KAMQAMGARIRKEGDTWIIDGVGNGGLLAPEAPLDFGNAATGCRLTMGLVGVYDFDSTFI
Sbjct 61	KAMQAMGARIRKEGDTWIIDGVGNGGLLAPEAPLDFGNAATGCRLTMGLVGVYDFDSTFI 120

Query 121 GDASLTKRPMGRVLNPLREMGVQVKSEDGDRLPVTLRGPKTPTPITYRVPMASAQVKSAV 180 GDASLTKRPMGRVLNPLREMGVQVKSEDGDRLPVTLRGPKTPTPITYRVPMASAQVKSAV
Sbjct 121 GDASLTKRPMGRVLNPLREMGVQVKSEDGDRLPVTLRGPKTPTPITYRVPMASAQVKSAV 180

Query 181 LLAGLNTPGITTVIEPIMTRDHTEKMLQGFGANLTVETDADGVRTIRLEGRGKLTGQVID 240 LLAGLNTPGITTVIEPIMTRDHTEKMLQGFGANLTVETDADGVRTIRLEGRGKLTGQVID
Sbjct 181 LLAGLNTPGITTVIEPIMTRDHTEKMLQGFGANLTVETDADGVRTIRLEGRGKLTGQVID 240

Query 241 VPGDPSSTAFPLVAALLVPGSDVTILNVLMNPTRTGLILTLQEMGADIEVINPRLAGGED 300 VPGDPSSTAFPLVAALLVPGSDVTILNVLMNPTRTGLILTLQEMGADIEVINPRLAGGED
Sbjct 241 VPGDPSSTAFPLVAALLVPGSDVTILNVLMNPTRTGLILTLQEMGADIEVINPRLAGGED 300

Query 301 VADLRVRSSTLKGVTVPEDRAPSMIDEYPILavaaafaegaTVMNGLEELRVKESDRLSA 360 VADLRVRSSTLKGVTVPEDRAPSMIDEYPILAVAAAFAEGATVMNGLEELRVKESDRLSA
Sbjct 301 VADLRVRSSTLKGVTVPEDRAPSMIDEYPILAVAAAFAEGATVMNGLEELRVKESDRLSA 360

Query 361 VANGLKLNGVDCDEGETSLVVRGRPDGKGLGNASGAAVATHLDHRIAMSFLVMGLVSENP 420 VANGLKLNGVDCDEGETSLVVRGRPDGKGLGNASGAAVATHLDHRIAMSFLVMGLVSENP
Sbjct 361 VANGLKLNGVDCDEGETSLVVRGRPDGKGLGNASGAAVATHLDHRIAMSFLVMGLVSENP 420

Query 421 VTVDDATMIATSFPEFMDLMAGLGAKIELSDTKAA 455 VTVDDATMIATSFPEFMDLMAGLGAKIELSDTKAA
Sbjct 421 VTVDDATMIATSFPEFMDLMAGLGAKIELSDTKAA 455

>AII71485.1 5-enolpyruvulshikimate-3-phosphate synthase [standard reference vector pMCS]
Length=455

Score = 882 bits (2280), Expect = 0.0, Method: Compositional matrix adjust.
```

| Identities | = 454/455 (99%), Positives = 454/455 (99%), Gaps = 0/455 (0%) |  |
| --- | --- | --- |
| Query 1 | MLHGASSRPATARKSSGLSGTVRIPGDKSISHRSFMFGGLASGETRITGLLEGEDVINTG | 60 |
| Sbjct 1 | M HGASSRPATARKSSGLSGTVRIPGDKSISHRSFMFGGLASGETRITGLLEGEDVINTG MAHGASSRPATARKSSGLSGTVRIPGDKSISHRSFMFGGLASGETRITGLLEGEDVINTG | 60 |
| Query 61 | KAMQAMGARIRKEGDTWIIDGVGNGGLLAPEAPLDFGNAATGCRLTMGLVGVYDFDSTFI | 120 |
| Sbjct 61 | KAMQAMGARIRKEGDTWIIDGVGNGGLLAPEAPLDFGNAATGCRLTMGLVGVYDFDSTFI KAMQAMGARIRKEGDTWIIDGVGNGGLLAPEAPLDFGNAATGCRLTMGLVGVYDFDSTFI | 120 |
| Query 121 | GDASLTKRPMGRVLNPLREMGVQVKSEDGDRLPVTLRGPKTPTPITYRVPMASAQVKSAV | 180 |
| Sbjct 121 | GDASLTKRPMGRVLNPLREMGVQVKSEDGDRLPVTLRGPKTPTPITYRVPMASAQVKSAV GDASLTKRPMGRVLNPLREMGVQVKSEDGDRLPVTLRGPKTPTPITYRVPMASAQVKSAV | 180 |
| Query 181 | LLAGLNTPGITTVIEPIMTRDHTEKMLQGFGANLTVETDADGVRTIRLEGRGKLTGQVID | 240 |
| Sbjct 181 | LLAGLNTPGITTVIEPIMTRDHTEKMLQGFGANLTVETDADGVRTIRLEGRGKLTGQVID LLAGLNTPGITTVIEPIMTRDHTEKMLQGFGANLTVETDADGVRTIRLEGRGKLTGQVID | 240 |
| Query 241 | VPGDPSSTAFPLVAALLVPGSDVTILNVLMNPTRTGLILTLQEMGADIEVINPRLAGGED | 300 |
| Sbjct 241 | VPGDPSSTAFPLVAALLVPGSDVTILNVLMNPTRTGLILTLQEMGADIEVINPRLAGGED VPGDPSSTAFPLVAALLVPGSDVTILNVLMNPTRTGLILTLQEMGADIEVINPRLAGGED | 300 |
| Query 301 | VADLRVRSSTLKGVTVPEDRAPSMIDEYPILavaaafaegaTVMNGLEELRVKESDRLSA | 360 |
| Sbjct 301 | VADLRVRSSTLKGVTVPEDRAPSMIDEYPILAVAAAFAEGATVMNGLEELRVKESDRLSA  VADLRVRSSTLKGVTVPEDRAPSMIDEYPILAVAAAFAEGATVMNGLEELRVKESDRLSA | 360 |

| Query | 361 | VANGLKLNGVDCDEGETSLVVRGRPDGKGLGNASGAAVATHLDHRIAMSFLVMGLVSENP 420 |
| --- | --- | --- |
| Sbjct | 361 | VANGLKLNGVDCDEGETSLVVRGRPDGKGLGNASGAAVATHLDHRIAMSFLVMGLVSENP VANGLKLNGVDCDEGETSLVVRGRPDGKGLGNASGAAVATHLDHRIAMSFLVMGLVSENP 420 |
| Query | 421 | VTVDDATMIATSFPEFMDLMAGLGAKIELSDTKAA 455 |
| Sbjct | 421 | VTVDDATMIATSFPEFMDLMAGLGAKIELSDTKAA  VTVDDATMIATSFPEFMDLMAGLGAKIELSDTKAA 455 |

>Q9R4E4.2 RecName: Full=3-phosphoshikimate 1-carboxyvinyltransferase; AltName:

Full=5-enolpyruvylshikimate-3-phosphate synthase; Short=EPSP synthase; Short=EPSPS [Agrobacterium sp. CP4]

2GG4_A CP4 EPSP synthase (unliganded) [Agrobacterium sp. CP4] 2GG6_A CP4 EPSP synthase liganded with S3P [Agrobacterium sp. CP4]

2GGA_A CP4 EPSP synthase liganded with S3P and Glyphosate [Agrobacterium sp. CP4]

Length=455

Score = 881 bits (2277), Expect = 0.0, Method: Compositional matrix adjust.

| Identities | = 454/455 (99%), Positives = 454/455 (99%), Gaps = 0/455 (0%) |  |
| --- | --- | --- |
| Query 1 | MLHGASSRPATARKSSGLSGTVRIPGDKSISHRSFMFGGLASGETRITGLLEGEDVINTG | 60 |
| Sbjct 1 | M HGASSRPATARKSSGLSGTVRIPGDKSISHRSFMFGGLASGETRITGLLEGEDVINTG MSHGASSRPATARKSSGLSGTVRIPGDKSISHRSFMFGGLASGETRITGLLEGEDVINTG | 60 |
| Query 61 | KAMQAMGARIRKEGDTWIIDGVGNGGLLAPEAPLDFGNAATGCRLTMGLVGVYDFDSTFI | 120 |
| Sbjct 61 | KAMQAMGARIRKEGDTWIIDGVGNGGLLAPEAPLDFGNAATGCRLTMGLVGVYDFDSTFI KAMQAMGARIRKEGDTWIIDGVGNGGLLAPEAPLDFGNAATGCRLTMGLVGVYDFDSTFI | 120 |
| Query 121 | GDASLTKRPMGRVLNPLREMGVQVKSEDGDRLPVTLRGPKTPTPITYRVPMASAQVKSAV | 180 |
| Sbjct 121 | GDASLTKRPMGRVLNPLREMGVQVKSEDGDRLPVTLRGPKTPTPITYRVPMASAQVKSAV GDASLTKRPMGRVLNPLREMGVQVKSEDGDRLPVTLRGPKTPTPITYRVPMASAQVKSAV | 180 |
| Query 181 | LLAGLNTPGITTVIEPIMTRDHTEKMLQGFGANLTVETDADGVRTIRLEGRGKLTGQVID | 240 |
| Sbjct 181 | LLAGLNTPGITTVIEPIMTRDHTEKMLQGFGANLTVETDADGVRTIRLEGRGKLTGQVID LLAGLNTPGITTVIEPIMTRDHTEKMLQGFGANLTVETDADGVRTIRLEGRGKLTGQVID | 240 |
| Query 241 | VPGDPSSTAFPLVAALLVPGSDVTILNVLMNPTRTGLILTLQEMGADIEVINPRLAGGED | 300 |
| Sbjct 241 | VPGDPSSTAFPLVAALLVPGSDVTILNVLMNPTRTGLILTLQEMGADIEVINPRLAGGED VPGDPSSTAFPLVAALLVPGSDVTILNVLMNPTRTGLILTLQEMGADIEVINPRLAGGED | 300 |
| Query 301 | VADLRVRSSTLKGVTVPEDRAPSMIDEYPILavaaafaegaTVMNGLEELRVKESDRLSA | 360 |
| Sbjct 301 | VADLRVRSSTLKGVTVPEDRAPSMIDEYPILAVAAAFAEGATVMNGLEELRVKESDRLSA VADLRVRSSTLKGVTVPEDRAPSMIDEYPILAVAAAFAEGATVMNGLEELRVKESDRLSA | 360 |
| Query 361 | VANGLKLNGVDCDEGETSLVVRGRPDGKGLGNASGAAVATHLDHRIAMSFLVMGLVSENP | 420 |
| Sbjct 361 | VANGLKLNGVDCDEGETSLVVRGRPDGKGLGNASGAAVATHLDHRIAMSFLVMGLVSENP VANGLKLNGVDCDEGETSLVVRGRPDGKGLGNASGAAVATHLDHRIAMSFLVMGLVSENP | 420 |
| Query 421  Sbjct 421 | VTVDDATMIATSFPEFMDLMAGLGAKIELSDTKAA 455 VTVDDATMIATSFPEFMDLMAGLGAKIELSDTKAA VTVDDATMIATSFPEFMDLMAGLGAKIELSDTKAA 455 |  |

>2GGD_A CP4 EPSP synthase Ala100Gly liganded with S3P and Glyphosate [Agrobacterium sp. CP4]

Length=455

Score = 879 bits (2271), Expect = 0.0, Method: Compositional matrix adjust.

| Identities | = 453/455 (99%), Positives = 453/455 (99%), Gaps = 0/455 (0%) |  |
| --- | --- | --- |
| Query 1 | MLHGASSRPATARKSSGLSGTVRIPGDKSISHRSFMFGGLASGETRITGLLEGEDVINTG | 60 |
| Sbjct 1 | M HGASSRPATARKSSGLSGTVRIPGDKSISHRSFMFGGLASGETRITGLLEGEDVINTG MSHGASSRPATARKSSGLSGTVRIPGDKSISHRSFMFGGLASGETRITGLLEGEDVINTG | 60 |
| Query 61 | KAMQAMGARIRKEGDTWIIDGVGNGGLLAPEAPLDFGNAATGCRLTMGLVGVYDFDSTFI | 120 |
| Sbjct 61 | KAMQAMGARIRKEGDTWIIDGVGNGGLLAPEAPLDFGNA TGCRLTMGLVGVYDFDSTFI KAMQAMGARIRKEGDTWIIDGVGNGGLLAPEAPLDFGNAGTGCRLTMGLVGVYDFDSTFI | 120 |
| Query 121 | GDASLTKRPMGRVLNPLREMGVQVKSEDGDRLPVTLRGPKTPTPITYRVPMASAQVKSAV | 180 |
| Sbjct 121 | GDASLTKRPMGRVLNPLREMGVQVKSEDGDRLPVTLRGPKTPTPITYRVPMASAQVKSAV  GDASLTKRPMGRVLNPLREMGVQVKSEDGDRLPVTLRGPKTPTPITYRVPMASAQVKSAV | 180 |

| Query | 181 | LLAGLNTPGITTVIEPIMTRDHTEKMLQGFGANLTVETDADGVRTIRLEGRGKLTGQVID | 240 |
| --- | --- | --- | --- |
| Sbjct | 181 | LLAGLNTPGITTVIEPIMTRDHTEKMLQGFGANLTVETDADGVRTIRLEGRGKLTGQVID LLAGLNTPGITTVIEPIMTRDHTEKMLQGFGANLTVETDADGVRTIRLEGRGKLTGQVID | 240 |
| Query | 241 | VPGDPSSTAFPLVAALLVPGSDVTILNVLMNPTRTGLILTLQEMGADIEVINPRLAGGED | 300 |
| Sbjct | 241 | VPGDPSSTAFPLVAALLVPGSDVTILNVLMNPTRTGLILTLQEMGADIEVINPRLAGGED VPGDPSSTAFPLVAALLVPGSDVTILNVLMNPTRTGLILTLQEMGADIEVINPRLAGGED | 300 |
| Query | 301 | VADLRVRSSTLKGVTVPEDRAPSMIDEYPILavaaafaegaTVMNGLEELRVKESDRLSA | 360 |
| Sbjct | 301 | VADLRVRSSTLKGVTVPEDRAPSMIDEYPILAVAAAFAEGATVMNGLEELRVKESDRLSA VADLRVRSSTLKGVTVPEDRAPSMIDEYPILAVAAAFAEGATVMNGLEELRVKESDRLSA | 360 |
| Query | 361 | VANGLKLNGVDCDEGETSLVVRGRPDGKGLGNASGAAVATHLDHRIAMSFLVMGLVSENP | 420 |
| Sbjct | 361 | VANGLKLNGVDCDEGETSLVVRGRPDGKGLGNASGAAVATHLDHRIAMSFLVMGLVSENP VANGLKLNGVDCDEGETSLVVRGRPDGKGLGNASGAAVATHLDHRIAMSFLVMGLVSENP | 420 |
| Query  Sbjct | 421  421 | VTVDDATMIATSFPEFMDLMAGLGAKIELSDTKAA 455 VTVDDATMIATSFPEFMDLMAGLGAKIELSDTKAA VTVDDATMIATSFPEFMDLMAGLGAKIELSDTKAA 455 |  |

>AII80555.1 5-enol-pyruvylshikimate-3-phospate synthase [synthetic construct] AQQ72801.1 EPSPS [synthetic construct]

BAD94823.1 5-enol-pyruvylshikimate-3-phospate synthase class 2 precursor [Glycine max]

Length=527

Score = 879 bits (2270), Expect = 0.0, Method: Compositional matrix adjust.

| Identities | = 452/455 (99%), Positives = 452/455 (99%), Gaps = 0/455 (0%) |  |
| --- | --- | --- |
| Query 1 | MLHGASSRPATARKSSGLSGTVRIPGDKSISHRSFMFGGLASGETRITGLLEGEDVINTG | 60 |
| Sbjct 73 | MLHGASSRPATARKSSGLSGTVRIPGDKSISHRSFMFGGLASGETRITGLLEGEDVINTG MLHGASSRPATARKSSGLSGTVRIPGDKSISHRSFMFGGLASGETRITGLLEGEDVINTG | 132 |
| Query 61 | KAMQAMGARIRKEGDTWIIDGVGNGGLLAPEAPLDFGNAATGCRLTMGLVGVYDFDSTFI | 120 |
| Sbjct 133 | KAMQAMGARIRKEGDTWIIDGVGNGGLLAPEAPLDFGNAATGCRLTMGLVGVYDFDSTFI KAMQAMGARIRKEGDTWIIDGVGNGGLLAPEAPLDFGNAATGCRLTMGLVGVYDFDSTFI | 192 |
| Query 121 | GDASLTKRPMGRVLNPLREMGVQVKSEDGDRLPVTLRGPKTPTPITYRVPMASAQVKSAV | 180 |
| Sbjct 193 | GDASLTKRPMGRVLNPLREMGVQVKSEDGDRLPVTLRGPKTPTPITYRVPMASAQVKSAV GDASLTKRPMGRVLNPLREMGVQVKSEDGDRLPVTLRGPKTPTPITYRVPMASAQVKSAV | 252 |
| Query 181 | LLAGLNTPGITTVIEPIMTRDHTEKMLQGFGANLTVETDADGVRTIRLEGRGKLTGQVID | 240 |
| Sbjct 253 | LLAGLNTPGITTVIEPIMT DHTEKMLQGFGANLTVETDADGVRTIRLEGRGKLTGQVID LLAGLNTPGITTVIEPIMTCDHTEKMLQGFGANLTVETDADGVRTIRLEGRGKLTGQVID | 312 |
| Query 241 | VPGDPSSTAFPLVAALLVPGSDVTILNVLMNPTRTGLILTLQEMGADIEVINPRLAGGED | 300 |
| Sbjct 313 | VPGDPSSTAFPLVAALLVPGSDVTILNVLMNPTRTGLILTLQEMGADIEVIN RLAGGED VPGDPSSTAFPLVAALLVPGSDVTILNVLMNPTRTGLILTLQEMGADIEVINLRLAGGED | 372 |
| Query 301 | VADLRVRSSTLKGVTVPEDRAPSMIDEYPILavaaafaegaTVMNGLEELRVKESDRLSA | 360 |
| Sbjct 373 | VADLRVRSSTLKGVTVPEDRAP MIDEYPILAVAAAFAEGATVMNGLEELRVKESDRLSA VADLRVRSSTLKGVTVPEDRAPPMIDEYPILAVAAAFAEGATVMNGLEELRVKESDRLSA | 432 |
| Query 361 | VANGLKLNGVDCDEGETSLVVRGRPDGKGLGNASGAAVATHLDHRIAMSFLVMGLVSENP | 420 |
| Sbjct 433 | VANGLKLNGVDCDEGETSLVVRGRPDGKGLGNASGAAVATHLDHRIAMSFLVMGLVSENP VANGLKLNGVDCDEGETSLVVRGRPDGKGLGNASGAAVATHLDHRIAMSFLVMGLVSENP | 492 |
| Query 421  Sbjct 493 | VTVDDATMIATSFPEFMDLMAGLGAKIELSDTKAA 455 VTVDDATMIATSFPEFMDLMAGLGAKIELSDTKAA VTVDDATMIATSFPEFMDLMAGLGAKIELSDTKAA 527 |  |

>ADZ55658.1 CP4EPSPS protein [synthetic construct] Length=482

Score = 877 bits (2267), Expect = 0.0, Method: Compositional matrix adjust. Identities = 452/455 (99%), Positives = 452/455 (99%), Gaps = 0/455 (0%)

Query 1	MLHGASSRPATARKSSGLSGTVRIPGDKSISHRSFMFGGLASGETRITGLLEGEDVINTG 60 MLHGASSRPATARKSSGLSGTVRIPGDKSISHRSFMFGGLASGETRITGLLEGEDVINTG

| Sbjct | 28 | MLHGASSRPATARKSSGLSGTVRIPGDKSISHRSFMFGGLASGETRITGLLEGEDVINTG | 87 |
| --- | --- | --- | --- |
| Query | 61 | KAMQAMGARIRKEGDTWIIDGVGNGGLLAPEAPLDFGNAATGCRLTMGLVGVYDFDSTFI | 120 |
| Sbjct | 88 | KAMQAMGARIRKEGDTWIIDGVGNGGLLAPEAPLDFGNAATGCRLTMGLVGVYDFDSTFI KAMQAMGARIRKEGDTWIIDGVGNGGLLAPEAPLDFGNAATGCRLTMGLVGVYDFDSTFI | 147 |
| Query | 121 | GDASLTKRPMGRVLNPLREMGVQVKSEDGDRLPVTLRGPKTPTPITYRVPMASAQVKSAV | 180 |
| Sbjct | 148 | GDASLTKRPMGRVLNPLREMGVQVKSEDGDRLPVTLRGPKTPTPITYRVPMASAQVKSAV GDASLTKRPMGRVLNPLREMGVQVKSEDGDRLPVTLRGPKTPTPITYRVPMASAQVKSAV | 207 |
| Query | 181 | LLAGLNTPGITTVIEPIMTRDHTEKMLQGFGANLTVETDADGVRTIRLEGRGKLTGQVID | 240 |
| Sbjct | 208 | LLAGLNTPGITTVIEPIMT DHTEKMLQGFGANLTVETDADGVRTIRLEGRGKLTGQVID LLAGLNTPGITTVIEPIMTCDHTEKMLQGFGANLTVETDADGVRTIRLEGRGKLTGQVID | 267 |
| Query | 241 | VPGDPSSTAFPLVAALLVPGSDVTILNVLMNPTRTGLILTLQEMGADIEVINPRLAGGED | 300 |
| Sbjct | 268 | VPGDPSSTAFPLVAALLVPGSDVTILNVLMNPTRTGLILTLQEMGADIEVIN RLAGGED VPGDPSSTAFPLVAALLVPGSDVTILNVLMNPTRTGLILTLQEMGADIEVINLRLAGGED | 327 |
| Query | 301 | VADLRVRSSTLKGVTVPEDRAPSMIDEYPILavaaafaegaTVMNGLEELRVKESDRLSA | 360 |
| Sbjct | 328 | VADLRVRSSTLKGVTVPEDRAP MIDEYPILAVAAAFAEGATVMNGLEELRVKESDRLSA VADLRVRSSTLKGVTVPEDRAPPMIDEYPILAVAAAFAEGATVMNGLEELRVKESDRLSA | 387 |
| Query | 361 | VANGLKLNGVDCDEGETSLVVRGRPDGKGLGNASGAAVATHLDHRIAMSFLVMGLVSENP | 420 |
| Sbjct | 388 | VANGLKLNGVDCDEGETSLVVRGRPDGKGLGNASGAAVATHLDHRIAMSFLVMGLVSENP VANGLKLNGVDCDEGETSLVVRGRPDGKGLGNASGAAVATHLDHRIAMSFLVMGLVSENP | 447 |
| Query  Sbjct | 421  448 | VTVDDATMIATSFPEFMDLMAGLGAKIELSDTKAA 455 VTVDDATMIATSFPEFMDLMAGLGAKIELSDTKAA VTVDDATMIATSFPEFMDLMAGLGAKIELSDTKAA 482 |  |

>AJW67920.1 CP4 EPSPS protein [synthetic construct] Length=455

Score = 874 bits (2259), Expect = 0.0, Method: Compositional matrix adjust.

| Identities | = 452/455 (99%), Positives = 452/455 (99%), Gaps = 0/455 (0%) |  |
| --- | --- | --- |
| Query 1 | MLHGASSRPATARKSSGLSGTVRIPGDKSISHRSFMFGGLASGETRITGLLEGEDVINTG | 60 |
| Sbjct 1 | MLHGASSRPATARKSSGLSGTVRIPGDKSISHRSFMFGGLASGETRITGLLEGEDVINTG MLHGASSRPATARKSSGLSGTVRIPGDKSISHRSFMFGGLASGETRITGLLEGEDVINTG | 60 |
| Query 61 | KAMQAMGARIRKEGDTWIIDGVGNGGLLAPEAPLDFGNAATGCRLTMGLVGVYDFDSTFI | 120 |
| Sbjct 61 | KAMQAMGARIRKEGDTWIIDGVGNGGLLAPEAPLDFGNAATGCRLTMGLVGVYDFDSTFI KAMQAMGARIRKEGDTWIIDGVGNGGLLAPEAPLDFGNAATGCRLTMGLVGVYDFDSTFI | 120 |
| Query 121 | GDASLTKRPMGRVLNPLREMGVQVKSEDGDRLPVTLRGPKTPTPITYRVPMASAQVKSAV | 180 |
| Sbjct 121 | GDASLTKRPMGRVLNPLREMGVQVKSEDGDRLPVTLRGPKTPTPITYRVPMASAQVKSAV GDASLTKRPMGRVLNPLREMGVQVKSEDGDRLPVTLRGPKTPTPITYRVPMASAQVKSAV | 180 |
| Query 181 | LLAGLNTPGITTVIEPIMTRDHTEKMLQGFGANLTVETDADGVRTIRLEGRGKLTGQVID | 240 |
| Sbjct 181 | LLAGLNTPGITTVIEPIMT DHTEKMLQGFGANLTVETDADGVRTIRLEGRGKLTGQVID LLAGLNTPGITTVIEPIMTCDHTEKMLQGFGANLTVETDADGVRTIRLEGRGKLTGQVID | 240 |
| Query 241 | VPGDPSSTAFPLVAALLVPGSDVTILNVLMNPTRTGLILTLQEMGADIEVINPRLAGGED | 300 |
| Sbjct 241 | VPGDPSSTAFPLVAALLVPGSDVTILNVLMNPTRTGLILTLQEMGADIEVIN RLAGGED VPGDPSSTAFPLVAALLVPGSDVTILNVLMNPTRTGLILTLQEMGADIEVINLRLAGGED | 300 |
| Query 301 | VADLRVRSSTLKGVTVPEDRAPSMIDEYPILavaaafaegaTVMNGLEELRVKESDRLSA | 360 |
| Sbjct 301 | VADLRVRSSTLKGVTVPEDRAP MIDEYPILAVAAAFAEGATVMNGLEELRVKESDRLSA VADLRVRSSTLKGVTVPEDRAPPMIDEYPILAVAAAFAEGATVMNGLEELRVKESDRLSA | 360 |
| Query 361 | VANGLKLNGVDCDEGETSLVVRGRPDGKGLGNASGAAVATHLDHRIAMSFLVMGLVSENP | 420 |
| Sbjct 361 | VANGLKLNGVDCDEGETSLVVRGRPDGKGLGNASGAAVATHLDHRIAMSFLVMGLVSENP VANGLKLNGVDCDEGETSLVVRGRPDGKGLGNASGAAVATHLDHRIAMSFLVMGLVSENP | 420 |
| Query 421  Sbjct 421 | VTVDDATMIATSFPEFMDLMAGLGAKIELSDTKAA 455 VTVDDATMIATSFPEFMDLMAGLGAKIELSDTKAA VTVDDATMIATSFPEFMDLMAGLGAKIELSDTKAA 455 |  |

>AEK64772.1 wheat CP4EPSPS protein [synthetic construct] Length=482

Score = 867 bits (2241), Expect = 0.0, Method: Compositional matrix adjust.

| Identities | = 448/455 (98%), Positives = 449/455 (99%), Gaps = 0/455 (0%) |  |
| --- | --- | --- |
| Query 1 | MLHGASSRPATARKSSGLSGTVRIPGDKSISHRSFMFGGLASGETRITGLLEGEDVINTG | 60 |
| Sbjct 28 | M HGASSRPATARKSSGLSGTVRIPGDKSISHRSFMFGGLASGETRITGLLEGEDVINTG MSHGASSRPATARKSSGLSGTVRIPGDKSISHRSFMFGGLASGETRITGLLEGEDVINTG | 87 |
| Query 61 | KAMQAMGARIRKEGDTWIIDGVGNGGLLAPEAPLDFGNAATGCRLTMGLVGVYDFDSTFI | 120 |
| Sbjct 88 | KAMQAMGARIRKEGDTWIIDGVGNGGLLAPEAPLDFGNAATGCRLTMGLVGVYDFDSTFI KAMQAMGARIRKEGDTWIIDGVGNGGLLAPEAPLDFGNAATGCRLTMGLVGVYDFDSTFI | 147 |
| Query 121 | GDASLTKRPMGRVLNPLREMGVQVKSEDGDRLPVTLRGPKTPTPITYRVPMASAQVKSAV | 180 |
| Sbjct 148 | GDASLTKRPMGRVLNPLREMGVQVKSEDGDRLPVTLRGPKTPTPITYRVPMASAQVKSAV GDASLTKRPMGRVLNPLREMGVQVKSEDGDRLPVTLRGPKTPTPITYRVPMASAQVKSAV | 207 |
| Query 181 | LLAGLNTPGITTVIEPIMTRDHTEKMLQGFGANLTVETDADGVRTIRLEGRGKLTGQVID | 240 |
| Sbjct 208 | LLAGLNTPGITTVIEPIMT DHTEKMLQGFGANLTVETDADGVRTIRLEGRGKLTGQVID LLAGLNTPGITTVIEPIMTCDHTEKMLQGFGANLTVETDADGVRTIRLEGRGKLTGQVID | 267 |
| Query 241 | VPGDPSSTAFPLVAALLVPGSDVTILNVLMNPTRTGLILTLQEMGADIEVINPRLAGGED | 300 |
| Sbjct 268 | VPGDPSSTAFPLVAALLVPGSDVTILNVLMNPTRTGLILTLQEMGADIEVIN RLAGGED VPGDPSSTAFPLVAALLVPGSDVTILNVLMNPTRTGLILTLQEMGADIEVINLRLAGGED | 327 |
| Query 301 | VADLRVRSSTLKGVTVPEDRAPSMIDEYPILavaaafaegaTVMNGLEELRVKESDRLSA | 360 |
| Sbjct 328 | VADLRVRSSTLKGVTVPEDRAP MIDEYPILAVAAAFAEGATVMNGLEELRVKESDRLSA VADLRVRSSTLKGVTVPEDRAPPMIDEYPILAVAAAFAEGATVMNGLEELRVKESDRLSA | 387 |
| Query 361 | VANGLKLNGVDCDEGETSLVVRGRPDGKGLGNASGAAVATHLDHRIAMSFLVMGLVSENP | 420 |
| Sbjct 388 | VANGLKLNGVDCDEGETSLVVRGRPDGKGLGNASGAAVATHLDHRIAMSFLVMGLVSENP VANGLKLNGVDCDEGETSLVVRGRPDGKGLGNASGAAVATHLDHRIAMSFLVMGLVSENP | 447 |
| Query 421  Sbjct 448 | VTVDDATMIATSFPEFMDLMAGLGAKIELSDTKAA 455 VTVDDATMIATSFPEFM LM GLGAKIELS+TKAA VTVDDATMIATSFPEFMGLMTGLGAKIELSETKAA 482 |  |

```
>2PQB_A CP4 EPSPS liganded with (R)-difluoromethyl tetrahedral intermediate analog [Agrobacterium sp. CP4]
2PQC_A CP4 EPSPS liganded with (R)-phosphonate tetrahedral reaction intermediate analog [Agrobacterium sp. CP4]
Length=445

Score = 863 bits (2229), Expect = 0.0, Method: Compositional matrix adjust. Identities = 445/445 (100%), Positives = 445/445 (100%), Gaps = 0/445 (0%)

Query 6	SSRPATARKSSGLSGTVRIPGDKSISHRSFMFGGLASGETRITGLLEGEDVINTGKAMQA 65 SSRPATARKSSGLSGTVRIPGDKSISHRSFMFGGLASGETRITGLLEGEDVINTGKAMQA
Sbjct 1	SSRPATARKSSGLSGTVRIPGDKSISHRSFMFGGLASGETRITGLLEGEDVINTGKAMQA 60

Query 66	MGARIRKEGDTWIIDGVGNGGLLAPEAPLDFGNAATGCRLTMGLVGVYDFDSTFIGDASL 125 MGARIRKEGDTWIIDGVGNGGLLAPEAPLDFGNAATGCRLTMGLVGVYDFDSTFIGDASL
Sbjct 61	MGARIRKEGDTWIIDGVGNGGLLAPEAPLDFGNAATGCRLTMGLVGVYDFDSTFIGDASL 120

Query 126 TKRPMGRVLNPLREMGVQVKSEDGDRLPVTLRGPKTPTPITYRVPMASAQVKSAVLLAGL 185 TKRPMGRVLNPLREMGVQVKSEDGDRLPVTLRGPKTPTPITYRVPMASAQVKSAVLLAGL
Sbjct 121 TKRPMGRVLNPLREMGVQVKSEDGDRLPVTLRGPKTPTPITYRVPMASAQVKSAVLLAGL 180

Query 186 NTPGITTVIEPIMTRDHTEKMLQGFGANLTVETDADGVRTIRLEGRGKLTGQVIDVPGDP 245 NTPGITTVIEPIMTRDHTEKMLQGFGANLTVETDADGVRTIRLEGRGKLTGQVIDVPGDP
Sbjct 181 NTPGITTVIEPIMTRDHTEKMLQGFGANLTVETDADGVRTIRLEGRGKLTGQVIDVPGDP 240

Query 246 SSTAFPLVAALLVPGSDVTILNVLMNPTRTGLILTLQEMGADIEVINPRLAGGEDVADLR 305 SSTAFPLVAALLVPGSDVTILNVLMNPTRTGLILTLQEMGADIEVINPRLAGGEDVADLR
Sbjct 241 SSTAFPLVAALLVPGSDVTILNVLMNPTRTGLILTLQEMGADIEVINPRLAGGEDVADLR 300

Query 306 VRSSTLKGVTVPEDRAPSMIDEYPILavaaafaegaTVMNGLEELRVKESDRLSAVANGL 365 VRSSTLKGVTVPEDRAPSMIDEYPILAVAAAFAEGATVMNGLEELRVKESDRLSAVANGL
Sbjct 301 VRSSTLKGVTVPEDRAPSMIDEYPILAVAAAFAEGATVMNGLEELRVKESDRLSAVANGL 360

Query 366 KLNGVDCDEGETSLVVRGRPDGKGLGNASGAAVATHLDHRIAMSFLVMGLVSENPVTVDD 425 KLNGVDCDEGETSLVVRGRPDGKGLGNASGAAVATHLDHRIAMSFLVMGLVSENPVTVDD
```

| Sbjct | 361 | KLNGVDCDEGETSLVVRGRPDGKGLGNASGAAVATHLDHRIAMSFLVMGLVSENPVTVDD 420 |
| --- | --- | --- |
| Query | 426 | ATMIATSFPEFMDLMAGLGAKIELS 450 |
| Sbjct | 421 | ATMIATSFPEFMDLMAGLGAKIELS  ATMIATSFPEFMDLMAGLGAKIELS 445 |

>2PQD_A A100G CP4 EPSPS liganded with (R)-difluoromethyl tetrahedral reaction intermediate analog [Agrobacterium sp.]

Length=445

Score = 860 bits (2223), Expect = 0.0, Method: Compositional matrix adjust.

| Identities | = 444/445 (99%), Positives = 444/445 (99%), Gaps = 0/445 (0%) |  |
| --- | --- | --- |
| Query 6 | SSRPATARKSSGLSGTVRIPGDKSISHRSFMFGGLASGETRITGLLEGEDVINTGKAMQA | 65 |
| Sbjct 1 | SSRPATARKSSGLSGTVRIPGDKSISHRSFMFGGLASGETRITGLLEGEDVINTGKAMQA SSRPATARKSSGLSGTVRIPGDKSISHRSFMFGGLASGETRITGLLEGEDVINTGKAMQA | 60 |
| Query 66 | MGARIRKEGDTWIIDGVGNGGLLAPEAPLDFGNAATGCRLTMGLVGVYDFDSTFIGDASL | 125 |
| Sbjct 61 | MGARIRKEGDTWIIDGVGNGGLLAPEAPLDFGNA TGCRLTMGLVGVYDFDSTFIGDASL MGARIRKEGDTWIIDGVGNGGLLAPEAPLDFGNAGTGCRLTMGLVGVYDFDSTFIGDASL | 120 |
| Query 126 | TKRPMGRVLNPLREMGVQVKSEDGDRLPVTLRGPKTPTPITYRVPMASAQVKSAVLLAGL | 185 |
| Sbjct 121 | TKRPMGRVLNPLREMGVQVKSEDGDRLPVTLRGPKTPTPITYRVPMASAQVKSAVLLAGL TKRPMGRVLNPLREMGVQVKSEDGDRLPVTLRGPKTPTPITYRVPMASAQVKSAVLLAGL | 180 |
| Query 186 | NTPGITTVIEPIMTRDHTEKMLQGFGANLTVETDADGVRTIRLEGRGKLTGQVIDVPGDP | 245 |
| Sbjct 181 | NTPGITTVIEPIMTRDHTEKMLQGFGANLTVETDADGVRTIRLEGRGKLTGQVIDVPGDP NTPGITTVIEPIMTRDHTEKMLQGFGANLTVETDADGVRTIRLEGRGKLTGQVIDVPGDP | 240 |
| Query 246 | SSTAFPLVAALLVPGSDVTILNVLMNPTRTGLILTLQEMGADIEVINPRLAGGEDVADLR | 305 |
| Sbjct 241 | SSTAFPLVAALLVPGSDVTILNVLMNPTRTGLILTLQEMGADIEVINPRLAGGEDVADLR SSTAFPLVAALLVPGSDVTILNVLMNPTRTGLILTLQEMGADIEVINPRLAGGEDVADLR | 300 |
| Query 306 | VRSSTLKGVTVPEDRAPSMIDEYPILavaaafaegaTVMNGLEELRVKESDRLSAVANGL | 365 |
| Sbjct 301 | VRSSTLKGVTVPEDRAPSMIDEYPILAVAAAFAEGATVMNGLEELRVKESDRLSAVANGL VRSSTLKGVTVPEDRAPSMIDEYPILAVAAAFAEGATVMNGLEELRVKESDRLSAVANGL | 360 |
| Query 366 | KLNGVDCDEGETSLVVRGRPDGKGLGNASGAAVATHLDHRIAMSFLVMGLVSENPVTVDD | 425 |
| Sbjct 361 | KLNGVDCDEGETSLVVRGRPDGKGLGNASGAAVATHLDHRIAMSFLVMGLVSENPVTVDD KLNGVDCDEGETSLVVRGRPDGKGLGNASGAAVATHLDHRIAMSFLVMGLVSENPVTVDD | 420 |
| Query 426  Sbjct 421 | ATMIATSFPEFMDLMAGLGAKIELS 450 ATMIATSFPEFMDLMAGLGAKIELS ATMIATSFPEFMDLMAGLGAKIELS 445 |  |

>WP_117368106.1 3-phosphoshikimate 1-carboxyvinyltransferase [Shinella sp. WSJ-2] RFZ88879.1 3-phosphoshikimate 1-carboxyvinyltransferase [Shinella sp. WSJ-2]

Length=451

Score = 854 bits (2206), Expect = 0.0, Method: Compositional matrix adjust.

| Identities | = 438/449 (98%), Positives = 446/449 (99%), Gaps = 0/449 (0%) |  |
| --- | --- | --- |
| Query 1 | MLHGASSRPATARKSSGLSGTVRIPGDKSISHRSFMFGGLASGETRITGLLEGEDVINTG | 60 |
| Sbjct 1 | M HGA++RPATARKSSGL+GTVRIPGDKSISHRSFMFGGLASGETRITGLLEGEDVINTG MSHGANARPATARKSSGLTGTVRIPGDKSISHRSFMFGGLASGETRITGLLEGEDVINTG | 60 |
| Query 61 | KAMQAMGARIRKEGDTWIIDGVGNGGLLAPEAPLDFGNAATGCRLTMGLVGVYDFDSTFI | 120 |
| Sbjct 61 | KAMQAMGA+IRKE DTWII+GVGNGGLLAPEAPLDFGNA TGCRLTMGLVGVYDFDSTFI KAMQAMGAKIRKERDTWIINGVGNGGLLAPEAPLDFGNAGTGCRLTMGLVGVYDFDSTFI | 120 |
| Query 121 | GDASLTKRPMGRVLNPLREMGVQVKSEDGDRLPVTLRGPKTPTPITYRVPMASAQVKSAV | 180 |
| Sbjct 121 | GDASLTKRPMGRVLNPLREMGVQVKSEDGDRLPVTLRGPKTPTPITYRVPMASAQVKSAV GDASLTKRPMGRVLNPLREMGVQVKSEDGDRLPVTLRGPKTPTPITYRVPMASAQVKSAV | 180 |
| Query 181 | LLAGLNTPGITTVIEPIMTRDHTEKMLQGFGANLTVETDADGVRTIRLEGRGKLTGQVID | 240 |
| Sbjct 181 | LLAGLNTPGITTVIEPIMTRDHTEKMLQGFGANLTVETDADGVRTIRLEGRGKLTGQVID LLAGLNTPGITTVIEPIMTRDHTEKMLQGFGANLTVETDADGVRTIRLEGRGKLTGQVID | 240 |
| Query 241 | VPGDPSSTAFPLVAALLVPGSDVTILNVLMNPTRTGLILTLQEMGADIEVINPRLAGGED VPGDPSSTAFPLVAALLVPGSDVTILNVLMNPTRTGLILTLQEMGADIEVINPRLAGGED | 300 |

| Sbjct | 241 | VPGDPSSTAFPLVAALLVPGSDVTILNVLMNPTRTGLILTLQEMGADIEVINPRLAGGED | 300 |
| --- | --- | --- | --- |
| Query | 301 | VADLRVRSSTLKGVTVPEDRAPSMIDEYPILavaaafaegaTVMNGLEELRVKESDRLSA | 360 |
| Sbjct | 301 | VADLRVRSSTLKGVTVPEDRAPSMIDEYP+LAVAAAFAEGATVMNGLEELRVKESDRLSA VADLRVRSSTLKGVTVPEDRAPSMIDEYPVLAVAAAFAEGATVMNGLEELRVKESDRLSA | 360 |
| Query | 361 | VANGLKLNGVDCDEGETSLVVRGRPDGKGLGNASGAAVATHLDHRIAMSFLVMGLVSENP | 420 |
| Sbjct | 361 | VANGLKLNGVDCDEGETSL+VRGRPDGKGLGNA+GAAVATHLDHRIAMSFLVMGLVSENP VANGLKLNGVDCDEGETSLIVRGRPDGKGLGNAAGAAVATHLDHRIAMSFLVMGLVSENP | 420 |
| Query  Sbjct | 421  421 | VTVDDATMIATSFPEFMDLMAGLGAKIEL 449 VTVDDATMIATSFPEFMDLMAGLGAKIEL VTVDDATMIATSFPEFMDLMAGLGAKIEL 449 |  |

>WP_160786970.1 3-phosphoshikimate 1-carboxyvinyltransferase [Shinella zoogloeoides] MXO01594.1 3-phosphoshikimate 1-carboxyvinyltransferase [Shinella zoogloeoides]

Length=455

Score = 848 bits (2192), Expect = 0.0, Method: Compositional matrix adjust.

| Identities | = 433/455 (95%), Positives = 444/455 (98%), Gaps = 0/455 (0%) |  |
| --- | --- | --- |
| Query 1 | MLHGASSRPATARKSSGLSGTVRIPGDKSISHRSFMFGGLASGETRITGLLEGEDVINTG | 60 |
| Sbjct 1 | M HGA +RPATARKSSGLSGTVRIPGDKSISHRSFMFGGLASGETRITGLLEGEDVINTG MSHGAIARPATARKSSGLSGTVRIPGDKSISHRSFMFGGLASGETRITGLLEGEDVINTG | 60 |
| Query 61 | KAMQAMGARIRKEGDTWIIDGVGNGGLLAPEAPLDFGNAATGCRLTMGLVGVYDFDSTFI | 120 |
| Sbjct 61 | KAMQAMGA+IRKEGDTWII+GVGNG LLAP+APLDFGNA TGCRLTMGLVGVYDFDSTFI KAMQAMGAKIRKEGDTWIINGVGNGALLAPQAPLDFGNAGTGCRLTMGLVGVYDFDSTFI | 120 |
| Query 121 | GDASLTKRPMGRVLNPLREMGVQVKSEDGDRLPVTLRGPKTPTPITYRVPMASAQVKSAV | 180 |
| Sbjct 121 | GDASLTKRPMGRVLNPLREMGVQV S +GDRLPVTLRGPKTPTPITYRVPMASAQVKSAV GDASLTKRPMGRVLNPLREMGVQVTSAEGDRLPVTLRGPKTPTPITYRVPMASAQVKSAV | 180 |
| Query 181 | LLAGLNTPGITTVIEPIMTRDHTEKMLQGFGANLTVETDADGVRTIRLEGRGKLTGQVID | 240 |
| Sbjct 181 | LLAGLNTPGITTVIEPIMTRDHTEKMLQGFGANLTV+TDADGVRTIRLEGRGKLTGQVID LLAGLNTPGITTVIEPIMTRDHTEKMLQGFGANLTVDTDADGVRTIRLEGRGKLTGQVID | 240 |
| Query 241 | VPGDPSSTAFPLVAALLVPGSDVTILNVLMNPTRTGLILTLQEMGADIEVINPRLAGGED | 300 |
| Sbjct 241 | VPGDPSSTAFPLVAALLVPGSDVTILNVLMNPTRTGLILTLQEMGADIE++N RLAGGED VPGDPSSTAFPLVAALLVPGSDVTILNVLMNPTRTGLILTLQEMGADIEILNARLAGGED | 300 |
| Query 301 | VADLRVRSSTLKGVTVPEDRAPSMIDEYPILavaaafaegaTVMNGLEELRVKESDRLSA | 360 |
| Sbjct 301 | +ADLRVRSS LKGVTVPEDRAPSMIDEYP+LAVAAAFAEG TVMNGLEELRVKESDRLSA MADLRVRSSALKGVTVPEDRAPSMIDEYPVLAVAAAFAEGTTVMNGLEELRVKESDRLSA | 360 |
| Query 361 | VANGLKLNGVDCDEGETSLVVRGRPDGKGLGNASGAAVATHLDHRIAMSFLVMGLVSENP | 420 |
| Sbjct 361 | VANGLKLNGVDCDEGETSLVVRGRPDGKGLGNASGAAVAT+LDHRIAMSFLVMGLVSENP VANGLKLNGVDCDEGETSLVVRGRPDGKGLGNASGAAVATYLDHRIAMSFLVMGLVSENP | 420 |
| Query 421  Sbjct 421 | VTVDDATMIATSFPEFMDLMAGLGAKIELSDTKAA 455 VTVDDATMIATSFPEFMDLM GLGAKIEL DTKAA VTVDDATMIATSFPEFMDLMKGLGAKIELHDTKAA 455 |  |

>WP_184145208.1 3-phosphoshikimate 1-carboxyvinyltransferase [Shinella fusca] MBB5043850.1 3-phosphoshikimate 1-carboxyvinyltransferase [Shinella fusca]

Length=455

Score = 847 bits (2188), Expect = 0.0, Method: Compositional matrix adjust.

| Identities | = 431/454 (95%), Positives = 441/454 (97%), Gaps = 0/454 (0%) |  |
| --- | --- | --- |
| Query 1 | MLHGASSRPATARKSSGLSGTVRIPGDKSISHRSFMFGGLASGETRITGLLEGEDVINTG | 60 |
| Sbjct 1 | M HGASSRPATARKSSGLSGTVRIPGDKSISHRSFMFGGLASGETRITGLLEGEDVINTG MSHGASSRPATARKSSGLSGTVRIPGDKSISHRSFMFGGLASGETRITGLLEGEDVINTG | 60 |
| Query 61 | KAMQAMGARIRKEGDTWIIDGVGNGGLLAPEAPLDFGNAATGCRLTMGLVGVYDFDSTFI | 120 |
| Sbjct 61 | KAMQAMGA+IRK+GDTWIIDGVGNG LLAPEAPLDFGNA TGCRLTMGLVGVYDFDSTFI KAMQAMGAKIRKDGDTWIIDGVGNGALLAPEAPLDFGNAGTGCRLTMGLVGVYDFDSTFI | 120 |
| Query 121 | GDASLTKRPMGRVLNPLREMGVQVKSEDGDRLPVTLRGPKTPTPITYRVPMASAQVKSAV GDASLTKRPMGRVLNPLREMGVQV S DGDRLP TL GPK+PTPITYRVPMASAQVKSAV | 180 |

| Sbjct | 121 | GDASLTKRPMGRVLNPLREMGVQVTSADGDRLPATLHGPKSPTPITYRVPMASAQVKSAV | 180 |
| --- | --- | --- | --- |
| Query | 181 | LLAGLNTPGITTVIEPIMTRDHTEKMLQGFGANLTVETDADGVRTIRLEGRGKLTGQVID | 240 |
| Sbjct | 181 | LLAGLNTPGITTVIEP+MTRDHTEKMLQGFGANLTVETDADGVRTIRLEGRGKLTGQVID LLAGLNTPGITTVIEPVMTRDHTEKMLQGFGANLTVETDADGVRTIRLEGRGKLTGQVID | 240 |
| Query | 241 | VPGDPSSTAFPLVAALLVPGSDVTILNVLMNPTRTGLILTLQEMGADIEVINPRLAGGED | 300 |
| Sbjct | 241 | VPGDPSSTAFPLVAALLVPGSDVTILNVLMNPTRTGLILTLQEMGADIEV+NPRLAGGED VPGDPSSTAFPLVAALLVPGSDVTILNVLMNPTRTGLILTLQEMGADIEVMNPRLAGGED | 300 |
| Query | 301 | VADLRVRSSTLKGVTVPEDRAPSMIDEYPILavaaafaegaTVMNGLEELRVKESDRLSA | 360 |
| Sbjct | 301 | VADLRVRSS+LKGVTVPEDRAPSMIDEYP+LAVAAAFAEG TVMNGLEELRVKESDRL+A VADLRVRSSSLKGVTVPEDRAPSMIDEYPVLAVAAAFAEGTTVMNGLEELRVKESDRLAA | 360 |
| Query | 361 | VANGLKLNGVDCDEGETSLVVRGRPDGKGLGNASGAAVATHLDHRIAMSFLVMGLVSENP | 420 |
| Sbjct | 361 | VA GLKLNGVDCDEGE SL+VRGRPDGKGLGNASGAAVATHLDHRIAMSFLVMGLVSENP VATGLKLNGVDCDEGEASLIVRGRPDGKGLGNASGAAVATHLDHRIAMSFLVMGLVSENP | 420 |
| Query  Sbjct | 421  421 | VTVDDATMIATSFPEFMDLMAGLGAKIELSDTKA 454 VTVDDATMIATSFPEFMDLMAGLGAKIEL + A VTVDDATMIATSFPEFMDLMAGLGAKIELHEASA 454 |  |

>WP_134649646.1 MULTISPECIES: 3-phosphoshikimate 1-carboxyvinyltransferase [unclassified Shinella]

TFE92897.1 3-phosphoshikimate 1-carboxyvinyltransferase [Shinella sp. MEC089] TFE97814.1 3-phosphoshikimate 1-carboxyvinyltransferase [Shinella sp. MEC087]

Length=455

Score = 847 bits (2187), Expect = 0.0, Method: Compositional matrix adjust.

| Identities | = 431/455 (95%), Positives = 443/455 (97%), Gaps = 0/455 (0%) |  |
| --- | --- | --- |
| Query 1 | MLHGASSRPATARKSSGLSGTVRIPGDKSISHRSFMFGGLASGETRITGLLEGEDVINTG | 60 |
| Sbjct 1 | M HGA +RPATARKSSGLSGTVRIPGDKSISHRSFMFGGLASGETRITGLLEGEDVINTG MSHGAHARPATARKSSGLSGTVRIPGDKSISHRSFMFGGLASGETRITGLLEGEDVINTG | 60 |
| Query 61 | KAMQAMGARIRKEGDTWIIDGVGNGGLLAPEAPLDFGNAATGCRLTMGLVGVYDFDSTFI | 120 |
| Sbjct 61 | KAMQAMGARIRK+GDTWII+GVGNG LLAPEAPLDFGNA TGCRLTMGLVGVYDFDSTFI KAMQAMGARIRKDGDTWIINGVGNGALLAPEAPLDFGNAGTGCRLTMGLVGVYDFDSTFI | 120 |
| Query 121 | GDASLTKRPMGRVLNPLREMGVQVKSEDGDRLPVTLRGPKTPTPITYRVPMASAQVKSAV | 180 |
| Sbjct 121 | GDASLTKRPMGRVLNPLREMGVQV S +GDRLPVTLRGPKTPTPITYRVPMASAQVKSAV GDASLTKRPMGRVLNPLREMGVQVTSAEGDRLPVTLRGPKTPTPITYRVPMASAQVKSAV | 180 |
| Query 181 | LLAGLNTPGITTVIEPIMTRDHTEKMLQGFGANLTVETDADGVRTIRLEGRGKLTGQVID | 240 |
| Sbjct 181 | LLAGLNTPGITTVIEPIMTRDHTEKMLQGFGANLTVETDADGVRTIRLEGRGKLTGQ+ID LLAGLNTPGITTVIEPIMTRDHTEKMLQGFGANLTVETDADGVRTIRLEGRGKLTGQIID | 240 |
| Query 241 | VPGDPSSTAFPLVAALLVPGSDVTILNVLMNPTRTGLILTLQEMGADIEVINPRLAGGED | 300 |
| Sbjct 241 | VPGDPSSTAFPLVAALLVPGSD+TILNVLMNPTRTGLILTLQEMGADIEV+NPRLAGGED VPGDPSSTAFPLVAALLVPGSDITILNVLMNPTRTGLILTLQEMGADIEVLNPRLAGGED | 300 |
| Query 301 | VADLRVRSSTLKGVTVPEDRAPSMIDEYPILavaaafaegaTVMNGLEELRVKESDRLSA | 360 |
| Sbjct 301 | VADLRVRSS LKGVTVPEDRAPSMIDEYP+LAVAA+FAEG TVMNGL+ELRVKESDRLSA VADLRVRSSVLKGVTVPEDRAPSMIDEYPVLAVAASFAEGTTVMNGLDELRVKESDRLSA | 360 |
| Query 361 | VANGLKLNGVDCDEGETSLVVRGRPDGKGLGNASGAAVATHLDHRIAMSFLVMGLVSENP | 420 |
| Sbjct 361 | VA GLKLNGVDCDEGE SLVVRGRPDG GLGNA+GAAVATHLDHRIAMSFLVMGLVSE+P VAEGLKLNGVDCDEGEASLVVRGRPDGNGLGNAAGAAVATHLDHRIAMSFLVMGLVSEHP | 420 |
| Query 421  Sbjct 421 | VTVDDATMIATSFPEFMDLMAGLGAKIELSDTKAA 455 VTVDDATMIATSFPEFMDLMAGLGAKIELSD KAA VTVDDATMIATSFPEFMDLMAGLGAKIELSDVKAA 455 |  |

>WP_160870076.1 3-phosphoshikimate 1-carboxyvinyltransferase [Shinella sp. AETb1-6] MXN50246.1 3-phosphoshikimate 1-carboxyvinyltransferase [Shinella sp. AETb1-6]

Length=455

Score = 846 bits (2186), Expect = 0.0, Method: Compositional matrix adjust. Identities = 431/455 (95%), Positives = 443/455 (97%), Gaps = 0/455 (0%)

| Query | 1 | MLHGASSRPATARKSSGLSGTVRIPGDKSISHRSFMFGGLASGETRITGLLEGEDVINTG | 60 |
| --- | --- | --- | --- |
| Sbjct | 1 | M HGA +RPATARKSSGLSGTVRIPGDKSISHRSFMFGGLASGETRITGLLEGEDVINTG MSHGAHARPATARKSSGLSGTVRIPGDKSISHRSFMFGGLASGETRITGLLEGEDVINTG | 60 |
| Query | 61 | KAMQAMGARIRKEGDTWIIDGVGNGGLLAPEAPLDFGNAATGCRLTMGLVGVYDFDSTFI | 120 |
| Sbjct | 61 | KAMQAMGARIRK+GDTWII+GVGNG LLAPEAPLDFGNA TGCRLTMGLVGVYDFDSTFI KAMQAMGARIRKDGDTWIINGVGNGALLAPEAPLDFGNAGTGCRLTMGLVGVYDFDSTFI | 120 |
| Query | 121 | GDASLTKRPMGRVLNPLREMGVQVKSEDGDRLPVTLRGPKTPTPITYRVPMASAQVKSAV | 180 |
| Sbjct | 121 | GDASLTKRPMGRVLNPLREMGVQV S +GDRLPVTLRGPKTPTPITYRVPMASAQVKSAV GDASLTKRPMGRVLNPLREMGVQVTSAEGDRLPVTLRGPKTPTPITYRVPMASAQVKSAV | 180 |
| Query | 181 | LLAGLNTPGITTVIEPIMTRDHTEKMLQGFGANLTVETDADGVRTIRLEGRGKLTGQVID | 240 |
| Sbjct | 181 | LLAGLNTPGITTVIEPIMTRDHTEKMLQGFGANLTVETDADGVRTIRLEGRGKLTGQ+ID LLAGLNTPGITTVIEPIMTRDHTEKMLQGFGANLTVETDADGVRTIRLEGRGKLTGQIID | 240 |
| Query | 241 | VPGDPSSTAFPLVAALLVPGSDVTILNVLMNPTRTGLILTLQEMGADIEVINPRLAGGED | 300 |
| Sbjct | 241 | VPGDPSSTAFPLVAALLVPGSD+TILNVLMNPTRTGLILTLQEMGADIEV+NPRLAGGED VPGDPSSTAFPLVAALLVPGSDITILNVLMNPTRTGLILTLQEMGADIEVLNPRLAGGED | 300 |
| Query | 301 | VADLRVRSSTLKGVTVPEDRAPSMIDEYPILavaaafaegaTVMNGLEELRVKESDRLSA | 360 |
| Sbjct | 301 | VADLRVRSS LKGVTVPEDRAPSMIDEYP+LAVAA+FAEG TVMNGL+ELRVKESDRLSA VADLRVRSSVLKGVTVPEDRAPSMIDEYPVLAVAASFAEGTTVMNGLDELRVKESDRLSA | 360 |
| Query | 361 | VANGLKLNGVDCDEGETSLVVRGRPDGKGLGNASGAAVATHLDHRIAMSFLVMGLVSENP | 420 |
| Sbjct | 361 | VA GLKLNGVDCDEGE SLVVRGRPDGKGLGNA+GAAV THLDHRIAMSFLVMGLVSE+P VAEGLKLNGVDCDEGEASLVVRGRPDGKGLGNAAGAAVDTHLDHRIAMSFLVMGLVSEHP | 420 |
| Query  Sbjct | 421  421 | VTVDDATMIATSFPEFMDLMAGLGAKIELSDTKAA 455 VTVDDATMIATSFPEFMDLMAGLGAKIELSD KAA VTVDDATMIATSFPEFMDLMAGLGAKIELSDVKAA 455 |  |

>WP_037405162.1 3-phosphoshikimate 1-carboxyvinyltransferase [Shinella sp. DD12] Length=448

Score = 844 bits (2180), Expect = 0.0, Method: Compositional matrix adjust.

| Identities | = 431/448 (96%), Positives = 439/448 (98%), Gaps = 0/448 (0%) |  |
| --- | --- | --- |
| Query 1 | MLHGASSRPATARKSSGLSGTVRIPGDKSISHRSFMFGGLASGETRITGLLEGEDVINTG | 60 |
| Sbjct 1 | M HGAS+RPATARKSSGLSGTVRIPGDKSISHRSFMFGGLASGETRITGLLEGEDVINTG MSHGASARPATARKSSGLSGTVRIPGDKSISHRSFMFGGLASGETRITGLLEGEDVINTG | 60 |
| Query 61 | KAMQAMGARIRKEGDTWIIDGVGNGGLLAPEAPLDFGNAATGCRLTMGLVGVYDFDSTFI | 120 |
| Sbjct 61 | KAMQAMGA+IRKEGDTWII+GVGNG LLAPEAPLDFGNA TGCRLTMGLVGVYDFDSTFI KAMQAMGAKIRKEGDTWIINGVGNGALLAPEAPLDFGNAGTGCRLTMGLVGVYDFDSTFI | 120 |
| Query 121 | GDASLTKRPMGRVLNPLREMGVQVKSEDGDRLPVTLRGPKTPTPITYRVPMASAQVKSAV | 180 |
| Sbjct 121 | GDASLTKRPMGRVLNPLREMGVQV S DGDRLPVTLRGPKTPTPITYRVPMASAQVKSAV GDASLTKRPMGRVLNPLREMGVQVTSADGDRLPVTLRGPKTPTPITYRVPMASAQVKSAV | 180 |
| Query 181 | LLAGLNTPGITTVIEPIMTRDHTEKMLQGFGANLTVETDADGVRTIRLEGRGKLTGQVID | 240 |
| Sbjct 181 | LLAGLNTPGITTVIEPIMTRDHTEKMLQGFGANLTVETD DGVRTIRLEG+GKLTGQVID LLAGLNTPGITTVIEPIMTRDHTEKMLQGFGANLTVETDTDGVRTIRLEGQGKLTGQVID | 240 |
| Query 241 | VPGDPSSTAFPLVAALLVPGSDVTILNVLMNPTRTGLILTLQEMGADIEVINPRLAGGED | 300 |
| Sbjct 241 | VPGDPSSTAFPLVAALLVPGSDVTILNVLMNPTRTGLILTLQEMGA+IEV+NPRLAGGED VPGDPSSTAFPLVAALLVPGSDVTILNVLMNPTRTGLILTLQEMGANIEVLNPRLAGGED | 300 |
| Query 301 | VADLRVRSSTLKGVTVPEDRAPSMIDEYPILavaaafaegaTVMNGLEELRVKESDRLSA | 360 |
| Sbjct 301 | VADLRVRSS LKGVTVPEDRAPSMIDEYP+LAVAAAFAEG TVMNGLEELRVKESDRLSA VADLRVRSSELKGVTVPEDRAPSMIDEYPVLAVAAAFAEGTTVMNGLEELRVKESDRLSA | 360 |
| Query 361 | VANGLKLNGVDCDEGETSLVVRGRPDGKGLGNASGAAVATHLDHRIAMSFLVMGLVSENP | 420 |
| Sbjct 361 | VA GLKLNGVDCDEGETSL+VRGRPDGKGLGNASGAAVATHLDHRIAMSFLVMGLVSENP VAVGLKLNGVDCDEGETSLIVRGRPDGKGLGNASGAAVATHLDHRIAMSFLVMGLVSENP | 420 |
| Query 421  Sbjct 421 | VTVDDATMIATSFPEFMDLMAGLGAKIE 448 VTVDDATMIATSFPEFMDLMAGLGAKIE VTVDDATMIATSFPEFMDLMAGLGAKIE 448 |  |

>EYR84130.1 3-phosphoshikimate 1-carboxyvinyltransferase AroA [Shinella sp. DD12]

Length=455

Score = 843 bits (2179), Expect = 0.0, Method: Compositional matrix adjust.

| Identities | = 431/448 (96%), Positives = 439/448 (98%), Gaps = 0/448 (0%) |  |
| --- | --- | --- |
| Query 1 | MLHGASSRPATARKSSGLSGTVRIPGDKSISHRSFMFGGLASGETRITGLLEGEDVINTG | 60 |
| Sbjct 8 | M HGAS+RPATARKSSGLSGTVRIPGDKSISHRSFMFGGLASGETRITGLLEGEDVINTG MSHGASARPATARKSSGLSGTVRIPGDKSISHRSFMFGGLASGETRITGLLEGEDVINTG | 67 |
| Query 61 | KAMQAMGARIRKEGDTWIIDGVGNGGLLAPEAPLDFGNAATGCRLTMGLVGVYDFDSTFI | 120 |
| Sbjct 68 | KAMQAMGA+IRKEGDTWII+GVGNG LLAPEAPLDFGNA TGCRLTMGLVGVYDFDSTFI KAMQAMGAKIRKEGDTWIINGVGNGALLAPEAPLDFGNAGTGCRLTMGLVGVYDFDSTFI | 127 |
| Query 121 | GDASLTKRPMGRVLNPLREMGVQVKSEDGDRLPVTLRGPKTPTPITYRVPMASAQVKSAV | 180 |
| Sbjct 128 | GDASLTKRPMGRVLNPLREMGVQV S DGDRLPVTLRGPKTPTPITYRVPMASAQVKSAV GDASLTKRPMGRVLNPLREMGVQVTSADGDRLPVTLRGPKTPTPITYRVPMASAQVKSAV | 187 |
| Query 181 | LLAGLNTPGITTVIEPIMTRDHTEKMLQGFGANLTVETDADGVRTIRLEGRGKLTGQVID | 240 |
| Sbjct 188 | LLAGLNTPGITTVIEPIMTRDHTEKMLQGFGANLTVETD DGVRTIRLEG+GKLTGQVID LLAGLNTPGITTVIEPIMTRDHTEKMLQGFGANLTVETDTDGVRTIRLEGQGKLTGQVID | 247 |
| Query 241 | VPGDPSSTAFPLVAALLVPGSDVTILNVLMNPTRTGLILTLQEMGADIEVINPRLAGGED | 300 |
| Sbjct 248 | VPGDPSSTAFPLVAALLVPGSDVTILNVLMNPTRTGLILTLQEMGA+IEV+NPRLAGGED VPGDPSSTAFPLVAALLVPGSDVTILNVLMNPTRTGLILTLQEMGANIEVLNPRLAGGED | 307 |
| Query 301 | VADLRVRSSTLKGVTVPEDRAPSMIDEYPILavaaafaegaTVMNGLEELRVKESDRLSA | 360 |
| Sbjct 308 | VADLRVRSS LKGVTVPEDRAPSMIDEYP+LAVAAAFAEG TVMNGLEELRVKESDRLSA VADLRVRSSELKGVTVPEDRAPSMIDEYPVLAVAAAFAEGTTVMNGLEELRVKESDRLSA | 367 |
| Query 361 | VANGLKLNGVDCDEGETSLVVRGRPDGKGLGNASGAAVATHLDHRIAMSFLVMGLVSENP | 420 |
| Sbjct 368 | VA GLKLNGVDCDEGETSL+VRGRPDGKGLGNASGAAVATHLDHRIAMSFLVMGLVSENP VAVGLKLNGVDCDEGETSLIVRGRPDGKGLGNASGAAVATHLDHRIAMSFLVMGLVSENP | 427 |
| Query 421  Sbjct 428 | VTVDDATMIATSFPEFMDLMAGLGAKIE 448 VTVDDATMIATSFPEFMDLMAGLGAKIE VTVDDATMIATSFPEFMDLMAGLGAKIE 455 |  |

>WP_050745479.1 MULTISPECIES: 3-phosphoshikimate 1-carboxyvinyltransferase [unclassified Shinella]

KNY14891.1 3-phosphoshikimate 1-carboxyvinyltransferase [Shinella sp. SUS2] KOC74544.1 3-phosphoshikimate 1-carboxyvinyltransferase [Shinella sp. GWS1]

Length=451

Score = 843 bits (2179), Expect = 0.0, Method: Compositional matrix adjust.

| Identities | = 433/450 (96%), Positives = 442/450 (98%), Gaps = 0/450 (0%) |  |
| --- | --- | --- |
| Query 1 | MLHGASSRPATARKSSGLSGTVRIPGDKSISHRSFMFGGLASGETRITGLLEGEDVINTG | 60 |
| Sbjct 1 | M HGAS+RPATARKSSGLSGTVRIPGDKSISHRSFMFGGLASGETRITGLLEGEDVINTG MSHGASARPATARKSSGLSGTVRIPGDKSISHRSFMFGGLASGETRITGLLEGEDVINTG | 60 |
| Query 61 | KAMQAMGARIRKEGDTWIIDGVGNGGLLAPEAPLDFGNAATGCRLTMGLVGVYDFDSTFI | 120 |
| Sbjct 61 | KAMQAMGA+IRKEGDTWII+GVGNG LLAPEAPLDFGNA TGCRLTMGLVGVYDFDSTFI KAMQAMGAKIRKEGDTWIINGVGNGALLAPEAPLDFGNAGTGCRLTMGLVGVYDFDSTFI | 120 |
| Query 121 | GDASLTKRPMGRVLNPLREMGVQVKSEDGDRLPVTLRGPKTPTPITYRVPMASAQVKSAV | 180 |
| Sbjct 121 | GDASLTKRPMGRVLNPLREMGVQV S DGDRLPVTLRGPKTPTPITYRVPMASAQVKSAV GDASLTKRPMGRVLNPLREMGVQVTSADGDRLPVTLRGPKTPTPITYRVPMASAQVKSAV | 180 |
| Query 181 | LLAGLNTPGITTVIEPIMTRDHTEKMLQGFGANLTVETDADGVRTIRLEGRGKLTGQVID | 240 |
| Sbjct 181 | LLAGLNTPGITTVIEPIMTRDHTEKMLQGFGANLTVETD DGVRTIRLEG+GKLTGQVID LLAGLNTPGITTVIEPIMTRDHTEKMLQGFGANLTVETDTDGVRTIRLEGQGKLTGQVID | 240 |
| Query 241 | VPGDPSSTAFPLVAALLVPGSDVTILNVLMNPTRTGLILTLQEMGADIEVINPRLAGGED | 300 |
| Sbjct 241 | VPGDPSSTAFPLVAALLVPGSDVTILNVLMNPTRTGLILTLQEMGA+IEV+NPRLAGGED VPGDPSSTAFPLVAALLVPGSDVTILNVLMNPTRTGLILTLQEMGANIEVLNPRLAGGED | 300 |
| Query 301 | VADLRVRSSTLKGVTVPEDRAPSMIDEYPILavaaafaegaTVMNGLEELRVKESDRLSA | 360 |
| Sbjct 301 | VADLRVRSS LKGVTVPEDRAPSMIDEYP+LAVAAAFAEGATVMNGLEELRVKESDRLSA  VADLRVRSSELKGVTVPEDRAPSMIDEYPVLAVAAAFAEGATVMNGLEELRVKESDRLSA | 360 |

| Query | 361 | VANGLKLNGVDCDEGETSLVVRGRPDGKGLGNASGAAVATHLDHRIAMSFLVMGLVSENP 420 |
| --- | --- | --- |
| Sbjct | 361 | VA GLKLNGVDCDEGETSL+VRGRPDGKGLGNASGAAVATHLDHRIAMSFLVMGLVSENP VAVGLKLNGVDCDEGETSLIVRGRPDGKGLGNASGAAVATHLDHRIAMSFLVMGLVSENP 420 |
| Query | 421 | VTVDDATMIATSFPEFMDLMAGLGAKIELS 450 |
| Sbjct | 421 | VTVDDATMIATSFPEFMDLMAGLGA+IELS  VTVDDATMIATSFPEFMDLMAGLGARIELS 450 |

>WP_130516341.1 3-phosphoshikimate 1-carboxyvinyltransferase [Shinella sp. JR1-6] TAA60711.1 3-phosphoshikimate 1-carboxyvinyltransferase [Shinella sp. JR1-6]

Length=448

Score = 842 bits (2174), Expect = 0.0, Method: Compositional matrix adjust.

| Identities | = 430/448 (96%), Positives = 438/448 (98%), Gaps = 0/448 (0%) |  |
| --- | --- | --- |
| Query 1 | MLHGASSRPATARKSSGLSGTVRIPGDKSISHRSFMFGGLASGETRITGLLEGEDVINTG | 60 |
| Sbjct 1 | M HGAS+RPATARKSSGLSGTVRIPGDKSISHRSFMFGGLASGETRITGLLEGEDVINTG MSHGASARPATARKSSGLSGTVRIPGDKSISHRSFMFGGLASGETRITGLLEGEDVINTG | 60 |
| Query 61 | KAMQAMGARIRKEGDTWIIDGVGNGGLLAPEAPLDFGNAATGCRLTMGLVGVYDFDSTFI | 120 |
| Sbjct 61 | KAMQAMGA+IRKEGDTWII+GVGNG LLAPEAPLDFGNA TGCRLTMGLVGVYDFDSTFI KAMQAMGAKIRKEGDTWIINGVGNGALLAPEAPLDFGNAGTGCRLTMGLVGVYDFDSTFI | 120 |
| Query 121 | GDASLTKRPMGRVLNPLREMGVQVKSEDGDRLPVTLRGPKTPTPITYRVPMASAQVKSAV | 180 |
| Sbjct 121 | GDASLTKRPMGRVLNPLREMGVQV S DGDRLPVTLRGPKTPTPITYRVPMASAQVKSAV GDASLTKRPMGRVLNPLREMGVQVTSADGDRLPVTLRGPKTPTPITYRVPMASAQVKSAV | 180 |
| Query 181 | LLAGLNTPGITTVIEPIMTRDHTEKMLQGFGANLTVETDADGVRTIRLEGRGKLTGQVID | 240 |
| Sbjct 181 | LLAGLNTPGITTVIEPIMTRDHTEKMLQGFGANLTVETD DGVRTIRLEG+GKLTGQVID LLAGLNTPGITTVIEPIMTRDHTEKMLQGFGANLTVETDTDGVRTIRLEGQGKLTGQVID | 240 |
| Query 241 | VPGDPSSTAFPLVAALLVPGSDVTILNVLMNPTRTGLILTLQEMGADIEVINPRLAGGED | 300 |
| Sbjct 241 | VPGDPSSTAFPLVAALLVPGSDVTILNVLMNPTRTGLILTLQEMGA+IEV+NPRLAGGED VPGDPSSTAFPLVAALLVPGSDVTILNVLMNPTRTGLILTLQEMGANIEVLNPRLAGGED | 300 |
| Query 301 | VADLRVRSSTLKGVTVPEDRAPSMIDEYPILavaaafaegaTVMNGLEELRVKESDRLSA | 360 |
| Sbjct 301 | VADLRVRSS LKGVTVPEDRAPSMIDEYP+LAVAAAFAEG TVMNGLEELRVKESDRLSA VADLRVRSSDLKGVTVPEDRAPSMIDEYPVLAVAAAFAEGTTVMNGLEELRVKESDRLSA | 360 |
| Query 361 | VANGLKLNGVDCDEGETSLVVRGRPDGKGLGNASGAAVATHLDHRIAMSFLVMGLVSENP | 420 |
| Sbjct 361 | VA GLKLNGVDCDEGETSL+VRGRPDGKGLGNASGAAVATHLDHRIAMSFLVMGLVSENP VAVGLKLNGVDCDEGETSLIVRGRPDGKGLGNASGAAVATHLDHRIAMSFLVMGLVSENP | 420 |
| Query 421  Sbjct 421 | VTVDDATMIATSFPEFMDLMAGLGAKIE 448 VTVDDATMIATSFPEFMDLM GLGAKIE VTVDDATMIATSFPEFMDLMTGLGAKIE 448 |  |

>WP_119257645.1 3-phosphoshikimate 1-carboxyvinyltransferase [Shinella zoogloeoides] Length=455

Score = 841 bits (2173), Expect = 0.0, Method: Compositional matrix adjust.

| Identities | = 431/455 (95%), Positives = 442/455 (97%), Gaps = 0/455 (0%) |  |
| --- | --- | --- |
| Query 1 | MLHGASSRPATARKSSGLSGTVRIPGDKSISHRSFMFGGLASGETRITGLLEGEDVINTG | 60 |
| Sbjct 1 | M HGA +RPA ARKSSGLSGTVRIPGDKSISHRSFMFGGLASGETRITGLLEGEDVINTG MSHGAVARPAIARKSSGLSGTVRIPGDKSISHRSFMFGGLASGETRITGLLEGEDVINTG | 60 |
| Query 61 | KAMQAMGARIRKEGDTWIIDGVGNGGLLAPEAPLDFGNAATGCRLTMGLVGVYDFDSTFI | 120 |
| Sbjct 61 | KAMQAMGARIRKEGDTWII+GVGNG LLAP+APLDFGNA TGCRLTMGLVGVYDFDSTFI KAMQAMGARIRKEGDTWIINGVGNGALLAPKAPLDFGNAGTGCRLTMGLVGVYDFDSTFI | 120 |
| Query 121 | GDASLTKRPMGRVLNPLREMGVQVKSEDGDRLPVTLRGPKTPTPITYRVPMASAQVKSAV | 180 |
| Sbjct 121 | GDASLTKRPMGRVLNPLREMGVQV S +GDRLPVTLRGPKTPTPITYRVPMASAQVKSAV GDASLTKRPMGRVLNPLREMGVQVTSAEGDRLPVTLRGPKTPTPITYRVPMASAQVKSAV | 180 |
| Query 181 | LLAGLNTPGITTVIEPIMTRDHTEKMLQGFGANLTVETDADGVRTIRLEGRGKLTGQVID | 240 |
| Sbjct 181 | LLAGLNTPGITTVIEPIMTRDHTEKMLQGFGANLTV+TDADGVRTIRLEGRGKLTGQVID  LLAGLNTPGITTVIEPIMTRDHTEKMLQGFGANLTVDTDADGVRTIRLEGRGKLTGQVID | 240 |

| Query | 241 | VPGDPSSTAFPLVAALLVPGSDVTILNVLMNPTRTGLILTLQEMGADIEVINPRLAGGED | 300 |
| --- | --- | --- | --- |
| Sbjct | 241 | VPGDPSSTAFPLVAALLVPGSDVTILNVLMNPTRTGLILTLQEMGADIEV+N RLAGGED VPGDPSSTAFPLVAALLVPGSDVTILNVLMNPTRTGLILTLQEMGADIEVLNARLAGGED | 300 |
| Query | 301 | VADLRVRSSTLKGVTVPEDRAPSMIDEYPILavaaafaegaTVMNGLEELRVKESDRLSA | 360 |
| Sbjct | 301 | +ADLRVRSS LKGVTVPEDRAPSMIDEYP+LAVAAAFAEG TVMNGL+ELRVKESDRLSA MADLRVRSSALKGVTVPEDRAPSMIDEYPVLAVAAAFAEGTTVMNGLDELRVKESDRLSA | 360 |
| Query | 361 | VANGLKLNGVDCDEGETSLVVRGRPDGKGLGNASGAAVATHLDHRIAMSFLVMGLVSENP | 420 |
| Sbjct | 361 | VA+GLKLNGVDCDEGE SLVVRGRPDGKGLGNASGAAVAT+LDHRIAMSFLVMGLVSENP VADGLKLNGVDCDEGEASLVVRGRPDGKGLGNASGAAVATYLDHRIAMSFLVMGLVSENP | 420 |
| Query  Sbjct | 421  421 | VTVDDATMIATSFPEFMDLMAGLGAKIELSDTKAA 455 VTVDDATMIATSFPEFMDLM GLGAKIEL DTKAA VTVDDATMIATSFPEFMDLMTGLGAKIELHDTKAA 455 |  |

>TCN38546.1 3-phosphoshikimate 1-carboxyvinyltransferase [Shinella granuli] Length=458

Score = 841 bits (2172), Expect = 0.0, Method: Compositional matrix adjust.

| Identities | = 428/451 (95%), Positives = 441/451 (98%), Gaps = 0/451 (0%) |  |
| --- | --- | --- |
| Query 1 | MLHGASSRPATARKSSGLSGTVRIPGDKSISHRSFMFGGLASGETRITGLLEGEDVINTG | 60 |
| Sbjct 8 | M HGAS+RPATARKSSGLSGTVRIPGDKSISHRSFMFGGLASGETRITGLLEGEDVINTG MSHGASARPATARKSSGLSGTVRIPGDKSISHRSFMFGGLASGETRITGLLEGEDVINTG | 67 |
| Query 61 | KAMQAMGARIRKEGDTWIIDGVGNGGLLAPEAPLDFGNAATGCRLTMGLVGVYDFDSTFI | 120 |
| Sbjct 68 | KAMQAMGA+IRK+GDTWII+G+GNG LLAPEAPLDFGNA TGCRLTMGLVGVYDFDSTF+ KAMQAMGAKIRKDGDTWIINGLGNGALLAPEAPLDFGNAGTGCRLTMGLVGVYDFDSTFV | 127 |
| Query 121 | GDASLTKRPMGRVLNPLREMGVQVKSEDGDRLPVTLRGPKTPTPITYRVPMASAQVKSAV | 180 |
| Sbjct 128 | GDASLTKRPMGRVLNPLREMGVQV S DGDRLPVTLRGPKTPTPITYRVPMASAQVKSAV GDASLTKRPMGRVLNPLREMGVQVTSADGDRLPVTLRGPKTPTPITYRVPMASAQVKSAV | 187 |
| Query 181 | LLAGLNTPGITTVIEPIMTRDHTEKMLQGFGANLTVETDADGVRTIRLEGRGKLTGQVID | 240 |
| Sbjct 188 | LLAGLNTPGITTVIEP+MTRDHTEKMLQGFGANL+VETDADGVRTIRLEG+GKLTGQVID LLAGLNTPGITTVIEPVMTRDHTEKMLQGFGANLSVETDADGVRTIRLEGQGKLTGQVID | 247 |
| Query 241 | VPGDPSSTAFPLVAALLVPGSDVTILNVLMNPTRTGLILTLQEMGADIEVINPRLAGGED | 300 |
| Sbjct 248 | VPGDPSSTAFPLVAALLVP SDVTILNVLMNPTRTGLILTLQEMGA+IEV+NPRLAGGED VPGDPSSTAFPLVAALLVPDSDVTILNVLMNPTRTGLILTLQEMGANIEVLNPRLAGGED | 307 |
| Query 301 | VADLRVRSSTLKGVTVPEDRAPSMIDEYPILavaaafaegaTVMNGLEELRVKESDRLSA | 360 |
| Sbjct 308 | VADLRVRSS LKGVTVPEDRAPSMIDEYP+LAVAAAFAEG TVMNGLEELRVKESDRLSA VADLRVRSSALKGVTVPEDRAPSMIDEYPVLAVAAAFAEGTTVMNGLEELRVKESDRLSA | 367 |
| Query 361 | VANGLKLNGVDCDEGETSLVVRGRPDGKGLGNASGAAVATHLDHRIAMSFLVMGLVSENP | 420 |
| Sbjct 368 | VA GLKLNGVDCDEGE SLVVRGRPDGKGLGNASGAAVATHLDHRIAMSFLVMGLVSENP VAVGLKLNGVDCDEGEASLVVRGRPDGKGLGNASGAAVATHLDHRIAMSFLVMGLVSENP | 427 |
| Query 421  Sbjct 428 | VTVDDATMIATSFPEFMDLMAGLGAKIELSD 451 VTVDDATMIATSFPEFMDLMAGLGAKIEL+D VTVDDATMIATSFPEFMDLMAGLGAKIELND 458 |  |

>WP_133035983.1 3-phosphoshikimate 1-carboxyvinyltransferase [Shinella granuli] Length=451

Score = 840 bits (2171), Expect = 0.0, Method: Compositional matrix adjust.

| Identities | = 428/451 (95%), Positives = 441/451 (98%), Gaps = 0/451 (0%) |  |
| --- | --- | --- |
| Query 1 | MLHGASSRPATARKSSGLSGTVRIPGDKSISHRSFMFGGLASGETRITGLLEGEDVINTG | 60 |
| Sbjct 1 | M HGAS+RPATARKSSGLSGTVRIPGDKSISHRSFMFGGLASGETRITGLLEGEDVINTG MSHGASARPATARKSSGLSGTVRIPGDKSISHRSFMFGGLASGETRITGLLEGEDVINTG | 60 |
| Query 61 | KAMQAMGARIRKEGDTWIIDGVGNGGLLAPEAPLDFGNAATGCRLTMGLVGVYDFDSTFI | 120 |
| Sbjct 61 | KAMQAMGA+IRK+GDTWII+G+GNG LLAPEAPLDFGNA TGCRLTMGLVGVYDFDSTF+ KAMQAMGAKIRKDGDTWIINGLGNGALLAPEAPLDFGNAGTGCRLTMGLVGVYDFDSTFV | 120 |
| Query 121 | GDASLTKRPMGRVLNPLREMGVQVKSEDGDRLPVTLRGPKTPTPITYRVPMASAQVKSAV GDASLTKRPMGRVLNPLREMGVQV S DGDRLPVTLRGPKTPTPITYRVPMASAQVKSAV | 180 |

| Sbjct | 121 | GDASLTKRPMGRVLNPLREMGVQVTSADGDRLPVTLRGPKTPTPITYRVPMASAQVKSAV | 180 |
| --- | --- | --- | --- |
| Query | 181 | LLAGLNTPGITTVIEPIMTRDHTEKMLQGFGANLTVETDADGVRTIRLEGRGKLTGQVID | 240 |
| Sbjct | 181 | LLAGLNTPGITTVIEP+MTRDHTEKMLQGFGANL+VETDADGVRTIRLEG+GKLTGQVID LLAGLNTPGITTVIEPVMTRDHTEKMLQGFGANLSVETDADGVRTIRLEGQGKLTGQVID | 240 |
| Query | 241 | VPGDPSSTAFPLVAALLVPGSDVTILNVLMNPTRTGLILTLQEMGADIEVINPRLAGGED | 300 |
| Sbjct | 241 | VPGDPSSTAFPLVAALLVP SDVTILNVLMNPTRTGLILTLQEMGA+IEV+NPRLAGGED VPGDPSSTAFPLVAALLVPDSDVTILNVLMNPTRTGLILTLQEMGANIEVLNPRLAGGED | 300 |
| Query | 301 | VADLRVRSSTLKGVTVPEDRAPSMIDEYPILavaaafaegaTVMNGLEELRVKESDRLSA | 360 |
| Sbjct | 301 | VADLRVRSS LKGVTVPEDRAPSMIDEYP+LAVAAAFAEG TVMNGLEELRVKESDRLSA VADLRVRSSALKGVTVPEDRAPSMIDEYPVLAVAAAFAEGTTVMNGLEELRVKESDRLSA | 360 |
| Query | 361 | VANGLKLNGVDCDEGETSLVVRGRPDGKGLGNASGAAVATHLDHRIAMSFLVMGLVSENP | 420 |
| Sbjct | 361 | VA GLKLNGVDCDEGE SLVVRGRPDGKGLGNASGAAVATHLDHRIAMSFLVMGLVSENP VAVGLKLNGVDCDEGEASLVVRGRPDGKGLGNASGAAVATHLDHRIAMSFLVMGLVSENP | 420 |
| Query  Sbjct | 421  421 | VTVDDATMIATSFPEFMDLMAGLGAKIELSD 451 VTVDDATMIATSFPEFMDLMAGLGAKIEL+D VTVDDATMIATSFPEFMDLMAGLGAKIELND 451 |  |

>WP_064328835.1 3-phosphoshikimate 1-carboxyvinyltransferase [Shinella sp. HZN7] ANH02812.1 3-phosphoshikimate 1-carboxyvinyltransferase [Shinella sp. HZN7]

Length=451

Score = 838 bits (2166), Expect = 0.0, Method: Compositional matrix adjust.

| Identities | = 427/451 (95%), Positives = 440/451 (98%), Gaps = 0/451 (0%) |  |
| --- | --- | --- |
| Query 1 | MLHGASSRPATARKSSGLSGTVRIPGDKSISHRSFMFGGLASGETRITGLLEGEDVINTG | 60 |
| Sbjct 1 | M HGAS+RPATARKSSGLSGTVRIPGDKSISHRSFMFGGLASGETRITGLLEGEDVINTG MSHGASARPATARKSSGLSGTVRIPGDKSISHRSFMFGGLASGETRITGLLEGEDVINTG | 60 |
| Query 61 | KAMQAMGARIRKEGDTWIIDGVGNGGLLAPEAPLDFGNAATGCRLTMGLVGVYDFDSTFI | 120 |
| Sbjct 61 | KAMQAMGA+IRK+GDTWII+G+GNG LLAPEAPLDFGNA TGCRLTMGLVGVYDFDSTF+ KAMQAMGAKIRKDGDTWIINGLGNGALLAPEAPLDFGNAGTGCRLTMGLVGVYDFDSTFV | 120 |
| Query 121 | GDASLTKRPMGRVLNPLREMGVQVKSEDGDRLPVTLRGPKTPTPITYRVPMASAQVKSAV | 180 |
| Sbjct 121 | GDASLTKRPMGRVLNPLREMGVQV S DGDRLPVTLRGPKTPTPITYRVPMASAQVKSAV GDASLTKRPMGRVLNPLREMGVQVTSADGDRLPVTLRGPKTPTPITYRVPMASAQVKSAV | 180 |
| Query 181 | LLAGLNTPGITTVIEPIMTRDHTEKMLQGFGANLTVETDADGVRTIRLEGRGKLTGQVID | 240 |
| Sbjct 181 | LLAGLNTPGITTVIEP+MTRDHTEKMLQGFGANL+VETD DGVRTIRLEG+GKLTGQVID LLAGLNTPGITTVIEPVMTRDHTEKMLQGFGANLSVETDTDGVRTIRLEGQGKLTGQVID | 240 |
| Query 241 | VPGDPSSTAFPLVAALLVPGSDVTILNVLMNPTRTGLILTLQEMGADIEVINPRLAGGED | 300 |
| Sbjct 241 | VPGDPSSTAFPLVAALLVP SDVTILNVLMNPTRTGLILTLQEMGA+IEV+NPRLAGGED VPGDPSSTAFPLVAALLVPDSDVTILNVLMNPTRTGLILTLQEMGANIEVLNPRLAGGED | 300 |
| Query 301 | VADLRVRSSTLKGVTVPEDRAPSMIDEYPILavaaafaegaTVMNGLEELRVKESDRLSA | 360 |
| Sbjct 301 | VADLRVRSS LKGVTVPEDRAPSMIDEYP+LAVAAAFAEG TVMNGLEELRVKESDRLSA VADLRVRSSALKGVTVPEDRAPSMIDEYPVLAVAAAFAEGTTVMNGLEELRVKESDRLSA | 360 |
| Query 361 | VANGLKLNGVDCDEGETSLVVRGRPDGKGLGNASGAAVATHLDHRIAMSFLVMGLVSENP | 420 |
| Sbjct 361 | VA GLKLNGVDCDEGE SLVVRGRPDGKGLGNASGAAVATHLDHRIAMSFLVMGLVSENP VAVGLKLNGVDCDEGEASLVVRGRPDGKGLGNASGAAVATHLDHRIAMSFLVMGLVSENP | 420 |
| Query 421  Sbjct 421 | VTVDDATMIATSFPEFMDLMAGLGAKIELSD 451 VTVDDATMIATSFPEFMDLMAGLGAKIEL+D VTVDDATMIATSFPEFMDLMAGLGAKIELND 451 |  |

>WP_069060003.1 3-phosphoshikimate 1-carboxyvinyltransferase [Sinorhizobium sp. RAC02]

AOF91760.1 3-phosphoshikimate 1-carboxyvinyltransferase [Sinorhizobium sp. RAC02]

Length=451

Score = 837 bits (2163), Expect = 0.0, Method: Compositional matrix adjust. Identities = 427/450 (95%), Positives = 441/450 (98%), Gaps = 0/450 (0%)

| Query | 1 | MLHGASSRPATARKSSGLSGTVRIPGDKSISHRSFMFGGLASGETRITGLLEGEDVINTG | 60 |
| --- | --- | --- | --- |
| Sbjct | 1 | M HGAS+RPATARKSSGLSGTVRIPGDKSISHRSFMFGGLASGETRITGLLEGEDVINTG MSHGASARPATARKSSGLSGTVRIPGDKSISHRSFMFGGLASGETRITGLLEGEDVINTG | 60 |
| Query | 61 | KAMQAMGARIRKEGDTWIIDGVGNGGLLAPEAPLDFGNAATGCRLTMGLVGVYDFDSTFI | 120 |
| Sbjct | 61 | KAMQAMGA+IRK+GDTWII+G+GNG LLAP+APLDFGNA TGCRLTMGLVGVYDFDSTFI KAMQAMGAKIRKDGDTWIINGLGNGALLAPQAPLDFGNAGTGCRLTMGLVGVYDFDSTFI | 120 |
| Query | 121 | GDASLTKRPMGRVLNPLREMGVQVKSEDGDRLPVTLRGPKTPTPITYRVPMASAQVKSAV | 180 |
| Sbjct | 121 | GDASLTKRPMGRVLNPLREMGVQV ++ GDRLPVTLRGPKTPTPITYRVPMASAQVKSAV GDASLTKRPMGRVLNPLREMGVQVTADAGDRLPVTLRGPKTPTPITYRVPMASAQVKSAV | 180 |
| Query | 181 | LLAGLNTPGITTVIEPIMTRDHTEKMLQGFGANLTVETDADGVRTIRLEGRGKLTGQVID | 240 |
| Sbjct | 181 | LLAGLNTPGITTVIEPIMTRDHTEKMLQGFGANL+VETDADGVRTIRLEGRGKLTGQVID LLAGLNTPGITTVIEPIMTRDHTEKMLQGFGANLSVETDADGVRTIRLEGRGKLTGQVID | 240 |
| Query | 241 | VPGDPSSTAFPLVAALLVPGSDVTILNVLMNPTRTGLILTLQEMGADIEVINPRLAGGED | 300 |
| Sbjct | 241 | VPGDPSSTAFPLVAALLVPGSDVTILNVLMNPTRTGLILTLQEMGA+I+V+NPRLAGGED VPGDPSSTAFPLVAALLVPGSDVTILNVLMNPTRTGLILTLQEMGANIDVLNPRLAGGED | 300 |
| Query | 301 | VADLRVRSSTLKGVTVPEDRAPSMIDEYPILavaaafaegaTVMNGLEELRVKESDRLSA | 360 |
| Sbjct | 301 | VADLRVRSS LKGVTVPEDRAPSMIDEYP+LAVAAAFAEG TVMNGLEELRVKESDRLSA VADLRVRSSELKGVTVPEDRAPSMIDEYPVLAVAAAFAEGTTVMNGLEELRVKESDRLSA | 360 |
| Query | 361 | VANGLKLNGVDCDEGETSLVVRGRPDGKGLGNASGAAVATHLDHRIAMSFLVMGLVSENP | 420 |
| Sbjct | 361 | VA GLKLNGVDCDEGE SLVVRGRPDGKGLGNASGAAVATHLDHRIAMSFLVMGLVSENP VAIGLKLNGVDCDEGEASLVVRGRPDGKGLGNASGAAVATHLDHRIAMSFLVMGLVSENP | 420 |
| Query  Sbjct | 421  421 | VTVDDATMIATSFPEFMDLMAGLGAKIELS 450 VTVDDATMIATSFPEFMDLMAGLGAKIEL+ VTVDDATMIATSFPEFMDLMAGLGAKIELN 450 |  |

>WP_160858055.1 3-phosphoshikimate 1-carboxyvinyltransferase [Shinella kummerowiae] MXN44578.1 3-phosphoshikimate 1-carboxyvinyltransferase [Shinella kummerowiae]

Length=451

Score = 836 bits (2160), Expect = 0.0, Method: Compositional matrix adjust.

| Identities | = 428/450 (95%), Positives = 440/450 (98%), Gaps = 0/450 (0%) |  |
| --- | --- | --- |
| Query 1 | MLHGASSRPATARKSSGLSGTVRIPGDKSISHRSFMFGGLASGETRITGLLEGEDVINTG | 60 |
| Sbjct 1 | M HGAS+RPA ARKSSGLSGTVRIPGDKSISHRSFMFGGLASGETRITGLLEGEDVINTG MSHGASARPAIARKSSGLSGTVRIPGDKSISHRSFMFGGLASGETRITGLLEGEDVINTG | 60 |
| Query 61 | KAMQAMGARIRKEGDTWIIDGVGNGGLLAPEAPLDFGNAATGCRLTMGLVGVYDFDSTFI | 120 |
| Sbjct 61 | KAMQAMGA+IRK+GDTWII+GVGNG LLAP+APLDFGNA TGCRLTMGLVGVYDFDSTFI KAMQAMGAKIRKDGDTWIINGVGNGALLAPQAPLDFGNAGTGCRLTMGLVGVYDFDSTFI | 120 |
| Query 121 | GDASLTKRPMGRVLNPLREMGVQVKSEDGDRLPVTLRGPKTPTPITYRVPMASAQVKSAV | 180 |
| Sbjct 121 | GDASLTKRPMGRVLNPLREMGVQV S+ GDRLPVTLRGPKTPTPITYRVPMASAQVKSAV GDASLTKRPMGRVLNPLREMGVQVTSDAGDRLPVTLRGPKTPTPITYRVPMASAQVKSAV | 180 |
| Query 181 | LLAGLNTPGITTVIEPIMTRDHTEKMLQGFGANLTVETDADGVRTIRLEGRGKLTGQVID | 240 |
| Sbjct 181 | LLAGLNTPGITTVIEPIMTRDHTEKMLQGFGANLTVETDADGVRTIRLEGRGKLTGQVID LLAGLNTPGITTVIEPIMTRDHTEKMLQGFGANLTVETDADGVRTIRLEGRGKLTGQVID | 240 |
| Query 241 | VPGDPSSTAFPLVAALLVPGSDVTILNVLMNPTRTGLILTLQEMGADIEVINPRLAGGED | 300 |
| Sbjct 241 | VPGDPSSTAFPLVAALLVPGSDVTILNVLMNPTRTGLILTLQEMGA+IEV+NPRLAGGED VPGDPSSTAFPLVAALLVPGSDVTILNVLMNPTRTGLILTLQEMGANIEVLNPRLAGGED | 300 |
| Query 301 | VADLRVRSSTLKGVTVPEDRAPSMIDEYPILavaaafaegaTVMNGLEELRVKESDRLSA | 360 |
| Sbjct 301 | VADLRVRSS LKGVTVPEDRAPSMIDEYP+LAVAAAFAEG TVMNGL+ELRVKESDRLSA VADLRVRSSELKGVTVPEDRAPSMIDEYPVLAVAAAFAEGTTVMNGLDELRVKESDRLSA | 360 |
| Query 361 | VANGLKLNGVDCDEGETSLVVRGRPDGKGLGNASGAAVATHLDHRIAMSFLVMGLVSENP | 420 |
| Sbjct 361 | VA GLKLNGVDCDEGE SLVVRGRPDGKGLGNASGAAVATHLDHRIAMSFLVMGLVSE+P VAIGLKLNGVDCDEGEASLVVRGRPDGKGLGNASGAAVATHLDHRIAMSFLVMGLVSEHP | 420 |
| Query 421  Sbjct 421 | VTVDDATMIATSFPEFMDLMAGLGAKIELS 450 VTVDDATMIATSFPEFMDLMAGLGAKIEL+ VTVDDATMIATSFPEFMDLMAGLGAKIELN 450 |  |

>WP_176950611.1 3-phosphoshikimate 1-carboxyvinyltransferase [Rhizobium sp. DBTS2] NVP56631.1 3-phosphoshikimate 1-carboxyvinyltransferase [Rhizobium sp. DBTS2]

Length=448

Score = 820 bits (2118), Expect = 0.0, Method: Compositional matrix adjust.

| Identities | = 417/448 (93%), Positives = 432/448 (96%), Gaps = 0/448 (0%) |  |
| --- | --- | --- |
| Query 1 | MLHGASSRPATARKSSGLSGTVRIPGDKSISHRSFMFGGLASGETRITGLLEGEDVINTG | 60 |
| Sbjct 1 | M HGA ++PATARKS+ LSGTVRIPGDKSISHRSFMFGGLASGETRITGLLEGEDVINTG MSHGAQAKPATARKSNALSGTVRIPGDKSISHRSFMFGGLASGETRITGLLEGEDVINTG | 60 |
| Query 61 | KAMQAMGARIRKEGDTWIIDGVGNGGLLAPEAPLDFGNAATGCRLTMGLVGVYDFDSTFI | 120 |
| Sbjct 61 | KAMQAMGA+IRKEGDTWII+GVGNG LLAPEA LDFGNA TGCRLTMGLVGVYDFDSTFI KAMQAMGAKIRKEGDTWIINGVGNGALLAPEASLDFGNAGTGCRLTMGLVGVYDFDSTFI | 120 |
| Query 121 | GDASLTKRPMGRVLNPLREMGVQVKSEDGDRLPVTLRGPKTPTPITYRVPMASAQVKSAV | 180 |
| Sbjct 121 | GDASLTKRPMGRVLNPLREMGVQVKS DGDRLPVTLRGP TPTPITYRVPMASAQVKSAV GDASLTKRPMGRVLNPLREMGVQVKSADGDRLPVTLRGPTTPTPITYRVPMASAQVKSAV | 180 |
| Query 181 | LLAGLNTPGITTVIEPIMTRDHTEKMLQGFGANLTVETDADGVRTIRLEGRGKLTGQVID | 240 |
| Sbjct 181 | LLAGLNTPGITTVIEP+MTRDHTEKMLQGFGANLTVETD DGVRTIRLEG+GKLTGQVID LLAGLNTPGITTVIEPVMTRDHTEKMLQGFGANLTVETDVDGVRTIRLEGQGKLTGQVID | 240 |
| Query 241 | VPGDPSSTAFPLVAALLVPGSDVTILNVLMNPTRTGLILTLQEMGADIEVINPRLAGGED | 300 |
| Sbjct 241 | VPGDPSSTAFPLVAALLVPGSDVTILNVLMNPTRTGLILTLQEMGA+IEV+N RLAGGED VPGDPSSTAFPLVAALLVPGSDVTILNVLMNPTRTGLILTLQEMGANIEVLNARLAGGED | 300 |
| Query 301 | VADLRVRSSTLKGVTVPEDRAPSMIDEYPILavaaafaegaTVMNGLEELRVKESDRLSA | 360 |
| Sbjct 301 | VAD+RVR S LKGVTVPE+RAPSMIDEYPILAVAAAFAEG TVMNGLEELRVKESDRLSA VADVRVRHSELKGVTVPEERAPSMIDEYPILAVAAAFAEGTTVMNGLEELRVKESDRLSA | 360 |
| Query 361 | VANGLKLNGVDCDEGETSLVVRGRPDGKGLGNASGAAVATHLDHRIAMSFLVMGLVSENP | 420 |
| Sbjct 361 | VA GLKLNGVDCDEGE SLVVRGRPDG+GLGNASGAAVATHLDHRIAMSFL+MGLVSE+P VAEGLKLNGVDCDEGEASLVVRGRPDGRGLGNASGAAVATHLDHRIAMSFLIMGLVSEHP | 420 |
| Query 421  Sbjct 421 | VTVDDATMIATSFPEFMDLMAGLGAKIE 448 VTVDDATMIATSFPEFM LMAGLGAKI+ VTVDDATMIATSFPEFMGLMAGLGAKID 448 |  |

>WP_163902602.1 3-phosphoshikimate 1-carboxyvinyltransferase [Rhizobium daejeonense] NGO65082.1 3-phosphoshikimate 1-carboxyvinyltransferase [Rhizobium daejeonense]

Length=455

Score = 820 bits (2118), Expect = 0.0, Method: Compositional matrix adjust.

| Identities | = 419/455 (92%), Positives = 435/455 (96%), Gaps = 0/455 (0%) |  |
| --- | --- | --- |
| Query 1 | MLHGASSRPATARKSSGLSGTVRIPGDKSISHRSFMFGGLASGETRITGLLEGEDVINTG | 60 |
| Sbjct 1 | M HGA S+PAT+RKS LSGT+RIPGDKSISHRSFMFGGLASG+TRITGLLEGEDVINTG MSHGALSKPATSRKSPALSGTIRIPGDKSISHRSFMFGGLASGQTRITGLLEGEDVINTG | 60 |
| Query 61 | KAMQAMGARIRKEGDTWIIDGVGNGGLLAPEAPLDFGNAATGCRLTMGLVGVYDFDSTFI | 120 |
| Sbjct 61 | KAMQAMGARI K GDTWIIDGVGNG LLAPEAPLDFGNA TGCRLTMGLVGVYDFDSTFI KAMQAMGARIVKVGDTWIIDGVGNGCLLAPEAPLDFGNAGTGCRLTMGLVGVYDFDSTFI | 120 |
| Query 121 | GDASLTKRPMGRVLNPLREMGVQVKSEDGDRLPVTLRGPKTPTPITYRVPMASAQVKSAV | 180 |
| Sbjct 121 | GDASLTKRPMGRVLNPLREMGVQVKS DGDRLPVTL GP+TP PITYRVPMASAQVKSAV GDASLTKRPMGRVLNPLREMGVQVKSADGDRLPVTLHGPRTPAPITYRVPMASAQVKSAV | 180 |
| Query 181 | LLAGLNTPGITTVIEPIMTRDHTEKMLQGFGANLTVETDADGVRTIRLEGRGKLTGQVID | 240 |
| Sbjct 181 | LLAGLNTPGITTVIEPIMTRDHTEKMLQGFGANLTVETDA+GVRTIR+EGRGKLTGQVID LLAGLNTPGITTVIEPIMTRDHTEKMLQGFGANLTVETDAEGVRTIRIEGRGKLTGQVID | 240 |
| Query 241 | VPGDPSSTAFPLVAALLVPGSDVTILNVLMNPTRTGLILTLQEMGADIEVINPRLAGGED | 300 |
| Sbjct 241 | VPGDPSSTAFPLVAALLVPGSDVTILNVLMNPTRTGLILTLQEMGADIE++NPRLAGGED VPGDPSSTAFPLVAALLVPGSDVTILNVLMNPTRTGLILTLQEMGADIEILNPRLAGGED | 300 |
| Query 301 | VADLRVRSSTLKGVTVPEDRAPSMIDEYPILavaaafaegaTVMNGLEELRVKESDRLSA | 360 |
| Sbjct 301 | VADLRVRSS+LKGVTVPEDRAPSMIDEYP+LAVAAAFAEG T M GLEELRVKESDRLSA  VADLRVRSSSLKGVTVPEDRAPSMIDEYPVLAVAAAFAEGTTTMLGLEELRVKESDRLSA | 360 |

| Query | 361 | VANGLKLNGVDCDEGETSLVVRGRPDGKGLGNASGAAVATHLDHRIAMSFLVMGLVSENP 420 |
| --- | --- | --- |
| Sbjct | 361 | VA GLKLNGVDCDEGE +LVVRGRPDGKGLGNASG AVATHLDHRIAMSFL+MGLVSE+P VAVGLKLNGVDCDEGEATLVVRGRPDGKGLGNASGEAVATHLDHRIAMSFLIMGLVSEHP 420 |
| Query | 421 | VTVDDATMIATSFPEFMDLMAGLGAKIELSDTKAA 455 |
| Sbjct | 421 | VTVDDATMIATSFPEFMDLM+GLGAKIE + KAA  VTVDDATMIATSFPEFMDLMSGLGAKIETHEGKAA 455 |

>WP_099056063.1 3-phosphoshikimate 1-carboxyvinyltransferase [Rhizobium sp. ACO-34A] ATN32384.1 3-phosphoshikimate 1-carboxyvinyltransferase [Rhizobium sp. ACO-34A]

Length=455

Score = 819 bits (2115), Expect = 0.0, Method: Compositional matrix adjust.

| Identities | = 418/455 (92%), Positives = 435/455 (96%), Gaps = 0/455 (0%) |  |
| --- | --- | --- |
| Query 1 | MLHGASSRPATARKSSGLSGTVRIPGDKSISHRSFMFGGLASGETRITGLLEGEDVINTG | 60 |
| Sbjct 1 | M HGA +PAT+RKS LSGT+RIPGDKSISHRSFMFGGLASG+TRITGLLEGEDVINTG MSHGALPKPATSRKSPALSGTIRIPGDKSISHRSFMFGGLASGQTRITGLLEGEDVINTG | 60 |
| Query 61 | KAMQAMGARIRKEGDTWIIDGVGNGGLLAPEAPLDFGNAATGCRLTMGLVGVYDFDSTFI | 120 |
| Sbjct 61 | KAMQAMGARI K+GDTWIIDGVGNG LLAPEAPLDFGNA TGCRLTMGLVGVYDFDSTFI KAMQAMGARIVKDGDTWIIDGVGNGCLLAPEAPLDFGNAGTGCRLTMGLVGVYDFDSTFI | 120 |
| Query 121 | GDASLTKRPMGRVLNPLREMGVQVKSEDGDRLPVTLRGPKTPTPITYRVPMASAQVKSAV | 180 |
| Sbjct 121 | GDASLTKRPMGRVLNPLREMGVQVKS +GDRLPVTL GPKTP PITYRVPMASAQVKSAV GDASLTKRPMGRVLNPLREMGVQVKSAEGDRLPVTLHGPKTPAPITYRVPMASAQVKSAV | 180 |
| Query 181 | LLAGLNTPGITTVIEPIMTRDHTEKMLQGFGANLTVETDADGVRTIRLEGRGKLTGQVID | 240 |
| Sbjct 181 | LLAGLNTPGITTVIEPIMTRDHTEKMLQGFGANLTVETDA+ VRTIR+EGRGKLTGQVID LLAGLNTPGITTVIEPIMTRDHTEKMLQGFGANLTVETDAESVRTIRIEGRGKLTGQVID | 240 |
| Query 241 | VPGDPSSTAFPLVAALLVPGSDVTILNVLMNPTRTGLILTLQEMGADIEVINPRLAGGED | 300 |
| Sbjct 241 | VPGDPSSTAFPLVAALLVPGSDVTILNVLMNPTRTGLILTLQEMGADIE++NPRLAGGED VPGDPSSTAFPLVAALLVPGSDVTILNVLMNPTRTGLILTLQEMGADIEILNPRLAGGED | 300 |
| Query 301 | VADLRVRSSTLKGVTVPEDRAPSMIDEYPILavaaafaegaTVMNGLEELRVKESDRLSA | 360 |
| Sbjct 301 | VADLRVRSS+LKGVTVPEDRAPSMIDEYP+LAVAAAFAEG T M GLEELRVKESDRLSA VADLRVRSSSLKGVTVPEDRAPSMIDEYPVLAVAAAFAEGTTTMLGLEELRVKESDRLSA | 360 |
| Query 361 | VANGLKLNGVDCDEGETSLVVRGRPDGKGLGNASGAAVATHLDHRIAMSFLVMGLVSENP | 420 |
| Sbjct 361 | VA GLKLNGVDCDEGE +LVVRGRPDGKGLGNASGAAVATHLDHRIAMSFL+MGLVSE+P VAVGLKLNGVDCDEGEATLVVRGRPDGKGLGNASGAAVATHLDHRIAMSFLIMGLVSEHP | 420 |
| Query 421  Sbjct 421 | VTVDDATMIATSFPEFMDLMAGLGAKIELSDTKAA 455 VTVDDATMIATSFPEFMDLM+GLGAKIE + KAA VTVDDATMIATSFPEFMDLMSGLGAKIETREGKAA 455 |  |

>WP_108001699.1 3-phosphoshikimate 1-carboxyvinyltransferase [Mycoplana dimorpha] PTM97418.1 3-phosphoshikimate 1-carboxyvinyltransferase [Mycoplana dimorpha]

Length=449

Score = 818 bits (2112), Expect = 0.0, Method: Compositional matrix adjust.

| Identities | = 414/448 (92%), Positives = 436/448 (97%), Gaps = 0/448 (0%) |  |
| --- | --- | --- |
| Query 1 | MLHGASSRPATARKSSGLSGTVRIPGDKSISHRSFMFGGLASGETRITGLLEGEDVINTG | 60 |
| Sbjct 1 | M HGA+++PATARKS+ LSGTVRIPGDKSISHR FMFGGLASGETRITGLLEGEDVINTG MSHGAAAKPATARKSNALSGTVRIPGDKSISHRCFMFGGLASGETRITGLLEGEDVINTG | 60 |
| Query 61 | KAMQAMGARIRKEGDTWIIDGVGNGGLLAPEAPLDFGNAATGCRLTMGLVGVYDFDSTFI | 120 |
| Sbjct 61 | KAMQAMGA+IRK+GDTWII+GVGNG LLAPEAPLDFGNA TGCRL MGLVGVYDFD+TFI KAMQAMGAKIRKDGDTWIINGVGNGALLAPEAPLDFGNAGTGCRLMMGLVGVYDFDTTFI | 120 |
| Query 121 | GDASLTKRPMGRVLNPLREMGVQVKSEDGDRLPVTLRGPKTPTPITYRVPMASAQVKSAV | 180 |
| Sbjct 121 | GDASLTKRPMGRVL+PLREMGVQVK+ DGDRLPVTLRGP+TP PITYRVPMASAQVKSAV GDASLTKRPMGRVLDPLREMGVQVKAADGDRLPVTLRGPRTPAPITYRVPMASAQVKSAV | 180 |
| Query 181 | LLAGLNTPGITTVIEPIMTRDHTEKMLQGFGANLTVETDADGVRTIRLEGRGKLTGQVID | 240 |
| Sbjct 181 | LLAGLNTPGITTVIEP+MTRDHTEKMLQGFGANLTVE+DADGVRTIRLEG+GKLTGQVID  LLAGLNTPGITTVIEPVMTRDHTEKMLQGFGANLTVESDADGVRTIRLEGQGKLTGQVID | 240 |

| Query | 241 | VPGDPSSTAFPLVAALLVPGSDVTILNVLMNPTRTGLILTLQEMGADIEVINPRLAGGED | 300 |
| --- | --- | --- | --- |
| Sbjct | 241 | VPGDPSSTAFPLVAALLVPGSDVTILNVLMNPTRTGLILTLQEMGA+IEV+N RLAGGED VPGDPSSTAFPLVAALLVPGSDVTILNVLMNPTRTGLILTLQEMGANIEVLNERLAGGED | 300 |
| Query | 301 | VADLRVRSSTLKGVTVPEDRAPSMIDEYPILavaaafaegaTVMNGLEELRVKESDRLSA | 360 |
| Sbjct | 301 | VADLRVR STLKGVTVPEDRAPSMIDEYPILA+AAAFAEG TVMNGLEELRVKESDRLSA VADLRVRHSTLKGVTVPEDRAPSMIDEYPILAIAAAFAEGTTVMNGLEELRVKESDRLSA | 360 |
| Query | 361 | VANGLKLNGVDCDEGETSLVVRGRPDGKGLGNASGAAVATHLDHRIAMSFLVMGLVSENP | 420 |
| Sbjct | 361 | VA+GLKLNGVDCDEGE+SLVVRGRPDGKGLGNASGAAVATHLDHRIAMSFLVMGLVSE+P VADGLKLNGVDCDEGESSLVVRGRPDGKGLGNASGAAVATHLDHRIAMSFLVMGLVSEHP | 420 |
| Query  Sbjct | 421  421 | VTVDDATMIATSFPEFMDLMAGLGAKIE 448 VTVDDATMIATSFPEFM LMAGLGA+I+ VTVDDATMIATSFPEFMGLMAGLGARID 448 |  |

>WP_183801805.1 3-phosphoshikimate 1-carboxyvinyltransferase [Rhizobium azooxidifex] MBB3976430.1 3-phosphoshikimate 1-carboxyvinyltransferase [Rhizobium azooxidifex]

Length=452

Score = 817 bits (2111), Expect = 0.0, Method: Compositional matrix adjust.

| Identities | = 415/448 (93%), Positives = 430/448 (96%), Gaps = 0/448 (0%) |  |
| --- | --- | --- |
| Query 1 | MLHGASSRPATARKSSGLSGTVRIPGDKSISHRSFMFGGLASGETRITGLLEGEDVINTG | 60 |
| Sbjct 1 | M HGA ++PATARKS LSGTVRIPGDKSISHRSFMFGGLASG+TRITGLLEGEDVINTG MSHGAQAKPATARKSQALSGTVRIPGDKSISHRSFMFGGLASGQTRITGLLEGEDVINTG | 60 |
| Query 61 | KAMQAMGARIRKEGDTWIIDGVGNGGLLAPEAPLDFGNAATGCRLTMGLVGVYDFDSTFI | 120 |
| Sbjct 61 | KAMQAMGA+IRKEGDTWIIDGVGNG LLAP+A LDFGNA TGCRLTMGLVGVYDFDSTF+ KAMQAMGAKIRKEGDTWIIDGVGNGALLAPQAALDFGNAGTGCRLTMGLVGVYDFDSTFV | 120 |
| Query 121 | GDASLTKRPMGRVLNPLREMGVQVKSEDGDRLPVTLRGPKTPTPITYRVPMASAQVKSAV | 180 |
| Sbjct 121 | GDASLTKRPMGRVLNPLREMGVQV + DGDRLPVTLRGP TPTPITYRVPMASAQVKSAV GDASLTKRPMGRVLNPLREMGVQVTAADGDRLPVTLRGPTTPTPITYRVPMASAQVKSAV | 180 |
| Query 181 | LLAGLNTPGITTVIEPIMTRDHTEKMLQGFGANLTVETDADGVRTIRLEGRGKLTGQVID | 240 |
| Sbjct 181 | LLAGLNTPGITTVIEPIMTRDHTEKMLQGFGANLTVETDADGVRTIRLEG+GKLTGQVID LLAGLNTPGITTVIEPIMTRDHTEKMLQGFGANLTVETDADGVRTIRLEGQGKLTGQVID | 240 |
| Query 241 | VPGDPSSTAFPLVAALLVPGSDVTILNVLMNPTRTGLILTLQEMGADIEVINPRLAGGED | 300 |
| Sbjct 241 | VPGDPSSTAFPLVAALLVPGSDVTILNVLMNPTRTGLILTLQEMGA+IEV+N RLAGGED VPGDPSSTAFPLVAALLVPGSDVTILNVLMNPTRTGLILTLQEMGANIEVLNARLAGGED | 300 |
| Query 301 | VADLRVRSSTLKGVTVPEDRAPSMIDEYPILavaaafaegaTVMNGLEELRVKESDRLSA | 360 |
| Sbjct 301 | VADLRVR S LKGVTVPE+RAPSMIDEYP+LAVAA+FAEG TVMNGLEELRVKESDRLSA VADLRVRHSELKGVTVPEERAPSMIDEYPVLAVAASFAEGTTVMNGLEELRVKESDRLSA | 360 |
| Query 361 | VANGLKLNGVDCDEGETSLVVRGRPDGKGLGNASGAAVATHLDHRIAMSFLVMGLVSENP | 420 |
| Sbjct 361 | VA+GLKLNGVDCDEGE SLVVRGRPDGKGLGNASGA VATHLDHRIAMSFLVMGLVSE+P VADGLKLNGVDCDEGEASLVVRGRPDGKGLGNASGAGVATHLDHRIAMSFLVMGLVSEHP | 420 |
| Query 421  Sbjct 421 | VTVDDATMIATSFPEFMDLMAGLGAKIE 448 VTVDDATMIATSFPEFM LMAGLGA IE VTVDDATMIATSFPEFMGLMAGLGATIE 448 |  |

>PZU81186.1 3-phosphoshikimate 1-carboxyvinyltransferase [Shinella sp.] Length=455

Score = 817 bits (2110), Expect = 0.0, Method: Compositional matrix adjust.

| Identities | = 417/455 (92%), Positives = 434/455 (95%), Gaps = 0/455 (0%) |  |
| --- | --- | --- |
| Query 1 | MLHGASSRPATARKSSGLSGTVRIPGDKSISHRSFMFGGLASGETRITGLLEGEDVINTG | 60 |
| Sbjct 1 | M HGA +PAT+RKS LSGT+RIPGDKSISHRSFMFGGLASG+TRITGLLEGEDVINTG MSHGALPKPATSRKSPALSGTIRIPGDKSISHRSFMFGGLASGQTRITGLLEGEDVINTG | 60 |
| Query 61 | KAMQAMGARIRKEGDTWIIDGVGNGGLLAPEAPLDFGNAATGCRLTMGLVGVYDFDSTFI | 120 |
| Sbjct 61 | KAMQAMGARI K+GDTWIIDGVGNG LLAPEAPLDFGNA TGCRLTMGLVGVYDFDSTFI KAMQAMGARIVKDGDTWIIDGVGNGCLLAPEAPLDFGNAGTGCRLTMGLVGVYDFDSTFI | 120 |
| Query 121 | GDASLTKRPMGRVLNPLREMGVQVKSEDGDRLPVTLRGPKTPTPITYRVPMASAQVKSAV | 180 |

| Sbjct | 121 | GDASLTKRPMGRVLNPLREMGVQVKS +GDRLPVTL GPKTP PITYRVPMASAQVKSAV GDASLTKRPMGRVLNPLREMGVQVKSAEGDRLPVTLHGPKTPAPITYRVPMASAQVKSAV | 180 |
| --- | --- | --- | --- |
| Query | 181 | LLAGLNTPGITTVIEPIMTRDHTEKMLQGFGANLTVETDADGVRTIRLEGRGKLTGQVID | 240 |
| Sbjct | 181 | LLAGLNTPGITTVIEPIMTRDHTEKMLQGFGANLTVETDA+GVRTIR+EGRGKLTGQVID LLAGLNTPGITTVIEPIMTRDHTEKMLQGFGANLTVETDAEGVRTIRIEGRGKLTGQVID | 240 |
| Query | 241 | VPGDPSSTAFPLVAALLVPGSDVTILNVLMNPTRTGLILTLQEMGADIEVINPRLAGGED | 300 |
| Sbjct | 241 | VPGDPSSTAFPLVAALLVPGSDVTILNVLMNPTRTGLILTLQEMGADIE++N RLAGGED VPGDPSSTAFPLVAALLVPGSDVTILNVLMNPTRTGLILTLQEMGADIEILNARLAGGED | 300 |
| Query | 301 | VADLRVRSSTLKGVTVPEDRAPSMIDEYPILavaaafaegaTVMNGLEELRVKESDRLSA | 360 |
| Sbjct | 301 | VADLRVRSS+LKGVTVPEDRAPSMIDEYP+LAVAAAFAEG T M GLEELRVKESDRLSA VADLRVRSSSLKGVTVPEDRAPSMIDEYPVLAVAAAFAEGTTTMLGLEELRVKESDRLSA | 360 |
| Query | 361 | VANGLKLNGVDCDEGETSLVVRGRPDGKGLGNASGAAVATHLDHRIAMSFLVMGLVSENP | 420 |
| Sbjct | 361 | VA GLKLNGVDCDEGE +LVVRGRPDGKGLGN SGAAVATHLDHRIAMSFL+MGLVSE+P VAVGLKLNGVDCDEGEATLVVRGRPDGKGLGNTSGAAVATHLDHRIAMSFLIMGLVSEHP | 420 |
| Query  Sbjct | 421  421 | VTVDDATMIATSFPEFMDLMAGLGAKIELSDTKAA 455 VTVDDATMIATSFPEFMDLM+GLGAKIE + KAA VTVDDATMIATSFPEFMDLMSGLGAKIETREGKAA 455 |  |

>WP_109458669.1 3-phosphoshikimate 1-carboxyvinyltransferase [Rhizobium album] PWE56351.1 3-phosphoshikimate 1-carboxyvinyltransferase [Rhizobium album]

Length=455

Score = 815 bits (2106), Expect = 0.0, Method: Compositional matrix adjust.

| Identities | = 416/455 (91%), Positives = 433/455 (95%), Gaps = 0/455 (0%) |  |
| --- | --- | --- |
| Query 1 | MLHGASSRPATARKSSGLSGTVRIPGDKSISHRSFMFGGLASGETRITGLLEGEDVINTG | 60 |
| Sbjct 1 | M HGA+++PATAR+SS LSGT RIPGDKSISHRSFMFGGLASGETRITGLLEGEDVINTG MSHGAAAKPATARRSSNLSGTARIPGDKSISHRSFMFGGLASGETRITGLLEGEDVINTG | 60 |
| Query 61 | KAMQAMGARIRKEGDTWIIDGVGNGGLLAPEAPLDFGNAATGCRLTMGLVGVYDFDSTFI | 120 |
| Sbjct 61 | KAMQAMGARIRK+GD WIIDG GNG LLAPEAPLDFGNA TGCRLTMGLVGVYDFDSTFI KAMQAMGARIRKDGDVWIIDGTGNGALLAPEAPLDFGNAGTGCRLTMGLVGVYDFDSTFI | 120 |
| Query 121 | GDASLTKRPMGRVLNPLREMGVQVKSEDGDRLPVTLRGPKTPTPITYRVPMASAQVKSAV | 180 |
| Sbjct 121 | GDASLTKRPMGRVL+PLR+MGVQV S DGDRLPVTL GP TP PITYRVPMASAQVKSAV GDASLTKRPMGRVLDPLRQMGVQVTSADGDRLPVTLHGPATPAPITYRVPMASAQVKSAV | 180 |
| Query 181 | LLAGLNTPGITTVIEPIMTRDHTEKMLQGFGANLTVETDADGVRTIRLEGRGKLTGQVID | 240 |
| Sbjct 181 | LLAGLNTPGITTVIEPIMTRDHTEKMLQGFGANLTVET +DGVRTIRLEGRGKLTGQ ID LLAGLNTPGITTVIEPIMTRDHTEKMLQGFGANLTVETGSDGVRTIRLEGRGKLTGQTID | 240 |
| Query 241 | VPGDPSSTAFPLVAALLVPGSDVTILNVLMNPTRTGLILTLQEMGADIEVINPRLAGGED | 300 |
| Sbjct 241 | VPGDPSSTAFPLVAALLVPGSDVTILNVLMNPTRTGLILTLQEMGADIEV+NPRLAGGED VPGDPSSTAFPLVAALLVPGSDVTILNVLMNPTRTGLILTLQEMGADIEVLNPRLAGGED | 300 |
| Query 301 | VADLRVRSSTLKGVTVPEDRAPSMIDEYPILavaaafaegaTVMNGLEELRVKESDRLSA | 360 |
| Sbjct 301 | VADLRVRSS LKGVTVPEDRAPSMIDEYP+LAVAA+FAEGATVMNGLEELRVKESDRLSA VADLRVRSSELKGVTVPEDRAPSMIDEYPVLAVAASFAEGATVMNGLEELRVKESDRLSA | 360 |
| Query 361 | VANGLKLNGVDCDEGETSLVVRGRPDGKGLGNASGAAVATHLDHRIAMSFLVMGLVSENP | 420 |
| Sbjct 361 | VANGLKLNGVDCDEGE++LVVRG P GKGLGNASGAAVATHLDHRIAMSFL+MGL SE+P VANGLKLNGVDCDEGESTLVVRGVPGGKGLGNASGAAVATHLDHRIAMSFLIMGLASEHP | 420 |
| Query 421  Sbjct 421 | VTVDDATMIATSFPEFMDLMAGLGAKIELSDTKAA 455 VTVDDATMIATSFPEFMDLMA LGAKIE+ + AA VTVDDATMIATSFPEFMDLMARLGAKIEVHEGAAA 455 |  |

>WP_097140341.1 3-phosphoshikimate 1-carboxyvinyltransferase [Rhizobium subbaraonis] SOC41324.1 3-phosphoshikimate 1-carboxyvinyltransferase [Rhizobium subbaraonis]

Length=448

Score = 815 bits (2104), Expect = 0.0, Method: Compositional matrix adjust. Identities = 417/448 (93%), Positives = 429/448 (96%), Gaps = 0/448 (0%)

Query 1	MLHGASSRPATARKSSGLSGTVRIPGDKSISHRSFMFGGLASGETRITGLLEGEDVINTG 60

| Sbjct | 1 | M HGA ++PATA KS+ LSGTVRIPGDKSISHRSFMFGGLASGETRITGLLEGEDVINTG MSHGAQAKPATASKSNALSGTVRIPGDKSISHRSFMFGGLASGETRITGLLEGEDVINTG | 60 |
| --- | --- | --- | --- |
| Query | 61 | KAMQAMGARIRKEGDTWIIDGVGNGGLLAPEAPLDFGNAATGCRLTMGLVGVYDFDSTFI | 120 |
| Sbjct | 61 | KAMQAMGA+IRKEGDTWII+GVGNG LLAPEAPLDFGNA TGCRLTMGLVGVYDFDSTFI KAMQAMGAKIRKEGDTWIINGVGNGALLAPEAPLDFGNAGTGCRLTMGLVGVYDFDSTFI | 120 |
| Query | 121 | GDASLTKRPMGRVLNPLREMGVQVKSEDGDRLPVTLRGPKTPTPITYRVPMASAQVKSAV | 180 |
| Sbjct | 121 | GDASLTKRPMGRVLNPLREMGVQV S DGDRLPVTLRGPKTPTPITYRVPMASAQVKSAV GDASLTKRPMGRVLNPLREMGVQVTSADGDRLPVTLRGPKTPTPITYRVPMASAQVKSAV | 180 |
| Query | 181 | LLAGLNTPGITTVIEPIMTRDHTEKMLQGFGANLTVETDADGVRTIRLEGRGKLTGQVID | 240 |
| Sbjct | 181 | LLAGLNTPGITTVIEP+MTRDHTEKMLQGFGANLTVETDA GVRTIRLEG+GKLTGQVID LLAGLNTPGITTVIEPVMTRDHTEKMLQGFGANLTVETDAAGVRTIRLEGQGKLTGQVID | 240 |
| Query | 241 | VPGDPSSTAFPLVAALLVPGSDVTILNVLMNPTRTGLILTLQEMGADIEVINPRLAGGED | 300 |
| Sbjct | 241 | VPGDPSSTAFPLVAALLVPGSDVTILNVLMNPTRTGLILTLQEMGA+IEV+N RLAGGED VPGDPSSTAFPLVAALLVPGSDVTILNVLMNPTRTGLILTLQEMGANIEVLNARLAGGED | 300 |
| Query | 301 | VADLRVRSSTLKGVTVPEDRAPSMIDEYPILavaaafaegaTVMNGLEELRVKESDRLSA | 360 |
| Sbjct | 301 | VADLRVR S LKGVTVPE+RAPSMIDEYPILAVAAAFAEG TVMNGLEELRVKESDRLSA VADLRVRHSELKGVTVPEERAPSMIDEYPILAVAAAFAEGTTVMNGLEELRVKESDRLSA | 360 |
| Query | 361 | VANGLKLNGVDCDEGETSLVVRGRPDGKGLGNASGAAVATHLDHRIAMSFLVMGLVSENP | 420 |
| Sbjct | 361 | VA GLKLNGVDCDEGE SLVV GRPDG GLGNASGAAVATHLDHRIAMSFLVMGLVSE+P VAEGLKLNGVDCDEGEASLVVHGRPDGLGLGNASGAAVATHLDHRIAMSFLVMGLVSEHP | 420 |
| Query  Sbjct | 421  421 | VTVDDATMIATSFPEFMDLMAGLGAKIE 448 VTVDDATMIATSFPEFM LM GLGAKI+ VTVDDATMIATSFPEFMGLMTGLGAKID 448 |  |

>OJT97432.1 3-phosphoshikimate 1-carboxyvinyltransferase [Rhizobium sp. 63-7] Length=455

Score = 815 bits (2104), Expect = 0.0, Method: Compositional matrix adjust.

| Identities | = 416/455 (91%), Positives = 432/455 (95%), Gaps = 0/455 (0%) |  |
| --- | --- | --- |
| Query 1 | MLHGASSRPATARKSSGLSGTVRIPGDKSISHRSFMFGGLASGETRITGLLEGEDVINTG | 60 |
| Sbjct 1 | M HGA+++PATAR+SS LSGT RIPGDKSISHRSFMFGGLASGETRITGLLEGEDVINTG MSHGAAAKPATARRSSNLSGTARIPGDKSISHRSFMFGGLASGETRITGLLEGEDVINTG | 60 |
| Query 61 | KAMQAMGARIRKEGDTWIIDGVGNGGLLAPEAPLDFGNAATGCRLTMGLVGVYDFDSTFI | 120 |
| Sbjct 61 | KAMQAMGARIRK+GD WIIDG GNG LLAPEAPLDFGNA TGCRLTMGLVGVYDFDSTFI KAMQAMGARIRKDGDVWIIDGTGNGALLAPEAPLDFGNAGTGCRLTMGLVGVYDFDSTFI | 120 |
| Query 121 | GDASLTKRPMGRVLNPLREMGVQVKSEDGDRLPVTLRGPKTPTPITYRVPMASAQVKSAV | 180 |
| Sbjct 121 | GDASLTKRPMGRVL+PLR+MGVQV S DGDRLPVTL GP TP PITYRVPMASAQVKSAV GDASLTKRPMGRVLDPLRQMGVQVTSADGDRLPVTLHGPATPAPITYRVPMASAQVKSAV | 180 |
| Query 181 | LLAGLNTPGITTVIEPIMTRDHTEKMLQGFGANLTVETDADGVRTIRLEGRGKLTGQVID | 240 |
| Sbjct 181 | LLAGLNTPGITTVIEPIMTRDHTEKMLQGFGANLTVET +DGVRTIRLEGRGKLTGQ ID LLAGLNTPGITTVIEPIMTRDHTEKMLQGFGANLTVETGSDGVRTIRLEGRGKLTGQTID | 240 |
| Query 241 | VPGDPSSTAFPLVAALLVPGSDVTILNVLMNPTRTGLILTLQEMGADIEVINPRLAGGED | 300 |
| Sbjct 241 | VPGDPSSTAFPLVAALLVPGSDVTILNVLMNPTRTGLILTLQEMGADIEV+NPRLAGGED VPGDPSSTAFPLVAALLVPGSDVTILNVLMNPTRTGLILTLQEMGADIEVLNPRLAGGED | 300 |
| Query 301 | VADLRVRSSTLKGVTVPEDRAPSMIDEYPILavaaafaegaTVMNGLEELRVKESDRLSA | 360 |
| Sbjct 301 | VADLRVRSS LKGVTVPEDRAPSMIDEYP+LAVAA+FAEGATVMNGLEELRVKESDRLSA VADLRVRSSELKGVTVPEDRAPSMIDEYPVLAVAASFAEGATVMNGLEELRVKESDRLSA | 360 |
| Query 361 | VANGLKLNGVDCDEGETSLVVRGRPDGKGLGNASGAAVATHLDHRIAMSFLVMGLVSENP | 420 |
| Sbjct 361 | VA GLKLNGVDCDEGE+SLVVRG P GKGLGNASGAAVATHLDHRIAMSFL+MGL SE+P VATGLKLNGVDCDEGESSLVVRGVPGGKGLGNASGAAVATHLDHRIAMSFLIMGLASEHP | 420 |
| Query 421  Sbjct 421 | VTVDDATMIATSFPEFMDLMAGLGAKIELSDTKAA 455 VTVDDATMIATSFPEFMDLMA LGAKIE+ + AA VTVDDATMIATSFPEFMDLMARLGAKIEVHEGAAA 455 |  |

>WP_153442469.1 3-phosphoshikimate 1-carboxyvinyltransferase [Sinorhizobium terangae]

MBB4183564.1 3-phosphoshikimate 1-carboxyvinyltransferase [Sinorhizobium terangae] MQX18698.1 3-phosphoshikimate 1-carboxyvinyltransferase [Sinorhizobium terangae]

Length=455

Score = 812 bits (2097), Expect = 0.0, Method: Compositional matrix adjust.

| Identities | = 414/455 (91%), Positives = 431/455 (95%), Gaps = 0/455 (0%) |  |
| --- | --- | --- |
| Query 1 | MLHGASSRPATARKSSGLSGTVRIPGDKSISHRSFMFGGLASGETRITGLLEGEDVINTG | 60 |
| Sbjct 1 | M HG+S RPATA KSS L GTVRIPGDKSISHRSFMFGGLA+GETRITGLLEGEDVINTG MSHGSSPRPATASKSSDLKGTVRIPGDKSISHRSFMFGGLAAGETRITGLLEGEDVINTG | 60 |
| Query 61 | KAMQAMGARIRKEGDTWIIDGVGNGGLLAPEAPLDFGNAATGCRLTMGLVGVYDFDSTFI | 120 |
| Sbjct 61 | KAMQAMGA+IRKEGDTWIIDGVGNG LLAPEAPLDFGNA TGCRLTMGLVGVYDFDSTFI KAMQAMGAKIRKEGDTWIIDGVGNGALLAPEAPLDFGNAGTGCRLTMGLVGVYDFDSTFI | 120 |
| Query 121 | GDASLTKRPMGRVLNPLREMGVQVKSEDGDRLPVTLRGPKTPTPITYRVPMASAQVKSAV | 180 |
| Sbjct 121 | GDASLTKRPMGRVLNPLREMGVQVKS +GDRLPVTL GPKTP P TYRVPMASAQVKSAV GDASLTKRPMGRVLNPLREMGVQVKSAEGDRLPVTLHGPKTPNPTTYRVPMASAQVKSAV | 180 |
| Query 181 | LLAGLNTPGITTVIEPIMTRDHTEKMLQGFGANLTVETDADGVRTIRLEGRGKLTGQVID | 240 |
| Sbjct 181 | LLAGLNTPGITTVIEP+MTRDHTEKMLQGFGANLTVETDA+GVRTIRLEGRG+LTGQVID LLAGLNTPGITTVIEPVMTRDHTEKMLQGFGANLTVETDAEGVRTIRLEGRGRLTGQVID | 240 |
| Query 241 | VPGDPSSTAFPLVAALLVPGSDVTILNVLMNPTRTGLILTLQEMGADIEVINPRLAGGED | 300 |
| Sbjct 241 | VPGDPSSTAFPLVAALLVPGSDVTILNVLMNPTRTGLILTLQEMGA+IEV+N RLAGGED VPGDPSSTAFPLVAALLVPGSDVTILNVLMNPTRTGLILTLQEMGANIEVMNARLAGGED | 300 |
| Query 301 | VADLRVRSSTLKGVTVPEDRAPSMIDEYPILavaaafaegaTVMNGLEELRVKESDRLSA | 360 |
| Sbjct 301 | VADLRVR S LKGVTVPE+RAPSMIDEYP+LAVAAAFAEG+TVMNGL+ELRVKESDRLSA VADLRVRYSELKGVTVPEERAPSMIDEYPVLAVAAAFAEGSTVMNGLDELRVKESDRLSA | 360 |
| Query 361 | VANGLKLNGVDCDEGETSLVVRGRPDGKGLGNASGAAVATHLDHRIAMSFLVMGLVSENP | 420 |
| Sbjct 361 | VA+GLKLNGVDCDEGE SLVVRGRP GKGLGNASG V THLDHRIAMSFLVMGL SE+P VADGLKLNGVDCDEGEASLVVRGRPGGKGLGNASGGKVKTHLDHRIAMSFLVMGLASEHP | 420 |
| Query 421  Sbjct 421 | VTVDDATMIATSFPEFMDLMAGLGAKIELSDTKAA 455 VTVDDATMIATSFPEFMDLM GLGAKIE ++ K A VTVDDATMIATSFPEFMDLMTGLGAKIEQTEAKIA 455 |  |

>WP_071024014.1 3-phosphoshikimate 1-carboxyvinyltransferase [Ensifer sp. LCM 4579]

OHV73072.1 3-phosphoshikimate 1-carboxyvinyltransferase [Ensifer sp. LCM 4579]

Length=455

Score = 811 bits (2096), Expect = 0.0, Method: Compositional matrix adjust.

| Identities | = 414/454 (91%), Positives = 431/454 (95%), Gaps = 0/454 (0%) |  |
| --- | --- | --- |
| Query 1 | MLHGASSRPATARKSSGLSGTVRIPGDKSISHRSFMFGGLASGETRITGLLEGEDVINTG | 60 |
| Sbjct 1 | M HG SSRPATARKSS L GTVRIPGDKSISHRSFMFGGLASGETRITGLLEGEDVINTG MSHGQSSRPATARKSSDLKGTVRIPGDKSISHRSFMFGGLASGETRITGLLEGEDVINTG | 60 |
| Query 61 | KAMQAMGARIRKEGDTWIIDGVGNGGLLAPEAPLDFGNAATGCRLTMGLVGVYDFDSTFI | 120 |
| Sbjct 61 | KAMQAMGA+IRKEGDTWIIDGVGNG LLAPEAPLDFGNA TGCRLTMGLVGVYDF STF+ KAMQAMGAKIRKEGDTWIIDGVGNGALLAPEAPLDFGNAGTGCRLTMGLVGVYDFASTFV | 120 |
| Query 121 | GDASLTKRPMGRVLNPLREMGVQVKSEDGDRLPVTLRGPKTPTPITYRVPMASAQVKSAV | 180 |
| Sbjct 121 | GDASL KRPMGRVL+PLREMGVQVKS +GDRLPVTL GPKTP PITYRVPMASAQVKSAV GDASLAKRPMGRVLDPLREMGVQVKSAEGDRLPVTLHGPKTPNPITYRVPMASAQVKSAV | 180 |
| Query 181 | LLAGLNTPGITTVIEPIMTRDHTEKMLQGFGANLTVETDADGVRTIRLEGRGKLTGQVID | 240 |
| Sbjct 181 | LLAGLNTPGITTV+EP+MTRDHTEKMLQGFGANL+VETD GVRTIRLEGRGKLTGQVID LLAGLNTPGITTVVEPVMTRDHTEKMLQGFGANLSVETDTQGVRTIRLEGRGKLTGQVID | 240 |
| Query 241 | VPGDPSSTAFPLVAALLVPGSDVTILNVLMNPTRTGLILTLQEMGADIEVINPRLAGGED | 300 |
| Sbjct 241 | VPGDPSSTAFPLVAAL+VPGSDVTILNVLMNPTRTGLILTLQEMGADIEV+N RLAGGED VPGDPSSTAFPLVAALIVPGSDVTILNVLMNPTRTGLILTLQEMGADIEVMNKRLAGGED | 300 |
| Query 301 | VADLRVRSSTLKGVTVPEDRAPSMIDEYPILavaaafaegaTVMNGLEELRVKESDRLSA | 360 |
| Sbjct 301 | VADLRVR S LKGVTVPE+RAPSMIDEYP+LAVAAAFAEGATVMNGL+ELRVKESDRLSA  VADLRVRHSELKGVTVPEERAPSMIDEYPVLAVAAAFAEGATVMNGLDELRVKESDRLSA | 360 |

| Query | 361 | VANGLKLNGVDCDEGETSLVVRGRPDGKGLGNASGAAVATHLDHRIAMSFLVMGLVSENP 420 |
| --- | --- | --- |
| Sbjct | 361 | VA+GLKLNGVDCDEGE SLVVRGRPDGKGLGNASG V THLDHRIAMSFLV+GL SE+P VADGLKLNGVDCDEGEASLVVRGRPDGKGLGNASGEKVKTHLDHRIAMSFLVLGLASEHP 420 |
| Query | 421 | VTVDDATMIATSFPEFMDLMAGLGAKIELSDTKA 454 |
| Sbjct | 421 | VTVDDATMIATSFPEFMDLM GLGAKIE ++TKA  VTVDDATMIATSFPEFMDLMTGLGAKIEQAETKA 454 |

>WP_034853513.1 3-phosphoshikimate 1-carboxyvinyltransferase [Ensifer sojae] ASY65004.1 5-Enolpyruvylshikimate-3-phosphate synthase [Ensifer sojae CCBAU

05684]

Length=455

Score = 811 bits (2096), Expect = 0.0, Method: Compositional matrix adjust.

| Identities | = 415/455 (91%), Positives = 432/455 (95%), Gaps = 0/455 (0%) |  |
| --- | --- | --- |
| Query 1 | MLHGASSRPATARKSSGLSGTVRIPGDKSISHRSFMFGGLASGETRITGLLEGEDVINTG | 60 |
| Sbjct 1 | M HG S RPATARKSS L GTVRIPGDKSISHRSFMFGGLASGETRITGLLEGEDVINTG MSHGQSPRPATARKSSDLKGTVRIPGDKSISHRSFMFGGLASGETRITGLLEGEDVINTG | 60 |
| Query 61 | KAMQAMGARIRKEGDTWIIDGVGNGGLLAPEAPLDFGNAATGCRLTMGLVGVYDFDSTFI | 120 |
| Sbjct 61 | KAMQAMGA+IRK GDTWIIDGVGNG LLAPEAPLDFGNA TGCRLTMGLVGVYDFDSTFI KAMQAMGAKIRKAGDTWIIDGVGNGALLAPEAPLDFGNAGTGCRLTMGLVGVYDFDSTFI | 120 |
| Query 121 | GDASLTKRPMGRVLNPLREMGVQVKSEDGDRLPVTLRGPKTPTPITYRVPMASAQVKSAV | 180 |
| Sbjct 121 | GDASLTKRPMGRVL+PLR+MGVQV+S +GDRLPVTL GPKTP PITYRVPMASAQVKSAV GDASLTKRPMGRVLDPLRKMGVQVQSAEGDRLPVTLHGPKTPNPITYRVPMASAQVKSAV | 180 |
| Query 181 | LLAGLNTPGITTVIEPIMTRDHTEKMLQGFGANLTVETDADGVRTIRLEGRGKLTGQVID | 240 |
| Sbjct 181 | LLAGLNTPGITTVIEP+MTRDHTEKMLQGFGANL+VETDA GVRTIRLEGRG+LTGQVID LLAGLNTPGITTVIEPVMTRDHTEKMLQGFGANLSVETDATGVRTIRLEGRGRLTGQVID | 240 |
| Query 241 | VPGDPSSTAFPLVAALLVPGSDVTILNVLMNPTRTGLILTLQEMGADIEVINPRLAGGED | 300 |
| Sbjct 241 | VPGDPSSTAFPLVAAL+VPGSDVTI NVLMNPTRTGLILTLQEMGADIEV+N RLAGGED VPGDPSSTAFPLVAALIVPGSDVTIFNVLMNPTRTGLILTLQEMGADIEVMNKRLAGGED | 300 |
| Query 301 | VADLRVRSSTLKGVTVPEDRAPSMIDEYPILavaaafaegaTVMNGLEELRVKESDRLSA | 360 |
| Sbjct 301 | VADLRVR S LKGVTVPE+RAPSMIDEYP+LAVAAAFAEGATVMNGL+ELRVKESDRLSA VADLRVRHSELKGVTVPEERAPSMIDEYPVLAVAAAFAEGATVMNGLDELRVKESDRLSA | 360 |
| Query 361 | VANGLKLNGVDCDEGETSLVVRGRPDGKGLGNASGAAVATHLDHRIAMSFLVMGLVSENP | 420 |
| Sbjct 361 | VA+GLKLNGVDCDEGE SLVVRGRPDGKGLGNASG V THLDHRIAMSFLVMGL SE+P VADGLKLNGVDCDEGEASLVVRGRPDGKGLGNASGEKVKTHLDHRIAMSFLVMGLASEHP | 420 |
| Query 421  Sbjct 421 | VTVDDATMIATSFPEFMDLMAGLGAKIELSDTKAA 455 VTVDDATMIATSFPEFMDLM GLGAKIE ++TKAA VTVDDATMIATSFPEFMDLMTGLGAKIEQAETKAA 455 |  |

>WP_066867978.1 3-phosphoshikimate 1-carboxyvinyltransferase [Sinorhizobium saheli] MQW88701.1 3-phosphoshikimate 1-carboxyvinyltransferase [Sinorhizobium saheli] OAP50369.1 3-phosphoshikimate 1-carboxyvinyltransferase [Sinorhizobium saheli]

Length=455

Score = 811 bits (2094), Expect = 0.0, Method: Compositional matrix adjust.

| Identities | = 415/454 (91%), Positives = 431/454 (95%), Gaps = 0/454 (0%) |  |
| --- | --- | --- |
| Query 1 | MLHGASSRPATARKSSGLSGTVRIPGDKSISHRSFMFGGLASGETRITGLLEGEDVINTG | 60 |
| Sbjct 1 | M HG+S RPATARKS L GTVRIPGDKSISHRSFMFGGLASGETRITGLLEGEDVINTG MSHGSSPRPATARKSLDLRGTVRIPGDKSISHRSFMFGGLASGETRITGLLEGEDVINTG | 60 |
| Query 61 | KAMQAMGARIRKEGDTWIIDGVGNGGLLAPEAPLDFGNAATGCRLTMGLVGVYDFDSTFI | 120 |
| Sbjct 61 | KAMQAMGARIRKEGDTWIIDGVGNG LLAPEAPLDFGNA TGCRLTMGLVGVYDFDSTFI KAMQAMGARIRKEGDTWIIDGVGNGALLAPEAPLDFGNAGTGCRLTMGLVGVYDFDSTFI | 120 |
| Query 121 | GDASLTKRPMGRVLNPLREMGVQVKSEDGDRLPVTLRGPKTPTPITYRVPMASAQVKSAV | 180 |
| Sbjct 121 | GDASLTKRPMGRVL+PLREMGVQVKS DGDRLPVTLRGP+TP PITYRVPMASAQVKSAV GDASLTKRPMGRVLDPLREMGVQVKSADGDRLPVTLRGPQTPNPITYRVPMASAQVKSAV | 180 |
| Query 181 | LLAGLNTPGITTVIEPIMTRDHTEKMLQGFGANLTVETDADGVRTIRLEGRGKLTGQVID | 240 |

| Sbjct | 181 | LLAGLNTPGITTV+EP+MTRDHTEKMLQGFGANL+VETDA+GVRTIRLEGRGKLTGQVID LLAGLNTPGITTVVEPVMTRDHTEKMLQGFGANLSVETDAEGVRTIRLEGRGKLTGQVID | 240 |
| --- | --- | --- | --- |
| Query | 241 | VPGDPSSTAFPLVAALLVPGSDVTILNVLMNPTRTGLILTLQEMGADIEVINPRLAGGED | 300 |
| Sbjct | 241 | VPGDPSSTAFPLVAALLVPGS+VTILNVLMNPTRTGLILTLQEMGADIEV+N RLAGGED VPGDPSSTAFPLVAALLVPGSEVTILNVLMNPTRTGLILTLQEMGADIEVMNKRLAGGED | 300 |
| Query | 301 | VADLRVRSSTLKGVTVPEDRAPSMIDEYPILavaaafaegaTVMNGLEELRVKESDRLSA | 360 |
| Sbjct | 301 | VADLRVR S LKGVTVPE+RAPSMIDEYP+LAVAAAFAEG TVMNGL+ELRVKESDRLSA VADLRVRHSDLKGVTVPEERAPSMIDEYPVLAVAAAFAEGTTVMNGLDELRVKESDRLSA | 360 |
| Query | 361 | VANGLKLNGVDCDEGETSLVVRGRPDGKGLGNASGAAVATHLDHRIAMSFLVMGLVSENP | 420 |
| Sbjct | 361 | VA+GLKLNGVDCDEGE SLVVRGRP GKGLGNASG V THLDHRIAMSFLVMGL SE+P VADGLKLNGVDCDEGEASLVVRGRPGGKGLGNASGGEVRTHLDHRIAMSFLVMGLASEHP | 420 |
| Query  Sbjct | 421  421 | VTVDDATMIATSFPEFMDLMAGLGAKIELSDTKA 454 VTVDDATMIATSFPEFMDLM GLGAKIE + K+ VTVDDATMIATSFPEFMDLMTGLGAKIEQTGAKS 454 |  |

>WP_026613614.1 MULTISPECIES: 3-phosphoshikimate 1-carboxyvinyltransferase [Sinorhizobium/Ensifer group]

Length=455

Score = 811 bits (2094), Expect = 0.0, Method: Compositional matrix adjust.

| Identities | = 413/455 (91%), Positives = 434/455 (95%), Gaps = 0/455 (0%) |  |
| --- | --- | --- |
| Query 1 | MLHGASSRPATARKSSGLSGTVRIPGDKSISHRSFMFGGLASGETRITGLLEGEDVINTG | 60 |
| Sbjct 1 | M HG+S RPATA+KSS L GTVRIPGDKSISHRSFMFGGLASGETRITGLLEGEDVINTG MSHGSSPRPATAKKSSDLKGTVRIPGDKSISHRSFMFGGLASGETRITGLLEGEDVINTG | 60 |
| Query 61 | KAMQAMGARIRKEGDTWIIDGVGNGGLLAPEAPLDFGNAATGCRLTMGLVGVYDFDSTFI | 120 |
| Sbjct 61 | KAMQAMGARIRKEGDTWII+GVGNG LLAPEAPLDFGNA TGCRLTMGLVGVYDFDSTFI KAMQAMGARIRKEGDTWIINGVGNGALLAPEAPLDFGNAGTGCRLTMGLVGVYDFDSTFI | 120 |
| Query 121 | GDASLTKRPMGRVLNPLREMGVQVKSEDGDRLPVTLRGPKTPTPITYRVPMASAQVKSAV | 180 |
| Sbjct 121 | GDASLTKRPMGRVL+PLREMGVQVKS +GDRLPVTL GP+TP PITYRVPMASAQVKSAV GDASLTKRPMGRVLDPLREMGVQVKSAEGDRLPVTLHGPQTPNPITYRVPMASAQVKSAV | 180 |
| Query 181 | LLAGLNTPGITTVIEPIMTRDHTEKMLQGFGANLTVETDADGVRTIRLEGRGKLTGQVID | 240 |
| Sbjct 181 | LLAGLNTPGITTVIEP+MTRDHTEKMLQGFGANLTVETDA+GVRTIRLEGRGKL GQVID LLAGLNTPGITTVIEPVMTRDHTEKMLQGFGANLTVETDAEGVRTIRLEGRGKLIGQVID | 240 |
| Query 241 | VPGDPSSTAFPLVAALLVPGSDVTILNVLMNPTRTGLILTLQEMGADIEVINPRLAGGED | 300 |
| Sbjct 241 | VPGDPSSTAFPLVAAL+VPGSD+TILNVLMNPTRTGLILTLQEMGA+IEV+N RLAGGED VPGDPSSTAFPLVAALIVPGSDITILNVLMNPTRTGLILTLQEMGAEIEVMNQRLAGGED | 300 |
| Query 301 | VADLRVRSSTLKGVTVPEDRAPSMIDEYPILavaaafaegaTVMNGLEELRVKESDRLSA | 360 |
| Sbjct 301 | VADLRVR S LKGV+VPE+RAPSMIDEYP+LAVAAAFAEGATVMNGLEELRVKESDRLSA VADLRVRYSELKGVSVPEERAPSMIDEYPVLAVAAAFAEGATVMNGLEELRVKESDRLSA | 360 |
| Query 361 | VANGLKLNGVDCDEGETSLVVRGRPDGKGLGNASGAAVATHLDHRIAMSFLVMGLVSENP | 420 |
| Sbjct 361 | VA+GLKLNG+DCDEGE SLVVRGRP GKGLGNASG V THLDHRIAMSFLVMGL SE+P VADGLKLNGLDCDEGEASLVVRGRPGGKGLGNASGEKVKTHLDHRIAMSFLVMGLASEHP | 420 |
| Query 421  Sbjct 421 | VTVDDATMIATSFPEFMDLMAGLGAKIELSDTKAA 455 VTVDDATMIATSFPEFMDLM GLGAKIE +++KAA VTVDDATMIATSFPEFMDLMTGLGAKIEQAESKAA 455 |  |

>WP_003534382.1 3-phosphoshikimate 1-carboxyvinyltransferase [Sinorhizobium meliloti] ASQ05367.1 3-phosphoshikimate 1-carboxyvinyltransferase [Sinorhizobium meliloti] EHK74715.1 3-phosphoshikimate 1-carboxyvinyltransferase [Sinorhizobium meliloti

CCNWSX0020]

MQU72260.1 3-phosphoshikimate 1-carboxyvinyltransferase [Sinorhizobium meliloti] MQV06715.1 3-phosphoshikimate 1-carboxyvinyltransferase [Sinorhizobium meliloti] MQV19824.1 3-phosphoshikimate 1-carboxyvinyltransferase [Sinorhizobium meliloti]

Length=455

Score = 810 bits (2093), Expect = 0.0, Method: Compositional matrix adjust. Identities = 413/455 (91%), Positives = 431/455 (95%), Gaps = 0/455 (0%)

| Query | 1 | MLHGASSRPATARKSSGLSGTVRIPGDKSISHRSFMFGGLASGETRITGLLEGEDVINTG | 60 |
| --- | --- | --- | --- |
| Sbjct | 1 | M HG++ RPATARKSS L GT+RIPGDKSISHRSFMFGGLA+GETRITGLLEGEDVINTG MSHGSNPRPATARKSSDLKGTLRIPGDKSISHRSFMFGGLAAGETRITGLLEGEDVINTG | 60 |
| Query | 61 | KAMQAMGARIRKEGDTWIIDGVGNGGLLAPEAPLDFGNAATGCRLTMGLVGVYDFDSTFI | 120 |
| Sbjct | 61 | KAMQAMGARIRKEGDTWIIDGVGNG LLAPEAPLDFGNA TGCRLTMGLVGVYDFDSTFI KAMQAMGARIRKEGDTWIIDGVGNGALLAPEAPLDFGNAGTGCRLTMGLVGVYDFDSTFI | 120 |
| Query | 121 | GDASLTKRPMGRVLNPLREMGVQVKSEDGDRLPVTLRGPKTPTPITYRVPMASAQVKSAV | 180 |
| Sbjct | 121 | GDASLTKRPMGRVL+PLREMGVQVKS +GDRLPVTLRGPKTP PITYRVPMASAQVKSAV GDASLTKRPMGRVLDPLREMGVQVKSAEGDRLPVTLRGPKTPNPITYRVPMASAQVKSAV | 180 |
| Query | 181 | LLAGLNTPGITTVIEPIMTRDHTEKMLQGFGANLTVETDADGVRTIRLEGRGKLTGQVID | 240 |
| Sbjct | 181 | LLAGLNTPGITTV+EP+MTRDHTEKMLQGFGANLTVETDA+GVRTIRLEGRGKLTGQVID LLAGLNTPGITTVVEPVMTRDHTEKMLQGFGANLTVETDAEGVRTIRLEGRGKLTGQVID | 240 |
| Query | 241 | VPGDPSSTAFPLVAALLVPGSDVTILNVLMNPTRTGLILTLQEMGADIEVINPRLAGGED | 300 |
| Sbjct | 241 | VPGDPSSTAFPLVA L+VPGSD+TILNVLMNPTRTGLILTLQEMGA+IEV+N RLAGGED VPGDPSSTAFPLVAGLIVPGSDITILNVLMNPTRTGLILTLQEMGANIEVMNKRLAGGED | 300 |
| Query | 301 | VADLRVRSSTLKGVTVPEDRAPSMIDEYPILavaaafaegaTVMNGLEELRVKESDRLSA | 360 |
| Sbjct | 301 | VADLRVR S LKGVTVPEDRAPSMIDEYP+LAVAAAFAEG TVMNGLEELRVKESDRLSA VADLRVRHSELKGVTVPEDRAPSMIDEYPVLAVAAAFAEGTTVMNGLEELRVKESDRLSA | 360 |
| Query | 361 | VANGLKLNGVDCDEGETSLVVRGRPDGKGLGNASGAAVATHLDHRIAMSFLVMGLVSENP | 420 |
| Sbjct | 361 | VA+GLKLNGVDCDEGE SLVVRGRP GKGLGN SG V THLDHRIAMSFLVMGL SE+P VADGLKLNGVDCDEGEASLVVRGRPGGKGLGNISGGQVKTHLDHRIAMSFLVMGLASEHP | 420 |
| Query  Sbjct | 421  421 | VTVDDATMIATSFPEFMDLMAGLGAKIELSDTKAA 455 VTVDDATMIATSFPEFM LM GLGAKIE ++ KAA VTVDDATMIATSFPEFMGLMTGLGAKIEEAENKAA 455 |  |

>WP_180939806.1 3-phosphoshikimate 1-carboxyvinyltransferase [Ensifer mexicanus] QLL60213.1 3-phosphoshikimate 1-carboxyvinyltransferase [Ensifer mexicanus]

Length=455

Score = 810 bits (2092), Expect = 0.0, Method: Compositional matrix adjust.

| Identities | = 414/455 (91%), Positives = 430/455 (95%), Gaps = 0/455 (0%) |  |
| --- | --- | --- |
| Query 1 | MLHGASSRPATARKSSGLSGTVRIPGDKSISHRSFMFGGLASGETRITGLLEGEDVINTG | 60 |
| Sbjct 1 | M HG+S RPATA+KSS L GTVRIPGDKSISHRSFMFGGLASGETRITGLLEGEDVINTG MSHGSSPRPATAKKSSDLKGTVRIPGDKSISHRSFMFGGLASGETRITGLLEGEDVINTG | 60 |
| Query 61 | KAMQAMGARIRKEGDTWIIDGVGNGGLLAPEAPLDFGNAATGCRLTMGLVGVYDFDSTFI | 120 |
| Sbjct 61 | KAMQAMGARIRKEGD WIIDGVGNG LLAPEAPLDFGNA TGCRLTMGLVGVYDFDSTFI KAMQAMGARIRKEGDAWIIDGVGNGALLAPEAPLDFGNAGTGCRLTMGLVGVYDFDSTFI | 120 |
| Query 121 | GDASLTKRPMGRVLNPLREMGVQVKSEDGDRLPVTLRGPKTPTPITYRVPMASAQVKSAV | 180 |
| Sbjct 121 | GDASLTKRPMGRVLNPLREMGVQV S +GDRLPVTLRGPKTP PITYRVPMASAQVKSAV GDASLTKRPMGRVLNPLREMGVQVNSAEGDRLPVTLRGPKTPNPITYRVPMASAQVKSAV | 180 |
| Query 181 | LLAGLNTPGITTVIEPIMTRDHTEKMLQGFGANLTVETDADGVRTIRLEGRGKLTGQVID | 240 |
| Sbjct 181 | LLAGLNTPGITTVIEP+MTRDHTEKMLQGFGA+LTVETDADGVRTIRLEGRGKLTGQVID LLAGLNTPGITTVIEPVMTRDHTEKMLQGFGASLTVETDADGVRTIRLEGRGKLTGQVID | 240 |
| Query 241 | VPGDPSSTAFPLVAALLVPGSDVTILNVLMNPTRTGLILTLQEMGADIEVINPRLAGGED | 300 |
| Sbjct 241 | VPGDPSSTAFPLVAALLVPGSDVTILNVLMNPTRTGLILTLQEMGA+IEV+N RLAGGED VPGDPSSTAFPLVAALLVPGSDVTILNVLMNPTRTGLILTLQEMGANIEVMNARLAGGED | 300 |
| Query 301 | VADLRVRSSTLKGVTVPEDRAPSMIDEYPILavaaafaegaTVMNGLEELRVKESDRLSA | 360 |
| Sbjct 301 | VADLRVR S L+GV VP++RAPSMIDEYP+LAVAAAFAEG TVMNGL+ELRVKESDRLSA VADLRVRFSELQGVMVPDERAPSMIDEYPVLAVAAAFAEGTTVMNGLDELRVKESDRLSA | 360 |
| Query 361 | VANGLKLNGVDCDEGETSLVVRGRPDGKGLGNASGAAVATHLDHRIAMSFLVMGLVSENP | 420 |
| Sbjct 361 | VA+GLKLNGVDCDEGE SLVVRGRP GKGLGNASG V THLDHRIAMSFLVMGL SE+P VADGLKLNGVDCDEGEASLVVRGRPGGKGLGNASGGEVKTHLDHRIAMSFLVMGLASEHP | 420 |
| Query 421  Sbjct 421 | VTVDDATMIATSFPEFMDLMAGLGAKIELSDTKAA 455 VTVDDATMIATSFPEFMDLM GLGAKIE ++ K A VTVDDATMIATSFPEFMDLMTGLGAKIEQTEAKVA 455 |  |

>WP_017271506.1 3-phosphoshikimate 1-carboxyvinyltransferase [Sinorhizobium meliloti] Length=455

Score = 810 bits (2092), Expect = 0.0, Method: Compositional matrix adjust.

| Identities | = 412/455 (91%), Positives = 430/455 (95%), Gaps = 0/455 (0%) |  |
| --- | --- | --- |
| Query 1 | MLHGASSRPATARKSSGLSGTVRIPGDKSISHRSFMFGGLASGETRITGLLEGEDVINTG | 60 |
| Sbjct 1 | M HG++ RPATARKSS L GT+RIPGDKSISHRSFMFGGLA+GETRITGLLEGEDVINTG MSHGSNQRPATARKSSDLKGTLRIPGDKSISHRSFMFGGLAAGETRITGLLEGEDVINTG | 60 |
| Query 61 | KAMQAMGARIRKEGDTWIIDGVGNGGLLAPEAPLDFGNAATGCRLTMGLVGVYDFDSTFI | 120 |
| Sbjct 61 | KAMQAMGARIRKEGDTWIIDGVGNG LLAPEAPLDFGNA TGCRLTMGLVGVYDFDSTFI KAMQAMGARIRKEGDTWIIDGVGNGALLAPEAPLDFGNAGTGCRLTMGLVGVYDFDSTFI | 120 |
| Query 121 | GDASLTKRPMGRVLNPLREMGVQVKSEDGDRLPVTLRGPKTPTPITYRVPMASAQVKSAV | 180 |
| Sbjct 121 | GDASLTKRPMGRVL+PLREMGVQVKS +GDRLPVTLRGPKTP PITYRVPMASAQVKSAV GDASLTKRPMGRVLDPLREMGVQVKSAEGDRLPVTLRGPKTPNPITYRVPMASAQVKSAV | 180 |
| Query 181 | LLAGLNTPGITTVIEPIMTRDHTEKMLQGFGANLTVETDADGVRTIRLEGRGKLTGQVID | 240 |
| Sbjct 181 | LLAGLNTPGITTV+EP+MTRDHTEKMLQGFGANLTVETDA+GVRTIRLEGRGKLTGQVID LLAGLNTPGITTVVEPVMTRDHTEKMLQGFGANLTVETDAEGVRTIRLEGRGKLTGQVID | 240 |
| Query 241 | VPGDPSSTAFPLVAALLVPGSDVTILNVLMNPTRTGLILTLQEMGADIEVINPRLAGGED | 300 |
| Sbjct 241 | VPGDPSSTAFPLVA L+VPGSD+TILNVLMNPTRTGLILTLQEMGA+IEV+N RLAGGED VPGDPSSTAFPLVAGLIVPGSDITILNVLMNPTRTGLILTLQEMGANIEVMNKRLAGGED | 300 |
| Query 301 | VADLRVRSSTLKGVTVPEDRAPSMIDEYPILavaaafaegaTVMNGLEELRVKESDRLSA | 360 |
| Sbjct 301 | VADLRVR S LKGVTVPEDRAPSMIDEYP+LAVAAAFAEG TVMNGLEELRVKESDRLSA VADLRVRHSELKGVTVPEDRAPSMIDEYPVLAVAAAFAEGTTVMNGLEELRVKESDRLSA | 360 |
| Query 361 | VANGLKLNGVDCDEGETSLVVRGRPDGKGLGNASGAAVATHLDHRIAMSFLVMGLVSENP | 420 |
| Sbjct 361 | VA+GLKLNGVDCDEGE SLVVRGRP GKGLG SG V THLDHRIAMSFLVMGL SE+P VADGLKLNGVDCDEGEASLVVRGRPGGKGLGKISGGQVKTHLDHRIAMSFLVMGLASEHP | 420 |
| Query 421  Sbjct 421 | VTVDDATMIATSFPEFMDLMAGLGAKIELSDTKAA 455 VTVDDATMIATSFPEFM LM GLGAKIE ++ KAA VTVDDATMIATSFPEFMGLMTGLGAKIEEAENKAA 455 |  |

>WP_153420160.1 3-phosphoshikimate 1-carboxyvinyltransferase [Sinorhizobium meliloti] MQW14514.1 3-phosphoshikimate 1-carboxyvinyltransferase [Sinorhizobium meliloti]

Length=455

Score = 809 bits (2089), Expect = 0.0, Method: Compositional matrix adjust.

| Identities | = 412/455 (91%), Positives = 430/455 (95%), Gaps = 0/455 (0%) |  |
| --- | --- | --- |
| Query 1 | MLHGASSRPATARKSSGLSGTVRIPGDKSISHRSFMFGGLASGETRITGLLEGEDVINTG | 60 |
| Sbjct 1 | M HG++ RPATARKSS L G +RIPGDKSISHRSFMFGGLA+GETRITGLLEGEDVINTG MSHGSNPRPATARKSSDLKGILRIPGDKSISHRSFMFGGLAAGETRITGLLEGEDVINTG | 60 |
| Query 61 | KAMQAMGARIRKEGDTWIIDGVGNGGLLAPEAPLDFGNAATGCRLTMGLVGVYDFDSTFI | 120 |
| Sbjct 61 | KAMQAMGARIRKEGDTWIIDGVGNG LLAPEAPLDFGNA TGCRLTMGLVGVYDFDSTFI KAMQAMGARIRKEGDTWIIDGVGNGALLAPEAPLDFGNAGTGCRLTMGLVGVYDFDSTFI | 120 |
| Query 121 | GDASLTKRPMGRVLNPLREMGVQVKSEDGDRLPVTLRGPKTPTPITYRVPMASAQVKSAV | 180 |
| Sbjct 121 | GDASLTKRPMGRVL+PLREMGVQVKS +GDRLPVTLRGPKTP PITYRVPMASAQVKSAV GDASLTKRPMGRVLDPLREMGVQVKSAEGDRLPVTLRGPKTPNPITYRVPMASAQVKSAV | 180 |
| Query 181 | LLAGLNTPGITTVIEPIMTRDHTEKMLQGFGANLTVETDADGVRTIRLEGRGKLTGQVID | 240 |
| Sbjct 181 | LLAGLNTPGITTV+EP+MTRDHTEKMLQGFGANLTVETDA+GVRTIRLEGRGKLTGQVID LLAGLNTPGITTVVEPVMTRDHTEKMLQGFGANLTVETDAEGVRTIRLEGRGKLTGQVID | 240 |
| Query 241 | VPGDPSSTAFPLVAALLVPGSDVTILNVLMNPTRTGLILTLQEMGADIEVINPRLAGGED | 300 |
| Sbjct 241 | VPGDPSSTAFPLVA L+VPGSD+TILNVLMNPTRTGLILTLQEMGA+IEV+N RLAGGED VPGDPSSTAFPLVAGLIVPGSDITILNVLMNPTRTGLILTLQEMGANIEVMNKRLAGGED | 300 |
| Query 301 | VADLRVRSSTLKGVTVPEDRAPSMIDEYPILavaaafaegaTVMNGLEELRVKESDRLSA | 360 |
| Sbjct 301 | VADLRVR S LKGVTVPEDRAPSMIDEYP+LAVAAAFAEG TVMNGLEELRVKESDRLSA VADLRVRHSELKGVTVPEDRAPSMIDEYPVLAVAAAFAEGTTVMNGLEELRVKESDRLSA | 360 |
| Query 361 | VANGLKLNGVDCDEGETSLVVRGRPDGKGLGNASGAAVATHLDHRIAMSFLVMGLVSENP | 420 |

| Sbjct | 361 | VA+GLKLNGVDCDEGE SLVVRGRP GKGLGN SG V THLDHRIAMSFLVMGL SE+P VADGLKLNGVDCDEGEASLVVRGRPGGKGLGNISGGQVKTHLDHRIAMSFLVMGLASEHP 420 |
| --- | --- | --- |
| Query | 421 | VTVDDATMIATSFPEFMDLMAGLGAKIELSDTKAA 455 |
| Sbjct | 421 | VTVDDATMIATSFPEFM LM GLGAKIE ++ KAA  VTVDDATMIATSFPEFMGLMTGLGAKIEEAENKAA 455 |

>WP_081157816.1 3-phosphoshikimate 1-carboxyvinyltransferase [Ensifer aridi] Length=455

Score = 808 bits (2088), Expect = 0.0, Method: Compositional matrix adjust.

| Identities | = 412/455 (91%), Positives = 433/455 (95%), Gaps = 0/455 (0%) |  |
| --- | --- | --- |
| Query 1 | MLHGASSRPATARKSSGLSGTVRIPGDKSISHRSFMFGGLASGETRITGLLEGEDVINTG | 60 |
| Sbjct 1 | M HG+S RPATA+KSS L GTVRIPGDKSISHRSFMFGGLASGETRITGLLEGEDVINTG MSHGSSPRPATAKKSSDLKGTVRIPGDKSISHRSFMFGGLASGETRITGLLEGEDVINTG | 60 |
| Query 61 | KAMQAMGARIRKEGDTWIIDGVGNGGLLAPEAPLDFGNAATGCRLTMGLVGVYDFDSTFI | 120 |
| Sbjct 61 | KAMQAMGARIRKEGDTWII+GVGNG LLAPEAPLDFGNA TGCRLTMGLVGVYDFDSTFI KAMQAMGARIRKEGDTWIINGVGNGALLAPEAPLDFGNAGTGCRLTMGLVGVYDFDSTFI | 120 |
| Query 121 | GDASLTKRPMGRVLNPLREMGVQVKSEDGDRLPVTLRGPKTPTPITYRVPMASAQVKSAV | 180 |
| Sbjct 121 | GDASLTKRPMGRVL+PLREMGVQVKS +GDRLPVTL GP+TP PITYRVPMASAQVKSAV GDASLTKRPMGRVLDPLREMGVQVKSAEGDRLPVTLHGPQTPNPITYRVPMASAQVKSAV | 180 |
| Query 181 | LLAGLNTPGITTVIEPIMTRDHTEKMLQGFGANLTVETDADGVRTIRLEGRGKLTGQVID | 240 |
| Sbjct 181 | LLAGLNTPGITTVIEP+MTRDHTEKMLQGFGANLTVETDA+GVRTIRLEGRGKL GQVID LLAGLNTPGITTVIEPVMTRDHTEKMLQGFGANLTVETDAEGVRTIRLEGRGKLIGQVID | 240 |
| Query 241 | VPGDPSSTAFPLVAALLVPGSDVTILNVLMNPTRTGLILTLQEMGADIEVINPRLAGGED | 300 |
| Sbjct 241 | VPGDPSSTAFPLVAAL+VPGSD+TILNVLMNPTRTGLILTLQEMGA+IEV+N RLAGGED VPGDPSSTAFPLVAALIVPGSDITILNVLMNPTRTGLILTLQEMGAEIEVMNQRLAGGED | 300 |
| Query 301 | VADLRVRSSTLKGVTVPEDRAPSMIDEYPILavaaafaegaTVMNGLEELRVKESDRLSA | 360 |
| Sbjct 301 | VADLRVR S LKGV+VPE+RAPSMIDEYP+LAVAAAFAEGATVMNGLEELRVKESDRLSA VADLRVRYSELKGVSVPEERAPSMIDEYPVLAVAAAFAEGATVMNGLEELRVKESDRLSA | 360 |
| Query 361 | VANGLKLNGVDCDEGETSLVVRGRPDGKGLGNASGAAVATHLDHRIAMSFLVMGLVSENP | 420 |
| Sbjct 361 | VA+GLKLNG+DCDEGE SL VRGRP GKGLGNASG V THLDHRIAMSFLVMGL SE+P VADGLKLNGLDCDEGEASLFVRGRPGGKGLGNASGEKVKTHLDHRIAMSFLVMGLASEHP | 420 |
| Query 421  Sbjct 421 | VTVDDATMIATSFPEFMDLMAGLGAKIELSDTKAA 455 VTVDDATMIATSFPEFMDLM GLGAKIE +++KAA VTVDDATMIATSFPEFMDLMTGLGAKIEQAESKAA 455 |  |

>WP_136509256.1 3-phosphoshikimate 1-carboxyvinyltransferase [Ensifer sp. MPMI2T] THK34821.1 3-phosphoshikimate 1-carboxyvinyltransferase [Ensifer sp. MPMI2T]

Length=455

Score = 808 bits (2087), Expect = 0.0, Method: Compositional matrix adjust.

| Identities | = 412/455 (91%), Positives = 428/455 (94%), Gaps = 0/455 (0%) |  |
| --- | --- | --- |
| Query 1 | MLHGASSRPATARKSSGLSGTVRIPGDKSISHRSFMFGGLASGETRITGLLEGEDVINTG | 60 |
| Sbjct 1 | M HG+S RPATA KSS L GTVRIPGDKSISHRSFMFGGLA+GETRITGLLEGEDVINTG MSHGSSPRPATATKSSDLKGTVRIPGDKSISHRSFMFGGLAAGETRITGLLEGEDVINTG | 60 |
| Query 61 | KAMQAMGARIRKEGDTWIIDGVGNGGLLAPEAPLDFGNAATGCRLTMGLVGVYDFDSTFI | 120 |
| Sbjct 61 | KAMQAMGA+IRKEGDTWIIDGVGNG LLAPEAPLDFGNA TGCRLTMGLVGVYDFDSTFI KAMQAMGAKIRKEGDTWIIDGVGNGALLAPEAPLDFGNAGTGCRLTMGLVGVYDFDSTFI | 120 |
| Query 121 | GDASLTKRPMGRVLNPLREMGVQVKSEDGDRLPVTLRGPKTPTPITYRVPMASAQVKSAV | 180 |
| Sbjct 121 | GDASLTKRPMGRVLNPLREMGVQVKS +GDRLPVTL GPKTP PITYRVPMASAQVKSAV GDASLTKRPMGRVLNPLREMGVQVKSAEGDRLPVTLHGPKTPNPITYRVPMASAQVKSAV | 180 |
| Query 181 | LLAGLNTPGITTVIEPIMTRDHTEKMLQGFGANLTVETDADGVRTIRLEGRGKLTGQVID | 240 |
| Sbjct 181 | LLAGLNTPGITTVIEP+MTRDHTEKMLQGFGANLTVETD GVRTIRLEGRGKL GQVID LLAGLNTPGITTVIEPVMTRDHTEKMLQGFGANLTVETDTQGVRTIRLEGRGKLAGQVID | 240 |
| Query 241 | VPGDPSSTAFPLVAALLVPGSDVTILNVLMNPTRTGLILTLQEMGADIEVINPRLAGGED VPGDPSSTAFPLVAALLVP SDVTILNVLMNPTRTGLILTLQEMGA+IEV+N RLAGGED | 300 |

| Sbjct | 241 | VPGDPSSTAFPLVAALLVPASDVTILNVLMNPTRTGLILTLQEMGANIEVMNARLAGGED | 300 |
| --- | --- | --- | --- |
| Query | 301 | VADLRVRSSTLKGVTVPEDRAPSMIDEYPILavaaafaegaTVMNGLEELRVKESDRLSA | 360 |
| Sbjct | 301 | VADLRVR S LKGVTVPE+RAPSMIDEYP+LAVAAAFAEG TVMNGL+ELRVKESDRLSA VADLRVRYSELKGVTVPEERAPSMIDEYPVLAVAAAFAEGTTVMNGLDELRVKESDRLSA | 360 |
| Query | 361 | VANGLKLNGVDCDEGETSLVVRGRPDGKGLGNASGAAVATHLDHRIAMSFLVMGLVSENP | 420 |
| Sbjct | 361 | VA+GLKLNGVDCDEGE SLVVRGRP GKGLGNASG+ V T+LDHRIAMSFLVMGL SE+P VADGLKLNGVDCDEGEASLVVRGRPGGKGLGNASGSEVKTYLDHRIAMSFLVMGLASEHP | 420 |
| Query  Sbjct | 421  421 | VTVDDATMIATSFPEFMDLMAGLGAKIELSDTKAA 455 VTVDDATMIATSFPEFMDLM GLGAKIE ++ K A VTVDDATMIATSFPEFMDLMTGLGAKIEQTEAKVA 455 |  |

>WP_010968452.1 MULTISPECIES: 3-phosphoshikimate 1-carboxyvinyltransferase [Rhizobiales] Q92SV5.1 RecName: Full=3-phosphoshikimate 1-carboxyvinyltransferase; AltName:

Full=5-enolpyruvylshikimate-3-phosphate synthase; Short=EPSP synthase; Short=EPSPS [Sinorhizobium meliloti 1021]

TWA92740.1 3-phosphoshikimate 1-carboxyvinyltransferase [Ensifer sp. SEMIA 134]

TWB28747.1 3-phosphoshikimate 1-carboxyvinyltransferase [Ensifer sp. SEMIA 135]

AEG06252.1 3-phosphoshikimate 1-carboxyvinyltransferase [Sinorhizobium meliloti BL225C]

AEH80956.1 3-phosphoshikimate 1-carboxyvinyltransferase [Sinorhizobium meliloti SM11]

AGA05240.1 3-phosphoshikimate 1-carboxyvinyltransferase [Sinorhizobium meliloti GR4]

Length=455

Score = 808 bits (2087), Expect = 0.0, Method: Compositional matrix adjust.

| Identities | = 412/455 (91%), Positives = 430/455 (95%), Gaps = 0/455 (0%) |  |
| --- | --- | --- |
| Query 1 | MLHGASSRPATARKSSGLSGTVRIPGDKSISHRSFMFGGLASGETRITGLLEGEDVINTG | 60 |
| Sbjct 1 | M HG++ RPATARKSS L GT+RIPGDKSISHRSFMFGGLA+GETRITGLLEGEDVINTG MSHGSNPRPATARKSSDLKGTLRIPGDKSISHRSFMFGGLAAGETRITGLLEGEDVINTG | 60 |
| Query 61 | KAMQAMGARIRKEGDTWIIDGVGNGGLLAPEAPLDFGNAATGCRLTMGLVGVYDFDSTFI | 120 |
| Sbjct 61 | KAMQAMGARIRKEGDTWIIDGVGNG LLAPEAPLDFGNA TGCRLTMGLVGVYDFDSTFI KAMQAMGARIRKEGDTWIIDGVGNGALLAPEAPLDFGNAGTGCRLTMGLVGVYDFDSTFI | 120 |
| Query 121 | GDASLTKRPMGRVLNPLREMGVQVKSEDGDRLPVTLRGPKTPTPITYRVPMASAQVKSAV | 180 |
| Sbjct 121 | GDASLTKRPMGRVL+PLREMGVQVKS +GDRLPVTLRGPKTP PITYRVPMASAQVKSAV GDASLTKRPMGRVLDPLREMGVQVKSAEGDRLPVTLRGPKTPNPITYRVPMASAQVKSAV | 180 |
| Query 181 | LLAGLNTPGITTVIEPIMTRDHTEKMLQGFGANLTVETDADGVRTIRLEGRGKLTGQVID | 240 |
| Sbjct 181 | LLAGLNTPGITTV+EP+MTRDHTEKMLQGFGANLTVETDA+GVRTIRLEGRGKLTGQVID LLAGLNTPGITTVVEPVMTRDHTEKMLQGFGANLTVETDAEGVRTIRLEGRGKLTGQVID | 240 |
| Query 241 | VPGDPSSTAFPLVAALLVPGSDVTILNVLMNPTRTGLILTLQEMGADIEVINPRLAGGED | 300 |
| Sbjct 241 | VPGDPSSTAFPLVA L+VPGSD+TILNVLMNPTRTGLILTLQEMGA+IEV+N RLAGGED VPGDPSSTAFPLVAGLIVPGSDITILNVLMNPTRTGLILTLQEMGANIEVMNKRLAGGED | 300 |
| Query 301 | VADLRVRSSTLKGVTVPEDRAPSMIDEYPILavaaafaegaTVMNGLEELRVKESDRLSA | 360 |
| Sbjct 301 | VADLRVR S LKGVTVPEDRAPSMIDEYP+LAVAAAFAEG TVMNGLEELRVKESDRLSA VADLRVRHSELKGVTVPEDRAPSMIDEYPVLAVAAAFAEGTTVMNGLEELRVKESDRLSA | 360 |
| Query 361 | VANGLKLNGVDCDEGETSLVVRGRPDGKGLGNASGAAVATHLDHRIAMSFLVMGLVSENP | 420 |
| Sbjct 361 | VA+GLKLNGVDCDEGE SLVVRGRP GKGLG SG V THLDHRIAMSFLVMGL SE+P VADGLKLNGVDCDEGEASLVVRGRPGGKGLGKISGGQVKTHLDHRIAMSFLVMGLASEHP | 420 |
| Query 421  Sbjct 421 | VTVDDATMIATSFPEFMDLMAGLGAKIELSDTKAA 455 VTVDDATMIATSFPEFM LM GLGAKIE ++ KAA VTVDDATMIATSFPEFMGLMTGLGAKIEEAENKAA 455 |  |

>WP_085031493.1 3-phosphoshikimate 1-carboxyvinyltransferase [Ensifer aridi] Length=455

Score = 808 bits (2087), Expect = 0.0, Method: Compositional matrix adjust. Identities = 412/455 (91%), Positives = 433/455 (95%), Gaps = 0/455 (0%)

| Query | 1 | MLHGASSRPATARKSSGLSGTVRIPGDKSISHRSFMFGGLASGETRITGLLEGEDVINTG | 60 |
| --- | --- | --- | --- |
| Sbjct | 1 | M HG+S RPA A+KSS L GTVRIPGDKSISHRSFMFGGLASGETRITGLLEGEDVINTG MSHGSSPRPAIAKKSSDLKGTVRIPGDKSISHRSFMFGGLASGETRITGLLEGEDVINTG | 60 |
| Query | 61 | KAMQAMGARIRKEGDTWIIDGVGNGGLLAPEAPLDFGNAATGCRLTMGLVGVYDFDSTFI | 120 |
| Sbjct | 61 | KAMQAMGARIRKEGDTWII+GVGNG LLAPEAPLDFGNA TGCRLTMGLVGVYDFDSTFI KAMQAMGARIRKEGDTWIINGVGNGALLAPEAPLDFGNAGTGCRLTMGLVGVYDFDSTFI | 120 |
| Query | 121 | GDASLTKRPMGRVLNPLREMGVQVKSEDGDRLPVTLRGPKTPTPITYRVPMASAQVKSAV | 180 |
| Sbjct | 121 | GDASLTKRPMGRVL+PLREMGVQVKS +GDRLPVTL GP+TP PITYRVPMASAQVKSAV GDASLTKRPMGRVLDPLREMGVQVKSAEGDRLPVTLHGPQTPNPITYRVPMASAQVKSAV | 180 |
| Query | 181 | LLAGLNTPGITTVIEPIMTRDHTEKMLQGFGANLTVETDADGVRTIRLEGRGKLTGQVID | 240 |
| Sbjct | 181 | LLAGLNTPGITTVIEP+MTRDHTEKMLQGFGANLTVETDA+GVRTIRLEGRGKL GQVID LLAGLNTPGITTVIEPVMTRDHTEKMLQGFGANLTVETDAEGVRTIRLEGRGKLIGQVID | 240 |
| Query | 241 | VPGDPSSTAFPLVAALLVPGSDVTILNVLMNPTRTGLILTLQEMGADIEVINPRLAGGED | 300 |
| Sbjct | 241 | VPGDPSSTAFPLVAAL+VPGSD+TILNVLMNPTRTGLILTLQEMGA+IEV+N RLAGGED VPGDPSSTAFPLVAALIVPGSDITILNVLMNPTRTGLILTLQEMGAEIEVMNQRLAGGED | 300 |
| Query | 301 | VADLRVRSSTLKGVTVPEDRAPSMIDEYPILavaaafaegaTVMNGLEELRVKESDRLSA | 360 |
| Sbjct | 301 | VADLRVR S LKGV+VPE+RAPSMIDEYP+LAVAAAFAEGATVMNGLEELRVKESDRLSA VADLRVRYSELKGVSVPEERAPSMIDEYPVLAVAAAFAEGATVMNGLEELRVKESDRLSA | 360 |
| Query | 361 | VANGLKLNGVDCDEGETSLVVRGRPDGKGLGNASGAAVATHLDHRIAMSFLVMGLVSENP | 420 |
| Sbjct | 361 | VA+GLKLNG+DCDEGE SLVVRGRP GKGLGNASG V THLDHRIAMSFLVMGL SE+P VADGLKLNGLDCDEGEASLVVRGRPGGKGLGNASGEKVKTHLDHRIAMSFLVMGLASEHP | 420 |
| Query  Sbjct | 421  421 | VTVDDATMIATSFPEFMDLMAGLGAKIELSDTKAA 455 VTVDDATMIATSFPEFMDLM GLGAKIE +++KAA VTVDDATMIATSFPEFMDLMTGLGAKIEQAESKAA 455 |  |

>WP_085024845.1 3-phosphoshikimate 1-carboxyvinyltransferase [Ensifer aridi] Length=455

Score = 808 bits (2087), Expect = 0.0, Method: Compositional matrix adjust.

| Identities | = 412/455 (91%), Positives = 433/455 (95%), Gaps = 0/455 (0%) |  |
| --- | --- | --- |
| Query 1 | MLHGASSRPATARKSSGLSGTVRIPGDKSISHRSFMFGGLASGETRITGLLEGEDVINTG | 60 |
| Sbjct 1 | M HG+S RPATA+KSS L GTVRIPGDKSISHRSFMFGGLASGETRITGLLEGEDVINTG MSHGSSPRPATAKKSSDLKGTVRIPGDKSISHRSFMFGGLASGETRITGLLEGEDVINTG | 60 |
| Query 61 | KAMQAMGARIRKEGDTWIIDGVGNGGLLAPEAPLDFGNAATGCRLTMGLVGVYDFDSTFI | 120 |
| Sbjct 61 | KAMQAMGARIRKEGDTWII+GVGNG LLAPEAPLDFGNA TGCRLTMGLVGVYDFDST I KAMQAMGARIRKEGDTWIINGVGNGALLAPEAPLDFGNAGTGCRLTMGLVGVYDFDSTLI | 120 |
| Query 121 | GDASLTKRPMGRVLNPLREMGVQVKSEDGDRLPVTLRGPKTPTPITYRVPMASAQVKSAV | 180 |
| Sbjct 121 | GDASLTKRPMGRVL+PLREMGVQVKS +GDRLPVTL GP+TP PITYRVPMASAQVKSAV GDASLTKRPMGRVLDPLREMGVQVKSAEGDRLPVTLHGPQTPNPITYRVPMASAQVKSAV | 180 |
| Query 181 | LLAGLNTPGITTVIEPIMTRDHTEKMLQGFGANLTVETDADGVRTIRLEGRGKLTGQVID | 240 |
| Sbjct 181 | LLAGLNTPGITTVIEP+MTRDHTEKMLQGFGANLTVETDA+GVRTIRLEGRGKL GQVID LLAGLNTPGITTVIEPVMTRDHTEKMLQGFGANLTVETDAEGVRTIRLEGRGKLIGQVID | 240 |
| Query 241 | VPGDPSSTAFPLVAALLVPGSDVTILNVLMNPTRTGLILTLQEMGADIEVINPRLAGGED | 300 |
| Sbjct 241 | VPGDPSSTAFPLVAAL+VPGSD+TILNVLMNPTRTGLILTLQEMGA+IEV+N RLAGGED VPGDPSSTAFPLVAALIVPGSDITILNVLMNPTRTGLILTLQEMGAEIEVMNQRLAGGED | 300 |
| Query 301 | VADLRVRSSTLKGVTVPEDRAPSMIDEYPILavaaafaegaTVMNGLEELRVKESDRLSA | 360 |
| Sbjct 301 | VADLRVR S LKGV+VPE+RAPSMIDEYP+LAVAAAFAEGATVMNGLEELRVKESDRLSA VADLRVRYSELKGVSVPEERAPSMIDEYPVLAVAAAFAEGATVMNGLEELRVKESDRLSA | 360 |
| Query 361 | VANGLKLNGVDCDEGETSLVVRGRPDGKGLGNASGAAVATHLDHRIAMSFLVMGLVSENP | 420 |
| Sbjct 361 | VA+GLKLNG+DCDEGE SLVVRGRP GKGLGNASG V THLDHRIAMSFLVMGL SE+P VADGLKLNGLDCDEGEASLVVRGRPGGKGLGNASGEKVKTHLDHRIAMSFLVMGLASEHP | 420 |
| Query 421  Sbjct 421 | VTVDDATMIATSFPEFMDLMAGLGAKIELSDTKAA 455 VTVDDATMIATSFPEFMDLM GLGAKIE +++KAA VTVDDATMIATSFPEFMDLMTGLGAKIEQAESKAA 455 |  |

>WP_127661569.1 3-phosphoshikimate 1-carboxyvinyltransferase [Sinorhizobium meliloti] RVH45886.1 3-phosphoshikimate 1-carboxyvinyltransferase [Sinorhizobium meliloti] RVM10816.1 3-phosphoshikimate 1-carboxyvinyltransferase [Sinorhizobium meliloti]

Length=455

Score = 808 bits (2086), Expect = 0.0, Method: Compositional matrix adjust.

| Identities | = 411/455 (90%), Positives = 430/455 (95%), Gaps = 0/455 (0%) |  |
| --- | --- | --- |
| Query 1 | MLHGASSRPATARKSSGLSGTVRIPGDKSISHRSFMFGGLASGETRITGLLEGEDVINTG | 60 |
| Sbjct 1 | M HG++ RPATARKSS L GT+RIPGDKSISHRSFMFGGLA+GETRITGLLEGEDVINTG MSHGSNPRPATARKSSDLKGTLRIPGDKSISHRSFMFGGLAAGETRITGLLEGEDVINTG | 60 |
| Query 61 | KAMQAMGARIRKEGDTWIIDGVGNGGLLAPEAPLDFGNAATGCRLTMGLVGVYDFDSTFI | 120 |
| Sbjct 61 | KAMQAMGARIRKEGDTWIIDGVGNG LLAPEAPLDFGNA TGCRLTMGLVGVYDFD+TFI KAMQAMGARIRKEGDTWIIDGVGNGALLAPEAPLDFGNAGTGCRLTMGLVGVYDFDTTFI | 120 |
| Query 121 | GDASLTKRPMGRVLNPLREMGVQVKSEDGDRLPVTLRGPKTPTPITYRVPMASAQVKSAV | 180 |
| Sbjct 121 | GDASLTKRPMGRVL+PLREMGVQVKS +GDRLPVTLRGPKTP PITYRVPMASAQVKSAV GDASLTKRPMGRVLDPLREMGVQVKSAEGDRLPVTLRGPKTPNPITYRVPMASAQVKSAV | 180 |
| Query 181 | LLAGLNTPGITTVIEPIMTRDHTEKMLQGFGANLTVETDADGVRTIRLEGRGKLTGQVID | 240 |
| Sbjct 181 | LLAGLNTPGITTV+EP+MTRDHTEKMLQGFGANLTVETDA+GVRTIRLEGRGKLTGQVID LLAGLNTPGITTVVEPVMTRDHTEKMLQGFGANLTVETDAEGVRTIRLEGRGKLTGQVID | 240 |
| Query 241 | VPGDPSSTAFPLVAALLVPGSDVTILNVLMNPTRTGLILTLQEMGADIEVINPRLAGGED | 300 |
| Sbjct 241 | VPGDPSSTAFPLVA L+VPGSD+TILNVLMNPTRTGLILTLQEMGA+IEV+N RLAGGED VPGDPSSTAFPLVAGLIVPGSDITILNVLMNPTRTGLILTLQEMGANIEVMNKRLAGGED | 300 |
| Query 301 | VADLRVRSSTLKGVTVPEDRAPSMIDEYPILavaaafaegaTVMNGLEELRVKESDRLSA | 360 |
| Sbjct 301 | VADLRVR S LKGVTVPEDRAPSMIDEYP+LAVAAAFAEG TVMNGLEELRVKESDRLSA VADLRVRHSELKGVTVPEDRAPSMIDEYPVLAVAAAFAEGTTVMNGLEELRVKESDRLSA | 360 |
| Query 361 | VANGLKLNGVDCDEGETSLVVRGRPDGKGLGNASGAAVATHLDHRIAMSFLVMGLVSENP | 420 |
| Sbjct 361 | VA+GLKLNGVDCDEGE SLVVRGRP GKGLG SG V THLDHRIAMSFLVMGL SE+P VADGLKLNGVDCDEGEASLVVRGRPGGKGLGKISGGQVKTHLDHRIAMSFLVMGLASEHP | 420 |
| Query 421  Sbjct 421 | VTVDDATMIATSFPEFMDLMAGLGAKIELSDTKAA 455 VTVDDATMIATSFPEFM LM GLGAKIE ++ KAA VTVDDATMIATSFPEFMGLMTGLGAKIEEAENKAA 455 |  |

>WP_018093872.1 MULTISPECIES: 3-phosphoshikimate 1-carboxyvinyltransferase [Sinorhizobium] PND22546.1 3-phosphoshikimate 1-carboxyvinyltransferase [Ensifer sp. MMN_5]

PND26824.1 3-phosphoshikimate 1-carboxyvinyltransferase [Sinorhizobium sp. M4_45]

RVQ04403.1 3-phosphoshikimate 1-carboxyvinyltransferase [Sinorhizobium meliloti] Length=455

Score = 807 bits (2085), Expect = 0.0, Method: Compositional matrix adjust.

| Identities | = 412/455 (91%), Positives = 431/455 (95%), Gaps = 0/455 (0%) |  |
| --- | --- | --- |
| Query 1 | MLHGASSRPATARKSSGLSGTVRIPGDKSISHRSFMFGGLASGETRITGLLEGEDVINTG | 60 |
| Sbjct 1 | M HG++ RPATARKSS L GT+RIPGDKSISHRSFMFGGLA+GETRITGLLEGEDVINTG MSHGSNPRPATARKSSDLKGTLRIPGDKSISHRSFMFGGLAAGETRITGLLEGEDVINTG | 60 |
| Query 61 | KAMQAMGARIRKEGDTWIIDGVGNGGLLAPEAPLDFGNAATGCRLTMGLVGVYDFDSTFI | 120 |
| Sbjct 61 | KAMQAMGARIRKEGDTWIIDGVGNG LLAPEAPLDFGNA TGCRLTMGLVGVYDFDSTFI KAMQAMGARIRKEGDTWIIDGVGNGALLAPEAPLDFGNAGTGCRLTMGLVGVYDFDSTFI | 120 |
| Query 121 | GDASLTKRPMGRVLNPLREMGVQVKSEDGDRLPVTLRGPKTPTPITYRVPMASAQVKSAV | 180 |
| Sbjct 121 | GDASLTKRPMGRVL+PLREMGVQVKS +GDRLPVTLRGPKTP PITYRVPMASAQVKSAV GDASLTKRPMGRVLDPLREMGVQVKSAEGDRLPVTLRGPKTPNPITYRVPMASAQVKSAV | 180 |
| Query 181 | LLAGLNTPGITTVIEPIMTRDHTEKMLQGFGANLTVETDADGVRTIRLEGRGKLTGQVID | 240 |
| Sbjct 181 | LLAGLNTPGITTV+EP+MTRDHTEKMLQGFGA LTVETDA+GVRTIRLEGRGKLTGQVID LLAGLNTPGITTVVEPVMTRDHTEKMLQGFGAKLTVETDAEGVRTIRLEGRGKLTGQVID | 240 |
| Query 241 | VPGDPSSTAFPLVAALLVPGSDVTILNVLMNPTRTGLILTLQEMGADIEVINPRLAGGED | 300 |
| Sbjct 241 | VPGDPSSTAFPLVAAL+VPGSD+TILNVLMNPTRTGLILTLQEMGA+IEV+N RLAGGED  VPGDPSSTAFPLVAALIVPGSDITILNVLMNPTRTGLILTLQEMGANIEVMNKRLAGGED | 300 |

| Query | 301 | VADLRVRSSTLKGVTVPEDRAPSMIDEYPILavaaafaegaTVMNGLEELRVKESDRLSA | 360 |
| --- | --- | --- | --- |
| Sbjct | 301 | VADLRV S LKGVTVPE+RAPSMIDEYP+LAVAAAFAEG TVMNGLEELRVKESDRLSA VADLRVCYSELKGVTVPEERAPSMIDEYPVLAVAAAFAEGTTVMNGLEELRVKESDRLSA | 360 |
| Query | 361 | VANGLKLNGVDCDEGETSLVVRGRPDGKGLGNASGAAVATHLDHRIAMSFLVMGLVSENP | 420 |
| Sbjct | 361 | VA+GLKLNGVDCDEGE SLVVRGRP GKGLGN SG V THLDHRIAMSFLVMGLVSE+P VADGLKLNGVDCDEGEASLVVRGRPGGKGLGNNSGGQVKTHLDHRIAMSFLVMGLVSEHP | 420 |
| Query  Sbjct | 421  421 | VTVDDATMIATSFPEFMDLMAGLGAKIELSDTKAA 455 VTVDDATMIATSFPEFM LM GLGAKIE ++ KAA VTVDDATMIATSFPEFMGLMTGLGAKIEEAENKAA 455 |  |

>WP_127622322.1 3-phosphoshikimate 1-carboxyvinyltransferase [Sinorhizobium meliloti] RVK35713.1 3-phosphoshikimate 1-carboxyvinyltransferase [Sinorhizobium meliloti]

Length=455

Score = 807 bits (2084), Expect = 0.0, Method: Compositional matrix adjust.

| Identities | = 411/455 (90%), Positives = 429/455 (94%), Gaps = 0/455 (0%) |  |
| --- | --- | --- |
| Query 1 | MLHGASSRPATARKSSGLSGTVRIPGDKSISHRSFMFGGLASGETRITGLLEGEDVINTG | 60 |
| Sbjct 1 | M HG++ RPATARKSS L GT+RIPGDKSISHRSFMFGGLA+GETRITGLLEGEDVINTG MSHGSNPRPATARKSSDLKGTLRIPGDKSISHRSFMFGGLAAGETRITGLLEGEDVINTG | 60 |
| Query 61 | KAMQAMGARIRKEGDTWIIDGVGNGGLLAPEAPLDFGNAATGCRLTMGLVGVYDFDSTFI | 120 |
| Sbjct 61 | K MQAMGARIRKEGDTWIIDGVGNG LLAPEAPLDFGNA TGCRLTMGLVGVYDFDSTFI KVMQAMGARIRKEGDTWIIDGVGNGALLAPEAPLDFGNAGTGCRLTMGLVGVYDFDSTFI | 120 |
| Query 121 | GDASLTKRPMGRVLNPLREMGVQVKSEDGDRLPVTLRGPKTPTPITYRVPMASAQVKSAV | 180 |
| Sbjct 121 | GDASLTKRPMGRVL+PLREMGVQVKS +GDRLPVTLRGPKTP PITYRVPMASAQVKSAV GDASLTKRPMGRVLDPLREMGVQVKSAEGDRLPVTLRGPKTPNPITYRVPMASAQVKSAV | 180 |
| Query 181 | LLAGLNTPGITTVIEPIMTRDHTEKMLQGFGANLTVETDADGVRTIRLEGRGKLTGQVID | 240 |
| Sbjct 181 | LLAGLNTPGITTV+EP+MTRDHTEKMLQGFGANLTVETDA+GVRTIRLEGRGKLTGQVID LLAGLNTPGITTVVEPVMTRDHTEKMLQGFGANLTVETDAEGVRTIRLEGRGKLTGQVID | 240 |
| Query 241 | VPGDPSSTAFPLVAALLVPGSDVTILNVLMNPTRTGLILTLQEMGADIEVINPRLAGGED | 300 |
| Sbjct 241 | VPGDPSSTAFPLVA L+VPGSD+TILNVLMNPTRTGLILTLQEMGA+IEV+N RLAGGED VPGDPSSTAFPLVAGLIVPGSDITILNVLMNPTRTGLILTLQEMGANIEVMNKRLAGGED | 300 |
| Query 301 | VADLRVRSSTLKGVTVPEDRAPSMIDEYPILavaaafaegaTVMNGLEELRVKESDRLSA | 360 |
| Sbjct 301 | VADLRVR S LKGVTVPEDRAPSMIDEYP+LAVAAAFAEG TVMNGLEELRVKESDRLSA VADLRVRHSELKGVTVPEDRAPSMIDEYPVLAVAAAFAEGTTVMNGLEELRVKESDRLSA | 360 |
| Query 361 | VANGLKLNGVDCDEGETSLVVRGRPDGKGLGNASGAAVATHLDHRIAMSFLVMGLVSENP | 420 |
| Sbjct 361 | VA+GLKLNGVDCDEGE SLVVRGRP GKGLG SG V THLDHRIAMSFLVMGL SE+P VADGLKLNGVDCDEGEASLVVRGRPGGKGLGKISGGQVKTHLDHRIAMSFLVMGLASEHP | 420 |
| Query 421  Sbjct 421 | VTVDDATMIATSFPEFMDLMAGLGAKIELSDTKAA 455 VTVDDATMIATSFPEFM LM GLGAKIE ++ KAA VTVDDATMIATSFPEFMGLMTGLGAKIEEAENKAA 455 |  |

>WP_127631404.1 3-phosphoshikimate 1-carboxyvinyltransferase [Sinorhizobium meliloti] RVK30177.1 3-phosphoshikimate 1-carboxyvinyltransferase [Sinorhizobium meliloti]

Length=455

Score = 807 bits (2084), Expect = 0.0, Method: Compositional matrix adjust.

| Identities | = 411/455 (90%), Positives = 429/455 (94%), Gaps = 0/455 (0%) |  |
| --- | --- | --- |
| Query 1 | MLHGASSRPATARKSSGLSGTVRIPGDKSISHRSFMFGGLASGETRITGLLEGEDVINTG | 60 |
| Sbjct 1 | M HG++ RPATARKSS L GT+RIPGDKSISHRSFMFGGLA+GETRITGLLEGEDVINTG MSHGSNPRPATARKSSDLKGTLRIPGDKSISHRSFMFGGLAAGETRITGLLEGEDVINTG | 60 |
| Query 61 | KAMQAMGARIRKEGDTWIIDGVGNGGLLAPEAPLDFGNAATGCRLTMGLVGVYDFDSTFI | 120 |
| Sbjct 61 | KAMQ MGARIRKEGDTWIIDGVGNG LLAPEAPLDFGNA TGCRLTMGLVGVYDFDSTFI KAMQVMGARIRKEGDTWIIDGVGNGALLAPEAPLDFGNAGTGCRLTMGLVGVYDFDSTFI | 120 |
| Query 121 | GDASLTKRPMGRVLNPLREMGVQVKSEDGDRLPVTLRGPKTPTPITYRVPMASAQVKSAV | 180 |
| Sbjct 121 | GDASLTKRPMGRVL+PLREMGVQVKS +GDRLPVTLRGPKTP PITYRVPMASAQVKSAV  GDASLTKRPMGRVLDPLREMGVQVKSAEGDRLPVTLRGPKTPNPITYRVPMASAQVKSAV | 180 |

| Query | 181 | LLAGLNTPGITTVIEPIMTRDHTEKMLQGFGANLTVETDADGVRTIRLEGRGKLTGQVID | 240 |
| --- | --- | --- | --- |
| Sbjct | 181 | LLAGLNTPGITTV+EP+MTRDHTEKMLQGFGANLTVETDA+GVRTIRLEGRGKLTGQVID LLAGLNTPGITTVVEPVMTRDHTEKMLQGFGANLTVETDAEGVRTIRLEGRGKLTGQVID | 240 |
| Query | 241 | VPGDPSSTAFPLVAALLVPGSDVTILNVLMNPTRTGLILTLQEMGADIEVINPRLAGGED | 300 |
| Sbjct | 241 | VPGDPSSTAFPLVA L+VPGSD+TILNVLMNPTRTGLILTLQEMGA+IEV+N RLAGGED VPGDPSSTAFPLVAGLIVPGSDITILNVLMNPTRTGLILTLQEMGANIEVMNKRLAGGED | 300 |
| Query | 301 | VADLRVRSSTLKGVTVPEDRAPSMIDEYPILavaaafaegaTVMNGLEELRVKESDRLSA | 360 |
| Sbjct | 301 | VADLRVR S LKGVTVPEDRAPSMIDEYP+LAVAAAFAEG TVMNGLEELRVKESDRLSA VADLRVRHSELKGVTVPEDRAPSMIDEYPVLAVAAAFAEGTTVMNGLEELRVKESDRLSA | 360 |
| Query | 361 | VANGLKLNGVDCDEGETSLVVRGRPDGKGLGNASGAAVATHLDHRIAMSFLVMGLVSENP | 420 |
| Sbjct | 361 | VA+GLKLNGVDCDEGE SLVVRGRP GKGLG SG V THLDHRIAMSFLVMGL SE+P VADGLKLNGVDCDEGEASLVVRGRPGGKGLGKISGGQVKTHLDHRIAMSFLVMGLASEHP | 420 |
| Query  Sbjct | 421  421 | VTVDDATMIATSFPEFMDLMAGLGAKIELSDTKAA 455 VTVDDATMIATSFPEFM LM GLGAKIE ++ KAA VTVDDATMIATSFPEFMGLMTGLGAKIEEAENKAA 455 |  |

>WP_192249075.1 3-phosphoshikimate 1-carboxyvinyltransferase [Rhizobium sp. ARZ01] MBD9374204.1 3-phosphoshikimate 1-carboxyvinyltransferase [Rhizobium sp. ARZ01]

Length=452

Score = 807 bits (2084), Expect = 0.0, Method: Compositional matrix adjust.

| Identities | = 414/449 (92%), Positives = 430/449 (96%), Gaps = 1/449 (0%) |  |
| --- | --- | --- |
| Query 1 | MLHGASSRPATARKSS-GLSGTVRIPGDKSISHRSFMFGGLASGETRITGLLEGEDVINT | 59 |
| Sbjct 1 | M HG +PATARKSS LSGT+RIPGDKSISHRSFMFGGLASGETRITGLLEGEDVINT MSHGVQPKPATARKSSKALSGTIRIPGDKSISHRSFMFGGLASGETRITGLLEGEDVINT | 60 |
| Query 60 | GKAMQAMGARIRKEGDTWIIDGVGNGGLLAPEAPLDFGNAATGCRLTMGLVGVYDFDSTF | 119 |
| Sbjct 61 | GKAMQAMGA+IRKEGDTWII+GVGNG LLAPEAPLDFGNA TGCRLTMGLVGVYDFDSTF GKAMQAMGAKIRKEGDTWIINGVGNGALLAPEAPLDFGNAGTGCRLTMGLVGVYDFDSTF | 120 |
| Query 120 | IGDASLTKRPMGRVLNPLREMGVQVKSEDGDRLPVTLRGPKTPTPITYRVPMASAQVKSA | 179 |
| Sbjct 121 | IGDASLTKRPMGRVLNPLREMGVQVK+ DGDRLPVTLRGP+ TPITYRVPMASAQVKSA IGDASLTKRPMGRVLNPLREMGVQVKAADGDRLPVTLRGPRAATPITYRVPMASAQVKSA | 180 |
| Query 180 | VLLAGLNTPGITTVIEPIMTRDHTEKMLQGFGANLTVETDADGVRTIRLEGRGKLTGQVI | 239 |
| Sbjct 181 | VLLAGLNTPGITTVIEPIMTRDHTEKMLQGFGANLTV+TDA GVRTIRLEG+GKLTGQVI VLLAGLNTPGITTVIEPIMTRDHTEKMLQGFGANLTVDTDAGGVRTIRLEGQGKLTGQVI | 240 |
| Query 240 | DVPGDPSSTAFPLVAALLVPGSDVTILNVLMNPTRTGLILTLQEMGADIEVINPRLAGGE | 299 |
| Sbjct 241 | DVPGDPSSTAFPLVAALLVPGSDVTILNVLMNPTRTGLILTLQEMGA+IEV++ RLAGGE DVPGDPSSTAFPLVAALLVPGSDVTILNVLMNPTRTGLILTLQEMGANIEVLDARLAGGE | 300 |
| Query 300 | DVADLRVRSSTLKGVTVPEDRAPSMIDEYPILavaaafaegaTVMNGLEELRVKESDRLS | 359 |
| Sbjct 301 | DVADLRVR S LKGVTVPEDRAPSMIDEYPILAVAAAFA GATVMNGLEELRVKESDRL+ DVADLRVRHSQLKGVTVPEDRAPSMIDEYPILAVAAAFANGATVMNGLEELRVKESDRLT | 360 |
| Query 360 | AVANGLKLNGVDCDEGETSLVVRGRPDGKGLGNASGAAVATHLDHRIAMSFLVMGLVSEN | 419 |
| Sbjct 361 | AVA+GLKLNGVDCDEGETSLVVRGRPDGKGLGNASGAAVATHLDHRIAMSFLVMGL SE+ AVADGLKLNGVDCDEGETSLVVRGRPDGKGLGNASGAAVATHLDHRIAMSFLVMGLASEH | 420 |
| Query 420  Sbjct 421 | PVTVDDATMIATSFPEFMDLMAGLGAKIE 448 PVTVDDATMIATSFPEFM +M GLGA+IE PVTVDDATMIATSFPEFMGMMTGLGAQIE 449 |  |

>WP_127510767.1 3-phosphoshikimate 1-carboxyvinyltransferase [Sinorhizobium meliloti] RVE90697.1 3-phosphoshikimate 1-carboxyvinyltransferase [Sinorhizobium meliloti] RVH45042.1 3-phosphoshikimate 1-carboxyvinyltransferase [Sinorhizobium meliloti] RVI47548.1 3-phosphoshikimate 1-carboxyvinyltransferase [Sinorhizobium meliloti] RVK13631.1 3-phosphoshikimate 1-carboxyvinyltransferase [Sinorhizobium meliloti] RVQ53677.1 3-phosphoshikimate 1-carboxyvinyltransferase [Sinorhizobium meliloti]

Length=455

Score = 806 bits (2081), Expect = 0.0, Method: Compositional matrix adjust. Identities = 411/455 (90%), Positives = 429/455 (94%), Gaps = 0/455 (0%)

| Query | 1 | MLHGASSRPATARKSSGLSGTVRIPGDKSISHRSFMFGGLASGETRITGLLEGEDVINTG | 60 |
| --- | --- | --- | --- |
| Sbjct | 1 | M HG++ RPATARKSS L GT+RIPGDKSISHRSFMFGGLA+GETRITGLLEGEDVINTG MSHGSNPRPATARKSSDLKGTLRIPGDKSISHRSFMFGGLAAGETRITGLLEGEDVINTG | 60 |
| Query | 61 | KAMQAMGARIRKEGDTWIIDGVGNGGLLAPEAPLDFGNAATGCRLTMGLVGVYDFDSTFI | 120 |
| Sbjct | 61 | KAMQAMGARIRKEGDTWIIDGVGNG LLAPEAPLDFGNA TGCRLTMGLVGVYDFDSTFI KAMQAMGARIRKEGDTWIIDGVGNGALLAPEAPLDFGNAGTGCRLTMGLVGVYDFDSTFI | 120 |
| Query | 121 | GDASLTKRPMGRVLNPLREMGVQVKSEDGDRLPVTLRGPKTPTPITYRVPMASAQVKSAV | 180 |
| Sbjct | 121 | GDASLTKRPMGRVL+PLREMGVQVKS +GDRLPVTLRGPKTP PITYRVPMASAQVKSAV GDASLTKRPMGRVLDPLREMGVQVKSAEGDRLPVTLRGPKTPNPITYRVPMASAQVKSAV | 180 |
| Query | 181 | LLAGLNTPGITTVIEPIMTRDHTEKMLQGFGANLTVETDADGVRTIRLEGRGKLTGQVID | 240 |
| Sbjct | 181 | LLAGLNTPGITTV+EP+MTRDHTEKMLQGFGANLTVETDA+GVRTIRLEGRGKLTGQVID LLAGLNTPGITTVVEPVMTRDHTEKMLQGFGANLTVETDAEGVRTIRLEGRGKLTGQVID | 240 |
| Query | 241 | VPGDPSSTAFPLVAALLVPGSDVTILNVLMNPTRTGLILTLQEMGADIEVINPRLAGGED | 300 |
| Sbjct | 241 | VPGDPSSTAFPLVA L+VPGSD+TILNVLMNPTRTGLILTLQEMGA+IEV+N RLAGGED VPGDPSSTAFPLVAGLIVPGSDITILNVLMNPTRTGLILTLQEMGANIEVMNKRLAGGED | 300 |
| Query | 301 | VADLRVRSSTLKGVTVPEDRAPSMIDEYPILavaaafaegaTVMNGLEELRVKESDRLSA | 360 |
| Sbjct | 301 | VADLRVR S LKGVTVPEDRAPSMIDEYP+LAVAAAFAEG TVMNGLEELRVKESDRLSA VADLRVRHSELKGVTVPEDRAPSMIDEYPVLAVAAAFAEGTTVMNGLEELRVKESDRLSA | 360 |
| Query | 361 | VANGLKLNGVDCDEGETSLVVRGRPDGKGLGNASGAAVATHLDHRIAMSFLVMGLVSENP | 420 |
| Sbjct | 361 | VA+GLKLNGVDCDEGE SLVVRGRP GKGLG SG V THLDHRIAMSFLVMGL SE+P VADGLKLNGVDCDEGEASLVVRGRPGGKGLGKISGGQVKTHLDHRIAMSFLVMGLASEHP | 420 |
| Query  Sbjct | 421  421 | VTVDDATMIATSFPEFMDLMAGLGAKIELSDTKAA 455 VTVDDATMIATSFPEFM LM GLGAKIE ++ AA VTVDDATMIATSFPEFMGLMTGLGAKIEEAENTAA 455 |  |

>WP_100669854.1 3-phosphoshikimate 1-carboxyvinyltransferase [Sinorhizobium meliloti] PJR17252.1 3-phosphoshikimate 1-carboxyvinyltransferase [Sinorhizobium meliloti]

Length=455

Score = 805 bits (2079), Expect = 0.0, Method: Compositional matrix adjust.

| Identities | = 410/455 (90%), Positives = 430/455 (95%), Gaps = 0/455 (0%) |  |
| --- | --- | --- |
| Query 1 | MLHGASSRPATARKSSGLSGTVRIPGDKSISHRSFMFGGLASGETRITGLLEGEDVINTG | 60 |
| Sbjct 1 | M HG++ RPATARKSS L GT+RIPGDKSISHRSFMFGGLA+GETRITGLLEGEDVINTG MSHGSNPRPATARKSSDLKGTLRIPGDKSISHRSFMFGGLAAGETRITGLLEGEDVINTG | 60 |
| Query 61 | KAMQAMGARIRKEGDTWIIDGVGNGGLLAPEAPLDFGNAATGCRLTMGLVGVYDFDSTFI | 120 |
| Sbjct 61 | KAMQAMGARIRKEGDTWIIDGVGNG LLAPEAPLDFGNA TGCRLTMGLVGVYDFDSTFI KAMQAMGARIRKEGDTWIIDGVGNGALLAPEAPLDFGNAGTGCRLTMGLVGVYDFDSTFI | 120 |
| Query 121 | GDASLTKRPMGRVLNPLREMGVQVKSEDGDRLPVTLRGPKTPTPITYRVPMASAQVKSAV | 180 |
| Sbjct 121 | GDASLTKRPMGRVL+PLREMGVQVKS +GDRLPVTLRGPKTP PITYRVPMASAQVKSAV GDASLTKRPMGRVLDPLREMGVQVKSAEGDRLPVTLRGPKTPNPITYRVPMASAQVKSAV | 180 |
| Query 181 | LLAGLNTPGITTVIEPIMTRDHTEKMLQGFGANLTVETDADGVRTIRLEGRGKLTGQVID | 240 |
| Sbjct 181 | LLAGLNTPGITTV+EP+MTRDHTEKMLQGFGA LTVETDA+GVRTIRLEGRGKLTGQVID LLAGLNTPGITTVVEPVMTRDHTEKMLQGFGAKLTVETDAEGVRTIRLEGRGKLTGQVID | 240 |
| Query 241 | VPGDPSSTAFPLVAALLVPGSDVTILNVLMNPTRTGLILTLQEMGADIEVINPRLAGGED | 300 |
| Sbjct 241 | VPGDPSSTAFPLVAAL+VPGSD+TILNVLMNPTRTGLILTLQEMGA+IEV+N RLAGGED VPGDPSSTAFPLVAALIVPGSDITILNVLMNPTRTGLILTLQEMGANIEVMNKRLAGGED | 300 |
| Query 301 | VADLRVRSSTLKGVTVPEDRAPSMIDEYPILavaaafaegaTVMNGLEELRVKESDRLSA | 360 |
| Sbjct 301 | VADLRV S LKGVTVPE+RAPSMIDEYP+LAVAAAFAEG TVMNGLEELRVKESDRLSA VADLRVCYSELKGVTVPEERAPSMIDEYPVLAVAAAFAEGTTVMNGLEELRVKESDRLSA | 360 |
| Query 361 | VANGLKLNGVDCDEGETSLVVRGRPDGKGLGNASGAAVATHLDHRIAMSFLVMGLVSENP | 420 |
| Sbjct 361 | VA+GLKLNGVDCDEGE SLVVRGRP GKGLGN +G V THLDHRIAMSFLVMGL SE+P VADGLKLNGVDCDEGEASLVVRGRPGGKGLGNIAGGQVKTHLDHRIAMSFLVMGLASEHP | 420 |
| Query 421  Sbjct 421 | VTVDDATMIATSFPEFMDLMAGLGAKIELSDTKAA 455 VTVDDATMIATSFPEFM LM GLGAKIE ++ KAA VTVDDATMIATSFPEFMGLMTGLGAKIEEAENKAA 455 |  |

>WP_013844999.1 3-phosphoshikimate 1-carboxyvinyltransferase [Sinorhizobium meliloti] AEG55285.1 3-phosphoshikimate 1-carboxyvinyltransferase [Sinorhizobium meliloti

AK83]

SEJ28950.1 3-phosphoshikimate 1-carboxyvinyltransferase [Sinorhizobium meliloti] Length=455

Score = 805 bits (2079), Expect = 0.0, Method: Compositional matrix adjust.

| Identities | = 411/455 (90%), Positives = 429/455 (94%), Gaps = 0/455 (0%) |  |
| --- | --- | --- |
| Query 1 | MLHGASSRPATARKSSGLSGTVRIPGDKSISHRSFMFGGLASGETRITGLLEGEDVINTG | 60 |
| Sbjct 1 | M HG++ RPATARKSS L GT+RIPGDKSISHRSFMFGGLA+GETRITGLLEGEDVINTG MSHGSNPRPATARKSSDLKGTLRIPGDKSISHRSFMFGGLAAGETRITGLLEGEDVINTG | 60 |
| Query 61 | KAMQAMGARIRKEGDTWIIDGVGNGGLLAPEAPLDFGNAATGCRLTMGLVGVYDFDSTFI | 120 |
| Sbjct 61 | KAMQAMGARIRKEG TWIIDGVGNG LLAPEAPLDFGNA TGCRLTMGLVGVYDFDSTFI KAMQAMGARIRKEGGTWIIDGVGNGALLAPEAPLDFGNAGTGCRLTMGLVGVYDFDSTFI | 120 |
| Query 121 | GDASLTKRPMGRVLNPLREMGVQVKSEDGDRLPVTLRGPKTPTPITYRVPMASAQVKSAV | 180 |
| Sbjct 121 | GDASLTKRPMGRVL+PLREMGVQVKS +GDRLPVTLRGPKTP PITYRVPMASAQVKSAV GDASLTKRPMGRVLDPLREMGVQVKSAEGDRLPVTLRGPKTPNPITYRVPMASAQVKSAV | 180 |
| Query 181 | LLAGLNTPGITTVIEPIMTRDHTEKMLQGFGANLTVETDADGVRTIRLEGRGKLTGQVID | 240 |
| Sbjct 181 | LLAGLNTPGITTV+EP+MTRDHTEKMLQGFGANLTVETDA+GVRTIRLEGRGKLTGQVID LLAGLNTPGITTVVEPVMTRDHTEKMLQGFGANLTVETDAEGVRTIRLEGRGKLTGQVID | 240 |
| Query 241 | VPGDPSSTAFPLVAALLVPGSDVTILNVLMNPTRTGLILTLQEMGADIEVINPRLAGGED | 300 |
| Sbjct 241 | VPGDPSSTAFPLVA L+VPGSD+TILNVLMNPTRTGLILTLQEMGA+IEV+N RLAGGED VPGDPSSTAFPLVAGLIVPGSDITILNVLMNPTRTGLILTLQEMGANIEVMNKRLAGGED | 300 |
| Query 301 | VADLRVRSSTLKGVTVPEDRAPSMIDEYPILavaaafaegaTVMNGLEELRVKESDRLSA | 360 |
| Sbjct 301 | VADLRVR S LKGVTVPEDRAPSMIDEYP+LAVAAAFAEG TVMNGLEELRVKESDRLSA VADLRVRHSELKGVTVPEDRAPSMIDEYPVLAVAAAFAEGTTVMNGLEELRVKESDRLSA | 360 |
| Query 361 | VANGLKLNGVDCDEGETSLVVRGRPDGKGLGNASGAAVATHLDHRIAMSFLVMGLVSENP | 420 |
| Sbjct 361 | VA+GLKLNGVDCDEGE SLVVRGRP GKGLG SG V THLDHRIAMSFLVMGL SE+P VADGLKLNGVDCDEGEASLVVRGRPGGKGLGKISGGQVKTHLDHRIAMSFLVMGLASEHP | 420 |
| Query 421  Sbjct 421 | VTVDDATMIATSFPEFMDLMAGLGAKIELSDTKAA 455 VTVDDATMIATSFPEFM LM GLGAKIE ++ KAA VTVDDATMIATSFPEFMGLMTGLGAKIEEAENKAA 455 |  |

>WP_127535276.1 3-phosphoshikimate 1-carboxyvinyltransferase [Sinorhizobium meliloti] RVH63306.1 3-phosphoshikimate 1-carboxyvinyltransferase [Sinorhizobium meliloti] RVK64950.1 3-phosphoshikimate 1-carboxyvinyltransferase [Sinorhizobium meliloti]

Length=455

Score = 805 bits (2079), Expect = 0.0, Method: Compositional matrix adjust.

| Identities | = 411/455 (90%), Positives = 429/455 (94%), Gaps = 0/455 (0%) |  |
| --- | --- | --- |
| Query 1 | MLHGASSRPATARKSSGLSGTVRIPGDKSISHRSFMFGGLASGETRITGLLEGEDVINTG | 60 |
| Sbjct 1 | M HG++ RPATARKSS L GT+RIPGDKSISHRSFMFGGLA+GETRITGLLEGEDVINTG MSHGSNPRPATARKSSDLKGTLRIPGDKSISHRSFMFGGLAAGETRITGLLEGEDVINTG | 60 |
| Query 61 | KAMQAMGARIRKEGDTWIIDGVGNGGLLAPEAPLDFGNAATGCRLTMGLVGVYDFDSTFI | 120 |
| Sbjct 61 | KAMQAMGARIRKEGDTWIIDGVGNG LLAPEAPLDFGNA TGCRLTMGLVGVYDFDSTFI KAMQAMGARIRKEGDTWIIDGVGNGALLAPEAPLDFGNAGTGCRLTMGLVGVYDFDSTFI | 120 |
| Query 121 | GDASLTKRPMGRVLNPLREMGVQVKSEDGDRLPVTLRGPKTPTPITYRVPMASAQVKSAV | 180 |
| Sbjct 121 | GDASLTKRPMGRVL+PLREMGVQVKS +GD LPVTLRGPKTP PITYRVPMASAQVKSAV GDASLTKRPMGRVLDPLREMGVQVKSAEGDWLPVTLRGPKTPNPITYRVPMASAQVKSAV | 180 |
| Query 181 | LLAGLNTPGITTVIEPIMTRDHTEKMLQGFGANLTVETDADGVRTIRLEGRGKLTGQVID | 240 |
| Sbjct 181 | LLAGLNTPGITTV+EP+MTRDHTEKMLQGFGANLTVETDA+GVRTIRLEGRGKLTGQVID LLAGLNTPGITTVVEPVMTRDHTEKMLQGFGANLTVETDAEGVRTIRLEGRGKLTGQVID | 240 |
| Query 241 | VPGDPSSTAFPLVAALLVPGSDVTILNVLMNPTRTGLILTLQEMGADIEVINPRLAGGED | 300 |
| Sbjct 241 | VPGDPSSTAFPLVA L+VPGSD+TILNVLMNPTRTGLILTLQEMGA+IEV+N RLAGGED VPGDPSSTAFPLVAGLIVPGSDITILNVLMNPTRTGLILTLQEMGANIEVMNKRLAGGED | 300 |
| Query 301 | VADLRVRSSTLKGVTVPEDRAPSMIDEYPILavaaafaegaTVMNGLEELRVKESDRLSA | 360 |

| Sbjct | 301 | VADLRVR S LKGVTVPEDRAPSMIDEYP+LAVAAAFAEG TVMNGLEELRVKESDRLSA VADLRVRHSELKGVTVPEDRAPSMIDEYPVLAVAAAFAEGTTVMNGLEELRVKESDRLSA | 360 |
| --- | --- | --- | --- |
| Query | 361 | VANGLKLNGVDCDEGETSLVVRGRPDGKGLGNASGAAVATHLDHRIAMSFLVMGLVSENP | 420 |
| Sbjct | 361 | VA+GLKLNGVDCDEGE SLVVRGRP GKGLG SG V THLDHRIAMSFLVMGL SE+P VADGLKLNGVDCDEGEASLVVRGRPGGKGLGKISGGQVKTHLDHRIAMSFLVMGLASEHP | 420 |
| Query  Sbjct | 421  421 | VTVDDATMIATSFPEFMDLMAGLGAKIELSDTKAA 455 VTVDDATMIATSFPEFM LM GLGAKIE ++ KAA VTVDDATMIATSFPEFMGLMTGLGAKIEEAENKAA 455 |  |

>WP_127597035.1 3-phosphoshikimate 1-carboxyvinyltransferase [Sinorhizobium meliloti] RVG71660.1 3-phosphoshikimate 1-carboxyvinyltransferase [Sinorhizobium meliloti] RVH38954.1 3-phosphoshikimate 1-carboxyvinyltransferase [Sinorhizobium meliloti] RVO65533.1 3-phosphoshikimate 1-carboxyvinyltransferase [Sinorhizobium meliloti]

Length=455

Score = 804 bits (2077), Expect = 0.0, Method: Compositional matrix adjust.

| Identities | = 411/455 (90%), Positives = 429/455 (94%), Gaps = 0/455 (0%) |  |
| --- | --- | --- |
| Query 1 | MLHGASSRPATARKSSGLSGTVRIPGDKSISHRSFMFGGLASGETRITGLLEGEDVINTG | 60 |
| Sbjct 1 | M HG++ RPATARKSS L GT+RIPGDKSISHRSFMFGGLA+GETRITGLLEGEDVINTG MSHGSNPRPATARKSSDLKGTLRIPGDKSISHRSFMFGGLAAGETRITGLLEGEDVINTG | 60 |
| Query 61 | KAMQAMGARIRKEGDTWIIDGVGNGGLLAPEAPLDFGNAATGCRLTMGLVGVYDFDSTFI | 120 |
| Sbjct 61 | KAMQAMGARIRKEGDTWIIDGVGNG LLAPEAPLDFGNA TGCRLTMGLVGVYDFDSTFI KAMQAMGARIRKEGDTWIIDGVGNGALLAPEAPLDFGNAGTGCRLTMGLVGVYDFDSTFI | 120 |
| Query 121 | GDASLTKRPMGRVLNPLREMGVQVKSEDGDRLPVTLRGPKTPTPITYRVPMASAQVKSAV | 180 |
| Sbjct 121 | GDASLTKRPMGRVL+PLREMGVQVKS +GDRLPVTLRGPKTP PITYRVPMASAQVKSAV GDASLTKRPMGRVLDPLREMGVQVKSAEGDRLPVTLRGPKTPNPITYRVPMASAQVKSAV | 180 |
| Query 181 | LLAGLNTPGITTVIEPIMTRDHTEKMLQGFGANLTVETDADGVRTIRLEGRGKLTGQVID | 240 |
| Sbjct 181 | LLAGLNTPGITTV+EP+MTRDHTEKMLQGFGANLTVETDA+GVRTIRLEGRGKLTGQVID LLAGLNTPGITTVVEPVMTRDHTEKMLQGFGANLTVETDAEGVRTIRLEGRGKLTGQVID | 240 |
| Query 241 | VPGDPSSTAFPLVAALLVPGSDVTILNVLMNPTRTGLILTLQEMGADIEVINPRLAGGED | 300 |
| Sbjct 241 | VPGDPSSTAFPLVA L+VPGSD+TILNVLMNPTRTGLILTLQEMGA+IEV+N RLAGGED VPGDPSSTAFPLVAGLIVPGSDITILNVLMNPTRTGLILTLQEMGANIEVMNKRLAGGED | 300 |
| Query 301 | VADLRVRSSTLKGVTVPEDRAPSMIDEYPILavaaafaegaTVMNGLEELRVKESDRLSA | 360 |
| Sbjct 301 | VADLRVR S LKGVTV EDRAPSMIDEYP+LAVAAAFAEG TVMNGLEELRVKESDRLSA VADLRVRHSELKGVTVLEDRAPSMIDEYPVLAVAAAFAEGTTVMNGLEELRVKESDRLSA | 360 |
| Query 361 | VANGLKLNGVDCDEGETSLVVRGRPDGKGLGNASGAAVATHLDHRIAMSFLVMGLVSENP | 420 |
| Sbjct 361 | VA+GLKLNGVDCDEGE SLVVRGRP GKGLGN S V THLDHRIAMSFLVMGL SE+P VADGLKLNGVDCDEGEASLVVRGRPGGKGLGNISVGQVKTHLDHRIAMSFLVMGLASEHP | 420 |
| Query 421  Sbjct 421 | VTVDDATMIATSFPEFMDLMAGLGAKIELSDTKAA 455 VTVDDATMIATSFPEFM LM GLGAKIE ++ KAA VTVDDATMIATSFPEFMGLMTGLGAKIEEAENKAA 455 |  |

>WP_037398737.1 3-phosphoshikimate 1-carboxyvinyltransferase [Sinorhizobium fredii] AWM27305.1 5-Enolpyruvylshikimate-3-phosphate synthase [Sinorhizobium fredii

CCBAU 25509]

MQW94742.1 3-phosphoshikimate 1-carboxyvinyltransferase [Sinorhizobium fredii] MQX11999.1 3-phosphoshikimate 1-carboxyvinyltransferase [Sinorhizobium fredii] GEC32713.1 3-phosphoshikimate 1-carboxyvinyltransferase [Sinorhizobium fredii]

Length=450

Score = 803 bits (2075), Expect = 0.0, Method: Compositional matrix adjust.

| Identities | = 411/447 (92%), Positives = 427/447 (96%), Gaps = 0/447 (0%) |  |
| --- | --- | --- |
| Query 1 | MLHGASSRPATARKSSGLSGTVRIPGDKSISHRSFMFGGLASGETRITGLLEGEDVINTG | 60 |
| Sbjct 1 | M HG S RPATA+KS+ L GTVRIPGDKSISHRSFMFGGLASGETRITGLLEGEDVINTG MSHGLSPRPATAKKSADLKGTVRIPGDKSISHRSFMFGGLASGETRITGLLEGEDVINTG | 60 |
| Query 61 | KAMQAMGARIRKEGDTWIIDGVGNGGLLAPEAPLDFGNAATGCRLTMGLVGVYDFDSTFI | 120 |
| Sbjct 61 | KAMQAMGA++RKEGDTWII+GVGNG LLAPEAPLDFGNA TGCRLTMGLVGVYDFDSTFI  KAMQAMGAKVRKEGDTWIINGVGNGALLAPEAPLDFGNAGTGCRLTMGLVGVYDFDSTFI | 120 |

| Query | 121 | GDASLTKRPMGRVLNPLREMGVQVKSEDGDRLPVTLRGPKTPTPITYRVPMASAQVKSAV | 180 |
| --- | --- | --- | --- |
| Sbjct | 121 | GDASLTKRPMGRVL+PLR+MGVQVKS +GDRLPVTLRGP TP PITYRVPMASAQVKSAV GDASLTKRPMGRVLDPLRQMGVQVKSAEGDRLPVTLRGPTTPNPITYRVPMASAQVKSAV | 180 |
| Query | 181 | LLAGLNTPGITTVIEPIMTRDHTEKMLQGFGANLTVETDADGVRTIRLEGRGKLTGQVID | 240 |
| Sbjct | 181 | LLAGLNTPGITTVIEP+MTRDHTEKMLQGFGANLTVETDA GVRTIRLEGRGKLTGQVID LLAGLNTPGITTVIEPVMTRDHTEKMLQGFGANLTVETDAQGVRTIRLEGRGKLTGQVID | 240 |
| Query | 241 | VPGDPSSTAFPLVAALLVPGSDVTILNVLMNPTRTGLILTLQEMGADIEVINPRLAGGED | 300 |
| Sbjct | 241 | VPGDPSSTAFPLVAALLVPGSDVTILNVLMNPTRTGLILTLQEMGA+IEV+N RLAGGED VPGDPSSTAFPLVAALLVPGSDVTILNVLMNPTRTGLILTLQEMGANIEVMNQRLAGGED | 300 |
| Query | 301 | VADLRVRSSTLKGVTVPEDRAPSMIDEYPILavaaafaegaTVMNGLEELRVKESDRLSA | 360 |
| Sbjct | 301 | VADLRVR S LKGVTVPEDRAPSMIDEYP+LAVAAAFAEGATVMNGL+ELRVKESDRLSA VADLRVRHSELKGVTVPEDRAPSMIDEYPVLAVAAAFAEGATVMNGLDELRVKESDRLSA | 360 |
| Query | 361 | VANGLKLNGVDCDEGETSLVVRGRPDGKGLGNASGAAVATHLDHRIAMSFLVMGLVSENP | 420 |
| Sbjct | 361 | VA+GLKLNGVDCDEGE SLVVRGRPDGKGLGNA+G V THLDHRIAMSFLVMGL SE+P VADGLKLNGVDCDEGEASLVVRGRPDGKGLGNAAGGQVKTHLDHRIAMSFLVMGLASEHP | 420 |
| Query  Sbjct | 421  421 | VTVDDATMIATSFPEFMDLMAGLGAKI 447 VTVDDATMIATSFPEFM LMAGLGAKI VTVDDATMIATSFPEFMGLMAGLGAKI 447 |  |

>WP_151611883.1 3-phosphoshikimate 1-carboxyvinyltransferase [Ensifer alkalisoli] QFI68367.1 5-Enolpyruvylshikimate-3-phosphate synthase [Ensifer alkalisoli]

Length=455

Score = 803 bits (2074), Expect = 0.0, Method: Compositional matrix adjust.

| Identities | = 409/454 (90%), Positives = 428/454 (94%), Gaps = 0/454 (0%) |  |
| --- | --- | --- |
| Query 1 | MLHGASSRPATARKSSGLSGTVRIPGDKSISHRSFMFGGLASGETRITGLLEGEDVINTG | 60 |
| Sbjct 1 | M HG SSRPATARKSS L GTVRIPGDKSISHRSFMFGGLASGETRITGLLEGEDVINTG MSHGQSSRPATARKSSDLKGTVRIPGDKSISHRSFMFGGLASGETRITGLLEGEDVINTG | 60 |
| Query 61 | KAMQAMGARIRKEGDTWIIDGVGNGGLLAPEAPLDFGNAATGCRLTMGLVGVYDFDSTFI | 120 |
| Sbjct 61 | KAMQAMGA+IRKEGD WIIDGVGNG LLAPEAPLDFGNA TGCRLTMGLVGVYDF STF+ KAMQAMGAKIRKEGDAWIIDGVGNGALLAPEAPLDFGNAGTGCRLTMGLVGVYDFASTFV | 120 |
| Query 121 | GDASLTKRPMGRVLNPLREMGVQVKSEDGDRLPVTLRGPKTPTPITYRVPMASAQVKSAV | 180 |
| Sbjct 121 | GDASLTKRPMGRVL+PLREMGVQVKS +GDRLPV L GPKTP PITYRVPMASAQVKSAV GDASLTKRPMGRVLDPLREMGVQVKSAEGDRLPVMLHGPKTPNPITYRVPMASAQVKSAV | 180 |
| Query 181 | LLAGLNTPGITTVIEPIMTRDHTEKMLQGFGANLTVETDADGVRTIRLEGRGKLTGQVID | 240 |
| Sbjct 181 | LLAGLNTPGITTVIEP+MTRDHTEKMLQGFGA+L+VETDA GVRTIRLEGRG+LTGQVID LLAGLNTPGITTVIEPVMTRDHTEKMLQGFGADLSVETDAAGVRTIRLEGRGRLTGQVID | 240 |
| Query 241 | VPGDPSSTAFPLVAALLVPGSDVTILNVLMNPTRTGLILTLQEMGADIEVINPRLAGGED | 300 |
| Sbjct 241 | VPGDPSSTAFPLVAAL+VPGSDVTILNVLMNPTRTGLILTLQEMGA+IEV+N RLAGGED VPGDPSSTAFPLVAALIVPGSDVTILNVLMNPTRTGLILTLQEMGANIEVMNKRLAGGED | 300 |
| Query 301 | VADLRVRSSTLKGVTVPEDRAPSMIDEYPILavaaafaegaTVMNGLEELRVKESDRLSA | 360 |
| Sbjct 301 | VADLRVR S LKGVTVPE+RAPSMIDEYP+LAVAAAFAEG TVMNGL+ELRVKESDRLSA VADLRVRHSELKGVTVPEERAPSMIDEYPVLAVAAAFAEGTTVMNGLDELRVKESDRLSA | 360 |
| Query 361 | VANGLKLNGVDCDEGETSLVVRGRPDGKGLGNASGAAVATHLDHRIAMSFLVMGLVSENP | 420 |
| Sbjct 361 | VA+GLKLNGVDCDEGE SLVVRGRPDGKGLGNA G V THLDHRIAMSFLV+GL SE+P VADGLKLNGVDCDEGEASLVVRGRPDGKGLGNAPGEKVKTHLDHRIAMSFLVLGLASEHP | 420 |
| Query 421  Sbjct 421 | VTVDDATMIATSFPEFMDLMAGLGAKIELSDTKA 454 VTVDDATMIATSFPEFM LM GLGAKIE ++TKA VTVDDATMIATSFPEFMGLMTGLGAKIEQTETKA 454 |  |

>WP_037456937.1 3-phosphoshikimate 1-carboxyvinyltransferase [Sinorhizobium fredii] AWI59675.1 hypothetical protein AB395_00004050 [Sinorhizobium fredii CCBAU

45436]

Length=448

Score = 802 bits (2072), Expect = 0.0, Method: Compositional matrix adjust.

| Identities | = 412/448 (92%), Positives = 427/448 (95%), Gaps = 0/448 (0%) |  |
| --- | --- | --- |
| Query 1 | MLHGASSRPATARKSSGLSGTVRIPGDKSISHRSFMFGGLASGETRITGLLEGEDVINTG | 60 |
| Sbjct 1 | M HG S RPATA+KS+ L GTVRIPGDKSISHRSFMFGGLASGETRITGLLEGEDVINTG MSHGLSPRPATAKKSADLKGTVRIPGDKSISHRSFMFGGLASGETRITGLLEGEDVINTG | 60 |
| Query 61 | KAMQAMGARIRKEGDTWIIDGVGNGGLLAPEAPLDFGNAATGCRLTMGLVGVYDFDSTFI | 120 |
| Sbjct 61 | KAMQAMGA++RKEGDTWII+GVGNG LLAPEAPLDFGNA TGCRLTMGLVGVYDFDSTFI KAMQAMGAKVRKEGDTWIINGVGNGALLAPEAPLDFGNAGTGCRLTMGLVGVYDFDSTFI | 120 |
| Query 121 | GDASLTKRPMGRVLNPLREMGVQVKSEDGDRLPVTLRGPKTPTPITYRVPMASAQVKSAV | 180 |
| Sbjct 121 | GDASLTKRPMGRVL+PLR+MGVQVKS +GDRLPVTLRGP TP PITYRVPMASAQVKSAV GDASLTKRPMGRVLDPLRQMGVQVKSAEGDRLPVTLRGPTTPNPITYRVPMASAQVKSAV | 180 |
| Query 181 | LLAGLNTPGITTVIEPIMTRDHTEKMLQGFGANLTVETDADGVRTIRLEGRGKLTGQVID | 240 |
| Sbjct 181 | LLAGLNTPGITTVIEP+MTRDHTEKMLQGFGANLTVETDA GVRTIRLEGRGKLTGQVID LLAGLNTPGITTVIEPVMTRDHTEKMLQGFGANLTVETDAQGVRTIRLEGRGKLTGQVID | 240 |
| Query 241 | VPGDPSSTAFPLVAALLVPGSDVTILNVLMNPTRTGLILTLQEMGADIEVINPRLAGGED | 300 |
| Sbjct 241 | VPGDPSSTAFPLVAALLVPGSDVTILNVLMNPTRTGLILTLQEMGA+IEV+N RLAGGED VPGDPSSTAFPLVAALLVPGSDVTILNVLMNPTRTGLILTLQEMGANIEVMNQRLAGGED | 300 |
| Query 301 | VADLRVRSSTLKGVTVPEDRAPSMIDEYPILavaaafaegaTVMNGLEELRVKESDRLSA | 360 |
| Sbjct 301 | VADLRVR S L GVTVPEDRAPSMIDEYP+LAVAAAFAEGATVMNGL+ELRVKESDRLSA VADLRVRYSELTGVTVPEDRAPSMIDEYPVLAVAAAFAEGATVMNGLDELRVKESDRLSA | 360 |
| Query 361 | VANGLKLNGVDCDEGETSLVVRGRPDGKGLGNASGAAVATHLDHRIAMSFLVMGLVSENP | 420 |
| Sbjct 361 | VA+GLKLNGVDCDEGE SLVVRGRPDGKGLGNASG V THLDHRIAMSFLVMGL SE+P VADGLKLNGVDCDEGEASLVVRGRPDGKGLGNASGDQVKTHLDHRIAMSFLVMGLASEHP | 420 |
| Query 421  Sbjct 421 | VTVDDATMIATSFPEFMDLMAGLGAKIE 448 VTVDDATMIATSFPEFM LMAGLGAKIE VTVDDATMIATSFPEFMGLMAGLGAKIE 448 |  |

>WP_014330677.1 3-phosphoshikimate 1-carboxyvinyltransferase [Sinorhizobium fredii] CCE98309.1 3-phosphoshikimate 1-carboxyvinyltransferase [Sinorhizobium fredii

HH103]

Length=450

Score = 801 bits (2069), Expect = 0.0, Method: Compositional matrix adjust.

| Identities | = 410/447 (92%), Positives = 426/447 (95%), Gaps = 0/447 (0%) |  |
| --- | --- | --- |
| Query 1 | MLHGASSRPATARKSSGLSGTVRIPGDKSISHRSFMFGGLASGETRITGLLEGEDVINTG | 60 |
| Sbjct 1 | M HG S RPATA+KS+ L GTVRIPGDKSISHRSFMFGGLASGETRITGLLEGEDVINTG MSHGLSPRPATAKKSADLKGTVRIPGDKSISHRSFMFGGLASGETRITGLLEGEDVINTG | 60 |
| Query 61 | KAMQAMGARIRKEGDTWIIDGVGNGGLLAPEAPLDFGNAATGCRLTMGLVGVYDFDSTFI | 120 |
| Sbjct 61 | KAMQAMGA++RKEGDTWII+GVGNG LLAPEAPLDFGNA TGCRLTMGLVGVYDFDSTFI KAMQAMGAKVRKEGDTWIINGVGNGALLAPEAPLDFGNAGTGCRLTMGLVGVYDFDSTFI | 120 |
| Query 121 | GDASLTKRPMGRVLNPLREMGVQVKSEDGDRLPVTLRGPKTPTPITYRVPMASAQVKSAV | 180 |
| Sbjct 121 | GDASL KRPMGRVL+PLR+MGVQVKS +GDRLPVTLRGP TP PITYRVPMASAQVKSAV GDASLAKRPMGRVLDPLRQMGVQVKSAEGDRLPVTLRGPTTPNPITYRVPMASAQVKSAV | 180 |
| Query 181 | LLAGLNTPGITTVIEPIMTRDHTEKMLQGFGANLTVETDADGVRTIRLEGRGKLTGQVID | 240 |
| Sbjct 181 | LLAGLNTPGITTVIEP+MTRDHTEKMLQGFGANLTVETDA GVRTIRLEGRGKLTGQVID LLAGLNTPGITTVIEPVMTRDHTEKMLQGFGANLTVETDAQGVRTIRLEGRGKLTGQVID | 240 |
| Query 241 | VPGDPSSTAFPLVAALLVPGSDVTILNVLMNPTRTGLILTLQEMGADIEVINPRLAGGED | 300 |
| Sbjct 241 | VPGDPSSTAFPLVAALLVPGSDVTILNVLMNPTRTGLILTLQEMGA+IEV+N RLAGGED VPGDPSSTAFPLVAALLVPGSDVTILNVLMNPTRTGLILTLQEMGANIEVMNQRLAGGED | 300 |
| Query 301 | VADLRVRSSTLKGVTVPEDRAPSMIDEYPILavaaafaegaTVMNGLEELRVKESDRLSA | 360 |
| Sbjct 301 | VADLRVR S LKGVTVPEDRAPSMIDEYP+LAVAAAFAEGATVMNGL+ELRVKESDRLSA VADLRVRHSELKGVTVPEDRAPSMIDEYPVLAVAAAFAEGATVMNGLDELRVKESDRLSA | 360 |
| Query 361 | VANGLKLNGVDCDEGETSLVVRGRPDGKGLGNASGAAVATHLDHRIAMSFLVMGLVSENP | 420 |
| Sbjct 361 | VA+GLKLNGVDCDEGE SLVVRGRPDGKGLGNA+G V THLDHRIAMSFLVMGL SE+P VADGLKLNGVDCDEGEASLVVRGRPDGKGLGNAAGGQVKTHLDHRIAMSFLVMGLASEHP | 420 |
| Query 421 | VTVDDATMIATSFPEFMDLMAGLGAKI 447 |  |

VTVDDATMIATSFPEFM LMAGLGAKI Sbjct 421 VTVDDATMIATSFPEFMGLMAGLGAKI 447

>OJU68221.1 3-phosphoshikimate 1-carboxyvinyltransferase [Rhizobiales bacterium 63-7]

Length=460

Score = 801 bits (2068), Expect = 0.0, Method: Compositional matrix adjust.

| Identities | = 404/453 (89%), Positives = 427/453 (94%), Gaps = 0/453 (0%) |  |
| --- | --- | --- |
| Query 3 | HGASSRPATARKSSGLSGTVRIPGDKSISHRSFMFGGLASGETRITGLLEGEDVINTGKA | 62 |
| Sbjct 8 | HGA+++PA AR+SS LSGT RIPGDKSISHRSFMFGGLASGETRITGLLEGEDVINTGKA HGAAAKPAIARRSSNLSGTARIPGDKSISHRSFMFGGLASGETRITGLLEGEDVINTGKA | 67 |
| Query 63 | MQAMGARIRKEGDTWIIDGVGNGGLLAPEAPLDFGNAATGCRLTMGLVGVYDFDSTFIGD | 122 |
| Sbjct 68 | MQAMGA+IRKEGD WII G GNG LLAPEAPLDFGNA TGCRLTMGLVGVYDFDSTFIGD MQAMGAKIRKEGDVWIIQGTGNGALLAPEAPLDFGNAGTGCRLTMGLVGVYDFDSTFIGD | 127 |
| Query 123 | ASLTKRPMGRVLNPLREMGVQVKSEDGDRLPVTLRGPKTPTPITYRVPMASAQVKSAVLL | 182 |
| Sbjct 128 | ASLTKRPMGRVL+PLR+MGVQVK+E GDRLPVTL GP TP PITYRVPMASAQVKSAVLL ASLTKRPMGRVLDPLRQMGVQVKAEAGDRLPVTLHGPLTPAPITYRVPMASAQVKSAVLL | 187 |
| Query 183 | AGLNTPGITTVIEPIMTRDHTEKMLQGFGANLTVETDADGVRTIRLEGRGKLTGQVIDVP | 242 |
| Sbjct 188 | AGLNTPGITTVIEP+MTRDHTEKMLQGFGA+LTVETDADGVRTIRL GRGKLTGQ IDVP AGLNTPGITTVIEPVMTRDHTEKMLQGFGADLTVETDADGVRTIRLAGRGKLTGQTIDVP | 247 |
| Query 243 | GDPSSTAFPLVAALLVPGSDVTILNVLMNPTRTGLILTLQEMGADIEVINPRLAGGEDVA | 302 |
| Sbjct 248 | GDPSSTAFPLVAALLVPGSD+TI+NVLMNPTRTGLILTLQ+MGADIEV+NPR AGGEDVA GDPSSTAFPLVAALLVPGSDITIVNVLMNPTRTGLILTLQDMGADIEVLNPRNAGGEDVA | 307 |
| Query 303 | DLRVRSSTLKGVTVPEDRAPSMIDEYPILavaaafaegaTVMNGLEELRVKESDRLSAVA | 362 |
| Sbjct 308 | DLRVR S LKGVTVPEDRAPSMIDEYP+LAVAAAFA+G TVMNGLEELRVKESDRLSAVA DLRVRHSELKGVTVPEDRAPSMIDEYPVLAVAAAFAKGETVMNGLEELRVKESDRLSAVA | 367 |
| Query 363 | NGLKLNGVDCDEGETSLVVRGRPDGKGLGNASGAAVATHLDHRIAMSFLVMGLVSENPVT | 422 |
| Sbjct 368 | NGLKLNG DCDEGE+SL+VRGRPDGKGLGNA+G AVATHLDHRIAMSFL+MGL SE+PVT NGLKLNGADCDEGESSLLVRGRPDGKGLGNAAGEAVATHLDHRIAMSFLIMGLASEHPVT | 427 |
| Query 423  Sbjct 428 | VDDATMIATSFPEFMDLMAGLGAKIELSDTKAA 455 VDDATMIATSFPEFMDLM LGAKIE+ D +A VDDATMIATSFPEFMDLMTRLGAKIEIKDGASA 460 |  |

>WP_037377818.1 MULTISPECIES: 3-phosphoshikimate 1-carboxyvinyltransferase [Sinorhizobium]

APG83057.1 3-phosphoshikimate 1-carboxyvinyltransferase AroA [Sinorhizobium americanum CCGM7]

APG89594.1 3-phosphoshikimate 1-carboxyvinyltransferase AroA [Sinorhizobium americanum]

OAP36081.1 3-phosphoshikimate 1-carboxyvinyltransferase [Sinorhizobium americanum] PDT41743.1 3-phosphoshikimate 1-carboxyvinyltransferase [Sinorhizobium sp.

FG01]

POH28557.1 3-phosphoshikimate 1-carboxyvinyltransferase [Sinorhizobium americanum] Length=448

Score = 800 bits (2066), Expect = 0.0, Method: Compositional matrix adjust.

| Identities | = 409/448 (91%), Positives = 426/448 (95%), Gaps = 0/448 (0%) |  |
| --- | --- | --- |
| Query 1 | MLHGASSRPATARKSSGLSGTVRIPGDKSISHRSFMFGGLASGETRITGLLEGEDVINTG | 60 |
| Sbjct 1 | M HG S RPATA+KS+ L GTVRIPGDKSISHRSFMFGGLASGETRITGLLEGEDVINTG MSHGLSPRPATAKKSADLKGTVRIPGDKSISHRSFMFGGLASGETRITGLLEGEDVINTG | 60 |
| Query 61 | KAMQAMGARIRKEGDTWIIDGVGNGGLLAPEAPLDFGNAATGCRLTMGLVGVYDFDSTFI | 120 |
| Sbjct 61 | KAMQAMGA IRKEGDTWII+GVGNG LLAPEAPLDFGNA TGCRLTMGLVGVYDFDSTFI KAMQAMGANIRKEGDTWIINGVGNGALLAPEAPLDFGNAGTGCRLTMGLVGVYDFDSTFI | 120 |
| Query 121 | GDASLTKRPMGRVLNPLREMGVQVKSEDGDRLPVTLRGPKTPTPITYRVPMASAQVKSAV | 180 |
| Sbjct 121 | GDASLTKRPMGRVL+PLR+MGVQVKS +GDRLPVTLRGP+TP PITYRVPMASAQVKSAV GDASLTKRPMGRVLDPLRQMGVQVKSAEGDRLPVTLRGPQTPNPITYRVPMASAQVKSAV | 180 |
| Query 181 | LLAGLNTPGITTVIEPIMTRDHTEKMLQGFGANLTVETDADGVRTIRLEGRGKLTGQVID LLAGLNTPGITTVIEP+MTRDHTEKMLQGFGANLTVETD GVRTIRLEGRGKLTGQVID | 240 |

| Sbjct | 181 | LLAGLNTPGITTVIEPVMTRDHTEKMLQGFGANLTVETDTQGVRTIRLEGRGKLTGQVID | 240 |
| --- | --- | --- | --- |
| Query | 241 | VPGDPSSTAFPLVAALLVPGSDVTILNVLMNPTRTGLILTLQEMGADIEVINPRLAGGED | 300 |
| Sbjct | 241 | VPGDPSSTAFPLVAALLVPGSDVTILNVLMNPTRTGLILTLQEMGA+IEV+N RLAGGED VPGDPSSTAFPLVAALLVPGSDVTILNVLMNPTRTGLILTLQEMGANIEVMNQRLAGGED | 300 |
| Query | 301 | VADLRVRSSTLKGVTVPEDRAPSMIDEYPILavaaafaegaTVMNGLEELRVKESDRLSA | 360 |
| Sbjct | 301 | VADLRVR S LKGVTVPE+RAPSMIDEYP+LAVAAAFAEGATVMNGL+ELRVKESDRLSA VADLRVRYSELKGVTVPEERAPSMIDEYPVLAVAAAFAEGATVMNGLDELRVKESDRLSA | 360 |
| Query | 361 | VANGLKLNGVDCDEGETSLVVRGRPDGKGLGNASGAAVATHLDHRIAMSFLVMGLVSENP | 420 |
| Sbjct | 361 | VA+GLKLNGVDCDEGE SLVVRGRPDGKGLGNA+G V THLDHRIAMSFLVMGL SE+P VADGLKLNGVDCDEGEASLVVRGRPDGKGLGNAAGEKVKTHLDHRIAMSFLVMGLASEHP | 420 |
| Query  Sbjct | 421  421 | VTVDDATMIATSFPEFMDLMAGLGAKIE 448 VT+DD TMIATSFPEFMDLM GLGAKIE VTIDDGTMIATSFPEFMDLMTGLGAKIE 448 |  |

>WP_012710050.1 3-phosphoshikimate 1-carboxyvinyltransferase [Sinorhizobium fredii] C3MC80.1 RecName: Full=3-phosphoshikimate 1-carboxyvinyltransferase; AltName:

Full=5-enolpyruvylshikimate-3-phosphate synthase; Short=EPSP synthase; Short=EPSPS [Sinorhizobium fredii NGR234]

ACP27305.1 3-phosphoshikimate 1-carboxyvinyltransferase [Sinorhizobium fredii NGR234]

Length=448

Score = 800 bits (2066), Expect = 0.0, Method: Compositional matrix adjust.

| Identities | = 410/448 (92%), Positives = 427/448 (95%), Gaps = 0/448 (0%) |  |
| --- | --- | --- |
| Query 1 | MLHGASSRPATARKSSGLSGTVRIPGDKSISHRSFMFGGLASGETRITGLLEGEDVINTG | 60 |
| Sbjct 1 | M HG S RPATA+KS+ L GTVRIPGDKSISHRSFMFGGLASGETRITGLLEGEDVINTG MSHGLSPRPATAKKSADLKGTVRIPGDKSISHRSFMFGGLASGETRITGLLEGEDVINTG | 60 |
| Query 61 | KAMQAMGARIRKEGDTWIIDGVGNGGLLAPEAPLDFGNAATGCRLTMGLVGVYDFDSTFI | 120 |
| Sbjct 61 | KAMQAMGA+IRKEGDTWII+GVGNG LLAPEAPLDFGNA TGCRLTMGLVGVYDFDSTFI KAMQAMGAKIRKEGDTWIINGVGNGALLAPEAPLDFGNAGTGCRLTMGLVGVYDFDSTFI | 120 |
| Query 121 | GDASLTKRPMGRVLNPLREMGVQVKSEDGDRLPVTLRGPKTPTPITYRVPMASAQVKSAV | 180 |
| Sbjct 121 | GDASLTKRPMGRVL+PLREMGVQVKS +GDRLPVTLRGPKTP PITYRVPMASAQVKSAV GDASLTKRPMGRVLDPLREMGVQVKSAEGDRLPVTLRGPKTPNPITYRVPMASAQVKSAV | 180 |
| Query 181 | LLAGLNTPGITTVIEPIMTRDHTEKMLQGFGANLTVETDADGVRTIRLEGRGKLTGQVID | 240 |
| Sbjct 181 | LLAGLNTPGITTVIEP+MTRDHTEKMLQGFGANL+VETD GVRTIRLEGRGKLTGQVID LLAGLNTPGITTVIEPVMTRDHTEKMLQGFGANLSVETDTAGVRTIRLEGRGKLTGQVID | 240 |
| Query 241 | VPGDPSSTAFPLVAALLVPGSDVTILNVLMNPTRTGLILTLQEMGADIEVINPRLAGGED | 300 |
| Sbjct 241 | VPGDPSSTAFPLVAALLVPGSDVTILNVLMNPTRTGLILTLQEMGA+IEV+NPRLAGGED VPGDPSSTAFPLVAALLVPGSDVTILNVLMNPTRTGLILTLQEMGANIEVMNPRLAGGED | 300 |
| Query 301 | VADLRVRSSTLKGVTVPEDRAPSMIDEYPILavaaafaegaTVMNGLEELRVKESDRLSA | 360 |
| Sbjct 301 | VADLRVR S LKGVTVPE+RAPSMIDEYP+LAVAAAFAEGATVMNGL+ELRVKESDRLSA VADLRVRYSELKGVTVPEERAPSMIDEYPVLAVAAAFAEGATVMNGLDELRVKESDRLSA | 360 |
| Query 361 | VANGLKLNGVDCDEGETSLVVRGRPDGKGLGNASGAAVATHLDHRIAMSFLVMGLVSENP | 420 |
| Sbjct 361 | VA+GLKLNGVDCDEGE SLVVRGRP GKGLGNA+G V THLDHRIAMSFLV+GL SE+P VADGLKLNGVDCDEGEASLVVRGRPGGKGLGNAAGGQVKTHLDHRIAMSFLVLGLASEHP | 420 |
| Query 421  Sbjct 421 | VTVDDATMIATSFPEFMDLMAGLGAKIE 448 VTVDDATMIATSFPEFMDLM GLGA IE VTVDDATMIATSFPEFMDLMTGLGATIE 448 |  |

```
>WP_040959218.1 3-phosphoshikimate 1-carboxyvinyltransferase [Sinorhizobium fredii] Length=450

Score = 800 bits (2065), Expect = 0.0, Method: Compositional matrix adjust. Identities = 408/447 (91%), Positives = 427/447 (96%), Gaps = 0/447 (0%)

Query 1	MLHGASSRPATARKSSGLSGTVRIPGDKSISHRSFMFGGLASGETRITGLLEGEDVINTG 60 M HG S RPATA+KS+ L GTVRIPGDKSISHRSFMFGGLASGETRITGLLEGEDVINTG
Sbjct 1	MSHGLSPRPATAKKSADLKGTVRIPGDKSISHRSFMFGGLASGETRITGLLEGEDVINTG 60
```

| Query | 61 | KAMQAMGARIRKEGDTWIIDGVGNGGLLAPEAPLDFGNAATGCRLTMGLVGVYDFDSTFI | 120 |
| --- | --- | --- | --- |
| Sbjct | 61 | KAMQAMGA +RK+GDTWII+GVGNG LLAPEAPLDFGNA TGCRLTMGLVGVYDFDSTF+ KAMQAMGANVRKDGDTWIINGVGNGALLAPEAPLDFGNAGTGCRLTMGLVGVYDFDSTFV | 120 |
| Query | 121 | GDASLTKRPMGRVLNPLREMGVQVKSEDGDRLPVTLRGPKTPTPITYRVPMASAQVKSAV | 180 |
| Sbjct | 121 | GDASLTKRPMGRVL+PLR+MGVQVKS +GDRLPVTL GPKTP PITYRVPMASAQVKSAV GDASLTKRPMGRVLDPLRQMGVQVKSAEGDRLPVTLHGPKTPNPITYRVPMASAQVKSAV | 180 |
| Query | 181 | LLAGLNTPGITTVIEPIMTRDHTEKMLQGFGANLTVETDADGVRTIRLEGRGKLTGQVID | 240 |
| Sbjct | 181 | LLAGLNTPGITTVIEP+MTRDHTEKMLQGFGANLTVETDA+GVRTIRLEGRGKLTGQVID LLAGLNTPGITTVIEPVMTRDHTEKMLQGFGANLTVETDAEGVRTIRLEGRGKLTGQVID | 240 |
| Query | 241 | VPGDPSSTAFPLVAALLVPGSDVTILNVLMNPTRTGLILTLQEMGADIEVINPRLAGGED | 300 |
| Sbjct | 241 | VPGDPSSTAFPLVAALLVPGSDVTILNVLMNPTRTGLILTLQEMGA+IEV+N RLAGGED VPGDPSSTAFPLVAALLVPGSDVTILNVLMNPTRTGLILTLQEMGANIEVMNQRLAGGED | 300 |
| Query | 301 | VADLRVRSSTLKGVTVPEDRAPSMIDEYPILavaaafaegaTVMNGLEELRVKESDRLSA | 360 |
| Sbjct | 301 | VADLRVR S LKGVTVPEDRAPSMIDEYP+LAVAAAFAEGATVMNGL+ELRVKESDRLSA VADLRVRYSELKGVTVPEDRAPSMIDEYPVLAVAAAFAEGATVMNGLDELRVKESDRLSA | 360 |
| Query | 361 | VANGLKLNGVDCDEGETSLVVRGRPDGKGLGNASGAAVATHLDHRIAMSFLVMGLVSENP | 420 |
| Sbjct | 361 | VA+GLKLNGVDCDEGE SLVVRGRPDGKGLGNA+G V THLDHRIAMSFLV+GL SE+P VADGLKLNGVDCDEGEASLVVRGRPDGKGLGNAAGGQVKTHLDHRIAMSFLVLGLASEHP | 420 |
| Query  Sbjct | 421  421 | VTVDDATMIATSFPEFMDLMAGLGAKI 447 VTVDDATMIATSFPEFMDLM GLGAKI VTVDDATMIATSFPEFMDLMTGLGAKI 447 |  |

>WP_069459855.1 3-phosphoshikimate 1-carboxyvinyltransferase [Ensifer alkalisoli] ODR90156.1 3-phosphoshikimate 1-carboxyvinyltransferase [Ensifer alkalisoli]

Length=455

Score = 800 bits (2065), Expect = 0.0, Method: Compositional matrix adjust.

| Identities | = 408/454 (90%), Positives = 427/454 (94%), Gaps = 0/454 (0%) |  |
| --- | --- | --- |
| Query 1 | MLHGASSRPATARKSSGLSGTVRIPGDKSISHRSFMFGGLASGETRITGLLEGEDVINTG | 60 |
| Sbjct 1 | M HG SSRPATARKSS L GTVRIPGDKSISHRSFMFGGLASGETRITGLLEGEDVINTG MSHGQSSRPATARKSSDLKGTVRIPGDKSISHRSFMFGGLASGETRITGLLEGEDVINTG | 60 |
| Query 61 | KAMQAMGARIRKEGDTWIIDGVGNGGLLAPEAPLDFGNAATGCRLTMGLVGVYDFDSTFI | 120 |
| Sbjct 61 | KAMQAMGA+IRKEGD WIIDGVGNG LLAPEAPLDFGNA TGCRLTMGLVGVYDF STF+ KAMQAMGAKIRKEGDAWIIDGVGNGALLAPEAPLDFGNAGTGCRLTMGLVGVYDFASTFV | 120 |
| Query 121 | GDASLTKRPMGRVLNPLREMGVQVKSEDGDRLPVTLRGPKTPTPITYRVPMASAQVKSAV | 180 |
| Sbjct 121 | GDASLTKRPMGRVL+PLREMGVQVKS +GDRLPV L GPKTP PITYRVPMASAQVKSAV GDASLTKRPMGRVLDPLREMGVQVKSAEGDRLPVMLHGPKTPNPITYRVPMASAQVKSAV | 180 |
| Query 181 | LLAGLNTPGITTVIEPIMTRDHTEKMLQGFGANLTVETDADGVRTIRLEGRGKLTGQVID | 240 |
| Sbjct 181 | LLAGLNTPGITTVIEP+MTRDHTEKMLQGFGA+L+VETDA GVRTIRLEGRG+LTGQVID LLAGLNTPGITTVIEPVMTRDHTEKMLQGFGADLSVETDAAGVRTIRLEGRGRLTGQVID | 240 |
| Query 241 | VPGDPSSTAFPLVAALLVPGSDVTILNVLMNPTRTGLILTLQEMGADIEVINPRLAGGED | 300 |
| Sbjct 241 | VPGDPSSTAFPLVAAL+VPGSDVTILNVLMNPTRTGLILTLQEMGA+IEV+N RLAGGED VPGDPSSTAFPLVAALIVPGSDVTILNVLMNPTRTGLILTLQEMGANIEVMNKRLAGGED | 300 |
| Query 301 | VADLRVRSSTLKGVTVPEDRAPSMIDEYPILavaaafaegaTVMNGLEELRVKESDRLSA | 360 |
| Sbjct 301 | VADLRVR S LKGVTVPE+RAPSMIDEYP+LAVAAAFAEG TVMNGL+ELRVKESDRLSA VADLRVRHSELKGVTVPEERAPSMIDEYPVLAVAAAFAEGTTVMNGLDELRVKESDRLSA | 360 |
| Query 361 | VANGLKLNGVDCDEGETSLVVRGRPDGKGLGNASGAAVATHLDHRIAMSFLVMGLVSENP | 420 |
| Sbjct 361 | VA+GLKLNGVDCDEGE SLVVRGRP GKGLGNA G V THLDHRIAMSFLV+GL SE+P VADGLKLNGVDCDEGEASLVVRGRPGGKGLGNAPGDKVKTHLDHRIAMSFLVLGLASEHP | 420 |
| Query 421  Sbjct 421 | VTVDDATMIATSFPEFMDLMAGLGAKIELSDTKA 454 VTVDDATMIATSFPEFM LM GLGAKIE ++TKA VTVDDATMIATSFPEFMGLMTGLGAKIEQAETKA 454 |  |

>WP_145161583.1 3-phosphoshikimate 1-carboxyvinyltransferase [Sinorhizobium medicae] MQU75128.1 3-phosphoshikimate 1-carboxyvinyltransferase [Sinorhizobium medicae]

TWA40570.1 3-phosphoshikimate 1-carboxyvinyltransferase [Sinorhizobium medicae] Length=456

Score = 799 bits (2064), Expect = 0.0, Method: Compositional matrix adjust.

| Identities | = 411/455 (90%), Positives = 428/455 (94%), Gaps = 0/455 (0%) |  |
| --- | --- | --- |
| Query 1 | MLHGASSRPATARKSSGLSGTVRIPGDKSISHRSFMFGGLASGETRITGLLEGEDVINTG | 60 |
| Sbjct 1 | M HG++ RPATARKSS L GTVRIPGDKSISHRSFMFGGLASGETRITGLLEGEDVINTG MSHGSNPRPATARKSSDLKGTVRIPGDKSISHRSFMFGGLASGETRITGLLEGEDVINTG | 60 |
| Query 61 | KAMQAMGARIRKEGDTWIIDGVGNGGLLAPEAPLDFGNAATGCRLTMGLVGVYDFDSTFI | 120 |
| Sbjct 61 | +AMQAMGA+IRKEGDTWIIDGVGNG LLAPEAPLDFGNA TGCRLTMGLVGVYDFDSTFI RAMQAMGAKIRKEGDTWIIDGVGNGALLAPEAPLDFGNAGTGCRLTMGLVGVYDFDSTFI | 120 |
| Query 121 | GDASLTKRPMGRVLNPLREMGVQVKSEDGDRLPVTLRGPKTPTPITYRVPMASAQVKSAV | 180 |
| Sbjct 121 | GDASLTKRPMGRVL+PLREMGVQVKS +GDRLPVTLRGPKTP PITYRVPMASAQVKSAV GDASLTKRPMGRVLDPLREMGVQVKSAEGDRLPVTLRGPKTPNPITYRVPMASAQVKSAV | 180 |
| Query 181 | LLAGLNTPGITTVIEPIMTRDHTEKMLQGFGANLTVETDADGVRTIRLEGRGKLTGQVID | 240 |
| Sbjct 181 | LLAGLNTPGITTV+EP+MTRDHTEKMLQGFGA+L+VETD DGVRTIRLEGRGKL GQVID LLAGLNTPGITTVVEPVMTRDHTEKMLQGFGADLSVETDRDGVRTIRLEGRGKLRGQVID | 240 |
| Query 241 | VPGDPSSTAFPLVAALLVPGSDVTILNVLMNPTRTGLILTLQEMGADIEVINPRLAGGED | 300 |
| Sbjct 241 | VPGDPSSTAFPLVAALLVPGSD+TILNVLMNPTRTGLILTLQEMGA IEV++ RLAGGED VPGDPSSTAFPLVAALLVPGSDLTILNVLMNPTRTGLILTLQEMGARIEVLSSRLAGGED | 300 |
| Query 301 | VADLRVRSSTLKGVTVPEDRAPSMIDEYPILavaaafaegaTVMNGLEELRVKESDRLSA | 360 |
| Sbjct 301 | VADLRVR S LKGVTVPE+RAPSMIDEYP+LAVAAAFAEGATVMNGLEELRVKESDRLSA VADLRVRYSELKGVTVPEERAPSMIDEYPVLAVAAAFAEGATVMNGLEELRVKESDRLSA | 360 |
| Query 361 | VANGLKLNGVDCDEGETSLVVRGRPDGKGLGNASGAAVATHLDHRIAMSFLVMGLVSENP | 420 |
| Sbjct 361 | VA GLKLNGVDCDEGE SLVVRGRP GKGLGN SG V THLDHRIAMSFLVMGL SE P VAEGLKLNGVDCDEGEASLVVRGRPGGKGLGNDSGGQVKTHLDHRIAMSFLVMGLASERP | 420 |
| Query 421  Sbjct 421 | VTVDDATMIATSFPEFMDLMAGLGAKIELSDTKAA 455 VTVDDATMIATSFPEFM LM GLGAKIE ++ KAA VTVDDATMIATSFPEFMGLMTGLGAKIEETENKAA 455 |  |

>WP_026621852.1 3-phosphoshikimate 1-carboxyvinyltransferase [Ensifer sp. WSM1721] Length=455

Score = 799 bits (2064), Expect = 0.0, Method: Compositional matrix adjust.

| Identities | = 410/455 (90%), Positives = 428/455 (94%), Gaps = 0/455 (0%) |  |
| --- | --- | --- |
| Query 1 | MLHGASSRPATARKSSGLSGTVRIPGDKSISHRSFMFGGLASGETRITGLLEGEDVINTG | 60 |
| Sbjct 1 | M HG+S RPATA+KSS L GTVRIPGDKSISHRSFMFGGLASGETRITGLLEGEDVINTG MSHGSSPRPATAKKSSDLKGTVRIPGDKSISHRSFMFGGLASGETRITGLLEGEDVINTG | 60 |
| Query 61 | KAMQAMGARIRKEGDTWIIDGVGNGGLLAPEAPLDFGNAATGCRLTMGLVGVYDFDSTFI | 120 |
| Sbjct 61 | KAMQAMGARIRKE DTWII+GVGNG LLAPEAPLDFGNA TGCRLTMGLVGVYDFDSTFI KAMQAMGARIRKESDTWIINGVGNGALLAPEAPLDFGNAGTGCRLTMGLVGVYDFDSTFI | 120 |
| Query 121 | GDASLTKRPMGRVLNPLREMGVQVKSEDGDRLPVTLRGPKTPTPITYRVPMASAQVKSAV | 180 |
| Sbjct 121 | GDASL+KRPMGRVL+PLREMGVQVKS +GDRLPVTL GP+TP PITYRVPMASAQVKSAV GDASLSKRPMGRVLDPLREMGVQVKSAEGDRLPVTLHGPQTPNPITYRVPMASAQVKSAV | 180 |
| Query 181 | LLAGLNTPGITTVIEPIMTRDHTEKMLQGFGANLTVETDADGVRTIRLEGRGKLTGQVID | 240 |
| Sbjct 181 | LLAGLNTPGITTVIEP+MTRDHTEKMLQGFGANL VETD GVRTIRLEG GKL GQVID LLAGLNTPGITTVIEPVMTRDHTEKMLQGFGANLAVETDPAGVRTIRLEGHGKLIGQVID | 240 |
| Query 241 | VPGDPSSTAFPLVAALLVPGSDVTILNVLMNPTRTGLILTLQEMGADIEVINPRLAGGED | 300 |
| Sbjct 241 | VPGDPSSTAFPLVAAL+VPGS +TILNVLMNPTRTGLILTLQEMGADIEV+N RLAGGED VPGDPSSTAFPLVAALIVPGSAITILNVLMNPTRTGLILTLQEMGADIEVMNRRLAGGED | 300 |
| Query 301 | VADLRVRSSTLKGVTVPEDRAPSMIDEYPILavaaafaegaTVMNGLEELRVKESDRLSA | 360 |
| Sbjct 301 | VADLRVR S LKGVTVPE+RAPSMIDEYP+LAVAAAFAEGATVMNGLEELRVKESDRLSA VADLRVRYSELKGVTVPEERAPSMIDEYPVLAVAAAFAEGATVMNGLEELRVKESDRLSA | 360 |
| Query 361 | VANGLKLNGVDCDEGETSLVVRGRPDGKGLGNASGAAVATHLDHRIAMSFLVMGLVSENP | 420 |
| Sbjct 361 | VA+GLKLNGVDCDEGE SLVVRGRP GKGLGNASG V THLDHRIAMSFLVMGL SE+P  VADGLKLNGVDCDEGEASLVVRGRPGGKGLGNASGEKVKTHLDHRIAMSFLVMGLASEHP | 420 |

Query 421 VTVDDATMIATSFPEFMDLMAGLGAKIELSDTKAA 455 VTVDDATMIATSFPEFMDLM GLGAKIE +++KAA

Sbjct 421 VTVDDATMIATSFPEFMDLMIGLGAKIEQAESKAA 455

>WP_037432760.1 3-phosphoshikimate 1-carboxyvinyltransferase [Sinorhizobium fredii] ASY71230.1 5-Enolpyruvylshikimate-3-phosphate synthase [Sinorhizobium fredii

CCBAU 83666]

PDT48513.1 3-phosphoshikimate 1-carboxyvinyltransferase [Sinorhizobium fredii] Length=448

Score = 799 bits (2063), Expect = 0.0, Method: Compositional matrix adjust.

| Identities | = 412/448 (92%), Positives = 427/448 (95%), Gaps = 0/448 (0%) |  |
| --- | --- | --- |
| Query 1 | MLHGASSRPATARKSSGLSGTVRIPGDKSISHRSFMFGGLASGETRITGLLEGEDVINTG | 60 |
| Sbjct 1 | M HG S RPATA+KS+ L GTVRIPGDKSISHRSFMFGGLASGETRITGLLEGEDVINTG MSHGLSPRPATAKKSADLKGTVRIPGDKSISHRSFMFGGLASGETRITGLLEGEDVINTG | 60 |
| Query 61 | KAMQAMGARIRKEGDTWIIDGVGNGGLLAPEAPLDFGNAATGCRLTMGLVGVYDFDSTFI | 120 |
| Sbjct 61 | KAMQAMGA IRK+GDTWII+GVGNG LLAPEAPLDFGNA TGCRLTMGLVGVYDFDSTFI KAMQAMGAGIRKDGDTWIINGVGNGALLAPEAPLDFGNAGTGCRLTMGLVGVYDFDSTFI | 120 |
| Query 121 | GDASLTKRPMGRVLNPLREMGVQVKSEDGDRLPVTLRGPKTPTPITYRVPMASAQVKSAV | 180 |
| Sbjct 121 | GDASLTKRPMGRVL+PLR+MGVQVKS +GDRLPVTLRGP TP PITYRVPMASAQVKSAV GDASLTKRPMGRVLDPLRQMGVQVKSAEGDRLPVTLRGPTTPNPITYRVPMASAQVKSAV | 180 |
| Query 181 | LLAGLNTPGITTVIEPIMTRDHTEKMLQGFGANLTVETDADGVRTIRLEGRGKLTGQVID | 240 |
| Sbjct 181 | LLAGLNTPGITTVIEP+MTRDHTEKMLQGFGANLTVETDA+GVRTIRLEGRGKLTGQVID LLAGLNTPGITTVIEPVMTRDHTEKMLQGFGANLTVETDAEGVRTIRLEGRGKLTGQVID | 240 |
| Query 241 | VPGDPSSTAFPLVAALLVPGSDVTILNVLMNPTRTGLILTLQEMGADIEVINPRLAGGED | 300 |
| Sbjct 241 | VPGDPSSTAFPLVAALLVPGSDVTILNVLMNPTRTGLILTLQEMGA+IEV+N RLAGGED VPGDPSSTAFPLVAALLVPGSDVTILNVLMNPTRTGLILTLQEMGANIEVMNQRLAGGED | 300 |
| Query 301 | VADLRVRSSTLKGVTVPEDRAPSMIDEYPILavaaafaegaTVMNGLEELRVKESDRLSA | 360 |
| Sbjct 301 | VADLRVR S L GVTVPEDRAPSMIDEYP+LAVAAAFAEGATVMNGL+ELRVKESDRLSA VADLRVRYSELTGVTVPEDRAPSMIDEYPVLAVAAAFAEGATVMNGLDELRVKESDRLSA | 360 |
| Query 361 | VANGLKLNGVDCDEGETSLVVRGRPDGKGLGNASGAAVATHLDHRIAMSFLVMGLVSENP | 420 |
| Sbjct 361 | VA+GLKLNGVDCDEGE SLVVRGRPDGKGLGNASG V THLDHRIAMSFLVMGL SE+P VADGLKLNGVDCDEGEASLVVRGRPDGKGLGNASGDQVKTHLDHRIAMSFLVMGLASEHP | 420 |
| Query 421  Sbjct 421 | VTVDDATMIATSFPEFMDLMAGLGAKIE 448 VTVDDATMIATSFPEFM LMAGLGAKIE VTVDDATMIATSFPEFMGLMAGLGAKIE 448 |  |

>WP_018209105.1 3-phosphoshikimate 1-carboxyvinyltransferase [Sinorhizobium medicae] MQV47850.1 3-phosphoshikimate 1-carboxyvinyltransferase [Sinorhizobium medicae] MQV52906.1 3-phosphoshikimate 1-carboxyvinyltransferase [Sinorhizobium medicae] MQV55765.1 3-phosphoshikimate 1-carboxyvinyltransferase [Sinorhizobium medicae] MQV71805.1 3-phosphoshikimate 1-carboxyvinyltransferase [Sinorhizobium medicae] MQV86606.1 3-phosphoshikimate 1-carboxyvinyltransferase [Sinorhizobium medicae]

Length=456

Score = 798 bits (2060), Expect = 0.0, Method: Compositional matrix adjust.

| Identities | = 410/455 (90%), Positives = 427/455 (94%), Gaps = 0/455 (0%) |  |
| --- | --- | --- |
| Query 1 | MLHGASSRPATARKSSGLSGTVRIPGDKSISHRSFMFGGLASGETRITGLLEGEDVINTG | 60 |
| Sbjct 1 | M HG++ RPATARKSS L GTVRIPGDKSISHRSFMFGGLASGETRITGLLEGEDVINTG MSHGSNPRPATARKSSDLKGTVRIPGDKSISHRSFMFGGLASGETRITGLLEGEDVINTG | 60 |
| Query 61 | KAMQAMGARIRKEGDTWIIDGVGNGGLLAPEAPLDFGNAATGCRLTMGLVGVYDFDSTFI | 120 |
| Sbjct 61 | +AMQAMGA+IRKEGDTWIIDGVGNG LLAPEAPLDFGNA TGCRLTMGLVGVYDFDSTFI RAMQAMGAKIRKEGDTWIIDGVGNGALLAPEAPLDFGNAGTGCRLTMGLVGVYDFDSTFI | 120 |
| Query 121 | GDASLTKRPMGRVLNPLREMGVQVKSEDGDRLPVTLRGPKTPTPITYRVPMASAQVKSAV | 180 |
| Sbjct 121 | GDASLTKRPMGRVL+PLREMGVQVKS +GDRLPVTLRGPKTP PITYRVPMASAQVKSAV GDASLTKRPMGRVLDPLREMGVQVKSAEGDRLPVTLRGPKTPNPITYRVPMASAQVKSAV | 180 |
| Query 181 | LLAGLNTPGITTVIEPIMTRDHTEKMLQGFGANLTVETDADGVRTIRLEGRGKLTGQVID | 240 |

| Sbjct | 181 | LLAGLNTPGITTV+EP+MTRDHTEKMLQGFGA+L+VETD DGVRTIRLEGRGKL GQVID LLAGLNTPGITTVVEPVMTRDHTEKMLQGFGADLSVETDRDGVRTIRLEGRGKLRGQVID | 240 |
| --- | --- | --- | --- |
| Query | 241 | VPGDPSSTAFPLVAALLVPGSDVTILNVLMNPTRTGLILTLQEMGADIEVINPRLAGGED | 300 |
| Sbjct | 241 | VPGDPSSTAFPLVAALLVPGSD+TI NVLMNPTRTGLILTLQEMGA IEV++ RLAGGED VPGDPSSTAFPLVAALLVPGSDLTIFNVLMNPTRTGLILTLQEMGARIEVLSSRLAGGED | 300 |
| Query | 301 | VADLRVRSSTLKGVTVPEDRAPSMIDEYPILavaaafaegaTVMNGLEELRVKESDRLSA | 360 |
| Sbjct | 301 | VADLRVR S LKGVTVPE+RAPSMIDEYP+LAVAAAFAEGATVMNGLEELRVKESDRLSA VADLRVRYSELKGVTVPEERAPSMIDEYPVLAVAAAFAEGATVMNGLEELRVKESDRLSA | 360 |
| Query | 361 | VANGLKLNGVDCDEGETSLVVRGRPDGKGLGNASGAAVATHLDHRIAMSFLVMGLVSENP | 420 |
| Sbjct | 361 | VA GLKLNGVDCDEGE SLVVRGRP GKGLGN SG V THLDHRIAMSFLVMGL SE P VAEGLKLNGVDCDEGEASLVVRGRPGGKGLGNDSGGQVKTHLDHRIAMSFLVMGLASERP | 420 |
| Query  Sbjct | 421  421 | VTVDDATMIATSFPEFMDLMAGLGAKIELSDTKAA 455 VTVDDATMIATSFPEFM LM GLGAKIE ++ KAA VTVDDATMIATSFPEFMGLMTGLGAKIEETENKAA 455 |  |

>HCL64536.1 3-phosphoshikimate 1-carboxyvinyltransferase [Rhizobium sp.] Length=455

Score = 797 bits (2058), Expect = 0.0, Method: Compositional matrix adjust.

| Identities | = 403/455 (89%), Positives = 428/455 (94%), Gaps = 0/455 (0%) |  |
| --- | --- | --- |
| Query 1 | MLHGASSRPATARKSSGLSGTVRIPGDKSISHRSFMFGGLASGETRITGLLEGEDVINTG | 60 |
| Sbjct 1 | M HGA +PATARKSSGLSGTVRIPGDKSISHRSFMFGGLASGETRITGLLEGEDVINTG MSHGAQPKPATARKSSGLSGTVRIPGDKSISHRSFMFGGLASGETRITGLLEGEDVINTG | 60 |
| Query 61 | KAMQAMGARIRKEGDTWIIDGVGNGGLLAPEAPLDFGNAATGCRLTMGLVGVYDFDSTFI | 120 |
| Sbjct 61 | KAMQAMGA+IRKEGD WII+GVGNG LL P PLDFGNA TGCRLTMGLVGVYDFDSTF+ KAMQAMGAQIRKEGDVWIINGVGNGCLLEPRGPLDFGNAGTGCRLTMGLVGVYDFDSTFV | 120 |
| Query 121 | GDASLTKRPMGRVLNPLREMGVQVKSEDGDRLPVTLRGPKTPTPITYRVPMASAQVKSAV | 180 |
| Sbjct 121 | GDASL+ RPMGRVLNPLREMGVQVK+ +GDRLPVTL GPKTP PITYRVPMASAQVKSAV GDASLSSRPMGRVLNPLREMGVQVKAAEGDRLPVTLHGPKTPNPITYRVPMASAQVKSAV | 180 |
| Query 181 | LLAGLNTPGITTVIEPIMTRDHTEKMLQGFGANLTVETDADGVRTIRLEGRGKLTGQVID | 240 |
| Sbjct 181 | LLAGLNTPG+TTVIEP+MTRDHTEKMLQGFGANLTVETD DGVRTIRLEGRGKLTGQVID LLAGLNTPGVTTVIEPVMTRDHTEKMLQGFGANLTVETDGDGVRTIRLEGRGKLTGQVID | 240 |
| Query 241 | VPGDPSSTAFPLVAALLVPGSDVTILNVLMNPTRTGLILTLQEMGADIEVINPRLAGGED | 300 |
| Sbjct 241 | VPGDPSSTAFPLVAA+LVPGSD+TILNVLMNPTRTGLILTLQEMGADIEV+N RLAGGED VPGDPSSTAFPLVAAILVPGSDITILNVLMNPTRTGLILTLQEMGADIEVLNRRLAGGED | 300 |
| Query 301 | VADLRVRSSTLKGVTVPEDRAPSMIDEYPILavaaafaegaTVMNGLEELRVKESDRLSA | 360 |
| Sbjct 301 | VADLRVRSS LKG+TVPEDRAPSMIDEYP+LAVAAAFAEG T M GL+ELRVKESDRL+A VADLRVRSSALKGITVPEDRAPSMIDEYPVLAVAAAFAEGTTAMPGLKELRVKESDRLTA | 360 |
| Query 361 | VANGLKLNGVDCDEGETSLVVRGRPDGKGLGNASGAAVATHLDHRIAMSFLVMGLVSENP | 420 |
| Sbjct 361 | VA+GLKLNGVDCDEGE +LVVRGRPDGKGLGN S V THLDHRIAMSFLVMGL SE+P VADGLKLNGVDCDEGEATLVVRGRPDGKGLGNPSLVPVRTHLDHRIAMSFLVMGLASEHP | 420 |
| Query 421  Sbjct 421 | VTVDDATMIATSFPEFMDLMAGLGAKIELSDTKAA 455 VTVDDATMIATSFPEFMDLM G+GA+IEL++++AA VTVDDATMIATSFPEFMDLMTGIGAQIELTESRAA 455 |  |

>WP_132072694.1 3-phosphoshikimate 1-carboxyvinyltransferase [Sinorhizobium americanum] TCN36071.1 3-phosphoshikimate 1-carboxyvinyltransferase [Sinorhizobium americanum]

Length=448

Score = 797 bits (2058), Expect = 0.0, Method: Compositional matrix adjust. Identities = 407/448 (91%), Positives = 424/448 (95%), Gaps = 0/448 (0%)

| Query | 1 | MLHGASSRPATARKSSGLSGTVRIPGDKSISHRSFMFGGLASGETRITGLLEGEDVINTG | 60 |
| --- | --- | --- | --- |
| Sbjct | 1 | M HG S RPATA+KS+ L GTVRIPGDKSISHRSFMFGGLASGETRITGLLEGEDVINTG MSHGLSPRPATAKKSADLKGTVRIPGDKSISHRSFMFGGLASGETRITGLLEGEDVINTG | 60 |
| Query | 61 | KAMQAMGARIRKEGDTWIIDGVGNGGLLAPEAPLDFGNAATGCRLTMGLVGVYDFDSTFI KAMQAMGA IRKEGDTWII+GVGNG LLAPEAPLDFGNA TGCRLTMGLVGVYDFDSTFI | 120 |

| Sbjct | 61 | KAMQAMGANIRKEGDTWIINGVGNGALLAPEAPLDFGNAGTGCRLTMGLVGVYDFDSTFI | 120 |
| --- | --- | --- | --- |
| Query | 121 | GDASLTKRPMGRVLNPLREMGVQVKSEDGDRLPVTLRGPKTPTPITYRVPMASAQVKSAV | 180 |
| Sbjct | 121 | GDASLTKRPMGRVL+PLR+MGVQVKS + DRLPVTLRGP+TP PITYRVPMASAQVKSAV GDASLTKRPMGRVLDPLRQMGVQVKSAESDRLPVTLRGPQTPNPITYRVPMASAQVKSAV | 180 |
| Query | 181 | LLAGLNTPGITTVIEPIMTRDHTEKMLQGFGANLTVETDADGVRTIRLEGRGKLTGQVID | 240 |
| Sbjct | 181 | LLAGLNTPGITTVIEP+MTRDHTEKMLQGFGANLTVETD GVRTIRLEGRGKLTGQVID LLAGLNTPGITTVIEPVMTRDHTEKMLQGFGANLTVETDTQGVRTIRLEGRGKLTGQVID | 240 |
| Query | 241 | VPGDPSSTAFPLVAALLVPGSDVTILNVLMNPTRTGLILTLQEMGADIEVINPRLAGGED | 300 |
| Sbjct | 241 | VPGDPSSTAFPLVAALLVPGSDVTILNVLMNPTRTGLILTLQEMGA+IEV+N RLAGGED VPGDPSSTAFPLVAALLVPGSDVTILNVLMNPTRTGLILTLQEMGANIEVMNQRLAGGED | 300 |
| Query | 301 | VADLRVRSSTLKGVTVPEDRAPSMIDEYPILavaaafaegaTVMNGLEELRVKESDRLSA | 360 |
| Sbjct | 301 | VADLRVR S LKGVTVPE+R PSMIDEYP+LAVAAAFAEGATVMNGL+ELRVKESDRLSA VADLRVRYSELKGVTVPEERVPSMIDEYPVLAVAAAFAEGATVMNGLDELRVKESDRLSA | 360 |
| Query | 361 | VANGLKLNGVDCDEGETSLVVRGRPDGKGLGNASGAAVATHLDHRIAMSFLVMGLVSENP | 420 |
| Sbjct | 361 | VA+GLKLNGVDCDEGE SLVVRGRPDGKGLGNA+G V THLDHRIAMSFLVMGL SE+P VADGLKLNGVDCDEGEASLVVRGRPDGKGLGNATGEKVKTHLDHRIAMSFLVMGLASEHP | 420 |
| Query  Sbjct | 421  421 | VTVDDATMIATSFPEFMDLMAGLGAKIE 448 VT+DD TMIATSFPEFMDLM GLGAKIE VTIDDGTMIATSFPEFMDLMTGLGAKIE 448 |  |

>WP_018235235.1 3-phosphoshikimate 1-carboxyvinyltransferase [Ensifer sp. BR816] Length=448

Score = 796 bits (2057), Expect = 0.0, Method: Compositional matrix adjust.

| Identities | = 407/448 (91%), Positives = 427/448 (95%), Gaps = 0/448 (0%) |  |
| --- | --- | --- |
| Query 1 | MLHGASSRPATARKSSGLSGTVRIPGDKSISHRSFMFGGLASGETRITGLLEGEDVINTG | 60 |
| Sbjct 1 | M HG S RPATA+KS+ L GTVRIPGDKSISHRSFMFGGLASGETRITGLLEGEDVINTG MSHGLSPRPATAKKSADLKGTVRIPGDKSISHRSFMFGGLASGETRITGLLEGEDVINTG | 60 |
| Query 61 | KAMQAMGARIRKEGDTWIIDGVGNGGLLAPEAPLDFGNAATGCRLTMGLVGVYDFDSTFI | 120 |
| Sbjct 61 | KAMQAMGA+IRK+GDTWIIDGVGNG LLAPEAPLDFGNA TGCRLTMGLVGVYDFDSTF+ KAMQAMGAKIRKDGDTWIIDGVGNGALLAPEAPLDFGNAGTGCRLTMGLVGVYDFDSTFV | 120 |
| Query 121 | GDASLTKRPMGRVLNPLREMGVQVKSEDGDRLPVTLRGPKTPTPITYRVPMASAQVKSAV | 180 |
| Sbjct 121 | GDASLTKRPMGRVL+PLREMGVQVKS +GDRLPVTLRGP+TP+PITYRVPMASAQVKSAV GDASLTKRPMGRVLDPLREMGVQVKSAEGDRLPVTLRGPQTPSPITYRVPMASAQVKSAV | 180 |
| Query 181 | LLAGLNTPGITTVIEPIMTRDHTEKMLQGFGANLTVETDADGVRTIRLEGRGKLTGQVID | 240 |
| Sbjct 181 | LLAGLNTPGITTVIEP+MTRDHTEKMLQGFGANLTVET +DGVRTIRLEGRGKL GQVID LLAGLNTPGITTVIEPVMTRDHTEKMLQGFGANLTVETGSDGVRTIRLEGRGKLIGQVID | 240 |
| Query 241 | VPGDPSSTAFPLVAALLVPGSDVTILNVLMNPTRTGLILTLQEMGADIEVINPRLAGGED | 300 |
| Sbjct 241 | VPGDPSSTAFPLVAALLVPGSDVTILNVLMNPTRTGLILTLQEMGA+IEV+N RLAGGED VPGDPSSTAFPLVAALLVPGSDVTILNVLMNPTRTGLILTLQEMGANIEVMNRRLAGGED | 300 |
| Query 301 | VADLRVRSSTLKGVTVPEDRAPSMIDEYPILavaaafaegaTVMNGLEELRVKESDRLSA | 360 |
| Sbjct 301 | VADLRVR S L GV VPE+RAPSMIDEYP+LAVAAAFAEGATVMNGL+ELRVKESDRLSA VADLRVRHSELTGVEVPEERAPSMIDEYPVLAVAAAFAEGATVMNGLDELRVKESDRLSA | 360 |
| Query 361 | VANGLKLNGVDCDEGETSLVVRGRPDGKGLGNASGAAVATHLDHRIAMSFLVMGLVSENP | 420 |
| Sbjct 361 | VA+GLKLNGVDCDEGE SLVVRGRPDGKGLGNA+G V THLDHRIAMSFLVMGL +E+P VADGLKLNGVDCDEGEASLVVRGRPDGKGLGNAAGGQVKTHLDHRIAMSFLVMGLAAEHP | 420 |
| Query 421  Sbjct 421 | VTVDDATMIATSFPEFMDLMAGLGAKIE 448 VTVDDATMIATSFPEFMDLM GLGAKIE VTVDDATMIATSFPEFMDLMTGLGAKIE 448 |  |

>WP_064241696.1 3-phosphoshikimate 1-carboxyvinyltransferase [Ensifer glycinis] OAP41407.1 3-phosphoshikimate 1-carboxyvinyltransferase [Ensifer glycinis]

Length=448

Score = 796 bits (2057), Expect = 0.0, Method: Compositional matrix adjust. Identities = 408/448 (91%), Positives = 425/448 (95%), Gaps = 0/448 (0%)

| Query | 1 | MLHGASSRPATARKSSGLSGTVRIPGDKSISHRSFMFGGLASGETRITGLLEGEDVINTG | 60 |
| --- | --- | --- | --- |
| Sbjct | 1 | M HG S RPATA+KS+ L GTVRIPGDKSISHRSFMFGGLASGETRITGLLEGEDVINTG MSHGLSPRPATAKKSADLKGTVRIPGDKSISHRSFMFGGLASGETRITGLLEGEDVINTG | 60 |
| Query | 61 | KAMQAMGARIRKEGDTWIIDGVGNGGLLAPEAPLDFGNAATGCRLTMGLVGVYDFDSTFI | 120 |
| Sbjct | 61 | KAMQAMGARIRKEG TWII+GVGNG LLAP+APLDFGNA TGCRLTMGLVGVYDFDSTFI KAMQAMGARIRKEGGTWIINGVGNGALLAPQAPLDFGNAGTGCRLTMGLVGVYDFDSTFI | 120 |
| Query | 121 | GDASLTKRPMGRVLNPLREMGVQVKSEDGDRLPVTLRGPKTPTPITYRVPMASAQVKSAV | 180 |
| Sbjct | 121 | GDASLTKRPMGRVL+PLREMGVQVKS +GDRLPVTLRGP+TP PITYRVPMASAQVKSAV GDASLTKRPMGRVLDPLREMGVQVKSAEGDRLPVTLRGPQTPNPITYRVPMASAQVKSAV | 180 |
| Query | 181 | LLAGLNTPGITTVIEPIMTRDHTEKMLQGFGANLTVETDADGVRTIRLEGRGKLTGQVID | 240 |
| Sbjct | 181 | LLAGLNTPGITTVIEP+MTRDHTEKMLQGFGANL+VETD GVRTIRLEGRGKLTGQVID LLAGLNTPGITTVIEPVMTRDHTEKMLQGFGANLSVETDTQGVRTIRLEGRGKLTGQVID | 240 |
| Query | 241 | VPGDPSSTAFPLVAALLVPGSDVTILNVLMNPTRTGLILTLQEMGADIEVINPRLAGGED | 300 |
| Sbjct | 241 | VPGDPSSTAFPLVAAL+VPGSDVTILNVLMNPTRTGLILTLQEMGA+IEV N RLAGGED VPGDPSSTAFPLVAALIVPGSDVTILNVLMNPTRTGLILTLQEMGANIEVTNQRLAGGED | 300 |
| Query | 301 | VADLRVRSSTLKGVTVPEDRAPSMIDEYPILavaaafaegaTVMNGLEELRVKESDRLSA | 360 |
| Sbjct | 301 | VADLRVR S LKGVTVPE+RAPSMIDEYP+LAVAAAFAEGATVMNGL+ELRVKESDRLSA VADLRVRYSELKGVTVPEERAPSMIDEYPVLAVAAAFAEGATVMNGLDELRVKESDRLSA | 360 |
| Query | 361 | VANGLKLNGVDCDEGETSLVVRGRPDGKGLGNASGAAVATHLDHRIAMSFLVMGLVSENP | 420 |
| Sbjct | 361 | VA+GLKLNGVDCDEGE SLVVRGRP GKGLGNA+G V THLDHRIAMSFLVMGL SE+P VADGLKLNGVDCDEGEASLVVRGRPGGKGLGNAAGGQVKTHLDHRIAMSFLVMGLASEHP | 420 |
| Query  Sbjct | 421  421 | VTVDDATMIATSFPEFMDLMAGLGAKIE 448 VTVDDATMIATSFPEFMDLM GLGAKIE VTVDDATMIATSFPEFMDLMTGLGAKIE 448 |  |

>WP_012067654.1 MULTISPECIES: 3-phosphoshikimate 1-carboxyvinyltransferase [Sinorhizobium] ABR62274.1 3-phosphoshikimate 1-carboxyvinyltransferase [Sinorhizobium medicae

WSM419]

PLT89960.1 3-phosphoshikimate 1-carboxyvinyltransferase [Sinorhizobium medicae] PLT93404.1 3-phosphoshikimate 1-carboxyvinyltransferase [Sinorhizobium medicae] PLU16441.1 3-phosphoshikimate 1-carboxyvinyltransferase [Sinorhizobium medicae] PLU24610.1 3-phosphoshikimate 1-carboxyvinyltransferase [Sinorhizobium medicae]

Length=456

Score = 796 bits (2057), Expect = 0.0, Method: Compositional matrix adjust.

| Identities | = 409/455 (90%), Positives = 427/455 (94%), Gaps = 0/455 (0%) |  |
| --- | --- | --- |
| Query 1 | MLHGASSRPATARKSSGLSGTVRIPGDKSISHRSFMFGGLASGETRITGLLEGEDVINTG | 60 |
| Sbjct 1 | M HG++ RPATARKSS L GTVRIPGDKSISHRSFMFGGLASGETRITGLLEGEDVINTG MSHGSNPRPATARKSSDLKGTVRIPGDKSISHRSFMFGGLASGETRITGLLEGEDVINTG | 60 |
| Query 61 | KAMQAMGARIRKEGDTWIIDGVGNGGLLAPEAPLDFGNAATGCRLTMGLVGVYDFDSTFI | 120 |
| Sbjct 61 | +AMQAMGA+IRKEGDTWIIDGVGNG LLAPEAPLDFGNA TGCRLTMGLVGVYDFDSTFI RAMQAMGAKIRKEGDTWIIDGVGNGALLAPEAPLDFGNAGTGCRLTMGLVGVYDFDSTFI | 120 |
| Query 121 | GDASLTKRPMGRVLNPLREMGVQVKSEDGDRLPVTLRGPKTPTPITYRVPMASAQVKSAV | 180 |
| Sbjct 121 | GDASLTKRPMGRVL+PLREMGVQVKS +GDRLPVTLRGPKTP PITYRVPMASAQVKSAV GDASLTKRPMGRVLDPLREMGVQVKSAEGDRLPVTLRGPKTPNPITYRVPMASAQVKSAV | 180 |
| Query 181 | LLAGLNTPGITTVIEPIMTRDHTEKMLQGFGANLTVETDADGVRTIRLEGRGKLTGQVID | 240 |
| Sbjct 181 | LLAGLNTPGITTV+EP+MTRDHTEKMLQGFGA+L+VETD DGVRTIRLEGRGKL GQVID LLAGLNTPGITTVVEPVMTRDHTEKMLQGFGADLSVETDRDGVRTIRLEGRGKLRGQVID | 240 |
| Query 241 | VPGDPSSTAFPLVAALLVPGSDVTILNVLMNPTRTGLILTLQEMGADIEVINPRLAGGED | 300 |
| Sbjct 241 | VPGDPSSTAFPLVAALLVPGSD++I NVLMNPTRTGLILTLQEMGA IEV++ RLAGGED VPGDPSSTAFPLVAALLVPGSDLSIFNVLMNPTRTGLILTLQEMGARIEVLSSRLAGGED | 300 |
| Query 301 | VADLRVRSSTLKGVTVPEDRAPSMIDEYPILavaaafaegaTVMNGLEELRVKESDRLSA | 360 |
| Sbjct 301 | VADLRVR S LKGVTVPE+RAPSMIDEYP+LAVAAAFAEGATVMNGLEELRVKESDRLSA VADLRVRYSELKGVTVPEERAPSMIDEYPVLAVAAAFAEGATVMNGLEELRVKESDRLSA | 360 |
| Query 361 | VANGLKLNGVDCDEGETSLVVRGRPDGKGLGNASGAAVATHLDHRIAMSFLVMGLVSENP VA GLKLNGVDCDEGE SLVVRGRP GKGLGN SG V THLDHRIAMSFLVMGL SE P | 420 |

| Sbjct | 361 | VAEGLKLNGVDCDEGEASLVVRGRPGGKGLGNDSGGQVKTHLDHRIAMSFLVMGLASERP 420 |
| --- | --- | --- |
| Query | 421 | VTVDDATMIATSFPEFMDLMAGLGAKIELSDTKAA 455 |
| Sbjct | 421 | VTVDDATMIATSFPEFM LM GLGAKIE ++ KAA  VTVDDATMIATSFPEFMGLMTGLGAKIEETENKAA 455 |

>WP_037422330.1 3-phosphoshikimate 1-carboxyvinyltransferase [Sinorhizobium sp. CCBAU 05631]

ASY58945.1 5-Enolpyruvylshikimate-3-phosphate synthase [Sinorhizobium sp. CCBAU 05631]

Length=448

Score = 796 bits (2056), Expect = 0.0, Method: Compositional matrix adjust.

| Identities | = 405/448 (90%), Positives = 428/448 (96%), Gaps = 0/448 (0%) |  |
| --- | --- | --- |
| Query 1 | MLHGASSRPATARKSSGLSGTVRIPGDKSISHRSFMFGGLASGETRITGLLEGEDVINTG | 60 |
| Sbjct 1 | M HG S RPATA+KS+ L GTVRIPGDKSISHRSFMFGGLASGETRITGLLEGEDVINTG MSHGLSPRPATAKKSADLKGTVRIPGDKSISHRSFMFGGLASGETRITGLLEGEDVINTG | 60 |
| Query 61 | KAMQAMGARIRKEGDTWIIDGVGNGGLLAPEAPLDFGNAATGCRLTMGLVGVYDFDSTFI | 120 |
| Sbjct 61 | KAMQAMGA+IRK+GDTWII+GVGNG LLAPEAPLDFGNA TGCRLTMGLVGVYDFDSTF+ KAMQAMGAKIRKDGDTWIINGVGNGALLAPEAPLDFGNAGTGCRLTMGLVGVYDFDSTFV | 120 |
| Query 121 | GDASLTKRPMGRVLNPLREMGVQVKSEDGDRLPVTLRGPKTPTPITYRVPMASAQVKSAV | 180 |
| Sbjct 121 | GDASLTKRPMGRVL+PLR+MGVQVKS +GDRLPVTLRGP+TP PITYRVPMASAQVKSAV GDASLTKRPMGRVLDPLRQMGVQVKSAEGDRLPVTLRGPQTPNPITYRVPMASAQVKSAV | 180 |
| Query 181 | LLAGLNTPGITTVIEPIMTRDHTEKMLQGFGANLTVETDADGVRTIRLEGRGKLTGQVID | 240 |
| Sbjct 181 | LLAGLNTPGITTVIEP+MTRDHTEKMLQGFGANLTVET +DGVRTIRLEGRGKLTGQVID LLAGLNTPGITTVIEPVMTRDHTEKMLQGFGANLTVETGSDGVRTIRLEGRGKLTGQVID | 240 |
| Query 241 | VPGDPSSTAFPLVAALLVPGSDVTILNVLMNPTRTGLILTLQEMGADIEVINPRLAGGED | 300 |
| Sbjct 241 | VPGDPSSTAFPLVAALLVPGSDVTILNVLMNPTRTGLILTLQEMGA+IEV+N RLAGGED VPGDPSSTAFPLVAALLVPGSDVTILNVLMNPTRTGLILTLQEMGANIEVMNRRLAGGED | 300 |
| Query 301 | VADLRVRSSTLKGVTVPEDRAPSMIDEYPILavaaafaegaTVMNGLEELRVKESDRLSA | 360 |
| Sbjct 301 | VADLRVR S L GVTVPE+RAPSMIDEYP+LAVAAAFA+GATVMNGL+ELRVKESDRLSA VADLRVRHSELTGVTVPEERAPSMIDEYPVLAVAAAFADGATVMNGLDELRVKESDRLSA | 360 |
| Query 361 | VANGLKLNGVDCDEGETSLVVRGRPDGKGLGNASGAAVATHLDHRIAMSFLVMGLVSENP | 420 |
| Sbjct 361 | VA+GLKLNGVDCDEGE SLVVRGRPDGK LG+A+GA V THLDHRIAMSFLVMGL SE+P VADGLKLNGVDCDEGEASLVVRGRPDGKSLGSAAGAQVKTHLDHRIAMSFLVMGLASEHP | 420 |
| Query 421  Sbjct 421 | VTVDDATMIATSFPEFMDLMAGLGAKIE 448 VT+DDATMIATSFPEFMDLM GLGAKIE VTIDDATMIATSFPEFMDLMTGLGAKIE 448 |  |

>WP_127617043.1 3-phosphoshikimate 1-carboxyvinyltransferase [Sinorhizobium medicae] RVI97482.1 3-phosphoshikimate 1-carboxyvinyltransferase [Sinorhizobium medicae]

Length=456

Score = 795 bits (2052), Expect = 0.0, Method: Compositional matrix adjust.

| Identities | = 409/455 (90%), Positives = 426/455 (94%), Gaps = 0/455 (0%) |  |
| --- | --- | --- |
| Query 1 | MLHGASSRPATARKSSGLSGTVRIPGDKSISHRSFMFGGLASGETRITGLLEGEDVINTG | 60 |
| Sbjct 1 | M HG++ RPATARKSS L GTVRIPGDKSISHRSFMFGGLASGETRITGLLEGEDVINTG MSHGSNPRPATARKSSDLKGTVRIPGDKSISHRSFMFGGLASGETRITGLLEGEDVINTG | 60 |
| Query 61 | KAMQAMGARIRKEGDTWIIDGVGNGGLLAPEAPLDFGNAATGCRLTMGLVGVYDFDSTFI | 120 |
| Sbjct 61 | +AMQAMGA+IRKEGDTWIIDGVGNG LLAPEAPLDFGNA TGCRLTMGLVGVYDFDSTFI RAMQAMGAKIRKEGDTWIIDGVGNGALLAPEAPLDFGNAGTGCRLTMGLVGVYDFDSTFI | 120 |
| Query 121 | GDASLTKRPMGRVLNPLREMGVQVKSEDGDRLPVTLRGPKTPTPITYRVPMASAQVKSAV | 180 |
| Sbjct 121 | GDASLTKRPMGRVL+PLREMGVQVKS +GDRLPVTLRGPKTP PITYRVPMASAQVKSAV GDASLTKRPMGRVLDPLREMGVQVKSAEGDRLPVTLRGPKTPNPITYRVPMASAQVKSAV | 180 |
| Query 181 | LLAGLNTPGITTVIEPIMTRDHTEKMLQGFGANLTVETDADGVRTIRLEGRGKLTGQVID | 240 |
| Sbjct 181 | LLAGLNTPGITTV+EP+MTRDHTEKMLQGFGA+L+VETD DGVRTIRLEGRGKL GQVID  LLAGLNTPGITTVVEPVMTRDHTEKMLQGFGADLSVETDRDGVRTIRLEGRGKLRGQVID | 240 |

| Query | 241 | VPGDPSSTAFPLVAALLVPGSDVTILNVLMNPTRTGLILTLQEMGADIEVINPRLAGGED | 300 |
| --- | --- | --- | --- |
| Sbjct | 241 | VPGDPSSTAFPLVAALLVPGSD+TI NVLMNPTRTGLILTLQEMGA IEV++ RL GGED VPGDPSSTAFPLVAALLVPGSDLTIFNVLMNPTRTGLILTLQEMGARIEVLSSRLDGGED | 300 |
| Query | 301 | VADLRVRSSTLKGVTVPEDRAPSMIDEYPILavaaafaegaTVMNGLEELRVKESDRLSA | 360 |
| Sbjct | 301 | VADLRVR S LKGVTVPE+RAPSMIDEYP+LAVAAAFAEGATVMNGLEELRVKESDRLSA VADLRVRYSELKGVTVPEERAPSMIDEYPVLAVAAAFAEGATVMNGLEELRVKESDRLSA | 360 |
| Query | 361 | VANGLKLNGVDCDEGETSLVVRGRPDGKGLGNASGAAVATHLDHRIAMSFLVMGLVSENP | 420 |
| Sbjct | 361 | VA GLKLNGVDCDEGE SLVVRGRP GKGLGN SG V THLDHRIAMSFLVMGL SE P VAEGLKLNGVDCDEGEASLVVRGRPGGKGLGNDSGGQVKTHLDHRIAMSFLVMGLASERP | 420 |
| Query  Sbjct | 421  421 | VTVDDATMIATSFPEFMDLMAGLGAKIELSDTKAA 455 VTVDDATMIATSFPEFM LM GLGAKIE ++ KAA VTVDDATMIATSFPEFMGLMTGLGAKIEETENKAA 455 |  |

>WP_018013258.1 3-phosphoshikimate 1-carboxyvinyltransferase [Sinorhizobium medicae] Length=456

Score = 795 bits (2052), Expect = 0.0, Method: Compositional matrix adjust.

| Identities | = 409/455 (90%), Positives = 426/455 (94%), Gaps = 0/455 (0%) |  |
| --- | --- | --- |
| Query 1 | MLHGASSRPATARKSSGLSGTVRIPGDKSISHRSFMFGGLASGETRITGLLEGEDVINTG | 60 |
| Sbjct 1 | M HG++ RPATARKSS L GTVRIPGDKSISHRSFMFGGLASGETRITGLLEGEDVINTG MSHGSNPRPATARKSSDLKGTVRIPGDKSISHRSFMFGGLASGETRITGLLEGEDVINTG | 60 |
| Query 61 | KAMQAMGARIRKEGDTWIIDGVGNGGLLAPEAPLDFGNAATGCRLTMGLVGVYDFDSTFI | 120 |
| Sbjct 61 | +AMQAMGA+IRKEGDTWIIDGVGNG LLAPEAPLDFGNA TGCRLTMGLVGVYDFDSTFI RAMQAMGAKIRKEGDTWIIDGVGNGALLAPEAPLDFGNAGTGCRLTMGLVGVYDFDSTFI | 120 |
| Query 121 | GDASLTKRPMGRVLNPLREMGVQVKSEDGDRLPVTLRGPKTPTPITYRVPMASAQVKSAV | 180 |
| Sbjct 121 | GDASLTKRPMGRVL+PLREMGVQVKS +GDRLPVTLRGPKTP PITYRVPMASAQVKSAV GDASLTKRPMGRVLDPLREMGVQVKSAEGDRLPVTLRGPKTPNPITYRVPMASAQVKSAV | 180 |
| Query 181 | LLAGLNTPGITTVIEPIMTRDHTEKMLQGFGANLTVETDADGVRTIRLEGRGKLTGQVID | 240 |
| Sbjct 181 | LLAGLNTPGITTV+EP+MTRDHTEKMLQGFGA+L+VETD DGVRTIRLEGRGKL GQVID LLAGLNTPGITTVVEPVMTRDHTEKMLQGFGADLSVETDRDGVRTIRLEGRGKLRGQVID | 240 |
| Query 241 | VPGDPSSTAFPLVAALLVPGSDVTILNVLMNPTRTGLILTLQEMGADIEVINPRLAGGED | 300 |
| Sbjct 241 | VPGDPSSTAFPLVAALLVPGSD+TI NVLMNPTRTGLI TLQEMGA IEV++ RLAGGED VPGDPSSTAFPLVAALLVPGSDLTIFNVLMNPTRTGLIPTLQEMGARIEVLSSRLAGGED | 300 |
| Query 301 | VADLRVRSSTLKGVTVPEDRAPSMIDEYPILavaaafaegaTVMNGLEELRVKESDRLSA | 360 |
| Sbjct 301 | VADLRVR S LKGVTVPE+RAPSMIDEYP+LAVAAAFAEGATVMNGLEELRVKESDRLSA VADLRVRYSELKGVTVPEERAPSMIDEYPVLAVAAAFAEGATVMNGLEELRVKESDRLSA | 360 |
| Query 361 | VANGLKLNGVDCDEGETSLVVRGRPDGKGLGNASGAAVATHLDHRIAMSFLVMGLVSENP | 420 |
| Sbjct 361 | VA GLKLNGVDCDEGE SLVVRGRP GKGLGN SG V THLDHRIAMSFLVMGL SE P VAEGLKLNGVDCDEGEASLVVRGRPGGKGLGNDSGGQVKTHLDHRIAMSFLVMGLASERP | 420 |
| Query 421  Sbjct 421 | VTVDDATMIATSFPEFMDLMAGLGAKIELSDTKAA 455 VTVDDATMIATSFPEFM LM GLGAKIE ++ KAA VTVDDATMIATSFPEFMGLMTGLGAKIEETENKAA 455 |  |

>WP_097519311.1 3-phosphoshikimate 1-carboxyvinyltransferase [Sinorhizobium sp. BJ1]

PDT86234.1 3-phosphoshikimate 1-carboxyvinyltransferase [Sinorhizobium sp. BJ1]

Length=448

Score = 794 bits (2051), Expect = 0.0, Method: Compositional matrix adjust. Identities = 406/448 (91%), Positives = 426/448 (95%), Gaps = 0/448 (0%)

| Query | 1 | MLHGASSRPATARKSSGLSGTVRIPGDKSISHRSFMFGGLASGETRITGLLEGEDVINTG | 60 |
| --- | --- | --- | --- |
| Sbjct | 1 | M HG S RPATA+KS+ L GTVRIPGDKSISHRSFMFGGLA+GETRITGLLEGEDVINTG MSHGLSPRPATAKKSADLKGTVRIPGDKSISHRSFMFGGLAAGETRITGLLEGEDVINTG | 60 |
| Query | 61 | KAMQAMGARIRKEGDTWIIDGVGNGGLLAPEAPLDFGNAATGCRLTMGLVGVYDFDSTFI | 120 |
| Sbjct | 61 | KAMQAMGA+IRKEGDTWII+GVGNG LLAPEAPLDFGNA TGCRLTMGLVGVYDFDSTFI  KAMQAMGAKIRKEGDTWIINGVGNGALLAPEAPLDFGNAGTGCRLTMGLVGVYDFDSTFI | 120 |

| Query | 121 | GDASLTKRPMGRVLNPLREMGVQVKSEDGDRLPVTLRGPKTPTPITYRVPMASAQVKSAV | 180 |
| --- | --- | --- | --- |
| Sbjct | 121 | GDASLTKRPMGRVL+PLREMGVQVKS DGDRLPVTLRGPKTP PITYRVPMASAQVKSAV GDASLTKRPMGRVLDPLREMGVQVKSADGDRLPVTLRGPKTPNPITYRVPMASAQVKSAV | 180 |
| Query | 181 | LLAGLNTPGITTVIEPIMTRDHTEKMLQGFGANLTVETDADGVRTIRLEGRGKLTGQVID | 240 |
| Sbjct | 181 | LLAGLNTPGITTVIEP++TRDHTEKMLQGFGANLTVETD VRTIRLEGRGKLTGQVID LLAGLNTPGITTVIEPVLTRDHTEKMLQGFGANLTVETDPQDVRTIRLEGRGKLTGQVID | 240 |
| Query | 241 | VPGDPSSTAFPLVAALLVPGSDVTILNVLMNPTRTGLILTLQEMGADIEVINPRLAGGED | 300 |
| Sbjct | 241 | VPGDPSSTAFPLVAAL+VPGS++TILNVLMNPTRTGLILTLQEMGADI+V+N RLAGGED VPGDPSSTAFPLVAALIVPGSEITILNVLMNPTRTGLILTLQEMGADIDVMNERLAGGED | 300 |
| Query | 301 | VADLRVRSSTLKGVTVPEDRAPSMIDEYPILavaaafaegaTVMNGLEELRVKESDRLSA | 360 |
| Sbjct | 301 | VADLRVR S LKGVTVPE+RAPSMIDEYP+LAVAAAFAEGATVMNGL+ELRVKESDRLSA VADLRVRYSELKGVTVPEERAPSMIDEYPVLAVAAAFAEGATVMNGLDELRVKESDRLSA | 360 |
| Query | 361 | VANGLKLNGVDCDEGETSLVVRGRPDGKGLGNASGAAVATHLDHRIAMSFLVMGLVSENP | 420 |
| Sbjct | 361 | VA+GLKLNGVDCDEGE SLVVRGRP GKGLGNA+G V THLDHRIAMSFLVMGL SE+P VADGLKLNGVDCDEGEASLVVRGRPGGKGLGNAAGGQVKTHLDHRIAMSFLVMGLASEHP | 420 |
| Query  Sbjct | 421  421 | VTVDDATMIATSFPEFMDLMAGLGAKIE 448 VTVDDA+MIATSFPEFMDLM GLGAKIE VTVDDASMIATSFPEFMDLMTGLGAKIE 448 |  |

>WP_104838674.1 3-phosphoshikimate 1-carboxyvinyltransferase [Sinorhizobium fredii] AUX74864.1 3-phosphoshikimate 1-carboxyvinyltransferase [Sinorhizobium fredii]

Length=448

Score = 794 bits (2050), Expect = 0.0, Method: Compositional matrix adjust.

| Identities | = 404/448 (90%), Positives = 427/448 (95%), Gaps = 0/448 (0%) |  |
| --- | --- | --- |
| Query 1 | MLHGASSRPATARKSSGLSGTVRIPGDKSISHRSFMFGGLASGETRITGLLEGEDVINTG | 60 |
| Sbjct 1 | M HG S RPATA+KS+ L GTVRIPGDKSISHRSFMFGGLASGETRITGLLEGEDVINTG MSHGLSPRPATAKKSADLKGTVRIPGDKSISHRSFMFGGLASGETRITGLLEGEDVINTG | 60 |
| Query 61 | KAMQAMGARIRKEGDTWIIDGVGNGGLLAPEAPLDFGNAATGCRLTMGLVGVYDFDSTFI | 120 |
| Sbjct 61 | KAMQAMGA+IRK+GDTWII+GVGNG LLAPEAPLDFGNA TGCRLTMGLVGVYDFDSTF+ KAMQAMGAKIRKDGDTWIINGVGNGALLAPEAPLDFGNAGTGCRLTMGLVGVYDFDSTFV | 120 |
| Query 121 | GDASLTKRPMGRVLNPLREMGVQVKSEDGDRLPVTLRGPKTPTPITYRVPMASAQVKSAV | 180 |
| Sbjct 121 | GDASLTKRPMGRVL+PLR+MGVQVKS +GDRLPVTLRGP+TP PITYRVPMASAQVKSAV GDASLTKRPMGRVLDPLRQMGVQVKSAEGDRLPVTLRGPQTPNPITYRVPMASAQVKSAV | 180 |
| Query 181 | LLAGLNTPGITTVIEPIMTRDHTEKMLQGFGANLTVETDADGVRTIRLEGRGKLTGQVID | 240 |
| Sbjct 181 | LLAGLNTPGITTVIEP+MTRDHTEKMLQGFGANLTVET +DGVRTIRLEGRGKLTGQVID LLAGLNTPGITTVIEPVMTRDHTEKMLQGFGANLTVETGSDGVRTIRLEGRGKLTGQVID | 240 |
| Query 241 | VPGDPSSTAFPLVAALLVPGSDVTILNVLMNPTRTGLILTLQEMGADIEVINPRLAGGED | 300 |
| Sbjct 241 | VPGDPSSTAFPLVAALLVPGSDV ILNVLMNPTRTGLILTLQEMGA+IEV+N RLAGGED VPGDPSSTAFPLVAALLVPGSDVIILNVLMNPTRTGLILTLQEMGANIEVMNRRLAGGED | 300 |
| Query 301 | VADLRVRSSTLKGVTVPEDRAPSMIDEYPILavaaafaegaTVMNGLEELRVKESDRLSA | 360 |
| Sbjct 301 | VADLRVR S L GVTVPE+RAPSMIDEYP+LAVAAAFA+GATVMNGL+ELRVKESDRLSA VADLRVRHSELTGVTVPEERAPSMIDEYPVLAVAAAFADGATVMNGLDELRVKESDRLSA | 360 |
| Query 361 | VANGLKLNGVDCDEGETSLVVRGRPDGKGLGNASGAAVATHLDHRIAMSFLVMGLVSENP | 420 |
| Sbjct 361 | VA+GLKLNGVDCDEGE SLVVRGRPDGK LG+A+GA V THLDHRIAMSFLVMGL SE+P VADGLKLNGVDCDEGEASLVVRGRPDGKSLGSAAGAQVKTHLDHRIAMSFLVMGLASEHP | 420 |
| Query 421  Sbjct 421 | VTVDDATMIATSFPEFMDLMAGLGAKIE 448 VT+DDATMIATSFPEFMDLM GLGAKIE VTIDDATMIATSFPEFMDLMTGLGAKIE 448 |  |

>WP_097537683.1 MULTISPECIES: 3-phosphoshikimate 1-carboxyvinyltransferase [Sinorhizobium] PDT53723.1 3-phosphoshikimate 1-carboxyvinyltransferase [Sinorhizobium sp.

NG07B]

POH30785.1 3-phosphoshikimate 1-carboxyvinyltransferase [Sinorhizobium americanum] Length=448

Score = 793 bits (2048), Expect = 0.0, Method: Compositional matrix adjust.

| Identities | = 404/448 (90%), Positives = 426/448 (95%), Gaps = 0/448 (0%) |  |
| --- | --- | --- |
| Query 1 | MLHGASSRPATARKSSGLSGTVRIPGDKSISHRSFMFGGLASGETRITGLLEGEDVINTG | 60 |
| Sbjct 1 | M HG S RPATA+KS+ L GTVRIPGDKSISHRSFMFGGLASGETRITGLLEGEDVINTG MSHGLSPRPATAKKSADLKGTVRIPGDKSISHRSFMFGGLASGETRITGLLEGEDVINTG | 60 |
| Query 61 | KAMQAMGARIRKEGDTWIIDGVGNGGLLAPEAPLDFGNAATGCRLTMGLVGVYDFDSTFI | 120 |
| Sbjct 61 | KAMQAMGA+IRK+GDTWII+GVGNG LLAPEAPLDFGNA TGCRLTMGLVGVYDFDSTF+ KAMQAMGAKIRKDGDTWIINGVGNGALLAPEAPLDFGNAGTGCRLTMGLVGVYDFDSTFV | 120 |
| Query 121 | GDASLTKRPMGRVLNPLREMGVQVKSEDGDRLPVTLRGPKTPTPITYRVPMASAQVKSAV | 180 |
| Sbjct 121 | GDASLTKRPMGRVL+PLR+MGVQVKS +GDRLPVTLRGP+TP PITYRVPMASAQVKSAV GDASLTKRPMGRVLDPLRQMGVQVKSAEGDRLPVTLRGPQTPNPITYRVPMASAQVKSAV | 180 |
| Query 181 | LLAGLNTPGITTVIEPIMTRDHTEKMLQGFGANLTVETDADGVRTIRLEGRGKLTGQVID | 240 |
| Sbjct 181 | LLAGLNTPGITTVIEP+MTRDHTEKMLQGFGANLTVET +DGVRTIRLEGRGKLTGQVID LLAGLNTPGITTVIEPVMTRDHTEKMLQGFGANLTVETGSDGVRTIRLEGRGKLTGQVID | 240 |
| Query 241 | VPGDPSSTAFPLVAALLVPGSDVTILNVLMNPTRTGLILTLQEMGADIEVINPRLAGGED | 300 |
| Sbjct 241 | VPGDPSSTAFPLVAALLVPGSDV ILNVLMNPTRTGLILTLQEMGA+IEV+N RLAGGED VPGDPSSTAFPLVAALLVPGSDVIILNVLMNPTRTGLILTLQEMGANIEVMNRRLAGGED | 300 |
| Query 301 | VADLRVRSSTLKGVTVPEDRAPSMIDEYPILavaaafaegaTVMNGLEELRVKESDRLSA | 360 |
| Sbjct 301 | VADLRVR S L GVTVPE+RAPSMIDEYP+LAVAAAFA+GATVMNGL+ELRVKESDRLSA VADLRVRHSELTGVTVPEERAPSMIDEYPVLAVAAAFADGATVMNGLDELRVKESDRLSA | 360 |
| Query 361 | VANGLKLNGVDCDEGETSLVVRGRPDGKGLGNASGAAVATHLDHRIAMSFLVMGLVSENP | 420 |
| Sbjct 361 | VA GLKLNGVDCDEGE SLVVRGRPDGK LG+A+GA V THLDHRIAMSFLVMGL SE+P VAGGLKLNGVDCDEGEASLVVRGRPDGKSLGSAAGAQVKTHLDHRIAMSFLVMGLASEHP | 420 |
| Query 421  Sbjct 421 | VTVDDATMIATSFPEFMDLMAGLGAKIE 448 VT+DDATMIATSFPEFMDLM GLGAKIE VTIDDATMIATSFPEFMDLMTGLGAKIE 448 |  |

>WP_014766697.1 3-phosphoshikimate 1-carboxyvinyltransferase [Sinorhizobium fredii]

AFL54595.1 3-phosphoshikimate 1-carboxyvinyltransferase AroA [Sinorhizobium fredii USDA 257]

Length=448

Score = 793 bits (2047), Expect = 0.0, Method: Compositional matrix adjust.

| Identities | = 405/448 (90%), Positives = 426/448 (95%), Gaps = 0/448 (0%) |  |
| --- | --- | --- |
| Query 1 | MLHGASSRPATARKSSGLSGTVRIPGDKSISHRSFMFGGLASGETRITGLLEGEDVINTG | 60 |
| Sbjct 1 | M HG S RPATA+KS+ L GTVRIPGDKSISHRSFMFGGLA+GETRITGLLEGEDVINTG MSHGLSPRPATAKKSADLKGTVRIPGDKSISHRSFMFGGLAAGETRITGLLEGEDVINTG | 60 |
| Query 61 | KAMQAMGARIRKEGDTWIIDGVGNGGLLAPEAPLDFGNAATGCRLTMGLVGVYDFDSTFI | 120 |
| Sbjct 61 | KAMQAMGA+IRKEGDTWII+GVGNG LLAPEAPLDFGNA TGCRLTMGLVGVYDFDSTFI KAMQAMGAKIRKEGDTWIINGVGNGALLAPEAPLDFGNAGTGCRLTMGLVGVYDFDSTFI | 120 |
| Query 121 | GDASLTKRPMGRVLNPLREMGVQVKSEDGDRLPVTLRGPKTPTPITYRVPMASAQVKSAV | 180 |
| Sbjct 121 | GDASLTKRPMGRVL+PLREMGVQVKS DGDRLPVTLRGPKTP PITYRVPMASAQVKSAV GDASLTKRPMGRVLDPLREMGVQVKSADGDRLPVTLRGPKTPNPITYRVPMASAQVKSAV | 180 |
| Query 181 | LLAGLNTPGITTVIEPIMTRDHTEKMLQGFGANLTVETDADGVRTIRLEGRGKLTGQVID | 240 |
| Sbjct 181 | LLAGLNTPGITTVIEP++TRDHTEKMLQGFGANLTVETD VRTIRLEGRGKLTGQVID LLAGLNTPGITTVIEPVLTRDHTEKMLQGFGANLTVETDPQDVRTIRLEGRGKLTGQVID | 240 |
| Query 241 | VPGDPSSTAFPLVAALLVPGSDVTILNVLMNPTRTGLILTLQEMGADIEVINPRLAGGED | 300 |
| Sbjct 241 | VPGDPSSTAFPLVAAL+VPGS++TILNVLMNPTRTGLILTLQEMGADI+++N RLAGGED VPGDPSSTAFPLVAALIVPGSEITILNVLMNPTRTGLILTLQEMGADIDLMNERLAGGED | 300 |
| Query 301 | VADLRVRSSTLKGVTVPEDRAPSMIDEYPILavaaafaegaTVMNGLEELRVKESDRLSA | 360 |
| Sbjct 301 | VADLRVR S LKGVTVPE+RAPSMIDEYP+LAVAAAFAEGATVMNGL+ELRVKESDRLSA VADLRVRYSELKGVTVPEERAPSMIDEYPVLAVAAAFAEGATVMNGLDELRVKESDRLSA | 360 |
| Query 361 | VANGLKLNGVDCDEGETSLVVRGRPDGKGLGNASGAAVATHLDHRIAMSFLVMGLVSENP | 420 |
| Sbjct 361 | VA+GLKLNGVDCDEGE SLVVRGRP GKGLGNA+G V THLDHRIAMSFLVMGL SE+P  VADGLKLNGVDCDEGEASLVVRGRPGGKGLGNAAGGQVKTHLDHRIAMSFLVMGLASEHP | 420 |

Query 421 VTVDDATMIATSFPEFMDLMAGLGAKIE 448 VTVDDA+MIATSFPEFMDLM GLGAKIE

Sbjct 421 VTVDDASMIATSFPEFMDLMTGLGAKIE 448

>WP_192119985.1 unnamed protein product

MBD8555406.1 3-phosphoshikimate 1-carboxyvinyltransferase [Rhizobium sp. CFBP 8762]

Length=452

Score = 789 bits (2038), Expect = 0.0, Method: Compositional matrix adjust.

| Identities | = 403/448 (90%), Positives = 424/448 (95%), Gaps = 1/448 (0%) |  |
| --- | --- | --- |
| Query 1 | MLHGASSRPATARKSSGLSGTVRIPGDKSISHRSFMFGGLASGETRITGLLEGEDVINTG | 60 |
| Sbjct 1 | M HG RPATARKS L+GTVRIPGDKSISHRSFMFGGLASG+TRITGLLEGEDVINTG MSHGVK-RPATARKSENLNGTVRIPGDKSISHRSFMFGGLASGQTRITGLLEGEDVINTG | 59 |
| Query 61 | KAMQAMGARIRKEGDTWIIDGVGNGGLLAPEAPLDFGNAATGCRLTMGLVGVYDFDSTFI | 120 |
| Sbjct 60 | +AMQAMGAR+RK+GDTWIIDGVGNG LLAPEAPLDFGNA TGCRLTMGLVGVYDFDSTFI RAMQAMGARVRKDGDTWIIDGVGNGALLAPEAPLDFGNAGTGCRLTMGLVGVYDFDSTFI | 119 |
| Query 121 | GDASLTKRPMGRVLNPLREMGVQVKSEDGDRLPVTLRGPKTPTPITYRVPMASAQVKSAV | 180 |
| Sbjct 120 | GDASLT RPMGRVLNPLREMGVQVK+ +GDRLPVTLRGPKT PITYRVPMASAQVKSAV GDASLTSRPMGRVLNPLREMGVQVKAAEGDRLPVTLRGPKTANPITYRVPMASAQVKSAV | 179 |
| Query 181 | LLAGLNTPGITTVIEPIMTRDHTEKMLQGFGANLTVETDADGVRTIRLEGRGKLTGQVID | 240 |
| Sbjct 180 | LLAGLNTPGITTVIEP+MTRDHTEKMLQGFGANLTVETDADGVRTIRLEGRG LTGQ ID LLAGLNTPGITTVIEPVMTRDHTEKMLQGFGANLTVETDADGVRTIRLEGRGTLTGQTID | 239 |
| Query 241 | VPGDPSSTAFPLVAALLVPGSDVTILNVLMNPTRTGLILTLQEMGADIEVINPRLAGGED | 300 |
| Sbjct 240 | VPGDPSSTAFPLVAALLVPGSDVTILNVLMNPTRTGLILTLQEMGA I+V+N RLAGGED VPGDPSSTAFPLVAALLVPGSDVTILNVLMNPTRTGLILTLQEMGAHIDVLNTRLAGGED | 299 |
| Query 301 | VADLRVRSSTLKGVTVPEDRAPSMIDEYPILavaaafaegaTVMNGLEELRVKESDRLSA | 360 |
| Sbjct 300 | VADLRVRSS LKGVTVPEDRAPSMIDEYP+LAVAAAFA+G T+MNGLEELRVKESDRL+A VADLRVRSSELKGVTVPEDRAPSMIDEYPVLAVAAAFADGVTIMNGLEELRVKESDRLAA | 359 |
| Query 361 | VANGLKLNGVDCDEGETSLVVRGRPDGKGLGNASGAAVATHLDHRIAMSFLVMGLVSENP | 420 |
| Sbjct 360 | VA+GLKLNGVDCDEGE+SL+VRGRP GK LGN +GAAV T LDHRIAMSFLVMGL SE+P VASGLKLNGVDCDEGESSLLVRGRPGGKELGNPAGAAVTTFLDHRIAMSFLVMGLASEHP | 419 |
| Query 421  Sbjct 420 | VTVDDATMIATSFPEFMDLMAGLGAKIE 448 VTVDDATMIATSFPEFMDLMAGLGA+I+ VTVDDATMIATSFPEFMDLMAGLGAQID 447 |  |

>AMM72836.1 5-enolypyruvylshikimate-3-phosphate synthase, partial [Agrobacterium sp. CP4] [Glycine max]

Length=417

Score = 781 bits (2017), Expect = 0.0, Method: Compositional matrix adjust.

| Identities | = 412/420 (98%), Positives = 412/420 (98%), Gaps = 3/420 (1%) |  |
| --- | --- | --- |
| Query 36 | MFGGLASGETRITGLLEGEDVINTGKAMQAMGARIRKEGDTWIIDGVGNGGLLAPEAPLD | 95 |
| Sbjct 1 | MFGGLASGETRITGLLEGEDVINTGKAMQAMGARIRKEGDTWIIDGVGNGGLLAPEAPLD MFGGLASGETRITGLLEGEDVINTGKAMQAMGARIRKEGDTWIIDGVGNGGLLAPEAPLD | 60 |
| Query 96 | FGNAATGCRLTMGLVGVYDFDSTFIGDASLTKRPMGRVLNPLREMGVQVKSEDGDRLPVT | 155 |
| Sbjct 61 | FGNAA GCRLTMGLVGVYDFDSTFIGDASLTKRPMGRVLNPLREMGVQVKSEDGDRLPVT FGNAA-GCRLTMGLVGVYDFDSTFIGDASLTKRPMGRVLNPLREMGVQVKSEDGDRLPVT | 119 |
| Query 156 | LRGPKTPTPITYRVPMASAQVKSAVLLAGLNTPGITTVIEPIMTRDHTEKMLQGFGANLT | 215 |
| Sbjct 120 | LRGPKTPTPITYRVPMASAQVKSAVLLAGLNTPGITTVIEPIMTRD TEKMLQGFGAN T LRGPKTPTPITYRVPMASAQVKSAVLLAGLNTPGITTVIEPIMTRDRTEKMLQGFGANPT | 179 |
| Query 216 | VETDADGVRTIRLEGRGKLTGQVIDVPGDPSSTAFPLVAALLVPGSDVTILNVLMNPTRT | 275 |
| Sbjct 180 | VETDADGVRTIR EGRGKLTGQVIDVPGDPSSTAFPLVAALLVPGSDVTILNVLMNPTRT VETDADGVRTIRPEGRGKLTGQVIDVPGDPSSTAFPLVAALLVPGSDVTILNVLMNPTRT | 239 |
| Query 276 | GLILTLQEMGADIEVINPRLAGGEDVADLRVRSSTLKGVTVPEDRAPSMIDEYPILavaa | 335 |
| Sbjct 240 | GLILTLQEMGADIEVINPRLAGGEDVADLRVRSSTLKGVTVPEDRAPSMIDEYPILAVAA  GLILTLQEMGADIEVINPRLAGGEDVADLRVRSSTLKGVTVPEDRAPSMIDEYPILAVAA | 299 |

| Query | 336 | afaegaTVMNGLEELRVKESDRLSAVANGLKLNGVDCDEGETSLVVRGRPDGKGLGNASG | 395 |
| --- | --- | --- | --- |
| Sbjct | 300 | AFAEGATVMNGLEEL KESDRLSAVANGLKLNGVDCDEGETSLVVRGRPDGKGLGNASG AFAEGATVMNGLEEL--KESDRLSAVANGLKLNGVDCDEGETSLVVRGRPDGKGLGNASG | 357 |
| Query | 396 | AAVATHLDHRIAMSFLVMGLVSENPVTVDDATMIATSFPEFMDLMAGLGAKIELSDTKAA | 455 |
| Sbjct | 358 | AAVATHLD RIAMSFLVMGLVSENPVTVDDATMIATSFPEFMD MAGLGAKIELSDTKAA  AAVATHLDRRIAMSFLVMGLVSENPVTVDDATMIATSFPEFMDPMAGLGAKIELSDTKAA | 417 |

>WP_173511260.1 3-phosphoshikimate 1-carboxyvinyltransferase [Ensifer psoraleae] NRP72087.1 3-phosphoshikimate 1-carboxyvinyltransferase [Ensifer psoraleae]

Length=450

Score = 780 bits (2013), Expect = 0.0, Method: Compositional matrix adjust.

| Identities | = 411/450 (91%), Positives = 425/450 (94%), Gaps = 0/450 (0%) |  |
| --- | --- | --- |
| Query 1 | MLHGASSRPATARKSSGLSGTVRIPGDKSISHRSFMFGGLASGETRITGLLEGEDVINTG | 60 |
| Sbjct 1 | M HG S +PATARKSS L GTVRIPGDKSISHRSFMFGGLASGETRITGLLEGEDVINTG MSHGLSPKPATARKSSDLKGTVRIPGDKSISHRSFMFGGLASGETRITGLLEGEDVINTG | 60 |
| Query 61 | KAMQAMGARIRKEGDTWIIDGVGNGGLLAPEAPLDFGNAATGCRLTMGLVGVYDFDSTFI | 120 |
| Sbjct 61 | KAMQAMGA+IRKEGDTWIIDGVGNG LLAPEAPLDFGNA TGCRLTMGLVGVYDF STFI KAMQAMGAKIRKEGDTWIIDGVGNGALLAPEAPLDFGNAGTGCRLTMGLVGVYDFGSTFI | 120 |
| Query 121 | GDASLTKRPMGRVLNPLREMGVQVKSEDGDRLPVTLRGPKTPTPITYRVPMASAQVKSAV | 180 |
| Sbjct 121 | GDASLTKRPMGRVLNPLREMGVQVKS +GDRLPVTL GPKTP PITYRVPMASAQVKSAV GDASLTKRPMGRVLNPLREMGVQVKSAEGDRLPVTLHGPKTPNPITYRVPMASAQVKSAV | 180 |
| Query 181 | LLAGLNTPGITTVIEPIMTRDHTEKMLQGFGANLTVETDADGVRTIRLEGRGKLTGQVID | 240 |
| Sbjct 181 | LLAGLNTPGITTVIEP+MTRDHTEKML+GFGANLTVETD DGVRTIRLEGRGKLTGQVID LLAGLNTPGITTVIEPVMTRDHTEKMLKGFGANLTVETDVDGVRTIRLEGRGKLTGQVID | 240 |
| Query 241 | VPGDPSSTAFPLVAALLVPGSDVTILNVLMNPTRTGLILTLQEMGADIEVINPRLAGGED | 300 |
| Sbjct 241 | VPGDPSSTAFPLVAALLVPGSD+TILNVLMNPTRTGLILTLQEMGA+IEV+N RLAGGED VPGDPSSTAFPLVAALLVPGSDITILNVLMNPTRTGLILTLQEMGANIEVMNARLAGGED | 300 |
| Query 301 | VADLRVRSSTLKGVTVPEDRAPSMIDEYPILavaaafaegaTVMNGLEELRVKESDRLSA | 360 |
| Sbjct 301 | VADLRVR S LKGVTVPE+RAPSMIDEYP+LAVAAAFAEGATVMNGL+ELRVKESDRLSA VADLRVRHSELKGVTVPEERAPSMIDEYPVLAVAAAFAEGATVMNGLDELRVKESDRLSA | 360 |
| Query 361 | VANGLKLNGVDCDEGETSLVVRGRPDGKGLGNASGAAVATHLDHRIAMSFLVMGLVSENP | 420 |
| Sbjct 361 | VA+GLKLNGVDCDEGE SLVVRGRP G+GLGNASG V THLDHRIAMSFLVMGL SE+P VADGLKLNGVDCDEGEASLVVRGRPGGRGLGNASGGEVKTHLDHRIAMSFLVMGLASEHP | 420 |
| Query 421  Sbjct 421 | VTVDDATMIATSFPEFMDLMAGLGAKIELS 450 VTVDDATMIATSFPEFMDLM GLGAK L VTVDDATMIATSFPEFMDLMTGLGAKFSLQ 450 |  |

>WP_168265554.1 3-phosphoshikimate 1-carboxyvinyltransferase [Rhizobium leguminosarum]

NKL08550.1 3-phosphoshikimate 1-carboxyvinyltransferase [Rhizobium leguminosarum bv. viciae]

NKL84045.1 3-phosphoshikimate 1-carboxyvinyltransferase [Rhizobium leguminosarum bv. viciae]

NKL90173.1 3-phosphoshikimate 1-carboxyvinyltransferase [Rhizobium leguminosarum bv. viciae]

NKM93800.1 3-phosphoshikimate 1-carboxyvinyltransferase [Rhizobium leguminosarum bv. viciae]

Length=452

Score = 776 bits (2005), Expect = 0.0, Method: Compositional matrix adjust.

| Identities | = 393/449 (88%), Positives = 418/449 (93%), Gaps = 0/449 (0%) |  |
| --- | --- | --- |
| Query 1 | MLHGASSRPATARKSSGLSGTVRIPGDKSISHRSFMFGGLASGETRITGLLEGEDVINTG | 60 |
| Sbjct 1 | MLHG++ +PATARKS+GL+G+VRIPGDKSISHRSFMFGGLASGETRITGLLEGEDVINTG MLHGSAPKPATARKSAGLAGSVRIPGDKSISHRSFMFGGLASGETRITGLLEGEDVINTG | 60 |
| Query 61 | KAMQAMGARIRKEGDTWIIDGVGNGGLLAPEAPLDFGNAATGCRLTMGLVGVYDFDSTFI | 120 |
| Sbjct 61 | +AMQAMGARIRK+G W+IDG GNG LLAP+APLDFGNA TG RLTMGLVG YDF STFI  RAMQAMGARIRKDGAQWVIDGTGNGALLAPDAPLDFGNAGTGVRLTMGLVGTYDFRSTFI | 120 |

| Query | 121 | GDASLTKRPMGRVLNPLREMGVQVKSEDGDRLPVTLRGPKTPTPITYRVPMASAQVKSAV | 180 |
| --- | --- | --- | --- |
| Sbjct | 121 | GDASL+KRPMGRVLNPLREMGVQV + +GDRLPVTLRGP TP+PI YRVPMASAQVKSAV GDASLSKRPMGRVLNPLREMGVQVSASEGDRLPVTLRGPGTPSPIRYRVPMASAQVKSAV | 180 |
| Query | 181 | LLAGLNTPGITTVIEPIMTRDHTEKMLQGFGANLTVETDADGVRTIRLEGRGKLTGQVID | 240 |
| Sbjct | 181 | LLAGLNTPG+TTVIEP+MTRDHTEKMLQGFGA L+VETD DGVRTIRLEGRGKL GQVID LLAGLNTPGVTTVIEPVMTRDHTEKMLQGFGAALSVETDGDGVRTIRLEGRGKLKGQVID | 240 |
| Query | 241 | VPGDPSSTAFPLVAALLVPGSDVTILNVLMNPTRTGLILTLQEMGADIEVINPRLAGGED | 300 |
| Sbjct | 241 | VPGDPSSTAFPLVAALLVPGSD+TI+NVLMNPTRTGLILTLQEMGADIEV N RLAGGED VPGDPSSTAFPLVAALLVPGSDITIVNVLMNPTRTGLILTLQEMGADIEVANARLAGGED | 300 |
| Query | 301 | VADLRVRSSTLKGVTVPEDRAPSMIDEYPILavaaafaegaTVMNGLEELRVKESDRLSA | 360 |
| Sbjct | 301 | VADLRVR S LKGVTVPE+RAPSMIDEYPILAVAA FAEG TVM GLEELRVKESDRLSA VADLRVRHSELKGVTVPEERAPSMIDEYPILAVAACFAEGVTVMKGLEELRVKESDRLSA | 360 |
| Query | 361 | VANGLKLNGVDCDEGETSLVVRGRPDGKGLGNASGAAVATHLDHRIAMSFLVMGLVSENP | 420 |
| Sbjct | 361 | VA+GLKLNGVDCDEGE LVVRGRPDGKGLGNA+G V+THLDHRIAMSFLVMGL SE+P VADGLKLNGVDCDEGEDFLVVRGRPDGKGLGNAAGDKVSTHLDHRIAMSFLVMGLASEHP | 420 |
| Query  Sbjct | 421  421 | VTVDDATMIATSFPEFMDLMAGLGAKIEL 449 VT+DDA MIATSFPEFM LM GLGAKIEL VTIDDAAMIATSFPEFMQLMTGLGAKIEL 449 |  |

>WP_180194384.1 3-phosphoshikimate 1-carboxyvinyltransferase [Rhizobium sp. WYCCWR 11128]

NYT30297.1 3-phosphoshikimate 1-carboxyvinyltransferase [Rhizobium sp. WYCCWR 11128]

Length=452

Score = 776 bits (2005), Expect = 0.0, Method: Compositional matrix adjust.

| Identities | = 391/448 (87%), Positives = 420/448 (94%), Gaps = 0/448 (0%) |  |
| --- | --- | --- |
| Query 1 | MLHGASSRPATARKSSGLSGTVRIPGDKSISHRSFMFGGLASGETRITGLLEGEDVINTG | 60 |
| Sbjct 1 | MLHG++ RPATARKS+GL+G+VRIPGDKSISHRSFMFGGLASGETRITGLLEGEDVINTG MLHGSAPRPATARKSAGLTGSVRIPGDKSISHRSFMFGGLASGETRITGLLEGEDVINTG | 60 |
| Query 61 | KAMQAMGARIRKEGDTWIIDGVGNGGLLAPEAPLDFGNAATGCRLTMGLVGVYDFDSTFI | 120 |
| Sbjct 61 | +AMQAMGARIRKEG W+IDG GNG LLAP+APLDFGNA TG RLTMGLVG YDF STFI RAMQAMGARIRKEGAQWVIDGTGNGALLAPDAPLDFGNAGTGVRLTMGLVGTYDFRSTFI | 120 |
| Query 121 | GDASLTKRPMGRVLNPLREMGVQVKSEDGDRLPVTLRGPKTPTPITYRVPMASAQVKSAV | 180 |
| Sbjct 121 | GDASL+KRPMGRVLNPLREMGVQV + +GDRLPVTLRGP TP+PI YRVPMASAQVKSAV GDASLSKRPMGRVLNPLREMGVQVSTSEGDRLPVTLRGPGTPSPIRYRVPMASAQVKSAV | 180 |
| Query 181 | LLAGLNTPGITTVIEPIMTRDHTEKMLQGFGANLTVETDADGVRTIRLEGRGKLTGQVID | 240 |
| Sbjct 181 | LLAGLNTPG+TTVIEP+MTRDHTEKMLQGFGA L+VETD++GVRTIRLEGRG+LTGQVID LLAGLNTPGVTTVIEPVMTRDHTEKMLQGFGAALSVETDSEGVRTIRLEGRGRLTGQVID | 240 |
| Query 241 | VPGDPSSTAFPLVAALLVPGSDVTILNVLMNPTRTGLILTLQEMGADIEVINPRLAGGED | 300 |
| Sbjct 241 | +PGDPSSTAFPLVAALLVPGSD+TI+NVLMNPTRTGLILTLQEMGADIEV+N RLAGGED IPGDPSSTAFPLVAALLVPGSDITIVNVLMNPTRTGLILTLQEMGADIEVVNARLAGGED | 300 |
| Query 301 | VADLRVRSSTLKGVTVPEDRAPSMIDEYPILavaaafaegaTVMNGLEELRVKESDRLSA | 360 |
| Sbjct 301 | VADLRVR S L+GVTVPEDRAPSMIDEYPILAVAA FAEGATVM GLEELRVKESDRLSA VADLRVRHSELRGVTVPEDRAPSMIDEYPILAVAACFAEGATVMKGLEELRVKESDRLSA | 360 |
| Query 361 | VANGLKLNGVDCDEGETSLVVRGRPDGKGLGNASGAAVATHLDHRIAMSFLVMGLVSENP | 420 |
| Sbjct 361 | VA+GLKLNGVDCDEGE L+VRGRPDGKGLGNA+ V+THLDHRIAMSFLVMGL SE+P VADGLKLNGVDCDEGEDFLIVRGRPDGKGLGNAADGRVSTHLDHRIAMSFLVMGLASEHP | 420 |
| Query 421  Sbjct 421 | VTVDDATMIATSFPEFMDLMAGLGAKIE 448 VT+DDA MIATSFPEFM LM GLGAKIE VTIDDAAMIATSFPEFMQLMTGLGAKIE 448 |  |

>WP_105007830.1 3-phosphoshikimate 1-carboxyvinyltransferase [Rhizobium leguminosarum] AUW45168.1 3-phosphoshikimate 1-carboxyvinyltransferase [Rhizobium leguminosarum]

Length=452

Score = 776 bits (2003), Expect = 0.0, Method: Compositional matrix adjust.

| Identities | = 392/448 (88%), Positives = 417/448 (93%), Gaps = 0/448 (0%) |  |
| --- | --- | --- |
| Query 1 | MLHGASSRPATARKSSGLSGTVRIPGDKSISHRSFMFGGLASGETRITGLLEGEDVINTG | 60 |
| Sbjct 1 | MLHG++ RPATARKS+GLSG+VRIPGDKSISHRSFMFGGLASGETRITGLLEGEDVINTG MLHGSAPRPATARKSAGLSGSVRIPGDKSISHRSFMFGGLASGETRITGLLEGEDVINTG | 60 |
| Query 61 | KAMQAMGARIRKEGDTWIIDGVGNGGLLAPEAPLDFGNAATGCRLTMGLVGVYDFDSTFI | 120 |
| Sbjct 61 | +AMQAMGARIRKEG+ W+I+G GNG LLAP+APLDFGNA TG RLTMGLVG YDF ST RAMQAMGARIRKEGEQWVIEGTGNGALLAPDAPLDFGNAGTGVRLTMGLVGTYDFRSTLT | 120 |
| Query 121 | GDASLTKRPMGRVLNPLREMGVQVKSEDGDRLPVTLRGPKTPTPITYRVPMASAQVKSAV | 180 |
| Sbjct 121 | GDASL+KRPMGRVLNPLREMGVQV + +GDRLPVTLRGP TP+PI YRVPMASAQVKSAV GDASLSKRPMGRVLNPLREMGVQVSASEGDRLPVTLRGPGTPSPIRYRVPMASAQVKSAV | 180 |
| Query 181 | LLAGLNTPGITTVIEPIMTRDHTEKMLQGFGANLTVETDADGVRTIRLEGRGKLTGQVID | 240 |
| Sbjct 181 | LLAGLNTPG+TTVIEP+MTRDHTEKMLQGFGA L+VETD DGVRTIRLEGRGKLTGQVID LLAGLNTPGVTTVIEPVMTRDHTEKMLQGFGAALSVETDEDGVRTIRLEGRGKLTGQVID | 240 |
| Query 241 | VPGDPSSTAFPLVAALLVPGSDVTILNVLMNPTRTGLILTLQEMGADIEVINPRLAGGED | 300 |
| Sbjct 241 | VPGDPSSTAFPLVAALLVPGSD+TI+NVLMNPTRTGLILTLQEMGADIEV+N RLAGGED VPGDPSSTAFPLVAALLVPGSDITIVNVLMNPTRTGLILTLQEMGADIEVLNTRLAGGED | 300 |
| Query 301 | VADLRVRSSTLKGVTVPEDRAPSMIDEYPILavaaafaegaTVMNGLEELRVKESDRLSA | 360 |
| Sbjct 301 | VADLRVR S LKGVTVPEDRAPSMIDEYPILAVAA FAEG T+M GLEELRVKESDRLSA VADLRVRHSELKGVTVPEDRAPSMIDEYPILAVAACFAEGTTIMKGLEELRVKESDRLSA | 360 |
| Query 361 | VANGLKLNGVDCDEGETSLVVRGRPDGKGLGNASGAAVATHLDHRIAMSFLVMGLVSENP | 420 |
| Sbjct 361 | VA+GLKLNGVDCDEGE LVVRGRPDGKGLGNA+ V+THLDHRIAMSFLVMGL SE+P VADGLKLNGVDCDEGEDFLVVRGRPDGKGLGNAADGRVSTHLDHRIAMSFLVMGLASEHP | 420 |
| Query 421  Sbjct 421 | VTVDDATMIATSFPEFMDLMAGLGAKIE 448 VT+DDA MIATSFPEFM LM GLGAKIE VTIDDAAMIATSFPEFMQLMTGLGAKIE 448 |  |

>WP_130653374.1 3-phosphoshikimate 1-carboxyvinyltransferase [Rhizobium leguminosarum] TAU23082.1 3-phosphoshikimate 1-carboxyvinyltransferase [Rhizobium leguminosarum] TAU43077.1 3-phosphoshikimate 1-carboxyvinyltransferase [Rhizobium leguminosarum] TAU98699.1 3-phosphoshikimate 1-carboxyvinyltransferase [Rhizobium leguminosarum] TAV13489.1 3-phosphoshikimate 1-carboxyvinyltransferase [Rhizobium leguminosarum] TAW54329.1 3-phosphoshikimate 1-carboxyvinyltransferase [Rhizobium leguminosarum]

Length=452

Score = 774 bits (1999), Expect = 0.0, Method: Compositional matrix adjust.

| Identities | = 390/448 (87%), Positives = 418/448 (93%), Gaps = 0/448 (0%) |  |
| --- | --- | --- |
| Query 1 | MLHGASSRPATARKSSGLSGTVRIPGDKSISHRSFMFGGLASGETRITGLLEGEDVINTG | 60 |
| Sbjct 1 | MLHG++ RPATARKS+GL+G+VRIPGDKSISHRSFMFGGLASGETRITGLLEGEDVINTG MLHGSAPRPATARKSAGLTGSVRIPGDKSISHRSFMFGGLASGETRITGLLEGEDVINTG | 60 |
| Query 61 | KAMQAMGARIRKEGDTWIIDGVGNGGLLAPEAPLDFGNAATGCRLTMGLVGVYDFDSTFI | 120 |
| Sbjct 61 | +AMQAMGARIRK G+ W+I+G GNG LLAP+APLDFGNA TG RLTMGLVG YDF STFI RAMQAMGARIRKVGEQWLIEGTGNGALLAPDAPLDFGNAGTGVRLTMGLVGTYDFRSTFI | 120 |
| Query 121 | GDASLTKRPMGRVLNPLREMGVQVKSEDGDRLPVTLRGPKTPTPITYRVPMASAQVKSAV | 180 |
| Sbjct 121 | GDASL+KRPMGRVLNPLREMGVQV + +GDRLPVTLRGP TP+PI YRVPMASAQVKSAV GDASLSKRPMGRVLNPLREMGVQVSASEGDRLPVTLRGPGTPSPIRYRVPMASAQVKSAV | 180 |
| Query 181 | LLAGLNTPGITTVIEPIMTRDHTEKMLQGFGANLTVETDADGVRTIRLEGRGKLTGQVID | 240 |
| Sbjct 181 | LLAGLNTPG+TTVIEP+MTRDHTEKMLQGFGA L+VETD +GVRTIRLEGRGKL GQVID LLAGLNTPGVTTVIEPVMTRDHTEKMLQGFGAALSVETDEEGVRTIRLEGRGKLAGQVID | 240 |
| Query 241 | VPGDPSSTAFPLVAALLVPGSDVTILNVLMNPTRTGLILTLQEMGADIEVINPRLAGGED | 300 |
| Sbjct 241 | +PGDPSSTAFPLVAALLVPGSD+TI+NVLMNPTRTGLILTLQEMGADIEV+N RLAGGED IPGDPSSTAFPLVAALLVPGSDITIVNVLMNPTRTGLILTLQEMGADIEVLNARLAGGED | 300 |
| Query 301 | VADLRVRSSTLKGVTVPEDRAPSMIDEYPILavaaafaegaTVMNGLEELRVKESDRLSA | 360 |
| Sbjct 301 | VADLRVR S LKGVTVPEDRAPSMIDEYPILAVAA FAEG TVM GLEELRVKESDRLSA VADLRVRHSELKGVTVPEDRAPSMIDEYPILAVAACFAEGVTVMKGLEELRVKESDRLSA | 360 |
| Query 361 | VANGLKLNGVDCDEGETSLVVRGRPDGKGLGNASGAAVATHLDHRIAMSFLVMGLVSENP VA+GLKLNGVDCDEGE L+VRGRPDGKGLGNA+G V+THLDHRIAMSFLVMGL SE+P | 420 |

| Sbjct | 361 | VADGLKLNGVDCDEGEDFLIVRGRPDGKGLGNAAGDKVSTHLDHRIAMSFLVMGLASEHP 420 |
| --- | --- | --- |
| Query | 421 | VTVDDATMIATSFPEFMDLMAGLGAKIE 448 |
| Sbjct | 421 | VT+DDA MIATSFPEFM LM GLGAKIE  VTIDDAAMIATSFPEFMQLMTGLGAKIE 448 |

>WP_065276731.1 3-phosphoshikimate 1-carboxyvinyltransferase [Rhizobium leguminosarum]

AOO90912.1 3-phosphoshikimate 1-carboxyvinyltransferase [Rhizobium leguminosarum bv. trifolii]

OBY06297.1 3-phosphoshikimate 1-carboxyvinyltransferase [Rhizobium leguminosarum bv. trifolii]

RWX41402.1 3-phosphoshikimate 1-carboxyvinyltransferase [Rhizobium leguminosarum] TBE57039.1 3-phosphoshikimate 1-carboxyvinyltransferase [Rhizobium leguminosarum]

TBZ96330.1 3-phosphoshikimate 1-carboxyvinyltransferase [Rhizobium leguminosarum bv. viciae]

Length=452

Score = 774 bits (1999), Expect = 0.0, Method: Compositional matrix adjust.

| Identities | = 390/449 (87%), Positives = 419/449 (93%), Gaps = 0/449 (0%) |  |
| --- | --- | --- |
| Query 1 | MLHGASSRPATARKSSGLSGTVRIPGDKSISHRSFMFGGLASGETRITGLLEGEDVINTG | 60 |
| Sbjct 1 | MLHG++ RPATARKS+GL+G+VRIPGDKSISHRSFMFGGLASGETRITGLLEGEDVINTG MLHGSAPRPATARKSAGLTGSVRIPGDKSISHRSFMFGGLASGETRITGLLEGEDVINTG | 60 |
| Query 61 | KAMQAMGARIRKEGDTWIIDGVGNGGLLAPEAPLDFGNAATGCRLTMGLVGVYDFDSTFI | 120 |
| Sbjct 61 | +AMQAMGARIRK G+ W+I+G GNG LLAP+APLDFGNA TG RLTMGLVG YDF STFI RAMQAMGARIRKVGEQWVIEGTGNGALLAPDAPLDFGNAGTGVRLTMGLVGTYDFRSTFI | 120 |
| Query 121 | GDASLTKRPMGRVLNPLREMGVQVKSEDGDRLPVTLRGPKTPTPITYRVPMASAQVKSAV | 180 |
| Sbjct 121 | GDASL+KRPMGRVLNPLREMGVQV + +GDRLPVTLRGP TP+PI YRVPMASAQVKSAV GDASLSKRPMGRVLNPLREMGVQVSASEGDRLPVTLRGPGTPSPIRYRVPMASAQVKSAV | 180 |
| Query 181 | LLAGLNTPGITTVIEPIMTRDHTEKMLQGFGANLTVETDADGVRTIRLEGRGKLTGQVID | 240 |
| Sbjct 181 | LLAGLNTPG+TTVIEP+MTRDHTEKMLQGFGA L+VETD++GVRTIRLEGRGKL GQVID LLAGLNTPGVTTVIEPVMTRDHTEKMLQGFGATLSVETDSEGVRTIRLEGRGKLAGQVID | 240 |
| Query 241 | VPGDPSSTAFPLVAALLVPGSDVTILNVLMNPTRTGLILTLQEMGADIEVINPRLAGGED | 300 |
| Sbjct 241 | VPGDPSSTAFPLVAALLVPGSD+TI+NVLMNPTRTGLILTLQEMGADIEV+N RLAGGED VPGDPSSTAFPLVAALLVPGSDITIVNVLMNPTRTGLILTLQEMGADIEVLNARLAGGED | 300 |
| Query 301 | VADLRVRSSTLKGVTVPEDRAPSMIDEYPILavaaafaegaTVMNGLEELRVKESDRLSA | 360 |
| Sbjct 301 | VADLRVR S LKGVTVPEDRAPSMIDEYPILAVAA FAEGAT+M GLEELRVKESDRLSA VADLRVRHSELKGVTVPEDRAPSMIDEYPILAVAACFAEGATIMKGLEELRVKESDRLSA | 360 |
| Query 361 | VANGLKLNGVDCDEGETSLVVRGRPDGKGLGNASGAAVATHLDHRIAMSFLVMGLVSENP | 420 |
| Sbjct 361 | VA+GLKLNGVDCDEGE L+VRGRPDG GLGNA+ V+THLDHRIAMSFLVMGL SE+P VADGLKLNGVDCDEGEDFLIVRGRPDGTGLGNAADGKVSTHLDHRIAMSFLVMGLASEHP | 420 |
| Query 421  Sbjct 421 | VTVDDATMIATSFPEFMDLMAGLGAKIEL 449 VT+DDA MIATSFPEFM LM GLGAKIEL VTIDDAAMIATSFPEFMQLMTGLGAKIEL 449 |  |

>WP_163878748.1 3-phosphoshikimate 1-carboxyvinyltransferase [Rhizobium laguerreae] NEH92263.1 3-phosphoshikimate 1-carboxyvinyltransferase [Rhizobium laguerreae]

Length=452

Score = 774 bits (1998), Expect = 0.0, Method: Compositional matrix adjust.

| Identities | = 392/448 (88%), Positives = 416/448 (93%), Gaps = 0/448 (0%) |  |
| --- | --- | --- |
| Query 1 | MLHGASSRPATARKSSGLSGTVRIPGDKSISHRSFMFGGLASGETRITGLLEGEDVINTG | 60 |
| Sbjct 1 | MLH ++ +PATARKS+GL+G+VRIPGDKSISHRSFMFGGLASGETRITGLLEGEDVINTG MLHDSAPKPATARKSAGLAGSVRIPGDKSISHRSFMFGGLASGETRITGLLEGEDVINTG | 60 |
| Query 61 | KAMQAMGARIRKEGDTWIIDGVGNGGLLAPEAPLDFGNAATGCRLTMGLVGVYDFDSTFI | 120 |
| Sbjct 61 | +AMQAMGARIRK+G W+IDG GNG LLAP+APLDFGNA TG RLTMGLVG YDF STFI RAMQAMGARIRKDGAQWVIDGTGNGALLAPDAPLDFGNAGTGVRLTMGLVGTYDFRSTFI | 120 |
| Query 121 | GDASLTKRPMGRVLNPLREMGVQVKSEDGDRLPVTLRGPKTPTPITYRVPMASAQVKSAV | 180 |
| Sbjct 121 | GDASL+KRPMGRVLNPLREMGVQV + +GDRLPVTLRGP TP+PI YRVPMASAQVKSAV  GDASLSKRPMGRVLNPLREMGVQVSASEGDRLPVTLRGPGTPSPIRYRVPMASAQVKSAV | 180 |

| Query | 181 | LLAGLNTPGITTVIEPIMTRDHTEKMLQGFGANLTVETDADGVRTIRLEGRGKLTGQVID | 240 |
| --- | --- | --- | --- |
| Sbjct | 181 | LLAGLNTPG+TTVIEP+MTRDHTEKMLQGFGA L+VETD DGVRTIRLEGRGKL GQVID LLAGLNTPGVTTVIEPVMTRDHTEKMLQGFGAALSVETDGDGVRTIRLEGRGKLKGQVID | 240 |
| Query | 241 | VPGDPSSTAFPLVAALLVPGSDVTILNVLMNPTRTGLILTLQEMGADIEVINPRLAGGED | 300 |
| Sbjct | 241 | VPGDPSSTAFPLVAALLVPGSD+TI+NVLMNPTRTGLILTLQEMGADIEV N RLAGGED VPGDPSSTAFPLVAALLVPGSDITIVNVLMNPTRTGLILTLQEMGADIEVANARLAGGED | 300 |
| Query | 301 | VADLRVRSSTLKGVTVPEDRAPSMIDEYPILavaaafaegaTVMNGLEELRVKESDRLSA | 360 |
| Sbjct | 301 | VADLRVR S LKGVTVPEDRAPSMIDEYPILAVAA FAEG TVM GLEELRVKESDRLSA VADLRVRHSELKGVTVPEDRAPSMIDEYPILAVAACFAEGVTVMKGLEELRVKESDRLSA | 360 |
| Query | 361 | VANGLKLNGVDCDEGETSLVVRGRPDGKGLGNASGAAVATHLDHRIAMSFLVMGLVSENP | 420 |
| Sbjct | 361 | VA+GLKLNGVDCDEGE LVVRGRPDGKGLGNA+G V+THLDHRIAMSFLVMGL SE+P VADGLKLNGVDCDEGEDFLVVRGRPDGKGLGNAAGDKVSTHLDHRIAMSFLVMGLASEHP | 420 |
| Query  Sbjct | 421  421 | VTVDDATMIATSFPEFMDLMAGLGAKIE 448 VT+DDA MIATSFPEFM LM GLGAKIE VTIDDAAMIATSFPEFMQLMTGLGAKIE 448 |  |

>WP_017989914.1 3-phosphoshikimate 1-carboxyvinyltransferase [Rhizobium leguminosarum] Length=452

Score = 774 bits (1998), Expect = 0.0, Method: Compositional matrix adjust.

| Identities | = 392/448 (88%), Positives = 418/448 (93%), Gaps = 0/448 (0%) |  |
| --- | --- | --- |
| Query 1 | MLHGASSRPATARKSSGLSGTVRIPGDKSISHRSFMFGGLASGETRITGLLEGEDVINTG | 60 |
| Sbjct 1 | MLHG++ RPATARKS+GL+G+VRIPGDKSISHRSFMFGGLASGETRITGLLEGEDVINTG MLHGSAPRPATARKSAGLAGSVRIPGDKSISHRSFMFGGLASGETRITGLLEGEDVINTG | 60 |
| Query 61 | KAMQAMGARIRKEGDTWIIDGVGNGGLLAPEAPLDFGNAATGCRLTMGLVGVYDFDSTFI | 120 |
| Sbjct 61 | +AMQAMGARIRKEG W+I+G GNG LLAP+APLDFGNA TG RLTMGLVG YDF STFI RAMQAMGARIRKEGTQWVIEGTGNGALLAPDAPLDFGNAGTGVRLTMGLVGTYDFRSTFI | 120 |
| Query 121 | GDASLTKRPMGRVLNPLREMGVQVKSEDGDRLPVTLRGPKTPTPITYRVPMASAQVKSAV | 180 |
| Sbjct 121 | GDASL+KRPMGRVL PLREMGVQV + +GDRLPVTLRGP TP+PI YRVPMASAQVKSAV GDASLSKRPMGRVLAPLREMGVQVSASEGDRLPVTLRGPGTPSPIRYRVPMASAQVKSAV | 180 |
| Query 181 | LLAGLNTPGITTVIEPIMTRDHTEKMLQGFGANLTVETDADGVRTIRLEGRGKLTGQVID | 240 |
| Sbjct 181 | LLAGLNTPG+TTVIEP+MTRDHTEKMLQGFGA L+VETD DGVRTIRLEGRGKLTGQVID LLAGLNTPGVTTVIEPVMTRDHTEKMLQGFGAALSVETDEDGVRTIRLEGRGKLTGQVID | 240 |
| Query 241 | VPGDPSSTAFPLVAALLVPGSDVTILNVLMNPTRTGLILTLQEMGADIEVINPRLAGGED | 300 |
| Sbjct 241 | VPGDPSSTAFPLVAALLVPGSD+TI+NVLMNPTRTGLILTLQEMGADI+V N RLAGGED VPGDPSSTAFPLVAALLVPGSDITIVNVLMNPTRTGLILTLQEMGADIQVANARLAGGED | 300 |
| Query 301 | VADLRVRSSTLKGVTVPEDRAPSMIDEYPILavaaafaegaTVMNGLEELRVKESDRLSA | 360 |
| Sbjct 301 | VADLRVR S LKGVTVPEDRAPSMIDEYPILA+AA+FAEGATVM GLEELRVKESDRLSA VADLRVRHSELKGVTVPEDRAPSMIDEYPILAIAASFAEGATVMKGLEELRVKESDRLSA | 360 |
| Query 361 | VANGLKLNGVDCDEGETSLVVRGRPDGKGLGNASGAAVATHLDHRIAMSFLVMGLVSENP | 420 |
| Sbjct 361 | VA+GLKLNGVDCDEGE LVVRGRPDGKGLGNA+ V+THLDHRIAMSFLVMGL SE+P VADGLKLNGVDCDEGEDFLVVRGRPDGKGLGNAADGRVSTHLDHRIAMSFLVMGLASEHP | 420 |
| Query 421  Sbjct 421 | VTVDDATMIATSFPEFMDLMAGLGAKIE 448 VT+DDA MIATSFPEFM LM GLGAKIE VTIDDAAMIATSFPEFMQLMTGLGAKIE 448 |  |

```
>WP_138388525.1 3-phosphoshikimate 1-carboxyvinyltransferase [Rhizobium indicum] QKK17245.1 3-phosphoshikimate 1-carboxyvinyltransferase [Rhizobium indicum]
Length=452

Score = 774 bits (1998), Expect = 0.0, Method: Compositional matrix adjust. Identities = 389/448 (87%), Positives = 420/448 (94%), Gaps = 0/448 (0%)

Query 1	MLHGASSRPATARKSSGLSGTVRIPGDKSISHRSFMFGGLASGETRITGLLEGEDVINTG 60 MLHG++ RPATARKS+GL+G+VRIPGDKSISHRSFMFGGLASGETRITGLLEGEDVINTG
Sbjct 1	MLHGSAPRPATARKSAGLTGSVRIPGDKSISHRSFMFGGLASGETRITGLLEGEDVINTG 60
```

| Query | 61 | KAMQAMGARIRKEGDTWIIDGVGNGGLLAPEAPLDFGNAATGCRLTMGLVGVYDFDSTFI | 120 |
| --- | --- | --- | --- |
| Sbjct | 61 | +AMQAMGARIRK+G W+IDG GNG LLAP+APLDFGNA TG RLTMGLVG YDF STFI RAMQAMGARIRKDGAQWVIDGTGNGALLAPDAPLDFGNAGTGARLTMGLVGTYDFRSTFI | 120 |
| Query | 121 | GDASLTKRPMGRVLNPLREMGVQVKSEDGDRLPVTLRGPKTPTPITYRVPMASAQVKSAV | 180 |
| Sbjct | 121 | GDASL+KRPMGRVLNPLREMGVQV + +GDRLPVTL+GP TP+PI YRVPMASAQVKSAV GDASLSKRPMGRVLNPLREMGVQVSASEGDRLPVTLKGPGTPSPIRYRVPMASAQVKSAV | 180 |
| Query | 181 | LLAGLNTPGITTVIEPIMTRDHTEKMLQGFGANLTVETDADGVRTIRLEGRGKLTGQVID | 240 |
| Sbjct | 181 | LLAGLNTPG+TTVIEP+MTRDHTEKMLQGFGA L+VETD++GVRTIRLEGRG+LTGQVID LLAGLNTPGVTTVIEPVMTRDHTEKMLQGFGAALSVETDSEGVRTIRLEGRGRLTGQVID | 240 |
| Query | 241 | VPGDPSSTAFPLVAALLVPGSDVTILNVLMNPTRTGLILTLQEMGADIEVINPRLAGGED | 300 |
| Sbjct | 241 | +PGDPSSTAFPLVAALLVPGSD+TI+NVLMNPTRTGLILTLQEMGADIEV+N RLAGGED IPGDPSSTAFPLVAALLVPGSDITIVNVLMNPTRTGLILTLQEMGADIEVVNARLAGGED | 300 |
| Query | 301 | VADLRVRSSTLKGVTVPEDRAPSMIDEYPILavaaafaegaTVMNGLEELRVKESDRLSA | 360 |
| Sbjct | 301 | VADLRVR S L+GVTVPEDRAPSMIDEYPILAVAA FAEGATVM GLEELRVKESDRLSA VADLRVRHSELRGVTVPEDRAPSMIDEYPILAVAACFAEGATVMKGLEELRVKESDRLSA | 360 |
| Query | 361 | VANGLKLNGVDCDEGETSLVVRGRPDGKGLGNASGAAVATHLDHRIAMSFLVMGLVSENP | 420 |
| Sbjct | 361 | VA+GLKLNGVDCDEGE L+VRGRPDGKGLGNA+ V+THLDHRIAMSFLVMGL SE+P VADGLKLNGVDCDEGEDFLIVRGRPDGKGLGNAADGRVSTHLDHRIAMSFLVMGLASEHP | 420 |
| Query  Sbjct | 421  421 | VTVDDATMIATSFPEFMDLMAGLGAKIE 448 VT+DDA MIATSFPEFM LM GLGAKIE VTIDDAAMIATSFPEFMQLMTGLGAKIE 448 |  |

>WP_168349154.1 3-phosphoshikimate 1-carboxyvinyltransferase [Rhizobium leguminosarum]

NKK67113.1 3-phosphoshikimate 1-carboxyvinyltransferase [Rhizobium leguminosarum bv. viciae]

Length=452

Score = 774 bits (1998), Expect = 0.0, Method: Compositional matrix adjust.

| Identities | = 392/449 (87%), Positives = 417/449 (93%), Gaps = 0/449 (0%) |  |
| --- | --- | --- |
| Query 1 | MLHGASSRPATARKSSGLSGTVRIPGDKSISHRSFMFGGLASGETRITGLLEGEDVINTG | 60 |
| Sbjct 1 | MLHG++ +PATARKS+GL+G+VRIPGDKSISHRSFMFGGLASGETRITGLLEGEDVINTG MLHGSAPKPATARKSAGLAGSVRIPGDKSISHRSFMFGGLASGETRITGLLEGEDVINTG | 60 |
| Query 61 | KAMQAMGARIRKEGDTWIIDGVGNGGLLAPEAPLDFGNAATGCRLTMGLVGVYDFDSTFI | 120 |
| Sbjct 61 | +AMQAMGARIRK+G W+IDG GNG LLAP+APLDFGNA TG RLTMGLVG YDF STFI RAMQAMGARIRKDGAQWVIDGTGNGALLAPDAPLDFGNAGTGVRLTMGLVGTYDFRSTFI | 120 |
| Query 121 | GDASLTKRPMGRVLNPLREMGVQVKSEDGDRLPVTLRGPKTPTPITYRVPMASAQVKSAV | 180 |
| Sbjct 121 | GDASL+KRPMGRVLNPLREMGVQV + +GDRLPVTLRGP TP+PI YRVPMASAQVKSAV GDASLSKRPMGRVLNPLREMGVQVSASEGDRLPVTLRGPGTPSPIRYRVPMASAQVKSAV | 180 |
| Query 181 | LLAGLNTPGITTVIEPIMTRDHTEKMLQGFGANLTVETDADGVRTIRLEGRGKLTGQVID | 240 |
| Sbjct 181 | LLAGLNTPG+TTVIEP+MTRDHTEKMLQGFGA L+VETD DGVRTIRLEGRGKL GQVID LLAGLNTPGVTTVIEPVMTRDHTEKMLQGFGAALSVETDGDGVRTIRLEGRGKLKGQVID | 240 |
| Query 241 | VPGDPSSTAFPLVAALLVPGSDVTILNVLMNPTRTGLILTLQEMGADIEVINPRLAGGED | 300 |
| Sbjct 241 | VPGDPSSTAFPLVAALLVPGSD+TI+NVLMNPTRTGLILTLQEMGADIEV N RLAGGED VPGDPSSTAFPLVAALLVPGSDITIVNVLMNPTRTGLILTLQEMGADIEVANARLAGGED | 300 |
| Query 301 | VADLRVRSSTLKGVTVPEDRAPSMIDEYPILavaaafaegaTVMNGLEELRVKESDRLSA | 360 |
| Sbjct 301 | VADLRVR S LKGVTVPE+RAPSMIDEYPILAVAA FAEG TVM GLEELRVKESDRLSA VADLRVRHSELKGVTVPEERAPSMIDEYPILAVAACFAEGVTVMKGLEELRVKESDRLSA | 360 |
| Query 361 | VANGLKLNGVDCDEGETSLVVRGRPDGKGLGNASGAAVATHLDHRIAMSFLVMGLVSENP | 420 |
| Sbjct 361 | VA+GLKLNGVDCDEGE LVVRGRPDGKGLGNA+G V+THLDHRIAMSFLVMGL SE+ VADGLKLNGVDCDEGEDFLVVRGRPDGKGLGNAAGDKVSTHLDHRIAMSFLVMGLASEHA | 420 |
| Query 421  Sbjct 421 | VTVDDATMIATSFPEFMDLMAGLGAKIEL 449 VT+DDA MIATSFPEFM LM GLGAKIEL VTIDDAAMIATSFPEFMQLMTGLGAKIEL 449 |  |

>WP_130759700.1 3-phosphoshikimate 1-carboxyvinyltransferase [Rhizobium leguminosarum] QIO73410.1 3-phosphoshikimate 1-carboxyvinyltransferase [Rhizobium leguminosarum

bv. trifolii]

QIO80429.1 3-phosphoshikimate 1-carboxyvinyltransferase [Rhizobium leguminosarum bv. trifolii]

TAZ64232.1 3-phosphoshikimate 1-carboxyvinyltransferase [Rhizobium leguminosarum] Length=452

Score = 774 bits (1998), Expect = 0.0, Method: Compositional matrix adjust.

| Identities | = 390/448 (87%), Positives = 418/448 (93%), Gaps = 0/448 (0%) |  |
| --- | --- | --- |
| Query 1 | MLHGASSRPATARKSSGLSGTVRIPGDKSISHRSFMFGGLASGETRITGLLEGEDVINTG | 60 |
| Sbjct 1 | MLHG++ RPATARKS+GL+G+VRIPGDKSISHRSFMFGGLASGETRITGLLEGEDVINTG MLHGSAPRPATARKSAGLTGSVRIPGDKSISHRSFMFGGLASGETRITGLLEGEDVINTG | 60 |
| Query 61 | KAMQAMGARIRKEGDTWIIDGVGNGGLLAPEAPLDFGNAATGCRLTMGLVGVYDFDSTFI | 120 |
| Sbjct 61 | +AMQAMGARIRK G+ W+I+G GNG LLAP+APLDFGNA TG RLTMGLVG YDF STFI RAMQAMGARIRKVGEQWLIEGTGNGALLAPDAPLDFGNAGTGVRLTMGLVGTYDFRSTFI | 120 |
| Query 121 | GDASLTKRPMGRVLNPLREMGVQVKSEDGDRLPVTLRGPKTPTPITYRVPMASAQVKSAV | 180 |
| Sbjct 121 | GDASL+KRPMGRVLNPLREMGVQV + +GDRLPVTLRGP TP+PI YRVPMASAQVKSAV GDASLSKRPMGRVLNPLREMGVQVSASEGDRLPVTLRGPGTPSPIRYRVPMASAQVKSAV | 180 |
| Query 181 | LLAGLNTPGITTVIEPIMTRDHTEKMLQGFGANLTVETDADGVRTIRLEGRGKLTGQVID | 240 |
| Sbjct 181 | LLAGLNTPG+TTVIEP+MTRDHTEKMLQGFGA L+VETD +GVRTIRLEGRGKL GQVID LLAGLNTPGVTTVIEPVMTRDHTEKMLQGFGAALSVETDEEGVRTIRLEGRGKLAGQVID | 240 |
| Query 241 | VPGDPSSTAFPLVAALLVPGSDVTILNVLMNPTRTGLILTLQEMGADIEVINPRLAGGED | 300 |
| Sbjct 241 | +PGDPSSTAFPLVAALLVPGSD+TI+NVLMNPTRTGLILTLQEMGADIEV+N RLAGGED IPGDPSSTAFPLVAALLVPGSDITIVNVLMNPTRTGLILTLQEMGADIEVLNARLAGGED | 300 |
| Query 301 | VADLRVRSSTLKGVTVPEDRAPSMIDEYPILavaaafaegaTVMNGLEELRVKESDRLSA | 360 |
| Sbjct 301 | VADLRVR S LKGVTVPEDRAPSMIDEYPILAVAA FAEG TVM GLEELRVKESDRLSA VADLRVRHSELKGVTVPEDRAPSMIDEYPILAVAACFAEGVTVMKGLEELRVKESDRLSA | 360 |
| Query 361 | VANGLKLNGVDCDEGETSLVVRGRPDGKGLGNASGAAVATHLDHRIAMSFLVMGLVSENP | 420 |
| Sbjct 361 | VA+GLKLNGVDCDEGE L+VRGRPDGKGLGNA+G V+THLDHRIAMSFLVMGL SE+P VADGLKLNGVDCDEGEDFLIVRGRPDGKGLGNAAGDEVSTHLDHRIAMSFLVMGLASEHP | 420 |
| Query 421  Sbjct 421 | VTVDDATMIATSFPEFMDLMAGLGAKIE 448 VT+DDA MIATSFPEFM LM GLGAKIE VTIDDAAMIATSFPEFMQLMTGLGAKIE 448 |  |

>WP_131702060.1 3-phosphoshikimate 1-carboxyvinyltransferase [Rhizobium leguminosarum]

NKJ90609.1 3-phosphoshikimate 1-carboxyvinyltransferase [Rhizobium leguminosarum bv. viciae]

QIO59230.1 3-phosphoshikimate 1-carboxyvinyltransferase [Rhizobium leguminosarum bv. trifolii]

TBZ72730.1 3-phosphoshikimate 1-carboxyvinyltransferase [Rhizobium leguminosarum bv. viciae]

Length=452

Score = 774 bits (1998), Expect = 0.0, Method: Compositional matrix adjust.

| Identities | = 390/448 (87%), Positives = 418/448 (93%), Gaps = 0/448 (0%) |  |
| --- | --- | --- |
| Query 1 | MLHGASSRPATARKSSGLSGTVRIPGDKSISHRSFMFGGLASGETRITGLLEGEDVINTG | 60 |
| Sbjct 1 | MLHG++ RPATARKS GL+G+VRIPGDKSISHRSFMFGGLASGETRITGLLEGEDVINTG MLHGSAPRPATARKSVGLTGSVRIPGDKSISHRSFMFGGLASGETRITGLLEGEDVINTG | 60 |
| Query 61 | KAMQAMGARIRKEGDTWIIDGVGNGGLLAPEAPLDFGNAATGCRLTMGLVGVYDFDSTFI | 120 |
| Sbjct 61 | +AMQAMGARIRK G+ W+I+G GNG LLAP+APLDFGNA TG RLTMGLVG YDF STFI RAMQAMGARIRKVGEQWVIEGTGNGALLAPDAPLDFGNAGTGVRLTMGLVGTYDFRSTFI | 120 |
| Query 121 | GDASLTKRPMGRVLNPLREMGVQVKSEDGDRLPVTLRGPKTPTPITYRVPMASAQVKSAV | 180 |
| Sbjct 121 | GDASL+KRPMGRVLNPLREMGVQV + +GDRLPVTLRGP TP+PI YRVPMASAQVKSAV GDASLSKRPMGRVLNPLREMGVQVSASEGDRLPVTLRGPGTPSPIRYRVPMASAQVKSAV | 180 |
| Query 181 | LLAGLNTPGITTVIEPIMTRDHTEKMLQGFGANLTVETDADGVRTIRLEGRGKLTGQVID | 240 |
| Sbjct 181 | LLAGLNTPG+TTVIEP+MTRDHTEKMLQGFGA L+VETD +GVRTIRLEGRGKLTGQVID LLAGLNTPGVTTVIEPVMTRDHTEKMLQGFGAALSVETDEEGVRTIRLEGRGKLTGQVID | 240 |
| Query 241 | VPGDPSSTAFPLVAALLVPGSDVTILNVLMNPTRTGLILTLQEMGADIEVINPRLAGGED  +PGDPSSTAFPLVAALLVPGSD+TI+NVLMNPTRTGLILTLQEMGADIEV+N RLAGGED | 300 |

| Sbjct | 241 | IPGDPSSTAFPLVAALLVPGSDITIVNVLMNPTRTGLILTLQEMGADIEVLNARLAGGED | 300 |
| --- | --- | --- | --- |
| Query | 301 | VADLRVRSSTLKGVTVPEDRAPSMIDEYPILavaaafaegaTVMNGLEELRVKESDRLSA | 360 |
| Sbjct | 301 | VADLRVR S LKGVTVPEDRAPSMIDEYPILAVAA FAEG TVM GLEELRVKESDRLSA VADLRVRHSELKGVTVPEDRAPSMIDEYPILAVAACFAEGVTVMKGLEELRVKESDRLSA | 360 |
| Query | 361 | VANGLKLNGVDCDEGETSLVVRGRPDGKGLGNASGAAVATHLDHRIAMSFLVMGLVSENP | 420 |
| Sbjct | 361 | VA+GLKLNGVDCDEGE L+VRGRPDGKGLGNA+G V+THLDHRIAMSFLVMGL SE+P VADGLKLNGVDCDEGEDFLIVRGRPDGKGLGNAAGDKVSTHLDHRIAMSFLVMGLASEHP | 420 |
| Query  Sbjct | 421  421 | VTVDDATMIATSFPEFMDLMAGLGAKIE 448 VT+DDA MIATSFPEFM LM GLGA+IE VTIDDAAMIATSFPEFMQLMTGLGARIE 448 |  |

>WP_128400242.1 3-phosphoshikimate 1-carboxyvinyltransferase [Rhizobium leguminosarum] RWX18948.1 3-phosphoshikimate 1-carboxyvinyltransferase [Rhizobium leguminosarum] RWX35520.1 3-phosphoshikimate 1-carboxyvinyltransferase [Rhizobium leguminosarum] TBC75416.1 3-phosphoshikimate 1-carboxyvinyltransferase [Rhizobium leguminosarum] TBE73293.1 3-phosphoshikimate 1-carboxyvinyltransferase [Rhizobium leguminosarum]

Length=452

Score = 773 bits (1996), Expect = 0.0, Method: Compositional matrix adjust.

| Identities | = 389/448 (87%), Positives = 417/448 (93%), Gaps = 0/448 (0%) |  |
| --- | --- | --- |
| Query 1 | MLHGASSRPATARKSSGLSGTVRIPGDKSISHRSFMFGGLASGETRITGLLEGEDVINTG | 60 |
| Sbjct 1 | MLHG++ RPATARKS+GL+G+VRIPGDKSISHRSFMFGGLASGETRITGLLEGEDVINTG MLHGSAPRPATARKSAGLTGSVRIPGDKSISHRSFMFGGLASGETRITGLLEGEDVINTG | 60 |
| Query 61 | KAMQAMGARIRKEGDTWIIDGVGNGGLLAPEAPLDFGNAATGCRLTMGLVGVYDFDSTFI | 120 |
| Sbjct 61 | +AMQAMGARIRKEG+ W+I+G GNG LLAP+APLDFGNA TG RLTMGLVG YDF STF RAMQAMGARIRKEGEQWVIEGTGNGALLAPDAPLDFGNAGTGVRLTMGLVGTYDFRSTFT | 120 |
| Query 121 | GDASLTKRPMGRVLNPLREMGVQVKSEDGDRLPVTLRGPKTPTPITYRVPMASAQVKSAV | 180 |
| Sbjct 121 | GDASL+KRPMGRVLNPLREMGVQV + +GDRLPVTLRGP TP+PI YRVPMASAQVKSAV GDASLSKRPMGRVLNPLREMGVQVSASEGDRLPVTLRGPGTPSPIRYRVPMASAQVKSAV | 180 |
| Query 181 | LLAGLNTPGITTVIEPIMTRDHTEKMLQGFGANLTVETDADGVRTIRLEGRGKLTGQVID | 240 |
| Sbjct 181 | LLAGLNTPG+TTVIEP+MTRDHTEKMLQGFGA L+VETD DGVRTIRLEGRGKL GQVID LLAGLNTPGVTTVIEPVMTRDHTEKMLQGFGAALSVETDEDGVRTIRLEGRGKLKGQVID | 240 |
| Query 241 | VPGDPSSTAFPLVAALLVPGSDVTILNVLMNPTRTGLILTLQEMGADIEVINPRLAGGED | 300 |
| Sbjct 241 | VPGDPSSTAFPLVAALLVPGSD+TI+NVLMNPTRTGLILTLQEMGADIEV+N RLAGGED VPGDPSSTAFPLVAALLVPGSDITIVNVLMNPTRTGLILTLQEMGADIEVLNTRLAGGED | 300 |
| Query 301 | VADLRVRSSTLKGVTVPEDRAPSMIDEYPILavaaafaegaTVMNGLEELRVKESDRLSA | 360 |
| Sbjct 301 | VADLRVR S LKGVTVPEDRAPSMIDEYPILAVAA FA+G TVM GLEELRVKESDRLSA VADLRVRHSELKGVTVPEDRAPSMIDEYPILAVAACFADGVTVMKGLEELRVKESDRLSA | 360 |
| Query 361 | VANGLKLNGVDCDEGETSLVVRGRPDGKGLGNASGAAVATHLDHRIAMSFLVMGLVSENP | 420 |
| Sbjct 361 | VA+GLKLNGVDCDEG+ L+VRGRPDGKGLGNA+ V+THLDHRIAMSFLVMGL SE+P VADGLKLNGVDCDEGKDFLIVRGRPDGKGLGNAADEKVSTHLDHRIAMSFLVMGLASEHP | 420 |
| Query 421  Sbjct 421 | VTVDDATMIATSFPEFMDLMAGLGAKIE 448 VT+DDA MIATSFPEFM LM GLGAKIE VTIDDAAMIATSFPEFMQLMTGLGAKIE 448 |  |

>WP_130784542.1 3-phosphoshikimate 1-carboxyvinyltransferase [Rhizobium leguminosarum] NEI95206.1 3-phosphoshikimate 1-carboxyvinyltransferase [Rhizobium leguminosarum] NEJ80441.1 3-phosphoshikimate 1-carboxyvinyltransferase [Rhizobium leguminosarum] TBF42868.1 3-phosphoshikimate 1-carboxyvinyltransferase [Rhizobium leguminosarum] TBF54453.1 3-phosphoshikimate 1-carboxyvinyltransferase [Rhizobium leguminosarum] TBF59137.1 3-phosphoshikimate 1-carboxyvinyltransferase [Rhizobium leguminosarum]

Length=452

Score = 773 bits (1995), Expect = 0.0, Method: Compositional matrix adjust. Identities = 390/449 (87%), Positives = 419/449 (93%), Gaps = 0/449 (0%)

Query 1	MLHGASSRPATARKSSGLSGTVRIPGDKSISHRSFMFGGLASGETRITGLLEGEDVINTG 60 ML+G++S+PATARKS+GL+G+VRIPGDKSISHRSFMFGGLASGETRITGLLEGEDVINTG

Sbjct 1	MLNGSASKPATARKSAGLTGSVRIPGDKSISHRSFMFGGLASGETRITGLLEGEDVINTG 60

| Query | 61 | KAMQAMGARIRKEGDTWIIDGVGNGGLLAPEAPLDFGNAATGCRLTMGLVGVYDFDSTFI | 120 |
| --- | --- | --- | --- |
| Sbjct | 61 | +AMQAMGARIRKEG W+I+G GNG LLAP+APLDFGNA TG RLTMGLVG YDF STFI RAMQAMGARIRKEGAQWVIEGTGNGALLAPDAPLDFGNAGTGVRLTMGLVGTYDFHSTFI | 120 |
| Query | 121 | GDASLTKRPMGRVLNPLREMGVQVKSEDGDRLPVTLRGPKTPTPITYRVPMASAQVKSAV | 180 |
| Sbjct | 121 | GDASL+KRPMGRVLNPLREMGVQV + +GDRLPVTLRGP TP+PI YRVPMASAQVKSAV GDASLSKRPMGRVLNPLREMGVQVSASEGDRLPVTLRGPGTPSPIRYRVPMASAQVKSAV | 180 |
| Query | 181 | LLAGLNTPGITTVIEPIMTRDHTEKMLQGFGANLTVETDADGVRTIRLEGRGKLTGQVID | 240 |
| Sbjct | 181 | LLAGLNTPG+TTVIEP+MTRDHTEKMLQGFGA L+VETD DGVRTIRLEGRGKL GQVID LLAGLNTPGVTTVIEPVMTRDHTEKMLQGFGAALSVETDGDGVRTIRLEGRGKLAGQVID | 240 |
| Query | 241 | VPGDPSSTAFPLVAALLVPGSDVTILNVLMNPTRTGLILTLQEMGADIEVINPRLAGGED | 300 |
| Sbjct | 241 | VPGDPSSTAFPLVAAL+VPGSD+TI+NVLMNPTRTGLILTLQEMGADIEV+N RLAGGED VPGDPSSTAFPLVAALIVPGSDITIVNVLMNPTRTGLILTLQEMGADIEVVNARLAGGED | 300 |
| Query | 301 | VADLRVRSSTLKGVTVPEDRAPSMIDEYPILavaaafaegaTVMNGLEELRVKESDRLSA | 360 |
| Sbjct | 301 | VADLRVR S LKGVTVPEDRAPSMIDEYPILAVAA FAEGATVM GLEELRVKESDRLSA VADLRVRHSELKGVTVPEDRAPSMIDEYPILAVAACFAEGATVMKGLEELRVKESDRLSA | 360 |
| Query | 361 | VANGLKLNGVDCDEGETSLVVRGRPDGKGLGNASGAAVATHLDHRIAMSFLVMGLVSENP | 420 |
| Sbjct | 361 | VA+GLKLNGVDCDEGE L+VRGRPDGKGLGNA+ V+THLDHRIAMSFLV+GL SE+ VADGLKLNGVDCDEGEDFLIVRGRPDGKGLGNAADGRVSTHLDHRIAMSFLVLGLASEHA | 420 |
| Query  Sbjct | 421  421 | VTVDDATMIATSFPEFMDLMAGLGAKIEL 449 VT+DDA MIATSFPEFM LM GLGAKIEL VTIDDAAMIATSFPEFMQLMTGLGAKIEL 449 |  |

>WP_164046176.1 3-phosphoshikimate 1-carboxyvinyltransferase [Rhizobium leguminosarum] NEK14559.1 3-phosphoshikimate 1-carboxyvinyltransferase [Rhizobium leguminosarum]

Length=452

Score = 773 bits (1995), Expect = 0.0, Method: Compositional matrix adjust.

| Identities | = 389/448 (87%), Positives = 419/448 (94%), Gaps = 0/448 (0%) |  |
| --- | --- | --- |
| Query 1 | MLHGASSRPATARKSSGLSGTVRIPGDKSISHRSFMFGGLASGETRITGLLEGEDVINTG | 60 |
| Sbjct 1 | ML+G++S+PATARKS+GL+G+VRIPGDKSISHRSFMFGGLASGETRITGLLEGEDVINTG MLNGSASKPATARKSAGLTGSVRIPGDKSISHRSFMFGGLASGETRITGLLEGEDVINTG | 60 |
| Query 61 | KAMQAMGARIRKEGDTWIIDGVGNGGLLAPEAPLDFGNAATGCRLTMGLVGVYDFDSTFI | 120 |
| Sbjct 61 | +AMQAMGARIRKEG+ W+IDG GNG LLAP+APLDFGNA TG RLTMGLVG YDF STF RAMQAMGARIRKEGEQWVIDGTGNGALLAPDAPLDFGNAGTGVRLTMGLVGTYDFRSTFT | 120 |
| Query 121 | GDASLTKRPMGRVLNPLREMGVQVKSEDGDRLPVTLRGPKTPTPITYRVPMASAQVKSAV | 180 |
| Sbjct 121 | GDASL+KRPMGRVLNPLREMGVQV + GDRLPVTLRGP TP+PI YRVPMASAQVKSAV GDASLSKRPMGRVLNPLREMGVQVSASQGDRLPVTLRGPGTPSPIRYRVPMASAQVKSAV | 180 |
| Query 181 | LLAGLNTPGITTVIEPIMTRDHTEKMLQGFGANLTVETDADGVRTIRLEGRGKLTGQVID | 240 |
| Sbjct 181 | LLAGLNTPGITTVIEP+MTRDHTEKMLQGFGA L+VETD++GVRTIRLEGRG+LTGQVID LLAGLNTPGITTVIEPVMTRDHTEKMLQGFGAALSVETDSEGVRTIRLEGRGRLTGQVID | 240 |
| Query 241 | VPGDPSSTAFPLVAALLVPGSDVTILNVLMNPTRTGLILTLQEMGADIEVINPRLAGGED | 300 |
| Sbjct 241 | VPGDPSSTAFPLVAALLVPGSD+TI+NVLMNPTRTGLILTLQEMGADIEV+N RLAGGED VPGDPSSTAFPLVAALLVPGSDITIVNVLMNPTRTGLILTLQEMGADIEVVNARLAGGED | 300 |
| Query 301 | VADLRVRSSTLKGVTVPEDRAPSMIDEYPILavaaafaegaTVMNGLEELRVKESDRLSA | 360 |
| Sbjct 301 | VADLRVR S LKGVTVPEDRAPSMIDEYPILAVAA FAEGAT+M GLEELRVKESDRLSA VADLRVRHSELKGVTVPEDRAPSMIDEYPILAVAACFAEGATIMKGLEELRVKESDRLSA | 360 |
| Query 361 | VANGLKLNGVDCDEGETSLVVRGRPDGKGLGNASGAAVATHLDHRIAMSFLVMGLVSENP | 420 |
| Sbjct 361 | VA+GLKLNGVDCDEGE L+VRGRPDGKGLGNA+G V+THLDHRIAMSFLV+G SE+ VADGLKLNGVDCDEGEDFLIVRGRPDGKGLGNAAGDKVSTHLDHRIAMSFLVLGFASEHA | 420 |
| Query 421  Sbjct 421 | VTVDDATMIATSFPEFMDLMAGLGAKIE 448 VT+DDA MIATSFPEFM LM GLGAKIE VTIDDAAMIATSFPEFMQLMTGLGAKIE 448 |  |

>WP_131605380.1 3-phosphoshikimate 1-carboxyvinyltransferase [Rhizobium leguminosarum] NKJ99691.1 3-phosphoshikimate 1-carboxyvinyltransferase [Rhizobium leguminosarum

bv. viciae]

TBZ72225.1 3-phosphoshikimate 1-carboxyvinyltransferase [Rhizobium leguminosarum bv. viciae]

TCA14762.1 3-phosphoshikimate 1-carboxyvinyltransferase [Rhizobium leguminosarum bv. viciae]

TCA23150.1 3-phosphoshikimate 1-carboxyvinyltransferase [Rhizobium leguminosarum bv. viciae]

Length=452

Score = 773 bits (1995), Expect = 0.0, Method: Compositional matrix adjust.

| Identities | = 389/448 (87%), Positives = 417/448 (93%), Gaps = 0/448 (0%) |  |
| --- | --- | --- |
| Query 1 | MLHGASSRPATARKSSGLSGTVRIPGDKSISHRSFMFGGLASGETRITGLLEGEDVINTG | 60 |
| Sbjct 1 | MLHG++ RPATARKS+GL+G+VRIPGDKSISHRSFMFGGLASGETRITGLLEGEDVINTG MLHGSAPRPATARKSAGLTGSVRIPGDKSISHRSFMFGGLASGETRITGLLEGEDVINTG | 60 |
| Query 61 | KAMQAMGARIRKEGDTWIIDGVGNGGLLAPEAPLDFGNAATGCRLTMGLVGVYDFDSTFI | 120 |
| Sbjct 61 | +AMQAMGARIRK G+ W+I+G GNG LLAP+APLDFGNA TG RLTMGLVG YDF STFI RAMQAMGARIRKVGEQWVIEGTGNGALLAPDAPLDFGNAGTGVRLTMGLVGTYDFRSTFI | 120 |
| Query 121 | GDASLTKRPMGRVLNPLREMGVQVKSEDGDRLPVTLRGPKTPTPITYRVPMASAQVKSAV | 180 |
| Sbjct 121 | GDASL+KRPMGRVLNPLREMGVQV + +GDRLPVTLRGP TP+PI YRVPMASAQVKSA+ GDASLSKRPMGRVLNPLREMGVQVSASEGDRLPVTLRGPGTPSPIRYRVPMASAQVKSAM | 180 |
| Query 181 | LLAGLNTPGITTVIEPIMTRDHTEKMLQGFGANLTVETDADGVRTIRLEGRGKLTGQVID | 240 |
| Sbjct 181 | LLAGLNTPG+TTVIEP+MTRDHTEKMLQGFGA L+VETD DGVRTIRLEGRGKL GQVID LLAGLNTPGVTTVIEPVMTRDHTEKMLQGFGAALSVETDEDGVRTIRLEGRGKLAGQVID | 240 |
| Query 241 | VPGDPSSTAFPLVAALLVPGSDVTILNVLMNPTRTGLILTLQEMGADIEVINPRLAGGED | 300 |
| Sbjct 241 | +PGDPSSTAFPLVAALLVPGSD+TI+NVLMNPTRTGLILTLQEMGADIEV+N RLAGGED IPGDPSSTAFPLVAALLVPGSDITIVNVLMNPTRTGLILTLQEMGADIEVLNARLAGGED | 300 |
| Query 301 | VADLRVRSSTLKGVTVPEDRAPSMIDEYPILavaaafaegaTVMNGLEELRVKESDRLSA | 360 |
| Sbjct 301 | VADLRVR S LKGVTVPEDRAPSMIDEYPILAVAA FAEG TVM GLEELRVKESDRLSA VADLRVRHSELKGVTVPEDRAPSMIDEYPILAVAACFAEGVTVMKGLEELRVKESDRLSA | 360 |
| Query 361 | VANGLKLNGVDCDEGETSLVVRGRPDGKGLGNASGAAVATHLDHRIAMSFLVMGLVSENP | 420 |
| Sbjct 361 | VA+GLKLNGVDCDEGE L+VRGRPDGKGLGNA+ V+THLDHRIAMSFLVMGL SE+P VADGLKLNGVDCDEGEDFLIVRGRPDGKGLGNAADGRVSTHLDHRIAMSFLVMGLASEHP | 420 |
| Query 421  Sbjct 421 | VTVDDATMIATSFPEFMDLMAGLGAKIE 448 VT+DDA MIATSFPEFM LM GLGAKIE VTIDDAAMIATSFPEFMQLMTGLGAKIE 448 |  |

>WP_047617633.1 3-phosphoshikimate 1-carboxyvinyltransferase [Rhizobium leguminosarum] Length=452

Score = 772 bits (1994), Expect = 0.0, Method: Compositional matrix adjust.

| Identities | = 388/448 (87%), Positives = 418/448 (93%), Gaps = 0/448 (0%) |  |
| --- | --- | --- |
| Query 1 | MLHGASSRPATARKSSGLSGTVRIPGDKSISHRSFMFGGLASGETRITGLLEGEDVINTG | 60 |
| Sbjct 1 | M HG++ RPATARKS+GL+G+VRIPGDKSISHRSFMFGGLASGETRITGLLEGEDVINTG MFHGSAPRPATARKSAGLAGSVRIPGDKSISHRSFMFGGLASGETRITGLLEGEDVINTG | 60 |
| Query 61 | KAMQAMGARIRKEGDTWIIDGVGNGGLLAPEAPLDFGNAATGCRLTMGLVGVYDFDSTFI | 120 |
| Sbjct 61 | +AMQAMGARIRK G+ W+I+G GNG LLAP+APLDFGNA TG RLTMGLVG YDF STFI RAMQAMGARIRKVGEQWLIEGTGNGALLAPDAPLDFGNAGTGVRLTMGLVGTYDFRSTFI | 120 |
| Query 121 | GDASLTKRPMGRVLNPLREMGVQVKSEDGDRLPVTLRGPKTPTPITYRVPMASAQVKSAV | 180 |
| Sbjct 121 | GDASL+KRPMGRVLNPL EMGVQV + +GDRLPVTLRGP TP+PI YRVPMASAQVKSAV GDASLSKRPMGRVLNPLLEMGVQVSASEGDRLPVTLRGPGTPSPIRYRVPMASAQVKSAV | 180 |
| Query 181 | LLAGLNTPGITTVIEPIMTRDHTEKMLQGFGANLTVETDADGVRTIRLEGRGKLTGQVID | 240 |
| Sbjct 181 | LLAGLNTPG+TTVIEP+MTRDHTEKMLQGFGA L+VETD +GVRTIRLEGRGKLTGQVID LLAGLNTPGVTTVIEPVMTRDHTEKMLQGFGAALSVETDEEGVRTIRLEGRGKLTGQVID | 240 |
| Query 241 | VPGDPSSTAFPLVAALLVPGSDVTILNVLMNPTRTGLILTLQEMGADIEVINPRLAGGED | 300 |
| Sbjct 241 | +PGDPSSTAFPLVAALLVPGSD+TI+NVLMNPTRTGLILTLQEMGADI+V+NPRLAGGED IPGDPSSTAFPLVAALLVPGSDITIVNVLMNPTRTGLILTLQEMGADIQVLNPRLAGGED | 300 |
| Query 301 | VADLRVRSSTLKGVTVPEDRAPSMIDEYPILavaaafaegaTVMNGLEELRVKESDRLSA | 360 |

| Sbjct | 301 | VADLRVR S LKGVTVPEDRAPSMIDEYPILAVAA FAEG TVM GLEELRVKESDRLSA VADLRVRHSELKGVTVPEDRAPSMIDEYPILAVAACFAEGVTVMKGLEELRVKESDRLSA | 360 |
| --- | --- | --- | --- |
| Query | 361 | VANGLKLNGVDCDEGETSLVVRGRPDGKGLGNASGAAVATHLDHRIAMSFLVMGLVSENP | 420 |
| Sbjct | 361 | VA+GLKLNGVDCDEGE L+VRGRPDGKGLGNA+G V+THLDHRIAMSFLVMGL SE+P VADGLKLNGVDCDEGEDFLIVRGRPDGKGLGNAAGDKVSTHLDHRIAMSFLVMGLASEHP | 420 |
| Query  Sbjct | 421  421 | VTVDDATMIATSFPEFMDLMAGLGAKIE 448 VT+DDA MIATSFPEFM LM GLGA+IE VTIDDAAMIATSFPEFMQLMTGLGARIE 448 |  |

>WP_003544289.1 3-phosphoshikimate 1-carboxyvinyltransferase [Rhizobium leguminosarum]

EJC68821.1 3-phosphoshikimate 1-carboxyvinyltransferase [Rhizobium leguminosarum bv. viciae WSM1455]

Length=452

Score = 772 bits (1994), Expect = 0.0, Method: Compositional matrix adjust.

| Identities | = 391/448 (87%), Positives = 418/448 (93%), Gaps = 0/448 (0%) |  |
| --- | --- | --- |
| Query 1 | MLHGASSRPATARKSSGLSGTVRIPGDKSISHRSFMFGGLASGETRITGLLEGEDVINTG | 60 |
| Sbjct 1 | MLHG++ RPATARKS+GL+G+VRIPGDKSISHRSFMFGGLASGETRITGLLEGEDVINTG MLHGSAPRPATARKSAGLAGSVRIPGDKSISHRSFMFGGLASGETRITGLLEGEDVINTG | 60 |
| Query 61 | KAMQAMGARIRKEGDTWIIDGVGNGGLLAPEAPLDFGNAATGCRLTMGLVGVYDFDSTFI | 120 |
| Sbjct 61 | +AMQAMGARIRKEG W+I+G GNG LLAP+APLDFGNA TG RLTMGLVG YDF STFI RAMQAMGARIRKEGTQWVIEGTGNGALLAPDAPLDFGNAGTGVRLTMGLVGTYDFRSTFI | 120 |
| Query 121 | GDASLTKRPMGRVLNPLREMGVQVKSEDGDRLPVTLRGPKTPTPITYRVPMASAQVKSAV | 180 |
| Sbjct 121 | GDASL+KRPMGRVL PLREMGVQV + +GDRLPVTLRGP TP+PI YRVPMASAQVKSAV GDASLSKRPMGRVLAPLREMGVQVSASEGDRLPVTLRGPGTPSPIRYRVPMASAQVKSAV | 180 |
| Query 181 | LLAGLNTPGITTVIEPIMTRDHTEKMLQGFGANLTVETDADGVRTIRLEGRGKLTGQVID | 240 |
| Sbjct 181 | LLAGLNTPG+TTVIEP+MTRDHTEKMLQGFGA L+VETD DGVRTIRLEGRGKLTGQVID LLAGLNTPGVTTVIEPVMTRDHTEKMLQGFGAALSVETDEDGVRTIRLEGRGKLTGQVID | 240 |
| Query 241 | VPGDPSSTAFPLVAALLVPGSDVTILNVLMNPTRTGLILTLQEMGADIEVINPRLAGGED | 300 |
| Sbjct 241 | VPGDPSSTAFPLVAALLVPGSD+TI+NVLMNPTRTGLILTLQEMGADI+V + RLAGGED VPGDPSSTAFPLVAALLVPGSDITIVNVLMNPTRTGLILTLQEMGADIQVAHARLAGGED | 300 |
| Query 301 | VADLRVRSSTLKGVTVPEDRAPSMIDEYPILavaaafaegaTVMNGLEELRVKESDRLSA | 360 |
| Sbjct 301 | VADLRVR S LKGVTVPEDRAPSMIDEYPILA+AA+FAEGATVM GLEELRVKESDRLSA VADLRVRHSELKGVTVPEDRAPSMIDEYPILAIAASFAEGATVMKGLEELRVKESDRLSA | 360 |
| Query 361 | VANGLKLNGVDCDEGETSLVVRGRPDGKGLGNASGAAVATHLDHRIAMSFLVMGLVSENP | 420 |
| Sbjct 361 | VA+GLKLNGVDCDEGE LVVRGRPDGKGLGNA+ V+THLDHRIAMSFLVMGL SE+P VADGLKLNGVDCDEGEDFLVVRGRPDGKGLGNAADGRVSTHLDHRIAMSFLVMGLASEHP | 420 |
| Query 421  Sbjct 421 | VTVDDATMIATSFPEFMDLMAGLGAKIE 448 VT+DDA MIATSFPEFM LM GLGAKIE VTIDDAAMIATSFPEFMQLMTGLGAKIE 448 |  |

>WP_072640868.1 3-phosphoshikimate 1-carboxyvinyltransferase [Rhizobium leguminosarum] API53844.1 3-phosphoshikimate 1-carboxyvinyltransferase [Rhizobium leguminosarum]

Length=452

Score = 772 bits (1994), Expect = 0.0, Method: Compositional matrix adjust.

| Identities | = 391/449 (87%), Positives = 415/449 (92%), Gaps = 0/449 (0%) |  |
| --- | --- | --- |
| Query 1 | MLHGASSRPATARKSSGLSGTVRIPGDKSISHRSFMFGGLASGETRITGLLEGEDVINTG | 60 |
| Sbjct 1 | MLHG++ +PATARKS+GL+G+VRIPGDKSISHRSFMFGGLASGETRITGLLEGEDVINTG MLHGSAPKPATARKSAGLAGSVRIPGDKSISHRSFMFGGLASGETRITGLLEGEDVINTG | 60 |
| Query 61 | KAMQAMGARIRKEGDTWIIDGVGNGGLLAPEAPLDFGNAATGCRLTMGLVGVYDFDSTFI | 120 |
| Sbjct 61 | +AMQAMGARIRK+G W+IDG GNG LLAP+APLDFGNA TG RLTMGLV YDF STFI RAMQAMGARIRKDGAQWVIDGTGNGALLAPDAPLDFGNAGTGVRLTMGLVSTYDFRSTFI | 120 |
| Query 121 | GDASLTKRPMGRVLNPLREMGVQVKSEDGDRLPVTLRGPKTPTPITYRVPMASAQVKSAV | 180 |
| Sbjct 121 | GDASL+KRPMGRVLNPLREMGVQV + +GDRLPVTLRGP TP+PI YRVPMASAQVKSAV  GDASLSKRPMGRVLNPLREMGVQVSASEGDRLPVTLRGPGTPSPIRYRVPMASAQVKSAV | 180 |

| Query | 181 | LLAGLNTPGITTVIEPIMTRDHTEKMLQGFGANLTVETDADGVRTIRLEGRGKLTGQVID | 240 |
| --- | --- | --- | --- |
| Sbjct | 181 | LLAGLNTPG+TTVIEP+MTRDHTEKMLQGFGA L+VETD DGVRTIRLEGRGKL GQVID LLAGLNTPGVTTVIEPVMTRDHTEKMLQGFGAALSVETDGDGVRTIRLEGRGKLKGQVID | 240 |
| Query | 241 | VPGDPSSTAFPLVAALLVPGSDVTILNVLMNPTRTGLILTLQEMGADIEVINPRLAGGED | 300 |
| Sbjct | 241 | VPGDPSSTAFPLVAALLVPGSD+TI+NVLMNPTRTGLILTLQEMGADIEV N RLAGGED VPGDPSSTAFPLVAALLVPGSDITIVNVLMNPTRTGLILTLQEMGADIEVANARLAGGED | 300 |
| Query | 301 | VADLRVRSSTLKGVTVPEDRAPSMIDEYPILavaaafaegaTVMNGLEELRVKESDRLSA | 360 |
| Sbjct | 301 | VADLRVR S LKGVTVPEDRAPSMIDEYPILAVAA FAEG TVM GLEELRVKESDRLS VADLRVRHSELKGVTVPEDRAPSMIDEYPILAVAACFAEGVTVMKGLEELRVKESDRLST | 360 |
| Query | 361 | VANGLKLNGVDCDEGETSLVVRGRPDGKGLGNASGAAVATHLDHRIAMSFLVMGLVSENP | 420 |
| Sbjct | 361 | VA+GLKLNGVDCDEGE LVVRGRPDGKGLGNA+G V+THLDHRIAMSFLVMGL SE+ VADGLKLNGVDCDEGEDFLVVRGRPDGKGLGNAAGDKVSTHLDHRIAMSFLVMGLASEHA | 420 |
| Query  Sbjct | 421  421 | VTVDDATMIATSFPEFMDLMAGLGAKIEL 449 VT+DDA MIATSFPEFM LM GLGAKIEL VTIDDAAMIATSFPEFMQLMTGLGAKIEL 449 |  |

>WP_130718486.1 3-phosphoshikimate 1-carboxyvinyltransferase [Rhizobium leguminosarum]

NKK42224.1 3-phosphoshikimate 1-carboxyvinyltransferase [Rhizobium leguminosarum bv. viciae]

TAY55065.1 3-phosphoshikimate 1-carboxyvinyltransferase [Rhizobium leguminosarum] Length=452

Score = 772 bits (1994), Expect = 0.0, Method: Compositional matrix adjust.

| Identities | = 388/448 (87%), Positives = 418/448 (93%), Gaps = 0/448 (0%) |  |
| --- | --- | --- |
| Query 1 | MLHGASSRPATARKSSGLSGTVRIPGDKSISHRSFMFGGLASGETRITGLLEGEDVINTG | 60 |
| Sbjct 1 | ML G++ RPATARKS+GL+G+VRIPGDKSISHRSFMFGGLASGETRITGLLEGEDVINTG MLQGSAPRPATARKSAGLTGSVRIPGDKSISHRSFMFGGLASGETRITGLLEGEDVINTG | 60 |
| Query 61 | KAMQAMGARIRKEGDTWIIDGVGNGGLLAPEAPLDFGNAATGCRLTMGLVGVYDFDSTFI | 120 |
| Sbjct 61 | +AMQAMGARIRKEG+ W+I+G GNG LLAP+APLDFGNA TG RLTMGLVG YDF STFI RAMQAMGARIRKEGEQWVIEGTGNGALLAPDAPLDFGNAGTGVRLTMGLVGTYDFRSTFI | 120 |
| Query 121 | GDASLTKRPMGRVLNPLREMGVQVKSEDGDRLPVTLRGPKTPTPITYRVPMASAQVKSAV | 180 |
| Sbjct 121 | GDASL+KRPMGRVLNPLREMGVQV + +GDRLPVTLRGP TP+PI YRVPMASAQVKSAV GDASLSKRPMGRVLNPLREMGVQVSASEGDRLPVTLRGPGTPSPIRYRVPMASAQVKSAV | 180 |
| Query 181 | LLAGLNTPGITTVIEPIMTRDHTEKMLQGFGANLTVETDADGVRTIRLEGRGKLTGQVID | 240 |
| Sbjct 181 | LLAGLNTPG+TTVIEP+MTRDHTEKMLQGFGA L+VETD +GVRTIRLEGRGKLTGQVI+ LLAGLNTPGVTTVIEPVMTRDHTEKMLQGFGAALSVETDEEGVRTIRLEGRGKLTGQVIN | 240 |
| Query 241 | VPGDPSSTAFPLVAALLVPGSDVTILNVLMNPTRTGLILTLQEMGADIEVINPRLAGGED | 300 |
| Sbjct 241 | +P DPSSTAFPLVAALLVPGSD+TI+NVLMNPTRTGLILTLQEMGADIEV+N RLAGGED IPSDPSSTAFPLVAALLVPGSDITIVNVLMNPTRTGLILTLQEMGADIEVLNARLAGGED | 300 |
| Query 301 | VADLRVRSSTLKGVTVPEDRAPSMIDEYPILavaaafaegaTVMNGLEELRVKESDRLSA | 360 |
| Sbjct 301 | VADLRVR S LKGVTVPEDRAPSMIDEYPILAVAA FAEG TVM GLEELRVKESDRLSA VADLRVRHSELKGVTVPEDRAPSMIDEYPILAVAACFAEGVTVMKGLEELRVKESDRLSA | 360 |
| Query 361 | VANGLKLNGVDCDEGETSLVVRGRPDGKGLGNASGAAVATHLDHRIAMSFLVMGLVSENP | 420 |
| Sbjct 361 | VA+GLKLNGVDCDEGE L+VRGRPDGKGLGNA+G V+THLDHRIAMSFLVMGL SE+P VADGLKLNGVDCDEGEDFLIVRGRPDGKGLGNAAGDKVSTHLDHRIAMSFLVMGLASEHP | 420 |
| Query 421  Sbjct 421 | VTVDDATMIATSFPEFMDLMAGLGAKIE 448 VT+DDA MIATSFPEFM LM GLGA+IE VTIDDAAMIATSFPEFMQLMTGLGARIE 448 |  |

>WP_145638220.1 3-phosphoshikimate 1-carboxyvinyltransferase [Neorhizobium alkalisoli] TWF52819.1 3-phosphoshikimate 1-carboxyvinyltransferase [Neorhizobium alkalisoli]

Length=451

Score = 772 bits (1993), Expect = 0.0, Method: Compositional matrix adjust. Identities = 394/448 (88%), Positives = 413/448 (92%), Gaps = 0/448 (0%)

Query 1	MLHGASSRPATARKSSGLSGTVRIPGDKSISHRSFMFGGLASGETRITGLLEGEDVINTG 60 M HG + +PA ++KS LSGTVRIPGDKSISHRSFMFGGLASGETRITGLLEGEDVINTG

| Sbjct | 1 | MSHGIALKPARSKKSENLSGTVRIPGDKSISHRSFMFGGLASGETRITGLLEGEDVINTG | 60 |
| --- | --- | --- | --- |
| Query | 61 | KAMQAMGARIRKEGDTWIIDGVGNGGLLAPEAPLDFGNAATGCRLTMGLVGVYDFDSTFI | 120 |
| Sbjct | 61 | KAM+AMGA+I K G WII G GNG LLAPEAPLDFGNA TGCRLTMGLVGVYDFDSTFI KAMRAMGAKIEKVGAEWIIQGTGNGALLAPEAPLDFGNAGTGCRLTMGLVGVYDFDSTFI | 120 |
| Query | 121 | GDASLTKRPMGRVLNPLREMGVQVKSEDGDRLPVTLRGPKTPTPITYRVPMASAQVKSAV | 180 |
| Sbjct | 121 | GDASLTKRPMGRVLNPLREMGVQVK+ DGDRLPVTLRGPKTP PI YRVPMASAQVKSAV GDASLTKRPMGRVLNPLREMGVQVKAADGDRLPVTLRGPKTPNPIVYRVPMASAQVKSAV | 180 |
| Query | 181 | LLAGLNTPGITTVIEPIMTRDHTEKMLQGFGANLTVETDADGVRTIRLEGRGKLTGQVID | 240 |
| Sbjct | 181 | LLAGLNTPGITTVIEP+MTRDHTEKMLQGFGANLTVETDADGVRTIRLEGRGKLTGQVID LLAGLNTPGITTVIEPVMTRDHTEKMLQGFGANLTVETDADGVRTIRLEGRGKLTGQVID | 240 |
| Query | 241 | VPGDPSSTAFPLVAALLVPGSDVTILNVLMNPTRTGLILTLQEMGADIEVINPRLAGGED | 300 |
| Sbjct | 241 | VPGDPSSTAFPLVAALLVPGSDVTI NVLMNPTRTGLILTLQEMGADIEV+N R AGGED VPGDPSSTAFPLVAALLVPGSDVTIKNVLMNPTRTGLILTLQEMGADIEVMNARSAGGED | 300 |
| Query | 301 | VADLRVRSSTLKGVTVPEDRAPSMIDEYPILavaaafaegaTVMNGLEELRVKESDRLSA | 360 |
| Sbjct | 301 | VADLRVR S L+GVTVP DRAPSMIDEYP+LAVAAA+AEG T M GLEELRVKESDRLSA VADLRVRGSELRGVTVPADRAPSMIDEYPVLAVAAAYAEGTTTMLGLEELRVKESDRLSA | 360 |
| Query | 361 | VANGLKLNGVDCDEGETSLVVRGRPDGKGLGNASGAAVATHLDHRIAMSFLVMGLVSENP | 420 |
| Sbjct | 361 | VA GLKLNGVDCDEG+ +LVVRGRP GKG GNASGAAV THLDHRIAMSFLV+GL SE+ VAEGLKLNGVDCDEGKETLVVRGRPGGKGYGNASGAAVVTHLDHRIAMSFLVLGLASEHE | 420 |
| Query  Sbjct | 421  421 | VTVDDATMIATSFPEFMDLMAGLGAKIE 448 VTVDD ++IATSFPEFMDLM GLGA+IE VTVDDTSIIATSFPEFMDLMTGLGARIE 448 |  |

>WP_131631191.1 3-phosphoshikimate 1-carboxyvinyltransferase [Rhizobium leguminosarum] MBA9030704.1 3-phosphoshikimate 1-carboxyvinyltransferase [Rhizobium leguminosarum]

NKK90249.1 3-phosphoshikimate 1-carboxyvinyltransferase [Rhizobium leguminosarum bv. viciae]

TBZ30767.1 3-phosphoshikimate 1-carboxyvinyltransferase [Rhizobium leguminosarum bv. viciae]

Length=452

Score = 772 bits (1993), Expect = 0.0, Method: Compositional matrix adjust.

| Identities | = 388/448 (87%), Positives = 417/448 (93%), Gaps = 0/448 (0%) |  |
| --- | --- | --- |
| Query 1 | MLHGASSRPATARKSSGLSGTVRIPGDKSISHRSFMFGGLASGETRITGLLEGEDVINTG | 60 |
| Sbjct 1 | MLHG++ RPATARKS GL+G+VRIPGDKSISHRSFMFGGLASGETRITGLLEGEDVINTG MLHGSAPRPATARKSPGLTGSVRIPGDKSISHRSFMFGGLASGETRITGLLEGEDVINTG | 60 |
| Query 61 | KAMQAMGARIRKEGDTWIIDGVGNGGLLAPEAPLDFGNAATGCRLTMGLVGVYDFDSTFI | 120 |
| Sbjct 61 | +AMQAMGARIRK G+ W+I+G GNG LLAP+APLDFGNA TG RLTMGLVG YDF STFI RAMQAMGARIRKVGEQWLIEGTGNGALLAPDAPLDFGNAGTGVRLTMGLVGAYDFRSTFI | 120 |
| Query 121 | GDASLTKRPMGRVLNPLREMGVQVKSEDGDRLPVTLRGPKTPTPITYRVPMASAQVKSAV | 180 |
| Sbjct 121 | GDASL+KRPMGRVLNPLREMGVQV + +GDRLPVTLRGP TP+PI YRVPMASAQVKSAV GDASLSKRPMGRVLNPLREMGVQVSASEGDRLPVTLRGPGTPSPIRYRVPMASAQVKSAV | 180 |
| Query 181 | LLAGLNTPGITTVIEPIMTRDHTEKMLQGFGANLTVETDADGVRTIRLEGRGKLTGQVID | 240 |
| Sbjct 181 | LLAGLNTPG+TTVIEP+MTRDHTEKMLQGFGA L+VETD +GVRTIRLEGRGKLTGQVID LLAGLNTPGVTTVIEPVMTRDHTEKMLQGFGAALSVETDEEGVRTIRLEGRGKLTGQVID | 240 |
| Query 241 | VPGDPSSTAFPLVAALLVPGSDVTILNVLMNPTRTGLILTLQEMGADIEVINPRLAGGED | 300 |
| Sbjct 241 | +PGDPSSTAFPLVAALLVPGSD+TI+NVLMNPTRTGLILTLQEMGADIEV+N RLAGGED IPGDPSSTAFPLVAALLVPGSDITIVNVLMNPTRTGLILTLQEMGADIEVLNARLAGGED | 300 |
| Query 301 | VADLRVRSSTLKGVTVPEDRAPSMIDEYPILavaaafaegaTVMNGLEELRVKESDRLSA | 360 |
| Sbjct 301 | VAD+RVR S LKGVTVPEDRAPSMIDEYPILAV A FAEG TVM GLEELRVKESDRLSA VADMRVRHSELKGVTVPEDRAPSMIDEYPILAVVACFAEGVTVMKGLEELRVKESDRLSA | 360 |
| Query 361 | VANGLKLNGVDCDEGETSLVVRGRPDGKGLGNASGAAVATHLDHRIAMSFLVMGLVSENP | 420 |
| Sbjct 361 | VA+GLKLNGVDCDEGE L+VRGRPDGKGLGNA+G V+THLDHRIAMSFLVMGL SE+P VADGLKLNGVDCDEGEDFLIVRGRPDGKGLGNAAGDKVSTHLDHRIAMSFLVMGLASEHP | 420 |
| Query 421 | VTVDDATMIATSFPEFMDLMAGLGAKIE 448 VT+DDA MIATSFPEFM LM GLGA+IE |  |

Sbjct 421 VTIDDAAMIATSFPEFMQLMTGLGARIE 448

>WP_112905641.1 3-phosphoshikimate 1-carboxyvinyltransferase [Rhizobium leguminosarum] AXA40661.1 3-phosphoshikimate 1-carboxyvinyltransferase [Rhizobium leguminosarum]

Length=452

Score = 772 bits (1993), Expect = 0.0, Method: Compositional matrix adjust.

| Identities | = 389/448 (87%), Positives = 417/448 (93%), Gaps = 0/448 (0%) |  |
| --- | --- | --- |
| Query 1 | MLHGASSRPATARKSSGLSGTVRIPGDKSISHRSFMFGGLASGETRITGLLEGEDVINTG | 60 |
| Sbjct 1 | ML G++ RPATARKS+GL+G+VRIPGDKSISHRSFMFGGLASGETRITGLLEGEDVINTG MLQGSAPRPATARKSAGLTGSVRIPGDKSISHRSFMFGGLASGETRITGLLEGEDVINTG | 60 |
| Query 61 | KAMQAMGARIRKEGDTWIIDGVGNGGLLAPEAPLDFGNAATGCRLTMGLVGVYDFDSTFI | 120 |
| Sbjct 61 | +AMQAMGARIRKEG+ W+I+G GNG LLAP+APLDFGNA TG RLTMGLVG YDF STFI RAMQAMGARIRKEGEQWVIEGTGNGALLAPDAPLDFGNAGTGVRLTMGLVGTYDFRSTFI | 120 |
| Query 121 | GDASLTKRPMGRVLNPLREMGVQVKSEDGDRLPVTLRGPKTPTPITYRVPMASAQVKSAV | 180 |
| Sbjct 121 | GDASL+KRPMGRVLNPLREMGVQV + +GDRLPVTLRGP TP+PI YRVPMASAQVKSAV GDASLSKRPMGRVLNPLREMGVQVSASEGDRLPVTLRGPGTPSPIRYRVPMASAQVKSAV | 180 |
| Query 181 | LLAGLNTPGITTVIEPIMTRDHTEKMLQGFGANLTVETDADGVRTIRLEGRGKLTGQVID | 240 |
| Sbjct 181 | LLAGLNTPG+TTVIEP+MTRDHTEKMLQGFGA L+VETD DGVRTIRLEGRGKL GQVID LLAGLNTPGVTTVIEPVMTRDHTEKMLQGFGAALSVETDEDGVRTIRLEGRGKLKGQVID | 240 |
| Query 241 | VPGDPSSTAFPLVAALLVPGSDVTILNVLMNPTRTGLILTLQEMGADIEVINPRLAGGED | 300 |
| Sbjct 241 | +PGDPSSTAFPLVAALLVPGSD+TI+NVLMNPTRTGLILTLQEMGADIEV+N RLAGGED IPGDPSSTAFPLVAALLVPGSDITIVNVLMNPTRTGLILTLQEMGADIEVLNARLAGGED | 300 |
| Query 301 | VADLRVRSSTLKGVTVPEDRAPSMIDEYPILavaaafaegaTVMNGLEELRVKESDRLSA | 360 |
| Sbjct 301 | VADLRVR S LKGVTVPEDRAPSMIDEYPILAVAA FAEG TVM GLEELRVKESDRLSA VADLRVRHSELKGVTVPEDRAPSMIDEYPILAVAACFAEGVTVMKGLEELRVKESDRLSA | 360 |
| Query 361 | VANGLKLNGVDCDEGETSLVVRGRPDGKGLGNASGAAVATHLDHRIAMSFLVMGLVSENP | 420 |
| Sbjct 361 | VA+GLKLNGVDCDEGE L+VRGRPDGKGLGNA+ V+THLDHRIAMSFLVMGL SE+P VADGLKLNGVDCDEGEDFLIVRGRPDGKGLGNATDGKVSTHLDHRIAMSFLVMGLASEHP | 420 |
| Query 421  Sbjct 421 | VTVDDATMIATSFPEFMDLMAGLGAKIE 448 VT+DDA MIATSFPEFM LM GLGAKI+ VTIDDAAMIATSFPEFMQLMTGLGAKID 448 |  |

>WP_028002217.1 3-phosphoshikimate 1-carboxyvinyltransferase [Sinorhizobium arboris] PST26943.1 3-phosphoshikimate 1-carboxyvinyltransferase [Mesorhizobium plurifarium]

Length=456

Score = 771 bits (1992), Expect = 0.0, Method: Compositional matrix adjust.

| Identities | = 405/454 (89%), Positives = 428/454 (94%), Gaps = 0/454 (0%) |  |
| --- | --- | --- |
| Query 1 | MLHGASSRPATARKSSGLSGTVRIPGDKSISHRSFMFGGLASGETRITGLLEGEDVINTG | 60 |
| Sbjct 1 | M HG++ RPATAR+SS L GTVRIPGDKSISHRSFMFGGLA+GETRITGLLEGEDVINTG MSHGSNPRPATARRSSDLKGTVRIPGDKSISHRSFMFGGLAAGETRITGLLEGEDVINTG | 60 |
| Query 61 | KAMQAMGARIRKEGDTWIIDGVGNGGLLAPEAPLDFGNAATGCRLTMGLVGVYDFDSTFI | 120 |
| Sbjct 61 | KAMQAMGARIRKEGDTWIIDGVGNG LLAPEAPLDFGNA TGCRLTMGLVGVYDFDSTFI KAMQAMGARIRKEGDTWIIDGVGNGALLAPEAPLDFGNAGTGCRLTMGLVGVYDFDSTFI | 120 |
| Query 121 | GDASLTKRPMGRVLNPLREMGVQVKSEDGDRLPVTLRGPKTPTPITYRVPMASAQVKSAV | 180 |
| Sbjct 121 | GDASLTKRPMGRVL+PLR+MGVQVKS +GDRLPVTLRGPKT PI YRVPMASAQVKSAV GDASLTKRPMGRVLDPLRQMGVQVKSAEGDRLPVTLRGPKTANPIIYRVPMASAQVKSAV | 180 |
| Query 181 | LLAGLNTPGITTVIEPIMTRDHTEKMLQGFGANLTVETDADGVRTIRLEGRGKLTGQVID | 240 |
| Sbjct 181 | LLAGLNTPGITTV+EP+MTRDHTE+MLQGFGA+L VETDA+GVRTIRL+GRGKLTGQVID LLAGLNTPGITTVVEPVMTRDHTERMLQGFGADLAVETDAEGVRTIRLQGRGKLTGQVID | 240 |
| Query 241 | VPGDPSSTAFPLVAALLVPGSDVTILNVLMNPTRTGLILTLQEMGADIEVINPRLAGGED | 300 |
| Sbjct 241 | VPGDPSSTAFPLVAALLVPGSDV I NVLMNPTRTGLILTLQEMGADIEV+N RLAGGED VPGDPSSTAFPLVAALLVPGSDVIIFNVLMNPTRTGLILTLQEMGADIEVLNRRLAGGED | 300 |
| Query 301 | VADLRVRSSTLKGVTVPEDRAPSMIDEYPILavaaafaegaTVMNGLEELRVKESDRLSA VADLRVR S LKGVTVPE+RAPSMIDEYP+LAVAAAFAEGATVMNGLEELRVKESDRLSA | 360 |

| Sbjct | 301 | VADLRVRYSELKGVTVPEERAPSMIDEYPVLAVAAAFAEGATVMNGLEELRVKESDRLSA | 360 |
| --- | --- | --- | --- |
| Query | 361 | VANGLKLNGVDCDEGETSLVVRGRPDGKGLGNASGAAVATHLDHRIAMSFLVMGLVSENP | 420 |
| Sbjct | 361 | VA+GLKLNGVDCDEGE SLVVRGRP G+GLGN SG V THLDHRIAMSFLVMGL SE+P VADGLKLNGVDCDEGEASLVVRGRPGGRGLGNISGGQVKTHLDHRIAMSFLVMGLASEHP | 420 |
| Query  Sbjct | 421  421 | VTVDDATMIATSFPEFMDLMAGLGAKIELSDTKA 454 VTVDDATMIATSFPEF+ LM GLGAKIE +++KA VTVDDATMIATSFPEFLGLMTGLGAKIEEAESKA 454 |  |

>WP_131719773.1 3-phosphoshikimate 1-carboxyvinyltransferase [Rhizobium leguminosarum]

NKN01289.1 3-phosphoshikimate 1-carboxyvinyltransferase [Rhizobium leguminosarum bv. viciae]

TCA02164.1 3-phosphoshikimate 1-carboxyvinyltransferase [Rhizobium leguminosarum bv. viciae]

Length=452

Score = 771 bits (1991), Expect = 0.0, Method: Compositional matrix adjust.

| Identities | = 388/448 (87%), Positives = 418/448 (93%), Gaps = 0/448 (0%) |  |
| --- | --- | --- |
| Query 1 | MLHGASSRPATARKSSGLSGTVRIPGDKSISHRSFMFGGLASGETRITGLLEGEDVINTG | 60 |
| Sbjct 1 | MLHG++ +PATARKS+GL+G+VRIPGDKSISHRSFMFGGLASGETRITGLLEGEDVINTG MLHGSAPKPATARKSAGLAGSVRIPGDKSISHRSFMFGGLASGETRITGLLEGEDVINTG | 60 |
| Query 61 | KAMQAMGARIRKEGDTWIIDGVGNGGLLAPEAPLDFGNAATGCRLTMGLVGVYDFDSTFI | 120 |
| Sbjct 61 | +AMQAMGARIRK+G W+IDG GNG LLAP+APLDFGNA TG RLTMGLVG YDF STFI RAMQAMGARIRKDGAQWVIDGTGNGALLAPDAPLDFGNAGTGVRLTMGLVGTYDFRSTFI | 120 |
| Query 121 | GDASLTKRPMGRVLNPLREMGVQVKSEDGDRLPVTLRGPKTPTPITYRVPMASAQVKSAV | 180 |
| Sbjct 121 | GDASL+KRPMGRVLNPLREMGVQV + +GDRLPVTLRGP TP+PI YRVPMASAQVKSAV GDASLSKRPMGRVLNPLREMGVQVSASEGDRLPVTLRGPGTPSPIRYRVPMASAQVKSAV | 180 |
| Query 181 | LLAGLNTPGITTVIEPIMTRDHTEKMLQGFGANLTVETDADGVRTIRLEGRGKLTGQVID | 240 |
| Sbjct 181 | LLAGLNTPG+TTVIEP+MTRDHTEKMLQGFGA L+VETD++GVRTIRLEGRGKL GQVID LLAGLNTPGVTTVIEPVMTRDHTEKMLQGFGAALSVETDSEGVRTIRLEGRGKLKGQVID | 240 |
| Query 241 | VPGDPSSTAFPLVAALLVPGSDVTILNVLMNPTRTGLILTLQEMGADIEVINPRLAGGED | 300 |
| Sbjct 241 | +PGDPSSTAFPLVAALLVPGSD+TI+NVLMNPTRTGLILTLQEMGA+IEV N RLAGGED IPGDPSSTAFPLVAALLVPGSDITIVNVLMNPTRTGLILTLQEMGANIEVANARLAGGED | 300 |
| Query 301 | VADLRVRSSTLKGVTVPEDRAPSMIDEYPILavaaafaegaTVMNGLEELRVKESDRLSA | 360 |
| Sbjct 301 | VADLRVR S LKGVTVPEDRAPSMIDEYPILAVAA FAEGAT+M GLEELRVKESDRLSA VADLRVRHSELKGVTVPEDRAPSMIDEYPILAVAACFAEGATIMKGLEELRVKESDRLSA | 360 |
| Query 361 | VANGLKLNGVDCDEGETSLVVRGRPDGKGLGNASGAAVATHLDHRIAMSFLVMGLVSENP | 420 |
| Sbjct 361 | VA+GLKLNGVDCDEGE L+VRGRPDGKGLGNA+ V+THLDHRIAMSFLVMGL SE+P VADGLKLNGVDCDEGEDFLIVRGRPDGKGLGNAADGRVSTHLDHRIAMSFLVMGLASEHP | 420 |
| Query 421  Sbjct 421 | VTVDDATMIATSFPEFMDLMAGLGAKIE 448 VT+DDA MIATSFPEFM LM GLGAKIE VTIDDAAMIATSFPEFMQLMTGLGAKIE 448 |  |

>WP_130712832.1 3-phosphoshikimate 1-carboxyvinyltransferase [Rhizobium leguminosarum] TAY69169.1 3-phosphoshikimate 1-carboxyvinyltransferase [Rhizobium leguminosarum]

Length=452

Score = 771 bits (1991), Expect = 0.0, Method: Compositional matrix adjust.

| Identities | = 384/449 (86%), Positives = 422/449 (94%), Gaps = 0/449 (0%) |  |
| --- | --- | --- |
| Query 1 | MLHGASSRPATARKSSGLSGTVRIPGDKSISHRSFMFGGLASGETRITGLLEGEDVINTG | 60 |
| Sbjct 1 | ML+G++ +PATARKS+GL+G+VRIPGDKSISHRSFMFGGLASG+TRITGLLEGEDVINTG MLNGSAPKPATARKSAGLAGSVRIPGDKSISHRSFMFGGLASGQTRITGLLEGEDVINTG | 60 |
| Query 61 | KAMQAMGARIRKEGDTWIIDGVGNGGLLAPEAPLDFGNAATGCRLTMGLVGVYDFDSTFI | 120 |
| Sbjct 61 | +AMQAMGARIRKEG W+I+G GNG LLAP+APLDFGNA TG RLTMGLVG YDF STF RAMQAMGARIRKEGAQWVIEGTGNGALLAPDAPLDFGNAGTGVRLTMGLVGTYDFHSTFT | 120 |
| Query 121 | GDASLTKRPMGRVLNPLREMGVQVKSEDGDRLPVTLRGPKTPTPITYRVPMASAQVKSAV | 180 |
| Sbjct 121 | GDASL+KRPMGRVLNPLREMGVQ+ + +GDRLPVTL+GP TP+PI YRVPMASAQVKSAV  GDASLSKRPMGRVLNPLREMGVQISASEGDRLPVTLKGPGTPSPIRYRVPMASAQVKSAV | 180 |

| Query | 181 | LLAGLNTPGITTVIEPIMTRDHTEKMLQGFGANLTVETDADGVRTIRLEGRGKLTGQVID | 240 |
| --- | --- | --- | --- |
| Sbjct | 181 | LLAGLNTPG+TTVIEP+MTRDHTEKMLQGFGA L+VETD++GVRTIRLEGRG+LTGQVID LLAGLNTPGVTTVIEPVMTRDHTEKMLQGFGAALSVETDSEGVRTIRLEGRGRLTGQVID | 240 |
| Query | 241 | VPGDPSSTAFPLVAALLVPGSDVTILNVLMNPTRTGLILTLQEMGADIEVINPRLAGGED | 300 |
| Sbjct | 241 | +PGDPSSTAFPLVAALLVPGSD+TI+NVLMNPTRTGLILTLQEMGADIEV+NPRLAGGED IPGDPSSTAFPLVAALLVPGSDITIVNVLMNPTRTGLILTLQEMGADIEVLNPRLAGGED | 300 |
| Query | 301 | VADLRVRSSTLKGVTVPEDRAPSMIDEYPILavaaafaegaTVMNGLEELRVKESDRLSA | 360 |
| Sbjct | 301 | VADLRVR S LKGVTVPE+RAPSMIDEYPILAVAA FAEGAT+M GLEELRVKESDRLSA VADLRVRHSELKGVTVPEERAPSMIDEYPILAVAACFAEGATIMKGLEELRVKESDRLSA | 360 |
| Query | 361 | VANGLKLNGVDCDEGETSLVVRGRPDGKGLGNASGAAVATHLDHRIAMSFLVMGLVSENP | 420 |
| Sbjct | 361 | VA+GLK+NGVDCDEGE L+VRGRPDGKGLGNA+G V+THLDHRIAMSFLVMGL S++P VADGLKINGVDCDEGEDFLIVRGRPDGKGLGNAAGGRVSTHLDHRIAMSFLVMGLASQHP | 420 |
| Query  Sbjct | 421  421 | VTVDDATMIATSFPEFMDLMAGLGAKIEL 449 VT+DDA MIATSFPEFM LM GLGAKIEL VTIDDAAMIATSFPEFMQLMTGLGAKIEL 449 |  |

>WP_017962713.1 3-phosphoshikimate 1-carboxyvinyltransferase [Rhizobium leguminosarum]

NKM44955.1 3-phosphoshikimate 1-carboxyvinyltransferase [Rhizobium leguminosarum bv. viciae]

TBY75579.1 3-phosphoshikimate 1-carboxyvinyltransferase [Rhizobium leguminosarum bv. viciae]

TBZ08871.1 3-phosphoshikimate 1-carboxyvinyltransferase [Rhizobium leguminosarum bv. viciae]

TBZ23168.1 3-phosphoshikimate 1-carboxyvinyltransferase [Rhizobium leguminosarum bv. viciae]

Length=452

Score = 771 bits (1991), Expect = 0.0, Method: Compositional matrix adjust.

| Identities | = 390/449 (87%), Positives = 417/449 (93%), Gaps = 0/449 (0%) |  |
| --- | --- | --- |
| Query 1 | MLHGASSRPATARKSSGLSGTVRIPGDKSISHRSFMFGGLASGETRITGLLEGEDVINTG | 60 |
| Sbjct 1 | ML+G++ +PATARKS+GL+G+VRIPGDKSISHRSFMFGGLASGETRITGLLEGEDVINTG MLNGSAPKPATARKSAGLAGSVRIPGDKSISHRSFMFGGLASGETRITGLLEGEDVINTG | 60 |
| Query 61 | KAMQAMGARIRKEGDTWIIDGVGNGGLLAPEAPLDFGNAATGCRLTMGLVGVYDFDSTFI | 120 |
| Sbjct 61 | +AMQAMGARIRK+G W+IDG GNG LLAP+APLDFGNA TG RLTMGLVG YDF STFI RAMQAMGARIRKDGAQWVIDGTGNGALLAPDAPLDFGNAGTGVRLTMGLVGTYDFHSTFI | 120 |
| Query 121 | GDASLTKRPMGRVLNPLREMGVQVKSEDGDRLPVTLRGPKTPTPITYRVPMASAQVKSAV | 180 |
| Sbjct 121 | GDASL+KRPMGRVLNPLREMGVQV + +GDRLPVTLRGP TP+PI YRVPMASAQVKSAV GDASLSKRPMGRVLNPLREMGVQVSASEGDRLPVTLRGPGTPSPIRYRVPMASAQVKSAV | 180 |
| Query 181 | LLAGLNTPGITTVIEPIMTRDHTEKMLQGFGANLTVETDADGVRTIRLEGRGKLTGQVID | 240 |
| Sbjct 181 | LLAGLNTPG+TTVIEP+MTRDHTEKMLQGFGA L+VETD DGVRTIRLEGRG+LTGQVID LLAGLNTPGVTTVIEPVMTRDHTEKMLQGFGAALSVETDGDGVRTIRLEGRGRLTGQVID | 240 |
| Query 241 | VPGDPSSTAFPLVAALLVPGSDVTILNVLMNPTRTGLILTLQEMGADIEVINPRLAGGED | 300 |
| Sbjct 241 | VPGDPSSTAFPLVAALLVPGSD+TI+NVLMNPTRTGLILTLQEMGADIEV N RLAGGED VPGDPSSTAFPLVAALLVPGSDITIVNVLMNPTRTGLILTLQEMGADIEVANARLAGGED | 300 |
| Query 301 | VADLRVRSSTLKGVTVPEDRAPSMIDEYPILavaaafaegaTVMNGLEELRVKESDRLSA | 360 |
| Sbjct 301 | VADLRVR S LKGVTVPEDRAPSMIDEYPILAVAA FAEG T+M GLEELRVKESDRLSA VADLRVRHSELKGVTVPEDRAPSMIDEYPILAVAACFAEGVTIMKGLEELRVKESDRLSA | 360 |
| Query 361 | VANGLKLNGVDCDEGETSLVVRGRPDGKGLGNASGAAVATHLDHRIAMSFLVMGLVSENP | 420 |
| Sbjct 361 | VA+GLKLNGVDCDEGE LVVRGRPDGKGLGNA+ V+THLDHRIAMSFLVMGL SE+ VADGLKLNGVDCDEGEDFLVVRGRPDGKGLGNAADEKVSTHLDHRIAMSFLVMGLASEHA | 420 |
| Query 421  Sbjct 421 | VTVDDATMIATSFPEFMDLMAGLGAKIEL 449 VT+DDA MIATSFPEFM LM GLGAKIEL VTIDDAAMIATSFPEFMQLMTGLGAKIEL 449 |  |

>WP_130809592.1 3-phosphoshikimate 1-carboxyvinyltransferase [Rhizobium leguminosarum]

NKL61645.1 3-phosphoshikimate 1-carboxyvinyltransferase [Rhizobium leguminosarum bv. viciae]

TBD07103.1 3-phosphoshikimate 1-carboxyvinyltransferase [Rhizobium leguminosarum] Length=452

Score = 771 bits (1991), Expect = 0.0, Method: Compositional matrix adjust.

| Identities | = 388/448 (87%), Positives = 416/448 (93%), Gaps = 0/448 (0%) |  |
| --- | --- | --- |
| Query 1 | MLHGASSRPATARKSSGLSGTVRIPGDKSISHRSFMFGGLASGETRITGLLEGEDVINTG | 60 |
| Sbjct 1 | MLHG++ RPATARKS+GL+G+VRIPGDKSISHRSFMFGGLASGETRITGLLEGEDVINTG MLHGSAPRPATARKSAGLTGSVRIPGDKSISHRSFMFGGLASGETRITGLLEGEDVINTG | 60 |
| Query 61 | KAMQAMGARIRKEGDTWIIDGVGNGGLLAPEAPLDFGNAATGCRLTMGLVGVYDFDSTFI | 120 |
| Sbjct 61 | +AMQAMGARIRKEG+ W+I+G GNG LLAP+ PLDFGNA TG RLTMGLVG YDF STF RAMQAMGARIRKEGEQWVIEGTGNGALLAPDTPLDFGNAGTGVRLTMGLVGTYDFRSTFT | 120 |
| Query 121 | GDASLTKRPMGRVLNPLREMGVQVKSEDGDRLPVTLRGPKTPTPITYRVPMASAQVKSAV | 180 |
| Sbjct 121 | GDASL+KRPMGRVLNPLREMGVQV + +GDRLPVTLRGP TP+PI YRVPMASAQVKSAV GDASLSKRPMGRVLNPLREMGVQVSASEGDRLPVTLRGPGTPSPIRYRVPMASAQVKSAV | 180 |
| Query 181 | LLAGLNTPGITTVIEPIMTRDHTEKMLQGFGANLTVETDADGVRTIRLEGRGKLTGQVID | 240 |
| Sbjct 181 | LLAGLNTPG+TTVIEP+MTRDHTEKMLQGFGA L+VETD DGVRTIRLEGRGKL GQVID LLAGLNTPGVTTVIEPVMTRDHTEKMLQGFGAALSVETDEDGVRTIRLEGRGKLKGQVID | 240 |
| Query 241 | VPGDPSSTAFPLVAALLVPGSDVTILNVLMNPTRTGLILTLQEMGADIEVINPRLAGGED | 300 |
| Sbjct 241 | VPGDPSSTAFPLVAALLVPGSD+TI+NVLMNPTRTGLILTLQEMGADIEV+N RLAGGED VPGDPSSTAFPLVAALLVPGSDITIVNVLMNPTRTGLILTLQEMGADIEVLNTRLAGGED | 300 |
| Query 301 | VADLRVRSSTLKGVTVPEDRAPSMIDEYPILavaaafaegaTVMNGLEELRVKESDRLSA | 360 |
| Sbjct 301 | VADLRVR S LKGVTVPEDRAPSMIDEYPILAVAA FA+G TVM GLEELRVKESDRLSA VADLRVRHSELKGVTVPEDRAPSMIDEYPILAVAACFADGVTVMKGLEELRVKESDRLSA | 360 |
| Query 361 | VANGLKLNGVDCDEGETSLVVRGRPDGKGLGNASGAAVATHLDHRIAMSFLVMGLVSENP | 420 |
| Sbjct 361 | VA+GLKLNGVDCDEG+ L+VRGRPDGKGLGNA+ V+THLDHRIAMSFLVMGL SE+P VADGLKLNGVDCDEGKDFLIVRGRPDGKGLGNAADEKVSTHLDHRIAMSFLVMGLASEHP | 420 |
| Query 421  Sbjct 421 | VTVDDATMIATSFPEFMDLMAGLGAKIE 448 VT+DDA MIATSFPEFM LM GLGAKIE VTIDDAAMIATSFPEFMQLMTGLGAKIE 448 |  |

>WP_065281752.1 3-phosphoshikimate 1-carboxyvinyltransferase [Rhizobium leguminosarum] ANP87819.1 3-phosphoshikimate 1-carboxyvinyltransferase [Rhizobium leguminosarum]

Length=452

Score = 771 bits (1990), Expect = 0.0, Method: Compositional matrix adjust.

| Identities | = 390/449 (87%), Positives = 417/449 (93%), Gaps = 0/449 (0%) |  |
| --- | --- | --- |
| Query 1 | MLHGASSRPATARKSSGLSGTVRIPGDKSISHRSFMFGGLASGETRITGLLEGEDVINTG | 60 |
| Sbjct 1 | MLHG++ +PATARKS+GL+G+VRIPGDKSISHRSFMFGGLASGETRITGLLEGEDVINTG MLHGSAPKPATARKSAGLAGSVRIPGDKSISHRSFMFGGLASGETRITGLLEGEDVINTG | 60 |
| Query 61 | KAMQAMGARIRKEGDTWIIDGVGNGGLLAPEAPLDFGNAATGCRLTMGLVGVYDFDSTFI | 120 |
| Sbjct 61 | +AMQAMGARIRK+G W+I+G GNG LLAP+APLDFGNA TG RLTMGLVG YDF STFI RAMQAMGARIRKDGAQWVIEGTGNGALLAPDAPLDFGNAGTGVRLTMGLVGTYDFRSTFI | 120 |
| Query 121 | GDASLTKRPMGRVLNPLREMGVQVKSEDGDRLPVTLRGPKTPTPITYRVPMASAQVKSAV | 180 |
| Sbjct 121 | GDASL+KRPMGRVLNPLREMGVQV +GDRLPVTLRGP TP+PI YRVPMASAQVKSAV GDASLSKRPMGRVLNPLREMGVQVSVSEGDRLPVTLRGPGTPSPIRYRVPMASAQVKSAV | 180 |
| Query 181 | LLAGLNTPGITTVIEPIMTRDHTEKMLQGFGANLTVETDADGVRTIRLEGRGKLTGQVID | 240 |
| Sbjct 181 | LLAGLNTPG+TTVIEP+MTRDHTEKMLQGFGA L+VETD DGVRTIRLEGRGKL GQVID LLAGLNTPGVTTVIEPVMTRDHTEKMLQGFGAALSVETDGDGVRTIRLEGRGKLKGQVID | 240 |
| Query 241 | VPGDPSSTAFPLVAALLVPGSDVTILNVLMNPTRTGLILTLQEMGADIEVINPRLAGGED | 300 |
| Sbjct 241 | +PGDPSSTAFPLVAALLVPGSD+TI+NVLMNP RTGLILTLQEMGADIEV+N RLAGGED IPGDPSSTAFPLVAALLVPGSDITIVNVLMNPIRTGLILTLQEMGADIEVLNTRLAGGED | 300 |
| Query 301 | VADLRVRSSTLKGVTVPEDRAPSMIDEYPILavaaafaegaTVMNGLEELRVKESDRLSA | 360 |
| Sbjct 301 | VADLRVR S LKGVTVPEDRAPSMIDEYPILAVAA FAEGAT+M GLEELRVKESDRLSA VADLRVRHSELKGVTVPEDRAPSMIDEYPILAVAACFAEGATIMKGLEELRVKESDRLSA | 360 |
| Query 361 | VANGLKLNGVDCDEGETSLVVRGRPDGKGLGNASGAAVATHLDHRIAMSFLVMGLVSENP VA+GLKLNGVDCDEGE LVVRGRPDGKGLGNA+G V+THLDHRIAMSFLVMGL SE+ | 420 |

| Sbjct | 361 | VADGLKLNGVDCDEGEDFLVVRGRPDGKGLGNAAGDKVSTHLDHRIAMSFLVMGLASEHA 420 |
| --- | --- | --- |
| Query | 421 | VTVDDATMIATSFPEFMDLMAGLGAKIEL 449 |
| Sbjct | 421 | VT+DDA MIATSFPEFM LM GLGAKIEL  VTIDDAAMIATSFPEFMQLMTGLGAKIEL 449 |

>WP_003555761.1 3-phosphoshikimate 1-carboxyvinyltransferase [Rhizobium leguminosarum] RWY90099.1 3-phosphoshikimate 1-carboxyvinyltransferase [Rhizobium leguminosarum]

Length=452

Score = 771 bits (1990), Expect = 0.0, Method: Compositional matrix adjust.

| Identities | = 386/449 (86%), Positives = 421/449 (94%), Gaps = 0/449 (0%) |  |
| --- | --- | --- |
| Query 1 | MLHGASSRPATARKSSGLSGTVRIPGDKSISHRSFMFGGLASGETRITGLLEGEDVINTG | 60 |
| Sbjct 1 | ML+G++ RPATARKS+GL+G+VRIPGDKSISHRSFMFGGLASG+TRITGLLEGEDVINTG MLNGSAPRPATARKSAGLTGSVRIPGDKSISHRSFMFGGLASGQTRITGLLEGEDVINTG | 60 |
| Query 61 | KAMQAMGARIRKEGDTWIIDGVGNGGLLAPEAPLDFGNAATGCRLTMGLVGVYDFDSTFI | 120 |
| Sbjct 61 | +AMQAMGARIRKEG W+IDG GNG LLAP+APLDFGNA TG RLTMGLVG YDF STFI RAMQAMGARIRKEGAQWVIDGTGNGALLAPDAPLDFGNAGTGVRLTMGLVGTYDFRSTFI | 120 |
| Query 121 | GDASLTKRPMGRVLNPLREMGVQVKSEDGDRLPVTLRGPKTPTPITYRVPMASAQVKSAV | 180 |
| Sbjct 121 | GDASL+KRPMGRVLNPLREMGVQ+ + +G+RLPVTL+GP TP+PI YRVPMASAQVKSAV GDASLSKRPMGRVLNPLREMGVQISASEGNRLPVTLKGPGTPSPIRYRVPMASAQVKSAV | 180 |
| Query 181 | LLAGLNTPGITTVIEPIMTRDHTEKMLQGFGANLTVETDADGVRTIRLEGRGKLTGQVID | 240 |
| Sbjct 181 | LLAGLNTPG+TTVIEP+MTRDHTEKMLQGFGA L+VETD++GVRTIRLEGRG+LTGQVID LLAGLNTPGVTTVIEPVMTRDHTEKMLQGFGAALSVETDSEGVRTIRLEGRGRLTGQVID | 240 |
| Query 241 | VPGDPSSTAFPLVAALLVPGSDVTILNVLMNPTRTGLILTLQEMGADIEVINPRLAGGED | 300 |
| Sbjct 241 | +PGDPSSTAFPLVAALLVPGSD+TI+NVLMNPTRTGLILTLQEMGADIEV+NPRLAGGED IPGDPSSTAFPLVAALLVPGSDITIVNVLMNPTRTGLILTLQEMGADIEVLNPRLAGGED | 300 |
| Query 301 | VADLRVRSSTLKGVTVPEDRAPSMIDEYPILavaaafaegaTVMNGLEELRVKESDRLSA | 360 |
| Sbjct 301 | V DLRVR S LKGVTVPE+RAPSMIDEYPILAVAA FAEGAT+M GLEELRVKESDRLSA VVDLRVRHSELKGVTVPEERAPSMIDEYPILAVAACFAEGATIMKGLEELRVKESDRLSA | 360 |
| Query 361 | VANGLKLNGVDCDEGETSLVVRGRPDGKGLGNASGAAVATHLDHRIAMSFLVMGLVSENP | 420 |
| Sbjct 361 | VA+GLKLNGVDCDEGE LVVRGRPDGKGLGNA+G V+THLDHRIAMSFLV+GL SE+ VADGLKLNGVDCDEGEDFLVVRGRPDGKGLGNAAGGKVSTHLDHRIAMSFLVLGLASEHA | 420 |
| Query 421  Sbjct 421 | VTVDDATMIATSFPEFMDLMAGLGAKIEL 449 VT+DDA MIATSFPEFM LM GLGAKIEL VTIDDAAMIATSFPEFMQLMTGLGAKIEL 449 |  |

>WP_164026695.1 3-phosphoshikimate 1-carboxyvinyltransferase [Rhizobium leguminosarum] NEH52973.1 3-phosphoshikimate 1-carboxyvinyltransferase [Rhizobium leguminosarum]

Length=452

Score = 771 bits (1990), Expect = 0.0, Method: Compositional matrix adjust.

| Identities | = 389/448 (87%), Positives = 419/448 (94%), Gaps = 0/448 (0%) |  |
| --- | --- | --- |
| Query 1 | MLHGASSRPATARKSSGLSGTVRIPGDKSISHRSFMFGGLASGETRITGLLEGEDVINTG | 60 |
| Sbjct 1 | ML+G++ +PATARKS+GL+G+VRIPGDKSISHRSFMFGGLASGETRITGLLEGEDVINTG MLNGSAPKPATARKSAGLTGSVRIPGDKSISHRSFMFGGLASGETRITGLLEGEDVINTG | 60 |
| Query 61 | KAMQAMGARIRKEGDTWIIDGVGNGGLLAPEAPLDFGNAATGCRLTMGLVGVYDFDSTFI | 120 |
| Sbjct 61 | +AMQAMGARIRKEG+ W+IDG GNG LLAP+APLDFGNA TG RLTMGLVG YDF STF RAMQAMGARIRKEGEQWVIDGTGNGALLAPDAPLDFGNAGTGVRLTMGLVGTYDFRSTFT | 120 |
| Query 121 | GDASLTKRPMGRVLNPLREMGVQVKSEDGDRLPVTLRGPKTPTPITYRVPMASAQVKSAV | 180 |
| Sbjct 121 | GDASL+KRPMGRVL PLREMGVQV + +GDRLPVTLRGP TP+PI YRVPMASAQVKSAV GDASLSKRPMGRVLAPLREMGVQVSASEGDRLPVTLRGPGTPSPIRYRVPMASAQVKSAV | 180 |
| Query 181 | LLAGLNTPGITTVIEPIMTRDHTEKMLQGFGANLTVETDADGVRTIRLEGRGKLTGQVID | 240 |
| Sbjct 181 | LLAGLNTPG+TTVIEP+MTRDHTEKMLQGFGA L+VETD++GVRTIRLEGRG+L GQVID LLAGLNTPGVTTVIEPVMTRDHTEKMLQGFGAALSVETDSEGVRTIRLEGRGRLMGQVID | 240 |
| Query 241 | VPGDPSSTAFPLVAALLVPGSDVTILNVLMNPTRTGLILTLQEMGADIEVINPRLAGGED VPGDPSSTAFPLVAALLVPGSD+TI+NVLMNPTRTGLILTLQEMGADIEV+N RLAGGED | 300 |

| Sbjct | 241 | VPGDPSSTAFPLVAALLVPGSDITIVNVLMNPTRTGLILTLQEMGADIEVVNARLAGGED | 300 |
| --- | --- | --- | --- |
| Query | 301 | VADLRVRSSTLKGVTVPEDRAPSMIDEYPILavaaafaegaTVMNGLEELRVKESDRLSA | 360 |
| Sbjct | 301 | VADLRVR S LKGVTVPEDRAPSMIDEYPILAVAA FAEGAT+MNGLEELRVKESDRLSA VADLRVRHSELKGVTVPEDRAPSMIDEYPILAVAACFAEGATIMNGLEELRVKESDRLSA | 360 |
| Query | 361 | VANGLKLNGVDCDEGETSLVVRGRPDGKGLGNASGAAVATHLDHRIAMSFLVMGLVSENP | 420 |
| Sbjct | 361 | VA+GLKLNGVDCDEGE LVVRGRPDGKGLGNA+ V+THLDHRIAMSFLVMGL SE+P VADGLKLNGVDCDEGEDFLVVRGRPDGKGLGNAADGRVSTHLDHRIAMSFLVMGLASEHP | 420 |
| Query  Sbjct | 421  421 | VTVDDATMIATSFPEFMDLMAGLGAKIE 448 VT+DDA MIATSFPEFM LM GLGAKIE VTIDDAAMIATSFPEFMQLMTGLGAKIE 448 |  |

>WP_131665415.1 3-phosphoshikimate 1-carboxyvinyltransferase [Rhizobium laguerreae] TBY03437.1 3-phosphoshikimate 1-carboxyvinyltransferase [Rhizobium laguerreae]

Length=452

Score = 770 bits (1989), Expect = 0.0, Method: Compositional matrix adjust.

| Identities | = 389/449 (87%), Positives = 418/449 (93%), Gaps = 0/449 (0%) |  |
| --- | --- | --- |
| Query 1 | MLHGASSRPATARKSSGLSGTVRIPGDKSISHRSFMFGGLASGETRITGLLEGEDVINTG | 60 |
| Sbjct 1 | MLHG++ RPATARKS+GL+G+VRIPGDKSISHRSFMFGGLASGETRITGLLEGEDVINTG MLHGSAPRPATARKSAGLAGSVRIPGDKSISHRSFMFGGLASGETRITGLLEGEDVINTG | 60 |
| Query 61 | KAMQAMGARIRKEGDTWIIDGVGNGGLLAPEAPLDFGNAATGCRLTMGLVGVYDFDSTFI | 120 |
| Sbjct 61 | +AMQAMGARIRK+G W+IDG GNG LLAP+APLDFGNA TG RLTMGLVG YDF STFI RAMQAMGARIRKDGAQWVIDGTGNGALLAPDAPLDFGNAGTGVRLTMGLVGTYDFRSTFI | 120 |
| Query 121 | GDASLTKRPMGRVLNPLREMGVQVKSEDGDRLPVTLRGPKTPTPITYRVPMASAQVKSAV | 180 |
| Sbjct 121 | GDASL+KRPMGRVLNPLREMGVQV + +GDRLPVTLRGP TP+PI YRVPMASAQVKSAV GDASLSKRPMGRVLNPLREMGVQVSASEGDRLPVTLRGPGTPSPIRYRVPMASAQVKSAV | 180 |
| Query 181 | LLAGLNTPGITTVIEPIMTRDHTEKMLQGFGANLTVETDADGVRTIRLEGRGKLTGQVID | 240 |
| Sbjct 181 | LLAGLNTPG+TTVIEP+MTRDHTEKMLQGFGA L+VETD DGVRTIRLEGRGKL GQ+ID LLAGLNTPGVTTVIEPVMTRDHTEKMLQGFGAALSVETDGDGVRTIRLEGRGKLKGQMID | 240 |
| Query 241 | VPGDPSSTAFPLVAALLVPGSDVTILNVLMNPTRTGLILTLQEMGADIEVINPRLAGGED | 300 |
| Sbjct 241 | +PGDPSSTAFPLVAALLVPGSD+TI+NVLMNPTRTGLILTLQEMGA+IEV+N RLAGGED IPGDPSSTAFPLVAALLVPGSDITIVNVLMNPTRTGLILTLQEMGANIEVLNTRLAGGED | 300 |
| Query 301 | VADLRVRSSTLKGVTVPEDRAPSMIDEYPILavaaafaegaTVMNGLEELRVKESDRLSA | 360 |
| Sbjct 301 | VADLRVR S LKGVTVPE+R PSMIDEYPILAVAA FAEGAT+M GLEELRVKESDRLSA VADLRVRHSELKGVTVPEERTPSMIDEYPILAVAACFAEGATIMKGLEELRVKESDRLSA | 360 |
| Query 361 | VANGLKLNGVDCDEGETSLVVRGRPDGKGLGNASGAAVATHLDHRIAMSFLVMGLVSENP | 420 |
| Sbjct 361 | VA+GLKLNGVDCDEGE LVVRGRPDGKGLGNA+G V+THLDHRIAMSFLVMGL SE+ VADGLKLNGVDCDEGEDFLVVRGRPDGKGLGNAAGGRVSTHLDHRIAMSFLVMGLASEHA | 420 |
| Query 421  Sbjct 421 | VTVDDATMIATSFPEFMDLMAGLGAKIEL 449 VT+DDA MIATSFPEFM LM GLGAKIEL VTIDDAAMIATSFPEFMQLMTGLGAKIEL 449 |  |

>WP_151517755.1 3-phosphoshikimate 1-carboxyvinyltransferase [Rhizobium sp. PEPV16] KAF5887251.1 3-phosphoshikimate 1-carboxyvinyltransferase [Rhizobium sp. PEPV16]

Length=452

Score = 770 bits (1989), Expect = 0.0, Method: Compositional matrix adjust.

| Identities | = 387/448 (86%), Positives = 418/448 (93%), Gaps = 0/448 (0%) |  |
| --- | --- | --- |
| Query 1 | MLHGASSRPATARKSSGLSGTVRIPGDKSISHRSFMFGGLASGETRITGLLEGEDVINTG | 60 |
| Sbjct 1 | MLHG++ +PATARKS+GL+G+VRIPGDKSISHRSFMFGGLASGETRITGLLEGEDVINTG MLHGSAPKPATARKSAGLAGSVRIPGDKSISHRSFMFGGLASGETRITGLLEGEDVINTG | 60 |
| Query 61 | KAMQAMGARIRKEGDTWIIDGVGNGGLLAPEAPLDFGNAATGCRLTMGLVGVYDFDSTFI | 120 |
| Sbjct 61 | +AMQAMGARIRK+G W+IDG GNG LLAP+APLDFGNA TG RLTMGLVG YDF STFI RAMQAMGARIRKDGAQWVIDGTGNGALLAPDAPLDFGNAGTGVRLTMGLVGTYDFRSTFI | 120 |
| Query 121 | GDASLTKRPMGRVLNPLREMGVQVKSEDGDRLPVTLRGPKTPTPITYRVPMASAQVKSAV GDASL+KRPMGRVLNPLREMGVQV + +GDRLPVTLRGP TP+PI YRVPMASAQVKSAV | 180 |

| Sbjct | 121 | GDASLSKRPMGRVLNPLREMGVQVSASEGDRLPVTLRGPGTPSPIRYRVPMASAQVKSAV | 180 |
| --- | --- | --- | --- |
| Query | 181 | LLAGLNTPGITTVIEPIMTRDHTEKMLQGFGANLTVETDADGVRTIRLEGRGKLTGQVID | 240 |
| Sbjct | 181 | LLAGLNTPG+TTVIEP+MTRDHTEKMLQGFGA ++VETD++GVRTIRLEGRGKL GQVID LLAGLNTPGVTTVIEPVMTRDHTEKMLQGFGAAVSVETDSEGVRTIRLEGRGKLKGQVID | 240 |
| Query | 241 | VPGDPSSTAFPLVAALLVPGSDVTILNVLMNPTRTGLILTLQEMGADIEVINPRLAGGED | 300 |
| Sbjct | 241 | +PGDPSSTAFPLVAALLVPGSD+TI+NVLMNPTRTGLILTLQEMGA+IEV N RLAGGED IPGDPSSTAFPLVAALLVPGSDITIVNVLMNPTRTGLILTLQEMGANIEVANARLAGGED | 300 |
| Query | 301 | VADLRVRSSTLKGVTVPEDRAPSMIDEYPILavaaafaegaTVMNGLEELRVKESDRLSA | 360 |
| Sbjct | 301 | VADLRVR S LKGVTVPEDRAPSMIDEYPILAVAA FAEGAT+M GLEELRVKESDRLSA VADLRVRHSELKGVTVPEDRAPSMIDEYPILAVAACFAEGATIMKGLEELRVKESDRLSA | 360 |
| Query | 361 | VANGLKLNGVDCDEGETSLVVRGRPDGKGLGNASGAAVATHLDHRIAMSFLVMGLVSENP | 420 |
| Sbjct | 361 | VA+GLKLNGVDCDEGE L+VRGRPDGKGLGNA+ V+THLDHRIAMSFLVMGL SE+P VADGLKLNGVDCDEGEDFLIVRGRPDGKGLGNAADGRVSTHLDHRIAMSFLVMGLASEHP | 420 |
| Query  Sbjct | 421  421 | VTVDDATMIATSFPEFMDLMAGLGAKIE 448 VT+DDA MIATSFPEFM LM GLGAKIE VTIDDAAMIATSFPEFMQLMTGLGAKIE 448 |  |

>WP_130872618.1 3-phosphoshikimate 1-carboxyvinyltransferase [Rhizobium leguminosarum] TBH44119.1 3-phosphoshikimate 1-carboxyvinyltransferase [Rhizobium leguminosarum]

Length=452

Score = 770 bits (1989), Expect = 0.0, Method: Compositional matrix adjust.

| Identities | = 389/449 (87%), Positives = 418/449 (93%), Gaps = 0/449 (0%) |  |
| --- | --- | --- |
| Query 1 | MLHGASSRPATARKSSGLSGTVRIPGDKSISHRSFMFGGLASGETRITGLLEGEDVINTG | 60 |
| Sbjct 1 | ML+G++S+PATARKS+GL+G+VRIPGDKSISHRSFMFGGLASGETRITGLLEGEDVINTG MLNGSASKPATARKSAGLTGSVRIPGDKSISHRSFMFGGLASGETRITGLLEGEDVINTG | 60 |
| Query 61 | KAMQAMGARIRKEGDTWIIDGVGNGGLLAPEAPLDFGNAATGCRLTMGLVGVYDFDSTFI | 120 |
| Sbjct 61 | +AMQAMGARIRKEG W+I+G GNG LLAP+APLDFGNA TG RLTMGLVG YDF STFI RAMQAMGARIRKEGAQWVIEGTGNGALLAPDAPLDFGNAGTGVRLTMGLVGTYDFHSTFI | 120 |
| Query 121 | GDASLTKRPMGRVLNPLREMGVQVKSEDGDRLPVTLRGPKTPTPITYRVPMASAQVKSAV | 180 |
| Sbjct 121 | GDASL+KRPMGRVLNPLREMGVQV + +GDRLPVTLRGP TP+PI YRVPMASAQVKSAV GDASLSKRPMGRVLNPLREMGVQVSASEGDRLPVTLRGPGTPSPIRYRVPMASAQVKSAV | 180 |
| Query 181 | LLAGLNTPGITTVIEPIMTRDHTEKMLQGFGANLTVETDADGVRTIRLEGRGKLTGQVID | 240 |
| Sbjct 181 | LLAGLNTPG+TTVIEP+MTRDHTEKMLQGFGA L+VETD DGV TIRLEGRGKL GQVID LLAGLNTPGVTTVIEPVMTRDHTEKMLQGFGAALSVETDGDGVHTIRLEGRGKLAGQVID | 240 |
| Query 241 | VPGDPSSTAFPLVAALLVPGSDVTILNVLMNPTRTGLILTLQEMGADIEVINPRLAGGED | 300 |
| Sbjct 241 | VPGDPSSTAFPLVAAL+VPGSD+TI+NVLMNPTRTGLILTLQEMGADIEV+N RLAGGED VPGDPSSTAFPLVAALIVPGSDITIVNVLMNPTRTGLILTLQEMGADIEVVNARLAGGED | 300 |
| Query 301 | VADLRVRSSTLKGVTVPEDRAPSMIDEYPILavaaafaegaTVMNGLEELRVKESDRLSA | 360 |
| Sbjct 301 | VADLRVR S LKGVTVPEDRAPSMIDEYPILAVAA FAEGATVM GLEELRVKESDRLSA VADLRVRHSELKGVTVPEDRAPSMIDEYPILAVAACFAEGATVMKGLEELRVKESDRLSA | 360 |
| Query 361 | VANGLKLNGVDCDEGETSLVVRGRPDGKGLGNASGAAVATHLDHRIAMSFLVMGLVSENP | 420 |
| Sbjct 361 | VA+GLKLNGVDCDEGE L+VRGRPDGKGLGNA+ V+THLDHRIAMSFLV+GL SE+ VADGLKLNGVDCDEGEDFLIVRGRPDGKGLGNAADGRVSTHLDHRIAMSFLVLGLASEHA | 420 |
| Query 421  Sbjct 421 | VTVDDATMIATSFPEFMDLMAGLGAKIEL 449 VT+DDA MIATSFPEFM LM GLGAKIEL VTIDDAAMIATSFPEFMQLMTGLGAKIEL 449 |  |

```
>WP_029618989.1 3-phosphoshikimate 1-carboxyvinyltransferase [Rhizobium marinum] Length=455

Score = 770 bits (1988), Expect = 0.0, Method: Compositional matrix adjust. Identities = 393/455 (86%), Positives = 418/455 (92%), Gaps = 0/455 (0%)

Query 1	MLHGASSRPATARKSSGLSGTVRIPGDKSISHRSFMFGGLASGETRITGLLEGEDVINTG 60 M HG + +PA + KS+ L GTVRIPGDKSISHRSFMFGGLASGETRITGLLEGEDVINTG
Sbjct 1	MSHGVALKPARSLKSADLKGTVRIPGDKSISHRSFMFGGLASGETRITGLLEGEDVINTG 60
```

| Query | 61 | KAMQAMGARIRKEGDTWIIDGVGNGGLLAPEAPLDFGNAATGCRLTMGLVGVYDFDSTFI | 120 |
| --- | --- | --- | --- |
| Sbjct | 61 | KAMQAMGA+I K G WII G GNG LLAPEAPLDFGNA TGCRLTMGLVGVYDFDSTFI KAMQAMGAKIEKIGAEWIIQGTGNGALLAPEAPLDFGNAGTGCRLTMGLVGVYDFDSTFI | 120 |
| Query | 121 | GDASLTKRPMGRVLNPLREMGVQVKSEDGDRLPVTLRGPKTPTPITYRVPMASAQVKSAV | 180 |
| Sbjct | 121 | GDASL+KRPMGRVL PLREMGVQV + +GDRLPVTLRGP TP PITYRVPMASAQVKSAV GDASLSKRPMGRVLAPLREMGVQVTAAEGDRLPVTLRGPATPNPITYRVPMASAQVKSAV | 180 |
| Query | 181 | LLAGLNTPGITTVIEPIMTRDHTEKMLQGFGANLTVETDADGVRTIRLEGRGKLTGQVID | 240 |
| Sbjct | 181 | LLAGLNTPGITTVIEP+MTRDHTEKMLQGFGA+LTVETDADGVRTIRL+GRGKLTGQVID LLAGLNTPGITTVIEPVMTRDHTEKMLQGFGADLTVETDADGVRTIRLQGRGKLTGQVID | 240 |
| Query | 241 | VPGDPSSTAFPLVAALLVPGSDVTILNVLMNPTRTGLILTLQEMGADIEVINPRLAGGED | 300 |
| Sbjct | 241 | VPGDPSSTAFPLVAALLVPGS+VTI NVLMNPTRTGLILTLQEMGADIEV+NPRLAGGED VPGDPSSTAFPLVAALLVPGSEVTIENVLMNPTRTGLILTLQEMGADIEVLNPRLAGGED | 300 |
| Query | 301 | VADLRVRSSTLKGVTVPEDRAPSMIDEYPILavaaafaegaTVMNGLEELRVKESDRLSA | 360 |
| Sbjct | 301 | VADLRVR S LKGVTVP +RAPSMIDEYP+LAVAAAFAEG T M G+EELRVKESDRLSA VADLRVRHSQLKGVTVPAERAPSMIDEYPVLAVAAAFAEGTTTMLGVEELRVKESDRLSA | 360 |
| Query | 361 | VANGLKLNGVDCDEGETSLVVRGRPDGKGLGNASGAAVATHLDHRIAMSFLVMGLVSENP | 420 |
| Sbjct | 361 | A GLKLNGVDCDEGE +L VRGRP GKG GNA+G AVATHLDHRIAMSFLV+GLVSE+P TAEGLKLNGVDCDEGEDTLTVRGRPGGKGYGNAAGEAVATHLDHRIAMSFLVLGLVSEHP | 420 |
| Query  Sbjct | 421  421 | VTVDDATMIATSFPEFMDLMAGLGAKIELSDTKAA 455 VTVDDA +IATSFPEFMDLMAGLGA+IE +++AA VTVDDAGIIATSFPEFMDLMAGLGARIEAVESRAA 455 |  |

>WP_011649931.1 3-phosphoshikimate 1-carboxyvinyltransferase [Rhizobium leguminosarum] Q1MN56.1 RecName: Full=3-phosphoshikimate 1-carboxyvinyltransferase; AltName:

Full=5-enolpyruvylshikimate-3-phosphate synthase; Short=EPSP synthase; Short=EPSPS [Rhizobium leguminosarum bv.

viciae 3841]

CAK05596.1 putative 3-phosphoshikimate 1-carboxyvinyltransferase (5-enolpyruvylshikimate-3- phosphate

synthase) (EPSP synthase) [Rhizobium leguminosarum bv. viciae 3841] Length=452

Score = 770 bits (1988), Expect = 0.0, Method: Compositional matrix adjust.

| Identities | = 389/449 (87%), Positives = 418/449 (93%), Gaps = 0/449 (0%) |  |
| --- | --- | --- |
| Query 1 | MLHGASSRPATARKSSGLSGTVRIPGDKSISHRSFMFGGLASGETRITGLLEGEDVINTG | 60 |
| Sbjct 1 | ML+G++S+PATARKS+GL+G+VRIPGDKSISHRSFM GGLASGETRITGLLEGEDVINTG MLNGSASKPATARKSAGLTGSVRIPGDKSISHRSFMIGGLASGETRITGLLEGEDVINTG | 60 |
| Query 61 | KAMQAMGARIRKEGDTWIIDGVGNGGLLAPEAPLDFGNAATGCRLTMGLVGVYDFDSTFI | 120 |
| Sbjct 61 | +AMQAMGARIRKEG W+I+G GNG LLAP+APLDFGNA TG RLTMGLVG YDF STFI RAMQAMGARIRKEGAQWVIEGTGNGALLAPDAPLDFGNAGTGVRLTMGLVGTYDFHSTFI | 120 |
| Query 121 | GDASLTKRPMGRVLNPLREMGVQVKSEDGDRLPVTLRGPKTPTPITYRVPMASAQVKSAV | 180 |
| Sbjct 121 | GDASL+KRPMGRVLNPLREMGVQV + +GDRLPVTLRGP TP+PI YRVPMASAQVKSAV GDASLSKRPMGRVLNPLREMGVQVSASEGDRLPVTLRGPGTPSPIRYRVPMASAQVKSAV | 180 |
| Query 181 | LLAGLNTPGITTVIEPIMTRDHTEKMLQGFGANLTVETDADGVRTIRLEGRGKLTGQVID | 240 |
| Sbjct 181 | LLAGLNTPG+TTVIEP+MTRDHTEKMLQGFGA L+VETD DGVRTIRLEGRGKL GQVID LLAGLNTPGVTTVIEPVMTRDHTEKMLQGFGAALSVETDGDGVRTIRLEGRGKLAGQVID | 240 |
| Query 241 | VPGDPSSTAFPLVAALLVPGSDVTILNVLMNPTRTGLILTLQEMGADIEVINPRLAGGED | 300 |
| Sbjct 241 | VPGDPSSTAFPLVAAL+VPGSD+TI+NVLMNPTRTGLILTLQEMGADIEV+N RLAGGED VPGDPSSTAFPLVAALIVPGSDITIVNVLMNPTRTGLILTLQEMGADIEVVNARLAGGED | 300 |
| Query 301 | VADLRVRSSTLKGVTVPEDRAPSMIDEYPILavaaafaegaTVMNGLEELRVKESDRLSA | 360 |
| Sbjct 301 | VADLRVR S LKGVTVPEDRAPSMIDEYPILAVAA FAEGATVM GLEELRVKESDRLSA VADLRVRHSELKGVTVPEDRAPSMIDEYPILAVAACFAEGATVMKGLEELRVKESDRLSA | 360 |
| Query 361 | VANGLKLNGVDCDEGETSLVVRGRPDGKGLGNASGAAVATHLDHRIAMSFLVMGLVSENP | 420 |
| Sbjct 361 | VA+GLKLNGVDCDEGE L+VRGRPDGKGLGNA+ V+THLDHRIAMSFLV+GL SE+  VADGLKLNGVDCDEGEDFLIVRGRPDGKGLGNAADGRVSTHLDHRIAMSFLVLGLASEHA | 420 |

Query 421 VTVDDATMIATSFPEFMDLMAGLGAKIEL 449 VT+DDA MIATSFPEFM LM GLGAKIEL

Sbjct 421 VTIDDAAMIATSFPEFMQLMTGLGAKIEL 449

>WP_033181070.1 3-phosphoshikimate 1-carboxyvinyltransferase [Rhizobium leguminosarum]

KPN25738.1 3-phosphoshikimate 1-carboxyvinyltransferase [Rhizobium leguminosarum bv. trifolii]

NZD51339.1 3-phosphoshikimate 1-carboxyvinyltransferase [Rhizobium leguminosarum]

QIO53908.1 3-phosphoshikimate 1-carboxyvinyltransferase [Rhizobium leguminosarum bv. trifolii]

QJX07577.1 3-phosphoshikimate 1-carboxyvinyltransferase [Rhizobium leguminosarum bv. trifolii]

TAV76106.1 3-phosphoshikimate 1-carboxyvinyltransferase [Rhizobium leguminosarum] Length=452

Score = 770 bits (1988), Expect = 0.0, Method: Compositional matrix adjust.

| Identities | = 386/448 (86%), Positives = 421/448 (94%), Gaps = 0/448 (0%) |  |
| --- | --- | --- |
| Query 1 | MLHGASSRPATARKSSGLSGTVRIPGDKSISHRSFMFGGLASGETRITGLLEGEDVINTG | 60 |
| Sbjct 1 | ML+G++S+PATARKS+GL+G+VRIPGDKSISHRSFMFGGLASGETRITGLLEGEDVINTG MLNGSASKPATARKSAGLTGSVRIPGDKSISHRSFMFGGLASGETRITGLLEGEDVINTG | 60 |
| Query 61 | KAMQAMGARIRKEGDTWIIDGVGNGGLLAPEAPLDFGNAATGCRLTMGLVGVYDFDSTFI | 120 |
| Sbjct 61 | +AMQAMGARIRKEG W+I+G GNG LLAP+APLDFGNA TG RLTMGLVG YDF STFI RAMQAMGARIRKEGAQWVIEGTGNGALLAPDAPLDFGNAGTGVRLTMGLVGTYDFRSTFI | 120 |
| Query 121 | GDASLTKRPMGRVLNPLREMGVQVKSEDGDRLPVTLRGPKTPTPITYRVPMASAQVKSAV | 180 |
| Sbjct 121 | GDASL+KRPMGRVL PLREMGVQ+ + +GDRLPVTL+GP TP+PI YRVPMASAQVKSAV GDASLSKRPMGRVLAPLREMGVQISASEGDRLPVTLKGPGTPSPIRYRVPMASAQVKSAV | 180 |
| Query 181 | LLAGLNTPGITTVIEPIMTRDHTEKMLQGFGANLTVETDADGVRTIRLEGRGKLTGQVID | 240 |
| Sbjct 181 | LLAGLNTPG+TTVIEP+MTRDHTEKMLQGFGA+L+VETD++GVRTIRLEGRG+LTGQ+ID LLAGLNTPGVTTVIEPVMTRDHTEKMLQGFGASLSVETDSEGVRTIRLEGRGRLTGQMID | 240 |
| Query 241 | VPGDPSSTAFPLVAALLVPGSDVTILNVLMNPTRTGLILTLQEMGADIEVINPRLAGGED | 300 |
| Sbjct 241 | VPGDPSSTAFPLVAALLVPGSD+TI+NVLMNPTRTGLILTLQEMGA+IEV+N RLAGGED VPGDPSSTAFPLVAALLVPGSDITIVNVLMNPTRTGLILTLQEMGANIEVLNARLAGGED | 300 |
| Query 301 | VADLRVRSSTLKGVTVPEDRAPSMIDEYPILavaaafaegaTVMNGLEELRVKESDRLSA | 360 |
| Sbjct 301 | VADLRVR S L+GVTVPEDRAPSMIDEYPILAVAA FAEG TVM GLEELRVKESDRLSA VADLRVRHSELRGVTVPEDRAPSMIDEYPILAVAACFAEGTTVMKGLEELRVKESDRLSA | 360 |
| Query 361 | VANGLKLNGVDCDEGETSLVVRGRPDGKGLGNASGAAVATHLDHRIAMSFLVMGLVSENP | 420 |
| Sbjct 361 | VA+GLKLNGVDCDEGE LVVRGRPDGKGLGNA+G V+THLDHRIAMSFLVMGL SE+P VADGLKLNGVDCDEGEDFLVVRGRPDGKGLGNAAGGRVSTHLDHRIAMSFLVMGLASEHP | 420 |
| Query 421  Sbjct 421 | VTVDDATMIATSFPEFMDLMAGLGAKIE 448 VT+DDA MIATSFPEFM LM GLGAKIE VTIDDAAMIATSFPEFMQLMTGLGAKIE 448 |  |

>WP_168277711.1 3-phosphoshikimate 1-carboxyvinyltransferase [Rhizobium leguminosarum]

NKL95488.1 3-phosphoshikimate 1-carboxyvinyltransferase [Rhizobium leguminosarum bv. viciae]

Length=452

Score = 770 bits (1988), Expect = 0.0, Method: Compositional matrix adjust.

| Identities | = 389/449 (87%), Positives = 418/449 (93%), Gaps = 0/449 (0%) |  |
| --- | --- | --- |
| Query 1 | MLHGASSRPATARKSSGLSGTVRIPGDKSISHRSFMFGGLASGETRITGLLEGEDVINTG | 60 |
| Sbjct 1 | MLHG++ +PATARKS+GL+G+VRIPGDKSISHRSFMFGGLASGETRITGLLEGEDVINTG MLHGSAPKPATARKSAGLAGSVRIPGDKSISHRSFMFGGLASGETRITGLLEGEDVINTG | 60 |
| Query 61 | KAMQAMGARIRKEGDTWIIDGVGNGGLLAPEAPLDFGNAATGCRLTMGLVGVYDFDSTFI | 120 |
| Sbjct 61 | +AMQAMGARIRK+G W+IDG GNG LLAP+APLDFGNA TG RLTMGLVG YDF STFI RAMQAMGARIRKDGAQWVIDGTGNGALLAPDAPLDFGNAGTGVRLTMGLVGTYDFRSTFI | 120 |
| Query 121 | GDASLTKRPMGRVLNPLREMGVQVKSEDGDRLPVTLRGPKTPTPITYRVPMASAQVKSAV | 180 |
| Sbjct 121 | GDASL+KRPMGRVLNPLREMGVQV + +GDRLPVTLRGP TP+PI YRVPMASAQVKSAV  GDASLSKRPMGRVLNPLREMGVQVSASEGDRLPVTLRGPGTPSPIRYRVPMASAQVKSAV | 180 |

| Query | 181 | LLAGLNTPGITTVIEPIMTRDHTEKMLQGFGANLTVETDADGVRTIRLEGRGKLTGQVID | 240 |
| --- | --- | --- | --- |
| Sbjct | 181 | LLAGLNTPG+TTVIEP+MTRDHTEKMLQGFGA L+VETD GVRTIRLEGRGKL GQVID LLAGLNTPGVTTVIEPVMTRDHTEKMLQGFGAALSVETDGVGVRTIRLEGRGKLKGQVID | 240 |
| Query | 241 | VPGDPSSTAFPLVAALLVPGSDVTILNVLMNPTRTGLILTLQEMGADIEVINPRLAGGED | 300 |
| Sbjct | 241 | +PGDPSSTAFPLVAALLVPGSD+TI+NVLMNPTRTGLILTLQEMGA+IEV+N RLAGGED IPGDPSSTAFPLVAALLVPGSDITIVNVLMNPTRTGLILTLQEMGANIEVLNTRLAGGED | 300 |
| Query | 301 | VADLRVRSSTLKGVTVPEDRAPSMIDEYPILavaaafaegaTVMNGLEELRVKESDRLSA | 360 |
| Sbjct | 301 | VADLRVR S LKGVTVPE+RAPSMIDEYPILAVAA FAEGAT+M GLEELRVKESDRLSA VADLRVRHSELKGVTVPEERAPSMIDEYPILAVAACFAEGATIMKGLEELRVKESDRLSA | 360 |
| Query | 361 | VANGLKLNGVDCDEGETSLVVRGRPDGKGLGNASGAAVATHLDHRIAMSFLVMGLVSENP | 420 |
| Sbjct | 361 | VA+GLKLNGVDCDEGE LVVRGRPDGKGLGNA+G V+THLDHRIAMSFLVMGL SE+ VADGLKLNGVDCDEGEDFLVVRGRPDGKGLGNAAGGRVSTHLDHRIAMSFLVMGLASEHA | 420 |
| Query  Sbjct | 421  421 | VTVDDATMIATSFPEFMDLMAGLGAKIEL 449 VT+DDA MIATSFPEFM LM GLGAKIEL VTIDDAAMIATSFPEFMQLMTGLGAKIEL 449 |  |

>WP_130659400.1 3-phosphoshikimate 1-carboxyvinyltransferase [Rhizobium leguminosarum] TAU55441.1 3-phosphoshikimate 1-carboxyvinyltransferase [Rhizobium leguminosarum] TBC96718.1 3-phosphoshikimate 1-carboxyvinyltransferase [Rhizobium leguminosarum]

Length=452

Score = 769 bits (1987), Expect = 0.0, Method: Compositional matrix adjust.

| Identities | = 387/448 (86%), Positives = 415/448 (93%), Gaps = 0/448 (0%) |  |
| --- | --- | --- |
| Query 1 | MLHGASSRPATARKSSGLSGTVRIPGDKSISHRSFMFGGLASGETRITGLLEGEDVINTG | 60 |
| Sbjct 1 | MLHG++ RPATARKS+GL+G+VRIPGDKSISHRSFMFGGLASGETRITGLLEGEDVINTG MLHGSAPRPATARKSAGLTGSVRIPGDKSISHRSFMFGGLASGETRITGLLEGEDVINTG | 60 |
| Query 61 | KAMQAMGARIRKEGDTWIIDGVGNGGLLAPEAPLDFGNAATGCRLTMGLVGVYDFDSTFI | 120 |
| Sbjct 61 | +AMQAMGARIRKEG+ W+I+G GNG LLAP+ PLDFGNA TG RLTMGLVG YDF STF RAMQAMGARIRKEGEQWVIEGTGNGALLAPDTPLDFGNAGTGVRLTMGLVGTYDFRSTFT | 120 |
| Query 121 | GDASLTKRPMGRVLNPLREMGVQVKSEDGDRLPVTLRGPKTPTPITYRVPMASAQVKSAV | 180 |
| Sbjct 121 | GDASL+KRPMGRVLNPLREMGVQV + +GDRLPVTLRGP TP+PI YRVPM SAQVKSAV GDASLSKRPMGRVLNPLREMGVQVSASEGDRLPVTLRGPGTPSPIRYRVPMTSAQVKSAV | 180 |
| Query 181 | LLAGLNTPGITTVIEPIMTRDHTEKMLQGFGANLTVETDADGVRTIRLEGRGKLTGQVID | 240 |
| Sbjct 181 | LLAGLNTPG+TTVIEP+MTRDHTEKMLQGFGA L+VETD DGVRTIRLEGRGKL GQVID LLAGLNTPGVTTVIEPVMTRDHTEKMLQGFGAALSVETDEDGVRTIRLEGRGKLKGQVID | 240 |
| Query 241 | VPGDPSSTAFPLVAALLVPGSDVTILNVLMNPTRTGLILTLQEMGADIEVINPRLAGGED | 300 |
| Sbjct 241 | VPGDPSSTAFPLVAALLVPGSD+TI+NVLMNPTRTGLILTLQEMGADIEV+N RLAGGED VPGDPSSTAFPLVAALLVPGSDITIVNVLMNPTRTGLILTLQEMGADIEVLNTRLAGGED | 300 |
| Query 301 | VADLRVRSSTLKGVTVPEDRAPSMIDEYPILavaaafaegaTVMNGLEELRVKESDRLSA | 360 |
| Sbjct 301 | VADLRVR S LKGVTVPEDRAPSMIDEYPILAVAA FA+G TVM GLEELRVKESDRLSA VADLRVRHSELKGVTVPEDRAPSMIDEYPILAVAACFADGVTVMKGLEELRVKESDRLSA | 360 |
| Query 361 | VANGLKLNGVDCDEGETSLVVRGRPDGKGLGNASGAAVATHLDHRIAMSFLVMGLVSENP | 420 |
| Sbjct 361 | VA+GLKLNGVDCDEG+ L+VRGRPDGKGLGNA+ V+THLDHRIAMSFLVMGL SE+P VADGLKLNGVDCDEGKDFLIVRGRPDGKGLGNAADEKVSTHLDHRIAMSFLVMGLASEHP | 420 |
| Query 421  Sbjct 421 | VTVDDATMIATSFPEFMDLMAGLGAKIE 448 VT+DDA MIATSFPEFM LM GLGAKIE VTIDDAAMIATSFPEFMQLMTGLGAKIE 448 |  |

```
>WP_139675970.1 3-phosphoshikimate 1-carboxyvinyltransferase [Rhizobium smilacinae] TNM66580.1 3-phosphoshikimate 1-carboxyvinyltransferase [Rhizobium smilacinae]
Length=455

Score = 769 bits (1987), Expect = 0.0, Method: Compositional matrix adjust. Identities = 390/455 (86%), Positives = 415/455 (91%), Gaps = 0/455 (0%)

Query 1	MLHGASSRPATARKSSGLSGTVRIPGDKSISHRSFMFGGLASGETRITGLLEGEDVINTG 60 M HG + +PA + +S L GT+RIPGDKSISHRSFMFGGLASGETRITGLLEGEDVINTG
Sbjct 1	MSHGTALKPAKSARSEALKGTIRIPGDKSISHRSFMFGGLASGETRITGLLEGEDVINTG 60
```

| Query | 61 | KAMQAMGARIRKEGDTWIIDGVGNGGLLAPEAPLDFGNAATGCRLTMGLVGVYDFDSTFI | 120 |
| --- | --- | --- | --- |
| Sbjct | 61 | KAM+AMGA+I K G WII G GNG LLAPEAPLDFGNA TGCRLTMGLVGVYDFDSTFI KAMRAMGAKIEKVGAEWIIQGTGNGALLAPEAPLDFGNAGTGCRLTMGLVGVYDFDSTFI | 120 |
| Query | 121 | GDASLTKRPMGRVLNPLREMGVQVKSEDGDRLPVTLRGPKTPTPITYRVPMASAQVKSAV | 180 |
| Sbjct | 121 | GDASLTKRPMGRVLNPLREMGVQV S DGDRLPVTLRGPKTP PITYRVPMASAQVKSAV GDASLTKRPMGRVLNPLREMGVQVTSHDGDRLPVTLRGPKTPNPITYRVPMASAQVKSAV | 180 |
| Query | 181 | LLAGLNTPGITTVIEPIMTRDHTEKMLQGFGANLTVETDADGVRTIRLEGRGKLTGQVID | 240 |
| Sbjct | 181 | LLAGLNTPGITTVIEP+MTRDHTEKMLQGFGA+L+VETDADGVRTIRL GRG+L GQVID LLAGLNTPGITTVIEPVMTRDHTEKMLQGFGADLSVETDADGVRTIRLVGRGQLKGQVID | 240 |
| Query | 241 | VPGDPSSTAFPLVAALLVPGSDVTILNVLMNPTRTGLILTLQEMGADIEVINPRLAGGED | 300 |
| Sbjct | 241 | VPGDPSSTAFPLVAALL PGSDVTILNVLMNPTRTGLILTLQEMGADIEV+N RLAGGED VPGDPSSTAFPLVAALLTPGSDVTILNVLMNPTRTGLILTLQEMGADIEVLNARLAGGED | 300 |
| Query | 301 | VADLRVRSSTLKGVTVPEDRAPSMIDEYPILavaaafaegaTVMNGLEELRVKESDRLSA | 360 |
| Sbjct | 301 | VADLRVR S LKGVTVP DRAPSMIDEYP+LAVAAA+A G T M GLEELRVKESDRLSA VADLRVRYSELKGVTVPADRAPSMIDEYPVLAVAAAYASGTTTMLGLEELRVKESDRLSA | 360 |
| Query | 361 | VANGLKLNGVDCDEGETSLVVRGRPDGKGLGNASGAAVATHLDHRIAMSFLVMGLVSENP | 420 |
| Sbjct | 361 | VA+GLKLNGVDCDEG+ +L VRGRP GKG GNA+GAAV THLDHRIAMSFLV+GL SE+P VADGLKLNGVDCDEGKETLTVRGRPGGKGYGNAAGAAVVTHLDHRIAMSFLVLGLASEHP | 420 |
| Query  Sbjct | 421  421 | VTVDDATMIATSFPEFMDLMAGLGAKIELSDTKAA 455 VTVDDA++IATSFPEFMDLM GLGAKI+L ++ A VTVDDASIIATSFPEFMDLMTGLGAKIDLVGSELA 455 |  |

>WP_018244244.1 3-phosphoshikimate 1-carboxyvinyltransferase [Rhizobium leguminosarum]

ASS55005.1 3-phosphoshikimate 1-carboxyvinyltransferase [Rhizobium leguminosarum bv. viciae]

MBB4327805.1 3-phosphoshikimate 1-carboxyvinyltransferase [Rhizobium leguminosarum] MBB4342012.1 3-phosphoshikimate 1-carboxyvinyltransferase [Rhizobium leguminosarum] MBB4353470.1 3-phosphoshikimate 1-carboxyvinyltransferase [Rhizobium leguminosarum] MBB4384792.1 3-phosphoshikimate 1-carboxyvinyltransferase [Rhizobium leguminosarum]

Length=452

Score = 769 bits (1987), Expect = 0.0, Method: Compositional matrix adjust.

| Identities | = 388/448 (87%), Positives = 416/448 (93%), Gaps = 0/448 (0%) |  |
| --- | --- | --- |
| Query 1 | MLHGASSRPATARKSSGLSGTVRIPGDKSISHRSFMFGGLASGETRITGLLEGEDVINTG | 60 |
| Sbjct 1 | MLHG++ RPATARKS+GL+G+VRIPGDKSISHRSFMFGGLASGETRITGLLEGEDVINTG MLHGSAPRPATARKSAGLTGSVRIPGDKSISHRSFMFGGLASGETRITGLLEGEDVINTG | 60 |
| Query 61 | KAMQAMGARIRKEGDTWIIDGVGNGGLLAPEAPLDFGNAATGCRLTMGLVGVYDFDSTFI | 120 |
| Sbjct 61 | +AMQAMGARIRK G+ W+I+G GNG LLAP+APLDFGNA TG RLTMGLV YDF STFI RAMQAMGARIRKVGEQWLIEGTGNGALLAPDAPLDFGNAGTGVRLTMGLVSTYDFRSTFI | 120 |
| Query 121 | GDASLTKRPMGRVLNPLREMGVQVKSEDGDRLPVTLRGPKTPTPITYRVPMASAQVKSAV | 180 |
| Sbjct 121 | GDASL+KRPMGRVLNPLREMGVQV + +GDRLPVTLRGP TP+PI YRVPMASAQVKSAV GDASLSKRPMGRVLNPLREMGVQVSASEGDRLPVTLRGPGTPSPIRYRVPMASAQVKSAV | 180 |
| Query 181 | LLAGLNTPGITTVIEPIMTRDHTEKMLQGFGANLTVETDADGVRTIRLEGRGKLTGQVID | 240 |
| Sbjct 181 | LLAGLNTPG+TTVIEP+MTRDHTEKMLQGFGA L+VETD DGVRTIRLEGRGKL GQVID LLAGLNTPGVTTVIEPVMTRDHTEKMLQGFGAALSVETDEDGVRTIRLEGRGKLKGQVID | 240 |
| Query 241 | VPGDPSSTAFPLVAALLVPGSDVTILNVLMNPTRTGLILTLQEMGADIEVINPRLAGGED | 300 |
| Sbjct 241 | +PGDPSSTAFPLVAALLVPGSD+TI+NVLMNPTRTGLILTLQEMGADIEV+N RLAGGED IPGDPSSTAFPLVAALLVPGSDITIVNVLMNPTRTGLILTLQEMGADIEVLNARLAGGED | 300 |
| Query 301 | VADLRVRSSTLKGVTVPEDRAPSMIDEYPILavaaafaegaTVMNGLEELRVKESDRLSA | 360 |
| Sbjct 301 | VADLRVR S LKGVTVPEDRAPSMIDEYPILAVAA FAEG TVM GLEELRVKESDRLSA VADLRVRHSELKGVTVPEDRAPSMIDEYPILAVAACFAEGVTVMKGLEELRVKESDRLSA | 360 |
| Query 361 | VANGLKLNGVDCDEGETSLVVRGRPDGKGLGNASGAAVATHLDHRIAMSFLVMGLVSENP | 420 |
| Sbjct 361 | VA+GLKLNGVDCDEGE L+VRGRPDGKGLGNA+ V+THLDHRIAMSFLVMGL SE+P VADGLKLNGVDCDEGEDFLIVRGRPDGKGLGNATDGRVSTHLDHRIAMSFLVMGLASEHP | 420 |
| Query 421 | VTVDDATMIATSFPEFMDLMAGLGAKIE 448 VT+DDA MIATSFPEFM LM GLGAKI+ |  |

Sbjct 421 VTIDDAAMIATSFPEFMQLMTGLGAKID 448

>WP_168331254.1 3-phosphoshikimate 1-carboxyvinyltransferase [Rhizobium leguminosarum]

NKK84144.1 3-phosphoshikimate 1-carboxyvinyltransferase [Rhizobium leguminosarum bv. viciae]

Length=452

Score = 769 bits (1987), Expect = 0.0, Method: Compositional matrix adjust.

| Identities | = 387/448 (86%), Positives = 416/448 (93%), Gaps = 0/448 (0%) |  |
| --- | --- | --- |
| Query 1 | MLHGASSRPATARKSSGLSGTVRIPGDKSISHRSFMFGGLASGETRITGLLEGEDVINTG | 60 |
| Sbjct 1 | MLHG++ RPATARKS+GL+G+VRIPGDKSISHRSFMFGGLASGETRITGLLEGEDVINTG MLHGSAPRPATARKSAGLTGSVRIPGDKSISHRSFMFGGLASGETRITGLLEGEDVINTG | 60 |
| Query 61 | KAMQAMGARIRKEGDTWIIDGVGNGGLLAPEAPLDFGNAATGCRLTMGLVGVYDFDSTFI | 120 |
| Sbjct 61 | +AMQAMGARIRKEG+ W+I+G GNG LLAP+APLDFGNA TG RLTMGLVG YDF STFI RAMQAMGARIRKEGEQWVIEGTGNGALLAPDAPLDFGNAGTGVRLTMGLVGTYDFRSTFI | 120 |
| Query 121 | GDASLTKRPMGRVLNPLREMGVQVKSEDGDRLPVTLRGPKTPTPITYRVPMASAQVKSAV | 180 |
| Sbjct 121 | GDASL+KRPMGRVLNPLREMGVQV + +GDRLPVTLRGP TP+ I YRVPMASAQVKSAV GDASLSKRPMGRVLNPLREMGVQVSASEGDRLPVTLRGPGTPSSIRYRVPMASAQVKSAV | 180 |
| Query 181 | LLAGLNTPGITTVIEPIMTRDHTEKMLQGFGANLTVETDADGVRTIRLEGRGKLTGQVID | 240 |
| Sbjct 181 | LLAGLNTPG+TTVIEP+MTRDHTEKMLQGFGA L+VETD DGVRTIRLEGRGKL GQVI+ LLAGLNTPGVTTVIEPVMTRDHTEKMLQGFGAALSVETDEDGVRTIRLEGRGKLKGQVIN | 240 |
| Query 241 | VPGDPSSTAFPLVAALLVPGSDVTILNVLMNPTRTGLILTLQEMGADIEVINPRLAGGED | 300 |
| Sbjct 241 | +P DPSSTAFPLVAALLVPGSD+TI+NVLMNPTRTGLILTLQEMGADIEV+N RLAGGED IPSDPSSTAFPLVAALLVPGSDITIVNVLMNPTRTGLILTLQEMGADIEVLNARLAGGED | 300 |
| Query 301 | VADLRVRSSTLKGVTVPEDRAPSMIDEYPILavaaafaegaTVMNGLEELRVKESDRLSA | 360 |
| Sbjct 301 | VADLRVR S LKGVTVPEDRAPSMIDEYPILAVAA FAEG TVM GLEELRVKESDRLSA VADLRVRHSELKGVTVPEDRAPSMIDEYPILAVAACFAEGVTVMKGLEELRVKESDRLSA | 360 |
| Query 361 | VANGLKLNGVDCDEGETSLVVRGRPDGKGLGNASGAAVATHLDHRIAMSFLVMGLVSENP | 420 |
| Sbjct 361 | VA+GLKLNGVDCDEGE L+VRGRPDGKGLGNA+ V+THLDHRIAMSFLVMGL SE+P VADGLKLNGVDCDEGEDFLIVRGRPDGKGLGNATDGKVSTHLDHRIAMSFLVMGLASEHP | 420 |
| Query 421  Sbjct 421 | VTVDDATMIATSFPEFMDLMAGLGAKIE 448 VT+DDA MIATSFPEFM LM GLGAKI+ VTIDDAAMIATSFPEFMQLMTGLGAKID 448 |  |

>WP_168268235.1 3-phosphoshikimate 1-carboxyvinyltransferase [Rhizobium leguminosarum]

NKL76097.1 3-phosphoshikimate 1-carboxyvinyltransferase [Rhizobium leguminosarum bv. viciae]

Length=452

Score = 769 bits (1987), Expect = 0.0, Method: Compositional matrix adjust.

| Identities | = 387/449 (86%), Positives = 416/449 (93%), Gaps = 0/449 (0%) |  |
| --- | --- | --- |
| Query 1 | MLHGASSRPATARKSSGLSGTVRIPGDKSISHRSFMFGGLASGETRITGLLEGEDVINTG | 60 |
| Sbjct 1 | MLHG++ RPATARKS+GL+G+VRIPGDKSISHRSFMFGGLASG+TRITGLLEGEDVINTG MLHGSAPRPATARKSAGLTGSVRIPGDKSISHRSFMFGGLASGQTRITGLLEGEDVINTG | 60 |
| Query 61 | KAMQAMGARIRKEGDTWIIDGVGNGGLLAPEAPLDFGNAATGCRLTMGLVGVYDFDSTFI | 120 |
| Sbjct 61 | +AMQAMGARIRK+G W+IDG GNG LLAP+APLDFGNA TG RLTMGLVG YDF STFI RAMQAMGARIRKDGAQWVIDGTGNGALLAPDAPLDFGNAGTGVRLTMGLVGTYDFRSTFI | 120 |
| Query 121 | GDASLTKRPMGRVLNPLREMGVQVKSEDGDRLPVTLRGPKTPTPITYRVPMASAQVKSAV | 180 |
| Sbjct 121 | GDASL+KRPMGRVLNPLREMGVQV + +GDRLPVTLRGP TP+PI YRVPMASAQVKSAV GDASLSKRPMGRVLNPLREMGVQVSASEGDRLPVTLRGPATPSPIRYRVPMASAQVKSAV | 180 |
| Query 181 | LLAGLNTPGITTVIEPIMTRDHTEKMLQGFGANLTVETDADGVRTIRLEGRGKLTGQVID | 240 |
| Sbjct 181 | LLAGLNTPG+TTVIEP+MTRDHTEKMLQGFGA L+VE D DGVRTIRLEGRGKL GQVID LLAGLNTPGVTTVIEPVMTRDHTEKMLQGFGAALSVEMDGDGVRTIRLEGRGKLKGQVID | 240 |
| Query 241 | VPGDPSSTAFPLVAALLVPGSDVTILNVLMNPTRTGLILTLQEMGADIEVINPRLAGGED | 300 |
| Sbjct 241 | +PGDPSSTAFPLVAALLVPGSD+TI+NVLMNPTRTGLILTLQEMGADIEV+N RLAGGED  IPGDPSSTAFPLVAALLVPGSDITIVNVLMNPTRTGLILTLQEMGADIEVVNARLAGGED | 300 |

| Query | 301 | VADLRVRSSTLKGVTVPEDRAPSMIDEYPILavaaafaegaTVMNGLEELRVKESDRLSA | 360 |
| --- | --- | --- | --- |
| Sbjct | 301 | VADLRVR S LKGVTVPE+RAPSMIDEYPILA+AA FAEGATVM GLEELRVKESDRLSA VADLRVRHSELKGVTVPEERAPSMIDEYPILAIAACFAEGATVMKGLEELRVKESDRLSA | 360 |
| Query | 361 | VANGLKLNGVDCDEGETSLVVRGRPDGKGLGNASGAAVATHLDHRIAMSFLVMGLVSENP | 420 |
| Sbjct | 361 | VA+GLKLNG DCDEGE L+VRGRPDGKGLGNA+G V THLDHRIAMSFLV+GL SE+ VADGLKLNGADCDEGEDFLIVRGRPDGKGLGNAAGGKVRTHLDHRIAMSFLVLGLASEHA | 420 |
| Query  Sbjct | 421  421 | VTVDDATMIATSFPEFMDLMAGLGAKIEL 449 VT+DDA MIATSFPEFM LM GLGAKIEL VTIDDAAMIATSFPEFMQLMTGLGAKIEL 449 |  |

>WP_062941108.1 3-phosphoshikimate 1-carboxyvinyltransferase [Rhizobium leguminosarum] KZB01403.1 3-phosphoshikimate 1-carboxyvinyltransferase [Rhizobium leguminosarum]

Length=452

Score = 769 bits (1986), Expect = 0.0, Method: Compositional matrix adjust.

| Identities | = 389/448 (87%), Positives = 416/448 (93%), Gaps = 0/448 (0%) |  |
| --- | --- | --- |
| Query 1 | MLHGASSRPATARKSSGLSGTVRIPGDKSISHRSFMFGGLASGETRITGLLEGEDVINTG | 60 |
| Sbjct 1 | ML+G++ +PATARKS+GL+G+VRIPGDKSISHRSFMFGGLASGETRITGLLEGEDVINTG MLNGSAPKPATARKSAGLTGSVRIPGDKSISHRSFMFGGLASGETRITGLLEGEDVINTG | 60 |
| Query 61 | KAMQAMGARIRKEGDTWIIDGVGNGGLLAPEAPLDFGNAATGCRLTMGLVGVYDFDSTFI | 120 |
| Sbjct 61 | +AMQAMGARIRK+G W+IDG GNG LLAP+APLDFGNA TG RLTMGLVG YDF STFI RAMQAMGARIRKDGAQWVIDGTGNGALLAPDAPLDFGNAGTGVRLTMGLVGTYDFRSTFI | 120 |
| Query 121 | GDASLTKRPMGRVLNPLREMGVQVKSEDGDRLPVTLRGPKTPTPITYRVPMASAQVKSAV | 180 |
| Sbjct 121 | GDASL+KRPMGRVLNPLREMGVQV + +GDRLPVTLRGP TP+PI YRVPMASAQVKSAV GDASLSKRPMGRVLNPLREMGVQVSASEGDRLPVTLRGPGTPSPIRYRVPMASAQVKSAV | 180 |
| Query 181 | LLAGLNTPGITTVIEPIMTRDHTEKMLQGFGANLTVETDADGVRTIRLEGRGKLTGQVID | 240 |
| Sbjct 181 | LLAGLNTPG+TTVIEP+MTRDHTEKMLQGFGA L+VETD DGVRTIRLEGRGKL GQVID LLAGLNTPGVTTVIEPVMTRDHTEKMLQGFGAALSVETDGDGVRTIRLEGRGKLKGQVID | 240 |
| Query 241 | VPGDPSSTAFPLVAALLVPGSDVTILNVLMNPTRTGLILTLQEMGADIEVINPRLAGGED | 300 |
| Sbjct 241 | VPGDPSSTAFPLV ALLVPGSD+TI+NVLMNPTRTGLILTLQEMGADIEV N RLAGGED VPGDPSSTAFPLVGALLVPGSDITIVNVLMNPTRTGLILTLQEMGADIEVANARLAGGED | 300 |
| Query 301 | VADLRVRSSTLKGVTVPEDRAPSMIDEYPILavaaafaegaTVMNGLEELRVKESDRLSA | 360 |
| Sbjct 301 | VADLRVR S LKGVTVPEDRAPSMIDEYPILA+AA FAEGAT+M GLEELRVKESDRLSA VADLRVRHSELKGVTVPEDRAPSMIDEYPILAIAACFAEGATIMKGLEELRVKESDRLSA | 360 |
| Query 361 | VANGLKLNGVDCDEGETSLVVRGRPDGKGLGNASGAAVATHLDHRIAMSFLVMGLVSENP | 420 |
| Sbjct 361 | VA+GLKLNGVDCDEGE LVVRGRPDGKGLGNA+ V+THLDHRIAMSFLVMGL SE+P VADGLKLNGVDCDEGEDFLVVRGRPDGKGLGNAADEKVSTHLDHRIAMSFLVMGLASEHP | 420 |
| Query 421  Sbjct 421 | VTVDDATMIATSFPEFMDLMAGLGAKIE 448 VT+DDA MIATSFPEFM LM GLGAKIE VTIDDAAMIATSFPEFMQLMTGLGAKIE 448 |  |

>WP_130709147.1 3-phosphoshikimate 1-carboxyvinyltransferase [Rhizobium leguminosarum] TAW32040.1 3-phosphoshikimate 1-carboxyvinyltransferase [Rhizobium leguminosarum] TAW45771.1 3-phosphoshikimate 1-carboxyvinyltransferase [Rhizobium leguminosarum] TAX32352.1 3-phosphoshikimate 1-carboxyvinyltransferase [Rhizobium leguminosarum] TAY35151.1 3-phosphoshikimate 1-carboxyvinyltransferase [Rhizobium leguminosarum]

Length=452

Score = 769 bits (1986), Expect = 0.0, Method: Compositional matrix adjust.

| Identities | = 389/449 (87%), Positives = 417/449 (93%), Gaps = 0/449 (0%) |  |
| --- | --- | --- |
| Query 1 | MLHGASSRPATARKSSGLSGTVRIPGDKSISHRSFMFGGLASGETRITGLLEGEDVINTG | 60 |
| Sbjct 1 | ML+G++ RPATARKS+GL+G+VRIPGDKSISHRSFMFGGLASGETRITGLLEGEDVINTG MLNGSAPRPATARKSAGLTGSVRIPGDKSISHRSFMFGGLASGETRITGLLEGEDVINTG | 60 |
| Query 61 | KAMQAMGARIRKEGDTWIIDGVGNGGLLAPEAPLDFGNAATGCRLTMGLVGVYDFDSTFI | 120 |
| Sbjct 61 | +AMQAMGARIR EG W+I+G GNG LLAP+APLDFGNA TG RLTMGLVG YDF STFI RAMQAMGARIRNEGAQWVIEGTGNGALLAPDAPLDFGNAGTGVRLTMGLVGTYDFRSTFI | 120 |
| Query 121 | GDASLTKRPMGRVLNPLREMGVQVKSEDGDRLPVTLRGPKTPTPITYRVPMASAQVKSAV | 180 |

| Sbjct | 121 | GDASL+KRPMGRVL+PLREMGVQV + +GDRLPVTL+GP TP+PI YRVPMASAQVKSAV GDASLSKRPMGRVLDPLREMGVQVSASEGDRLPVTLKGPGTPSPIRYRVPMASAQVKSAV | 180 |
| --- | --- | --- | --- |
| Query | 181 | LLAGLNTPGITTVIEPIMTRDHTEKMLQGFGANLTVETDADGVRTIRLEGRGKLTGQVID | 240 |
| Sbjct | 181 | LLAGLNTPG+TTVIEP+MTRDHTEKMLQGFGA L+VETD DGVRTIRLEGRG+LTGQVID LLAGLNTPGVTTVIEPVMTRDHTEKMLQGFGAALSVETDEDGVRTIRLEGRGRLTGQVID | 240 |
| Query | 241 | VPGDPSSTAFPLVAALLVPGSDVTILNVLMNPTRTGLILTLQEMGADIEVINPRLAGGED | 300 |
| Sbjct | 241 | VPGDPSSTAFPLVAALLVPGSD+TI+NVLMNPTRTGLILTLQEMGADIEV N RLAGGED VPGDPSSTAFPLVAALLVPGSDITIVNVLMNPTRTGLILTLQEMGADIEVANARLAGGED | 300 |
| Query | 301 | VADLRVRSSTLKGVTVPEDRAPSMIDEYPILavaaafaegaTVMNGLEELRVKESDRLSA | 360 |
| Sbjct | 301 | VADLRVR S LKGVTVPE RAPSMIDEYPILAVAA FA+GAT+M GLEELRVKESDRLSA VADLRVRHSELKGVTVPEQRAPSMIDEYPILAVAACFADGATIMKGLEELRVKESDRLSA | 360 |
| Query | 361 | VANGLKLNGVDCDEGETSLVVRGRPDGKGLGNASGAAVATHLDHRIAMSFLVMGLVSENP | 420 |
| Sbjct | 361 | VANGLKLNGVDCDEGE LVVRGRPDGKGLGNA+G V+THLDHRIAMSFLVMGL SE+ VANGLKLNGVDCDEGEDFLVVRGRPDGKGLGNAAGGRVSTHLDHRIAMSFLVMGLASEHA | 420 |
| Query  Sbjct | 421  421 | VTVDDATMIATSFPEFMDLMAGLGAKIEL 449 VT+DDA MIATSFPEFM LM GLGAKIEL VTIDDAAMIATSFPEFMQLMTGLGAKIEL 449 |  |

>WP_130711011.1 3-phosphoshikimate 1-carboxyvinyltransferase [Rhizobium leguminosarum] TAX12193.1 3-phosphoshikimate 1-carboxyvinyltransferase [Rhizobium leguminosarum] TAY14760.1 3-phosphoshikimate 1-carboxyvinyltransferase [Rhizobium leguminosarum]

Length=452

Score = 769 bits (1986), Expect = 0.0, Method: Compositional matrix adjust.

| Identities | = 386/448 (86%), Positives = 418/448 (93%), Gaps = 0/448 (0%) |  |
| --- | --- | --- |
| Query 1 | MLHGASSRPATARKSSGLSGTVRIPGDKSISHRSFMFGGLASGETRITGLLEGEDVINTG | 60 |
| Sbjct 1 | ML+G++ +P TARKS+ L+G+VRIPGDKSISHRSFMFGGLASGETRITGLLEGEDVINTG MLNGSAPKPVTARKSASLTGSVRIPGDKSISHRSFMFGGLASGETRITGLLEGEDVINTG | 60 |
| Query 61 | KAMQAMGARIRKEGDTWIIDGVGNGGLLAPEAPLDFGNAATGCRLTMGLVGVYDFDSTFI | 120 |
| Sbjct 61 | +AMQAMGARIRKEG W+IDG GNG LLAP+APLDFGNA TG RLTMGLVG YDF STFI RAMQAMGARIRKEGSQWVIDGTGNGALLAPDAPLDFGNAGTGVRLTMGLVGTYDFHSTFI | 120 |
| Query 121 | GDASLTKRPMGRVLNPLREMGVQVKSEDGDRLPVTLRGPKTPTPITYRVPMASAQVKSAV | 180 |
| Sbjct 121 | GDASL+KRPMGRVLNPLREMGVQV + +GDRLPVTL+GP TP+PI YRVPMASAQVKSAV GDASLSKRPMGRVLNPLREMGVQVSASEGDRLPVTLKGPGTPSPIRYRVPMASAQVKSAV | 180 |
| Query 181 | LLAGLNTPGITTVIEPIMTRDHTEKMLQGFGANLTVETDADGVRTIRLEGRGKLTGQVID | 240 |
| Sbjct 181 | LLAGLNTPG+TTVIEP+MTRDHTEKMLQGFGA L+VET+++GVRTIRLEGRGKLTGQVID LLAGLNTPGVTTVIEPVMTRDHTEKMLQGFGAALSVETNSEGVRTIRLEGRGKLTGQVID | 240 |
| Query 241 | VPGDPSSTAFPLVAALLVPGSDVTILNVLMNPTRTGLILTLQEMGADIEVINPRLAGGED | 300 |
| Sbjct 241 | VPGDPSSTAFPLVAALLVPGSD+TI+NVLMNPTRTGLILTLQEMGADIEV+NPRLAGGED VPGDPSSTAFPLVAALLVPGSDITIVNVLMNPTRTGLILTLQEMGADIEVLNPRLAGGED | 300 |
| Query 301 | VADLRVRSSTLKGVTVPEDRAPSMIDEYPILavaaafaegaTVMNGLEELRVKESDRLSA | 360 |
| Sbjct 301 | VADLRVR S LKGVTVPE+RAPSMIDEYPILAVAA FAEGAT+M GLEELRVKESDRLSA VADLRVRHSELKGVTVPEERAPSMIDEYPILAVAACFAEGATIMKGLEELRVKESDRLSA | 360 |
| Query 361 | VANGLKLNGVDCDEGETSLVVRGRPDGKGLGNASGAAVATHLDHRIAMSFLVMGLVSENP | 420 |
| Sbjct 361 | VA GLKLNGVDCDEGE L+VRGRPDGKGLGNA+ V+THLDHRIAMSFLVMGL S++P VAEGLKLNGVDCDEGEDFLIVRGRPDGKGLGNAADGRVSTHLDHRIAMSFLVMGLASQHP | 420 |
| Query 421  Sbjct 421 | VTVDDATMIATSFPEFMDLMAGLGAKIE 448 VT+DDA MIATSFPEFM LM GLGAKIE VTIDDAAMIATSFPEFMQLMTGLGAKIE 448 |  |

>WP_131640109.1 3-phosphoshikimate 1-carboxyvinyltransferase [Rhizobium laguerreae] NKM13018.1 3-phosphoshikimate 1-carboxyvinyltransferase [Rhizobium laguerreae] NKM34424.1 3-phosphoshikimate 1-carboxyvinyltransferase [Rhizobium laguerreae] NKM40653.1 3-phosphoshikimate 1-carboxyvinyltransferase [Rhizobium laguerreae] NKM88978.1 3-phosphoshikimate 1-carboxyvinyltransferase [Rhizobium laguerreae] NKN09143.1 3-phosphoshikimate 1-carboxyvinyltransferase [Rhizobium laguerreae]

Length=452

Score = 769 bits (1986), Expect = 0.0, Method: Compositional matrix adjust.

| Identities | = 388/448 (87%), Positives = 417/448 (93%), Gaps = 0/448 (0%) |  |
| --- | --- | --- |
| Query 1 | MLHGASSRPATARKSSGLSGTVRIPGDKSISHRSFMFGGLASGETRITGLLEGEDVINTG | 60 |
| Sbjct 1 | MLHG++ RPATARKS+GL+G+VRIPGDKSISHRSFMFGGLASGETRITGLLEGEDVINTG MLHGSAPRPATARKSAGLAGSVRIPGDKSISHRSFMFGGLASGETRITGLLEGEDVINTG | 60 |
| Query 61 | KAMQAMGARIRKEGDTWIIDGVGNGGLLAPEAPLDFGNAATGCRLTMGLVGVYDFDSTFI | 120 |
| Sbjct 61 | +AMQAMGARIRK+G W+IDG GNG LLAP+APLDFGNA TG RLTMGLVG YDF STF RAMQAMGARIRKDGAQWVIDGTGNGALLAPDAPLDFGNAGTGVRLTMGLVGTYDFRSTFT | 120 |
| Query 121 | GDASLTKRPMGRVLNPLREMGVQVKSEDGDRLPVTLRGPKTPTPITYRVPMASAQVKSAV | 180 |
| Sbjct 121 | GDASL+KRPMGRVLNPLREMGVQV + +GDRLPVTLRGP TP+PI YRVPMASAQVKSAV GDASLSKRPMGRVLNPLREMGVQVSASEGDRLPVTLRGPGTPSPIRYRVPMASAQVKSAV | 180 |
| Query 181 | LLAGLNTPGITTVIEPIMTRDHTEKMLQGFGANLTVETDADGVRTIRLEGRGKLTGQVID | 240 |
| Sbjct 181 | LLAGLNTPG+TTVIEP+MTRDHTEKMLQGFGA L+VETD DGVRTIRLEGRGKL GQVID LLAGLNTPGVTTVIEPVMTRDHTEKMLQGFGAALSVETDGDGVRTIRLEGRGKLKGQVID | 240 |
| Query 241 | VPGDPSSTAFPLVAALLVPGSDVTILNVLMNPTRTGLILTLQEMGADIEVINPRLAGGED | 300 |
| Sbjct 241 | +PGDPSSTAFPLVAALLVPGSD+TI+NVLMNPTRTGLILTLQEMGA+IEV+N RLAGGED IPGDPSSTAFPLVAALLVPGSDITIVNVLMNPTRTGLILTLQEMGANIEVLNTRLAGGED | 300 |
| Query 301 | VADLRVRSSTLKGVTVPEDRAPSMIDEYPILavaaafaegaTVMNGLEELRVKESDRLSA | 360 |
| Sbjct 301 | VADLRVR S LKGVTVPE+RAPSMIDEYPILAVAA FAEGAT+M GLEELRVKESDRLSA VADLRVRHSELKGVTVPEERAPSMIDEYPILAVAACFAEGATIMKGLEELRVKESDRLSA | 360 |
| Query 361 | VANGLKLNGVDCDEGETSLVVRGRPDGKGLGNASGAAVATHLDHRIAMSFLVMGLVSENP | 420 |
| Sbjct 361 | VA+GLKLNG+DCDEGE LVVRGRPDGKGLGNA+G V+THLDHRIAMSFLVMGL SE+ VADGLKLNGIDCDEGEDFLVVRGRPDGKGLGNAAGGRVSTHLDHRIAMSFLVMGLASEHA | 420 |
| Query 421  Sbjct 421 | VTVDDATMIATSFPEFMDLMAGLGAKIE 448 VT+DDA MIATSFPEFM LM GLGAKIE VTIDDAAMIATSFPEFMQLMTGLGAKIE 448 |  |

>WP_164070240.1 3-phosphoshikimate 1-carboxyvinyltransferase [Rhizobium leguminosarum] NEH60239.1 3-phosphoshikimate 1-carboxyvinyltransferase [Rhizobium leguminosarum]

Length=452

Score = 769 bits (1986), Expect = 0.0, Method: Compositional matrix adjust.

| Identities | = 386/448 (86%), Positives = 419/448 (94%), Gaps = 0/448 (0%) |  |
| --- | --- | --- |
| Query 1 | MLHGASSRPATARKSSGLSGTVRIPGDKSISHRSFMFGGLASGETRITGLLEGEDVINTG | 60 |
| Sbjct 1 | ML+G++S+PATARKS+GL+G+VRIPGDKSISHRSFMFGGLASGETRITGLLEGEDVINTG MLNGSASKPATARKSAGLTGSVRIPGDKSISHRSFMFGGLASGETRITGLLEGEDVINTG | 60 |
| Query 61 | KAMQAMGARIRKEGDTWIIDGVGNGGLLAPEAPLDFGNAATGCRLTMGLVGVYDFDSTFI | 120 |
| Sbjct 61 | +AMQAMGARIRKEG W+I+G GNG LLAP+APLDFGNA TG RLTMGLVG YDF STFI RAMQAMGARIRKEGAQWVIEGTGNGALLAPDAPLDFGNAGTGVRLTMGLVGTYDFHSTFI | 120 |
| Query 121 | GDASLTKRPMGRVLNPLREMGVQVKSEDGDRLPVTLRGPKTPTPITYRVPMASAQVKSAV | 180 |
| Sbjct 121 | GDASL+KRPMGRVLNPLREMGVQ+ + +GDRLPVTL+GP TP+PI YRVPMASAQVKSAV GDASLSKRPMGRVLNPLREMGVQISASEGDRLPVTLKGPGTPSPIRYRVPMASAQVKSAV | 180 |
| Query 181 | LLAGLNTPGITTVIEPIMTRDHTEKMLQGFGANLTVETDADGVRTIRLEGRGKLTGQVID | 240 |
| Sbjct 181 | LLAGLNTPG+TTVIEP+MTRDHTEKMLQGFGA L+VETD +GVRTIRLEGRG+LTGQVID LLAGLNTPGVTTVIEPVMTRDHTEKMLQGFGAALSVETDNEGVRTIRLEGRGRLTGQVID | 240 |
| Query 241 | VPGDPSSTAFPLVAALLVPGSDVTILNVLMNPTRTGLILTLQEMGADIEVINPRLAGGED | 300 |
| Sbjct 241 | VPGDPSSTAFPLVAALLVPGSD+TI+NVLMNPTRTGLILTLQEMGA+IEV+N RLAGGED VPGDPSSTAFPLVAALLVPGSDITIVNVLMNPTRTGLILTLQEMGANIEVLNARLAGGED | 300 |
| Query 301 | VADLRVRSSTLKGVTVPEDRAPSMIDEYPILavaaafaegaTVMNGLEELRVKESDRLSA | 360 |
| Sbjct 301 | VADLRVR S L+GVTVPEDRAPSMIDEYPILAVAA FAEG TVM GLEELRVKESDRLSA VADLRVRHSELRGVTVPEDRAPSMIDEYPILAVAACFAEGTTVMKGLEELRVKESDRLSA | 360 |
| Query 361 | VANGLKLNGVDCDEGETSLVVRGRPDGKGLGNASGAAVATHLDHRIAMSFLVMGLVSENP | 420 |
| Sbjct 361 | VA+GLKLNGVDCDEGE L VRGRPDG+GLGNA+G V+THLDHRIAMSFLVMGL SE+P  VADGLKLNGVDCDEGEDFLTVRGRPDGRGLGNAAGGRVSTHLDHRIAMSFLVMGLASEHP | 420 |

Query 421 VTVDDATMIATSFPEFMDLMAGLGAKIE 448 VT+DDA MIATSFPEFM LM GLGAKIE

Sbjct 421 VTIDDAAMIATSFPEFMQLMTGLGAKIE 448

>WP_168283252.1 3-phosphoshikimate 1-carboxyvinyltransferase [Rhizobium laguerreae] NKM25740.1 3-phosphoshikimate 1-carboxyvinyltransferase [Rhizobium laguerreae]

Length=452

Score = 769 bits (1986), Expect = 0.0, Method: Compositional matrix adjust.

| Identities | = 388/449 (86%), Positives = 417/449 (93%), Gaps = 0/449 (0%) |  |
| --- | --- | --- |
| Query 1 | MLHGASSRPATARKSSGLSGTVRIPGDKSISHRSFMFGGLASGETRITGLLEGEDVINTG | 60 |
| Sbjct 1 | MLHG++ RPATARKS+GL+G+VRIPGDKSISHRSFMFGGLASGETRITGLLEGEDVINTG MLHGSAPRPATARKSAGLAGSVRIPGDKSISHRSFMFGGLASGETRITGLLEGEDVINTG | 60 |
| Query 61 | KAMQAMGARIRKEGDTWIIDGVGNGGLLAPEAPLDFGNAATGCRLTMGLVGVYDFDSTFI | 120 |
| Sbjct 61 | +AMQAMGARIRK+G W+IDG GNG LLAP+APLDFGNA TG RLTMGLVG YDF STF RAMQAMGARIRKDGAQWVIDGTGNGALLAPDAPLDFGNAGTGVRLTMGLVGTYDFRSTFT | 120 |
| Query 121 | GDASLTKRPMGRVLNPLREMGVQVKSEDGDRLPVTLRGPKTPTPITYRVPMASAQVKSAV | 180 |
| Sbjct 121 | GDASL+KRPMGRVL+PLREMGVQV + +GDRLPVTLRGP TP+PI YRVPMASAQVKSAV GDASLSKRPMGRVLDPLREMGVQVSASEGDRLPVTLRGPGTPSPIRYRVPMASAQVKSAV | 180 |
| Query 181 | LLAGLNTPGITTVIEPIMTRDHTEKMLQGFGANLTVETDADGVRTIRLEGRGKLTGQVID | 240 |
| Sbjct 181 | LLAGLNTPG+TTVIEP+MTRDHTEKMLQGFGA L+VETD DGVRTIRLEGRGKL GQVID LLAGLNTPGVTTVIEPVMTRDHTEKMLQGFGAALSVETDGDGVRTIRLEGRGKLKGQVID | 240 |
| Query 241 | VPGDPSSTAFPLVAALLVPGSDVTILNVLMNPTRTGLILTLQEMGADIEVINPRLAGGED | 300 |
| Sbjct 241 | +PGDPSSTAFPLVAALLVPGSD+TI+NVLMNPTRTGLI+TLQEMGADIEV+N RLAGGED IPGDPSSTAFPLVAALLVPGSDITIVNVLMNPTRTGLIVTLQEMGADIEVLNTRLAGGED | 300 |
| Query 301 | VADLRVRSSTLKGVTVPEDRAPSMIDEYPILavaaafaegaTVMNGLEELRVKESDRLSA | 360 |
| Sbjct 301 | VADLRVR S LKGVTVPE+RAPSMIDEYPILAVAA FAEG T+M GLEELRVKESDRLSA VADLRVRHSELKGVTVPEERAPSMIDEYPILAVAACFAEGVTIMKGLEELRVKESDRLSA | 360 |
| Query 361 | VANGLKLNGVDCDEGETSLVVRGRPDGKGLGNASGAAVATHLDHRIAMSFLVMGLVSENP | 420 |
| Sbjct 361 | VA+GLKLNGVDCDEGE LVVRGRPDGKGLGNA+G V+THLDHRIAMSFLVMGL SE+ VADGLKLNGVDCDEGEDFLVVRGRPDGKGLGNAAGDKVSTHLDHRIAMSFLVMGLASEHA | 420 |
| Query 421  Sbjct 421 | VTVDDATMIATSFPEFMDLMAGLGAKIEL 449 VT+DDA MIATSFPEFM LM GLGAKIEL VTIDDAAMIATSFPEFMQLMTGLGAKIEL 449 |  |

>WP_168290947.1 3-phosphoshikimate 1-carboxyvinyltransferase [Rhizobium leguminosarum]

NKK79279.1 3-phosphoshikimate 1-carboxyvinyltransferase [Rhizobium leguminosarum bv. viciae]

Length=452

Score = 769 bits (1986), Expect = 0.0, Method: Compositional matrix adjust.

| Identities | = 389/449 (87%), Positives = 416/449 (93%), Gaps = 0/449 (0%) |  |
| --- | --- | --- |
| Query 1 | MLHGASSRPATARKSSGLSGTVRIPGDKSISHRSFMFGGLASGETRITGLLEGEDVINTG | 60 |
| Sbjct 1 | MLHG++ +PATARKS+GL+G+VRIPGDKSISHRSFMFGGLASG+TRITGLLEGEDVINTG MLHGSAPKPATARKSAGLAGSVRIPGDKSISHRSFMFGGLASGQTRITGLLEGEDVINTG | 60 |
| Query 61 | KAMQAMGARIRKEGDTWIIDGVGNGGLLAPEAPLDFGNAATGCRLTMGLVGVYDFDSTFI | 120 |
| Sbjct 61 | +AMQAMGARIRK+G W+IDG GNG LLAP+APLDFGNA TG RLTMGLVG YDF STFI RAMQAMGARIRKDGAQWVIDGTGNGALLAPDAPLDFGNAGTGVRLTMGLVGTYDFRSTFI | 120 |
| Query 121 | GDASLTKRPMGRVLNPLREMGVQVKSEDGDRLPVTLRGPKTPTPITYRVPMASAQVKSAV | 180 |
| Sbjct 121 | GDASL+KRPMGRVLNPLREMGVQV + +GDRLPVTLRGP TP+PI YRVPMASAQVKSAV GDASLSKRPMGRVLNPLREMGVQVSASEGDRLPVTLRGPGTPSPIRYRVPMASAQVKSAV | 180 |
| Query 181 | LLAGLNTPGITTVIEPIMTRDHTEKMLQGFGANLTVETDADGVRTIRLEGRGKLTGQVID | 240 |
| Sbjct 181 | LLAGLNTPG+TTVIEP+MTRDHTEKMLQGFGA L+VETD DGVRTIRLEGRGKL GQVID LLAGLNTPGVTTVIEPVMTRDHTEKMLQGFGAALSVETDGDGVRTIRLEGRGKLKGQVID | 240 |
| Query 241 | VPGDPSSTAFPLVAALLVPGSDVTILNVLMNPTRTGLILTLQEMGADIEVINPRLAGGED | 300 |
| Sbjct 241 | VPGDPSSTAFPLVAALLVPGSD+TI+NVLMNPTRTGLILTL+EMGADI V N RLAGGED  VPGDPSSTAFPLVAALLVPGSDITIVNVLMNPTRTGLILTLEEMGADIAVANARLAGGED | 300 |

| Query | 301 | VADLRVRSSTLKGVTVPEDRAPSMIDEYPILavaaafaegaTVMNGLEELRVKESDRLSA | 360 |
| --- | --- | --- | --- |
| Sbjct | 301 | VADLRVR S LKGVTVPE+RAPSMIDEYPILAVAA FAEG TVM GLEELRVKESDRLSA VADLRVRHSELKGVTVPEERAPSMIDEYPILAVAACFAEGVTVMKGLEELRVKESDRLSA | 360 |
| Query | 361 | VANGLKLNGVDCDEGETSLVVRGRPDGKGLGNASGAAVATHLDHRIAMSFLVMGLVSENP | 420 |
| Sbjct | 361 | VA+GLKLNGVDCDEGE LVVRGRPDGKGLGNA+G V+THLDHRIAMSFLVMGL SE+ VADGLKLNGVDCDEGEDFLVVRGRPDGKGLGNAAGDKVSTHLDHRIAMSFLVMGLASEHA | 420 |
| Query  Sbjct | 421  421 | VTVDDATMIATSFPEFMDLMAGLGAKIEL 449 VT+DDA MIATSFPEFM LM GLGAKIEL VTIDDAAMIATSFPEFMQLMTGLGAKIEL 449 |  |

>WP_138332068.1 3-phosphoshikimate 1-carboxyvinyltransferase [Rhizobium indicum] QKK31371.1 3-phosphoshikimate 1-carboxyvinyltransferase [Rhizobium indicum]

Length=452

Score = 769 bits (1985), Expect = 0.0, Method: Compositional matrix adjust.

| Identities | = 387/448 (86%), Positives = 419/448 (94%), Gaps = 0/448 (0%) |  |
| --- | --- | --- |
| Query 1 | MLHGASSRPATARKSSGLSGTVRIPGDKSISHRSFMFGGLASGETRITGLLEGEDVINTG | 60 |
| Sbjct 1 | MLHG++ RPATARKS+GL+G+VRIPGDKSISHRSFMFGGLASGETRITGLLEGEDVINTG MLHGSAPRPATARKSAGLTGSVRIPGDKSISHRSFMFGGLASGETRITGLLEGEDVINTG | 60 |
| Query 61 | KAMQAMGARIRKEGDTWIIDGVGNGGLLAPEAPLDFGNAATGCRLTMGLVGVYDFDSTFI | 120 |
| Sbjct 61 | +AMQAMGARIRK+G W+IDG GNG LLAP+APLDFGNA TG RLTMGLVG YDF STFI RAMQAMGARIRKDGAQWVIDGTGNGALLAPDAPLDFGNAGTGVRLTMGLVGTYDFRSTFI | 120 |
| Query 121 | GDASLTKRPMGRVLNPLREMGVQVKSEDGDRLPVTLRGPKTPTPITYRVPMASAQVKSAV | 180 |
| Sbjct 121 | GDASL+KRPMGRVLNPLREMGVQV + +GDRLPVTL+GP TP+PI YRVPMASAQVKSAV GDASLSKRPMGRVLNPLREMGVQVSASEGDRLPVTLKGPGTPSPIRYRVPMASAQVKSAV | 180 |
| Query 181 | LLAGLNTPGITTVIEPIMTRDHTEKMLQGFGANLTVETDADGVRTIRLEGRGKLTGQVID | 240 |
| Sbjct 181 | LLAGLNTPG+TTVIEP+MTRDHTEKMLQGFGA L+VETD++G RTIRLEGRG+LTGQVID LLAGLNTPGVTTVIEPVMTRDHTEKMLQGFGAALSVETDSEGARTIRLEGRGRLTGQVID | 240 |
| Query 241 | VPGDPSSTAFPLVAALLVPGSDVTILNVLMNPTRTGLILTLQEMGADIEVINPRLAGGED | 300 |
| Sbjct 241 | +PGDPSSTAFPLVAALLVPGSD+TI+NVLMNPTRTGLILTLQEMGADIEV N RLAGGED IPGDPSSTAFPLVAALLVPGSDITIVNVLMNPTRTGLILTLQEMGADIEVANARLAGGED | 300 |
| Query 301 | VADLRVRSSTLKGVTVPEDRAPSMIDEYPILavaaafaegaTVMNGLEELRVKESDRLSA | 360 |
| Sbjct 301 | VADLRVR S LKGVTVPEDRAPSMIDEYPILAVAA+FAEGAT+M GLEELRVKESDRLSA VADLRVRHSKLKGVTVPEDRAPSMIDEYPILAVAASFAEGATIMKGLEELRVKESDRLSA | 360 |
| Query 361 | VANGLKLNGVDCDEGETSLVVRGRPDGKGLGNASGAAVATHLDHRIAMSFLVMGLVSENP | 420 |
| Sbjct 361 | VA+GLKLNGVDCDEGE L+VRGRPDGKGLGNA+ V+THLDHRIAMSFLVMGL SE+P VADGLKLNGVDCDEGEDFLIVRGRPDGKGLGNAADDRVSTHLDHRIAMSFLVMGLASEHP | 420 |
| Query 421  Sbjct 421 | VTVDDATMIATSFPEFMDLMAGLGAKIE 448 VT+DDA MIATSFPEFM LM GLGA+IE VTIDDAAMIATSFPEFMTLMKGLGARIE 448 |  |

>WP_181293776.1 3-phosphoshikimate 1-carboxyvinyltransferase [Rhizobium sp. WYCCWR 11146]

MBA1348874.1 3-phosphoshikimate 1-carboxyvinyltransferase [Rhizobium sp. WYCCWR 11146]

Length=452

Score = 769 bits (1985), Expect = 0.0, Method: Compositional matrix adjust. Identities = 387/448 (86%), Positives = 418/448 (93%), Gaps = 0/448 (0%)

| Query | 1 | MLHGASSRPATARKSSGLSGTVRIPGDKSISHRSFMFGGLASGETRITGLLEGEDVINTG | 60 |
| --- | --- | --- | --- |
| Sbjct | 1 | MLHG++ RPATARKS+GL+G+VRIPGDKSISHRSFMFGGLASGETRITGLLEGEDVINT MLHGSAPRPATARKSAGLTGSVRIPGDKSISHRSFMFGGLASGETRITGLLEGEDVINTS | 60 |
| Query | 61 | KAMQAMGARIRKEGDTWIIDGVGNGGLLAPEAPLDFGNAATGCRLTMGLVGVYDFDSTFI | 120 |
| Sbjct | 61 | +AMQAMGARIRK+G W+IDG GNG LLAP+APLDFGNA TG RLTMGLVG YDF STFI RAMQAMGARIRKDGAQWVIDGTGNGALLAPDAPLDFGNAGTGVRLTMGLVGTYDFRSTFI | 120 |
| Query | 121 | GDASLTKRPMGRVLNPLREMGVQVKSEDGDRLPVTLRGPKTPTPITYRVPMASAQVKSAV | 180 |

| Sbjct | 121 | GDASL+KRPMGRVLNPLREMGVQV + +GDRLPVTL+GP TP+PI YRVPMASAQVKSAV GDASLSKRPMGRVLNPLREMGVQVSASEGDRLPVTLKGPGTPSPIRYRVPMASAQVKSAV | 180 |
| --- | --- | --- | --- |
| Query | 181 | LLAGLNTPGITTVIEPIMTRDHTEKMLQGFGANLTVETDADGVRTIRLEGRGKLTGQVID | 240 |
| Sbjct | 181 | LLAGLNTPG+TTVIEP+MTRDHTEKMLQGFGA L+VETD++G RTIRLEGRG+LTGQVID LLAGLNTPGVTTVIEPVMTRDHTEKMLQGFGAALSVETDSEGARTIRLEGRGRLTGQVID | 240 |
| Query | 241 | VPGDPSSTAFPLVAALLVPGSDVTILNVLMNPTRTGLILTLQEMGADIEVINPRLAGGED | 300 |
| Sbjct | 241 | +PGDPSSTAFPLVAALLVPGSD+TI+NVLMNPTRTGLILTLQEMGADIEV N RLAGGED IPGDPSSTAFPLVAALLVPGSDITIVNVLMNPTRTGLILTLQEMGADIEVANARLAGGED | 300 |
| Query | 301 | VADLRVRSSTLKGVTVPEDRAPSMIDEYPILavaaafaegaTVMNGLEELRVKESDRLSA | 360 |
| Sbjct | 301 | VADLRVR S LKGVTVPEDRAPSMIDEYPILAVAA FAEGAT+M GLEELRVKESDRLSA VADLRVRHSELKGVTVPEDRAPSMIDEYPILAVAACFAEGATIMKGLEELRVKESDRLSA | 360 |
| Query | 361 | VANGLKLNGVDCDEGETSLVVRGRPDGKGLGNASGAAVATHLDHRIAMSFLVMGLVSENP | 420 |
| Sbjct | 361 | VA+GLKLNGVDCDEGE L+VRGRPDGKGLGNA+G V+THLDHRIAMSFLVMGL SE+P VADGLKLNGVDCDEGEDFLIVRGRPDGKGLGNAAGDRVSTHLDHRIAMSFLVMGLASEHP | 420 |
| Query  Sbjct | 421  421 | VTVDDATMIATSFPEFMDLMAGLGAKIE 448 VT+DDA MIATSFPEFM LM GLGA+IE VTIDDAAMIATSFPEFMALMKGLGARIE 448 |  |

>WP_094087019.1 3-phosphoshikimate 1-carboxyvinyltransferase [Rhizobium leguminosarum]

ASR08455.1 3-phosphoshikimate 1-carboxyvinyltransferase [Rhizobium leguminosarum bv. viciae]

Length=452

Score = 769 bits (1985), Expect = 0.0, Method: Compositional matrix adjust.

| Identities | = 389/448 (87%), Positives = 418/448 (93%), Gaps = 0/448 (0%) |  |
| --- | --- | --- |
| Query 1 | MLHGASSRPATARKSSGLSGTVRIPGDKSISHRSFMFGGLASGETRITGLLEGEDVINTG | 60 |
| Sbjct 1 | ML+G++ +PATARKS+GL+G+VRIPGDKSISHRSFMFGGLASGETRITGLLEGEDVINTG MLNGSAPKPATARKSAGLTGSVRIPGDKSISHRSFMFGGLASGETRITGLLEGEDVINTG | 60 |
| Query 61 | KAMQAMGARIRKEGDTWIIDGVGNGGLLAPEAPLDFGNAATGCRLTMGLVGVYDFDSTFI | 120 |
| Sbjct 61 | +AMQAMGARIRKEG+ W+IDG GNG LLAP+APLDFGNA TG RLTMGLVG YDF STF RAMQAMGARIRKEGEQWVIDGTGNGALLAPDAPLDFGNAGTGVRLTMGLVGTYDFRSTFT | 120 |
| Query 121 | GDASLTKRPMGRVLNPLREMGVQVKSEDGDRLPVTLRGPKTPTPITYRVPMASAQVKSAV | 180 |
| Sbjct 121 | GDASL+KRPMGRVLNPLREMGVQV + GDRLPVTLRGP TP+PI YRVPMASAQVKSAV GDASLSKRPMGRVLNPLREMGVQVSASQGDRLPVTLRGPGTPSPIRYRVPMASAQVKSAV | 180 |
| Query 181 | LLAGLNTPGITTVIEPIMTRDHTEKMLQGFGANLTVETDADGVRTIRLEGRGKLTGQVID | 240 |
| Sbjct 181 | LLAGLNTPGITTVIEP+MTRDHTEKMLQGFGA L+V+TD++GVRTIRLEGRG+LTGQVID LLAGLNTPGITTVIEPVMTRDHTEKMLQGFGAALSVKTDSEGVRTIRLEGRGRLTGQVID | 240 |
| Query 241 | VPGDPSSTAFPLVAALLVPGSDVTILNVLMNPTRTGLILTLQEMGADIEVINPRLAGGED | 300 |
| Sbjct 241 | VPGDPSSTAFPLVAALLVPGSD+TI+NVLMNPTRTGLILTLQEMGADIEV N RLAGGED VPGDPSSTAFPLVAALLVPGSDITIVNVLMNPTRTGLILTLQEMGADIEVANARLAGGED | 300 |
| Query 301 | VADLRVRSSTLKGVTVPEDRAPSMIDEYPILavaaafaegaTVMNGLEELRVKESDRLSA | 360 |
| Sbjct 301 | VADLRVR S LKGVTVPEDRAPSMIDEYPILAVAA FAEGAT+M GLEELRVKESDRLSA VADLRVRHSELKGVTVPEDRAPSMIDEYPILAVAACFAEGATIMKGLEELRVKESDRLSA | 360 |
| Query 361 | VANGLKLNGVDCDEGETSLVVRGRPDGKGLGNASGAAVATHLDHRIAMSFLVMGLVSENP | 420 |
| Sbjct 361 | VA+GLKLNGVDCDEGE LVVRGRPDGKGLGNA+G V+THLDHRIAMSFLVMGL SE+ VADGLKLNGVDCDEGEDFLVVRGRPDGKGLGNAAGGRVSTHLDHRIAMSFLVMGLASEHA | 420 |
| Query 421  Sbjct 421 | VTVDDATMIATSFPEFMDLMAGLGAKIE 448 VT+DDA MIATSFPEFM LM GLGA+IE VTIDDAAMIATSFPEFMQLMKGLGARIE 448 |  |

>WP_129422250.1 3-phosphoshikimate 1-carboxyvinyltransferase [Rhizobium leguminosarum] RXT16514.1 3-phosphoshikimate 1-carboxyvinyltransferase [Rhizobium leguminosarum]

Length=452

Score = 768 bits (1984), Expect = 0.0, Method: Compositional matrix adjust. Identities = 387/448 (86%), Positives = 417/448 (93%), Gaps = 0/448 (0%)

| Query | 1 | MLHGASSRPATARKSSGLSGTVRIPGDKSISHRSFMFGGLASGETRITGLLEGEDVINTG | 60 |
| --- | --- | --- | --- |
| Sbjct | 1 | MLHG++ RPATARKS+GL+G+VRIPGDKSISHRSFMFGGLASG+TRITGLLEGEDVINTG MLHGSAPRPATARKSAGLTGSVRIPGDKSISHRSFMFGGLASGQTRITGLLEGEDVINTG | 60 |
| Query | 61 | KAMQAMGARIRKEGDTWIIDGVGNGGLLAPEAPLDFGNAATGCRLTMGLVGVYDFDSTFI | 120 |
| Sbjct | 61 | +AM+AMGARIRKEG W+IDG GNG LLAP+APLDFGNA TG RLTMGLVG YDF STFI RAMRAMGARIRKEGAQWVIDGTGNGALLAPDAPLDFGNAGTGVRLTMGLVGTYDFHSTFI | 120 |
| Query | 121 | GDASLTKRPMGRVLNPLREMGVQVKSEDGDRLPVTLRGPKTPTPITYRVPMASAQVKSAV | 180 |
| Sbjct | 121 | GDASL+KRPMGRVLNPLREMGVQV + +GDRLPVTLRGP TP+PI YRVPMASAQVKSAV GDASLSKRPMGRVLNPLREMGVQVSASEGDRLPVTLRGPGTPSPIRYRVPMASAQVKSAV | 180 |
| Query | 181 | LLAGLNTPGITTVIEPIMTRDHTEKMLQGFGANLTVETDADGVRTIRLEGRGKLTGQVID | 240 |
| Sbjct | 181 | LLAGLN PG+TTVIEP+MTRDHTEKMLQGFGA L+VETD++GVRTIRLEGRGKLTGQVID LLAGLNAPGVTTVIEPVMTRDHTEKMLQGFGAALSVETDSEGVRTIRLEGRGKLTGQVID | 240 |
| Query | 241 | VPGDPSSTAFPLVAALLVPGSDVTILNVLMNPTRTGLILTLQEMGADIEVINPRLAGGED | 300 |
| Sbjct | 241 | VPGDPSSTAFPLVAALLVPGSD+TI+NVLMNPTRTGLILTLQEMGADIEV N RLAGGED VPGDPSSTAFPLVAALLVPGSDITIVNVLMNPTRTGLILTLQEMGADIEVANARLAGGED | 300 |
| Query | 301 | VADLRVRSSTLKGVTVPEDRAPSMIDEYPILavaaafaegaTVMNGLEELRVKESDRLSA | 360 |
| Sbjct | 301 | VADLRVR S L+GVTVPEDRAPSMIDEYPILAVAA FAEG TVM GLEELRVKESDRLSA VADLRVRHSELRGVTVPEDRAPSMIDEYPILAVAACFAEGVTVMKGLEELRVKESDRLSA | 360 |
| Query | 361 | VANGLKLNGVDCDEGETSLVVRGRPDGKGLGNASGAAVATHLDHRIAMSFLVMGLVSENP | 420 |
| Sbjct | 361 | VA+GLKLNGVDCDEGE L+VRGRPDGKGLGNA+G V+THLDHRIAMSFLVMGL +E+ VADGLKLNGVDCDEGEDFLIVRGRPDGKGLGNAAGGRVSTHLDHRIAMSFLVMGLAAEHA | 420 |
| Query  Sbjct | 421  421 | VTVDDATMIATSFPEFMDLMAGLGAKIE 448 VT+DDA MIATSFPEFM LM GLGA+IE VTIDDAAMIATSFPEFMTLMKGLGARIE 448 |  |

>WP_193446494.1 3-phosphoshikimate 1-carboxyvinyltransferase [Rhizobium indigoferae] NNU57824.1 3-phosphoshikimate 1-carboxyvinyltransferase [Rhizobium indigoferae]

Length=452

Score = 768 bits (1984), Expect = 0.0, Method: Compositional matrix adjust.

| Identities | = 386/448 (86%), Positives = 417/448 (93%), Gaps = 0/448 (0%) |  |
| --- | --- | --- |
| Query 1 | MLHGASSRPATARKSSGLSGTVRIPGDKSISHRSFMFGGLASGETRITGLLEGEDVINTG | 60 |
| Sbjct 1 | ML G++ RPATARKS+GL+G+VRIPGDKSISHRSFMFGGLASGETRITGLLEGEDVINTG MLQGSAPRPATARKSAGLTGSVRIPGDKSISHRSFMFGGLASGETRITGLLEGEDVINTG | 60 |
| Query 61 | KAMQAMGARIRKEGDTWIIDGVGNGGLLAPEAPLDFGNAATGCRLTMGLVGVYDFDSTFI | 120 |
| Sbjct 61 | +AMQAMGARIRKEG+ W+I+G GNG LLAP+APLDFGNA TG RLTMGLVG YDF STFI RAMQAMGARIRKEGEQWLIEGTGNGALLAPDAPLDFGNAGTGVRLTMGLVGTYDFRSTFI | 120 |
| Query 121 | GDASLTKRPMGRVLNPLREMGVQVKSEDGDRLPVTLRGPKTPTPITYRVPMASAQVKSAV | 180 |
| Sbjct 121 | GDASL+KRPMGRVLNPLREMGVQV + +GDRLPVTLRGP TP+PI YRVPMASAQVKSAV GDASLSKRPMGRVLNPLREMGVQVSASEGDRLPVTLRGPGTPSPIRYRVPMASAQVKSAV | 180 |
| Query 181 | LLAGLNTPGITTVIEPIMTRDHTEKMLQGFGANLTVETDADGVRTIRLEGRGKLTGQVID | 240 |
| Sbjct 181 | LLAGLNTPG+TTVIEP+MTRDHTEKMLQGFGA L+VETD +GVRTIRLEGRGKL GQVID LLAGLNTPGVTTVIEPVMTRDHTEKMLQGFGAALSVETDEEGVRTIRLEGRGKLAGQVID | 240 |
| Query 241 | VPGDPSSTAFPLVAALLVPGSDVTILNVLMNPTRTGLILTLQEMGADIEVINPRLAGGED | 300 |
| Sbjct 241 | +PGDPSSTAFPLVAALLVPGSD+TI+NVLMNPTRTGLILTLQEMGADI+V+N RLAGGED IPGDPSSTAFPLVAALLVPGSDITIVNVLMNPTRTGLILTLQEMGADIQVLNARLAGGED | 300 |
| Query 301 | VADLRVRSSTLKGVTVPEDRAPSMIDEYPILavaaafaegaTVMNGLEELRVKESDRLSA | 360 |
| Sbjct 301 | VADLRVR S LKGVTVPEDRAPSMIDEYPILAVAA FAEG TVM GLEELRVKESDRLSA VADLRVRHSELKGVTVPEDRAPSMIDEYPILAVAACFAEGVTVMKGLEELRVKESDRLSA | 360 |
| Query 361 | VANGLKLNGVDCDEGETSLVVRGRPDGKGLGNASGAAVATHLDHRIAMSFLVMGLVSENP | 420 |
| Sbjct 361 | VA+GLKLNGVDCDEGE L+VRGRPDGKGLGNA+ V+THLDHRIAMSFLVMGL SE+P VADGLKLNGVDCDEGEDFLIVRGRPDGKGLGNATDGKVSTHLDHRIAMSFLVMGLASEHP | 420 |
| Query 421  Sbjct 421 | VTVDDATMIATSFPEFMDLMAGLGAKIE 448 VT+DDA MIATSFPEFM LM GLGA+I+ VTIDDAAMIATSFPEFMQLMTGLGARID 448 |  |

>WP_179038807.1 3-phosphoshikimate 1-carboxyvinyltransferase [Rhizobium leguminosarum] Length=452

Score = 768 bits (1984), Expect = 0.0, Method: Compositional matrix adjust.

| Identities | = 388/449 (86%), Positives = 417/449 (93%), Gaps = 0/449 (0%) |  |
| --- | --- | --- |
| Query 1 | MLHGASSRPATARKSSGLSGTVRIPGDKSISHRSFMFGGLASGETRITGLLEGEDVINTG | 60 |
| Sbjct 1 | ML+G++ +PATARKS+GL+G+VRIPGDKSISHRSFMFGGLASGETRITGLLEGEDVINTG MLNGSAPKPATARKSAGLTGSVRIPGDKSISHRSFMFGGLASGETRITGLLEGEDVINTG | 60 |
| Query 61 | KAMQAMGARIRKEGDTWIIDGVGNGGLLAPEAPLDFGNAATGCRLTMGLVGVYDFDSTFI | 120 |
| Sbjct 61 | +AMQAMGARIRK+G W+IDG GNG LLAP+APLDFGNA TG RLTMGLVG YDF STFI RAMQAMGARIRKDGAQWVIDGTGNGALLAPDAPLDFGNAGTGVRLTMGLVGTYDFRSTFI | 120 |
| Query 121 | GDASLTKRPMGRVLNPLREMGVQVKSEDGDRLPVTLRGPKTPTPITYRVPMASAQVKSAV | 180 |
| Sbjct 121 | GDASL+KRPMGRVLNPLREMGVQV + +GDRLPVTLR P TP+PI YRVPMASAQVKSAV GDASLSKRPMGRVLNPLREMGVQVSASEGDRLPVTLRSPGTPSPIRYRVPMASAQVKSAV | 180 |
| Query 181 | LLAGLNTPGITTVIEPIMTRDHTEKMLQGFGANLTVETDADGVRTIRLEGRGKLTGQVID | 240 |
| Sbjct 181 | LLAGLNTPG+TTVIEP+MTRDHTEKMLQGFGA L+VETD++GVRTIRLEGRG+LTGQVID LLAGLNTPGVTTVIEPVMTRDHTEKMLQGFGAALSVETDSEGVRTIRLEGRGRLTGQVID | 240 |
| Query 241 | VPGDPSSTAFPLVAALLVPGSDVTILNVLMNPTRTGLILTLQEMGADIEVINPRLAGGED | 300 |
| Sbjct 241 | VPGDPSSTAFPLVAALLVPGSD+TI+NVLMNPTRTGLILTLQEMGADIEV N RLAGGED VPGDPSSTAFPLVAALLVPGSDITIVNVLMNPTRTGLILTLQEMGADIEVANARLAGGED | 300 |
| Query 301 | VADLRVRSSTLKGVTVPEDRAPSMIDEYPILavaaafaegaTVMNGLEELRVKESDRLSA | 360 |
| Sbjct 301 | VADLRVR S LKGVTVPEDRAPSMIDEYPILAVAA FAEG T+M GLEELRVKESDRLSA VADLRVRHSELKGVTVPEDRAPSMIDEYPILAVAACFAEGVTIMKGLEELRVKESDRLSA | 360 |
| Query 361 | VANGLKLNGVDCDEGETSLVVRGRPDGKGLGNASGAAVATHLDHRIAMSFLVMGLVSENP | 420 |
| Sbjct 361 | VA+GLKLNGVDCDEGE LVVRGRPDGKGLGNA+ V+THLDHRIAMSFLVMGL SE+ VADGLKLNGVDCDEGEDFLVVRGRPDGKGLGNAADEKVSTHLDHRIAMSFLVMGLASEHA | 420 |
| Query 421  Sbjct 421 | VTVDDATMIATSFPEFMDLMAGLGAKIEL 449 VT+DDA MIATSFPEFM LM GLGAKIEL VTIDDAAMIATSFPEFMQLMTGLGAKIEL 449 |  |

>WP_180694191.1 3-phosphoshikimate 1-carboxyvinyltransferase [Rhizobium sp. WYCCWR 11290]

NZD61169.1 3-phosphoshikimate 1-carboxyvinyltransferase [Rhizobium sp. WYCCWR 11290]

Length=452

Score = 768 bits (1984), Expect = 0.0, Method: Compositional matrix adjust.

| Identities | = 387/448 (86%), Positives = 417/448 (93%), Gaps = 0/448 (0%) |  |
| --- | --- | --- |
| Query 1 | MLHGASSRPATARKSSGLSGTVRIPGDKSISHRSFMFGGLASGETRITGLLEGEDVINTG | 60 |
| Sbjct 1 | MLHG++ RPATARKS+GLSG+VRIPGDKSISHRSFMFGGLASG+TRITGLLEGEDVINTG MLHGSAPRPATARKSAGLSGSVRIPGDKSISHRSFMFGGLASGQTRITGLLEGEDVINTG | 60 |
| Query 61 | KAMQAMGARIRKEGDTWIIDGVGNGGLLAPEAPLDFGNAATGCRLTMGLVGVYDFDSTFI | 120 |
| Sbjct 61 | +AMQAMGARIRKEG W+IDG GNG LLAP+APLDFGNA TG RLTMGLVG YDF S F RAMQAMGARIRKEGAQWVIDGTGNGALLAPDAPLDFGNAGTGVRLTMGLVGTYDFHSIFT | 120 |
| Query 121 | GDASLTKRPMGRVLNPLREMGVQVKSEDGDRLPVTLRGPKTPTPITYRVPMASAQVKSAV | 180 |
| Sbjct 121 | GDASL+KRPMGRVLNPLREMGVQV + +GDRLPVTLRGP TP+PI YRVPMASAQVKSAV GDASLSKRPMGRVLNPLREMGVQVSASEGDRLPVTLRGPGTPSPIRYRVPMASAQVKSAV | 180 |
| Query 181 | LLAGLNTPGITTVIEPIMTRDHTEKMLQGFGANLTVETDADGVRTIRLEGRGKLTGQVID | 240 |
| Sbjct 181 | LLAGLNTPG+TTVIEP+MTRDHTEKMLQGFGA L+VETD++GVRTIRLEGRGKLTGQVID LLAGLNTPGVTTVIEPVMTRDHTEKMLQGFGAALSVETDSEGVRTIRLEGRGKLTGQVID | 240 |
| Query 241 | VPGDPSSTAFPLVAALLVPGSDVTILNVLMNPTRTGLILTLQEMGADIEVINPRLAGGED | 300 |
| Sbjct 241 | VPGDPSSTAFPLVAALLVPGSD+TI+NVLMNPTRTGLILTLQEMGADIEV+N RLAGGED VPGDPSSTAFPLVAALLVPGSDITIVNVLMNPTRTGLILTLQEMGADIEVLNARLAGGED | 300 |
| Query 301 | VADLRVRSSTLKGVTVPEDRAPSMIDEYPILavaaafaegaTVMNGLEELRVKESDRLSA | 360 |
| Sbjct 301 | VADLRVR S L+GV+VPEDRAPSMIDEYPILAVAA FAEGAT+M GLEELRVKESDRLSA  VADLRVRHSELRGVSVPEDRAPSMIDEYPILAVAACFAEGATIMKGLEELRVKESDRLSA | 360 |

| Query | 361 | VANGLKLNGVDCDEGETSLVVRGRPDGKGLGNASGAAVATHLDHRIAMSFLVMGLVSENP 420 |
| --- | --- | --- |
| Sbjct | 361 | VA+GLKLNGVDCDEGE L+VRGRPDGKGLGNA+ V+THLDHRIAMSFLVMGL SE+P VADGLKLNGVDCDEGEDFLIVRGRPDGKGLGNAASGRVSTHLDHRIAMSFLVMGLASEHP 420 |
| Query | 421 | VTVDDATMIATSFPEFMDLMAGLGAKIE 448 |
| Sbjct | 421 | VT+DDA MIATSFPEFM LM LGA+IE  VTIDDAAMIATSFPEFMSLMKRLGARIE 448 |

>WP_168336420.1 3-phosphoshikimate 1-carboxyvinyltransferase [Rhizobium laguerreae] NKM21568.1 3-phosphoshikimate 1-carboxyvinyltransferase [Rhizobium laguerreae]

Length=452

Score = 768 bits (1984), Expect = 0.0, Method: Compositional matrix adjust.

| Identities | = 387/448 (86%), Positives = 417/448 (93%), Gaps = 0/448 (0%) |  |
| --- | --- | --- |
| Query 1 | MLHGASSRPATARKSSGLSGTVRIPGDKSISHRSFMFGGLASGETRITGLLEGEDVINTG | 60 |
| Sbjct 1 | MLHG++ RPATARKS+GL+G+VRIPGDKSISHRSFMFGGLASGETRITGLLEGEDVINTG MLHGSAPRPATARKSAGLAGSVRIPGDKSISHRSFMFGGLASGETRITGLLEGEDVINTG | 60 |
| Query 61 | KAMQAMGARIRKEGDTWIIDGVGNGGLLAPEAPLDFGNAATGCRLTMGLVGVYDFDSTFI | 120 |
| Sbjct 61 | +AMQAMGARIRK+G W+IDG GNG LLAP+APLDFGNA TG RLTMGLVG YDF STF RAMQAMGARIRKDGAQWVIDGTGNGALLAPDAPLDFGNAGTGVRLTMGLVGTYDFRSTFT | 120 |
| Query 121 | GDASLTKRPMGRVLNPLREMGVQVKSEDGDRLPVTLRGPKTPTPITYRVPMASAQVKSAV | 180 |
| Sbjct 121 | GDASL+KRPMGRVLNPLREMGVQV + +GDRLPVTLRGP TP+PI YRVPMASAQVKSAV GDASLSKRPMGRVLNPLREMGVQVSASEGDRLPVTLRGPGTPSPIRYRVPMASAQVKSAV | 180 |
| Query 181 | LLAGLNTPGITTVIEPIMTRDHTEKMLQGFGANLTVETDADGVRTIRLEGRGKLTGQVID | 240 |
| Sbjct 181 | LLAGLNTPG+TTVIEP+MTRDHTEKMLQGFGA L+VETD DGVRTIRLEGRGKL GQVID LLAGLNTPGVTTVIEPVMTRDHTEKMLQGFGAALSVETDGDGVRTIRLEGRGKLKGQVID | 240 |
| Query 241 | VPGDPSSTAFPLVAALLVPGSDVTILNVLMNPTRTGLILTLQEMGADIEVINPRLAGGED | 300 |
| Sbjct 241 | +PGDPSSTAFPLVAALLVPGSD+TI+NVLMNPTRTGLILTLQEMGA+IEV+N RLAGGED IPGDPSSTAFPLVAALLVPGSDITIVNVLMNPTRTGLILTLQEMGANIEVLNTRLAGGED | 300 |
| Query 301 | VADLRVRSSTLKGVTVPEDRAPSMIDEYPILavaaafaegaTVMNGLEELRVKESDRLSA | 360 |
| Sbjct 301 | VADLRVR S LKGVTVPE+RAPSMIDEYPILAVAA FAEGAT+M GLEELRVKESDRLSA VADLRVRHSELKGVTVPEERAPSMIDEYPILAVAACFAEGATIMKGLEELRVKESDRLSA | 360 |
| Query 361 | VANGLKLNGVDCDEGETSLVVRGRPDGKGLGNASGAAVATHLDHRIAMSFLVMGLVSENP | 420 |
| Sbjct 361 | VA+GLKLNG+DCDEGE LVVRGRPDGKGLGNA+G V+THLDHRIAMSFLVMGL SE+ VAHGLKLNGIDCDEGEDFLVVRGRPDGKGLGNAAGGRVSTHLDHRIAMSFLVMGLASEHA | 420 |
| Query 421  Sbjct 421 | VTVDDATMIATSFPEFMDLMAGLGAKIE 448 VT+DDA MIATSFPEFM LM GLGAK+E VTIDDAAMIATSFPEFMQLMTGLGAKVE 448 |  |

>WP_152336385.1 3-phosphoshikimate 1-carboxyvinyltransferase [Rhizobium sp. TCK] Length=455

Score = 768 bits (1983), Expect = 0.0, Method: Compositional matrix adjust.

| Identities | = 393/455 (86%), Positives = 417/455 (92%), Gaps = 0/455 (0%) |  |
| --- | --- | --- |
| Query 1 | MLHGASSRPATARKSSGLSGTVRIPGDKSISHRSFMFGGLASGETRITGLLEGEDVINTG | 60 |
| Sbjct 1 | M HG + +PA + KS+ L GTVRIPGDKSISHRSFMFGGLA+GETRITGLLEGEDVINTG MSHGVALKPARSLKSADLRGTVRIPGDKSISHRSFMFGGLAAGETRITGLLEGEDVINTG | 60 |
| Query 61 | KAMQAMGARIRKEGDTWIIDGVGNGGLLAPEAPLDFGNAATGCRLTMGLVGVYDFDSTFI | 120 |
| Sbjct 61 | KAM AMGARI K GD WII G GNG LLAPEAPLDFGNA TGCRLTMGLVGVYDF+STFI KAMHAMGARIEKVGDEWIIQGTGNGALLAPEAPLDFGNAGTGCRLTMGLVGVYDFESTFI | 120 |
| Query 121 | GDASLTKRPMGRVLNPLREMGVQVKSEDGDRLPVTLRGPKTPTPITYRVPMASAQVKSAV | 180 |
| Sbjct 121 | GDASLTKRPMGRVLNPLREMGVQVK+ +GDRLPVTLRGP P PITYRVPMASAQVKSAV GDASLTKRPMGRVLNPLREMGVQVKAAEGDRLPVTLRGPSIPNPITYRVPMASAQVKSAV | 180 |
| Query 181 | LLAGLNTPGITTVIEPIMTRDHTEKMLQGFGANLTVETDADGVRTIRLEGRGKLTGQVID | 240 |
| Sbjct 181 | LLAGLNTPG+TTVIEP+MTRDHTEKMLQGFGANL V+TDADGVRTIRLEGRG+L GQVID  LLAGLNTPGVTTVIEPVMTRDHTEKMLQGFGANLMVDTDADGVRTIRLEGRGQLIGQVID | 240 |

| Query | 241 | VPGDPSSTAFPLVAALLVPGSDVTILNVLMNPTRTGLILTLQEMGADIEVINPRLAGGED | 300 |
| --- | --- | --- | --- |
| Sbjct | 241 | VPGDPSSTAFPLVAALLVPGSDVTI NVLMNPTRTGLILTLQEMGADIEVINPRLAGGED VPGDPSSTAFPLVAALLVPGSDVTIENVLMNPTRTGLILTLQEMGADIEVINPRLAGGED | 300 |
| Query | 301 | VADLRVRSSTLKGVTVPEDRAPSMIDEYPILavaaafaegaTVMNGLEELRVKESDRLSA | 360 |
| Sbjct | 301 | VADLRVR S LKGVTVP DRAPSMIDEYP+LAVAAAFAEG T M GLEELRVKESDRLSA VADLRVRHSDLKGVTVPADRAPSMIDEYPVLAVAAAFAEGTTTMLGLEELRVKESDRLSA | 360 |
| Query | 361 | VANGLKLNGVDCDEGETSLVVRGRPDGKGLGNASGAAVATHLDHRIAMSFLVMGLVSENP | 420 |
| Sbjct | 361 | VA+GLKLNGVDCDEGE +LVVRGRP GKG GNA+G +V THLDHRIAMSFL+MGLVSE+ VADGLKLNGVDCDEGEDTLVVRGRPGGKGYGNANGLSVVTHLDHRIAMSFLIMGLVSEHA | 420 |
| Query  Sbjct | 421  421 | VTVDDATMIATSFPEFMDLMAGLGAKIELSDTKAA 455 VTVDDA +IATSFPEFMDLM GLGA+IE +++AA VTVDDAGIIATSFPEFMDLMTGLGARIEPVESRAA 455 |  |

>WP_168323482.1 3-phosphoshikimate 1-carboxyvinyltransferase [Rhizobium leguminosarum]

NKL02125.1 3-phosphoshikimate 1-carboxyvinyltransferase [Rhizobium leguminosarum bv. viciae]

Length=452

Score = 768 bits (1983), Expect = 0.0, Method: Compositional matrix adjust.

| Identities | = 388/449 (86%), Positives = 415/449 (92%), Gaps = 0/449 (0%) |  |
| --- | --- | --- |
| Query 1 | MLHGASSRPATARKSSGLSGTVRIPGDKSISHRSFMFGGLASGETRITGLLEGEDVINTG | 60 |
| Sbjct 1 | MLHG++SRPATARKS+ LSG+VRIPGDKSISHRSFMFGGLASGETRITGLLEGEDVINTG MLHGSASRPATARKSASLSGSVRIPGDKSISHRSFMFGGLASGETRITGLLEGEDVINTG | 60 |
| Query 61 | KAMQAMGARIRKEGDTWIIDGVGNGGLLAPEAPLDFGNAATGCRLTMGLVGVYDFDSTFI | 120 |
| Sbjct 61 | +AMQAMGARIRK+G W+IDG GNG LLAP+APLDFGNA TG RLTMGLVG YDF STFI RAMQAMGARIRKDGAQWVIDGTGNGALLAPDAPLDFGNAGTGVRLTMGLVGTYDFRSTFI | 120 |
| Query 121 | GDASLTKRPMGRVLNPLREMGVQVKSEDGDRLPVTLRGPKTPTPITYRVPMASAQVKSAV | 180 |
| Sbjct 121 | GDASL+KRPM RVL PLREMGVQV + +GDR+PVTLRGP TP+PI YRVPMASAQVKSAV GDASLSKRPMVRVLAPLREMGVQVSASEGDRIPVTLRGPGTPSPIRYRVPMASAQVKSAV | 180 |
| Query 181 | LLAGLNTPGITTVIEPIMTRDHTEKMLQGFGANLTVETDADGVRTIRLEGRGKLTGQVID | 240 |
| Sbjct 181 | LLAGLNTPG+TTVIEP+MTRDHTEKMLQGFGA L+VETD DGVRTIRLEGRGKL GQVID LLAGLNTPGVTTVIEPVMTRDHTEKMLQGFGAALSVETDGDGVRTIRLEGRGKLKGQVID | 240 |
| Query 241 | VPGDPSSTAFPLVAALLVPGSDVTILNVLMNPTRTGLILTLQEMGADIEVINPRLAGGED | 300 |
| Sbjct 241 | +PGDPSSTAFPLVAALLVPGSD+TI+NVLMNPTRTGLILTLQEMGADIEV+N RLAGGED IPGDPSSTAFPLVAALLVPGSDITIVNVLMNPTRTGLILTLQEMGADIEVVNARLAGGED | 300 |
| Query 301 | VADLRVRSSTLKGVTVPEDRAPSMIDEYPILavaaafaegaTVMNGLEELRVKESDRLSA | 360 |
| Sbjct 301 | VADLRVR S LKGVTVPE+RAPSMIDEYPILAVAA FAEGATVM GLEELRVKESDRLSA VADLRVRHSELKGVTVPEERAPSMIDEYPILAVAACFAEGATVMKGLEELRVKESDRLSA | 360 |
| Query 361 | VANGLKLNGVDCDEGETSLVVRGRPDGKGLGNASGAAVATHLDHRIAMSFLVMGLVSENP | 420 |
| Sbjct 361 | VA+GLKLNG DCDEGE L+VRGRPDGKGLGNA+G V THLDHRIAMSFLV+GL SE+ VADGLKLNGADCDEGEDFLIVRGRPDGKGLGNAAGGKVRTHLDHRIAMSFLVLGLASEHA | 420 |
| Query 421  Sbjct 421 | VTVDDATMIATSFPEFMDLMAGLGAKIEL 449 VT+DDA MIATSFPEFM LM GLGAKIEL VTIDDAAMIATSFPEFMQLMTGLGAKIEL 449 |  |

```
>WP_012759631.1 3-phosphoshikimate 1-carboxyvinyltransferase [Rhizobium leguminosarum]
ACS58602.1 3-phosphoshikimate 1-carboxyvinyltransferase [Rhizobium leguminosarum bv. trifolii WSM1325]
RWY75638.1 3-phosphoshikimate 1-carboxyvinyltransferase [Rhizobium leguminosarum] RWY78285.1 3-phosphoshikimate 1-carboxyvinyltransferase [Rhizobium leguminosarum]
Length=452

Score = 768 bits (1983), Expect = 0.0, Method: Compositional matrix adjust. Identities = 388/447 (87%), Positives = 417/447 (93%), Gaps = 0/447 (0%)

Query 1	MLHGASSRPATARKSSGLSGTVRIPGDKSISHRSFMFGGLASGETRITGLLEGEDVINTG 60 ML+G++ +PATARKS+GL+G+VRIPGDKSISHRSFMFGGLASGETRITGLLEGEDVINTG
Sbjct 1	MLNGSAPKPATARKSAGLTGSVRIPGDKSISHRSFMFGGLASGETRITGLLEGEDVINTG 60
```

| Query | 61 | KAMQAMGARIRKEGDTWIIDGVGNGGLLAPEAPLDFGNAATGCRLTMGLVGVYDFDSTFI | 120 |
| --- | --- | --- | --- |
| Sbjct | 61 | +AMQAMGARIRKEG+ W+IDG GNG LLAP+APLDFGNA TG RLTMGLVG YDF STF RAMQAMGARIRKEGEQWVIDGTGNGALLAPDAPLDFGNAGTGVRLTMGLVGTYDFRSTFT | 120 |
| Query | 121 | GDASLTKRPMGRVLNPLREMGVQVKSEDGDRLPVTLRGPKTPTPITYRVPMASAQVKSAV | 180 |
| Sbjct | 121 | GDASL+KRPMGRVLNPLREMGVQV + +GDRLPVTLRGP TP+PI YRVPMASAQVKSAV GDASLSKRPMGRVLNPLREMGVQVSASEGDRLPVTLRGPGTPSPIRYRVPMASAQVKSAV | 180 |
| Query | 181 | LLAGLNTPGITTVIEPIMTRDHTEKMLQGFGANLTVETDADGVRTIRLEGRGKLTGQVID | 240 |
| Sbjct | 181 | LLAGLNTPGITTVIEP+MTRDHTEKMLQGFGA L+VETD++GVRTIRLEGRGKL GQVID LLAGLNTPGITTVIEPVMTRDHTEKMLQGFGAALSVETDSEGVRTIRLEGRGKLAGQVID | 240 |
| Query | 241 | VPGDPSSTAFPLVAALLVPGSDVTILNVLMNPTRTGLILTLQEMGADIEVINPRLAGGED | 300 |
| Sbjct | 241 | VPGDPSSTAFPLVAALLVPGSD+TI+NVLMNPTRTGLILTLQEMGADIEV N RLAGGED VPGDPSSTAFPLVAALLVPGSDITIVNVLMNPTRTGLILTLQEMGADIEVANARLAGGED | 300 |
| Query | 301 | VADLRVRSSTLKGVTVPEDRAPSMIDEYPILavaaafaegaTVMNGLEELRVKESDRLSA | 360 |
| Sbjct | 301 | VADLRVR S LKGVTVPE+RAPSMIDEYPILAVAA FAEGAT+M GLEELRVKESDRLSA VADLRVRHSELKGVTVPEERAPSMIDEYPILAVAACFAEGATIMKGLEELRVKESDRLSA | 360 |
| Query | 361 | VANGLKLNGVDCDEGETSLVVRGRPDGKGLGNASGAAVATHLDHRIAMSFLVMGLVSENP | 420 |
| Sbjct | 361 | VA+GLKLNGVDCDEGE L+VRGRPDGKGLGNA+ V+THLDHRIAMSFLVMGL SE+P VADGLKLNGVDCDEGEDFLIVRGRPDGKGLGNAADGRVSTHLDHRIAMSFLVMGLASEHP | 420 |
| Query  Sbjct | 421  421 | VTVDDATMIATSFPEFMDLMAGLGAKI 447 VT+DDA MIATSFPEFM LM GLGAKI VTIDDAAMIATSFPEFMQLMTGLGAKI 447 |  |

>WP_183906586.1 3-phosphoshikimate 1-carboxyvinyltransferase [Rhizobium sp. BK008] MBB4255904.1 3-phosphoshikimate 1-carboxyvinyltransferase [Rhizobium sp. BK008]

Length=452

Score = 768 bits (1982), Expect = 0.0, Method: Compositional matrix adjust.

| Identities | = 385/448 (86%), Positives = 418/448 (93%), Gaps = 0/448 (0%) |  |
| --- | --- | --- |
| Query 1 | MLHGASSRPATARKSSGLSGTVRIPGDKSISHRSFMFGGLASGETRITGLLEGEDVINTG | 60 |
| Sbjct 1 | MLHG++ RPATARKS+GL+G VRIPGDKSISHRSFMFGGLASG+TRITGLLEGEDVINTG MLHGSAPRPATARKSAGLTGRVRIPGDKSISHRSFMFGGLASGQTRITGLLEGEDVINTG | 60 |
| Query 61 | KAMQAMGARIRKEGDTWIIDGVGNGGLLAPEAPLDFGNAATGCRLTMGLVGVYDFDSTFI | 120 |
| Sbjct 61 | +AMQAMGARIRK+G+ W+I+G GNG LLAP+APLDFGNA TG RLTMGLVG YDF STFI RAMQAMGARIRKQGEEWVIEGTGNGALLAPDAPLDFGNAGTGVRLTMGLVGTYDFHSTFI | 120 |
| Query 121 | GDASLTKRPMGRVLNPLREMGVQVKSEDGDRLPVTLRGPKTPTPITYRVPMASAQVKSAV | 180 |
| Sbjct 121 | GDASL+KRPMGRVLNPLR+MGVQV + +GDRLPVTLRGP TP+PI YRVPMASAQVKSAV GDASLSKRPMGRVLNPLRDMGVQVSASEGDRLPVTLRGPGTPSPILYRVPMASAQVKSAV | 180 |
| Query 181 | LLAGLNTPGITTVIEPIMTRDHTEKMLQGFGANLTVETDADGVRTIRLEGRGKLTGQVID | 240 |
| Sbjct 181 | LLAGLNTPGITTVIEP+MTRDHTEKMLQGFGA L VETDADG+RTIRLEGRG+LTGQVID LLAGLNTPGITTVIEPVMTRDHTEKMLQGFGAALAVETDADGIRTIRLEGRGRLTGQVID | 240 |
| Query 241 | VPGDPSSTAFPLVAALLVPGSDVTILNVLMNPTRTGLILTLQEMGADIEVINPRLAGGED | 300 |
| Sbjct 241 | VPGDPSSTAFPLVAAL+VPGSD+TI+NVLMNPTRTGLILTLQEMGADIEV+N RLAGGED VPGDPSSTAFPLVAALIVPGSDITIVNVLMNPTRTGLILTLQEMGADIEVLNARLAGGED | 300 |
| Query 301 | VADLRVRSSTLKGVTVPEDRAPSMIDEYPILavaaafaegaTVMNGLEELRVKESDRLSA | 360 |
| Sbjct 301 | VADLRVR S LKGV VPE+RAPSMIDEYPILAVAA FAEG TVM GLEELRVKESDRLSA VADLRVRHSELKGVAVPEERAPSMIDEYPILAVAACFAEGTTVMKGLEELRVKESDRLSA | 360 |
| Query 361 | VANGLKLNGVDCDEGETSLVVRGRPDGKGLGNASGAAVATHLDHRIAMSFLVMGLVSENP | 420 |
| Sbjct 361 | VA+GLKLNGVDCDEG+ L+VRGRPDGKGLG+A G+ V+THLDHRIAMSFLV+GL SE+ VADGLKLNGVDCDEGKDFLIVRGRPDGKGLGSAGGSKVSTHLDHRIAMSFLVLGLASEHA | 420 |
| Query 421  Sbjct 421 | VTVDDATMIATSFPEFMDLMAGLGAKIE 448 VT+DDA MIATSFPEFM LMAGLGAKIE VTIDDAAMIATSFPEFMQLMAGLGAKIE 448 |  |

>WP_152341413.1 3-phosphoshikimate 1-carboxyvinyltransferase [Rhizobium sp. Khangiran2] Length=455

Score = 768 bits (1982), Expect = 0.0, Method: Compositional matrix adjust.

| Identities | = 394/455 (87%), Positives = 420/455 (92%), Gaps = 0/455 (0%) |  |
| --- | --- | --- |
| Query 1 | MLHGASSRPATARKSSGLSGTVRIPGDKSISHRSFMFGGLASGETRITGLLEGEDVINTG | 60 |
| Sbjct 1 | M HG + +PA + +S+ L GTVRIPGDKSISHRSFMFGGLA+GETRITGLLEGEDVINTG MSHGVALKPARSLQSADLKGTVRIPGDKSISHRSFMFGGLAAGETRITGLLEGEDVINTG | 60 |
| Query 61 | KAMQAMGARIRKEGDTWIIDGVGNGGLLAPEAPLDFGNAATGCRLTMGLVGVYDFDSTFI | 120 |
| Sbjct 61 | KAM+AMGA+I K GD WII G GNG LLAPEAPLDFGNA TGCRLTMGLVGVYDF+STFI KAMRAMGAKIDKVGDEWIIQGTGNGALLAPEAPLDFGNAGTGCRLTMGLVGVYDFESTFI | 120 |
| Query 121 | GDASLTKRPMGRVLNPLREMGVQVKSEDGDRLPVTLRGPKTPTPITYRVPMASAQVKSAV | 180 |
| Sbjct 121 | GDASLTKRPMGRVLNPLREMGVQVK+ DGDRLPVTLRGP P PITYRVPMASAQVKSAV GDASLTKRPMGRVLNPLREMGVQVKAADGDRLPVTLRGPSIPNPITYRVPMASAQVKSAV | 180 |
| Query 181 | LLAGLNTPGITTVIEPIMTRDHTEKMLQGFGANLTVETDADGVRTIRLEGRGKLTGQVID | 240 |
| Sbjct 181 | LLAGLNTPG+TTVIEP+MTRDHTEKMLQGFGANL VETDADGVRTIRLEGRG+L GQVID LLAGLNTPGVTTVIEPVMTRDHTEKMLQGFGANLMVETDADGVRTIRLEGRGQLIGQVID | 240 |
| Query 241 | VPGDPSSTAFPLVAALLVPGSDVTILNVLMNPTRTGLILTLQEMGADIEVINPRLAGGED | 300 |
| Sbjct 241 | VPGDPSSTAFPLVAALLVPGSDVTI NVLMNPTRTGLILTLQEMGADIEVINPRLAGGED VPGDPSSTAFPLVAALLVPGSDVTIENVLMNPTRTGLILTLQEMGADIEVINPRLAGGED | 300 |
| Query 301 | VADLRVRSSTLKGVTVPEDRAPSMIDEYPILavaaafaegaTVMNGLEELRVKESDRLSA | 360 |
| Sbjct 301 | VADLRVR S LKG+TVP DRAPSMIDEYP+LAVAAAFAEGAT M GLEELRVKESDRLSA VADLRVRHSDLKGITVPADRAPSMIDEYPVLAVAAAFAEGATTMLGLEELRVKESDRLSA | 360 |
| Query 361 | VANGLKLNGVDCDEGETSLVVRGRPDGKGLGNASGAAVATHLDHRIAMSFLVMGLVSENP | 420 |
| Sbjct 361 | VA+GLKLNGVDCDEGE +LVVRGRP GKG GNA+G++V THLDHRIAMSFLVMGLVSE+ VADGLKLNGVDCDEGEDTLVVRGRPGGKGYGNATGSSVVTHLDHRIAMSFLVMGLVSEHA | 420 |
| Query 421  Sbjct 421 | VTVDDATMIATSFPEFMDLMAGLGAKIELSDTKAA 455 VTVDDA +IATSFPEFMDLM GLGA+IE +++AA VTVDDAGIIATSFPEFMDLMTGLGARIEPVESRAA 455 |  |

>WP_132576056.1 3-phosphoshikimate 1-carboxyvinyltransferase [Rhizobium sp. BK398] TCS12540.1 3-phosphoshikimate 1-carboxyvinyltransferase [Rhizobium sp. BK398]

Length=452

Score = 768 bits (1982), Expect = 0.0, Method: Compositional matrix adjust.

| Identities | = 386/448 (86%), Positives = 417/448 (93%), Gaps = 0/448 (0%) |  |
| --- | --- | --- |
| Query 1 | MLHGASSRPATARKSSGLSGTVRIPGDKSISHRSFMFGGLASGETRITGLLEGEDVINTG | 60 |
| Sbjct 1 | MLHG++ RPATARKS+GLSG VRIPGDKSISHRSFMFGGLASG+TRITGLLEGEDVINTG MLHGSAPRPATARKSAGLSGRVRIPGDKSISHRSFMFGGLASGQTRITGLLEGEDVINTG | 60 |
| Query 61 | KAMQAMGARIRKEGDTWIIDGVGNGGLLAPEAPLDFGNAATGCRLTMGLVGVYDFDSTFI | 120 |
| Sbjct 61 | +AMQAMGARIRK+G+ W+I+G GNG LLAP+APLDFGNA TG RLTMGLVG YDF STFI RAMQAMGARIRKQGEEWVIEGTGNGALLAPDAPLDFGNAGTGVRLTMGLVGTYDFHSTFI | 120 |
| Query 121 | GDASLTKRPMGRVLNPLREMGVQVKSEDGDRLPVTLRGPKTPTPITYRVPMASAQVKSAV | 180 |
| Sbjct 121 | GDASL+KRPMGRVLNPLR+MGVQV + GDRLPVTLRGP TP+PI YRVPMASAQVKSAV GDASLSKRPMGRVLNPLRDMGVQVSASAGDRLPVTLRGPGTPSPIRYRVPMASAQVKSAV | 180 |
| Query 181 | LLAGLNTPGITTVIEPIMTRDHTEKMLQGFGANLTVETDADGVRTIRLEGRGKLTGQVID | 240 |
| Sbjct 181 | LLAGLNTPGITTVIEP+MTRDHTEKMLQGFGA L VETDADG+RTIRLEGRG+LTGQVID LLAGLNTPGITTVIEPVMTRDHTEKMLQGFGAALAVETDADGIRTIRLEGRGRLTGQVID | 240 |
| Query 241 | VPGDPSSTAFPLVAALLVPGSDVTILNVLMNPTRTGLILTLQEMGADIEVINPRLAGGED | 300 |
| Sbjct 241 | VPGDPSSTAFPLVAAL+VPGSD+TI+NVLMNPTRTGLILTLQEMGADIEV+N RLAGGED VPGDPSSTAFPLVAALIVPGSDITIVNVLMNPTRTGLILTLQEMGADIEVLNARLAGGED | 300 |
| Query 301 | VADLRVRSSTLKGVTVPEDRAPSMIDEYPILavaaafaegaTVMNGLEELRVKESDRLSA | 360 |
| Sbjct 301 | VADLRVR S LKGV VPE+RAPSMIDEYPILAVAA FAEG TVM GLEELRVKESDRLSA VADLRVRHSELKGVAVPEERAPSMIDEYPILAVAACFAEGTTVMKGLEELRVKESDRLSA | 360 |
| Query 361 | VANGLKLNGVDCDEGETSLVVRGRPDGKGLGNASGAAVATHLDHRIAMSFLVMGLVSENP | 420 |
| Sbjct 361 | VA+GLKLNGVDCDEG+ L+VRGRPDGKGLG+A G+ V+THLDHRIAMSFLV+GL SE+ VADGLKLNGVDCDEGKDFLIVRGRPDGKGLGSAGGSKVSTHLDHRIAMSFLVLGLASEHA | 420 |
| Query 421 | VTVDDATMIATSFPEFMDLMAGLGAKIE 448 |  |

VT+DDA MIATSFPEFM LMAGLGAKIE Sbjct 421 VTIDDAAMIATSFPEFMQLMAGLGAKIE 448

>WP_063473421.1 MULTISPECIES: 3-phosphoshikimate 1-carboxyvinyltransferase [Rhizobium]

KZS57196.1 3-phosphoshikimate 1-carboxyvinyltransferase [Rhizobium anhuiense bv. trifolii]

MBB4117537.1 3-phosphoshikimate 1-carboxyvinyltransferase [Rhizobium sp. BK226] PDS34088.1 3-phosphoshikimate 1-carboxyvinyltransferase [Rhizobium anhuiense] PDS54690.1 3-phosphoshikimate 1-carboxyvinyltransferase [Rhizobium anhuiense] PDS61706.1 3-phosphoshikimate 1-carboxyvinyltransferase [Rhizobium anhuiense]

Length=452

Score = 768 bits (1982), Expect = 0.0, Method: Compositional matrix adjust.

| Identities | = 385/448 (86%), Positives = 418/448 (93%), Gaps = 0/448 (0%) |  |
| --- | --- | --- |
| Query 1 | MLHGASSRPATARKSSGLSGTVRIPGDKSISHRSFMFGGLASGETRITGLLEGEDVINTG | 60 |
| Sbjct 1 | MLHG++ RPATARKS+GL+G VRIPGDKSISHRSFMFGGLASG+TRITGLLEGEDVINTG MLHGSAPRPATARKSAGLTGRVRIPGDKSISHRSFMFGGLASGQTRITGLLEGEDVINTG | 60 |
| Query 61 | KAMQAMGARIRKEGDTWIIDGVGNGGLLAPEAPLDFGNAATGCRLTMGLVGVYDFDSTFI | 120 |
| Sbjct 61 | +AMQAMGARIRK+G+ W+I+G GNG LLAP+APLDFGNA TG RLTMGLVG YDF STFI RAMQAMGARIRKQGEEWVIEGTGNGALLAPDAPLDFGNAGTGVRLTMGLVGTYDFHSTFI | 120 |
| Query 121 | GDASLTKRPMGRVLNPLREMGVQVKSEDGDRLPVTLRGPKTPTPITYRVPMASAQVKSAV | 180 |
| Sbjct 121 | GDASL+KRPMGRVLNPLR+MGVQV + +GDRLPVTLRGP TP+PI YRVPMASAQVKSAV GDASLSKRPMGRVLNPLRDMGVQVSASEGDRLPVTLRGPGTPSPIRYRVPMASAQVKSAV | 180 |
| Query 181 | LLAGLNTPGITTVIEPIMTRDHTEKMLQGFGANLTVETDADGVRTIRLEGRGKLTGQVID | 240 |
| Sbjct 181 | LLAGLNTPGITTVIEP+MTRDHTEKMLQGFGA L VETDADG+RTIRLEGRG+LTGQVID LLAGLNTPGITTVIEPVMTRDHTEKMLQGFGAALAVETDADGIRTIRLEGRGRLTGQVID | 240 |
| Query 241 | VPGDPSSTAFPLVAALLVPGSDVTILNVLMNPTRTGLILTLQEMGADIEVINPRLAGGED | 300 |
| Sbjct 241 | VPGDPSSTAFPLVAAL+VPGSD+TI+NVLMNPTRTGLILTLQEMGADIEV+N RLAGGED VPGDPSSTAFPLVAALIVPGSDITIVNVLMNPTRTGLILTLQEMGADIEVLNARLAGGED | 300 |
| Query 301 | VADLRVRSSTLKGVTVPEDRAPSMIDEYPILavaaafaegaTVMNGLEELRVKESDRLSA | 360 |
| Sbjct 301 | VADLRVR S LKGV VPE+RAPSMIDEYPILAVAA FAEG TVM GLEELRVKESDRLSA VADLRVRHSELKGVAVPEERAPSMIDEYPILAVAACFAEGTTVMKGLEELRVKESDRLSA | 360 |
| Query 361 | VANGLKLNGVDCDEGETSLVVRGRPDGKGLGNASGAAVATHLDHRIAMSFLVMGLVSENP | 420 |
| Sbjct 361 | VA+GLKLNGVDCDEG+ L+VRGRPDGKGLG+A G+ V+THLDHRIAMSFLV+GL SE+ VADGLKLNGVDCDEGKDFLIVRGRPDGKGLGSAGGSKVSTHLDHRIAMSFLVLGLASEHA | 420 |
| Query 421  Sbjct 421 | VTVDDATMIATSFPEFMDLMAGLGAKIE 448 VT+DDA MIATSFPEFM LMAGLGAKIE VTIDDAAMIATSFPEFMQLMAGLGAKIE 448 |  |

>WP_130678697.1 3-phosphoshikimate 1-carboxyvinyltransferase [Rhizobium leguminosarum] TAU86017.1 3-phosphoshikimate 1-carboxyvinyltransferase [Rhizobium leguminosarum] TAV51124.1 3-phosphoshikimate 1-carboxyvinyltransferase [Rhizobium leguminosarum] TAV60484.1 3-phosphoshikimate 1-carboxyvinyltransferase [Rhizobium leguminosarum] TAV71531.1 3-phosphoshikimate 1-carboxyvinyltransferase [Rhizobium leguminosarum] TAX58039.1 3-phosphoshikimate 1-carboxyvinyltransferase [Rhizobium leguminosarum]

Length=452

Score = 767 bits (1981), Expect = 0.0, Method: Compositional matrix adjust.

| Identities | = 387/448 (86%), Positives = 419/448 (94%), Gaps = 0/448 (0%) |  |
| --- | --- | --- |
| Query 1 | MLHGASSRPATARKSSGLSGTVRIPGDKSISHRSFMFGGLASGETRITGLLEGEDVINTG | 60 |
| Sbjct 1 | ML+G++ +P TARKS+GL+G+VRIPGDKSISHRSFMFGGLASGETRITGLLEGEDVINTG MLNGSAPKPVTARKSAGLTGSVRIPGDKSISHRSFMFGGLASGETRITGLLEGEDVINTG | 60 |
| Query 61 | KAMQAMGARIRKEGDTWIIDGVGNGGLLAPEAPLDFGNAATGCRLTMGLVGVYDFDSTFI | 120 |
| Sbjct 61 | +AMQAMGARIRKEG W+IDG GNG LLAP+APLDFGN TG RLTMGLVG YDF STFI RAMQAMGARIRKEGPQWVIDGTGNGALLAPDAPLDFGNTGTGVRLTMGLVGTYDFRSTFI | 120 |
| Query 121 | GDASLTKRPMGRVLNPLREMGVQVKSEDGDRLPVTLRGPKTPTPITYRVPMASAQVKSAV | 180 |
| Sbjct 121 | GDASL+KRPMGRVLNPLREMGVQV + +GDRLPVTL+GP TP+PI YRVPMASAQVKSAV  GDASLSKRPMGRVLNPLREMGVQVSASEGDRLPVTLKGPGTPSPIRYRVPMASAQVKSAV | 180 |

| Query | 181 | LLAGLNTPGITTVIEPIMTRDHTEKMLQGFGANLTVETDADGVRTIRLEGRGKLTGQVID | 240 |
| --- | --- | --- | --- |
| Sbjct | 181 | LLAGLNTPG+TTVIEP+MTRDHTEKMLQGFGA L+VET+++GVRTIRLEGRGKLTGQVID LLAGLNTPGVTTVIEPVMTRDHTEKMLQGFGAALSVETNSEGVRTIRLEGRGKLTGQVID | 240 |
| Query | 241 | VPGDPSSTAFPLVAALLVPGSDVTILNVLMNPTRTGLILTLQEMGADIEVINPRLAGGED | 300 |
| Sbjct | 241 | VPGDPSSTAFPLVAALL+PGSD+TI+NVLMNPTRTGLILTLQEMGA+I+V+N RLAGGED VPGDPSSTAFPLVAALLIPGSDITIVNVLMNPTRTGLILTLQEMGANIQVLNTRLAGGED | 300 |
| Query | 301 | VADLRVRSSTLKGVTVPEDRAPSMIDEYPILavaaafaegaTVMNGLEELRVKESDRLSA | 360 |
| Sbjct | 301 | VADLRVR S LKGVTVPEDRAPSMIDEYPILAVAA FAEGATVMNGLEELRVKESDRLSA VADLRVRHSELKGVTVPEDRAPSMIDEYPILAVAACFAEGATVMNGLEELRVKESDRLSA | 360 |
| Query | 361 | VANGLKLNGVDCDEGETSLVVRGRPDGKGLGNASGAAVATHLDHRIAMSFLVMGLVSENP | 420 |
| Sbjct | 361 | VA+GLKLNGVDCDEGE LVVRGRPDGKGLGNA+ V+THLDHRIAMSFLVMGL SE+P VADGLKLNGVDCDEGEDFLVVRGRPDGKGLGNAADDKVSTHLDHRIAMSFLVMGLASEHP | 420 |
| Query  Sbjct | 421  421 | VTVDDATMIATSFPEFMDLMAGLGAKIE 448 VT+DDA MIATSFPEFM LM GLGAKIE VTIDDAAMIATSFPEFMQLMTGLGAKIE 448 |  |

>WP_052641111.1 3-phosphoshikimate 1-carboxyvinyltransferase [Rhizobium sp. NT-26]

CAD6595846.1 3-phosphoshikimate 1-carboxyvinyltransferase [arsenite-oxidising bacterium NT-25]

CCF21737.1 3-phosphoshikimate 1-carboxyvinyltransferase (5-enolpyruvylshikimate-3-phosphate synthase) (EPSP synthase) (EPSPS) [Rhizobium

sp. NT-26]

Length=455

Score = 767 bits (1981), Expect = 0.0, Method: Compositional matrix adjust.

| Identities | = 395/455 (87%), Positives = 418/455 (92%), Gaps = 0/455 (0%) |  |
| --- | --- | --- |
| Query 1 | MLHGASSRPATARKSSGLSGTVRIPGDKSISHRSFMFGGLASGETRITGLLEGEDVINTG | 60 |
| Sbjct 1 | M HG + +PA + KS+ L GTVRIPGDKSISHRSFMFGGLA+GETRITGLLEGEDVINTG MSHGVALKPARSLKSADLRGTVRIPGDKSISHRSFMFGGLAAGETRITGLLEGEDVINTG | 60 |
| Query 61 | KAMQAMGARIRKEGDTWIIDGVGNGGLLAPEAPLDFGNAATGCRLTMGLVGVYDFDSTFI | 120 |
| Sbjct 61 | KAM AMGARI K GD WII G GNG LLAPEAPLDFGNA TGCRLTMGLVGVYDF+STFI KAMHAMGARIEKVGDEWIIQGTGNGALLAPEAPLDFGNAGTGCRLTMGLVGVYDFESTFI | 120 |
| Query 121 | GDASLTKRPMGRVLNPLREMGVQVKSEDGDRLPVTLRGPKTPTPITYRVPMASAQVKSAV | 180 |
| Sbjct 121 | GDASLTKRPMGRVLNPLREMGVQVK+ +GDRLPVTLRGP P PITYRVPMASAQVKSAV GDASLTKRPMGRVLNPLREMGVQVKAAEGDRLPVTLRGPSIPNPITYRVPMASAQVKSAV | 180 |
| Query 181 | LLAGLNTPGITTVIEPIMTRDHTEKMLQGFGANLTVETDADGVRTIRLEGRGKLTGQVID | 240 |
| Sbjct 181 | LLAGLNTPG+TTVIEP+MTRDHTEKMLQGFGANL VE DADGVRTIRLEGRG+L GQVID LLAGLNTPGVTTVIEPVMTRDHTEKMLQGFGANLMVELDADGVRTIRLEGRGQLIGQVID | 240 |
| Query 241 | VPGDPSSTAFPLVAALLVPGSDVTILNVLMNPTRTGLILTLQEMGADIEVINPRLAGGED | 300 |
| Sbjct 241 | VPGDPSSTAFPLVAALLVPGSDVTI NVLMNPTRTGLILTLQEMGADIEVINPRLAGGED VPGDPSSTAFPLVAALLVPGSDVTIENVLMNPTRTGLILTLQEMGADIEVINPRLAGGED | 300 |
| Query 301 | VADLRVRSSTLKGVTVPEDRAPSMIDEYPILavaaafaegaTVMNGLEELRVKESDRLSA | 360 |
| Sbjct 301 | VADLRVR S LKGVTVP DRAPSMIDEYP+LAVAAAFAEGAT M GLEELRVKESDRLSA VADLRVRHSELKGVTVPADRAPSMIDEYPVLAVAAAFAEGATTMLGLEELRVKESDRLSA | 360 |
| Query 361 | VANGLKLNGVDCDEGETSLVVRGRPDGKGLGNASGAAVATHLDHRIAMSFLVMGLVSENP | 420 |
| Sbjct 361 | VA+GLKLNGVDCDEGE +LVVRGRP GKG GNA+G++V THLDHRIAMSFLVMGLVSE+ VADGLKLNGVDCDEGEDTLVVRGRPGGKGYGNATGSSVVTHLDHRIAMSFLVMGLVSEHA | 420 |
| Query 421  Sbjct 421 | VTVDDATMIATSFPEFMDLMAGLGAKIELSDTKAA 455 VTVDDA +IATSFPEFMDLM GLGA+IE +++AA VTVDDAGIIATSFPEFMDLMTGLGARIEPVESRAA 455 |  |

>WP_168345505.1 3-phosphoshikimate 1-carboxyvinyltransferase [Rhizobium laguerreae] NKN14821.1 3-phosphoshikimate 1-carboxyvinyltransferase [Rhizobium laguerreae]

Length=452

Score = 767 bits (1981), Expect = 0.0, Method: Compositional matrix adjust. Identities = 387/448 (86%), Positives = 416/448 (93%), Gaps = 0/448 (0%)

| Query | 1 | MLHGASSRPATARKSSGLSGTVRIPGDKSISHRSFMFGGLASGETRITGLLEGEDVINTG | 60 |
| --- | --- | --- | --- |
| Sbjct | 1 | MLHG++ RPATARKS+GL+G+VRIPGDKSISHRSFMFGGLASGETRITGLLEGEDVINTG MLHGSAPRPATARKSAGLAGSVRIPGDKSISHRSFMFGGLASGETRITGLLEGEDVINTG | 60 |
| Query | 61 | KAMQAMGARIRKEGDTWIIDGVGNGGLLAPEAPLDFGNAATGCRLTMGLVGVYDFDSTFI | 120 |
| Sbjct | 61 | +AMQAMGARIRK+G W+IDG GNG LLAP+APLDFGNA TG RLTMGLVG YDF STF RAMQAMGARIRKDGAQWVIDGTGNGALLAPDAPLDFGNAGTGVRLTMGLVGTYDFRSTFT | 120 |
| Query | 121 | GDASLTKRPMGRVLNPLREMGVQVKSEDGDRLPVTLRGPKTPTPITYRVPMASAQVKSAV | 180 |
| Sbjct | 121 | GDASL+KRPMGRVLNPLREMGVQV + +GDRLPVTLRGP TP+PI YRVPMASAQVKSAV GDASLSKRPMGRVLNPLREMGVQVSASEGDRLPVTLRGPGTPSPIRYRVPMASAQVKSAV | 180 |
| Query | 181 | LLAGLNTPGITTVIEPIMTRDHTEKMLQGFGANLTVETDADGVRTIRLEGRGKLTGQVID | 240 |
| Sbjct | 181 | LLAGLNTPG+TTVIEP+MTRDHTEKMLQGFGA L+VETD DGVRTIRLEGRGKL GQVID LLAGLNTPGVTTVIEPVMTRDHTEKMLQGFGAALSVETDGDGVRTIRLEGRGKLKGQVID | 240 |
| Query | 241 | VPGDPSSTAFPLVAALLVPGSDVTILNVLMNPTRTGLILTLQEMGADIEVINPRLAGGED | 300 |
| Sbjct | 241 | +PGDPSSTAFPLVAALLVPGSD+TI+NVLMNPTRTGLILTLQEMGA+IEV+N RLAGGED IPGDPSSTAFPLVAALLVPGSDITIVNVLMNPTRTGLILTLQEMGANIEVLNTRLAGGED | 300 |
| Query | 301 | VADLRVRSSTLKGVTVPEDRAPSMIDEYPILavaaafaegaTVMNGLEELRVKESDRLSA | 360 |
| Sbjct | 301 | VADLRVR S LKGVTVPE+RAPSMIDEYPILAVAA FAEGAT+M GLEELRVKESDRLSA VADLRVRHSELKGVTVPEERAPSMIDEYPILAVAACFAEGATIMKGLEELRVKESDRLSA | 360 |
| Query | 361 | VANGLKLNGVDCDEGETSLVVRGRPDGKGLGNASGAAVATHLDHRIAMSFLVMGLVSENP | 420 |
| Sbjct | 361 | VA+GLKLNG+DCDEGE LVVRGRPDGKGLGNA+ V+THLDHRIAMSFLVMGL SE+ VADGLKLNGIDCDEGEDFLVVRGRPDGKGLGNAADEKVSTHLDHRIAMSFLVMGLASEHA | 420 |
| Query  Sbjct | 421  421 | VTVDDATMIATSFPEFMDLMAGLGAKIE 448 VT+DDA MIATSFPEFM LM GLGAKIE VTIDDAAMIATSFPEFMQLMTGLGAKIE 448 |  |

>WP_131707667.1 3-phosphoshikimate 1-carboxyvinyltransferase [Rhizobium leguminosarum]

TBY85655.1 3-phosphoshikimate 1-carboxyvinyltransferase [Rhizobium leguminosarum bv. viciae]

Length=452

Score = 767 bits (1981), Expect = 0.0, Method: Compositional matrix adjust.

| Identities | = 387/449 (86%), Positives = 417/449 (93%), Gaps = 0/449 (0%) |  |
| --- | --- | --- |
| Query 1 | MLHGASSRPATARKSSGLSGTVRIPGDKSISHRSFMFGGLASGETRITGLLEGEDVINTG | 60 |
| Sbjct 1 | ML+G++ +PATARKS+GL+G+VRIPGDKSISHRSFMFGGLASGETRITGLLEGEDVINTG MLNGSAPKPATARKSAGLAGSVRIPGDKSISHRSFMFGGLASGETRITGLLEGEDVINTG | 60 |
| Query 61 | KAMQAMGARIRKEGDTWIIDGVGNGGLLAPEAPLDFGNAATGCRLTMGLVGVYDFDSTFI | 120 |
| Sbjct 61 | +AMQAMGARIRK+G W+IDG GNG LLAP+APLDFGNA TG RLTMGLVG YDF STFI RAMQAMGARIRKDGAQWVIDGTGNGALLAPDAPLDFGNAGTGVRLTMGLVGTYDFHSTFI | 120 |
| Query 121 | GDASLTKRPMGRVLNPLREMGVQVKSEDGDRLPVTLRGPKTPTPITYRVPMASAQVKSAV | 180 |
| Sbjct 121 | GDASL+KRPMGRVLNPLREMGVQV + +GDRLPVTLRGP TP+PI YRVPMASAQVKSAV GDASLSKRPMGRVLNPLREMGVQVSASEGDRLPVTLRGPGTPSPIRYRVPMASAQVKSAV | 180 |
| Query 181 | LLAGLNTPGITTVIEPIMTRDHTEKMLQGFGANLTVETDADGVRTIRLEGRGKLTGQVID | 240 |
| Sbjct 181 | LLAGLNTPG+TTVIEP+MTRDHTEKMLQGFGA L+VETD DGVRTIRLEGRG+LTGQVID LLAGLNTPGVTTVIEPVMTRDHTEKMLQGFGAALSVETDGDGVRTIRLEGRGRLTGQVID | 240 |
| Query 241 | VPGDPSSTAFPLVAALLVPGSDVTILNVLMNPTRTGLILTLQEMGADIEVINPRLAGGED | 300 |
| Sbjct 241 | +PGDPSSTAFPLVAALLVPGSD+TI+NVLMNPTRTGLILTLQEMGA+IEV N RLAGGED IPGDPSSTAFPLVAALLVPGSDLTIVNVLMNPTRTGLILTLQEMGANIEVANARLAGGED | 300 |
| Query 301 | VADLRVRSSTLKGVTVPEDRAPSMIDEYPILavaaafaegaTVMNGLEELRVKESDRLSA | 360 |
| Sbjct 301 | VADLRVR S LKGVTVPE+RAPSMIDEYPILAVAA FAEG T+M GLEELRVKESDRLSA VADLRVRHSELKGVTVPEERAPSMIDEYPILAVAACFAEGVTIMKGLEELRVKESDRLSA | 360 |
| Query 361 | VANGLKLNGVDCDEGETSLVVRGRPDGKGLGNASGAAVATHLDHRIAMSFLVMGLVSENP | 420 |
| Sbjct 361 | VA+GLKLNGVDCDEGE LVVRGRPDGKGLGNA+ V+THLDHRIAMSFLVMGL SE+ VADGLKLNGVDCDEGEDFLVVRGRPDGKGLGNAADEKVSTHLDHRIAMSFLVMGLASEHA | 420 |
| Query 421  Sbjct 421 | VTVDDATMIATSFPEFMDLMAGLGAKIEL 449 VT+DDA MIATSFPEFM LM GLGAKIEL VTIDDAAMIATSFPEFMQLMTGLGAKIEL 449 |  |

>WP_018448039.1 MULTISPECIES: 3-phosphoshikimate 1-carboxyvinyltransferase [Rhizobium] MBA8832195.1 3-phosphoshikimate 1-carboxyvinyltransferase [Rhizobium leguminosarum]

MVO91886.1 3-phosphoshikimate 1-carboxyvinyltransferase [Rhizobium leguminosarum bv. phaseoli]

Length=452

Score = 767 bits (1981), Expect = 0.0, Method: Compositional matrix adjust.

| Identities | = 386/448 (86%), Positives = 415/448 (93%), Gaps = 0/448 (0%) |  |
| --- | --- | --- |
| Query 1 | MLHGASSRPATARKSSGLSGTVRIPGDKSISHRSFMFGGLASGETRITGLLEGEDVINTG | 60 |
| Sbjct 1 | MLHG++ RPATARKS+GL+G+VRIPGDKSISHRSFMFGGLASGETRITGLLEGEDVINTG MLHGSAPRPATARKSAGLTGSVRIPGDKSISHRSFMFGGLASGETRITGLLEGEDVINTG | 60 |
| Query 61 | KAMQAMGARIRKEGDTWIIDGVGNGGLLAPEAPLDFGNAATGCRLTMGLVGVYDFDSTFI | 120 |
| Sbjct 61 | +AMQAMGARIRK G+ W+I+G GNG LLAP+APLDFGNA TG RLTMGLVG YDF STFI RAMQAMGARIRKVGEQWVIEGTGNGALLAPDAPLDFGNAGTGVRLTMGLVGTYDFRSTFI | 120 |
| Query 121 | GDASLTKRPMGRVLNPLREMGVQVKSEDGDRLPVTLRGPKTPTPITYRVPMASAQVKSAV | 180 |
| Sbjct 121 | GDASL+KRPMGRVLNPLREMGVQV + +GDRLPVTLRGP TP+PI YRVPMASAQVKSAV GDASLSKRPMGRVLNPLREMGVQVSASEGDRLPVTLRGPGTPSPIRYRVPMASAQVKSAV | 180 |
| Query 181 | LLAGLNTPGITTVIEPIMTRDHTEKMLQGFGANLTVETDADGVRTIRLEGRGKLTGQVID | 240 |
| Sbjct 181 | LLAGLNTPG+TTVIEP+MTRDHTEKMLQGFGA L+VETD DGVRTIRLEGRGKL GQVID LLAGLNTPGVTTVIEPVMTRDHTEKMLQGFGAALSVETDEDGVRTIRLEGRGKLKGQVID | 240 |
| Query 241 | VPGDPSSTAFPLVAALLVPGSDVTILNVLMNPTRTGLILTLQEMGADIEVINPRLAGGED | 300 |
| Sbjct 241 | +P DPSSTAFPLVAALLVPGSD+TI+NVLMNPTRTGLILTLQEMGADIEV+N RLAGGED IPSDPSSTAFPLVAALLVPGSDITIVNVLMNPTRTGLILTLQEMGADIEVLNARLAGGED | 300 |
| Query 301 | VADLRVRSSTLKGVTVPEDRAPSMIDEYPILavaaafaegaTVMNGLEELRVKESDRLSA | 360 |
| Sbjct 301 | VADLRVR S LKGVT+PEDRAPSMIDEYPILAVAA FAEG TVM GLEELRVKESDRLSA VADLRVRHSELKGVTIPEDRAPSMIDEYPILAVAACFAEGVTVMKGLEELRVKESDRLSA | 360 |
| Query 361 | VANGLKLNGVDCDEGETSLVVRGRPDGKGLGNASGAAVATHLDHRIAMSFLVMGLVSENP | 420 |
| Sbjct 361 | VA+GLKLNGVDCDEGE L+VRGRPDGKGLGNA+ V+THLDHRIAMSFLVMGL SE+P VADGLKLNGVDCDEGEDFLIVRGRPDGKGLGNATDGKVSTHLDHRIAMSFLVMGLASEHP | 420 |
| Query 421  Sbjct 421 | VTVDDATMIATSFPEFMDLMAGLGAKIE 448 VT+DD MIATSFPEFM LM GLGAKI+ VTIDDTAMIATSFPEFMQLMTGLGAKID 448 |  |

>WP_171599944.1 3-phosphoshikimate 1-carboxyvinyltransferase [Rhizobium sp. WYCCWR 11152]

NNU68590.1 3-phosphoshikimate 1-carboxyvinyltransferase [Rhizobium sp. WYCCWR 11152]

Length=452

Score = 767 bits (1981), Expect = 0.0, Method: Compositional matrix adjust.

| Identities | = 387/448 (86%), Positives = 418/448 (93%), Gaps = 0/448 (0%) |  |
| --- | --- | --- |
| Query 1 | MLHGASSRPATARKSSGLSGTVRIPGDKSISHRSFMFGGLASGETRITGLLEGEDVINTG | 60 |
| Sbjct 1 | MLHG++ RPATARKS+GL+G+VRIPGDKSISHRSFMFGGLASGETRITGLLEGEDVINTG MLHGSAPRPATARKSAGLTGSVRIPGDKSISHRSFMFGGLASGETRITGLLEGEDVINTG | 60 |
| Query 61 | KAMQAMGARIRKEGDTWIIDGVGNGGLLAPEAPLDFGNAATGCRLTMGLVGVYDFDSTFI | 120 |
| Sbjct 61 | +AMQAMGARIRK+G W+IDG GNG LLAP+APLDFGNA TG RLTMGLVG YDF STFI RAMQAMGARIRKDGAQWVIDGTGNGALLAPDAPLDFGNAGTGVRLTMGLVGTYDFRSTFI | 120 |
| Query 121 | GDASLTKRPMGRVLNPLREMGVQVKSEDGDRLPVTLRGPKTPTPITYRVPMASAQVKSAV | 180 |
| Sbjct 121 | GDASL+KRPMGRVLNPLREMGVQV + +GDRLPVTL+GP TP+PI YRVPMASAQVKSAV GDASLSKRPMGRVLNPLREMGVQVSASEGDRLPVTLKGPGTPSPIRYRVPMASAQVKSAV | 180 |
| Query 181 | LLAGLNTPGITTVIEPIMTRDHTEKMLQGFGANLTVETDADGVRTIRLEGRGKLTGQVID | 240 |
| Sbjct 181 | LLAGLNTPG+TTVIEP+MTRDHTEKMLQGFGA L+VETD++G RTIRLEGRG+LTGQVID LLAGLNTPGVTTVIEPVMTRDHTEKMLQGFGAALSVETDSEGARTIRLEGRGRLTGQVID | 240 |
| Query 241 | VPGDPSSTAFPLVAALLVPGSDVTILNVLMNPTRTGLILTLQEMGADIEVINPRLAGGED | 300 |
| Sbjct 241 | +PGDPSSTAFPLVAALLVPGSD+TI+NVLMNPTRTGLILTLQEMGADIEV N RLAGGED  IPGDPSSTAFPLVAALLVPGSDITIVNVLMNPTRTGLILTLQEMGADIEVANARLAGGED | 300 |

| Query | 301 | VADLRVRSSTLKGVTVPEDRAPSMIDEYPILavaaafaegaTVMNGLEELRVKESDRLSA | 360 |
| --- | --- | --- | --- |
| Sbjct | 301 | VADLRVR S LKGVTVPEDRAPSMIDEYPILAVAA+FAEGAT M GLEELRVKESDRLSA VADLRVRHSKLKGVTVPEDRAPSMIDEYPILAVAASFAEGATTMKGLEELRVKESDRLSA | 360 |
| Query | 361 | VANGLKLNGVDCDEGETSLVVRGRPDGKGLGNASGAAVATHLDHRIAMSFLVMGLVSENP | 420 |
| Sbjct | 361 | VA+GLKLNGVDCDEGE L+VRGRPDGKGLGNA+ V+THLDHRIAMSFLVMGL SE+P VADGLKLNGVDCDEGEDFLIVRGRPDGKGLGNAADDRVSTHLDHRIAMSFLVMGLASEHP | 420 |
| Query  Sbjct | 421  421 | VTVDDATMIATSFPEFMDLMAGLGAKIE 448 VT+DDA MIATSFPEFM LM GLGA+IE VTIDDAAMIATSFPEFMTLMKGLGARIE 448 |  |

>WP_130703641.1 3-phosphoshikimate 1-carboxyvinyltransferase [Rhizobium leguminosarum] TAY19336.1 3-phosphoshikimate 1-carboxyvinyltransferase [Rhizobium leguminosarum]

Length=452

Score = 767 bits (1980), Expect = 0.0, Method: Compositional matrix adjust.

| Identities | = 385/448 (86%), Positives = 419/448 (94%), Gaps = 0/448 (0%) |  |
| --- | --- | --- |
| Query 1 | MLHGASSRPATARKSSGLSGTVRIPGDKSISHRSFMFGGLASGETRITGLLEGEDVINTG | 60 |
| Sbjct 1 | ML+G++ +PATARKS+GL+G+VRIPGDKSISHRSFMFGGLASGETRITGLLEGEDVINTG MLNGSAPKPATARKSAGLTGSVRIPGDKSISHRSFMFGGLASGETRITGLLEGEDVINTG | 60 |
| Query 61 | KAMQAMGARIRKEGDTWIIDGVGNGGLLAPEAPLDFGNAATGCRLTMGLVGVYDFDSTFI | 120 |
| Sbjct 61 | +AMQAMGARIRKEG W+I+G GNG LLAP+APLDFGNA TG RLTMGLVG YDF STF RAMQAMGARIRKEGAQWVIEGTGNGALLAPDAPLDFGNAGTGVRLTMGLVGTYDFHSTFT | 120 |
| Query 121 | GDASLTKRPMGRVLNPLREMGVQVKSEDGDRLPVTLRGPKTPTPITYRVPMASAQVKSAV | 180 |
| Sbjct 121 | GDASL+KRPMGRVLNPLREMGVQ+ + +GDRLPVTL+GP TP+PI YRVPMASAQVKSAV GDASLSKRPMGRVLNPLREMGVQISASEGDRLPVTLKGPGTPSPIRYRVPMASAQVKSAV | 180 |
| Query 181 | LLAGLNTPGITTVIEPIMTRDHTEKMLQGFGANLTVETDADGVRTIRLEGRGKLTGQVID | 240 |
| Sbjct 181 | LLAGLNTPG+TTVIEP+MTRDHTEKMLQGFGA L+VETD++GVRTIRLEGRGKLTGQVID LLAGLNTPGVTTVIEPVMTRDHTEKMLQGFGAALSVETDSEGVRTIRLEGRGKLTGQVID | 240 |
| Query 241 | VPGDPSSTAFPLVAALLVPGSDVTILNVLMNPTRTGLILTLQEMGADIEVINPRLAGGED | 300 |
| Sbjct 241 | +P DPSSTAFPLVAALL+PGSD+TI+NVLMNPTRTGLILTLQEMGA+I+V+N RLAGGED IPSDPSSTAFPLVAALLIPGSDITIVNVLMNPTRTGLILTLQEMGANIQVLNTRLAGGED | 300 |
| Query 301 | VADLRVRSSTLKGVTVPEDRAPSMIDEYPILavaaafaegaTVMNGLEELRVKESDRLSA | 360 |
| Sbjct 301 | VADLRVR S LKGVTVPEDRAPSMIDEYPILAVAA FAEGATVMNGLEELRVKESDRLSA VADLRVRHSELKGVTVPEDRAPSMIDEYPILAVAACFAEGATVMNGLEELRVKESDRLSA | 360 |
| Query 361 | VANGLKLNGVDCDEGETSLVVRGRPDGKGLGNASGAAVATHLDHRIAMSFLVMGLVSENP | 420 |
| Sbjct 361 | VA+GLKLNGVDCDEGE LVVRGRPDGKGLGNA+ V+THLDHRIAMSFLVMGL SE+P VADGLKLNGVDCDEGEDFLVVRGRPDGKGLGNAADDKVSTHLDHRIAMSFLVMGLASEHP | 420 |
| Query 421  Sbjct 421 | VTVDDATMIATSFPEFMDLMAGLGAKIE 448 VT+DDA MIATSFPEFM LM GLGAKIE VTIDDAAMIATSFPEFMQLMTGLGAKIE 448 |  |

>WP_027669080.1 3-phosphoshikimate 1-carboxyvinyltransferase [Rhizobium leguminosarum] Length=452

Score = 767 bits (1980), Expect = 0.0, Method: Compositional matrix adjust.

| Identities | = 388/449 (86%), Positives = 414/449 (92%), Gaps = 0/449 (0%) |  |
| --- | --- | --- |
| Query 1 | MLHGASSRPATARKSSGLSGTVRIPGDKSISHRSFMFGGLASGETRITGLLEGEDVINTG | 60 |
| Sbjct 1 | MLH ++ +PATARKS+GL+G+VRIPGDKSISHRSFMFGGLASGETRITGLLEGEDVINTG MLHDSAPKPATARKSAGLAGSVRIPGDKSISHRSFMFGGLASGETRITGLLEGEDVINTG | 60 |
| Query 61 | KAMQAMGARIRKEGDTWIIDGVGNGGLLAPEAPLDFGNAATGCRLTMGLVGVYDFDSTFI | 120 |
| Sbjct 61 | +AMQAMGARIRK+G W+IDG GNG LLAP+APLDFGNA TG RLTMGLVG YDF STFI RAMQAMGARIRKDGAQWVIDGTGNGALLAPDAPLDFGNAGTGVRLTMGLVGTYDFRSTFI | 120 |
| Query 121 | GDASLTKRPMGRVLNPLREMGVQVKSEDGDRLPVTLRGPKTPTPITYRVPMASAQVKSAV | 180 |
| Sbjct 121 | GDASL+KRPMGRVLNPLREMGVQV + +GDRLPVTLRGP TP+PI YRVPMASAQVKSAV  GDASLSKRPMGRVLNPLREMGVQVSASEGDRLPVTLRGPGTPSPIRYRVPMASAQVKSAV | 180 |

| Query | 181 | LLAGLNTPGITTVIEPIMTRDHTEKMLQGFGANLTVETDADGVRTIRLEGRGKLTGQVID | 240 |
| --- | --- | --- | --- |
| Sbjct | 181 | LLAGLNTPG+TTVIEP+MTRDHTEKMLQGFGA L+VETD DGVRTIRLEGRG L GQVID LLAGLNTPGVTTVIEPVMTRDHTEKMLQGFGAALSVETDGDGVRTIRLEGRGNLKGQVID | 240 |
| Query | 241 | VPGDPSSTAFPLVAALLVPGSDVTILNVLMNPTRTGLILTLQEMGADIEVINPRLAGGED | 300 |
| Sbjct | 241 | +PGDPSSTAFPLVAALLVPGSD+TI+NVLMNPTRTGLILTLQEMGADI V N RLAGGED IPGDPSSTAFPLVAALLVPGSDITIVNVLMNPTRTGLILTLQEMGADIAVANARLAGGED | 300 |
| Query | 301 | VADLRVRSSTLKGVTVPEDRAPSMIDEYPILavaaafaegaTVMNGLEELRVKESDRLSA | 360 |
| Sbjct | 301 | VADLRVR S LKGVTVPE+RAPSMIDEYPILAVAA FAEG TVM GLEELRVKESDRLSA VADLRVRHSELKGVTVPEERAPSMIDEYPILAVAACFAEGVTVMKGLEELRVKESDRLSA | 360 |
| Query | 361 | VANGLKLNGVDCDEGETSLVVRGRPDGKGLGNASGAAVATHLDHRIAMSFLVMGLVSENP | 420 |
| Sbjct | 361 | VA+GLKLNGVDCDEGE LVVRGRPDGKGLGNA+G V+THLDHRIAMSFLVMGL SE+ VADGLKLNGVDCDEGEDFLVVRGRPDGKGLGNAAGDKVSTHLDHRIAMSFLVMGLASEHA | 420 |
| Query  Sbjct | 421  421 | VTVDDATMIATSFPEFMDLMAGLGAKIEL 449 VT+DDA MIATSFPEFM LM GLGAKIEL VTIDDAAMIATSFPEFMQLMTGLGAKIEL 449 |  |

>WP_168287538.1 3-phosphoshikimate 1-carboxyvinyltransferase [Rhizobium laguerreae] NKM71821.1 3-phosphoshikimate 1-carboxyvinyltransferase [Rhizobium laguerreae]

Length=452

Score = 767 bits (1980), Expect = 0.0, Method: Compositional matrix adjust.

| Identities | = 385/449 (86%), Positives = 415/449 (92%), Gaps = 0/449 (0%) |  |
| --- | --- | --- |
| Query 1 | MLHGASSRPATARKSSGLSGTVRIPGDKSISHRSFMFGGLASGETRITGLLEGEDVINTG | 60 |
| Sbjct 1 | MLHG++ RPATARKS+GL+G+VRIPGDKSISHRSFMFGGLASG+TRITGLLEGEDVINTG MLHGSAPRPATARKSAGLTGSVRIPGDKSISHRSFMFGGLASGQTRITGLLEGEDVINTG | 60 |
| Query 61 | KAMQAMGARIRKEGDTWIIDGVGNGGLLAPEAPLDFGNAATGCRLTMGLVGVYDFDSTFI | 120 |
| Sbjct 61 | +AMQAMGARIRK+G W+IDG GNG LLAP+APLDFGNA TG RLTMGLVG YDF STFI RAMQAMGARIRKDGAQWVIDGTGNGALLAPDAPLDFGNAGTGVRLTMGLVGTYDFRSTFI | 120 |
| Query 121 | GDASLTKRPMGRVLNPLREMGVQVKSEDGDRLPVTLRGPKTPTPITYRVPMASAQVKSAV | 180 |
| Sbjct 121 | GDASL+KRPMGRVLNPLREMGVQV + +GDRLPVTLRGP TP+PI YRVPMASAQVKSAV GDASLSKRPMGRVLNPLREMGVQVSASEGDRLPVTLRGPATPSPIRYRVPMASAQVKSAV | 180 |
| Query 181 | LLAGLNTPGITTVIEPIMTRDHTEKMLQGFGANLTVETDADGVRTIRLEGRGKLTGQVID | 240 |
| Sbjct 181 | LLAGLNTPG+TTVIEP+MTRDHTEKMLQGFGA L+VET DG+RTIRLEGRGKL GQVID LLAGLNTPGVTTVIEPVMTRDHTEKMLQGFGAALSVETGGDGIRTIRLEGRGKLKGQVID | 240 |
| Query 241 | VPGDPSSTAFPLVAALLVPGSDVTILNVLMNPTRTGLILTLQEMGADIEVINPRLAGGED | 300 |
| Sbjct 241 | +PGDPSSTAFPLVAALLVPGSD+TI+NVLMNPTRTGLILTLQEMGADIEV+N RLAGGED IPGDPSSTAFPLVAALLVPGSDITIVNVLMNPTRTGLILTLQEMGADIEVVNTRLAGGED | 300 |
| Query 301 | VADLRVRSSTLKGVTVPEDRAPSMIDEYPILavaaafaegaTVMNGLEELRVKESDRLSA | 360 |
| Sbjct 301 | VADLRVR S L GVTVPE+RAPSMIDEYPILA+AA FAEGATVM GLEELRVKESDRLSA VADLRVRHSELNGVTVPEERAPSMIDEYPILAIAACFAEGATVMKGLEELRVKESDRLSA | 360 |
| Query 361 | VANGLKLNGVDCDEGETSLVVRGRPDGKGLGNASGAAVATHLDHRIAMSFLVMGLVSENP | 420 |
| Sbjct 361 | VA+GLKLNG DCDEGE L+VRGRPDGKGLGNA+G V THLDHRIAMSFLV+GL SE+ VADGLKLNGADCDEGEDFLIVRGRPDGKGLGNAAGGKVRTHLDHRIAMSFLVLGLASEHA | 420 |
| Query 421  Sbjct 421 | VTVDDATMIATSFPEFMDLMAGLGAKIEL 449 VT+DDA MIATSFPEFM LM GLGAKIEL VTIDDAAMIATSFPEFMQLMTGLGAKIEL 449 |  |

>WP_018068678.1 3-phosphoshikimate 1-carboxyvinyltransferase [Rhizobium leguminosarum] NEH67284.1 3-phosphoshikimate 1-carboxyvinyltransferase [Rhizobium leguminosarum] NEI24423.1 3-phosphoshikimate 1-carboxyvinyltransferase [Rhizobium leguminosarum] NEI98830.1 3-phosphoshikimate 1-carboxyvinyltransferase [Rhizobium leguminosarum] NEJ40688.1 3-phosphoshikimate 1-carboxyvinyltransferase [Rhizobium leguminosarum]

QJS30042.1 3-phosphoshikimate 1-carboxyvinyltransferase [Rhizobium leguminosarum bv. trifolii TA1]

Length=452

Score = 767 bits (1980), Expect = 0.0, Method: Compositional matrix adjust. Identities = 386/449 (86%), Positives = 415/449 (92%), Gaps = 0/449 (0%)

| Query | 1 | MLHGASSRPATARKSSGLSGTVRIPGDKSISHRSFMFGGLASGETRITGLLEGEDVINTG | 60 |
| --- | --- | --- | --- |
| Sbjct | 1 | MLHG++ RPATARKS+ L+G+VRIPGDKSISHRSFMFGGLASGETRITGLLEGEDVINTG MLHGSAPRPATARKSASLTGSVRIPGDKSISHRSFMFGGLASGETRITGLLEGEDVINTG | 60 |
| Query | 61 | KAMQAMGARIRKEGDTWIIDGVGNGGLLAPEAPLDFGNAATGCRLTMGLVGVYDFDSTFI | 120 |
| Sbjct | 61 | +AMQAMGARIRKEG W+I+G GNG LLAP+APLDFGNA TG RLTMGLVG YDF STF RAMQAMGARIRKEGAQWVIEGTGNGALLAPDAPLDFGNAGTGVRLTMGLVGTYDFHSTFT | 120 |
| Query | 121 | GDASLTKRPMGRVLNPLREMGVQVKSEDGDRLPVTLRGPKTPTPITYRVPMASAQVKSAV | 180 |
| Sbjct | 121 | GDASL+KRPMGRVL PLREMGVQV + +GDRLPVTLRGP TP+PI YRVPMASAQVKSAV GDASLSKRPMGRVLAPLREMGVQVSASEGDRLPVTLRGPGTPSPIRYRVPMASAQVKSAV | 180 |
| Query | 181 | LLAGLNTPGITTVIEPIMTRDHTEKMLQGFGANLTVETDADGVRTIRLEGRGKLTGQVID | 240 |
| Sbjct | 181 | LLAGLNTPG+TTVIEP+MTR+HTEKMLQGFGA L+VETD DGVRTIRLEGRGKL GQVID LLAGLNTPGVTTVIEPVMTRNHTEKMLQGFGAALSVETDGDGVRTIRLEGRGKLAGQVID | 240 |
| Query | 241 | VPGDPSSTAFPLVAALLVPGSDVTILNVLMNPTRTGLILTLQEMGADIEVINPRLAGGED | 300 |
| Sbjct | 241 | +PGDPSSTAFPLVAAL+VPGSD+TI+NVLMNPTRTGLILTLQEMGADIEV+N RLAGGED IPGDPSSTAFPLVAALIVPGSDITIVNVLMNPTRTGLILTLQEMGADIEVVNARLAGGED | 300 |
| Query | 301 | VADLRVRSSTLKGVTVPEDRAPSMIDEYPILavaaafaegaTVMNGLEELRVKESDRLSA | 360 |
| Sbjct | 301 | VADLRVR S LKGVTVPEDRAPSMIDEYPILA+AA FAEGATVM GLEELRVKESDRLSA VADLRVRHSELKGVTVPEDRAPSMIDEYPILAIAACFAEGATVMKGLEELRVKESDRLSA | 360 |
| Query | 361 | VANGLKLNGVDCDEGETSLVVRGRPDGKGLGNASGAAVATHLDHRIAMSFLVMGLVSENP | 420 |
| Sbjct | 361 | VA+GLKLNGVDCDEGE L+VRGRPDGKGLGNA+G V THLDHRIAMSFLV+GL SE+ VADGLKLNGVDCDEGEDFLIVRGRPDGKGLGNAAGGQVRTHLDHRIAMSFLVLGLASEHA | 420 |
| Query  Sbjct | 421  421 | VTVDDATMIATSFPEFMDLMAGLGAKIEL 449 VT+DDA MIATSFPEFM LM GLGAKIEL VTIDDAAMIATSFPEFMQLMTGLGAKIEL 449 |  |

>WP_168312063.1 MULTISPECIES: 3-phosphoshikimate 1-carboxyvinyltransferase [Rhizobium]

NKK73914.1 3-phosphoshikimate 1-carboxyvinyltransferase [Rhizobium leguminosarum bv. viciae]

NNU37191.1 3-phosphoshikimate 1-carboxyvinyltransferase [Rhizobium sophorae] Length=452

Score = 766 bits (1979), Expect = 0.0, Method: Compositional matrix adjust.

| Identities | = 386/448 (86%), Positives = 415/448 (93%), Gaps = 0/448 (0%) |  |
| --- | --- | --- |
| Query 1 | MLHGASSRPATARKSSGLSGTVRIPGDKSISHRSFMFGGLASGETRITGLLEGEDVINTG | 60 |
| Sbjct 1 | MLHG++ RPATARKS+GL+G+VRIPGDKSISHRSFMFGGLASG+TRITGLLEGEDVINTG MLHGSAPRPATARKSAGLTGSVRIPGDKSISHRSFMFGGLASGQTRITGLLEGEDVINTG | 60 |
| Query 61 | KAMQAMGARIRKEGDTWIIDGVGNGGLLAPEAPLDFGNAATGCRLTMGLVGVYDFDSTFI | 120 |
| Sbjct 61 | +AMQAMGARIRKEG W+IDG GNG LLAP+APLDFGNA TG RLTMGLVG YDF S F RAMQAMGARIRKEGAQWVIDGTGNGALLAPDAPLDFGNAGTGVRLTMGLVGTYDFHSIFT | 120 |
| Query 121 | GDASLTKRPMGRVLNPLREMGVQVKSEDGDRLPVTLRGPKTPTPITYRVPMASAQVKSAV | 180 |
| Sbjct 121 | GDASL+KRPMGRVLNPLREMGVQV + +GDRLPVTLRGP TP+PI YRVPMASAQVKSAV GDASLSKRPMGRVLNPLREMGVQVSASEGDRLPVTLRGPGTPSPIRYRVPMASAQVKSAV | 180 |
| Query 181 | LLAGLNTPGITTVIEPIMTRDHTEKMLQGFGANLTVETDADGVRTIRLEGRGKLTGQVID | 240 |
| Sbjct 181 | LLAGLNTPG+TTVIEP+MTRDHTEKMLQGFGA L+VETD++GVRTIRLEGRGKLTGQVID LLAGLNTPGVTTVIEPVMTRDHTEKMLQGFGAALSVETDSEGVRTIRLEGRGKLTGQVID | 240 |
| Query 241 | VPGDPSSTAFPLVAALLVPGSDVTILNVLMNPTRTGLILTLQEMGADIEVINPRLAGGED | 300 |
| Sbjct 241 | VPGDPSSTAFPLVAALLVPGSD+TI+NVLMNPTRTGLILTLQEMGADIEV+N RLAGGED VPGDPSSTAFPLVAALLVPGSDITIVNVLMNPTRTGLILTLQEMGADIEVLNARLAGGED | 300 |
| Query 301 | VADLRVRSSTLKGVTVPEDRAPSMIDEYPILavaaafaegaTVMNGLEELRVKESDRLSA | 360 |
| Sbjct 301 | VADLRVR S L+GVTVPEDRAPSMIDEYPILAVAA FAEG T M GLEELRVKESDRLSA VADLRVRHSELRGVTVPEDRAPSMIDEYPILAVAACFAEGVTTMKGLEELRVKESDRLSA | 360 |
| Query 361 | VANGLKLNGVDCDEGETSLVVRGRPDGKGLGNASGAAVATHLDHRIAMSFLVMGLVSENP | 420 |
| Sbjct 361 | VA+GLKLNGVDCDEGE L+VRGRPDGKGLGNA+ V+THLDHRIAMSFLVMGL SE+ VADGLKLNGVDCDEGEDFLIVRGRPDGKGLGNAADGRVSTHLDHRIAMSFLVMGLASEHA | 420 |
| Query 421 | VTVDDATMIATSFPEFMDLMAGLGAKIE 448 |  |

VT+DDA MIATSFPEFM LM GLGA+IE Sbjct 421 VTIDDAAMIATSFPEFMTLMKGLGARIE 448

>WP_130684197.1 3-phosphoshikimate 1-carboxyvinyltransferase [Rhizobium leguminosarum] TAU91150.1 3-phosphoshikimate 1-carboxyvinyltransferase [Rhizobium leguminosarum] TAV55795.1 3-phosphoshikimate 1-carboxyvinyltransferase [Rhizobium leguminosarum] TAX74330.1 3-phosphoshikimate 1-carboxyvinyltransferase [Rhizobium leguminosarum]

Length=452

Score = 766 bits (1979), Expect = 0.0, Method: Compositional matrix adjust.

| Identities | = 386/449 (86%), Positives = 418/449 (93%), Gaps = 0/449 (0%) |  |
| --- | --- | --- |
| Query 1 | MLHGASSRPATARKSSGLSGTVRIPGDKSISHRSFMFGGLASGETRITGLLEGEDVINTG | 60 |
| Sbjct 1 | ML+G++ +PATARKS+GL+G+VRIPGDKSISHRSFMFGGLASGETRITGLLEGEDVINTG MLNGSAPKPATARKSAGLTGSVRIPGDKSISHRSFMFGGLASGETRITGLLEGEDVINTG | 60 |
| Query 61 | KAMQAMGARIRKEGDTWIIDGVGNGGLLAPEAPLDFGNAATGCRLTMGLVGVYDFDSTFI | 120 |
| Sbjct 61 | +AMQAMGARIRKEG W+I G GNG LLAP+APLDFGNA TG RLT+GLVG YDF STF RAMQAMGARIRKEGAQWVIGGTGNGALLAPDAPLDFGNAGTGVRLTIGLVGTYDFRSTFT | 120 |
| Query 121 | GDASLTKRPMGRVLNPLREMGVQVKSEDGDRLPVTLRGPKTPTPITYRVPMASAQVKSAV | 180 |
| Sbjct 121 | GDASL+KRPMGRVLNPLREMGVQ+ + +GDRLPVTL+G TP+PI YRVPMASAQVKSAV GDASLSKRPMGRVLNPLREMGVQISASEGDRLPVTLKGSGTPSPIRYRVPMASAQVKSAV | 180 |
| Query 181 | LLAGLNTPGITTVIEPIMTRDHTEKMLQGFGANLTVETDADGVRTIRLEGRGKLTGQVID | 240 |
| Sbjct 181 | LLAGLNTPG+TTVIEP+MTRDHTEKMLQGFGA+L+VETD++GVRTIRLEGRG+LTGQ+ID LLAGLNTPGVTTVIEPVMTRDHTEKMLQGFGASLSVETDSEGVRTIRLEGRGRLTGQMID | 240 |
| Query 241 | VPGDPSSTAFPLVAALLVPGSDVTILNVLMNPTRTGLILTLQEMGADIEVINPRLAGGED | 300 |
| Sbjct 241 | VPGDPSSTAFPLVAALLVPGSD+TI+NVLMNPTRTGLILTLQEMGADIEV N RLAGGED VPGDPSSTAFPLVAALLVPGSDITIVNVLMNPTRTGLILTLQEMGADIEVANARLAGGED | 300 |
| Query 301 | VADLRVRSSTLKGVTVPEDRAPSMIDEYPILavaaafaegaTVMNGLEELRVKESDRLSA | 360 |
| Sbjct 301 | VADLRVR S LKGVTVPE RAPSMIDEYPILAVAA FAEGATVM GLEELRVKESDRLSA VADLRVRHSELKGVTVPEQRAPSMIDEYPILAVAACFAEGATVMKGLEELRVKESDRLSA | 360 |
| Query 361 | VANGLKLNGVDCDEGETSLVVRGRPDGKGLGNASGAAVATHLDHRIAMSFLVMGLVSENP | 420 |
| Sbjct 361 | VANGLKLNGVDCDEGE L+VRGRPDGKGLGNA+G V+THLDHRIAMSFLVMGL SE+P VANGLKLNGVDCDEGEDFLIVRGRPDGKGLGNAAGGRVSTHLDHRIAMSFLVMGLASEHP | 420 |
| Query 421  Sbjct 421 | VTVDDATMIATSFPEFMDLMAGLGAKIEL 449 VT+DDA MIATSFPEFM LM GLGAKIEL VTIDDAAMIATSFPEFMQLMTGLGAKIEL 449 |  |

>WP_077977105.1 3-phosphoshikimate 1-carboxyvinyltransferase [Rhizobium laguerreae] MBB3162816.1 3-phosphoshikimate 1-carboxyvinyltransferase [Rhizobium laguerreae] OOO50203.1 3-phosphoshikimate 1-carboxyvinyltransferase [Rhizobium laguerreae]

Length=452

Score = 766 bits (1979), Expect = 0.0, Method: Compositional matrix adjust.

| Identities | = 386/448 (86%), Positives = 416/448 (93%), Gaps = 0/448 (0%) |  |
| --- | --- | --- |
| Query 1 | MLHGASSRPATARKSSGLSGTVRIPGDKSISHRSFMFGGLASGETRITGLLEGEDVINTG | 60 |
| Sbjct 1 | MLHG++ RPATARKS+GL+G+VRIPGDKSISHRSFMFGGLASGETRITGLLEGEDVINTG MLHGSAPRPATARKSAGLAGSVRIPGDKSISHRSFMFGGLASGETRITGLLEGEDVINTG | 60 |
| Query 61 | KAMQAMGARIRKEGDTWIIDGVGNGGLLAPEAPLDFGNAATGCRLTMGLVGVYDFDSTFI | 120 |
| Sbjct 61 | +AMQAMGARIRK+G W+IDG GNG LLAP+APLDFGNA TG RLTMGLVG YDF STF RAMQAMGARIRKDGAQWVIDGTGNGALLAPDAPLDFGNAGTGVRLTMGLVGTYDFRSTFT | 120 |
| Query 121 | GDASLTKRPMGRVLNPLREMGVQVKSEDGDRLPVTLRGPKTPTPITYRVPMASAQVKSAV | 180 |
| Sbjct 121 | GDASL+KRPMGRVLNPLREMGVQV + +GDRLPVTLRGP TP+PI YRVPMASAQVKSAV GDASLSKRPMGRVLNPLREMGVQVSASEGDRLPVTLRGPGTPSPIRYRVPMASAQVKSAV | 180 |
| Query 181 | LLAGLNTPGITTVIEPIMTRDHTEKMLQGFGANLTVETDADGVRTIRLEGRGKLTGQVID | 240 |
| Sbjct 181 | LLAGLNTPG+TTVIEP+MTRDHTEKMLQGFGA L+VETD DGVRTIRLEGRGKL GQVID LLAGLNTPGVTTVIEPVMTRDHTEKMLQGFGAALSVETDGDGVRTIRLEGRGKLKGQVID | 240 |
| Query 241 | VPGDPSSTAFPLVAALLVPGSDVTILNVLMNPTRTGLILTLQEMGADIEVINPRLAGGED  +PGDPSSTAFPLVAALLVPGSD+TI+NVLMNPTRTGLILTLQEMGA+IEV+N RLAGGED | 300 |

| Sbjct | 241 | IPGDPSSTAFPLVAALLVPGSDITIVNVLMNPTRTGLILTLQEMGANIEVLNTRLAGGED | 300 |
| --- | --- | --- | --- |
| Query | 301 | VADLRVRSSTLKGVTVPEDRAPSMIDEYPILavaaafaegaTVMNGLEELRVKESDRLSA | 360 |
| Sbjct | 301 | VADLRVR S LKGVTVPE+RAPSMIDEYPILAVAA FAEGAT+M GLEELRVKESDRLSA VADLRVRHSELKGVTVPEERAPSMIDEYPILAVAACFAEGATIMKGLEELRVKESDRLSA | 360 |
| Query | 361 | VANGLKLNGVDCDEGETSLVVRGRPDGKGLGNASGAAVATHLDHRIAMSFLVMGLVSENP | 420 |
| Sbjct | 361 | VA+GLKLNG+DCDEGE LVVRGRPDGKGLGNA+G V+THLDHRIAMSFLVMGL SE+ VADGLKLNGIDCDEGEDFLVVRGRPDGKGLGNAAGGRVSTHLDHRIAMSFLVMGLASEHA | 420 |
| Query  Sbjct | 421  421 | VTVDDATMIATSFPEFMDLMAGLGAKIE 448 V +DDA MIATSFPEFM LM GLGAK+E VMIDDAAMIATSFPEFMQLMTGLGAKVE 448 |  |

>WP_028731871.1 3-phosphoshikimate 1-carboxyvinyltransferase [Rhizobium leguminosarum] Length=452

Score = 766 bits (1979), Expect = 0.0, Method: Compositional matrix adjust.

| Identities | = 386/448 (86%), Positives = 416/448 (93%), Gaps = 0/448 (0%) |  |
| --- | --- | --- |
| Query 1 | MLHGASSRPATARKSSGLSGTVRIPGDKSISHRSFMFGGLASGETRITGLLEGEDVINTG | 60 |
| Sbjct 1 | ML+G++ R ATARKS+GL+G+VRIPGDKSISHRSFMFGGLASGETRITGLLEGEDVINTG MLYGSAPRRATARKSAGLTGSVRIPGDKSISHRSFMFGGLASGETRITGLLEGEDVINTG | 60 |
| Query 61 | KAMQAMGARIRKEGDTWIIDGVGNGGLLAPEAPLDFGNAATGCRLTMGLVGVYDFDSTFI | 120 |
| Sbjct 61 | +AMQAMGARIRKEG+ W+I+G GNG LLAP+APLDFGNA TG RLTMGLVG YDF STFI RAMQAMGARIRKEGEQWVIEGTGNGALLAPDAPLDFGNAGTGVRLTMGLVGTYDFRSTFI | 120 |
| Query 121 | GDASLTKRPMGRVLNPLREMGVQVKSEDGDRLPVTLRGPKTPTPITYRVPMASAQVKSAV | 180 |
| Sbjct 121 | GDASL+KRPMGRVLNPLREMGVQV + +GDRLPVTL+GP TP+PI YRVPMASAQVKSAV GDASLSKRPMGRVLNPLREMGVQVSASEGDRLPVTLKGPGTPSPIRYRVPMASAQVKSAV | 180 |
| Query 181 | LLAGLNTPGITTVIEPIMTRDHTEKMLQGFGANLTVETDADGVRTIRLEGRGKLTGQVID | 240 |
| Sbjct 181 | LLAGLNTPG+TTVIEP+MTRDHTEKMLQGFGA L+ ETD DGVRTIRLEGRGKL GQVID LLAGLNTPGVTTVIEPVMTRDHTEKMLQGFGAALSAETDEDGVRTIRLEGRGKLKGQVID | 240 |
| Query 241 | VPGDPSSTAFPLVAALLVPGSDVTILNVLMNPTRTGLILTLQEMGADIEVINPRLAGGED | 300 |
| Sbjct 241 | +PGDPSSTAFPLVAALLVPGSD+TI+NVLMNPTRTGLILTLQEMGADI+V+NPRLAGGED IPGDPSSTAFPLVAALLVPGSDITIVNVLMNPTRTGLILTLQEMGADIQVLNPRLAGGED | 300 |
| Query 301 | VADLRVRSSTLKGVTVPEDRAPSMIDEYPILavaaafaegaTVMNGLEELRVKESDRLSA | 360 |
| Sbjct 301 | VADLRVR S LKGVTVPEDRAPSMIDEYPILAVAA FAEG TVM GLEELRVKESDRLSA VADLRVRHSELKGVTVPEDRAPSMIDEYPILAVAACFAEGVTVMKGLEELRVKESDRLSA | 360 |
| Query 361 | VANGLKLNGVDCDEGETSLVVRGRPDGKGLGNASGAAVATHLDHRIAMSFLVMGLVSENP | 420 |
| Sbjct 361 | VA+GLKLNGVDCDEGE L+V GRPDGKGLGNA+ V+THLDHRIAMSFLVMGL SE+P VADGLKLNGVDCDEGEDFLIVCGRPDGKGLGNAADGRVSTHLDHRIAMSFLVMGLASEHP | 420 |
| Query 421  Sbjct 421 | VTVDDATMIATSFPEFMDLMAGLGAKIE 448 VT+DDA MIATSFPEFM LM GLGAKIE VTIDDAAMIATSFPEFMQLMTGLGAKIE 448 |  |

>WP_162115601.1 3-phosphoshikimate 1-carboxyvinyltransferase [Rhizobium laguerreae] NDK48709.1 3-phosphoshikimate 1-carboxyvinyltransferase [Rhizobium laguerreae]

Length=452

Score = 766 bits (1979), Expect = 0.0, Method: Compositional matrix adjust.

| Identities | = 387/449 (86%), Positives = 414/449 (92%), Gaps = 0/449 (0%) |  |
| --- | --- | --- |
| Query 1 | MLHGASSRPATARKSSGLSGTVRIPGDKSISHRSFMFGGLASGETRITGLLEGEDVINTG | 60 |
| Sbjct 1 | MLHG++ RPATARKS+ L+G+VRIPGDKSISHRSFMFGGLASGETRITGLLEGEDVINTG MLHGSAPRPATARKSASLTGSVRIPGDKSISHRSFMFGGLASGETRITGLLEGEDVINTG | 60 |
| Query 61 | KAMQAMGARIRKEGDTWIIDGVGNGGLLAPEAPLDFGNAATGCRLTMGLVGVYDFDSTFI | 120 |
| Sbjct 61 | +AMQAMGARIRKEG W+I+G GNG LLAP+APLDFGNA TG RLTMGLVG YDF STF RAMQAMGARIRKEGAQWVIEGTGNGALLAPDAPLDFGNAGTGVRLTMGLVGTYDFHSTFT | 120 |
| Query 121 | GDASLTKRPMGRVLNPLREMGVQVKSEDGDRLPVTLRGPKTPTPITYRVPMASAQVKSAV | 180 |
| Sbjct 121 | GDASL+KRPMGRVL PLREMGVQV + +GDRLPVTLRGP TP+PI YRVPMASAQVKSAV  GDASLSKRPMGRVLAPLREMGVQVSASEGDRLPVTLRGPGTPSPIRYRVPMASAQVKSAV | 180 |

| Query | 181 | LLAGLNTPGITTVIEPIMTRDHTEKMLQGFGANLTVETDADGVRTIRLEGRGKLTGQVID | 240 |
| --- | --- | --- | --- |
| Sbjct | 181 | LLAGLNTPG+TTVIEP+MTRDHTEKMLQGFGA L+VET DGVRTIRLEGRGKL GQVID LLAGLNTPGVTTVIEPVMTRDHTEKMLQGFGAALSVETAGDGVRTIRLEGRGKLAGQVID | 240 |
| Query | 241 | VPGDPSSTAFPLVAALLVPGSDVTILNVLMNPTRTGLILTLQEMGADIEVINPRLAGGED | 300 |
| Sbjct | 241 | VPGDPSSTAFPLVAALLVPGSD+TI+NVLMNPTRTGLILTLQEMGADIEV+N RLAGGED VPGDPSSTAFPLVAALLVPGSDITIVNVLMNPTRTGLILTLQEMGADIEVVNARLAGGED | 300 |
| Query | 301 | VADLRVRSSTLKGVTVPEDRAPSMIDEYPILavaaafaegaTVMNGLEELRVKESDRLSA | 360 |
| Sbjct | 301 | VADLRVR S LKGVT+PEDRAPSMIDEYPILA+AA FAEGATVM GLEELRVKESDRLSA VADLRVRHSELKGVTIPEDRAPSMIDEYPILAIAACFAEGATVMKGLEELRVKESDRLSA | 360 |
| Query | 361 | VANGLKLNGVDCDEGETSLVVRGRPDGKGLGNASGAAVATHLDHRIAMSFLVMGLVSENP | 420 |
| Sbjct | 361 | VA+GLKLNGVDCDEGE L+VRGRPDGKGLGNA+G V THLDHRIAMSFLV+GL SE+ VADGLKLNGVDCDEGEDFLIVRGRPDGKGLGNAAGGQVRTHLDHRIAMSFLVLGLASEHA | 420 |
| Query  Sbjct | 421  421 | VTVDDATMIATSFPEFMDLMAGLGAKIEL 449 VT+DDA MIATSFPEFM LM GLGAKIEL VTIDDAAMIATSFPEFMQLMTGLGAKIEL 449 |  |

>WP_027682251.1 3-phosphoshikimate 1-carboxyvinyltransferase [Rhizobium leguminosarum] Length=452

Score = 766 bits (1979), Expect = 0.0, Method: Compositional matrix adjust.

| Identities | = 387/448 (86%), Positives = 419/448 (94%), Gaps = 0/448 (0%) |  |
| --- | --- | --- |
| Query 1 | MLHGASSRPATARKSSGLSGTVRIPGDKSISHRSFMFGGLASGETRITGLLEGEDVINTG | 60 |
| Sbjct 1 | ML+G++ RPATARKS+GL+G+VRIPGDKSISHRSFMFGGLASG+TRITGLLEGEDVINTG MLNGSAPRPATARKSAGLTGSVRIPGDKSISHRSFMFGGLASGQTRITGLLEGEDVINTG | 60 |
| Query 61 | KAMQAMGARIRKEGDTWIIDGVGNGGLLAPEAPLDFGNAATGCRLTMGLVGVYDFDSTFI | 120 |
| Sbjct 61 | +AMQAMGARIRKEG W+IDG GNG LLAP+APLDFGNA TG RLTMGLVG YDF STF RAMQAMGARIRKEGAQWVIDGTGNGALLAPDAPLDFGNAGTGVRLTMGLVGTYDFRSTFT | 120 |
| Query 121 | GDASLTKRPMGRVLNPLREMGVQVKSEDGDRLPVTLRGPKTPTPITYRVPMASAQVKSAV | 180 |
| Sbjct 121 | GDASL+KRPMGRVLNPLREMGVQV + +G RLPVTLRGP TP+PI YRVPMASAQVKSAV GDASLSKRPMGRVLNPLREMGVQVSASEGGRLPVTLRGPGTPSPIRYRVPMASAQVKSAV | 180 |
| Query 181 | LLAGLNTPGITTVIEPIMTRDHTEKMLQGFGANLTVETDADGVRTIRLEGRGKLTGQVID | 240 |
| Sbjct 181 | LLAGLNTPG+TTVIEP+MTRDHTEKMLQGFGA L+VETD++GVRTIRLEGRG+LTGQVID LLAGLNTPGVTTVIEPVMTRDHTEKMLQGFGAALSVETDSEGVRTIRLEGRGRLTGQVID | 240 |
| Query 241 | VPGDPSSTAFPLVAALLVPGSDVTILNVLMNPTRTGLILTLQEMGADIEVINPRLAGGED | 300 |
| Sbjct 241 | VPGDPSSTAFPLVAALLVPGSD+TI+NVLMNPTRTGLILTLQEMGA+I+V+N RLAGGED VPGDPSSTAFPLVAALLVPGSDITIVNVLMNPTRTGLILTLQEMGANIQVLNTRLAGGED | 300 |
| Query 301 | VADLRVRSSTLKGVTVPEDRAPSMIDEYPILavaaafaegaTVMNGLEELRVKESDRLSA | 360 |
| Sbjct 301 | VADLRVR S LKGVTVPEDRAPSMIDEYPILAVAA FAEGAT+M GLEELRVKESDRLSA VADLRVRHSELKGVTVPEDRAPSMIDEYPILAVAACFAEGATIMKGLEELRVKESDRLSA | 360 |
| Query 361 | VANGLKLNGVDCDEGETSLVVRGRPDGKGLGNASGAAVATHLDHRIAMSFLVMGLVSENP | 420 |
| Sbjct 361 | VA+GLKLNGVDCDEGE LVVRGRPDGKGLGNA+G V+THLDHRIAMSFLV+GL SE+P VADGLKLNGVDCDEGEDFLVVRGRPDGKGLGNAAGDKVSTHLDHRIAMSFLVLGLASEHP | 420 |
| Query 421  Sbjct 421 | VTVDDATMIATSFPEFMDLMAGLGAKIE 448 VT+DDA MIATSFPEFM LM GLGAKIE VTIDDAAMIATSFPEFMQLMTGLGAKIE 448 |  |

>WP_020052313.1 3-phosphoshikimate 1-carboxyvinyltransferase [Rhizobium leguminosarum]

NKM63599.1 3-phosphoshikimate 1-carboxyvinyltransferase [Rhizobium leguminosarum bv. viciae]

QHW27107.1 3-phosphoshikimate 1-carboxyvinyltransferase [Rhizobium leguminosarum bv. viciae 248]

Length=452

Score = 766 bits (1978), Expect = 0.0, Method: Compositional matrix adjust. Identities = 385/449 (86%), Positives = 414/449 (92%), Gaps = 0/449 (0%)

Query 1	MLHGASSRPATARKSSGLSGTVRIPGDKSISHRSFMFGGLASGETRITGLLEGEDVINTG 60

| Sbjct | 1 | MLHG++ RPATARKS+GL+G+ RIPGDKSISHRSFMFGGLASGETRITGLLEGEDVINTG MLHGSAPRPATARKSAGLAGSARIPGDKSISHRSFMFGGLASGETRITGLLEGEDVINTG | 60 |
| --- | --- | --- | --- |
| Query | 61 | KAMQAMGARIRKEGDTWIIDGVGNGGLLAPEAPLDFGNAATGCRLTMGLVGVYDFDSTFI | 120 |
| Sbjct | 61 | +AMQAMGARIRK+G W+IDG GNG LLAP+ PLDFGNA TG RLTMGLVG YDF STFI RAMQAMGARIRKDGAQWVIDGTGNGALLAPDEPLDFGNAGTGVRLTMGLVGTYDFRSTFI | 120 |
| Query | 121 | GDASLTKRPMGRVLNPLREMGVQVKSEDGDRLPVTLRGPKTPTPITYRVPMASAQVKSAV | 180 |
| Sbjct | 121 | GDASL+KRPMGRVL PLREMGVQV + +GDR+PVTLRGP TP+PI YRVPMASAQVKSAV GDASLSKRPMGRVLAPLREMGVQVSASEGDRIPVTLRGPATPSPIRYRVPMASAQVKSAV | 180 |
| Query | 181 | LLAGLNTPGITTVIEPIMTRDHTEKMLQGFGANLTVETDADGVRTIRLEGRGKLTGQVID | 240 |
| Sbjct | 181 | LLAGLNTPG+TTVIEP+MTRDHTEKMLQGFGA L+VETD DGVRTIRLEGRGKL GQVID LLAGLNTPGVTTVIEPVMTRDHTEKMLQGFGAALSVETDGDGVRTIRLEGRGKLKGQVID | 240 |
| Query | 241 | VPGDPSSTAFPLVAALLVPGSDVTILNVLMNPTRTGLILTLQEMGADIEVINPRLAGGED | 300 |
| Sbjct | 241 | +PGDPSSTAFPLVAALLVPGSD+TI+NVLMNPTRTGLILTLQEMGADIEV+N RLAGGED IPGDPSSTAFPLVAALLVPGSDITIVNVLMNPTRTGLILTLQEMGADIEVVNARLAGGED | 300 |
| Query | 301 | VADLRVRSSTLKGVTVPEDRAPSMIDEYPILavaaafaegaTVMNGLEELRVKESDRLSA | 360 |
| Sbjct | 301 | VADLRVR S LKGVTVPE+RAPSMIDEYPILA+AA FAEGATVM GLEELRVKESDRLSA VADLRVRHSELKGVTVPEERAPSMIDEYPILAIAACFAEGATVMKGLEELRVKESDRLSA | 360 |
| Query | 361 | VANGLKLNGVDCDEGETSLVVRGRPDGKGLGNASGAAVATHLDHRIAMSFLVMGLVSENP | 420 |
| Sbjct | 361 | VA+GLKLNG DCDEGE L+VRGRPDGKGLGNA+G V THLDHRIAMSFLV+GL SE+ VADGLKLNGADCDEGEDFLIVRGRPDGKGLGNAAGGKVRTHLDHRIAMSFLVLGLASEHA | 420 |
| Query  Sbjct | 421  421 | VTVDDATMIATSFPEFMDLMAGLGAKIEL 449 VT+DDA MIATSFPEFM LM GLGAKIEL VTIDDAAMIATSFPEFMQLMTGLGAKIEL 449 |  |

>WP_164578062.1 3-phosphoshikimate 1-carboxyvinyltransferase [Rhizobium leguminosarum] NEI37060.1 3-phosphoshikimate 1-carboxyvinyltransferase [Rhizobium leguminosarum] NEI43536.1 3-phosphoshikimate 1-carboxyvinyltransferase [Rhizobium leguminosarum]

Length=452

Score = 766 bits (1977), Expect = 0.0, Method: Compositional matrix adjust.

| Identities | = 386/449 (86%), Positives = 413/449 (92%), Gaps = 0/449 (0%) |  |
| --- | --- | --- |
| Query 1 | MLHGASSRPATARKSSGLSGTVRIPGDKSISHRSFMFGGLASGETRITGLLEGEDVINTG | 60 |
| Sbjct 1 | MLHG++ RPATARKS+ L+G+VRIPGDKSISHRSFMFGGLASGETRITGLLEGEDVINTG MLHGSAPRPATARKSTSLTGSVRIPGDKSISHRSFMFGGLASGETRITGLLEGEDVINTG | 60 |
| Query 61 | KAMQAMGARIRKEGDTWIIDGVGNGGLLAPEAPLDFGNAATGCRLTMGLVGVYDFDSTFI | 120 |
| Sbjct 61 | +AMQAMGARIRKEG W+I+G GNG LLAPEAPLDFGNA TG RLTMGLVG YDF STF RAMQAMGARIRKEGAQWVIEGTGNGALLAPEAPLDFGNAGTGVRLTMGLVGTYDFHSTFT | 120 |
| Query 121 | GDASLTKRPMGRVLNPLREMGVQVKSEDGDRLPVTLRGPKTPTPITYRVPMASAQVKSAV | 180 |
| Sbjct 121 | GDASL+KRPMGRVL PLREMGVQV + +GDRLPVTLRGP TP+PI YRVPMASAQVKSAV GDASLSKRPMGRVLAPLREMGVQVSASEGDRLPVTLRGPGTPSPIRYRVPMASAQVKSAV | 180 |
| Query 181 | LLAGLNTPGITTVIEPIMTRDHTEKMLQGFGANLTVETDADGVRTIRLEGRGKLTGQVID | 240 |
| Sbjct 181 | LLAGLNTPG+TTVIEP+MTRDHTEKMLQGFGA L+VETD DGVRTIRLEGRGKL GQVID LLAGLNTPGVTTVIEPVMTRDHTEKMLQGFGAALSVETDGDGVRTIRLEGRGKLKGQVID | 240 |
| Query 241 | VPGDPSSTAFPLVAALLVPGSDVTILNVLMNPTRTGLILTLQEMGADIEVINPRLAGGED | 300 |
| Sbjct 241 | +PGDPSSTAFPLVAAL+VPGSD+TI+NVLMNPTRTGLILTLQEMGADIEV+N RLAGGED IPGDPSSTAFPLVAALIVPGSDITIVNVLMNPTRTGLILTLQEMGADIEVLNARLAGGED | 300 |
| Query 301 | VADLRVRSSTLKGVTVPEDRAPSMIDEYPILavaaafaegaTVMNGLEELRVKESDRLSA | 360 |
| Sbjct 301 | VADLRVR S LKGVTVPEDR PSMIDEYPILA+AA FAEGATVM GLEELRVKESDRLSA VADLRVRHSELKGVTVPEDRVPSMIDEYPILAIAACFAEGATVMKGLEELRVKESDRLSA | 360 |
| Query 361 | VANGLKLNGVDCDEGETSLVVRGRPDGKGLGNASGAAVATHLDHRIAMSFLVMGLVSENP | 420 |
| Sbjct 361 | VA GLKLNGVDCDEGE L+VRGRPDGKGLGNA+G V THLDHRIAMSFLV+GL SE+ VAGGLKLNGVDCDEGEDFLIVRGRPDGKGLGNAAGGQVRTHLDHRIAMSFLVLGLASEHA | 420 |
| Query 421  Sbjct 421 | VTVDDATMIATSFPEFMDLMAGLGAKIEL 449 VT+D+A MIATSFPEFM LM GLGAKIEL VTIDNAAMIATSFPEFMQLMTGLGAKIEL 449 |  |

>WP_130765412.1 3-phosphoshikimate 1-carboxyvinyltransferase [Rhizobium leguminosarum] TBA23692.1 3-phosphoshikimate 1-carboxyvinyltransferase [Rhizobium leguminosarum]

Length=452

Score = 766 bits (1977), Expect = 0.0, Method: Compositional matrix adjust.

| Identities | = 385/449 (86%), Positives = 415/449 (92%), Gaps = 0/449 (0%) |  |
| --- | --- | --- |
| Query 1 | MLHGASSRPATARKSSGLSGTVRIPGDKSISHRSFMFGGLASGETRITGLLEGEDVINTG | 60 |
| Sbjct 1 | MLHG++ RPATARKS+ L+G+VRIPGDKSISHRSFMFGGLASGETRITGLLEGEDVINTG MLHGSAPRPATARKSASLTGSVRIPGDKSISHRSFMFGGLASGETRITGLLEGEDVINTG | 60 |
| Query 61 | KAMQAMGARIRKEGDTWIIDGVGNGGLLAPEAPLDFGNAATGCRLTMGLVGVYDFDSTFI | 120 |
| Sbjct 61 | +AMQAMGARIRKEG W+I+G GNG LLAP+APLDFGNA TG RLTMGLVG YDF STF RAMQAMGARIRKEGAQWVIEGTGNGALLAPDAPLDFGNAGTGVRLTMGLVGTYDFHSTFT | 120 |
| Query 121 | GDASLTKRPMGRVLNPLREMGVQVKSEDGDRLPVTLRGPKTPTPITYRVPMASAQVKSAV | 180 |
| Sbjct 121 | GDASL+KRPMGRVL PLREMGVQV + +GDRLPVTLRGP TP+PI YRVPMASAQVKSAV GDASLSKRPMGRVLAPLREMGVQVSASEGDRLPVTLRGPGTPSPIRYRVPMASAQVKSAV | 180 |
| Query 181 | LLAGLNTPGITTVIEPIMTRDHTEKMLQGFGANLTVETDADGVRTIRLEGRGKLTGQVID | 240 |
| Sbjct 181 | LLAGLNTPG+TTVIEP+MTR+HTEKMLQGFGA L+VETD DGVRTIRLEGRGKL GQVID LLAGLNTPGVTTVIEPVMTRNHTEKMLQGFGAALSVETDGDGVRTIRLEGRGKLAGQVID | 240 |
| Query 241 | VPGDPSSTAFPLVAALLVPGSDVTILNVLMNPTRTGLILTLQEMGADIEVINPRLAGGED | 300 |
| Sbjct 241 | +PGDPSSTAFPLVAAL+VPGSD+TI+NVLMNPTRTGLILTLQEMGADIEV+N RLAGGED IPGDPSSTAFPLVAALIVPGSDITIVNVLMNPTRTGLILTLQEMGADIEVVNARLAGGED | 300 |
| Query 301 | VADLRVRSSTLKGVTVPEDRAPSMIDEYPILavaaafaegaTVMNGLEELRVKESDRLSA | 360 |
| Sbjct 301 | VADLR+R S LKGVTVPEDRAPSMIDEYPILA+AA FAEGATVM GLEELRVKESDRLSA VADLRMRHSELKGVTVPEDRAPSMIDEYPILAIAACFAEGATVMKGLEELRVKESDRLSA | 360 |
| Query 361 | VANGLKLNGVDCDEGETSLVVRGRPDGKGLGNASGAAVATHLDHRIAMSFLVMGLVSENP | 420 |
| Sbjct 361 | VA+GLKLNGVDCDEGE L+VRGRPDGKGLGNA+G V THLDHRIAMSFLV+GL SE+ VADGLKLNGVDCDEGEDFLIVRGRPDGKGLGNAAGGQVRTHLDHRIAMSFLVLGLASEHA | 420 |
| Query 421  Sbjct 421 | VTVDDATMIATSFPEFMDLMAGLGAKIEL 449 VT+DDA MIATSFPEFM LM GLGAKIEL VTIDDAAMIATSFPEFMQLMTGLGAKIEL 449 |  |

>WP_029873774.1 3-phosphoshikimate 1-carboxyvinyltransferase [Rhizobium leguminosarum] Length=452

Score = 765 bits (1976), Expect = 0.0, Method: Compositional matrix adjust.

| Identities | = 385/448 (86%), Positives = 419/448 (94%), Gaps = 0/448 (0%) |  |
| --- | --- | --- |
| Query 1 | MLHGASSRPATARKSSGLSGTVRIPGDKSISHRSFMFGGLASGETRITGLLEGEDVINTG | 60 |
| Sbjct 1 | ML+G++ +PATARKS+GL+G+VRIPGDKSISHRSFMFGGLASGETRITGLLEGEDVINTG MLNGSAPKPATARKSAGLTGSVRIPGDKSISHRSFMFGGLASGETRITGLLEGEDVINTG | 60 |
| Query 61 | KAMQAMGARIRKEGDTWIIDGVGNGGLLAPEAPLDFGNAATGCRLTMGLVGVYDFDSTFI | 120 |
| Sbjct 61 | +AMQAMGARIRKEG W+I+G GNG LLAP+APLDFGNA TG RLTMGLVG YDF STF RAMQAMGARIRKEGAQWVIEGTGNGALLAPDAPLDFGNAGTGVRLTMGLVGTYDFHSTFT | 120 |
| Query 121 | GDASLTKRPMGRVLNPLREMGVQVKSEDGDRLPVTLRGPKTPTPITYRVPMASAQVKSAV | 180 |
| Sbjct 121 | GDASL+KRPMGRVL PLREMGVQV + +GDRLPVTLRGP TP+PI YRVPMASAQVKSAV GDASLSKRPMGRVLAPLREMGVQVSASEGDRLPVTLRGPGTPSPIRYRVPMASAQVKSAV | 180 |
| Query 181 | LLAGLNTPGITTVIEPIMTRDHTEKMLQGFGANLTVETDADGVRTIRLEGRGKLTGQVID | 240 |
| Sbjct 181 | LLAGLNTPG+TTVIEP+MTRDHTEKMLQGFGA L+VETD++GVRTIRLEGRG+L GQVID LLAGLNTPGVTTVIEPVMTRDHTEKMLQGFGAALSVETDSEGVRTIRLEGRGRLMGQVID | 240 |
| Query 241 | VPGDPSSTAFPLVAALLVPGSDVTILNVLMNPTRTGLILTLQEMGADIEVINPRLAGGED | 300 |
| Sbjct 241 | VPGDPSSTAFPLVAALLVPGSD+TI+NVLMNPTRTGLILTLQEMGADIEV+N RLAGGED VPGDPSSTAFPLVAALLVPGSDITIVNVLMNPTRTGLILTLQEMGADIEVVNARLAGGED | 300 |
| Query 301 | VADLRVRSSTLKGVTVPEDRAPSMIDEYPILavaaafaegaTVMNGLEELRVKESDRLSA | 360 |
| Sbjct 301 | VADLRVR S LKGVTVPE+RAPSMIDEYPILAVAA FAEGAT+M GLEELRVKESDRLSA VADLRVRHSELKGVTVPEERAPSMIDEYPILAVAACFAEGATIMKGLEELRVKESDRLSA | 360 |
| Query 361 | VANGLKLNGVDCDEGETSLVVRGRPDGKGLGNASGAAVATHLDHRIAMSFLVMGLVSENP | 420 |

| Sbjct | 361 | VA+GLKLNGVDCDEGE L+VRGRPDGKGLGNA+G +V+THLDHRIAMSFLVMGL SE+P VADGLKLNGVDCDEGEDFLIVRGRPDGKGLGNAAGGSVSTHLDHRIAMSFLVMGLASEHP 420 |
| --- | --- | --- |
| Query | 421 | VTVDDATMIATSFPEFMDLMAGLGAKIE 448 |
| Sbjct | 421 | VT+DDA MIATSFPEFM LM GLGA+IE  VTIDDAAMIATSFPEFMTLMKGLGARIE 448 |

>WP_130670010.1 3-phosphoshikimate 1-carboxyvinyltransferase [Rhizobium leguminosarum] TAV91824.1 3-phosphoshikimate 1-carboxyvinyltransferase [Rhizobium leguminosarum] TAV96431.1 3-phosphoshikimate 1-carboxyvinyltransferase [Rhizobium leguminosarum] TAW37510.1 3-phosphoshikimate 1-carboxyvinyltransferase [Rhizobium leguminosarum] TAZ16813.1 3-phosphoshikimate 1-carboxyvinyltransferase [Rhizobium leguminosarum]

Length=452

Score = 765 bits (1976), Expect = 0.0, Method: Compositional matrix adjust.

| Identities | = 384/448 (86%), Positives = 416/448 (93%), Gaps = 0/448 (0%) |  |
| --- | --- | --- |
| Query 1 | MLHGASSRPATARKSSGLSGTVRIPGDKSISHRSFMFGGLASGETRITGLLEGEDVINTG | 60 |
| Sbjct 1 | ML+G++ +P TARKS+GL+G+VR+PGDKSISHRSFMFGGLASGETRITGLLEGEDVINTG MLNGSAPKPVTARKSAGLTGSVRVPGDKSISHRSFMFGGLASGETRITGLLEGEDVINTG | 60 |
| Query 61 | KAMQAMGARIRKEGDTWIIDGVGNGGLLAPEAPLDFGNAATGCRLTMGLVGVYDFDSTFI | 120 |
| Sbjct 61 | +AMQAMGARIRKEG W+IDG GNG LLAP+APLDFGN TG RLTMGLVG YDF STFI RAMQAMGARIRKEGSQWVIDGTGNGALLAPDAPLDFGNTGTGVRLTMGLVGTYDFRSTFI | 120 |
| Query 121 | GDASLTKRPMGRVLNPLREMGVQVKSEDGDRLPVTLRGPKTPTPITYRVPMASAQVKSAV | 180 |
| Sbjct 121 | GDASL+KRPMGRVLNPLREMGVQV + +GDRLPVTL+GP TP+PI YRVPMASAQVKSAV GDASLSKRPMGRVLNPLREMGVQVSASEGDRLPVTLKGPGTPSPIRYRVPMASAQVKSAV | 180 |
| Query 181 | LLAGLNTPGITTVIEPIMTRDHTEKMLQGFGANLTVETDADGVRTIRLEGRGKLTGQVID | 240 |
| Sbjct 181 | LLAGLNTPG+TTVIEP+MTRDHTEKMLQGFGA L+VETD++GVRTIRLEGRGKLTGQVID LLAGLNTPGVTTVIEPVMTRDHTEKMLQGFGAALSVETDSEGVRTIRLEGRGKLTGQVID | 240 |
| Query 241 | VPGDPSSTAFPLVAALLVPGSDVTILNVLMNPTRTGLILTLQEMGADIEVINPRLAGGED | 300 |
| Sbjct 241 | VPGDPSSTAFPLVAALLVPGSD+TI+NVLMNPTRTGLILTLQEMGA I+V+N RLAGGED VPGDPSSTAFPLVAALLVPGSDITIVNVLMNPTRTGLILTLQEMGAKIQVLNTRLAGGED | 300 |
| Query 301 | VADLRVRSSTLKGVTVPEDRAPSMIDEYPILavaaafaegaTVMNGLEELRVKESDRLSA | 360 |
| Sbjct 301 | VADLRVR S LKGVTVPEDRAPSMIDEYPILAVAA FAEGAT+M GLEELRVKESDRLSA VADLRVRHSELKGVTVPEDRAPSMIDEYPILAVAACFAEGATIMKGLEELRVKESDRLSA | 360 |
| Query 361 | VANGLKLNGVDCDEGETSLVVRGRPDGKGLGNASGAAVATHLDHRIAMSFLVMGLVSENP | 420 |
| Sbjct 361 | VA GLKLNGVDCDEGE L+VRGRPDGKGLGNA+ V+THLDHRIAMSFLVMGL S++P VAEGLKLNGVDCDEGEDFLIVRGRPDGKGLGNAADGRVSTHLDHRIAMSFLVMGLASQHP | 420 |
| Query 421  Sbjct 421 | VTVDDATMIATSFPEFMDLMAGLGAKIE 448 VT+DDA MIATSFPEFM LM GLGAKIE VTIDDAAMIATSFPEFMQLMTGLGAKIE 448 |  |

>WP_130665327.1 3-phosphoshikimate 1-carboxyvinyltransferase [Rhizobium leguminosarum] TAU29002.1 3-phosphoshikimate 1-carboxyvinyltransferase [Rhizobium leguminosarum] TAU50747.1 3-phosphoshikimate 1-carboxyvinyltransferase [Rhizobium leguminosarum] TAU65819.1 3-phosphoshikimate 1-carboxyvinyltransferase [Rhizobium leguminosarum] TAU78741.1 3-phosphoshikimate 1-carboxyvinyltransferase [Rhizobium leguminosarum] TAV18137.1 3-phosphoshikimate 1-carboxyvinyltransferase [Rhizobium leguminosarum]

Length=452

Score = 765 bits (1976), Expect = 0.0, Method: Compositional matrix adjust.

| Identities | = 386/449 (86%), Positives = 414/449 (92%), Gaps = 0/449 (0%) |  |
| --- | --- | --- |
| Query 1 | MLHGASSRPATARKSSGLSGTVRIPGDKSISHRSFMFGGLASGETRITGLLEGEDVINTG | 60 |
| Sbjct 1 | MLHG++ RPATARKS+ L+G+VRIPGDKSISHRSFMFGGLASGETRITGLLEGEDVINTG MLHGSAPRPATARKSASLTGSVRIPGDKSISHRSFMFGGLASGETRITGLLEGEDVINTG | 60 |
| Query 61 | KAMQAMGARIRKEGDTWIIDGVGNGGLLAPEAPLDFGNAATGCRLTMGLVGVYDFDSTFI | 120 |
| Sbjct 61 | +AMQAMGARIRKEG W+I+G GNG LLAP+APLDFGNA TG RLTMGLVG YDF STF RAMQAMGARIRKEGAQWVIEGTGNGALLAPDAPLDFGNAGTGVRLTMGLVGTYDFHSTFT | 120 |
| Query 121 | GDASLTKRPMGRVLNPLREMGVQVKSEDGDRLPVTLRGPKTPTPITYRVPMASAQVKSAV GDASL+KRPMGRVL PLREMGVQV + +GDRLPVTLRGP TP+PI YRVPMASAQVKSAV | 180 |

| Sbjct | 121 | GDASLSKRPMGRVLAPLREMGVQVSASEGDRLPVTLRGPGTPSPIRYRVPMASAQVKSAV | 180 |
| --- | --- | --- | --- |
| Query | 181 | LLAGLNTPGITTVIEPIMTRDHTEKMLQGFGANLTVETDADGVRTIRLEGRGKLTGQVID | 240 |
| Sbjct | 181 | LLAGLNTPG+TTVIEP+MTRDHTEKMLQGFGA L+VETD DGVRTIRLEGRGKL GQVID LLAGLNTPGVTTVIEPVMTRDHTEKMLQGFGAALSVETDGDGVRTIRLEGRGKLKGQVID | 240 |
| Query | 241 | VPGDPSSTAFPLVAALLVPGSDVTILNVLMNPTRTGLILTLQEMGADIEVINPRLAGGED | 300 |
| Sbjct | 241 | +PGDPSSTAFPLVAALLVPGSD+TI+NVLMNPTRTGLILTLQEMGA+IEV+N RLAGGED IPGDPSSTAFPLVAALLVPGSDITIVNVLMNPTRTGLILTLQEMGANIEVLNARLAGGED | 300 |
| Query | 301 | VADLRVRSSTLKGVTVPEDRAPSMIDEYPILavaaafaegaTVMNGLEELRVKESDRLSA | 360 |
| Sbjct | 301 | VADLRVR S LKGV VPEDRAPSMIDEYPILA+AA FAEGATVM GLEELRVKESDRLSA VADLRVRHSELKGVGVPEDRAPSMIDEYPILAIAACFAEGATVMKGLEELRVKESDRLSA | 360 |
| Query | 361 | VANGLKLNGVDCDEGETSLVVRGRPDGKGLGNASGAAVATHLDHRIAMSFLVMGLVSENP | 420 |
| Sbjct | 361 | VA+GLKLNGVDCDEGE L+VRGRPDGKGLGNA+G V THLDHRIAMSFLV+GL SE+ VADGLKLNGVDCDEGEDFLIVRGRPDGKGLGNAAGGQVRTHLDHRIAMSFLVLGLASEHA | 420 |
| Query  Sbjct | 421  421 | VTVDDATMIATSFPEFMDLMAGLGAKIEL 449 VT+DDA MIATSFPEFM LM GLGAKIEL VTIDDAAMIATSFPEFMQLMTGLGAKIEL 449 |  |

Score = 765 bits (1976), Expect = 0.0, Method: Compositional matrix adjust. Identities = 386/449 (86%), Positives = 414/449 (92%), Gaps = 0/449 (0%)

| Query | 1 | MLHGASSRPATARKSSGLSGTVRIPGDKSISHRSFMFGGLASGETRITGLLEGEDVINTG | 60 |
| --- | --- | --- | --- |
| Sbjct | 1 | MLHG++ RPATARKS+ L+G+VRIPGDKSISHRSFMFGGLASGETRITGLLEGEDVINTG MLHGSAPRPATARKSASLTGSVRIPGDKSISHRSFMFGGLASGETRITGLLEGEDVINTG | 60 |
| Query | 61 | KAMQAMGARIRKEGDTWIIDGVGNGGLLAPEAPLDFGNAATGCRLTMGLVGVYDFDSTFI | 120 |
| Sbjct | 61 | +AMQAMGARIRKEG W+I+G GNG LLAP+APLDFGNA TG RLTMGLVG YDF STF RAMQAMGARIRKEGAQWVIEGTGNGALLAPDAPLDFGNAGTGVRLTMGLVGTYDFHSTFT | 120 |
| Query | 121 | GDASLTKRPMGRVLNPLREMGVQVKSEDGDRLPVTLRGPKTPTPITYRVPMASAQVKSAV | 180 |
| Sbjct | 121 | GDASL+KRPMGRVL PLREMGVQV + +GDRLPVTLRGP TP+PI YRVPMASAQVKSAV GDASLSKRPMGRVLAPLREMGVQVSASEGDRLPVTLRGPGTPSPIRYRVPMASAQVKSAV | 180 |
| Query | 181 | LLAGLNTPGITTVIEPIMTRDHTEKMLQGFGANLTVETDADGVRTIRLEGRGKLTGQVID | 240 |
| Sbjct | 181 | LLAGLNTPG+TTVIEP+MTRDHTEKMLQGFGA L+VETD DGVRTIRLEGRGKL GQVID LLAGLNTPGVTTVIEPVMTRDHTEKMLQGFGAALSVETDGDGVRTIRLEGRGKLKGQVID | 240 |
| Query | 241 | VPGDPSSTAFPLVAALLVPGSDVTILNVLMNPTRTGLILTLQEMGADIEVINPRLAGGED | 300 |
| Sbjct | 241 | +PGDPSSTAFPLVAALLVPGSD+TI+NVLMNPTRTGLILTLQEMGA+IEV+N RLAGGED IPGDPSSTAFPLVAALLVPGSDITIVNVLMNPTRTGLILTLQEMGANIEVLNARLAGGED | 300 |
| Query | 301 | VADLRVRSSTLKGVTVPEDRAPSMIDEYPILavaaafaegaTVMNGLEELRVKESDRLSA | 360 |
| Sbjct | 301 | VADLRVR S LKGV VPEDRAPSMIDEYPILA+AA FAEGATVM GLEELRVKESDRLSA VADLRVRHSELKGVGVPEDRAPSMIDEYPILAIAACFAEGATVMKGLEELRVKESDRLSA | 360 |
| Query | 361 | VANGLKLNGVDCDEGETSLVVRGRPDGKGLGNASGAAVATHLDHRIAMSFLVMGLVSENP | 420 |
| Sbjct | 361 | VA+GLKLNGVDCDEGE L+VRGRPDGKGLGNA+G V THLDHRIAMSFLV+GL SE+ VADGLKLNGVDCDEGEDFLIVRGRPDGKGLGNAAGGQVRTHLDHRIAMSFLVLGLASEHA | 420 |
| Query  Sbjct | 421  421 | VTVDDATMIATSFPEFMDLMAGLGAKIEL 449 VT+DDA MIATSFPEFM LM GLGAKIEL VTIDDAAMIATSFPEFMQLMTGLGAKIEL 449 |  |

>WP_184499397.1 3-phosphoshikimate 1-carboxyvinyltransferase [Rhizobium esperanzae] MBB4439131.1 3-phosphoshikimate 1-carboxyvinyltransferase [Rhizobium esperanzae]

Length=452

Score = 765 bits (1976), Expect = 0.0, Method: Compositional matrix adjust. Identities = 386/448 (86%), Positives = 417/448 (93%), Gaps = 0/448 (0%)

| Query | 1 | MLHGASSRPATARKSSGLSGTVRIPGDKSISHRSFMFGGLASGETRITGLLEGEDVINTG | 60 |
| --- | --- | --- | --- |
| Sbjct | 1 | ML+G++ RPATARKS GL+G+VRIPGDKSISHRSFMFGGLASGETRITGLLEGEDVINTG MLNGSAPRPATARKSGGLTGSVRIPGDKSISHRSFMFGGLASGETRITGLLEGEDVINTG | 60 |
| Query | 61 | KAMQAMGARIRKEGDTWIIDGVGNGGLLAPEAPLDFGNAATGCRLTMGLVGVYDFDSTFI  +AMQAMGARIRKEG W+IDG GNG LLAP+APLDFGNA TG RLTMGLVG YDF STFI | 120 |

| Sbjct | 61 | RAMQAMGARIRKEGAQWVIDGTGNGALLAPDAPLDFGNAGTGVRLTMGLVGTYDFRSTFI | 120 |
| --- | --- | --- | --- |
| Query | 121 | GDASLTKRPMGRVLNPLREMGVQVKSEDGDRLPVTLRGPKTPTPITYRVPMASAQVKSAV | 180 |
| Sbjct | 121 | GDASL+KRPMGRVLNPL EMGVQ+ + +GDRLPVTL+GP TP+PI YRVPMASAQVKSAV GDASLSKRPMGRVLNPLSEMGVQISASEGDRLPVTLKGPGTPSPIRYRVPMASAQVKSAV | 180 |
| Query | 181 | LLAGLNTPGITTVIEPIMTRDHTEKMLQGFGANLTVETDADGVRTIRLEGRGKLTGQVID | 240 |
| Sbjct | 181 | LLAGLNTPG+TTVIEP+MTRDHTEKMLQGFGA L+VETD++GVRTIRLEGRGKL GQVID LLAGLNTPGVTTVIEPVMTRDHTEKMLQGFGATLSVETDSEGVRTIRLEGRGKLAGQVID | 240 |
| Query | 241 | VPGDPSSTAFPLVAALLVPGSDVTILNVLMNPTRTGLILTLQEMGADIEVINPRLAGGED | 300 |
| Sbjct | 241 | VPGDPSSTAFPLVAALLVPGSD+TI+NVLMNPTRTGLILTLQEMGADIEV+N RLAGGED VPGDPSSTAFPLVAALLVPGSDITIVNVLMNPTRTGLILTLQEMGADIEVLNTRLAGGED | 300 |
| Query | 301 | VADLRVRSSTLKGVTVPEDRAPSMIDEYPILavaaafaegaTVMNGLEELRVKESDRLSA | 360 |
| Sbjct | 301 | VADLRVR S LKGVTVPEDRAPSMIDEYPILAVAA FAEGAT+M GLEELRVKESDRLSA VADLRVRHSELKGVTVPEDRAPSMIDEYPILAVAACFAEGATIMKGLEELRVKESDRLSA | 360 |
| Query | 361 | VANGLKLNGVDCDEGETSLVVRGRPDGKGLGNASGAAVATHLDHRIAMSFLVMGLVSENP | 420 |
| Sbjct | 361 | VA+GLKLNGVDCDEGE L+VRGRPDGKGLG+A+G V+THLDHRIAMSFLV+GL SE+ VADGLKLNGVDCDEGEDFLIVRGRPDGKGLGSAAGGRVSTHLDHRIAMSFLVLGLASEHA | 420 |
| Query  Sbjct | 421  421 | VTVDDATMIATSFPEFMDLMAGLGAKIE 448 VT+DDA MIATSFPEFM LM GLGAKIE VTIDDAAMIATSFPEFMQLMTGLGAKIE 448 |  |

>WP_130822769.1 3-phosphoshikimate 1-carboxyvinyltransferase [Rhizobium leguminosarum] NEH81313.1 3-phosphoshikimate 1-carboxyvinyltransferase [Rhizobium leguminosarum] NEI81233.1 3-phosphoshikimate 1-carboxyvinyltransferase [Rhizobium leguminosarum] TBB25057.1 3-phosphoshikimate 1-carboxyvinyltransferase [Rhizobium leguminosarum]

Length=452

Score = 765 bits (1976), Expect = 0.0, Method: Compositional matrix adjust.

| Identities | = 386/449 (86%), Positives = 418/449 (93%), Gaps = 0/449 (0%) |  |
| --- | --- | --- |
| Query 1 | MLHGASSRPATARKSSGLSGTVRIPGDKSISHRSFMFGGLASGETRITGLLEGEDVINTG | 60 |
| Sbjct 1 | ML+G++ +PATARKS+GL+G+VRIPGDKSISHRSFMFGGLASGETRITGLLEGEDVINTG MLNGSAPKPATARKSAGLTGSVRIPGDKSISHRSFMFGGLASGETRITGLLEGEDVINTG | 60 |
| Query 61 | KAMQAMGARIRKEGDTWIIDGVGNGGLLAPEAPLDFGNAATGCRLTMGLVGVYDFDSTFI | 120 |
| Sbjct 61 | +AMQAMGA+IRKEG W+I+G GNG LLAP+APLDFGNA TG RLTMGLVG YDF S F RAMQAMGAKIRKEGAQWVIEGTGNGALLAPDAPLDFGNAGTGVRLTMGLVGTYDFRSNFT | 120 |
| Query 121 | GDASLTKRPMGRVLNPLREMGVQVKSEDGDRLPVTLRGPKTPTPITYRVPMASAQVKSAV | 180 |
| Sbjct 121 | GDASL+KRPMGRVL PLREMGVQ+ + +GDRLPVTLRGP TP+PI YRVPMASAQVKSAV GDASLSKRPMGRVLAPLREMGVQISASEGDRLPVTLRGPGTPSPIRYRVPMASAQVKSAV | 180 |
| Query 181 | LLAGLNTPGITTVIEPIMTRDHTEKMLQGFGANLTVETDADGVRTIRLEGRGKLTGQVID | 240 |
| Sbjct 181 | LLAGLNTPG+TTVIEP+MTR+HTEKMLQGFGA L+VETD DGVRTIRLEGRGKL GQVID LLAGLNTPGVTTVIEPVMTRNHTEKMLQGFGAALSVETDEDGVRTIRLEGRGKLAGQVID | 240 |
| Query 241 | VPGDPSSTAFPLVAALLVPGSDVTILNVLMNPTRTGLILTLQEMGADIEVINPRLAGGED | 300 |
| Sbjct 241 | VPGDPSSTAFPLVAALLVPGSD+TI+NVLMNPTRTGLILTLQEMGA+IEV+NPRLAGGED VPGDPSSTAFPLVAALLVPGSDITIVNVLMNPTRTGLILTLQEMGANIEVLNPRLAGGED | 300 |
| Query 301 | VADLRVRSSTLKGVTVPEDRAPSMIDEYPILavaaafaegaTVMNGLEELRVKESDRLSA | 360 |
| Sbjct 301 | VADLRVR S LKGVTVPEDRAPSMIDEYPILAVAA FAEGATVM GLEELRVKESDRLSA VADLRVRHSELKGVTVPEDRAPSMIDEYPILAVAACFAEGATVMKGLEELRVKESDRLSA | 360 |
| Query 361 | VANGLKLNGVDCDEGETSLVVRGRPDGKGLGNASGAAVATHLDHRIAMSFLVMGLVSENP | 420 |
| Sbjct 361 | VA+GLKLNGVDCDEGE L+VRGRPDGKGLGNA+G V+THLDHRIAMSFLVMGL SE+ VADGLKLNGVDCDEGEDFLIVRGRPDGKGLGNAAGGRVSTHLDHRIAMSFLVMGLASEHA | 420 |
| Query 421  Sbjct 421 | VTVDDATMIATSFPEFMDLMAGLGAKIEL 449 VT+DDA MIATSFPEFM LM+GLGAKIEL VTIDDAAMIATSFPEFMQLMSGLGAKIEL 449 |  |

>WP_017995641.1 3-phosphoshikimate 1-carboxyvinyltransferase [Rhizobium leguminosarum] Length=452

Score = 765 bits (1976), Expect = 0.0, Method: Compositional matrix adjust.

| Identities | = 387/448 (86%), Positives = 417/448 (93%), Gaps = 0/448 (0%) |  |
| --- | --- | --- |
| Query 1 | MLHGASSRPATARKSSGLSGTVRIPGDKSISHRSFMFGGLASGETRITGLLEGEDVINTG | 60 |
| Sbjct 1 | ML+G++ +PATARKS+GL+G+VRIPGDKSISHRSFMFGGLASGETRITGLLEGEDVINTG MLNGSAPKPATARKSAGLTGSVRIPGDKSISHRSFMFGGLASGETRITGLLEGEDVINTG | 60 |
| Query 61 | KAMQAMGARIRKEGDTWIIDGVGNGGLLAPEAPLDFGNAATGCRLTMGLVGVYDFDSTFI | 120 |
| Sbjct 61 | +AMQAMGARIRK+G W+I+G GNG LLAP+APLDFGNA TG RLTMGLVG YDF STF RAMQAMGARIRKDGAQWVIEGTGNGALLAPDAPLDFGNAGTGVRLTMGLVGTYDFRSTFT | 120 |
| Query 121 | GDASLTKRPMGRVLNPLREMGVQVKSEDGDRLPVTLRGPKTPTPITYRVPMASAQVKSAV | 180 |
| Sbjct 121 | GDASL+KRPMGRVLNPLREMGVQV + +GDRLPVTLRGP TP+PI YRVPMASAQVKSAV GDASLSKRPMGRVLNPLREMGVQVSASEGDRLPVTLRGPGTPSPIRYRVPMASAQVKSAV | 180 |
| Query 181 | LLAGLNTPGITTVIEPIMTRDHTEKMLQGFGANLTVETDADGVRTIRLEGRGKLTGQVID | 240 |
| Sbjct 181 | LLAGLNTPGITTVIEP+MTRDHTEKMLQGFGA L+VETD++GVRTIRLEGRGKL GQVID LLAGLNTPGITTVIEPVMTRDHTEKMLQGFGAALSVETDSEGVRTIRLEGRGKLAGQVID | 240 |
| Query 241 | VPGDPSSTAFPLVAALLVPGSDVTILNVLMNPTRTGLILTLQEMGADIEVINPRLAGGED | 300 |
| Sbjct 241 | VPGDPSSTAFPLVAALLVPGSD+TI+NVLMNPTRTGLILTLQEMGADIEV N RLAGGED VPGDPSSTAFPLVAALLVPGSDITIVNVLMNPTRTGLILTLQEMGADIEVANARLAGGED | 300 |
| Query 301 | VADLRVRSSTLKGVTVPEDRAPSMIDEYPILavaaafaegaTVMNGLEELRVKESDRLSA | 360 |
| Sbjct 301 | VADLRVR S LKGVTVPE+RAPSMIDEYPILAVAA FAEGAT+M GLEELRVKESDRLSA VADLRVRHSELKGVTVPEERAPSMIDEYPILAVAACFAEGATIMKGLEELRVKESDRLSA | 360 |
| Query 361 | VANGLKLNGVDCDEGETSLVVRGRPDGKGLGNASGAAVATHLDHRIAMSFLVMGLVSENP | 420 |
| Sbjct 361 | VA+GLKLNGVDCDEGE L+VRGRPDGKGLGNA+ V+THLDHRIAMSFLVMGL SE+P VADGLKLNGVDCDEGEDFLIVRGRPDGKGLGNAADGRVSTHLDHRIAMSFLVMGLASEHP | 420 |
| Query 421  Sbjct 421 | VTVDDATMIATSFPEFMDLMAGLGAKIE 448 VT+DDA MIATSFPEFM LM GLGAKIE VTIDDAAMIATSFPEFMQLMTGLGAKIE 448 |  |

>WP_168317284.1 3-phosphoshikimate 1-carboxyvinyltransferase [Rhizobium leguminosarum]

NKL38189.1 3-phosphoshikimate 1-carboxyvinyltransferase [Rhizobium leguminosarum bv. viciae]

Length=452

Score = 765 bits (1975), Expect = 0.0, Method: Compositional matrix adjust.

| Identities | = 386/448 (86%), Positives = 415/448 (93%), Gaps = 0/448 (0%) |  |
| --- | --- | --- |
| Query 1 | MLHGASSRPATARKSSGLSGTVRIPGDKSISHRSFMFGGLASGETRITGLLEGEDVINTG | 60 |
| Sbjct 1 | MLHG++ RPATARKS+GL+G+VRIPGDKSISHRSFMFGGLASG+TRITGLLEGEDVINTG MLHGSAPRPATARKSAGLTGSVRIPGDKSISHRSFMFGGLASGQTRITGLLEGEDVINTG | 60 |
| Query 61 | KAMQAMGARIRKEGDTWIIDGVGNGGLLAPEAPLDFGNAATGCRLTMGLVGVYDFDSTFI | 120 |
| Sbjct 61 | +AMQAMGARIRKEG W+IDG GNG LLAP+APLDFGNA TG RLTMGLVG YDF S F RAMQAMGARIRKEGAQWVIDGTGNGALLAPDAPLDFGNAGTGVRLTMGLVGTYDFHSIFT | 120 |
| Query 121 | GDASLTKRPMGRVLNPLREMGVQVKSEDGDRLPVTLRGPKTPTPITYRVPMASAQVKSAV | 180 |
| Sbjct 121 | GDASL+KRPMGRVLNPLREMGVQV + +GDRLPVTLRGP TP+PI YRVPMASAQVKSAV GDASLSKRPMGRVLNPLREMGVQVSASEGDRLPVTLRGPGTPSPIRYRVPMASAQVKSAV | 180 |
| Query 181 | LLAGLNTPGITTVIEPIMTRDHTEKMLQGFGANLTVETDADGVRTIRLEGRGKLTGQVID | 240 |
| Sbjct 181 | LLAGLNTPG+TTVIEP+MTRDHTEKMLQGFGA L+VETD++GVRTIRLEGRGKLTGQVID LLAGLNTPGVTTVIEPVMTRDHTEKMLQGFGAVLSVETDSEGVRTIRLEGRGKLTGQVID | 240 |
| Query 241 | VPGDPSSTAFPLVAALLVPGSDVTILNVLMNPTRTGLILTLQEMGADIEVINPRLAGGED | 300 |
| Sbjct 241 | VPGDPSSTAFPLVAALLVPGSD+TI+NVLMNPTRTGLILTLQEMGADIEV+N RLAGGED VPGDPSSTAFPLVAALLVPGSDITIVNVLMNPTRTGLILTLQEMGADIEVLNARLAGGED | 300 |
| Query 301 | VADLRVRSSTLKGVTVPEDRAPSMIDEYPILavaaafaegaTVMNGLEELRVKESDRLSA | 360 |
| Sbjct 301 | VADLRVR S L+GVTVPEDRAPSMIDEYPILAVAA FAEG T M GLEELRVKESDRLSA VADLRVRHSELRGVTVPEDRAPSMIDEYPILAVAACFAEGVTTMKGLEELRVKESDRLSA | 360 |
| Query 361 | VANGLKLNGVDCDEGETSLVVRGRPDGKGLGNASGAAVATHLDHRIAMSFLVMGLVSENP | 420 |
| Sbjct 361 | VA+GLKLNGVDCDEGE L+VRGRPDGKGLGNA+ V+THLDHRIAMSFLVMGL SE+  VADGLKLNGVDCDEGEDFLIVRGRPDGKGLGNAADGRVSTHLDHRIAMSFLVMGLASEHA | 420 |

Query 421 VTVDDATMIATSFPEFMDLMAGLGAKIE 448 VT+DDA MIATSFPEFM LM GLGA+IE

Sbjct 421 VTIDDAAMIATSFPEFMTLMKGLGARIE 448

>WP_130676013.1 3-phosphoshikimate 1-carboxyvinyltransferase [Rhizobium leguminosarum] TAW01282.1 3-phosphoshikimate 1-carboxyvinyltransferase [Rhizobium leguminosarum] TAW18723.1 3-phosphoshikimate 1-carboxyvinyltransferase [Rhizobium leguminosarum] TAZ54400.1 3-phosphoshikimate 1-carboxyvinyltransferase [Rhizobium leguminosarum]

Length=452

Score = 765 bits (1975), Expect = 0.0, Method: Compositional matrix adjust.

| Identities | = 384/449 (86%), Positives = 412/449 (92%), Gaps = 0/449 (0%) |  |
| --- | --- | --- |
| Query 1 | MLHGASSRPATARKSSGLSGTVRIPGDKSISHRSFMFGGLASGETRITGLLEGEDVINTG | 60 |
| Sbjct 1 | MLHG++ RPATARKS+ L G+VRIPGDKSISHRSFMFGGLASGETRITGLLEGEDVINTG MLHGSAPRPATARKSASLKGSVRIPGDKSISHRSFMFGGLASGETRITGLLEGEDVINTG | 60 |
| Query 61 | KAMQAMGARIRKEGDTWIIDGVGNGGLLAPEAPLDFGNAATGCRLTMGLVGVYDFDSTFI | 120 |
| Sbjct 61 | +AMQAMGARIRKEG W+I+G GNG LLAP+APLDFGNA TG RLTMGLVG YDF STF RAMQAMGARIRKEGAQWVIEGTGNGALLAPDAPLDFGNAGTGVRLTMGLVGTYDFHSTFT | 120 |
| Query 121 | GDASLTKRPMGRVLNPLREMGVQVKSEDGDRLPVTLRGPKTPTPITYRVPMASAQVKSAV | 180 |
| Sbjct 121 | GDASL+KRPMGRVL PLREMGVQV + +GDRLPVTLRGP TP+PI YRVPMASAQVKSAV GDASLSKRPMGRVLAPLREMGVQVSASEGDRLPVTLRGPGTPSPIRYRVPMASAQVKSAV | 180 |
| Query 181 | LLAGLNTPGITTVIEPIMTRDHTEKMLQGFGANLTVETDADGVRTIRLEGRGKLTGQVID | 240 |
| Sbjct 181 | LLAGLNTPG+TTVIEP+MTRDHTEKMLQGFGA L+VETD DGVRTIRLEGRGKL GQVID LLAGLNTPGVTTVIEPVMTRDHTEKMLQGFGAALSVETDGDGVRTIRLEGRGKLKGQVID | 240 |
| Query 241 | VPGDPSSTAFPLVAALLVPGSDVTILNVLMNPTRTGLILTLQEMGADIEVINPRLAGGED | 300 |
| Sbjct 241 | +PGDPSSTAFPLVAALLVPGSD+TI+NVLMNPTRTGLILTLQEMGADIEV+N RLAGGED IPGDPSSTAFPLVAALLVPGSDITIVNVLMNPTRTGLILTLQEMGADIEVLNARLAGGED | 300 |
| Query 301 | VADLRVRSSTLKGVTVPEDRAPSMIDEYPILavaaafaegaTVMNGLEELRVKESDRLSA | 360 |
| Sbjct 301 | VADLRVR S LKGVTVPE+RAPSMIDEYPILA+AA FA+G TVM GLEELRVKESDRLSA VADLRVRHSELKGVTVPEERAPSMIDEYPILAIAACFADGTTVMKGLEELRVKESDRLSA | 360 |
| Query 361 | VANGLKLNGVDCDEGETSLVVRGRPDGKGLGNASGAAVATHLDHRIAMSFLVMGLVSENP | 420 |
| Sbjct 361 | VA+GLKLNG DCDEGE L+VRGRPDGKGLGNA+G V THLDHRIAMSFLV+GL SE+ VADGLKLNGADCDEGEDFLIVRGRPDGKGLGNAAGGQVRTHLDHRIAMSFLVLGLASEHA | 420 |
| Query 421  Sbjct 421 | VTVDDATMIATSFPEFMDLMAGLGAKIEL 449 VT+DDA MIATSFPEFM LM GLGAKIEL VTIDDAAMIATSFPEFMQLMTGLGAKIEL 449 |  |

>WP_164564939.1 3-phosphoshikimate 1-carboxyvinyltransferase [Rhizobium leguminosarum] NEI09718.1 3-phosphoshikimate 1-carboxyvinyltransferase [Rhizobium leguminosarum]

Length=452

Score = 764 bits (1974), Expect = 0.0, Method: Compositional matrix adjust.

| Identities | = 387/449 (86%), Positives = 413/449 (92%), Gaps = 0/449 (0%) |  |
| --- | --- | --- |
| Query 1 | MLHGASSRPATARKSSGLSGTVRIPGDKSISHRSFMFGGLASGETRITGLLEGEDVINTG | 60 |
| Sbjct 1 | MLHG++ RPATARKS+GL+G VRIPGDKSISHRSFMFGGLASGETRITGLLEGEDVINTG MLHGSAPRPATARKSAGLTGRVRIPGDKSISHRSFMFGGLASGETRITGLLEGEDVINTG | 60 |
| Query 61 | KAMQAMGARIRKEGDTWIIDGVGNGGLLAPEAPLDFGNAATGCRLTMGLVGVYDFDSTFI | 120 |
| Sbjct 61 | +AMQAMGARIRKEG W+I+G GNG LLAP+APLDFGNA TG RLTMGLVG YDF STF RAMQAMGARIRKEGAQWVIEGTGNGALLAPDAPLDFGNAGTGVRLTMGLVGTYDFHSTFT | 120 |
| Query 121 | GDASLTKRPMGRVLNPLREMGVQVKSEDGDRLPVTLRGPKTPTPITYRVPMASAQVKSAV | 180 |
| Sbjct 121 | GDASL+KRPMGRVL PLREMGVQV + +GDRLPVTLRGP TP+PI YRVPMASAQVKSAV GDASLSKRPMGRVLAPLREMGVQVSASEGDRLPVTLRGPGTPSPIRYRVPMASAQVKSAV | 180 |
| Query 181 | LLAGLNTPGITTVIEPIMTRDHTEKMLQGFGANLTVETDADGVRTIRLEGRGKLTGQVID | 240 |
| Sbjct 181 | LLAGLNTPG+TTVIEP+MTRDHTEKMLQGFGA L+VET DGVRTIRLEGRGKL GQVID LLAGLNTPGVTTVIEPVMTRDHTEKMLQGFGAALSVETGGDGVRTIRLEGRGKLAGQVID | 240 |
| Query 241 | VPGDPSSTAFPLVAALLVPGSDVTILNVLMNPTRTGLILTLQEMGADIEVINPRLAGGED VPGDPSSTAFPLVAAL+VPGSD+TI+NVLMNPTRTGLILTLQEMGADIEV+N RLAGGED | 300 |

| Sbjct | 241 | VPGDPSSTAFPLVAALIVPGSDITIVNVLMNPTRTGLILTLQEMGADIEVVNARLAGGED | 300 |
| --- | --- | --- | --- |
| Query | 301 | VADLRVRSSTLKGVTVPEDRAPSMIDEYPILavaaafaegaTVMNGLEELRVKESDRLSA | 360 |
| Sbjct | 301 | VADLRVR S LKGV VPEDRAPSMIDEYPILA+AA FAEGATVM GLEELRVKESDRLSA VADLRVRHSELKGVGVPEDRAPSMIDEYPILAIAACFAEGATVMKGLEELRVKESDRLSA | 360 |
| Query | 361 | VANGLKLNGVDCDEGETSLVVRGRPDGKGLGNASGAAVATHLDHRIAMSFLVMGLVSENP | 420 |
| Sbjct | 361 | VA+GLKLNGVDCDEGE L+VRGRPDGKGLGNA+G V THLDHRIAMSFLV+GL SE+ VADGLKLNGVDCDEGEDFLIVRGRPDGKGLGNAAGGQVRTHLDHRIAMSFLVLGLASEHA | 420 |
| Query  Sbjct | 421  421 | VTVDDATMIATSFPEFMDLMAGLGAKIEL 449 VT+DDA MIATSFPEFM LM GLGAKIEL VTIDDAAMIATSFPEFMQLMTGLGAKIEL 449 |  |

>WP_130769267.1 3-phosphoshikimate 1-carboxyvinyltransferase [Rhizobium leguminosarum] TBE94631.1 3-phosphoshikimate 1-carboxyvinyltransferase [Rhizobium leguminosarum]

TCA45890.1 3-phosphoshikimate 1-carboxyvinyltransferase [Rhizobium leguminosarum bv. viciae]

Length=452

Score = 764 bits (1974), Expect = 0.0, Method: Compositional matrix adjust.

| Identities | = 385/448 (86%), Positives = 414/448 (92%), Gaps = 0/448 (0%) |  |
| --- | --- | --- |
| Query 1 | MLHGASSRPATARKSSGLSGTVRIPGDKSISHRSFMFGGLASGETRITGLLEGEDVINTG | 60 |
| Sbjct 1 | MLHG++ RPATARKS+GL+G+VRIPGDKSISHRSFMFGGLASGETRITGLLEGEDVINTG MLHGSAPRPATARKSAGLTGSVRIPGDKSISHRSFMFGGLASGETRITGLLEGEDVINTG | 60 |
| Query 61 | KAMQAMGARIRKEGDTWIIDGVGNGGLLAPEAPLDFGNAATGCRLTMGLVGVYDFDSTFI | 120 |
| Sbjct 61 | +AMQAMGARIRK G+ W+I+G GNG LLAP+APLDFGNA TG RLTMGLVG YDF STFI RAMQAMGARIRKVGEQWLIEGTGNGALLAPDAPLDFGNAGTGVRLTMGLVGTYDFRSTFI | 120 |
| Query 121 | GDASLTKRPMGRVLNPLREMGVQVKSEDGDRLPVTLRGPKTPTPITYRVPMASAQVKSAV | 180 |
| Sbjct 121 | GDASL+KRPMGRVLNPLREMGVQV + +GDRLPVTL+GP TP+PI YRVPMASAQVKSAV GDASLSKRPMGRVLNPLREMGVQVSASEGDRLPVTLKGPGTPSPIRYRVPMASAQVKSAV | 180 |
| Query 181 | LLAGLNTPGITTVIEPIMTRDHTEKMLQGFGANLTVETDADGVRTIRLEGRGKLTGQVID | 240 |
| Sbjct 181 | LLAGLNTPG+TTVIEP+MTRDHTEKMLQGFGA L+VETD DGVRTIRLEGRGKL GQVID LLAGLNTPGVTTVIEPVMTRDHTEKMLQGFGAALSVETDEDGVRTIRLEGRGKLKGQVID | 240 |
| Query 241 | VPGDPSSTAFPLVAALLVPGSDVTILNVLMNPTRTGLILTLQEMGADIEVINPRLAGGED | 300 |
| Sbjct 241 | +P DPSSTAFPLVAALLVPGSD+TI+NVLMNPTRTGLILTLQEMGADIEV+N RLAGGED IPSDPSSTAFPLVAALLVPGSDITIVNVLMNPTRTGLILTLQEMGADIEVLNARLAGGED | 300 |
| Query 301 | VADLRVRSSTLKGVTVPEDRAPSMIDEYPILavaaafaegaTVMNGLEELRVKESDRLSA | 360 |
| Sbjct 301 | VADLRVR S LKGVT+PEDRAPSMIDEYPILAVAA FAEG TVM GLEELRVKESDRLSA VADLRVRHSELKGVTIPEDRAPSMIDEYPILAVAACFAEGVTVMKGLEELRVKESDRLSA | 360 |
| Query 361 | VANGLKLNGVDCDEGETSLVVRGRPDGKGLGNASGAAVATHLDHRIAMSFLVMGLVSENP | 420 |
| Sbjct 361 | VA+GLKLNGVDCDEGE L+VRGR DGKGLGNA+ V+THLDHRIAMSFLVMGL SE+P VADGLKLNGVDCDEGEDFLIVRGRTDGKGLGNATDGRVSTHLDHRIAMSFLVMGLASEHP | 420 |
| Query 421  Sbjct 421 | VTVDDATMIATSFPEFMDLMAGLGAKIE 448 VT+DD MIATSFPEFM LM GLGAKIE VTIDDTAMIATSFPEFMQLMTGLGAKIE 448 |  |

```
>WP_130826003.1 3-phosphoshikimate 1-carboxyvinyltransferase [Rhizobium leguminosarum]
NKK51138.1 3-phosphoshikimate 1-carboxyvinyltransferase [Rhizobium leguminosarum bv. viciae]
TBG01523.1 3-phosphoshikimate 1-carboxyvinyltransferase [Rhizobium leguminosarum] TBG87096.1 3-phosphoshikimate 1-carboxyvinyltransferase [Rhizobium leguminosarum]
TBY99882.1 3-phosphoshikimate 1-carboxyvinyltransferase [Rhizobium leguminosarum bv. viciae]
Length=452

Score = 764 bits (1974), Expect = 0.0, Method: Compositional matrix adjust. Identities = 383/449 (85%), Positives = 417/449 (93%), Gaps = 0/449 (0%)

Query 1	MLHGASSRPATARKSSGLSGTVRIPGDKSISHRSFMFGGLASGETRITGLLEGEDVINTG 60 ML+G++ +PATARKS+GL+G+VRIPGDKSISHRSFMFGGLASGETRITGLLEGEDVINTG
Sbjct 1	MLNGSAPKPATARKSAGLTGSVRIPGDKSISHRSFMFGGLASGETRITGLLEGEDVINTG 60
```

| Query | 61 | KAMQAMGARIRKEGDTWIIDGVGNGGLLAPEAPLDFGNAATGCRLTMGLVGVYDFDSTFI | 120 |
| --- | --- | --- | --- |
| Sbjct | 61 | +AMQAMGARIRKEG W+I+G GNG LLAP+APLDFGNA TG RLTMGLVG YDF STF RAMQAMGARIRKEGAQWVIEGTGNGALLAPDAPLDFGNAGTGVRLTMGLVGTYDFHSTFT | 120 |
| Query | 121 | GDASLTKRPMGRVLNPLREMGVQVKSEDGDRLPVTLRGPKTPTPITYRVPMASAQVKSAV | 180 |
| Sbjct | 121 | GDASL+KRPMGRVLNPLREMGVQ+ + +GDRLPVTLRGP TP+PI YRVPMASAQVKSAV GDASLSKRPMGRVLNPLREMGVQISASEGDRLPVTLRGPGTPSPIRYRVPMASAQVKSAV | 180 |
| Query | 181 | LLAGLNTPGITTVIEPIMTRDHTEKMLQGFGANLTVETDADGVRTIRLEGRGKLTGQVID | 240 |
| Sbjct | 181 | LLAGLNTPG+TTVIEP+MTRDHTE+MLQGFGA L+VETD++GVRTIRLEGRGKL GQVID LLAGLNTPGVTTVIEPVMTRDHTERMLQGFGAALSVETDSEGVRTIRLEGRGKLAGQVID | 240 |
| Query | 241 | VPGDPSSTAFPLVAALLVPGSDVTILNVLMNPTRTGLILTLQEMGADIEVINPRLAGGED | 300 |
| Sbjct | 241 | VPGDPSSTAFPLVAAL+VPGSD+TI+NVLMNPTRTGLILTLQEMGADIEV+N RLAGGED VPGDPSSTAFPLVAALIVPGSDITIVNVLMNPTRTGLILTLQEMGADIEVVNARLAGGED | 300 |
| Query | 301 | VADLRVRSSTLKGVTVPEDRAPSMIDEYPILavaaafaegaTVMNGLEELRVKESDRLSA | 360 |
| Sbjct | 301 | VADLRVR S LKGVTVPEDRAPSMIDEYPILAVAA FAEGAT+M GLEELRVKESDRLSA VADLRVRHSELKGVTVPEDRAPSMIDEYPILAVAACFAEGATIMKGLEELRVKESDRLSA | 360 |
| Query | 361 | VANGLKLNGVDCDEGETSLVVRGRPDGKGLGNASGAAVATHLDHRIAMSFLVMGLVSENP | 420 |
| Sbjct | 361 | VA+GLKLNGVDCDEGE L+VRGRPDG+ LGNA+G V THLDHRIAMSFLV+GL SE+ VADGLKLNGVDCDEGEDFLIVRGRPDGRSLGNAAGGQVRTHLDHRIAMSFLVLGLASEHA | 420 |
| Query  Sbjct | 421  421 | VTVDDATMIATSFPEFMDLMAGLGAKIEL 449 VT+DDA MIATSFPEFM LM GLGAKIEL VTIDDAAMIATSFPEFMQLMTGLGAKIEL 449 |  |

>WP_183637345.1 3-phosphoshikimate 1-carboxyvinyltransferase [Rhizobium leguminosarum] MBB4506531.1 3-phosphoshikimate 1-carboxyvinyltransferase [Rhizobium leguminosarum]

Length=452

Score = 764 bits (1973), Expect = 0.0, Method: Compositional matrix adjust.

| Identities | = 384/449 (86%), Positives = 418/449 (93%), Gaps = 0/449 (0%) |  |
| --- | --- | --- |
| Query 1 | MLHGASSRPATARKSSGLSGTVRIPGDKSISHRSFMFGGLASGETRITGLLEGEDVINTG | 60 |
| Sbjct 1 | ML+G++S+PATARKS+GL+G+VRIPGDKSISHRSFMFGGLASGETRITGLLEGEDVINTG MLNGSASKPATARKSAGLTGSVRIPGDKSISHRSFMFGGLASGETRITGLLEGEDVINTG | 60 |
| Query 61 | KAMQAMGARIRKEGDTWIIDGVGNGGLLAPEAPLDFGNAATGCRLTMGLVGVYDFDSTFI | 120 |
| Sbjct 61 | +AMQAMGARIRKEG W+I+G GNG LLAP+APLDFGNA TG RLTMGLVG YDF STFI RAMQAMGARIRKEGAQWVIEGTGNGALLAPDAPLDFGNAGTGVRLTMGLVGTYDFHSTFI | 120 |
| Query 121 | GDASLTKRPMGRVLNPLREMGVQVKSEDGDRLPVTLRGPKTPTPITYRVPMASAQVKSAV | 180 |
| Sbjct 121 | GDASL+KRPMGRVLNPLREMGVQ+ + +GDRLPVTL+GP TP+PI YRVPMASAQVKSAV GDASLSKRPMGRVLNPLREMGVQISASEGDRLPVTLKGPGTPSPIRYRVPMASAQVKSAV | 180 |
| Query 181 | LLAGLNTPGITTVIEPIMTRDHTEKMLQGFGANLTVETDADGVRTIRLEGRGKLTGQVID | 240 |
| Sbjct 181 | LLAGLNTPG+TTVIEP+MTRDHTEKMLQGFGA L+VETD +GVRTIRLEGRG+LTGQVID LLAGLNTPGVTTVIEPVMTRDHTEKMLQGFGAALSVETDNEGVRTIRLEGRGRLTGQVID | 240 |
| Query 241 | VPGDPSSTAFPLVAALLVPGSDVTILNVLMNPTRTGLILTLQEMGADIEVINPRLAGGED | 300 |
| Sbjct 241 | VPGDPSSTAFPLVAALLVPGSD+TI+NVLMNPTRTGLILTLQEMGA+IEV+N RLAGGED VPGDPSSTAFPLVAALLVPGSDITIVNVLMNPTRTGLILTLQEMGANIEVLNARLAGGED | 300 |
| Query 301 | VADLRVRSSTLKGVTVPEDRAPSMIDEYPILavaaafaegaTVMNGLEELRVKESDRLSA | 360 |
| Sbjct 301 | VADLRVR S L+GVTVPEDRAPSMIDEYPILAVAA FAEG TVM GLEELRVKESDRLSA VADLRVRHSELRGVTVPEDRAPSMIDEYPILAVAACFAEGTTVMKGLEELRVKESDRLSA | 360 |
| Query 361 | VANGLKLNGVDCDEGETSLVVRGRPDGKGLGNASGAAVATHLDHRIAMSFLVMGLVSENP | 420 |
| Sbjct 361 | VA+GLKLNGV CDEGE L VRGRPDG+GLGNA+G V+THLDHRIAMSFLV+GL SE+ VADGLKLNGVLCDEGEDFLTVRGRPDGRGLGNAAGGRVSTHLDHRIAMSFLVLGLASEHA | 420 |
| Query 421  Sbjct 421 | VTVDDATMIATSFPEFMDLMAGLGAKIEL 449 VT+DDA MIATSFPEFM LM GLGAKIEL VTIDDAAMIATSFPEFMQLMTGLGAKIEL 449 |  |

>WP_092995433.1 3-phosphoshikimate 1-carboxyvinyltransferase [Rhizobium sp. NFR07] SFA90342.1 3-phosphoshikimate 1-carboxyvinyltransferase [Rhizobium sp. NFR07]

Length=454

Score = 764 bits (1973), Expect = 0.0, Method: Compositional matrix adjust.

| Identities | = 384/454 (85%), Positives = 419/454 (92%), Gaps = 0/454 (0%) |  |
| --- | --- | --- |
| Query 1 | MLHGASSRPATARKSSGLSGTVRIPGDKSISHRSFMFGGLASGETRITGLLEGEDVINTG | 60 |
| Sbjct 1 | M HG + +PA + +S L+GT++IPGDKSISHRSFMFGGLASGETRITGLLEGEDVINTG MSHGIALKPARSTRSEALNGTIKIPGDKSISHRSFMFGGLASGETRITGLLEGEDVINTG | 60 |
| Query 61 | KAMQAMGARIRKEGDTWIIDGVGNGGLLAPEAPLDFGNAATGCRLTMGLVGVYDFDSTFI | 120 |
| Sbjct 61 | +AM+AMGA I K G W+I G GNG LLAP+APLDFGNA TGCRLTMGLVGVYDFDSTFI RAMRAMGATIEKVGAEWVIQGTGNGALLAPQAPLDFGNAGTGCRLTMGLVGVYDFDSTFI | 120 |
| Query 121 | GDASLTKRPMGRVLNPLREMGVQVKSEDGDRLPVTLRGPKTPTPITYRVPMASAQVKSAV | 180 |
| Sbjct 121 | GDASLTKRPMGRVLNPLREMGVQV S +GDRLPVTLRGPKTP PITYRVPMASAQVKSAV GDASLTKRPMGRVLNPLREMGVQVTSTEGDRLPVTLRGPKTPNPITYRVPMASAQVKSAV | 180 |
| Query 181 | LLAGLNTPGITTVIEPIMTRDHTEKMLQGFGANLTVETDADGVRTIRLEGRGKLTGQVID | 240 |
| Sbjct 181 | LLAGLNTPGITTVIEP+MTRDHTEKMLQGFGA+L+V+TDA+GVRTIRL GRG+L GQVID LLAGLNTPGITTVIEPVMTRDHTEKMLQGFGADLSVDTDAEGVRTIRLVGRGQLKGQVID | 240 |
| Query 241 | VPGDPSSTAFPLVAALLVPGSDVTILNVLMNPTRTGLILTLQEMGADIEVINPRLAGGED | 300 |
| Sbjct 241 | VPGDPSSTAFPLVAALLVPGSDVTILNVLMNPTRTGLILTLQEMGADIE++NPRLAGGED VPGDPSSTAFPLVAALLVPGSDVTILNVLMNPTRTGLILTLQEMGADIEILNPRLAGGED | 300 |
| Query 301 | VADLRVRSSTLKGVTVPEDRAPSMIDEYPILavaaafaegaTVMNGLEELRVKESDRLSA | 360 |
| Sbjct 301 | VADLR+R+S LKGVTVP DRAPSMIDEYP+LAVAAA+A G+T M GLEELRVKESDRLSA VADLRIRASELKGVTVPADRAPSMIDEYPVLAVAAAYATGSTTMLGLEELRVKESDRLSA | 360 |
| Query 361 | VANGLKLNGVDCDEGETSLVVRGRPDGKGLGNASGAAVATHLDHRIAMSFLVMGLVSENP | 420 |
| Sbjct 361 | VA GLKLNGVDCDEG+ +LVVRGRPDGKG GNA+GAAV THLDHRIAMSFLV+GLVSE+ VAEGLKLNGVDCDEGKETLVVRGRPDGKGYGNAAGAAVVTHLDHRIAMSFLVLGLVSEHA | 420 |
| Query 421  Sbjct 421 | VTVDDATMIATSFPEFMDLMAGLGAKIELSDTKA 454 VTVDDA++IATSFPEFMDLM GLGA+I+L + A VTVDDASIIATSFPEFMDLMTGLGARIDLVEEAA 454 |  |

>WP_047619821.1 MULTISPECIES: 3-phosphoshikimate 1-carboxyvinyltransferase [Rhizobium] MBB3303098.1 3-phosphoshikimate 1-carboxyvinyltransferase [Rhizobium sp. BK112] MBB3371991.1 3-phosphoshikimate 1-carboxyvinyltransferase [Rhizobium sp. BK077] MBB3747408.1 3-phosphoshikimate 1-carboxyvinyltransferase [Rhizobium sp. BK591] MBB4182957.1 3-phosphoshikimate 1-carboxyvinyltransferase [Rhizobium sp. BK109]

Length=452

Score = 764 bits (1973), Expect = 0.0, Method: Compositional matrix adjust.

| Identities | = 384/448 (86%), Positives = 416/448 (93%), Gaps = 0/448 (0%) |  |
| --- | --- | --- |
| Query 1 | MLHGASSRPATARKSSGLSGTVRIPGDKSISHRSFMFGGLASGETRITGLLEGEDVINTG | 60 |
| Sbjct 1 | MLHG++ RPATARKS+GLSG VRIPGDKSISHRSFMFGGLASG+TRITGLLEGEDVINTG MLHGSAPRPATARKSAGLSGRVRIPGDKSISHRSFMFGGLASGQTRITGLLEGEDVINTG | 60 |
| Query 61 | KAMQAMGARIRKEGDTWIIDGVGNGGLLAPEAPLDFGNAATGCRLTMGLVGVYDFDSTFI | 120 |
| Sbjct 61 | +AMQAMGARIRK+G+ W+I+G GNG LLAP+APLDFGNA TG RLTMGLVG YDF STFI RAMQAMGARIRKQGEEWVIEGTGNGALLAPDAPLDFGNAGTGVRLTMGLVGTYDFHSTFI | 120 |
| Query 121 | GDASLTKRPMGRVLNPLREMGVQVKSEDGDRLPVTLRGPKTPTPITYRVPMASAQVKSAV | 180 |
| Sbjct 121 | GDASL+KRPMGRVLNPLR+MGVQV + GDRLPVTLRGP TP+PI YRVPMASAQVKSAV GDASLSKRPMGRVLNPLRDMGVQVSASAGDRLPVTLRGPGTPSPIRYRVPMASAQVKSAV | 180 |
| Query 181 | LLAGLNTPGITTVIEPIMTRDHTEKMLQGFGANLTVETDADGVRTIRLEGRGKLTGQVID | 240 |
| Sbjct 181 | LLAGLNTPGITTVIEP+ TRDHTEKMLQGFGA L+VETDADG+R IRLEGRG+LTGQVID LLAGLNTPGITTVIEPVKTRDHTEKMLQGFGAALSVETDADGIRMIRLEGRGRLTGQVID | 240 |
| Query 241 | VPGDPSSTAFPLVAALLVPGSDVTILNVLMNPTRTGLILTLQEMGADIEVINPRLAGGED | 300 |
| Sbjct 241 | VPGDPSSTAFPLVAAL+VPGSD+TI+NVLMNPTRTGLILTLQEMGADIEV+N RLAGGED VPGDPSSTAFPLVAALIVPGSDITIVNVLMNPTRTGLILTLQEMGADIEVLNARLAGGED | 300 |
| Query 301 | VADLRVRSSTLKGVTVPEDRAPSMIDEYPILavaaafaegaTVMNGLEELRVKESDRLSA | 360 |
| Sbjct 301 | VADLRVR S LKGV VPE+RAPSMIDEYPILAVAA FAEG TVM GLEELRVKESDRLSA  VADLRVRHSELKGVAVPEERAPSMIDEYPILAVAACFAEGTTVMKGLEELRVKESDRLSA | 360 |

| Query | 361 | VANGLKLNGVDCDEGETSLVVRGRPDGKGLGNASGAAVATHLDHRIAMSFLVMGLVSENP 420 |
| --- | --- | --- |
| Sbjct | 361 | VA+GLKLNGVDCDEG+ L+VRGRPDGKGLG+A G+ V+THLDHRIAMSFLV+GL SE+ VADGLKLNGVDCDEGKDFLIVRGRPDGKGLGSAGGSKVSTHLDHRIAMSFLVLGLASEHA 420 |
| Query | 421 | VTVDDATMIATSFPEFMDLMAGLGAKIE 448 |
| Sbjct | 421 | VT+DDA MIATSFPEFM LMAGLGAKIE  VTIDDAAMIATSFPEFMQLMAGLGAKIE 448 |

>WP_024323105.1 3-phosphoshikimate 1-carboxyvinyltransferase [Rhizobium leguminosarum] NEH37009.1 3-phosphoshikimate 1-carboxyvinyltransferase [Rhizobium leguminosarum] NEH88995.1 3-phosphoshikimate 1-carboxyvinyltransferase [Rhizobium leguminosarum] NEI16959.1 3-phosphoshikimate 1-carboxyvinyltransferase [Rhizobium leguminosarum] NEJ31791.1 3-phosphoshikimate 1-carboxyvinyltransferase [Rhizobium leguminosarum] NEJ61107.1 3-phosphoshikimate 1-carboxyvinyltransferase [Rhizobium leguminosarum]

Length=452

Score = 764 bits (1973), Expect = 0.0, Method: Compositional matrix adjust.

| Identities | = 386/449 (86%), Positives = 413/449 (92%), Gaps = 0/449 (0%) |  |
| --- | --- | --- |
| Query 1 | MLHGASSRPATARKSSGLSGTVRIPGDKSISHRSFMFGGLASGETRITGLLEGEDVINTG | 60 |
| Sbjct 1 | MLHG++ RPATARKS+ L+G+VRIPGDKSISHRSFMFGGLASGETRITGLLEGEDVINTG MLHGSAPRPATARKSASLTGSVRIPGDKSISHRSFMFGGLASGETRITGLLEGEDVINTG | 60 |
| Query 61 | KAMQAMGARIRKEGDTWIIDGVGNGGLLAPEAPLDFGNAATGCRLTMGLVGVYDFDSTFI | 120 |
| Sbjct 61 | +AMQAMGARIRKEG W+I+G GNG LLAP+APLDFGNA TG RLTMGLVG YDF STF RAMQAMGARIRKEGAQWVIEGTGNGALLAPDAPLDFGNAGTGVRLTMGLVGTYDFHSTFT | 120 |
| Query 121 | GDASLTKRPMGRVLNPLREMGVQVKSEDGDRLPVTLRGPKTPTPITYRVPMASAQVKSAV | 180 |
| Sbjct 121 | GDASL+KRPMGRVL PLREMGVQV + +GDRLPVTLRGP TP+PI YRVPMASAQVKSAV GDASLSKRPMGRVLAPLREMGVQVSASEGDRLPVTLRGPGTPSPIRYRVPMASAQVKSAV | 180 |
| Query 181 | LLAGLNTPGITTVIEPIMTRDHTEKMLQGFGANLTVETDADGVRTIRLEGRGKLTGQVID | 240 |
| Sbjct 181 | LLAGLNTPG+TTVIEP+MTRDHTEKMLQGFGA L+VET DGVRTIRLEGRGKL GQVID LLAGLNTPGVTTVIEPVMTRDHTEKMLQGFGAALSVETGGDGVRTIRLEGRGKLAGQVID | 240 |
| Query 241 | VPGDPSSTAFPLVAALLVPGSDVTILNVLMNPTRTGLILTLQEMGADIEVINPRLAGGED | 300 |
| Sbjct 241 | VPGDPSSTAFPLVAAL+VPGSD+TI+NVLMNPTRTGLILTLQEMGADIEV+N RLAGGED VPGDPSSTAFPLVAALIVPGSDITIVNVLMNPTRTGLILTLQEMGADIEVVNARLAGGED | 300 |
| Query 301 | VADLRVRSSTLKGVTVPEDRAPSMIDEYPILavaaafaegaTVMNGLEELRVKESDRLSA | 360 |
| Sbjct 301 | VADLRVR S LKGV VPEDRAPSMIDEYPILA+AA FAEGATVM GLEELRVKESDRLSA VADLRVRHSELKGVGVPEDRAPSMIDEYPILAIAACFAEGATVMKGLEELRVKESDRLSA | 360 |
| Query 361 | VANGLKLNGVDCDEGETSLVVRGRPDGKGLGNASGAAVATHLDHRIAMSFLVMGLVSENP | 420 |
| Sbjct 361 | VA+GLKLNGVDCDEGE L+VRGRPDGKGLGNA+G V THLDHRIAMSFLV+GL SE+ VADGLKLNGVDCDEGEDFLIVRGRPDGKGLGNAAGGQVRTHLDHRIAMSFLVLGLASEHA | 420 |
| Query 421  Sbjct 421 | VTVDDATMIATSFPEFMDLMAGLGAKIEL 449 VT+DDA MIATSFPEFM LM GLGAKIEL VTIDDAAMIATSFPEFMQLMTGLGAKIEL 449 |  |

>WP_064653653.1 3-phosphoshikimate 1-carboxyvinyltransferase [Rhizobium sp. WYCCWR10014] OAV50952.1 3-phosphoshikimate 1-carboxyvinyltransferase [Rhizobium sp. WYCCWR10014]

Length=452

Score = 764 bits (1973), Expect = 0.0, Method: Compositional matrix adjust.

| Identities | = 385/448 (86%), Positives = 416/448 (93%), Gaps = 0/448 (0%) |  |
| --- | --- | --- |
| Query 1 | MLHGASSRPATARKSSGLSGTVRIPGDKSISHRSFMFGGLASGETRITGLLEGEDVINTG | 60 |
| Sbjct 1 | ML+G++ RPATARKS+GL+G+VRIPGDKSISHRSFMFGGLASG+TRITGLLEGEDVINTG MLNGSAPRPATARKSAGLTGSVRIPGDKSISHRSFMFGGLASGQTRITGLLEGEDVINTG | 60 |
| Query 61 | KAMQAMGARIRKEGDTWIIDGVGNGGLLAPEAPLDFGNAATGCRLTMGLVGVYDFDSTFI | 120 |
| Sbjct 61 | +AMQAMGARIRKEG W+IDG GNG L AP+APLDFGNA TG RLTMGLVG YDF STFI RAMQAMGARIRKEGAQWVIDGTGNGALFAPDAPLDFGNAGTGVRLTMGLVGTYDFRSTFI | 120 |
| Query 121 | GDASLTKRPMGRVLNPLREMGVQVKSEDGDRLPVTLRGPKTPTPITYRVPMASAQVKSAV | 180 |
| Sbjct 121 | GDASL+KRPMGRVLNPLREMGVQ+ + +GDRLPVTL+GP TP+PI YRVPMASAQVKSAV  GDASLSKRPMGRVLNPLREMGVQISASEGDRLPVTLKGPGTPSPIRYRVPMASAQVKSAV | 180 |

| Query | 181 | LLAGLNTPGITTVIEPIMTRDHTEKMLQGFGANLTVETDADGVRTIRLEGRGKLTGQVID | 240 |
| --- | --- | --- | --- |
| Sbjct | 181 | LLAGLNTPG+TTVIEP+MTRDHTEKMLQGFGA L+VETD++GVRTIRLEGRG+LTGQVID LLAGLNTPGVTTVIEPVMTRDHTEKMLQGFGAALSVETDSEGVRTIRLEGRGRLTGQVID | 240 |
| Query | 241 | VPGDPSSTAFPLVAALLVPGSDVTILNVLMNPTRTGLILTLQEMGADIEVINPRLAGGED | 300 |
| Sbjct | 241 | VPGDPSSTAFPLVAALLVPGSD+TI+NVLMNPTRTGLILTLQEMGADIEV+N RLAGGED VPGDPSSTAFPLVAALLVPGSDITIVNVLMNPTRTGLILTLQEMGADIEVLNTRLAGGED | 300 |
| Query | 301 | VADLRVRSSTLKGVTVPEDRAPSMIDEYPILavaaafaegaTVMNGLEELRVKESDRLSA | 360 |
| Sbjct | 301 | VADLRVR S LKGV VPEDRAPSMIDEYPILAVAA FAEGAT+M GLEELRVKESDRLSA VADLRVRHSELKGVIVPEDRAPSMIDEYPILAVAACFAEGATIMKGLEELRVKESDRLSA | 360 |
| Query | 361 | VANGLKLNGVDCDEGETSLVVRGRPDGKGLGNASGAAVATHLDHRIAMSFLVMGLVSENP | 420 |
| Sbjct | 361 | VA+GLKLNGVDCDEGE L+VRGRPDGKGLG A V+THLDHRIAMSFLVMGL SE+P VADGLKLNGVDCDEGEDFLIVRGRPDGKGLGTAVDGRVSTHLDHRIAMSFLVMGLASEHP | 420 |
| Query  Sbjct | 421  421 | VTVDDATMIATSFPEFMDLMAGLGAKIE 448 VT+DDA MIATSFPEFM LM GLGAKIE VTIDDAAMIATSFPEFMQLMTGLGAKIE 448 |  |

>WP_164055205.1 3-phosphoshikimate 1-carboxyvinyltransferase [Rhizobium leguminosarum] NEK38764.1 3-phosphoshikimate 1-carboxyvinyltransferase [Rhizobium leguminosarum]

Length=452

Score = 764 bits (1973), Expect = 0.0, Method: Compositional matrix adjust.

| Identities | = 385/449 (86%), Positives = 416/449 (93%), Gaps = 0/449 (0%) |  |
| --- | --- | --- |
| Query 1 | MLHGASSRPATARKSSGLSGTVRIPGDKSISHRSFMFGGLASGETRITGLLEGEDVINTG | 60 |
| Sbjct 1 | ML+G++ +PATARKS+GL+G+VRIPGDKSISHRSFMFGGLASGETRITGLLEGEDVINTG MLNGSAPKPATARKSAGLTGSVRIPGDKSISHRSFMFGGLASGETRITGLLEGEDVINTG | 60 |
| Query 61 | KAMQAMGARIRKEGDTWIIDGVGNGGLLAPEAPLDFGNAATGCRLTMGLVGVYDFDSTFI | 120 |
| Sbjct 61 | +AMQAMGARIRKEG+ W+IDG GNG LLAP+APLDFGNA TG RLTMGLVG YDF STF RAMQAMGARIRKEGEQWVIDGTGNGALLAPDAPLDFGNAGTGVRLTMGLVGTYDFRSTFT | 120 |
| Query 121 | GDASLTKRPMGRVLNPLREMGVQVKSEDGDRLPVTLRGPKTPTPITYRVPMASAQVKSAV | 180 |
| Sbjct 121 | GDASL+KRPMGRVL PLREMGVQV + GDRLPVTLRGP TP+PI YRVPMASAQVKSAV GDASLSKRPMGRVLAPLREMGVQVSASQGDRLPVTLRGPGTPSPIRYRVPMASAQVKSAV | 180 |
| Query 181 | LLAGLNTPGITTVIEPIMTRDHTEKMLQGFGANLTVETDADGVRTIRLEGRGKLTGQVID | 240 |
| Sbjct 181 | LLAGLNTPG+TTVIEP+MTRDHTEKMLQGFGA L+VETD++GVRTIRLEGRG+L GQVID LLAGLNTPGVTTVIEPVMTRDHTEKMLQGFGAALSVETDSEGVRTIRLEGRGRLMGQVID | 240 |
| Query 241 | VPGDPSSTAFPLVAALLVPGSDVTILNVLMNPTRTGLILTLQEMGADIEVINPRLAGGED | 300 |
| Sbjct 241 | VPGDPSSTAFPLVAALLVPGSD+TI+NVLMNPTRTGLILTLQEMGADIEV+N RLAGGED VPGDPSSTAFPLVAALLVPGSDITIVNVLMNPTRTGLILTLQEMGADIEVVNARLAGGED | 300 |
| Query 301 | VADLRVRSSTLKGVTVPEDRAPSMIDEYPILavaaafaegaTVMNGLEELRVKESDRLSA | 360 |
| Sbjct 301 | VADLRVR S LKGVTVPE+RAPSMIDEYPILAVAA FAEGAT+M GLEELRVKESDRLSA VADLRVRHSELKGVTVPEERAPSMIDEYPILAVAACFAEGATIMKGLEELRVKESDRLSA | 360 |
| Query 361 | VANGLKLNGVDCDEGETSLVVRGRPDGKGLGNASGAAVATHLDHRIAMSFLVMGLVSENP | 420 |
| Sbjct 361 | VA+GLKLNGVDCDEGE L+VRGRPDGKGLGNA+G V THLDHRIAMSFLV+G SE+ VADGLKLNGVDCDEGEDFLIVRGRPDGKGLGNAAGGQVRTHLDHRIAMSFLVLGFASEHA | 420 |
| Query 421  Sbjct 421 | VTVDDATMIATSFPEFMDLMAGLGAKIEL 449 VT+DDA MIATSFPEFM LM GLGAKIEL VTIDDAAMIATSFPEFMQLMTGLGAKIEL 449 |  |

>WP_130763894.1 3-phosphoshikimate 1-carboxyvinyltransferase [Rhizobium leguminosarum] NEJ15280.1 3-phosphoshikimate 1-carboxyvinyltransferase [Rhizobium leguminosarum] NEK29355.1 3-phosphoshikimate 1-carboxyvinyltransferase [Rhizobium leguminosarum] TAX79009.1 3-phosphoshikimate 1-carboxyvinyltransferase [Rhizobium leguminosarum]

Length=452

Score = 764 bits (1973), Expect = 0.0, Method: Compositional matrix adjust. Identities = 386/449 (86%), Positives = 413/449 (92%), Gaps = 0/449 (0%)

Query 1	MLHGASSRPATARKSSGLSGTVRIPGDKSISHRSFMFGGLASGETRITGLLEGEDVINTG 60 MLHG++ RPATARKS+ L+G+VRIPGDKSISHRSFMFGGLASGETRITGLLEGEDVINTG

| Sbjct | 1 | MLHGSAPRPATARKSASLTGSVRIPGDKSISHRSFMFGGLASGETRITGLLEGEDVINTG | 60 |
| --- | --- | --- | --- |
| Query | 61 | KAMQAMGARIRKEGDTWIIDGVGNGGLLAPEAPLDFGNAATGCRLTMGLVGVYDFDSTFI | 120 |
| Sbjct | 61 | +AMQAMGARIRKEG W+I+G GNG LLAP+APLDFGNA TG RLTMGLVG YDF STF RAMQAMGARIRKEGAQWVIEGTGNGALLAPDAPLDFGNAGTGVRLTMGLVGTYDFHSTFT | 120 |
| Query | 121 | GDASLTKRPMGRVLNPLREMGVQVKSEDGDRLPVTLRGPKTPTPITYRVPMASAQVKSAV | 180 |
| Sbjct | 121 | GDASL+KRPMGRVL PLREMGVQV + +GDRLPVTLRGP TP+PI YRVPMASAQVKSAV GDASLSKRPMGRVLAPLREMGVQVSASEGDRLPVTLRGPGTPSPIRYRVPMASAQVKSAV | 180 |
| Query | 181 | LLAGLNTPGITTVIEPIMTRDHTEKMLQGFGANLTVETDADGVRTIRLEGRGKLTGQVID | 240 |
| Sbjct | 181 | LLAGLNTPG+TTVIEP+MTRDHTEKMLQGFGA L+VET DGVRTIRLEGRGKL GQVID LLAGLNTPGVTTVIEPVMTRDHTEKMLQGFGAALSVETGGDGVRTIRLEGRGKLAGQVID | 240 |
| Query | 241 | VPGDPSSTAFPLVAALLVPGSDVTILNVLMNPTRTGLILTLQEMGADIEVINPRLAGGED | 300 |
| Sbjct | 241 | VPGDPSSTAFPLVAAL+VPGSD+TI+NVLMNPTRTGLILTLQEMGADIEV+N RLAGGED VPGDPSSTAFPLVAALIVPGSDITIINVLMNPTRTGLILTLQEMGADIEVVNARLAGGED | 300 |
| Query | 301 | VADLRVRSSTLKGVTVPEDRAPSMIDEYPILavaaafaegaTVMNGLEELRVKESDRLSA | 360 |
| Sbjct | 301 | VADLRVR S LKGV VPEDRAPSMIDEYPILA+AA FAEGATVM GLEELRVKESDRLSA VADLRVRHSELKGVGVPEDRAPSMIDEYPILAIAACFAEGATVMKGLEELRVKESDRLSA | 360 |
| Query | 361 | VANGLKLNGVDCDEGETSLVVRGRPDGKGLGNASGAAVATHLDHRIAMSFLVMGLVSENP | 420 |
| Sbjct | 361 | VA+GLKLNGVDCDEGE L+VRGRPDGKGLGNA+G V THLDHRIAMSFLV+GL SE+ VADGLKLNGVDCDEGEDFLIVRGRPDGKGLGNAAGGQVRTHLDHRIAMSFLVLGLASEHA | 420 |
| Query  Sbjct | 421  421 | VTVDDATMIATSFPEFMDLMAGLGAKIEL 449 VT+DDA MIATSFPEFM LM GLGAKIEL VTIDDAAMIATSFPEFMQLMTGLGAKIEL 449 |  |

>WP_168334042.1 3-phosphoshikimate 1-carboxyvinyltransferase [Rhizobium anhuiense] NKM58942.1 3-phosphoshikimate 1-carboxyvinyltransferase [Rhizobium anhuiense]

Length=452

Score = 764 bits (1972), Expect = 0.0, Method: Compositional matrix adjust.

| Identities | = 384/448 (86%), Positives = 416/448 (93%), Gaps = 0/448 (0%) |  |
| --- | --- | --- |
| Query 1 | MLHGASSRPATARKSSGLSGTVRIPGDKSISHRSFMFGGLASGETRITGLLEGEDVINTG | 60 |
| Sbjct 1 | MLHG++ RPATARKS+GLSG VRIPGDKSISHRSFMFGGLASG+TRITGLLEGEDVINTG MLHGSAPRPATARKSAGLSGRVRIPGDKSISHRSFMFGGLASGQTRITGLLEGEDVINTG | 60 |
| Query 61 | KAMQAMGARIRKEGDTWIIDGVGNGGLLAPEAPLDFGNAATGCRLTMGLVGVYDFDSTFI | 120 |
| Sbjct 61 | +AMQAMGARIRK+G+ W+I+G GNG LLAP+APLDFGNA TG RLTMGLVG YDF STFI RAMQAMGARIRKQGEEWVIEGTGNGALLAPDAPLDFGNAGTGVRLTMGLVGTYDFHSTFI | 120 |
| Query 121 | GDASLTKRPMGRVLNPLREMGVQVKSEDGDRLPVTLRGPKTPTPITYRVPMASAQVKSAV | 180 |
| Sbjct 121 | GDASL+KRPMGRVLNPLR+MGVQV + GDRLPVTLRGP TP+PI YRVPMASAQVKSAV GDASLSKRPMGRVLNPLRDMGVQVSASAGDRLPVTLRGPGTPSPIRYRVPMASAQVKSAV | 180 |
| Query 181 | LLAGLNTPGITTVIEPIMTRDHTEKMLQGFGANLTVETDADGVRTIRLEGRGKLTGQVID | 240 |
| Sbjct 181 | LLAGLNTPGITTVIEP+ TRDHTEKMLQGFGA L+VETDADG+R IRLEGRG+LTGQVID LLAGLNTPGITTVIEPVKTRDHTEKMLQGFGAALSVETDADGIRMIRLEGRGRLTGQVID | 240 |
| Query 241 | VPGDPSSTAFPLVAALLVPGSDVTILNVLMNPTRTGLILTLQEMGADIEVINPRLAGGED | 300 |
| Sbjct 241 | VPGDPSSTAFPLVAAL+VPGSD+TI+NVLMNPTRTGLILTLQEMGADIEV+N RLAGGED VPGDPSSTAFPLVAALIVPGSDITIVNVLMNPTRTGLILTLQEMGADIEVLNARLAGGED | 300 |
| Query 301 | VADLRVRSSTLKGVTVPEDRAPSMIDEYPILavaaafaegaTVMNGLEELRVKESDRLSA | 360 |
| Sbjct 301 | VADLRVR S LKGV VPE+RAPSMIDEYPILAVAA FAEG TVM GLEELRVKESDRLSA VADLRVRHSELKGVAVPEERAPSMIDEYPILAVAACFAEGTTVMKGLEELRVKESDRLSA | 360 |
| Query 361 | VANGLKLNGVDCDEGETSLVVRGRPDGKGLGNASGAAVATHLDHRIAMSFLVMGLVSENP | 420 |
| Sbjct 361 | VA+GLKLNGVDCDEG+ L+VRGRPDGKGLG+A G+ V+THLDHRIAMSFLV+GL SE+ VADGLKLNGVDCDEGKDFLIVRGRPDGKGLGSAGGSKVSTHLDHRIAMSFLVLGLASEHA | 420 |
| Query 421  Sbjct 421 | VTVDDATMIATSFPEFMDLMAGLGAKIE 448 VT+DDA MIATSFPEFM LMAGLGAKIE VTIDDAAMIATSFPEFMQLMAGLGAKIE 448 |  |

>WP_037167849.1 3-phosphoshikimate 1-carboxyvinyltransferase [Pseudorhizobium

pelagicum]

KEQ04362.1 3-phosphoshikimate 1-carboxyvinyltransferase [Pseudorhizobium pelagicum]

KEQ07272.1 3-phosphoshikimate 1-carboxyvinyltransferase [Pseudorhizobium pelagicum]

Length=455

Score = 763 bits (1971), Expect = 0.0, Method: Compositional matrix adjust.

| Identities | = 390/455 (86%), Positives = 416/455 (91%), Gaps = 0/455 (0%) |  |
| --- | --- | --- |
| Query 1 | MLHGASSRPATARKSSGLSGTVRIPGDKSISHRSFMFGGLASGETRITGLLEGEDVINTG | 60 |
| Sbjct 1 | M HG + +PA + KS+ L GTVRIPGDKSISHRSFMFGGLASGETRITGLLEGEDVINTG MSHGVALKPARSLKSADLKGTVRIPGDKSISHRSFMFGGLASGETRITGLLEGEDVINTG | 60 |
| Query 61 | KAMQAMGARIRKEGDTWIIDGVGNGGLLAPEAPLDFGNAATGCRLTMGLVGVYDFDSTFI | 120 |
| Sbjct 61 | KAMQAMGA+I K G WII G GNG LLAPEAPLDFGNA TGCRLTMGLVGVYDFDSTFI KAMQAMGAKIEKVGAEWIIQGTGNGALLAPEAPLDFGNAGTGCRLTMGLVGVYDFDSTFI | 120 |
| Query 121 | GDASLTKRPMGRVLNPLREMGVQVKSEDGDRLPVTLRGPKTPTPITYRVPMASAQVKSAV | 180 |
| Sbjct 121 | GDASL+KRPMGRVL PLREMGVQV + +GDRLPVTLRGP TP PITYRVPMASAQVKSAV GDASLSKRPMGRVLAPLREMGVQVTAAEGDRLPVTLRGPATPNPITYRVPMASAQVKSAV | 180 |
| Query 181 | LLAGLNTPGITTVIEPIMTRDHTEKMLQGFGANLTVETDADGVRTIRLEGRGKLTGQVID | 240 |
| Sbjct 181 | LLAGLNTPG TTVIEP+MTRDHTEKMLQGFGA+LTVETDADGVRTIRL+GRG+LTGQVID LLAGLNTPGTTTVIEPVMTRDHTEKMLQGFGADLTVETDADGVRTIRLQGRGRLTGQVID | 240 |
| Query 241 | VPGDPSSTAFPLVAALLVPGSDVTILNVLMNPTRTGLILTLQEMGADIEVINPRLAGGED | 300 |
| Sbjct 241 | VPGDPSSTAFPLVAALLVPGS+VTI NVLMNPTRTGLILTLQEMGADIEV+N RLAGGED VPGDPSSTAFPLVAALLVPGSEVTIENVLMNPTRTGLILTLQEMGADIEVLNARLAGGED | 300 |
| Query 301 | VADLRVRSSTLKGVTVPEDRAPSMIDEYPILavaaafaegaTVMNGLEELRVKESDRLSA | 360 |
| Sbjct 301 | VADLRVR S LKGVTVP +RAPSMIDEYP+LAVAAAFAEG T M G+EELRVKESDRLSA VADLRVRHSQLKGVTVPAERAPSMIDEYPVLAVAAAFAEGTTTMLGVEELRVKESDRLSA | 360 |
| Query 361 | VANGLKLNGVDCDEGETSLVVRGRPDGKGLGNASGAAVATHLDHRIAMSFLVMGLVSENP | 420 |
| Sbjct 361 | A GLKLNGVDCDEGE +L VRGRP GKG GNA+G AVATHLDHRIAMSFLV+GLVSE+P TAAGLKLNGVDCDEGEDTLTVRGRPGGKGYGNAAGEAVATHLDHRIAMSFLVLGLVSEHP | 420 |
| Query 421  Sbjct 421 | VTVDDATMIATSFPEFMDLMAGLGAKIELSDTKAA 455 VTVDDA +IATSFPEFMDLMAGLGA+IE +++AA VTVDDAGIIATSFPEFMDLMAGLGARIEAVESRAA 455 |  |

>WP_064250148.1 3-phosphoshikimate 1-carboxyvinyltransferase [Rhizobium leguminosarum] OAP89721.1 3-phosphoshikimate 1-carboxyvinyltransferase [Rhizobium leguminosarum]

Length=452

Score = 763 bits (1971), Expect = 0.0, Method: Compositional matrix adjust.

| Identities | = 383/449 (85%), Positives = 412/449 (92%), Gaps = 0/449 (0%) |  |
| --- | --- | --- |
| Query 1 | MLHGASSRPATARKSSGLSGTVRIPGDKSISHRSFMFGGLASGETRITGLLEGEDVINTG | 60 |
| Sbjct 1 | MLHG++ RPATARKS+ L G+VRIPGDKSISHRSFMFGGLASGETRITGLLEGEDVINTG MLHGSAPRPATARKSASLKGSVRIPGDKSISHRSFMFGGLASGETRITGLLEGEDVINTG | 60 |
| Query 61 | KAMQAMGARIRKEGDTWIIDGVGNGGLLAPEAPLDFGNAATGCRLTMGLVGVYDFDSTFI | 120 |
| Sbjct 61 | +AMQAMGARIRKEG W+I+G GNG LLAP+APLDFGNA TG RLTMGLVG YDF STF RAMQAMGARIRKEGAQWVIEGTGNGALLAPDAPLDFGNAGTGVRLTMGLVGTYDFHSTFT | 120 |
| Query 121 | GDASLTKRPMGRVLNPLREMGVQVKSEDGDRLPVTLRGPKTPTPITYRVPMASAQVKSAV | 180 |
| Sbjct 121 | GDASL+KRPMGRVL PLREMGVQV + +GDRLPVTLRGP TP+PI YRVPMASAQVKSAV GDASLSKRPMGRVLAPLREMGVQVSASEGDRLPVTLRGPGTPSPIRYRVPMASAQVKSAV | 180 |
| Query 181 | LLAGLNTPGITTVIEPIMTRDHTEKMLQGFGANLTVETDADGVRTIRLEGRGKLTGQVID | 240 |
| Sbjct 181 | LLAGLNTPG+TTVIEP+MTRDHTEKMLQGFGA L+VETD DGVRTIRLEGRGKL GQVID LLAGLNTPGVTTVIEPVMTRDHTEKMLQGFGAALSVETDGDGVRTIRLEGRGKLKGQVID | 240 |
| Query 241 | VPGDPSSTAFPLVAALLVPGSDVTILNVLMNPTRTGLILTLQEMGADIEVINPRLAGGED | 300 |
| Sbjct 241 | +PGDPSSTAFPLVAALLVPGSD+TI+NVLMNPTRTGLILTLQEMGA+IEV+N RLAGGED IPGDPSSTAFPLVAALLVPGSDITIVNVLMNPTRTGLILTLQEMGANIEVLNARLAGGED | 300 |
| Query 301 | VADLRVRSSTLKGVTVPEDRAPSMIDEYPILavaaafaegaTVMNGLEELRVKESDRLSA VADLRVR S LKGVTVPE+RAPSMIDEYPILA+AA FA+G TVM GLEELRVKESDRLSA | 360 |

| Sbjct | 301 | VADLRVRHSELKGVTVPEERAPSMIDEYPILAIAACFADGTTVMKGLEELRVKESDRLSA | 360 |
| --- | --- | --- | --- |
| Query | 361 | VANGLKLNGVDCDEGETSLVVRGRPDGKGLGNASGAAVATHLDHRIAMSFLVMGLVSENP | 420 |
| Sbjct | 361 | VA+GLKLNG DCDEGE L+VRGRPDGKGLGNA+G V THLDHRIAMSFLV+GL SE+ VADGLKLNGADCDEGEDFLIVRGRPDGKGLGNAAGGQVRTHLDHRIAMSFLVLGLASEHA | 420 |
| Query  Sbjct | 421  421 | VTVDDATMIATSFPEFMDLMAGLGAKIEL 449 VT+DDA MIATSFPEFM LM GLGAKIEL VTIDDAAMIATSFPEFMQLMTGLGAKIEL 449 |  |

>WP_128405682.1 3-phosphoshikimate 1-carboxyvinyltransferase [Rhizobium leguminosarum] RWX40163.1 3-phosphoshikimate 1-carboxyvinyltransferase [Rhizobium leguminosarum]

Length=452

Score = 763 bits (1971), Expect = 0.0, Method: Compositional matrix adjust.

| Identities | = 386/447 (86%), Positives = 416/447 (93%), Gaps = 0/447 (0%) |  |
| --- | --- | --- |
| Query 1 | MLHGASSRPATARKSSGLSGTVRIPGDKSISHRSFMFGGLASGETRITGLLEGEDVINTG | 60 |
| Sbjct 1 | ML+G++ +PATARKS+GL+G+VRIPGDKSISHRSFMFGGLASGETRITGLLEGEDVINTG MLNGSAPKPATARKSAGLTGSVRIPGDKSISHRSFMFGGLASGETRITGLLEGEDVINTG | 60 |
| Query 61 | KAMQAMGARIRKEGDTWIIDGVGNGGLLAPEAPLDFGNAATGCRLTMGLVGVYDFDSTFI | 120 |
| Sbjct 61 | +AMQAMGARIRK+G W+I+G GNG LLAP+APLDFGNA TG RLTMGLVG YDF STF RAMQAMGARIRKDGAQWVIEGTGNGALLAPDAPLDFGNAGTGVRLTMGLVGTYDFRSTFT | 120 |
| Query 121 | GDASLTKRPMGRVLNPLREMGVQVKSEDGDRLPVTLRGPKTPTPITYRVPMASAQVKSAV | 180 |
| Sbjct 121 | GDASL+KRPMGRVLNPLREMGVQV + +GDRLPVTLRGP TP+PI YRVPMASAQVKSAV GDASLSKRPMGRVLNPLREMGVQVSASEGDRLPVTLRGPGTPSPIRYRVPMASAQVKSAV | 180 |
| Query 181 | LLAGLNTPGITTVIEPIMTRDHTEKMLQGFGANLTVETDADGVRTIRLEGRGKLTGQVID | 240 |
| Sbjct 181 | LLAGLNTPGITTVIEP+MTRDHTEKMLQGFGA L+VETD++GVRTIRLEGRGKL GQVID LLAGLNTPGITTVIEPVMTRDHTEKMLQGFGAALSVETDSEGVRTIRLEGRGKLAGQVID | 240 |
| Query 241 | VPGDPSSTAFPLVAALLVPGSDVTILNVLMNPTRTGLILTLQEMGADIEVINPRLAGGED | 300 |
| Sbjct 241 | VPGDPSSTAFPLVAALLVPGSD+TI+NVLMNPTRTGLILTLQEMGADIEV N RLAGGED VPGDPSSTAFPLVAALLVPGSDITIVNVLMNPTRTGLILTLQEMGADIEVANARLAGGED | 300 |
| Query 301 | VADLRVRSSTLKGVTVPEDRAPSMIDEYPILavaaafaegaTVMNGLEELRVKESDRLSA | 360 |
| Sbjct 301 | VADLRVR S LKGVTVPE+RAPSMIDEYPILAVAA FAEGAT+M GLEELRVKESDRLSA VADLRVRHSELKGVTVPEERAPSMIDEYPILAVAACFAEGATIMKGLEELRVKESDRLSA | 360 |
| Query 361 | VANGLKLNGVDCDEGETSLVVRGRPDGKGLGNASGAAVATHLDHRIAMSFLVMGLVSENP | 420 |
| Sbjct 361 | VA+GLKLNGVDCDEGE L+VRGRPDGKGLGNA+ V+THLDHRIAMSFLVMGL SE+P VADGLKLNGVDCDEGEDFLIVRGRPDGKGLGNAADGRVSTHLDHRIAMSFLVMGLASEHP | 420 |
| Query 421  Sbjct 421 | VTVDDATMIATSFPEFMDLMAGLGAKI 447 VT+DDA MIATSFPEFM LM GLGAKI VTIDDAAMIATSFPEFMQLMTGLGAKI 447 |  |

>WP_168304617.1 3-phosphoshikimate 1-carboxyvinyltransferase [Rhizobium leguminosarum]

NKM79091.1 3-phosphoshikimate 1-carboxyvinyltransferase [Rhizobium leguminosarum bv. viciae]

Length=452

Score = 763 bits (1971), Expect = 0.0, Method: Compositional matrix adjust.

| Identities | = 384/449 (86%), Positives = 415/449 (92%), Gaps = 0/449 (0%) |  |
| --- | --- | --- |
| Query 1 | MLHGASSRPATARKSSGLSGTVRIPGDKSISHRSFMFGGLASGETRITGLLEGEDVINTG | 60 |
| Sbjct 1 | MLHG++ RPATARKS+ L+G+VRIPGDKSISHRSFMFGGLASGETRITGLLEGEDVINTG MLHGSAPRPATARKSASLNGSVRIPGDKSISHRSFMFGGLASGETRITGLLEGEDVINTG | 60 |
| Query 61 | KAMQAMGARIRKEGDTWIIDGVGNGGLLAPEAPLDFGNAATGCRLTMGLVGVYDFDSTFI | 120 |
| Sbjct 61 | +AMQAMGARIRK+G W+IDG GNG LLAP+APLDFGNA TG RLTMGLVG YDF STF RAMQAMGARIRKDGAQWVIDGTGNGALLAPDAPLDFGNAGTGVRLTMGLVGTYDFRSTFT | 120 |
| Query 121 | GDASLTKRPMGRVLNPLREMGVQVKSEDGDRLPVTLRGPKTPTPITYRVPMASAQVKSAV | 180 |
| Sbjct 121 | GDASL+KRPMGRVL PLREMGVQV + +GDRLPVTLRGP TP+PI YRVPMASAQVKSAV GDASLSKRPMGRVLAPLREMGVQVSASEGDRLPVTLRGPGTPSPIRYRVPMASAQVKSAV | 180 |
| Query 181 | LLAGLNTPGITTVIEPIMTRDHTEKMLQGFGANLTVETDADGVRTIRLEGRGKLTGQVID | 240 |

| Sbjct | 181 | LLAGLNTPG+TTVIEP+MTRDHTEKMLQGFGA L+VETD++GVRTIRLEGRG+LTGQVID LLAGLNTPGVTTVIEPVMTRDHTEKMLQGFGAALSVETDSEGVRTIRLEGRGRLTGQVID | 240 |
| --- | --- | --- | --- |
| Query | 241 | VPGDPSSTAFPLVAALLVPGSDVTILNVLMNPTRTGLILTLQEMGADIEVINPRLAGGED | 300 |
| Sbjct | 241 | +PGDPSSTAFPLVAALLVPGSD+TI+NVLMNPTRTGLILTLQEMGADIEV+N RLAGGED IPGDPSSTAFPLVAALLVPGSDITIVNVLMNPTRTGLILTLQEMGADIEVVNTRLAGGED | 300 |
| Query | 301 | VADLRVRSSTLKGVTVPEDRAPSMIDEYPILavaaafaegaTVMNGLEELRVKESDRLSA | 360 |
| Sbjct | 301 | VADLRVR S LKGVTVPE+RAPSMIDEYPILA+AA FAEGATVM GLEELRVKESDRLSA VADLRVRHSELKGVTVPEERAPSMIDEYPILAIAACFAEGATVMKGLEELRVKESDRLSA | 360 |
| Query | 361 | VANGLKLNGVDCDEGETSLVVRGRPDGKGLGNASGAAVATHLDHRIAMSFLVMGLVSENP | 420 |
| Sbjct | 361 | VA+GLKLNG DCDEGE L+VRGRPDGKGLGNA+G V THLDHRIAMSFLV+GL SE+ VADGLKLNGADCDEGEDFLIVRGRPDGKGLGNAAGGKVRTHLDHRIAMSFLVLGLASEHA | 420 |
| Query  Sbjct | 421  421 | VTVDDATMIATSFPEFMDLMAGLGAKIEL 449 VT+DDA MI TSFPEFM LM GLGAKIEL VTIDDAAMITTSFPEFMQLMTGLGAKIEL 449 |  |

>WP_168330041.1 3-phosphoshikimate 1-carboxyvinyltransferase [Rhizobium leguminosarum]

NKM07511.1 3-phosphoshikimate 1-carboxyvinyltransferase [Rhizobium leguminosarum bv. viciae]

Length=452

Score = 763 bits (1970), Expect = 0.0, Method: Compositional matrix adjust.

| Identities | = 384/449 (86%), Positives = 416/449 (93%), Gaps = 0/449 (0%) |  |
| --- | --- | --- |
| Query 1 | MLHGASSRPATARKSSGLSGTVRIPGDKSISHRSFMFGGLASGETRITGLLEGEDVINTG | 60 |
| Sbjct 1 | MLHG++ RPATARKS+ L+G+VRIPGDKSISHRSFMFGGLASG+TRITGLLEGEDVINTG MLHGSAPRPATARKSASLNGSVRIPGDKSISHRSFMFGGLASGQTRITGLLEGEDVINTG | 60 |
| Query 61 | KAMQAMGARIRKEGDTWIIDGVGNGGLLAPEAPLDFGNAATGCRLTMGLVGVYDFDSTFI | 120 |
| Sbjct 61 | +AMQAMGARIRK+G W+IDG GNG LLAP+APLDFGNA TG RLTMGLVG YDF STF RAMQAMGARIRKDGAQWVIDGNGNGALLAPDAPLDFGNAGTGVRLTMGLVGTYDFHSTFT | 120 |
| Query 121 | GDASLTKRPMGRVLNPLREMGVQVKSEDGDRLPVTLRGPKTPTPITYRVPMASAQVKSAV | 180 |
| Sbjct 121 | GDASL+KRPMGRVL PLREMGVQV + +GDRLPVTLRGP TP+PI YRVPMASAQVKSAV GDASLSKRPMGRVLAPLREMGVQVSASEGDRLPVTLRGPATPSPIRYRVPMASAQVKSAV | 180 |
| Query 181 | LLAGLNTPGITTVIEPIMTRDHTEKMLQGFGANLTVETDADGVRTIRLEGRGKLTGQVID | 240 |
| Sbjct 181 | LLAGLNTPG+TTVIEP+MTRDHTEKMLQGFGA L+VETD++GVRTIRLEGRG+LTGQ+ID LLAGLNTPGVTTVIEPVMTRDHTEKMLQGFGAALSVETDSEGVRTIRLEGRGRLTGQMID | 240 |
| Query 241 | VPGDPSSTAFPLVAALLVPGSDVTILNVLMNPTRTGLILTLQEMGADIEVINPRLAGGED | 300 |
| Sbjct 241 | +PGDPSSTAFPLVAALLVPGSD+TI+NVLMNPTRTGLILTLQEMGADIEV+N RLAGGED IPGDPSSTAFPLVAALLVPGSDITIVNVLMNPTRTGLILTLQEMGADIEVVNARLAGGED | 300 |
| Query 301 | VADLRVRSSTLKGVTVPEDRAPSMIDEYPILavaaafaegaTVMNGLEELRVKESDRLSA | 360 |
| Sbjct 301 | VADLRVR S LKGVTVPE+RAPSMIDEYPILAVAA FAEGATVM GLEELRVKESDRLSA VADLRVRHSELKGVTVPEERAPSMIDEYPILAVAACFAEGATVMKGLEELRVKESDRLSA | 360 |
| Query 361 | VANGLKLNGVDCDEGETSLVVRGRPDGKGLGNASGAAVATHLDHRIAMSFLVMGLVSENP | 420 |
| Sbjct 361 | VA+GLKLNG DCDEGE L+VRGRPDGKGLGNA+G V THLDHRIAMSFLV+GL SE+ VADGLKLNGADCDEGEDFLIVRGRPDGKGLGNAAGGKVRTHLDHRIAMSFLVLGLASEHA | 420 |
| Query 421  Sbjct 421 | VTVDDATMIATSFPEFMDLMAGLGAKIEL 449 VT+DDA MIATSFPEFM LM GLGAKIEL VTIDDAAMIATSFPEFMQLMTGLGAKIEL 449 |  |

>WP_028743594.1 3-phosphoshikimate 1-carboxyvinyltransferase [Rhizobium leguminosarum] NEH41099.1 3-phosphoshikimate 1-carboxyvinyltransferase [Rhizobium leguminosarum] NEH74259.1 3-phosphoshikimate 1-carboxyvinyltransferase [Rhizobium leguminosarum] NEH99452.1 3-phosphoshikimate 1-carboxyvinyltransferase [Rhizobium leguminosarum] NEJ42536.1 3-phosphoshikimate 1-carboxyvinyltransferase [Rhizobium leguminosarum] NEJ48655.1 3-phosphoshikimate 1-carboxyvinyltransferase [Rhizobium leguminosarum]

Length=452

Score = 763 bits (1970), Expect = 0.0, Method: Compositional matrix adjust. Identities = 386/449 (86%), Positives = 416/449 (93%), Gaps = 0/449 (0%)

| Query | 1 | MLHGASSRPATARKSSGLSGTVRIPGDKSISHRSFMFGGLASGETRITGLLEGEDVINTG | 60 |
| --- | --- | --- | --- |
| Sbjct | 1 | ML+G++ +PATARKS+GL+G+VRIPGDKSISHRSFMFGGLASGETRITGLLEGEDVINTG MLNGSAPKPATARKSAGLTGSVRIPGDKSISHRSFMFGGLASGETRITGLLEGEDVINTG | 60 |
| Query | 61 | KAMQAMGARIRKEGDTWIIDGVGNGGLLAPEAPLDFGNAATGCRLTMGLVGVYDFDSTFI | 120 |
| Sbjct | 61 | +AMQAMGARIRK+G W+I+G GNG LLAP+APLDFGNA TG RLTMGLVG YDF STF RAMQAMGARIRKDGAQWVIEGTGNGALLAPDAPLDFGNAGTGVRLTMGLVGTYDFHSTFT | 120 |
| Query | 121 | GDASLTKRPMGRVLNPLREMGVQVKSEDGDRLPVTLRGPKTPTPITYRVPMASAQVKSAV | 180 |
| Sbjct | 121 | GDASL+KRPMGRVL PLREMGVQV + +GDRLPVTLRGP TP+PI YRVPMASAQVKSAV GDASLSKRPMGRVLAPLREMGVQVSASEGDRLPVTLRGPGTPSPIRYRVPMASAQVKSAV | 180 |
| Query | 181 | LLAGLNTPGITTVIEPIMTRDHTEKMLQGFGANLTVETDADGVRTIRLEGRGKLTGQVID | 240 |
| Sbjct | 181 | LLAGLNTPG+TTVIEP+MTRDHTEKMLQGFGA L+VETD DGVRTIRLEGRGKL GQVID LLAGLNTPGVTTVIEPVMTRDHTEKMLQGFGAALSVETDGDGVRTIRLEGRGKLAGQVID | 240 |
| Query | 241 | VPGDPSSTAFPLVAALLVPGSDVTILNVLMNPTRTGLILTLQEMGADIEVINPRLAGGED | 300 |
| Sbjct | 241 | VPGDPSSTAFPLVAAL+VPGSD+TI+NVLMNPTRTGLILTLQEMGADIEV+N RLAGGED VPGDPSSTAFPLVAALIVPGSDITIVNVLMNPTRTGLILTLQEMGADIEVVNARLAGGED | 300 |
| Query | 301 | VADLRVRSSTLKGVTVPEDRAPSMIDEYPILavaaafaegaTVMNGLEELRVKESDRLSA | 360 |
| Sbjct | 301 | VADLRVR S LKGVTVPEDRAPSMIDEYPILAVAA FAEGATVM GLEELRVKESDRLSA VADLRVRHSELKGVTVPEDRAPSMIDEYPILAVAACFAEGATVMKGLEELRVKESDRLSA | 360 |
| Query | 361 | VANGLKLNGVDCDEGETSLVVRGRPDGKGLGNASGAAVATHLDHRIAMSFLVMGLVSENP | 420 |
| Sbjct | 361 | VA+GLKLNGVDCDEGE L+VRGRPDGKGLGNA+ V+THLDHRIAMSFLV+GL SE+ VADGLKLNGVDCDEGEDFLIVRGRPDGKGLGNAADGRVSTHLDHRIAMSFLVLGLASEHA | 420 |
| Query  Sbjct | 421  421 | VTVDDATMIATSFPEFMDLMAGLGAKIEL 449 VT+DDA MIATSFPEFM LM GLGAKIEL VTIDDAAMIATSFPEFMQLMTGLGAKIEL 449 |  |

>WP_164580775.1 3-phosphoshikimate 1-carboxyvinyltransferase [Rhizobium leguminosarum] NEI57369.1 3-phosphoshikimate 1-carboxyvinyltransferase [Rhizobium leguminosarum] NEI86229.1 3-phosphoshikimate 1-carboxyvinyltransferase [Rhizobium leguminosarum]

Length=452

Score = 763 bits (1970), Expect = 0.0, Method: Compositional matrix adjust.

| Identities | = 382/449 (85%), Positives = 417/449 (93%), Gaps = 0/449 (0%) |  |
| --- | --- | --- |
| Query 1 | MLHGASSRPATARKSSGLSGTVRIPGDKSISHRSFMFGGLASGETRITGLLEGEDVINTG | 60 |
| Sbjct 1 | ML+G++ +PATARKS+GL+G+VRIPGDKSISHRSFMFGGLASGETRITGLLEGEDVINTG MLNGSAPKPATARKSAGLTGSVRIPGDKSISHRSFMFGGLASGETRITGLLEGEDVINTG | 60 |
| Query 61 | KAMQAMGARIRKEGDTWIIDGVGNGGLLAPEAPLDFGNAATGCRLTMGLVGVYDFDSTFI | 120 |
| Sbjct 61 | +AMQAMGARIRKEG W+I+G GNG LLAP+APLDFGNA TG RLTMGLVG YDF STF RAMQAMGARIRKEGAQWVIEGTGNGALLAPDAPLDFGNAGTGVRLTMGLVGTYDFHSTFT | 120 |
| Query 121 | GDASLTKRPMGRVLNPLREMGVQVKSEDGDRLPVTLRGPKTPTPITYRVPMASAQVKSAV | 180 |
| Sbjct 121 | GDASL+KRPMGRVLNPLREMGVQ+ + +GDRLPVTLRGP TP+PI YRVPMASAQVKSAV GDASLSKRPMGRVLNPLREMGVQISASEGDRLPVTLRGPGTPSPIRYRVPMASAQVKSAV | 180 |
| Query 181 | LLAGLNTPGITTVIEPIMTRDHTEKMLQGFGANLTVETDADGVRTIRLEGRGKLTGQVID | 240 |
| Sbjct 181 | LLAGLNTPG+TTVIEP+MTRDHTE+MLQGFGA L+VETD++GVRTIRLEGRGKL GQVID LLAGLNTPGVTTVIEPVMTRDHTERMLQGFGAALSVETDSEGVRTIRLEGRGKLAGQVID | 240 |
| Query 241 | VPGDPSSTAFPLVAALLVPGSDVTILNVLMNPTRTGLILTLQEMGADIEVINPRLAGGED | 300 |
| Sbjct 241 | VPGDPSSTAFPLVAAL+VPGSD+TI+NVLMNPTRTGLILTLQEMGADIEV+N RLAGGED VPGDPSSTAFPLVAALIVPGSDITIVNVLMNPTRTGLILTLQEMGADIEVVNARLAGGED | 300 |
| Query 301 | VADLRVRSSTLKGVTVPEDRAPSMIDEYPILavaaafaegaTVMNGLEELRVKESDRLSA | 360 |
| Sbjct 301 | VADLRVR S L+GVTVPEDRAPSMIDEYPILAVAA FAEGAT+M GLEELRVKESDRLSA VADLRVRHSELRGVTVPEDRAPSMIDEYPILAVAACFAEGATIMKGLEELRVKESDRLSA | 360 |
| Query 361 | VANGLKLNGVDCDEGETSLVVRGRPDGKGLGNASGAAVATHLDHRIAMSFLVMGLVSENP | 420 |
| Sbjct 361 | VA+GLKLNGVDCDEGE L+VRGRPDG+ LGNA+G V THLDHRIAMSFLV+GL SE+ VADGLKLNGVDCDEGEDFLIVRGRPDGRSLGNAAGGQVRTHLDHRIAMSFLVLGLASEHA | 420 |
| Query 421  Sbjct 421 | VTVDDATMIATSFPEFMDLMAGLGAKIEL 449 VT+DDA MIATSFPEFM LM GLGAKIEL VTIDDAAMIATSFPEFMQLMTGLGAKIEL 449 |  |

>WP_130786092.1 3-phosphoshikimate 1-carboxyvinyltransferase [Rhizobium leguminosarum] NEI50939.1 3-phosphoshikimate 1-carboxyvinyltransferase [Rhizobium leguminosarum] TBD02317.1 3-phosphoshikimate 1-carboxyvinyltransferase [Rhizobium leguminosarum] TBD18461.1 3-phosphoshikimate 1-carboxyvinyltransferase [Rhizobium leguminosarum] TBE35601.1 3-phosphoshikimate 1-carboxyvinyltransferase [Rhizobium leguminosarum] TBE99729.1 3-phosphoshikimate 1-carboxyvinyltransferase [Rhizobium leguminosarum]

Length=452

Score = 763 bits (1970), Expect = 0.0, Method: Compositional matrix adjust.

| Identities | = 385/449 (86%), Positives = 413/449 (92%), Gaps = 0/449 (0%) |  |
| --- | --- | --- |
| Query 1 | MLHGASSRPATARKSSGLSGTVRIPGDKSISHRSFMFGGLASGETRITGLLEGEDVINTG | 60 |
| Sbjct 1 | MLHG++ RPATARKS+ L+G+V IPGDKSISHRSFMFGGLASGETRITGLLE EDVINTG MLHGSAPRPATARKSASLTGSVCIPGDKSISHRSFMFGGLASGETRITGLLESEDVINTG | 60 |
| Query 61 | KAMQAMGARIRKEGDTWIIDGVGNGGLLAPEAPLDFGNAATGCRLTMGLVGVYDFDSTFI | 120 |
| Sbjct 61 | +AMQAMGARIRKEG W+I+G GNG LLAP+APLDFGNA TG RLTMGLVG YDF STF RAMQAMGARIRKEGAQWVIEGTGNGALLAPDAPLDFGNAGTGVRLTMGLVGTYDFHSTFT | 120 |
| Query 121 | GDASLTKRPMGRVLNPLREMGVQVKSEDGDRLPVTLRGPKTPTPITYRVPMASAQVKSAV | 180 |
| Sbjct 121 | GDASL+KRPMGRVL PLREMGVQV + +GDRLPVTLRGP TP+PI YRVPMASAQVKSAV GDASLSKRPMGRVLAPLREMGVQVSASEGDRLPVTLRGPGTPSPIRYRVPMASAQVKSAV | 180 |
| Query 181 | LLAGLNTPGITTVIEPIMTRDHTEKMLQGFGANLTVETDADGVRTIRLEGRGKLTGQVID | 240 |
| Sbjct 181 | LLAGLNTPG+TTVIEP+MTRDHTEKMLQGFGA L+VETD DGVRTIRLEGRGKL GQVID LLAGLNTPGVTTVIEPVMTRDHTEKMLQGFGAALSVETDGDGVRTIRLEGRGKLAGQVID | 240 |
| Query 241 | VPGDPSSTAFPLVAALLVPGSDVTILNVLMNPTRTGLILTLQEMGADIEVINPRLAGGED | 300 |
| Sbjct 241 | +PGDPSSTAFPLVAALLVPGSD+TI+NVLMNPTRTGLILTLQEMGADIEV+N RLAGGED IPGDPSSTAFPLVAALLVPGSDITIVNVLMNPTRTGLILTLQEMGADIEVVNARLAGGED | 300 |
| Query 301 | VADLRVRSSTLKGVTVPEDRAPSMIDEYPILavaaafaegaTVMNGLEELRVKESDRLSA | 360 |
| Sbjct 301 | VADLRVR S LKGVTVPEDRAPSMIDEYPILA+AA FAEGATVM GLEELRVKESDRLSA VADLRVRHSELKGVTVPEDRAPSMIDEYPILAIAACFAEGATVMKGLEELRVKESDRLSA | 360 |
| Query 361 | VANGLKLNGVDCDEGETSLVVRGRPDGKGLGNASGAAVATHLDHRIAMSFLVMGLVSENP | 420 |
| Sbjct 361 | VA+GLKLNGV+CDEGE L+VRGRPDGKGLGNA+G V THLDHRIAMSFLV+GL SE+ VADGLKLNGVNCDEGEDFLIVRGRPDGKGLGNAAGGQVRTHLDHRIAMSFLVLGLASEHA | 420 |
| Query 421  Sbjct 421 | VTVDDATMIATSFPEFMDLMAGLGAKIEL 449 VT+DDA MIATSFPEFM LM GLGAKIEL VTIDDAAMIATSFPEFMQLMTGLGAKIEL 449 |  |

>WP_018483104.1 3-phosphoshikimate 1-carboxyvinyltransferase [Rhizobium leguminosarum] NEJ24653.1 3-phosphoshikimate 1-carboxyvinyltransferase [Rhizobium leguminosarum]

NKK58978.1 3-phosphoshikimate 1-carboxyvinyltransferase [Rhizobium leguminosarum bv. viciae]

Length=452

Score = 763 bits (1970), Expect = 0.0, Method: Compositional matrix adjust.

| Identities | = 386/449 (86%), Positives = 412/449 (92%), Gaps = 0/449 (0%) |  |
| --- | --- | --- |
| Query 1 | MLHGASSRPATARKSSGLSGTVRIPGDKSISHRSFMFGGLASGETRITGLLEGEDVINTG | 60 |
| Sbjct 1 | MLHG++ RPATARKS+ L+G+VRIPGDKSISHRSFMFGGLASGETRITGLLEGEDVINTG MLHGSAPRPATARKSASLTGSVRIPGDKSISHRSFMFGGLASGETRITGLLEGEDVINTG | 60 |
| Query 61 | KAMQAMGARIRKEGDTWIIDGVGNGGLLAPEAPLDFGNAATGCRLTMGLVGVYDFDSTFI | 120 |
| Sbjct 61 | +AM AMGARIRKEG W+I+G GNG LLAP+APLDFGNA TG RLTMGLVG YDF STF RAMHAMGARIRKEGAQWVIEGTGNGALLAPDAPLDFGNAGTGVRLTMGLVGTYDFHSTFT | 120 |
| Query 121 | GDASLTKRPMGRVLNPLREMGVQVKSEDGDRLPVTLRGPKTPTPITYRVPMASAQVKSAV | 180 |
| Sbjct 121 | GDASL+KRPMGRVL PLREMGVQV + +GDRLPVTLRGP TP+PI YRVPM SAQVKSAV GDASLSKRPMGRVLAPLREMGVQVSASEGDRLPVTLRGPGTPSPIRYRVPMVSAQVKSAV | 180 |
| Query 181 | LLAGLNTPGITTVIEPIMTRDHTEKMLQGFGANLTVETDADGVRTIRLEGRGKLTGQVID | 240 |
| Sbjct 181 | LLAGLNTPG+TTVIEP+MTRDHTEKMLQGFGA L+VET DGVRTIRLEGRGKL GQVID LLAGLNTPGVTTVIEPVMTRDHTEKMLQGFGAALSVETAGDGVRTIRLEGRGKLAGQVID | 240 |
| Query 241 | VPGDPSSTAFPLVAALLVPGSDVTILNVLMNPTRTGLILTLQEMGADIEVINPRLAGGED | 300 |

| Sbjct | 241 | VPGDPSSTAFPLVAALLVPGSD+TI+NVLMNPTRTGLILTLQEMGADIEV+N RLAGGED VPGDPSSTAFPLVAALLVPGSDITIVNVLMNPTRTGLILTLQEMGADIEVVNARLAGGED | 300 |
| --- | --- | --- | --- |
| Query | 301 | VADLRVRSSTLKGVTVPEDRAPSMIDEYPILavaaafaegaTVMNGLEELRVKESDRLSA | 360 |
| Sbjct | 301 | VADLRVR S LKGVTVPEDRAPSMIDEYPILA+AA FAEGATVM GLEELRVKESDRLSA VADLRVRHSELKGVTVPEDRAPSMIDEYPILAIAACFAEGATVMKGLEELRVKESDRLSA | 360 |
| Query | 361 | VANGLKLNGVDCDEGETSLVVRGRPDGKGLGNASGAAVATHLDHRIAMSFLVMGLVSENP | 420 |
| Sbjct | 361 | VA+GLKLNGVDCDEGE L+VRGRPDGKGLGNA+G V THLDHRIAMSFLV+GL SE+ VADGLKLNGVDCDEGEDFLIVRGRPDGKGLGNAAGGQVRTHLDHRIAMSFLVLGLASEHA | 420 |
| Query  Sbjct | 421  421 | VTVDDATMIATSFPEFMDLMAGLGAKIEL 449 VT+DDA MIATSFPEFM LM GLGAKIEL VTIDDAAMIATSFPEFMQLMTGLGAKIEL 449 |  |

>WP_182209093.1 3-phosphoshikimate 1-carboxyvinyltransferase [Rhizobium sp. WYCCWR 11317]

MBA5802298.1 3-phosphoshikimate 1-carboxyvinyltransferase [Rhizobium sp. WYCCWR 11317]

Length=452

Score = 763 bits (1970), Expect = 0.0, Method: Compositional matrix adjust.

| Identities | = 385/448 (86%), Positives = 415/448 (93%), Gaps = 0/448 (0%) |  |
| --- | --- | --- |
| Query 1 | MLHGASSRPATARKSSGLSGTVRIPGDKSISHRSFMFGGLASGETRITGLLEGEDVINTG | 60 |
| Sbjct 1 | MLHG++ RPATARKS+GL+G+VRIPGDKSISHRSFMFGGLASG+TRITGLLEGEDVINTG MLHGSAPRPATARKSAGLTGSVRIPGDKSISHRSFMFGGLASGQTRITGLLEGEDVINTG | 60 |
| Query 61 | KAMQAMGARIRKEGDTWIIDGVGNGGLLAPEAPLDFGNAATGCRLTMGLVGVYDFDSTFI | 120 |
| Sbjct 61 | +AMQAMGARIRKEG W+IDG GNG LLAP+APLDFGNA TG RLTMGLVG YDF S F RAMQAMGARIRKEGAQWVIDGTGNGALLAPDAPLDFGNAGTGVRLTMGLVGTYDFHSIFT | 120 |
| Query 121 | GDASLTKRPMGRVLNPLREMGVQVKSEDGDRLPVTLRGPKTPTPITYRVPMASAQVKSAV | 180 |
| Sbjct 121 | GDASL+KRPMGRVLNPLREMGVQV + +GDRLPVTLRGP TP+PI YRVPMASAQVKSAV GDASLSKRPMGRVLNPLREMGVQVSASEGDRLPVTLRGPGTPSPIRYRVPMASAQVKSAV | 180 |
| Query 181 | LLAGLNTPGITTVIEPIMTRDHTEKMLQGFGANLTVETDADGVRTIRLEGRGKLTGQVID | 240 |
| Sbjct 181 | LLAGLNTPG+TTVIEP+MTRDHTEKMLQGFGA L+VETD++GVRTIRLEGRGKLTGQVID LLAGLNTPGVTTVIEPVMTRDHTEKMLQGFGAALSVETDSEGVRTIRLEGRGKLTGQVID | 240 |
| Query 241 | VPGDPSSTAFPLVAALLVPGSDVTILNVLMNPTRTGLILTLQEMGADIEVINPRLAGGED | 300 |
| Sbjct 241 | VPGDPSSTAFPLVAALLVPGSD+TI+NVLMNPTRTGLILTLQEMGADIEV+N RLAGGED VPGDPSSTAFPLVAALLVPGSDITIVNVLMNPTRTGLILTLQEMGADIEVLNARLAGGED | 300 |
| Query 301 | VADLRVRSSTLKGVTVPEDRAPSMIDEYPILavaaafaegaTVMNGLEELRVKESDRLSA | 360 |
| Sbjct 301 | VADLRVR S L+GVTVPEDRAPSMIDEYPILAVAA FAEGAT+M GLEELRVKESDRLSA VADLRVRHSELRGVTVPEDRAPSMIDEYPILAVAACFAEGATIMKGLEELRVKESDRLSA | 360 |
| Query 361 | VANGLKLNGVDCDEGETSLVVRGRPDGKGLGNASGAAVATHLDHRIAMSFLVMGLVSENP | 420 |
| Sbjct 361 | VA+GLKLNG DCDEG L+VRGRPDGKGLGNA+ V+THLDHRIAMSFLVMGL SE+ VADGLKLNGADCDEGGDFLIVRGRPDGKGLGNAADGRVSTHLDHRIAMSFLVMGLASEHA | 420 |
| Query 421  Sbjct 421 | VTVDDATMIATSFPEFMDLMAGLGAKIE 448 VT+DDA MIATSFPEFM LM GLGA+IE VTIDDAAMIATSFPEFMTLMKGLGARIE 448 |  |

>WP_142589029.1 3-phosphoshikimate 1-carboxyvinyltransferase [Rhizobium halotolerans] MBB5249210.1 3-phosphoshikimate 1-carboxyvinyltransferase [Rhizobium halotolerans] CAD6434158.1 3-phosphoshikimate 1-carboxyvinyltransferase [Rhizobium halotolerans]

Length=455

Score = 763 bits (1969), Expect = 0.0, Method: Compositional matrix adjust. Identities = 391/455 (86%), Positives = 419/455 (92%), Gaps = 0/455 (0%)

| Query | 1 | MLHGASSRPATARKSSGLSGTVRIPGDKSISHRSFMFGGLASGETRITGLLEGEDVINTG | 60 |
| --- | --- | --- | --- |
| Sbjct | 1 | M HG + +PA + +S+ L GTVRIPGDKSISHRSFMFGGLA+GETRITGLLEGEDVINTG MSHGVALKPARSLQSADLKGTVRIPGDKSISHRSFMFGGLAAGETRITGLLEGEDVINTG | 60 |
| Query | 61 | KAMQAMGARIRKEGDTWIIDGVGNGGLLAPEAPLDFGNAATGCRLTMGLVGVYDFDSTFI KAM+AMGA+I K GD WII G GNG LLAPEAPLDFGNA TGCRLTMGLVGVYDF+STFI | 120 |

| Sbjct | 61 | KAMRAMGAKIDKVGDEWIIQGTGNGALLAPEAPLDFGNAGTGCRLTMGLVGVYDFESTFI | 120 |
| --- | --- | --- | --- |
| Query | 121 | GDASLTKRPMGRVLNPLREMGVQVKSEDGDRLPVTLRGPKTPTPITYRVPMASAQVKSAV | 180 |
| Sbjct | 121 | GDASLTKRPMGRVLNPLREMGVQVK+ +GDRLPVTLRGP P PITYRVPMASAQVKSAV GDASLTKRPMGRVLNPLREMGVQVKAAEGDRLPVTLRGPSIPNPITYRVPMASAQVKSAV | 180 |
| Query | 181 | LLAGLNTPGITTVIEPIMTRDHTEKMLQGFGANLTVETDADGVRTIRLEGRGKLTGQVID | 240 |
| Sbjct | 181 | LLAGLNTPG+TTVIEP+MTRDHTEKMLQGFGANL VETDADG+RTIRLEGRG+L GQVID LLAGLNTPGVTTVIEPVMTRDHTEKMLQGFGANLMVETDADGLRTIRLEGRGQLIGQVID | 240 |
| Query | 241 | VPGDPSSTAFPLVAALLVPGSDVTILNVLMNPTRTGLILTLQEMGADIEVINPRLAGGED | 300 |
| Sbjct | 241 | VPGDPSSTAFPLVAALL PGSDVTI NVLMNPTRTGLILTLQEMGADIEV+NPRLAGGED VPGDPSSTAFPLVAALLAPGSDVTIENVLMNPTRTGLILTLQEMGADIEVVNPRLAGGED | 300 |
| Query | 301 | VADLRVRSSTLKGVTVPEDRAPSMIDEYPILavaaafaegaTVMNGLEELRVKESDRLSA | 360 |
| Sbjct | 301 | VADLRVR S LKGVTVP DRAPSMIDEYP+LAVAAAFAEGAT M GLEELRVKESDRLSA VADLRVRHSELKGVTVPADRAPSMIDEYPVLAVAAAFAEGATTMLGLEELRVKESDRLSA | 360 |
| Query | 361 | VANGLKLNGVDCDEGETSLVVRGRPDGKGLGNASGAAVATHLDHRIAMSFLVMGLVSENP | 420 |
| Sbjct | 361 | VA+GLKLNGVDCDEGE +LVVRGRP GKG GNA+G++V THLDHRIAMSFLVMGLVSE+ VADGLKLNGVDCDEGEDTLVVRGRPGGKGYGNATGSSVVTHLDHRIAMSFLVMGLVSEHA | 420 |
| Query  Sbjct | 421  421 | VTVDDATMIATSFPEFMDLMAGLGAKIELSDTKAA 455 VTVDDA +IATSFPEFMDLM GLGA+IE +++AA VTVDDAGIIATSFPEFMDLMTGLGARIEPVESRAA 455 |  |

>WP_127431863.1 3-phosphoshikimate 1-carboxyvinyltransferase [Rhizobium anhuiense] RUL95706.1 3-phosphoshikimate 1-carboxyvinyltransferase [Rhizobium anhuiense] GGE13066.1 3-phosphoshikimate 1-carboxyvinyltransferase [Rhizobium anhuiense]

Length=452

Score = 763 bits (1969), Expect = 0.0, Method: Compositional matrix adjust.

| Identities | = 383/448 (85%), Positives = 416/448 (93%), Gaps = 0/448 (0%) |  |
| --- | --- | --- |
| Query 1 | MLHGASSRPATARKSSGLSGTVRIPGDKSISHRSFMFGGLASGETRITGLLEGEDVINTG | 60 |
| Sbjct 1 | MLHG++ RPATARKS+GL+G VRIPGDKSISHRSFMFGGLASG+TRITGLLEGEDVINTG MLHGSAPRPATARKSAGLTGRVRIPGDKSISHRSFMFGGLASGQTRITGLLEGEDVINTG | 60 |
| Query 61 | KAMQAMGARIRKEGDTWIIDGVGNGGLLAPEAPLDFGNAATGCRLTMGLVGVYDFDSTFI | 120 |
| Sbjct 61 | +AMQAMGARIRK+G+ W+I+G GNG LLAP+APLDFGNA TG RLTMGLVG YDF STFI RAMQAMGARIRKQGEEWVIEGTGNGALLAPDAPLDFGNAGTGVRLTMGLVGTYDFHSTFI | 120 |
| Query 121 | GDASLTKRPMGRVLNPLREMGVQVKSEDGDRLPVTLRGPKTPTPITYRVPMASAQVKSAV | 180 |
| Sbjct 121 | GDASL+KRPMGRVLNPLR+MGVQV + GDRLPVTLRGP +P+PI YRVPMASAQVKSAV GDASLSKRPMGRVLNPLRDMGVQVSASAGDRLPVTLRGPGSPSPIRYRVPMASAQVKSAV | 180 |
| Query 181 | LLAGLNTPGITTVIEPIMTRDHTEKMLQGFGANLTVETDADGVRTIRLEGRGKLTGQVID | 240 |
| Sbjct 181 | LLAGLNTPG TTVIEP+MTRDHTEKMLQGFGA L VETDADG+RTIRLEGRG+LTGQVID LLAGLNTPGTTTVIEPVMTRDHTEKMLQGFGAALAVETDADGIRTIRLEGRGRLTGQVID | 240 |
| Query 241 | VPGDPSSTAFPLVAALLVPGSDVTILNVLMNPTRTGLILTLQEMGADIEVINPRLAGGED | 300 |
| Sbjct 241 | VPGDPSSTAFPLVAAL+VPGSD+TI+NVLMNPTRTGLILTLQEMGADIEV+N RLAGGED VPGDPSSTAFPLVAALIVPGSDITIVNVLMNPTRTGLILTLQEMGADIEVLNARLAGGED | 300 |
| Query 301 | VADLRVRSSTLKGVTVPEDRAPSMIDEYPILavaaafaegaTVMNGLEELRVKESDRLSA | 360 |
| Sbjct 301 | VADLRVR S LKGV VPE+RAPSMIDEYPILAVAA FAEG TVM GLEELRVKESDRLSA VADLRVRHSELKGVAVPEERAPSMIDEYPILAVAACFAEGTTVMKGLEELRVKESDRLSA | 360 |
| Query 361 | VANGLKLNGVDCDEGETSLVVRGRPDGKGLGNASGAAVATHLDHRIAMSFLVMGLVSENP | 420 |
| Sbjct 361 | VA+GLKLNGVDCDEG+ L+VRGRPDGKGLG+A G+ V+THLDHRIAMSFLV+GL SE+ VADGLKLNGVDCDEGKDFLIVRGRPDGKGLGSAGGSRVSTHLDHRIAMSFLVLGLASEHA | 420 |
| Query 421  Sbjct 421 | VTVDDATMIATSFPEFMDLMAGLGAKIE 448 VT+DDA MIATSFPEFM LMAGLGAKIE VTIDDAAMIATSFPEFMQLMAGLGAKIE 448 |  |

>WP_183904688.1 3-phosphoshikimate 1-carboxyvinyltransferase [Rhizobium sp. BK212] MBB4219117.1 3-phosphoshikimate 1-carboxyvinyltransferase [Rhizobium sp. BK212]

Length=452

Score = 763 bits (1969), Expect = 0.0, Method: Compositional matrix adjust.

| Identities | = 385/448 (86%), Positives = 416/448 (93%), Gaps = 0/448 (0%) |  |
| --- | --- | --- |
| Query 1 | MLHGASSRPATARKSSGLSGTVRIPGDKSISHRSFMFGGLASGETRITGLLEGEDVINTG | 60 |
| Sbjct 1 | MLHG++ RPATARKS+GLSG VRIPGDKSISHRSFMFGGLASG+TRITGLLEGEDVINTG MLHGSAPRPATARKSAGLSGRVRIPGDKSISHRSFMFGGLASGQTRITGLLEGEDVINTG | 60 |
| Query 61 | KAMQAMGARIRKEGDTWIIDGVGNGGLLAPEAPLDFGNAATGCRLTMGLVGVYDFDSTFI | 120 |
| Sbjct 61 | +AMQAMGARIRK+G+ W+I+G GNG LLAP+APLDFGNA TG RLTMGLVG YDF STFI RAMQAMGARIRKQGEEWVIEGTGNGALLAPDAPLDFGNAGTGVRLTMGLVGTYDFHSTFI | 120 |
| Query 121 | GDASLTKRPMGRVLNPLREMGVQVKSEDGDRLPVTLRGPKTPTPITYRVPMASAQVKSAV | 180 |
| Sbjct 121 | GDASL+KRPMGRVLNPLR+MGVQV + GDRLPVTLRGP TP+PI YRVPMASAQVKSAV GDASLSKRPMGRVLNPLRDMGVQVSASAGDRLPVTLRGPGTPSPIRYRVPMASAQVKSAV | 180 |
| Query 181 | LLAGLNTPGITTVIEPIMTRDHTEKMLQGFGANLTVETDADGVRTIRLEGRGKLTGQVID | 240 |
| Sbjct 181 | LLAGLNTPGITTVIEP+MTRDHTEKMLQGFGA L VETDADG+RTIRLEGRG+LTGQVID LLAGLNTPGITTVIEPVMTRDHTEKMLQGFGAALAVETDADGIRTIRLEGRGRLTGQVID | 240 |
| Query 241 | VPGDPSSTAFPLVAALLVPGSDVTILNVLMNPTRTGLILTLQEMGADIEVINPRLAGGED | 300 |
| Sbjct 241 | VPGDPSSTAFPLVAAL+VPGSD+TI+NVLMNPTRTGLILTLQEMGADIEV+N RLAGGED VPGDPSSTAFPLVAALIVPGSDITIVNVLMNPTRTGLILTLQEMGADIEVLNARLAGGED | 300 |
| Query 301 | VADLRVRSSTLKGVTVPEDRAPSMIDEYPILavaaafaegaTVMNGLEELRVKESDRLSA | 360 |
| Sbjct 301 | VADLRVR S LKGV VPE+RAPSMIDEYPILAVAA FAEG TVM GLEELRVKESDRLSA VADLRVRHSELKGVAVPEERAPSMIDEYPILAVAACFAEGTTVMKGLEELRVKESDRLSA | 360 |
| Query 361 | VANGLKLNGVDCDEGETSLVVRGRPDGKGLGNASGAAVATHLDHRIAMSFLVMGLVSENP | 420 |
| Sbjct 361 | VA+GLKLNGVD DEG+ L+VRGRPDGKGLG+A G+ V+THLDHRIAMSFLV+GL SE+ VADGLKLNGVDRDEGKDFLIVRGRPDGKGLGSAGGSKVSTHLDHRIAMSFLVLGLASEHA | 420 |
| Query 421  Sbjct 421 | VTVDDATMIATSFPEFMDLMAGLGAKIE 448 VT+DDA MIATSFPEFM LMAGLGAKIE VTIDDAAMIATSFPEFMQLMAGLGAKIE 448 |  |

>WP_130697986.1 3-phosphoshikimate 1-carboxyvinyltransferase [Rhizobium leguminosarum] NEJ98090.1 3-phosphoshikimate 1-carboxyvinyltransferase [Rhizobium leguminosarum] TAT80922.1 3-phosphoshikimate 1-carboxyvinyltransferase [Rhizobium leguminosarum] TAT90892.1 3-phosphoshikimate 1-carboxyvinyltransferase [Rhizobium leguminosarum] TAZ75392.1 3-phosphoshikimate 1-carboxyvinyltransferase [Rhizobium leguminosarum] TBA02369.1 3-phosphoshikimate 1-carboxyvinyltransferase [Rhizobium leguminosarum]

Length=452

Score = 763 bits (1969), Expect = 0.0, Method: Compositional matrix adjust.

| Identities | = 385/449 (86%), Positives = 416/449 (93%), Gaps = 0/449 (0%) |  |
| --- | --- | --- |
| Query 1 | MLHGASSRPATARKSSGLSGTVRIPGDKSISHRSFMFGGLASGETRITGLLEGEDVINTG | 60 |
| Sbjct 1 | ML+G++ +P TARKS+GL+G+VRIPGDKSISHRSFMFGGLASGETRITGLLEGEDVINTG MLNGSAPKPVTARKSAGLTGSVRIPGDKSISHRSFMFGGLASGETRITGLLEGEDVINTG | 60 |
| Query 61 | KAMQAMGARIRKEGDTWIIDGVGNGGLLAPEAPLDFGNAATGCRLTMGLVGVYDFDSTFI | 120 |
| Sbjct 61 | +AMQAMGARIRKEG W+I+G GNG LLAP+APLDFGNA TG RLTMGLVG YDF STF RAMQAMGARIRKEGAQWVIEGTGNGALLAPDAPLDFGNAGTGVRLTMGLVGTYDFRSTFT | 120 |
| Query 121 | GDASLTKRPMGRVLNPLREMGVQVKSEDGDRLPVTLRGPKTPTPITYRVPMASAQVKSAV | 180 |
| Sbjct 121 | GDASL+KRPMGRVL PLREMGVQV + +G+RLPVTLRGP TP+PI YRVPMASAQVKSAV GDASLSKRPMGRVLAPLREMGVQVSASEGNRLPVTLRGPGTPSPIRYRVPMASAQVKSAV | 180 |
| Query 181 | LLAGLNTPGITTVIEPIMTRDHTEKMLQGFGANLTVETDADGVRTIRLEGRGKLTGQVID | 240 |
| Sbjct 181 | LLAGLNTPG+TTVIEP+MTRDHTEKMLQGFGA L+VETD++GVRTIRLEGRG+L GQVID LLAGLNTPGVTTVIEPVMTRDHTEKMLQGFGAALSVETDSEGVRTIRLEGRGRLMGQVID | 240 |
| Query 241 | VPGDPSSTAFPLVAALLVPGSDVTILNVLMNPTRTGLILTLQEMGADIEVINPRLAGGED | 300 |
| Sbjct 241 | VPGDPSSTAFPLVAALLVPGSD+TI+NVLMNPTRTGLILTLQEMGADIEV+N RLAGGED VPGDPSSTAFPLVAALLVPGSDITIVNVLMNPTRTGLILTLQEMGADIEVVNARLAGGED | 300 |
| Query 301 | VADLRVRSSTLKGVTVPEDRAPSMIDEYPILavaaafaegaTVMNGLEELRVKESDRLSA | 360 |
| Sbjct 301 | VADLRVR S LKGVTVPEDRAPSMIDEYPILAVAA FAEG TVM GLEELRVKESDRLSA VADLRVRHSELKGVTVPEDRAPSMIDEYPILAVAACFAEGTTVMKGLEELRVKESDRLSA | 360 |
| Query 361 | VANGLKLNGVDCDEGETSLVVRGRPDGKGLGNASGAAVATHLDHRIAMSFLVMGLVSENP | 420 |

| Sbjct | 361 | VA+GLKLNGVDCDEGE LVVRGRPDGKGLGNA+G V+THLDHRIAMSFLV+GL SE+ VADGLKLNGVDCDEGEDFLVVRGRPDGKGLGNAAGGRVSTHLDHRIAMSFLVLGLASEHA 420 |
| --- | --- | --- |
| Query | 421 | VTVDDATMIATSFPEFMDLMAGLGAKIEL 449 |
| Sbjct | 421 | VT+DDA MIATSFPEFM LM GLGAKIEL  VTIDDAAMIATSFPEFMQLMTGLGAKIEL 449 |

>WP_077548496.1 3-phosphoshikimate 1-carboxyvinyltransferase [Rhizobium flavum] MBB6180426.1 3-phosphoshikimate 1-carboxyvinyltransferase [Rhizobium flavum] CAD6595423.1 3-phosphoshikimate 1-carboxyvinyltransferase [Rhizobium flavum]

Length=455

Score = 762 bits (1968), Expect = 0.0, Method: Compositional matrix adjust.

| Identities | = 392/455 (86%), Positives = 417/455 (92%), Gaps = 0/455 (0%) |  |
| --- | --- | --- |
| Query 1 | MLHGASSRPATARKSSGLSGTVRIPGDKSISHRSFMFGGLASGETRITGLLEGEDVINTG | 60 |
| Sbjct 1 | M HG + +PA + KS+ L GTVRIPGDKSISHRSFMFGGLA+GETRITGLLEGEDVINTG MSHGVALKPARSLKSADLRGTVRIPGDKSISHRSFMFGGLAAGETRITGLLEGEDVINTG | 60 |
| Query 61 | KAMQAMGARIRKEGDTWIIDGVGNGGLLAPEAPLDFGNAATGCRLTMGLVGVYDFDSTFI | 120 |
| Sbjct 61 | KAM+AMGA I K GD WII G GNG LLAPEAPLDFGNA TGCRLTMGLVGVYDF+STFI KAMRAMGASIEKVGDEWIIQGTGNGALLAPEAPLDFGNAGTGCRLTMGLVGVYDFESTFI | 120 |
| Query 121 | GDASLTKRPMGRVLNPLREMGVQVKSEDGDRLPVTLRGPKTPTPITYRVPMASAQVKSAV | 180 |
| Sbjct 121 | GDASLTKRPMGRVLNPLREMGVQVK+ +GDRLPVTLRGP P PITYRVPMASAQVKSAV GDASLTKRPMGRVLNPLREMGVQVKAAEGDRLPVTLRGPSIPNPITYRVPMASAQVKSAV | 180 |
| Query 181 | LLAGLNTPGITTVIEPIMTRDHTEKMLQGFGANLTVETDADGVRTIRLEGRGKLTGQVID | 240 |
| Sbjct 181 | LLAGLNTPG+TTVIEP+MTRDHTEKMLQGFGANL VETDADGVRTIRLEGRG+L GQVID LLAGLNTPGVTTVIEPVMTRDHTEKMLQGFGANLMVETDADGVRTIRLEGRGQLIGQVID | 240 |
| Query 241 | VPGDPSSTAFPLVAALLVPGSDVTILNVLMNPTRTGLILTLQEMGADIEVINPRLAGGED | 300 |
| Sbjct 241 | VPGDPSSTAFPLVAALLVPGSDVTI NVLMNPTRTGLILTLQEMGADIEVINPRL GGED VPGDPSSTAFPLVAALLVPGSDVTIENVLMNPTRTGLILTLQEMGADIEVINPRLTGGED | 300 |
| Query 301 | VADLRVRSSTLKGVTVPEDRAPSMIDEYPILavaaafaegaTVMNGLEELRVKESDRLSA | 360 |
| Sbjct 301 | VADLRVR S LKGVTVP DRAPSMIDEYP+LAVAAAFAEGAT M GLEELRVKESDRLSA VADLRVRHSDLKGVTVPADRAPSMIDEYPVLAVAAAFAEGATTMLGLEELRVKESDRLSA | 360 |
| Query 361 | VANGLKLNGVDCDEGETSLVVRGRPDGKGLGNASGAAVATHLDHRIAMSFLVMGLVSENP | 420 |
| Sbjct 361 | VA+GLKLNGVDCDEGE +LVVRGRP GKG GNA+G++V THLDHRIAMSFL+MGLVSE+ VADGLKLNGVDCDEGEDTLVVRGRPGGKGYGNATGSSVVTHLDHRIAMSFLIMGLVSEHA | 420 |
| Query 421  Sbjct 421 | VTVDDATMIATSFPEFMDLMAGLGAKIELSDTKAA 455 VTVDDA +I TSFPEFMDLM GLGA+IE +++AA VTVDDAGIITTSFPEFMDLMTGLGARIEPVESRAA 455 |  |

>WP_168574935.1 3-phosphoshikimate 1-carboxyvinyltransferase [Rhizobium ruizarguesonis] NKQ83259.1 3-phosphoshikimate 1-carboxyvinyltransferase [Rhizobium ruizarguesonis]

Length=452

Score = 762 bits (1968), Expect = 0.0, Method: Compositional matrix adjust.

| Identities | = 384/449 (86%), Positives = 413/449 (92%), Gaps = 0/449 (0%) |  |
| --- | --- | --- |
| Query 1 | MLHGASSRPATARKSSGLSGTVRIPGDKSISHRSFMFGGLASGETRITGLLEGEDVINTG | 60 |
| Sbjct 1 | MLHG++ RPATARKS+ L+G+VRIPGDKSISHRSFMFGGLASGETRITGLLEGEDVINTG MLHGSAPRPATARKSASLTGSVRIPGDKSISHRSFMFGGLASGETRITGLLEGEDVINTG | 60 |
| Query 61 | KAMQAMGARIRKEGDTWIIDGVGNGGLLAPEAPLDFGNAATGCRLTMGLVGVYDFDSTFI | 120 |
| Sbjct 61 | +AMQAMGARIRKEG W+I+G GNG LLAP+APLDFGNA TG RLTMGLVG YDF STF RAMQAMGARIRKEGAQWVIEGTGNGALLAPDAPLDFGNAGTGVRLTMGLVGTYDFHSTFT | 120 |
| Query 121 | GDASLTKRPMGRVLNPLREMGVQVKSEDGDRLPVTLRGPKTPTPITYRVPMASAQVKSAV | 180 |
| Sbjct 121 | GDASL+KRPMGRVL PLREMGVQV + +GDRLPVTLRGP TP+PI YRVPMASAQVKSAV GDASLSKRPMGRVLAPLREMGVQVSASEGDRLPVTLRGPGTPSPIRYRVPMASAQVKSAV | 180 |
| Query 181 | LLAGLNTPGITTVIEPIMTRDHTEKMLQGFGANLTVETDADGVRTIRLEGRGKLTGQVID | 240 |
| Sbjct 181 | LLAGLNTPG+TTVIEP+MTR+HTEKMLQGFGA L+VETD DGVRTIRLEGRGKL G VID  LLAGLNTPGVTTVIEPVMTRNHTEKMLQGFGAALSVETDGDGVRTIRLEGRGKLAGHVID | 240 |

| Query | 241 | VPGDPSSTAFPLVAALLVPGSDVTILNVLMNPTRTGLILTLQEMGADIEVINPRLAGGED | 300 |
| --- | --- | --- | --- |
| Sbjct | 241 | +PGDPSSTAFPLVAAL+VPGSD+TI+NVLMNPTRTGLILTLQEMGADIEV+N RLAGGED IPGDPSSTAFPLVAALIVPGSDITIVNVLMNPTRTGLILTLQEMGADIEVVNARLAGGED | 300 |
| Query | 301 | VADLRVRSSTLKGVTVPEDRAPSMIDEYPILavaaafaegaTVMNGLEELRVKESDRLSA | 360 |
| Sbjct | 301 | VADLRVR S LKGVTVPEDRAPSMIDEYPILA+AA FAEGATVM GLEELRVKESDRLSA VADLRVRHSELKGVTVPEDRAPSMIDEYPILAIAACFAEGATVMKGLEELRVKESDRLSA | 360 |
| Query | 361 | VANGLKLNGVDCDEGETSLVVRGRPDGKGLGNASGAAVATHLDHRIAMSFLVMGLVSENP | 420 |
| Sbjct | 361 | VA+GLKLNGVDCDEGE L+VRGRP GKGLGNA+G V THLDHRIAMSFLV+GL SE+ VADGLKLNGVDCDEGEDFLIVRGRPGGKGLGNAAGGQVRTHLDHRIAMSFLVLGLASEHA | 420 |
| Query  Sbjct | 421  421 | VTVDDATMIATSFPEFMDLMAGLGAKIEL 449 VT+DDA MIATSFPEFM LM GLGAKIEL VTIDDAAMIATSFPEFMQLMTGLGAKIEL 449 |  |

>WP_133317022.1 3-phosphoshikimate 1-carboxyvinyltransferase [Rhizobium deserti] TDK35601.1 3-phosphoshikimate 1-carboxyvinyltransferase [Rhizobium deserti]

Length=455

Score = 762 bits (1967), Expect = 0.0, Method: Compositional matrix adjust.

| Identities | = 389/455 (85%), Positives = 413/455 (91%), Gaps = 0/455 (0%) |  |
| --- | --- | --- |
| Query 1 | MLHGASSRPATARKSSGLSGTVRIPGDKSISHRSFMFGGLASGETRITGLLEGEDVINTG | 60 |
| Sbjct 1 | M HG + +PA +RKS+ LSGTVRIPGDKSISHRSFMFGGLASGETRITGLLEGEDVINTG MSHGIALKPAKSRKSTNLSGTVRIPGDKSISHRSFMFGGLASGETRITGLLEGEDVINTG | 60 |
| Query 61 | KAMQAMGARIRKEGDTWIIDGVGNGGLLAPEAPLDFGNAATGCRLTMGLVGVYDFDSTFI | 120 |
| Sbjct 61 | KAMQAMGARI K G WII G GNG LLAPEAPLDFGNA TGCRLTMGLVGVYDFDSTFI KAMQAMGARIEKVGAEWIIQGTGNGALLAPEAPLDFGNAGTGCRLTMGLVGVYDFDSTFI | 120 |
| Query 121 | GDASLTKRPMGRVLNPLREMGVQVKSEDGDRLPVTLRGPKTPTPITYRVPMASAQVKSAV | 180 |
| Sbjct 121 | GD SL+KRPMGRVL+PLREMGVQV S GDRLPVTLRGP+TP PITYRVPMASAQVKSAV GDGSLSKRPMGRVLDPLREMGVQVTSTAGDRLPVTLRGPRTPNPITYRVPMASAQVKSAV | 180 |
| Query 181 | LLAGLNTPGITTVIEPIMTRDHTEKMLQGFGANLTVETDADGVRTIRLEGRGKLTGQVID | 240 |
| Sbjct 181 | LLAGLNTPGITTVIEP+MTRDHTEKMLQGFGANLTVETD DGVRTIRLEGRG+L GQVID LLAGLNTPGITTVIEPVMTRDHTEKMLQGFGANLTVETDGDGVRTIRLEGRGRLVGQVID | 240 |
| Query 241 | VPGDPSSTAFPLVAALLVPGSDVTILNVLMNPTRTGLILTLQEMGADIEVINPRLAGGED | 300 |
| Sbjct 241 | VPGDPSSTAFPLVAALLVPGSD+ I NVLMNPTRTGLILTLQEMGADI+V+N R AGGED VPGDPSSTAFPLVAALLVPGSDLVIENVLMNPTRTGLILTLQEMGADIDVMNARSAGGED | 300 |
| Query 301 | VADLRVRSSTLKGVTVPEDRAPSMIDEYPILavaaafaegaTVMNGLEELRVKESDRLSA | 360 |
| Sbjct 301 | VADLRVR S LKGVTVP DRAPSMIDEYP+LA+AAAFAEG T M GLEELRVKESDRLSA VADLRVRGSELKGVTVPADRAPSMIDEYPVLAIAAAFAEGTTTMLGLEELRVKESDRLSA | 360 |
| Query 361 | VANGLKLNGVDCDEGETSLVVRGRPDGKGLGNASGAAVATHLDHRIAMSFLVMGLVSENP | 420 |
| Sbjct 361 | A GLKLNGVDCDEG+ +LVVRGRP GKG GNA+G AV THLDHRIAMSFLVMGL SE+P TAAGLKLNGVDCDEGKETLVVRGRPGGKGYGNAAGKAVVTHLDHRIAMSFLVMGLASEHP | 420 |
| Query 421  Sbjct 421 | VTVDDATMIATSFPEFMDLMAGLGAKIELSDTKAA 455 V+VDDA +IATSFPEFMDLM GLGA+IE +++AA VSVDDAGIIATSFPEFMDLMTGLGARIESDESQAA 455 |  |

>WP_130656708.1 3-phosphoshikimate 1-carboxyvinyltransferase [Rhizobium leguminosarum] TAU70997.1 3-phosphoshikimate 1-carboxyvinyltransferase [Rhizobium leguminosarum]

Length=452

Score = 762 bits (1967), Expect = 0.0, Method: Compositional matrix adjust. Identities = 385/449 (86%), Positives = 413/449 (92%), Gaps = 0/449 (0%)

| Query | 1 | MLHGASSRPATARKSSGLSGTVRIPGDKSISHRSFMFGGLASGETRITGLLEGEDVINTG | 60 |
| --- | --- | --- | --- |
| Sbjct | 1 | MLHG++ RPATARKS+ L+G+VRIPGDKSISHRSFMFGGLASGETRITGLLEGEDVINTG MLHGSAPRPATARKSASLTGSVRIPGDKSISHRSFMFGGLASGETRITGLLEGEDVINTG | 60 |
| Query | 61 | KAMQAMGARIRKEGDTWIIDGVGNGGLLAPEAPLDFGNAATGCRLTMGLVGVYDFDSTFI | 120 |
| Sbjct | 61 | +AMQAMGARIRKEG W+I+G GNG LLAP+APLDFGNA TG RLTMGLVG YDF STF  RAMQAMGARIRKEGAQWVIEGTGNGALLAPDAPLDFGNAGTGVRLTMGLVGTYDFHSTFT | 120 |

| Query | 121 | GDASLTKRPMGRVLNPLREMGVQVKSEDGDRLPVTLRGPKTPTPITYRVPMASAQVKSAV | 180 |
| --- | --- | --- | --- |
| Sbjct | 121 | GDASL+KRPMGRVL PLREMGVQV + +GDRLPVTLRGP TP+PI YRVPMASAQVKSAV GDASLSKRPMGRVLAPLREMGVQVSASEGDRLPVTLRGPGTPSPIRYRVPMASAQVKSAV | 180 |
| Query | 181 | LLAGLNTPGITTVIEPIMTRDHTEKMLQGFGANLTVETDADGVRTIRLEGRGKLTGQVID | 240 |
| Sbjct | 181 | LLAGLNTPG+TTVIEP+MTRDHTEKMLQGFGA L+VETD DGVRTIRLEGRGKL GQVID LLAGLNTPGVTTVIEPVMTRDHTEKMLQGFGAALSVETDGDGVRTIRLEGRGKLKGQVID | 240 |
| Query | 241 | VPGDPSSTAFPLVAALLVPGSDVTILNVLMNPTRTGLILTLQEMGADIEVINPRLAGGED | 300 |
| Sbjct | 241 | +PGDPSSTAFPLVAALLVPGSD+TI+NVLMNPTRTGLILTLQEMGA+IEV+N RLAGGED IPGDPSSTAFPLVAALLVPGSDITIVNVLMNPTRTGLILTLQEMGANIEVLNARLAGGED | 300 |
| Query | 301 | VADLRVRSSTLKGVTVPEDRAPSMIDEYPILavaaafaegaTVMNGLEELRVKESDRLSA | 360 |
| Sbjct | 301 | VADLRVR S LKGV VPE RAPSMIDEYPILA+AA FAEGATVM GLEELRVKESDRLSA VADLRVRHSELKGVGVPEGRAPSMIDEYPILAIAACFAEGATVMKGLEELRVKESDRLSA | 360 |
| Query | 361 | VANGLKLNGVDCDEGETSLVVRGRPDGKGLGNASGAAVATHLDHRIAMSFLVMGLVSENP | 420 |
| Sbjct | 361 | VA+GLKLNGVDCDEGE L+VRGRPDGKGLGNA+G V THLDHRIAMSFLV+GL SE+ VADGLKLNGVDCDEGEDFLIVRGRPDGKGLGNAAGGQVRTHLDHRIAMSFLVLGLASEHA | 420 |
| Query  Sbjct | 421  421 | VTVDDATMIATSFPEFMDLMAGLGAKIEL 449 VT+DDA MIATSFPEFM LM GLGAKIEL VTIDDAAMIATSFPEFMQLMTGLGAKIEL 449 |  |

>WP_018494545.1 3-phosphoshikimate 1-carboxyvinyltransferase [Rhizobium leguminosarum] Length=452

Score = 762 bits (1967), Expect = 0.0, Method: Compositional matrix adjust.

| Identities | = 386/449 (86%), Positives = 412/449 (92%), Gaps = 0/449 (0%) |  |
| --- | --- | --- |
| Query 1 | MLHGASSRPATARKSSGLSGTVRIPGDKSISHRSFMFGGLASGETRITGLLEGEDVINTG | 60 |
| Sbjct 1 | MLHG++ RPATARKS+ L+G+VRIPGDKSISHRSFMFGGLASGETRITGLLEGEDVINTG MLHGSAPRPATARKSASLTGSVRIPGDKSISHRSFMFGGLASGETRITGLLEGEDVINTG | 60 |
| Query 61 | KAMQAMGARIRKEGDTWIIDGVGNGGLLAPEAPLDFGNAATGCRLTMGLVGVYDFDSTFI | 120 |
| Sbjct 61 | +AM AMGARIRKEG W+I+G GNG LLAP+APLDFGNA TG RLTMGLVG YDF STF RAMHAMGARIRKEGAQWVIEGTGNGALLAPDAPLDFGNAGTGVRLTMGLVGTYDFHSTFT | 120 |
| Query 121 | GDASLTKRPMGRVLNPLREMGVQVKSEDGDRLPVTLRGPKTPTPITYRVPMASAQVKSAV | 180 |
| Sbjct 121 | GDASL+KRPMGRVL PLREMGVQV + +GDRLPVTLRGP TP+PI YRVPMASAQVKSAV GDASLSKRPMGRVLAPLREMGVQVSASEGDRLPVTLRGPGTPSPIRYRVPMASAQVKSAV | 180 |
| Query 181 | LLAGLNTPGITTVIEPIMTRDHTEKMLQGFGANLTVETDADGVRTIRLEGRGKLTGQVID | 240 |
| Sbjct 181 | LLAGLNTPG+TTVIEP+MTRDHTEKMLQGFGA L+VET DGVRTIRLEGRGKL GQVID LLAGLNTPGVTTVIEPVMTRDHTEKMLQGFGAALSVETAGDGVRTIRLEGRGKLAGQVID | 240 |
| Query 241 | VPGDPSSTAFPLVAALLVPGSDVTILNVLMNPTRTGLILTLQEMGADIEVINPRLAGGED | 300 |
| Sbjct 241 | VPGDPSSTAFPLVAALLVPGSD+TI+NVLMNPTRTGLILTLQEMGADIEV+N RLAGGED VPGDPSSTAFPLVAALLVPGSDITIVNVLMNPTRTGLILTLQEMGADIEVVNARLAGGED | 300 |
| Query 301 | VADLRVRSSTLKGVTVPEDRAPSMIDEYPILavaaafaegaTVMNGLEELRVKESDRLSA | 360 |
| Sbjct 301 | VADLRVR S LKGVTVPEDRAPSMIDEYPILA+AA FAEGATVM GLEELRVKESDRLSA VADLRVRHSELKGVTVPEDRAPSMIDEYPILAIAACFAEGATVMKGLEELRVKESDRLSA | 360 |
| Query 361 | VANGLKLNGVDCDEGETSLVVRGRPDGKGLGNASGAAVATHLDHRIAMSFLVMGLVSENP | 420 |
| Sbjct 361 | VA+GLKLNGVDCDEGE L+VRGR DGKGLGNA+G V THLDHRIAMSFLV+GL SE+ VADGLKLNGVDCDEGEDFLIVRGRRDGKGLGNAAGGQVRTHLDHRIAMSFLVLGLASEHA | 420 |
| Query 421  Sbjct 421 | VTVDDATMIATSFPEFMDLMAGLGAKIEL 449 VT+DDA MIATSFPEFM LM GLGAKIEL VTIDDAAMIATSFPEFMQLMTGLGAKIEL 449 |  |

>WP_148146226.1 3-phosphoshikimate 1-carboxyvinyltransferase [Rhizobium cellulosilyticum] MBB2876355.1 3-phosphoshikimate 1-carboxyvinyltransferase [Rhizobium cellulosilyticum]

Length=455

Score = 761 bits (1966), Expect = 0.0, Method: Compositional matrix adjust. Identities = 387/455 (85%), Positives = 412/455 (91%), Gaps = 0/455 (0%)

Query 1	MLHGASSRPATARKSSGLSGTVRIPGDKSISHRSFMFGGLASGETRITGLLEGEDVINTG 60

| Sbjct | 1 | M HG + +PA + +S L GT+RIPGDKSISHRSFMFGGLASGETRITGLLEGEDVINTG MSHGIALKPAKSARSEALKGTIRIPGDKSISHRSFMFGGLASGETRITGLLEGEDVINTG | 60 |
| --- | --- | --- | --- |
| Query | 61 | KAMQAMGARIRKEGDTWIIDGVGNGGLLAPEAPLDFGNAATGCRLTMGLVGVYDFDSTFI | 120 |
| Sbjct | 61 | KAM+AMGA+I K G WII G GNG LLAPEAPLDFGNA TGCRLTMGLVGVYDF STFI KAMRAMGAQIEKVGAEWIIQGTGNGALLAPEAPLDFGNAGTGCRLTMGLVGVYDFASTFI | 120 |
| Query | 121 | GDASLTKRPMGRVLNPLREMGVQVKSEDGDRLPVTLRGPKTPTPITYRVPMASAQVKSAV | 180 |
| Sbjct | 121 | GDASL+KRPMGRVLNPLREMGVQV S +GDRLPVTL GPKTP PITYRVPMASAQVKSAV GDASLSKRPMGRVLNPLREMGVQVSSTEGDRLPVTLHGPKTPNPITYRVPMASAQVKSAV | 180 |
| Query | 181 | LLAGLNTPGITTVIEPIMTRDHTEKMLQGFGANLTVETDADGVRTIRLEGRGKLTGQVID | 240 |
| Sbjct | 181 | LLAGLNTPGITTVIEP+MTRDHTEKMLQGFGA+L+VETDA+GVRTIRL G+G+L GQVID LLAGLNTPGITTVIEPVMTRDHTEKMLQGFGADLSVETDAEGVRTIRLVGQGQLKGQVID | 240 |
| Query | 241 | VPGDPSSTAFPLVAALLVPGSDVTILNVLMNPTRTGLILTLQEMGADIEVINPRLAGGED | 300 |
| Sbjct | 241 | VPGDPSSTAFPLVAALL PGSDVTILNVLMNPTRTGLILTLQEMGADIEV N RLAGGED VPGDPSSTAFPLVAALLTPGSDVTILNVLMNPTRTGLILTLQEMGADIEVQNARLAGGED | 300 |
| Query | 301 | VADLRVRSSTLKGVTVPEDRAPSMIDEYPILavaaafaegaTVMNGLEELRVKESDRLSA | 360 |
| Sbjct | 301 | VADLRVR S LKGVTVP DRAPSMIDEYP+LAVAAA+A G T M GLEELRVKESDRLSA VADLRVRYSELKGVTVPADRAPSMIDEYPVLAVAAAYASGTTTMLGLEELRVKESDRLSA | 360 |
| Query | 361 | VANGLKLNGVDCDEGETSLVVRGRPDGKGLGNASGAAVATHLDHRIAMSFLVMGLVSENP | 420 |
| Sbjct | 361 | VA GLKLNGVDCDEG+ +LVVRGRP GKG GNA+G AV THLDHRIAMSFLV+GLVSE+ VAEGLKLNGVDCDEGKETLVVRGRPGGKGYGNAAGKAVVTHLDHRIAMSFLVLGLVSEHA | 420 |
| Query  Sbjct | 421  421 | VTVDDATMIATSFPEFMDLMAGLGAKIELSDTKAA 455 VTVDDA++IATSFPEFMDLM GLGAKIEL D +AA VTVDDASIIATSFPEFMDLMTGLGAKIELVDGEAA 455 |  |

>WP_097545160.1 3-phosphoshikimate 1-carboxyvinyltransferase [Rhizobium anhuiense] PDS40874.1 3-phosphoshikimate 1-carboxyvinyltransferase [Rhizobium anhuiense] PDS47846.1 3-phosphoshikimate 1-carboxyvinyltransferase [Rhizobium anhuiense]

Length=452

Score = 761 bits (1966), Expect = 0.0, Method: Compositional matrix adjust.

| Identities | = 383/448 (85%), Positives = 415/448 (93%), Gaps = 0/448 (0%) |  |
| --- | --- | --- |
| Query 1 | MLHGASSRPATARKSSGLSGTVRIPGDKSISHRSFMFGGLASGETRITGLLEGEDVINTG | 60 |
| Sbjct 1 | MLHG++ RPATARKS+GLSG VRIPGDKSISHRSFMFGGLASG+TRITGLLEGEDVINTG MLHGSAPRPATARKSAGLSGRVRIPGDKSISHRSFMFGGLASGQTRITGLLEGEDVINTG | 60 |
| Query 61 | KAMQAMGARIRKEGDTWIIDGVGNGGLLAPEAPLDFGNAATGCRLTMGLVGVYDFDSTFI | 120 |
| Sbjct 61 | +AMQAMGARIRK+G+ W+I+G GNG LLAP+APLDFGNA TG RL MGLVG YDF STFI RAMQAMGARIRKQGEEWVIEGTGNGALLAPDAPLDFGNAGTGVRLIMGLVGTYDFHSTFI | 120 |
| Query 121 | GDASLTKRPMGRVLNPLREMGVQVKSEDGDRLPVTLRGPKTPTPITYRVPMASAQVKSAV | 180 |
| Sbjct 121 | GDASL+KRPMGRVLNPLR+MGVQV + GDRLPVTLRGP TP+PI YRVPMASAQVKSAV GDASLSKRPMGRVLNPLRDMGVQVSASAGDRLPVTLRGPGTPSPIRYRVPMASAQVKSAV | 180 |
| Query 181 | LLAGLNTPGITTVIEPIMTRDHTEKMLQGFGANLTVETDADGVRTIRLEGRGKLTGQVID | 240 |
| Sbjct 181 | LLAGLNTPGITTVIEP+ TRDHTEKMLQGFGA L+VETDADG+R IRLEGRG+LTGQVID LLAGLNTPGITTVIEPVKTRDHTEKMLQGFGAALSVETDADGIRMIRLEGRGRLTGQVID | 240 |
| Query 241 | VPGDPSSTAFPLVAALLVPGSDVTILNVLMNPTRTGLILTLQEMGADIEVINPRLAGGED | 300 |
| Sbjct 241 | VPGDPSSTAFPLVAAL+VPGSD+TI+NVLMNPTRTGLILTLQEMGADIEV+N RLAGGED VPGDPSSTAFPLVAALIVPGSDITIVNVLMNPTRTGLILTLQEMGADIEVLNARLAGGED | 300 |
| Query 301 | VADLRVRSSTLKGVTVPEDRAPSMIDEYPILavaaafaegaTVMNGLEELRVKESDRLSA | 360 |
| Sbjct 301 | VADLRVR S LKGV VPE+RAPSMIDEYPILAVAA FAEG TVM GLEELRVKESDRLSA VADLRVRHSELKGVAVPEERAPSMIDEYPILAVAACFAEGTTVMKGLEELRVKESDRLSA | 360 |
| Query 361 | VANGLKLNGVDCDEGETSLVVRGRPDGKGLGNASGAAVATHLDHRIAMSFLVMGLVSENP | 420 |
| Sbjct 361 | VA+GLKLNGVDCDEG+ L+VRGRPDGKGLG+A G+ V+THLDHRIAMSFLV+GL SE+ VADGLKLNGVDCDEGKDFLIVRGRPDGKGLGSAGGSKVSTHLDHRIAMSFLVLGLASEHA | 420 |
| Query 421  Sbjct 421 | VTVDDATMIATSFPEFMDLMAGLGAKIE 448 VT+DDA MIATSFPEFM LMAGLGAKIE VTIDDAAMIATSFPEFMQLMAGLGAKIE 448 |  |

>WP_025396586.1 3-phosphoshikimate 1-carboxyvinyltransferase [Rhizobium leguminosarum]

AHF86319.1 3-phosphoshikimate 1-carboxyvinyltransferase [Rhizobium leguminosarum bv. trifolii WSM1689]

Length=452

Score = 761 bits (1966), Expect = 0.0, Method: Compositional matrix adjust.

| Identities | = 384/448 (86%), Positives = 417/448 (93%), Gaps = 0/448 (0%) |  |
| --- | --- | --- |
| Query 1 | MLHGASSRPATARKSSGLSGTVRIPGDKSISHRSFMFGGLASGETRITGLLEGEDVINTG | 60 |
| Sbjct 1 | MLHG++ +PATARKS+GL+G+VRIPGDKSISHRSFMFGGLASGETRITGLLEGEDVINTG MLHGSAPKPATARKSAGLTGSVRIPGDKSISHRSFMFGGLASGETRITGLLEGEDVINTG | 60 |
| Query 61 | KAMQAMGARIRKEGDTWIIDGVGNGGLLAPEAPLDFGNAATGCRLTMGLVGVYDFDSTFI | 120 |
| Sbjct 61 | +AMQAMGA IRKEG+ WII+G GNG LLAP+APLDFGNA TG RLTMGLVG YDF STF RAMQAMGASIRKEGEQWIINGTGNGALLAPDAPLDFGNAGTGVRLTMGLVGTYDFHSTFT | 120 |
| Query 121 | GDASLTKRPMGRVLNPLREMGVQVKSEDGDRLPVTLRGPKTPTPITYRVPMASAQVKSAV | 180 |
| Sbjct 121 | GDASL+KRPMGRVL+PLREMGVQV + +GDRLPVTLRGP TP+PI YRVPMASAQVKSAV GDASLSKRPMGRVLDPLREMGVQVSASEGDRLPVTLRGPGTPSPIRYRVPMASAQVKSAV | 180 |
| Query 181 | LLAGLNTPGITTVIEPIMTRDHTEKMLQGFGANLTVETDADGVRTIRLEGRGKLTGQVID | 240 |
| Sbjct 181 | LLAGLNTPG+TTVIEP+MTRDHTEKMLQGFGA L+VETD++GVRTIRLEGRG+LTGQVID LLAGLNTPGVTTVIEPVMTRDHTEKMLQGFGAALSVETDSEGVRTIRLEGRGRLTGQVID | 240 |
| Query 241 | VPGDPSSTAFPLVAALLVPGSDVTILNVLMNPTRTGLILTLQEMGADIEVINPRLAGGED | 300 |
| Sbjct 241 | +PGDPSSTAFPLVAALLVPGSD+TI+NVLMNPTRTGLILTLQEMGA+IEV+N RLAGGED IPGDPSSTAFPLVAALLVPGSDITIVNVLMNPTRTGLILTLQEMGANIEVLNTRLAGGED | 300 |
| Query 301 | VADLRVRSSTLKGVTVPEDRAPSMIDEYPILavaaafaegaTVMNGLEELRVKESDRLSA | 360 |
| Sbjct 301 | VADLRVR S LKGVTVPEDRAPSMIDEYPILAVAA FAEGAT+M GLEELRVKESDRLSA VADLRVRHSELKGVTVPEDRAPSMIDEYPILAVAACFAEGATIMKGLEELRVKESDRLSA | 360 |
| Query 361 | VANGLKLNGVDCDEGETSLVVRGRPDGKGLGNASGAAVATHLDHRIAMSFLVMGLVSENP | 420 |
| Sbjct 361 | VA+GLKLNGVDCDEGE L+VRGRP GKGLGNA+G V+THL HRIAMSFLVMGL SE+ VADGLKLNGVDCDEGEDFLIVRGRPGGKGLGNAAGDKVSTHLVHRIAMSFLVMGLASEHA | 420 |
| Query 421  Sbjct 421 | VTVDDATMIATSFPEFMDLMAGLGAKIE 448 VT+DDA MIATSFPEFM LM GLGAKIE VTIDDAAMIATSFPEFMQLMTGLGAKIE 448 |  |

>WP_185827687.1 3-phosphoshikimate 1-carboxyvinyltransferase [Rhizobium ruizarguesonis] MBC2801623.1 3-phosphoshikimate 1-carboxyvinyltransferase [Rhizobium ruizarguesonis]

Length=452

Score = 761 bits (1966), Expect = 0.0, Method: Compositional matrix adjust.

| Identities | = 384/449 (86%), Positives = 414/449 (92%), Gaps = 0/449 (0%) |  |
| --- | --- | --- |
| Query 1 | MLHGASSRPATARKSSGLSGTVRIPGDKSISHRSFMFGGLASGETRITGLLEGEDVINTG | 60 |
| Sbjct 1 | MLHG++ RPATARKS+ L+G+VRIPGDKSISHRSFMFGGLASGETRITGLLEGEDVINTG MLHGSAPRPATARKSASLTGSVRIPGDKSISHRSFMFGGLASGETRITGLLEGEDVINTG | 60 |
| Query 61 | KAMQAMGARIRKEGDTWIIDGVGNGGLLAPEAPLDFGNAATGCRLTMGLVGVYDFDSTFI | 120 |
| Sbjct 61 | +AMQAMGARIRKEG W+I+G GNG LLAP+APLDFGNA TG RLTMGLVG YDF STF RAMQAMGARIRKEGAQWVIEGTGNGALLAPDAPLDFGNAGTGVRLTMGLVGTYDFHSTFT | 120 |
| Query 121 | GDASLTKRPMGRVLNPLREMGVQVKSEDGDRLPVTLRGPKTPTPITYRVPMASAQVKSAV | 180 |
| Sbjct 121 | GDASL+KRPMGRVL PLREMGVQV + +G+RLPVTLRG TP+PI YRVPMASAQVKSAV GDASLSKRPMGRVLAPLREMGVQVSASEGNRLPVTLRGLGTPSPIRYRVPMASAQVKSAV | 180 |
| Query 181 | LLAGLNTPGITTVIEPIMTRDHTEKMLQGFGANLTVETDADGVRTIRLEGRGKLTGQVID | 240 |
| Sbjct 181 | LLAGLNTPG+TTVIEP+MTRDHTEKMLQGFGA L+VETD DGVRTIRLEGRGKL GQVID LLAGLNTPGVTTVIEPVMTRDHTEKMLQGFGAALSVETDGDGVRTIRLEGRGKLKGQVID | 240 |
| Query 241 | VPGDPSSTAFPLVAALLVPGSDVTILNVLMNPTRTGLILTLQEMGADIEVINPRLAGGED | 300 |
| Sbjct 241 | +PGDPSSTAFPLVAAL+VPGSD+TI+NVLMNPTRTGLILTLQEMGADIEV+N RLAGGED IPGDPSSTAFPLVAALIVPGSDITIVNVLMNPTRTGLILTLQEMGADIEVVNARLAGGED | 300 |
| Query 301 | VADLRVRSSTLKGVTVPEDRAPSMIDEYPILavaaafaegaTVMNGLEELRVKESDRLSA | 360 |
| Sbjct 301 | VADLRVR S LKGVTVPEDRAPSMIDEYPILA+AA FAEGATVM GLEELRVKESDRLSA  VADLRVRHSELKGVTVPEDRAPSMIDEYPILAIAACFAEGATVMKGLEELRVKESDRLSA | 360 |

| Query | 361 | VANGLKLNGVDCDEGETSLVVRGRPDGKGLGNASGAAVATHLDHRIAMSFLVMGLVSENP 420 |
| --- | --- | --- |
| Sbjct | 361 | VA+GLKLNGV+CDEGE L+VRGRPDGKGLGNA+G V THLDHRIAMSFLV+GL SE+ VADGLKLNGVNCDEGEDFLIVRGRPDGKGLGNAAGGQVRTHLDHRIAMSFLVLGLASEHA 420 |
| Query | 421 | VTVDDATMIATSFPEFMDLMAGLGAKIEL 449 |
| Sbjct | 421 | VT+DDA MIATSFPEFM LM GLGAKIEL  VTIDDAAMIATSFPEFMQLMTGLGAKIEL 449 |

>WP_166478212.1 3-phosphoshikimate 1-carboxyvinyltransferase [Rhizobium leguminosarum]

QIO66756.1 3-phosphoshikimate 1-carboxyvinyltransferase [Rhizobium leguminosarum bv. trifolii]

Length=452

Score = 761 bits (1965), Expect = 0.0, Method: Compositional matrix adjust.

| Identities | = 385/449 (86%), Positives = 415/449 (92%), Gaps = 0/449 (0%) |  |
| --- | --- | --- |
| Query 1 | MLHGASSRPATARKSSGLSGTVRIPGDKSISHRSFMFGGLASGETRITGLLEGEDVINTG | 60 |
| Sbjct 1 | ML+G++ +PATARKS+GL+G+VRIPGDKSISHRSFMFGGLASGETRITGLLEGEDVINTG MLNGSAPKPATARKSAGLTGSVRIPGDKSISHRSFMFGGLASGETRITGLLEGEDVINTG | 60 |
| Query 61 | KAMQAMGARIRKEGDTWIIDGVGNGGLLAPEAPLDFGNAATGCRLTMGLVGVYDFDSTFI | 120 |
| Sbjct 61 | +AMQAMGARIRK+G W+I+G GNG LLAP+APLDFGNA TG RLTMGLVG YDF STF RAMQAMGARIRKDGAQWVIEGTGNGALLAPDAPLDFGNAGTGVRLTMGLVGTYDFHSTFT | 120 |
| Query 121 | GDASLTKRPMGRVLNPLREMGVQVKSEDGDRLPVTLRGPKTPTPITYRVPMASAQVKSAV | 180 |
| Sbjct 121 | GDASL+KRPMGRVL PLREMGVQV + +GDRLPVTLRGP TP+PI YRVPMASAQVKSAV GDASLSKRPMGRVLAPLREMGVQVSASEGDRLPVTLRGPGTPSPIRYRVPMASAQVKSAV | 180 |
| Query 181 | LLAGLNTPGITTVIEPIMTRDHTEKMLQGFGANLTVETDADGVRTIRLEGRGKLTGQVID | 240 |
| Sbjct 181 | LLAGLNTPG+TTVIEP+MTRDHTEKMLQGFGA L+VETD DGVRTIRLEGRGKL GQVID LLAGLNTPGVTTVIEPVMTRDHTEKMLQGFGAALSVETDGDGVRTIRLEGRGKLAGQVID | 240 |
| Query 241 | VPGDPSSTAFPLVAALLVPGSDVTILNVLMNPTRTGLILTLQEMGADIEVINPRLAGGED | 300 |
| Sbjct 241 | VPGDPSSTAFPLVAAL+VPGSD+TI+NVLMNPTRTGLILTLQEMGADIEV+N RLAGGED VPGDPSSTAFPLVAALIVPGSDITIVNVLMNPTRTGLILTLQEMGADIEVVNARLAGGED | 300 |
| Query 301 | VADLRVRSSTLKGVTVPEDRAPSMIDEYPILavaaafaegaTVMNGLEELRVKESDRLSA | 360 |
| Sbjct 301 | VADLRVR S LKGVTVPEDRAPSMIDEYPILAVAA FAEGATVM GLEELRVKESDRLSA VADLRVRHSELKGVTVPEDRAPSMIDEYPILAVAACFAEGATVMKGLEELRVKESDRLSA | 360 |
| Query 361 | VANGLKLNGVDCDEGETSLVVRGRPDGKGLGNASGAAVATHLDHRIAMSFLVMGLVSENP | 420 |
| Sbjct 361 | VA+GLKLNGVDCDEGE L+VRGRPDGKGLGN + V+THLDHRIAMSFLV+GL SE+ VADGLKLNGVDCDEGEDFLIVRGRPDGKGLGNTADGRVSTHLDHRIAMSFLVLGLASEHA | 420 |
| Query 421  Sbjct 421 | VTVDDATMIATSFPEFMDLMAGLGAKIEL 449 VT+DDA MIATSFPEFM LM GLGAKIEL VTIDDAAMIATSFPEFMQLMTGLGAKIEL 449 |  |

>WP_170282642.1 3-phosphoshikimate 1-carboxyvinyltransferase [Rhizobium laguerreae] NNH67434.1 3-phosphoshikimate 1-carboxyvinyltransferase [Rhizobium laguerreae]

Length=452

Score = 761 bits (1965), Expect = 0.0, Method: Compositional matrix adjust.

| Identities | = 383/449 (85%), Positives = 413/449 (92%), Gaps = 0/449 (0%) |  |
| --- | --- | --- |
| Query 1 | MLHGASSRPATARKSSGLSGTVRIPGDKSISHRSFMFGGLASGETRITGLLEGEDVINTG | 60 |
| Sbjct 1 | MLHG++ RPATARKS+GL G+VRIPGDKSISHRSFMFGGLASGETRITGLLEGEDVINTG MLHGSAPRPATARKSAGLKGSVRIPGDKSISHRSFMFGGLASGETRITGLLEGEDVINTG | 60 |
| Query 61 | KAMQAMGARIRKEGDTWIIDGVGNGGLLAPEAPLDFGNAATGCRLTMGLVGVYDFDSTFI | 120 |
| Sbjct 61 | +AM+AMGA IRKEG W+I+G GNG LLAPEAPLDFGNA TG RLTMGLVG YDF STF RAMRAMGAGIRKEGAQWVIEGTGNGALLAPEAPLDFGNAGTGVRLTMGLVGTYDFHSTFT | 120 |
| Query 121 | GDASLTKRPMGRVLNPLREMGVQVKSEDGDRLPVTLRGPKTPTPITYRVPMASAQVKSAV | 180 |
| Sbjct 121 | GDASL+KRPMGRVL PLREMGVQV + +GDRLPVTLRGP TP+PI YRVPMASAQVKSAV GDASLSKRPMGRVLAPLREMGVQVSASEGDRLPVTLRGPGTPSPIRYRVPMASAQVKSAV | 180 |
| Query 181 | LLAGLNTPGITTVIEPIMTRDHTEKMLQGFGANLTVETDADGVRTIRLEGRGKLTGQVID LLAGLNTPG+TTVIEP+MTRDHTEKMLQGFGA L+VETD DGVRTIRLEGRGKL GQVID | 240 |

| Sbjct | 181 | LLAGLNTPGVTTVIEPVMTRDHTEKMLQGFGAALSVETDGDGVRTIRLEGRGKLKGQVID | 240 |
| --- | --- | --- | --- |
| Query | 241 | VPGDPSSTAFPLVAALLVPGSDVTILNVLMNPTRTGLILTLQEMGADIEVINPRLAGGED | 300 |
| Sbjct | 241 | +PGDPSSTAFPLVAAL+VPGSD+TI+NVLMNPTRTGLILTLQEMGADIEV+N RLAGGED IPGDPSSTAFPLVAALIVPGSDITIVNVLMNPTRTGLILTLQEMGADIEVVNARLAGGED | 300 |
| Query | 301 | VADLRVRSSTLKGVTVPEDRAPSMIDEYPILavaaafaegaTVMNGLEELRVKESDRLSA | 360 |
| Sbjct | 301 | VADLRVR S LKGVTVPEDRAPSMIDEYPILA+AA FA GATVM GLEELRVKESDRLSA VADLRVRHSELKGVTVPEDRAPSMIDEYPILAIAACFAAGATVMKGLEELRVKESDRLSA | 360 |
| Query | 361 | VANGLKLNGVDCDEGETSLVVRGRPDGKGLGNASGAAVATHLDHRIAMSFLVMGLVSENP | 420 |
| Sbjct | 361 | VA+GLKLNGVDCDEG+ L+VRGRPDG+G+GNA+G V THLDHRIAMSFLV+GL SE+ VADGLKLNGVDCDEGKDFLIVRGRPDGRGIGNAAGGQVRTHLDHRIAMSFLVLGLASEHA | 420 |
| Query  Sbjct | 421  421 | VTVDDATMIATSFPEFMDLMAGLGAKIEL 449 VT+DDA MIATSFPEFM LM GLGAKIEL VTIDDAAMIATSFPEFMQLMTGLGAKIEL 449 |  |

>WP_183367161.1 3-phosphoshikimate 1-carboxyvinyltransferase [Gellertiella hungarica] MBB4065873.1 3-phosphoshikimate 1-carboxyvinyltransferase [Gellertiella hungarica]

Length=451

Score = 761 bits (1965), Expect = 0.0, Method: Compositional matrix adjust.

| Identities | = 385/448 (86%), Positives = 411/448 (92%), Gaps = 0/448 (0%) |  |
| --- | --- | --- |
| Query 1 | MLHGASSRPATARKSSGLSGTVRIPGDKSISHRSFMFGGLASGETRITGLLEGEDVINTG | 60 |
| Sbjct 1 | M HGA+ +PATAR+S L+G+V IPGDKSISHRSFMFGGLASG+TRITGLLEGEDVINTG MSHGAAPKPATARRSENLAGSVTIPGDKSISHRSFMFGGLASGQTRITGLLEGEDVINTG | 60 |
| Query 61 | KAMQAMGARIRKEGDTWIIDGVGNGGLLAPEAPLDFGNAATGCRLTMGLVGVYDFDSTFI | 120 |
| Sbjct 61 | +AM AMGA+IRK+GDTWIIDG GNG LLAPEAPLDFGNA TGCRLTMGLVGVYDF STFI RAMSAMGAKIRKDGDTWIIDGTGNGALLAPEAPLDFGNAGTGCRLTMGLVGVYDFASTFI | 120 |
| Query 121 | GDASLTKRPMGRVLNPLREMGVQVKSEDGDRLPVTLRGPKTPTPITYRVPMASAQVKSAV | 180 |
| Sbjct 121 | GDASLT RPMGRVLNPLREMGVQV++ GDRLPVTLRGPKTP PITYRVPMASAQVKSAV GDASLTARPMGRVLNPLREMGVQVEAAPGDRLPVTLRGPKTPAPITYRVPMASAQVKSAV | 180 |
| Query 181 | LLAGLNTPGITTVIEPIMTRDHTEKMLQGFGANLTVETDADGVRTIRLEGRGKLTGQVID | 240 |
| Sbjct 181 | LLAGLNTPGITTVIEP+MTRDHTEKML GFGA L+VETDA GVRTIRLEGRGKLTGQ I LLAGLNTPGITTVIEPVMTRDHTEKMLAGFGAALSVETDAAGVRTIRLEGRGKLTGQTIA | 240 |
| Query 241 | VPGDPSSTAFPLVAALLVPGSDVTILNVLMNPTRTGLILTLQEMGADIEVINPRLAGGED | 300 |
| Sbjct 241 | VPGDPSSTAFPLVAA+LVPGSD+TI NVLMNPTRTGLILTLQEMGA IE+++PRLAGGED VPGDPSSTAFPLVAAILVPGSDITIRNVLMNPTRTGLILTLQEMGASIEILDPRLAGGED | 300 |
| Query 301 | VADLRVRSSTLKGVTVPEDRAPSMIDEYPILavaaafaegaTVMNGLEELRVKESDRLSA | 360 |
| Sbjct 301 | VADLRVR S L+GVTVPE+RAPSMIDEYPILA+AAAFA+G TVM+GLEELRVKESDRLSA VADLRVRHSELRGVTVPEERAPSMIDEYPILAIAAAFAKGDTVMHGLEELRVKESDRLSA | 360 |
| Query 361 | VANGLKLNGVDCDEGETSLVVRGRPDGKGLGNASGAAVATHLDHRIAMSFLVMGLVSENP | 420 |
| Sbjct 361 | VANGLKLNGV C EGE SL V GRPDGKG G G AV THLDHRIAMSFLVMGL +ENP VANGLKLNGVACVEGEASLTVTGRPDGKGYGTPGGQAVKTHLDHRIAMSFLVMGLAAENP | 420 |
| Query 421  Sbjct 421 | VTVDDATMIATSFPEFMDLMAGLGAKIE 448 VTVDDATMIATSFPEFMDLM GLGA+I+ VTVDDATMIATSFPEFMDLMTGLGARID 448 |  |

>WP_130662575.1 3-phosphoshikimate 1-carboxyvinyltransferase [Rhizobium leguminosarum] TAT86176.1 3-phosphoshikimate 1-carboxyvinyltransferase [Rhizobium leguminosarum] TAU33802.1 3-phosphoshikimate 1-carboxyvinyltransferase [Rhizobium leguminosarum] TAW23986.1 3-phosphoshikimate 1-carboxyvinyltransferase [Rhizobium leguminosarum]

Length=452

Score = 761 bits (1965), Expect = 0.0, Method: Compositional matrix adjust. Identities = 384/449 (86%), Positives = 412/449 (92%), Gaps = 0/449 (0%)

Query 1	MLHGASSRPATARKSSGLSGTVRIPGDKSISHRSFMFGGLASGETRITGLLEGEDVINTG 60 MLHG++ RPATARKS+ L+G+V IPGDKSISHRSFMFGGLASGETRITGLLE EDVINTG

Sbjct 1	MLHGSAPRPATARKSASLTGSVCIPGDKSISHRSFMFGGLASGETRITGLLESEDVINTG 60

| Query | 61 | KAMQAMGARIRKEGDTWIIDGVGNGGLLAPEAPLDFGNAATGCRLTMGLVGVYDFDSTFI | 120 |
| --- | --- | --- | --- |
| Sbjct | 61 | +AMQAMGARIRKEG W+I+G GNG LLAP+APLDFGNA TG RLTMGLVG YDF STF RAMQAMGARIRKEGAQWVIEGTGNGALLAPDAPLDFGNAGTGVRLTMGLVGTYDFHSTFT | 120 |
| Query | 121 | GDASLTKRPMGRVLNPLREMGVQVKSEDGDRLPVTLRGPKTPTPITYRVPMASAQVKSAV | 180 |
| Sbjct | 121 | GDASL+KRPMGRVL PLREMGVQV + +GDRLPV LRGP TP+PI YRVPMASAQVKSAV GDASLSKRPMGRVLAPLREMGVQVSASEGDRLPVMLRGPGTPSPIRYRVPMASAQVKSAV | 180 |
| Query | 181 | LLAGLNTPGITTVIEPIMTRDHTEKMLQGFGANLTVETDADGVRTIRLEGRGKLTGQVID | 240 |
| Sbjct | 181 | LLAGLNTPG+TTVIEP+MTRDHTEKMLQGFGA L+VETD DGVRTIRLEGRGKL GQVID LLAGLNTPGVTTVIEPVMTRDHTEKMLQGFGAALSVETDGDGVRTIRLEGRGKLAGQVID | 240 |
| Query | 241 | VPGDPSSTAFPLVAALLVPGSDVTILNVLMNPTRTGLILTLQEMGADIEVINPRLAGGED | 300 |
| Sbjct | 241 | +PGDPSSTAFPLVAALLVPGSD+TI+NVLMNPTRTGLILTLQEMGADIEV+N RLAGGED IPGDPSSTAFPLVAALLVPGSDITIVNVLMNPTRTGLILTLQEMGADIEVVNARLAGGED | 300 |
| Query | 301 | VADLRVRSSTLKGVTVPEDRAPSMIDEYPILavaaafaegaTVMNGLEELRVKESDRLSA | 360 |
| Sbjct | 301 | VADLRVR S LKGVTVPEDRAPSMIDEYPILA+AA FAEGATVM GLEELRVKESDRLSA VADLRVRHSELKGVTVPEDRAPSMIDEYPILAIAACFAEGATVMKGLEELRVKESDRLSA | 360 |
| Query | 361 | VANGLKLNGVDCDEGETSLVVRGRPDGKGLGNASGAAVATHLDHRIAMSFLVMGLVSENP | 420 |
| Sbjct | 361 | VA+GLKLNGV+CDEGE L+VRGRPDGKGLGNA+G V THLDHRIAMSFLV+GL SE+ VADGLKLNGVNCDEGEDFLIVRGRPDGKGLGNAAGGQVRTHLDHRIAMSFLVLGLASEHA | 420 |
| Query  Sbjct | 421  421 | VTVDDATMIATSFPEFMDLMAGLGAKIEL 449 VT+DDA MIATSFPEFM LM GLGAKIEL VTIDDAAMIATSFPEFMQLMTGLGAKIEL 449 |  |

>WP_130745589.1 3-phosphoshikimate 1-carboxyvinyltransferase [Rhizobium leguminosarum] TAY76857.1 3-phosphoshikimate 1-carboxyvinyltransferase [Rhizobium leguminosarum]

Length=452

Score = 761 bits (1965), Expect = 0.0, Method: Compositional matrix adjust.

| Identities | = 384/449 (86%), Positives = 413/449 (92%), Gaps = 0/449 (0%) |  |
| --- | --- | --- |
| Query 1 | MLHGASSRPATARKSSGLSGTVRIPGDKSISHRSFMFGGLASGETRITGLLEGEDVINTG | 60 |
| Sbjct 1 | MLHG++ RPATARKS+ L+G+VRIPGDKSISHRSFMFGGLASGETRITGLLEGEDVINTG MLHGSAPRPATARKSASLTGSVRIPGDKSISHRSFMFGGLASGETRITGLLEGEDVINTG | 60 |
| Query 61 | KAMQAMGARIRKEGDTWIIDGVGNGGLLAPEAPLDFGNAATGCRLTMGLVGVYDFDSTFI | 120 |
| Sbjct 61 | +AMQAMGARIRKEG W+I+G GNG LLAP+APLDFGNA TG RLTMGLVG YDF STF RAMQAMGARIRKEGAQWVIEGTGNGALLAPDAPLDFGNAGTGVRLTMGLVGTYDFHSTFT | 120 |
| Query 121 | GDASLTKRPMGRVLNPLREMGVQVKSEDGDRLPVTLRGPKTPTPITYRVPMASAQVKSAV | 180 |
| Sbjct 121 | GDASL+KRPMGRVL PLREMGVQV + +GDRLPVTLRGP TP+PI YRVPMASAQVKSAV GDASLSKRPMGRVLAPLREMGVQVSASEGDRLPVTLRGPGTPSPIRYRVPMASAQVKSAV | 180 |
| Query 181 | LLAGLNTPGITTVIEPIMTRDHTEKMLQGFGANLTVETDADGVRTIRLEGRGKLTGQVID | 240 |
| Sbjct 181 | LLAGLNTPG+TTVIEP+MTRDHTEKMLQGFGA L+VET DGVRTIRLEGRGKL GQVID LLAGLNTPGVTTVIEPVMTRDHTEKMLQGFGAALSVETAGDGVRTIRLEGRGKLAGQVID | 240 |
| Query 241 | VPGDPSSTAFPLVAALLVPGSDVTILNVLMNPTRTGLILTLQEMGADIEVINPRLAGGED | 300 |
| Sbjct 241 | VPGDPSSTAFPLVAALLVPGSD+TI+NVLMNPTRTGLILTLQEMGADIEV+N RLAGGED VPGDPSSTAFPLVAALLVPGSDITIVNVLMNPTRTGLILTLQEMGADIEVVNARLAGGED | 300 |
| Query 301 | VADLRVRSSTLKGVTVPEDRAPSMIDEYPILavaaafaegaTVMNGLEELRVKESDRLSA | 360 |
| Sbjct 301 | VADLRVR S LKGV+VPEDRAPSMIDEYPILA+AA FAEGATVM GLEELRVKESDRLSA VADLRVRHSELKGVSVPEDRAPSMIDEYPILAIAACFAEGATVMKGLEELRVKESDRLSA | 360 |
| Query 361 | VANGLKLNGVDCDEGETSLVVRGRPDGKGLGNASGAAVATHLDHRIAMSFLVMGLVSENP | 420 |
| Sbjct 361 | VA+GLKLNGVDC+EGE L+VRGRPDG+GLG A+G V THLDHRIAMSFLV+GL SE+ VADGLKLNGVDCNEGEDFLIVRGRPDGRGLGTAAGGQVRTHLDHRIAMSFLVLGLASEHA | 420 |
| Query 421  Sbjct 421 | VTVDDATMIATSFPEFMDLMAGLGAKIEL 449 VT+DDA MIATSFPEFM LM GLGAKIEL VTIDDAAMIATSFPEFMQLMTGLGAKIEL 449 |  |

>WP_168251672.1 MULTISPECIES: 3-phosphoshikimate 1-carboxyvinyltransferase [Rhizobium]

NKJ74586.1 3-phosphoshikimate 1-carboxyvinyltransferase [Rhizobium leguminosarum bv. viciae]

NKQ73779.1 3-phosphoshikimate 1-carboxyvinyltransferase [Rhizobium ruizarguesonis] NKQ79731.1 3-phosphoshikimate 1-carboxyvinyltransferase [Rhizobium ruizarguesonis]

Length=452

Score = 760 bits (1963), Expect = 0.0, Method: Compositional matrix adjust.

| Identities | = 385/449 (86%), Positives = 412/449 (92%), Gaps = 0/449 (0%) |  |
| --- | --- | --- |
| Query 1 | MLHGASSRPATARKSSGLSGTVRIPGDKSISHRSFMFGGLASGETRITGLLEGEDVINTG | 60 |
| Sbjct 1 | MLHG++ RPATARKS+ L+G+VRIPGDKSISHRSFMFGGLASGETRITGLLEGEDVINTG MLHGSAPRPATARKSASLTGSVRIPGDKSISHRSFMFGGLASGETRITGLLEGEDVINTG | 60 |
| Query 61 | KAMQAMGARIRKEGDTWIIDGVGNGGLLAPEAPLDFGNAATGCRLTMGLVGVYDFDSTFI | 120 |
| Sbjct 61 | +AMQAMGARIRKEG W+I+G GNG LLAP+APLDFGNA TG RLTMGLVG YDF STF RAMQAMGARIRKEGAQWVIEGTGNGALLAPDAPLDFGNAGTGVRLTMGLVGTYDFHSTFT | 120 |
| Query 121 | GDASLTKRPMGRVLNPLREMGVQVKSEDGDRLPVTLRGPKTPTPITYRVPMASAQVKSAV | 180 |
| Sbjct 121 | GDASL+KRPMGRVL PLREMGVQV + +GDRLPVTLRGP TP+PI YRVPMASAQVKSAV GDASLSKRPMGRVLAPLREMGVQVSASEGDRLPVTLRGPGTPSPIRYRVPMASAQVKSAV | 180 |
| Query 181 | LLAGLNTPGITTVIEPIMTRDHTEKMLQGFGANLTVETDADGVRTIRLEGRGKLTGQVID | 240 |
| Sbjct 181 | LLAGLNTPG+TTVIEP+MTRDHTEKMLQGFGA L+VET DGVRTIRLEGRG L G+VID LLAGLNTPGVTTVIEPVMTRDHTEKMLQGFGAALSVETAGDGVRTIRLEGRGNLKGRVID | 240 |
| Query 241 | VPGDPSSTAFPLVAALLVPGSDVTILNVLMNPTRTGLILTLQEMGADIEVINPRLAGGED | 300 |
| Sbjct 241 | VPGDPSSTAFPLVAALLVPGSD+TI+NVLMNPTRTGLILTLQEMGADIEV+N RLAGGED VPGDPSSTAFPLVAALLVPGSDITIVNVLMNPTRTGLILTLQEMGADIEVVNARLAGGED | 300 |
| Query 301 | VADLRVRSSTLKGVTVPEDRAPSMIDEYPILavaaafaegaTVMNGLEELRVKESDRLSA | 360 |
| Sbjct 301 | VADLRVR S LKGVTVPEDRAPSMIDEYPILA+AA FAEGATVM GLEELRVKESDRLSA VADLRVRHSELKGVTVPEDRAPSMIDEYPILAIAACFAEGATVMKGLEELRVKESDRLSA | 360 |
| Query 361 | VANGLKLNGVDCDEGETSLVVRGRPDGKGLGNASGAAVATHLDHRIAMSFLVMGLVSENP | 420 |
| Sbjct 361 | VA+GLKLNGVDCDEGE L+VRGRP GKGLGNA+G V THLDHRIAMSFLV+GL SE+ VADGLKLNGVDCDEGEDFLIVRGRPGGKGLGNAAGGQVRTHLDHRIAMSFLVLGLASEHA | 420 |
| Query 421  Sbjct 421 | VTVDDATMIATSFPEFMDLMAGLGAKIEL 449 VT+DDA MIATSFPEFM LM GLGAKIEL VTIDDAAMIATSFPEFMQLMTGLGAKIEL 449 |  |

>WP_168282289.1 3-phosphoshikimate 1-carboxyvinyltransferase [Rhizobium leguminosarum]

NKL58608.1 3-phosphoshikimate 1-carboxyvinyltransferase [Rhizobium leguminosarum bv. viciae]

Length=452

Score = 760 bits (1963), Expect = 0.0, Method: Compositional matrix adjust.

| Identities | = 381/449 (85%), Positives = 416/449 (93%), Gaps = 0/449 (0%) |  |
| --- | --- | --- |
| Query 1 | MLHGASSRPATARKSSGLSGTVRIPGDKSISHRSFMFGGLASGETRITGLLEGEDVINTG | 60 |
| Sbjct 1 | ML+G++ +PATARKS+GL+G+VRIPGDKSISHRSFMFGGLASGETRITGLLEGEDVINTG MLNGSAPKPATARKSAGLTGSVRIPGDKSISHRSFMFGGLASGETRITGLLEGEDVINTG | 60 |
| Query 61 | KAMQAMGARIRKEGDTWIIDGVGNGGLLAPEAPLDFGNAATGCRLTMGLVGVYDFDSTFI | 120 |
| Sbjct 61 | +AMQAMGARIRKEG W+I+G GNG LLAP+APLDFGNA TG RLTMGLVG YDF STF RAMQAMGARIRKEGAQWVIEGTGNGALLAPDAPLDFGNAGTGVRLTMGLVGTYDFHSTFT | 120 |
| Query 121 | GDASLTKRPMGRVLNPLREMGVQVKSEDGDRLPVTLRGPKTPTPITYRVPMASAQVKSAV | 180 |
| Sbjct 121 | GDASL+KRPMGRVLNPLREMGVQ+ + +GDRLPVTLRGP TP+PI YRVPMASAQVKSAV GDASLSKRPMGRVLNPLREMGVQISASEGDRLPVTLRGPGTPSPIRYRVPMASAQVKSAV | 180 |
| Query 181 | LLAGLNTPGITTVIEPIMTRDHTEKMLQGFGANLTVETDADGVRTIRLEGRGKLTGQVID | 240 |
| Sbjct 181 | LLAGLNTPG+TTVIEP+MTRDHTE+MLQGFGA L+VETD++GVRTIRLEGRGKL GQVI LLAGLNTPGVTTVIEPVMTRDHTERMLQGFGAALSVETDSEGVRTIRLEGRGKLAGQVIA | 240 |
| Query 241 | VPGDPSSTAFPLVAALLVPGSDVTILNVLMNPTRTGLILTLQEMGADIEVINPRLAGGED | 300 |
| Sbjct 241 | VPGDPSSTAFPLVAAL+VPGSD+TI+NVLMNPTRTGLILTLQEMGADIEV+N RLAGGED VPGDPSSTAFPLVAALIVPGSDITIVNVLMNPTRTGLILTLQEMGADIEVVNARLAGGED | 300 |
| Query 301 | VADLRVRSSTLKGVTVPEDRAPSMIDEYPILavaaafaegaTVMNGLEELRVKESDRLSA | 360 |
| Sbjct 301 | VADLRVR S L+GVTVPEDRAPSMIDEYPILAVAA FAEGAT+M GLEELRVKESDRLSA  VADLRVRHSELRGVTVPEDRAPSMIDEYPILAVAACFAEGATIMKGLEELRVKESDRLSA | 360 |

| Query | 361 | VANGLKLNGVDCDEGETSLVVRGRPDGKGLGNASGAAVATHLDHRIAMSFLVMGLVSENP 420 |
| --- | --- | --- |
| Sbjct | 361 | VA+GLKLNGVDCDEGE L+VRGRPDG+ LGNA+G V THLDHRIAMSFLV+GL SE+ VADGLKLNGVDCDEGEDFLIVRGRPDGRSLGNAAGGQVRTHLDHRIAMSFLVLGLASEHA 420 |
| Query | 421 | VTVDDATMIATSFPEFMDLMAGLGAKIEL 449 |
| Sbjct | 421 | VT+DDA MIATSFPEFM LM GLGAKIEL  VTIDDAAMIATSFPEFMQLMTGLGAKIEL 449 |

>QND16088.1 3-phosphoshikimate 1-carboxyvinyltransferase [Rhizobium leguminosarum bv. trifolii]

Length=452

Score = 760 bits (1963), Expect = 0.0, Method: Compositional matrix adjust.

| Identities | = 382/448 (85%), Positives = 418/448 (93%), Gaps = 0/448 (0%) |  |
| --- | --- | --- |
| Query 1 | MLHGASSRPATARKSSGLSGTVRIPGDKSISHRSFMFGGLASGETRITGLLEGEDVINTG | 60 |
| Sbjct 1 | ML+G++ RPATARKS+GL+G+VRIPGDKSISHRSFMFGGLASG+TRITGLLEGEDVINTG MLNGSAPRPATARKSAGLTGSVRIPGDKSISHRSFMFGGLASGQTRITGLLEGEDVINTG | 60 |
| Query 61 | KAMQAMGARIRKEGDTWIIDGVGNGGLLAPEAPLDFGNAATGCRLTMGLVGVYDFDSTFI | 120 |
| Sbjct 61 | +AMQAMGARIRKEG W+IDG GNG LLAP+APLDFGNA TG RLTMGLVG YDF STFI RAMQAMGARIRKEGAQWVIDGTGNGALLAPDAPLDFGNAGTGVRLTMGLVGTYDFRSTFI | 120 |
| Query 121 | GDASLTKRPMGRVLNPLREMGVQVKSEDGDRLPVTLRGPKTPTPITYRVPMASAQVKSAV | 180 |
| Sbjct 121 | GDASL+KRPMGRVLNPLREMGVQ+ + +G+RLPVTL+GP TP+PI YRVPMASAQVKSAV GDASLSKRPMGRVLNPLREMGVQISASEGNRLPVTLKGPGTPSPIRYRVPMASAQVKSAV | 180 |
| Query 181 | LLAGLNTPGITTVIEPIMTRDHTEKMLQGFGANLTVETDADGVRTIRLEGRGKLTGQVID | 240 |
| Sbjct 181 | LLAGLNTPG+TTVIEP+MTRDHTEKMLQGFGA L+VETD++GVRTIRLEGRG+LTG+VID LLAGLNTPGVTTVIEPVMTRDHTEKMLQGFGAALSVETDSEGVRTIRLEGRGRLTGRVID | 240 |
| Query 241 | VPGDPSSTAFPLVAALLVPGSDVTILNVLMNPTRTGLILTLQEMGADIEVINPRLAGGED | 300 |
| Sbjct 241 | VPGDPSSTAFPLVAALLVPGSD+TI++VLMNPTRTGL+LTLQEMGADIEV+N RLAGGED VPGDPSSTAFPLVAALLVPGSDITIVHVLMNPTRTGLMLTLQEMGADIEVLNTRLAGGED | 300 |
| Query 301 | VADLRVRSSTLKGVTVPEDRAPSMIDEYPILavaaafaegaTVMNGLEELRVKESDRLSA | 360 |
| Sbjct 301 | VADLRVR S LKGVTVPEDRAPSMIDEYPILAVAA FA+GAT+M GLEELRVKESDRLSA VADLRVRHSELKGVTVPEDRAPSMIDEYPILAVAACFAKGATIMKGLEELRVKESDRLSA | 360 |
| Query 361 | VANGLKLNGVDCDEGETSLVVRGRPDGKGLGNASGAAVATHLDHRIAMSFLVMGLVSENP | 420 |
| Sbjct 361 | VA+GLKLNGVDCDEGE L+VRGRPDGKGLG A V+THLDHRIAMSFLVMGL SE+P VADGLKLNGVDCDEGEDFLIVRGRPDGKGLGTAVDGRVSTHLDHRIAMSFLVMGLASEHP | 420 |
| Query 421  Sbjct 421 | VTVDDATMIATSFPEFMDLMAGLGAKIE 448 VT+DDA MIATSFPEFM LM GLGAKIE VTIDDAAMIATSFPEFMQLMTGLGAKIE 448 |  |

>QND21961.1 3-phosphoshikimate 1-carboxyvinyltransferase [Rhizobium leguminosarum bv. viciae]

Length=452

Score = 760 bits (1962), Expect = 0.0, Method: Compositional matrix adjust.

| Identities | = 383/449 (85%), Positives = 414/449 (92%), Gaps = 0/449 (0%) |  |
| --- | --- | --- |
| Query 1 | MLHGASSRPATARKSSGLSGTVRIPGDKSISHRSFMFGGLASGETRITGLLEGEDVINTG | 60 |
| Sbjct 1 | MLHG++ RPATARKS+ L+G+VRIPGDKSISHRSFMFGGLASGETRITGLLEGEDVINTG MLHGSAPRPATARKSTSLTGSVRIPGDKSISHRSFMFGGLASGETRITGLLEGEDVINTG | 60 |
| Query 61 | KAMQAMGARIRKEGDTWIIDGVGNGGLLAPEAPLDFGNAATGCRLTMGLVGVYDFDSTFI | 120 |
| Sbjct 61 | +AMQAMGARIRKEG W+I+G GNG LLAP+APLDFGNA TG RLTMGLVG YDF STF RAMQAMGARIRKEGAQWVIEGTGNGALLAPDAPLDFGNAGTGVRLTMGLVGTYDFHSTFT | 120 |
| Query 121 | GDASLTKRPMGRVLNPLREMGVQVKSEDGDRLPVTLRGPKTPTPITYRVPMASAQVKSAV | 180 |
| Sbjct 121 | GDASL+KRPMGRVL PLREMGVQV + +GDRLPVTLRGP TP+PI YRVPMASAQVKSAV GDASLSKRPMGRVLAPLREMGVQVSASEGDRLPVTLRGPGTPSPIRYRVPMASAQVKSAV | 180 |
| Query 181 | LLAGLNTPGITTVIEPIMTRDHTEKMLQGFGANLTVETDADGVRTIRLEGRGKLTGQVID | 240 |
| Sbjct 181 | LLAGLNTPG+TTVIEP+MTRDHTEKMLQGFGA L+VETD DGVRTIRLEGRGKL G+VID  LLAGLNTPGVTTVIEPVMTRDHTEKMLQGFGAALSVETDGDGVRTIRLEGRGKLKGRVID | 240 |

| Query | 241 | VPGDPSSTAFPLVAALLVPGSDVTILNVLMNPTRTGLILTLQEMGADIEVINPRLAGGED | 300 |
| --- | --- | --- | --- |
| Sbjct | 241 | +PGDPSSTAFPLVAALLVPGSD+TI+NVL NPTRTGLILTLQEMGADIEV+N RLAGGED IPGDPSSTAFPLVAALLVPGSDITIVNVLTNPTRTGLILTLQEMGADIEVLNARLAGGED | 300 |
| Query | 301 | VADLRVRSSTLKGVTVPEDRAPSMIDEYPILavaaafaegaTVMNGLEELRVKESDRLSA | 360 |
| Sbjct | 301 | VADLRVR S LKGVTVPE+RAPSMIDEYPILA+AA FAEGATVM GLEELRVKESDRLSA VADLRVRHSELKGVTVPEERAPSMIDEYPILAIAACFAEGATVMKGLEELRVKESDRLSA | 360 |
| Query | 361 | VANGLKLNGVDCDEGETSLVVRGRPDGKGLGNASGAAVATHLDHRIAMSFLVMGLVSENP | 420 |
| Sbjct | 361 | VA+GLKLNGVDCDEG+ L+VRGRPDGKG+GNA+G V THLDHRIAMSFLV+GL SE+ VADGLKLNGVDCDEGKDFLIVRGRPDGKGIGNAAGGQVRTHLDHRIAMSFLVLGLASEHA | 420 |
| Query  Sbjct | 421  421 | VTVDDATMIATSFPEFMDLMAGLGAKIEL 449 VT+DDA MIATSFPEFM LM GLGAKIEL VTIDDAAMIATSFPEFMQLMTGLGAKIEL 449 |  |

>WP_164593118.1 3-phosphoshikimate 1-carboxyvinyltransferase [Rhizobium leguminosarum] NEJ88773.1 3-phosphoshikimate 1-carboxyvinyltransferase [Rhizobium leguminosarum]

Length=452

Score = 760 bits (1962), Expect = 0.0, Method: Compositional matrix adjust.

| Identities | = 385/449 (86%), Positives = 413/449 (92%), Gaps = 0/449 (0%) |  |
| --- | --- | --- |
| Query 1 | MLHGASSRPATARKSSGLSGTVRIPGDKSISHRSFMFGGLASGETRITGLLEGEDVINTG | 60 |
| Sbjct 1 | ML+G++ RPATARKS+GL+G VRIPGDKSISHRSFMFGGLASGETRITGLLEGEDVINTG MLNGSAPRPATARKSAGLTGRVRIPGDKSISHRSFMFGGLASGETRITGLLEGEDVINTG | 60 |
| Query 61 | KAMQAMGARIRKEGDTWIIDGVGNGGLLAPEAPLDFGNAATGCRLTMGLVGVYDFDSTFI | 120 |
| Sbjct 61 | +AMQAMGARIRKEG W+I+G GNG LLAP+APLDFGNA TG RLTMGLVG YDF STF RAMQAMGARIRKEGAQWVIEGTGNGALLAPDAPLDFGNAGTGVRLTMGLVGTYDFHSTFT | 120 |
| Query 121 | GDASLTKRPMGRVLNPLREMGVQVKSEDGDRLPVTLRGPKTPTPITYRVPMASAQVKSAV | 180 |
| Sbjct 121 | GDASL+KRPMGRVL PLREMGVQV + +GDRLPVTLRGP TP+PI YRVPMASAQVKSAV GDASLSKRPMGRVLAPLREMGVQVSASEGDRLPVTLRGPGTPSPIRYRVPMASAQVKSAV | 180 |
| Query 181 | LLAGLNTPGITTVIEPIMTRDHTEKMLQGFGANLTVETDADGVRTIRLEGRGKLTGQVID | 240 |
| Sbjct 181 | LLAGLNTPG+TTVIEP+MTRDHTEK+LQGFGA L+VET DGVRTIRLEGRGKL GQVID LLAGLNTPGVTTVIEPVMTRDHTEKILQGFGAALSVETGGDGVRTIRLEGRGKLAGQVID | 240 |
| Query 241 | VPGDPSSTAFPLVAALLVPGSDVTILNVLMNPTRTGLILTLQEMGADIEVINPRLAGGED | 300 |
| Sbjct 241 | VPGDPSSTAFPLVAAL+VPGSD+TI+NVLMNPTRTGLILTLQEMGADIEV+N RLAGGED VPGDPSSTAFPLVAALIVPGSDITIVNVLMNPTRTGLILTLQEMGADIEVVNARLAGGED | 300 |
| Query 301 | VADLRVRSSTLKGVTVPEDRAPSMIDEYPILavaaafaegaTVMNGLEELRVKESDRLSA | 360 |
| Sbjct 301 | VADLRVR S LKGV VPEDRAPSMIDEYPILA+AA FAEGATVM GLEELRVKESDRLSA VADLRVRHSELKGVGVPEDRAPSMIDEYPILAIAACFAEGATVMKGLEELRVKESDRLSA | 360 |
| Query 361 | VANGLKLNGVDCDEGETSLVVRGRPDGKGLGNASGAAVATHLDHRIAMSFLVMGLVSENP | 420 |
| Sbjct 361 | VA+GLKLNGVDCDEGE L+VRGRPDGKGLGNA+G V THLDHRIAMSFLV+GL SE+ VADGLKLNGVDCDEGEDFLIVRGRPDGKGLGNAAGGQVRTHLDHRIAMSFLVLGLASEHA | 420 |
| Query 421  Sbjct 421 | VTVDDATMIATSFPEFMDLMAGLGAKIEL 449 VT+DDA MIATSFPEFM LM GLGAKIEL VTIDDAAMIATSFPEFMQLMTGLGAKIEL 449 |  |

>WP_017966433.1 3-phosphoshikimate 1-carboxyvinyltransferase [Rhizobium leguminosarum] Length=452

Score = 759 bits (1961), Expect = 0.0, Method: Compositional matrix adjust. Identities = 382/449 (85%), Positives = 412/449 (92%), Gaps = 0/449 (0%)

| Query | 1 | MLHGASSRPATARKSSGLSGTVRIPGDKSISHRSFMFGGLASGETRITGLLEGEDVINTG | 60 |
| --- | --- | --- | --- |
| Sbjct | 1 | MLHG++ RPATARKS+ L+G+VRIPGDKSISHRSFMFGGLASGETRITGLLEGEDVINTG MLHGSAPRPATARKSASLTGSVRIPGDKSISHRSFMFGGLASGETRITGLLEGEDVINTG | 60 |
| Query | 61 | KAMQAMGARIRKEGDTWIIDGVGNGGLLAPEAPLDFGNAATGCRLTMGLVGVYDFDSTFI | 120 |
| Sbjct | 61 | +AMQAMGARIRK+G W+I+G GNG LLAP+APLDFGNA TG RLTMGLVG YDF STF RAMQAMGARIRKDGAQWVIEGTGNGALLAPDAPLDFGNAGTGVRLTMGLVGTYDFHSTFT | 120 |
| Query | 121 | GDASLTKRPMGRVLNPLREMGVQVKSEDGDRLPVTLRGPKTPTPITYRVPMASAQVKSAV | 180 |

| Sbjct | 121 | GDASL+KRPMGRVL PLREMGVQV + GDRLPVTLRGP TP+PI YRVPMASAQVKSAV GDASLSKRPMGRVLAPLREMGVQVSASQGDRLPVTLRGPGTPSPIRYRVPMASAQVKSAV | 180 |
| --- | --- | --- | --- |
| Query | 181 | LLAGLNTPGITTVIEPIMTRDHTEKMLQGFGANLTVETDADGVRTIRLEGRGKLTGQVID | 240 |
| Sbjct | 181 | LLAGLNTPG+TTVIEP+MTRDHTEKMLQGFGA L+VETD DGVRTIRLEGRGKL GQVID LLAGLNTPGVTTVIEPVMTRDHTEKMLQGFGAALSVETDGDGVRTIRLEGRGKLKGQVID | 240 |
| Query | 241 | VPGDPSSTAFPLVAALLVPGSDVTILNVLMNPTRTGLILTLQEMGADIEVINPRLAGGED | 300 |
| Sbjct | 241 | +PGDPSSTAFPLVAAL+VPGSD+TI+NVLMNPTRTGLILTLQEMGADIEV+N RLAGGED IPGDPSSTAFPLVAALIVPGSDITIVNVLMNPTRTGLILTLQEMGADIEVVNARLAGGED | 300 |
| Query | 301 | VADLRVRSSTLKGVTVPEDRAPSMIDEYPILavaaafaegaTVMNGLEELRVKESDRLSA | 360 |
| Sbjct | 301 | VADLRVR S LKGVTVPEDRAPSMIDEYPILAVAA FAEGATVM GLEELRVKESDRLSA VADLRVRHSELKGVTVPEDRAPSMIDEYPILAVAACFAEGATVMKGLEELRVKESDRLSA | 360 |
| Query | 361 | VANGLKLNGVDCDEGETSLVVRGRPDGKGLGNASGAAVATHLDHRIAMSFLVMGLVSENP | 420 |
| Sbjct | 361 | VA+GL LNGVDCDEG+ L+VRGRPDG+G+GNA+G THLDHRIAMSFLV+GL SE+ VADGLTLNGVDCDEGKDFLIVRGRPDGRGIGNAAGGQAQTHLDHRIAMSFLVLGLASEHA | 420 |
| Query  Sbjct | 421  421 | VTVDDATMIATSFPEFMDLMAGLGAKIEL 449 VT+DDA MIATSFPEFM LM GLGAKIEL VTIDDAAMIATSFPEFMQLMTGLGAKIEL 449 |  |

>WP_130673329.1 3-phosphoshikimate 1-carboxyvinyltransferase [Rhizobium leguminosarum] NEH32660.1 3-phosphoshikimate 1-carboxyvinyltransferase [Rhizobium leguminosarum] NEJ09466.1 3-phosphoshikimate 1-carboxyvinyltransferase [Rhizobium leguminosarum] NEK06973.1 3-phosphoshikimate 1-carboxyvinyltransferase [Rhizobium leguminosarum] QIJ42879.1 3-phosphoshikimate 1-carboxyvinyltransferase [Rhizobium leguminosarum] TAU12360.1 3-phosphoshikimate 1-carboxyvinyltransferase [Rhizobium leguminosarum]

Length=452

Score = 759 bits (1960), Expect = 0.0, Method: Compositional matrix adjust.

| Identities | = 384/449 (86%), Positives = 411/449 (92%), Gaps = 0/449 (0%) |  |
| --- | --- | --- |
| Query 1 | MLHGASSRPATARKSSGLSGTVRIPGDKSISHRSFMFGGLASGETRITGLLEGEDVINTG | 60 |
| Sbjct 1 | MLHG++ RPATARKS+ L+G+V IPGDKSISHRSFMFGGLASGETRITGLLE EDVINTG MLHGSAPRPATARKSASLTGSVCIPGDKSISHRSFMFGGLASGETRITGLLESEDVINTG | 60 |
| Query 61 | KAMQAMGARIRKEGDTWIIDGVGNGGLLAPEAPLDFGNAATGCRLTMGLVGVYDFDSTFI | 120 |
| Sbjct 61 | +AMQAMGARIRKEG W+I+G GNG LLAP+APLDFGNA TG RLTMGLVG YDF STF RAMQAMGARIRKEGAQWVIEGTGNGALLAPDAPLDFGNAGTGVRLTMGLVGTYDFHSTFT | 120 |
| Query 121 | GDASLTKRPMGRVLNPLREMGVQVKSEDGDRLPVTLRGPKTPTPITYRVPMASAQVKSAV | 180 |
| Sbjct 121 | GDASL+KRPMGRVL PLREMGVQV + +GDRLPVTLRGP TP+PI YRVPMASAQVKSAV GDASLSKRPMGRVLAPLREMGVQVSASEGDRLPVTLRGPGTPSPIRYRVPMASAQVKSAV | 180 |
| Query 181 | LLAGLNTPGITTVIEPIMTRDHTEKMLQGFGANLTVETDADGVRTIRLEGRGKLTGQVID | 240 |
| Sbjct 181 | LLAGLNTPG+TTVIEP+MTRDHTEKMLQGFGA L+VET DGVRTIRLEGRGKL GQVID LLAGLNTPGVTTVIEPVMTRDHTEKMLQGFGAALSVETGGDGVRTIRLEGRGKLAGQVID | 240 |
| Query 241 | VPGDPSSTAFPLVAALLVPGSDVTILNVLMNPTRTGLILTLQEMGADIEVINPRLAGGED | 300 |
| Sbjct 241 | VPGDPSSTAFPLVAAL+VPGSD+TI+NVLMNPTRTGLILTLQEMGADIEV+N RLAGGED VPGDPSSTAFPLVAALIVPGSDITIVNVLMNPTRTGLILTLQEMGADIEVVNARLAGGED | 300 |
| Query 301 | VADLRVRSSTLKGVTVPEDRAPSMIDEYPILavaaafaegaTVMNGLEELRVKESDRLSA | 360 |
| Sbjct 301 | VADLRVR S LKGV VPEDRAPSMIDEYPILA+AA FAEGATVM GLEELRVKESDRLSA VADLRVRHSELKGVGVPEDRAPSMIDEYPILAIAACFAEGATVMKGLEELRVKESDRLSA | 360 |
| Query 361 | VANGLKLNGVDCDEGETSLVVRGRPDGKGLGNASGAAVATHLDHRIAMSFLVMGLVSENP | 420 |
| Sbjct 361 | VA+GLKLNGVDCDEGE L+VRGRPDGKGLGNA+G V THLDHRIAMSFLV+GL SE+ VADGLKLNGVDCDEGEDFLIVRGRPDGKGLGNAAGGQVRTHLDHRIAMSFLVLGLASEHA | 420 |
| Query 421  Sbjct 421 | VTVDDATMIATSFPEFMDLMAGLGAKIEL 449 VT+DDA MIATSFPEFM LM GLGAKIEL VTIDDAAMIATSFPEFMQLMTGLGAKIEL 449 |  |

>WP_170269375.1 3-phosphoshikimate 1-carboxyvinyltransferase [Rhizobium laguerreae] NNH84794.1 3-phosphoshikimate 1-carboxyvinyltransferase [Rhizobium laguerreae]

Length=452

Score = 758 bits (1958), Expect = 0.0, Method: Compositional matrix adjust.

| Identities | = 383/448 (85%), Positives = 412/448 (92%), Gaps = 0/448 (0%) |  |
| --- | --- | --- |
| Query 1 | MLHGASSRPATARKSSGLSGTVRIPGDKSISHRSFMFGGLASGETRITGLLEGEDVINTG | 60 |
| Sbjct 1 | MLHG++ +PATARKS+GL+G+VRIPGDKSISHRSFMFGGLASGETRITGLLEGEDVINTG MLHGSAPKPATARKSAGLTGSVRIPGDKSISHRSFMFGGLASGETRITGLLEGEDVINTG | 60 |
| Query 61 | KAMQAMGARIRKEGDTWIIDGVGNGGLLAPEAPLDFGNAATGCRLTMGLVGVYDFDSTFI | 120 |
| Sbjct 61 | +AMQAMGA IRKEG+ WII+G GNG LLAP+APLDFGNA TG RLTMGLVG YDF STF RAMQAMGASIRKEGEQWIINGTGNGALLAPDAPLDFGNAGTGVRLTMGLVGTYDFHSTFT | 120 |
| Query 121 | GDASLTKRPMGRVLNPLREMGVQVKSEDGDRLPVTLRGPKTPTPITYRVPMASAQVKSAV | 180 |
| Sbjct 121 | GDASL+KRPMGRVL+PLREMGVQV + +GDRLPVTLRGP TP+PI YRVPMASAQVKSAV GDASLSKRPMGRVLDPLREMGVQVSASEGDRLPVTLRGPGTPSPIRYRVPMASAQVKSAV | 180 |
| Query 181 | LLAGLNTPGITTVIEPIMTRDHTEKMLQGFGANLTVETDADGVRTIRLEGRGKLTGQVID | 240 |
| Sbjct 181 | LLAGLNTPGIT VIEP+MTRDHTEKMLQGFGA L+VETD DGVRTIRLEGRGKL GQVID LLAGLNTPGITMVIEPVMTRDHTEKMLQGFGAALSVETDKDGVRTIRLEGRGKLAGQVID | 240 |
| Query 241 | VPGDPSSTAFPLVAALLVPGSDVTILNVLMNPTRTGLILTLQEMGADIEVINPRLAGGED | 300 |
| Sbjct 241 | +PGDPSSTAFPLVAALLVPGSD+TI+NVLMNPTRTGLILTLQEMGA+IEV+N RLAGGED IPGDPSSTAFPLVAALLVPGSDITIVNVLMNPTRTGLILTLQEMGANIEVLNTRLAGGED | 300 |
| Query 301 | VADLRVRSSTLKGVTVPEDRAPSMIDEYPILavaaafaegaTVMNGLEELRVKESDRLSA | 360 |
| Sbjct 301 | VADLRVR S LKGV VP +RAPSMIDEYP+LAVAA FAEG TVM GLEELRVKESDRLSA VADLRVRHSELKGVNVPAERAPSMIDEYPVLAVAACFAEGVTVMKGLEELRVKESDRLSA | 360 |
| Query 361 | VANGLKLNGVDCDEGETSLVVRGRPDGKGLGNASGAAVATHLDHRIAMSFLVMGLVSENP | 420 |
| Sbjct 361 | VA+GLKLNGVDCDEGE LVVRGRPDGKGLGNA+G V+THLDHRIAMSFLVMGL SE+ VADGLKLNGVDCDEGEDFLVVRGRPDGKGLGNAAGDKVSTHLDHRIAMSFLVMGLASEHA | 420 |
| Query 421  Sbjct 421 | VTVDDATMIATSFPEFMDLMAGLGAKIE 448 VT+DDA MIATSFPEF LM GLGAKIE VTIDDAAMIATSFPEFRQLMTGLGAKIE 448 |  |

>WP_183823914.1 3-phosphoshikimate 1-carboxyvinyltransferase [Rhizobium cellulosilyticum] MBB4349144.1 3-phosphoshikimate 1-carboxyvinyltransferase [Rhizobium cellulosilyticum] MBB4412635.1 3-phosphoshikimate 1-carboxyvinyltransferase [Rhizobium cellulosilyticum] MBB4447267.1 3-phosphoshikimate 1-carboxyvinyltransferase [Rhizobium cellulosilyticum]

Length=455

Score = 758 bits (1957), Expect = 0.0, Method: Compositional matrix adjust.

| Identities | = 385/455 (85%), Positives = 410/455 (90%), Gaps = 0/455 (0%) |  |
| --- | --- | --- |
| Query 1 | MLHGASSRPATARKSSGLSGTVRIPGDKSISHRSFMFGGLASGETRITGLLEGEDVINTG | 60 |
| Sbjct 1 | M HG + +PA + +S L GT+RIPGDKSISHRSFMFGGLASGETRITGLLEGEDVINTG MSHGIALKPAKSARSEALKGTIRIPGDKSISHRSFMFGGLASGETRITGLLEGEDVINTG | 60 |
| Query 61 | KAMQAMGARIRKEGDTWIIDGVGNGGLLAPEAPLDFGNAATGCRLTMGLVGVYDFDSTFI | 120 |
| Sbjct 61 | KAM+AMGA+I K G WII G GNG LLAPEAPLDFGNA TGCRLTMGLVGVYDF STFI KAMRAMGAQIEKVGAEWIIQGTGNGALLAPEAPLDFGNAGTGCRLTMGLVGVYDFASTFI | 120 |
| Query 121 | GDASLTKRPMGRVLNPLREMGVQVKSEDGDRLPVTLRGPKTPTPITYRVPMASAQVKSAV | 180 |
| Sbjct 121 | GDASL+KRPMGRVLNPLREMGVQV S +GDRLPVTL GPK P PITYRVPMASAQVKSAV GDASLSKRPMGRVLNPLREMGVQVTSVEGDRLPVTLHGPKVPNPITYRVPMASAQVKSAV | 180 |
| Query 181 | LLAGLNTPGITTVIEPIMTRDHTEKMLQGFGANLTVETDADGVRTIRLEGRGKLTGQVID | 240 |
| Sbjct 181 | LLAGLNTPGITTVIEP+MTRDHTEKMLQGFGA+L+VETDA+GVRTIRL G+G+L GQVID LLAGLNTPGITTVIEPVMTRDHTEKMLQGFGADLSVETDAEGVRTIRLVGQGQLKGQVID | 240 |
| Query 241 | VPGDPSSTAFPLVAALLVPGSDVTILNVLMNPTRTGLILTLQEMGADIEVINPRLAGGED | 300 |
| Sbjct 241 | VPGDPSSTAFPLVAALL PGSDVTILNVLMNPTRTGLILTLQEMGADIEV N RLAGGED VPGDPSSTAFPLVAALLTPGSDVTILNVLMNPTRTGLILTLQEMGADIEVQNARLAGGED | 300 |
| Query 301 | VADLRVRSSTLKGVTVPEDRAPSMIDEYPILavaaafaegaTVMNGLEELRVKESDRLSA | 360 |
| Sbjct 301 | VADLRVR S LKGVTVP DRAPSMIDEYP+LAVAAA+A G T M GLEELRVKESDRLSA VADLRVRYSELKGVTVPADRAPSMIDEYPVLAVAAAYASGTTTMLGLEELRVKESDRLSA | 360 |
| Query 361 | VANGLKLNGVDCDEGETSLVVRGRPDGKGLGNASGAAVATHLDHRIAMSFLVMGLVSENP | 420 |
| Sbjct 361 | VA GLKLNGVDCDEG+ +LVVRGRP GKG GNASG AV THLDHRIAMSFLV+GL SE+P  VAEGLKLNGVDCDEGQETLVVRGRPGGKGYGNASGKAVVTHLDHRIAMSFLVLGLASEHP | 420 |

Query 421 VTVDDATMIATSFPEFMDLMAGLGAKIELSDTKAA 455 VTVDDA++IATSFPEFMDLM GLGAKI+ D +AA

Sbjct 421 VTVDDASIIATSFPEFMDLMTGLGAKIDAVDGEAA 455

>WP_142592023.1 3-phosphoshikimate 1-carboxyvinyltransferase [Rhizobium endolithicum] CAD6414249.1 3-phosphoshikimate 1-carboxyvinyltransferase [Rhizobium endolithicum]

Length=455

Score = 758 bits (1957), Expect = 0.0, Method: Compositional matrix adjust.

| Identities | = 384/455 (84%), Positives = 416/455 (91%), Gaps = 0/455 (0%) |  |
| --- | --- | --- |
| Query 1 | MLHGASSRPATARKSSGLSGTVRIPGDKSISHRSFMFGGLASGETRITGLLEGEDVINTG | 60 |
| Sbjct 1 | M HG + +PA + KS+ L GTVRIPGDKSISHR FMFGGLASGETRITGLLEGEDVINTG MSHGVALKPARSLKSADLKGTVRIPGDKSISHRCFMFGGLASGETRITGLLEGEDVINTG | 60 |
| Query 61 | KAMQAMGARIRKEGDTWIIDGVGNGGLLAPEAPLDFGNAATGCRLTMGLVGVYDFDSTFI | 120 |
| Sbjct 61 | KAM+AMGA+I K GD WII G GNG LLAPEAPLDFGNA TGCRLTMGLVGVYDF+STFI KAMRAMGAKIEKVGDEWIIQGTGNGALLAPEAPLDFGNAGTGCRLTMGLVGVYDFESTFI | 120 |
| Query 121 | GDASLTKRPMGRVLNPLREMGVQVKSEDGDRLPVTLRGPKTPTPITYRVPMASAQVKSAV | 180 |
| Sbjct 121 | GDASL+KRPM RVLNPLREMGVQVK+ +GDRLPVTLRGP P PITYRVPMASAQVKSAV GDASLSKRPMARVLNPLREMGVQVKAAEGDRLPVTLRGPSIPNPITYRVPMASAQVKSAV | 180 |
| Query 181 | LLAGLNTPGITTVIEPIMTRDHTEKMLQGFGANLTVETDADGVRTIRLEGRGKLTGQVID | 240 |
| Sbjct 181 | LLAGLNTPG+TTVIEP+MTRDHTEKMLQGFGANL VETDA+GVRTIRLEGRG+L GQVID LLAGLNTPGVTTVIEPVMTRDHTEKMLQGFGANLMVETDAEGVRTIRLEGRGQLIGQVID | 240 |
| Query 241 | VPGDPSSTAFPLVAALLVPGSDVTILNVLMNPTRTGLILTLQEMGADIEVINPRLAGGED | 300 |
| Sbjct 241 | VPGDPSSTAFPLVAA+LVPGSDVTI NVLMNPTRTGLILTLQEMGADIE+++PRLAGGED VPGDPSSTAFPLVAAILVPGSDVTIENVLMNPTRTGLILTLQEMGADIEILDPRLAGGED | 300 |
| Query 301 | VADLRVRSSTLKGVTVPEDRAPSMIDEYPILavaaafaegaTVMNGLEELRVKESDRLSA | 360 |
| Sbjct 301 | VADLRV+ S LKGVTVP DRAPSMIDEYP+LAVAAAFAEG T M GLEELRVKESDRLSA VADLRVKHSELKGVTVPADRAPSMIDEYPVLAVAAAFAEGTTTMLGLEELRVKESDRLSA | 360 |
| Query 361 | VANGLKLNGVDCDEGETSLVVRGRPDGKGLGNASGAAVATHLDHRIAMSFLVMGLVSENP | 420 |
| Sbjct 361 | VA+GLK+NGVDCDEGE +LVVRGRP GKG GN +GAAV THLDHRIAMSFLVMGLV+E+ VADGLKVNGVDCDEGEDTLVVRGRPGGKGYGNTAGAAVVTHLDHRIAMSFLVMGLVAEHA | 420 |
| Query 421  Sbjct 421 | VTVDDATMIATSFPEFMDLMAGLGAKIELSDTKAA 455 VTVD+A +IATSFPEFMDLM GLGA+IE +++AA VTVDNAGIIATSFPEFMDLMTGLGARIEPVESRAA 455 |  |

>WP_130853502.1 3-phosphoshikimate 1-carboxyvinyltransferase [Rhizobium leguminosarum] TBH56445.1 3-phosphoshikimate 1-carboxyvinyltransferase [Rhizobium leguminosarum]

Length=452

Score = 757 bits (1955), Expect = 0.0, Method: Compositional matrix adjust.

| Identities | = 381/449 (85%), Positives = 415/449 (92%), Gaps = 0/449 (0%) |  |
| --- | --- | --- |
| Query 1 | MLHGASSRPATARKSSGLSGTVRIPGDKSISHRSFMFGGLASGETRITGLLEGEDVINTG | 60 |
| Sbjct 1 | ML+G++ +PATARKS+GL+G+VRIPGDKSISHRSFMFGGLASGETRITGLLEGEDVINTG MLNGSAPKPATARKSAGLTGSVRIPGDKSISHRSFMFGGLASGETRITGLLEGEDVINTG | 60 |
| Query 61 | KAMQAMGARIRKEGDTWIIDGVGNGGLLAPEAPLDFGNAATGCRLTMGLVGVYDFDSTFI | 120 |
| Sbjct 61 | +AMQAMGARIRK+G W+I+G GNG LLAP+APLDFGNA TG RLTMGLVG YDF STF RAMQAMGARIRKDGAQWVIEGTGNGALLAPDAPLDFGNAGTGVRLTMGLVGTYDFHSTFT | 120 |
| Query 121 | GDASLTKRPMGRVLNPLREMGVQVKSEDGDRLPVTLRGPKTPTPITYRVPMASAQVKSAV | 180 |
| Sbjct 121 | GDASL+KRPMGRVLNPLREMGVQV + +GDRLPVTLRG TP+PI YRVPMASAQVKSAV GDASLSKRPMGRVLNPLREMGVQVSASEGDRLPVTLRGLGTPSPIRYRVPMASAQVKSAV | 180 |
| Query 181 | LLAGLNTPGITTVIEPIMTRDHTEKMLQGFGANLTVETDADGVRTIRLEGRGKLTGQVID | 240 |
| Sbjct 181 | LLAGLNTPG+TTVIEP+MTRDHTEKMLQGFGA L+VETD++GVRTIRLEGRGKL GQVID LLAGLNTPGVTTVIEPVMTRDHTEKMLQGFGAALSVETDSEGVRTIRLEGRGKLAGQVID | 240 |
| Query 241 | VPGDPSSTAFPLVAALLVPGSDVTILNVLMNPTRTGLILTLQEMGADIEVINPRLAGGED | 300 |
| Sbjct 241 | +PGDPSSTAFPLVAAL+VPGSD+TI+NVLMNPTRTGLILTLQEMGADIEV+N RLAGGED  IPGDPSSTAFPLVAALIVPGSDITIVNVLMNPTRTGLILTLQEMGADIEVVNARLAGGED | 300 |

| Query | 301 | VADLRVRSSTLKGVTVPEDRAPSMIDEYPILavaaafaegaTVMNGLEELRVKESDRLSA | 360 |
| --- | --- | --- | --- |
| Sbjct | 301 | VADLRVR S LKGVTVPE+R PSMIDEYPILA+AA FAEGATVM GLEELRVKESDRLSA VADLRVRHSELKGVTVPEERTPSMIDEYPILAIAACFAEGATVMKGLEELRVKESDRLSA | 360 |
| Query | 361 | VANGLKLNGVDCDEGETSLVVRGRPDGKGLGNASGAAVATHLDHRIAMSFLVMGLVSENP | 420 |
| Sbjct | 361 | VA+GLKLNGVDCDEGE L+VRGRPDGKGLGN + V+THLDHRIAMSFLVMGL SE+ VADGLKLNGVDCDEGEDFLIVRGRPDGKGLGNPADGRVSTHLDHRIAMSFLVMGLASEHA | 420 |
| Query  Sbjct | 421  421 | VTVDDATMIATSFPEFMDLMAGLGAKIEL 449 VT+DDA MIATSFPEFM LM GLGAKIEL VTIDDAAMIATSFPEFMQLMTGLGAKIEL 449 |  |

>WP_171602083.1 3-phosphoshikimate 1-carboxyvinyltransferase [Rhizobium sp. WYCCWR 11279]

NNU47293.1 3-phosphoshikimate 1-carboxyvinyltransferase [Rhizobium sp. WYCCWR 11279]

Length=452

Score = 756 bits (1953), Expect = 0.0, Method: Compositional matrix adjust.

| Identities | = 379/448 (85%), Positives = 415/448 (93%), Gaps = 0/448 (0%) |  |
| --- | --- | --- |
| Query 1 | MLHGASSRPATARKSSGLSGTVRIPGDKSISHRSFMFGGLASGETRITGLLEGEDVINTG | 60 |
| Sbjct 1 | ML G++ +PATARKS+GL+G+VRIPGDKSISHRSFMFGGLASG+TRITGLLEGEDVINTG MLKGSAPKPATARKSAGLTGSVRIPGDKSISHRSFMFGGLASGQTRITGLLEGEDVINTG | 60 |
| Query 61 | KAMQAMGARIRKEGDTWIIDGVGNGGLLAPEAPLDFGNAATGCRLTMGLVGVYDFDSTFI | 120 |
| Sbjct 61 | +AMQAMGARIRK+G W+IDG GNG LLAP+APLDFG+A TG RLTMGLVG YDF STFI RAMQAMGARIRKDGAQWVIDGTGNGALLAPDAPLDFGSAGTGVRLTMGLVGTYDFRSTFI | 120 |
| Query 121 | GDASLTKRPMGRVLNPLREMGVQVKSEDGDRLPVTLRGPKTPTPITYRVPMASAQVKSAV | 180 |
| Sbjct 121 | GDASL+KRPMGRVLNPLREMGVQV + +GDRLPVTL+GP TP+PI YRVPMASAQVKSAV GDASLSKRPMGRVLNPLREMGVQVSASEGDRLPVTLKGPGTPSPIRYRVPMASAQVKSAV | 180 |
| Query 181 | LLAGLNTPGITTVIEPIMTRDHTEKMLQGFGANLTVETDADGVRTIRLEGRGKLTGQVID | 240 |
| Sbjct 181 | LLAGLNTPG+TTVIEP+MTRDHTEKMLQGFGA L+VETD++GVRTIRLEGRG+LTGQVID LLAGLNTPGVTTVIEPVMTRDHTEKMLQGFGAALSVETDSEGVRTIRLEGRGRLTGQVID | 240 |
| Query 241 | VPGDPSSTAFPLVAALLVPGSDVTILNVLMNPTRTGLILTLQEMGADIEVINPRLAGGED | 300 |
| Sbjct 241 | VPGDPSSTAFPLVAALLVPGSD+TI+NVLMNPTRTGLILTLQEMGADIEV+NPRLAGGED VPGDPSSTAFPLVAALLVPGSDITIVNVLMNPTRTGLILTLQEMGADIEVLNPRLAGGED | 300 |
| Query 301 | VADLRVRSSTLKGVTVPEDRAPSMIDEYPILavaaafaegaTVMNGLEELRVKESDRLSA | 360 |
| Sbjct 301 | VADLRVR S LKGVTVPEDRAPSMIDEYPILAVAA FAEG T+M GLEELRVKESDRL+A VADLRVRHSELKGVTVPEDRAPSMIDEYPILAVAACFAEGTTIMKGLEELRVKESDRLAA | 360 |
| Query 361 | VANGLKLNGVDCDEGETSLVVRGRPDGKGLGNASGAAVATHLDHRIAMSFLVMGLVSENP | 420 |
| Sbjct 361 | VA+GLKLNG CDEGE L+VRGRPDG GLGNA+ V++HLDHRIAMSFLVMGL SE+P VADGLKLNGAYCDEGEDFLIVRGRPDGTGLGNAADGKVSSHLDHRIAMSFLVMGLASEHP | 420 |
| Query 421  Sbjct 421 | VTVDDATMIATSFPEFMDLMAGLGAKIE 448 VT+DDA MIATSFPEFM LM LGA+IE VTIDDAAMIATSFPEFMSLMKRLGARIE 448 |  |

>WP_183992976.1 3-phosphoshikimate 1-carboxyvinyltransferase [Rhizobium wenxiniae] MBB6163238.1 3-phosphoshikimate 1-carboxyvinyltransferase [Rhizobium wenxiniae] GGF92408.1 3-phosphoshikimate 1-carboxyvinyltransferase [Rhizobium wenxiniae]

Length=455

Score = 756 bits (1952), Expect = 0.0, Method: Compositional matrix adjust. Identities = 384/455 (84%), Positives = 409/455 (90%), Gaps = 0/455 (0%)

| Query | 1 | MLHGASSRPATARKSSGLSGTVRIPGDKSISHRSFMFGGLASGETRITGLLEGEDVINTG | 60 |
| --- | --- | --- | --- |
| Sbjct | 1 | M HG + +PA + +S L GT+RIPGDKSISHRSFMFGGLASGETRITGLLEGEDVINTG MSHGIALKPAKSARSEALKGTIRIPGDKSISHRSFMFGGLASGETRITGLLEGEDVINTG | 60 |
| Query | 61 | KAMQAMGARIRKEGDTWIIDGVGNGGLLAPEAPLDFGNAATGCRLTMGLVGVYDFDSTFI | 120 |
| Sbjct | 61 | KAM+AMGARI K G WII G GNG LLAPEAPLDFGNA TGCRLTMGLVGVYDF STFI  KAMRAMGARIEKVGAEWIIQGTGNGALLAPEAPLDFGNAGTGCRLTMGLVGVYDFASTFI | 120 |

| Query | 121 | GDASLTKRPMGRVLNPLREMGVQVKSEDGDRLPVTLRGPKTPTPITYRVPMASAQVKSAV | 180 |
| --- | --- | --- | --- |
| Sbjct | 121 | GDASL+KRPMGRVLNPLREMGVQV S +GDRLPVTL GPKTP PITYRVPMASAQVKSAV GDASLSKRPMGRVLNPLREMGVQVSSTEGDRLPVTLHGPKTPNPITYRVPMASAQVKSAV | 180 |
| Query | 181 | LLAGLNTPGITTVIEPIMTRDHTEKMLQGFGANLTVETDADGVRTIRLEGRGKLTGQVID | 240 |
| Sbjct | 181 | LLAGLNTPGITTVIEP+MTRDHTEKMLQGFGA+LTVETDA+GVRTIRL GRG+L GQVID LLAGLNTPGITTVIEPVMTRDHTEKMLQGFGADLTVETDAEGVRTIRLVGRGQLKGQVID | 240 |
| Query | 241 | VPGDPSSTAFPLVAALLVPGSDVTILNVLMNPTRTGLILTLQEMGADIEVINPRLAGGED | 300 |
| Sbjct | 241 | VPGDPSSTAFPLVAA+L PGSDVTILNVLMNPTRTGLILTLQEMGADIEV N RLAGGED VPGDPSSTAFPLVAAILTPGSDVTILNVLMNPTRTGLILTLQEMGADIEVQNARLAGGED | 300 |
| Query | 301 | VADLRVRSSTLKGVTVPEDRAPSMIDEYPILavaaafaegaTVMNGLEELRVKESDRLSA | 360 |
| Sbjct | 301 | VADLRVR S LKGVTVP DRAPSMIDEYP+LAVAAA+A G T M GLEELRVKESDRLSA VADLRVRYSELKGVTVPADRAPSMIDEYPVLAVAAAYASGTTTMLGLEELRVKESDRLSA | 360 |
| Query | 361 | VANGLKLNGVDCDEGETSLVVRGRPDGKGLGNASGAAVATHLDHRIAMSFLVMGLVSENP | 420 |
| Sbjct | 361 | VA GLKLNGVDCDEG+ +LVVRGRP GKG GNA G +V THLDHRIAMSFLV+GL SE+P VAEGLKLNGVDCDEGKETLVVRGRPGGKGYGNAQGKSVVTHLDHRIAMSFLVLGLASEHP | 420 |
| Query  Sbjct | 421  421 | VTVDDATMIATSFPEFMDLMAGLGAKIELSDTKAA 455 VTVDDA++IATSFPEFMDLM GLGAKI+ + + A VTVDDASIIATSFPEFMDLMTGLGAKIDAVEGEGA 455 |  |

>WP_131718266.1 3-phosphoshikimate 1-carboxyvinyltransferase [Rhizobium leguminosarum]

TCA14578.1 3-phosphoshikimate 1-carboxyvinyltransferase [Rhizobium leguminosarum bv. viciae]

Length=452

Score = 755 bits (1950), Expect = 0.0, Method: Compositional matrix adjust.

| Identities | = 382/449 (85%), Positives = 410/449 (91%), Gaps = 0/449 (0%) |  |
| --- | --- | --- |
| Query 1 | MLHGASSRPATARKSSGLSGTVRIPGDKSISHRSFMFGGLASGETRITGLLEGEDVINTG | 60 |
| Sbjct 1 | MLHG++ RPATARKS+ L G+VRIPGDKSISHRSFMFGGLASGETRITGLLEGEDVINTG MLHGSAPRPATARKSASLKGSVRIPGDKSISHRSFMFGGLASGETRITGLLEGEDVINTG | 60 |
| Query 61 | KAMQAMGARIRKEGDTWIIDGVGNGGLLAPEAPLDFGNAATGCRLTMGLVGVYDFDSTFI | 120 |
| Sbjct 61 | +AMQAMGARIRKEG W+I+G GNG LLAP+APLDFGNA TG RLTMGLVG YDF STF RAMQAMGARIRKEGAQWVIEGTGNGALLAPDAPLDFGNAGTGVRLTMGLVGTYDFHSTFT | 120 |
| Query 121 | GDASLTKRPMGRVLNPLREMGVQVKSEDGDRLPVTLRGPKTPTPITYRVPMASAQVKSAV | 180 |
| Sbjct 121 | GDASL+ RPMGRVL PLREMGVQV S +G RLPVTLRGP TP+PI YRVPMASAQVKSAV GDASLSNRPMGRVLAPLREMGVQVSSSEGGRLPVTLRGPGTPSPIRYRVPMASAQVKSAV | 180 |
| Query 181 | LLAGLNTPGITTVIEPIMTRDHTEKMLQGFGANLTVETDADGVRTIRLEGRGKLTGQVID | 240 |
| Sbjct 181 | LLAGLNTPG+TTVIEP+MTRDHTEKMLQGFGA L+VET + GVRTIRLEGRG+LTGQVID LLAGLNTPGVTTVIEPVMTRDHTEKMLQGFGAALSVETGSQGVRTIRLEGRGRLTGQVID | 240 |
| Query 241 | VPGDPSSTAFPLVAALLVPGSDVTILNVLMNPTRTGLILTLQEMGADIEVINPRLAGGED | 300 |
| Sbjct 241 | VPGDPSSTAFPLVAALLVP SD+TI+NVLMNPTRTGLILTLQEMGADIEV+N RLAGGED VPGDPSSTAFPLVAALLVPDSDITIVNVLMNPTRTGLILTLQEMGADIEVVNARLAGGED | 300 |
| Query 301 | VADLRVRSSTLKGVTVPEDRAPSMIDEYPILavaaafaegaTVMNGLEELRVKESDRLSA | 360 |
| Sbjct 301 | VADLRVR S LKGVTVPEDRAPSMIDEYPILA+AA FAEGATVM GLEELRVKESDRLSA VADLRVRHSELKGVTVPEDRAPSMIDEYPILAIAACFAEGATVMKGLEELRVKESDRLSA | 360 |
| Query 361 | VANGLKLNGVDCDEGETSLVVRGRPDGKGLGNASGAAVATHLDHRIAMSFLVMGLVSENP | 420 |
| Sbjct 361 | VA+GLKLNGVDCDEGE L+V GRPDG+G+GNA+G V THLDHRIAMSFLV+GL SE+ VADGLKLNGVDCDEGEDFLIVHGRPDGRGIGNAAGGQVRTHLDHRIAMSFLVLGLASEHA | 420 |
| Query 421  Sbjct 421 | VTVDDATMIATSFPEFMDLMAGLGAKIEL 449 VT+DDA MIATSFPEFM LM GLGAKIEL VTIDDAAMIATSFPEFMQLMTGLGAKIEL 449 |  |

>WP_170277187.1 3-phosphoshikimate 1-carboxyvinyltransferase [Rhizobium laguerreae] NNH57715.1 3-phosphoshikimate 1-carboxyvinyltransferase [Rhizobium laguerreae]

Length=452

Score = 755 bits (1949), Expect = 0.0, Method: Compositional matrix adjust. Identities = 382/449 (85%), Positives = 411/449 (92%), Gaps = 0/449 (0%)

| Query | 1 | MLHGASSRPATARKSSGLSGTVRIPGDKSISHRSFMFGGLASGETRITGLLEGEDVINTG | 60 |
| --- | --- | --- | --- |
| Sbjct | 1 | M +G++ RPATARKS+GL G+VRIPGDKSISHRSFMFGGLASGETRITGLLEGEDVINTG MPNGSAPRPATARKSAGLKGSVRIPGDKSISHRSFMFGGLASGETRITGLLEGEDVINTG | 60 |
| Query | 61 | KAMQAMGARIRKEGDTWIIDGVGNGGLLAPEAPLDFGNAATGCRLTMGLVGVYDFDSTFI | 120 |
| Sbjct | 61 | +AMQAMGARIRK+G W+I+G GNG LLAP+APLDFGNA TG RLTMGLVG YDF STF RAMQAMGARIRKDGAQWVIEGTGNGALLAPDAPLDFGNAGTGVRLTMGLVGTYDFHSTFT | 120 |
| Query | 121 | GDASLTKRPMGRVLNPLREMGVQVKSEDGDRLPVTLRGPKTPTPITYRVPMASAQVKSAV | 180 |
| Sbjct | 121 | GDASL+KRPMGRVL PLREMGVQV + GDRLPVTLRGP TP+PI YRVPMASAQVKSAV GDASLSKRPMGRVLAPLREMGVQVSASQGDRLPVTLRGPGTPSPIRYRVPMASAQVKSAV | 180 |
| Query | 181 | LLAGLNTPGITTVIEPIMTRDHTEKMLQGFGANLTVETDADGVRTIRLEGRGKLTGQVID | 240 |
| Sbjct | 181 | LLAGLNTPG+TTVIEP+MTRDHTEKMLQGFGA L+VETD DGVRTIRLEGRGKL GQVID LLAGLNTPGVTTVIEPVMTRDHTEKMLQGFGAALSVETDGDGVRTIRLEGRGKLKGQVID | 240 |
| Query | 241 | VPGDPSSTAFPLVAALLVPGSDVTILNVLMNPTRTGLILTLQEMGADIEVINPRLAGGED | 300 |
| Sbjct | 241 | +PGDPSSTAFPLVA L+VPGSD+TI+NVLMNPTRTGLILTLQEMGADIEV+N RLAGGED IPGDPSSTAFPLVATLIVPGSDITIVNVLMNPTRTGLILTLQEMGADIEVVNARLAGGED | 300 |
| Query | 301 | VADLRVRSSTLKGVTVPEDRAPSMIDEYPILavaaafaegaTVMNGLEELRVKESDRLSA | 360 |
| Sbjct | 301 | VADLRVR S LKGVTVPEDRAPSMIDEYPILAVAA FAEGATVM GLEELRVKESDRLSA VADLRVRHSELKGVTVPEDRAPSMIDEYPILAVAACFAEGATVMKGLEELRVKESDRLSA | 360 |
| Query | 361 | VANGLKLNGVDCDEGETSLVVRGRPDGKGLGNASGAAVATHLDHRIAMSFLVMGLVSENP | 420 |
| Sbjct | 361 | VA+GLKLNGVDCDEG+ L+VRGRPDG+GLGNA+G V THLDHRIAMSFLV+GL SE+ VADGLKLNGVDCDEGKDFLIVRGRPDGRGLGNAAGGQVRTHLDHRIAMSFLVLGLASEHA | 420 |
| Query  Sbjct | 421  421 | VTVDDATMIATSFPEFMDLMAGLGAKIEL 449 VT+DDA MIATSFPEFM LM GLGA IEL VTIDDAAMIATSFPEFMQLMTGLGATIEL 449 |  |

>WP_192078030.1 unnamed protein product

MBD8652248.1 3-phosphoshikimate 1-carboxyvinyltransferase [Rhizobium sp. CFBP 13726]

Length=455

Score = 753 bits (1945), Expect = 0.0, Method: Compositional matrix adjust.

| Identities | = 383/448 (85%), Positives = 412/448 (92%), Gaps = 0/448 (0%) |  |
| --- | --- | --- |
| Query 1 | MLHGASSRPATARKSSGLSGTVRIPGDKSISHRSFMFGGLASGETRITGLLEGEDVINTG | 60 |
| Sbjct 1 | M HG + +PA +R+S+ L+GTVRIPGDKSISHRSFMFGGLA+GETRITGLLEGEDVINTG MSHGIALKPAKSRRSTNLTGTVRIPGDKSISHRSFMFGGLAAGETRITGLLEGEDVINTG | 60 |
| Query 61 | KAMQAMGARIRKEGDTWIIDGVGNGGLLAPEAPLDFGNAATGCRLTMGLVGVYDFDSTFI | 120 |
| Sbjct 61 | KAM+AMGA+I K G WII G GNG LLAP+APLDFGNA TGCRLTMGLVGVYDF+S FI KAMRAMGAKIDKVGAEWIIQGTGNGALLAPQAPLDFGNAGTGCRLTMGLVGVYDFESAFI | 120 |
| Query 121 | GDASLTKRPMGRVLNPLREMGVQVKSEDGDRLPVTLRGPKTPTPITYRVPMASAQVKSAV | 180 |
| Sbjct 121 | GDASL+KRPMGRVL+PLREMGVQV S DGDRLPVTLRGP+TP PI YRVPMASAQVKSAV GDASLSKRPMGRVLDPLREMGVQVTSMDGDRLPVTLRGPRTPNPIVYRVPMASAQVKSAV | 180 |
| Query 181 | LLAGLNTPGITTVIEPIMTRDHTEKMLQGFGANLTVETDADGVRTIRLEGRGKLTGQVID | 240 |
| Sbjct 181 | LLAGLNTPG+TTVIEP+MTRDHTEKMLQGFGA+LTVETDADGVRTIRLEGRG L GQVID LLAGLNTPGVTTVIEPVMTRDHTEKMLQGFGADLTVETDADGVRTIRLEGRGNLKGQVID | 240 |
| Query 241 | VPGDPSSTAFPLVAALLVPGSDVTILNVLMNPTRTGLILTLQEMGADIEVINPRLAGGED | 300 |
| Sbjct 241 | VPGDPSSTAFPLVAA+LVPGSDVTI+NVLMNPTRTGLILTLQEMGADIEV+N R AGGED VPGDPSSTAFPLVAAILVPGSDVTIMNVLMNPTRTGLILTLQEMGADIEVMNARSAGGED | 300 |
| Query 301 | VADLRVRSSTLKGVTVPEDRAPSMIDEYPILavaaafaegaTVMNGLEELRVKESDRLSA | 360 |
| Sbjct 301 | VADLRVR S LKGVTVP DRAPSMIDEYP+LAVAAAFAEG T M GLEELRVKESDRLSA VADLRVRGSDLKGVTVPADRAPSMIDEYPVLAVAAAFAEGTTTMLGLEELRVKESDRLSA | 360 |
| Query 361 | VANGLKLNGVDCDEGETSLVVRGRPDGKGLGNASGAAVATHLDHRIAMSFLVMGLVSENP | 420 |
| Sbjct 361 | VA GLKLNGVDCDEG+ +LVVRGRP GKG GNASGAAV T+LDHRIAMSFLV+GL SE+ VAEGLKLNGVDCDEGKETLVVRGRPGGKGYGNASGAAVVTYLDHRIAMSFLVLGLASEHE | 420 |
| Query 421 | VTVDDATMIATSFPEFMDLMAGLGAKIE 448 VTVDDA++IATSFPEFMDLM GLGA+IE |  |

Sbjct 421 VTVDDASIIATSFPEFMDLMTGLGAQIE 448

>WP_170263476.1 3-phosphoshikimate 1-carboxyvinyltransferase [Rhizobium laguerreae] NNG74524.1 3-phosphoshikimate 1-carboxyvinyltransferase [Rhizobium laguerreae]

Length=452

Score = 753 bits (1945), Expect = 0.0, Method: Compositional matrix adjust.

| Identities | = 380/449 (85%), Positives = 412/449 (92%), Gaps = 0/449 (0%) |  |
| --- | --- | --- |
| Query 1 | MLHGASSRPATARKSSGLSGTVRIPGDKSISHRSFMFGGLASGETRITGLLEGEDVINTG | 60 |
| Sbjct 1 | M +G++ RPATARKS+GL G+VRIPGDKSISHRSFMFGGLASGETRITGLLEGEDVINTG MPNGSAPRPATARKSAGLKGSVRIPGDKSISHRSFMFGGLASGETRITGLLEGEDVINTG | 60 |
| Query 61 | KAMQAMGARIRKEGDTWIIDGVGNGGLLAPEAPLDFGNAATGCRLTMGLVGVYDFDSTFI | 120 |
| Sbjct 61 | +AMQAMGARIRK+G W+I+G GNG LLAP+APLDFGNA TG RLTMGLVG YDF STF RAMQAMGARIRKDGAQWVIEGTGNGALLAPDAPLDFGNAGTGVRLTMGLVGTYDFHSTFT | 120 |
| Query 121 | GDASLTKRPMGRVLNPLREMGVQVKSEDGDRLPVTLRGPKTPTPITYRVPMASAQVKSAV | 180 |
| Sbjct 121 | GDASL+KRPMGRVL PLREMGVQV + GDRLPVTLRGP TP+PI YRVPMASAQVKSAV GDASLSKRPMGRVLAPLREMGVQVSASQGDRLPVTLRGPGTPSPIRYRVPMASAQVKSAV | 180 |
| Query 181 | LLAGLNTPGITTVIEPIMTRDHTEKMLQGFGANLTVETDADGVRTIRLEGRGKLTGQVID | 240 |
| Sbjct 181 | LLAGLNTPG+TTVIEP+MTRDHTEKMLQGFGA L+VETD DGVRTIRLEGRGKL GQVID LLAGLNTPGVTTVIEPVMTRDHTEKMLQGFGAALSVETDGDGVRTIRLEGRGKLKGQVID | 240 |
| Query 241 | VPGDPSSTAFPLVAALLVPGSDVTILNVLMNPTRTGLILTLQEMGADIEVINPRLAGGED | 300 |
| Sbjct 241 | +PGDPSSTAFPLVAAL+VPGSD+TI+NVLMNPTRTGLILTLQEMGADIEV+N RLAGGED IPGDPSSTAFPLVAALIVPGSDITIVNVLMNPTRTGLILTLQEMGADIEVVNARLAGGED | 300 |
| Query 301 | VADLRVRSSTLKGVTVPEDRAPSMIDEYPILavaaafaegaTVMNGLEELRVKESDRLSA | 360 |
| Sbjct 301 | VADLRVR S L+GV+VPE RAPSMIDEYPILA+AA FAEGATVM GLEELRVKESDRLSA VADLRVRHSELRGVSVPEGRAPSMIDEYPILAIAACFAEGATVMKGLEELRVKESDRLSA | 360 |
| Query 361 | VANGLKLNGVDCDEGETSLVVRGRPDGKGLGNASGAAVATHLDHRIAMSFLVMGLVSENP | 420 |
| Sbjct 361 | VA+GLKLNGVDCDEG+ L+VRGRPDG+GLGNA+G V THLDHRIAMSFLV+GL SE+ VADGLKLNGVDCDEGKDFLIVRGRPDGRGLGNAAGGQVRTHLDHRIAMSFLVLGLASEHA | 420 |
| Query 421  Sbjct 421 | VTVDDATMIATSFPEFMDLMAGLGAKIEL 449 VT+DDA MIATSFPEFM LM GLGAKIEL VTIDDAAMIATSFPEFMQLMTGLGAKIEL 449 |  |

>WP_092664007.1 3-phosphoshikimate 1-carboxyvinyltransferase [Rhizobium sp. NFR12] SEH29168.1 3-phosphoshikimate 1-carboxyvinyltransferase [Rhizobium sp. NFR12]

Length=455

Score = 752 bits (1942), Expect = 0.0, Method: Compositional matrix adjust.

| Identities | = 383/448 (85%), Positives = 412/448 (92%), Gaps = 0/448 (0%) |  |
| --- | --- | --- |
| Query 1 | MLHGASSRPATARKSSGLSGTVRIPGDKSISHRSFMFGGLASGETRITGLLEGEDVINTG | 60 |
| Sbjct 1 | M HG + +PA +R+S+ L+GTVRIPGDKSISHRSFMFGGLA+GETRITGLLEGEDVINTG MSHGIALKPAKSRRSTNLTGTVRIPGDKSISHRSFMFGGLAAGETRITGLLEGEDVINTG | 60 |
| Query 61 | KAMQAMGARIRKEGDTWIIDGVGNGGLLAPEAPLDFGNAATGCRLTMGLVGVYDFDSTFI | 120 |
| Sbjct 61 | KAM+AMGA+I K G WII G GNG LLAP+APLDFGNA TGCRLTMGLVGVYDF+STFI KAMRAMGAKIDKVGAEWIIQGTGNGALLAPQAPLDFGNAGTGCRLTMGLVGVYDFESTFI | 120 |
| Query 121 | GDASLTKRPMGRVLNPLREMGVQVKSEDGDRLPVTLRGPKTPTPITYRVPMASAQVKSAV | 180 |
| Sbjct 121 | GDASL+KRPMGRVL+PLREMGVQV S GDRLPVTLRGP+TP PI YRVPMASAQVKSAV GDASLSKRPMGRVLDPLREMGVQVTSMAGDRLPVTLRGPRTPNPIVYRVPMASAQVKSAV | 180 |
| Query 181 | LLAGLNTPGITTVIEPIMTRDHTEKMLQGFGANLTVETDADGVRTIRLEGRGKLTGQVID | 240 |
| Sbjct 181 | LLAGLNTPG+TTVIEP+MTRDHTEKMLQGFGA+LTVETDADGVRTIRLEGRG L GQVID LLAGLNTPGVTTVIEPVMTRDHTEKMLQGFGADLTVETDADGVRTIRLEGRGNLKGQVID | 240 |
| Query 241 | VPGDPSSTAFPLVAALLVPGSDVTILNVLMNPTRTGLILTLQEMGADIEVINPRLAGGED | 300 |
| Sbjct 241 | VPGDPSSTAFPLVAA+LVPGSDVTI+NVLMNPTRTGLILTLQEMGADIEV+N R AGGED VPGDPSSTAFPLVAAILVPGSDVTIMNVLMNPTRTGLILTLQEMGADIEVMNARSAGGED | 300 |
| Query 301 | VADLRVRSSTLKGVTVPEDRAPSMIDEYPILavaaafaegaTVMNGLEELRVKESDRLSA VADLRVR S LKGVTVP DRAPSMIDEYP+LAVAAAFAEG T M GLEELRVKESDRLSA | 360 |

| Sbjct | 301 | VADLRVRGSDLKGVTVPADRAPSMIDEYPVLAVAAAFAEGTTTMLGLEELRVKESDRLSA | 360 |
| --- | --- | --- | --- |
| Query | 361 | VANGLKLNGVDCDEGETSLVVRGRPDGKGLGNASGAAVATHLDHRIAMSFLVMGLVSENP | 420 |
| Sbjct | 361 | VA GLKLNGVDCDEG+ +LVVRGRP GKG GNASGAAV T+LDHRIAMSFLV+GL SE+ VAEGLKLNGVDCDEGKETLVVRGRPGGKGYGNASGAAVITYLDHRIAMSFLVLGLASEHE | 420 |
| Query  Sbjct | 421  421 | VTVDDATMIATSFPEFMDLMAGLGAKIE 448 VTVDDA++IATSFPEFMDLM GLGA+IE VTVDDASIIATSFPEFMDLMTGLGAQIE 448 |  |

>WP_174195395.1 3-phosphoshikimate 1-carboxyvinyltransferase [Neorhizobium sp. AL 9.2.2]

NSY15779.1 3-phosphoshikimate 1-carboxyvinyltransferase [Neorhizobium sp. AL 9.2.2]

Length=455

Score = 752 bits (1941), Expect = 0.0, Method: Compositional matrix adjust.

| Identities | = 382/448 (85%), Positives = 412/448 (92%), Gaps = 0/448 (0%) |  |
| --- | --- | --- |
| Query 1 | MLHGASSRPATARKSSGLSGTVRIPGDKSISHRSFMFGGLASGETRITGLLEGEDVINTG | 60 |
| Sbjct 1 | M HG + +PA +R+S+ L+GTVRIPGDKSISHRSFMFGGLA+GETRITGLLEGEDVINTG MSHGIALKPAKSRRSTNLTGTVRIPGDKSISHRSFMFGGLAAGETRITGLLEGEDVINTG | 60 |
| Query 61 | KAMQAMGARIRKEGDTWIIDGVGNGGLLAPEAPLDFGNAATGCRLTMGLVGVYDFDSTFI | 120 |
| Sbjct 61 | +AM+AMGA+I K G WII G GNG LLAP+APLDFGNA TGCRLTMGLVGVYDF+STFI RAMRAMGAKIEKVGAEWIIQGTGNGALLAPQAPLDFGNAGTGCRLTMGLVGVYDFESTFI | 120 |
| Query 121 | GDASLTKRPMGRVLNPLREMGVQVKSEDGDRLPVTLRGPKTPTPITYRVPMASAQVKSAV | 180 |
| Sbjct 121 | GDASL+KRPMGRVL+PLREMGVQV S DGDRLPVTLRGP+TP PI YRVPMASAQVKSAV GDASLSKRPMGRVLDPLREMGVQVTSMDGDRLPVTLRGPRTPNPIVYRVPMASAQVKSAV | 180 |
| Query 181 | LLAGLNTPGITTVIEPIMTRDHTEKMLQGFGANLTVETDADGVRTIRLEGRGKLTGQVID | 240 |
| Sbjct 181 | LLAGLNTPG+TTVIEP+MTRDHTEKMLQGFGA+LTVETDA GVRTIRLEGRG L GQVID LLAGLNTPGVTTVIEPVMTRDHTEKMLQGFGADLTVETDAHGVRTIRLEGRGALKGQVID | 240 |
| Query 241 | VPGDPSSTAFPLVAALLVPGSDVTILNVLMNPTRTGLILTLQEMGADIEVINPRLAGGED | 300 |
| Sbjct 241 | VPGDPSSTAFPLVAA+LVPGSDVTI+NVLMNPTRTGLILTLQEMGADIEV+N R AGGED VPGDPSSTAFPLVAAILVPGSDVTIMNVLMNPTRTGLILTLQEMGADIEVMNARSAGGED | 300 |
| Query 301 | VADLRVRSSTLKGVTVPEDRAPSMIDEYPILavaaafaegaTVMNGLEELRVKESDRLSA | 360 |
| Sbjct 301 | VADLRVR S LKGVTVP DRAPSMIDEYP+LAVAAAFAEG T M GLEELRVKESDRLSA VADLRVRGSDLKGVTVPADRAPSMIDEYPVLAVAAAFAEGTTTMLGLEELRVKESDRLSA | 360 |
| Query 361 | VANGLKLNGVDCDEGETSLVVRGRPDGKGLGNASGAAVATHLDHRIAMSFLVMGLVSENP | 420 |
| Sbjct 361 | VA GLKLNGVDCDEG+ +LVVRGRP GKG GNASGAAV T+LDHRIAMSFLV+GL SE+ VAEGLKLNGVDCDEGKETLVVRGRPGGKGYGNASGAAVVTYLDHRIAMSFLVLGLASEHE | 420 |
| Query 421  Sbjct 421 | VTVDDATMIATSFPEFMDLMAGLGAKIE 448 VTVDDA++IATSFPEFMDLM GLGA+IE VTVDDASIIATSFPEFMDLMTGLGAQIE 448 |  |

>WP_062578552.1 3-phosphoshikimate 1-carboxyvinyltransferase [Rhizobium sp. Leaf321] KQQ73313.1 3-phosphoshikimate 1-carboxyvinyltransferase [Rhizobium sp. Leaf321]

Length=455

Score = 750 bits (1936), Expect = 0.0, Method: Compositional matrix adjust.

| Identities | = 382/448 (85%), Positives = 411/448 (92%), Gaps = 0/448 (0%) |  |
| --- | --- | --- |
| Query 1 | MLHGASSRPATARKSSGLSGTVRIPGDKSISHRSFMFGGLASGETRITGLLEGEDVINTG | 60 |
| Sbjct 1 | M HG + +PA +R+S+ L+GTVRIPGDKSISHRSFMFGGLA+GETRITGLLEGEDVINTG MSHGIALKPAKSRRSTNLTGTVRIPGDKSISHRSFMFGGLAAGETRITGLLEGEDVINTG | 60 |
| Query 61 | KAMQAMGARIRKEGDTWIIDGVGNGGLLAPEAPLDFGNAATGCRLTMGLVGVYDFDSTFI | 120 |
| Sbjct 61 | KAM+AMGA+I K G WII G GNG LLAP+APLDFGNA TGCRLTMGLVGVYDF+STFI KAMRAMGAKIDKVGAEWIIQGTGNGALLAPQAPLDFGNAGTGCRLTMGLVGVYDFESTFI | 120 |
| Query 121 | GDASLTKRPMGRVLNPLREMGVQVKSEDGDRLPVTLRGPKTPTPITYRVPMASAQVKSAV | 180 |
| Sbjct 121 | GDASL+KRPMGRVL+PLREMGVQV S GDRLPVTLRGP+TP PI YRVPMASAQVKSAV  GDASLSKRPMGRVLDPLREMGVQVTSMAGDRLPVTLRGPRTPNPIVYRVPMASAQVKSAV | 180 |

| Query | 181 | LLAGLNTPGITTVIEPIMTRDHTEKMLQGFGANLTVETDADGVRTIRLEGRGKLTGQVID | 240 |
| --- | --- | --- | --- |
| Sbjct | 181 | LLAGLNTPG+TTVIEP+MTRDHTEKMLQGFGA+LTVETDADGVRTIRLEGRG L GQVID LLAGLNTPGVTTVIEPVMTRDHTEKMLQGFGADLTVETDADGVRTIRLEGRGALKGQVID | 240 |
| Query | 241 | VPGDPSSTAFPLVAALLVPGSDVTILNVLMNPTRTGLILTLQEMGADIEVINPRLAGGED | 300 |
| Sbjct | 241 | VPGDPSSTAFPLVAA+LVPGSDVTI+NVLMNPTRTGLILTLQEMGADIEV+N R AGGED VPGDPSSTAFPLVAAILVPGSDVTIMNVLMNPTRTGLILTLQEMGADIEVMNARSAGGED | 300 |
| Query | 301 | VADLRVRSSTLKGVTVPEDRAPSMIDEYPILavaaafaegaTVMNGLEELRVKESDRLSA | 360 |
| Sbjct | 301 | VADLRVR S LKGVTVP DRAPSMIDEYP+LAVAAAFAEG T M GLEELRVKESDRLSA VADLRVRGSDLKGVTVPADRAPSMIDEYPVLAVAAAFAEGTTTMLGLEELRVKESDRLSA | 360 |
| Query | 361 | VANGLKLNGVDCDEGETSLVVRGRPDGKGLGNASGAAVATHLDHRIAMSFLVMGLVSENP | 420 |
| Sbjct | 361 | VA GLKLNGVDCDEG+ +LVVRGRP GKG GN SGAAV T+LDHRIAMSFLV+GL SE+ VAEGLKLNGVDCDEGKETLVVRGRPGGKGYGNPSGAAVVTYLDHRIAMSFLVLGLASEHE | 420 |
| Query  Sbjct | 421  421 | VTVDDATMIATSFPEFMDLMAGLGAKIE 448 VTVDDA++IATSFPEFMDLM GLGA+IE VTVDDASIIATSFPEFMDLMTGLGAQIE 448 |  |

>WP_183837182.1 3-phosphoshikimate 1-carboxyvinyltransferase [Rhizobium sp. BK007] MBB3252058.1 3-phosphoshikimate 1-carboxyvinyltransferase [Rhizobium sp. BK007]

Length=452

Score = 750 bits (1936), Expect = 0.0, Method: Compositional matrix adjust.

| Identities | = 380/448 (85%), Positives = 411/448 (92%), Gaps = 0/448 (0%) |  |
| --- | --- | --- |
| Query 1 | MLHGASSRPATARKSSGLSGTVRIPGDKSISHRSFMFGGLASGETRITGLLEGEDVINTG | 60 |
| Sbjct 1 | M HG + RPA ARKS+GL+G+VRIPGDKSISHRSFMFGGLASGETRITGLLEGEDVINTG MPHGPAPRPAIARKSAGLTGSVRIPGDKSISHRSFMFGGLASGETRITGLLEGEDVINTG | 60 |
| Query 61 | KAMQAMGARIRKEGDTWIIDGVGNGGLLAPEAPLDFGNAATGCRLTMGLVGVYDFDSTFI | 120 |
| Sbjct 61 | +AMQAMGARIRKEG+ W+I+G GNG LLAP+APLDFGNA TG RLTMGLVG YDF STF RAMQAMGARIRKEGEQWVINGTGNGALLAPDAPLDFGNAGTGVRLTMGLVGPYDFPSTFT | 120 |
| Query 121 | GDASLTKRPMGRVLNPLREMGVQVKSEDGDRLPVTLRGPKTPTPITYRVPMASAQVKSAV | 180 |
| Sbjct 121 | GDASL+KRPMGRVL+PLREMGVQV + GDRLPVTLRGP TP+PI YRVPMASAQVKSAV GDASLSKRPMGRVLDPLREMGVQVSASAGDRLPVTLRGPATPSPIRYRVPMASAQVKSAV | 180 |
| Query 181 | LLAGLNTPGITTVIEPIMTRDHTEKMLQGFGANLTVETDADGVRTIRLEGRGKLTGQVID | 240 |
| Sbjct 181 | LLAGLNTPG+TTVIEP+MTRDHTEKMLQGFGA L+VETD G RTIRLEGRG+LTGQVID LLAGLNTPGVTTVIEPVMTRDHTEKMLQGFGAALSVETDRQGARTIRLEGRGRLTGQVID | 240 |
| Query 241 | VPGDPSSTAFPLVAALLVPGSDVTILNVLMNPTRTGLILTLQEMGADIEVINPRLAGGED | 300 |
| Sbjct 241 | VPGDPSSTAFPLVAALLVPGSD++I+NVLMNPTRTGLILTLQEMGADIEV+N RLAGGED VPGDPSSTAFPLVAALLVPGSDISIVNVLMNPTRTGLILTLQEMGADIEVLNARLAGGED | 300 |
| Query 301 | VADLRVRSSTLKGVTVPEDRAPSMIDEYPILavaaafaegaTVMNGLEELRVKESDRLSA | 360 |
| Sbjct 301 | VADLRVR S LKGVTVPEDRAPSMIDEYP+LAVAA FAEGATVM GLEELRVKESDRLSA VADLRVRHSELKGVTVPEDRAPSMIDEYPVLAVAACFAEGATVMKGLEELRVKESDRLSA | 360 |
| Query 361 | VANGLKLNGVDCDEGETSLVVRGRPDGKGLGNASGAAVATHLDHRIAMSFLVMGLVSENP | 420 |
| Sbjct 361 | VA+GLKLNGVDCDEG+ L+VRGRP G GLG+A+G V THLDHRIAMSFLVMGL SE+P VADGLKLNGVDCDEGKDFLIVRGRPGGTGLGSAAGGKVRTHLDHRIAMSFLVMGLASEHP | 420 |
| Query 421  Sbjct 421 | VTVDDATMIATSFPEFMDLMAGLGAKIE 448 VT+DDA MI+TSFPEFM LM GLGA+IE VTIDDAAMISTSFPEFMALMTGLGARIE 448 |  |

```
>WP_168827834.1 3-phosphoshikimate 1-carboxyvinyltransferase [Rhizobium sp. P32RR-XVIII] NLS01783.1 3-phosphoshikimate 1-carboxyvinyltransferase [Rhizobium sp. P32RR-XVIII]
Length=450

Score = 749 bits (1935), Expect = 0.0, Method: Compositional matrix adjust. Identities = 383/448 (85%), Positives = 409/448 (91%), Gaps = 0/448 (0%)

Query 1	MLHGASSRPATARKSSGLSGTVRIPGDKSISHRSFMFGGLASGETRITGLLEGEDVINTG 60 M HG++ +PATARKS+ L+GTV IPGDKSISHR+FM GGLASGETRITGLLEGEDVINTG
Sbjct 1	MSHGSAPKPATARKSAALTGTVHIPGDKSISHRAFMLGGLASGETRITGLLEGEDVINTG 60
```

| Query | 61 | KAMQAMGARIRKEGDTWIIDGVGNGGLLAPEAPLDFGNAATGCRLTMGLVGVYDFDSTFI | 120 |
| --- | --- | --- | --- |
| Sbjct | 61 | +AM AMGA IRK+GD WII G GNG LLAPE PLDFGNA TGCRLTMGLVGVYDF+STFI RAMHAMGATIRKDGDEWIIRGTGNGALLAPETPLDFGNAGTGCRLTMGLVGVYDFESTFI | 120 |
| Query | 121 | GDASLTKRPMGRVLNPLREMGVQVKSEDGDRLPVTLRGPKTPTPITYRVPMASAQVKSAV | 180 |
| Sbjct | 121 | GDASL+KRPMGRVL+PLREMGVQVKS GD+LPVTL GP TP P+ YRVPMASAQVKSAV GDASLSKRPMGRVLDPLREMGVQVKSTAGDKLPVTLHGPGTPGPLIYRVPMASAQVKSAV | 180 |
| Query | 181 | LLAGLNTPGITTVIEPIMTRDHTEKMLQGFGANLTVETDADGVRTIRLEGRGKLTGQVID | 240 |
| Sbjct | 181 | LLAGLNTPGITTVIEP+MTRDHTEKMLQGFGA L+VETD GVRTIRLEGRG+LTGQVID LLAGLNTPGITTVIEPVMTRDHTEKMLQGFGAELSVETDNAGVRTIRLEGRGRLTGQVID | 240 |
| Query | 241 | VPGDPSSTAFPLVAALLVPGSDVTILNVLMNPTRTGLILTLQEMGADIEVINPRLAGGED | 300 |
| Sbjct | 241 | VPGDPSSTAFPLVAALLVPGSD+TI NVLMNPTRTGLILTL+EMGADIEV+N RLAGGED VPGDPSSTAFPLVAALLVPGSDITIRNVLMNPTRTGLILTLREMGADIEVLNARLAGGED | 300 |
| Query | 301 | VADLRVRSSTLKGVTVPEDRAPSMIDEYPILavaaafaegaTVMNGLEELRVKESDRLSA | 360 |
| Sbjct | 301 | VADLRVR+S LKGVTVPE+RAPSMIDEYP+LAVAA FAEGATVM GLEELRVKESDRLSA VADLRVRASELKGVTVPEERAPSMIDEYPVLAVAACFAEGATVMKGLEELRVKESDRLSA | 360 |
| Query | 361 | VANGLKLNGVDCDEGETSLVVRGRPDGKGLGNASGAAVATHLDHRIAMSFLVMGLVSENP | 420 |
| Sbjct | 361 | VA+GLKLNG DCDEG LVVRG+P GK LG+ASG V THLDHRIAMSFLVMGL SE+ VADGLKLNGADCDEGRGFLVVRGKPGGKALGSASGGRVKTHLDHRIAMSFLVMGLASEHA | 420 |
| Query  Sbjct | 421  421 | VTVDDATMIATSFPEFMDLMAGLGAKIE 448 VTVDDATMIATSFPEFMDLM GLGAKIE VTVDDATMIATSFPEFMDLMTGLGAKIE 448 |  |

>WP_126925121.1 3-phosphoshikimate 1-carboxyvinyltransferase [Rhizobium vallis] RUM18421.1 3-phosphoshikimate 1-carboxyvinyltransferase [Rhizobium vallis]

Length=451

Score = 749 bits (1935), Expect = 0.0, Method: Compositional matrix adjust.

| Identities | = 382/448 (85%), Positives = 409/448 (91%), Gaps = 1/448 (0%) |  |
| --- | --- | --- |
| Query 1 | MLHGASSRPATARKSSGLSGTVRIPGDKSISHRSFMFGGLASGETRITGLLEGEDVINTG | 60 |
| Sbjct 1 | M HG++ RPATARKS+GLSG VRIPGDKSISHRSFMFGGLASGETRITGLLEGEDVINTG MSHGSAPRPATARKSAGLSGRVRIPGDKSISHRSFMFGGLASGETRITGLLEGEDVINTG | 60 |
| Query 61 | KAMQAMGARIRKEGDTWIIDGVGNGGLLAPEAPLDFGNAATGCRLTMGLVGVYDFDSTFI | 120 |
| Sbjct 61 | +AMQAMGARIRKEG+ W+I+G GNG LLAP+APLDFGNA TG RLTMGLVG YDF STFI RAMQAMGARIRKEGEQWVIEGTGNGALLAPDAPLDFGNAGTGVRLTMGLVGTYDFHSTFI | 120 |
| Query 121 | GDASLTKRPMGRVLNPLREMGVQVKSEDGDRLPVTLRGPKTPTPITYRVPMASAQVKSAV | 180 |
| Sbjct 121 | GDASL+KRPMGRVL+PLREMGVQV + GDRLPVTLRG TP+ I YRVPMASAQVKSAV GDASLSKRPMGRVLDPLREMGVQVSASAGDRLPVTLRG-GTPSAIRYRVPMASAQVKSAV | 179 |
| Query 181 | LLAGLNTPGITTVIEPIMTRDHTEKMLQGFGANLTVETDADGVRTIRLEGRGKLTGQVID | 240 |
| Sbjct 180 | LLAGLNTPGITTVIEP+MTRDHTEKMLQGFGA L+V+TD DGVRTIRLEGRG+LTGQVID LLAGLNTPGITTVIEPVMTRDHTEKMLQGFGAALSVDTDKDGVRTIRLEGRGRLTGQVID | 239 |
| Query 241 | VPGDPSSTAFPLVAALLVPGSDVTILNVLMNPTRTGLILTLQEMGADIEVINPRLAGGED | 300 |
| Sbjct 240 | VPGDPSSTAFPLVAAL+ P SD+TILNVLMNPTRTGLILTLQEMGADIEV+N RLAGGED VPGDPSSTAFPLVAALIAPDSDITILNVLMNPTRTGLILTLQEMGADIEVLNARLAGGED | 299 |
| Query 301 | VADLRVRSSTLKGVTVPEDRAPSMIDEYPILavaaafaegaTVMNGLEELRVKESDRLSA | 360 |
| Sbjct 300 | VADLRVR S LKGV VPE+R PSMIDEYP+LAVAA FAEG TVM GLEELRVKESDRLSA VADLRVRHSELKGVAVPEERGPSMIDEYPVLAVAACFAEGTTVMKGLEELRVKESDRLSA | 359 |
| Query 361 | VANGLKLNGVDCDEGETSLVVRGRPDGKGLGNASGAAVATHLDHRIAMSFLVMGLVSENP | 420 |
| Sbjct 360 | VANGLKLNG DCDEG+ LVVRGRPDGKGLGNA+G V+THLDHRIAMSFLVMGL +E+P VANGLKLNGADCDEGKDFLVVRGRPDGKGLGNAAGDKVSTHLDHRIAMSFLVMGLAAEHP | 419 |
| Query 421  Sbjct 420 | VTVDDATMIATSFPEFMDLMAGLGAKIE 448 VT+DDA MIATSFPEFM LM GLGA IE VTIDDAAMIATSFPEFMPLMTGLGAWIE 447 |  |

>WP_184654285.1 3-phosphoshikimate 1-carboxyvinyltransferase [Rhizobium soli] MBB6508152.1 3-phosphoshikimate 1-carboxyvinyltransferase [Rhizobium soli]

Length=455

Score = 749 bits (1934), Expect = 0.0, Method: Compositional matrix adjust.

| Identities | = 382/448 (85%), Positives = 411/448 (92%), Gaps = 0/448 (0%) |  |
| --- | --- | --- |
| Query 1 | MLHGASSRPATARKSSGLSGTVRIPGDKSISHRSFMFGGLASGETRITGLLEGEDVINTG | 60 |
| Sbjct 1 | M HG + +PA +R+S+ L+GTVRIPGDKSISHRSFMFGGLA+GETRITGLLEGEDVINTG MSHGIALKPAKSRRSTNLTGTVRIPGDKSISHRSFMFGGLAAGETRITGLLEGEDVINTG | 60 |
| Query 61 | KAMQAMGARIRKEGDTWIIDGVGNGGLLAPEAPLDFGNAATGCRLTMGLVGVYDFDSTFI | 120 |
| Sbjct 61 | KAM+AMGA+I K G WII G GNG LLAP+APLDFGNA TGCRLTMGLVGVYDF+STFI KAMRAMGAKIDKVGAEWIIQGTGNGALLAPQAPLDFGNAGTGCRLTMGLVGVYDFESTFI | 120 |
| Query 121 | GDASLTKRPMGRVLNPLREMGVQVKSEDGDRLPVTLRGPKTPTPITYRVPMASAQVKSAV | 180 |
| Sbjct 121 | GDASL+KRPMGRVL+PLREMGVQV S GDRLPVTLRGP+TP PI YRVPMASAQVKSAV GDASLSKRPMGRVLDPLREMGVQVTSMAGDRLPVTLRGPRTPNPIVYRVPMASAQVKSAV | 180 |
| Query 181 | LLAGLNTPGITTVIEPIMTRDHTEKMLQGFGANLTVETDADGVRTIRLEGRGKLTGQVID | 240 |
| Sbjct 181 | LLAGLNTPG+TTVIEP+MTRDHTEKMLQGFGA+LTVETDADGVRTIRLEGRG L GQVID LLAGLNTPGVTTVIEPVMTRDHTEKMLQGFGADLTVETDADGVRTIRLEGRGALKGQVID | 240 |
| Query 241 | VPGDPSSTAFPLVAALLVPGSDVTILNVLMNPTRTGLILTLQEMGADIEVINPRLAGGED | 300 |
| Sbjct 241 | VPGDPSSTAFPLVAA+LVPGSDVTI+NVLMNPTRTGLILTLQEMGADIEV+N R AGGED VPGDPSSTAFPLVAAILVPGSDVTIMNVLMNPTRTGLILTLQEMGADIEVMNARSAGGED | 300 |
| Query 301 | VADLRVRSSTLKGVTVPEDRAPSMIDEYPILavaaafaegaTVMNGLEELRVKESDRLSA | 360 |
| Sbjct 301 | VADLRVR S LKGVTVP DRAPSMIDEYP+LAVAAAFAEG T M GLEELRVKESDRLSA VADLRVRGSDLKGVTVPADRAPSMIDEYPVLAVAAAFAEGTTTMLGLEELRVKESDRLSA | 360 |
| Query 361 | VANGLKLNGVDCDEGETSLVVRGRPDGKGLGNASGAAVATHLDHRIAMSFLVMGLVSENP | 420 |
| Sbjct 361 | VA GLKLNGVDCDEG+ +LVVRGRP GKG GN SGAAV T+LDHRIAMSFLV+GL SE+ VAEGLKLNGVDCDEGKETLVVRGRPGGKGYGNPSGAAVVTYLDHRIAMSFLVLGLASEHE | 420 |
| Query 421  Sbjct 421 | VTVDDATMIATSFPEFMDLMAGLGAKIE 448 VTVDDA++IATSFPEFMDLM GLGA+IE VTVDDASIIATSFPEFMDLMTGLGAQIE 448 |  |

>WP_170257465.1 3-phosphoshikimate 1-carboxyvinyltransferase [Rhizobium laguerreae] NNH44170.1 3-phosphoshikimate 1-carboxyvinyltransferase [Rhizobium laguerreae]

Length=452

Score = 749 bits (1933), Expect = 0.0, Method: Compositional matrix adjust.

| Identities | = 378/449 (84%), Positives = 411/449 (92%), Gaps = 0/449 (0%) |  |
| --- | --- | --- |
| Query 1 | MLHGASSRPATARKSSGLSGTVRIPGDKSISHRSFMFGGLASGETRITGLLEGEDVINTG | 60 |
| Sbjct 1 | M +G++ RPA ARKS+GL G+VRIPGDKSISHRSFMFGGLASGETRITGLLEGEDVINTG MPNGSAPRPAAARKSAGLKGSVRIPGDKSISHRSFMFGGLASGETRITGLLEGEDVINTG | 60 |
| Query 61 | KAMQAMGARIRKEGDTWIIDGVGNGGLLAPEAPLDFGNAATGCRLTMGLVGVYDFDSTFI | 120 |
| Sbjct 61 | +AMQAMGARIRK+G W+I+G GNG LLAP+APLDFGNA TG RLTMGLVG YDF STFI RAMQAMGARIRKDGAQWVIEGTGNGALLAPDAPLDFGNAGTGVRLTMGLVGTYDFHSTFI | 120 |
| Query 121 | GDASLTKRPMGRVLNPLREMGVQVKSEDGDRLPVTLRGPKTPTPITYRVPMASAQVKSAV | 180 |
| Sbjct 121 | GDASL+KRPMGRVL PLREMGVQV + GDRLPVTLRGP TP+PI YRVPMASAQVKSAV GDASLSKRPMGRVLAPLREMGVQVSASQGDRLPVTLRGPGTPSPIRYRVPMASAQVKSAV | 180 |
| Query 181 | LLAGLNTPGITTVIEPIMTRDHTEKMLQGFGANLTVETDADGVRTIRLEGRGKLTGQVID | 240 |
| Sbjct 181 | LLAGLNTPG+TTVIEP+MTRDHTE+MLQGFGA L+VETD DGVRTIRLEGRGKL GQVID LLAGLNTPGVTTVIEPVMTRDHTEEMLQGFGAALSVETDGDGVRTIRLEGRGKLKGQVID | 240 |
| Query 241 | VPGDPSSTAFPLVAALLVPGSDVTILNVLMNPTRTGLILTLQEMGADIEVINPRLAGGED | 300 |
| Sbjct 241 | +PGDPSSTAFPLVAAL+VPGSD+TI+NVLMNPTRTGLILTLQEMGADIEV+N RLAGGED IPGDPSSTAFPLVAALIVPGSDITIVNVLMNPTRTGLILTLQEMGADIEVVNARLAGGED | 300 |
| Query 301 | VADLRVRSSTLKGVTVPEDRAPSMIDEYPILavaaafaegaTVMNGLEELRVKESDRLSA | 360 |
| Sbjct 301 | VADLRVR S L+GV+VPE RA SMIDEYPILA+AA FAEGATVM GLEELRVKESDRLSA VADLRVRHSELRGVSVPEGRALSMIDEYPILAIAACFAEGATVMKGLEELRVKESDRLSA | 360 |
| Query 361 | VANGLKLNGVDCDEGETSLVVRGRPDGKGLGNASGAAVATHLDHRIAMSFLVMGLVSENP | 420 |
| Sbjct 361 | VA+GLKLNGVDCDEG+ L+VRGRPDG+GLGNA+G V THLDHRIAMSFLV+GL SE+  VADGLKLNGVDCDEGKDFLIVRGRPDGRGLGNAAGGQVRTHLDHRIAMSFLVLGLASEHA | 420 |

Query 421 VTVDDATMIATSFPEFMDLMAGLGAKIEL 449 VT+DDA MIATSFPEFM LM GLGAKIEL

Sbjct 421 VTIDDAAMIATSFPEFMQLMTGLGAKIEL 449

>MBA4785050.1 3-phosphoshikimate 1-carboxyvinyltransferase [Rhizobiales bacterium] Length=455

Score = 749 bits (1933), Expect = 0.0, Method: Compositional matrix adjust.

| Identities | = 392/455 (86%), Positives = 415/455 (91%), Gaps = 0/455 (0%) |  |
| --- | --- | --- |
| Query 1 | MLHGASSRPATARKSSGLSGTVRIPGDKSISHRSFMFGGLASGETRITGLLEGEDVINTG | 60 |
| Sbjct 1 | M HG + +PA + KS+ L GTVRIPGDKSISHRSFMFGGLASGETRITGLLEGEDVINTG MSHGVALKPARSLKSADLKGTVRIPGDKSISHRSFMFGGLASGETRITGLLEGEDVINTG | 60 |
| Query 61 | KAMQAMGARIRKEGDTWIIDGVGNGGLLAPEAPLDFGNAATGCRLTMGLVGVYDFDSTFI | 120 |
| Sbjct 61 | KAMQAMGA+I K G WII G GNG LLAPEAPLDFGNA TGCRLTMGLVGVYDFDSTFI KAMQAMGAKIEKVGAEWIIQGTGNGALLAPEAPLDFGNAGTGCRLTMGLVGVYDFDSTFI | 120 |
| Query 121 | GDASLTKRPMGRVLNPLREMGVQVKSEDGDRLPVTLRGPKTPTPITYRVPMASAQVKSAV | 180 |
| Sbjct 121 | GDASL+KRPMGRVL PLREMGVQV + +GDRLPVTLRGP TP PITYRVPMASAQVKSAV GDASLSKRPMGRVLAPLREMGVQVTAAEGDRLPVTLRGPATPNPITYRVPMASAQVKSAV | 180 |
| Query 181 | LLAGLNTPGITTVIEPIMTRDHTEKMLQGFGANLTVETDADGVRTIRLEGRGKLTGQVID | 240 |
| Sbjct 181 | LLAGLNTPG TTVIEP+MTRDHTEKMLQGFGA+LTVETDADGVRTIRL+GRG+LTGQVID LLAGLNTPGTTTVIEPVMTRDHTEKMLQGFGADLTVETDADGVRTIRLQGRGRLTGQVID | 240 |
| Query 241 | VPGDPSSTAFPLVAALLVPGSDVTILNVLMNPTRTGLILTLQEMGADIEVINPRLAGGED | 300 |
| Sbjct 241 | VPGDPSSTAFPLVAALLVPGSDVTI NVLMNPTRTGLILTLQEMGADIEV+NPRLAGGED VPGDPSSTAFPLVAALLVPGSDVTIENVLMNPTRTGLILTLQEMGADIEVLNPRLAGGED | 300 |
| Query 301 | VADLRVRSSTLKGVTVPEDRAPSMIDEYPILavaaafaegaTVMNGLEELRVKESDRLSA | 360 |
| Sbjct 301 | VADLRVR S LKGVTVP +RAPSMIDEYP+LAVAAAFAEG T M G+EELRVKESDRLSA VADLRVRHSQLKGVTVPAERAPSMIDEYPVLAVAAAFAEGTTTMLGVEELRVKESDRLSA | 360 |
| Query 361 | VANGLKLNGVDCDEGETSLVVRGRPDGKGLGNASGAAVATHLDHRIAMSFLVMGLVSENP | 420 |
| Sbjct 361 | A GLKLNGVDCDEGE L VRGRP GKG GNA G AVATHLDHRIAMSFLV+GLVSE+P TAAGLKLNGVDCDEGEDRLTVRGRPGGKGYGNAGGEAVATHLDHRIAMSFLVLGLVSEHP | 420 |
| Query 421  Sbjct 421 | VTVDDATMIATSFPEFMDLMAGLGAKIELSDTKAA 455 VTVDDA +IATSFPEFMDLMAGLGA+IE +++AA VTVDDAGIIATSFPEFMDLMAGLGARIEAVESRAA 455 |  |

>WP_192087715.1 3-phosphoshikimate 1-carboxyvinyltransferase [Rhizobium sp. CFBP 8752]

MBD8665167.1 3-phosphoshikimate 1-carboxyvinyltransferase [Rhizobium sp. CFBP 8752]

Length=455

Score = 749 bits (1933), Expect = 0.0, Method: Compositional matrix adjust.

| Identities | = 381/448 (85%), Positives = 411/448 (92%), Gaps = 0/448 (0%) |  |
| --- | --- | --- |
| Query 1 | MLHGASSRPATARKSSGLSGTVRIPGDKSISHRSFMFGGLASGETRITGLLEGEDVINTG | 60 |
| Sbjct 1 | M HG + +PA +R+S+ L+GTVRIPGDKSISHRSFMFGGLA+GETRITGLLEGEDVINTG MSHGIALKPAKSRRSTNLTGTVRIPGDKSISHRSFMFGGLAAGETRITGLLEGEDVINTG | 60 |
| Query 61 | KAMQAMGARIRKEGDTWIIDGVGNGGLLAPEAPLDFGNAATGCRLTMGLVGVYDFDSTFI | 120 |
| Sbjct 61 | KAM+AMGA+I K G WII G GNG LLAP+APLDFGNA TGCRLTMGLVGVYDF+STFI KAMRAMGAKIDKVGAEWIIQGTGNGALLAPQAPLDFGNAGTGCRLTMGLVGVYDFESTFI | 120 |
| Query 121 | GDASLTKRPMGRVLNPLREMGVQVKSEDGDRLPVTLRGPKTPTPITYRVPMASAQVKSAV | 180 |
| Sbjct 121 | GDASL+KRPMGRVL+PLREMGVQV S GDRLPVTLRGP+TP PI YRVPMASAQVKSAV GDASLSKRPMGRVLDPLREMGVQVTSMAGDRLPVTLRGPRTPNPIVYRVPMASAQVKSAV | 180 |
| Query 181 | LLAGLNTPGITTVIEPIMTRDHTEKMLQGFGANLTVETDADGVRTIRLEGRGKLTGQVID | 240 |
| Sbjct 181 | LLAGLNTPG+TTVIEP+MTRDHTEKMLQGFGA+LTVETDADGVRTIRLEGRG L GQVID LLAGLNTPGVTTVIEPVMTRDHTEKMLQGFGADLTVETDADGVRTIRLEGRGALKGQVID | 240 |
| Query 241 | VPGDPSSTAFPLVAALLVPGSDVTILNVLMNPTRTGLILTLQEMGADIEVINPRLAGGED | 300 |
| Sbjct 241 | VPGDPSSTAFPLVAA+LVPGSDVTI+NVLMNPTRTGLILTLQEMGADIEV+N R AGGED  VPGDPSSTAFPLVAAILVPGSDVTIMNVLMNPTRTGLILTLQEMGADIEVMNARSAGGED | 300 |

| Query | 301 | VADLRVRSSTLKGVTVPEDRAPSMIDEYPILavaaafaegaTVMNGLEELRVKESDRLSA | 360 |
| --- | --- | --- | --- |
| Sbjct | 301 | VADLRVR + LKGVTVP DRAPSMIDEYP+LAVAAAFAEG T M GLEELRVKESDRLSA VADLRVRGADLKGVTVPADRAPSMIDEYPVLAVAAAFAEGTTTMLGLEELRVKESDRLSA | 360 |
| Query | 361 | VANGLKLNGVDCDEGETSLVVRGRPDGKGLGNASGAAVATHLDHRIAMSFLVMGLVSENP | 420 |
| Sbjct | 361 | VA GLKLNGVDCDEG+ +LVVRGRP GKG GN SGAAV T+LDHRIAMSFLV+GL SE+ VAEGLKLNGVDCDEGKETLVVRGRPGGKGYGNPSGAAVVTYLDHRIAMSFLVLGLASEHE | 420 |
| Query  Sbjct | 421  421 | VTVDDATMIATSFPEFMDLMAGLGAKIE 448 VTVDDA++IATSFPEFMDLM GLGA+IE VTVDDASIIATSFPEFMDLMTGLGAQIE 448 |  |

>WP_133282785.1 3-phosphoshikimate 1-carboxyvinyltransferase [Pseudohoeflea suaedae] TDH37948.1 3-phosphoshikimate 1-carboxyvinyltransferase [Pseudohoeflea suaedae]

Length=452

Score = 749 bits (1933), Expect = 0.0, Method: Compositional matrix adjust.

| Identities | = 382/451 (85%), Positives = 409/451 (91%), Gaps = 0/451 (0%) |  |
| --- | --- | --- |
| Query 1 | MLHGASSRPATARKSSGLSGTVRIPGDKSISHRSFMFGGLASGETRITGLLEGEDVINTG | 60 |
| Sbjct 1 | M H RPATAR+SS L G +RIPGDKSISHRSFMFGGLA GETRITGLLEGEDVINTG MNHVNKLRPATARRSSALKGDIRIPGDKSISHRSFMFGGLARGETRITGLLEGEDVINTG | 60 |
| Query 61 | KAMQAMGARIRKEGDTWIIDGVGNGGLLAPEAPLDFGNAATGCRLTMGLVGVYDFDSTFI | 120 |
| Sbjct 61 | KAMQAMGA +RKEGDTWI+ G GNG LLAPEAPLDFGNA TGCRLTMGLVGVYDFDSTFI KAMQAMGASVRKEGDTWIVTGTGNGCLLAPEAPLDFGNAGTGCRLTMGLVGVYDFDSTFI | 120 |
| Query 121 | GDASLTKRPMGRVLNPLREMGVQVKSEDGDRLPVTLRGPKTPTPITYRVPMASAQVKSAV | 180 |
| Sbjct 121 | GDASL++RPMGRVLNPLREMGVQVKSE GDRLPVTL GP TP PITYRVPMASAQVKSAV GDASLSRRPMGRVLNPLREMGVQVKSEPGDRLPVTLHGPATPAPITYRVPMASAQVKSAV | 180 |
| Query 181 | LLAGLNTPGITTVIEPIMTRDHTEKMLQGFGANLTVETDADGVRTIRLEGRGKLTGQVID | 240 |
| Sbjct 181 | LLAGLNTPG+TTVIEP+MTRDHTEKML+GFGA+L VETDADGVRTIRL G+G+LTGQVID LLAGLNTPGVTTVIEPVMTRDHTEKMLRGFGADLEVETDADGVRTIRLIGQGELTGQVID | 240 |
| Query 241 | VPGDPSSTAFPLVAALLVPGSDVTILNVLMNPTRTGLILTLQEMGADIEVINPRLAGGED | 300 |
| Sbjct 241 | VPGDPSSTAFPLVAALLVPGS++ I NVLMNPTRTGLI TLQEMG DIE+++ RLAGGED VPGDPSSTAFPLVAALLVPGSEILIRNVLMNPTRTGLIQTLQEMGGDIEILDARLAGGED | 300 |
| Query 301 | VADLRVRSSTLKGVTVPEDRAPSMIDEYPILavaaafaegaTVMNGLEELRVKESDRLSA | 360 |
| Sbjct 301 | VADLRVR S+L+GVTVP DRAPSMIDEYP+LAVAA+FAEG TVM+GLEELRVKESDRL+A VADLRVRGSSLRGVTVPPDRAPSMIDEYPVLAVAASFAEGETVMHGLEELRVKESDRLAA | 360 |
| Query 361 | VANGLKLNGVDCDEGETSLVVRGRPDGKGLGNASGAAVATHLDHRIAMSFLVMGLVSENP | 420 |
| Sbjct 361 | VA GL+LNGVDC EGE L VRGRPDGKGLGNA G AV THLDHRIAMSFLVMGLVSE+P VARGLELNGVDCTEGEDWLTVRGRPDGKGLGNAKGEAVVTHLDHRIAMSFLVMGLVSEHP | 420 |
| Query 421  Sbjct 421 | VTVDDATMIATSFPEFMDLMAGLGAKIELSD 451 VTVDD+ +IATSFPEFMDLM GLGA I D VTVDDSAIIATSFPEFMDLMTGLGATIAARD 451 |  |

>WP_062454213.1 3-phosphoshikimate 1-carboxyvinyltransferase [Rhizobium sp. Leaf306] KQQ38598.1 3-phosphoshikimate 1-carboxyvinyltransferase [Rhizobium sp. Leaf306]

Length=455

Score = 748 bits (1932), Expect = 0.0, Method: Compositional matrix adjust.

| Identities | = 381/448 (85%), Positives = 410/448 (92%), Gaps = 0/448 (0%) |  |
| --- | --- | --- |
| Query 1 | MLHGASSRPATARKSSGLSGTVRIPGDKSISHRSFMFGGLASGETRITGLLEGEDVINTG | 60 |
| Sbjct 1 | M HG + +PA +R+S+ L+GTVRIPGDKSISHRSFMFGGLA+GETRITGLLEGEDVINTG MSHGIALKPAKSRRSTNLTGTVRIPGDKSISHRSFMFGGLAAGETRITGLLEGEDVINTG | 60 |
| Query 61 | KAMQAMGARIRKEGDTWIIDGVGNGGLLAPEAPLDFGNAATGCRLTMGLVGVYDFDSTFI | 120 |
| Sbjct 61 | KAM+AMGA+I K G WII G GNG LLAP+APLDFGNA TGCRLTMGLVGVYDF+S FI KAMRAMGAKIDKVGAEWIIQGTGNGALLAPQAPLDFGNAGTGCRLTMGLVGVYDFESAFI | 120 |
| Query 121 | GDASLTKRPMGRVLNPLREMGVQVKSEDGDRLPVTLRGPKTPTPITYRVPMASAQVKSAV | 180 |
| Sbjct 121 | GDASL+KRPMGRVL+PLREMGVQV S GDRLPVTLRGP+TP PI YRVPMASAQVKSAV  GDASLSKRPMGRVLDPLREMGVQVTSMAGDRLPVTLRGPRTPNPIVYRVPMASAQVKSAV | 180 |

| Query | 181 | LLAGLNTPGITTVIEPIMTRDHTEKMLQGFGANLTVETDADGVRTIRLEGRGKLTGQVID | 240 |
| --- | --- | --- | --- |
| Sbjct | 181 | LLAGLNTPG+TTVIEP+MTRDHTEKMLQGFGA+LTVETDADGVRTIRLEGRG L GQVID LLAGLNTPGVTTVIEPVMTRDHTEKMLQGFGADLTVETDADGVRTIRLEGRGNLKGQVID | 240 |
| Query | 241 | VPGDPSSTAFPLVAALLVPGSDVTILNVLMNPTRTGLILTLQEMGADIEVINPRLAGGED | 300 |
| Sbjct | 241 | VPGDPSSTAFPLVAA+LVPGSDVTI+NVLMNPTRTGLILTLQEMGADIEV+N R AGGED VPGDPSSTAFPLVAAILVPGSDVTIMNVLMNPTRTGLILTLQEMGADIEVMNARSAGGED | 300 |
| Query | 301 | VADLRVRSSTLKGVTVPEDRAPSMIDEYPILavaaafaegaTVMNGLEELRVKESDRLSA | 360 |
| Sbjct | 301 | VADLRVR S L GVTVP DRAPSMIDEYP+LAVAAAFAEG T M GLEELRVKESDRLSA VADLRVRGSDLNGVTVPADRAPSMIDEYPVLAVAAAFAEGTTTMLGLEELRVKESDRLSA | 360 |
| Query | 361 | VANGLKLNGVDCDEGETSLVVRGRPDGKGLGNASGAAVATHLDHRIAMSFLVMGLVSENP | 420 |
| Sbjct | 361 | VA GLKLNGVDCDEG+ +LVVRGRP GKG GNASGAAV T+LDHRIAMSFLV+GL SE+ VAEGLKLNGVDCDEGKETLVVRGRPGGKGYGNASGAAVITYLDHRIAMSFLVLGLASEHE | 420 |
| Query  Sbjct | 421  421 | VTVDDATMIATSFPEFMDLMAGLGAKIE 448 VTVDDA++IATSFPEFMDLM GLGA+IE VTVDDASIIATSFPEFMDLMTGLGAQIE 448 |  |

>WP_119942315.1 3-phosphoshikimate 1-carboxyvinyltransferase [Neorhizobium sp. NCHU2750]

AYD03249.1 3-phosphoshikimate 1-carboxyvinyltransferase [Neorhizobium sp. NCHU2750]

Length=454

Score = 747 bits (1929), Expect = 0.0, Method: Compositional matrix adjust.

| Identities | = 384/454 (85%), Positives = 407/454 (90%), Gaps = 0/454 (0%) |  |
| --- | --- | --- |
| Query 1 | MLHGASSRPATARKSSGLSGTVRIPGDKSISHRSFMFGGLASGETRITGLLEGEDVINTG | 60 |
| Sbjct 1 | M HG + +PA + +S L GT+RIPGDKSISHRSFMFGGLASGETRITGLLEGEDVINTG MSHGIALKPARSARSEALKGTIRIPGDKSISHRSFMFGGLASGETRITGLLEGEDVINTG | 60 |
| Query 61 | KAMQAMGARIRKEGDTWIIDGVGNGGLLAPEAPLDFGNAATGCRLTMGLVGVYDFDSTFI | 120 |
| Sbjct 61 | KAM+AMGA+I K G WII G GNG LLAPEAPLDFGNA TGCRLTMGLVGVYDF STFI KAMRAMGAKIDKAGAEWIIQGTGNGALLAPEAPLDFGNAGTGCRLTMGLVGVYDFPSTFI | 120 |
| Query 121 | GDASLTKRPMGRVLNPLREMGVQVKSEDGDRLPVTLRGPKTPTPITYRVPMASAQVKSAV | 180 |
| Sbjct 121 | GDASL+KRPMGRVLNPLREMGVQV S +GDRLPVTL GPKTP PITYRVPMASAQVKSAV GDASLSKRPMGRVLNPLREMGVQVSSTEGDRLPVTLHGPKTPNPITYRVPMASAQVKSAV | 180 |
| Query 181 | LLAGLNTPGITTVIEPIMTRDHTEKMLQGFGANLTVETDADGVRTIRLEGRGKLTGQVID | 240 |
| Sbjct 181 | LLAGLNTPGITTVIEP+MTRDHTEKMLQGFGA+LTV+TDA GVRTIRL GRG L GQ+ID LLAGLNTPGITTVIEPVMTRDHTEKMLQGFGADLTVDTDAQGVRTIRLVGRGDLKGQIID | 240 |
| Query 241 | VPGDPSSTAFPLVAALLVPGSDVTILNVLMNPTRTGLILTLQEMGADIEVINPRLAGGED | 300 |
| Sbjct 241 | VPGDPSSTAFPLVAALL PGSDVTILNVLMNPTRTGLILTLQEMGADIEV N RLAGGED VPGDPSSTAFPLVAALLTPGSDVTILNVLMNPTRTGLILTLQEMGADIEVQNARLAGGED | 300 |
| Query 301 | VADLRVRSSTLKGVTVPEDRAPSMIDEYPILavaaafaegaTVMNGLEELRVKESDRLSA | 360 |
| Sbjct 301 | VADLRVR S LKGVTVP DRAPSMIDEYP+LAVAAA+A G T M GLEELRVKESDRLSA VADLRVRYSELKGVTVPADRAPSMIDEYPVLAVAAAYATGTTTMLGLEELRVKESDRLSA | 360 |
| Query 361 | VANGLKLNGVDCDEGETSLVVRGRPDGKGLGNASGAAVATHLDHRIAMSFLVMGLVSENP | 420 |
| Sbjct 361 | VA GLKLNGVDCDEG+ +LVVRGRP GKG GNASG AV THLDHRIAMSFLV+GL SE+ VAEGLKLNGVDCDEGKETLVVRGRPGGKGYGNASGKAVVTHLDHRIAMSFLVLGLASEHA | 420 |
| Query 421  Sbjct 421 | VTVDDATMIATSFPEFMDLMAGLGAKIELSDTKA 454 VTVDDA++IATSFPEFMDLM GLGAKIE + +A VTVDDASIIATSFPEFMDLMTGLGAKIEQVEGEA 454 |  |

>WP_128443486.1 3-phosphoshikimate 1-carboxyvinyltransferase [Rhizobium sp. 24NR] RWX76568.1 3-phosphoshikimate 1-carboxyvinyltransferase [Rhizobium sp. 24NR]

Length=455

Score = 746 bits (1927), Expect = 0.0, Method: Compositional matrix adjust. Identities = 379/448 (85%), Positives = 409/448 (91%), Gaps = 0/448 (0%)

Query 1	MLHGASSRPATARKSSGLSGTVRIPGDKSISHRSFMFGGLASGETRITGLLEGEDVINTG 60

| Sbjct | 1 | MLHG + +PA +RKSS LSGTVRIPGDKSISHRSFMFGGLASGETRITGLLEGEDVINTG MLHGVALKPAKSRKSSTLSGTVRIPGDKSISHRSFMFGGLASGETRITGLLEGEDVINTG | 60 |
| --- | --- | --- | --- |
| Query | 61 | KAMQAMGARIRKEGDTWIIDGVGNGGLLAPEAPLDFGNAATGCRLTMGLVGVYDFDSTFI | 120 |
| Sbjct | 61 | KAMQAMGARI K G WII G GNG LLAPEAPLDFGNA TGCRLTMGLVGVYDF+STF+ KAMQAMGARIEKLGAEWIIHGTGNGALLAPEAPLDFGNAGTGCRLTMGLVGVYDFESTFV | 120 |
| Query | 121 | GDASLTKRPMGRVLNPLREMGVQVKSEDGDRLPVTLRGPKTPTPITYRVPMASAQVKSAV | 180 |
| Sbjct | 121 | GDASL++RPMGRVL+PLR+MGVQV+S +GDRLPVTL GP+TP PI YRVPMASAQVKSAV GDASLSRRPMGRVLDPLRDMGVQVRSSEGDRLPVTLYGPRTPNPIVYRVPMASAQVKSAV | 180 |
| Query | 181 | LLAGLNTPGITTVIEPIMTRDHTEKMLQGFGANLTVETDADGVRTIRLEGRGKLTGQVID | 240 |
| Sbjct | 181 | LLAGLNTPG+TTVIEPIMTRDHTEKMLQGFGA L+VETD +GVRTIRLEGRG+LTGQ ID LLAGLNTPGVTTVIEPIMTRDHTEKMLQGFGAELSVETDGNGVRTIRLEGRGRLTGQRID | 240 |
| Query | 241 | VPGDPSSTAFPLVAALLVPGSDVTILNVLMNPTRTGLILTLQEMGADIEVINPRLAGGED | 300 |
| Sbjct | 241 | VPGDPSSTAFPLVAALLVPGSDVTI NVLMNPTRTGLILTLQEMGA+IEV+NPR AGGED VPGDPSSTAFPLVAALLVPGSDVTIENVLMNPTRTGLILTLQEMGANIEVLNPRSAGGED | 300 |
| Query | 301 | VADLRVRSSTLKGVTVPEDRAPSMIDEYPILavaaafaegaTVMNGLEELRVKESDRLSA | 360 |
| Sbjct | 301 | VADLRVR+S LKGVTVP +RAP MIDEYP+LAVA+AFAEG T M GLEELRVKESDRL+A VADLRVRASELKGVTVPAERAPFMIDEYPVLAVASAFAEGTTTMLGLEELRVKESDRLTA | 360 |
| Query | 361 | VANGLKLNGVDCDEGETSLVVRGRPDGKGLGNASGAAVATHLDHRIAMSFLVMGLVSENP | 420 |
| Sbjct | 361 | A GLKLNGVDCDEG +LVVRGRP GKG GN +G AV THLDHRIAMSFLVMGLVSE+ TATGLKLNGVDCDEGRDTLVVRGRPGGKGYGNPAGGAVETHLDHRIAMSFLVMGLVSEHE | 420 |
| Query  Sbjct | 421  421 | VTVDDATMIATSFPEFMDLMAGLGAKIE 448 VTVDDA++I TSFPEFMDLM GLGA+I+ VTVDDASIITTSFPEFMDLMTGLGARID 448 |  |

>MAZ85107.1 3-phosphoshikimate 1-carboxyvinyltransferase [Hoeflea sp.] Length=452

Score = 742 bits (1915), Expect = 0.0, Method: Compositional matrix adjust.

| Identities | = 376/447 (84%), Positives = 407/447 (91%), Gaps = 0/447 (0%) |  |
| --- | --- | --- |
| Query 1 | MLHGASSRPATARKSSGLSGTVRIPGDKSISHRSFMFGGLASGETRITGLLEGEDVINTG | 60 |
| Sbjct 1 | M H RPATAR+SS L G +RIPGDKSISHRSFMFGGLA GETRITGLLEGEDVINTG MNHVNKLRPATARRSSALKGDIRIPGDKSISHRSFMFGGLARGETRITGLLEGEDVINTG | 60 |
| Query 61 | KAMQAMGARIRKEGDTWIIDGVGNGGLLAPEAPLDFGNAATGCRLTMGLVGVYDFDSTFI | 120 |
| Sbjct 61 | KAMQAMGA +RKEGDTWI+ G GNG LLAPEAPLDFGNA TGCRLTMGLVGVYDFDSTFI KAMQAMGASVRKEGDTWIVTGTGNGCLLAPEAPLDFGNAGTGCRLTMGLVGVYDFDSTFI | 120 |
| Query 121 | GDASLTKRPMGRVLNPLREMGVQVKSEDGDRLPVTLRGPKTPTPITYRVPMASAQVKSAV | 180 |
| Sbjct 121 | GDASL++RPMGRVL+PLREMGVQVKSE GDRLPVTL GP TP PITYRVPMASAQVKSAV GDASLSRRPMGRVLDPLREMGVQVKSEPGDRLPVTLHGPATPAPITYRVPMASAQVKSAV | 180 |
| Query 181 | LLAGLNTPGITTVIEPIMTRDHTEKMLQGFGANLTVETDADGVRTIRLEGRGKLTGQVID | 240 |
| Sbjct 181 | LLAGLNTPG+TTVIEP+MTRDHTEKML+GFGA+L VETD+DGVRTIRL G+G+LTGQVID LLAGLNTPGVTTVIEPVMTRDHTEKMLKGFGADLEVETDSDGVRTIRLVGQGELTGQVID | 240 |
| Query 241 | VPGDPSSTAFPLVAALLVPGSDVTILNVLMNPTRTGLILTLQEMGADIEVINPRLAGGED | 300 |
| Sbjct 241 | VPGDPSSTAFPLVAALLVPGS+V I NVLMNPTRTGLI TLQEMG DIE+++PRLAGGED VPGDPSSTAFPLVAALLVPGSEVLIRNVLMNPTRTGLIQTLQEMGGDIEILDPRLAGGED | 300 |
| Query 301 | VADLRVRSSTLKGVTVPEDRAPSMIDEYPILavaaafaegaTVMNGLEELRVKESDRLSA | 360 |
| Sbjct 301 | VADLRVR S+LKGVTVP +RAP+MIDEYP+LAVAA+FAEG TVM+GLEELRVKESDRL+A VADLRVRGSSLKGVTVPPERAPAMIDEYPVLAVAASFAEGETVMHGLEELRVKESDRLAA | 360 |
| Query 361 | VANGLKLNGVDCDEGETSLVVRGRPDGKGLGNASGAAVATHLDHRIAMSFLVMGLVSENP | 420 |
| Sbjct 361 | VA GL+LNGVDC EGE L VRGRPDGKG+GN AV THLDHRIAMSFLVMGLVSE+P VARGLELNGVDCTEGEDWLTVRGRPDGKGIGNGKNEAVVTHLDHRIAMSFLVMGLVSEHP | 420 |
| Query 421  Sbjct 421 | VTVDDATMIATSFPEFMDLMAGLGAKI 447 VTVDD+ +IATSFPEFMD+M GLGA I VTVDDSAIIATSFPEFMDIMTGLGAAI 447 |  |

>WP_126828979.1 3-phosphoshikimate 1-carboxyvinyltransferase [Rhizobium fabae]

MBB3917058.1 3-phosphoshikimate 1-carboxyvinyltransferase [Rhizobium fabae] RUM10573.1 3-phosphoshikimate 1-carboxyvinyltransferase [Rhizobium fabae]

Length=452

Score = 741 bits (1914), Expect = 0.0, Method: Compositional matrix adjust.

| Identities | = 374/448 (83%), Positives = 404/448 (90%), Gaps = 0/448 (0%) |  |
| --- | --- | --- |
| Query 1 | MLHGASSRPATARKSSGLSGTVRIPGDKSISHRSFMFGGLASGETRITGLLEGEDVINTG | 60 |
| Sbjct 1 | M HG+ RP TARKS+ L+G VRIPGDKSISHR FMFGGLASG+TRITGLLEGEDVINTG MSHGSRPRPVTARKSANLAGVVRIPGDKSISHRCFMFGGLASGQTRITGLLEGEDVINTG | 60 |
| Query 61 | KAMQAMGARIRKEGDTWIIDGVGNGGLLAPEAPLDFGNAATGCRLTMGLVGVYDFDSTFI | 120 |
| Sbjct 61 | +AMQAMGARIR++G W+IDG GNG LLAPEAPLDFGNA TG RLTMGLVG YDF STF RAMQAMGARIRRQGAEWVIDGTGNGALLAPEAPLDFGNAGTGVRLTMGLVGAYDFSSTFT | 120 |
| Query 121 | GDASLTKRPMGRVLNPLREMGVQVKSEDGDRLPVTLRGPKTPTPITYRVPMASAQVKSAV | 180 |
| Sbjct 121 | GDASL+KRPMGRVL+PLREMGVQV + GD+LPVTLRGPKTP+ I YRVPMASAQVKSAV GDASLSKRPMGRVLDPLREMGVQVSASAGDQLPVTLRGPKTPSAIRYRVPMASAQVKSAV | 180 |
| Query 181 | LLAGLNTPGITTVIEPIMTRDHTEKMLQGFGANLTVETDADGVRTIRLEGRGKLTGQVID | 240 |
| Sbjct 181 | LLAGLNTPGITTVIEP+MTRDHTEKMLQGFGA L+VETD GVRTIRLEGRG+LTGQVID LLAGLNTPGITTVIEPVMTRDHTEKMLQGFGAALSVETDKGGVRTIRLEGRGRLTGQVID | 240 |
| Query 241 | VPGDPSSTAFPLVAALLVPGSDVTILNVLMNPTRTGLILTLQEMGADIEVINPRLAGGED | 300 |
| Sbjct 241 | VPGDPSSTAFPLVAAL+VPGSD+TI NVLMNPTRTGLILTLQEMGADIEV+N LAGGED VPGDPSSTAFPLVAALIVPGSDITIHNVLMNPTRTGLILTLQEMGADIEVLNAHLAGGED | 300 |
| Query 301 | VADLRVRSSTLKGVTVPEDRAPSMIDEYPILavaaafaegaTVMNGLEELRVKESDRLSA | 360 |
| Sbjct 301 | VADLRVR S LKGVTVPE+RAPSMIDEYPILAVAA FAEG T M GLEELRVKESDRLSA VADLRVRHSELKGVTVPEERAPSMIDEYPILAVAACFAEGTTAMKGLEELRVKESDRLSA | 360 |
| Query 361 | VANGLKLNGVDCDEGETSLVVRGRPDGKGLGNASGAAVATHLDHRIAMSFLVMGLVSENP | 420 |
| Sbjct 361 | VA+GLKLNG DC+EG+ L+VRGRPDGKGLG+A+G V THLDHRIAMSFLVMGL +E+ VADGLKLNGADCEEGKDFLIVRGRPDGKGLGSAAGGEVRTHLDHRIAMSFLVMGLAAEHA | 420 |
| Query 421  Sbjct 421 | VTVDDATMIATSFPEFMDLMAGLGAKIE 448 VT+DDA MIATSFPEFM LM GLGA+IE VTIDDAAMIATSFPEFMTLMTGLGARIE 448 |  |

>WP_125846735.1 3-phosphoshikimate 1-carboxyvinyltransferase [Rhizobium pisi] MBB3136214.1 3-phosphoshikimate 1-carboxyvinyltransferase [Rhizobium pisi] RSB75527.1 3-phosphoshikimate 1-carboxyvinyltransferase [Rhizobium pisi] TCA53209.1 3-phosphoshikimate 1-carboxyvinyltransferase [Rhizobium pisi]

Length=452

Score = 741 bits (1914), Expect = 0.0, Method: Compositional matrix adjust.

| Identities | = 376/448 (84%), Positives = 402/448 (90%), Gaps = 0/448 (0%) |  |
| --- | --- | --- |
| Query 1 | MLHGASSRPATARKSSGLSGTVRIPGDKSISHRSFMFGGLASGETRITGLLEGEDVINTG | 60 |
| Sbjct 1 | M HG+ RP TARKS L+G VRIPGDKSISHRSFMFGGLASG+TRITGLLEGEDVINTG MSHGSRPRPVTARKSVNLAGVVRIPGDKSISHRSFMFGGLASGQTRITGLLEGEDVINTG | 60 |
| Query 61 | KAMQAMGARIRKEGDTWIIDGVGNGGLLAPEAPLDFGNAATGCRLTMGLVGVYDFDSTFI | 120 |
| Sbjct 61 | +AMQAMGARI ++G W+IDG GNG LLAPEAPLDFGNA TG RLTMGLVG YDF STF RAMQAMGARIHRQGAEWVIDGTGNGALLAPEAPLDFGNAGTGVRLTMGLVGTYDFSSTFT | 120 |
| Query 121 | GDASLTKRPMGRVLNPLREMGVQVKSEDGDRLPVTLRGPKTPTPITYRVPMASAQVKSAV | 180 |
| Sbjct 121 | GDASL+KRPMGRVL+PLREMGVQV + GDRLPVTLRGPK P+ I YRVPMASAQVKSAV GDASLSKRPMGRVLDPLREMGVQVSASAGDRLPVTLRGPKMPSAIRYRVPMASAQVKSAV | 180 |
| Query 181 | LLAGLNTPGITTVIEPIMTRDHTEKMLQGFGANLTVETDADGVRTIRLEGRGKLTGQVID | 240 |
| Sbjct 181 | LLAGLNTPGITTVIEP+MTRDHTEKMLQGFGA L+VETD GVR IRLEGRG+LTGQVID LLAGLNTPGITTVIEPVMTRDHTEKMLQGFGAALSVETDKGGVRAIRLEGRGRLTGQVID | 240 |
| Query 241 | VPGDPSSTAFPLVAALLVPGSDVTILNVLMNPTRTGLILTLQEMGADIEVINPRLAGGED | 300 |
| Sbjct 241 | VPGDPSSTAFPLVAAL+VPGSD+TILNVLMNPTRTGLILTLQEMGADIEV+N RLAGGED VPGDPSSTAFPLVAALIVPGSDITILNVLMNPTRTGLILTLQEMGADIEVLNARLAGGED | 300 |
| Query 301 | VADLRVRSSTLKGVTVPEDRAPSMIDEYPILavaaafaegaTVMNGLEELRVKESDRLSA | 360 |
| Sbjct 301 | VADLRVR S LKGVTVPE+RAPSMIDEYPILAVAA FAEG T M GLEELRVKESDRLSA  VADLRVRHSELKGVTVPEERAPSMIDEYPILAVAACFAEGTTAMKGLEELRVKESDRLSA | 360 |

| Query | 361 | VANGLKLNGVDCDEGETSLVVRGRPDGKGLGNASGAAVATHLDHRIAMSFLVMGLVSENP 420 |
| --- | --- | --- |
| Sbjct | 361 | VA+GLKLNG DC+EG+ LVVRGRPDGKGLGNA+G V THLDHRIAM FLVMGL +E+ VADGLKLNGADCEEGKDFLVVRGRPDGKGLGNAAGGEVRTHLDHRIAMGFLVMGLAAEHA 420 |
| Query | 421 | VTVDDATMIATSFPEFMDLMAGLGAKIE 448 |
| Sbjct | 421 | VT+DDA MIATSFPEFM LM GLGA+IE  VTIDDAAMIATSFPEFMTLMTGLGARIE 448 |

>WP_121981347.1 MULTISPECIES: 3-phosphoshikimate 1-carboxyvinyltransferase [Ochrobactrum] KAB2682631.1 3-phosphoshikimate 1-carboxyvinyltransferase [Ochrobactrum pseudintermedium] NKE76126.1 3-phosphoshikimate 1-carboxyvinyltransferase [Brucella intermedia]

TWH00301.1 3-phosphoshikimate 1-carboxyvinyltransferase [Ochrobactrum sp. J50]

Length=450

Score = 738 bits (1904), Expect = 0.0, Method: Compositional matrix adjust.

| Identities | = 375/450 (83%), Positives = 408/450 (91%), Gaps = 3/450 (1%) |  |
| --- | --- | --- |
| Query 1 | MLHGASSRPATARKSSGLSGTVRIPGDKSISHRSFMFGGLASGETRITGLLEGEDVINTG | 60 |
| Sbjct 1 | M H AS +PATAR+S L+G +RIPGDKSISHRSFMFGGLASGETRITGLLEGEDVINTG MSHSASPKPATARRSEALTGEIRIPGDKSISHRSFMFGGLASGETRITGLLEGEDVINTG | 60 |
| Query 61 | KAMQAMGARIRKEGDTWIIDGVGNGGLLAPEAPLDFGNAATGCRLTMGLVGVYDFDSTFI | 120 |
| Sbjct 61 | +AMQAMGA+IRKEGD WII+GVGNG LL PEA LDFGNA TG RLTMGLVG YD ++FI RAMQAMGAKIRKEGDVWIINGVGNGCLLQPEAALDFGNAGTGARLTMGLVGTYDMKTSFI | 120 |
| Query 121 | GDASLTKRPMGRVLNPLREMGVQVKSEDGDRLPVTLRGPKTPTPITYRVPMASAQVKSAV | 180 |
| Sbjct 121 | GDASL+KRPMGRVLNPLREMGVQV++ +GDR+P+TL GPKT PITYRVPMASAQVKSAV GDASLSKRPMGRVLNPLREMGVQVEAAEGDRMPLTLIGPKTANPITYRVPMASAQVKSAV | 180 |
| Query 181 | LLAGLNTPGITTVIEPIMTRDHTEKMLQGFGANLTVETDADGVRTIRLEGRGKLTGQVID | 240 |
| Sbjct 181 | LLAGLNTPG+TTVIEP+MTRDHTEKMLQGFGA+LTVETD DGVR IR+ G+GKLTGQ ID LLAGLNTPGVTTVIEPVMTRDHTEKMLQGFGADLTVETDKDGVRHIRIVGQGKLTGQTID | 240 |
| Query 241 | VPGDPSSTAFPLVAALLVPGSDVTILNVLMNPTRTGLILTLQEMGADIEVINPRLAGGED | 300 |
| Sbjct 241 | VPGDPSSTAFPLVAALLV GSDVTI NVLMNPTRTGLILTLQEMGADIEV+N RLAGGED VPGDPSSTAFPLVAALLVEGSDVTIRNVLMNPTRTGLILTLQEMGADIEVLNARLAGGED | 300 |
| Query 301 | VADLRVRSSTLKGVTVPEDRAPSMIDEYPILavaaafaegaTVMNGLEELRVKESDRLSA | 360 |
| Sbjct 301 | VADLRV++S LKGVTVP +RAPSMIDEYP+LA+AA+FAEG TVM+GL+ELRVKESDRL+A VADLRVKASKLKGVTVPPERAPSMIDEYPVLAIAASFAEGETVMDGLDELRVKESDRLAA | 360 |
| Query 361 | VANGLKLNGVDCDEGETSLVVRGRPDGKGLGNASGAAVATHLDHRIAMSFLVMGLVSENP | 420 |
| Sbjct 361 | VA GL+ NGVDC EGE SL VRGRPDGKGLG G VATHLDHRIAMSFLVMGL SE P VARGLEANGVDCTEGEMSLTVRGRPDGKGLG---GGTVATHLDHRIAMSFLVMGLASEKP | 417 |
| Query 421  Sbjct 418 | VTVDDATMIATSFPEFMDLMAGLGAKIELS 450 VTVDD+TMIATSFPEFMD+M GLGAKIELS VTVDDSTMIATSFPEFMDMMPGLGAKIELS 447 |  |

>WP_094506051.1 3-phosphoshikimate 1-carboxyvinyltransferase [Ochrobactrum thiophenivorans] OYR19826.1 3-phosphoshikimate 1-carboxyvinyltransferase [Ochrobactrum thiophenivorans]

Length=450

Score = 737 bits (1903), Expect = 0.0, Method: Compositional matrix adjust.

| Identities | = 374/453 (83%), Positives = 408/453 (90%), Gaps = 3/453 (1%) |  |
| --- | --- | --- |
| Query 1 | MLHGASSRPATARKSSGLSGTVRIPGDKSISHRSFMFGGLASGETRITGLLEGEDVINTG | 60 |
| Sbjct 1 | M H AS +PA AR+S L G +RIPGDKSISHRSFMFGGLASGETRITGLLEGEDVINTG MSHSASPKPAIARRSEALKGEIRIPGDKSISHRSFMFGGLASGETRITGLLEGEDVINTG | 60 |
| Query 61 | KAMQAMGARIRKEGDTWIIDGVGNGGLLAPEAPLDFGNAATGCRLTMGLVGVYDFDSTFI | 120 |
| Sbjct 61 | +AMQAMGA+IRK+GD WII+GVGNG LL PE+PLDFGNA TG RLTMGLVG YD +TFI RAMQAMGAKIRKDGDVWIINGVGNGCLLQPESPLDFGNAGTGARLTMGLVGTYDMTTTFI | 120 |
| Query 121 | GDASLTKRPMGRVLNPLREMGVQVKSEDGDRLPVTLRGPKTPTPITYRVPMASAQVKSAV | 180 |
| Sbjct 121 | GDASLT RPMGRVLNPLREMGVQVK+ +GDRLP+TL GPKT PI+YRVPMASAQVKSAV  GDASLTSRPMGRVLNPLREMGVQVKAAEGDRLPLTLTGPKTANPISYRVPMASAQVKSAV | 180 |

| Query | 181 | LLAGLNTPGITTVIEPIMTRDHTEKMLQGFGANLTVETDADGVRTIRLEGRGKLTGQVID | 240 |
| --- | --- | --- | --- |
| Sbjct | 181 | LLAGLNTPG+TTVIEP+MTRDHTEKMLQGFGA+LTVETD DGVR IR+ G+GKLTGQ ID LLAGLNTPGVTTVIEPVMTRDHTEKMLQGFGADLTVETDKDGVRHIRIVGQGKLTGQTID | 240 |
| Query | 241 | VPGDPSSTAFPLVAALLVPGSDVTILNVLMNPTRTGLILTLQEMGADIEVINPRLAGGED | 300 |
| Sbjct | 241 | VPGDPSSTAFPLVAALLV GSDVTILNVLMNPTRTGLILTLQEMGADIE+INPRLAGGED VPGDPSSTAFPLVAALLVEGSDVTILNVLMNPTRTGLILTLQEMGADIEIINPRLAGGED | 300 |
| Query | 301 | VADLRVRSSTLKGVTVPEDRAPSMIDEYPILavaaafaegaTVMNGLEELRVKESDRLSA | 360 |
| Sbjct | 301 | VADLRV+SS LKGV VP +RAPSMIDEYP+LA+AA+FAEG TVM+GL+ELRVKESDRL+A VADLRVKSSKLKGVVVPPERAPSMIDEYPVLAIAASFAEGETVMDGLDELRVKESDRLAA | 360 |
| Query | 361 | VANGLKLNGVDCDEGETSLVVRGRPDGKGLGNASGAAVATHLDHRIAMSFLVMGLVSENP | 420 |
| Sbjct | 361 | VA GL+ NGVDC EGE SL VRGRPDGKGLG G VATHLDHRIAMSFLVMGL +E P VARGLEANGVDCTEGEMSLTVRGRPDGKGLG---GGTVATHLDHRIAMSFLVMGLAAEKP | 417 |
| Query  Sbjct | 421  418 | VTVDDATMIATSFPEFMDLMAGLGAKIELSDTK 453 VTVDD+TMIATSFPEFM +M GLGAKIE+S+ + VTVDDSTMIATSFPEFMTMMPGLGAKIEVSEPQ 450 |  |

>WP_105518014.1 MULTISPECIES: 3-phosphoshikimate 1-carboxyvinyltransferase [Ochrobactrum] MQP39834.1 3-phosphoshikimate 1-carboxyvinyltransferase [Ochrobactrum sp.

MYb237]

PQZ42429.1 3-phosphoshikimate 1-carboxyvinyltransferase [Ochrobactrum pseudogrignonense] PRA41859.1 3-phosphoshikimate 1-carboxyvinyltransferase [Ochrobactrum pseudogrignonense] PRA70715.1 3-phosphoshikimate 1-carboxyvinyltransferase [Ochrobactrum pseudogrignonense]

Length=450

Score = 737 bits (1902), Expect = 0.0, Method: Compositional matrix adjust.

| Identities | = 373/453 (82%), Positives = 410/453 (91%), Gaps = 3/453 (1%) |  |
| --- | --- | --- |
| Query 1 | MLHGASSRPATARKSSGLSGTVRIPGDKSISHRSFMFGGLASGETRITGLLEGEDVINTG | 60 |
| Sbjct 1 | M H A+ +PATAR+S L+G +RIPGDKSISHRSFMFGGLASGETRITGLLEGEDVINTG MSHSATPKPATARRSEALTGEIRIPGDKSISHRSFMFGGLASGETRITGLLEGEDVINTG | 60 |
| Query 61 | KAMQAMGARIRKEGDTWIIDGVGNGGLLAPEAPLDFGNAATGCRLTMGLVGVYDFDSTFI | 120 |
| Sbjct 61 | +AMQAMGA+IRK+GD W+I+GVGNG LL PEAPLDFGNA TG RLTMGLVG YD +TFI RAMQAMGAKIRKDGDIWVINGVGNGCLLQPEAPLDFGNAGTGARLTMGLVGTYDMTTTFI | 120 |
| Query 121 | GDASLTKRPMGRVLNPLREMGVQVKSEDGDRLPVTLRGPKTPTPITYRVPMASAQVKSAV | 180 |
| Sbjct 121 | GDASLT RPMGRVLNPLREMGVQVK+ +GDRLP+TL GPKT PI+YRVPMASAQVKSAV GDASLTSRPMGRVLNPLREMGVQVKAAEGDRLPLTLTGPKTANPISYRVPMASAQVKSAV | 180 |
| Query 181 | LLAGLNTPGITTVIEPIMTRDHTEKMLQGFGANLTVETDADGVRTIRLEGRGKLTGQVID | 240 |
| Sbjct 181 | LLAGLNTPG+TTVIEP+MTRDHTEKMLQGFGA+LTVETD DGVR IR+ G+GKLTGQ ID LLAGLNTPGVTTVIEPVMTRDHTEKMLQGFGADLTVETDKDGVRHIRIVGQGKLTGQTID | 240 |
| Query 241 | VPGDPSSTAFPLVAALLVPGSDVTILNVLMNPTRTGLILTLQEMGADIEVINPRLAGGED | 300 |
| Sbjct 241 | VPGDPSSTAFPLVAALLV GSDVTI NVLMNPTRTGLILTLQEMGADIE+I+PRLAGGED VPGDPSSTAFPLVAALLVKGSDVTIRNVLMNPTRTGLILTLQEMGADIEIIDPRLAGGED | 300 |
| Query 301 | VADLRVRSSTLKGVTVPEDRAPSMIDEYPILavaaafaegaTVMNGLEELRVKESDRLSA | 360 |
| Sbjct 301 | VADLRV+SS LKGVTVP +RAPSMIDEYP+LA+AA+FAEG TVM+GL+ELRVKESDRL+A VADLRVKSSKLKGVTVPPERAPSMIDEYPVLAIAASFAEGETVMDGLDELRVKESDRLAA | 360 |
| Query 361 | VANGLKLNGVDCDEGETSLVVRGRPDGKGLGNASGAAVATHLDHRIAMSFLVMGLVSENP | 420 |
| Sbjct 361 | VA GL+ NGVDC EGE SL VRGRPDGKGLG G VATHLDHRIAMSFLVMGL +E P VARGLEANGVDCTEGEMSLTVRGRPDGKGLG---GGTVATHLDHRIAMSFLVMGLAAEKP | 417 |
| Query 421  Sbjct 418 | VTVDDATMIATSFPEFMDLMAGLGAKIELSDTK 453 VTVDD+TMIATSFPEFM +M GLGAKIE+S+ + VTVDDSTMIATSFPEFMTMMPGLGAKIEVSEQQ 450 |  |

>WP_064322336.1 MULTISPECIES: 3-phosphoshikimate 1-carboxyvinyltransferase [Ochrobactrum] ANG98270.1 3-phosphoshikimate 1-carboxyvinyltransferase [Ochrobactrum pseudogrignonense]

Length=450

Score = 736 bits (1901), Expect = 0.0, Method: Compositional matrix adjust. Identities = 373/453 (82%), Positives = 410/453 (91%), Gaps = 3/453 (1%)

| Query | 1 | MLHGASSRPATARKSSGLSGTVRIPGDKSISHRSFMFGGLASGETRITGLLEGEDVINTG | 60 |
| --- | --- | --- | --- |
| Sbjct | 1 | M H A+ +PATAR+S L+G +RIPGDKSISHRSFMFGGLASGETRITGLLEGEDVINTG MSHSATPKPATARRSEALTGEIRIPGDKSISHRSFMFGGLASGETRITGLLEGEDVINTG | 60 |
| Query | 61 | KAMQAMGARIRKEGDTWIIDGVGNGGLLAPEAPLDFGNAATGCRLTMGLVGVYDFDSTFI | 120 |
| Sbjct | 61 | +AMQAMGA+IRK+GD W+I+GVGNG LL PEAPLDFGNA TG RLTMGLVG YD +TFI RAMQAMGAKIRKDGDIWVINGVGNGCLLQPEAPLDFGNAGTGARLTMGLVGTYDMTTTFI | 120 |
| Query | 121 | GDASLTKRPMGRVLNPLREMGVQVKSEDGDRLPVTLRGPKTPTPITYRVPMASAQVKSAV | 180 |
| Sbjct | 121 | GDASLT RPMGRVLNPLREMGVQVK+ +GDRLP+TL GPKT PI+YRVPMASAQVKSAV GDASLTSRPMGRVLNPLREMGVQVKAAEGDRLPLTLTGPKTANPISYRVPMASAQVKSAV | 180 |
| Query | 181 | LLAGLNTPGITTVIEPIMTRDHTEKMLQGFGANLTVETDADGVRTIRLEGRGKLTGQVID | 240 |
| Sbjct | 181 | LLAGLNTPG+TTVIEP+MTRDHTEKMLQGFGA+LTVETD DGVR IR+ G+GKLTGQ ID LLAGLNTPGVTTVIEPVMTRDHTEKMLQGFGADLTVETDKDGVRHIRIVGQGKLTGQTID | 240 |
| Query | 241 | VPGDPSSTAFPLVAALLVPGSDVTILNVLMNPTRTGLILTLQEMGADIEVINPRLAGGED | 300 |
| Sbjct | 241 | VPGDPSSTAFPLVAALLV GSDVTI NVLMNPTRTGLILTLQEMGADIE+I+PRLAGGED VPGDPSSTAFPLVAALLVEGSDVTIRNVLMNPTRTGLILTLQEMGADIEIIDPRLAGGED | 300 |
| Query | 301 | VADLRVRSSTLKGVTVPEDRAPSMIDEYPILavaaafaegaTVMNGLEELRVKESDRLSA | 360 |
| Sbjct | 301 | VADLRV+SS LKGVTVP +RAPSMIDEYP+LA+AA+FAEG TVM+GL+ELRVKESDRL+A VADLRVKSSKLKGVTVPPERAPSMIDEYPVLAIAASFAEGETVMDGLDELRVKESDRLAA | 360 |
| Query | 361 | VANGLKLNGVDCDEGETSLVVRGRPDGKGLGNASGAAVATHLDHRIAMSFLVMGLVSENP | 420 |
| Sbjct | 361 | VA GL+ NGVDC EGE SL VRGRPDGKGLG G VATHLDHRIAMSFLVMGL +E P VARGLEANGVDCTEGEMSLTVRGRPDGKGLG---GGTVATHLDHRIAMSFLVMGLTAEKP | 417 |
| Query  Sbjct | 421  418 | VTVDDATMIATSFPEFMDLMAGLGAKIELSDTK 453 VTVDD+TMIATSFPEFM +M GLGAKIE+S+ + VTVDDSTMIATSFPEFMTMMPGLGAKIEVSEQQ 450 |  |

>WP_113485490.1 3-phosphoshikimate 1-carboxyvinyltransferase [Ochrobactrum pseudogrignonense] KAB2690707.1 3-phosphoshikimate 1-carboxyvinyltransferase [Ochrobactrum pseudogrignonense]

Length=450

Score = 736 bits (1900), Expect = 0.0, Method: Compositional matrix adjust.

| Identities | = 373/453 (82%), Positives = 409/453 (90%), Gaps = 3/453 (1%) |  |
| --- | --- | --- |
| Query 1 | MLHGASSRPATARKSSGLSGTVRIPGDKSISHRSFMFGGLASGETRITGLLEGEDVINTG | 60 |
| Sbjct 1 | M H A+ +PATAR+S L G +RIPGDKSISHRSFMFGGLASGETRITGLLEGEDVINTG MSHSATPKPATARRSEALMGAIRIPGDKSISHRSFMFGGLASGETRITGLLEGEDVINTG | 60 |
| Query 61 | KAMQAMGARIRKEGDTWIIDGVGNGGLLAPEAPLDFGNAATGCRLTMGLVGVYDFDSTFI | 120 |
| Sbjct 61 | +AMQAMGA+IRK+GD W+I+GVGNG LL PEAPLDFGNA TG RLTMGLVG YD +TFI RAMQAMGAKIRKDGDIWVINGVGNGCLLQPEAPLDFGNAGTGARLTMGLVGTYDMTTTFI | 120 |
| Query 121 | GDASLTKRPMGRVLNPLREMGVQVKSEDGDRLPVTLRGPKTPTPITYRVPMASAQVKSAV | 180 |
| Sbjct 121 | GDASLT RPMGRVLNPLREMGVQVK+ +GDRLP+TL GPKT PI+YRVPMASAQVKSAV GDASLTSRPMGRVLNPLREMGVQVKAAEGDRLPLTLTGPKTANPISYRVPMASAQVKSAV | 180 |
| Query 181 | LLAGLNTPGITTVIEPIMTRDHTEKMLQGFGANLTVETDADGVRTIRLEGRGKLTGQVID | 240 |
| Sbjct 181 | LLAGLNTPG+TTVIEP+MTRDHTEKMLQGFGA+LTVETD DGVR IR+ G+GKLTGQ ID LLAGLNTPGVTTVIEPVMTRDHTEKMLQGFGADLTVETDKDGVRHIRIVGQGKLTGQTID | 240 |
| Query 241 | VPGDPSSTAFPLVAALLVPGSDVTILNVLMNPTRTGLILTLQEMGADIEVINPRLAGGED | 300 |
| Sbjct 241 | VPGDPSSTAFPLVAALLV GSDVTI NVLMNPTRTGLILTLQEMGADIE+I+PRLAGGED VPGDPSSTAFPLVAALLVEGSDVTIRNVLMNPTRTGLILTLQEMGADIEIIDPRLAGGED | 300 |
| Query 301 | VADLRVRSSTLKGVTVPEDRAPSMIDEYPILavaaafaegaTVMNGLEELRVKESDRLSA | 360 |
| Sbjct 301 | VADLRV+SS LKGVTVP +RAPSMIDEYP+LA+AA+FAEG TVM+GL+ELRVKESDRL+A VADLRVKSSKLKGVTVPPERAPSMIDEYPVLAIAASFAEGETVMDGLDELRVKESDRLAA | 360 |
| Query 361 | VANGLKLNGVDCDEGETSLVVRGRPDGKGLGNASGAAVATHLDHRIAMSFLVMGLVSENP | 420 |
| Sbjct 361 | VA GL+ NGVDC EGE SL VRGRPDGKGLG G VATHLDHRIAMSFLVMGL +E P VARGLEANGVDCTEGEMSLTVRGRPDGKGLG---GGTVATHLDHRIAMSFLVMGLAAEKP | 417 |
| Query 421  Sbjct 418 | VTVDDATMIATSFPEFMDLMAGLGAKIELSDTK 453 VTVDD+TMIATSFPEFM +M GLGAKIE+S+ + VTVDDSTMIATSFPEFMTMMPGLGAKIEVSEQQ 450 |  |

>WP_100647755.1 MULTISPECIES: 3-phosphoshikimate 1-carboxyvinyltransferase [unclassified Ochrobactrum]

PJR90710.1 3-phosphoshikimate 1-carboxyvinyltransferase [Ochrobactrum sp. 721/2009]

PJT16003.1 3-phosphoshikimate 1-carboxyvinyltransferase [Ochrobactrum sp. 720/2009]

PJT25823.1 3-phosphoshikimate 1-carboxyvinyltransferase [Ochrobactrum sp. 715/2009]

PJT29429.1 3-phosphoshikimate 1-carboxyvinyltransferase [Ochrobactrum sp. 695/2009]

PJT35344.1 3-phosphoshikimate 1-carboxyvinyltransferase [Ochrobactrum sp. 689/2009]

Length=450

Score = 735 bits (1898), Expect = 0.0, Method: Compositional matrix adjust.

| Identities | = 373/453 (82%), Positives = 407/453 (90%), Gaps = 3/453 (1%) |  |
| --- | --- | --- |
| Query 1 | MLHGASSRPATARKSSGLSGTVRIPGDKSISHRSFMFGGLASGETRITGLLEGEDVINTG | 60 |
| Sbjct 1 | M H AS +PATAR+S L+G +RIPGDKSISHRSFMFGGLASGETRITGLLEGEDVINTG MSHSASPKPATARRSEALTGEIRIPGDKSISHRSFMFGGLASGETRITGLLEGEDVINTG | 60 |
| Query 61 | KAMQAMGARIRKEGDTWIIDGVGNGGLLAPEAPLDFGNAATGCRLTMGLVGVYDFDSTFI | 120 |
| Sbjct 61 | +AMQAMGA+IRKEGD WII+GVGNG LL PEA LDFGNA TG RLTMGLVG YD ++FI RAMQAMGAKIRKEGDVWIINGVGNGCLLQPEAALDFGNAGTGARLTMGLVGTYDMKTSFI | 120 |
| Query 121 | GDASLTKRPMGRVLNPLREMGVQVKSEDGDRLPVTLRGPKTPTPITYRVPMASAQVKSAV | 180 |
| Sbjct 121 | GDASL+KRPMGRVLNPLREMGVQV++ DGDR+P+TL GPKT PITYRVPMASAQVKSAV GDASLSKRPMGRVLNPLREMGVQVEAADGDRMPLTLIGPKTANPITYRVPMASAQVKSAV | 180 |
| Query 181 | LLAGLNTPGITTVIEPIMTRDHTEKMLQGFGANLTVETDADGVRTIRLEGRGKLTGQVID | 240 |
| Sbjct 181 | LLAGLNTPG+TTVIEP+MTRDHTEKMLQGFGA+LTVETD DGVR IR+ G+GKL GQ ID LLAGLNTPGVTTVIEPVMTRDHTEKMLQGFGADLTVETDKDGVRHIRITGQGKLVGQTID | 240 |
| Query 241 | VPGDPSSTAFPLVAALLVPGSDVTILNVLMNPTRTGLILTLQEMGADIEVINPRLAGGED | 300 |
| Sbjct 241 | VPGDPSSTAFPLVAALLV GSDVTI NVLMNPTRTGLILTLQEMGADIEV+N RLAGGED VPGDPSSTAFPLVAALLVEGSDVTIRNVLMNPTRTGLILTLQEMGADIEVLNARLAGGED | 300 |
| Query 301 | VADLRVRSSTLKGVTVPEDRAPSMIDEYPILavaaafaegaTVMNGLEELRVKESDRLSA | 360 |
| Sbjct 301 | VADLRVR+S LKGV VP +RAPSMIDEYP+LA+AA+FAEG TVM+GL+ELRVKESDRL+A VADLRVRASKLKGVAVPPERAPSMIDEYPVLAIAASFAEGETVMDGLDELRVKESDRLAA | 360 |
| Query 361 | VANGLKLNGVDCDEGETSLVVRGRPDGKGLGNASGAAVATHLDHRIAMSFLVMGLVSENP | 420 |
| Sbjct 361 | VA GL+ NGVDC EGE SL VRGRPDGKGLG G VATHLDHRIAMSFLVMGL +E P VARGLEANGVDCTEGEMSLTVRGRPDGKGLG---GGTVATHLDHRIAMSFLVMGLAAEKP | 417 |
| Query 421  Sbjct 418 | VTVDDATMIATSFPEFMDLMAGLGAKIELSDTK 453 VTVDD+ MIATSFPEFMD+M GLGAKIELS+ + VTVDDSNMIATSFPEFMDMMPGLGAKIELSNAQ 450 |  |

>WP_006465745.1 MULTISPECIES: 3-phosphoshikimate 1-carboxyvinyltransferase [Brucellaceae] EEQ94708.1 3-phosphoshikimate 1-carboxyvinyltransferase [Ochrobactrum intermedium

LMG 3301]

ELT49755.1 3-phosphoshikimate 1-carboxyvinyltransferase [Ochrobactrum intermedium M86]

KAB2672884.1 3-phosphoshikimate 1-carboxyvinyltransferase [Ochrobactrum sp. LMG 5442]

KAB2720180.1 3-phosphoshikimate 1-carboxyvinyltransferase [Brucella intermedia] OOC64721.1 3-phosphoshikimate 1-carboxyvinyltransferase [Ochrobactrum intermedium

M86]

Length=450

Score = 735 bits (1898), Expect = 0.0, Method: Compositional matrix adjust. Identities = 373/453 (82%), Positives = 407/453 (90%), Gaps = 3/453 (1%)

| Query | 1 | MLHGASSRPATARKSSGLSGTVRIPGDKSISHRSFMFGGLASGETRITGLLEGEDVINTG | 60 |
| --- | --- | --- | --- |
| Sbjct | 1 | M H AS +PATAR+S L+G +RIPGDKSISHRSFMFGGLASGETRITGLLEGEDVINTG MSHSASPKPATARRSEALTGEIRIPGDKSISHRSFMFGGLASGETRITGLLEGEDVINTG | 60 |
| Query | 61 | KAMQAMGARIRKEGDTWIIDGVGNGGLLAPEAPLDFGNAATGCRLTMGLVGVYDFDSTFI  +AMQAMGA+IRKEGD WII+GVGNG LL PEA LDFGNA TG RLTMGLVG YD ++FI | 120 |

| Sbjct | 61 | RAMQAMGAKIRKEGDVWIINGVGNGCLLQPEAALDFGNAGTGARLTMGLVGTYDMKTSFI | 120 |
| --- | --- | --- | --- |
| Query | 121 | GDASLTKRPMGRVLNPLREMGVQVKSEDGDRLPVTLRGPKTPTPITYRVPMASAQVKSAV | 180 |
| Sbjct | 121 | GDASL+KRPMGRVLNPLREMGVQV++ DGDR+P+TL GPKT PITYRVPMASAQVKSAV GDASLSKRPMGRVLNPLREMGVQVEAADGDRMPLTLIGPKTANPITYRVPMASAQVKSAV | 180 |
| Query | 181 | LLAGLNTPGITTVIEPIMTRDHTEKMLQGFGANLTVETDADGVRTIRLEGRGKLTGQVID | 240 |
| Sbjct | 181 | LLAGLNTPG+TTVIEP+MTRDHTEKMLQGFGA+LTVETD DGVR IR+ G+GKL GQ ID LLAGLNTPGVTTVIEPVMTRDHTEKMLQGFGADLTVETDKDGVRHIRITGQGKLVGQTID | 240 |
| Query | 241 | VPGDPSSTAFPLVAALLVPGSDVTILNVLMNPTRTGLILTLQEMGADIEVINPRLAGGED | 300 |
| Sbjct | 241 | VPGDPSSTAFPLVAALLV GSDVTI NVLMNPTRTGLILTLQEMGADIEV+N RLAGGED VPGDPSSTAFPLVAALLVEGSDVTIRNVLMNPTRTGLILTLQEMGADIEVLNARLAGGED | 300 |
| Query | 301 | VADLRVRSSTLKGVTVPEDRAPSMIDEYPILavaaafaegaTVMNGLEELRVKESDRLSA | 360 |
| Sbjct | 301 | VADLRVR+S LKGV VP +RAPSMIDEYP+LA+AA+FAEG TVM+GL+ELRVKESDRL+A VADLRVRASKLKGVVVPPERAPSMIDEYPVLAIAASFAEGETVMDGLDELRVKESDRLAA | 360 |
| Query | 361 | VANGLKLNGVDCDEGETSLVVRGRPDGKGLGNASGAAVATHLDHRIAMSFLVMGLVSENP | 420 |
| Sbjct | 361 | VA GL+ NGVDC EGE SL VRGRPDGKGLG G VATHLDHRIAMSFLVMGL +E P VARGLEANGVDCTEGEMSLTVRGRPDGKGLG---GGTVATHLDHRIAMSFLVMGLAAEKP | 417 |
| Query  Sbjct | 421  418 | VTVDDATMIATSFPEFMDLMAGLGAKIELSDTK 453 VTVDD+ MIATSFPEFMD+M GLGAKIELS+ + VTVDDSNMIATSFPEFMDMMPGLGAKIELSNAQ 450 |  |

>WP_134153901.1 3-phosphoshikimate 1-carboxyvinyltransferase [Ochrobactrum sp. LP_5_YM]

TDV65079.1 3-phosphoshikimate 1-carboxyvinyltransferase [Ochrobactrum sp. LP_5_YM]

Length=450

Score = 735 bits (1897), Expect = 0.0, Method: Compositional matrix adjust.

| Identities | = 372/453 (82%), Positives = 407/453 (90%), Gaps = 3/453 (1%) |  |
| --- | --- | --- |
| Query 1 | MLHGASSRPATARKSSGLSGTVRIPGDKSISHRSFMFGGLASGETRITGLLEGEDVINTG | 60 |
| Sbjct 1 | M H AS +PA AR S L G +RIPGDKSISHRSFMFGGLASGETRITGLLEGEDVINTG MSHSASPKPAIARHSEALKGEIRIPGDKSISHRSFMFGGLASGETRITGLLEGEDVINTG | 60 |
| Query 61 | KAMQAMGARIRKEGDTWIIDGVGNGGLLAPEAPLDFGNAATGCRLTMGLVGVYDFDSTFI | 120 |
| Sbjct 61 | +AMQAMGA+IRK+GD WII+GVGNG LL PEAPLDFGNA TG RLTMGLVG YD +TFI RAMQAMGAKIRKDGDVWIINGVGNGCLLQPEAPLDFGNAGTGARLTMGLVGTYDMTTTFI | 120 |
| Query 121 | GDASLTKRPMGRVLNPLREMGVQVKSEDGDRLPVTLRGPKTPTPITYRVPMASAQVKSAV | 180 |
| Sbjct 121 | GDASLT RPMGRVLNPLREMGVQVK+ +GDRLP+TL GPKT PI+YRVPMASAQVKSAV GDASLTSRPMGRVLNPLREMGVQVKAAEGDRLPLTLTGPKTANPISYRVPMASAQVKSAV | 180 |
| Query 181 | LLAGLNTPGITTVIEPIMTRDHTEKMLQGFGANLTVETDADGVRTIRLEGRGKLTGQVID | 240 |
| Sbjct 181 | LLAGLNTPG+TTVIEP+MTRDHTEKMLQGFGA+L+VETD DGVR IR+ G+GKLTGQ ID LLAGLNTPGVTTVIEPVMTRDHTEKMLQGFGADLSVETDKDGVRHIRITGQGKLTGQTID | 240 |
| Query 241 | VPGDPSSTAFPLVAALLVPGSDVTILNVLMNPTRTGLILTLQEMGADIEVINPRLAGGED | 300 |
| Sbjct 241 | VPGDPSSTAFPLVAALLV GSDVTILNVLMNPTRTGLILTLQEMGA+IE+I+PRLAGGED VPGDPSSTAFPLVAALLVEGSDVTILNVLMNPTRTGLILTLQEMGANIEIIDPRLAGGED | 300 |
| Query 301 | VADLRVRSSTLKGVTVPEDRAPSMIDEYPILavaaafaegaTVMNGLEELRVKESDRLSA | 360 |
| Sbjct 301 | VADLRV+SS LKGV VP +RAPSMIDEYP+LA+AA+FAEG T+M+GL+ELRVKESDRL+A VADLRVKSSKLKGVVVPPERAPSMIDEYPVLAIAASFAEGETIMDGLDELRVKESDRLAA | 360 |
| Query 361 | VANGLKLNGVDCDEGETSLVVRGRPDGKGLGNASGAAVATHLDHRIAMSFLVMGLVSENP | 420 |
| Sbjct 361 | VA GL+ NGVDC EGE SL VRGRPDGKGLG G VATHLDHRIAMSFLVMGL SE P VARGLEANGVDCTEGEMSLTVRGRPDGKGLG---GGTVATHLDHRIAMSFLVMGLASEKP | 417 |
| Query 421  Sbjct 418 | VTVDDATMIATSFPEFMDLMAGLGAKIELSDTK 453 VTVDD+TMIATSFPEFM +M GLGAKIE+S+ + VTVDDSTMIATSFPEFMTMMPGLGAKIEVSEQQ 450 |  |

>WP_007872980.1 MULTISPECIES: 3-phosphoshikimate 1-carboxyvinyltransferase [Ochrobactrum] EMG55307.1 3-phosphoshikimate 1-carboxyvinyltransferase [Ochrobactrum sp.

CDB2]

NNV21378.1 3-phosphoshikimate 1-carboxyvinyltransferase [Ochrobactrum pseudogrignonense] Length=450

Score = 735 bits (1897), Expect = 0.0, Method: Compositional matrix adjust.

| Identities | = 372/453 (82%), Positives = 410/453 (91%), Gaps = 3/453 (1%) |  |
| --- | --- | --- |
| Query 1 | MLHGASSRPATARKSSGLSGTVRIPGDKSISHRSFMFGGLASGETRITGLLEGEDVINTG | 60 |
| Sbjct 1 | M H A+ +PATAR+S L+G +RIPGDKSISHRSFMFGGLASGETRITGLLEGEDVINTG MSHSATPKPATARRSEALTGEIRIPGDKSISHRSFMFGGLASGETRITGLLEGEDVINTG | 60 |
| Query 61 | KAMQAMGARIRKEGDTWIIDGVGNGGLLAPEAPLDFGNAATGCRLTMGLVGVYDFDSTFI | 120 |
| Sbjct 61 | +AMQAMGA+IRK+GD W+I+GVGNG LL PEAPLDFGNA TG RLTMGLVG YD +TFI RAMQAMGAKIRKDGDIWVINGVGNGCLLQPEAPLDFGNAGTGARLTMGLVGTYDMTTTFI | 120 |
| Query 121 | GDASLTKRPMGRVLNPLREMGVQVKSEDGDRLPVTLRGPKTPTPITYRVPMASAQVKSAV | 180 |
| Sbjct 121 | GDASLT RPMGRVLNPLREMGVQVK+ +GDRLP+TL GPKT PI+YRVPMASAQVKSAV GDASLTSRPMGRVLNPLREMGVQVKAAEGDRLPLTLTGPKTANPISYRVPMASAQVKSAV | 180 |
| Query 181 | LLAGLNTPGITTVIEPIMTRDHTEKMLQGFGANLTVETDADGVRTIRLEGRGKLTGQVID | 240 |
| Sbjct 181 | LLAGLNTPG+TTVIEP+MTRDHTEKMLQGFGA+LTVETD DGVR IR+ G+GKLTGQ ID LLAGLNTPGVTTVIEPVMTRDHTEKMLQGFGADLTVETDKDGVRHIRIVGQGKLTGQTID | 240 |
| Query 241 | VPGDPSSTAFPLVAALLVPGSDVTILNVLMNPTRTGLILTLQEMGADIEVINPRLAGGED | 300 |
| Sbjct 241 | VPGDPSSTAFPLVAALLV GSDVTI NVLMNPTRTGLILTLQEMGADIE+I+PRLAGGED VPGDPSSTAFPLVAALLVEGSDVTIRNVLMNPTRTGLILTLQEMGADIEIIDPRLAGGED | 300 |
| Query 301 | VADLRVRSSTLKGVTVPEDRAPSMIDEYPILavaaafaegaTVMNGLEELRVKESDRLSA | 360 |
| Sbjct 301 | VADLRV+SS LKGVTVP +RAPSMIDEYP+LA+AA+FAEG TVM+GL+ELRVKESDRL+A VADLRVKSSKLKGVTVPPERAPSMIDEYPVLAIAASFAEGETVMDGLDELRVKESDRLAA | 360 |
| Query 361 | VANGLKLNGVDCDEGETSLVVRGRPDGKGLGNASGAAVATHLDHRIAMSFLVMGLVSENP | 420 |
| Sbjct 361 | VA GL+ NGVDC EGE SL VRGRPDGKGLG G VATHLDHRIAMSFLVMGL ++ P VARGLEANGVDCTEGEMSLTVRGRPDGKGLG---GGTVATHLDHRIAMSFLVMGLAADKP | 417 |
| Query 421  Sbjct 418 | VTVDDATMIATSFPEFMDLMAGLGAKIELSDTK 453 VTVDD+TMIATSFPEFM +M GLGAKIE+S+ + VTVDDSTMIATSFPEFMTMMPGLGAKIEVSEQQ 450 |  |

>AVC52229.1 3-phosphoshikimate 1-carboxyvinyltransferase [Rhizobium leguminosarum bv. viciae]

Length=431

Score = 734 bits (1896), Expect = 0.0, Method: Compositional matrix adjust.

| Identities | = 372/427 (87%), Positives = 396/427 (93%), Gaps = 0/427 (0%) |  |
| --- | --- | --- |
| Query 22 | VRIPGDKSISHRSFMFGGLASGETRITGLLEGEDVINTGKAMQAMGARIRKEGDTWIIDG | 81 |
| Sbjct 1 | +RIPGDKSISHRSFMFGGLASGETRITGLLEGEDVINTG+AMQAMGARIRK G+ W+I+G MRIPGDKSISHRSFMFGGLASGETRITGLLEGEDVINTGRAMQAMGARIRKVGEQWLIEG | 60 |
| Query 82 | VGNGGLLAPEAPLDFGNAATGCRLTMGLVGVYDFDSTFIGDASLTKRPMGRVLNPLREMG | 141 |
| Sbjct 61 | GNG LLAP+APLDFGNA TG RLTMGLV YDF STFIGDASL+KRPMGRVLNPLREMG TGNGALLAPDAPLDFGNAGTGVRLTMGLVSTYDFRSTFIGDASLSKRPMGRVLNPLREMG | 120 |
| Query 142 | VQVKSEDGDRLPVTLRGPKTPTPITYRVPMASAQVKSAVLLAGLNTPGITTVIEPIMTRD | 201 |
| Sbjct 121 | VQV + +GDRLPVTLRGP TP+PI YRVPMASAQVKSAVLLAGLNTPG+TTVIEP+MTRD VQVSASEGDRLPVTLRGPGTPSPIRYRVPMASAQVKSAVLLAGLNTPGVTTVIEPVMTRD | 180 |
| Query 202 | HTEKMLQGFGANLTVETDADGVRTIRLEGRGKLTGQVIDVPGDPSSTAFPLVAALLVPGS | 261 |
| Sbjct 181 | HTEKMLQGFGA L+VETD DGVRTIRLEGRGKL GQVID+PGDPSSTAFPLVAALLVPGS HTEKMLQGFGAALSVETDEDGVRTIRLEGRGKLKGQVIDIPGDPSSTAFPLVAALLVPGS | 240 |
| Query 262 | DVTILNVLMNPTRTGLILTLQEMGADIEVINPRLAGGEDVADLRVRSSTLKGVTVPEDRA | 321 |
| Sbjct 241 | D+TI+NVLMNPTRTGLILTLQEMGADIEV+N RLAGGEDVADLRVR S LKGVTVPEDRA DITIVNVLMNPTRTGLILTLQEMGADIEVLNARLAGGEDVADLRVRHSELKGVTVPEDRA | 300 |
| Query 322 | PSMIDEYPILavaaafaegaTVMNGLEELRVKESDRLSAVANGLKLNGVDCDEGETSLVV | 381 |
| Sbjct 301 | PSMIDEYPILAVAA FAEG TVM GLEELRVKESDRLSAVA+GLKLNGVDCDEGE L+V PSMIDEYPILAVAACFAEGVTVMKGLEELRVKESDRLSAVADGLKLNGVDCDEGEDFLIV | 360 |
| Query 382 | RGRPDGKGLGNASGAAVATHLDHRIAMSFLVMGLVSENPVTVDDATMIATSFPEFMDLMA RGRPDGKGLGNA+ V+THLDHRIAMSFLVMGL SE+PVT+DDA MIATSFPEFM LM | 441 |

| Sbjct | 361 | RGRPDGKGLGNATDGRVSTHLDHRIAMSFLVMGLASEHPVTIDDAAMIATSFPEFMQLMT 420 |
| --- | --- | --- |
| Query | 442 | GLGAKIE 448 |
| Sbjct | 421 | GLGAKI+  GLGAKID 427 |

>WP_151645746.1 3-phosphoshikimate 1-carboxyvinyltransferase [Ochrobactrum tritici] KAB2657025.1 3-phosphoshikimate 1-carboxyvinyltransferase [Ochrobactrum tritici]

Length=450

Score = 734 bits (1896), Expect = 0.0, Method: Compositional matrix adjust.

| Identities | = 373/453 (82%), Positives = 408/453 (90%), Gaps = 3/453 (1%) |  |
| --- | --- | --- |
| Query 1 | MLHGASSRPATARKSSGLSGTVRIPGDKSISHRSFMFGGLASGETRITGLLEGEDVINTG | 60 |
| Sbjct 1 | M H AS +PATAR+S L+G +RIPGDKSISHRSFMFGGLASGETRITGLLEGEDVINTG MSHSASPKPATARRSEALTGEIRIPGDKSISHRSFMFGGLASGETRITGLLEGEDVINTG | 60 |
| Query 61 | KAMQAMGARIRKEGDTWIIDGVGNGGLLAPEAPLDFGNAATGCRLTMGLVGVYDFDSTFI | 120 |
| Sbjct 61 | +AMQAMGA+IRK+GD WII+GVGNG LL PEA LDFGNA TG RLTMGLVG YD ++FI RAMQAMGAKIRKDGDVWIINGVGNGCLLQPEAALDFGNAGTGARLTMGLVGTYDMKTSFI | 120 |
| Query 121 | GDASLTKRPMGRVLNPLREMGVQVKSEDGDRLPVTLRGPKTPTPITYRVPMASAQVKSAV | 180 |
| Sbjct 121 | GDASL+KRPMGRVLNPLREMGVQV++ DGDR+P+TL GP+T PITYRVPMASAQVKSAV GDASLSKRPMGRVLNPLREMGVQVEAADGDRMPLTLIGPRTANPITYRVPMASAQVKSAV | 180 |
| Query 181 | LLAGLNTPGITTVIEPIMTRDHTEKMLQGFGANLTVETDADGVRTIRLEGRGKLTGQVID | 240 |
| Sbjct 181 | LLAGLNTPG+TTVIEP+MTRDHTEKMLQGFGA+L+VETD DGVR IR+ G+GKL GQ ID LLAGLNTPGVTTVIEPVMTRDHTEKMLQGFGADLSVETDKDGVRHIRITGQGKLIGQTID | 240 |
| Query 241 | VPGDPSSTAFPLVAALLVPGSDVTILNVLMNPTRTGLILTLQEMGADIEVINPRLAGGED | 300 |
| Sbjct 241 | VPGDPSSTAFPLVAALLV GSDVTI NVLMNPTRTGLILTLQEMGADIEV+N RLAGGED VPGDPSSTAFPLVAALLVEGSDVTIHNVLMNPTRTGLILTLQEMGADIEVLNARLAGGED | 300 |
| Query 301 | VADLRVRSSTLKGVTVPEDRAPSMIDEYPILavaaafaegaTVMNGLEELRVKESDRLSA | 360 |
| Sbjct 301 | VADLRV++S LKGV VP +RAPSMIDEYP+LA+AAAFAEG TVM+GL+ELRVKESDRL+A VADLRVKASKLKGVVVPPERAPSMIDEYPVLAIAAAFAEGETVMDGLDELRVKESDRLAA | 360 |
| Query 361 | VANGLKLNGVDCDEGETSLVVRGRPDGKGLGNASGAAVATHLDHRIAMSFLVMGLVSENP | 420 |
| Sbjct 361 | VA GL+ NGVDC EGE SL VRGRPDGKGLG G VATHLDHRIAMSFLVMGL SE P VARGLEANGVDCTEGEMSLTVRGRPDGKGLG---GGTVATHLDHRIAMSFLVMGLASEKP | 417 |
| Query 421  Sbjct 418 | VTVDDATMIATSFPEFMDLMAGLGAKIELSDTK 453 VTVDD+TMIATSFPEFMD+M GLGAKIELSD + VTVDDSTMIATSFPEFMDMMPGLGAKIELSDAR 450 |  |

>WP_128092818.1 3-phosphoshikimate 1-carboxyvinyltransferase [Ochrobactrum pituitosum] KAB0572424.1 3-phosphoshikimate 1-carboxyvinyltransferase [Ochrobactrum pituitosum]

Length=450

Score = 734 bits (1896), Expect = 0.0, Method: Compositional matrix adjust.

| Identities | = 373/453 (82%), Positives = 407/453 (90%), Gaps = 3/453 (1%) |  |
| --- | --- | --- |
| Query 1 | MLHGASSRPATARKSSGLSGTVRIPGDKSISHRSFMFGGLASGETRITGLLEGEDVINTG | 60 |
| Sbjct 1 | M H AS +PA AR S L G +RIPGDKSISHRSFMFGGLASGETRITGLLEGEDVINTG MSHSASPKPAIARHSEALKGEIRIPGDKSISHRSFMFGGLASGETRITGLLEGEDVINTG | 60 |
| Query 61 | KAMQAMGARIRKEGDTWIIDGVGNGGLLAPEAPLDFGNAATGCRLTMGLVGVYDFDSTFI | 120 |
| Sbjct 61 | +AMQAMGA+IRK+G+ WII+GVGNG LL PEAPLDFGNA TG RLTMGLVG YD +TFI RAMQAMGAKIRKDGNVWIINGVGNGCLLQPEAPLDFGNAGTGARLTMGLVGTYDMTTTFI | 120 |
| Query 121 | GDASLTKRPMGRVLNPLREMGVQVKSEDGDRLPVTLRGPKTPTPITYRVPMASAQVKSAV | 180 |
| Sbjct 121 | GDASLT RPMGRVLNPLREMGVQVK+ +GDRLP+TL GPKT PI+YRVPMASAQVKSAV GDASLTSRPMGRVLNPLREMGVQVKAAEGDRLPLTLTGPKTANPISYRVPMASAQVKSAV | 180 |
| Query 181 | LLAGLNTPGITTVIEPIMTRDHTEKMLQGFGANLTVETDADGVRTIRLEGRGKLTGQVID | 240 |
| Sbjct 181 | LLAGLNTPG+TTVIEP+MTRDHTEKMLQGFGA+LTVETD DGVR IR+ G+GKLTGQ ID LLAGLNTPGVTTVIEPVMTRDHTEKMLQGFGADLTVETDKDGVRHIRITGQGKLTGQTID | 240 |
| Query 241 | VPGDPSSTAFPLVAALLVPGSDVTILNVLMNPTRTGLILTLQEMGADIEVINPRLAGGED VPGDPSSTAFPLVAALLV GSDVTI NVLMNPTRTGLILTLQEMGADIE+I+PRLAGGED | 300 |

| Sbjct | 241 | VPGDPSSTAFPLVAALLVEGSDVTIRNVLMNPTRTGLILTLQEMGADIEIIDPRLAGGED | 300 |
| --- | --- | --- | --- |
| Query | 301 | VADLRVRSSTLKGVTVPEDRAPSMIDEYPILavaaafaegaTVMNGLEELRVKESDRLSA | 360 |
| Sbjct | 301 | VADLRV+SS LKGVTVP +RAPSMIDEYP+LA+AA+FAEG T+M+GL+ELRVKESDRL+A VADLRVKSSRLKGVTVPPERAPSMIDEYPVLAIAASFAEGETIMDGLDELRVKESDRLAA | 360 |
| Query | 361 | VANGLKLNGVDCDEGETSLVVRGRPDGKGLGNASGAAVATHLDHRIAMSFLVMGLVSENP | 420 |
| Sbjct | 361 | VA GL+ NGVDC EGE SL VRGRPDG+GLG G VATHLDHRIAMSFLVMGL SE P VARGLEANGVDCTEGEMSLTVRGRPDGRGLG---GGTVATHLDHRIAMSFLVMGLASEKP | 417 |
| Query  Sbjct | 421  418 | VTVDDATMIATSFPEFMDLMAGLGAKIELSDTK 453 VTVDD+TMIATSFPEFM +M GLGAKIE+SD + VTVDDSTMIATSFPEFMTMMPGLGAKIEVSDVR 450 |  |

>WP_151688841.1 3-phosphoshikimate 1-carboxyvinyltransferase [Ochrobactrum anthropi] KAB2750322.1 3-phosphoshikimate 1-carboxyvinyltransferase [Ochrobactrum anthropi]

Length=450

Score = 734 bits (1895), Expect = 0.0, Method: Compositional matrix adjust.

| Identities | = 372/453 (82%), Positives = 408/453 (90%), Gaps = 3/453 (1%) |  |
| --- | --- | --- |
| Query 1 | MLHGASSRPATARKSSGLSGTVRIPGDKSISHRSFMFGGLASGETRITGLLEGEDVINTG | 60 |
| Sbjct 1 | M H AS +PATAR+S L+G +RIPGDKSISHRSFMFGGLASGETRITGLLEGEDVINTG MSHSASPKPATARRSEALTGEIRIPGDKSISHRSFMFGGLASGETRITGLLEGEDVINTG | 60 |
| Query 61 | KAMQAMGARIRKEGDTWIIDGVGNGGLLAPEAPLDFGNAATGCRLTMGLVGVYDFDSTFI | 120 |
| Sbjct 61 | +AMQAMGA+IRK+GD WII+GVGNG LL PEA LDFGNA TG RLTMGLVG YD ++FI RAMQAMGAKIRKDGDAWIINGVGNGCLLQPEAALDFGNAGTGARLTMGLVGTYDMKTSFI | 120 |
| Query 121 | GDASLTKRPMGRVLNPLREMGVQVKSEDGDRLPVTLRGPKTPTPITYRVPMASAQVKSAV | 180 |
| Sbjct 121 | GDASL+KRPMGRVLNPLREMGVQV++ +GDR+P+TL GPKT PITYRVPMASAQVKSAV GDASLSKRPMGRVLNPLREMGVQVEAAEGDRMPLTLIGPKTANPITYRVPMASAQVKSAV | 180 |
| Query 181 | LLAGLNTPGITTVIEPIMTRDHTEKMLQGFGANLTVETDADGVRTIRLEGRGKLTGQVID | 240 |
| Sbjct 181 | LLAGLNTPG+TTVIEP+MTRDHTEKMLQGFGA+L+VETD DGVR IR+ G+GKL GQ ID LLAGLNTPGVTTVIEPVMTRDHTEKMLQGFGADLSVETDKDGVRHIRITGQGKLIGQTID | 240 |
| Query 241 | VPGDPSSTAFPLVAALLVPGSDVTILNVLMNPTRTGLILTLQEMGADIEVINPRLAGGED | 300 |
| Sbjct 241 | VPGDPSSTAFPLVAALLV GSDVTI NVLMNPTRTGLILTLQEMGADIEV+N RLAGGED VPGDPSSTAFPLVAALLVEGSDVTIRNVLMNPTRTGLILTLQEMGADIEVLNARLAGGED | 300 |
| Query 301 | VADLRVRSSTLKGVTVPEDRAPSMIDEYPILavaaafaegaTVMNGLEELRVKESDRLSA | 360 |
| Sbjct 301 | VADLRV++S LKGV VP +RAPSMIDEYP+LA+AA+FAEG TVM+GL+ELRVKESDRL+A VADLRVKASKLKGVVVPPERAPSMIDEYPVLAIAASFAEGETVMDGLDELRVKESDRLAA | 360 |
| Query 361 | VANGLKLNGVDCDEGETSLVVRGRPDGKGLGNASGAAVATHLDHRIAMSFLVMGLVSENP | 420 |
| Sbjct 361 | VA GL+ NGVDC EGE SL VRGRPDGKGLG G VATHLDHRIAMSFLVMGL SE P VARGLEANGVDCTEGEMSLTVRGRPDGKGLG---GGTVATHLDHRIAMSFLVMGLASEKP | 417 |
| Query 421  Sbjct 418 | VTVDDATMIATSFPEFMDLMAGLGAKIELSDTK 453 VTVDD+TMIATSFPEFMD+M GLGAKIELSD + VTVDDSTMIATSFPEFMDMMPGLGAKIELSDAR 450 |  |

>WP_105734053.1 3-phosphoshikimate 1-carboxyvinyltransferase [Phyllobacterium myrsinacearum]

PRD53039.1 3-phosphoshikimate 1-carboxyvinyltransferase [Phyllobacterium myrsinacearum]

PWV94123.1 3-phosphoshikimate 1-carboxyvinyltransferase [Phyllobacterium myrsinacearum]

RZS82577.1 3-phosphoshikimate 1-carboxyvinyltransferase [Phyllobacterium myrsinacearum]

RZV07438.1 3-phosphoshikimate 1-carboxyvinyltransferase [Phyllobacterium myrsinacearum]

Length=451

Score = 734 bits (1895), Expect = 0.0, Method: Compositional matrix adjust. Identities = 372/454 (82%), Positives = 403/454 (89%), Gaps = 3/454 (1%)

Query 1	MLHGASSRPATARKSSGLSGTVRIPGDKSISHRSFMFGGLASGETRITGLLEGEDVINTG 60 M H A +PATAR+S LSG +RIPGDKSISHRSFMFGGLASGETRITGLLEGEDVINTG

| Sbjct | 1 | MSHSALPKPATARRSKALSGDIRIPGDKSISHRSFMFGGLASGETRITGLLEGEDVINTG | 60 |
| --- | --- | --- | --- |
| Query | 61 | KAMQAMGARIRKEGDTWIIDGVGNGGLLAPEAPLDFGNAATGCRLTMGLVGVYDFDSTFI | 120 |
| Sbjct | 61 | +AMQAMGA+I +EGD WII G GNG LL PEAPLDFGNA TG RLTMGLVGVY ++TFI RAMQAMGAKIAREGDVWIIQGTGNGCLLQPEAPLDFGNAGTGARLTMGLVGVYAMETTFI | 120 |
| Query | 121 | GDASLTKRPMGRVLNPLREMGVQVKSEDGDRLPVTLRGPKTPTPITYRVPMASAQVKSAV | 180 |
| Sbjct | 121 | GDASL+KRPMGRVLNPLREMGVQV + DGDR+P+TLRGPK PITYRVPMASAQVKSAV GDASLSKRPMGRVLNPLREMGVQVTASDGDRMPLTLRGPKVAAPITYRVPMASAQVKSAV | 180 |
| Query | 181 | LLAGLNTPGITTVIEPIMTRDHTEKMLQGFGANLTVETDADGVRTIRLEGRGKLTGQVID | 240 |
| Sbjct | 181 | LLAGLNTPGITTVIEP+MTRDHTEKMLQGFGANL+VETD D VR IR+EG+GKLTGQ+ID LLAGLNTPGITTVIEPVMTRDHTEKMLQGFGANLSVETDKDNVRHIRIEGQGKLTGQIID | 240 |
| Query | 241 | VPGDPSSTAFPLVAALLVPGSDVTILNVLMNPTRTGLILTLQEMGADIEVINPRLAGGED | 300 |
| Sbjct | 241 | VPGDPSSTAFPLVAALLVPGSD+TI NVLMNPTRTGLILTLQ+MGA IE++N RLAGGED VPGDPSSTAFPLVAALLVPGSDITIRNVLMNPTRTGLILTLQDMGASIEILNARLAGGED | 300 |
| Query | 301 | VADLRVRSSTLKGVTVPEDRAPSMIDEYPILavaaafaegaTVMNGLEELRVKESDRLSA | 360 |
| Sbjct | 301 | VADLRVR+S LKGV VP DRAPSMIDEYP+LAVAAAFA G TVMNGLEELRVKESDRLSA VADLRVRASELKGVIVPADRAPSMIDEYPVLAVAAAFATGETVMNGLEELRVKESDRLSA | 360 |
| Query | 361 | VANGLKLNGVDCDEGETSLVVRGRPDGKGLGNASGAAVATHLDHRIAMSFLVMGLVSENP | 420 |
| Sbjct | 361 | VANGLK NGVDC EG +L VRGRPDGK LG G V THLDHRIAMSFLVMGLV+E P VANGLKANGVDCTEGPETLSVRGRPDGKALG---GGTVETHLDHRIAMSFLVMGLVAEKP | 417 |
| Query  Sbjct | 421  418 | VTVDDATMIATSFPEFMDLMAGLGAKIELSDTKA 454 VTVDD+TMIATSFPEFM +M G+GA+I + +T A VTVDDSTMIATSFPEFMGMMTGMGAEISVQETPA 451 |  |

>WP_176036429.1 3-phosphoshikimate 1-carboxyvinyltransferase [Ochrobactrum tritici] Length=450

Score = 734 bits (1894), Expect = 0.0, Method: Compositional matrix adjust.

| Identities | = 372/453 (82%), Positives = 408/453 (90%), Gaps = 3/453 (1%) |  |
| --- | --- | --- |
| Query 1 | MLHGASSRPATARKSSGLSGTVRIPGDKSISHRSFMFGGLASGETRITGLLEGEDVINTG | 60 |
| Sbjct 1 | M H AS +PATAR+S L+G +RIPGDKSISHRSFMFGGLASGETRITGLLEGEDVINTG MSHSASPKPATARRSEALTGEIRIPGDKSISHRSFMFGGLASGETRITGLLEGEDVINTG | 60 |
| Query 61 | KAMQAMGARIRKEGDTWIIDGVGNGGLLAPEAPLDFGNAATGCRLTMGLVGVYDFDSTFI | 120 |
| Sbjct 61 | +AMQAMGA+IRK+GD WII+GVGNG LL PEA LDFGNA TG RLTMGLVG YD ++FI RAMQAMGAKIRKDGDVWIINGVGNGCLLQPEAALDFGNAGTGARLTMGLVGTYDMKTSFI | 120 |
| Query 121 | GDASLTKRPMGRVLNPLREMGVQVKSEDGDRLPVTLRGPKTPTPITYRVPMASAQVKSAV | 180 |
| Sbjct 121 | GDASLT RPMGRVLNPLREMGVQV++ +GDR+P+TL GP+T PITYRVPMASAQVKSAV GDASLTSRPMGRVLNPLREMGVQVEAAEGDRMPLTLIGPRTANPITYRVPMASAQVKSAV | 180 |
| Query 181 | LLAGLNTPGITTVIEPIMTRDHTEKMLQGFGANLTVETDADGVRTIRLEGRGKLTGQVID | 240 |
| Sbjct 181 | LLAGLNTPG+TTVIEP+MTRDHTEKMLQGFGA+L+VETD DGVR IR+ G+GKLTGQ ID LLAGLNTPGVTTVIEPVMTRDHTEKMLQGFGADLSVETDKDGVRHIRITGQGKLTGQTID | 240 |
| Query 241 | VPGDPSSTAFPLVAALLVPGSDVTILNVLMNPTRTGLILTLQEMGADIEVINPRLAGGED | 300 |
| Sbjct 241 | VPGDPSSTAFPLVAALLV GSDVTI NVLMNPTRTGLILTLQEMGADIEV+N RLAGGED VPGDPSSTAFPLVAALLVEGSDVTIHNVLMNPTRTGLILTLQEMGADIEVLNARLAGGED | 300 |
| Query 301 | VADLRVRSSTLKGVTVPEDRAPSMIDEYPILavaaafaegaTVMNGLEELRVKESDRLSA | 360 |
| Sbjct 301 | VADLRV++S LKGV VP +RAPSMIDEYP+LA+AA+FAEG TVM+GL+ELRVKESDRL+A VADLRVKASKLKGVVVPPERAPSMIDEYPVLAIAASFAEGETVMDGLDELRVKESDRLAA | 360 |
| Query 361 | VANGLKLNGVDCDEGETSLVVRGRPDGKGLGNASGAAVATHLDHRIAMSFLVMGLVSENP | 420 |
| Sbjct 361 | VA GL+ NGVDC EGE SL VRGRPDGKGLG G VATHLDHRIAMSFLVMGL SE P VARGLEANGVDCTEGEMSLTVRGRPDGKGLG---GGTVATHLDHRIAMSFLVMGLASEKP | 417 |
| Query 421  Sbjct 418 | VTVDDATMIATSFPEFMDLMAGLGAKIELSDTK 453 VTVDD+TMIATSFPEFMD+M GLGAKIELSD + VTVDDSTMIATSFPEFMDMMPGLGAKIELSDAR 450 |  |

>WP_025090682.1 3-phosphoshikimate 1-carboxyvinyltransferase [Brucella intermedia] Length=450

Score = 734 bits (1894), Expect = 0.0, Method: Compositional matrix adjust.

| Identities | = 373/453 (82%), Positives = 406/453 (90%), Gaps = 3/453 (1%) |  |
| --- | --- | --- |
| Query 1 | MLHGASSRPATARKSSGLSGTVRIPGDKSISHRSFMFGGLASGETRITGLLEGEDVINTG | 60 |
| Sbjct 1 | M H AS +PATAR+S L+G +RIPGDKSISHRSFMFGGLASGETRITGLLEGEDVINTG MSHSASPKPATARRSEALTGEIRIPGDKSISHRSFMFGGLASGETRITGLLEGEDVINTG | 60 |
| Query 61 | KAMQAMGARIRKEGDTWIIDGVGNGGLLAPEAPLDFGNAATGCRLTMGLVGVYDFDSTFI | 120 |
| Sbjct 61 | +AMQAMGA+IRKEGD WII+GVGNG LL PEA LDFGNA TG RLTMGLVG YD ++FI RAMQAMGAKIRKEGDVWIINGVGNGCLLQPEAALDFGNAGTGARLTMGLVGTYDMKTSFI | 120 |
| Query 121 | GDASLTKRPMGRVLNPLREMGVQVKSEDGDRLPVTLRGPKTPTPITYRVPMASAQVKSAV | 180 |
| Sbjct 121 | GDASL+KRPMGRVLNPLREMGVQV + DGDR+P+TL GPKT PITYRVPMASAQVKSAV GDASLSKRPMGRVLNPLREMGVQVGAADGDRMPLTLIGPKTANPITYRVPMASAQVKSAV | 180 |
| Query 181 | LLAGLNTPGITTVIEPIMTRDHTEKMLQGFGANLTVETDADGVRTIRLEGRGKLTGQVID | 240 |
| Sbjct 181 | LLAGLNTPG+TTVIEP+MTRDHTEKMLQGFGA+LTVETD DGVR IR+ G+GKL GQ ID LLAGLNTPGVTTVIEPVMTRDHTEKMLQGFGADLTVETDKDGVRHIRITGQGKLVGQTID | 240 |
| Query 241 | VPGDPSSTAFPLVAALLVPGSDVTILNVLMNPTRTGLILTLQEMGADIEVINPRLAGGED | 300 |
| Sbjct 241 | VPGDPSSTAFPLVAALLV GSDVTI NVLMNPTRTGLILTLQEMGADIEV+N RLAGGED VPGDPSSTAFPLVAALLVEGSDVTIRNVLMNPTRTGLILTLQEMGADIEVLNARLAGGED | 300 |
| Query 301 | VADLRVRSSTLKGVTVPEDRAPSMIDEYPILavaaafaegaTVMNGLEELRVKESDRLSA | 360 |
| Sbjct 301 | VADLRVR+S LKGV VP +RAPSMIDEYP+LA+AA+FAEG TVM+GL+ELRVKESDRL+A VADLRVRASKLKGVIVPPERAPSMIDEYPVLAIAASFAEGETVMDGLDELRVKESDRLAA | 360 |
| Query 361 | VANGLKLNGVDCDEGETSLVVRGRPDGKGLGNASGAAVATHLDHRIAMSFLVMGLVSENP | 420 |
| Sbjct 361 | VA GL+ NGVDC EGE SL VRGRPDGKGLG G VATHLDHRIAMSFLVMGL +E P VARGLEANGVDCTEGEMSLTVRGRPDGKGLG---GGTVATHLDHRIAMSFLVMGLAAEKP | 417 |
| Query 421  Sbjct 418 | VTVDDATMIATSFPEFMDLMAGLGAKIELSDTK 453 VTVDD+ MIATSFPEFMD+M GLGAKIELS+ + VTVDDSNMIATSFPEFMDMMPGLGAKIELSNAQ 450 |  |

>WP_094541214.1 3-phosphoshikimate 1-carboxyvinyltransferase [Ochrobactrum grignonense] OYR10481.1 3-phosphoshikimate 1-carboxyvinyltransferase [Ochrobactrum grignonense]

Length=450

Score = 734 bits (1894), Expect = 0.0, Method: Compositional matrix adjust.

| Identities | = 373/453 (82%), Positives = 405/453 (89%), Gaps = 3/453 (1%) |  |
| --- | --- | --- |
| Query 1 | MLHGASSRPATARKSSGLSGTVRIPGDKSISHRSFMFGGLASGETRITGLLEGEDVINTG | 60 |
| Sbjct 1 | M H AS +PA AR S L G +RIPGDKSISHRSFMFGGLASGETRITGLLEGEDVINTG MSHSASPKPAIARHSEALKGEIRIPGDKSISHRSFMFGGLASGETRITGLLEGEDVINTG | 60 |
| Query 61 | KAMQAMGARIRKEGDTWIIDGVGNGGLLAPEAPLDFGNAATGCRLTMGLVGVYDFDSTFI | 120 |
| Sbjct 61 | +AMQAMGA+IRK+GD WII+GVGNG LL PEAPLDFGNA TG RLTMGLVG YD +TFI RAMQAMGAKIRKDGDIWIINGVGNGCLLQPEAPLDFGNAGTGARLTMGLVGTYDMTTTFI | 120 |
| Query 121 | GDASLTKRPMGRVLNPLREMGVQVKSEDGDRLPVTLRGPKTPTPITYRVPMASAQVKSAV | 180 |
| Sbjct 121 | GDASLT RPMGRVLNPLREMGVQVK+ +GDRLP+TL GPKT PI+YRVPMASAQVKSAV GDASLTSRPMGRVLNPLREMGVQVKAAEGDRLPLTLTGPKTANPISYRVPMASAQVKSAV | 180 |
| Query 181 | LLAGLNTPGITTVIEPIMTRDHTEKMLQGFGANLTVETDADGVRTIRLEGRGKLTGQVID | 240 |
| Sbjct 181 | LLAGLNTPG+TTVIEP+MTRDHTEKMLQGFGA+L VETD DGVR IR+ G+GKLTGQ ID LLAGLNTPGVTTVIEPVMTRDHTEKMLQGFGADLIVETDKDGVRHIRITGQGKLTGQTID | 240 |
| Query 241 | VPGDPSSTAFPLVAALLVPGSDVTILNVLMNPTRTGLILTLQEMGADIEVINPRLAGGED | 300 |
| Sbjct 241 | VPGDPSSTAFPLVAALLV GSDVTI NVLMNPTRTGLILTLQEMGADIE+I+PRLAGGED VPGDPSSTAFPLVAALLVEGSDVTIRNVLMNPTRTGLILTLQEMGADIEIIDPRLAGGED | 300 |
| Query 301 | VADLRVRSSTLKGVTVPEDRAPSMIDEYPILavaaafaegaTVMNGLEELRVKESDRLSA | 360 |
| Sbjct 301 | VADLRV+SS LKGV VP +RAPSMIDEYP+LA+AA+FAEG T+M+GL+ELRVKESDRL+A VADLRVKSSKLKGVVVPPERAPSMIDEYPVLAIAASFAEGETIMDGLDELRVKESDRLAA | 360 |
| Query 361 | VANGLKLNGVDCDEGETSLVVRGRPDGKGLGNASGAAVATHLDHRIAMSFLVMGLVSENP | 420 |
| Sbjct 361 | VA GL+ NGVDC EGE SL VRGRPDGKGLG G VATHLDHRIAMSFLVMGL SE P  VARGLEANGVDCTEGEMSLTVRGRPDGKGLG---GGTVATHLDHRIAMSFLVMGLASEKP | 417 |

Query 421 VTVDDATMIATSFPEFMDLMAGLGAKIELSDTK 453 VTVDD+TMIATSFPEFM +M GLGAKIE+SD +

Sbjct 418 VTVDDSTMIATSFPEFMTMMPGLGAKIEVSDAR 450

>WP_176073658.1 3-phosphoshikimate 1-carboxyvinyltransferase [Brucella intermedia] Length=450

Score = 733 bits (1893), Expect = 0.0, Method: Compositional matrix adjust.

| Identities | = 373/453 (82%), Positives = 406/453 (90%), Gaps = 3/453 (1%) |  |
| --- | --- | --- |
| Query 1 | MLHGASSRPATARKSSGLSGTVRIPGDKSISHRSFMFGGLASGETRITGLLEGEDVINTG | 60 |
| Sbjct 1 | M H AS +PATAR+S L+G +RIPGDKSISHRSFMFGGLASGETRITGLLEGEDVINTG MSHSASPKPATARRSEALTGEIRIPGDKSISHRSFMFGGLASGETRITGLLEGEDVINTG | 60 |
| Query 61 | KAMQAMGARIRKEGDTWIIDGVGNGGLLAPEAPLDFGNAATGCRLTMGLVGVYDFDSTFI | 120 |
| Sbjct 61 | +AMQAMGA+IRKEGD WII+GVGNG LL PEA LDFGNA TG RLTMGLVG YD S+FI RAMQAMGAKIRKEGDVWIINGVGNGCLLQPEAALDFGNAGTGARLTMGLVGTYDMKSSFI | 120 |
| Query 121 | GDASLTKRPMGRVLNPLREMGVQVKSEDGDRLPVTLRGPKTPTPITYRVPMASAQVKSAV | 180 |
| Sbjct 121 | GDASL+KRPMGRVL PLREMGVQV+S +GDR+P+TL GPKT PITYRVPMASAQVKSAV GDASLSKRPMGRVLTPLREMGVQVESAEGDRMPLTLIGPKTANPITYRVPMASAQVKSAV | 180 |
| Query 181 | LLAGLNTPGITTVIEPIMTRDHTEKMLQGFGANLTVETDADGVRTIRLEGRGKLTGQVID | 240 |
| Sbjct 181 | LLAGLNTPG+TTVIEP+MTRDHTEKMLQGFGA+LTVETD DGVR IR+ G+GKL GQ ID LLAGLNTPGVTTVIEPVMTRDHTEKMLQGFGADLTVETDKDGVRHIRITGQGKLVGQTID | 240 |
| Query 241 | VPGDPSSTAFPLVAALLVPGSDVTILNVLMNPTRTGLILTLQEMGADIEVINPRLAGGED | 300 |
| Sbjct 241 | VPGDPSSTAFPLVAALLV GSDVTI NVLMNPTRTGLILTLQEMGADIEV+N RLAGGED VPGDPSSTAFPLVAALLVEGSDVTIRNVLMNPTRTGLILTLQEMGADIEVLNARLAGGED | 300 |
| Query 301 | VADLRVRSSTLKGVTVPEDRAPSMIDEYPILavaaafaegaTVMNGLEELRVKESDRLSA | 360 |
| Sbjct 301 | VADLRVR+S LKGV VP +RAPSMIDEYP+LA+AA+FAEG TVM+GL+ELRVKESDRL+A VADLRVRASKLKGVIVPPERAPSMIDEYPVLAIAASFAEGETVMDGLDELRVKESDRLAA | 360 |
| Query 361 | VANGLKLNGVDCDEGETSLVVRGRPDGKGLGNASGAAVATHLDHRIAMSFLVMGLVSENP | 420 |
| Sbjct 361 | VA GL+ NGVDC EGE SL VRGRPDGKGLG G VATHLDHRIAMSFLVMGL +E P VARGLEANGVDCTEGEMSLTVRGRPDGKGLG---GGTVATHLDHRIAMSFLVMGLAAEKP | 417 |
| Query 421  Sbjct 418 | VTVDDATMIATSFPEFMDLMAGLGAKIELSDTK 453 VTVDD+ MIATSFPEFMD+M GLGAKIELS+ + VTVDDSNMIATSFPEFMDMMPGLGAKIELSNAQ 450 |  |

>WP_125300483.1 3-phosphoshikimate 1-carboxyvinyltransferase [Ochrobactrum anthropi] RRY20504.1 3-phosphoshikimate 1-carboxyvinyltransferase [Ochrobactrum anthropi]

Length=450

Score = 733 bits (1893), Expect = 0.0, Method: Compositional matrix adjust.

| Identities | = 371/453 (82%), Positives = 408/453 (90%), Gaps = 3/453 (1%) |  |
| --- | --- | --- |
| Query 1 | MLHGASSRPATARKSSGLSGTVRIPGDKSISHRSFMFGGLASGETRITGLLEGEDVINTG | 60 |
| Sbjct 1 | M H AS +PATAR+S L+G +RIPGDKSISHRSFMFGGLASGETRITGLLEGEDVINTG MSHSASPKPATARRSEALTGEIRIPGDKSISHRSFMFGGLASGETRITGLLEGEDVINTG | 60 |
| Query 61 | KAMQAMGARIRKEGDTWIIDGVGNGGLLAPEAPLDFGNAATGCRLTMGLVGVYDFDSTFI | 120 |
| Sbjct 61 | +AMQAMGA+IRK+GD WII+GVGNG LL PEA LDFGNA TG RLTMGLVG YD ++FI RAMQAMGAKIRKDGDVWIINGVGNGCLLQPEAALDFGNAGTGARLTMGLVGTYDMKTSFI | 120 |
| Query 121 | GDASLTKRPMGRVLNPLREMGVQVKSEDGDRLPVTLRGPKTPTPITYRVPMASAQVKSAV | 180 |
| Sbjct 121 | GDASL+KRPMGRVLNPLREMGVQV++ +GDR+P+TL GPKT PITYRVPMASAQVKSAV GDASLSKRPMGRVLNPLREMGVQVEAAEGDRMPLTLIGPKTANPITYRVPMASAQVKSAV | 180 |
| Query 181 | LLAGLNTPGITTVIEPIMTRDHTEKMLQGFGANLTVETDADGVRTIRLEGRGKLTGQVID | 240 |
| Sbjct 181 | LLAGLNTPG+TTVIEP+MTRDHTEKMLQGFGA+L+VETD DGVR IR+ G+GKL GQ ID LLAGLNTPGVTTVIEPVMTRDHTEKMLQGFGADLSVETDKDGVRHIRITGQGKLIGQTID | 240 |
| Query 241 | VPGDPSSTAFPLVAALLVPGSDVTILNVLMNPTRTGLILTLQEMGADIEVINPRLAGGED | 300 |
| Sbjct 241 | VPGDPSSTAFPLVAALLV GSDVTI NVLMNPTRTGLILTLQEMGADIEV+N RLAGGED VPGDPSSTAFPLVAALLVEGSDVTIRNVLMNPTRTGLILTLQEMGADIEVLNARLAGGED | 300 |
| Query 301 | VADLRVRSSTLKGVTVPEDRAPSMIDEYPILavaaafaegaTVMNGLEELRVKESDRLSA | 360 |

| Sbjct | 301 | VADLRV++S LKGV VP +RAPSMIDEYP+LA+AA+FAEG TVM+GL+ELRVKESDRL+A VADLRVKASKLKGVVVPPERAPSMIDEYPVLAIAASFAEGETVMDGLDELRVKESDRLAA | 360 |
| --- | --- | --- | --- |
| Query | 361 | VANGLKLNGVDCDEGETSLVVRGRPDGKGLGNASGAAVATHLDHRIAMSFLVMGLVSENP | 420 |
| Sbjct | 361 | VA GL+ NGVDC EGE SL VRGRPDGKGLG G VATHLDHRIAMSFLVMGL SE P VARGLEANGVDCTEGEMSLTVRGRPDGKGLG---GGTVATHLDHRIAMSFLVMGLASEKP | 417 |
| Query  Sbjct | 421  418 | VTVDDATMIATSFPEFMDLMAGLGAKIELSDTK 453 VT+DD+TMIATSFPEFMD+M GLGAKIELSD + VTIDDSTMIATSFPEFMDMMPGLGAKIELSDAR 450 |  |

>WP_183515843.1 MULTISPECIES: 3-phosphoshikimate 1-carboxyvinyltransferase [unclassified Ochrobactrum]

MBB3218801.1 3-phosphoshikimate 1-carboxyvinyltransferase [Ochrobactrum sp. RC6B]

MBB3341734.1 3-phosphoshikimate 1-carboxyvinyltransferase [Ochrobactrum sp. RC4RE]

Length=450

Score = 733 bits (1893), Expect = 0.0, Method: Compositional matrix adjust.

| Identities | = 372/453 (82%), Positives = 406/453 (90%), Gaps = 3/453 (1%) |  |
| --- | --- | --- |
| Query 1 | MLHGASSRPATARKSSGLSGTVRIPGDKSISHRSFMFGGLASGETRITGLLEGEDVINTG | 60 |
| Sbjct 1 | M H AS +PATAR+S L+G +RIPGDKSISHRSFMFGGLASGETRITGLLEGEDVINTG MSHSASPKPATARRSEALTGEIRIPGDKSISHRSFMFGGLASGETRITGLLEGEDVINTG | 60 |
| Query 61 | KAMQAMGARIRKEGDTWIIDGVGNGGLLAPEAPLDFGNAATGCRLTMGLVGVYDFDSTFI | 120 |
| Sbjct 61 | +AMQAMGA+IRKEGD WII+GVGNG LL PEA LDFGNA TG RLTMGLVG YD ++FI RAMQAMGAKIRKEGDVWIINGVGNGCLLQPEAALDFGNAGTGARLTMGLVGTYDMKTSFI | 120 |
| Query 121 | GDASLTKRPMGRVLNPLREMGVQVKSEDGDRLPVTLRGPKTPTPITYRVPMASAQVKSAV | 180 |
| Sbjct 121 | GDASL+KRPMGRVLNPLREMGVQV++ DGDR+P+TL GPKT PITYRVPMASAQVKSAV GDASLSKRPMGRVLNPLREMGVQVEAADGDRMPLTLIGPKTANPITYRVPMASAQVKSAV | 180 |
| Query 181 | LLAGLNTPGITTVIEPIMTRDHTEKMLQGFGANLTVETDADGVRTIRLEGRGKLTGQVID | 240 |
| Sbjct 181 | LLAGLNTPG+TTVIEP+MTRDHTEKMLQGFGA+L VETD DGVR IR+ G+GKL GQ ID LLAGLNTPGVTTVIEPVMTRDHTEKMLQGFGADLAVETDKDGVRHIRITGQGKLVGQTID | 240 |
| Query 241 | VPGDPSSTAFPLVAALLVPGSDVTILNVLMNPTRTGLILTLQEMGADIEVINPRLAGGED | 300 |
| Sbjct 241 | VPGDPSSTAFPLVAALLV GSDVTI NVLMNPTRTGLILTLQEMGADIEV+N RLAGGED VPGDPSSTAFPLVAALLVEGSDVTIRNVLMNPTRTGLILTLQEMGADIEVLNARLAGGED | 300 |
| Query 301 | VADLRVRSSTLKGVTVPEDRAPSMIDEYPILavaaafaegaTVMNGLEELRVKESDRLSA | 360 |
| Sbjct 301 | VADLRVR+S LKGV VP +RAPSMIDEYP+LA+AA+FAEG TVM+GL+ELRVKESDRL+A VADLRVRASKLKGVVVPPERAPSMIDEYPVLAIAASFAEGETVMDGLDELRVKESDRLAA | 360 |
| Query 361 | VANGLKLNGVDCDEGETSLVVRGRPDGKGLGNASGAAVATHLDHRIAMSFLVMGLVSENP | 420 |
| Sbjct 361 | VA GL+ NGVDC EGE SL VRGRPDGKGLG G VATHLDHRIAMSFLVMGL +E P VARGLEANGVDCTEGEMSLTVRGRPDGKGLG---GGTVATHLDHRIAMSFLVMGLAAEKP | 417 |
| Query 421  Sbjct 418 | VTVDDATMIATSFPEFMDLMAGLGAKIELSDTK 453 VTVDD+ MIATSFPEFMD+M GLGAKIELS+ + VTVDDSNMIATSFPEFMDMMPGLGAKIELSNAQ 450 |  |

>WP_021587681.1 MULTISPECIES: 3-phosphoshikimate 1-carboxyvinyltransferase [Brucellaceae] P0A2Y4.1 RecName: Full=3-phosphoshikimate 1-carboxyvinyltransferase; AltName:

Full=5-enolpyruvylshikimate-3-phosphate synthase; Short=EPSP synthase; Short=EPSPS [Pseudomonas sp. PG2982]

P0A2Y5.1 RecName: Full=3-phosphoshikimate 1-carboxyvinyltransferase; AltName:

Full=5-enolpyruvylshikimate-3-phosphate synthase; Short=EPSP synthase; Short=EPSPS [Achromobacter sp. LBAA]

ERI13118.1 3-phosphoshikimate 1-carboxyvinyltransferase [Ochrobactrum sp. EGD-AQ16]

KAB2695809.1 3-phosphoshikimate 1-carboxyvinyltransferase [Brucella intermedia] MPR62540.1 3-phosphoshikimate 1-carboxyvinyltransferase [Brucella intermedia] NVM40047.1 3-phosphoshikimate 1-carboxyvinyltransferase [Brucella intermedia]

Length=449

Score = 733 bits (1893), Expect = 0.0, Method: Compositional matrix adjust. Identities = 373/450 (83%), Positives = 405/450 (90%), Gaps = 3/450 (1%)

| Query | 1 | MLHGASSRPATARKSSGLSGTVRIPGDKSISHRSFMFGGLASGETRITGLLEGEDVINTG | 60 |
| --- | --- | --- | --- |
| Sbjct | 1 | M H AS +PATAR+S L+G +RIPGDKSISHRSFMFGGLASGETRITGLLEGEDVINTG MSHSASPKPATARRSEALTGEIRIPGDKSISHRSFMFGGLASGETRITGLLEGEDVINTG | 60 |
| Query | 61 | KAMQAMGARIRKEGDTWIIDGVGNGGLLAPEAPLDFGNAATGCRLTMGLVGVYDFDSTFI | 120 |
| Sbjct | 61 | +AMQAMGA+IRKEGD WII+GVGNG LL PEA LDFGNA TG RLTMGLVG YD ++FI RAMQAMGAKIRKEGDVWIINGVGNGCLLQPEAALDFGNAGTGARLTMGLVGTYDMKTSFI | 120 |
| Query | 121 | GDASLTKRPMGRVLNPLREMGVQVKSEDGDRLPVTLRGPKTPTPITYRVPMASAQVKSAV | 180 |
| Sbjct | 121 | GDASL+KRPMGRVLNPLREMGVQV++ DGDR+P+TL GPKT PITYRVPMASAQVKSAV GDASLSKRPMGRVLNPLREMGVQVEAADGDRMPLTLIGPKTANPITYRVPMASAQVKSAV | 180 |
| Query | 181 | LLAGLNTPGITTVIEPIMTRDHTEKMLQGFGANLTVETDADGVRTIRLEGRGKLTGQVID | 240 |
| Sbjct | 181 | LLAGLNTPG+TTVIEP+MTRDHTEKMLQGFGA+LTVETD DGVR IR+ G+GKL GQ ID LLAGLNTPGVTTVIEPVMTRDHTEKMLQGFGADLTVETDKDGVRHIRITGQGKLVGQTID | 240 |
| Query | 241 | VPGDPSSTAFPLVAALLVPGSDVTILNVLMNPTRTGLILTLQEMGADIEVINPRLAGGED | 300 |
| Sbjct | 241 | VPGDPSSTAFPLVAALLV GSDVTI NVLMNPTRTGLILTLQEMGADIEV+N RLAGGED VPGDPSSTAFPLVAALLVEGSDVTIRNVLMNPTRTGLILTLQEMGADIEVLNARLAGGED | 300 |
| Query | 301 | VADLRVRSSTLKGVTVPEDRAPSMIDEYPILavaaafaegaTVMNGLEELRVKESDRLSA | 360 |
| Sbjct | 301 | VADLRVR+S LKGV VP +RAPSMIDEYP+LA+AA+FAEG TVM+GL+ELRVKESDRL+A VADLRVRASKLKGVVVPPERAPSMIDEYPVLAIAASFAEGETVMDGLDELRVKESDRLAA | 360 |
| Query | 361 | VANGLKLNGVDCDEGETSLVVRGRPDGKGLGNASGAAVATHLDHRIAMSFLVMGLVSENP | 420 |
| Sbjct | 361 | VA GL+ NGVDC EGE SL VRGRPDGKGLG G VATHLDHRIAMSFLVMGL +E P VARGLEANGVDCTEGEMSLTVRGRPDGKGLG---GGTVATHLDHRIAMSFLVMGLAAEKP | 417 |
| Query  Sbjct | 421  418 | VTVDDATMIATSFPEFMDLMAGLGAKIELS 450 VTVDD+ MIATSFPEFMD+M GLGAKIELS VTVDDSNMIATSFPEFMDMMPGLGAKIELS 447 |  |

>WP_151660289.1 3-phosphoshikimate 1-carboxyvinyltransferase [Brucella intermedia] KAB2732196.1 3-phosphoshikimate 1-carboxyvinyltransferase [Brucella intermedia]

Length=450

Score = 733 bits (1893), Expect = 0.0, Method: Compositional matrix adjust.

| Identities | = 373/453 (82%), Positives = 405/453 (89%), Gaps = 3/453 (1%) |  |
| --- | --- | --- |
| Query 1 | MLHGASSRPATARKSSGLSGTVRIPGDKSISHRSFMFGGLASGETRITGLLEGEDVINTG | 60 |
| Sbjct 1 | M H AS +PATAR+S L+G +RIPGDKSISHRSFMFGGLASGETRITGLLEGEDVINTG MSHSASPKPATARRSEALTGEIRIPGDKSISHRSFMFGGLASGETRITGLLEGEDVINTG | 60 |
| Query 61 | KAMQAMGARIRKEGDTWIIDGVGNGGLLAPEAPLDFGNAATGCRLTMGLVGVYDFDSTFI | 120 |
| Sbjct 61 | +AMQAMGA+IRKEGD WII+GVGNG LL PEA LDFGNA TG RLTMGLVG YD ++FI RAMQAMGAKIRKEGDVWIINGVGNGCLLQPEAALDFGNAGTGARLTMGLVGTYDMKTSFI | 120 |
| Query 121 | GDASLTKRPMGRVLNPLREMGVQVKSEDGDRLPVTLRGPKTPTPITYRVPMASAQVKSAV | 180 |
| Sbjct 121 | GDASL+KRPMGRVLNPLREMGVQV++ DGDR+P+TL GPKT PITYRVPMASAQVKSAV GDASLSKRPMGRVLNPLREMGVQVEAADGDRMPLTLIGPKTANPITYRVPMASAQVKSAV | 180 |
| Query 181 | LLAGLNTPGITTVIEPIMTRDHTEKMLQGFGANLTVETDADGVRTIRLEGRGKLTGQVID | 240 |
| Sbjct 181 | LLAGLNTPG+TTVIEP+MTRDHTEKMLQGFGA LTVETD DGVR IR+ G+GKL GQ ID LLAGLNTPGVTTVIEPVMTRDHTEKMLQGFGAGLTVETDKDGVRHIRITGQGKLVGQTID | 240 |
| Query 241 | VPGDPSSTAFPLVAALLVPGSDVTILNVLMNPTRTGLILTLQEMGADIEVINPRLAGGED | 300 |
| Sbjct 241 | VPGDPSSTAFPLVAALLV GSDVTI NVLMNPTRTGLILTLQEMGADIEV+N RLAGGED VPGDPSSTAFPLVAALLVEGSDVTIRNVLMNPTRTGLILTLQEMGADIEVLNARLAGGED | 300 |
| Query 301 | VADLRVRSSTLKGVTVPEDRAPSMIDEYPILavaaafaegaTVMNGLEELRVKESDRLSA | 360 |
| Sbjct 301 | VADLRVR+S LKGV VP +RAPSMIDEYP+LA+AA+FAEG TVM+GL+ELRVKESDRL+A VADLRVRASKLKGVVVPPERAPSMIDEYPVLAIAASFAEGETVMDGLDELRVKESDRLAA | 360 |
| Query 361 | VANGLKLNGVDCDEGETSLVVRGRPDGKGLGNASGAAVATHLDHRIAMSFLVMGLVSENP | 420 |
| Sbjct 361 | VA GL+ NGVDC EGE SL VRG PDGKGLG G VATHLDHRIAMSFLVMGL SE P VARGLEANGVDCTEGEMSLTVRGSPDGKGLG---GGTVATHLDHRIAMSFLVMGLASEKP | 417 |
| Query 421  Sbjct 418 | VTVDDATMIATSFPEFMDLMAGLGAKIELSDTK 453 VTVDD+ MIATSFPEFMD+M GLGAKIELS+ + VTVDDSNMIATSFPEFMDMMPGLGAKIELSNAQ 450 |  |

>ADG03677.1 5-enolpyruvylshikimate 3-phosphate synthase [Ochrobactrum anthropi] Length=450

Score = 733 bits (1893), Expect = 0.0, Method: Compositional matrix adjust.

| Identities | = 372/453 (82%), Positives = 408/453 (90%), Gaps = 3/453 (1%) |  |
| --- | --- | --- |
| Query 1 | MLHGASSRPATARKSSGLSGTVRIPGDKSISHRSFMFGGLASGETRITGLLEGEDVINTG | 60 |
| Sbjct 1 | M H AS +PATAR+S L+G +RIPGDKSISHRSFMFGGLASGETRITGLLEGEDVINTG MSHSASPKPATARRSEALTGEIRIPGDKSISHRSFMFGGLASGETRITGLLEGEDVINTG | 60 |
| Query 61 | KAMQAMGARIRKEGDTWIIDGVGNGGLLAPEAPLDFGNAATGCRLTMGLVGVYDFDSTFI | 120 |
| Sbjct 61 | +AMQAMGA+IRK+GD WII+GVGNG LL PEA LDFGNA TG RLTMGLVG YD ++FI RAMQAMGAKIRKDGDAWIINGVGNGCLLQPEAALDFGNAGTGARLTMGLVGTYDMRTSFI | 120 |
| Query 121 | GDASLTKRPMGRVLNPLREMGVQVKSEDGDRLPVTLRGPKTPTPITYRVPMASAQVKSAV | 180 |
| Sbjct 121 | GDASL+KRPMGRVLNPLREMGVQV++ +GDR+P+TL GPKT PITYRVPMASAQVKSAV GDASLSKRPMGRVLNPLREMGVQVEAAEGDRMPLTLIGPKTANPITYRVPMASAQVKSAV | 180 |
| Query 181 | LLAGLNTPGITTVIEPIMTRDHTEKMLQGFGANLTVETDADGVRTIRLEGRGKLTGQVID | 240 |
| Sbjct 181 | LLAGLNTPG+TTVIEP+MTRDHTEKMLQGFGA+L+VETD DGVR IR+ G+GKL GQ ID LLAGLNTPGVTTVIEPVMTRDHTEKMLQGFGADLSVETDKDGVRHIRITGQGKLIGQTID | 240 |
| Query 241 | VPGDPSSTAFPLVAALLVPGSDVTILNVLMNPTRTGLILTLQEMGADIEVINPRLAGGED | 300 |
| Sbjct 241 | VPGDPSSTAFPLVAALLV GSDVTI NVLMNPTRTGLILTLQEMGADIEV+N RLAGGED VPGDPSSTAFPLVAALLVEGSDVTIRNVLMNPTRTGLILTLQEMGADIEVLNARLAGGED | 300 |
| Query 301 | VADLRVRSSTLKGVTVPEDRAPSMIDEYPILavaaafaegaTVMNGLEELRVKESDRLSA | 360 |
| Sbjct 301 | VADLRV++S LKGV VP +RAPSMIDEYP+LA+AA+FAEG TVM+GL+ELRVKESDRL+A VADLRVKASKLKGVVVPPERAPSMIDEYPVLAIAASFAEGETVMDGLDELRVKESDRLAA | 360 |
| Query 361 | VANGLKLNGVDCDEGETSLVVRGRPDGKGLGNASGAAVATHLDHRIAMSFLVMGLVSENP | 420 |
| Sbjct 361 | VA GL+ NGVDC EGE SL VRGRPDGKGLG G VATHLDHRIAMSFLVMGL SE P VARGLEANGVDCTEGEMSLTVRGRPDGKGLG---GGTVATHLDHRIAMSFLVMGLASEKP | 417 |
| Query 421  Sbjct 418 | VTVDDATMIATSFPEFMDLMAGLGAKIELSDTK 453 VTVDD+TMIATSFPEFMD+M GLGAKIELSD + VTVDDSTMIATSFPEFMDMMPGLGAKIELSDAR 450 |  |

>WP_133704665.1 MULTISPECIES: 3-phosphoshikimate 1-carboxyvinyltransferase [unclassified Rhizobium]

MBB3459052.1 3-phosphoshikimate 1-carboxyvinyltransferase [Rhizobium sp. BK313] TDP26032.1 3-phosphoshikimate 1-carboxyvinyltransferase [Rhizobium sp. BK333]

Length=452

Score = 733 bits (1891), Expect = 0.0, Method: Compositional matrix adjust.

| Identities | = 369/446 (83%), Positives = 404/446 (91%), Gaps = 0/446 (0%) |  |
| --- | --- | --- |
| Query 7 | SRPATARKSSGLSGTVRIPGDKSISHRSFMFGGLASGETRITGLLEGEDVINTGKAMQAM | 66 |
| Sbjct 7 | SRPATAR+S+ LSGT+R+PGDKSISHR+ MFGGLA+GETRITGLLEGEDV+NTG+AMQAM SRPATARRSTNLSGTIRVPGDKSISHRALMFGGLAAGETRITGLLEGEDVLNTGRAMQAM | 66 |
| Query 67 | GARIRKEGDTWIIDGVGNGGLLAPEAPLDFGNAATGCRLTMGLVGVYDFDSTFIGDASLT | 126 |
| Sbjct 67 | GARI K G WII GVGNG LLAPEAPLDFGNA TG RLTMGLVGVY+F+STFIGDASL+ GARIEKTGAEWIIKGVGNGALLAPEAPLDFGNAGTGSRLTMGLVGVYNFESTFIGDASLS | 126 |
| Query 127 | KRPMGRVLNPLREMGVQVKSEDGDRLPVTLRGPKTPTPITYRVPMASAQVKSAVLLAGLN | 186 |
| Sbjct 127 | KRPMGR+L+PLR+MGVQVKS +GDRLPVTL GP+T +PI YRVPMASAQVKSAVLLAGLN KRPMGRILDPLRQMGVQVKSSEGDRLPVTLHGPETASPIIYRVPMASAQVKSAVLLAGLN | 186 |
| Query 187 | TPGITTVIEPIMTRDHTEKMLQGFGANLTVETDADGVRTIRLEGRGKLTGQVIDVPGDPS | 246 |
| Sbjct 187 | PGITTVIEP+MTRDHTEKML GFGA L+VETD DGVRTI LEG+G+L+GQ+IDVPGDPS APGITTVIEPVMTRDHTEKMLAGFGAALSVETDRDGVRTISLEGQGRLSGQIIDVPGDPS | 246 |
| Query 247 | STAFPLVAALLVPGSDVTILNVLMNPTRTGLILTLQEMGADIEVINPRLAGGEDVADLRV | 306 |
| Sbjct 247 | STAFPLVAAL+VPGSD+TI NVLMNPTRTGLILTLQEMGADIE++ RLAGGEDVADLRV STAFPLVAALIVPGSDITIGNVLMNPTRTGLILTLQEMGADIEILERRLAGGEDVADLRV | 306 |
| Query 307 | RSSTLKGVTVPEDRAPSMIDEYPILavaaafaegaTVMNGLEELRVKESDRLSAVANGLK RSS LKG+TVP +RAPSMIDEYP+LAVAAAFA+G TVM GLEELRVKESDRLSAVA GLK | 366 |

| Sbjct | 307 | RSSALKGITVPAERAPSMIDEYPVLAVAAAFADGTTVMLGLEELRVKESDRLSAVAEGLK | 366 |
| --- | --- | --- | --- |
| Query | 367 | LNGVDCDEGETSLVVRGRPDGKGLGNASGAAVATHLDHRIAMSFLVMGLVSENPVTVDDA | 426 |
| Sbjct | 367 | LNGVDC EGE SL VRGRPDGKGLGNA G+ + THLDHRIAMSFLVMGL SE+PV VDD+ LNGVDCAEGEASLTVRGRPDGKGLGNAGGSQIRTHLDHRIAMSFLVMGLASEHPVAVDDS | 426 |
| Query  Sbjct | 427  427 | TMIATSFPEFMDLMAGLGAKIELSDT 452 TMIATSFPEF DLM LGA IE D TMIATSFPEFFDLMTRLGATIEAEDC 452 |  |

>ANN25048.1 5-enolpyruvylshikimate-3-phosphate synthase [Brucella intermedia] Length=450

Score = 733 bits (1891), Expect = 0.0, Method: Compositional matrix adjust.

| Identities | = 371/453 (82%), Positives = 406/453 (90%), Gaps = 3/453 (1%) |  |
| --- | --- | --- |
| Query 1 | MLHGASSRPATARKSSGLSGTVRIPGDKSISHRSFMFGGLASGETRITGLLEGEDVINTG | 60 |
| Sbjct 1 | M H AS +PATAR+S L+G +RIPGDKSISHRSFMFGGLASGETRITGLLEGEDVINTG MSHSASPKPATARRSEALAGEIRIPGDKSISHRSFMFGGLASGETRITGLLEGEDVINTG | 60 |
| Query 61 | KAMQAMGARIRKEGDTWIIDGVGNGGLLAPEAPLDFGNAATGCRLTMGLVGVYDFDSTFI | 120 |
| Sbjct 61 | +AMQAMGA+IRK+GD WII+GVGNG LL PEA LDFGNA TG RLTMGLVG YD ++FI RAMQAMGAKIRKDGDVWIINGVGNGCLLQPEAALDFGNAGTGARLTMGLVGTYDMKTSFI | 120 |
| Query 121 | GDASLTKRPMGRVLNPLREMGVQVKSEDGDRLPVTLRGPKTPTPITYRVPMASAQVKSAV | 180 |
| Sbjct 121 | GDASL+KRPMGRVLNPLREMGVQV++ DGDR+P+TL GPKT PITYRVPMASAQ KSAV GDASLSKRPMGRVLNPLREMGVQVEAADGDRMPLTLIGPKTANPITYRVPMASAQAKSAV | 180 |
| Query 181 | LLAGLNTPGITTVIEPIMTRDHTEKMLQGFGANLTVETDADGVRTIRLEGRGKLTGQVID | 240 |
| Sbjct 181 | LLAGLNTPG+TTVIEP+MTRDHTEKMLQGFGA+LTVETD +GVR IR+ G+GKL GQ ID LLAGLNTPGVTTVIEPVMTRDHTEKMLQGFGADLTVETDKEGVRHIRITGQGKLVGQTID | 240 |
| Query 241 | VPGDPSSTAFPLVAALLVPGSDVTILNVLMNPTRTGLILTLQEMGADIEVINPRLAGGED | 300 |
| Sbjct 241 | VPGDPSSTAFPLVAALLV GSDVTI NVLMNPTRTGLILTLQEMGADIEV+N RLAGGED VPGDPSSTAFPLVAALLVDGSDVTIRNVLMNPTRTGLILTLQEMGADIEVLNARLAGGED | 300 |
| Query 301 | VADLRVRSSTLKGVTVPEDRAPSMIDEYPILavaaafaegaTVMNGLEELRVKESDRLSA | 360 |
| Sbjct 301 | VADLRVR+S LKGV VP +RAPSMIDEYP+LA+AA+FA+G TVM+GL+ELRVKESDRL+A VADLRVRASKLKGVVVPPERAPSMIDEYPVLAIAASFADGETVMDGLDELRVKESDRLTA | 360 |
| Query 361 | VANGLKLNGVDCDEGETSLVVRGRPDGKGLGNASGAAVATHLDHRIAMSFLVMGLVSENP | 420 |
| Sbjct 361 | VA GL+ NGVDC EGE SL VRGRPDGKGLG G VATHLDHRIAMSFLVMGL SE P VARGLEANGVDCTEGEMSLTVRGRPDGKGLG---GGTVATHLDHRIAMSFLVMGLASEKP | 417 |
| Query 421  Sbjct 418 | VTVDDATMIATSFPEFMDLMAGLGAKIELSDTK 453 VTVDD+ MIATSFPEFMD+M GLGAKIELSD + VTVDDSNMIATSFPEFMDMMPGLGAKIELSDAR 450 |  |

>WP_061344725.1 3-phosphoshikimate 1-carboxyvinyltransferase [Ochrobactrum anthropi] KXO79387.1 3-phosphoshikimate 1-carboxyvinyltransferase [Ochrobactrum anthropi]

Length=450

Score = 733 bits (1891), Expect = 0.0, Method: Compositional matrix adjust.

| Identities | = 372/453 (82%), Positives = 408/453 (90%), Gaps = 3/453 (1%) |  |
| --- | --- | --- |
| Query 1 | MLHGASSRPATARKSSGLSGTVRIPGDKSISHRSFMFGGLASGETRITGLLEGEDVINTG | 60 |
| Sbjct 1 | M H AS +PATAR+S L+G +RIPGDKSISHRSFMFGGLASGETRITGLLEGEDVINTG MSHSASPKPATARRSEALTGEIRIPGDKSISHRSFMFGGLASGETRITGLLEGEDVINTG | 60 |
| Query 61 | KAMQAMGARIRKEGDTWIIDGVGNGGLLAPEAPLDFGNAATGCRLTMGLVGVYDFDSTFI | 120 |
| Sbjct 61 | +AMQAMGA+IRK+GD WII+GVGNG LL PEA LDFGNA TG RLTMGLVG YD ++FI RAMQAMGAKIRKDGDVWIINGVGNGCLLQPEAALDFGNAGTGARLTMGLVGTYDMKTSFI | 120 |
| Query 121 | GDASLTKRPMGRVLNPLREMGVQVKSEDGDRLPVTLRGPKTPTPITYRVPMASAQVKSAV | 180 |
| Sbjct 121 | GDASL+KRPMGRVLNPLREMGVQV++ DGDR+P+TL GP+T PITYRVPMASAQVKSAV GDASLSKRPMGRVLNPLREMGVQVEAADGDRMPLTLIGPRTANPITYRVPMASAQVKSAV | 180 |
| Query 181 | LLAGLNTPGITTVIEPIMTRDHTEKMLQGFGANLTVETDADGVRTIRLEGRGKLTGQVID | 240 |
| Sbjct 181 | LLAGLNTPG+TTVIEP+MTRDHTEKMLQGFGA+L+VETD +GVR IR+ G+GKL GQ ID  LLAGLNTPGVTTVIEPVMTRDHTEKMLQGFGADLSVETDEEGVRHIRITGQGKLIGQTID | 240 |

| Query | 241 | VPGDPSSTAFPLVAALLVPGSDVTILNVLMNPTRTGLILTLQEMGADIEVINPRLAGGED | 300 |
| --- | --- | --- | --- |
| Sbjct | 241 | VPGDPSSTAFPLVAALLV GSDVTI NVLMNPTRTGLILTLQEMGADIEV+N RLAGGED VPGDPSSTAFPLVAALLVEGSDVTIHNVLMNPTRTGLILTLQEMGADIEVLNARLAGGED | 300 |
| Query | 301 | VADLRVRSSTLKGVTVPEDRAPSMIDEYPILavaaafaegaTVMNGLEELRVKESDRLSA | 360 |
| Sbjct | 301 | VADLRV++S LKGV VP +RAPSMIDEYP+LA+AAAFAEG TVM+GL+ELRVKESDRL+A VADLRVKASKLKGVVVPPERAPSMIDEYPVLAIAAAFAEGETVMDGLDELRVKESDRLAA | 360 |
| Query | 361 | VANGLKLNGVDCDEGETSLVVRGRPDGKGLGNASGAAVATHLDHRIAMSFLVMGLVSENP | 420 |
| Sbjct | 361 | VA GL+ NGVDC EGE SL VRGRPDGKGLG G VATHLDHRIAMSFLVMGL SE P VARGLEANGVDCTEGEMSLTVRGRPDGKGLG---GGTVATHLDHRIAMSFLVMGLASEKP | 417 |
| Query  Sbjct | 421  418 | VTVDDATMIATSFPEFMDLMAGLGAKIELSDTK 453 VTVDD+TMIATSFPEFMD+M GLGAKIELSD + VTVDDSTMIATSFPEFMDMMPGLGAKIELSDAR 450 |  |

>WP_132226789.1 3-phosphoshikimate 1-carboxyvinyltransferase [Ochrobactrum sp. BH3]

TCQ81935.1 3-phosphoshikimate 1-carboxyvinyltransferase [Ochrobactrum sp. BH3]

Length=450

Score = 733 bits (1891), Expect = 0.0, Method: Compositional matrix adjust.

| Identities | = 372/453 (82%), Positives = 406/453 (90%), Gaps = 3/453 (1%) |  |
| --- | --- | --- |
| Query 1 | MLHGASSRPATARKSSGLSGTVRIPGDKSISHRSFMFGGLASGETRITGLLEGEDVINTG | 60 |
| Sbjct 1 | M H AS +PA AR S L G +RIPGDKSISHRSFMFGGLASGETRITGLLEGEDVINTG MSHSASPKPAIARHSEALKGEIRIPGDKSISHRSFMFGGLASGETRITGLLEGEDVINTG | 60 |
| Query 61 | KAMQAMGARIRKEGDTWIIDGVGNGGLLAPEAPLDFGNAATGCRLTMGLVGVYDFDSTFI | 120 |
| Sbjct 61 | +AMQAMGA+IRK+G+ WII+GVGNG LL PEAPLDFGNA TG RLTMGLVG YD +TFI RAMQAMGAKIRKDGNVWIINGVGNGCLLQPEAPLDFGNAGTGARLTMGLVGTYDMTTTFI | 120 |
| Query 121 | GDASLTKRPMGRVLNPLREMGVQVKSEDGDRLPVTLRGPKTPTPITYRVPMASAQVKSAV | 180 |
| Sbjct 121 | GDASLT RPMGRVLNPLREMGVQVK+ +GDRLP+TL GPKT PI+YRVPMASAQVKSAV GDASLTSRPMGRVLNPLREMGVQVKAAEGDRLPLTLTGPKTANPISYRVPMASAQVKSAV | 180 |
| Query 181 | LLAGLNTPGITTVIEPIMTRDHTEKMLQGFGANLTVETDADGVRTIRLEGRGKLTGQVID | 240 |
| Sbjct 181 | LLAGLNTPG+TTVIEP+MTRDHTEKMLQGFGA+LTVETD DGVR IR+ G+GKL GQ ID LLAGLNTPGVTTVIEPVMTRDHTEKMLQGFGADLTVETDKDGVRHIRITGQGKLIGQTID | 240 |
| Query 241 | VPGDPSSTAFPLVAALLVPGSDVTILNVLMNPTRTGLILTLQEMGADIEVINPRLAGGED | 300 |
| Sbjct 241 | VPGDPSSTAFPLVAALLV GSDVTI NVLMNPTRTGLILTLQEMGADIE+I+PRLAGGED VPGDPSSTAFPLVAALLVEGSDVTIRNVLMNPTRTGLILTLQEMGADIEIIDPRLAGGED | 300 |
| Query 301 | VADLRVRSSTLKGVTVPEDRAPSMIDEYPILavaaafaegaTVMNGLEELRVKESDRLSA | 360 |
| Sbjct 301 | VADLRV+SS LKGVTVP +RAPSMIDEYP+LA+AA+FAEG T+M+GL+ELRVKESDRL+A VADLRVKSSRLKGVTVPPERAPSMIDEYPVLAIAASFAEGETIMDGLDELRVKESDRLAA | 360 |
| Query 361 | VANGLKLNGVDCDEGETSLVVRGRPDGKGLGNASGAAVATHLDHRIAMSFLVMGLVSENP | 420 |
| Sbjct 361 | VA GL+ NGVDC EGE SL VRGRPDG+GLG G VATHLDHRIAMSFLVMGL SE P VARGLEANGVDCTEGEMSLTVRGRPDGRGLG---GGTVATHLDHRIAMSFLVMGLASEKP | 417 |
| Query 421  Sbjct 418 | VTVDDATMIATSFPEFMDLMAGLGAKIELSDTK 453 VTVDD+TMIATSFPEFM +M GLGAKIE+SD + VTVDDSTMIATSFPEFMTMMPGLGAKIEVSDVR 450 |  |

>WP_063619892.1 MULTISPECIES: 3-phosphoshikimate 1-carboxyvinyltransferase [Brucellaceae] KAB2710533.1 3-phosphoshikimate 1-carboxyvinyltransferase [Brucella intermedia] MBA8844692.1 3-phosphoshikimate 1-carboxyvinyltransferase [Ochrobactrum sp.

RH1CCR137]

MBA8856170.1 3-phosphoshikimate 1-carboxyvinyltransferase [Ochrobactrum sp. RH1CCR134]

OAB85530.1 3-phosphoshikimate 1-carboxyvinyltransferase [Brucella intermedia] OAE46274.1 3-phosphoshikimate 1-carboxyvinyltransferase [Brucella intermedia]

Length=450

Score = 732 bits (1890), Expect = 0.0, Method: Compositional matrix adjust. Identities = 371/453 (82%), Positives = 407/453 (90%), Gaps = 3/453 (1%)

| Query | 1 | MLHGASSRPATARKSSGLSGTVRIPGDKSISHRSFMFGGLASGETRITGLLEGEDVINTG | 60 |
| --- | --- | --- | --- |
| Sbjct | 1 | M H AS +PATAR+S L+G +RIPGDKSISHRSFMFGGLASGETRITGLLEGEDVINTG MSHSASPKPATARRSEALTGEIRIPGDKSISHRSFMFGGLASGETRITGLLEGEDVINTG | 60 |
| Query | 61 | KAMQAMGARIRKEGDTWIIDGVGNGGLLAPEAPLDFGNAATGCRLTMGLVGVYDFDSTFI | 120 |
| Sbjct | 61 | +AMQAMGA+IRK+GD WII+GVGNG LL PEA LDFGNA TG RLTMGLVG YD ++FI RAMQAMGAKIRKDGDVWIINGVGNGCLLQPEAALDFGNAGTGARLTMGLVGTYDMKTSFI | 120 |
| Query | 121 | GDASLTKRPMGRVLNPLREMGVQVKSEDGDRLPVTLRGPKTPTPITYRVPMASAQVKSAV | 180 |
| Sbjct | 121 | GDASL+KRPMGRVLNPLREMGVQV++ DGDR+P+TL GPKT PITYRVPMASAQVKSAV GDASLSKRPMGRVLNPLREMGVQVEAADGDRMPLTLIGPKTANPITYRVPMASAQVKSAV | 180 |
| Query | 181 | LLAGLNTPGITTVIEPIMTRDHTEKMLQGFGANLTVETDADGVRTIRLEGRGKLTGQVID | 240 |
| Sbjct | 181 | LLAGLNTPG+TTVIEP+MTRDHTEKMLQGFGA+LTVETD +GVR IR+ G+GKL GQ ID LLAGLNTPGVTTVIEPVMTRDHTEKMLQGFGADLTVETDKEGVRHIRITGQGKLVGQTID | 240 |
| Query | 241 | VPGDPSSTAFPLVAALLVPGSDVTILNVLMNPTRTGLILTLQEMGADIEVINPRLAGGED | 300 |
| Sbjct | 241 | VPGDPSSTAFPLVAALLV GSDVTI NVLMNPTRTGLILTLQEMGADIEV+N RLAGGED VPGDPSSTAFPLVAALLVDGSDVTIRNVLMNPTRTGLILTLQEMGADIEVLNARLAGGED | 300 |
| Query | 301 | VADLRVRSSTLKGVTVPEDRAPSMIDEYPILavaaafaegaTVMNGLEELRVKESDRLSA | 360 |
| Sbjct | 301 | VADLRVR+S LKGV VP +RAPSMIDEYP+LA+AA+FA+G TVM+GL+ELRVKESDRL+A VADLRVRASKLKGVVVPPERAPSMIDEYPVLAIAASFADGETVMDGLDELRVKESDRLAA | 360 |
| Query | 361 | VANGLKLNGVDCDEGETSLVVRGRPDGKGLGNASGAAVATHLDHRIAMSFLVMGLVSENP | 420 |
| Sbjct | 361 | VA GL+ NGVDC EGE SL VRGRPDGKGLG G VATHLDHRIAMSFLVMGL SE P VARGLEANGVDCTEGEMSLTVRGRPDGKGLG---GGTVATHLDHRIAMSFLVMGLASEKP | 417 |
| Query  Sbjct | 421  418 | VTVDDATMIATSFPEFMDLMAGLGAKIELSDTK 453 VTVDD+ MIATSFPEFMD+M GLGAKIELS+ + VTVDDSNMIATSFPEFMDMMPGLGAKIELSNAQ 450 |  |

>WP_151662889.1 3-phosphoshikimate 1-carboxyvinyltransferase [Ochrobactrum anthropi] KAB2773360.1 3-phosphoshikimate 1-carboxyvinyltransferase [Ochrobactrum anthropi] KAB2793901.1 3-phosphoshikimate 1-carboxyvinyltransferase [Ochrobactrum anthropi]

Length=450

Score = 732 bits (1890), Expect = 0.0, Method: Compositional matrix adjust.

| Identities | = 372/453 (82%), Positives = 408/453 (90%), Gaps = 3/453 (1%) |  |
| --- | --- | --- |
| Query 1 | MLHGASSRPATARKSSGLSGTVRIPGDKSISHRSFMFGGLASGETRITGLLEGEDVINTG | 60 |
| Sbjct 1 | M H AS +PATAR+S L+G +RIPGDKSISHRSFMFGGLASGETRITGLLEGEDVINTG MSHSASPKPATARRSEALTGEIRIPGDKSISHRSFMFGGLASGETRITGLLEGEDVINTG | 60 |
| Query 61 | KAMQAMGARIRKEGDTWIIDGVGNGGLLAPEAPLDFGNAATGCRLTMGLVGVYDFDSTFI | 120 |
| Sbjct 61 | +AMQAMGA+IRK+GD WII+GVGNG LL PEA LDFGNA TG RLTMGLVG YD ++FI RAMQAMGAKIRKDGDAWIINGVGNGCLLQPEAALDFGNAGTGARLTMGLVGTYDMRTSFI | 120 |
| Query 121 | GDASLTKRPMGRVLNPLREMGVQVKSEDGDRLPVTLRGPKTPTPITYRVPMASAQVKSAV | 180 |
| Sbjct 121 | GDASL+KRPMGRVLNPLREMGVQV++ +GDR+P+TL GPKT PITYRVPMASAQVKSAV GDASLSKRPMGRVLNPLREMGVQVEAAEGDRMPLTLIGPKTANPITYRVPMASAQVKSAV | 180 |
| Query 181 | LLAGLNTPGITTVIEPIMTRDHTEKMLQGFGANLTVETDADGVRTIRLEGRGKLTGQVID | 240 |
| Sbjct 181 | LLAGLNTPG+TTVIEP+MTRDHTEKMLQGFGA+L+VETD DGVR IR+ G+GKL GQ ID LLAGLNTPGVTTVIEPVMTRDHTEKMLQGFGADLSVETDKDGVRHIRITGQGKLIGQTID | 240 |
| Query 241 | VPGDPSSTAFPLVAALLVPGSDVTILNVLMNPTRTGLILTLQEMGADIEVINPRLAGGED | 300 |
| Sbjct 241 | VPGDPSSTAFPLVAALLV GSDVTI NVLMNPTRTGLILTLQEMGA+IEV+N RLAGGED VPGDPSSTAFPLVAALLVEGSDVTIRNVLMNPTRTGLILTLQEMGANIEVLNARLAGGED | 300 |
| Query 301 | VADLRVRSSTLKGVTVPEDRAPSMIDEYPILavaaafaegaTVMNGLEELRVKESDRLSA | 360 |
| Sbjct 301 | VADLRV++S LKGV VP +RAPSMIDEYP+LA+AAAFAEG TVM+GL+ELRVKESDRL+A VADLRVKASKLKGVVVPPERAPSMIDEYPVLAIAAAFAEGETVMDGLDELRVKESDRLAA | 360 |
| Query 361 | VANGLKLNGVDCDEGETSLVVRGRPDGKGLGNASGAAVATHLDHRIAMSFLVMGLVSENP | 420 |
| Sbjct 361 | VA GL+ NGVDC EGE SL VRGRPDGKGLG G VATHLDHRIAMSFLVMGL SE P VARGLETNGVDCTEGEMSLTVRGRPDGKGLG---GGTVATHLDHRIAMSFLVMGLASEKP | 417 |
| Query 421 | VTVDDATMIATSFPEFMDLMAGLGAKIELSDTK 453 VTVDD+TMIATSFPEFMD+M GLGAKIELSD + |  |

Sbjct 418 VTVDDSTMIATSFPEFMDMMPGLGAKIELSDAR 450

>WP_094578647.1 3-phosphoshikimate 1-carboxyvinyltransferase [Ochrobactrum rhizosphaerae] OYR09644.1 3-phosphoshikimate 1-carboxyvinyltransferase [Ochrobactrum rhizosphaerae]

Length=450

Score = 732 bits (1890), Expect = 0.0, Method: Compositional matrix adjust.

| Identities | = 373/453 (82%), Positives = 404/453 (89%), Gaps = 3/453 (1%) |  |
| --- | --- | --- |
| Query 1 | MLHGASSRPATARKSSGLSGTVRIPGDKSISHRSFMFGGLASGETRITGLLEGEDVINTG | 60 |
| Sbjct 1 | M H AS +PA AR S L G +RIPGDKSISHRSFMFGGLASGETRITGLLEGEDVINTG MSHSASPKPAIARHSEALKGEIRIPGDKSISHRSFMFGGLASGETRITGLLEGEDVINTG | 60 |
| Query 61 | KAMQAMGARIRKEGDTWIIDGVGNGGLLAPEAPLDFGNAATGCRLTMGLVGVYDFDSTFI | 120 |
| Sbjct 61 | +AMQAMGA IRK+G+ WII+GVGNG LL PEAPLDFGNA TG RLTMGLVG YD +TFI RAMQAMGANIRKDGNVWIINGVGNGCLLQPEAPLDFGNAGTGARLTMGLVGTYDMTTTFI | 120 |
| Query 121 | GDASLTKRPMGRVLNPLREMGVQVKSEDGDRLPVTLRGPKTPTPITYRVPMASAQVKSAV | 180 |
| Sbjct 121 | GDASLT RPMGRVLNPLREMGVQVK+ +GDRLP+TL GPKT PI+YRVPMASAQVKSAV GDASLTSRPMGRVLNPLREMGVQVKAAEGDRLPLTLTGPKTANPISYRVPMASAQVKSAV | 180 |
| Query 181 | LLAGLNTPGITTVIEPIMTRDHTEKMLQGFGANLTVETDADGVRTIRLEGRGKLTGQVID | 240 |
| Sbjct 181 | LLAGLNTPG+TTVIEP+MTRDHTEKMLQGFGA+LTVETD DGVR IR+ G GKLTGQ ID LLAGLNTPGVTTVIEPVMTRDHTEKMLQGFGADLTVETDKDGVRHIRITGHGKLTGQTID | 240 |
| Query 241 | VPGDPSSTAFPLVAALLVPGSDVTILNVLMNPTRTGLILTLQEMGADIEVINPRLAGGED | 300 |
| Sbjct 241 | VPGDPSSTAFPLVAALLV GSDVTI NVLMNPTRTGLILTLQEMGADIE+I+PRLAGGED VPGDPSSTAFPLVAALLVEGSDVTIRNVLMNPTRTGLILTLQEMGADIEIIDPRLAGGED | 300 |
| Query 301 | VADLRVRSSTLKGVTVPEDRAPSMIDEYPILavaaafaegaTVMNGLEELRVKESDRLSA | 360 |
| Sbjct 301 | VADLRV+SS LKGVTVP +RAPSMIDEYP+LA+AA+FAEG TVM+GL+ELRVKESDRL+A VADLRVKSSKLKGVTVPPERAPSMIDEYPVLAIAASFAEGETVMDGLDELRVKESDRLAA | 360 |
| Query 361 | VANGLKLNGVDCDEGETSLVVRGRPDGKGLGNASGAAVATHLDHRIAMSFLVMGLVSENP | 420 |
| Sbjct 361 | VA GL+ NGV+C EGE SL VRGRPDGKGLG G VATHLDHRIAMSFLVMGL SE P VARGLEANGVNCTEGEMSLTVRGRPDGKGLG---GGTVATHLDHRIAMSFLVMGLASEKP | 417 |
| Query 421  Sbjct 418 | VTVDDATMIATSFPEFMDLMAGLGAKIELSDTK 453 VTVDD+ MIATSFPEFM +M GLGAKIE+SD + VTVDDSAMIATSFPEFMTMMPGLGAKIEVSDAQ 450 |  |

>WP_094514590.1 MULTISPECIES: 3-phosphoshikimate 1-carboxyvinyltransferase [Ochrobactrum] KAB2705684.1 3-phosphoshikimate 1-carboxyvinyltransferase [Ochrobactrum lupini] KAB2726831.1 3-phosphoshikimate 1-carboxyvinyltransferase [Ochrobactrum anthropi] KAB2743993.1 3-phosphoshikimate 1-carboxyvinyltransferase [Ochrobactrum anthropi] KAB2796969.1 3-phosphoshikimate 1-carboxyvinyltransferase [Ochrobactrum anthropi] KAB2804739.1 3-phosphoshikimate 1-carboxyvinyltransferase [Ochrobactrum anthropi]

Length=450

Score = 732 bits (1889), Expect = 0.0, Method: Compositional matrix adjust.

| Identities | = 372/453 (82%), Positives = 408/453 (90%), Gaps = 3/453 (1%) |  |
| --- | --- | --- |
| Query 1 | MLHGASSRPATARKSSGLSGTVRIPGDKSISHRSFMFGGLASGETRITGLLEGEDVINTG | 60 |
| Sbjct 1 | M H AS +PATAR+S L+G +RIPGDKSISHRSFMFGGLASGETRITGLLEGEDVINTG MSHSASPKPATARRSEALTGEIRIPGDKSISHRSFMFGGLASGETRITGLLEGEDVINTG | 60 |
| Query 61 | KAMQAMGARIRKEGDTWIIDGVGNGGLLAPEAPLDFGNAATGCRLTMGLVGVYDFDSTFI | 120 |
| Sbjct 61 | +AMQAMGA+IRK+GD WII+GVGNG LL PEA LDFGNA TG RLTMGLVG YD ++FI RAMQAMGAKIRKDGDAWIINGVGNGCLLQPEAALDFGNAGTGARLTMGLVGTYDMRTSFI | 120 |
| Query 121 | GDASLTKRPMGRVLNPLREMGVQVKSEDGDRLPVTLRGPKTPTPITYRVPMASAQVKSAV | 180 |
| Sbjct 121 | GDASL+KRPMGRVLNPLREMGVQV++ +GDR+P+TL GPKT PITYRVPMASAQVKSAV GDASLSKRPMGRVLNPLREMGVQVEAAEGDRMPLTLIGPKTANPITYRVPMASAQVKSAV | 180 |
| Query 181 | LLAGLNTPGITTVIEPIMTRDHTEKMLQGFGANLTVETDADGVRTIRLEGRGKLTGQVID | 240 |
| Sbjct 181 | LLAGLNTPG+TTVIEP+MTRDHTEKMLQGFGA+L+VETD DGVR IR+ G+GKL GQ ID LLAGLNTPGVTTVIEPVMTRDHTEKMLQGFGADLSVETDKDGVRHIRITGQGKLIGQTID | 240 |
| Query 241 | VPGDPSSTAFPLVAALLVPGSDVTILNVLMNPTRTGLILTLQEMGADIEVINPRLAGGED VPGDPSSTAFPLVAALLV GSDVTI NVLMNPTRTGLILTLQEMGA+IEV+N RLAGGED | 300 |

| Sbjct | 241 | VPGDPSSTAFPLVAALLVEGSDVTIRNVLMNPTRTGLILTLQEMGANIEVLNARLAGGED | 300 |
| --- | --- | --- | --- |
| Query | 301 | VADLRVRSSTLKGVTVPEDRAPSMIDEYPILavaaafaegaTVMNGLEELRVKESDRLSA | 360 |
| Sbjct | 301 | VADLRV++S LKGV VP +RAPSMIDEYP+LA+AAAFAEG TVM+GL+ELRVKESDRL+A VADLRVKASKLKGVVVPPERAPSMIDEYPVLAIAAAFAEGETVMDGLDELRVKESDRLAA | 360 |
| Query | 361 | VANGLKLNGVDCDEGETSLVVRGRPDGKGLGNASGAAVATHLDHRIAMSFLVMGLVSENP | 420 |
| Sbjct | 361 | VA GL+ NGVDC EGE SL VRGRPDGKGLG G VATHLDHRIAMSFLVMGL SE P VARGLEANGVDCTEGEMSLTVRGRPDGKGLG---GGTVATHLDHRIAMSFLVMGLASEKP | 417 |
| Query  Sbjct | 421  418 | VTVDDATMIATSFPEFMDLMAGLGAKIELSDTK 453 VTVDD+TMIATSFPEFMD+M GLGAKIELSD + VTVDDSTMIATSFPEFMDMMPGLGAKIELSDAR 450 |  |

>WP_024898290.1 MULTISPECIES: 3-phosphoshikimate 1-carboxyvinyltransferase [Ochrobactrum] PQZ49076.1 3-phosphoshikimate 1-carboxyvinyltransferase [Ochrobactrum sp.

MYb19]

PRA67105.1 3-phosphoshikimate 1-carboxyvinyltransferase [Ochrobactrum sp. MYb18]

PRA75864.1 3-phosphoshikimate 1-carboxyvinyltransferase [Ochrobactrum thiophenivorans] PRA92116.1 3-phosphoshikimate 1-carboxyvinyltransferase [Ochrobactrum sp.

MYb14]

PRA97871.1 3-phosphoshikimate 1-carboxyvinyltransferase [Ochrobactrum sp. MYb15]

Length=450

Score = 732 bits (1889), Expect = 0.0, Method: Compositional matrix adjust.

| Identities | = 372/453 (82%), Positives = 406/453 (90%), Gaps = 3/453 (1%) |  |
| --- | --- | --- |
| Query 1 | MLHGASSRPATARKSSGLSGTVRIPGDKSISHRSFMFGGLASGETRITGLLEGEDVINTG | 60 |
| Sbjct 1 | M H AS +PA AR S L G +RIPGDKSISHRSFMFGGLASGETRITGLLEGEDVINTG MSHSASPKPAIARHSEALKGEIRIPGDKSISHRSFMFGGLASGETRITGLLEGEDVINTG | 60 |
| Query 61 | KAMQAMGARIRKEGDTWIIDGVGNGGLLAPEAPLDFGNAATGCRLTMGLVGVYDFDSTFI | 120 |
| Sbjct 61 | +AMQAMGA+IRK+G+ WII+GVGNG LL PEAPLDFGNA TG RLTMGLVG YD +TFI RAMQAMGAKIRKDGNVWIINGVGNGCLLQPEAPLDFGNAGTGARLTMGLVGTYDMTTTFI | 120 |
| Query 121 | GDASLTKRPMGRVLNPLREMGVQVKSEDGDRLPVTLRGPKTPTPITYRVPMASAQVKSAV | 180 |
| Sbjct 121 | GDASLT RPMGRVLNPLREMGVQVK+ +GDRLP+TL GPKT PI+YRVPMASAQVKSAV GDASLTSRPMGRVLNPLREMGVQVKAAEGDRLPLTLTGPKTANPISYRVPMASAQVKSAV | 180 |
| Query 181 | LLAGLNTPGITTVIEPIMTRDHTEKMLQGFGANLTVETDADGVRTIRLEGRGKLTGQVID | 240 |
| Sbjct 181 | LLAGLNTPG+TTVIEPIMTRDHTEKMLQGFGA+LTVE+D DGVR IR+ G+GKL GQ ID LLAGLNTPGVTTVIEPIMTRDHTEKMLQGFGADLTVESDKDGVRHIRITGQGKLIGQTID | 240 |
| Query 241 | VPGDPSSTAFPLVAALLVPGSDVTILNVLMNPTRTGLILTLQEMGADIEVINPRLAGGED | 300 |
| Sbjct 241 | VPGDPSSTAFPLVAALLV GSDVTI NVLMNPTRTGLILTLQEMGADIE+I+PRLAGGED VPGDPSSTAFPLVAALLVEGSDVTIRNVLMNPTRTGLILTLQEMGADIEIIDPRLAGGED | 300 |
| Query 301 | VADLRVRSSTLKGVTVPEDRAPSMIDEYPILavaaafaegaTVMNGLEELRVKESDRLSA | 360 |
| Sbjct 301 | VADLRV+SS LKGVTVP +RAPSMIDEYP+LA+AA+FAEG T+M+GL+ELRVKESDRL+A VADLRVKSSRLKGVTVPPERAPSMIDEYPVLAIAASFAEGETIMDGLDELRVKESDRLAA | 360 |
| Query 361 | VANGLKLNGVDCDEGETSLVVRGRPDGKGLGNASGAAVATHLDHRIAMSFLVMGLVSENP | 420 |
| Sbjct 361 | VA GL+ NGVDC EGE SL VRGRPDG+GLG G VATHLDHRIAMSFLVMGL SE P VARGLEANGVDCTEGEMSLTVRGRPDGRGLG---GGTVATHLDHRIAMSFLVMGLASEKP | 417 |
| Query 421  Sbjct 418 | VTVDDATMIATSFPEFMDLMAGLGAKIELSDTK 453 VTVDD+TMIATSFPEFM +M GLGAKIE+SD + VTVDDSTMIATSFPEFMTMMPGLGAKIEVSDVR 450 |  |

>WP_106712475.1 3-phosphoshikimate 1-carboxyvinyltransferase [Phyllobacterium brassicacearum]

PSH66809.1 3-phosphoshikimate 1-carboxyvinyltransferase [Phyllobacterium brassicacearum]

Length=451

Score = 732 bits (1889), Expect = 0.0, Method: Compositional matrix adjust. Identities = 367/447 (82%), Positives = 397/447 (89%), Gaps = 3/447 (1%)

| Query | 1 | MLHGASSRPATARKSSGLSGTVRIPGDKSISHRSFMFGGLASGETRITGLLEGEDVINTG | 60 |
| --- | --- | --- | --- |
| Sbjct | 1 | M H A RPA AR+S LSG +RIPGDKSISHRSFMFGGLA+GETRITGLLEGEDVINTG MSHSALPRPAIARRSKALSGDIRIPGDKSISHRSFMFGGLAAGETRITGLLEGEDVINTG | 60 |
| Query | 61 | KAMQAMGARIRKEGDTWIIDGVGNGGLLAPEAPLDFGNAATGCRLTMGLVGVYDFDSTFI | 120 |
| Sbjct | 61 | +AMQAMGA+I KEGD W+I G GNG LL PEAPLDFGNA TG RLTMGLVG YD ++TFI RAMQAMGAKITKEGDVWVIQGTGNGCLLQPEAPLDFGNAGTGARLTMGLVGTYDMETTFI | 120 |
| Query | 121 | GDASLTKRPMGRVLNPLREMGVQVKSEDGDRLPVTLRGPKTPTPITYRVPMASAQVKSAV | 180 |
| Sbjct | 121 | GDASL+KRPMGRVLNPLREMGVQV + +GDR+P+TLRGPK PITYRVPMASAQVKSAV GDASLSKRPMGRVLNPLREMGVQVTASEGDRMPLTLRGPKVAAPITYRVPMASAQVKSAV | 180 |
| Query | 181 | LLAGLNTPGITTVIEPIMTRDHTEKMLQGFGANLTVETDADGVRTIRLEGRGKLTGQVID | 240 |
| Sbjct | 181 | LLAGLNTPGITTVIEP+MTRDHTEKMLQGFGANL+VETD D VR IR+EG+GKLTGQ ID LLAGLNTPGITTVIEPVMTRDHTEKMLQGFGANLSVETDRDNVRHIRIEGQGKLTGQTID | 240 |
| Query | 241 | VPGDPSSTAFPLVAALLVPGSDVTILNVLMNPTRTGLILTLQEMGADIEVINPRLAGGED | 300 |
| Sbjct | 241 | VPGDPSSTAFPLVAALLVPGSD+TI NVLMNPTRTGLILTLQEMGA+IE++N RLAGGED VPGDPSSTAFPLVAALLVPGSDITIRNVLMNPTRTGLILTLQEMGANIEILNARLAGGED | 300 |
| Query | 301 | VADLRVRSSTLKGVTVPEDRAPSMIDEYPILavaaafaegaTVMNGLEELRVKESDRLSA | 360 |
| Sbjct | 301 | VADLRVR+S LKGV VP +RAPSMIDEYP+LAVAA A G TVMNGLEELRVKESDRLSA VADLRVRASELKGVNVPAERAPSMIDEYPVLAVAACLASGETVMNGLEELRVKESDRLSA | 360 |
| Query | 361 | VANGLKLNGVDCDEGETSLVVRGRPDGKGLGNASGAAVATHLDHRIAMSFLVMGLVSENP | 420 |
| Sbjct | 361 | VANGLK NGVDC EG SL VRGRPDGKG+G G V THLDHRIAMSFLVMGL +E P VANGLKANGVDCTEGPESLSVRGRPDGKGIG---GGTVETHLDHRIAMSFLVMGLAAEKP | 417 |
| Query  Sbjct | 421  418 | VTVDDATMIATSFPEFMDLMAGLGAKI 447 V+VDD+TMIATSFPEFMD+M G+GAKI VSVDDSTMIATSFPEFMDMMTGMGAKI 444 |  |

>WP_104756599.1 3-phosphoshikimate 1-carboxyvinyltransferase [Ochrobactrum oryzae] PQA72563.1 3-phosphoshikimate 1-carboxyvinyltransferase [Ochrobactrum oryzae]

Length=450

Score = 732 bits (1889), Expect = 0.0, Method: Compositional matrix adjust.

| Identities | = 371/453 (82%), Positives = 407/453 (90%), Gaps = 3/453 (1%) |  |
| --- | --- | --- |
| Query 1 | MLHGASSRPATARKSSGLSGTVRIPGDKSISHRSFMFGGLASGETRITGLLEGEDVINTG | 60 |
| Sbjct 1 | M H AS +PATAR+S L+G +RIPGDKSISHRSFMFGGLASGETRITGLLEGEDVINTG MSHSASPKPATARRSEALTGEIRIPGDKSISHRSFMFGGLASGETRITGLLEGEDVINTG | 60 |
| Query 61 | KAMQAMGARIRKEGDTWIIDGVGNGGLLAPEAPLDFGNAATGCRLTMGLVGVYDFDSTFI | 120 |
| Sbjct 61 | +AMQAMGA+IRK+GD WII+GVGNG LL PEA LDFGNA TG RLTMGLVG YD ++FI RAMQAMGAKIRKDGDVWIINGVGNGCLLQPEAALDFGNAGTGARLTMGLVGTYDMKTSFI | 120 |
| Query 121 | GDASLTKRPMGRVLNPLREMGVQVKSEDGDRLPVTLRGPKTPTPITYRVPMASAQVKSAV | 180 |
| Sbjct 121 | GDASLT RPMGRVLNPLREMGVQV++ +GDR+P+TL GP+T PITYRVPMASAQVKSAV GDASLTSRPMGRVLNPLREMGVQVEAAEGDRMPLTLIGPRTANPITYRVPMASAQVKSAV | 180 |
| Query 181 | LLAGLNTPGITTVIEPIMTRDHTEKMLQGFGANLTVETDADGVRTIRLEGRGKLTGQVID | 240 |
| Sbjct 181 | LLAGLNTPG+TTVIEP+MTRDHTEKMLQGFGA+L+VETD DGVR IR+ G+GKLTGQ ID LLAGLNTPGVTTVIEPVMTRDHTEKMLQGFGADLSVETDKDGVRHIRITGQGKLTGQTID | 240 |
| Query 241 | VPGDPSSTAFPLVAALLVPGSDVTILNVLMNPTRTGLILTLQEMGADIEVINPRLAGGED | 300 |
| Sbjct 241 | VPGDPSSTAFPLVAALLV GSDVTI NVLMNPTRTGL++TLQEMGADIEV N RLAGGED VPGDPSSTAFPLVAALLVEGSDVTIRNVLMNPTRTGLVMTLQEMGADIEVFNERLAGGED | 300 |
| Query 301 | VADLRVRSSTLKGVTVPEDRAPSMIDEYPILavaaafaegaTVMNGLEELRVKESDRLSA | 360 |
| Sbjct 301 | VADLRVR+S LKGV VP +RAPSMIDEYP+LA+AA+FAEG TVM+GL+ELRVKESDRL+A VADLRVRASRLKGVVVPPERAPSMIDEYPVLAIAASFAEGETVMDGLDELRVKESDRLAA | 360 |
| Query 361 | VANGLKLNGVDCDEGETSLVVRGRPDGKGLGNASGAAVATHLDHRIAMSFLVMGLVSENP | 420 |
| Sbjct 361 | VA GL+ NGVDC EGE SL VRGRPDGKGLG G VATHLDHRIAMSFLVMGL SE P VARGLEANGVDCTEGEMSLTVRGRPDGKGLG---GGTVATHLDHRIAMSFLVMGLASEKP | 417 |
| Query 421  Sbjct 418 | VTVDDATMIATSFPEFMDLMAGLGAKIELSDTK 453 VTVDD+TMIATSFPEFMD+M GLGAKIELSD + VTVDDSTMIATSFPEFMDMMPGLGAKIELSDAR 450 |  |

>WP_071632039.1 3-phosphoshikimate 1-carboxyvinyltransferase [Ochrobactrum cytisi] OIS93083.1 3-phosphoshikimate 1-carboxyvinyltransferase [Ochrobactrum cytisi]

Length=450

Score = 732 bits (1889), Expect = 0.0, Method: Compositional matrix adjust.

| Identities | = 371/453 (82%), Positives = 406/453 (90%), Gaps = 3/453 (1%) |  |
| --- | --- | --- |
| Query 1 | MLHGASSRPATARKSSGLSGTVRIPGDKSISHRSFMFGGLASGETRITGLLEGEDVINTG | 60 |
| Sbjct 1 | M H AS +PATAR+S L+G +RIPGDKSISHRSFMFGGLASGET ITGLLEGEDVINTG MSHSASPKPATARRSEALTGEIRIPGDKSISHRSFMFGGLASGETHITGLLEGEDVINTG | 60 |
| Query 61 | KAMQAMGARIRKEGDTWIIDGVGNGGLLAPEAPLDFGNAATGCRLTMGLVGVYDFDSTFI | 120 |
| Sbjct 61 | +AMQAMGA+IRK+GD WII+GVGNG LL PEA LDFGNA TG RLTMGLVG YD ++FI RAMQAMGAKIRKDGDAWIINGVGNGCLLQPEAALDFGNAGTGARLTMGLVGTYDMKTSFI | 120 |
| Query 121 | GDASLTKRPMGRVLNPLREMGVQVKSEDGDRLPVTLRGPKTPTPITYRVPMASAQVKSAV | 180 |
| Sbjct 121 | GDASL+KRPMGRVLNPLREMGVQV++ +GDR+P+TL GPKT PITYRVPMASAQVKSAV GDASLSKRPMGRVLNPLREMGVQVEAAEGDRMPLTLIGPKTANPITYRVPMASAQVKSAV | 180 |
| Query 181 | LLAGLNTPGITTVIEPIMTRDHTEKMLQGFGANLTVETDADGVRTIRLEGRGKLTGQVID | 240 |
| Sbjct 181 | LLAGLNTPG+TTVIEP+MTRDHTEKMLQGFGA+L+VETD DGVR IR+ G+GKL GQ ID LLAGLNTPGVTTVIEPVMTRDHTEKMLQGFGADLSVETDKDGVRHIRITGQGKLIGQTID | 240 |
| Query 241 | VPGDPSSTAFPLVAALLVPGSDVTILNVLMNPTRTGLILTLQEMGADIEVINPRLAGGED | 300 |
| Sbjct 241 | VPGDPSSTAFPLVAALLV GSDVTI NVLMNPTRTGLILTLQEMGADIEV+N RLAGGED VPGDPSSTAFPLVAALLVEGSDVTIRNVLMNPTRTGLILTLQEMGADIEVLNARLAGGED | 300 |
| Query 301 | VADLRVRSSTLKGVTVPEDRAPSMIDEYPILavaaafaegaTVMNGLEELRVKESDRLSA | 360 |
| Sbjct 301 | VADLRV++S LKGV VP RAPSMIDEYP+LA+AA+FAEG TVM+GL+ELRVKESDRL+A VADLRVKASKLKGVVVPSGRAPSMIDEYPVLAIAASFAEGETVMDGLDELRVKESDRLAA | 360 |
| Query 361 | VANGLKLNGVDCDEGETSLVVRGRPDGKGLGNASGAAVATHLDHRIAMSFLVMGLVSENP | 420 |
| Sbjct 361 | VA GL+ NGVDC EGE SL VRGRPDGKGLG G VATHLDHRIAMSFLVMGL SE P VARGLEANGVDCTEGEMSLTVRGRPDGKGLG---GGTVATHLDHRIAMSFLVMGLASEKP | 417 |
| Query 421  Sbjct 418 | VTVDDATMIATSFPEFMDLMAGLGAKIELSDTK 453 VTVDD+TMIATSFPEFMD+M GLGAKIELSD + VTVDDSTMIATSFPEFMDMMPGLGAKIELSDAR 450 |  |

>WP_151658442.1 3-phosphoshikimate 1-carboxyvinyltransferase [Brucella intermedia] KAB2713336.1 3-phosphoshikimate 1-carboxyvinyltransferase [Brucella intermedia] MBA8851700.1 3-phosphoshikimate 1-carboxyvinyltransferase [Brucella intermedia] NYD83074.1 3-phosphoshikimate 1-carboxyvinyltransferase [Brucella intermedia]

Length=449

Score = 731 bits (1888), Expect = 0.0, Method: Compositional matrix adjust.

| Identities | = 372/450 (83%), Positives = 404/450 (90%), Gaps = 3/450 (1%) |  |
| --- | --- | --- |
| Query 1 | MLHGASSRPATARKSSGLSGTVRIPGDKSISHRSFMFGGLASGETRITGLLEGEDVINTG | 60 |
| Sbjct 1 | M H AS +PATAR+S L+G +RIPGDKSISHRSFMFGGLASGETRITGLLEGEDVINTG MSHSASPKPATARRSEALTGEIRIPGDKSISHRSFMFGGLASGETRITGLLEGEDVINTG | 60 |
| Query 61 | KAMQAMGARIRKEGDTWIIDGVGNGGLLAPEAPLDFGNAATGCRLTMGLVGVYDFDSTFI | 120 |
| Sbjct 61 | +AMQAMGA+IRKEGD WII+GVGNG LL PEA LDFGNA TG RLTMGLVG YD ++FI RAMQAMGAKIRKEGDVWIINGVGNGCLLQPEAALDFGNAGTGARLTMGLVGTYDMKTSFI | 120 |
| Query 121 | GDASLTKRPMGRVLNPLREMGVQVKSEDGDRLPVTLRGPKTPTPITYRVPMASAQVKSAV | 180 |
| Sbjct 121 | GDASL+KRPMGRVLNPLREMGVQV++ DGDR+P+TL GPKT PITYRVPMASAQVKSAV GDASLSKRPMGRVLNPLREMGVQVEAADGDRMPLTLIGPKTANPITYRVPMASAQVKSAV | 180 |
| Query 181 | LLAGLNTPGITTVIEPIMTRDHTEKMLQGFGANLTVETDADGVRTIRLEGRGKLTGQVID | 240 |
| Sbjct 181 | LLAGLNTPG+TTVIEP+MTRDHTEKMLQGFGA+LTVETD DGVR IR+ +GKL GQ ID LLAGLNTPGVTTVIEPVMTRDHTEKMLQGFGADLTVETDKDGVRHIRITSQGKLVGQTID | 240 |
| Query 241 | VPGDPSSTAFPLVAALLVPGSDVTILNVLMNPTRTGLILTLQEMGADIEVINPRLAGGED | 300 |
| Sbjct 241 | VPGDPSSTAFPLVAALLV GSDVTI NVLMNPTRTGLILTLQEMGADIEV+N RLAGGED VPGDPSSTAFPLVAALLVEGSDVTIRNVLMNPTRTGLILTLQEMGADIEVLNARLAGGED | 300 |
| Query 301 | VADLRVRSSTLKGVTVPEDRAPSMIDEYPILavaaafaegaTVMNGLEELRVKESDRLSA VADLRVR+S LKGV VP +RAPSMIDEYP+LA+AA+FAEG TVM+GL+ELRVKESDRL+A | 360 |

| Sbjct | 301 | VADLRVRASKLKGVVVPPERAPSMIDEYPVLAIAASFAEGETVMDGLDELRVKESDRLAA | 360 |
| --- | --- | --- | --- |
| Query | 361 | VANGLKLNGVDCDEGETSLVVRGRPDGKGLGNASGAAVATHLDHRIAMSFLVMGLVSENP | 420 |
| Sbjct | 361 | VA GL+ NGVDC EGE SL VRGRPDGKGLG G VATHLDHRIAMSFLVMGL +E P VARGLEANGVDCTEGEMSLTVRGRPDGKGLG---GGTVATHLDHRIAMSFLVMGLAAEKP | 417 |
| Query  Sbjct | 421  418 | VTVDDATMIATSFPEFMDLMAGLGAKIELS 450 VTVDD+ MIATSFPEFMD+M GLGAKIELS VTVDDSNMIATSFPEFMDMMPGLGAKIELS 447 |  |

>TDQ32008.1 3-phosphoshikimate 1-carboxyvinyltransferase [Phyllobacterium brassicacearum]

Length=470

Score = 731 bits (1888), Expect = 0.0, Method: Compositional matrix adjust.

| Identities | = 367/447 (82%), Positives = 397/447 (89%), Gaps = 3/447 (1%) |  |
| --- | --- | --- |
| Query 1 | MLHGASSRPATARKSSGLSGTVRIPGDKSISHRSFMFGGLASGETRITGLLEGEDVINTG | 60 |
| Sbjct 20 | M H A RPA AR+S LSG +RIPGDKSISHRSFMFGGLA+GETRITGLLEGEDVINTG MSHSALPRPAIARRSKALSGDIRIPGDKSISHRSFMFGGLAAGETRITGLLEGEDVINTG | 79 |
| Query 61 | KAMQAMGARIRKEGDTWIIDGVGNGGLLAPEAPLDFGNAATGCRLTMGLVGVYDFDSTFI | 120 |
| Sbjct 80 | +AMQAMGA+I KEGD W+I G GNG LL PEAPLDFGNA TG RLTMGLVG YD ++TFI RAMQAMGAKITKEGDVWVIQGTGNGCLLQPEAPLDFGNAGTGARLTMGLVGTYDMETTFI | 139 |
| Query 121 | GDASLTKRPMGRVLNPLREMGVQVKSEDGDRLPVTLRGPKTPTPITYRVPMASAQVKSAV | 180 |
| Sbjct 140 | GDASL+KRPMGRVLNPLREMGVQV + +GDR+P+TLRGPK PITYRVPMASAQVKSAV GDASLSKRPMGRVLNPLREMGVQVTASEGDRMPLTLRGPKVAAPITYRVPMASAQVKSAV | 199 |
| Query 181 | LLAGLNTPGITTVIEPIMTRDHTEKMLQGFGANLTVETDADGVRTIRLEGRGKLTGQVID | 240 |
| Sbjct 200 | LLAGLNTPGITTVIEP+MTRDHTEKMLQGFGANL+VETD D VR IR+EG+GKLTGQ ID LLAGLNTPGITTVIEPVMTRDHTEKMLQGFGANLSVETDRDNVRHIRIEGQGKLTGQTID | 259 |
| Query 241 | VPGDPSSTAFPLVAALLVPGSDVTILNVLMNPTRTGLILTLQEMGADIEVINPRLAGGED | 300 |
| Sbjct 260 | VPGDPSSTAFPLVAALLVPGSD+TI NVLMNPTRTGLILTLQEMGA+IE++N RLAGGED VPGDPSSTAFPLVAALLVPGSDITIRNVLMNPTRTGLILTLQEMGANIEILNARLAGGED | 319 |
| Query 301 | VADLRVRSSTLKGVTVPEDRAPSMIDEYPILavaaafaegaTVMNGLEELRVKESDRLSA | 360 |
| Sbjct 320 | VADLRVR+S LKGV VP +RAPSMIDEYP+LAVAA A G TVMNGLEELRVKESDRLSA VADLRVRASELKGVNVPAERAPSMIDEYPVLAVAACLASGETVMNGLEELRVKESDRLSA | 379 |
| Query 361 | VANGLKLNGVDCDEGETSLVVRGRPDGKGLGNASGAAVATHLDHRIAMSFLVMGLVSENP | 420 |
| Sbjct 380 | VANGLK NGVDC EG SL VRGRPDGKG+G G V THLDHRIAMSFLVMGL +E P VANGLKANGVDCTEGPESLSVRGRPDGKGIG---GGTVETHLDHRIAMSFLVMGLAAEKP | 436 |
| Query 421  Sbjct 437 | VTVDDATMIATSFPEFMDLMAGLGAKI 447 V+VDD+TMIATSFPEFMD+M G+GAKI VSVDDSTMIATSFPEFMDMMTGMGAKI 463 |  |

>WP_010657837.1 MULTISPECIES: 3-phosphoshikimate 1-carboxyvinyltransferase [Ochrobactrum] EXL07608.1 3-phosphoshikimate 1-carboxyvinyltransferase [Ochrobactrum anthropi] KAB2741199.1 3-phosphoshikimate 1-carboxyvinyltransferase [Ochrobactrum anthropi] KAB2759104.1 3-phosphoshikimate 1-carboxyvinyltransferase [Ochrobactrum anthropi] KAB2768735.1 3-phosphoshikimate 1-carboxyvinyltransferase [Ochrobactrum anthropi] KAB2801448.1 3-phosphoshikimate 1-carboxyvinyltransferase [Ochrobactrum anthropi]

Length=450

Score = 731 bits (1887), Expect = 0.0, Method: Compositional matrix adjust.

| Identities | = 371/453 (82%), Positives = 407/453 (90%), Gaps = 3/453 (1%) |  |
| --- | --- | --- |
| Query 1 | MLHGASSRPATARKSSGLSGTVRIPGDKSISHRSFMFGGLASGETRITGLLEGEDVINTG | 60 |
| Sbjct 1 | M H A +PATAR+S L+G +RIPGDKSISHRSFMFGGLASGETRITGLLEGEDVINTG MSHSAPPKPATARRSEALTGEIRIPGDKSISHRSFMFGGLASGETRITGLLEGEDVINTG | 60 |
| Query 61 | KAMQAMGARIRKEGDTWIIDGVGNGGLLAPEAPLDFGNAATGCRLTMGLVGVYDFDSTFI | 120 |
| Sbjct 61 | +AMQAMGA+IRK+GD WII+GVGNG LL PEA LDFGNA TG RLTMGLVG YD ++FI RAMQAMGAKIRKDGDAWIINGVGNGCLLQPEAALDFGNAGTGARLTMGLVGTYDMRTSFI | 120 |
| Query 121 | GDASLTKRPMGRVLNPLREMGVQVKSEDGDRLPVTLRGPKTPTPITYRVPMASAQVKSAV GDASL+KRPMGRVLNPLREMGVQV++ +GDR+P+TL GPKT PITYRVPMASAQVKSAV | 180 |

| Sbjct | 121 | GDASLSKRPMGRVLNPLREMGVQVEAAEGDRMPLTLIGPKTANPITYRVPMASAQVKSAV | 180 |
| --- | --- | --- | --- |
| Query | 181 | LLAGLNTPGITTVIEPIMTRDHTEKMLQGFGANLTVETDADGVRTIRLEGRGKLTGQVID | 240 |
| Sbjct | 181 | LLAGLNTPG+TTVIEP+MTRDHTEKMLQGFGA+L+VETD DGVR IR+ G+GKL GQ ID LLAGLNTPGVTTVIEPVMTRDHTEKMLQGFGADLSVETDKDGVRHIRITGQGKLIGQTID | 240 |
| Query | 241 | VPGDPSSTAFPLVAALLVPGSDVTILNVLMNPTRTGLILTLQEMGADIEVINPRLAGGED | 300 |
| Sbjct | 241 | VPGDPSSTAFPLVAALLV GSDVTI NVLMNPTRTGLILTLQEMGADIEV+N RLAGGED VPGDPSSTAFPLVAALLVEGSDVTIRNVLMNPTRTGLILTLQEMGADIEVLNARLAGGED | 300 |
| Query | 301 | VADLRVRSSTLKGVTVPEDRAPSMIDEYPILavaaafaegaTVMNGLEELRVKESDRLSA | 360 |
| Sbjct | 301 | VADLRV++S LKGV VP +RAPSMIDEYP+LA+AA+FAEG TVM+GL+ELRVKESDRL+A VADLRVKASKLKGVVVPPERAPSMIDEYPVLAIAASFAEGETVMDGLDELRVKESDRLAA | 360 |
| Query | 361 | VANGLKLNGVDCDEGETSLVVRGRPDGKGLGNASGAAVATHLDHRIAMSFLVMGLVSENP | 420 |
| Sbjct | 361 | VA GL+ NGVDC EGE SL VRGRPDGKGLG G VATHLDHRIAMSFLVMGL SE P VARGLEANGVDCTEGEMSLTVRGRPDGKGLG---GGTVATHLDHRIAMSFLVMGLASEKP | 417 |
| Query  Sbjct | 421  418 | VTVDDATMIATSFPEFMDLMAGLGAKIELSDTK 453 VTVDD+TMIATSFPEFMD+M GLGAKIELSD + VTVDDSTMIATSFPEFMDMMPGLGAKIELSDAR 450 |  |

>WP_105705525.1 3-phosphoshikimate 1-carboxyvinyltransferase [Ochrobactrum sp. MYb68]

PRA57719.1 3-phosphoshikimate 1-carboxyvinyltransferase [Ochrobactrum sp. MYb68]

Length=450

Score = 731 bits (1887), Expect = 0.0, Method: Compositional matrix adjust.

| Identities | = 371/453 (82%), Positives = 406/453 (90%), Gaps = 3/453 (1%) |  |
| --- | --- | --- |
| Query 1 | MLHGASSRPATARKSSGLSGTVRIPGDKSISHRSFMFGGLASGETRITGLLEGEDVINTG | 60 |
| Sbjct 1 | M H AS +PA AR S L G +RIPGDKSISHRSFMFGGLASGETRITGLLEGEDVINTG MSHSASPKPAIARHSEALKGEIRIPGDKSISHRSFMFGGLASGETRITGLLEGEDVINTG | 60 |
| Query 61 | KAMQAMGARIRKEGDTWIIDGVGNGGLLAPEAPLDFGNAATGCRLTMGLVGVYDFDSTFI | 120 |
| Sbjct 61 | +AMQAMGA+IRK+G+ WII+GVGNG LL PEAPLDFGNA TG RLTMGLVG YD +TFI RAMQAMGAKIRKDGNVWIINGVGNGCLLQPEAPLDFGNAGTGARLTMGLVGTYDMTTTFI | 120 |
| Query 121 | GDASLTKRPMGRVLNPLREMGVQVKSEDGDRLPVTLRGPKTPTPITYRVPMASAQVKSAV | 180 |
| Sbjct 121 | GDASLT RPMGRVLNPLREMGVQVK+ +GDRLP+TL GPKT PI+YRVPMASAQVKSAV GDASLTSRPMGRVLNPLREMGVQVKAAEGDRLPLTLTGPKTANPISYRVPMASAQVKSAV | 180 |
| Query 181 | LLAGLNTPGITTVIEPIMTRDHTEKMLQGFGANLTVETDADGVRTIRLEGRGKLTGQVID | 240 |
| Sbjct 181 | LLAGLNTPG+TTVIEP+MTRDHTEKMLQGFGA+LTVE+D DGVR IR+ G+GKL GQ ID LLAGLNTPGVTTVIEPVMTRDHTEKMLQGFGADLTVESDKDGVRHIRITGQGKLIGQTID | 240 |
| Query 241 | VPGDPSSTAFPLVAALLVPGSDVTILNVLMNPTRTGLILTLQEMGADIEVINPRLAGGED | 300 |
| Sbjct 241 | VPGDPSSTAFPLVAALLV GSDVTI NVLMNPTRTGLILTLQEMGADIE+I+PRLAGGED VPGDPSSTAFPLVAALLVEGSDVTIRNVLMNPTRTGLILTLQEMGADIEIIDPRLAGGED | 300 |
| Query 301 | VADLRVRSSTLKGVTVPEDRAPSMIDEYPILavaaafaegaTVMNGLEELRVKESDRLSA | 360 |
| Sbjct 301 | VADLRV+SS LKGVTVP +RAPSMIDEYP+LA+AA+FAEG T+M+GL+ELRVKESDRL+A VADLRVKSSRLKGVTVPPERAPSMIDEYPVLAIAASFAEGETIMDGLDELRVKESDRLAA | 360 |
| Query 361 | VANGLKLNGVDCDEGETSLVVRGRPDGKGLGNASGAAVATHLDHRIAMSFLVMGLVSENP | 420 |
| Sbjct 361 | VA GL+ NGVDC EGE SL VRGRPDG+GLG G VATHLDHRIAMSFLVMGL SE P VARGLEANGVDCTEGEMSLTVRGRPDGRGLG---GGTVATHLDHRIAMSFLVMGLASEKP | 417 |
| Query 421  Sbjct 418 | VTVDDATMIATSFPEFMDLMAGLGAKIELSDTK 453 VTVDD+TMIATSFPEFM +M GLGAKIE+SD + VTVDDSTMIATSFPEFMTMMPGLGAKIEVSDVR 450 |  |

>WP_187543617.1 3-phosphoshikimate 1-carboxyvinyltransferase [Ochrobactrum sp. Marseille-Q0166]

MBC8716207.1 3-phosphoshikimate 1-carboxyvinyltransferase [Ochrobactrum sp. Marseille-Q0166]

Length=450

Score = 731 bits (1887), Expect = 0.0, Method: Compositional matrix adjust.

| Identities | = 369/453 (81%), Positives = 409/453 (90%), Gaps = 3/453 (1%) |  |
| --- | --- | --- |
| Query 1 | MLHGASSRPATARKSSGLSGTVRIPGDKSISHRSFMFGGLASGETRITGLLEGEDVINTG | 60 |
| Sbjct 1 | M H A+ +PATAR+S L+G +RIPGDKSISHRSFMFGGLA+GETRITGLLEGEDVINTG MSHSATPKPATARRSEALTGEIRIPGDKSISHRSFMFGGLAAGETRITGLLEGEDVINTG | 60 |
| Query 61 | KAMQAMGARIRKEGDTWIIDGVGNGGLLAPEAPLDFGNAATGCRLTMGLVGVYDFDSTFI | 120 |
| Sbjct 61 | +AMQAMGA+IRK+GD WII+GVGNG LL PE+PLDFGNA G RLTMGLVG YD +TF+ RAMQAMGAKIRKDGDIWIINGVGNGCLLQPESPLDFGNAGPGARLTMGLVGTYDMTTTFV | 120 |
| Query 121 | GDASLTKRPMGRVLNPLREMGVQVKSEDGDRLPVTLRGPKTPTPITYRVPMASAQVKSAV | 180 |
| Sbjct 121 | GDASLT RPMGRVLNPLREMGVQVK+ +GDRLP+TL GPKT PI+YRVPMASAQVKSAV GDASLTSRPMGRVLNPLREMGVQVKAAEGDRLPLTLTGPKTANPISYRVPMASAQVKSAV | 180 |
| Query 181 | LLAGLNTPGITTVIEPIMTRDHTEKMLQGFGANLTVETDADGVRTIRLEGRGKLTGQVID | 240 |
| Sbjct 181 | LLAGLNTPG+TTVIEP+MTRDHTEKMLQGFGA+LTVETD DGVR IR+ G+GKLTGQ ID LLAGLNTPGVTTVIEPVMTRDHTEKMLQGFGADLTVETDKDGVRHIRIVGQGKLTGQTID | 240 |
| Query 241 | VPGDPSSTAFPLVAALLVPGSDVTILNVLMNPTRTGLILTLQEMGADIEVINPRLAGGED | 300 |
| Sbjct 241 | VPGDPSSTAFPLVAALLV GSDVTI NVLMNPTRTGLILTLQEMGADIE+I+PRLAGGED VPGDPSSTAFPLVAALLVEGSDVTIRNVLMNPTRTGLILTLQEMGADIEIIDPRLAGGED | 300 |
| Query 301 | VADLRVRSSTLKGVTVPEDRAPSMIDEYPILavaaafaegaTVMNGLEELRVKESDRLSA | 360 |
| Sbjct 301 | VADLRV+SS LKG+ VP +RAPSMIDEYP+LA+AA+FAEG TVM+GL+ELRVKESDRL+A VADLRVKSSKLKGIVVPPERAPSMIDEYPVLAIAASFAEGETVMDGLDELRVKESDRLAA | 360 |
| Query 361 | VANGLKLNGVDCDEGETSLVVRGRPDGKGLGNASGAAVATHLDHRIAMSFLVMGLVSENP | 420 |
| Sbjct 361 | VA GL+ NGVDC EGE SL VRGRPDGKGLG G VATHLDHRIAMSFLVMGL +E P VARGLEANGVDCTEGEMSLTVRGRPDGKGLG---GGTVATHLDHRIAMSFLVMGLAAEKP | 417 |
| Query 421  Sbjct 418 | VTVDDATMIATSFPEFMDLMAGLGAKIELSDTK 453 VTVDD+TMIATSFPEFM +M GLGAKIE+S++K VTVDDSTMIATSFPEFMTMMPGLGAKIEVSESK 450 |  |

>WP_011982268.1 3-phosphoshikimate 1-carboxyvinyltransferase [Ochrobactrum anthropi] A6WUV8.1 RecName: Full=3-phosphoshikimate 1-carboxyvinyltransferase; AltName:

Full=5-enolpyruvylshikimate-3-phosphate synthase; Short=EPSP synthase; Short=EPSPS [Ochrobactrum anthropi ATCC 49188]

ABS12762.1 3-phosphoshikimate 1-carboxyvinyltransferase [Ochrobactrum anthropi ATCC 49188]

AIK43744.1 3-phosphoshikimate 1-carboxyvinyltransferase [Ochrobactrum anthropi] KAB2737101.1 3-phosphoshikimate 1-carboxyvinyltransferase [Ochrobactrum anthropi] KAB2753087.1 3-phosphoshikimate 1-carboxyvinyltransferase [Ochrobactrum anthropi] KAB2763587.1 3-phosphoshikimate 1-carboxyvinyltransferase [Ochrobactrum anthropi]

Length=450

Score = 731 bits (1886), Expect = 0.0, Method: Compositional matrix adjust.

| Identities | = 372/453 (82%), Positives = 407/453 (90%), Gaps = 3/453 (1%) |  |
| --- | --- | --- |
| Query 1 | MLHGASSRPATARKSSGLSGTVRIPGDKSISHRSFMFGGLASGETRITGLLEGEDVINTG | 60 |
| Sbjct 1 | M H A +PATAR+S L+G +RIPGDKSISHRSFMFGGLASGETRITGLLEGEDVINTG MSHSAPPKPATARRSEALTGEIRIPGDKSISHRSFMFGGLASGETRITGLLEGEDVINTG | 60 |
| Query 61 | KAMQAMGARIRKEGDTWIIDGVGNGGLLAPEAPLDFGNAATGCRLTMGLVGVYDFDSTFI | 120 |
| Sbjct 61 | +AMQAMGA+IRK+GD WII+GVGNG LL PEA LDFGNA TG RLTMGLVG YD ++FI RAMQAMGAKIRKDGDAWIINGVGNGCLLQPEAALDFGNAGTGARLTMGLVGTYDMRTSFI | 120 |
| Query 121 | GDASLTKRPMGRVLNPLREMGVQVKSEDGDRLPVTLRGPKTPTPITYRVPMASAQVKSAV | 180 |
| Sbjct 121 | GDASL+KRPMGRVLNPLREMGVQV++ +GDR+P+TL GPKT PITYRVPMASAQVKSAV GDASLSKRPMGRVLNPLREMGVQVEAAEGDRMPLTLIGPKTANPITYRVPMASAQVKSAV | 180 |
| Query 181 | LLAGLNTPGITTVIEPIMTRDHTEKMLQGFGANLTVETDADGVRTIRLEGRGKLTGQVID | 240 |
| Sbjct 181 | LLAGLNTPG+TTVIEP+MTRDHTEKMLQGFGA+L+VETD DGVR IR+ G+GKL GQ ID LLAGLNTPGVTTVIEPVMTRDHTEKMLQGFGADLSVETDKDGVRHIRITGQGKLIGQTID | 240 |
| Query 241 | VPGDPSSTAFPLVAALLVPGSDVTILNVLMNPTRTGLILTLQEMGADIEVINPRLAGGED | 300 |
| Sbjct 241 | VPGDPSSTAFPLVAALLV GSDVTI NVLMNPTRTGLILTLQEMGADIEV+N RLAGGED VPGDPSSTAFPLVAALLVEGSDVTIRNVLMNPTRTGLILTLQEMGADIEVLNARLAGGED | 300 |
| Query 301 | VADLRVRSSTLKGVTVPEDRAPSMIDEYPILavaaafaegaTVMNGLEELRVKESDRLSA VADLRV++S LKGV VP +RAPSMIDEYP+LA+AAAFAEG TVM+GL+ELRVKESDRL+A | 360 |

| Sbjct | 301 | VADLRVKASKLKGVVVPPERAPSMIDEYPVLAIAAAFAEGETVMDGLDELRVKESDRLAA | 360 |
| --- | --- | --- | --- |
| Query | 361 | VANGLKLNGVDCDEGETSLVVRGRPDGKGLGNASGAAVATHLDHRIAMSFLVMGLVSENP | 420 |
| Sbjct | 361 | VA GL+ NGVDC EGE SL VRGRPDGKGLG G VATHLDHRIAMSFLVMGL SE P VARGLEANGVDCTEGEMSLTVRGRPDGKGLG---GGTVATHLDHRIAMSFLVMGLASEKP | 417 |
| Query  Sbjct | 421  418 | VTVDDATMIATSFPEFMDLMAGLGAKIELSDTK 453 VTVDD+TMIATSFPEFMD+M GLGAKIELSD + VTVDDSTMIATSFPEFMDMMPGLGAKIELSDAR 450 |  |

>WP_196108699.1 MULTISPECIES: 3-phosphoshikimate 1-carboxyvinyltransferase [unclassified Ochrobactrum]

Length=450

Score = 730 bits (1885), Expect = 0.0, Method: Compositional matrix adjust.

| Identities | = 371/453 (82%), Positives = 406/453 (90%), Gaps = 3/453 (1%) |  |
| --- | --- | --- |
| Query 1 | MLHGASSRPATARKSSGLSGTVRIPGDKSISHRSFMFGGLASGETRITGLLEGEDVINTG | 60 |
| Sbjct 1 | M H AS +PATAR+S L+G +RIPGDKSISHRSFMFGGLASGETRITGLLEGEDVINTG MSHSASPKPATARRSEALTGEIRIPGDKSISHRSFMFGGLASGETRITGLLEGEDVINTG | 60 |
| Query 61 | KAMQAMGARIRKEGDTWIIDGVGNGGLLAPEAPLDFGNAATGCRLTMGLVGVYDFDSTFI | 120 |
| Sbjct 61 | +AMQAMGA+IRK+GD WII+GVGNG LL EA LDFGNA TG RLTMGLVG YD ++FI RAMQAMGAKIRKDGDVWIINGVGNGCLLQAEAALDFGNAGTGARLTMGLVGTYDMKTSFI | 120 |
| Query 121 | GDASLTKRPMGRVLNPLREMGVQVKSEDGDRLPVTLRGPKTPTPITYRVPMASAQVKSAV | 180 |
| Sbjct 121 | GDASL+KRPMGRVLNPLREMGVQV++ DGDR+P+TL GPKT PITYRVPMASAQVKSAV GDASLSKRPMGRVLNPLREMGVQVEAADGDRMPLTLIGPKTANPITYRVPMASAQVKSAV | 180 |
| Query 181 | LLAGLNTPGITTVIEPIMTRDHTEKMLQGFGANLTVETDADGVRTIRLEGRGKLTGQVID | 240 |
| Sbjct 181 | LLAGLNTPG+TTVIEP+MTRDHTEKMLQGFGA+LTVETD DGVR IR+ G+GKL GQ ID LLAGLNTPGVTTVIEPVMTRDHTEKMLQGFGADLTVETDKDGVRHIRITGQGKLVGQTID | 240 |
| Query 241 | VPGDPSSTAFPLVAALLVPGSDVTILNVLMNPTRTGLILTLQEMGADIEVINPRLAGGED | 300 |
| Sbjct 241 | VPGDPSSTAFPLVAALLV GSDVTI NVLMNPTRTGLILTLQEMGADIEV+N RLAGGED VPGDPSSTAFPLVAALLVEGSDVTIRNVLMNPTRTGLILTLQEMGADIEVLNARLAGGED | 300 |
| Query 301 | VADLRVRSSTLKGVTVPEDRAPSMIDEYPILavaaafaegaTVMNGLEELRVKESDRLSA | 360 |
| Sbjct 301 | VADLRVR+S LKGV VP +RAPSMIDEYP+LA+AA+FAEG TVM+GL+ELRVKESDRL+A VADLRVRASKLKGVVVPPERAPSMIDEYPVLAIAASFAEGETVMDGLDELRVKESDRLAA | 360 |
| Query 361 | VANGLKLNGVDCDEGETSLVVRGRPDGKGLGNASGAAVATHLDHRIAMSFLVMGLVSENP | 420 |
| Sbjct 361 | VA GL+ NGVDC EGE SL VRGRPDGKGLG G VATHLDHRIAMSFLVMGL +E P VARGLEANGVDCTEGEMSLTVRGRPDGKGLG---GGTVATHLDHRIAMSFLVMGLAAEKP | 417 |
| Query 421  Sbjct 418 | VTVDDATMIATSFPEFMDLMAGLGAKIELSDTK 453 VTVDD+ MIATSFPEFMD+M GLGAKIELS+ + VTVDDSNMIATSFPEFMDMMPGLGAKIELSNAQ 450 |  |

>AWH63006.1 5-enolpyruvylshikimate-3-phosphate (EPSP) synthase [synthetic construct]

Length=475

Score = 730 bits (1885), Expect = 0.0, Method: Compositional matrix adjust.

| Identities | = 371/449 (83%), Positives = 403/449 (90%), Gaps = 1/449 (0%) |  |
| --- | --- | --- |
| Query 1 | MLHGASSRPATARKSSGLSGTVRIPGDKSISHRSFMFGGLASGETRITGLLEGEDVINTG | 60 |
| Sbjct 22 | M H SRPATAR+S+ LSGT+R+PGDKSISHR+FMFGGLASGETRITGLLEGEDV+NTG MPHDTHSRPATARRSTNLSGTIRVPGDKSISHRAFMFGGLASGETRITGLLEGEDVLNTG | 81 |
| Query 61 | KAMQAMGARIRKEGDTWIIDGVGNGGLLAPEAPLDFGNAATGCRLTMGLVGVYDFDSTFI | 120 |
| Sbjct 82 | KAM+AMGA+I K GD WII GVGNG LLAPEAPLDFGNA TG RLTMGLVGVY F+STFI KAMRAMGAKIEKAGDEWIIKGVGNGALLAPEAPLDFGNAGTGSRLTMGLVGVYHFESTFI | 141 |
| Query 121 | GDASLTKRPMGRVLNPLREMGVQVKSEDGDRLPVTLRGPKTPTPITYRVPMASAQVKSAV | 180 |
| Sbjct 142 | GDASL+KRPMGR+L+PLR+MGVQVKS +GDRLPVTL GP+T PITYRVPMASAQVKSAV GDASLSKRPMGRILDPLRQMGVQVKSSEGDRLPVTLHGPETANPITYRVPMASAQVKSAV | 201 |
| Query 181 | LLAGLNTPGITTVIEPIMTRDHTEKMLQGFGANLTVETDADGVRTIRLEGRGKLTGQVID LLAGLN PGIT+VIEP+MTRDHTEKML GFGA L+VETD DGVR IRL+G+GKL GQ ID | 240 |

| Sbjct | 202 | LLAGLNAPGITSVIEPVMTRDHTEKMLAGFGAELSVETDRDGVRIIRLQGQGKLFGQTID | 261 |
| --- | --- | --- | --- |
| Query | 241 | VPGDPSSTAFPLVAALLVPGSDVTILNVLMNPTRTGLILTLQEMGADIEVINPRLAGGED | 300 |
| Sbjct | 262 | VPGDPSSTAFPLVAAL+V GSD+TI NVLMNPTRTGLILTLQEMGADIE+++ RLAGGED VPGDPSSTAFPLVAALIVAGSDITIRNVLMNPTRTGLILTLQEMGADIEILDKRLAGGED | 321 |
| Query | 301 | VADLRVRSSTLKGVTVPEDRAPSMIDEYPILavaaafaegaTVMNGLEELRVKESDRLSA | 360 |
| Sbjct | 322 | VADLRVRSS LKGVTVP +RAPSMIDEYP+LAVAAAFAEG TVM GLEELRVKESDRLSA VADLRVRSSALKGVTVPAERAPSMIDEYPVLAVAAAFAEGTTVMLGLEELRVKESDRLSA | 381 |
| Query | 361 | VANGLKLNGVDCDEGETSLVVRGRPDGKGLGNASGAAVATHLDHRIAMSFLVMGLVSENP | 420 |
| Sbjct | 382 | VA GL LNGVDC EGE SL VRGRPDGKGLGNA G V THLDHRIAMSFLVMGL SE+P VAEGLTLNGVDCTEGEASLTVRGRPDGKGLGNA-GGEVKTHLDHRIAMSFLVMGLASEHP | 440 |
| Query  Sbjct | 421  441 | VTVDDATMIATSFPEFMDLMAGLGAKIEL 449 V VDD+ MIATSFPEF DLMA LGA +++ VKVDDSAMIATSFPEFFDLMAELGADMDV 469 |  |

>WP_139975008.1 3-phosphoshikimate 1-carboxyvinyltransferase [Ochrobactrum sp. CGA5]

Length=450

Score = 730 bits (1884), Expect = 0.0, Method: Compositional matrix adjust.

| Identities | = 368/453 (81%), Positives = 409/453 (90%), Gaps = 3/453 (1%) |  |
| --- | --- | --- |
| Query 1 | MLHGASSRPATARKSSGLSGTVRIPGDKSISHRSFMFGGLASGETRITGLLEGEDVINTG | 60 |
| Sbjct 1 | M H AS +PATAR+S L+G +RIPGDKSISHR+FMFGGLASGETRITGLLEGEDVINTG MSHSASPKPATARRSEALTGEIRIPGDKSISHRAFMFGGLASGETRITGLLEGEDVINTG | 60 |
| Query 61 | KAMQAMGARIRKEGDTWIIDGVGNGGLLAPEAPLDFGNAATGCRLTMGLVGVYDFDSTFI | 120 |
| Sbjct 61 | +AMQAMGA+IRK+GD WII+GVGNG LL PE LDFGNA TG RLTMGLVG YD +TFI RAMQAMGAKIRKDGDVWIINGVGNGCLLQPETALDFGNAGTGARLTMGLVGTYDMTTTFI | 120 |
| Query 121 | GDASLTKRPMGRVLNPLREMGVQVKSEDGDRLPVTLRGPKTPTPITYRVPMASAQVKSAV | 180 |
| Sbjct 121 | GDASL+KRPMGRVLNPLREMGVQV++ +GDR+P+TL GPKT PITYRVPMASAQVKSAV GDASLSKRPMGRVLNPLREMGVQVEAAEGDRMPLTLIGPKTANPITYRVPMASAQVKSAV | 180 |
| Query 181 | LLAGLNTPGITTVIEPIMTRDHTEKMLQGFGANLTVETDADGVRTIRLEGRGKLTGQVID | 240 |
| Sbjct 181 | LLAGLNTPG+TTVIEP+MTRDHTEKMLQGFGA+L+V+TD DGVR IR+ G+GKLTGQ ID LLAGLNTPGVTTVIEPVMTRDHTEKMLQGFGADLSVDTDKDGVRHIRITGQGKLTGQTID | 240 |
| Query 241 | VPGDPSSTAFPLVAALLVPGSDVTILNVLMNPTRTGLILTLQEMGADIEVINPRLAGGED | 300 |
| Sbjct 241 | VPGDPSSTAFPLVAALLV GSD+TI NVLMNPTRTGLILTLQEMGA+IE+I+PRLAGGED VPGDPSSTAFPLVAALLVEGSDITIRNVLMNPTRTGLILTLQEMGANIEIIDPRLAGGED | 300 |
| Query 301 | VADLRVRSSTLKGVTVPEDRAPSMIDEYPILavaaafaegaTVMNGLEELRVKESDRLSA | 360 |
| Sbjct 301 | VADLRV++S LKGV VP +RAPSMIDEYP+LA+AA+FAEG TVM+GL+ELRVKESDRL+A VADLRVKASKLKGVVVPPERAPSMIDEYPVLAIAASFAEGETVMDGLDELRVKESDRLAA | 360 |
| Query 361 | VANGLKLNGVDCDEGETSLVVRGRPDGKGLGNASGAAVATHLDHRIAMSFLVMGLVSENP | 420 |
| Sbjct 361 | VA GL+ NGVDC EGE SL VRGRPDG+GLG G VATHLDHRIAMSFLVMGL SE P VARGLEANGVDCTEGEMSLTVRGRPDGRGLG---GGTVATHLDHRIAMSFLVMGLASEKP | 417 |
| Query 421  Sbjct 418 | VTVDDATMIATSFPEFMDLMAGLGAKIELSDTK 453 VTVDD+TMIATSFPEFMD+M GLGAKIELSD + VTVDDSTMIATSFPEFMDMMPGLGAKIELSDAQ 450 |  |

>WP_100558340.1 MULTISPECIES: 3-phosphoshikimate 1-carboxyvinyltransferase [Ochrobactrum] PJO45788.1 3-phosphoshikimate 1-carboxyvinyltransferase [Ochrobactrum pituitosum] PRA88862.1 3-phosphoshikimate 1-carboxyvinyltransferase [Ochrobactrum sp.

MYb29]

Length=450

Score = 730 bits (1884), Expect = 0.0, Method: Compositional matrix adjust. Identities = 371/453 (82%), Positives = 405/453 (89%), Gaps = 3/453 (1%)

Query 1	MLHGASSRPATARKSSGLSGTVRIPGDKSISHRSFMFGGLASGETRITGLLEGEDVINTG 60 M H AS +PA AR S L G + IPGDKSISHRSFMFGGLASGETRITGLLEGEDVINTG

Sbjct 1	MSHSASPKPAIARHSEALKGEIHIPGDKSISHRSFMFGGLASGETRITGLLEGEDVINTG 60

| Query | 61 | KAMQAMGARIRKEGDTWIIDGVGNGGLLAPEAPLDFGNAATGCRLTMGLVGVYDFDSTFI | 120 |
| --- | --- | --- | --- |
| Sbjct | 61 | +AMQAMGA+IRK+G+ WII+GVGNG LL PEAPLDFGNA TG RLTMGLVG YD +TFI RAMQAMGAKIRKDGNVWIINGVGNGCLLQPEAPLDFGNAGTGARLTMGLVGTYDMTTTFI | 120 |
| Query | 121 | GDASLTKRPMGRVLNPLREMGVQVKSEDGDRLPVTLRGPKTPTPITYRVPMASAQVKSAV | 180 |
| Sbjct | 121 | GDASLT RPMGRVLNPLREMGVQVK+ +GDRLP+TL GPKT PI+YRVPMASAQVKSAV GDASLTSRPMGRVLNPLREMGVQVKAAEGDRLPLTLTGPKTANPISYRVPMASAQVKSAV | 180 |
| Query | 181 | LLAGLNTPGITTVIEPIMTRDHTEKMLQGFGANLTVETDADGVRTIRLEGRGKLTGQVID | 240 |
| Sbjct | 181 | LLAGLNTPG+TTVIEPIMTRDHTEKMLQGFGA+LTVE+D DGVR IR+ G+GKL GQ ID LLAGLNTPGVTTVIEPIMTRDHTEKMLQGFGADLTVESDKDGVRHIRITGQGKLIGQTID | 240 |
| Query | 241 | VPGDPSSTAFPLVAALLVPGSDVTILNVLMNPTRTGLILTLQEMGADIEVINPRLAGGED | 300 |
| Sbjct | 241 | VPGDPSSTAFPLVAALLV GSDVTI NVLMNPTRTGLILTLQEMGADIE+I+PRLAGGED VPGDPSSTAFPLVAALLVEGSDVTIRNVLMNPTRTGLILTLQEMGADIEIIDPRLAGGED | 300 |
| Query | 301 | VADLRVRSSTLKGVTVPEDRAPSMIDEYPILavaaafaegaTVMNGLEELRVKESDRLSA | 360 |
| Sbjct | 301 | VADLRV+SS LKGVTVP +RAPSMIDEYP+LA+AA+FAEG T+M+GL+ELRVKESDRL+A VADLRVKSSRLKGVTVPPERAPSMIDEYPVLAIAASFAEGETIMDGLDELRVKESDRLAA | 360 |
| Query | 361 | VANGLKLNGVDCDEGETSLVVRGRPDGKGLGNASGAAVATHLDHRIAMSFLVMGLVSENP | 420 |
| Sbjct | 361 | VA GL+ NGVDC EGE SL VRGRPDG+GLG G VATHLDHRIAMSFLVMGL SE P VARGLEANGVDCTEGEMSLTVRGRPDGRGLG---GGTVATHLDHRIAMSFLVMGLASEKP | 417 |
| Query  Sbjct | 421  418 | VTVDDATMIATSFPEFMDLMAGLGAKIELSDTK 453 VTVDD+TMIATSFPEFM +M GLGAKIE+SD + VTVDDSTMIATSFPEFMTMMPGLGAKIEVSDVR 450 |  |

>MAY63235.1 3-phosphoshikimate 1-carboxyvinyltransferase [Rhizobiales bacterium] MBA69987.1 3-phosphoshikimate 1-carboxyvinyltransferase [Rhizobiales bacterium]

Length=452

Score = 730 bits (1884), Expect = 0.0, Method: Compositional matrix adjust.

| Identities | = 371/451 (82%), Positives = 404/451 (90%), Gaps = 0/451 (0%) |  |
| --- | --- | --- |
| Query 1 | MLHGASSRPATARKSSGLSGTVRIPGDKSISHRSFMFGGLASGETRITGLLEGEDVINTG | 60 |
| Sbjct 1 | M H + RPA+AR+ + L G +RIPGDKSISHRSFMFGGLA GETRITGLLEGEDVINTG MNHISKLRPASARRGAALKGDIRIPGDKSISHRSFMFGGLARGETRITGLLEGEDVINTG | 60 |
| Query 61 | KAMQAMGARIRKEGDTWIIDGVGNGGLLAPEAPLDFGNAATGCRLTMGLVGVYDFDSTFI | 120 |
| Sbjct 61 | KAMQAMGA +RK+GDTWI+ G GNG LLAP+APLDFGNA TGCRLTMGLVGVYDFDSTFI KAMQAMGASVRKDGDTWIVTGTGNGCLLAPDAPLDFGNAGTGCRLTMGLVGVYDFDSTFI | 120 |
| Query 121 | GDASLTKRPMGRVLNPLREMGVQVKSEDGDRLPVTLRGPKTPTPITYRVPMASAQVKSAV | 180 |
| Sbjct 121 | GDASL++RPMGRVL+PLREMGVQV SE GDRLPVTL GP+ PITYRVPMASAQVKSAV GDASLSRRPMGRVLDPLREMGVQVTSEPGDRLPVTLHGPRVAAPITYRVPMASAQVKSAV | 180 |
| Query 181 | LLAGLNTPGITTVIEPIMTRDHTEKMLQGFGANLTVETDADGVRTIRLEGRGKLTGQVID | 240 |
| Sbjct 181 | LLAGLNTPG+T VIEP+MTRDHTEKML+GFGA L VETDA+GVRTIRL G+G+L GQVID LLAGLNTPGVTIVIEPVMTRDHTEKMLRGFGAELEVETDAEGVRTIRLTGQGELKGQVID | 240 |
| Query 241 | VPGDPSSTAFPLVAALLVPGSDVTILNVLMNPTRTGLILTLQEMGADIEVINPRLAGGED | 300 |
| Sbjct 241 | VPGDPSSTAFPLVAALLVPGS+V I NVLMNPTRTGLI TLQEMG DIE+++PRLAGGED VPGDPSSTAFPLVAALLVPGSEVLIRNVLMNPTRTGLIQTLQEMGGDIEILDPRLAGGED | 300 |
| Query 301 | VADLRVRSSTLKGVTVPEDRAPSMIDEYPILavaaafaegaTVMNGLEELRVKESDRLSA | 360 |
| Sbjct 301 | VADLRVR S LKGVTVP +RAPSMIDEYP+LAVAA+FAEG TVM+GL+ELRVKESDRL+A VADLRVRGSDLKGVTVPPERAPSMIDEYPVLAVAASFAEGETVMHGLDELRVKESDRLAA | 360 |
| Query 361 | VANGLKLNGVDCDEGETSLVVRGRPDGKGLGNASGAAVATHLDHRIAMSFLVMGLVSENP | 420 |
| Sbjct 361 | VA GL+LNGVDC EGE L VRGRPDGKGLGNA G AV THLDHRIAMSFLVMGL+SE P VARGLELNGVDCTEGEDWLTVRGRPDGKGLGNAKGEAVVTHLDHRIAMSFLVMGLMSEQP | 420 |
| Query 421  Sbjct 421 | VTVDDATMIATSFPEFMDLMAGLGAKIELSD 451 VTVDD+ +IATSFPEFMDLMAGLGA I D VTVDDSAIIATSFPEFMDLMAGLGADISSGD 451 |  |

>WP_140020666.1 3-phosphoshikimate 1-carboxyvinyltransferase [Ochrobactrum pecoris] MBB4093646.1 3-phosphoshikimate 1-carboxyvinyltransferase [Ochrobactrum pecoris] TNV12975.1 3-phosphoshikimate 1-carboxyvinyltransferase [Ochrobactrum pecoris]

Length=450

Score = 729 bits (1883), Expect = 0.0, Method: Compositional matrix adjust.

| Identities | = 370/453 (82%), Positives = 404/453 (89%), Gaps = 3/453 (1%) |  |
| --- | --- | --- |
| Query 1 | MLHGASSRPATARKSSGLSGTVRIPGDKSISHRSFMFGGLASGETRITGLLEGEDVINTG | 60 |
| Sbjct 1 | M H AS +PA AR+S L G +RIPGDKSISHRSFMFGGLASGETRITGLLEGEDVINTG MSHSASPKPAIARRSEALKGEIRIPGDKSISHRSFMFGGLASGETRITGLLEGEDVINTG | 60 |
| Query 61 | KAMQAMGARIRKEGDTWIIDGVGNGGLLAPEAPLDFGNAATGCRLTMGLVGVYDFDSTFI | 120 |
| Sbjct 61 | +AMQAMGA+IRK+GD WII+GVGNG LL PEA LDFGNA TG RLTMGLVG YD + FI RAMQAMGAKIRKDGDVWIINGVGNGCLLQPEAALDFGNAGTGARLTMGLVGTYDMKTVFI | 120 |
| Query 121 | GDASLTKRPMGRVLNPLREMGVQVKSEDGDRLPVTLRGPKTPTPITYRVPMASAQVKSAV | 180 |
| Sbjct 121 | GDASLT RPMGRVLNPLREMGVQV++ +GDR+P+TL GP+T PITYRVPMASAQVKSAV GDASLTSRPMGRVLNPLREMGVQVEAAEGDRMPLTLIGPRTANPITYRVPMASAQVKSAV | 180 |
| Query 181 | LLAGLNTPGITTVIEPIMTRDHTEKMLQGFGANLTVETDADGVRTIRLEGRGKLTGQVID | 240 |
| Sbjct 181 | LLAGLN PG+TTVIEP+MTRDHTEKMLQGFGA+L+VETD DGVR IR+ G+GKLTGQ ID LLAGLNAPGVTTVIEPVMTRDHTEKMLQGFGADLSVETDKDGVRHIRITGQGKLTGQTID | 240 |
| Query 241 | VPGDPSSTAFPLVAALLVPGSDVTILNVLMNPTRTGLILTLQEMGADIEVINPRLAGGED | 300 |
| Sbjct 241 | VPGDPSSTAFPLVAALLV GSDVTI NVLMNPTRTGLILTLQEMGADIEV+N RLAGGED VPGDPSSTAFPLVAALLVEGSDVTIRNVLMNPTRTGLILTLQEMGADIEVLNARLAGGED | 300 |
| Query 301 | VADLRVRSSTLKGVTVPEDRAPSMIDEYPILavaaafaegaTVMNGLEELRVKESDRLSA | 360 |
| Sbjct 301 | VADLRV++S LKGV VP +RAPSMIDEYP+LA+AA+FAEG TVM+GL+ELRVKESDRL+A VADLRVKASKLKGVVVPPERAPSMIDEYPVLAIAASFAEGETVMDGLDELRVKESDRLAA | 360 |
| Query 361 | VANGLKLNGVDCDEGETSLVVRGRPDGKGLGNASGAAVATHLDHRIAMSFLVMGLVSENP | 420 |
| Sbjct 361 | VA GL+ NGVDC EGE SL VRGRPDGKGLG G VATHLDHRIAMSFLVMGL SE P VARGLEANGVDCTEGEMSLTVRGRPDGKGLG---GGTVATHLDHRIAMSFLVMGLASEKP | 417 |
| Query 421  Sbjct 418 | VTVDDATMIATSFPEFMDLMAGLGAKIELSDTK 453 VTVDD+TMIATSFPEFMD+M GLGAKIELSD + VTVDDSTMIATSFPEFMDMMPGLGAKIELSDAQ 450 |  |

>WP_151677450.1 3-phosphoshikimate 1-carboxyvinyltransferase [Ochrobactrum tritici] KAB2666225.1 3-phosphoshikimate 1-carboxyvinyltransferase [Ochrobactrum tritici]

Length=450

Score = 729 bits (1883), Expect = 0.0, Method: Compositional matrix adjust.

| Identities | = 370/453 (82%), Positives = 407/453 (90%), Gaps = 3/453 (1%) |  |
| --- | --- | --- |
| Query 1 | MLHGASSRPATARKSSGLSGTVRIPGDKSISHRSFMFGGLASGETRITGLLEGEDVINTG | 60 |
| Sbjct 1 | M H AS +PATAR+S L+G +RIPGDKSISHRSFMFGGLASGETRITGLLEGEDVINTG MSHSASPKPATARRSEALTGEIRIPGDKSISHRSFMFGGLASGETRITGLLEGEDVINTG | 60 |
| Query 61 | KAMQAMGARIRKEGDTWIIDGVGNGGLLAPEAPLDFGNAATGCRLTMGLVGVYDFDSTFI | 120 |
| Sbjct 61 | +AMQAMGA+IRK+GD WII+GVGNG LL PEA LDFGNA TG RLTMGLVG YD ++FI RAMQAMGAKIRKDGDVWIINGVGNGCLLQPEAALDFGNAGTGARLTMGLVGTYDMKTSFI | 120 |
| Query 121 | GDASLTKRPMGRVLNPLREMGVQVKSEDGDRLPVTLRGPKTPTPITYRVPMASAQVKSAV | 180 |
| Sbjct 121 | GDASL+KRPMGRVLNPLREMGVQV++ DGDR+P+TL GP+T PITYRVPMASAQVKSAV GDASLSKRPMGRVLNPLREMGVQVEAADGDRMPLTLIGPRTANPITYRVPMASAQVKSAV | 180 |
| Query 181 | LLAGLNTPGITTVIEPIMTRDHTEKMLQGFGANLTVETDADGVRTIRLEGRGKLTGQVID | 240 |
| Sbjct 181 | LLAGLNTPG+TTVIEP+MTRDHTEKMLQGFGA+L+VETD DGVR IR+ G+GKL GQ ID LLAGLNTPGVTTVIEPVMTRDHTEKMLQGFGADLSVETDKDGVRHIRITGQGKLIGQTID | 240 |
| Query 241 | VPGDPSSTAFPLVAALLVPGSDVTILNVLMNPTRTGLILTLQEMGADIEVINPRLAGGED | 300 |
| Sbjct 241 | VPGDPSSTAFPLVAALLV GSDVTI NVLMNPTRTGLILTLQEMGA+IEV+N RLAGGED VPGDPSSTAFPLVAALLVEGSDVTIHNVLMNPTRTGLILTLQEMGANIEVLNARLAGGED | 300 |
| Query 301 | VADLRVRSSTLKGVTVPEDRAPSMIDEYPILavaaafaegaTVMNGLEELRVKESDRLSA | 360 |
| Sbjct 301 | VADLRV++S LKGV VP +RAPSMIDEYP+LA+AAAFAEG TVM+GL+ELRVKESDRL+A VADLRVKASKLKGVVVPPERAPSMIDEYPVLAIAAAFAEGETVMDGLDELRVKESDRLAA | 360 |
| Query 361 | VANGLKLNGVDCDEGETSLVVRGRPDGKGLGNASGAAVATHLDHRIAMSFLVMGLVSENP | 420 |
| Sbjct 361 | VA GL+ NGVDC EGE SL VRGRPDG+GLG G VATHLDHRIAMSFLVMGL SE P  VARGLEANGVDCTEGEMSLTVRGRPDGRGLG---GGTVATHLDHRIAMSFLVMGLASEKP | 417 |

Query 421 VTVDDATMIATSFPEFMDLMAGLGAKIELSDTK 453 VTVDD+TMIATSFPEFMD+M GLGAKIE SD +

Sbjct 418 VTVDDSTMIATSFPEFMDMMPGLGAKIEQSDAR 450

>WP_174049715.1 3-phosphoshikimate 1-carboxyvinyltransferase [Agrobacterium rhizogenes] NTF85517.1 3-phosphoshikimate 1-carboxyvinyltransferase [Agrobacterium rhizogenes]

Length=454

Score = 729 bits (1882), Expect = 0.0, Method: Compositional matrix adjust.

| Identities | = 371/449 (83%), Positives = 403/449 (90%), Gaps = 1/449 (0%) |  |
| --- | --- | --- |
| Query 1 | MLHGASSRPATARKSSGLSGTVRIPGDKSISHRSFMFGGLASGETRITGLLEGEDVINTG | 60 |
| Sbjct 1 | M H SRPATAR+S+ LSGT+R+PGDKSISHR+FMFGGLASGETRI+GLLEGEDV+NTG MPHDTHSRPATARRSTNLSGTIRVPGDKSISHRAFMFGGLASGETRISGLLEGEDVLNTG | 60 |
| Query 61 | KAMQAMGARIRKEGDTWIIDGVGNGGLLAPEAPLDFGNAATGCRLTMGLVGVYDFDSTFI | 120 |
| Sbjct 61 | KAMQAMGA+I K G WII GVGNG LLAP+APLDFGNA TG RLTMGLVG Y F+STFI KAMQAMGAKIEKIGSEWIIKGVGNGALLAPQAPLDFGNAGTGSRLTMGLVGAYHFESTFI | 120 |
| Query 121 | GDASLTKRPMGRVLNPLREMGVQVKSEDGDRLPVTLRGPKTPTPITYRVPMASAQVKSAV | 180 |
| Sbjct 121 | GDASL+KRPMGR+L+PLR+MGVQVKS +GDRLPVTL GP+T PITYRVPMASAQVKSAV GDASLSKRPMGRILDPLRQMGVQVKSSEGDRLPVTLHGPETANPITYRVPMASAQVKSAV | 180 |
| Query 181 | LLAGLNTPGITTVIEPIMTRDHTEKMLQGFGANLTVETDADGVRTIRLEGRGKLTGQVID | 240 |
| Sbjct 181 | LLAGLN PGITTVIEP+MTRDHTEKML GFGA L+VETD DGVR IRL+G+ KL+GQ ID LLAGLNAPGITTVIEPVMTRDHTEKMLAGFGAELSVETDRDGVRIIRLQGQSKLSGQTID | 240 |
| Query 241 | VPGDPSSTAFPLVAALLVPGSDVTILNVLMNPTRTGLILTLQEMGADIEVINPRLAGGED | 300 |
| Sbjct 241 | VPGDPSSTAFPLVAAL+VPGSD+TI NVLMNPTRTGLILTLQEMGADIEV++ RLAGGED VPGDPSSTAFPLVAALIVPGSDITIRNVLMNPTRTGLILTLQEMGADIEVLDKRLAGGED | 300 |
| Query 301 | VADLRVRSSTLKGVTVPEDRAPSMIDEYPILavaaafaegaTVMNGLEELRVKESDRLSA | 360 |
| Sbjct 301 | VADLRVRSS LKGVTVP +RAPSMIDEYP+LAVAAAFAEG TVM GLEELRVKESDRLSA VADLRVRSSALKGVTVPAERAPSMIDEYPVLAVAAAFAEGTTVMLGLEELRVKESDRLSA | 360 |
| Query 361 | VANGLKLNGVDCDEGETSLVVRGRPDGKGLGNASGAAVATHLDHRIAMSFLVMGLVSENP | 420 |
| Sbjct 361 | VA GL LNGVDC EGE SL VRGRPDGKGLGNA G V THLDHRIAMSFLVMGL SE+P VAEGLTLNGVDCTEGEASLTVRGRPDGKGLGNA-GGEVKTHLDHRIAMSFLVMGLASEHP | 419 |
| Query 421  Sbjct 420 | VTVDDATMIATSFPEFMDLMAGLGAKIEL 449 V VDD+TMIATSFPEF DLMA LGA +++ VRVDDSTMIATSFPEFFDLMAKLGADMDV 448 |  |

>WP_109987137.1 3-phosphoshikimate 1-carboxyvinyltransferase [Ochrobactrum sp. POC9]

PWU75313.1 3-phosphoshikimate 1-carboxyvinyltransferase [Ochrobactrum sp. POC9]

Length=450

Score = 729 bits (1882), Expect = 0.0, Method: Compositional matrix adjust.

| Identities | = 370/453 (82%), Positives = 406/453 (90%), Gaps = 3/453 (1%) |  |
| --- | --- | --- |
| Query 1 | MLHGASSRPATARKSSGLSGTVRIPGDKSISHRSFMFGGLASGETRITGLLEGEDVINTG | 60 |
| Sbjct 1 | M H AS +PATAR+S L+G +RIPGDKSISHRSFMFGGLASGETRITGLLEGEDVINTG MSHSASPKPATARRSEALTGEIRIPGDKSISHRSFMFGGLASGETRITGLLEGEDVINTG | 60 |
| Query 61 | KAMQAMGARIRKEGDTWIIDGVGNGGLLAPEAPLDFGNAATGCRLTMGLVGVYDFDSTFI | 120 |
| Sbjct 61 | +AMQAMGA+IRK+GD WII+GVGNG LL PEA LDFGNA TG RLTMGLVG YD ++FI RAMQAMGAKIRKDGDVWIINGVGNGCLLQPEAALDFGNAGTGARLTMGLVGTYDMKTSFI | 120 |
| Query 121 | GDASLTKRPMGRVLNPLREMGVQVKSEDGDRLPVTLRGPKTPTPITYRVPMASAQVKSAV | 180 |
| Sbjct 121 | GDASLT RPMGRVLNPLREMGVQV++ +GDR+P+TL GP+T PITYRVPMASAQVKSAV GDASLTSRPMGRVLNPLREMGVQVEAAEGDRMPLTLIGPRTANPITYRVPMASAQVKSAV | 180 |
| Query 181 | LLAGLNTPGITTVIEPIMTRDHTEKMLQGFGANLTVETDADGVRTIRLEGRGKLTGQVID | 240 |
| Sbjct 181 | LLAGLNTPG+TTVIEP+MTRDHTEKMLQGFGA+L+VETD DGVR IR+ G+GKLTGQ ID LLAGLNTPGVTTVIEPVMTRDHTEKMLQGFGADLSVETDKDGVRHIRITGQGKLTGQTID | 240 |
| Query 241 | VPGDPSSTAFPLVAALLVPGSDVTILNVLMNPTRTGLILTLQEMGADIEVINPRLAGGED | 300 |

| Sbjct | 241 | VPGDPSSTAFPLVAALLV GSDVTI NVLMNPTRTGLILTLQEMGADIEV+N RLAGGED VPGDPSSTAFPLVAALLVEGSDVTIHNVLMNPTRTGLILTLQEMGADIEVLNARLAGGED | 300 |
| --- | --- | --- | --- |
| Query | 301 | VADLRVRSSTLKGVTVPEDRAPSMIDEYPILavaaafaegaTVMNGLEELRVKESDRLSA | 360 |
| Sbjct | 301 | VADL V++S LKGV VP +RAPSMIDEYP+LA+AA+FAEG TVM+GL+ELRVKESDRL+A VADLHVKASKLKGVVVPPERAPSMIDEYPVLAIAASFAEGETVMDGLDELRVKESDRLAA | 360 |
| Query | 361 | VANGLKLNGVDCDEGETSLVVRGRPDGKGLGNASGAAVATHLDHRIAMSFLVMGLVSENP | 420 |
| Sbjct | 361 | VA GL+ NGVDC EGE SL VRGRPDGKGLG G VATHLDHRIAMSFLVMGL SE P VARGLEANGVDCTEGEMSLTVRGRPDGKGLG---GGTVATHLDHRIAMSFLVMGLASEKP | 417 |
| Query  Sbjct | 421  418 | VTVDDATMIATSFPEFMDLMAGLGAKIELSDTK 453 VTVDD+TMIATSFPEFMD+M LGAKIELSD + VTVDDSTMIATSFPEFMDMMPRLGAKIELSDAQ 450 |  |

>WP_114214379.1 3-phosphoshikimate 1-carboxyvinyltransferase [Ochrobactrum sp. 3-3]

Length=449

Score = 729 bits (1882), Expect = 0.0, Method: Compositional matrix adjust.

| Identities | = 370/450 (82%), Positives = 405/450 (90%), Gaps = 3/450 (1%) |  |
| --- | --- | --- |
| Query 1 | MLHGASSRPATARKSSGLSGTVRIPGDKSISHRSFMFGGLASGETRITGLLEGEDVINTG | 60 |
| Sbjct 1 | M H AS +PATAR+S L G +RIPGDKSISHRSFMFGGLASGETRITGLLEGEDVINTG MSHSASPKPATARRSEALVGEIRIPGDKSISHRSFMFGGLASGETRITGLLEGEDVINTG | 60 |
| Query 61 | KAMQAMGARIRKEGDTWIIDGVGNGGLLAPEAPLDFGNAATGCRLTMGLVGVYDFDSTFI | 120 |
| Sbjct 61 | +AMQ+MGA+IRKEGD WII+GVGNG LL PEAPLDFGNA TG RLTMGLVG YD ++FI RAMQSMGAKIRKEGDVWIINGVGNGCLLQPEAPLDFGNAGTGARLTMGLVGTYDMKTSFI | 120 |
| Query 121 | GDASLTKRPMGRVLNPLREMGVQVKSEDGDRLPVTLRGPKTPTPITYRVPMASAQVKSAV | 180 |
| Sbjct 121 | GDASL+KRPMGRVLNPLREMGVQV++ +GDR+P+TL GPKT PITYRVPMASAQVKSAV GDASLSKRPMGRVLNPLREMGVQVEAAEGDRMPLTLIGPKTANPITYRVPMASAQVKSAV | 180 |
| Query 181 | LLAGLNTPGITTVIEPIMTRDHTEKMLQGFGANLTVETDADGVRTIRLEGRGKLTGQVID | 240 |
| Sbjct 181 | LLAGLNTPG+TTVIEP+MTRDHTEKMLQGFGA+L+VETD DGVR IR+ G+GKL GQ ID LLAGLNTPGVTTVIEPVMTRDHTEKMLQGFGADLSVETDKDGVRHIRIVGQGKLVGQTID | 240 |
| Query 241 | VPGDPSSTAFPLVAALLVPGSDVTILNVLMNPTRTGLILTLQEMGADIEVINPRLAGGED | 300 |
| Sbjct 241 | VPGDPSSTAFPLVAALLV GSDVTI NVLMNPTRTGLILTLQEMGADIEV+N RLAGGED VPGDPSSTAFPLVAALLVEGSDVTIRNVLMNPTRTGLILTLQEMGADIEVLNARLAGGED | 300 |
| Query 301 | VADLRVRSSTLKGVTVPEDRAPSMIDEYPILavaaafaegaTVMNGLEELRVKESDRLSA | 360 |
| Sbjct 301 | VADLRV++S LKGV VP +RAPSMIDEYP+LA+AA+FAEG TVM+GL+ELRVKESDRL+A VADLRVKASKLKGVVVPPERAPSMIDEYPVLAIAASFAEGETVMDGLDELRVKESDRLAA | 360 |
| Query 361 | VANGLKLNGVDCDEGETSLVVRGRPDGKGLGNASGAAVATHLDHRIAMSFLVMGLVSENP | 420 |
| Sbjct 361 | VA GL+ NGVDC EGE SL VRGRPDGKGLG G VATHLDHRIAMSFLVMGL SE P VARGLEANGVDCTEGEMSLTVRGRPDGKGLG---GGTVATHLDHRIAMSFLVMGLASEKP | 417 |
| Query 421  Sbjct 418 | VTVDDATMIATSFPEFMDLMAGLGAKIELS 450 VTVDD+ MIATSFPEFMD+M GLGAKIEL+ VTVDDSNMIATSFPEFMDMMPGLGAKIELN 447 |  |

>WP_091947273.1 MULTISPECIES: 3-phosphoshikimate 1-carboxyvinyltransferase [unclassified Phyllobacterium]

NTS32814.1 3-phosphoshikimate 1-carboxyvinyltransferase [Phyllobacterium sp. BT25]

SDP34793.1 3-phosphoshikimate 1-carboxyvinyltransferase [Phyllobacterium sp. YR620]

Length=451

Score = 729 bits (1882), Expect = 0.0, Method: Compositional matrix adjust. Identities = 370/447 (83%), Positives = 401/447 (90%), Gaps = 3/447 (1%)

| Query | 1 | MLHGASSRPATARKSSGLSGTVRIPGDKSISHRSFMFGGLASGETRITGLLEGEDVINTG | 60 |
| --- | --- | --- | --- |
|  |  | M H + RPATAR+S LSG +RIPGDKSISHRSFMFGGLASGETRITGLLEGEDVINTG |  |
| Sbjct | 1 | MSHTSLPRPATARRSKALSGDIRIPGDKSISHRSFMFGGLASGETRITGLLEGEDVINTG | 60 |
| Query | 61 | KAMQAMGARIRKEGDTWIIDGVGNGGLLAPEAPLDFGNAATGCRLTMGLVGVYDFDSTFI | 120 |

| Sbjct | 61 | +AMQAMGA+I K+GD WII G GNG LL PEAPLDFGNA TG RLTMGLVG Y ++TFI RAMQAMGAKIAKDGDVWIIQGTGNGCLLEPEAPLDFGNAGTGARLTMGLVGTYAMETTFI | 120 |
| --- | --- | --- | --- |
| Query | 121 | GDASLTKRPMGRVLNPLREMGVQVKSEDGDRLPVTLRGPKTPTPITYRVPMASAQVKSAV | 180 |
| Sbjct | 121 | GDASL+KRPMGRVLNPLREMGVQV + DGDRLP+TLRGPK PITYRVPMASAQVKSAV GDASLSKRPMGRVLNPLREMGVQVSAADGDRLPLTLRGPKAAAPITYRVPMASAQVKSAV | 180 |
| Query | 181 | LLAGLNTPGITTVIEPIMTRDHTEKMLQGFGANLTVETDADGVRTIRLEGRGKLTGQVID | 240 |
| Sbjct | 181 | LLAGLNTPGITTV+EP+MTRDHTEKMLQGFGANL+VETD DGVR IR+EG+GKLTGQVID LLAGLNTPGITTVVEPVMTRDHTEKMLQGFGANLSVETDKDGVRHIRIEGQGKLTGQVID | 240 |
| Query | 241 | VPGDPSSTAFPLVAALLVPGSDVTILNVLMNPTRTGLILTLQEMGADIEVINPRLAGGED | 300 |
| Sbjct | 241 | VPGDPSSTAFPLVAALLVPGSD+TI NVLMNPTRTGLILTLQEMGA+IEV+N RLAGGED VPGDPSSTAFPLVAALLVPGSDITIRNVLMNPTRTGLILTLQEMGANIEVLNARLAGGED | 300 |
| Query | 301 | VADLRVRSSTLKGVTVPEDRAPSMIDEYPILavaaafaegaTVMNGLEELRVKESDRLSA | 360 |
| Sbjct | 301 | VADLRVRSS LKGV+VP +RAPSMIDEYP+LAVAA+FA G T+M+GL+ELRVKESDRLSA VADLRVRSSELKGVSVPPERAPSMIDEYPVLAVAASFAMGETLMHGLDELRVKESDRLSA | 360 |
| Query | 361 | VANGLKLNGVDCDEGETSLVVRGRPDGKGLGNASGAAVATHLDHRIAMSFLVMGLVSENP | 420 |
| Sbjct | 361 | VANGLK NGVDC EG SL VRGRPDGKGLG G V THLDHRIAMSFLVMGL +E P VANGLKTNGVDCTEGPESLSVRGRPDGKGLG---GGTVETHLDHRIAMSFLVMGLAAEKP | 417 |
| Query  Sbjct | 421  418 | VTVDDATMIATSFPEFMDLMAGLGAKI 447 VTVDDATMIATSFPEFM++M +GA+I VTVDDATMIATSFPEFMEMMTRMGAEI 444 |  |

>WP_119044001.1 3-phosphoshikimate 1-carboxyvinyltransferase [Ochrobactrum haematophilum] RIA07566.1 3-phosphoshikimate 1-carboxyvinyltransferase [Ochrobactrum haematophilum]

Length=450

Score = 729 bits (1881), Expect = 0.0, Method: Compositional matrix adjust.

| Identities | = 371/453 (82%), Positives = 405/453 (89%), Gaps = 3/453 (1%) |  |
| --- | --- | --- |
| Query 1 | MLHGASSRPATARKSSGLSGTVRIPGDKSISHRSFMFGGLASGETRITGLLEGEDVINTG | 60 |
| Sbjct 1 | M H AS +PA AR+S LSG +RIPGDKSISHRSFMFGGLASGETRITGLLEGEDVINTG MSHSASPKPAIARRSEALSGEIRIPGDKSISHRSFMFGGLASGETRITGLLEGEDVINTG | 60 |
| Query 61 | KAMQAMGARIRKEGDTWIIDGVGNGGLLAPEAPLDFGNAATGCRLTMGLVGVYDFDSTFI | 120 |
| Sbjct 61 | +AMQAMGA IRK+GD WII+GVGNG LL PEAPLDFGNA TG RLTMGLVG YD + FI RAMQAMGATIRKDGDVWIINGVGNGCLLEPEAPLDFGNAGTGARLTMGLVGTYDMTTAFI | 120 |
| Query 121 | GDASLTKRPMGRVLNPLREMGVQVKSEDGDRLPVTLRGPKTPTPITYRVPMASAQVKSAV | 180 |
| Sbjct 121 | GDASL++RPMGRVL+PLR+MGVQVKS DGDR+P+TL GP PITYRVPMASAQVKSAV GDASLSRRPMGRVLDPLRQMGVQVKSTDGDRMPLTLVGPSVANPITYRVPMASAQVKSAV | 180 |
| Query 181 | LLAGLNTPGITTVIEPIMTRDHTEKMLQGFGANLTVETDADGVRTIRLEGRGKLTGQVID | 240 |
| Sbjct 181 | LLAGLNTPG+TTVIEP+MTRDHTEKMLQGFGA+LTVETD DGVR IR+ G+GKLTGQ ID LLAGLNTPGVTTVIEPVMTRDHTEKMLQGFGADLTVETDHDGVRHIRITGQGKLTGQTID | 240 |
| Query 241 | VPGDPSSTAFPLVAALLVPGSDVTILNVLMNPTRTGLILTLQEMGADIEVINPRLAGGED | 300 |
| Sbjct 241 | VPGDPSSTAFPLVAALLV GSDVTI NVLMNPTRTGLILTLQEMGA+IEV+N RLAGGED VPGDPSSTAFPLVAALLVEGSDVTIRNVLMNPTRTGLILTLQEMGAEIEVLNARLAGGED | 300 |
| Query 301 | VADLRVRSSTLKGVTVPEDRAPSMIDEYPILavaaafaegaTVMNGLEELRVKESDRLSA | 360 |
| Sbjct 301 | VADLRV+SS LKGVTVP +RAPSMIDEYP+LA+AA+FAEG T+M+GL+ELRVKESDRL+A VADLRVKSSKLKGVTVPPERAPSMIDEYPVLAIAASFAEGETIMDGLDELRVKESDRLAA | 360 |
| Query 361 | VANGLKLNGVDCDEGETSLVVRGRPDGKGLGNASGAAVATHLDHRIAMSFLVMGLVSENP | 420 |
| Sbjct 361 | VA GL+ NGVDC EGE SL VRGRPDGKGLG G VATHLDHRIAMSFLVMGL SE P VARGLEANGVDCTEGEMSLTVRGRPDGKGLG---GGTVATHLDHRIAMSFLVMGLASEKP | 417 |
| Query 421  Sbjct 418 | VTVDDATMIATSFPEFMDLMAGLGAKIELSDTK 453 VTVDD+TMIATSFPEFMDLMAGLG +IE S+ + VTVDDSTMIATSFPEFMDLMAGLGGRIEQSEAE 450 |  |

>WP_043062739.1 3-phosphoshikimate 1-carboxyvinyltransferase [Ochrobactrum anthropi] KIU67185.1 3-phosphoshikimate 1-carboxyvinyltransferase [Ochrobactrum anthropi]

Length=450

Score = 728 bits (1880), Expect = 0.0, Method: Compositional matrix adjust.

| Identities | = 370/453 (82%), Positives = 406/453 (90%), Gaps = 3/453 (1%) |  |
| --- | --- | --- |
| Query 1 | MLHGASSRPATARKSSGLSGTVRIPGDKSISHRSFMFGGLASGETRITGLLEGEDVINTG | 60 |
| Sbjct 1 | M H AS +PATAR S L+G +RIPGDKSISHRSFMFGGLASGETRITGLLEGEDVINTG MSHSASPKPATARHSEALTGEIRIPGDKSISHRSFMFGGLASGETRITGLLEGEDVINTG | 60 |
| Query 61 | KAMQAMGARIRKEGDTWIIDGVGNGGLLAPEAPLDFGNAATGCRLTMGLVGVYDFDSTFI | 120 |
| Sbjct 61 | +AMQAMGA+IRK+GD WII+GVGNG LL PEA LDFGNA TG RLTMGLVG YD ++FI RAMQAMGAKIRKDGDVWIINGVGNGCLLQPEAALDFGNAGTGARLTMGLVGTYDMRTSFI | 120 |
| Query 121 | GDASLTKRPMGRVLNPLREMGVQVKSEDGDRLPVTLRGPKTPTPITYRVPMASAQVKSAV | 180 |
| Sbjct 121 | GDASL+KRPMGRVLNPLREMGVQV++ +GDR+P+ L GPKT PITYRVPMASAQVKSAV GDASLSKRPMGRVLNPLREMGVQVEAAEGDRMPLMLIGPKTANPITYRVPMASAQVKSAV | 180 |
| Query 181 | LLAGLNTPGITTVIEPIMTRDHTEKMLQGFGANLTVETDADGVRTIRLEGRGKLTGQVID | 240 |
| Sbjct 181 | LLAGLNTPG+TTVIEP+MTRDHTEKMLQGFGA+L+VETD +GVR IR+ G+GKL GQ ID LLAGLNTPGVTTVIEPVMTRDHTEKMLQGFGADLSVETDKEGVRHIRITGQGKLIGQTID | 240 |
| Query 241 | VPGDPSSTAFPLVAALLVPGSDVTILNVLMNPTRTGLILTLQEMGADIEVINPRLAGGED | 300 |
| Sbjct 241 | VPGDPSSTAFPLVAALLV GSDVTI NVLMNPTRTGLILTLQEMGADIEV+N RLAGGED VPGDPSSTAFPLVAALLVEGSDVTIRNVLMNPTRTGLILTLQEMGADIEVLNARLAGGED | 300 |
| Query 301 | VADLRVRSSTLKGVTVPEDRAPSMIDEYPILavaaafaegaTVMNGLEELRVKESDRLSA | 360 |
| Sbjct 301 | VADLRV++S LKGV VP +RAPSMIDEYP+LA+AA+FAEG TVM+GL+ELRVKESDRL+A VADLRVKASKLKGVVVPPERAPSMIDEYPVLAIAASFAEGETVMDGLDELRVKESDRLAA | 360 |
| Query 361 | VANGLKLNGVDCDEGETSLVVRGRPDGKGLGNASGAAVATHLDHRIAMSFLVMGLVSENP | 420 |
| Sbjct 361 | VA GL+ NGVDC EGE SL VRGRPDGKGLG G VATHLDHRIAMSFLVMGL SE P VARGLEANGVDCTEGEMSLTVRGRPDGKGLG---GGTVATHLDHRIAMSFLVMGLASEKP | 417 |
| Query 421  Sbjct 418 | VTVDDATMIATSFPEFMDLMAGLGAKIELSDTK 453 VTVDD+TMIATSFPEFMD+M GLGAKIELSD + VTVDDSTMIATSFPEFMDMMPGLGAKIELSDAR 450 |  |

>WP_027230834.1 3-phosphoshikimate 1-carboxyvinyltransferase [Phyllobacterium sp. UNC302MFCol5.2]

Length=451

Score = 728 bits (1880), Expect = 0.0, Method: Compositional matrix adjust.

| Identities | = 370/447 (83%), Positives = 401/447 (90%), Gaps = 3/447 (1%) |  |
| --- | --- | --- |
| Query 1 | MLHGASSRPATARKSSGLSGTVRIPGDKSISHRSFMFGGLASGETRITGLLEGEDVINTG | 60 |
| Sbjct 1 | M H + RPATAR+S LSG +RIPGDKSISHRSFMFGGLASGETRITGLLEGEDVINTG MSHTSLPRPATARRSKALSGDIRIPGDKSISHRSFMFGGLASGETRITGLLEGEDVINTG | 60 |
| Query 61 | KAMQAMGARIRKEGDTWIIDGVGNGGLLAPEAPLDFGNAATGCRLTMGLVGVYDFDSTFI | 120 |
| Sbjct 61 | +AMQAMGA+I K+GD WII G GNG LL PEAPLDFGNA TG RLTMGLVG Y ++TFI RAMQAMGAKIAKDGDVWIIQGTGNGCLLEPEAPLDFGNAGTGARLTMGLVGTYAMETTFI | 120 |
| Query 121 | GDASLTKRPMGRVLNPLREMGVQVKSEDGDRLPVTLRGPKTPTPITYRVPMASAQVKSAV | 180 |
| Sbjct 121 | GDASL+KRPMGRVLNPLREMGVQV + DGDRLP+TLRGPK PITYRVPMASAQVKSAV GDASLSKRPMGRVLNPLREMGVQVSAADGDRLPLTLRGPKAAAPITYRVPMASAQVKSAV | 180 |
| Query 181 | LLAGLNTPGITTVIEPIMTRDHTEKMLQGFGANLTVETDADGVRTIRLEGRGKLTGQVID | 240 |
| Sbjct 181 | LLAGLNTPGITTV+EP+MTRDHTEKMLQGFGANL+VETD DGVR IR+EG+GKLTGQVID LLAGLNTPGITTVVEPVMTRDHTEKMLQGFGANLSVETDKDGVRHIRIEGQGKLTGQVID | 240 |
| Query 241 | VPGDPSSTAFPLVAALLVPGSDVTILNVLMNPTRTGLILTLQEMGADIEVINPRLAGGED | 300 |
| Sbjct 241 | VPGDPSSTAFPLVAALLVPGSD+TI NVLMNPTRTGLILTLQEMGA+IEV+N RLAGGED VPGDPSSTAFPLVAALLVPGSDITIRNVLMNPTRTGLILTLQEMGANIEVLNARLAGGED | 300 |
| Query 301 | VADLRVRSSTLKGVTVPEDRAPSMIDEYPILavaaafaegaTVMNGLEELRVKESDRLSA | 360 |
| Sbjct 301 | VADLRVRSS LKGV+VP +RAPSMIDEYP+LAVAA+FA G T+M+GL+ELRVKESDRLSA VADLRVRSSELKGVSVPPERAPSMIDEYPVLAVAASFAMGETLMHGLDELRVKESDRLSA | 360 |
| Query 361 | VANGLKLNGVDCDEGETSLVVRGRPDGKGLGNASGAAVATHLDHRIAMSFLVMGLVSENP | 420 |
| Sbjct 361 | VANGLK NGVDC EG SL VRGRPDGKGLG G V THLDHRIAMSFLVMGL +E P VANGLKANGVDCTEGPESLSVRGRPDGKGLG---GGTVETHLDHRIAMSFLVMGLAAEKP | 417 |
| Query 421 | VTVDDATMIATSFPEFMDLMAGLGAKI 447 |  |

VTVDDATMIATSFPEFM++M +GA+I Sbjct 418 VTVDDATMIATSFPEFMEMMTRMGAEI 444

>WP_151557108.1 3-phosphoshikimate 1-carboxyvinyltransferase [Ochrobactrum tritici] KAB2674317.1 3-phosphoshikimate 1-carboxyvinyltransferase [Ochrobactrum tritici] KAB2690232.1 3-phosphoshikimate 1-carboxyvinyltransferase [Ochrobactrum tritici]

Length=450

Score = 728 bits (1879), Expect = 0.0, Method: Compositional matrix adjust.

| Identities | = 370/453 (82%), Positives = 405/453 (89%), Gaps = 3/453 (1%) |  |
| --- | --- | --- |
| Query 1 | MLHGASSRPATARKSSGLSGTVRIPGDKSISHRSFMFGGLASGETRITGLLEGEDVINTG | 60 |
| Sbjct 1 | M H AS +PATAR+S L+G +RIPGDKSISHRSFMFGGLASGETRITGLLEGEDVINTG MSHSASPKPATARRSEALTGEIRIPGDKSISHRSFMFGGLASGETRITGLLEGEDVINTG | 60 |
| Query 61 | KAMQAMGARIRKEGDTWIIDGVGNGGLLAPEAPLDFGNAATGCRLTMGLVGVYDFDSTFI | 120 |
| Sbjct 61 | +AMQAMGA+I K+GD WII+GVGNG LL PEA LDFGNA TG RLTMGLVG YD ++FI RAMQAMGAKIHKDGDVWIINGVGNGCLLQPEAALDFGNAGTGARLTMGLVGTYDMKTSFI | 120 |
| Query 121 | GDASLTKRPMGRVLNPLREMGVQVKSEDGDRLPVTLRGPKTPTPITYRVPMASAQVKSAV | 180 |
| Sbjct 121 | GDASL+KRPMGRVLNPLREMGVQV++ DGDR+P+TL GP+T PITYRVPMASAQVKSAV GDASLSKRPMGRVLNPLREMGVQVEAADGDRMPLTLIGPRTANPITYRVPMASAQVKSAV | 180 |
| Query 181 | LLAGLNTPGITTVIEPIMTRDHTEKMLQGFGANLTVETDADGVRTIRLEGRGKLTGQVID | 240 |
| Sbjct 181 | LLAGLNTPG+TTVIEP+MTRDHTEKMLQGFGA+L+VETD DGVR IR+ G+GKL GQ ID LLAGLNTPGVTTVIEPVMTRDHTEKMLQGFGADLSVETDKDGVRHIRITGQGKLIGQTID | 240 |
| Query 241 | VPGDPSSTAFPLVAALLVPGSDVTILNVLMNPTRTGLILTLQEMGADIEVINPRLAGGED | 300 |
| Sbjct 241 | VPGDPSSTAFPLVAALLV GSDVTI NVLMNPTRTGLILTLQEMGADIEV+N RLAGGED VPGDPSSTAFPLVAALLVEGSDVTIHNVLMNPTRTGLILTLQEMGADIEVLNARLAGGED | 300 |
| Query 301 | VADLRVRSSTLKGVTVPEDRAPSMIDEYPILavaaafaegaTVMNGLEELRVKESDRLSA | 360 |
| Sbjct 301 | VADLRV++S L GV VP +RAPSMIDEYP+LA+AAAFAEG TVM+GL+ELRVKESDRL+A VADLRVKASKLNGVVVPPERAPSMIDEYPVLAIAAAFAEGETVMDGLDELRVKESDRLAA | 360 |
| Query 361 | VANGLKLNGVDCDEGETSLVVRGRPDGKGLGNASGAAVATHLDHRIAMSFLVMGLVSENP | 420 |
| Sbjct 361 | VA GL+ NGVDC EGE SL VRGRPDGKGLG G VATHLDHRIAMSFLVMGL SE P VARGLEANGVDCTEGEMSLTVRGRPDGKGLG---GGTVATHLDHRIAMSFLVMGLASEKP | 417 |
| Query 421  Sbjct 418 | VTVDDATMIATSFPEFMDLMAGLGAKIELSDTK 453 VTVDD+TMIATSFPEFMD+M GLGAKIE SD + VTVDDSTMIATSFPEFMDMMPGLGAKIEQSDAR 450 |  |

>WP_028754674.1 3-phosphoshikimate 1-carboxyvinyltransferase [Rhizobium leucaenae] MBB4571270.1 3-phosphoshikimate 1-carboxyvinyltransferase [Rhizobium leucaenae] MBB6304893.1 3-phosphoshikimate 1-carboxyvinyltransferase [Rhizobium leucaenae]

Length=455

Score = 728 bits (1879), Expect = 0.0, Method: Compositional matrix adjust.

| Identities | = 369/449 (82%), Positives = 405/449 (90%), Gaps = 0/449 (0%) |  |
| --- | --- | --- |
| Query 1 | MLHGASSRPATARKSSGLSGTVRIPGDKSISHRSFMFGGLASGETRITGLLEGEDVINTG | 60 |
| Sbjct 1 | M H R ATAR+S+ LSGT+R+PGDKSISHR+ MFGGLA+GETRITGLLEGEDV+NTG MPHDIQPRLATARRSTNLSGTIRVPGDKSISHRALMFGGLAAGETRITGLLEGEDVLNTG | 60 |
| Query 61 | KAMQAMGARIRKEGDTWIIDGVGNGGLLAPEAPLDFGNAATGCRLTMGLVGVYDFDSTFI | 120 |
| Sbjct 61 | KAMQAMGA+I K G WII G+GNG LLAPEAPLDFGNA TG RLTMGLVGVYDF+STFI KAMQAMGAKIEKIGAEWIIRGIGNGALLAPEAPLDFGNAGTGSRLTMGLVGVYDFESTFI | 120 |
| Query 121 | GDASLTKRPMGRVLNPLREMGVQVKSEDGDRLPVTLRGPKTPTPITYRVPMASAQVKSAV | 180 |
| Sbjct 121 | GDASL+KRPMGR+L+PLR MGVQVKS +GDRLPVTL GP+T +PITYRVPMASAQVKSAV GDASLSKRPMGRILDPLRRMGVQVKSSEGDRLPVTLHGPETASPITYRVPMASAQVKSAV | 180 |
| Query 181 | LLAGLNTPGITTVIEPIMTRDHTEKMLQGFGANLTVETDADGVRTIRLEGRGKLTGQVID | 240 |
| Sbjct 181 | LLAGLN PGITTVIEP+MTRDHTEKML GFGA+L+VETD DGVRTIRL+G+G+L+GQ+ID LLAGLNAPGITTVIEPVMTRDHTEKMLAGFGADLSVETDRDGVRTIRLQGQGRLSGQIID | 240 |
| Query 241 | VPGDPSSTAFPLVAALLVPGSDVTILNVLMNPTRTGLILTLQEMGADIEVINPRLAGGED | 300 |
| Sbjct 241 | VPGDPSSTAF LVAAL+VPGSD+TI NVLMNPTRTGLILTLQEMG DIEV++ RLAGGED  VPGDPSSTAFSLVAALIVPGSDITIRNVLMNPTRTGLILTLQEMGGDIEVLDRRLAGGED | 300 |

| Query | 301 | VADLRVRSSTLKGVTVPEDRAPSMIDEYPILavaaafaegaTVMNGLEELRVKESDRLSA | 360 |
| --- | --- | --- | --- |
| Sbjct | 301 | VADLRV SS LKGVTVP +RAPSMIDEYP+LAVAAAFAEG T+M GLEELRVKESDRLSA VADLRVHSSALKGVTVPAERAPSMIDEYPVLAVAAAFAEGTTIMLGLEELRVKESDRLSA | 360 |
| Query | 361 | VANGLKLNGVDCDEGETSLVVRGRPDGKGLGNASGAAVATHLDHRIAMSFLVMGLVSENP | 420 |
| Sbjct | 361 | VA GL LNGVDC EGE SL V GRPDGKGLGNA+G AV THLDHRIAMSFLVMGL SE+P VAEGLALNGVDCTEGEASLTVCGRPDGKGLGNAAGGAVKTHLDHRIAMSFLVMGLASEHP | 420 |
| Query  Sbjct | 421  421 | VTVDDATMIATSFPEFMDLMAGLGAKIEL 449 V VDD+TMIATSFPEF DLMAGLGA +E+ VAVDDSTMIATSFPEFFDLMAGLGANMEV 449 |  |

>WP_091879195.1 3-phosphoshikimate 1-carboxyvinyltransferase [Phyllobacterium sp. OV277]

SDO56081.1 3-phosphoshikimate 1-carboxyvinyltransferase [Phyllobacterium sp. OV277]

Length=451

Score = 728 bits (1878), Expect = 0.0, Method: Compositional matrix adjust.

| Identities | = 369/454 (81%), Positives = 400/454 (88%), Gaps = 3/454 (1%) |  |
| --- | --- | --- |
| Query 1 | MLHGASSRPATARKSSGLSGTVRIPGDKSISHRSFMFGGLASGETRITGLLEGEDVINTG | 60 |
| Sbjct 1 | M H A +PATAR+S L+G +RIPGDKSISHRSFMFGGLASGETRITGLLEGEDVINTG MSHSALPKPATARRSKALTGDIRIPGDKSISHRSFMFGGLASGETRITGLLEGEDVINTG | 60 |
| Query 61 | KAMQAMGARIRKEGDTWIIDGVGNGGLLAPEAPLDFGNAATGCRLTMGLVGVYDFDSTFI | 120 |
| Sbjct 61 | +AMQAMGA I KEG+ WII G GNG LL PEAPLDFGNA TG RLTMGLVG Y ++TFI RAMQAMGATIVKEGEVWIIKGTGNGCLLQPEAPLDFGNAGTGARLTMGLVGTYAMETTFI | 120 |
| Query 121 | GDASLTKRPMGRVLNPLREMGVQVKSEDGDRLPVTLRGPKTPTPITYRVPMASAQVKSAV | 180 |
| Sbjct 121 | GDASL+KRPMGRVLNPLREMGVQV + +GDR+P+TLRGPK PITYRVPMASAQVKSAV GDASLSKRPMGRVLNPLREMGVQVTASEGDRMPLTLRGPKVAAPITYRVPMASAQVKSAV | 180 |
| Query 181 | LLAGLNTPGITTVIEPIMTRDHTEKMLQGFGANLTVETDADGVRTIRLEGRGKLTGQVID | 240 |
| Sbjct 181 | LLAGLNTPGITTVIEP+MTRDHTEKMLQGFGANLTVETD D VR IR+EG+GKLTGQ ID LLAGLNTPGITTVIEPVMTRDHTEKMLQGFGANLTVETDRDNVRHIRIEGQGKLTGQTID | 240 |
| Query 241 | VPGDPSSTAFPLVAALLVPGSDVTILNVLMNPTRTGLILTLQEMGADIEVINPRLAGGED | 300 |
| Sbjct 241 | VPGDPSSTAFPLVAALLVPGSD+TI NVLMNPTRTGLILTLQEMGA IE++N RLAGGED VPGDPSSTAFPLVAALLVPGSDITIRNVLMNPTRTGLILTLQEMGASIEILNARLAGGED | 300 |
| Query 301 | VADLRVRSSTLKGVTVPEDRAPSMIDEYPILavaaafaegaTVMNGLEELRVKESDRLSA | 360 |
| Sbjct 301 | VADLRVR+S LKG+ VP DRAPSMIDEYP+LAVAA+FA G TVMNGLEELRVKESDRLSA VADLRVRASELKGIAVPADRAPSMIDEYPVLAVAASFATGETVMNGLEELRVKESDRLSA | 360 |
| Query 361 | VANGLKLNGVDCDEGETSLVVRGRPDGKGLGNASGAAVATHLDHRIAMSFLVMGLVSENP | 420 |
| Sbjct 361 | VANGLK+NGVDC EG +L VRGRPDGKG+G G V THLDHRIAMSFLVMGLV+E P VANGLKVNGVDCTEGPETLSVRGRPDGKGIG---GGTVETHLDHRIAMSFLVMGLVAEKP | 417 |
| Query 421  Sbjct 418 | VTVDDATMIATSFPEFMDLMAGLGAKIELSDTKA 454 VTVDD+TMIATSFPEFM +M G+GA+I T A VTVDDSTMIATSFPEFMGMMTGMGAEISEQGTPA 451 |  |

>WP_007690336.1 MULTISPECIES: 3-phosphoshikimate 1-carboxyvinyltransferase [Agrobacterium] EJK87587.1 3-phosphoshikimate 1-carboxyvinyltransferase [Rhizobium sp. AP16]

ACM24911.1 3-phosphoshikimate 1-carboxyvinyltransferase [Agrobacterium radiobacter K84]

KEA07980.1 3-phosphoshikimate 1-carboxyvinyltransferase [Agrobacterium rhizogenes] MQB34614.1 3-phosphoshikimate 1-carboxyvinyltransferase [Agrobacterium rhizogenes] NTF66333.1 3-phosphoshikimate 1-carboxyvinyltransferase [Agrobacterium rhizogenes]

Length=454

Score = 728 bits (1878), Expect = 0.0, Method: Compositional matrix adjust. Identities = 371/449 (83%), Positives = 403/449 (90%), Gaps = 1/449 (0%)

Query 1	MLHGASSRPATARKSSGLSGTVRIPGDKSISHRSFMFGGLASGETRITGLLEGEDVINTG 60 M H	SRPATAR+S+ LSGT+R+PGDKSISHR+FMFGGLASGETRITGLLEGEDV+NTG

Sbjct 1	MPHDTHSRPATARRSTNLSGTIRVPGDKSISHRAFMFGGLASGETRITGLLEGEDVLNTG 60

| Query | 61 | KAMQAMGARIRKEGDTWIIDGVGNGGLLAPEAPLDFGNAATGCRLTMGLVGVYDFDSTFI | 120 |
| --- | --- | --- | --- |
| Sbjct | 61 | KAM+AMGA+I K GD WII GVGNG LLAPEAPLDFGNA TG RLTMGLVGVY F+STFI KAMRAMGAKIEKAGDEWIIKGVGNGALLAPEAPLDFGNAGTGSRLTMGLVGVYHFESTFI | 120 |
| Query | 121 | GDASLTKRPMGRVLNPLREMGVQVKSEDGDRLPVTLRGPKTPTPITYRVPMASAQVKSAV | 180 |
| Sbjct | 121 | GDASL+KRPMGR+L+PLR+MGVQVKS +GDRLPVTL GP+T PITYRVPMASAQVKSAV GDASLSKRPMGRILDPLRQMGVQVKSSEGDRLPVTLHGPETANPITYRVPMASAQVKSAV | 180 |
| Query | 181 | LLAGLNTPGITTVIEPIMTRDHTEKMLQGFGANLTVETDADGVRTIRLEGRGKLTGQVID | 240 |
| Sbjct | 181 | LLAGLN PGIT+VIEP+MTRDHTEKML GFGA L+VETD DGVR IRL+G+GKL GQ ID LLAGLNAPGITSVIEPVMTRDHTEKMLAGFGAELSVETDRDGVRIIRLQGQGKLFGQTID | 240 |
| Query | 241 | VPGDPSSTAFPLVAALLVPGSDVTILNVLMNPTRTGLILTLQEMGADIEVINPRLAGGED | 300 |
| Sbjct | 241 | VPGDPSSTAFPLVAAL+V GSD+TI NVLMNPTRTGLILTLQEMGADIE+++ RLAGGED VPGDPSSTAFPLVAALIVAGSDITIRNVLMNPTRTGLILTLQEMGADIEILDKRLAGGED | 300 |
| Query | 301 | VADLRVRSSTLKGVTVPEDRAPSMIDEYPILavaaafaegaTVMNGLEELRVKESDRLSA | 360 |
| Sbjct | 301 | VADLRVRSS LKGVTVP +RAPSMIDEYP+LAVAAAFAEG TVM GLEELRVKESDRLSA VADLRVRSSALKGVTVPAERAPSMIDEYPVLAVAAAFAEGTTVMLGLEELRVKESDRLSA | 360 |
| Query | 361 | VANGLKLNGVDCDEGETSLVVRGRPDGKGLGNASGAAVATHLDHRIAMSFLVMGLVSENP | 420 |
| Sbjct | 361 | VA GL LNGVDC EGE SL VRGRPDGKGLGNA G V THLDHRIAMSFLVMGL SE+P VAEGLTLNGVDCTEGEASLTVRGRPDGKGLGNA-GGEVKTHLDHRIAMSFLVMGLASEHP | 419 |
| Query  Sbjct | 421  420 | VTVDDATMIATSFPEFMDLMAGLGAKIEL 449 V VDD+ MIATSFPEF DLMA LGA +++ VKVDDSAMIATSFPEFFDLMAELGADMDV 448 |  |

>WP_029926097.1 MULTISPECIES: 3-phosphoshikimate 1-carboxyvinyltransferase [unclassified Ochrobactrum]

QOD63587.1 3-phosphoshikimate 1-carboxyvinyltransferase [Ochrobactrum sp. MT180101]

Length=450

Score = 728 bits (1878), Expect = 0.0, Method: Compositional matrix adjust.

| Identities | = 369/453 (81%), Positives = 406/453 (90%), Gaps = 3/453 (1%) |  |
| --- | --- | --- |
| Query 1 | MLHGASSRPATARKSSGLSGTVRIPGDKSISHRSFMFGGLASGETRITGLLEGEDVINTG | 60 |
| Sbjct 1 | M H AS +PATAR S L+G +RIPGDKSISHRSFMFGGLASGETRITGLLEGEDVINTG MSHSASPKPATARHSEALTGEIRIPGDKSISHRSFMFGGLASGETRITGLLEGEDVINTG | 60 |
| Query 61 | KAMQAMGARIRKEGDTWIIDGVGNGGLLAPEAPLDFGNAATGCRLTMGLVGVYDFDSTFI | 120 |
| Sbjct 61 | +AMQAMGA+IRK+GD WII+GVGNG LL PEA LDFGNA TG RLTMGLVG YD ++FI RAMQAMGAKIRKDGDVWIINGVGNGCLLQPEAALDFGNAGTGARLTMGLVGTYDMRTSFI | 120 |
| Query 121 | GDASLTKRPMGRVLNPLREMGVQVKSEDGDRLPVTLRGPKTPTPITYRVPMASAQVKSAV | 180 |
| Sbjct 121 | GDASL+KRPMGRVLNPLREMGVQV++ +GDR+P+ L GPKT PITYRVPMASAQVKSAV GDASLSKRPMGRVLNPLREMGVQVEAAEGDRMPLMLIGPKTANPITYRVPMASAQVKSAV | 180 |
| Query 181 | LLAGLNTPGITTVIEPIMTRDHTEKMLQGFGANLTVETDADGVRTIRLEGRGKLTGQVID | 240 |
| Sbjct 181 | LLAGLNTPG+TTVIEP+MTRDHTEKMLQGFGA+L+VETD +GVR IR+ G+GKL GQ +D LLAGLNTPGVTTVIEPVMTRDHTEKMLQGFGADLSVETDKEGVRHIRITGQGKLIGQTLD | 240 |
| Query 241 | VPGDPSSTAFPLVAALLVPGSDVTILNVLMNPTRTGLILTLQEMGADIEVINPRLAGGED | 300 |
| Sbjct 241 | VPGDPSSTAFPLVAALLV GSDVTI NVLMNPTRTGLILTLQEMGADIEV+N RLAGGED VPGDPSSTAFPLVAALLVEGSDVTIRNVLMNPTRTGLILTLQEMGADIEVLNARLAGGED | 300 |
| Query 301 | VADLRVRSSTLKGVTVPEDRAPSMIDEYPILavaaafaegaTVMNGLEELRVKESDRLSA | 360 |
| Sbjct 301 | VADLRV++S LKGV VP +RAPSMIDEYP+LA+AA+FAEG TVM+GL+ELRVKESDRL+A VADLRVKASKLKGVVVPPERAPSMIDEYPVLAIAASFAEGETVMDGLDELRVKESDRLAA | 360 |
| Query 361 | VANGLKLNGVDCDEGETSLVVRGRPDGKGLGNASGAAVATHLDHRIAMSFLVMGLVSENP | 420 |
| Sbjct 361 | VA GL+ NGVDC EGE SL VRGRPDGKGLG G VATHLDHRIAMSFLVMGL SE P VARGLEANGVDCTEGEMSLTVRGRPDGKGLG---GGTVATHLDHRIAMSFLVMGLASEKP | 417 |
| Query 421  Sbjct 418 | VTVDDATMIATSFPEFMDLMAGLGAKIELSDTK 453 VTVDD+TMIATSFPEFMD+M GLGAKIELSD + VTVDDSTMIATSFPEFMDMMPGLGAKIELSDAR 450 |  |

>WP_047466864.1 3-phosphoshikimate 1-carboxyvinyltransferase [Agrobacterium rhizogenes]

Length=454

Score = 728 bits (1878), Expect = 0.0, Method: Compositional matrix adjust.

| Identities | = 371/449 (83%), Positives = 403/449 (90%), Gaps = 1/449 (0%) |  |
| --- | --- | --- |
| Query 1 | MLHGASSRPATARKSSGLSGTVRIPGDKSISHRSFMFGGLASGETRITGLLEGEDVINTG | 60 |
| Sbjct 1 | M H SRPATAR+S+ LSGT+R+PGDKSISHR+FMFGGLASGETRITGLLEGEDV+NTG MPHDTHSRPATARRSTNLSGTIRVPGDKSISHRAFMFGGLASGETRITGLLEGEDVLNTG | 60 |
| Query 61 | KAMQAMGARIRKEGDTWIIDGVGNGGLLAPEAPLDFGNAATGCRLTMGLVGVYDFDSTFI | 120 |
| Sbjct 61 | KAM+AMGA+I K GD WII GVGNG LLAPEAPLDFGNA TG RLTMGLVGVY F+STFI KAMRAMGAKIEKAGDEWIIKGVGNGALLAPEAPLDFGNAGTGSRLTMGLVGVYHFESTFI | 120 |
| Query 121 | GDASLTKRPMGRVLNPLREMGVQVKSEDGDRLPVTLRGPKTPTPITYRVPMASAQVKSAV | 180 |
| Sbjct 121 | GDASL+KRPMGR+L+PLR+MGVQVKS +GDRLPVTL GP+T PITYRVPMASAQVKSAV GDASLSKRPMGRILDPLRQMGVQVKSSEGDRLPVTLHGPETANPITYRVPMASAQVKSAV | 180 |
| Query 181 | LLAGLNTPGITTVIEPIMTRDHTEKMLQGFGANLTVETDADGVRTIRLEGRGKLTGQVID | 240 |
| Sbjct 181 | LLAGLN PGIT+VIEP+MTRDHTEKML GFGA L+VETD DGVR IRL+G+GKL GQ ID LLAGLNAPGITSVIEPVMTRDHTEKMLAGFGAELSVETDRDGVRIIRLQGQGKLFGQTID | 240 |
| Query 241 | VPGDPSSTAFPLVAALLVPGSDVTILNVLMNPTRTGLILTLQEMGADIEVINPRLAGGED | 300 |
| Sbjct 241 | VPGDPSSTAFPLVAAL+V GSD+TI NVLMNPTRTGLILTLQEMGADIE+++ RLAGGED VPGDPSSTAFPLVAALIVAGSDITIRNVLMNPTRTGLILTLQEMGADIEILDKRLAGGED | 300 |
| Query 301 | VADLRVRSSTLKGVTVPEDRAPSMIDEYPILavaaafaegaTVMNGLEELRVKESDRLSA | 360 |
| Sbjct 301 | VADLRVRSS LKGVTVP +RAPSMIDEYP+LAVAAAFAEG TVM GLEELRVKESDRLSA VADLRVRSSALKGVTVPAERAPSMIDEYPVLAVAAAFAEGTTVMLGLEELRVKESDRLSA | 360 |
| Query 361 | VANGLKLNGVDCDEGETSLVVRGRPDGKGLGNASGAAVATHLDHRIAMSFLVMGLVSENP | 420 |
| Sbjct 361 | VA GL LNGVDC EGE SL VRGRPDGKGLGNA G V THLDHRIAMSFLVMGL SE+P VAEGLTLNGVDCTEGEASLTVRGRPDGKGLGNA-GGEVKTHLDHRIAMSFLVMGLTSEHP | 419 |
| Query 421  Sbjct 420 | VTVDDATMIATSFPEFMDLMAGLGAKIEL 449 V VDD+ MIATSFPEF DLMA LGA +++ VKVDDSAMIATSFPEFFDLMAELGADMDV 448 |  |

>WP_110032593.1 3-phosphoshikimate 1-carboxyvinyltransferase [Hoeflea marina] PWW00368.1 3-phosphoshikimate 1-carboxyvinyltransferase [Hoeflea marina]

Length=452

Score = 727 bits (1877), Expect = 0.0, Method: Compositional matrix adjust.

| Identities | = 375/443 (85%), Positives = 401/443 (91%), Gaps = 1/443 (0%) |  |
| --- | --- | --- |
| Query 9 | PATARKSSGLSGTVRIPGDKSISHRSFMFGGLASGETRITGLLEGEDVINTGKAMQAMGA | 68 |
| Sbjct 10 | PATAR+SS LSG +RIPGDKSISHRSFMFGGLASGETRITGLLEGEDVINTG+AMQAMGA PATARRSSALSGDIRIPGDKSISHRSFMFGGLASGETRITGLLEGEDVINTGRAMQAMGA | 69 |
| Query 69 | RIRKEGDTWIIDGVGNGGLLAPEAPLDFGNAATGCRLTMGLVGVYDFDSTFIGDASLTKR | 128 |
| Sbjct 70 | IRK+GDTWII G GNG LLAPEAPLDFGNA TGCRLTMGLVGVYDF STFIGDASL++R SIRKDGDTWIIQGTGNGCLLAPEAPLDFGNAGTGCRLTMGLVGVYDFPSTFIGDASLSRR | 129 |
| Query 129 | PMGRVLNPLREMGVQVKSEDGDRLPVTLRGPKTPTPITYRVPMASAQVKSAVLLAGLNTP | 188 |
| Sbjct 130 | PMGRVL+PLR MGVQV SE GDRLPVTLRGP PTPI+YRVPM SAQVKSAVLLAGLNTP PMGRVLDPLRLMGVQVTSEPGDRLPVTLRGPAIPTPISYRVPMPSAQVKSAVLLAGLNTP | 189 |
| Query 189 | GITTVIEPIMTRDHTEKMLQGFGANLTVETDADGVRTIRLEGRGKLTGQVIDVPGDPSST | 248 |
| Sbjct 190 | G+TTVIEP+MTRDHTEKML GFGA+LTVETDA GVRTIRLEGRG+LTGQVIDVPGDPSST GVTTVIEPVMTRDHTEKMLAGFGADLTVETDAAGVRTIRLEGRGRLTGQVIDVPGDPSST | 249 |
| Query 249 | AFPLVAALLVPGSDVTILNVLMNPTRTGLILTLQEMGADIEVINPRLAGGEDVADLRVRS | 308 |
| Sbjct 250 | AFPLVAALLVPGSDVTI NVLMNPTRTGLILTL+EMGADIE+++PRLAGGEDVADLRVR+ AFPLVAALLVPGSDVTIRNVLMNPTRTGLILTLREMGADIEILDPRLAGGEDVADLRVRA | 309 |
| Query 309 | STLKGVTVPEDRAPSMIDEYPILavaaafaegaTVMNGLEELRVKESDRLSAVANGLKLN | 368 |
| Sbjct 310 | S L GVTVP +RAPSMIDEYP+LAVAA FA G TVM GL+ELRVKESDRL+AVA GL+LN SELTGVTVPAERAPSMIDEYPVLAVAACFARGETVMTGLDELRVKESDRLAAVARGLELN | 369 |
| Query 369 | GVDCDEGETSLVVRGRPDGKGLGNAS-GAAVATHLDHRIAMSFLVMGLVSENPVTVDDAT | 427 |
| Sbjct 370 | G DC EG L VRGRPDG+GLGN S A V T LDHRIAMSFLVMGL SE+PVTVDDA  GADCTEGPDWLSVRGRPDGRGLGNPSPDARVVTWLDHRIAMSFLVMGLASEHPVTVDDAA | 429 |

Query 428 MIATSFPEFMDLMAGLGAKIELS 450

+IATSFPEFM LMAGLGA I+ S Sbjct 430 IIATSFPEFMQLMAGLGADIQQS 452

>WP_151614879.1 3-phosphoshikimate 1-carboxyvinyltransferase [Ochrobactrum anthropi] KAB2789144.1 3-phosphoshikimate 1-carboxyvinyltransferase [Ochrobactrum anthropi]

Length=450

Score = 727 bits (1876), Expect = 0.0, Method: Compositional matrix adjust.

| Identities | = 370/453 (82%), Positives = 406/453 (90%), Gaps = 3/453 (1%) |  |
| --- | --- | --- |
| Query 1 | MLHGASSRPATARKSSGLSGTVRIPGDKSISHRSFMFGGLASGETRITGLLEGEDVINTG | 60 |
| Sbjct 1 | M H A +PATAR+S L+G +RIPGDKSISHRSFMFGGLASGETRITGLLEGEDVINTG MSHSAPPKPATARRSEALTGEIRIPGDKSISHRSFMFGGLASGETRITGLLEGEDVINTG | 60 |
| Query 61 | KAMQAMGARIRKEGDTWIIDGVGNGGLLAPEAPLDFGNAATGCRLTMGLVGVYDFDSTFI | 120 |
| Sbjct 61 | +AMQAMGA+IRK+GD WII+GVGNG LL PEA LDFGNA TG RLTMGLVG YD ++FI RAMQAMGAKIRKDGDAWIINGVGNGCLLQPEAALDFGNAGTGARLTMGLVGTYDMRTSFI | 120 |
| Query 121 | GDASLTKRPMGRVLNPLREMGVQVKSEDGDRLPVTLRGPKTPTPITYRVPMASAQVKSAV | 180 |
| Sbjct 121 | GDASL+KRPMGRVLNPLREMGVQV++ +GDR+P+TL GPKT PITYRVPMASAQVKSAV GDASLSKRPMGRVLNPLREMGVQVEAAEGDRMPLTLIGPKTANPITYRVPMASAQVKSAV | 180 |
| Query 181 | LLAGLNTPGITTVIEPIMTRDHTEKMLQGFGANLTVETDADGVRTIRLEGRGKLTGQVID | 240 |
| Sbjct 181 | LLAGLNTPG+TTVIEP+MTRDHTEKMLQGFGA+L+VETD DGVR IR+ G+GKL GQ ID LLAGLNTPGVTTVIEPVMTRDHTEKMLQGFGADLSVETDKDGVRHIRITGQGKLIGQTID | 240 |
| Query 241 | VPGDPSSTAFPLVAALLVPGSDVTILNVLMNPTRTGLILTLQEMGADIEVINPRLAGGED | 300 |
| Sbjct 241 | VPGDPSSTAFPLVAALLV GSDVTI NVLMNPTRTGLILTLQEMGA+IEV+N RLAGGED VPGDPSSTAFPLVAALLVEGSDVTIRNVLMNPTRTGLILTLQEMGANIEVLNARLAGGED | 300 |
| Query 301 | VADLRVRSSTLKGVTVPEDRAPSMIDEYPILavaaafaegaTVMNGLEELRVKESDRLSA | 360 |
| Sbjct 301 | VADLRV++S LKGV VP +RAPSMIDEYP+LA+AAAFAEG TVM+GL+ELRVKESDRL+A VADLRVKASKLKGVVVPPERAPSMIDEYPVLAIAAAFAEGETVMDGLDELRVKESDRLAA | 360 |
| Query 361 | VANGLKLNGVDCDEGETSLVVRGRPDGKGLGNASGAAVATHLDHRIAMSFLVMGLVSENP | 420 |
| Sbjct 361 | VA GL+ NGVDC EGE SL VRGRPDGK LG G VATHLDHRIAMSFLVMGL SE P VARGLEANGVDCTEGEMSLTVRGRPDGKELG---GGTVATHLDHRIAMSFLVMGLASEKP | 417 |
| Query 421  Sbjct 418 | VTVDDATMIATSFPEFMDLMAGLGAKIELSDTK 453 VTVDD+TMIATSFPEFMD+M GLGAKIELSD + VTVDDSTMIATSFPEFMDMMPGLGAKIELSDAR 450 |  |

>WP_031352532.1 3-phosphoshikimate 1-carboxyvinyltransferase [Brucella intermedia] Length=446

Score = 727 bits (1876), Expect = 0.0, Method: Compositional matrix adjust.

| Identities | = 369/446 (83%), Positives = 403/446 (90%), Gaps = 3/446 (1%) |  |
| --- | --- | --- |
| Query 8 | RPATARKSSGLSGTVRIPGDKSISHRSFMFGGLASGETRITGLLEGEDVINTGKAMQAMG | 67 |
| Sbjct 4 | +PATAR+S L+G +RIPGDKSISHRSFMFGGLASGETRITGLLEGEDVINTG+AMQAMG KPATARRSEALTGEIRIPGDKSISHRSFMFGGLASGETRITGLLEGEDVINTGRAMQAMG | 63 |
| Query 68 | ARIRKEGDTWIIDGVGNGGLLAPEAPLDFGNAATGCRLTMGLVGVYDFDSTFIGDASLTK | 127 |
| Sbjct 64 | A+IRKEGD WII+GVGNG LL PEA LDFGNA TG RLTMGLVG YD ++FIGDASL+K AKIRKEGDVWIINGVGNGCLLQPEAALDFGNAGTGARLTMGLVGTYDMKTSFIGDASLSK | 123 |
| Query 128 | RPMGRVLNPLREMGVQVKSEDGDRLPVTLRGPKTPTPITYRVPMASAQVKSAVLLAGLNT | 187 |
| Sbjct 124 | RPMGRVLNPLREMGVQV++ DGDR+P+TL GPKT PITYRVPMASAQVKSAVLLAGLNT RPMGRVLNPLREMGVQVEAADGDRMPLTLIGPKTANPITYRVPMASAQVKSAVLLAGLNT | 183 |
| Query 188 | PGITTVIEPIMTRDHTEKMLQGFGANLTVETDADGVRTIRLEGRGKLTGQVIDVPGDPSS | 247 |
| Sbjct 184 | PG+TTVIEP+MTRDHTEKMLQGFGA+LTVETD DGVR IR+ G+GKL GQ IDVPGDPSS PGVTTVIEPVMTRDHTEKMLQGFGADLTVETDKDGVRHIRITGQGKLVGQTIDVPGDPSS | 243 |
| Query 248 | TAFPLVAALLVPGSDVTILNVLMNPTRTGLILTLQEMGADIEVINPRLAGGEDVADLRVR | 307 |
| Sbjct 244 | TAFPLVAALLV GSDVTI NVLMNPTRTGLILTLQEMGADIEV+N RLAGGEDVADLRVR  TAFPLVAALLVEGSDVTIRNVLMNPTRTGLILTLQEMGADIEVLNARLAGGEDVADLRVR | 303 |

| Query | 308 | SSTLKGVTVPEDRAPSMIDEYPILavaaafaegaTVMNGLEELRVKESDRLSAVANGLKL | 367 |
| --- | --- | --- | --- |
| Sbjct | 304 | +S LKGV VP +RAPSMIDEYP+LA+AA+FAEG TVM+GL+ELRVKESDRL+AVA GL+ ASKLKGVVVPPERAPSMIDEYPVLAIAASFAEGETVMDGLDELRVKESDRLAAVARGLEA | 363 |
| Query | 368 | NGVDCDEGETSLVVRGRPDGKGLGNASGAAVATHLDHRIAMSFLVMGLVSENPVTVDDAT | 427 |
| Sbjct | 364 | NGVDC EGE SL VRGRPDGKGLG G VATHLDHRIAMSFLVMGL +E PVTVDD+ NGVDCTEGEMSLTVRGRPDGKGLG---GGTVATHLDHRIAMSFLVMGLAAEKPVTVDDSN | 420 |
| Query  Sbjct | 428  421 | MIATSFPEFMDLMAGLGAKIELSDTK 453 MIATSFPEFMD+M GLGAKIELS+ + MIATSFPEFMDMMPGLGAKIELSNAQ 446 |  |

>WP_105540776.1 3-phosphoshikimate 1-carboxyvinyltransferase [Ochrobactrum sp. MYb71]

PQZ28763.1 3-phosphoshikimate 1-carboxyvinyltransferase [Ochrobactrum sp. MYb71]

Length=450

Score = 727 bits (1876), Expect = 0.0, Method: Compositional matrix adjust.

| Identities | = 370/453 (82%), Positives = 404/453 (89%), Gaps = 3/453 (1%) |  |
| --- | --- | --- |
| Query 1 | MLHGASSRPATARKSSGLSGTVRIPGDKSISHRSFMFGGLASGETRITGLLEGEDVINTG | 60 |
| Sbjct 1 | M H AS +PA AR+S L G +RIPGDKSISHRSFMFGGLASGETRITGLLEGEDVINTG MSHSASPKPAIARRSEALKGEIRIPGDKSISHRSFMFGGLASGETRITGLLEGEDVINTG | 60 |
| Query 61 | KAMQAMGARIRKEGDTWIIDGVGNGGLLAPEAPLDFGNAATGCRLTMGLVGVYDFDSTFI | 120 |
| Sbjct 61 | +AMQAMGA+IRK+GD WII+GVGNG LL PEA LDFGNA TG RLTMGLVG YD + FI RAMQAMGAKIRKDGDVWIINGVGNGCLLQPEAALDFGNAGTGARLTMGLVGTYDMKTAFI | 120 |
| Query 121 | GDASLTKRPMGRVLNPLREMGVQVKSEDGDRLPVTLRGPKTPTPITYRVPMASAQVKSAV | 180 |
| Sbjct 121 | GDASLT RPMGRVLNPLREMGVQV++ GDR+P+TL GP+T PITYRVPMASAQVKSAV GDASLTSRPMGRVLNPLREMGVQVEAAAGDRMPLTLIGPRTANPITYRVPMASAQVKSAV | 180 |
| Query 181 | LLAGLNTPGITTVIEPIMTRDHTEKMLQGFGANLTVETDADGVRTIRLEGRGKLTGQVID | 240 |
| Sbjct 181 | LLAGLNTPG+TTVIEP+MTRDHTEKMLQGFGA+L+VETD DGVR IR+ G+GKLTGQ ID LLAGLNTPGVTTVIEPVMTRDHTEKMLQGFGADLSVETDKDGVRHIRITGQGKLTGQTID | 240 |
| Query 241 | VPGDPSSTAFPLVAALLVPGSDVTILNVLMNPTRTGLILTLQEMGADIEVINPRLAGGED | 300 |
| Sbjct 241 | VPGDPSSTAFPLVAALLV GSDVTI NVLMNPTRTGLILTLQEMGADIE+I+PRLAGGED VPGDPSSTAFPLVAALLVEGSDVTIRNVLMNPTRTGLILTLQEMGADIEIIDPRLAGGED | 300 |
| Query 301 | VADLRVRSSTLKGVTVPEDRAPSMIDEYPILavaaafaegaTVMNGLEELRVKESDRLSA | 360 |
| Sbjct 301 | VADLRV++S LKGV VP +RAPSMIDEYP+LAVAA+FAEG T+M+GL+ELRVKESDRL+A VADLRVKASKLKGVVVPPERAPSMIDEYPVLAVAASFAEGETLMDGLDELRVKESDRLAA | 360 |
| Query 361 | VANGLKLNGVDCDEGETSLVVRGRPDGKGLGNASGAAVATHLDHRIAMSFLVMGLVSENP | 420 |
| Sbjct 361 | VA GL+ NGVDC EGE SL VRGRPDGKGLG G VATHLDHRIAMSFLVMGL SE P VARGLEANGVDCTEGEMSLTVRGRPDGKGLG---GGTVATHLDHRIAMSFLVMGLASEKP | 417 |
| Query 421  Sbjct 418 | VTVDDATMIATSFPEFMDLMAGLGAKIELSDTK 453 VTVDD+TMIATSFPEFM +M GLGAKIELSD + VTVDDSTMIATSFPEFMSMMPGLGAKIELSDAR 450 |  |

>WP_182519086.1 3-phosphoshikimate 1-carboxyvinyltransferase [Ochrobactrum sp. RH2CCR150]

MBA8837077.1 3-phosphoshikimate 1-carboxyvinyltransferase [Ochrobactrum sp. RH2CCR150]

Length=450

Score = 727 bits (1876), Expect = 0.0, Method: Compositional matrix adjust. Identities = 370/453 (82%), Positives = 404/453 (89%), Gaps = 3/453 (1%)

| Query | 1 | MLHGASSRPATARKSSGLSGTVRIPGDKSISHRSFMFGGLASGETRITGLLEGEDVINTG | 60 |
| --- | --- | --- | --- |
| Sbjct | 1 | M H AS +PA AR+S L+G +RIPGDKSISHRSFMFGGLASGETRITGLLEGEDVINTG MSHSASPKPAVARRSEALAGEIRIPGDKSISHRSFMFGGLASGETRITGLLEGEDVINTG | 60 |
| Query | 61 | KAMQAMGARIRKEGDTWIIDGVGNGGLLAPEAPLDFGNAATGCRLTMGLVGVYDFDSTFI | 120 |
| Sbjct | 61 | +AMQAMGA+I K+GD WII+GVGNG LL PEAPLDFGNA TG RLTMGLVG YD + FI  RAMQAMGAKIEKDGDVWIINGVGNGCLLEPEAPLDFGNAGTGARLTMGLVGTYDMTTAFI | 120 |

| Query | 121 | GDASLTKRPMGRVLNPLREMGVQVKSEDGDRLPVTLRGPKTPTPITYRVPMASAQVKSAV | 180 |
| --- | --- | --- | --- |
| Sbjct | 121 | GDASL++RPMGRVL+PLR+MGVQVKS DGDR+P+TL GP PITYRVPMASAQVKSAV GDASLSRRPMGRVLDPLRQMGVQVKSTDGDRMPLTLVGPSVANPITYRVPMASAQVKSAV | 180 |
| Query | 181 | LLAGLNTPGITTVIEPIMTRDHTEKMLQGFGANLTVETDADGVRTIRLEGRGKLTGQVID | 240 |
| Sbjct | 181 | LLAGLNTPG+TTVIEP+MTRDHTEKMLQGFGA+LTVETD DGVR IR+ G+GKL GQ ID LLAGLNTPGVTTVIEPVMTRDHTEKMLQGFGADLTVETDKDGVRHIRITGQGKLIGQTID | 240 |
| Query | 241 | VPGDPSSTAFPLVAALLVPGSDVTILNVLMNPTRTGLILTLQEMGADIEVINPRLAGGED | 300 |
| Sbjct | 241 | VPGDPSSTAFPLVAALLV GSDVTI NVLMNPTRTGLILTLQEMGADIEV N RLAGGED VPGDPSSTAFPLVAALLVEGSDVTIRNVLMNPTRTGLILTLQEMGADIEVFNERLAGGED | 300 |
| Query | 301 | VADLRVRSSTLKGVTVPEDRAPSMIDEYPILavaaafaegaTVMNGLEELRVKESDRLSA | 360 |
| Sbjct | 301 | VADLRV+SS LKGVTVP +RAPSMIDEYP+LA+AA+FAEG TVM+GL+ELRVKESDRL+A VADLRVKSSRLKGVTVPPERAPSMIDEYPVLAIAASFAEGETVMDGLDELRVKESDRLAA | 360 |
| Query | 361 | VANGLKLNGVDCDEGETSLVVRGRPDGKGLGNASGAAVATHLDHRIAMSFLVMGLVSENP | 420 |
| Sbjct | 361 | VA GL++NGVDC EGE SL VRGRPDGKGLG G VATHLDHRIAMSFLVMGL SE P VARGLEVNGVDCTEGEMSLTVRGRPDGKGLG---GGVVATHLDHRIAMSFLVMGLASEKP | 417 |
| Query  Sbjct | 421  418 | VTVDDATMIATSFPEFMDLMAGLGAKIELSDTK 453 VTVDD+TMIATSFPEFMDLM G+GAKIE S+ + VTVDDSTMIATSFPEFMDLMTGMGAKIEPSEAE 450 |  |

>OJY06829.1 3-phosphoshikimate 1-carboxyvinyltransferase [Rhizobiales bacterium 63-22]

Length=450

Score = 726 bits (1875), Expect = 0.0, Method: Compositional matrix adjust.

| Identities | = 372/450 (83%), Positives = 405/450 (90%), Gaps = 3/450 (1%) |  |
| --- | --- | --- |
| Query 1 | MLHGASSRPATARKSSGLSGTVRIPGDKSISHRSFMFGGLASGETRITGLLEGEDVINTG | 60 |
| Sbjct 1 | M H AS +PATAR+S+ LSG +RIPGDKSISHRSFMFGGLASG+TRITGLLEGEDVINTG MSHSASPKPATARRSTALSGDIRIPGDKSISHRSFMFGGLASGQTRITGLLEGEDVINTG | 60 |
| Query 61 | KAMQAMGARIRKEGDTWIIDGVGNGGLLAPEAPLDFGNAATGCRLTMGLVGVYDFDSTFI | 120 |
| Sbjct 61 | +AMQAMGA+IRKEGD WIIDGVGNG LL P+ PLDFGNA TG RLTMGLVG D +TF+ RAMQAMGAKIRKEGDVWIIDGVGNGCLLEPKGPLDFGNAGTGARLTMGLVGTCDMTTTFV | 120 |
| Query 121 | GDASLTKRPMGRVLNPLREMGVQVKSEDGDRLPVTLRGPKTPTPITYRVPMASAQVKSAV | 180 |
| Sbjct 121 | GDASL++RPMGRVLNPLR+MGVQVK+ +GDRLP+TL GPKT PI+YRVPMASAQVKSAV GDASLSRRPMGRVLNPLRQMGVQVKAAEGDRLPLTLTGPKTANPISYRVPMASAQVKSAV | 180 |
| Query 181 | LLAGLNTPGITTVIEPIMTRDHTEKMLQGFGANLTVETDADGVRTIRLEGRGKLTGQVID | 240 |
| Sbjct 181 | LLAGLNTPG+TTVIEP+MTRDHTEKMLQGFGA+LTVETD DGVR IR+ G+GKLTGQ ID LLAGLNTPGVTTVIEPVMTRDHTEKMLQGFGADLTVETDKDGVRHIRIVGQGKLTGQTID | 240 |
| Query 241 | VPGDPSSTAFPLVAALLVPGSDVTILNVLMNPTRTGLILTLQEMGADIEVINPRLAGGED | 300 |
| Sbjct 241 | VPGDPSSTAFPLVAALL GSDVTI NVLMNPTRTGLILTLQEMGA+IEV+N RLAGGED VPGDPSSTAFPLVAALLTEGSDVTIRNVLMNPTRTGLILTLQEMGAEIEVMNARLAGGED | 300 |
| Query 301 | VADLRVRSSTLKGVTVPEDRAPSMIDEYPILavaaafaegaTVMNGLEELRVKESDRLSA | 360 |
| Sbjct 301 | VADLRV++S LKGVTVP +RAPSMIDEYP+LAVAAAFAEG TVM+GL+ELRVKESDRL+A VADLRVKASKLKGVTVPPERAPSMIDEYPVLAVAAAFAEGETVMDGLDELRVKESDRLAA | 360 |
| Query 361 | VANGLKLNGVDCDEGETSLVVRGRPDGKGLGNASGAAVATHLDHRIAMSFLVMGLVSENP | 420 |
| Sbjct 361 | VA GL+ NGVDC EG SL VRGRPDGKGLG G VATHLDHRIAMSFLVMGL SE P VARGLEANGVDCTEGAMSLTVRGRPDGKGLG---GGTVATHLDHRIAMSFLVMGLASEKP | 417 |
| Query 421  Sbjct 418 | VTVDDATMIATSFPEFMDLMAGLGAKIELS 450 VTVDD+TMIATSFPEFM LMAGLGAKIE S VTVDDSTMIATSFPEFMGLMAGLGAKIEQS 447 |  |

>WP_171316908.1 3-phosphoshikimate 1-carboxyvinyltransferase [Ochrobactrum soli] NNU59081.1 3-phosphoshikimate 1-carboxyvinyltransferase [Ochrobactrum soli]

Length=450

Score = 726 bits (1875), Expect = 0.0, Method: Compositional matrix adjust. Identities = 368/453 (81%), Positives = 404/453 (89%), Gaps = 3/453 (1%)

| Query | 1 | MLHGASSRPATARKSSGLSGTVRIPGDKSISHRSFMFGGLASGETRITGLLEGEDVINTG | 60 |
| --- | --- | --- | --- |
| Sbjct | 1 | M H AS +PA AR+S L+G +RIPGDKSISHRSFMFGGLASGETRITGLLEGEDVINTG MSHSASPKPAVARRSEALAGEIRIPGDKSISHRSFMFGGLASGETRITGLLEGEDVINTG | 60 |
| Query | 61 | KAMQAMGARIRKEGDTWIIDGVGNGGLLAPEAPLDFGNAATGCRLTMGLVGVYDFDSTFI | 120 |
| Sbjct | 61 | +AMQAMGA+I K+GDTWII GVGNG LL PEAPLDFGNA TG RLTMGLVG YD + FI RAMQAMGAKIEKDGDTWIIHGVGNGCLLEPEAPLDFGNAGTGARLTMGLVGTYDMTTAFI | 120 |
| Query | 121 | GDASLTKRPMGRVLNPLREMGVQVKSEDGDRLPVTLRGPKTPTPITYRVPMASAQVKSAV | 180 |
| Sbjct | 121 | GDASL++RPMGRVL+PLR+MGVQVKS DGDR+P+TL GP PITYRVPMASAQVKSAV GDASLSRRPMGRVLDPLRQMGVQVKSTDGDRMPLTLVGPSVANPITYRVPMASAQVKSAV | 180 |
| Query | 181 | LLAGLNTPGITTVIEPIMTRDHTEKMLQGFGANLTVETDADGVRTIRLEGRGKLTGQVID | 240 |
| Sbjct | 181 | LLAGLNTPG+TTVIEP+MTRDHTEKMLQGFGA+LTVETD DGVR IR+ G+GKL GQ ID LLAGLNTPGVTTVIEPVMTRDHTEKMLQGFGADLTVETDKDGVRHIRITGQGKLIGQTID | 240 |
| Query | 241 | VPGDPSSTAFPLVAALLVPGSDVTILNVLMNPTRTGLILTLQEMGADIEVINPRLAGGED | 300 |
| Sbjct | 241 | VPGDPSSTAFPLVAALLV GSD+TI NVLMNPTRTGLILTLQEMGADIEV+N RLAGGED VPGDPSSTAFPLVAALLVEGSDITIRNVLMNPTRTGLILTLQEMGADIEVLNERLAGGED | 300 |
| Query | 301 | VADLRVRSSTLKGVTVPEDRAPSMIDEYPILavaaafaegaTVMNGLEELRVKESDRLSA | 360 |
| Sbjct | 301 | VADLRV+SS LKGVTVP +RAPSMIDEYP+LA+AA FAEG T+M+GL+ELRVKESDRL+A VADLRVKSSKLKGVTVPPERAPSMIDEYPVLAIAATFAEGETIMDGLDELRVKESDRLAA | 360 |
| Query | 361 | VANGLKLNGVDCDEGETSLVVRGRPDGKGLGNASGAAVATHLDHRIAMSFLVMGLVSENP | 420 |
| Sbjct | 361 | VA GL++NGVDC EGE SL VRGRPDGKG+G G VATHLDHRIAMSFLVMGL SE P VARGLEVNGVDCTEGEMSLTVRGRPDGKGIG---GGVVATHLDHRIAMSFLVMGLASEKP | 417 |
| Query  Sbjct | 421  418 | VTVDDATMIATSFPEFMDLMAGLGAKIELSDTK 453 VTVDD+TMIATSFPEFMDLM G+GAKIE S+ + VTVDDSTMIATSFPEFMDLMTGMGAKIEPSEAE 450 |  |

>WP_174041607.1 3-phosphoshikimate 1-carboxyvinyltransferase [Agrobacterium rhizogenes] NTH43191.1 3-phosphoshikimate 1-carboxyvinyltransferase [Agrobacterium rhizogenes] NTH56067.1 3-phosphoshikimate 1-carboxyvinyltransferase [Agrobacterium rhizogenes] NTH87696.1 3-phosphoshikimate 1-carboxyvinyltransferase [Agrobacterium rhizogenes]

Length=454

Score = 726 bits (1874), Expect = 0.0, Method: Compositional matrix adjust.

| Identities | = 370/449 (82%), Positives = 403/449 (90%), Gaps = 1/449 (0%) |  |
| --- | --- | --- |
| Query 1 | MLHGASSRPATARKSSGLSGTVRIPGDKSISHRSFMFGGLASGETRITGLLEGEDVINTG | 60 |
| Sbjct 1 | M H SRPATAR+S+ LSGT+R+PGDKSISHR+FMFGGLASGETRITGLLEGEDV+NTG MPHDTHSRPATARRSTNLSGTIRVPGDKSISHRAFMFGGLASGETRITGLLEGEDVLNTG | 60 |
| Query 61 | KAMQAMGARIRKEGDTWIIDGVGNGGLLAPEAPLDFGNAATGCRLTMGLVGVYDFDSTFI | 120 |
| Sbjct 61 | KAM+AMGA+I K GD WII GVGNG LLAPEAPLDFGNA TG RLTMGLVGVY F+STFI KAMRAMGAKIEKAGDEWIIKGVGNGALLAPEAPLDFGNAGTGSRLTMGLVGVYHFESTFI | 120 |
| Query 121 | GDASLTKRPMGRVLNPLREMGVQVKSEDGDRLPVTLRGPKTPTPITYRVPMASAQVKSAV | 180 |
| Sbjct 121 | GDASL+KRPMGR+L+PLR+MGVQVKS +GDRLPVTL GP+T PITYRVPMASAQVKSA+ GDASLSKRPMGRILDPLRQMGVQVKSSEGDRLPVTLHGPETANPITYRVPMASAQVKSAM | 180 |
| Query 181 | LLAGLNTPGITTVIEPIMTRDHTEKMLQGFGANLTVETDADGVRTIRLEGRGKLTGQVID | 240 |
| Sbjct 181 | LLAGLN PGIT+VIEP+MTRDHTEKML GFGA L+VETD DGVR IRL+G+GKL GQ ID LLAGLNAPGITSVIEPVMTRDHTEKMLAGFGAELSVETDRDGVRIIRLQGQGKLFGQTID | 240 |
| Query 241 | VPGDPSSTAFPLVAALLVPGSDVTILNVLMNPTRTGLILTLQEMGADIEVINPRLAGGED | 300 |
| Sbjct 241 | VPGDPSSTAFPLVAAL+V GSD+TI NVLMNPTRTGLILTLQEMGADIE+++ RLAGGED VPGDPSSTAFPLVAALIVAGSDITIRNVLMNPTRTGLILTLQEMGADIEILDKRLAGGED | 300 |
| Query 301 | VADLRVRSSTLKGVTVPEDRAPSMIDEYPILavaaafaegaTVMNGLEELRVKESDRLSA | 360 |
| Sbjct 301 | VADLRVRSS LKGVTVP +RAPSMIDEYP+LAVAAAFAEG TVM GLEELRVKESDRLSA VADLRVRSSALKGVTVPAERAPSMIDEYPVLAVAAAFAEGTTVMLGLEELRVKESDRLSA | 360 |
| Query 361 | VANGLKLNGVDCDEGETSLVVRGRPDGKGLGNASGAAVATHLDHRIAMSFLVMGLVSENP | 420 |
| Sbjct 361 | VA GL LNGVDC EGE SL VRGRPDGKGLGNA G V THLDHRIAMSFLVMGL SE+P VAEGLTLNGVDCTEGEASLTVRGRPDGKGLGNA-GGEVKTHLDHRIAMSFLVMGLASEHP | 419 |
| Query 421 | VTVDDATMIATSFPEFMDLMAGLGAKIEL 449 V VDD+ MIATSFPEF DLMA LGA +++ |  |

Sbjct 420 VKVDDSAMIATSFPEFFDLMAELGADMDV 448

>WP_109368078.1 3-phosphoshikimate 1-carboxyvinyltransferase [Ochrobactrum soli] RLL75079.1 3-phosphoshikimate 1-carboxyvinyltransferase [Ochrobactrum soli] RRD27770.1 3-phosphoshikimate 1-carboxyvinyltransferase [Brucellaceae bacterium

VT-16-1752]

SPL64162.1 5-Enolpyruvylshikimate-3-phosphate synthase [Ochrobactrum soli] Length=450

Score = 726 bits (1874), Expect = 0.0, Method: Compositional matrix adjust.

| Identities | = 368/453 (81%), Positives = 403/453 (89%), Gaps = 3/453 (1%) |  |
| --- | --- | --- |
| Query 1 | MLHGASSRPATARKSSGLSGTVRIPGDKSISHRSFMFGGLASGETRITGLLEGEDVINTG | 60 |
| Sbjct 1 | M H AS +PA AR+S L+G +RIPGDKSISHRSFMFGGLASGETRITGLLEGEDVINTG MSHSASPKPAVARRSEALAGEIRIPGDKSISHRSFMFGGLASGETRITGLLEGEDVINTG | 60 |
| Query 61 | KAMQAMGARIRKEGDTWIIDGVGNGGLLAPEAPLDFGNAATGCRLTMGLVGVYDFDSTFI | 120 |
| Sbjct 61 | +AMQAMGA+I K+GDTWII GVGNG LL PEAPLDFGNA TG RLTMGLVG YD + FI RAMQAMGAKIEKDGDTWIIHGVGNGCLLEPEAPLDFGNAGTGARLTMGLVGTYDMTTAFI | 120 |
| Query 121 | GDASLTKRPMGRVLNPLREMGVQVKSEDGDRLPVTLRGPKTPTPITYRVPMASAQVKSAV | 180 |
| Sbjct 121 | GDASL++RPMGRVL+PLR+MGVQVKS DGDR+P+TL GP PITYRVPMASAQVKSAV GDASLSRRPMGRVLDPLRQMGVQVKSTDGDRMPLTLVGPSVANPITYRVPMASAQVKSAV | 180 |
| Query 181 | LLAGLNTPGITTVIEPIMTRDHTEKMLQGFGANLTVETDADGVRTIRLEGRGKLTGQVID | 240 |
| Sbjct 181 | LLAGLNTPG+TTVIEP+MTRDHTEKMLQGFGA+LTVETD DGVR IR+ G+GKL GQ ID LLAGLNTPGVTTVIEPVMTRDHTEKMLQGFGADLTVETDKDGVRHIRITGQGKLIGQTID | 240 |
| Query 241 | VPGDPSSTAFPLVAALLVPGSDVTILNVLMNPTRTGLILTLQEMGADIEVINPRLAGGED | 300 |
| Sbjct 241 | VPGDPSSTAFPLVAALLV GSD+TI NVLMNPTRTGLILTLQEMGADIEV N RLAGGED VPGDPSSTAFPLVAALLVEGSDITIRNVLMNPTRTGLILTLQEMGADIEVFNERLAGGED | 300 |
| Query 301 | VADLRVRSSTLKGVTVPEDRAPSMIDEYPILavaaafaegaTVMNGLEELRVKESDRLSA | 360 |
| Sbjct 301 | VADLRV+SS LKGVTVP +RAPSMIDEYP+LA+AA FAEG T+M+GL+ELRVKESDRL+A VADLRVKSSKLKGVTVPPERAPSMIDEYPVLAIAATFAEGETIMDGLDELRVKESDRLAA | 360 |
| Query 361 | VANGLKLNGVDCDEGETSLVVRGRPDGKGLGNASGAAVATHLDHRIAMSFLVMGLVSENP | 420 |
| Sbjct 361 | VA GL++NGVDC EGE SL VRGRPDGKG+G G VATHLDHRIAMSFLVMGL SE P VARGLEVNGVDCTEGEMSLTVRGRPDGKGIG---GGVVATHLDHRIAMSFLVMGLASEKP | 417 |
| Query 421  Sbjct 418 | VTVDDATMIATSFPEFMDLMAGLGAKIELSDTK 453 VTVDD+TMIATSFPEFMDLM G+GAKIE S+ + VTVDDSTMIATSFPEFMDLMTGMGAKIEPSEAE 450 |  |

>WP_174015292.1 3-phosphoshikimate 1-carboxyvinyltransferase [Agrobacterium rhizogenes] NTG84580.1 3-phosphoshikimate 1-carboxyvinyltransferase [Agrobacterium rhizogenes] NTI14068.1 3-phosphoshikimate 1-carboxyvinyltransferase [Agrobacterium rhizogenes] NTI26980.1 3-phosphoshikimate 1-carboxyvinyltransferase [Agrobacterium rhizogenes] NTI59934.1 3-phosphoshikimate 1-carboxyvinyltransferase [Agrobacterium rhizogenes]

Length=454

Score = 726 bits (1874), Expect = 0.0, Method: Compositional matrix adjust.

| Identities | = 370/449 (82%), Positives = 402/449 (90%), Gaps = 1/449 (0%) |  |
| --- | --- | --- |
| Query 1 | MLHGASSRPATARKSSGLSGTVRIPGDKSISHRSFMFGGLASGETRITGLLEGEDVINTG | 60 |
| Sbjct 1 | M H SRPATAR+S+ LSGT+R+PGDKSISHR+FMFGGLASGETRITGLLEGEDV+NTG MPHDTHSRPATARRSTNLSGTIRVPGDKSISHRAFMFGGLASGETRITGLLEGEDVLNTG | 60 |
| Query 61 | KAMQAMGARIRKEGDTWIIDGVGNGGLLAPEAPLDFGNAATGCRLTMGLVGVYDFDSTFI | 120 |
| Sbjct 61 | KAM+AMGA+I K GD WII GVGNG LLAPEAPLDFGNA TG RLTMGLVGVY F+STFI KAMRAMGAKIEKAGDEWIIKGVGNGALLAPEAPLDFGNAGTGSRLTMGLVGVYHFESTFI | 120 |
| Query 121 | GDASLTKRPMGRVLNPLREMGVQVKSEDGDRLPVTLRGPKTPTPITYRVPMASAQVKSAV | 180 |
| Sbjct 121 | GD SL+KRPMGR+L+PLR+MGVQVKS +GDRLPVTL GP+T PITYRVPMASAQVKSAV GDVSLSKRPMGRILDPLRQMGVQVKSSEGDRLPVTLHGPETANPITYRVPMASAQVKSAV | 180 |
| Query 181 | LLAGLNTPGITTVIEPIMTRDHTEKMLQGFGANLTVETDADGVRTIRLEGRGKLTGQVID | 240 |
| Sbjct 181 | LLAGLN PGIT+VIEP+MTRDHTEKML GFGA L+VETD DGVR IRL+G+GKL GQ ID  LLAGLNAPGITSVIEPVMTRDHTEKMLAGFGAELSVETDRDGVRIIRLQGQGKLFGQTID | 240 |

| Query | 241 | VPGDPSSTAFPLVAALLVPGSDVTILNVLMNPTRTGLILTLQEMGADIEVINPRLAGGED | 300 |
| --- | --- | --- | --- |
| Sbjct | 241 | VPGDPSSTAFPLVAAL+V GSD+TI NVLMNPTRTGLILTLQEMGADIE+++ RLAGGED VPGDPSSTAFPLVAALIVAGSDITIRNVLMNPTRTGLILTLQEMGADIEILDKRLAGGED | 300 |
| Query | 301 | VADLRVRSSTLKGVTVPEDRAPSMIDEYPILavaaafaegaTVMNGLEELRVKESDRLSA | 360 |
| Sbjct | 301 | VADLRVRSS LKGVTVP +RAPSMIDEYP+LAVAAAFAEG TVM GLEELRVKESDRLSA VADLRVRSSALKGVTVPAERAPSMIDEYPVLAVAAAFAEGTTVMLGLEELRVKESDRLSA | 360 |
| Query | 361 | VANGLKLNGVDCDEGETSLVVRGRPDGKGLGNASGAAVATHLDHRIAMSFLVMGLVSENP | 420 |
| Sbjct | 361 | VA GL LNGVDC EGE SL VRGRPDGKGLGNA G V THLDHRIAMSFLVMGL SE+P VAEGLTLNGVDCTEGEASLTVRGRPDGKGLGNA-GGEVKTHLDHRIAMSFLVMGLASEHP | 419 |
| Query  Sbjct | 421  420 | VTVDDATMIATSFPEFMDLMAGLGAKIEL 449 V VDD+ MIATSFPEF DLMA LGA +++ VKVDDSAMIATSFPEFFDLMAELGADMDV 448 |  |

>WP_174013348.1 3-phosphoshikimate 1-carboxyvinyltransferase [Agrobacterium rhizogenes] NTF53319.1 3-phosphoshikimate 1-carboxyvinyltransferase [Agrobacterium rhizogenes] NTF72900.1 3-phosphoshikimate 1-carboxyvinyltransferase [Agrobacterium rhizogenes] NTG12182.1 3-phosphoshikimate 1-carboxyvinyltransferase [Agrobacterium rhizogenes] NTG25588.1 3-phosphoshikimate 1-carboxyvinyltransferase [Agrobacterium rhizogenes] NTH62275.1 3-phosphoshikimate 1-carboxyvinyltransferase [Agrobacterium rhizogenes]

Length=454

Score = 726 bits (1874), Expect = 0.0, Method: Compositional matrix adjust.

| Identities | = 370/449 (82%), Positives = 402/449 (90%), Gaps = 1/449 (0%) |  |
| --- | --- | --- |
| Query 1 | MLHGASSRPATARKSSGLSGTVRIPGDKSISHRSFMFGGLASGETRITGLLEGEDVINTG | 60 |
| Sbjct 1 | M H SRPATAR+S+ LSGT+R+PGDKSISHR+FMFGGLASGETRITGLLEGEDV+NTG MPHDTHSRPATARRSTNLSGTIRVPGDKSISHRAFMFGGLASGETRITGLLEGEDVLNTG | 60 |
| Query 61 | KAMQAMGARIRKEGDTWIIDGVGNGGLLAPEAPLDFGNAATGCRLTMGLVGVYDFDSTFI | 120 |
| Sbjct 61 | KAM+AMGA+I K GD WII GVGNG LLAPEAPLDFGNA TG RLTMGLVGVY F+STFI KAMRAMGAKIEKAGDEWIIKGVGNGALLAPEAPLDFGNAGTGSRLTMGLVGVYHFESTFI | 120 |
| Query 121 | GDASLTKRPMGRVLNPLREMGVQVKSEDGDRLPVTLRGPKTPTPITYRVPMASAQVKSAV | 180 |
| Sbjct 121 | GDASL+KRPMGR+L+P R+MGVQVKS +GDRLPVTL GP+T PITYRVPMASAQVKSAV GDASLSKRPMGRILDPFRQMGVQVKSSEGDRLPVTLHGPETANPITYRVPMASAQVKSAV | 180 |
| Query 181 | LLAGLNTPGITTVIEPIMTRDHTEKMLQGFGANLTVETDADGVRTIRLEGRGKLTGQVID | 240 |
| Sbjct 181 | LLAGLN PGIT+VIEP+MTRDHTEKML GFGA L+VETD DGVR IRL+G+GKL GQ ID LLAGLNAPGITSVIEPVMTRDHTEKMLAGFGAELSVETDRDGVRIIRLQGQGKLFGQTID | 240 |
| Query 241 | VPGDPSSTAFPLVAALLVPGSDVTILNVLMNPTRTGLILTLQEMGADIEVINPRLAGGED | 300 |
| Sbjct 241 | VPGDPSSTAFPLVAAL+V GSD+TI NVLMNPTRTGLILTLQEMGADIE+++ RLAGGED VPGDPSSTAFPLVAALIVAGSDITIRNVLMNPTRTGLILTLQEMGADIEILDKRLAGGED | 300 |
| Query 301 | VADLRVRSSTLKGVTVPEDRAPSMIDEYPILavaaafaegaTVMNGLEELRVKESDRLSA | 360 |
| Sbjct 301 | VADLRVRSS LKGVTVP +RAPSMIDEYP+LAVAAAFAEG TVM GLEELRVKESDRLSA VADLRVRSSALKGVTVPAERAPSMIDEYPVLAVAAAFAEGTTVMLGLEELRVKESDRLSA | 360 |
| Query 361 | VANGLKLNGVDCDEGETSLVVRGRPDGKGLGNASGAAVATHLDHRIAMSFLVMGLVSENP | 420 |
| Sbjct 361 | VA GL LNGVDC EGE SL VRGRPDGKGLGNA G V THLDHRIAMSFLVMGL SE+P VAEGLTLNGVDCTEGEASLTVRGRPDGKGLGNA-GGEVKTHLDHRIAMSFLVMGLASEHP | 419 |
| Query 421  Sbjct 420 | VTVDDATMIATSFPEFMDLMAGLGAKIEL 449 V VDD+ MIATSFPEF DLMA LGA +++ VKVDDSAMIATSFPEFFDLMAELGADMDV 448 |  |

```
>WP_174017532.1 3-phosphoshikimate 1-carboxyvinyltransferase [Agrobacterium rhizogenes] NTG39439.1 3-phosphoshikimate 1-carboxyvinyltransferase [Agrobacterium rhizogenes]
Length=454

Score = 726 bits (1873), Expect = 0.0, Method: Compositional matrix adjust. Identities = 370/449 (82%), Positives = 402/449 (90%), Gaps = 1/449 (0%)

Query 1	MLHGASSRPATARKSSGLSGTVRIPGDKSISHRSFMFGGLASGETRITGLLEGEDVINTG 60 M H	SRPATAR+S+ LSGT+R+PGDKSISHR+FMFGGLASGETRITGLLEGEDV+NTG
Sbjct 1	MPHDTHSRPATARRSTNLSGTIRVPGDKSISHRAFMFGGLASGETRITGLLEGEDVLNTG 60
```

| Query | 61 | KAMQAMGARIRKEGDTWIIDGVGNGGLLAPEAPLDFGNAATGCRLTMGLVGVYDFDSTFI | 120 |
| --- | --- | --- | --- |
| Sbjct | 61 | KAM+AMGA+I K GD WII GVGNG LLAPEAPLDFGNA TG RLTMGLVGVY F+STFI KAMRAMGAKIEKAGDEWIIKGVGNGALLAPEAPLDFGNAGTGSRLTMGLVGVYHFESTFI | 120 |
| Query | 121 | GDASLTKRPMGRVLNPLREMGVQVKSEDGDRLPVTLRGPKTPTPITYRVPMASAQVKSAV | 180 |
| Sbjct | 121 | GDASL+KRPMGR+L+PLR+MGVQVKS +GDRLPVTL GP+T PITYRVPMASAQVKSAV GDASLSKRPMGRILDPLRQMGVQVKSSEGDRLPVTLHGPETANPITYRVPMASAQVKSAV | 180 |
| Query | 181 | LLAGLNTPGITTVIEPIMTRDHTEKMLQGFGANLTVETDADGVRTIRLEGRGKLTGQVID | 240 |
| Sbjct | 181 | LLAGLN PGIT+VIEP+MTRDHTEKML GFGA L+VETD DGVR IRL+G+GKL GQ ID LLAGLNAPGITSVIEPVMTRDHTEKMLAGFGAELSVETDRDGVRIIRLQGQGKLFGQTID | 240 |
| Query | 241 | VPGDPSSTAFPLVAALLVPGSDVTILNVLMNPTRTGLILTLQEMGADIEVINPRLAGGED | 300 |
| Sbjct | 241 | VPGDPSSTAFPLVAAL+V GSD+TI NVLMNP RTGLILTLQEMGADIE+++ RLAGGED VPGDPSSTAFPLVAALIVAGSDITIRNVLMNPARTGLILTLQEMGADIEILDKRLAGGED | 300 |
| Query | 301 | VADLRVRSSTLKGVTVPEDRAPSMIDEYPILavaaafaegaTVMNGLEELRVKESDRLSA | 360 |
| Sbjct | 301 | VADLRVRSS LKGVTVP +RAPSMIDEYP+LAVAAAFAEG TVM GLEELRVKESDRLSA VADLRVRSSALKGVTVPAERAPSMIDEYPVLAVAAAFAEGTTVMLGLEELRVKESDRLSA | 360 |
| Query | 361 | VANGLKLNGVDCDEGETSLVVRGRPDGKGLGNASGAAVATHLDHRIAMSFLVMGLVSENP | 420 |
| Sbjct | 361 | VA GL LNGVDC EGE SL VRGRPDGKGLGNA G V THLDHRIAMSFLVMGL SE+P VAEGLTLNGVDCTEGEASLTVRGRPDGKGLGNA-GGEVKTHLDHRIAMSFLVMGLASEHP | 419 |
| Query  Sbjct | 421  420 | VTVDDATMIATSFPEFMDLMAGLGAKIEL 449 V VDD+ MIATSFPEF DLMA LGA +++ VRVDDSAMIATSFPEFFDLMAELGADMDV 448 |  |

>WP_091920605.1 3-phosphoshikimate 1-carboxyvinyltransferase [Phyllobacterium sp. CL33Tsu]

SFI72059.1 3-phosphoshikimate 1-carboxyvinyltransferase [Phyllobacterium sp. CL33Tsu]

Length=451

Score = 726 bits (1873), Expect = 0.0, Method: Compositional matrix adjust.

| Identities | = 369/447 (83%), Positives = 400/447 (89%), Gaps = 3/447 (1%) |  |
| --- | --- | --- |
| Query 1 | MLHGASSRPATARKSSGLSGTVRIPGDKSISHRSFMFGGLASGETRITGLLEGEDVINTG | 60 |
| Sbjct 1 | M H + RPATAR+S LSG +RIPGDKSISHRSFMFGGLASGETRITGLLEGEDVINTG MSHTSLPRPATARRSKALSGDIRIPGDKSISHRSFMFGGLASGETRITGLLEGEDVINTG | 60 |
| Query 61 | KAMQAMGARIRKEGDTWIIDGVGNGGLLAPEAPLDFGNAATGCRLTMGLVGVYDFDSTFI | 120 |
| Sbjct 61 | +AMQAMGA+I K+GD WII G GNG LL PEAPLDFGNA TG RLTMGLVG Y ++ FI RAMQAMGAKIAKDGDVWIIQGTGNGCLLEPEAPLDFGNAGTGARLTMGLVGTYAMETKFI | 120 |
| Query 121 | GDASLTKRPMGRVLNPLREMGVQVKSEDGDRLPVTLRGPKTPTPITYRVPMASAQVKSAV | 180 |
| Sbjct 121 | GDASL+KRPMGRVLNPLREMGVQV + DGDRLP+TLRGPK PITYRVPMASAQVKSAV GDASLSKRPMGRVLNPLREMGVQVSAADGDRLPLTLRGPKAAAPITYRVPMASAQVKSAV | 180 |
| Query 181 | LLAGLNTPGITTVIEPIMTRDHTEKMLQGFGANLTVETDADGVRTIRLEGRGKLTGQVID | 240 |
| Sbjct 181 | LLAGLNTPGITTV+EP+MTRDHTEKMLQGFGANL+VETD DGVR IR+EG+GKLTGQVID LLAGLNTPGITTVVEPVMTRDHTEKMLQGFGANLSVETDKDGVRHIRIEGQGKLTGQVID | 240 |
| Query 241 | VPGDPSSTAFPLVAALLVPGSDVTILNVLMNPTRTGLILTLQEMGADIEVINPRLAGGED | 300 |
| Sbjct 241 | VPGDPSSTAFPLVAALLVPGSD+TI NVLMNPTRTGLILTLQEMGA+IEV+N RLAGGED VPGDPSSTAFPLVAALLVPGSDITIRNVLMNPTRTGLILTLQEMGANIEVLNARLAGGED | 300 |
| Query 301 | VADLRVRSSTLKGVTVPEDRAPSMIDEYPILavaaafaegaTVMNGLEELRVKESDRLSA | 360 |
| Sbjct 301 | VADLRVRSS LKGV+VP +RAPSMIDEYP+LAVAA+FA G T+M+GL+ELRVKESDRLSA VADLRVRSSELKGVSVPPERAPSMIDEYPVLAVAASFAMGETLMHGLDELRVKESDRLSA | 360 |
| Query 361 | VANGLKLNGVDCDEGETSLVVRGRPDGKGLGNASGAAVATHLDHRIAMSFLVMGLVSENP | 420 |
| Sbjct 361 | VANGLK NGVDC EG SL VRGRPDGKGLG G V THLDHRIAMSFLVMGL +E P VANGLKANGVDCTEGPESLNVRGRPDGKGLG---GGTVETHLDHRIAMSFLVMGLAAEKP | 417 |
| Query 421  Sbjct 418 | VTVDDATMIATSFPEFMDLMAGLGAKI 447 VTVDDATMIATSFPEFM++M +GA+I VTVDDATMIATSFPEFMEMMTRMGAEI 444 |  |

>WP_042476496.1 3-phosphoshikimate 1-carboxyvinyltransferase [Agrobacterium rhizogenes]

NTF59895.1 3-phosphoshikimate 1-carboxyvinyltransferase [Agrobacterium rhizogenes] NTG32699.1 3-phosphoshikimate 1-carboxyvinyltransferase [Agrobacterium rhizogenes] NTG51964.1 3-phosphoshikimate 1-carboxyvinyltransferase [Agrobacterium rhizogenes] NTG58705.1 3-phosphoshikimate 1-carboxyvinyltransferase [Agrobacterium rhizogenes] NTG65243.1 3-phosphoshikimate 1-carboxyvinyltransferase [Agrobacterium rhizogenes]

Length=454

Score = 726 bits (1873), Expect = 0.0, Method: Compositional matrix adjust.

| Identities | = 370/449 (82%), Positives = 402/449 (90%), Gaps = 1/449 (0%) |  |
| --- | --- | --- |
| Query 1 | MLHGASSRPATARKSSGLSGTVRIPGDKSISHRSFMFGGLASGETRITGLLEGEDVINTG | 60 |
| Sbjct 1 | M H SRPATAR+S+ LSGT+R+PGDKSISHR+FMFGGLASGETRITGLLEGEDV+NTG MPHDTHSRPATARRSTNLSGTIRVPGDKSISHRAFMFGGLASGETRITGLLEGEDVLNTG | 60 |
| Query 61 | KAMQAMGARIRKEGDTWIIDGVGNGGLLAPEAPLDFGNAATGCRLTMGLVGVYDFDSTFI | 120 |
| Sbjct 61 | KAM+AMGA+I K GD WII GVGNG LLAPEAPLDFGNA TG RLTMGLVGVY F+STFI KAMRAMGAKIEKAGDEWIIKGVGNGALLAPEAPLDFGNAGTGSRLTMGLVGVYHFESTFI | 120 |
| Query 121 | GDASLTKRPMGRVLNPLREMGVQVKSEDGDRLPVTLRGPKTPTPITYRVPMASAQVKSAV | 180 |
| Sbjct 121 | GDASL+KRPMGR+L+PLR+MGVQVKS +GDRLPVTL GP+T PITYRVPMASAQVKSAV GDASLSKRPMGRILDPLRQMGVQVKSSEGDRLPVTLHGPETANPITYRVPMASAQVKSAV | 180 |
| Query 181 | LLAGLNTPGITTVIEPIMTRDHTEKMLQGFGANLTVETDADGVRTIRLEGRGKLTGQVID | 240 |
| Sbjct 181 | LLAGLN PGIT+VIEP+MTRDHTEKML GFGA L+VETD DGVR IRL+G+GKL GQ ID LLAGLNAPGITSVIEPVMTRDHTEKMLAGFGAELSVETDRDGVRIIRLQGQGKLFGQTID | 240 |
| Query 241 | VPGDPSSTAFPLVAALLVPGSDVTILNVLMNPTRTGLILTLQEMGADIEVINPRLAGGED | 300 |
| Sbjct 241 | VPGDPSS AFPLVAAL+V GSD+TI NVLMNPTRTGLILTLQEMGADIE+++ RLAGGED VPGDPSSNAFPLVAALIVAGSDITIRNVLMNPTRTGLILTLQEMGADIEILDKRLAGGED | 300 |
| Query 301 | VADLRVRSSTLKGVTVPEDRAPSMIDEYPILavaaafaegaTVMNGLEELRVKESDRLSA | 360 |
| Sbjct 301 | VADLRVRSS LKGVTVP +RAPSMIDEYP+LAVAAAFAEG TVM GLEELRVKESDRLSA VADLRVRSSALKGVTVPAERAPSMIDEYPVLAVAAAFAEGTTVMLGLEELRVKESDRLSA | 360 |
| Query 361 | VANGLKLNGVDCDEGETSLVVRGRPDGKGLGNASGAAVATHLDHRIAMSFLVMGLVSENP | 420 |
| Sbjct 361 | VA GL LNGVDC EGE SL VRGRPDGKGLGNA G V THLDHRIAMSFLVMGL SE+P VAEGLTLNGVDCTEGEASLTVRGRPDGKGLGNA-GGEVKTHLDHRIAMSFLVMGLASEHP | 419 |
| Query 421  Sbjct 420 | VTVDDATMIATSFPEFMDLMAGLGAKIEL 449 V VDD+ MIATSFPEF DLMA LGA +++ VKVDDSAMIATSFPEFFDLMAELGADMDV 448 |  |

>WP_153666806.1 3-phosphoshikimate 1-carboxyvinyltransferase [Phyllobacterium sp. SYP-B3895]

MRG54542.1 3-phosphoshikimate 1-carboxyvinyltransferase [Phyllobacterium sp. SYP-B3895]

Length=451

Score = 726 bits (1873), Expect = 0.0, Method: Compositional matrix adjust.

| Identities | = 369/447 (83%), Positives = 401/447 (90%), Gaps = 3/447 (1%) |  |
| --- | --- | --- |
| Query 1 | MLHGASSRPATARKSSGLSGTVRIPGDKSISHRSFMFGGLASGETRITGLLEGEDVINTG | 60 |
| Sbjct 1 | M H + RPATAR+S LSG +RIPGDKSISHRSFMFGGLASGETRITGLLEGEDVINTG MSHTSLPRPATARRSKALSGDIRIPGDKSISHRSFMFGGLASGETRITGLLEGEDVINTG | 60 |
| Query 61 | KAMQAMGARIRKEGDTWIIDGVGNGGLLAPEAPLDFGNAATGCRLTMGLVGVYDFDSTFI | 120 |
| Sbjct 61 | +AMQAMGA+I K+GD+WII G GNG LL PEAPLDFGNA TG RLTMGLVG Y ++TFI RAMQAMGAKIAKDGDSWIIHGTGNGCLLEPEAPLDFGNAGTGARLTMGLVGTYAMETTFI | 120 |
| Query 121 | GDASLTKRPMGRVLNPLREMGVQVKSEDGDRLPVTLRGPKTPTPITYRVPMASAQVKSAV | 180 |
| Sbjct 121 | GDASL+KRPMGRVLNPLREMGVQV + DGDRLP+TLRGPK TPITYRVPMASAQVKSAV GDASLSKRPMGRVLNPLREMGVQVSAADGDRLPLTLRGPKAATPITYRVPMASAQVKSAV | 180 |
| Query 181 | LLAGLNTPGITTVIEPIMTRDHTEKMLQGFGANLTVETDADGVRTIRLEGRGKLTGQVID | 240 |
| Sbjct 181 | LLAGLNTPGITTVIEP+MTRDHTEKMLQGFGANL+VET DGVR IR+EG+GKL GQVID LLAGLNTPGITTVIEPVMTRDHTEKMLQGFGANLSVETGTDGVRHIRIEGQGKLVGQVID | 240 |
| Query 241 | VPGDPSSTAFPLVAALLVPGSDVTILNVLMNPTRTGLILTLQEMGADIEVINPRLAGGED | 300 |
| Sbjct 241 | VPGDPSSTAFPLVAALLVPGSD+TI NVLMNPTRTGLILTLQEMGA+IEV+N RLAGGED  VPGDPSSTAFPLVAALLVPGSDITIRNVLMNPTRTGLILTLQEMGANIEVLNARLAGGED | 300 |

| Query | 301 | VADLRVRSSTLKGVTVPEDRAPSMIDEYPILavaaafaegaTVMNGLEELRVKESDRLSA | 360 |
| --- | --- | --- | --- |
| Sbjct | 301 | VADLRVRSS LKGV+VP +RAPSMIDEYP+LAVAA+FA G T+M+GL+ELRVKESDRLSA VADLRVRSSELKGVSVPPERAPSMIDEYPVLAVAASFAAGETLMHGLDELRVKESDRLSA | 360 |
| Query | 361 | VANGLKLNGVDCDEGETSLVVRGRPDGKGLGNASGAAVATHLDHRIAMSFLVMGLVSENP | 420 |
| Sbjct | 361 | VANGLK N VDC EG SL VRGRPDGKG+G G V THLDHRIAMSFLVMGL +E P VANGLKANRVDCTEGPESLSVRGRPDGKGVG---GGTVETHLDHRIAMSFLVMGLAAEKP | 417 |
| Query  Sbjct | 421  418 | VTVDDATMIATSFPEFMDLMAGLGAKI 447 VTVDDATMIATSFPEFM++M G+GA+I VTVDDATMIATSFPEFMEMMTGMGAEI 444 |  |

>WP_140024540.1 3-phosphoshikimate 1-carboxyvinyltransferase [Ochrobactrum sp. LCB8]

TNV17162.1 3-phosphoshikimate 1-carboxyvinyltransferase [Ochrobactrum sp. LCB8]

Length=450

Score = 725 bits (1872), Expect = 0.0, Method: Compositional matrix adjust.

| Identities | = 367/453 (81%), Positives = 403/453 (89%), Gaps = 3/453 (1%) |  |
| --- | --- | --- |
| Query 1 | MLHGASSRPATARKSSGLSGTVRIPGDKSISHRSFMFGGLASGETRITGLLEGEDVINTG | 60 |
| Sbjct 1 | M H AS +PA AR+S L+G +RIPGDKSISHRSFMFGGLASGETRITGLLEGEDVINTG MSHSASPKPAVARRSEALAGEIRIPGDKSISHRSFMFGGLASGETRITGLLEGEDVINTG | 60 |
| Query 61 | KAMQAMGARIRKEGDTWIIDGVGNGGLLAPEAPLDFGNAATGCRLTMGLVGVYDFDSTFI | 120 |
| Sbjct 61 | +AMQAMGA+I K+GDTWII GVGNG LL PEAPLDFGNA TG RLTMGLVG YD + FI RAMQAMGAKIEKDGDTWIIHGVGNGCLLEPEAPLDFGNAGTGARLTMGLVGTYDMTTAFI | 120 |
| Query 121 | GDASLTKRPMGRVLNPLREMGVQVKSEDGDRLPVTLRGPKTPTPITYRVPMASAQVKSAV | 180 |
| Sbjct 121 | GDASL++RPMGRVL+PLR+MGVQVKS DGDR+P+TL GP PITYRVPMASAQVKSAV GDASLSRRPMGRVLDPLRQMGVQVKSTDGDRMPLTLVGPSVANPITYRVPMASAQVKSAV | 180 |
| Query 181 | LLAGLNTPGITTVIEPIMTRDHTEKMLQGFGANLTVETDADGVRTIRLEGRGKLTGQVID | 240 |
| Sbjct 181 | LLAGLNTPG+TTVIEP+MTRDHTEKMLQGFGA+LTVETD DGVR IR+ G+GKL GQ ID LLAGLNTPGVTTVIEPVMTRDHTEKMLQGFGADLTVETDKDGVRHIRITGQGKLVGQTID | 240 |
| Query 241 | VPGDPSSTAFPLVAALLVPGSDVTILNVLMNPTRTGLILTLQEMGADIEVINPRLAGGED | 300 |
| Sbjct 241 | VPGDPSSTAFPLVAALLV GSD+TI NVLMNPTRTGLILTLQEMGA+IEV N RLAGGED VPGDPSSTAFPLVAALLVEGSDITIRNVLMNPTRTGLILTLQEMGANIEVFNERLAGGED | 300 |
| Query 301 | VADLRVRSSTLKGVTVPEDRAPSMIDEYPILavaaafaegaTVMNGLEELRVKESDRLSA | 360 |
| Sbjct 301 | VADLRV+SS LKGVTVP +RAPSMIDEYP+LA+A +FAEG TVM+GL+ELRVKESDRL+A VADLRVKSSKLKGVTVPPERAPSMIDEYPVLAIAGSFAEGETVMDGLDELRVKESDRLAA | 360 |
| Query 361 | VANGLKLNGVDCDEGETSLVVRGRPDGKGLGNASGAAVATHLDHRIAMSFLVMGLVSENP | 420 |
| Sbjct 361 | VA GL++NGVDC EGE SL VRGRPDGKG+G G VATHLDHRIAMSFLVMGL SE P VARGLEVNGVDCTEGEMSLTVRGRPDGKGIG---GGVVATHLDHRIAMSFLVMGLASEKP | 417 |
| Query 421  Sbjct 418 | VTVDDATMIATSFPEFMDLMAGLGAKIELSDTK 453 VTVDD+TMIATSFPEFMDLM G+GAKIE S+ + VTVDDSTMIATSFPEFMDLMTGMGAKIEPSEAE 450 |  |

>WP_151588586.1 3-phosphoshikimate 1-carboxyvinyltransferase [Ochrobactrum sp. Kaboul]

KAB2700926.1 3-phosphoshikimate 1-carboxyvinyltransferase [Ochrobactrum sp. Kaboul]

Length=450

Score = 725 bits (1872), Expect = 0.0, Method: Compositional matrix adjust. Identities = 370/453 (82%), Positives = 403/453 (89%), Gaps = 3/453 (1%)

| Query | 1 | MLHGASSRPATARKSSGLSGTVRIPGDKSISHRSFMFGGLASGETRITGLLEGEDVINTG | 60 |
| --- | --- | --- | --- |
| Sbjct | 1 | M H AS +PA AR+S L G +RIPGDKSISHRSFMFGGLASGETRITGLLEGEDVINTG MSHSASPKPAVARRSEALVGEIRIPGDKSISHRSFMFGGLASGETRITGLLEGEDVINTG | 60 |
| Query | 61 | KAMQAMGARIRKEGDTWIIDGVGNGGLLAPEAPLDFGNAATGCRLTMGLVGVYDFDSTFI | 120 |
| Sbjct | 61 | +AMQAMGA+I K+GD WII+GVGNG LL PEAPLDFGNA TG RLTMGLVG YD + FI  RAMQAMGAKIEKDGDVWIINGVGNGCLLEPEAPLDFGNAGTGARLTMGLVGTYDMTTAFI | 120 |

| Query | 121 | GDASLTKRPMGRVLNPLREMGVQVKSEDGDRLPVTLRGPKTPTPITYRVPMASAQVKSAV | 180 |
| --- | --- | --- | --- |
| Sbjct | 121 | GDASL++RPMGRVL+PLR+MGVQVKS DGDR+P+TL GP PITYRVPMASAQVKSAV GDASLSRRPMGRVLDPLRQMGVQVKSTDGDRMPLTLVGPSVANPITYRVPMASAQVKSAV | 180 |
| Query | 181 | LLAGLNTPGITTVIEPIMTRDHTEKMLQGFGANLTVETDADGVRTIRLEGRGKLTGQVID | 240 |
| Sbjct | 181 | LLAGLNTPG+TTVIEP+MTRDHTEKMLQGFGA+LTVETD DGVR IR+ G+GKL GQ ID LLAGLNTPGVTTVIEPVMTRDHTEKMLQGFGADLTVETDKDGVRHIRITGQGKLIGQTID | 240 |
| Query | 241 | VPGDPSSTAFPLVAALLVPGSDVTILNVLMNPTRTGLILTLQEMGADIEVINPRLAGGED | 300 |
| Sbjct | 241 | VPGDPSSTAFPLVAALLV GSDVTI NVLMNPTRTGLILTLQEMGADIEV N RLAGGED VPGDPSSTAFPLVAALLVEGSDVTIRNVLMNPTRTGLILTLQEMGADIEVFNERLAGGED | 300 |
| Query | 301 | VADLRVRSSTLKGVTVPEDRAPSMIDEYPILavaaafaegaTVMNGLEELRVKESDRLSA | 360 |
| Sbjct | 301 | VADLRV+SS LKGVTVP +RAPSMIDEYP+LA+AA+FAEG TVM+GL+ELRVKESDRL+A VADLRVKSSRLKGVTVPPERAPSMIDEYPVLAIAASFAEGETVMDGLDELRVKESDRLAA | 360 |
| Query | 361 | VANGLKLNGVDCDEGETSLVVRGRPDGKGLGNASGAAVATHLDHRIAMSFLVMGLVSENP | 420 |
| Sbjct | 361 | VA GL++NGVDC EGE SL VRGRPDGKGLG G VATHLDHRIAMSFLVMGL SE P VARGLEVNGVDCTEGEMSLTVRGRPDGKGLG---GGVVATHLDHRIAMSFLVMGLASEKP | 417 |
| Query  Sbjct | 421  418 | VTVDDATMIATSFPEFMDLMAGLGAKIELSDTK 453 VTVDD+TMIATSFPEFMDLM G+GAKIE S+ + VTVDDSTMIATSFPEFMDLMTGMGAKIEPSEAE 450 |  |

>WP_185917167.1 3-phosphoshikimate 1-carboxyvinyltransferase [Ochrobactrum sp. CM-21-5]

MBC2884103.1 3-phosphoshikimate 1-carboxyvinyltransferase [Ochrobactrum sp. CM-21-5]

Length=450

Score = 725 bits (1872), Expect = 0.0, Method: Compositional matrix adjust.

| Identities | = 369/453 (81%), Positives = 403/453 (89%), Gaps = 3/453 (1%) |  |
| --- | --- | --- |
| Query 1 | MLHGASSRPATARKSSGLSGTVRIPGDKSISHRSFMFGGLASGETRITGLLEGEDVINTG | 60 |
| Sbjct 1 | M H S +PATA +S GLSG +RIPGDKSISHRSFMFGGLASGETRITGLLEGEDVINTG MSHSFSPKPATAHRSKGLSGDIRIPGDKSISHRSFMFGGLASGETRITGLLEGEDVINTG | 60 |
| Query 61 | KAMQAMGARIRKEGDTWIIDGVGNGGLLAPEAPLDFGNAATGCRLTMGLVGVYDFDSTFI | 120 |
| Sbjct 61 | +AM+AMGA+IRKEG WII+GVGNG LL PEAPLDFGNA TG RLTMGLVG YD +TFI RAMEAMGAKIRKEGHAWIINGVGNGCLLQPEAPLDFGNAGTGARLTMGLVGTYDMTTTFI | 120 |
| Query 121 | GDASLTKRPMGRVLNPLREMGVQVKSEDGDRLPVTLRGPKTPTPITYRVPMASAQVKSAV | 180 |
| Sbjct 121 | GDASL+KRPMGRVLNPLREMGVQVK+ +GDR+P+TL GPKT PITYRVPMASAQVKSAV GDASLSKRPMGRVLNPLREMGVQVKAAEGDRMPLTLIGPKTANPITYRVPMASAQVKSAV | 180 |
| Query 181 | LLAGLNTPGITTVIEPIMTRDHTEKMLQGFGANLTVETDADGVRTIRLEGRGKLTGQVID | 240 |
| Sbjct 181 | LLAGLNTPGI+TVIEP+MTRDHTEKMLQGFGA+L+VETD DGVR IR+ G+GKLTGQ ID LLAGLNTPGISTVIEPVMTRDHTEKMLQGFGADLSVETDRDGVRHIRIVGQGKLTGQTID | 240 |
| Query 241 | VPGDPSSTAFPLVAALLVPGSDVTILNVLMNPTRTGLILTLQEMGADIEVINPRLAGGED | 300 |
| Sbjct 241 | VPGDPSSTAFPLVAALLV GSDVTI NVLMNPTRTGLILTLQEMGADIE+I+PRLAGGED VPGDPSSTAFPLVAALLVEGSDVTIRNVLMNPTRTGLILTLQEMGADIEIIDPRLAGGED | 300 |
| Query 301 | VADLRVRSSTLKGVTVPEDRAPSMIDEYPILavaaafaegaTVMNGLEELRVKESDRLSA | 360 |
| Sbjct 301 | VADLRV+SS LKG+ VP DRAPSMIDEYP+LA+AA FAEG TVM+GL+ELRVKESDRL+A VADLRVKSSKLKGIVVPPDRAPSMIDEYPVLAIAATFAEGETVMDGLDELRVKESDRLAA | 360 |
| Query 361 | VANGLKLNGVDCDEGETSLVVRGRPDGKGLGNASGAAVATHLDHRIAMSFLVMGLVSENP | 420 |
| Sbjct 361 | VA GL+ NGVDC EG SL VRGRPDGKG+G G ATHLDHRIAMSFLVMGL SE P VARGLEANGVDCTEGAMSLTVRGRPDGKGIG---GGTAATHLDHRIAMSFLVMGLASEKP | 417 |
| Query 421  Sbjct 418 | VTVDDATMIATSFPEFMDLMAGLGAKIELSDTK 453 VTVDD +IATSFPEFMD+M GLGA+IE SD + VTVDDYKIIATSFPEFMDMMGGLGARIERSDAE 450 |  |

>WP_174005577.1 3-phosphoshikimate 1-carboxyvinyltransferase [Agrobacterium rhizogenes] NTF79454.1 3-phosphoshikimate 1-carboxyvinyltransferase [Agrobacterium rhizogenes] NTI72409.1 3-phosphoshikimate 1-carboxyvinyltransferase [Agrobacterium rhizogenes]

Length=454

Score = 725 bits (1872), Expect = 0.0, Method: Compositional matrix adjust.

| Identities | = 369/449 (82%), Positives = 402/449 (90%), Gaps = 1/449 (0%) |  |
| --- | --- | --- |
| Query 1 | MLHGASSRPATARKSSGLSGTVRIPGDKSISHRSFMFGGLASGETRITGLLEGEDVINTG | 60 |
| Sbjct 1 | M H SRPATAR+S+ LSGT+R+PGDKSISHR+FMFGGLASGETRITGLLEGEDV+NTG MPHDTHSRPATARRSTNLSGTIRVPGDKSISHRAFMFGGLASGETRITGLLEGEDVLNTG | 60 |
| Query 61 | KAMQAMGARIRKEGDTWIIDGVGNGGLLAPEAPLDFGNAATGCRLTMGLVGVYDFDSTFI | 120 |
| Sbjct 61 | KAM+AMGA+I K GD WII GVGNG LLAPEAPLDFGNA TG RLTMGLVGVY F+STFI KAMRAMGAKIEKAGDEWIIKGVGNGALLAPEAPLDFGNAGTGSRLTMGLVGVYHFESTFI | 120 |
| Query 121 | GDASLTKRPMGRVLNPLREMGVQVKSEDGDRLPVTLRGPKTPTPITYRVPMASAQVKSAV | 180 |
| Sbjct 121 | GDASL+KRPMGR+L+PLR+MGVQVKS +GDRLPVTL GP+T PITYRVPMASAQVKSAV GDASLSKRPMGRILDPLRQMGVQVKSSEGDRLPVTLHGPETANPITYRVPMASAQVKSAV | 180 |
| Query 181 | LLAGLNTPGITTVIEPIMTRDHTEKMLQGFGANLTVETDADGVRTIRLEGRGKLTGQVID | 240 |
| Sbjct 181 | LLAGLN PGIT+VIEP+MTRDHTEKML GFGA L++ETD DGVR IRL+G+GKL GQ ID LLAGLNAPGITSVIEPVMTRDHTEKMLAGFGAELSIETDRDGVRIIRLQGQGKLFGQTID | 240 |
| Query 241 | VPGDPSSTAFPLVAALLVPGSDVTILNVLMNPTRTGLILTLQEMGADIEVINPRLAGGED | 300 |
| Sbjct 241 | VPGDPSS AFPLVAAL+V GSD+TI NVLMNPTRTGLILTLQEMGADIE+++ RLAGGED VPGDPSSNAFPLVAALIVAGSDITIRNVLMNPTRTGLILTLQEMGADIEILDKRLAGGED | 300 |
| Query 301 | VADLRVRSSTLKGVTVPEDRAPSMIDEYPILavaaafaegaTVMNGLEELRVKESDRLSA | 360 |
| Sbjct 301 | VADLRVRSS LKGVTVP +RAPSMIDEYP+LAVAAAFAEG TVM GLEELRVKESDRLSA VADLRVRSSALKGVTVPAERAPSMIDEYPVLAVAAAFAEGTTVMLGLEELRVKESDRLSA | 360 |
| Query 361 | VANGLKLNGVDCDEGETSLVVRGRPDGKGLGNASGAAVATHLDHRIAMSFLVMGLVSENP | 420 |
| Sbjct 361 | VA GL LNGVDC EGE SL VRGRPDGKGLGNA G V THLDHRIAMSFLVMGL SE+P VAEGLTLNGVDCTEGEASLTVRGRPDGKGLGNA-GGEVKTHLDHRIAMSFLVMGLASEHP | 419 |
| Query 421  Sbjct 420 | VTVDDATMIATSFPEFMDLMAGLGAKIEL 449 V VDD+ MIATSFPEF DLMA LGA +++ VKVDDSAMIATSFPEFFDLMAELGADMDV 448 |  |

>NKC47702.1 3-phosphoshikimate 1-carboxyvinyltransferase [Ochrobactrum anthropi ATCC 49188]

Length=451

Score = 725 bits (1871), Expect = 0.0, Method: Compositional matrix adjust.

| Identities | | = 369/449 (82%), Positives = 405/449 (90%), Gaps = 3/449 (1%) |  |
| --- | --- | --- | --- |
| Query 5  Sbjct 6 | | ASSRPATARKSSGLSGTVRIPGDKSISHRSFMFGGLASGETRITGLLEGEDVINTGKAMQ  + +PATAR+S L+G +RIPGDKSISHRSFMFGGLASGETRITGLLEGEDVINTG+AMQ CTPKPATARRSEALTGEIRIPGDKSISHRSFMFGGLASGETRITGLLEGEDVINTGRAMQ | 64  65 |
| Query 65  Sbjct 66 | | AMGARIRKEGDTWIIDGVGNGGLLAPEAPLDFGNAATGCRLTMGLVGVYDFDSTFIGDAS AMGA+IRK+GD WII+GVGNG LL PEA LDFGNA TG RLTMGLVG YD ++FIGDAS AMGAKIRKDGDAWIINGVGNGCLLQPEAALDFGNAGTGARLTMGLVGTYDMRTSFIGDAS | 124  125 |
| Query 125  Sbjct 126 | | LTKRPMGRVLNPLREMGVQVKSEDGDRLPVTLRGPKTPTPITYRVPMASAQVKSAVLLAG L+KRPMGRVLNPLREMGVQV++ +GDR+P+TL GPKT PITYRVPMASAQVKSAVLLAG LSKRPMGRVLNPLREMGVQVEAAEGDRMPLTLIGPKTANPITYRVPMASAQVKSAVLLAG | 184  185 |
| Query 185  Sbjct 186 | | LNTPGITTVIEPIMTRDHTEKMLQGFGANLTVETDADGVRTIRLEGRGKLTGQVIDVPGD LNTPG+TTVIEP+MTRDHTEKMLQGFGA+L+VETD DGVR IR+ G+GKL GQ IDVPGD LNTPGVTTVIEPVMTRDHTEKMLQGFGADLSVETDKDGVRHIRITGQGKLIGQTIDVPGD | 244  245 |
| Query 245  Sbjct 246 | | PSSTAFPLVAALLVPGSDVTILNVLMNPTRTGLILTLQEMGADIEVINPRLAGGEDVADL PSSTAFPLVAALLV GSDVTI NVLMNPTRTGLILTLQEMGADIEV+N RLAGGEDVADL PSSTAFPLVAALLVEGSDVTIRNVLMNPTRTGLILTLQEMGADIEVLNARLAGGEDVADL | 304  305 |
| Query 305  Sbjct 306 | | RVRSSTLKGVTVPEDRAPSMIDEYPILavaaafaegaTVMNGLEELRVKESDRLSAVANG RV++S LKGV VP +RAPSMIDEYP+LA+AAAFAEG TVM+GL+ELRVKESDRL+AVA G RVKASKLKGVVVPPERAPSMIDEYPVLAIAAAFAEGETVMDGLDELRVKESDRLAAVARG | 364  365 |
| Query 365  Sbjct 366 | | LKLNGVDCDEGETSLVVRGRPDGKGLGNASGAAVATHLDHRIAMSFLVMGLVSENPVTVD L+ NGVDC EGE SL VRGRPDGKGLG G VATHLDHRIAMSFLVMGL SE PVTVD LEANGVDCTEGEMSLTVRGRPDGKGLG---GGTVATHLDHRIAMSFLVMGLASEKPVTVD | 424  422 |
| Query | 425 DATMIATSFPEFMDLMAGLGAKIELSDTK 453 | | |

D+TMIATSFPEFMD+M GLGAKIELSD + Sbjct 423 DSTMIATSFPEFMDMMPGLGAKIELSDAR 451

>WP_174031631.1 3-phosphoshikimate 1-carboxyvinyltransferase [Agrobacterium rhizogenes] NTG71884.1 3-phosphoshikimate 1-carboxyvinyltransferase [Agrobacterium rhizogenes]

Length=454

Score = 725 bits (1871), Expect = 0.0, Method: Compositional matrix adjust.

| Identities | = 370/449 (82%), Positives = 402/449 (90%), Gaps = 1/449 (0%) |  |
| --- | --- | --- |
| Query 1 | MLHGASSRPATARKSSGLSGTVRIPGDKSISHRSFMFGGLASGETRITGLLEGEDVINTG | 60 |
| Sbjct 1 | M H SRPATAR+S+ LSGT+R+PGDKSISHR+FMFGGLASGETRITGLLEGEDV+NTG MPHDTHSRPATARRSTNLSGTIRVPGDKSISHRAFMFGGLASGETRITGLLEGEDVLNTG | 60 |
| Query 61 | KAMQAMGARIRKEGDTWIIDGVGNGGLLAPEAPLDFGNAATGCRLTMGLVGVYDFDSTFI | 120 |
| Sbjct 61 | KAM+AMGA+I K GD WII GVGNG LLAPEAPLDFGNA TG RLTMGLVGVY F+STFI KAMRAMGAKIEKAGDEWIIKGVGNGALLAPEAPLDFGNAGTGSRLTMGLVGVYHFESTFI | 120 |
| Query 121 | GDASLTKRPMGRVLNPLREMGVQVKSEDGDRLPVTLRGPKTPTPITYRVPMASAQVKSAV | 180 |
| Sbjct 121 | GDASL+KRPMGR+L+PLR+MGVQVKS +GDRLPVTL GP+T PITYRVPMASAQVKSAV GDASLSKRPMGRILDPLRQMGVQVKSSEGDRLPVTLHGPETANPITYRVPMASAQVKSAV | 180 |
| Query 181 | LLAGLNTPGITTVIEPIMTRDHTEKMLQGFGANLTVETDADGVRTIRLEGRGKLTGQVID | 240 |
| Sbjct 181 | LLAGLN PGIT+VIEP+MTRDHTEKML GFGA L+VETD DGVR IRL+G+GKL GQ ID LLAGLNAPGITSVIEPVMTRDHTEKMLAGFGAELSVETDRDGVRIIRLQGQGKLFGQTID | 240 |
| Query 241 | VPGDPSSTAFPLVAALLVPGSDVTILNVLMNPTRTGLILTLQEMGADIEVINPRLAGGED | 300 |
| Sbjct 241 | VPGDPSSTAFPLVAAL+V GSD+TI NVLMNPTRTGLILTLQEMGADIE+++ RLAGGED VPGDPSSTAFPLVAALIVAGSDITIRNVLMNPTRTGLILTLQEMGADIEILDKRLAGGED | 300 |
| Query 301 | VADLRVRSSTLKGVTVPEDRAPSMIDEYPILavaaafaegaTVMNGLEELRVKESDRLSA | 360 |
| Sbjct 301 | VADLRVRSS LKGVTVP +RAPSMIDEYP+LAVAAAFAEG TVM GLEELRVKESDRLSA VADLRVRSSALKGVTVPAERAPSMIDEYPVLAVAAAFAEGTTVMLGLEELRVKESDRLSA | 360 |
| Query 361 | VANGLKLNGVDCDEGETSLVVRGRPDGKGLGNASGAAVATHLDHRIAMSFLVMGLVSENP | 420 |
| Sbjct 361 | VA L LNGVDC EGE SL VRGRPDGKGLGNA G V THLDHRIAMSFLVMGL SE+P VAEELTLNGVDCTEGEASLTVRGRPDGKGLGNA-GGEVKTHLDHRIAMSFLVMGLASEHP | 419 |
| Query 421  Sbjct 420 | VTVDDATMIATSFPEFMDLMAGLGAKIEL 449 V VDD+ MIATSFPEF DLMA LGA +++ VKVDDSAMIATSFPEFFDLMAELGADMDV 448 |  |

>WP_138783991.1 3-phosphoshikimate 1-carboxyvinyltransferase [Ochrobactrum haematophilum] TMV05152.1 3-phosphoshikimate 1-carboxyvinyltransferase [Ochrobactrum haematophilum]

Length=450

Score = 724 bits (1870), Expect = 0.0, Method: Compositional matrix adjust.

| Identities | = 369/453 (81%), Positives = 403/453 (89%), Gaps = 3/453 (1%) |  |
| --- | --- | --- |
| Query 1 | MLHGASSRPATARKSSGLSGTVRIPGDKSISHRSFMFGGLASGETRITGLLEGEDVINTG | 60 |
| Sbjct 1 | M H AS +PA AR+S L G +RIPGDKSISHRSFMFGGLASGETRITGLLEGEDVINTG MSHSASPKPAVARRSEALVGEIRIPGDKSISHRSFMFGGLASGETRITGLLEGEDVINTG | 60 |
| Query 61 | KAMQAMGARIRKEGDTWIIDGVGNGGLLAPEAPLDFGNAATGCRLTMGLVGVYDFDSTFI | 120 |
| Sbjct 61 | +AMQAMGA+I K+GD WII+GVGNG LL PEAPLDFGNA TG RLTMGLVG YD + FI RAMQAMGAKIEKDGDVWIINGVGNGCLLEPEAPLDFGNAGTGARLTMGLVGTYDMTTAFI | 120 |
| Query 121 | GDASLTKRPMGRVLNPLREMGVQVKSEDGDRLPVTLRGPKTPTPITYRVPMASAQVKSAV | 180 |
| Sbjct 121 | GDASL++RPMGRVL+PLR+MGVQVKS DGDR+P+TL GP PITYRVPMASAQVKSAV GDASLSRRPMGRVLDPLRQMGVQVKSTDGDRMPLTLVGPSVANPITYRVPMASAQVKSAV | 180 |
| Query 181 | LLAGLNTPGITTVIEPIMTRDHTEKMLQGFGANLTVETDADGVRTIRLEGRGKLTGQVID | 240 |
| Sbjct 181 | LLAGLNTPG+TTVIEP+MTRDHTEKMLQGFGA+LTVETD DGVR IR+ G+GKL GQ ID LLAGLNTPGVTTVIEPVMTRDHTEKMLQGFGADLTVETDKDGVRHIRITGQGKLIGQTID | 240 |
| Query 241 | VPGDPSSTAFPLVAALLVPGSDVTILNVLMNPTRTGLILTLQEMGADIEVINPRLAGGED | 300 |
| Sbjct 241 | VPGDPSSTAFPLVAALLV GSDVTI NVLMNPTRTGLILTLQEMGADIEV N R+AGGED VPGDPSSTAFPLVAALLVEGSDVTIHNVLMNPTRTGLILTLQEMGADIEVFNERIAGGED | 300 |
| Query 301 | VADLRVRSSTLKGVTVPEDRAPSMIDEYPILavaaafaegaTVMNGLEELRVKESDRLSA | 360 |

| Sbjct | 301 | VADLRV+SS LKGVTVP +RAPSMIDEYP+LA+AA+FAEG TVM+GL+ELRVKESDRL+A VADLRVKSSRLKGVTVPPERAPSMIDEYPVLAIAASFAEGETVMDGLDELRVKESDRLAA | 360 |
| --- | --- | --- | --- |
| Query | 361 | VANGLKLNGVDCDEGETSLVVRGRPDGKGLGNASGAAVATHLDHRIAMSFLVMGLVSENP | 420 |
| Sbjct | 361 | VA GL++NGVDC EGE SL VRGRPDGKGLG G VATHLDHRIAMSFLVMGL SE P VARGLEVNGVDCTEGEMSLTVRGRPDGKGLG---GGVVATHLDHRIAMSFLVMGLASEKP | 417 |
| Query  Sbjct | 421  418 | VTVDDATMIATSFPEFMDLMAGLGAKIELSDTK 453 VTVDD+TMIATSFPEFMDLM G+GAKIE S+ + VTVDDSTMIATSFPEFMDLMTGMGAKIEPSEAE 450 |  |

>WP_169592424.1 3-phosphoshikimate 1-carboxyvinyltransferase [Rhizobium sp. S-51] NML75373.1 3-phosphoshikimate 1-carboxyvinyltransferase [Rhizobium sp. S-51]

Length=455

Score = 724 bits (1869), Expect = 0.0, Method: Compositional matrix adjust.

| Identities | = 371/455 (82%), Positives = 406/455 (89%), Gaps = 0/455 (0%) |  |
| --- | --- | --- |
| Query 1 | MLHGASSRPATARKSSGLSGTVRIPGDKSISHRSFMFGGLASGETRITGLLEGEDVINTG | 60 |
| Sbjct 1 | M H + +PA AR+S LSG++RIPGD SIS+RS MFGGLA+GETRITGL E EDV+ T MSHASPPKPAIARRSPALSGSIRIPGDTSISYRSLMFGGLAAGETRITGLSEDEDVVITS | 60 |
| Query 61 | KAMQAMGARIRKEGDTWIIDGVGNGGLLAPEAPLDFGNAATGCRLTMGLVGVYDFDSTFI | 120 |
| Sbjct 61 | +AMQAMGA IRK+GDTWII+GVGNG L+APEAPLDFGN GCRL MGLVGVYDFDSTF+ RAMQAMGAAIRKDGDTWIINGVGNGCLIAPEAPLDFGNVGAGCRLVMGLVGVYDFDSTFV | 120 |
| Query 121 | GDASLTKRPMGRVLNPLREMGVQVKSEDGDRLPVTLRGPKTPTPITYRVPMASAQVKSAV | 180 |
| Sbjct 121 | GDASL +RP GRVL+PLR+MGVQV+S +GDRLP+TLRGP+T TPITYRVPM SAQVKSAV GDASLARRPRGRVLDPLRDMGVQVRSAEGDRLPITLRGPRTATPITYRVPMPSAQVKSAV | 180 |
| Query 181 | LLAGLNTPGITTVIEPIMTRDHTEKMLQGFGANLTVETDADGVRTIRLEGRGKLTGQVID | 240 |
| Sbjct 181 | LLAGLNTPGITTVIEP TRDHTEKML+GFGA+L VETDA G RTIRLEGRG+LTGQ ID LLAGLNTPGITTVIEPATTRDHTEKMLKGFGADLAVETDARGGRTIRLEGRGRLTGQAID | 240 |
| Query 241 | VPGDPSSTAFPLVAALLVPGSDVTILNVLMNPTRTGLILTLQEMGADIEVINPRLAGGED | 300 |
| Sbjct 241 | VPGDPSSTAFPLVAALLVP SDVTI+NVLMNPTRTGLILTLQ MGA IEVINPRLAGGED VPGDPSSTAFPLVAALLVPDSDVTIVNVLMNPTRTGLILTLQAMGARIEVINPRLAGGED | 300 |
| Query 301 | VADLRVRSSTLKGVTVPEDRAPSMIDEYPILavaaafaegaTVMNGLEELRVKESDRLSA | 360 |
| Sbjct 301 | VADLRVRSS LKGVTVPE+RAPSMIDEYP+LAVAAAFAEG T M GL+ELR KE DRL+A VADLRVRSSRLKGVTVPEERAPSMIDEYPVLAVAAAFAEGTTTMLGLDELRGKEPDRLAA | 360 |
| Query 361 | VANGLKLNGVDCDEGETSLVVRGRPDGKGLGNASGAAVATHLDHRIAMSFLVMGLVSENP | 420 |
| Sbjct 361 | VA GLKLNGVDCDEG +SL+VRGRPDGKGLGNA+G AVATHLDHRIAMSFLVMGLVS++P VAAGLKLNGVDCDEGHSSLIVRGRPDGKGLGNAAGDAVATHLDHRIAMSFLVMGLVSQHP | 420 |
| Query 421  Sbjct 421 | VTVDDATMIATSFPEFMDLMAGLGAKIELSDTKAA 455 VTVDD TMIAT FP FMDLM GLGA+IE + KAA VTVDDTTMIATRFPSFMDLMTGLGARIEPIEGKAA 455 |  |

>WP_100000714.1 3-phosphoshikimate 1-carboxyvinyltransferase [Phyllobacterium zundukense]

ATU93308.1 3-phosphoshikimate 1-carboxyvinyltransferase [Phyllobacterium zundukense]

PIO42739.1 3-phosphoshikimate 1-carboxyvinyltransferase [Phyllobacterium zundukense]

Length=451

Score = 724 bits (1868), Expect = 0.0, Method: Compositional matrix adjust.

| Identities | = 365/447 (82%), Positives = 396/447 (89%), Gaps = 3/447 (1%) |  |
| --- | --- | --- |
| Query 1 | MLHGASSRPATARKSSGLSGTVRIPGDKSISHRSFMFGGLASGETRITGLLEGEDVINTG | 60 |
| Sbjct 1 | M H +PATAR+S LSG +RIPGDKSISHRSFMFGGLASGETRITGLLEGEDVINTG MSHSVLPKPATARRSKALSGDIRIPGDKSISHRSFMFGGLASGETRITGLLEGEDVINTG | 60 |
| Query 61 | KAMQAMGARIRKEGDTWIIDGVGNGGLLAPEAPLDFGNAATGCRLTMGLVGVYDFDSTFI | 120 |
| Sbjct 61 | +AMQAMGA+I K+GD WII G GNG LLAPEAPLDFGNA TG RLTMGLVG YD ++TF+ RAMQAMGAKIAKDGDVWIIQGTGNGCLLAPEAPLDFGNAGTGARLTMGLVGTYDMETTFV | 120 |
| Query 121 | GDASLTKRPMGRVLNPLREMGVQVKSEDGDRLPVTLRGPKTPTPITYRVPMASAQVKSAV | 180 |

GDASL+KRPMGRVLNPLREMGVQV + +GDRLP+TLRGPK	PITYRVPMASAQVKSAV

| Sbjct | 121 | GDASLSKRPMGRVLNPLREMGVQVTAAEGDRLPLTLRGPKVAAPITYRVPMASAQVKSAV | 180 |
| --- | --- | --- | --- |
| Query | 181 | LLAGLNTPGITTVIEPIMTRDHTEKMLQGFGANLTVETDADGVRTIRLEGRGKLTGQVID | 240 |
| Sbjct | 181 | LLAGLNTPGITTVIEP+MTRDHTEKMLQGFGANL+V+TD VR IR+EG+GKLTGQ ID LLAGLNTPGITTVIEPVMTRDHTEKMLQGFGANLSVKTDKANVRHIRIEGQGKLTGQTID | 240 |
| Query | 241 | VPGDPSSTAFPLVAALLVPGSDVTILNVLMNPTRTGLILTLQEMGADIEVINPRLAGGED | 300 |
| Sbjct | 241 | VPGDPSSTAFPLVAALLVPGSD+TI NVLMNPTRTGLILTLQEMGADIE++ RLAGGED VPGDPSSTAFPLVAALLVPGSDITIRNVLMNPTRTGLILTLQEMGADIEILGARLAGGED | 300 |
| Query | 301 | VADLRVRSSTLKGVTVPEDRAPSMIDEYPILavaaafaegaTVMNGLEELRVKESDRLSA | 360 |
| Sbjct | 301 | VADLRVR+S L GV VP DRAPSMIDEYP+LAVAA FA G TVMNGLEELRVKESDRLSA VADLRVRASELTGVNVPADRAPSMIDEYPVLAVAACFAAGETVMNGLEELRVKESDRLSA | 360 |
| Query | 361 | VANGLKLNGVDCDEGETSLVVRGRPDGKGLGNASGAAVATHLDHRIAMSFLVMGLVSENP | 420 |
| Sbjct | 361 | VANGLK NGVDC EG +L VRGRPDGKG+G G V THLDHRIAMSFLVMGL +E P VANGLKANGVDCTEGPETLSVRGRPDGKGIG---GGTVETHLDHRIAMSFLVMGLAAEKP | 417 |
| Query  Sbjct | 421  418 | VTVDDATMIATSFPEFMDLMAGLGAKI 447 V+VDD+TMIATSFPEFMD+M G+GA+I VSVDDSTMIATSFPEFMDMMTGMGAEI 444 |  |

>WP_174167749.1 3-phosphoshikimate 1-carboxyvinyltransferase [Agrobacterium rhizogenes] NTI33084.1 3-phosphoshikimate 1-carboxyvinyltransferase [Agrobacterium rhizogenes]

Length=454

Score = 724 bits (1868), Expect = 0.0, Method: Compositional matrix adjust.

| Identities | = 369/449 (82%), Positives = 401/449 (89%), Gaps = 1/449 (0%) |  |
| --- | --- | --- |
| Query 1 | MLHGASSRPATARKSSGLSGTVRIPGDKSISHRSFMFGGLASGETRITGLLEGEDVINTG | 60 |
| Sbjct 1 | M H SRPATAR+S+ LSGT+ +PGDKSISHR+FMFGGLASGETRITGLLEGEDV+NTG MPHDTYSRPATARRSTNLSGTICVPGDKSISHRAFMFGGLASGETRITGLLEGEDVLNTG | 60 |
| Query 61 | KAMQAMGARIRKEGDTWIIDGVGNGGLLAPEAPLDFGNAATGCRLTMGLVGVYDFDSTFI | 120 |
| Sbjct 61 | KAM+AMGA+I K GD WII GVGNG LLAPEAPLDFGNA TG RLTMGLVGVY F+STFI KAMRAMGAKIEKAGDEWIIKGVGNGALLAPEAPLDFGNAGTGSRLTMGLVGVYHFESTFI | 120 |
| Query 121 | GDASLTKRPMGRVLNPLREMGVQVKSEDGDRLPVTLRGPKTPTPITYRVPMASAQVKSAV | 180 |
| Sbjct 121 | GD SL+KRPMGR+L+PLR+MGVQVKS +GDRLPVTL GP+T PITYRVPMASAQVKSAV GDVSLSKRPMGRILDPLRQMGVQVKSSEGDRLPVTLHGPETANPITYRVPMASAQVKSAV | 180 |
| Query 181 | LLAGLNTPGITTVIEPIMTRDHTEKMLQGFGANLTVETDADGVRTIRLEGRGKLTGQVID | 240 |
| Sbjct 181 | LLAGLN PGIT+VIEP+MTRDHTEKML GFGA L+VETD DGVR IRL+G+GKL GQ ID LLAGLNAPGITSVIEPVMTRDHTEKMLAGFGAELSVETDRDGVRIIRLQGQGKLFGQTID | 240 |
| Query 241 | VPGDPSSTAFPLVAALLVPGSDVTILNVLMNPTRTGLILTLQEMGADIEVINPRLAGGED | 300 |
| Sbjct 241 | VPGDPSS AFPLVAAL+VPGSD+TI NVLMNPTRTGLILTLQEMGADIE+++ RLAGGED VPGDPSSAAFPLVAALIVPGSDITIRNVLMNPTRTGLILTLQEMGADIEILDKRLAGGED | 300 |
| Query 301 | VADLRVRSSTLKGVTVPEDRAPSMIDEYPILavaaafaegaTVMNGLEELRVKESDRLSA | 360 |
| Sbjct 301 | VADLRVRSS LKGVTVP +RAPSMIDEYP+LAVAAAFAEG TVM GLEELRVKESDRLSA VADLRVRSSALKGVTVPAERAPSMIDEYPVLAVAAAFAEGTTVMLGLEELRVKESDRLSA | 360 |
| Query 361 | VANGLKLNGVDCDEGETSLVVRGRPDGKGLGNASGAAVATHLDHRIAMSFLVMGLVSENP | 420 |
| Sbjct 361 | VA GL LNGVDC EGE SL VRGRPDGKGLGNA G V THLDHRIAMSFLVMGL SE+P VAEGLTLNGVDCTEGEASLTVRGRPDGKGLGNA-GGEVKTHLDHRIAMSFLVMGLASEHP | 419 |
| Query 421  Sbjct 420 | VTVDDATMIATSFPEFMDLMAGLGAKIEL 449 V VDD+ MIATSFPEF DLMA LGA +++ VKVDDSAMIATSFPEFFDLMAELGADMDV 448 |  |

>WP_174038288.1 3-phosphoshikimate 1-carboxyvinyltransferase [Agrobacterium rhizogenes] NTH75547.1 3-phosphoshikimate 1-carboxyvinyltransferase [Agrobacterium rhizogenes] NTH81553.1 3-phosphoshikimate 1-carboxyvinyltransferase [Agrobacterium rhizogenes]

Length=454

Score = 724 bits (1868), Expect = 0.0, Method: Compositional matrix adjust. Identities = 369/449 (82%), Positives = 401/449 (89%), Gaps = 1/449 (0%)

| Query | 1 | MLHGASSRPATARKSSGLSGTVRIPGDKSISHRSFMFGGLASGETRITGLLEGEDVINTG | 60 |
| --- | --- | --- | --- |
| Sbjct | 1 | M H SRPATAR+S+ LSGT+R+PGDKSISHR+FMFGGLASGETRITGLLEGEDV+NTG MPHDTHSRPATARRSTNLSGTIRVPGDKSISHRAFMFGGLASGETRITGLLEGEDVLNTG | 60 |
| Query | 61 | KAMQAMGARIRKEGDTWIIDGVGNGGLLAPEAPLDFGNAATGCRLTMGLVGVYDFDSTFI | 120 |
| Sbjct | 61 | AM+AMGA+I K GD WII GVGNG LLAPEAPLDFGNA TG RLTMGLVGVY F+STFI NAMRAMGAKIEKAGDEWIIKGVGNGALLAPEAPLDFGNAGTGSRLTMGLVGVYHFESTFI | 120 |
| Query | 121 | GDASLTKRPMGRVLNPLREMGVQVKSEDGDRLPVTLRGPKTPTPITYRVPMASAQVKSAV | 180 |
| Sbjct | 121 | GD SL+KRPMGR+L+PLR+MGVQVKS +GDRLPVTL GP+T PITYRVPMASAQVKSAV GDVSLSKRPMGRILDPLRQMGVQVKSSEGDRLPVTLHGPETANPITYRVPMASAQVKSAV | 180 |
| Query | 181 | LLAGLNTPGITTVIEPIMTRDHTEKMLQGFGANLTVETDADGVRTIRLEGRGKLTGQVID | 240 |
| Sbjct | 181 | LLAGLN PGIT+VIEP+MTRDHTEKML GFGA L+VETD DGVR IRL+G+GKL GQ ID LLAGLNAPGITSVIEPVMTRDHTEKMLAGFGAELSVETDRDGVRIIRLQGQGKLFGQTID | 240 |
| Query | 241 | VPGDPSSTAFPLVAALLVPGSDVTILNVLMNPTRTGLILTLQEMGADIEVINPRLAGGED | 300 |
| Sbjct | 241 | VPGDPSSTAFPLVAAL+V GSD+TI NVLMNPTRTGLILTLQEMGADIE+++ RLAGGED VPGDPSSTAFPLVAALIVAGSDITIRNVLMNPTRTGLILTLQEMGADIEILDKRLAGGED | 300 |
| Query | 301 | VADLRVRSSTLKGVTVPEDRAPSMIDEYPILavaaafaegaTVMNGLEELRVKESDRLSA | 360 |
| Sbjct | 301 | VADLRVRSS LKGVTVP +RAPSMIDEYP+LAVAAAFAEG TVM GLEELRVKESDRLSA VADLRVRSSALKGVTVPAERAPSMIDEYPVLAVAAAFAEGTTVMLGLEELRVKESDRLSA | 360 |
| Query | 361 | VANGLKLNGVDCDEGETSLVVRGRPDGKGLGNASGAAVATHLDHRIAMSFLVMGLVSENP | 420 |
| Sbjct | 361 | VA GL LNGVDC EGE SL VRGRPDGKGLGNA G V THLDHRIAMSFLVMGL SE+P VAEGLTLNGVDCTEGEASLTVRGRPDGKGLGNA-GGEVKTHLDHRIAMSFLVMGLASEHP | 419 |
| Query  Sbjct | 421  420 | VTVDDATMIATSFPEFMDLMAGLGAKIEL 449 V VDD+ MIATSFPEF DLMA LGA +++ VKVDDSAMIATSFPEFFDLMAELGADMDV 448 |  |

>WP_095445804.1 MULTISPECIES: 3-phosphoshikimate 1-carboxyvinyltransferase [Ochrobactrum] ASV87196.1 3-phosphoshikimate 1-carboxyvinyltransferase [Ochrobactrum quorumnocens] KAA9366625.1 3-phosphoshikimate 1-carboxyvinyltransferase [Ochrobactrum quorumnocens] MBD7992514.1 3-phosphoshikimate 1-carboxyvinyltransferase [Ochrobactrum sp.

Sa2BUA5]

Length=450

Score = 724 bits (1868), Expect = 0.0, Method: Compositional matrix adjust.

| Identities | = 366/453 (81%), Positives = 404/453 (89%), Gaps = 3/453 (1%) |  |
| --- | --- | --- |
| Query 1 | MLHGASSRPATARKSSGLSGTVRIPGDKSISHRSFMFGGLASGETRITGLLEGEDVINTG | 60 |
| Sbjct 1 | M H AS +PA AR S L G +RIPGDKSISHRSFMFGGLASGETRITGLLEGEDVINTG MSHSASPKPAIARHSEALKGEIRIPGDKSISHRSFMFGGLASGETRITGLLEGEDVINTG | 60 |
| Query 61 | KAMQAMGARIRKEGDTWIIDGVGNGGLLAPEAPLDFGNAATGCRLTMGLVGVYDFDSTFI | 120 |
| Sbjct 61 | +AMQAMGA+IRK+G+ W+I+GVGNG LL PEAPLDFGNA TG RLTMGLVG YD +TFI RAMQAMGAKIRKDGNVWVINGVGNGCLLQPEAPLDFGNAGTGARLTMGLVGTYDMTTTFI | 120 |
| Query 121 | GDASLTKRPMGRVLNPLREMGVQVKSEDGDRLPVTLRGPKTPTPITYRVPMASAQVKSAV | 180 |
| Sbjct 121 | GDASLT RPMGRVLNPLREMGVQVK+ +GDRLP+TL GPKT PI+YRVPMASAQVKSAV GDASLTSRPMGRVLNPLREMGVQVKASEGDRLPLTLTGPKTANPISYRVPMASAQVKSAV | 180 |
| Query 181 | LLAGLNTPGITTVIEPIMTRDHTEKMLQGFGANLTVETDADGVRTIRLEGRGKLTGQVID | 240 |
| Sbjct 181 | LLAGLNTPG+TTVIEP+MTRDHTEKMLQGFGA+L+VETD +GVR IR+ G+GKL GQ ID LLAGLNTPGVTTVIEPVMTRDHTEKMLQGFGADLSVETDNEGVRHIRITGQGKLIGQTID | 240 |
| Query 241 | VPGDPSSTAFPLVAALLVPGSDVTILNVLMNPTRTGLILTLQEMGADIEVINPRLAGGED | 300 |
| Sbjct 241 | VPGDPSSTAFPLVAALLV GSDVTI NVLMNPTRTGLILTLQEMGADIE+I+PRLAGGED VPGDPSSTAFPLVAALLVEGSDVTIRNVLMNPTRTGLILTLQEMGADIEIIDPRLAGGED | 300 |
| Query 301 | VADLRVRSSTLKGVTVPEDRAPSMIDEYPILavaaafaegaTVMNGLEELRVKESDRLSA | 360 |
| Sbjct 301 | VADLRV+SS LKGVTVP +RAPSMIDEYP+LA+AA+FAEG T M+GL+ELRVKESDRL+A VADLRVKSSKLKGVTVPPERAPSMIDEYPVLAIAASFAEGETTMDGLDELRVKESDRLAA | 360 |
| Query 361 | VANGLKLNGVDCDEGETSLVVRGRPDGKGLGNASGAAVATHLDHRIAMSFLVMGLVSENP | 420 |
| Sbjct 361 | VA GL+ NGVDC EGE SL VRGRPDGKGLG G VATHLDHRIAMSFLVMGL SE P VARGLEANGVDCTEGEMSLTVRGRPDGKGLG---GGTVATHLDHRIAMSFLVMGLASEKP | 417 |
| Query 421 | VTVDDATMIATSFPEFMDLMAGLGAKIELSDTK 453 |  |

VTVDD+TMIATSFPEFM +M +GAKI++S+ + Sbjct 418 VTVDDSTMIATSFPEFMTMMPDIGAKIKVSEQQ 450

>WP_065706665.1 3-phosphoshikimate 1-carboxyvinyltransferase [Agrobacterium sp. 13-626]

OCJ03433.1 3-phosphoshikimate 1-carboxyvinyltransferase [Agrobacterium sp. 13-626]

Length=454

Score = 724 bits (1868), Expect = 0.0, Method: Compositional matrix adjust.

| Identities | = 369/449 (82%), Positives = 401/449 (89%), Gaps = 1/449 (0%) |  |
| --- | --- | --- |
| Query 1 | MLHGASSRPATARKSSGLSGTVRIPGDKSISHRSFMFGGLASGETRITGLLEGEDVINTG | 60 |
| Sbjct 1 | M H SRPATAR+S+ LSGT+R+PGDKSISHR+FMFGGLASGETRITGLLEGEDV+NTG MPHDTHSRPATARRSTNLSGTIRVPGDKSISHRAFMFGGLASGETRITGLLEGEDVLNTG | 60 |
| Query 61 | KAMQAMGARIRKEGDTWIIDGVGNGGLLAPEAPLDFGNAATGCRLTMGLVGVYDFDSTFI | 120 |
| Sbjct 61 | KAM+AMGA+I K GD WII GVGNG LLAPEAPLDFGNA TG RLTMGLVGVY F+STFI KAMRAMGAKIEKAGDEWIIKGVGNGALLAPEAPLDFGNAGTGSRLTMGLVGVYHFESTFI | 120 |
| Query 121 | GDASLTKRPMGRVLNPLREMGVQVKSEDGDRLPVTLRGPKTPTPITYRVPMASAQVKSAV | 180 |
| Sbjct 121 | GD SL+ RPMGR+L+PLR+MGVQVKS +GDRLPVTL GP+T PITYRVPMASAQVKSAV GDVSLSNRPMGRILDPLRQMGVQVKSSEGDRLPVTLHGPETANPITYRVPMASAQVKSAV | 180 |
| Query 181 | LLAGLNTPGITTVIEPIMTRDHTEKMLQGFGANLTVETDADGVRTIRLEGRGKLTGQVID | 240 |
| Sbjct 181 | LLAGLN PGIT+VIEP+MTRDHTEKML GFGA L+VETD DGVR IRL+G+GKL GQ ID LLAGLNAPGITSVIEPVMTRDHTEKMLAGFGAELSVETDRDGVRIIRLQGQGKLFGQTID | 240 |
| Query 241 | VPGDPSSTAFPLVAALLVPGSDVTILNVLMNPTRTGLILTLQEMGADIEVINPRLAGGED | 300 |
| Sbjct 241 | VPGDPSSTAFPLVAAL+V GSD+TI NVLMNPTRTGLILTLQEMGADIE+++ RLAGGED VPGDPSSTAFPLVAALIVAGSDITIRNVLMNPTRTGLILTLQEMGADIEILDKRLAGGED | 300 |
| Query 301 | VADLRVRSSTLKGVTVPEDRAPSMIDEYPILavaaafaegaTVMNGLEELRVKESDRLSA | 360 |
| Sbjct 301 | VADLRVRSS LKGVTVP +RAPSMIDEYP+LAVAAAFAEG TVM GLEELRVKESDRLSA VADLRVRSSALKGVTVPAERAPSMIDEYPVLAVAAAFAEGTTVMLGLEELRVKESDRLSA | 360 |
| Query 361 | VANGLKLNGVDCDEGETSLVVRGRPDGKGLGNASGAAVATHLDHRIAMSFLVMGLVSENP | 420 |
| Sbjct 361 | VA GL LNGVDC EGE SL VRGRPDGKGLGNA G V THLDHRIAMSFLVMGL SE+P VAEGLTLNGVDCTEGEASLTVRGRPDGKGLGNA-GGEVKTHLDHRIAMSFLVMGLASEHP | 419 |
| Query 421  Sbjct 420 | VTVDDATMIATSFPEFMDLMAGLGAKIEL 449 V VDD+ MIATSFPEF DLMA LGA +++ VKVDDSAMIATSFPEFFDLMAELGADMDV 448 |  |

>WP_182514632.1 3-phosphoshikimate 1-carboxyvinyltransferase [Ochrobactrum sp. P6BSIII]

MBA8817883.1 3-phosphoshikimate 1-carboxyvinyltransferase [Ochrobactrum sp. P6BSIII]

Length=450

Score = 723 bits (1867), Expect = 0.0, Method: Compositional matrix adjust.

| Identities | = 369/453 (81%), Positives = 403/453 (89%), Gaps = 3/453 (1%) |  |
| --- | --- | --- |
| Query 1 | MLHGASSRPATARKSSGLSGTVRIPGDKSISHRSFMFGGLASGETRITGLLEGEDVINTG | 60 |
| Sbjct 1 | M H AS +PA AR+S L G +RIPGDKSISHRSFMFGGLASGETRITGLLEGEDVINTG MSHSASPKPAVARRSEALVGEIRIPGDKSISHRSFMFGGLASGETRITGLLEGEDVINTG | 60 |
| Query 61 | KAMQAMGARIRKEGDTWIIDGVGNGGLLAPEAPLDFGNAATGCRLTMGLVGVYDFDSTFI | 120 |
| Sbjct 61 | +AMQAMGA+I K+GD WII+GVGNG LL PEAPLDFGNA TG RLTMGLVG YD + FI RAMQAMGAKIEKDGDVWIINGVGNGCLLEPEAPLDFGNAGTGARLTMGLVGTYDMTTAFI | 120 |
| Query 121 | GDASLTKRPMGRVLNPLREMGVQVKSEDGDRLPVTLRGPKTPTPITYRVPMASAQVKSAV | 180 |
| Sbjct 121 | GDASL++RPMGRVL+PLR+MGVQVKS DGDR+P+TL GP PITYRVPMASAQVKSAV GDASLSRRPMGRVLDPLRQMGVQVKSTDGDRMPLTLVGPSVANPITYRVPMASAQVKSAV | 180 |
| Query 181 | LLAGLNTPGITTVIEPIMTRDHTEKMLQGFGANLTVETDADGVRTIRLEGRGKLTGQVID | 240 |
| Sbjct 181 | LLAGLNTPG+TTVIEP+MTRDHTEKMLQGFGA+LTVETD +GVR IR+ G+GKL GQ ID LLAGLNTPGVTTVIEPVMTRDHTEKMLQGFGADLTVETDKNGVRHIRITGQGKLIGQTID | 240 |
| Query 241 | VPGDPSSTAFPLVAALLVPGSDVTILNVLMNPTRTGLILTLQEMGADIEVINPRLAGGED | 300 |

| Sbjct | 241 | VPGDPSSTAFPLVAALLV GSDVTI NVLMNPTRTGLILTLQEMGADIEV N RLAGGED VPGDPSSTAFPLVAALLVEGSDVTIRNVLMNPTRTGLILTLQEMGADIEVFNERLAGGED | 300 |
| --- | --- | --- | --- |
| Query | 301 | VADLRVRSSTLKGVTVPEDRAPSMIDEYPILavaaafaegaTVMNGLEELRVKESDRLSA | 360 |
| Sbjct | 301 | VADLRV+SS LKGVTVP +RAPSMIDEYP+LA+AA+FAEG TVM+GL+ELRVKESDRL+A VADLRVKSSRLKGVTVPPERAPSMIDEYPVLAIAASFAEGETVMDGLDELRVKESDRLAA | 360 |
| Query | 361 | VANGLKLNGVDCDEGETSLVVRGRPDGKGLGNASGAAVATHLDHRIAMSFLVMGLVSENP | 420 |
| Sbjct | 361 | VA GL++NGVDC EGE SL VRGRPDGKGLG G VATHLDHRIAMSFLVMGL SE P VARGLEVNGVDCTEGEMSLTVRGRPDGKGLG---GGVVATHLDHRIAMSFLVMGLASEKP | 417 |
| Query  Sbjct | 421  418 | VTVDDATMIATSFPEFMDLMAGLGAKIELSDTK 453 VTVDD+TMIATSFPEFMDLM G+GAKIE S+ + VTVDDSTMIATSFPEFMDLMTGMGAKIEPSEAE 450 |  |

>WP_182548377.1 3-phosphoshikimate 1-carboxyvinyltransferase [Phyllobacterium myrsinacearum]

MBA8877747.1 3-phosphoshikimate 1-carboxyvinyltransferase [Phyllobacterium myrsinacearum]

Length=451

Score = 723 bits (1866), Expect = 0.0, Method: Compositional matrix adjust.

| Identities | = 366/452 (81%), Positives = 399/452 (88%), Gaps = 3/452 (1%) |  |
| --- | --- | --- |
| Query 1 | MLHGASSRPATARKSSGLSGTVRIPGDKSISHRSFMFGGLASGETRITGLLEGEDVINTG | 60 |
| Sbjct 1 | M H A +PATAR+S+ LSG +RIPGDKSISHRSFMFGGLASGETRITGLLEGEDVINTG MSHSALPKPATARRSNALSGDIRIPGDKSISHRSFMFGGLASGETRITGLLEGEDVINTG | 60 |
| Query 61 | KAMQAMGARIRKEGDTWIIDGVGNGGLLAPEAPLDFGNAATGCRLTMGLVGVYDFDSTFI | 120 |
| Sbjct 61 | +AM+AMGA+I KEGD WII G GNG LL PEAPLDFGNA TG RLTMGLVG Y ++TFI RAMRAMGAKIAKEGDVWIIHGTGNGCLLQPEAPLDFGNAGTGARLTMGLVGTYAMETTFI | 120 |
| Query 121 | GDASLTKRPMGRVLNPLREMGVQVKSEDGDRLPVTLRGPKTPTPITYRVPMASAQVKSAV | 180 |
| Sbjct 121 | GDASL+KRPMGRVLNPLREMGVQV + DGDR+P+TLRGPK PITYRVPMASAQVKSAV GDASLSKRPMGRVLNPLREMGVQVTASDGDRMPLTLRGPKVAAPITYRVPMASAQVKSAV | 180 |
| Query 181 | LLAGLNTPGITTVIEPIMTRDHTEKMLQGFGANLTVETDADGVRTIRLEGRGKLTGQVID | 240 |
| Sbjct 181 | LLAGLN PGITTVIEP+MTRDHTEKMLQGFGANL+VETD D VR IR+EG+GKL GQ+ID LLAGLNAPGITTVIEPVMTRDHTEKMLQGFGANLSVETDKDNVRHIRIEGQGKLIGQIID | 240 |
| Query 241 | VPGDPSSTAFPLVAALLVPGSDVTILNVLMNPTRTGLILTLQEMGADIEVINPRLAGGED | 300 |
| Sbjct 241 | VPGDPSSTAFPLVAALLVPGSD+TI NVLMNPTRTGLILTLQEMGA IE++N RLAGGED VPGDPSSTAFPLVAALLVPGSDITIRNVLMNPTRTGLILTLQEMGASIEILNARLAGGED | 300 |
| Query 301 | VADLRVRSSTLKGVTVPEDRAPSMIDEYPILavaaafaegaTVMNGLEELRVKESDRLSA | 360 |
| Sbjct 301 | VADLRVR+S LKGV+VP RAPSMIDEYP+LAVAA+FA G TVMNG+EELRVKESDRLSA VADLRVRASELKGVSVPAHRAPSMIDEYPVLAVAASFATGETVMNGIEELRVKESDRLSA | 360 |
| Query 361 | VANGLKLNGVDCDEGETSLVVRGRPDGKGLGNASGAAVATHLDHRIAMSFLVMGLVSENP | 420 |
| Sbjct 361 | VANGLK NGVDC EG +L VRGRPDG GLG G V THLDHRIAMSFLVMGLV+E P VANGLKANGVDCTEGLETLSVRGRPDGTGLG---GGMVETHLDHRIAMSFLVMGLVAEKP | 417 |
| Query 421  Sbjct 418 | VTVDDATMIATSFPEFMDLMAGLGAKIELSDT 452 VTVDD+TMIATSFPEFM +M G+GA+I +T VTVDDSTMIATSFPEFMGMMTGMGAEISEQET 449 |  |

>WP_142889431.1 MULTISPECIES: 3-phosphoshikimate 1-carboxyvinyltransferase [Agrobacterium] KAA6475509.1 3-phosphoshikimate 1-carboxyvinyltransferase [Agrobacterium sp.

ICMP 7243]

NTF46725.1 3-phosphoshikimate 1-carboxyvinyltransferase [Agrobacterium rhizogenes] NTF91751.1 3-phosphoshikimate 1-carboxyvinyltransferase [Agrobacterium rhizogenes] NTF98543.1 3-phosphoshikimate 1-carboxyvinyltransferase [Agrobacterium rhizogenes] NTG18863.1 3-phosphoshikimate 1-carboxyvinyltransferase [Agrobacterium rhizogenes]

Length=454

Score = 723 bits (1865), Expect = 0.0, Method: Compositional matrix adjust. Identities = 369/449 (82%), Positives = 401/449 (89%), Gaps = 1/449 (0%)

Query 1	MLHGASSRPATARKSSGLSGTVRIPGDKSISHRSFMFGGLASGETRITGLLEGEDVINTG 60 M H	SRPATAR+S+ LSGT+R+PGDKSISHR+FMFGGLASGETRITGLLEGEDV+NTG

| Sbjct | 1 | MPHDTHSRPATARRSTNLSGTIRVPGDKSISHRAFMFGGLASGETRITGLLEGEDVLNTG | 60 |
| --- | --- | --- | --- |
| Query | 61 | KAMQAMGARIRKEGDTWIIDGVGNGGLLAPEAPLDFGNAATGCRLTMGLVGVYDFDSTFI | 120 |
| Sbjct | 61 | KAM+AMGA+I K GD WII GVGNG LLAPEAPLDFGNA TG RLTMGLVGVY F+STFI KAMRAMGAKIEKAGDEWIIKGVGNGALLAPEAPLDFGNAGTGSRLTMGLVGVYHFESTFI | 120 |
| Query | 121 | GDASLTKRPMGRVLNPLREMGVQVKSEDGDRLPVTLRGPKTPTPITYRVPMASAQVKSAV | 180 |
| Sbjct | 121 | GD SL+KRPMGR+L+PLR+MGVQVKS +GDRLPVTL GP+T PITYRVPMASAQVKSAV GDVSLSKRPMGRILDPLRQMGVQVKSSEGDRLPVTLHGPETANPITYRVPMASAQVKSAV | 180 |
| Query | 181 | LLAGLNTPGITTVIEPIMTRDHTEKMLQGFGANLTVETDADGVRTIRLEGRGKLTGQVID | 240 |
| Sbjct | 181 | LLAGLN PGIT+VIEP+MTRDHTEKML GFGA L+VETD DGVR IRL+G+GKL GQ ID LLAGLNAPGITSVIEPVMTRDHTEKMLAGFGAELSVETDRDGVRIIRLQGQGKLFGQTID | 240 |
| Query | 241 | VPGDPSSTAFPLVAALLVPGSDVTILNVLMNPTRTGLILTLQEMGADIEVINPRLAGGED | 300 |
| Sbjct | 241 | VPGDPSSTAFPLVAAL+V GSD+TI NVLMNPTRTGLILTLQEMGADIE+++ RLAGGED VPGDPSSTAFPLVAALIVAGSDITIRNVLMNPTRTGLILTLQEMGADIEILDKRLAGGED | 300 |
| Query | 301 | VADLRVRSSTLKGVTVPEDRAPSMIDEYPILavaaafaegaTVMNGLEELRVKESDRLSA | 360 |
| Sbjct | 301 | VADLRVRSS LKGVTVP +RAPSMIDEYP+LAVAAAFAEG TVM GLEELRVKESDRLSA VADLRVRSSALKGVTVPAERAPSMIDEYPVLAVAAAFAEGTTVMLGLEELRVKESDRLSA | 360 |
| Query | 361 | VANGLKLNGVDCDEGETSLVVRGRPDGKGLGNASGAAVATHLDHRIAMSFLVMGLVSENP | 420 |
| Sbjct | 361 | VA GL LNGVDC EGE SL VRGRPDGKGLGNA G V THLDHRIAMSFLVMGL SE+ VAEGLTLNGVDCTEGEASLTVRGRPDGKGLGNA-GGEVKTHLDHRIAMSFLVMGLASEHL | 419 |
| Query  Sbjct | 421  420 | VTVDDATMIATSFPEFMDLMAGLGAKIEL 449 V VDD+ MIATSFPEF DLMA LGA +++ VKVDDSAMIATSFPEFFDLMAELGADMDV 448 |  |

>WP_109704805.1 3-phosphoshikimate 1-carboxyvinyltransferase [Falsochrobactrum shanghaiense]

PWL19416.1 3-phosphoshikimate 1-carboxyvinyltransferase [Falsochrobactrum shanghaiense]

Length=450

Score = 723 bits (1865), Expect = 0.0, Method: Compositional matrix adjust.

| Identities | = 366/453 (81%), Positives = 404/453 (89%), Gaps = 3/453 (1%) |  |
| --- | --- | --- |
| Query 1 | MLHGASSRPATARKSSGLSGTVRIPGDKSISHRSFMFGGLASGETRITGLLEGEDVINTG | 60 |
| Sbjct 1 | M H + +PA+A +S GLSG +RIPGDKSISHRSFMFGGLASGETRITGLLEGEDVINTG MSHSSPLKPASAHRSEGLSGDIRIPGDKSISHRSFMFGGLASGETRITGLLEGEDVINTG | 60 |
| Query 61 | KAMQAMGARIRKEGDTWIIDGVGNGGLLAPEAPLDFGNAATGCRLTMGLVGVYDFDSTFI | 120 |
| Sbjct 61 | +AM+AMGA+IRKEGD WII+G GNG LL PEAPLDFGNA TG RLTMGLVG YD +TFI RAMEAMGAKIRKEGDVWIINGTGNGCLLQPEAPLDFGNAGTGARLTMGLVGTYDMTTTFI | 120 |
| Query 121 | GDASLTKRPMGRVLNPLREMGVQVKSEDGDRLPVTLRGPKTPTPITYRVPMASAQVKSAV | 180 |
| Sbjct 121 | GDASL+KRPMGRVL+PLR+MGVQVKS +GDR+P+TL GPKT PI+YRVPMASAQVKSAV GDASLSKRPMGRVLDPLRQMGVQVKSAEGDRMPLTLTGPKTANPISYRVPMASAQVKSAV | 180 |
| Query 181 | LLAGLNTPGITTVIEPIMTRDHTEKMLQGFGANLTVETDADGVRTIRLEGRGKLTGQVID | 240 |
| Sbjct 181 | LLAGLNTPGITTVIEP+MTRDHTEKMLQGFGA+L+VETD +GVR I + G+GKL+GQ ID LLAGLNTPGITTVIEPVMTRDHTEKMLQGFGADLSVETDTEGVRHIHIVGQGKLSGQSID | 240 |
| Query 241 | VPGDPSSTAFPLVAALLVPGSDVTILNVLMNPTRTGLILTLQEMGADIEVINPRLAGGED | 300 |
| Sbjct 241 | VPGDPSSTAFPLVAALLV GSDVTI NVLMNPTRTGLILTLQEMGADIEV+N RLAGGED VPGDPSSTAFPLVAALLVEGSDVTIRNVLMNPTRTGLILTLQEMGADIEVLNERLAGGED | 300 |
| Query 301 | VADLRVRSSTLKGVTVPEDRAPSMIDEYPILavaaafaegaTVMNGLEELRVKESDRLSA | 360 |
| Sbjct 301 | VADLRV+SS LKG+ VP +RAPSMIDEYP+LA+AA FAEG TVM+GL+ELRVKESDRL+A VADLRVKSSKLKGIVVPPERAPSMIDEYPVLAIAATFAEGETVMDGLDELRVKESDRLAA | 360 |
| Query 361 | VANGLKLNGVDCDEGETSLVVRGRPDGKGLGNASGAAVATHLDHRIAMSFLVMGLVSENP | 420 |
| Sbjct 361 | VA GL+ NGVDC EGE SL VRGRPDGKG+G G V THLDHRIAMSFLVMGL SE P VARGLEANGVDCTEGEMSLTVRGRPDGKGIG---GGTVETHLDHRIAMSFLVMGLASEKP | 417 |
| Query 421  Sbjct 418 | VTVDDATMIATSFPEFMDLMAGLGAKIELSDTK 453 VTVDD T+IATSFPEFMD+MAGLGAKIE SD + VTVDDYTIIATSFPEFMDMMAGLGAKIERSDAQ 450 |  |

>WP_047453994.1 3-phosphoshikimate 1-carboxyvinyltransferase [Agrobacterium rhizogenes] Length=453

Score = 722 bits (1864), Expect = 0.0, Method: Compositional matrix adjust.

| Identities | = 368/449 (82%), Positives = 403/449 (90%), Gaps = 1/449 (0%) |  |
| --- | --- | --- |
| Query 1 | MLHGASSRPATARKSSGLSGTVRIPGDKSISHRSFMFGGLASGETRITGLLEGEDVINTG | 60 |
| Sbjct 1 | M H SRP TAR+S+ LSGT+R+PGDKSISHR+FMFGGLASGETRI+GLLEGEDV+NTG MPHDTHSRPVTARRSTNLSGTIRVPGDKSISHRAFMFGGLASGETRISGLLEGEDVLNTG | 60 |
| Query 61 | KAMQAMGARIRKEGDTWIIDGVGNGGLLAPEAPLDFGNAATGCRLTMGLVGVYDFDSTFI | 120 |
| Sbjct 61 | +AMQAMGA I K GD WII GVGNG LLAP+APLDFGNA TG RLTMGLVGVY F+STFI RAMQAMGAGIEKIGDEWIIRGVGNGALLAPQAPLDFGNAGTGSRLTMGLVGVYHFESTFI | 120 |
| Query 121 | GDASLTKRPMGRVLNPLREMGVQVKSEDGDRLPVTLRGPKTPTPITYRVPMASAQVKSAV | 180 |
| Sbjct 121 | GDASL+KRPMGR+L+PLR+MGVQVKS DGDRLPVTL GP+T PITYRVPMASAQVKSAV GDASLSKRPMGRILDPLRQMGVQVKSSDGDRLPVTLHGPETANPITYRVPMASAQVKSAV | 180 |
| Query 181 | LLAGLNTPGITTVIEPIMTRDHTEKMLQGFGANLTVETDADGVRTIRLEGRGKLTGQVID | 240 |
| Sbjct 181 | LLAGLN PGITTVIEP+MTRDHTEKML GFGA L+VETD DGVRTIRL+G+G+L+GQ ID LLAGLNAPGITTVIEPVMTRDHTEKMLAGFGAELSVETDRDGVRTIRLQGQGRLSGQTID | 240 |
| Query 241 | VPGDPSSTAFPLVAALLVPGSDVTILNVLMNPTRTGLILTLQEMGADIEVINPRLAGGED | 300 |
| Sbjct 241 | VPGDPSSTAFPLVAAL+ PGSD+TI NVLMNPTRTGLILTLQEMG DIEV++ RLAGGED VPGDPSSTAFPLVAALIAPGSDITIRNVLMNPTRTGLILTLQEMGGDIEVLDKRLAGGED | 300 |
| Query 301 | VADLRVRSSTLKGVTVPEDRAPSMIDEYPILavaaafaegaTVMNGLEELRVKESDRLSA | 360 |
| Sbjct 301 | VADLRVR+S LKGVTVP +RAPSMIDEYP+LAVAAAFAEG TVM GLEELRVKESDRLSA VADLRVRASALKGVTVPAERAPSMIDEYPVLAVAAAFAEGVTVMLGLEELRVKESDRLSA | 360 |
| Query 361 | VANGLKLNGVDCDEGETSLVVRGRPDGKGLGNASGAAVATHLDHRIAMSFLVMGLVSENP | 420 |
| Sbjct 361 | VA GL LNGVDC EG+ SL VRGRPDGKGLGNA G+ V THLDHRIAMSFLVMGL SE+P VAEGLTLNGVDCTEGKDSLTVRGRPDGKGLGNA-GSEVKTHLDHRIAMSFLVMGLASEHP | 419 |
| Query 421  Sbjct 420 | VTVDDATMIATSFPEFMDLMAGLGAKIEL 449 V VDD+ MIATSFPEF DLMA LGA +++ VRVDDSAMIATSFPEFFDLMAKLGADMDV 448 |  |

>WP_174059048.1 3-phosphoshikimate 1-carboxyvinyltransferase [Agrobacterium rhizogenes] NTH10543.1 3-phosphoshikimate 1-carboxyvinyltransferase [Agrobacterium rhizogenes]

Length=454

Score = 722 bits (1864), Expect = 0.0, Method: Compositional matrix adjust.

| Identities | = 368/449 (82%), Positives = 400/449 (89%), Gaps = 1/449 (0%) |  |
| --- | --- | --- |
| Query 1 | MLHGASSRPATARKSSGLSGTVRIPGDKSISHRSFMFGGLASGETRITGLLEGEDVINTG | 60 |
| Sbjct 1 | M H SRPAT R+S+ LSGT+R+PGDKSISHR+FMFGGLASGETRITGLLEGEDV+NTG MPHDTHSRPATTRRSTNLSGTIRVPGDKSISHRAFMFGGLASGETRITGLLEGEDVLNTG | 60 |
| Query 61 | KAMQAMGARIRKEGDTWIIDGVGNGGLLAPEAPLDFGNAATGCRLTMGLVGVYDFDSTFI | 120 |
| Sbjct 61 | AM+AMGA+I K GD WII GVGNG LLAPEAPLDFGNA TG RLTMGLVGVY F+STFI NAMRAMGAKIEKAGDEWIIKGVGNGALLAPEAPLDFGNAGTGSRLTMGLVGVYHFESTFI | 120 |
| Query 121 | GDASLTKRPMGRVLNPLREMGVQVKSEDGDRLPVTLRGPKTPTPITYRVPMASAQVKSAV | 180 |
| Sbjct 121 | GD SL+KRPMGR+L+PLR+MGVQVKS +GDRLPVTL GP+T PITYRVPMASAQVKSAV GDVSLSKRPMGRILDPLRQMGVQVKSSEGDRLPVTLHGPETANPITYRVPMASAQVKSAV | 180 |
| Query 181 | LLAGLNTPGITTVIEPIMTRDHTEKMLQGFGANLTVETDADGVRTIRLEGRGKLTGQVID | 240 |
| Sbjct 181 | LLAGLN PGIT+VIEP+MTRDHTEKML GFGA L+VETD DGVR IRL+G+GKL GQ ID LLAGLNAPGITSVIEPVMTRDHTEKMLAGFGAELSVETDRDGVRIIRLQGQGKLFGQTID | 240 |
| Query 241 | VPGDPSSTAFPLVAALLVPGSDVTILNVLMNPTRTGLILTLQEMGADIEVINPRLAGGED | 300 |
| Sbjct 241 | VPGDPSSTAFPLVAAL+V GSD+TI NVLMNPTRTGLILTLQEMGADIE+++ RLAGGED VPGDPSSTAFPLVAALIVAGSDITIRNVLMNPTRTGLILTLQEMGADIEILDKRLAGGED | 300 |
| Query 301 | VADLRVRSSTLKGVTVPEDRAPSMIDEYPILavaaafaegaTVMNGLEELRVKESDRLSA | 360 |
| Sbjct 301 | VADLRVRSS LKGVTVP +RAPSMIDEYP+LAVAAAFAEG TVM GLEELRVKESDRLSA VADLRVRSSALKGVTVPAERAPSMIDEYPVLAVAAAFAEGTTVMLGLEELRVKESDRLSA | 360 |
| Query 361 | VANGLKLNGVDCDEGETSLVVRGRPDGKGLGNASGAAVATHLDHRIAMSFLVMGLVSENP | 420 |

| Sbjct | 361 | VA GL LNGVDC EGE SL VRGRPDGKGLGNA G V THLDHRIAMSFLVMGL SE+P VAEGLTLNGVDCTEGEASLTVRGRPDGKGLGNA-GGEVKTHLDHRIAMSFLVMGLASEHP 419 |
| --- | --- | --- |
| Query | 421 | VTVDDATMIATSFPEFMDLMAGLGAKIEL 449 |
| Sbjct | 420 | V VDD+ MIATSFPEF DLMA LGA +++  VKVDDSAMIATSFPEFFDLMAELGADMDV 448 |

>WP_182509161.1 3-phosphoshikimate 1-carboxyvinyltransferase [Phyllobacterium sp. P30BS-XVII]

MBA8899692.1 3-phosphoshikimate 1-carboxyvinyltransferase [Phyllobacterium sp. P30BS-XVII]

Length=451

Score = 722 bits (1864), Expect = 0.0, Method: Compositional matrix adjust.

| Identities | = 367/454 (81%), Positives = 399/454 (88%), Gaps = 3/454 (1%) |  |
| --- | --- | --- |
| Query 1 | MLHGASSRPATARKSSGLSGTVRIPGDKSISHRSFMFGGLASGETRITGLLEGEDVINTG | 60 |
| Sbjct 1 | M H A +PATAR+S+ L+G +RIPGDKSISHRSFMFGGLASGETRITGLLEGEDVINTG MSHSALPKPATARRSNALTGDIRIPGDKSISHRSFMFGGLASGETRITGLLEGEDVINTG | 60 |
| Query 61 | KAMQAMGARIRKEGDTWIIDGVGNGGLLAPEAPLDFGNAATGCRLTMGLVGVYDFDSTFI | 120 |
| Sbjct 61 | +AMQAMGA+I KEG+ WII G GNG LL PEAPLDFGNA TG RLTMGLVG Y ++TFI RAMQAMGAKIVKEGNVWIIKGTGNGCLLQPEAPLDFGNAGTGARLTMGLVGTYAMETTFI | 120 |
| Query 121 | GDASLTKRPMGRVLNPLREMGVQVKSEDGDRLPVTLRGPKTPTPITYRVPMASAQVKSAV | 180 |
| Sbjct 121 | GDASL+KRPMGRVLNPLREMGVQV + +GDR+P+TLRGPK PITYRVPMASAQVKSAV GDASLSKRPMGRVLNPLREMGVQVTASEGDRMPLTLRGPKVAAPITYRVPMASAQVKSAV | 180 |
| Query 181 | LLAGLNTPGITTVIEPIMTRDHTEKMLQGFGANLTVETDADGVRTIRLEGRGKLTGQVID | 240 |
| Sbjct 181 | LLAGLN PGITTVIEP+MTRDHTEKMLQGFGANLTVETD D VR IR+EG+GKLTGQ ID LLAGLNAPGITTVIEPVMTRDHTEKMLQGFGANLTVETDRDNVRHIRIEGQGKLTGQTID | 240 |
| Query 241 | VPGDPSSTAFPLVAALLVPGSDVTILNVLMNPTRTGLILTLQEMGADIEVINPRLAGGED | 300 |
| Sbjct 241 | VPGDPSSTAFPLVAALLVPGSD+ I NVLMNPTRTGLILTLQEMGA IE++N RLAGGED VPGDPSSTAFPLVAALLVPGSDIIIRNVLMNPTRTGLILTLQEMGASIEILNARLAGGED | 300 |
| Query 301 | VADLRVRSSTLKGVTVPEDRAPSMIDEYPILavaaafaegaTVMNGLEELRVKESDRLSA | 360 |
| Sbjct 301 | VADLRVR+S LKG+ VP DRAPSMIDEYP+LAVAA+FA G TVMNGLEELRVKESDRLSA VADLRVRASELKGIDVPADRAPSMIDEYPVLAVAASFATGETVMNGLEELRVKESDRLSA | 360 |
| Query 361 | VANGLKLNGVDCDEGETSLVVRGRPDGKGLGNASGAAVATHLDHRIAMSFLVMGLVSENP | 420 |
| Sbjct 361 | VANGLK NGVDC EG +L VRGRPDGKG+G G V THLDHRIAMSFLVMGLV+E P VANGLKANGVDCMEGPETLSVRGRPDGKGIG---GGTVETHLDHRIAMSFLVMGLVAEKP | 417 |
| Query 421  Sbjct 418 | VTVDDATMIATSFPEFMDLMAGLGAKIELSDTKA 454 VTVDD+TMIATSFPEFM +M G+GA+I T A VTVDDSTMIATSFPEFMGMMTGMGAEISEQGTPA 451 |  |

>WP_107106441.1 MULTISPECIES: 3-phosphoshikimate 1-carboxyvinyltransferase [Rhizobium] MBB5577560.1 3-phosphoshikimate 1-carboxyvinyltransferase [Rhizobium paranaense] PST64791.1 3-phosphoshikimate 1-carboxyvinyltransferase [Rhizobium sp. SEMIA4064]

Length=451

Score = 722 bits (1863), Expect = 0.0, Method: Compositional matrix adjust.

| Identities | = 368/449 (82%), Positives = 405/449 (90%), Gaps = 0/449 (0%) |  |
| --- | --- | --- |
| Query 1 | MLHGASSRPATARKSSGLSGTVRIPGDKSISHRSFMFGGLASGETRITGLLEGEDVINTG | 60 |
| Sbjct 1 | M H RPATAR S+ LSGT+R+PGDKSISHR+ MFGGLA+GET ITGLLEGEDV+NTG MPHDIHPRPATARHSTNLSGTIRVPGDKSISHRALMFGGLAAGETHITGLLEGEDVLNTG | 60 |
| Query 61 | KAMQAMGARIRKEGDTWIIDGVGNGGLLAPEAPLDFGNAATGCRLTMGLVGVYDFDSTFI | 120 |
| Sbjct 61 | KAMQAMGARI K G WII GVGNG LLAPEAPLDFGNA TG RLTMGLVGVYDF+STFI KAMQAMGARIEKIGAEWIIKGVGNGALLAPEAPLDFGNAGTGSRLTMGLVGVYDFESTFI | 120 |
| Query 121 | GDASLTKRPMGRVLNPLREMGVQVKSEDGDRLPVTLRGPKTPTPITYRVPMASAQVKSAV | 180 |
| Sbjct 121 | GDASL+KRPMGR+L+PLR+MGVQVKS +GDRLPVTL GP+T +PIT+RVPMASAQVKSAV GDASLSKRPMGRILDPLRQMGVQVKSSEGDRLPVTLHGPETASPITHRVPMASAQVKSAV | 180 |
| Query 181 | LLAGLNTPGITTVIEPIMTRDHTEKMLQGFGANLTVETDADGVRTIRLEGRGKLTGQVID LLAGLN PGITTVIEP+MTRDHTEKML GFGA+L+VE D DGVRTIRL+G+G+L+GQ+ID | 240 |

| Sbjct | 181 | LLAGLNAPGITTVIEPVMTRDHTEKMLAGFGADLSVEIDRDGVRTIRLQGQGRLSGQIID | 240 |
| --- | --- | --- | --- |
| Query | 241 | VPGDPSSTAFPLVAALLVPGSDVTILNVLMNPTRTGLILTLQEMGADIEVINPRLAGGED | 300 |
| Sbjct | 241 | VPGDPSSTAFPLVAAL+VPGSD+TI NVLMNPTRTGLILTLQEMG DIEV++ RLAGGED VPGDPSSTAFPLVAALIVPGSDITIRNVLMNPTRTGLILTLQEMGGDIEVLDRRLAGGED | 300 |
| Query | 301 | VADLRVRSSTLKGVTVPEDRAPSMIDEYPILavaaafaegaTVMNGLEELRVKESDRLSA | 360 |
| Sbjct | 301 | VADLRVRSS LKGVTVP +RAPSMIDEYP+LAVAAAFAEGATVM GLEELRVKESDRLSA VADLRVRSSALKGVTVPAERAPSMIDEYPVLAVAAAFAEGATVMLGLEELRVKESDRLSA | 360 |
| Query | 361 | VANGLKLNGVDCDEGETSLVVRGRPDGKGLGNASGAAVATHLDHRIAMSFLVMGLVSENP | 420 |
| Sbjct | 361 | VA GL LNGVDC EG+ SL V GRP+GKGLGNA G+ V THLDHRIAMSFLVMGL SE+P VAEGLTLNGVDCAEGKDSLTVHGRPNGKGLGNARGSQVKTHLDHRIAMSFLVMGLASEHP | 420 |
| Query  Sbjct | 421  421 | VTVDDATMIATSFPEFMDLMAGLGAKIEL 449 V++DD+TMIATSFPEF DLM LGA IE+ VSIDDSTMIATSFPEFFDLMTRLGATIEV 449 |  |

>WP_184364822.1 3-phosphoshikimate 1-carboxyvinyltransferase [Rhizobium sp. BK602] MBB3612968.1 3-phosphoshikimate 1-carboxyvinyltransferase [Rhizobium sp. BK602]

Length=455

Score = 721 bits (1861), Expect = 0.0, Method: Compositional matrix adjust.

| Identities | = 366/449 (82%), Positives = 398/449 (89%), Gaps = 0/449 (0%) |  |
| --- | --- | --- |
| Query 1 | MLHGASSRPATARKSSGLSGTVRIPGDKSISHRSFMFGGLASGETRITGLLEGEDVINTG | 60 |
| Sbjct 1 | M H RPATAR+S+ LSGT+R+PGDKSISHRSFMFGGLASGETRITGLLEGEDV+NTG MPHDNHPRPATARRSANLSGTIRVPGDKSISHRSFMFGGLASGETRITGLLEGEDVLNTG | 60 |
| Query 61 | KAMQAMGARIRKEGDTWIIDGVGNGGLLAPEAPLDFGNAATGCRLTMGLVGVYDFDSTFI | 120 |
| Sbjct 61 | KAMQAMGA I K G WII GVGNG LLAPEAPLDFGNA TG RLTMGLVGVYDF +TFI KAMQAMGAAIEKVGAEWIIKGVGNGALLAPEAPLDFGNAGTGSRLTMGLVGVYDFSTTFI | 120 |
| Query 121 | GDASLTKRPMGRVLNPLREMGVQVKSEDGDRLPVTLRGPKTPTPITYRVPMASAQVKSAV | 180 |
| Sbjct 121 | GDASL++RPM R+L+PLR+MGV+VKS +GDRLPVTLRGP PI+YRVPMASAQVKSAV GDASLSRRPMARILDPLRQMGVEVKSSEGDRLPVTLRGPAVANPISYRVPMASAQVKSAV | 180 |
| Query 181 | LLAGLNTPGITTVIEPIMTRDHTEKMLQGFGANLTVETDADGVRTIRLEGRGKLTGQVID | 240 |
| Sbjct 181 | LLAGLN PG+TTVIEP+MTRDHTEKML GFGA L+VETD DGVRTIRL+GR KL+GQ ID LLAGLNAPGVTTVIEPVMTRDHTEKMLAGFGAELSVETDRDGVRTIRLQGRTKLSGQTID | 240 |
| Query 241 | VPGDPSSTAFPLVAALLVPGSDVTILNVLMNPTRTGLILTLQEMGADIEVINPRLAGGED | 300 |
| Sbjct 241 | VPGDPSSTAFPLVAAL+ PGSD+TI NVLMNPTRTGLILTLQEMGADIEV++ RLAGGED VPGDPSSTAFPLVAALITPGSDITISNVLMNPTRTGLILTLQEMGADIEVLDKRLAGGED | 300 |
| Query 301 | VADLRVRSSTLKGVTVPEDRAPSMIDEYPILavaaafaegaTVMNGLEELRVKESDRLSA | 360 |
| Sbjct 301 | VADLRVRSS LKGVTVP +RAPSMIDEYP+LAVAAAFAEG T+M GLEELRVKESDRL+A VADLRVRSSALKGVTVPAERAPSMIDEYPVLAVAAAFAEGTTLMLGLEELRVKESDRLAA | 360 |
| Query 361 | VANGLKLNGVDCDEGETSLVVRGRPDGKGLGNASGAAVATHLDHRIAMSFLVMGLVSENP | 420 |
| Sbjct 361 | VA GL LNGVDC E SL VRGRPDGKGLGNA+G V THLDHR+AMSFLVMGL SE+P VAEGLTLNGVDCTETRDSLSVRGRPDGKGLGNAAGGTVKTHLDHRVAMSFLVMGLASEHP | 420 |
| Query 421  Sbjct 421 | VTVDDATMIATSFPEFMDLMAGLGAKIEL 449 V VDD TMIATSFPEF DLMA LGA +E+ VAVDDCTMIATSFPEFFDLMADLGADMEV 449 |  |

```
>WP_111573814.1 3-phosphoshikimate 1-carboxyvinyltransferase [Falsochrobactrum ovis]
RAK33953.1 3-phosphoshikimate 1-carboxyvinyltransferase [Falsochrobactrum ovis]
Length=450

Score = 721 bits (1861), Expect = 0.0, Method: Compositional matrix adjust. Identities = 364/453 (80%), Positives = 404/453 (89%), Gaps = 3/453 (1%)

Query 1	MLHGASSRPATARKSSGLSGTVRIPGDKSISHRSFMFGGLASGETRITGLLEGEDVINTG 60 M H + +PATA +S GLSG +RIPGDKSISHRSFMFGGLASGETRITGLLEGEDVINTG
Sbjct 1	MSHSSPLKPATAHRSEGLSGDIRIPGDKSISHRSFMFGGLASGETRITGLLEGEDVINTG 60
```

| Query | 61 | KAMQAMGARIRKEGDTWIIDGVGNGGLLAPEAPLDFGNAATGCRLTMGLVGVYDFDSTFI | 120 |
| --- | --- | --- | --- |
| Sbjct | 61 | +AM+AMGA+IRKEGD WII+GVGNG LL PEAPLDFGNA TG RLTMGLVG YD +TFI RAMEAMGAKIRKEGDVWIINGVGNGCLLQPEAPLDFGNAGTGARLTMGLVGTYDMTTTFI | 120 |
| Query | 121 | GDASLTKRPMGRVLNPLREMGVQVKSEDGDRLPVTLRGPKTPTPITYRVPMASAQVKSAV | 180 |
| Sbjct | 121 | GDASL+ RPMGRVLNPLREMGVQVK+ +GDR+P+TL GPKT PI+YRVPMASAQVKSA+ GDASLSSRPMGRVLNPLREMGVQVKAAEGDRMPLTLTGPKTANPISYRVPMASAQVKSAI | 180 |
| Query | 181 | LLAGLNTPGITTVIEPIMTRDHTEKMLQGFGANLTVETDADGVRTIRLEGRGKLTGQVID | 240 |
| Sbjct | 181 | LLAGLNTPG+TTVIEP+MTRDHTEKMLQGFGA+LTVETD+ GVR I + G+G+LTGQ+ID LLAGLNTPGVTTVIEPVMTRDHTEKMLQGFGADLTVETDSAGVRHIHIVGQGQLTGQIID | 240 |
| Query | 241 | VPGDPSSTAFPLVAALLVPGSDVTILNVLMNPTRTGLILTLQEMGADIEVINPRLAGGED | 300 |
| Sbjct | 241 | VPGDPSSTAFPLVAA+LV GSD+TI NVLMNPTRTGLILTLQEMGADIEV+N RLAGGED VPGDPSSTAFPLVAAILVEGSDLTIRNVLMNPTRTGLILTLQEMGADIEVLNARLAGGED | 300 |
| Query | 301 | VADLRVRSSTLKGVTVPEDRAPSMIDEYPILavaaafaegaTVMNGLEELRVKESDRLSA | 360 |
| Sbjct | 301 | VADLRVRSS LKG+ VP +RAPSMIDEYPILA+AAAFAEG T+M+GL+ELRVKESDRL+A VADLRVRSSKLKGIVVPPERAPSMIDEYPILAIAAAFAEGETIMDGLDELRVKESDRLAA | 360 |
| Query | 361 | VANGLKLNGVDCDEGETSLVVRGRPDGKGLGNASGAAVATHLDHRIAMSFLVMGLVSENP | 420 |
| Sbjct | 361 | VA GL+ NGVDC EGE SL VRGRPDGKG+G G THLDHRIAMSFLVMGL SE P VARGLEANGVDCTEGEMSLSVRGRPDGKGIG---GGTAKTHLDHRIAMSFLVMGLASEKP | 417 |
| Query  Sbjct | 421  418 | VTVDDATMIATSFPEFMDLMAGLGAKIELSDTK 453 V+VDD +IATSFPEFMD+MAGLGAKIE SD + VSVDDYKIIATSFPEFMDMMAGLGAKIEQSDAE 450 |  |

>WP_058326193.1 3-phosphoshikimate 1-carboxyvinyltransferase [Sinorhizobium sp. Sb3]

KSV80933.1 3-phosphoshikimate 1-carboxyvinyltransferase [Sinorhizobium sp. Sb3]

Length=448

Score = 720 bits (1859), Expect = 0.0, Method: Compositional matrix adjust.

| Identities | = 367/448 (82%), Positives = 401/448 (90%), Gaps = 0/448 (0%) |  |
| --- | --- | --- |
| Query 1 | MLHGASSRPATARKSSGLSGTVRIPGDKSISHRSFMFGGLASGETRITGLLEGEDVINTG | 60 |
| Sbjct 1 | M G PATARKS GL GTVRIPGDKSISHRS M GGLA+GETRITGLLEGEDVI TG MSQGLGQSPATARKSDGLKGTVRIPGDKSISHRSLMLGGLAAGETRITGLLEGEDVIATG | 60 |
| Query 61 | KAMQAMGARIRKEGDTWIIDGVGNGGLLAPEAPLDFGNAATGCRLTMGLVGVYDFDSTFI | 120 |
| Sbjct 61 | +AMQAMGARIRKEGDTWI++GVGNG LLAPEAPLDFGNA TGCRL MGLVGVYDF++TFI RAMQAMGARIRKEGDTWIVNGVGNGALLAPEAPLDFGNAGTGCRLIMGLVGVYDFETTFI | 120 |
| Query 121 | GDASLTKRPMGRVLNPLREMGVQVKSEDGDRLPVTLRGPKTPTPITYRVPMASAQVKSAV | 180 |
| Sbjct 121 | GDASL++RPM RVL+PLREMGVQVK+ GD LPVTLRGPKTPTP+TYRVP+ASAQVKSAV GDASLSRRPMARVLDPLREMGVQVKAAMGDGLPVTLRGPKTPTPVTYRVPVASAQVKSAV | 180 |
| Query 181 | LLAGLNTPGITTVIEPIMTRDHTEKMLQGFGANLTVETDADGVRTIRLEGRGKLTGQVID | 240 |
| Sbjct 181 | L AGLN PGITTVIEP++TRDH E+ML+GFGA L+VETDA GVRTIRLEGRG+L Q ID LFAGLNAPGITTVIEPVITRDHAERMLRGFGAELSVETDATGVRTIRLEGRGRLVAQEID | 240 |
| Query 241 | VPGDPSSTAFPLVAALLVPGSDVTILNVLMNPTRTGLILTLQEMGADIEVINPRLAGGED | 300 |
| Sbjct 241 | VPGDPSS AFPLVAAL+VPGSD+TI NVLMNPTRTGLILTLQEMG DIEVI R AGGED VPGDPSSAAFPLVAALVVPGSDITIRNVLMNPTRTGLILTLQEMGGDIEVIEIRQAGGED | 300 |
| Query 301 | VADLRVRSSTLKGVTVPEDRAPSMIDEYPILavaaafaegaTVMNGLEELRVKESDRLSA | 360 |
| Sbjct 301 | +ADLRVR S LKGVTVPE+RA SMIDEYPILA+AAAFAEGAT+M+GL+ELRVKESDRL+A LADLRVRHSELKGVTVPEERAASMIDEYPILAIAAAFAEGATIMHGLDELRVKESDRLAA | 360 |
| Query 361 | VANGLKLNGVDCDEGETSLVVRGRPDGKGLGNASGAAVATHLDHRIAMSFLVMGLVSENP | 420 |
| Sbjct 361 | +A GLKLNGVDCDEG +SLVVRGRP GKGLGNASG V THLDHRIAMSFLVMGLVSE P IAAGLKLNGVDCDEGRSSLVVRGRPGGKGLGNASGGVVRTHLDHRIAMSFLVMGLVSEYP | 420 |
| Query 421  Sbjct 421 | VTVDDATMIATSFPEFMDLMAGLGAKIE 448 V VDDA+MIATSFP +++LM LGA +E VAVDDASMIATSFPGYIELMTTLGAVVE 448 |  |

>WP_008127565.1 3-phosphoshikimate 1-carboxyvinyltransferase [Phyllobacterium

sp. YR531]

EJN00041.1 3-phosphoshikimate 1-carboxyvinyltransferase [Phyllobacterium sp. YR531]

Length=451

Score = 720 bits (1858), Expect = 0.0, Method: Compositional matrix adjust.

| Identities | = 363/447 (81%), Positives = 394/447 (88%), Gaps = 3/447 (1%) |  |
| --- | --- | --- |
| Query 1 | MLHGASSRPATARKSSGLSGTVRIPGDKSISHRSFMFGGLASGETRITGLLEGEDVINTG | 60 |
| Sbjct 1 | M H + +PAT+R+S+ L+G +RIPGDKSISHRSFMFGGLASGETRITGLLEGEDVINTG MSHSSLPKPATSRRSNALNGDIRIPGDKSISHRSFMFGGLASGETRITGLLEGEDVINTG | 60 |
| Query 61 | KAMQAMGARIRKEGDTWIIDGVGNGGLLAPEAPLDFGNAATGCRLTMGLVGVYDFDSTFI | 120 |
| Sbjct 61 | +AMQAMGA+I KEGD WII G GNG LL PEAPLDFGNA TG RLTMGLVG Y ++ FI RAMQAMGAKIAKEGDVWIIKGTGNGCLLQPEAPLDFGNAGTGARLTMGLVGTYAMETIFI | 120 |
| Query 121 | GDASLTKRPMGRVLNPLREMGVQVKSEDGDRLPVTLRGPKTPTPITYRVPMASAQVKSAV | 180 |
| Sbjct 121 | GDASL+KRPMGRVLNPLREMGVQV + DGDR+P+TLRGPK PI YRVPMASAQVKSAV GDASLSKRPMGRVLNPLREMGVQVSAADGDRMPLTLRGPKVAAPIAYRVPMASAQVKSAV | 180 |
| Query 181 | LLAGLNTPGITTVIEPIMTRDHTEKMLQGFGANLTVETDADGVRTIRLEGRGKLTGQVID | 240 |
| Sbjct 181 | LLAGLNTPGITTVIEP+MTRDHTEKMLQGFGANL+VETD D VR IR+EG+GKLTGQ ID LLAGLNTPGITTVIEPVMTRDHTEKMLQGFGANLSVETDKDNVRHIRIEGQGKLTGQTID | 240 |
| Query 241 | VPGDPSSTAFPLVAALLVPGSDVTILNVLMNPTRTGLILTLQEMGADIEVINPRLAGGED | 300 |
| Sbjct 241 | VPGDPSSTAFPLVAALLVPGSDVTI NVLMNPTRTGLILTLQEMGA IE++N RLAGGED VPGDPSSTAFPLVAALLVPGSDVTIRNVLMNPTRTGLILTLQEMGAKIEIMNARLAGGED | 300 |
| Query 301 | VADLRVRSSTLKGVTVPEDRAPSMIDEYPILavaaafaegaTVMNGLEELRVKESDRLSA | 360 |
| Sbjct 301 | VADLRV S LKGV VP DRAPSMIDEYP+LAVAA+FA G TVMNGLEELRVKESDRL+A VADLRVMHSELKGVNVPADRAPSMIDEYPVLAVAASFASGETVMNGLEELRVKESDRLAA | 360 |
| Query 361 | VANGLKLNGVDCDEGETSLVVRGRPDGKGLGNASGAAVATHLDHRIAMSFLVMGLVSENP | 420 |
| Sbjct 361 | VANGLK NGVDC EG +L VRGRPDGK +G G V THLDHRIAMSFLVMGL +E P VANGLKANGVDCTEGVETLSVRGRPDGKAIG---GGTVETHLDHRIAMSFLVMGLAAEKP | 417 |
| Query 421  Sbjct 418 | VTVDDATMIATSFPEFMDLMAGLGAKI 447 VTVDD+TMIATSFPEFM +MAG+GA+I VTVDDSTMIATSFPEFMAMMAGMGAEI 444 |  |

>WP_112529618.1 3-phosphoshikimate 1-carboxyvinyltransferase [Phyllobacterium trifolii]

MBB3147729.1 3-phosphoshikimate 1-carboxyvinyltransferase [Phyllobacterium trifolii]

Length=449

Score = 719 bits (1856), Expect = 0.0, Method: Compositional matrix adjust.

| Identities | = 358/447 (80%), Positives = 394/447 (88%), Gaps = 3/447 (1%) |  |
| --- | --- | --- |
| Query 1 | MLHGASSRPATARKSSGLSGTVRIPGDKSISHRSFMFGGLASGETRITGLLEGEDVINTG | 60 |
| Sbjct 1 | M H A +PATA +S LSG +RIPGDKSISHRSFMFGGLASGETRITGLLEGEDVINTG MSHSAPPKPATAHRSQALSGDIRIPGDKSISHRSFMFGGLASGETRITGLLEGEDVINTG | 60 |
| Query 61 | KAMQAMGARIRKEGDTWIIDGVGNGGLLAPEAPLDFGNAATGCRLTMGLVGVYDFDSTFI | 120 |
| Sbjct 61 | +AMQAMGA+I KEGD W+I G GNG LLAPEAPLDFGNA TG RLTMGLVG Y ++TF+ RAMQAMGAKIAKEGDVWVIQGTGNGCLLAPEAPLDFGNAGTGARLTMGLVGTYAMETTFV | 120 |
| Query 121 | GDASLTKRPMGRVLNPLREMGVQVKSEDGDRLPVTLRGPKTPTPITYRVPMASAQVKSAV | 180 |
| Sbjct 121 | GDASL+KRPMGRVLNPLREMGVQV + +GDR+P+TLRGPK PITYRVPMASAQVKSAV GDASLSKRPMGRVLNPLREMGVQVSAAEGDRMPLTLRGPKVAAPITYRVPMASAQVKSAV | 180 |
| Query 181 | LLAGLNTPGITTVIEPIMTRDHTEKMLQGFGANLTVETDADGVRTIRLEGRGKLTGQVID | 240 |
| Sbjct 181 | LLAGLNTPGITTV+EP+MTRDHTEKMLQGFGANL+V+TD D VR IR+EG+GKLTGQ ID LLAGLNTPGITTVVEPVMTRDHTEKMLQGFGANLSVDTDRDNVRHIRIEGQGKLTGQTID | 240 |
| Query 241 | VPGDPSSTAFPLVAALLVPGSDVTILNVLMNPTRTGLILTLQEMGADIEVINPRLAGGED | 300 |
| Sbjct 241 | VPGDPSSTAFPLVAA++VPGSD+TI NVLMNPTRTGLILTLQEMGA IE++N RLAGGED VPGDPSSTAFPLVAAIIVPGSDITIRNVLMNPTRTGLILTLQEMGAHIEILNARLAGGED | 300 |
| Query 301 | VADLRVRSSTLKGVTVPEDRAPSMIDEYPILavaaafaegaTVMNGLEELRVKESDRLSA VADLRVR+S L GV VP RAPSMIDEYP+LAVAA FA G TVMNGLEELRVKESDRLSA | 360 |

| Sbjct | 301 | VADLRVRASELNGVHVPASRAPSMIDEYPVLAVAACFATGETVMNGLEELRVKESDRLSA | 360 |
| --- | --- | --- | --- |
| Query | 361 | VANGLKLNGVDCDEGETSLVVRGRPDGKGLGNASGAAVATHLDHRIAMSFLVMGLVSENP | 420 |
| Sbjct | 361 | VANGLK NG DC EG +L VRGRPDGKGLG G +V THLDHRIAMSFLV+GL +E P VANGLKANGADCTEGHDTLTVRGRPDGKGLG---GGSVETHLDHRIAMSFLVLGLAAEKP | 417 |
| Query  Sbjct | 421  418 | VTVDDATMIATSFPEFMDLMAGLGAKI 447 VT+DD+TMI TSFPEFMD+M G+GA+I VTIDDSTMIVTSFPEFMDMMTGMGAEI 444 |  |

>WP_183690442.1 3-phosphoshikimate 1-carboxyvinyltransferase [Rhizobium lusitanum] QND49047.1 3-phosphoshikimate 1-carboxyvinyltransferase [Rhizobium lusitanum]

Length=455

Score = 719 bits (1855), Expect = 0.0, Method: Compositional matrix adjust.

| Identities | = 366/449 (82%), Positives = 401/449 (89%), Gaps = 0/449 (0%) |  |
| --- | --- | --- |
| Query 1 | MLHGASSRPATARKSSGLSGTVRIPGDKSISHRSFMFGGLASGETRITGLLEGEDVINTG | 60 |
| Sbjct 1 | M H SRPATAR+S+ LSGT+R+PGDKSISHR+FMF GLASGETRITGLLEGEDV+NTG MPHDTHSRPATARRSTNLSGTIRVPGDKSISHRAFMFAGLASGETRITGLLEGEDVLNTG | 60 |
| Query 61 | KAMQAMGARIRKEGDTWIIDGVGNGGLLAPEAPLDFGNAATGCRLTMGLVGVYDFDSTFI | 120 |
| Sbjct 61 | KAMQAMGA+I K G WII GVGNG LLAP+APLDFGNA TG RLTMGLVGVY+F+STFI KAMQAMGAKIEKIGGEWIIKGVGNGALLAPQAPLDFGNAGTGSRLTMGLVGVYNFESTFI | 120 |
| Query 121 | GDASLTKRPMGRVLNPLREMGVQVKSEDGDRLPVTLRGPKTPTPITYRVPMASAQVKSAV | 180 |
| Sbjct 121 | GDASL+KRPMGR+L+PLR+MGVQVKS GDRLPVTL GP+T PITYRVPMASAQVKSAV GDASLSKRPMGRILDPLRQMGVQVKSSAGDRLPVTLHGPETANPITYRVPMASAQVKSAV | 180 |
| Query 181 | LLAGLNTPGITTVIEPIMTRDHTEKMLQGFGANLTVETDADGVRTIRLEGRGKLTGQVID | 240 |
| Sbjct 181 | LLAGLN PGITTVIEP+MTRDHTEKML GFGA L+VETD DGVRTIRL+G GKL+GQ ID LLAGLNAPGITTVIEPVMTRDHTEKMLAGFGAELSVETDRDGVRTIRLQGEGKLSGQTID | 240 |
| Query 241 | VPGDPSSTAFPLVAALLVPGSDVTILNVLMNPTRTGLILTLQEMGADIEVINPRLAGGED | 300 |
| Sbjct 241 | VPGDPSSTAFPLVAAL+VPGSD+TI NVLMNPTRTGLILTL EMGADI++++ RLAGGED VPGDPSSTAFPLVAALIVPGSDITIRNVLMNPTRTGLILTLLEMGADIDILDERLAGGED | 300 |
| Query 301 | VADLRVRSSTLKGVTVPEDRAPSMIDEYPILavaaafaegaTVMNGLEELRVKESDRLSA | 360 |
| Sbjct 301 | VADLRVRSS L+GVTVP +RAPSMIDEYP+LAVAAAFAEG T M GLEELRVKESDRLSA VADLRVRSSALRGVTVPTERAPSMIDEYPVLAVAAAFAEGTTDMLGLEELRVKESDRLSA | 360 |
| Query 361 | VANGLKLNGVDCDEGETSLVVRGRPDGKGLGNASGAAVATHLDHRIAMSFLVMGLVSENP | 420 |
| Sbjct 361 | VA GL LNGVDC EG+ SL VRGRPDGKGLGNA G V T+LDHRIAMSFLVMGL SE+P VAEGLALNGVDCTEGDASLTVRGRPDGKGLGNAGGERVRTYLDHRIAMSFLVMGLASEHP | 420 |
| Query 421  Sbjct 421 | VTVDDATMIATSFPEFMDLMAGLGAKIEL 449 V +DD+ MIATSFPEF DLMA LGA +E+ VRIDDSAMIATSFPEFFDLMAELGADMEV 449 |  |

>WP_104821503.1 3-phosphoshikimate 1-carboxyvinyltransferase [Rhizobium sp. NXC24] AVA19769.1 3-phosphoshikimate 1-carboxyvinyltransferase 1 [Rhizobium sp.

NXC24]

Length=451

Score = 718 bits (1854), Expect = 0.0, Method: Compositional matrix adjust.

| Identities | = 367/449 (82%), Positives = 401/449 (89%), Gaps = 0/449 (0%) |  |
| --- | --- | --- |
| Query 1 | MLHGASSRPATARKSSGLSGTVRIPGDKSISHRSFMFGGLASGETRITGLLEGEDVINTG | 60 |
| Sbjct 1 | M H RPATAR+S+ LSGT+R+PGDKSISHR+ MFGGLA+GETRITGLLEGEDV+NTG MPHDIHPRPATARRSTNLSGTIRVPGDKSISHRALMFGGLAAGETRITGLLEGEDVLNTG | 60 |
| Query 61 | KAMQAMGARIRKEGDTWIIDGVGNGGLLAPEAPLDFGNAATGCRLTMGLVGVYDFDSTFI | 120 |
| Sbjct 61 | KAMQAMGA I K G WII GVGNG LLAP+APLDFGNA TG RLTMGLVGVYDF++TFI KAMQAMGAAIEKVGAEWIIKGVGNGALLAPQAPLDFGNAGTGSRLTMGLVGVYDFETTFI | 120 |
| Query 121 | GDASLTKRPMGRVLNPLREMGVQVKSEDGDRLPVTLRGPKTPTPITYRVPMASAQVKSAV | 180 |
| Sbjct 121 | GDASL+KRPMGR+L+PLR MGVQV+S +GDRLPVTL GP+T +PITYRVPMASAQVKSAV GDASLSKRPMGRILDPLRRMGVQVQSSEGDRLPVTLHGPETASPITYRVPMASAQVKSAV | 180 |
| Query 181 | LLAGLNTPGITTVIEPIMTRDHTEKMLQGFGANLTVETDADGVRTIRLEGRGKLTGQVID | 240 |

| Sbjct | 181 | LLAGLN PGITTVIEP+MTRDHTEKML GFGA L+VETD DGVRTIRL+G+GKL+GQ+ID LLAGLNAPGITTVIEPVMTRDHTEKMLVGFGAELSVETDRDGVRTIRLQGQGKLSGQIID | 240 |
| --- | --- | --- | --- |
| Query | 241 | VPGDPSSTAFPLVAALLVPGSDVTILNVLMNPTRTGLILTLQEMGADIEVINPRLAGGED | 300 |
| Sbjct | 241 | VPGDPSSTAFPLVAAL+VPGSD+TI NVLMNPTRTGLILTLQEMGADIE+++ RLAGGED VPGDPSSTAFPLVAALIVPGSDITIRNVLMNPTRTGLILTLQEMGADIEILDRRLAGGED | 300 |
| Query | 301 | VADLRVRSSTLKGVTVPEDRAPSMIDEYPILavaaafaegaTVMNGLEELRVKESDRLSA | 360 |
| Sbjct | 301 | VADLRVRSS LKGVTVP +RAP MIDEYP+LAVAAAFAEG TVM GLEELRVKESDRLSA VADLRVRSSPLKGVTVPAERAPRMIDEYPVLAVAAAFAEGTTVMLGLEELRVKESDRLSA | 360 |
| Query | 361 | VANGLKLNGVDCDEGETSLVVRGRPDGKGLGNASGAAVATHLDHRIAMSFLVMGLVSENP | 420 |
| Sbjct | 361 | VA GL LNGVDC EG SL VRGRPDGKGLG A G V THLDHRIAMSFLVMGL S++P VAEGLMLNGVDCTEGADSLTVRGRPDGKGLGRADGGEVRTHLDHRIAMSFLVMGLASDHP | 420 |
| Query  Sbjct | 421  421 | VTVDDATMIATSFPEFMDLMAGLGAKIEL 449 V VDD+TMIATSFPEF DLM LGA IE+ VKVDDSTMIATSFPEFFDLMTRLGATIEV 449 |  |

>WP_095436112.1 3-phosphoshikimate 1-carboxyvinyltransferase [Rhizobium sp. 11515TR] ASW07963.1 3-phosphoshikimate 1-carboxyvinyltransferase [Rhizobium sp. 11515TR]

Length=451

Score = 718 bits (1854), Expect = 0.0, Method: Compositional matrix adjust.

| Identities | = 360/451 (80%), Positives = 402/451 (89%), Gaps = 0/451 (0%) |  |
| --- | --- | --- |
| Query 1 | MLHGASSRPATARKSSGLSGTVRIPGDKSISHRSFMFGGLASGETRITGLLEGEDVINTG | 60 |
| Sbjct 1 | M H +PA AR+S+GLSGT+RIPGDKSISHRS MFGGLA+GETRITGLLEGEDV+NTG MPHDTLPQPAMARRSAGLSGTIRIPGDKSISHRSLMFGGLAAGETRITGLLEGEDVLNTG | 60 |
| Query 61 | KAMQAMGARIRKEGDTWIIDGVGNGGLLAPEAPLDFGNAATGCRLTMGLVGVYDFDSTFI | 120 |
| Sbjct 61 | KAMQA+GA IRK+ D WI+DG GNGGLLAPEAPLDFGNA TGCRLTMGLVG YDF++T I KAMQALGAAIRKDDDVWIVDGTGNGGLLAPEAPLDFGNAGTGCRLTMGLVGAYDFETTLI | 120 |
| Query 121 | GDASLTKRPMGRVLNPLREMGVQVKSEDGDRLPVTLRGPKTPTPITYRVPMASAQVKSAV | 180 |
| Sbjct 121 | GDASL+KRPMGR+L+PLR MG Q+++ GD+LPVTLRGP +P+TYRVPMASAQVKSAV GDASLSKRPMGRILDPLRLMGTQIRAVAGDKLPVTLRGPGVASPLTYRVPMASAQVKSAV | 180 |
| Query 181 | LLAGLNTPGITTVIEPIMTRDHTEKMLQGFGANLTVETDADGVRTIRLEGRGKLTGQVID | 240 |
| Sbjct 181 | LLAGLN PGITTVIEP+MTRDHTE+ML GFGA+L+VETDA GVRTIRLEG+GKLTGQ ID LLAGLNAPGITTVIEPVMTRDHTERMLAGFGASLSVETDAAGVRTIRLEGQGKLTGQTID | 240 |
| Query 241 | VPGDPSSTAFPLVAALLVPGSDVTILNVLMNPTRTGLILTLQEMGADIEVINPRLAGGED | 300 |
| Sbjct 241 | VPGDPSS AFPLVAAL+VP SD+TI NVLMNPTRTGLILTLQEMGADIE+++ R+ GGED VPGDPSSAAFPLVAALIVPSSDITIRNVLMNPTRTGLILTLQEMGADIEILDRRMTGGED | 300 |
| Query 301 | VADLRVRSSTLKGVTVPEDRAPSMIDEYPILavaaafaegaTVMNGLEELRVKESDRLSA | 360 |
| Sbjct 301 | VADLRVRSSTL GVTVP +RAPSMIDEYP+LAVAA FAEG T+M GLEELRVKESDRLSA VADLRVRSSTLNGVTVPAERAPSMIDEYPVLAVAATFAEGTTIMQGLEELRVKESDRLSA | 360 |
| Query 361 | VANGLKLNGVDCDEGETSLVVRGRPDGKGLGNASGAAVATHLDHRIAMSFLVMGLVSENP | 420 |
| Sbjct 361 | VA GLKLNGVDC EG+ +L V+GRPDGKGLGNA GA V+THLDHRIAMSFLVMGLVSE+P VAEGLKLNGVDCIEGKDTLTVQGRPDGKGLGNAGGAKVSTHLDHRIAMSFLVMGLVSEHP | 420 |
| Query 421  Sbjct 421 | VTVDDATMIATSFPEFMDLMAGLGAKIELSD 451 VT+DD+ MIATSFPEF +M LGA IE D VTIDDSAMIATSFPEFFAMMTRLGATIETLD 451 |  |

```
>WP_114429810.1 3-phosphoshikimate 1-carboxyvinyltransferase [Phyllobacterium bourgognense]
RCW84548.1 3-phosphoshikimate 1-carboxyvinyltransferase [Phyllobacterium bourgognense]
Length=449

Score = 717 bits (1851), Expect = 0.0, Method: Compositional matrix adjust. Identities = 357/447 (80%), Positives = 395/447 (88%), Gaps = 3/447 (1%)

Query 1	MLHGASSRPATARKSSGLSGTVRIPGDKSISHRSFMFGGLASGETRITGLLEGEDVINTG 60 M H A +PATAR+S LSG +RIPGDKSISHR+FMFGGLASGETRITGLLEGEDVINTG
Sbjct 1	MSHSAPPKPATARRSQALSGDIRIPGDKSISHRAFMFGGLASGETRITGLLEGEDVINTG 60
```

| Query | 61 | KAMQAMGARIRKEGDTWIIDGVGNGGLLAPEAPLDFGNAATGCRLTMGLVGVYDFDSTFI | 120 |
| --- | --- | --- | --- |
| Sbjct | 61 | +AMQAMGA+I KEGD W+I G GNG LLAPEAPLDFGNA TG RLTMGLVG Y ++TF+ RAMQAMGAKIVKEGDVWVIQGTGNGCLLAPEAPLDFGNAGTGARLTMGLVGTYAMETTFV | 120 |
| Query | 121 | GDASLTKRPMGRVLNPLREMGVQVKSEDGDRLPVTLRGPKTPTPITYRVPMASAQVKSAV | 180 |
| Sbjct | 121 | GDASL+KRPMGRVL+PLREMGVQV + +GDR+P+TLRGPK PITYRVPMASAQVKSAV GDASLSKRPMGRVLDPLREMGVQVSAAEGDRMPLTLRGPKVAAPITYRVPMASAQVKSAV | 180 |
| Query | 181 | LLAGLNTPGITTVIEPIMTRDHTEKMLQGFGANLTVETDADGVRTIRLEGRGKLTGQVID | 240 |
| Sbjct | 181 | LLAGLNTPGITTVIEP+MTRDHTEKMLQGFGANL+VETD D R IR+EG+GKL GQ ID LLAGLNTPGITTVIEPVMTRDHTEKMLQGFGANLSVETDRDNARHIRIEGQGKLIGQTID | 240 |
| Query | 241 | VPGDPSSTAFPLVAALLVPGSDVTILNVLMNPTRTGLILTLQEMGADIEVINPRLAGGED | 300 |
| Sbjct | 241 | VPGDPSSTAFPLVAA++VPGSD+TI NVLMNP+RTGLILTLQEMGA +E++N RLAGGED VPGDPSSTAFPLVAAIIVPGSDITIRNVLMNPSRTGLILTLQEMGAHVEILNARLAGGED | 300 |
| Query | 301 | VADLRVRSSTLKGVTVPEDRAPSMIDEYPILavaaafaegaTVMNGLEELRVKESDRLSA | 360 |
| Sbjct | 301 | VADLRVR+S LKGV VP DRAPSMIDEYP+LAVAA FA G TVMNGLEELRVKESDRLSA VADLRVRASELKGVHVPADRAPSMIDEYPVLAVAACFATGETVMNGLEELRVKESDRLSA | 360 |
| Query | 361 | VANGLKLNGVDCDEGETSLVVRGRPDGKGLGNASGAAVATHLDHRIAMSFLVMGLVSENP | 420 |
| Sbjct | 361 | VANGLK NG DC EG +L VRGRPDGKGLG G +V THLDHRIAMSFLV+GL +E P VANGLKANGADCTEGHDTLTVRGRPDGKGLG---GGSVETHLDHRIAMSFLVLGLAAEKP | 417 |
| Query  Sbjct | 421  418 | VTVDDATMIATSFPEFMDLMAGLGAKI 447 VT+DD+TMI TSFPEFMD+M G+GA+I VTIDDSTMIVTSFPEFMDMMKGMGAEI 444 |  |

>WP_188344663.1 unnamed protein product

QND51305.1 3-phosphoshikimate 1-carboxyvinyltransferase [Phyllobacterium sp. 628]

Length=451

Score = 717 bits (1850), Expect = 0.0, Method: Compositional matrix adjust.

| Identities | = 360/454 (79%), Positives = 402/454 (89%), Gaps = 3/454 (1%) |  |
| --- | --- | --- |
| Query 1 | MLHGASSRPATARKSSGLSGTVRIPGDKSISHRSFMFGGLASGETRITGLLEGEDVINTG | 60 |
| Sbjct 1 | M H A +PATAR+S+ L+G +RIPGDKSISHRSFMFGGLASGETRITGLLEGEDVINTG MSHSALLKPATARRSNALTGDIRIPGDKSISHRSFMFGGLASGETRITGLLEGEDVINTG | 60 |
| Query 61 | KAMQAMGARIRKEGDTWIIDGVGNGGLLAPEAPLDFGNAATGCRLTMGLVGVYDFDSTFI | 120 |
| Sbjct 61 | +AM+AMGA+I +EG WII+G GNG LL PEAPLDFGNA TG RLTMGLVG Y ++TFI RAMRAMGAKISQEGKVWIIEGTGNGCLLQPEAPLDFGNAGTGARLTMGLVGTYAMETTFI | 120 |
| Query 121 | GDASLTKRPMGRVLNPLREMGVQVKSEDGDRLPVTLRGPKTPTPITYRVPMASAQVKSAV | 180 |
| Sbjct 121 | GDASL+KRPM RVL+PLREMGVQV + DGDR+P+TLRGPK PITYRVPMASAQVKSAV GDASLSKRPMARVLDPLREMGVQVTAADGDRMPLTLRGPKVAAPITYRVPMASAQVKSAV | 180 |
| Query 181 | LLAGLNTPGITTVIEPIMTRDHTEKMLQGFGANLTVETDADGVRTIRLEGRGKLTGQVID | 240 |
| Sbjct 181 | LLAGLNTPGITTVIEP+MTRDHTEKMLQGFGANL+VETD D VR IR+EG+GKLTGQ+ID LLAGLNTPGITTVIEPVMTRDHTEKMLQGFGANLSVETDKDNVRHIRIEGQGKLTGQIID | 240 |
| Query 241 | VPGDPSSTAFPLVAALLVPGSDVTILNVLMNPTRTGLILTLQEMGADIEVINPRLAGGED | 300 |
| Sbjct 241 | VPGDPSSTAFPLVAALLVPGSD+TI NVLMNPTRTGLILTLQEMGA IE++N RLAGGED VPGDPSSTAFPLVAALLVPGSDITIRNVLMNPTRTGLILTLQEMGASIEILNARLAGGED | 300 |
| Query 301 | VADLRVRSSTLKGVTVPEDRAPSMIDEYPILavaaafaegaTVMNGLEELRVKESDRLSA | 360 |
| Sbjct 301 | VADL VR+S L GV VP +RAPSMIDEYP+LAVAA+FA+G TVMNGLEELRVKESDRLSA VADLHVRASELTGVNVPSERAPSMIDEYPVLAVAASFAKGETVMNGLEELRVKESDRLSA | 360 |
| Query 361 | VANGLKLNGVDCDEGETSLVVRGRPDGKGLGNASGAAVATHLDHRIAMSFLVMGLVSENP | 420 |
| Sbjct 361 | VA GLK+NGVDC EG+ +L VRGRPDGKGLG G V THLDHRIAMSFLVMGLV+E P VAEGLKINGVDCTEGKETLSVRGRPDGKGLG---GGTVETHLDHRIAMSFLVMGLVAERP | 417 |
| Query 421  Sbjct 418 | VTVDDATMIATSFPEFMDLMAGLGAKIELSDTKA 454 V+VDD+ +IATSFPEFMD+M G+GA+I + + A VSVDDSAIIATSFPEFMDMMTGMGAEISVHGSPA 451 |  |

>WP_093234324.1 3-phosphoshikimate 1-carboxyvinyltransferase [Sinorhizobium sp.

NFACC03]

SDA88722.1 3-phosphoshikimate 1-carboxyvinyltransferase [Sinorhizobium sp. NFACC03]

Length=448

Score = 717 bits (1850), Expect = 0.0, Method: Compositional matrix adjust.

| Identities | = 366/448 (82%), Positives = 400/448 (89%), Gaps = 0/448 (0%) |  |
| --- | --- | --- |
| Query 1 | MLHGASSRPATARKSSGLSGTVRIPGDKSISHRSFMFGGLASGETRITGLLEGEDVINTG | 60 |
| Sbjct 1 | M G PATARKS GL GTVRIPGDKSISHRS M GGLA+GETRITGLLEGEDVI TG MSQGLGQSPATARKSDGLKGTVRIPGDKSISHRSLMLGGLAAGETRITGLLEGEDVIATG | 60 |
| Query 61 | KAMQAMGARIRKEGDTWIIDGVGNGGLLAPEAPLDFGNAATGCRLTMGLVGVYDFDSTFI | 120 |
| Sbjct 61 | +AMQAMGARIRKEGDTWI++GVGNG LLAPEAPLDFGNA TGCRL MGLVGVYDF++TFI RAMQAMGARIRKEGDTWIVNGVGNGALLAPEAPLDFGNAGTGCRLIMGLVGVYDFETTFI | 120 |
| Query 121 | GDASLTKRPMGRVLNPLREMGVQVKSEDGDRLPVTLRGPKTPTPITYRVPMASAQVKSAV | 180 |
| Sbjct 121 | GDASL++RPM RVL+PLREMGVQVK+ GD LPVTLRGPKTPTP+TYRVP+ASAQVKSAV GDASLSRRPMARVLDPLREMGVQVKAAMGDGLPVTLRGPKTPTPVTYRVPVASAQVKSAV | 180 |
| Query 181 | LLAGLNTPGITTVIEPIMTRDHTEKMLQGFGANLTVETDADGVRTIRLEGRGKLTGQVID | 240 |
| Sbjct 181 | L AGLN PGITTVIEP++TRDH E+ML+GFGA L+VETDA GVRTI LEGRG+L Q ID LFAGLNAPGITTVIEPVITRDHAERMLRGFGAELSVETDATGVRTILLEGRGRLVAQEID | 240 |
| Query 241 | VPGDPSSTAFPLVAALLVPGSDVTILNVLMNPTRTGLILTLQEMGADIEVINPRLAGGED | 300 |
| Sbjct 241 | VPGDPSS AFPLVAAL+VPGSD+TI NVLMNPTRTGLILTLQEMG DIEVI R AGGED VPGDPSSAAFPLVAALVVPGSDITIRNVLMNPTRTGLILTLQEMGGDIEVIEIRQAGGED | 300 |
| Query 301 | VADLRVRSSTLKGVTVPEDRAPSMIDEYPILavaaafaegaTVMNGLEELRVKESDRLSA | 360 |
| Sbjct 301 | +ADLRVR S LKGVTVPE+RA SMIDEYPILA+AAAFAEGAT+M+GL+ELRVKESDRL+A LADLRVRHSELKGVTVPEERAASMIDEYPILAIAAAFAEGATIMHGLDELRVKESDRLAA | 360 |
| Query 361 | VANGLKLNGVDCDEGETSLVVRGRPDGKGLGNASGAAVATHLDHRIAMSFLVMGLVSENP | 420 |
| Sbjct 361 | +A GLKLNGVDCDEG +SLVVRGRP GKGLGNASG V THLDHRIAMSFLVMGLVSE P IAAGLKLNGVDCDEGRSSLVVRGRPGGKGLGNASGGVVRTHLDHRIAMSFLVMGLVSEYP | 420 |
| Query 421  Sbjct 421 | VTVDDATMIATSFPEFMDLMAGLGAKIE 448 V VDDA+MIATSFP +++LM LGA +E VAVDDASMIATSFPGYIELMTTLGAVVE 448 |  |

>WP_106662274.1 3-phosphoshikimate 1-carboxyvinyltransferase [Phyllobacterium sophorae]

PSH67563.1 3-phosphoshikimate 1-carboxyvinyltransferase [Phyllobacterium sophorae]

Length=451

Score = 716 bits (1847), Expect = 0.0, Method: Compositional matrix adjust.

| Identities | = 361/447 (81%), Positives = 396/447 (89%), Gaps = 3/447 (1%) |  |
| --- | --- | --- |
| Query 1 | MLHGASSRPATARKSSGLSGTVRIPGDKSISHRSFMFGGLASGETRITGLLEGEDVINTG | 60 |
| Sbjct 1 | M H A +PATAR+S LSG +RIPGDKSISHRSFMFGGLASGETRITGLLEGEDVINTG MSHSALPKPATARRSRALSGDIRIPGDKSISHRSFMFGGLASGETRITGLLEGEDVINTG | 60 |
| Query 61 | KAMQAMGARIRKEGDTWIIDGVGNGGLLAPEAPLDFGNAATGCRLTMGLVGVYDFDSTFI | 120 |
| Sbjct 61 | +AMQAMGARI K+G+ WII G GNG LL PEAPLDFGNA TG RLTMGLVG Y ++TF+ RAMQAMGARISKDGNVWIIQGTGNGCLLQPEAPLDFGNAGTGARLTMGLVGTYAMETTFV | 120 |
| Query 121 | GDASLTKRPMGRVLNPLREMGVQVKSEDGDRLPVTLRGPKTPTPITYRVPMASAQVKSAV | 180 |
| Sbjct 121 | GDASL+KRPMGRVL+PLREMGVQV + +GDR+P+TLRGPK PITYRVPMASAQVKSAV GDASLSKRPMGRVLDPLREMGVQVTAAEGDRMPLTLRGPKVAAPITYRVPMASAQVKSAV | 180 |
| Query 181 | LLAGLNTPGITTVIEPIMTRDHTEKMLQGFGANLTVETDADGVRTIRLEGRGKLTGQVID | 240 |
| Sbjct 181 | LLAGLN PGITTVIEP+MTRDHTEKMLQGFGANL+VETD D VR IR+EG+G+LTGQ ID LLAGLNAPGITTVIEPVMTRDHTEKMLQGFGANLSVETDKDNVRHIRIEGQGRLTGQTID | 240 |
| Query 241 | VPGDPSSTAFPLVAALLVPGSDVTILNVLMNPTRTGLILTLQEMGADIEVINPRLAGGED | 300 |
| Sbjct 241 | VPGDPSSTAFPLVAALLVPGSD+TI NVLMNPTRTGLILTLQEMGA I ++N RLAGGED VPGDPSSTAFPLVAALLVPGSDITIRNVLMNPTRTGLILTLQEMGASIGILNARLAGGED | 300 |
| Query 301 | VADLRVRSSTLKGVTVPEDRAPSMIDEYPILavaaafaegaTVMNGLEELRVKESDRLSA VADLRVR+S LKGV VP DRAPSMIDEYP+LAVAA+FA G TVMNGL ELRVKESDRLSA | 360 |

| Sbjct | 301 | VADLRVRASELKGVNVPADRAPSMIDEYPVLAVAASFATGETVMNGLGELRVKESDRLSA | 360 |
| --- | --- | --- | --- |
| Query | 361 | VANGLKLNGVDCDEGETSLVVRGRPDGKGLGNASGAAVATHLDHRIAMSFLVMGLVSENP | 420 |
| Sbjct | 361 | VA+GLK NGVDC EG +L VRGRPDGKG+G G V THLDHRIAMSFLVMGL +E P VADGLKANGVDCTEGPETLSVRGRPDGKGIG---GGTVETHLDHRIAMSFLVMGLAAEKP | 417 |
| Query  Sbjct | 421  418 | VTVDDATMIATSFPEFMDLMAGLGAKI 447 V+VDD+TMIATSFPEFMD+MAG+GA+I VSVDDSTMIATSFPEFMDMMAGMGAEI 444 |  |

>WP_153370279.1 3-phosphoshikimate 1-carboxyvinyltransferase [Rhizobium sp. ICMP 5592]

MQB46354.1 3-phosphoshikimate 1-carboxyvinyltransferase [Rhizobium sp. ICMP 5592]

Length=454

Score = 715 bits (1845), Expect = 0.0, Method: Compositional matrix adjust.

| Identities | = 367/454 (81%), Positives = 400/454 (88%), Gaps = 1/454 (0%) |  |
| --- | --- | --- |
| Query 1 | MLHGASSRPATARKSSGLSGTVRIPGDKSISHRSFMFGGLASGETRITGLLEGEDVINTG | 60 |
| Sbjct 1 | M H SRPATAR+S+ LSGT+R+PGDKSISHR+FMFGGLASGETRI+GLLEGEDV+NTG MPHDTHSRPATARRSTNLSGTIRVPGDKSISHRAFMFGGLASGETRISGLLEGEDVLNTG | 60 |
| Query 61 | KAMQAMGARIRKEGDTWIIDGVGNGGLLAPEAPLDFGNAATGCRLTMGLVGVYDFDSTFI | 120 |
| Sbjct 61 | KAMQAMGA+I K WII GVGNG LLAPE PLDFGNA TG RLTMGLVGVY F+STFI KAMQAMGAKIEKIDGEWIIKGVGNGALLAPETPLDFGNAGTGSRLTMGLVGVYHFESTFI | 120 |
| Query 121 | GDASLTKRPMGRVLNPLREMGVQVKSEDGDRLPVTLRGPKTPTPITYRVPMASAQVKSAV | 180 |
| Sbjct 121 | GDASL+KRPMGR+L+PLR+MGVQVKS DGDRLPVTL GP+T PITYRVPMASAQVKSAV GDASLSKRPMGRILDPLRQMGVQVKSSDGDRLPVTLHGPETANPITYRVPMASAQVKSAV | 180 |
| Query 181 | LLAGLNTPGITTVIEPIMTRDHTEKMLQGFGANLTVETDADGVRTIRLEGRGKLTGQVID | 240 |
| Sbjct 181 | LLAGLN PG TTVIEP+MTRDHTEKML GFGA L+VET DGVRTIRL G+GKL+GQ ID LLAGLNAPGTTTVIEPVMTRDHTEKMLAGFGAELSVETGRDGVRTIRLLGQGKLSGQTID | 240 |
| Query 241 | VPGDPSSTAFPLVAALLVPGSDVTILNVLMNPTRTGLILTLQEMGADIEVINPRLAGGED | 300 |
| Sbjct 241 | VPGDPSSTAFPLVAAL+VPGSD+ I NVLMNPTRTGLILTLQ+MGA+IEV++ RLAGGED VPGDPSSTAFPLVAALIVPGSDIIISNVLMNPTRTGLILTLQDMGANIEVLDKRLAGGED | 300 |
| Query 301 | VADLRVRSSTLKGVTVPEDRAPSMIDEYPILavaaafaegaTVMNGLEELRVKESDRLSA | 360 |
| Sbjct 301 | VADLRVRSS LKG+TVP +RAPSMIDEYP+LAVAAAFAEG TVM GLEELRVKESDRLSA VADLRVRSSALKGITVPAERAPSMIDEYPVLAVAAAFAEGTTVMLGLEELRVKESDRLSA | 360 |
| Query 361 | VANGLKLNGVDCDEGETSLVVRGRPDGKGLGNASGAAVATHLDHRIAMSFLVMGLVSENP | 420 |
| Sbjct 361 | VA GL LNGVDC EGE SL VRGRPDGKGLGNA G V THLDHRIAMSFLVMGL SE+P VAEGLTLNGVDCAEGEASLTVRGRPDGKGLGNA-GGEVKTHLDHRIAMSFLVMGLASEHP | 419 |
| Query 421  Sbjct 420 | VTVDDATMIATSFPEFMDLMAGLGAKIELSDTKA 454 V VDD+ MIATSFPEF DLM LGA +++ + A VKVDDSAMIATSFPEFFDLMTELGADMDVEGSIA 453 |  |

>WP_113232730.1 3-phosphoshikimate 1-carboxyvinyltransferase [Rhizobium sp. BK463] NIK86269.1 3-phosphoshikimate 1-carboxyvinyltransferase [Rhizobium sp. BK463]

Length=449

Score = 714 bits (1843), Expect = 0.0, Method: Compositional matrix adjust.

| Identities | = 359/448 (80%), Positives = 400/448 (89%), Gaps = 0/448 (0%) |  |
| --- | --- | --- |
| Query 1 | MLHGASSRPATARKSSGLSGTVRIPGDKSISHRSFMFGGLASGETRITGLLEGEDVINTG | 60 |
| Sbjct 1 | M H +PA AR+S+GLSGT+RIPGDKSISHRS MFGGLA+GETRITGLLEGEDV+NTG MPHDTLPQPAVARRSAGLSGTIRIPGDKSISHRSLMFGGLAAGETRITGLLEGEDVLNTG | 60 |
| Query 61 | KAMQAMGARIRKEGDTWIIDGVGNGGLLAPEAPLDFGNAATGCRLTMGLVGVYDFDSTFI | 120 |
| Sbjct 61 | +AMQA+GA IRK+GD WI+ G GNGGLLAPEAPLDFGNA TG RLTMGLVG YDF++T I RAMQALGAAIRKDGDVWIVGGTGNGGLLAPEAPLDFGNAGTGSRLTMGLVGAYDFETTII | 120 |
| Query 121 | GDASLTKRPMGRVLNPLREMGVQVKSEDGDRLPVTLRGPKTPTPITYRVPMASAQVKSAV | 180 |
| Sbjct 121 | GDASL+KRPMGR+L+PLR MG QV++ +GD+LPVTLRGP +P+TYRVPMASAQVKSAV  GDASLSKRPMGRILHPLRLMGTQVRAAEGDKLPVTLRGPGVASPLTYRVPMASAQVKSAV | 180 |

| Query | 181 | LLAGLNTPGITTVIEPIMTRDHTEKMLQGFGANLTVETDADGVRTIRLEGRGKLTGQVID | 240 |
| --- | --- | --- | --- |
| Sbjct | 181 | LLAGLN PGITTVIEP+MTRDHTE+ML GFGA L+VETDA GVRTIRLEG+GKLTGQ ID LLAGLNAPGITTVIEPVMTRDHTERMLAGFGAALSVETDATGVRTIRLEGQGKLTGQTID | 240 |
| Query | 241 | VPGDPSSTAFPLVAALLVPGSDVTILNVLMNPTRTGLILTLQEMGADIEVINPRLAGGED | 300 |
| Sbjct | 241 | VPGDPSS AFPLVAAL+VPGSD+TI NVLMNPTRTGLIL LQEMGADIEV++ R+ GGED VPGDPSSAAFPLVAALIVPGSDITIRNVLMNPTRTGLILMLQEMGADIEVLDRRMTGGED | 300 |
| Query | 301 | VADLRVRSSTLKGVTVPEDRAPSMIDEYPILavaaafaegaTVMNGLEELRVKESDRLSA | 360 |
| Sbjct | 301 | VADLRVRSS LKGVTVP +RAPSMIDEYP+LAVAAAFAEG T+M GLEELRVKESDRLSA VADLRVRSSALKGVTVPAERAPSMIDEYPVLAVAAAFAEGTTIMQGLEELRVKESDRLSA | 360 |
| Query | 361 | VANGLKLNGVDCDEGETSLVVRGRPDGKGLGNASGAAVATHLDHRIAMSFLVMGLVSENP | 420 |
| Sbjct | 361 | VA+GLKLNGVDC EG+ SL+VRGRPDGKGLGNA G V+THLDHRIAMSFLVMGL +E P VADGLKLNGVDCSEGKDSLIVRGRPDGKGLGNAVGRKVSTHLDHRIAMSFLVMGLAAERP | 420 |
| Query  Sbjct | 421  421 | VTVDDATMIATSFPEFMDLMAGLGAKIE 448 VT+DD+ MIATSFPEF +M LGA IE VTIDDSAMIATSFPEFFAMMTRLGATIE 448 |  |

>WP_065372074.1 3-phosphoshikimate 1-carboxyvinyltransferase [Ensifer adhaerens] Length=448

Score = 714 bits (1842), Expect = 0.0, Method: Compositional matrix adjust.

| Identities | = 360/447 (81%), Positives = 403/447 (90%), Gaps = 0/447 (0%) |  |
| --- | --- | --- |
| Query 1 | MLHGASSRPATARKSSGLSGTVRIPGDKSISHRSFMFGGLASGETRITGLLEGEDVINTG | 60 |
| Sbjct 1 | M G P TARKS GL GTVRIPGDKSISHRS M GGLA+GETRITGLLEGEDVI TG MSQGLGQSPVTARKSDGLKGTVRIPGDKSISHRSLMLGGLAAGETRITGLLEGEDVIATG | 60 |
| Query 61 | KAMQAMGARIRKEGDTWIIDGVGNGGLLAPEAPLDFGNAATGCRLTMGLVGVYDFDSTFI | 120 |
| Sbjct 61 | +AMQAMGARIRKEGDTWI++GVGNG LLAPEAPLDFGNA TGCRL MGLVGVYDF++TFI RAMQAMGARIRKEGDTWIVNGVGNGALLAPEAPLDFGNAGTGCRLIMGLVGVYDFETTFI | 120 |
| Query 121 | GDASLTKRPMGRVLNPLREMGVQVKSEDGDRLPVTLRGPKTPTPITYRVPMASAQVKSAV | 180 |
| Sbjct 121 | GDASL++RPM RVL+PLREMGVQV++ GDRLPVTLRGPKTPTP+TYRVP+ASAQVKSAV GDASLSRRPMARVLDPLREMGVQVEAATGDRLPVTLRGPKTPTPVTYRVPVASAQVKSAV | 180 |
| Query 181 | LLAGLNTPGITTVIEPIMTRDHTEKMLQGFGANLTVETDADGVRTIRLEGRGKLTGQVID | 240 |
| Sbjct 181 | L AGLN PGITTVIEP ++RDHTE+ML+GFGA L+VETDA GVRTIRLEGRG+L Q ID LFAGLNAPGITTVIEPAISRDHTERMLRGFGAELSVETDATGVRTIRLEGRGRLVAQEID | 240 |
| Query 241 | VPGDPSSTAFPLVAALLVPGSDVTILNVLMNPTRTGLILTLQEMGADIEVINPRLAGGED | 300 |
| Sbjct 241 | VPGDPSS AFPLVAAL+VPGSD+TI NVLMNPTR+GLILTLQEMG DIEVI+ R GGED VPGDPSSAAFPLVAALVVPGSDITIRNVLMNPTRSGLILTLQEMGGDIEVIDIRQVGGED | 300 |
| Query 301 | VADLRVRSSTLKGVTVPEDRAPSMIDEYPILavaaafaegaTVMNGLEELRVKESDRLSA | 360 |
| Sbjct 301 | VADLRVR S LKGVTVPE+RA SMIDEYPILA+AAAFAEGAT+M+GL+ELRVKESDRL+A VADLRVRHSDLKGVTVPEERAASMIDEYPILAIAAAFAEGATIMHGLDELRVKESDRLAA | 360 |
| Query 361 | VANGLKLNGVDCDEGETSLVVRGRPDGKGLGNASGAAVATHLDHRIAMSFLVMGLVSENP | 420 |
| Sbjct 361 | +A GLKLNG+DC++G++SLVVRGRP GKGLGNASG V THLDHRIAMSFLV+GL SE+P IAAGLKLNGLDCEQGKSSLVVRGRPGGKGLGNASGGVVKTHLDHRIAMSFLVLGLASEHP | 420 |
| Query 421  Sbjct 421 | VTVDDATMIATSFPEFMDLMAGLGAKI 447 V VDDA+MIATSFP +++LM+ LGA+I VAVDDASMIATSFPGYIELMSRLGARI 447 |  |

>WP_053251884.1 3-phosphoshikimate 1-carboxyvinyltransferase [Ensifer adhaerens] KOF14483.1 3-phosphoshikimate 1-carboxyvinyltransferase [Ensifer adhaerens]

Length=448

Score = 712 bits (1837), Expect = 0.0, Method: Compositional matrix adjust. Identities = 357/448 (80%), Positives = 397/448 (89%), Gaps = 0/448 (0%)

| Query | 1 | MLHGASSRPATARKSSGLSGTVRIPGDKSISHRSFMFGGLASGETRITGLLEGEDVINTG | 60 |
| --- | --- | --- | --- |
|  |  | M G ++PA ARKS L GTVRIPGDKSISHR+ M GGLA+G TRI GLLEGED+ +T |  |
| Sbjct | 1 | MSAGVIAKPALARKSMALRGTVRIPGDKSISHRALMLGGLAAGRTRIAGLLEGEDIFSTA | 60 |
| Query | 61 | KAMQAMGARIRKEGDTWIIDGVGNGGLLAPEAPLDFGNAATGCRLTMGLVGVYDFDSTFI | 120 |

| Sbjct | 61 | +AMQA+GA +RKEGDTWI+DGVGNG LLAPEAPLDFGNA TGCRLTMGLVGVYDF +TF+ RAMQALGATVRKEGDTWIVDGVGNGALLAPEAPLDFGNAGTGCRLTMGLVGVYDFTTTFV | 120 |
| --- | --- | --- | --- |
| Query | 121 | GDASLTKRPMGRVLNPLREMGVQVKSEDGDRLPVTLRGPKTPTPITYRVPMASAQVKSAV | 180 |
| Sbjct | 121 | GDASL +RPMGRVL+PL +MGVQV S +G RLPV L GPKT P++YRVPMASAQVKSAV GDASLARRPMGRVLDPLSQMGVQVTSAEGGRLPVALSGPKTANPVSYRVPMASAQVKSAV | 180 |
| Query | 181 | LLAGLNTPGITTVIEPIMTRDHTEKMLQGFGANLTVETDADGVRTIRLEGRGKLTGQVID | 240 |
| Sbjct | 181 | LLAGLNTPG+TTVIEP+MTRDHTE+MLQ FGANLTVE ++G R IRLEG+G+LTGQ ID LLAGLNTPGVTTVIEPVMTRDHTERMLQAFGANLTVEAGSEGERIIRLEGQGRLTGQDID | 240 |
| Query | 241 | VPGDPSSTAFPLVAALLVPGSDVTILNVLMNPTRTGLILTLQEMGADIEVINPRLAGGED | 300 |
| Sbjct | 241 | VPGDPSS AFPLVAAL+VPGSD+TILNVLMN TRTGLI+TLQEMGA I+V+N R+AGGED VPGDPSSAAFPLVAALIVPGSDITILNVLMNATRTGLIVTLQEMGARIDVLNARIAGGED | 300 |
| Query | 301 | VADLRVRSSTLKGVTVPEDRAPSMIDEYPILavaaafaegaTVMNGLEELRVKESDRLSA | 360 |
| Sbjct | 301 | VADLRVRSS LKG+TVPE RAPSMIDEYP+LAVAAAFAEG TVM GLEELRVKESDRLSA VADLRVRSSELKGITVPEGRAPSMIDEYPVLAVAAAFAEGQTVMRGLEELRVKESDRLSA | 360 |
| Query | 361 | VANGLKLNGVDCDEGETSLVVRGRPDGKGLGNASGAAVATHLDHRIAMSFLVMGLVSENP | 420 |
| Sbjct | 361 | +A GLKLNGVDC+E SLVV GRP GKG+GNA GAAVATHLDHRIAMSFLVMGL SE+P IAAGLKLNGVDCEEAPASLVVHGRPGGKGIGNAVGAAVATHLDHRIAMSFLVMGLASEHP | 420 |
| Query  Sbjct | 421  421 | VTVDDATMIATSFPEFMDLMAGLGAKIE 448 VTVDD TMIATSFPEF+++MAGLGA IE VTVDDTTMIATSFPEFVEVMAGLGASIE 448 |  |

>WP_009364909.1 3-phosphoshikimate 1-carboxyvinyltransferase [Brucella sp. BO2] EFM58630.1 3-phosphoshikimate 1-carboxyvinyltransferase [Brucella sp. BO2]

Length=449

Score = 712 bits (1837), Expect = 0.0, Method: Compositional matrix adjust.

| Identities | = 368/450 (82%), Positives = 400/450 (89%), Gaps = 3/450 (1%) |  |
| --- | --- | --- |
| Query 1 | MLHGASSRPATARKSSGLSGTVRIPGDKSISHRSFMFGGLASGETRITGLLEGEDVINTG | 60 |
| Sbjct 1 | M H A +PATAR S L+G +RIPGDKSISHRSFMFGGLASGETRITGLLEGEDVINTG MSHSACPKPATARHSQALTGEIRIPGDKSISHRSFMFGGLASGETRITGLLEGEDVINTG | 60 |
| Query 61 | KAMQAMGARIRKEGDTWIIDGVGNGGLLAPEAPLDFGNAATGCRLTMGLVGVYDFDSTFI | 120 |
| Sbjct 61 | +AMQAMGARIRKEGD WII+GVGNG LL PEAPLDFGNA TG RLTMGLVG YD ++FI RAMQAMGARIRKEGDVWIINGVGNGCLLQPEAPLDFGNAGTGARLTMGLVGTYDMKTSFI | 120 |
| Query 121 | GDASLTKRPMGRVLNPLREMGVQVKSEDGDRLPVTLRGPKTPTPITYRVPMASAQVKSAV | 180 |
| Sbjct 121 | GDASL+KRPMGRVLNPLREMGVQV++ +GDR+P+TL GP+T PITYRVPMASAQVKSAV GDASLSKRPMGRVLNPLREMGVQVEAAEGDRMPLTLIGPRTANPITYRVPMASAQVKSAV | 180 |
| Query 181 | LLAGLNTPGITTVIEPIMTRDHTEKMLQGFGANLTVETDADGVRTIRLEGRGKLTGQVID | 240 |
| Sbjct 181 | LLAGLNTPG+TTVIEP+MTRDHTEKMLQGFGA+LTVETD DGVR IR+ G+GKLTGQ ID LLAGLNTPGVTTVIEPVMTRDHTEKMLQGFGADLTVETDKDGVRHIRIVGQGKLTGQTID | 240 |
| Query 241 | VPGDPSSTAFPLVAALLVPGSDVTILNVLMNPTRTGLILTLQEMGADIEVINPRLAGGED | 300 |
| Sbjct 241 | VPGDPSSTAFPLVAALLV GSDVTI NVLMNPTRTGLILTLQEMGADIE+I+PRLAGGED VPGDPSSTAFPLVAALLVEGSDVTIRNVLMNPTRTGLILTLQEMGADIEIIDPRLAGGED | 300 |
| Query 301 | VADLRVRSSTLKGVTVPEDRAPSMIDEYPILavaaafaegaTVMNGLEELRVKESDRLSA | 360 |
| Sbjct 301 | VADLRVR+S LKGV VP +RAPSMIDEYP+LA+AA+FAEG TVM+GL+ELRVKESDRL+A VADLRVRASKLKGVVVPPERAPSMIDEYPVLAIAASFAEGETVMDGLDELRVKESDRLAA | 360 |
| Query 361 | VANGLKLNGVDCDEGETSLVVRGRPDGKGLGNASGAAVATHLDHRIAMSFLVMGLVSENP | 420 |
| Sbjct 361 | VA GL+ NGVDC EGE SL VRGR G G VATHLDHRIAMSFLVMGL SE P VARGLEANGVDCTEGEMSLTVRGR---PGGKGLGGGTVATHLDHRIAMSFLVMGLASEKP | 417 |
| Query 421  Sbjct 418 | VTVDDATMIATSFPEFMDLMAGLGAKIELS 450 VTVDD+TMIATSFPEFM +MAGL AKIELS VTVDDSTMIATSFPEFMGMMAGLSAKIELS 447 |  |

>WP_183814360.1 3-phosphoshikimate 1-carboxyvinyltransferase [Rhizobium sp. BK386] MBB3509896.1 3-phosphoshikimate 1-carboxyvinyltransferase [Rhizobium sp. BK386]

Length=449

Score = 710 bits (1832), Expect = 0.0, Method: Compositional matrix adjust.

| Identities | = 356/448 (79%), Positives = 397/448 (89%), Gaps = 0/448 (0%) |  |
| --- | --- | --- |
| Query 1 | MLHGASSRPATARKSSGLSGTVRIPGDKSISHRSFMFGGLASGETRITGLLEGEDVINTG | 60 |
| Sbjct 1 | M H +PA AR+S+GLSGT+RIPGDKSISHRS MFGGLA+GETRITGLLEGEDV+NTG MPHDTLPQPAVARRSAGLSGTIRIPGDKSISHRSLMFGGLAAGETRITGLLEGEDVLNTG | 60 |
| Query 61 | KAMQAMGARIRKEGDTWIIDGVGNGGLLAPEAPLDFGNAATGCRLTMGLVGVYDFDSTFI | 120 |
| Sbjct 61 | KAMQA+GA IRK D WI+ G GNGGLLA EAPLDFGNA TGCRLTMGLVG YDF +TF+ KAMQALGATIRKIDDEWIVRGTGNGGLLASEAPLDFGNAGTGCRLTMGLVGSYDFKTTFV | 120 |
| Query 121 | GDASLTKRPMGRVLNPLREMGVQVKSEDGDRLPVTLRGPKTPTPITYRVPMASAQVKSAV | 180 |
| Sbjct 121 | GDASL+KRPMGR+L+PLR MG QV + GDRLPVTL GP +P+TYRVPMASAQVKSAV GDASLSKRPMGRILDPLRRMGTQVSAAAGDRLPVTLHGPGVASPLTYRVPMASAQVKSAV | 180 |
| Query 181 | LLAGLNTPGITTVIEPIMTRDHTEKMLQGFGANLTVETDADGVRTIRLEGRGKLTGQVID | 240 |
| Sbjct 181 | LLAGLN PGITTVIEP+MTRDHTEKML GFGA L+VETDA GVRTIRLEG+GKLTG+ ID LLAGLNAPGITTVIEPVMTRDHTEKMLAGFGAALSVETDAAGVRTIRLEGQGKLTGRTID | 240 |
| Query 241 | VPGDPSSTAFPLVAALLVPGSDVTILNVLMNPTRTGLILTLQEMGADIEVINPRLAGGED | 300 |
| Sbjct 241 | VPGDPSS AFPLVAAL+VPGSD+TI NVLMNPTRTGLILTLQEMGADIE+++ R+AGGED VPGDPSSAAFPLVAALIVPGSDITIRNVLMNPTRTGLILTLQEMGADIEILDRRMAGGED | 300 |
| Query 301 | VADLRVRSSTLKGVTVPEDRAPSMIDEYPILavaaafaegaTVMNGLEELRVKESDRLSA | 360 |
| Sbjct 301 | VADLRVRSS LKG+TVP +RAPSMIDEYP+LAVAAAFAEG T+M GL+ELRVKESDRL+A VADLRVRSSALKGITVPAERAPSMIDEYPVLAVAAAFAEGTTIMQGLDELRVKESDRLAA | 360 |
| Query 361 | VANGLKLNGVDCDEGETSLVVRGRPDGKGLGNASGAAVATHLDHRIAMSFLVMGLVSENP | 420 |
| Sbjct 361 | VA GLKLNGVDC EG+ SL VRGRPDGKGLGNA G+ V+THLDHRIAMSFL+MGL +E+P VAEGLKLNGVDCSEGKDSLAVRGRPDGKGLGNAGGSEVSTHLDHRIAMSFLIMGLAAEHP | 420 |
| Query 421  Sbjct 421 | VTVDDATMIATSFPEFMDLMAGLGAKIE 448 VT+DD+ MIATSFPEF +M LGA IE VTIDDSAMIATSFPEFFAMMTRLGATIE 448 |  |

>WP_077962779.1 3-phosphoshikimate 1-carboxyvinyltransferase [Ensifer adhaerens] Length=448

Score = 709 bits (1831), Expect = 0.0, Method: Compositional matrix adjust.

| Identities | = 364/448 (81%), Positives = 397/448 (89%), Gaps = 0/448 (0%) |  |
| --- | --- | --- |
| Query 1 | MLHGASSRPATARKSSGLSGTVRIPGDKSISHRSFMFGGLASGETRITGLLEGEDVINTG | 60 |
| Sbjct 1 | M HG+ RPATARKS+ LSGT+RIPGDKSISHR+ M GGLASGETRITGLLEG DV+ TG MSHGSGPRPATARKSADLSGTIRIPGDKSISHRALMLGGLASGETRITGLLEGADVLATG | 60 |
| Query 61 | KAMQAMGARIRKEGDTWIIDGVGNGGLLAPEAPLDFGNAATGCRLTMGLVGVYDFDSTFI | 120 |
| Sbjct 61 | +AMQAMGA IRKE D W+++GVGNG LLAPEAPLDFGNA TGCRLTMGL GVYDF++ F RAMQAMGAGIRKEADGWVVNGVGNGALLAPEAPLDFGNAGTGCRLTMGLTGVYDFETVFT | 120 |
| Query 121 | GDASLTKRPMGRVLNPLREMGVQVKSEDGDRLPVTLRGPKTPTPITYRVPMASAQVKSAV | 180 |
| Sbjct 121 | GDASL++RPM RVL PLREMGVQV++ GDRLPVTLRGPKTPTPIT RVP+ASAQVKSAV GDASLSRRPMARVLGPLREMGVQVRAAAGDRLPVTLRGPKTPTPITCRVPVASAQVKSAV | 180 |
| Query 181 | LLAGLNTPGITTVIEPIMTRDHTEKMLQGFGANLTVETDADGVRTIRLEGRGKLTGQVID | 240 |
| Sbjct 181 | LLAGLN PGITTVIEPIMTRDHTE+ML+GFGA L+VETD GVR IRLEGRGKLTGQ ID LLAGLNAPGITTVIEPIMTRDHTERMLKGFGAALSVETDVSGVRKIRLEGRGKLTGQEID | 240 |
| Query 241 | VPGDPSSTAFPLVAALLVPGSDVTILNVLMNPTRTGLILTLQEMGADIEVINPRLAGGED | 300 |
| Sbjct 241 | VPGDPSS AFPLVAAL+VPGSD+TI NVLMNPTRTGL+LTL+EMG +IEVI+ R GGED VPGDPSSAAFPLVAALIVPGSDITITNVLMNPTRTGLVLTLKEMGGEIEVIDSRQVGGED | 300 |
| Query 301 | VADLRVRSSTLKGVTVPEDRAPSMIDEYPILavaaafaegaTVMNGLEELRVKESDRLSA | 360 |
| Sbjct 301 | VADLRVR S L GVTVPE+RA SMIDEYP LA+AAAFAEGATVM GL ELRVKESDRLSA VADLRVRHSELSGVTVPEERAASMIDEYPALAIAAAFAEGATVMAGLGELRVKESDRLSA | 360 |
| Query 361 | VANGLKLNGVDCDEGETSLVVRGRPDGKGLGNASGAAVATHLDHRIAMSFLVMGLVSENP | 420 |
| Sbjct 361 | VA GL LNGVDC+EG SLVV GRP GKGLG+ASGAAV THLDHRIAMSFLVMGL SE+P VAEGLTLNGVDCEEGAASLVVCGRPGGKGLGSASGAAVKTHLDHRIAMSFLVMGLASEHP | 420 |
| Query 421 | VTVDDATMIATSFPEFMDLMAGLGAKIE 448 VTVDD +MIATSFPEF ++M GLGAKIE |  |

Sbjct 421 VTVDDVSMIATSFPEFTEMMTGLGAKIE 448

>WP_187959664.1 3-phosphoshikimate 1-carboxyvinyltransferase [Brucella sp. 6810] QNQ61617.1 3-phosphoshikimate 1-carboxyvinyltransferase [Brucella sp. 6810]

Length=445

Score = 709 bits (1831), Expect = 0.0, Method: Compositional matrix adjust.

| Identities | = 367/448 (82%), Positives = 399/448 (89%), Gaps = 3/448 (1%) |  |
| --- | --- | --- |
| Query 1 | MLHGASSRPATARKSSGLSGTVRIPGDKSISHRSFMFGGLASGETRITGLLEGEDVINTG | 60 |
| Sbjct 1 | M H A +PATAR S L+G +RIPGDKSISHRSFMFGGLASGETRITGLLEGEDVINTG MSHSACPKPATARHSEALTGEIRIPGDKSISHRSFMFGGLASGETRITGLLEGEDVINTG | 60 |
| Query 61 | KAMQAMGARIRKEGDTWIIDGVGNGGLLAPEAPLDFGNAATGCRLTMGLVGVYDFDSTFI | 120 |
| Sbjct 61 | +AMQAMGARIRKEGD WII+GVGNG LL PEAPLDFGNA TG RLTMGLVG YD ++FI RAMQAMGARIRKEGDVWIINGVGNGCLLQPEAPLDFGNAGTGARLTMGLVGTYDMKTSFI | 120 |
| Query 121 | GDASLTKRPMGRVLNPLREMGVQVKSEDGDRLPVTLRGPKTPTPITYRVPMASAQVKSAV | 180 |
| Sbjct 121 | GDASL+KRPMGRVLNPLREMGVQV++ +GDR+P+TL GP+T PITYRVPMASAQVKSAV GDASLSKRPMGRVLNPLREMGVQVEAAEGDRMPLTLIGPRTANPITYRVPMASAQVKSAV | 180 |
| Query 181 | LLAGLNTPGITTVIEPIMTRDHTEKMLQGFGANLTVETDADGVRTIRLEGRGKLTGQVID | 240 |
| Sbjct 181 | LLAGLNTPG+TTVIEP+MTRDHTEKMLQGFGA+LTVETD DGVR IR+ G+GKLTGQ ID LLAGLNTPGVTTVIEPVMTRDHTEKMLQGFGADLTVETDKDGVRHIRIVGQGKLTGQTID | 240 |
| Query 241 | VPGDPSSTAFPLVAALLVPGSDVTILNVLMNPTRTGLILTLQEMGADIEVINPRLAGGED | 300 |
| Sbjct 241 | VPGDPSSTAFPLVAALLV GSDVTI NVLMNPTRTGLILTLQEMGADIE+I+PRLAGGED VPGDPSSTAFPLVAALLVEGSDVTIRNVLMNPTRTGLILTLQEMGADIEIIDPRLAGGED | 300 |
| Query 301 | VADLRVRSSTLKGVTVPEDRAPSMIDEYPILavaaafaegaTVMNGLEELRVKESDRLSA | 360 |
| Sbjct 301 | VADLRVR+S LKGV VP +RAPSMIDEYP+LA+AA+FAEG TVM+GL+ELRVKESDRL+A VADLRVRASKLKGVVVPPERAPSMIDEYPVLAIAASFAEGETVMDGLDELRVKESDRLAA | 360 |
| Query 361 | VANGLKLNGVDCDEGETSLVVRGRPDGKGLGNASGAAVATHLDHRIAMSFLVMGLVSENP | 420 |
| Sbjct 361 | VA GL+ NGVDC EGE SL VRGR G G VATHLDHRIAMSFLVMGL SE P VARGLEANGVDCTEGEMSLTVRGR---PGGKGLGGGTVATHLDHRIAMSFLVMGLASEKP | 417 |
| Query 421  Sbjct 418 | VTVDDATMIATSFPEFMDLMAGLGAKIE 448 VTVDD+TMIATSFPEFM +MAGLGAKIE VTVDDSTMIATSFPEFMGMMAGLGAKIE 445 |  |

>WP_040448875.1 3-phosphoshikimate 1-carboxyvinyltransferase [Hoeflea phototrophica] EDQ35464.2 3-phosphoshikimate 1-carboxyvinyltransferase [Hoeflea phototrophica

DFL-43]

Length=456

Score = 709 bits (1829), Expect = 0.0, Method: Compositional matrix adjust.

| Identities | = 363/447 (81%), Positives = 395/447 (88%), Gaps = 1/447 (0%) |  |
| --- | --- | --- |
| Query 8 | RPATARKSSGLSGTVRIPGDKSISHRSFMFGGLASGETRITGLLEGEDVINTGKAMQAMG | 67 |
| Sbjct 9 | RPA+A KS L G +RIPGDKSISHRSFMFGGLA+GETRITGLLEGEDVINTGKAMQAMG RPASASKSRSLKGDIRIPGDKSISHRSFMFGGLANGETRITGLLEGEDVINTGKAMQAMG | 68 |
| Query 68 | ARIRKEGDTWIIDGVGNGGLLAPEAPLDFGNAATGCRLTMGLVGVYDFDSTFIGDASLTK | 127 |
| Sbjct 69 | A IRKEGD W+I+GVGNG LLAPE PLDFGNA TGCRLTMGLVG YDFD+TF+GDASL++ ASIRKEGDAWVINGVGNGCLLAPEKPLDFGNAGTGCRLTMGLVGPYDFDTTFVGDASLSR | 128 |
| Query 128 | RPMGRVLNPLREMGVQVKSEDGDRLPVTLRGPKTPTPITYRVPMASAQVKSAVLLAGLNT | 187 |
| Sbjct 129 | RPMGRVL+PLR MGVQV+SEDGDRLPVTL GP P PI+YRVPMASAQVKSAVLLAGLN RPMGRVLDPLRLMGVQVRSEDGDRLPVTLHGPLAPAPISYRVPMASAQVKSAVLLAGLNV | 188 |
| Query 188 | PGITTVIEPIMTRDHTEKMLQGFGANLTVETDADGVRTIRLEGRGKLTGQVIDVPGDPSS | 247 |
| Sbjct 189 | PG+TTVIEP+MTRDHTEKML GFGA L VET ADGVRTIRLEGRG LT Q+IDVPGDPSS PGVTTVIEPVMTRDHTEKMLAGFGAQLEVETGADGVRTIRLEGRGTLTAQIIDVPGDPSS | 248 |
| Query 248 | TAFPLVAALLVPGSDVTILNVLMNPTRTGLILTLQEMGADIEVINPRLAGGEDVADLRVR | 307 |
| Sbjct 249 | TAFPLVAALLVP SDVTI NVLMN TRTGLILTLQEMGA+IE+I+PR AGGEDVADLRVR TAFPLVAALLVPDSDVTIRNVLMNETRTGLILTLQEMGANIEIIDPRQAGGEDVADLRVR | 308 |
| Query 308 | SSTLKGVTVPEDRAPSMIDEYPILavaaafaegaTVMNGLEELRVKESDRLSAVANGLKL | 367 |

| Sbjct | 309 | SS L GV VP DRAPSMIDEYP+LAVAA+FAEG TVM GL+ELRVKESDRLSAVA GL++ SSELTGVNVPADRAPSMIDEYPVLAVAASFAEGETVMTGLDELRVKESDRLSAVARGLEI | 368 |
| --- | --- | --- | --- |
| Query | 368 | NGVDCDEGETSLVVRGRPDGKGLGNAS-GAAVATHLDHRIAMSFLVMGLVSENPVTVDDA | 426 |
| Sbjct | 369 | NGVDC EG+ L VRGRPDGKGLG S A V T LDHRIAMSFLVMGL SE+PV VDD+ NGVDCTEGDDWLSVRGRPDGKGLGARSEKAVVKTWLDHRIAMSFLVMGLASEHPVHVDDS | 428 |
| Query | 427 | TMIATSFPEFMDLMAGLGAKIELSDTK 453  +IATSFPEFMD+M GLGA+I++ + |  |
| Sbjct | 429 | AIIATSFPEFMDMMTGLGARIDIGSAR 455 | |

>WP_140904801.1 3-phosphoshikimate 1-carboxyvinyltransferase [Ochrobactrum gallinifaecis] TPF75363.1 3-phosphoshikimate 1-carboxyvinyltransferase [Ochrobactrum gallinifaecis]

Length=450

Score = 708 bits (1828), Expect = 0.0, Method: Compositional matrix adjust.

| Identities | = 363/453 (80%), Positives = 402/453 (89%), Gaps = 3/453 (1%) |  |
| --- | --- | --- |
| Query 1 | MLHGASSRPATARKSSGLSGTVRIPGDKSISHRSFMFGGLASGETRITGLLEGEDVINTG | 60 |
| Sbjct 1 | M H A+ +PATAR+S L+G +RIPGDKSISHRSFMFGGLA+GETRITGLLEGEDVINTG MSHSATPKPATARRSEALTGEIRIPGDKSISHRSFMFGGLAAGETRITGLLEGEDVINTG | 60 |
| Query 61 | KAMQAMGARIRKEGDTWIIDGVGNGGLLAPEAPLDFGNAATGCRLTMGLVGVYDFDSTFI | 120 |
| Sbjct 61 | +AMQAMGARIRK+GD W+I+GVGNG LL PEAPLDFGNA TG RLTMGLVG YD ++FI RAMQAMGARIRKDGDVWVINGVGNGCLLQPEAPLDFGNAGTGARLTMGLVGTYDMTTSFI | 120 |
| Query 121 | GDASLTKRPMGRVLNPLREMGVQVKSEDGDRLPVTLRGPKTPTPITYRVPMASAQVKSAV | 180 |
| Sbjct 121 | GDASLT RPMGRVLNPLREMGVQVK+ +GDR+P+TL GPKT PI+YRVPMASAQVKSAV GDASLTSRPMGRVLNPLREMGVQVKAAEGDRMPLTLTGPKTANPISYRVPMASAQVKSAV | 180 |
| Query 181 | LLAGLNTPGITTVIEPIMTRDHTEKMLQGFGANLTVETDADGVRTIRLEGRGKLTGQVID | 240 |
| Sbjct 181 | LLAGLN PG+TTVIEP+MTRDHTEKMLQGFGA+LTVETD +GVR IR+ G+GKLTGQ ID LLAGLNAPGVTTVIEPVMTRDHTEKMLQGFGADLTVETDKEGVRHIRIVGQGKLTGQTID | 240 |
| Query 241 | VPGDPSSTAFPLVAALLVPGSDVTILNVLMNPTRTGLILTLQEMGADIEVINPRLAGGED | 300 |
| Sbjct 241 | VPGDPSSTAFPLVAALLV GSDVTILNVLMNPTRTGLILTLQEMGADIE+INPRLAGGED VPGDPSSTAFPLVAALLVEGSDVTILNVLMNPTRTGLILTLQEMGADIEIINPRLAGGED | 300 |
| Query 301 | VADLRVRSSTLKGVTVPEDRAPSMIDEYPILavaaafaegaTVMNGLEELRVKESDRLSA | 360 |
| Sbjct 301 | VADLRV+SS LKGV VP +RAPSMIDEYP+LA+AA+FAEG TVM+GL+ELRVKESDRL+A VADLRVKSSKLKGVVVPPERAPSMIDEYPVLAIAASFAEGETVMDGLDELRVKESDRLAA | 360 |
| Query 361 | VANGLKLNGVDCDEGETSLVVRGRPDGKGLGNASGAAVATHLDHRIAMSFLVMGLVSENP | 420 |
| Sbjct 361 | VA GL+ NGVDC EGE SL VRGR G G VATHLDHRIAMSFLVMGL +E P VARGLEANGVDCTEGEMSLTVRGR---PGGKGLGGGTVATHLDHRIAMSFLVMGLAAEKP | 417 |
| Query 421  Sbjct 418 | VTVDDATMIATSFPEFMDLMAGLGAKIELSDTK 453 VTVDD+ MIATSFPEFM +M GLGAKIE+S+ + VTVDDSLMIATSFPEFMTMMPGLGAKIEVSEKQ 450 |  |

>WP_153527654.1 3-phosphoshikimate 1-carboxyvinyltransferase [Brucella sp. 2280] QGA57008.1 3-phosphoshikimate 1-carboxyvinyltransferase [Brucella sp. 2280]

Length=450

Score = 708 bits (1828), Expect = 0.0, Method: Compositional matrix adjust.

| Identities | = 366/447 (82%), Positives = 398/447 (89%), Gaps = 3/447 (1%) |  |
| --- | --- | --- |
| Query 1 | MLHGASSRPATARKSSGLSGTVRIPGDKSISHRSFMFGGLASGETRITGLLEGEDVINTG | 60 |
| Sbjct 1 | M H A +PATAR S L+G +RIPGDKSISHRSFMFGGLASGETRITGLLEGEDVINTG MSHSACPKPATARHSEALTGEIRIPGDKSISHRSFMFGGLASGETRITGLLEGEDVINTG | 60 |
| Query 61 | KAMQAMGARIRKEGDTWIIDGVGNGGLLAPEAPLDFGNAATGCRLTMGLVGVYDFDSTFI | 120 |
| Sbjct 61 | +AMQAMGARIRKEGD WII+GVGNG LL PEAPLDFGNA TG RLTMGLVG YD ++FI RAMQAMGARIRKEGDVWIINGVGNGCLLQPEAPLDFGNAGTGARLTMGLVGTYDMKTSFI | 120 |
| Query 121 | GDASLTKRPMGRVLNPLREMGVQVKSEDGDRLPVTLRGPKTPTPITYRVPMASAQVKSAV | 180 |
| Sbjct 121 | GDASL+KRPMGRVLNPLREMGVQV++ +GDR+P+TL GP+T PITYRVPMASAQVKSAV GDASLSKRPMGRVLNPLREMGVQVEAAEGDRMPLTLIGPRTANPITYRVPMASAQVKSAV | 180 |
| Query 181 | LLAGLNTPGITTVIEPIMTRDHTEKMLQGFGANLTVETDADGVRTIRLEGRGKLTGQVID | 240 |

| Sbjct | 181 | LLAGLNTPG+TTVIEP+MTRDHTEKMLQGFGA+LTVETD DGVR IR+ G+GKLTGQ ID LLAGLNTPGVTTVIEPVMTRDHTEKMLQGFGADLTVETDKDGVRHIRIVGQGKLTGQTID | 240 |
| --- | --- | --- | --- |
| Query | 241 | VPGDPSSTAFPLVAALLVPGSDVTILNVLMNPTRTGLILTLQEMGADIEVINPRLAGGED | 300 |
| Sbjct | 241 | VPGDPSSTAFPLVAALLV GSDVTI NVLMNPTRTGLILTLQEMGADIE+I+PRLAGGED VPGDPSSTAFPLVAALLVEGSDVTIRNVLMNPTRTGLILTLQEMGADIEIIDPRLAGGED | 300 |
| Query | 301 | VADLRVRSSTLKGVTVPEDRAPSMIDEYPILavaaafaegaTVMNGLEELRVKESDRLSA | 360 |
| Sbjct | 301 | VADLRVR+S LKGV VP +RAPSMIDEYP+LA+AA+FAEG TVM+GL+ELRVKESDRL+A VADLRVRASKLKGVVVPPERAPSMIDEYPVLAIAASFAEGETVMDGLDELRVKESDRLAA | 360 |
| Query | 361 | VANGLKLNGVDCDEGETSLVVRGRPDGKGLGNASGAAVATHLDHRIAMSFLVMGLVSENP | 420 |
| Sbjct | 361 | VA GL+ NGVDC EGE SL VRGR G G VATHLDHRIAMSFLVMGL SE P VARGLEANGVDCTEGEMSLTVRGR---PGGKGLGGGTVATHLDHRIAMSFLVMGLASEKP | 417 |
| Query  Sbjct | 421  418 | VTVDDATMIATSFPEFMDLMAGLGAKI 447 VTVDD+TMIATSFPEFM +MAGLGAKI VTVDDSTMIATSFPEFMGMMAGLGAKI 444 |  |

>WP_076780315.1 MULTISPECIES: 3-phosphoshikimate 1-carboxyvinyltransferase [unclassified Brucella]

APX69790.1 3-phosphoshikimate 1-carboxyvinyltransferase [Brucella sp. 09RB8471] MRN78465.1 3-phosphoshikimate 1-carboxyvinyltransferase [Brucella sp. 10RB9210] SBW15046.1 3-phosphoshikimate 1-carboxyvinyltransferase [Brucella sp. 10RB9215]

Length=450

Score = 708 bits (1828), Expect = 0.0, Method: Compositional matrix adjust.

| Identities | = 366/447 (82%), Positives = 398/447 (89%), Gaps = 3/447 (1%) |  |
| --- | --- | --- |
| Query 1 | MLHGASSRPATARKSSGLSGTVRIPGDKSISHRSFMFGGLASGETRITGLLEGEDVINTG | 60 |
| Sbjct 1 | M H A +PATAR S L+G +RIPGDKSISHRSFMFGGLASGETRITGLLEGEDVINTG MSHSACPKPATARHSQALTGEIRIPGDKSISHRSFMFGGLASGETRITGLLEGEDVINTG | 60 |
| Query 61 | KAMQAMGARIRKEGDTWIIDGVGNGGLLAPEAPLDFGNAATGCRLTMGLVGVYDFDSTFI | 120 |
| Sbjct 61 | +AMQAMGARIRKEGD WII+GVGNG LL PEAPLDFGNA TG RLTMGLVG YD ++FI RAMQAMGARIRKEGDVWIINGVGNGCLLQPEAPLDFGNAGTGARLTMGLVGTYDMKTSFI | 120 |
| Query 121 | GDASLTKRPMGRVLNPLREMGVQVKSEDGDRLPVTLRGPKTPTPITYRVPMASAQVKSAV | 180 |
| Sbjct 121 | GDASL+KRPMGRVLNPLREMGVQV++ +GDR+P+TL GP+T PITYRVPMASAQVKSAV GDASLSKRPMGRVLNPLREMGVQVEAAEGDRMPLTLIGPRTANPITYRVPMASAQVKSAV | 180 |
| Query 181 | LLAGLNTPGITTVIEPIMTRDHTEKMLQGFGANLTVETDADGVRTIRLEGRGKLTGQVID | 240 |
| Sbjct 181 | LLAGLNTPG+TTVIEP+MTRDHTEKMLQGFGA+LTVETD DGVR IR+ G+GKLTGQ ID LLAGLNTPGVTTVIEPVMTRDHTEKMLQGFGADLTVETDKDGVRHIRIVGQGKLTGQTID | 240 |
| Query 241 | VPGDPSSTAFPLVAALLVPGSDVTILNVLMNPTRTGLILTLQEMGADIEVINPRLAGGED | 300 |
| Sbjct 241 | VPGDPSSTAFPLVAALLV GSDVTI NVLMNPTRTGLILTLQEMGADIE+I+PRLAGGED VPGDPSSTAFPLVAALLVEGSDVTIRNVLMNPTRTGLILTLQEMGADIEIIDPRLAGGED | 300 |
| Query 301 | VADLRVRSSTLKGVTVPEDRAPSMIDEYPILavaaafaegaTVMNGLEELRVKESDRLSA | 360 |
| Sbjct 301 | VADLRVR+S LKGV VP +RAPSMIDEYP+LA+AA+FAEG TVM+GL+ELRVKESDRL+A VADLRVRASKLKGVVVPPERAPSMIDEYPVLAIAASFAEGETVMDGLDELRVKESDRLAA | 360 |
| Query 361 | VANGLKLNGVDCDEGETSLVVRGRPDGKGLGNASGAAVATHLDHRIAMSFLVMGLVSENP | 420 |
| Sbjct 361 | VA GL+ NGVDC EGE SL VRGR G G VATHLDHRIAMSFLVMGL SE P VARGLEANGVDCTEGEMSLTVRGR---PGGKGLGGGTVATHLDHRIAMSFLVMGLASEKP | 417 |
| Query 421  Sbjct 418 | VTVDDATMIATSFPEFMDLMAGLGAKI 447 VTVDD+TMIATSFPEFM +MAGLGAKI VTVDDSTMIATSFPEFMGMMAGLGAKI 444 |  |

```
>WP_104662475.1 3-phosphoshikimate 1-carboxyvinyltransferase [Ensifer adhaerens] Length=448

Score = 708 bits (1827), Expect = 0.0, Method: Compositional matrix adjust. Identities = 358/448 (80%), Positives = 399/448 (89%), Gaps = 0/448 (0%)

Query 1	MLHGASSRPATARKSSGLSGTVRIPGDKSISHRSFMFGGLASGETRITGLLEGEDVINTG 60 M HG+ RPATARKS+ LSGT+RIPGDKSISHR+ + GGLASGETRITGLLEG DV+ TG
Sbjct 1	MSHGSGPRPATARKSADLSGTIRIPGDKSISHRALILGGLASGETRITGLLEGVDVLATG 60
```

| Query | 61 | KAMQAMGARIRKEGDTWIIDGVGNGGLLAPEAPLDFGNAATGCRLTMGLVGVYDFDSTFI | 120 |
| --- | --- | --- | --- |
| Sbjct | 61 | +AMQAMGA IRKE D WI++GVGNG LLAPEAPLDFGNA TGCRLTMGL GVYDF++ F RAMQAMGATIRKEADAWIVNGVGNGALLAPEAPLDFGNAGTGCRLTMGLTGVYDFEAVFT | 120 |
| Query | 121 | GDASLTKRPMGRVLNPLREMGVQVKSEDGDRLPVTLRGPKTPTPITYRVPMASAQVKSAV | 180 |
| Sbjct | 121 | GDASL++RPM RV+ PLREMGVQV++ GDRLPVTLRGPKTPTPITYRVP+ASAQVKSAV GDASLSRRPMARVIAPLREMGVQVRAAAGDRLPVTLRGPKTPTPITYRVPVASAQVKSAV | 180 |
| Query | 181 | LLAGLNTPGITTVIEPIMTRDHTEKMLQGFGANLTVETDADGVRTIRLEGRGKLTGQVID | 240 |
| Sbjct | 181 | LLAGLN PGITTVIEPIMTRDHTE+ML+GFG L+VE+D GVRTI LEGRGKL GQ ID LLAGLNAPGITTVIEPIMTRDHTERMLKGFGVELSVESDGSGVRTIHLEGRGKLVGQQID | 240 |
| Query | 241 | VPGDPSSTAFPLVAALLVPGSDVTILNVLMNPTRTGLILTLQEMGADIEVINPRLAGGED | 300 |
| Sbjct | 241 | VPGDPSS AFPLVAAL+VPGSD+TI NVLMNPTRTGLILTL+EMG DIEV++ R GGED VPGDPSSAAFPLVAALIVPGSDITIKNVLMNPTRTGLILTLREMGGDIEVVDSRQVGGED | 300 |
| Query | 301 | VADLRVRSSTLKGVTVPEDRAPSMIDEYPILavaaafaegaTVMNGLEELRVKESDRLSA | 360 |
| Sbjct | 301 | VADLRVR S LKGVTVP +RA SMIDEYP LA+AAAFAEGATVM GL+ELRVKESDRL+A VADLRVRHSELKGVTVPRERAASMIDEYPALAIAAAFAEGATVMTGLDELRVKESDRLAA | 360 |
| Query | 361 | VANGLKLNGVDCDEGETSLVVRGRPDGKGLGNASGAAVATHLDHRIAMSFLVMGLVSENP | 420 |
| Sbjct | 361 | +A GL LNGVDC+EG++SLVVRGR DGKGLGNA+GAAV THLDHRIAMSFLV+GL SE P IAAGLTLNGVDCEEGKSSLVVRGRRDGKGLGNAAGAAVKTHLDHRIAMSFLVLGLASEQP | 420 |
| Query  Sbjct | 421  421 | VTVDDATMIATSFPEFMDLMAGLGAKIE 448 VTVDD +MIATSFPEF ++M GLGA++E VTVDDISMIATSFPEFTEMMTGLGARLE 448 |  |

>WP_069714071.1 MULTISPECIES: 3-phosphoshikimate 1-carboxyvinyltransferase [unclassified Brucella]

OEI84321.1 3-phosphoshikimate 1-carboxyvinyltransferase [Brucella sp. B13-0095] QMV25425.1 3-phosphoshikimate 1-carboxyvinyltransferase [Brucella sp. BO3]

Length=450

Score = 707 bits (1826), Expect = 0.0, Method: Compositional matrix adjust.

| Identities | = 365/447 (82%), Positives = 398/447 (89%), Gaps = 3/447 (1%) |  |
| --- | --- | --- |
| Query 1 | MLHGASSRPATARKSSGLSGTVRIPGDKSISHRSFMFGGLASGETRITGLLEGEDVINTG | 60 |
| Sbjct 1 | M H A +PATAR S L+G +RIPGDKSISHRSFMFGGLASGETRITGLLEGEDVINTG MSHSACPKPATARHSQALTGEIRIPGDKSISHRSFMFGGLASGETRITGLLEGEDVINTG | 60 |
| Query 61 | KAMQAMGARIRKEGDTWIIDGVGNGGLLAPEAPLDFGNAATGCRLTMGLVGVYDFDSTFI | 120 |
| Sbjct 61 | +AMQAMGARIRKEGD WII+GVGNG LL PEAPLDFGNA TG RLTMGLVG YD ++FI RAMQAMGARIRKEGDVWIINGVGNGCLLQPEAPLDFGNAGTGARLTMGLVGTYDMKTSFI | 120 |
| Query 121 | GDASLTKRPMGRVLNPLREMGVQVKSEDGDRLPVTLRGPKTPTPITYRVPMASAQVKSAV | 180 |
| Sbjct 121 | GDASL+KRPMGRVLNPLREMGVQV++ +GDR+P+TL GP+T PITYRVPMASAQVKSAV GDASLSKRPMGRVLNPLREMGVQVEAAEGDRMPLTLIGPRTANPITYRVPMASAQVKSAV | 180 |
| Query 181 | LLAGLNTPGITTVIEPIMTRDHTEKMLQGFGANLTVETDADGVRTIRLEGRGKLTGQVID | 240 |
| Sbjct 181 | LLAGLNTPG+TTVIEP+MTRDHTEKMLQGFGA+LTVETD DGVR IR+ G+GKLTGQ ID LLAGLNTPGVTTVIEPVMTRDHTEKMLQGFGADLTVETDKDGVRHIRIVGQGKLTGQTID | 240 |
| Query 241 | VPGDPSSTAFPLVAALLVPGSDVTILNVLMNPTRTGLILTLQEMGADIEVINPRLAGGED | 300 |
| Sbjct 241 | VPGDPSSTAFPLVAALLV GSDVTI NVLMNPTRTGLILTLQEMGADIE+I+PRLAGGED VPGDPSSTAFPLVAALLVEGSDVTIRNVLMNPTRTGLILTLQEMGADIEIIDPRLAGGED | 300 |
| Query 301 | VADLRVRSSTLKGVTVPEDRAPSMIDEYPILavaaafaegaTVMNGLEELRVKESDRLSA | 360 |
| Sbjct 301 | VADLRV++S LKGV VP +RAPSMIDEYP+LA+AA+FAEG TVM+GL+ELRVKESDRL+A VADLRVKASKLKGVVVPPERAPSMIDEYPVLAIAASFAEGETVMDGLDELRVKESDRLAA | 360 |
| Query 361 | VANGLKLNGVDCDEGETSLVVRGRPDGKGLGNASGAAVATHLDHRIAMSFLVMGLVSENP | 420 |
| Sbjct 361 | VA GL+ NGVDC EGE SL VRGR G G VATHLDHRIAMSFLVMGL SE P VARGLEANGVDCTEGEMSLTVRGR---PGGKGLGGGTVATHLDHRIAMSFLVMGLASEKP | 417 |
| Query 421  Sbjct 418 | VTVDDATMIATSFPEFMDLMAGLGAKI 447 VTVDD+TMIATSFPEFM +MAGLGAKI VTVDDSTMIATSFPEFMGMMAGLGAKI 444 |  |

>MBC7281698.1 3-phosphoshikimate 1-carboxyvinyltransferase [Hoeflea sp.] Length=452

Score = 707 bits (1826), Expect = 0.0, Method: Compositional matrix adjust.

| Identities | = 360/443 (81%), Positives = 395/443 (89%), Gaps = 1/443 (0%) |  |
| --- | --- | --- |
| Query 6 | SSRPATARKSSGLSGTVRIPGDKSISHRSFMFGGLASGETRITGLLEGEDVINTGKAMQA | 65 |
| Sbjct 7 | + RPATA +S L G +RIPGDKSISHRSFMFGGLA GETRITGLLEGEDVINTGKAMQA TPRPATAARSPALRGDIRIPGDKSISHRSFMFGGLAEGETRITGLLEGEDVINTGKAMQA | 66 |
| Query 66 | MGARIRKEGDTWIIDGVGNGGLLAPEAPLDFGNAATGCRLTMGLVGVYDFDSTFIGDASL | 125 |
| Sbjct 67 | MGAR+RK+G TWII+GVGNG LLAPEAPLDFGNA TGCRLTMGL+G YDF++TFIGDASL MGARVRKDGATWIINGVGNGCLLAPEAPLDFGNAGTGCRLTMGLMGPYDFETTFIGDASL | 126 |
| Query 126 | TKRPMGRVLNPLREMGVQVKSEDGDRLPVTLRGPKTPTPITYRVPMASAQVKSAVLLAGL | 185 |
| Sbjct 127 | ++RPMGRVL+PLR+MGVQV +E GDRLPVTLRGP+ P PI+YRVPMASAQVKSAVLLAGL SRRPMGRVLDPLRQMGVQVTAEAGDRLPVTLRGPRAPAPISYRVPMASAQVKSAVLLAGL | 186 |
| Query 186 | NTPGITTVIEPIMTRDHTEKMLQGFGANLTVETDADGVRTIRLEGRGKLTGQVIDVPGDP | 245 |
| Sbjct 187 | N PGITTVIEP+MTRDHTEKML+GFGA L VETDADGVRTIRLEGRG L GQ IDVPGDP NIPGITTVIEPVMTRDHTEKMLKGFGAALDVETDADGVRTIRLEGRGVLKGQTIDVPGDP | 246 |
| Query 246 | SSTAFPLVAALLVPGSDVTILNVLMNPTRTGLILTLQEMGADIEVINPRLAGGEDVADLR | 305 |
| Sbjct 247 | SSTAFPLVAAL+VPGSDV I NVLMNPTRTGLILTLQEMGADIEV++PR AGGEDVADLR SSTAFPLVAALIVPGSDVIIRNVLMNPTRTGLILTLQEMGADIEVLDPRQAGGEDVADLR | 306 |
| Query 306 | VRSSTLKGVTVPEDRAPSMIDEYPILavaaafaegaTVMNGLEELRVKESDRLSAVANGL | 365 |
| Sbjct 307 | VR+S LKGV VP DRAPSMIDEYP+LA+AA FA G T+M GL ELRVKESDRL+AVA GL VRASELKGVRVPVDRAPSMIDEYPVLAIAACFATGETMMPGLVELRVKESDRLAAVARGL | 366 |
| Query 366 | KLNGVDCDEGETSLVVRGRPDGKGLG-NASGAAVATHLDHRIAMSFLVMGLVSENPVTVD | 424 |
| Sbjct 367 | ++NGVDC EGE L VRGRPDGKG G +S A VAT LDHRIAMSFLV+GL SE+PV VD EINGVDCTEGEDWLSVRGRPDGKGFGARSSDAIVATWLDHRIAMSFLVLGLASEHPVHVD | 426 |
| Query 425  Sbjct 427 | DATMIATSFPEFMDLMAGLGAKI 447 D +IATSFPEFMD+M G+GA+I DCAIIATSFPEFMDMMTGMGAEI 449 |  |

>WP_134885619.1 3-phosphoshikimate 1-carboxyvinyltransferase [Ensifer adhaerens] OKP80823.1 3-phosphoshikimate 1-carboxyvinyltransferase [Ensifer adhaerens]

Length=448

Score = 707 bits (1824), Expect = 0.0, Method: Compositional matrix adjust.

| Identities | = 356/447 (80%), Positives = 395/447 (88%), Gaps = 0/447 (0%) |  |
| --- | --- | --- |
| Query 1 | MLHGASSRPATARKSSGLSGTVRIPGDKSISHRSFMFGGLASGETRITGLLEGEDVINTG | 60 |
| Sbjct 1 | M HG+ RPATARKS+ L G+VRIPGDKSISHR+ M GGLASGETRITGLL+G DV+ TG MSHGSGPRPATARKSADLRGSVRIPGDKSISHRALMLGGLASGETRITGLLQGADVLATG | 60 |
| Query 61 | KAMQAMGARIRKEGDTWIIDGVGNGGLLAPEAPLDFGNAATGCRLTMGLVGVYDFDSTFI | 120 |
| Sbjct 61 | +AMQAMGARIRKE DT+I+DGVGNG LLAPEAPLDFGNA TGCRL MGL GVYDF++TF+ RAMQAMGARIRKEADTFIVDGVGNGALLAPEAPLDFGNAGTGCRLAMGLAGVYDFETTFL | 120 |
| Query 121 | GDASLTKRPMGRVLNPLREMGVQVKSEDGDRLPVTLRGPKTPTPITYRVPMASAQVKSAV | 180 |
| Sbjct 121 | GDASL++RPM RVL PLREMG QV++ GDRLPVTLRGPKTP PITYRVP+ASAQVKSAV GDASLSRRPMARVLEPLREMGAQVRAAAGDRLPVTLRGPKTPAPITYRVPVASAQVKSAV | 180 |
| Query 181 | LLAGLNTPGITTVIEPIMTRDHTEKMLQGFGANLTVETDADGVRTIRLEGRGKLTGQVID | 240 |
| Sbjct 181 | LLAGLN PGITTVIEP+MTRDHTE+ML+ FGA L+VETD GVRTIRL+GRGKLTGQ ID LLAGLNAPGITTVIEPVMTRDHTERMLEAFGAALSVETDVSGVRTIRLDGRGKLTGQEID | 240 |
| Query 241 | VPGDPSSTAFPLVAALLVPGSDVTILNVLMNPTRTGLILTLQEMGADIEVINPRLAGGED | 300 |
| Sbjct 241 | VPGDPSS AFPLVAAL+VPGSD+TI NVLMNPTRTGLILTL+EMG DIEVI R GGED VPGDPSSAAFPLVAALIVPGSDITITNVLMNPTRTGLILTLKEMGGDIEVIGSRQVGGED | 300 |
| Query 301 | VADLRVRSSTLKGVTVPEDRAPSMIDEYPILavaaafaegaTVMNGLEELRVKESDRLSA | 360 |
| Sbjct 301 | VADLRVR S L GVTVPE+RA SMIDEYP LA+AAAFAEG TVM GL+ELRVKESDRL+A VADLRVRHSELSGVTVPEERAASMIDEYPPLAIAAAFAEGNTVMAGLDELRVKESDRLAA | 360 |
| Query 361 | VANGLKLNGVDCDEGETSLVVRGRPDGKGLGNASGAAVATHLDHRIAMSFLVMGLVSENP  +A GLK NGVDC+EG +SLVVRGR DGKGLG+A+GA V THLDHRIAMSFL+ GL SE+P | 420 |

| Sbjct | 361 | IAAGLKRNGVDCEEGASSLVVRGRRDGKGLGSAAGATVNTHLDHRIAMSFLIFGLASEHP 420 |
| --- | --- | --- |
| Query | 421 | VTVDDATMIATSFPEFMDLMAGLGAKI 447 |
| Sbjct | 421 | VTVDD +M+ATSFPEFM LM LGA+I  VTVDDVSMVATSFPEFMHLMTSLGARI 447 |

>WP_183646676.1 3-phosphoshikimate 1-carboxyvinyltransferase [Ochrobactrum daejeonense] MBB5700331.1 3-phosphoshikimate 1-carboxyvinyltransferase [Ochrobactrum daejeonense]

Length=450

Score = 706 bits (1823), Expect = 0.0, Method: Compositional matrix adjust.

| Identities | = 367/448 (82%), Positives = 397/448 (89%), Gaps = 3/448 (1%) |  |
| --- | --- | --- |
| Query 1 | MLHGASSRPATARKSSGLSGTVRIPGDKSISHRSFMFGGLASGETRITGLLEGEDVINTG | 60 |
| Sbjct 1 | M H AS +PATAR+S L G +RIPGDKSISHRSFMFGGLASGETRITGLLEGEDVINTG MSHSASPKPATARRSEALVGEIRIPGDKSISHRSFMFGGLASGETRITGLLEGEDVINTG | 60 |
| Query 61 | KAMQAMGARIRKEGDTWIIDGVGNGGLLAPEAPLDFGNAATGCRLTMGLVGVYDFDSTFI | 120 |
| Sbjct 61 | +AMQAMGA+IRK+GD WIIDGVGNG LL PEAPLDFGNA TG RLTMGLVG YD ++FI RAMQAMGAKIRKDGDVWIIDGVGNGCLLEPEAPLDFGNAGTGARLTMGLVGTYDMKTSFI | 120 |
| Query 121 | GDASLTKRPMGRVLNPLREMGVQVKSEDGDRLPVTLRGPKTPTPITYRVPMASAQVKSAV | 180 |
| Sbjct 121 | GDASL+KRPMGRVLNPLREMGVQV++ DGDR+P+TL GP+T PITYRVPMASAQVKSAV GDASLSKRPMGRVLNPLREMGVQVEAADGDRMPLTLIGPRTANPITYRVPMASAQVKSAV | 180 |
| Query 181 | LLAGLNTPGITTVIEPIMTRDHTEKMLQGFGANLTVETDADGVRTIRLEGRGKLTGQVID | 240 |
| Sbjct 181 | LLAGLNTPG+TTV+EP+MTRDHTEKMLQGFGA+LTVETD DGVR IR+ G+GKL GQVID LLAGLNTPGVTTVVEPVMTRDHTEKMLQGFGADLTVETDKDGVRHIRIAGQGKLAGQVID | 240 |
| Query 241 | VPGDPSSTAFPLVAALLVPGSDVTILNVLMNPTRTGLILTLQEMGADIEVINPRLAGGED | 300 |
| Sbjct 241 | VPGDPSSTAFPLVAALLV GSDVTI NVLMNPTRTGLILTLQEMGADIEV+N RLAGGED VPGDPSSTAFPLVAALLVEGSDVTIRNVLMNPTRTGLILTLQEMGADIEVLNERLAGGED | 300 |
| Query 301 | VADLRVRSSTLKGVTVPEDRAPSMIDEYPILavaaafaegaTVMNGLEELRVKESDRLSA | 360 |
| Sbjct 301 | VADLRVRSS LKGVTVP DRAPSMIDEYP+LAVAA+FAEG T+M+GL+ELRVKESDRL+A VADLRVRSSDLKGVTVPPDRAPSMIDEYPVLAVAASFAEGETIMDGLDELRVKESDRLAA | 360 |
| Query 361 | VANGLKLNGVDCDEGETSLVVRGRPDGKGLGNASGAAVATHLDHRIAMSFLVMGLVSENP | 420 |
| Sbjct 361 | VA GL+ NGVDC EGE SL VRGR G G VATHLDHRIAMSFLVMGL SE P VARGLEANGVDCTEGEMSLTVRGR---PGGKGLGGGTVATHLDHRIAMSFLVMGLASEKP | 417 |
| Query 421  Sbjct 418 | VTVDDATMIATSFPEFMDLMAGLGAKIE 448 VTVDD+ MIATSFPEFM +M LGAKIE VTVDDSNMIATSFPEFMTMMPDLGAKIE 445 |  |

>WP_070996949.1 MULTISPECIES: 3-phosphoshikimate 1-carboxyvinyltransferase [Brucella] MBB5237777.1 3-phosphoshikimate 1-carboxyvinyltransferase [Brucella sp. DSM

103976]

MRN43848.1 3-phosphoshikimate 1-carboxyvinyltransferase [Brucella sp. 09RB8913] MRN60158.1 3-phosphoshikimate 1-carboxyvinyltransferase [Brucella sp. 09RB8918] CAB4326079.1 3-phosphoshikimate 1-carboxyvinyltransferase [Brucella sp. 191011898] SCD22976.1 3-phosphoshikimate 1-carboxyvinyltransferase [Brucella inopinata]

Length=450

Score = 706 bits (1821), Expect = 0.0, Method: Compositional matrix adjust.

| Identities | = 364/447 (81%), Positives = 398/447 (89%), Gaps = 3/447 (1%) |  |
| --- | --- | --- |
| Query 1 | MLHGASSRPATARKSSGLSGTVRIPGDKSISHRSFMFGGLASGETRITGLLEGEDVINTG | 60 |
| Sbjct 1 | M H A +PATAR S L+G +RIPGDKSISHRSFMFGGLASGETRITGLLEGEDVINTG MSHSACPKPATARHSEALTGEIRIPGDKSISHRSFMFGGLASGETRITGLLEGEDVINTG | 60 |
| Query 61 | KAMQAMGARIRKEGDTWIIDGVGNGGLLAPEAPLDFGNAATGCRLTMGLVGVYDFDSTFI | 120 |
| Sbjct 61 | +AMQAMGARIRKEGD WII+GVGNG LL PEAPLDFGNA TG RLTMGLVG YD ++FI RAMQAMGARIRKEGDVWIINGVGNGCLLQPEAPLDFGNAGTGARLTMGLVGTYDMKTSFI | 120 |
| Query 121 | GDASLTKRPMGRVLNPLREMGVQVKSEDGDRLPVTLRGPKTPTPITYRVPMASAQVKSAV | 180 |
| Sbjct 121 | GDASL+KRPMGRVLNPLREMGVQV++ +GDR+P+TL GP+T PITYRVPMASAQVKSAV GDASLSKRPMGRVLNPLREMGVQVEAAEGDRMPLTLIGPRTANPITYRVPMASAQVKSAV | 180 |
| Query 181 | LLAGLNTPGITTVIEPIMTRDHTEKMLQGFGANLTVETDADGVRTIRLEGRGKLTGQVID | 240 |

| Sbjct | 181 | LLAGLNTPG+TTVIEP+MTRDHTEKMLQGFGA+LTVETD DGVR IR+ G+GKLTGQ ID LLAGLNTPGVTTVIEPVMTRDHTEKMLQGFGADLTVETDKDGVRHIRIVGQGKLTGQTID | 240 |
| --- | --- | --- | --- |
| Query | 241 | VPGDPSSTAFPLVAALLVPGSDVTILNVLMNPTRTGLILTLQEMGADIEVINPRLAGGED | 300 |
| Sbjct | 241 | VPGDPSSTAFPLVAA+LV GSDVTI NVLMNPTRTGLILTLQEMGADIE+I+PRLAGGED VPGDPSSTAFPLVAAILVEGSDVTIRNVLMNPTRTGLILTLQEMGADIEIIDPRLAGGED | 300 |
| Query | 301 | VADLRVRSSTLKGVTVPEDRAPSMIDEYPILavaaafaegaTVMNGLEELRVKESDRLSA | 360 |
| Sbjct | 301 | VADLRV++S LKGV VP +RAPSMIDEYP+LA+AA+FAEG TVM+GL+ELRVKESDRL+A VADLRVKASKLKGVVVPPERAPSMIDEYPVLAIAASFAEGETVMDGLDELRVKESDRLAA | 360 |
| Query | 361 | VANGLKLNGVDCDEGETSLVVRGRPDGKGLGNASGAAVATHLDHRIAMSFLVMGLVSENP | 420 |
| Sbjct | 361 | VA GL+ NGVDC EGE SL VRGR G G VATHLDHRIAMSFLVMGL SE P VARGLEANGVDCTEGEMSLTVRGR---PGGKGLGGGTVATHLDHRIAMSFLVMGLASEKP | 417 |
| Query  Sbjct | 421  418 | VTVDDATMIATSFPEFMDLMAGLGAKI 447 VTVDD+TMIATSFPEFM +MAGLGAKI VTVDDSTMIATSFPEFMGMMAGLGAKI 444 |  |

>EEH15273.1 3-phosphoshikimate 1-carboxyvinyltransferase [Brucella ceti str. Cudo]

Length=480

Score = 705 bits (1820), Expect = 0.0, Method: Compositional matrix adjust.

| Identities | = 363/447 (81%), Positives = 397/447 (89%), Gaps = 3/447 (1%) |  |
| --- | --- | --- |
| Query 1 | MLHGASSRPATARKSSGLSGTVRIPGDKSISHRSFMFGGLASGETRITGLLEGEDVINTG | 60 |
| Sbjct 31 | M H A +PATAR S L+G +RIPGDKSISHRSFMFGGLASG+TRITGLLEGEDVINTG MSHSACPKPATARHSQALTGEIRIPGDKSISHRSFMFGGLASGKTRITGLLEGEDVINTG | 90 |
| Query 61 | KAMQAMGARIRKEGDTWIIDGVGNGGLLAPEAPLDFGNAATGCRLTMGLVGVYDFDSTFI | 120 |
| Sbjct 91 | +AMQAMGARIRKEGD WII+GVGNG LL PEAPLDFGNA TG RLTMGLVG YD ++FI RAMQAMGARIRKEGDVWIINGVGNGCLLQPEAPLDFGNAGTGARLTMGLVGTYDMKTSFI | 150 |
| Query 121 | GDASLTKRPMGRVLNPLREMGVQVKSEDGDRLPVTLRGPKTPTPITYRVPMASAQVKSAV | 180 |
| Sbjct 151 | GDASL+KRPMGRVLNPLREMGVQV++ +GDR+P+TL GP+T PI YRVPMASAQVKSAV GDASLSKRPMGRVLNPLREMGVQVEAAEGDRMPLTLIGPRTANPIAYRVPMASAQVKSAV | 210 |
| Query 181 | LLAGLNTPGITTVIEPIMTRDHTEKMLQGFGANLTVETDADGVRTIRLEGRGKLTGQVID | 240 |
| Sbjct 211 | LLAGLNTPG+TTVIEP+MTRDHTEKMLQGFGA+LTVETD DGVR IR+ G+GKLTGQ ID LLAGLNTPGVTTVIEPVMTRDHTEKMLQGFGADLTVETDKDGVRHIRIVGQGKLTGQTID | 270 |
| Query 241 | VPGDPSSTAFPLVAALLVPGSDVTILNVLMNPTRTGLILTLQEMGADIEVINPRLAGGED | 300 |
| Sbjct 271 | VPGDPSSTAFPLVAALLV GSDVTI NVLMNPTRTGLILTLQEMGADIE+I+PRLAGGED VPGDPSSTAFPLVAALLVEGSDVTIRNVLMNPTRTGLILTLQEMGADIEIIDPRLAGGED | 330 |
| Query 301 | VADLRVRSSTLKGVTVPEDRAPSMIDEYPILavaaafaegaTVMNGLEELRVKESDRLSA | 360 |
| Sbjct 331 | VADLRV++S LKGV VP +RAPSMIDEYP+LA+AA+FAEG TVM+GL+ELRVKESDRL+A VADLRVKASKLKGVVVPPERAPSMIDEYPVLAIAASFAEGETVMDGLDELRVKESDRLAA | 390 |
| Query 361 | VANGLKLNGVDCDEGETSLVVRGRPDGKGLGNASGAAVATHLDHRIAMSFLVMGLVSENP | 420 |
| Sbjct 391 | VA GL+ NGVDC EGE SL VRGR G G VATHLDHRIAMSFLVMGL SE P VARGLEANGVDCTEGEMSLTVRGR---PGGKGLGGGTVATHLDHRIAMSFLVMGLASEKP | 447 |
| Query 421  Sbjct 448 | VTVDDATMIATSFPEFMDLMAGLGAKI 447 VTVDD+TMIATSFPEFM +MAGLGAKI VTVDDSTMIATSFPEFMGMMAGLGAKI 474 |  |

>WP_008935122.1 MULTISPECIES: 3-phosphoshikimate 1-carboxyvinyltransferase [unclassified Brucella]

EEZ32059.1 3-phosphoshikimate 1-carboxyvinyltransferase [Brucella sp. 83/13] EFM61237.1 3-phosphoshikimate 1-carboxyvinyltransferase [Brucella sp. NF

2653]

Length=450

Score = 705 bits (1820), Expect = 0.0, Method: Compositional matrix adjust. Identities = 365/447 (82%), Positives = 396/447 (89%), Gaps = 3/447 (1%)

Query 1	MLHGASSRPATARKSSGLSGTVRIPGDKSISHRSFMFGGLASGETRITGLLEGEDVINTG 60 M H A +PATAR S L+G +RIPGDKSISHRSFMFGGLASGETRITGLLEGEDVINTG

| Sbjct | 1 | MSHSACPKPATARHSQALTGEIRIPGDKSISHRSFMFGGLASGETRITGLLEGEDVINTG | 60 |
| --- | --- | --- | --- |
| Query | 61 | KAMQAMGARIRKEGDTWIIDGVGNGGLLAPEAPLDFGNAATGCRLTMGLVGVYDFDSTFI | 120 |
| Sbjct | 61 | +AMQAMGARIRKEGD WII+GVGNG LL PEAPLDFGNA TG RLTMGLVG YD ++FI RAMQAMGARIRKEGDVWIINGVGNGCLLQPEAPLDFGNAGTGARLTMGLVGTYDMKTSFI | 120 |
| Query | 121 | GDASLTKRPMGRVLNPLREMGVQVKSEDGDRLPVTLRGPKTPTPITYRVPMASAQVKSAV | 180 |
| Sbjct | 121 | GDASL+KRPMGRVLNPLREMGVQV++ +GDR+P+TL GP+T PITYRVPMASAQVKSAV GDASLSKRPMGRVLNPLREMGVQVEAAEGDRMPLTLIGPRTANPITYRVPMASAQVKSAV | 180 |
| Query | 181 | LLAGLNTPGITTVIEPIMTRDHTEKMLQGFGANLTVETDADGVRTIRLEGRGKLTGQVID | 240 |
| Sbjct | 181 | LLAGLNTPG+TTVIEP+MTRDHTEKMLQGFGA+LTVETD DGVR I + G+GKLTGQ ID LLAGLNTPGVTTVIEPVMTRDHTEKMLQGFGADLTVETDKDGVRHIHIVGQGKLTGQTID | 240 |
| Query | 241 | VPGDPSSTAFPLVAALLVPGSDVTILNVLMNPTRTGLILTLQEMGADIEVINPRLAGGED | 300 |
| Sbjct | 241 | VPGDPSSTAFPLVAALLV GSDVTI NVLMNPTRTGLILTLQEMGADIE+I+PRLAGGED VPGDPSSTAFPLVAALLVEGSDVTIRNVLMNPTRTGLILTLQEMGADIEIIDPRLAGGED | 300 |
| Query | 301 | VADLRVRSSTLKGVTVPEDRAPSMIDEYPILavaaafaegaTVMNGLEELRVKESDRLSA | 360 |
| Sbjct | 301 | VADLRVR+S LKGV VP +RAPSMIDEYP+LA+AA FAEG TVM+GL+ELRVKESDRL+A VADLRVRASKLKGVVVPPERAPSMIDEYPVLAIAAPFAEGETVMDGLDELRVKESDRLAA | 360 |
| Query | 361 | VANGLKLNGVDCDEGETSLVVRGRPDGKGLGNASGAAVATHLDHRIAMSFLVMGLVSENP | 420 |
| Sbjct | 361 | VA GL+ NGVDC EGE SL VRGR G G VATHLDHRIAMSFLVMGL SE P VARGLEANGVDCTEGEMSLTVRGR---PGGKGLGGGTVATHLDHRIAMSFLVMGLASEKP | 417 |
| Query  Sbjct | 421  418 | VTVDDATMIATSFPEFMDLMAGLGAKI 447 VTVDD+TMIATSFPEFM +MAGLGAKI VTVDDSTMIATSFPEFMGMMAGLGAKI 444 |  |

>WP_004691209.1 MULTISPECIES: 3-phosphoshikimate 1-carboxyvinyltransferase [Brucella] A9M6N0.1 RecName: Full=3-phosphoshikimate 1-carboxyvinyltransferase; AltName:

Full=5-enolpyruvylshikimate-3-phosphate synthase; Short=EPSP synthase; Short=EPSPS [Brucella canis ATCC 23365]

Q8G3C4.1 RecName: Full=3-phosphoshikimate 1-carboxyvinyltransferase; AltName:

Full=5-enolpyruvylshikimate-3-phosphate synthase; Short=EPSP synthase; Short=EPSPS [Brucella suis 1330]

KEX97622.1 3-phosphoshikimate 1-carboxyvinyltransferase [Brucella inopinata BO1]

AAN28982.1 3-phosphoshikimate 1-carboxyvinyltransferase [Brucella suis 1330] ABX61131.1 3-phosphoshikimate 1-carboxyvinyltransferase [Brucella canis

ATCC 23365]

AEM17394.1 3-phosphoshikimate 1-carboxyvinyltransferase [Brucella suis 1330] AEU05062.1 3-phosphoshikimate 1-carboxyvinyltransferase [Brucella suis VBI22]

Length=450

Score = 705 bits (1819), Expect = 0.0, Method: Compositional matrix adjust.

| Identities | = 365/450 (81%), Positives = 398/450 (88%), Gaps = 3/450 (1%) |  |
| --- | --- | --- |
| Query 1 | MLHGASSRPATARKSSGLSGTVRIPGDKSISHRSFMFGGLASGETRITGLLEGEDVINTG | 60 |
| Sbjct 1 | M H A +PATAR S L+G +RIPGDKSISHRSFMFGGLASG+TRITGLLEGEDVINTG MSHSACPKPATARHSQALTGEIRIPGDKSISHRSFMFGGLASGKTRITGLLEGEDVINTG | 60 |
| Query 61 | KAMQAMGARIRKEGDTWIIDGVGNGGLLAPEAPLDFGNAATGCRLTMGLVGVYDFDSTFI | 120 |
| Sbjct 61 | +AMQAMGARIRKEGD WII+GVGNG LL PEAPLDFGNA TG RLTMGLVG YD ++FI RAMQAMGARIRKEGDVWIINGVGNGCLLQPEAPLDFGNAGTGARLTMGLVGTYDMKTSFI | 120 |
| Query 121 | GDASLTKRPMGRVLNPLREMGVQVKSEDGDRLPVTLRGPKTPTPITYRVPMASAQVKSAV | 180 |
| Sbjct 121 | GDASL+KRPMGRVLNPLREMGVQV++ +GDR+P+TL GP+T PI YRVPMASAQVKSAV GDASLSKRPMGRVLNPLREMGVQVEAAEGDRMPLTLIGPRTANPIAYRVPMASAQVKSAV | 180 |
| Query 181 | LLAGLNTPGITTVIEPIMTRDHTEKMLQGFGANLTVETDADGVRTIRLEGRGKLTGQVID | 240 |
| Sbjct 181 | LLAGLNTPG+TTVIEP+MTRDHTEKMLQGFGA+LTVETD DGVR IR+ G+GKLTGQ ID LLAGLNTPGVTTVIEPVMTRDHTEKMLQGFGADLTVETDKDGVRHIRIVGQGKLTGQTID | 240 |
| Query 241 | VPGDPSSTAFPLVAALLVPGSDVTILNVLMNPTRTGLILTLQEMGADIEVINPRLAGGED | 300 |
| Sbjct 241 | VPGDPSSTAFPLVAALLV GSDVTI NVLMNPTRTGLILTLQEMGADIE+I+PRLAGGED VPGDPSSTAFPLVAALLVEGSDVTIRNVLMNPTRTGLILTLQEMGADIEIIDPRLAGGED | 300 |
| Query 301 | VADLRVRSSTLKGVTVPEDRAPSMIDEYPILavaaafaegaTVMNGLEELRVKESDRLSA VADLRVR+S LKGV VP +RAPSMIDEYP+LA+AA+FAEG TVM+GL+ELRVKESDRL+A | 360 |

| Sbjct | 301 | VADLRVRASKLKGVVVPPERAPSMIDEYPVLAIAASFAEGETVMDGLDELRVKESDRLAA | 360 |
| --- | --- | --- | --- |
| Query | 361 | VANGLKLNGVDCDEGETSLVVRGRPDGKGLGNASGAAVATHLDHRIAMSFLVMGLVSENP | 420 |
| Sbjct | 361 | VA GL+ NGVDC EGE SL VRGR G G VATHLDHRIAMSFLVMGL SE P VARGLEANGVDCTEGEMSLTVRGR---PGGKGLGGGTVATHLDHRIAMSFLVMGLASEKP | 417 |
| Query  Sbjct | 421  418 | VTVDDATMIATSFPEFMDLMAGLGAKIELS 450 VTVDD+TMIATSFPEFM +MAGLGAKI S VTVDDSTMIATSFPEFMGMMAGLGAKIAES 447 |  |

>WP_068880270.1 3-phosphoshikimate 1-carboxyvinyltransferase [Paramesorhizobium deserti]

KXF79000.1 3-phosphoshikimate 1-carboxyvinyltransferase [Paramesorhizobium deserti]

Length=447

Score = 705 bits (1819), Expect = 0.0, Method: Compositional matrix adjust.

| Identities | = 364/450 (81%), Positives = 399/450 (89%), Gaps = 3/450 (1%) |  |
| --- | --- | --- |
| Query 1 | MLHGASSRPATARKSSGLSGTVRIPGDKSISHRSFMFGGLASGETRITGLLEGEDVINTG | 60 |
| Sbjct 1 | M H AS RPATA +S L+G +RIPGDKSISHRSFMFGGLASGETRITGLLEGEDVINTG MSHSASPRPATAHRSRDLTGDIRIPGDKSISHRSFMFGGLASGETRITGLLEGEDVINTG | 60 |
| Query 61 | KAMQAMGARIRKEGDTWIIDGVGNGGLLAPEAPLDFGNAATGCRLTMGLVGVYDFDSTFI | 120 |
| Sbjct 61 | +AMQAMGARI+K+GDTWII G GNG LL PEAPLDFGNA TG RLTMGLVG YD ++TFI RAMQAMGARIKKDGDTWIIQGTGNGCLLKPEAPLDFGNAGTGARLTMGLVGTYDMETTFI | 120 |
| Query 121 | GDASLTKRPMGRVLNPLREMGVQVKSEDGDRLPVTLRGPKTPTPITYRVPMASAQVKSAV | 180 |
| Sbjct 121 | GDASL+KRPMGRVLNPLREMGVQVK+ +GDR+P+TL GPK PITYRVPMASAQVKSAV GDASLSKRPMGRVLNPLREMGVQVKAAEGDRMPLTLHGPKVAAPITYRVPMASAQVKSAV | 180 |
| Query 181 | LLAGLNTPGITTVIEPIMTRDHTEKMLQGFGANLTVETDADGVRTIRLEGRGKLTGQVID | 240 |
| Sbjct 181 | LLAGLNTPGITTVIEP+MTRDHTEKML GFGA+LTVET+ +GVR IR+ G+G+L GQ ID LLAGLNTPGITTVIEPVMTRDHTEKMLAGFGADLTVETNREGVRHIRIVGQGELKGQTID | 240 |
| Query 241 | VPGDPSSTAFPLVAALLVPGSDVTILNVLMNPTRTGLILTLQEMGADIEVINPRLAGGED | 300 |
| Sbjct 241 | VPGDPSSTAFPL+AALLVPGSDVTI NVLMNPTRTGLILTLQEMGA+I+++N RLAGGED VPGDPSSTAFPLMAALLVPGSDVTIRNVLMNPTRTGLILTLQEMGANIDILNARLAGGED | 300 |
| Query 301 | VADLRVRSSTLKGVTVPEDRAPSMIDEYPILavaaafaegaTVMNGLEELRVKESDRLSA | 360 |
| Sbjct 301 | VADLRVR+S LKGV+VP +RAPSMIDEYP+LAVAAAFAEG T+M+GLEELRVKESDRLSA VADLRVRASELKGVSVPPERAPSMIDEYPVLAVAAAFAEGETIMDGLEELRVKESDRLSA | 360 |
| Query 361 | VANGLKLNGVDCDEGETSLVVRGRPDGKGLGNASGAAVATHLDHRIAMSFLVMGLVSENP | 420 |
| Sbjct 361 | VA+GLK NGVDC EGE SL VRG G G VATHLDHRIAMSFLVMGL +E P VADGLKANGVDCTEGEASLTVRGI---PGGKGLGGGTVATHLDHRIAMSFLVMGLAAEKP | 417 |
| Query 421  Sbjct 418 | VTVDDATMIATSFPEFMDLMAGLGAKIELS 450 VT+DD+ MIATSFPEFMDLMAGLGAKIE S VTIDDSAMIATSFPEFMDLMAGLGAKIERS 447 |  |

>KSV66437.1 3-phosphoshikimate 1-carboxyvinyltransferase [Sinorhizobium sp. GW3]

Length=448

Score = 705 bits (1819), Expect = 0.0, Method: Compositional matrix adjust.

| Identities | = 355/447 (79%), Positives = 396/447 (89%), Gaps = 0/447 (0%) |  |
| --- | --- | --- |
| Query 1 | MLHGASSRPATARKSSGLSGTVRIPGDKSISHRSFMFGGLASGETRITGLLEGEDVINTG | 60 |
| Sbjct 1 | M HG+ RPATARKS+ L G++RIPGDKSISHR+ M GGLASGETRITGLL+G DV+ TG MSHGSGPRPATARKSADLRGSIRIPGDKSISHRALMLGGLASGETRITGLLQGADVLATG | 60 |
| Query 61 | KAMQAMGARIRKEGDTWIIDGVGNGGLLAPEAPLDFGNAATGCRLTMGLVGVYDFDSTFI | 120 |
| Sbjct 61 | +AMQAMGARIRKE DT+I+DGVGNG LLAPEAPLDFGNA TGCRL MGL GVYDF++TF+ RAMQAMGARIRKEADTFIVDGVGNGALLAPEAPLDFGNAGTGCRLAMGLAGVYDFETTFL | 120 |
| Query 121 | GDASLTKRPMGRVLNPLREMGVQVKSEDGDRLPVTLRGPKTPTPITYRVPMASAQVKSAV | 180 |
| Sbjct 121 | GDASL++RPM RVL PLREMG QV++ GDRLPVTLRGPKTP+PITYRVP+ASAQVKSAV  GDASLSRRPMARVLEPLREMGAQVRAAAGDRLPVTLRGPKTPSPITYRVPVASAQVKSAV | 180 |

| Query | 181 | LLAGLNTPGITTVIEPIMTRDHTEKMLQGFGANLTVETDADGVRTIRLEGRGKLTGQVID | 240 |
| --- | --- | --- | --- |
| Sbjct | 181 | LLAGLN PGITTVIEP+MTRDHTE+ML+ FGA L+VETD GVRTIRL+GRGKLTGQ ID LLAGLNAPGITTVIEPVMTRDHTERMLEAFGAALSVETDVSGVRTIRLDGRGKLTGQEID | 240 |
| Query | 241 | VPGDPSSTAFPLVAALLVPGSDVTILNVLMNPTRTGLILTLQEMGADIEVINPRLAGGED | 300 |
| Sbjct | 241 | VPGDPSS AFPLVAAL+VPGSD+TI NVLMNPTRTGLILTL+EMG DIEVI R GGED VPGDPSSAAFPLVAALIVPGSDITITNVLMNPTRTGLILTLKEMGGDIEVIGSRQVGGED | 300 |
| Query | 301 | VADLRVRSSTLKGVTVPEDRAPSMIDEYPILavaaafaegaTVMNGLEELRVKESDRLSA | 360 |
| Sbjct | 301 | VADLRVR S L GVTVPE+RA SMIDEYP LA+AAAFAEG TVM GL+ELRVKESDRL+A VADLRVRHSELSGVTVPEERAASMIDEYPALAIAAAFAEGNTVMAGLDELRVKESDRLAA | 360 |
| Query | 361 | VANGLKLNGVDCDEGETSLVVRGRPDGKGLGNASGAAVATHLDHRIAMSFLVMGLVSENP | 420 |
| Sbjct | 361 | +A GLK NGVDC+EG +SLVVRGR DGKGLG+A+GA V THLDHRIAMSFL+ GL SE+P IAAGLKRNGVDCEEGASSLVVRGRRDGKGLGSAAGATVNTHLDHRIAMSFLIFGLASEHP | 420 |
| Query  Sbjct | 421  421 | VTVDDATMIATSFPEFMDLMAGLGAKI 447 VTVDD +M+ATSFPEFM LM LGA+I VTVDDVSMVATSFPEFMHLMTSLGARI 447 |  |

>WP_092720367.1 3-phosphoshikimate 1-carboxyvinyltransferase [Rhizobium multihospitium] SCB50759.1 3-phosphoshikimate 1-carboxyvinyltransferase [Rhizobium multihospitium]

Length=449

Score = 704 bits (1818), Expect = 0.0, Method: Compositional matrix adjust.

| Identities | = 354/448 (79%), Positives = 395/448 (88%), Gaps = 0/448 (0%) |  |
| --- | --- | --- |
| Query 1 | MLHGASSRPATARKSSGLSGTVRIPGDKSISHRSFMFGGLASGETRITGLLEGEDVINTG | 60 |
| Sbjct 1 | M H +PA AR+S+GLSG +RIPGDKSISHRS MFGGLA+GETRITGLLEGEDV+NTG MPHDTLPQPAVARRSAGLSGAIRIPGDKSISHRSLMFGGLAAGETRITGLLEGEDVLNTG | 60 |
| Query 61 | KAMQAMGARIRKEGDTWIIDGVGNGGLLAPEAPLDFGNAATGCRLTMGLVGVYDFDSTFI | 120 |
| Sbjct 61 | KAMQA+GA IRK GD WI+ G GNG LLAPEAPLDFGNA TGCRLTMGLVG YDF++T + KAMQALGATIRKIGDEWIVGGTGNGALLAPEAPLDFGNAGTGCRLTMGLVGSYDFETTLV | 120 |
| Query 121 | GDASLTKRPMGRVLNPLREMGVQVKSEDGDRLPVTLRGPKTPTPITYRVPMASAQVKSAV | 180 |
| Sbjct 121 | GDASL+KRPMGR+L+PLR MG QVK+ D+LPVTLRGP+ +P+TYRVPMASAQVKSAV GDASLSKRPMGRILDPLRLMGTQVKAAKADKLPVTLRGPRVASPLTYRVPMASAQVKSAV | 180 |
| Query 181 | LLAGLNTPGITTVIEPIMTRDHTEKMLQGFGANLTVETDADGVRTIRLEGRGKLTGQVID | 240 |
| Sbjct 181 | LLAGLN PGITTVIEP+MTRDHTE+ML GFGA L+VETDA GVRTIRLEG+GKLTGQ ID LLAGLNAPGITTVIEPVMTRDHTERMLAGFGAALSVETDAAGVRTIRLEGQGKLTGQTID | 240 |
| Query 241 | VPGDPSSTAFPLVAALLVPGSDVTILNVLMNPTRTGLILTLQEMGADIEVINPRLAGGED | 300 |
| Sbjct 241 | VPGDPSS AFPLVAAL+VPGSD+TI +VLMNPTRTGLILTLQEMGADIE+++ + GGED VPGDPSSAAFPLVAALIVPGSDITIRDVLMNPTRTGLILTLQEMGADIEILDRHMTGGED | 300 |
| Query 301 | VADLRVRSSTLKGVTVPEDRAPSMIDEYPILavaaafaegaTVMNGLEELRVKESDRLSA | 360 |
| Sbjct 301 | VADLRVRSSTLKGVTVP +RAPSMIDEYP+LAVAAAFAEG T+M GL+ELRVKESDRLSA VADLRVRSSTLKGVTVPAERAPSMIDEYPVLAVAAAFAEGTTIMQGLDELRVKESDRLSA | 360 |
| Query 361 | VANGLKLNGVDCDEGETSLVVRGRPDGKGLGNASGAAVATHLDHRIAMSFLVMGLVSENP | 420 |
| Sbjct 361 | VA GLKLNGVDC EG L V+G+PDGKGLGNA A V+THLDHRIAMSFLVMGL +E+P VAEGLKLNGVDCIEGRDMLTVQGKPDGKGLGNAGSAKVSTHLDHRIAMSFLVMGLAAEHP | 420 |
| Query 421  Sbjct 421 | VTVDDATMIATSFPEFMDLMAGLGAKIE 448 VTVDD+ MIATSFPEF +M LGA IE VTVDDSAMIATSFPEFFAMMTRLGATIE 448 |  |

>WP_114439432.1 3-phosphoshikimate 1-carboxyvinyltransferase [Phyllobacterium salinisoli]

RCS24986.1 3-phosphoshikimate 1-carboxyvinyltransferase [Phyllobacterium salinisoli]

Length=451

Score = 704 bits (1818), Expect = 0.0, Method: Compositional matrix adjust. Identities = 364/448 (81%), Positives = 399/448 (89%), Gaps = 3/448 (1%)

Query 1	MLHGASSRPATARKSSGLSGTVRIPGDKSISHRSFMFGGLASGETRITGLLEGEDVINTG 60 M H A +PATA +S L+G +RIPGDKSISHRSFMFGGLASGETRITGLLEGEDVINTG

| Sbjct | 1 | MSHSALPKPATAHRSRDLAGDIRIPGDKSISHRSFMFGGLASGETRITGLLEGEDVINTG | 60 |
| --- | --- | --- | --- |
| Query | 61 | KAMQAMGARIRKEGDTWIIDGVGNGGLLAPEAPLDFGNAATGCRLTMGLVGVYDFDSTFI | 120 |
| Sbjct | 61 | +AMQAMGA IRK+G TWII G GNG LL PEAPLDFGNA TG RLTMGLVG YD ++TFI RAMQAMGANIRKDGATWIIQGTGNGCLLQPEAPLDFGNAGTGARLTMGLVGTYDMETTFI | 120 |
| Query | 121 | GDASLTKRPMGRVLNPLREMGVQVKSEDGDRLPVTLRGPKTPTPITYRVPMASAQVKSAV | 180 |
| Sbjct | 121 | GDASL+KRPMGRVLNPLREMGVQVK+ +GDRLP+TL GPK P+ YRVPMASAQVKSAV GDASLSKRPMGRVLNPLREMGVQVKAAEGDRLPLTLHGPKVAAPVIYRVPMASAQVKSAV | 180 |
| Query | 181 | LLAGLNTPGITTVIEPIMTRDHTEKMLQGFGANLTVETDADGVRTIRLEGRGKLTGQVID | 240 |
| Sbjct | 181 | LLAGLNTPGITTVIEP+MTRDHTEKMLQGFGA+LTVETD +GVR IR+ G+G+L GQ ID LLAGLNTPGITTVIEPVMTRDHTEKMLQGFGADLTVETDREGVRHIRIVGQGELKGQTID | 240 |
| Query | 241 | VPGDPSSTAFPLVAALLVPGSDVTILNVLMNPTRTGLILTLQEMGADIEVINPRLAGGED | 300 |
| Sbjct | 241 | VPGDPSSTAFPLVAALLVPGSDVTI NVLMNPTRTGLILTLQEMGA+I+++N RLAGGED VPGDPSSTAFPLVAALLVPGSDVTIRNVLMNPTRTGLILTLQEMGANIDILNARLAGGED | 300 |
| Query | 301 | VADLRVRSSTLKGVTVPEDRAPSMIDEYPILavaaafaegaTVMNGLEELRVKESDRLSA | 360 |
| Sbjct | 301 | VADLRVR+S LKGV+VP +RAPSMIDEYP+LAVAAAFAEG TVM GL+ELRVKESDRLSA VADLRVRASELKGVSVPPERAPSMIDEYPVLAVAAAFAEGDTVMEGLDELRVKESDRLSA | 360 |
| Query | 361 | VANGLKLNGVDCDEGETSLVVRGRPDGKGLGNASGAAVATHLDHRIAMSFLVMGLVSENP | 420 |
| Sbjct | 361 | VA+GLK NGVDC EGE SLVVRG+P G GLG + V THLDHRIAMSFLVMG V+E P VASGLKANGVDCTEGEASLVVRGKPGGLGLGGGT---VETHLDHRIAMSFLVMGFVTEKP | 417 |
| Query  Sbjct | 421  418 | VTVDDATMIATSFPEFMDLMAGLGAKIE 448 VT+DD+ MIATSFPEFMDLMAGLGA+IE VTIDDSAMIATSFPEFMDLMAGLGARIE 445 |  |

>EEP63548.1 3-phosphoshikimate 1-carboxyvinyltransferase [Brucella abortus str. 2308 A]

EFH33949.1 3-phosphoshikimate 1-carboxyvinyltransferase [Brucella abortus bv. 5 str. B3196]

Length=480

Score = 704 bits (1818), Expect = 0.0, Method: Compositional matrix adjust.

| Identities | = 362/447 (81%), Positives = 397/447 (89%), Gaps = 3/447 (1%) |  |
| --- | --- | --- |
| Query 1 | MLHGASSRPATARKSSGLSGTVRIPGDKSISHRSFMFGGLASGETRITGLLEGEDVINTG | 60 |
| Sbjct 31 | M H A +PATAR S L+G +RIPGDKSISHRSFMFGGLASG+TRITGLLEGEDVINTG MSHSACPKPATARHSQALTGEIRIPGDKSISHRSFMFGGLASGKTRITGLLEGEDVINTG | 90 |
| Query 61 | KAMQAMGARIRKEGDTWIIDGVGNGGLLAPEAPLDFGNAATGCRLTMGLVGVYDFDSTFI | 120 |
| Sbjct 91 | +AMQAMGARIRKEGD WII+GVGNG LL PEAPLDFGNA TG RLTMGLVG YD ++FI RAMQAMGARIRKEGDVWIINGVGNGCLLQPEAPLDFGNAGTGARLTMGLVGTYDMKTSFI | 150 |
| Query 121 | GDASLTKRPMGRVLNPLREMGVQVKSEDGDRLPVTLRGPKTPTPITYRVPMASAQVKSAV | 180 |
| Sbjct 151 | GDASL+KRPMGRVLNPLREMGVQV++ +GDR+P+TL GP+T PI YRVPMASAQVKSAV GDASLSKRPMGRVLNPLREMGVQVEAAEGDRMPLTLIGPRTANPIAYRVPMASAQVKSAV | 210 |
| Query 181 | LLAGLNTPGITTVIEPIMTRDHTEKMLQGFGANLTVETDADGVRTIRLEGRGKLTGQVID | 240 |
| Sbjct 211 | LLAGLNTPG+TTVIEP+MTRDHTEKMLQGFGA+LTVETD DGVR IR+ G+GKLTGQ ID LLAGLNTPGVTTVIEPVMTRDHTEKMLQGFGADLTVETDKDGVRHIRIVGQGKLTGQTID | 270 |
| Query 241 | VPGDPSSTAFPLVAALLVPGSDVTILNVLMNPTRTGLILTLQEMGADIEVINPRLAGGED | 300 |
| Sbjct 271 | VPGDPSSTAFPLVAALLV GS+VTI NVLMNPTRTGLILTLQEMGADIE+I+PRLAGGED VPGDPSSTAFPLVAALLVEGSEVTIRNVLMNPTRTGLILTLQEMGADIEIIDPRLAGGED | 330 |
| Query 301 | VADLRVRSSTLKGVTVPEDRAPSMIDEYPILavaaafaegaTVMNGLEELRVKESDRLSA | 360 |
| Sbjct 331 | VADLRV++S LKGV VP +RAPSMIDEYP+LA+AA+FAEG TVM+GL+ELRVKESDRL+A VADLRVKASKLKGVVVPPERAPSMIDEYPVLAIAASFAEGETVMDGLDELRVKESDRLAA | 390 |
| Query 361 | VANGLKLNGVDCDEGETSLVVRGRPDGKGLGNASGAAVATHLDHRIAMSFLVMGLVSENP | 420 |
| Sbjct 391 | VA GL+ NGVDC EGE SL VRGR G G VATHLDHRIAMSFLVMGL SE P VARGLEANGVDCTEGEMSLTVRGR---PGGKGLGGGTVATHLDHRIAMSFLVMGLASEKP | 447 |
| Query 421  Sbjct 448 | VTVDDATMIATSFPEFMDLMAGLGAKI 447 VTVDD+TMIATSFPEFM +MAGLGAKI VTVDDSTMIATSFPEFMGMMAGLGAKI 474 |  |

>WP_004685324.1 MULTISPECIES: 3-phosphoshikimate 1-carboxyvinyltransferase [Brucella] B0CI45.1 RecName: Full=3-phosphoshikimate 1-carboxyvinyltransferase; AltName:

Full=5-enolpyruvylshikimate-3-phosphate synthase; Short=EPSP synthase; Short=EPSPS [Brucella suis ATCC 23445]

C0RG90.1 RecName: Full=3-phosphoshikimate 1-carboxyvinyltransferase; AltName:

Full=5-enolpyruvylshikimate-3-phosphate synthase; Short=EPSP synthase; Short=EPSPS [Brucella melitensis ATCC 23457]

ERU00249.1 3-phosphoshikimate 1-carboxyvinyltransferase [Brucella abortus 07-0994-2411]

EXU84234.1 3-phosphoshikimate 1-carboxyvinyltransferase [Brucella melitensis 548]

MUJ51313.1 3-phosphoshikimate 1-carboxyvinyltransferase [Brucella abortus] ABY37135.1 3-phosphoshikimate 1-carboxyvinyltransferase [Brucella suis ATCC

23445]

ACN99847.1 3-phosphoshikimate 1-carboxyvinyltransferase [Brucella melitensis ATCC 23457]

Length=450

Score = 704 bits (1817), Expect = 0.0, Method: Compositional matrix adjust.

| Identities | = 364/450 (81%), Positives = 398/450 (88%), Gaps = 3/450 (1%) |  |
| --- | --- | --- |
| Query 1 | MLHGASSRPATARKSSGLSGTVRIPGDKSISHRSFMFGGLASGETRITGLLEGEDVINTG | 60 |
| Sbjct 1 | M H A +PATAR S L+G +RIPGDKSISHRSFMFGGLASG+TRITGLLEGEDVINTG MSHSACPKPATARHSQALTGEIRIPGDKSISHRSFMFGGLASGKTRITGLLEGEDVINTG | 60 |
| Query 61 | KAMQAMGARIRKEGDTWIIDGVGNGGLLAPEAPLDFGNAATGCRLTMGLVGVYDFDSTFI | 120 |
| Sbjct 61 | +AMQAMGARIRKEGD WII+GVGNG LL PEAPLDFGNA TG RLTMGLVG YD ++FI RAMQAMGARIRKEGDVWIINGVGNGCLLQPEAPLDFGNAGTGARLTMGLVGTYDMKTSFI | 120 |
| Query 121 | GDASLTKRPMGRVLNPLREMGVQVKSEDGDRLPVTLRGPKTPTPITYRVPMASAQVKSAV | 180 |
| Sbjct 121 | GDASL+KRPMGRVLNPLREMGVQV++ +GDR+P+TL GP+T PI YRVPMASAQVKSAV GDASLSKRPMGRVLNPLREMGVQVEAAEGDRMPLTLIGPRTANPIAYRVPMASAQVKSAV | 180 |
| Query 181 | LLAGLNTPGITTVIEPIMTRDHTEKMLQGFGANLTVETDADGVRTIRLEGRGKLTGQVID | 240 |
| Sbjct 181 | LLAGLNTPG+TTVIEP+MTRDHTEKMLQGFGA+LTVETD DGVR IR+ G+GKLTGQ ID LLAGLNTPGVTTVIEPVMTRDHTEKMLQGFGADLTVETDKDGVRHIRIVGQGKLTGQTID | 240 |
| Query 241 | VPGDPSSTAFPLVAALLVPGSDVTILNVLMNPTRTGLILTLQEMGADIEVINPRLAGGED | 300 |
| Sbjct 241 | VPGDPSSTAFPLVAALLV GSDVTI NVLMNPTRTGLILTLQEMGADIE+I+PRLAGGED VPGDPSSTAFPLVAALLVEGSDVTIRNVLMNPTRTGLILTLQEMGADIEIIDPRLAGGED | 300 |
| Query 301 | VADLRVRSSTLKGVTVPEDRAPSMIDEYPILavaaafaegaTVMNGLEELRVKESDRLSA | 360 |
| Sbjct 301 | VADLRV++S LKGV VP +RAPSMIDEYP+LA+AA+FAEG TVM+GL+ELRVKESDRL+A VADLRVKASKLKGVVVPPERAPSMIDEYPVLAIAASFAEGETVMDGLDELRVKESDRLAA | 360 |
| Query 361 | VANGLKLNGVDCDEGETSLVVRGRPDGKGLGNASGAAVATHLDHRIAMSFLVMGLVSENP | 420 |
| Sbjct 361 | VA GL+ NGVDC EGE SL VRGR G G VATHLDHRIAMSFLVMGL SE P VARGLEANGVDCTEGEMSLTVRGR---PGGKGLGGGTVATHLDHRIAMSFLVMGLASEKP | 417 |
| Query 421  Sbjct 418 | VTVDDATMIATSFPEFMDLMAGLGAKIELS 450 VTVDD+TMIATSFPEFM +MAGLGAKI S VTVDDSTMIATSFPEFMGMMAGLGAKIAES 447 |  |

>CUW43595.1 3-phosphoshikimate 1-carboxyvinyltransferase [Brucella vulpis] CUW49728.1 3-phosphoshikimate 1-carboxyvinyltransferase [Brucella vulpis]

Length=450

Score = 704 bits (1817), Expect = 0.0, Method: Compositional matrix adjust.

| Identities | = 365/447 (82%), Positives = 396/447 (89%), Gaps = 3/447 (1%) |  |
| --- | --- | --- |
| Query 1 | MLHGASSRPATARKSSGLSGTVRIPGDKSISHRSFMFGGLASGETRITGLLEGEDVINTG | 60 |
| Sbjct 1 | M H A +PATAR S L+G +RIPGDKSISHRSFMFGGLASGETRITGLLEGEDVINTG MSHSACPKPATARHSEALTGEIRIPGDKSISHRSFMFGGLASGETRITGLLEGEDVINTG | 60 |
| Query 61 | KAMQAMGARIRKEGDTWIIDGVGNGGLLAPEAPLDFGNAATGCRLTMGLVGVYDFDSTFI | 120 |
| Sbjct 61 | +AMQAMGARIRKEGD WII+GVGNG LL PEAPLDFGNA TG RLTMGLVG YD ++FI RAMQAMGARIRKEGDVWIINGVGNGCLLQPEAPLDFGNAGTGARLTMGLVGTYDMKTSFI | 120 |
| Query 121 | GDASLTKRPMGRVLNPLREMGVQVKSEDGDRLPVTLRGPKTPTPITYRVPMASAQVKSAV GDASL+KRPMGRVLNPLREMGVQV + +GD +P+TL GP+T PITYRVPMASAQVKSAV | 180 |

| Sbjct | 121 | GDASLSKRPMGRVLNPLREMGVQVDAAEGDWMPLTLIGPRTANPITYRVPMASAQVKSAV | 180 |
| --- | --- | --- | --- |
| Query | 181 | LLAGLNTPGITTVIEPIMTRDHTEKMLQGFGANLTVETDADGVRTIRLEGRGKLTGQVID | 240 |
| Sbjct | 181 | LLAGLNTPG+TTVIEP+MTRDHTEKMLQGFGA+LTVETD DGVR IR+ G+GKLTGQ ID LLAGLNTPGVTTVIEPVMTRDHTEKMLQGFGADLTVETDKDGVRHIRIVGQGKLTGQTID | 240 |
| Query | 241 | VPGDPSSTAFPLVAALLVPGSDVTILNVLMNPTRTGLILTLQEMGADIEVINPRLAGGED | 300 |
| Sbjct | 241 | VPGDPSSTAFPLVAALLV GSDVTI NVLMNPTRTGLILTLQEMGADIE+I+PRLAGGED VPGDPSSTAFPLVAALLVEGSDVTIRNVLMNPTRTGLILTLQEMGADIEIIDPRLAGGED | 300 |
| Query | 301 | VADLRVRSSTLKGVTVPEDRAPSMIDEYPILavaaafaegaTVMNGLEELRVKESDRLSA | 360 |
| Sbjct | 301 | VADLRVR+S LKGV VP +RAPSMIDEYP+LA+AA+FAEG TVM+GL+ELRVKESDRL+A VADLRVRASKLKGVVVPPERAPSMIDEYPVLAIAASFAEGETVMDGLDELRVKESDRLAA | 360 |
| Query | 361 | VANGLKLNGVDCDEGETSLVVRGRPDGKGLGNASGAAVATHLDHRIAMSFLVMGLVSENP | 420 |
| Sbjct | 361 | VA GL+ NGVDC EGE SL VRGR G G VATHLDHRIAMSFLVMGL SE P VARGLEANGVDCTEGEMSLTVRGR---PGGKGLGGGTVATHLDHRIAMSFLVMGLASEKP | 417 |
| Query  Sbjct | 421  418 | VTVDDATMIATSFPEFMDLMAGLGAKI 447 VTVDD+TMIATSFPEFM +MAGLGAKI VTVDDSTMIATSFPEFMGMMAGLGAKI 444 |  |

>WP_192437361.1 3-phosphoshikimate 1-carboxyvinyltransferase [Ensifer sp. ENS03] MBD9554916.1 3-phosphoshikimate 1-carboxyvinyltransferase [Ensifer sp. ENS03]

Length=448

Score = 704 bits (1817), Expect = 0.0, Method: Compositional matrix adjust.

| Identities | = 355/447 (79%), Positives = 396/447 (89%), Gaps = 0/447 (0%) |  |
| --- | --- | --- |
| Query 1 | MLHGASSRPATARKSSGLSGTVRIPGDKSISHRSFMFGGLASGETRITGLLEGEDVINTG | 60 |
| Sbjct 1 | M HG+ RPATARKS+ L G++RIPGDKSISHR+ M GGLASGETRITGLL+G DV+ TG MSHGSGPRPATARKSADLRGSIRIPGDKSISHRALMLGGLASGETRITGLLQGADVLATG | 60 |
| Query 61 | KAMQAMGARIRKEGDTWIIDGVGNGGLLAPEAPLDFGNAATGCRLTMGLVGVYDFDSTFI | 120 |
| Sbjct 61 | +AMQAMGARIRKE DT+I+DGVGNG LLAPEAPLDFGNA TGCRL MGL GVYDF++TF+ RAMQAMGARIRKEADTFIVDGVGNGALLAPEAPLDFGNAGTGCRLAMGLAGVYDFETTFL | 120 |
| Query 121 | GDASLTKRPMGRVLNPLREMGVQVKSEDGDRLPVTLRGPKTPTPITYRVPMASAQVKSAV | 180 |
| Sbjct 121 | GDASL++RPM RVL PLREMG QV++ GDRLPVTLRGPKTP PITYRVP+ASAQVKSAV GDASLSRRPMARVLEPLREMGTQVRAAAGDRLPVTLRGPKTPAPITYRVPVASAQVKSAV | 180 |
| Query 181 | LLAGLNTPGITTVIEPIMTRDHTEKMLQGFGANLTVETDADGVRTIRLEGRGKLTGQVID | 240 |
| Sbjct 181 | LLAGLN PGITTVIEP+MTRDHTE+ML+ FGA L+VETD GVRTIRL+GRGKLTGQ ID LLAGLNAPGITTVIEPVMTRDHTERMLEAFGAALSVETDVSGVRTIRLDGRGKLTGQEID | 240 |
| Query 241 | VPGDPSSTAFPLVAALLVPGSDVTILNVLMNPTRTGLILTLQEMGADIEVINPRLAGGED | 300 |
| Sbjct 241 | VPGDPSS AFPLVAAL+VPGSD+TI NVLMNPTRTGLILTL+EMG DIEVI R GGED VPGDPSSAAFPLVAALIVPGSDITITNVLMNPTRTGLILTLKEMGGDIEVIGSRQVGGED | 300 |
| Query 301 | VADLRVRSSTLKGVTVPEDRAPSMIDEYPILavaaafaegaTVMNGLEELRVKESDRLSA | 360 |
| Sbjct 301 | VADLRVR S L GVTVPE+RA S+IDEYP LA+AAAFAEG TVM GL+ELRVKESDRL+A VADLRVRHSELSGVTVPEERAASLIDEYPALAIAAAFAEGNTVMAGLDELRVKESDRLAA | 360 |
| Query 361 | VANGLKLNGVDCDEGETSLVVRGRPDGKGLGNASGAAVATHLDHRIAMSFLVMGLVSENP | 420 |
| Sbjct 361 | +A GLK NGVDC+EG +SLVVRGR DGKGLG+A+GA V THLDHRIAMSFL+ GL SE+P IAAGLKRNGVDCEEGASSLVVRGRRDGKGLGSAAGATVNTHLDHRIAMSFLIFGLASEHP | 420 |
| Query 421  Sbjct 421 | VTVDDATMIATSFPEFMDLMAGLGAKI 447 VTVDD +M+ATSFPEFMDLM LGA+I VTVDDVSMVATSFPEFMDLMTSLGARI 447 |  |

>ENR88457.1 3-phosphoshikimate 1-carboxyvinyltransferase [Brucella abortus 78/32]

ENS13733.1 3-phosphoshikimate 1-carboxyvinyltransferase [Brucella abortus F1/06-B21]

Length=489

Score = 704 bits (1816), Expect = 0.0, Method: Compositional matrix adjust. Identities = 361/447 (81%), Positives = 396/447 (89%), Gaps = 3/447 (1%)

| Query | 1 | MLHGASSRPATARKSSGLSGTVRIPGDKSISHRSFMFGGLASGETRITGLLEGEDVINTG | 60 |
| --- | --- | --- | --- |
| Sbjct | 40 | M H A +PATAR S L+G +RIPGDKSISHRSFMFGGLASG+TRITGLLEGEDVINTG MSHSACPKPATARHSQALTGEIRIPGDKSISHRSFMFGGLASGKTRITGLLEGEDVINTG | 99 |
| Query | 61 | KAMQAMGARIRKEGDTWIIDGVGNGGLLAPEAPLDFGNAATGCRLTMGLVGVYDFDSTFI | 120 |
| Sbjct | 100 | +AMQAMGARIRKEGD WII+GVGNG LL PEAPLDFGNA TG RLTMGLVG YD ++FI RAMQAMGARIRKEGDVWIINGVGNGCLLQPEAPLDFGNAGTGARLTMGLVGTYDMKTSFI | 159 |
| Query | 121 | GDASLTKRPMGRVLNPLREMGVQVKSEDGDRLPVTLRGPKTPTPITYRVPMASAQVKSAV | 180 |
| Sbjct | 160 | GDASL+KRPMGRVLNPLREMGVQV++ +GDR+P+TL GP+T PI YRVPMASAQVKSAV GDASLSKRPMGRVLNPLREMGVQVEAAEGDRMPLTLIGPRTANPIAYRVPMASAQVKSAV | 219 |
| Query | 181 | LLAGLNTPGITTVIEPIMTRDHTEKMLQGFGANLTVETDADGVRTIRLEGRGKLTGQVID | 240 |
| Sbjct | 220 | LLAGLNTPG+TTVIEP+MTRDHTEKMLQGFGA+LTVETD DGVR IR+ G+GKLTGQ ID LLAGLNTPGVTTVIEPVMTRDHTEKMLQGFGADLTVETDKDGVRHIRIVGQGKLTGQTID | 279 |
| Query | 241 | VPGDPSSTAFPLVAALLVPGSDVTILNVLMNPTRTGLILTLQEMGADIEVINPRLAGGED | 300 |
| Sbjct | 280 | VPGDPSSTAFPLVAALLV GS+VTI NVLMNPTRTGLILTLQEMGADIE+I+P LAGGED VPGDPSSTAFPLVAALLVEGSEVTIRNVLMNPTRTGLILTLQEMGADIEIIDPHLAGGED | 339 |
| Query | 301 | VADLRVRSSTLKGVTVPEDRAPSMIDEYPILavaaafaegaTVMNGLEELRVKESDRLSA | 360 |
| Sbjct | 340 | VADLRV++S LKGV VP +RAPSMIDEYP+LA+AA+FAEG TVM+GL+ELRVKESDRL+A VADLRVKASKLKGVVVPPERAPSMIDEYPVLAIAASFAEGETVMDGLDELRVKESDRLAA | 399 |
| Query | 361 | VANGLKLNGVDCDEGETSLVVRGRPDGKGLGNASGAAVATHLDHRIAMSFLVMGLVSENP | 420 |
| Sbjct | 400 | VA GL+ NGVDC EGE SL VRGR G G VATHLDHRIAMSFLVMGL SE P VARGLEANGVDCTEGEMSLTVRGR---PGGKGLGGGTVATHLDHRIAMSFLVMGLASEKP | 456 |
| Query  Sbjct | 421  457 | VTVDDATMIATSFPEFMDLMAGLGAKI 447 VTVDD+TMIATSFPEFM +MAGLGAKI VTVDDSTMIATSFPEFMGMMAGLGAKI 483 |  |

>WP_147836101.1 3-phosphoshikimate 1-carboxyvinyltransferase [Phyllobacterium endophyticum]

TXR48208.1 3-phosphoshikimate 1-carboxyvinyltransferase [Phyllobacterium endophyticum]

Length=452

Score = 704 bits (1816), Expect = 0.0, Method: Compositional matrix adjust.

| Identities | = 354/452 (78%), Positives = 395/452 (87%), Gaps = 3/452 (1%) |  |
| --- | --- | --- |
| Query 1 | MLHGASSRPATARKSSGLSGTVRIPGDKSISHRSFMFGGLASGETRITGLLEGEDVINTG | 60 |
| Sbjct 1 | M H A +PAT+R+S LSG VRIPGDKSISHRSFMFGGLASGETRITGLLEGEDVINTG MSHSALPKPATSRRSKALSGDVRIPGDKSISHRSFMFGGLASGETRITGLLEGEDVINTG | 60 |
| Query 61 | KAMQAMGARIRKEGDTWIIDGVGNGGLLAPEAPLDFGNAATGCRLTMGLVGVYDFDSTFI | 120 |
| Sbjct 61 | +AMQAMGA+I KEGD WII G GNG LL PE+PLDFGNA TG RLTMGLVG Y ++TF+ RAMQAMGAKIAKEGDIWIIQGTGNGCLLQPESPLDFGNAGTGARLTMGLVGTYAMETTFV | 120 |
| Query 121 | GDASLTKRPMGRVLNPLREMGVQVKSEDGDRLPVTLRGPKTPTPITYRVPMASAQVKSAV | 180 |
| Sbjct 121 | GDASL+KRPMGRVL+PLR+MGVQV + +GDR+P+TLRGPK PITYRVPMASAQVKSAV GDASLSKRPMGRVLDPLRQMGVQVSAAEGDRMPLTLRGPKVAAPITYRVPMASAQVKSAV | 180 |
| Query 181 | LLAGLNTPGITTVIEPIMTRDHTEKMLQGFGANLTVETDADGVRTIRLEGRGKLTGQVID | 240 |
| Sbjct 181 | LLAGLNTPGITTVIEP+MTRDHTEKML GFGANL+VETD +GVR IR+EG+GKLTGQ ID LLAGLNTPGITTVIEPVMTRDHTEKMLAGFGANLSVETDREGVRHIRIEGQGKLTGQTID | 240 |
| Query 241 | VPGDPSSTAFPLVAALLVPGSDVTILNVLMNPTRTGLILTLQEMGADIEVINPRLAGGED | 300 |
| Sbjct 241 | VPGDPSSTAFPLVAA++VPGSD+TI NVLMNPTRTGLILTLQEMGA+IE+++ RLAGGED VPGDPSSTAFPLVAAIIVPGSDITIRNVLMNPTRTGLILTLQEMGANIEIVDERLAGGED | 300 |
| Query 301 | VADLRVRSSTLKGVTVPEDRAPSMIDEYPILavaaafaegaTVMNGLEELRVKESDRLSA | 360 |
| Sbjct 301 | VADLRV++S LKGV VP DRAPSMIDEYP+LAVAA FA G TVMNGLEELRVKESDRL+A VADLRVQASELKGVNVPADRAPSMIDEYPVLAVAACFATGETVMNGLEELRVKESDRLAA | 360 |
| Query 361 | VANGLKLNGVDCDEGETSLVVRGRPDGKGLGNASGAAVATHLDHRIAMSFLVMGLVSENP | 420 |
| Sbjct 361 | VA GLK NGVDC EG L VRGRPDGKG+G G V THLDHRIAMSFLV+GL ++ VAAGLKANGVDCTEGHDRLSVRGRPDGKGIG---GGRVETHLDHRIAMSFLVLGLAADKA | 417 |
| Query 421 | VTVDDATMIATSFPEFMDLMAGLGAKIELSDT 452 VT+DDA MIATSFPEFM +M G+GA+I D+ |  |

Sbjct 418 VTIDDAAMIATSFPEFMGMMTGMGAEIAEFDS 449

>WP_008503805.1 3-phosphoshikimate 1-carboxyvinyltransferase [Brucella inopinata] EFM58016.1 3-phosphoshikimate 1-carboxyvinyltransferase [Brucella inopinata

BO1]

Length=450

Score = 704 bits (1816), Expect = 0.0, Method: Compositional matrix adjust.

| Identities | = 363/447 (81%), Positives = 396/447 (89%), Gaps = 3/447 (1%) |  |
| --- | --- | --- |
| Query 1 | MLHGASSRPATARKSSGLSGTVRIPGDKSISHRSFMFGGLASGETRITGLLEGEDVINTG | 60 |
| Sbjct 1 | M H A +PATAR S L+G +RIPGDKSISHRSFMFGGLASGETRITGLLEGEDVINTG MSHSACPKPATARHSQALTGEIRIPGDKSISHRSFMFGGLASGETRITGLLEGEDVINTG | 60 |
| Query 61 | KAMQAMGARIRKEGDTWIIDGVGNGGLLAPEAPLDFGNAATGCRLTMGLVGVYDFDSTFI | 120 |
| Sbjct 61 | +AMQAMGARIRKEGD WII+GVGNG LL PEAPLDFGNA TG RLTMGLVG YD ++FI RAMQAMGARIRKEGDVWIINGVGNGCLLQPEAPLDFGNAGTGARLTMGLVGTYDMKTSFI | 120 |
| Query 121 | GDASLTKRPMGRVLNPLREMGVQVKSEDGDRLPVTLRGPKTPTPITYRVPMASAQVKSAV | 180 |
| Sbjct 121 | GDASL+KRPMGRVLNPLREMGVQV++ +GDR+P+TL GP+T PITYRVPMASAQVKSAV GDASLSKRPMGRVLNPLREMGVQVEAAEGDRMPLTLIGPRTANPITYRVPMASAQVKSAV | 180 |
| Query 181 | LLAGLNTPGITTVIEPIMTRDHTEKMLQGFGANLTVETDADGVRTIRLEGRGKLTGQVID | 240 |
| Sbjct 181 | LLAGLNTPG+TTVIEP+MTRDHTEKMLQGFGA+LTVETD DGVR IR+ G+GKLTGQ ID LLAGLNTPGVTTVIEPVMTRDHTEKMLQGFGADLTVETDKDGVRHIRIVGQGKLTGQTID | 240 |
| Query 241 | VPGDPSSTAFPLVAALLVPGSDVTILNVLMNPTRTGLILTLQEMGADIEVINPRLAGGED | 300 |
| Sbjct 241 | VPGDPSSTAFPLVAALLV GSDVTI NVLMNPTRTGLILTLQEMGADIE+I+PRLAGGED VPGDPSSTAFPLVAALLVEGSDVTIRNVLMNPTRTGLILTLQEMGADIEIIDPRLAGGED | 300 |
| Query 301 | VADLRVRSSTLKGVTVPEDRAPSMIDEYPILavaaafaegaTVMNGLEELRVKESDRLSA | 360 |
| Sbjct 301 | VADLRV++S LKGV VP +RAPSMIDEYP+LA+AA+FAEG TVM+GL+ELRVKESDRL+A VADLRVKASKLKGVVVPPERAPSMIDEYPVLAIAASFAEGETVMDGLDELRVKESDRLAA | 360 |
| Query 361 | VANGLKLNGVDCDEGETSLVVRGRPDGKGLGNASGAAVATHLDHRIAMSFLVMGLVSENP | 420 |
| Sbjct 361 | VA GL+ NG DC EGE SL VRGR G G V THLDHRIAMSFLVMGL SE P VARGLEANGADCTEGEMSLTVRGR---PGGKGLGGGTVETHLDHRIAMSFLVMGLASEKP | 417 |
| Query 421  Sbjct 418 | VTVDDATMIATSFPEFMDLMAGLGAKI 447 VTVDD+TMIATSFPEFM +MAGLGAKI VTVDDSTMIATSFPEFMGMMAGLGAKI 444 |  |

>WP_060519521.1 MULTISPECIES: 3-phosphoshikimate 1-carboxyvinyltransferase [unclassified Ensifer]

KQX54105.1 3-phosphoshikimate 1-carboxyvinyltransferase [Ensifer sp. Root1298] KQX85793.1 3-phosphoshikimate 1-carboxyvinyltransferase [Ensifer sp. Root1312] KRC22851.1 3-phosphoshikimate 1-carboxyvinyltransferase [Ensifer sp. Root74] KRD57378.1 3-phosphoshikimate 1-carboxyvinyltransferase [Ensifer sp. Root954]

Length=448

Score = 703 bits (1815), Expect = 0.0, Method: Compositional matrix adjust.

| Identities | = 355/447 (79%), Positives = 395/447 (88%), Gaps = 0/447 (0%) |  |
| --- | --- | --- |
| Query 1 | MLHGASSRPATARKSSGLSGTVRIPGDKSISHRSFMFGGLASGETRITGLLEGEDVINTG | 60 |
| Sbjct 1 | M HG+ RPATARKS+ L G++RIPGDKSISHR+ M GGLASGETRITGLL+G DV+ TG MSHGSGPRPATARKSADLRGSIRIPGDKSISHRALMLGGLASGETRITGLLQGADVMATG | 60 |
| Query 61 | KAMQAMGARIRKEGDTWIIDGVGNGGLLAPEAPLDFGNAATGCRLTMGLVGVYDFDSTFI | 120 |
| Sbjct 61 | +AMQAMGARIRKE DT+I+DGVGNG LLAPEAPLDFGNA TGCRL MGL GVYDF++TF+ RAMQAMGARIRKEADTFIVDGVGNGALLAPEAPLDFGNAGTGCRLAMGLAGVYDFETTFL | 120 |
| Query 121 | GDASLTKRPMGRVLNPLREMGVQVKSEDGDRLPVTLRGPKTPTPITYRVPMASAQVKSAV | 180 |
| Sbjct 121 | GDASL++RPM RVL PLREMG QV++ GDRLPVTLRGPKTP PITYRVP+ASAQVKSAV GDASLSRRPMARVLEPLREMGTQVRAAAGDRLPVTLRGPKTPAPITYRVPVASAQVKSAV | 180 |
| Query 181 | LLAGLNTPGITTVIEPIMTRDHTEKMLQGFGANLTVETDADGVRTIRLEGRGKLTGQVID | 240 |
| Sbjct 181 | LLAGLN PGITTVIEP+MTRDHTE+ML+ FGA L+VETD GVRTIRL+GRGKLTGQ ID LLAGLNAPGITTVIEPVMTRDHTERMLEAFGAALSVETDVSGVRTIRLDGRGKLTGQEID | 240 |
| Query 241 | VPGDPSSTAFPLVAALLVPGSDVTILNVLMNPTRTGLILTLQEMGADIEVINPRLAGGED | 300 |

| Sbjct | 241 | VPGDPSS AFPLVAAL+VPGSD+TI NVLMNPTRTGLILTL+EMG DIEVI R GGED VPGDPSSAAFPLVAALIVPGSDITITNVLMNPTRTGLILTLKEMGGDIEVIGSRQVGGED | 300 |
| --- | --- | --- | --- |
| Query | 301 | VADLRVRSSTLKGVTVPEDRAPSMIDEYPILavaaafaegaTVMNGLEELRVKESDRLSA | 360 |
| Sbjct | 301 | VADLRVR S L GVTVPE+RA SMIDEYP LA+AAAFAEG TVM GL+ELRVKESDRL+A VADLRVRHSELSGVTVPEERAASMIDEYPALAIAAAFAEGNTVMAGLDELRVKESDRLAA | 360 |
| Query | 361 | VANGLKLNGVDCDEGETSLVVRGRPDGKGLGNASGAAVATHLDHRIAMSFLVMGLVSENP | 420 |
| Sbjct | 361 | +A GLK NGVDC+EG +SLVVRGR DGKGLG+A+GA V THLDHRIAMSFL+ GL SE+P IAAGLKRNGVDCEEGASSLVVRGRRDGKGLGSAAGATVNTHLDHRIAMSFLIFGLASEHP | 420 |
| Query  Sbjct | 421  421 | VTVDDATMIATSFPEFMDLMAGLGAKI 447 VTVDD +M+ATSFPEFM LM LGA+I VTVDDVSMVATSFPEFMHLMTSLGARI 447 |  |

>WP_069615366.1 3-phosphoshikimate 1-carboxyvinyltransferase [Rhizobium sp. YK2] OEC94390.1 3-phosphoshikimate 1-carboxyvinyltransferase [Rhizobium sp. YK2]

Length=451

Score = 703 bits (1815), Expect = 0.0, Method: Compositional matrix adjust.

| Identities | = 357/447 (80%), Positives = 392/447 (88%), Gaps = 0/447 (0%) |  |
| --- | --- | --- |
| Query 1 | MLHGASSRPATARKSSGLSGTVRIPGDKSISHRSFMFGGLASGETRITGLLEGEDVINTG | 60 |
| Sbjct 1 | M H +PA AR+S+GLSGT+RIPGDKSISHRS MFGGLA+GETRITGLLEGEDV+NTG MPHDTLPQPAAARRSAGLSGTIRIPGDKSISHRSLMFGGLAAGETRITGLLEGEDVLNTG | 60 |
| Query 61 | KAMQAMGARIRKEGDTWIIDGVGNGGLLAPEAPLDFGNAATGCRLTMGLVGVYDFDSTFI | 120 |
| Sbjct 61 | KAMQA+GA IRK D WI+ G GNGGLLAP APLDFGNA TGCRLTMGLVG YDF +TFI KAMQALGATIRKIDDEWIVGGTGNGGLLAPAAPLDFGNAGTGCRLTMGLVGSYDFKTTFI | 120 |
| Query 121 | GDASLTKRPMGRVLNPLREMGVQVKSEDGDRLPVTLRGPKTPTPITYRVPMASAQVKSAV | 180 |
| Sbjct 121 | GDASL+KRPMGR+L+PLR MG Q+K+ GD+LPVTLRGP +P+TYRVPMASAQVKSAV GDASLSKRPMGRILDPLRLMGTQIKAAAGDKLPVTLRGPGVASPLTYRVPMASAQVKSAV | 180 |
| Query 181 | LLAGLNTPGITTVIEPIMTRDHTEKMLQGFGANLTVETDADGVRTIRLEGRGKLTGQVID | 240 |
| Sbjct 181 | LLAGLN PGITTVIEP+MTRDHTEKML GFGA L+VETDA GVRTIRLEG+GKLTGQ ID LLAGLNAPGITTVIEPVMTRDHTEKMLAGFGAALSVETDAAGVRTIRLEGQGKLTGQTID | 240 |
| Query 241 | VPGDPSSTAFPLVAALLVPGSDVTILNVLMNPTRTGLILTLQEMGADIEVINPRLAGGED | 300 |
| Sbjct 241 | VPGDPSS AFPLVAAL+VPGS +TI NVLMNPTRTGLILTLQEM ADIE+++ R+ GGED VPGDPSSAAFPLVAALIVPGSYITIRNVLMNPTRTGLILTLQEMDADIEILDRRMTGGED | 300 |
| Query 301 | VADLRVRSSTLKGVTVPEDRAPSMIDEYPILavaaafaegaTVMNGLEELRVKESDRLSA | 360 |
| Sbjct 301 | VADLRVRSS LKGV VP +RAPSMIDEYP+LAVAAAFAEG TVM GLEELRVKESDRLSA VADLRVRSSALKGVIVPAERAPSMIDEYPVLAVAAAFAEGTTVMQGLEELRVKESDRLSA | 360 |
| Query 361 | VANGLKLNGVDCDEGETSLVVRGRPDGKGLGNASGAAVATHLDHRIAMSFLVMGLVSENP | 420 |
| Sbjct 361 | VA GLKLNGVDC EG SL VRGRPDGKGLG++SG V THLDHRIAMSFLVMGL SE+P VAEGLKLNGVDCSEGRDSLTVRGRPDGKGLGSSSGTEVRTHLDHRIAMSFLVMGLASEHP | 420 |
| Query 421  Sbjct 421 | VTVDDATMIATSFPEFMDLMAGLGAKI 447 V++DD+ MIATSFPEF +M LGA I VSIDDSAMIATSFPEFFAMMTRLGATI 447 |  |

>WP_112770687.1 3-phosphoshikimate 1-carboxyvinyltransferase [Ensifer adhaerens] RAS18669.1 3-phosphoshikimate 1-carboxyvinyltransferase [Ensifer adhaerens]

Length=448

Score = 703 bits (1815), Expect = 0.0, Method: Compositional matrix adjust.

| Identities | = 355/447 (79%), Positives = 395/447 (88%), Gaps = 0/447 (0%) |  |
| --- | --- | --- |
| Query 1 | MLHGASSRPATARKSSGLSGTVRIPGDKSISHRSFMFGGLASGETRITGLLEGEDVINTG | 60 |
| Sbjct 1 | M HG+ RPATARKS+ L G++RIPGDKSISHR+ M GGLASGETRITGLL+G DV+ TG MSHGSGPRPATARKSADLRGSIRIPGDKSISHRALMLGGLASGETRITGLLQGADVLATG | 60 |
| Query 61 | KAMQAMGARIRKEGDTWIIDGVGNGGLLAPEAPLDFGNAATGCRLTMGLVGVYDFDSTFI | 120 |
| Sbjct 61 | +AMQAMGARIRKE DT+I+DGVGNG LLAPEAPLDFGNA TGCRL MGL GVYDF++TF+ RAMQAMGARIRKEADTFIVDGVGNGALLAPEAPLDFGNAGTGCRLAMGLAGVYDFETTFL | 120 |
| Query 121 | GDASLTKRPMGRVLNPLREMGVQVKSEDGDRLPVTLRGPKTPTPITYRVPMASAQVKSAV | 180 |

| Sbjct | 121 | GDASL++RPM RVL PLREMG QV++ GDRLPVTLRGPKTP PITYRVP+ASAQVKSAV GDASLSRRPMARVLEPLREMGAQVRAAAGDRLPVTLRGPKTPAPITYRVPVASAQVKSAV | 180 |
| --- | --- | --- | --- |
| Query | 181 | LLAGLNTPGITTVIEPIMTRDHTEKMLQGFGANLTVETDADGVRTIRLEGRGKLTGQVID | 240 |
| Sbjct | 181 | LLAGLN PGITTVIEP+MTRDHTE+ML+ FGA L+VETD GVRTIRL+GRGKLTGQ ID LLAGLNAPGITTVIEPVMTRDHTERMLEAFGAALSVETDVSGVRTIRLDGRGKLTGQEID | 240 |
| Query | 241 | VPGDPSSTAFPLVAALLVPGSDVTILNVLMNPTRTGLILTLQEMGADIEVINPRLAGGED | 300 |
| Sbjct | 241 | VPGDPSS AFPLVAAL+VPGSD+TI NVLMNPTRTGLILTL+EMG DIEVI R GGED VPGDPSSAAFPLVAALIVPGSDITITNVLMNPTRTGLILTLKEMGGDIEVIGSRQVGGED | 300 |
| Query | 301 | VADLRVRSSTLKGVTVPEDRAPSMIDEYPILavaaafaegaTVMNGLEELRVKESDRLSA | 360 |
| Sbjct | 301 | VADLRVR S L GVTVPE+RA SMIDEYP LA+AAAFAEG TVM GL+ELRVKESDRL+A VADLRVRHSELSGVTVPEERAASMIDEYPALAIAAAFAEGNTVMAGLDELRVKESDRLAA | 360 |
| Query | 361 | VANGLKLNGVDCDEGETSLVVRGRPDGKGLGNASGAAVATHLDHRIAMSFLVMGLVSENP | 420 |
| Sbjct | 361 | +A GLK NGVDC+EG +SLVVRGR DGKGLG+A+GA V THLDHRIAMSFL+ GL SE+P IAAGLKRNGVDCEEGASSLVVRGRRDGKGLGSAAGATVNTHLDHRIAMSFLIFGLASEHP | 420 |
| Query  Sbjct | 421  421 | VTVDDATMIATSFPEFMDLMAGLGAKI 447 VTVDD +M+ATSFPEF DLM LGA+I VTVDDVSMVATSFPEFTDLMTSLGARI 447 |  |

>WP_012783809.1 3-phosphoshikimate 1-carboxyvinyltransferase [Brucella microti] ACU47047.1 3-phosphoshikimate 1-carboxyvinyltransferase [Brucella microti

CCM 4915]

Length=450

Score = 703 bits (1815), Expect = 0.0, Method: Compositional matrix adjust.

| Identities | = 363/450 (81%), Positives = 398/450 (88%), Gaps = 3/450 (1%) |  |
| --- | --- | --- |
| Query 1 | MLHGASSRPATARKSSGLSGTVRIPGDKSISHRSFMFGGLASGETRITGLLEGEDVINTG | 60 |
| Sbjct 1 | M H A +PATAR S L+G +RIPGDKSISHRSFMFGGLASG+TRITGLLEGEDVINTG MSHSACPKPATARHSQALTGEIRIPGDKSISHRSFMFGGLASGKTRITGLLEGEDVINTG | 60 |
| Query 61 | KAMQAMGARIRKEGDTWIIDGVGNGGLLAPEAPLDFGNAATGCRLTMGLVGVYDFDSTFI | 120 |
| Sbjct 61 | +AMQAMGARIRKEGD WII+GVGNG LL PEAPLDFGNA TG RLTMGLVG YD ++FI RAMQAMGARIRKEGDVWIINGVGNGCLLQPEAPLDFGNAGTGARLTMGLVGTYDMKTSFI | 120 |
| Query 121 | GDASLTKRPMGRVLNPLREMGVQVKSEDGDRLPVTLRGPKTPTPITYRVPMASAQVKSAV | 180 |
| Sbjct 121 | GDASL+KRPMGRVLNPLREMGVQV++ +GDR+P+TL GP+T PI YRVPMASAQVKSA+ GDASLSKRPMGRVLNPLREMGVQVEAAEGDRMPLTLIGPRTANPIAYRVPMASAQVKSAI | 180 |
| Query 181 | LLAGLNTPGITTVIEPIMTRDHTEKMLQGFGANLTVETDADGVRTIRLEGRGKLTGQVID | 240 |
| Sbjct 181 | LLAGLNTPG+TTVIEP+MTRDHTEKMLQGFGA+LTVETD DGVR IR+ G+GKLTGQ ID LLAGLNTPGVTTVIEPVMTRDHTEKMLQGFGADLTVETDKDGVRHIRIVGQGKLTGQTID | 240 |
| Query 241 | VPGDPSSTAFPLVAALLVPGSDVTILNVLMNPTRTGLILTLQEMGADIEVINPRLAGGED | 300 |
| Sbjct 241 | VPGDPSSTAFPLVAALLV GSDVTI NVLMNPTRTGLILTLQEMGADIE+I+PRLAGGED VPGDPSSTAFPLVAALLVEGSDVTIRNVLMNPTRTGLILTLQEMGADIEIIDPRLAGGED | 300 |
| Query 301 | VADLRVRSSTLKGVTVPEDRAPSMIDEYPILavaaafaegaTVMNGLEELRVKESDRLSA | 360 |
| Sbjct 301 | VADLRV++S LKGV VP +RAPSMIDEYP+LA+AA+FAEG TVM+GL+ELRVKESDRL+A VADLRVKASKLKGVVVPPERAPSMIDEYPVLAIAASFAEGETVMDGLDELRVKESDRLAA | 360 |
| Query 361 | VANGLKLNGVDCDEGETSLVVRGRPDGKGLGNASGAAVATHLDHRIAMSFLVMGLVSENP | 420 |
| Sbjct 361 | VA GL+ NGVDC EGE SL VRGR G G VATHLDHRIAMSFLVMGL SE P VARGLEANGVDCTEGEMSLTVRGR---PGGKGLGGGTVATHLDHRIAMSFLVMGLASEKP | 417 |
| Query 421  Sbjct 418 | VTVDDATMIATSFPEFMDLMAGLGAKIELS 450 VTVDD+TMIATSFPEFM +MAGLGAKI S VTVDDSTMIATSFPEFMGMMAGLGAKIAES 447 |  |

>WP_105985360.1 3-phosphoshikimate 1-carboxyvinyltransferase [Brucella sp. BCCN 84-3]

Length=450

Score = 703 bits (1815), Expect = 0.0, Method: Compositional matrix adjust. Identities = 364/450 (81%), Positives = 398/450 (88%), Gaps = 3/450 (1%)

| Query | 1 | MLHGASSRPATARKSSGLSGTVRIPGDKSISHRSFMFGGLASGETRITGLLEGEDVINTG | 60 |
| --- | --- | --- | --- |
| Sbjct | 1 | M H A +PATAR S L+G +RIPGDKSISHRSFMFGGLASG+TRITGLLEGEDVINTG MSHSACPKPATARHSRALTGEIRIPGDKSISHRSFMFGGLASGKTRITGLLEGEDVINTG | 60 |
| Query | 61 | KAMQAMGARIRKEGDTWIIDGVGNGGLLAPEAPLDFGNAATGCRLTMGLVGVYDFDSTFI | 120 |
| Sbjct | 61 | +AMQAMGARIRKEGD WII+GVGNG LL PEAPLDFGNA TG RLTMGLVG YD ++FI RAMQAMGARIRKEGDVWIINGVGNGCLLQPEAPLDFGNAGTGARLTMGLVGTYDMKTSFI | 120 |
| Query | 121 | GDASLTKRPMGRVLNPLREMGVQVKSEDGDRLPVTLRGPKTPTPITYRVPMASAQVKSAV | 180 |
| Sbjct | 121 | GDASL+KRPMGRVLNPLREMGVQV++ +GDR+P+TL GP+T PI YRVPMASAQVKSAV GDASLSKRPMGRVLNPLREMGVQVEAAEGDRMPLTLIGPRTANPIAYRVPMASAQVKSAV | 180 |
| Query | 181 | LLAGLNTPGITTVIEPIMTRDHTEKMLQGFGANLTVETDADGVRTIRLEGRGKLTGQVID | 240 |
| Sbjct | 181 | LLAGLNTPG+TTVIEP+MTRDHTEKMLQGFGA+LTVETD DGVR IR+ G+GKLTGQ ID LLAGLNTPGVTTVIEPVMTRDHTEKMLQGFGADLTVETDKDGVRHIRIVGQGKLTGQTID | 240 |
| Query | 241 | VPGDPSSTAFPLVAALLVPGSDVTILNVLMNPTRTGLILTLQEMGADIEVINPRLAGGED | 300 |
| Sbjct | 241 | VPGDPSSTAFPLVAALLV GSDVTI NVLMNPTRTGLILTLQEMGADIE+I+PRLAGGED VPGDPSSTAFPLVAALLVEGSDVTIRNVLMNPTRTGLILTLQEMGADIEIIDPRLAGGED | 300 |
| Query | 301 | VADLRVRSSTLKGVTVPEDRAPSMIDEYPILavaaafaegaTVMNGLEELRVKESDRLSA | 360 |
| Sbjct | 301 | VADLRV++S LKGV VP +RAPSMIDEYP+LA+AA+FAEG TVM+GL+ELRVKESDRL+A VADLRVKASKLKGVVVPPERAPSMIDEYPVLAIAASFAEGETVMDGLDELRVKESDRLAA | 360 |
| Query | 361 | VANGLKLNGVDCDEGETSLVVRGRPDGKGLGNASGAAVATHLDHRIAMSFLVMGLVSENP | 420 |
| Sbjct | 361 | VA GL+ NGVDC EGE SL VRGR G G VATHLDHRIAMSFLVMGL SE P VARGLEANGVDCTEGEMSLTVRGR---PGGKGLGGGTVATHLDHRIAMSFLVMGLASEKP | 417 |
| Query  Sbjct | 421  418 | VTVDDATMIATSFPEFMDLMAGLGAKIELS 450 VTVDD+TMIATSFPEFM +MAGLGAKI S VTVDDSTMIATSFPEFMGMMAGLGAKIAES 447 |  |

>WP_076770992.1 MULTISPECIES: 3-phosphoshikimate 1-carboxyvinyltransferase [unclassified Brucella]

APY13751.1 3-phosphoshikimate 1-carboxyvinyltransferase [Brucella sp. 09RB8910] MRN47881.1 3-phosphoshikimate 1-carboxyvinyltransferase [Brucella sp. 10RB9212] MRN48644.1 3-phosphoshikimate 1-carboxyvinyltransferase [Brucella sp. 10RB9214]

Length=450

Score = 703 bits (1814), Expect = 0.0, Method: Compositional matrix adjust.

| Identities | = 363/447 (81%), Positives = 397/447 (89%), Gaps = 3/447 (1%) |  |
| --- | --- | --- |
| Query 1 | MLHGASSRPATARKSSGLSGTVRIPGDKSISHRSFMFGGLASGETRITGLLEGEDVINTG | 60 |
| Sbjct 1 | M H A +PATAR S L+G +RIPGDKSISHRSFMFGGLASGETRITGLLEGEDVINTG MSHSACPKPATARHSQALTGEIRIPGDKSISHRSFMFGGLASGETRITGLLEGEDVINTG | 60 |
| Query 61 | KAMQAMGARIRKEGDTWIIDGVGNGGLLAPEAPLDFGNAATGCRLTMGLVGVYDFDSTFI | 120 |
| Sbjct 61 | +AMQAMGARIRKEG+ WII+GVGNG LL PEA LDFGNA TG RLTMGLVG YD ++FI RAMQAMGARIRKEGNVWIINGVGNGCLLQPEASLDFGNAGTGARLTMGLVGTYDMKTSFI | 120 |
| Query 121 | GDASLTKRPMGRVLNPLREMGVQVKSEDGDRLPVTLRGPKTPTPITYRVPMASAQVKSAV | 180 |
| Sbjct 121 | GDASL+KRPMGRVLNPLREMGVQV++ +GDR+P+TL GP+T PITYRVPMASAQVKSAV GDASLSKRPMGRVLNPLREMGVQVEAAEGDRMPLTLIGPRTANPITYRVPMASAQVKSAV | 180 |
| Query 181 | LLAGLNTPGITTVIEPIMTRDHTEKMLQGFGANLTVETDADGVRTIRLEGRGKLTGQVID | 240 |
| Sbjct 181 | LLAGLNTPG+TTVIEP+MTRDHTEKMLQGFGA+LTVETD DGVR IR+ G+GKLTGQ ID LLAGLNTPGVTTVIEPVMTRDHTEKMLQGFGADLTVETDKDGVRHIRIVGQGKLTGQTID | 240 |
| Query 241 | VPGDPSSTAFPLVAALLVPGSDVTILNVLMNPTRTGLILTLQEMGADIEVINPRLAGGED | 300 |
| Sbjct 241 | VPGDPSSTAFPLVAALLV GSDVTI NVLMNPTRTGLILTLQEMGADIE+I+PRLAGGED VPGDPSSTAFPLVAALLVEGSDVTIRNVLMNPTRTGLILTLQEMGADIEIIDPRLAGGED | 300 |
| Query 301 | VADLRVRSSTLKGVTVPEDRAPSMIDEYPILavaaafaegaTVMNGLEELRVKESDRLSA | 360 |
| Sbjct 301 | VADLRV++S LKGV VP +RAPSMIDEYP+LA+AA+FAEG TVM+GL+ELRVKESDRL+A VADLRVKASKLKGVVVPPERAPSMIDEYPVLAIAASFAEGETVMDGLDELRVKESDRLAA | 360 |
| Query 361 | VANGLKLNGVDCDEGETSLVVRGRPDGKGLGNASGAAVATHLDHRIAMSFLVMGLVSENP | 420 |
| Sbjct 361 | VA GL+ NGVDC EGE SL VRGR G G VATHLDHRIAMSFLVMGL SE P VARGLEANGVDCTEGEMSLTVRGR---PGGKGLGGGTVATHLDHRIAMSFLVMGLASEKP | 417 |
| Query 421 | VTVDDATMIATSFPEFMDLMAGLGAKI 447 |  |

VTVDD+TMIATSFPEFM +MAGLGAKI Sbjct 418 VTVDDSTMIATSFPEFMGMMAGLGAKI 444

>EEW80290.1 5-enolpyruvyl shikimate 3-phosphate synthase [Brucella abortus NCTC 8038]

Length=461

Score = 703 bits (1814), Expect = 0.0, Method: Compositional matrix adjust.

| Identities | = 362/447 (81%), Positives = 397/447 (89%), Gaps = 3/447 (1%) |  |
| --- | --- | --- |
| Query 1 | MLHGASSRPATARKSSGLSGTVRIPGDKSISHRSFMFGGLASGETRITGLLEGEDVINTG | 60 |
| Sbjct 12 | M H A +PATAR S L+G +RIPGDKSISHRSFMFGGLASG+TRITGLLEGEDVINTG MSHSACPKPATARHSQALTGEIRIPGDKSISHRSFMFGGLASGKTRITGLLEGEDVINTG | 71 |
| Query 61 | KAMQAMGARIRKEGDTWIIDGVGNGGLLAPEAPLDFGNAATGCRLTMGLVGVYDFDSTFI | 120 |
| Sbjct 72 | +AMQAMGARIRKEGD WII+GVGNG LL PEAPLDFGNA TG RLTMGLVG YD ++FI RAMQAMGARIRKEGDVWIINGVGNGCLLQPEAPLDFGNAGTGARLTMGLVGTYDMKTSFI | 131 |
| Query 121 | GDASLTKRPMGRVLNPLREMGVQVKSEDGDRLPVTLRGPKTPTPITYRVPMASAQVKSAV | 180 |
| Sbjct 132 | GDASL+KRPMGRVLNPLREMGVQV++ +GDR+P+TL GP+T PI YRVPMASAQVKSAV GDASLSKRPMGRVLNPLREMGVQVEAAEGDRMPLTLIGPRTANPIAYRVPMASAQVKSAV | 191 |
| Query 181 | LLAGLNTPGITTVIEPIMTRDHTEKMLQGFGANLTVETDADGVRTIRLEGRGKLTGQVID | 240 |
| Sbjct 192 | LLAGLNTPG+TTVIEP+MTRDHTEKMLQGFGA+LTVETD DGVR IR+ G+GKLTGQ ID LLAGLNTPGVTTVIEPVMTRDHTEKMLQGFGADLTVETDKDGVRHIRIVGQGKLTGQTID | 251 |
| Query 241 | VPGDPSSTAFPLVAALLVPGSDVTILNVLMNPTRTGLILTLQEMGADIEVINPRLAGGED | 300 |
| Sbjct 252 | VPGDPSSTAFPLVAALLV GS+VTI NVLMNPTRTGLILTLQEMGADIE+I+PRLAGGED VPGDPSSTAFPLVAALLVEGSEVTIRNVLMNPTRTGLILTLQEMGADIEIIDPRLAGGED | 311 |
| Query 301 | VADLRVRSSTLKGVTVPEDRAPSMIDEYPILavaaafaegaTVMNGLEELRVKESDRLSA | 360 |
| Sbjct 312 | VADLRV++S LKGV VP +RAPSMIDEYP+LA+AA+FAEG TVM+GL+ELRVKESDRL+A VADLRVKASKLKGVVVPPERAPSMIDEYPVLAIAASFAEGETVMDGLDELRVKESDRLAA | 371 |
| Query 361 | VANGLKLNGVDCDEGETSLVVRGRPDGKGLGNASGAAVATHLDHRIAMSFLVMGLVSENP | 420 |
| Sbjct 372 | VA GL+ NGVDC EGE SL VRGR G G VATHLDHRIAMSFLVMGL SE P VARGLEANGVDCTEGEMSLTVRGR---PGGKGLGGGTVATHLDHRIAMSFLVMGLASEKP | 428 |
| Query 421  Sbjct 429 | VTVDDATMIATSFPEFMDLMAGLGAKI 447 VTVDD+TMIATSFPEFM +MAGLGAKI VTVDDSTMIATSFPEFMGMMAGLGAKI 455 |  |

>OWZ94747.1 3-phosphoshikimate 1-carboxyvinyltransferase [Sinorhizobium sp. LM21]

Length=448

Score = 703 bits (1814), Expect = 0.0, Method: Compositional matrix adjust.

| Identities | = 355/447 (79%), Positives = 395/447 (88%), Gaps = 0/447 (0%) |  |
| --- | --- | --- |
| Query 1 | MLHGASSRPATARKSSGLSGTVRIPGDKSISHRSFMFGGLASGETRITGLLEGEDVINTG | 60 |
| Sbjct 1 | M HG+ RPATARKS+ L G++RIPGDKSISHR+ M GGLASGETRITGLL+G DV+ TG MSHGSGPRPATARKSADLRGSIRIPGDKSISHRALMLGGLASGETRITGLLQGADVLATG | 60 |
| Query 61 | KAMQAMGARIRKEGDTWIIDGVGNGGLLAPEAPLDFGNAATGCRLTMGLVGVYDFDSTFI | 120 |
| Sbjct 61 | +AMQAMGARIRKE DT+I+DGVGNG LLAPEAPLDFGNA TGCRL MGL GVYDF++TF+ RAMQAMGARIRKEADTFIVDGVGNGALLAPEAPLDFGNAGTGCRLAMGLAGVYDFETTFL | 120 |
| Query 121 | GDASLTKRPMGRVLNPLREMGVQVKSEDGDRLPVTLRGPKTPTPITYRVPMASAQVKSAV | 180 |
| Sbjct 121 | GDASL++RPM RVL PLREMG QV++ GDRLPVTLRGPKTP PITYRVP+ASAQVKSAV GDASLSRRPMARVLEPLREMGAQVRAAAGDRLPVTLRGPKTPAPITYRVPVASAQVKSAV | 180 |
| Query 181 | LLAGLNTPGITTVIEPIMTRDHTEKMLQGFGANLTVETDADGVRTIRLEGRGKLTGQVID | 240 |
| Sbjct 181 | LLAGLN PGITTVIEP+MTRDHTE+ML+ FGA L+VETD GVRTIRL+GRGKLTGQ ID LLAGLNAPGITTVIEPVMTRDHTERMLEAFGAALSVETDVSGVRTIRLDGRGKLTGQEID | 240 |
| Query 241 | VPGDPSSTAFPLVAALLVPGSDVTILNVLMNPTRTGLILTLQEMGADIEVINPRLAGGED | 300 |
| Sbjct 241 | VPGDPSS AFPLVAAL+VPGSD+TI NVLMNPTRTGLILTL+EMG DIEVI R GGED VPGDPSSAAFPLVAALIVPGSDITITNVLMNPTRTGLILTLKEMGGDIEVIGSRQVGGED | 300 |
| Query 301 | VADLRVRSSTLKGVTVPEDRAPSMIDEYPILavaaafaegaTVMNGLEELRVKESDRLSA | 360 |

| Sbjct | 301 | VADLRVR S L GVTVPE+RA SMIDEYP LA+AAAFAEG TVM GL+ELRVKESDRL+A VADLRVRHSELSGVTVPEERAASMIDEYPALAIAAAFAEGNTVMAGLDELRVKESDRLAA | 360 |
| --- | --- | --- | --- |
| Query | 361 | VANGLKLNGVDCDEGETSLVVRGRPDGKGLGNASGAAVATHLDHRIAMSFLVMGLVSENP | 420 |
| Sbjct | 361 | +A GLK NGVDC+E +SLVVRGR DGKGLG+A+GA V THLDHRIAMSFL+ GL SE+P IAAGLKRNGVDCEERASSLVVRGRRDGKGLGSAAGATVNTHLDHRIAMSFLIFGLASEHP | 420 |
| Query  Sbjct | 421  421 | VTVDDATMIATSFPEFMDLMAGLGAKI 447 VTVDD +M+ATSFPEFMDLM LGA+I VTVDDVSMVATSFPEFMDLMTSLGARI 447 |  |

>WP_110818568.1 MULTISPECIES: 3-phosphoshikimate 1-carboxyvinyltransferase [Ensifer] MBD9637727.1 3-phosphoshikimate 1-carboxyvinyltransferase [Ensifer sp. ENS07] PYE77606.1 3-phosphoshikimate 1-carboxyvinyltransferase [Ensifer adhaerens]

Length=448

Score = 703 bits (1814), Expect = 0.0, Method: Compositional matrix adjust.

| Identities | = 355/447 (79%), Positives = 394/447 (88%), Gaps = 0/447 (0%) |  |
| --- | --- | --- |
| Query 1 | MLHGASSRPATARKSSGLSGTVRIPGDKSISHRSFMFGGLASGETRITGLLEGEDVINTG | 60 |
| Sbjct 1 | M HG+ RPATARKS+ L G++RIPGDKSISHR+ M GGLASGETRITGLL+G DV+ TG MSHGSGPRPATARKSADLRGSIRIPGDKSISHRALMLGGLASGETRITGLLQGADVLATG | 60 |
| Query 61 | KAMQAMGARIRKEGDTWIIDGVGNGGLLAPEAPLDFGNAATGCRLTMGLVGVYDFDSTFI | 120 |
| Sbjct 61 | +AMQAMGARIRKE DT+I+DGVGNG LLAPEAPLDFGNA TGCRL MGL GVYDF++TF+ RAMQAMGARIRKEADTFIVDGVGNGALLAPEAPLDFGNAGTGCRLAMGLAGVYDFETTFL | 120 |
| Query 121 | GDASLTKRPMGRVLNPLREMGVQVKSEDGDRLPVTLRGPKTPTPITYRVPMASAQVKSAV | 180 |
| Sbjct 121 | GDASL++RPM RVL PLREMG QV++ GDRLPVTLRGPKTP PITYRVP+ASAQVKSAV GDASLSRRPMARVLEPLREMGAQVRAAAGDRLPVTLRGPKTPAPITYRVPVASAQVKSAV | 180 |
| Query 181 | LLAGLNTPGITTVIEPIMTRDHTEKMLQGFGANLTVETDADGVRTIRLEGRGKLTGQVID | 240 |
| Sbjct 181 | LLAGLN PGITTVIEP+MTRDHTE+ML+ FGA LTVETD GVRTIRL+GRGKLTGQ ID LLAGLNAPGITTVIEPVMTRDHTERMLEAFGAALTVETDVSGVRTIRLDGRGKLTGQEID | 240 |
| Query 241 | VPGDPSSTAFPLVAALLVPGSDVTILNVLMNPTRTGLILTLQEMGADIEVINPRLAGGED | 300 |
| Sbjct 241 | VPGDPSS AFPLVAAL+VPGSD+TI NVLMNPTRTGLILTL+EMG DIEVI R GGED VPGDPSSAAFPLVAALIVPGSDITITNVLMNPTRTGLILTLKEMGGDIEVIGSRQVGGED | 300 |
| Query 301 | VADLRVRSSTLKGVTVPEDRAPSMIDEYPILavaaafaegaTVMNGLEELRVKESDRLSA | 360 |
| Sbjct 301 | V DLRVR S L GVTVPE+RA SMIDEYP LA+AAAFAEG TVM GL+ELRVKESDR +A VGDLRVRHSELSGVTVPEERAASMIDEYPALAIAAAFAEGNTVMAGLDELRVKESDRFAA | 360 |
| Query 361 | VANGLKLNGVDCDEGETSLVVRGRPDGKGLGNASGAAVATHLDHRIAMSFLVMGLVSENP | 420 |
| Sbjct 361 | +A GLK NGVDC+EG +SLVVRGR DGKGLG+A+GA V THLDHRIAMSFL+ GL SE+P IAGGLKRNGVDCEEGASSLVVRGRRDGKGLGSAAGATVNTHLDHRIAMSFLIFGLASEHP | 420 |
| Query 421  Sbjct 421 | VTVDDATMIATSFPEFMDLMAGLGAKI 447 VTVDD +M+ATSFPEFMDLM LGA+I VTVDDVSMVATSFPEFMDLMTSLGARI 447 |  |

>WP_127889705.1 MULTISPECIES: 3-phosphoshikimate 1-carboxyvinyltransferase [Sinorhizobium/Ensifer group]

THA65906.1 3-phosphoshikimate 1-carboxyvinyltransferase [Ensifer adhaerens] Length=448

Score = 702 bits (1813), Expect = 0.0, Method: Compositional matrix adjust.

| Identities | = 354/447 (79%), Positives = 395/447 (88%), Gaps = 0/447 (0%) |  |
| --- | --- | --- |
| Query 1 | MLHGASSRPATARKSSGLSGTVRIPGDKSISHRSFMFGGLASGETRITGLLEGEDVINTG | 60 |
| Sbjct 1 | M HG+ RPA ARKS+ L G++RIPGDKSISHR+ M GGLASGETRITGLL+G DV+ TG MSHGSGPRPANARKSADLRGSIRIPGDKSISHRALMLGGLASGETRITGLLQGADVLATG | 60 |
| Query 61 | KAMQAMGARIRKEGDTWIIDGVGNGGLLAPEAPLDFGNAATGCRLTMGLVGVYDFDSTFI | 120 |
| Sbjct 61 | +AMQAMGARIRKE DT+I+DGVGNG LLAPEAPLDFGNA TGCRL MGL GVYDF++TF+ RAMQAMGARIRKEADTFIVDGVGNGALLAPEAPLDFGNAGTGCRLAMGLAGVYDFETTFL | 120 |
| Query 121 | GDASLTKRPMGRVLNPLREMGVQVKSEDGDRLPVTLRGPKTPTPITYRVPMASAQVKSAV | 180 |
| Sbjct 121 | GDASL++RPM RVL PLREMG QV++ GDRLPVTLRGPKTP PITYRVP+ASAQVKSA+  GDASLSRRPMARVLEPLREMGTQVRAAAGDRLPVTLRGPKTPAPITYRVPVASAQVKSAL | 180 |

| Query | 181 | LLAGLNTPGITTVIEPIMTRDHTEKMLQGFGANLTVETDADGVRTIRLEGRGKLTGQVID | 240 |
| --- | --- | --- | --- |
| Sbjct | 181 | LLAGLN PGITTVIEP+MTRDHTE+ML+ FGA L+VETD GVRTIRL+GRGKLTGQ ID LLAGLNAPGITTVIEPVMTRDHTERMLEAFGAALSVETDVSGVRTIRLDGRGKLTGQEID | 240 |
| Query | 241 | VPGDPSSTAFPLVAALLVPGSDVTILNVLMNPTRTGLILTLQEMGADIEVINPRLAGGED | 300 |
| Sbjct | 241 | VPGDPSS AFPLVAAL+VPGSD+TI NVLMNPTRTGLILTL+EMG DIEVI R GGED VPGDPSSAAFPLVAALIVPGSDITITNVLMNPTRTGLILTLKEMGGDIEVIGSRQVGGED | 300 |
| Query | 301 | VADLRVRSSTLKGVTVPEDRAPSMIDEYPILavaaafaegaTVMNGLEELRVKESDRLSA | 360 |
| Sbjct | 301 | VADLRVR S L GVTVPE+RA SMIDEYP LA+AAAFAEG TVM GL+ELRVKESDRL+A VADLRVRQSELSGVTVPEERAASMIDEYPALAIAAAFAEGNTVMAGLDELRVKESDRLAA | 360 |
| Query | 361 | VANGLKLNGVDCDEGETSLVVRGRPDGKGLGNASGAAVATHLDHRIAMSFLVMGLVSENP | 420 |
| Sbjct | 361 | +A GL LNGVDC+EG +SLVVRGR DGKGLG+A+GA V THLDHRIAMSFL+ GL SE+P IAAGLWLNGVDCEEGASSLVVRGRRDGKGLGSAAGATVNTHLDHRIAMSFLIFGLASEHP | 420 |
| Query  Sbjct | 421  421 | VTVDDATMIATSFPEFMDLMAGLGAKI 447 VTVDD +M+ATSFPEFMDLM LGA+I VTVDDVSMVATSFPEFMDLMTSLGARI 447 |  |

>WP_043611868.1 3-phosphoshikimate 1-carboxyvinyltransferase [Ensifer sp. ZNC0028] Length=448

Score = 702 bits (1813), Expect = 0.0, Method: Compositional matrix adjust.

| Identities | = 352/447 (79%), Positives = 395/447 (88%), Gaps = 0/447 (0%) |  |
| --- | --- | --- |
| Query 1 | MLHGASSRPATARKSSGLSGTVRIPGDKSISHRSFMFGGLASGETRITGLLEGEDVINTG | 60 |
| Sbjct 1 | M HG+ RPATARKS+ L G++RIPGDKSISHR+ M GGLA GETRITGLLEG DV+ TG MSHGSGPRPATARKSADLRGSIRIPGDKSISHRALMLGGLAGGETRITGLLEGADVLATG | 60 |
| Query 61 | KAMQAMGARIRKEGDTWIIDGVGNGGLLAPEAPLDFGNAATGCRLTMGLVGVYDFDSTFI | 120 |
| Sbjct 61 | +AMQAMGARIRKEG+T+++DGVGNG LLAPEAPLDFGNA TGCRL MGL GVYDF++TF+ RAMQAMGARIRKEGNTFVVDGVGNGALLAPEAPLDFGNAGTGCRLVMGLAGVYDFETTFV | 120 |
| Query 121 | GDASLTKRPMGRVLNPLREMGVQVKSEDGDRLPVTLRGPKTPTPITYRVPMASAQVKSAV | 180 |
| Sbjct 121 | GDASL++RPM RVL+PLREMGVQV++ GDRLPVTLRGPKTPTPI YRVP+ASAQ KSAV GDASLSRRPMARVLDPLREMGVQVRAAAGDRLPVTLRGPKTPTPIAYRVPVASAQAKSAV | 180 |
| Query 181 | LLAGLNTPGITTVIEPIMTRDHTEKMLQGFGANLTVETDADGVRTIRLEGRGKLTGQVID | 240 |
| Sbjct 181 | LLAGLN PGITTVIEP+MTRDHTE+ML+ FGA L+VETD GVRTI LEGRGKLTGQ ID LLAGLNAPGITTVIEPVMTRDHTERMLEAFGAALSVETDVSGVRTICLEGRGKLTGQEID | 240 |
| Query 241 | VPGDPSSTAFPLVAALLVPGSDVTILNVLMNPTRTGLILTLQEMGADIEVINPRLAGGED | 300 |
| Sbjct 241 | VPGDPSS AFPLVAAL+VPGSD+TI NVLMNPTRTGLILTLQEMG DIEVI+ GGED VPGDPSSAAFPLVAALIVPGSDITITNVLMNPTRTGLILTLQEMGGDIEVIDSSQVGGED | 300 |
| Query 301 | VADLRVRSSTLKGVTVPEDRAPSMIDEYPILavaaafaegaTVMNGLEELRVKESDRLSA | 360 |
| Sbjct 301 | VADLRVR S L GVTVPE+RA SMIDEYP LA+AA FAEG TVM GL+ELR KESDRL+A VADLRVRHSELSGVTVPEERAASMIDEYPALAIAAVFAEGKTVMTGLDELRPKESDRLAA | 360 |
| Query 361 | VANGLKLNGVDCDEGETSLVVRGRPDGKGLGNASGAAVATHLDHRIAMSFLVMGLVSENP | 420 |
| Sbjct 361 | + GL LNGV+C+EG +SL+VRGR DGKGLG+A+GA+V THLDHRIAMSFLV+GL +E+P ITAGLTLNGVECEEGASSLMVRGRRDGKGLGSAAGASVKTHLDHRIAMSFLVLGLAAEHP | 420 |
| Query 421  Sbjct 421 | VTVDDATMIATSFPEFMDLMAGLGAKI 447 VTVDD +MIATSFP FMDLMAGLGA I VTVDDVSMIATSFPGFMDLMAGLGASI 447 |  |

>WP_002965272.1 MULTISPECIES: 3-phosphoshikimate 1-carboxyvinyltransferase [Brucella] B2S7T0.1 RecName: Full=3-phosphoshikimate 1-carboxyvinyltransferase; AltName:

Full=5-enolpyruvylshikimate-3-phosphate synthase; Short=EPSP synthase; Short=EPSPS [Brucella abortus S19]

Q2YPN6.1 RecName: Full=3-phosphoshikimate 1-carboxyvinyltransferase; AltName:

Full=5-enolpyruvylshikimate-3-phosphate synthase; Short=EPSP synthase; Short=EPSPS [Brucella abortus 2308]

ERT86002.1 3-phosphoshikimate 1-carboxyvinyltransferase [Brucella abortus 90-12178]

ERT97089.1 3-phosphoshikimate 1-carboxyvinyltransferase [Brucella abortus 99-9971-135]

KFH21427.1 3-phosphoshikimate 1-carboxyvinyltransferase [Brucella abortus LMN1]

KFH23900.1 3-phosphoshikimate 1-carboxyvinyltransferase [Brucella abortus LMN2]

ACD71584.1 3-phosphoshikimate 1-carboxyvinyltransferase [Brucella abortus S19]

Length=450

Score = 702 bits (1813), Expect = 0.0, Method: Compositional matrix adjust.

| Identities | = 362/447 (81%), Positives = 397/447 (89%), Gaps = 3/447 (1%) |  |
| --- | --- | --- |
| Query 1 | MLHGASSRPATARKSSGLSGTVRIPGDKSISHRSFMFGGLASGETRITGLLEGEDVINTG | 60 |
| Sbjct 1 | M H A +PATAR S L+G +RIPGDKSISHRSFMFGGLASG+TRITGLLEGEDVINTG MSHSACPKPATARHSQALTGEIRIPGDKSISHRSFMFGGLASGKTRITGLLEGEDVINTG | 60 |
| Query 61 | KAMQAMGARIRKEGDTWIIDGVGNGGLLAPEAPLDFGNAATGCRLTMGLVGVYDFDSTFI | 120 |
| Sbjct 61 | +AMQAMGARIRKEGD WII+GVGNG LL PEAPLDFGNA TG RLTMGLVG YD ++FI RAMQAMGARIRKEGDVWIINGVGNGCLLQPEAPLDFGNAGTGARLTMGLVGTYDMKTSFI | 120 |
| Query 121 | GDASLTKRPMGRVLNPLREMGVQVKSEDGDRLPVTLRGPKTPTPITYRVPMASAQVKSAV | 180 |
| Sbjct 121 | GDASL+KRPMGRVLNPLREMGVQV++ +GDR+P+TL GP+T PI YRVPMASAQVKSAV GDASLSKRPMGRVLNPLREMGVQVEAAEGDRMPLTLIGPRTANPIAYRVPMASAQVKSAV | 180 |
| Query 181 | LLAGLNTPGITTVIEPIMTRDHTEKMLQGFGANLTVETDADGVRTIRLEGRGKLTGQVID | 240 |
| Sbjct 181 | LLAGLNTPG+TTVIEP+MTRDHTEKMLQGFGA+LTVETD DGVR IR+ G+GKLTGQ ID LLAGLNTPGVTTVIEPVMTRDHTEKMLQGFGADLTVETDKDGVRHIRIVGQGKLTGQTID | 240 |
| Query 241 | VPGDPSSTAFPLVAALLVPGSDVTILNVLMNPTRTGLILTLQEMGADIEVINPRLAGGED | 300 |
| Sbjct 241 | VPGDPSSTAFPLVAALLV GS+VTI NVLMNPTRTGLILTLQEMGADIE+I+PRLAGGED VPGDPSSTAFPLVAALLVEGSEVTIRNVLMNPTRTGLILTLQEMGADIEIIDPRLAGGED | 300 |
| Query 301 | VADLRVRSSTLKGVTVPEDRAPSMIDEYPILavaaafaegaTVMNGLEELRVKESDRLSA | 360 |
| Sbjct 301 | VADLRV++S LKGV VP +RAPSMIDEYP+LA+AA+FAEG TVM+GL+ELRVKESDRL+A VADLRVKASKLKGVVVPPERAPSMIDEYPVLAIAASFAEGETVMDGLDELRVKESDRLAA | 360 |
| Query 361 | VANGLKLNGVDCDEGETSLVVRGRPDGKGLGNASGAAVATHLDHRIAMSFLVMGLVSENP | 420 |
| Sbjct 361 | VA GL+ NGVDC EGE SL VRGR G G VATHLDHRIAMSFLVMGL SE P VARGLEANGVDCTEGEMSLTVRGR---PGGKGLGGGTVATHLDHRIAMSFLVMGLASEKP | 417 |
| Query 421  Sbjct 418 | VTVDDATMIATSFPEFMDLMAGLGAKI 447 VTVDD+TMIATSFPEFM +MAGLGAKI VTVDDSTMIATSFPEFMGMMAGLGAKI 444 |  |

>WP_008933634.1 3-phosphoshikimate 1-carboxyvinyltransferase [Brucella sp. NVSL 07-0026]

EFG37020.1 3-phosphoshikimate 1-carboxyvinyltransferase [Brucella sp. NVSL 07-0026]

Length=450

Score = 702 bits (1812), Expect = 0.0, Method: Compositional matrix adjust.

| Identities | = 362/447 (81%), Positives = 396/447 (89%), Gaps = 3/447 (1%) |  |
| --- | --- | --- |
| Query 1 | MLHGASSRPATARKSSGLSGTVRIPGDKSISHRSFMFGGLASGETRITGLLEGEDVINTG | 60 |
| Sbjct 1 | M H A +PATAR S L+G +RIPGDKSISHRSFMFGGLASG+TRITGLLEGEDVINTG MSHSACPKPATARHSQALTGEIRIPGDKSISHRSFMFGGLASGKTRITGLLEGEDVINTG | 60 |
| Query 61 | KAMQAMGARIRKEGDTWIIDGVGNGGLLAPEAPLDFGNAATGCRLTMGLVGVYDFDSTFI | 120 |
| Sbjct 61 | +AMQAMGARIRKEGD WII+GVGNG LL PEAPLDFGNA TG RLTMGLVG YD ++FI RAMQAMGARIRKEGDVWIINGVGNGCLLQPEAPLDFGNAGTGARLTMGLVGTYDMKTSFI | 120 |
| Query 121 | GDASLTKRPMGRVLNPLREMGVQVKSEDGDRLPVTLRGPKTPTPITYRVPMASAQVKSAV | 180 |
| Sbjct 121 | GDASL+KRPMGRVLNPLREMGVQV++ +GDR+P+TL GP+T PI YRVPMASAQVKSAV GDASLSKRPMGRVLNPLREMGVQVEAAEGDRMPLTLIGPRTANPIAYRVPMASAQVKSAV | 180 |
| Query 181 | LLAGLNTPGITTVIEPIMTRDHTEKMLQGFGANLTVETDADGVRTIRLEGRGKLTGQVID | 240 |
| Sbjct 181 | LLAGLNTPG+TTVIEP+MTRDHTEKMLQGFGA+LTVETD DGVR IR+ G+GKLTGQ ID LLAGLNTPGVTTVIEPVMTRDHTEKMLQGFGADLTVETDKDGVRHIRIVGQGKLTGQTID | 240 |
| Query 241 | VPGDPSSTAFPLVAALLVPGSDVTILNVLMNPTRTGLILTLQEMGADIEVINPRLAGGED | 300 |
| Sbjct 241 | VPGDPSSTAFPLVAALLV GSDVTI NVLMNPTRTGLILTLQEMGADIE+I+PRL GGED  VPGDPSSTAFPLVAALLVEGSDVTIRNVLMNPTRTGLILTLQEMGADIEIIDPRLTGGED | 300 |

| Query | 301 | VADLRVRSSTLKGVTVPEDRAPSMIDEYPILavaaafaegaTVMNGLEELRVKESDRLSA | 360 |
| --- | --- | --- | --- |
| Sbjct | 301 | VADLRV++S LKGV VP +RAPSMIDEYP+LA+AA+FAEG TVM+GL+ELRVKESDRL+A VADLRVKASKLKGVVVPPERAPSMIDEYPVLAIAASFAEGETVMDGLDELRVKESDRLAA | 360 |
| Query | 361 | VANGLKLNGVDCDEGETSLVVRGRPDGKGLGNASGAAVATHLDHRIAMSFLVMGLVSENP | 420 |
| Sbjct | 361 | VA GL+ NGVDC EGE SL VRGR G G VATHLDHRIAMSFLVMGL SE P VARGLEANGVDCTEGEMSLTVRGR---PGGKGLGGGTVATHLDHRIAMSFLVMGLASEKP | 417 |
| Query  Sbjct | 421  418 | VTVDDATMIATSFPEFMDLMAGLGAKI 447 VTVDD+TMIATSFPEFM +MAGLGAKI VTVDDSTMIATSFPEFMGMMAGLGAKI 444 |  |

>WP_065874153.1 3-phosphoshikimate 1-carboxyvinyltransferase [Brucella melitensis] ARY55332.1 3-phosphoshikimate 1-carboxyvinyltransferase [Brucella melitensis] ARY71203.1 3-phosphoshikimate 1-carboxyvinyltransferase [Brucella melitensis] ARY77552.1 3-phosphoshikimate 1-carboxyvinyltransferase [Brucella melitensis] OCW05807.1 3-phosphoshikimate 1-carboxyvinyltransferase [Brucella melitensis]

Length=450

Score = 702 bits (1812), Expect = 0.0, Method: Compositional matrix adjust.

| Identities | = 364/450 (81%), Positives = 397/450 (88%), Gaps = 3/450 (1%) |  |
| --- | --- | --- |
| Query 1 | MLHGASSRPATARKSSGLSGTVRIPGDKSISHRSFMFGGLASGETRITGLLEGEDVINTG | 60 |
| Sbjct 1 | M H A +PATAR S L+G +RIPGDKSISHRSFMFGGLASG+TRITGLLEGEDVINTG MSHSACPKPATARHSQALTGEIRIPGDKSISHRSFMFGGLASGKTRITGLLEGEDVINTG | 60 |
| Query 61 | KAMQAMGARIRKEGDTWIIDGVGNGGLLAPEAPLDFGNAATGCRLTMGLVGVYDFDSTFI | 120 |
| Sbjct 61 | +AMQAMGARIRKEGD WII+GVGNG LL PEAPLDFGNA TG RLTMGLVG YD ++FI RAMQAMGARIRKEGDVWIINGVGNGCLLQPEAPLDFGNAGTGARLTMGLVGTYDMKTSFI | 120 |
| Query 121 | GDASLTKRPMGRVLNPLREMGVQVKSEDGDRLPVTLRGPKTPTPITYRVPMASAQVKSAV | 180 |
| Sbjct 121 | GDASL+KRPMGRVLNPLREMGVQV + +GDR+P+TL GP+T PI YRVPMASAQVKSAV GDASLSKRPMGRVLNPLREMGVQVGAAEGDRMPLTLIGPRTANPIAYRVPMASAQVKSAV | 180 |
| Query 181 | LLAGLNTPGITTVIEPIMTRDHTEKMLQGFGANLTVETDADGVRTIRLEGRGKLTGQVID | 240 |
| Sbjct 181 | LLAGLNTPG+TTVIEP+MTRDHTEKMLQGFGA+LTVETD DGVR IR+ G+GKLTGQ ID LLAGLNTPGVTTVIEPVMTRDHTEKMLQGFGADLTVETDKDGVRHIRIVGQGKLTGQTID | 240 |
| Query 241 | VPGDPSSTAFPLVAALLVPGSDVTILNVLMNPTRTGLILTLQEMGADIEVINPRLAGGED | 300 |
| Sbjct 241 | VPGDPSSTAFPLVAALLV GSDVTI NVLMNPTRTGLILTLQEMGADIE+I+PRLAGGED VPGDPSSTAFPLVAALLVEGSDVTIRNVLMNPTRTGLILTLQEMGADIEIIDPRLAGGED | 300 |
| Query 301 | VADLRVRSSTLKGVTVPEDRAPSMIDEYPILavaaafaegaTVMNGLEELRVKESDRLSA | 360 |
| Sbjct 301 | VADLRV++S LKGV VP +RAPSMIDEYP+LA+AA+FAEG TVM+GL+ELRVKESDRL+A VADLRVKASKLKGVVVPPERAPSMIDEYPVLAIAASFAEGETVMDGLDELRVKESDRLAA | 360 |
| Query 361 | VANGLKLNGVDCDEGETSLVVRGRPDGKGLGNASGAAVATHLDHRIAMSFLVMGLVSENP | 420 |
| Sbjct 361 | VA GL+ NGVDC EGE SL VRGR G G VATHLDHRIAMSFLVMGL SE P VARGLEANGVDCTEGEMSLTVRGR---PGGKGLGGGTVATHLDHRIAMSFLVMGLASEKP | 417 |
| Query 421  Sbjct 418 | VTVDDATMIATSFPEFMDLMAGLGAKIELS 450 VTVDD+TMIATSFPEFM +MAGLGAKI S VTVDDSTMIATSFPEFMGMMAGLGAKIAES 447 |  |

```
>WP_090296221.1 MULTISPECIES: 3-phosphoshikimate 1-carboxyvinyltransferase [unclassified Ensifer]
AIY27529.1 glyphosate resistant 5-enolpyruvylshikimate-3-phosphate synthase [uncultured bacterium]
MBD9592854.1 3-phosphoshikimate 1-carboxyvinyltransferase [Ensifer sp. ENS05] SDM25134.1 3-phosphoshikimate 1-carboxyvinyltransferase [Ensifer sp. YR511]
Length=448

Score = 702 bits (1811), Expect = 0.0, Method: Compositional matrix adjust. Identities = 354/447 (79%), Positives = 395/447 (88%), Gaps = 0/447 (0%)

Query 1	MLHGASSRPATARKSSGLSGTVRIPGDKSISHRSFMFGGLASGETRITGLLEGEDVINTG 60 M HG+ RPATARKS+ L G++RIPGDKSISHR+ M GGLASGETRITGLL+G DV+ TG
Sbjct 1	MSHGSGPRPATARKSADLRGSIRIPGDKSISHRALMLGGLASGETRITGLLQGADVLATG 60
```

| Query | 61 | KAMQAMGARIRKEGDTWIIDGVGNGGLLAPEAPLDFGNAATGCRLTMGLVGVYDFDSTFI | 120 |
| --- | --- | --- | --- |
| Sbjct | 61 | +AMQAMGARIRKE DT+I+DGVGNG LLAPEAPLDFGNA TGCRL MGL GVYDF++TF+ RAMQAMGARIRKEADTFIVDGVGNGALLAPEAPLDFGNAGTGCRLAMGLAGVYDFETTFL | 120 |
| Query | 121 | GDASLTKRPMGRVLNPLREMGVQVKSEDGDRLPVTLRGPKTPTPITYRVPMASAQVKSAV | 180 |
| Sbjct | 121 | GDASL++RPM RVL PLREMG QV++ GDRLPVTLRGPKTP PITYRVP+ASAQVKSAV GDASLSRRPMARVLEPLREMGTQVRAAAGDRLPVTLRGPKTPAPITYRVPVASAQVKSAV | 180 |
| Query | 181 | LLAGLNTPGITTVIEPIMTRDHTEKMLQGFGANLTVETDADGVRTIRLEGRGKLTGQVID | 240 |
| Sbjct | 181 | LLAGLN PGITTVIEP+MTRDHTE+ML+ FGA L+VETD GVRTIRL+GRGKLTGQ ID LLAGLNAPGITTVIEPVMTRDHTERMLEAFGAALSVETDVSGVRTIRLDGRGKLTGQEID | 240 |
| Query | 241 | VPGDPSSTAFPLVAALLVPGSDVTILNVLMNPTRTGLILTLQEMGADIEVINPRLAGGED | 300 |
| Sbjct | 241 | VPGDPSS AFPLVAAL+VPGSD+TI NVLMNPTRTGLILTL+EMG DIEVI R GGED VPGDPSSAAFPLVAALIVPGSDITITNVLMNPTRTGLILTLKEMGGDIEVIGSRQVGGED | 300 |
| Query | 301 | VADLRVRSSTLKGVTVPEDRAPSMIDEYPILavaaafaegaTVMNGLEELRVKESDRLSA | 360 |
| Sbjct | 301 | VADLRVR S L GVTVPE+RA S+IDEYP LA+AAAFAEG TVM GL+ELRVKESDRL+A VADLRVRHSELSGVTVPEERAASLIDEYPALAIAAAFAEGNTVMAGLDELRVKESDRLAA | 360 |
| Query | 361 | VANGLKLNGVDCDEGETSLVVRGRPDGKGLGNASGAAVATHLDHRIAMSFLVMGLVSENP | 420 |
| Sbjct | 361 | +A GLK NGVDC+EG +SLVVRGR DGKGLG+A+GA V THLDHRIAMSFL+ GL SE+P IAAGLKRNGVDCEEGASSLVVRGRRDGKGLGSAAGATVNTHLDHRIAMSFLIFGLASEHP | 420 |
| Query  Sbjct | 421  421 | VTVDDATMIATSFPEFMDLMAGLGAKI 447 VTVDD +M+ATSFPEFM LM LGA+I VTVDDVSMVATSFPEFMHLMTSLGARI 447 |  |

>AAL53098.1 3-phosphoshikimate 1-carboxyvinyltransferase [Brucella melitensis bv. 1 str. 16M]

Length=480

Score = 702 bits (1811), Expect = 0.0, Method: Compositional matrix adjust.

| Identities | = 362/447 (81%), Positives = 396/447 (89%), Gaps = 3/447 (1%) |  |
| --- | --- | --- |
| Query 1 | MLHGASSRPATARKSSGLSGTVRIPGDKSISHRSFMFGGLASGETRITGLLEGEDVINTG | 60 |
| Sbjct 31 | M H A +PATAR S L+G +RIPGDKSISHRSFMFGGLASG+TRITGLLEGEDVINTG MSHSACPKPATARHSQALTGEIRIPGDKSISHRSFMFGGLASGKTRITGLLEGEDVINTG | 90 |
| Query 61 | KAMQAMGARIRKEGDTWIIDGVGNGGLLAPEAPLDFGNAATGCRLTMGLVGVYDFDSTFI | 120 |
| Sbjct 91 | +AMQAMGARIRKEGD WII+GVGNG LL PEAPLDFGNA TG RLTMGLVG YD ++FI RAMQAMGARIRKEGDVWIINGVGNGCLLQPEAPLDFGNAGTGARLTMGLVGTYDMKTSFI | 150 |
| Query 121 | GDASLTKRPMGRVLNPLREMGVQVKSEDGDRLPVTLRGPKTPTPITYRVPMASAQVKSAV | 180 |
| Sbjct 151 | GDASL+KRPMGRVLNPLREMGVQV++ +GDR+P+TL GP+T PI YRVPMASAQVKSAV GDASLSKRPMGRVLNPLREMGVQVEAAEGDRMPLTLIGPRTANPIAYRVPMASAQVKSAV | 210 |
| Query 181 | LLAGLNTPGITTVIEPIMTRDHTEKMLQGFGANLTVETDADGVRTIRLEGRGKLTGQVID | 240 |
| Sbjct 211 | LLAGLNTPG+TTVIEP+MTRDHTEKMLQGFGA+LTVETD DGVR IR+ G+GKLTGQ ID LLAGLNTPGVTTVIEPVMTRDHTEKMLQGFGADLTVETDKDGVRHIRIVGQGKLTGQTID | 270 |
| Query 241 | VPGDPSSTAFPLVAALLVPGSDVTILNVLMNPTRTGLILTLQEMGADIEVINPRLAGGED | 300 |
| Sbjct 271 | VPGDPSSTAFPLVAALLV GSDVTI NVLMNPTRTGLILTLQEMGADIE+I+PRLAGGED VPGDPSSTAFPLVAALLVEGSDVTIRNVLMNPTRTGLILTLQEMGADIEIIDPRLAGGED | 330 |
| Query 301 | VADLRVRSSTLKGVTVPEDRAPSMIDEYPILavaaafaegaTVMNGLEELRVKESDRLSA | 360 |
| Sbjct 331 | VADLRV++S LKGV VP +RAPSMIDEYP+LA+AA+FAEG TVM+GL+ELRVKESDRL+A VADLRVKASKLKGVVVPPERAPSMIDEYPVLAIAASFAEGETVMDGLDELRVKESDRLAA | 390 |
| Query 361 | VANGLKLNGVDCDEGETSLVVRGRPDGKGLGNASGAAVATHLDHRIAMSFLVMGLVSENP | 420 |
| Sbjct 391 | VA GL+ NGVDC EGE SL VRGR G G V THLDHRIAMSFLVMGL SE P VARGLEANGVDCTEGEMSLTVRGR---PGGKGLGGGTVGTHLDHRIAMSFLVMGLASEKP | 447 |
| Query 421  Sbjct 448 | VTVDDATMIATSFPEFMDLMAGLGAKI 447 VTVDD+TMIATSFPEFM +MAGLGAKI VTVDDSTMIATSFPEFMGMMAGLGAKI 474 |  |

>WP_154718478.1 3-phosphoshikimate 1-carboxyvinyltransferase [Brucella melitensis] SPU62950.1 3-phosphoshikimate 1-carboxyvinyltransferase [Brucella melitensis]

Length=450

Score = 702 bits (1811), Expect = 0.0, Method: Compositional matrix adjust.

| Identities | = 363/450 (81%), Positives = 397/450 (88%), Gaps = 3/450 (1%) |  |
| --- | --- | --- |
| Query 1 | MLHGASSRPATARKSSGLSGTVRIPGDKSISHRSFMFGGLASGETRITGLLEGEDVINTG | 60 |
| Sbjct 1 | M H A +PATAR S L+G +RIPGDKSISHRSFMFGGLASG+TRITGLLEGEDVINTG MSHSACPKPATARHSQALTGEIRIPGDKSISHRSFMFGGLASGKTRITGLLEGEDVINTG | 60 |
| Query 61 | KAMQAMGARIRKEGDTWIIDGVGNGGLLAPEAPLDFGNAATGCRLTMGLVGVYDFDSTFI | 120 |
| Sbjct 61 | +AMQAMGARIRKEGD WII+GVGNG LL PEAPLDFGNA TG RLTMGLVG YD ++FI RAMQAMGARIRKEGDVWIINGVGNGCLLQPEAPLDFGNAGTGARLTMGLVGTYDMKTSFI | 120 |
| Query 121 | GDASLTKRPMGRVLNPLREMGVQVKSEDGDRLPVTLRGPKTPTPITYRVPMASAQVKSAV | 180 |
| Sbjct 121 | GDASL+KRPMGRVLNPLREMGVQV++ +GDR+P+TL GP+T PI YRVPMASAQVKSAV GDASLSKRPMGRVLNPLREMGVQVEAAEGDRMPLTLIGPRTANPIAYRVPMASAQVKSAV | 180 |
| Query 181 | LLAGLNTPGITTVIEPIMTRDHTEKMLQGFGANLTVETDADGVRTIRLEGRGKLTGQVID | 240 |
| Sbjct 181 | LLAGLNTPG+TTVIEP+MTRDHTE MLQGFGA+LTVETD DGVR IR+ G+GKLTGQ ID LLAGLNTPGVTTVIEPVMTRDHTENMLQGFGADLTVETDKDGVRHIRIVGQGKLTGQTID | 240 |
| Query 241 | VPGDPSSTAFPLVAALLVPGSDVTILNVLMNPTRTGLILTLQEMGADIEVINPRLAGGED | 300 |
| Sbjct 241 | VPGDPSSTAFPLVAALLV GSDVTI NVLMNPTRTGLILTLQEMGADIE+I+PRLAGGED VPGDPSSTAFPLVAALLVEGSDVTIRNVLMNPTRTGLILTLQEMGADIEIIDPRLAGGED | 300 |
| Query 301 | VADLRVRSSTLKGVTVPEDRAPSMIDEYPILavaaafaegaTVMNGLEELRVKESDRLSA | 360 |
| Sbjct 301 | VADLRV++S LKGV VP +RAPSMIDEYP+LA+AA+FAEG TVM+GL+ELRVKESDRL+A VADLRVKASKLKGVVVPPERAPSMIDEYPVLAIAASFAEGETVMDGLDELRVKESDRLAA | 360 |
| Query 361 | VANGLKLNGVDCDEGETSLVVRGRPDGKGLGNASGAAVATHLDHRIAMSFLVMGLVSENP | 420 |
| Sbjct 361 | VA GL+ NGVDC EGE SL VRGR G G VATHLDHRIAMSFLVMGL SE P VARGLEANGVDCTEGEMSLTVRGR---PGGKGLGGGTVATHLDHRIAMSFLVMGLASEKP | 417 |
| Query 421  Sbjct 418 | VTVDDATMIATSFPEFMDLMAGLGAKIELS 450 VTVDD+TMIATSFPEFM +MAGLGAKI S VTVDDSTMIATSFPEFMGMMAGLGAKIAES 447 |  |

>WP_106715944.1 3-phosphoshikimate 1-carboxyvinyltransferase [Phyllobacterium endophyticum]

MBB3235772.1 3-phosphoshikimate 1-carboxyvinyltransferase [Phyllobacterium endophyticum]

PSH59626.1 3-phosphoshikimate 1-carboxyvinyltransferase [Phyllobacterium endophyticum]

TYR41766.1 3-phosphoshikimate 1-carboxyvinyltransferase [Phyllobacterium endophyticum]

Length=452

Score = 702 bits (1811), Expect = 0.0, Method: Compositional matrix adjust.

| Identities | = 354/455 (78%), Positives = 395/455 (87%), Gaps = 3/455 (1%) |  |
| --- | --- | --- |
| Query 1 | MLHGASSRPATARKSSGLSGTVRIPGDKSISHRSFMFGGLASGETRITGLLEGEDVINTG | 60 |
| Sbjct 1 | M H A +PAT+R+S LSG VRIPGDKSISHRSFMFGGLASGETRITGLLEGEDVINTG MSHSALPKPATSRRSKALSGDVRIPGDKSISHRSFMFGGLASGETRITGLLEGEDVINTG | 60 |
| Query 61 | KAMQAMGARIRKEGDTWIIDGVGNGGLLAPEAPLDFGNAATGCRLTMGLVGVYDFDSTFI | 120 |
| Sbjct 61 | +AMQAMGA+I KEGD WII G GNG LL PE+PLDFGNA TG RLTMGLVG Y ++ F+ RAMQAMGAKIAKEGDIWIIQGTGNGCLLQPESPLDFGNAGTGARLTMGLVGTYAMETRFV | 120 |
| Query 121 | GDASLTKRPMGRVLNPLREMGVQVKSEDGDRLPVTLRGPKTPTPITYRVPMASAQVKSAV | 180 |
| Sbjct 121 | GDASL+KRPMGRVL+PLR+MGVQV + +GDR+P+TLRGPK PITYRVPMASAQVKSAV GDASLSKRPMGRVLDPLRQMGVQVSAAEGDRMPLTLRGPKVAAPITYRVPMASAQVKSAV | 180 |
| Query 181 | LLAGLNTPGITTVIEPIMTRDHTEKMLQGFGANLTVETDADGVRTIRLEGRGKLTGQVID | 240 |
| Sbjct 181 | LLAGLNTPGITTVIEP+MTRDHTEKML GFGANL+VETD +GVR IR+EG+GKLTGQ ID LLAGLNTPGITTVIEPVMTRDHTEKMLAGFGANLSVETDREGVRHIRIEGQGKLTGQTID | 240 |
| Query 241 | VPGDPSSTAFPLVAALLVPGSDVTILNVLMNPTRTGLILTLQEMGADIEVINPRLAGGED | 300 |
| Sbjct 241 | VPGDPSSTAFPLVAA++VPGSD+TI NVLMNPTRTGLILTLQEMGA+IE+++ RLAGGED VPGDPSSTAFPLVAAIIVPGSDITIRNVLMNPTRTGLILTLQEMGANIEIVDERLAGGED | 300 |
| Query 301 | VADLRVRSSTLKGVTVPEDRAPSMIDEYPILavaaafaegaTVMNGLEELRVKESDRLSA VADLRV++S LKGV VP DRAPSMIDEYP+LAVAA FA G TVMNGLEELRVKESDRL+A | 360 |

| Sbjct | 301 | VADLRVQASELKGVNVPADRAPSMIDEYPVLAVAACFATGETVMNGLEELRVKESDRLAA | 360 |
| --- | --- | --- | --- |
| Query | 361 | VANGLKLNGVDCDEGETSLVVRGRPDGKGLGNASGAAVATHLDHRIAMSFLVMGLVSENP | 420 |
| Sbjct | 361 | VA GLK NGVDC EG L VRGRPDGKG+G G V THLDHRIAMSFLV+GL ++ VAAGLKANGVDCTEGHDRLSVRGRPDGKGIG---GGRVETHLDHRIAMSFLVLGLAADKA | 417 |
| Query  Sbjct | 421  418 | VTVDDATMIATSFPEFMDLMAGLGAKIELSDTKAA 455 VT+DDA MIATSFPEFM +M G+GA+I D+ A VTIDDAAMIATSFPEFMGMMTGMGAEIAEFDSPVA 452 |  |

>ABQ60885.1 3-phosphoshikimate 1-carboxyvinyltransferase [Brucella ovis ATCC 25840]

Length=461

Score = 702 bits (1811), Expect = 0.0, Method: Compositional matrix adjust.

| Identities | = 362/447 (81%), Positives = 396/447 (89%), Gaps = 3/447 (1%) |  |
| --- | --- | --- |
| Query 1 | MLHGASSRPATARKSSGLSGTVRIPGDKSISHRSFMFGGLASGETRITGLLEGEDVINTG | 60 |
| Sbjct 12 | M H A +PATAR S L+G +RIPGDKSISHRSFMFGGLASG+TRITGLLEGEDVINTG MSHSACPKPATARHSQALTGEIRIPGDKSISHRSFMFGGLASGKTRITGLLEGEDVINTG | 71 |
| Query 61 | KAMQAMGARIRKEGDTWIIDGVGNGGLLAPEAPLDFGNAATGCRLTMGLVGVYDFDSTFI | 120 |
| Sbjct 72 | +AMQAMGARIRKEGD WII+GVGNG LL PEAPLDFGNA TG RLTMGLVG YD ++FI RAMQAMGARIRKEGDVWIINGVGNGCLLQPEAPLDFGNAGTGARLTMGLVGTYDMKTSFI | 131 |
| Query 121 | GDASLTKRPMGRVLNPLREMGVQVKSEDGDRLPVTLRGPKTPTPITYRVPMASAQVKSAV | 180 |
| Sbjct 132 | GDASL+KRPMGRVLNPLREMGVQV++ +GD +P+TL GP+T PI YRVPMASAQVKSAV GDASLSKRPMGRVLNPLREMGVQVEAAEGDWMPLTLIGPRTANPIAYRVPMASAQVKSAV | 191 |
| Query 181 | LLAGLNTPGITTVIEPIMTRDHTEKMLQGFGANLTVETDADGVRTIRLEGRGKLTGQVID | 240 |
| Sbjct 192 | LLAGLNTPG+TTVIEP+MTRDHTEKMLQGFGA+LTVETD DGVR IR+ G+GKLTGQ ID LLAGLNTPGVTTVIEPVMTRDHTEKMLQGFGADLTVETDKDGVRHIRIVGQGKLTGQTID | 251 |
| Query 241 | VPGDPSSTAFPLVAALLVPGSDVTILNVLMNPTRTGLILTLQEMGADIEVINPRLAGGED | 300 |
| Sbjct 252 | VPGDPSSTAFPLVAALLV GSDVTI NVLMNPTRTGLILTLQEMGADIE+I+PRLAGGED VPGDPSSTAFPLVAALLVEGSDVTIRNVLMNPTRTGLILTLQEMGADIEIIDPRLAGGED | 311 |
| Query 301 | VADLRVRSSTLKGVTVPEDRAPSMIDEYPILavaaafaegaTVMNGLEELRVKESDRLSA | 360 |
| Sbjct 312 | VADLRV++S LKGV VP +RAPSMIDEYP+LA+AA+FAEG TVM+GL+ELRVKESDRL+A VADLRVKASKLKGVVVPPERAPSMIDEYPVLAIAASFAEGETVMDGLDELRVKESDRLAA | 371 |
| Query 361 | VANGLKLNGVDCDEGETSLVVRGRPDGKGLGNASGAAVATHLDHRIAMSFLVMGLVSENP | 420 |
| Sbjct 372 | VA GL+ NGVDC EGE SL VRGR G G VATHLDHRIAMSFLVMGL SE P VARGLEANGVDCTEGEMSLTVRGR---PGGKGLGGGTVATHLDHRIAMSFLVMGLASEKP | 428 |
| Query 421  Sbjct 429 | VTVDDATMIATSFPEFMDLMAGLGAKI 447 VTVDD+TMIATSFPEFM +MAGLGAKI VTVDDSTMIATSFPEFMGMMAGLGAKI 455 |  |

>WP_174164335.1 3-phosphoshikimate 1-carboxyvinyltransferase [Agrobacterium rhizogenes] NTG50240.1 3-phosphoshikimate 1-carboxyvinyltransferase [Agrobacterium rhizogenes]

Length=453

Score = 701 bits (1810), Expect = 0.0, Method: Compositional matrix adjust.

| Identities | = 359/449 (80%), Positives = 394/449 (88%), Gaps = 1/449 (0%) |  |
| --- | --- | --- |
| Query 1 | MLHGASSRPATARKSSGLSGTVRIPGDKSISHRSFMFGGLASGETRITGLLEGEDVINTG | 60 |
| Sbjct 1 | M H SRPATAR+S+ L GT+R+PGDKSISHR+FMFGGLASGETRI+GLLEGEDV+NTG MPHDTHSRPATARRSTNLFGTIRVPGDKSISHRAFMFGGLASGETRISGLLEGEDVLNTG | 60 |
| Query 61 | KAMQAMGARIRKEGDTWIIDGVGNGGLLAPEAPLDFGNAATGCRLTMGLVGVYDFDSTFI | 120 |
| Sbjct 61 | KAMQAMGA+I K G WII GVGNG LLAP+APLDFGNA TG RLTMGLVGVY F+STFI KAMQAMGAKIEKIGSEWIIKGVGNGALLAPQAPLDFGNAGTGSRLTMGLVGVYHFESTFI | 120 |
| Query 121 | GDASLTKRPMGRVLNPLREMGVQVKSEDGDRLPVTLRGPKTPTPITYRVPMASAQVKSAV | 180 |
| Sbjct 121 | GDASL+KRPMGR+L+PLR+MGVQVKS +GDRLPVTL GP+T PITYRVPMASAQVKSAV GDASLSKRPMGRILDPLRQMGVQVKSSEGDRLPVTLHGPETANPITYRVPMASAQVKSAV | 180 |
| Query 181 | LLAGLNTPGITTVIEPIMTRDHTEKMLQGFGANLTVETDADGVRTIRLEGRGKLTGQVID LLAGLN PGITTVIEP+MTRDHTEKML GFGA L+VETD DGVR IRL+G+GKL+GQ ID | 240 |

| Sbjct | 181 | LLAGLNAPGITTVIEPVMTRDHTEKMLAGFGAALSVETDRDGVRIIRLQGQGKLSGQTID | 240 |
| --- | --- | --- | --- |
| Query | 241 | VPGDPSSTAFPLVAALLVPGSDVTILNVLMNPTRTGLILTLQEMGADIEVINPRLAGGED | 300 |
| Sbjct | 241 | VPGDPSSTAFPLVAAL+VPGSD+TI NVLMNP RTGLILTLQEMGADIE+++ RLAGGED VPGDPSSTAFPLVAALIVPGSDITIRNVLMNPARTGLILTLQEMGADIEILDKRLAGGED | 300 |
| Query | 301 | VADLRVRSSTLKGVTVPEDRAPSMIDEYPILavaaafaegaTVMNGLEELRVKESDRLSA | 360 |
| Sbjct | 301 | VADLRVRSS LKGVTVP +RAPSMIDEYP+LA AAAFAEG T+M GLEELRVKESDRLSA VADLRVRSSALKGVTVPAERAPSMIDEYPVLAAAAAFAEGTTLMLGLEELRVKESDRLSA | 360 |
| Query | 361 | VANGLKLNGVDCDEGETSLVVRGRPDGKGLGNASGAAVATHLDHRIAMSFLVMGLVSENP | 420 |
| Sbjct | 361 | V GL LNGVDC EGE SL VRGRP +G V THLDHRIAMSFLVMGL SE+P VTEGLTLNGVDCTEGEASLTVRGRPG-GKGLGNAGGEVKTHLDHRIAMSFLVMGLASEHP | 419 |
| Query  Sbjct | 421  420 | VTVDDATMIATSFPEFMDLMAGLGAKIEL 449 V VDD+TMIATSFPEF DLMA LGA +++ VRVDDSTMIATSFPEFFDLMAKLGADMDV 448 |  |

>WP_058324529.1 MULTISPECIES: 3-phosphoshikimate 1-carboxyvinyltransferase [Sinorhizobium/Ensifer group]

KSV86665.1 3-phosphoshikimate 1-carboxyvinyltransferase [Sinorhizobium sp. GL28]

PYF60316.1 3-phosphoshikimate 1-carboxyvinyltransferase [Ensifer sp. AP48] Length=448

Score = 701 bits (1810), Expect = 0.0, Method: Compositional matrix adjust.

| Identities | = 366/447 (82%), Positives = 403/447 (90%), Gaps = 0/447 (0%) |  |
| --- | --- | --- |
| Query 1 | MLHGASSRPATARKSSGLSGTVRIPGDKSISHRSFMFGGLASGETRITGLLEGEDVINTG | 60 |
| Sbjct 1 | M G PATARKS GL GTVRIPGDKSISHRS M GGLA+GETRITGLLEGEDVI TG MSQGLGQSPATARKSDGLKGTVRIPGDKSISHRSLMLGGLAAGETRITGLLEGEDVIATG | 60 |
| Query 61 | KAMQAMGARIRKEGDTWIIDGVGNGGLLAPEAPLDFGNAATGCRLTMGLVGVYDFDSTFI | 120 |
| Sbjct 61 | +AMQAMGARIRKEGDTWI++GVGNG LLAPEAPLDFGNA T CRL MGLVGVYDF++TFI RAMQAMGARIRKEGDTWIVNGVGNGALLAPEAPLDFGNAGTSCRLIMGLVGVYDFETTFI | 120 |
| Query 121 | GDASLTKRPMGRVLNPLREMGVQVKSEDGDRLPVTLRGPKTPTPITYRVPMASAQVKSAV | 180 |
| Sbjct 121 | GDASL++RPM RVL+PLREMGVQV++ GDRLPVTLRGPKTPTP+TYRVP+ASAQVKSAV GDASLSRRPMARVLDPLREMGVQVEAATGDRLPVTLRGPKTPTPVTYRVPVASAQVKSAV | 180 |
| Query 181 | LLAGLNTPGITTVIEPIMTRDHTEKMLQGFGANLTVETDADGVRTIRLEGRGKLTGQVID | 240 |
| Sbjct 181 | L AGLN PGITTVIEP +TRDHTE+ML+GFGA L+VETDA GVRTIRLEGRG+L Q ID LFAGLNAPGITTVIEPAITRDHTERMLRGFGAELSVETDATGVRTIRLEGRGRLVAQEID | 240 |
| Query 241 | VPGDPSSTAFPLVAALLVPGSDVTILNVLMNPTRTGLILTLQEMGADIEVINPRLAGGED | 300 |
| Sbjct 241 | VPGDPSSTAFPLVAAL+VPGSD+TI NVLMNPTRTGLILTLQEMG DIEVI+ R GGED VPGDPSSTAFPLVAALVVPGSDITIRNVLMNPTRTGLILTLQEMGGDIEVIDIRQVGGED | 300 |
| Query 301 | VADLRVRSSTLKGVTVPEDRAPSMIDEYPILavaaafaegaTVMNGLEELRVKESDRLSA | 360 |
| Sbjct 301 | VADLRVR S LKGVTVPE+RA SMIDEYPILA+AAAFAEGAT+M+GL+ELRVKESDRL+A VADLRVRHSELKGVTVPEERAASMIDEYPILAIAAAFAEGATIMHGLDELRVKESDRLAA | 360 |
| Query 361 | VANGLKLNGVDCDEGETSLVVRGRPDGKGLGNASGAAVATHLDHRIAMSFLVMGLVSENP | 420 |
| Sbjct 361 | +A GLKLNGVDC+EG +SLVVRGR GKGLGNASG V THLDHRIAMSFLV+GL +E+P IAAGLKLNGVDCEEGRSSLVVRGRRGGKGLGNASGGVVKTHLDHRIAMSFLVLGLAAEHP | 420 |
| Query 421  Sbjct 421 | VTVDDATMIATSFPEFMDLMAGLGAKI 447 V VDD+TMIATSFP ++DLMAGLGA+I VKVDDSTMIATSFPGYIDLMAGLGARI 447 |  |

>QOK51506.1 3-phosphoshikimate 1-carboxyvinyltransferase [Brucella suis bv. 2]

QOK72011.1 3-phosphoshikimate 1-carboxyvinyltransferase [Brucella suis bv. 2]

Length=450

Score = 701 bits (1810), Expect = 0.0, Method: Compositional matrix adjust. Identities = 362/447 (81%), Positives = 396/447 (89%), Gaps = 3/447 (1%)

Query 1	MLHGASSRPATARKSSGLSGTVRIPGDKSISHRSFMFGGLASGETRITGLLEGEDVINTG 60

| Sbjct | 1 | M H A +PATAR S L+G +RIPGDKSISHRSFMFGGLASG+TRITGLLEGEDVINTG MSHSACPKPATARHSQALTGEIRIPGDKSISHRSFMFGGLASGKTRITGLLEGEDVINTG | 60 |
| --- | --- | --- | --- |
| Query | 61 | KAMQAMGARIRKEGDTWIIDGVGNGGLLAPEAPLDFGNAATGCRLTMGLVGVYDFDSTFI | 120 |
| Sbjct | 61 | +AMQAMGARIRKEGD WII+GVGNG LL PEAPLDFGNA TG RLTMGLVG YD ++FI RAMQAMGARIRKEGDVWIINGVGNGCLLQPEAPLDFGNAGTGARLTMGLVGTYDMKTSFI | 120 |
| Query | 121 | GDASLTKRPMGRVLNPLREMGVQVKSEDGDRLPVTLRGPKTPTPITYRVPMASAQVKSAV | 180 |
| Sbjct | 121 | GDASL+KRPMGRVLNPLREMGVQV++ +GDR+P+TL GP+T PI YRVPMASAQVKSAV GDASLSKRPMGRVLNPLREMGVQVEAAEGDRMPLTLIGPRTANPIAYRVPMASAQVKSAV | 180 |
| Query | 181 | LLAGLNTPGITTVIEPIMTRDHTEKMLQGFGANLTVETDADGVRTIRLEGRGKLTGQVID | 240 |
| Sbjct | 181 | LLAGLNTPG+TTVIEP+MTRDHTEKMLQGFGA+LTVETD DGVR IR+ G+GKL GQ ID LLAGLNTPGVTTVIEPVMTRDHTEKMLQGFGADLTVETDKDGVRHIRIVGQGKLIGQTID | 240 |
| Query | 241 | VPGDPSSTAFPLVAALLVPGSDVTILNVLMNPTRTGLILTLQEMGADIEVINPRLAGGED | 300 |
| Sbjct | 241 | VPGDPSSTAFPLVAALLV GSDVTI NVLMNPTRTGLILTLQEMGADIE+I+PRLAGGED VPGDPSSTAFPLVAALLVEGSDVTIRNVLMNPTRTGLILTLQEMGADIEIIDPRLAGGED | 300 |
| Query | 301 | VADLRVRSSTLKGVTVPEDRAPSMIDEYPILavaaafaegaTVMNGLEELRVKESDRLSA | 360 |
| Sbjct | 301 | VADLRV++S LKGV VP +RAPSMIDEYP+LA+AA+FAEG TVM+GL+ELRVKESDRL+A VADLRVKASKLKGVVVPPERAPSMIDEYPVLAIAASFAEGETVMDGLDELRVKESDRLAA | 360 |
| Query | 361 | VANGLKLNGVDCDEGETSLVVRGRPDGKGLGNASGAAVATHLDHRIAMSFLVMGLVSENP | 420 |
| Sbjct | 361 | VA GL+ NGVDC EGE SL VRGR G G VATHLDHRIAMSFLVMGL SE P VARGLEANGVDCTEGEMSLTVRGR---PGGKGLGGGTVATHLDHRIAMSFLVMGLASEKP | 417 |
| Query  Sbjct | 421  418 | VTVDDATMIATSFPEFMDLMAGLGAKI 447 VTVDD+TMIATSFPEFM +MAGLGAKI VTVDDSTMIATSFPEFMGMMAGLGAKI 444 |  |

>WP_117346186.1 3-phosphoshikimate 1-carboxyvinyltransferase [Brucella melitensis] RFZ73763.1 3-phosphoshikimate 1-carboxyvinyltransferase [Brucella melitensis]

Length=450

Score = 701 bits (1809), Expect = 0.0, Method: Compositional matrix adjust.

| Identities | = 362/447 (81%), Positives = 396/447 (89%), Gaps = 3/447 (1%) |  |
| --- | --- | --- |
| Query 1 | MLHGASSRPATARKSSGLSGTVRIPGDKSISHRSFMFGGLASGETRITGLLEGEDVINTG | 60 |
| Sbjct 1 | M H A +PATAR S L+G +RIPGDKSISHRSFMFGGLASG+TRITGLLEGEDVINTG MSHSACPKPATARHSQALTGEIRIPGDKSISHRSFMFGGLASGKTRITGLLEGEDVINTG | 60 |
| Query 61 | KAMQAMGARIRKEGDTWIIDGVGNGGLLAPEAPLDFGNAATGCRLTMGLVGVYDFDSTFI | 120 |
| Sbjct 61 | +AMQAMGARIRKEGD WII+GVGNG LL PEAPLDFGNA TG RLTMGLVG YD ++FI RAMQAMGARIRKEGDVWIINGVGNGCLLQPEAPLDFGNAGTGARLTMGLVGTYDMKTSFI | 120 |
| Query 121 | GDASLTKRPMGRVLNPLREMGVQVKSEDGDRLPVTLRGPKTPTPITYRVPMASAQVKSAV | 180 |
| Sbjct 121 | GDASL+KRPMGRVLNPLREMGVQV++ +GDR+P+TL GP+T P YRVPMASAQVKSAV GDASLSKRPMGRVLNPLREMGVQVEAAEGDRMPLTLIGPRTANPSAYRVPMASAQVKSAV | 180 |
| Query 181 | LLAGLNTPGITTVIEPIMTRDHTEKMLQGFGANLTVETDADGVRTIRLEGRGKLTGQVID | 240 |
| Sbjct 181 | LLAGLNTPG+TTVIEP+MTRDHTEKMLQGFGA+LTVETD DGVR IR+ G+GKLTGQ ID LLAGLNTPGVTTVIEPVMTRDHTEKMLQGFGADLTVETDKDGVRHIRIVGQGKLTGQTID | 240 |
| Query 241 | VPGDPSSTAFPLVAALLVPGSDVTILNVLMNPTRTGLILTLQEMGADIEVINPRLAGGED | 300 |
| Sbjct 241 | VPGDPSSTAFPLVAALLV GSDVTI NVLMNPTRTGLILTLQEMGADIE+I+PRLAGGED VPGDPSSTAFPLVAALLVEGSDVTIRNVLMNPTRTGLILTLQEMGADIEIIDPRLAGGED | 300 |
| Query 301 | VADLRVRSSTLKGVTVPEDRAPSMIDEYPILavaaafaegaTVMNGLEELRVKESDRLSA | 360 |
| Sbjct 301 | VADLRV++S LKGV VP +RAPSMIDEYP+LA+AA+FAEG TVM+GL+ELRVKESDRL+A VADLRVKASKLKGVVVPPERAPSMIDEYPVLAIAASFAEGETVMDGLDELRVKESDRLAA | 360 |
| Query 361 | VANGLKLNGVDCDEGETSLVVRGRPDGKGLGNASGAAVATHLDHRIAMSFLVMGLVSENP | 420 |
| Sbjct 361 | VA GL+ NGVDC EGE SL VRGR G G VATHLDHRIAMSFLVMGL SE P VARGLEANGVDCTEGEMSLTVRGR---PGGKGLGGGTVATHLDHRIAMSFLVMGLASEKP | 417 |
| Query 421  Sbjct 418 | VTVDDATMIATSFPEFMDLMAGLGAKI 447 VTVDD+TMIATSFPEFM +MAGLGAKI VTVDDSTMIATSFPEFMGMMAGLGAKI 444 |  |

>WP_006206089.1 3-phosphoshikimate 1-carboxyvinyltransferase [Brucella suis] ENT64172.1 3-phosphoshikimate 1-carboxyvinyltransferase [Brucella suis F8/06-3]

Length=450

Score = 701 bits (1809), Expect = 0.0, Method: Compositional matrix adjust.

| Identities | = 362/447 (81%), Positives = 396/447 (89%), Gaps = 3/447 (1%) |  |
| --- | --- | --- |
| Query 1 | MLHGASSRPATARKSSGLSGTVRIPGDKSISHRSFMFGGLASGETRITGLLEGEDVINTG | 60 |
| Sbjct 1 | M H A +PATAR S L+G +RIPGDKSISHRSFMFGGLASG+TRITGLLEGEDVINTG MSHSACPKPATARHSQALTGEIRIPGDKSISHRSFMFGGLASGKTRITGLLEGEDVINTG | 60 |
| Query 61 | KAMQAMGARIRKEGDTWIIDGVGNGGLLAPEAPLDFGNAATGCRLTMGLVGVYDFDSTFI | 120 |
| Sbjct 61 | +AMQAMGARIRKEGD WII+GVGNG LL PEAPLDFGNA TG RLTMGLVG YD ++FI RAMQAMGARIRKEGDVWIINGVGNGCLLQPEAPLDFGNAGTGARLTMGLVGTYDMKTSFI | 120 |
| Query 121 | GDASLTKRPMGRVLNPLREMGVQVKSEDGDRLPVTLRGPKTPTPITYRVPMASAQVKSAV | 180 |
| Sbjct 121 | GDASL+KRPMGRVLNPLREMGVQV++ +GDR+P+TL GP+T PI YRVPMASAQVKSAV GDASLSKRPMGRVLNPLREMGVQVEAAEGDRMPLTLIGPRTANPIAYRVPMASAQVKSAV | 180 |
| Query 181 | LLAGLNTPGITTVIEPIMTRDHTEKMLQGFGANLTVETDADGVRTIRLEGRGKLTGQVID | 240 |
| Sbjct 181 | LLAGLNTPG+TTVIEP+MTRDHTEKMLQGFGA+LTVETD DGVR IR+ G+GKLTGQ ID LLAGLNTPGVTTVIEPVMTRDHTEKMLQGFGADLTVETDKDGVRHIRIVGQGKLTGQTID | 240 |
| Query 241 | VPGDPSSTAFPLVAALLVPGSDVTILNVLMNPTRTGLILTLQEMGADIEVINPRLAGGED | 300 |
| Sbjct 241 | VPGDPSSTAFPLVAALLV GSDVTI NVLMNPTRTGLILTLQEMGADIE+I+PRLAGGED VPGDPSSTAFPLVAALLVEGSDVTIRNVLMNPTRTGLILTLQEMGADIEIIDPRLAGGED | 300 |
| Query 301 | VADLRVRSSTLKGVTVPEDRAPSMIDEYPILavaaafaegaTVMNGLEELRVKESDRLSA | 360 |
| Sbjct 301 | VADLRV++S LKGV VP +RAPSMIDEYP+LA+AA+FAEG TVM+GL+ELRVKESDRL+A VADLRVKASKLKGVVVPPERAPSMIDEYPVLAIAASFAEGETVMDGLDELRVKESDRLAA | 360 |
| Query 361 | VANGLKLNGVDCDEGETSLVVRGRPDGKGLGNASGAAVATHLDHRIAMSFLVMGLVSENP | 420 |
| Sbjct 361 | VA GL+ NGVDC EGE SL VRGR G G VATHLDHRIA SFLVMGL SE P VARGLEANGVDCTEGEMSLTVRGR---PGGKGLGGGTVATHLDHRIAKSFLVMGLASEKP | 417 |
| Query 421  Sbjct 418 | VTVDDATMIATSFPEFMDLMAGLGAKI 447 VTVDD+TMIATSFPEFM +MAGLGAKI VTVDDSTMIATSFPEFMGMMAGLGAKI 444 |  |

>WP_006014665.1 3-phosphoshikimate 1-carboxyvinyltransferase [Brucella ovis] ENR02868.1 3-phosphoshikimate 1-carboxyvinyltransferase [Brucella ovis 80/125] ENR07187.1 3-phosphoshikimate 1-carboxyvinyltransferase [Brucella ovis F8/05B] ENS97487.1 3-phosphoshikimate 1-carboxyvinyltransferase [Brucella ovis 63/96] ENT01774.1 3-phosphoshikimate 1-carboxyvinyltransferase [Brucella ovis 81/8]

ENT77180.1 3-phosphoshikimate 1-carboxyvinyltransferase [Brucella ovis IntaBari-2009-88-4] Length=450

Score = 701 bits (1809), Expect = 0.0, Method: Compositional matrix adjust.

| Identities | = 363/450 (81%), Positives = 397/450 (88%), Gaps = 3/450 (1%) |  |
| --- | --- | --- |
| Query 1 | MLHGASSRPATARKSSGLSGTVRIPGDKSISHRSFMFGGLASGETRITGLLEGEDVINTG | 60 |
| Sbjct 1 | M H A +PATAR S L+G +RIPGDKSISHRSFMFGGLASG+TRITGLLEGEDVINTG MSHSACPKPATARHSQALTGEIRIPGDKSISHRSFMFGGLASGKTRITGLLEGEDVINTG | 60 |
| Query 61 | KAMQAMGARIRKEGDTWIIDGVGNGGLLAPEAPLDFGNAATGCRLTMGLVGVYDFDSTFI | 120 |
| Sbjct 61 | +AMQAMGARIRKEGD WII+GVGNG LL PEAPLDFGNA TG RLTMGLVG YD ++FI RAMQAMGARIRKEGDVWIINGVGNGCLLQPEAPLDFGNAGTGARLTMGLVGTYDMKTSFI | 120 |
| Query 121 | GDASLTKRPMGRVLNPLREMGVQVKSEDGDRLPVTLRGPKTPTPITYRVPMASAQVKSAV | 180 |
| Sbjct 121 | GDASL+KRPMGRVLNPLREMGVQV++ +GD +P+TL GP+T PI YRVPMASAQVKSAV GDASLSKRPMGRVLNPLREMGVQVEAAEGDWMPLTLIGPRTANPIAYRVPMASAQVKSAV | 180 |
| Query 181 | LLAGLNTPGITTVIEPIMTRDHTEKMLQGFGANLTVETDADGVRTIRLEGRGKLTGQVID | 240 |
| Sbjct 181 | LLAGLNTPG+TTVIEP+MTRDHTEKMLQGFGA+LTVETD DGVR IR+ G+GKLTGQ ID LLAGLNTPGVTTVIEPVMTRDHTEKMLQGFGADLTVETDKDGVRHIRIVGQGKLTGQTID | 240 |
| Query 241 | VPGDPSSTAFPLVAALLVPGSDVTILNVLMNPTRTGLILTLQEMGADIEVINPRLAGGED | 300 |
| Sbjct 241 | VPGDPSSTAFPLVAALLV GSDVTI NVLMNPTRTGLILTLQEMGADIE+I+PRLAGGED VPGDPSSTAFPLVAALLVEGSDVTIRNVLMNPTRTGLILTLQEMGADIEIIDPRLAGGED | 300 |
| Query 301 | VADLRVRSSTLKGVTVPEDRAPSMIDEYPILavaaafaegaTVMNGLEELRVKESDRLSA | 360 |

| Sbjct | 301 | VADLRV++S LKGV VP +RAPSMIDEYP+LA+AA+FAEG TVM+GL+ELRVKESDRL+A VADLRVKASKLKGVVVPPERAPSMIDEYPVLAIAASFAEGETVMDGLDELRVKESDRLAA | 360 |
| --- | --- | --- | --- |
| Query | 361 | VANGLKLNGVDCDEGETSLVVRGRPDGKGLGNASGAAVATHLDHRIAMSFLVMGLVSENP | 420 |
| Sbjct | 361 | VA GL+ NGVDC EGE SL VRGR G G VATHLDHRIAMSFLVMGL SE P VARGLEANGVDCTEGEMSLTVRGR---PGGKGLGGGTVATHLDHRIAMSFLVMGLASEKP | 417 |
| Query  Sbjct | 421  418 | VTVDDATMIATSFPEFMDLMAGLGAKIELS 450 VTVDD+TMIATSFPEFM +MAGLGAKI S VTVDDSTMIATSFPEFMGMMAGLGAKIAES 447 |  |

>WP_002968066.1 3-phosphoshikimate 1-carboxyvinyltransferase [Brucella abortus] AIJ55523.1 3-phosphoshikimate 1-carboxyvinyltransferase [Brucella abortus] AIJ77387.1 3-phosphoshikimate 1-carboxyvinyltransferase [Brucella abortus]

EEX83624.1 5-enolpyruvylshikimate-3-phosphate synthase [Brucella abortus bv. 3 str. Tulya]

ENP42052.1 3-phosphoshikimate 1-carboxyvinyltransferase [Brucella abortus 78/36]

ENR92002.1 3-phosphoshikimate 1-carboxyvinyltransferase [Brucella abortus 80/101]

Length=450

Score = 701 bits (1808), Expect = 0.0, Method: Compositional matrix adjust.

| Identities | = 361/447 (81%), Positives = 396/447 (89%), Gaps = 3/447 (1%) |  |
| --- | --- | --- |
| Query 1 | MLHGASSRPATARKSSGLSGTVRIPGDKSISHRSFMFGGLASGETRITGLLEGEDVINTG | 60 |
| Sbjct 1 | M H A +PATAR S L+G +RIPGDKSISHRSFMFGGLASG+TRITGLLEGEDVINTG MSHSACPKPATARHSQALTGEIRIPGDKSISHRSFMFGGLASGKTRITGLLEGEDVINTG | 60 |
| Query 61 | KAMQAMGARIRKEGDTWIIDGVGNGGLLAPEAPLDFGNAATGCRLTMGLVGVYDFDSTFI | 120 |
| Sbjct 61 | +AMQAMGARIRKEGD WII+GVGNG LL PEAPLDFGNA TG RLTMGLVG YD ++FI RAMQAMGARIRKEGDVWIINGVGNGCLLQPEAPLDFGNAGTGARLTMGLVGTYDMKTSFI | 120 |
| Query 121 | GDASLTKRPMGRVLNPLREMGVQVKSEDGDRLPVTLRGPKTPTPITYRVPMASAQVKSAV | 180 |
| Sbjct 121 | GDASL+KRPMGRVLNPLREMGVQV++ +GDR+P+TL GP+T PI YRVPMASAQVKSAV GDASLSKRPMGRVLNPLREMGVQVEAAEGDRMPLTLIGPRTANPIAYRVPMASAQVKSAV | 180 |
| Query 181 | LLAGLNTPGITTVIEPIMTRDHTEKMLQGFGANLTVETDADGVRTIRLEGRGKLTGQVID | 240 |
| Sbjct 181 | LLAGLNTPG+TTVIEP+MTRDHTEKMLQGFGA+LTVETD DGVR IR+ G+GKLTGQ ID LLAGLNTPGVTTVIEPVMTRDHTEKMLQGFGADLTVETDKDGVRHIRIVGQGKLTGQTID | 240 |
| Query 241 | VPGDPSSTAFPLVAALLVPGSDVTILNVLMNPTRTGLILTLQEMGADIEVINPRLAGGED | 300 |
| Sbjct 241 | VPGDPSSTAFPLVAALLV GS+VTI NVLMNPTRTGLILTLQEMGADIE+I+P LAGGED VPGDPSSTAFPLVAALLVEGSEVTIRNVLMNPTRTGLILTLQEMGADIEIIDPHLAGGED | 300 |
| Query 301 | VADLRVRSSTLKGVTVPEDRAPSMIDEYPILavaaafaegaTVMNGLEELRVKESDRLSA | 360 |
| Sbjct 301 | VADLRV++S LKGV VP +RAPSMIDEYP+LA+AA+FAEG TVM+GL+ELRVKESDRL+A VADLRVKASKLKGVVVPPERAPSMIDEYPVLAIAASFAEGETVMDGLDELRVKESDRLAA | 360 |
| Query 361 | VANGLKLNGVDCDEGETSLVVRGRPDGKGLGNASGAAVATHLDHRIAMSFLVMGLVSENP | 420 |
| Sbjct 361 | VA GL+ NGVDC EGE SL VRGR G G VATHLDHRIAMSFLVMGL SE P VARGLEANGVDCTEGEMSLTVRGR---PGGKGLGGGTVATHLDHRIAMSFLVMGLASEKP | 417 |
| Query 421  Sbjct 418 | VTVDDATMIATSFPEFMDLMAGLGAKI 447 VTVDD+TMIATSFPEFM +MAGLGAKI VTVDDSTMIATSFPEFMGMMAGLGAKI 444 |  |

>WP_145324223.1 MULTISPECIES: 3-phosphoshikimate 1-carboxyvinyltransferase [unclassified Rhizobium]

TWB45631.1 3-phosphoshikimate 1-carboxyvinyltransferase [Rhizobium sp. ERR 922]

TWB88510.1 3-phosphoshikimate 1-carboxyvinyltransferase [Rhizobium sp. ERR 942]

Length=449

Score = 701 bits (1808), Expect = 0.0, Method: Compositional matrix adjust. Identities = 352/448 (79%), Positives = 393/448 (88%), Gaps = 0/448 (0%)

Query 1	MLHGASSRPATARKSSGLSGTVRIPGDKSISHRSFMFGGLASGETRITGLLEGEDVINTG 60 M H	+PA AR+S+GLSGT+RIPGDKSISHRS MFGGLA+GETRITGLLEGEDV+NTG

| Sbjct | 1 | MPHDTLPQPAVARRSAGLSGTIRIPGDKSISHRSLMFGGLAAGETRITGLLEGEDVLNTG | 60 |
| --- | --- | --- | --- |
| Query | 61 | KAMQAMGARIRKEGDTWIIDGVGNGGLLAPEAPLDFGNAATGCRLTMGLVGVYDFDSTFI | 120 |
| Sbjct | 61 | KAMQA+GA IRK D WI+ G GNGGLLAPE PLDFGNA TGCRLTMGLVG YDF +TF+ KAMQALGATIRKIDDEWIVRGTGNGGLLAPETPLDFGNAGTGCRLTMGLVGSYDFKTTFV | 120 |
| Query | 121 | GDASLTKRPMGRVLNPLREMGVQVKSEDGDRLPVTLRGPKTPTPITYRVPMASAQVKSAV | 180 |
| Sbjct | 121 | GDASL+KRPMGR+L+PLR MG Q+ + GDRLPV+L GP +P+TYRVPMASAQVKSAV GDASLSKRPMGRILDPLRRMGTQISAATGDRLPVSLHGPGVASPLTYRVPMASAQVKSAV | 180 |
| Query | 181 | LLAGLNTPGITTVIEPIMTRDHTEKMLQGFGANLTVETDADGVRTIRLEGRGKLTGQVID | 240 |
| Sbjct | 181 | LLAGLN PG+TTVIEP+MTRDHTEKML GFGA L+VE+DA GVRTIRLEG+GKLTGQ ID LLAGLNAPGMTTVIEPVMTRDHTEKMLAGFGAALSVESDAAGVRTIRLEGQGKLTGQTID | 240 |
| Query | 241 | VPGDPSSTAFPLVAALLVPGSDVTILNVLMNPTRTGLILTLQEMGADIEVINPRLAGGED | 300 |
| Sbjct | 241 | VPGDPSS AFPLVAAL+VPGSD+TI NVLMNPTRTGLILTLQEMGADIE+++ R+ GGED VPGDPSSAAFPLVAALIVPGSDITIRNVLMNPTRTGLILTLQEMGADIEMLDRRMTGGED | 300 |
| Query | 301 | VADLRVRSSTLKGVTVPEDRAPSMIDEYPILavaaafaegaTVMNGLEELRVKESDRLSA | 360 |
| Sbjct | 301 | VADLRVRSS LKGVTVP +RAPSMIDEYP+LAVAA FAEG T+M GL+ELRVKESDRLSA VADLRVRSSALKGVTVPAERAPSMIDEYPVLAVAATFAEGTTIMQGLDELRVKESDRLSA | 360 |
| Query | 361 | VANGLKLNGVDCDEGETSLVVRGRPDGKGLGNASGAAVATHLDHRIAMSFLVMGLVSENP | 420 |
| Sbjct | 361 | VA GLKLNGVDC EG L V+GRPDGKGLGNA GA V+THLDHRIAMSFLVMGL SE+P VAEGLKLNGVDCIEGIDMLTVQGRPDGKGLGNAGGAKVSTHLDHRIAMSFLVMGLASEHP | 420 |
| Query  Sbjct | 421  421 | VTVDDATMIATSFPEFMDLMAGLGAKIE 448 VT+DD+ MIATSFP+F ++ LGA IE VTIDDSAMIATSFPDFFAMITRLGATIE 448 |  |

>WP_127664702.1 3-phosphoshikimate 1-carboxyvinyltransferase, partial [Sinorhizobium meliloti]

RVK01776.1 3-phosphoshikimate 1-carboxyvinyltransferase, partial [Sinorhizobium meliloti]

RVL45423.1 3-phosphoshikimate 1-carboxyvinyltransferase, partial [Sinorhizobium meliloti]

Length=387

Score = 700 bits (1807), Expect = 0.0, Method: Compositional matrix adjust.

| Identities | = 357/387 (92%), Positives = 372/387 (96%), Gaps = 0/387 (0%) |  |
| --- | --- | --- |
| Query 1 | MLHGASSRPATARKSSGLSGTVRIPGDKSISHRSFMFGGLASGETRITGLLEGEDVINTG | 60 |
| Sbjct 1 | M HG++ RPATARKSS L GT+RIPGDKSISHRSFMFGGLA+GETRITGLLEGEDVINTG MSHGSNPRPATARKSSDLKGTLRIPGDKSISHRSFMFGGLAAGETRITGLLEGEDVINTG | 60 |
| Query 61 | KAMQAMGARIRKEGDTWIIDGVGNGGLLAPEAPLDFGNAATGCRLTMGLVGVYDFDSTFI | 120 |
| Sbjct 61 | KAMQAMGARIRKEGDTWIIDGVGNG LLAPEAPLDFGNA TGCRLTMGLVGVYDFDSTFI KAMQAMGARIRKEGDTWIIDGVGNGALLAPEAPLDFGNAGTGCRLTMGLVGVYDFDSTFI | 120 |
| Query 121 | GDASLTKRPMGRVLNPLREMGVQVKSEDGDRLPVTLRGPKTPTPITYRVPMASAQVKSAV | 180 |
| Sbjct 121 | GDASLTKRPMGRVL+PLREMGVQVKS +GDRLPVTLRGPKTP PITYRVPMASAQVKSAV GDASLTKRPMGRVLDPLREMGVQVKSAEGDRLPVTLRGPKTPNPITYRVPMASAQVKSAV | 180 |
| Query 181 | LLAGLNTPGITTVIEPIMTRDHTEKMLQGFGANLTVETDADGVRTIRLEGRGKLTGQVID | 240 |
| Sbjct 181 | LLAGLNTPGITTV+EP+MTRDHTEKMLQGFGANLTVETDA+GVRTIRLEGRGKLTGQVID LLAGLNTPGITTVVEPVMTRDHTEKMLQGFGANLTVETDAEGVRTIRLEGRGKLTGQVID | 240 |
| Query 241 | VPGDPSSTAFPLVAALLVPGSDVTILNVLMNPTRTGLILTLQEMGADIEVINPRLAGGED | 300 |
| Sbjct 241 | VPGDPSSTAFPLVA L+VPGSD+TILNVLMNPTRTGLILTLQEMGA+IEV+N RLAGGED VPGDPSSTAFPLVAGLIVPGSDITILNVLMNPTRTGLILTLQEMGANIEVMNKRLAGGED | 300 |
| Query 301 | VADLRVRSSTLKGVTVPEDRAPSMIDEYPILavaaafaegaTVMNGLEELRVKESDRLSA | 360 |
| Sbjct 301 | VADLRVR S LKGVTVPEDRAPSMIDEYP+LAVAAAFAEG TVMNGLEELRVKESDRLSA VADLRVRHSELKGVTVPEDRAPSMIDEYPVLAVAAAFAEGTTVMNGLEELRVKESDRLSA | 360 |
| Query 361  Sbjct 361 | VANGLKLNGVDCDEGETSLVVRGRPDG 387 VA+GLKLNGVDCDEGE SLVVRGRP G VADGLKLNGVDCDEGEASLVVRGRPGG 387 |  |

>WP_034804733.1 MULTISPECIES: 3-phosphoshikimate 1-carboxyvinyltransferase [Ensifer]

ANK74483.1 3-phosphoshikimate 1-carboxyvinyltransferase [Ensifer adhaerens] KDP70738.1 3-phosphoshikimate 1-carboxyvinyltransferase [Ensifer adhaerens] KQX04738.1 3-phosphoshikimate 1-carboxyvinyltransferase [Ensifer sp. Root423] KQZ51273.1 3-phosphoshikimate 1-carboxyvinyltransferase [Ensifer sp. Root558] MBD9492444.1 3-phosphoshikimate 1-carboxyvinyltransferase [Ensifer sp. ENS01]

Length=448

Score = 700 bits (1807), Expect = 0.0, Method: Compositional matrix adjust.

| Identities | = 354/447 (79%), Positives = 394/447 (88%), Gaps = 0/447 (0%) |  |
| --- | --- | --- |
| Query 1 | MLHGASSRPATARKSSGLSGTVRIPGDKSISHRSFMFGGLASGETRITGLLEGEDVINTG | 60 |
| Sbjct 1 | M HG+ RPATARKS+ L G++RIPGDKSISHR+ M GGLASGETRITGLL+G DV+ TG MSHGSGPRPATARKSADLRGSIRIPGDKSISHRALMLGGLASGETRITGLLQGADVLATG | 60 |
| Query 61 | KAMQAMGARIRKEGDTWIIDGVGNGGLLAPEAPLDFGNAATGCRLTMGLVGVYDFDSTFI | 120 |
| Sbjct 61 | +AMQAMGARIRKE DT+I+DGVGNG LLAPEAPLDFGNA TGCRL MGL GVYDF++TF+ RAMQAMGARIRKEADTFIVDGVGNGALLAPEAPLDFGNAGTGCRLAMGLAGVYDFETTFL | 120 |
| Query 121 | GDASLTKRPMGRVLNPLREMGVQVKSEDGDRLPVTLRGPKTPTPITYRVPMASAQVKSAV | 180 |
| Sbjct 121 | GDASL +RPM RVL PLREMG QV++ GDRLPVTLRGPKTP PITYRVP+ASAQVKSA+ GDASLGRRPMARVLEPLREMGTQVRAAAGDRLPVTLRGPKTPAPITYRVPVASAQVKSAL | 180 |
| Query 181 | LLAGLNTPGITTVIEPIMTRDHTEKMLQGFGANLTVETDADGVRTIRLEGRGKLTGQVID | 240 |
| Sbjct 181 | LLAGLN PGITTVIEP+MTRDHTE+ML+ FGA L+VETD GVRTIRL+GRGKLTGQ ID LLAGLNAPGITTVIEPVMTRDHTERMLEAFGAALSVETDVSGVRTIRLDGRGKLTGQEID | 240 |
| Query 241 | VPGDPSSTAFPLVAALLVPGSDVTILNVLMNPTRTGLILTLQEMGADIEVINPRLAGGED | 300 |
| Sbjct 241 | VPGDPSS AFPLVAAL+VPGSD+TI NVLMNPTRTGLILTL+EMG DIEVI R GGED VPGDPSSAAFPLVAALIVPGSDITITNVLMNPTRTGLILTLKEMGGDIEVIGSRQVGGED | 300 |
| Query 301 | VADLRVRSSTLKGVTVPEDRAPSMIDEYPILavaaafaegaTVMNGLEELRVKESDRLSA | 360 |
| Sbjct 301 | VADLRVR S L GVTVPE+RA SMIDEYP LA+AAAFAEG TVM GL+ELRVKESDRL+A VADLRVRHSELSGVTVPEERAASMIDEYPALAIAAAFAEGNTVMAGLDELRVKESDRLAA | 360 |
| Query 361 | VANGLKLNGVDCDEGETSLVVRGRPDGKGLGNASGAAVATHLDHRIAMSFLVMGLVSENP | 420 |
| Sbjct 361 | +A GLK NGVDC+EG +SLVVRGR DGKGLG+A+GA V THLDHRIAMSFL+ GL SE+P IAAGLKRNGVDCEEGASSLVVRGRRDGKGLGSAAGATVNTHLDHRIAMSFLIFGLASEHP | 420 |
| Query 421  Sbjct 421 | VTVDDATMIATSFPEFMDLMAGLGAKI 447 VTVDD +M+ATSFPEFM LM LGA+I VTVDDVSMVATSFPEFMHLMTSLGARI 447 |  |

>WP_037100378.1 3-phosphoshikimate 1-carboxyvinyltransferase [Rhizobium alamii] Length=450

Score = 700 bits (1807), Expect = 0.0, Method: Compositional matrix adjust.

| Identities | = 350/448 (78%), Positives = 395/448 (88%), Gaps = 0/448 (0%) |  |
| --- | --- | --- |
| Query 1 | MLHGASSRPATARKSSGLSGTVRIPGDKSISHRSFMFGGLASGETRITGLLEGEDVINTG | 60 |
| Sbjct 1 | M G++S+PA AR+SSGLSGTVRIPGDKSISHR+ + GGLASGETR+ GLLEGEDV+NTG MSQGSASKPAIARRSSGLSGTVRIPGDKSISHRALILGGLASGETRVAGLLEGEDVLNTG | 60 |
| Query 61 | KAMQAMGARIRKEGDTWIIDGVGNGGLLAPEAPLDFGNAATGCRLTMGLVGVYDFDSTFI | 120 |
| Sbjct 61 | KAMQ MGA I KEGD WII+G GNG LLAPE PLDFGN+ TG RLTMGL GVYDFD+ F KAMQTMGAVIHKEGDVWIINGTGNGALLAPEVPLDFGNSGTGARLTMGLAGVYDFDTVFT | 120 |
| Query 121 | GDASLTKRPMGRVLNPLREMGVQVKSEDGDRLPVTLRGPKTPTPITYRVPMASAQVKSAV | 180 |
| Sbjct 121 | GDASL++RPMGRVL+PLR MGVQVK+ +GDRLPV LRGP+TP PI Y +P+ASAQVKSAV GDASLSRRPMGRVLDPLRRMGVQVKAVEGDRLPVMLRGPETPAPIRYTLPVASAQVKSAV | 180 |
| Query 181 | LLAGLNTPGITTVIEPIMTRDHTEKMLQGFGANLTVETDADGVRTIRLEGRGKLTGQVID | 240 |
| Sbjct 181 | LLAGLN PG+TTVIEP +TRDHTEKML GFGA L+++TD DG RTIRL+GRG+LTGQ +D LLAGLNIPGVTTVIEPAITRDHTEKMLAGFGAELSIKTDKDGTRTIRLQGRGRLTGQTLD | 240 |
| Query 241 | VPGDPSSTAFPLVAALLVPGSDVTILNVLMNPTRTGLILTLQEMGADIEVINPRLAGGED | 300 |
| Sbjct 241 | VPGDPSS AFPLVAALLVPGSDVTI NVLMNPTRTGLILTLQEMGADI++++PRLAGGE+ VPGDPSSAAFPLVAALLVPGSDVTISNVLMNPTRTGLILTLQEMGADIDILDPRLAGGEN | 300 |
| Query 301 | VADLRVRSSTLKGVTVPEDRAPSMIDEYPILavaaafaegaTVMNGLEELRVKESDRLSA | 360 |
| Sbjct 301 | VADLRVR S L GVTVP +RAPSMIDEYP+LAVAAAFA+G TVMNGL+ELRVKESDRL A  VADLRVRYSELTGVTVPTERAPSMIDEYPVLAVAAAFAKGRTVMNGLQELRVKESDRLPA | 360 |

| Query | 361 | VANGLKLNGVDCDEGETSLVVRGRPDGKGLGNASGAAVATHLDHRIAMSFLVMGLVSENP 420 |
| --- | --- | --- |
| Sbjct | 361 | VA GLKLNGVDC+EG+ L V GRPDG GLGN +G V THLDHRIAMSFLVMGL SE+P VAEGLKLNGVDCEEGKDWLSVTGRPDGLGLGNTAGEQVVTHLDHRIAMSFLVMGLASEHP 420 |
| Query | 421 | VTVDDATMIATSFPEFMDLMAGLGAKIE 448 |
| Sbjct | 421 | V ++D++MIATSFP+FMDLM LGAKIE  VGINDSSMIATSFPQFMDLMESLGAKIE 448 |

>WP_060580572.1 3-phosphoshikimate 1-carboxyvinyltransferase [Ensifer sp. ENS08] KSV82432.1 3-phosphoshikimate 1-carboxyvinyltransferase [Sinorhizobium sp.

GL2]

MBD9567849.1 3-phosphoshikimate 1-carboxyvinyltransferase [Ensifer sp. ENS08] Length=448

Score = 700 bits (1807), Expect = 0.0, Method: Compositional matrix adjust.

| Identities | = 354/447 (79%), Positives = 394/447 (88%), Gaps = 0/447 (0%) |  |
| --- | --- | --- |
| Query 1 | MLHGASSRPATARKSSGLSGTVRIPGDKSISHRSFMFGGLASGETRITGLLEGEDVINTG | 60 |
| Sbjct 1 | M HG+ RPATARKS+ L G++RIPGDKSISHR+ M GGLASGETRITGLL+G DV+ TG MSHGSGPRPATARKSADLRGSIRIPGDKSISHRALMLGGLASGETRITGLLQGADVLATG | 60 |
| Query 61 | KAMQAMGARIRKEGDTWIIDGVGNGGLLAPEAPLDFGNAATGCRLTMGLVGVYDFDSTFI | 120 |
| Sbjct 61 | +AMQAMGARIRKE DT+I+DGVGNG LLAPEAPLDFGNA TGCRL MGL GVYDF++TF+ RAMQAMGARIRKEADTFIVDGVGNGALLAPEAPLDFGNAGTGCRLAMGLAGVYDFETTFL | 120 |
| Query 121 | GDASLTKRPMGRVLNPLREMGVQVKSEDGDRLPVTLRGPKTPTPITYRVPMASAQVKSAV | 180 |
| Sbjct 121 | GDASL +RPM RVL PLREMG QV++ GDRLPVTLRGPKTP PITYRVP+ASAQVKSA+ GDASLGRRPMARVLEPLREMGTQVRAAAGDRLPVTLRGPKTPAPITYRVPVASAQVKSAL | 180 |
| Query 181 | LLAGLNTPGITTVIEPIMTRDHTEKMLQGFGANLTVETDADGVRTIRLEGRGKLTGQVID | 240 |
| Sbjct 181 | LLAGLN PGITTVIEP+MTRDHTE+ML+ FGA L+VETD GVRTIRL+GRGKLTGQ ID LLAGLNAPGITTVIEPVMTRDHTERMLEAFGAALSVETDVSGVRTIRLDGRGKLTGQEID | 240 |
| Query 241 | VPGDPSSTAFPLVAALLVPGSDVTILNVLMNPTRTGLILTLQEMGADIEVINPRLAGGED | 300 |
| Sbjct 241 | VPGDPSS AFPLVAAL+VPGSD+TI NVLMNPTRTGLILTL+EMG DIEVI R GGED VPGDPSSAAFPLVAALIVPGSDITITNVLMNPTRTGLILTLKEMGGDIEVIGSRQVGGED | 300 |
| Query 301 | VADLRVRSSTLKGVTVPEDRAPSMIDEYPILavaaafaegaTVMNGLEELRVKESDRLSA | 360 |
| Sbjct 301 | VADLRVR S L GVTVPE+RA SMIDEYP LA+AAAFAEG TVM GL+ELRVKESDRL+A VADLRVRQSELSGVTVPEERAASMIDEYPALAIAAAFAEGNTVMAGLDELRVKESDRLAA | 360 |
| Query 361 | VANGLKLNGVDCDEGETSLVVRGRPDGKGLGNASGAAVATHLDHRIAMSFLVMGLVSENP | 420 |
| Sbjct 361 | +A GLK NGVDC+EG +SLVVRGR DGKGLG+A+GA V THLDHRIAMSFL+ GL SE+P IAAGLKRNGVDCEEGASSLVVRGRRDGKGLGSAAGATVNTHLDHRIAMSFLIFGLASEHP | 420 |
| Query 421  Sbjct 421 | VTVDDATMIATSFPEFMDLMAGLGAKI 447 VTVDD +M+ATSFPEF DLM LGA+I VTVDDVSMVATSFPEFTDLMTSLGARI 447 |  |

>WP_004684607.1 MULTISPECIES: 3-phosphoshikimate 1-carboxyvinyltransferase [Brucella] Q8YEG1.2 RecName: Full=3-phosphoshikimate 1-carboxyvinyltransferase; AltName:

Full=5-enolpyruvylshikimate-3-phosphate synthase; Short=EPSP synthase; Short=EPSPS [Brucella melitensis bv. 1 str.

16M]

EPZ75835.1 3-phosphoshikimate 1-carboxyvinyltransferase [Brucella melitensis ADMAS-G1]

AIJ88899.1 3-phosphoshikimate 1-carboxyvinyltransferase [Brucella melitensis bv. 1 str. 16M]

AVM31089.1 3-phosphoshikimate 1-carboxyvinyltransferase [Brucella melitensis]

EEW88214.1 3-phosphoshikimate 1-carboxyvinyltransferase [Brucella melitensis bv. 1 str. 16M]

EEZ15589.1 3-phosphoshikimate 1-carboxyvinyltransferase [Brucella melitensis bv. 1 str. Rev.1]

Length=450

Score = 700 bits (1806), Expect = 0.0, Method: Compositional matrix adjust. Identities = 362/447 (81%), Positives = 396/447 (89%), Gaps = 3/447 (1%)

Query 1	MLHGASSRPATARKSSGLSGTVRIPGDKSISHRSFMFGGLASGETRITGLLEGEDVINTG 60

| Sbjct | 1 | M H A +PATAR S L+G +RIPGDKSISHRSFMFGGLASG+TRITGLLEGEDVINTG MSHSACPKPATARHSQALTGEIRIPGDKSISHRSFMFGGLASGKTRITGLLEGEDVINTG | 60 |
| --- | --- | --- | --- |
| Query | 61 | KAMQAMGARIRKEGDTWIIDGVGNGGLLAPEAPLDFGNAATGCRLTMGLVGVYDFDSTFI | 120 |
| Sbjct | 61 | +AMQAMGARIRKEGD WII+GVGNG LL PEAPLDFGNA TG RLTMGLVG YD ++FI RAMQAMGARIRKEGDVWIINGVGNGCLLQPEAPLDFGNAGTGARLTMGLVGTYDMKTSFI | 120 |
| Query | 121 | GDASLTKRPMGRVLNPLREMGVQVKSEDGDRLPVTLRGPKTPTPITYRVPMASAQVKSAV | 180 |
| Sbjct | 121 | GDASL+KRPMGRVLNPLREMGVQV++ +GDR+P+TL GP+T PI YRVPMASAQVKSAV GDASLSKRPMGRVLNPLREMGVQVEAAEGDRMPLTLIGPRTANPIAYRVPMASAQVKSAV | 180 |
| Query | 181 | LLAGLNTPGITTVIEPIMTRDHTEKMLQGFGANLTVETDADGVRTIRLEGRGKLTGQVID | 240 |
| Sbjct | 181 | LLAGLNTPG+TTVIEP+MTRDHTEKMLQGFGA+LTVETD DGVR IR+ G+GKLTGQ ID LLAGLNTPGVTTVIEPVMTRDHTEKMLQGFGADLTVETDKDGVRHIRIVGQGKLTGQTID | 240 |
| Query | 241 | VPGDPSSTAFPLVAALLVPGSDVTILNVLMNPTRTGLILTLQEMGADIEVINPRLAGGED | 300 |
| Sbjct | 241 | VPGDPSSTAFPLVAALLV GSDVTI NVLMNPTRTGLILTLQEMGADIE+I+PRLAGGED VPGDPSSTAFPLVAALLVEGSDVTIRNVLMNPTRTGLILTLQEMGADIEIIDPRLAGGED | 300 |
| Query | 301 | VADLRVRSSTLKGVTVPEDRAPSMIDEYPILavaaafaegaTVMNGLEELRVKESDRLSA | 360 |
| Sbjct | 301 | VADLRV++S LKGV VP +RAPSMIDEYP+LA+AA+FAEG TVM+GL+ELRVKESDRL+A VADLRVKASKLKGVVVPPERAPSMIDEYPVLAIAASFAEGETVMDGLDELRVKESDRLAA | 360 |
| Query | 361 | VANGLKLNGVDCDEGETSLVVRGRPDGKGLGNASGAAVATHLDHRIAMSFLVMGLVSENP | 420 |
| Sbjct | 361 | VA GL+ NGVDC EGE SL VRGR G G V THLDHRIAMSFLVMGL SE P VARGLEANGVDCTEGEMSLTVRGR---PGGKGLGGGTVGTHLDHRIAMSFLVMGLASEKP | 417 |
| Query  Sbjct | 421  418 | VTVDDATMIATSFPEFMDLMAGLGAKI 447 VTVDD+TMIATSFPEFM +MAGLGAKI VTVDDSTMIATSFPEFMGMMAGLGAKI 444 |  |

>WP_011265281.1 3-phosphoshikimate 1-carboxyvinyltransferase [Brucella abortus] P0C100.1 RecName: Full=3-phosphoshikimate 1-carboxyvinyltransferase; AltName:

Full=5-enolpyruvylshikimate-3-phosphate synthase; Short=EPSP synthase; Short=EPSPS [Brucella abortus bv. 1 str.

9-941]

AAX73446.1 AroA, 3-phosphoshikimate 1-carboxyvinyltransferase [Brucella abortus bv. 1 str. 9-941]

Length=450

Score = 700 bits (1806), Expect = 0.0, Method: Compositional matrix adjust.

| Identities | = 361/447 (81%), Positives = 396/447 (89%), Gaps = 3/447 (1%) |  |
| --- | --- | --- |
| Query 1 | MLHGASSRPATARKSSGLSGTVRIPGDKSISHRSFMFGGLASGETRITGLLEGEDVINTG | 60 |
| Sbjct 1 | M H A +PATAR S L+G +RIPGDKSISHRSFMFGGLASG+TRITGLLEGEDVINTG MSHSACPKPATARHSQALTGEIRIPGDKSISHRSFMFGGLASGKTRITGLLEGEDVINTG | 60 |
| Query 61 | KAMQAMGARIRKEGDTWIIDGVGNGGLLAPEAPLDFGNAATGCRLTMGLVGVYDFDSTFI | 120 |
| Sbjct 61 | +AMQAMGARIRKEGD WII+GVGNG LL PEAPLDFGNA TG RLTMGLVG YD ++FI RAMQAMGARIRKEGDVWIINGVGNGCLLQPEAPLDFGNAGTGARLTMGLVGTYDMKTSFI | 120 |
| Query 121 | GDASLTKRPMGRVLNPLREMGVQVKSEDGDRLPVTLRGPKTPTPITYRVPMASAQVKSAV | 180 |
| Sbjct 121 | GDASL+KRPMGRVLNPLREMGVQV++ +GDR+P+TL GP+T PI YRVPMASAQVKSAV GDASLSKRPMGRVLNPLREMGVQVEAAEGDRMPLTLIGPRTANPIAYRVPMASAQVKSAV | 180 |
| Query 181 | LLAGLNTPGITTVIEPIMTRDHTEKMLQGFGANLTVETDADGVRTIRLEGRGKLTGQVID | 240 |
| Sbjct 181 | LLAGLNTPG+TTVIEP+MTRDHTEKMLQ FGA+LTVETD DGVR IR+ G+GKLTGQ ID LLAGLNTPGVTTVIEPVMTRDHTEKMLQDFGADLTVETDKDGVRHIRIVGQGKLTGQTID | 240 |
| Query 241 | VPGDPSSTAFPLVAALLVPGSDVTILNVLMNPTRTGLILTLQEMGADIEVINPRLAGGED | 300 |
| Sbjct 241 | VPGDPSSTAFPLVAALLV GS+VTI NVLMNPTRTGLILTLQEMGADIE+I+PRLAGGED VPGDPSSTAFPLVAALLVEGSEVTIRNVLMNPTRTGLILTLQEMGADIEIIDPRLAGGED | 300 |
| Query 301 | VADLRVRSSTLKGVTVPEDRAPSMIDEYPILavaaafaegaTVMNGLEELRVKESDRLSA | 360 |
| Sbjct 301 | VADLRV++S LKGV VP +RAPSMIDEYP+LA+AA+FAEG TVM+GL+ELRVKESDRL+A VADLRVKASKLKGVVVPPERAPSMIDEYPVLAIAASFAEGETVMDGLDELRVKESDRLAA | 360 |
| Query 361 | VANGLKLNGVDCDEGETSLVVRGRPDGKGLGNASGAAVATHLDHRIAMSFLVMGLVSENP | 420 |
| Sbjct 361 | VA GL+ NGVDC EGE SL VRGR G G VATHLDHRIAMSFLVMGL SE P  VARGLEANGVDCTEGEMSLTVRGR---PGGKGLGGGTVATHLDHRIAMSFLVMGLASEKP | 417 |

Query 421 VTVDDATMIATSFPEFMDLMAGLGAKI 447 VTVDD+TMIATSFPEFM +MAGLGAKI

Sbjct 418 VTVDDSTMIATSFPEFMGMMAGLGAKI 444

>WP_113397470.1 MULTISPECIES: 3-phosphoshikimate 1-carboxyvinyltransferase [unclassified Rhizobium]

TWB08473.1 3-phosphoshikimate 1-carboxyvinyltransferase [Rhizobium sp. ERR1071] GES53022.1 3-phosphoshikimate 1-carboxyvinyltransferase [Rhizobium sp. S-93]

Length=449

Score = 700 bits (1806), Expect = 0.0, Method: Compositional matrix adjust.

| Identities | = 353/448 (79%), Positives = 392/448 (88%), Gaps = 0/448 (0%) |  |
| --- | --- | --- |
| Query 1 | MLHGASSRPATARKSSGLSGTVRIPGDKSISHRSFMFGGLASGETRITGLLEGEDVINTG | 60 |
| Sbjct 1 | M H +PA AR+S+GLSGT+RIPGDKSISHRS MFGGLA+GETRITGLLEGEDV+NTG MPHDTLPQPAVARRSAGLSGTIRIPGDKSISHRSLMFGGLAAGETRITGLLEGEDVLNTG | 60 |
| Query 61 | KAMQAMGARIRKEGDTWIIDGVGNGGLLAPEAPLDFGNAATGCRLTMGLVGVYDFDSTFI | 120 |
| Sbjct 61 | KAMQA+GA IRK D WI+ G GNGGLLAPE PLDFGNA TGCRLTMGLVG YDF +TF+ KAMQALGATIRKIDDEWIVRGTGNGGLLAPETPLDFGNAGTGCRLTMGLVGSYDFKTTFV | 120 |
| Query 121 | GDASLTKRPMGRVLNPLREMGVQVKSEDGDRLPVTLRGPKTPTPITYRVPMASAQVKSAV | 180 |
| Sbjct 121 | GDASL+KRPMGR+L+PLR MG QV + GDRLPV+L GP +P+TYRVPMASAQVKSAV GDASLSKRPMGRILDPLRRMGTQVSAATGDRLPVSLHGPGVASPLTYRVPMASAQVKSAV | 180 |
| Query 181 | LLAGLNTPGITTVIEPIMTRDHTEKMLQGFGANLTVETDADGVRTIRLEGRGKLTGQVID | 240 |
| Sbjct 181 | LLAGLN PG+TTVIEP+MTRDHTEKML GFGA L+VE DA GVRTIRLEG+GKLTGQ ID LLAGLNAPGMTTVIEPVMTRDHTEKMLAGFGAALSVEIDAAGVRTIRLEGQGKLTGQTID | 240 |
| Query 241 | VPGDPSSTAFPLVAALLVPGSDVTILNVLMNPTRTGLILTLQEMGADIEVINPRLAGGED | 300 |
| Sbjct 241 | VPGDPSS AFPLVAAL+VPGSD+TI NVLMNPTRTGLILTLQEMGADIE+++ R+ GGED VPGDPSSAAFPLVAALIVPGSDITIRNVLMNPTRTGLILTLQEMGADIEMLDRRMTGGED | 300 |
| Query 301 | VADLRVRSSTLKGVTVPEDRAPSMIDEYPILavaaafaegaTVMNGLEELRVKESDRLSA | 360 |
| Sbjct 301 | VADLRVRSS LKGVTVP +RAPSMIDEYP+LAVAA FAEG T+M GL+ELRVKESDRLSA VADLRVRSSALKGVTVPAERAPSMIDEYPVLAVAATFAEGTTIMQGLDELRVKESDRLSA | 360 |
| Query 361 | VANGLKLNGVDCDEGETSLVVRGRPDGKGLGNASGAAVATHLDHRIAMSFLVMGLVSENP | 420 |
| Sbjct 361 | VA GLKLNGVDC EG L V+GRPDGKGLGNA GA V+THLDHRIAMSFLVMGL SE+P VAEGLKLNGVDCIEGIDMLTVQGRPDGKGLGNAGGAKVSTHLDHRIAMSFLVMGLASEHP | 420 |
| Query 421  Sbjct 421 | VTVDDATMIATSFPEFMDLMAGLGAKIE 448 VT+DD+ MIATSFP+F ++ LGA IE VTIDDSAMIATSFPDFFAMITRLGATIE 448 |  |

>WP_192338922.1 unnamed protein product

MBD9621464.1 3-phosphoshikimate 1-carboxyvinyltransferase [Ensifer sp. ENS06] Length=448

Score = 700 bits (1806), Expect = 0.0, Method: Compositional matrix adjust.

| Identities | = 353/447 (79%), Positives = 395/447 (88%), Gaps = 0/447 (0%) |  |
| --- | --- | --- |
| Query 1 | MLHGASSRPATARKSSGLSGTVRIPGDKSISHRSFMFGGLASGETRITGLLEGEDVINTG | 60 |
| Sbjct 1 | M HG+ RPATARKS+ L G++RIPGDKSISHR+ M GGLASGETRITGLL+G DV+ TG MSHGSGPRPATARKSADLRGSIRIPGDKSISHRALMLGGLASGETRITGLLQGADVLATG | 60 |
| Query 61 | KAMQAMGARIRKEGDTWIIDGVGNGGLLAPEAPLDFGNAATGCRLTMGLVGVYDFDSTFI | 120 |
| Sbjct 61 | +AM+AMGARIRKE DT+I+DGVGNG LLAPEAPLDFGNA TGCRL +GL GVYDF++TF+ RAMRAMGARIRKEADTFIVDGVGNGALLAPEAPLDFGNAGTGCRLAIGLAGVYDFETTFL | 120 |
| Query 121 | GDASLTKRPMGRVLNPLREMGVQVKSEDGDRLPVTLRGPKTPTPITYRVPMASAQVKSAV | 180 |
| Sbjct 121 | GDASL++RPM RVL PLREMG QV++ GDRLPVTLRGPKTP PITYRVP+ASAQVKSAV GDASLSRRPMARVLEPLREMGAQVRAAAGDRLPVTLRGPKTPAPITYRVPVASAQVKSAV | 180 |
| Query 181 | LLAGLNTPGITTVIEPIMTRDHTEKMLQGFGANLTVETDADGVRTIRLEGRGKLTGQVID | 240 |
| Sbjct 181 | LLAGLN PGITTVIEP+MTRDHTE+ML+ FGA L+VETD GVRTIRL+GRGKLTGQ ID LLAGLNAPGITTVIEPVMTRDHTERMLEAFGAALSVETDVSGVRTIRLDGRGKLTGQEID | 240 |
| Query 241 | VPGDPSSTAFPLVAALLVPGSDVTILNVLMNPTRTGLILTLQEMGADIEVINPRLAGGED VPGDPSS AFPLVAAL+VPGSD+TI NVLMNPTRTGLILTL+EMG DIEVI R GGED | 300 |

| Sbjct | 241 | VPGDPSSAAFPLVAALIVPGSDITITNVLMNPTRTGLILTLKEMGGDIEVIGSRQVGGED | 300 |
| --- | --- | --- | --- |
| Query | 301 | VADLRVRSSTLKGVTVPEDRAPSMIDEYPILavaaafaegaTVMNGLEELRVKESDRLSA | 360 |
| Sbjct | 301 | VADLRVR S L GVTVPE+RA SMIDEYP LA+AAAFAEG TVM GL+ELRVKESDRL+A VADLRVRHSELSGVTVPEERAASMIDEYPALAIAAAFAEGNTVMAGLDELRVKESDRLAA | 360 |
| Query | 361 | VANGLKLNGVDCDEGETSLVVRGRPDGKGLGNASGAAVATHLDHRIAMSFLVMGLVSENP | 420 |
| Sbjct | 361 | +A GLK NGVDC+EG +SLVVRGR DGKGLG+A+GA V THLDHRIAMSFL+ GL SE+P IAAGLKRNGVDCEEGASSLVVRGRRDGKGLGSAAGATVNTHLDHRIAMSFLIFGLASEHP | 420 |
| Query  Sbjct | 421  421 | VTVDDATMIATSFPEFMDLMAGLGAKI 447 VTVDD +M+ATSFPEFM LM LGA+I VTVDDVSMVATSFPEFMHLMTSLGARI 447 |  |

>WP_154167325.1 3-phosphoshikimate 1-carboxyvinyltransferase [Brucella sp. 10RB9213] MRN65196.1 3-phosphoshikimate 1-carboxyvinyltransferase [Brucella sp. 10RB9213]

Length=450

Score = 700 bits (1806), Expect = 0.0, Method: Compositional matrix adjust.

| Identities | = 362/447 (81%), Positives = 395/447 (88%), Gaps = 3/447 (1%) |  |
| --- | --- | --- |
| Query 1 | MLHGASSRPATARKSSGLSGTVRIPGDKSISHRSFMFGGLASGETRITGLLEGEDVINTG | 60 |
| Sbjct 1 | M H A +PATAR S L+G +RIPGDKSISHRSFMFGGLASGETRITGLLEGEDVINTG MSHSACPKPATARHSEALTGEIRIPGDKSISHRSFMFGGLASGETRITGLLEGEDVINTG | 60 |
| Query 61 | KAMQAMGARIRKEGDTWIIDGVGNGGLLAPEAPLDFGNAATGCRLTMGLVGVYDFDSTFI | 120 |
| Sbjct 61 | +AMQAMGARIRKEGD WII+GVGNG LL PEAPLDFGNA TG RLTMGLVG YD ++FI RAMQAMGARIRKEGDVWIINGVGNGCLLQPEAPLDFGNAGTGARLTMGLVGTYDMKTSFI | 120 |
| Query 121 | GDASLTKRPMGRVLNPLREMGVQVKSEDGDRLPVTLRGPKTPTPITYRVPMASAQVKSAV | 180 |
| Sbjct 121 | GDASL+KRPMGRVLNPLREMGVQV++ +GDR+P+TL GP+T PI YRVPMASAQVKSAV GDASLSKRPMGRVLNPLREMGVQVEAAEGDRMPLTLIGPRTANPIAYRVPMASAQVKSAV | 180 |
| Query 181 | LLAGLNTPGITTVIEPIMTRDHTEKMLQGFGANLTVETDADGVRTIRLEGRGKLTGQVID | 240 |
| Sbjct 181 | LLAGLNTPG+TTVIEP+MTRDHTEKMLQGFGA+LTVETD GVR IR+ G+GKLTGQ ID LLAGLNTPGVTTVIEPVMTRDHTEKMLQGFGADLTVETDKGGVRHIRIVGQGKLTGQTID | 240 |
| Query 241 | VPGDPSSTAFPLVAALLVPGSDVTILNVLMNPTRTGLILTLQEMGADIEVINPRLAGGED | 300 |
| Sbjct 241 | VPGDPSSTAFPLVAALLV GSDVTI NVLMNPTRTGLILTLQEMGADIE+I+PRLAGGED VPGDPSSTAFPLVAALLVEGSDVTIRNVLMNPTRTGLILTLQEMGADIEIIDPRLAGGED | 300 |
| Query 301 | VADLRVRSSTLKGVTVPEDRAPSMIDEYPILavaaafaegaTVMNGLEELRVKESDRLSA | 360 |
| Sbjct 301 | VADLRV++S LKGV VP +RAPSMIDEYP+LA+AA+FAEG TVM+GL+ELRVKESDRL+A VADLRVKASKLKGVVVPPERAPSMIDEYPVLAIAASFAEGETVMDGLDELRVKESDRLAA | 360 |
| Query 361 | VANGLKLNGVDCDEGETSLVVRGRPDGKGLGNASGAAVATHLDHRIAMSFLVMGLVSENP | 420 |
| Sbjct 361 | VA GL+ NGVDC EGE SL VRGR G G VATHLDHRIAMSFLVMGL SE P VARGLEANGVDCTEGEMSLTVRGR---PGGKGLGGGTVATHLDHRIAMSFLVMGLASEKP | 417 |
| Query 421  Sbjct 418 | VTVDDATMIATSFPEFMDLMAGLGAKI 447 VTVDD+TMIATSFPEFM +MAGLGA I VTVDDSTMIATSFPEFMGMMAGLGANI 444 |  |

>WP_188823230.1 3-phosphoshikimate 1-carboxyvinyltransferase [Ochrobactrum endophyticum] Length=451

Score = 699 bits (1805), Expect = 0.0, Method: Compositional matrix adjust.

| Identities | = 361/452 (80%), Positives = 397/452 (88%), Gaps = 3/452 (1%) |  |
| --- | --- | --- |
| Query 1 | MLHGASSRPATARKSSGLSGTVRIPGDKSISHRSFMFGGLASGETRITGLLEGEDVINTG | 60 |
| Sbjct 1 | M H AS +PATA +S L G +RIPGDKSISHRSFMFGGLASGETRITGLLEGEDVINTG MSHSASLKPATAHRSRDLKGDIRIPGDKSISHRSFMFGGLASGETRITGLLEGEDVINTG | 60 |
| Query 61 | KAMQAMGARIRKEGDTWIIDGVGNGGLLAPEAPLDFGNAATGCRLTMGLVGVYDFDSTFI | 120 |
| Sbjct 61 | +AMQAMGA IRKEG TWII+G GNG LL PEAPLDFGNA TG RLTMGLVG YD +TFI RAMQAMGANIRKEGATWIIEGTGNGCLLEPEAPLDFGNAGTGARLTMGLVGTYDMRTTFI | 120 |
| Query 121 | GDASLTKRPMGRVLNPLREMGVQVKSEDGDRLPVTLRGPKTPTPITYRVPMASAQVKSAV | 180 |
| Sbjct 121 | GDASL+KRPMGRVL+PLR MGVQV++ +GDR+P+TLRGPK TPI+YRVPMASAQVKSAV  GDASLSKRPMGRVLDPLRLMGVQVEAAEGDRVPLTLRGPKAATPISYRVPMASAQVKSAV | 180 |

| Query | 181 | LLAGLNTPGITTVIEPIMTRDHTEKMLQGFGANLTVETDADGVRTIRLEGRGKLTGQVID | 240 |
| --- | --- | --- | --- |
| Sbjct | 181 | LLAGLNTPGITTVIEP+MTRDHTEKMLQGFGA+LTVETD +GVR IR+ G+G+L GQ+ID LLAGLNTPGITTVIEPVMTRDHTEKMLQGFGADLTVETDREGVRHIRIVGQGQLKGQIID | 240 |
| Query | 241 | VPGDPSSTAFPLVAALLVPGSDVTILNVLMNPTRTGLILTLQEMGADIEVINPRLAGGED | 300 |
| Sbjct | 241 | VPGDPSSTAFPLVAAL+VPGSDVTI NVLMNPTRTGLILTLQEMGADIE++N RLAGGED VPGDPSSTAFPLVAALIVPGSDVTIRNVLMNPTRTGLILTLQEMGADIEILNARLAGGED | 300 |
| Query | 301 | VADLRVRSSTLKGVTVPEDRAPSMIDEYPILavaaafaegaTVMNGLEELRVKESDRLSA | 360 |
| Sbjct | 301 | VADLRVR+S LKGV+VP +RAPSMIDEYP+LA+AA+FAEG TVM+GLEELRVKESDRLSA VADLRVRASELKGVSVPPERAPSMIDEYPVLAIAASFAEGETVMDGLEELRVKESDRLSA | 360 |
| Query | 361 | VANGLKLNGVDCDEGETSLVVRGRPDGKGLGNASGAAVATHLDHRIAMSFLVMGLVSENP | 420 |
| Sbjct | 361 | VA GLK NGVDC EG SL VRG G G VATHLDHRIAMSFLVMGL +E P VATGLKANGVDCTEGAASLAVRGV---PGGKGLGGGTVATHLDHRIAMSFLVMGLAAEKP | 417 |
| Query  Sbjct | 421  418 | VTVDDATMIATSFPEFMDLMAGLGAKIELSDT 452 VTVDD+ +IATSFPEFMDLM GLGAKI+ +T VTVDDSAIIATSFPEFMDLMTGLGAKIDTVET 449 |  |

>WP_007814132.1 3-phosphoshikimate 1-carboxyvinyltransferase [Rhizobium sp. CF142] EJJ30666.1 3-phosphoshikimate 1-carboxyvinyltransferase [Rhizobium sp. CF142]

Length=450

Score = 699 bits (1804), Expect = 0.0, Method: Compositional matrix adjust.

| Identities | = 350/448 (78%), Positives = 397/448 (89%), Gaps = 0/448 (0%) |  |
| --- | --- | --- |
| Query 1 | MLHGASSRPATARKSSGLSGTVRIPGDKSISHRSFMFGGLASGETRITGLLEGEDVINTG | 60 |
| Sbjct 1 | M G++S+PA ARKSSGLSGTV IPGDKSISHR+ + GGLASGETR+TGLLEGEDV+NTG MSQGSASKPAIARKSSGLSGTVLIPGDKSISHRALILGGLASGETRVTGLLEGEDVLNTG | 60 |
| Query 61 | KAMQAMGARIRKEGDTWIIDGVGNGGLLAPEAPLDFGNAATGCRLTMGLVGVYDFDSTFI | 120 |
| Sbjct 61 | KA++A+GA IRKEGD WII+G GNG LLAPE PLDFGN+ TG RLTMGL VYDFD+ F KAIRAIGAVIRKEGDAWIINGTGNGALLAPEVPLDFGNSGTGARLTMGLASVYDFDTVFT | 120 |
| Query 121 | GDASLTKRPMGRVLNPLREMGVQVKSEDGDRLPVTLRGPKTPTPITYRVPMASAQVKSAV | 180 |
| Sbjct 121 | GDASL++RPMGRVL+PLR MGVQVK+ +GDRLPV LRGP+TP PI Y +P+ASAQVKSAV GDASLSRRPMGRVLDPLRRMGVQVKAVEGDRLPVMLRGPETPAPIRYTLPVASAQVKSAV | 180 |
| Query 181 | LLAGLNTPGITTVIEPIMTRDHTEKMLQGFGANLTVETDADGVRTIRLEGRGKLTGQVID | 240 |
| Sbjct 181 | LLAGLN PG+TTVIEP +TRDHTEKML GFGA L+++TD DG RTIRL+GRG+LTGQ +D LLAGLNIPGVTTVIEPAITRDHTEKMLAGFGAELSIKTDKDGTRTIRLQGRGRLTGQTLD | 240 |
| Query 241 | VPGDPSSTAFPLVAALLVPGSDVTILNVLMNPTRTGLILTLQEMGADIEVINPRLAGGED | 300 |
| Sbjct 241 | VPGDPSS AFPLVAALLVPGSDVTI NVLMNPTRTGLILTLQEMGADI++++PRLAGGE+ VPGDPSSAAFPLVAALLVPGSDVTISNVLMNPTRTGLILTLQEMGADIDILDPRLAGGEN | 300 |
| Query 301 | VADLRVRSSTLKGVTVPEDRAPSMIDEYPILavaaafaegaTVMNGLEELRVKESDRLSA | 360 |
| Sbjct 301 | VADLRVR S L GVTVP +RAPSMIDEYP+LAVAAAFA+G TVMNGL+ELRVKESDRLSA VADLRVRYSELTGVTVPTERAPSMIDEYPVLAVAAAFAKGRTVMNGLQELRVKESDRLSA | 360 |
| Query 361 | VANGLKLNGVDCDEGETSLVVRGRPDGKGLGNASGAAVATHLDHRIAMSFLVMGLVSENP | 420 |
| Sbjct 361 | VA GLKLNGVDC+EG+ L V GRPDG GLGN +G V THLDHRIAMSFLVMGL SE+P VAEGLKLNGVDCEEGKDWLSVTGRPDGLGLGNTAGEQVVTHLDHRIAMSFLVMGLASEHP | 420 |
| Query 421  Sbjct 421 | VTVDDATMIATSFPEFMDLMAGLGAKIE 448 V ++D++MIATSFP+FMDLM LGAKIE VGINDSSMIATSFPQFMDLMESLGAKIE 448 |  |

>WP_132653128.1 MULTISPECIES: 3-phosphoshikimate 1-carboxyvinyltransferase [unclassified Rhizobium]

MBB3593044.1 3-phosphoshikimate 1-carboxyvinyltransferase [Rhizobium sp. BK529] TCS07425.1 3-phosphoshikimate 1-carboxyvinyltransferase [Rhizobium sp. BK418]

Length=450

Score = 699 bits (1803), Expect = 0.0, Method: Compositional matrix adjust. Identities = 346/448 (77%), Positives = 397/448 (89%), Gaps = 0/448 (0%)

Query 1	MLHGASSRPATARKSSGLSGTVRIPGDKSISHRSFMFGGLASGETRITGLLEGEDVINTG 60

| Sbjct | 1 | M G++ +PA ARKSSGLSGTVRIPGDKSISHR+ M GGLASGETRITGLLEGEDV+NTG MSQGSTPKPAIARKSSGLSGTVRIPGDKSISHRALMLGGLASGETRITGLLEGEDVLNTG | 60 |
| --- | --- | --- | --- |
| Query | 61 | KAMQAMGARIRKEGDTWIIDGVGNGGLLAPEAPLDFGNAATGCRLTMGLVGVYDFDSTFI | 120 |
| Sbjct | 61 | KAMQAMGA IRK+G WI++G GNG LLA E+PLDFGN+ TG RL+MGL GVYDFD+ + KAMQAMGAVIRKDGGEWIVNGTGNGALLASESPLDFGNSGTGARLSMGLAGVYDFDTVLV | 120 |
| Query | 121 | GDASLTKRPMGRVLNPLREMGVQVKSEDGDRLPVTLRGPKTPTPITYRVPMASAQVKSAV | 180 |
| Sbjct | 121 | GDASL++RPMGRVL+PLR MGVQVK+ +GDRLPV LRGP+TP PI Y +P+ASAQVKSAV GDASLSRRPMGRVLDPLRRMGVQVKAVEGDRLPVMLRGPETPAPIHYTLPVASAQVKSAV | 180 |
| Query | 181 | LLAGLNTPGITTVIEPIMTRDHTEKMLQGFGANLTVETDADGVRTIRLEGRGKLTGQVID | 240 |
| Sbjct | 181 | LLAGLN PG+TTVIEP +TRDHTEKML GFGA L++ETD DG RTIRL+GRG+LTGQVI+ LLAGLNIPGVTTVIEPAITRDHTEKMLVGFGAELSIETDKDGTRTIRLQGRGRLTGQVIE | 240 |
| Query | 241 | VPGDPSSTAFPLVAALLVPGSDVTILNVLMNPTRTGLILTLQEMGADIEVINPRLAGGED | 300 |
| Sbjct | 241 | VPGDPSS AFPL+AALLVPGSD+TI NVLMNPTRTGLILTLQEMGADI+++N RLAGGED VPGDPSSAAFPLIAALLVPGSDITISNVLMNPTRTGLILTLQEMGADIQILNLRLAGGED | 300 |
| Query | 301 | VADLRVRSSTLKGVTVPEDRAPSMIDEYPILavaaafaegaTVMNGLEELRVKESDRLSA | 360 |
| Sbjct | 301 | VADLRVR L GV+VP +RAP+MIDEYP+LAVAAAFA+G ++MNGL+ELRVKESDRLSA VADLRVRHCELTGVSVPAERAPAMIDEYPVLAVAAAFAKGKSIMNGLQELRVKESDRLSA | 360 |
| Query | 361 | VANGLKLNGVDCDEGETSLVVRGRPDGKGLGNASGAAVATHLDHRIAMSFLVMGLVSENP | 420 |
| Sbjct | 361 | VA+GLKLNG +C+EG+ L V GRPDGKGLGNA+G V THLDHRIAMSFLVMGL SE+P VADGLKLNGAECEEGQDWLSVTGRPDGKGLGNAAGERVVTHLDHRIAMSFLVMGLASEHP | 420 |
| Query  Sbjct | 421  421 | VTVDDATMIATSFPEFMDLMAGLGAKIE 448 V +DD+ MIATSFP+FMDLM LGA+IE VGIDDSNMIATSFPQFMDLMKSLGARIE 448 |  |

>WP_006104394.1 3-phosphoshikimate 1-carboxyvinyltransferase [Brucella abortus] ENP35200.1 3-phosphoshikimate 1-carboxyvinyltransferase [Brucella abortus

65/110]

ENQ04005.1 3-phosphoshikimate 1-carboxyvinyltransferase [Brucella abortus F6/05-2]

ENR85372.1 3-phosphoshikimate 1-carboxyvinyltransferase [Brucella abortus 78/14]

ENS04381.1 3-phosphoshikimate 1-carboxyvinyltransferase [Brucella abortus 87/28]

ENS28667.1 3-phosphoshikimate 1-carboxyvinyltransferase [Brucella abortus F6/05-4]

Length=450

Score = 699 bits (1803), Expect = 0.0, Method: Compositional matrix adjust.

| Identities | = 360/447 (81%), Positives = 395/447 (88%), Gaps = 3/447 (1%) |  |
| --- | --- | --- |
| Query 1 | MLHGASSRPATARKSSGLSGTVRIPGDKSISHRSFMFGGLASGETRITGLLEGEDVINTG | 60 |
| Sbjct 1 | M H A +PATAR S L+G +RIPGDKSISHRSFMFGGLASG+TRITGLLEGEDVINTG MSHSACPKPATARHSQALTGEIRIPGDKSISHRSFMFGGLASGKTRITGLLEGEDVINTG | 60 |
| Query 61 | KAMQAMGARIRKEGDTWIIDGVGNGGLLAPEAPLDFGNAATGCRLTMGLVGVYDFDSTFI | 120 |
| Sbjct 61 | +AMQAMGARIRKEGD WII+GVGNG LL PEAPLDFGNA TG RLTMGLVG YD ++FI RAMQAMGARIRKEGDVWIINGVGNGCLLQPEAPLDFGNAGTGARLTMGLVGTYDMKTSFI | 120 |
| Query 121 | GDASLTKRPMGRVLNPLREMGVQVKSEDGDRLPVTLRGPKTPTPITYRVPMASAQVKSAV | 180 |
| Sbjct 121 | GDASL+KRPMGRVLNPLREMGVQV++ +GDR+P+TL GP+T PI YRVPMASAQVKSAV GDASLSKRPMGRVLNPLREMGVQVEAAEGDRMPLTLIGPRTANPIAYRVPMASAQVKSAV | 180 |
| Query 181 | LLAGLNTPGITTVIEPIMTRDHTEKMLQGFGANLTVETDADGVRTIRLEGRGKLTGQVID | 240 |
| Sbjct 181 | LLAGLNTPG+TTVIEP+MTRDHTEKMLQGFGA+LTVETD DGVR IR+ G+GKLTGQ ID LLAGLNTPGVTTVIEPVMTRDHTEKMLQGFGADLTVETDKDGVRHIRIVGQGKLTGQTID | 240 |
| Query 241 | VPGDPSSTAFPLVAALLVPGSDVTILNVLMNPTRTGLILTLQEMGADIEVINPRLAGGED | 300 |
| Sbjct 241 | VPGDPSSTAFPLVAALLV GS+VTI NVLMNPTRTGLILTLQEMGADIE+I+P LAGGED VPGDPSSTAFPLVAALLVEGSEVTIRNVLMNPTRTGLILTLQEMGADIEIIDPHLAGGED | 300 |
| Query 301 | VADLRVRSSTLKGVTVPEDRAPSMIDEYPILavaaafaegaTVMNGLEELRVKESDRLSA | 360 |
| Sbjct 301 | VADLRV++S LKGV VP +RAPSMIDEYP+LA+AA+FAEG TVM+GL+ELR KESDRL+A  VADLRVKASKLKGVVVPPERAPSMIDEYPVLAIAASFAEGETVMDGLDELRAKESDRLAA | 360 |

| Query | 361 | VANGLKLNGVDCDEGETSLVVRGRPDGKGLGNASGAAVATHLDHRIAMSFLVMGLVSENP 420 |
| --- | --- | --- |
| Sbjct | 361 | VA GL+ NGVDC EGE SL VRGR G G VATHLDHRIAMSFLVMGL SE P VARGLEANGVDCTEGEMSLTVRGR---PGGKGLGGGTVATHLDHRIAMSFLVMGLASEKP 417 |
| Query | 421 | VTVDDATMIATSFPEFMDLMAGLGAKI 447 |
| Sbjct | 418 | VTVDD+TMIATSFPEFM +MAGLGAKI  VTVDDSTMIATSFPEFMGMMAGLGAKI 444 |

>WP_110749612.1 3-phosphoshikimate 1-carboxyvinyltransferase [Phyllobacterium leguminum]

PYE89281.1 3-phosphoshikimate 1-carboxyvinyltransferase [Phyllobacterium leguminum]

Length=451

Score = 698 bits (1802), Expect = 0.0, Method: Compositional matrix adjust.

| Identities | = 363/454 (80%), Positives = 398/454 (88%), Gaps = 3/454 (1%) |  |
| --- | --- | --- |
| Query 1 | MLHGASSRPATARKSSGLSGTVRIPGDKSISHRSFMFGGLASGETRITGLLEGEDVINTG | 60 |
| Sbjct 1 | M H A+ +PATA +S L+G +RIPGDKSISHRSFMFGGLASGETRITGLLEGEDVINTG MSHSATPKPATAGRSRDLAGDIRIPGDKSISHRSFMFGGLASGETRITGLLEGEDVINTG | 60 |
| Query 61 | KAMQAMGARIRKEGDTWIIDGVGNGGLLAPEAPLDFGNAATGCRLTMGLVGVYDFDSTFI | 120 |
| Sbjct 61 | +AMQAMGA IRK+G+TWII G GNG LL PE PLDFGNA TG RLTMGLVG YD ++TFI RAMQAMGADIRKDGETWIIQGTGNGCLLEPETPLDFGNAGTGARLTMGLVGTYDMETTFI | 120 |
| Query 121 | GDASLTKRPMGRVLNPLREMGVQVKSEDGDRLPVTLRGPKTPTPITYRVPMASAQVKSAV | 180 |
| Sbjct 121 | GDASL+KRPMGRVL PLREMGVQVKS +GDRLP+TL GPKT PI YRVPMASAQVKSAV GDASLSKRPMGRVLGPLREMGVQVKSAEGDRLPLTLHGPKTAAPIIYRVPMASAQVKSAV | 180 |
| Query 181 | LLAGLNTPGITTVIEPIMTRDHTEKMLQGFGANLTVETDADGVRTIRLEGRGKLTGQVID | 240 |
| Sbjct 181 | LLAGLNTPGITTVIEPIMTRDHTEKMLQGFGA+LTVETD +GVR IR+ G+G+L GQ ID LLAGLNTPGITTVIEPIMTRDHTEKMLQGFGADLTVETDREGVRHIRIVGQGELKGQTID | 240 |
| Query 241 | VPGDPSSTAFPLVAALLVPGSDVTILNVLMNPTRTGLILTLQEMGADIEVINPRLAGGED | 300 |
| Sbjct 241 | VPGDPSSTAFPLVAAL+VPGSDVTI NVLMNPTRTGLILTLQEMGA+IE++N RLAGGED VPGDPSSTAFPLVAALIVPGSDVTIRNVLMNPTRTGLILTLQEMGANIEILNARLAGGED | 300 |
| Query 301 | VADLRVRSSTLKGVTVPEDRAPSMIDEYPILavaaafaegaTVMNGLEELRVKESDRLSA | 360 |
| Sbjct 301 | VADLRVR+S LKGV+VP +RAPSMIDEYP+LAVAAAFAEG T+M+GLEELRVKESDRLSA VADLRVRASELKGVSVPPERAPSMIDEYPVLAVAAAFAEGETIMDGLEELRVKESDRLSA | 360 |
| Query 361 | VANGLKLNGVDCDEGETSLVVRGRPDGKGLGNASGAAVATHLDHRIAMSFLVMGLVSENP | 420 |
| Sbjct 361 | VA+GLK NGV+C EGE SL VRG G G VATHLDHRIAMSFLVMGL +E P VADGLKANGVECAEGEASLTVRGT---PGGKGIGGGTVATHLDHRIAMSFLVMGLAAEKP | 417 |
| Query 421  Sbjct 418 | VTVDDATMIATSFPEFMDLMAGLGAKIELSDTKA 454 VT+DD+ MIATSFPEFMDLM LGAKIE ++T A VTIDDSAMIATSFPEFMDLMKDLGAKIETAETVA 451 |  |

>WP_183431406.1 3-phosphoshikimate 1-carboxyvinyltransferase [Mesorhizobium sp. RMAD-H1]

MBB2972013.1 3-phosphoshikimate 1-carboxyvinyltransferase [Mesorhizobium sp. RMAD-H1]

Length=447

Score = 698 bits (1802), Expect = 0.0, Method: Compositional matrix adjust.

| Identities | = 361/450 (80%), Positives = 395/450 (88%), Gaps = 3/450 (1%) |  |
| --- | --- | --- |
| Query 1 | MLHGASSRPATARKSSGLSGTVRIPGDKSISHRSFMFGGLASGETRITGLLEGEDVINTG | 60 |
| Sbjct 1 | M H AS +PATA +S L+G +RIPGDKSISHR+FMFGGLASGETRITGLLEGEDVINTG MSHTASPKPATAHRSRDLAGDIRIPGDKSISHRAFMFGGLASGETRITGLLEGEDVINTG | 60 |
| Query 61 | KAMQAMGARIRKEGDTWIIDGVGNGGLLAPEAPLDFGNAATGCRLTMGLVGVYDFDSTFI | 120 |
| Sbjct 61 | +AMQ MGARI+K+G TWII G GNG LL PEAPLDFGNA TG RLTMGLVG YD +TFI RAMQTMGARIKKDGGTWIIQGTGNGCLLEPEAPLDFGNAGTGARLTMGLVGTYDMKTTFI | 120 |
| Query 121 | GDASLTKRPMGRVLNPLREMGVQVKSEDGDRLPVTLRGPKTPTPITYRVPMASAQVKSAV | 180 |
| Sbjct 121 | GDASL+KRPMGRVL+PLREMGVQVK+ +GDRLP+TL GPK PITYRVPMASAQVKSAV  GDASLSKRPMGRVLDPLREMGVQVKAAEGDRLPLTLHGPKVAAPITYRVPMASAQVKSAV | 180 |

| Query | 181 | LLAGLNTPGITTVIEPIMTRDHTEKMLQGFGANLTVETDADGVRTIRLEGRGKLTGQVID | 240 |
| --- | --- | --- | --- |
| Sbjct | 181 | LLAGLNTPG+TTVIEP+MTRDHTEKMLQGFGA+LTVETD DGVR IR+ G+G+L GQ ID LLAGLNTPGVTTVIEPVMTRDHTEKMLQGFGADLTVETDRDGVRHIRIVGQGELKGQTID | 240 |
| Query | 241 | VPGDPSSTAFPLVAALLVPGSDVTILNVLMNPTRTGLILTLQEMGADIEVINPRLAGGED | 300 |
| Sbjct | 241 | VPGDPSSTAFPLVAAL+VPGSDVTI NVLMNPTRTGLILTL+EMGA I+++N RLAGGED VPGDPSSTAFPLVAALIVPGSDVTIRNVLMNPTRTGLILTLREMGAKIDILNARLAGGED | 300 |
| Query | 301 | VADLRVRSSTLKGVTVPEDRAPSMIDEYPILavaaafaegaTVMNGLEELRVKESDRLSA | 360 |
| Sbjct | 301 | VADLRV +S LKGV+VP +RAPSMIDEYP+LAVAA FAEG TVM+GLEELRVKESDRLSA VADLRVCASELKGVSVPPERAPSMIDEYPVLAVAATFAEGETVMDGLEELRVKESDRLSA | 360 |
| Query | 361 | VANGLKLNGVDCDEGETSLVVRGRPDGKGLGNASGAAVATHLDHRIAMSFLVMGLVSENP | 420 |
| Sbjct | 361 | VANGLK NGVDC EGE SL+VRG G G VATHLDHRIAMSFLVMGL +E P VANGLKANGVDCTEGEASLIVRGV---PGGKGIGGGTVATHLDHRIAMSFLVMGLAAEKP | 417 |
| Query  Sbjct | 421  418 | VTVDDATMIATSFPEFMDLMAGLGAKIELS 450 VT+DD+ MIATSFPEFMDLMAGLGAKIE S VTIDDSAMIATSFPEFMDLMAGLGAKIERS 447 |  |

>GGA89126.1 3-phosphoshikimate 1-carboxyvinyltransferase [Ochrobactrum endophyticum] Length=461

Score = 698 bits (1801), Expect = 0.0, Method: Compositional matrix adjust.

| Identities | = 361/452 (80%), Positives = 397/452 (88%), Gaps = 3/452 (1%) |  |
| --- | --- | --- |
| Query 1 | MLHGASSRPATARKSSGLSGTVRIPGDKSISHRSFMFGGLASGETRITGLLEGEDVINTG | 60 |
| Sbjct 11 | M H AS +PATA +S L G +RIPGDKSISHRSFMFGGLASGETRITGLLEGEDVINTG MSHSASLKPATAHRSRDLKGDIRIPGDKSISHRSFMFGGLASGETRITGLLEGEDVINTG | 70 |
| Query 61 | KAMQAMGARIRKEGDTWIIDGVGNGGLLAPEAPLDFGNAATGCRLTMGLVGVYDFDSTFI | 120 |
| Sbjct 71 | +AMQAMGA IRKEG TWII+G GNG LL PEAPLDFGNA TG RLTMGLVG YD +TFI RAMQAMGANIRKEGATWIIEGTGNGCLLEPEAPLDFGNAGTGARLTMGLVGTYDMRTTFI | 130 |
| Query 121 | GDASLTKRPMGRVLNPLREMGVQVKSEDGDRLPVTLRGPKTPTPITYRVPMASAQVKSAV | 180 |
| Sbjct 131 | GDASL+KRPMGRVL+PLR MGVQV++ +GDR+P+TLRGPK TPI+YRVPMASAQVKSAV GDASLSKRPMGRVLDPLRLMGVQVEAAEGDRVPLTLRGPKAATPISYRVPMASAQVKSAV | 190 |
| Query 181 | LLAGLNTPGITTVIEPIMTRDHTEKMLQGFGANLTVETDADGVRTIRLEGRGKLTGQVID | 240 |
| Sbjct 191 | LLAGLNTPGITTVIEP+MTRDHTEKMLQGFGA+LTVETD +GVR IR+ G+G+L GQ+ID LLAGLNTPGITTVIEPVMTRDHTEKMLQGFGADLTVETDREGVRHIRIVGQGQLKGQIID | 250 |
| Query 241 | VPGDPSSTAFPLVAALLVPGSDVTILNVLMNPTRTGLILTLQEMGADIEVINPRLAGGED | 300 |
| Sbjct 251 | VPGDPSSTAFPLVAAL+VPGSDVTI NVLMNPTRTGLILTLQEMGADIE++N RLAGGED VPGDPSSTAFPLVAALIVPGSDVTIRNVLMNPTRTGLILTLQEMGADIEILNARLAGGED | 310 |
| Query 301 | VADLRVRSSTLKGVTVPEDRAPSMIDEYPILavaaafaegaTVMNGLEELRVKESDRLSA | 360 |
| Sbjct 311 | VADLRVR+S LKGV+VP +RAPSMIDEYP+LA+AA+FAEG TVM+GLEELRVKESDRLSA VADLRVRASELKGVSVPPERAPSMIDEYPVLAIAASFAEGETVMDGLEELRVKESDRLSA | 370 |
| Query 361 | VANGLKLNGVDCDEGETSLVVRGRPDGKGLGNASGAAVATHLDHRIAMSFLVMGLVSENP | 420 |
| Sbjct 371 | VA GLK NGVDC EG SL VRG G G VATHLDHRIAMSFLVMGL +E P VATGLKANGVDCTEGAASLAVRGV---PGGKGLGGGTVATHLDHRIAMSFLVMGLAAEKP | 427 |
| Query 421  Sbjct 428 | VTVDDATMIATSFPEFMDLMAGLGAKIELSDT 452 VTVDD+ +IATSFPEFMDLM GLGAKI+ +T VTVDDSAIIATSFPEFMDLMTGLGAKIDTVET 459 |  |

```
>WP_184221298.1 3-phosphoshikimate 1-carboxyvinyltransferase [Paenochrobactrum gallinarii]
MBB6260669.1 3-phosphoshikimate 1-carboxyvinyltransferase [Paenochrobactrum gallinarii]
Length=446

Score = 698 bits (1801), Expect = 0.0, Method: Compositional matrix adjust. Identities = 354/448 (79%), Positives = 392/448 (88%), Gaps = 3/448 (1%)

Query 1	MLHGASSRPATARKSSGLSGTVRIPGDKSISHRSFMFGGLASGETRITGLLEGEDVINTG 60 M H AS +PATAR+SS L G +RIPGDKSISHRSFMFGGLASGETRITGLLEGED+INTG
Sbjct 1	MSHSASPKPATARRSSELKGEIRIPGDKSISHRSFMFGGLASGETRITGLLEGEDIINTG 60
```

| Query | 61 | KAMQAMGARIRKEGDTWIIDGVGNGGLLAPEAPLDFGNAATGCRLTMGLVGVYDFDSTFI | 120 |
| --- | --- | --- | --- |
| Sbjct | 61 | +AMQAMGA+IRKEGD WII+G GNG LL PEAP DFGNA TG RLTMGLVGVYD +TF RAMQAMGAQIRKEGDEWIINGTGNGCLLEPEAPFDFGNAGTGARLTMGLVGVYDMKTTFT | 120 |
| Query | 121 | GDASLTKRPMGRVLNPLREMGVQVKSEDGDRLPVTLRGPKTPTPITYRVPMASAQVKSAV | 180 |
| Sbjct | 121 | GDASLT RPM RVL+PLR MGVQV++ GDR+P+TLRGPK PI YRVPMASAQVKSAV GDASLTSRPMARVLDPLRLMGVQVEAASGDRMPLTLRGPKLAAPIRYRVPMASAQVKSAV | 180 |
| Query | 181 | LLAGLNTPGITTVIEPIMTRDHTEKMLQGFGANLTVETDADGVRTIRLEGRGKLTGQVID | 240 |
| Sbjct | 181 | LLAGLNTPGITTVIEP+MTRDHTEKMLQGFGA+L+VETD DGVR I + G+GKLTGQ ID LLAGLNTPGITTVIEPVMTRDHTEKMLQGFGADLSVETDNDGVRHIHIVGQGKLTGQTID | 240 |
| Query | 241 | VPGDPSSTAFPLVAALLVPGSDVTILNVLMNPTRTGLILTLQEMGADIEVINPRLAGGED | 300 |
| Sbjct | 241 | VPGDPSSTAFPLVAALLV GSDVTI NVLMNPTRTGLI TLQEMGA+IE++N RLAGGED VPGDPSSTAFPLVAALLVEGSDVTIRNVLMNPTRTGLITTLQEMGANIEILNARLAGGED | 300 |
| Query | 301 | VADLRVRSSTLKGVTVPEDRAPSMIDEYPILavaaafaegaTVMNGLEELRVKESDRLSA | 360 |
| Sbjct | 301 | VADLRVR+S LKGVTVP +RAPSMIDEYP+LA+AA+FAEG T+M+GL+ELRVKESDRL+A VADLRVRASDLKGVTVPPERAPSMIDEYPVLAIAASFAEGETIMDGLDELRVKESDRLAA | 360 |
| Query | 361 | VANGLKLNGVDCDEGETSLVVRGRPDGKGLGNASGAAVATHLDHRIAMSFLVMGLVSENP | 420 |
| Sbjct | 361 | VA GL+ NGVDC EGE SL VRG P GKG+G G VATHLDHRIAMSFLVMG+ +E P VARGLEANGVDCTEGEMSLTVRGTPSGKGIG---GGTVATHLDHRIAMSFLVMGMAAEKP | 417 |
| Query  Sbjct | 421  418 | VTVDDATMIATSFPEFMDLMAGLGAKIE 448 VTVDD MIATSFPEF ++M GLGA+ + VTVDDGNMIATSFPEFFEMMRGLGAQFD 445 |  |

>WP_183823742.1 3-phosphoshikimate 1-carboxyvinyltransferase [Rhizobium sp. BK377] MBB3461087.1 3-phosphoshikimate 1-carboxyvinyltransferase [Rhizobium sp. BK377]

Length=450

Score = 697 bits (1800), Expect = 0.0, Method: Compositional matrix adjust.

| Identities | = 346/448 (77%), Positives = 393/448 (88%), Gaps = 0/448 (0%) |  |
| --- | --- | --- |
| Query 1 | MLHGASSRPATARKSSGLSGTVRIPGDKSISHRSFMFGGLASGETRITGLLEGEDVINTG | 60 |
| Sbjct 1 | M G++S+PA ARKS+GLSGTVRIPGDKSISHR+ + GGLASGETRITGLLEGEDV+NTG MSQGSASKPAIARKSAGLSGTVRIPGDKSISHRALILGGLASGETRITGLLEGEDVLNTG | 60 |
| Query 61 | KAMQAMGARIRKEGDTWIIDGVGNGGLLAPEAPLDFGNAATGCRLTMGLVGVYDFDSTFI | 120 |
| Sbjct 61 | KAMQAMGA IRKE D W ++G GNG LLAPE PLDFGN+ TG RL MGL GVYDFD+ F KAMQAMGAVIRKEDDAWTVNGTGNGALLAPEVPLDFGNSGTGARLIMGLAGVYDFDTVFT | 120 |
| Query 121 | GDASLTKRPMGRVLNPLREMGVQVKSEDGDRLPVTLRGPKTPTPITYRVPMASAQVKSAV | 180 |
| Sbjct 121 | GDASL++RPMGRVL+PLR MGVQVK+ +GDRLPV LRGP+TP PI Y +P+ASAQVKSA GDASLSRRPMGRVLDPLRRMGVQVKAVEGDRLPVMLRGPETPAPIRYTLPVASAQVKSAA | 180 |
| Query 181 | LLAGLNTPGITTVIEPIMTRDHTEKMLQGFGANLTVETDADGVRTIRLEGRGKLTGQVID | 240 |
| Sbjct 181 | LLAGLN PG+TTVIEP +TRDHTEKML GFGA L+++TD DG RTIRL+GRGKLTGQ+ID LLAGLNIPGVTTVIEPAITRDHTEKMLAGFGAELSIKTDKDGTRTIRLQGRGKLTGQIID | 240 |
| Query 241 | VPGDPSSTAFPLVAALLVPGSDVTILNVLMNPTRTGLILTLQEMGADIEVINPRLAGGED | 300 |
| Sbjct 241 | VPGDPSS AFPLVAALLVPGSD+TI NVLMN TRTGLILTLQEMGADIE+++PRLAGGED VPGDPSSAAFPLVAALLVPGSDITISNVLMNQTRTGLILTLQEMGADIEILDPRLAGGED | 300 |
| Query 301 | VADLRVRSSTLKGVTVPEDRAPSMIDEYPILavaaafaegaTVMNGLEELRVKESDRLSA | 360 |
| Sbjct 301 | +ADLRVR S L G++VP +RAPSMIDEYP+LAVAAAFA+G TVMNGL+ELR+KESDRL+ IADLRVRHSELTGISVPTERAPSMIDEYPVLAVAAAFAKGRTVMNGLQELRIKESDRLTT | 360 |
| Query 361 | VANGLKLNGVDCDEGETSLVVRGRPDGKGLGNASGAAVATHLDHRIAMSFLVMGLVSENP | 420 |
| Sbjct 361 | V GLKLNGV+C+EG+ L V GRPDGKGLGNA G V THLDHRIAMSFLVMGL SE+P VVEGLKLNGVECEEGKDWLSVTGRPDGKGLGNAGGEQVITHLDHRIAMSFLVMGLASEHP | 420 |
| Query 421  Sbjct 421 | VTVDDATMIATSFPEFMDLMAGLGAKIE 448 V +DD ++IATSFP+FMDLM LGAKIE VGIDDGSVIATSFPQFMDLMESLGAKIE 448 |  |

>WP_173515182.1 3-phosphoshikimate 1-carboxyvinyltransferase [Ensifer sesbaniae] NRQ13150.1 3-phosphoshikimate 1-carboxyvinyltransferase [Ensifer sesbaniae]

Length=448

Score = 697 bits (1800), Expect = 0.0, Method: Compositional matrix adjust.

| Identities | = 358/447 (80%), Positives = 397/447 (89%), Gaps = 0/447 (0%) |  |
| --- | --- | --- |
| Query 1 | MLHGASSRPATARKSSGLSGTVRIPGDKSISHRSFMFGGLASGETRITGLLEGEDVINTG | 60 |
| Sbjct 1 | M G PATARKS GL G+ RIPGDKSISHRS M G LA+GETR+TGLLEG+DVI TG MSQGLGQSPATARKSDGLKGSTRIPGDKSISHRSLMLGALAAGETRVTGLLEGDDVIATG | 60 |
| Query 61 | KAMQAMGARIRKEGDTWIIDGVGNGGLLAPEAPLDFGNAATGCRLTMGLVGVYDFDSTFI | 120 |
| Sbjct 61 | AMQAMGA IRK+GDTWI++GVGNG LLAPEAPLDFGNA TGCRL MGL GVYDF++TFI HAMQAMGAGIRKDGDTWIVNGVGNGALLAPEAPLDFGNAGTGCRLIMGLAGVYDFETTFI | 120 |
| Query 121 | GDASLTKRPMGRVLNPLREMGVQVKSEDGDRLPVTLRGPKTPTPITYRVPMASAQVKSAV | 180 |
| Sbjct 121 | GD+SL +RPM RVL+PLREMGVQVK+ GDRLPV LRGPKTPTPITYRVP+ASAQVKSAV GDSSLGRRPMARVLDPLREMGVQVKAAAGDRLPVILRGPKTPTPITYRVPVASAQVKSAV | 180 |
| Query 181 | LLAGLNTPGITTVIEPIMTRDHTEKMLQGFGANLTVETDADGVRTIRLEGRGKLTGQVID | 240 |
| Sbjct 181 | LLAGL+ PGITTVIEP++TRDHTE+ML+GFGA L+VETDA GVRTIRLEGRG+L GQ I+ LLAGLDAPGITTVIEPVLTRDHTERMLRGFGAELSVETDAAGVRTIRLEGRGRLVGQEIE | 240 |
| Query 241 | VPGDPSSTAFPLVAALLVPGSDVTILNVLMNPTRTGLILTLQEMGADIEVINPRLAGGED | 300 |
| Sbjct 241 | VPGDPSS AFPLVAAL+VPGSD+TI +VLMNPTRTGLILTLQEMGADIEVI+ R AGGED VPGDPSSAAFPLVAALVVPGSDITIRSVLMNPTRTGLILTLQEMGADIEVIDIRQAGGED | 300 |
| Query 301 | VADLRVRSSTLKGVTVPEDRAPSMIDEYPILavaaafaegaTVMNGLEELRVKESDRLSA | 360 |
| Sbjct 301 | VADLRVR S LKGVTVPE+RA SMIDEYP+LAVAAA AEGATVM+GL+ELRVKESDRL+A VADLRVRHSELKGVTVPEERAASMIDEYPVLAVAAALAEGATVMSGLDELRVKESDRLAA | 360 |
| Query 361 | VANGLKLNGVDCDEGETSLVVRGRPDGKGLGNASGAAVATHLDHRIAMSFLVMGLVSENP | 420 |
| Sbjct 361 | +A GLK +GVDCD G++SLVVRGRP GKGLG ASGA V THLDHRIAMSFLV+GL SE+P IAAGLKRSGVDCDGGKSSLVVRGRPGGKGLGAASGAVVKTHLDHRIAMSFLVLGLASEHP | 420 |
| Query 421  Sbjct 421 | VTVDDATMIATSFPEFMDLMAGLGAKI 447 V VDD +MIATSFP F DLM GLGA+I VQVDDVSMIATSFPGFTDLMTGLGAEI 447 |  |

>AAK27445.1 5-enolpyruvyl shikimate 3-phosphate synthase [Brucella abortus] Length=480

Score = 697 bits (1800), Expect = 0.0, Method: Compositional matrix adjust.

| Identities | = 359/447 (80%), Positives = 394/447 (88%), Gaps = 3/447 (1%) |  |
| --- | --- | --- |
| Query 1 | MLHGASSRPATARKSSGLSGTVRIPGDKSISHRSFMFGGLASGETRITGLLEGEDVINTG | 60 |
| Sbjct 31 | M H A +PATAR S L+G +RIPGDKSIS RSFMFGGLASG+TRITGLLEGEDVINTG MSHSACPKPATARHSQALTGEIRIPGDKSISTRSFMFGGLASGKTRITGLLEGEDVINTG | 90 |
| Query 61 | KAMQAMGARIRKEGDTWIIDGVGNGGLLAPEAPLDFGNAATGCRLTMGLVGVYDFDSTFI | 120 |
| Sbjct 91 | +AMQAMGARIRKEGD WII+GVGNG LL PEAPLDFGNA TG RLTMGLVG YD ++FI RAMQAMGARIRKEGDVWIINGVGNGCLLQPEAPLDFGNAGTGARLTMGLVGTYDMKTSFI | 150 |
| Query 121 | GDASLTKRPMGRVLNPLREMGVQVKSEDGDRLPVTLRGPKTPTPITYRVPMASAQVKSAV | 180 |
| Sbjct 151 | GDASL+KRPMGRVLNPLREMGVQV++ +GDR+P+TL GP+T PI YRVPMASAQVKSAV GDASLSKRPMGRVLNPLREMGVQVEAAEGDRMPLTLIGPRTANPIAYRVPMASAQVKSAV | 210 |
| Query 181 | LLAGLNTPGITTVIEPIMTRDHTEKMLQGFGANLTVETDADGVRTIRLEGRGKLTGQVID | 240 |
| Sbjct 211 | LLAGLNTPG+TTVIEP+MTRDHTEKMLQGFGA+LTVETD DGVR IR+ G+GKLTGQ ID LLAGLNTPGVTTVIEPVMTRDHTEKMLQGFGADLTVETDKDGVRHIRIVGQGKLTGQTID | 270 |
| Query 241 | VPGDPSSTAFPLVAALLVPGSDVTILNVLMNPTRTGLILTLQEMGADIEVINPRLAGGED | 300 |
| Sbjct 271 | VPGDPSSTAFPLV ALLV GS+VTI NVLMNPTRTGLILTLQEMGADIE+I+PRLAGGED VPGDPSSTAFPLVPALLVEGSEVTIRNVLMNPTRTGLILTLQEMGADIEIIDPRLAGGED | 330 |
| Query 301 | VADLRVRSSTLKGVTVPEDRAPSMIDEYPILavaaafaegaTVMNGLEELRVKESDRLSA | 360 |
| Sbjct 331 | VADLRV++S LKGV VP +RAPSMIDEYP+LA+ A+FAEG TVM+GL+ELRVKESDRL+A VADLRVKASKLKGVVVPPERAPSMIDEYPVLAIXASFAEGETVMDGLDELRVKESDRLAA | 390 |
| Query 361 | VANGLKLNGVDCDEGETSLVVRGRPDGKGLGNASGAAVATHLDHRIAMSFLVMGLVSENP | 420 |
| Sbjct 391 | VA GL+ NGVDC EGE SL VRGR G G VATHLDHRIAMSFLVMGL SE P  VARGLEANGVDCTEGEMSLTVRGR---PGGKGLGGGTVATHLDHRIAMSFLVMGLASEKP | 447 |

Query 421 VTVDDATMIATSFPEFMDLMAGLGAKI 447 VTVDD+TMIATSFPEFM +MAGLGAKI

Sbjct 448 VTVDDSTMIATSFPEFMGMMAGLGAKI 474

>NUS20768.1 3-phosphoshikimate 1-carboxyvinyltransferase [Mesorhizobium sp.] Length=452

Score = 696 bits (1797), Expect = 0.0, Method: Compositional matrix adjust.

| Identities | = 357/452 (79%), Positives = 391/452 (87%), Gaps = 4/452 (1%) |  |
| --- | --- | --- |
| Query 1 | MLHGASSRPATARKSSGLSGTVRIPGDKSISHRSFMFGGLASGETRITGLLEGEDVINTG | 60 |
| Sbjct 1 | M H A+++PATARKS L+GT R+PGDKSISHRSFMFGGLASGETRITGLLEGEDV+ TG MSHSAAAKPATARKSPALAGTARVPGDKSISHRSFMFGGLASGETRITGLLEGEDVMRTG | 60 |
| Query 61 | KAMQAMGARIRKEGDTWIIDGVGNGGLLAPEAPLDFGNAATGCRLTMGLVGVYDFDSTFI | 120 |
| Sbjct 61 | AM+AMGA I K G W+I G GNG LL PE PLDFGNA TG RLTMGLVG YD ++TFI AAMKAMGAHIEKNGAEWVIRGTGNGALLQPEGPLDFGNAGTGSRLTMGLVGTYDMETTFI | 120 |
| Query 121 | GDASLTKRPMGRVLNPLREMGVQV-KSEDGDRLPVTLRGPKTPTPITYRVPMASAQVKSA | 179 |
| Sbjct 121 | GDASL+ RPMGRVL+PLR+MGVQV K+ GDR+P+TLRGPK PITYRVPMASAQVKSA GDASLSGRPMGRVLDPLRQMGVQVLKAAPGDRMPITLRGPKHAAPITYRVPMASAQVKSA | 180 |
| Query 180 | VLLAGLNTPGITTVIEPIMTRDHTEKMLQGFGANLTVETDADGVRTIRLEGRGKLTGQVI | 239 |
| Sbjct 181 | VLLAGLNTPGITTVIEP+MTRDHTEKML+GFGANLTVETD GVR I +EG+GKLTGQ I VLLAGLNTPGITTVIEPVMTRDHTEKMLKGFGANLTVETDERGVRHIFIEGQGKLTGQTI | 240 |
| Query 240 | DVPGDPSSTAFPLVAALLVPGSDVTILNVLMNPTRTGLILTLQEMGADIEVINPRLAGGE | 299 |
| Sbjct 241 | VPGDPSS FPLVAAL+VPGSD+TI NVLMNPTRTGL+LTLQEMG I+++NPR AGGE AVPGDPSSAGFPLVAALIVPGSDITIENVLMNPTRTGLLLTLQEMGGKIDILNPRNAGGE | 300 |
| Query 300 | DVADLRVRSSTLKGVTVPEDRAPSMIDEYPILavaaafaegaTVMNGLEELRVKESDRLS | 359 |
| Sbjct 301 | DVADLRVRSS LKGV VP +RAPSMIDEYP+LAVAA+FAEG T+M GLEELRVKESDRLS DVADLRVRSSDLKGVVVPPERAPSMIDEYPVLAVAASFAEGETLMQGLEELRVKESDRLS | 360 |
| Query 360 | AVANGLKLNGVDCDEGETSLVVRGRPDGKGLG---NASGAAVATHLDHRIAMSFLVMGLV | 416 |
| Sbjct 361 | AVANGLKLNGVDC EGE SL VRGRP GKGLG N AV THLDHRIAMSFLVMGL AVANGLKLNGVDCTEGEASLAVRGRPGGKGLGAHPNGLDTAVKTHLDHRIAMSFLVMGLA | 420 |
| Query 417  Sbjct 421 | SENPVTVDDATMIATSFPEFMDLMAGLGAKIE 448  +E PVT+DDA+MIATSFPEFM LM GLGA+IE  TEKPVTIDDASMIATSFPEFMGLMKGLGAEIE 452 |  |

>WP_152096677.1 3-phosphoshikimate 1-carboxyvinyltransferase [Rhizobium sp. S-62] GES47138.1 3-phosphoshikimate 1-carboxyvinyltransferase [Rhizobium sp. S-62]

Length=449

Score = 696 bits (1796), Expect = 0.0, Method: Compositional matrix adjust.

| Identities | = 351/448 (78%), Positives = 392/448 (88%), Gaps = 0/448 (0%) |  |
| --- | --- | --- |
| Query 1 | MLHGASSRPATARKSSGLSGTVRIPGDKSISHRSFMFGGLASGETRITGLLEGEDVINTG | 60 |
| Sbjct 1 | M H +PA AR+S+GLSGT+RIPGDKSISHRS MFGGLA+GETRITGLLEGEDV+NTG MPHDTLPQPAVARRSAGLSGTIRIPGDKSISHRSLMFGGLAAGETRITGLLEGEDVLNTG | 60 |
| Query 61 | KAMQAMGARIRKEGDTWIIDGVGNGGLLAPEAPLDFGNAATGCRLTMGLVGVYDFDSTFI | 120 |
| Sbjct 61 | KAMQA+GA IRK D WI+ G GNGGLLAPE PLDFGNA TGCRLTMGLVG YDF +TF+ KAMQALGATIRKIDDEWIVRGTGNGGLLAPETPLDFGNAGTGCRLTMGLVGSYDFKTTFV | 120 |
| Query 121 | GDASLTKRPMGRVLNPLREMGVQVKSEDGDRLPVTLRGPKTPTPITYRVPMASAQVKSAV | 180 |
| Sbjct 121 | GDASL+KRPMGR+L+PLR MG QV + GDRLPV+L GP +P+TYRVPMASAQVKSAV GDASLSKRPMGRILDPLRRMGTQVSAATGDRLPVSLHGPGVASPLTYRVPMASAQVKSAV | 180 |
| Query 181 | LLAGLNTPGITTVIEPIMTRDHTEKMLQGFGANLTVETDADGVRTIRLEGRGKLTGQVID | 240 |
| Sbjct 181 | LLAGLN PG+TTVIEP+MTRDHTEKML GFGA L+VE+DA GVRTIRLEG+GKLTGQ ID LLAGLNAPGMTTVIEPVMTRDHTEKMLAGFGAALSVESDAAGVRTIRLEGQGKLTGQTID | 240 |
| Query 241 | VPGDPSSTAFPLVAALLVPGSDVTILNVLMNPTRTGLILTLQEMGADIEVINPRLAGGED | 300 |
| Sbjct 241 | VPGDPSS AFPLVAAL+VPGSD+TI NVLMN TRTGLILTLQEMGADIE+++ R+ GED VPGDPSSAAFPLVAALIVPGSDITIRNVLMNLTRTGLILTLQEMGADIEILDRRMTSGED | 300 |
| Query 301 | VADLRVRSSTLKGVTVPEDRAPSMIDEYPILavaaafaegaTVMNGLEELRVKESDRLSA | 360 |

| Sbjct | 301 | VADLRVRSS LKG+TVP +RAPSMIDEYP+LAVAAAFAEG T+M GL+ELRVKESDRLSA VADLRVRSSALKGITVPAERAPSMIDEYPVLAVAAAFAEGTTIMQGLDELRVKESDRLSA | 360 |
| --- | --- | --- | --- |
| Query | 361 | VANGLKLNGVDCDEGETSLVVRGRPDGKGLGNASGAAVATHLDHRIAMSFLVMGLVSENP | 420 |
| Sbjct | 361 | VA GLKLNGVDC EG L V+GRPDGKGLGNA GA V+THLDHRIAMSFLVMGL SE+P VAEGLKLNGVDCIEGMDMLTVQGRPDGKGLGNAGGAKVSTHLDHRIAMSFLVMGLASEHP | 420 |
| Query  Sbjct | 421  421 | VTVDDATMIATSFPEFMDLMAGLGAKIE 448 VT+DD+ MIATSFP+F ++ LGA IE VTIDDSAMIATSFPDFFAMITRLGATIE 448 |  |

>WP_099303422.1 3-phosphoshikimate 1-carboxyvinyltransferase [Zhengella mangrovi] PHP68899.1 3-phosphoshikimate 1-carboxyvinyltransferase [Zhengella mangrovi]

Length=450

Score = 696 bits (1796), Expect = 0.0, Method: Compositional matrix adjust.

| Identities | = 356/448 (79%), Positives = 389/448 (87%), Gaps = 3/448 (1%) |  |
| --- | --- | --- |
| Query 1 | MLHGASSRPATARKSSGLSGTVRIPGDKSISHRSFMFGGLASGETRITGLLEGEDVINTG | 60 |
| Sbjct 1 | M H ++RPA +RKS+ LSGT R+PGDKSISHRSFMFGGLASGETRITGLLEGEDV+ TG MSHSGTARPALSRKSNALSGTARVPGDKSISHRSFMFGGLASGETRITGLLEGEDVLATG | 60 |
| Query 61 | KAMQAMGARIRKEGDTWIIDGVGNGGLLAPEAPLDFGNAATGCRLTMGLVGVYDFDSTFI | 120 |
| Sbjct 61 | +AM AMGARI K GDTWII G GNG LL PE PLDFGNA TGCRLTMGL GVYD + F RAMSAMGARISKVGDTWIIQGAGNGALLEPEGPLDFGNAGTGCRLTMGLAGVYDMQTVFT | 120 |
| Query 121 | GDASLTKRPMGRVLNPLREMGVQVKSEDGDRLPVTLRGPKTPTPITYRVPMASAQVKSAV | 180 |
| Sbjct 121 | GDASL++RPMGRVL+PLR MGVQV+++DGDRLPVTL GPKTPTPITYRVPMASAQVKSAV GDASLSRRPMGRVLDPLRLMGVQVQAQDGDRLPVTLHGPKTPTPITYRVPMASAQVKSAV | 180 |
| Query 181 | LLAGLNTPGITTVIEPIMTRDHTEKMLQGFGANLTVETDADGVRTIRLEGRGKLTGQVID | 240 |
| Sbjct 181 | LLAGLNTPG+TTV+EP+MTRDHTEKML GFGA+L VE DADGVRTIRL+GR LTG+VID LLAGLNTPGVTTVVEPVMTRDHTEKMLAGFGAHLEVEEDADGVRTIRLQGRPTLTGRVID | 240 |
| Query 241 | VPGDPSSTAFPLVAALLVPGSDVTILNVLMNPTRTGLILTLQEMGADIEVINPRLAGGED | 300 |
| Sbjct 241 | VPGDPSS AFPLVAAL+VPGSDV I NVLMNPTR G++ TLQEMG I++IN R AGGED VPGDPSSAAFPLVAALIVPGSDVVIENVLMNPTRNGIVTTLQEMGGQIDIINWRDAGGED | 300 |
| Query 301 | VADLRVRSSTLKGVTVPEDRAPSMIDEYPILavaaafaegaTVMNGLEELRVKESDRLSA | 360 |
| Sbjct 301 | VADLRVR+S LKGVTVP +RAPSMIDEYP LA+AAA A+G TVM GL+ELRVKESDRLSA VADLRVRASDLKGVTVPPERAPSMIDEYPALAMAAALADGETVMEGLDELRVKESDRLSA | 360 |
| Query 361 | VANGLKLNGVDCDEGETSLVVRGRPDGKGLGNASGAAVATHLDHRIAMSFLVMGLVSENP | 420 |
| Sbjct 361 | V +GLK NGVDC EGE SL VRGRPDGKGLG G VATHLDHRIAMSFLV+GL +E P VTDGLKANGVDCTEGEASLSVRGRPDGKGLG---GGTVATHLDHRIAMSFLVLGLATEKP | 417 |
| Query 421  Sbjct 418 | VTVDDATMIATSFPEFMDLMAGLGAKIE 448 VTVDDATMIATSFPEF LM GLGA IE VTVDDATMIATSFPEFFGLMGGLGAAIE 445 |  |

>WP_065797057.1 3-phosphoshikimate 1-carboxyvinyltransferase [Ensifer sp. LC163] OCP34566.1 3-phosphoshikimate 1-carboxyvinyltransferase [Ensifer sp. LC163]

Length=448

Score = 696 bits (1796), Expect = 0.0, Method: Compositional matrix adjust.

| Identities | = 355/448 (79%), Positives = 393/448 (88%), Gaps = 0/448 (0%) |  |
| --- | --- | --- |
| Query 1 | MLHGASSRPATARKSSGLSGTVRIPGDKSISHRSFMFGGLASGETRITGLLEGEDVINTG | 60 |
| Sbjct 1 | M G ++PA AR+S L GTVRIPGDKSISHR+ M GGLA+GETRI GLLEGEDV NT MSAGVIAKPAIARRSGALGGTVRIPGDKSISHRALMLGGLAAGETRIAGLLEGEDVFNTA | 60 |
| Query 61 | KAMQAMGARIRKEGDTWIIDGVGNGGLLAPEAPLDFGNAATGCRLTMGLVGVYDFDSTFI | 120 |
| Sbjct 61 | +AMQA+GA +RKEGDTWI+DGVGNG LLAPEAPL+FGNA TGCRLTMGLVGVYDF +TF+ RAMQALGATVRKEGDTWIVDGVGNGALLAPEAPLNFGNAGTGCRLTMGLVGVYDFATTFV | 120 |
| Query 121 | GDASLTKRPMGRVLNPLREMGVQVKSEDGDRLPVTLRGPKTPTPITYRVPMASAQVKSAV | 180 |
| Sbjct 121 | GDASL++RPMGRVL+PLR+MGVQV S +G RLPVTLRGPKT P++YRVPMASAQVKSAV GDASLSRRPMGRVLDPLRQMGVQVTSAEGGRLPVTLRGPKTANPVSYRVPMASAQVKSAV | 180 |
| Query 181 | LLAGLNTPGITTVIEPIMTRDHTEKMLQGFGANLTVETDADGVRTIRLEGRGKLTGQVID | 240 |

LL GLN PGITTVIEP+MTRDHTE+MLQ FG NLTVE	+ R IRLEG+G+LTGQ ID

| Sbjct | 181 | LLGGLNAPGITTVIEPVMTRDHTERMLQAFGVNLTVEAGNERERIIRLEGQGRLTGQDID | 240 |
| --- | --- | --- | --- |
| Query | 241 | VPGDPSSTAFPLVAALLVPGSDVTILNVLMNPTRTGLILTLQEMGADIEVINPRLAGGED | 300 |
| Sbjct | 241 | VPGDPSS AFPLVAALLVPGSDVTILNVLMN TRTGLI+TLQ+MGA I+V N R+AGGED VPGDPSSAAFPLVAALLVPGSDVTILNVLMNATRTGLIVTLQQMGARIDVRNTRIAGGED | 300 |
| Query | 301 | VADLRVRSSTLKGVTVPEDRAPSMIDEYPILavaaafaegaTVMNGLEELRVKESDRLSA | 360 |
| Sbjct | 301 | V DLRVRSS LKG+TVP+DRAPSMIDEYP+LAVAAAFAEG TVM GLEELRVKESDRLSA VVDLRVRSSELKGITVPDDRAPSMIDEYPVLAVAAAFAEGRTVMRGLEELRVKESDRLSA | 360 |
| Query | 361 | VANGLKLNGVDCDEGETSLVVRGRPDGKGLGNASGAAVATHLDHRIAMSFLVMGLVSENP | 420 |
| Sbjct | 361 | V GLKLNGVDC++G + LVV GRP GKGLGNA+G AVAT LDHRIAMSFLVMGL SE+P VVAGLKLNGVDCEDGPSFLVVHGRPGGKGLGNAAGTAVATDLDHRIAMSFLVMGLASEHP | 420 |
| Query  Sbjct | 421  421 | VTVDDATMIATSFPEFMDLMAGLGAKIE 448 VTVDD TMIATSFP F++LM GLGA IE VTVDDTTMIATSFPGFVELMTGLGATIE 448 |  |

>WP_105741050.1 3-phosphoshikimate 1-carboxyvinyltransferase [Phyllobacterium phragmitis]

PRD44165.1 3-phosphoshikimate 1-carboxyvinyltransferase [Phyllobacterium phragmitis]

Length=451

Score = 696 bits (1796), Expect = 0.0, Method: Compositional matrix adjust.

| Identities | = 361/452 (80%), Positives = 396/452 (88%), Gaps = 3/452 (1%) |  |
| --- | --- | --- |
| Query 1 | MLHGASSRPATARKSSGLSGTVRIPGDKSISHRSFMFGGLASGETRITGLLEGEDVINTG | 60 |
| Sbjct 1 | M H AS +PATAR+S LSG +RIPGDKSISHRSFMFGGLASGETRITGLLEGEDVINTG MSHSASPKPATARRSRDLSGDIRIPGDKSISHRSFMFGGLASGETRITGLLEGEDVINTG | 60 |
| Query 61 | KAMQAMGARIRKEGDTWIIDGVGNGGLLAPEAPLDFGNAATGCRLTMGLVGVYDFDSTFI | 120 |
| Sbjct 61 | +AMQAMGA+IRK+G TWII G GNG LL PEAPLDFGNA TG RLTMGLVG YD ++ FI RAMQAMGAQIRKDGATWIIQGTGNGCLLEPEAPLDFGNAGTGARLTMGLVGTYDMETIFI | 120 |
| Query 121 | GDASLTKRPMGRVLNPLREMGVQVKSEDGDRLPVTLRGPKTPTPITYRVPMASAQVKSAV | 180 |
| Sbjct 121 | GDASL+KRPMGRVLNPLREMGVQVK+ +GDRLP+TL GPK PI+YRVPMASAQVKSAV GDASLSKRPMGRVLNPLREMGVQVKAAEGDRLPLTLHGPKVAAPISYRVPMASAQVKSAV | 180 |
| Query 181 | LLAGLNTPGITTVIEPIMTRDHTEKMLQGFGANLTVETDADGVRTIRLEGRGKLTGQVID | 240 |
| Sbjct 181 | LLAGLNTPGITTVIEPIMTRDHTEKMLQGFGA+++VETD DGVR I++ G+G+L GQ ID LLAGLNTPGITTVIEPIMTRDHTEKMLQGFGADISVETDRDGVRHIQIVGQGELKGQTID | 240 |
| Query 241 | VPGDPSSTAFPLVAALLVPGSDVTILNVLMNPTRTGLILTLQEMGADIEVINPRLAGGED | 300 |
| Sbjct 241 | VPGDPSSTAFPLVAAL+VPGSDVTI NVLMNPTRTGLILTLQEMGA+I+++N RLAGGED VPGDPSSTAFPLVAALIVPGSDVTIRNVLMNPTRTGLILTLQEMGAEIDILNARLAGGED | 300 |
| Query 301 | VADLRVRSSTLKGVTVPEDRAPSMIDEYPILavaaafaegaTVMNGLEELRVKESDRLSA | 360 |
| Sbjct 301 | VADLRVR S LKGV VP +RAPSMIDEYP+LAVAAAFAEG TVM+GLEELRVKESDRLSA VADLRVRGSDLKGVGVPPERAPSMIDEYPVLAVAAAFAEGETVMDGLEELRVKESDRLSA | 360 |
| Query 361 | VANGLKLNGVDCDEGETSLVVRGRPDGKGLGNASGAAVATHLDHRIAMSFLVMGLVSENP | 420 |
| Sbjct 361 | VANGLK NGVDC EGE SL VRG G G V THLDHRIAMSFLVMGL +E P VANGLKANGVDCTEGEASLTVRGT---PGGKGIGGGTVETHLDHRIAMSFLVMGLAAEKP | 417 |
| Query 421  Sbjct 418 | VTVDDATMIATSFPEFMDLMAGLGAKIELSDT 452 VT+DD+ MIATSFPEFM LM G+GA+IE ++T VTIDDSAMIATSFPEFMGLMRGVGARIEAAET 449 |  |

>WP_025425507.1 MULTISPECIES: 3-phosphoshikimate 1-carboxyvinyltransferase [Ensifer] AHK43039.1 3-phosphoshikimate 1-carboxyvinyltransferase [Ensifer adhaerens

OV14]

KQW63226.1 3-phosphoshikimate 1-carboxyvinyltransferase [Ensifer sp. Root1252] KQW85241.1 3-phosphoshikimate 1-carboxyvinyltransferase [Ensifer sp. Root127] KQY75634.1 3-phosphoshikimate 1-carboxyvinyltransferase [Ensifer sp. Root142] KRC84046.1 3-phosphoshikimate 1-carboxyvinyltransferase [Ensifer sp. Root231]

Length=447

Score = 695 bits (1794), Expect = 0.0, Method: Compositional matrix adjust.

| Identities | = 354/448 (79%), Positives = 393/448 (88%), Gaps = 1/448 (0%) |  |
| --- | --- | --- |
| Query 1 | MLHGASSRPATARKSSGLSGTVRIPGDKSISHRSFMFGGLASGETRITGLLEGEDVINTG | 60 |
| Sbjct 1 | M G ++PA AR+S L GTVRI GDKSISHR+ M GGLA+G TRI GLLEGEDVINT MSAGLIAKPAIARRSKALGGTVRIRGDKSISHRALMLGGLAAGRTRIAGLLEGEDVINTA | 60 |
| Query 61 | KAMQAMGARIRKEGDTWIIDGVGNGGLLAPEAPLDFGNAATGCRLTMGLVGVYDFDSTFI | 120 |
| Sbjct 61 | +AM+A+GA +R+EG TWI+DG+GNG LL PEAPLDFGNA TGCRLTMGLVG YDF +TF+ RAMRALGATVREEGGTWIVDGLGNGALLEPEAPLDFGNAGTGCRLTMGLVGAYDFTTTFV | 120 |
| Query 121 | GDASLTKRPMGRVLNPLREMGVQVKSEDGDRLPVTLRGPKTPTPITYRVPMASAQVKSAV | 180 |
| Sbjct 121 | GDASL+KRPMGRVL+PLR+MGVQV S +G RLPVTLRGPKT P+ YRVPMASAQVKSAV GDASLSKRPMGRVLDPLRQMGVQVASAEGGRLPVTLRGPKTANPVNYRVPMASAQVKSAV | 180 |
| Query 181 | LLAGLNTPGITTVIEPIMTRDHTEKMLQGFGANLTVETDADGVRTIRLEGRGKLTGQVID | 240 |
| Sbjct 181 | LLAGLN PGITTVIEP++TRDHTE+MLQ FGANL VET +G R IRLEG+G+LTGQ ID LLAGLNAPGITTVIEPVITRDHTERMLQAFGANLAVETGNEGERIIRLEGQGRLTGQDID | 240 |
| Query 241 | VPGDPSSTAFPLVAALLVPGSDVTILNVLMNPTRTGLILTLQEMGADIEVINPRLAGGED | 300 |
| Sbjct 241 | VPGDPSS AFPLVAALLVPGSD+TILNVLMNP RTGLI+TL EMGA I+V+N R AGGED VPGDPSSAAFPLVAALLVPGSDITILNVLMNPIRTGLIVTLLEMGAQIDVLNARTAGGED | 300 |
| Query 301 | VADLRVRSSTLKGVTVPEDRAPSMIDEYPILavaaafaegaTVMNGLEELRVKESDRLSA | 360 |
| Sbjct 301 | VADLRVR S LKG+TVPEDRAPSMIDEYP+LAVAAAFAEG T+M+GLEELRVKESDRLSA VADLRVRYSELKGITVPEDRAPSMIDEYPVLAVAAAFAEGRTIMSGLEELRVKESDRLSA | 360 |
| Query 361 | VANGLKLNGVDCDEGETSLVVRGRPDGKGLGNASGAAVATHLDHRIAMSFLVMGLVSENP | 420 |
| Sbjct 361 | VA GLKL GVDC+E +LVV GRP GKGLGNA+ AAVATHLDHRIAMSFLVMGL SE+P VAAGLKLTGVDCEEAPAALVVHGRPGGKGLGNAA-AAVATHLDHRIAMSFLVMGLASEHP | 419 |
| Query 421  Sbjct 420 | VTVDDATMIATSFPEFMDLMAGLGAKIE 448 VTVDDATMIATSFP F++LMAGLGA IE VTVDDATMIATSFPGFLELMAGLGAAIE 447 |  |

>WP_057247832.1 MULTISPECIES: 3-phosphoshikimate 1-carboxyvinyltransferase [unclassified Ensifer]

KRD72961.1 3-phosphoshikimate 1-carboxyvinyltransferase [Ensifer sp. Root278] MBD9505296.1 3-phosphoshikimate 1-carboxyvinyltransferase [Ensifer sp. ENS10]

Length=448

Score = 695 bits (1793), Expect = 0.0, Method: Compositional matrix adjust.

| Identities | = 363/447 (81%), Positives = 402/447 (90%), Gaps = 0/447 (0%) |  |
| --- | --- | --- |
| Query 1 | MLHGASSRPATARKSSGLSGTVRIPGDKSISHRSFMFGGLASGETRITGLLEGEDVINTG | 60 |
| Sbjct 1 | M G PATARKS GL GTVRIPGDKSISHRS M GGLA+GETRITGLL+G DVI TG MSQGLGQSPATARKSDGLKGTVRIPGDKSISHRSLMLGGLAAGETRITGLLQGADVIATG | 60 |
| Query 61 | KAMQAMGARIRKEGDTWIIDGVGNGGLLAPEAPLDFGNAATGCRLTMGLVGVYDFDSTFI | 120 |
| Sbjct 61 | +AMQA+GARIRKEGDTWI++GVGNG LLAPEAPLDFGNA TGCRL MGLVGVYDF++TFI RAMQAIGARIRKEGDTWIVNGVGNGALLAPEAPLDFGNAGTGCRLIMGLVGVYDFETTFI | 120 |
| Query 121 | GDASLTKRPMGRVLNPLREMGVQVKSEDGDRLPVTLRGPKTPTPITYRVPMASAQVKSAV | 180 |
| Sbjct 121 | GDASL++RPM RVL+PLREMGVQV++ GDRLPVTLRGPKTPTP+TYRVP+ASAQVKSAV GDASLSRRPMARVLDPLREMGVQVEAATGDRLPVTLRGPKTPTPVTYRVPVASAQVKSAV | 180 |
| Query 181 | LLAGLNTPGITTVIEPIMTRDHTEKMLQGFGANLTVETDADGVRTIRLEGRGKLTGQVID | 240 |
| Sbjct 181 | L AGLN PGITTVIEP +TRDHTE+ML+GFGA L+VETDA GVRTIRLEGRG+L Q ID LFAGLNAPGITTVIEPAITRDHTERMLRGFGAELSVETDATGVRTIRLEGRGRLVAQEID | 240 |
| Query 241 | VPGDPSSTAFPLVAALLVPGSDVTILNVLMNPTRTGLILTLQEMGADIEVINPRLAGGED | 300 |
| Sbjct 241 | VPGDPSSTAFPLVAAL+VPGSD+TI NVLMNPTRTGLILTLQEMG DIEVI+ GGED VPGDPSSTAFPLVAALVVPGSDITIRNVLMNPTRTGLILTLQEMGGDIEVIDIGQVGGED | 300 |
| Query 301 | VADLRVRSSTLKGVTVPEDRAPSMIDEYPILavaaafaegaTVMNGLEELRVKESDRLSA | 360 |
| Sbjct 301 | VADLRVR S LKGVTVPE+RA SMIDEYPILA+AAAFAEGAT+M+GL+ELRVKESDRL+A VADLRVRHSELKGVTVPEERAASMIDEYPILAIAAAFAEGATIMHGLDELRVKESDRLAA | 360 |
| Query 361 | VANGLKLNGVDCDEGETSLVVRGRPDGKGLGNASGAAVATHLDHRIAMSFLVMGLVSENP | 420 |
| Sbjct 361 | +A GLKLNGVDC+EG +SLVVRGR GKGLGNASG V THLDHRIAMSFLV+GL +E+P  IAAGLKLNGVDCEEGRSSLVVRGRRGGKGLGNASGGVVKTHLDHRIAMSFLVLGLAAEHP | 420 |

Query 421 VTVDDATMIATSFPEFMDLMAGLGAKI 447 V VDD+TMIATSFP ++DLMAGLGA+I

Sbjct 421 VKVDDSTMIATSFPGYIDLMAGLGARI 447

>WP_113943644.1 3-phosphoshikimate 1-carboxyvinyltransferase [Pseudochrobactrum asaccharolyticum]

RBO97671.1 3-phosphoshikimate 1-carboxyvinyltransferase [Pseudochrobactrum asaccharolyticum]

Length=450

Score = 694 bits (1792), Expect = 0.0, Method: Compositional matrix adjust.

| Identities | = 352/453 (78%), Positives = 391/453 (86%), Gaps = 3/453 (1%) |  |
| --- | --- | --- |
| Query 1 | MLHGASSRPATARKSSGLSGTVRIPGDKSISHRSFMFGGLASGETRITGLLEGEDVINTG | 60 |
| Sbjct 1 | M H S+PATAR+S L G +RIPGDKSISHRSFMFGGLASGETRI+GLLEGED++ TG MSHSQVSKPATARRSRDLKGDIRIPGDKSISHRSFMFGGLASGETRISGLLEGEDILATG | 60 |
| Query 61 | KAMQAMGARIRKEGDTWIIDGVGNGGLLAPEAPLDFGNAATGCRLTMGLVGVYDFDSTFI | 120 |
| Sbjct 61 | KAMQAMGA+IRKEGD WII+G GNG LL E PLDFGNA TG RLTMGLVG YD ++F+ KAMQAMGAQIRKEGDIWIINGTGNGCLLESETPLDFGNAGTGVRLTMGLVGSYDMKTSFL | 120 |
| Query 121 | GDASLTKRPMGRVLNPLREMGVQVKSEDGDRLPVTLRGPKTPTPITYRVPMASAQVKSAV | 180 |
| Sbjct 121 | GDASLT RPM RVLNPLREMGVQV++ +GDRLP+TL GP+T PI YRVPMASAQVKSAV GDASLTSRPMARVLNPLREMGVQVEAAEGDRLPLTLHGPRTANPIRYRVPMASAQVKSAV | 180 |
| Query 181 | LLAGLNTPGITTVIEPIMTRDHTEKMLQGFGANLTVETDADGVRTIRLEGRGKLTGQVID | 240 |
| Sbjct 181 | LLAGLNTPG+TTVIEPIMTRDHTEKMLQGFGA+L+VETD DGVR I + G+GKLTGQ ID LLAGLNTPGVTTVIEPIMTRDHTEKMLQGFGADLSVETDKDGVRHIHIAGQGKLTGQTID | 240 |
| Query 241 | VPGDPSSTAFPLVAALLVPGSDVTILNVLMNPTRTGLILTLQEMGADIEVINPRLAGGED | 300 |
| Sbjct 241 | VPGDPSSTAFPLVAAL+ GSD+ I NVLMNPTRTGLI TLQEMGA I++++PRLAGGED VPGDPSSTAFPLVAALITEGSDLIIRNVLMNPTRTGLITTLQEMGAQIDILDPRLAGGED | 300 |
| Query 301 | VADLRVRSSTLKGVTVPEDRAPSMIDEYPILavaaafaegaTVMNGLEELRVKESDRLSA | 360 |
| Sbjct 301 | VADLRVRSS L G+TVP DRAPSMIDEYP+LA+AAAFAEG T+M+GL+ELRVKESDRL+A VADLRVRSSKLTGITVPVDRAPSMIDEYPVLAIAAAFAEGETIMDGLDELRVKESDRLAA | 360 |
| Query 361 | VANGLKLNGVDCDEGETSLVVRGRPDGKGLGNASGAAVATHLDHRIAMSFLVMGLVSENP | 420 |
| Sbjct 361 | VA GL+ NGVDC EGE SL VRG P GKG G G VATHLDHRIAMSFLV+GL SE P VARGLEANGVDCTEGEMSLTVRGNPSGKGFG---GGTVATHLDHRIAMSFLVLGLASEKP | 417 |
| Query 421  Sbjct 418 | VTVDDATMIATSFPEFMDLMAGLGAKIELSDTK 453 VTVDDATMIATSFPEFMDLM LGA I +++ K VTVDDATMIATSFPEFMDLMRNLGADITVAEAK 450 |  |

>WP_120702574.1 3-phosphoshikimate 1-carboxyvinyltransferase [Rhizobium jaguaris] AYG57473.1 3-phosphoshikimate 1-carboxyvinyltransferase [Rhizobium jaguaris]

Length=451

Score = 694 bits (1792), Expect = 0.0, Method: Compositional matrix adjust.

| Identities | = 361/449 (80%), Positives = 397/449 (88%), Gaps = 0/449 (0%) |  |
| --- | --- | --- |
| Query 1 | MLHGASSRPATARKSSGLSGTVRIPGDKSISHRSFMFGGLASGETRITGLLEGEDVINTG | 60 |
| Sbjct 1 | M H RPATAR+S+ LSGT+R+PGDKSISHR+ MFGGLASGETRI+GLLEGEDV+NTG MPHDIHPRPATARRSTNLSGTIRVPGDKSISHRALMFGGLASGETRISGLLEGEDVLNTG | 60 |
| Query 61 | KAMQAMGARIRKEGDTWIIDGVGNGGLLAPEAPLDFGNAATGCRLTMGLVGVYDFDSTFI | 120 |
| Sbjct 61 | KAMQAMGA+I K G WII GVGNG LLAPE PLDFGN TG RLTMGLVGVY+F+S FI KAMQAMGAKIEKIGAEWIIKGVGNGALLAPEVPLDFGNTGTGSRLTMGLVGVYNFESIFI | 120 |
| Query 121 | GDASLTKRPMGRVLNPLREMGVQVKSEDGDRLPVTLRGPKTPTPITYRVPMASAQVKSAV | 180 |
| Sbjct 121 | GDASL+KRPMGR+L+PLR+MG QVKS +GDRLPVTL GP T +PITYRVPMASAQVKSAV GDASLSKRPMGRILDPLRQMGAQVKSSEGDRLPVTLHGPDTASPITYRVPMASAQVKSAV | 180 |
| Query 181 | LLAGLNTPGITTVIEPIMTRDHTEKMLQGFGANLTVETDADGVRTIRLEGRGKLTGQVID | 240 |
| Sbjct 181 | LLAGLN PG+TTVIEP+MTRDHTEKML GFGA+L+VET DGVRTIRLEG+GKL+GQ+ID LLAGLNAPGVTTVIEPVMTRDHTEKMLAGFGADLSVETGQDGVRTIRLEGQGKLSGQLID | 240 |
| Query 241 | VPGDPSSTAFPLVAALLVPGSDVTILNVLMNPTRTGLILTLQEMGADIEVINPRLAGGED VPGDPSSTAFPLVAAL+V GSD+TI NVLMNPTRTGLILTLQEMG DIE+++ RLAGGED | 300 |

| Sbjct | 241 | VPGDPSSTAFPLVAALIVSGSDITIRNVLMNPTRTGLILTLQEMGGDIEILDKRLAGGED | 300 |
| --- | --- | --- | --- |
| Query | 301 | VADLRVRSSTLKGVTVPEDRAPSMIDEYPILavaaafaegaTVMNGLEELRVKESDRLSA | 360 |
| Sbjct | 301 | VADLRVRSS LKG+TVP +RAPSMIDEYP+LAVAAAFAEG TVM GLEELRVKESDRLSA VADLRVRSSALKGITVPAERAPSMIDEYPVLAVAAAFAEGTTVMLGLEELRVKESDRLSA | 360 |
| Query | 361 | VANGLKLNGVDCDEGETSLVVRGRPDGKGLGNASGAAVATHLDHRIAMSFLVMGLVSENP | 420 |
| Sbjct | 361 | VA GLKLNGVDC EG SL VRGRPDGKGLG A G V T+LDHRIAMSFLVMGL SE+ VAEGLKLNGVDCTEGRDSLAVRGRPDGKGLGGAGGGEVKTYLDHRIAMSFLVMGLASEHA | 420 |
| Query  Sbjct | 421  421 | VTVDDATMIATSFPEFMDLMAGLGAKIEL 449 V VDD+TMIATSFPEF +LM LGA IE+ VRVDDSTMIATSFPEFFELMTRLGATIEV 449 |  |

>WP_022710242.1 MULTISPECIES: 3-phosphoshikimate 1-carboxyvinyltransferase [unclassified Pseudochrobactrum]

Length=450

Score = 694 bits (1792), Expect = 0.0, Method: Compositional matrix adjust.

| Identities | = 350/453 (77%), Positives = 393/453 (87%), Gaps = 3/453 (1%) |  |
| --- | --- | --- |
| Query 1 | MLHGASSRPATARKSSGLSGTVRIPGDKSISHRSFMFGGLASGETRITGLLEGEDVINTG | 60 |
| Sbjct 1 | M H S+PAT+R+S L G +RIPGDKSISHRSFMFGGLASGETRI+GLLEGED++ TG MSHSQVSKPATSRRSRDLKGDIRIPGDKSISHRSFMFGGLASGETRISGLLEGEDILATG | 60 |
| Query 61 | KAMQAMGARIRKEGDTWIIDGVGNGGLLAPEAPLDFGNAATGCRLTMGLVGVYDFDSTFI | 120 |
| Sbjct 61 | KAMQAMGA+IRKEGD WII+G GNG LL E PLDFGNA TG RLTMGLVG YD ++F+ KAMQAMGAQIRKEGDIWIINGTGNGCLLESETPLDFGNAGTGVRLTMGLVGTYDMKTSFL | 120 |
| Query 121 | GDASLTKRPMGRVLNPLREMGVQVKSEDGDRLPVTLRGPKTPTPITYRVPMASAQVKSAV | 180 |
| Sbjct 121 | GDASLT RPM RVLNPLREMGVQV++ +GDRLP+TLRGP+T PI YRVPMASAQVKSAV GDASLTSRPMARVLNPLREMGVQVEAAEGDRLPLTLRGPRTANPIRYRVPMASAQVKSAV | 180 |
| Query 181 | LLAGLNTPGITTVIEPIMTRDHTEKMLQGFGANLTVETDADGVRTIRLEGRGKLTGQVID | 240 |
| Sbjct 181 | LLAGLNTPG+TTVIEP+MTRDHTEKMLQGFGA+L+VETD DGVR I + G+GKLTGQ+ID LLAGLNTPGVTTVIEPVMTRDHTEKMLQGFGADLSVETDKDGVRHIHITGQGKLTGQIID | 240 |
| Query 241 | VPGDPSSTAFPLVAALLVPGSDVTILNVLMNPTRTGLILTLQEMGADIEVINPRLAGGED | 300 |
| Sbjct 241 | VPGDPSSTAFPLVAAL+ GSD+ I NVLMNPTRTGLI TLQEMGA I++++PRLAGGED VPGDPSSTAFPLVAALITEGSDIIIRNVLMNPTRTGLITTLQEMGAQIDILDPRLAGGED | 300 |
| Query 301 | VADLRVRSSTLKGVTVPEDRAPSMIDEYPILavaaafaegaTVMNGLEELRVKESDRLSA | 360 |
| Sbjct 301 | VADLRVRSS L G+TVP +RAPSMIDEYP+LA+AAAFAEG T+M+GL+ELRVKESDRL+A VADLRVRSSKLTGITVPVERAPSMIDEYPVLAIAAAFAEGETIMDGLDELRVKESDRLAA | 360 |
| Query 361 | VANGLKLNGVDCDEGETSLVVRGRPDGKGLGNASGAAVATHLDHRIAMSFLVMGLVSENP | 420 |
| Sbjct 361 | VA GL+ NGVDC EGE SL VRG P GKG G G VATHLDHRIAMSFLV+GL S+ P VARGLEANGVDCTEGEMSLSVRGNPSGKGFG---GGTVATHLDHRIAMSFLVLGLASQKP | 417 |
| Query 421  Sbjct 418 | VTVDDATMIATSFPEFMDLMAGLGAKIELSDTK 453 VTVDDATMIATSFPEFMDLM GLGA I ++ K VTVDDATMIATSFPEFMDLMRGLGADISMAGAK 450 |  |

>WP_127861800.1 MULTISPECIES: 3-phosphoshikimate 1-carboxyvinyltransferase [unclassified Mesorhizobium]

TGS95016.1 3-phosphoshikimate 1-carboxyvinyltransferase [bacterium M00.F.Ca.ET.177.01.1.1] TGQ51355.1 3-phosphoshikimate 1-carboxyvinyltransferase [Mesorhizobium sp.

M1C.F.Ca.ET.210.01.1.1]

TGQ67145.1 3-phosphoshikimate 1-carboxyvinyltransferase [Mesorhizobium sp. M1C.F.Ca.ET.212.01.1.1]

TGR01799.1 3-phosphoshikimate 1-carboxyvinyltransferase [Mesorhizobium sp. M1C.F.Ca.ET.204.01.1.1]

TGR22482.1 3-phosphoshikimate 1-carboxyvinyltransferase [Mesorhizobium sp. M1C.F.Ca.ET.196.01.1.1]

Length=452

Score = 694 bits (1792), Expect = 0.0, Method: Compositional matrix adjust. Identities = 354/452 (78%), Positives = 389/452 (86%), Gaps = 4/452 (1%)

Query 1	MLHGASSRPATARKSSGLSGTVRIPGDKSISHRSFMFGGLASGETRITGLLEGEDVINTG 60

| Sbjct | 1 | M H A+++PATARKS L+GT R+PGDKSISHRSFMFGGLASGETRITGLLEGEDV+ TG MSHSAAAKPATARKSPALAGTARVPGDKSISHRSFMFGGLASGETRITGLLEGEDVMRTG | 60 |
| --- | --- | --- | --- |
| Query | 61 | KAMQAMGARIRKEGDTWIIDGVGNGGLLAPEAPLDFGNAATGCRLTMGLVGVYDFDSTFI | 120 |
| Sbjct | 61 | AM+AMGA I K+G W+I G GNG LL PE PLDFGNA TG RLTMGLVG YD ++TFI AAMKAMGAHIEKKGAEWVIRGTGNGALLEPEGPLDFGNAGTGSRLTMGLVGTYDMETTFI | 120 |
| Query | 121 | GDASLTKRPMGRVLNPLREMGVQV-KSEDGDRLPVTLRGPKTPTPITYRVPMASAQVKSA | 179 |
| Sbjct | 121 | GDASL+ RPMGRVL PLR+MGVQV K+ GDR+P+TLRGPK PITYRVPMASAQVKSA GDASLSGRPMGRVLEPLRQMGVQVLKAASGDRMPITLRGPKHAAPITYRVPMASAQVKSA | 180 |
| Query | 180 | VLLAGLNTPGITTVIEPIMTRDHTEKMLQGFGANLTVETDADGVRTIRLEGRGKLTGQVI | 239 |
| Sbjct | 181 | VLLAGLNTPGITTVIEP+MTRDHTEKML+GFGANLTVETD GVR I +EG+GKLTGQ I VLLAGLNTPGITTVIEPVMTRDHTEKMLKGFGANLTVETDERGVRHIFIEGQGKLTGQTI | 240 |
| Query | 240 | DVPGDPSSTAFPLVAALLVPGSDVTILNVLMNPTRTGLILTLQEMGADIEVINPRLAGGE | 299 |
| Sbjct | 241 | VPGDPSS FPLVAAL+VPGSD+ I NVLMNPTRTGL+LTLQEMG I+++NPR AGGE AVPGDPSSAGFPLVAALIVPGSDIVIENVLMNPTRTGLLLTLQEMGGRIDILNPRNAGGE | 300 |
| Query | 300 | DVADLRVRSSTLKGVTVPEDRAPSMIDEYPILavaaafaegaTVMNGLEELRVKESDRLS | 359 |
| Sbjct | 301 | DVADLRVRSS LKGV VP +RAPSMIDEYP+LAVAA+FAEG T+M GLEELRVKESDRLS DVADLRVRSSELKGVVVPPERAPSMIDEYPVLAVAASFAEGETLMQGLEELRVKESDRLS | 360 |
| Query | 360 | AVANGLKLNGVDCDEGETSLVVRGRPDGKGLG---NASGAAVATHLDHRIAMSFLVMGLV | 416 |
| Sbjct | 361 | AVANGLKLNGVDC EGE SL VRG+P GKGLG N AV THLDHRIAMSFL+MGL AVANGLKLNGVDCSEGEASLAVRGKPGGKGLGAHPNGQDTAVKTHLDHRIAMSFLIMGLA | 420 |
| Query  Sbjct | 417  421 | SENPVTVDDATMIATSFPEFMDLMAGLGAKIE 448  +E PVT+DDA MIATSFPEFM LM GLGA+IE  TEKPVTIDDANMIATSFPEFMGLMKGLGAEIE 452 |  |

>WP_183801150.1 3-phosphoshikimate 1-carboxyvinyltransferase [Rhizobium sp. BK316] MBB3407869.1 3-phosphoshikimate 1-carboxyvinyltransferase [Rhizobium sp. BK316]

Length=450

Score = 694 bits (1791), Expect = 0.0, Method: Compositional matrix adjust.

| Identities | = 348/448 (78%), Positives = 393/448 (88%), Gaps = 0/448 (0%) |  |
| --- | --- | --- |
| Query 1 | MLHGASSRPATARKSSGLSGTVRIPGDKSISHRSFMFGGLASGETRITGLLEGEDVINTG | 60 |
| Sbjct 1 | M G++S+PA AR+SSGLSGTVRIPGDKSISHR+ M GGLASGETRI GLLEGEDV+NTG MSQGSASKPAIARRSSGLSGTVRIPGDKSISHRALMLGGLASGETRIAGLLEGEDVLNTG | 60 |
| Query 61 | KAMQAMGARIRKEGDTWIIDGVGNGGLLAPEAPLDFGNAATGCRLTMGLVGVYDFDSTFI | 120 |
| Sbjct 61 | KAMQAMGA IRKEG WII+G GNG LLAPE PLDFGN+ TG RL MGL GVYDFD+ KAMQAMGAVIRKEGGVWIINGTGNGALLAPEVPLDFGNSGTGARLIMGLAGVYDFDTVLT | 120 |
| Query 121 | GDASLTKRPMGRVLNPLREMGVQVKSEDGDRLPVTLRGPKTPTPITYRVPMASAQVKSAV | 180 |
| Sbjct 121 | GDASL++RPMGRVL+PLR MGVQVK+ +GDRLPV LRGP+TP PI Y +P+ASAQVKSAV GDASLSRRPMGRVLDPLRRMGVQVKTVEGDRLPVMLRGPETPAPIRYTLPVASAQVKSAV | 180 |
| Query 181 | LLAGLNTPGITTVIEPIMTRDHTEKMLQGFGANLTVETDADGVRTIRLEGRGKLTGQVID | 240 |
| Sbjct 181 | LLAGLN PG+TTVIEP +TRDHTEKML GFGA L+VE D G RT+RL+GRG+LTGQ+ID LLAGLNIPGVTTVIEPAITRDHTEKMLAGFGAELSVEADKGGTRTVRLQGRGRLTGQIID | 240 |
| Query 241 | VPGDPSSTAFPLVAALLVPGSDVTILNVLMNPTRTGLILTLQEMGADIEVINPRLAGGED | 300 |
| Sbjct 241 | VPGDPSS AFPLVAALLVPGSD+ I NVLMNPTRTGL+LTL+EMGADIE++NPRLAGGED VPGDPSSAAFPLVAALLVPGSDIIIPNVLMNPTRTGLVLTLREMGADIEILNPRLAGGED | 300 |
| Query 301 | VADLRVRSSTLKGVTVPEDRAPSMIDEYPILavaaafaegaTVMNGLEELRVKESDRLSA | 360 |
| Sbjct 301 | VADLRVR S L GV+VP +R PSMIDEYP+LAVAAAFA+G TVM+GL+ELRVKESDRLS VADLRVRHSELTGVSVPAERVPSMIDEYPVLAVAAAFAKGRTVMSGLQELRVKESDRLST | 360 |
| Query 361 | VANGLKLNGVDCDEGETSLVVRGRPDGKGLGNASGAAVATHLDHRIAMSFLVMGLVSENP | 420 |
| Sbjct 361 | VA+GLKLNGV+C+EG+ L V GRPDGKGLGNA+ VATHLDHRIAMSFLVMGL SE+P VADGLKLNGVECEEGKDWLSVTGRPDGKGLGNAAVKWVATHLDHRIAMSFLVMGLASEHP | 420 |
| Query 421  Sbjct 421 | VTVDDATMIATSFPEFMDLMAGLGAKIE 448 V +DD++MIATSFP+FMDLM LGA IE VGIDDSSMIATSFPQFMDLMKSLGAGIE 448 |  |

>WP_168843475.1 MULTISPECIES: 3-phosphoshikimate 1-carboxyvinyltransferase [unclassified Rhizobium]

NLR88897.1 3-phosphoshikimate 1-carboxyvinyltransferase [Rhizobium sp. P28RR-XV] NLS20625.1 3-phosphoshikimate 1-carboxyvinyltransferase [Rhizobium sp. P40RR-XXII]

Length=451

Score = 694 bits (1790), Expect = 0.0, Method: Compositional matrix adjust.

| Identities | = 347/448 (77%), Positives = 388/448 (87%), Gaps = 0/448 (0%) |  |
| --- | --- | --- |
| Query 1 | MLHGASSRPATARKSSGLSGTVRIPGDKSISHRSFMFGGLASGETRITGLLEGEDVINTG | 60 |
| Sbjct 1 | M H RPA AR+S+GL GT+RIPGDKSISHRS MFGGLASGET ITGLLEGEDV+NTG MPHDTLPRPAVARRSAGLFGTIRIPGDKSISHRSLMFGGLASGETHITGLLEGEDVLNTG | 60 |
| Query 61 | KAMQAMGARIRKEGDTWIIDGVGNGGLLAPEAPLDFGNAATGCRLTMGLVGVYDFDSTFI | 120 |
| Sbjct 61 | + MQA+GA IR++GD WI+ G GNG LLAP+ PLDFGNA TG RLTMGLVG YDF +TF+ RVMQALGAAIRRDGDAWIVSGTGNGALLAPQTPLDFGNAGTGSRLTMGLVGSYDFKTTFV | 120 |
| Query 121 | GDASLTKRPMGRVLNPLREMGVQVKSEDGDRLPVTLRGPKTPTPITYRVPMASAQVKSAV | 180 |
| Sbjct 121 | GDASL+KRPMGR+L+PLR MG QV + GDRLPVTL GP+ +P+TY VPMASAQVKSAV GDASLSKRPMGRILDPLRRMGTQVSAAAGDRLPVTLHGPRVASPLTYHVPMASAQVKSAV | 180 |
| Query 181 | LLAGLNTPGITTVIEPIMTRDHTEKMLQGFGANLTVETDADGVRTIRLEGRGKLTGQVID | 240 |
| Sbjct 181 | LLAGLN PGITTVIEP+MTRDHTE+ML GFGA+L VETDA G+RTIRLEG+G+L GQ ID LLAGLNAPGITTVIEPVMTRDHTERMLVGFGASLAVETDAAGIRTIRLEGQGRLAGQTID | 240 |
| Query 241 | VPGDPSSTAFPLVAALLVPGSDVTILNVLMNPTRTGLILTLQEMGADIEVINPRLAGGED | 300 |
| Sbjct 241 | VPGDPSS AFPLVAAL+VPGSD+TI NVLMNPTRTGLILTLQEMGADIEVI+ R+ GGED VPGDPSSAAFPLVAALIVPGSDITICNVLMNPTRTGLILTLQEMGADIEVIDRRVTGGED | 300 |
| Query 301 | VADLRVRSSTLKGVTVPEDRAPSMIDEYPILavaaafaegaTVMNGLEELRVKESDRLSA | 360 |
| Sbjct 301 | VADLRVRS LKGVTVP +RAPSMIDEYP+LAVAAAFA+G T+M GLEELRVKESDRLSA VADLRVRSGALKGVTVPAERAPSMIDEYPVLAVAAAFADGTTIMQGLEELRVKESDRLSA | 360 |
| Query 361 | VANGLKLNGVDCDEGETSLVVRGRPDGKGLGNASGAAVATHLDHRIAMSFLVMGLVSENP | 420 |
| Sbjct 361 | VA GLKLNGV+C EG SL V+GRPDGKGLG +S AV THLDHRIAMSFLVMGL SE+P VAEGLKLNGVECSEGRDSLTVQGRPDGKGLGRSSAGAVKTHLDHRIAMSFLVMGLASEHP | 420 |
| Query 421  Sbjct 421 | VTVDDATMIATSFPEFMDLMAGLGAKIE 448 V+VDD+ MI TSFP+F +M GLGA IE VSVDDSAMIPTSFPDFFAMMTGLGATIE 448 |  |

>WP_151158754.1 3-phosphoshikimate 1-carboxyvinyltransferase [Pseudochrobactrum saccharolyticum]

KAB0540717.1 3-phosphoshikimate 1-carboxyvinyltransferase [Pseudochrobactrum saccharolyticum]

MBB5090296.1 3-phosphoshikimate 1-carboxyvinyltransferase [Pseudochrobactrum saccharolyticum]

Length=450

Score = 694 bits (1790), Expect = 0.0, Method: Compositional matrix adjust.

| Identities | = 350/453 (77%), Positives = 393/453 (87%), Gaps = 3/453 (1%) |  |
| --- | --- | --- |
| Query 1 | MLHGASSRPATARKSSGLSGTVRIPGDKSISHRSFMFGGLASGETRITGLLEGEDVINTG | 60 |
| Sbjct 1 | M H S+PAT+R+S L G +RIPGDKSISHRSFMFGGLASGETRI+GLLEGED++ TG MSHSQVSKPATSRRSRDLKGDIRIPGDKSISHRSFMFGGLASGETRISGLLEGEDILATG | 60 |
| Query 61 | KAMQAMGARIRKEGDTWIIDGVGNGGLLAPEAPLDFGNAATGCRLTMGLVGVYDFDSTFI | 120 |
| Sbjct 61 | KAMQAMGA+IRK+GD WII+G GNG LL E PLDFGNA TG RLTMGLVG YD ++F+ KAMQAMGAQIRKDGDIWIINGTGNGCLLESETPLDFGNAGTGVRLTMGLVGTYDMKTSFL | 120 |
| Query 121 | GDASLTKRPMGRVLNPLREMGVQVKSEDGDRLPVTLRGPKTPTPITYRVPMASAQVKSAV | 180 |
| Sbjct 121 | GDASLT RPM RVLNPLREMGVQV++ +GDRLP+TLRGP+T PI YRVPMASAQVKSAV GDASLTSRPMARVLNPLREMGVQVEAAEGDRLPLTLRGPRTANPIRYRVPMASAQVKSAV | 180 |
| Query 181 | LLAGLNTPGITTVIEPIMTRDHTEKMLQGFGANLTVETDADGVRTIRLEGRGKLTGQVID | 240 |
| Sbjct 181 | LLAGLNTPG+TTVIEPIMTRDHTEKMLQGFGA+L+VETD DGVR I + G+GKLTGQ+ID LLAGLNTPGVTTVIEPIMTRDHTEKMLQGFGADLSVETDKDGVRHIHITGQGKLTGQIID | 240 |
| Query 241 | VPGDPSSTAFPLVAALLVPGSDVTILNVLMNPTRTGLILTLQEMGADIEVINPRLAGGED | 300 |
| Sbjct 241 | VPGDPSSTAFPLVAAL+ GSD+ I NVLMNPTRTGLI TLQEMGA I++++PRLAGGED  VPGDPSSTAFPLVAALITEGSDIIIRNVLMNPTRTGLITTLQEMGAQIDILDPRLAGGED | 300 |

| Query | 301 | VADLRVRSSTLKGVTVPEDRAPSMIDEYPILavaaafaegaTVMNGLEELRVKESDRLSA | 360 |
| --- | --- | --- | --- |
| Sbjct | 301 | VADLRVRSS L G+TVP +RAPSMIDEYP+LA+AAAFAEG T+M+GL+ELRVKESDRL+A VADLRVRSSKLTGITVPVERAPSMIDEYPVLAIAAAFAEGETIMDGLDELRVKESDRLAA | 360 |
| Query | 361 | VANGLKLNGVDCDEGETSLVVRGRPDGKGLGNASGAAVATHLDHRIAMSFLVMGLVSENP | 420 |
| Sbjct | 361 | VA GL+ NGVDC EGE SL VRG P GKG G G VATHLDHRIAMSFLV+GL S+ P VARGLEANGVDCTEGEMSLSVRGNPSGKGFG---GGTVATHLDHRIAMSFLVLGLASQKP | 417 |
| Query  Sbjct | 421  418 | VTVDDATMIATSFPEFMDLMAGLGAKIELSDTK 453 VTVDDATMIATSFPEFMDLM GLGA I ++ K VTVDDATMIATSFPEFMDLMRGLGADISMAGAK 450 |  |

>RWE74684.1 3-phosphoshikimate 1-carboxyvinyltransferase [Mesorhizobium sp.] TJW63937.1 3-phosphoshikimate 1-carboxyvinyltransferase [Mesorhizobium sp.]

Length=452

Score = 694 bits (1790), Expect = 0.0, Method: Compositional matrix adjust.

| Identities | = 355/452 (79%), Positives = 389/452 (86%), Gaps = 4/452 (1%) |  |
| --- | --- | --- |
| Query 1 | MLHGASSRPATARKSSGLSGTVRIPGDKSISHRSFMFGGLASGETRITGLLEGEDVINTG | 60 |
| Sbjct 1 | M H A+++PATARKS L+GT R+PGDKSISHRSFMFGGLASGETRITGLLEGEDV+ TG MSHSAAAKPATARKSPALAGTARVPGDKSISHRSFMFGGLASGETRITGLLEGEDVMRTG | 60 |
| Query 61 | KAMQAMGARIRKEGDTWIIDGVGNGGLLAPEAPLDFGNAATGCRLTMGLVGVYDFDSTFI | 120 |
| Sbjct 61 | AM+AMGA I K G W+I G GNG LL PE PLDFGNA TG RLTMGLVG YD ++TFI AAMKAMGAHIEKNGAEWVIRGTGNGALLQPEGPLDFGNAGTGSRLTMGLVGTYDMETTFI | 120 |
| Query 121 | GDASLTKRPMGRVLNPLREMGVQV-KSEDGDRLPVTLRGPKTPTPITYRVPMASAQVKSA | 179 |
| Sbjct 121 | GDASL+ RPMGRVL+PLR+MGVQV K+ GDR+P+TLRGPK PITYRVPMASAQVKSA GDASLSGRPMGRVLDPLRQMGVQVLKAAPGDRMPITLRGPKHAAPITYRVPMASAQVKSA | 180 |
| Query 180 | VLLAGLNTPGITTVIEPIMTRDHTEKMLQGFGANLTVETDADGVRTIRLEGRGKLTGQVI | 239 |
| Sbjct 181 | VLLAGLNTPGITTVIEP+MTRDHTEKML+GFGANLTVETD GVR I +EG+GKLTGQ I VLLAGLNTPGITTVIEPVMTRDHTEKMLKGFGANLTVETDERGVRHIFIEGQGKLTGQTI | 240 |
| Query 240 | DVPGDPSSTAFPLVAALLVPGSDVTILNVLMNPTRTGLILTLQEMGADIEVINPRLAGGE | 299 |
| Sbjct 241 | VPGDPSS FPLVAAL+VPGSD+ I NVLMNPTRTGL+LTLQEMG I+++NPR AGGE AVPGDPSSAGFPLVAALIVPGSDIVIENVLMNPTRTGLLLTLQEMGGRIDILNPRNAGGE | 300 |
| Query 300 | DVADLRVRSSTLKGVTVPEDRAPSMIDEYPILavaaafaegaTVMNGLEELRVKESDRLS | 359 |
| Sbjct 301 | DVADLRVR S LKGVTVP +RAPSMIDEYP+LAVAA+FAEG T+M GLEELRVKESDRLS DVADLRVRYSELKGVTVPPERAPSMIDEYPVLAVAASFAEGETLMQGLEELRVKESDRLS | 360 |
| Query 360 | AVANGLKLNGVDCDEGETSLVVRGRPDGKGLG---NASGAAVATHLDHRIAMSFLVMGLV | 416 |
| Sbjct 361 | AVANGLKLNGVDC EGE SL VRGRP GKGLG N AV THLDHRIAMSFL+MGL AVANGLKLNGVDCTEGEASLAVRGRPGGKGLGAHPNGPDTAVKTHLDHRIAMSFLIMGLA | 420 |
| Query 417  Sbjct 421 | SENPVTVDDATMIATSFPEFMDLMAGLGAKIE 448  +E PVT+DDA MIATSFPEFM LM GLGA+IE  TEKPVTIDDANMIATSFPEFMGLMKGLGAEIE 452 |  |

>WP_149635011.1 3-phosphoshikimate 1-carboxyvinyltransferase [Rhizobium tropici] KAA1181195.1 3-phosphoshikimate 1-carboxyvinyltransferase [Rhizobium tropici]

Length=451

Score = 693 bits (1789), Expect = 0.0, Method: Compositional matrix adjust.

| Identities | = 350/448 (78%), Positives = 391/448 (87%), Gaps = 0/448 (0%) |  |
| --- | --- | --- |
| Query 1 | MLHGASSRPATARKSSGLSGTVRIPGDKSISHRSFMFGGLASGETRITGLLEGEDVINTG | 60 |
| Sbjct 1 | M H PA AR+S+GL GT+RIPGDKSISHRS MFGGLASGETRITGLLEGEDV++TG MPHDTLPHPAVARRSAGLFGTIRIPGDKSISHRSLMFGGLASGETRITGLLEGEDVLHTG | 60 |
| Query 61 | KAMQAMGARIRKEGDTWIIDGVGNGGLLAPEAPLDFGNAATGCRLTMGLVGVYDFDSTFI | 120 |
| Sbjct 61 | +AMQA+GA I+++GD WI++G GNG LL PEAPLDFGNA TG RLTMGLVG YDF +TF+ RAMQALGATIQRDGDAWIVNGTGNGALLTPEAPLDFGNAGTGSRLTMGLVGSYDFKTTFL | 120 |
| Query 121 | GDASLTKRPMGRVLNPLREMGVQVKSEDGDRLPVTLRGPKTPTPITYRVPMASAQVKSAV | 180 |
| Sbjct 121 | GDASL+KRPMGR+L+PLR MG QV + GDRLPVTL GP +P+TYRVPMASAQVKSAV  GDASLSKRPMGRILDPLRRMGTQVSAATGDRLPVTLHGPGVASPLTYRVPMASAQVKSAV | 180 |

| Query | 181 | LLAGLNTPGITTVIEPIMTRDHTEKMLQGFGANLTVETDADGVRTIRLEGRGKLTGQVID | 240 |
| --- | --- | --- | --- |
| Sbjct | 181 | LLAGLN PGITTVIEP+MTRDHTE+ML GFGA+L VETD GVRTIRLEG+GKLTGQ ID LLAGLNAPGITTVIEPVMTRDHTERMLVGFGASLAVETDVAGVRTIRLEGQGKLTGQTID | 240 |
| Query | 241 | VPGDPSSTAFPLVAALLVPGSDVTILNVLMNPTRTGLILTLQEMGADIEVINPRLAGGED | 300 |
| Sbjct | 241 | VPGDPSS AFPLVAAL+VP SD+TI NVLMNPTRTGLILTLQEMGA+IEVI+ R+ GGED VPGDPSSAAFPLVAALIVPNSDITIRNVLMNPTRTGLILTLQEMGANIEVIDRRVTGGED | 300 |
| Query | 301 | VADLRVRSSTLKGVTVPEDRAPSMIDEYPILavaaafaegaTVMNGLEELRVKESDRLSA | 360 |
| Sbjct | 301 | VADL VRSSTLKGV+VP +RAPSMIDEYP+LAVAAAFA+G T+M GLEELRVKESDRLSA VADLGVRSSTLKGVSVPAERAPSMIDEYPVLAVAAAFAKGTTIMQGLEELRVKESDRLSA | 360 |
| Query | 361 | VANGLKLNGVDCDEGETSLVVRGRPDGKGLGNASGAAVATHLDHRIAMSFLVMGLVSENP | 420 |
| Sbjct | 361 | VA GLKLNGVDC EG SL VRGRPDGKGLG++S AV THLDHR+AMSFLVMGL SE+P VAEGLKLNGVDCSEGRDSLTVRGRPDGKGLGSSSRGAVKTHLDHRLAMSFLVMGLASEHP | 420 |
| Query  Sbjct | 421  421 | VTVDDATMIATSFPEFMDLMAGLGAKIE 448 V+VDD+ MIATSFPEF +M LGA IE VSVDDSAMIATSFPEFFAMMTRLGATIE 448 |  |

>WP_127424639.1 MULTISPECIES: 3-phosphoshikimate 1-carboxyvinyltransferase [unclassified Mesorhizobium]

TGP24036.1 3-phosphoshikimate 1-carboxyvinyltransferase [Mesorhizobium sp. M1D.F.Ca.ET.231.01.1.1]

TGP35377.1 3-phosphoshikimate 1-carboxyvinyltransferase [Mesorhizobium sp. M1D.F.Ca.ET.234.01.1.1]

TGS49399.1 3-phosphoshikimate 1-carboxyvinyltransferase [Mesorhizobium sp. M1D.F.Ca.ET.184.01.1.1]

TGS63596.1 3-phosphoshikimate 1-carboxyvinyltransferase [Mesorhizobium sp. M1D.F.Ca.ET.183.01.1.1]

Length=452

Score = 693 bits (1789), Expect = 0.0, Method: Compositional matrix adjust.

| Identities | = 356/452 (79%), Positives = 389/452 (86%), Gaps = 4/452 (1%) |  |
| --- | --- | --- |
| Query 1 | MLHGASSRPATARKSSGLSGTVRIPGDKSISHRSFMFGGLASGETRITGLLEGEDVINTG | 60 |
| Sbjct 1 | M H A+++PATARKS L+GT R+PGDKSISHRSFMFGGLASGETRITGLLEGEDV+ TG MSHSAAAKPATARKSPALAGTARVPGDKSISHRSFMFGGLASGETRITGLLEGEDVMRTG | 60 |
| Query 61 | KAMQAMGARIRKEGDTWIIDGVGNGGLLAPEAPLDFGNAATGCRLTMGLVGVYDFDSTFI | 120 |
| Sbjct 61 | AM+AMGA I K+G W+I G GNG LL PE PLDFGNA TG RLTMGLVG YD ++TFI AAMKAMGAHIEKKGAEWVIRGTGNGALLEPEGPLDFGNAGTGSRLTMGLVGTYDMETTFI | 120 |
| Query 121 | GDASLTKRPMGRVLNPLREMGVQV-KSEDGDRLPVTLRGPKTPTPITYRVPMASAQVKSA | 179 |
| Sbjct 121 | GDASL+ RPMGRVL PLR+MGVQV K+ GDR+P+TLRGPK PITYRVPMASAQVKSA GDASLSGRPMGRVLEPLRQMGVQVLKAAPGDRMPITLRGPKHAAPITYRVPMASAQVKSA | 180 |
| Query 180 | VLLAGLNTPGITTVIEPIMTRDHTEKMLQGFGANLTVETDADGVRTIRLEGRGKLTGQVI | 239 |
| Sbjct 181 | VLLAGLNTPGITTVIEP+MTRDHTEKMLQGFGANL+VETD GVR I +EG+GKLTGQ I VLLAGLNTPGITTVIEPVMTRDHTEKMLQGFGANLSVETDERGVRHIFIEGQGKLTGQTI | 240 |
| Query 240 | DVPGDPSSTAFPLVAALLVPGSDVTILNVLMNPTRTGLILTLQEMGADIEVINPRLAGGE | 299 |
| Sbjct 241 | VPGDPSS FPLVAAL+VPGSD+ I NVLMNPTRTGL+LTLQEMG I+++NPR AGGE AVPGDPSSAGFPLVAALIVPGSDIVIENVLMNPTRTGLLLTLQEMGGRIDILNPRNAGGE | 300 |
| Query 300 | DVADLRVRSSTLKGVTVPEDRAPSMIDEYPILavaaafaegaTVMNGLEELRVKESDRLS | 359 |
| Sbjct 301 | DVADLRVRSS LKGV VP +RAPSMIDEYP+LAVAA+FAEG T+M GLEELRVKESDRLS DVADLRVRSSDLKGVVVPPERAPSMIDEYPVLAVAASFAEGETLMQGLEELRVKESDRLS | 360 |
| Query 360 | AVANGLKLNGVDCDEGETSLVVRGRPDGKGLG---NASGAAVATHLDHRIAMSFLVMGLV | 416 |
| Sbjct 361 | AVANGLKLNGVDC EGE SL VRGRP GKGLG N AV THLDHRIAMSFLVMGL AVANGLKLNGVDCTEGEASLAVRGRPGGKGLGAHPNGPDTAVRTHLDHRIAMSFLVMGLA | 420 |
| Query 417  Sbjct 421 | SENPVTVDDATMIATSFPEFMDLMAGLGAKIE 448  +E PVT+DDA MIATSFPEFM LM GLGA+IE  TEKPVTIDDANMIATSFPEFMGLMKGLGAEIE 452 |  |

>WP_059187322.1 3-phosphoshikimate 1-carboxyvinyltransferase [Mesorhizobium loti] KUM25968.1 3-phosphoshikimate 1-carboxyvinyltransferase [Mesorhizobium loti]

Length=452

Score = 693 bits (1789), Expect = 0.0, Method: Compositional matrix adjust.

| Identities | = 354/452 (78%), Positives = 389/452 (86%), Gaps = 4/452 (1%) |  |
| --- | --- | --- |
| Query 1 | MLHGASSRPATARKSSGLSGTVRIPGDKSISHRSFMFGGLASGETRITGLLEGEDVINTG | 60 |
| Sbjct 1 | M H A+ +PATARKS L+GT R+PGDKSISHRSFMFGGLASGETRITGLLEGEDV+ TG MSHSAAPKPATARKSPALAGTARVPGDKSISHRSFMFGGLASGETRITGLLEGEDVMRTG | 60 |
| Query 61 | KAMQAMGARIRKEGDTWIIDGVGNGGLLAPEAPLDFGNAATGCRLTMGLVGVYDFDSTFI | 120 |
| Sbjct 61 | AM+AMGA + K+G W+I G GNG LL PE PLDFGNA TG RLTMGLVG YD ++TFI AAMKAMGAHVEKKGAEWVIRGTGNGALLKPEGPLDFGNAGTGSRLTMGLVGTYDMETTFI | 120 |
| Query 121 | GDASLTKRPMGRVLNPLREMGVQV-KSEDGDRLPVTLRGPKTPTPITYRVPMASAQVKSA | 179 |
| Sbjct 121 | GDASL+ RPMGRVL PLR+MGVQV K+ GDR+P+TLRGPK PITYRVPMASAQVKSA GDASLSGRPMGRVLEPLRQMGVQVLKAAPGDRMPITLRGPKHAAPITYRVPMASAQVKSA | 180 |
| Query 180 | VLLAGLNTPGITTVIEPIMTRDHTEKMLQGFGANLTVETDADGVRTIRLEGRGKLTGQVI | 239 |
| Sbjct 181 | VLLAGLNTPGITTVIEP+MTRDHTEKML+GFGANLTVETD GVR I +EG+GKLTGQ I VLLAGLNTPGITTVIEPVMTRDHTEKMLKGFGANLTVETDERGVRHIFIEGQGKLTGQTI | 240 |
| Query 240 | DVPGDPSSTAFPLVAALLVPGSDVTILNVLMNPTRTGLILTLQEMGADIEVINPRLAGGE | 299 |
| Sbjct 241 | VPGDPSS FPLVAAL+VPGSD+TI NVLMNPTRTGL+LTLQEMG I+++NPR AGGE AVPGDPSSAGFPLVAALIVPGSDITIENVLMNPTRTGLLLTLQEMGGKIDILNPRSAGGE | 300 |
| Query 300 | DVADLRVRSSTLKGVTVPEDRAPSMIDEYPILavaaafaegaTVMNGLEELRVKESDRLS | 359 |
| Sbjct 301 | DVADLRVR S LKGVTVP +RAPSMIDEYP+LAVAA+FAEG T+M GLEELRVKESDRL+ DVADLRVRYSELKGVTVPPERAPSMIDEYPVLAVAASFAEGETLMQGLEELRVKESDRLA | 360 |
| Query 360 | AVANGLKLNGVDCDEGETSLVVRGRPDGKGLG---NASGAAVATHLDHRIAMSFLVMGLV | 416 |
| Sbjct 361 | AVANGLKLNGVDC EGE SL VRGRP GKGLG N AV THLDHRIAMSFL+MGL AVANGLKLNGVDCTEGEASLAVRGRPGGKGLGAHPNGPDTAVKTHLDHRIAMSFLIMGLA | 420 |
| Query 417  Sbjct 421 | SENPVTVDDATMIATSFPEFMDLMAGLGAKIE 448  +E PVT+DDA MIATSFPEFM LM GLGA+IE  TEKPVTIDDANMIATSFPEFMGLMKGLGAEIE 452 |  |

>RWK01831.1 3-phosphoshikimate 1-carboxyvinyltransferase [Mesorhizobium sp.] Length=452

Score = 693 bits (1789), Expect = 0.0, Method: Compositional matrix adjust.

| Identities | = 354/452 (78%), Positives = 389/452 (86%), Gaps = 4/452 (1%) |  |
| --- | --- | --- |
| Query 1 | MLHGASSRPATARKSSGLSGTVRIPGDKSISHRSFMFGGLASGETRITGLLEGEDVINTG | 60 |
| Sbjct 1 | M H A+++PATARKS L+GT R+PGDKSISHRSFMFGGLASGETRITGLLEGEDV+ TG MSHSAAAKPATARKSPALAGTARVPGDKSISHRSFMFGGLASGETRITGLLEGEDVMRTG | 60 |
| Query 61 | KAMQAMGARIRKEGDTWIIDGVGNGGLLAPEAPLDFGNAATGCRLTMGLVGVYDFDSTFI | 120 |
| Sbjct 61 | AM+AMGA I K+G W+I G GNG LL PE PLDFGNA TG RLTMGLVG YD ++TFI AAMKAMGAHIEKKGAEWVIRGTGNGALLEPEGPLDFGNAGTGSRLTMGLVGTYDMETTFI | 120 |
| Query 121 | GDASLTKRPMGRVLNPLREMGVQV-KSEDGDRLPVTLRGPKTPTPITYRVPMASAQVKSA | 179 |
| Sbjct 121 | GDASL+ RPMGRVL PLR+MGVQV K+ GDR+P+TLRGPK PITYRVPMASAQVKSA GDASLSGRPMGRVLEPLRQMGVQVLKAAPGDRMPITLRGPKHAAPITYRVPMASAQVKSA | 180 |
| Query 180 | VLLAGLNTPGITTVIEPIMTRDHTEKMLQGFGANLTVETDADGVRTIRLEGRGKLTGQVI | 239 |
| Sbjct 181 | VLLAGLNTPGITTVIEP+MTRDHTEKML+GFGANLTVETD GVR I +EG+GKLTGQ I VLLAGLNTPGITTVIEPVMTRDHTEKMLKGFGANLTVETDERGVRHIFIEGQGKLTGQTI | 240 |
| Query 240 | DVPGDPSSTAFPLVAALLVPGSDVTILNVLMNPTRTGLILTLQEMGADIEVINPRLAGGE | 299 |
| Sbjct 241 | VPGDPSS FPLVAAL+VPGSD+ I NVLMNPTRTGL+LTLQEMG I+++NPR AGGE AVPGDPSSAGFPLVAALIVPGSDIVIENVLMNPTRTGLLLTLQEMGGRIDILNPRNAGGE | 300 |
| Query 300 | DVADLRVRSSTLKGVTVPEDRAPSMIDEYPILavaaafaegaTVMNGLEELRVKESDRLS | 359 |
| Sbjct 301 | DVADLRVRSS LKGV VP +RAPSMIDEYP+LAVAA+FAEG T+M GLEELRVKESDRLS DVADLRVRSSELKGVVVPPERAPSMIDEYPVLAVAASFAEGETLMQGLEELRVKESDRLS | 360 |
| Query 360 | AVANGLKLNGVDCDEGETSLVVRGRPDGKGLG---NASGAAVATHLDHRIAMSFLVMGLV | 416 |
| Sbjct 361 | AVANGLKLNGVDC EGE SL VRG+P GKGLG N AV THLDHRIAMSFL+MGL  AVANGLKLNGVDCSEGEASLAVRGKPGGKGLGAHPNGQDTAVKTHLDHRIAMSFLIMGLA | 420 |

Query 417 SENPVTVDDATMIATSFPEFMDLMAGLGAKIE 448

+E PVT+DDA MIATSFPEFM LM GLGA+IE Sbjct 421 TEKPVTIDDANMIATSFPEFMGLMKGLGAEIE 452

>WP_127866572.1 3-phosphoshikimate 1-carboxyvinyltransferase [Mesorhizobium sp. M2E.F.Ca.ET.219.01.1.1]

TGQ06748.1 3-phosphoshikimate 1-carboxyvinyltransferase [Mesorhizobium sp. M2E.F.Ca.ET.219.01.1.1]

Length=452

Score = 693 bits (1788), Expect = 0.0, Method: Compositional matrix adjust.

| Identities | = 355/452 (79%), Positives = 388/452 (86%), Gaps = 4/452 (1%) |  |
| --- | --- | --- |
| Query 1 | MLHGASSRPATARKSSGLSGTVRIPGDKSISHRSFMFGGLASGETRITGLLEGEDVINTG | 60 |
| Sbjct 1 | M H A+++PATARKS L+GT R+PGDKSISHRSFMFGGLASGETRITGLLEGEDV+ TG MSHSAAAKPATARKSPALAGTARVPGDKSISHRSFMFGGLASGETRITGLLEGEDVMRTG | 60 |
| Query 61 | KAMQAMGARIRKEGDTWIIDGVGNGGLLAPEAPLDFGNAATGCRLTMGLVGVYDFDSTFI | 120 |
| Sbjct 61 | AM+AMGA I K G W+I G GNG LL PE PLDFGNA TG RLTMGLVG YD ++TFI AAMKAMGAHIEKNGAEWVIRGTGNGALLQPEGPLDFGNAGTGSRLTMGLVGTYDMETTFI | 120 |
| Query 121 | GDASLTKRPMGRVLNPLREMGVQV-KSEDGDRLPVTLRGPKTPTPITYRVPMASAQVKSA | 179 |
| Sbjct 121 | GDASL+ RPMGRVL+PLR+MGVQV K+ GDR+P+TLRGPK PITYRVPMASAQVKSA GDASLSGRPMGRVLDPLRQMGVQVLKAAPGDRMPITLRGPKHAAPITYRVPMASAQVKSA | 180 |
| Query 180 | VLLAGLNTPGITTVIEPIMTRDHTEKMLQGFGANLTVETDADGVRTIRLEGRGKLTGQVI | 239 |
| Sbjct 181 | VLLAGLNTPGITTVIEP+MTRDHTEKML+GFGANLTVETD GVR I +EG+GKLTGQ I VLLAGLNTPGITTVIEPVMTRDHTEKMLKGFGANLTVETDERGVRHIFIEGQGKLTGQTI | 240 |
| Query 240 | DVPGDPSSTAFPLVAALLVPGSDVTILNVLMNPTRTGLILTLQEMGADIEVINPRLAGGE | 299 |
| Sbjct 241 | VPGDPSS FPLVAAL+VPGSD+ I NVLMNPTRTGL+LTLQEMG I+++NPR AGGE AVPGDPSSAGFPLVAALIVPGSDIVIENVLMNPTRTGLLLTLQEMGGRIDILNPRNAGGE | 300 |
| Query 300 | DVADLRVRSSTLKGVTVPEDRAPSMIDEYPILavaaafaegaTVMNGLEELRVKESDRLS | 359 |
| Sbjct 301 | DVADLRVRSS LKGV VP +RAPSMIDEYP+LAVAA+FAEG T+M GLEELRVKESDRLS DVADLRVRSSDLKGVVVPPERAPSMIDEYPVLAVAASFAEGETLMQGLEELRVKESDRLS | 360 |
| Query 360 | AVANGLKLNGVDCDEGETSLVVRGRPDGKGLG---NASGAAVATHLDHRIAMSFLVMGLV | 416 |
| Sbjct 361 | AVANGLKLNGVDC EGE SL VRGRP GKGLG N V THLDHRIAMSFLVMGL AVANGLKLNGVDCTEGEASLAVRGRPGGKGLGAHPNGQDTVVKTHLDHRIAMSFLVMGLA | 420 |
| Query 417  Sbjct 421 | SENPVTVDDATMIATSFPEFMDLMAGLGAKIE 448  +E PVT+DDA MIATSFPEFM LM GLGA+IE  TEKPVTIDDANMIATSFPEFMGLMKGLGAEIE 452 |  |

>WP_095799189.1 3-phosphoshikimate 1-carboxyvinyltransferase [Mesorhizobium sp. WSM3866]

PBB45258.1 3-phosphoshikimate 1-carboxyvinyltransferase [Mesorhizobium sp. WSM3866]

Length=452

Score = 693 bits (1788), Expect = 0.0, Method: Compositional matrix adjust.

| Identities | = 355/452 (79%), Positives = 387/452 (86%), Gaps = 4/452 (1%) |  |
| --- | --- | --- |
| Query 1 | MLHGASSRPATARKSSGLSGTVRIPGDKSISHRSFMFGGLASGETRITGLLEGEDVINTG | 60 |
| Sbjct 1 | M H A+ +PATARKS L+GT R+PGDKSISHRSFMFGGLASGETRITGLLEGEDV+ TG MSHAAAPKPATARKSPALAGTARVPGDKSISHRSFMFGGLASGETRITGLLEGEDVMRTG | 60 |
| Query 61 | KAMQAMGARIRKEGDTWIIDGVGNGGLLAPEAPLDFGNAATGCRLTMGLVGVYDFDSTFI | 120 |
| Sbjct 61 | AM+AMGA I K+G W+I G GNG LL PE PLDFGNA TG RLTMGLVG YD ++TFI AAMKAMGAHIEKKGAEWVIRGTGNGALLEPEGPLDFGNAGTGSRLTMGLVGTYDMETTFI | 120 |
| Query 121 | GDASLTKRPMGRVLNPLREMGVQV-KSEDGDRLPVTLRGPKTPTPITYRVPMASAQVKSA | 179 |
| Sbjct 121 | GDASL+ RPMGRVL PLR+MGVQV K+ GDR+P+TLRGPK PITYRVPMASAQVKSA GDASLSGRPMGRVLEPLRQMGVQVLKAAPGDRMPITLRGPKHAAPITYRVPMASAQVKSA | 180 |
| Query 180 | VLLAGLNTPGITTVIEPIMTRDHTEKMLQGFGANLTVETDADGVRTIRLEGRGKLTGQVI | 239 |
| Sbjct 181 | VLLAGLNTPGITTVIEP+MTRDHTEKML+GFGANLTVETD GVR I +EG+GKLTGQ I  VLLAGLNTPGITTVIEPVMTRDHTEKMLKGFGANLTVETDERGVRHIFIEGQGKLTGQTI | 240 |

| Query | 240 | DVPGDPSSTAFPLVAALLVPGSDVTILNVLMNPTRTGLILTLQEMGADIEVINPRLAGGE | 299 |
| --- | --- | --- | --- |
| Sbjct | 241 | VPGDPSS FPLVAAL+VPGSD+ I NVLMNPTRTGL LTLQEMG I+++NPR+AGGE AVPGDPSSAGFPLVAALIVPGSDIVIENVLMNPTRTGLFLTLQEMGGRIDILNPRIAGGE | 300 |
| Query | 300 | DVADLRVRSSTLKGVTVPEDRAPSMIDEYPILavaaafaegaTVMNGLEELRVKESDRLS | 359 |
| Sbjct | 301 | DVADLRVRSS LKGV VP +RAPSMIDEYP+LAVAA FAEG T+M GLEELRVKESDRLS DVADLRVRSSDLKGVVVPPERAPSMIDEYPVLAVAAVFAEGETLMQGLEELRVKESDRLS | 360 |
| Query | 360 | AVANGLKLNGVDCDEGETSLVVRGRPDGKGLG---NASGAAVATHLDHRIAMSFLVMGLV | 416 |
| Sbjct | 361 | AVANGLKLNGVDC EGE SL VRG+P GKGLG N AV THLDHRIAMSFLVMGL AVANGLKLNGVDCSEGEASLAVRGKPGGKGLGAHPNGPDTAVKTHLDHRIAMSFLVMGLA | 420 |
| Query  Sbjct | 417  421 | SENPVTVDDATMIATSFPEFMDLMAGLGAKIE 448  +E PVT+DDA MIATSFPEFM LM GLGA+IE  TEKPVTIDDANMIATSFPEFMGLMKGLGAEIE 452 |  |

>RWK26142.1 3-phosphoshikimate 1-carboxyvinyltransferase [Mesorhizobium sp.] RWK36788.1 3-phosphoshikimate 1-carboxyvinyltransferase [Mesorhizobium sp.]

Length=452

Score = 693 bits (1788), Expect = 0.0, Method: Compositional matrix adjust.

| Identities | = 354/452 (78%), Positives = 389/452 (86%), Gaps = 4/452 (1%) |  |
| --- | --- | --- |
| Query 1 | MLHGASSRPATARKSSGLSGTVRIPGDKSISHRSFMFGGLASGETRITGLLEGEDVINTG | 60 |
| Sbjct 1 | M H A+++PATARKS L+GT R+PGDKSISHRSFMFGGLASGETRITGLLEGEDV+ TG MSHSAAAKPATARKSPALAGTARVPGDKSISHRSFMFGGLASGETRITGLLEGEDVMRTG | 60 |
| Query 61 | KAMQAMGARIRKEGDTWIIDGVGNGGLLAPEAPLDFGNAATGCRLTMGLVGVYDFDSTFI | 120 |
| Sbjct 61 | AM+AMGA I K+G W+I G GNG LL PE PLDFGNA TG RLTMGLVG YD ++TFI AAMKAMGAHIEKKGAEWVIRGTGNGALLEPEGPLDFGNAGTGSRLTMGLVGTYDMETTFI | 120 |
| Query 121 | GDASLTKRPMGRVLNPLREMGVQV-KSEDGDRLPVTLRGPKTPTPITYRVPMASAQVKSA | 179 |
| Sbjct 121 | GDASL+ RPMGRVL PLR+MGVQV K+ GDR+P+TLRGPK PITYRVPMASAQVKSA GDASLSGRPMGRVLEPLRQMGVQVLKAAPGDRMPITLRGPKHAAPITYRVPMASAQVKSA | 180 |
| Query 180 | VLLAGLNTPGITTVIEPIMTRDHTEKMLQGFGANLTVETDADGVRTIRLEGRGKLTGQVI | 239 |
| Sbjct 181 | VLLAGLNTPGITTVIEP+MTRDHTEKML+GFGANLTVETD GVR I +EG+GKLTGQ I VLLAGLNTPGITTVIEPVMTRDHTEKMLKGFGANLTVETDERGVRHIFIEGQGKLTGQTI | 240 |
| Query 240 | DVPGDPSSTAFPLVAALLVPGSDVTILNVLMNPTRTGLILTLQEMGADIEVINPRLAGGE | 299 |
| Sbjct 241 | VPGDPSS FPLVAAL+VPGSD+ I NVLMNPTRTGL+LTLQEMG I+++NPR AGGE AVPGDPSSAGFPLVAALIVPGSDIVIENVLMNPTRTGLLLTLQEMGGRIDILNPRNAGGE | 300 |
| Query 300 | DVADLRVRSSTLKGVTVPEDRAPSMIDEYPILavaaafaegaTVMNGLEELRVKESDRLS | 359 |
| Sbjct 301 | DVADLRVRSS LKGV VP +RAPSMIDEYP+LAVAA+FAEG T+M GLEELRVKESDRLS DVADLRVRSSELKGVVVPPERAPSMIDEYPVLAVAASFAEGETLMQGLEELRVKESDRLS | 360 |
| Query 360 | AVANGLKLNGVDCDEGETSLVVRGRPDGKGLG---NASGAAVATHLDHRIAMSFLVMGLV | 416 |
| Sbjct 361 | AVANGLKLNGVDC EGE SL VRG+P GKGLG N AV THLDHRIAMSFL+MGL AVANGLKLNGVDCTEGEASLAVRGKPGGKGLGAHPNGQDTAVKTHLDHRIAMSFLIMGLA | 420 |
| Query 417  Sbjct 421 | SENPVTVDDATMIATSFPEFMDLMAGLGAKIE 448  +E PVT+DDA MIATSFPEFM LM GLGA+IE  TEKPVTIDDANMIATSFPEFMGLMKGLGAEIE 452 |  |

>WP_127311342.1 MULTISPECIES: 3-phosphoshikimate 1-carboxyvinyltransferase [unclassified Mesorhizobium]

RUW68437.1 3-phosphoshikimate 1-carboxyvinyltransferase [Mesorhizobium sp. M4B.F.Ca.ET.049.02.1.2]

TGQ45547.1 3-phosphoshikimate 1-carboxyvinyltransferase [Mesorhizobium sp. M4B.F.Ca.ET.214.01.1.1]

TGQ63176.1 3-phosphoshikimate 1-carboxyvinyltransferase [Mesorhizobium sp. M4B.F.Ca.ET.211.01.1.1]

TGU40814.1 3-phosphoshikimate 1-carboxyvinyltransferase [Mesorhizobium sp. M4B.F.Ca.ET.150.01.1.1]

Length=455

Score = 693 bits (1788), Expect = 0.0, Method: Compositional matrix adjust. Identities = 356/454 (78%), Positives = 389/454 (86%), Gaps = 4/454 (1%)

| Query | 1 | MLHGASSRPATARKSSGLSGTVRIPGDKSISHRSFMFGGLASGETRITGLLEGEDVINTG | 60 |
| --- | --- | --- | --- |
| Sbjct | 1 | M H A+ +PATARKSS LSGT R+PGDKSISHRSFMFGGLASGETRITGLLEGEDV+ TG MSHAAAPKPATARKSSALSGTARVPGDKSISHRSFMFGGLASGETRITGLLEGEDVMRTG | 60 |
| Query | 61 | KAMQAMGARIRKEGDTWIIDGVGNGGLLAPEAPLDFGNAATGCRLTMGLVGVYDFDSTFI | 120 |
| Sbjct | 61 | AM+AMGA I K G W+I G GNG LL PE PLDFGNA TG RLTMGLVG YD ++TFI AAMKAMGAHIEKRGAEWVIRGTGNGALLQPEGPLDFGNAGTGSRLTMGLVGTYDMETTFI | 120 |
| Query | 121 | GDASLTKRPMGRVLNPLREMGVQV-KSEDGDRLPVTLRGPKTPTPITYRVPMASAQVKSA | 179 |
| Sbjct | 121 | GDASL+ RPMGRVL PLR+MGVQV K+ GDR+P+TLRGPK PITYRVPMASAQVKSA GDASLSGRPMGRVLEPLRQMGVQVLKATPGDRMPITLRGPKHAAPITYRVPMASAQVKSA | 180 |
| Query | 180 | VLLAGLNTPGITTVIEPIMTRDHTEKMLQGFGANLTVETDADGVRTIRLEGRGKLTGQVI | 239 |
| Sbjct | 181 | VLLAGLNTPGITTVIEP+MTRDHTEKML+GFGANLTVETD GVR I +EG+GKL+GQ I VLLAGLNTPGITTVIEPVMTRDHTEKMLKGFGANLTVETDERGVRHIFIEGQGKLSGQTI | 240 |
| Query | 240 | DVPGDPSSTAFPLVAALLVPGSDVTILNVLMNPTRTGLILTLQEMGADIEVINPRLAGGE | 299 |
| Sbjct | 241 | VPGDPSS FPLVAAL+VPGSD+ I NVLMNPTRTGL+LTLQEMG I+++NPR AGGE AVPGDPSSAGFPLVAALIVPGSDIVIENVLMNPTRTGLLLTLQEMGGRIDILNPRNAGGE | 300 |
| Query | 300 | DVADLRVRSSTLKGVTVPEDRAPSMIDEYPILavaaafaegaTVMNGLEELRVKESDRLS | 359 |
| Sbjct | 301 | DVADLRVRSS LKGVTVP +RAPSMIDEYP+LAVAA+FAEG T+M GLEELRVKESDRLS DVADLRVRSSELKGVTVPPERAPSMIDEYPVLAVAASFAEGETLMQGLEELRVKESDRLS | 360 |
| Query | 360 | AVANGLKLNGVDCDEGETSLVVRGRPDGKGLG---NASGAAVATHLDHRIAMSFLVMGLV | 416 |
| Sbjct | 361 | AVANGLKLNGVDC EGE +L VRGRP GKGLG N V THLDHRIAMSFLVMGL AVANGLKLNGVDCTEGEATLAVRGRPGGKGLGRHPNGPDTTVKTHLDHRIAMSFLVMGLA | 420 |
| Query  Sbjct | 417  421 | SENPVTVDDATMIATSFPEFMDLMAGLGAKIELS 450 SE PVT+DDA MIATSFPEFM LM GLGA+I++ SEKPVTIDDANMIATSFPEFMGLMKGLGAEIDVQ 454 |  |

>RWM20009.1 3-phosphoshikimate 1-carboxyvinyltransferase [Mesorhizobium sp.] Length=452

Score = 692 bits (1787), Expect = 0.0, Method: Compositional matrix adjust.

| Identities | = 355/452 (79%), Positives = 389/452 (86%), Gaps = 4/452 (1%) |  |
| --- | --- | --- |
| Query 1 | MLHGASSRPATARKSSGLSGTVRIPGDKSISHRSFMFGGLASGETRITGLLEGEDVINTG | 60 |
| Sbjct 1 | M H A+++PATARKS L+GT R+PGDKSISHRSFMFGGLASGETRITGLLEGEDV+ TG MSHSAAAKPATARKSPALAGTARVPGDKSISHRSFMFGGLASGETRITGLLEGEDVMRTG | 60 |
| Query 61 | KAMQAMGARIRKEGDTWIIDGVGNGGLLAPEAPLDFGNAATGCRLTMGLVGVYDFDSTFI | 120 |
| Sbjct 61 | AM+AMGA I K G W+I G GNG LL PE PLDFGNA TG RLTMGLVG YD ++TFI AAMKAMGAHIEKNGAEWVIRGTGNGALLQPEGPLDFGNAGTGSRLTMGLVGTYDMETTFI | 120 |
| Query 121 | GDASLTKRPMGRVLNPLREMGVQV-KSEDGDRLPVTLRGPKTPTPITYRVPMASAQVKSA | 179 |
| Sbjct 121 | GDASL+ RPMGRVL+PLR+MGVQV K+ GDR+P+TLRGPK PITYRVPMASAQVKSA GDASLSGRPMGRVLDPLRQMGVQVLKAAPGDRMPITLRGPKHAAPITYRVPMASAQVKSA | 180 |
| Query 180 | VLLAGLNTPGITTVIEPIMTRDHTEKMLQGFGANLTVETDADGVRTIRLEGRGKLTGQVI | 239 |
| Sbjct 181 | VLLAGLNTPGITTVIEP+MTRDHTEKML+GFGANLTVETD GVR I +EG+GKLTGQ I VLLAGLNTPGITTVIEPVMTRDHTEKMLKGFGANLTVETDERGVRHIFIEGQGKLTGQTI | 240 |
| Query 240 | DVPGDPSSTAFPLVAALLVPGSDVTILNVLMNPTRTGLILTLQEMGADIEVINPRLAGGE | 299 |
| Sbjct 241 | VPGDPSS FPLVAAL+VPGSD+ I NVLMNPTRTGL+LTLQEMG I+++NPR AGGE AVPGDPSSAGFPLVAALIVPGSDIVIENVLMNPTRTGLLLTLQEMGGRIDILNPRNAGGE | 300 |
| Query 300 | DVADLRVRSSTLKGVTVPEDRAPSMIDEYPILavaaafaegaTVMNGLEELRVKESDRLS | 359 |
| Sbjct 301 | DVADLRVRSS LKGV VP +RAPSMIDEYP+LAVAA+FAEG T+M GLEELRVKESDRL+ DVADLRVRSSDLKGVVVPPERAPSMIDEYPVLAVAASFAEGETLMQGLEELRVKESDRLA | 360 |
| Query 360 | AVANGLKLNGVDCDEGETSLVVRGRPDGKGLG---NASGAAVATHLDHRIAMSFLVMGLV | 416 |
| Sbjct 361 | AVANGLKLNGVDC EGE SL VRGRP GKGLG N AV THLDHRIAMSFLVMGL AVANGLKLNGVDCTEGEASLAVRGRPGGKGLGAHPNGPDTAVKTHLDHRIAMSFLVMGLA | 420 |
| Query 417  Sbjct 421 | SENPVTVDDATMIATSFPEFMDLMAGLGAKIE 448  +E PVT+DDA MIATSFPEFM LM GLGA+IE  TEKPVTIDDANMIATSFPEFMGLMKGLGAEIE 452 |  |

>WP_140866714.1 3-phosphoshikimate 1-carboxyvinyltransferase [Mesorhizobium sp. B2-8-3]

TPJ33345.1 3-phosphoshikimate 1-carboxyvinyltransferase [Mesorhizobium sp. B2-8-3]

Length=452

Score = 692 bits (1787), Expect = 0.0, Method: Compositional matrix adjust.

| Identities | = 354/452 (78%), Positives = 389/452 (86%), Gaps = 4/452 (1%) |  |
| --- | --- | --- |
| Query 1 | MLHGASSRPATARKSSGLSGTVRIPGDKSISHRSFMFGGLASGETRITGLLEGEDVINTG | 60 |
| Sbjct 1 | M H A+++PATARKS L+GT R+PGDKSISHRSFMFGGLASGETRITGLLEGEDV+ TG MSHSAAAKPATARKSPALAGTARVPGDKSISHRSFMFGGLASGETRITGLLEGEDVMRTG | 60 |
| Query 61 | KAMQAMGARIRKEGDTWIIDGVGNGGLLAPEAPLDFGNAATGCRLTMGLVGVYDFDSTFI | 120 |
| Sbjct 61 | AM+AMGA + K G W+I G GNG LL PE PLDFGNA TG RLTMGLVG YD +STFI AAMKAMGAHVEKNGAEWVIRGTGNGALLQPEGPLDFGNAGTGSRLTMGLVGTYDMESTFI | 120 |
| Query 121 | GDASLTKRPMGRVLNPLREMGVQV-KSEDGDRLPVTLRGPKTPTPITYRVPMASAQVKSA | 179 |
| Sbjct 121 | GDASL+ RPMGRVL+PLR+MGVQV K+ GDR+P+TLRGPK PITYRVPMASAQVKSA GDASLSGRPMGRVLDPLRQMGVQVLKAAPGDRMPITLRGPKHAAPITYRVPMASAQVKSA | 180 |
| Query 180 | VLLAGLNTPGITTVIEPIMTRDHTEKMLQGFGANLTVETDADGVRTIRLEGRGKLTGQVI | 239 |
| Sbjct 181 | VLLAGLNTPGITTVIEP+MTRDHTEKML+GFGANLTVETD GVR I +EG+GKLTGQ I VLLAGLNTPGITTVIEPVMTRDHTEKMLKGFGANLTVETDERGVRHIFIEGQGKLTGQTI | 240 |
| Query 240 | DVPGDPSSTAFPLVAALLVPGSDVTILNVLMNPTRTGLILTLQEMGADIEVINPRLAGGE | 299 |
| Sbjct 241 | VPGDPSS FPLVAAL+VPGSD+TI NVLMNPTRTGL+LTLQEMG I+++NPR AGGE AVPGDPSSAGFPLVAALIVPGSDITIENVLMNPTRTGLLLTLQEMGGKIDILNPRNAGGE | 300 |
| Query 300 | DVADLRVRSSTLKGVTVPEDRAPSMIDEYPILavaaafaegaTVMNGLEELRVKESDRLS | 359 |
| Sbjct 301 | DVADLRVRSS LKGV VP +RAPSMIDEYP+LAVAA+FAEG T+M GLEELRVKESDRLS DVADLRVRSSELKGVVVPPERAPSMIDEYPVLAVAASFAEGETLMQGLEELRVKESDRLS | 360 |
| Query 360 | AVANGLKLNGVDCDEGETSLVVRGRPDGKGLG---NASGAAVATHLDHRIAMSFLVMGLV | 416 |
| Sbjct 361 | AVANGLKLNGVDC EGE SL VRG+P GK LG N AV T+LDHRIAMSFLVMGL AVANGLKLNGVDCTEGEASLAVRGKPGGKALGAHPNGQDTAVKTYLDHRIAMSFLVMGLA | 420 |
| Query 417  Sbjct 421 | SENPVTVDDATMIATSFPEFMDLMAGLGAKIE 448  +E PVT+DDA MIATSFPEFM LM GLGA+IE  TEKPVTIDDANMIATSFPEFMGLMKGLGAEIE 452 |  |

>WP_140684744.1 MULTISPECIES: 3-phosphoshikimate 1-carboxyvinyltransferase [unclassified Mesorhizobium]

TPK90382.1 3-phosphoshikimate 1-carboxyvinyltransferase [Mesorhizobium sp. B2-4-16]

TPL73214.1 3-phosphoshikimate 1-carboxyvinyltransferase [Mesorhizobium sp. B2-4-3]

Length=452

Score = 692 bits (1786), Expect = 0.0, Method: Compositional matrix adjust.

| Identities | = 354/452 (78%), Positives = 391/452 (87%), Gaps = 4/452 (1%) |  |
| --- | --- | --- |
| Query 1 | MLHGASSRPATARKSSGLSGTVRIPGDKSISHRSFMFGGLASGETRITGLLEGEDVINTG | 60 |
| Sbjct 1 | M H A+++PATARKS L+GT R+PGDKSISHRSFMFGGLASGETRITGLLEGEDV+ TG MSHSAAAKPATARKSPALAGTARVPGDKSISHRSFMFGGLASGETRITGLLEGEDVMRTG | 60 |
| Query 61 | KAMQAMGARIRKEGDTWIIDGVGNGGLLAPEAPLDFGNAATGCRLTMGLVGVYDFDSTFI | 120 |
| Sbjct 61 | AM+AMGA I K+G W+I G GNG LL PE PLDFGNA TG RLTMGLVG YD ++TFI AAMKAMGAHIEKKGAEWVIRGTGNGALLQPEGPLDFGNAGTGSRLTMGLVGTYDMETTFI | 120 |
| Query 121 | GDASLTKRPMGRVLNPLREMGVQV-KSEDGDRLPVTLRGPKTPTPITYRVPMASAQVKSA | 179 |
| Sbjct 121 | GDASL+ RPMGRVL+PLR+MGVQV K+ GDR+P+TLRGPK PITYRVPMASAQVKSA GDASLSGRPMGRVLDPLRQMGVQVLKAAPGDRMPITLRGPKHAAPITYRVPMASAQVKSA | 180 |
| Query 180 | VLLAGLNTPGITTVIEPIMTRDHTEKMLQGFGANLTVETDADGVRTIRLEGRGKLTGQVI | 239 |
| Sbjct 181 | VLLAGLNTPGITTVIEP+MTRDHTEKML+GFGANL+VETD GVR I +EG+GKLTGQ I VLLAGLNTPGITTVIEPVMTRDHTEKMLKGFGANLSVETDERGVRHIFIEGQGKLTGQTI | 240 |
| Query 240 | DVPGDPSSTAFPLVAALLVPGSDVTILNVLMNPTRTGLILTLQEMGADIEVINPRLAGGE | 299 |
| Sbjct 241 | VPGDPSS FPLVAAL+VPGSD+TI NVLMNPTRTGL+LTLQEMG I+++NPR AGGE  AVPGDPSSAGFPLVAALIVPGSDITIENVLMNPTRTGLLLTLQEMGGKIDILNPRNAGGE | 300 |

| Query | 300 | DVADLRVRSSTLKGVTVPEDRAPSMIDEYPILavaaafaegaTVMNGLEELRVKESDRLS | 359 |
| --- | --- | --- | --- |
| Sbjct | 301 | DVADLRVRSS LKGV VP +RAPSMIDEYP+LAVAA+FAEG T+M GLEELRVKESDRL+ DVADLRVRSSELKGVVVPPERAPSMIDEYPVLAVAASFAEGETLMQGLEELRVKESDRLA | 360 |
| Query | 360 | AVANGLKLNGVDCDEGETSLVVRGRPDGKGLG---NASGAAVATHLDHRIAMSFLVMGLV | 416 |
| Sbjct | 361 | AVANGLKLNGVDC EGE SL VRGRP GKGLG N AV T+LDHRIAMSFLVMGL AVANGLKLNGVDCAEGEASLAVRGRPGGKGLGAHPNGHDTAVKTYLDHRIAMSFLVMGLA | 420 |
| Query  Sbjct | 417  421 | SENPVTVDDATMIATSFPEFMDLMAGLGAKIE 448  +E PVT+DDA MIATSFPEFM LM GLGA+IE  TEKPVTIDDANMIATSFPEFMGLMKGLGAEIE 452 |  |

>WP_183745483.1 3-phosphoshikimate 1-carboxyvinyltransferase [Rhizobium sp. BK196] MBB3311396.1 3-phosphoshikimate 1-carboxyvinyltransferase [Rhizobium sp. BK196]

Length=450

Score = 692 bits (1785), Expect = 0.0, Method: Compositional matrix adjust.

| Identities | = 345/448 (77%), Positives = 391/448 (87%), Gaps = 0/448 (0%) |  |
| --- | --- | --- |
| Query 1 | MLHGASSRPATARKSSGLSGTVRIPGDKSISHRSFMFGGLASGETRITGLLEGEDVINTG | 60 |
| Sbjct 1 | M G++S+PA A KS+GLSGTVRIPGDKSISHR+ + GGLASGETRITGLLEGEDV+NTG MSQGSASKPAIAHKSAGLSGTVRIPGDKSISHRALILGGLASGETRITGLLEGEDVLNTG | 60 |
| Query 61 | KAMQAMGARIRKEGDTWIIDGVGNGGLLAPEAPLDFGNAATGCRLTMGLVGVYDFDSTFI | 120 |
| Sbjct 61 | KAMQAMGA IRKE D W ++G GNG LLAPE PLDFGN+ T RL MGL GVYDFD+ F KAMQAMGAVIRKEDDAWTVNGTGNGALLAPEVPLDFGNSGTDARLIMGLAGVYDFDTVFT | 120 |
| Query 121 | GDASLTKRPMGRVLNPLREMGVQVKSEDGDRLPVTLRGPKTPTPITYRVPMASAQVKSAV | 180 |
| Sbjct 121 | GDASL++R MGRVL+PLR MGVQVK+ +GDRLPV LRGP+TP PI Y +P+ASAQVKSAV GDASLSRRTMGRVLDPLRRMGVQVKAVEGDRLPVMLRGPETPAPIRYTLPVASAQVKSAV | 180 |
| Query 181 | LLAGLNTPGITTVIEPIMTRDHTEKMLQGFGANLTVETDADGVRTIRLEGRGKLTGQVID | 240 |
| Sbjct 181 | LLAGLN PG+TTVIEP +TRDHTEKML GFGA L+++TD DG RTIRL+GRGKLTGQ+ID LLAGLNIPGVTTVIEPAITRDHTEKMLAGFGAELSIKTDKDGTRTIRLQGRGKLTGQIID | 240 |
| Query 241 | VPGDPSSTAFPLVAALLVPGSDVTILNVLMNPTRTGLILTLQEMGADIEVINPRLAGGED | 300 |
| Sbjct 241 | VPGDPSS AFPLVAALLVPGSD+TI NVLMN TRTGLILTLQEMGADIE+++PRLAGGED VPGDPSSAAFPLVAALLVPGSDITISNVLMNQTRTGLILTLQEMGADIEILDPRLAGGED | 300 |
| Query 301 | VADLRVRSSTLKGVTVPEDRAPSMIDEYPILavaaafaegaTVMNGLEELRVKESDRLSA | 360 |
| Sbjct 301 | +ADLRVR S L G++VP +RAPSMIDEYP+LAVAAAFA+G TVMNGL+ELRVKESDRL+ IADLRVRHSELTGISVPTERAPSMIDEYPVLAVAAAFAKGRTVMNGLQELRVKESDRLTT | 360 |
| Query 361 | VANGLKLNGVDCDEGETSLVVRGRPDGKGLGNASGAAVATHLDHRIAMSFLVMGLVSENP | 420 |
| Sbjct 361 | V GLKLNGV+C+EG+ L V GRPDGKGLGNA G V THLDHRIAMSFLVMGL SE+P VVEGLKLNGVECEEGKDWLSVTGRPDGKGLGNAGGEQVITHLDHRIAMSFLVMGLASEHP | 420 |
| Query 421  Sbjct 421 | VTVDDATMIATSFPEFMDLMAGLGAKIE 448 V +DD ++IATSFP+FMDLM LGAKIE VGIDDGSVIATSFPQFMDLMESLGAKIE 448 |  |

>WP_192250998.1 3-phosphoshikimate 1-carboxyvinyltransferase [Mesorhizobium silamurunense] Length=452

Score = 692 bits (1785), Expect = 0.0, Method: Compositional matrix adjust.

| Identities | = 355/452 (79%), Positives = 388/452 (86%), Gaps = 4/452 (1%) |  |
| --- | --- | --- |
| Query 1 | MLHGASSRPATARKSSGLSGTVRIPGDKSISHRSFMFGGLASGETRITGLLEGEDVINTG | 60 |
| Sbjct 1 | M H A+ +PATARKS LSGT R+PGDKSISHRSFMFGGLASGETRITGLLEGEDV+ TG MSHSAAPKPATARKSDALSGTARVPGDKSISHRSFMFGGLASGETRITGLLEGEDVMRTG | 60 |
| Query 61 | KAMQAMGARIRKEGDTWIIDGVGNGGLLAPEAPLDFGNAATGCRLTMGLVGVYDFDSTFI | 120 |
| Sbjct 61 | AM+AMGA I K+G W+I G GNG LL PEAPLDFGNA TG RLTMGLVG YD ++TFI AAMKAMGAHIEKKGTEWVIRGTGNGALLQPEAPLDFGNAGTGSRLTMGLVGTYDMETTFI | 120 |
| Query 121 | GDASLTKRPMGRVLNPLREMGVQV-KSEDGDRLPVTLRGPKTPTPITYRVPMASAQVKSA | 179 |
| Sbjct 121 | GDASL+ RPMGRVL PLR+MGVQV K+ GDR+P+TLRGPK PITYRVPMASAQVKSA  GDASLSGRPMGRVLEPLRQMGVQVLKAAPGDRMPITLRGPKHAAPITYRVPMASAQVKSA | 180 |

| Query | 180 | VLLAGLNTPGITTVIEPIMTRDHTEKMLQGFGANLTVETDADGVRTIRLEGRGKLTGQVI | 239 |
| --- | --- | --- | --- |
| Sbjct | 181 | VLLAGLNTPGITTV+EP+MTRDHTEKML+GFGANLTVETD GVR I +EG+GKLTGQ I VLLAGLNTPGITTVVEPVMTRDHTEKMLKGFGANLTVETDERGVRHIFIEGQGKLTGQTI | 240 |
| Query | 240 | DVPGDPSSTAFPLVAALLVPGSDVTILNVLMNPTRTGLILTLQEMGADIEVINPRLAGGE | 299 |
| Sbjct | 241 | VPGDPSS FPLVAAL+VPGSD+ I NVLMNPTRTGL+LTLQEMG I+++NPR AGGE AVPGDPSSAGFPLVAALIVPGSDIVIENVLMNPTRTGLLLTLQEMGGRIDILNPRNAGGE | 300 |
| Query | 300 | DVADLRVRSSTLKGVTVPEDRAPSMIDEYPILavaaafaegaTVMNGLEELRVKESDRLS | 359 |
| Sbjct | 301 | DVADLRVRSS LKGV VP +RAPSMIDEYP+LAVAA+FAEG T+M GLEELRVKESDRLS DVADLRVRSSDLKGVVVPPERAPSMIDEYPVLAVAASFAEGETLMQGLEELRVKESDRLS | 360 |
| Query | 360 | AVANGLKLNGVDCDEGETSLVVRGRPDGKGLG---NASGAAVATHLDHRIAMSFLVMGLV | 416 |
| Sbjct | 361 | AVANGLKLNGVDC EGE SL VRG+P GKGLG N AV THLDHRIAMSFLVMGL AVANGLKLNGVDCTEGEASLAVRGKPGGKGLGAHPNGPDTAVKTHLDHRIAMSFLVMGLA | 420 |
| Query  Sbjct | 417  421 | SENPVTVDDATMIATSFPEFMDLMAGLGAKIE 448  +E PVT+DDA MIATSFPEFM LM GL A+IE  TEKPVTIDDANMIATSFPEFMGLMKGLAAEIE 452 |  |

>WP_127239465.1 MULTISPECIES: 3-phosphoshikimate 1-carboxyvinyltransferase [unclassified Mesorhizobium]

RWF73304.1 3-phosphoshikimate 1-carboxyvinyltransferase [Mesorhizobium sp.] RUW02965.1 3-phosphoshikimate 1-carboxyvinyltransferase [Mesorhizobium sp.

M1A.F.Ca.IN.020.04.1.1]

RUW16059.1 3-phosphoshikimate 1-carboxyvinyltransferase [Mesorhizobium sp. M1A.F.Ca.IN.020.03.1.1]

RWG18754.1 3-phosphoshikimate 1-carboxyvinyltransferase [Mesorhizobium sp.] RWG56813.1 3-phosphoshikimate 1-carboxyvinyltransferase [Mesorhizobium sp.]

Length=452

Score = 692 bits (1785), Expect = 0.0, Method: Compositional matrix adjust.

| Identities | = 355/452 (79%), Positives = 387/452 (86%), Gaps = 4/452 (1%) |  |
| --- | --- | --- |
| Query 1 | MLHGASSRPATARKSSGLSGTVRIPGDKSISHRSFMFGGLASGETRITGLLEGEDVINTG | 60 |
| Sbjct 1 | M H A+ +PATARKS L+GT R+PGDKSISHRSFMFGGLASGETRITGLLEGEDV+ TG MSHAAAPKPATARKSPALAGTARVPGDKSISHRSFMFGGLASGETRITGLLEGEDVMRTG | 60 |
| Query 61 | KAMQAMGARIRKEGDTWIIDGVGNGGLLAPEAPLDFGNAATGCRLTMGLVGVYDFDSTFI | 120 |
| Sbjct 61 | AM+AMGA I K+G W+I G GNG LL PE PLDFGNA TG RLTMGLVG YD ++TFI AAMKAMGAHIEKKGAEWVIRGTGNGALLEPEGPLDFGNAGTGSRLTMGLVGTYDMETTFI | 120 |
| Query 121 | GDASLTKRPMGRVLNPLREMGVQV-KSEDGDRLPVTLRGPKTPTPITYRVPMASAQVKSA | 179 |
| Sbjct 121 | GDASL+ RPMGRVL PLR+MGVQV K+ GDR+P+TLRGPK PITYRVPMASAQVKSA GDASLSGRPMGRVLEPLRQMGVQVLKAAPGDRMPITLRGPKHAAPITYRVPMASAQVKSA | 180 |
| Query 180 | VLLAGLNTPGITTVIEPIMTRDHTEKMLQGFGANLTVETDADGVRTIRLEGRGKLTGQVI | 239 |
| Sbjct 181 | VLLAGLNTPGITTVIEP+MTRDHTEKML+GFGANLTVETD GVR I +EG+GKLTGQ I VLLAGLNTPGITTVIEPVMTRDHTEKMLKGFGANLTVETDERGVRHIFIEGQGKLTGQTI | 240 |
| Query 240 | DVPGDPSSTAFPLVAALLVPGSDVTILNVLMNPTRTGLILTLQEMGADIEVINPRLAGGE | 299 |
| Sbjct 241 | VPGDPSS FPLVAAL+VPGSD+ I NVLMNPTRTGL+LTLQEMG I+++NPR AGGE AVPGDPSSAGFPLVAALIVPGSDIVIENVLMNPTRTGLLLTLQEMGGRIDILNPRNAGGE | 300 |
| Query 300 | DVADLRVRSSTLKGVTVPEDRAPSMIDEYPILavaaafaegaTVMNGLEELRVKESDRLS | 359 |
| Sbjct 301 | DVADLRVRSS LKGV VP +RAPSMIDEYP+LAVAA FAEG T+M GLEELRVKESDRLS DVADLRVRSSDLKGVVVPPERAPSMIDEYPVLAVAAVFAEGETLMQGLEELRVKESDRLS | 360 |
| Query 360 | AVANGLKLNGVDCDEGETSLVVRGRPDGKGLG---NASGAAVATHLDHRIAMSFLVMGLV | 416 |
| Sbjct 361 | AVANGLKLNGVDC EGE SL VRG+P GKGLG N AV THLDHRIAMSFLVMGL AVANGLKLNGVDCSEGEASLAVRGKPGGKGLGAHPNGPDTAVKTHLDHRIAMSFLVMGLA | 420 |
| Query 417  Sbjct 421 | SENPVTVDDATMIATSFPEFMDLMAGLGAKIE 448  +E PVT+DDA MIATSFPEFM LM GLGA+IE  TEKPVTIDDANMIATSFPEFMGLMKGLGAEIE 452 |  |

>WP_169646177.1 3-phosphoshikimate 1-carboxyvinyltransferase [Rhizobium sp. SG526] NMN55403.1 3-phosphoshikimate 1-carboxyvinyltransferase [Rhizobium sp. SG526]

Length=451

Score = 691 bits (1784), Expect = 0.0, Method: Compositional matrix adjust.

| Identities | = 348/447 (78%), Positives = 388/447 (87%), Gaps = 0/447 (0%) |  |
| --- | --- | --- |
| Query 1 | MLHGASSRPATARKSSGLSGTVRIPGDKSISHRSFMFGGLASGETRITGLLEGEDVINTG | 60 |
| Sbjct 1 | M H +PA AR+S+GLSGT+RIPGDKSISHRS MFGGLASGET I GLLEGEDV+NT MPHDTLPQPAVARRSAGLSGTIRIPGDKSISHRSLMFGGLASGETHIKGLLEGEDVLNTA | 60 |
| Query 61 | KAMQAMGARIRKEGDTWIIDGVGNGGLLAPEAPLDFGNAATGCRLTMGLVGVYDFDSTFI | 120 |
| Sbjct 61 | AMQA+GA IR++GD WI++G GNG LLAPEAPLDFGNA TG RLTMGLVG YDF +TF+ GAMQALGATIRRDGDAWIVNGTGNGALLAPEAPLDFGNAGTGSRLTMGLVGSYDFKTTFV | 120 |
| Query 121 | GDASLTKRPMGRVLNPLREMGVQVKSEDGDRLPVTLRGPKTPTPITYRVPMASAQVKSAV | 180 |
| Sbjct 121 | GDASL+KRPMGR+L+PLR MG QV + GDRLPVTL GP +P+TYRVPMASAQVKSAV GDASLSKRPMGRILDPLRRMGTQVSAAKGDRLPVTLHGPGVASPLTYRVPMASAQVKSAV | 180 |
| Query 181 | LLAGLNTPGITTVIEPIMTRDHTEKMLQGFGANLTVETDADGVRTIRLEGRGKLTGQVID | 240 |
| Sbjct 181 | LLAGLN PGITTVIEP+MTRDHTE+ML GFGA L+VETDA GVRTIRLEG+G+LTGQ ID LLAGLNAPGITTVIEPVMTRDHTERMLAGFGAALSVETDAAGVRTIRLEGQGRLTGQTID | 240 |
| Query 241 | VPGDPSSTAFPLVAALLVPGSDVTILNVLMNPTRTGLILTLQEMGADIEVINPRLAGGED | 300 |
| Sbjct 241 | VPGDPSS AFPLVAAL+VPGSD+TI NVLMN TRTGLILTLQEMGADIE+++ R+ GGED VPGDPSSAAFPLVAALIVPGSDITIRNVLMNTTRTGLILTLQEMGADIEILDRRVTGGED | 300 |
| Query 301 | VADLRVRSSTLKGVTVPEDRAPSMIDEYPILavaaafaegaTVMNGLEELRVKESDRLSA | 360 |
| Sbjct 301 | VADL VRSS LKGVTVP +R PSMIDEYP+LAVAAAFAEG T+M GL+ELRVKESDRLSA VADLHVRSSALKGVTVPAERVPSMIDEYPVLAVAAAFAEGTTIMQGLDELRVKESDRLSA | 360 |
| Query 361 | VANGLKLNGVDCDEGETSLVVRGRPDGKGLGNASGAAVATHLDHRIAMSFLVMGLVSENP | 420 |
| Sbjct 361 | VA GLKLNGVDC EG SL VRGRPDGKGLG++SG V THLDHRIAMSFLVMGL SE+P VAEGLKLNGVDCSEGRDSLTVRGRPDGKGLGSSSGTEVRTHLDHRIAMSFLVMGLASEHP | 420 |
| Query 421  Sbjct 421 | VTVDDATMIATSFPEFMDLMAGLGAKI 447 V++DD+ MIATSFPEF +M LGA I VSIDDSAMIATSFPEFFAMMTRLGATI 447 |  |

>WP_145646366.1 3-phosphoshikimate 1-carboxyvinyltransferase [Rhizobium miluonense] TWE84062.1 3-phosphoshikimate 1-carboxyvinyltransferase [Rhizobium miluonense]

Length=451

Score = 691 bits (1784), Expect = 0.0, Method: Compositional matrix adjust.

| Identities | = 348/447 (78%), Positives = 388/447 (87%), Gaps = 0/447 (0%) |  |
| --- | --- | --- |
| Query 1 | MLHGASSRPATARKSSGLSGTVRIPGDKSISHRSFMFGGLASGETRITGLLEGEDVINTG | 60 |
| Sbjct 1 | M H +PA AR+S+GLSGT+RIPGDKSISHRS MFGGLASGET I GLLEGEDV+NT MPHDTLPQPAVARRSAGLSGTIRIPGDKSISHRSLMFGGLASGETHIKGLLEGEDVLNTA | 60 |
| Query 61 | KAMQAMGARIRKEGDTWIIDGVGNGGLLAPEAPLDFGNAATGCRLTMGLVGVYDFDSTFI | 120 |
| Sbjct 61 | AMQA+GA IR++ D WI++G GNG LLAPEAPLDFGNA TG RLTMGLVG YDF +TF+ GAMQALGATIRRDSDAWIVNGTGNGALLAPEAPLDFGNAGTGSRLTMGLVGSYDFKTTFV | 120 |
| Query 121 | GDASLTKRPMGRVLNPLREMGVQVKSEDGDRLPVTLRGPKTPTPITYRVPMASAQVKSAV | 180 |
| Sbjct 121 | GDASL+KRPMGR+L+PLR MG QV + GDRLPVTL GP +P+TYRVPMASAQVKSAV GDASLSKRPMGRILDPLRRMGTQVSAAKGDRLPVTLHGPGVASPLTYRVPMASAQVKSAV | 180 |
| Query 181 | LLAGLNTPGITTVIEPIMTRDHTEKMLQGFGANLTVETDADGVRTIRLEGRGKLTGQVID | 240 |
| Sbjct 181 | LLAGLN PGITTVIEP+MTRDHTE+ML GFGA L+VETDA GVRTIRLEG+G+LTGQ ID LLAGLNAPGITTVIEPVMTRDHTERMLAGFGAALSVETDAAGVRTIRLEGQGRLTGQTID | 240 |
| Query 241 | VPGDPSSTAFPLVAALLVPGSDVTILNVLMNPTRTGLILTLQEMGADIEVINPRLAGGED | 300 |
| Sbjct 241 | VPGDPSS AFPLVAAL+VPGSD+TI NVLMN TRTGLILTLQEMGADIE+++ R+ GGED VPGDPSSAAFPLVAALIVPGSDITIRNVLMNTTRTGLILTLQEMGADIEILDRRVTGGED | 300 |
| Query 301 | VADLRVRSSTLKGVTVPEDRAPSMIDEYPILavaaafaegaTVMNGLEELRVKESDRLSA | 360 |
| Sbjct 301 | VADL VRSS LKGVTVP +RAPSMIDEYP+LAVAAAFAEG T+M GL+ELRVKESDRLSA VADLHVRSSALKGVTVPAERAPSMIDEYPVLAVAAAFAEGTTIMQGLDELRVKESDRLSA | 360 |
| Query 361 | VANGLKLNGVDCDEGETSLVVRGRPDGKGLGNASGAAVATHLDHRIAMSFLVMGLVSENP | 420 |
| Sbjct 361 | VA GLKLNGVDC EG SL VRGRPDGKGLG++SG V THLDHRIAMSFLVMGL SE+P VAEGLKLNGVDCSEGRDSLTVRGRPDGKGLGSSSGTEVRTHLDHRIAMSFLVMGLASEHP | 420 |
| Query 421 | VTVDDATMIATSFPEFMDLMAGLGAKI 447 |  |

V++DD+ MIATSFPEF +M LGA I Sbjct 421 VSIDDSAMIATSFPEFFAMMTRLGATI 447

>WP_112128512.1 3-phosphoshikimate 1-carboxyvinyltransferase [Mesorhizobium sp. CNPSo 3140]

RAZ76271.1 3-phosphoshikimate 1-carboxyvinyltransferase [Mesorhizobium sp. CNPSo 3140]

Length=452

Score = 691 bits (1784), Expect = 0.0, Method: Compositional matrix adjust.

| Identities | = 354/452 (78%), Positives = 388/452 (86%), Gaps = 4/452 (1%) |  |
| --- | --- | --- |
| Query 1 | MLHGASSRPATARKSSGLSGTVRIPGDKSISHRSFMFGGLASGETRITGLLEGEDVINTG | 60 |
| Sbjct 1 | M H A+++PATARKS L+GT R+PGDKSISHRSFMFGGLASGETRITGLLEGEDV+ TG MSHSAAAKPATARKSPALAGTARVPGDKSISHRSFMFGGLASGETRITGLLEGEDVMRTG | 60 |
| Query 61 | KAMQAMGARIRKEGDTWIIDGVGNGGLLAPEAPLDFGNAATGCRLTMGLVGVYDFDSTFI | 120 |
| Sbjct 61 | AM+AMGA I K G W+I G GNG LL PE PLDFGNA TG RLTMGLVG YD ++TF+ AAMKAMGAHIEKNGAEWVIRGTGNGALLQPEEPLDFGNAGTGSRLTMGLVGTYDMETTFM | 120 |
| Query 121 | GDASLTKRPMGRVLNPLREMGVQV-KSEDGDRLPVTLRGPKTPTPITYRVPMASAQVKSA | 179 |
| Sbjct 121 | GDASL+ RPMGRVL+PLR+MGVQV K+ GDR+P+TLRGPK PITYRVPMASAQVKSA GDASLSGRPMGRVLDPLRQMGVQVLKAAPGDRMPITLRGPKHAAPITYRVPMASAQVKSA | 180 |
| Query 180 | VLLAGLNTPGITTVIEPIMTRDHTEKMLQGFGANLTVETDADGVRTIRLEGRGKLTGQVI | 239 |
| Sbjct 181 | VLLAGLNTPGITTVIEP+MTRDHTEKML+GFGANLTVETD GVR I +EG+GKLTGQ I VLLAGLNTPGITTVIEPVMTRDHTEKMLKGFGANLTVETDERGVRHIFIEGQGKLTGQTI | 240 |
| Query 240 | DVPGDPSSTAFPLVAALLVPGSDVTILNVLMNPTRTGLILTLQEMGADIEVINPRLAGGE | 299 |
| Sbjct 241 | VPGDPSS FPLVAAL+VPGSD+ I NVLMNPTRTGL+LTLQEMG I+++NPR AGGE AVPGDPSSAGFPLVAALIVPGSDIVIENVLMNPTRTGLLLTLQEMGGRIDILNPRNAGGE | 300 |
| Query 300 | DVADLRVRSSTLKGVTVPEDRAPSMIDEYPILavaaafaegaTVMNGLEELRVKESDRLS | 359 |
| Sbjct 301 | DVADLRVRSS LKGV VP +RAPSMIDEYP+LAVAA+FAEG T+M GLEELRVKESDRLS DVADLRVRSSDLKGVVVPPERAPSMIDEYPVLAVAASFAEGETLMQGLEELRVKESDRLS | 360 |
| Query 360 | AVANGLKLNGVDCDEGETSLVVRGRPDGKGLG---NASGAAVATHLDHRIAMSFLVMGLV | 416 |
| Sbjct 361 | AVANGLKLNGVDC EGE SL VRGRP GKGLG N V THLDHRIAMSFLVMGL AVANGLKLNGVDCTEGEASLAVRGRPGGKGLGAHPNGQDTVVKTHLDHRIAMSFLVMGLA | 420 |
| Query 417  Sbjct 421 | SENPVTVDDATMIATSFPEFMDLMAGLGAKIE 448  +E PVT+DDA MIATSFPEFM LM GLGA+IE  TEKPVTIDDANMIATSFPEFMGLMKGLGAEIE 452 |  |

>RWF25187.1 3-phosphoshikimate 1-carboxyvinyltransferase [Mesorhizobium sp.] TIX17326.1 3-phosphoshikimate 1-carboxyvinyltransferase [Mesorhizobium sp.] TJW07936.1 3-phosphoshikimate 1-carboxyvinyltransferase [Mesorhizobium sp.]

Length=452

Score = 691 bits (1784), Expect = 0.0, Method: Compositional matrix adjust.

| Identities | = 356/452 (79%), Positives = 387/452 (86%), Gaps = 4/452 (1%) |  |
| --- | --- | --- |
| Query 1 | MLHGASSRPATARKSSGLSGTVRIPGDKSISHRSFMFGGLASGETRITGLLEGEDVINTG | 60 |
| Sbjct 1 | M H A+ +PATARKSS LSGT R+PGDKSISHRSFMFGGLASGETRITGLLEGEDV+ TG MSHAAAPKPATARKSSALSGTARVPGDKSISHRSFMFGGLASGETRITGLLEGEDVMRTG | 60 |
| Query 61 | KAMQAMGARIRKEGDTWIIDGVGNGGLLAPEAPLDFGNAATGCRLTMGLVGVYDFDSTFI | 120 |
| Sbjct 61 | AM+AMGA I K G W+I G GNG LL PE PLDFGNA TG RLTMGLVG YD ++ FI AAMKAMGAHIEKRGAEWVIRGTGNGALLQPEGPLDFGNAGTGSRLTMGLVGTYDMETRFI | 120 |
| Query 121 | GDASLTKRPMGRVLNPLREMGVQV-KSEDGDRLPVTLRGPKTPTPITYRVPMASAQVKSA | 179 |
| Sbjct 121 | GDASL+ RPMGRVL PLR+MGVQV K+ GDR+P+TLRGPK PITYRVPMASAQVKSA GDASLSGRPMGRVLEPLRQMGVQVLKATPGDRMPITLRGPKHAAPITYRVPMASAQVKSA | 180 |
| Query 180 | VLLAGLNTPGITTVIEPIMTRDHTEKMLQGFGANLTVETDADGVRTIRLEGRGKLTGQVI | 239 |
| Sbjct 181 | VLLAGLNTPGITTVIEP+MTRDHTEKML+GFGANLTVETD GVR I +EG+GKL+GQ I VLLAGLNTPGITTVIEPVMTRDHTEKMLKGFGANLTVETDERGVRHIFIEGQGKLSGQTI | 240 |
| Query 240 | DVPGDPSSTAFPLVAALLVPGSDVTILNVLMNPTRTGLILTLQEMGADIEVINPRLAGGE VPGDPSS FPLVAAL+VPGSD+ I NVLMNPTRTGL+LTLQEMG I+++NPR AGGE | 299 |

| Sbjct | 241 | AVPGDPSSAGFPLVAALIVPGSDIVIENVLMNPTRTGLLLTLQEMGGRIDILNPRNAGGE | 300 |
| --- | --- | --- | --- |
| Query | 300 | DVADLRVRSSTLKGVTVPEDRAPSMIDEYPILavaaafaegaTVMNGLEELRVKESDRLS | 359 |
| Sbjct | 301 | DVADLRVRSS LKGVTVP +RAPSMIDEYP+LAVAA+FAEG T+M GLEELRVKESDRLS DVADLRVRSSELKGVTVPPERAPSMIDEYPVLAVAASFAEGETLMQGLEELRVKESDRLS | 360 |
| Query | 360 | AVANGLKLNGVDCDEGETSLVVRGRPDGKGLG---NASGAAVATHLDHRIAMSFLVMGLV | 416 |
| Sbjct | 361 | AVANGLKLNGVDC EGE +L VRGRP GKGLG N V THLDHRIAMSFLVMGL AVANGLKLNGVDCTEGEATLAVRGRPGGKGLGRHPNGPDTTVKTHLDHRIAMSFLVMGLA | 420 |
| Query  Sbjct | 417  421 | SENPVTVDDATMIATSFPEFMDLMAGLGAKIE 448 SE PVT+DDA MIATSFPEFM LM GLGA+IE SEKPVTIDDANMIATSFPEFMGLMKGLGAEIE 452 |  |

>WP_140753761.1 3-phosphoshikimate 1-carboxyvinyltransferase [Mesorhizobium sp. B4-1-3]

TPI11777.1 3-phosphoshikimate 1-carboxyvinyltransferase [Mesorhizobium sp. B4-1-3]

Length=452

Score = 691 bits (1784), Expect = 0.0, Method: Compositional matrix adjust.

| Identities | = 354/452 (78%), Positives = 388/452 (86%), Gaps = 4/452 (1%) |  |
| --- | --- | --- |
| Query 1 | MLHGASSRPATARKSSGLSGTVRIPGDKSISHRSFMFGGLASGETRITGLLEGEDVINTG | 60 |
| Sbjct 1 | M H A+++PATARKS L+GT R+PGDKSISHRSFMFGGLASGETRITGLLEGEDV+ TG MSHSAAAKPATARKSPALAGTARVPGDKSISHRSFMFGGLASGETRITGLLEGEDVMRTG | 60 |
| Query 61 | KAMQAMGARIRKEGDTWIIDGVGNGGLLAPEAPLDFGNAATGCRLTMGLVGVYDFDSTFI | 120 |
| Sbjct 61 | AM+AMGA I K G W+I G GNG LL PE PLDFGNA TG RLTMGLVG YD ++TFI AAMKAMGAHIEKNGAEWVIRGTGNGALLEPEGPLDFGNAGTGSRLTMGLVGTYDMETTFI | 120 |
| Query 121 | GDASLTKRPMGRVLNPLREMGVQV-KSEDGDRLPVTLRGPKTPTPITYRVPMASAQVKSA | 179 |
| Sbjct 121 | GDASL+ RPMGRVL+PLR+MGVQV K+ GDR+P+TLRGPK PITYRVPMASAQVKSA GDASLSGRPMGRVLDPLRQMGVQVLKAAPGDRMPITLRGPKHAAPITYRVPMASAQVKSA | 180 |
| Query 180 | VLLAGLNTPGITTVIEPIMTRDHTEKMLQGFGANLTVETDADGVRTIRLEGRGKLTGQVI | 239 |
| Sbjct 181 | VLLAGLNTPGITTVIEP+MTRDHTEKML+GFGANLTVETD GVR I +EG+GKLTGQ I VLLAGLNTPGITTVIEPVMTRDHTEKMLKGFGANLTVETDERGVRHIFIEGQGKLTGQTI | 240 |
| Query 240 | DVPGDPSSTAFPLVAALLVPGSDVTILNVLMNPTRTGLILTLQEMGADIEVINPRLAGGE | 299 |
| Sbjct 241 | VPGDPSS FPLVAAL+VPGSD+TI NVLMNPTRTGL+LTLQEMG I+++NPR AGGE AVPGDPSSAGFPLVAALIVPGSDITIENVLMNPTRTGLLLTLQEMGGRIDILNPRNAGGE | 300 |
| Query 300 | DVADLRVRSSTLKGVTVPEDRAPSMIDEYPILavaaafaegaTVMNGLEELRVKESDRLS | 359 |
| Sbjct 301 | DVADLRVR S LKGV VP +RAPSMIDEYP+LAVAA+FAEG T+M GLEELRVKESDRLS DVADLRVRYSELKGVVVPPERAPSMIDEYPVLAVAASFAEGETLMQGLEELRVKESDRLS | 360 |
| Query 360 | AVANGLKLNGVDCDEGETSLVVRGRPDGKGLG---NASGAAVATHLDHRIAMSFLVMGLV | 416 |
| Sbjct 361 | AVANGLKLNGVDC EGE SL VRG+P GKGLG N V THLDHRIAMSFLVMGL AVANGLKLNGVDCTEGEASLAVRGKPGGKGLGAHPNGQDTVVKTHLDHRIAMSFLVMGLA | 420 |
| Query 417  Sbjct 421 | SENPVTVDDATMIATSFPEFMDLMAGLGAKIE 448  +E PVT+DDA MIATSFPEFM LM GLGA+IE  TEKPVTIDDANMIATSFPEFMGLMKGLGAEIE 452 |  |

>RWA83849.1 3-phosphoshikimate 1-carboxyvinyltransferase [Mesorhizobium sp.] RWB23139.1 3-phosphoshikimate 1-carboxyvinyltransferase [Mesorhizobium sp.] TIS49352.1 3-phosphoshikimate 1-carboxyvinyltransferase [Mesorhizobium sp.]

Length=452

Score = 691 bits (1784), Expect = 0.0, Method: Compositional matrix adjust.

| Identities | = 353/452 (78%), Positives = 388/452 (86%), Gaps = 4/452 (1%) |  |
| --- | --- | --- |
| Query 1 | MLHGASSRPATARKSSGLSGTVRIPGDKSISHRSFMFGGLASGETRITGLLEGEDVINTG | 60 |
| Sbjct 1 | M H A+ +PATARKS LSGT R+PGDKSISHRSFMFGGLASGETRITGLLEGEDV+ TG MSHSAAVKPATARKSPALSGTARVPGDKSISHRSFMFGGLASGETRITGLLEGEDVMRTG | 60 |
| Query 61 | KAMQAMGARIRKEGDTWIIDGVGNGGLLAPEAPLDFGNAATGCRLTMGLVGVYDFDSTFI | 120 |
| Sbjct 61 | AM+AMGA I K+G W+I G GNG LL PE PLDFGNA TG RLTMGLVG YD ++TFI  AAMKAMGAHIEKKGAEWVIRGTGNGALLEPEGPLDFGNAGTGSRLTMGLVGTYDMETTFI | 120 |

| Query | 121 | GDASLTKRPMGRVLNPLREMGVQV-KSEDGDRLPVTLRGPKTPTPITYRVPMASAQVKSA | 179 |
| --- | --- | --- | --- |
| Sbjct | 121 | GDASL+ RPMGRVL PLR+MGVQV K+ GDR+P+TLRGPK PITYRVPMASAQVKSA GDASLSGRPMGRVLEPLRQMGVQVLKAAPGDRMPITLRGPKHAAPITYRVPMASAQVKSA | 180 |
| Query | 180 | VLLAGLNTPGITTVIEPIMTRDHTEKMLQGFGANLTVETDADGVRTIRLEGRGKLTGQVI | 239 |
| Sbjct | 181 | VLLAGLNTPGITTVIEP+MTRDHTEKML+GFGANL+VETD GVR I +EG+GKL GQ I VLLAGLNTPGITTVIEPVMTRDHTEKMLKGFGANLSVETDERGVRHIFIEGQGKLIGQTI | 240 |
| Query | 240 | DVPGDPSSTAFPLVAALLVPGSDVTILNVLMNPTRTGLILTLQEMGADIEVINPRLAGGE | 299 |
| Sbjct | 241 | VPGDPSS FPLVAAL+VPGSD+TI NVLMNPTRTGL+LTLQEMG I+++NPR AGGE AVPGDPSSAGFPLVAALIVPGSDITIENVLMNPTRTGLLLTLQEMGGRIDILNPRNAGGE | 300 |
| Query | 300 | DVADLRVRSSTLKGVTVPEDRAPSMIDEYPILavaaafaegaTVMNGLEELRVKESDRLS | 359 |
| Sbjct | 301 | DVADLRVRSS LKGVTVP +RAPSMIDEYP+LAVAA+FAEG T+M GLEELRVKESDRLS DVADLRVRSSELKGVTVPPERAPSMIDEYPVLAVAASFAEGETLMQGLEELRVKESDRLS | 360 |
| Query | 360 | AVANGLKLNGVDCDEGETSLVVRGRPDGKGLG---NASGAAVATHLDHRIAMSFLVMGLV | 416 |
| Sbjct | 361 | AVANGLKLNGVDC EGE SL VRG+P GKGLG + V THLDHRIAMSFL+MGL AVANGLKLNGVDCSEGEASLAVRGKPSGKGLGAHPDGQDTKVKTHLDHRIAMSFLIMGLA | 420 |
| Query  Sbjct | 417  421 | SENPVTVDDATMIATSFPEFMDLMAGLGAKIE 448  +E PVT+DDA MIATSFPEFM LM GLGA+IE  TEKPVTIDDANMIATSFPEFMGLMKGLGAEIE 452 |  |

>WP_135908800.1 3-phosphoshikimate 1-carboxyvinyltransferase [Mesorhizobium sp. M4B.F.Ca.ET.143.01.1.1]

TGV24272.1 3-phosphoshikimate 1-carboxyvinyltransferase [Mesorhizobium sp. M4B.F.Ca.ET.143.01.1.1]

Length=455

Score = 691 bits (1784), Expect = 0.0, Method: Compositional matrix adjust.

| Identities | = 355/454 (78%), Positives = 389/454 (86%), Gaps = 4/454 (1%) |  |
| --- | --- | --- |
| Query 1 | MLHGASSRPATARKSSGLSGTVRIPGDKSISHRSFMFGGLASGETRITGLLEGEDVINTG | 60 |
| Sbjct 1 | M H A+ +PATARKSS LSGT R+PGDKSISHRSFMFGGLASGETRITGLLEGEDV+ TG MSHAAAPKPATARKSSALSGTARVPGDKSISHRSFMFGGLASGETRITGLLEGEDVMRTG | 60 |
| Query 61 | KAMQAMGARIRKEGDTWIIDGVGNGGLLAPEAPLDFGNAATGCRLTMGLVGVYDFDSTFI | 120 |
| Sbjct 61 | AM+AMGA I K G W+I G GNG LL PE PLDFGNA TG RLTMGLVG YD ++TFI AAMKAMGAHIEKRGAEWVIRGTGNGALLQPEGPLDFGNAGTGSRLTMGLVGTYDMETTFI | 120 |
| Query 121 | GDASLTKRPMGRVLNPLREMGVQV-KSEDGDRLPVTLRGPKTPTPITYRVPMASAQVKSA | 179 |
| Sbjct 121 | GDASL+ RPMGRVL PLR+MGVQV K+ GDR+P+TLRGPK PITYRVPMASAQVKSA GDASLSGRPMGRVLEPLRQMGVQVLKATPGDRMPITLRGPKHAAPITYRVPMASAQVKSA | 180 |
| Query 180 | VLLAGLNTPGITTVIEPIMTRDHTEKMLQGFGANLTVETDADGVRTIRLEGRGKLTGQVI | 239 |
| Sbjct 181 | VLLAGLNTPGITTVIEP+MTRDHTEKML+GFGANLTVETD GVR I +EG+GKL+GQ I VLLAGLNTPGITTVIEPVMTRDHTEKMLKGFGANLTVETDERGVRHIFIEGQGKLSGQTI | 240 |
| Query 240 | DVPGDPSSTAFPLVAALLVPGSDVTILNVLMNPTRTGLILTLQEMGADIEVINPRLAGGE | 299 |
| Sbjct 241 | VPGDPSS FPLVAAL+VPGSD+ I NVLMNPTRTG++LTLQEMG I+++NPR AGGE AVPGDPSSAGFPLVAALIVPGSDIVIENVLMNPTRTGVLLTLQEMGGRIDILNPRNAGGE | 300 |
| Query 300 | DVADLRVRSSTLKGVTVPEDRAPSMIDEYPILavaaafaegaTVMNGLEELRVKESDRLS | 359 |
| Sbjct 301 | DVADLRVRSS LKGVTVP +RAPSMIDEYP+LAVAA+FAEG T+M GLEELRVKESDRLS DVADLRVRSSELKGVTVPPERAPSMIDEYPVLAVAASFAEGETLMQGLEELRVKESDRLS | 360 |
| Query 360 | AVANGLKLNGVDCDEGETSLVVRGRPDGKGLG---NASGAAVATHLDHRIAMSFLVMGLV | 416 |
| Sbjct 361 | AVANGLKLNGVDC EGE +L VRGRP GKGLG N V THLDHRIAMSFLVMGL AVANGLKLNGVDCTEGEATLAVRGRPGGKGLGRHPNGPDTTVKTHLDHRIAMSFLVMGLA | 420 |
| Query 417  Sbjct 421 | SENPVTVDDATMIATSFPEFMDLMAGLGAKIELS 450 SE PVT+DDA MIATSFPEFM LM GLGA+I++ SEKPVTIDDANMIATSFPEFMGLMKGLGAEIDVQ 454 |  |

>RWM09552.1 3-phosphoshikimate 1-carboxyvinyltransferase [Mesorhizobium sp.] TIO49638.1 3-phosphoshikimate 1-carboxyvinyltransferase [Mesorhizobium sp.] TIO59062.1 3-phosphoshikimate 1-carboxyvinyltransferase [Mesorhizobium sp.] TJV62553.1 3-phosphoshikimate 1-carboxyvinyltransferase [Mesorhizobium sp.]

Length=452

Score = 691 bits (1783), Expect = 0.0, Method: Compositional matrix adjust.

| Identities | = 355/451 (79%), Positives = 387/451 (86%), Gaps = 4/451 (1%) |  |
| --- | --- | --- |
| Query 1 | MLHGASSRPATARKSSGLSGTVRIPGDKSISHRSFMFGGLASGETRITGLLEGEDVINTG | 60 |
| Sbjct 1 | M H A+ +PATARKS L+GT R+PGDKSISHRSFMFGGLASGETRITGLLEGEDV+ TG MSHAAAPKPATARKSPALAGTARVPGDKSISHRSFMFGGLASGETRITGLLEGEDVMRTG | 60 |
| Query 61 | KAMQAMGARIRKEGDTWIIDGVGNGGLLAPEAPLDFGNAATGCRLTMGLVGVYDFDSTFI | 120 |
| Sbjct 61 | AM+AMGA I K+G W+I G GNG LL PE PLDFGNA TG RLTMGLVG YD ++TFI AAMKAMGAHIEKKGAEWVICGTGNGALLEPEGPLDFGNAGTGSRLTMGLVGTYDMETTFI | 120 |
| Query 121 | GDASLTKRPMGRVLNPLREMGVQV-KSEDGDRLPVTLRGPKTPTPITYRVPMASAQVKSA | 179 |
| Sbjct 121 | GDASL+ RPMGRVL PLR+MGVQV K+ GDR+P+TLRGPK PITYRVPMASAQVKSA GDASLSGRPMGRVLEPLRQMGVQVLKAAPGDRMPITLRGPKHAAPITYRVPMASAQVKSA | 180 |
| Query 180 | VLLAGLNTPGITTVIEPIMTRDHTEKMLQGFGANLTVETDADGVRTIRLEGRGKLTGQVI | 239 |
| Sbjct 181 | VLLAGLNTPGITTVIEP+MTRDHTEKML+GFGANLTVETD GVR I +EG+GKLTGQ I VLLAGLNTPGITTVIEPVMTRDHTEKMLKGFGANLTVETDERGVRHIFIEGQGKLTGQTI | 240 |
| Query 240 | DVPGDPSSTAFPLVAALLVPGSDVTILNVLMNPTRTGLILTLQEMGADIEVINPRLAGGE | 299 |
| Sbjct 241 | VPGDPSS FPLVAAL+VPGSD+ I NVLMNPTRTGL+LTLQEMG I+++NPR AGGE AVPGDPSSAGFPLVAALIVPGSDIVIENVLMNPTRTGLLLTLQEMGGRIDILNPRNAGGE | 300 |
| Query 300 | DVADLRVRSSTLKGVTVPEDRAPSMIDEYPILavaaafaegaTVMNGLEELRVKESDRLS | 359 |
| Sbjct 301 | DVADLRVRSS LKGV VP DRAPSMIDEYP+LAVAA+FAEG T+M GLEELRVKESDRLS DVADLRVRSSDLKGVVVPPDRAPSMIDEYPVLAVAASFAEGETLMQGLEELRVKESDRLS | 360 |
| Query 360 | AVANGLKLNGVDCDEGETSLVVRGRPDGKGLG---NASGAAVATHLDHRIAMSFLVMGLV | 416 |
| Sbjct 361 | AVANGLKLNGVDC EGE SL VRG+P GKGLG N AV THLDHRIAMSFLVMGL AVANGLKLNGVDCTEGEASLAVRGKPGGKGLGAHPNGQDTAVKTHLDHRIAMSFLVMGLA | 420 |
| Query 417  Sbjct 421 | SENPVTVDDATMIATSFPEFMDLMAGLGAKI 447  +E PVT+DDA MIATSFPEFM LM GLGA+I  TEKPVTIDDANMIATSFPEFMGLMTGLGAEI 451 |  |

>WP_127876384.1 3-phosphoshikimate 1-carboxyvinyltransferase [Mesorhizobium sp. M2E.F.Ca.ET.209.01.1.1]

TGS12946.1 3-phosphoshikimate 1-carboxyvinyltransferase [Mesorhizobium sp. M2E.F.Ca.ET.209.01.1.1]

Length=452

Score = 691 bits (1783), Expect = 0.0, Method: Compositional matrix adjust.

| Identities | = 353/452 (78%), Positives = 388/452 (86%), Gaps = 4/452 (1%) |  |
| --- | --- | --- |
| Query 1 | MLHGASSRPATARKSSGLSGTVRIPGDKSISHRSFMFGGLASGETRITGLLEGEDVINTG | 60 |
| Sbjct 1 | M H A+++PATARKS L+GT R+PGDKSISHRSFMFGGLASGETRITGLLEGEDV+ TG MSHSAAAKPATARKSPALAGTARVPGDKSISHRSFMFGGLASGETRITGLLEGEDVMRTG | 60 |
| Query 61 | KAMQAMGARIRKEGDTWIIDGVGNGGLLAPEAPLDFGNAATGCRLTMGLVGVYDFDSTFI | 120 |
| Sbjct 61 | AM+AMGA I K G W+I G GNG LL PE PLDFGNA TG RLTMGLVG YD ++TFI AAMKAMGAHIEKNGAEWVIRGTGNGALLQPEGPLDFGNAGTGSRLTMGLVGTYDMETTFI | 120 |
| Query 121 | GDASLTKRPMGRVLNPLREMGVQV-KSEDGDRLPVTLRGPKTPTPITYRVPMASAQVKSA | 179 |
| Sbjct 121 | GDASL+ RPMGRVL+PLR+MGVQV K+ GDR+P+TLRGPK PITYRVPMASAQVKSA GDASLSGRPMGRVLDPLRQMGVQVLKAAPGDRMPITLRGPKHAAPITYRVPMASAQVKSA | 180 |
| Query 180 | VLLAGLNTPGITTVIEPIMTRDHTEKMLQGFGANLTVETDADGVRTIRLEGRGKLTGQVI | 239 |
| Sbjct 181 | VLLAGLNTPGITTVIEP+MTRDHTEKML+GFGANLTVETD GVR I +EG+GKLTGQ I VLLAGLNTPGITTVIEPVMTRDHTEKMLKGFGANLTVETDERGVRHIFIEGQGKLTGQTI | 240 |
| Query 240 | DVPGDPSSTAFPLVAALLVPGSDVTILNVLMNPTRTGLILTLQEMGADIEVINPRLAGGE | 299 |
| Sbjct 241 | VPGDPSS FPLVAAL+VPGSD+ I NVLMNPTRTGL+LTLQEMG I+++NPR AGGE AVPGDPSSAGFPLVAALIVPGSDIVIENVLMNPTRTGLLLTLQEMGGRIDILNPRNAGGE | 300 |
| Query 300 | DVADLRVRSSTLKGVTVPEDRAPSMIDEYPILavaaafaegaTVMNGLEELRVKESDRLS | 359 |
| Sbjct 301 | DVADLRVRSS LKGV VP +RAPSMIDEYP+LA+AA+FAEG T+M GLEELRVKESDRL+ DVADLRVRSSDLKGVVVPPERAPSMIDEYPVLAIAASFAEGETLMQGLEELRVKESDRLA | 360 |
| Query 360 | AVANGLKLNGVDCDEGETSLVVRGRPDGKGLG---NASGAAVATHLDHRIAMSFLVMGLV | 416 |

AVANGLKLNGVDC EGE SL VRGRP GKGLG	N	V THLDHRIAMSFLVMGL

| Sbjct | 361 | AVANGLKLNGVDCTEGEASLAVRGRPGGKGLGAHPNGQDTVVKTHLDHRIAMSFLVMGLA 420 |
| --- | --- | --- |
| Query | 417 | SENPVTVDDATMIATSFPEFMDLMAGLGAKIE 448 |
| Sbjct | 421 | +E PVT+DDA MIATSFPEFM LM GLGA+IE  TEKPVTIDDANMIATSFPEFMGLMKGLGAEIE 452 |

>WP_067003404.1 MULTISPECIES: 3-phosphoshikimate 1-carboxyvinyltransferase [unclassified Mesorhizobium]

AZO60078.1 3-phosphoshikimate 1-carboxyvinyltransferase [Mesorhizobium sp. M1A.F.Ca.IN.022.06.1.1]

RWG06413.1 3-phosphoshikimate 1-carboxyvinyltransferase [Mesorhizobium sp.] OBQ77197.1 3-phosphoshikimate 1-carboxyvinyltransferase [Mesorhizobium sp.

WSM3873]

PBB33561.1 3-phosphoshikimate 1-carboxyvinyltransferase [Mesorhizobium sp. WSM3882]

PBB39124.1 3-phosphoshikimate 1-carboxyvinyltransferase [Mesorhizobium sp. WSM3868]

Length=452

Score = 691 bits (1782), Expect = 0.0, Method: Compositional matrix adjust.

| Identities | = 355/452 (79%), Positives = 386/452 (85%), Gaps = 4/452 (1%) |  |
| --- | --- | --- |
| Query 1 | MLHGASSRPATARKSSGLSGTVRIPGDKSISHRSFMFGGLASGETRITGLLEGEDVINTG | 60 |
| Sbjct 1 | M H A+ +PATARKS L+GT R+PGDKSISHRSFMFGGLASGETRITGLLEGEDV+ TG MSHAAAPKPATARKSPALAGTARVPGDKSISHRSFMFGGLASGETRITGLLEGEDVMRTG | 60 |
| Query 61 | KAMQAMGARIRKEGDTWIIDGVGNGGLLAPEAPLDFGNAATGCRLTMGLVGVYDFDSTFI | 120 |
| Sbjct 61 | AM+AMGA I K G W+I G GNG LL PE PLDFGNA TG RLTMGLVG YD ++TFI AAMKAMGAHIEKTGAEWVIRGTGNGALLEPEGPLDFGNAGTGSRLTMGLVGTYDMETTFI | 120 |
| Query 121 | GDASLTKRPMGRVLNPLREMGVQV-KSEDGDRLPVTLRGPKTPTPITYRVPMASAQVKSA | 179 |
| Sbjct 121 | GDASL+ RPMGRVL PLR+MGVQV K+ GDR+P+TLRGPK PITYRVPMASAQVKSA GDASLSGRPMGRVLEPLRQMGVQVLKAAPGDRMPITLRGPKHAAPITYRVPMASAQVKSA | 180 |
| Query 180 | VLLAGLNTPGITTVIEPIMTRDHTEKMLQGFGANLTVETDADGVRTIRLEGRGKLTGQVI | 239 |
| Sbjct 181 | VLLAGLNTPGITTVIEP+MTRDHTEKML+GFGANLTVETD GVR I +EG+GKLTGQ I VLLAGLNTPGITTVIEPVMTRDHTEKMLKGFGANLTVETDERGVRHIFIEGQGKLTGQTI | 240 |
| Query 240 | DVPGDPSSTAFPLVAALLVPGSDVTILNVLMNPTRTGLILTLQEMGADIEVINPRLAGGE | 299 |
| Sbjct 241 | VPGDPSS FPLVAAL+VPGSD+ I NVLMNPTRTGL+LTLQEMG I+++NPR AGGE AVPGDPSSAGFPLVAALIVPGSDIVIENVLMNPTRTGLLLTLQEMGGRIDILNPRNAGGE | 300 |
| Query 300 | DVADLRVRSSTLKGVTVPEDRAPSMIDEYPILavaaafaegaTVMNGLEELRVKESDRLS | 359 |
| Sbjct 301 | DVADLRVRSS LKGV VP +RAPSMIDEYP+LAVAA FAEG T+M GLEELRVKESDRLS DVADLRVRSSDLKGVVVPPERAPSMIDEYPVLAVAAVFAEGETLMQGLEELRVKESDRLS | 360 |
| Query 360 | AVANGLKLNGVDCDEGETSLVVRGRPDGKGLG---NASGAAVATHLDHRIAMSFLVMGLV | 416 |
| Sbjct 361 | AVANGLKLNGVDC EGE SL VRG+P GKGLG N AV THLDHRIAMSFLVMGL AVANGLKLNGVDCSEGEASLAVRGKPGGKGLGAHPNGPDTAVKTHLDHRIAMSFLVMGLA | 420 |
| Query 417  Sbjct 421 | SENPVTVDDATMIATSFPEFMDLMAGLGAKIE 448  +E PVT+DDA MIATSFPEFM LM GLGA+IE  TEKPVTIDDANMIATSFPEFMGLMKGLGAEIE 452 |  |

>WP_127256349.1 MULTISPECIES: 3-phosphoshikimate 1-carboxyvinyltransferase [unclassified Mesorhizobium]

RUY11277.1 3-phosphoshikimate 1-carboxyvinyltransferase [Mesorhizobium sp. M2A.F.Ca.ET.040.01.1.1]

RWA93181.1 3-phosphoshikimate 1-carboxyvinyltransferase [Mesorhizobium sp.] AZO04788.1 3-phosphoshikimate 1-carboxyvinyltransferase [Mesorhizobium sp.

M2A.F.Ca.ET.043.02.1.1]

RUW81106.1 3-phosphoshikimate 1-carboxyvinyltransferase [Mesorhizobium sp. M2A.F.Ca.ET.067.02.1.1]

RUX18890.1 3-phosphoshikimate 1-carboxyvinyltransferase [Mesorhizobium sp. M2A.F.Ca.ET.037.01.1.1]

Length=452

Score = 691 bits (1782), Expect = 0.0, Method: Compositional matrix adjust. Identities = 354/452 (78%), Positives = 388/452 (86%), Gaps = 4/452 (1%)

| Query | 1 | MLHGASSRPATARKSSGLSGTVRIPGDKSISHRSFMFGGLASGETRITGLLEGEDVINTG | 60 |
| --- | --- | --- | --- |
| Sbjct | 1 | M H A+++ ATARKS L+GT R+PGDKSISHRSFMFGGLASGETRITGLLEGEDV+ TG MSHSAAAKQATARKSPALAGTARVPGDKSISHRSFMFGGLASGETRITGLLEGEDVMRTG | 60 |
| Query | 61 | KAMQAMGARIRKEGDTWIIDGVGNGGLLAPEAPLDFGNAATGCRLTMGLVGVYDFDSTFI | 120 |
| Sbjct | 61 | AM+AMGA I K G W+I G GNG LL PE PLDFGNA TG RLTMGLVG YD ++TFI AAMKAMGAHIEKNGAEWVIRGTGNGALLQPEGPLDFGNAGTGSRLTMGLVGTYDMETTFI | 120 |
| Query | 121 | GDASLTKRPMGRVLNPLREMGVQV-KSEDGDRLPVTLRGPKTPTPITYRVPMASAQVKSA | 179 |
| Sbjct | 121 | GDASL+ RPMGRVL+PLR+MGVQV K+ GDR+P+TLRGPK PITYRVPMASAQVKSA GDASLSGRPMGRVLDPLRQMGVQVLKAAPGDRMPITLRGPKHAAPITYRVPMASAQVKSA | 180 |
| Query | 180 | VLLAGLNTPGITTVIEPIMTRDHTEKMLQGFGANLTVETDADGVRTIRLEGRGKLTGQVI | 239 |
| Sbjct | 181 | VLLAGLNTPGITTVIEP+MTRDHTEKML+GFGANLTVETD GVR I +EG+GKLTGQ I VLLAGLNTPGITTVIEPVMTRDHTEKMLKGFGANLTVETDERGVRHIFIEGQGKLTGQTI | 240 |
| Query | 240 | DVPGDPSSTAFPLVAALLVPGSDVTILNVLMNPTRTGLILTLQEMGADIEVINPRLAGGE | 299 |
| Sbjct | 241 | VPGDPSS FPLVAAL+VPGSD+ I NVLMNPTRTGL+LTLQEMG I+++NPR AGGE AVPGDPSSAGFPLVAALIVPGSDIVIENVLMNPTRTGLLLTLQEMGGRIDILNPRNAGGE | 300 |
| Query | 300 | DVADLRVRSSTLKGVTVPEDRAPSMIDEYPILavaaafaegaTVMNGLEELRVKESDRLS | 359 |
| Sbjct | 301 | DVADLRVR S LKGVTVP +RAPSMIDEYP+LAVAA+FAEG T+M GLEELRVKESDRLS DVADLRVRYSELKGVTVPPERAPSMIDEYPVLAVAASFAEGETLMQGLEELRVKESDRLS | 360 |
| Query | 360 | AVANGLKLNGVDCDEGETSLVVRGRPDGKGLG---NASGAAVATHLDHRIAMSFLVMGLV | 416 |
| Sbjct | 361 | AVANGLKLNGVDC EGE SL VRGRP GKGLG N AV THLDHRIAMSFL+MGL AVANGLKLNGVDCTEGEASLAVRGRPGGKGLGAHPNGPDTAVKTHLDHRIAMSFLIMGLA | 420 |
| Query  Sbjct | 417  421 | SENPVTVDDATMIATSFPEFMDLMAGLGAKIE 448  +E PVT+DDA MIATSFPEFM LM GLGA+IE  TEKPVTIDDANMIATSFPEFMGLMKGLGAEIE 452 |  |

>RWG25457.1 3-phosphoshikimate 1-carboxyvinyltransferase [Mesorhizobium sp.] Length=466

Score = 691 bits (1782), Expect = 0.0, Method: Compositional matrix adjust.

| Identities | = 355/452 (79%), Positives = 386/452 (85%), Gaps = 4/452 (1%) |  |
| --- | --- | --- |
| Query 1 | MLHGASSRPATARKSSGLSGTVRIPGDKSISHRSFMFGGLASGETRITGLLEGEDVINTG | 60 |
| Sbjct 15 | M H A+ +PATARKS L+GT R+PGDKSISHRSFMFGGLASGETRITGLLEGEDV+ TG MSHAAAPKPATARKSPALAGTARVPGDKSISHRSFMFGGLASGETRITGLLEGEDVMRTG | 74 |
| Query 61 | KAMQAMGARIRKEGDTWIIDGVGNGGLLAPEAPLDFGNAATGCRLTMGLVGVYDFDSTFI | 120 |
| Sbjct 75 | AM+AMGA I K G W+I G GNG LL PE PLDFGNA TG RLTMGLVG YD ++TFI AAMKAMGAHIEKTGAEWVIRGTGNGALLEPEGPLDFGNAGTGSRLTMGLVGTYDMETTFI | 134 |
| Query 121 | GDASLTKRPMGRVLNPLREMGVQV-KSEDGDRLPVTLRGPKTPTPITYRVPMASAQVKSA | 179 |
| Sbjct 135 | GDASL+ RPMGRVL PLR+MGVQV K+ GDR+P+TLRGPK PITYRVPMASAQVKSA GDASLSGRPMGRVLEPLRQMGVQVLKAAPGDRMPITLRGPKHAAPITYRVPMASAQVKSA | 194 |
| Query 180 | VLLAGLNTPGITTVIEPIMTRDHTEKMLQGFGANLTVETDADGVRTIRLEGRGKLTGQVI | 239 |
| Sbjct 195 | VLLAGLNTPGITTVIEP+MTRDHTEKML+GFGANLTVETD GVR I +EG+GKLTGQ I VLLAGLNTPGITTVIEPVMTRDHTEKMLKGFGANLTVETDERGVRHIFIEGQGKLTGQTI | 254 |
| Query 240 | DVPGDPSSTAFPLVAALLVPGSDVTILNVLMNPTRTGLILTLQEMGADIEVINPRLAGGE | 299 |
| Sbjct 255 | VPGDPSS FPLVAAL+VPGSD+ I NVLMNPTRTGL+LTLQEMG I+++NPR AGGE AVPGDPSSAGFPLVAALIVPGSDIVIENVLMNPTRTGLLLTLQEMGGRIDILNPRNAGGE | 314 |
| Query 300 | DVADLRVRSSTLKGVTVPEDRAPSMIDEYPILavaaafaegaTVMNGLEELRVKESDRLS | 359 |
| Sbjct 315 | DVADLRVRSS LKGV VP +RAPSMIDEYP+LAVAA FAEG T+M GLEELRVKESDRLS DVADLRVRSSDLKGVVVPPERAPSMIDEYPVLAVAAVFAEGETLMQGLEELRVKESDRLS | 374 |
| Query 360 | AVANGLKLNGVDCDEGETSLVVRGRPDGKGLG---NASGAAVATHLDHRIAMSFLVMGLV | 416 |
| Sbjct 375 | AVANGLKLNGVDC EGE SL VRG+P GKGLG N AV THLDHRIAMSFLVMGL AVANGLKLNGVDCSEGEASLAVRGKPGGKGLGAHPNGPDTAVKTHLDHRIAMSFLVMGLA | 434 |
| Query 417  Sbjct 435 | SENPVTVDDATMIATSFPEFMDLMAGLGAKIE 448  +E PVT+DDA MIATSFPEFM LM GLGA+IE  TEKPVTIDDANMIATSFPEFMGLMKGLGAEIE 466 |  |

>WP_040974942.1 3-phosphoshikimate 1-carboxyvinyltransferase [Mesorhizobium sp. ORS 3324]

Length=452

Score = 691 bits (1782), Expect = 0.0, Method: Compositional matrix adjust.

| Identities | = 355/452 (79%), Positives = 387/452 (86%), Gaps = 4/452 (1%) |  |
| --- | --- | --- |
| Query 1 | MLHGASSRPATARKSSGLSGTVRIPGDKSISHRSFMFGGLASGETRITGLLEGEDVINTG | 60 |
| Sbjct 1 | M H A+ +PATARKS L+GT R+PGDKSISHRSFMFGGLASGETRITGLLEGEDV+ TG MSHAAAPKPATARKSPALAGTARVPGDKSISHRSFMFGGLASGETRITGLLEGEDVMRTG | 60 |
| Query 61 | KAMQAMGARIRKEGDTWIIDGVGNGGLLAPEAPLDFGNAATGCRLTMGLVGVYDFDSTFI | 120 |
| Sbjct 61 | AM+AMGA I K G W+I G GNG LL PE PLDFGNA TG RLTMGLVG YD ++TFI AAMKAMGAHIEKRGAEWVIRGTGNGALLEPEGPLDFGNAGTGSRLTMGLVGTYDMETTFI | 120 |
| Query 121 | GDASLTKRPMGRVLNPLREMGVQV-KSEDGDRLPVTLRGPKTPTPITYRVPMASAQVKSA | 179 |
| Sbjct 121 | GDASL+ RPMGRVL PLR+MGVQV K+ GDR+P+TLRGPK PITYRVPMASAQVKSA GDASLSGRPMGRVLEPLRQMGVQVLKAAPGDRMPITLRGPKHAAPITYRVPMASAQVKSA | 180 |
| Query 180 | VLLAGLNTPGITTVIEPIMTRDHTEKMLQGFGANLTVETDADGVRTIRLEGRGKLTGQVI | 239 |
| Sbjct 181 | VLLAGLNTPGITTVIEP+MTRDHTEKML+GFGANLTVETD GVR I +EG+GKLTGQ I VLLAGLNTPGITTVIEPVMTRDHTEKMLKGFGANLTVETDERGVRHIFIEGQGKLTGQTI | 240 |
| Query 240 | DVPGDPSSTAFPLVAALLVPGSDVTILNVLMNPTRTGLILTLQEMGADIEVINPRLAGGE | 299 |
| Sbjct 241 | VPGDPSS FPLVAAL+VPGSD+TI NVLMNPTRTGL+ TLQEMG I+++NPR AGGE AVPGDPSSAGFPLVAALIVPGSDITIENVLMNPTRTGLLSTLQEMGGRIDILNPRNAGGE | 300 |
| Query 300 | DVADLRVRSSTLKGVTVPEDRAPSMIDEYPILavaaafaegaTVMNGLEELRVKESDRLS | 359 |
| Sbjct 301 | DVADLRVRSS LKGV VP +RAPSMIDEYP+LAVAA+FAEG T+M GLEELRVKESDRLS DVADLRVRSSELKGVVVPPERAPSMIDEYPVLAVAASFAEGETLMQGLEELRVKESDRLS | 360 |
| Query 360 | AVANGLKLNGVDCDEGETSLVVRGRPDGKGLG---NASGAAVATHLDHRIAMSFLVMGLV | 416 |
| Sbjct 361 | AVANGLKLNGVDC EGE SL VRG+P GKGLG N AV THLDHRIAMSFLVMGL AVANGLKLNGVDCTEGEASLAVRGKPGGKGLGAHPNGPDTAVKTHLDHRIAMSFLVMGLA | 420 |
| Query 417  Sbjct 421 | SENPVTVDDATMIATSFPEFMDLMAGLGAKIE 448  +E PVT+DDA MIATSFPEFM LM GLGA+IE  TEKPVTIDDANMIATSFPEFMGLMKGLGAEIE 452 |  |

>WP_192728618.1 3-phosphoshikimate 1-carboxyvinyltransferase [Rhizobium viscosum] MBE1504612.1 3-phosphoshikimate 1-carboxyvinyltransferase [Rhizobium viscosum]

Length=450

Score = 691 bits (1782), Expect = 0.0, Method: Compositional matrix adjust.

| Identities | = 346/448 (77%), Positives = 391/448 (87%), Gaps = 0/448 (0%) |  |
| --- | --- | --- |
| Query 1 | MLHGASSRPATARKSSGLSGTVRIPGDKSISHRSFMFGGLASGETRITGLLEGEDVINTG | 60 |
| Sbjct 1 | M G++S+PA ARKSSGLSGTVRIPGDKSISHR+ + GGLASGETRI GLL+GEDV+NTG MSQGSASKPAIARKSSGLSGTVRIPGDKSISHRALILGGLASGETRIFGLLKGEDVLNTG | 60 |
| Query 61 | KAMQAMGARIRKEGDTWIIDGVGNGGLLAPEAPLDFGNAATGCRLTMGLVGVYDFDSTFI | 120 |
| Sbjct 61 | K MQAMGA IRK+G WII+G GNG LLAPE LDFGN+ TG RLTMGL GVYDFD+ F KVMQAMGAVIRKDGGAWIINGTGNGALLAPEVSLDFGNSGTGVRLTMGLAGVYDFDTVFT | 120 |
| Query 121 | GDASLTKRPMGRVLNPLREMGVQVKSEDGDRLPVTLRGPKTPTPITYRVPMASAQVKSAV | 180 |
| Sbjct 121 | GDASL++RPMGRVL+PLR MGVQVK+ +GDRLPV LRGP+TP PI Y + +ASAQVKSAV GDASLSQRPMGRVLDPLRRMGVQVKAVEGDRLPVILRGPETPAPIRYTLSVASAQVKSAV | 180 |
| Query 181 | LLAGLNTPGITTVIEPIMTRDHTEKMLQGFGANLTVETDADGVRTIRLEGRGKLTGQVID | 240 |
| Sbjct 181 | LLAGLN PG+TTVIEP +TRDHTEKML GFGA L+V+TD G RTI+L+GRG+LTGQ+ID LLAGLNVPGVTTVIEPAITRDHTEKMLAGFGAELSVKTDKHGTRTIQLQGRGRLTGQIID | 240 |
| Query 241 | VPGDPSSTAFPLVAALLVPGSDVTILNVLMNPTRTGLILTLQEMGADIEVINPRLAGGED | 300 |
| Sbjct 241 | VPGDPSS AFPLVAALLVPGSD+TI NVLM+PTRTGLILTLQEM ADIE++N RLAGGED VPGDPSSAAFPLVAALLVPGSDITISNVLMSPTRTGLILTLQEMDADIEILNRRLAGGED | 300 |
| Query 301 | VADLRVRSSTLKGVTVPEDRAPSMIDEYPILavaaafaegaTVMNGLEELRVKESDRLSA | 360 |
| Sbjct 301 | VADLRVR S L GVTVP +RAPSMIDEYP+LAVAAAFA+G TVMNGL+ELR KESDRLSA  VADLRVRHSELTGVTVPAERAPSMIDEYPVLAVAAAFAKGRTVMNGLQELRFKESDRLSA | 360 |

| Query | 361 | VANGLKLNGVDCDEGETSLVVRGRPDGKGLGNASGAAVATHLDHRIAMSFLVMGLVSENP 420 |
| --- | --- | --- |
| Sbjct | 361 | +A GLKLNGV+C+EG+ L V GRPDGKG GNA+G V THLDHRIAMSFLVMGL SE+P IAQGLKLNGVECEEGQDWLSVTGRPDGKGFGNAAGERVVTHLDHRIAMSFLVMGLASEHP 420 |
| Query | 421 | VTVDDATMIATSFPEFMDLMAGLGAKIE 448 |
| Sbjct | 421 | V +DD++MI TSFP+FMDLM LGAKIE  VGIDDSSMITTSFPQFMDLMESLGAKIE 448 |

>WP_135860344.1 MULTISPECIES: 3-phosphoshikimate 1-carboxyvinyltransferase [unclassified Mesorhizobium]

RWA59632.1 3-phosphoshikimate 1-carboxyvinyltransferase [Mesorhizobium sp.] TGR09155.1 3-phosphoshikimate 1-carboxyvinyltransferase [Mesorhizobium sp.

M4B.F.Ca.ET.200.01.1.1]

TGS18634.1 3-phosphoshikimate 1-carboxyvinyltransferase [Mesorhizobium sp. M4B.F.Ca.ET.190.01.1.1]

TGT30446.1 3-phosphoshikimate 1-carboxyvinyltransferase [Mesorhizobium sp. M4B.F.Ca.ET.172.01.1.1]

Length=452

Score = 691 bits (1782), Expect = 0.0, Method: Compositional matrix adjust.

| Identities | = 355/451 (79%), Positives = 387/451 (86%), Gaps = 4/451 (1%) |  |
| --- | --- | --- |
| Query 1 | MLHGASSRPATARKSSGLSGTVRIPGDKSISHRSFMFGGLASGETRITGLLEGEDVINTG | 60 |
| Sbjct 1 | M H A+ +PATARKSS LSGT R+PGDKSISHRSFMFGGLASGETRITGLLEGEDV+ TG MSHAAAPKPATARKSSALSGTARVPGDKSISHRSFMFGGLASGETRITGLLEGEDVMRTG | 60 |
| Query 61 | KAMQAMGARIRKEGDTWIIDGVGNGGLLAPEAPLDFGNAATGCRLTMGLVGVYDFDSTFI | 120 |
| Sbjct 61 | AM+AMGA I K G W+I G GNG LL PE PLDFGNA TG RLTMGLVG YD ++TFI AAMKAMGAHIEKRGAEWVIRGTGNGALLQPEGPLDFGNAGTGSRLTMGLVGTYDMETTFI | 120 |
| Query 121 | GDASLTKRPMGRVLNPLREMGVQV-KSEDGDRLPVTLRGPKTPTPITYRVPMASAQVKSA | 179 |
| Sbjct 121 | GDASL+ RPMGRVL PLR+MGVQV K+ GDR+P+TLRGPK PITYRVPMASAQVKSA GDASLSGRPMGRVLEPLRQMGVQVLKATPGDRMPITLRGPKHAAPITYRVPMASAQVKSA | 180 |
| Query 180 | VLLAGLNTPGITTVIEPIMTRDHTEKMLQGFGANLTVETDADGVRTIRLEGRGKLTGQVI | 239 |
| Sbjct 181 | VLLAGLNTPGITTVIEP+MTRDHTEKML+GFGANLTVETD GVR I +EG+GKL+GQ I VLLAGLNTPGITTVIEPVMTRDHTEKMLKGFGANLTVETDERGVRHIFIEGQGKLSGQTI | 240 |
| Query 240 | DVPGDPSSTAFPLVAALLVPGSDVTILNVLMNPTRTGLILTLQEMGADIEVINPRLAGGE | 299 |
| Sbjct 241 | VPGDPSS FPLVAAL+VPGSD+ I NVLMNPTRTGL+LTLQEMG I+++NPR AGGE AVPGDPSSAGFPLVAALIVPGSDIVIENVLMNPTRTGLLLTLQEMGGRIDILNPRNAGGE | 300 |
| Query 300 | DVADLRVRSSTLKGVTVPEDRAPSMIDEYPILavaaafaegaTVMNGLEELRVKESDRLS | 359 |
| Sbjct 301 | DVADLRVRSS LKGVTVP +RAPSMIDEYP+LAVAA+FAEG T+M GLEELRVKESDRLS DVADLRVRSSELKGVTVPPERAPSMIDEYPVLAVAASFAEGETLMQGLEELRVKESDRLS | 360 |
| Query 360 | AVANGLKLNGVDCDEGETSLVVRGRPDGKGLG---NASGAAVATHLDHRIAMSFLVMGLV | 416 |
| Sbjct 361 | AVANGLKLNGVDC EG+ +L VRGRP GKGLG N V THLDHRIAMSFLVMGL AVANGLKLNGVDCTEGDATLAVRGRPGGKGLGRHPNGPDTTVKTHLDHRIAMSFLVMGLA | 420 |
| Query 417  Sbjct 421 | SENPVTVDDATMIATSFPEFMDLMAGLGAKI 447 SE PVT+DDA MIATSFPEFM LM GLGA+I SEKPVTIDDANMIATSFPEFMGLMKGLGAEI 451 |  |

>WP_127284415.1 3-phosphoshikimate 1-carboxyvinyltransferase [Mesorhizobium sp. M4B.F.Ca.ET.017.02.2.1]

RVD24595.1 3-phosphoshikimate 1-carboxyvinyltransferase [Mesorhizobium sp. M4B.F.Ca.ET.017.02.2.1]

Length=452

Score = 691 bits (1782), Expect = 0.0, Method: Compositional matrix adjust. Identities = 355/451 (79%), Positives = 387/451 (86%), Gaps = 4/451 (1%)

| Query | 1 | MLHGASSRPATARKSSGLSGTVRIPGDKSISHRSFMFGGLASGETRITGLLEGEDVINTG | 60 |
| --- | --- | --- | --- |
| Sbjct | 1 | M H A+ +PATARKSS LSGT R+PGDKSISHRSFMFGGLASGETRITGLLEGEDV+ TG MSHAAAPKPATARKSSALSGTARVPGDKSISHRSFMFGGLASGETRITGLLEGEDVMRTG | 60 |
| Query | 61 | KAMQAMGARIRKEGDTWIIDGVGNGGLLAPEAPLDFGNAATGCRLTMGLVGVYDFDSTFI | 120 |
| Sbjct | 61 | AM+AMGA I K G W+I G GNG LL P+ PLDFGNA TG RLTMGLVG YD ++TFI  AAMKAMGAHIEKRGAEWVIRGTGNGALLQPDGPLDFGNAGTGSRLTMGLVGTYDMETTFI | 120 |

| Query | 121 | GDASLTKRPMGRVLNPLREMGVQV-KSEDGDRLPVTLRGPKTPTPITYRVPMASAQVKSA | 179 |
| --- | --- | --- | --- |
| Sbjct | 121 | GDASL+ RPMGRVL PLR+MGVQV K+ GDR+P+TLRGPK PITYRVPMASAQVKSA GDASLSGRPMGRVLEPLRQMGVQVLKATPGDRMPITLRGPKHAAPITYRVPMASAQVKSA | 180 |
| Query | 180 | VLLAGLNTPGITTVIEPIMTRDHTEKMLQGFGANLTVETDADGVRTIRLEGRGKLTGQVI | 239 |
| Sbjct | 181 | VLLAGLNTPGITTVIEP+MTRDHTEKML+GFGANLTVETD GVR I +EG+GKL+GQ I VLLAGLNTPGITTVIEPVMTRDHTEKMLKGFGANLTVETDERGVRHIFIEGQGKLSGQTI | 240 |
| Query | 240 | DVPGDPSSTAFPLVAALLVPGSDVTILNVLMNPTRTGLILTLQEMGADIEVINPRLAGGE | 299 |
| Sbjct | 241 | VPGDPSS FPLVAAL+VPGSD+ I NVLMNPTRTGL+LTLQEMG I+++NPR AGGE AVPGDPSSAGFPLVAALIVPGSDIVIENVLMNPTRTGLLLTLQEMGGRIDILNPRNAGGE | 300 |
| Query | 300 | DVADLRVRSSTLKGVTVPEDRAPSMIDEYPILavaaafaegaTVMNGLEELRVKESDRLS | 359 |
| Sbjct | 301 | DVADLRVRSS LKGVTVP +RAPSMIDEYP+LAVAA+FAEG T+M GLEELRVKESDRLS DVADLRVRSSELKGVTVPPERAPSMIDEYPVLAVAASFAEGETLMQGLEELRVKESDRLS | 360 |
| Query | 360 | AVANGLKLNGVDCDEGETSLVVRGRPDGKGLG---NASGAAVATHLDHRIAMSFLVMGLV | 416 |
| Sbjct | 361 | AVANGLKLNGVDC EGE +L VRGRP GKGLG N V THLDHRIAMSFLVMGL AVANGLKLNGVDCTEGEATLAVRGRPGGKGLGRHPNGPDTTVKTHLDHRIAMSFLVMGLA | 420 |
| Query  Sbjct | 417  421 | SENPVTVDDATMIATSFPEFMDLMAGLGAKI 447 SE PVT+DDA MIATSFPEFM LM GLGA+I SEKPVTIDDANMIATSFPEFMGLMKGLGAEI 451 |  |

>RWC96807.1 3-phosphoshikimate 1-carboxyvinyltransferase [Mesorhizobium sp.] Length=455

Score = 690 bits (1781), Expect = 0.0, Method: Compositional matrix adjust.

| Identities | = 355/454 (78%), Positives = 388/454 (85%), Gaps = 4/454 (1%) |  |
| --- | --- | --- |
| Query 1 | MLHGASSRPATARKSSGLSGTVRIPGDKSISHRSFMFGGLASGETRITGLLEGEDVINTG | 60 |
| Sbjct 1 | M H A+ +PATARKSS LSGT R+PGDKSISHRSFMFGGLASGETRITGLLEGEDV+ TG MSHAAAPKPATARKSSALSGTARVPGDKSISHRSFMFGGLASGETRITGLLEGEDVMRTG | 60 |
| Query 61 | KAMQAMGARIRKEGDTWIIDGVGNGGLLAPEAPLDFGNAATGCRLTMGLVGVYDFDSTFI | 120 |
| Sbjct 61 | AM+AMGA I K G W+I G GNG LL PE PLDFGNA TG RLTMGLVG YD ++TFI AAMKAMGAHIEKRGAEWVIRGTGNGALLQPEGPLDFGNAGTGSRLTMGLVGTYDMETTFI | 120 |
| Query 121 | GDASLTKRPMGRVLNPLREMGVQV-KSEDGDRLPVTLRGPKTPTPITYRVPMASAQVKSA | 179 |
| Sbjct 121 | GDASL+ RPMGRVL PLR+MGVQV K+ GDR+P+TLRGPK PITYRVPMASAQVKSA GDASLSGRPMGRVLEPLRQMGVQVLKATPGDRMPITLRGPKHAAPITYRVPMASAQVKSA | 180 |
| Query 180 | VLLAGLNTPGITTVIEPIMTRDHTEKMLQGFGANLTVETDADGVRTIRLEGRGKLTGQVI | 239 |
| Sbjct 181 | VLLAGLNTPGITTVIEP+MTRDHTEKML+GFGANLTVETD GVR I +EG+GKL+GQ I VLLAGLNTPGITTVIEPVMTRDHTEKMLKGFGANLTVETDERGVRHIFIEGQGKLSGQTI | 240 |
| Query 240 | DVPGDPSSTAFPLVAALLVPGSDVTILNVLMNPTRTGLILTLQEMGADIEVINPRLAGGE | 299 |
| Sbjct 241 | VPGDPSS FPLVAAL+VPGSD+ I NVLMNPTRTGL+LTLQEMG I+++NPR AGGE AVPGDPSSAGFPLVAALIVPGSDIVIENVLMNPTRTGLLLTLQEMGGRIDILNPRNAGGE | 300 |
| Query 300 | DVADLRVRSSTLKGVTVPEDRAPSMIDEYPILavaaafaegaTVMNGLEELRVKESDRLS | 359 |
| Sbjct 301 | VADLRVRSS LKGVTVP +RAPSMIDEYP+LAVAA+FAEG T+M GLEELRVKESDRLS GVADLRVRSSELKGVTVPPERAPSMIDEYPVLAVAASFAEGETLMQGLEELRVKESDRLS | 360 |
| Query 360 | AVANGLKLNGVDCDEGETSLVVRGRPDGKGLG---NASGAAVATHLDHRIAMSFLVMGLV | 416 |
| Sbjct 361 | AVANGLKLNGVDC EGE +L VRGRP GKGLG N V THLDHRIAMSFLVMGL AVANGLKLNGVDCTEGEATLAVRGRPGGKGLGRHPNGPDTTVKTHLDHRIAMSFLVMGLA | 420 |
| Query 417  Sbjct 421 | SENPVTVDDATMIATSFPEFMDLMAGLGAKIELS 450 SE PVT+DDA MIATSFPEFM LM GLGA+I++ SEKPVTIDDANMIATSFPEFMGLMKGLGAEIDVQ 454 |  |

>WP_095831201.1 3-phosphoshikimate 1-carboxyvinyltransferase [Mesorhizobium sp. WSM3864]

PBB91963.1 3-phosphoshikimate 1-carboxyvinyltransferase [Mesorhizobium sp. WSM3864]

Length=452

Score = 690 bits (1781), Expect = 0.0, Method: Compositional matrix adjust.

| Identities | = 354/452 (78%), Positives = 385/452 (85%), Gaps = 4/452 (1%) |  |
| --- | --- | --- |
| Query 1 | MLHGASSRPATARKSSGLSGTVRIPGDKSISHRSFMFGGLASGETRITGLLEGEDVINTG | 60 |
| Sbjct 1 | M H A+ +PATARKS L+G R+PGDKSISHRSFMFGGLASGETRITGLLEGEDV+ TG MSHAAAPKPATARKSPALAGAARVPGDKSISHRSFMFGGLASGETRITGLLEGEDVMRTG | 60 |
| Query 61 | KAMQAMGARIRKEGDTWIIDGVGNGGLLAPEAPLDFGNAATGCRLTMGLVGVYDFDSTFI | 120 |
| Sbjct 61 | AM+AMGA I K G W+I G GNG LL PE PLDFGNA TG RLTMGLVG YD ++TFI AAMKAMGAHIEKTGAEWVIRGTGNGALLEPEGPLDFGNAGTGSRLTMGLVGTYDMETTFI | 120 |
| Query 121 | GDASLTKRPMGRVLNPLREMGVQV-KSEDGDRLPVTLRGPKTPTPITYRVPMASAQVKSA | 179 |
| Sbjct 121 | GDASL+ RPMGRVL PLR+MGVQV K+ GDR+P+TLRGPK PITYRVPMASAQVKSA GDASLSGRPMGRVLEPLRQMGVQVLKAAPGDRMPITLRGPKHAAPITYRVPMASAQVKSA | 180 |
| Query 180 | VLLAGLNTPGITTVIEPIMTRDHTEKMLQGFGANLTVETDADGVRTIRLEGRGKLTGQVI | 239 |
| Sbjct 181 | VLLAGLNTPGITTVIEP+MTRDHTEKML+GFGANLTVETD GVR I +EG+GKLTGQ I VLLAGLNTPGITTVIEPVMTRDHTEKMLKGFGANLTVETDERGVRHIFIEGQGKLTGQTI | 240 |
| Query 240 | DVPGDPSSTAFPLVAALLVPGSDVTILNVLMNPTRTGLILTLQEMGADIEVINPRLAGGE | 299 |
| Sbjct 241 | VPGDPSS FPLVAAL+VPGSD+ I NVLMNPTRTGL+LTLQEMG I+++NPR AGGE AVPGDPSSAGFPLVAALIVPGSDIVIENVLMNPTRTGLLLTLQEMGGRIDILNPRNAGGE | 300 |
| Query 300 | DVADLRVRSSTLKGVTVPEDRAPSMIDEYPILavaaafaegaTVMNGLEELRVKESDRLS | 359 |
| Sbjct 301 | DVADLRVRSS LKGV VP +RAPSMIDEYP+LAVAA FAEG T+M GLEELRVKESDRLS DVADLRVRSSDLKGVVVPPERAPSMIDEYPVLAVAAVFAEGETLMQGLEELRVKESDRLS | 360 |
| Query 360 | AVANGLKLNGVDCDEGETSLVVRGRPDGKGLG---NASGAAVATHLDHRIAMSFLVMGLV | 416 |
| Sbjct 361 | AVANGLKLNGVDC EGE SL VRG+P GKGLG N AV THLDHRIAMSFLVMGL AVANGLKLNGVDCSEGEASLAVRGKPGGKGLGAHPNGQDTAVKTHLDHRIAMSFLVMGLA | 420 |
| Query 417  Sbjct 421 | SENPVTVDDATMIATSFPEFMDLMAGLGAKIE 448  +E PVT+DDA MIATSFPEFM LM GLGA+IE  TEKPVTIDDANMIATSFPEFMGLMKGLGAEIE 452 |  |

>WP_095782258.1 3-phosphoshikimate 1-carboxyvinyltransferase [Mesorhizobium sp. WSM3862]

PBB97919.1 3-phosphoshikimate 1-carboxyvinyltransferase [Mesorhizobium sp. WSM3862]

Length=452

Score = 690 bits (1780), Expect = 0.0, Method: Compositional matrix adjust.

| Identities | = 354/452 (78%), Positives = 386/452 (85%), Gaps = 4/452 (1%) |  |
| --- | --- | --- |
| Query 1 | MLHGASSRPATARKSSGLSGTVRIPGDKSISHRSFMFGGLASGETRITGLLEGEDVINTG | 60 |
| Sbjct 1 | M H A+ +PATARKS L+GT R+PGDKSISHRSFMFGGLASGETRITGLLEGEDV+ TG MSHAAAPKPATARKSPALAGTARVPGDKSISHRSFMFGGLASGETRITGLLEGEDVMRTG | 60 |
| Query 61 | KAMQAMGARIRKEGDTWIIDGVGNGGLLAPEAPLDFGNAATGCRLTMGLVGVYDFDSTFI | 120 |
| Sbjct 61 | AM+AMGA I K G W+I G GNG LL PE PLDFGNA TG RLTMGLVG YD ++TFI AAMKAMGAHIEKTGAEWVIRGTGNGALLEPEGPLDFGNAGTGSRLTMGLVGTYDMETTFI | 120 |
| Query 121 | GDASLTKRPMGRVLNPLREMGVQV-KSEDGDRLPVTLRGPKTPTPITYRVPMASAQVKSA | 179 |
| Sbjct 121 | GDASL+ RPMGRVL PLR+MGVQV K+ GDR+P+TLRGPK PITYRVPMASAQVKSA GDASLSGRPMGRVLEPLRQMGVQVLKASPGDRMPITLRGPKHAAPITYRVPMASAQVKSA | 180 |
| Query 180 | VLLAGLNTPGITTVIEPIMTRDHTEKMLQGFGANLTVETDADGVRTIRLEGRGKLTGQVI | 239 |
| Sbjct 181 | VLLAGLNTPGITTVIEP+MTRDHTEKML+GFGANLTVETD GVR I +EG+GKLTGQ I VLLAGLNTPGITTVIEPVMTRDHTEKMLKGFGANLTVETDERGVRHIFIEGQGKLTGQTI | 240 |
| Query 240 | DVPGDPSSTAFPLVAALLVPGSDVTILNVLMNPTRTGLILTLQEMGADIEVINPRLAGGE | 299 |
| Sbjct 241 | VPGDPSS FPLVAAL+VPGSD+ I NVLMNPTRTGL+LTLQEMG I+++NPR AGGE AVPGDPSSAGFPLVAALIVPGSDIVIENVLMNPTRTGLLLTLQEMGGRIDILNPRNAGGE | 300 |
| Query 300 | DVADLRVRSSTLKGVTVPEDRAPSMIDEYPILavaaafaegaTVMNGLEELRVKESDRLS | 359 |
| Sbjct 301 | DVADLRVRSS LKGV VP +RAPSMIDEYP+LAVAA FAEG T+M GLEELRVKESDRLS DVADLRVRSSDLKGVVVPPERAPSMIDEYPVLAVAAVFAEGETLMQGLEELRVKESDRLS | 360 |
| Query 360 | AVANGLKLNGVDCDEGETSLVVRGRPDGKGLG---NASGAAVATHLDHRIAMSFLVMGLV | 416 |
| Sbjct 361 | AVANGLKLNGVDC EGE SL VRG+P GKGLG N AV THLDHR+AMSFLVMGL  AVANGLKLNGVDCSEGEASLAVRGKPGGKGLGAHPNGPDTAVKTHLDHRLAMSFLVMGLA | 420 |

Query 417 SENPVTVDDATMIATSFPEFMDLMAGLGAKIE 448

+E PVT+DDA MIATSFPEFM LM GLGA+IE Sbjct 421 TEKPVTIDDANMIATSFPEFMGLMKGLGAEIE 452

>WP_067324710.1 3-phosphoshikimate 1-carboxyvinyltransferase [Mesorhizobium sp. AA23]

OBQ94986.1 3-phosphoshikimate 1-carboxyvinyltransferase [Mesorhizobium sp. AA23]

Length=452

Score = 690 bits (1780), Expect = 0.0, Method: Compositional matrix adjust.

| Identities | = 353/452 (78%), Positives = 386/452 (85%), Gaps = 4/452 (1%) |  |
| --- | --- | --- |
| Query 1 | MLHGASSRPATARKSSGLSGTVRIPGDKSISHRSFMFGGLASGETRITGLLEGEDVINTG | 60 |
| Sbjct 1 | M H A+ +PATARKS L+G R+PGDKSISHRSFMFGGLASGETRITGLLEGEDV+ TG MSHAAAPKPATARKSPALAGAARVPGDKSISHRSFMFGGLASGETRITGLLEGEDVMRTG | 60 |
| Query 61 | KAMQAMGARIRKEGDTWIIDGVGNGGLLAPEAPLDFGNAATGCRLTMGLVGVYDFDSTFI | 120 |
| Sbjct 61 | AM+AMGA I K+G W+I G GNG LL PE PLDFGNA TG RLTMGLVG YD ++TFI AAMKAMGAHIEKKGAEWVIRGTGNGALLEPEGPLDFGNAGTGSRLTMGLVGTYDMETTFI | 120 |
| Query 121 | GDASLTKRPMGRVLNPLREMGVQV-KSEDGDRLPVTLRGPKTPTPITYRVPMASAQVKSA | 179 |
| Sbjct 121 | GDASL+ RPMGRVL PLR+MGVQV K+ GDR+P+TLRGPK PITYRVPMASAQVKSA GDASLSGRPMGRVLEPLRQMGVQVLKAAPGDRMPITLRGPKHAAPITYRVPMASAQVKSA | 180 |
| Query 180 | VLLAGLNTPGITTVIEPIMTRDHTEKMLQGFGANLTVETDADGVRTIRLEGRGKLTGQVI | 239 |
| Sbjct 181 | VLLAGLNTPGITTVIEP+MTRDHTEKML+GFGANLTVETD GVR I +EG+GKLTGQ I VLLAGLNTPGITTVIEPVMTRDHTEKMLKGFGANLTVETDERGVRHIFIEGQGKLTGQTI | 240 |
| Query 240 | DVPGDPSSTAFPLVAALLVPGSDVTILNVLMNPTRTGLILTLQEMGADIEVINPRLAGGE | 299 |
| Sbjct 241 | VPGDPSS FPLVAAL+VPGSD+ I NVLMNPTRTGL+LTLQEMG I+++NPR AGGE AVPGDPSSAGFPLVAALIVPGSDIVIENVLMNPTRTGLLLTLQEMGGRIDILNPRNAGGE | 300 |
| Query 300 | DVADLRVRSSTLKGVTVPEDRAPSMIDEYPILavaaafaegaTVMNGLEELRVKESDRLS | 359 |
| Sbjct 301 | DVADLRVRSS LKGV VP +RAPSMIDEYP+LAVAA FAEG T+M GLEELR+KESDRLS DVADLRVRSSDLKGVVVPPERAPSMIDEYPVLAVAAVFAEGETLMQGLEELRIKESDRLS | 360 |
| Query 360 | AVANGLKLNGVDCDEGETSLVVRGRPDGKGLG---NASGAAVATHLDHRIAMSFLVMGLV | 416 |
| Sbjct 361 | AVANGLKLNGVDC EGE SL VRG+P GKGLG N AV THLDHRIAMSFLVMGL AVANGLKLNGVDCSEGEASLAVRGKPGGKGLGAHPNGQDTAVKTHLDHRIAMSFLVMGLA | 420 |
| Query 417  Sbjct 421 | SENPVTVDDATMIATSFPEFMDLMAGLGAKIE 448  +E PVT+DDA MIATSFPEFM LM GLGA+IE  TEKPVTIDDANMIATSFPEFMGLMKGLGAEIE 452 |  |

>WP_183727425.1 3-phosphoshikimate 1-carboxyvinyltransferase [Rhizobium sp. BK275] MBB3388524.1 3-phosphoshikimate 1-carboxyvinyltransferase [Rhizobium sp. BK275]

Length=450

Score = 690 bits (1780), Expect = 0.0, Method: Compositional matrix adjust.

| Identities | = 347/448 (77%), Positives = 392/448 (88%), Gaps = 0/448 (0%) |  |
| --- | --- | --- |
| Query 1 | MLHGASSRPATARKSSGLSGTVRIPGDKSISHRSFMFGGLASGETRITGLLEGEDVINTG | 60 |
| Sbjct 1 | M G++S+PA AR+SSGLSGTVRIPGDKSISHR+ M GGLASGETRI GLLEGEDV+NTG MSQGSASKPAIARRSSGLSGTVRIPGDKSISHRALMLGGLASGETRIAGLLEGEDVLNTG | 60 |
| Query 61 | KAMQAMGARIRKEGDTWIIDGVGNGGLLAPEAPLDFGNAATGCRLTMGLVGVYDFDSTFI | 120 |
| Sbjct 61 | KAMQAMGA IRKEG WII+G GNG LLAPE PLDFGN+ TG RL MGL GVYDFD+ KAMQAMGAVIRKEGGVWIINGTGNGALLAPEVPLDFGNSGTGARLIMGLAGVYDFDTVLT | 120 |
| Query 121 | GDASLTKRPMGRVLNPLREMGVQVKSEDGDRLPVTLRGPKTPTPITYRVPMASAQVKSAV | 180 |
| Sbjct 121 | GDASL++RPMGRVL+PLR MGVQVK+ +GDRLPV LRGP+TP PI Y +P+ASAQVKSAV GDASLSRRPMGRVLDPLRRMGVQVKTVEGDRLPVMLRGPETPAPIRYTLPVASAQVKSAV | 180 |
| Query 181 | LLAGLNTPGITTVIEPIMTRDHTEKMLQGFGANLTVETDADGVRTIRLEGRGKLTGQVID | 240 |
| Sbjct 181 | LLAGLN PG+TTVIEP +TRDHTEKML GFGA L+VE D G RT+RL+GRG+LTGQ+ID LLAGLNIPGVTTVIEPAITRDHTEKMLAGFGAELSVEADKGGTRTVRLQGRGRLTGQIID | 240 |
| Query 241 | VPGDPSSTAFPLVAALLVPGSDVTILNVLMNPTRTGLILTLQEMGADIEVINPRLAGGED VPGD SS AFPLVAALLVPGSD+ I NVLMNPTRTGL+LTL+EMGADIE++NPRLAGGED | 300 |

| Sbjct | 241 | VPGDLSSAAFPLVAALLVPGSDIIIPNVLMNPTRTGLVLTLREMGADIEILNPRLAGGED | 300 |
| --- | --- | --- | --- |
| Query | 301 | VADLRVRSSTLKGVTVPEDRAPSMIDEYPILavaaafaegaTVMNGLEELRVKESDRLSA | 360 |
| Sbjct | 301 | VADLRVR S L GV+VP +R PSMIDEYP+LAVAAAFA+G TVM+GL+ELRVKESDRLS VADLRVRHSELTGVSVPAERVPSMIDEYPVLAVAAAFAKGRTVMSGLQELRVKESDRLST | 360 |
| Query | 361 | VANGLKLNGVDCDEGETSLVVRGRPDGKGLGNASGAAVATHLDHRIAMSFLVMGLVSENP | 420 |
| Sbjct | 361 | VA+GLKLNGV+C+EG+ L V GRPDGKGLGNA+ VATHLDHRIAMSFLVMGL SE+P VADGLKLNGVECEEGKDWLSVTGRPDGKGLGNAAVKWVATHLDHRIAMSFLVMGLASEHP | 420 |
| Query  Sbjct | 421  421 | VTVDDATMIATSFPEFMDLMAGLGAKIE 448 V +DD++MIATSFP+FMDLM LGA IE VGIDDSSMIATSFPQFMDLMKSLGAGIE 448 |  |

>RWE17666.1 3-phosphoshikimate 1-carboxyvinyltransferase [Mesorhizobium sp.] Length=452

Score = 690 bits (1780), Expect = 0.0, Method: Compositional matrix adjust.

| Identities | = 354/452 (78%), Positives = 388/452 (86%), Gaps = 4/452 (1%) |  |
| --- | --- | --- |
| Query 1 | MLHGASSRPATARKSSGLSGTVRIPGDKSISHRSFMFGGLASGETRITGLLEGEDVINTG | 60 |
| Sbjct 1 | M H A+++PATARKS L+GT R+PGDKSISHRSFMFGGLASGETRITGLLEGEDV+ TG MSHSAAAKPATARKSPALAGTARVPGDKSISHRSFMFGGLASGETRITGLLEGEDVMRTG | 60 |
| Query 61 | KAMQAMGARIRKEGDTWIIDGVGNGGLLAPEAPLDFGNAATGCRLTMGLVGVYDFDSTFI | 120 |
| Sbjct 61 | AM+AMGA I K+G W+I G GNG LL PE PLDFGNA TG RLTMGLVG YD ++TFI AAMKAMGAHIEKKGAEWVIRGTGNGALLEPEGPLDFGNAGTGSRLTMGLVGTYDMETTFI | 120 |
| Query 121 | GDASLTKRPMGRVLNPLREMGVQV-KSEDGDRLPVTLRGPKTPTPITYRVPMASAQVKSA | 179 |
| Sbjct 121 | GDASL+ RPMGRVL PLR+MGVQV K+ GDR+P+TLRGPK PITYRVPMASAQVKSA GDASLSGRPMGRVLEPLRQMGVQVLKAAPGDRMPITLRGPKHAAPITYRVPMASAQVKSA | 180 |
| Query 180 | VLLAGLNTPGITTVIEPIMTRDHTEKMLQGFGANLTVETDADGVRTIRLEGRGKLTGQVI | 239 |
| Sbjct 181 | VLLAGLNTPGITTVIEP+MTRDHTEKMLQGFGANL+VETD GVR I +EG+GKLTGQ I VLLAGLNTPGITTVIEPVMTRDHTEKMLQGFGANLSVETDERGVRHIFIEGQGKLTGQTI | 240 |
| Query 240 | DVPGDPSSTAFPLVAALLVPGSDVTILNVLMNPTRTGLILTLQEMGADIEVINPRLAGGE | 299 |
| Sbjct 241 | VPGDPSS FPLVA L+VPGSD+ I NVLMNPTRTGL+LTLQEMG I+++NPR AGGE AVPGDPSSAGFPLVATLIVPGSDIVIENVLMNPTRTGLLLTLQEMGGRIDILNPRNAGGE | 300 |
| Query 300 | DVADLRVRSSTLKGVTVPEDRAPSMIDEYPILavaaafaegaTVMNGLEELRVKESDRLS | 359 |
| Sbjct 301 | DVADLRVRSS LKGV VP +RAPSMIDEYP+LAVAA+FAEG T+M GLEELRVKESDRLS DVADLRVRSSDLKGVVVPPERAPSMIDEYPVLAVAASFAEGETLMQGLEELRVKESDRLS | 360 |
| Query 360 | AVANGLKLNGVDCDEGETSLVVRGRPDGKGLG---NASGAAVATHLDHRIAMSFLVMGLV | 416 |
| Sbjct 361 | AVANGLKLNGVDC EGE SL VRGRP GKGLG + AV THLDHRIAMSFLVMGL AVANGLKLNGVDCTEGEASLAVRGRPGGKGLGAHPDGLDTAVKTHLDHRIAMSFLVMGLA | 420 |
| Query 417  Sbjct 421 | SENPVTVDDATMIATSFPEFMDLMAGLGAKIE 448  +E PVT+DDA MIATSFPEFM LM GLGA+IE  TEKPVTIDDANMIATSFPEFMGLMKGLGAEIE 452 |  |

>PBB80549.1 3-phosphoshikimate 1-carboxyvinyltransferase [Mesorhizobium sp. WSM3879]

Length=481

Score = 690 bits (1780), Expect = 0.0, Method: Compositional matrix adjust.

| Identities | = 355/452 (79%), Positives = 386/452 (85%), Gaps = 4/452 (1%) |  |
| --- | --- | --- |
| Query 1 | MLHGASSRPATARKSSGLSGTVRIPGDKSISHRSFMFGGLASGETRITGLLEGEDVINTG | 60 |
| Sbjct 30 | M H A+ +PATARKS L+GT R+PGDKSISHRSFMFGGLASGETRITGLLEGEDV+ TG MSHAAAPKPATARKSPALAGTARVPGDKSISHRSFMFGGLASGETRITGLLEGEDVMRTG | 89 |
| Query 61 | KAMQAMGARIRKEGDTWIIDGVGNGGLLAPEAPLDFGNAATGCRLTMGLVGVYDFDSTFI | 120 |
| Sbjct 90 | AM+AMGA I K G W+I G GNG LL PE PLDFGNA TG RLTMGLVG YD ++TFI AAMKAMGAHIEKTGAEWVIRGTGNGALLEPEGPLDFGNAGTGSRLTMGLVGTYDMETTFI | 149 |
| Query 121 | GDASLTKRPMGRVLNPLREMGVQV-KSEDGDRLPVTLRGPKTPTPITYRVPMASAQVKSA | 179 |
| Sbjct 150 | GDASL+ RPMGRVL PLR+MGVQV K+ GDR+P+TLRGPK PITYRVPMASAQVKSA  GDASLSGRPMGRVLEPLRQMGVQVLKAAPGDRMPITLRGPKHAAPITYRVPMASAQVKSA | 209 |

| Query | 180 | VLLAGLNTPGITTVIEPIMTRDHTEKMLQGFGANLTVETDADGVRTIRLEGRGKLTGQVI | 239 |
| --- | --- | --- | --- |
| Sbjct | 210 | VLLAGLNTPGITTVIEP+MTRDHTEKML+GFGANLTVETD GVR I +EG+GKLTGQ I VLLAGLNTPGITTVIEPVMTRDHTEKMLKGFGANLTVETDERGVRHIFIEGQGKLTGQTI | 269 |
| Query | 240 | DVPGDPSSTAFPLVAALLVPGSDVTILNVLMNPTRTGLILTLQEMGADIEVINPRLAGGE | 299 |
| Sbjct | 270 | VPGDPSS FPLVAAL+VPGSD+ I NVLMNPTRTGL+LTLQEMG I+++NPR AGGE AVPGDPSSAGFPLVAALIVPGSDIVIENVLMNPTRTGLLLTLQEMGGRIDILNPRNAGGE | 329 |
| Query | 300 | DVADLRVRSSTLKGVTVPEDRAPSMIDEYPILavaaafaegaTVMNGLEELRVKESDRLS | 359 |
| Sbjct | 330 | DVADLRVRSS LKGV VP +RAPSMIDEYP+LAVAA FAEG T+M GLEELRVKESDRLS DVADLRVRSSDLKGVVVPPERAPSMIDEYPVLAVAAVFAEGETLMQGLEELRVKESDRLS | 389 |
| Query | 360 | AVANGLKLNGVDCDEGETSLVVRGRPDGKGLG---NASGAAVATHLDHRIAMSFLVMGLV | 416 |
| Sbjct | 390 | AVANGLKLNGVDC EGE SL VRG+P GKGLG N AV THLDHRIAMSFLVMGL AVANGLKLNGVDCSEGEASLAVRGKPGGKGLGAHPNGPDTAVKTHLDHRIAMSFLVMGLA | 449 |
| Query  Sbjct | 417  450 | SENPVTVDDATMIATSFPEFMDLMAGLGAKIE 448  +E PVT+DDA MIATSFPEFM LM GLGA+IE  TEKPVTIDDANMIATSFPEFMGLMKGLGAEIE 481 |  |

>WP_126096578.1 3-phosphoshikimate 1-carboxyvinyltransferase [Mesorhizobium sp. M1B.F.Ca.ET.045.04.1.1]

AZO26384.1 3-phosphoshikimate 1-carboxyvinyltransferase [Mesorhizobium sp. M1B.F.Ca.ET.045.04.1.1]

RWE03843.1 3-phosphoshikimate 1-carboxyvinyltransferase [Mesorhizobium sp.] Length=452

Score = 690 bits (1780), Expect = 0.0, Method: Compositional matrix adjust.

| Identities | = 353/452 (78%), Positives = 387/452 (86%), Gaps = 4/452 (1%) |  |
| --- | --- | --- |
| Query 1 | MLHGASSRPATARKSSGLSGTVRIPGDKSISHRSFMFGGLASGETRITGLLEGEDVINTG | 60 |
| Sbjct 1 | M H A+ +PATARKS LSGT R+PGDKSISHRSFMFGGLASGETRITGLLEGEDV+ TG MSHSAAVKPATARKSPALSGTARVPGDKSISHRSFMFGGLASGETRITGLLEGEDVMRTG | 60 |
| Query 61 | KAMQAMGARIRKEGDTWIIDGVGNGGLLAPEAPLDFGNAATGCRLTMGLVGVYDFDSTFI | 120 |
| Sbjct 61 | AM+AMGA I K+G W+I G GNG LL PE PLDFGNA TG RLTMGLVG YD ++TFI AAMKAMGAHIEKKGAEWVIRGTGNGALLEPEGPLDFGNAGTGSRLTMGLVGTYDMETTFI | 120 |
| Query 121 | GDASLTKRPMGRVLNPLREMGVQV-KSEDGDRLPVTLRGPKTPTPITYRVPMASAQVKSA | 179 |
| Sbjct 121 | GDASL+ RPMGRVL PLR+MGVQV K+ GDR+P+TLRGPK PITYRVPMASAQVKSA GDASLSGRPMGRVLEPLRQMGVQVLKAAPGDRMPITLRGPKHAAPITYRVPMASAQVKSA | 180 |
| Query 180 | VLLAGLNTPGITTVIEPIMTRDHTEKMLQGFGANLTVETDADGVRTIRLEGRGKLTGQVI | 239 |
| Sbjct 181 | VLLAGLNTPGITTVIEP+MTRDHTEKML+GFGANLTVETD GVR I +EG+GKL GQ I VLLAGLNTPGITTVIEPVMTRDHTEKMLKGFGANLTVETDERGVRHIFIEGQGKLIGQTI | 240 |
| Query 240 | DVPGDPSSTAFPLVAALLVPGSDVTILNVLMNPTRTGLILTLQEMGADIEVINPRLAGGE | 299 |
| Sbjct 241 | VPGDPSS FPLVAAL+VPGSD+TI NVLMNPTRTGL+LTLQEMG I+++NPR AGGE AVPGDPSSAGFPLVAALIVPGSDITIENVLMNPTRTGLLLTLQEMGGRIDILNPRNAGGE | 300 |
| Query 300 | DVADLRVRSSTLKGVTVPEDRAPSMIDEYPILavaaafaegaTVMNGLEELRVKESDRLS | 359 |
| Sbjct 301 | DVADLRVRSS LKGV VP +RAPSMIDEYP+LAVAA+FAEG T+M GLEELRVKESDRLS DVADLRVRSSELKGVVVPAERAPSMIDEYPVLAVAASFAEGETLMQGLEELRVKESDRLS | 360 |
| Query 360 | AVANGLKLNGVDCDEGETSLVVRGRPDGKGLG---NASGAAVATHLDHRIAMSFLVMGLV | 416 |
| Sbjct 361 | AVANGLKLNGVDC EGE SL VRG+P GKGLG + V THLDHRIAMSFL+MGL AVANGLKLNGVDCSEGEASLAVRGKPGGKGLGAHPDGQDTKVKTHLDHRIAMSFLIMGLA | 420 |
| Query 417  Sbjct 421 | SENPVTVDDATMIATSFPEFMDLMAGLGAKIE 448  +E PVT+DDA MIATSFPEFM LM GLGA+IE  TEKPVTIDDANMIATSFPEFMGLMKGLGAEIE 452 |  |

>WP_127391261.1 MULTISPECIES: 3-phosphoshikimate 1-carboxyvinyltransferase [unclassified Mesorhizobium]

RUW24195.1 3-phosphoshikimate 1-carboxyvinyltransferase [Mesorhizobium sp. M4B.F.Ca.ET.013.02.1.1]

RVD45628.1 3-phosphoshikimate 1-carboxyvinyltransferase [Mesorhizobium sp. M4B.F.Ca.ET.019.03.1.1]

TGQ15439.1 3-phosphoshikimate 1-carboxyvinyltransferase [Mesorhizobium sp.

M4B.F.Ca.ET.215.01.1.1]

TGQ48352.1 3-phosphoshikimate 1-carboxyvinyltransferase [Mesorhizobium sp. M00.F.Ca.ET.220.01.1.1]

TGR11503.1 3-phosphoshikimate 1-carboxyvinyltransferase [Mesorhizobium sp. M4B.F.Ca.ET.203.01.1.1]

Length=452

Score = 689 bits (1779), Expect = 0.0, Method: Compositional matrix adjust.

| Identities | = 355/452 (79%), Positives = 387/452 (86%), Gaps = 4/452 (1%) |  |
| --- | --- | --- |
| Query 1 | MLHGASSRPATARKSSGLSGTVRIPGDKSISHRSFMFGGLASGETRITGLLEGEDVINTG | 60 |
| Sbjct 1 | M H A+ +PATARKSS LSGT R+PGDKSISHRSFMFGGLASGETRITGLLEGEDV+ TG MSHAAAPKPATARKSSALSGTARVPGDKSISHRSFMFGGLASGETRITGLLEGEDVMRTG | 60 |
| Query 61 | KAMQAMGARIRKEGDTWIIDGVGNGGLLAPEAPLDFGNAATGCRLTMGLVGVYDFDSTFI | 120 |
| Sbjct 61 | AM+AMGA I K G W+I G GNG LL P+ PLDFGNA TG RLTMGLVG YD ++TFI AAMKAMGAHIEKRGAEWVIRGTGNGALLQPDGPLDFGNAGTGSRLTMGLVGTYDMETTFI | 120 |
| Query 121 | GDASLTKRPMGRVLNPLREMGVQV-KSEDGDRLPVTLRGPKTPTPITYRVPMASAQVKSA | 179 |
| Sbjct 121 | GDASL+ RPMGRVL PLR+MGVQV K+ GDR+P+TLRGPK PITYRVPMASAQVKSA GDASLSGRPMGRVLEPLRQMGVQVLKATPGDRMPITLRGPKHAAPITYRVPMASAQVKSA | 180 |
| Query 180 | VLLAGLNTPGITTVIEPIMTRDHTEKMLQGFGANLTVETDADGVRTIRLEGRGKLTGQVI | 239 |
| Sbjct 181 | VLLAGLNTPGITTVIEP+MTRDHTEKML+GFGANLTVETD GVR I +EG+GKL+GQ I VLLAGLNTPGITTVIEPVMTRDHTEKMLKGFGANLTVETDERGVRHIFIEGQGKLSGQTI | 240 |
| Query 240 | DVPGDPSSTAFPLVAALLVPGSDVTILNVLMNPTRTGLILTLQEMGADIEVINPRLAGGE | 299 |
| Sbjct 241 | VPGDPSS FPLVAAL+VPGSD+ I NVLMNPTRTGL+LTLQEMG I+++NPR AGGE AVPGDPSSAGFPLVAALIVPGSDIVIENVLMNPTRTGLLLTLQEMGGRIDILNPRNAGGE | 300 |
| Query 300 | DVADLRVRSSTLKGVTVPEDRAPSMIDEYPILavaaafaegaTVMNGLEELRVKESDRLS | 359 |
| Sbjct 301 | VADLRVRSS LKGVTVP +RAPSMIDEYP+LAVAA+FAEG T+M GLEELRVKESDRLS GVADLRVRSSELKGVTVPPERAPSMIDEYPVLAVAASFAEGETLMQGLEELRVKESDRLS | 360 |
| Query 360 | AVANGLKLNGVDCDEGETSLVVRGRPDGKGLG---NASGAAVATHLDHRIAMSFLVMGLV | 416 |
| Sbjct 361 | AVANGLKLNGVDC EGE +L VRGRP GKGLG N V THLDHRIAMSFLVMGL AVANGLKLNGVDCTEGEATLAVRGRPGGKGLGRHPNGPDTTVKTHLDHRIAMSFLVMGLA | 420 |
| Query 417  Sbjct 421 | SENPVTVDDATMIATSFPEFMDLMAGLGAKIE 448 SE PVT+DDA MIATSFPEFM LM GLGA+IE SEKPVTIDDANMIATSFPEFMGLMKGLGAEIE 452 |  |

>WP_127247776.1 3-phosphoshikimate 1-carboxyvinyltransferase [Mesorhizobium sp. M1A.T.Ca.IN.004.03.1.1]

RWG13382.1 3-phosphoshikimate 1-carboxyvinyltransferase [Mesorhizobium sp.] RUV41661.1 3-phosphoshikimate 1-carboxyvinyltransferase [Mesorhizobium sp.

M1A.T.Ca.IN.004.03.1.1]

RWI95125.1 3-phosphoshikimate 1-carboxyvinyltransferase [Mesorhizobium sp.] RWK37701.1 3-phosphoshikimate 1-carboxyvinyltransferase [Mesorhizobium sp.] RWK88772.1 3-phosphoshikimate 1-carboxyvinyltransferase [Mesorhizobium sp.]

Length=452

Score = 689 bits (1779), Expect = 0.0, Method: Compositional matrix adjust.

| Identities | = 354/452 (78%), Positives = 386/452 (85%), Gaps = 4/452 (1%) |  |
| --- | --- | --- |
| Query 1 | MLHGASSRPATARKSSGLSGTVRIPGDKSISHRSFMFGGLASGETRITGLLEGEDVINTG | 60 |
| Sbjct 1 | M H A+ +PATARKS L+GT R+PGDKSISHRSFMFGGLASGETRITGLLEGEDV+ TG MSHAAAPKPATARKSPALAGTARVPGDKSISHRSFMFGGLASGETRITGLLEGEDVMRTG | 60 |
| Query 61 | KAMQAMGARIRKEGDTWIIDGVGNGGLLAPEAPLDFGNAATGCRLTMGLVGVYDFDSTFI | 120 |
| Sbjct 61 | AM+AMGA I K+G W+I G GNG LL PE PLDFGNA TG RLTMGLVG YD ++TFI AAMKAMGAHIEKKGAEWVIRGTGNGALLEPEGPLDFGNAGTGSRLTMGLVGTYDMETTFI | 120 |
| Query 121 | GDASLTKRPMGRVLNPLREMGVQV-KSEDGDRLPVTLRGPKTPTPITYRVPMASAQVKSA | 179 |
| Sbjct 121 | GDASL+ RPMGRVL PLR+MGVQV K+ GDR+P+TLRGPK PITYRVPMASAQVKSA GDASLSGRPMGRVLEPLRQMGVQVLKAAPGDRMPITLRGPKHAAPITYRVPMASAQVKSA | 180 |
| Query 180 | VLLAGLNTPGITTVIEPIMTRDHTEKMLQGFGANLTVETDADGVRTIRLEGRGKLTGQVI | 239 |
| Sbjct 181 | VLLAGLNTPGITTVIEP+MTRDHTEKML+GFGANLTVETD GVR I +EG+GKLTGQ I  VLLAGLNTPGITTVIEPVMTRDHTEKMLKGFGANLTVETDERGVRHIFIEGQGKLTGQTI | 240 |

| Query | 240 | DVPGDPSSTAFPLVAALLVPGSDVTILNVLMNPTRTGLILTLQEMGADIEVINPRLAGGE | 299 |
| --- | --- | --- | --- |
| Sbjct | 241 | VPGDPSS FPLVAAL+VPGSD+ I NVLMNPTRTGL+LTLQEMG I+++NPR AGGE AVPGDPSSAGFPLVAALIVPGSDIVIENVLMNPTRTGLLLTLQEMGGRIDILNPRNAGGE | 300 |
| Query | 300 | DVADLRVRSSTLKGVTVPEDRAPSMIDEYPILavaaafaegaTVMNGLEELRVKESDRLS | 359 |
| Sbjct | 301 | DVADLRVRSS LKGV VP +RAPSMIDEYP+LAVAA FAEG T+M GLEELRVKESDRLS DVADLRVRSSDLKGVVVPPERAPSMIDEYPVLAVAAVFAEGETLMQGLEELRVKESDRLS | 360 |
| Query | 360 | AVANGLKLNGVDCDEGETSLVVRGRPDGKGLG---NASGAAVATHLDHRIAMSFLVMGLV | 416 |
| Sbjct | 361 | AVANGLKLNGVDC EGE SL VRG+P GKGLG N AV THLDHRIAMSFLVMGL AVANGLKLNGVDCSEGEASLAVRGKPGGKGLGAHPNGPDTAVKTHLDHRIAMSFLVMGLA | 420 |
| Query  Sbjct | 417  421 | SENPVTVDDATMIATSFPEFMDLMAGLGAKIE 448  +E PVT DDA M+ATSFPEFM LM GLGA+IE  TEKPVTTDDANMVATSFPEFMGLMKGLGAEIE 452 |  |

>RWH72139.1 3-phosphoshikimate 1-carboxyvinyltransferase [Mesorhizobium sp.] RWL33147.1 3-phosphoshikimate 1-carboxyvinyltransferase [Mesorhizobium sp.] RWL34154.1 3-phosphoshikimate 1-carboxyvinyltransferase [Mesorhizobium sp.] RWL40247.1 3-phosphoshikimate 1-carboxyvinyltransferase [Mesorhizobium sp.] RWL51882.1 3-phosphoshikimate 1-carboxyvinyltransferase [Mesorhizobium sp.]

Length=452

Score = 689 bits (1779), Expect = 0.0, Method: Compositional matrix adjust.

| Identities | = 353/452 (78%), Positives = 388/452 (86%), Gaps = 4/452 (1%) |  |
| --- | --- | --- |
| Query 1 | MLHGASSRPATARKSSGLSGTVRIPGDKSISHRSFMFGGLASGETRITGLLEGEDVINTG | 60 |
| Sbjct 1 | M H A+++PATARKS L+GT R+PGDKSISHRSFMFGGLASGETRITGLLEGEDV+ TG MSHSAAAKPATARKSPALAGTARVPGDKSISHRSFMFGGLASGETRITGLLEGEDVMRTG | 60 |
| Query 61 | KAMQAMGARIRKEGDTWIIDGVGNGGLLAPEAPLDFGNAATGCRLTMGLVGVYDFDSTFI | 120 |
| Sbjct 61 | AM+AMGA I K G W+I G GNG LL PE PLDFGNA TG RLTMGLVG YD ++TFI AAMKAMGAHIEKNGAEWVIRGTGNGALLQPEGPLDFGNAGTGSRLTMGLVGTYDMETTFI | 120 |
| Query 121 | GDASLTKRPMGRVLNPLREMGVQV-KSEDGDRLPVTLRGPKTPTPITYRVPMASAQVKSA | 179 |
| Sbjct 121 | GDASL+ RPMGRVL+PLR+MGVQV K+ GDR+P+TLRGPK PITYRVPMASAQVKSA GDASLSGRPMGRVLDPLRQMGVQVLKAAPGDRMPITLRGPKHAAPITYRVPMASAQVKSA | 180 |
| Query 180 | VLLAGLNTPGITTVIEPIMTRDHTEKMLQGFGANLTVETDADGVRTIRLEGRGKLTGQVI | 239 |
| Sbjct 181 | VLLAGLNTPGITTVIEP+MTRDHTEKML+GFGANLTVETD GVR I +EG+GKL GQ I VLLAGLNTPGITTVIEPVMTRDHTEKMLKGFGANLTVETDERGVRHIFIEGQGKLIGQTI | 240 |
| Query 240 | DVPGDPSSTAFPLVAALLVPGSDVTILNVLMNPTRTGLILTLQEMGADIEVINPRLAGGE | 299 |
| Sbjct 241 | VPGDPSS FPLVAAL+VPGSD+ I NVLMNPTRTGL+LTLQEMG I+++NPR AGGE AVPGDPSSAGFPLVAALIVPGSDIVIENVLMNPTRTGLLLTLQEMGGRIDILNPRNAGGE | 300 |
| Query 300 | DVADLRVRSSTLKGVTVPEDRAPSMIDEYPILavaaafaegaTVMNGLEELRVKESDRLS | 359 |
| Sbjct 301 | DVADLRVRSS LKGV VP +RAPSMIDEYP+LAVAA+FAEG T+M GLEELRVKESDRLS DVADLRVRSSDLKGVVVPPERAPSMIDEYPVLAVAASFAEGETLMQGLEELRVKESDRLS | 360 |
| Query 360 | AVANGLKLNGVDCDEGETSLVVRGRPDGKGLG---NASGAAVATHLDHRIAMSFLVMGLV | 416 |
| Sbjct 361 | AVANGL+LNGVDC EGE SL VRG+P GKGLG N AV THLDHRIAMSFLVMGL AVANGLELNGVDCTEGEASLAVRGKPGGKGLGAHPNGQDTAVKTHLDHRIAMSFLVMGLA | 420 |
| Query 417  Sbjct 421 | SENPVTVDDATMIATSFPEFMDLMAGLGAKIE 448  +E PVT+DDA MIATSFPEFM LM GLGA+IE  TEKPVTIDDANMIATSFPEFMGLMKGLGAEIE 452 |  |

>WP_112340061.1 3-phosphoshikimate 1-carboxyvinyltransferase [Rhizobium tropici] RAX43300.1 3-phosphoshikimate 1-carboxyvinyltransferase [Rhizobium tropici]

Length=451

Score = 689 bits (1779), Expect = 0.0, Method: Compositional matrix adjust. Identities = 348/447 (78%), Positives = 387/447 (87%), Gaps = 0/447 (0%)

| Query | 1 | MLHGASSRPATARKSSGLSGTVRIPGDKSISHRSFMFGGLASGETRITGLLEGEDVINTG | 60 |
| --- | --- | --- | --- |
|  |  | M H +PA AR+S+GLSGT+RIPGDKSISHRS MFGGLASGET I GLLEGEDV+NT |  |
| Sbjct | 1 | MPHDTLPQPAVARRSAGLSGTIRIPGDKSISHRSLMFGGLASGETHIKGLLEGEDVLNTA | 60 |
| Query | 61 | KAMQAMGARIRKEGDTWIIDGVGNGGLLAPEAPLDFGNAATGCRLTMGLVGVYDFDSTFI | 120 |

| Sbjct | 61 | AMQA+GA IR+ D WI++G GNG LLAPEAPLDFGNA TG RLTMGLVG YDF +TF+ GAMQALGATIRRFSDAWIVNGTGNGALLAPEAPLDFGNAGTGSRLTMGLVGSYDFKTTFV | 120 |
| --- | --- | --- | --- |
| Query | 121 | GDASLTKRPMGRVLNPLREMGVQVKSEDGDRLPVTLRGPKTPTPITYRVPMASAQVKSAV | 180 |
| Sbjct | 121 | GDASL+KRPMGR+L+PLR MG QV + GDRLPVTL GP +P+TYRVPMASAQVKSAV GDASLSKRPMGRILDPLRRMGTQVSAATGDRLPVTLHGPGVTSPLTYRVPMASAQVKSAV | 180 |
| Query | 181 | LLAGLNTPGITTVIEPIMTRDHTEKMLQGFGANLTVETDADGVRTIRLEGRGKLTGQVID | 240 |
| Sbjct | 181 | LLAGLN PGITTVIEP+MTRDHTE+ML GFGA L+VETDA GVRTIRLEG+G+LTGQ ID LLAGLNAPGITTVIEPVMTRDHTERMLAGFGAALSVETDAAGVRTIRLEGQGRLTGQTID | 240 |
| Query | 241 | VPGDPSSTAFPLVAALLVPGSDVTILNVLMNPTRTGLILTLQEMGADIEVINPRLAGGED | 300 |
| Sbjct | 241 | VPGDPSS AFPLVAAL+VPGSD+TI NVLMN TRTGLILTLQEMGADIE+++ R+ GGED VPGDPSSAAFPLVAALIVPGSDITIRNVLMNTTRTGLILTLQEMGADIEILDRRVTGGED | 300 |
| Query | 301 | VADLRVRSSTLKGVTVPEDRAPSMIDEYPILavaaafaegaTVMNGLEELRVKESDRLSA | 360 |
| Sbjct | 301 | VADL VRSS LKGVTVP +RAPSMIDEYP+LAVAAAFAEG T+M GL+ELRVKESDRLSA VADLHVRSSALKGVTVPAERAPSMIDEYPVLAVAAAFAEGTTIMQGLDELRVKESDRLSA | 360 |
| Query | 361 | VANGLKLNGVDCDEGETSLVVRGRPDGKGLGNASGAAVATHLDHRIAMSFLVMGLVSENP | 420 |
| Sbjct | 361 | VA GLKLNGVDC EG SL VRGRPDGKGLG++SG V THLDHRIAMSFLVMGL SE+P VAEGLKLNGVDCSEGRDSLTVRGRPDGKGLGSSSGTEVRTHLDHRIAMSFLVMGLASEHP | 420 |
| Query  Sbjct | 421  421 | VTVDDATMIATSFPEFMDLMAGLGAKI 447 V++DD+ MIATSFPEF +M LGA I VSIDDSAMIATSFPEFFAMMTRLGATI 447 |  |

>WP_057204358.1 3-phosphoshikimate 1-carboxyvinyltransferase [Ensifer sp. Root31] KQU98467.1 3-phosphoshikimate 1-carboxyvinyltransferase [Ensifer sp. Root31]

Length=447

Score = 689 bits (1779), Expect = 0.0, Method: Compositional matrix adjust.

| Identities | = 352/448 (79%), Positives = 391/448 (87%), Gaps = 1/448 (0%) |  |
| --- | --- | --- |
| Query 1 | MLHGASSRPATARKSSGLSGTVRIPGDKSISHRSFMFGGLASGETRITGLLEGEDVINTG | 60 |
| Sbjct 1 | M G ++PA AR+S L GTVRI GDKSISHR+ M GGLA+G TRI GLLEGEDVINT MSAGLIAKPAIARRSKALGGTVRIRGDKSISHRALMLGGLAAGRTRIAGLLEGEDVINTA | 60 |
| Query 61 | KAMQAMGARIRKEGDTWIIDGVGNGGLLAPEAPLDFGNAATGCRLTMGLVGVYDFDSTFI | 120 |
| Sbjct 61 | +AM+A+GA +R+EG TWI+DG+GNG LL PEAPLDFGNA T CRLTMGLVG YDF +TF+ RAMRALGATVREEGGTWIVDGLGNGALLEPEAPLDFGNAGTSCRLTMGLVGAYDFTTTFV | 120 |
| Query 121 | GDASLTKRPMGRVLNPLREMGVQVKSEDGDRLPVTLRGPKTPTPITYRVPMASAQVKSAV | 180 |
| Sbjct 121 | GDASL+KRPMGRVL+PLR+MGVQV S +G RLPVTLRGPKT P+ YRVPMASA VKSAV GDASLSKRPMGRVLDPLRQMGVQVASAEGGRLPVTLRGPKTANPVNYRVPMASAPVKSAV | 180 |
| Query 181 | LLAGLNTPGITTVIEPIMTRDHTEKMLQGFGANLTVETDADGVRTIRLEGRGKLTGQVID | 240 |
| Sbjct 181 | LLAGLN PGITTVIEP++TRDHTE+MLQ FGANL VET +G R IRLEG+G+LTGQ ID LLAGLNAPGITTVIEPVITRDHTERMLQAFGANLAVETGNEGERIIRLEGQGRLTGQDID | 240 |
| Query 241 | VPGDPSSTAFPLVAALLVPGSDVTILNVLMNPTRTGLILTLQEMGADIEVINPRLAGGED | 300 |
| Sbjct 241 | VPGDPSS AFPLVAALLVPGSD+TILNVLMNP RTGLI+TL EMGA I+V+N R AGGED VPGDPSSAAFPLVAALLVPGSDITILNVLMNPIRTGLIVTLLEMGAQIDVLNARTAGGED | 300 |
| Query 301 | VADLRVRSSTLKGVTVPEDRAPSMIDEYPILavaaafaegaTVMNGLEELRVKESDRLSA | 360 |
| Sbjct 301 | VADLRVR S LKG+TVPEDRAPSMIDEYP+LAVAAAFAEG T+M+GLEELRVKESDRLSA VADLRVRYSELKGITVPEDRAPSMIDEYPVLAVAAAFAEGRTIMSGLEELRVKESDRLSA | 360 |
| Query 361 | VANGLKLNGVDCDEGETSLVVRGRPDGKGLGNASGAAVATHLDHRIAMSFLVMGLVSENP | 420 |
| Sbjct 361 | VA GLKL GVDC+E +LVV GRP GKGLGNA+ AAVATHLDHRIAMSFLVMGL SE+P VAAGLKLTGVDCEEAPAALVVHGRPGGKGLGNAA-AAVATHLDHRIAMSFLVMGLASEHP | 419 |
| Query 421  Sbjct 420 | VTVDDATMIATSFPEFMDLMAGLGAKIE 448 VTVDDATMIATSFP F++LMAGLGA IE VTVDDATMIATSFPGFLELMAGLGAAIE 447 |  |

>RWD72893.1 3-phosphoshikimate 1-carboxyvinyltransferase [Mesorhizobium sp.] RWE71894.1 3-phosphoshikimate 1-carboxyvinyltransferase [Mesorhizobium sp.] TIV57188.1 3-phosphoshikimate 1-carboxyvinyltransferase [Mesorhizobium sp.]

Length=452

Score = 689 bits (1779), Expect = 0.0, Method: Compositional matrix adjust.

| Identities | = 353/452 (78%), Positives = 387/452 (86%), Gaps = 4/452 (1%) |  |
| --- | --- | --- |
| Query 1 | MLHGASSRPATARKSSGLSGTVRIPGDKSISHRSFMFGGLASGETRITGLLEGEDVINTG | 60 |
| Sbjct 1 | M H A+++PATARKS L+GT R+PGDKSISHRSFMFGGLASGETRITGLLEGEDV+ TG MSHSAAAKPATARKSPALAGTARVPGDKSISHRSFMFGGLASGETRITGLLEGEDVMRTG | 60 |
| Query 61 | KAMQAMGARIRKEGDTWIIDGVGNGGLLAPEAPLDFGNAATGCRLTMGLVGVYDFDSTFI | 120 |
| Sbjct 61 | AM+AMGA I K G W+I G GNG LL PE PLDFGNA TG RLTMGLVG YD ++TFI AAMKAMGAHIEKNGAEWVIRGTGNGALLQPEGPLDFGNAGTGSRLTMGLVGTYDMETTFI | 120 |
| Query 121 | GDASLTKRPMGRVLNPLREMGVQV-KSEDGDRLPVTLRGPKTPTPITYRVPMASAQVKSA | 179 |
| Sbjct 121 | GDASL+ RPMGRVL+PLR+MGVQV K+ GDR+P+TLRGPK PITYRVPMASAQVKSA GDASLSGRPMGRVLDPLRQMGVQVLKAAPGDRMPITLRGPKHAAPITYRVPMASAQVKSA | 180 |
| Query 180 | VLLAGLNTPGITTVIEPIMTRDHTEKMLQGFGANLTVETDADGVRTIRLEGRGKLTGQVI | 239 |
| Sbjct 181 | VLLAGLNTPGITTVIEP+MTRDHTEKML+GFGANLTVETD GVR I +EG+GKLTGQ I VLLAGLNTPGITTVIEPVMTRDHTEKMLKGFGANLTVETDERGVRHIFIEGQGKLTGQTI | 240 |
| Query 240 | DVPGDPSSTAFPLVAALLVPGSDVTILNVLMNPTRTGLILTLQEMGADIEVINPRLAGGE | 299 |
| Sbjct 241 | VPGDPSS FPLVAAL+VPGSD+ I NVLMNPTRTGL+LTLQEMG I+++NPR AGGE AVPGDPSSAGFPLVAALIVPGSDIVIENVLMNPTRTGLLLTLQEMGGRIDILNPRNAGGE | 300 |
| Query 300 | DVADLRVRSSTLKGVTVPEDRAPSMIDEYPILavaaafaegaTVMNGLEELRVKESDRLS | 359 |
| Sbjct 301 | DVADLRVR S LKGV VP +RAPSMIDEYP+LAVAA+FAEG T+M GLEELRVKESDRLS DVADLRVRYSELKGVVVPPERAPSMIDEYPVLAVAASFAEGETLMQGLEELRVKESDRLS | 360 |
| Query 360 | AVANGLKLNGVDCDEGETSLVVRGRPDGKGLG---NASGAAVATHLDHRIAMSFLVMGLV | 416 |
| Sbjct 361 | AVANGLKLN VDC EGE SL VRGRP GKGLG N AV THLDHRIAMSFL+MGL AVANGLKLNSVDCTEGEASLAVRGRPGGKGLGAHPNGPDTAVKTHLDHRIAMSFLIMGLA | 420 |
| Query 417  Sbjct 421 | SENPVTVDDATMIATSFPEFMDLMAGLGAKIE 448  +E PVT+DDA MIATSFPEFM LM GLGA+IE  TEKPVTIDDANMIATSFPEFMGLMKGLGAEIE 452 |  |

>TIW94727.1 3-phosphoshikimate 1-carboxyvinyltransferase [Mesorhizobium sp.] Length=452

Score = 689 bits (1779), Expect = 0.0, Method: Compositional matrix adjust.

| Identities | = 355/452 (79%), Positives = 387/452 (86%), Gaps = 4/452 (1%) |  |
| --- | --- | --- |
| Query 1 | MLHGASSRPATARKSSGLSGTVRIPGDKSISHRSFMFGGLASGETRITGLLEGEDVINTG | 60 |
| Sbjct 1 | M H A+ +PATARKSS LSGT R+PGDKSISHRSFMFGGLASGETRITGLLEGEDV+ TG MSHAAAPKPATARKSSALSGTARVPGDKSISHRSFMFGGLASGETRITGLLEGEDVMRTG | 60 |
| Query 61 | KAMQAMGARIRKEGDTWIIDGVGNGGLLAPEAPLDFGNAATGCRLTMGLVGVYDFDSTFI | 120 |
| Sbjct 61 | AM+AMGA I K G W+I G GNG LL P+ PLDFGNA TG RLTMGLVG YD ++TFI AAMKAMGAHIEKRGAEWVIRGTGNGALLQPDGPLDFGNAGTGSRLTMGLVGTYDMETTFI | 120 |
| Query 121 | GDASLTKRPMGRVLNPLREMGVQV-KSEDGDRLPVTLRGPKTPTPITYRVPMASAQVKSA | 179 |
| Sbjct 121 | GDASL+ RPMGRVL PLR+MGVQV K+ GDR+P+TLRGPK PITYRVPMASAQVKSA GDASLSGRPMGRVLEPLRQMGVQVLKAAPGDRMPITLRGPKHAAPITYRVPMASAQVKSA | 180 |
| Query 180 | VLLAGLNTPGITTVIEPIMTRDHTEKMLQGFGANLTVETDADGVRTIRLEGRGKLTGQVI | 239 |
| Sbjct 181 | VLLAGLNTPGITTVIEP+MTRDHTEKML+GFGANLTVETD GVR I +EG+GKL+GQ I VLLAGLNTPGITTVIEPVMTRDHTEKMLKGFGANLTVETDERGVRHIFIEGQGKLSGQTI | 240 |
| Query 240 | DVPGDPSSTAFPLVAALLVPGSDVTILNVLMNPTRTGLILTLQEMGADIEVINPRLAGGE | 299 |
| Sbjct 241 | VPGDPSS FPLVAAL+VPGSD+ I NVLMNPTRTGL+LTLQEMG I+++NPR AGGE AVPGDPSSAGFPLVAALIVPGSDIVIENVLMNPTRTGLLLTLQEMGGRIDILNPRNAGGE | 300 |
| Query 300 | DVADLRVRSSTLKGVTVPEDRAPSMIDEYPILavaaafaegaTVMNGLEELRVKESDRLS | 359 |
| Sbjct 301 | VADLRVRSS LKGVTVP +RAPSMIDEYP+LAVAA+FAEG T+M GLEELRVKESDRLS GVADLRVRSSELKGVTVPPERAPSMIDEYPVLAVAASFAEGETLMQGLEELRVKESDRLS | 360 |
| Query 360 | AVANGLKLNGVDCDEGETSLVVRGRPDGKGLG---NASGAAVATHLDHRIAMSFLVMGLV | 416 |
| Sbjct 361 | AVANGLKLNGVDC EGE +L VRGRP GKGLG N V THLDHRIAMSFLVMGL AVANGLKLNGVDCTEGEATLAVRGRPGGKGLGRHPNGPDTTVKTHLDHRIAMSFLVMGLA | 420 |
| Query 417 | SENPVTVDDATMIATSFPEFMDLMAGLGAKIE 448 |  |

SE PVT+DDA MIATSFPEFM LM GLGA+IE Sbjct 421 SEKPVTIDDANMIATSFPEFMGLMKGLGAEIE 452

>CDX26639.1 3-phosphoshikimate 1-carboxyvinyltransferase [Mesorhizobium sp. ORS 3324]

Length=481

Score = 689 bits (1779), Expect = 0.0, Method: Compositional matrix adjust.

| Identities | = 355/452 (79%), Positives = 387/452 (86%), Gaps = 4/452 (1%) |  |
| --- | --- | --- |
| Query 1 | MLHGASSRPATARKSSGLSGTVRIPGDKSISHRSFMFGGLASGETRITGLLEGEDVINTG | 60 |
| Sbjct 30 | M H A+ +PATARKS L+GT R+PGDKSISHRSFMFGGLASGETRITGLLEGEDV+ TG MSHAAAPKPATARKSPALAGTARVPGDKSISHRSFMFGGLASGETRITGLLEGEDVMRTG | 89 |
| Query 61 | KAMQAMGARIRKEGDTWIIDGVGNGGLLAPEAPLDFGNAATGCRLTMGLVGVYDFDSTFI | 120 |
| Sbjct 90 | AM+AMGA I K G W+I G GNG LL PE PLDFGNA TG RLTMGLVG YD ++TFI AAMKAMGAHIEKRGAEWVIRGTGNGALLEPEGPLDFGNAGTGSRLTMGLVGTYDMETTFI | 149 |
| Query 121 | GDASLTKRPMGRVLNPLREMGVQV-KSEDGDRLPVTLRGPKTPTPITYRVPMASAQVKSA | 179 |
| Sbjct 150 | GDASL+ RPMGRVL PLR+MGVQV K+ GDR+P+TLRGPK PITYRVPMASAQVKSA GDASLSGRPMGRVLEPLRQMGVQVLKAAPGDRMPITLRGPKHAAPITYRVPMASAQVKSA | 209 |
| Query 180 | VLLAGLNTPGITTVIEPIMTRDHTEKMLQGFGANLTVETDADGVRTIRLEGRGKLTGQVI | 239 |
| Sbjct 210 | VLLAGLNTPGITTVIEP+MTRDHTEKML+GFGANLTVETD GVR I +EG+GKLTGQ I VLLAGLNTPGITTVIEPVMTRDHTEKMLKGFGANLTVETDERGVRHIFIEGQGKLTGQTI | 269 |
| Query 240 | DVPGDPSSTAFPLVAALLVPGSDVTILNVLMNPTRTGLILTLQEMGADIEVINPRLAGGE | 299 |
| Sbjct 270 | VPGDPSS FPLVAAL+VPGSD+TI NVLMNPTRTGL+ TLQEMG I+++NPR AGGE AVPGDPSSAGFPLVAALIVPGSDITIENVLMNPTRTGLLSTLQEMGGRIDILNPRNAGGE | 329 |
| Query 300 | DVADLRVRSSTLKGVTVPEDRAPSMIDEYPILavaaafaegaTVMNGLEELRVKESDRLS | 359 |
| Sbjct 330 | DVADLRVRSS LKGV VP +RAPSMIDEYP+LAVAA+FAEG T+M GLEELRVKESDRLS DVADLRVRSSELKGVVVPPERAPSMIDEYPVLAVAASFAEGETLMQGLEELRVKESDRLS | 389 |
| Query 360 | AVANGLKLNGVDCDEGETSLVVRGRPDGKGLG---NASGAAVATHLDHRIAMSFLVMGLV | 416 |
| Sbjct 390 | AVANGLKLNGVDC EGE SL VRG+P GKGLG N AV THLDHRIAMSFLVMGL AVANGLKLNGVDCTEGEASLAVRGKPGGKGLGAHPNGPDTAVKTHLDHRIAMSFLVMGLA | 449 |
| Query 417  Sbjct 450 | SENPVTVDDATMIATSFPEFMDLMAGLGAKIE 448  +E PVT+DDA MIATSFPEFM LM GLGA+IE  TEKPVTIDDANMIATSFPEFMGLMKGLGAEIE 481 |  |

>WP_126086286.1 3-phosphoshikimate 1-carboxyvinyltransferase [Mesorhizobium sp. M1D.F.Ca.ET.043.01.1.1]

AZO72172.1 3-phosphoshikimate 1-carboxyvinyltransferase [Mesorhizobium sp. M1D.F.Ca.ET.043.01.1.1]

RWA94955.1 3-phosphoshikimate 1-carboxyvinyltransferase [Mesorhizobium sp.] Length=452

Score = 689 bits (1778), Expect = 0.0, Method: Compositional matrix adjust.

| Identities | = 353/452 (78%), Positives = 388/452 (86%), Gaps = 4/452 (1%) |  |
| --- | --- | --- |
| Query 1 | MLHGASSRPATARKSSGLSGTVRIPGDKSISHRSFMFGGLASGETRITGLLEGEDVINTG | 60 |
| Sbjct 1 | M H A+++PATARKS L+GT R+PGDKSISHRSFMFGGLASGETRITGLLEGEDV+ TG MSHSAAAKPATARKSPALAGTARVPGDKSISHRSFMFGGLASGETRITGLLEGEDVMRTG | 60 |
| Query 61 | KAMQAMGARIRKEGDTWIIDGVGNGGLLAPEAPLDFGNAATGCRLTMGLVGVYDFDSTFI | 120 |
| Sbjct 61 | AM+AMGA I K+G W+I G GNG LL PE PLDFGNA TG RLTMGLVG YD ++TFI AAMKAMGAHIEKKGAEWVIRGTGNGALLEPEGPLDFGNAGTGSRLTMGLVGTYDMETTFI | 120 |
| Query 121 | GDASLTKRPMGRVLNPLREMGVQV-KSEDGDRLPVTLRGPKTPTPITYRVPMASAQVKSA | 179 |
| Sbjct 121 | GDASL+ RPMGRVL PLR+MGVQV K+ GDR+P+TLRGPK PITYRVPMASAQVKSA GDASLSGRPMGRVLEPLRQMGVQVLKAAPGDRMPITLRGPKHAAPITYRVPMASAQVKSA | 180 |
| Query 180 | VLLAGLNTPGITTVIEPIMTRDHTEKMLQGFGANLTVETDADGVRTIRLEGRGKLTGQVI | 239 |
| Sbjct 181 | VLLAGLNTPGITTVIEP+MTRDHTEKMLQGFGANL+VETD GVR I +EG+GKLTGQ I VLLAGLNTPGITTVIEPVMTRDHTEKMLQGFGANLSVETDERGVRHIFIEGQGKLTGQTI | 240 |
| Query 240 | DVPGDPSSTAFPLVAALLVPGSDVTILNVLMNPTRTGLILTLQEMGADIEVINPRLAGGE VPGDPSS FPLVA L+VPGSD+ I NVLMNPTRTGL+LTLQEMG I+++NPR AGGE | 299 |

| Sbjct | 241 | AVPGDPSSAGFPLVATLIVPGSDIVIENVLMNPTRTGLLLTLQEMGGRIDILNPRNAGGE | 300 |
| --- | --- | --- | --- |
| Query | 300 | DVADLRVRSSTLKGVTVPEDRAPSMIDEYPILavaaafaegaTVMNGLEELRVKESDRLS | 359 |
| Sbjct | 301 | DVADLRVRSS LKG+ VP +RAPSMIDEYP+LAVAA+FAEG T+M GLEELRVKESDRLS DVADLRVRSSDLKGIVVPPERAPSMIDEYPVLAVAASFAEGETLMQGLEELRVKESDRLS | 360 |
| Query | 360 | AVANGLKLNGVDCDEGETSLVVRGRPDGKGLG---NASGAAVATHLDHRIAMSFLVMGLV | 416 |
| Sbjct | 361 | AVANGLKLNGVDC EGE SL VRGRP GKGLG + AV THLDHRIAMSFLVMGL AVANGLKLNGVDCTEGEASLAVRGRPGGKGLGAHPDGLDTAVKTHLDHRIAMSFLVMGLA | 420 |
| Query  Sbjct | 417  421 | SENPVTVDDATMIATSFPEFMDLMAGLGAKIE 448  +E PVT+DDA MIATSFPEFM LM GLGA+IE  TEKPVTIDDANMIATSFPEFMGLMKGLGAEIE 452 |  |

>WP_095806734.1 MULTISPECIES: 3-phosphoshikimate 1-carboxyvinyltransferase [unclassified Mesorhizobium]

RWB75785.1 3-phosphoshikimate 1-carboxyvinyltransferase [Mesorhizobium sp.] AZO10098.1 3-phosphoshikimate 1-carboxyvinyltransferase [Mesorhizobium sp.

M3A.F.Ca.ET.080.04.2.1]

PBB86555.1 3-phosphoshikimate 1-carboxyvinyltransferase [Mesorhizobium sp. WSM3876]

RWB91536.1 3-phosphoshikimate 1-carboxyvinyltransferase [Mesorhizobium sp.] RWE23588.1 3-phosphoshikimate 1-carboxyvinyltransferase [Mesorhizobium sp.]

Length=452

Score = 689 bits (1778), Expect = 0.0, Method: Compositional matrix adjust.

| Identities | = 353/452 (78%), Positives = 386/452 (85%), Gaps = 4/452 (1%) |  |
| --- | --- | --- |
| Query 1 | MLHGASSRPATARKSSGLSGTVRIPGDKSISHRSFMFGGLASGETRITGLLEGEDVINTG | 60 |
| Sbjct 1 | M H A+ +PATARKS L+G R+PGDKSISHRSFMFGGLASGETRITGLLEGEDV+ TG MSHAAAPKPATARKSPALAGVARVPGDKSISHRSFMFGGLASGETRITGLLEGEDVMRTG | 60 |
| Query 61 | KAMQAMGARIRKEGDTWIIDGVGNGGLLAPEAPLDFGNAATGCRLTMGLVGVYDFDSTFI | 120 |
| Sbjct 61 | AM+AMGA I K+GD W+I G GNG LL PE PLDFGNA TG RLTMGLVG YD ++TFI AAMKAMGAHIEKKGDEWVIRGTGNGALLQPEGPLDFGNAGTGSRLTMGLVGTYDMETTFI | 120 |
| Query 121 | GDASLTKRPMGRVLNPLREMGVQV-KSEDGDRLPVTLRGPKTPTPITYRVPMASAQVKSA | 179 |
| Sbjct 121 | GDASL+ RPM RVL PLR+MGVQV K+ GDR+P+TLRGPK PITYRVPMASAQVKSA GDASLSGRPMARVLEPLRQMGVQVLKASPGDRMPITLRGPKHAAPITYRVPMASAQVKSA | 180 |
| Query 180 | VLLAGLNTPGITTVIEPIMTRDHTEKMLQGFGANLTVETDADGVRTIRLEGRGKLTGQVI | 239 |
| Sbjct 181 | VLLAGLNT GITTVIEP+MTRDHTEKML+GFGANLTVETD GVR I +EG+GKLTGQ I VLLAGLNTAGITTVIEPVMTRDHTEKMLKGFGANLTVETDERGVRHIFIEGQGKLTGQTI | 240 |
| Query 240 | DVPGDPSSTAFPLVAALLVPGSDVTILNVLMNPTRTGLILTLQEMGADIEVINPRLAGGE | 299 |
| Sbjct 241 | VPGDPSS FPLVAAL+VPGSD+ I NVLMNPTRTGL+LTLQEMG I+++NPR AGGE AVPGDPSSAGFPLVAALIVPGSDIVIENVLMNPTRTGLLLTLQEMGGRIDILNPRNAGGE | 300 |
| Query 300 | DVADLRVRSSTLKGVTVPEDRAPSMIDEYPILavaaafaegaTVMNGLEELRVKESDRLS | 359 |
| Sbjct 301 | DVADLRVRSS LKGV VP +RAPSMIDEYP+LAVAA+FAEG T+M GLEELRVKESDRLS DVADLRVRSSKLKGVAVPAERAPSMIDEYPVLAVAASFAEGETLMQGLEELRVKESDRLS | 360 |
| Query 360 | AVANGLKLNGVDCDEGETSLVVRGRPDGKGLG---NASGAAVATHLDHRIAMSFLVMGLV | 416 |
| Sbjct 361 | AVANGLKLNGVDC EGE SL VRG+P GKGLG N AV THLDHRIAMSFLVMGL AVANGLKLNGVDCTEGEASLAVRGKPGGKGLGAHPNGLDTAVKTHLDHRIAMSFLVMGLA | 420 |
| Query 417  Sbjct 421 | SENPVTVDDATMIATSFPEFMDLMAGLGAKIE 448  +E PVT+DDA MIATSFPEFM LM GLGA+IE  TEKPVTIDDANMIATSFPEFMGLMKGLGAEIE 452 |  |

```
>CDX50135.1 3-phosphoshikimate 1-carboxyvinyltransferase [Mesorhizobium plurifarium] Length=478

Score = 689 bits (1778), Expect = 0.0, Method: Compositional matrix adjust. Identities = 354/452 (78%), Positives = 388/452 (86%), Gaps = 4/452 (1%)

Query 1	MLHGASSRPATARKSSGLSGTVRIPGDKSISHRSFMFGGLASGETRITGLLEGEDVINTG 60 M H A+++PATARKS L+GT R+PGDKSISHRSFMFGGLASGETRITGLLEGEDV+ TG
Sbjct 27	MSHSAAAKPATARKSPALAGTARVPGDKSISHRSFMFGGLASGETRITGLLEGEDVMRTG 86
```

| Query | 61 | KAMQAMGARIRKEGDTWIIDGVGNGGLLAPEAPLDFGNAATGCRLTMGLVGVYDFDSTFI | 120 |
| --- | --- | --- | --- |
| Sbjct | 87 | AM+AMGA I K+ W+I G GNG LL PE PLDFGNA TG RLTMGLVG YD ++TF+ AAMKAMGAHIEKKDGEWVIRGTGNGALLEPEGPLDFGNAGTGSRLTMGLVGTYDMETTFV | 146 |
| Query | 121 | GDASLTKRPMGRVLNPLREMGVQV-KSEDGDRLPVTLRGPKTPTPITYRVPMASAQVKSA | 179 |
| Sbjct | 147 | GDASL+ RPMGRVL+PLR+MGVQV K+ GDR+P+TLRGPK PITYRVPMASAQVKSA GDASLSGRPMGRVLDPLRQMGVQVLKAAPGDRMPITLRGPKHAAPITYRVPMASAQVKSA | 206 |
| Query | 180 | VLLAGLNTPGITTVIEPIMTRDHTEKMLQGFGANLTVETDADGVRTIRLEGRGKLTGQVI | 239 |
| Sbjct | 207 | VLLAGLNTPGITTVIEP+MTRDHTEKML+GFGANLTVETD GVR I +EG+GKLTGQ I VLLAGLNTPGITTVIEPVMTRDHTEKMLKGFGANLTVETDERGVRHIFIEGQGKLTGQTI | 266 |
| Query | 240 | DVPGDPSSTAFPLVAALLVPGSDVTILNVLMNPTRTGLILTLQEMGADIEVINPRLAGGE | 299 |
| Sbjct | 267 | VPGDPSS FPLVAAL+VPGSDVTI NVLMNPTRTGL+LTLQEMG I+++NPR AGGE AVPGDPSSAGFPLVAALIVPGSDVTIENVLMNPTRTGLLLTLQEMGGRIDILNPRNAGGE | 326 |
| Query | 300 | DVADLRVRSSTLKGVTVPEDRAPSMIDEYPILavaaafaegaTVMNGLEELRVKESDRLS | 359 |
| Sbjct | 327 | DVADLRVRSS LKGV VP +RAPSMIDEYP+LAVAA+FAEG T+M GLEELRVKESDRLS DVADLRVRSSELKGVVVPPERAPSMIDEYPVLAVAASFAEGETLMQGLEELRVKESDRLS | 386 |
| Query | 360 | AVANGLKLNGVDCDEGETSLVVRGRPDGKGLG---NASGAAVATHLDHRIAMSFLVMGLV | 416 |
| Sbjct | 387 | AVANGLKLNGVDC EGE SL VRG+P GKGLG N V THLDHRIAMSFLVMGL AVANGLKLNGVDCTEGEASLAVRGKPGGKGLGAHPNGQDTVVKTHLDHRIAMSFLVMGLA | 446 |
| Query  Sbjct | 417  447 | SENPVTVDDATMIATSFPEFMDLMAGLGAKIE 448  +E PVT+DDA MIATSFPEFM LM GLGA IE  TEKPVTIDDANMIATSFPEFMGLMKGLGADIE 478 |  |

>WP_149790164.1 3-phosphoshikimate 1-carboxyvinyltransferase [Mesorhizobium sp. NFR06]

SFN67856.1 3-phosphoshikimate 1-carboxyvinyltransferase [Mesorhizobium sp. NFR06]

Length=452

Score = 689 bits (1778), Expect = 0.0, Method: Compositional matrix adjust.

| Identities | = 351/452 (78%), Positives = 389/452 (86%), Gaps = 4/452 (1%) |  |
| --- | --- | --- |
| Query 1 | MLHGASSRPATARKSSGLSGTVRIPGDKSISHRSFMFGGLASGETRITGLLEGEDVINTG | 60 |
| Sbjct 1 | M H A+++PATARKS L+GT R+PGDKSISHRSFMFGGLASGETRITGLLEGEDV+ TG MSHSAAAKPATARKSPALTGTARVPGDKSISHRSFMFGGLASGETRITGLLEGEDVMRTG | 60 |
| Query 61 | KAMQAMGARIRKEGDTWIIDGVGNGGLLAPEAPLDFGNAATGCRLTMGLVGVYDFDSTFI | 120 |
| Sbjct 61 | AM+AMGA + K+G W+I G GNG LL PE PLDFGNA TG RLTMGLVG YD ++TFI AAMKAMGAHVEKKGAEWVIRGTGNGALLQPEGPLDFGNAGTGSRLTMGLVGTYDMETTFI | 120 |
| Query 121 | GDASLTKRPMGRVLNPLREMGVQV-KSEDGDRLPVTLRGPKTPTPITYRVPMASAQVKSA | 179 |
| Sbjct 121 | GD SL+ RPMGRVL+PLR+MGVQV K+ GDR+P+TLRGPK PITYRVPMASAQVKSA GDTSLSGRPMGRVLDPLRQMGVQVLKAAPGDRMPITLRGPKHTAPITYRVPMASAQVKSA | 180 |
| Query 180 | VLLAGLNTPGITTVIEPIMTRDHTEKMLQGFGANLTVETDADGVRTIRLEGRGKLTGQVI | 239 |
| Sbjct 181 | VLLAGLNTPGITTVIEP+MTRDHTEKML+GFGANLTVETD GVR I +EG+G+LTGQ I VLLAGLNTPGITTVIEPVMTRDHTEKMLKGFGANLTVETDERGVRHIFIEGQGRLTGQTI | 240 |
| Query 240 | DVPGDPSSTAFPLVAALLVPGSDVTILNVLMNPTRTGLILTLQEMGADIEVINPRLAGGE | 299 |
| Sbjct 241 | VPGDPSS FPLVAAL+VPGSD+TI NVLMNPTRTGL+LTLQEMG I+++NPR AGGE AVPGDPSSAGFPLVAALIVPGSDITIENVLMNPTRTGLLLTLQEMGGRIDILNPRNAGGE | 300 |
| Query 300 | DVADLRVRSSTLKGVTVPEDRAPSMIDEYPILavaaafaegaTVMNGLEELRVKESDRLS | 359 |
| Sbjct 301 | DVADLRVR S L+GVTVP +RAPSMIDEYP+LAVAA+FAEG T+M GLEELRVKESDRLS DVADLRVRYSELQGVTVPPERAPSMIDEYPVLAVAASFAEGETLMQGLEELRVKESDRLS | 360 |
| Query 360 | AVANGLKLNGVDCDEGETSLVVRGRPDGKGLG---NASGAAVATHLDHRIAMSFLVMGLV | 416 |
| Sbjct 361 | AVANGLKLNGVDC EGE SL VRG+P GKGLG N G V THLDHRIAMSFLVMGL AVANGLKLNGVDCTEGEASLAVRGKPGGKGLGQHPNGPGTIVQTHLDHRIAMSFLVMGLA | 420 |
| Query 417  Sbjct 421 | SENPVTVDDATMIATSFPEFMDLMAGLGAKIE 448  +E PVT+DD MIATSFPEFM LM GLGA+IE  TEKPVTIDDQAMIATSFPEFMGLMTGLGAEIE 452 |  |

>WP_124999662.1 3-phosphoshikimate 1-carboxyvinyltransferase [Mesorhizobium tamadayense]

RRI01122.1 3-phosphoshikimate 1-carboxyvinyltransferase [Mesorhizobium tamadayense] Length=452

Score = 689 bits (1778), Expect = 0.0, Method: Compositional matrix adjust.

| Identities | = 353/452 (78%), Positives = 388/452 (86%), Gaps = 4/452 (1%) |  |
| --- | --- | --- |
| Query 1 | MLHGASSRPATARKSSGLSGTVRIPGDKSISHRSFMFGGLASGETRITGLLEGEDVINTG | 60 |
| Sbjct 1 | M H A+ +PATARKS LSGT R+PGDKSISHRSFMFGGLASGETRITGLLEGEDV+ TG MSHSAAPKPATARKSDALSGTARVPGDKSISHRSFMFGGLASGETRITGLLEGEDVMRTG | 60 |
| Query 61 | KAMQAMGARIRKEGDTWIIDGVGNGGLLAPEAPLDFGNAATGCRLTMGLVGVYDFDSTFI | 120 |
| Sbjct 61 | AM+AMGA + K+G W+I G GNG LL PE PLDFGNA TG RLTMGLVG YD ++TFI AAMKAMGAHVEKKGAEWVIRGTGNGALLRPEGPLDFGNAGTGSRLTMGLVGTYDMETTFI | 120 |
| Query 121 | GDASLTKRPMGRVLNPLREMGVQV-KSEDGDRLPVTLRGPKTPTPITYRVPMASAQVKSA | 179 |
| Sbjct 121 | GDASL+ RPMGRVL PLR+MGVQV + GDR+P+TLRGPK PITYRVPMASAQVKSA GDASLSGRPMGRVLEPLRQMGVQVLNAAPGDRMPITLRGPKHAAPITYRVPMASAQVKSA | 180 |
| Query 180 | VLLAGLNTPGITTVIEPIMTRDHTEKMLQGFGANLTVETDADGVRTIRLEGRGKLTGQVI | 239 |
| Sbjct 181 | VLLAGLNTPGITTVIEP+MTRDHTEKML+GFGANL+VETD GVR I +EG+GKLTGQ I VLLAGLNTPGITTVIEPVMTRDHTEKMLKGFGANLSVETDERGVRHIFIEGQGKLTGQTI | 240 |
| Query 240 | DVPGDPSSTAFPLVAALLVPGSDVTILNVLMNPTRTGLILTLQEMGADIEVINPRLAGGE | 299 |
| Sbjct 241 | VPGDPSS FPLVAAL+VPGSD+ I NVLMNPTRTGL+LTLQEMG I+V+NPR AGGE AVPGDPSSAGFPLVAALIVPGSDIVIENVLMNPTRTGLLLTLQEMGGRIDVLNPRNAGGE | 300 |
| Query 300 | DVADLRVRSSTLKGVTVPEDRAPSMIDEYPILavaaafaegaTVMNGLEELRVKESDRLS | 359 |
| Sbjct 301 | DVADLRVRSS LKGV VP +RAPSMIDEYP+LAVAA+FAEG T+M GLEELRVKESDRLS DVADLRVRSSDLKGVAVPPERAPSMIDEYPVLAVAASFAEGETLMQGLEELRVKESDRLS | 360 |
| Query 360 | AVANGLKLNGVDCDEGETSLVVRGRPDGKGLG---NASGAAVATHLDHRIAMSFLVMGLV | 416 |
| Sbjct 361 | AVANGLKLNGVDC EGE SL VRG+P GKGLG N AV THLDHRIAMSFL+MGL AVANGLKLNGVDCTEGEASLAVRGKPGGKGLGAHPNGPDTAVKTHLDHRIAMSFLIMGLA | 420 |
| Query 417  Sbjct 421 | SENPVTVDDATMIATSFPEFMDLMAGLGAKIE 448  +E PVT+DDA+MIATSFPEFM LM GLGA+IE  TEKPVTIDDASMIATSFPEFMGLMTGLGAEIE 452 |  |

>RWM41150.1 3-phosphoshikimate 1-carboxyvinyltransferase [Mesorhizobium sp.] TJV50088.1 3-phosphoshikimate 1-carboxyvinyltransferase [Mesorhizobium sp.]

Length=452

Score = 689 bits (1777), Expect = 0.0, Method: Compositional matrix adjust.

| Identities | = 352/451 (78%), Positives = 388/451 (86%), Gaps = 4/451 (1%) |  |
| --- | --- | --- |
| Query 1 | MLHGASSRPATARKSSGLSGTVRIPGDKSISHRSFMFGGLASGETRITGLLEGEDVINTG | 60 |
| Sbjct 1 | M H A+++PATARKS L+GT R+PGDKSISHRSFMFGGLASGETRITGLLEGEDV+ TG MSHSAAAKPATARKSPALAGTARVPGDKSISHRSFMFGGLASGETRITGLLEGEDVMRTG | 60 |
| Query 61 | KAMQAMGARIRKEGDTWIIDGVGNGGLLAPEAPLDFGNAATGCRLTMGLVGVYDFDSTFI | 120 |
| Sbjct 61 | AM+AMGA I K+G W+I G GNG LL PE PLDFGNA TG RLTMGLVG YD ++TFI AAMKAMGAHIEKKGAEWVIRGTGNGALLEPEGPLDFGNAGTGSRLTMGLVGTYDMETTFI | 120 |
| Query 121 | GDASLTKRPMGRVLNPLREMGVQV-KSEDGDRLPVTLRGPKTPTPITYRVPMASAQVKSA | 179 |
| Sbjct 121 | GDASL+ RPMGRVL PLR+MGVQV K+ GDR+P+TLRGPK PITYRVPMASAQVKSA GDASLSGRPMGRVLEPLRQMGVQVLKAAPGDRMPITLRGPKHAAPITYRVPMASAQVKSA | 180 |
| Query 180 | VLLAGLNTPGITTVIEPIMTRDHTEKMLQGFGANLTVETDADGVRTIRLEGRGKLTGQVI | 239 |
| Sbjct 181 | VLLAGLNTPGITTVIEP+MTRDHTEKML+GFGANLTVETD GVR I +EG+GKLTGQ I VLLAGLNTPGITTVIEPVMTRDHTEKMLKGFGANLTVETDERGVRHIFIEGQGKLTGQTI | 240 |
| Query 240 | DVPGDPSSTAFPLVAALLVPGSDVTILNVLMNPTRTGLILTLQEMGADIEVINPRLAGGE | 299 |
| Sbjct 241 | VPGDPSS FPLVAAL+VPGSD+ I NVLMNPTRTGL+LTLQEMG I+++NPR AGGE AVPGDPSSAGFPLVAALIVPGSDIVIENVLMNPTRTGLLLTLQEMGGRIDILNPRNAGGE | 300 |
| Query 300 | DVADLRVRSSTLKGVTVPEDRAPSMIDEYPILavaaafaegaTVMNGLEELRVKESDRLS | 359 |
| Sbjct 301 | DVADLRVRSS LKGV VP +RAPSMIDEYP+LAVAA+FA+G T+M GLEELRVKESDRL+ DVADLRVRSSDLKGVVVPPERAPSMIDEYPVLAVAASFADGETLMQGLEELRVKESDRLA | 360 |
| Query 360 | AVANGLKLNGVDCDEGETSLVVRGRPDGKGLG---NASGAAVATHLDHRIAMSFLVMGLV AVANGLKLNGVDC EGE SL VRG+P GKGLG N AV THLDHRIAMSFLVMGL | 416 |

| Sbjct | 361 | AVANGLKLNGVDCTEGEASLAVRGKPGGKGLGAHPNGPDTAVKTHLDHRIAMSFLVMGLA 420 |
| --- | --- | --- |
| Query | 417 | SENPVTVDDATMIATSFPEFMDLMAGLGAKI 447 |
| Sbjct | 421 | +E PVT+DDA MIATSFPEFM LM GLGA+I  TEKPVTIDDANMIATSFPEFMGLMTGLGAEI 451 |

>TIT78119.1 3-phosphoshikimate 1-carboxyvinyltransferase [Mesorhizobium sp.] Length=452

Score = 689 bits (1777), Expect = 0.0, Method: Compositional matrix adjust.

| Identities | = 354/452 (78%), Positives = 387/452 (86%), Gaps = 4/452 (1%) |  |
| --- | --- | --- |
| Query 1 | MLHGASSRPATARKSSGLSGTVRIPGDKSISHRSFMFGGLASGETRITGLLEGEDVINTG | 60 |
| Sbjct 1 | M H A+++PATARKS L+GT R+PGDKSISHRSFMFGGLAS ETRITGLLEGEDV+ TG MSHSAAAKPATARKSPALAGTARVPGDKSISHRSFMFGGLASDETRITGLLEGEDVMRTG | 60 |
| Query 61 | KAMQAMGARIRKEGDTWIIDGVGNGGLLAPEAPLDFGNAATGCRLTMGLVGVYDFDSTFI | 120 |
| Sbjct 61 | AM+AMGA I K+G W+I G GNG LL PE PLDFGNA TG RLTMGLVG YD ++TFI AAMKAMGAHIEKKGAEWVIRGTGNGALLEPEGPLDFGNAGTGSRLTMGLVGTYDMETTFI | 120 |
| Query 121 | GDASLTKRPMGRVLNPLREMGVQV-KSEDGDRLPVTLRGPKTPTPITYRVPMASAQVKSA | 179 |
| Sbjct 121 | GDASL+ RPMGRVL PLR+MGVQV K+ GDR+P+TLRGPK PITYRVPMASAQVKSA GDASLSGRPMGRVLEPLRQMGVQVLKAAPGDRMPITLRGPKHAAPITYRVPMASAQVKSA | 180 |
| Query 180 | VLLAGLNTPGITTVIEPIMTRDHTEKMLQGFGANLTVETDADGVRTIRLEGRGKLTGQVI | 239 |
| Sbjct 181 | VLLAGLN PGITTVIEP+MTRDHTEKMLQGFGANL+VETD GVR I +EG+GKLTGQ I VLLAGLNAPGITTVIEPVMTRDHTEKMLQGFGANLSVETDERGVRYIFIEGQGKLTGQTI | 240 |
| Query 240 | DVPGDPSSTAFPLVAALLVPGSDVTILNVLMNPTRTGLILTLQEMGADIEVINPRLAGGE | 299 |
| Sbjct 241 | VPGDPSS FPLVAAL+VPGSD+ I NVLMNPTRTGL+LTLQEMG I+++NPR AGGE AVPGDPSSAGFPLVAALIVPGSDIVIENVLMNPTRTGLLLTLQEMGGRIDILNPRNAGGE | 300 |
| Query 300 | DVADLRVRSSTLKGVTVPEDRAPSMIDEYPILavaaafaegaTVMNGLEELRVKESDRLS | 359 |
| Sbjct 301 | DVADLRVRSS LKGV VP +RAPSMIDEYP+LAVAA+FAEG T+M GLEELRVKESDRLS DVADLRVRSSDLKGVVVPPERAPSMIDEYPVLAVAASFAEGETLMQGLEELRVKESDRLS | 360 |
| Query 360 | AVANGLKLNGVDCDEGETSLVVRGRPDGKGLG---NASGAAVATHLDHRIAMSFLVMGLV | 416 |
| Sbjct 361 | AVANGLKLNGVDC EGE SL VRGRP GKGLG N AV THLDHRIAMSFLVMGL AVANGLKLNGVDCTEGEASLAVRGRPGGKGLGAHPNGPDTAVRTHLDHRIAMSFLVMGLA | 420 |
| Query 417  Sbjct 421 | SENPVTVDDATMIATSFPEFMDLMAGLGAKIE 448  +E PVT+DDA MIATSFPEFM LM GLGA+IE  TEKPVTIDDANMIATSFPEFMGLMKGLGAEIE 452 |  |

>WP_127878736.1 MULTISPECIES: 3-phosphoshikimate 1-carboxyvinyltransferase [unclassified Mesorhizobium]

TGT73399.1 3-phosphoshikimate 1-carboxyvinyltransferase [Mesorhizobium sp. M2E.F.Ca.ET.166.01.1.1]

TGV99915.1 3-phosphoshikimate 1-carboxyvinyltransferase [Mesorhizobium sp. M2E.F.Ca.ET.154.01.1.1]

Length=452

Score = 689 bits (1777), Expect = 0.0, Method: Compositional matrix adjust.

| Identities | = 353/452 (78%), Positives = 388/452 (86%), Gaps = 4/452 (1%) |  |
| --- | --- | --- |
| Query 1 | MLHGASSRPATARKSSGLSGTVRIPGDKSISHRSFMFGGLASGETRITGLLEGEDVINTG | 60 |
| Sbjct 1 | M H A+++PATARKS L+GT R+PGDKSISHRSFMFGGLASGETRITGLLEGEDV+ TG MSHSAAAKPATARKSPALAGTARVPGDKSISHRSFMFGGLASGETRITGLLEGEDVMRTG | 60 |
| Query 61 | KAMQAMGARIRKEGDTWIIDGVGNGGLLAPEAPLDFGNAATGCRLTMGLVGVYDFDSTFI | 120 |
| Sbjct 61 | AM+AMGA I K G W+I G GNG LL PE PLDFGNA TG RLTMGLVG YD ++TFI AAMKAMGAHIEKNGAEWVIRGTGNGALLQPEGPLDFGNAGTGSRLTMGLVGTYDMETTFI | 120 |
| Query 121 | GDASLTKRPMGRVLNPLREMGVQV-KSEDGDRLPVTLRGPKTPTPITYRVPMASAQVKSA | 179 |
| Sbjct 121 | GDASL+ RPMGRVL+PLR+MGVQV K+ GDR+P+TLRGPK PITYRVPMASAQVKSA GDASLSGRPMGRVLDPLRQMGVQVLKAAPGDRMPITLRGPKHAAPITYRVPMASAQVKSA | 180 |
| Query 180 | VLLAGLNTPGITTVIEPIMTRDHTEKMLQGFGANLTVETDADGVRTIRLEGRGKLTGQVI | 239 |
| Sbjct 181 | VLLAGLNTPGITTVIEP+MTRDHTEKML+GFGANLTVETD GVR I +EG+GKLTGQ I  VLLAGLNTPGITTVIEPVMTRDHTEKMLKGFGANLTVETDERGVRHIFIEGQGKLTGQTI | 240 |

| Query | 240 | DVPGDPSSTAFPLVAALLVPGSDVTILNVLMNPTRTGLILTLQEMGADIEVINPRLAGGE | 299 |
| --- | --- | --- | --- |
| Sbjct | 241 | VPGDPSS FPLVAAL+VPGSD+ I NVLMNPTRTGL+LTLQEMG I+++NPR AGGE AVPGDPSSAGFPLVAALIVPGSDIVIENVLMNPTRTGLLLTLQEMGGRIDILNPRNAGGE | 300 |
| Query | 300 | DVADLRVRSSTLKGVTVPEDRAPSMIDEYPILavaaafaegaTVMNGLEELRVKESDRLS | 359 |
| Sbjct | 301 | DVADLRVRSS LKGV VP +RAPSMIDEYP+LAVAA+FAEG T+M GLEELRVKESDRLS DVADLRVRSSDLKGVVVPPERAPSMIDEYPVLAVAASFAEGETLMQGLEELRVKESDRLS | 360 |
| Query | 360 | AVANGLKLNGVDCDEGETSLVVRGRPDGKGLG---NASGAAVATHLDHRIAMSFLVMGLV | 416 |
| Sbjct | 361 | AVANGLKLNGVDC EGE SL VRG+P GKGLG N V THLDHRIAMSF+VMGL AVANGLKLNGVDCTEGEASLAVRGKPGGKGLGTHPNGPDTVVKTHLDHRIAMSFMVMGLA | 420 |
| Query  Sbjct | 417  421 | SENPVTVDDATMIATSFPEFMDLMAGLGAKIE 448  +E PVT+DDA MIATSFPEFM LM GLGA+IE  TEKPVTIDDANMIATSFPEFMGLMKGLGAEIE 452 |  |

>RWB78626.1 3-phosphoshikimate 1-carboxyvinyltransferase [Mesorhizobium sp.] Length=452

Score = 688 bits (1776), Expect = 0.0, Method: Compositional matrix adjust.

| Identities | = 352/452 (78%), Positives = 388/452 (86%), Gaps = 4/452 (1%) |  |
| --- | --- | --- |
| Query 1 | MLHGASSRPATARKSSGLSGTVRIPGDKSISHRSFMFGGLASGETRITGLLEGEDVINTG | 60 |
| Sbjct 1 | M H A+++PATARKS L+GT R+PGDKSISHRSFMFGGLASGETRITGLLEGEDV+ TG MSHSAAAKPATARKSPALAGTARVPGDKSISHRSFMFGGLASGETRITGLLEGEDVMRTG | 60 |
| Query 61 | KAMQAMGARIRKEGDTWIIDGVGNGGLLAPEAPLDFGNAATGCRLTMGLVGVYDFDSTFI | 120 |
| Sbjct 61 | AM+AMGA I K G W+I G GNG LL PE PLDFGNA TG RLTMGLVG YD ++TFI AAMKAMGAHIEKNGAEWVIRGTGNGALLQPEGPLDFGNAGTGSRLTMGLVGTYDMETTFI | 120 |
| Query 121 | GDASLTKRPMGRVLNPLREMGVQV-KSEDGDRLPVTLRGPKTPTPITYRVPMASAQVKSA | 179 |
| Sbjct 121 | GDASL+ RPMGRVL+PLR+MGVQV K+ GDR+P+TLRGPK PITYRVPMASAQVKSA GDASLSGRPMGRVLDPLRQMGVQVLKAAPGDRMPITLRGPKHAAPITYRVPMASAQVKSA | 180 |
| Query 180 | VLLAGLNTPGITTVIEPIMTRDHTEKMLQGFGANLTVETDADGVRTIRLEGRGKLTGQVI | 239 |
| Sbjct 181 | VLLAGLNTPGITTVIEP+MTRDHTEKML+GFGANLTVETD GVR I +EG+GKLTGQ I VLLAGLNTPGITTVIEPVMTRDHTEKMLKGFGANLTVETDERGVRHIFIEGQGKLTGQTI | 240 |
| Query 240 | DVPGDPSSTAFPLVAALLVPGSDVTILNVLMNPTRTGLILTLQEMGADIEVINPRLAGGE | 299 |
| Sbjct 241 | VPGDPSS FPLVAAL+VPGSD+ I NVLMNPTRTGL+LTLQEMG I+++NPR AGGE AVPGDPSSAGFPLVAALIVPGSDIVIENVLMNPTRTGLLLTLQEMGGRIDILNPRNAGGE | 300 |
| Query 300 | DVADLRVRSSTLKGVTVPEDRAPSMIDEYPILavaaafaegaTVMNGLEELRVKESDRLS | 359 |
| Sbjct 301 | DVADLRVRSS LKGV VP +RAPSMIDEYP+LAVAA+FAEG T+M GLEELRVKESDRLS DVADLRVRSSELKGVVVPPERAPSMIDEYPVLAVAASFAEGETLMQGLEELRVKESDRLS | 360 |
| Query 360 | AVANGLKLNGVDCDEGETSLVVRGRPDGKGLG---NASGAAVATHLDHRIAMSFLVMGLV | 416 |
| Sbjct 361 | AVANGLKLNGVDC EGE +L V G+P GKGLG N AV THLDHRIAMSFL+MGL AVANGLKLNGVDCTEGEATLAVSGKPGGKGLGAHPNGPDTAVKTHLDHRIAMSFLIMGLA | 420 |
| Query 417  Sbjct 421 | SENPVTVDDATMIATSFPEFMDLMAGLGAKIE 448  +E PVT+DDA MIATSFPEFM LM GLGA+IE  TEKPVTIDDANMIATSFPEFMGLMKGLGAEIE 452 |  |

>WP_040994398.1 3-phosphoshikimate 1-carboxyvinyltransferase [Mesorhizobium sp. SOD10]

Length=452

Score = 688 bits (1776), Expect = 0.0, Method: Compositional matrix adjust. Identities = 352/452 (78%), Positives = 387/452 (86%), Gaps = 4/452 (1%)

| Query | 1 | MLHGASSRPATARKSSGLSGTVRIPGDKSISHRSFMFGGLASGETRITGLLEGEDVINTG | 60 |
| --- | --- | --- | --- |
| Sbjct | 1 | M H A+++PATARKS L+GT R+PGDKSISHRSFMFGGLASGETRITGLLEGEDV+ TG MSHSAAAKPATARKSPALAGTARVPGDKSISHRSFMFGGLASGETRITGLLEGEDVMRTG | 60 |
| Query | 61 | KAMQAMGARIRKEGDTWIIDGVGNGGLLAPEAPLDFGNAATGCRLTMGLVGVYDFDSTFI | 120 |
| Sbjct | 61 | AM+AMGA I K+G W+I G GNG LL PE PLDFGNA TG RLTMGLVG YD ++TFI  AAMKAMGAHIEKKGAEWVIRGTGNGALLQPEGPLDFGNAGTGSRLTMGLVGTYDMETTFI | 120 |

| Query | 121 | GDASLTKRPMGRVLNPLREMGVQV-KSEDGDRLPVTLRGPKTPTPITYRVPMASAQVKSA | 179 |
| --- | --- | --- | --- |
| Sbjct | 121 | GDASL+ RPMGRVL+PLR+MGVQV K+ GDR+P+TLRGPK PITYRVPMASAQVKSA GDASLSGRPMGRVLDPLRQMGVQVLKAAPGDRMPITLRGPKHAAPITYRVPMASAQVKSA | 180 |
| Query | 180 | VLLAGLNTPGITTVIEPIMTRDHTEKMLQGFGANLTVETDADGVRTIRLEGRGKLTGQVI | 239 |
| Sbjct | 181 | VLLAGLNTPGITTVIEP+MTRDHTEKML+GFGANLTVETD GVR I +EG+GKLTGQ I VLLAGLNTPGITTVIEPVMTRDHTEKMLKGFGANLTVETDERGVRHIFIEGQGKLTGQTI | 240 |
| Query | 240 | DVPGDPSSTAFPLVAALLVPGSDVTILNVLMNPTRTGLILTLQEMGADIEVINPRLAGGE | 299 |
| Sbjct | 241 | VPGDPSS FPLVAAL+VP SD+ I NVLMNPTRTGL+LTLQEMG I+++NPR AGGE AVPGDPSSAGFPLVAALIVPDSDIIIENVLMNPTRTGLLLTLQEMGGKIDILNPRNAGGE | 300 |
| Query | 300 | DVADLRVRSSTLKGVTVPEDRAPSMIDEYPILavaaafaegaTVMNGLEELRVKESDRLS | 359 |
| Sbjct | 301 | DVADLRVR S LKGV VP +RAPSMIDEYP+LAVAA+FAEG T+M GLEELRVKESDRLS DVADLRVRYSELKGVVVPAERAPSMIDEYPVLAVAASFAEGETLMQGLEELRVKESDRLS | 360 |
| Query | 360 | AVANGLKLNGVDCDEGETSLVVRGRPDGKGLG---NASGAAVATHLDHRIAMSFLVMGLV | 416 |
| Sbjct | 361 | AVANGLKLNGVDC EGE SL VRG+P GKGLG N V THLDHRIAMSFLVMGL AVANGLKLNGVDCTEGEASLAVRGKPGGKGLGAHPNGQDTVVKTHLDHRIAMSFLVMGLA | 420 |
| Query  Sbjct | 417  421 | SENPVTVDDATMIATSFPEFMDLMAGLGAKIE 448  +E PVT+DDA MIATSFPEFM LM GLGA+IE  TEKPVTIDDANMIATSFPEFMGLMKGLGAEIE 452 |  |

>WP_127302779.1 MULTISPECIES: 3-phosphoshikimate 1-carboxyvinyltransferase [unclassified Mesorhizobium]

RUX34297.1 3-phosphoshikimate 1-carboxyvinyltransferase [Mesorhizobium sp. M2A.F.Ca.ET.042.01.1.1]

Length=452

Score = 688 bits (1776), Expect = 0.0, Method: Compositional matrix adjust.

| Identities | = 353/452 (78%), Positives = 387/452 (86%), Gaps = 4/452 (1%) |  |
| --- | --- | --- |
| Query 1 | MLHGASSRPATARKSSGLSGTVRIPGDKSISHRSFMFGGLASGETRITGLLEGEDVINTG | 60 |
| Sbjct 1 | M H A+++PATARKS L+GT R+PGDKSISHRSFMFGGLASGETRITGLLEGEDV+ TG MSHSAAAKPATARKSPALAGTARVPGDKSISHRSFMFGGLASGETRITGLLEGEDVMRTG | 60 |
| Query 61 | KAMQAMGARIRKEGDTWIIDGVGNGGLLAPEAPLDFGNAATGCRLTMGLVGVYDFDSTFI | 120 |
| Sbjct 61 | AM+AMGA I K G W+I G GNG LL PE PLDFGNA TG RLTMGLVG YD ++TFI AAMKAMGAHIEKNGAEWVIRGTGNGALLQPEGPLDFGNAGTGSRLTMGLVGTYDMETTFI | 120 |
| Query 121 | GDASLTKRPMGRVLNPLREMGVQV-KSEDGDRLPVTLRGPKTPTPITYRVPMASAQVKSA | 179 |
| Sbjct 121 | GDASL+ RPMGRVL+PLR+MGVQV K+ GDR+P+TLRGPK PITYRVPMASAQVKSA GDASLSGRPMGRVLDPLRQMGVQVLKAAPGDRMPITLRGPKHAAPITYRVPMASAQVKSA | 180 |
| Query 180 | VLLAGLNTPGITTVIEPIMTRDHTEKMLQGFGANLTVETDADGVRTIRLEGRGKLTGQVI | 239 |
| Sbjct 181 | VLLAGLNTPGITTVIEP+MTRDHTEKML+GFGANLTVETD GVR I +EG+GKLTGQ I VLLAGLNTPGITTVIEPVMTRDHTEKMLKGFGANLTVETDERGVRHIFIEGQGKLTGQTI | 240 |
| Query 240 | DVPGDPSSTAFPLVAALLVPGSDVTILNVLMNPTRTGLILTLQEMGADIEVINPRLAGGE | 299 |
| Sbjct 241 | VPGDPSS FPLVAAL+VPGSD+ I NVLMNPTRTGL+LTLQEMG I+++NPR AGGE AVPGDPSSAGFPLVAALIVPGSDIVIENVLMNPTRTGLLLTLQEMGGRIDILNPRNAGGE | 300 |
| Query 300 | DVADLRVRSSTLKGVTVPEDRAPSMIDEYPILavaaafaegaTVMNGLEELRVKESDRLS | 359 |
| Sbjct 301 | DVADLRVR S LKGV VP +RAPSMIDEYP+LAVAA+FAEG T+M GLEELRVKESDRL+ DVADLRVRYSELKGVVVPPERAPSMIDEYPVLAVAASFAEGETLMQGLEELRVKESDRLA | 360 |
| Query 360 | AVANGLKLNGVDCDEGETSLVVRGRPDGKGLG---NASGAAVATHLDHRIAMSFLVMGLV | 416 |
| Sbjct 361 | AVA GLKLNGVDC EGE SL VRGRP GKGLG N AV THLDHRIAMSFLVMGL AVATGLKLNGVDCTEGEASLAVRGRPGGKGLGAHPNGPDTAVKTHLDHRIAMSFLVMGLA | 420 |
| Query 417  Sbjct 421 | SENPVTVDDATMIATSFPEFMDLMAGLGAKIE 448  +E PVT+DDA MIATSFPEFM LM GLGA+IE  TEKPVTIDDANMIATSFPEFMGLMKGLGAEIE 452 |  |

>WP_071036801.1 3-phosphoshikimate 1-carboxyvinyltransferase [Mesorhizobium sp. LCM 4576]

OHV58609.1 3-phosphoshikimate 1-carboxyvinyltransferase [Mesorhizobium sp. LCM 4576]

Length=452

Score = 688 bits (1776), Expect = 0.0, Method: Compositional matrix adjust.

| Identities | = 353/452 (78%), Positives = 388/452 (86%), Gaps = 4/452 (1%) |  |
| --- | --- | --- |
| Query 1 | MLHGASSRPATARKSSGLSGTVRIPGDKSISHRSFMFGGLASGETRITGLLEGEDVINTG | 60 |
| Sbjct 1 | M H A+++PATARKS L+GT R+PGDKSISHRSFMFGGLASGETRITGLLEGEDV+ TG MSHSAAAKPATARKSPALAGTARVPGDKSISHRSFMFGGLASGETRITGLLEGEDVMRTG | 60 |
| Query 61 | KAMQAMGARIRKEGDTWIIDGVGNGGLLAPEAPLDFGNAATGCRLTMGLVGVYDFDSTFI | 120 |
| Sbjct 61 | AM+AMGA I K+ W+I G GNG LL PE PLDFGNA TG RLTMGLVG YD ++TFI AAMKAMGAHIEKKDGEWVIRGTGNGALLQPEGPLDFGNAGTGSRLTMGLVGTYDMETTFI | 120 |
| Query 121 | GDASLTKRPMGRVLNPLREMGVQV-KSEDGDRLPVTLRGPKTPTPITYRVPMASAQVKSA | 179 |
| Sbjct 121 | GDASL+ RPMGRVL+PLR+MGVQV K+ GDR+P+TLRGPK PITYRVPMASAQVKSA GDASLSGRPMGRVLDPLRQMGVQVLKAAPGDRMPITLRGPKHAAPITYRVPMASAQVKSA | 180 |
| Query 180 | VLLAGLNTPGITTVIEPIMTRDHTEKMLQGFGANLTVETDADGVRTIRLEGRGKLTGQVI | 239 |
| Sbjct 181 | VLLAGLNTPGITTVIEP+MTRDHTEKML+GFGANLTVETD GVR I +EG+GKLTGQ I VLLAGLNTPGITTVIEPVMTRDHTEKMLKGFGANLTVETDERGVRHIFIEGQGKLTGQTI | 240 |
| Query 240 | DVPGDPSSTAFPLVAALLVPGSDVTILNVLMNPTRTGLILTLQEMGADIEVINPRLAGGE | 299 |
| Sbjct 241 | VPGDPSS FPLVAAL+VPGSDVTI NVLMNPTRTGL+LTLQEMG I+++NPR AGGE AVPGDPSSAGFPLVAALIVPGSDVTIENVLMNPTRTGLLLTLQEMGGRIDILNPRNAGGE | 300 |
| Query 300 | DVADLRVRSSTLKGVTVPEDRAPSMIDEYPILavaaafaegaTVMNGLEELRVKESDRLS | 359 |
| Sbjct 301 | DVADLRVR S LKGV VP +RAPSMIDEYP+LAVAA+FAEG T+M GLEELRVKESDRL+ DVADLRVRYSELKGVVVPPERAPSMIDEYPVLAVAASFAEGETLMQGLEELRVKESDRLA | 360 |
| Query 360 | AVANGLKLNGVDCDEGETSLVVRGRPDGKGLG---NASGAAVATHLDHRIAMSFLVMGLV | 416 |
| Sbjct 361 | AVANGLKLNGVDC EGE SL VRG+P GKGLG N V THLDHRIAMSFLVMGL AVANGLKLNGVDCTEGEASLAVRGKPGGKGLGAHPNGQDTVVKTHLDHRIAMSFLVMGLA | 420 |
| Query 417  Sbjct 421 | SENPVTVDDATMIATSFPEFMDLMAGLGAKIE 448  +E PVT+DDA MIATSFPEFM LM GLGA+IE  TEKPVTIDDANMIATSFPEFMGLMKGLGAEIE 452 |  |

>WP_040583313.1 3-phosphoshikimate 1-carboxyvinyltransferase [Mesorhizobium amorphae] ANT49582.1 3-phosphoshikimate 1-carboxyvinyltransferase [Mesorhizobium amorphae

CCNWGS0123]

Length=452

Score = 688 bits (1776), Expect = 0.0, Method: Compositional matrix adjust.

| Identities | = 353/451 (78%), Positives = 384/451 (85%), Gaps = 4/451 (1%) |  |
| --- | --- | --- |
| Query 1 | MLHGASSRPATARKSSGLSGTVRIPGDKSISHRSFMFGGLASGETRITGLLEGEDVINTG | 60 |
| Sbjct 1 | M H A+ +PATARKS LSG R+PGDKSISHRSFMFGGLASGETRITGLLEGEDV+ TG MSHAAAPKPATARKSPALSGAARVPGDKSISHRSFMFGGLASGETRITGLLEGEDVMRTG | 60 |
| Query 61 | KAMQAMGARIRKEGDTWIIDGVGNGGLLAPEAPLDFGNAATGCRLTMGLVGVYDFDSTFI | 120 |
| Sbjct 61 | AM+AMGA I K D W+I G GNG LL PE PLDFGNA TG RLTMGLVG YD ++TFI AAMKAMGAHIEKHDDEWVIRGTGNGALLQPEGPLDFGNAGTGSRLTMGLVGTYDMETTFI | 120 |
| Query 121 | GDASLTKRPMGRVLNPLREMGVQV-KSEDGDRLPVTLRGPKTPTPITYRVPMASAQVKSA | 179 |
| Sbjct 121 | GDASL+ RPMGRVL PLR+MGVQV K+ GDR+P+TLRGPK PITYRVPMASAQVKSA GDASLSGRPMGRVLEPLRQMGVQVLKATPGDRMPITLRGPKHAAPITYRVPMASAQVKSA | 180 |
| Query 180 | VLLAGLNTPGITTVIEPIMTRDHTEKMLQGFGANLTVETDADGVRTIRLEGRGKLTGQVI | 239 |
| Sbjct 181 | VLLAGLNTPGITTVIEP+MTRDHTEKML+GFGANLTVETD GVR I +EG+GKLTGQ I VLLAGLNTPGITTVIEPVMTRDHTEKMLKGFGANLTVETDERGVRHIFIEGQGKLTGQTI | 240 |
| Query 240 | DVPGDPSSTAFPLVAALLVPGSDVTILNVLMNPTRTGLILTLQEMGADIEVINPRLAGGE | 299 |
| Sbjct 241 | VPGDPSS FPLVAAL+VPGSD+TI NVLMNPTRTGL+LTLQEMG I+++NPR AGGE AVPGDPSSAGFPLVAALIVPGSDITIENVLMNPTRTGLLLTLQEMGGRIDIVNPRNAGGE | 300 |
| Query 300 | DVADLRVRSSTLKGVTVPEDRAPSMIDEYPILavaaafaegaTVMNGLEELRVKESDRLS | 359 |
| Sbjct 301 | DVADLRVR S LKGV VP +RAPSMIDEYP+LAVAA+FAEG T+M GLEELRVKESDRLS DVADLRVRYSELKGVIVPPERAPSMIDEYPVLAVAASFAEGETLMQGLEELRVKESDRLS | 360 |
| Query 360 | AVANGLKLNGVDCDEGETSLVVRGRPDGKGLG---NASGAAVATHLDHRIAMSFLVMGLV | 416 |
| Sbjct 361 | AVANGLKLNGVDC EGE +L VRGRP GKGLG N G V THLDHRIAMSFLVMGL  AVANGLKLNGVDCTEGEATLAVRGRPGGKGLGQHPNGQGTTVQTHLDHRIAMSFLVMGLA | 420 |

Query 417 SENPVTVDDATMIATSFPEFMDLMAGLGAKI 447

+E PVT+DD MIATSFPEFM LM GLGA+I Sbjct 421 TEKPVTIDDQAMIATSFPEFMGLMKGLGAEI 451

>RWL45835.1 3-phosphoshikimate 1-carboxyvinyltransferase [Mesorhizobium sp.] Length=452

Score = 688 bits (1775), Expect = 0.0, Method: Compositional matrix adjust.

| Identities | = 353/452 (78%), Positives = 387/452 (86%), Gaps = 4/452 (1%) |  |
| --- | --- | --- |
| Query 1 | MLHGASSRPATARKSSGLSGTVRIPGDKSISHRSFMFGGLASGETRITGLLEGEDVINTG | 60 |
| Sbjct 1 | M H A+++PATARKS L+GT R+PGDKSISHRSFMFGGLASGETRITGLLEGEDV+ TG MSHSAAAKPATARKSPALAGTARVPGDKSISHRSFMFGGLASGETRITGLLEGEDVMRTG | 60 |
| Query 61 | KAMQAMGARIRKEGDTWIIDGVGNGGLLAPEAPLDFGNAATGCRLTMGLVGVYDFDSTFI | 120 |
| Sbjct 61 | AM+AMGA I K G W+I G GNG LL PE PLDFGNA TG RLTMGLVG YD ++TFI AAMKAMGAHIEKNGAEWVIRGTGNGALLQPEGPLDFGNAGTGSRLTMGLVGTYDMETTFI | 120 |
| Query 121 | GDASLTKRPMGRVLNPLREMGVQV-KSEDGDRLPVTLRGPKTPTPITYRVPMASAQVKSA | 179 |
| Sbjct 121 | GDASL+ RPMGRVL+PLR+MGVQV K+ GDR+P+TLRGPK PITYRVPMASAQVKSA GDASLSGRPMGRVLDPLRQMGVQVLKAAPGDRMPITLRGPKHAAPITYRVPMASAQVKSA | 180 |
| Query 180 | VLLAGLNTPGITTVIEPIMTRDHTEKMLQGFGANLTVETDADGVRTIRLEGRGKLTGQVI | 239 |
| Sbjct 181 | VLLAGLNTPGITTVIEP+MTRDHTEKML+GFGANLTVETD GVR I + G+GKLTGQ I VLLAGLNTPGITTVIEPVMTRDHTEKMLKGFGANLTVETDERGVRHIFIGGQGKLTGQTI | 240 |
| Query 240 | DVPGDPSSTAFPLVAALLVPGSDVTILNVLMNPTRTGLILTLQEMGADIEVINPRLAGGE | 299 |
| Sbjct 241 | VPGDPSS FPLVAAL+VPGSD+ I NVLMNPTRTGL+LTLQEMG I+++NPR AGGE AVPGDPSSAGFPLVAALIVPGSDIVIENVLMNPTRTGLLLTLQEMGGRIDILNPRNAGGE | 300 |
| Query 300 | DVADLRVRSSTLKGVTVPEDRAPSMIDEYPILavaaafaegaTVMNGLEELRVKESDRLS | 359 |
| Sbjct 301 | DVADLRVRSS LKGV VP +RAPSMIDEYP+LAVAA+FAEG T+M GLEELRVKESDRL+ DVADLRVRSSDLKGVVVPPERAPSMIDEYPVLAVAASFAEGETLMQGLEELRVKESDRLA | 360 |
| Query 360 | AVANGLKLNGVDCDEGETSLVVRGRPDGKGLG---NASGAAVATHLDHRIAMSFLVMGLV | 416 |
| Sbjct 361 | AVANGLKLNGVDC EGE SL VRGRP GKGLG N V THLDHRIAMSFLVMGL AVANGLKLNGVDCTEGEASLAVRGRPGGKGLGAHPNGQDTVVKTHLDHRIAMSFLVMGLA | 420 |
| Query 417  Sbjct 421 | SENPVTVDDATMIATSFPEFMDLMAGLGAKIE 448  +E PVT+DDA MIATSFPEFM LM GLGA+IE  TEKPVTIDDANMIATSFPEFMGLMKGLGAEIE 452 |  |

>RWC46869.1 3-phosphoshikimate 1-carboxyvinyltransferase [Mesorhizobium sp.] RWF01541.1 3-phosphoshikimate 1-carboxyvinyltransferase [Mesorhizobium sp.] RWF70558.1 3-phosphoshikimate 1-carboxyvinyltransferase [Mesorhizobium sp.]

Length=452

Score = 687 bits (1774), Expect = 0.0, Method: Compositional matrix adjust.

| Identities | = 353/452 (78%), Positives = 386/452 (85%), Gaps = 4/452 (1%) |  |
| --- | --- | --- |
| Query 1 | MLHGASSRPATARKSSGLSGTVRIPGDKSISHRSFMFGGLASGETRITGLLEGEDVINTG | 60 |
| Sbjct 1 | M H A+ +PATARKSS LSG R+PGDKSISHRSFMFGGLASGETRITGLLEGEDV+ TG MSHTAAPKPATARKSSALSGAARVPGDKSISHRSFMFGGLASGETRITGLLEGEDVMRTG | 60 |
| Query 61 | KAMQAMGARIRKEGDTWIIDGVGNGGLLAPEAPLDFGNAATGCRLTMGLVGVYDFDSTFI | 120 |
| Sbjct 61 | AM+AMGA I K G W+I G GNG LL PE PLDFGNA TG RLTMGLVG YD ++TFI AAMKAMGAHIEKLGTEWVIRGTGNGALLQPEGPLDFGNAGTGSRLTMGLVGTYDMETTFI | 120 |
| Query 121 | GDASLTKRPMGRVLNPLREMGVQV-KSEDGDRLPVTLRGPKTPTPITYRVPMASAQVKSA | 179 |
| Sbjct 121 | GDASL+ RPMGRVL PLR+MGVQV K+ GDR+P+TLRGPK PITYRVPMASAQVKSA GDASLSGRPMGRVLEPLRQMGVQVLKATPGDRMPITLRGPKHAAPITYRVPMASAQVKSA | 180 |
| Query 180 | VLLAGLNTPGITTVIEPIMTRDHTEKMLQGFGANLTVETDADGVRTIRLEGRGKLTGQVI | 239 |
| Sbjct 181 | VLLAGLNTPGITTVIEP+MTRDHTEKML+GFGANL+VETD GVR I +EG+GKLTGQ I VLLAGLNTPGITTVIEPVMTRDHTEKMLRGFGANLSVETDERGVRHIFIEGQGKLTGQTI | 240 |
| Query 240 | DVPGDPSSTAFPLVAALLVPGSDVTILNVLMNPTRTGLILTLQEMGADIEVINPRLAGGE | 299 |
| Sbjct 241 | VPGDPSS FPLVAAL+VPGSD+TI NVLMNPTRTGL+LTLQEMGA I++++PR GGE  AVPGDPSSAGFPLVAALIVPGSDITIENVLMNPTRTGLLLTLQEMGARIDIVDPRNEGGE | 300 |

| Query | 300 | DVADLRVRSSTLKGVTVPEDRAPSMIDEYPILavaaafaegaTVMNGLEELRVKESDRLS | 359 |
| --- | --- | --- | --- |
| Sbjct | 301 | DVADLRVR S LK VTVP +RAP+MIDEYP+LAVAA+FAEG T+M GLEELRVKESDRLS DVADLRVRHSELKAVTVPPERAPTMIDEYPVLAVAASFAEGETLMQGLEELRVKESDRLS | 360 |
| Query | 360 | AVANGLKLNGVDCDEGETSLVVRGRPDGKGLG---NASGAAVATHLDHRIAMSFLVMGLV | 416 |
| Sbjct | 361 | AVA GLKLNGVDC EGE SL VRGRP GKGLG N G V THLDHRIAMSFLVMGL AVAEGLKLNGVDCTEGEASLAVRGRPGGKGLGQHPNGQGTIVQTHLDHRIAMSFLVMGLA | 420 |
| Query  Sbjct | 417  421 | SENPVTVDDATMIATSFPEFMDLMAGLGAKIE 448  +E PVT+DD TMIATSFPEFM LM GLGA+IE  TEKPVTIDDQTMIATSFPEFMGLMKGLGAEIE 452 |  |

>WP_127249697.1 MULTISPECIES: 3-phosphoshikimate 1-carboxyvinyltransferase [unclassified Mesorhizobium]

RWB41376.1 3-phosphoshikimate 1-carboxyvinyltransferase [Mesorhizobium sp.] RUW42509.1 3-phosphoshikimate 1-carboxyvinyltransferase [Mesorhizobium sp.

M2A.F.Ca.ET.015.02.1.1]

RVC93023.1 3-phosphoshikimate 1-carboxyvinyltransferase [Mesorhizobium sp. M2A.F.Ca.ET.017.03.2.1]

RVD00153.1 3-phosphoshikimate 1-carboxyvinyltransferase [Mesorhizobium sp. M2A.F.Ca.ET.029.05.1.1]

RWB59409.1 3-phosphoshikimate 1-carboxyvinyltransferase [Mesorhizobium sp.] Length=452

Score = 687 bits (1774), Expect = 0.0, Method: Compositional matrix adjust.

| Identities | = 353/452 (78%), Positives = 387/452 (86%), Gaps = 4/452 (1%) |  |
| --- | --- | --- |
| Query 1 | MLHGASSRPATARKSSGLSGTVRIPGDKSISHRSFMFGGLASGETRITGLLEGEDVINTG | 60 |
| Sbjct 1 | M H A+++ ATARKS L+GT R+PGDKSISHRSFMFGGLASGETRITGLLEGEDV+ TG MSHSAAAKQATARKSPALAGTARVPGDKSISHRSFMFGGLASGETRITGLLEGEDVMRTG | 60 |
| Query 61 | KAMQAMGARIRKEGDTWIIDGVGNGGLLAPEAPLDFGNAATGCRLTMGLVGVYDFDSTFI | 120 |
| Sbjct 61 | AM+AMGA I K G W+I G GNG LL PE PLDFGNA TG RLTMGLVG YD ++TFI AAMKAMGAHIEKNGAEWVIRGTGNGALLQPEGPLDFGNAGTGSRLTMGLVGTYDMETTFI | 120 |
| Query 121 | GDASLTKRPMGRVLNPLREMGVQV-KSEDGDRLPVTLRGPKTPTPITYRVPMASAQVKSA | 179 |
| Sbjct 121 | GDASL+ RPMGRVL+PLR+MGVQV K+ GDR+P+TLRGPK PITYRVPMASAQVKSA GDASLSGRPMGRVLDPLRQMGVQVLKAAPGDRMPITLRGPKHAAPITYRVPMASAQVKSA | 180 |
| Query 180 | VLLAGLNTPGITTVIEPIMTRDHTEKMLQGFGANLTVETDADGVRTIRLEGRGKLTGQVI | 239 |
| Sbjct 181 | VLLAGLNT GITTVIEP+MTRDHTEKML+GFGANLTVETD GVR I +EG+GKLTGQ I VLLAGLNTAGITTVIEPVMTRDHTEKMLKGFGANLTVETDERGVRHIFIEGQGKLTGQTI | 240 |
| Query 240 | DVPGDPSSTAFPLVAALLVPGSDVTILNVLMNPTRTGLILTLQEMGADIEVINPRLAGGE | 299 |
| Sbjct 241 | VPGDPSS FPLVAAL+VPGSD+ I NVLMNPTRTGL+LTLQEMG I+++NPR AGGE AVPGDPSSAGFPLVAALIVPGSDIVIENVLMNPTRTGLLLTLQEMGGRIDILNPRNAGGE | 300 |
| Query 300 | DVADLRVRSSTLKGVTVPEDRAPSMIDEYPILavaaafaegaTVMNGLEELRVKESDRLS | 359 |
| Sbjct 301 | DVADLRVR S LKGVTVP +RAPSMIDEYP+LAVAA+FAEG T+M GLEELRVKESDRLS DVADLRVRYSELKGVTVPPERAPSMIDEYPVLAVAASFAEGETLMQGLEELRVKESDRLS | 360 |
| Query 360 | AVANGLKLNGVDCDEGETSLVVRGRPDGKGLG---NASGAAVATHLDHRIAMSFLVMGLV | 416 |
| Sbjct 361 | AVANGLKLNGVDC EGE SL VRGRP GKGLG N AV THLDHRIAMSFL+MGL AVANGLKLNGVDCTEGEASLAVRGRPGGKGLGAHPNGPDTAVKTHLDHRIAMSFLIMGLA | 420 |
| Query 417  Sbjct 421 | SENPVTVDDATMIATSFPEFMDLMAGLGAKIE 448  +E PVT+DDA MIATSFPEFM LM GLGA+IE  TEKPVTIDDANMIATSFPEFMGLMKGLGAEIE 452 |  |

```
>CDX12151.1 3-phosphoshikimate 1-carboxyvinyltransferase [Mesorhizobium sp. SOD10]
Length=478

Score = 687 bits (1774), Expect = 0.0, Method: Compositional matrix adjust. Identities = 352/452 (78%), Positives = 387/452 (86%), Gaps = 4/452 (1%)

Query 1	MLHGASSRPATARKSSGLSGTVRIPGDKSISHRSFMFGGLASGETRITGLLEGEDVINTG 60 M H A+++PATARKS L+GT R+PGDKSISHRSFMFGGLASGETRITGLLEGEDV+ TG
Sbjct 27	MSHSAAAKPATARKSPALAGTARVPGDKSISHRSFMFGGLASGETRITGLLEGEDVMRTG 86
```

| Query | 61 | KAMQAMGARIRKEGDTWIIDGVGNGGLLAPEAPLDFGNAATGCRLTMGLVGVYDFDSTFI | 120 |
| --- | --- | --- | --- |
| Sbjct | 87 | AM+AMGA I K+G W+I G GNG LL PE PLDFGNA TG RLTMGLVG YD ++TFI AAMKAMGAHIEKKGAEWVIRGTGNGALLQPEGPLDFGNAGTGSRLTMGLVGTYDMETTFI | 146 |
| Query | 121 | GDASLTKRPMGRVLNPLREMGVQV-KSEDGDRLPVTLRGPKTPTPITYRVPMASAQVKSA | 179 |
| Sbjct | 147 | GDASL+ RPMGRVL+PLR+MGVQV K+ GDR+P+TLRGPK PITYRVPMASAQVKSA GDASLSGRPMGRVLDPLRQMGVQVLKAAPGDRMPITLRGPKHAAPITYRVPMASAQVKSA | 206 |
| Query | 180 | VLLAGLNTPGITTVIEPIMTRDHTEKMLQGFGANLTVETDADGVRTIRLEGRGKLTGQVI | 239 |
| Sbjct | 207 | VLLAGLNTPGITTVIEP+MTRDHTEKML+GFGANLTVETD GVR I +EG+GKLTGQ I VLLAGLNTPGITTVIEPVMTRDHTEKMLKGFGANLTVETDERGVRHIFIEGQGKLTGQTI | 266 |
| Query | 240 | DVPGDPSSTAFPLVAALLVPGSDVTILNVLMNPTRTGLILTLQEMGADIEVINPRLAGGE | 299 |
| Sbjct | 267 | VPGDPSS FPLVAAL+VP SD+ I NVLMNPTRTGL+LTLQEMG I+++NPR AGGE AVPGDPSSAGFPLVAALIVPDSDIIIENVLMNPTRTGLLLTLQEMGGKIDILNPRNAGGE | 326 |
| Query | 300 | DVADLRVRSSTLKGVTVPEDRAPSMIDEYPILavaaafaegaTVMNGLEELRVKESDRLS | 359 |
| Sbjct | 327 | DVADLRVR S LKGV VP +RAPSMIDEYP+LAVAA+FAEG T+M GLEELRVKESDRLS DVADLRVRYSELKGVVVPAERAPSMIDEYPVLAVAASFAEGETLMQGLEELRVKESDRLS | 386 |
| Query | 360 | AVANGLKLNGVDCDEGETSLVVRGRPDGKGLG---NASGAAVATHLDHRIAMSFLVMGLV | 416 |
| Sbjct | 387 | AVANGLKLNGVDC EGE SL VRG+P GKGLG N V THLDHRIAMSFLVMGL AVANGLKLNGVDCTEGEASLAVRGKPGGKGLGAHPNGQDTVVKTHLDHRIAMSFLVMGLA | 446 |
| Query  Sbjct | 417  447 | SENPVTVDDATMIATSFPEFMDLMAGLGAKIE 448  +E PVT+DDA MIATSFPEFM LM GLGA+IE  TEKPVTIDDANMIATSFPEFMGLMKGLGAEIE 478 |  |

>RWM27419.1 3-phosphoshikimate 1-carboxyvinyltransferase [Mesorhizobium sp.] TIO77774.1 3-phosphoshikimate 1-carboxyvinyltransferase [Mesorhizobium sp.] TIO85634.1 3-phosphoshikimate 1-carboxyvinyltransferase [Mesorhizobium sp.]

Length=452

Score = 687 bits (1774), Expect = 0.0, Method: Compositional matrix adjust.

| Identities | = 351/451 (78%), Positives = 388/451 (86%), Gaps = 4/451 (1%) |  |
| --- | --- | --- |
| Query 1 | MLHGASSRPATARKSSGLSGTVRIPGDKSISHRSFMFGGLASGETRITGLLEGEDVINTG | 60 |
| Sbjct 1 | M H A+++PATARKS L+GT R+PGDKSISHRSFMFGGLASGETRITGLLEGEDV+ TG MSHSAAAKPATARKSPALAGTARVPGDKSISHRSFMFGGLASGETRITGLLEGEDVMRTG | 60 |
| Query 61 | KAMQAMGARIRKEGDTWIIDGVGNGGLLAPEAPLDFGNAATGCRLTMGLVGVYDFDSTFI | 120 |
| Sbjct 61 | AM+AMGA I K+G W+I G GNG LL PE PLDFGNA TG RLTMGLVG YD ++TFI AAMKAMGAHIEKKGAEWVIRGTGNGALLEPEGPLDFGNAGTGSRLTMGLVGTYDMETTFI | 120 |
| Query 121 | GDASLTKRPMGRVLNPLREMGVQV-KSEDGDRLPVTLRGPKTPTPITYRVPMASAQVKSA | 179 |
| Sbjct 121 | GDASL+ RPMGRVL PLR+MGVQV K+ GDR+P+TLRGPK PITYRVPMASAQVKSA GDASLSGRPMGRVLEPLRQMGVQVLKAAPGDRMPITLRGPKHAAPITYRVPMASAQVKSA | 180 |
| Query 180 | VLLAGLNTPGITTVIEPIMTRDHTEKMLQGFGANLTVETDADGVRTIRLEGRGKLTGQVI | 239 |
| Sbjct 181 | VLLAGLNTPGITTVIEP+MTRDHTEKML+GFGANLTVETD GVR I +EG+GKLTGQ I VLLAGLNTPGITTVIEPVMTRDHTEKMLKGFGANLTVETDERGVRHIFIEGQGKLTGQTI | 240 |
| Query 240 | DVPGDPSSTAFPLVAALLVPGSDVTILNVLMNPTRTGLILTLQEMGADIEVINPRLAGGE | 299 |
| Sbjct 241 | VPGDPSS FPLVAAL+VPGSD+ I N+LMNPTRTGL+LTLQEMG I+++NPR AGGE AVPGDPSSAGFPLVAALIVPGSDIVIENMLMNPTRTGLLLTLQEMGGRIDILNPRNAGGE | 300 |
| Query 300 | DVADLRVRSSTLKGVTVPEDRAPSMIDEYPILavaaafaegaTVMNGLEELRVKESDRLS | 359 |
| Sbjct 301 | DVADLRVRSS LKGV VP +RAPSMIDEYP+LAVAA+FA+G T+M GLEELRVKESDRL+ DVADLRVRSSDLKGVVVPPERAPSMIDEYPVLAVAASFADGETLMQGLEELRVKESDRLA | 360 |
| Query 360 | AVANGLKLNGVDCDEGETSLVVRGRPDGKGLG---NASGAAVATHLDHRIAMSFLVMGLV | 416 |
| Sbjct 361 | AVANGLKLNGVDC EGE SL VRG+P GKGLG N AV THLDHRIAMSFLVMGL AVANGLKLNGVDCTEGEASLAVRGKPGGKGLGAHPNGPDTAVKTHLDHRIAMSFLVMGLA | 420 |
| Query 417  Sbjct 421 | SENPVTVDDATMIATSFPEFMDLMAGLGAKI 447  +E PVT+DDA MIATSFPEFM LM GLGA+I  TEKPVTIDDANMIATSFPEFMGLMTGLGAEI 451 |  |

Score = 687 bits (1774), Expect = 0.0, Method: Compositional matrix adjust.

| Identities | = 351/451 (78%), Positives = 388/451 (86%), Gaps = 4/451 (1%) |  |
| --- | --- | --- |
| Query 1 | MLHGASSRPATARKSSGLSGTVRIPGDKSISHRSFMFGGLASGETRITGLLEGEDVINTG | 60 |
| Sbjct 1 | M H A+++PATARKS L+GT R+PGDKSISHRSFMFGGLASGETRITGLLEGEDV+ TG MSHSAAAKPATARKSPALAGTARVPGDKSISHRSFMFGGLASGETRITGLLEGEDVMRTG | 60 |
| Query 61 | KAMQAMGARIRKEGDTWIIDGVGNGGLLAPEAPLDFGNAATGCRLTMGLVGVYDFDSTFI | 120 |
| Sbjct 61 | AM+AMGA I K+G W+I G GNG LL PE PLDFGNA TG RLTMGLVG YD ++TFI AAMKAMGAHIEKKGAEWVIRGTGNGALLEPEGPLDFGNAGTGSRLTMGLVGTYDMETTFI | 120 |
| Query 121 | GDASLTKRPMGRVLNPLREMGVQV-KSEDGDRLPVTLRGPKTPTPITYRVPMASAQVKSA | 179 |
| Sbjct 121 | GDASL+ RPMGRVL PLR+MGVQV K+ GDR+P+TLRGPK PITYRVPMASAQVKSA GDASLSGRPMGRVLEPLRQMGVQVLKAAPGDRMPITLRGPKHAAPITYRVPMASAQVKSA | 180 |
| Query 180 | VLLAGLNTPGITTVIEPIMTRDHTEKMLQGFGANLTVETDADGVRTIRLEGRGKLTGQVI | 239 |
| Sbjct 181 | VLLAGLNTPGITTVIEP+MTRDHTEKML+GFGANLTVETD GVR I +EG+GKLTGQ I VLLAGLNTPGITTVIEPVMTRDHTEKMLKGFGANLTVETDERGVRHIFIEGQGKLTGQTI | 240 |
| Query 240 | DVPGDPSSTAFPLVAALLVPGSDVTILNVLMNPTRTGLILTLQEMGADIEVINPRLAGGE | 299 |
| Sbjct 241 | VPGDPSS FPLVAAL+VPGSD+ I N+LMNPTRTGL+LTLQEMG I+++NPR AGGE AVPGDPSSAGFPLVAALIVPGSDIVIENMLMNPTRTGLLLTLQEMGGRIDILNPRNAGGE | 300 |
| Query 300 | DVADLRVRSSTLKGVTVPEDRAPSMIDEYPILavaaafaegaTVMNGLEELRVKESDRLS | 359 |
| Sbjct 301 | DVADLRVRSS LKGV VP +RAPSMIDEYP+LAVAA+FA+G T+M GLEELRVKESDRL+ DVADLRVRSSDLKGVVVPPERAPSMIDEYPVLAVAASFADGETLMQGLEELRVKESDRLA | 360 |
| Query 360 | AVANGLKLNGVDCDEGETSLVVRGRPDGKGLG---NASGAAVATHLDHRIAMSFLVMGLV | 416 |
| Sbjct 361 | AVANGLKLNGVDC EGE SL VRG+P GKGLG N AV THLDHRIAMSFLVMGL AVANGLKLNGVDCTEGEASLAVRGKPGGKGLGAHPNGPDTAVKTHLDHRIAMSFLVMGLA | 420 |
| Query 417  Sbjct 421 | SENPVTVDDATMIATSFPEFMDLMAGLGAKI 447  +E PVT+DDA MIATSFPEFM LM GLGA+I  TEKPVTIDDANMIATSFPEFMGLMTGLGAEI 451 |  |

>WP_065784613.1 MULTISPECIES: 3-phosphoshikimate 1-carboxyvinyltransferase [unclassified Ensifer]

OCP20976.1 3-phosphoshikimate 1-carboxyvinyltransferase [Ensifer sp. LC384] OCP21217.1 3-phosphoshikimate 1-carboxyvinyltransferase [Ensifer sp. LC54]

Length=448

Score = 687 bits (1773), Expect = 0.0, Method: Compositional matrix adjust.

| Identities | = 355/448 (79%), Positives = 396/448 (88%), Gaps = 0/448 (0%) |  |
| --- | --- | --- |
| Query 1 | MLHGASSRPATARKSSGLSGTVRIPGDKSISHRSFMFGGLASGETRITGLLEGEDVINTG | 60 |
| Sbjct 1 | M G ++PA AR+S L GTVRIPGDKSISHR+ + GGLA+GETRI GLLEGEDV NT MSAGVIAKPAIARRSGALGGTVRIPGDKSISHRALLLGGLAAGETRIAGLLEGEDVFNTA | 60 |
| Query 61 | KAMQAMGARIRKEGDTWIIDGVGNGGLLAPEAPLDFGNAATGCRLTMGLVGVYDFDSTFI | 120 |
| Sbjct 61 | +AMQA+G +RKEGDTWI+ GVGNG LLAPEAPL+FGNA TGCRLTMGLVGVYDF +TFI RAMQALGTTVRKEGDTWIVHGVGNGALLAPEAPLNFGNAGTGCRLTMGLVGVYDFATTFI | 120 |
| Query 121 | GDASLTKRPMGRVLNPLREMGVQVKSEDGDRLPVTLRGPKTPTPITYRVPMASAQVKSAV | 180 |
| Sbjct 121 | GDASL++RPMGRVL+PLR+MGVQV S +G RLPVTLRGPKT P++YRVPMASAQ+KSAV GDASLSRRPMGRVLDPLRQMGVQVTSAEGGRLPVTLRGPKTANPVSYRVPMASAQMKSAV | 180 |
| Query 181 | LLAGLNTPGITTVIEPIMTRDHTEKMLQGFGANLTVETDADGVRTIRLEGRGKLTGQVID | 240 |
| Sbjct 181 | LL GLN PGITTVIEP+MTRDHTE+MLQ FGANLTVET +G R IRLEG+G+LTGQ ID LLGGLNAPGITTVIEPVMTRDHTERMLQAFGANLTVETGNEGERIIRLEGQGRLTGQDID | 240 |
| Query 241 | VPGDPSSTAFPLVAALLVPGSDVTILNVLMNPTRTGLILTLQEMGADIEVINPRLAGGED | 300 |
| Sbjct 241 | VPGDPSS AFPLVAALLVPGSD+TILNVLMN TRTGLI+TLQ+MGA I+V+N R+AGGED VPGDPSSAAFPLVAALLVPGSDITILNVLMNATRTGLIVTLQQMGARIDVLNARIAGGED | 300 |
| Query 301 | VADLRVRSSTLKGVTVPEDRAPSMIDEYPILavaaafaegaTVMNGLEELRVKESDRLSA | 360 |
| Sbjct 301 | V DLRVRSS LKG+TVP+DRAPSMIDEYP+LAVAAAFAEG TVM GLEELRVKESDRLSA VVDLRVRSSELKGITVPDDRAPSMIDEYPVLAVAAAFAEGRTVMRGLEELRVKESDRLSA | 360 |
| Query 361 | VANGLKLNGVDCDEGETSLVVRGRPDGKGLGNASGAAVATHLDHRIAMSFLVMGLVSENP | 420 |
| Sbjct 361 | V GLKLNGVDC++G + LVV GRPDGKGLGNA+G AVAT LDHRIAMSFLVMGL SE+P  VVAGLKLNGVDCEDGSSFLVVHGRPDGKGLGNAAGTAVATDLDHRIAMSFLVMGLASEHP | 420 |

Query 421 VTVDDATMIATSFPEFMDLMAGLGAKIE 448 VTVDD TMIATSFP F++LM GLGA IE

Sbjct 421 VTVDDTTMIATSFPGFVELMTGLGATIE 448

>RWA68673.1 3-phosphoshikimate 1-carboxyvinyltransferase [Mesorhizobium sp.] Length=452

Score = 687 bits (1773), Expect = 0.0, Method: Compositional matrix adjust.

| Identities | = 351/452 (78%), Positives = 387/452 (86%), Gaps = 4/452 (1%) |  |
| --- | --- | --- |
| Query 1 | MLHGASSRPATARKSSGLSGTVRIPGDKSISHRSFMFGGLASGETRITGLLEGEDVINTG | 60 |
| Sbjct 1 | M H A+ +PATARKS LSGT R+PGDKSISHRSFMFGGLASGETRITGLLEGEDV+ TG MSHSAAVKPATARKSPALSGTARVPGDKSISHRSFMFGGLASGETRITGLLEGEDVMRTG | 60 |
| Query 61 | KAMQAMGARIRKEGDTWIIDGVGNGGLLAPEAPLDFGNAATGCRLTMGLVGVYDFDSTFI | 120 |
| Sbjct 61 | AM+AMGA I K+G W+I G GNG LL PE PLDFGNA TG RLTMGLVG YD ++TFI AAMKAMGAHIEKKGAEWVIRGTGNGALLEPEGPLDFGNAGTGSRLTMGLVGTYDMETTFI | 120 |
| Query 121 | GDASLTKRPMGRVLNPLREMGVQV-KSEDGDRLPVTLRGPKTPTPITYRVPMASAQVKSA | 179 |
| Sbjct 121 | GDASL+ RPMGRVL PLR+MGVQV K+ GDR+P+TLRGPK PITYRVPMASAQVKSA GDASLSGRPMGRVLEPLRQMGVQVLKAAPGDRMPITLRGPKHAAPITYRVPMASAQVKSA | 180 |
| Query 180 | VLLAGLNTPGITTVIEPIMTRDHTEKMLQGFGANLTVETDADGVRTIRLEGRGKLTGQVI | 239 |
| Sbjct 181 | VLLAGLNTPGITTVIEP+MTRDHTEKML+GFGANL+VETD GVR I +EG+GKL GQ I VLLAGLNTPGITTVIEPVMTRDHTEKMLKGFGANLSVETDERGVRHIFIEGQGKLIGQTI | 240 |
| Query 240 | DVPGDPSSTAFPLVAALLVPGSDVTILNVLMNPTRTGLILTLQEMGADIEVINPRLAGGE | 299 |
| Sbjct 241 | VPGDPSS FPLVAAL+VPGSD+TI NVLMNPTRTGL+LTLQEMG I+++NPR AGGE AVPGDPSSAGFPLVAALIVPGSDITIENVLMNPTRTGLLLTLQEMGGRIDILNPRNAGGE | 300 |
| Query 300 | DVADLRVRSSTLKGVTVPEDRAPSMIDEYPILavaaafaegaTVMNGLEELRVKESDRLS | 359 |
| Sbjct 301 | DVADLRVR S LKGVTVP +RAPSMIDEYP+LAVAA+FAEG T+M GLEELRVKESDRLS DVADLRVRYSELKGVTVPPERAPSMIDEYPVLAVAASFAEGETLMQGLEELRVKESDRLS | 360 |
| Query 360 | AVANGLKLNGVDCDEGETSLVVRGRPDGKGLG---NASGAAVATHLDHRIAMSFLVMGLV | 416 |
| Sbjct 361 | AVANGLKLNGVDC EG+ SL VRG+P GKGLG + V THLDHRIAMSFL+MGL AVANGLKLNGVDCSEGDASLAVRGKPSGKGLGAHPDGQDTKVKTHLDHRIAMSFLIMGLA | 420 |
| Query 417  Sbjct 421 | SENPVTVDDATMIATSFPEFMDLMAGLGAKIE 448  +E PVT+DDA MIATSFPEFM LM GLGA+IE  TEKPVTIDDANMIATSFPEFMGLMKGLGAEIE 452 |  |

>WP_145718824.1 3-phosphoshikimate 1-carboxyvinyltransferase [Mesorhizobium tianshanense] TWI35488.1 3-phosphoshikimate 1-carboxyvinyltransferase [Mesorhizobium tianshanense]

Length=452

Score = 687 bits (1773), Expect = 0.0, Method: Compositional matrix adjust.

| Identities | = 352/452 (78%), Positives = 385/452 (85%), Gaps = 4/452 (1%) |  |
| --- | --- | --- |
| Query 1 | MLHGASSRPATARKSSGLSGTVRIPGDKSISHRSFMFGGLASGETRITGLLEGEDVINTG | 60 |
| Sbjct 1 | M H A+ +PATARKS LSG R+PGDKSISHRSFMFGGLASGETRITGLLEGEDV+ TG MSHTAAPKPATARKSPALSGAARVPGDKSISHRSFMFGGLASGETRITGLLEGEDVMRTG | 60 |
| Query 61 | KAMQAMGARIRKEGDTWIIDGVGNGGLLAPEAPLDFGNAATGCRLTMGLVGVYDFDSTFI | 120 |
| Sbjct 61 | AM+AMGA I K G W+I G GNG LL PE PLDFGNA TG RLTMGLVG YD ++TFI TAMKAMGAHIEKHGAEWVIRGTGNGALLQPEGPLDFGNAGTGARLTMGLVGTYDMETTFI | 120 |
| Query 121 | GDASLTKRPMGRVLNPLREMGVQV-KSEDGDRLPVTLRGPKTPTPITYRVPMASAQVKSA | 179 |
| Sbjct 121 | GDASL+ RPMGRVL PLR+MGVQV K+ GDR+P+TLRGPK PITYRVPMASAQVKSA GDASLSGRPMGRVLEPLRQMGVQVLKATPGDRMPITLRGPKHAAPITYRVPMASAQVKSA | 180 |
| Query 180 | VLLAGLNTPGITTVIEPIMTRDHTEKMLQGFGANLTVETDADGVRTIRLEGRGKLTGQVI | 239 |
| Sbjct 181 | VL+AGLNTPGITTVIEP+MTRDHTEKML+GFGANL+VETD GVR I +EG+GKLTGQ I VLIAGLNTPGITTVIEPVMTRDHTEKMLRGFGANLSVETDERGVRHIFIEGQGKLTGQTI | 240 |
| Query 240 | DVPGDPSSTAFPLVAALLVPGSDVTILNVLMNPTRTGLILTLQEMGADIEVINPRLAGGE | 299 |
| Sbjct 241 | VPGDPSS FPLVAAL+VPGSDVTI NVLMNPTRTGL+LTLQEMGA I+++NPR GGE AVPGDPSSAGFPLVAALIVPGSDVTIENVLMNPTRTGLLLTLQEMGARIDIVNPRKEGGE | 300 |
| Query 300 | DVADLRVRSSTLKGVTVPEDRAPSMIDEYPILavaaafaegaTVMNGLEELRVKESDRLS | 359 |

| Sbjct | 301 | DVADLRVR S LKGVTVP +RAP+MIDEYP+LAVAA+FAEG T+M GLEELRVKESDRLS DVADLRVRHSELKGVTVPPERAPTMIDEYPVLAVAASFAEGETLMQGLEELRVKESDRLS | 360 |
| --- | --- | --- | --- |
| Query | 360 | AVANGLKLNGVDCDEGETSLVVRGRPDGKGLG---NASGAAVATHLDHRIAMSFLVMGLV | 416 |
| Sbjct | 361 | AVANGLKLNGVDC EGE +L VRGRP GKGLG N V THLDHRIAMSFLVMGL AVANGLKLNGVDCTEGEATLAVRGRPGGKGLGSHPNGQDTPVKTHLDHRIAMSFLVMGLA | 420 |
| Query  Sbjct | 417  421 | SENPVTVDDATMIATSFPEFMDLMAGLGAKIE 448  +E PVT+DD MIATSFPEFM LM GLGA+IE  TEKPVTIDDQAMIATSFPEFMGLMKGLGAEIE 452 |  |

>TIQ33697.1 3-phosphoshikimate 1-carboxyvinyltransferase [Mesorhizobium sp.] Length=452

Score = 687 bits (1772), Expect = 0.0, Method: Compositional matrix adjust.

| Identities | = 352/452 (78%), Positives = 387/452 (86%), Gaps = 4/452 (1%) |  |
| --- | --- | --- |
| Query 1 | MLHGASSRPATARKSSGLSGTVRIPGDKSISHRSFMFGGLASGETRITGLLEGEDVINTG | 60 |
| Sbjct 1 | M H A+ +PATARKS LSGT R+PGDKSISHRSFMFGGLASGETRITGLLEGEDV+ TG MSHAAAPKPATARKSPALSGTARVPGDKSISHRSFMFGGLASGETRITGLLEGEDVMRTG | 60 |
| Query 61 | KAMQAMGARIRKEGDTWIIDGVGNGGLLAPEAPLDFGNAATGCRLTMGLVGVYDFDSTFI | 120 |
| Sbjct 61 | AM+AMGA I K+G W+I G GNG LL PE PLDFGNA TG RLTMGLVG YD ++TFI AAMKAMGAHIEKKGAEWVIRGTGNGALLQPEGPLDFGNAGTGSRLTMGLVGTYDMETTFI | 120 |
| Query 121 | GDASLTKRPMGRVLNPLREMGVQV-KSEDGDRLPVTLRGPKTPTPITYRVPMASAQVKSA | 179 |
| Sbjct 121 | GDASL+ RPMGRVL PLR+MGVQV K+ GDR+P+TLRGPK PITYRVPMASAQVKSA GDASLSGRPMGRVLEPLRQMGVQVLKAAPGDRMPITLRGPKHAAPITYRVPMASAQVKSA | 180 |
| Query 180 | VLLAGLNTPGITTVIEPIMTRDHTEKMLQGFGANLTVETDADGVRTIRLEGRGKLTGQVI | 239 |
| Sbjct 181 | VLLAGLNTPGITTV+EP+MTRDHTEKML+GFGANLTV+TD GVR I +EG+GKLTGQ I VLLAGLNTPGITTVVEPVMTRDHTEKMLKGFGANLTVDTDERGVRHIFIEGQGKLTGQTI | 240 |
| Query 240 | DVPGDPSSTAFPLVAALLVPGSDVTILNVLMNPTRTGLILTLQEMGADIEVINPRLAGGE | 299 |
| Sbjct 241 | VPGDPSS FPLVAAL+VPGSD+ I NVLMNPTRTGL+LTLQEMG I+++NPR AGGE AVPGDPSSAGFPLVAALIVPGSDIVIENVLMNPTRTGLLLTLQEMGGKIDILNPRNAGGE | 300 |
| Query 300 | DVADLRVRSSTLKGVTVPEDRAPSMIDEYPILavaaafaegaTVMNGLEELRVKESDRLS | 359 |
| Sbjct 301 | DVADLRVRSS LKGV VP +RAPSMIDEYP+LAVAA+FAEG T+M GLEELRVKESDRL+ DVADLRVRSSDLKGVVVPPERAPSMIDEYPVLAVAASFAEGETLMQGLEELRVKESDRLA | 360 |
| Query 360 | AVANGLKLNGVDCDEGETSLVVRGRPDGKGLG---NASGAAVATHLDHRIAMSFLVMGLV | 416 |
| Sbjct 361 | AVANGLKLNGVDC EG SL VRG+P GKGLG N AV THLDHRIAMSFLVMGL AVANGLKLNGVDCTEGGASLAVRGKPGGKGLGAHPNGPDTAVKTHLDHRIAMSFLVMGLA | 420 |
| Query 417  Sbjct 421 | SENPVTVDDATMIATSFPEFMDLMAGLGAKIE 448  +E PVT+DDA MIATSFPEFM LM GLGA+IE  TEKPVTIDDANMIATSFPEFMGLMKGLGAEIE 452 |  |

>WP_071069874.1 3-phosphoshikimate 1-carboxyvinyltransferase [Mesorhizobium sp. LCM 4577]

OHV71448.1 3-phosphoshikimate 1-carboxyvinyltransferase [Mesorhizobium sp. LCM 4577]

Length=452

Score = 687 bits (1772), Expect = 0.0, Method: Compositional matrix adjust.

| Identities | = 352/452 (78%), Positives = 387/452 (86%), Gaps = 4/452 (1%) |  |
| --- | --- | --- |
| Query 1 | MLHGASSRPATARKSSGLSGTVRIPGDKSISHRSFMFGGLASGETRITGLLEGEDVINTG | 60 |
| Sbjct 1 | M H A+++PATARKS L+GT R+PGDKSISHRSFMFGGLASGETRITGLLEGEDV+ TG MSHSAAAKPATARKSPALAGTARVPGDKSISHRSFMFGGLASGETRITGLLEGEDVMRTG | 60 |
| Query 61 | KAMQAMGARIRKEGDTWIIDGVGNGGLLAPEAPLDFGNAATGCRLTMGLVGVYDFDSTFI | 120 |
| Sbjct 61 | AM+AMGA I K+ W+I G GNG LL PE PLDFGNA TG RLTMGLVG YD ++TFI AAMKAMGAHIEKKDGEWVIRGTGNGALLQPEGPLDFGNAGTGSRLTMGLVGTYDMETTFI | 120 |
| Query 121 | GDASLTKRPMGRVLNPLREMGVQV-KSEDGDRLPVTLRGPKTPTPITYRVPMASAQVKSA | 179 |
| Sbjct 121 | GDASL+ RPMGRVL+PLR+MGVQV K+ GDR+P+TLRGPK PITYRVPMAS QVKSA  GDASLSGRPMGRVLDPLRQMGVQVLKAAPGDRMPITLRGPKHAAPITYRVPMASXQVKSA | 180 |

| Query | 180 | VLLAGLNTPGITTVIEPIMTRDHTEKMLQGFGANLTVETDADGVRTIRLEGRGKLTGQVI | 239 |
| --- | --- | --- | --- |
| Sbjct | 181 | VLLAGLNTPGITTVIEP+MTRDHTEKML+GFGANLTVETD GVR I +EG+GKLTGQ I VLLAGLNTPGITTVIEPVMTRDHTEKMLKGFGANLTVETDERGVRHIFIEGQGKLTGQTI | 240 |
| Query | 240 | DVPGDPSSTAFPLVAALLVPGSDVTILNVLMNPTRTGLILTLQEMGADIEVINPRLAGGE | 299 |
| Sbjct | 241 | VPGDPSS FPLVAAL+VPGSDVTI NVLMNPTRTGL+LTLQEMG I+++NPR AGGE AVPGDPSSAGFPLVAALIVPGSDVTIENVLMNPTRTGLLLTLQEMGGRIDILNPRNAGGE | 300 |
| Query | 300 | DVADLRVRSSTLKGVTVPEDRAPSMIDEYPILavaaafaegaTVMNGLEELRVKESDRLS | 359 |
| Sbjct | 301 | DVADLRVR S LKGV VP +RAPSMIDEYP+LAVAA+FAEG T+M GLEELRVKESDRL+ DVADLRVRYSELKGVVVPPERAPSMIDEYPVLAVAASFAEGETLMQGLEELRVKESDRLA | 360 |
| Query | 360 | AVANGLKLNGVDCDEGETSLVVRGRPDGKGLG---NASGAAVATHLDHRIAMSFLVMGLV | 416 |
| Sbjct | 361 | AVANGLKLNGVDC EGE SL VRG+P GKGLG N V THLDHRIAMSFLVMGL AVANGLKLNGVDCTEGEASLAVRGKPGGKGLGAHPNGQDTVVKTHLDHRIAMSFLVMGLA | 420 |
| Query  Sbjct | 417  421 | SENPVTVDDATMIATSFPEFMDLMAGLGAKIE 448  +E PVT+DDA MIATSFPEFM LM GLGA+IE  TEKPVTIDDANMIATSFPEFMGLMKGLGAEIE 452 |  |

>CDX14990.1 3-phosphoshikimate 1-carboxyvinyltransferase [Mesorhizobium plurifarium] Length=478

Score = 687 bits (1772), Expect = 0.0, Method: Compositional matrix adjust.

| Identities | = 353/452 (78%), Positives = 387/452 (86%), Gaps = 4/452 (1%) |  |
| --- | --- | --- |
| Query 1 | MLHGASSRPATARKSSGLSGTVRIPGDKSISHRSFMFGGLASGETRITGLLEGEDVINTG | 60 |
| Sbjct 27 | M H A+++PATARKS L+GT R+PGDKSISHRSFMFGGLASGETRITGLLEGEDV+ TG MSHSAAAKPATARKSPALAGTARVPGDKSISHRSFMFGGLASGETRITGLLEGEDVMRTG | 86 |
| Query 61 | KAMQAMGARIRKEGDTWIIDGVGNGGLLAPEAPLDFGNAATGCRLTMGLVGVYDFDSTFI | 120 |
| Sbjct 87 | AM+AMGA I K+ W+I G GNG LL PE PLDFGNA TG RLTMGLVG YD ++TFI AAMKAMGAHIEKKDGEWVIRGTGNGALLQPEGPLDFGNAGTGSRLTMGLVGTYDMETTFI | 146 |
| Query 121 | GDASLTKRPMGRVLNPLREMGVQV-KSEDGDRLPVTLRGPKTPTPITYRVPMASAQVKSA | 179 |
| Sbjct 147 | GDASL+ RPMGRVL+PLR+MGVQV K+ GDR+P+TLRGPK PITYRVPMASAQVKSA GDASLSGRPMGRVLDPLRQMGVQVLKAAPGDRMPITLRGPKHAAPITYRVPMASAQVKSA | 206 |
| Query 180 | VLLAGLNTPGITTVIEPIMTRDHTEKMLQGFGANLTVETDADGVRTIRLEGRGKLTGQVI | 239 |
| Sbjct 207 | VLLAGLNTPGITTVIEP+MTRDHTEKML+GFGANLTVE D GVR I +EG+GKLTGQ I VLLAGLNTPGITTVIEPVMTRDHTEKMLKGFGANLTVEADERGVRHIFIEGQGKLTGQTI | 266 |
| Query 240 | DVPGDPSSTAFPLVAALLVPGSDVTILNVLMNPTRTGLILTLQEMGADIEVINPRLAGGE | 299 |
| Sbjct 267 | VPGDPSS FPLVAAL+VPGSDVTI NVLMNPTRTGL+LTLQEMG I+++NPR AGGE AVPGDPSSAGFPLVAALIVPGSDVTIENVLMNPTRTGLLLTLQEMGGRIDILNPRNAGGE | 326 |
| Query 300 | DVADLRVRSSTLKGVTVPEDRAPSMIDEYPILavaaafaegaTVMNGLEELRVKESDRLS | 359 |
| Sbjct 327 | DVADLRVR S LKGV VP +RAPSMIDEYP+LAVAA+FAEG T+M GLEELRVKESDRLS DVADLRVRYSELKGVVVPPERAPSMIDEYPVLAVAASFAEGETLMQGLEELRVKESDRLS | 386 |
| Query 360 | AVANGLKLNGVDCDEGETSLVVRGRPDGKGLG---NASGAAVATHLDHRIAMSFLVMGLV | 416 |
| Sbjct 387 | AVANGLKLNGVDC EGE SL VRG+P GKGLG N V THLDHRIAMSFLVMGL AVANGLKLNGVDCTEGEASLAVRGKPGGKGLGAHPNGQDTVVKTHLDHRIAMSFLVMGLA | 446 |
| Query 417  Sbjct 447 | SENPVTVDDATMIATSFPEFMDLMAGLGAKIE 448  +E PVT+DDA MIATSFPEFM LM GLGA+IE  TEKPVTIDDANMIATSFPEFMGLMKGLGAEIE 478 |  |

>WP_128342427.1 3-phosphoshikimate 1-carboxyvinyltransferase [Mesorhizobium sp. M5C.F.Ca.IN.020.32.2.1]

RUV94994.1 3-phosphoshikimate 1-carboxyvinyltransferase [Mesorhizobium sp. M5C.F.Ca.IN.020.14.1.1]

RWG46347.1 3-phosphoshikimate 1-carboxyvinyltransferase [Mesorhizobium sp.] RUV29443.1 3-phosphoshikimate 1-carboxyvinyltransferase [Mesorhizobium sp.

M5C.F.Ca.IN.020.32.2.1]

RWH37966.1 3-phosphoshikimate 1-carboxyvinyltransferase [Mesorhizobium sp.] RWH51766.1 3-phosphoshikimate 1-carboxyvinyltransferase [Mesorhizobium sp.]

Length=452

Score = 686 bits (1771), Expect = 0.0, Method: Compositional matrix adjust.

| Identities | = 353/452 (78%), Positives = 386/452 (85%), Gaps = 4/452 (1%) |  |
| --- | --- | --- |
| Query 1 | MLHGASSRPATARKSSGLSGTVRIPGDKSISHRSFMFGGLASGETRITGLLEGEDVINTG | 60 |
| Sbjct 1 | M H A+ +PATARKSS LSG R+PGDKSISHRSFMFGGLASGETRITGLLEGEDV+ TG MSHTAAPKPATARKSSALSGAARVPGDKSISHRSFMFGGLASGETRITGLLEGEDVMRTG | 60 |
| Query 61 | KAMQAMGARIRKEGDTWIIDGVGNGGLLAPEAPLDFGNAATGCRLTMGLVGVYDFDSTFI | 120 |
| Sbjct 61 | AM+AMGA I K G W+I G GNG LL PE PLDFGNA TG RLTMGLVG YD ++TFI AAMKAMGAHIEKLGTEWVIRGTGNGALLQPEGPLDFGNAGTGSRLTMGLVGTYDMETTFI | 120 |
| Query 121 | GDASLTKRPMGRVLNPLREMGVQV-KSEDGDRLPVTLRGPKTPTPITYRVPMASAQVKSA | 179 |
| Sbjct 121 | GDASL+ RPMGRVL PLR+MGVQV K+ GDR+P+TLRGPK PITYRVPMASAQVKSA GDASLSGRPMGRVLEPLRQMGVQVLKAIPGDRMPITLRGPKHAAPITYRVPMASAQVKSA | 180 |
| Query 180 | VLLAGLNTPGITTVIEPIMTRDHTEKMLQGFGANLTVETDADGVRTIRLEGRGKLTGQVI | 239 |
| Sbjct 181 | VLLAGLNTPGITTVIEP+MTRDHTEKML+GFGANL+VETD GVR I +EG+GKLTGQ I VLLAGLNTPGITTVIEPVMTRDHTEKMLRGFGANLSVETDERGVRHIFIEGQGKLTGQTI | 240 |
| Query 240 | DVPGDPSSTAFPLVAALLVPGSDVTILNVLMNPTRTGLILTLQEMGADIEVINPRLAGGE | 299 |
| Sbjct 241 | VPGDPSS FPLVAAL+VPGSD+TI NVLMNPTRTGL+LTLQEMGA I++++PR GGE AVPGDPSSAGFPLVAALIVPGSDITIENVLMNPTRTGLLLTLQEMGARIDIVDPRNEGGE | 300 |
| Query 300 | DVADLRVRSSTLKGVTVPEDRAPSMIDEYPILavaaafaegaTVMNGLEELRVKESDRLS | 359 |
| Sbjct 301 | DVADLRVR S LK VTVP +RAP+MIDEYP+LAVAA+FAEG T+M GLEELRVKESDRLS DVADLRVRHSELKAVTVPPERAPTMIDEYPVLAVAASFAEGETLMQGLEELRVKESDRLS | 360 |
| Query 360 | AVANGLKLNGVDCDEGETSLVVRGRPDGKGLG---NASGAAVATHLDHRIAMSFLVMGLV | 416 |
| Sbjct 361 | AVA GLKLNGVDC EGE SL VRGRP GKGLG N G V THLDHRIAMSFLVMGL AVAEGLKLNGVDCTEGEASLAVRGRPGGKGLGQHPNGQGTIVQTHLDHRIAMSFLVMGLA | 420 |
| Query 417  Sbjct 421 | SENPVTVDDATMIATSFPEFMDLMAGLGAKIE 448  +E PVT+DD TMIATSFPEFM LM GLGA+IE  TEKPVTIDDQTMIATSFPEFMGLMKGLGAEIE 452 |  |

>WP_135920479.1 3-phosphoshikimate 1-carboxyvinyltransferase [Mesorhizobium sp. M3A.F.Ca.ET.201.01.1.1]

TGS63971.1 3-phosphoshikimate 1-carboxyvinyltransferase [Mesorhizobium sp. M3A.F.Ca.ET.201.01.1.1]

Length=452

Score = 686 bits (1771), Expect = 0.0, Method: Compositional matrix adjust.

| Identities | = 352/452 (78%), Positives = 385/452 (85%), Gaps = 4/452 (1%) |  |
| --- | --- | --- |
| Query 1 | MLHGASSRPATARKSSGLSGTVRIPGDKSISHRSFMFGGLASGETRITGLLEGEDVINTG | 60 |
| Sbjct 1 | M H A+ +PATARKS L+G R+PGDKSISHRSFMFGGLASGETRITGLLEGEDV+ TG MSHAAAPKPATARKSPALAGVARVPGDKSISHRSFMFGGLASGETRITGLLEGEDVMRTG | 60 |
| Query 61 | KAMQAMGARIRKEGDTWIIDGVGNGGLLAPEAPLDFGNAATGCRLTMGLVGVYDFDSTFI | 120 |
| Sbjct 61 | AM+AMGA I K+GD W+I G GNG LL PE PLDFGNA TG RLTMGLVG YD ++TFI AAMKAMGAHIEKKGDEWVIRGTGNGALLQPEGPLDFGNAGTGSRLTMGLVGTYDMETTFI | 120 |
| Query 121 | GDASLTKRPMGRVLNPLREMGVQV-KSEDGDRLPVTLRGPKTPTPITYRVPMASAQVKSA | 179 |
| Sbjct 121 | GDASL+ RPM RVL PLR+MGVQV K+ GDR+P+TLRGPK PITYRVPMASAQVKSA GDASLSGRPMARVLEPLRQMGVQVLKASPGDRMPITLRGPKHAAPITYRVPMASAQVKSA | 180 |
| Query 180 | VLLAGLNTPGITTVIEPIMTRDHTEKMLQGFGANLTVETDADGVRTIRLEGRGKLTGQVI | 239 |
| Sbjct 181 | VLLAGLNT GITTVIEP+MTRDHTEKML+GFGANLTVETD GVR I +EG+GKLTGQ I VLLAGLNTAGITTVIEPVMTRDHTEKMLKGFGANLTVETDERGVRHIFIEGQGKLTGQTI | 240 |
| Query 240 | DVPGDPSSTAFPLVAALLVPGSDVTILNVLMNPTRTGLILTLQEMGADIEVINPRLAGGE | 299 |
| Sbjct 241 | VPGDPSS FPLVAAL+VPGSD+ I NVLMNPTRTGL+LTLQEMG I+++NPR AGGE AVPGDPSSAGFPLVAALIVPGSDIVIENVLMNPTRTGLLLTLQEMGGRIDILNPRNAGGE | 300 |
| Query 300 | DVADLRVRSSTLKGVTVPEDRAPSMIDEYPILavaaafaegaTVMNGLEELRVKESDRLS | 359 |
| Sbjct 301 | DVADLRVRSS LKGV VP +RAPSMIDEYP+LAVAA+FAEG T+M GLEELRVKESDRLS DVADLRVRSSKLKGVAVPAERAPSMIDEYPVLAVAASFAEGETLMQGLEELRVKESDRLS | 360 |
| Query 360 | AVANGLKLNGVDCDEGETSLVVRGRPDGKGLG---NASGAAVATHLDHRIAMSFLVMGLV | 416 |
| Sbjct 361 | AVANGLKLNGVDC EGE L VRG+P GKGLG N AV THLDHRIAMSFLVMGL  AVANGLKLNGVDCTEGEAWLAVRGKPGGKGLGAHPNGLDTAVKTHLDHRIAMSFLVMGLA | 420 |

Query 417 SENPVTVDDATMIATSFPEFMDLMAGLGAKIE 448

+E PVT+DDA MIATSFPEFM LM GLGA+IE Sbjct 421 TEKPVTIDDANMIATSFPEFMGLMKGLGAEIE 452

>TIS77745.1 3-phosphoshikimate 1-carboxyvinyltransferase [Mesorhizobium sp.] Length=452

Score = 686 bits (1770), Expect = 0.0, Method: Compositional matrix adjust.

| Identities | = 352/452 (78%), Positives = 386/452 (85%), Gaps = 4/452 (1%) |  |
| --- | --- | --- |
| Query 1 | MLHGASSRPATARKSSGLSGTVRIPGDKSISHRSFMFGGLASGETRITGLLEGEDVINTG | 60 |
| Sbjct 1 | M H A+ +PATARKSS LSG R+PGDKSISHRSFMFGGLASGETRITGLLEGEDV+ TG MSHSAAPKPATARKSSALSGAARVPGDKSISHRSFMFGGLASGETRITGLLEGEDVMRTG | 60 |
| Query 61 | KAMQAMGARIRKEGDTWIIDGVGNGGLLAPEAPLDFGNAATGCRLTMGLVGVYDFDSTFI | 120 |
| Sbjct 61 | AM+AMGA I K W+I G GNG LL PE PLDFGNA TG RLTMGLVG YD ++TFI AAMKAMGAHIEKLDTEWVIRGTGNGALLQPEGPLDFGNAGTGSRLTMGLVGTYDMETTFI | 120 |
| Query 121 | GDASLTKRPMGRVLNPLREMGVQV-KSEDGDRLPVTLRGPKTPTPITYRVPMASAQVKSA | 179 |
| Sbjct 121 | GDASL+ RPMGRVL PLR+MGVQV K+ GDR+P+TLRGPK PITYRVPMASAQVKSA GDASLSSRPMGRVLEPLRQMGVQVLKATPGDRMPITLRGPKHAAPITYRVPMASAQVKSA | 180 |
| Query 180 | VLLAGLNTPGITTVIEPIMTRDHTEKMLQGFGANLTVETDADGVRTIRLEGRGKLTGQVI | 239 |
| Sbjct 181 | VLLAGLNTPGITTVIEP+MTRDHTEKML+GFGANL+VETD GVR I +EG+GKLTGQ I VLLAGLNTPGITTVIEPVMTRDHTEKMLRGFGANLSVETDERGVRHIFIEGQGKLTGQTI | 240 |
| Query 240 | DVPGDPSSTAFPLVAALLVPGSDVTILNVLMNPTRTGLILTLQEMGADIEVINPRLAGGE | 299 |
| Sbjct 241 | VPGDPSS FPLVAAL+VPGSD+TI NVLMNPTRTGL+LTLQEMGA I++++PR GGE AVPGDPSSAGFPLVAALIVPGSDITIENVLMNPTRTGLLLTLQEMGARIDIVDPRNEGGE | 300 |
| Query 300 | DVADLRVRSSTLKGVTVPEDRAPSMIDEYPILavaaafaegaTVMNGLEELRVKESDRLS | 359 |
| Sbjct 301 | DVADLRVR S LKGV VP +RAP+MIDEYP+LAVAA+FAEG T+M GLEELRVKESDRLS DVADLRVRYSELKGVAVPPERAPTMIDEYPVLAVAASFAEGETLMQGLEELRVKESDRLS | 360 |
| Query 360 | AVANGLKLNGVDCDEGETSLVVRGRPDGKGLG---NASGAAVATHLDHRIAMSFLVMGLV | 416 |
| Sbjct 361 | AVA+GLKLNGVDC EGETSL V GRP GKGLG N G V THLDHRIAMSFLVMGL AVADGLKLNGVDCTEGETSLAVHGRPGGKGLGQHPNGQGTIVQTHLDHRIAMSFLVMGLA | 420 |
| Query 417  Sbjct 421 | SENPVTVDDATMIATSFPEFMDLMAGLGAKIE 448  +E PVT+DD TMIATSFPEFM LM GLGA+IE  TEKPVTIDDQTMIATSFPEFMGLMKGLGAEIE 452 |  |

>WP_188256751.1 3-phosphoshikimate 1-carboxyvinyltransferase [Pseudaminobacter sp. HC 19]

Length=446

Score = 686 bits (1770), Expect = 0.0, Method: Compositional matrix adjust.

| Identities | = 354/449 (79%), Positives = 388/449 (86%), Gaps = 4/449 (1%) |  |
| --- | --- | --- |
| Query 1 | MLHGASSRPATARKSSGLSGTVRIPGDKSISHRSFMFGGLASGETRITGLLEGEDVINTG | 60 |
| Sbjct 1 | M H +RPATA++SS L GT R+PGDKSISHRSFMFGGLASGETRITGLLEGEDV+ TG MSHQEIARPATAKRSSALKGTARMPGDKSISHRSFMFGGLASGETRITGLLEGEDVLRTG | 60 |
| Query 61 | KAMQAMGARIRKEGDTWIIDGVGNGGLLAPEAPLDFGNAATGCRLTMGLVGVYDFDSTFI | 120 |
| Sbjct 61 | +AM+AMGARI K GD WII G GNG LL P APLDFGNA TG RLTMGLVG YD ++TFI EAMKAMGARIEKHGDEWIIQGTGNGCLLEPRAPLDFGNAGTGSRLTMGLVGTYDMETTFI | 120 |
| Query 121 | GDASLTKRPMGRVLNPLREMGVQV-KSEDGDRLPVTLRGPKTPTPITYRVPMASAQVKSA | 179 |
| Sbjct 121 | GDASL+KRPM RVL+PLREMGVQV KS GDR+P+TLRGP+ PITYRVPMASAQVKSA GDASLSKRPMARVLDPLREMGVQVLKSAPGDRMPLTLRGPRQAAPITYRVPMASAQVKSA | 180 |
| Query 180 | VLLAGLNTPGITTVIEPIMTRDHTEKMLQGFGANLTVETDADGVRTIRLEGRGKLTGQVI | 239 |
| Sbjct 181 | VLLAGLNTPG+TTVIE ++TRDHTEKMLQGFGANL VETDADGVR IR+EG+GKL+GQ I VLLAGLNTPGVTTVIEAVITRDHTEKMLQGFGANLEVETDADGVRHIRIEGQGKLSGQTI | 240 |
| Query 240 | DVPGDPSSTAFPLVAALLVPGSDVTILNVLMNPTRTGLILTLQEMGADIEVINPRLAGGE | 299 |
| Sbjct 241 | VPGDPSS AFPLVAALLVPGSD+ I NVLMNPTRTGLI TL EMGA+IE++N R AGGE AVPGDPSSAAFPLVAALLVPGSDILIENVLMNPTRTGLITTLLEMGAEIELLNQRNAGGE | 300 |
| Query 300 | DVADLRVRSSTLKGVTVPEDRAPSMIDEYPILavaaafaegaTVMNGLEELRVKESDRLS | 359 |

| Sbjct | 301 | DVADLRVRSS LKGVTVP +RAPSMIDEYP+LAVAAAFAEG T+M GL+ELRVKESDRL+ DVADLRVRSSELKGVTVPAERAPSMIDEYPVLAVAAAFAEGETLMQGLDELRVKESDRLA | 360 |
| --- | --- | --- | --- |
| Query | 360 | AVANGLKLNGVDCDEGETSLVVRGRPDGKGLGNASGAAVATHLDHRIAMSFLVMGLVSEN | 419 |
| Sbjct | 361 | AVA GL+ NGVDC EG L VRGRPDGKGLG G VATHLDHRIAMSFLVMG +E AVARGLETNGVDCSEGHDWLSVRGRPDGKGLG---GGTVATHLDHRIAMSFLVMGFAAEK | 417 |
| Query  Sbjct | 420  418 | PVTVDDATMIATSFPEFMDLMAGLGAKIE 448 PVTVDD +MIATSFPEFM LMA LGA+I+ PVTVDDQSMIATSFPEFMGLMAELGAEID 446 |  |

>WP_140628796.1 3-phosphoshikimate 1-carboxyvinyltransferase [Mesorhizobium sp. B2-9-1]

TPI48763.1 3-phosphoshikimate 1-carboxyvinyltransferase [Mesorhizobium sp. B2-9-1]

Length=452

Score = 686 bits (1770), Expect = 0.0, Method: Compositional matrix adjust.

| Identities | = 351/451 (78%), Positives = 387/451 (86%), Gaps = 4/451 (1%) |  |
| --- | --- | --- |
| Query 1 | MLHGASSRPATARKSSGLSGTVRIPGDKSISHRSFMFGGLASGETRITGLLEGEDVINTG | 60 |
| Sbjct 1 | M H A+++PATARKS L+GT R+PGDKSISHRSFMFGGLASGETRITGLLEGEDV+ TG MSHSAAAKPATARKSPALAGTARVPGDKSISHRSFMFGGLASGETRITGLLEGEDVMRTG | 60 |
| Query 61 | KAMQAMGARIRKEGDTWIIDGVGNGGLLAPEAPLDFGNAATGCRLTMGLVGVYDFDSTFI | 120 |
| Sbjct 61 | AM+AMGA I K G W+I G GNG LL PE PLDFGNA TG RLTMGLVG YD ++TFI AAMKAMGAHIEKNGGEWVIRGTGNGALLQPEGPLDFGNAGTGSRLTMGLVGTYDMETTFI | 120 |
| Query 121 | GDASLTKRPMGRVLNPLREMGVQV-KSEDGDRLPVTLRGPKTPTPITYRVPMASAQVKSA | 179 |
| Sbjct 121 | GDASL+ RPMGRVL+PLR+MGVQV K+ GDR+P+TLRGPK PITYRVPMASAQVKSA GDASLSGRPMGRVLDPLRQMGVQVLKAAPGDRMPITLRGPKHAAPITYRVPMASAQVKSA | 180 |
| Query 180 | VLLAGLNTPGITTVIEPIMTRDHTEKMLQGFGANLTVETDADGVRTIRLEGRGKLTGQVI | 239 |
| Sbjct 181 | VLLAGLNTPGITTVIEP+MTRDHTEKML+GFGANL+VETD GVR I +EG+GKLTGQ I VLLAGLNTPGITTVIEPVMTRDHTEKMLKGFGANLSVETDERGVRHIFIEGQGKLTGQTI | 240 |
| Query 240 | DVPGDPSSTAFPLVAALLVPGSDVTILNVLMNPTRTGLILTLQEMGADIEVINPRLAGGE | 299 |
| Sbjct 241 | VPGDPSS FPLVAAL+VPGSD+ I NVLMNPTRTGL+LTLQEMG I+++NPR AGGE AVPGDPSSAGFPLVAALIVPGSDIVIENVLMNPTRTGLLLTLQEMGGKIDILNPRNAGGE | 300 |
| Query 300 | DVADLRVRSSTLKGVTVPEDRAPSMIDEYPILavaaafaegaTVMNGLEELRVKESDRLS | 359 |
| Sbjct 301 | DVADLRVRSS LKGV VP +RAPSMIDEYP+LAVAA+FAEG T+M GLEELRVKESDRL+ DVADLRVRSSELKGVAVPPERAPSMIDEYPVLAVAASFAEGETLMQGLEELRVKESDRLA | 360 |
| Query 360 | AVANGLKLNGVDCDEGETSLVVRGRPDGKGLG---NASGAAVATHLDHRIAMSFLVMGLV | 416 |
| Sbjct 361 | AVA GL+LNGVDC EGE SL VRGRP GKGLG N AV THLDHRIAMSFLVMGL AVARGLELNGVDCTEGEASLAVRGRPGGKGLGAHPNGPDTAVKTHLDHRIAMSFLVMGLA | 420 |
| Query 417  Sbjct 421 | SENPVTVDDATMIATSFPEFMDLMAGLGAKI 447  +E PVT+DDA MIATSFPEFM LM GLGA+I  TEKPVTIDDANMIATSFPEFMGLMKGLGAEI 451 |  |

>WP_127229749.1 MULTISPECIES: 3-phosphoshikimate 1-carboxyvinyltransferase [unclassified Mesorhizobium]

TGP82262.1 3-phosphoshikimate 1-carboxyvinyltransferase [bacterium M00.F.Ca.ET.227.01.1.1] TGP91854.1 3-phosphoshikimate 1-carboxyvinyltransferase [bacterium M00.F.Ca.ET.221.01.1.1] TGP95360.1 3-phosphoshikimate 1-carboxyvinyltransferase [bacterium M00.F.Ca.ET.222.01.1.1] TGT71386.1 3-phosphoshikimate 1-carboxyvinyltransferase [bacterium M00.F.Ca.ET.159.01.1.1] TGT83563.1 3-phosphoshikimate 1-carboxyvinyltransferase [bacterium M00.F.Ca.ET.157.01.1.1] TGU03645.1 3-phosphoshikimate 1-carboxyvinyltransferase [bacterium M00.F.Ca.ET.163.01.1.1] TGU38710.1 3-phosphoshikimate 1-carboxyvinyltransferase [bacterium M00.F.Ca.ET.156.01.1.1] TGU47944.1 3-phosphoshikimate 1-carboxyvinyltransferase [bacterium M00.F.Ca.ET.146.01.1.1] TGV66353.1 3-phosphoshikimate 1-carboxyvinyltransferase [Mesorhizobium sp.

M2D.F.Ca.ET.160.01.1.1]

TGW11878.1 3-phosphoshikimate 1-carboxyvinyltransferase [Mesorhizobium sp. M2D.F.Ca.ET.145.01.1.1]

Length=452

Score = 686 bits (1770), Expect = 0.0, Method: Compositional matrix adjust. Identities = 351/452 (78%), Positives = 385/452 (85%), Gaps = 4/452 (1%)

| Query | 1 | MLHGASSRPATARKSSGLSGTVRIPGDKSISHRSFMFGGLASGETRITGLLEGEDVINTG | 60 |
| --- | --- | --- | --- |
| Sbjct | 1 | M H A+++PATARKS L+GT R+PGDKSISHRSFMFGGLASGETRITGLLEGEDV+ TG MSHSAAAKPATARKSPALAGTARVPGDKSISHRSFMFGGLASGETRITGLLEGEDVMRTG | 60 |
| Query | 61 | KAMQAMGARIRKEGDTWIIDGVGNGGLLAPEAPLDFGNAATGCRLTMGLVGVYDFDSTFI | 120 |
| Sbjct | 61 | AM+AMGA I K G W+I G GNG LL PE PLDFGNA TG RLTMGLVG YD ++TFI AAMKAMGAHIEKNGAEWVIRGTGNGALLQPEGPLDFGNAGTGSRLTMGLVGTYDMETTFI | 120 |
| Query | 121 | GDASLTKRPMGRVLNPLREMGVQV-KSEDGDRLPVTLRGPKTPTPITYRVPMASAQVKSA | 179 |
| Sbjct | 121 | GDASL+ RPMGRVL+PLR+MGVQV K+ GDR+P+TLRGPK PITYRVPMASAQVKSA GDASLSGRPMGRVLDPLRQMGVQVLKAAPGDRMPITLRGPKHAAPITYRVPMASAQVKSA | 180 |
| Query | 180 | VLLAGLNTPGITTVIEPIMTRDHTEKMLQGFGANLTVETDADGVRTIRLEGRGKLTGQVI | 239 |
| Sbjct | 181 | VLLAGLNTPGITTVIEP+MTRDHTEKML+GFGANLTVETD GVR I +EG+GKLTGQ I VLLAGLNTPGITTVIEPVMTRDHTEKMLKGFGANLTVETDERGVRHIFIEGQGKLTGQTI | 240 |
| Query | 240 | DVPGDPSSTAFPLVAALLVPGSDVTILNVLMNPTRTGLILTLQEMGADIEVINPRLAGGE | 299 |
| Sbjct | 241 | VPGDPSS FPLVAAL+VPGSD+ I NVLMNPTRTGL+LTLQEMG I+++NPR AGGE AVPGDPSSAGFPLVAALIVPGSDIVIENVLMNPTRTGLLLTLQEMGGKIDILNPRTAGGE | 300 |
| Query | 300 | DVADLRVRSSTLKGVTVPEDRAPSMIDEYPILavaaafaegaTVMNGLEELRVKESDRLS | 359 |
| Sbjct | 301 | DVADLRV S LKGV VP +RAPSMIDEYP+LAVAA+FAEG T+M GLEELRVKESDRL+ DVADLRVCFSELKGVVVPPERAPSMIDEYPVLAVAASFAEGETLMQGLEELRVKESDRLA | 360 |
| Query | 360 | AVANGLKLNGVDCDEGETSLVVRGRPDGKGLG---NASGAAVATHLDHRIAMSFLVMGLV | 416 |
| Sbjct | 361 | AVANGLKLNG DC EGE SL VRGRP GKGLG N V THLDHRIAMSFLVMGL AVANGLKLNGADCTEGEASLAVRGRPGGKGLGAHPNGQDTVVKTHLDHRIAMSFLVMGLA | 420 |
| Query  Sbjct | 417  421 | SENPVTVDDATMIATSFPEFMDLMAGLGAKIE 448  +E PVT+DDA MIATSFPEFM LM GLGA+IE  TEKPVTIDDANMIATSFPEFMGLMKGLGAEIE 452 |  |

>WP_140782444.1 MULTISPECIES: 3-phosphoshikimate 1-carboxyvinyltransferase [unclassified Mesorhizobium]

TPI42490.1 3-phosphoshikimate 1-carboxyvinyltransferase [Mesorhizobium sp. B3-1-9]

TPI63938.1 3-phosphoshikimate 1-carboxyvinyltransferase [Mesorhizobium sp. B3-1-7]

Length=452

Score = 686 bits (1770), Expect = 0.0, Method: Compositional matrix adjust.

| Identities | = 351/452 (78%), Positives = 388/452 (86%), Gaps = 4/452 (1%) |  |
| --- | --- | --- |
| Query 1 | MLHGASSRPATARKSSGLSGTVRIPGDKSISHRSFMFGGLASGETRITGLLEGEDVINTG | 60 |
| Sbjct 1 | M H A+++PATARKS L+GT R+PGDKSISHRSFMFGGLASGETRITGLLEGEDV+ TG MSHSAAAKPATARKSPALAGTARVPGDKSISHRSFMFGGLASGETRITGLLEGEDVMRTG | 60 |
| Query 61 | KAMQAMGARIRKEGDTWIIDGVGNGGLLAPEAPLDFGNAATGCRLTMGLVGVYDFDSTFI | 120 |
| Sbjct 61 | AM+AMGA + K+G W+I G GNG LL PE PLDFGNA TG RLTMGLVG YD ++TFI AAMKAMGAHVEKKGAEWVIRGTGNGALLQPEGPLDFGNAGTGSRLTMGLVGTYDMETTFI | 120 |
| Query 121 | GDASLTKRPMGRVLNPLREMGVQV-KSEDGDRLPVTLRGPKTPTPITYRVPMASAQVKSA | 179 |
| Sbjct 121 | GDASL+ RPMGRVL+PLR+MGVQV K+ GDR+P+ LRGPK PITYRVPMASAQVKSA GDASLSGRPMGRVLDPLRQMGVQVLKAAPGDRMPIALRGPKHAAPITYRVPMASAQVKSA | 180 |
| Query 180 | VLLAGLNTPGITTVIEPIMTRDHTEKMLQGFGANLTVETDADGVRTIRLEGRGKLTGQVI | 239 |
| Sbjct 181 | VLLAGLNTPGITTVIEP+MTRDHTEKML+GFGANL+VETD GVR I +EG+GKLTGQ I VLLAGLNTPGITTVIEPVMTRDHTEKMLKGFGANLSVETDERGVRHIFIEGQGKLTGQTI | 240 |
| Query 240 | DVPGDPSSTAFPLVAALLVPGSDVTILNVLMNPTRTGLILTLQEMGADIEVINPRLAGGE | 299 |
| Sbjct 241 | VPGDPSS FPLVAAL+VPGSD+TI NVLMNPTRTGL+LTLQEMG I+++NPR AGGE AVPGDPSSAGFPLVAALIVPGSDITIENVLMNPTRTGLLLTLQEMGGKIDILNPRNAGGE | 300 |
| Query 300 | DVADLRVRSSTLKGVTVPEDRAPSMIDEYPILavaaafaegaTVMNGLEELRVKESDRLS | 359 |
| Sbjct 301 | DVADLRVR S LKGVTVP +RAPSMIDEYP+LAVAA+FAEG T+M GLEELRVKESDRLS DVADLRVRYSELKGVTVPPERAPSMIDEYPVLAVAASFAEGETLMQGLEELRVKESDRLS | 360 |
| Query 360 | AVANGLKLNGVDCDEGETSLVVRGRPDGKGLG---NASGAAVATHLDHRIAMSFLVMGLV | 416 |
| Sbjct 361 | AVANGLKLNGVDC E E SL VRG+P GKGLG N AV T LDHRIAMSFLVMGL  AVANGLKLNGVDCTEDEASLAVRGKPGGKGLGAHPNGQDTAVKTDLDHRIAMSFLVMGLA | 420 |

Query 417 SENPVTVDDATMIATSFPEFMDLMAGLGAKIE 448

+E PVT+DDA MIATSFPEFM LM GLGA+IE Sbjct 421 TEKPVTIDDANMIATSFPEFMGLMKGLGAEIE 452

>RWD84160.1 3-phosphoshikimate 1-carboxyvinyltransferase [Mesorhizobium sp.] RWD92093.1 3-phosphoshikimate 1-carboxyvinyltransferase [Mesorhizobium sp.] TIV48713.1 3-phosphoshikimate 1-carboxyvinyltransferase [Mesorhizobium sp.]

Length=452

Score = 686 bits (1770), Expect = 0.0, Method: Compositional matrix adjust.

| Identities | = 351/452 (78%), Positives = 387/452 (86%), Gaps = 4/452 (1%) |  |
| --- | --- | --- |
| Query 1 | MLHGASSRPATARKSSGLSGTVRIPGDKSISHRSFMFGGLASGETRITGLLEGEDVINTG | 60 |
| Sbjct 1 | M H A+ +PATARKS L+G R+PGDKSISHRSFMFGGLASGETRITGLLEGEDV+ TG MSHSAAPKPATARKSPALTGVARVPGDKSISHRSFMFGGLASGETRITGLLEGEDVMRTG | 60 |
| Query 61 | KAMQAMGARIRKEGDTWIIDGVGNGGLLAPEAPLDFGNAATGCRLTMGLVGVYDFDSTFI | 120 |
| Sbjct 61 | AM+AMGA I K+G W+I G GNG LL PE PLDFGNA TG RLTMGLVG YD ++TFI AAMKAMGAHIEKKGAEWVIRGTGNGALLEPEGPLDFGNAGTGSRLTMGLVGTYDMETTFI | 120 |
| Query 121 | GDASLTKRPMGRVLNPLREMGVQV-KSEDGDRLPVTLRGPKTPTPITYRVPMASAQVKSA | 179 |
| Sbjct 121 | GDASL+ RPMGRVL PLR+MGVQV K+ GDR+P+TLRGPK PITYRVPMASAQVKSA GDASLSGRPMGRVLEPLRQMGVQVLKAAPGDRMPITLRGPKHAAPITYRVPMASAQVKSA | 180 |
| Query 180 | VLLAGLNTPGITTVIEPIMTRDHTEKMLQGFGANLTVETDADGVRTIRLEGRGKLTGQVI | 239 |
| Sbjct 181 | VLLAGLNTPGITTVIEP+MTRDHTEKML+GFGANLTVETD GVR I ++G+GKLTGQ I VLLAGLNTPGITTVIEPVMTRDHTEKMLKGFGANLTVETDERGVRHIFIDGQGKLTGQTI | 240 |
| Query 240 | DVPGDPSSTAFPLVAALLVPGSDVTILNVLMNPTRTGLILTLQEMGADIEVINPRLAGGE | 299 |
| Sbjct 241 | VPGDPSS FPLVAAL+VPGSD+ I NVLMNPTRTGL+LTLQEMG I+++NPR AGGE AVPGDPSSAGFPLVAALIVPGSDIIIENVLMNPTRTGLLLTLQEMGGRIDILNPRNAGGE | 300 |
| Query 300 | DVADLRVRSSTLKGVTVPEDRAPSMIDEYPILavaaafaegaTVMNGLEELRVKESDRLS | 359 |
| Sbjct 301 | DVADLRVRSS LKGV VP +RAPSMIDEYP+LAVAA+FAEG T+M GLEELRVKESDRL+ DVADLRVRSSDLKGVVVPPERAPSMIDEYPVLAVAASFAEGETLMQGLEELRVKESDRLA | 360 |
| Query 360 | AVANGLKLNGVDCDEGETSLVVRGRPDGKGLG---NASGAAVATHLDHRIAMSFLVMGLV | 416 |
| Sbjct 361 | AVANGLKLNGVDC EGE SL VRG+P GKGLG N AV THLDHRIAMSFL+MGL AVANGLKLNGVDCTEGEASLAVRGKPGGKGLGAHPNGQDTAVKTHLDHRIAMSFLIMGLA | 420 |
| Query 417  Sbjct 421 | SENPVTVDDATMIATSFPEFMDLMAGLGAKIE 448  +E PVT+DDA MIATSFPEFM LM GLGA+IE  TEKPVTIDDANMIATSFPEFMGLMKGLGAEIE 452 |  |

>WP_183765354.1 MULTISPECIES: 3-phosphoshikimate 1-carboxyvinyltransferase [unclassified Rhizobium]

MBB3290684.1 3-phosphoshikimate 1-carboxyvinyltransferase [Rhizobium sp. BK252] MBB3405464.1 3-phosphoshikimate 1-carboxyvinyltransferase [Rhizobium sp. BK289] MBB3418033.1 3-phosphoshikimate 1-carboxyvinyltransferase [Rhizobium sp. BK284] MBB3485890.1 3-phosphoshikimate 1-carboxyvinyltransferase [Rhizobium sp. BK347] MBB3492208.1 3-phosphoshikimate 1-carboxyvinyltransferase [Rhizobium sp. BK338]

Length=451

Score = 686 bits (1770), Expect = 0.0, Method: Compositional matrix adjust.

| Identities | = 346/447 (77%), Positives = 387/447 (87%), Gaps = 0/447 (0%) |  |
| --- | --- | --- |
| Query 1 | MLHGASSRPATARKSSGLSGTVRIPGDKSISHRSFMFGGLASGETRITGLLEGEDVINTG | 60 |
| Sbjct 1 | M H +PA AR+S+GLSGT+RIPGDKSISHRS +FGGLASGET I GLLEGEDV+NT MPHDTLPQPAVARRSAGLSGTIRIPGDKSISHRSLVFGGLASGETHIKGLLEGEDVLNTA | 60 |
| Query 61 | KAMQAMGARIRKEGDTWIIDGVGNGGLLAPEAPLDFGNAATGCRLTMGLVGVYDFDSTFI | 120 |
| Sbjct 61 | AMQA+GA IR++ D WI++G GNG LLAPEAPLDFGNA TG RLTMGLVG YDF +TF+ GAMQALGATIRRDSDAWIVNGTGNGALLAPEAPLDFGNAGTGSRLTMGLVGSYDFKTTFV | 120 |
| Query 121 | GDASLTKRPMGRVLNPLREMGVQVKSEDGDRLPVTLRGPKTPTPITYRVPMASAQVKSAV | 180 |
| Sbjct 121 | GDASL+KRPMGR+L+PL MG QV + GDRLPVTL GP +P+TYRVPMASAQVKSAV GDASLSKRPMGRILDPLCRMGTQVSAATGDRLPVTLHGPGVTSPLTYRVPMASAQVKSAV | 180 |
| Query 181 | LLAGLNTPGITTVIEPIMTRDHTEKMLQGFGANLTVETDADGVRTIRLEGRGKLTGQVID | 240 |

| Sbjct | 181 | LLAGLN PGITTVIEP+MTRDHTE+ML GFGA L+VETDA GVRTIRLEG+G+LTGQ ID LLAGLNAPGITTVIEPVMTRDHTERMLAGFGAALSVETDAAGVRTIRLEGQGRLTGQTID | 240 |
| --- | --- | --- | --- |
| Query | 241 | VPGDPSSTAFPLVAALLVPGSDVTILNVLMNPTRTGLILTLQEMGADIEVINPRLAGGED | 300 |
| Sbjct | 241 | VPGDPSS AFPLVAAL+VPGSD+TI NVLMN TRTGLILTLQEMGADIE+++ R+ GGED VPGDPSSAAFPLVAALIVPGSDITIRNVLMNTTRTGLILTLQEMGADIEILDRRVTGGED | 300 |
| Query | 301 | VADLRVRSSTLKGVTVPEDRAPSMIDEYPILavaaafaegaTVMNGLEELRVKESDRLSA | 360 |
| Sbjct | 301 | VADL VRSS LKGVTVP +RAPSMIDEYP+LAVAAAFAEG T+M GL+ELRVKESDRLSA VADLHVRSSALKGVTVPAERAPSMIDEYPVLAVAAAFAEGTTIMQGLDELRVKESDRLSA | 360 |
| Query | 361 | VANGLKLNGVDCDEGETSLVVRGRPDGKGLGNASGAAVATHLDHRIAMSFLVMGLVSENP | 420 |
| Sbjct | 361 | VA GLKLNGVDC EG SL VRGRPDGKGLG++SG V THLDHRIAMSFLVMGL SE+P VAEGLKLNGVDCSEGRDSLTVRGRPDGKGLGSSSGTEVRTHLDHRIAMSFLVMGLASEHP | 420 |
| Query  Sbjct | 421  421 | VTVDDATMIATSFPEFMDLMAGLGAKI 447 V++DD+ MIATSFPEF +M LGA I VSIDDSAMIATSFPEFFAMMTRLGATI 447 |  |

>WP_126063101.1 3-phosphoshikimate 1-carboxyvinyltransferase [Mesorhizobium sp. M2A.F.Ca.ET.043.05.1.1]

AZO17153.1 3-phosphoshikimate 1-carboxyvinyltransferase [Mesorhizobium sp. M2A.F.Ca.ET.043.05.1.1]

Length=452

Score = 686 bits (1770), Expect = 0.0, Method: Compositional matrix adjust.

| Identities | = 351/451 (78%), Positives = 387/451 (86%), Gaps = 4/451 (1%) |  |
| --- | --- | --- |
| Query 1 | MLHGASSRPATARKSSGLSGTVRIPGDKSISHRSFMFGGLASGETRITGLLEGEDVINTG | 60 |
| Sbjct 1 | M H A+++PATARKS L+GT R+PGDKSISHRSFMFGGLASGETRITGLLEGEDV+ TG MSHSAAAKPATARKSPALAGTARVPGDKSISHRSFMFGGLASGETRITGLLEGEDVMRTG | 60 |
| Query 61 | KAMQAMGARIRKEGDTWIIDGVGNGGLLAPEAPLDFGNAATGCRLTMGLVGVYDFDSTFI | 120 |
| Sbjct 61 | AM+AMGA I K G W+I G GNG LL PE PLDFGNA TG RLTMGLVG YD ++TFI AAMKAMGAHIEKNGAEWVIRGTGNGALLQPEGPLDFGNAGTGSRLTMGLVGTYDMETTFI | 120 |
| Query 121 | GDASLTKRPMGRVLNPLREMGVQV-KSEDGDRLPVTLRGPKTPTPITYRVPMASAQVKSA | 179 |
| Sbjct 121 | GDASL+ RPMGRVL+PLR+MGVQV K+ GDR+P+TLRGPK PITYRVPMASAQVKSA GDASLSGRPMGRVLDPLRQMGVQVLKTAPGDRMPITLRGPKHAAPITYRVPMASAQVKSA | 180 |
| Query 180 | VLLAGLNTPGITTVIEPIMTRDHTEKMLQGFGANLTVETDADGVRTIRLEGRGKLTGQVI | 239 |
| Sbjct 181 | VLLAGLNTPGITTVIEP+MTRDHTEKML+GFGANLTVETD GVR I +EG+GKLTGQ I VLLAGLNTPGITTVIEPVMTRDHTEKMLKGFGANLTVETDERGVRHIFIEGQGKLTGQTI | 240 |
| Query 240 | DVPGDPSSTAFPLVAALLVPGSDVTILNVLMNPTRTGLILTLQEMGADIEVINPRLAGGE | 299 |
| Sbjct 241 | VPGDPSS FPLVAAL+VPGSD+ I NVLMNPTRTGL+LTLQEMG I+++NPR AGGE AVPGDPSSAGFPLVAALIVPGSDIVIENVLMNPTRTGLLLTLQEMGGRIDILNPRNAGGE | 300 |
| Query 300 | DVADLRVRSSTLKGVTVPEDRAPSMIDEYPILavaaafaegaTVMNGLEELRVKESDRLS | 359 |
| Sbjct 301 | DVADLRVR S LKGV VP +RAPSMIDEYP+LAVAA+FAEG T+M GLEELRVKESDRL+ DVADLRVRYSELKGVVVPPERAPSMIDEYPVLAVAASFAEGETLMQGLEELRVKESDRLA | 360 |
| Query 360 | AVANGLKLNGVDCDEGETSLVVRGRPDGKGLG---NASGAAVATHLDHRIAMSFLVMGLV | 416 |
| Sbjct 361 | AVA+GLKLNGVDC EGE SL VRGRP GKGLG N AV THLDHRIAMSFL+MGL AVASGLKLNGVDCTEGEASLAVRGRPGGKGLGAHPNGPDTAVKTHLDHRIAMSFLIMGLA | 420 |
| Query 417  Sbjct 421 | SENPVTVDDATMIATSFPEFMDLMAGLGAKI 447  +E PVT+DDA MIATSFPEFM LM GLGA+I  TEKPVTIDDANMIATSFPEFMGLMKGLGAEI 451 |  |

```
>RVD73587.1 3-phosphoshikimate 1-carboxyvinyltransferase [Mesorhizobium sp. M4A.F.Ca.ET.029.04.2.1]
Length=452

Score = 686 bits (1769), Expect = 0.0, Method: Compositional matrix adjust. Identities = 352/452 (78%), Positives = 385/452 (85%), Gaps = 4/452 (1%)

Query 1	MLHGASSRPATARKSSGLSGTVRIPGDKSISHRSFMFGGLASGETRITGLLEGEDVINTG 60 M H A+ +PATARKS LSG R+PGDKSISHRSFMFGGLASGETRITGLLEGEDV+ TG
Sbjct 1	MSHAAAPKPATARKSPALSGAARVPGDKSISHRSFMFGGLASGETRITGLLEGEDVMRTG 60
```

| Query | 61 | KAMQAMGARIRKEGDTWIIDGVGNGGLLAPEAPLDFGNAATGCRLTMGLVGVYDFDSTFI | 120 |
| --- | --- | --- | --- |
| Sbjct | 61 | AM+AMGA I K G W+I G GNG LL PE PLDFGNA TG RLTMGLVG YD ++TFI AAMKAMGAHIEKRGAEWVIRGTGNGALLEPEGPLDFGNAGTGSRLTMGLVGTYDMETTFI | 120 |
| Query | 121 | GDASLTKRPMGRVLNPLREMGVQV-KSEDGDRLPVTLRGPKTPTPITYRVPMASAQVKSA | 179 |
| Sbjct | 121 | GDASL+ RPMGRVL PLR+MGVQV K+ GDR+P+TLRGPK PITYRVPMASAQVKSA GDASLSGRPMGRVLEPLRQMGVQVLKATPGDRMPITLRGPKHAAPITYRVPMASAQVKSA | 180 |
| Query | 180 | VLLAGLNTPGITTVIEPIMTRDHTEKMLQGFGANLTVETDADGVRTIRLEGRGKLTGQVI | 239 |
| Sbjct | 181 | VLLAGLNTPGITTVIEP+MTRDHTEKML+GFGANLTVETD GVR I +EG+GKL+GQ I VLLAGLNTPGITTVIEPVMTRDHTEKMLKGFGANLTVETDERGVRHIFIEGQGKLSGQTI | 240 |
| Query | 240 | DVPGDPSSTAFPLVAALLVPGSDVTILNVLMNPTRTGLILTLQEMGADIEVINPRLAGGE | 299 |
| Sbjct | 241 | VPGDPSS FPLVAAL+VPGSD+ I NVLMNPTRTGL+LTLQEMG I+++NPR AGGE AVPGDPSSAGFPLVAALIVPGSDIVIENVLMNPTRTGLLLTLQEMGGRIDILNPRNAGGE | 300 |
| Query | 300 | DVADLRVRSSTLKGVTVPEDRAPSMIDEYPILavaaafaegaTVMNGLEELRVKESDRLS | 359 |
| Sbjct | 301 | DVADLRVRSS LKGV VP +RAPSMIDEYP+LAVAA+FAEG T+M GLEELRVKESDRLS DVADLRVRSSELKGVVVPPERAPSMIDEYPVLAVAASFAEGETLMQGLEELRVKESDRLS | 360 |
| Query | 360 | AVANGLKLNGVDCDEGETSLVVRGRPDGKGLG---NASGAAVATHLDHRIAMSFLVMGLV | 416 |
| Sbjct | 361 | AVANGLKLNGVDC EGE +L VRG+P GKGLG N V THLDHRIAMSFLVMGL AVANGLKLNGVDCTEGEATLAVRGKPGGKGLGRHPNGPDTTVQTHLDHRIAMSFLVMGLA | 420 |
| Query  Sbjct | 417  421 | SENPVTVDDATMIATSFPEFMDLMAGLGAKIE 448  +E PVT+DDA MIATSFPEFM LM GLGA+IE  AEKPVTIDDANMIATSFPEFMGLMKGLGAEIE 452 |  |

>RWB57618.1 3-phosphoshikimate 1-carboxyvinyltransferase [Mesorhizobium sp.] Length=452

Score = 686 bits (1769), Expect = 0.0, Method: Compositional matrix adjust.

| Identities | = 350/452 (77%), Positives = 387/452 (86%), Gaps = 4/452 (1%) |  |
| --- | --- | --- |
| Query 1 | MLHGASSRPATARKSSGLSGTVRIPGDKSISHRSFMFGGLASGETRITGLLEGEDVINTG | 60 |
| Sbjct 1 | M H A+ +PATARKS L+G R+PGDKSISHRSFMFGGLASGETRITGLLEGEDV+ TG MSHSAAPKPATARKSPALTGVARVPGDKSISHRSFMFGGLASGETRITGLLEGEDVMRTG | 60 |
| Query 61 | KAMQAMGARIRKEGDTWIIDGVGNGGLLAPEAPLDFGNAATGCRLTMGLVGVYDFDSTFI | 120 |
| Sbjct 61 | AM+AMGA+I ++G W+I G GNG LL PE PLDFGNA TG RLTMGLVG YD ++TFI AAMKAMGAQIERKGAEWVIRGTGNGALLEPEGPLDFGNAGTGSRLTMGLVGTYDMETTFI | 120 |
| Query 121 | GDASLTKRPMGRVLNPLREMGVQV-KSEDGDRLPVTLRGPKTPTPITYRVPMASAQVKSA | 179 |
| Sbjct 121 | GDASL+ RPMGRVL PLR+MGVQV K+ GDR+P+TL GPK PITYRVPMASAQVKSA GDASLSGRPMGRVLEPLRQMGVQVLKAAPGDRMPITLHGPKHAAPITYRVPMASAQVKSA | 180 |
| Query 180 | VLLAGLNTPGITTVIEPIMTRDHTEKMLQGFGANLTVETDADGVRTIRLEGRGKLTGQVI | 239 |
| Sbjct 181 | VLLAGLNTPGITTVIEP+MTRDHTEKML+GFGANLTVETD GVR I +EG+GKLTGQ I VLLAGLNTPGITTVIEPVMTRDHTEKMLKGFGANLTVETDERGVRHIFIEGQGKLTGQTI | 240 |
| Query 240 | DVPGDPSSTAFPLVAALLVPGSDVTILNVLMNPTRTGLILTLQEMGADIEVINPRLAGGE | 299 |
| Sbjct 241 | VPGDPSS FPLVAAL+VPGSD+ I NVLMNPTRTGL+LTLQEMG I+++NPR AGGE AVPGDPSSAGFPLVAALIVPGSDIIIENVLMNPTRTGLLLTLQEMGGRIDILNPRNAGGE | 300 |
| Query 300 | DVADLRVRSSTLKGVTVPEDRAPSMIDEYPILavaaafaegaTVMNGLEELRVKESDRLS | 359 |
| Sbjct 301 | DVADLRVRSS LKGV VP +RAPSMIDEYP+LAVAA+FAEG T+M GLEELRVKESDRL+ DVADLRVRSSELKGVVVPPERAPSMIDEYPVLAVAASFAEGETLMQGLEELRVKESDRLA | 360 |
| Query 360 | AVANGLKLNGVDCDEGETSLVVRGRPDGKGLG---NASGAAVATHLDHRIAMSFLVMGLV | 416 |
| Sbjct 361 | AVANGLKLNGVDC EGE SL VRG+P GKGLG N AV THLDHRIAMSFL+MGL AVANGLKLNGVDCTEGEASLAVRGKPGGKGLGAHPNGQDTAVKTHLDHRIAMSFLIMGLA | 420 |
| Query 417  Sbjct 421 | SENPVTVDDATMIATSFPEFMDLMAGLGAKIE 448  +E PVT+DDA MIATSFPEFM LM GLGA+IE  TEKPVTIDDANMIATSFPEFMGLMKGLGAEIE 452 |  |

>RWL90264.1 3-phosphoshikimate 1-carboxyvinyltransferase [Mesorhizobium sp.] TIP49503.1 3-phosphoshikimate 1-carboxyvinyltransferase [Mesorhizobium sp.]

Length=452

Score = 686 bits (1769), Expect = 0.0, Method: Compositional matrix adjust.

| Identities | = 351/452 (78%), Positives = 387/452 (86%), Gaps = 4/452 (1%) |  |
| --- | --- | --- |
| Query 1 | MLHGASSRPATARKSSGLSGTVRIPGDKSISHRSFMFGGLASGETRITGLLEGEDVINTG | 60 |
| Sbjct 1 | M H A+++ ATARKS L+GT R+PGDKSISHRSFMFGGLASGETRITGLLEGEDV+ TG MSHSAAAKSATARKSPALAGTARVPGDKSISHRSFMFGGLASGETRITGLLEGEDVMRTG | 60 |
| Query 61 | KAMQAMGARIRKEGDTWIIDGVGNGGLLAPEAPLDFGNAATGCRLTMGLVGVYDFDSTFI | 120 |
| Sbjct 61 | AM+AMGA I K G W+I G GNG LL PE PLDFGNA TG RLTMGLVG YD ++TFI AAMKAMGAHIEKNGAEWVIRGTGNGALLQPEGPLDFGNAGTGSRLTMGLVGTYDMETTFI | 120 |
| Query 121 | GDASLTKRPMGRVLNPLREMGVQV-KSEDGDRLPVTLRGPKTPTPITYRVPMASAQVKSA | 179 |
| Sbjct 121 | GDASL+ RPMGRVL+PLR+MGVQV K+ GDR+P+TLRGPK PITYRVPMASAQVKSA GDASLSGRPMGRVLDPLRQMGVQVLKAAPGDRMPITLRGPKHAAPITYRVPMASAQVKSA | 180 |
| Query 180 | VLLAGLNTPGITTVIEPIMTRDHTEKMLQGFGANLTVETDADGVRTIRLEGRGKLTGQVI | 239 |
| Sbjct 181 | VLLAGLNTPGITTVIEP+MTRDHTEKML+GFGANLTVETD GVR I +EG+GKLTGQ I VLLAGLNTPGITTVIEPVMTRDHTEKMLKGFGANLTVETDERGVRHIFIEGQGKLTGQTI | 240 |
| Query 240 | DVPGDPSSTAFPLVAALLVPGSDVTILNVLMNPTRTGLILTLQEMGADIEVINPRLAGGE | 299 |
| Sbjct 241 | VPGDPSS FPLVAAL+VPGSD+ I NVLMNPTRTGL+LTLQEMG I+++NPR AGGE AVPGDPSSAGFPLVAALIVPGSDIVIENVLMNPTRTGLLLTLQEMGGRIDILNPRNAGGE | 300 |
| Query 300 | DVADLRVRSSTLKGVTVPEDRAPSMIDEYPILavaaafaegaTVMNGLEELRVKESDRLS | 359 |
| Sbjct 301 | DVADLRVRSS LKGV VP +RAPSMIDEYP+LAVAA+FAEG T+M GLEELRVKESDRLS DVADLRVRSSELKGVVVPPERAPSMIDEYPVLAVAASFAEGETLMQGLEELRVKESDRLS | 360 |
| Query 360 | AVANGLKLNGVDCDEGETSLVVRGRPDGKGLG---NASGAAVATHLDHRIAMSFLVMGLV | 416 |
| Sbjct 361 | AVANGLKLNGVDC EGE +L V G+P GKGLG N AV THLDHRIAMSFL+MGL AVANGLKLNGVDCTEGEATLAVSGKPGGKGLGAHPNGPDTAVKTHLDHRIAMSFLIMGLA | 420 |
| Query 417  Sbjct 421 | SENPVTVDDATMIATSFPEFMDLMAGLGAKIE 448  +E PVT+DDA MIATSFPEFM LM GLGA+IE  TEKPVTIDDANMIATSFPEFMGLMKGLGAEIE 452 |  |

>WP_192448167.1 3-phosphoshikimate 1-carboxyvinyltransferase [Ensifer sp. ENS09] MBD9649537.1 3-phosphoshikimate 1-carboxyvinyltransferase [Ensifer sp. ENS09]

Length=448

Score = 685 bits (1768), Expect = 0.0, Method: Compositional matrix adjust.

| Identities | = 351/447 (79%), Positives = 396/447 (89%), Gaps = 0/447 (0%) |  |
| --- | --- | --- |
| Query 1 | MLHGASSRPATARKSSGLSGTVRIPGDKSISHRSFMFGGLASGETRITGLLEGEDVINTG | 60 |
| Sbjct 1 | M HG+ RPATARKS+ L G++RIPGDKSISHR+ M GGLA GETRITGLLEG DV+ TG MSHGSGPRPATARKSADLRGSIRIPGDKSISHRALMLGGLAGGETRITGLLEGADVLATG | 60 |
| Query 61 | KAMQAMGARIRKEGDTWIIDGVGNGGLLAPEAPLDFGNAATGCRLTMGLVGVYDFDSTFI | 120 |
| Sbjct 61 | +AMQAMGARIRKEG+T+++DGVGNG LLAPE PLDFGNA TGCRL MGL GVYDF++TF+ RAMQAMGARIRKEGNTFVVDGVGNGALLAPEVPLDFGNAGTGCRLVMGLAGVYDFETTFV | 120 |
| Query 121 | GDASLTKRPMGRVLNPLREMGVQVKSEDGDRLPVTLRGPKTPTPITYRVPMASAQVKSAV | 180 |
| Sbjct 121 | GD SL++RPM RVL+PLREMGVQV++ GDRLPVTLRGPKTPTPI YRVP+ASAQ KSAV GDTSLSRRPMARVLDPLREMGVQVRAAAGDRLPVTLRGPKTPTPIAYRVPVASAQAKSAV | 180 |
| Query 181 | LLAGLNTPGITTVIEPIMTRDHTEKMLQGFGANLTVETDADGVRTIRLEGRGKLTGQVID | 240 |
| Sbjct 181 | LLAGLN PGITTVIEP+MTRDHTE+MLQ FGA L+VET+ GVRTIRLEGRGKLTGQ ID LLAGLNAPGITTVIEPVMTRDHTERMLQAFGAALSVETEISGVRTIRLEGRGKLTGQEID | 240 |
| Query 241 | VPGDPSSTAFPLVAALLVPGSDVTILNVLMNPTRTGLILTLQEMGADIEVINPRLAGGED | 300 |
| Sbjct 241 | VPGDPSS AFPLVAAL+VPGSD+TI NVLMNPTRTGLILTLQEMG DIEVI+ GGED VPGDPSSAAFPLVAALIVPGSDITITNVLMNPTRTGLILTLQEMGGDIEVIDSSQVGGED | 300 |
| Query 301 | VADLRVRSSTLKGVTVPEDRAPSMIDEYPILavaaafaegaTVMNGLEELRVKESDRLSA | 360 |
| Sbjct 301 | VADLRVR S L GVTVPE+RA SMIDEYP LA+AAAFAEG TVM GL+ELRVKESDRL+A VADLRVRHSELSGVTVPEERAASMIDEYPALAIAAAFAEGKTVMAGLDELRVKESDRLAA | 360 |
| Query 361 | VANGLKLNGVDCDEGETSLVVRGRPDGKGLGNASGAAVATHLDHRIAMSFLVMGLVSENP | 420 |
| Sbjct 361 | +A L LNGV+C+E +SL+VRGR DGKGLGNA+GA+V THLDHRIAMSFLV+GL +E+P  IAASLALNGVECEEEASSLMVRGRRDGKGLGNAAGASVKTHLDHRIAMSFLVLGLAAEHP | 420 |

Query 421 VTVDDATMIATSFPEFMDLMAGLGAKI 447 VTVDD +MIATSFP F+DLM+GLGA+I

Sbjct 421 VTVDDVSMIATSFPGFLDLMSGLGARI 447

>WP_149759219.1 3-phosphoshikimate 1-carboxyvinyltransferase [Mesorhizobium albiziae] SFK11591.1 3-phosphoshikimate 1-carboxyvinyltransferase [Mesorhizobium albiziae]

Length=446

Score = 685 bits (1768), Expect = 0.0, Method: Compositional matrix adjust.

| Identities | = 350/448 (78%), Positives = 387/448 (86%), Gaps = 4/448 (1%) |  |
| --- | --- | --- |
| Query 1 | MLHGASSRPATARKSSGLSGTVRIPGDKSISHRSFMFGGLASGETRITGLLEGEDVINTG | 60 |
| Sbjct 1 | M H A PATAR+S L+GT R+PGDKSISHRSFMFGGLASGETRITGLLEGEDV+ TG MAHDAKPHPATARRSPALTGTARVPGDKSISHRSFMFGGLASGETRITGLLEGEDVMRTG | 60 |
| Query 61 | KAMQAMGARIRKEGDTWIIDGVGNGGLLAPEAPLDFGNAATGCRLTMGLVGVYDFDSTFI | 120 |
| Sbjct 61 | +AM+AMGA I K GD W+I G GNG LL P+APLDFGNA TG RLTMGLVGVYD ++TF+ EAMKAMGAHITKTGDEWVIRGTGNGALLQPQAPLDFGNAGTGSRLTMGLVGVYDMETTFV | 120 |
| Query 121 | GDASLTKRPMGRVLNPLREMGVQV-KSEDGDRLPVTLRGPKTPTPITYRVPMASAQVKSA | 179 |
| Sbjct 121 | GDASL RPM RVL+PLREMG+QV KS GDR+P+TLRGPK PI+YRVPMASAQVKSA GDASLQSRPMARVLDPLREMGMQVLKSSPGDRMPLTLRGPKVAAPISYRVPMASAQVKSA | 180 |
| Query 180 | VLLAGLNTPGITTVIEPIMTRDHTEKMLQGFGANLTVETDADGVRTIRLEGRGKLTGQVI | 239 |
| Sbjct 181 | VLLAGLNTPG+TTVIEP+MTRDHTEKML+GFGANL VETD+ GVR IR+EG+G+LTGQ I VLLAGLNTPGVTTVIEPVMTRDHTEKMLKGFGANLEVETDSLGVRHIRIEGQGRLTGQTI | 240 |
| Query 240 | DVPGDPSSTAFPLVAALLVPGSDVTILNVLMNPTRTGLILTLQEMGADIEVINPRLAGGE | 299 |
| Sbjct 241 | VPGDPSS FPLVAAL+VPGSD+TI NVLMNPTRTGL+LTLQEMG IE++NPR AGGE AVPGDPSSAGFPLVAALIVPGSDITIENVLMNPTRTGLLLTLQEMGGRIEILNPRNAGGE | 300 |
| Query 300 | DVADLRVRSSTLKGVTVPEDRAPSMIDEYPILavaaafaegaTVMNGLEELRVKESDRLS | 359 |
| Sbjct 301 | DVADLRVR S L+GV VP +RAPSMIDEYP+LAVAAAFAEG T+M GL+ELRVKESDRLS DVADLRVRGSELRGVKVPAERAPSMIDEYPVLAVAAAFAEGETLMEGLDELRVKESDRLS | 360 |
| Query 360 | AVANGLKLNGVDCDEGETSLVVRGRPDGKGLGNASGAAVATHLDHRIAMSFLVMGLVSEN | 419 |
| Sbjct 361 | AVANGLK NGVDC EGE SL VRGRPDGKGLG G VATHLDHRIAMSFLV+GL +E AVANGLKANGVDCTEGEASLSVRGRPDGKGLG---GGTVATHLDHRIAMSFLVLGLATEK | 417 |
| Query 420  Sbjct 418 | PVTVDDATMIATSFPEFMDLMAGLGAKI 447 PV++DDA MIATSFPEFM LM GLGA I PVSIDDANMIATSFPEFMGLMKGLGAAI 445 |  |

>WP_095769357.1 3-phosphoshikimate 1-carboxyvinyltransferase [Mesorhizobium sp. WSM4313]

PBB17119.1 3-phosphoshikimate 1-carboxyvinyltransferase [Mesorhizobium sp. WSM4313]

Length=452

Score = 685 bits (1768), Expect = 0.0, Method: Compositional matrix adjust.

| Identities | = 351/451 (78%), Positives = 387/451 (86%), Gaps = 4/451 (1%) |  |
| --- | --- | --- |
| Query 1 | MLHGASSRPATARKSSGLSGTVRIPGDKSISHRSFMFGGLASGETRITGLLEGEDVINTG | 60 |
| Sbjct 1 | M H A+++PATARKS L+GT R+PGDKSISHRSFMFGGLASGETRITGLLEGEDV+ TG MSHSAAAKPATARKSPALAGTARVPGDKSISHRSFMFGGLASGETRITGLLEGEDVMRTG | 60 |
| Query 61 | KAMQAMGARIRKEGDTWIIDGVGNGGLLAPEAPLDFGNAATGCRLTMGLVGVYDFDSTFI | 120 |
| Sbjct 61 | AM+AMGA I K G W+I G GNG LL PE PLDFGNA TG RLTMGLVG YD ++TFI AAMKAMGAHIEKNGGEWVIRGTGNGALLQPEGPLDFGNAGTGSRLTMGLVGTYDMETTFI | 120 |
| Query 121 | GDASLTKRPMGRVLNPLREMGVQV-KSEDGDRLPVTLRGPKTPTPITYRVPMASAQVKSA | 179 |
| Sbjct 121 | GDASL+ RPMGRVL+PLR+MGVQV K+ GDR+P+TLRGPK PITYRVPMASAQVKSA GDASLSGRPMGRVLDPLRQMGVQVLKAAPGDRMPITLRGPKHAAPITYRVPMASAQVKSA | 180 |
| Query 180 | VLLAGLNTPGITTVIEPIMTRDHTEKMLQGFGANLTVETDADGVRTIRLEGRGKLTGQVI | 239 |
| Sbjct 181 | VLLAGLNTPGITTVIEP+MTRDHTEKML+GFGANL+VETD GVR I +EG+GKLTGQ I VLLAGLNTPGITTVIEPVMTRDHTEKMLKGFGANLSVETDERGVRHIFIEGQGKLTGQTI | 240 |
| Query 240 | DVPGDPSSTAFPLVAALLVPGSDVTILNVLMNPTRTGLILTLQEMGADIEVINPRLAGGE VPGDPSS FPLVAAL+VPGSD+ I NVLMNPTRTGL+LTLQEMG I+++NPR AGGE | 299 |

| Sbjct | 241 | AVPGDPSSAGFPLVAALVVPGSDIVIENVLMNPTRTGLLLTLQEMGGKIDILNPRNAGGE | 300 |
| --- | --- | --- | --- |
| Query | 300 | DVADLRVRSSTLKGVTVPEDRAPSMIDEYPILavaaafaegaTVMNGLEELRVKESDRLS | 359 |
| Sbjct | 301 | DVADLRVRSS LKGV VP +RAPSMIDEYP+LAVAA+FAEG T+M GLEELRVKESDRL+ DVADLRVRSSELKGVVVPPERAPSMIDEYPVLAVAASFAEGETLMQGLEELRVKESDRLA | 360 |
| Query | 360 | AVANGLKLNGVDCDEGETSLVVRGRPDGKGLG---NASGAAVATHLDHRIAMSFLVMGLV | 416 |
| Sbjct | 361 | AVA GL+LNGVDC EGE SL VRGRP GKGLG N AV THLDHRIAMSFLVMGL AVARGLELNGVDCTEGEASLAVRGRPGGKGLGAHPNGPDTAVKTHLDHRIAMSFLVMGLA | 420 |
| Query  Sbjct | 417  421 | SENPVTVDDATMIATSFPEFMDLMAGLGAKI 447  +E PVT+DDA MIATSFPEFM LM GLGA+I  TEKPVTIDDANMIATSFPEFMGLMKGLGAEI 451 |  |

>RTM07576.1 3-phosphoshikimate 1-carboxyvinyltransferase [Rhizobiales bacterium] Length=452

Score = 685 bits (1768), Expect = 0.0, Method: Compositional matrix adjust.

| Identities | = 351/451 (78%), Positives = 387/451 (86%), Gaps = 4/451 (1%) |  |
| --- | --- | --- |
| Query 1 | MLHGASSRPATARKSSGLSGTVRIPGDKSISHRSFMFGGLASGETRITGLLEGEDVINTG | 60 |
| Sbjct 1 | M H A+++PATARKS L+GT R+PGDKSISHRSFMFGGLASGETRITGLLEGEDV+ TG MSHSAAAKPATARKSPALAGTARVPGDKSISHRSFMFGGLASGETRITGLLEGEDVMRTG | 60 |
| Query 61 | KAMQAMGARIRKEGDTWIIDGVGNGGLLAPEAPLDFGNAATGCRLTMGLVGVYDFDSTFI | 120 |
| Sbjct 61 | AM+AMGA I K G W+I G GNG LL PE PLDFGNA TG RLTMGLVG YD ++TFI AAMKAMGAHIEKNGAEWVIRGTGNGALLQPEGPLDFGNAGTGSRLTMGLVGTYDMETTFI | 120 |
| Query 121 | GDASLTKRPMGRVLNPLREMGVQV-KSEDGDRLPVTLRGPKTPTPITYRVPMASAQVKSA | 179 |
| Sbjct 121 | GDASL+ RPMGRVL+PLR+MGVQV K+ GDR+P+TLRGPK PITYRVPMASAQVKSA GDASLSGRPMGRVLDPLRQMGVQVLKAAPGDRMPITLRGPKHAAPITYRVPMASAQVKSA | 180 |
| Query 180 | VLLAGLNTPGITTVIEPIMTRDHTEKMLQGFGANLTVETDADGVRTIRLEGRGKLTGQVI | 239 |
| Sbjct 181 | VLLAGLNTPGITTVIEP+MTRDHTEKML+GFGANLTVETD GVR I +EG+GKLTGQ I VLLAGLNTPGITTVIEPVMTRDHTEKMLKGFGANLTVETDERGVRHIFIEGQGKLTGQTI | 240 |
| Query 240 | DVPGDPSSTAFPLVAALLVPGSDVTILNVLMNPTRTGLILTLQEMGADIEVINPRLAGGE | 299 |
| Sbjct 241 | VPGDPSS FPLVAAL+VPGS++ I NVLMNPTRTGL+LTLQEMG I+++N R AGGE AVPGDPSSAGFPLVAALIVPGSEIVIENVLMNPTRTGLLLTLQEMGGRIDILNSRNAGGE | 300 |
| Query 300 | DVADLRVRSSTLKGVTVPEDRAPSMIDEYPILavaaafaegaTVMNGLEELRVKESDRLS | 359 |
| Sbjct 301 | DV+DLRVR S LKGVTVP +RAPSMIDEYP+LAVAA+FAEG T+M GLEELRVKESDRL+ DVSDLRVRYSELKGVTVPPERAPSMIDEYPVLAVAASFAEGETLMQGLEELRVKESDRLA | 360 |
| Query 360 | AVANGLKLNGVDCDEGETSLVVRGRPDGKGLG---NASGAAVATHLDHRIAMSFLVMGLV | 416 |
| Sbjct 361 | AVANGLKLNGVDC EGE SL VRGRP GKGLG N AV THLDHRIAMSFLVMGL AVANGLKLNGVDCTEGEASLAVRGRPGGKGLGAHPNGPDTAVKTHLDHRIAMSFLVMGLA | 420 |
| Query 417  Sbjct 421 | SENPVTVDDATMIATSFPEFMDLMAGLGAKI 447  +E PVT+DDA MIATSFPEFM LM GLGA+I  TEKPVTIDDANMIATSFPEFMGLMTGLGAEI 451 |  |

>TGP51891.1 3-phosphoshikimate 1-carboxyvinyltransferase [bacterium M00.F.Ca.ET.230.01.1.1] Length=452

Score = 685 bits (1768), Expect = 0.0, Method: Compositional matrix adjust.

| Identities | = 351/452 (78%), Positives = 385/452 (85%), Gaps = 4/452 (1%) |  |
| --- | --- | --- |
| Query 1 | MLHGASSRPATARKSSGLSGTVRIPGDKSISHRSFMFGGLASGETRITGLLEGEDVINTG | 60 |
| Sbjct 1 | M H A+++PATARKS L+GT R+PGDKSISHRSFMFGGLASGETRITGLLEGEDV+ TG MSHSAAAKPATARKSPALAGTARVPGDKSISHRSFMFGGLASGETRITGLLEGEDVMRTG | 60 |
| Query 61 | KAMQAMGARIRKEGDTWIIDGVGNGGLLAPEAPLDFGNAATGCRLTMGLVGVYDFDSTFI | 120 |
| Sbjct 61 | AM+AMGA I K G W+I G GNG LL PE PLDFGNA TG RLTMGLVG YD ++ FI AAMKAMGAHIEKNGAEWVIRGTGNGALLQPEGPLDFGNAGTGSRLTMGLVGTYDMETAFI | 120 |
| Query 121 | GDASLTKRPMGRVLNPLREMGVQV-KSEDGDRLPVTLRGPKTPTPITYRVPMASAQVKSA | 179 |
| Sbjct 121 | GDASL+ RPMGRVL+PLR+MGVQV K+ GDR+P+TLRGPK PITYRVPMASAQVKSA  GDASLSGRPMGRVLDPLRQMGVQVLKAAPGDRMPITLRGPKHAAPITYRVPMASAQVKSA | 180 |

| Query | 180 | VLLAGLNTPGITTVIEPIMTRDHTEKMLQGFGANLTVETDADGVRTIRLEGRGKLTGQVI | 239 |
| --- | --- | --- | --- |
| Sbjct | 181 | VLLAGLNTPGITTVIEP+MTRDHTEKML+GFGANLTVETD GVR I +EG+GKLTGQ I VLLAGLNTPGITTVIEPVMTRDHTEKMLKGFGANLTVETDERGVRHIFIEGQGKLTGQTI | 240 |
| Query | 240 | DVPGDPSSTAFPLVAALLVPGSDVTILNVLMNPTRTGLILTLQEMGADIEVINPRLAGGE | 299 |
| Sbjct | 241 | VPGDPSS FPLVAAL+VPGSD+ I NVLMNPTRTGL+LTLQEMG I+++NPR AGGE AVPGDPSSAGFPLVAALIVPGSDIVIENVLMNPTRTGLLLTLQEMGGKIDILNPRTAGGE | 300 |
| Query | 300 | DVADLRVRSSTLKGVTVPEDRAPSMIDEYPILavaaafaegaTVMNGLEELRVKESDRLS | 359 |
| Sbjct | 301 | DVADLRV S LKGV VP +RAPSMIDEYP+LAVAA+FAEG T+M GLEELRVKESDRL+ DVADLRVCFSELKGVVVPPERAPSMIDEYPVLAVAASFAEGETLMQGLEELRVKESDRLA | 360 |
| Query | 360 | AVANGLKLNGVDCDEGETSLVVRGRPDGKGLG---NASGAAVATHLDHRIAMSFLVMGLV | 416 |
| Sbjct | 361 | AVANGLKLNGVDC EGE SL VRGRP GKGLG N V THLDHRIAMSFLVMGL AVANGLKLNGVDCTEGEASLAVRGRPGGKGLGAHPNGQDTVVKTHLDHRIAMSFLVMGLA | 420 |
| Query  Sbjct | 417  421 | SENPVTVDDATMIATSFPEFMDLMAGLGAKIE 448  +E PVT+DDA MIATSFPEFM LM GLGA+IE  TEKPVTIDDANMIATSFPEFMGLMKGLGAEIE 452 |  |

>WP_126090586.1 3-phosphoshikimate 1-carboxyvinyltransferase [Mesorhizobium sp. M1E.F.Ca.ET.045.02.1.1]

AZO20342.1 3-phosphoshikimate 1-carboxyvinyltransferase [Mesorhizobium sp. M1E.F.Ca.ET.045.02.1.1]

TKB07353.1 3-phosphoshikimate 1-carboxyvinyltransferase [Mesorhizobium sp.] Length=452

Score = 685 bits (1768), Expect = 0.0, Method: Compositional matrix adjust.

| Identities | = 350/452 (77%), Positives = 386/452 (85%), Gaps = 4/452 (1%) |  |
| --- | --- | --- |
| Query 1 | MLHGASSRPATARKSSGLSGTVRIPGDKSISHRSFMFGGLASGETRITGLLEGEDVINTG | 60 |
| Sbjct 1 | M H A+ +PATARKS L+G R+PGDKSISHRSFMFGGLASGETRITGLLEGEDV+ TG MSHSAAPKPATARKSPALTGVARVPGDKSISHRSFMFGGLASGETRITGLLEGEDVMRTG | 60 |
| Query 61 | KAMQAMGARIRKEGDTWIIDGVGNGGLLAPEAPLDFGNAATGCRLTMGLVGVYDFDSTFI | 120 |
| Sbjct 61 | AM+AMGA I ++G W+I G GNG LL PE PLDFGNA TG RLTMGLVG YD ++TFI AAMKAMGAHIERKGAEWVIRGTGNGALLEPEGPLDFGNAGTGSRLTMGLVGTYDMETTFI | 120 |
| Query 121 | GDASLTKRPMGRVLNPLREMGVQV-KSEDGDRLPVTLRGPKTPTPITYRVPMASAQVKSA | 179 |
| Sbjct 121 | GDASL+ RPMGRVL PLR+MGVQV K+ GDR+P+TL GPK PITYRVPMASAQVKSA GDASLSGRPMGRVLEPLRQMGVQVLKAAPGDRMPITLHGPKHAAPITYRVPMASAQVKSA | 180 |
| Query 180 | VLLAGLNTPGITTVIEPIMTRDHTEKMLQGFGANLTVETDADGVRTIRLEGRGKLTGQVI | 239 |
| Sbjct 181 | VLLAGLNTPGITTVIEP+MTRDHTEKML+GFGANLTVETD GVR I +EG+GKLTGQ I VLLAGLNTPGITTVIEPVMTRDHTEKMLKGFGANLTVETDERGVRHIFIEGQGKLTGQTI | 240 |
| Query 240 | DVPGDPSSTAFPLVAALLVPGSDVTILNVLMNPTRTGLILTLQEMGADIEVINPRLAGGE | 299 |
| Sbjct 241 | VPGDPSS FPLVAAL+VPGSD+ I NVLMNPTRTGL+LTLQEMG I+++NPR AGGE AVPGDPSSAGFPLVAALIVPGSDIIIENVLMNPTRTGLLLTLQEMGGRIDILNPRNAGGE | 300 |
| Query 300 | DVADLRVRSSTLKGVTVPEDRAPSMIDEYPILavaaafaegaTVMNGLEELRVKESDRLS | 359 |
| Sbjct 301 | DVADLRVRSS LKGV VP +RAPSMIDEYP+LAVAA+FAEG T+M GLEELRVKESDRL+ DVADLRVRSSELKGVVVPPERAPSMIDEYPVLAVAASFAEGETLMQGLEELRVKESDRLA | 360 |
| Query 360 | AVANGLKLNGVDCDEGETSLVVRGRPDGKGLG---NASGAAVATHLDHRIAMSFLVMGLV | 416 |
| Sbjct 361 | AVANGLKLNGVDC EGE SL VRG+P GKGLG N AV THLDHRIAMSFL+MGL AVANGLKLNGVDCTEGEASLAVRGKPGGKGLGAHPNGQDTAVKTHLDHRIAMSFLIMGLA | 420 |
| Query 417  Sbjct 421 | SENPVTVDDATMIATSFPEFMDLMAGLGAKIE 448  +E PVT+DDA MIATSFPEFM LM GLGA+IE  TEKPVTIDDANMIATSFPEFMGLMKGLGAEIE 452 |  |

>WP_127236872.1 3-phosphoshikimate 1-carboxyvinyltransferase [Mesorhizobium sp. M2C.T.Ca.TU.002.02.1.1]

RUU54461.1 3-phosphoshikimate 1-carboxyvinyltransferase [Mesorhizobium sp. M2C.T.Ca.TU.002.02.1.1]

RUU63907.1 3-phosphoshikimate 1-carboxyvinyltransferase [Mesorhizobium sp. M2C.T.Ca.TU.009.01.2.1]

Length=452

Score = 685 bits (1768), Expect = 0.0, Method: Compositional matrix adjust.

| Identities | = 350/452 (77%), Positives = 387/452 (86%), Gaps = 4/452 (1%) |  |
| --- | --- | --- |
| Query 1 | MLHGASSRPATARKSSGLSGTVRIPGDKSISHRSFMFGGLASGETRITGLLEGEDVINTG | 60 |
| Sbjct 1 | M H A+++PATARKS L+GT R+PGDKSISHRSFMFGGLASGETRITGLLEGEDV+ TG MSHSAAAKPATARKSPALAGTARVPGDKSISHRSFMFGGLASGETRITGLLEGEDVMRTG | 60 |
| Query 61 | KAMQAMGARIRKEGDTWIIDGVGNGGLLAPEAPLDFGNAATGCRLTMGLVGVYDFDSTFI | 120 |
| Sbjct 61 | AM+AMGA I K+ W+I G GNG LL PE PLDFGNA TG RLTMGLVG YD ++TF+ AAMKAMGAHIEKKDGEWVIRGTGNGALLEPEGPLDFGNAGTGSRLTMGLVGTYDMETTFV | 120 |
| Query 121 | GDASLTKRPMGRVLNPLREMGVQV-KSEDGDRLPVTLRGPKTPTPITYRVPMASAQVKSA | 179 |
| Sbjct 121 | GDASL+ RPMGRVL+PLR+MGVQV K+ GDR+P+TLRGPK PITYRVPMASAQVKSA GDASLSGRPMGRVLDPLRQMGVQVLKAAPGDRMPITLRGPKHAAPITYRVPMASAQVKSA | 180 |
| Query 180 | VLLAGLNTPGITTVIEPIMTRDHTEKMLQGFGANLTVETDADGVRTIRLEGRGKLTGQVI | 239 |
| Sbjct 181 | VLLAGLNTPGITTVIEP+MTRDHTEKML+GFGANLTVETD GVR I +EG+GKLTGQ I VLLAGLNTPGITTVIEPVMTRDHTEKMLKGFGANLTVETDERGVRHIFIEGQGKLTGQTI | 240 |
| Query 240 | DVPGDPSSTAFPLVAALLVPGSDVTILNVLMNPTRTGLILTLQEMGADIEVINPRLAGGE | 299 |
| Sbjct 241 | VPGDPSS FPLVAAL+VPGSD+ I NVLMNPTRTGL+LTLQEMG I+++NPR AGGE AVPGDPSSAGFPLVAALIVPGSDIVIENVLMNPTRTGLLLTLQEMGGRIDILNPRNAGGE | 300 |
| Query 300 | DVADLRVRSSTLKGVTVPEDRAPSMIDEYPILavaaafaegaTVMNGLEELRVKESDRLS | 359 |
| Sbjct 301 | DVADLRVR S LKGV VP +RAPSMIDEYP+LAVAA+FAEG T+M GLEELRVKESDRL+ DVADLRVRYSELKGVVVPPERAPSMIDEYPVLAVAASFAEGETLMQGLEELRVKESDRLA | 360 |
| Query 360 | AVANGLKLNGVDCDEGETSLVVRGRPDGKGLG---NASGAAVATHLDHRIAMSFLVMGLV | 416 |
| Sbjct 361 | AVANGLKLNGVDC EGE SL VRG+P GKGLG N V THLDHRIAMSFLVMGL AVANGLKLNGVDCTEGEASLAVRGKPGGKGLGAHPNGQDTVVKTHLDHRIAMSFLVMGLA | 420 |
| Query 417  Sbjct 421 | SENPVTVDDATMIATSFPEFMDLMAGLGAKIE 448  +E PVT+DDA MIATSFPEFM LM GLGA+IE  TEKPVTIDDANMIATSFPEFMGLMKGLGAEIE 452 |  |

>WP_127328864.1 3-phosphoshikimate 1-carboxyvinyltransferase [Mesorhizobium sp. M7A.F.Ca.MR.245.00.0.0]

RUV19808.1 3-phosphoshikimate 1-carboxyvinyltransferase [Mesorhizobium sp. M7A.F.Ca.MR.245.00.0.0]

Length=452

Score = 685 bits (1767), Expect = 0.0, Method: Compositional matrix adjust.

| Identities | = 349/451 (77%), Positives = 384/451 (85%), Gaps = 4/451 (1%) |  |
| --- | --- | --- |
| Query 1 | MLHGASSRPATARKSSGLSGTVRIPGDKSISHRSFMFGGLASGETRITGLLEGEDVINTG | 60 |
| Sbjct 1 | M H A+ +PATAR+S LSG R+PGDKSISHRSFMFGGLASGETRITGLLEGEDV+ TG MSHAAAPKPATARQSPALSGIARVPGDKSISHRSFMFGGLASGETRITGLLEGEDVMRTG | 60 |
| Query 61 | KAMQAMGARIRKEGDTWIIDGVGNGGLLAPEAPLDFGNAATGCRLTMGLVGVYDFDSTFI | 120 |
| Sbjct 61 | AM+AMGA I K GD W+I G GNG LL PE PLDFGNA TG RLTMGLVG YD ++TFI AAMKAMGAHIEKNGDEWVIRGTGNGALLQPEGPLDFGNAGTGSRLTMGLVGTYDMETTFI | 120 |
| Query 121 | GDASLTKRPMGRVLNPLREMGVQV-KSEDGDRLPVTLRGPKTPTPITYRVPMASAQVKSA | 179 |
| Sbjct 121 | GDASL+ RPMGRVL PLR+MGVQV K+ GDR+P+TL GPK PITYRVPMASAQVKSA GDASLSGRPMGRVLEPLRQMGVQVLKATPGDRMPITLHGPKHAAPITYRVPMASAQVKSA | 180 |
| Query 180 | VLLAGLNTPGITTVIEPIMTRDHTEKMLQGFGANLTVETDADGVRTIRLEGRGKLTGQVI | 239 |
| Sbjct 181 | VLLAGLNTPGITTVIEP+MTRDHTEKML+GFGANL+VETD GVR I +EG+G+LTGQ I VLLAGLNTPGITTVIEPVMTRDHTEKMLKGFGANLSVETDERGVRHIFIEGQGRLTGQTI | 240 |
| Query 240 | DVPGDPSSTAFPLVAALLVPGSDVTILNVLMNPTRTGLILTLQEMGADIEVINPRLAGGE | 299 |
| Sbjct 241 | VPGDPSS FPLVAAL+VPGSD+TI NVLMNPTRTGL+LTLQEMG +I+++NPR AGGE AVPGDPSSAGFPLVAALIVPGSDITIENVLMNPTRTGLLLTLQEMGGNIDILNPRNAGGE | 300 |
| Query 300 | DVADLRVRSSTLKGVTVPEDRAPSMIDEYPILavaaafaegaTVMNGLEELRVKESDRLS | 359 |
| Sbjct 301 | DVADLRVR S LKGV VP +RAPSMIDEYP+LAVAA+FAEG T+M GLEELRVKESDRLS DVADLRVRYSELKGVIVPPERAPSMIDEYPVLAVAASFAEGETLMQGLEELRVKESDRLS | 360 |
| Query 360 | AVANGLKLNGVDCDEGETSLVVRGRPDGKGLG---NASGAAVATHLDHRIAMSFLVMGLV | 416 |
| Sbjct 361 | AVANGLKLNGVDC EGE SL VRG+P GKGLG N G V THLDHRIAMSFLVMGL  AVANGLKLNGVDCTEGEASLAVRGKPSGKGLGGHPNGQGTTVQTHLDHRIAMSFLVMGLA | 420 |

Query 417 SENPVTVDDATMIATSFPEFMDLMAGLGAKI 447

+E PVT+DD MIATSFPEFM LM LGA+I Sbjct 421 TEKPVTIDDQAMIATSFPEFMGLMKALGAEI 451

>WP_073989321.1 3-phosphoshikimate 1-carboxyvinyltransferase [Mesorhizobium plurifarium] Length=452

Score = 685 bits (1767), Expect = 0.0, Method: Compositional matrix adjust.

| Identities | = 350/452 (77%), Positives = 386/452 (85%), Gaps = 4/452 (1%) |  |
| --- | --- | --- |
| Query 1 | MLHGASSRPATARKSSGLSGTVRIPGDKSISHRSFMFGGLASGETRITGLLEGEDVINTG | 60 |
| Sbjct 1 | M H A+++PATARKS L+GT R+PGDKSISHRSFMFGGLASGETRITGLLEGEDV+ TG MSHSAAAKPATARKSPALAGTARVPGDKSISHRSFMFGGLASGETRITGLLEGEDVMRTG | 60 |
| Query 61 | KAMQAMGARIRKEGDTWIIDGVGNGGLLAPEAPLDFGNAATGCRLTMGLVGVYDFDSTFI | 120 |
| Sbjct 61 | AM+AMGA I K+ D W+I G GNG LL PE PLDFGNA TG RLTMGLVG YD ++TFI AAMKAMGAHIEKKDDEWVIRGTGNGALLEPEGPLDFGNAGTGSRLTMGLVGTYDMETTFI | 120 |
| Query 121 | GDASLTKRPMGRVLNPLREMGVQV-KSEDGDRLPVTLRGPKTPTPITYRVPMASAQVKSA | 179 |
| Sbjct 121 | GDASL+ RPMGRVL+PLR+MGVQV K+ GDR+P+TLRGPK PITYRVPMASAQVKSA GDASLSGRPMGRVLDPLRQMGVQVLKAAPGDRMPITLRGPKHAAPITYRVPMASAQVKSA | 180 |
| Query 180 | VLLAGLNTPGITTVIEPIMTRDHTEKMLQGFGANLTVETDADGVRTIRLEGRGKLTGQVI | 239 |
| Sbjct 181 | VLLAGLNTPGITTVIEP+MTRDHTEKML+GFGANLTVETD GVR I +EG+G TGQ I VLLAGLNTPGITTVIEPVMTRDHTEKMLKGFGANLTVETDERGVRHIFIEGQGNFTGQTI | 240 |
| Query 240 | DVPGDPSSTAFPLVAALLVPGSDVTILNVLMNPTRTGLILTLQEMGADIEVINPRLAGGE | 299 |
| Sbjct 241 | VPGDPSS FPLVAAL+VPGSD+ I NVLMNPTRTGL+LTLQEMG I+++NPR AGGE AVPGDPSSAGFPLVAALIVPGSDIIIENVLMNPTRTGLLLTLQEMGGRIDILNPRNAGGE | 300 |
| Query 300 | DVADLRVRSSTLKGVTVPEDRAPSMIDEYPILavaaafaegaTVMNGLEELRVKESDRLS | 359 |
| Sbjct 301 | DVADLRVR S LKGV VP +RAPSMIDEYP+LAVAA+FAEG T+M GLEELRVKESDRL+ DVADLRVRYSELKGVVVPPERAPSMIDEYPVLAVAASFAEGETLMQGLEELRVKESDRLA | 360 |
| Query 360 | AVANGLKLNGVDCDEGETSLVVRGRPDGKGLG---NASGAAVATHLDHRIAMSFLVMGLV | 416 |
| Sbjct 361 | AVANGLKLNGVDC EGE SL VRG+P GKGLG N V THLDHRIAMSFLVMGL AVANGLKLNGVDCTEGEASLAVRGKPGGKGLGAHPNGQDTVVKTHLDHRIAMSFLVMGLA | 420 |
| Query 417  Sbjct 421 | SENPVTVDDATMIATSFPEFMDLMAGLGAKIE 448  +E PVT+DDA MIATSFPEFM LM GLGA+IE  TEKPVTIDDANMIATSFPEFMGLMKGLGAEIE 452 |  |

>WP_135933667.1 3-phosphoshikimate 1-carboxyvinyltransferase [Mesorhizobium sp. M5C.F.Ca.ET.164.01.1.1]

RWB33727.1 3-phosphoshikimate 1-carboxyvinyltransferase [Mesorhizobium sp.] RWB51573.1 3-phosphoshikimate 1-carboxyvinyltransferase [Mesorhizobium sp.] RWC14249.1 3-phosphoshikimate 1-carboxyvinyltransferase [Mesorhizobium sp.] RWC36646.1 3-phosphoshikimate 1-carboxyvinyltransferase [Mesorhizobium sp.] RWD21430.1 3-phosphoshikimate 1-carboxyvinyltransferase [Mesorhizobium sp.]

Length=452

Score = 685 bits (1767), Expect = 0.0, Method: Compositional matrix adjust.

| Identities | = 352/452 (78%), Positives = 386/452 (85%), Gaps = 4/452 (1%) |  |
| --- | --- | --- |
| Query 1 | MLHGASSRPATARKSSGLSGTVRIPGDKSISHRSFMFGGLASGETRITGLLEGEDVINTG | 60 |
| Sbjct 1 | M H A+ +PATARKSS LSG R+PGDKSISHRSFMFGGLASGETRITGLLEGEDV+ TG MSHTAAPKPATARKSSALSGAARVPGDKSISHRSFMFGGLASGETRITGLLEGEDVMRTG | 60 |
| Query 61 | KAMQAMGARIRKEGDTWIIDGVGNGGLLAPEAPLDFGNAATGCRLTMGLVGVYDFDSTFI | 120 |
| Sbjct 61 | AM+AMGA I K G W+I G GNG LL PE PLDFGNA TG RLTMGLVG YD ++TFI AAMKAMGAHIEKLGTEWVIRGTGNGALLQPEGPLDFGNAGTGSRLTMGLVGTYDMETTFI | 120 |
| Query 121 | GDASLTKRPMGRVLNPLREMGVQV-KSEDGDRLPVTLRGPKTPTPITYRVPMASAQVKSA | 179 |
| Sbjct 121 | GDASL+ RPMGRVL PLR+MGVQV K+ GDR+P+TLRGPK PITYRVPMASAQVKSA GDASLSGRPMGRVLEPLRQMGVQVLKATPGDRMPITLRGPKHAAPITYRVPMASAQVKSA | 180 |
| Query 180 | VLLAGLNTPGITTVIEPIMTRDHTEKMLQGFGANLTVETDADGVRTIRLEGRGKLTGQVI | 239 |
| Sbjct 181 | VLLAGLNTPGITTVIEP+MTRDHTEKML+GFGANL+VETD GVR I +EG+GKLTGQ I  VLLAGLNTPGITTVIEPVMTRDHTEKMLRGFGANLSVETDERGVRHIFIEGQGKLTGQTI | 240 |

| Query | 240 | DVPGDPSSTAFPLVAALLVPGSDVTILNVLMNPTRTGLILTLQEMGADIEVINPRLAGGE | 299 |
| --- | --- | --- | --- |
| Sbjct | 241 | VPGDPSS FPLVAAL+VPGSD+TI NVLMNPTRTGL+LTLQEMGA I++++PR GGE AVPGDPSSAGFPLVAALIVPGSDITIENVLMNPTRTGLLLTLQEMGARIDIVDPRNEGGE | 300 |
| Query | 300 | DVADLRVRSSTLKGVTVPEDRAPSMIDEYPILavaaafaegaTVMNGLEELRVKESDRLS | 359 |
| Sbjct | 301 | DVADLRVR S LKGV VP +RAP+MIDEYP+LAVAA+FAEG T+M GLEELRVKESDRLS DVADLRVRYSELKGVAVPPERAPTMIDEYPVLAVAASFAEGETLMQGLEELRVKESDRLS | 360 |
| Query | 360 | AVANGLKLNGVDCDEGETSLVVRGRPDGKGLG---NASGAAVATHLDHRIAMSFLVMGLV | 416 |
| Sbjct | 361 | AVA+GLKLNGVDC EGE SL V GRP GKGLG N G V THLDHRIAMSFLVMGL AVADGLKLNGVDCTEGEASLAVHGRPGGKGLGQHPNGQGTIVQTHLDHRIAMSFLVMGLA | 420 |
| Query  Sbjct | 417  421 | SENPVTVDDATMIATSFPEFMDLMAGLGAKIE 448  +E PVT+DD TMIATSFPEFM LM GLGA+IE  TEKPVTIDDQTMIATSFPEFMGLMKGLGAEIE 452 |  |

>RWD50221.1 3-phosphoshikimate 1-carboxyvinyltransferase [Mesorhizobium sp.] RWE13203.1 3-phosphoshikimate 1-carboxyvinyltransferase [Mesorhizobium sp.] RWE53007.1 3-phosphoshikimate 1-carboxyvinyltransferase [Mesorhizobium sp.] RWE84670.1 3-phosphoshikimate 1-carboxyvinyltransferase [Mesorhizobium sp.] RWF10317.1 3-phosphoshikimate 1-carboxyvinyltransferase [Mesorhizobium sp.]

Length=452

Score = 685 bits (1767), Expect = 0.0, Method: Compositional matrix adjust.

| Identities | = 352/452 (78%), Positives = 386/452 (85%), Gaps = 4/452 (1%) |  |
| --- | --- | --- |
| Query 1 | MLHGASSRPATARKSSGLSGTVRIPGDKSISHRSFMFGGLASGETRITGLLEGEDVINTG | 60 |
| Sbjct 1 | M H A+ +PATARKSS LSG R+PGDKSISHRSFMFGGLASGETRITGLLEGEDV+ TG MSHTAAPKPATARKSSALSGAARVPGDKSISHRSFMFGGLASGETRITGLLEGEDVMRTG | 60 |
| Query 61 | KAMQAMGARIRKEGDTWIIDGVGNGGLLAPEAPLDFGNAATGCRLTMGLVGVYDFDSTFI | 120 |
| Sbjct 61 | AM+AMGA I K G W+I G GNG LL PE PLDFGNA TG RLTMGLVG YD ++TFI AAMKAMGAHIEKLGTEWVIRGTGNGALLQPEGPLDFGNAGTGSRLTMGLVGTYDMETTFI | 120 |
| Query 121 | GDASLTKRPMGRVLNPLREMGVQV-KSEDGDRLPVTLRGPKTPTPITYRVPMASAQVKSA | 179 |
| Sbjct 121 | GDASL+ RPMGRVL PLR+MGVQV K+ GDR+P+TLRGPK PITYRVPMASAQVKSA GDASLSGRPMGRVLEPLRQMGVQVLKAIPGDRMPITLRGPKHAAPITYRVPMASAQVKSA | 180 |
| Query 180 | VLLAGLNTPGITTVIEPIMTRDHTEKMLQGFGANLTVETDADGVRTIRLEGRGKLTGQVI | 239 |
| Sbjct 181 | VLLAGLNTPGITTVIEP+MTRDHTEKML+GFGANL+VETD GVR I +EG+GKLTGQ I VLLAGLNTPGITTVIEPVMTRDHTEKMLRGFGANLSVETDERGVRHIFIEGQGKLTGQTI | 240 |
| Query 240 | DVPGDPSSTAFPLVAALLVPGSDVTILNVLMNPTRTGLILTLQEMGADIEVINPRLAGGE | 299 |
| Sbjct 241 | VPGDPSS FPLVAAL+VPGSD+TI NVLMNPTRTGL+LTLQEMGA I++++PR GGE AVPGDPSSAGFPLVAALIVPGSDITIENVLMNPTRTGLLLTLQEMGARIDIVDPRNEGGE | 300 |
| Query 300 | DVADLRVRSSTLKGVTVPEDRAPSMIDEYPILavaaafaegaTVMNGLEELRVKESDRLS | 359 |
| Sbjct 301 | DVADLRVR S LK VTVP +RAP+MIDEYP+LAVAA+FAEG T+M GLEELRVKESDRLS DVADLRVRHSELKAVTVPPERAPTMIDEYPVLAVAASFAEGETLMQGLEELRVKESDRLS | 360 |
| Query 360 | AVANGLKLNGVDCDEGETSLVVRGRPDGKGLG---NASGAAVATHLDHRIAMSFLVMGLV | 416 |
| Sbjct 361 | AVA GLKLNGVDC EGE SL VRGRP GKGLG N G V THLDHRIAMSFLVMGL AVAEGLKLNGVDCTEGEASLAVRGRPGGKGLGQHPNGQGTIVQTHLDHRIAMSFLVMGLA | 420 |
| Query 417  Sbjct 421 | SENPVTVDDATMIATSFPEFMDLMAGLGAKIE 448  +E PVT+DD TMI+TSFPEFM LM GLGA+IE  TEKPVTIDDQTMISTSFPEFMGLMKGLGAEIE 452 |  |

>RWF54871.1 3-phosphoshikimate 1-carboxyvinyltransferase [Mesorhizobium sp.] Length=452

Score = 685 bits (1767), Expect = 0.0, Method: Compositional matrix adjust.

| Identities | = 352/452 (78%), Positives = 386/452 (85%), Gaps = 4/452 (1%) |  |
| --- | --- | --- |
| Query 1 | MLHGASSRPATARKSSGLSGTVRIPGDKSISHRSFMFGGLASGETRITGLLEGEDVINTG | 60 |
| Sbjct 1 | M H A+ +PATARKSS LSG R+PGDKSISHRSFMFGGLASGETRITGLLEGEDV+ TG MSHTAAPKPATARKSSALSGAARVPGDKSISHRSFMFGGLASGETRITGLLEGEDVMRTG | 60 |
| Query 61 | KAMQAMGARIRKEGDTWIIDGVGNGGLLAPEAPLDFGNAATGCRLTMGLVGVYDFDSTFI | 120 |

| Sbjct | 61 | AM+AMGA I K G W+I G GNG LL PE PLDFGNA TG RLTMGLVG YD ++TFI AAMKAMGAHIEKLGTEWVIRGTGNGALLQPEGPLDFGNAGTGSRLTMGLVGTYDMETTFI | 120 |
| --- | --- | --- | --- |
| Query | 121 | GDASLTKRPMGRVLNPLREMGVQV-KSEDGDRLPVTLRGPKTPTPITYRVPMASAQVKSA | 179 |
| Sbjct | 121 | GDASL+ RPMGRVL PLR+MGVQV K+ GDR+P+TLRGPK PITYRVPMASAQVKSA GDASLSGRPMGRVLEPLRQMGVQVLKATPGDRMPITLRGPKHAAPITYRVPMASAQVKSA | 180 |
| Query | 180 | VLLAGLNTPGITTVIEPIMTRDHTEKMLQGFGANLTVETDADGVRTIRLEGRGKLTGQVI | 239 |
| Sbjct | 181 | VLLAGLNTPGITTVIEP+MTRDHTEKML+GFGANL+VETD GVR I +EG+GKLTGQ I VLLAGLNTPGITTVIEPVMTRDHTEKMLRGFGANLSVETDERGVRHIFIEGQGKLTGQTI | 240 |
| Query | 240 | DVPGDPSSTAFPLVAALLVPGSDVTILNVLMNPTRTGLILTLQEMGADIEVINPRLAGGE | 299 |
| Sbjct | 241 | VPGDPSS FPLVAAL+VPGSD+TI NVLMNPTRTGL+LTLQEMGA I++++PR GGE AVPGDPSSAGFPLVAALIVPGSDITIENVLMNPTRTGLLLTLQEMGARIDIVDPRNEGGE | 300 |
| Query | 300 | DVADLRVRSSTLKGVTVPEDRAPSMIDEYPILavaaafaegaTVMNGLEELRVKESDRLS | 359 |
| Sbjct | 301 | DVADLRVR S LKGV VP +RAP+MIDEYP+LAVAA+FAEG T+M GLEELRVKESDRLS DVADLRVRYSELKGVAVPPERAPTMIDEYPVLAVAASFAEGETLMQGLEELRVKESDRLS | 360 |
| Query | 360 | AVANGLKLNGVDCDEGETSLVVRGRPDGKGLG---NASGAAVATHLDHRIAMSFLVMGLV | 416 |
| Sbjct | 361 | AVA+GLKLNGVDC EGE SL V GRP GKGLG N G V THLDHRIAMSFLVMGL AVADGLKLNGVDCTEGEASLAVHGRPGGKGLGQHPNGQGIIVQTHLDHRIAMSFLVMGLA | 420 |
| Query  Sbjct | 417  421 | SENPVTVDDATMIATSFPEFMDLMAGLGAKIE 448  +E PVT+DD TMIATSFPEFM LM GLGA+IE  TEKPVTIDDQTMIATSFPEFMGLMKGLGAEIE 452 |  |

>WP_006325315.1 3-phosphoshikimate 1-carboxyvinyltransferase [Mesorhizobium sp. STM 4661]

CCV09810.1 3-phosphoshikimate 1-carboxyvinyltransferase [Mesorhizobium sp. STM 4661]

Length=452

Score = 685 bits (1767), Expect = 0.0, Method: Compositional matrix adjust.

| Identities | = 352/451 (78%), Positives = 385/451 (85%), Gaps = 4/451 (1%) |  |
| --- | --- | --- |
| Query 1 | MLHGASSRPATARKSSGLSGTVRIPGDKSISHRSFMFGGLASGETRITGLLEGEDVINTG | 60 |
| Sbjct 1 | M H A+ +PATARKS LSGT R+PGDKSISHRSFMFGGLASGETRITGLLEGEDV+ TG MSHPAAPQPATARKSPALSGTARVPGDKSISHRSFMFGGLASGETRITGLLEGEDVMRTG | 60 |
| Query 61 | KAMQAMGARIRKEGDTWIIDGVGNGGLLAPEAPLDFGNAATGCRLTMGLVGVYDFDSTFI | 120 |
| Sbjct 61 | AM+AMGA I K G W+I G GNG LL PE PLDFGNA TG RLTMGLVG YD ++TFI AAMKAMGAYIEKRGTEWVIRGTGNGALLQPEGPLDFGNAGTGSRLTMGLVGTYDMETTFI | 120 |
| Query 121 | GDASLTKRPMGRVLNPLREMGVQV-KSEDGDRLPVTLRGPKTPTPITYRVPMASAQVKSA | 179 |
| Sbjct 121 | GDASL+ RPMGRVL PLR+MGVQV K+ GDR+P+TLRGPK PITYRVPMASAQVKSA GDASLSGRPMGRVLEPLRQMGVQVLKATPGDRMPITLRGPKHAAPITYRVPMASAQVKSA | 180 |
| Query 180 | VLLAGLNTPGITTVIEPIMTRDHTEKMLQGFGANLTVETDADGVRTIRLEGRGKLTGQVI | 239 |
| Sbjct 181 | VLLAGLNTPG+TTVIEP+MTRDHTEKML+GFGANL+VETD G R I +EG+GKLTGQ I VLLAGLNTPGVTTVIEPVMTRDHTEKMLKGFGANLSVETDERGGRHIFIEGQGKLTGQTI | 240 |
| Query 240 | DVPGDPSSTAFPLVAALLVPGSDVTILNVLMNPTRTGLILTLQEMGADIEVINPRLAGGE | 299 |
| Sbjct 241 | VPGDPSS FPLVAAL+VPGSDVTI NVLMNPTRTGL+LTLQEMG I+++NPR AGGE AVPGDPSSAGFPLVAALIVPGSDVTIENVLMNPTRTGLLLTLQEMGGKIDILNPRNAGGE | 300 |
| Query 300 | DVADLRVRSSTLKGVTVPEDRAPSMIDEYPILavaaafaegaTVMNGLEELRVKESDRLS | 359 |
| Sbjct 301 | DVADLRVR S LKGVTVP +RAPSMIDEYP+LAVAA+FAEG T+M GLEELRVKESDRLS DVADLRVRYSELKGVTVPPERAPSMIDEYPVLAVAASFAEGETLMQGLEELRVKESDRLS | 360 |
| Query 360 | AVANGLKLNGVDCDEGETSLVVRGRPDGKGLG---NASGAAVATHLDHRIAMSFLVMGLV | 416 |
| Sbjct 361 | AVANGLKLNGVDC EGE SL VRG+P+GKGLG N V THLDHRIAMSFLVMGL AVANGLKLNGVDCTEGEASLAVRGKPEGKGLGGHPNGQDTTVQTHLDHRIAMSFLVMGLA | 420 |
| Query 417  Sbjct 421 | SENPVTVDDATMIATSFPEFMDLMAGLGAKI 447  +E PVT+DD MIATSFPEFM LM GLGA+I  TEKPVTIDDQAMIATSFPEFMGLMKGLGAEI 451 |  |

>WP_095810358.1 3-phosphoshikimate 1-carboxyvinyltransferase [Mesorhizobium sp. WSM3860]

PBC05195.1 3-phosphoshikimate 1-carboxyvinyltransferase [Mesorhizobium sp. WSM3860]

Length=452

Score = 685 bits (1767), Expect = 0.0, Method: Compositional matrix adjust.

| Identities | = 353/452 (78%), Positives = 384/452 (85%), Gaps = 4/452 (1%) |  |
| --- | --- | --- |
| Query 1 | MLHGASSRPATARKSSGLSGTVRIPGDKSISHRSFMFGGLASGETRITGLLEGEDVINTG | 60 |
| Sbjct 1 | M H A+ +PATARKS L+GT R+PGDKSISHRSFMFGGLASGETRITGLLEGEDV+ TG MSHAAAPKPATARKSPALAGTARVPGDKSISHRSFMFGGLASGETRITGLLEGEDVMRTG | 60 |
| Query 61 | KAMQAMGARIRKEGDTWIIDGVGNGGLLAPEAPLDFGNAATGCRLTMGLVGVYDFDSTFI | 120 |
| Sbjct 61 | AM+AMGA I K W+I G GNG LL PE PLDFGNA TG RLTMGLVG YD ++TFI AAMKAMGAHIEKRDAEWVIRGTGNGALLEPEGPLDFGNAGTGSRLTMGLVGTYDMETTFI | 120 |
| Query 121 | GDASLTKRPMGRVLNPLREMGVQV-KSEDGDRLPVTLRGPKTPTPITYRVPMASAQVKSA | 179 |
| Sbjct 121 | GDASL+ RPMGRVL PLR+MGVQV + GDR+P+TLRGPK PITYRVPMASAQVKSA GDASLSGRPMGRVLEPLRQMGVQVLNAAPGDRMPITLRGPKHAAPITYRVPMASAQVKSA | 180 |
| Query 180 | VLLAGLNTPGITTVIEPIMTRDHTEKMLQGFGANLTVETDADGVRTIRLEGRGKLTGQVI | 239 |
| Sbjct 181 | VLLAGLNTPGITTVIEP+MTRDHTEKML+GFGANLTVETD G R I +EG+GKLTGQ I VLLAGLNTPGITTVIEPVMTRDHTEKMLKGFGANLTVETDERGARHIFVEGQGKLTGQTI | 240 |
| Query 240 | DVPGDPSSTAFPLVAALLVPGSDVTILNVLMNPTRTGLILTLQEMGADIEVINPRLAGGE | 299 |
| Sbjct 241 | VPGDPSS FPLVAAL+VPGSD+ I NVLMNPTRTGL+LTLQEMG I+++NPR AGGE AVPGDPSSAGFPLVAALIVPGSDIVIENVLMNPTRTGLLLTLQEMGGRIDILNPRNAGGE | 300 |
| Query 300 | DVADLRVRSSTLKGVTVPEDRAPSMIDEYPILavaaafaegaTVMNGLEELRVKESDRLS | 359 |
| Sbjct 301 | DVADLRVRSS LKGV VP +RAPSMIDEYP+LAVAA+FAEG T+M GLEELRVKESDRLS DVADLRVRSSDLKGVVVPPERAPSMIDEYPVLAVAASFAEGETLMQGLEELRVKESDRLS | 360 |
| Query 360 | AVANGLKLNGVDCDEGETSLVVRGRPDGKGLG---NASGAAVATHLDHRIAMSFLVMGLV | 416 |
| Sbjct 361 | AVANGLKLNGVDC EGE SL VRGRP GKGLG N AV THLDHRIAMSFLVMGL AVANGLKLNGVDCSEGEASLAVRGRPGGKGLGAHPNGPDTAVKTHLDHRIAMSFLVMGLA | 420 |
| Query 417  Sbjct 421 | SENPVTVDDATMIATSFPEFMDLMAGLGAKIE 448  +E PVT+DDA MIATSFPEFM LM GLGA+IE  TEKPVTIDDANMIATSFPEFMGLMKGLGAEIE 452 |  |

>WP_140900106.1 3-phosphoshikimate 1-carboxyvinyltransferase [Mesorhizobium sp. B4-1-1]

TPI20506.1 3-phosphoshikimate 1-carboxyvinyltransferase [Mesorhizobium sp. B4-1-1]

Length=452

Score = 684 bits (1766), Expect = 0.0, Method: Compositional matrix adjust.

| Identities | = 350/452 (77%), Positives = 388/452 (86%), Gaps = 4/452 (1%) |  |
| --- | --- | --- |
| Query 1 | MLHGASSRPATARKSSGLSGTVRIPGDKSISHRSFMFGGLASGETRITGLLEGEDVINTG | 60 |
| Sbjct 1 | M H A+++PATARKS L+G R+PGDKSISHRSFMFGGLASGETRITGLLEGEDV+ TG MSHSAAAKPATARKSPPLAGIARVPGDKSISHRSFMFGGLASGETRITGLLEGEDVMRTG | 60 |
| Query 61 | KAMQAMGARIRKEGDTWIIDGVGNGGLLAPEAPLDFGNAATGCRLTMGLVGVYDFDSTFI | 120 |
| Sbjct 61 | A++AMGA + K+G W+I G GNG LL PE PLDFGNA TG RLTMGLVG YD ++TFI AAVKAMGAHVEKKGAEWVIRGTGNGALLQPEGPLDFGNAGTGSRLTMGLVGTYDMETTFI | 120 |
| Query 121 | GDASLTKRPMGRVLNPLREMGVQV-KSEDGDRLPVTLRGPKTPTPITYRVPMASAQVKSA | 179 |
| Sbjct 121 | GDASL+ RPMGRVL+PLR+MGVQV K+ GDR+P+TLRG K PITYRVPMASAQVKSA GDASLSGRPMGRVLDPLRQMGVQVLKAAPGDRMPITLRGSKHAAPITYRVPMASAQVKSA | 180 |
| Query 180 | VLLAGLNTPGITTVIEPIMTRDHTEKMLQGFGANLTVETDADGVRTIRLEGRGKLTGQVI | 239 |
| Sbjct 181 | VLLAGLNTPGITTVIEP+MTRDHTEKML+GFGANL+VETD GVR I +EG+GKLTGQ I VLLAGLNTPGITTVIEPVMTRDHTEKMLKGFGANLSVETDERGVRHIFIEGQGKLTGQTI | 240 |
| Query 240 | DVPGDPSSTAFPLVAALLVPGSDVTILNVLMNPTRTGLILTLQEMGADIEVINPRLAGGE | 299 |
| Sbjct 241 | VPGDPSS FPLVAAL+VPGSD+ I NVLMNPTRTGL+LTLQEMG IE++NPR AGGE AVPGDPSSAGFPLVAALIVPGSDIVIENVLMNPTRTGLLLTLQEMGGKIEILNPRNAGGE | 300 |
| Query 300 | DVADLRVRSSTLKGVTVPEDRAPSMIDEYPILavaaafaegaTVMNGLEELRVKESDRLS | 359 |
| Sbjct 301 | DVADLRVR S LKGVTVP +RAPSMIDEYP+LAVAA+FAEG T+M GLEELRVKESDRLS  DVADLRVRYSELKGVTVPPERAPSMIDEYPVLAVAASFAEGETLMQGLEELRVKESDRLS | 360 |

| Query | 360 | AVANGLKLNGVDCDEGETSLVVRGRPDGKGLG---NASGAAVATHLDHRIAMSFLVMGLV 416 |
| --- | --- | --- |
| Sbjct | 361 | AVANGLKLNGVDC EGE SL VRG+P GKGLG N AV T+LDHRIAMSFLVMGL AVANGLKLNGVDCTEGEASLAVRGKPGGKGLGAHPNGQDTAVKTYLDHRIAMSFLVMGLA 420 |
| Query | 417 | SENPVTVDDATMIATSFPEFMDLMAGLGAKIE 448 |
| Sbjct | 421 | +E PVT+DDA MIATSFPEFM LM GLGA+IE  TEKPVTIDDANMIATSFPEFMGLMKGLGAEIE 452 |

>RWO84248.1 3-phosphoshikimate 1-carboxyvinyltransferase [Mesorhizobium sp.] Length=452

Score = 684 bits (1766), Expect = 0.0, Method: Compositional matrix adjust.

| Identities | = 349/451 (77%), Positives = 383/451 (85%), Gaps = 4/451 (1%) |  |
| --- | --- | --- |
| Query 1 | MLHGASSRPATARKSSGLSGTVRIPGDKSISHRSFMFGGLASGETRITGLLEGEDVINTG | 60 |
| Sbjct 1 | M H A+ +PATAR+S LSG R+PGDKSISHRSFMFGGLASGETRITGLLEGEDV+ TG MSHAAAPKPATARQSPALSGIARVPGDKSISHRSFMFGGLASGETRITGLLEGEDVMRTG | 60 |
| Query 61 | KAMQAMGARIRKEGDTWIIDGVGNGGLLAPEAPLDFGNAATGCRLTMGLVGVYDFDSTFI | 120 |
| Sbjct 61 | AM+AMGA I K GD W+I G GNG LL PE PLDFGNA TG RLTMGLVG YD ++TFI AAMKAMGAHIEKHGDEWVIRGTGNGALLQPEGPLDFGNAGTGSRLTMGLVGTYDMETTFI | 120 |
| Query 121 | GDASLTKRPMGRVLNPLREMGVQV-KSEDGDRLPVTLRGPKTPTPITYRVPMASAQVKSA | 179 |
| Sbjct 121 | GDASL+ RPMGRVL PLR+MGVQV K+ GDR+P+TL GPK PITYRVPMASAQVKSA GDASLSGRPMGRVLEPLRQMGVQVLKATPGDRMPITLHGPKHAAPITYRVPMASAQVKSA | 180 |
| Query 180 | VLLAGLNTPGITTVIEPIMTRDHTEKMLQGFGANLTVETDADGVRTIRLEGRGKLTGQVI | 239 |
| Sbjct 181 | VLLAGLNTPGITTVIEP+MTRDHTEKML+GFGANL+VETD GVR I +EG+G+LTGQ I VLLAGLNTPGITTVIEPVMTRDHTEKMLKGFGANLSVETDERGVRHIFIEGQGRLTGQTI | 240 |
| Query 240 | DVPGDPSSTAFPLVAALLVPGSDVTILNVLMNPTRTGLILTLQEMGADIEVINPRLAGGE | 299 |
| Sbjct 241 | VPGDPSS FPLVAAL+VPGSD+TI NVLMNPTRTGL+LTLQEMG I+++NPR AGGE AVPGDPSSAGFPLVAALIVPGSDITIENVLMNPTRTGLLLTLQEMGGKIDILNPRNAGGE | 300 |
| Query 300 | DVADLRVRSSTLKGVTVPEDRAPSMIDEYPILavaaafaegaTVMNGLEELRVKESDRLS | 359 |
| Sbjct 301 | DVADLRVR S LKGV VP +RAPSMIDEYP+LAVAA+FAEG T+M GLEELRVKESDRLS DVADLRVRYSELKGVIVPPERAPSMIDEYPVLAVAASFAEGETLMQGLEELRVKESDRLS | 360 |
| Query 360 | AVANGLKLNGVDCDEGETSLVVRGRPDGKGLG---NASGAAVATHLDHRIAMSFLVMGLV | 416 |
| Sbjct 361 | AVANGLKLNGVDC EGE SL VRG+P GKGLG N G V THLDHRIAMSFLVMGL AVANGLKLNGVDCTEGEASLAVRGKPSGKGLGGHPNGQGTTVQTHLDHRIAMSFLVMGLA | 420 |
| Query 417  Sbjct 421 | SENPVTVDDATMIATSFPEFMDLMAGLGAKI 447  +E PVT+DD MIATSFPEFM LM LGA+I  TEKPVTIDDQAMIATSFPEFMGLMKALGAEI 451 |  |

>RWO81408.1 3-phosphoshikimate 1-carboxyvinyltransferase [Mesorhizobium sp.] Length=452

Score = 684 bits (1766), Expect = 0.0, Method: Compositional matrix adjust.

| Identities | = 352/452 (78%), Positives = 384/452 (85%), Gaps = 4/452 (1%) |  |
| --- | --- | --- |
| Query 1 | MLHGASSRPATARKSSGLSGTVRIPGDKSISHRSFMFGGLASGETRITGLLEGEDVINTG | 60 |
| Sbjct 1 | M H A+ +PATARKS LSGT R+PGDKSISHRSFMFGGLASGETRITGLLEGEDV+ TG MSHTAAPKPATARKSPALSGTARVPGDKSISHRSFMFGGLASGETRITGLLEGEDVMRTG | 60 |
| Query 61 | KAMQAMGARIRKEGDTWIIDGVGNGGLLAPEAPLDFGNAATGCRLTMGLVGVYDFDSTFI | 120 |
| Sbjct 61 | AM+AMGA I K G W+I G GNG LL PE PLDFGNA TG RLTMGLVG YD ++TFI AAMKAMGAHIEKRGAEWVIRGTGNGALLQPEGPLDFGNAGTGSRLTMGLVGTYDMETTFI | 120 |
| Query 121 | GDASLTKRPMGRVLNPLREMGVQV-KSEDGDRLPVTLRGPKTPTPITYRVPMASAQVKSA | 179 |
| Sbjct 121 | GDASL+ RPMGRVL PLR+MGVQV K+ GDR+P+TLRGPK PITYRVPMASAQVKSA GDASLSGRPMGRVLEPLRQMGVQVLKATPGDRMPITLRGPKHAAPITYRVPMASAQVKSA | 180 |
| Query 180 | VLLAGLNTPGITTVIEPIMTRDHTEKMLQGFGANLTVETDADGVRTIRLEGRGKLTGQVI | 239 |
| Sbjct 181 | VLLAGLNTPGITTVIEP+MTRDHTEKMLQGFGANL+VETD GVR I +EG+GKLTGQ I VLLAGLNTPGITTVIEPVMTRDHTEKMLQGFGANLSVETDERGVRHIFIEGQGKLTGQTI | 240 |
| Query 240 | DVPGDPSSTAFPLVAALLVPGSDVTILNVLMNPTRTGLILTLQEMGADIEVINPRLAGGE | 299 |

| Sbjct | 241 | VPGDPSS FPLVAAL+VPGSD+TI NVLMNPTRTGL+LTLQEMGA I+V+ PR GGE AVPGDPSSAGFPLVAALIVPGSDITIENVLMNPTRTGLLLTLQEMGARIDVLEPRNEGGE | 300 |
| --- | --- | --- | --- |
| Query | 300 | DVADLRVRSSTLKGVTVPEDRAPSMIDEYPILavaaafaegaTVMNGLEELRVKESDRLS | 359 |
| Sbjct | 301 | DVADLRVR S LKGVTVP +RAP+MIDEYP+LAVAA+FAEG T+M GLEELRVKESDRLS DVADLRVRYSELKGVTVPPERAPTMIDEYPVLAVAASFAEGETLMQGLEELRVKESDRLS | 360 |
| Query | 360 | AVANGLKLNGVDCDEGETSLVVRGRPDGKGLG---NASGAAVATHLDHRIAMSFLVMGLV | 416 |
| Sbjct | 361 | AVA GLKLNGVDC EGE +L VRG+P GKGLG N V THLDHRIAMSFLVMGL AVAEGLKLNGVDCTEGEATLAVRGKPGGKGLGQHPNGKSTIVQTHLDHRIAMSFLVMGLA | 420 |
| Query  Sbjct | 417  421 | SENPVTVDDATMIATSFPEFMDLMAGLGAKIE 448  +E PVT+DD MIATSFPEFM LM GLGA+IE  TEKPVTIDDQAMIATSFPEFMGLMKGLGAEIE 452 |  |

>RUU13362.1 3-phosphoshikimate 1-carboxyvinyltransferase [Mesorhizobium sp. USDA-HM6]

Length=452

Score = 684 bits (1766), Expect = 0.0, Method: Compositional matrix adjust.

| Identities | = 350/452 (77%), Positives = 386/452 (85%), Gaps = 4/452 (1%) |  |
| --- | --- | --- |
| Query 1 | MLHGASSRPATARKSSGLSGTVRIPGDKSISHRSFMFGGLASGETRITGLLEGEDVINTG | 60 |
| Sbjct 1 | M H A+ +PA ARKS L+GT R+PGDKSISHRSFMFGGLASGETRITGLLEGEDV+ TG MSHSAAPKPAIARKSPALAGTARVPGDKSISHRSFMFGGLASGETRITGLLEGEDVMRTG | 60 |
| Query 61 | KAMQAMGARIRKEGDTWIIDGVGNGGLLAPEAPLDFGNAATGCRLTMGLVGVYDFDSTFI | 120 |
| Sbjct 61 | AM+AMGA I K+G W+I G GNG LL PE PLDFGNA TG RLTMGLVG YD ++TFI AAMKAMGAHIEKKGAEWVIRGTGNGALLEPEGPLDFGNAGTGSRLTMGLVGTYDMETTFI | 120 |
| Query 121 | GDASLTKRPMGRVLNPLREMGVQV-KSEDGDRLPVTLRGPKTPTPITYRVPMASAQVKSA | 179 |
| Sbjct 121 | GDASL+ RPMGRVL PLR+MGVQV K+ GDR+P+TLRGPK PITYRVPMASAQVKSA GDASLSGRPMGRVLEPLRQMGVQVLKAAPGDRMPITLRGPKHAAPITYRVPMASAQVKSA | 180 |
| Query 180 | VLLAGLNTPGITTVIEPIMTRDHTEKMLQGFGANLTVETDADGVRTIRLEGRGKLTGQVI | 239 |
| Sbjct 181 | VLLAGLNTPGITTVIEP+MTRDHTEKML+GFGANLTVETD GVR I +EG+GKL GQ I VLLAGLNTPGITTVIEPVMTRDHTEKMLKGFGANLTVETDERGVRHIFIEGQGKLIGQTI | 240 |
| Query 240 | DVPGDPSSTAFPLVAALLVPGSDVTILNVLMNPTRTGLILTLQEMGADIEVINPRLAGGE | 299 |
| Sbjct 241 | VPGDPSS FPLVAAL+VPGSD+ I NVLMNPTRTGL+LTLQEMG I+++NPR AGGE AVPGDPSSAGFPLVAALIVPGSDIVIENVLMNPTRTGLLLTLQEMGGRIDILNPRNAGGE | 300 |
| Query 300 | DVADLRVRSSTLKGVTVPEDRAPSMIDEYPILavaaafaegaTVMNGLEELRVKESDRLS | 359 |
| Sbjct 301 | DVADLRVRSS LKGV VP +RAPSMIDEYP+LAVAA+FAEG T+M GLEELRVKESDRLS DVADLRVRSSELKGVVVPPERAPSMIDEYPVLAVAASFAEGETLMQGLEELRVKESDRLS | 360 |
| Query 360 | AVANGLKLNGVDCDEGETSLVVRGRPDGKGLG---NASGAAVATHLDHRIAMSFLVMGLV | 416 |
| Sbjct 361 | AVA+GLKLNGVDC EGE +L VRG+P GKGLG N AV THLDHRIAMSFL+MGL AVADGLKLNGVDCSEGEATLAVRGKPGGKGLGAHPNGQDTAVKTHLDHRIAMSFLIMGLA | 420 |
| Query 417  Sbjct 421 | SENPVTVDDATMIATSFPEFMDLMAGLGAKIE 448  +E PVT+DDA MIATSFPEFM LM GLGA+IE  TEKPVTIDDANMIATSFPEFMGLMKGLGAEIE 452 |  |

>WP_127395335.1 3-phosphoshikimate 1-carboxyvinyltransferase [Mesorhizobium sp. M7A.F.Ca.ET.027.03.2.1]

RVD66977.1 3-phosphoshikimate 1-carboxyvinyltransferase [Mesorhizobium sp. M7A.F.Ca.ET.027.03.2.1]

RWB03709.1 3-phosphoshikimate 1-carboxyvinyltransferase [Mesorhizobium sp.] RWB14247.1 3-phosphoshikimate 1-carboxyvinyltransferase [Mesorhizobium sp.]

Length=452

Score = 684 bits (1765), Expect = 0.0, Method: Compositional matrix adjust. Identities = 349/451 (77%), Positives = 383/451 (85%), Gaps = 4/451 (1%)

| Query | 1 | MLHGASSRPATARKSSGLSGTVRIPGDKSISHRSFMFGGLASGETRITGLLEGEDVINTG | 60 |
| --- | --- | --- | --- |
|  |  | M H A+ +PATAR+S LSG R+PGDKSISHRSFMFGGLASGETRITGLLEGEDV+ TG |  |
| Sbjct | 1 | MSHAAAPKPATARQSPALSGIARVPGDKSISHRSFMFGGLASGETRITGLLEGEDVMRTG | 60 |
| Query | 61 | KAMQAMGARIRKEGDTWIIDGVGNGGLLAPEAPLDFGNAATGCRLTMGLVGVYDFDSTFI | 120 |

| Sbjct | 61 | AM+AMGA I K GD W+I G GNG LL PE PLDFGNA TG RLTMGLVG YD ++TFI AAMKAMGAHIEKNGDEWVIRGTGNGALLQPEGPLDFGNAGTGSRLTMGLVGTYDMETTFI | 120 |
| --- | --- | --- | --- |
| Query | 121 | GDASLTKRPMGRVLNPLREMGVQV-KSEDGDRLPVTLRGPKTPTPITYRVPMASAQVKSA | 179 |
| Sbjct | 121 | GDASL+ RPMGRVL PLR+MGVQV K+ GDR+P+TL GPK PITYRVPMASAQVKSA GDASLSGRPMGRVLEPLRQMGVQVLKATPGDRMPITLHGPKHAAPITYRVPMASAQVKSA | 180 |
| Query | 180 | VLLAGLNTPGITTVIEPIMTRDHTEKMLQGFGANLTVETDADGVRTIRLEGRGKLTGQVI | 239 |
| Sbjct | 181 | VLLAGLNTPGITTVIEP+MTRDHTEKML+GFGANL+VETD GVR I +EG+G+LTGQ I VLLAGLNTPGITTVIEPVMTRDHTEKMLKGFGANLSVETDERGVRHIFIEGQGRLTGQTI | 240 |
| Query | 240 | DVPGDPSSTAFPLVAALLVPGSDVTILNVLMNPTRTGLILTLQEMGADIEVINPRLAGGE | 299 |
| Sbjct | 241 | VPGDPSS FPLVAAL+VPGSD+TI NVLMNPTRTGL+LTLQEMG I+++NPR AGGE AVPGDPSSAGFPLVAALIVPGSDITIENVLMNPTRTGLLLTLQEMGGKIDILNPRNAGGE | 300 |
| Query | 300 | DVADLRVRSSTLKGVTVPEDRAPSMIDEYPILavaaafaegaTVMNGLEELRVKESDRLS | 359 |
| Sbjct | 301 | DVADLRVR S LKGV VP +RAPSMIDEYP+LAVAA+FAEG T+M GLEELRVKESDRLS DVADLRVRYSELKGVIVPPERAPSMIDEYPVLAVAASFAEGETLMQGLEELRVKESDRLS | 360 |
| Query | 360 | AVANGLKLNGVDCDEGETSLVVRGRPDGKGLG---NASGAAVATHLDHRIAMSFLVMGLV | 416 |
| Sbjct | 361 | AVANGLKLNGVDC EGE SL VRG+P GKGLG N G V THLDHRIAMSFLVMGL AVANGLKLNGVDCTEGEASLAVRGKPSGKGLGGHPNGQGTTVQTHLDHRIAMSFLVMGLA | 420 |
| Query  Sbjct | 417  421 | SENPVTVDDATMIATSFPEFMDLMAGLGAKI 447  +E PVT+DD MIATSFPEFM LM LGA+I  TEKPVTIDDQAMIATSFPEFMGLMKALGAEI 451 |  |

>WP_107649170.1 3-phosphoshikimate 1-carboxyvinyltransferase [Mesorhizobium helmanticense] PTE10571.1 3-phosphoshikimate 1-carboxyvinyltransferase [Mesorhizobium helmanticense]

Length=452

Score = 684 bits (1765), Expect = 0.0, Method: Compositional matrix adjust.

| Identities | = 349/451 (77%), Positives = 383/451 (85%), Gaps = 4/451 (1%) |  |
| --- | --- | --- |
| Query 1 | MLHGASSRPATARKSSGLSGTVRIPGDKSISHRSFMFGGLASGETRITGLLEGEDVINTG | 60 |
| Sbjct 1 | M H A+ +PATARKS LSGT R+PGDKSISHRSFMFGGLASGETRITGLLEGEDV+ TG MSHSAAPKPATARKSPALSGTARVPGDKSISHRSFMFGGLASGETRITGLLEGEDVMRTG | 60 |
| Query 61 | KAMQAMGARIRKEGDTWIIDGVGNGGLLAPEAPLDFGNAATGCRLTMGLVGVYDFDSTFI | 120 |
| Sbjct 61 | AM+AMGA I K G W+I G GNG LL PE PLDFGNA TG RLTMGLVG YD ++TFI AAMKAMGAHIEKRGAEWLIRGTGNGALLQPEGPLDFGNAGTGSRLTMGLVGTYDMETTFI | 120 |
| Query 121 | GDASLTKRPMGRVLNPLREMGVQV-KSEDGDRLPVTLRGPKTPTPITYRVPMASAQVKSA | 179 |
| Sbjct 121 | GDASL+ RPMGRVL PLR+MGVQV K+ GDR+P+TL GPK PITYRVPMASAQVKSA GDASLSGRPMGRVLEPLRQMGVQVLKATPGDRMPITLHGPKHAAPITYRVPMASAQVKSA | 180 |
| Query 180 | VLLAGLNTPGITTVIEPIMTRDHTEKMLQGFGANLTVETDADGVRTIRLEGRGKLTGQVI | 239 |
| Sbjct 181 | VLLAGLNTPG+TTVIEP+MTRDHTEKML+GFGANL+VETD GVR I +EG+GKLTGQ I VLLAGLNTPGVTTVIEPVMTRDHTEKMLKGFGANLSVETDERGVRHIFIEGQGKLTGQTI | 240 |
| Query 240 | DVPGDPSSTAFPLVAALLVPGSDVTILNVLMNPTRTGLILTLQEMGADIEVINPRLAGGE | 299 |
| Sbjct 241 | VPGDPSS FPLVAAL+VPGSD+TI NVLMNPTRTGL+LTLQEMG I+++NPR AGGE AVPGDPSSAGFPLVAALIVPGSDITIENVLMNPTRTGLLLTLQEMGGKIDILNPRNAGGE | 300 |
| Query 300 | DVADLRVRSSTLKGVTVPEDRAPSMIDEYPILavaaafaegaTVMNGLEELRVKESDRLS | 359 |
| Sbjct 301 | DVADLRVR S LKGV VP +RAPSMIDEYP+LAVAA+FAEG T+M GLEELRVKESDRLS DVADLRVRYSELKGVIVPPERAPSMIDEYPVLAVAASFAEGETLMQGLEELRVKESDRLS | 360 |
| Query 360 | AVANGLKLNGVDCDEGETSLVVRGRPDGKGLG---NASGAAVATHLDHRIAMSFLVMGLV | 416 |
| Sbjct 361 | AV NGLKLNGVDC EGE SL VRG+P+GKGLG N V THLDHRIAMSFLVMGL AVTNGLKLNGVDCSEGEASLAVRGKPEGKGLGGHPNGQDTTVQTHLDHRIAMSFLVMGLA | 420 |
| Query 417  Sbjct 421 | SENPVTVDDATMIATSFPEFMDLMAGLGAKI 447  +E PVT+DD MIATSFPEFM LM GLGA+I  TEKPVTIDDQAMIATSFPEFMGLMKGLGAEI 451 |  |

>WP_140832121.1 3-phosphoshikimate 1-carboxyvinyltransferase [Mesorhizobium sp. B2-4-6]

TPL48203.1 3-phosphoshikimate 1-carboxyvinyltransferase [Mesorhizobium sp. B2-4-6]

Length=452

Score = 684 bits (1765), Expect = 0.0, Method: Compositional matrix adjust.

| Identities | = 351/452 (78%), Positives = 387/452 (86%), Gaps = 4/452 (1%) |  |
| --- | --- | --- |
| Query 1 | MLHGASSRPATARKSSGLSGTVRIPGDKSISHRSFMFGGLASGETRITGLLEGEDVINTG | 60 |
| Sbjct 1 | M H A+++PATARKS L+G R+PGDKSISHRSFMFGGLASGETRITGLLEGEDV+ TG MSHSAAAKPATARKSPPLAGIARVPGDKSISHRSFMFGGLASGETRITGLLEGEDVMRTG | 60 |
| Query 61 | KAMQAMGARIRKEGDTWIIDGVGNGGLLAPEAPLDFGNAATGCRLTMGLVGVYDFDSTFI | 120 |
| Sbjct 61 | AM+AMGA + K+G W+I G GNG LL PE PLDFGNA TG RLTMGLVG YD ++TFI AAMKAMGAHVEKKGAEWVIRGTGNGALLQPEGPLDFGNAGTGSRLTMGLVGTYDMETTFI | 120 |
| Query 121 | GDASLTKRPMGRVLNPLREMGVQV-KSEDGDRLPVTLRGPKTPTPITYRVPMASAQVKSA | 179 |
| Sbjct 121 | GDASL+ RPMGRVL+PLR+MGVQV K+ GDR+P+TLRG K PITYRVPMASAQVKSA GDASLSGRPMGRVLDPLRQMGVQVLKAAPGDRMPITLRGSKHAAPITYRVPMASAQVKSA | 180 |
| Query 180 | VLLAGLNTPGITTVIEPIMTRDHTEKMLQGFGANLTVETDADGVRTIRLEGRGKLTGQVI | 239 |
| Sbjct 181 | VLLAGLNTPGITTVIEP+MTRDHTEKML+GFGANLTVETD GVR I +EG+GKLTGQ I VLLAGLNTPGITTVIEPVMTRDHTEKMLKGFGANLTVETDERGVRHIFIEGQGKLTGQTI | 240 |
| Query 240 | DVPGDPSSTAFPLVAALLVPGSDVTILNVLMNPTRTGLILTLQEMGADIEVINPRLAGGE | 299 |
| Sbjct 241 | VPGDPSS FPLVAAL+VPGSD+ I NVLMNPTRTGL+LTLQEMG IE++NPR AGGE AVPGDPSSAGFPLVAALIVPGSDIVIENVLMNPTRTGLLLTLQEMGGKIEILNPRNAGGE | 300 |
| Query 300 | DVADLRVRSSTLKGVTVPEDRAPSMIDEYPILavaaafaegaTVMNGLEELRVKESDRLS | 359 |
| Sbjct 301 | DVADLRVR S LKGVTVP +RAPSMIDEYP+LAVAA+FAEG T+M GLEELRVKESDRLS DVADLRVRYSELKGVTVPPERAPSMIDEYPVLAVAASFAEGETLMQGLEELRVKESDRLS | 360 |
| Query 360 | AVANGLKLNGVDCDEGETSLVVRGRPDGKGLG---NASGAAVATHLDHRIAMSFLVMGLV | 416 |
| Sbjct 361 | AVANGLKLNGVDC E E SL VRG+P GKGLG N AV T+LDHRIAMSFLVMGL AVANGLKLNGVDCTEDEASLAVRGKPGGKGLGAHPNGQDTAVKTYLDHRIAMSFLVMGLA | 420 |
| Query 417  Sbjct 421 | SENPVTVDDATMIATSFPEFMDLMAGLGAKIE 448  +E PVT+DDA MIATSFPEFM LM GLGA+IE  TEKPVTIDDANMIATSFPEFMGLMKGLGAEIE 452 |  |

>TIR86484.1 3-phosphoshikimate 1-carboxyvinyltransferase [Mesorhizobium sp.] Length=458

Score = 684 bits (1765), Expect = 0.0, Method: Compositional matrix adjust.

| Identities | = 355/458 (78%), Positives = 386/458 (84%), Gaps = 10/458 (2%) |  |
| --- | --- | --- |
| Query 1 | MLHGASSRPATARKSSGLSGTVRIPGDKSISHRSFMFGGLASGETRITGLLEGEDVINTG | 60 |
| Sbjct 1 | M H A+ +PATARKS L+GT R+PGDKSISHRSFMFGGLASGETRITGLLEGEDV+ TG MSHAAAPKPATARKSPALAGTARVPGDKSISHRSFMFGGLASGETRITGLLEGEDVMRTG | 60 |
| Query 61 | KAMQAMGARIRKEGDTWIIDGVGNGGLLAPEAPLDFGNAATGCRLTMGLVGVYDFDSTFI | 120 |
| Sbjct 61 | AM+AMGA I K G W+I G GNG LL PE PLDFGNA TG RLTMGLVG YD ++TFI AAMKAMGAHIEKTGAEWVIRGTGNGALLEPEGPLDFGNAGTGSRLTMGLVGTYDMETTFI | 120 |
| Query 121 | GDASLTKRPMGRVLNPLREMGVQV-KSEDGDRLPVTLRGPKTPTPITYRVPMASAQVKSA | 179 |
| Sbjct 121 | GDASL+ RPMGRVL PLR+MGVQV K+ GDR+P+TLRGPK PITYRVPMASAQVKSA GDASLSGRPMGRVLEPLRQMGVQVLKAAPGDRMPITLRGPKHAAPITYRVPMASAQVKSA | 180 |
| Query 180 | VLLAGLNTPGITTVIEPIMTRDHTEKMLQGFGANLTVETDADGVRTIRLEGRGKLTGQVI | 239 |
| Sbjct 181 | VLLAGLNTPGITTVIEP+MTRDHTEKML+GFGANLTVETD GVR I +EG+GKLTGQ I VLLAGLNTPGITTVIEPVMTRDHTEKMLKGFGANLTVETDERGVRHIFIEGQGKLTGQTI | 240 |
| Query 240 | DVPGDPSSTAFPLVAALLVPGSDVTILNVLMNPTRTGLILTLQEMGADIEVINPRLAGGE | 299 |
| Sbjct 241 | VPGDPSS FPLVAAL+VPGSD+ I NVLMNPTRTGL+LTLQEMG I+++NPR AGGE AVPGDPSSAGFPLVAALIVPGSDIVIENVLMNPTRTGLLLTLQEMGGRIDILNPRNAGGE | 300 |
| Query 300 | DVADLRVRSSTLKGVTVPEDRAPSMIDEYPILavaaafaegaTVMNGLEELRVKESDRLS | 359 |
| Sbjct 301 | DVADLRVRSS LKGV VP +RAPSMIDEYP+LAVAA FAEG T+M GLEELRVKESDRLS DVADLRVRSSDLKGVVVPPERAPSMIDEYPVLAVAAVFAEGETLMQGLEELRVKESDRLS | 360 |
| Query 360 | AVANGLKLNGVDCDEGE------TSLVVRGRPDGKGLG---NASGAAVATHLDHRIAMSF | 410 |
| Sbjct 361 | AVANGLKLNGVDC EGE SL VRG+P GKGLG N AV THLDHRIAMSF  AVANGLKLNGVDCSEGEASLXXXASLAVRGKPGGKGLGAHPNGPDTAVKTHLDHRIAMSF | 420 |

Query 411 LVMGLVSENPVTVDDATMIATSFPEFMDLMAGLGAKIE 448 LVMGL +E PVT+DDA MIATSFPEFM LM GLGA+IE

Sbjct 421 LVMGLATEKPVTIDDANMIATSFPEFMGLMKGLGAEIE 458

>WP_151983503.1 3-phosphoshikimate 1-carboxyvinyltransferase [Rhizobium sp. EC-SD404] VVS98699.1 3-phosphoshikimate 1-carboxyvinyltransferase [Rhizobium sp. EC-SD404]

Length=456

Score = 684 bits (1765), Expect = 0.0, Method: Compositional matrix adjust.

| Identities | = 350/449 (78%), Positives = 391/449 (87%), Gaps = 1/449 (0%) |  |
| --- | --- | --- |
| Query 1 | MLHGAS-SRPATARKSSGLSGTVRIPGDKSISHRSFMFGGLASGETRITGLLEGEDVINT | 59 |
| Sbjct 1 | M HG+ + PATAR+S LSGTVRIPGDKSISHRSFMFGGLA+GETRITGLLEGEDVI T MAHGSQGAIPATARRSDPLSGTVRIPGDKSISHRSFMFGGLAAGETRITGLLEGEDVIAT | 60 |
| Query 60 | GKAMQAMGARIRKEGDTWIIDGVGNGGLLAPEAPLDFGNAATGCRLTMGLVGVYDFDSTF | 119 |
| Sbjct 61 | G AM+AMGA +RK+GD WII+G GNG LL P+APLDFGNA TGCRLTMGL G YDF++TF GNAMRAMGASLRKDGDVWIIEGTGNGCLLEPKAPLDFGNAGTGCRLTMGLAGTYDFETTF | 120 |
| Query 120 | IGDASLTKRPMGRVLNPLREMGVQVKSEDGDRLPVTLRGPKTPTPITYRVPMASAQVKSA | 179 |
| Sbjct 121 | IGDASL+ RPMGRVL+PL+ MGVQVK+ +G +LPVTLRGP+ PI Y VPMASAQVKSA IGDASLSARPMGRVLDPLKLMGVQVKAAEGGKLPVTLRGPRIAAPIHYTVPMASAQVKSA | 180 |
| Query 180 | VLLAGLNTPGITTVIEPIMTRDHTEKMLQGFGANLTVETDADGVRTIRLEGRGKLTGQVI | 239 |
| Sbjct 181 | VL+AGLN PG+TTV E +MTRDHTEKML GFGA+L ++TDA+G+R IR+EG+ KL G VI VLIAGLNAPGVTTVTEKVMTRDHTEKMLAGFGASLDIQTDANGIRHIRIEGQPKLKGCVI | 240 |
| Query 240 | DVPGDPSSTAFPLVAALLVPGSDVTILNVLMNPTRTGLILTLQEMGADIEVINPRLAGGE | 299 |
| Sbjct 241 | DVPGDPSSTAFPLVAALLVPGSDVTI+NVLMNPTRTGLI TL EMG I+++NPR AGGE DVPGDPSSTAFPLVAALLVPGSDVTIMNVLMNPTRTGLITTLIEMGGRIDILNPRSAGGE | 300 |
| Query 300 | DVADLRVRSSTLKGVTVPEDRAPSMIDEYPILavaaafaegaTVMNGLEELRVKESDRLS | 359 |
| Sbjct 301 | DVADLRVR S LKGV VP DRA SMIDEYP+LAVAA+FAEG TVM+G+EELRVKESDRL+ DVADLRVRHSELKGVVVPPDRAASMIDEYPVLAVAASFAEGETVMDGIEELRVKESDRLA | 360 |
| Query 360 | AVANGLKLNGVDCDEGETSLVVRGRPDGKGLGNASGAAVATHLDHRIAMSFLVMGLVSEN | 419 |
| Sbjct 361 | AVA GL+LNGVDC EGE L VRG P GKG+G+ G AVATHLDHRIAMSFLVMGL SE+ AVAAGLRLNGVDCTEGEAILTVRGTPSGKGIGSTRGNAVATHLDHRIAMSFLVMGLASEH | 420 |
| Query 420  Sbjct 421 | PVTVDDATMIATSFPEFMDLMAGLGAKIE 448 PVTVDDA MIATSFPEFMDLMAGLGA IE PVTVDDARMIATSFPEFMDLMAGLGATIE 449 |  |

>RWE58108.1 3-phosphoshikimate 1-carboxyvinyltransferase [Mesorhizobium sp.] Length=452

Score = 684 bits (1765), Expect = 0.0, Method: Compositional matrix adjust.

| Identities | = 351/452 (78%), Positives = 386/452 (85%), Gaps = 4/452 (1%) |  |
| --- | --- | --- |
| Query 1 | MLHGASSRPATARKSSGLSGTVRIPGDKSISHRSFMFGGLASGETRITGLLEGEDVINTG | 60 |
| Sbjct 1 | M H A+ +PATARKSS LSG R+PGDKSISHRSFMFGGLASGETRITGLLEGEDV+ TG MSHTAAPKPATARKSSALSGAARVPGDKSISHRSFMFGGLASGETRITGLLEGEDVMRTG | 60 |
| Query 61 | KAMQAMGARIRKEGDTWIIDGVGNGGLLAPEAPLDFGNAATGCRLTMGLVGVYDFDSTFI | 120 |
| Sbjct 61 | AM+AMGA I K G W+I G GNG LL PE PLDFGNA TG RLTMGLVG YD ++TFI AAMKAMGAHIEKRGAEWVIRGTGNGALLQPEGPLDFGNAGTGSRLTMGLVGTYDMETTFI | 120 |
| Query 121 | GDASLTKRPMGRVLNPLREMGVQV-KSEDGDRLPVTLRGPKTPTPITYRVPMASAQVKSA | 179 |
| Sbjct 121 | GDASL+ RPMGRVL PLR+MGVQV K+ GDR+P+TL GPK +PITYRVPMASAQVKSA GDASLSGRPMGRVLEPLRQMGVQVLKATPGDRMPITLHGPKHASPITYRVPMASAQVKSA | 180 |
| Query 180 | VLLAGLNTPGITTVIEPIMTRDHTEKMLQGFGANLTVETDADGVRTIRLEGRGKLTGQVI | 239 |
| Sbjct 181 | VLLAGLNTPG+TTVIEP+MTRDHTEKML+GFGANL+VETD GVR I +EG+GKLTGQ I VLLAGLNTPGVTTVIEPVMTRDHTEKMLKGFGANLSVETDERGVRHIFIEGQGKLTGQTI | 240 |
| Query 240 | DVPGDPSSTAFPLVAALLVPGSDVTILNVLMNPTRTGLILTLQEMGADIEVINPRLAGGE | 299 |
| Sbjct 241 | VPGDPSS FPLVAAL+VPGSD+TI NVLMNPTRTGL+LTLQEMGA I++++PR GGE AVPGDPSSAGFPLVAALIVPGSDITIENVLMNPTRTGLLLTLQEMGARIDIVDPRNEGGE | 300 |
| Query 300 | DVADLRVRSSTLKGVTVPEDRAPSMIDEYPILavaaafaegaTVMNGLEELRVKESDRLS | 359 |

| Sbjct | 301 | DVADLRVR S LKGVTVP +RAP+MIDEYP+LAVAA+FAEG T+M GLEELRVKESDRLS DVADLRVRYSELKGVTVPPERAPTMIDEYPVLAVAASFAEGETMMLGLEELRVKESDRLS | 360 |
| --- | --- | --- | --- |
| Query | 360 | AVANGLKLNGVDCDEGETSLVVRGRPDGKGLG---NASGAAVATHLDHRIAMSFLVMGLV | 416 |
| Sbjct | 361 | AVA GLKLNGVDC EGE SL V GRP GKGLG N G V THLDHRIAMSFLVMGL AVAEGLKLNGVDCTEGEASLAVHGRPGGKGLGQHPNGQGTIVQTHLDHRIAMSFLVMGLA | 420 |
| Query  Sbjct | 417  421 | SENPVTVDDATMIATSFPEFMDLMAGLGAKIE 448  +E PVT+DD TMIATSFPEFM LM GLGA+IE  TEKPVTIDDQTMIATSFPEFMGLMKGLGAEIE 452 |  |

>WP_123146362.1 3-phosphoshikimate 1-carboxyvinyltransferase [Mesorhizobium delmotii] SJM28632.1 3-phosphoshikimate 1-carboxyvinyltransferase [Mesorhizobium delmotii]

Length=452

Score = 684 bits (1765), Expect = 0.0, Method: Compositional matrix adjust.

| Identities | = 349/452 (77%), Positives = 385/452 (85%), Gaps = 4/452 (1%) |  |
| --- | --- | --- |
| Query 1 | MLHGASSRPATARKSSGLSGTVRIPGDKSISHRSFMFGGLASGETRITGLLEGEDVINTG | 60 |
| Sbjct 1 | M H A+ +PATARKSS LSG R+PGDKSISHRSFMFGGLASGETRITGLLEGEDV+ TG MSHTAAPKPATARKSSALSGAARVPGDKSISHRSFMFGGLASGETRITGLLEGEDVMRTG | 60 |
| Query 61 | KAMQAMGARIRKEGDTWIIDGVGNGGLLAPEAPLDFGNAATGCRLTMGLVGVYDFDSTFI | 120 |
| Sbjct 61 | AM+AMGA + K G W+I G GNG LL PE PLDFGNA TG RLTMGLVG YD ++TFI AAMKAMGAHVEKRGAEWVIRGTGNGALLQPEGPLDFGNAGTGSRLTMGLVGTYDMETTFI | 120 |
| Query 121 | GDASLTKRPMGRVLNPLREMGVQV-KSEDGDRLPVTLRGPKTPTPITYRVPMASAQVKSA | 179 |
| Sbjct 121 | GDASL+ RPMGRVL PLR+MGVQV ++ GDR+P+TL GPK PITYRVPMASAQVKSA GDASLSGRPMGRVLEPLRQMGVQVLRATPGDRMPITLHGPKHAAPITYRVPMASAQVKSA | 180 |
| Query 180 | VLLAGLNTPGITTVIEPIMTRDHTEKMLQGFGANLTVETDADGVRTIRLEGRGKLTGQVI | 239 |
| Sbjct 181 | VLLAGLNTPG+TTVIEP+MTRDHTEKML+GFGANL+VETD GVR I +EG+GKLTGQ I VLLAGLNTPGVTTVIEPVMTRDHTEKMLKGFGANLSVETDERGVRHIFIEGQGKLTGQTI | 240 |
| Query 240 | DVPGDPSSTAFPLVAALLVPGSDVTILNVLMNPTRTGLILTLQEMGADIEVINPRLAGGE | 299 |
| Sbjct 241 | VPGDPSS FPLVAAL+VPGSD+TI NVLMNPTRTGL+LTLQEMGA I+V++PR GGE AVPGDPSSAGFPLVAALIVPGSDITIENVLMNPTRTGLLLTLQEMGARIDVLDPRNEGGE | 300 |
| Query 300 | DVADLRVRSSTLKGVTVPEDRAPSMIDEYPILavaaafaegaTVMNGLEELRVKESDRLS | 359 |
| Sbjct 301 | DVADLRVR S LKGVTVP +RAP+MIDEYP+LAVAA+FAEG T+M GLEELRVKESDRLS DVADLRVRHSELKGVTVPPERAPTMIDEYPVLAVAASFAEGETLMQGLEELRVKESDRLS | 360 |
| Query 360 | AVANGLKLNGVDCDEGETSLVVRGRPDGKGLG---NASGAAVATHLDHRIAMSFLVMGLV | 416 |
| Sbjct 361 | AVANGLK+NGVDC EGE SL VRG+P GKGLG N V THLDHRIAMSFLVMGL AVANGLKVNGVDCTEGEASLAVRGKPGGKGLGQHPNGQSTIVQTHLDHRIAMSFLVMGLA | 420 |
| Query 417  Sbjct 421 | SENPVTVDDATMIATSFPEFMDLMAGLGAKIE 448  +E PVT+DD MIATSFPEFM LM GLGAKIE  TEKPVTIDDQAMIATSFPEFMGLMKGLGAKIE 452 |  |

>CDX61544.1 3-phosphoshikimate 1-carboxyvinyltransferase [Mesorhizobium plurifarium] Length=478

Score = 684 bits (1765), Expect = 0.0, Method: Compositional matrix adjust.

| Identities | = 350/452 (77%), Positives = 386/452 (85%), Gaps = 4/452 (1%) |  |
| --- | --- | --- |
| Query 1 | MLHGASSRPATARKSSGLSGTVRIPGDKSISHRSFMFGGLASGETRITGLLEGEDVINTG | 60 |
| Sbjct 27 | M H A+++PATARKS L+GT R+PGDKSISHRSFMFGGLASGETRITGLLEGEDV+ TG MSHSAAAKPATARKSPALAGTARVPGDKSISHRSFMFGGLASGETRITGLLEGEDVMRTG | 86 |
| Query 61 | KAMQAMGARIRKEGDTWIIDGVGNGGLLAPEAPLDFGNAATGCRLTMGLVGVYDFDSTFI | 120 |
| Sbjct 87 | AM+AMGA I K+ D W+I G GNG LL PE PLDFGNA TG RLTMGLVG YD ++TFI AAMKAMGAHIEKKDDEWVIRGTGNGALLEPEGPLDFGNAGTGSRLTMGLVGTYDMETTFI | 146 |
| Query 121 | GDASLTKRPMGRVLNPLREMGVQV-KSEDGDRLPVTLRGPKTPTPITYRVPMASAQVKSA | 179 |
| Sbjct 147 | GDASL+ RPMGRVL+PLR+MGVQV K+ GDR+P+TLRGPK PITYRVPMASAQVKSA GDASLSGRPMGRVLDPLRQMGVQVLKAAPGDRMPITLRGPKHAAPITYRVPMASAQVKSA | 206 |
| Query 180 | VLLAGLNTPGITTVIEPIMTRDHTEKMLQGFGANLTVETDADGVRTIRLEGRGKLTGQVI VLLAGLNTPGITTVIEP+MTRDHTEKML+GFGANLTVETD GVR I +EG+G TGQ I | 239 |

| Sbjct | 207 | VLLAGLNTPGITTVIEPVMTRDHTEKMLKGFGANLTVETDERGVRHIFIEGQGNFTGQTI | 266 |
| --- | --- | --- | --- |
| Query | 240 | DVPGDPSSTAFPLVAALLVPGSDVTILNVLMNPTRTGLILTLQEMGADIEVINPRLAGGE | 299 |
| Sbjct | 267 | VPGDPSS FPLVAAL+VPGSD+ I NVLMNPTRTGL+LTLQEMG I+++NPR AGGE AVPGDPSSAGFPLVAALIVPGSDIIIENVLMNPTRTGLLLTLQEMGGRIDILNPRNAGGE | 326 |
| Query | 300 | DVADLRVRSSTLKGVTVPEDRAPSMIDEYPILavaaafaegaTVMNGLEELRVKESDRLS | 359 |
| Sbjct | 327 | DVADLRVR S LKGV VP +RAPSMIDEYP+LAVAA+FAEG T+M GLEELRVKESDRL+ DVADLRVRYSELKGVVVPPERAPSMIDEYPVLAVAASFAEGETLMQGLEELRVKESDRLA | 386 |
| Query | 360 | AVANGLKLNGVDCDEGETSLVVRGRPDGKGLG---NASGAAVATHLDHRIAMSFLVMGLV | 416 |
| Sbjct | 387 | AVANGLKLNGVDC EGE SL VRG+P GKGLG N V THLDHRIAMSFLVMGL AVANGLKLNGVDCTEGEASLAVRGKPGGKGLGAHPNGQDTVVKTHLDHRIAMSFLVMGLA | 446 |
| Query  Sbjct | 417  447 | SENPVTVDDATMIATSFPEFMDLMAGLGAKIE 448  +E PVT+DDA MIATSFPEFM LM GLGA+IE  TEKPVTIDDANMIATSFPEFMGLMKGLGAEIE 478 |  |

>WP_095817868.1 3-phosphoshikimate 1-carboxyvinyltransferase [Mesorhizobium sp. WSM3859]

PBC09798.1 3-phosphoshikimate 1-carboxyvinyltransferase [Mesorhizobium sp. WSM3859]

Length=452

Score = 684 bits (1765), Expect = 0.0, Method: Compositional matrix adjust.

| Identities | = 350/451 (78%), Positives = 385/451 (85%), Gaps = 4/451 (1%) |  |
| --- | --- | --- |
| Query 1 | MLHGASSRPATARKSSGLSGTVRIPGDKSISHRSFMFGGLASGETRITGLLEGEDVINTG | 60 |
| Sbjct 1 | M H A+++PATARKS L+GT R+PGDKSISHRSFMFGGLASGETRITGLLEGEDV+ TG MSHSAAAKPATARKSPALAGTARVPGDKSISHRSFMFGGLASGETRITGLLEGEDVMRTG | 60 |
| Query 61 | KAMQAMGARIRKEGDTWIIDGVGNGGLLAPEAPLDFGNAATGCRLTMGLVGVYDFDSTFI | 120 |
| Sbjct 61 | AM+AMGA I K G W+I G GNG LL PE PLDFGNA TG RLTMGLVG YD ++TFI AAMKAMGAHIEKNGAEWVIRGTGNGALLQPEEPLDFGNAGTGSRLTMGLVGTYDMETTFI | 120 |
| Query 121 | GDASLTKRPMGRVLNPLREMGVQV-KSEDGDRLPVTLRGPKTPTPITYRVPMASAQVKSA | 179 |
| Sbjct 121 | GDASL+ RPMGRVL+PLR+MGVQV K+ GDR+P+TLRGPK PITYRVPMASAQVKSA GDASLSGRPMGRVLDPLRQMGVQVLKAAPGDRMPITLRGPKHAAPITYRVPMASAQVKSA | 180 |
| Query 180 | VLLAGLNTPGITTVIEPIMTRDHTEKMLQGFGANLTVETDADGVRTIRLEGRGKLTGQVI | 239 |
| Sbjct 181 | VLLA LNTPGITTVIEP+MTRDHTEKML+GFGANLTVETD GVR I +EG+GKLTGQ I VLLAALNTPGITTVIEPVMTRDHTEKMLKGFGANLTVETDERGVRHIFIEGQGKLTGQTI | 240 |
| Query 240 | DVPGDPSSTAFPLVAALLVPGSDVTILNVLMNPTRTGLILTLQEMGADIEVINPRLAGGE | 299 |
| Sbjct 241 | VPGDPSS FPLVAAL+VPGS++ I NVLMNPTRTGL+LTLQEMG I+++NPR AGGE AVPGDPSSAGFPLVAALIVPGSNIVIENVLMNPTRTGLLLTLQEMGGRIDILNPRNAGGE | 300 |
| Query 300 | DVADLRVRSSTLKGVTVPEDRAPSMIDEYPILavaaafaegaTVMNGLEELRVKESDRLS | 359 |
| Sbjct 301 | DVADLRVRSS LKGV VP +RAPSMIDEYP+LAVAA FAEG T+M GLEELRVKESDRL+ DVADLRVRSSDLKGVVVPPERAPSMIDEYPVLAVAAVFAEGETLMQGLEELRVKESDRLA | 360 |
| Query 360 | AVANGLKLNGVDCDEGETSLVVRGRPDGKGLG---NASGAAVATHLDHRIAMSFLVMGLV | 416 |
| Sbjct 361 | AVANGLKLNGVDC EGE SL VRG+P GKGLG N V THLDHRIAMSFLVMGL AVANGLKLNGVDCTEGEASLAVRGKPGGKGLGAHPNGQDTVVKTHLDHRIAMSFLVMGLA | 420 |
| Query 417  Sbjct 421 | SENPVTVDDATMIATSFPEFMDLMAGLGAKI 447  +E PVT+DDA MIATSFPEFM LM GLGA+I  TEKPVTIDDANMIATSFPEFMGLMKGLGAEI 451 |  |

>TIN70018.1 3-phosphoshikimate 1-carboxyvinyltransferase [Mesorhizobium sp.] Length=452

Score = 684 bits (1764), Expect = 0.0, Method: Compositional matrix adjust.

| Identities | = 349/451 (77%), Positives = 383/451 (85%), Gaps = 4/451 (1%) |  |
| --- | --- | --- |
| Query 1 | MLHGASSRPATARKSSGLSGTVRIPGDKSISHRSFMFGGLASGETRITGLLEGEDVINTG | 60 |
| Sbjct 1 | M H A+ +PATAR+S LSG R+PGDKSISHRSFMFGGLASGETRITGLLEGEDV+ TG MSHAAAPKPATARQSPALSGIARVPGDKSISHRSFMFGGLASGETRITGLLEGEDVMRTG | 60 |
| Query 61 | KAMQAMGARIRKEGDTWIIDGVGNGGLLAPEAPLDFGNAATGCRLTMGLVGVYDFDSTFI | 120 |

| Sbjct | 61 | AM+AMGA I K GD W+I G GNG LL PE PLDFGNA TG RLTMGLVG YD ++TFI AAMKAMGAHIGKHGDEWVIRGTGNGALLQPEGPLDFGNAGTGSRLTMGLVGTYDMETTFI | 120 |
| --- | --- | --- | --- |
| Query | 121 | GDASLTKRPMGRVLNPLREMGVQV-KSEDGDRLPVTLRGPKTPTPITYRVPMASAQVKSA | 179 |
| Sbjct | 121 | GDASL+ RPMGRVL PLR+MGVQV K+ GDR+P+TL GPK PITYRVPMASAQVKSA GDASLSGRPMGRVLEPLRQMGVQVLKATPGDRMPITLHGPKHAAPITYRVPMASAQVKSA | 180 |
| Query | 180 | VLLAGLNTPGITTVIEPIMTRDHTEKMLQGFGANLTVETDADGVRTIRLEGRGKLTGQVI | 239 |
| Sbjct | 181 | VLLAGLNTPGITTVIEP+MTRDHTEKML+GFGANL+VETD GVR I +EG+G+LTGQ I VLLAGLNTPGITTVIEPVMTRDHTEKMLKGFGANLSVETDERGVRHIFIEGQGRLTGQTI | 240 |
| Query | 240 | DVPGDPSSTAFPLVAALLVPGSDVTILNVLMNPTRTGLILTLQEMGADIEVINPRLAGGE | 299 |
| Sbjct | 241 | VPGDPSS FPLVAAL+VPGSD+TI NVLMNPTRTGL+LTLQEMG I+++NPR AGGE AVPGDPSSAGFPLVAALIVPGSDITIENVLMNPTRTGLLLTLQEMGGKIDILNPRNAGGE | 300 |
| Query | 300 | DVADLRVRSSTLKGVTVPEDRAPSMIDEYPILavaaafaegaTVMNGLEELRVKESDRLS | 359 |
| Sbjct | 301 | DVADLRVR S LKGV VP +RAPSMIDEYP+LAVAA+FAEG T+M GLEELRVKESDRLS DVADLRVRYSELKGVIVPPERAPSMIDEYPVLAVAASFAEGETLMQGLEELRVKESDRLS | 360 |
| Query | 360 | AVANGLKLNGVDCDEGETSLVVRGRPDGKGLG---NASGAAVATHLDHRIAMSFLVMGLV | 416 |
| Sbjct | 361 | AVANGLKLNGVDC EGE SL VRG+P GKGLG N G V THLDHRIAMSFLVMGL AVANGLKLNGVDCTEGEASLAVRGKPSGKGLGGHPNGQGTTVQTHLDHRIAMSFLVMGLA | 420 |
| Query  Sbjct | 417  421 | SENPVTVDDATMIATSFPEFMDLMAGLGAKI 447  +E PVT+DD MIATSFPEFM LM LGA+I  TEKPVTIDDQAMIATSFPEFMGLMKALGAEI 451 |  |

>RWB99656.1 3-phosphoshikimate 1-carboxyvinyltransferase [Mesorhizobium sp.] RWO10313.1 3-phosphoshikimate 1-carboxyvinyltransferase [Mesorhizobium sp.] RWO21064.1 3-phosphoshikimate 1-carboxyvinyltransferase [Mesorhizobium sp.] RWO96732.1 3-phosphoshikimate 1-carboxyvinyltransferase [Mesorhizobium sp.] RWP08662.1 3-phosphoshikimate 1-carboxyvinyltransferase [Mesorhizobium sp.]

Length=452

Score = 684 bits (1764), Expect = 0.0, Method: Compositional matrix adjust.

| Identities | = 350/452 (77%), Positives = 385/452 (85%), Gaps = 4/452 (1%) |  |
| --- | --- | --- |
| Query 1 | MLHGASSRPATARKSSGLSGTVRIPGDKSISHRSFMFGGLASGETRITGLLEGEDVINTG | 60 |
| Sbjct 1 | M H A+ +PATARKS LSGT R+PGDKSISHRSFMFGGLASGETRITGLLEGEDV+ TG MSHTAAPKPATARKSPALSGTARVPGDKSISHRSFMFGGLASGETRITGLLEGEDVMRTG | 60 |
| Query 61 | KAMQAMGARIRKEGDTWIIDGVGNGGLLAPEAPLDFGNAATGCRLTMGLVGVYDFDSTFI | 120 |
| Sbjct 61 | AM+AMGA I K G W+I G GNG LL PE PLDFGNA TG RLTMGLVG YD ++TFI AAMKAMGAHIEKRGAEWVIRGTGNGALLQPEGPLDFGNAGTGSRLTMGLVGTYDMETTFI | 120 |
| Query 121 | GDASLTKRPMGRVLNPLREMGVQV-KSEDGDRLPVTLRGPKTPTPITYRVPMASAQVKSA | 179 |
| Sbjct 121 | GDASL+ RPMGRVL PLR+MGVQV K+ GDR+P+TLRGPK PITYRVPMASAQVKSA GDASLSGRPMGRVLEPLRQMGVQVLKATPGDRMPITLRGPKHAAPITYRVPMASAQVKSA | 180 |
| Query 180 | VLLAGLNTPGITTVIEPIMTRDHTEKMLQGFGANLTVETDADGVRTIRLEGRGKLTGQVI | 239 |
| Sbjct 181 | VLLAGLNTPGITTVIEP+MTRDHTEKMLQGFGANL+VETD GVR I +EG+GKLTGQ I VLLAGLNTPGITTVIEPVMTRDHTEKMLQGFGANLSVETDERGVRHIFIEGQGKLTGQTI | 240 |
| Query 240 | DVPGDPSSTAFPLVAALLVPGSDVTILNVLMNPTRTGLILTLQEMGADIEVINPRLAGGE | 299 |
| Sbjct 241 | VPGDPSS FPLVAAL+VPGSD+TI NVLMNPTRTGL+LTLQEMGA I++++PR GGE AVPGDPSSAGFPLVAALIVPGSDITIENVLMNPTRTGLLLTLQEMGARIDIVDPRNEGGE | 300 |
| Query 300 | DVADLRVRSSTLKGVTVPEDRAPSMIDEYPILavaaafaegaTVMNGLEELRVKESDRLS | 359 |
| Sbjct 301 | DVADLRVR S L+GVTVP +RAP+MIDEYP+LAVAA+FAEG T+M GLEELRVKESDRLS DVADLRVRYSELRGVTVPPERAPTMIDEYPVLAVAASFAEGETLMQGLEELRVKESDRLS | 360 |
| Query 360 | AVANGLKLNGVDCDEGETSLVVRGRPDGKGLG---NASGAAVATHLDHRIAMSFLVMGLV | 416 |
| Sbjct 361 | AVA GLKLNGVDC EGE +L VRG+P GKGLG N V THLDHRIAMSFLVMGL AVAEGLKLNGVDCTEGEATLAVRGKPGGKGLGQHPNGKSTIVQTHLDHRIAMSFLVMGLA | 420 |
| Query 417  Sbjct 421 | SENPVTVDDATMIATSFPEFMDLMAGLGAKIE 448  +E PVT+DD MIATSFPEFM LM GLGA+IE  TEKPVTIDDQAMIATSFPEFMGLMKGLGAEIE 452 |  |

>TIV34104.1 3-phosphoshikimate 1-carboxyvinyltransferase [Mesorhizobium sp.]

Length=458

Score = 684 bits (1764), Expect = 0.0, Method: Compositional matrix adjust.

| Identities | = 354/458 (77%), Positives = 388/458 (85%), Gaps = 10/458 (2%) |  |
| --- | --- | --- |
| Query 1 | MLHGASSRPATARKSSGLSGTVRIPGDKSISHRSFMFGGLASGETRITGLLEGEDVINTG | 60 |
| Sbjct 1 | M H A+++ ATARKS L+GT R+PGDKSISHRSFMFGGLASGETRITGLLEGEDV+ TG MSHSAAAKQATARKSPALAGTARVPGDKSISHRSFMFGGLASGETRITGLLEGEDVMRTG | 60 |
| Query 61 | KAMQAMGARIRKEGDTWIIDGVGNGGLLAPEAPLDFGNAATGCRLTMGLVGVYDFDSTFI | 120 |
| Sbjct 61 | AM+AMGA I K G W+I G GNG LL PE PLDFGNA TG RLTMGLVG YD ++TFI AAMKAMGAHIEKNGAEWVIRGTGNGALLQPEGPLDFGNAGTGSRLTMGLVGTYDMETTFI | 120 |
| Query 121 | GDASLTKRPMGRVLNPLREMGVQV-KSEDGDRLPVTLRGPKTPTPITYRVPMASAQVKSA | 179 |
| Sbjct 121 | GDASL+ RPMGRVL+PLR+MGVQV K+ GDR+P+TLRGPK PITYRVPMASAQVKSA GDASLSGRPMGRVLDPLRQMGVQVLKAAPGDRMPITLRGPKHAAPITYRVPMASAQVKSA | 180 |
| Query 180 | VLLAGLNTPGITTVIEPIMTRDHTEKMLQGFGANLTVETDADGVRTIRLEGRGKLTGQVI | 239 |
| Sbjct 181 | VLLAGLNTPGITTVIEP+MTRDHTEKML+GFGANLTVETD GVR I +EG+GKLTGQ I VLLAGLNTPGITTVIEPVMTRDHTEKMLKGFGANLTVETDERGVRHIFIEGQGKLTGQTI | 240 |
| Query 240 | DVPGDPSSTAFPLVAALLVPGSDVTILNVLMNPTRTGLILTLQEMGADIEVINPRLAGGE | 299 |
| Sbjct 241 | VPGDPSS FPLVAAL+VPGSD+ I NVLMNPTRTGL+LTLQEMG I+++NPR AGGE AVPGDPSSAGFPLVAALIVPGSDIVIENVLMNPTRTGLLLTLQEMGGRIDILNPRNAGGE | 300 |
| Query 300 | DVADLRVRSSTLKGVTVPEDRAPSMIDEYPILavaaafaegaTVMNGLEELRVKESDR-- | 357 |
| Sbjct 301 | DVADLRVR S LKGVTVP +RAPSMIDEYP+LAVAA+FAEG T+M GLEELRVKESDR DVADLRVRYSELKGVTVPPERAPSMIDEYPVLAVAASFAEGETLMQGLEELRVKESDRLS | 360 |
| Query 358 | ----LSAVANGLKLNGVDCDEGETSLVVRGRPDGKGLG---NASGAAVATHLDHRIAMSF | 410 |
| Sbjct 361 | LSAVANGLKLNGVDC EGE SL VRGRP GKGLG N AV THLDHRIAMSF AXXXLSAVANGLKLNGVDCTEGEASLAVRGRPGGKGLGAHPNGPDTAVKTHLDHRIAMSF | 420 |
| Query 411  Sbjct 421 | LVMGLVSENPVTVDDATMIATSFPEFMDLMAGLGAKIE 448 L+MGL +E PVT+DDA MIATSFPEFM LM GLGA+IE LIMGLATEKPVTIDDANMIATSFPEFMGLMKGLGAEIE 458 |  |

>WP_140722107.1 3-phosphoshikimate 1-carboxyvinyltransferase [Mesorhizobium sp. B2-7-2]

TPJ31045.1 3-phosphoshikimate 1-carboxyvinyltransferase [Mesorhizobium sp. B2-7-2]

Length=452

Score = 684 bits (1764), Expect = 0.0, Method: Compositional matrix adjust.

| Identities | = 350/451 (78%), Positives = 386/451 (86%), Gaps = 4/451 (1%) |  |
| --- | --- | --- |
| Query 1 | MLHGASSRPATARKSSGLSGTVRIPGDKSISHRSFMFGGLASGETRITGLLEGEDVINTG | 60 |
| Sbjct 1 | M H A+++PATARKS L+GT R+PGDKSISHRSFMFGGLASGETRITGLLEGEDV+ TG MSHSAAAKPATARKSPALAGTARVPGDKSISHRSFMFGGLASGETRITGLLEGEDVMRTG | 60 |
| Query 61 | KAMQAMGARIRKEGDTWIIDGVGNGGLLAPEAPLDFGNAATGCRLTMGLVGVYDFDSTFI | 120 |
| Sbjct 61 | AM+AMGA I K G W+I G GNG LL PE PLDFGNA TG RLTMGLVG YD ++TFI AAMKAMGAHIEKNGGEWVIRGTGNGALLQPEGPLDFGNAGTGSRLTMGLVGTYDMETTFI | 120 |
| Query 121 | GDASLTKRPMGRVLNPLREMGVQV-KSEDGDRLPVTLRGPKTPTPITYRVPMASAQVKSA | 179 |
| Sbjct 121 | GDASL+ RPMGRVL+PLR+MGVQV K+ GDR+P+TLRGPK PITYRVPMASAQVKSA GDASLSGRPMGRVLDPLRQMGVQVLKAAPGDRMPITLRGPKHAAPITYRVPMASAQVKSA | 180 |
| Query 180 | VLLAGLNTPGITTVIEPIMTRDHTEKMLQGFGANLTVETDADGVRTIRLEGRGKLTGQVI | 239 |
| Sbjct 181 | VLLAGLNTPGI TVIEP+MTRDHTEKML+GFGANL+VETD GVR I +EG+GKLTGQ I VLLAGLNTPGIITVIEPVMTRDHTEKMLKGFGANLSVETDERGVRHIFIEGQGKLTGQTI | 240 |
| Query 240 | DVPGDPSSTAFPLVAALLVPGSDVTILNVLMNPTRTGLILTLQEMGADIEVINPRLAGGE | 299 |
| Sbjct 241 | VPGDPSS FPLVAAL+VPGSD+ I NVLMNPTRTGL+LTLQEMG I+++NPR AGGE AVPGDPSSAGFPLVAALIVPGSDIVIENVLMNPTRTGLLLTLQEMGGKIDILNPRNAGGE | 300 |
| Query 300 | DVADLRVRSSTLKGVTVPEDRAPSMIDEYPILavaaafaegaTVMNGLEELRVKESDRLS | 359 |
| Sbjct 301 | DVADLRVRSS LKGV VP +RAPSMIDEYP+LAVAA+FAEG T+M GLEELRVKESDRL+ DVADLRVRSSELKGVAVPPERAPSMIDEYPVLAVAASFAEGETLMQGLEELRVKESDRLA | 360 |
| Query 360 | AVANGLKLNGVDCDEGETSLVVRGRPDGKGLG---NASGAAVATHLDHRIAMSFLVMGLV | 416 |

AVA GL+LNGVDC EGE SL VRGRP GKGLG	N	AV THLDHRIAMSFLVMGL

| Sbjct | 361 | AVARGLELNGVDCTEGEASLAVRGRPGGKGLGAHPNGPDTAVKTHLDHRIAMSFLVMGLA 420 |
| --- | --- | --- |
| Query | 417 | SENPVTVDDATMIATSFPEFMDLMAGLGAKI 447 |
| Sbjct | 421 | +E PVT+DDA MIATSFPEFM LM GLGA+I  TEKPVTIDDANMIATSFPEFMGLMKGLGAEI 451 |

>RWO34190.1 3-phosphoshikimate 1-carboxyvinyltransferase [Mesorhizobium sp.] RWO44405.1 3-phosphoshikimate 1-carboxyvinyltransferase [Mesorhizobium sp.] TIN78588.1 3-phosphoshikimate 1-carboxyvinyltransferase [Mesorhizobium sp.]

Length=452

Score = 684 bits (1764), Expect = 0.0, Method: Compositional matrix adjust.

| Identities | = 352/452 (78%), Positives = 383/452 (85%), Gaps = 4/452 (1%) |  |
| --- | --- | --- |
| Query 1 | MLHGASSRPATARKSSGLSGTVRIPGDKSISHRSFMFGGLASGETRITGLLEGEDVINTG | 60 |
| Sbjct 1 | M H A+ PATARKS LSGT R+PGDKSISHRSFMFGGLASGETRITGLLEGEDV+ TG MSHTAAPSPATARKSPALSGTARVPGDKSISHRSFMFGGLASGETRITGLLEGEDVMRTG | 60 |
| Query 61 | KAMQAMGARIRKEGDTWIIDGVGNGGLLAPEAPLDFGNAATGCRLTMGLVGVYDFDSTFI | 120 |
| Sbjct 61 | AM+AMGA I K G W+I G GNG LL PE PLDFGNA TG RLTMGLVG YD ++TFI AAMKAMGAHIEKRGAEWVIRGTGNGALLQPEGPLDFGNAGTGSRLTMGLVGTYDMETTFI | 120 |
| Query 121 | GDASLTKRPMGRVLNPLREMGVQV-KSEDGDRLPVTLRGPKTPTPITYRVPMASAQVKSA | 179 |
| Sbjct 121 | GDASL+ RPMGRVL PLR+MGVQV K+ GDR+P+TLRGPK PITYRVPMASAQVKSA GDASLSGRPMGRVLEPLRQMGVQVLKATPGDRMPITLRGPKHAAPITYRVPMASAQVKSA | 180 |
| Query 180 | VLLAGLNTPGITTVIEPIMTRDHTEKMLQGFGANLTVETDADGVRTIRLEGRGKLTGQVI | 239 |
| Sbjct 181 | VLLAGLNTPGITTVIEP+MTRDHTEKMLQGFGANL+VETD GVR I +EG+GKLTGQ I VLLAGLNTPGITTVIEPVMTRDHTEKMLQGFGANLSVETDERGVRHIFIEGQGKLTGQTI | 240 |
| Query 240 | DVPGDPSSTAFPLVAALLVPGSDVTILNVLMNPTRTGLILTLQEMGADIEVINPRLAGGE | 299 |
| Sbjct 241 | VPGDPSS FPLVAAL+VPGSD+TI NVLMNPTRTGL+LTLQEMGA I+V+ PR GGE AVPGDPSSAGFPLVAALIVPGSDITIENVLMNPTRTGLLLTLQEMGARIDVLEPRNEGGE | 300 |
| Query 300 | DVADLRVRSSTLKGVTVPEDRAPSMIDEYPILavaaafaegaTVMNGLEELRVKESDRLS | 359 |
| Sbjct 301 | DVADLRVR S LKGVTVP +RAP+MIDEYP+LAVAA+FAEG T+M GLEELRVKESDRLS DVADLRVRYSELKGVTVPPERAPTMIDEYPVLAVAASFAEGETLMQGLEELRVKESDRLS | 360 |
| Query 360 | AVANGLKLNGVDCDEGETSLVVRGRPDGKGLG---NASGAAVATHLDHRIAMSFLVMGLV | 416 |
| Sbjct 361 | AVA GLKLNGVDC EGE +L VRG+P GKGLG N V THLDHRIAMSFLVMGL AVAEGLKLNGVDCTEGEATLAVRGKPGGKGLGQHPNGKSTIVQTHLDHRIAMSFLVMGLA | 420 |
| Query 417  Sbjct 421 | SENPVTVDDATMIATSFPEFMDLMAGLGAKIE 448  +E PVT+DD MIATSFPEFM LM GLGA+IE  TEKPVTIDDQAMIATSFPEFMGLMKGLGAEIE 452 |  |

>WP_140845573.1 3-phosphoshikimate 1-carboxyvinyltransferase [Mesorhizobium sp. B2-8-9]

TPI74214.1 3-phosphoshikimate 1-carboxyvinyltransferase [Mesorhizobium sp. B2-8-9]

Length=452

Score = 684 bits (1764), Expect = 0.0, Method: Compositional matrix adjust.

| Identities | = 350/451 (78%), Positives = 387/451 (86%), Gaps = 4/451 (1%) |  |
| --- | --- | --- |
| Query 1 | MLHGASSRPATARKSSGLSGTVRIPGDKSISHRSFMFGGLASGETRITGLLEGEDVINTG | 60 |
| Sbjct 1 | M H A+++PATARKS L+GT R+PGDKSISHRSFMFGGLASGETRITGLLEGEDV+ TG MSHSAAAKPATARKSPALAGTARVPGDKSISHRSFMFGGLASGETRITGLLEGEDVMRTG | 60 |
| Query 61 | KAMQAMGARIRKEGDTWIIDGVGNGGLLAPEAPLDFGNAATGCRLTMGLVGVYDFDSTFI | 120 |
| Sbjct 61 | AM+AMGA I K G W+I G GNG LL PE PLDFGNA TG RLTMGLVG YD ++TFI AAMKAMGAHIEKNGGEWVIRGTGNGALLQPEGPLDFGNAGTGSRLTMGLVGTYDMETTFI | 120 |
| Query 121 | GDASLTKRPMGRVLNPLREMGVQV-KSEDGDRLPVTLRGPKTPTPITYRVPMASAQVKSA | 179 |
| Sbjct 121 | GDASL+ RPMGRVL+PLR+MGVQV K+ GDR+P+TLRGPK PITYRVPMASAQVKSA GDASLSGRPMGRVLDPLRQMGVQVLKAAPGDRMPITLRGPKHAAPITYRVPMASAQVKSA | 180 |
| Query 180 | VLLAGLNTPGITTVIEPIMTRDHTEKMLQGFGANLTVETDADGVRTIRLEGRGKLTGQVI VLLAGLNTPGITTVIEP+MTRDHTEKML+GFGANL+VETD GVR I +EG+GKLTGQ I | 239 |

| Sbjct | 181 | VLLAGLNTPGITTVIEPVMTRDHTEKMLKGFGANLSVETDERGVRHIFIEGQGKLTGQTI | 240 |
| --- | --- | --- | --- |
| Query | 240 | DVPGDPSSTAFPLVAALLVPGSDVTILNVLMNPTRTGLILTLQEMGADIEVINPRLAGGE | 299 |
| Sbjct | 241 | VPGDPSS FPLVAAL+VPGSD+ I NVLMNPTRTGL+LTLQEMG I+++NPR AGGE AVPGDPSSAGFPLVAALIVPGSDIVIENVLMNPTRTGLLLTLQEMGGRIDILNPRNAGGE | 300 |
| Query | 300 | DVADLRVRSSTLKGVTVPEDRAPSMIDEYPILavaaafaegaTVMNGLEELRVKESDRLS | 359 |
| Sbjct | 301 | DVADLRVRSS LKGV VP +RAPSMIDEYP+LAVAA+FAEG T+M GLEELRVKESDRL+ DVADLRVRSSELKGVIVPPERAPSMIDEYPVLAVAASFAEGETLMQGLEELRVKESDRLA | 360 |
| Query | 360 | AVANGLKLNGVDCDEGETSLVVRGRPDGKGLG---NASGAAVATHLDHRIAMSFLVMGLV | 416 |
| Sbjct | 361 | AVA GL+LNGVDC EGE SL VRG+P GKGLG N AV THLDHRIAMSFLVMGL AVARGLELNGVDCTEGEASLAVRGKPGGKGLGAHPNGPDTAVKTHLDHRIAMSFLVMGLA | 420 |
| Query  Sbjct | 417  421 | SENPVTVDDATMIATSFPEFMDLMAGLGAKI 447  +E PVT+DDA MIATSFPEFM LM GLGA+I  TEKPVTIDDANMIATSFPEFMGLMKGLGAEI 451 |  |

>WP_140687181.1 3-phosphoshikimate 1-carboxyvinyltransferase [Mesorhizobium sp. B2-6-2]

TPJ82648.1 3-phosphoshikimate 1-carboxyvinyltransferase [Mesorhizobium sp. B2-6-2]

Length=452

Score = 683 bits (1763), Expect = 0.0, Method: Compositional matrix adjust.

| Identities | = 350/451 (78%), Positives = 387/451 (86%), Gaps = 4/451 (1%) |  |
| --- | --- | --- |
| Query 1 | MLHGASSRPATARKSSGLSGTVRIPGDKSISHRSFMFGGLASGETRITGLLEGEDVINTG | 60 |
| Sbjct 1 | M H A+++PATARKS L+GT R+PGDKSISHRSFMFGGLASGETRITGLLEGEDV+ TG MSHSAAAKPATARKSPALAGTARVPGDKSISHRSFMFGGLASGETRITGLLEGEDVMRTG | 60 |
| Query 61 | KAMQAMGARIRKEGDTWIIDGVGNGGLLAPEAPLDFGNAATGCRLTMGLVGVYDFDSTFI | 120 |
| Sbjct 61 | AM+AMGA I K GD W+I G GNG LL PE PLDFGNA TG RLTMGLVG YD ++TFI AAMKAMGAHIEKSGDEWVIRGTGNGALLQPEGPLDFGNAGTGSRLTMGLVGTYDMETTFI | 120 |
| Query 121 | GDASLTKRPMGRVLNPLREMGVQV-KSEDGDRLPVTLRGPKTPTPITYRVPMASAQVKSA | 179 |
| Sbjct 121 | GDASL+ RPMGRVL+PLR+MGVQV K+ GDR+P+TLRGPK PITYRVPMASAQVKSA GDASLSGRPMGRVLDPLRQMGVQVLKAAPGDRMPITLRGPKHAAPITYRVPMASAQVKSA | 180 |
| Query 180 | VLLAGLNTPGITTVIEPIMTRDHTEKMLQGFGANLTVETDADGVRTIRLEGRGKLTGQVI | 239 |
| Sbjct 181 | VLLAGLNTPGITTVIEP+MTRDHTEKML+GFGANL+VE D GVR I +EG+GKLTGQ I VLLAGLNTPGITTVIEPVMTRDHTEKMLKGFGANLSVEADERGVRHIFIEGQGKLTGQTI | 240 |
| Query 240 | DVPGDPSSTAFPLVAALLVPGSDVTILNVLMNPTRTGLILTLQEMGADIEVINPRLAGGE | 299 |
| Sbjct 241 | VPGDPSS FPLVAAL+VP SD+ I NVLMNPTRTGL+LTLQEMG I+++NPR AGGE AVPGDPSSAGFPLVAALIVPASDIVIENVLMNPTRTGLLLTLQEMGGKIDILNPRNAGGE | 300 |
| Query 300 | DVADLRVRSSTLKGVTVPEDRAPSMIDEYPILavaaafaegaTVMNGLEELRVKESDRLS | 359 |
| Sbjct 301 | DVADLRVRSS L+GV VP +RAPSMIDEYP+LAVAA+FAEG T+M GLEELRVKESDRL+ DVADLRVRSSDLRGVIVPPERAPSMIDEYPVLAVAASFAEGETLMQGLEELRVKESDRLA | 360 |
| Query 360 | AVANGLKLNGVDCDEGETSLVVRGRPDGKGLG---NASGAAVATHLDHRIAMSFLVMGLV | 416 |
| Sbjct 361 | AVA GL+LNGVDC EGE SL VRGRP GKGLG N AV THLDHRIAMSFLVMGL AVARGLELNGVDCTEGEASLAVRGRPGGKGLGAHPNGPDTAVETHLDHRIAMSFLVMGLA | 420 |
| Query 417  Sbjct 421 | SENPVTVDDATMIATSFPEFMDLMAGLGAKI 447  +E PVTVDDA+MIATSFPEFM LM GLGA+I  TEKPVTVDDASMIATSFPEFMGLMKGLGAEI 451 |  |

```
>RWE69018.1 3-phosphoshikimate 1-carboxyvinyltransferase [Mesorhizobium sp.] RWE99951.1 3-phosphoshikimate 1-carboxyvinyltransferase [Mesorhizobium sp.]
Length=452

Score = 683 bits (1763), Expect = 0.0, Method: Compositional matrix adjust. Identities = 351/452 (78%), Positives = 385/452 (85%), Gaps = 4/452 (1%)

Query 1	MLHGASSRPATARKSSGLSGTVRIPGDKSISHRSFMFGGLASGETRITGLLEGEDVINTG 60 M H A+ +PATARKSS LSG R+PGDKSISHRSFMFGGLASGETRITGLLEGEDV+ TG
Sbjct 1	MSHSAAPKPATARKSSALSGAARVPGDKSISHRSFMFGGLASGETRITGLLEGEDVMRTG 60
```

| Query | 61 | KAMQAMGARIRKEGDTWIIDGVGNGGLLAPEAPLDFGNAATGCRLTMGLVGVYDFDSTFI | 120 |
| --- | --- | --- | --- |
| Sbjct | 61 | AM+AMGA I K W+I G GNG LL PE PLDFGNA TG RLTMGLVG YD ++TFI AAMKAMGAHIEKLDTEWVIRGTGNGALLQPEGPLDFGNAGTGSRLTMGLVGTYDMETTFI | 120 |
| Query | 121 | GDASLTKRPMGRVLNPLREMGVQV-KSEDGDRLPVTLRGPKTPTPITYRVPMASAQVKSA | 179 |
| Sbjct | 121 | GDASL+ RPMGRVL PLR+MGVQV K+ GDR+P+TLRGPK PITYRVPMASAQVKSA GDASLSGRPMGRVLEPLRQMGVQVLKATPGDRMPITLRGPKHAAPITYRVPMASAQVKSA | 180 |
| Query | 180 | VLLAGLNTPGITTVIEPIMTRDHTEKMLQGFGANLTVETDADGVRTIRLEGRGKLTGQVI | 239 |
| Sbjct | 181 | VLLAGLNTPGITTVIEP+MTRDHTEKML+GFGANL+VETD GVR I +EG+GKLTGQ I VLLAGLNTPGITTVIEPVMTRDHTEKMLRGFGANLSVETDERGVRHIFIEGQGKLTGQTI | 240 |
| Query | 240 | DVPGDPSSTAFPLVAALLVPGSDVTILNVLMNPTRTGLILTLQEMGADIEVINPRLAGGE | 299 |
| Sbjct | 241 | VPGDPSS FPLVAAL+VPGSD+TI NVLMNPTRTGL+LTLQEMGA I++++PR GGE AVPGDPSSAGFPLVAALIVPGSDITIENVLMNPTRTGLLLTLQEMGARIDIVDPRNEGGE | 300 |
| Query | 300 | DVADLRVRSSTLKGVTVPEDRAPSMIDEYPILavaaafaegaTVMNGLEELRVKESDRLS | 359 |
| Sbjct | 301 | DVADLRVR S LKGV VP +RAP+MIDEYP+LAVAA+FAEG T+M GLEELRVKESDRLS DVADLRVRYSELKGVAVPPERAPTMIDEYPVLAVAASFAEGETLMQGLEELRVKESDRLS | 360 |
| Query | 360 | AVANGLKLNGVDCDEGETSLVVRGRPDGKGLG---NASGAAVATHLDHRIAMSFLVMGLV | 416 |
| Sbjct | 361 | AVA+GLKLNGVDC EGE SL V GRP GKGLG N G V THLDHRIAMSFLVMGL AVADGLKLNGVDCTEGEASLAVHGRPGGKGLGQHPNGQGTIVQTHLDHRIAMSFLVMGLA | 420 |
| Query  Sbjct | 417  421 | SENPVTVDDATMIATSFPEFMDLMAGLGAKIE 448  +E PVT+DD TMIATSFPEFM LM GLGA+IE  TEKPVTIDDQTMIATSFPEFMGLMKGLGAEIE 452 |  |

>TIM06126.1 3-phosphoshikimate 1-carboxyvinyltransferase [Mesorhizobium sp.] Length=452

Score = 683 bits (1763), Expect = 0.0, Method: Compositional matrix adjust.

| Identities | = 350/452 (77%), Positives = 385/452 (85%), Gaps = 4/452 (1%) |  |
| --- | --- | --- |
| Query 1 | MLHGASSRPATARKSSGLSGTVRIPGDKSISHRSFMFGGLASGETRITGLLEGEDVINTG | 60 |
| Sbjct 1 | M H A+ +PATARKS LSGT R+PGDKSISHRSFMFGGLASGETRITGLLEGEDV+ TG MSHTAAPKPATARKSPALSGTARVPGDKSISHRSFMFGGLASGETRITGLLEGEDVMRTG | 60 |
| Query 61 | KAMQAMGARIRKEGDTWIIDGVGNGGLLAPEAPLDFGNAATGCRLTMGLVGVYDFDSTFI | 120 |
| Sbjct 61 | AM+AMGA I K G W+I G GNG LL PE PLDFGNA TG RLTMGLVG YD ++TFI AAMKAMGAHIEKRGAEWVIRGTGNGALLQPEGPLDFGNAGTGSRLTMGLVGTYDMETTFI | 120 |
| Query 121 | GDASLTKRPMGRVLNPLREMGVQV-KSEDGDRLPVTLRGPKTPTPITYRVPMASAQVKSA | 179 |
| Sbjct 121 | GDASL+ RPMGRVL PLR+MGVQV K+ GDR+P+TLRGPK PITYRVPMASAQVKSA GDASLSGRPMGRVLEPLRQMGVQVLKATPGDRMPITLRGPKHAAPITYRVPMASAQVKSA | 180 |
| Query 180 | VLLAGLNTPGITTVIEPIMTRDHTEKMLQGFGANLTVETDADGVRTIRLEGRGKLTGQVI | 239 |
| Sbjct 181 | VLLAGLNTPGITTVIEP+MTRDHTEKMLQGFGANL+VETD GVR I +EG+GKLTGQ I VLLAGLNTPGITTVIEPVMTRDHTEKMLQGFGANLSVETDERGVRHIFIEGQGKLTGQTI | 240 |
| Query 240 | DVPGDPSSTAFPLVAALLVPGSDVTILNVLMNPTRTGLILTLQEMGADIEVINPRLAGGE | 299 |
| Sbjct 241 | VPGDPSS FPLVAAL+VPGSD+TI NVLMNPTRTGL+LTLQEMGA I++++PR GGE AVPGDPSSAGFPLVAALIVPGSDITIENVLMNPTRTGLLLTLQEMGARIDIVDPRNEGGE | 300 |
| Query 300 | DVADLRVRSSTLKGVTVPEDRAPSMIDEYPILavaaafaegaTVMNGLEELRVKESDRLS | 359 |
| Sbjct 301 | DVADLRVR S L+GVTVP +RAP+MIDEYP+LAVAA+FAEG T+M GLEELRVKESDRLS DVADLRVRYSELRGVTVPPERAPTMIDEYPVLAVAASFAEGETLMQGLEELRVKESDRLS | 360 |
| Query 360 | AVANGLKLNGVDCDEGETSLVVRGRPDGKGLG---NASGAAVATHLDHRIAMSFLVMGLV | 416 |
| Sbjct 361 | AVA GLKLNGVDC EGE +L VRG+P GKGLG N V THLDHRIAMSFLVMGL AVAEGLKLNGVDCTEGEATLAVRGKPVGKGLGQHPNGKSTIVQTHLDHRIAMSFLVMGLA | 420 |
| Query 417  Sbjct 421 | SENPVTVDDATMIATSFPEFMDLMAGLGAKIE 448  +E PVT+DD MIATSFPEFM LM GLGA+IE  TEKPVTIDDQAMIATSFPEFMGLMKGLGAEIE 452 |  |

>TIR30984.1 3-phosphoshikimate 1-carboxyvinyltransferase [Mesorhizobium sp.] TIS18889.1 3-phosphoshikimate 1-carboxyvinyltransferase [Mesorhizobium sp.]

Length=452

Score = 683 bits (1763), Expect = 0.0, Method: Compositional matrix adjust.

| Identities | = 352/452 (78%), Positives = 385/452 (85%), Gaps = 4/452 (1%) |  |
| --- | --- | --- |
| Query 1 | MLHGASSRPATARKSSGLSGTVRIPGDKSISHRSFMFGGLASGETRITGLLEGEDVINTG | 60 |
| Sbjct 1 | M H A+ +PATARKSS L G R+PGDKSISHRSFMFGGLASGETRITGLLEGEDV+ TG MSHTAAPKPATARKSSALLGAARVPGDKSISHRSFMFGGLASGETRITGLLEGEDVMRTG | 60 |
| Query 61 | KAMQAMGARIRKEGDTWIIDGVGNGGLLAPEAPLDFGNAATGCRLTMGLVGVYDFDSTFI | 120 |
| Sbjct 61 | AM+AMGA I K G W+I G GNG LL PE PLDFGNA TG RLTMGLVG YD ++TFI AAMKAMGAHIEKLGTEWVIRGTGNGALLQPEGPLDFGNAGTGSRLTMGLVGTYDMETTFI | 120 |
| Query 121 | GDASLTKRPMGRVLNPLREMGVQV-KSEDGDRLPVTLRGPKTPTPITYRVPMASAQVKSA | 179 |
| Sbjct 121 | GDASL+ RPMGRVL PLR+MGVQV K+ GDR+P+TLRGPK PITYRVPMASAQVKSA GDASLSGRPMGRVLEPLRQMGVQVLKAIPGDRMPITLRGPKHAAPITYRVPMASAQVKSA | 180 |
| Query 180 | VLLAGLNTPGITTVIEPIMTRDHTEKMLQGFGANLTVETDADGVRTIRLEGRGKLTGQVI | 239 |
| Sbjct 181 | VLLAGLNTPGITTVIEP+MTRDHTEKML+GFGANL+VETD GVR I +EG+GKLTGQ I VLLAGLNTPGITTVIEPVMTRDHTEKMLRGFGANLSVETDERGVRHIFIEGQGKLTGQTI | 240 |
| Query 240 | DVPGDPSSTAFPLVAALLVPGSDVTILNVLMNPTRTGLILTLQEMGADIEVINPRLAGGE | 299 |
| Sbjct 241 | VPGDPSS FPLVAAL+VPGSD+TI NVLMNPTRTGL+LTLQEMGA I++++PR GGE AVPGDPSSAGFPLVAALIVPGSDITIENVLMNPTRTGLLLTLQEMGARIDIVDPRNEGGE | 300 |
| Query 300 | DVADLRVRSSTLKGVTVPEDRAPSMIDEYPILavaaafaegaTVMNGLEELRVKESDRLS | 359 |
| Sbjct 301 | DVADLRVR S LK VTVP +RAP+MIDEYP+LAVAA+FAEG T+M GLEELRVKESDRLS DVADLRVRHSELKAVTVPPERAPTMIDEYPVLAVAASFAEGETLMQGLEELRVKESDRLS | 360 |
| Query 360 | AVANGLKLNGVDCDEGETSLVVRGRPDGKGLG---NASGAAVATHLDHRIAMSFLVMGLV | 416 |
| Sbjct 361 | AVA GLKLNGVDC EGE SL VRGRP GKGLG N G V THLDHRIAMSFLVMGL AVAEGLKLNGVDCTEGEASLAVRGRPGGKGLGQHPNGQGTIVQTHLDHRIAMSFLVMGLA | 420 |
| Query 417  Sbjct 421 | SENPVTVDDATMIATSFPEFMDLMAGLGAKIE 448  +E PVT+DD TMIATSFPEFM LM GLGA+IE  TEKPVTIDDQTMIATSFPEFMGLMKGLGAEIE 452 |  |

>TIM83740.1 3-phosphoshikimate 1-carboxyvinyltransferase [Mesorhizobium sp.] Length=452

Score = 683 bits (1763), Expect = 0.0, Method: Compositional matrix adjust.

| Identities | = 351/452 (78%), Positives = 385/452 (85%), Gaps = 4/452 (1%) |  |
| --- | --- | --- |
| Query 1 | MLHGASSRPATARKSSGLSGTVRIPGDKSISHRSFMFGGLASGETRITGLLEGEDVINTG | 60 |
| Sbjct 1 | M H A+ +PATARKSS LSG R+PGDKSISHRSFMFGGLASGETRITGLLEGEDV+ TG MSHTAAPKPATARKSSALSGAARVPGDKSISHRSFMFGGLASGETRITGLLEGEDVMRTG | 60 |
| Query 61 | KAMQAMGARIRKEGDTWIIDGVGNGGLLAPEAPLDFGNAATGCRLTMGLVGVYDFDSTFI | 120 |
| Sbjct 61 | AM+AMGA I K G W+I G GNG LL PE PLDFGNA TG RLTMGLVG YD ++TFI AAMKAMGAHIEKLGTEWVIRGTGNGALLQPEGPLDFGNAGTGSRLTMGLVGTYDMETTFI | 120 |
| Query 121 | GDASLTKRPMGRVLNPLREMGVQV-KSEDGDRLPVTLRGPKTPTPITYRVPMASAQVKSA | 179 |
| Sbjct 121 | GDASL+ RPMGRVL PLR+MGVQV K+ GDR+P+TL GPK PITYRVPMASAQVKSA GDASLSGRPMGRVLEPLRQMGVQVLKATPGDRMPITLHGPKHAAPITYRVPMASAQVKSA | 180 |
| Query 180 | VLLAGLNTPGITTVIEPIMTRDHTEKMLQGFGANLTVETDADGVRTIRLEGRGKLTGQVI | 239 |
| Sbjct 181 | VLLAGLNTPGITTVIEP+MTRDHTEKML+GFGANL+VETD GVR I +EG+GKLTGQ I VLLAGLNTPGITTVIEPVMTRDHTEKMLRGFGANLSVETDERGVRHIFIEGQGKLTGQTI | 240 |
| Query 240 | DVPGDPSSTAFPLVAALLVPGSDVTILNVLMNPTRTGLILTLQEMGADIEVINPRLAGGE | 299 |
| Sbjct 241 | VPGDPSS FPLVAAL+VPGSD+TI NVLMNPTRTGL+LTLQEMGA I++++PR GGE AVPGDPSSAGFPLVAALIVPGSDITIENVLMNPTRTGLLLTLQEMGARIDIVDPRNEGGE | 300 |
| Query 300 | DVADLRVRSSTLKGVTVPEDRAPSMIDEYPILavaaafaegaTVMNGLEELRVKESDRLS | 359 |
| Sbjct 301 | DVADLRVR S LK VTVP +RAP+MIDEYP+LAVAA+FAEG T+M GLEELRVKESDRLS DVADLRVRHSELKAVTVPPERAPTMIDEYPVLAVAASFAEGETLMQGLEELRVKESDRLS | 360 |
| Query 360 | AVANGLKLNGVDCDEGETSLVVRGRPDGKGLG---NASGAAVATHLDHRIAMSFLVMGLV | 416 |
| Sbjct 361 | AVA GLKLNGVDC EGE SL VRGRP GKGLG N G V THLDHRIAMSFLVMGL AVAEGLKLNGVDCTEGEASLAVRGRPGGKGLGQHPNGQGTIVQTHLDHRIAMSFLVMGLA | 420 |
| Query 417 | SENPVTVDDATMIATSFPEFMDLMAGLGAKIE 448  +E PVT+DD TMIATSFPEF+ LM GLGA+IE |  |

Sbjct 421 TEKPVTIDDQTMIATSFPEFVGLMKGLGAEIE 452

>TKD39474.1 3-phosphoshikimate 1-carboxyvinyltransferase [Mesorhizobium sp.] Length=452

Score = 683 bits (1763), Expect = 0.0, Method: Compositional matrix adjust.

| Identities | = 352/452 (78%), Positives = 385/452 (85%), Gaps = 4/452 (1%) |  |
| --- | --- | --- |
| Query 1 | MLHGASSRPATARKSSGLSGTVRIPGDKSISHRSFMFGGLASGETRITGLLEGEDVINTG | 60 |
| Sbjct 1 | M H A+ +PATARKSS LSG R+PGDKSISHRSFMFGGLASGETRITGLLEGEDV+ TG MSHTAAPKPATARKSSALSGAARVPGDKSISHRSFMFGGLASGETRITGLLEGEDVMRTG | 60 |
| Query 61 | KAMQAMGARIRKEGDTWIIDGVGNGGLLAPEAPLDFGNAATGCRLTMGLVGVYDFDSTFI | 120 |
| Sbjct 61 | AM+AMGA I K G W+I G GNG LL PE PLDFGNA TG RLTMGLVG YD ++TFI AAMKAMGAHIEKLGTEWVIRGTGNGALLQPEGPLDFGNAGTGSRLTMGLVGTYDMETTFI | 120 |
| Query 121 | GDASLTKRPMGRVLNPLREMGVQV-KSEDGDRLPVTLRGPKTPTPITYRVPMASAQVKSA | 179 |
| Sbjct 121 | GDASL+ RPMGRVL PLR+MGVQV K+ GDR+P+TLRGPK PITYRVPMASAQVKSA GDASLSGRPMGRVLEPLRQMGVQVLKATPGDRMPITLRGPKHAAPITYRVPMASAQVKSA | 180 |
| Query 180 | VLLAGLNTPGITTVIEPIMTRDHTEKMLQGFGANLTVETDADGVRTIRLEGRGKLTGQVI | 239 |
| Sbjct 181 | VLLAGLNTPGITTVIEP MTRDHTEKML+GFGANL+VETD GVR I +EG+GKLTGQ I VLLAGLNTPGITTVIEPAMTRDHTEKMLRGFGANLSVETDERGVRHIFIEGQGKLTGQTI | 240 |
| Query 240 | DVPGDPSSTAFPLVAALLVPGSDVTILNVLMNPTRTGLILTLQEMGADIEVINPRLAGGE | 299 |
| Sbjct 241 | VPGDPSS FPLVAAL+VPGSD+TI NVLMNPTRTGL+LTLQEMGA I++++PR GGE AVPGDPSSAGFPLVAALIVPGSDITIENVLMNPTRTGLLLTLQEMGARIDIVDPRNEGGE | 300 |
| Query 300 | DVADLRVRSSTLKGVTVPEDRAPSMIDEYPILavaaafaegaTVMNGLEELRVKESDRLS | 359 |
| Sbjct 301 | DVADLRVR S LKGV VP +RAP+MIDEYP+LAVAA+FAEG T+M GLEELRVKESDRLS DVADLRVRYSELKGVAVPPERAPTMIDEYPVLAVAASFAEGETLMQGLEELRVKESDRLS | 360 |
| Query 360 | AVANGLKLNGVDCDEGETSLVVRGRPDGKGLG---NASGAAVATHLDHRIAMSFLVMGLV | 416 |
| Sbjct 361 | AVA+GLKLNGVDC EGE SL V GRP GKGLG N G V THLDHRIAMSFLVMGL AVADGLKLNGVDCTEGEASLAVHGRPGGKGLGQHPNGQGTIVQTHLDHRIAMSFLVMGLA | 420 |
| Query 417  Sbjct 421 | SENPVTVDDATMIATSFPEFMDLMAGLGAKIE 448  +E PVT+DD TMIATSFPEFM LM GLGA+IE  TEKPVTIDDQTMIATSFPEFMGLMKGLGAEIE 452 |  |

>WP_023796773.1 3-phosphoshikimate 1-carboxyvinyltransferase [Mesorhizobium sp. L48C026A00]

ESZ22504.1 3-phosphoshikimate 1-carboxyvinyltransferase [Mesorhizobium sp. L48C026A00]

Length=452

Score = 683 bits (1762), Expect = 0.0, Method: Compositional matrix adjust.

| Identities | = 351/452 (78%), Positives = 384/452 (85%), Gaps = 4/452 (1%) |  |
| --- | --- | --- |
| Query 1 | MLHGASSRPATARKSSGLSGTVRIPGDKSISHRSFMFGGLASGETRITGLLEGEDVINTG | 60 |
| Sbjct 1 | M H A+ +PATARKS LSGT R+PGDKSISHRSFMFGGLASGETRITGLLEGEDV+ TG MSHTAAPKPATARKSPALSGTARVPGDKSISHRSFMFGGLASGETRITGLLEGEDVMRTG | 60 |
| Query 61 | KAMQAMGARIRKEGDTWIIDGVGNGGLLAPEAPLDFGNAATGCRLTMGLVGVYDFDSTFI | 120 |
| Sbjct 61 | AM+AMGA I K G W+I G GNG LL PE PLDFGNA TG RLTMGLVG YD ++TFI AAMKAMGAHIEKRGAEWVIRGTGNGALLQPEGPLDFGNAGTGSRLTMGLVGTYDMETTFI | 120 |
| Query 121 | GDASLTKRPMGRVLNPLREMGVQV-KSEDGDRLPVTLRGPKTPTPITYRVPMASAQVKSA | 179 |
| Sbjct 121 | GDASL+ RPMGRVL PLR+MGVQV K+ GDR+P+TLRGPK PITYRVPMASAQVKSA GDASLSGRPMGRVLEPLRQMGVQVLKATPGDRMPITLRGPKHAAPITYRVPMASAQVKSA | 180 |
| Query 180 | VLLAGLNTPGITTVIEPIMTRDHTEKMLQGFGANLTVETDADGVRTIRLEGRGKLTGQVI | 239 |
| Sbjct 181 | VLLAGLNTPGITTVIEP+MTRDHTEKMLQGFGANL+VETD GVR I +EG+GKLTGQ I VLLAGLNTPGITTVIEPVMTRDHTEKMLQGFGANLSVETDERGVRHIFIEGQGKLTGQTI | 240 |
| Query 240 | DVPGDPSSTAFPLVAALLVPGSDVTILNVLMNPTRTGLILTLQEMGADIEVINPRLAGGE | 299 |
| Sbjct 241 | VPGDPSS FPLVAAL+VPGSD+TI NVLMNPTRTGL+LTLQEMGA I+V+ PR GGE AVPGDPSSAGFPLVAALIVPGSDITIENVLMNPTRTGLLLTLQEMGARIDVLEPRNEGGE | 300 |
| Query 300 | DVADLRVRSSTLKGVTVPEDRAPSMIDEYPILavaaafaegaTVMNGLEELRVKESDRLS | 359 |

| Sbjct | 301 | DVADLRVR S LKGVTVP +RAP+MIDEYP+LAVAA+FAEG T+M GLEELRVKESDRLS DVADLRVRYSELKGVTVPPERAPTMIDEYPVLAVAASFAEGETLMQGLEELRVKESDRLS | 360 |
| --- | --- | --- | --- |
| Query | 360 | AVANGLKLNGVDCDEGETSLVVRGRPDGKGLG---NASGAAVATHLDHRIAMSFLVMGLV | 416 |
| Sbjct | 361 | AVA GLKLNGVDC EGE +L VRG+P GKGLG N V THLDHRIAMSFLVMGL AVAEGLKLNGVDCTEGEATLAVRGKPGGKGLGQHPNGKSTIVQTHLDHRIAMSFLVMGLA | 420 |
| Query  Sbjct | 417  421 | SENPVTVDDATMIATSFPEFMDLMAGLGAKIE 448  +E PVT+DD MIATSFPEFM LM GLGA+I+  TEKPVTIDDQAMIATSFPEFMGLMKGLGAEID 452 |  |

>WP_140545274.1 3-phosphoshikimate 1-carboxyvinyltransferase [Mesorhizobium sp. B1-1-5]

TPO02199.1 3-phosphoshikimate 1-carboxyvinyltransferase [Mesorhizobium sp. B1-1-5]

Length=452

Score = 683 bits (1762), Expect = 0.0, Method: Compositional matrix adjust.

| Identities | = 350/451 (78%), Positives = 387/451 (86%), Gaps = 4/451 (1%) |  |
| --- | --- | --- |
| Query 1 | MLHGASSRPATARKSSGLSGTVRIPGDKSISHRSFMFGGLASGETRITGLLEGEDVINTG | 60 |
| Sbjct 1 | M H A+++PATARKS L+GT R+PGDKSISHRSFMFGGLASGETRITGLLEGEDV+ TG MSHSAAAKPATARKSPALAGTARVPGDKSISHRSFMFGGLASGETRITGLLEGEDVMRTG | 60 |
| Query 61 | KAMQAMGARIRKEGDTWIIDGVGNGGLLAPEAPLDFGNAATGCRLTMGLVGVYDFDSTFI | 120 |
| Sbjct 61 | AM+AMGA I K G W+I G GNG LL PE PLDFGNA TG RLTMGLVG YD ++TFI AAMKAMGAHIEKNGGEWVIRGTGNGALLQPEGPLDFGNAGTGSRLTMGLVGTYDMETTFI | 120 |
| Query 121 | GDASLTKRPMGRVLNPLREMGVQV-KSEDGDRLPVTLRGPKTPTPITYRVPMASAQVKSA | 179 |
| Sbjct 121 | GDASL+ RPMGRVL+PLR+MGVQV K+ GDR+P+TLRGPK PITYRVPMASAQVKSA GDASLSGRPMGRVLDPLRQMGVQVLKAAPGDRMPITLRGPKHAAPITYRVPMASAQVKSA | 180 |
| Query 180 | VLLAGLNTPGITTVIEPIMTRDHTEKMLQGFGANLTVETDADGVRTIRLEGRGKLTGQVI | 239 |
| Sbjct 181 | VLLAGLNTPGITTVIEP+MTRDHTEKML+GFGANL+VETD GVR I +EG+GKLTGQ I VLLAGLNTPGITTVIEPVMTRDHTEKMLKGFGANLSVETDERGVRHIFIEGQGKLTGQTI | 240 |
| Query 240 | DVPGDPSSTAFPLVAALLVPGSDVTILNVLMNPTRTGLILTLQEMGADIEVINPRLAGGE | 299 |
| Sbjct 241 | VPGDPSS FPLVAAL+VPGSD+ I NVLMNPTRTGL+LTLQEMG I+++NPR AGGE AVPGDPSSAGFPLVAALIVPGSDIVIENVLMNPTRTGLLLTLQEMGGKIDILNPRNAGGE | 300 |
| Query 300 | DVADLRVRSSTLKGVTVPEDRAPSMIDEYPILavaaafaegaTVMNGLEELRVKESDRLS | 359 |
| Sbjct 301 | DVADLRVRSS L+GV VP +RAPSMIDEYP+LAVAA+FAEG T+M GLEELRVKESDRL+ DVADLRVRSSDLRGVIVPPERAPSMIDEYPVLAVAASFAEGETLMQGLEELRVKESDRLA | 360 |
| Query 360 | AVANGLKLNGVDCDEGETSLVVRGRPDGKGLG---NASGAAVATHLDHRIAMSFLVMGLV | 416 |
| Sbjct 361 | AVA GL+LNGVDC EGE SL VRGRP GKGLG N AV THLDHRIAMSFLVMGL AVARGLELNGVDCTEGEASLAVRGRPGGKGLGAHPNGPDTAVKTHLDHRIAMSFLVMGLA | 420 |
| Query 417  Sbjct 421 | SENPVTVDDATMIATSFPEFMDLMAGLGAKI 447  +E PVT+DDA MIATSFPEFM LM GLGA+I  TEKPVTIDDANMIATSFPEFMGLMKGLGAEI 451 |  |

```
>WP_126056883.1 MULTISPECIES: 3-phosphoshikimate 1-carboxyvinyltransferase [unclassified Mesorhizobium]
RWC53862.1 3-phosphoshikimate 1-carboxyvinyltransferase [Mesorhizobium sp.] AZO49423.1 3-phosphoshikimate 1-carboxyvinyltransferase [Mesorhizobium sp.
M4B.F.Ca.ET.058.02.1.1]
RVC40337.1 3-phosphoshikimate 1-carboxyvinyltransferase [Mesorhizobium sp. M4A.F.Ca.ET.090.04.2.1]
RVC83386.1 3-phosphoshikimate 1-carboxyvinyltransferase [Mesorhizobium sp. M4A.F.Ca.ET.022.05.2.1]
RWD05100.1 3-phosphoshikimate 1-carboxyvinyltransferase [Mesorhizobium sp.] Length=452

Score = 683 bits (1762), Expect = 0.0, Method: Compositional matrix adjust. Identities = 351/451 (78%), Positives = 384/451 (85%), Gaps = 4/451 (1%)

Query 1	MLHGASSRPATARKSSGLSGTVRIPGDKSISHRSFMFGGLASGETRITGLLEGEDVINTG 60 M H A+ +PATARKS LSG R+PGDKSISHRSFMFGGLASGETRITGLLEGEDV+ TG
Sbjct 1	MSHAAAPKPATARKSPALSGAARVPGDKSISHRSFMFGGLASGETRITGLLEGEDVMRTG 60
```

| Query | 61 | KAMQAMGARIRKEGDTWIIDGVGNGGLLAPEAPLDFGNAATGCRLTMGLVGVYDFDSTFI | 120 |
| --- | --- | --- | --- |
| Sbjct | 61 | AM+AMGA I K G W+I G GNG LL PE PLDFGNA TG RLTMGLVG YD ++TFI AAMKAMGAHIEKRGAEWVIRGTGNGALLEPEGPLDFGNAGTGSRLTMGLVGTYDMETTFI | 120 |
| Query | 121 | GDASLTKRPMGRVLNPLREMGVQV-KSEDGDRLPVTLRGPKTPTPITYRVPMASAQVKSA | 179 |
| Sbjct | 121 | GDASL+ RPMGRVL PLR+MGVQV K+ GDR+P+TLRGPK PITYRVPMASAQVKSA GDASLSGRPMGRVLEPLRQMGVQVLKATPGDRMPITLRGPKHAAPITYRVPMASAQVKSA | 180 |
| Query | 180 | VLLAGLNTPGITTVIEPIMTRDHTEKMLQGFGANLTVETDADGVRTIRLEGRGKLTGQVI | 239 |
| Sbjct | 181 | VLLAGLNTPGITTVIEP+MTRDHTEKML+GFGANLTVETD GVR I +EG+GKL+GQ I VLLAGLNTPGITTVIEPVMTRDHTEKMLKGFGANLTVETDERGVRHIFIEGQGKLSGQTI | 240 |
| Query | 240 | DVPGDPSSTAFPLVAALLVPGSDVTILNVLMNPTRTGLILTLQEMGADIEVINPRLAGGE | 299 |
| Sbjct | 241 | VPGDPSS FPLVAAL+VPGSD+ I NVLMNPTRTGL+LTLQEMG I+++NPR AGGE AVPGDPSSAGFPLVAALIVPGSDIVIENVLMNPTRTGLLLTLQEMGGRIDILNPRNAGGE | 300 |
| Query | 300 | DVADLRVRSSTLKGVTVPEDRAPSMIDEYPILavaaafaegaTVMNGLEELRVKESDRLS | 359 |
| Sbjct | 301 | DVADLRVRSS LKGV VP +RAPSMIDEYP+LAVAA+FAEG T+M GLEELRVKESDRLS DVADLRVRSSELKGVVVPPERAPSMIDEYPVLAVAASFAEGETLMQGLEELRVKESDRLS | 360 |
| Query | 360 | AVANGLKLNGVDCDEGETSLVVRGRPDGKGLG---NASGAAVATHLDHRIAMSFLVMGLV | 416 |
| Sbjct | 361 | AVANGL+LNGVDC EGE +L VRGRP GKGLG N V THLDHRIAMSFLVMGL AVANGLRLNGVDCTEGEATLAVRGRPGGKGLGRHPNGPDTTVKTHLDHRIAMSFLVMGLA | 420 |
| Query  Sbjct | 417  421 | SENPVTVDDATMIATSFPEFMDLMAGLGAKI 447  +E PVT+DDA MIATSFPEFM LM GLGA+I  TEKPVTIDDANMIATSFPEFMGLMKGLGAEI 451 |  |

>RWO92749.1 3-phosphoshikimate 1-carboxyvinyltransferase [Mesorhizobium sp.] RWQ57347.1 3-phosphoshikimate 1-carboxyvinyltransferase [Mesorhizobium sp.]

Length=452

Score = 683 bits (1762), Expect = 0.0, Method: Compositional matrix adjust.

| Identities | = 351/451 (78%), Positives = 382/451 (85%), Gaps = 4/451 (1%) |  |
| --- | --- | --- |
| Query 1 | MLHGASSRPATARKSSGLSGTVRIPGDKSISHRSFMFGGLASGETRITGLLEGEDVINTG | 60 |
| Sbjct 1 | M H A+ +PATARKS LSGT R+PGDKSISHRSFMFGGLASGETRITGLLEGEDV+ TG MSHTAAPKPATARKSPALSGTARVPGDKSISHRSFMFGGLASGETRITGLLEGEDVMRTG | 60 |
| Query 61 | KAMQAMGARIRKEGDTWIIDGVGNGGLLAPEAPLDFGNAATGCRLTMGLVGVYDFDSTFI | 120 |
| Sbjct 61 | AM+AMGA I K G W+I G GNG LL PE PLDFGNA TG RLTMGLVG YD ++TFI AAMKAMGAHIEKRGAEWVIRGTGNGALLQPEGPLDFGNAGTGSRLTMGLVGTYDMETTFI | 120 |
| Query 121 | GDASLTKRPMGRVLNPLREMGVQV-KSEDGDRLPVTLRGPKTPTPITYRVPMASAQVKSA | 179 |
| Sbjct 121 | GDASL+ RPMGRVL PLR+MGVQV K+ GDR+P+TLRGPK PITYRVPMASAQVKSA GDASLSGRPMGRVLEPLRQMGVQVLKATPGDRMPITLRGPKHAAPITYRVPMASAQVKSA | 180 |
| Query 180 | VLLAGLNTPGITTVIEPIMTRDHTEKMLQGFGANLTVETDADGVRTIRLEGRGKLTGQVI | 239 |
| Sbjct 181 | VLLAGLNTPGITTVIEP+MTRDHTEKMLQGFGANL+VETD GVR I +EG+GKLTGQ I VLLAGLNTPGITTVIEPVMTRDHTEKMLQGFGANLSVETDERGVRHIFIEGQGKLTGQTI | 240 |
| Query 240 | DVPGDPSSTAFPLVAALLVPGSDVTILNVLMNPTRTGLILTLQEMGADIEVINPRLAGGE | 299 |
| Sbjct 241 | VPGDPSS FPLVAAL+VPGSD+TI NVLMNPTRTGL+ TLQEMGA I+V+ PR GGE AVPGDPSSAGFPLVAALIVPGSDITIENVLMNPTRTGLLFTLQEMGARIDVLEPRNEGGE | 300 |
| Query 300 | DVADLRVRSSTLKGVTVPEDRAPSMIDEYPILavaaafaegaTVMNGLEELRVKESDRLS | 359 |
| Sbjct 301 | DVADLRVR S LKGVTVP +RAP+MIDEYP+LAVAA+FAEG T+M GLEELRVKESDRLS DVADLRVRYSELKGVTVPPERAPTMIDEYPVLAVAASFAEGETLMQGLEELRVKESDRLS | 360 |
| Query 360 | AVANGLKLNGVDCDEGETSLVVRGRPDGKGLG---NASGAAVATHLDHRIAMSFLVMGLV | 416 |
| Sbjct 361 | AVA GLKLNGVDC EGE SL VRG+P GKGLG N V THLDHRIAMSFLVMGL AVAEGLKLNGVDCTEGEASLAVRGKPGGKGLGQHPNGKSTIVQTHLDHRIAMSFLVMGLA | 420 |
| Query 417  Sbjct 421 | SENPVTVDDATMIATSFPEFMDLMAGLGAKI 447  +E PVT+DD MIATSFPEFM LM GLGA+I  TEKPVTIDDQAMIATSFPEFMGLMKGLGAEI 451 |  |

>RUU39409.1 3-phosphoshikimate 1-carboxyvinyltransferase [Mesorhizobium sp. M6A.T.Ca.TU.002.02.2.1]

Length=452

Score = 683 bits (1762), Expect = 0.0, Method: Compositional matrix adjust.

| Identities | = 348/452 (77%), Positives = 385/452 (85%), Gaps = 4/452 (1%) |  |
| --- | --- | --- |
| Query 1 | MLHGASSRPATARKSSGLSGTVRIPGDKSISHRSFMFGGLASGETRITGLLEGEDVINTG | 60 |
| Sbjct 1 | M H A+ +PATARKSS LSGT R+PGDKSISHRSFMFGGLASGETRITGLLEGEDV+ TG MSHTAAPKPATARKSSALSGTARVPGDKSISHRSFMFGGLASGETRITGLLEGEDVMRTG | 60 |
| Query 61 | KAMQAMGARIRKEGDTWIIDGVGNGGLLAPEAPLDFGNAATGCRLTMGLVGVYDFDSTFI | 120 |
| Sbjct 61 | AM+AMGA I K G W+I G GNG LL PE PLDFGNA TG RLTMGLVG YD ++TFI AAMKAMGAHIEKHGTEWVIRGTGNGALLQPEGPLDFGNAGTGSRLTMGLVGTYDMETTFI | 120 |
| Query 121 | GDASLTKRPMGRVLNPLREMGVQV-KSEDGDRLPVTLRGPKTPTPITYRVPMASAQVKSA | 179 |
| Sbjct 121 | GDASL+ RPMGRVL PLR+MGVQV K+ GDR+P+TL GPK PITYRVPMASAQVKSA GDASLSGRPMGRVLEPLRQMGVQVLKATPGDRMPITLHGPKHAAPITYRVPMASAQVKSA | 180 |
| Query 180 | VLLAGLNTPGITTVIEPIMTRDHTEKMLQGFGANLTVETDADGVRTIRLEGRGKLTGQVI | 239 |
| Sbjct 181 | VLLAGLNTPG+TTVIEP+MTRDHTEKML+GFGANL+VETD GVR I +EG+GKLTGQ I VLLAGLNTPGVTTVIEPVMTRDHTEKMLKGFGANLSVETDERGVRHIFIEGQGKLTGQTI | 240 |
| Query 240 | DVPGDPSSTAFPLVAALLVPGSDVTILNVLMNPTRTGLILTLQEMGADIEVINPRLAGGE | 299 |
| Sbjct 241 | VPGDPSS FPLVAAL+VPGSD+TI NVLMNPTRTGL+LTLQEMGA I++++PR GGE AVPGDPSSAGFPLVAALIVPGSDITIENVLMNPTRTGLLLTLQEMGARIDILDPRNEGGE | 300 |
| Query 300 | DVADLRVRSSTLKGVTVPEDRAPSMIDEYPILavaaafaegaTVMNGLEELRVKESDRLS | 359 |
| Sbjct 301 | DVADLRVR S LKGVTVP +RAP+MIDEYP+LAVAA+FAEG T+M GLEELRVKESDRLS DVADLRVRYSELKGVTVPPERAPTMIDEYPVLAVAASFAEGETLMQGLEELRVKESDRLS | 360 |
| Query 360 | AVANGLKLNGVDCDEGETSLVVRGRPDGKGLG---NASGAAVATHLDHRIAMSFLVMGLV | 416 |
| Sbjct 361 | AVA GLKLNGVDC EGE +L VRG+P GKGLG N V THLDHRIAMSFL+MGL AVAEGLKLNGVDCTEGEATLAVRGKPGGKGLGQHPNGHNTVVQTHLDHRIAMSFLIMGLA | 420 |
| Query 417  Sbjct 421 | SENPVTVDDATMIATSFPEFMDLMAGLGAKIE 448  +E PVT+DD MIATSFPEFM LM GLGA+IE  TEKPVTIDDQAMIATSFPEFMGLMKGLGAEIE 452 |  |

>WP_136618322.1 MULTISPECIES: 3-phosphoshikimate 1-carboxyvinyltransferase [Mesorhizobium] Length=452

Score = 683 bits (1762), Expect = 0.0, Method: Compositional matrix adjust.

| Identities | = 349/451 (77%), Positives = 381/451 (84%), Gaps = 4/451 (1%) |  |
| --- | --- | --- |
| Query 1 | MLHGASSRPATARKSSGLSGTVRIPGDKSISHRSFMFGGLASGETRITGLLEGEDVINTG | 60 |
| Sbjct 1 | M H A+ +PATARK LSG R+PGDKSISHRSFMFGGLASGETRITGLLEGEDV+ TG MSHAAAPKPATARKCQALSGIARVPGDKSISHRSFMFGGLASGETRITGLLEGEDVMRTG | 60 |
| Query 61 | KAMQAMGARIRKEGDTWIIDGVGNGGLLAPEAPLDFGNAATGCRLTMGLVGVYDFDSTFI | 120 |
| Sbjct 61 | AM+AMGA I K G W+I G GNG LL PE PLDFGNA TG RLTMGLVG YD ++TFI AAMKAMGAHIEKRGAEWVIRGTGNGALLQPEGPLDFGNAGTGSRLTMGLVGTYDMETTFI | 120 |
| Query 121 | GDASLTKRPMGRVLNPLREMGVQV-KSEDGDRLPVTLRGPKTPTPITYRVPMASAQVKSA | 179 |
| Sbjct 121 | GDASL+ RPMGRVL PLR+MGVQV K+ GDR+P+TL GPK PITYRVPMASAQVKSA GDASLSGRPMGRVLEPLRQMGVQVLKATPGDRMPITLHGPKHAAPITYRVPMASAQVKSA | 180 |
| Query 180 | VLLAGLNTPGITTVIEPIMTRDHTEKMLQGFGANLTVETDADGVRTIRLEGRGKLTGQVI | 239 |
| Sbjct 181 | VLLAGLNTPGITTVIEP+MTRDHTEKML+GFGANL+VETD GVR I +EG+GKLTGQ I VLLAGLNTPGITTVIEPVMTRDHTEKMLKGFGANLSVETDERGVRHIFIEGQGKLTGQTI | 240 |
| Query 240 | DVPGDPSSTAFPLVAALLVPGSDVTILNVLMNPTRTGLILTLQEMGADIEVINPRLAGGE | 299 |
| Sbjct 241 | VPGDPSS FPLVAAL+VPGSD+TI NVLMNPTRTGL+LTLQEMG I+++NPR AGGE AVPGDPSSAGFPLVAALIVPGSDITIENVLMNPTRTGLLLTLQEMGGKIDILNPRNAGGE | 300 |
| Query 300 | DVADLRVRSSTLKGVTVPEDRAPSMIDEYPILavaaafaegaTVMNGLEELRVKESDRLS | 359 |
| Sbjct 301 | DVADLRVR S LKGV VP +RAPSMIDEYP+LAVAA+FAEG T+M GLEELRVKESDRLS DVADLRVRYSELKGVAVPPERAPSMIDEYPVLAVAASFAEGETLMQGLEELRVKESDRLS | 360 |
| Query 360 | AVANGLKLNGVDCDEGETSLVVRGRPDGKGLG---NASGAAVATHLDHRIAMSFLVMGLV | 416 |
| Sbjct 361 | AVANGLKLNGVDC EGE SL VRG+P GKGLG N G V THLDHRIAMSFLVMGL  AVANGLKLNGVDCTEGEASLAVRGKPGGKGLGQHPNGQGTIVETHLDHRIAMSFLVMGLA | 420 |

Query 417 SENPVTVDDATMIATSFPEFMDLMAGLGAKI 447

+E PVT+DD MIATSFPEFM LM LGA+I Sbjct 421 TEKPVTIDDQAMIATSFPEFMGLMKALGAEI 451

>RWN54766.1 3-phosphoshikimate 1-carboxyvinyltransferase [Mesorhizobium sp.] RWN75053.1 3-phosphoshikimate 1-carboxyvinyltransferase [Mesorhizobium sp.] RWN78659.1 3-phosphoshikimate 1-carboxyvinyltransferase [Mesorhizobium sp.] RWN90153.1 3-phosphoshikimate 1-carboxyvinyltransferase [Mesorhizobium sp.] RWO14093.1 3-phosphoshikimate 1-carboxyvinyltransferase [Mesorhizobium sp.]

Length=452

Score = 682 bits (1761), Expect = 0.0, Method: Compositional matrix adjust.

| Identities | = 349/452 (77%), Positives = 385/452 (85%), Gaps = 4/452 (1%) |  |
| --- | --- | --- |
| Query 1 | MLHGASSRPATARKSSGLSGTVRIPGDKSISHRSFMFGGLASGETRITGLLEGEDVINTG | 60 |
| Sbjct 1 | M H A+ +PATARKS LSGT R+PGDKSISHRSFMFGGLASGETRITGLLEGEDV+ TG MSHTAAPKPATARKSPALSGTARVPGDKSISHRSFMFGGLASGETRITGLLEGEDVMRTG | 60 |
| Query 61 | KAMQAMGARIRKEGDTWIIDGVGNGGLLAPEAPLDFGNAATGCRLTMGLVGVYDFDSTFI | 120 |
| Sbjct 61 | AM+AMGA I K G W+I G GNG LL PE PLDFGNA TG RLTMGLVG YD ++TFI AAMKAMGAHIEKRGAEWVIRGTGNGALLQPEGPLDFGNAGTGSRLTMGLVGTYDMETTFI | 120 |
| Query 121 | GDASLTKRPMGRVLNPLREMGVQV-KSEDGDRLPVTLRGPKTPTPITYRVPMASAQVKSA | 179 |
| Sbjct 121 | GDASL+ RPMGRVL PLR+MGVQV K+ GDR+P+TLRGPK PITYRVPMASAQVKSA GDASLSGRPMGRVLEPLRQMGVQVLKATPGDRMPITLRGPKHAAPITYRVPMASAQVKSA | 180 |
| Query 180 | VLLAGLNTPGITTVIEPIMTRDHTEKMLQGFGANLTVETDADGVRTIRLEGRGKLTGQVI | 239 |
| Sbjct 181 | VLLAGLNTPGITTVIEP+MTRDHTEKMLQGFGANL+VETD GVR I +EG+G+LTGQ I VLLAGLNTPGITTVIEPVMTRDHTEKMLQGFGANLSVETDERGVRHIFIEGQGRLTGQTI | 240 |
| Query 240 | DVPGDPSSTAFPLVAALLVPGSDVTILNVLMNPTRTGLILTLQEMGADIEVINPRLAGGE | 299 |
| Sbjct 241 | VPGDPSS FPLVAAL+VPGSD+TI NVLMNPTRTGL+LTLQEMGA I++++PR GGE AVPGDPSSAGFPLVAALIVPGSDITIENVLMNPTRTGLLLTLQEMGARIDIVDPRNEGGE | 300 |
| Query 300 | DVADLRVRSSTLKGVTVPEDRAPSMIDEYPILavaaafaegaTVMNGLEELRVKESDRLS | 359 |
| Sbjct 301 | DVADLRVR S L+GVTVP +RAP+MIDEYP+LAVAA+FAEG T+M GLEELRVKESDRLS DVADLRVRYSELRGVTVPPERAPTMIDEYPVLAVAASFAEGETLMQGLEELRVKESDRLS | 360 |
| Query 360 | AVANGLKLNGVDCDEGETSLVVRGRPDGKGLG---NASGAAVATHLDHRIAMSFLVMGLV | 416 |
| Sbjct 361 | AVA GLKLNGVDC EGE +L VRG+P GKGLG N V THLDHRIAMSFLVMGL AVAEGLKLNGVDCTEGEATLAVRGKPGGKGLGQHPNGKSTIVQTHLDHRIAMSFLVMGLA | 420 |
| Query 417  Sbjct 421 | SENPVTVDDATMIATSFPEFMDLMAGLGAKIE 448  +E PVT+DD MIATSFPEFM LM GLGA+IE  TEKPVTIDDQAMIATSFPEFMGLMKGLGAEIE 452 |  |

>WP_192179333.1 3-phosphoshikimate 1-carboxyvinyltransferase [Mesorhizobium amorphae] Length=452

Score = 682 bits (1761), Expect = 0.0, Method: Compositional matrix adjust.

| Identities | = 352/451 (78%), Positives = 382/451 (85%), Gaps = 4/451 (1%) |  |
| --- | --- | --- |
| Query 1 | MLHGASSRPATARKSSGLSGTVRIPGDKSISHRSFMFGGLASGETRITGLLEGEDVINTG | 60 |
| Sbjct 1 | M H A+ +PATARKS LSG +PGDKSISHRSFMFGGLASGETRITGLLEGEDV+ TG MSHAAAPKPATARKSPALSGAACVPGDKSISHRSFMFGGLASGETRITGLLEGEDVMRTG | 60 |
| Query 61 | KAMQAMGARIRKEGDTWIIDGVGNGGLLAPEAPLDFGNAATGCRLTMGLVGVYDFDSTFI | 120 |
| Sbjct 61 | AM+AMGA I K G WII G GNG LL PE PLDFGNA TG RLTMGLVG YD ++TFI AAMKAMGAHIEKHGAEWIIRGTGNGALLQPEGPLDFGNAGTGSRLTMGLVGTYDVETTFI | 120 |
| Query 121 | GDASLTKRPMGRVLNPLREMGVQV-KSEDGDRLPVTLRGPKTPTPITYRVPMASAQVKSA | 179 |
| Sbjct 121 | GDASL+ RPMGRVL PLR+MGVQV K+ GDR+P+TLRGPK PITYRVPMASAQVKSA GDASLSGRPMGRVLEPLRQMGVQVLKATPGDRMPITLRGPKHAAPITYRVPMASAQVKSA | 180 |
| Query 180 | VLLAGLNTPGITTVIEPIMTRDHTEKMLQGFGANLTVETDADGVRTIRLEGRGKLTGQVI | 239 |
| Sbjct 181 | VLLAGLNTPGITTVIEP+MTRDHTEKML+GFGANLTVETD GVR I +EG+GKLTGQ I VLLAGLNTPGITTVIEPVMTRDHTEKMLKGFGANLTVETDERGVRHIFIEGQGKLTGQTI | 240 |
| Query 240 | DVPGDPSSTAFPLVAALLVPGSDVTILNVLMNPTRTGLILTLQEMGADIEVINPRLAGGE VPGDPSS FPLVAAL+VPGSD+ I NVLMNPTRTGL+LTLQEMG I+++NPR AGGE | 299 |

| Sbjct | 241 | AVPGDPSSAGFPLVAALIVPGSDIVIENVLMNPTRTGLLLTLQEMGGRIDILNPRNAGGE | 300 |
| --- | --- | --- | --- |
| Query | 300 | DVADLRVRSSTLKGVTVPEDRAPSMIDEYPILavaaafaegaTVMNGLEELRVKESDRLS | 359 |
| Sbjct | 301 | DVADLRVR S LKGV VP +RAPSMIDEYP+LAVAA+FAEG T+M GLEELRVKESDRLS DVADLRVRFSELKGVIVPPERAPSMIDEYPVLAVAASFAEGETLMQGLEELRVKESDRLS | 360 |
| Query | 360 | AVANGLKLNGVDCDEGETSLVVRGRPDGKGLG---NASGAAVATHLDHRIAMSFLVMGLV | 416 |
| Sbjct | 361 | AVANGLKLNGVDC EGE +L VRGRP GKGLG N G V THLDHRIAMSFLVMGL AVANGLKLNGVDCTEGEATLAVRGRPGGKGLGQHPNGQGTIVQTHLDHRIAMSFLVMGLA | 420 |
| Query  Sbjct | 417  421 | SENPVTVDDATMIATSFPEFMDLMAGLGAKI 447  +E PVT+DD MIATSFPEFM LM GLGA+I  TEKPVTIDDQAMIATSFPEFMGLMTGLGAEI 451 |  |

>TIP95793.1 3-phosphoshikimate 1-carboxyvinyltransferase [Mesorhizobium sp.] Length=452

Score = 682 bits (1761), Expect = 0.0, Method: Compositional matrix adjust.

| Identities | = 350/452 (77%), Positives = 384/452 (85%), Gaps = 4/452 (1%) |  |
| --- | --- | --- |
| Query 1 | MLHGASSRPATARKSSGLSGTVRIPGDKSISHRSFMFGGLASGETRITGLLEGEDVINTG | 60 |
| Sbjct 1 | M H A+ +PATARKSS LSG R+PGDKSISHRSFMFGGLASGETRITGLLEGEDV+ TG MSHTAAPKPATARKSSALSGAARVPGDKSISHRSFMFGGLASGETRITGLLEGEDVMRTG | 60 |
| Query 61 | KAMQAMGARIRKEGDTWIIDGVGNGGLLAPEAPLDFGNAATGCRLTMGLVGVYDFDSTFI | 120 |
| Sbjct 61 | AM+AMGA I K G W+I G GNG LL PEAPLDFGNA TG RLTMGLVG YD ++TFI AAMKAMGAHIEKRGAEWVIRGTGNGALLQPEAPLDFGNAGTGSRLTMGLVGTYDMETTFI | 120 |
| Query 121 | GDASLTKRPMGRVLNPLREMGVQV-KSEDGDRLPVTLRGPKTPTPITYRVPMASAQVKSA | 179 |
| Sbjct 121 | GDASL+ RPMGRVL PLR+MGVQV K+ GDR+P+TL GPK PITYRVPMASAQVKSA GDASLSGRPMGRVLEPLRQMGVQVLKATPGDRMPITLHGPKHAAPITYRVPMASAQVKSA | 180 |
| Query 180 | VLLAGLNTPGITTVIEPIMTRDHTEKMLQGFGANLTVETDADGVRTIRLEGRGKLTGQVI | 239 |
| Sbjct 181 | VLLAGLNTPGITTVIEP+MTRDHTEKML+GFGANL+VETD GVR I +EG+GKLTGQ I VLLAGLNTPGITTVIEPVMTRDHTEKMLKGFGANLSVETDERGVRHIFIEGQGKLTGQTI | 240 |
| Query 240 | DVPGDPSSTAFPLVAALLVPGSDVTILNVLMNPTRTGLILTLQEMGADIEVINPRLAGGE | 299 |
| Sbjct 241 | VPGDPSS FPLVAAL+VPGSD+TI NVLMNPTRTGL+LTLQEMGA I+V++PR GGE AVPGDPSSAGFPLVAALIVPGSDITIENVLMNPTRTGLLLTLQEMGARIDVLDPRNEGGE | 300 |
| Query 300 | DVADLRVRSSTLKGVTVPEDRAPSMIDEYPILavaaafaegaTVMNGLEELRVKESDRLS | 359 |
| Sbjct 301 | DVADLR R S LKGV VP +RAP+MIDEYP+LAVAA+FAEG T+M GLEELRVKESDRLS DVADLRARYSELKGVAVPPERAPTMIDEYPVLAVAASFAEGETLMQGLEELRVKESDRLS | 360 |
| Query 360 | AVANGLKLNGVDCDEGETSLVVRGRPDGKGLG---NASGAAVATHLDHRIAMSFLVMGLV | 416 |
| Sbjct 361 | AVANGLK+NGVDC EGE SL VRGRP GKGLG N V THLDHRIAMSFLVMGL AVANGLKVNGVDCTEGEASLAVRGRPGGKGLGQHPNGQNTVVQTHLDHRIAMSFLVMGLA | 420 |
| Query 417  Sbjct 421 | SENPVTVDDATMIATSFPEFMDLMAGLGAKIE 448  +E PVT+DD MIATSFPEFM LM GLGA+I+  TEKPVTIDDQAMIATSFPEFMGLMKGLGAEID 452 |  |

>TIO06699.1 3-phosphoshikimate 1-carboxyvinyltransferase [Mesorhizobium sp.] TIO31795.1 3-phosphoshikimate 1-carboxyvinyltransferase [Mesorhizobium sp.] TIP08544.1 3-phosphoshikimate 1-carboxyvinyltransferase [Mesorhizobium sp.]

Length=452

Score = 682 bits (1761), Expect = 0.0, Method: Compositional matrix adjust.

| Identities | = 350/451 (78%), Positives = 384/451 (85%), Gaps = 4/451 (1%) |  |
| --- | --- | --- |
| Query 1 | MLHGASSRPATARKSSGLSGTVRIPGDKSISHRSFMFGGLASGETRITGLLEGEDVINTG | 60 |
| Sbjct 1 | M H A+ +PATARKSS LSG R+PGDKSISHRSFMFGGLASGETRITGLLEGEDV+ TG MSHTAAPKPATARKSSALSGAARVPGDKSISHRSFMFGGLASGETRITGLLEGEDVMRTG | 60 |
| Query 61 | KAMQAMGARIRKEGDTWIIDGVGNGGLLAPEAPLDFGNAATGCRLTMGLVGVYDFDSTFI | 120 |
| Sbjct 61 | AM+AMGA I K G W+I G GNG LL PE PLDFGNA TG RLTMGLVG YD ++TFI TAMKAMGAHIEKHGAEWVIRGTGNGALLQPEGPLDFGNAGTGARLTMGLVGTYDMETTFI | 120 |
| Query 121 | GDASLTKRPMGRVLNPLREMGVQV-KSEDGDRLPVTLRGPKTPTPITYRVPMASAQVKSA GDASL+ RPMGRVL PLR+MGVQV K+ GDR+P+TLRGPK PITYRVPMASAQVKSA | 179 |

| Sbjct | 121 | GDASLSGRPMGRVLEPLRQMGVQVLKATPGDRMPITLRGPKHAAPITYRVPMASAQVKSA | 180 |
| --- | --- | --- | --- |
| Query | 180 | VLLAGLNTPGITTVIEPIMTRDHTEKMLQGFGANLTVETDADGVRTIRLEGRGKLTGQVI | 239 |
| Sbjct | 181 | VL+AGLNTPGITTVIEP+MTRDHTEKML GFGANL+VETD GVR I +EG+GKL GQ I VLIAGLNTPGITTVIEPVMTRDHTEKMLIGFGANLSVETDERGVRHIFIEGQGKLIGQTI | 240 |
| Query | 240 | DVPGDPSSTAFPLVAALLVPGSDVTILNVLMNPTRTGLILTLQEMGADIEVINPRLAGGE | 299 |
| Sbjct | 241 | VPGDPSS FPLVAAL+VPGSD+TI NVLMNPTRTGL+LTLQEMGA I+++NPR GGE AVPGDPSSAGFPLVAALIVPGSDITIENVLMNPTRTGLLLTLQEMGARIDIVNPRNEGGE | 300 |
| Query | 300 | DVADLRVRSSTLKGVTVPEDRAPSMIDEYPILavaaafaegaTVMNGLEELRVKESDRLS | 359 |
| Sbjct | 301 | DVADLRVR S LKGVTVP +RAP+MIDEYP+LAVAA+FAEG T+M GLEELRVKESDRLS DVADLRVRHSDLKGVTVPPERAPTMIDEYPVLAVAASFAEGETLMQGLEELRVKESDRLS | 360 |
| Query | 360 | AVANGLKLNGVDCDEGETSLVVRGRPDGKGLG---NASGAAVATHLDHRIAMSFLVMGLV | 416 |
| Sbjct | 361 | AVANGLKLNGVDC EGE +L VRGRP GKGLG N +V THLDHRIAMSFLVMGL AVANGLKLNGVDCTEGEATLAVRGRPGGKGLGSHPNGQDTSVKTHLDHRIAMSFLVMGLA | 420 |
| Query  Sbjct | 417  421 | SENPVTVDDATMIATSFPEFMDLMAGLGAKI 447  +E PVT+DD MIATSFPEFM LM GLGA+I  TEKPVTIDDQAMIATSFPEFMGLMKGLGAEI 451 |  |

>RWB01057.1 3-phosphoshikimate 1-carboxyvinyltransferase [Mesorhizobium sp.] Length=452

Score = 682 bits (1761), Expect = 0.0, Method: Compositional matrix adjust.

| Identities | = 351/452 (78%), Positives = 384/452 (85%), Gaps = 4/452 (1%) |  |
| --- | --- | --- |
| Query 1 | MLHGASSRPATARKSSGLSGTVRIPGDKSISHRSFMFGGLASGETRITGLLEGEDVINTG | 60 |
| Sbjct 1 | M H A+ +PATARKS LSGT R+PGDKSISHRSFMFGGLASGETRITGLLEGEDV+ TG MSHTAAPKPATARKSPALSGTARVPGDKSISHRSFMFGGLASGETRITGLLEGEDVMRTG | 60 |
| Query 61 | KAMQAMGARIRKEGDTWIIDGVGNGGLLAPEAPLDFGNAATGCRLTMGLVGVYDFDSTFI | 120 |
| Sbjct 61 | AM+AMGA I K G W+I G GNG LL PE PLDFGNA TG RLTMGLVG YD ++TFI AAMKAMGAHIEKRGAEWVIRGTGNGALLQPEGPLDFGNAGTGSRLTMGLVGTYDMETTFI | 120 |
| Query 121 | GDASLTKRPMGRVLNPLREMGVQV-KSEDGDRLPVTLRGPKTPTPITYRVPMASAQVKSA | 179 |
| Sbjct 121 | GDASL+ RPMGRVL PLR+MGVQV K+ GDR+P+TLRGPK PITYRVPMASAQVKSA GDASLSGRPMGRVLEPLRQMGVQVLKATPGDRMPITLRGPKHAAPITYRVPMASAQVKSA | 180 |
| Query 180 | VLLAGLNTPGITTVIEPIMTRDHTEKMLQGFGANLTVETDADGVRTIRLEGRGKLTGQVI | 239 |
| Sbjct 181 | VLLAGLNTPGITTVIEP+MTRDHTEKMLQGFGANL+VETD GVR I +EG+GKLTGQ I VLLAGLNTPGITTVIEPVMTRDHTEKMLQGFGANLSVETDERGVRHIFIEGQGKLTGQTI | 240 |
| Query 240 | DVPGDPSSTAFPLVAALLVPGSDVTILNVLMNPTRTGLILTLQEMGADIEVINPRLAGGE | 299 |
| Sbjct 241 | VPGDPSS FPLVAAL+VP SD+TI NVLMNPTRTGL+LTLQEMGA I+V+ PR GGE AVPGDPSSAGFPLVAALIVPDSDITIENVLMNPTRTGLLLTLQEMGARIDVLEPRNEGGE | 300 |
| Query 300 | DVADLRVRSSTLKGVTVPEDRAPSMIDEYPILavaaafaegaTVMNGLEELRVKESDRLS | 359 |
| Sbjct 301 | DVADLRVR S LKGVTVP +RAP+MIDEYP+LAVAA+FAEG T+M GLEELRVKESDRLS DVADLRVRYSELKGVTVPPERAPTMIDEYPVLAVAASFAEGETLMQGLEELRVKESDRLS | 360 |
| Query 360 | AVANGLKLNGVDCDEGETSLVVRGRPDGKGLG---NASGAAVATHLDHRIAMSFLVMGLV | 416 |
| Sbjct 361 | AVA GLKLNGVDC EGE +L VRG+P GKGLG N V THLDHRIAMSFLVMGL AVAEGLKLNGVDCTEGEATLAVRGKPGGKGLGQHPNGKSTIVQTHLDHRIAMSFLVMGLA | 420 |
| Query 417  Sbjct 421 | SENPVTVDDATMIATSFPEFMDLMAGLGAKIE 448  +E PVT+DD +MIATSFPEFM LM GLGA+IE  TEKPVTIDDQSMIATSFPEFMGLMKGLGAEIE 452 |  |

>WP_128364101.1 3-phosphoshikimate 1-carboxyvinyltransferase [Mesorhizobium sp. M7A.F.Ca.ET.027.02.1.1]

RVD13323.1 3-phosphoshikimate 1-carboxyvinyltransferase [Mesorhizobium sp. M7A.F.Ca.ET.027.02.1.1]

RWD03896.1 3-phosphoshikimate 1-carboxyvinyltransferase [Mesorhizobium sp.] Length=452

Score = 682 bits (1761), Expect = 0.0, Method: Compositional matrix adjust. Identities = 348/451 (77%), Positives = 382/451 (85%), Gaps = 4/451 (1%)

| Query | 1 | MLHGASSRPATARKSSGLSGTVRIPGDKSISHRSFMFGGLASGETRITGLLEGEDVINTG | 60 |
| --- | --- | --- | --- |
| Sbjct | 1 | M H A+ +PAT R+S LSG R+PGDKSISHRSFMFGGLASGETRITGLLEGEDV+ TG MSHAAAPKPATGRQSPALSGIARVPGDKSISHRSFMFGGLASGETRITGLLEGEDVMRTG | 60 |
| Query | 61 | KAMQAMGARIRKEGDTWIIDGVGNGGLLAPEAPLDFGNAATGCRLTMGLVGVYDFDSTFI | 120 |
| Sbjct | 61 | AM+AMGA I K GD W+I G GNG LL PE PLDFGNA TG RLTMGLVG YD ++TFI AAMKAMGAHIEKNGDEWVIRGTGNGALLQPEGPLDFGNAGTGSRLTMGLVGTYDMETTFI | 120 |
| Query | 121 | GDASLTKRPMGRVLNPLREMGVQV-KSEDGDRLPVTLRGPKTPTPITYRVPMASAQVKSA | 179 |
| Sbjct | 121 | GDASL+ RPMGRVL PLR+MGVQV K+ GDR+P+TL GPK PITYRVPMASAQVKSA GDASLSGRPMGRVLEPLRQMGVQVLKATPGDRMPITLHGPKHAAPITYRVPMASAQVKSA | 180 |
| Query | 180 | VLLAGLNTPGITTVIEPIMTRDHTEKMLQGFGANLTVETDADGVRTIRLEGRGKLTGQVI | 239 |
| Sbjct | 181 | VLLAGLNTPGITTVIEP+MTRDHTEKML+GFGANL+VETD GVR I +EG+G+LTGQ I VLLAGLNTPGITTVIEPVMTRDHTEKMLKGFGANLSVETDERGVRHIFIEGQGRLTGQTI | 240 |
| Query | 240 | DVPGDPSSTAFPLVAALLVPGSDVTILNVLMNPTRTGLILTLQEMGADIEVINPRLAGGE | 299 |
| Sbjct | 241 | VPGDPSS FPLVAAL+VPGSD+TI NVLMNPTRTGL+LTLQEMG I+++NPR AGGE AVPGDPSSAGFPLVAALIVPGSDITIENVLMNPTRTGLLLTLQEMGGKIDILNPRNAGGE | 300 |
| Query | 300 | DVADLRVRSSTLKGVTVPEDRAPSMIDEYPILavaaafaegaTVMNGLEELRVKESDRLS | 359 |
| Sbjct | 301 | DVADLRVR S LKGV VP +RAPSMIDEYP+LAVAA+FAEG T+M GLEELRVKESDRLS DVADLRVRYSELKGVIVPPERAPSMIDEYPVLAVAASFAEGETLMQGLEELRVKESDRLS | 360 |
| Query | 360 | AVANGLKLNGVDCDEGETSLVVRGRPDGKGLG---NASGAAVATHLDHRIAMSFLVMGLV | 416 |
| Sbjct | 361 | AVANGLKLNGVDC EGE SL VRG+P GKGLG N G V THLDHRIAMSFLVMGL AVANGLKLNGVDCTEGEASLAVRGKPSGKGLGGHPNGQGTTVQTHLDHRIAMSFLVMGLA | 420 |
| Query  Sbjct | 417  421 | SENPVTVDDATMIATSFPEFMDLMAGLGAKI 447  +E PVT+DD MIATSFPEFM LM LGA+I  TEKPVTIDDQVMIATSFPEFMGLMKALGAEI 451 |  |

>OYR27757.1 3-phosphoshikimate 1-carboxyvinyltransferase [Ochrobactrum pseudogrignonense] Length=415

Score = 682 bits (1761), Expect = 0.0, Method: Compositional matrix adjust.

| Identities | = 348/418 (83%), Positives = 381/418 (91%), Gaps = 3/418 (1%) |  |
| --- | --- | --- |
| Query 36 | MFGGLASGETRITGLLEGEDVINTGKAMQAMGARIRKEGDTWIIDGVGNGGLLAPEAPLD | 95 |
| Sbjct 1 | MFGGLASGETRITGLLEGEDVINTG+AMQAMGA+IRK+GD W+I+GVGNG LL PEAPLD MFGGLASGETRITGLLEGEDVINTGRAMQAMGAKIRKDGDIWVINGVGNGCLLQPEAPLD | 60 |
| Query 96 | FGNAATGCRLTMGLVGVYDFDSTFIGDASLTKRPMGRVLNPLREMGVQVKSEDGDRLPVT | 155 |
| Sbjct 61 | FGNA TG RLTMGLVG YD +TFIGDASLT RPMGRVLNPLREMGVQVK+ +GDRLP+T FGNAGTGARLTMGLVGTYDMTTTFIGDASLTSRPMGRVLNPLREMGVQVKAAEGDRLPLT | 120 |
| Query 156 | LRGPKTPTPITYRVPMASAQVKSAVLLAGLNTPGITTVIEPIMTRDHTEKMLQGFGANLT | 215 |
| Sbjct 121 | L GPKT PI+YRVPMASAQVKSAVLLAGLNTPG+TTVIEP+MTRDHTEKMLQGFGA+LT LTGPKTANPISYRVPMASAQVKSAVLLAGLNTPGVTTVIEPVMTRDHTEKMLQGFGADLT | 180 |
| Query 216 | VETDADGVRTIRLEGRGKLTGQVIDVPGDPSSTAFPLVAALLVPGSDVTILNVLMNPTRT | 275 |
| Sbjct 181 | VETD DGVR IR+ G+GKLTGQ IDVPGDPSSTAFPLVAALLV GSDVTI NVLMNPTRT VETDKDGVRHIRIVGQGKLTGQTIDVPGDPSSTAFPLVAALLVEGSDVTIRNVLMNPTRT | 240 |
| Query 276 | GLILTLQEMGADIEVINPRLAGGEDVADLRVRSSTLKGVTVPEDRAPSMIDEYPILavaa | 335 |
| Sbjct 241 | GLILTLQEMGADIE+I+PRLAGGEDVADLRV+SS LKGVTVP +RAPSMIDEYP+LA+AA GLILTLQEMGADIEIIDPRLAGGEDVADLRVKSSKLKGVTVPPERAPSMIDEYPVLAIAA | 300 |
| Query 336 | afaegaTVMNGLEELRVKESDRLSAVANGLKLNGVDCDEGETSLVVRGRPDGKGLGNASG | 395 |
| Sbjct 301 | +FAEG TVM+GL+ELRVKESDRL+AVA GL+ NGVDC EGE SL VRGRPDGKGLG G  SFAEGETVMDGLDELRVKESDRLAAVARGLEANGVDCTEGEMSLTVRGRPDGKGLG---G | 357 |

Query 396 AAVATHLDHRIAMSFLVMGLVSENPVTVDDATMIATSFPEFMDLMAGLGAKIELSDTK 453 VATHLDHRIAMSFLVMGL ++ PVTVDD+TMIATSFPEFM +M GLGAKIE+S+ +

Sbjct 358 GTVATHLDHRIAMSFLVMGLAADKPVTVDDSTMIATSFPEFMTMMPGLGAKIEVSEQQ 415

>RWQ18689.1 3-phosphoshikimate 1-carboxyvinyltransferase [Mesorhizobium sp.] Length=452

Score = 682 bits (1761), Expect = 0.0, Method: Compositional matrix adjust.

| Identities | = 349/451 (77%), Positives = 383/451 (85%), Gaps = 4/451 (1%) |  |
| --- | --- | --- |
| Query 1 | MLHGASSRPATARKSSGLSGTVRIPGDKSISHRSFMFGGLASGETRITGLLEGEDVINTG | 60 |
| Sbjct 1 | M H A+ +PATAR+S LSG R+PGDKSISHRSFMFGGLASGETRITGLLEGEDV+ TG MSHAAAPKPATARQSPALSGIARVPGDKSISHRSFMFGGLASGETRITGLLEGEDVMRTG | 60 |
| Query 61 | KAMQAMGARIRKEGDTWIIDGVGNGGLLAPEAPLDFGNAATGCRLTMGLVGVYDFDSTFI | 120 |
| Sbjct 61 | AM+AMGA I K GD W+I G GNG LL PE PLDFGNA TG RLTMGLVG YD ++TFI AAMKAMGALIEKNGDEWVIRGTGNGALLQPEGPLDFGNAGTGSRLTMGLVGTYDMETTFI | 120 |
| Query 121 | GDASLTKRPMGRVLNPLREMGVQV-KSEDGDRLPVTLRGPKTPTPITYRVPMASAQVKSA | 179 |
| Sbjct 121 | GDASL+ RPMGRVL PLR+MGVQV K+ GDR+P+TL GPK PITYRVPMASAQVKSA GDASLSGRPMGRVLEPLRQMGVQVLKATPGDRMPITLHGPKHAAPITYRVPMASAQVKSA | 180 |
| Query 180 | VLLAGLNTPGITTVIEPIMTRDHTEKMLQGFGANLTVETDADGVRTIRLEGRGKLTGQVI | 239 |
| Sbjct 181 | VLLAGLNTPGITTVIEP+MTRDHTEKML+GFGANL+VETD GVR I +EG+G+LTGQ I VLLAGLNTPGITTVIEPVMTRDHTEKMLKGFGANLSVETDERGVRHIFIEGQGRLTGQTI | 240 |
| Query 240 | DVPGDPSSTAFPLVAALLVPGSDVTILNVLMNPTRTGLILTLQEMGADIEVINPRLAGGE | 299 |
| Sbjct 241 | VPGDPSS FPLVAAL+VPGSD+TI NVLMNPTRTGL+LTLQEMG I+++NPR AGGE AVPGDPSSAGFPLVAALIVPGSDITIENVLMNPTRTGLLLTLQEMGGKIDILNPRNAGGE | 300 |
| Query 300 | DVADLRVRSSTLKGVTVPEDRAPSMIDEYPILavaaafaegaTVMNGLEELRVKESDRLS | 359 |
| Sbjct 301 | DVADLRVR S LKGV VP +RAPSMIDEYP+LAVAA+FAEG T+M GLEELRVKESDRLS DVADLRVRYSELKGVIVPPERAPSMIDEYPVLAVAASFAEGETLMQGLEELRVKESDRLS | 360 |
| Query 360 | AVANGLKLNGVDCDEGETSLVVRGRPDGKGLG---NASGAAVATHLDHRIAMSFLVMGLV | 416 |
| Sbjct 361 | AVANGLKLNGVDC EGE SL VRG+P GKGLG N G V THLDHRIAMSFLVMGL AVANGLKLNGVDCTEGEASLAVRGKPSGKGLGGHPNGQGTTVQTHLDHRIAMSFLVMGLA | 420 |
| Query 417  Sbjct 421 | SENPVTVDDATMIATSFPEFMDLMAGLGAKI 447  +E PVT+DD MIATSFPEFM LM LGA+I  TEKPVTIDDQAMIATSFPEFMGLMKALGAEI 451 |  |

>RWB27226.1 3-phosphoshikimate 1-carboxyvinyltransferase [Mesorhizobium sp.] RWD34759.1 3-phosphoshikimate 1-carboxyvinyltransferase [Mesorhizobium sp.] RWD42927.1 3-phosphoshikimate 1-carboxyvinyltransferase [Mesorhizobium sp.] RWD78511.1 3-phosphoshikimate 1-carboxyvinyltransferase [Mesorhizobium sp.]

Length=452

Score = 682 bits (1761), Expect = 0.0, Method: Compositional matrix adjust.

| Identities | = 351/452 (78%), Positives = 385/452 (85%), Gaps = 4/452 (1%) |  |
| --- | --- | --- |
| Query 1 | MLHGASSRPATARKSSGLSGTVRIPGDKSISHRSFMFGGLASGETRITGLLEGEDVINTG | 60 |
| Sbjct 1 | M H A+ +PATARKSS LSG R+PGDKSISHRSFMFGGLASGETRITGLLEGEDV+ TG MSHTAAPKPATARKSSALSGAARVPGDKSISHRSFMFGGLASGETRITGLLEGEDVMRTG | 60 |
| Query 61 | KAMQAMGARIRKEGDTWIIDGVGNGGLLAPEAPLDFGNAATGCRLTMGLVGVYDFDSTFI | 120 |
| Sbjct 61 | AM+AMGA I G W+I G GNG LL PE PLDFGNA TG RLTMGLVG YD ++TFI AAMKAMGAHIEMLGTEWVIRGTGNGALLQPEGPLDFGNAGTGSRLTMGLVGTYDMETTFI | 120 |
| Query 121 | GDASLTKRPMGRVLNPLREMGVQV-KSEDGDRLPVTLRGPKTPTPITYRVPMASAQVKSA | 179 |
| Sbjct 121 | GDASL+ RPMGRVL PLR+MGVQV K+ GDR+P+TLRGPK PITYRVPMASAQVKSA GDASLSGRPMGRVLEPLRQMGVQVLKATPGDRMPITLRGPKHAAPITYRVPMASAQVKSA | 180 |
| Query 180 | VLLAGLNTPGITTVIEPIMTRDHTEKMLQGFGANLTVETDADGVRTIRLEGRGKLTGQVI | 239 |
| Sbjct 181 | VLLAGLNTPGITTVIEP+MTRDHTEKML+GFGANL+VETD GVR I +EG+GKLTGQ I VLLAGLNTPGITTVIEPVMTRDHTEKMLRGFGANLSVETDERGVRHIFIEGQGKLTGQTI | 240 |
| Query 240 | DVPGDPSSTAFPLVAALLVPGSDVTILNVLMNPTRTGLILTLQEMGADIEVINPRLAGGE | 299 |
| Sbjct 241 | VPGDPSS FPLVAAL+VPGSD+TI NVLMNPTRTGL+LTLQEMGA I++++PR GGE AVPGDPSSAGFPLVAALIVPGSDITIENVLMNPTRTGLLLTLQEMGARIDIVDPRNEGGE | 300 |
| Query 300 | DVADLRVRSSTLKGVTVPEDRAPSMIDEYPILavaaafaegaTVMNGLEELRVKESDRLS | 359 |
| Sbjct 301 | DVADLRVR S LKGV VP +RAP+MIDEYP+LAVAA+FAEG T+M GLEELRVKESDRLS DVADLRVRYSELKGVAVPPERAPTMIDEYPVLAVAASFAEGETLMQGLEELRVKESDRLS | 360 |
| Query 360 | AVANGLKLNGVDCDEGETSLVVRGRPDGKGLG---NASGAAVATHLDHRIAMSFLVMGLV | 416 |
| Sbjct 361 | AVA+GLKLNGVDC EGE SL V GRP GKGLG N G V THLDHRIAMSFLVMGL  AVADGLKLNGVDCTEGEASLAVHGRPGGKGLGQHPNGQGTIVQTHLDHRIAMSFLVMGLA | 420 |

Query 417 SENPVTVDDATMIATSFPEFMDLMAGLGAKIE 448

+E PVT+DD TMIATSFPEFM LM GLGA+IE Sbjct 421 TEKPVTIDDQTMIATSFPEFMGLMKGLGAEIE 452

>RWN67225.1 3-phosphoshikimate 1-carboxyvinyltransferase [Mesorhizobium sp.] Length=452

Score = 682 bits (1760), Expect = 0.0, Method: Compositional matrix adjust.

| Identities | = 348/452 (77%), Positives = 385/452 (85%), Gaps = 4/452 (1%) |  |
| --- | --- | --- |
| Query 1 | MLHGASSRPATARKSSGLSGTVRIPGDKSISHRSFMFGGLASGETRITGLLEGEDVINTG | 60 |
| Sbjct 1 | M H A+ +PATARKSS LSGT R+PGDKSISHRSFMFGGLASGETRITGLLEGEDV+ TG MSHTAAPKPATARKSSALSGTARMPGDKSISHRSFMFGGLASGETRITGLLEGEDVMRTG | 60 |
| Query 61 | KAMQAMGARIRKEGDTWIIDGVGNGGLLAPEAPLDFGNAATGCRLTMGLVGVYDFDSTFI | 120 |
| Sbjct 61 | AM+AMGA I K G W+I G GNG LL PE PLDFGNA TG RLTMGLVG YD ++TFI AAMKAMGAHIEKHGTEWVIRGTGNGALLQPEGPLDFGNAGTGSRLTMGLVGTYDMETTFI | 120 |
| Query 121 | GDASLTKRPMGRVLNPLREMGVQV-KSEDGDRLPVTLRGPKTPTPITYRVPMASAQVKSA | 179 |
| Sbjct 121 | GDASL+ RPMGRVL PLR+MGVQV K+ GDR+P+TL GPK PITYRVPMASAQVKSA GDASLSGRPMGRVLEPLRQMGVQVLKATPGDRMPITLHGPKHAAPITYRVPMASAQVKSA | 180 |
| Query 180 | VLLAGLNTPGITTVIEPIMTRDHTEKMLQGFGANLTVETDADGVRTIRLEGRGKLTGQVI | 239 |
| Sbjct 181 | VLLAGLNTPG+TTVIEP+MTRDHTEKML+GFGANL+VETD GVR I +EG+GKLTGQ I VLLAGLNTPGVTTVIEPVMTRDHTEKMLKGFGANLSVETDERGVRHIFIEGQGKLTGQTI | 240 |
| Query 240 | DVPGDPSSTAFPLVAALLVPGSDVTILNVLMNPTRTGLILTLQEMGADIEVINPRLAGGE | 299 |
| Sbjct 241 | VPGDPSS FPLVAAL+VPGSD+TI NVLMNPTRTGL+LTLQEMGA I++++PR GGE AVPGDPSSAGFPLVAALIVPGSDITIENVLMNPTRTGLLLTLQEMGARIDILDPRNEGGE | 300 |
| Query 300 | DVADLRVRSSTLKGVTVPEDRAPSMIDEYPILavaaafaegaTVMNGLEELRVKESDRLS | 359 |
| Sbjct 301 | DVADLRVR S LKGVTVP +RAP+MIDEYP+LAVAA+FAEG T+M GLEELRVKESDRLS DVADLRVRYSELKGVTVPPERAPTMIDEYPVLAVAASFAEGETLMQGLEELRVKESDRLS | 360 |
| Query 360 | AVANGLKLNGVDCDEGETSLVVRGRPDGKGLG---NASGAAVATHLDHRIAMSFLVMGLV | 416 |
| Sbjct 361 | AVA GLKLNGVDC EGE +L VRG+P GKGLG N V THLDHRIAMSFL+MGL AVAEGLKLNGVDCTEGEATLAVRGKPGGKGLGQHPNGHNTVVQTHLDHRIAMSFLIMGLA | 420 |
| Query 417  Sbjct 421 | SENPVTVDDATMIATSFPEFMDLMAGLGAKIE 448  +E PVT+DD MIATSFPEFM LM GLGA+IE  TEKPVTIDDQAMIATSFPEFMGLMKGLGAEIE 452 |  |

>RWH78490.1 3-phosphoshikimate 1-carboxyvinyltransferase [Mesorhizobium sp.] RWH80890.1 3-phosphoshikimate 1-carboxyvinyltransferase [Mesorhizobium sp.] RWH90488.1 3-phosphoshikimate 1-carboxyvinyltransferase [Mesorhizobium sp.] RWH97351.1 3-phosphoshikimate 1-carboxyvinyltransferase [Mesorhizobium sp.] RWI01049.1 3-phosphoshikimate 1-carboxyvinyltransferase [Mesorhizobium sp.]

Length=452

Score = 682 bits (1760), Expect = 0.0, Method: Compositional matrix adjust.

| Identities | = 349/452 (77%), Positives = 384/452 (85%), Gaps = 4/452 (1%) |  |
| --- | --- | --- |
| Query 1 | MLHGASSRPATARKSSGLSGTVRIPGDKSISHRSFMFGGLASGETRITGLLEGEDVINTG | 60 |
| Sbjct 1 | M H A+ +PATARKSS LSG R+PGDKSISHRSFMFGGLASGETRITGLLEGEDV+ TG MSHTAAPKPATARKSSALSGAARVPGDKSISHRSFMFGGLASGETRITGLLEGEDVMRTG | 60 |
| Query 61 | KAMQAMGARIRKEGDTWIIDGVGNGGLLAPEAPLDFGNAATGCRLTMGLVGVYDFDSTFI | 120 |
| Sbjct 61 | AM+AMGA I K G W+I G GNG LL PEAPLDFGNA TG RLTMGLVG YD ++TFI AAMKAMGAHIEKRGAEWVIRGTGNGALLQPEAPLDFGNAGTGSRLTMGLVGTYDMETTFI | 120 |
| Query 121 | GDASLTKRPMGRVLNPLREMGVQV-KSEDGDRLPVTLRGPKTPTPITYRVPMASAQVKSA | 179 |
| Sbjct 121 | GDASL+ RPMGRVL PLR+MGVQV K+ GDR+P+TL GPK PITYRVPMASAQVKSA GDASLSGRPMGRVLEPLRQMGVQVLKATPGDRMPITLHGPKHAAPITYRVPMASAQVKSA | 180 |
| Query 180 | VLLAGLNTPGITTVIEPIMTRDHTEKMLQGFGANLTVETDADGVRTIRLEGRGKLTGQVI | 239 |
| Sbjct 181 | VLLAGLNTPG+TTVIEP+MTRDHTEKML+GFGANL+VETD GVR I +EG+GKLTGQ I VLLAGLNTPGVTTVIEPVMTRDHTEKMLKGFGANLSVETDERGVRHIFIEGQGKLTGQTI | 240 |
| Query 240 | DVPGDPSSTAFPLVAALLVPGSDVTILNVLMNPTRTGLILTLQEMGADIEVINPRLAGGE VPGDPSS FPLVAAL+VPGSD+TI NVLMNPTRTGL+LTLQEMGA I+V++PR GGE | 299 |

| Sbjct | 241 | AVPGDPSSAGFPLVAALIVPGSDITIENVLMNPTRTGLLLTLQEMGARIDVLDPRNEGGE | 300 |
| --- | --- | --- | --- |
| Query | 300 | DVADLRVRSSTLKGVTVPEDRAPSMIDEYPILavaaafaegaTVMNGLEELRVKESDRLS | 359 |
| Sbjct | 301 | DVADLR R S LKGV VP +RAP+MIDEYP+LAVAA+FAEG T+M GLEELRVKESDRLS DVADLRARYSELKGVAVPPERAPTMIDEYPVLAVAASFAEGETLMQGLEELRVKESDRLS | 360 |
| Query | 360 | AVANGLKLNGVDCDEGETSLVVRGRPDGKGLG---NASGAAVATHLDHRIAMSFLVMGLV | 416 |
| Sbjct | 361 | AVANGLK+NGVDC EGE SL VRGRP GKGLG N V THLDHRIAMSFLVMGL AVANGLKVNGVDCTEGEASLAVRGRPGGKGLGQHPNGQNTVVQTHLDHRIAMSFLVMGLA | 420 |
| Query  Sbjct | 417  421 | SENPVTVDDATMIATSFPEFMDLMAGLGAKIE 448  +E PVT+DD MIATSFPEFM LM GLGA+I+  TEKPVTIDDQAMIATSFPEFMGLMKGLGAEID 452 |  |

>RWO02711.1 3-phosphoshikimate 1-carboxyvinyltransferase [Mesorhizobium sp.] RWO52172.1 3-phosphoshikimate 1-carboxyvinyltransferase [Mesorhizobium sp.] TIN28765.1 3-phosphoshikimate 1-carboxyvinyltransferase [Mesorhizobium sp.] TIN37416.1 3-phosphoshikimate 1-carboxyvinyltransferase [Mesorhizobium sp.] TJU86019.1 3-phosphoshikimate 1-carboxyvinyltransferase [Mesorhizobium sp.]

Length=452

Score = 682 bits (1760), Expect = 0.0, Method: Compositional matrix adjust.

| Identities | = 349/452 (77%), Positives = 384/452 (85%), Gaps = 4/452 (1%) |  |
| --- | --- | --- |
| Query 1 | MLHGASSRPATARKSSGLSGTVRIPGDKSISHRSFMFGGLASGETRITGLLEGEDVINTG | 60 |
| Sbjct 1 | M H A+ +PATARKS LSGT R+PGDKSISHRSFMFGGLASGETRITGLLEGEDV+ TG MSHTAAEKPATARKSPALSGTARVPGDKSISHRSFMFGGLASGETRITGLLEGEDVMRTG | 60 |
| Query 61 | KAMQAMGARIRKEGDTWIIDGVGNGGLLAPEAPLDFGNAATGCRLTMGLVGVYDFDSTFI | 120 |
| Sbjct 61 | AM+AMGA I K G W+I G GNG LL PE PLDFGNA TG RLTMGLVG YD ++TFI AAMKAMGAHIEKRGAEWVIRGTGNGALLQPEGPLDFGNAGTGSRLTMGLVGTYDMETTFI | 120 |
| Query 121 | GDASLTKRPMGRVLNPLREMGVQV-KSEDGDRLPVTLRGPKTPTPITYRVPMASAQVKSA | 179 |
| Sbjct 121 | GDASL+ RPMGRVL PLR+MGVQV K+ GDR+P+TLRGPK PI+YRVPMASAQVKSA GDASLSGRPMGRVLEPLRQMGVQVLKATPGDRMPITLRGPKHAAPISYRVPMASAQVKSA | 180 |
| Query 180 | VLLAGLNTPGITTVIEPIMTRDHTEKMLQGFGANLTVETDADGVRTIRLEGRGKLTGQVI | 239 |
| Sbjct 181 | VLLAGLNTPGITTVIEP+MTRDHTEKML+GFGANL+VETD GVR I +EG+GKLTGQ I VLLAGLNTPGITTVIEPVMTRDHTEKMLKGFGANLSVETDERGVRHIFIEGQGKLTGQTI | 240 |
| Query 240 | DVPGDPSSTAFPLVAALLVPGSDVTILNVLMNPTRTGLILTLQEMGADIEVINPRLAGGE | 299 |
| Sbjct 241 | VPGDPSS FPLVAAL+VPGSD+TI NVLMNPTRTGL+LTLQEMGA I++++PR GGE AVPGDPSSAGFPLVAALIVPGSDITIENVLMNPTRTGLLLTLQEMGARIDIVDPRNEGGE | 300 |
| Query 300 | DVADLRVRSSTLKGVTVPEDRAPSMIDEYPILavaaafaegaTVMNGLEELRVKESDRLS | 359 |
| Sbjct 301 | DVADLRVR S LK VTVP +RAP+MIDEYP+LAVAA+FAEG T+M GLEELRVKESDRLS DVADLRVRYSELKAVTVPPERAPTMIDEYPVLAVAASFAEGETLMQGLEELRVKESDRLS | 360 |
| Query 360 | AVANGLKLNGVDCDEGETSLVVRGRPDGKGLG---NASGAAVATHLDHRIAMSFLVMGLV | 416 |
| Sbjct 361 | AVA GLKLNGVDC EGE SL VRG+P GKGLG N V THLDHRIAMSFLVMGL AVAEGLKLNGVDCTEGEASLAVRGKPGGKGLGQHPNGKSTIVQTHLDHRIAMSFLVMGLA | 420 |
| Query 417  Sbjct 421 | SENPVTVDDATMIATSFPEFMDLMAGLGAKIE 448  +E PVT+DD MIATSFPEFM LM GLGA+IE  TEKPVTIDDQAMIATSFPEFMGLMKGLGAEIE 452 |  |

>WP_023718021.1 3-phosphoshikimate 1-carboxyvinyltransferase [Mesorhizobium sp. LSHC420B00]

ESX81476.1 3-phosphoshikimate 1-carboxyvinyltransferase [Mesorhizobium sp. LSHC420B00]

Length=452

Score = 682 bits (1760), Expect = 0.0, Method: Compositional matrix adjust. Identities = 351/452 (78%), Positives = 386/452 (85%), Gaps = 4/452 (1%)

| Query | 1 | MLHGASSRPATARKSSGLSGTVRIPGDKSISHRSFMFGGLASGETRITGLLEGEDVINTG | 60 |
| --- | --- | --- | --- |
|  |  | M H A+ + ATARKSSGLSG R+PGDKSISHRSFMFGGLASGETRITGLLEGEDV+ TG |  |
| Sbjct | 1 | MSHTAAPKQATARKSSGLSGAARVPGDKSISHRSFMFGGLASGETRITGLLEGEDVMRTG | 60 |
| Query | 61 | KAMQAMGARIRKEGDTWIIDGVGNGGLLAPEAPLDFGNAATGCRLTMGLVGVYDFDSTFI | 120 |

| Sbjct | 61 | AM+AMGA I K G W+I G GNG LL PE PLDFGNA TG RLTMGLVG YD ++TFI AAMKAMGAHIEKHGTEWVIRGTGNGALLQPEGPLDFGNAGTGSRLTMGLVGTYDMETTFI | 120 |
| --- | --- | --- | --- |
| Query | 121 | GDASLTKRPMGRVLNPLREMGVQV-KSEDGDRLPVTLRGPKTPTPITYRVPMASAQVKSA | 179 |
| Sbjct | 121 | GDASL+ RPMGRVL PLR+MGVQV K+ GDR+P+TLRGPK PITYRVPMASAQVKSA GDASLSGRPMGRVLEPLRQMGVQVLKATPGDRMPITLRGPKHAAPITYRVPMASAQVKSA | 180 |
| Query | 180 | VLLAGLNTPGITTVIEPIMTRDHTEKMLQGFGANLTVETDADGVRTIRLEGRGKLTGQVI | 239 |
| Sbjct | 181 | VLLAGLNTPGITTVIEP+MTRDHTEKML+GFGANL+VETD GVR I +EG+GKLTGQ I VLLAGLNTPGITTVIEPVMTRDHTEKMLKGFGANLSVETDERGVRHIFIEGQGKLTGQTI | 240 |
| Query | 240 | DVPGDPSSTAFPLVAALLVPGSDVTILNVLMNPTRTGLILTLQEMGADIEVINPRLAGGE | 299 |
| Sbjct | 241 | VPGDPSS FPLVAAL+VPGSD+TI NVLMNPTRTGL+LTLQEMGA I++++ R GGE AVPGDPSSAGFPLVAALIVPGSDITIENVLMNPTRTGLLLTLQEMGARIDILDRRNEGGE | 300 |
| Query | 300 | DVADLRVRSSTLKGVTVPEDRAPSMIDEYPILavaaafaegaTVMNGLEELRVKESDRLS | 359 |
| Sbjct | 301 | DVADLRVR S LKGVTVP +RAP+MIDEYP+LAVAA+FAEG T+M GLEELRVKESDRLS DVADLRVRYSELKGVTVPPERAPTMIDEYPVLAVAASFAEGETLMLGLEELRVKESDRLS | 360 |
| Query | 360 | AVANGLKLNGVDCDEGETSLVVRGRPDGKGLG---NASGAAVATHLDHRIAMSFLVMGLV | 416 |
| Sbjct | 361 | AVANGLKLNGVDC EGE +L VRG+P GKGLG N G V THLDHRIAMSFLVMGL AVANGLKLNGVDCSEGEATLAVRGKPGGKGLGQHPNGQGTIVQTHLDHRIAMSFLVMGLA | 420 |
| Query  Sbjct | 417  421 | SENPVTVDDATMIATSFPEFMDLMAGLGAKIE 448  +E PVT+DD MIATSFPEFM LM GLGA+IE  TEKPVTIDDQAMIATSFPEFMGLMKGLGAEIE 452 |  |

>RWL83793.1 3-phosphoshikimate 1-carboxyvinyltransferase [Mesorhizobium sp.] RWL85178.1 3-phosphoshikimate 1-carboxyvinyltransferase [Mesorhizobium sp.] RWM02859.1 3-phosphoshikimate 1-carboxyvinyltransferase [Mesorhizobium sp.]

Length=452

Score = 682 bits (1760), Expect = 0.0, Method: Compositional matrix adjust.

| Identities | = 349/452 (77%), Positives = 385/452 (85%), Gaps = 4/452 (1%) |  |
| --- | --- | --- |
| Query 1 | MLHGASSRPATARKSSGLSGTVRIPGDKSISHRSFMFGGLASGETRITGLLEGEDVINTG | 60 |
| Sbjct 1 | M H A+++ ATAR S L+GT R+PGDKSISHRSFMFGGLASGETRITGLLEGEDV+ TG MSHSAAAKSATARNSPALAGTARVPGDKSISHRSFMFGGLASGETRITGLLEGEDVMRTG | 60 |
| Query 61 | KAMQAMGARIRKEGDTWIIDGVGNGGLLAPEAPLDFGNAATGCRLTMGLVGVYDFDSTFI | 120 |
| Sbjct 61 | AM+AMGA I K G W+I G GNG LL PE PLDFGNA TG RLTMGLVG YD ++TFI AAMKAMGAHIEKNGAEWVIRGTGNGALLQPEGPLDFGNAGTGSRLTMGLVGTYDMETTFI | 120 |
| Query 121 | GDASLTKRPMGRVLNPLREMGVQV-KSEDGDRLPVTLRGPKTPTPITYRVPMASAQVKSA | 179 |
| Sbjct 121 | GDASL+ RPMGRVL+PLR+MGVQV K+ GDR+P+TLRGPK PITYRVPMASAQVKSA GDASLSGRPMGRVLDPLRQMGVQVLKAAPGDRMPITLRGPKHAAPITYRVPMASAQVKSA | 180 |
| Query 180 | VLLAGLNTPGITTVIEPIMTRDHTEKMLQGFGANLTVETDADGVRTIRLEGRGKLTGQVI | 239 |
| Sbjct 181 | VLLAGLNTPGITTVIEP+MTRDHTEKML+GFGANLTVETD GVR I +EG+GKLTGQ I VLLAGLNTPGITTVIEPVMTRDHTEKMLKGFGANLTVETDERGVRHIFIEGQGKLTGQTI | 240 |
| Query 240 | DVPGDPSSTAFPLVAALLVPGSDVTILNVLMNPTRTGLILTLQEMGADIEVINPRLAGGE | 299 |
| Sbjct 241 | VPGDPSS FPLVAAL+VPGSD+ I NVLMNPTRTGL+LTL EMG I+++NPR AGGE AVPGDPSSAGFPLVAALIVPGSDIVIENVLMNPTRTGLLLTLHEMGGRIDILNPRNAGGE | 300 |
| Query 300 | DVADLRVRSSTLKGVTVPEDRAPSMIDEYPILavaaafaegaTVMNGLEELRVKESDRLS | 359 |
| Sbjct 301 | DVADLRVRSS LKGV VP +RAPSMIDEYP+LAVAA+FAEG T+M GLEELRVKESDRLS DVADLRVRSSELKGVVVPPERAPSMIDEYPVLAVAASFAEGETLMQGLEELRVKESDRLS | 360 |
| Query 360 | AVANGLKLNGVDCDEGETSLVVRGRPDGKGLG---NASGAAVATHLDHRIAMSFLVMGLV | 416 |
| Sbjct 361 | AVANGLKLNGVDC EGE +L V G+P GKGLG N AV THLDHRIAMSFL+MGL AVANGLKLNGVDCTEGEATLAVSGKPGGKGLGAHPNGPDTAVKTHLDHRIAMSFLIMGLA | 420 |
| Query 417  Sbjct 421 | SENPVTVDDATMIATSFPEFMDLMAGLGAKIE 448  +E PVT+DDA MIATSFPEFM LM GLGA+IE  TEKPVTIDDANMIATSFPEFMGLMKGLGAEIE 452 |  |

>RWN61857.1 3-phosphoshikimate 1-carboxyvinyltransferase [Mesorhizobium sp.] Length=452

Score = 682 bits (1760), Expect = 0.0, Method: Compositional matrix adjust.

| Identities | = 351/452 (78%), Positives = 382/452 (85%), Gaps = 4/452 (1%) |  |
| --- | --- | --- |
| Query 1 | MLHGASSRPATARKSSGLSGTVRIPGDKSISHRSFMFGGLASGETRITGLLEGEDVINTG | 60 |
| Sbjct 1 | M H A+ PATARKS LSGT R+PGDKSISHRSFMFGGLASGETRITGLLEGEDV+ TG MSHTAAPSPATARKSPALSGTARVPGDKSISHRSFMFGGLASGETRITGLLEGEDVMRTG | 60 |
| Query 61 | KAMQAMGARIRKEGDTWIIDGVGNGGLLAPEAPLDFGNAATGCRLTMGLVGVYDFDSTFI | 120 |
| Sbjct 61 | AM+AMGA I K G W+I G GNG LL PE PLDFGNA TG RLTMGLVG YD ++TFI AAMKAMGAHIEKRGAEWVIRGTGNGALLQPEGPLDFGNAGTGSRLTMGLVGTYDMETTFI | 120 |
| Query 121 | GDASLTKRPMGRVLNPLREMGVQV-KSEDGDRLPVTLRGPKTPTPITYRVPMASAQVKSA | 179 |
| Sbjct 121 | GDASL+ RPMGRVL PLR+MGVQV K+ GDR+P+TLRGPK PITYRVPMASAQVKSA GDASLSGRPMGRVLEPLRQMGVQVLKATPGDRMPITLRGPKHAAPITYRVPMASAQVKSA | 180 |
| Query 180 | VLLAGLNTPGITTVIEPIMTRDHTEKMLQGFGANLTVETDADGVRTIRLEGRGKLTGQVI | 239 |
| Sbjct 181 | VLLAGLNTPGITTVIEP+MTRDHTEKMLQGFGANL+VETD GVR I +EG+GKLTGQ I VLLAGLNTPGITTVIEPVMTRDHTEKMLQGFGANLSVETDERGVRHIFIEGQGKLTGQTI | 240 |
| Query 240 | DVPGDPSSTAFPLVAALLVPGSDVTILNVLMNPTRTGLILTLQEMGADIEVINPRLAGGE | 299 |
| Sbjct 241 | VPGDPSS FPLVAAL+VPGSD+TI NVLMNPTRTG +LTLQEMGA I+V+ PR GGE AVPGDPSSAGFPLVAALIVPGSDITIENVLMNPTRTGFLLTLQEMGARIDVLEPRNEGGE | 300 |
| Query 300 | DVADLRVRSSTLKGVTVPEDRAPSMIDEYPILavaaafaegaTVMNGLEELRVKESDRLS | 359 |
| Sbjct 301 | DVADLRVR S LKGVTVP +RAP+MIDEYP+LAVAA+FAEG T+M GLEELRVKESDRLS DVADLRVRYSELKGVTVPPERAPTMIDEYPVLAVAASFAEGETLMQGLEELRVKESDRLS | 360 |
| Query 360 | AVANGLKLNGVDCDEGETSLVVRGRPDGKGLG---NASGAAVATHLDHRIAMSFLVMGLV | 416 |
| Sbjct 361 | AVA GLKLNGVDC EGE +L VRG+P GKGLG N V THLDHRIAMSFLVMGL AVAEGLKLNGVDCTEGEATLAVRGKPGGKGLGQHPNGKSTIVQTHLDHRIAMSFLVMGLA | 420 |
| Query 417  Sbjct 421 | SENPVTVDDATMIATSFPEFMDLMAGLGAKIE 448  +E PVT+DD MIATSFPEFM LM GLGA+IE  TEKPVTIDDQAMIATSFPEFMGLMKGLGAEIE 452 |  |

>WP_027151509.1 3-phosphoshikimate 1-carboxyvinyltransferase [Mesorhizobium sp. WSM2561]

Length=452

Score = 682 bits (1760), Expect = 0.0, Method: Compositional matrix adjust.

| Identities | = 351/452 (78%), Positives = 383/452 (85%), Gaps = 4/452 (1%) |  |
| --- | --- | --- |
| Query 1 | MLHGASSRPATARKSSGLSGTVRIPGDKSISHRSFMFGGLASGETRITGLLEGEDVINTG | 60 |
| Sbjct 1 | M H A+ +PATARKS LSGT R+PGDKSISHRS MFGGLASGETRITGLLEGEDV+ TG MSHTAAPKPATARKSPALSGTARVPGDKSISHRSLMFGGLASGETRITGLLEGEDVMRTG | 60 |
| Query 61 | KAMQAMGARIRKEGDTWIIDGVGNGGLLAPEAPLDFGNAATGCRLTMGLVGVYDFDSTFI | 120 |
| Sbjct 61 | AM+AMGA I K G W+I G GNG LL PE PLDFGNA TG RLTMGLVG YD ++TFI AAMKAMGAHIEKRGAEWVIRGTGNGALLQPEGPLDFGNAGTGSRLTMGLVGTYDMETTFI | 120 |
| Query 121 | GDASLTKRPMGRVLNPLREMGVQV-KSEDGDRLPVTLRGPKTPTPITYRVPMASAQVKSA | 179 |
| Sbjct 121 | GDASL+ RPMGRVL PLR+MGVQV K+ GDR+P+TLRGPK PITYRVPMASAQVKSA GDASLSGRPMGRVLEPLRQMGVQVLKATPGDRMPITLRGPKHAAPITYRVPMASAQVKSA | 180 |
| Query 180 | VLLAGLNTPGITTVIEPIMTRDHTEKMLQGFGANLTVETDADGVRTIRLEGRGKLTGQVI | 239 |
| Sbjct 181 | VLLAGLNTPGITTVIEP+MTRDHTEKMLQGFGANL+VETD GVR I +EG+GKLTGQ I VLLAGLNTPGITTVIEPVMTRDHTEKMLQGFGANLSVETDERGVRHIFIEGQGKLTGQTI | 240 |
| Query 240 | DVPGDPSSTAFPLVAALLVPGSDVTILNVLMNPTRTGLILTLQEMGADIEVINPRLAGGE | 299 |
| Sbjct 241 | VPGDPSS FPLVAAL+VPGSD+TI NVLMNPTRTGL+LTLQEMGA I+V+ PR GGE AVPGDPSSAGFPLVAALIVPGSDITIENVLMNPTRTGLLLTLQEMGARIDVLEPRNEGGE | 300 |
| Query 300 | DVADLRVRSSTLKGVTVPEDRAPSMIDEYPILavaaafaegaTVMNGLEELRVKESDRLS | 359 |
| Sbjct 301 | DVADLRVR S LKGVTVP +RAP+MIDEYP+LAVAA+FAEG T+M GLEELRVKESDRLS DVADLRVRYSELKGVTVPPERAPTMIDEYPVLAVAASFAEGETLMQGLEELRVKESDRLS | 360 |
| Query 360 | AVANGLKLNGVDCDEGETSLVVRGRPDGKGLG---NASGAAVATHLDHRIAMSFLVMGLV | 416 |
| Sbjct 361 | AVA GLKLNGVDC EGE +L VRG+P GKGLG N V THLDHRIAMSFLVMGL AVAEGLKLNGVDCTEGEATLAVRGKPGGKGLGQHPNGKSTIVQTHLDHRIAMSFLVMGLA | 420 |
| Query 417 | SENPVTVDDATMIATSFPEFMDLMAGLGAKIE 448 |  |

+E PVT+DD MIATSFPEFM LM GLGA+IE Sbjct 421 TEKPVTIDDQAMIATSFPEFMGLMKGLGAEIE 452

>WP_127220727.1 3-phosphoshikimate 1-carboxyvinyltransferase [Mesorhizobium sp. M1E.F.Ca.ET.063.01.1.1]

RUW84693.1 3-phosphoshikimate 1-carboxyvinyltransferase [Mesorhizobium sp. M1E.F.Ca.ET.063.01.1.1]

Length=452

Score = 682 bits (1760), Expect = 0.0, Method: Compositional matrix adjust.

| Identities | = 349/452 (77%), Positives = 385/452 (85%), Gaps = 4/452 (1%) |  |
| --- | --- | --- |
| Query 1 | MLHGASSRPATARKSSGLSGTVRIPGDKSISHRSFMFGGLASGETRITGLLEGEDVINTG | 60 |
| Sbjct 1 | M H A+ +PATARKS L+G R+PGDKSISHRSFMFGGLASGETRITGLLEGEDV+ TG MSHSAAPKPATARKSPALTGVARVPGDKSISHRSFMFGGLASGETRITGLLEGEDVMRTG | 60 |
| Query 61 | KAMQAMGARIRKEGDTWIIDGVGNGGLLAPEAPLDFGNAATGCRLTMGLVGVYDFDSTFI | 120 |
| Sbjct 61 | AM+AMGA I ++G W+I G GNG LL PE PLDFGNA TG RLTMGLVG YD ++TFI AAMKAMGAHIERKGAEWVIRGTGNGALLEPEGPLDFGNAGTGSRLTMGLVGTYDMETTFI | 120 |
| Query 121 | GDASLTKRPMGRVLNPLREMGVQV-KSEDGDRLPVTLRGPKTPTPITYRVPMASAQVKSA | 179 |
| Sbjct 121 | GDASL+ RPMGRVL PLR+MGVQV K+ GDR+P+TL GPK PITYRVPMASAQVKSA GDASLSGRPMGRVLEPLRQMGVQVLKAAPGDRMPITLHGPKHAAPITYRVPMASAQVKSA | 180 |
| Query 180 | VLLAGLNTPGITTVIEPIMTRDHTEKMLQGFGANLTVETDADGVRTIRLEGRGKLTGQVI | 239 |
| Sbjct 181 | VLLAGLNTPGITTVIEP+MTRDHTEKML+GFGANLTVETD GVR I +EG+GKLTGQ I VLLAGLNTPGITTVIEPVMTRDHTEKMLKGFGANLTVETDERGVRHIFIEGQGKLTGQTI | 240 |
| Query 240 | DVPGDPSSTAFPLVAALLVPGSDVTILNVLMNPTRTGLILTLQEMGADIEVINPRLAGGE | 299 |
| Sbjct 241 | VP DPSS FPLVAAL+VPGSD+ I NVLMNPTRTGL+LTLQEMG I+++NPR AGGE AVPVDPSSAGFPLVAALIVPGSDIIIENVLMNPTRTGLLLTLQEMGGRIDILNPRNAGGE | 300 |
| Query 300 | DVADLRVRSSTLKGVTVPEDRAPSMIDEYPILavaaafaegaTVMNGLEELRVKESDRLS | 359 |
| Sbjct 301 | DVADLRVRSS LKGV VP +RAPSMIDEYP+LAVAA+FAEG T+M GLEELRVKESDRL+ DVADLRVRSSELKGVVVPPERAPSMIDEYPVLAVAASFAEGETLMQGLEELRVKESDRLA | 360 |
| Query 360 | AVANGLKLNGVDCDEGETSLVVRGRPDGKGLG---NASGAAVATHLDHRIAMSFLVMGLV | 416 |
| Sbjct 361 | AVANGLKLNGVDC EGE SL VRG+P GKGLG N AV THLDHRIAMSFL+MGL AVANGLKLNGVDCTEGEASLAVRGKPGGKGLGAHPNGQDTAVKTHLDHRIAMSFLIMGLA | 420 |
| Query 417  Sbjct 421 | SENPVTVDDATMIATSFPEFMDLMAGLGAKIE 448  +E PVT+DDA MIATSFPEFM LM GLGA+IE  TEKPVTIDDANMIATSFPEFMGLMKGLGAEIE 452 |  |

>WP_184394089.1 3-phosphoshikimate 1-carboxyvinyltransferase [Rhizobium sp. BK650] MBB3655702.1 3-phosphoshikimate 1-carboxyvinyltransferase [Rhizobium sp. BK650]

Length=450

Score = 682 bits (1759), Expect = 0.0, Method: Compositional matrix adjust.

| Identities | = 342/448 (76%), Positives = 389/448 (87%), Gaps = 0/448 (0%) |  |
| --- | --- | --- |
| Query 1 | MLHGASSRPATARKSSGLSGTVRIPGDKSISHRSFMFGGLASGETRITGLLEGEDVINTG | 60 |
| Sbjct 1 | M G++ +PA ARKSS LSGTVRIPGDKSISHR+ M GGLASGETRI+GLLEGEDV+NTG MSQGSTLKPAIARKSSSLSGTVRIPGDKSISHRALMLGGLASGETRISGLLEGEDVLNTG | 60 |
| Query 61 | KAMQAMGARIRKEGDTWIIDGVGNGGLLAPEAPLDFGNAATGCRLTMGLVGVYDFDSTFI | 120 |
| Sbjct 61 | KAMQAMGA IRK+GD WI++G GNG LLAPEAPLDFGNA TG RL MGL GVYDFD+ F+ KAMQAMGAVIRKDGDKWIVNGTGNGALLAPEAPLDFGNAGTGARLIMGLAGVYDFDTVFV | 120 |
| Query 121 | GDASLTKRPMGRVLNPLREMGVQVKSEDGDRLPVTLRGPKTPTPITYRVPMASAQVKSAV | 180 |
| Sbjct 121 | GDASL++RP GRVL+PL MGVQVK+ +GDRLPV LRGP+TP PI Y +P+ASAQVKSA+ GDASLSRRPTGRVLDPLHRMGVQVKAMEGDRLPVMLRGPETPAPIRYTLPVASAQVKSAI | 180 |
| Query 181 | LLAGLNTPGITTVIEPIMTRDHTEKMLQGFGANLTVETDADGVRTIRLEGRGKLTGQVID | 240 |
| Sbjct 181 | LLAGLN PG+TTVIEP +TRDHTEKML GFGA L+ ET DG RTIRL+GRG+LTGQ+I+ LLAGLNIPGVTTVIEPAITRDHTEKMLAGFGAELSAETGEDGARTIRLQGRGRLTGQIIE | 240 |
| Query 241 | VPGDPSSTAFPLVAALLVPGSDVTILNVLMNPTRTGLILTLQEMGADIEVINPRLAGGED | 300 |
| Sbjct 241 | VPGDPSS AFPLVAALLVPGSD+ I NVLMNPTRTGLILTLQEMGADIE+ +PR AGGED  VPGDPSSAAFPLVAALLVPGSDIIISNVLMNPTRTGLILTLQEMGADIEIRDPRFAGGED | 300 |

| Query | 301 | VADLRVRSSTLKGVTVPEDRAPSMIDEYPILavaaafaegaTVMNGLEELRVKESDRLSA | 360 |
| --- | --- | --- | --- |
| Sbjct | 301 | VADLRVR S L GV+VP +RAP+MIDEYP LAVAAAFA+G T+MNGL+ELR KESDRLSA VADLRVRHSELTGVSVPAERAPTMIDEYPALAVAAAFAKGKTIMNGLQELRGKESDRLSA | 360 |
| Query | 361 | VANGLKLNGVDCDEGETSLVVRGRPDGKGLGNASGAAVATHLDHRIAMSFLVMGLVSENP | 420 |
| Sbjct | 361 | +A+GLKLNGV+C+EG L V GRPDGKGLGNA+G V THLDHRIAMSFLVMGL SE+P IADGLKLNGVECEEGRDWLSVTGRPDGKGLGNAAGERVITHLDHRIAMSFLVMGLASEHP | 420 |
| Query  Sbjct | 421  421 | VTVDDATMIATSFPEFMDLMAGLGAKIE 448 V +DD+ MI TSFP+FM+LM LG +IE VGIDDSNMITTSFPQFMNLMKSLGGRIE 448 |  |

>WP_126007218.1 3-phosphoshikimate 1-carboxyvinyltransferase [Georhizobium profundi] AZN70237.1 3-phosphoshikimate 1-carboxyvinyltransferase [Georhizobium profundi]

Length=456

Score = 682 bits (1759), Expect = 0.0, Method: Compositional matrix adjust.

| Identities | = 353/450 (78%), Positives = 389/450 (86%), Gaps = 1/450 (0%) |  |
| --- | --- | --- |
| Query 1 | MLHGAS-SRPATARKSSGLSGTVRIPGDKSISHRSFMFGGLASGETRITGLLEGEDVINT | 59 |
| Sbjct 1 | M HG+ + PATAR+S LSGTVRIPGDKSISHRSFMFGGLA+GETRITGLLEGEDVI T MAHGSQGAIPATARRSEPLSGTVRIPGDKSISHRSFMFGGLAAGETRITGLLEGEDVIAT | 60 |
| Query 60 | GKAMQAMGARIRKEGDTWIIDGVGNGGLLAPEAPLDFGNAATGCRLTMGLVGVYDFDSTF | 119 |
| Sbjct 61 | G AM+AMGA IRKEGDTWII+G GNG LL P+APLDFGNA TGCRLTMGL G YDF++TF GNAMRAMGASIRKEGDTWIIEGTGNGCLLEPKAPLDFGNAGTGCRLTMGLAGTYDFETTF | 120 |
| Query 120 | IGDASLTKRPMGRVLNPLREMGVQVKSEDGDRLPVTLRGPKTPTPITYRVPMASAQVKSA | 179 |
| Sbjct 121 | IGDASL+ RPMGRVL+PL+ MGVQVKS +G +LPVTLRGP+ PI Y VPMASAQVKSA IGDASLSARPMGRVLDPLKLMGVQVKSVEGGKLPVTLRGPRMAAPIHYTVPMASAQVKSA | 180 |
| Query 180 | VLLAGLNTPGITTVIEPIMTRDHTEKMLQGFGANLTVETDADGVRTIRLEGRGKLTGQVI | 239 |
| Sbjct 181 | VL+AGLN PG+TTV E IMTRDHTEKML GFGA+L +ETDADG R I +EG+ KL G VI VLIAGLNAPGVTTVTEKIMTRDHTEKMLAGFGASLDIETDADGTRHISIEGQPKLKGCVI | 240 |
| Query 240 | DVPGDPSSTAFPLVAALLVPGSDVTILNVLMNPTRTGLILTLQEMGADIEVINPRLAGGE | 299 |
| Sbjct 241 | DVPGDPSSTAFPLVAALLVPGSD+TILNVLMNPTRTGLI TL EMG I++++ R AGGE DVPGDPSSTAFPLVAALLVPGSDITILNVLMNPTRTGLITTLIEMGGRIDILDARSAGGE | 300 |
| Query 300 | DVADLRVRSSTLKGVTVPEDRAPSMIDEYPILavaaafaegaTVMNGLEELRVKESDRLS | 359 |
| Sbjct 301 | DVADLRVR S LKGV VP +RA SMIDEYP+LAVAAAFAEG TVM+G+EELRVKESDRL+ DVADLRVRHSELKGVVVPPERAASMIDEYPVLAVAAAFAEGETVMDGIEELRVKESDRLA | 360 |
| Query 360 | AVANGLKLNGVDCDEGETSLVVRGRPDGKGLGNASGAAVATHLDHRIAMSFLVMGLVSEN | 419 |
| Sbjct 361 | AVA GL+LN VDC EGE L VRG P G+G+G+A G AVATHLDHRIAMSFLVMGL SE+ AVAAGLRLNNVDCTEGEAILTVRGTPSGRGIGSARGDAVATHLDHRIAMSFLVMGLASEH | 420 |
| Query 420  Sbjct 421 | PVTVDDATMIATSFPEFMDLMAGLGAKIEL 449 PVTVDDA MIATSFPEFMDLMAGLGA IE PVTVDDARMIATSFPEFMDLMAGLGATIEC 450 |  |

>TIX55651.1 3-phosphoshikimate 1-carboxyvinyltransferase [Mesorhizobium sp.] Length=452

Score = 682 bits (1759), Expect = 0.0, Method: Compositional matrix adjust.

| Identities | = 351/452 (78%), Positives = 383/452 (85%), Gaps = 4/452 (1%) |  |
| --- | --- | --- |
| Query 1 | MLHGASSRPATARKSSGLSGTVRIPGDKSISHRSFMFGGLASGETRITGLLEGEDVINTG | 60 |
| Sbjct 1 | M H A+ +PATARKSS LSG R+PGDKSISHRSFMFGGLASGETRITGLLEGEDV+ TG MSHTAAPKPATARKSSALSGAARVPGDKSISHRSFMFGGLASGETRITGLLEGEDVMRTG | 60 |
| Query 61 | KAMQAMGARIRKEGDTWIIDGVGNGGLLAPEAPLDFGNAATGCRLTMGLVGVYDFDSTFI | 120 |
| Sbjct 61 | AM+AMGA I K G W+I G GNG LL PE PLDFGNA TG RLTMGLVG YD ++TFI AAMKAMGAHIEKLGTEWVIRGTGNGALLQPEGPLDFGNAGTGSRLTMGLVGTYDMETTFI | 120 |
| Query 121 | GDASLTKRPMGRVLNPLREMGVQV-KSEDGDRLPVTLRGPKTPTPITYRVPMASAQVKSA | 179 |
| Sbjct 121 | GDASL+ RPMGRVL PLR+MGVQV K+ GDR+P+TL GPK PITYRVPMASAQVKSA  GDASLSGRPMGRVLEPLRQMGVQVLKATPGDRMPITLHGPKHAAPITYRVPMASAQVKSA | 180 |

| Query | 180 | VLLAGLNTPGITTVIEPIMTRDHTEKMLQGFGANLTVETDADGVRTIRLEGRGKLTGQVI | 239 |
| --- | --- | --- | --- |
| Sbjct | 181 | VLLAGLNTPG+TTVIEP MTRDHTEKML GFGANL+VETD GVR I +EG+GKLTGQ I VLLAGLNTPGVTTVIEPAMTRDHTEKMLMGFGANLSVETDERGVRHIFIEGQGKLTGQTI | 240 |
| Query | 240 | DVPGDPSSTAFPLVAALLVPGSDVTILNVLMNPTRTGLILTLQEMGADIEVINPRLAGGE | 299 |
| Sbjct | 241 | VPGDPSS FPLVAAL+VPGSD+TI NVLMNPTRTGL+LTLQEMGA I++++PR GGE AVPGDPSSAGFPLVAALIVPGSDITIENVLMNPTRTGLLLTLQEMGARIDILDPRNEGGE | 300 |
| Query | 300 | DVADLRVRSSTLKGVTVPEDRAPSMIDEYPILavaaafaegaTVMNGLEELRVKESDRLS | 359 |
| Sbjct | 301 | DVADLRVR S LK VTVP +RAP+MIDEYP+LAVAA+FAEG T+M GLEELRVKESDRLS DVADLRVRHSELKAVTVPPERAPTMIDEYPVLAVAASFAEGETLMQGLEELRVKESDRLS | 360 |
| Query | 360 | AVANGLKLNGVDCDEGETSLVVRGRPDGKGLG---NASGAAVATHLDHRIAMSFLVMGLV | 416 |
| Sbjct | 361 | AVA GLKLNGVDC EGE SL VRGRP GKGLG N G V THLDHRIAMSFLVMGL AVAEGLKLNGVDCTEGEASLAVRGRPGGKGLGQHPNGQGTIVQTHLDHRIAMSFLVMGLA | 420 |
| Query  Sbjct | 417  421 | SENPVTVDDATMIATSFPEFMDLMAGLGAKIE 448  +E PVT+DD TMIATSFPEFM LM GLGA+IE  TEKPVTIDDQTMIATSFPEFMGLMKGLGAEIE 452 |  |

>RWO74798.1 3-phosphoshikimate 1-carboxyvinyltransferase [Mesorhizobium sp.] Length=452

Score = 681 bits (1758), Expect = 0.0, Method: Compositional matrix adjust.

| Identities | = 348/451 (77%), Positives = 382/451 (85%), Gaps = 4/451 (1%) |  |
| --- | --- | --- |
| Query 1 | MLHGASSRPATARKSSGLSGTVRIPGDKSISHRSFMFGGLASGETRITGLLEGEDVINTG | 60 |
| Sbjct 1 | M H A+ +PATAR+S LSG R+PGDKSISHRSFMFGGLASGETRITGLLEGEDV+ TG MSHAAAPKPATARQSPALSGIARVPGDKSISHRSFMFGGLASGETRITGLLEGEDVMRTG | 60 |
| Query 61 | KAMQAMGARIRKEGDTWIIDGVGNGGLLAPEAPLDFGNAATGCRLTMGLVGVYDFDSTFI | 120 |
| Sbjct 61 | AM+AMGA I K GD W+I G GNG LL PE PLDFGNA TG RLTMGLVG YD ++TFI AAMKAMGAHIEKNGDEWVIRGTGNGALLQPEGPLDFGNAGTGSRLTMGLVGTYDMETTFI | 120 |
| Query 121 | GDASLTKRPMGRVLNPLREMGVQV-KSEDGDRLPVTLRGPKTPTPITYRVPMASAQVKSA | 179 |
| Sbjct 121 | GDASL+ RPMGRVL PLR+MGVQV K+ GDR+P+TL GPK PITYRVPMASAQVKSA GDASLSGRPMGRVLEPLRQMGVQVLKATPGDRMPITLHGPKHAAPITYRVPMASAQVKSA | 180 |
| Query 180 | VLLAGLNTPGITTVIEPIMTRDHTEKMLQGFGANLTVETDADGVRTIRLEGRGKLTGQVI | 239 |
| Sbjct 181 | VLLAGLNTPGITTVIEP+MTRDHTEKML+GFGANL+VETD GVR I +EG+G+LTGQ I VLLAGLNTPGITTVIEPVMTRDHTEKMLKGFGANLSVETDERGVRHIFIEGQGRLTGQTI | 240 |
| Query 240 | DVPGDPSSTAFPLVAALLVPGSDVTILNVLMNPTRTGLILTLQEMGADIEVINPRLAGGE | 299 |
| Sbjct 241 | VPGDPSS FPLVAAL+VPGSD+TI NVLMNPTRTGL+LTLQEMG I+++NPR AGGE AVPGDPSSAGFPLVAALIVPGSDITIENVLMNPTRTGLLLTLQEMGGKIDILNPRNAGGE | 300 |
| Query 300 | DVADLRVRSSTLKGVTVPEDRAPSMIDEYPILavaaafaegaTVMNGLEELRVKESDRLS | 359 |
| Sbjct 301 | DVADLRVR S LKGV VP +RAPSMIDEYP+LAVAA+FAEG T+M GLEELRVKESDRLS DVADLRVRYSELKGVIVPPERAPSMIDEYPVLAVAASFAEGETLMQGLEELRVKESDRLS | 360 |
| Query 360 | AVANGLKLNGVDCDEGETSLVVRGRPDGKGLG---NASGAAVATHLDHRIAMSFLVMGLV | 416 |
| Sbjct 361 | AVANGLKLNGVDC EGE SL VRG+P GKGLG N V THLDHRIAMSFLVMGL AVANGLKLNGVDCTEGEASLAVRGKPSGKGLGGHPNGQDTTVQTHLDHRIAMSFLVMGLA | 420 |
| Query 417  Sbjct 421 | SENPVTVDDATMIATSFPEFMDLMAGLGAKI 447  +E PVT+DD MIATSFPEFM LM LGA+I  TEKPVTIDDQAMIATSFPEFMGLMKALGAEI 451 |  |

>RWN01664.1 3-phosphoshikimate 1-carboxyvinyltransferase [Mesorhizobium sp.] Length=452

Score = 681 bits (1758), Expect = 0.0, Method: Compositional matrix adjust.

| Identities | = 350/451 (78%), Positives = 383/451 (85%), Gaps = 4/451 (1%) |  |
| --- | --- | --- |
| Query 1 | MLHGASSRPATARKSSGLSGTVRIPGDKSISHRSFMFGGLASGETRITGLLEGEDVINTG | 60 |
| Sbjct 1 | M H A+ +PATARKS LSGT R+PGDKSISHRSFMFGGLASGETRITGLLEGEDV+ TG MSHTAAPKPATARKSPALSGTARVPGDKSISHRSFMFGGLASGETRITGLLEGEDVMRTG | 60 |
| Query 61 | KAMQAMGARIRKEGDTWIIDGVGNGGLLAPEAPLDFGNAATGCRLTMGLVGVYDFDSTFI AM+AMGA I K G W+I G GNG LL PE PLDFGNA TG RLTMGLVG YD ++TFI | 120 |

| Sbjct | 61 | AAMKAMGAHIEKRGTEWVIRGTGNGALLQPEGPLDFGNAGTGSRLTMGLVGTYDMETTFI | 120 |
| --- | --- | --- | --- |
| Query | 121 | GDASLTKRPMGRVLNPLREMGVQV-KSEDGDRLPVTLRGPKTPTPITYRVPMASAQVKSA | 179 |
| Sbjct | 121 | GDASL+ RPMGRVL PLR+MGVQV K+ GDR+P+TLRGPK PITYRVPMASAQVKSA GDASLSGRPMGRVLEPLRQMGVQVLKATPGDRMPITLRGPKHAAPITYRVPMASAQVKSA | 180 |
| Query | 180 | VLLAGLNTPGITTVIEPIMTRDHTEKMLQGFGANLTVETDADGVRTIRLEGRGKLTGQVI | 239 |
| Sbjct | 181 | VLLAGLNTPGITTVIEP+MTRDHTEKML+GFGANL+VETD GVR I +EG+ KLTGQ I VLLAGLNTPGITTVIEPVMTRDHTEKMLKGFGANLSVETDERGVRHIFIEGQAKLTGQTI | 240 |
| Query | 240 | DVPGDPSSTAFPLVAALLVPGSDVTILNVLMNPTRTGLILTLQEMGADIEVINPRLAGGE | 299 |
| Sbjct | 241 | VPGDPSS FPLVAAL+VPGSD+TI NVLMNPTRTGL+LTLQEMGA I++++PR GGE AVPGDPSSAGFPLVAALIVPGSDITIENVLMNPTRTGLLLTLQEMGARIDIVDPRSEGGE | 300 |
| Query | 300 | DVADLRVRSSTLKGVTVPEDRAPSMIDEYPILavaaafaegaTVMNGLEELRVKESDRLS | 359 |
| Sbjct | 301 | DVADLRVR S LKGVTVP +RAP+MIDEYP+LAVAA FAEG T+M GLEELRVKESDRLS DVADLRVRYSELKGVTVPPERAPTMIDEYPVLAVAAGFAEGETLMQGLEELRVKESDRLS | 360 |
| Query | 360 | AVANGLKLNGVDCDEGETSLVVRGRPDGKGLG---NASGAAVATHLDHRIAMSFLVMGLV | 416 |
| Sbjct | 361 | AVA GLKLNGVDC EGE SLVVRG+P GKGLG N V THLDHRIAMSFLVMGL AVAEGLKLNGVDCTEGEASLVVRGKPGGKGLGQHPNGKSTIVQTHLDHRIAMSFLVMGLA | 420 |
| Query  Sbjct | 417  421 | SENPVTVDDATMIATSFPEFMDLMAGLGAKI 447  +E PVT+DD MIATSFPEFM LM GLGA+I  TEKPVTIDDQAMIATSFPEFMGLMKGLGAEI 451 |  |

>ERM02521.1 3-phosphoshikimate 1-carboxyvinyltransferase [Ochrobactrum intermedium 229E]

Length=415

Score = 681 bits (1758), Expect = 0.0, Method: Compositional matrix adjust.

| Identities | = 348/418 (83%), Positives = 378/418 (90%), Gaps = 3/418 (1%) |  |
| --- | --- | --- |
| Query 36 | MFGGLASGETRITGLLEGEDVINTGKAMQAMGARIRKEGDTWIIDGVGNGGLLAPEAPLD | 95 |
| Sbjct 1 | MFGGLASGETRITGLLEGEDVINTG+AMQAMGA+IRKEGD WII+GVGNG LL PEA LD MFGGLASGETRITGLLEGEDVINTGRAMQAMGAKIRKEGDVWIINGVGNGCLLQPEAALD | 60 |
| Query 96 | FGNAATGCRLTMGLVGVYDFDSTFIGDASLTKRPMGRVLNPLREMGVQVKSEDGDRLPVT | 155 |
| Sbjct 61 | FGNA TG RLTMGLVG YD ++FIGDASL+KRPMGRVLNPLREMGVQV++ DGDR+P+T FGNAGTGARLTMGLVGTYDMKTSFIGDASLSKRPMGRVLNPLREMGVQVEAADGDRMPLT | 120 |
| Query 156 | LRGPKTPTPITYRVPMASAQVKSAVLLAGLNTPGITTVIEPIMTRDHTEKMLQGFGANLT | 215 |
| Sbjct 121 | L GPKT PITYRVPMASAQVKSAVLLAGLNTPG+TTVIEP+MTRDHTEKMLQGFGA+LT LIGPKTANPITYRVPMASAQVKSAVLLAGLNTPGVTTVIEPVMTRDHTEKMLQGFGADLT | 180 |
| Query 216 | VETDADGVRTIRLEGRGKLTGQVIDVPGDPSSTAFPLVAALLVPGSDVTILNVLMNPTRT | 275 |
| Sbjct 181 | VETD DGVR IR+ G+GKL GQ IDVPGDPSSTAFPLVAALLV GSDVTI NVLMNPTRT VETDKDGVRHIRITGQGKLVGQTIDVPGDPSSTAFPLVAALLVEGSDVTIRNVLMNPTRT | 240 |
| Query 276 | GLILTLQEMGADIEVINPRLAGGEDVADLRVRSSTLKGVTVPEDRAPSMIDEYPILavaa | 335 |
| Sbjct 241 | GLILTLQEMGADIEV+N RLAGGEDVADLRVR+S LKGV VP +RAPSMIDEYP+LA+AA GLILTLQEMGADIEVLNARLAGGEDVADLRVRASKLKGVVVPPERAPSMIDEYPVLAIAA | 300 |
| Query 336 | afaegaTVMNGLEELRVKESDRLSAVANGLKLNGVDCDEGETSLVVRGRPDGKGLGNASG | 395 |
| Sbjct 301 | +FAEG TVM+GL+ELRVKESDRL+AVA GL+ NGVDC EGE SL VRGRPDGKGLG G  SFAEGETVMDGLDELRVKESDRLAAVARGLEANGVDCTEGEMSLTVRGRPDGKGLG---G | 357 |

Query 396 AAVATHLDHRIAMSFLVMGLVSENPVTVDDATMIATSFPEFMDLMAGLGAKIELSDTK 453 VATHLDHRIAMSFLVMGL +E PVTVDD+ MIATSFPEFMD+M GLGAKIELS+ +

Sbjct 358 GTVATHLDHRIAMSFLVMGLAAEKPVTVDDSNMIATSFPEFMDMMPGLGAKIELSNAQ 415

>TIS60377.1 3-phosphoshikimate 1-carboxyvinyltransferase [Mesorhizobium sp.] TIS91449.1 3-phosphoshikimate 1-carboxyvinyltransferase [Mesorhizobium sp.]

Length=452

Score = 681 bits (1758), Expect = 0.0, Method: Compositional matrix adjust. Identities = 348/452 (77%), Positives = 384/452 (85%), Gaps = 4/452 (1%)

Query 1	MLHGASSRPATARKSSGLSGTVRIPGDKSISHRSFMFGGLASGETRITGLLEGEDVINTG 60 M H A+ +PATARKS LSG R+PGDKSISHRSFMFGGLASGETRITGLLEGEDV+ TG

| Sbjct | 1 | MSHTAAPKPATARKSPALSGAARVPGDKSISHRSFMFGGLASGETRITGLLEGEDVMRTG | 60 |
| --- | --- | --- | --- |
| Query | 61 | KAMQAMGARIRKEGDTWIIDGVGNGGLLAPEAPLDFGNAATGCRLTMGLVGVYDFDSTFI | 120 |
| Sbjct | 61 | AM+AMGA I K G W+I G GNG LL PE PLDFGNA TG RLTMGLVG YD ++TFI AAMKAMGAHIEKRGAEWVIRGTGNGALLQPEGPLDFGNAGTGSRLTMGLVGTYDMETTFI | 120 |
| Query | 121 | GDASLTKRPMGRVLNPLREMGVQV-KSEDGDRLPVTLRGPKTPTPITYRVPMASAQVKSA | 179 |
| Sbjct | 121 | GDASL+ RPMGRVL PLR+MGVQV K+ GDR+P+TLRGPK PITYRVPMASAQVKSA GDASLSGRPMGRVLEPLRQMGVQVLKATPGDRMPITLRGPKHAAPITYRVPMASAQVKSA | 180 |
| Query | 180 | VLLAGLNTPGITTVIEPIMTRDHTEKMLQGFGANLTVETDADGVRTIRLEGRGKLTGQVI | 239 |
| Sbjct | 181 | VLLAGLNTPG+TTV+EP+MTRDHTEKML+GFGANL+VETD GVR I +EG+GKLTGQ I VLLAGLNTPGVTTVVEPVMTRDHTEKMLRGFGANLSVETDERGVRHIFIEGQGKLTGQTI | 240 |
| Query | 240 | DVPGDPSSTAFPLVAALLVPGSDVTILNVLMNPTRTGLILTLQEMGADIEVINPRLAGGE | 299 |
| Sbjct | 241 | VPGDPSS FPLVAAL+VPGSD+TI NVLMNPTRTGL+LTLQEMG I+V+NPR GGE AVPGDPSSAGFPLVAALIVPGSDITIQNVLMNPTRTGLLLTLQEMGGRIDVLNPRNEGGE | 300 |
| Query | 300 | DVADLRVRSSTLKGVTVPEDRAPSMIDEYPILavaaafaegaTVMNGLEELRVKESDRLS | 359 |
| Sbjct | 301 | DVADLRVR S L GVTVP +RAP+MIDEYP+LAVAA+FAEG T+M GLEELRVKESDRLS DVADLRVRYSELTGVTVPPERAPTMIDEYPVLAVAASFAEGETLMQGLEELRVKESDRLS | 360 |
| Query | 360 | AVANGLKLNGVDCDEGETSLVVRGRPDGKGLG---NASGAAVATHLDHRIAMSFLVMGLV | 416 |
| Sbjct | 361 | AVANGLKLNGVDC EGE++L VRG+P GKGLG N V THLDHRIAMSFLVMGL AVANGLKLNGVDCTEGESTLAVRGKPGGKGLGQHPNGQSTIVQTHLDHRIAMSFLVMGLA | 420 |
| Query  Sbjct | 417  421 | SENPVTVDDATMIATSFPEFMDLMAGLGAKIE 448  +E PVT+DD MIATSFPEFM LM GLGA+IE  TEKPVTIDDQAMIATSFPEFMGLMKGLGAEIE 452 |  |

>RWM21195.1 3-phosphoshikimate 1-carboxyvinyltransferase [Mesorhizobium sp.] TIP69727.1 3-phosphoshikimate 1-carboxyvinyltransferase [Mesorhizobium sp.] TIQ13451.1 3-phosphoshikimate 1-carboxyvinyltransferase [Mesorhizobium sp.] TIR53304.1 3-phosphoshikimate 1-carboxyvinyltransferase [Mesorhizobium sp.] TJV99281.1 3-phosphoshikimate 1-carboxyvinyltransferase [Mesorhizobium sp.]

Length=452

Score = 681 bits (1758), Expect = 0.0, Method: Compositional matrix adjust.

| Identities | = 349/452 (77%), Positives = 383/452 (85%), Gaps = 4/452 (1%) |  |
| --- | --- | --- |
| Query 1 | MLHGASSRPATARKSSGLSGTVRIPGDKSISHRSFMFGGLASGETRITGLLEGEDVINTG | 60 |
| Sbjct 1 | M H A+ +PATARKSS LSG R+PGDKSISHRSFMFGGLASGETRITGLLEGEDV+ TG MSHTAAPKPATARKSSALSGVARVPGDKSISHRSFMFGGLASGETRITGLLEGEDVMRTG | 60 |
| Query 61 | KAMQAMGARIRKEGDTWIIDGVGNGGLLAPEAPLDFGNAATGCRLTMGLVGVYDFDSTFI | 120 |
| Sbjct 61 | AM+AMGA I K G W+I G GNG LL PE PLDFGNA TG RLTMGLVG YD ++TFI AAMKAMGAHIEKHGTEWVIRGTGNGALLQPEGPLDFGNAGTGSRLTMGLVGTYDMETTFI | 120 |
| Query 121 | GDASLTKRPMGRVLNPLREMGVQV-KSEDGDRLPVTLRGPKTPTPITYRVPMASAQVKSA | 179 |
| Sbjct 121 | GDASL+ RPMGRVL PLR+MGVQV K+ GDR+P+TL GPK PITYRVPMASAQVKSA GDASLSGRPMGRVLEPLRQMGVQVLKATPGDRMPITLHGPKHAAPITYRVPMASAQVKSA | 180 |
| Query 180 | VLLAGLNTPGITTVIEPIMTRDHTEKMLQGFGANLTVETDADGVRTIRLEGRGKLTGQVI | 239 |
| Sbjct 181 | VLLAGLNTPG+TTVIEP+MTRDHTEKML+GFGANL+VETD GVR I +EG+GKLTGQ I VLLAGLNTPGVTTVIEPVMTRDHTEKMLKGFGANLSVETDERGVRHIFIEGQGKLTGQTI | 240 |
| Query 240 | DVPGDPSSTAFPLVAALLVPGSDVTILNVLMNPTRTGLILTLQEMGADIEVINPRLAGGE | 299 |
| Sbjct 241 | VPGDPSS FPLVAAL+VPGSD+TI NVLMNPTRTGL+LTLQEMGA I++++PR GGE AVPGDPSSAGFPLVAALIVPGSDITIENVLMNPTRTGLLLTLQEMGARIDIVDPRNEGGE | 300 |
| Query 300 | DVADLRVRSSTLKGVTVPEDRAPSMIDEYPILavaaafaegaTVMNGLEELRVKESDRLS | 359 |
| Sbjct 301 | DVADLRVR S LKGVTVP +RAP+MIDEYP+LAVAA+FAEG T+M GLEELRVKESDRLS DVADLRVRYSELKGVTVPPERAPTMIDEYPVLAVAASFAEGETLMQGLEELRVKESDRLS | 360 |
| Query 360 | AVANGLKLNGVDCDEGETSLVVRGRPDGKGLG---NASGAAVATHLDHRIAMSFLVMGLV | 416 |
| Sbjct 361 | AVA GLKLNGVDC EGE SL VRG+P GKGLG N V THLDHRIAMSFLVMGL AVAEGLKLNGVDCTEGEASLAVRGKPGGKGLGQHPNGPNTVVQTHLDHRIAMSFLVMGLA | 420 |
| Query 417  Sbjct 421 | SENPVTVDDATMIATSFPEFMDLMAGLGAKIE 448  +E PVT+DD MIATSFPEFM LM GLGA IE  TEKPVTIDDQAMIATSFPEFMGLMKGLGAAIE 452 |  |

>RWQ24501.1 3-phosphoshikimate 1-carboxyvinyltransferase [Mesorhizobium sp.] Length=452

Score = 681 bits (1758), Expect = 0.0, Method: Compositional matrix adjust.

| Identities | = 350/452 (77%), Positives = 384/452 (85%), Gaps = 4/452 (1%) |  |
| --- | --- | --- |
| Query 1 | MLHGASSRPATARKSSGLSGTVRIPGDKSISHRSFMFGGLASGETRITGLLEGEDVINTG | 60 |
| Sbjct 1 | M H A+ +PATARKS LSGT R+PGDKSISHRSFMFGGLASGETRITGLLEGEDV+ TG MSHTAAPKPATARKSPALSGTARVPGDKSISHRSFMFGGLASGETRITGLLEGEDVMRTG | 60 |
| Query 61 | KAMQAMGARIRKEGDTWIIDGVGNGGLLAPEAPLDFGNAATGCRLTMGLVGVYDFDSTFI | 120 |
| Sbjct 61 | AM+AMGA I K G W+I G GNG LL PE PLDFGNA TG RLTMGLVG YD ++TFI AAMKAMGAHIEKRGAEWVIRGTGNGALLQPEGPLDFGNAGTGSRLTMGLVGTYDMETTFI | 120 |
| Query 121 | GDASLTKRPMGRVLNPLREMGVQV-KSEDGDRLPVTLRGPKTPTPITYRVPMASAQVKSA | 179 |
| Sbjct 121 | GDASL+ RPMGRVL PLR+MGVQV K+ GDR+P+TLRGPK PITYRVPMASAQVKSA GDASLSGRPMGRVLEPLRQMGVQVLKATPGDRMPITLRGPKHAAPITYRVPMASAQVKSA | 180 |
| Query 180 | VLLAGLNTPGITTVIEPIMTRDHTEKMLQGFGANLTVETDADGVRTIRLEGRGKLTGQVI | 239 |
| Sbjct 181 | VLLAGLNTPGITTVIEP+MTRDHTEKMLQGFGANL+VETD GVR I +EG+GKLTGQ I VLLAGLNTPGITTVIEPVMTRDHTEKMLQGFGANLSVETDERGVRHIFIEGQGKLTGQTI | 240 |
| Query 240 | DVPGDPSSTAFPLVAALLVPGSDVTILNVLMNPTRTGLILTLQEMGADIEVINPRLAGGE | 299 |
| Sbjct 241 | VPGDPSS FPLVAAL+VPGSD+TI NVLMNPTRTGL+LTLQEMGA I++++PR GGE AVPGDPSSAGFPLVAALIVPGSDITIENVLMNPTRTGLLLTLQEMGARIDIVDPRNEGGE | 300 |
| Query 300 | DVADLRVRSSTLKGVTVPEDRAPSMIDEYPILavaaafaegaTVMNGLEELRVKESDRLS | 359 |
| Sbjct 301 | DVADLRVR S LKGVTVP +RAP+MIDEYP+LAVAA+FAEG T+M GLEELRVKESDRLS DVADLRVRYSELKGVTVPPERAPTMIDEYPVLAVAASFAEGETLMQGLEELRVKESDRLS | 360 |
| Query 360 | AVANGLKLNGVDCDEGETSLVVRGRPDGKGLG---NASGAAVATHLDHRIAMSFLVMGLV | 416 |
| Sbjct 361 | AVA GLKLNGVDC EGE +L VRG+P GKGLG N V T LDHRIAMSFLVMGL AVAEGLKLNGVDCTEGEATLAVRGKPGGKGLGQHPNGKSTIVQTRLDHRIAMSFLVMGLA | 420 |
| Query 417  Sbjct 421 | SENPVTVDDATMIATSFPEFMDLMAGLGAKIE 448  +E PVT+DD MIATSFPEFM LM GLGA+IE  TEKPVTIDDQAMIATSFPEFMGLMKGLGAEIE 452 |  |

>CDX42125.1 3-phosphoshikimate 1-carboxyvinyltransferase [Mesorhizobium plurifarium] Length=478

Score = 681 bits (1758), Expect = 0.0, Method: Compositional matrix adjust.

| Identities | = 349/452 (77%), Positives = 386/452 (85%), Gaps = 4/452 (1%) |  |
| --- | --- | --- |
| Query 1 | MLHGASSRPATARKSSGLSGTVRIPGDKSISHRSFMFGGLASGETRITGLLEGEDVINTG | 60 |
| Sbjct 27 | M H A+++PATARKS L+G R+PGDKSISHRSFMFGGLASGETRITGLLEGEDV+ TG MSHSAAAKPATARKSPALAGIARVPGDKSISHRSFMFGGLASGETRITGLLEGEDVMRTG | 86 |
| Query 61 | KAMQAMGARIRKEGDTWIIDGVGNGGLLAPEAPLDFGNAATGCRLTMGLVGVYDFDSTFI | 120 |
| Sbjct 87 | AM+AMGA I K+ W+I G GNG LL PE PLDFGNA TG RLTMGLVG YD ++TF+ AAMKAMGAHIEKKDGEWVIRGTGNGALLEPEGPLDFGNAGTGSRLTMGLVGTYDMETTFV | 146 |
| Query 121 | GDASLTKRPMGRVLNPLREMGVQV-KSEDGDRLPVTLRGPKTPTPITYRVPMASAQVKSA | 179 |
| Sbjct 147 | GDASL+ RPMGRVL+PLR+MGVQV K+ GDR+P+TLRGPK PITYRVPMASAQVKSA GDASLSGRPMGRVLDPLRQMGVQVLKAAPGDRMPITLRGPKHAAPITYRVPMASAQVKSA | 206 |
| Query 180 | VLLAGLNTPGITTVIEPIMTRDHTEKMLQGFGANLTVETDADGVRTIRLEGRGKLTGQVI | 239 |
| Sbjct 207 | VLLAGLNTPGITTVIEP+MTRDHTEKML+GFGANLTVETD GVR I +EG+GKLTGQ I VLLAGLNTPGITTVIEPVMTRDHTEKMLKGFGANLTVETDERGVRHIFIEGQGKLTGQTI | 266 |
| Query 240 | DVPGDPSSTAFPLVAALLVPGSDVTILNVLMNPTRTGLILTLQEMGADIEVINPRLAGGE | 299 |
| Sbjct 267 | VPGDPSS FPLVAAL+VPGSD+TI NVLMNPTRTGL+LTLQEMG I+++NPR AGGE AVPGDPSSAGFPLVAALIVPGSDITIENVLMNPTRTGLLLTLQEMGGRIDILNPRNAGGE | 326 |
| Query 300 | DVADLRVRSSTLKGVTVPEDRAPSMIDEYPILavaaafaegaTVMNGLEELRVKESDRLS | 359 |
| Sbjct 327 | DVADL VR S LKGV VP +RAPSMIDEYP+LAVAA+FAEG T+M GLEELRVKESDRL+ DVADLHVRYSELKGVVVPPERAPSMIDEYPVLAVAASFAEGETLMQGLEELRVKESDRLA | 386 |
| Query 360 | AVANGLKLNGVDCDEGETSLVVRGRPDGKGLG---NASGAAVATHLDHRIAMSFLVMGLV | 416 |

AVANGLKLNGVDC EGE SL VRG+P GKGLG	N	V THLDHRIAMSFLVMGL

| Sbjct | 387 | AVANGLKLNGVDCTEGEASLAVRGKPGGKGLGAHPNDQDTVVKTHLDHRIAMSFLVMGLA 446 |
| --- | --- | --- |
| Query | 417 | SENPVTVDDATMIATSFPEFMDLMAGLGAKIE 448 |
| Sbjct | 447 | +E PVT+DDA MIATSFPEFM LM GLGA+IE  TEKPVTIDDANMIATSFPEFMGLMKGLGAEIE 478 |

>WP_091592754.1 3-phosphoshikimate 1-carboxyvinyltransferase [Mesorhizobium muleiense] SDJ08993.1 3-phosphoshikimate 1-carboxyvinyltransferase [Mesorhizobium muleiense]

Length=452

Score = 681 bits (1757), Expect = 0.0, Method: Compositional matrix adjust.

| Identities | = 350/451 (78%), Positives = 383/451 (85%), Gaps = 4/451 (1%) |  |
| --- | --- | --- |
| Query 1 | MLHGASSRPATARKSSGLSGTVRIPGDKSISHRSFMFGGLASGETRITGLLEGEDVINTG | 60 |
| Sbjct 1 | M H A+ +PATARKS LSGT R+PGDKSISHRSFMFGGLASGETRITGLLEGEDV+ TG MSHTAAPKPATARKSPALSGTARVPGDKSISHRSFMFGGLASGETRITGLLEGEDVMRTG | 60 |
| Query 61 | KAMQAMGARIRKEGDTWIIDGVGNGGLLAPEAPLDFGNAATGCRLTMGLVGVYDFDSTFI | 120 |
| Sbjct 61 | AM+AMGA I K G W+I G GNG LL PE PLDFGNA TG RLTMGLVG YD ++TFI AAMKAMGAHIEKRGAEWVIRGTGNGALLQPEGPLDFGNAGTGSRLTMGLVGTYDMETTFI | 120 |
| Query 121 | GDASLTKRPMGRVLNPLREMGVQV-KSEDGDRLPVTLRGPKTPTPITYRVPMASAQVKSA | 179 |
| Sbjct 121 | GDASL+ RPMGRVL PLR+MGVQV K+ GDR+P+TLRGPK PITYRVPMASAQVKSA GDASLSGRPMGRVLEPLRQMGVQVLKATPGDRMPITLRGPKHAAPITYRVPMASAQVKSA | 180 |
| Query 180 | VLLAGLNTPGITTVIEPIMTRDHTEKMLQGFGANLTVETDADGVRTIRLEGRGKLTGQVI | 239 |
| Sbjct 181 | VLLAGLNTPGITTVIEP+MTRDHTEKMLQGFGANL+VETD GVR I +EG+GKLTGQ I VLLAGLNTPGITTVIEPVMTRDHTEKMLQGFGANLSVETDERGVRHIFIEGQGKLTGQTI | 240 |
| Query 240 | DVPGDPSSTAFPLVAALLVPGSDVTILNVLMNPTRTGLILTLQEMGADIEVINPRLAGGE | 299 |
| Sbjct 241 | VPGDPSS FPLVAAL+VPGSD+ I NVLMNPTRTGL+LTLQEMGA I+V++PR GGE AVPGDPSSAGFPLVAALIVPGSDIKIENVLMNPTRTGLLLTLQEMGARIDVLDPRNEGGE | 300 |
| Query 300 | DVADLRVRSSTLKGVTVPEDRAPSMIDEYPILavaaafaegaTVMNGLEELRVKESDRLS | 359 |
| Sbjct 301 | DVADLRVR S LKGVTVP +RAP+MIDEYP+LAVAA+FAEG T+M GLEELRVKESDRLS DVADLRVRYSELKGVTVPPERAPTMIDEYPVLAVAASFAEGETLMQGLEELRVKESDRLS | 360 |
| Query 360 | AVANGLKLNGVDCDEGETSLVVRGRPDGKGLG---NASGAAVATHLDHRIAMSFLVMGLV | 416 |
| Sbjct 361 | AVA GLKLNGVDC EGE +L VRG+P GKGLG N V THLDHRIAMSFLVMGL AVAEGLKLNGVDCTEGEATLAVRGKPGGKGLGQHPNGKSTIVQTHLDHRIAMSFLVMGLA | 420 |
| Query 417  Sbjct 421 | SENPVTVDDATMIATSFPEFMDLMAGLGAKI 447  +E PVT+DD MIATSFPEFM LM GLGA+I  TEKPVTIDDQAMIATSFPEFMGLMKGLGAEI 451 |  |

>RWM82345.1 3-phosphoshikimate 1-carboxyvinyltransferase [Mesorhizobium sp.] Length=452

Score = 681 bits (1757), Expect = 0.0, Method: Compositional matrix adjust.

| Identities | = 348/452 (77%), Positives = 385/452 (85%), Gaps = 4/452 (1%) |  |
| --- | --- | --- |
| Query 1 | MLHGASSRPATARKSSGLSGTVRIPGDKSISHRSFMFGGLASGETRITGLLEGEDVINTG | 60 |
| Sbjct 1 | M H A+ +PATARKSS LSG R+PGDKSISHRSFMFGGLASGETRITGLLEGEDV+ TG MSHTAAPKPATARKSSALSGAARVPGDKSISHRSFMFGGLASGETRITGLLEGEDVMRTG | 60 |
| Query 61 | KAMQAMGARIRKEGDTWIIDGVGNGGLLAPEAPLDFGNAATGCRLTMGLVGVYDFDSTFI | 120 |
| Sbjct 61 | AM+AMGA I K G W+I G GNG LL PE PLDFGNA TG RLTMGLVG YD ++TFI AAMKAMGAHIEKRGAEWVIRGTGNGALLQPEGPLDFGNAGTGSRLTMGLVGTYDMETTFI | 120 |
| Query 121 | GDASLTKRPMGRVLNPLREMGVQV-KSEDGDRLPVTLRGPKTPTPITYRVPMASAQVKSA | 179 |
| Sbjct 121 | GDASL+ RPMGRVL PLR+MGVQV K+ GDR+P+TL GPK PITYRVPMASAQVKSA GDASLSGRPMGRVLEPLRQMGVQVLKATPGDRMPITLHGPKHAAPITYRVPMASAQVKSA | 180 |
| Query 180 | VLLAGLNTPGITTVIEPIMTRDHTEKMLQGFGANLTVETDADGVRTIRLEGRGKLTGQVI | 239 |
| Sbjct 181 | VLLAGLNTPG+TTVIEP+MTRDHTEKML+GFGANL+VETD GVR I +EG+GKLTGQ I VLLAGLNTPGVTTVIEPVMTRDHTEKMLKGFGANLSVETDERGVRHIFIEGQGKLTGQTI | 240 |
| Query 240 | DVPGDPSSTAFPLVAALLVPGSDVTILNVLMNPTRTGLILTLQEMGADIEVINPRLAGGE VPGDPSS FPLVAAL+VPGSD+TI NVLMNPTRTGL+LTLQEMGA I++++PR GGE | 299 |

| Sbjct | 241 | AVPGDPSSAGFPLVAALIVPGSDITIENVLMNPTRTGLLLTLQEMGARIDILDPRNEGGE | 300 |
| --- | --- | --- | --- |
| Query | 300 | DVADLRVRSSTLKGVTVPEDRAPSMIDEYPILavaaafaegaTVMNGLEELRVKESDRLS | 359 |
| Sbjct | 301 | DVADLRVR S LKGVTVP +RAP+MIDEYP+LAVAA+FAEG T+M GLEELRVKESDRLS DVADLRVRYSELKGVTVPPERAPTMIDEYPVLAVAASFAEGETLMQGLEELRVKESDRLS | 360 |
| Query | 360 | AVANGLKLNGVDCDEGETSLVVRGRPDGKGLG---NASGAAVATHLDHRIAMSFLVMGLV | 416 |
| Sbjct | 361 | AVANGLK+NGVDC EGE SL VRG+P GKGLG N V THLDHRIAMSFLVMGL AVANGLKVNGVDCTEGEASLAVRGKPGGKGLGQHPNGQSTIVQTHLDHRIAMSFLVMGLA | 420 |
| Query  Sbjct | 417  421 | SENPVTVDDATMIATSFPEFMDLMAGLGAKIE 448  +E PVT+DD MIATSFPEFM LM GLGA+I+  TEKPVTIDDQAMIATSFPEFMGLMRGLGAEID 452 |  |

>WP_127405880.1 MULTISPECIES: 3-phosphoshikimate 1-carboxyvinyltransferase [unclassified Mesorhizobium]

RWC19432.1 3-phosphoshikimate 1-carboxyvinyltransferase [Mesorhizobium sp.] RUX48941.1 3-phosphoshikimate 1-carboxyvinyltransferase [Mesorhizobium sp.

M4A.F.Ca.ET.050.02.1.1]

RVD38251.1 3-phosphoshikimate 1-carboxyvinyltransferase [Mesorhizobium sp. M4A.F.Ca.ET.020.02.1.1]

RWD27792.1 3-phosphoshikimate 1-carboxyvinyltransferase [Mesorhizobium sp.] TIW25076.1 3-phosphoshikimate 1-carboxyvinyltransferase [Mesorhizobium sp.]

Length=452

Score = 681 bits (1757), Expect = 0.0, Method: Compositional matrix adjust.

| Identities | = 350/451 (78%), Positives = 383/451 (85%), Gaps = 4/451 (1%) |  |
| --- | --- | --- |
| Query 1 | MLHGASSRPATARKSSGLSGTVRIPGDKSISHRSFMFGGLASGETRITGLLEGEDVINTG | 60 |
| Sbjct 1 | M H A+ +PATARKS LSG R+PGDKSISHRSFMFGGLASGETRITGLLEGEDV+ TG MSHAAAPKPATARKSPALSGAARVPGDKSISHRSFMFGGLASGETRITGLLEGEDVMRTG | 60 |
| Query 61 | KAMQAMGARIRKEGDTWIIDGVGNGGLLAPEAPLDFGNAATGCRLTMGLVGVYDFDSTFI | 120 |
| Sbjct 61 | AM+AMGA I K G W+I G GNG LL PE PLDFGNA TG RLTMGLVG YD ++TFI AAMKAMGAHIEKRGAEWVIRGTGNGALLEPEGPLDFGNAGTGSRLTMGLVGTYDMETTFI | 120 |
| Query 121 | GDASLTKRPMGRVLNPLREMGVQV-KSEDGDRLPVTLRGPKTPTPITYRVPMASAQVKSA | 179 |
| Sbjct 121 | GDASL+ RPMGRVL PLR+MGVQV K+ GDR+P+TLRGPK PITYRVPMASAQVKSA GDASLSGRPMGRVLEPLRQMGVQVLKATPGDRMPITLRGPKHAAPITYRVPMASAQVKSA | 180 |
| Query 180 | VLLAGLNTPGITTVIEPIMTRDHTEKMLQGFGANLTVETDADGVRTIRLEGRGKLTGQVI | 239 |
| Sbjct 181 | VLLAGLNTPGITTVIEP+MTRDHTEKML+GFGANLTVETD GVR I +EG+GKL+GQ I VLLAGLNTPGITTVIEPVMTRDHTEKMLKGFGANLTVETDERGVRHIFIEGQGKLSGQTI | 240 |
| Query 240 | DVPGDPSSTAFPLVAALLVPGSDVTILNVLMNPTRTGLILTLQEMGADIEVINPRLAGGE | 299 |
| Sbjct 241 | VPGDPSS FPLVAAL+V GSD+ I NVLMNPTRTGL+LTLQEMG I+++NPR AGGE AVPGDPSSAGFPLVAALIVSGSDIVIENVLMNPTRTGLLLTLQEMGGRIDILNPRNAGGE | 300 |
| Query 300 | DVADLRVRSSTLKGVTVPEDRAPSMIDEYPILavaaafaegaTVMNGLEELRVKESDRLS | 359 |
| Sbjct 301 | DVADLRVRSS LKGV VP +RAPSMIDEYP+LAVAA+FAEG T+M GLEELRVKESDRLS DVADLRVRSSELKGVVVPPERAPSMIDEYPVLAVAASFAEGETLMQGLEELRVKESDRLS | 360 |
| Query 360 | AVANGLKLNGVDCDEGETSLVVRGRPDGKGLG---NASGAAVATHLDHRIAMSFLVMGLV | 416 |
| Sbjct 361 | AVANGLKLNGVDC EGE +L VRG+P GKGLG N V THLDHRIAMSFLVMGL AVANGLKLNGVDCTEGEATLAVRGKPGGKGLGRHPNGPDTTVQTHLDHRIAMSFLVMGLA | 420 |
| Query 417  Sbjct 421 | SENPVTVDDATMIATSFPEFMDLMAGLGAKI 447  +E PVT+DDA MIATSFPEFM LM GLGA+I  AEKPVTIDDANMIATSFPEFMGLMKGLGAEI 451 |  |

>TIL28789.1 3-phosphoshikimate 1-carboxyvinyltransferase [Mesorhizobium sp.] TIL50561.1 3-phosphoshikimate 1-carboxyvinyltransferase [Mesorhizobium sp.] TIL60961.1 3-phosphoshikimate 1-carboxyvinyltransferase [Mesorhizobium sp.] TIM09282.1 3-phosphoshikimate 1-carboxyvinyltransferase [Mesorhizobium sp.] TIM44653.1 3-phosphoshikimate 1-carboxyvinyltransferase [Mesorhizobium sp.]

Length=452

Score = 681 bits (1757), Expect = 0.0, Method: Compositional matrix adjust. Identities = 349/452 (77%), Positives = 384/452 (85%), Gaps = 4/452 (1%)

| Query | 1 | MLHGASSRPATARKSSGLSGTVRIPGDKSISHRSFMFGGLASGETRITGLLEGEDVINTG | 60 |
| --- | --- | --- | --- |
| Sbjct | 1 | M H A+ +PATARKS LSGT R+PGDKSISHRSFMFGGLA+GETRITGLLEGEDV+ TG MSHTAAEKPATARKSPALSGTARVPGDKSISHRSFMFGGLAAGETRITGLLEGEDVMRTG | 60 |
| Query | 61 | KAMQAMGARIRKEGDTWIIDGVGNGGLLAPEAPLDFGNAATGCRLTMGLVGVYDFDSTFI | 120 |
| Sbjct | 61 | AM+AMGA I K G W+I G GNG LL PE PLDFGNA TG RLTMGLVG YD ++TFI AAMKAMGAHIEKRGAEWVIRGTGNGALLQPEGPLDFGNAGTGSRLTMGLVGTYDMETTFI | 120 |
| Query | 121 | GDASLTKRPMGRVLNPLREMGVQV-KSEDGDRLPVTLRGPKTPTPITYRVPMASAQVKSA | 179 |
| Sbjct | 121 | GDASL+ RPMGRVL PLR+MGVQV K+ GDR+P+TLRGPK PI+YRVPMASAQVKSA GDASLSGRPMGRVLEPLRQMGVQVLKATPGDRMPITLRGPKHAAPISYRVPMASAQVKSA | 180 |
| Query | 180 | VLLAGLNTPGITTVIEPIMTRDHTEKMLQGFGANLTVETDADGVRTIRLEGRGKLTGQVI | 239 |
| Sbjct | 181 | VLLAGLNTPGITTVIEP+MTRDHTEKML+GFGANL+VETD GVR I +EG+GKLTGQ I VLLAGLNTPGITTVIEPVMTRDHTEKMLKGFGANLSVETDERGVRHIFIEGQGKLTGQTI | 240 |
| Query | 240 | DVPGDPSSTAFPLVAALLVPGSDVTILNVLMNPTRTGLILTLQEMGADIEVINPRLAGGE | 299 |
| Sbjct | 241 | VPGDPSS FPLVAAL+VPGSD+TI NVLMNPTRTGL+LTLQEMGA I+V+ PR GGE AVPGDPSSAGFPLVAALIVPGSDITIENVLMNPTRTGLLLTLQEMGARIDVLEPRNEGGE | 300 |
| Query | 300 | DVADLRVRSSTLKGVTVPEDRAPSMIDEYPILavaaafaegaTVMNGLEELRVKESDRLS | 359 |
| Sbjct | 301 | DVADLRVR S LKGVTVP +RAP+MIDEYP+LAVAA+FAEG T+M GLEELRVKESDRLS DVADLRVRYSELKGVTVPPERAPTMIDEYPVLAVAASFAEGETLMQGLEELRVKESDRLS | 360 |
| Query | 360 | AVANGLKLNGVDCDEGETSLVVRGRPDGKGLG---NASGAAVATHLDHRIAMSFLVMGLV | 416 |
| Sbjct | 361 | AVA GLKLNGVDC EGE +L VRG+P GKGLG N V THLDHRIAMSFLVMGL AVAEGLKLNGVDCTEGEATLAVRGKPGGKGLGQHPNGKSTIVQTHLDHRIAMSFLVMGLA | 420 |
| Query  Sbjct | 417  421 | SENPVTVDDATMIATSFPEFMDLMAGLGAKIE 448  +E PVT+DD MIATSFPEFM LM GLGA+IE  TEKPVTIDDQAMIATSFPEFMGLMKGLGAEIE 452 |  |

>RWK64962.1 3-phosphoshikimate 1-carboxyvinyltransferase [Mesorhizobium sp.] RWM46252.1 3-phosphoshikimate 1-carboxyvinyltransferase [Mesorhizobium sp.] RWM56541.1 3-phosphoshikimate 1-carboxyvinyltransferase [Mesorhizobium sp.] RWM61065.1 3-phosphoshikimate 1-carboxyvinyltransferase [Mesorhizobium sp.] RWN05439.1 3-phosphoshikimate 1-carboxyvinyltransferase [Mesorhizobium sp.]

Length=452

Score = 681 bits (1757), Expect = 0.0, Method: Compositional matrix adjust.

| Identities | = 348/452 (77%), Positives = 385/452 (85%), Gaps = 4/452 (1%) |  |
| --- | --- | --- |
| Query 1 | MLHGASSRPATARKSSGLSGTVRIPGDKSISHRSFMFGGLASGETRITGLLEGEDVINTG | 60 |
| Sbjct 1 | M H A+ +PATARKSS LSG R+PGDKSISHRSFMFGGLASGETRITGLLEGEDV+ TG MSHTAAPKPATARKSSALSGAARVPGDKSISHRSFMFGGLASGETRITGLLEGEDVMRTG | 60 |
| Query 61 | KAMQAMGARIRKEGDTWIIDGVGNGGLLAPEAPLDFGNAATGCRLTMGLVGVYDFDSTFI | 120 |
| Sbjct 61 | AM+AMGA I K G W+I G GNG LL PE PLDFGNA TG RLTMGLVG YD ++TFI AAMKAMGAHIEKRGAEWVIRGAGNGALLQPEGPLDFGNAGTGSRLTMGLVGTYDMETTFI | 120 |
| Query 121 | GDASLTKRPMGRVLNPLREMGVQV-KSEDGDRLPVTLRGPKTPTPITYRVPMASAQVKSA | 179 |
| Sbjct 121 | GDASL+ RPMGRVL PLR+MGVQV K+ GDR+P+TL GPK PITYRVPMASAQVKSA GDASLSGRPMGRVLEPLRQMGVQVLKATPGDRMPITLHGPKHAAPITYRVPMASAQVKSA | 180 |
| Query 180 | VLLAGLNTPGITTVIEPIMTRDHTEKMLQGFGANLTVETDADGVRTIRLEGRGKLTGQVI | 239 |
| Sbjct 181 | VLLAGLNTPG+TTVIEP+MTRDHTEKML+GFGANL+VETD GVR I +EG+GKLTGQ I VLLAGLNTPGVTTVIEPVMTRDHTEKMLKGFGANLSVETDERGVRHIFIEGQGKLTGQTI | 240 |
| Query 240 | DVPGDPSSTAFPLVAALLVPGSDVTILNVLMNPTRTGLILTLQEMGADIEVINPRLAGGE | 299 |
| Sbjct 241 | VPGDPSS FPLVAAL+VPGSD+TI NVLMNPTRTGL+LTLQEMGA I++++PR GGE AVPGDPSSAGFPLVAALIVPGSDITIENVLMNPTRTGLLLTLQEMGARIDILDPRNEGGE | 300 |
| Query 300 | DVADLRVRSSTLKGVTVPEDRAPSMIDEYPILavaaafaegaTVMNGLEELRVKESDRLS | 359 |
| Sbjct 301 | DVADLRVR S LKGVTVP +RAP+MIDEYP+LAVAA+FAEG T+M GLEELRVKESDRLS DVADLRVRYSELKGVTVPPERAPTMIDEYPVLAVAASFAEGETLMQGLEELRVKESDRLS | 360 |
| Query 360 | AVANGLKLNGVDCDEGETSLVVRGRPDGKGLG---NASGAAVATHLDHRIAMSFLVMGLV | 416 |
| Sbjct 361 | AVANGLK+NGVDC EGE SL VRG+P GKGLG N V THLDHRIAMSFLVMGL AVANGLKVNGVDCTEGEASLAVRGKPGGKGLGQHPNGQSTIVQTHLDHRIAMSFLVMGLA | 420 |
| Query 417 | SENPVTVDDATMIATSFPEFMDLMAGLGAKIE 448 |  |

+E PVT+DD MIATSFPEFM LM GLGA+I+ Sbjct 421 TEKPVTIDDQAMIATSFPEFMGLMRGLGAEID 452

>WP_095082926.1 3-phosphoshikimate 1-carboxyvinyltransferase [Mesorhizobium sophorae] Length=452

Score = 681 bits (1757), Expect = 0.0, Method: Compositional matrix adjust.

| Identities | = 349/452 (77%), Positives = 382/452 (85%), Gaps = 4/452 (1%) |  |
| --- | --- | --- |
| Query 1 | MLHGASSRPATARKSSGLSGTVRIPGDKSISHRSFMFGGLASGETRITGLLEGEDVINTG | 60 |
| Sbjct 1 | M H A+ + ATARKS L+GT R+PGDKSISHRSFMFGGLASGETRITGLLEGEDV+ TG MSHAAAPKSATARKSPALAGTARVPGDKSISHRSFMFGGLASGETRITGLLEGEDVMRTG | 60 |
| Query 61 | KAMQAMGARIRKEGDTWIIDGVGNGGLLAPEAPLDFGNAATGCRLTMGLVGVYDFDSTFI | 120 |
| Sbjct 61 | AM+AMGA I K G W+I G GNG LL PE PLDFGNA TG RLTMGLVG YD ++TFI AAMKAMGAHIEKRGAEWVIRGTGNGALLQPEGPLDFGNAGTGSRLTMGLVGTYDMETTFI | 120 |
| Query 121 | GDASLTKRPMGRVLNPLREMGVQV-KSEDGDRLPVTLRGPKTPTPITYRVPMASAQVKSA | 179 |
| Sbjct 121 | GDASL+ RPMGRVL PLR+MGVQV K+ GDR+P+TL GPK PITYRVPMASAQVKSA GDASLSGRPMGRVLEPLRQMGVQVLKATPGDRMPITLHGPKHAAPITYRVPMASAQVKSA | 180 |
| Query 180 | VLLAGLNTPGITTVIEPIMTRDHTEKMLQGFGANLTVETDADGVRTIRLEGRGKLTGQVI | 239 |
| Sbjct 181 | VLLAGLNTPGITTVIEP+MTRDHTEKML+GFGANL+VETD GVR I +EG+GKLTGQ I VLLAGLNTPGITTVIEPVMTRDHTEKMLKGFGANLSVETDERGVRHIFIEGQGKLTGQTI | 240 |
| Query 240 | DVPGDPSSTAFPLVAALLVPGSDVTILNVLMNPTRTGLILTLQEMGADIEVINPRLAGGE | 299 |
| Sbjct 241 | VPGDPSS FPLVAAL+VPGSD+ I NVLMNPTRTGL+LTLQEMG IE++NPR AGGE AVPGDPSSAGFPLVAALIVPGSDIVIENVLMNPTRTGLLLTLQEMGGKIEILNPRNAGGE | 300 |
| Query 300 | DVADLRVRSSTLKGVTVPEDRAPSMIDEYPILavaaafaegaTVMNGLEELRVKESDRLS | 359 |
| Sbjct 301 | DVADLRVR S L GVTVP +RAPSMIDEYP+LAVAA+FAEG T+M GLEELRVKESDRLS DVADLRVRYSELNGVTVPPERAPSMIDEYPVLAVAASFAEGETLMQGLEELRVKESDRLS | 360 |
| Query 360 | AVANGLKLNGVDCDEGETSLVVRGRPDGKGLG---NASGAAVATHLDHRIAMSFLVMGLV | 416 |
| Sbjct 361 | AVANGLK+NGVDC EGE SL VRG+P GKGLG N V THLDHRIAMSFLVMGL AVANGLKINGVDCTEGEASLAVRGKPGGKGLGRHPNGQDTTVQTHLDHRIAMSFLVMGLA | 420 |
| Query 417  Sbjct 421 | SENPVTVDDATMIATSFPEFMDLMAGLGAKIE 448  +E PVT+DD MIATSFPEFM LM GLGA+IE  TEKPVTIDDQAMIATSFPEFMGLMKGLGAEIE 452 |  |

>TIL45112.1 3-phosphoshikimate 1-carboxyvinyltransferase [Mesorhizobium sp.] Length=452

Score = 681 bits (1756), Expect = 0.0, Method: Compositional matrix adjust.

| Identities | = 350/452 (77%), Positives = 383/452 (85%), Gaps = 4/452 (1%) |  |
| --- | --- | --- |
| Query 1 | MLHGASSRPATARKSSGLSGTVRIPGDKSISHRSFMFGGLASGETRITGLLEGEDVINTG | 60 |
| Sbjct 1 | M H A+ +PATARKS LSGT R+PGDKSISHRSFMFGGLASGETRITGLLEGEDV+ TG MSHTAAPKPATARKSPALSGTARVPGDKSISHRSFMFGGLASGETRITGLLEGEDVMRTG | 60 |
| Query 61 | KAMQAMGARIRKEGDTWIIDGVGNGGLLAPEAPLDFGNAATGCRLTMGLVGVYDFDSTFI | 120 |
| Sbjct 61 | AM+AMGA I K G W+I G GNG LL PE PLDFGNA TG RLTMGLVG YD ++TFI AAMKAMGAHIEKRGAEWVIRGTGNGALLQPEGPLDFGNAGTGSRLTMGLVGTYDMETTFI | 120 |
| Query 121 | GDASLTKRPMGRVLNPLREMGVQV-KSEDGDRLPVTLRGPKTPTPITYRVPMASAQVKSA | 179 |
| Sbjct 121 | GDASL+ RPMGRVL PLR+MGVQV K+ GDR+P+TLRGPK PITYRVPMASAQVKSA GDASLSGRPMGRVLEPLRQMGVQVLKATPGDRMPITLRGPKHAAPITYRVPMASAQVKSA | 180 |
| Query 180 | VLLAGLNTPGITTVIEPIMTRDHTEKMLQGFGANLTVETDADGVRTIRLEGRGKLTGQVI | 239 |
| Sbjct 181 | VLLAGLNTPGITTVIEP+MTRDHTEKMLQGFGANL+VETD GVR I +EG+GKLTGQ I VLLAGLNTPGITTVIEPVMTRDHTEKMLQGFGANLSVETDERGVRHIFIEGQGKLTGQTI | 240 |
| Query 240 | DVPGDPSSTAFPLVAALLVPGSDVTILNVLMNPTRTGLILTLQEMGADIEVINPRLAGGE | 299 |
| Sbjct 241 | VPGDPSS FPLVAAL+VPGSD+TI NVLMNPTRTGL+LTLQEMGA I+V+ PR GGE AVPGDPSSAGFPLVAALIVPGSDITIENVLMNPTRTGLLLTLQEMGARIDVLEPRNEGGE | 300 |
| Query 300 | DVADLRVRSSTLKGVTVPEDRAPSMIDEYPILavaaafaegaTVMNGLEELRVKESDRLS | 359 |
| Sbjct 301 | DVADLRVR S LKGVTVP +RAP+MIDEYP+LAVAA+FAEG T+M GLEELRVKESDRLS  DVADLRVRYSELKGVTVPPERAPTMIDEYPVLAVAASFAEGETLMQGLEELRVKESDRLS | 360 |

| Query | 360 | AVANGLKLNGVDCDEGETSLVVRGRPDGKGLG---NASGAAVATHLDHRIAMSFLVMGLV 416 |
| --- | --- | --- |
| Sbjct | 361 | AVA GLKLNGVDC EGE +L VRG+P GKGLG N V T LDHRIAMSFLVMGL AVAEGLKLNGVDCTEGEATLSVRGKPAGKGLGQHPNGKSTIVQTQLDHRIAMSFLVMGLA 420 |
| Query | 417 | SENPVTVDDATMIATSFPEFMDLMAGLGAKIE 448 |
| Sbjct | 421 | +E PVT+DD MIATSFP+FM LM GLGA+IE  TEKPVTIDDQAMIATSFPQFMGLMKGLGAEIE 452 |

>RWP32998.1 3-phosphoshikimate 1-carboxyvinyltransferase [Mesorhizobium sp.] RWP56505.1 3-phosphoshikimate 1-carboxyvinyltransferase [Mesorhizobium sp.] TIM25312.1 3-phosphoshikimate 1-carboxyvinyltransferase [Mesorhizobium sp.]

Length=452

Score = 680 bits (1755), Expect = 0.0, Method: Compositional matrix adjust.

| Identities | = 349/452 (77%), Positives = 383/452 (85%), Gaps = 4/452 (1%) |  |
| --- | --- | --- |
| Query 1 | MLHGASSRPATARKSSGLSGTVRIPGDKSISHRSFMFGGLASGETRITGLLEGEDVINTG | 60 |
| Sbjct 1 | M H A+ +PATARKS LSGT R+PGDKSISHRSFMFGGLASGETRITGLLEGEDV+ TG MSHTAAPKPATARKSPALSGTARVPGDKSISHRSFMFGGLASGETRITGLLEGEDVMRTG | 60 |
| Query 61 | KAMQAMGARIRKEGDTWIIDGVGNGGLLAPEAPLDFGNAATGCRLTMGLVGVYDFDSTFI | 120 |
| Sbjct 61 | AM AMGA I K G W+I G GNG LL PE PLDFGNA TG RLTMGLVG YD ++TFI AAMTAMGAHIEKRGAEWVIRGTGNGALLQPEGPLDFGNAGTGSRLTMGLVGTYDMETTFI | 120 |
| Query 121 | GDASLTKRPMGRVLNPLREMGVQV-KSEDGDRLPVTLRGPKTPTPITYRVPMASAQVKSA | 179 |
| Sbjct 121 | GDASL+ RPMGRVL PLR+MGVQV K+ GDR+P+TLRGPK PITYRVPMASAQVKSA GDASLSGRPMGRVLEPLRQMGVQVLKATPGDRMPITLRGPKHAAPITYRVPMASAQVKSA | 180 |
| Query 180 | VLLAGLNTPGITTVIEPIMTRDHTEKMLQGFGANLTVETDADGVRTIRLEGRGKLTGQVI | 239 |
| Sbjct 181 | VLLAGLNTPGITTVIEP+MTRDHTEKML+GFGANL+VETD GVR I +EG+GKLTGQ I VLLAGLNTPGITTVIEPVMTRDHTEKMLKGFGANLSVETDERGVRHIFIEGQGKLTGQTI | 240 |
| Query 240 | DVPGDPSSTAFPLVAALLVPGSDVTILNVLMNPTRTGLILTLQEMGADIEVINPRLAGGE | 299 |
| Sbjct 241 | VPGDPSS FPLVAAL+VPGSD+ I NVLMNPTRTGL+LTLQEMGA I++++PR GGE AVPGDPSSAGFPLVAALIVPGSDIAIENVLMNPTRTGLLLTLQEMGARIDIVDPRNEGGE | 300 |
| Query 300 | DVADLRVRSSTLKGVTVPEDRAPSMIDEYPILavaaafaegaTVMNGLEELRVKESDRLS | 359 |
| Sbjct 301 | DVADLRVR S LKGVTVP +RAP+MIDEYP+LAVAA+FAEG T+M GLEELRVKESDRLS DVADLRVRYSELKGVTVPPERAPTMIDEYPVLAVAASFAEGETLMQGLEELRVKESDRLS | 360 |
| Query 360 | AVANGLKLNGVDCDEGETSLVVRGRPDGKGLG---NASGAAVATHLDHRIAMSFLVMGLV | 416 |
| Sbjct 361 | AVA GLKLNGVDC EGE +L VRG+P GKGLG N V THLDHRIAMSFLVMGL AVAEGLKLNGVDCTEGEATLAVRGKPGGKGLGQHPNGKSTIVQTHLDHRIAMSFLVMGLA | 420 |
| Query 417  Sbjct 421 | SENPVTVDDATMIATSFPEFMDLMAGLGAKIE 448  +E PVT+DD MIATSFPEFM LM GLGA+IE  TEKPVTIDDQAMIATSFPEFMGLMKGLGAEIE 452 |  |

>WP_109665793.1 3-phosphoshikimate 1-carboxyvinyltransferase [Mesorhizobium loti] PWJ90744.1 3-phosphoshikimate 1-carboxyvinyltransferase [Mesorhizobium loti]

Length=452

Score = 680 bits (1755), Expect = 0.0, Method: Compositional matrix adjust.

| Identities | = 349/451 (77%), Positives = 382/451 (85%), Gaps = 4/451 (1%) |  |
| --- | --- | --- |
| Query 1 | MLHGASSRPATARKSSGLSGTVRIPGDKSISHRSFMFGGLASGETRITGLLEGEDVINTG | 60 |
| Sbjct 1 | M H A+ +PATAR+S LSGT R+PGDKSISHRSFMFGGLASGETRITGLLEGEDV+ TG MSHAAAPKPATARRSPALSGTARVPGDKSISHRSFMFGGLASGETRITGLLEGEDVMRTG | 60 |
| Query 61 | KAMQAMGARIRKEGDTWIIDGVGNGGLLAPEAPLDFGNAATGCRLTMGLVGVYDFDSTFI | 120 |
| Sbjct 61 | AM+AMGA I K G W+I G GNG LL PE PLDFGNA TG RLTMGLVG YD ++TFI AAMKAMGAHIEKRGAEWVIRGTGNGALLQPEGPLDFGNAGTGSRLTMGLVGTYDMETTFI | 120 |
| Query 121 | GDASLTKRPMGRVLNPLREMGVQV-KSEDGDRLPVTLRGPKTPTPITYRVPMASAQVKSA | 179 |
| Sbjct 121 | GDASL+ RPMGRVL PLR+MGVQV K+ GDR+P+TL GPK PITYRVPMASAQVKSA GDASLSGRPMGRVLEPLRQMGVQVLKATPGDRMPITLHGPKHAAPITYRVPMASAQVKSA | 180 |
| Query 180 | VLLAGLNTPGITTVIEPIMTRDHTEKMLQGFGANLTVETDADGVRTIRLEGRGKLTGQVI VLLAGLNTPGITTVIEP+MTRDHTEKML+GFGANL+VETD GVR I +EG+GKLTGQ I | 239 |

| Sbjct | 181 | VLLAGLNTPGITTVIEPVMTRDHTEKMLKGFGANLSVETDERGVRHIFIEGQGKLTGQTI | 240 |
| --- | --- | --- | --- |
| Query | 240 | DVPGDPSSTAFPLVAALLVPGSDVTILNVLMNPTRTGLILTLQEMGADIEVINPRLAGGE | 299 |
| Sbjct | 241 | VPGDPSS FPLVAAL+VPGSD+ I NVLMNPTRTGL+LTLQEMG I+++N R AGGE AVPGDPSSAGFPLVAALIVPGSDIVIENVLMNPTRTGLLLTLQEMGGKIDILNARNAGGE | 300 |
| Query | 300 | DVADLRVRSSTLKGVTVPEDRAPSMIDEYPILavaaafaegaTVMNGLEELRVKESDRLS | 359 |
| Sbjct | 301 | DVADLRVR S LKGVTVP +RAPSMIDEYP+LAVAA+FAEG T+M GLEELRVKESDRLS DVADLRVRYSELKGVTVPPERAPSMIDEYPVLAVAASFAEGETLMQGLEELRVKESDRLS | 360 |
| Query | 360 | AVANGLKLNGVDCDEGETSLVVRGRPDGKGLG---NASGAAVATHLDHRIAMSFLVMGLV | 416 |
| Sbjct | 361 | AVANGLKLNGVDC EGE SL VRG+P GKGLG N V THLDHRIAMSFLVMGL AVANGLKLNGVDCTEGEASLAVRGKPRGKGLGGHPNGQDTTVQTHLDHRIAMSFLVMGLA | 420 |
| Query  Sbjct | 417  421 | SENPVTVDDATMIATSFPEFMDLMAGLGAKI 447  +E PVT+DD MIATSFPEFM LM GLGA+I  TEKPVTIDDQAMIATSFPEFMGLMTGLGAEI 451 |  |

>TIL95137.1 3-phosphoshikimate 1-carboxyvinyltransferase [Mesorhizobium sp.] Length=452

Score = 680 bits (1755), Expect = 0.0, Method: Compositional matrix adjust.

| Identities | = 350/452 (77%), Positives = 383/452 (85%), Gaps = 4/452 (1%) |  |
| --- | --- | --- |
| Query 1 | MLHGASSRPATARKSSGLSGTVRIPGDKSISHRSFMFGGLASGETRITGLLEGEDVINTG | 60 |
| Sbjct 1 | M H A+ +PATARKS LSGT R+PGDKSISHRSFMFGGLASGETRITGLLEGEDV+ TG MSHTAAPKPATARKSPALSGTARVPGDKSISHRSFMFGGLASGETRITGLLEGEDVMRTG | 60 |
| Query 61 | KAMQAMGARIRKEGDTWIIDGVGNGGLLAPEAPLDFGNAATGCRLTMGLVGVYDFDSTFI | 120 |
| Sbjct 61 | AM+AMGA I K G W+I G GNG LL PE PLDFGNA TG RLTMGLVG YD ++TFI AAMKAMGAHIEKRGAEWVIRGTGNGALLQPEGPLDFGNAGTGSRLTMGLVGTYDMETTFI | 120 |
| Query 121 | GDASLTKRPMGRVLNPLREMGVQV-KSEDGDRLPVTLRGPKTPTPITYRVPMASAQVKSA | 179 |
| Sbjct 121 | GDASL+ RPMGRVL PLR+MGVQV K+ GDR+P+TLRGPK PITYRVPMASAQVKSA GDASLSGRPMGRVLEPLRQMGVQVLKATPGDRMPITLRGPKHAAPITYRVPMASAQVKSA | 180 |
| Query 180 | VLLAGLNTPGITTVIEPIMTRDHTEKMLQGFGANLTVETDADGVRTIRLEGRGKLTGQVI | 239 |
| Sbjct 181 | VLLAGLNTPGITTVIEP+MTRDHTEKMLQGFGANL+VETD GVR I +EG+GKLTGQ I VLLAGLNTPGITTVIEPVMTRDHTEKMLQGFGANLSVETDERGVRHIFIEGQGKLTGQTI | 240 |
| Query 240 | DVPGDPSSTAFPLVAALLVPGSDVTILNVLMNPTRTGLILTLQEMGADIEVINPRLAGGE | 299 |
| Sbjct 241 | VPGDPSS FPLVAAL+VPGSD+TI NVLMNPTRTGL+LTLQEMGA I+V+ PR GGE AVPGDPSSAGFPLVAALIVPGSDITIENVLMNPTRTGLLLTLQEMGARIDVLEPRNEGGE | 300 |
| Query 300 | DVADLRVRSSTLKGVTVPEDRAPSMIDEYPILavaaafaegaTVMNGLEELRVKESDRLS | 359 |
| Sbjct 301 | DVADLRVR S LKGVTVP +RAP+MIDEYP+LAVAA+FAEG T+M GLEELRVKESDRLS DVADLRVRYSELKGVTVPPERAPAMIDEYPVLAVAASFAEGETLMQGLEELRVKESDRLS | 360 |
| Query 360 | AVANGLKLNGVDCDEGETSLVVRGRPDGKGLG---NASGAAVATHLDHRIAMSFLVMGLV | 416 |
| Sbjct 361 | AVA GLKLNGVDC EGE +L VRG+P GKGLG N V T LDHRIAMSFLVMGL AVAEGLKLNGVDCTEGEATLSVRGKPGGKGLGQHPNGKSTIVQTQLDHRIAMSFLVMGLA | 420 |
| Query 417  Sbjct 421 | SENPVTVDDATMIATSFPEFMDLMAGLGAKIE 448  +E PVT+DD MIATSFP+FM LM GLGA+IE  TEKPVTIDDQAMIATSFPQFMGLMKGLGAEIE 452 |  |

>WP_029347642.1 3-phosphoshikimate 1-carboxyvinyltransferase [Mesorhizobium ciceri] RWL22880.1 3-phosphoshikimate 1-carboxyvinyltransferase [Mesorhizobium sp.] RWM71370.1 3-phosphoshikimate 1-carboxyvinyltransferase [Mesorhizobium sp.] TIO24489.1 3-phosphoshikimate 1-carboxyvinyltransferase [Mesorhizobium sp.] TJV59604.1 3-phosphoshikimate 1-carboxyvinyltransferase [Mesorhizobium sp.]

Length=452

Score = 680 bits (1754), Expect = 0.0, Method: Compositional matrix adjust. Identities = 349/452 (77%), Positives = 383/452 (85%), Gaps = 4/452 (1%)

Query 1	MLHGASSRPATARKSSGLSGTVRIPGDKSISHRSFMFGGLASGETRITGLLEGEDVINTG 60 M H A+ +PATARKSS LSG R+PGDKSISHRSFMFGGLASGETRITGLLEGEDV+ TG

Sbjct 1	MSHTAAPKPATARKSSALSGAARVPGDKSISHRSFMFGGLASGETRITGLLEGEDVMRTG 60

| Query | 61 | KAMQAMGARIRKEGDTWIIDGVGNGGLLAPEAPLDFGNAATGCRLTMGLVGVYDFDSTFI | 120 |
| --- | --- | --- | --- |
| Sbjct | 61 | AM+AMGA I K G W+I G GNG LL PE PLDFGNA TG RLTMGLVG YD ++TFI AAMKAMGAHIEKLGTEWVIRGTGNGALLQPEGPLDFGNAGTGSRLTMGLVGTYDMETTFI | 120 |
| Query | 121 | GDASLTKRPMGRVLNPLREMGVQV-KSEDGDRLPVTLRGPKTPTPITYRVPMASAQVKSA | 179 |
| Sbjct | 121 | GDASL+ RPMGRVL PLR+MGVQV K+ GDR+P+TL GPK PITYRVPMASAQVKSA GDASLSGRPMGRVLEPLRQMGVQVLKATPGDRMPITLHGPKHAAPITYRVPMASAQVKSA | 180 |
| Query | 180 | VLLAGLNTPGITTVIEPIMTRDHTEKMLQGFGANLTVETDADGVRTIRLEGRGKLTGQVI | 239 |
| Sbjct | 181 | VLLAGLNTPG+TTVIEP MTRDHTEKML+GFGANL+VETD GVR I +EG+GKLTGQ I VLLAGLNTPGVTTVIEPAMTRDHTEKMLKGFGANLSVETDERGVRHIFIEGQGKLTGQTI | 240 |
| Query | 240 | DVPGDPSSTAFPLVAALLVPGSDVTILNVLMNPTRTGLILTLQEMGADIEVINPRLAGGE | 299 |
| Sbjct | 241 | VPGDPSS FPLVAAL+VPGSD+TI NVLMNPTRTGL+LTLQEMGA I++++PR GGE AVPGDPSSAGFPLVAALIVPGSDITIENVLMNPTRTGLLLTLQEMGARIDIVDPRNEGGE | 300 |
| Query | 300 | DVADLRVRSSTLKGVTVPEDRAPSMIDEYPILavaaafaegaTVMNGLEELRVKESDRLS | 359 |
| Sbjct | 301 | DVADLRVR S LKGVTVP +RAP+MIDEYP+LAVAA+FAEG T+M GLEELRVKESDRLS DVADLRVRYSELKGVTVPPERAPTMIDEYPVLAVAASFAEGETLMQGLEELRVKESDRLS | 360 |
| Query | 360 | AVANGLKLNGVDCDEGETSLVVRGRPDGKGLG---NASGAAVATHLDHRIAMSFLVMGLV | 416 |
| Sbjct | 361 | AVA GLKLNGVDC EGE SL VRG+P GKGLG N V THLDHRIAMSFLVMGL AVAEGLKLNGVDCTEGEASLAVRGKPGGKGLGRHPNGHNTVVQTHLDHRIAMSFLVMGLA | 420 |
| Query  Sbjct | 417  421 | SENPVTVDDATMIATSFPEFMDLMAGLGAKIE 448  +E PVT+DD MIATSFPEFM LM GLGA+IE  TEKPVTIDDQAMIATSFPEFMGLMKGLGAEIE 452 |  |

>WP_095517360.1 3-phosphoshikimate 1-carboxyvinyltransferase [Mesorhizobium wenxiniae] PAP96655.1 3-phosphoshikimate 1-carboxyvinyltransferase [Mesorhizobium wenxiniae]

Length=452

Score = 680 bits (1754), Expect = 0.0, Method: Compositional matrix adjust.

| Identities | = 349/452 (77%), Positives = 383/452 (85%), Gaps = 4/452 (1%) |  |
| --- | --- | --- |
| Query 1 | MLHGASSRPATARKSSGLSGTVRIPGDKSISHRSFMFGGLASGETRITGLLEGEDVINTG | 60 |
| Sbjct 1 | M H A+ +PATARKSS LSG R+PGDKSISHRSFMFGGLASGETRITGLLEGEDV+ TG MSHTAAPKPATARKSSALSGAARVPGDKSISHRSFMFGGLASGETRITGLLEGEDVMRTG | 60 |
| Query 61 | KAMQAMGARIRKEGDTWIIDGVGNGGLLAPEAPLDFGNAATGCRLTMGLVGVYDFDSTFI | 120 |
| Sbjct 61 | AM+AMGA I K G W+I G GNG LL PE PLDFGNA TG RLTMGLVG YD ++TFI AAMKAMGAHIEKLGTEWVIRGTGNGALLQPEGPLDFGNAGTGSRLTMGLVGTYDMETTFI | 120 |
| Query 121 | GDASLTKRPMGRVLNPLREMGVQV-KSEDGDRLPVTLRGPKTPTPITYRVPMASAQVKSA | 179 |
| Sbjct 121 | GDASL+ RPMGRVL PLR+MGVQV K+ GDR+P+TL GPK PITYRVPMASAQVKSA GDASLSGRPMGRVLEPLRQMGVQVLKATPGDRMPITLHGPKHAAPITYRVPMASAQVKSA | 180 |
| Query 180 | VLLAGLNTPGITTVIEPIMTRDHTEKMLQGFGANLTVETDADGVRTIRLEGRGKLTGQVI | 239 |
| Sbjct 181 | VLLAGLNTPG+TTVIEP MTRDHTEKML+GFGANL+VETD GVR I +EG+GKLTGQ I VLLAGLNTPGVTTVIEPAMTRDHTEKMLKGFGANLSVETDERGVRHIFIEGQGKLTGQTI | 240 |
| Query 240 | DVPGDPSSTAFPLVAALLVPGSDVTILNVLMNPTRTGLILTLQEMGADIEVINPRLAGGE | 299 |
| Sbjct 241 | VPGDPSS FPLVAAL+VPGSD+TI NVLMNPTRTGL+LTLQEMGA I++++PR GGE AVPGDPSSAGFPLVAALIVPGSDITIENVLMNPTRTGLLLTLQEMGARIDIVDPRNEGGE | 300 |
| Query 300 | DVADLRVRSSTLKGVTVPEDRAPSMIDEYPILavaaafaegaTVMNGLEELRVKESDRLS | 359 |
| Sbjct 301 | DVADLRVR S LKGVTVP +RAP+MIDEYP+LAVAA+FAEG T+M GLEELRVKESDRLS DVADLRVRYSELKGVTVPPERAPTMIDEYPVLAVAASFAEGETLMQGLEELRVKESDRLS | 360 |
| Query 360 | AVANGLKLNGVDCDEGETSLVVRGRPDGKGLG---NASGAAVATHLDHRIAMSFLVMGLV | 416 |
| Sbjct 361 | AVA GLKLNGVDC EGE SL VRG+P GKGLG N V THLDHRIAMSFLVMGL AVAEGLKLNGVDCTEGEASLAVRGKPGGKGLGRHPNGHNIVVQTHLDHRIAMSFLVMGLA | 420 |
| Query 417  Sbjct 421 | SENPVTVDDATMIATSFPEFMDLMAGLGAKIE 448  +E PVT+DD MIATSFPEFM LM GLGA+IE  TEKPVTIDDQAMIATSFPEFMGLMKGLGAEIE 452 |  |

>WP_127853519.1 3-phosphoshikimate 1-carboxyvinyltransferase [Mesorhizobium sp. M00.F.Ca.ET.216.01.1.1]

TGQ40664.1 3-phosphoshikimate 1-carboxyvinyltransferase [Mesorhizobium sp.

M00.F.Ca.ET.216.01.1.1]

TJW13996.1 3-phosphoshikimate 1-carboxyvinyltransferase [Mesorhizobium sp.] TJW44644.1 3-phosphoshikimate 1-carboxyvinyltransferase [Mesorhizobium sp.]

Length=452

Score = 680 bits (1754), Expect = 0.0, Method: Compositional matrix adjust.

| Identities | = 347/452 (77%), Positives = 383/452 (85%), Gaps = 4/452 (1%) |  |
| --- | --- | --- |
| Query 1 | MLHGASSRPATARKSSGLSGTVRIPGDKSISHRSFMFGGLASGETRITGLLEGEDVINTG | 60 |
| Sbjct 1 | M H A+ +PATARKS LSG R+PGDKSISHRSFMFGGLASGETRITGLLEGEDV+ TG MSHTAAPKPATARKSPALSGAARVPGDKSISHRSFMFGGLASGETRITGLLEGEDVMRTG | 60 |
| Query 61 | KAMQAMGARIRKEGDTWIIDGVGNGGLLAPEAPLDFGNAATGCRLTMGLVGVYDFDSTFI | 120 |
| Sbjct 61 | AM+AMGA + K G W+I G GNG LL PE PLDFGNA TG RLTMGLVG YD ++TFI AAMKAMGAHMEKRGAEWVIRGTGNGALLQPEGPLDFGNAGTGSRLTMGLVGTYDMETTFI | 120 |
| Query 121 | GDASLTKRPMGRVLNPLREMGVQV-KSEDGDRLPVTLRGPKTPTPITYRVPMASAQVKSA | 179 |
| Sbjct 121 | GDASL+ RPMGRVL PLR+MGVQV K+ GDR+P+TLRGPK PITYRVPMASAQVKSA GDASLSGRPMGRVLEPLRQMGVQVLKATPGDRMPITLRGPKHAAPITYRVPMASAQVKSA | 180 |
| Query 180 | VLLAGLNTPGITTVIEPIMTRDHTEKMLQGFGANLTVETDADGVRTIRLEGRGKLTGQVI | 239 |
| Sbjct 181 | VLLAGLNTPG+TTV+EP+MTRDHTEKML+GFGANL+VETD GVR I +EG+GKLTGQ I VLLAGLNTPGVTTVVEPVMTRDHTEKMLRGFGANLSVETDERGVRHIFIEGQGKLTGQTI | 240 |
| Query 240 | DVPGDPSSTAFPLVAALLVPGSDVTILNVLMNPTRTGLILTLQEMGADIEVINPRLAGGE | 299 |
| Sbjct 241 | VPGDPSS FPLVAAL+VPGSD+TI NVLMNPTRTGL+LTLQEMG I+V+NPR GGE AVPGDPSSAGFPLVAALIVPGSDITIQNVLMNPTRTGLLLTLQEMGGRIDVLNPRSEGGE | 300 |
| Query 300 | DVADLRVRSSTLKGVTVPEDRAPSMIDEYPILavaaafaegaTVMNGLEELRVKESDRLS | 359 |
| Sbjct 301 | DVADLRVR S L GVTVP +RAP+MIDEYP+LAVAA+FAEG T+M GLEELRVKESDRLS DVADLRVRYSELTGVTVPPERAPTMIDEYPVLAVAASFAEGETLMQGLEELRVKESDRLS | 360 |
| Query 360 | AVANGLKLNGVDCDEGETSLVVRGRPDGKGLG---NASGAAVATHLDHRIAMSFLVMGLV | 416 |
| Sbjct 361 | AVANGLKLNGVDC EGE +L VRG+P GKGLG N V THLDHRIAMSFLVMGL AVANGLKLNGVDCTEGEATLAVRGKPGGKGLGQHPNGQSTIVQTHLDHRIAMSFLVMGLA | 420 |
| Query 417  Sbjct 421 | SENPVTVDDATMIATSFPEFMDLMAGLGAKIE 448  +E PVT+DD MIATSFPEFM LM GLGA+IE  TEKPVTIDDQAMIATSFPEFMGLMKGLGAEIE 452 |  |

>RWO22386.1 3-phosphoshikimate 1-carboxyvinyltransferase [Mesorhizobium sp.] Length=452

Score = 680 bits (1754), Expect = 0.0, Method: Compositional matrix adjust.

| Identities | = 348/452 (77%), Positives = 384/452 (85%), Gaps = 4/452 (1%) |  |
| --- | --- | --- |
| Query 1 | MLHGASSRPATARKSSGLSGTVRIPGDKSISHRSFMFGGLASGETRITGLLEGEDVINTG | 60 |
| Sbjct 1 | M H A+ PATARKS LSGT R+PGDKSISHRSFMFGGLASGETRITGLLEGEDV+ TG MSHTAAPSPATARKSPALSGTARVPGDKSISHRSFMFGGLASGETRITGLLEGEDVMRTG | 60 |
| Query 61 | KAMQAMGARIRKEGDTWIIDGVGNGGLLAPEAPLDFGNAATGCRLTMGLVGVYDFDSTFI | 120 |
| Sbjct 61 | AM+AMGA I K G W+I G GNG LL PE PLDFGNA TG RLTMGLVG YD ++TFI AAMKAMGAHIEKRGTEWVIRGTGNGALLQPEGPLDFGNAGTGSRLTMGLVGTYDMETTFI | 120 |
| Query 121 | GDASLTKRPMGRVLNPLREMGVQV-KSEDGDRLPVTLRGPKTPTPITYRVPMASAQVKSA | 179 |
| Sbjct 121 | GDASL+ RPMGRVL PLR+MGVQV K+ GDR+P+TLRGPK PITYRVPMASAQVKSA GDASLSGRPMGRVLEPLRQMGVQVLKATPGDRMPITLRGPKHAAPITYRVPMASAQVKSA | 180 |
| Query 180 | VLLAGLNTPGITTVIEPIMTRDHTEKMLQGFGANLTVETDADGVRTIRLEGRGKLTGQVI | 239 |
| Sbjct 181 | VLLAGLNTPGITTVIEP+MTRDHTEKML+GFGANL+VETD GVR I +EG+GKLTGQ I VLLAGLNTPGITTVIEPVMTRDHTEKMLKGFGANLSVETDERGVRHIFIEGQGKLTGQTI | 240 |
| Query 240 | DVPGDPSSTAFPLVAALLVPGSDVTILNVLMNPTRTGLILTLQEMGADIEVINPRLAGGE | 299 |
| Sbjct 241 | VPGDPSS FPLVAAL+VPGSD+TI NVLMNPTRTGL+LTLQEMGA I++++PR GGE AVPGDPSSAGFPLVAALIVPGSDITIENVLMNPTRTGLLLTLQEMGARIDIVDPRSEGGE | 300 |
| Query 300 | DVADLRVRSSTLKGVTVPEDRAPSMIDEYPILavaaafaegaTVMNGLEELRVKESDRLS | 359 |
| Sbjct 301 | DVADLRVR S LKGVTVP +RAP+MIDEYP+LAVAA+FA+G T+M GLEELRVKESDRLS DVADLRVRYSELKGVTVPPERAPTMIDEYPVLAVAASFADGETLMQGLEELRVKESDRLS | 360 |
| Query 360 | AVANGLKLNGVDCDEGETSLVVRGRPDGKGLG---NASGAAVATHLDHRIAMSFLVMGLV | 416 |

AVA GLKLNGVDC EGE +L VRG+P GKGLG	N	V THLDHRIAMSFLVMGL

| Sbjct | 361 | AVAEGLKLNGVDCTEGEATLAVRGKPGGKGLGQHPNGKSTIVQTHLDHRIAMSFLVMGLA 420 |
| --- | --- | --- |
| Query | 417 | SENPVTVDDATMIATSFPEFMDLMAGLGAKIE 448 |
| Sbjct | 421 | +E PVT+DD MIATSFPEFM LM GLGA+I+  TEKPVTIDDQAMIATSFPEFMGLMMGLGAEID 452 |

>WP_095493879.1 3-phosphoshikimate 1-carboxyvinyltransferase [Mesorhizobium temperatum] PAQ07933.1 3-phosphoshikimate 1-carboxyvinyltransferase [Mesorhizobium temperatum]

Length=452

Score = 680 bits (1754), Expect = 0.0, Method: Compositional matrix adjust.

| Identities | = 348/452 (77%), Positives = 383/452 (85%), Gaps = 4/452 (1%) |  |
| --- | --- | --- |
| Query 1 | MLHGASSRPATARKSSGLSGTVRIPGDKSISHRSFMFGGLASGETRITGLLEGEDVINTG | 60 |
| Sbjct 1 | M H A +PATARKS LSG R+PGDKSISHRSFMFGGLASGETRITGLLEGEDV+ TG MSHTAVPKPATARKSPALSGAARVPGDKSISHRSFMFGGLASGETRITGLLEGEDVMRTG | 60 |
| Query 61 | KAMQAMGARIRKEGDTWIIDGVGNGGLLAPEAPLDFGNAATGCRLTMGLVGVYDFDSTFI | 120 |
| Sbjct 61 | AM+AMGA I K G W+I G GNG LL PE PLDFGNA TG RLTMGLVG YD ++TFI AAMKAMGANIEKRGAEWVIRGTGNGALLQPEGPLDFGNAGTGSRLTMGLVGTYDMETTFI | 120 |
| Query 121 | GDASLTKRPMGRVLNPLREMGVQV-KSEDGDRLPVTLRGPKTPTPITYRVPMASAQVKSA | 179 |
| Sbjct 121 | GDASL+ RPMGRVL PLR+MGVQV K+ GDR+P+TL GPK PITYRVPMASAQVKSA GDASLSGRPMGRVLEPLRQMGVQVLKATPGDRMPITLHGPKHAAPITYRVPMASAQVKSA | 180 |
| Query 180 | VLLAGLNTPGITTVIEPIMTRDHTEKMLQGFGANLTVETDADGVRTIRLEGRGKLTGQVI | 239 |
| Sbjct 181 | VLLAGLNTPG+TTVIEP+MTRDHTEKML+GFGANL+VETD GVR I +EG+GKLTGQ I VLLAGLNTPGVTTVIEPVMTRDHTEKMLKGFGANLSVETDERGVRHIFIEGQGKLTGQTI | 240 |
| Query 240 | DVPGDPSSTAFPLVAALLVPGSDVTILNVLMNPTRTGLILTLQEMGADIEVINPRLAGGE | 299 |
| Sbjct 241 | VPGDPSS FPLVAAL+VPGSD+TI NVLMNPTRTGL+LTLQEMGA I++++PR GGE AVPGDPSSAGFPLVAALIVPGSDITIENVLMNPTRTGLLLTLQEMGARIDIVDPRNEGGE | 300 |
| Query 300 | DVADLRVRSSTLKGVTVPEDRAPSMIDEYPILavaaafaegaTVMNGLEELRVKESDRLS | 359 |
| Sbjct 301 | DVADLRVR S LKGVTVP +RAP+MIDEYP+LAVAA+FAEG T+M GLEELRVKESDRLS DVADLRVRYSELKGVTVPPERAPTMIDEYPVLAVAASFAEGETLMQGLEELRVKESDRLS | 360 |
| Query 360 | AVANGLKLNGVDCDEGETSLVVRGRPDGKGLG---NASGAAVATHLDHRIAMSFLVMGLV | 416 |
| Sbjct 361 | AVANGLK+NGVDC EGE SL VRG+P GKGLG N V THLDHRIAMSFLVMGL AVANGLKVNGVDCTEGEASLAVRGKPGGKGLGQHPNGQSTIVQTHLDHRIAMSFLVMGLA | 420 |
| Query 417  Sbjct 421 | SENPVTVDDATMIATSFPEFMDLMAGLGAKIE 448  +E PVT+DD MIATSFPEFM LM GLGA+IE  TEKPVTIDDQAMIATSFPEFMGLMKGLGAEIE 452 |  |

>TJV72134.1 3-phosphoshikimate 1-carboxyvinyltransferase [Mesorhizobium sp.] Length=452

Score = 679 bits (1753), Expect = 0.0, Method: Compositional matrix adjust.

| Identities | = 348/452 (77%), Positives = 384/452 (85%), Gaps = 4/452 (1%) |  |
| --- | --- | --- |
| Query 1 | MLHGASSRPATARKSSGLSGTVRIPGDKSISHRSFMFGGLASGETRITGLLEGEDVINTG | 60 |
| Sbjct 1 | M H A+++ ATAR S L+GT R+PGDKSISHRSFMFGGLASGETRITGLLEGEDV+ TG MSHSAAAKSATARNSPALAGTARVPGDKSISHRSFMFGGLASGETRITGLLEGEDVMRTG | 60 |
| Query 61 | KAMQAMGARIRKEGDTWIIDGVGNGGLLAPEAPLDFGNAATGCRLTMGLVGVYDFDSTFI | 120 |
| Sbjct 61 | AM+AMGA I K G W+I G GNG LL PE PLDFGNA TG RLTMGLVG YD ++TFI AAMKAMGAHIEKNGAEWVIRGTGNGALLQPEGPLDFGNAGTGSRLTMGLVGTYDMETTFI | 120 |
| Query 121 | GDASLTKRPMGRVLNPLREMGVQV-KSEDGDRLPVTLRGPKTPTPITYRVPMASAQVKSA | 179 |
| Sbjct 121 | GDASL+ RPMGRVL+PLR+MGVQV K+ GDR+P+TLRGPK PITYRVPMASAQVKSA GDASLSGRPMGRVLDPLRQMGVQVLKAAPGDRMPITLRGPKHAAPITYRVPMASAQVKSA | 180 |
| Query 180 | VLLAGLNTPGITTVIEPIMTRDHTEKMLQGFGANLTVETDADGVRTIRLEGRGKLTGQVI | 239 |
| Sbjct 181 | VLLAGLNTPGITTVIEP+MTRDHTEKML+GFGANLTVETD GVR I +EG+GKLTGQ I VLLAGLNTPGITTVIEPVMTRDHTEKMLKGFGANLTVETDERGVRHIFIEGQGKLTGQTI | 240 |
| Query 240 | DVPGDPSSTAFPLVAALLVPGSDVTILNVLMNPTRTGLILTLQEMGADIEVINPRLAGGE VPGDPSS FPLVAAL+VPGSD+ I NVLMNPTRTGL+LTLQEMG I+++NPR AGGE | 299 |

| Sbjct | 241 | AVPGDPSSAGFPLVAALIVPGSDIVIENVLMNPTRTGLLLTLQEMGGRIDILNPRNAGGE | 300 |
| --- | --- | --- | --- |
| Query | 300 | DVADLRVRSSTLKGVTVPEDRAPSMIDEYPILavaaafaegaTVMNGLEELRVKESDRLS | 359 |
| Sbjct | 301 | DVADLRVR S L GV VP +RAPSMIDEYP+LAVAA+FAEG T+M GLEELRVKESDRLS DVADLRVRYSELTGVVVPPERAPSMIDEYPVLAVAASFAEGETLMQGLEELRVKESDRLS | 360 |
| Query | 360 | AVANGLKLNGVDCDEGETSLVVRGRPDGKGLG---NASGAAVATHLDHRIAMSFLVMGLV | 416 |
| Sbjct | 361 | AVANGLKLNGVDC EGE +L V G+P GKGLG N AV THLDHRIAMSFL+MGL AVANGLKLNGVDCTEGEATLAVSGKPGGKGLGAHPNGPDTAVKTHLDHRIAMSFLIMGLA | 420 |
| Query  Sbjct | 417  421 | SENPVTVDDATMIATSFPEFMDLMAGLGAKIE 448  +E PVT+DDA MIATSFPEFM LM GLGA+IE  TEKPVTIDDANMIATSFPEFMGLMKGLGAEIE 452 |  |

>WP_023676281.1 3-phosphoshikimate 1-carboxyvinyltransferase [Mesorhizobium sp. LSJC280B00]

ESW86941.1 3-phosphoshikimate 1-carboxyvinyltransferase [Mesorhizobium sp. LSJC280B00]

Length=452

Score = 679 bits (1753), Expect = 0.0, Method: Compositional matrix adjust.

| Identities | = 352/452 (78%), Positives = 383/452 (85%), Gaps = 4/452 (1%) |  |
| --- | --- | --- |
| Query 1 | MLHGASSRPATARKSSGLSGTVRIPGDKSISHRSFMFGGLASGETRITGLLEGEDVINTG | 60 |
| Sbjct 1 | M H A+ + ATARKSSGLSG R+PGDKSISHRSFMFGGLASGETRITGLLEGEDV+ TG MSHTAAPKQATARKSSGLSGAARVPGDKSISHRSFMFGGLASGETRITGLLEGEDVMRTG | 60 |
| Query 61 | KAMQAMGARIRKEGDTWIIDGVGNGGLLAPEAPLDFGNAATGCRLTMGLVGVYDFDSTFI | 120 |
| Sbjct 61 | AM+AMGA I K G W+I G GNG LL P PLDFGNA TG RLTMGLVG YD +TFI AAMKAMGAHIEKHGAEWVIRGTGNGALLQPVGPLDFGNAGTGSRLTMGLVGTYDMKTTFI | 120 |
| Query 121 | GDASLTKRPMGRVLNPLREMGVQV-KSEDGDRLPVTLRGPKTPTPITYRVPMASAQVKSA | 179 |
| Sbjct 121 | GDASL+ RPMGRVL PLR+MGVQV K+ GDR+P+TL GPK PITYRVPMASAQVKSA GDASLSGRPMGRVLEPLRQMGVQVLKATPGDRMPITLHGPKHAAPITYRVPMASAQVKSA | 180 |
| Query 180 | VLLAGLNTPGITTVIEPIMTRDHTEKMLQGFGANLTVETDADGVRTIRLEGRGKLTGQVI | 239 |
| Sbjct 181 | VLLAGLNTPGITTVIEP+MTRDHTEKML+GFGANL+VETD GVR I +EG+GKLTGQ I VLLAGLNTPGITTVIEPVMTRDHTEKMLKGFGANLSVETDERGVRHIFIEGQGKLTGQTI | 240 |
| Query 240 | DVPGDPSSTAFPLVAALLVPGSDVTILNVLMNPTRTGLILTLQEMGADIEVINPRLAGGE | 299 |
| Sbjct 241 | VPGDPSS FPLVAAL+VPGSD+TI NVLMNPTRTGL+LTLQEMGA I++I+PR GGE AVPGDPSSAGFPLVAALIVPGSDITIENVLMNPTRTGLLLTLQEMGAWIDIIDPRNEGGE | 300 |
| Query 300 | DVADLRVRSSTLKGVTVPEDRAPSMIDEYPILavaaafaegaTVMNGLEELRVKESDRLS | 359 |
| Sbjct 301 | DVADLRVR S LKGVTVP RAP+MIDEYP+LAVAA+FAEG T+M GLEELRVKESDRLS DVADLRVRYSELKGVTVPPGRAPTMIDEYPVLAVAASFAEGETLMLGLEELRVKESDRLS | 360 |
| Query 360 | AVANGLKLNGVDCDEGETSLVVRGRPDGKGLG---NASGAAVATHLDHRIAMSFLVMGLV | 416 |
| Sbjct 361 | AVA GLKLNGVDC EGE SLVVRG+P GKGLG N G V THLDHRIAMSFLVMGL AVAEGLKLNGVDCSEGEASLVVRGKPGGKGLGQHPNGQGTIVQTHLDHRIAMSFLVMGLA | 420 |
| Query 417  Sbjct 421 | SENPVTVDDATMIATSFPEFMDLMAGLGAKIE 448  +E PVT+DD MIATSFPEFM LM GLGA+IE  TEKPVTIDDQAMIATSFPEFMGLMKGLGAEIE 452 |  |

>TIS36796.1 3-phosphoshikimate 1-carboxyvinyltransferase [Mesorhizobium sp.] Length=452

Score = 679 bits (1753), Expect = 0.0, Method: Compositional matrix adjust.

| Identities | = 350/452 (77%), Positives = 384/452 (85%), Gaps = 4/452 (1%) |  |
| --- | --- | --- |
| Query 1 | MLHGASSRPATARKSSGLSGTVRIPGDKSISHRSFMFGGLASGETRITGLLEGEDVINTG | 60 |
| Sbjct 1 | M H A+ +PATARKSS LSG R+PGDKSISHRSFMFGGLASGETRITGLLEGEDV+ TG MSHTAAPKPATARKSSALSGAARVPGDKSISHRSFMFGGLASGETRITGLLEGEDVMRTG | 60 |
| Query 61 | KAMQAMGARIRKEGDTWIIDGVGNGGLLAPEAPLDFGNAATGCRLTMGLVGVYDFDSTFI | 120 |
| Sbjct 61 | AM+AMGA I G W+I G GNG LL PE PLDFGNA TG RLTMGLVG YD ++TFI AAMKAMGAHIEMLGTEWVIRGTGNGALLQPEGPLDFGNAGTGSRLTMGLVGTYDMETTFI | 120 |
| Query 121 | GDASLTKRPMGRVLNPLREMGVQV-KSEDGDRLPVTLRGPKTPTPITYRVPMASAQVKSA | 179 |

GDASL+ RPMGRVL PLR+MGVQV K+ GDR+P+TLRGPK	PITYRVPMASAQVKSA

| Sbjct | 121 | GDASLSGRPMGRVLEPLRQMGVQVLKATPGDRMPITLRGPKHAAPITYRVPMASAQVKSA | 180 |
| --- | --- | --- | --- |
| Query | 180 | VLLAGLNTPGITTVIEPIMTRDHTEKMLQGFGANLTVETDADGVRTIRLEGRGKLTGQVI | 239 |
| Sbjct | 181 | VLLAGLNTPGITTVIEP+MTRDHTEKML+GFGANL+VETD GVR I +EG+GKLTGQ I VLLAGLNTPGITTVIEPVMTRDHTEKMLRGFGANLSVETDERGVRHIFIEGQGKLTGQTI | 240 |
| Query | 240 | DVPGDPSSTAFPLVAALLVPGSDVTILNVLMNPTRTGLILTLQEMGADIEVINPRLAGGE | 299 |
| Sbjct | 241 | VPGDPSS FPLVAAL+VPGSD+TI NVLMN TRTGL+LTLQEMGA I++++PR GGE AVPGDPSSAGFPLVAALIVPGSDITIENVLMNSTRTGLLLTLQEMGARIDIVDPRNEGGE | 300 |
| Query | 300 | DVADLRVRSSTLKGVTVPEDRAPSMIDEYPILavaaafaegaTVMNGLEELRVKESDRLS | 359 |
| Sbjct | 301 | DVADLRVR S LKGV VP +RAP+MIDEYP+LAVAA+FAEG T+M GLEELRVKESDRLS DVADLRVRYSELKGVAVPPERAPTMIDEYPVLAVAASFAEGETLMQGLEELRVKESDRLS | 360 |
| Query | 360 | AVANGLKLNGVDCDEGETSLVVRGRPDGKGLG---NASGAAVATHLDHRIAMSFLVMGLV | 416 |
| Sbjct | 361 | AVA+GLKLNGVDC EGE SL V GRP GKGLG N G V THLDHRIAMSFLVMGL AVADGLKLNGVDCTEGEASLAVHGRPGGKGLGQHPNGQGTIVQTHLDHRIAMSFLVMGLA | 420 |
| Query  Sbjct | 417  421 | SENPVTVDDATMIATSFPEFMDLMAGLGAKIE 448  +E PVT+DD TMIATSFPEFM LM GLGA+IE  TEKPVTIDDQTMIATSFPEFMGLMKGLGAEIE 452 |  |

>WP_144702376.1 3-phosphoshikimate 1-carboxyvinyltransferase [Aminobacter sp. J44]

TWG65596.1 3-phosphoshikimate 1-carboxyvinyltransferase [Aminobacter sp. J44]

Length=446

Score = 679 bits (1753), Expect = 0.0, Method: Compositional matrix adjust.

| Identities | = 351/442 (79%), Positives = 382/442 (86%), Gaps = 4/442 (1%) |  |
| --- | --- | --- |
| Query 8 | RPATARKSSGLSGTVRIPGDKSISHRSFMFGGLASGETRITGLLEGEDVINTGKAMQAMG | 67 |
| Sbjct 8 | +PATA KS LSGTVR+PGDKSISHRSFMFGGLASG TRITGLLEGEDV+ TG+AM+AMG KPATASKSEALSGTVRVPGDKSISHRSFMFGGLASGVTRITGLLEGEDVMRTGEAMKAMG | 67 |
| Query 68 | ARIRKEGDTWIIDGVGNGGLLAPEAPLDFGNAATGCRLTMGLVGVYDFDSTFIGDASLTK | 127 |
| Sbjct 68 | A+I K+GD WII G GNG LL P+ PLDFGNA TG RLTMGLVG YD +TFIGDASL AKIEKQGDEWIIHGTGNGALLEPQGPLDFGNAGTGSRLTMGLVGTYDMTTTFIGDASLQS | 127 |
| Query 128 | RPMGRVLNPLREMGVQV-KSEDGDRLPVTLRGPKTPTPITYRVPMASAQVKSAVLLAGLN | 186 |
| Sbjct 128 | RPMGRVLNPLREMGVQV + GDR+P+T+RGPK PITYRVPMASAQVKSAVLLAGLN RPMGRVLNPLREMGVQVVDAAPGDRMPLTIRGPKRANPITYRVPMASAQVKSAVLLAGLN | 187 |
| Query 187 | TPGITTVIEPIMTRDHTEKMLQGFGANLTVETDADGVRTIRLEGRGKLTGQVIDVPGDPS | 246 |
| Sbjct 188 | TPGITTVIEPIMTRDHTEKML+GFGANL VETD DGVR IR+EG GKLTGQVI VPGDPS TPGITTVIEPIMTRDHTEKMLKGFGANLEVETDKDGVRHIRIEGEGKLTGQVIAVPGDPS | 247 |
| Query 247 | STAFPLVAALLVPGSDVTILNVLMNPTRTGLILTLQEMGADIEVINPRLAGGEDVADLRV | 306 |
| Sbjct 248 | S AFPLVAAL+VPGSD+ I NVLMNPTRTGLI TL EMGA IE++N R AGGE+VADLRV SAAFPLVAALIVPGSDIVIENVLMNPTRTGLIDTLLEMGASIEILNRRDAGGEEVADLRV | 307 |
| Query 307 | RSSTLKGVTVPEDRAPSMIDEYPILavaaafaegaTVMNGLEELRVKESDRLSAVANGLK | 366 |
| Sbjct 308 | R S LKGV+VP RAP MIDEYPILA+AA+ AEG TVM+GLEELRVKESDRL+AVA GL+ RHSELKGVSVPPARAPFMIDEYPILAIAASLAEGETVMDGLEELRVKESDRLAAVARGLE | 367 |
| Query 367 | LNGVDCDEGETSLVVRGRPDGKGLGNASGAAVATHLDHRIAMSFLVMGLVSENPVTVDDA | 426 |
| Sbjct 368 | +NGVDC EGE SL VRGRPDGKGLG G VATHLDHRIAMSFLV+GL +E PVTVDD VNGVDCTEGEASLTVRGRPDGKGLG---GGTVATHLDHRIAMSFLVLGLAAEKPVTVDDR | 424 |
| Query 427  Sbjct 425 | TMIATSFPEFMDLMAGLGAKIE 448  +MIATSFPEFM LM GLGAKI+  SMIATSFPEFMGLMTGLGAKID 446 |  |

>WP_035025239.1 3-phosphoshikimate 1-carboxyvinyltransferase [Aquamicrobium defluvii] EXL09205.1 3-phosphoshikimate 1-carboxyvinyltransferase [Aquamicrobium defluvii] EZQ17398.1 3-phosphoshikimate 1-carboxyvinyltransferase [Pseudomonas bauzanensis] TDR37635.1 3-phosphoshikimate 1-carboxyvinyltransferase [Aquamicrobium defluvii]

Length=453

Score = 679 bits (1753), Expect = 0.0, Method: Compositional matrix adjust.

| Identities | = 352/455 (77%), Positives = 387/455 (85%), Gaps = 3/455 (1%) |  |
| --- | --- | --- |
| Query 1 | MLHGASSRPATARKSSGLSGTVRIPGDKSISHRSFMFGGLASGETRITGLLEGEDVINTG | 60 |
| Sbjct 1 | M H A+ +PA AR+SS L G R+PGDKSISHRSFMFGGLASGETRITGLLEGEDV+ TG MSHTAAPQPAIARRSSPLRGEARVPGDKSISHRSFMFGGLASGETRITGLLEGEDVLRTG | 60 |
| Query 61 | KAMQAMGARIRKEGDTWIIDGVGNGGLLAPEAPLDFGNAATGCRLTMGLVGVYDFDSTFI | 120 |
| Sbjct 61 | +AM+AMGA I K+GD WII G GNG LL PE PLDFGNA TG RLTMGLVG YD ++TFI EAMKAMGAHIEKKGDEWIIRGTGNGALLQPEGPLDFGNAGTGSRLTMGLVGTYDMETTFI | 120 |
| Query 121 | GDASLTKRPMGRVLNPLREMGVQVKSE-DGDRLPVTLRGPKTPTPITYRVPMASAQVKSA | 179 |
| Sbjct 121 | GDASL RPMGRVL+PLR+MGVQV S GDR+P+TLRGPK PITYRVPMASAQVKSA GDASLQSRPMGRVLDPLRQMGVQVLSAAPGDRMPLTLRGPKHAAPITYRVPMASAQVKSA | 180 |
| Query 180 | VLLAGLNTPGITTVIEPIMTRDHTEKMLQGFGANLTVETDADGVRTIRLEGRGKLTGQVI | 239 |
| Sbjct 181 | VLLA LNTPGITTVIEP+MTRDHTEKML+GFGA++ VETD GVR I +EG+G+L GQ I VLLAALNTPGITTVIEPVMTRDHTEKMLKGFGASIDVETDERGVRHIFIEGQGRLAGQTI | 240 |
| Query 240 | DVPGDPSSTAFPLVAALLVPGSDVTILNVLMNPTRTGLILTLQEMGADIEVINPRLAGGE | 299 |
| Sbjct 241 | VPGDPSS AFPLVAAL+VPGSD+ I NVLMNPTRTGLILTLQEMGADIEV N R GGE RVPGDPSSAAFPLVAALIVPGSDIVIRNVLMNPTRTGLILTLQEMGADIEVQNTRNEGGE | 300 |
| Query 300 | DVADLRVRSSTLKGVTVPEDRAPSMIDEYPILavaaafaegaTVMNGLEELRVKESDRLS | 359 |
| Sbjct 301 | DVADLRVR S LKGVTVP +RAPSMIDEYP+LAVAAAFAEG T+M GLEELRVKESDRL+ DVADLRVRHSQLKGVTVPAERAPSMIDEYPVLAVAAAFAEGETLMQGLEELRVKESDRLA | 360 |
| Query 360 | AVANGLKLNGVDCDEGETSLVVRGRPDGKGLGNASGAAVATHLDHRIAMSFLVMGLVSEN | 419 |
| Sbjct 361 | AVA GL+LNGVDC EGE +L VRG P+GKGLG G AV THLDHRIAMSFLVMGL SE+ AVAAGLRLNGVDCTEGEATLAVRGVPEGKGLGG--GEAVKTHLDHRIAMSFLVMGLASEH | 418 |
| Query 420  Sbjct 419 | PVTVDDATMIATSFPEFMDLMAGLGAKIELSDTKA 454 PVT+DD MIATSFPEFMDLM GLGA+I+ +D A PVTIDDRAMIATSFPEFMDLMTGLGARIDAADKAA 453 |  |

>WP_024847074.1 3-phosphoshikimate 1-carboxyvinyltransferase [Aminobacter sp. J41]

Length=446

Score = 679 bits (1752), Expect = 0.0, Method: Compositional matrix adjust.

| Identities | = 351/442 (79%), Positives = 381/442 (86%), Gaps = 4/442 (1%) |  |
| --- | --- | --- |
| Query 8 | RPATARKSSGLSGTVRIPGDKSISHRSFMFGGLASGETRITGLLEGEDVINTGKAMQAMG | 67 |
| Sbjct 8 | +PATA KS LSGTVR+PGDKSISHRSFMFGGLASG TRITGLLEGEDV+ TG+AM+AMG KPATASKSEALSGTVRVPGDKSISHRSFMFGGLASGVTRITGLLEGEDVLRTGEAMKAMG | 67 |
| Query 68 | ARIRKEGDTWIIDGVGNGGLLAPEAPLDFGNAATGCRLTMGLVGVYDFDSTFIGDASLTK | 127 |
| Sbjct 68 | A+I K+GD WII G GNG LL P+ PLDFGNA TG RLTMGLVG YD +TFIGD SL AKIEKQGDEWIIHGTGNGALLEPQGPLDFGNAGTGSRLTMGLVGTYDMTTTFIGDTSLQS | 127 |
| Query 128 | RPMGRVLNPLREMGVQV-KSEDGDRLPVTLRGPKTPTPITYRVPMASAQVKSAVLLAGLN | 186 |
| Sbjct 128 | RPMGRVLNPLREMGVQV + GDR+P+T+RGPK PITYRVPMASAQVKSAVLLAGLN RPMGRVLNPLREMGVQVVDAAPGDRMPLTIRGPKRANPITYRVPMASAQVKSAVLLAGLN | 187 |
| Query 187 | TPGITTVIEPIMTRDHTEKMLQGFGANLTVETDADGVRTIRLEGRGKLTGQVIDVPGDPS | 246 |
| Sbjct 188 | TPGITTVIEPIMTRDHTEKML+GFGANL VETD DGVR IR+EG GKLTGQVI VPGDPS TPGITTVIEPIMTRDHTEKMLKGFGANLQVETDKDGVRHIRIEGEGKLTGQVIAVPGDPS | 247 |
| Query 247 | STAFPLVAALLVPGSDVTILNVLMNPTRTGLILTLQEMGADIEVINPRLAGGEDVADLRV | 306 |
| Sbjct 248 | S AFPLVAAL+VPGSD+ I NVLMNPTRTGLI TL EMGA IE++N R AGGE+VADLRV SAAFPLVAALIVPGSDIVIENVLMNPTRTGLIDTLLEMGASIEILNRRDAGGEEVADLRV | 307 |
| Query 307 | RSSTLKGVTVPEDRAPSMIDEYPILavaaafaegaTVMNGLEELRVKESDRLSAVANGLK | 366 |
| Sbjct 308 | R S LKGVTVP RAP MIDEYPILA+AA+ AEG TVM+GLEELRVKESDRL+AVA GL+ RHSELKGVTVPPARAPFMIDEYPILAIAASLAEGETVMDGLEELRVKESDRLAAVARGLE | 367 |
| Query 367 | LNGVDCDEGETSLVVRGRPDGKGLGNASGAAVATHLDHRIAMSFLVMGLVSENPVTVDDA | 426 |
| Sbjct 368 | +NGVDC EGE SL VRGRPDGKGLG G VATHLDHRIAMSFLV+GL +E PVTVDD VNGVDCTEGEASLTVRGRPDGKGLG---GGTVATHLDHRIAMSFLVLGLAAEKPVTVDDR | 424 |
| Query 427 | TMIATSFPEFMDLMAGLGAKIE 448 |  |

+MIATSFPEFM LM GLGAKI+ Sbjct 425 SMIATSFPEFMGLMTGLGAKID 446

>WP_162654266.1 3-phosphoshikimate 1-carboxyvinyltransferase [Lentilitoribacter sp. Alg239-R112]

Length=462

Score = 679 bits (1752), Expect = 0.0, Method: Compositional matrix adjust.

| Identities | = 346/441 (78%), Positives = 378/441 (86%), Gaps = 0/441 (0%) |  |
| --- | --- | --- |
| Query 8 | RPATARKSSGLSGTVRIPGDKSISHRSFMFGGLASGETRITGLLEGEDVINTGKAMQAMG | 67 |
| Sbjct 19 | RP +A KSS L GT+RIPGDKSISHRSFMFGGLASGETRI GLLEGEDVI TG AM+AMG RPTSAHKSSSLKGTIRIPGDKSISHRSFMFGGLASGETRIRGLLEGEDVIATGNAMRAMG | 78 |
| Query 68 | ARIRKEGDTWIIDGVGNGGLLAPEAPLDFGNAATGCRLTMGLVGVYDFDSTFIGDASLTK | 127 |
| Sbjct 79 | A+I K+G+ WI+ G GNG LLAPE PLDFGNA TGCRLTMGLVGVYDF STFIGDASL+ AKIEKQGEEWIVTGTGNGCLLAPEQPLDFGNAGTGCRLTMGLVGVYDFKSTFIGDASLSS | 138 |
| Query 128 | RPMGRVLNPLREMGVQVKSEDGDRLPVTLRGPKTPTPITYRVPMASAQVKSAVLLAGLNT | 187 |
| Sbjct 139 | RPMGRVLNPLREMGVQV++E GDRLP TLRGP+ PITY VPMASAQVKSAVLLAGLNT RPMGRVLNPLREMGVQVEAESGDRLPATLRGPRIAAPITYTVPMASAQVKSAVLLAGLNT | 198 |
| Query 188 | PGITTVIEPIMTRDHTEKMLQGFGANLTVETDADGVRTIRLEGRGKLTGQVIDVPGDPSS | 247 |
| Sbjct 199 | PG T VIE + TRDHTEKMLQGFGA L V+TD +GVRTI LEG+G+L GQ IDVPGDPSS PGTTVVIENVYTRDHTEKMLQGFGAKLDVQTDTNGVRTISLEGQGELRGQNIDVPGDPSS | 258 |
| Query 248 | TAFPLVAALLVPGSDVTILNVLMNPTRTGLILTLQEMGADIEVINPRLAGGEDVADLRVR | 307 |
| Sbjct 259 | TAFPLVAALL P SD+TI NVLMNPTRTGLI TL EMGA I+++NPR GGEDVADLRVR TAFPLVAALLTPDSDITIENVLMNPTRTGLITTLIEMGAKIDILNPRQNGGEDVADLRVR | 318 |
| Query 308 | SSTLKGVTVPEDRAPSMIDEYPILavaaafaegaTVMNGLEELRVKESDRLSAVANGLKL | 367 |
| Sbjct 319 | SS LKG+ VP +RA SMIDEYPILAVAAA+AEG T M+GL+ELRVKESDRL+AVANGLKL SSDLKGIIVPPERAASMIDEYPILAVAAAYAEGETFMDGLDELRVKESDRLAAVANGLKL | 378 |
| Query 368 | NGVDCDEGETSLVVRGRPDGKGLGNASGAAVATHLDHRIAMSFLVMGLVSENPVTVDDAT | 427 |
| Sbjct 379 | N V C+EGE SL V GRPDGKG GN +G V THLDHRIAMSFLV+GL SE+PV VDD NDVSCEEGEMSLKVYGRPDGKGYGNPNGDQVTTHLDHRIAMSFLVLGLASEHPVQVDDGN | 438 |
| Query 428  Sbjct 439 | MIATSFPEFMDLMAGLGAKIE 448  +I TSFPEFMDLM GLGA I+  VIKTSFPEFMDLMTGLGADIK 459 |  |

>RWA77644.1 3-phosphoshikimate 1-carboxyvinyltransferase [Mesorhizobium sp.] RWC05917.1 3-phosphoshikimate 1-carboxyvinyltransferase [Mesorhizobium sp.] RWG87955.1 3-phosphoshikimate 1-carboxyvinyltransferase [Mesorhizobium sp.] RWK12881.1 3-phosphoshikimate 1-carboxyvinyltransferase [Mesorhizobium sp.] TIQ44621.1 3-phosphoshikimate 1-carboxyvinyltransferase [Mesorhizobium sp.]

Length=452

Score = 679 bits (1752), Expect = 0.0, Method: Compositional matrix adjust.

| Identities | = 354/452 (78%), Positives = 388/452 (86%), Gaps = 4/452 (1%) |  |
| --- | --- | --- |
| Query 1 | MLHGASSRPATARKSSGLSGTVRIPGDKSISHRSFMFGGLASGETRITGLLEGEDVINTG | 60 |
| Sbjct 1 | M H A++ PATARKS L+GT R+PGDKSISHRSFMFGGLASGETRITGLLEGEDV+ TG MSHSAAAAPATARKSPALAGTARVPGDKSISHRSFMFGGLASGETRITGLLEGEDVMRTG | 60 |
| Query 61 | KAMQAMGARIRKEGDTWIIDGVGNGGLLAPEAPLDFGNAATGCRLTMGLVGVYDFDSTFI | 120 |
| Sbjct 61 | AM+AMGA I K+G W+I G GNG LL PE PLDFGNA TG RLTMGLVG YD ++TFI AAMKAMGAHIEKKGAEWVIRGTGNGALLEPEGPLDFGNAGTGSRLTMGLVGTYDMETTFI | 120 |
| Query 121 | GDASLTKRPMGRVLNPLREMGVQV-KSEDGDRLPVTLRGPKTPTPITYRVPMASAQVKSA | 179 |
| Sbjct 121 | GDASL+ RPMGRVL PLR+MGVQV K+ GDR+P+TLRGPK PITYRVPMASAQVKSA GDASLSGRPMGRVLEPLRQMGVQVLKAASGDRMPITLRGPKHAAPITYRVPMASAQVKSA | 180 |
| Query 180 | VLLAGLNTPGITTVIEPIMTRDHTEKMLQGFGANLTVETDADGVRTIRLEGRGKLTGQVI | 239 |
| Sbjct 181 | VLLAGLNTPGITTVIEP+MTRDHTEKML+GFGANLTVETD GVR I +EG+GKLTGQ I VLLAGLNTPGITTVIEPVMTRDHTEKMLKGFGANLTVETDERGVRHIFIEGQGKLTGQTI | 240 |
| Query 240 | DVPGDPSSTAFPLVAALLVPGSDVTILNVLMNPTRTGLILTLQEMGADIEVINPRLAGGE VPGDPSS FPLVAAL+VPGSD+ I NVLMNPTRTGL+LTLQEMG I+++NPR AGGE | 299 |

| Sbjct | 241 | AVPGDPSSAGFPLVAALIVPGSDIVIENVLMNPTRTGLLLTLQEMGGRIDILNPRNAGGE | 300 |
| --- | --- | --- | --- |
| Query | 300 | DVADLRVRSSTLKGVTVPEDRAPSMIDEYPILavaaafaegaTVMNGLEELRVKESDRLS | 359 |
| Sbjct | 301 | DVADLRVRSS LKGV VP +RAPSMIDEYP+LAVAA+FAEG T+M GLEELRVKESDRLS DVADLRVRSSELKGVVVPPERAPSMIDEYPVLAVAASFAEGETLMQGLEELRVKESDRLS | 360 |
| Query | 360 | AVANGLKLNGVDCDEGETSLVVRGRPDGKGLG---NASGAAVATHLDHRIAMSFLVMGLV | 416 |
| Sbjct | 361 | AVANGLKLNGVDC EGE SL VRG+P GKGLG N AV THLDHRIAMSFL+MGL AVANGLKLNGVDCTEGEASLAVRGKPGGKGLGAHPNGQDTAVKTHLDHRIAMSFLIMGLA | 420 |
| Query  Sbjct | 417  421 | SENPVTVDDATMIATSFPEFMDLMAGLGAKIE 448  +E PVT+DDA MIATSFPEFM LM GLGA+IE  TEKPVTIDDANMIATSFPEFMGLMKGLGAEIE 452 |  |

>RWO60206.1 3-phosphoshikimate 1-carboxyvinyltransferase [Mesorhizobium sp.] Length=452

Score = 679 bits (1752), Expect = 0.0, Method: Compositional matrix adjust.

| Identities | = 350/451 (78%), Positives = 381/451 (84%), Gaps = 4/451 (1%) |  |
| --- | --- | --- |
| Query 1 | MLHGASSRPATARKSSGLSGTVRIPGDKSISHRSFMFGGLASGETRITGLLEGEDVINTG | 60 |
| Sbjct 1 | M H A+ PATAR S LSGT R+PGDKSISHRSFMFGGLASGETRITGLLEGEDV+ TG MSHTAAPSPATARTSPALSGTARVPGDKSISHRSFMFGGLASGETRITGLLEGEDVMRTG | 60 |
| Query 61 | KAMQAMGARIRKEGDTWIIDGVGNGGLLAPEAPLDFGNAATGCRLTMGLVGVYDFDSTFI | 120 |
| Sbjct 61 | AM+AMGA I K G W+I G GNG LL PE PLDFGNA TG RLTMGLVG YD ++TFI AAMKAMGAHIEKRGTEWVIRGTGNGALLQPEGPLDFGNAGTGSRLTMGLVGTYDMETTFI | 120 |
| Query 121 | GDASLTKRPMGRVLNPLREMGVQV-KSEDGDRLPVTLRGPKTPTPITYRVPMASAQVKSA | 179 |
| Sbjct 121 | GDASL+ RPMGRVL PLR+MGVQV K+ GDR+P+TLRGPK PITYRVPMASAQVKSA GDASLSGRPMGRVLEPLRQMGVQVLKATPGDRMPITLRGPKHAAPITYRVPMASAQVKSA | 180 |
| Query 180 | VLLAGLNTPGITTVIEPIMTRDHTEKMLQGFGANLTVETDADGVRTIRLEGRGKLTGQVI | 239 |
| Sbjct 181 | VLLAGLNTPGITTVIEP+MTRDHTEKMLQGFGANL+VETD GVR I +EG+GKLTGQ I VLLAGLNTPGITTVIEPVMTRDHTEKMLQGFGANLSVETDERGVRHIFIEGQGKLTGQTI | 240 |
| Query 240 | DVPGDPSSTAFPLVAALLVPGSDVTILNVLMNPTRTGLILTLQEMGADIEVINPRLAGGE | 299 |
| Sbjct 241 | VPGDPSS FPLVAAL+VPGSD+TI NVLMNPTRTGL+LTLQEMGA I+V+ PR GGE AVPGDPSSAGFPLVAALIVPGSDITIENVLMNPTRTGLLLTLQEMGARIDVLEPRNEGGE | 300 |
| Query 300 | DVADLRVRSSTLKGVTVPEDRAPSMIDEYPILavaaafaegaTVMNGLEELRVKESDRLS | 359 |
| Sbjct 301 | DVADLRVR S L+GVTVP +RAP+MIDEYP+LAVAA+FAEG T+M GLEELRVKESDRLS DVADLRVRYSELRGVTVPPERAPTMIDEYPVLAVAASFAEGETLMQGLEELRVKESDRLS | 360 |
| Query 360 | AVANGLKLNGVDCDEGETSLVVRGRPDGKGLG---NASGAAVATHLDHRIAMSFLVMGLV | 416 |
| Sbjct 361 | AVA GLKLNGVDC EGE SL VRG+P GKGLG N V THLDHRIAMSFLVMGL AVAEGLKLNGVDCTEGEASLAVRGKPGGKGLGQHPNGKSTIVQTHLDHRIAMSFLVMGLA | 420 |
| Query 417  Sbjct 421 | SENPVTVDDATMIATSFPEFMDLMAGLGAKI 447  +E PVT+DD MIATSFPEFM LM GLGA+I  TEKPVTIDDQAMIATSFPEFMGLMKGLGAEI 451 |  |

>TIP26977.1 3-phosphoshikimate 1-carboxyvinyltransferase [Mesorhizobium sp.] Length=452

Score = 679 bits (1752), Expect = 0.0, Method: Compositional matrix adjust.

| Identities | = 349/452 (77%), Positives = 384/452 (85%), Gaps = 4/452 (1%) |  |
| --- | --- | --- |
| Query 1 | MLHGASSRPATARKSSGLSGTVRIPGDKSISHRSFMFGGLASGETRITGLLEGEDVINTG | 60 |
| Sbjct 1 | M H A+ +PATARKS LSGT R+PGDKSISHRSFMFGGLASGETRITGLLEGEDV+ TG MSHTAAPKPATARKSPALSGTARVPGDKSISHRSFMFGGLASGETRITGLLEGEDVMRTG | 60 |
| Query 61 | KAMQAMGARIRKEGDTWIIDGVGNGGLLAPEAPLDFGNAATGCRLTMGLVGVYDFDSTFI | 120 |
| Sbjct 61 | AM+AMGA + K G W+I G GNG LL PE PLDFGNA TG RLTMGLVG YD ++TFI AAMKAMGAHVEKRGTEWVIRGTGNGALLQPEGPLDFGNAGTGSRLTMGLVGTYDMETTFI | 120 |
| Query 121 | GDASLTKRPMGRVLNPLREMGVQV-KSEDGDRLPVTLRGPKTPTPITYRVPMASAQVKSA | 179 |
| Sbjct 121 | GDASL+ RPMGRVL PLR+MGVQV K+ GDR+P+TLRGPK PITYRVPMASAQVKSA  GDASLSGRPMGRVLEPLRQMGVQVLKATPGDRMPITLRGPKHTAPITYRVPMASAQVKSA | 180 |

| Query | 180 | VLLAGLNTPGITTVIEPIMTRDHTEKMLQGFGANLTVETDADGVRTIRLEGRGKLTGQVI | 239 |
| --- | --- | --- | --- |
| Sbjct | 181 | VLLAGLNTPGITTVIEP+MTRDHTEKML+GFGANL+VETD GVR I +EG+GKLTGQ I VLLAGLNTPGITTVIEPVMTRDHTEKMLKGFGANLSVETDERGVRHIFIEGQGKLTGQTI | 240 |
| Query | 240 | DVPGDPSSTAFPLVAALLVPGSDVTILNVLMNPTRTGLILTLQEMGADIEVINPRLAGGE | 299 |
| Sbjct | 241 | VPGDPSS FPLVAAL+VPGSD+TI NVLMNPTRTGL+LTLQEMGA I++++PR GGE AVPGDPSSAGFPLVAALIVPGSDITIENVLMNPTRTGLLLTLQEMGARIDIVDPRNEGGE | 300 |
| Query | 300 | DVADLRVRSSTLKGVTVPEDRAPSMIDEYPILavaaafaegaTVMNGLEELRVKESDRLS | 359 |
| Sbjct | 301 | DVADLRVR S LKGVTVP +RAP+MIDEYP+LAVAA+FAEG T+M GLEELRVKESDRLS DVADLRVRYSELKGVTVPPERAPTMIDEYPVLAVAASFAEGKTLMEGLEELRVKESDRLS | 360 |
| Query | 360 | AVANGLKLNGVDCDEGETSLVVRGRPDGKGLG---NASGAAVATHLDHRIAMSFLVMGLV | 416 |
| Sbjct | 361 | AVA GLKLNGVDC EGE SL VRG+P GKGLG N V T LDHRIAMSFLVMGL AVAEGLKLNGVDCTEGEASLAVRGKPGGKGLGQHPNGPNTVVQTQLDHRIAMSFLVMGLA | 420 |
| Query  Sbjct | 417  421 | SENPVTVDDATMIATSFPEFMDLMAGLGAKIE 448  +E PVT+DD MIATSFPEFM LM GLGA+IE  TEKPVTIDDHAMIATSFPEFMGLMKGLGAEIE 452 |  |

>RWN38360.1 3-phosphoshikimate 1-carboxyvinyltransferase [Mesorhizobium sp.] RWP00638.1 3-phosphoshikimate 1-carboxyvinyltransferase [Mesorhizobium sp.] RWP45455.1 3-phosphoshikimate 1-carboxyvinyltransferase [Mesorhizobium sp.] RWP78204.1 3-phosphoshikimate 1-carboxyvinyltransferase [Mesorhizobium sp.] RWQ39055.1 3-phosphoshikimate 1-carboxyvinyltransferase [Mesorhizobium sp.]

Length=452

Score = 679 bits (1751), Expect = 0.0, Method: Compositional matrix adjust.

| Identities | = 349/452 (77%), Positives = 384/452 (85%), Gaps = 4/452 (1%) |  |
| --- | --- | --- |
| Query 1 | MLHGASSRPATARKSSGLSGTVRIPGDKSISHRSFMFGGLASGETRITGLLEGEDVINTG | 60 |
| Sbjct 1 | M H A+ +PATARKSS LSGT R+PGDKSISHRSFMFGGLASGETRITGLLEGEDV+ TG MSHTAAPKPATARKSSALSGTARVPGDKSISHRSFMFGGLASGETRITGLLEGEDVMRTG | 60 |
| Query 61 | KAMQAMGARIRKEGDTWIIDGVGNGGLLAPEAPLDFGNAATGCRLTMGLVGVYDFDSTFI | 120 |
| Sbjct 61 | AM+AMGA I K G W+I G GNG LL PE PLDFGNA TG RLTMGLVG YD ++TFI AAMKAMGAHIEKHGTEWVIRGTGNGALLQPEGPLDFGNAGTGSRLTMGLVGTYDMETTFI | 120 |
| Query 121 | GDASLTKRPMGRVLNPLREMGVQV-KSEDGDRLPVTLRGPKTPTPITYRVPMASAQVKSA | 179 |
| Sbjct 121 | GDASL+ RPMGRVL PLR+MGVQV K+ GDR+P+TL GPK PITYRVPMASAQVKSA GDASLSGRPMGRVLEPLRQMGVQVLKATPGDRMPITLHGPKHAAPITYRVPMASAQVKSA | 180 |
| Query 180 | VLLAGLNTPGITTVIEPIMTRDHTEKMLQGFGANLTVETDADGVRTIRLEGRGKLTGQVI | 239 |
| Sbjct 181 | VLLAGLNTPG+TTVIEP+MTRDHTEKML+GFGANL+VETD GVR I +EG+GKLTGQ I VLLAGLNTPGVTTVIEPVMTRDHTEKMLKGFGANLSVETDERGVRHIFIEGQGKLTGQTI | 240 |
| Query 240 | DVPGDPSSTAFPLVAALLVPGSDVTILNVLMNPTRTGLILTLQEMGADIEVINPRLAGGE | 299 |
| Sbjct 241 | VPGDPSS FPLVAAL+VPGSD+TI NVLMNPTRTGL+LTLQEMGA I++++PR GGE AVPGDPSSAGFPLVAALIVPGSDITIENVLMNPTRTGLLLTLQEMGARIDILDPRNEGGE | 300 |
| Query 300 | DVADLRVRSSTLKGVTVPEDRAPSMIDEYPILavaaafaegaTVMNGLEELRVKESDRLS | 359 |
| Sbjct 301 | DVADLRVR S LKGVTVP +RAP+MIDEYP+LAVAA+FAEG T+M GLEELRVKESDRLS DVADLRVRYSELKGVTVPPERAPTMIDEYPVLAVAASFAEGETLMLGLEELRVKESDRLS | 360 |
| Query 360 | AVANGLKLNGVDCDEGETSLVVRGRPDGKGLG---NASGAAVATHLDHRIAMSFLVMGLV | 416 |
| Sbjct 361 | AVA GLKLNGVDC EGE +L VRG+P GKGLG N V THLDHRIAMSFLVMGL AVAEGLKLNGVDCAEGEATLAVRGKPGGKGLGRHPNGHNTVVQTHLDHRIAMSFLVMGLA | 420 |
| Query 417  Sbjct 421 | SENPVTVDDATMIATSFPEFMDLMAGLGAKIE 448  +E PVTVDD MIATSFPEFM LM LGA+IE  AEKPVTVDDQAMIATSFPEFMGLMKRLGAEIE 452 |  |

>TIR25212.1 3-phosphoshikimate 1-carboxyvinyltransferase [Mesorhizobium sp.] Length=452

Score = 678 bits (1750), Expect = 0.0, Method: Compositional matrix adjust. Identities = 353/452 (78%), Positives = 388/452 (86%), Gaps = 4/452 (1%)

Query 1	MLHGASSRPATARKSSGLSGTVRIPGDKSISHRSFMFGGLASGETRITGLLEGEDVINTG 60 M H A+++ ATARKS L+GT R+PGDKSISHRSFMFGGLASGETRITGLLEGEDV+ TG

| Sbjct | 1 | MSHSAAAKAATARKSPALAGTARVPGDKSISHRSFMFGGLASGETRITGLLEGEDVMRTG | 60 |
| --- | --- | --- | --- |
| Query | 61 | KAMQAMGARIRKEGDTWIIDGVGNGGLLAPEAPLDFGNAATGCRLTMGLVGVYDFDSTFI | 120 |
| Sbjct | 61 | AM+AMGA I K G W+I G GNG LL PE PLDFGNA TG RLTMGLVG YD ++TFI AAMKAMGAHIEKNGAEWVIRGTGNGALLQPEGPLDFGNAGTGSRLTMGLVGTYDMETTFI | 120 |
| Query | 121 | GDASLTKRPMGRVLNPLREMGVQV-KSEDGDRLPVTLRGPKTPTPITYRVPMASAQVKSA | 179 |
| Sbjct | 121 | GDASL+ RPMGRVL+PLR+MGVQV K+ GDR+P+TLRGPK PITYRVPMASAQVKSA GDASLSGRPMGRVLDPLRQMGVQVLKAAPGDRMPITLRGPKHAAPITYRVPMASAQVKSA | 180 |
| Query | 180 | VLLAGLNTPGITTVIEPIMTRDHTEKMLQGFGANLTVETDADGVRTIRLEGRGKLTGQVI | 239 |
| Sbjct | 181 | VLLAGLNTPGITTVIEP+MTRDHTEKML+GFGANLTVETD GVR I +EG+GKL+GQ I VLLAGLNTPGITTVIEPVMTRDHTEKMLKGFGANLTVETDERGVRHIFIEGQGKLSGQTI | 240 |
| Query | 240 | DVPGDPSSTAFPLVAALLVPGSDVTILNVLMNPTRTGLILTLQEMGADIEVINPRLAGGE | 299 |
| Sbjct | 241 | VPGDPSS FPLVAAL+VPGSD+ I NVLMNPTRTGL+LTLQEMG I+++NPR AGGE AVPGDPSSAGFPLVAALIVPGSDIVIENVLMNPTRTGLLLTLQEMGGRIDILNPRNAGGE | 300 |
| Query | 300 | DVADLRVRSSTLKGVTVPEDRAPSMIDEYPILavaaafaegaTVMNGLEELRVKESDRLS | 359 |
| Sbjct | 301 | DVADLRVRSS LKGV VP +RAPSMIDEYP+LAVAA+FAEG T+M GLEELRVKESDRLS DVADLRVRSSELKGVVVPPERAPSMIDEYPVLAVAASFAEGETLMQGLEELRVKESDRLS | 360 |
| Query | 360 | AVANGLKLNGVDCDEGETSLVVRGRPDGKGLG---NASGAAVATHLDHRIAMSFLVMGLV | 416 |
| Sbjct | 361 | AVANGLKLNGVDC EGE SL VRGRP GKGLG N AV THLDHRIAMSFL+MGL AVANGLKLNGVDCTEGEASLAVRGRPGGKGLGAHPNGQDTAVKTHLDHRIAMSFLIMGLA | 420 |
| Query  Sbjct | 417  421 | SENPVTVDDATMIATSFPEFMDLMAGLGAKIE 448  +E PVT+DDA MIATSFPEFM LM GLGA+IE  TEKPVTIDDANMIATSFPEFMGLMKGLGAEIE 452 |  |

>WP_129412169.1 3-phosphoshikimate 1-carboxyvinyltransferase [Mesorhizobium sp. Pch-S]

QAZ43313.1 3-phosphoshikimate 1-carboxyvinyltransferase [Mesorhizobium sp. Pch-S]

Length=450

Score = 678 bits (1750), Expect = 0.0, Method: Compositional matrix adjust.

| Identities | | = 352/450 (78%), Positives = 385/450 (86%), Gaps = 2/450 (0%) |  |
| --- | --- | --- | --- |
| Query 1  Sbjct 1 | | MLHGASSRPATARKSSGLSGTVRIPGDKSISHRSFMFGGLASGETRITGLLEGEDVINTG M H A+ +PATAR+S LSGT R+PGDKSISHRSFMFGGLASGETRITGLLEGEDV+ TG MAHDATPQPATARRSPPLSGTARVPGDKSISHRSFMFGGLASGETRITGLLEGEDVMRTG | 60  60 |
| Query 61  Sbjct 61 | | KAMQAMGARIRKEGDTWIIDGVGNGGLLAPEAPLDFGNAATGCRLTMGLVGVYDFDSTFI  +AM+AMGA I K GD W+I G GNG LL PEAPLDFGNA TG RLTMGLVG YD ++TFI QAMKAMGAHIEKHGDEWVIRGTGNGALLQPEAPLDFGNAGTGSRLTMGLVGTYDMETTFI | 120  120 |
| Query 121  Sbjct 121 | | GDASLTKRPMGRVLNPLREMGVQV-KSEDGDRLPVTLRGPKTPTPITYRVPMASAQVKSA GDASL+ RPMGRVL PLR+MGVQV ++ GDR+P+TLRGPK PITYRVPMASAQVKSA GDASLSSRPMGRVLEPLRQMGVQVLQAAPGDRMPITLRGPKHAAPITYRVPMASAQVKSA | 179  180 |
| Query 180  Sbjct 181 | | VLLAGLNTPGITTVIEPIMTRDHTEKMLQGFGANLTVETDADGVRTIRLEGRGKLTGQVI VLLAGLNTPGITTVIEP+MTRDHTEKML+GFGANLTVETD GVR I +EG+GKLTGQVI VLLAGLNTPGITTVIEPVMTRDHTEKMLKGFGANLTVETDERGVRHIFIEGQGKLTGQVI | 239  240 |
| Query 240  Sbjct 241 | | DVPGDPSSTAFPLVAALLVPGSDVTILNVLMNPTRTGLILTLQEMGADIEVINPRLAGGE VPGDPSS FPLVAAL+VPGSD+TI NVLMNPTRTGL+LTLQEMG I+++NPR AGGE AVPGDPSSAGFPLVAALIVPGSDITIENVLMNPTRTGLLLTLQEMGGQIDILNPRNAGGE | 299  300 |
| Query 300  Sbjct 301 | | DVADLRVRSSTLKGVTVPEDRAPSMIDEYPILavaaafaegaTVMNGLEELRVKESDRLS DVADLRVRSS LKGVTVP +RAPSMIDEYP+LAVAAAFAEG TVM GLEELRVKESDRL+ DVADLRVRSSELKGVTVPAERAPSMIDEYPVLAVAAAFAEGETVMQGLEELRVKESDRLA | 359  360 |
| Query 360  Sbjct 361 | | AVANGLKLNGVDCDEGETSLVVR-GRPDGKGLGNASGAAVATHLDHRIAMSFLVMGLVSE AVANGLKLNGVDC EGE SL VR G A G AV THLDHRIAMSFL+MGL +E AVANGLKLNGVDCTEGEVSLSVRGVPGGKGLGGEAKGEAVKTHLDHRIAMSFLIMGLATE | 418  420 |
| Query 419 | | NPVTVDDATMIATSFPEFMDLMAGLGAKIE 448 PVTVDD MIATSFPEFM LM LGA+I+ |  |
| Sbjct | 421 | KPVTVDDRAMIATSFPEFMGLMTRLGAEID 450 | |

>WP_077375103.1 3-phosphoshikimate 1-carboxyvinyltransferase [Mesorhizobium prunaredense] SIT54318.1 3-phosphoshikimate 1-carboxyvinyltransferase [Mesorhizobium prunaredense]

Length=452

Score = 678 bits (1750), Expect = 0.0, Method: Compositional matrix adjust.

| Identities | = 348/451 (77%), Positives = 382/451 (85%), Gaps = 4/451 (1%) |  |
| --- | --- | --- |
| Query 1 | MLHGASSRPATARKSSGLSGTVRIPGDKSISHRSFMFGGLASGETRITGLLEGEDVINTG | 60 |
| Sbjct 1 | M H A+ +PATARKS LSGT R+PGDKSISHRSFMFGGLASGETRITGLLEGEDV+ TG MSHTAAPKPATARKSPALSGTARVPGDKSISHRSFMFGGLASGETRITGLLEGEDVMRTG | 60 |
| Query 61 | KAMQAMGARIRKEGDTWIIDGVGNGGLLAPEAPLDFGNAATGCRLTMGLVGVYDFDSTFI | 120 |
| Sbjct 61 | AM+AMGA I K G W+I G GNG LL PE PLDFGNA TG RLTMGLVG YD ++TFI AAMKAMGAHIEKRGTEWVIRGTGNGALLQPEGPLDFGNAGTGSRLTMGLVGTYDMETTFI | 120 |
| Query 121 | GDASLTKRPMGRVLNPLREMGVQV-KSEDGDRLPVTLRGPKTPTPITYRVPMASAQVKSA | 179 |
| Sbjct 121 | GDASL+ RPMGRVL PLR+MGVQV K+ GDR+P+TLRGPK PITYRVPMASAQVKSA GDASLSGRPMGRVLEPLRQMGVQVLKATPGDRMPITLRGPKHAAPITYRVPMASAQVKSA | 180 |
| Query 180 | VLLAGLNTPGITTVIEPIMTRDHTEKMLQGFGANLTVETDADGVRTIRLEGRGKLTGQVI | 239 |
| Sbjct 181 | VLLAGLNTPGITTVIEP+MTRDHTEKML+GFGANL+VETD GVR I +EG+GKL Q I VLLAGLNTPGITTVIEPVMTRDHTEKMLKGFGANLSVETDERGVRHIFIEGQGKLVAQTI | 240 |
| Query 240 | DVPGDPSSTAFPLVAALLVPGSDVTILNVLMNPTRTGLILTLQEMGADIEVINPRLAGGE | 299 |
| Sbjct 241 | VPGDPSS FPLVAAL+VPGSD+TI NVLMNPTRTGL+LTLQEMGA I++++PR GGE AVPGDPSSAGFPLVAALIVPGSDITIENVLMNPTRTGLLLTLQEMGARIDIVDPRNEGGE | 300 |
| Query 300 | DVADLRVRSSTLKGVTVPEDRAPSMIDEYPILavaaafaegaTVMNGLEELRVKESDRLS | 359 |
| Sbjct 301 | DVADLRVR S LKGVTVP +RAP+MIDEYP+LAVAA+FAEG T+M GLEELRVKESDRLS DVADLRVRYSELKGVTVPPERAPAMIDEYPVLAVAASFAEGETLMQGLEELRVKESDRLS | 360 |
| Query 360 | AVANGLKLNGVDCDEGETSLVVRGRPDGKGLG---NASGAAVATHLDHRIAMSFLVMGLV | 416 |
| Sbjct 361 | AVA GLKLNGVDC EGE SL VRG+P GKGLG N V THLDHRIAMSFLVMGL AVAEGLKLNGVDCTEGEASLAVRGKPGGKGLGQHPNGKSTIVQTHLDHRIAMSFLVMGLA | 420 |
| Query 417  Sbjct 421 | SENPVTVDDATMIATSFPEFMDLMAGLGAKI 447  +E PVT+DD MIATSFPEFM LM GLGA+I  TEKPVTIDDQAMIATSFPEFMGLMKGLGAEI 451 |  |

>TIL70542.1 3-phosphoshikimate 1-carboxyvinyltransferase [Mesorhizobium sp.] TIL89707.1 3-phosphoshikimate 1-carboxyvinyltransferase [Mesorhizobium sp.] TIL97747.1 3-phosphoshikimate 1-carboxyvinyltransferase [Mesorhizobium sp.]

Length=452

Score = 678 bits (1750), Expect = 0.0, Method: Compositional matrix adjust.

| Identities | = 349/451 (77%), Positives = 383/451 (85%), Gaps = 4/451 (1%) |  |
| --- | --- | --- |
| Query 1 | MLHGASSRPATARKSSGLSGTVRIPGDKSISHRSFMFGGLASGETRITGLLEGEDVINTG | 60 |
| Sbjct 1 | M H A+ PATARKS LSGT R+PGDKSISHRSFMFGGLASGETRITGLLEGEDV+ TG MSHTAAPSPATARKSPALSGTARVPGDKSISHRSFMFGGLASGETRITGLLEGEDVMRTG | 60 |
| Query 61 | KAMQAMGARIRKEGDTWIIDGVGNGGLLAPEAPLDFGNAATGCRLTMGLVGVYDFDSTFI | 120 |
| Sbjct 61 | AM+AMGA I K G W+I G GNG LL PE PLDFGNA TG RLTMGLVG YD ++TFI AAMKAMGAHIEKRGTEWVIRGTGNGALLQPEGPLDFGNAGTGSRLTMGLVGTYDMETTFI | 120 |
| Query 121 | GDASLTKRPMGRVLNPLREMGVQV-KSEDGDRLPVTLRGPKTPTPITYRVPMASAQVKSA | 179 |
| Sbjct 121 | GDASL+ RPMGRVL PLR+MGVQV K+ GDR+P+TLRGPK PITYRVPMASAQVKSA GDASLSGRPMGRVLEPLRQMGVQVLKATPGDRMPITLRGPKHAAPITYRVPMASAQVKSA | 180 |
| Query 180 | VLLAGLNTPGITTVIEPIMTRDHTEKMLQGFGANLTVETDADGVRTIRLEGRGKLTGQVI | 239 |
| Sbjct 181 | VLLAGLNTPGITTVIEP+MTRDHTEKML+GFGANL+VETD GVR I +EG+GKLTGQ I VLLAGLNTPGITTVIEPVMTRDHTEKMLKGFGANLSVETDERGVRHIFIEGQGKLTGQTI | 240 |
| Query 240 | DVPGDPSSTAFPLVAALLVPGSDVTILNVLMNPTRTGLILTLQEMGADIEVINPRLAGGE | 299 |
| Sbjct 241 | VPGDPSS FPLVAAL+VPGSD+TI NVLMNPTRTGL+LTLQEMGA I+V++PR GGE AVPGDPSSAGFPLVAALIVPGSDITIENVLMNPTRTGLLLTLQEMGARIDVLDPRNEGGE | 300 |
| Query 300 | DVADLRVRSSTLKGVTVPEDRAPSMIDEYPILavaaafaegaTVMNGLEELRVKESDRLS | 359 |
| Sbjct 301 | DVADLRVR S LKGVTVP +RAP+MIDEYP+LAVAA+FAEG T+M GLEELRVKESDRLS  DVADLRVRYSELKGVTVPPERAPTMIDEYPVLAVAASFAEGETLMQGLEELRVKESDRLS | 360 |

| Query | 360 | AVANGLKLNGVDCDEGETSLVVRGRPDGKGLG---NASGAAVATHLDHRIAMSFLVMGLV 416 |
| --- | --- | --- |
| Sbjct | 361 | AVA GLKLNGVDC EGE +L VRG+P GKGLG N V THLDHRIAMSFLVMGL AVAEGLKLNGVDCTEGEATLAVRGKPGGKGLGQHPNGKSTIVQTHLDHRIAMSFLVMGLA 420 |
| Query | 417 | SENPVTVDDATMIATSFPEFMDLMAGLGAKI 447 |
| Sbjct | 421 | +E PVT+DD MIATSFP+FM LM GLGA+I  TEKPVTIDDQAMIATSFPQFMGLMKGLGAEI 451 |

>WP_065006055.1 3-phosphoshikimate 1-carboxyvinyltransferase [Mesorhizobium sp. AA22]

QIA20545.1 3-phosphoshikimate 1-carboxyvinyltransferase [Mesorhizobium sp. AA22]

Length=452

Score = 678 bits (1750), Expect = 0.0, Method: Compositional matrix adjust.

| Identities | = 348/452 (77%), Positives = 382/452 (85%), Gaps = 4/452 (1%) |  |
| --- | --- | --- |
| Query 1 | MLHGASSRPATARKSSGLSGTVRIPGDKSISHRSFMFGGLASGETRITGLLEGEDVINTG | 60 |
| Sbjct 1 | M H A+ +PATARKSS LSG R+PGDKSISHRSFMFGGLASGETRITGLLEGEDV+ TG MSHTAAPKPATARKSSALSGAARVPGDKSISHRSFMFGGLASGETRITGLLEGEDVMRTG | 60 |
| Query 61 | KAMQAMGARIRKEGDTWIIDGVGNGGLLAPEAPLDFGNAATGCRLTMGLVGVYDFDSTFI | 120 |
| Sbjct 61 | AM+AMGA I K G W+I G GNG LL PE PLDFGNA TG RLTMGLVG YD ++TFI AAMKAMGAHIEKLGTEWVIRGTGNGALLQPEGPLDFGNAGTGSRLTMGLVGTYDMETTFI | 120 |
| Query 121 | GDASLTKRPMGRVLNPLREMGVQV-KSEDGDRLPVTLRGPKTPTPITYRVPMASAQVKSA | 179 |
| Sbjct 121 | GDASL+ RPMGRVL PLR+MGVQV K+ GDR+P+TL GPK PITYRVPMASAQVKSA GDASLSGRPMGRVLEPLRQMGVQVLKATPGDRMPITLHGPKHAAPITYRVPMASAQVKSA | 180 |
| Query 180 | VLLAGLNTPGITTVIEPIMTRDHTEKMLQGFGANLTVETDADGVRTIRLEGRGKLTGQVI | 239 |
| Sbjct 181 | VLLAGLNTPG+TTVIEP MTRDHTEKML+GFGANL+VETD GVR I +EG+GKLTGQ I VLLAGLNTPGVTTVIEPAMTRDHTEKMLKGFGANLSVETDERGVRHIFIEGQGKLTGQTI | 240 |
| Query 240 | DVPGDPSSTAFPLVAALLVPGSDVTILNVLMNPTRTGLILTLQEMGADIEVINPRLAGGE | 299 |
| Sbjct 241 | VPGDPSS FPLVAAL+VPGSD+TI NVLMNPTRTGL+LTLQEMGA I++++PR GGE AVPGDPSSAGFPLVAALIVPGSDITIENVLMNPTRTGLLLTLQEMGARIDILDPRNEGGE | 300 |
| Query 300 | DVADLRVRSSTLKGVTVPEDRAPSMIDEYPILavaaafaegaTVMNGLEELRVKESDRLS | 359 |
| Sbjct 301 | DVADLRVR S LK VTVP +RAP+MIDEYP+LAVAA+FAEG T+M GLEELRVKESDRLS DVADLRVRHSELKAVTVPPERAPTMIDEYPVLAVAASFAEGETLMQGLEELRVKESDRLS | 360 |
| Query 360 | AVANGLKLNGVDCDEGETSLVVRGRPDGKGLG---NASGAAVATHLDHRIAMSFLVMGLV | 416 |
| Sbjct 361 | AVA GLKLNGVDC EGE SL VRG+P GKGLG N V THLDHRIAMSFLVMGL AVAEGLKLNGVDCTEGEASLAVRGKPGGKGLGRHPNGHNTVVQTHLDHRIAMSFLVMGLA | 420 |
| Query 417  Sbjct 421 | SENPVTVDDATMIATSFPEFMDLMAGLGAKIE 448  +E PVT+DD MIATSFPEFM LM GLGA+IE  TEKPVTIDDQAMIATSFPEFMGLMKGLGAEIE 452 |  |

>RWK53918.1 3-phosphoshikimate 1-carboxyvinyltransferase [Mesorhizobium sp.] Length=452

Score = 678 bits (1749), Expect = 0.0, Method: Compositional matrix adjust.

| Identities | = 348/452 (77%), Positives = 383/452 (85%), Gaps = 4/452 (1%) |  |
| --- | --- | --- |
| Query 1 | MLHGASSRPATARKSSGLSGTVRIPGDKSISHRSFMFGGLASGETRITGLLEGEDVINTG | 60 |
| Sbjct 1 | M H A+ +PATARKS LSGT R+PGDKSISHRSFMFGGLASGETR+TGLLEGEDV+ TG MSHTAAPKPATARKSPALSGTARVPGDKSISHRSFMFGGLASGETRVTGLLEGEDVMRTG | 60 |
| Query 61 | KAMQAMGARIRKEGDTWIIDGVGNGGLLAPEAPLDFGNAATGCRLTMGLVGVYDFDSTFI | 120 |
| Sbjct 61 | AM+AMGA I K G W+I G GNG LL PE PLDFGNA TG RLTMGLVG YD ++TFI AAMKAMGAHIEKRGTEWVIRGTGNGALLQPEGPLDFGNAGTGSRLTMGLVGTYDMETTFI | 120 |
| Query 121 | GDASLTKRPMGRVLNPLREMGVQV-KSEDGDRLPVTLRGPKTPTPITYRVPMASAQVKSA | 179 |
| Sbjct 121 | GDASL+ RPMGRVL PLR+MGVQV K+ GDR+P+TLRGPK PITYRVPMASAQVKSA GDASLSGRPMGRVLEPLRQMGVQVLKATPGDRMPITLRGPKHAAPITYRVPMASAQVKSA | 180 |
| Query 180 | VLLAGLNTPGITTVIEPIMTRDHTEKMLQGFGANLTVETDADGVRTIRLEGRGKLTGQVI VLLAGLNTPGITTVIEP+MTRDHTEKML+GFGANL+VETD GVR I +EG+GKLTGQ I | 239 |

| Sbjct | 181 | VLLAGLNTPGITTVIEPVMTRDHTEKMLKGFGANLSVETDERGVRHIFIEGQGKLTGQTI | 240 |
| --- | --- | --- | --- |
| Query | 240 | DVPGDPSSTAFPLVAALLVPGSDVTILNVLMNPTRTGLILTLQEMGADIEVINPRLAGGE | 299 |
| Sbjct | 241 | VPGDPSS FPLVAAL+VPGSD+TI NVLMNPTRTGL+LTLQEMGA I++++PR GGE AVPGDPSSAGFPLVAALIVPGSDITIENVLMNPTRTGLLLTLQEMGARIDIVDPRNEGGE | 300 |
| Query | 300 | DVADLRVRSSTLKGVTVPEDRAPSMIDEYPILavaaafaegaTVMNGLEELRVKESDRLS | 359 |
| Sbjct | 301 | DVADLRVR S LKGV VP +RAP+MIDEYP+LAVAA+FAEG T+M GLEELRVKESDRLS DVADLRVRYSELKGVAVPPERAPTMIDEYPVLAVAASFAEGETLMEGLEELRVKESDRLS | 360 |
| Query | 360 | AVANGLKLNGVDCDEGETSLVVRGRPDGKGLG---NASGAAVATHLDHRIAMSFLVMGLV | 416 |
| Sbjct | 361 | AVA GLKLNGVDC EGE SL VRG+P GKGLG N V T LDHRIAMSFLVMGL AVAEGLKLNGVDCTEGEASLAVRGKPGGKGLGQHPNGPNTVVQTQLDHRIAMSFLVMGLA | 420 |
| Query  Sbjct | 417  421 | SENPVTVDDATMIATSFPEFMDLMAGLGAKIE 448  +E PVT+DD MIATSFPEFM LM GLGA+IE  TEKPVTIDDQAMIATSFPEFMGLMKGLGAEIE 452 |  |

>TIP80571.1 3-phosphoshikimate 1-carboxyvinyltransferase [Mesorhizobium sp.] Length=452

Score = 678 bits (1749), Expect = 0.0, Method: Compositional matrix adjust.

| Identities | = 347/452 (77%), Positives = 384/452 (85%), Gaps = 4/452 (1%) |  |
| --- | --- | --- |
| Query 1 | MLHGASSRPATARKSSGLSGTVRIPGDKSISHRSFMFGGLASGETRITGLLEGEDVINTG | 60 |
| Sbjct 1 | M H A+ +PATARKS LSGT R+PGDKSISHRSFMFGGLASGETRITGLLEGEDV+ TG MSHTAAPKPATARKSPALSGTARVPGDKSISHRSFMFGGLASGETRITGLLEGEDVMRTG | 60 |
| Query 61 | KAMQAMGARIRKEGDTWIIDGVGNGGLLAPEAPLDFGNAATGCRLTMGLVGVYDFDSTFI | 120 |
| Sbjct 61 | AM+AMGA I K G W+I G GNG LL PE PLDFGNA TG RLTMGLVG YD ++TFI AAMKAMGAHIEKRGAEWVIRGTGNGALLQPEGPLDFGNAGTGSRLTMGLVGTYDMETTFI | 120 |
| Query 121 | GDASLTKRPMGRVLNPLREMGVQV-KSEDGDRLPVTLRGPKTPTPITYRVPMASAQVKSA | 179 |
| Sbjct 121 | GDASL+ RPMGRVL PLR+MGVQV K+ GDR+P+TLRGPK PITYRVPMASAQVKSA GDASLSGRPMGRVLEPLRQMGVQVLKATPGDRMPITLRGPKHTAPITYRVPMASAQVKSA | 180 |
| Query 180 | VLLAGLNTPGITTVIEPIMTRDHTEKMLQGFGANLTVETDADGVRTIRLEGRGKLTGQVI | 239 |
| Sbjct 181 | VLLAGLNTPG+TTVIEP+MTRDHTEKML+GFGANL+VETD GVR I +EG+GKLTGQ I VLLAGLNTPGVTTVIEPVMTRDHTEKMLKGFGANLSVETDERGVRHIFIEGQGKLTGQTI | 240 |
| Query 240 | DVPGDPSSTAFPLVAALLVPGSDVTILNVLMNPTRTGLILTLQEMGADIEVINPRLAGGE | 299 |
| Sbjct 241 | VPGDPSS FPLVAAL+VPGSD+TI NVLMNPTRTGL+LTLQEMGA I++++PR GGE AVPGDPSSAGFPLVAALIVPGSDITIENVLMNPTRTGLLLTLQEMGARIDIVDPRNEGGE | 300 |
| Query 300 | DVADLRVRSSTLKGVTVPEDRAPSMIDEYPILavaaafaegaTVMNGLEELRVKESDRLS | 359 |
| Sbjct 301 | DVADLRVR S LKGV VP +RAP+MIDEYP+LAVAA+FAEG T+M GLEELRVKESDRLS DVADLRVRYSELKGVAVPPERAPTMIDEYPVLAVAASFAEGETLMEGLEELRVKESDRLS | 360 |
| Query 360 | AVANGLKLNGVDCDEGETSLVVRGRPDGKGLG---NASGAAVATHLDHRIAMSFLVMGLV | 416 |
| Sbjct 361 | AVA GLK+NGVDC EGE SL VRGRP GKGLG N V THLDHRIAMSFLVMGL AVAEGLKVNGVDCTEGEASLAVRGRPGGKGLGQHPNGQNTVVQTHLDHRIAMSFLVMGLA | 420 |
| Query 417  Sbjct 421 | SENPVTVDDATMIATSFPEFMDLMAGLGAKIE 448  +E PVT+DD MIATSFPEF+ LM GLGA+I+  TEKPVTIDDQAMIATSFPEFVGLMKGLGAEID 452 |  |

>RWG78543.1 3-phosphoshikimate 1-carboxyvinyltransferase [Mesorhizobium sp.] RWK22539.1 3-phosphoshikimate 1-carboxyvinyltransferase [Mesorhizobium sp.]

Length=452

Score = 678 bits (1749), Expect = 0.0, Method: Compositional matrix adjust.

| Identities | = 354/452 (78%), Positives = 388/452 (86%), Gaps = 4/452 (1%) |  |
| --- | --- | --- |
| Query 1 | MLHGASSRPATARKSSGLSGTVRIPGDKSISHRSFMFGGLASGETRITGLLEGEDVINTG | 60 |
| Sbjct 1 | M H A++ PATARKS L+GT R+PGDKSISHRSFMFGGLASGETRITGLLEGEDV+ TG MSHSAAAAPATARKSPALAGTARVPGDKSISHRSFMFGGLASGETRITGLLEGEDVMRTG | 60 |
| Query 61 | KAMQAMGARIRKEGDTWIIDGVGNGGLLAPEAPLDFGNAATGCRLTMGLVGVYDFDSTFI | 120 |
| Sbjct 61 | AM+AMGA I K+G W+I G GNG LL PE PLDFGNA TG RLTMGLVG YD ++TFI  AAMKAMGAHIEKKGAEWVIRGTGNGALLEPEGPLDFGNAGTGSRLTMGLVGTYDMETTFI | 120 |

| Query | 121 | GDASLTKRPMGRVLNPLREMGVQV-KSEDGDRLPVTLRGPKTPTPITYRVPMASAQVKSA | 179 |
| --- | --- | --- | --- |
| Sbjct | 121 | GDASL+ RPMGRVL PLR+MGVQV K+ GDR+P+TLRGPK PITYRVPMASAQVKSA GDASLSGRPMGRVLEPLRQMGVQVLKAAPGDRMPITLRGPKHAAPITYRVPMASAQVKSA | 180 |
| Query | 180 | VLLAGLNTPGITTVIEPIMTRDHTEKMLQGFGANLTVETDADGVRTIRLEGRGKLTGQVI | 239 |
| Sbjct | 181 | VLLAGLNTPGITTVIEP+MTRDHTEKML+GFGANLTVETD GVR I +EG+GKLTGQ I VLLAGLNTPGITTVIEPVMTRDHTEKMLKGFGANLTVETDERGVRHIFIEGQGKLTGQTI | 240 |
| Query | 240 | DVPGDPSSTAFPLVAALLVPGSDVTILNVLMNPTRTGLILTLQEMGADIEVINPRLAGGE | 299 |
| Sbjct | 241 | VPGDPSS FPLVAAL+VPGSD+ I NVLMNPTRTGL+LTLQEMG I+++NPR AGGE AVPGDPSSAGFPLVAALIVPGSDIVIENVLMNPTRTGLLLTLQEMGGRIDILNPRNAGGE | 300 |
| Query | 300 | DVADLRVRSSTLKGVTVPEDRAPSMIDEYPILavaaafaegaTVMNGLEELRVKESDRLS | 359 |
| Sbjct | 301 | DVADLRVRSS LKGV VP +RAPSMIDEYP+LAVAA+FAEG T+M GLEELRVKESDRLS DVADLRVRSSELKGVVVPPERAPSMIDEYPVLAVAASFAEGETLMQGLEELRVKESDRLS | 360 |
| Query | 360 | AVANGLKLNGVDCDEGETSLVVRGRPDGKGLG---NASGAAVATHLDHRIAMSFLVMGLV | 416 |
| Sbjct | 361 | AVANGLKLNGVDC EGE SL VRG+P GKGLG N AV THLDHRIAMSFL+MGL AVANGLKLNGVDCTEGEASLAVRGKPGGKGLGAHPNGQDTAVKTHLDHRIAMSFLIMGLA | 420 |
| Query  Sbjct | 417  421 | SENPVTVDDATMIATSFPEFMDLMAGLGAKIE 448  +E PVT+DDA MIATSFPEFM LM GLGA+IE  TEKPVTIDDANMIATSFPEFMGLMKGLGAEIE 452 |  |

>RWK33405.1 3-phosphoshikimate 1-carboxyvinyltransferase [Mesorhizobium sp.] RWK63693.1 3-phosphoshikimate 1-carboxyvinyltransferase [Mesorhizobium sp.] RWK72353.1 3-phosphoshikimate 1-carboxyvinyltransferase [Mesorhizobium sp.] RWK75247.1 3-phosphoshikimate 1-carboxyvinyltransferase [Mesorhizobium sp.] RWL00329.1 3-phosphoshikimate 1-carboxyvinyltransferase [Mesorhizobium sp.]

Length=452

Score = 677 bits (1748), Expect = 0.0, Method: Compositional matrix adjust.

| Identities | = 348/452 (77%), Positives = 384/452 (85%), Gaps = 4/452 (1%) |  |
| --- | --- | --- |
| Query 1 | MLHGASSRPATARKSSGLSGTVRIPGDKSISHRSFMFGGLASGETRITGLLEGEDVINTG | 60 |
| Sbjct 1 | M H A+ +PATARKS LSGT R+PGDKSISHRSFMFGGLASGETRITGLLEGEDV+ TG MSHTAAPKPATARKSPALSGTARVPGDKSISHRSFMFGGLASGETRITGLLEGEDVMRTG | 60 |
| Query 61 | KAMQAMGARIRKEGDTWIIDGVGNGGLLAPEAPLDFGNAATGCRLTMGLVGVYDFDSTFI | 120 |
| Sbjct 61 | AM+AMGA + K G W+I G GNG LL PE PLDFGNA TG RLTMGLVG YD ++TFI AAMKAMGAHVEKRGTEWVIRGTGNGALLQPEGPLDFGNAGTGSRLTMGLVGTYDMETTFI | 120 |
| Query 121 | GDASLTKRPMGRVLNPLREMGVQV-KSEDGDRLPVTLRGPKTPTPITYRVPMASAQVKSA | 179 |
| Sbjct 121 | GDASL+ RPMGRVL PLR+MGVQV K+ GDR+P+TLRGPK PITYRVPMASAQVKSA GDASLSGRPMGRVLEPLRQMGVQVLKATPGDRMPITLRGPKHTAPITYRVPMASAQVKSA | 180 |
| Query 180 | VLLAGLNTPGITTVIEPIMTRDHTEKMLQGFGANLTVETDADGVRTIRLEGRGKLTGQVI | 239 |
| Sbjct 181 | VLLAGLNTPGITTVIEP+MTRDHTEKML+GFGANL+VETD GVR I +EG+GKLTGQ I VLLAGLNTPGITTVIEPVMTRDHTEKMLKGFGANLSVETDERGVRHIFIEGQGKLTGQTI | 240 |
| Query 240 | DVPGDPSSTAFPLVAALLVPGSDVTILNVLMNPTRTGLILTLQEMGADIEVINPRLAGGE | 299 |
| Sbjct 241 | VPGDPSS FPLVAAL+VPGSD+TI NVLMNPTRTGL+LTLQEMGA I++++PR GGE AVPGDPSSAGFPLVAALIVPGSDITIENVLMNPTRTGLLLTLQEMGARIDIVDPRNEGGE | 300 |
| Query 300 | DVADLRVRSSTLKGVTVPEDRAPSMIDEYPILavaaafaegaTVMNGLEELRVKESDRLS | 359 |
| Sbjct 301 | DVADLRVR S LKGVTVP +RAP+MIDEYP+LAVAA+FAEG T+M GLEELRVKESDRLS DVADLRVRYSELKGVTVPPERAPTMIDEYPVLAVAASFAEGETLMEGLEELRVKESDRLS | 360 |
| Query 360 | AVANGLKLNGVDCDEGETSLVVRGRPDGKGLG---NASGAAVATHLDHRIAMSFLVMGLV | 416 |
| Sbjct 361 | AVA GLKLNGVDC EGE SL VRG+P GKGLG N V T LDHRIAMSFLVMGL AVAEGLKLNGVDCTEGEASLAVRGKPGGKGLGQHPNGPNTVVQTQLDHRIAMSFLVMGLA | 420 |
| Query 417  Sbjct 421 | SENPVTVDDATMIATSFPEFMDLMAGLGAKIE 448  +E PVT+DD MIATSFPEF+ LM GLGA+IE  TEKPVTIDDQAMIATSFPEFVGLMKGLGAEIE 452 |  |

>WP_095484342.1 MULTISPECIES: 3-phosphoshikimate 1-carboxyvinyltransferase [Mesorhizobium] PAQ01722.1 3-phosphoshikimate 1-carboxyvinyltransferase [Mesorhizobium mediterraneum] RUU30091.1 3-phosphoshikimate 1-carboxyvinyltransferase [Mesorhizobium sp.

M6A.T.Ce.TU.016.01.1.1]

RUU99345.1 3-phosphoshikimate 1-carboxyvinyltransferase [Mesorhizobium sp. M6A.T.Cr.TU.017.01.1.1]

RWN39742.1 3-phosphoshikimate 1-carboxyvinyltransferase [Mesorhizobium sp.] Length=452

Score = 677 bits (1748), Expect = 0.0, Method: Compositional matrix adjust.

| Identities | = 347/452 (77%), Positives = 384/452 (85%), Gaps = 4/452 (1%) |  |
| --- | --- | --- |
| Query 1 | MLHGASSRPATARKSSGLSGTVRIPGDKSISHRSFMFGGLASGETRITGLLEGEDVINTG | 60 |
| Sbjct 1 | M H A+ +PATARKSS LSGT R+PGDKSISHRSFMFGGLASGETRITGLLEGEDV+ TG MSHTAAPKPATARKSSALSGTARVPGDKSISHRSFMFGGLASGETRITGLLEGEDVMRTG | 60 |
| Query 61 | KAMQAMGARIRKEGDTWIIDGVGNGGLLAPEAPLDFGNAATGCRLTMGLVGVYDFDSTFI | 120 |
| Sbjct 61 | AM+AMGA I K G W+I G GNG LL PE PLDFGNA TG RLTMGLVG YD ++TFI AAMKAMGAHIEKHGTEWVIRGTGNGALLQPEGPLDFGNAGTGSRLTMGLVGTYDMETTFI | 120 |
| Query 121 | GDASLTKRPMGRVLNPLREMGVQV-KSEDGDRLPVTLRGPKTPTPITYRVPMASAQVKSA | 179 |
| Sbjct 121 | GDASL+ RPMGRVL PLR+MGVQV K+ GDR+P+TL GPK PITYRVPMASAQVKSA GDASLSGRPMGRVLEPLRQMGVQVLKATPGDRMPITLHGPKHAAPITYRVPMASAQVKSA | 180 |
| Query 180 | VLLAGLNTPGITTVIEPIMTRDHTEKMLQGFGANLTVETDADGVRTIRLEGRGKLTGQVI | 239 |
| Sbjct 181 | VLLAGLNTPG+TTVIEP+MTRDHTEKML+GFGANL+VETD GVR I +EG+GKLTGQ I VLLAGLNTPGVTTVIEPVMTRDHTEKMLKGFGANLSVETDERGVRHIFIEGQGKLTGQTI | 240 |
| Query 240 | DVPGDPSSTAFPLVAALLVPGSDVTILNVLMNPTRTGLILTLQEMGADIEVINPRLAGGE | 299 |
| Sbjct 241 | VPGDPSS FPLVAAL+VPGSD+TI NVLMNPTRTGL+LTLQEMGA I++++PR GGE AVPGDPSSAGFPLVAALIVPGSDITIENVLMNPTRTGLLLTLQEMGARIDILDPRNEGGE | 300 |
| Query 300 | DVADLRVRSSTLKGVTVPEDRAPSMIDEYPILavaaafaegaTVMNGLEELRVKESDRLS | 359 |
| Sbjct 301 | DVADLRVR S LKGVTVP +RAP+MIDEYP+LAVAA+FAEG T+M GLEELRVKESDRLS DVADLRVRYSELKGVTVPPERAPTMIDEYPVLAVAASFAEGETLMLGLEELRVKESDRLS | 360 |
| Query 360 | AVANGLKLNGVDCDEGETSLVVRGRPDGKGLG---NASGAAVATHLDHRIAMSFLVMGLV | 416 |
| Sbjct 361 | AVA GLKLNGVDC EGE +L VRG+P GKGLG N V THLDHRIAMSFL+MGL AVAEGLKLNGVDCTEGEATLAVRGKPGGKGLGQHPNGHNTVVQTHLDHRIAMSFLIMGLA | 420 |
| Query 417  Sbjct 421 | SENPVTVDDATMIATSFPEFMDLMAGLGAKIE 448  +E PVT+DD MIATSFPEFM LM LGA+IE  TEKPVTIDDQAMIATSFPEFMGLMKRLGAEIE 452 |  |

>WP_126079852.1 3-phosphoshikimate 1-carboxyvinyltransferase [Mesorhizobium sp. M2A.F.Ca.ET.046.03.2.1]

AZO35067.1 3-phosphoshikimate 1-carboxyvinyltransferase [Mesorhizobium sp. M2A.F.Ca.ET.046.03.2.1]

RVC70736.1 3-phosphoshikimate 1-carboxyvinyltransferase [Mesorhizobium sp. M00.F.Ca.ET.038.03.1.1]

RWB49984.1 3-phosphoshikimate 1-carboxyvinyltransferase [Mesorhizobium sp.] RWE11616.1 3-phosphoshikimate 1-carboxyvinyltransferase [Mesorhizobium sp.]

Length=452

Score = 677 bits (1748), Expect = 0.0, Method: Compositional matrix adjust.

| Identities | = 353/452 (78%), Positives = 388/452 (86%), Gaps = 4/452 (1%) |  |
| --- | --- | --- |
| Query 1 | MLHGASSRPATARKSSGLSGTVRIPGDKSISHRSFMFGGLASGETRITGLLEGEDVINTG | 60 |
| Sbjct 1 | M H A+++ ATARKS L+GT R+PGDKSISHRSFMFGGLASGETRITGLLEGEDV+ TG MSHSAAAKAATARKSPALAGTARVPGDKSISHRSFMFGGLASGETRITGLLEGEDVMRTG | 60 |
| Query 61 | KAMQAMGARIRKEGDTWIIDGVGNGGLLAPEAPLDFGNAATGCRLTMGLVGVYDFDSTFI | 120 |
| Sbjct 61 | AM+AMGA I K G W+I G GNG LL PE PLDFGNA TG RLTMGLVG YD ++TFI AAMKAMGAHIEKNGAEWVIRGTGNGALLQPEGPLDFGNAGTGSRLTMGLVGTYDMETTFI | 120 |
| Query 121 | GDASLTKRPMGRVLNPLREMGVQV-KSEDGDRLPVTLRGPKTPTPITYRVPMASAQVKSA | 179 |
| Sbjct 121 | GDASL+ RPMGRVL+PLR+MGVQV K+ GDR+P+TLRGPK PITYRVPMASAQVKSA GDASLSGRPMGRVLDPLRQMGVQVLKAAPGDRMPITLRGPKHAAPITYRVPMASAQVKSA | 180 |
| Query 180 | VLLAGLNTPGITTVIEPIMTRDHTEKMLQGFGANLTVETDADGVRTIRLEGRGKLTGQVI | 239 |
| Sbjct 181 | VLLAGLNTPGITTVIEP+MTRDHTEKML+GFGANLTVETD GVR I +EG+GKLTGQ I VLLAGLNTPGITTVIEPVMTRDHTEKMLKGFGANLTVETDERGVRHIFIEGQGKLTGQTI | 240 |
| Query 240 | DVPGDPSSTAFPLVAALLVPGSDVTILNVLMNPTRTGLILTLQEMGADIEVINPRLAGGE | 299 |

| Sbjct | 241 | VPGDPSS FPLVAAL+VPGSD+ I NVLMNPTRTGL+LTLQEMG I+++NPR AGGE AVPGDPSSAGFPLVAALIVPGSDIVIENVLMNPTRTGLLLTLQEMGGRIDILNPRNAGGE | 300 |
| --- | --- | --- | --- |
| Query | 300 | DVADLRVRSSTLKGVTVPEDRAPSMIDEYPILavaaafaegaTVMNGLEELRVKESDRLS | 359 |
| Sbjct | 301 | DVADLRVR S LKGVTVP +RAPSMIDEYP+LAVAA+FAEG T+M GLEELRVKESDRLS DVADLRVRYSELKGVTVPPERAPSMIDEYPVLAVAASFAEGETLMQGLEELRVKESDRLS | 360 |
| Query | 360 | AVANGLKLNGVDCDEGETSLVVRGRPDGKGLG---NASGAAVATHLDHRIAMSFLVMGLV | 416 |
| Sbjct | 361 | AVANGLKLNGVDC EGE SL VRGRP GKGLG N +V THLDHRIAMSFL+MGL AVANGLKLNGVDCTEGEASLAVRGRPGGKGLGAHPNGPDTSVKTHLDHRIAMSFLIMGLA | 420 |
| Query  Sbjct | 417  421 | SENPVTVDDATMIATSFPEFMDLMAGLGAKIE 448  +E PVT+DDA MIATSFPEFM LM GLGA+IE  TEKPVTIDDANMIATSFPEFMGLMKGLGAEIE 452 |  |

>TIP01447.1 3-phosphoshikimate 1-carboxyvinyltransferase [Mesorhizobium sp.] Length=452

Score = 677 bits (1748), Expect = 0.0, Method: Compositional matrix adjust.

| Identities | = 347/452 (77%), Positives = 383/452 (85%), Gaps = 4/452 (1%) |  |
| --- | --- | --- |
| Query 1 | MLHGASSRPATARKSSGLSGTVRIPGDKSISHRSFMFGGLASGETRITGLLEGEDVINTG | 60 |
| Sbjct 1 | M H A+++ ATAR S L+GT R+PGDKSISHRSFMFGGLASGETRITGLLEGEDV+ TG MSHSAAAKSATARNSPALAGTARVPGDKSISHRSFMFGGLASGETRITGLLEGEDVMRTG | 60 |
| Query 61 | KAMQAMGARIRKEGDTWIIDGVGNGGLLAPEAPLDFGNAATGCRLTMGLVGVYDFDSTFI | 120 |
| Sbjct 61 | AM+AMGA I K G W+I G GNG LL PE PLDFGNA TG RLTMGLVG YD ++TFI AAMKAMGAHIEKNGAEWVIRGTGNGALLQPEGPLDFGNAGTGSRLTMGLVGTYDMETTFI | 120 |
| Query 121 | GDASLTKRPMGRVLNPLREMGVQV-KSEDGDRLPVTLRGPKTPTPITYRVPMASAQVKSA | 179 |
| Sbjct 121 | GDASL+ RPMGRVL+PLR+MGVQV K+ GDR+P+TLRGPK PITYRVPMASAQVKSA GDASLSGRPMGRVLDPLRQMGVQVLKAAPGDRMPITLRGPKHAAPITYRVPMASAQVKSA | 180 |
| Query 180 | VLLAGLNTPGITTVIEPIMTRDHTEKMLQGFGANLTVETDADGVRTIRLEGRGKLTGQVI | 239 |
| Sbjct 181 | VLLAGLNTPGITTVIEP+MTRDHTEKML+GFGANLTVETD GVR I +EG+GKLTGQ I VLLAGLNTPGITTVIEPVMTRDHTEKMLKGFGANLTVETDERGVRHIFIEGQGKLTGQTI | 240 |
| Query 240 | DVPGDPSSTAFPLVAALLVPGSDVTILNVLMNPTRTGLILTLQEMGADIEVINPRLAGGE | 299 |
| Sbjct 241 | VPGDPSS FPLVAAL+VPGSD+ I NVLMNPTRTGL+LTL EMG I+++NPR AGGE AVPGDPSSAGFPLVAALIVPGSDIVIENVLMNPTRTGLLLTLHEMGGRIDILNPRNAGGE | 300 |
| Query 300 | DVADLRVRSSTLKGVTVPEDRAPSMIDEYPILavaaafaegaTVMNGLEELRVKESDRLS | 359 |
| Sbjct 301 | DVADLRVR S L GV VP +RAPSMIDEYP+LAVAA+FAEG T+M GLEELRVKESDRLS DVADLRVRYSELTGVVVPPERAPSMIDEYPVLAVAASFAEGETLMQGLEELRVKESDRLS | 360 |
| Query 360 | AVANGLKLNGVDCDEGETSLVVRGRPDGKGLG---NASGAAVATHLDHRIAMSFLVMGLV | 416 |
| Sbjct 361 | AVANGLKLNGVDC EGE +L V G+P GKGLG N AV THLDHRIAMSFL+MGL AVANGLKLNGVDCTEGEATLAVSGKPGGKGLGAHPNGPDTAVKTHLDHRIAMSFLIMGLA | 420 |
| Query 417  Sbjct 421 | SENPVTVDDATMIATSFPEFMDLMAGLGAKIE 448  +E PVT+DDA MIATSFPEFM LM GLGA+IE  TEKPVTIDDANMIATSFPEFMGLMKGLGAEIE 452 |  |

>WP_144708598.1 3-phosphoshikimate 1-carboxyvinyltransferase [Aminobacter sp. J15]

TWH36306.1 3-phosphoshikimate 1-carboxyvinyltransferase [Aminobacter sp. J15]

Length=446

Score = 677 bits (1746), Expect = 0.0, Method: Compositional matrix adjust. Identities = 350/444 (79%), Positives = 381/444 (86%), Gaps = 4/444 (1%)

| Query | 6 | SSRPATARKSSGLSGTVRIPGDKSISHRSFMFGGLASGETRITGLLEGEDVINTGKAMQA | 65 |
| --- | --- | --- | --- |
| Sbjct | 6 | + +PATA KS LSGTVR+PGDKSISHRSFMFGGLASG TRITGLLEGEDV+ TG+AM+A TPKPATASKSEALSGTVRVPGDKSISHRSFMFGGLASGVTRITGLLEGEDVLRTGEAMKA | 65 |
| Query | 66 | MGARIRKEGDTWIIDGVGNGGLLAPEAPLDFGNAATGCRLTMGLVGVYDFDSTFIGDASL | 125 |
| Sbjct | 66 | MGA+I K+GD WII G GNG LL P+ PLDFGNA TG LTMGLVG YD +TFIGD SL  MGAKIEKQGDEWIIHGTGNGALLEPQGPLDFGNAGTGSXLTMGLVGTYDMTTTFIGDTSL | 125 |

| Query | 126 | TKRPMGRVLNPLREMGVQV-KSEDGDRLPVTLRGPKTPTPITYRVPMASAQVKSAVLLAG | 184 |
| --- | --- | --- | --- |
| Sbjct | 126 | RPMGRVLNPLREMGVQV + GDR+P+T+RGPK PITYRVPMASAQVKSAVLLAG QSRPMGRVLNPLREMGVQVVDAAPGDRMPLTIRGPKRANPITYRVPMASAQVKSAVLLAG | 185 |
| Query | 185 | LNTPGITTVIEPIMTRDHTEKMLQGFGANLTVETDADGVRTIRLEGRGKLTGQVIDVPGD | 244 |
| Sbjct | 186 | LNTPGITTVIEPIMTRDHTEKML+GFGANL VETD DGVR IR+EG GKLTGQVI VPGD LNTPGITTVIEPIMTRDHTEKMLKGFGANLQVETDKDGVRHIRIEGEGKLTGQVIAVPGD | 245 |
| Query | 245 | PSSTAFPLVAALLVPGSDVTILNVLMNPTRTGLILTLQEMGADIEVINPRLAGGEDVADL | 304 |
| Sbjct | 246 | PSS AFPLVAAL+VPGSD+ I NVLMNPTRTGLI TL EMGA IE++N R AGGE+VADL PSSAAFPLVAALIVPGSDIVIENVLMNPTRTGLIDTLLEMGASIEILNRRDAGGEEVADL | 305 |
| Query | 305 | RVRSSTLKGVTVPEDRAPSMIDEYPILavaaafaegaTVMNGLEELRVKESDRLSAVANG | 364 |
| Sbjct | 306 | RVR S LKGVTVP RAP MIDEYPILA+AA+ AEG TVM+GLEELRVKESDRL+AVA G RVRHSELKGVTVPPARAPFMIDEYPILAIAASLAEGETVMDGLEELRVKESDRLAAVARG | 365 |
| Query | 365 | LKLNGVDCDEGETSLVVRGRPDGKGLGNASGAAVATHLDHRIAMSFLVMGLVSENPVTVD | 424 |
| Sbjct | 366 | L++NGVDC EGE SL VRGRPDGKGLG G VATHLDHRIAMSFLV+GL +E PVTVD LEVNGVDCTEGEASLTVRGRPDGKGLG---GGTVATHLDHRIAMSFLVLGLAAEKPVTVD | 422 |
| Query  Sbjct | 425  423 | DATMIATSFPEFMDLMAGLGAKIE 448 D +MIATSFPEFM LM GLGAKI+ DRSMIATSFPEFMGLMTGLGAKID 446 |  |

>TIT21188.1 3-phosphoshikimate 1-carboxyvinyltransferase [Mesorhizobium sp.] Length=452

Score = 677 bits (1746), Expect = 0.0, Method: Compositional matrix adjust.

| Identities | = 349/452 (77%), Positives = 383/452 (85%), Gaps = 4/452 (1%) |  |
| --- | --- | --- |
| Query 1 | MLHGASSRPATARKSSGLSGTVRIPGDKSISHRSFMFGGLASGETRITGLLEGEDVINTG | 60 |
| Sbjct 1 | M H A+ + ATARKSSGLSG R+PGDKSISHRSFMFGGLASGETRITGLLEGEDV+ TG MSHTAAPKQATARKSSGLSGAARVPGDKSISHRSFMFGGLASGETRITGLLEGEDVMRTG | 60 |
| Query 61 | KAMQAMGARIRKEGDTWIIDGVGNGGLLAPEAPLDFGNAATGCRLTMGLVGVYDFDSTFI | 120 |
| Sbjct 61 | AM+AMGA I K G W+I G GNG LL PE PLDFGNA TG RLTMGLVG YD ++TFI AAMKAMGAHIEKHGAEWVIRGTGNGALLQPEGPLDFGNAGTGSRLTMGLVGTYDMETTFI | 120 |
| Query 121 | GDASLTKRPMGRVLNPLREMGVQV-KSEDGDRLPVTLRGPKTPTPITYRVPMASAQVKSA | 179 |
| Sbjct 121 | GDASL+ RPMGRVL PLR+MGVQV K+ GDR+P+TL GPK PITYRVPMASAQVKSA GDASLSGRPMGRVLEPLRQMGVQVLKATPGDRMPITLHGPKHAAPITYRVPMASAQVKSA | 180 |
| Query 180 | VLLAGLNTPGITTVIEPIMTRDHTEKMLQGFGANLTVETDADGVRTIRLEGRGKLTGQVI | 239 |
| Sbjct 181 | VLLAGLNTPGITTVIEP+MTRDHTEKML+GFGANL+VETD GVR I +EG+GKL GQ I VLLAGLNTPGITTVIEPVMTRDHTEKMLKGFGANLSVETDERGVRHIFIEGQGKLMGQTI | 240 |
| Query 240 | DVPGDPSSTAFPLVAALLVPGSDVTILNVLMNPTRTGLILTLQEMGADIEVINPRLAGGE | 299 |
| Sbjct 241 | VPGDPSS FPLVAAL+VPGSD+TI NVLMNPTRTGL+LTLQEMGA I++++ R GGE AVPGDPSSAGFPLVAALIVPGSDITIENVLMNPTRTGLLLTLQEMGARIDILDRRNEGGE | 300 |
| Query 300 | DVADLRVRSSTLKGVTVPEDRAPSMIDEYPILavaaafaegaTVMNGLEELRVKESDRLS | 359 |
| Sbjct 301 | DVADLRV S LKGVTVP +RAP+MIDEYP+LAVAA+FAEG T+M GLEELRVKESDRLS DVADLRVLYSELKGVTVPPERAPTMIDEYPVLAVAASFAEGETLMLGLEELRVKESDRLS | 360 |
| Query 360 | AVANGLKLNGVDCDEGETSLVVRGRPDGKGLG---NASGAAVATHLDHRIAMSFLVMGLV | 416 |
| Sbjct 361 | AVA GLKLNGVDC EGE SLVVRG+P GKGLG N G V THLDHRIAMSFLVMGL AVAEGLKLNGVDCSEGEASLVVRGKPGGKGLGQHPNGQGTVVQTHLDHRIAMSFLVMGLA | 420 |
| Query 417  Sbjct 421 | SENPVTVDDATMIATSFPEFMDLMAGLGAKIE 448  +E PVT+DD MIATSFPEFM LM GLGA+IE  TEKPVTIDDQAMIATSFPEFMGLMKGLGAEIE 452 |  |

>WP_008834206.1 3-phosphoshikimate 1-carboxyvinyltransferase [Mesorhizobium alhagi] EHK58814.1 3-phosphoshikimate 1-carboxyvinyltransferase [Mesorhizobium alhagi

CCNWXJ12-2]

Length=446

Score = 676 bits (1745), Expect = 0.0, Method: Compositional matrix adjust. Identities = 346/449 (77%), Positives = 385/449 (86%), Gaps = 4/449 (1%)

| Query | 1 | MLHGASSRPATARKSSGLSGTVRIPGDKSISHRSFMFGGLASGETRITGLLEGEDVINTG | 60 |
| --- | --- | --- | --- |
| Sbjct | 1 | M H + ++PA A+KS L+GTVR+PGDKSISHRSFMFGGLASG+TRITGLLEGEDV+ TG MAHNSPAQPAIAKKSEALAGTVRMPGDKSISHRSFMFGGLASGQTRITGLLEGEDVMRTG | 60 |
| Query | 61 | KAMQAMGARIRKEGDTWIIDGVGNGGLLAPEAPLDFGNAATGCRLTMGLVGVYDFDSTFI | 120 |
| Sbjct | 61 | +AM+ MGARI K G WIIDG GNG LL P APLDFGNA TG RLTMGLVG YD ++TF+ EAMKTMGARIEKRGAEWIIDGTGNGCLLEPTAPLDFGNAGTGSRLTMGLVGTYDMETTFV | 120 |
| Query | 121 | GDASLTKRPMGRVLNPLREMGVQV-KSEDGDRLPVTLRGPKTPTPITYRVPMASAQVKSA | 179 |
| Sbjct | 121 | GDASL+ RPMGRVL PLREMGVQV K+ GDR+P+TLRGP T PI+YRVPMASAQVKSA GDASLSGRPMGRVLGPLREMGVQVLKASPGDRMPITLRGPNTAAPISYRVPMASAQVKSA | 180 |
| Query | 180 | VLLAGLNTPGITTVIEPIMTRDHTEKMLQGFGANLTVETDADGVRTIRLEGRGKLTGQVI | 239 |
| Sbjct | 181 | VLLAGLNTPGITTVIEP+MTRDHTEKML+GFGAN+ VETD DGVR IR+EG+GKL GQ I VLLAGLNTPGITTVIEPVMTRDHTEKMLKGFGANIEVETDRDGVRHIRIEGQGKLFGQTI | 240 |
| Query | 240 | DVPGDPSSTAFPLVAALLVPGSDVTILNVLMNPTRTGLILTLQEMGADIEVINPRLAGGE | 299 |
| Sbjct | 241 | VPGDPSS FPLVAAL+VPGSD+ I NVLMNPTRTGLI TL EMGA I+++NPR AGGE AVPGDPSSAGFPLVAALIVPGSDIIIENVLMNPTRTGLITTLLEMGASIDLLNPRNAGGE | 300 |
| Query | 300 | DVADLRVRSSTLKGVTVPEDRAPSMIDEYPILavaaafaegaTVMNGLEELRVKESDRLS | 359 |
| Sbjct | 301 | DVADLRVRSS LKGV+VP +RAPSMIDEYP+LAVAA+FAEG T+M GLEELRVKESDRLS DVADLRVRSSQLKGVSVPAERAPSMIDEYPVLAVAASFAEGETLMQGLEELRVKESDRLS | 360 |
| Query | 360 | AVANGLKLNGVDCDEGETSLVVRGRPDGKGLGNASGAAVATHLDHRIAMSFLVMGLVSEN | 419 |
| Sbjct | 361 | AVANGLK NGVDC EGE SL VRG+P GK LG G V THLDHRIAMSFL+MGL +E AVANGLKANGVDCTEGEASLAVRGKPGGKELG---GGMVKTHLDHRIAMSFLIMGLATEK | 417 |
| Query  Sbjct | 420  418 | PVTVDDATMIATSFPEFMDLMAGLGAKIE 448 PVTVDD MIATSFPEFM LM G+GA+I+ PVTVDDRAMIATSFPEFMGLMTGMGAEID 446 |  |

>WP_140497241.1 MULTISPECIES: 3-phosphoshikimate 1-carboxyvinyltransferase [unclassified Mesorhizobium]

TPJ49242.1 3-phosphoshikimate 1-carboxyvinyltransferase [Mesorhizobium sp. B2-6-6]

TPK35998.1 3-phosphoshikimate 1-carboxyvinyltransferase [Mesorhizobium sp. B2-5-3]

TPK63142.1 3-phosphoshikimate 1-carboxyvinyltransferase [Mesorhizobium sp. B2-5-1]

TPL34993.1 3-phosphoshikimate 1-carboxyvinyltransferase [Mesorhizobium sp. B2-4-8]

TPL64830.1 3-phosphoshikimate 1-carboxyvinyltransferase [Mesorhizobium sp. B2-4-1]

Length=452

Score = 676 bits (1744), Expect = 0.0, Method: Compositional matrix adjust.

| Identities | = 355/451 (79%), Positives = 386/451 (86%), Gaps = 4/451 (1%) |  |
| --- | --- | --- |
| Query 1 | MLHGASSRPATARKSSGLSGTVRIPGDKSISHRSFMFGGLASGETRITGLLEGEDVINTG | 60 |
| Sbjct 1 | M H A+S+PATARK LSGT R+PGDKSISHRSFMFGGLASGETRITGLLEGEDV+ TG MSHAAASKPATARKCQALSGTARVPGDKSISHRSFMFGGLASGETRITGLLEGEDVMRTG | 60 |
| Query 61 | KAMQAMGARIRKEGDTWIIDGVGNGGLLAPEAPLDFGNAATGCRLTMGLVGVYDFDSTFI | 120 |
| Sbjct 61 | AM+AMGA I K GD W+I G GNG LL PE PLDFGNA TG RLTMGLVG YD ++TFI AAMKAMGAHIEKRGDEWVIRGTGNGALLQPEGPLDFGNAGTGSRLTMGLVGTYDMETTFI | 120 |
| Query 121 | GDASLTKRPMGRVLNPLREMGVQV-KSEDGDRLPVTLRGPKTPTPITYRVPMASAQVKSA | 179 |
| Sbjct 121 | GDASL+ RPMGRVL PLR+MGVQV K+ GDR+P+TL GPK PITYRVPMASAQVKSA GDASLSGRPMGRVLEPLRQMGVQVLKATPGDRMPITLHGPKHAAPITYRVPMASAQVKSA | 180 |
| Query 180 | VLLAGLNTPGITTVIEPIMTRDHTEKMLQGFGANLTVETDADGVRTIRLEGRGKLTGQVI | 239 |
| Sbjct 181 | VLLAGLNTPGITTVIEP+MTRDHTEKML+GFGANL+VETD GVR I +EG+GKLTGQ I VLLAGLNTPGITTVIEPVMTRDHTEKMLKGFGANLSVETDERGVRHIFIEGQGKLTGQTI | 240 |
| Query 240 | DVPGDPSSTAFPLVAALLVPGSDVTILNVLMNPTRTGLILTLQEMGADIEVINPRLAGGE | 299 |
| Sbjct 241 | VPGDPSS FPLVAAL+VPGSD+ I NVLMNPTRTGL+LTLQEMG IE++NPR AGGE AVPGDPSSAGFPLVAALIVPGSDIVIENVLMNPTRTGLLLTLQEMGGQIEILNPRNAGGE | 300 |
| Query 300 | DVADLRVRSSTLKGVTVPEDRAPSMIDEYPILavaaafaegaTVMNGLEELRVKESDRLS DVADLRVR S LKGVTVP +RAPSMIDEYP+LAVAA+FAEG T+M GLEELRVKESDRLS | 359 |

| Sbjct | 301 | DVADLRVRYSELKGVTVPAERAPSMIDEYPVLAVAASFAEGETLMQGLEELRVKESDRLS | 360 |
| --- | --- | --- | --- |
| Query | 360 | AVANGLKLNGVDCDEGETSLVVRGRPDGKGLG---NASGAAVATHLDHRIAMSFLVMGLV | 416 |
| Sbjct | 361 | AVANGLKLNGVDC EGETSL VRG+P GKGLG N V THLDHRIAMSFLVMGL AVANGLKLNGVDCTEGETSLAVRGKPGGKGLGGHPNGPDTTVQTHLDHRIAMSFLVMGLA | 420 |
| Query  Sbjct | 417  421 | SENPVTVDDATMIATSFPEFMDLMAGLGAKI 447  +E PVT+DDA MIATSFPEFM LM GLGA+I  TEKPVTIDDANMIATSFPEFMSLMTGLGAEI 451 |  |

>WP_095200843.1 3-phosphoshikimate 1-carboxyvinyltransferase [Mesorhizobium sp. ICMP 18942]

Length=452

Score = 676 bits (1744), Expect = 0.0, Method: Compositional matrix adjust.

| Identities | = 354/452 (78%), Positives = 386/452 (85%), Gaps = 4/452 (1%) |  |
| --- | --- | --- |
| Query 1 | MLHGASSRPATARKSSGLSGTVRIPGDKSISHRSFMFGGLASGETRITGLLEGEDVINTG | 60 |
| Sbjct 1 | M H A+++PATARKS LSGT R+PGDKSISHRSFMFGGLASGETRITGLLEGEDV+ TG MSHAAAAKPATARKSQALSGTARVPGDKSISHRSFMFGGLASGETRITGLLEGEDVMRTG | 60 |
| Query 61 | KAMQAMGARIRKEGDTWIIDGVGNGGLLAPEAPLDFGNAATGCRLTMGLVGVYDFDSTFI | 120 |
| Sbjct 61 | AM+AMGA I K G W+I G GNG LL PE PLDFGNA TG RLTMGLVG YD ++TFI AAMKAMGAHIEKRGAEWVIRGTGNGALLQPEGPLDFGNAGTGSRLTMGLVGTYDMETTFI | 120 |
| Query 121 | GDASLTKRPMGRVLNPLREMGVQV-KSEDGDRLPVTLRGPKTPTPITYRVPMASAQVKSA | 179 |
| Sbjct 121 | GDASL+ RPMGRVL PLR+MGVQV K+ GDR+P+TL GPK PITYRVPMASAQVKSA GDASLSGRPMGRVLEPLRQMGVQVLKATPGDRMPITLHGPKHAAPITYRVPMASAQVKSA | 180 |
| Query 180 | VLLAGLNTPGITTVIEPIMTRDHTEKMLQGFGANLTVETDADGVRTIRLEGRGKLTGQVI | 239 |
| Sbjct 181 | VLLAGLNTPGITTVIEP+MTRDHTEKML+GFGANL+VETD GVR I +EG+GKLTGQ I VLLAGLNTPGITTVIEPVMTRDHTEKMLKGFGANLSVETDERGVRHIFIEGQGKLTGQTI | 240 |
| Query 240 | DVPGDPSSTAFPLVAALLVPGSDVTILNVLMNPTRTGLILTLQEMGADIEVINPRLAGGE | 299 |
| Sbjct 241 | VPGDPSS FPLVAAL+VPGSD+TI NVLMNPTRTGL+LTLQEMG I+++NPR AGGE AVPGDPSSAGFPLVAALIVPGSDITIENVLMNPTRTGLLLTLQEMGGQIDILNPRNAGGE | 300 |
| Query 300 | DVADLRVRSSTLKGVTVPEDRAPSMIDEYPILavaaafaegaTVMNGLEELRVKESDRLS | 359 |
| Sbjct 301 | DVADLRVR S LKGV VP +RAPSMIDEYP+LAVAA+FAEG T+M GLEELRVKESDRLS DVADLRVRYSELKGVAVPPERAPSMIDEYPVLAVAASFAEGETLMQGLEELRVKESDRLS | 360 |
| Query 360 | AVANGLKLNGVDCDEGETSLVVRGRPDGKGLG---NASGAAVATHLDHRIAMSFLVMGLV | 416 |
| Sbjct 361 | AVANGLKLNGVDC EGE SL VRGRP GKGLG N V THLDHRIAMSFLVMGL AVANGLKLNGVDCTEGEASLAVRGRPGGKGLGRHPNGLDTTVKTHLDHRIAMSFLVMGLA | 420 |
| Query 417  Sbjct 421 | SENPVTVDDATMIATSFPEFMDLMAGLGAKIE 448  +E PVT+DDA MIATSFPEFM LM GLGA+IE  TEKPVTIDDANMIATSFPEFMGLMKGLGAEIE 452 |  |

>WP_184873211.1 3-phosphoshikimate 1-carboxyvinyltransferase [Mesorhizobium sangaii] MBB6410303.1 3-phosphoshikimate 1-carboxyvinyltransferase [Mesorhizobium sangaii]

Length=452

Score = 676 bits (1744), Expect = 0.0, Method: Compositional matrix adjust.

| Identities | = 346/451 (77%), Positives = 380/451 (84%), Gaps = 4/451 (1%) |  |
| --- | --- | --- |
| Query 1 | MLHGASSRPATARKSSGLSGTVRIPGDKSISHRSFMFGGLASGETRITGLLEGEDVINTG | 60 |
| Sbjct 1 | M H A+ + ATARKS L+GT R+PGDKSISHRSFMFGGLASGETRITGLLEGEDV+ TG MSHAAAPKSATARKSPALAGTARVPGDKSISHRSFMFGGLASGETRITGLLEGEDVMRTG | 60 |
| Query 61 | KAMQAMGARIRKEGDTWIIDGVGNGGLLAPEAPLDFGNAATGCRLTMGLVGVYDFDSTFI | 120 |
| Sbjct 61 | AM+AMGA I K G W+I G GNG LL PE PLDFGNA TG RLTMGLVG YD ++TFI AAMKAMGAHIEKRGAEWVIRGTGNGALLQPEGPLDFGNAGTGSRLTMGLVGTYDMETTFI | 120 |
| Query 121 | GDASLTKRPMGRVLNPLREMGVQV-KSEDGDRLPVTLRGPKTPTPITYRVPMASAQVKSA | 179 |
| Sbjct 121 | GDASL+ RPMGRVL PLR+MGVQV K+ GDR+P+TL GPK PITYRVPMASAQVKSA GDASLSGRPMGRVLEPLRQMGVQVLKATPGDRMPITLHGPKHAAPITYRVPMASAQVKSA | 180 |
| Query 180 | VLLAGLNTPGITTVIEPIMTRDHTEKMLQGFGANLTVETDADGVRTIRLEGRGKLTGQVI VLLAGLNTPGITTVIEP+MTRDHTEKML+GFGANL+VETD GVR I +EG+ +LTGQ I | 239 |

| Sbjct | 181 | VLLAGLNTPGITTVIEPVMTRDHTEKMLKGFGANLSVETDERGVRHIFIEGQCRLTGQTI | 240 |
| --- | --- | --- | --- |
| Query | 240 | DVPGDPSSTAFPLVAALLVPGSDVTILNVLMNPTRTGLILTLQEMGADIEVINPRLAGGE | 299 |
| Sbjct | 241 | VPGDPSS FPLVAAL+VPGSD+ I NVLMNPTRTGL+LTLQEMG IE++NPR AGGE AVPGDPSSAGFPLVAALIVPGSDIVIENVLMNPTRTGLLLTLQEMGGKIEILNPRNAGGE | 300 |
| Query | 300 | DVADLRVRSSTLKGVTVPEDRAPSMIDEYPILavaaafaegaTVMNGLEELRVKESDRLS | 359 |
| Sbjct | 301 | DVADLRVR S LKGV VP +RAPSMIDEYP+LAVAA+FAEG T+M GLEELRVKESDRLS DVADLRVRYSELKGVIVPPERAPSMIDEYPVLAVAASFAEGETLMQGLEELRVKESDRLS | 360 |
| Query | 360 | AVANGLKLNGVDCDEGETSLVVRGRPDGKGLG---NASGAAVATHLDHRIAMSFLVMGLV | 416 |
| Sbjct | 361 | AVANGLK+NGVDC EGE SL VRG+P GKGLG N V THLDHRIAMSFLVMGL AVANGLKINGVDCTEGEASLAVRGKPGGKGLGRHPNGQDTTVQTHLDHRIAMSFLVMGLA | 420 |
| Query  Sbjct | 417  421 | SENPVTVDDATMIATSFPEFMDLMAGLGAKI 447  +E PVT+DD MIATSFPEFM LM GLGA+I  TEKPVTIDDQAMIATSFPEFMGLMKGLGAEI 451 |  |

>WP_126103907.1 3-phosphoshikimate 1-carboxyvinyltransferase [Mesorhizobium sp. M6A.T.Cr.TU.016.01.1.1]

AZO64830.1 3-phosphoshikimate 1-carboxyvinyltransferase [Mesorhizobium sp. M6A.T.Cr.TU.016.01.1.1]

RWP54073.1 3-phosphoshikimate 1-carboxyvinyltransferase [Mesorhizobium sp.] RWQ76364.1 3-phosphoshikimate 1-carboxyvinyltransferase [Mesorhizobium sp.]

Length=452

Score = 676 bits (1744), Expect = 0.0, Method: Compositional matrix adjust.

| Identities | = 346/452 (77%), Positives = 383/452 (85%), Gaps = 4/452 (1%) |  |
| --- | --- | --- |
| Query 1 | MLHGASSRPATARKSSGLSGTVRIPGDKSISHRSFMFGGLASGETRITGLLEGEDVINTG | 60 |
| Sbjct 1 | M H A+ +PATARKSS LSGT R+PGDKSISHRSFMFGGLASGETRITGLLEGEDV+ TG MSHTAAPKPATARKSSALSGTARVPGDKSISHRSFMFGGLASGETRITGLLEGEDVMRTG | 60 |
| Query 61 | KAMQAMGARIRKEGDTWIIDGVGNGGLLAPEAPLDFGNAATGCRLTMGLVGVYDFDSTFI | 120 |
| Sbjct 61 | AM+AMGA I K G W+I G GNG LL PE PLDFGNA TG RLTMGLVG YD ++TFI AAMKAMGAHIEKHGTEWVIRGTGNGALLQPEGPLDFGNAGTGSRLTMGLVGTYDMETTFI | 120 |
| Query 121 | GDASLTKRPMGRVLNPLREMGVQV-KSEDGDRLPVTLRGPKTPTPITYRVPMASAQVKSA | 179 |
| Sbjct 121 | GDASL+ RPMGRVL PLR+MGVQV K+ GDR+P+TL GPK PITYRVPMASAQVKSA GDASLSGRPMGRVLEPLRQMGVQVLKATPGDRMPITLHGPKHAAPITYRVPMASAQVKSA | 180 |
| Query 180 | VLLAGLNTPGITTVIEPIMTRDHTEKMLQGFGANLTVETDADGVRTIRLEGRGKLTGQVI | 239 |
| Sbjct 181 | VLLAGLNTPG+TTVIEP+MTRDHTEKML+GFGANL+VETD GVR I +EG+GKLTGQ I VLLAGLNTPGVTTVIEPVMTRDHTEKMLKGFGANLSVETDERGVRHIFIEGQGKLTGQTI | 240 |
| Query 240 | DVPGDPSSTAFPLVAALLVPGSDVTILNVLMNPTRTGLILTLQEMGADIEVINPRLAGGE | 299 |
| Sbjct 241 | VPGDPSS FPLVAAL+VPGSD+TI NVLMNPTRTGL+LTLQEMGA I++++PR GGE AVPGDPSSAGFPLVAALIVPGSDITIENVLMNPTRTGLLLTLQEMGARIDILDPRNEGGE | 300 |
| Query 300 | DVADLRVRSSTLKGVTVPEDRAPSMIDEYPILavaaafaegaTVMNGLEELRVKESDRLS | 359 |
| Sbjct 301 | DVADLRVR S LKGVTVP +RAP+MIDEYP+LAVAA+FAEG T+M GLEELRVKESDRLS DVADLRVRYSELKGVTVPPERAPTMIDEYPVLAVAASFAEGETLMLGLEELRVKESDRLS | 360 |
| Query 360 | AVANGLKLNGVDCDEGETSLVVRGRPDGKGLG---NASGAAVATHLDHRIAMSFLVMGLV | 416 |
| Sbjct 361 | AVA GLKLNGVDC EGE +L VRG+P GKGLG N V THLDHRIAMSFL+MGL AVAEGLKLNGVDCTEGEATLAVRGKPGGKGLGQHPNGHNTVVQTHLDHRIAMSFLIMGLA | 420 |
| Query 417  Sbjct 421 | SENPVTVDDATMIATSFPEFMDLMAGLGAKIE 448  +E PVT+DD MIATSFPEFM LM LG +IE  TEKPVTIDDQAMIATSFPEFMGLMKRLGVEIE 452 |  |

Score = 676 bits (1744), Expect = 0.0, Method: Compositional matrix adjust. Identities = 346/452 (77%), Positives = 383/452 (85%), Gaps = 4/452 (1%)

| Query | 1 | MLHGASSRPATARKSSGLSGTVRIPGDKSISHRSFMFGGLASGETRITGLLEGEDVINTG | 60 |
| --- | --- | --- | --- |
| Sbjct | 1 | M H A+ +PATARKSS LSGT R+PGDKSISHRSFMFGGLASGETRITGLLEGEDV+ TG MSHTAAPKPATARKSSALSGTARVPGDKSISHRSFMFGGLASGETRITGLLEGEDVMRTG | 60 |
| Query | 61 | KAMQAMGARIRKEGDTWIIDGVGNGGLLAPEAPLDFGNAATGCRLTMGLVGVYDFDSTFI AM+AMGA I K G W+I G GNG LL PE PLDFGNA TG RLTMGLVG YD ++TFI | 120 |

| Sbjct | 61 | AAMKAMGAHIEKHGTEWVIRGTGNGALLQPEGPLDFGNAGTGSRLTMGLVGTYDMETTFI | 120 |
| --- | --- | --- | --- |
| Query | 121 | GDASLTKRPMGRVLNPLREMGVQV-KSEDGDRLPVTLRGPKTPTPITYRVPMASAQVKSA | 179 |
| Sbjct | 121 | GDASL+ RPMGRVL PLR+MGVQV K+ GDR+P+TL GPK PITYRVPMASAQVKSA GDASLSGRPMGRVLEPLRQMGVQVLKATPGDRMPITLHGPKHAAPITYRVPMASAQVKSA | 180 |
| Query | 180 | VLLAGLNTPGITTVIEPIMTRDHTEKMLQGFGANLTVETDADGVRTIRLEGRGKLTGQVI | 239 |
| Sbjct | 181 | VLLAGLNTPG+TTVIEP+MTRDHTEKML+GFGANL+VETD GVR I +EG+GKLTGQ I VLLAGLNTPGVTTVIEPVMTRDHTEKMLKGFGANLSVETDERGVRHIFIEGQGKLTGQTI | 240 |
| Query | 240 | DVPGDPSSTAFPLVAALLVPGSDVTILNVLMNPTRTGLILTLQEMGADIEVINPRLAGGE | 299 |
| Sbjct | 241 | VPGDPSS FPLVAAL+VPGSD+TI NVLMNPTRTGL+LTLQEMGA I++++PR GGE AVPGDPSSAGFPLVAALIVPGSDITIENVLMNPTRTGLLLTLQEMGARIDILDPRNEGGE | 300 |
| Query | 300 | DVADLRVRSSTLKGVTVPEDRAPSMIDEYPILavaaafaegaTVMNGLEELRVKESDRLS | 359 |
| Sbjct | 301 | DVADLRVR S LKGVTVP +RAP+MIDEYP+LAVAA+FAEG T+M GLEELRVKESDRLS DVADLRVRYSELKGVTVPPERAPTMIDEYPVLAVAASFAEGETLMLGLEELRVKESDRLS | 360 |
| Query | 360 | AVANGLKLNGVDCDEGETSLVVRGRPDGKGLG---NASGAAVATHLDHRIAMSFLVMGLV | 416 |
| Sbjct | 361 | AVA GLKLNGVDC EGE +L VRG+P GKGLG N V THLDHRIAMSFL+MGL AVAEGLKLNGVDCTEGEATLAVRGKPGGKGLGQHPNGHNTVVQTHLDHRIAMSFLIMGLA | 420 |
| Query  Sbjct | 417  421 | SENPVTVDDATMIATSFPEFMDLMAGLGAKIE 448  +E PVT+DD MIATSFPEFM LM LG +IE  TEKPVTIDDQAMIATSFPEFMGLMKRLGVEIE 452 |  |

>WP_140802303.1 3-phosphoshikimate 1-carboxyvinyltransferase [Mesorhizobium sp. B2-7-1]

TPJ72692.1 3-phosphoshikimate 1-carboxyvinyltransferase [Mesorhizobium sp. B2-7-1]

Length=452

Score = 676 bits (1743), Expect = 0.0, Method: Compositional matrix adjust.

| Identities | = 355/452 (79%), Positives = 388/452 (86%), Gaps = 4/452 (1%) |  |
| --- | --- | --- |
| Query 1 | MLHGASSRPATARKSSGLSGTVRIPGDKSISHRSFMFGGLASGETRITGLLEGEDVINTG | 60 |
| Sbjct 1 | M H A ++PATARKS LSGT R+PGDKSISHRSFMFGGLASGETRITGLLEGEDV+ TG MSHSAGAKPATARKSPALSGTARVPGDKSISHRSFMFGGLASGETRITGLLEGEDVMRTG | 60 |
| Query 61 | KAMQAMGARIRKEGDTWIIDGVGNGGLLAPEAPLDFGNAATGCRLTMGLVGVYDFDSTFI | 120 |
| Sbjct 61 | AMQAMGA + K+G W+I G GNG LL PE PLDFGNA TG RLTMGLVG YD ++TFI AAMQAMGAHVEKKGAEWVIRGTGNGALLQPEGPLDFGNAGTGSRLTMGLVGTYDMETTFI | 120 |
| Query 121 | GDASLTKRPMGRVLNPLREMGVQV-KSEDGDRLPVTLRGPKTPTPITYRVPMASAQVKSA | 179 |
| Sbjct 121 | GDASL+ RPMGRVL+PLR+MGVQV K+ GDR+P+TLRGPK PITYRVPMASAQVKSA GDASLSGRPMGRVLDPLRQMGVQVLKAAPGDRMPITLRGPKHAAPITYRVPMASAQVKSA | 180 |
| Query 180 | VLLAGLNTPGITTVIEPIMTRDHTEKMLQGFGANLTVETDADGVRTIRLEGRGKLTGQVI | 239 |
| Sbjct 181 | VLLAGLNTPGITTVIEP+MTRDHTEKML+GFGANLTVETD GVR I +EG+GKLTGQ I VLLAGLNTPGITTVIEPVMTRDHTEKMLKGFGANLTVETDERGVRHIFIEGQGKLTGQTI | 240 |
| Query 240 | DVPGDPSSTAFPLVAALLVPGSDVTILNVLMNPTRTGLILTLQEMGADIEVINPRLAGGE | 299 |
| Sbjct 241 | VPGDPSS FPLVAAL+VPGSD+TI NVLMNPTRTGL+LTLQEMG I+++NPR AGGE AVPGDPSSAGFPLVAALIVPGSDITIENVLMNPTRTGLLLTLQEMGGRIDILNPRNAGGE | 300 |
| Query 300 | DVADLRVRSSTLKGVTVPEDRAPSMIDEYPILavaaafaegaTVMNGLEELRVKESDRLS | 359 |
| Sbjct 301 | DVADLRVRSS LKGV VP +RAPSMIDEYP+LAVAA+FAEG T+M GLEELRVKESDRL+ DVADLRVRSSDLKGVVVPAERAPSMIDEYPVLAVAASFAEGETLMQGLEELRVKESDRLA | 360 |
| Query 360 | AVANGLKLNGVDCDEGETSLVVRGRPDGKGLG---NASGAAVATHLDHRIAMSFLVMGLV | 416 |
| Sbjct 361 | AVANGLKLNGVDC EGE SL VRG P GKGLG N V THLDHRIAMSFLVMGL AVANGLKLNGVDCSEGEASLAVRGTPGGKGLGGHPNGQDTKVQTHLDHRIAMSFLVMGLA | 420 |
| Query 417  Sbjct 421 | SENPVTVDDATMIATSFPEFMDLMAGLGAKIE 448  +E PVT+DDA MIATSFPEFM LM GLGA+IE  TEKPVTIDDANMIATSFPEFMGLMKGLGAEIE 452 |  |

>WP_140506683.1 3-phosphoshikimate 1-carboxyvinyltransferase [Mesorhizobium sp. B2-3-13]

TPL81543.1 3-phosphoshikimate 1-carboxyvinyltransferase [Mesorhizobium sp.

B2-3-13]

Length=452

Score = 676 bits (1743), Expect = 0.0, Method: Compositional matrix adjust.

| Identities | = 353/452 (78%), Positives = 384/452 (85%), Gaps = 4/452 (1%) |  |
| --- | --- | --- |
| Query 1 | MLHGASSRPATARKSSGLSGTVRIPGDKSISHRSFMFGGLASGETRITGLLEGEDVINTG | 60 |
| Sbjct 1 | M H A+++PA ARKS LSGT R+PGDKSISHRSFMFGGLASGETRITGLLEGEDV+ TG MSHAAAAKPAIARKSPALSGTARVPGDKSISHRSFMFGGLASGETRITGLLEGEDVMRTG | 60 |
| Query 61 | KAMQAMGARIRKEGDTWIIDGVGNGGLLAPEAPLDFGNAATGCRLTMGLVGVYDFDSTFI | 120 |
| Sbjct 61 | AM+AMGA I K G W+I G GNG LL PE PLDFGNA TG RLTMGLVG YD ++TFI AAMKAMGAHIEKHGAEWVIRGTGNGALLQPEGPLDFGNAGTGSRLTMGLVGTYDMETTFI | 120 |
| Query 121 | GDASLTKRPMGRVLNPLREMGVQV-KSEDGDRLPVTLRGPKTPTPITYRVPMASAQVKSA | 179 |
| Sbjct 121 | GDASL+ RPMGRVL PLR+MGVQV K+ GDR+P+TL GPK PITYRVPMASAQVKSA GDASLSGRPMGRVLEPLRQMGVQVLKATPGDRMPITLHGPKHAAPITYRVPMASAQVKSA | 180 |
| Query 180 | VLLAGLNTPGITTVIEPIMTRDHTEKMLQGFGANLTVETDADGVRTIRLEGRGKLTGQVI | 239 |
| Sbjct 181 | VLLAGLNTPGITTVIEP+MTRDHTEKML+GFGANL+VETD GVR I +EG+GKLTGQ I VLLAGLNTPGITTVIEPVMTRDHTEKMLKGFGANLSVETDERGVRHIFIEGQGKLTGQTI | 240 |
| Query 240 | DVPGDPSSTAFPLVAALLVPGSDVTILNVLMNPTRTGLILTLQEMGADIEVINPRLAGGE | 299 |
| Sbjct 241 | VPGDPSS FPLVAAL+VPGSD+TI NVLMNPTRTGL+LTLQEMG I+++NPR AGGE AVPGDPSSAGFPLVAALIVPGSDITIENVLMNPTRTGLLLTLQEMGGQIDILNPRNAGGE | 300 |
| Query 300 | DVADLRVRSSTLKGVTVPEDRAPSMIDEYPILavaaafaegaTVMNGLEELRVKESDRLS | 359 |
| Sbjct 301 | DVADLRVR S LKGVTVP +RAPSMIDEYP+LAVAA+FAEG T+M GLEELRVKESDRLS DVADLRVRYSELKGVTVPPERAPSMIDEYPVLAVAASFAEGETLMQGLEELRVKESDRLS | 360 |
| Query 360 | AVANGLKLNGVDCDEGETSLVVRGRPDGKGLG---NASGAAVATHLDHRIAMSFLVMGLV | 416 |
| Sbjct 361 | AVA GLKLNGVDC E E SL VRGRPDGKGLG N V THLDHRIAMSFLVMGL AVATGLKLNGVDCTESEASLAVRGRPDGKGLGGHPNGRDTTVETHLDHRIAMSFLVMGLA | 420 |
| Query 417  Sbjct 421 | SENPVTVDDATMIATSFPEFMDLMAGLGAKIE 448  +E PVT+DD MIATSFPEFM LM GLGAKIE  TEKPVTIDDQAMIATSFPEFMGLMKGLGAKIE 452 |  |

>WP_140624910.1 3-phosphoshikimate 1-carboxyvinyltransferase [Mesorhizobium sp. B2-3-6]

TPM18551.1 3-phosphoshikimate 1-carboxyvinyltransferase [Mesorhizobium sp. B2-3-6]

Length=452

Score = 676 bits (1743), Expect = 0.0, Method: Compositional matrix adjust.

| Identities | = 354/451 (78%), Positives = 385/451 (85%), Gaps = 4/451 (1%) |  |
| --- | --- | --- |
| Query 1 | MLHGASSRPATARKSSGLSGTVRIPGDKSISHRSFMFGGLASGETRITGLLEGEDVINTG | 60 |
| Sbjct 1 | M H A+S+PATARK LSGT R+PGDKSISHRSFMFGGLASGETRITGLLEGEDV+ TG MSHAAASKPATARKCQALSGTARVPGDKSISHRSFMFGGLASGETRITGLLEGEDVMRTG | 60 |
| Query 61 | KAMQAMGARIRKEGDTWIIDGVGNGGLLAPEAPLDFGNAATGCRLTMGLVGVYDFDSTFI | 120 |
| Sbjct 61 | AM+AMGA I K GD W+I G GNG LL PE PLDFGNA TG RLTMGLVG YD ++TFI AAMKAMGAHIEKRGDEWVIRGTGNGALLQPEGPLDFGNAGTGSRLTMGLVGTYDMETTFI | 120 |
| Query 121 | GDASLTKRPMGRVLNPLREMGVQV-KSEDGDRLPVTLRGPKTPTPITYRVPMASAQVKSA | 179 |
| Sbjct 121 | GDASL+ RPMGRVL PLR+MGVQV K+ GDR+P+TL GPK PITYRVPMASAQVKSA GDASLSGRPMGRVLEPLRQMGVQVLKATPGDRMPITLHGPKHAAPITYRVPMASAQVKSA | 180 |
| Query 180 | VLLAGLNTPGITTVIEPIMTRDHTEKMLQGFGANLTVETDADGVRTIRLEGRGKLTGQVI | 239 |
| Sbjct 181 | VLLAGLNTPGITTVIEP+MTRDHTEKMLQGFGANL+VETD GVR I +EG+GKLTGQ I VLLAGLNTPGITTVIEPVMTRDHTEKMLQGFGANLSVETDERGVRHIFIEGQGKLTGQTI | 240 |
| Query 240 | DVPGDPSSTAFPLVAALLVPGSDVTILNVLMNPTRTGLILTLQEMGADIEVINPRLAGGE | 299 |
| Sbjct 241 | VPGDPSS FPLVAAL+VPGSD+ I NVLMNPTRTGL+LTLQEMG IE++NPR AGGE AVPGDPSSAGFPLVAALIVPGSDIVIENVLMNPTRTGLLLTLQEMGGQIEILNPRNAGGE | 300 |
| Query 300 | DVADLRVRSSTLKGVTVPEDRAPSMIDEYPILavaaafaegaTVMNGLEELRVKESDRLS | 359 |
| Sbjct 301 | DVADLRVR S LKGVTVP +RAPSMIDEYP+LAVAA+FAEG T+M GLEELRVKESDRLS  DVADLRVRYSELKGVTVPAERAPSMIDEYPVLAVAASFAEGETLMQGLEELRVKESDRLS | 360 |

| Query | 360 | AVANGLKLNGVDCDEGETSLVVRGRPDGKGLG---NASGAAVATHLDHRIAMSFLVMGLV 416 |
| --- | --- | --- |
| Sbjct | 361 | AVANGLKLNGVDC EGE SL VRG+P GKGLG N V THLDHRIAMSFL+MGL AVANGLKLNGVDCTEGEASLAVRGKPGGKGLGGHPNGPDTTVQTHLDHRIAMSFLIMGLA 420 |
| Query | 417 | SENPVTVDDATMIATSFPEFMDLMAGLGAKI 447 |
| Sbjct | 421 | +E PVT+DDA MIATSFPEFM LM GLGA+I  TEKPVTIDDANMIATSFPEFMSLMTGLGAEI 451 |

>WP_140657796.1 MULTISPECIES: 3-phosphoshikimate 1-carboxyvinyltransferase [unclassified Mesorhizobium]

TPM01721.1 3-phosphoshikimate 1-carboxyvinyltransferase [Mesorhizobium sp. B2-3-8]

TPM12363.1 3-phosphoshikimate 1-carboxyvinyltransferase [Mesorhizobium sp. B2-3-7]

Length=452

Score = 676 bits (1743), Expect = 0.0, Method: Compositional matrix adjust.

| Identities | = 355/451 (79%), Positives = 386/451 (86%), Gaps = 4/451 (1%) |  |
| --- | --- | --- |
| Query 1 | MLHGASSRPATARKSSGLSGTVRIPGDKSISHRSFMFGGLASGETRITGLLEGEDVINTG | 60 |
| Sbjct 1 | M H A+S+PATARK LSGT R+PGDKSISHRSFMFGGLASGETRITGLLEGEDV+ TG MSHAAASKPATARKCQALSGTARVPGDKSISHRSFMFGGLASGETRITGLLEGEDVMRTG | 60 |
| Query 61 | KAMQAMGARIRKEGDTWIIDGVGNGGLLAPEAPLDFGNAATGCRLTMGLVGVYDFDSTFI | 120 |
| Sbjct 61 | AM+AMGA I K GD W+I G GNG LL PE PLDFGNA TG RLTMGLVG YD ++TFI AAMKAMGAHIEKRGDEWVIRGTGNGALLQPEGPLDFGNAGTGSRLTMGLVGTYDMETTFI | 120 |
| Query 121 | GDASLTKRPMGRVLNPLREMGVQV-KSEDGDRLPVTLRGPKTPTPITYRVPMASAQVKSA | 179 |
| Sbjct 121 | GDASL+ RPMGRVL PLR+MGVQV K+ GDR+P+TL GPK PITYRVPMASAQVKSA GDASLSGRPMGRVLEPLRQMGVQVLKATPGDRMPITLHGPKHAAPITYRVPMASAQVKSA | 180 |
| Query 180 | VLLAGLNTPGITTVIEPIMTRDHTEKMLQGFGANLTVETDADGVRTIRLEGRGKLTGQVI | 239 |
| Sbjct 181 | VLLAGLNTPGITTVIEP+MTRDHTEKML+GFGANL+VETD GVR I +EG+GKLTGQ I VLLAGLNTPGITTVIEPVMTRDHTEKMLKGFGANLSVETDERGVRHIFIEGQGKLTGQTI | 240 |
| Query 240 | DVPGDPSSTAFPLVAALLVPGSDVTILNVLMNPTRTGLILTLQEMGADIEVINPRLAGGE | 299 |
| Sbjct 241 | VPGDPSS FPLVAAL+VPGSD+ I NVLMNPTRTGL+LTLQEMG IE++NPR AGGE AVPGDPSSAGFPLVAALIVPGSDILIENVLMNPTRTGLLLTLQEMGGQIEILNPRNAGGE | 300 |
| Query 300 | DVADLRVRSSTLKGVTVPEDRAPSMIDEYPILavaaafaegaTVMNGLEELRVKESDRLS | 359 |
| Sbjct 301 | DVADLRVR S LKGVTVP +RAPSMIDEYP+LAVAA+FAEG T+M GLEELRVKESDRLS DVADLRVRYSELKGVTVPAERAPSMIDEYPVLAVAASFAEGETLMQGLEELRVKESDRLS | 360 |
| Query 360 | AVANGLKLNGVDCDEGETSLVVRGRPDGKGLG---NASGAAVATHLDHRIAMSFLVMGLV | 416 |
| Sbjct 361 | AVANGLKLNGVDC EGETSL VRG+P GKGLG N V THLDHRIAMSFLVMGL AVANGLKLNGVDCTEGETSLAVRGKPGGKGLGGHPNGPDTTVQTHLDHRIAMSFLVMGLA | 420 |
| Query 417  Sbjct 421 | SENPVTVDDATMIATSFPEFMDLMAGLGAKI 447  +E PVT+DDA MIATSFPEFM LM GLGA+I  TEKPVTIDDANMIATSFPEFMSLMTGLGAEI 451 |  |

>WP_128242333.1 3-phosphoshikimate 1-carboxyvinyltransferase [Mesorhizobium sp. M6A.T.Cr.TU.014.01.1.1]

RVB76890.1 3-phosphoshikimate 1-carboxyvinyltransferase [Mesorhizobium sp. M6A.T.Cr.TU.014.01.1.1]

RWP78050.1 3-phosphoshikimate 1-carboxyvinyltransferase [Mesorhizobium sp.] RWQ05749.1 3-phosphoshikimate 1-carboxyvinyltransferase [Mesorhizobium sp.] RWQ07152.1 3-phosphoshikimate 1-carboxyvinyltransferase [Mesorhizobium sp.]

Length=452

Score = 675 bits (1742), Expect = 0.0, Method: Compositional matrix adjust.

| Identities | = 348/452 (77%), Positives = 383/452 (85%), Gaps = 4/452 (1%) |  |
| --- | --- | --- |
| Query 1 | MLHGASSRPATARKSSGLSGTVRIPGDKSISHRSFMFGGLASGETRITGLLEGEDVINTG | 60 |
| Sbjct 1 | M H A+ +PATARKS LSGT R+PGDKSISHRSFMFGGLASGETRITGLLEGEDV+ TG MSHTAAPKPATARKSLALSGTARVPGDKSISHRSFMFGGLASGETRITGLLEGEDVMRTG | 60 |
| Query 61 | KAMQAMGARIRKEGDTWIIDGVGNGGLLAPEAPLDFGNAATGCRLTMGLVGVYDFDSTFI | 120 |
| Sbjct 61 | AM+AMGA I K G W+I G GNG LL PE PLDFGNA TG RLTMGLVG YD ++TFI  AAMKAMGAHIEKHGTEWVIRGTGNGALLQPEGPLDFGNAGTGSRLTMGLVGTYDMETTFI | 120 |

| Query | 121 | GDASLTKRPMGRVLNPLREMGVQV-KSEDGDRLPVTLRGPKTPTPITYRVPMASAQVKSA | 179 |
| --- | --- | --- | --- |
| Sbjct | 121 | GDASL+ RPMGRVL PLR+MGVQV K+ GDR+P+TL GPK PITYRVPMASAQVKSA GDASLSGRPMGRVLEPLRQMGVQVLKATPGDRMPITLHGPKHAAPITYRVPMASAQVKSA | 180 |
| Query | 180 | VLLAGLNTPGITTVIEPIMTRDHTEKMLQGFGANLTVETDADGVRTIRLEGRGKLTGQVI | 239 |
| Sbjct | 181 | VLLAGLNTPG+TTVIEP+MTRDHTEKML+GFGANL+VETD GVR I +EG+GKLTGQ I VLLAGLNTPGVTTVIEPVMTRDHTEKMLKGFGANLSVETDERGVRHIFIEGQGKLTGQTI | 240 |
| Query | 240 | DVPGDPSSTAFPLVAALLVPGSDVTILNVLMNPTRTGLILTLQEMGADIEVINPRLAGGE | 299 |
| Sbjct | 241 | VPGDPSS FPLVAAL+VPGSD+TI NVLMNPTRTGL+LTLQEMGA I++++PR GGE AVPGDPSSAGFPLVAALIVPGSDITIENVLMNPTRTGLLLTLQEMGARIDILDPRNEGGE | 300 |
| Query | 300 | DVADLRVRSSTLKGVTVPEDRAPSMIDEYPILavaaafaegaTVMNGLEELRVKESDRLS | 359 |
| Sbjct | 301 | DVADLRVR S LKGVTVP +RAP+MIDEYP+LAVAA+FAEG T+M GLEELRVKESDRLS DVADLRVRYSELKGVTVPPERAPTMIDEYPVLAVAASFAEGETLMLGLEELRVKESDRLS | 360 |
| Query | 360 | AVANGLKLNGVDCDEGETSLVVRGRPDGKGLG---NASGAAVATHLDHRIAMSFLVMGLV | 416 |
| Sbjct | 361 | AVA GLKLNGVDC EGE +L VRG+P GKGLG N V THLDHRIAMSFLVMGL AVAEGLKLNGVDCAEGEATLAVRGKPGGKGLGRHPNGRNTVVQTHLDHRIAMSFLVMGLA | 420 |
| Query  Sbjct | 417  421 | SENPVTVDDATMIATSFPEFMDLMAGLGAKIE 448  +E PVTVDD MIATSFPEFM LM LGA+IE  AEKPVTVDDQAMIATSFPEFMGLMKRLGAEIE 452 |  |

>RWQ67813.1 3-phosphoshikimate 1-carboxyvinyltransferase [Mesorhizobium sp.] Length=452

Score = 675 bits (1742), Expect = 0.0, Method: Compositional matrix adjust.

| Identities | = 348/452 (77%), Positives = 383/452 (85%), Gaps = 4/452 (1%) |  |
| --- | --- | --- |
| Query 1 | MLHGASSRPATARKSSGLSGTVRIPGDKSISHRSFMFGGLASGETRITGLLEGEDVINTG | 60 |
| Sbjct 1 | M H A+ +PATARKS LSGT R+PGDKSISHRSFMFGGLASGETRITGLLEGEDV+ TG MSHTAAPKPATARKSLALSGTARVPGDKSISHRSFMFGGLASGETRITGLLEGEDVMRTG | 60 |
| Query 61 | KAMQAMGARIRKEGDTWIIDGVGNGGLLAPEAPLDFGNAATGCRLTMGLVGVYDFDSTFI | 120 |
| Sbjct 61 | AM+AMGA I K G W+I G GNG LL PE PLDFGNA TG RLTMGLVG YD ++TFI AAMKAMGAHIEKHGTEWVIRGTGNGALLQPEGPLDFGNAGTGSRLTMGLVGTYDMETTFI | 120 |
| Query 121 | GDASLTKRPMGRVLNPLREMGVQV-KSEDGDRLPVTLRGPKTPTPITYRVPMASAQVKSA | 179 |
| Sbjct 121 | GDASL+ RPMGRVL PLR+MGVQV K+ GDR+P+TL GPK PITYRVPMASAQVKSA GDASLSGRPMGRVLEPLRQMGVQVLKATPGDRMPITLHGPKHAAPITYRVPMASAQVKSA | 180 |
| Query 180 | VLLAGLNTPGITTVIEPIMTRDHTEKMLQGFGANLTVETDADGVRTIRLEGRGKLTGQVI | 239 |
| Sbjct 181 | VLLAGLNTPG+TTVIEP+MTRDHTEKML+GFGANL+VETD GVR I +EG+GKLTGQ I VLLAGLNTPGVTTVIEPVMTRDHTEKMLKGFGANLSVETDERGVRHIFIEGQGKLTGQTI | 240 |
| Query 240 | DVPGDPSSTAFPLVAALLVPGSDVTILNVLMNPTRTGLILTLQEMGADIEVINPRLAGGE | 299 |
| Sbjct 241 | VPGDPSS FPLVAAL+VPGSD+TI NVLMNPTRTGL+LTLQEMGA I++++PR GGE AVPGDPSSAGFPLVAALIVPGSDITIENVLMNPTRTGLLLTLQEMGARIDILDPRNEGGE | 300 |
| Query 300 | DVADLRVRSSTLKGVTVPEDRAPSMIDEYPILavaaafaegaTVMNGLEELRVKESDRLS | 359 |
| Sbjct 301 | DVADLRVR S LKGVTVP +RAP+MIDEYP+LAVAA+FAEG T+M GLEELRVKESDRLS DVADLRVRYSELKGVTVPPERAPTMIDEYPVLAVAASFAEGETLMLGLEELRVKESDRLS | 360 |
| Query 360 | AVANGLKLNGVDCDEGETSLVVRGRPDGKGLG---NASGAAVATHLDHRIAMSFLVMGLV | 416 |
| Sbjct 361 | AVA GLKLNGVDC EGE +L VRG+P GKGLG N V THLDHRIAMSFLVMGL AVAEGLKLNGVDCAEGEATLAVRGKPGGKGLGRHPNGHNTVVQTHLDHRIAMSFLVMGLA | 420 |
| Query 417  Sbjct 421 | SENPVTVDDATMIATSFPEFMDLMAGLGAKIE 448  +E PVTVDD MIATSFPEFM LM LGA+IE  AEKPVTVDDQAMIATSFPEFMGLMKRLGAEIE 452 |  |

>WP_140550305.1 3-phosphoshikimate 1-carboxyvinyltransferase [Mesorhizobium sp. B2-3-2]

TPM47953.1 3-phosphoshikimate 1-carboxyvinyltransferase [Mesorhizobium sp. B2-3-2]

Length=452

Score = 675 bits (1741), Expect = 0.0, Method: Compositional matrix adjust.

| Identities | = 354/452 (78%), Positives = 386/452 (85%), Gaps = 4/452 (1%) |  |
| --- | --- | --- |
| Query 1 | MLHGASSRPATARKSSGLSGTVRIPGDKSISHRSFMFGGLASGETRITGLLEGEDVINTG | 60 |
| Sbjct 1 | M H A+S+PATARK LSGT R+PGDKSISHRSFMFGGLASGETRITGLLEGEDV+ TG MSHAAASKPATARKCQALSGTARVPGDKSISHRSFMFGGLASGETRITGLLEGEDVMRTG | 60 |
| Query 61 | KAMQAMGARIRKEGDTWIIDGVGNGGLLAPEAPLDFGNAATGCRLTMGLVGVYDFDSTFI | 120 |
| Sbjct 61 | AM+AMGA I K GD W+I G GNG LL PE PLDFGNA TG RLTMGLVG YD ++TF+ AAMKAMGAHIEKRGDEWVIRGTGNGALLQPEGPLDFGNAGTGSRLTMGLVGTYDMETTFV | 120 |
| Query 121 | GDASLTKRPMGRVLNPLREMGVQV-KSEDGDRLPVTLRGPKTPTPITYRVPMASAQVKSA | 179 |
| Sbjct 121 | GDASL+ RPMGRVL PLR+MGVQV K+ GDR+P+TL GPK PITYRVPMASAQVKSA GDASLSGRPMGRVLEPLRQMGVQVLKATPGDRMPITLHGPKHAAPITYRVPMASAQVKSA | 180 |
| Query 180 | VLLAGLNTPGITTVIEPIMTRDHTEKMLQGFGANLTVETDADGVRTIRLEGRGKLTGQVI | 239 |
| Sbjct 181 | VLLAGLNTPGITTVIEP+MTRDHTEKML+GFGANL+VETD GVR I +EG+GKLTGQ I VLLAGLNTPGITTVIEPVMTRDHTEKMLKGFGANLSVETDERGVRHIFIEGQGKLTGQTI | 240 |
| Query 240 | DVPGDPSSTAFPLVAALLVPGSDVTILNVLMNPTRTGLILTLQEMGADIEVINPRLAGGE | 299 |
| Sbjct 241 | VPGDPSS FPLVAAL+VPGSD+ I NVLMNPTRTGL+LTLQEMG IE++NPR AGGE AVPGDPSSAGFPLVAALIVPGSDIVIENVLMNPTRTGLLLTLQEMGGQIEILNPRNAGGE | 300 |
| Query 300 | DVADLRVRSSTLKGVTVPEDRAPSMIDEYPILavaaafaegaTVMNGLEELRVKESDRLS | 359 |
| Sbjct 301 | DVADLRVR S LKGVTVP +RAPSMIDEYP+LAVAA+FAEG T+M GLEELRVKESDRLS DVADLRVRYSELKGVTVPAERAPSMIDEYPVLAVAASFAEGETLMQGLEELRVKESDRLS | 360 |
| Query 360 | AVANGLKLNGVDCDEGETSLVVRGRPDGKGLG---NASGAAVATHLDHRIAMSFLVMGLV | 416 |
| Sbjct 361 | AVANGLKLNGVDC EGE SL VRG+P GKGLG N V THLDHRIAMSFLVMGL AVANGLKLNGVDCTEGEASLAVRGKPGGKGLGGHPNGPDTTVQTHLDHRIAMSFLVMGLA | 420 |
| Query 417  Sbjct 421 | SENPVTVDDATMIATSFPEFMDLMAGLGAKIE 448  +E PVT+DDA MIATSFPEFM LM GLGA+IE  TEKPVTIDDANMIATSFPEFMGLMTGLGAEIE 452 |  |

>WP_140815185.1 MULTISPECIES: 3-phosphoshikimate 1-carboxyvinyltransferase [unclassified Mesorhizobium]

TPN89835.1 3-phosphoshikimate 1-carboxyvinyltransferase [Mesorhizobium sp. CU3]

TPO17918.1 3-phosphoshikimate 1-carboxyvinyltransferase [Mesorhizobium sp. CU2]

Length=450

Score = 675 bits (1741), Expect = 0.0, Method: Compositional matrix adjust.

| Identities | = 355/450 (79%), Positives = 390/450 (87%), Gaps = 2/450 (0%) |  |
| --- | --- | --- |
| Query 1 | MLHGASSRPATARKSSGLSGTVRIPGDKSISHRSFMFGGLASGETRITGLLEGEDVINTG | 60 |
| Sbjct 1 | M H A+++PATARKS LSGT R+PGDKSISHRSFMFGGLASGETRITGLLEGEDV+ TG MSHSAAAKPATARKSPALSGTARVPGDKSISHRSFMFGGLASGETRITGLLEGEDVMRTG | 60 |
| Query 61 | KAMQAMGARIRKEGDTWIIDGVGNGGLLAPEAPLDFGNAATGCRLTMGLVGVYDFDSTFI | 120 |
| Sbjct 61 | AM+AMGA I K G W+I G GNG LL PEAPLDFGNA TG RLTMGLVG YD ++TFI AAMKAMGAHIEKNGTEWVIRGTGNGALLQPEAPLDFGNAGTGSRLTMGLVGTYDMETTFI | 120 |
| Query 121 | GDASLTKRPMGRVLNPLREMGVQV-KSEDGDRLPVTLRGPKTPTPITYRVPMASAQVKSA | 179 |
| Sbjct 121 | GDASL+ RPMGRVL+PLR+MGVQV K+ GDR+P+TLRGPK PITYRVPMASAQVKSA GDASLSGRPMGRVLDPLRQMGVQVLKATPGDRMPITLRGPKHAAPITYRVPMASAQVKSA | 180 |
| Query 180 | VLLAGLNTPGITTVIEPIMTRDHTEKMLQGFGANLTVETDADGVRTIRLEGRGKLTGQVI | 239 |
| Sbjct 181 | VLLAGLNTPGITTVIEP+MTRDHTEKML+GFGANLTVETD GVR I +EG+GKLTGQ I VLLAGLNTPGITTVIEPVMTRDHTEKMLKGFGANLTVETDERGVRHIFIEGQGKLTGQTI | 240 |
| Query 240 | DVPGDPSSTAFPLVAALLVPGSDVTILNVLMNPTRTGLILTLQEMGADIEVINPRLAGGE | 299 |
| Sbjct 241 | VPGDPSS FPLVAAL+VPGSD+TI NVLMNPTRTGL+LTLQEMG I+++NPR AGGE AVPGDPSSAGFPLVAALIVPGSDITIENVLMNPTRTGLLLTLQEMGGKIDILNPRNAGGE | 300 |
| Query 300 | DVADLRVRSSTLKGVTVPEDRAPSMIDEYPILavaaafaegaTVMNGLEELRVKESDRLS | 359 |
| Sbjct 301 | DVADLRV S LKGV VP +RAPSMIDEYP+LAVAA+FAEG T+M GLEELRVKESDRL+ DVADLRVWYSELKGVAVPPERAPSMIDEYPVLAVAASFAEGETLMQGLEELRVKESDRLA | 360 |
| Query 360 | AVANGLKLNGVDCDEGETSLVVRGRPDGKGLGNAS-GAAVATHLDHRIAMSFLVMGLVSE AVANGLKLNGVDC EGE SL VRG+P GKGLG + AAV THLDHRIAMSFLVMGL +E | 418 |

| Sbjct | 361 | AVANGLKLNGVDCTEGEASLAVRGKPGGKGLGGSDRDAAVKTHLDHRIAMSFLVMGLATE 420 |
| --- | --- | --- |
| Query | 419 | NPVTVDDATMIATSFPEFMDLMAGLGAKIE 448 |
| Sbjct | 421 | PVT+DDA MIATSFPEFM LM GLGA+IE  KPVTIDDANMIATSFPEFMGLMKGLGAEIE 450 |

>TJX01309.1 3-phosphoshikimate 1-carboxyvinyltransferase [Mesorhizobium sp.] Length=452

Score = 674 bits (1740), Expect = 0.0, Method: Compositional matrix adjust.

| Identities | = 347/452 (77%), Positives = 382/452 (85%), Gaps = 4/452 (1%) |  |
| --- | --- | --- |
| Query 1 | MLHGASSRPATARKSSGLSGTVRIPGDKSISHRSFMFGGLASGETRITGLLEGEDVINTG | 60 |
| Sbjct 1 | M H A+ +PATARKS LSGT R+PGDKSISHRSFMFGGLASGETRITGLLEGEDV+ TG MSHTAAPKPATARKSPALSGTARVPGDKSISHRSFMFGGLASGETRITGLLEGEDVMRTG | 60 |
| Query 61 | KAMQAMGARIRKEGDTWIIDGVGNGGLLAPEAPLDFGNAATGCRLTMGLVGVYDFDSTFI | 120 |
| Sbjct 61 | AM+AMGA I K G W+I G GNG LL PE PLDFGNA TG RLTMGLVG YD ++TFI AAMKAMGAHIEKRGTEWVIRGTGNGALLQPEGPLDFGNAGTGSRLTMGLVGTYDMETTFI | 120 |
| Query 121 | GDASLTKRPMGRVLNPLREMGVQV-KSEDGDRLPVTLRGPKTPTPITYRVPMASAQVKSA | 179 |
| Sbjct 121 | GDASL+ RPMGRVL PLR+MGVQV K+ GDR+P+TLRGPK PITYRVPMASAQVKSA GDASLSGRPMGRVLEPLRQMGVQVLKATPGDRMPITLRGPKHAAPITYRVPMASAQVKSA | 180 |
| Query 180 | VLLAGLNTPGITTVIEPIMTRDHTEKMLQGFGANLTVETDADGVRTIRLEGRGKLTGQVI | 239 |
| Sbjct 181 | VLLAGLNTPGITTVIEP+MTRDHTEKML+GFGANL+VE+D GVR I +EG+GKLTGQ I VLLAGLNTPGITTVIEPVMTRDHTEKMLKGFGANLSVESDERGVRHIFIEGQGKLTGQTI | 240 |
| Query 240 | DVPGDPSSTAFPLVAALLVPGSDVTILNVLMNPTRTGLILTLQEMGADIEVINPRLAGGE | 299 |
| Sbjct 241 | VPGDPSS FPLVAAL+VPGSD+TI NVLMNPTRTGL+LTLQEMGA I++++PR GGE AVPGDPSSAGFPLVAALIVPGSDITIENVLMNPTRTGLLLTLQEMGARIDIVDPRNEGGE | 300 |
| Query 300 | DVADLRVRSSTLKGVTVPEDRAPSMIDEYPILavaaafaegaTVMNGLEELRVKESDRLS | 359 |
| Sbjct 301 | DVADLRVR S LKGV VP +RAP+MIDEYP+LAVAA+FAEG T+M GLEELRVKESDRLS DVADLRVRYSELKGVAVPPERAPTMIDEYPVLAVAASFAEGETLMEGLEELRVKESDRLS | 360 |
| Query 360 | AVANGLKLNGVDCDEGETSLVVRGRPDGKGLG---NASGAAVATHLDHRIAMSFLVMGLV | 416 |
| Sbjct 361 | AVA GLKLNGVDC EGE SL VRG+P GKGLG N V T LDHRIAMSFLVMGL AVAEGLKLNGVDCTEGEASLAVRGKPGGKGLGQHPNGPNTVVQTQLDHRIAMSFLVMGLA | 420 |
| Query 417  Sbjct 421 | SENPVTVDDATMIATSFPEFMDLMAGLGAKIE 448  +E PVT+DD MIATSFPEFM LM LGA+IE  TEKPVTIDDQAMIATSFPEFMGLMRRLGAEIE 452 |  |

>TIP47873.1 3-phosphoshikimate 1-carboxyvinyltransferase [Mesorhizobium sp.] Length=452

Score = 674 bits (1739), Expect = 0.0, Method: Compositional matrix adjust.

| Identities | = 347/452 (77%), Positives = 382/452 (85%), Gaps = 4/452 (1%) |  |
| --- | --- | --- |
| Query 1 | MLHGASSRPATARKSSGLSGTVRIPGDKSISHRSFMFGGLASGETRITGLLEGEDVINTG | 60 |
| Sbjct 1 | M H A+ +PATARKS LSGT R+PGDKSISHRSFMFGGLASGETRITGLLEGEDV+ TG MSHTAAPKPATARKSPALSGTARVPGDKSISHRSFMFGGLASGETRITGLLEGEDVMRTG | 60 |
| Query 61 | KAMQAMGARIRKEGDTWIIDGVGNGGLLAPEAPLDFGNAATGCRLTMGLVGVYDFDSTFI | 120 |
| Sbjct 61 | AM+AMGA I K G W+I G GNG LL PE PLDFGNA TG RLTMGLVG YD ++TFI AAMKAMGAHIEKRGTEWVIRGTGNGALLQPEGPLDFGNAGTGSRLTMGLVGTYDMETTFI | 120 |
| Query 121 | GDASLTKRPMGRVLNPLREMGVQV-KSEDGDRLPVTLRGPKTPTPITYRVPMASAQVKSA | 179 |
| Sbjct 121 | GDASL+ RPMGRVL PLR+MGVQV K+ GDR+P+TLRGPK PITYRVPMASAQVKSA GDASLSGRPMGRVLEPLRQMGVQVLKATPGDRMPITLRGPKHAAPITYRVPMASAQVKSA | 180 |
| Query 180 | VLLAGLNTPGITTVIEPIMTRDHTEKMLQGFGANLTVETDADGVRTIRLEGRGKLTGQVI | 239 |
| Sbjct 181 | VLLAGLNTPGITTVIEP+MTRDHTEKML+GFGANL+VETD GVR I +EG+GKLTGQ I VLLAGLNTPGITTVIEPVMTRDHTEKMLKGFGANLSVETDERGVRHIFIEGQGKLTGQTI | 240 |
| Query 240 | DVPGDPSSTAFPLVAALLVPGSDVTILNVLMNPTRTGLILTLQEMGADIEVINPRLAGGE | 299 |
| Sbjct 241 | VPGDPSS FPLVAAL+VPGSD+TI NVLMNPTRTGL+LTLQEMGA I++++PR GGE  AVPGDPSSAGFPLVAALIVPGSDITIENVLMNPTRTGLLLTLQEMGARIDIVDPRNEGGE | 300 |

| Query | 300 | DVADLRVRSSTLKGVTVPEDRAPSMIDEYPILavaaafaegaTVMNGLEELRVKESDRLS | 359 |
| --- | --- | --- | --- |
| Sbjct | 301 | DVADLRVR S LKGV VP +RAP+MIDEYP+LAVAA+FAEG T+M GLEELRVKESDRLS DVADLRVRYSELKGVAVPPERAPTMIDEYPVLAVAASFAEGETLMEGLEELRVKESDRLS | 360 |
| Query | 360 | AVANGLKLNGVDCDEGETSLVVRGRPDGKGLG---NASGAAVATHLDHRIAMSFLVMGLV | 416 |
| Sbjct | 361 | A+A GLKLNGVDC EGE SL VRG+P GKGLG N V T LDHRIAMSFLVMGL AMAEGLKLNGVDCTEGEASLAVRGKPGGKGLGQHPNGPNTVVQTQLDHRIAMSFLVMGLA | 420 |
| Query  Sbjct | 417  421 | SENPVTVDDATMIATSFPEFMDLMAGLGAKIE 448  +E PVT+DD MIATSFPEFM LM LGA+IE  TEKPVTIDDQAMIATSFPEFMGLMRRLGAEIE 452 |  |

>RWI29033.1 3-phosphoshikimate 1-carboxyvinyltransferase [Mesorhizobium sp.] RWK52847.1 3-phosphoshikimate 1-carboxyvinyltransferase [Mesorhizobium sp.] RWK97755.1 3-phosphoshikimate 1-carboxyvinyltransferase [Mesorhizobium sp.] RWL02772.1 3-phosphoshikimate 1-carboxyvinyltransferase [Mesorhizobium sp.] TIP61559.1 3-phosphoshikimate 1-carboxyvinyltransferase [Mesorhizobium sp.]

Length=452

Score = 674 bits (1739), Expect = 0.0, Method: Compositional matrix adjust.

| Identities | = 347/452 (77%), Positives = 382/452 (85%), Gaps = 4/452 (1%) |  |
| --- | --- | --- |
| Query 1 | MLHGASSRPATARKSSGLSGTVRIPGDKSISHRSFMFGGLASGETRITGLLEGEDVINTG | 60 |
| Sbjct 1 | M H A+ +PATARKS LSGT R+PGDKSISHRSFMFGGLASGETRITGLLEGEDV+ TG MSHPAAPKPATARKSPALSGTARVPGDKSISHRSFMFGGLASGETRITGLLEGEDVMRTG | 60 |
| Query 61 | KAMQAMGARIRKEGDTWIIDGVGNGGLLAPEAPLDFGNAATGCRLTMGLVGVYDFDSTFI | 120 |
| Sbjct 61 | AM+AMGA I K G W+I G GNG LL PE PLDFGNA TG RLTMGLVG YD ++TFI AAMKAMGAHIEKRGTEWVIRGTGNGALLQPEGPLDFGNAGTGSRLTMGLVGTYDMETTFI | 120 |
| Query 121 | GDASLTKRPMGRVLNPLREMGVQV-KSEDGDRLPVTLRGPKTPTPITYRVPMASAQVKSA | 179 |
| Sbjct 121 | GDASL+ RPMGRVL PLR+MGVQV K+ GDR+P+TLRG K PITYRVPMASAQVKSA GDASLSGRPMGRVLEPLRQMGVQVLKATPGDRMPITLRGSKHAAPITYRVPMASAQVKSA | 180 |
| Query 180 | VLLAGLNTPGITTVIEPIMTRDHTEKMLQGFGANLTVETDADGVRTIRLEGRGKLTGQVI | 239 |
| Sbjct 181 | VLLAGLNTPGITTVIEP+MTRDHTEKML+GFGANL+VE+D GVR I +EG+GKLTGQ I VLLAGLNTPGITTVIEPVMTRDHTEKMLKGFGANLSVESDERGVRHIFIEGQGKLTGQTI | 240 |
| Query 240 | DVPGDPSSTAFPLVAALLVPGSDVTILNVLMNPTRTGLILTLQEMGADIEVINPRLAGGE | 299 |
| Sbjct 241 | VPGDPSS FPLVAAL+VPGSD+TI NVLMNPTRTGL+LTLQEMGA I++++PR GGE AVPGDPSSAGFPLVAALIVPGSDITIENVLMNPTRTGLLLTLQEMGARIDIVDPRNEGGE | 300 |
| Query 300 | DVADLRVRSSTLKGVTVPEDRAPSMIDEYPILavaaafaegaTVMNGLEELRVKESDRLS | 359 |
| Sbjct 301 | DVADLRVR S LKGV VP +RAP+MIDEYP+LAVAA+FAEG T+M GLEELRVKESDRLS DVADLRVRYSELKGVAVPPERAPTMIDEYPVLAVAASFAEGETLMEGLEELRVKESDRLS | 360 |
| Query 360 | AVANGLKLNGVDCDEGETSLVVRGRPDGKGLG---NASGAAVATHLDHRIAMSFLVMGLV | 416 |
| Sbjct 361 | AVA GLKLNGVDC EGE SL VRG+P GKGLG N V T LDHRIAMSFLVMGL AVAEGLKLNGVDCTEGEASLAVRGKPGGKGLGQHPNGPNTVVQTQLDHRIAMSFLVMGLA | 420 |
| Query 417  Sbjct 421 | SENPVTVDDATMIATSFPEFMDLMAGLGAKIE 448  +E PVT+DD MIATSFPEFM LM GLGA+IE  TEKPVTIDDQAMIATSFPEFMGLMKGLGAEIE 452 |  |

```
>WP_140580572.1 MULTISPECIES: 3-phosphoshikimate 1-carboxyvinyltransferase [unclassified Mesorhizobium]
TPI96024.1 3-phosphoshikimate 1-carboxyvinyltransferase [Mesorhizobium sp. B2-8-1]
TPL15270.1 3-phosphoshikimate 1-carboxyvinyltransferase [Mesorhizobium sp. B2-4-11]
TPN40692.1 3-phosphoshikimate 1-carboxyvinyltransferase [Mesorhizobium sp. B1-1-6]
TPN68306.1 3-phosphoshikimate 1-carboxyvinyltransferase [Mesorhizobium sp. B1-1-1]
Length=452

Score = 674 bits (1738), Expect = 0.0, Method: Compositional matrix adjust. Identities = 353/451 (78%), Positives = 385/451 (85%), Gaps = 4/451 (1%)

Query 1	MLHGASSRPATARKSSGLSGTVRIPGDKSISHRSFMFGGLASGETRITGLLEGEDVINTG 60

M H A+S+PATARK	LSGT R+PGDKSISHRSFMFGGLASGETRITGLLEGEDV+ TG
```

| Sbjct | 1 | MSHAAASKPATARKCQALSGTARVPGDKSISHRSFMFGGLASGETRITGLLEGEDVMRTG | 60 |
| --- | --- | --- | --- |
| Query | 61 | KAMQAMGARIRKEGDTWIIDGVGNGGLLAPEAPLDFGNAATGCRLTMGLVGVYDFDSTFI | 120 |
| Sbjct | 61 | AM+AMGA I K GD W+I G GNG LL PE PLDFGNA TG RLTMGLVG YD ++TFI AAMKAMGAHIEKRGDEWVIRGTGNGALLQPEGPLDFGNAGTGSRLTMGLVGTYDMETTFI | 120 |
| Query | 121 | GDASLTKRPMGRVLNPLREMGVQV-KSEDGDRLPVTLRGPKTPTPITYRVPMASAQVKSA | 179 |
| Sbjct | 121 | GDASL+ RPMGRVL PLR+MGVQV K+ GDR+P+TL GPK PITYRVPMASAQVKSA GDASLSGRPMGRVLEPLRQMGVQVLKATPGDRMPITLHGPKHAAPITYRVPMASAQVKSA | 180 |
| Query | 180 | VLLAGLNTPGITTVIEPIMTRDHTEKMLQGFGANLTVETDADGVRTIRLEGRGKLTGQVI | 239 |
| Sbjct | 181 | VLLAGLNTPGITTVIEP+MTRDHTEKML+GFGANL+VETD GVR I +EG+GKLTGQ I VLLAGLNTPGITTVIEPVMTRDHTEKMLKGFGANLSVETDERGVRHIFIEGQGKLTGQTI | 240 |
| Query | 240 | DVPGDPSSTAFPLVAALLVPGSDVTILNVLMNPTRTGLILTLQEMGADIEVINPRLAGGE | 299 |
| Sbjct | 241 | VPGDPSS FPLVAAL+VPGSD+ I NVLMNPTRTGL+LTLQEMG IE++NPR AGGE AVPGDPSSAGFPLVAALIVPGSDIVIENVLMNPTRTGLLLTLQEMGGQIEILNPRNAGGE | 300 |
| Query | 300 | DVADLRVRSSTLKGVTVPEDRAPSMIDEYPILavaaafaegaTVMNGLEELRVKESDRLS | 359 |
| Sbjct | 301 | DVADLRVR S LKGVTVP +RAPSMIDEYP+LAVAA+FAEG T+M GLEELRVKESDRLS DVADLRVRYSELKGVTVPAERAPSMIDEYPVLAVAASFAEGETLMQGLEELRVKESDRLS | 360 |
| Query | 360 | AVANGLKLNGVDCDEGETSLVVRGRPDGKGLG---NASGAAVATHLDHRIAMSFLVMGLV | 416 |
| Sbjct | 361 | AVANGLKLNGVDC EGE SL VRG+P GKGLG N V THLDHRIAMSFL+MGL AVANGLKLNGVDCTEGEASLAVRGKPGGKGLGGHPNGPDTTVQTHLDHRIAMSFLIMGLA | 420 |
| Query  Sbjct | 417  421 | SENPVTVDDATMIATSFPEFMDLMAGLGAKI 447  +E PVT+DDA MIATSFPEFM LM GLGA+I  TEKPVTIDDANMIATSFPEFMSLMTGLGAEI 451 |  |

>WP_140768585.1 3-phosphoshikimate 1-carboxyvinyltransferase [Mesorhizobium sp. B2-5-4]

TPK48862.1 3-phosphoshikimate 1-carboxyvinyltransferase [Mesorhizobium sp. B2-5-4]

Length=452

Score = 673 bits (1737), Expect = 0.0, Method: Compositional matrix adjust.

| Identities | = 352/451 (78%), Positives = 383/451 (85%), Gaps = 4/451 (1%) |  |
| --- | --- | --- |
| Query 1 | MLHGASSRPATARKSSGLSGTVRIPGDKSISHRSFMFGGLASGETRITGLLEGEDVINTG | 60 |
| Sbjct 1 | M H A+++PA ARKS LSGT R+PGDKSISHRSFMFGGLASGETRITGLLEGEDV+ TG MSHAAAAKPAIARKSPALSGTARVPGDKSISHRSFMFGGLASGETRITGLLEGEDVMRTG | 60 |
| Query 61 | KAMQAMGARIRKEGDTWIIDGVGNGGLLAPEAPLDFGNAATGCRLTMGLVGVYDFDSTFI | 120 |
| Sbjct 61 | AM+AMGA I K G W+I G GNG LL PE PLDFGNA TG RLTMGLVG YD ++TFI AAMKAMGAHIEKHGAEWVIRGTGNGALLQPEGPLDFGNAGTGSRLTMGLVGTYDMETTFI | 120 |
| Query 121 | GDASLTKRPMGRVLNPLREMGVQV-KSEDGDRLPVTLRGPKTPTPITYRVPMASAQVKSA | 179 |
| Sbjct 121 | GDASL+ RPMGRVL PLR+MGVQV K+ GDR+P+TL GPK PITYRVPMASAQVKSA GDASLSGRPMGRVLEPLRQMGVQVLKATPGDRMPITLHGPKHAAPITYRVPMASAQVKSA | 180 |
| Query 180 | VLLAGLNTPGITTVIEPIMTRDHTEKMLQGFGANLTVETDADGVRTIRLEGRGKLTGQVI | 239 |
| Sbjct 181 | VLLAGLNTPGITTVIEP+MTRDHTEKML+GFGANL+VETD GVR I +EG+GKLTGQ I VLLAGLNTPGITTVIEPVMTRDHTEKMLKGFGANLSVETDERGVRHIFIEGQGKLTGQTI | 240 |
| Query 240 | DVPGDPSSTAFPLVAALLVPGSDVTILNVLMNPTRTGLILTLQEMGADIEVINPRLAGGE | 299 |
| Sbjct 241 | VPGDPSS FPLVAAL+VPGSD+TI NVLMNPTRTGL+LTLQEMG I+++NPR AGGE AVPGDPSSAGFPLVAALIVPGSDITIENVLMNPTRTGLLLTLQEMGGQIDILNPRNAGGE | 300 |
| Query 300 | DVADLRVRSSTLKGVTVPEDRAPSMIDEYPILavaaafaegaTVMNGLEELRVKESDRLS | 359 |
| Sbjct 301 | DVADLRVR S LKGVTVP +RAPSMIDEYP+LAVAA+FAEG T+M GLEELRVKESDRLS DVADLRVRYSELKGVTVPPERAPSMIDEYPVLAVAASFAEGETLMQGLEELRVKESDRLS | 360 |
| Query 360 | AVANGLKLNGVDCDEGETSLVVRGRPDGKGLG---NASGAAVATHLDHRIAMSFLVMGLV | 416 |
| Sbjct 361 | AVA GLKLNGVDC E E SL VRGRPDGKGLG N V THLDHRIAMSFLVMGL AVATGLKLNGVDCTESEASLAVRGRPDGKGLGGHPNGRDTTVETHLDHRIAMSFLVMGLA | 420 |
| Query 417  Sbjct 421 | SENPVTVDDATMIATSFPEFMDLMAGLGAKI 447  +E PVT+DD MIATSFPEFM LM GLGAKI  TEKPVTIDDQAMIATSFPEFMGLMKGLGAKI 451 |  |

>WP_140643738.1 3-phosphoshikimate 1-carboxyvinyltransferase [Mesorhizobium sp. B2-2-2]

TPM24177.1 3-phosphoshikimate 1-carboxyvinyltransferase [Mesorhizobium sp. B2-2-2]

Length=452

Score = 673 bits (1736), Expect = 0.0, Method: Compositional matrix adjust.

| Identities | = 352/452 (78%), Positives = 385/452 (85%), Gaps = 4/452 (1%) |  |
| --- | --- | --- |
| Query 1 | MLHGASSRPATARKSSGLSGTVRIPGDKSISHRSFMFGGLASGETRITGLLEGEDVINTG | 60 |
| Sbjct 1 | M H A+S+PATARK LSGT R+PGDKSISHRSFMFGGLASGETRITGLLEGEDV+ TG MSHAAASKPATARKCQALSGTARVPGDKSISHRSFMFGGLASGETRITGLLEGEDVMRTG | 60 |
| Query 61 | KAMQAMGARIRKEGDTWIIDGVGNGGLLAPEAPLDFGNAATGCRLTMGLVGVYDFDSTFI | 120 |
| Sbjct 61 | AM+AMGA I K GD W+I G GNG LL PE PLDFGNA TG RLTMGLVG YD ++TFI AAMKAMGAHIEKRGDEWVIRGTGNGALLQPEGPLDFGNAGTGSRLTMGLVGTYDMETTFI | 120 |
| Query 121 | GDASLTKRPMGRVLNPLREMGVQV-KSEDGDRLPVTLRGPKTPTPITYRVPMASAQVKSA | 179 |
| Sbjct 121 | GDASL+ RPMGRVL PLR+MGVQV K+ GDR+P+TL GPK PITYRVPMASAQVKSA GDASLSGRPMGRVLEPLRQMGVQVLKATPGDRMPITLHGPKHAAPITYRVPMASAQVKSA | 180 |
| Query 180 | VLLAGLNTPGITTVIEPIMTRDHTEKMLQGFGANLTVETDADGVRTIRLEGRGKLTGQVI | 239 |
| Sbjct 181 | VLLAGLNTPGITTVIEP+MTRDHTEKML+GFGANL+VETD GVR I +EG+GKLTGQ I VLLAGLNTPGITTVIEPVMTRDHTEKMLKGFGANLSVETDERGVRHIFIEGQGKLTGQTI | 240 |
| Query 240 | DVPGDPSSTAFPLVAALLVPGSDVTILNVLMNPTRTGLILTLQEMGADIEVINPRLAGGE | 299 |
| Sbjct 241 | VPGDPSS FPLVAAL+VPGSD+ I NVLMNPTRTGL+LTLQEMG IE++NPR AGGE AVPGDPSSAGFPLVAALIVPGSDIVIENVLMNPTRTGLLLTLQEMGGQIEILNPRNAGGE | 300 |
| Query 300 | DVADLRVRSSTLKGVTVPEDRAPSMIDEYPILavaaafaegaTVMNGLEELRVKESDRLS | 359 |
| Sbjct 301 | DVADLRVR S LKG+TVP +RAPSMIDEYP+LAVAA+FAEG T+M GLEELRVKESDRLS DVADLRVRYSELKGITVPAERAPSMIDEYPVLAVAASFAEGETLMQGLEELRVKESDRLS | 360 |
| Query 360 | AVANGLKLNGVDCDEGETSLVVRGRPDGKGLG---NASGAAVATHLDHRIAMSFLVMGLV | 416 |
| Sbjct 361 | AVANGLKLNGVDC EGE SL VRG+P GKGLG N V THLDHRIAMSFL+MGL AVANGLKLNGVDCTEGEASLAVRGKPGGKGLGGHPNGPDTTVQTHLDHRIAMSFLIMGLA | 420 |
| Query 417  Sbjct 421 | SENPVTVDDATMIATSFPEFMDLMAGLGAKIE 448  +E PVT+DD MIATSFPEFM LM GLGA+IE  TEKPVTIDDQAMIATSFPEFMGLMKGLGAEIE 452 |  |

>RWF32751.1 3-phosphoshikimate 1-carboxyvinyltransferase, partial [Mesorhizobium sp.]

Length=439

Score = 673 bits (1736), Expect = 0.0, Method: Compositional matrix adjust.

| Identities | = 346/439 (79%), Positives = 376/439 (86%), Gaps = 4/439 (1%) |  |
| --- | --- | --- |
| Query 1 | MLHGASSRPATARKSSGLSGTVRIPGDKSISHRSFMFGGLASGETRITGLLEGEDVINTG | 60 |
| Sbjct 1 | M H A+ +PATARKSS LSGT R+PGDKSISHRSFMFGGLASGETRITGLLEGEDV+ TG MSHAAAPKPATARKSSALSGTARVPGDKSISHRSFMFGGLASGETRITGLLEGEDVMRTG | 60 |
| Query 61 | KAMQAMGARIRKEGDTWIIDGVGNGGLLAPEAPLDFGNAATGCRLTMGLVGVYDFDSTFI | 120 |
| Sbjct 61 | AM+AMGA I K G W+I G GNG LL PE PLDFGNA TG RLTMGLVG YD ++ FI AAMKAMGAHIEKRGAEWVIRGTGNGALLQPEGPLDFGNAGTGSRLTMGLVGTYDMETRFI | 120 |
| Query 121 | GDASLTKRPMGRVLNPLREMGVQV-KSEDGDRLPVTLRGPKTPTPITYRVPMASAQVKSA | 179 |
| Sbjct 121 | GDASL+ RPMGRVL PLR+MGVQV K+ GDR+P+TLRGPK PITYRVPMASAQVKSA GDASLSGRPMGRVLEPLRQMGVQVLKATPGDRMPITLRGPKHAAPITYRVPMASAQVKSA | 180 |
| Query 180 | VLLAGLNTPGITTVIEPIMTRDHTEKMLQGFGANLTVETDADGVRTIRLEGRGKLTGQVI | 239 |
| Sbjct 181 | VLLAGLNTPGITTVIEP+MTRDHTEKML+GFGANLTVETD GVR I +EG+GKL+GQ I VLLAGLNTPGITTVIEPVMTRDHTEKMLKGFGANLTVETDERGVRHIFIEGQGKLSGQTI | 240 |
| Query 240 | DVPGDPSSTAFPLVAALLVPGSDVTILNVLMNPTRTGLILTLQEMGADIEVINPRLAGGE | 299 |
| Sbjct 241 | VPGDPSS FPLVAAL+VPGSD+ I NVLMNPTRTGL+LTLQEMG I+++NPR AGGE AVPGDPSSAGFPLVAALIVPGSDIVIENVLMNPTRTGLLLTLQEMGGRIDILNPRNAGGE | 300 |
| Query 300 | DVADLRVRSSTLKGVTVPEDRAPSMIDEYPILavaaafaegaTVMNGLEELRVKESDRLS | 359 |

| Sbjct | 301 | DVADLRVRSS LKGVTVP +RAPSMIDEYP+LAVAA+FAEG T+M GLEELRVKESDRLS DVADLRVRSSELKGVTVPPERAPSMIDEYPVLAVAASFAEGETLMQGLEELRVKESDRLS | 360 |
| --- | --- | --- | --- |
| Query | 360 | AVANGLKLNGVDCDEGETSLVVRGRPDGKGLG---NASGAAVATHLDHRIAMSFLVMGLV | 416 |
| Sbjct | 361 | AVANGLKLNGVDC EGE +L VRGRP GKGLG N V THLDHRIAMSFLVMGL AVANGLKLNGVDCTEGEATLAVRGRPGGKGLGRHPNGPDTTVKTHLDHRIAMSFLVMGLA | 420 |
| Query  Sbjct | 417  421 | SENPVTVDDATMIATSFPE 435 SE PVT+DDA MIATSFPE SEKPVTIDDANMIATSFPE 439 |  |

>WP_140559770.1 MULTISPECIES: 3-phosphoshikimate 1-carboxyvinyltransferase [unclassified Mesorhizobium]

TPJ56473.1 3-phosphoshikimate 1-carboxyvinyltransferase [Mesorhizobium sp. B2-6-4]

TPN00277.1 3-phosphoshikimate 1-carboxyvinyltransferase [Mesorhizobium sp. B2-1-5]

Length=452

Score = 673 bits (1736), Expect = 0.0, Method: Compositional matrix adjust.

| Identities | = 349/451 (77%), Positives = 380/451 (84%), Gaps = 4/451 (1%) |  |
| --- | --- | --- |
| Query 1 | MLHGASSRPATARKSSGLSGTVRIPGDKSISHRSFMFGGLASGETRITGLLEGEDVINTG | 60 |
| Sbjct 1 | M H A+S+PATARK LSGT R+PGDKSISHRSFMFGGLASGETRITGLLEGEDV+ TG MSHAAASKPATARKCQALSGTARVPGDKSISHRSFMFGGLASGETRITGLLEGEDVMRTG | 60 |
| Query 61 | KAMQAMGARIRKEGDTWIIDGVGNGGLLAPEAPLDFGNAATGCRLTMGLVGVYDFDSTFI | 120 |
| Sbjct 61 | AM+AMGA I K GD W+I G GNG LL PE PLDFGNA TG RLTMGLVG YD ++TFI AAMKAMGAHIEKRGDEWVIRGTGNGALLQPEGPLDFGNAGTGSRLTMGLVGTYDMETTFI | 120 |
| Query 121 | GDASLTKRPMGRVLNPLREMGVQV-KSEDGDRLPVTLRGPKTPTPITYRVPMASAQVKSA | 179 |
| Sbjct 121 | GDASL+ RPMGRVL PLR+MGVQV K+ GDR+P+TL GPK PITYRVPMASAQVKSA GDASLSGRPMGRVLEPLRQMGVQVLKATPGDRMPITLHGPKHAAPITYRVPMASAQVKSA | 180 |
| Query 180 | VLLAGLNTPGITTVIEPIMTRDHTEKMLQGFGANLTVETDADGVRTIRLEGRGKLTGQVI | 239 |
| Sbjct 181 | VLLAGLNTPGITTVIEP+MTRDHTEKML+GFGANL+VETD GVR I +EG+GKLTGQ I VLLAGLNTPGITTVIEPVMTRDHTEKMLKGFGANLSVETDERGVRHIFIEGQGKLTGQTI | 240 |
| Query 240 | DVPGDPSSTAFPLVAALLVPGSDVTILNVLMNPTRTGLILTLQEMGADIEVINPRLAGGE | 299 |
| Sbjct 241 | VPGDPSS FPLVAAL+VPGSD+ I NVLMNPTRTGL+LTLQEMG IE++NPR AGGE AVPGDPSSAGFPLVAALIVPGSDIVIENVLMNPTRTGLLLTLQEMGGQIEILNPRNAGGE | 300 |
| Query 300 | DVADLRVRSSTLKGVTVPEDRAPSMIDEYPILavaaafaegaTVMNGLEELRVKESDRLS | 359 |
| Sbjct 301 | DVADLRVR S LKGVTVP +RAPSMIDEYP+LAVAA+FAEG T+M GLEELRVKESDRLS DVADLRVRYSELKGVTVPAERAPSMIDEYPVLAVAASFAEGETLMQGLEELRVKESDRLS | 360 |
| Query 360 | AVANGLKLNGVDCDEGETSLVVR-GRPDGKGLGNASG--AAVATHLDHRIAMSFLVMGLV | 416 |
| Sbjct 361 | AVANGLKLNGVDC EGE SL VR G+ SG V THLDHRIAMSFLVMGL AVANGLKLNGVDCTEGEASLAVRGKPGGKGLGGHPSGPDTTVQTHLDHRIAMSFLVMGLA | 420 |
| Query 417  Sbjct 421 | SENPVTVDDATMIATSFPEFMDLMAGLGAKI 447  +E PVT+DDA MIATSFPEFM LM GLGA+I  TEKPVTIDDANMIATSFPEFMSLMTGLGAEI 451 |  |

```
>WP_140649465.1 MULTISPECIES: 3-phosphoshikimate 1-carboxyvinyltransferase [unclassified Mesorhizobium]
TPJ63786.1 3-phosphoshikimate 1-carboxyvinyltransferase [Mesorhizobium sp. B2-6-1]
TPL20660.1 3-phosphoshikimate 1-carboxyvinyltransferase [Mesorhizobium sp. B2-4-10]
TPL58985.1 3-phosphoshikimate 1-carboxyvinyltransferase [Mesorhizobium sp. B2-4-2]
TPN57970.1 3-phosphoshikimate 1-carboxyvinyltransferase [Mesorhizobium sp. B1-1-4]
Length=452

Score = 672 bits (1735), Expect = 0.0, Method: Compositional matrix adjust. Identities = 352/451 (78%), Positives = 385/451 (85%), Gaps = 4/451 (1%)

Query 1	MLHGASSRPATARKSSGLSGTVRIPGDKSISHRSFMFGGLASGETRITGLLEGEDVINTG 60

M H A+S+PATARK	LSGT R+PGDKSISHRSFMFGGLASGETRITGLLEGEDV+ TG
```

| Sbjct | 1 | MSHAAASKPATARKCQALSGTARVPGDKSISHRSFMFGGLASGETRITGLLEGEDVMRTG | 60 |
| --- | --- | --- | --- |
| Query | 61 | KAMQAMGARIRKEGDTWIIDGVGNGGLLAPEAPLDFGNAATGCRLTMGLVGVYDFDSTFI | 120 |
| Sbjct | 61 | AM+AMGA I K GD W+I G GNG LL PE PLDFGNA TG RLTMGLVG YD ++TFI AAMKAMGAHIEKRGDEWVIRGTGNGALLQPEGPLDFGNAGTGSRLTMGLVGTYDMETTFI | 120 |
| Query | 121 | GDASLTKRPMGRVLNPLREMGVQV-KSEDGDRLPVTLRGPKTPTPITYRVPMASAQVKSA | 179 |
| Sbjct | 121 | GDASL+ RPMGRVL PLR+MGVQV K+ GDR+P+TL GPK PITYRVPMASAQVKSA GDASLSGRPMGRVLEPLRQMGVQVLKATPGDRMPITLHGPKHAAPITYRVPMASAQVKSA | 180 |
| Query | 180 | VLLAGLNTPGITTVIEPIMTRDHTEKMLQGFGANLTVETDADGVRTIRLEGRGKLTGQVI | 239 |
| Sbjct | 181 | VLLAGLNTPGITTVIEP+MTRDHTEKML+GFGANL+VETD GVR I +EG+GKLTGQ I VLLAGLNTPGITTVIEPVMTRDHTEKMLKGFGANLSVETDERGVRHIFIEGQGKLTGQTI | 240 |
| Query | 240 | DVPGDPSSTAFPLVAALLVPGSDVTILNVLMNPTRTGLILTLQEMGADIEVINPRLAGGE | 299 |
| Sbjct | 241 | VPGDPSS FPLVAAL+VPGS++ I NVLMNPTRTGL+LTLQEMG IE++NPR AGGE AVPGDPSSAGFPLVAALIVPGSEIVIENVLMNPTRTGLLLTLQEMGGQIEILNPRNAGGE | 300 |
| Query | 300 | DVADLRVRSSTLKGVTVPEDRAPSMIDEYPILavaaafaegaTVMNGLEELRVKESDRLS | 359 |
| Sbjct | 301 | DVADLRVR S LKGVTVP +RAPSMIDEYP+LAVAA+FAEG T+M GLEELRVKESDRLS DVADLRVRYSELKGVTVPAERAPSMIDEYPVLAVAASFAEGETLMQGLEELRVKESDRLS | 360 |
| Query | 360 | AVANGLKLNGVDCDEGETSLVVRGRPDGKGLG---NASGAAVATHLDHRIAMSFLVMGLV | 416 |
| Sbjct | 361 | AVANGLKLNGVDC EGE SL VRG+P GKGLG N V THLDHRIAMSFL+MGL AVANGLKLNGVDCTEGEASLAVRGKPGGKGLGGHPNGPDTTVQTHLDHRIAMSFLIMGLA | 420 |
| Query  Sbjct | 417  421 | SENPVTVDDATMIATSFPEFMDLMAGLGAKI 447  +E PVT+DDA MIATSFPEFM LM GLGA+I  TEKPVTIDDANMIATSFPEFMSLMTGLGAEI 451 |  |

>WP_140512883.1 3-phosphoshikimate 1-carboxyvinyltransferase [Mesorhizobium sp. B2-2-3]

TPM53496.1 3-phosphoshikimate 1-carboxyvinyltransferase [Mesorhizobium sp. B2-2-3]

Length=452

Score = 672 bits (1735), Expect = 0.0, Method: Compositional matrix adjust.

| Identities | = 352/451 (78%), Positives = 385/451 (85%), Gaps = 4/451 (1%) |  |
| --- | --- | --- |
| Query 1 | MLHGASSRPATARKSSGLSGTVRIPGDKSISHRSFMFGGLASGETRITGLLEGEDVINTG | 60 |
| Sbjct 1 | M H A+S+PATARK LSGT R+PGDKSISHRSFMFGGLASGETRITGLLEGEDV+ TG MSHAAASKPATARKCQALSGTARVPGDKSISHRSFMFGGLASGETRITGLLEGEDVMRTG | 60 |
| Query 61 | KAMQAMGARIRKEGDTWIIDGVGNGGLLAPEAPLDFGNAATGCRLTMGLVGVYDFDSTFI | 120 |
| Sbjct 61 | AM+AMGA I K GD W+I G GNG LL PE PLDFGNA TG RLTMGLVG YD ++TFI AAMKAMGAHIEKRGDVWVIRGTGNGALLQPEGPLDFGNAGTGSRLTMGLVGTYDMETTFI | 120 |
| Query 121 | GDASLTKRPMGRVLNPLREMGVQV-KSEDGDRLPVTLRGPKTPTPITYRVPMASAQVKSA | 179 |
| Sbjct 121 | GDASL+ RPMGRVL PLR+MGVQV K+ GDR+P+TL GPK PITYRVPMASAQVKSA GDASLSGRPMGRVLEPLRQMGVQVLKATPGDRMPITLHGPKHAAPITYRVPMASAQVKSA | 180 |
| Query 180 | VLLAGLNTPGITTVIEPIMTRDHTEKMLQGFGANLTVETDADGVRTIRLEGRGKLTGQVI | 239 |
| Sbjct 181 | VLLAGLNTPGITTVIEP+MTRDHTEKML+GFGANL+VETD GVR I +EG+GKLTGQ I VLLAGLNTPGITTVIEPVMTRDHTEKMLKGFGANLSVETDERGVRHIFIEGQGKLTGQTI | 240 |
| Query 240 | DVPGDPSSTAFPLVAALLVPGSDVTILNVLMNPTRTGLILTLQEMGADIEVINPRLAGGE | 299 |
| Sbjct 241 | VPGDPSS FPLVAAL+VPGS++ I NVLMNPTRTGL+LTLQEMG IE++NPR AGGE AVPGDPSSAGFPLVAALIVPGSEIVIENVLMNPTRTGLLLTLQEMGGQIEILNPRNAGGE | 300 |
| Query 300 | DVADLRVRSSTLKGVTVPEDRAPSMIDEYPILavaaafaegaTVMNGLEELRVKESDRLS | 359 |
| Sbjct 301 | DVADLRVR S LKGVTVP +RAPSMIDEYP+LAVAA+FAEG T+M GLEELRVKESDRLS DVADLRVRYSELKGVTVPAERAPSMIDEYPVLAVAASFAEGETLMQGLEELRVKESDRLS | 360 |
| Query 360 | AVANGLKLNGVDCDEGETSLVVRGRPDGKGLG---NASGAAVATHLDHRIAMSFLVMGLV | 416 |
| Sbjct 361 | AVANGLKLNGVDC EGE SL VRG+P GKGLG N V THLDHRIAMSFL+MGL AVANGLKLNGVDCTEGEASLAVRGKPGGKGLGGHPNGPDTTVQTHLDHRIAMSFLIMGLA | 420 |
| Query 417  Sbjct 421 | SENPVTVDDATMIATSFPEFMDLMAGLGAKI 447  +E PVT+DDA MIATSFPEFM LM GLGA+I  TEKPVTIDDANMIATSFPEFMSLMTGLGAEI 451 |  |

>WP_096452931.1 3-phosphoshikimate 1-carboxyvinyltransferase [Mesorhizobium loti] BAV49602.1 3-phosphoshikimate 1-carboxyvinyltransferase [Mesorhizobium loti]

Length=452

Score = 672 bits (1734), Expect = 0.0, Method: Compositional matrix adjust.

| Identities | = 351/452 (78%), Positives = 385/452 (85%), Gaps = 4/452 (1%) |  |
| --- | --- | --- |
| Query 1 | MLHGASSRPATARKSSGLSGTVRIPGDKSISHRSFMFGGLASGETRITGLLEGEDVINTG | 60 |
| Sbjct 1 | M H A+++PATARKS LSGT R+PGDKSISHRSFMFGGLASGETRITGLLEGEDV+ TG MSHAAAAKPATARKSQALSGTARVPGDKSISHRSFMFGGLASGETRITGLLEGEDVMRTG | 60 |
| Query 61 | KAMQAMGARIRKEGDTWIIDGVGNGGLLAPEAPLDFGNAATGCRLTMGLVGVYDFDSTFI | 120 |
| Sbjct 61 | AM+AMGA I K G W+I G GNG LL PE PLDFGNA TG RLTMGLVG YD ++TFI AAMKAMGAHIEKRGTEWVIRGTGNGALLQPEGPLDFGNAGTGSRLTMGLVGTYDMETTFI | 120 |
| Query 121 | GDASLTKRPMGRVLNPLREMGVQV-KSEDGDRLPVTLRGPKTPTPITYRVPMASAQVKSA | 179 |
| Sbjct 121 | GDASL+ RPMGRVL PLR+MGVQV K+ GDR+P+TL GPK PITYRVPMASAQVKSA GDASLSGRPMGRVLEPLRQMGVQVLKATPGDRMPITLHGPKHAAPITYRVPMASAQVKSA | 180 |
| Query 180 | VLLAGLNTPGITTVIEPIMTRDHTEKMLQGFGANLTVETDADGVRTIRLEGRGKLTGQVI | 239 |
| Sbjct 181 | VLLAGLNTPGITTVIEP+MTRDHTEKML+GFGANL+VETD GVR I +EG+G+LTGQ I VLLAGLNTPGITTVIEPVMTRDHTEKMLKGFGANLSVETDERGVRHIFIEGQGRLTGQTI | 240 |
| Query 240 | DVPGDPSSTAFPLVAALLVPGSDVTILNVLMNPTRTGLILTLQEMGADIEVINPRLAGGE | 299 |
| Sbjct 241 | VPGDPSS FPLVAAL+VPGSD+ I NVLMNPTRTGL+LTLQEMG I+++NPR AGGE AVPGDPSSAGFPLVAALIVPGSDIVIENVLMNPTRTGLLLTLQEMGGQIDILNPRNAGGE | 300 |
| Query 300 | DVADLRVRSSTLKGVTVPEDRAPSMIDEYPILavaaafaegaTVMNGLEELRVKESDRLS | 359 |
| Sbjct 301 | DVADLRVR S LKGV VP +RAPSMIDEYP+LAVAA+FAEG T+M GLEELRVKESDRLS DVADLRVRYSELKGVAVPPERAPSMIDEYPVLAVAASFAEGETLMQGLEELRVKESDRLS | 360 |
| Query 360 | AVANGLKLNGVDCDEGETSLVVRGRPDGKGLG---NASGAAVATHLDHRIAMSFLVMGLV | 416 |
| Sbjct 361 | AVANGLKLNGVDC EGE SL VRG+P GKGLG N V THLDHRIAMSFLVMGL AVANGLKLNGVDCTEGEASLAVRGKPGGKGLGRHPNGLDTTVKTHLDHRIAMSFLVMGLA | 420 |
| Query 417  Sbjct 421 | SENPVTVDDATMIATSFPEFMDLMAGLGAKIE 448  +E PVT+DDA MIATSFPEFM LM GLGA+IE  TEKPVTIDDANMIATSFPEFMGLMTGLGAEIE 452 |  |

>WP_173418650.1 3-phosphoshikimate 1-carboxyvinyltransferase [Ensifer adhaerens] NRP17362.1 3-phosphoshikimate 1-carboxyvinyltransferase [Ensifer adhaerens]

Length=448

Score = 672 bits (1734), Expect = 0.0, Method: Compositional matrix adjust.

| Identities | = 339/448 (76%), Positives = 388/448 (87%), Gaps = 0/448 (0%) |  |
| --- | --- | --- |
| Query 1 | MLHGASSRPATARKSSGLSGTVRIPGDKSISHRSFMFGGLASGETRITGLLEGEDVINTG | 60 |
| Sbjct 1 | M G ++PA AR+S L G VRIPGDKS+SHR+ M GGLA+G+TRI GLLEGEDV NT MSAGVIAKPAVARRSGALRGEVRIPGDKSMSHRALMLGGLAAGQTRIGGLLEGEDVFNTA | 60 |
| Query 61 | KAMQAMGARIRKEGDTWIIDGVGNGGLLAPEAPLDFGNAATGCRLTMGLVGVYDFDSTFI | 120 |
| Sbjct 61 | +AMQA+GA +RKE +WI+DGVGNG LLAPEAPLDF NA +GC L MGLVGVYDF +TF+ RAMQALGATVRKEDGSWIVDGVGNGALLAPEAPLDFANAGSGCHLIMGLVGVYDFATTFV | 120 |
| Query 121 | GDASLTKRPMGRVLNPLREMGVQVKSEDGDRLPVTLRGPKTPTPITYRVPMASAQVKSAV | 180 |
| Sbjct 121 | GDA+L++RPM VL+PLR+MGVQV S +G RLPVTL GPKT ++YR+P+ASA+VKSAV GDAALSRRPMDCVLDPLRQMGVQVTSAEGGRLPVTLSGPKTANSVSYRLPIASAEVKSAV | 180 |
| Query 181 | LLAGLNTPGITTVIEPIMTRDHTEKMLQGFGANLTVETDADGVRTIRLEGRGKLTGQVID | 240 |
| Sbjct 181 | LLAGLN PGITT+IEP+ T DHTE+MLQ FGANLTV+ A+G R IRLEG+G+LTGQ ID LLAGLNAPGITTLIEPVPTGDHTERMLQAFGANLTVDAGAEGERIIRLEGQGRLTGQDID | 240 |
| Query 241 | VPGDPSSTAFPLVAALLVPGSDVTILNVLMNPTRTGLILTLQEMGADIEVINPRLAGGED | 300 |
| Sbjct 241 | +PGDPSS AFPLVAALLVPGSDVT+LNVLMNPTR GLI+TLQEMG I+V+N R+ GGED IPGDPSSAAFPLVAALLVPGSDVTLLNVLMNPTRAGLIVTLQEMGGKIDVLNARIQGGED | 300 |
| Query 301 | VADLRVRSSTLKGVTVPEDRAPSMIDEYPILavaaafaegaTVMNGLEELRVKESDRLSA | 360 |
| Sbjct 301 | VADLRVRSS LKG+TVPEDRAPSMIDEY +LAVAAAFA+G TVM GLEELR+KESDRL+A  VADLRVRSSELKGITVPEDRAPSMIDEYQVLAVAAAFADGHTVMRGLEELRMKESDRLAA | 360 |

| Query | 361 | VANGLKLNGVDCDEGETSLVVRGRPDGKGLGNASGAAVATHLDHRIAMSFLVMGLVSENP 420 |
| --- | --- | --- |
| Sbjct | 361 | V +GLKLNGVDCDEG SLVV GRP GKGLGNA+GAAVAT LDHR+AMSFLVMGL SE+P VLSGLKLNGVDCDEGPASLVVHGRPGGKGLGNAAGAAVATRLDHRVAMSFLVMGLASEHP 420 |
| Query | 421 | VTVDDATMIATSFPEFMDLMAGLGAKIE 448 |
| Sbjct | 421 | VTVDDATMIATSFP FM+LMAGLGA IE  VTVDDATMIATSFPGFMELMAGLGATIE 448 |

>WP_054310093.1 3-phosphoshikimate 1-carboxyvinyltransferase [Mesorhizobium sp. 1M-11]

Length=450

Score = 672 bits (1734), Expect = 0.0, Method: Compositional matrix adjust.

| Identities | | = 347/450 (77%), Positives = 385/450 (86%), Gaps = 2/450 (0%) |  |
| --- | --- | --- | --- |
| Query 1  Sbjct 1 | | MLHGASSRPATARKSSGLSGTVRIPGDKSISHRSFMFGGLASGETRITGLLEGEDVINTG M H A+ +PATAR+S LSGT R+PGDKSISHRSFMFGGLASGETRITGLLEGEDV+ TG MAHDATPQPATARRSPPLSGTARVPGDKSISHRSFMFGGLASGETRITGLLEGEDVMRTG | 60  60 |
| Query 61  Sbjct 61 | | KAMQAMGARIRKEGDTWIIDGVGNGGLLAPEAPLDFGNAATGCRLTMGLVGVYDFDSTFI  +AM+AMGA + K G+ W+I G GNG LL PEAPLDFGNA TG RLTMGLVG YD ++TFI QAMKAMGAHVEKHGNEWVIRGTGNGALLQPEAPLDFGNAGTGSRLTMGLVGTYDMETTFI | 120  120 |
| Query 121  Sbjct 121 | | GDASLTKRPMGRVLNPLREMGVQV-KSEDGDRLPVTLRGPKTPTPITYRVPMASAQVKSA GDASL+ RPMGRVL PLR+MGVQV ++ GDR+P+TLRGPK PITYRVPMASAQVKSA GDASLSSRPMGRVLEPLRQMGVQVLQAAPGDRMPITLRGPKHAAPITYRVPMASAQVKSA | 179  180 |
| Query 180  Sbjct 181 | | VLLAGLNTPGITTVIEPIMTRDHTEKMLQGFGANLTVETDADGVRTIRLEGRGKLTGQVI VLLAGLNTPGITTVIEP+MTRDHTEKML+GFGANL+VETD GVR I +EG+GKLTGQ+I VLLAGLNTPGITTVIEPVMTRDHTEKMLKGFGANLSVETDERGVRHIFIEGQGKLTGQII | 239  240 |
| Query 240  Sbjct 241 | | DVPGDPSSTAFPLVAALLVPGSDVTILNVLMNPTRTGLILTLQEMGADIEVINPRLAGGE VPGDPSS FPLVAAL+VPGSD+TI NVLMNPTRTGL+LTLQEMG I+++NPR AGGE AVPGDPSSAGFPLVAALIVPGSDITIENVLMNPTRTGLLLTLQEMGGRIDILNPRNAGGE | 299  300 |
| Query 300  Sbjct 301 | | DVADLRVRSSTLKGVTVPEDRAPSMIDEYPILavaaafaegaTVMNGLEELRVKESDRLS DVADLRVR S LKGVTVP +RAPSMIDEYP+LAVAAAFAEG TVM GLEELRVKESDRL+ DVADLRVRYSELKGVTVPAERAPSMIDEYPVLAVAAAFAEGETVMQGLEELRVKESDRLA | 359  360 |
| Query 360  Sbjct 361 | | AVANGLKLNGVDCDEGETSLVVR-GRPDGKGLGNASGAAVATHLDHRIAMSFLVMGLVSE AVANGLKLNGVDC EGE SL VR G A G AV THLDHRIAMSFL++GL +E AVANGLKLNGVDCTEGEASLTVRGVPGGKGLGGEAKGEAVRTHLDHRIAMSFLILGLATE | 418  420 |
| Query 419 | | NPVTVDDATMIATSFPEFMDLMAGLGAKIE 448 PVTVDD MIATSFPEFM LMA LGA+I+ |  |
| Sbjct | 421 | KPVTVDDRAMIATSFPEFMGLMARLGAEID 450 | |

>WP_011582919.1 MULTISPECIES: 3-phosphoshikimate 1-carboxyvinyltransferase [Chelativorans] Q11C97.1 RecName: Full=3-phosphoshikimate 1-carboxyvinyltransferase; AltName:

Full=5-enolpyruvylshikimate-3-phosphate synthase; Short=EPSP synthase; Short=EPSPS [Chelativorans sp. BNC1]

ABG64978.1 3-phosphoshikimate 1-carboxyvinyltransferase [Chelativorans sp. BNC1]

Length=449

Score = 672 bits (1734), Expect = 0.0, Method: Compositional matrix adjust.

| Identities | = 345/452 (76%), Positives = 384/452 (85%), Gaps = 4/452 (1%) |  |
| --- | --- | --- |
| Query 1 | MLHGASSRPATARKSSGLSGTVRIPGDKSISHRSFMFGGLASGETRITGLLEGEDVINTG | 60 |
| Sbjct 1 | M H SRPA +R+S GL G +R+PGDKSISHR+ MFGGLASGETRI+GLLEGEDV+ T MNHHLPSRPARSRQSQGLKGNLRVPGDKSISHRALMFGGLASGETRISGLLEGEDVLRTA | 60 |
| Query 61 | KAMQAMGARIRKEGDTWIIDGVGNGGLLAPEAPLDFGNAATGCRLTMGLVGVYDFDSTFI | 120 |
| Sbjct 61 | +AM+AMGA+I + TW I GVGNG LL PEAPLDFGNA TG RLTMGLVG YD + F EAMRAMGAQIDRRDGTWTIRGVGNGCLLEPEAPLDFGNAGTGSRLTMGLVGTYDMTTRFT | 120 |
| Query 121 | GDASLTKRPMGRVLNPLREMGVQV-KSEDGDRLPVTLRGPKTPTPITYRVPMASAQVKSA | 179 |
| Sbjct 121 | GDASL+KRPMGRVLNPLREMG QV ++E GDRLP+TLRGPK TPI YRVPMASAQVKSA  GDASLSKRPMGRVLNPLREMGTQVLQAEPGDRLPITLRGPKHATPINYRVPMASAQVKSA | 180 |

| Query | 180 | VLLAGLNTPGITTVIEPIMTRDHTEKMLQGFGANLTVETDADGVRTIRLEGRGKLTGQVI | 239 |
| --- | --- | --- | --- |
| Sbjct | 181 | VLLAGLNTPG+TTVIEP+MTRDHTEKML GFGA + +ETD +G R I ++G+G L GQVI VLLAGLNTPGVTTVIEPVMTRDHTEKMLSGFGAAIQIETDKEGARHISIQGQGTLKGQVI | 240 |
| Query | 240 | DVPGDPSSTAFPLVAALLVPGSDVTILNVLMNPTRTGLILTLQEMGADIEVINPRLAGGE | 299 |
| Sbjct | 241 | VPGDPSS AFPLVAAL+VPGSD+ I NVLMNPTRTGLILTLQEMGADIE++N R AGGE AVPGDPSSAAFPLVAALIVPGSDILIENVLMNPTRTGLILTLQEMGADIELLNRRSAGGE | 300 |
| Query | 300 | DVADLRVRSSTLKGVTVPEDRAPSMIDEYPILavaaafaegaTVMNGLEELRVKESDRLS | 359 |
| Sbjct | 301 | DVADLRVRSS LKGVTVP RAPSMIDEYPILAVAA+ AEG TVM GLEELRVKESDRLS DVADLRVRSSALKGVTVPASRAPSMIDEYPILAVAASLAEGETVMLGLEELRVKESDRLS | 360 |
| Query | 360 | AVANGLKLNGVDCDEGETSLVVRGRPDGKGLGNASGAAVATHLDHRIAMSFLVMGLVSEN | 419 |
| Sbjct | 361 | AVA GLK+NG+DC EG+ +L VRGRP+GKGLG GA V THLDHRIAM+FLV+GL SE AVAEGLKVNGIDCTEGKDTLTVRGRPEGKGLG---GATVTTHLDHRIAMAFLVLGLASER | 417 |
| Query  Sbjct | 420  418 | PVTVDDATMIATSFPEFMDLMAGLGAKIELSD 451 PV+VDD +MIATSFPEFMDLM GLGA+IE D PVSVDDQSMIATSFPEFMDLMTGLGAEIEDRD 449 |  |

>WP_101939518.1 3-phosphoshikimate 1-carboxyvinyltransferase [Mesorhizobium loti] PLP56180.1 3-phosphoshikimate 1-carboxyvinyltransferase [Mesorhizobium loti]

Length=450

Score = 672 bits (1733), Expect = 0.0, Method: Compositional matrix adjust.

| Identities | | = 350/450 (78%), Positives = 382/450 (85%), Gaps = 2/450 (0%) |  |
| --- | --- | --- | --- |
| Query 1  Sbjct 1 | | MLHGASSRPATARKSSGLSGTVRIPGDKSISHRSFMFGGLASGETRITGLLEGEDVINTG M H A +PATAR+S LSGT R+PGDKSISHRSFMFGGLASGETRITGLLEGEDV+ TG MAHDAKPQPATARRSPPLSGTARVPGDKSISHRSFMFGGLASGETRITGLLEGEDVMRTG | 60  60 |
| Query 61  Sbjct 61 | | KAMQAMGARIRKEGDTWIIDGVGNGGLLAPEAPLDFGNAATGCRLTMGLVGVYDFDSTFI  +AM+AMGA I K D W+I G GNG LL PEAPLDFGNA TG RLTMGLVG YD ++TFI QAMKAMGAHIEKHDDEWVIRGTGNGALLQPEAPLDFGNAGTGSRLTMGLVGTYDMETTFI | 120  120 |
| Query 121  Sbjct 121 | | GDASLTKRPMGRVLNPLREMGVQV-KSEDGDRLPVTLRGPKTPTPITYRVPMASAQVKSA GDASL+ RPMGRVL PLR+MGVQV ++ GDR+P+TLRGPK PITYRVPMASAQVKSA GDASLSSRPMGRVLEPLRQMGVQVVQAAPGDRMPITLRGPKHAAPITYRVPMASAQVKSA | 179  180 |
| Query 180  Sbjct 181 | | VLLAGLNTPGITTVIEPIMTRDHTEKMLQGFGANLTVETDADGVRTIRLEGRGKLTGQVI VLLAGLNTPGITTVIEP+MTRDHTEKML+GFGANL+VETD GVR I +EG+GKLTGQVI VLLAGLNTPGITTVIEPVMTRDHTEKMLKGFGANLSVETDERGVRHIFIEGQGKLTGQVI | 239  240 |
| Query 240  Sbjct 241 | | DVPGDPSSTAFPLVAALLVPGSDVTILNVLMNPTRTGLILTLQEMGADIEVINPRLAGGE VPGDPSS FPLVAAL+VPGSD+TI NVLMNPTRTGL+LTLQEMG I+++NPR AGGE AVPGDPSSAGFPLVAALIVPGSDITIENVLMNPTRTGLLLTLQEMGGRIDILNPRNAGGE | 299  300 |
| Query 300  Sbjct 301 | | DVADLRVRSSTLKGVTVPEDRAPSMIDEYPILavaaafaegaTVMNGLEELRVKESDRLS DVADLRVRSS LKGVTVP +RAPSMIDEYP+LAVAAAFAEG TVM GLEELRVKESDRL+ DVADLRVRSSELKGVTVPAERAPSMIDEYPVLAVAAAFAEGETVMQGLEELRVKESDRLA | 359  360 |
| Query 360  Sbjct 361 | | AVANGLKLNGVDCDEGETSLVVR-GRPDGKGLGNASGAAVATHLDHRIAMSFLVMGLVSE AVANGLKLNGVDC EGE SL VR G A G AV THLDHRIAMSFLV+GL +E AVANGLKLNGVDCSEGEASLTVRGMPGGKGLGGEAKGEAVKTHLDHRIAMSFLVLGLATE | 418  420 |
| Query 419 | | NPVTVDDATMIATSFPEFMDLMAGLGAKIE 448 PVTVDD MIATSFPEFM LM LGA I+ |  |
| Sbjct | 421 | KPVTVDDRAMIATSFPEFMGLMTRLGAVID 450 | |

>MAW88510.1 3-phosphoshikimate 1-carboxyvinyltransferase [Phyllobacteriaceae bacterium]

MBA90187.1 3-phosphoshikimate 1-carboxyvinyltransferase [Phyllobacteriaceae bacterium]

Length=450

Score = 671 bits (1732), Expect = 0.0, Method: Compositional matrix adjust. Identities = 350/448 (78%), Positives = 383/448 (85%), Gaps = 3/448 (1%)

Query 1	MLHGASSRPATARKSSGLSGTVRIPGDKSISHRSFMFGGLASGETRITGLLEGEDVINTG 60

| Sbjct | 1 | M H + RPA ARKS+ LSGT R+PGDKSISHRSFMFGGLASGETRITGLLEGEDV+ TG MSHSGAPRPALARKSNALSGTARVPGDKSISHRSFMFGGLASGETRITGLLEGEDVLATG | 60 |
| --- | --- | --- | --- |
| Query | 61 | KAMQAMGARIRKEGDTWIIDGVGNGGLLAPEAPLDFGNAATGCRLTMGLVGVYDFDSTFI | 120 |
| Sbjct | 61 | +AM AMGA+I K GDTWII G GNG LL PE PLDFGNA TGCRLTMGL GVYD + F RAMGAMGAQISKLGDTWIIQGAGNGALLEPEGPLDFGNAGTGCRLTMGLAGVYDMQTVFT | 120 |
| Query | 121 | GDASLTKRPMGRVLNPLREMGVQVKSEDGDRLPVTLRGPKTPTPITYRVPMASAQVKSAV | 180 |
| Sbjct | 121 | GDASL++RPMGRVL+PLR MGVQV+++DGDRLPVTL GPKTP+PITYRVPMASAQVKSAV GDASLSRRPMGRVLDPLRLMGVQVQAQDGDRLPVTLHGPKTPSPITYRVPMASAQVKSAV | 180 |
| Query | 181 | LLAGLNTPGITTVIEPIMTRDHTEKMLQGFGANLTVETDADGVRTIRLEGRGKLTGQVID | 240 |
| Sbjct | 181 | LLAGLNTPG+TTVIEP+MTRDHTEKML GFGA L VE DA+GVRTIRL+GR KLTG+VID LLAGLNTPGVTTVIEPVMTRDHTEKMLAGFGAQLEVEEDANGVRTIRLQGRPKLTGRVID | 240 |
| Query | 241 | VPGDPSSTAFPLVAALLVPGSDVTILNVLMNPTRTGLILTLQEMGADIEVINPRLAGGED | 300 |
| Sbjct | 241 | VPGDPSS AFPLVAAL+VPGSD+TI NVLMNPTR G++ TLQEMG I++IN R AGGED VPGDPSSAAFPLVAALIVPGSDITIENVLMNPTRNGIVTTLQEMGGQIDIINWRDAGGED | 300 |
| Query | 301 | VADLRVRSSTLKGVTVPEDRAPSMIDEYPILavaaafaegaTVMNGLEELRVKESDRLSA | 360 |
| Sbjct | 301 | VADLRVR+S LKGVTVP +RAPSMIDEYP LA+AAA AEG TVM GLEELRVKESDRLSA VADLRVRASDLKGVTVPPERAPSMIDEYPALAMAAALAEGETVMEGLEELRVKESDRLSA | 360 |
| Query | 361 | VANGLKLNGVDCDEGETSLVVRGRPDGKGLGNASGAAVATHLDHRIAMSFLVMGLVSENP | 420 |
| Sbjct | 361 | VA GLK NGVDC EGET+L VRGR G G V THLDHRIAMSFLV+GL +E P VAEGLKANGVDCTEGETTLSVRGR---PGGKGLGGGTVETHLDHRIAMSFLVLGLATEKP | 417 |
| Query  Sbjct | 421  418 | VTVDDATMIATSFPEFMDLMAGLGAKIE 448 V VDDATMIATSFPEF LM GLGA++E VAVDDATMIATSFPEFFTLMGGLGAEME 445 |  |

>WP_077499323.1 3-phosphoshikimate 1-carboxyvinyltransferase [Sinorhizobium sp. A49]

OOG74381.1 3-phosphoshikimate 1-carboxyvinyltransferase [Sinorhizobium sp. A49]

Length=448

Score = 671 bits (1732), Expect = 0.0, Method: Compositional matrix adjust.

| Identities | = 342/448 (76%), Positives = 382/448 (85%), Gaps = 0/448 (0%) |  |
| --- | --- | --- |
| Query 1 | MLHGASSRPATARKSSGLSGTVRIPGDKSISHRSFMFGGLASGETRITGLLEGEDVINTG | 60 |
| Sbjct 1 | M G ++PA AR+S L GTVRIPGDKSISHR+ M GGLA+G TRI GLLEGED+ T MSAGLIAKPAIARRSVALRGTVRIPGDKSISHRALMLGGLAAGRTRIAGLLEGEDIFKTA | 60 |
| Query 61 | KAMQAMGARIRKEGDTWIIDGVGNGGLLAPEAPLDFGNAATGCRLTMGLVGVYDFDSTFI | 120 |
| Sbjct 61 | AMQA+GA + KEG TWI+DGVGNG LL PEAPLDFGNA TGC L MGLVGVYDF +TF+ WAMQALGATVHKEGGTWIVDGVGNGALLEPEAPLDFGNAGTGCCLIMGLVGVYDFATTFV | 120 |
| Query 121 | GDASLTKRPMGRVLNPLREMGVQVKSEDGDRLPVTLRGPKTPTPITYRVPMASAQVKSAV | 180 |
| Sbjct 121 | GD SL RPM RVL+PLR+MGVQV S DG RLPVTLRGPKT P++YR+P+ASAQVKSAV GDPSLVMRPMNRVLDPLRQMGVQVTSADGGRLPVTLRGPKTANPVSYRLPIASAQVKSAV | 180 |
| Query 181 | LLAGLNTPGITTVIEPIMTRDHTEKMLQGFGANLTVETDADGVRTIRLEGRGKLTGQVID | 240 |
| Sbjct 181 | LLAGLN PGITTVIEP+ TRDHTE+MLQ FG NL VE A+G IRLEG+G+LTGQ ID LLAGLNAPGITTVIEPVATRDHTERMLQAFGVNLMVEAGAEGEHIIRLEGQGRLTGQDID | 240 |
| Query 241 | VPGDPSSTAFPLVAALLVPGSDVTILNVLMNPTRTGLILTLQEMGADIEVINPRLAGGED | 300 |
| Sbjct 241 | +PGDPSS AFPLVAAL+VPGSD+T+L+VLMNPTRTGLI TLQEMGA I+V+N RL GGE+ IPGDPSSAAFPLVAALVVPGSDLTLLDVLMNPTRTGLIATLQEMGARIDVLNERLQGGEE | 300 |
| Query 301 | VADLRVRSSTLKGVTVPEDRAPSMIDEYPILavaaafaegaTVMNGLEELRVKESDRLSA | 360 |
| Sbjct 301 | VADLRVRSS LKGVTVP+DR PSMID YP+LAVAAAFAEG TVM+GLE L KESDRLSA VADLRVRSSELKGVTVPQDRTPSMIDAYPVLAVAAAFAEGRTVMSGLEALCAKESDRLSA | 360 |
| Query 361 | VANGLKLNGVDCDEGETSLVVRGRPDGKGLGNASGAAVATHLDHRIAMSFLVMGLVSENP | 420 |
| Sbjct 361 | V GLKLNGVDC+EG SLVV+GRP GKGLGNA+GAAVATHLDHRIAMSFLVMGL SE+P VTAGLKLNGVDCEEGPGSLVVQGRPGGKGLGNAAGAAVATHLDHRIAMSFLVMGLASEHP | 420 |
| Query 421  Sbjct 421 | VTVDDATMIATSFPEFMDLMAGLGAKIE 448 VT+DDATMIATSFP F++LM GLGA IE VTIDDATMIATSFPGFIELMTGLGATIE 448 |  |

>WP_112097278.1 3-phosphoshikimate 1-carboxyvinyltransferase [Mesorhizobium hawassense] RAZ91648.1 3-phosphoshikimate 1-carboxyvinyltransferase [Mesorhizobium hawassense]

Length=450

Score = 671 bits (1732), Expect = 0.0, Method: Compositional matrix adjust.

| Identities | | = 354/450 (79%), Positives = 389/450 (86%), Gaps = 2/450 (0%) |  |
| --- | --- | --- | --- |
| Query 1  Sbjct 1 | | MLHGASSRPATARKSSGLSGTVRIPGDKSISHRSFMFGGLASGETRITGLLEGEDVINTG M H A+++PATARKS L+GT R+PGDKSISHRSFMFGGLASGETRITGLLEGEDV+ TG MSHSAAAKPATARKSPALAGTARVPGDKSISHRSFMFGGLASGETRITGLLEGEDVMRTG | 60  60 |
| Query 61  Sbjct 61 | | KAMQAMGARIRKEGDTWIIDGVGNGGLLAPEAPLDFGNAATGCRLTMGLVGVYDFDSTFI AM+AMGA I K G W+I G GNG LL PE PLDFGNA TG RLTMGLVG YD ++TFI AAMKAMGAHIEKRGAEWVIRGTGNGALLQPEGPLDFGNAGTGSRLTMGLVGTYDMETTFI | 120  120 |
| Query 121  Sbjct 121 | | GDASLTKRPMGRVLNPLREMGVQV-KSEDGDRLPVTLRGPKTPTPITYRVPMASAQVKSA GDASL+ RPMGRVL+PLR+MGVQV K+ GDR+P+TLRGPK PITYRVPMASAQVKSA GDASLSGRPMGRVLDPLRQMGVQVLKAAPGDRMPITLRGPKHAAPITYRVPMASAQVKSA | 179  180 |
| Query 180  Sbjct 181 | | VLLAGLNTPGITTVIEPIMTRDHTEKMLQGFGANLTVETDADGVRTIRLEGRGKLTGQVI VLLAGLNTPGITTVIEP+MTRDHTEKML+GFGANLTVETD GVR I +EG+GKLTGQ I VLLAGLNTPGITTVIEPVMTRDHTEKMLKGFGANLTVETDERGVRHIFIEGQGKLTGQAI | 239  240 |
| Query 240  Sbjct 241 | | DVPGDPSSTAFPLVAALLVPGSDVTILNVLMNPTRTGLILTLQEMGADIEVINPRLAGGE VPGDPSS FPLVAAL+VPGSD+TI NVLMNPTRTGL+LTLQEMG I+++NPR AGGE AVPGDPSSAGFPLVAALIVPGSDITIENVLMNPTRTGLLLTLQEMGGRIDILNPRNAGGE | 299  300 |
| Query 300  Sbjct 301 | | DVADLRVRSSTLKGVTVPEDRAPSMIDEYPILavaaafaegaTVMNGLEELRVKESDRLS DVADLRVR S LKGV VP +RAPSMIDEYP+LAVAA+FAEG T+M GLEELRVKESDRL+ DVADLRVRYSELKGVIVPPERAPSMIDEYPVLAVAASFAEGETLMQGLEELRVKESDRLA | 359  360 |
| Query 360  Sbjct 361 | | AVANGLKLNGVDCDEGETSLVVRGRPDGKGLGNASGA-AVATHLDHRIAMSFLVMGLVSE AVANGLKLNGVDC EGE SL VRGRP GKGLG + AV THLDHRIAMSFLVMGL +E AVANGLKLNGVDCSEGEASLAVRGRPGGKGLGGSGQEIAVKTHLDHRIAMSFLVMGLATE | 418  420 |
| Query | 419 NPVTVDDATMIATSFPEFMDLMAGLGAKIE 448 | | |
|  |  | PVT+DDA MIATSFPEFM LM GLGA+IE |  |
| Sbjct | 421 | KPVTIDDANMIATSFPEFMGLMKGLGAEIE 450 | |

>WP_195164173.1 3-phosphoshikimate 1-carboxyvinyltransferase [Mesorhizobium sp. NBSH29]

Length=446

Score = 671 bits (1731), Expect = 0.0, Method: Compositional matrix adjust.

| Identities | = 339/448 (76%), Positives = 385/448 (86%), Gaps = 4/448 (1%) |  |
| --- | --- | --- |
| Query 1 | MLHGASSRPATARKSSGLSGTVRIPGDKSISHRSFMFGGLASGETRITGLLEGEDVINTG | 60 |
| Sbjct 1 | M H + RPATAR+S LSGTVR+PGDKSISHRSFMFGGLASGETRITGLLEGEDV+ TG MSHSHAPRPATARRSRALSGTVRVPGDKSISHRSFMFGGLASGETRITGLLEGEDVMRTG | 60 |
| Query 61 | KAMQAMGARIRKEGDTWIIDGVGNGGLLAPEAPLDFGNAATGCRLTMGLVGVYDFDSTFI | 120 |
| Sbjct 61 | +AM+AMGA I+K G W+I G GNG LL P+APLDFGNA TG RLTMGLVG YD ++TFI EAMKAMGAHIQKTGTEWVIRGTGNGALLEPQAPLDFGNAGTGSRLTMGLVGTYDMETTFI | 120 |
| Query 121 | GDASLTKRPMGRVLNPLREMGVQV-KSEDGDRLPVTLRGPKTPTPITYRVPMASAQVKSA | 179 |
| Sbjct 121 | GDASL+ RPMGRVLNPLREMGVQV ++ GDR+P+ L GPK PI YRVPMASAQVKSA GDASLSGRPMGRVLNPLREMGVQVLEASPGDRMPIRLHGPKVAAPIEYRVPMASAQVKSA | 180 |
| Query 180 | VLLAGLNTPGITTVIEPIMTRDHTEKMLQGFGANLTVETDADGVRTIRLEGRGKLTGQVI | 239 |
| Sbjct 181 | VLLAGLNTPG+TTVIEP+MTRDHTEKML+GFGANL VET+ GVR IR+EG+GKLTGQ I VLLAGLNTPGVTTVIEPVMTRDHTEKMLKGFGANLEVETNRQGVRHIRIEGQGKLTGQTI | 240 |
| Query 240 | DVPGDPSSTAFPLVAALLVPGSDVTILNVLMNPTRTGLILTLQEMGADIEVINPRLAGGE | 299 |
| Sbjct 241 | +PGDPSS +FPLVAAL+VPGSD+TI NVLMNPTRTGL+LTLQEMG IE++N R GGE AIPGDPSSASFPLVAALIVPGSDITIENVLMNPTRTGLLLTLQEMGGKIEILNLRNEGGE | 300 |
| Query 300 | DVADLRVRSSTLKGVTVPEDRAPSMIDEYPILavaaafaegaTVMNGLEELRVKESDRLS | 359 |
| Sbjct 301 | +VADLRVRSS LKGVTVP +RAPSMIDEYP+LAVAAAFA G TVM+GL+ELRVKESDRL+  EVADLRVRSSELKGVTVPPERAPSMIDEYPVLAVAAAFARGETVMDGLDELRVKESDRLA | 360 |

| Query | 360 | AVANGLKLNGVDCDEGETSLVVRGRPDGKGLGNASGAAVATHLDHRIAMSFLVMGLVSEN 419 |
| --- | --- | --- |
| Sbjct | 361 | AVA GL+ NG+DC EGE SL VRG PDGKGLG G V THLDHRIAM+FLV+G+ ++ AVARGLEANGIDCTEGEMSLTVRGHPDGKGLG---GGTVKTHLDHRIAMAFLVLGMATDK 417 |
| Query | 420 | PVTVDDATMIATSFPEFMDLMAGLGAKI 447 |
| Sbjct | 418 | PVT+DDA MI TSFPEFM LM+GLGA++  PVTIDDAAMIVTSFPEFMGLMSGLGAEV 445 |

>RWP91139.1 3-phosphoshikimate 1-carboxyvinyltransferase [Mesorhizobium sp.] Length=458

Score = 671 bits (1731), Expect = 0.0, Method: Compositional matrix adjust.

| Identities | = 342/439 (78%), Positives = 376/439 (86%), Gaps = 4/439 (1%) |  |
| --- | --- | --- |
| Query 13 | RKSSGLSGTVRIPGDKSISHRSFMFGGLASGETRITGLLEGEDVINTGKAMQAMGARIRK | 72 |
| Sbjct 19 | R+ LSG R+PGDKSISHRSFMFGGLASGETRITGLLEGEDV+ TG AM+AMGA I K RRPQALSGIARVPGDKSISHRSFMFGGLASGETRITGLLEGEDVMRTGAAMKAMGAHIEK | 78 |
| Query 73 | EGDTWIIDGVGNGGLLAPEAPLDFGNAATGCRLTMGLVGVYDFDSTFIGDASLTKRPMGR | 132 |
| Sbjct 79 | GD W+I G GNG LL PE PLDFGNA TG RLTMGLVG YD ++TFIGDASL+ RPMGR NGDEWVIRGTGNGALLQPEGPLDFGNAGTGSRLTMGLVGTYDMETTFIGDASLSGRPMGR | 138 |
| Query 133 | VLNPLREMGVQV-KSEDGDRLPVTLRGPKTPTPITYRVPMASAQVKSAVLLAGLNTPGIT | 191 |
| Sbjct 139 | VL PLR+MGVQV K+ GDR+P+TL GPK PITYRVPMASAQVKSAVLLAGLNTPG+T VLEPLRQMGVQVLKATPGDRMPITLHGPKHAAPITYRVPMASAQVKSAVLLAGLNTPGMT | 198 |
| Query 192 | TVIEPIMTRDHTEKMLQGFGANLTVETDADGVRTIRLEGRGKLTGQVIDVPGDPSSTAFP | 251 |
| Sbjct 199 | TVIEP+MTRDHTEKML+GFGANL+VETD GVR I +EG+G+LTGQ I VPGDPSS FP TVIEPVMTRDHTEKMLKGFGANLSVETDERGVRHIFIEGQGRLTGQTIAVPGDPSSAGFP | 258 |
| Query 252 | LVAALLVPGSDVTILNVLMNPTRTGLILTLQEMGADIEVINPRLAGGEDVADLRVRSSTL | 311 |
| Sbjct 259 | LVAAL+VPGSD+TI NVLMNPTRTGL+LTLQEMG +I+++NPR AGGEDVADLRVR S L LVAALIVPGSDITIENVLMNPTRTGLLLTLQEMGGNIDILNPRNAGGEDVADLRVRYSEL | 318 |
| Query 312 | KGVTVPEDRAPSMIDEYPILavaaafaegaTVMNGLEELRVKESDRLSAVANGLKLNGVD | 371 |
| Sbjct 319 | KGVTVP +RAPSMIDEYP+LAVAA+FAEG T+M GLEELRVKESDRLSAVANGLKLNGVD KGVTVPPERAPSMIDEYPVLAVAASFAEGETLMQGLEELRVKESDRLSAVANGLKLNGVD | 378 |
| Query 372 | CDEGETSLVVRGRPDGKGLG---NASGAAVATHLDHRIAMSFLVMGLVSENPVTVDDATM | 428 |
| Sbjct 379 | C EGE SL VRG+P GKGLG N G V THLDHRIAMSFLVMGL +E PVT+DD M CTEGEASLAVRGKPSGKGLGGHPNGQGTTVQTHLDHRIAMSFLVMGLATEKPVTIDDQAM | 438 |
| Query 429  Sbjct 439 | IATSFPEFMDLMAGLGAKI 447 IATSFPEFM LM GLGA+I IATSFPEFMGLMMGLGAEI 457 |  |

>WP_106726316.1 3-phosphoshikimate 1-carboxyvinyltransferase [Mesorhizobium soli] PSJ57352.1 3-phosphoshikimate 1-carboxyvinyltransferase [Mesorhizobium soli]

Length=452

Score = 671 bits (1730), Expect = 0.0, Method: Compositional matrix adjust.

| Identities | = 350/455 (77%), Positives = 382/455 (84%), Gaps = 4/455 (1%) |  |
| --- | --- | --- |
| Query 1 | MLHGASSRPATARKSSGLSGTVRIPGDKSISHRSFMFGGLASGETRITGLLEGEDVINTG | 60 |
| Sbjct 1 | M H A +PA A +S LSG VR+PGDKSISHRSFMFGGLASGETRITGLLEGEDV+ TG MSHQALPKPAVANRSPALSGKVRMPGDKSISHRSFMFGGLASGETRITGLLEGEDVLRTG | 60 |
| Query 61 | KAMQAMGARIRKEGDTWIIDGVGNGGLLAPEAPLDFGNAATGCRLTMGLVGVYDFDSTFI | 120 |
| Sbjct 61 | +AM+AMGARI K G+ WII G GNG LL P+APLDFGNA TG RLTMGLVG YD ++TFI EAMKAMGARIEKNGEEWIIQGTGNGCLLEPKAPLDFGNAGTGSRLTMGLVGTYDMETTFI | 120 |
| Query 121 | GDASLTKRPMGRVLNPLREMGVQV-KSEDGDRLPVTLRGPKTPTPITYRVPMASAQVKSA | 179 |
| Sbjct 121 | GDASL+ RPMGRVLNPLREMGVQV + GDR+P+TLRGPK PI+YRVPMASAQVKSA GDASLSGRPMGRVLNPLREMGVQVINAAPGDRMPLTLRGPKVAAPISYRVPMASAQVKSA | 180 |
| Query 180 | VLLAGLNTPGITTVIEPIMTRDHTEKMLQGFGANLTVETDADGVRTIRLEGRGKLTGQVI | 239 |
| Sbjct 181 | VLLAGLNTPGITTVIEP+MTRDHTEKMLQGFGANL VETD DGVR IR+EG+GKL+GQVI  VLLAGLNTPGITTVIEPVMTRDHTEKMLQGFGANLEVETDKDGVRHIRIEGQGKLSGQVI | 240 |

| Query | 240 | DVPGDPSSTAFPLVAALLVPGSDVTILNVLMNPTRTGLILTLQEMGADIEVINPRLAGGE | 299 |
| --- | --- | --- | --- |
| Sbjct | 241 | VPGDPSS FPLVAAL+VPGSD+ I NVLMNPTRTGLI TL EMGA IE++NPR AGGE AVPGDPSSAGFPLVAALIVPGSDIVIENVLMNPTRTGLITTLIEMGAQIELMNPRNAGGE | 300 |
| Query | 300 | DVADLRVRSSTLKGVTVPEDRAPSMIDEYPILavaaafaegaTVMNGLEELRVKESDRLS | 359 |
| Sbjct | 301 | DVADLRVRSS LKGVTVP +RAPSMIDEYP+LAVAA+FAEG T+M GLEELRVKESDRL+ DVADLRVRSSELKGVTVPAERAPSMIDEYPVLAVAASFAEGETLMQGLEELRVKESDRLA | 360 |
| Query | 360 | AVANGLKLNGVDCDEGETSLVVRGRPDGKGLGNASGAAVATHLDHRIAMSFLVMGLVSEN | 419 |
| Sbjct | 361 | AVA GL+ NGVDC EGE +L VR G G V THLDHRIAMSFLVMGL +E AVARGLEANGVDCTEGEVTLAVR---GKPGGKGLGGGTVKTHLDHRIAMSFLVMGLAAEK | 417 |
| Query  Sbjct | 420  418 | PVTVDDATMIATSFPEFMDLMAGLGAKIELSDTKA 454 PVTVDD TMIATSFPEFM LM G+GAKIE D A PVTVDDQTMIATSFPEFMGLMTGMGAKIETMDAAA 452 |  |

>WP_140556906.1 MULTISPECIES: 3-phosphoshikimate 1-carboxyvinyltransferase [unclassified Mesorhizobium]

TPI53116.1 3-phosphoshikimate 1-carboxyvinyltransferase [Mesorhizobium sp. B3-1-1]

TPJ71142.1 3-phosphoshikimate 1-carboxyvinyltransferase [Mesorhizobium sp. B2-6-7]

TPJ85672.1 3-phosphoshikimate 1-carboxyvinyltransferase [Mesorhizobium sp. B2-6-3]

TPJ99541.1 3-phosphoshikimate 1-carboxyvinyltransferase [Mesorhizobium sp. B2-5-10]

TPK07882.1 3-phosphoshikimate 1-carboxyvinyltransferase [Mesorhizobium sp. B2-5-11]

Length=452

Score = 671 bits (1730), Expect = 0.0, Method: Compositional matrix adjust.

| Identities | = 351/451 (78%), Positives = 384/451 (85%), Gaps = 4/451 (1%) |  |
| --- | --- | --- |
| Query 1 | MLHGASSRPATARKSSGLSGTVRIPGDKSISHRSFMFGGLASGETRITGLLEGEDVINTG | 60 |
| Sbjct 1 | M H A+S+PATARK LSGT R+PGDKSISHRSFMFGGLASGETRITGLLEGEDV+ TG MSHAAASKPATARKCQALSGTARVPGDKSISHRSFMFGGLASGETRITGLLEGEDVMRTG | 60 |
| Query 61 | KAMQAMGARIRKEGDTWIIDGVGNGGLLAPEAPLDFGNAATGCRLTMGLVGVYDFDSTFI | 120 |
| Sbjct 61 | AM+AMGA I K GD W+I G GNG LL PE PLDFGNA TG RLTMGLVG YD ++TFI AAMKAMGAHIEKRGDEWVIRGTGNGALLQPEGPLDFGNAGTGSRLTMGLVGTYDMETTFI | 120 |
| Query 121 | GDASLTKRPMGRVLNPLREMGVQV-KSEDGDRLPVTLRGPKTPTPITYRVPMASAQVKSA | 179 |
| Sbjct 121 | GDASL+ RPMGRVL PLR+MGVQV K+ GDR+P+TL GPK PITYRVPMASAQVKSA GDASLSGRPMGRVLEPLRQMGVQVLKATPGDRMPITLHGPKHAAPITYRVPMASAQVKSA | 180 |
| Query 180 | VLLAGLNTPGITTVIEPIMTRDHTEKMLQGFGANLTVETDADGVRTIRLEGRGKLTGQVI | 239 |
| Sbjct 181 | VLLAGLNTPGITTVIEP+MTRDHTEKML+GFGANL+VETD GVR I +EG+GKLTGQ I VLLAGLNTPGITTVIEPVMTRDHTEKMLKGFGANLSVETDERGVRHIFIEGQGKLTGQTI | 240 |
| Query 240 | DVPGDPSSTAFPLVAALLVPGSDVTILNVLMNPTRTGLILTLQEMGADIEVINPRLAGGE | 299 |
| Sbjct 241 | VPGDPSS FPLVAAL+VPGS++ I NVLMNPTRTGL+LTLQEMG IE++NPR AGGE AVPGDPSSAGFPLVAALIVPGSEIVIENVLMNPTRTGLLLTLQEMGGQIEILNPRNAGGE | 300 |
| Query 300 | DVADLRVRSSTLKGVTVPEDRAPSMIDEYPILavaaafaegaTVMNGLEELRVKESDRLS | 359 |
| Sbjct 301 | DVADLRV S LKGVTVP +RAPSMIDEYP+LAVAA+FAEG T+M GLEELRVKESDRLS DVADLRVHYSELKGVTVPAERAPSMIDEYPVLAVAASFAEGETLMQGLEELRVKESDRLS | 360 |
| Query 360 | AVANGLKLNGVDCDEGETSLVVRGRPDGKGLG---NASGAAVATHLDHRIAMSFLVMGLV | 416 |
| Sbjct 361 | AVANGLKLNGVDC EGE SL VRG+P GKGLG N V THLDHRIAMSFL+MGL AVANGLKLNGVDCTEGEASLAVRGKPGGKGLGGHPNGPDTTVQTHLDHRIAMSFLIMGLA | 420 |
| Query 417  Sbjct 421 | SENPVTVDDATMIATSFPEFMDLMAGLGAKI 447  +E PVT+DDA MIATSFPEFM LM GLGA+I  TEKPVTIDDANMIATSFPEFMSLMTGLGAEI 451 |  |

>WP_056243951.1 3-phosphoshikimate 1-carboxyvinyltransferase [Mesorhizobium sp. Root552]

KQZ18550.1 3-phosphoshikimate 1-carboxyvinyltransferase [Mesorhizobium sp. Root552]

Length=450

Score = 671 bits (1730), Expect = 0.0, Method: Compositional matrix adjust.

| Identities | = 349/450 (78%), Positives = 382/450 (85%), Gaps = 2/450 (0%) |  |
| --- | --- | --- |
| Query 1 | MLHGASSRPATARKSSGLSGTVRIPGDKSISHRSFMFGGLASGETRITGLLEGEDVINTG | 60 |
| Sbjct 1 | M H A+ +PATAR+S+GL GTVR+PGDKSISHRSFMFGGLASGETRITGLLEGEDV+ TG MSHDAAPQPATARRSAGLVGTVRVPGDKSISHRSFMFGGLASGETRITGLLEGEDVLRTG | 60 |
| Query 61 | KAMQAMGARIRKEGDTWIIDGVGNGGLLAPEAPLDFGNAATGCRLTMGLVGVYDFDSTFI | 120 |
| Sbjct 61 | +AM+AMGA I+K+G W+I G GNG LL PE PLDFGNA TG RLTMGLVG YD ++TFI EAMKAMGAHIQKQGGEWMIRGTGNGALLEPEGPLDFGNAGTGSRLTMGLVGTYDMETTFI | 120 |
| Query 121 | GDASLTKRPMGRVLNPLREMGVQV-KSEDGDRLPVTLRGPKTPTPITYRVPMASAQVKSA | 179 |
| Sbjct 121 | GDASL+ RPMGRVL+PLR+MGVQV K+ GDR+P+TLRGPK PITYRVPMASAQVKSA GDASLSGRPMGRVLDPLRQMGVQVLKAAPGDRMPITLRGPKHSAPITYRVPMASAQVKSA | 180 |
| Query 180 | VLLAGLNTPGITTVIEPIMTRDHTEKMLQGFGANLTVETDADGVRTIRLEGRGKLTGQVI | 239 |
| Sbjct 181 | VLLA LNTPGITTVIEP+MTRDHTEKML+GFGANL VETD GVR I +EG+GKLTGQ I VLLAALNTPGITTVIEPVMTRDHTEKMLKGFGANLQVETDERGVRHIFIEGQGKLTGQTI | 240 |
| Query 240 | DVPGDPSSTAFPLVAALLVPGSDVTILNVLMNPTRTGLILTLQEMGADIEVINPRLAGGE | 299 |
| Sbjct 241 | VPGDPSS AFPLVAAL+VPGSD+ I N LMNPTRTGLILTLQEMGA IEV+N R AGGE AVPGDPSSAAFPLVAALIVPGSDIVIQNALMNPTRTGLILTLQEMGAQIEVLNERNAGGE | 300 |
| Query 300 | DVADLRVRSSTLKGVTVPEDRAPSMIDEYPILavaaafaegaTVMNGLEELRVKESDRLS | 359 |
| Sbjct 301 | DVADL VR S LKGV VP +RAPSMIDEYP+LAVAA+FAEG T+M GLEELRVKESDRLS DVADLHVRHSELKGVVVPAERAPSMIDEYPVLAVAASFAEGETLMQGLEELRVKESDRLS | 360 |
| Query 360 | AVANGLKLNGVDCDEGETSLVVR-GRPDGKGLGNASGAAVATHLDHRIAMSFLVMGLVSE | 418 |
| Sbjct 361 | AVANGLKLNGVDC EGETSL VR G G AV THLDHRIAMSFLVMGL SE AVANGLKLNGVDCTEGETSLAVRGTPGGKGLGGREKGEAVKTHLDHRIAMSFLVMGLASE | 420 |
| Query 419  Sbjct 421 | NPVTVDDATMIATSFPEFMDLMAGLGAKIE 448  +PVTVDD +MIATSFPEFM LM LGA I+  HPVTVDDRSMIATSFPEFMGLMERLGAHID 450 |  |

>WP_140564979.1 MULTISPECIES: 3-phosphoshikimate 1-carboxyvinyltransferase [unclassified Mesorhizobium]

TPK54216.1 3-phosphoshikimate 1-carboxyvinyltransferase [Mesorhizobium sp. B2-5-2]

TPL25383.1 3-phosphoshikimate 1-carboxyvinyltransferase [Mesorhizobium sp. B2-4-7]

TPL29329.1 3-phosphoshikimate 1-carboxyvinyltransferase [Mesorhizobium sp. B2-4-9]

TPL41148.1 3-phosphoshikimate 1-carboxyvinyltransferase [Mesorhizobium sp. B2-4-5]

TPM75037.1 3-phosphoshikimate 1-carboxyvinyltransferase [Mesorhizobium sp. B2-1-6]

Length=452

Score = 670 bits (1729), Expect = 0.0, Method: Compositional matrix adjust.

| Identities | = 351/451 (78%), Positives = 384/451 (85%), Gaps = 4/451 (1%) |  |
| --- | --- | --- |
| Query 1 | MLHGASSRPATARKSSGLSGTVRIPGDKSISHRSFMFGGLASGETRITGLLEGEDVINTG | 60 |
| Sbjct 1 | M H A+S+PATARK LSGT R+PGDKSISHRSFMFGGLASGETRITGLLEGEDV+ TG MSHAAASKPATARKCQALSGTARVPGDKSISHRSFMFGGLASGETRITGLLEGEDVMRTG | 60 |
| Query 61 | KAMQAMGARIRKEGDTWIIDGVGNGGLLAPEAPLDFGNAATGCRLTMGLVGVYDFDSTFI | 120 |
| Sbjct 61 | AM+AMGA I K GD W+I G GNG LL PE PLDFGNA TG RLTMGLVG YD ++TFI AAMKAMGAHIEKRGDEWVIRGTGNGALLQPEGPLDFGNAGTGSRLTMGLVGTYDMETTFI | 120 |
| Query 121 | GDASLTKRPMGRVLNPLREMGVQV-KSEDGDRLPVTLRGPKTPTPITYRVPMASAQVKSA | 179 |
| Sbjct 121 | GDASL+ RPMGRVL PLR+MGVQV K+ GDR+P+TL GPK PITYRVPMASAQVKSA GDASLSGRPMGRVLEPLRQMGVQVLKATPGDRMPITLHGPKHAAPITYRVPMASAQVKSA | 180 |
| Query 180 | VLLAGLNTPGITTVIEPIMTRDHTEKMLQGFGANLTVETDADGVRTIRLEGRGKLTGQVI | 239 |
| Sbjct 181 | VLLAGLNTPGITTVIEP+MTRDHTEKML+GFGANL+VETD GVR I +EG+GKLTGQ I VLLAGLNTPGITTVIEPVMTRDHTEKMLKGFGANLSVETDERGVRHIFIEGQGKLTGQTI | 240 |
| Query 240 | DVPGDPSSTAFPLVAALLVPGSDVTILNVLMNPTRTGLILTLQEMGADIEVINPRLAGGE VPGDPSS FPLVAAL+VPGS++ I NVLMNPTRTGL+LTLQEMG IE++NPR AGGE | 299 |

| Sbjct | 241 | AVPGDPSSAGFPLVAALIVPGSEIVIENVLMNPTRTGLLLTLQEMGGQIEILNPRNAGGE | 300 |
| --- | --- | --- | --- |
| Query | 300 | DVADLRVRSSTLKGVTVPEDRAPSMIDEYPILavaaafaegaTVMNGLEELRVKESDRLS | 359 |
| Sbjct | 301 | DVADLRV S LKGVTVP +RAPSMIDEYP+LAVAA+FAEG T+M GLEELRVKESDRLS DVADLRVHYSELKGVTVPAERAPSMIDEYPVLAVAASFAEGETLMQGLEELRVKESDRLS | 360 |
| Query | 360 | AVANGLKLNGVDCDEGETSLVVRGRPDGKGLG---NASGAAVATHLDHRIAMSFLVMGLV | 416 |
| Sbjct | 361 | AVANGLKLNGVDC EGE SL VRG+P GKGLG N V THLDHRIAMSFL+MGL AVANGLKLNGVDCTEGEASLAVRGKPGGKGLGGHPNGPDTTVQTHLDHRIAMSFLIMGLA | 420 |
| Query  Sbjct | 417  421 | SENPVTVDDATMIATSFPEFMDLMAGLGAKI 447  +E PVT+DDA MIATSFPEFM LM GLGA+I  TEEPVTIDDANMIATSFPEFMSLMTGLGAEI 451 |  |

>WP_027166167.1 3-phosphoshikimate 1-carboxyvinyltransferase [Mesorhizobium sp. WSM3224]

Length=452

Score = 670 bits (1729), Expect = 0.0, Method: Compositional matrix adjust.

| Identities | = 354/452 (78%), Positives = 388/452 (86%), Gaps = 4/452 (1%) |  |
| --- | --- | --- |
| Query 1 | MLHGASSRPATARKSSGLSGTVRIPGDKSISHRSFMFGGLASGETRITGLLEGEDVINTG | 60 |
| Sbjct 1 | M H A+++PATARKS LSGT R+PGDKSISHRSFMFGGLASGETRITGLLEGEDV+ TG MSHSAAAKPATARKSPALSGTARVPGDKSISHRSFMFGGLASGETRITGLLEGEDVMRTG | 60 |
| Query 61 | KAMQAMGARIRKEGDTWIIDGVGNGGLLAPEAPLDFGNAATGCRLTMGLVGVYDFDSTFI | 120 |
| Sbjct 61 | AMQAMGA I K+G W+I G GNG LL PE PLDFGNA TG RLTMGLVG YD ++TFI AAMQAMGAHIEKKGAEWVIRGTGNGALLQPEGPLDFGNAGTGSRLTMGLVGPYDMETTFI | 120 |
| Query 121 | GDASLTKRPMGRVLNPLREMGVQV-KSEDGDRLPVTLRGPKTPTPITYRVPMASAQVKSA | 179 |
| Sbjct 121 | GDASL+ RPMGRVL+PLR+MGVQV K+ GDR+P+TLRGPK PITYRVPMASAQVKSA GDASLSGRPMGRVLDPLRQMGVQVLKAAPGDRMPITLRGPKHTAPITYRVPMASAQVKSA | 180 |
| Query 180 | VLLAGLNTPGITTVIEPIMTRDHTEKMLQGFGANLTVETDADGVRTIRLEGRGKLTGQVI | 239 |
| Sbjct 181 | VLLAGLNTPGITTV+EP+MTRDHTEKML+GFGANLTVETD GVR I +EG+GKLTGQ I VLLAGLNTPGITTVVEPVMTRDHTEKMLRGFGANLTVETDERGVRHIFIEGQGKLTGQTI | 240 |
| Query 240 | DVPGDPSSTAFPLVAALLVPGSDVTILNVLMNPTRTGLILTLQEMGADIEVINPRLAGGE | 299 |
| Sbjct 241 | VPGDPSS FPLVAAL+VPGSD+ I NVLMNPTRTGL+LTLQEMG I+V+NPR AGGE AVPGDPSSAGFPLVAALIVPGSDIVIENVLMNPTRTGLLLTLQEMGGRIDVLNPRNAGGE | 300 |
| Query 300 | DVADLRVRSSTLKGVTVPEDRAPSMIDEYPILavaaafaegaTVMNGLEELRVKESDRLS | 359 |
| Sbjct 301 | DVADLRVRSS LKGV VP +RAPSMIDEYP+LAVAA+FAEG T+M GLEELRVKESDRL+ DVADLRVRSSDLKGVVVPPERAPSMIDEYPVLAVAASFAEGETLMQGLEELRVKESDRLA | 360 |
| Query 360 | AVANGLKLNGVDCDEGETSLVVRGRPDGKGLG---NASGAAVATHLDHRIAMSFLVMGLV | 416 |
| Sbjct 361 | AVA GLKLNGVDC EGE SL VRG+P GKGLG N V THLDHRIAMSFLVMGL AVATGLKLNGVDCSEGEASLAVRGKPGGKGLGGHPNGQDTTVQTHLDHRIAMSFLVMGLA | 420 |
| Query 417  Sbjct 421 | SENPVTVDDATMIATSFPEFMDLMAGLGAKIE 448  +E PVT+DDA MIATSFPEFM LM GLGA+IE  TEKPVTIDDANMIATSFPEFMGLMTGLGAEIE 452 |  |

>WP_140702313.1 3-phosphoshikimate 1-carboxyvinyltransferase [Mesorhizobium sp. B2-8-5]

TPJ12508.1 3-phosphoshikimate 1-carboxyvinyltransferase [Mesorhizobium sp. B2-8-5]

Length=452

Score = 670 bits (1728), Expect = 0.0, Method: Compositional matrix adjust. Identities = 351/452 (78%), Positives = 390/452 (86%), Gaps = 4/452 (1%)

| Query | 1 | MLHGASSRPATARKSSGLSGTVRIPGDKSISHRSFMFGGLASGETRITGLLEGEDVINTG | 60 |
| --- | --- | --- | --- |
| Sbjct | 1 | M H A+++PATARKS L+GT R+PGDKSISHRSFMFGGLASGETRITGLLEGEDV+ TG MSHSAAAKPATARKSPALAGTARVPGDKSISHRSFMFGGLASGETRITGLLEGEDVMRTG | 60 |
| Query | 61 | KAMQAMGARIRKEGDTWIIDGVGNGGLLAPEAPLDFGNAATGCRLTMGLVGVYDFDSTFI | 120 |
| Sbjct | 61 | AM+AMGA + K+G W+I G GNG LL PE PLDFGNA TG RLTMGLVG YD ++TFI  AAMKAMGAHVEKKGAEWVIRGTGNGALLQPEGPLDFGNAGTGSRLTMGLVGTYDMETTFI | 120 |

| Query | 121 | GDASLTKRPMGRVLNPLREMGVQV-KSEDGDRLPVTLRGPKTPTPITYRVPMASAQVKSA | 179 |
| --- | --- | --- | --- |
| Sbjct | 121 | GDASL+ RPMGRVL+PLR+MGVQV K+ GDR+P+TLRGPK PITYRVPMASAQVKSA GDASLSGRPMGRVLDPLRQMGVQVLKAAPGDRMPITLRGPKHAAPITYRVPMASAQVKSA | 180 |
| Query | 180 | VLLAGLNTPGITTVIEPIMTRDHTEKMLQGFGANLTVETDADGVRTIRLEGRGKLTGQVI | 239 |
| Sbjct | 181 | VLLAGLNTPGITTVIEP+MTRDHTEKML+GFGANL+VETD GVR I +EG+GKLTGQ I VLLAGLNTPGITTVIEPVMTRDHTEKMLKGFGANLSVETDERGVRHIFIEGQGKLTGQTI | 240 |
| Query | 240 | DVPGDPSSTAFPLVAALLVPGSDVTILNVLMNPTRTGLILTLQEMGADIEVINPRLAGGE | 299 |
| Sbjct | 241 | VPGDPSS FPLVAAL+VPGSD+TI NVLMNPTRTGL+LTLQEMG I+++NPR AGGE AVPGDPSSAGFPLVAALIVPGSDITIENVLMNPTRTGLLLTLQEMGGRIDILNPRNAGGE | 300 |
| Query | 300 | DVADLRVRSSTLKGVTVPEDRAPSMIDEYPILavaaafaegaTVMNGLEELRVKESDRLS | 359 |
| Sbjct | 301 | DVADLRVR S LKGVTVP +RAPSMIDEYP+LAVAA+FAEG T+M GLEELRVKESDRLS DVADLRVRYSELKGVTVPPERAPSMIDEYPVLAVAASFAEGETLMRGLEELRVKESDRLS | 360 |
| Query | 360 | AVANGLKLNGVDCDEGETSLVVRGRPDGKGLG---NASGAAVATHLDHRIAMSFLVMGLV | 416 |
| Sbjct | 361 | AVANGLKLNGVDC EGE SL V G+P G+GLG N AV T+LDHRIAMSFLVMGL AVANGLKLNGVDCTEGEASLAVHGKPGGRGLGGHPNGQDTAVKTYLDHRIAMSFLVMGLA | 420 |
| Query  Sbjct | 417  421 | SENPVTVDDATMIATSFPEFMDLMAGLGAKIE 448  +E PVT+DDA MIATSFPEFM LM GLGA+IE  TEKPVTIDDANMIATSFPEFMGLMKGLGAEIE 452 |  |

>OJX77951.1 3-phosphoshikimate 1-carboxyvinyltransferase [Mesorhizobium sp. 65-26]

Length=450

Score = 670 bits (1728), Expect = 0.0, Method: Compositional matrix adjust.

| Identities | | = 354/449 (79%), Positives = 388/449 (86%), Gaps = 2/449 (0%) |  |
| --- | --- | --- | --- |
| Query 1  Sbjct 1 | | MLHGASSRPATARKSSGLSGTVRIPGDKSISHRSFMFGGLASGETRITGLLEGEDVINTG M H A+ +PATARKS L+G R+PGDKSISHRSFMFGGLASGETRITGLLEGEDV+ TG MSHAAAPKPATARKSPALAGKARVPGDKSISHRSFMFGGLASGETRITGLLEGEDVMRTG | 60  60 |
| Query 61  Sbjct 61 | | KAMQAMGARIRKEGDTWIIDGVGNGGLLAPEAPLDFGNAATGCRLTMGLVGVYDFDSTFI AM+AMGA I K+GD WII G GNG LL PE PLDFGNA TG RLTMGLVG YD ++TFI AAMKAMGAHIEKKGDEWIIRGTGNGALLQPEGPLDFGNAGTGSRLTMGLVGTYDMETTFI | 120  120 |
| Query 121  Sbjct 121 | | GDASLTKRPMGRVLNPLREMGVQV-KSEDGDRLPVTLRGPKTPTPITYRVPMASAQVKSA GDASL+ RPMGRVL+PLR+MGVQV K+ GDR+P+TLRGPK PITYRVPMASAQVKSA GDASLSGRPMGRVLDPLRQMGVQVLKASPGDRMPITLRGPKHAAPITYRVPMASAQVKSA | 179  180 |
| Query 180  Sbjct 181 | | VLLAGLNTPGITTVIEPIMTRDHTEKMLQGFGANLTVETDADGVRTIRLEGRGKLTGQVI VLLAGLNTPGITTVIEP+MTRDHTEKML+GFGANLTVETD GVR I +EG+GKL GQ I VLLAGLNTPGITTVIEPVMTRDHTEKMLKGFGANLTVETDERGVRHIFIEGQGKLKGQTI | 239  240 |
| Query 240  Sbjct 241 | | DVPGDPSSTAFPLVAALLVPGSDVTILNVLMNPTRTGLILTLQEMGADIEVINPRLAGGE VPGDPSS FPLVAAL+VPGSD+ I NVLMNPTRTGL+LTLQEMG I+++NPR AGGE AVPGDPSSAGFPLVAALIVPGSDIVIENVLMNPTRTGLLLTLQEMGGRIDILNPRNAGGE | 299  300 |
| Query 300  Sbjct 301 | | DVADLRVRSSTLKGVTVPEDRAPSMIDEYPILavaaafaegaTVMNGLEELRVKESDRLS DVADLRVRSS L+GVTVP +RAPSMIDEYP+LAVAA+FAEG T+M GLEELRVKESDRLS DVADLRVRSSDLRGVTVPAERAPSMIDEYPVLAVAASFAEGETLMQGLEELRVKESDRLS | 359  360 |
| Query 360  Sbjct 361 | | AVANGLKLNGVDCDEGETSLVVRGRPDGKGLGNAS-GAAVATHLDHRIAMSFLVMGLVSE AVANGLKLNGVDC EGE SL VRGRP GKGLG + AV THLDHRIAMSFLVMGL +E AVANGLKLNGVDCTEGEASLAVRGRPGGKGLGGSGPETAVQTHLDHRIAMSFLVMGLATE | 418  420 |
| Query | 419 NPVTVDDATMIATSFPEFMDLMAGLGAKI 447 | | |
|  |  | PVT+DDA MIATSFPEFM LM GLGA+I |  |
| Sbjct | 421 | KPVTIDDANMIATSFPEFMGLMKGLGAEI 449 | |

>WP_106772857.1 3-phosphoshikimate 1-carboxyvinyltransferase [Mesorhizobium ephedrae] PSJ59173.1 3-phosphoshikimate 1-carboxyvinyltransferase [Mesorhizobium ephedrae]

Length=449

Score = 670 bits (1728), Expect = 0.0, Method: Compositional matrix adjust. Identities = 349/452 (77%), Positives = 383/452 (85%), Gaps = 4/452 (1%)

| Query | 1 | MLHGASSRPATARKSSGLSGTVRIPGDKSISHRSFMFGGLASGETRITGLLEGEDVINTG | 60 |
| --- | --- | --- | --- |
| Sbjct | 1 | M H S+RPATA +S L+G VR+PGDKSISHRSFMFGGLASGETRITGLLEGEDV+ TG MSHHGSARPATASRSPALNGRVRMPGDKSISHRSFMFGGLASGETRITGLLEGEDVMRTG | 60 |
| Query | 61 | KAMQAMGARIRKEGDTWIIDGVGNGGLLAPEAPLDFGNAATGCRLTMGLVGVYDFDSTFI | 120 |
| Sbjct | 61 | +AM+AMGA I K G+ WII G GNG LL P APLDFGNA TG RLTMGLVG YD +TFI EAMKAMGAAIEKRGNEWIIQGTGNGCLLEPAAPLDFGNAGTGSRLTMGLVGTYDMTTTFI | 120 |
| Query | 121 | GDASLTKRPMGRVLNPLREMGVQV-KSEDGDRLPVTLRGPKTPTPITYRVPMASAQVKSA | 179 |
| Sbjct | 121 | GDASL+KRPMGRVL+PLREMGVQV + GDR+P+TLRGP+ PI YRVPMASAQVKSA GDASLSKRPMGRVLDPLREMGVQVIDAAPGDRMPLTLRGPRQAAPIAYRVPMASAQVKSA | 180 |
| Query | 180 | VLLAGLNTPGITTVIEPIMTRDHTEKMLQGFGANLTVETDADGVRTIRLEGRGKLTGQVI | 239 |
| Sbjct | 181 | VLLAGLNTPG+TTVIEP+MTRDHTEKML+GFGA+L VETD DGVR IR+EG+GKL+GQVI VLLAGLNTPGVTTVIEPVMTRDHTEKMLKGFGASLEVETDRDGVRHIRIEGQGKLSGQVI | 240 |
| Query | 240 | DVPGDPSSTAFPLVAALLVPGSDVTILNVLMNPTRTGLILTLQEMGADIEVINPRLAGGE | 299 |
| Sbjct | 241 | VPGDPSS FPLVAAL+VPGSD+ I NVLMNPTRTGLI TL EMGA IE++NPR AGGE AVPGDPSSAGFPLVAALIVPGSDIIIENVLMNPTRTGLIATLLEMGASIELVNPRNAGGE | 300 |
| Query | 300 | DVADLRVRSSTLKGVTVPEDRAPSMIDEYPILavaaafaegaTVMNGLEELRVKESDRLS | 359 |
| Sbjct | 301 | DVADLRVRSS LKGVTVP +RAPSMIDEYP+LAVAA+FAEG TVM GLEELRVKESDRL+ DVADLRVRSSELKGVTVPAERAPSMIDEYPVLAVAASFAEGETVMLGLEELRVKESDRLA | 360 |
| Query | 360 | AVANGLKLNGVDCDEGETSLVVRGRPDGKGLGNASGAAVATHLDHRIAMSFLVMGLVSEN | 419 |
| Sbjct | 361 | AVA GL NGVDC EG+ +L VRG P GK LG G V THLDHRIAMSFLVMGL +E AVARGLAANGVDCTEGKDTLAVRGVPGGKNLG---GGTVTTHLDHRIAMSFLVMGLAAEK | 417 |
| Query  Sbjct | 420  418 | PVTVDDATMIATSFPEFMDLMAGLGAKIELSD 451 PVTVDD TMIATSFPEFM LM GLG KI +D PVTVDDQTMIATSFPEFMGLMTGLGGKIGEAD 449 |  |

>WP_140738324.1 MULTISPECIES: 3-phosphoshikimate 1-carboxyvinyltransferase [unclassified Mesorhizobium]

TPK00286.1 3-phosphoshikimate 1-carboxyvinyltransferase [Mesorhizobium sp. B2-5-12]

TPK27750.1 3-phosphoshikimate 1-carboxyvinyltransferase [Mesorhizobium sp. B2-5-6]

Length=451

Score = 669 bits (1727), Expect = 0.0, Method: Compositional matrix adjust.

| Identities | = 351/447 (79%), Positives = 383/447 (86%), Gaps = 4/447 (1%) |  |
| --- | --- | --- |
| Query 5 | ASSRPATARKSSGLSGTVRIPGDKSISHRSFMFGGLASGETRITGLLEGEDVINTGKAMQ | 64 |
| Sbjct 4 | A+S+PATARK LSGT R+PGDKSISHRSFMFGGLASGETRITGLLEGEDV+ TG AM+ AASKPATARKCQALSGTARVPGDKSISHRSFMFGGLASGETRITGLLEGEDVMRTGAAMK | 63 |
| Query 65 | AMGARIRKEGDTWIIDGVGNGGLLAPEAPLDFGNAATGCRLTMGLVGVYDFDSTFIGDAS | 124 |
| Sbjct 64 | AMGA I K GD W+I G GNG LL PE PLDFGNA TG RLTMGLVG YD ++TFIGDAS AMGAHIEKRGDEWVIRGTGNGALLQPEGPLDFGNAGTGSRLTMGLVGTYDMETTFIGDAS | 123 |
| Query 125 | LTKRPMGRVLNPLREMGVQV-KSEDGDRLPVTLRGPKTPTPITYRVPMASAQVKSAVLLA | 183 |
| Sbjct 124 | L+ RPMGRVL PLR+MGVQV K+ GDR+P+TL GPK PITYRVPMASAQVKSAVLLA LSGRPMGRVLEPLRQMGVQVLKATPGDRMPITLHGPKHAAPITYRVPMASAQVKSAVLLA | 183 |
| Query 184 | GLNTPGITTVIEPIMTRDHTEKMLQGFGANLTVETDADGVRTIRLEGRGKLTGQVIDVPG | 243 |
| Sbjct 184 | GLNTPGITTVIEP+MTRDHTEKML+GFGANL+VETD GVR I +EG+GKLTGQ I VPG GLNTPGITTVIEPVMTRDHTEKMLKGFGANLSVETDERGVRHIFIEGQGKLTGQTIAVPG | 243 |
| Query 244 | DPSSTAFPLVAALLVPGSDVTILNVLMNPTRTGLILTLQEMGADIEVINPRLAGGEDVAD | 303 |
| Sbjct 244 | DPSS FPLVAAL+VPGSD+ I NVLMNPTRTGL+LTLQEMG IE++NPR AGGEDVAD DPSSAGFPLVAALIVPGSDIVIENVLMNPTRTGLLLTLQEMGGQIEILNPRNAGGEDVAD | 303 |
| Query 304 | LRVRSSTLKGVTVPEDRAPSMIDEYPILavaaafaegaTVMNGLEELRVKESDRLSAVAN | 363 |
| Sbjct 304 | LRVR S LKGVTVP +RAPSMIDEYP+LAVAA+FAEG T+M GLEELRVKESDRLSAVAN LRVRYSELKGVTVPAERAPSMIDEYPVLAVAASFAEGETLMQGLEELRVKESDRLSAVAN | 363 |
| Query 364 | GLKLNGVDCDEGETSLVVRGRPDGKGLG---NASGAAVATHLDHRIAMSFLVMGLVSENP | 420 |
| Sbjct 364 | GLKLNGVDC EGE SL VRG+P GKGLG N V THLDHRIAMSFL+MGL +E P  GLKLNGVDCTEGEASLAVRGKPGGKGLGGHPNGPDTTVQTHLDHRIAMSFLIMGLATEKP | 423 |

Query 421 VTVDDATMIATSFPEFMDLMAGLGAKI 447 VT+DDA MIATSFPEFM LM GLGA+I

Sbjct 424 VTIDDANMIATSFPEFMSLMTGLGAEI 450

>WP_042645218.1 3-phosphoshikimate 1-carboxyvinyltransferase [Mesorhizobium sp. ORS 3359]

Length=452

Score = 669 bits (1727), Expect = 0.0, Method: Compositional matrix adjust.

| Identities | = 354/452 (78%), Positives = 387/452 (86%), Gaps = 4/452 (1%) |  |
| --- | --- | --- |
| Query 1 | MLHGASSRPATARKSSGLSGTVRIPGDKSISHRSFMFGGLASGETRITGLLEGEDVINTG | 60 |
| Sbjct 1 | M H A+++PATARKS L+GT R+PGDKSISHRSFMFGGLASGETRITGLLEGEDV+ TG MSHSAAAKPATARKSPALAGTARVPGDKSISHRSFMFGGLASGETRITGLLEGEDVMRTG | 60 |
| Query 61 | KAMQAMGARIRKEGDTWIIDGVGNGGLLAPEAPLDFGNAATGCRLTMGLVGVYDFDSTFI | 120 |
| Sbjct 61 | AM+AMGA I K G W+I G GNG LL PE PLDFGNA TG RLTMGLVG YD ++TFI AAMKAMGAHIEKNGAEWVIRGTGNGALLQPEGPLDFGNAGTGSRLTMGLVGTYDMETTFI | 120 |
| Query 121 | GDASLTKRPMGRVLNPLREMGVQV-KSEDGDRLPVTLRGPKTPTPITYRVPMASAQVKSA | 179 |
| Sbjct 121 | GDASL+ RPMGRVL+PLR+MGVQV K+ GDR+P+TLRGPK PITYRVPMASAQVKSA GDASLSGRPMGRVLDPLRQMGVQVLKAAPGDRMPITLRGPKHAAPITYRVPMASAQVKSA | 180 |
| Query 180 | VLLAGLNTPGITTVIEPIMTRDHTEKMLQGFGANLTVETDADGVRTIRLEGRGKLTGQVI | 239 |
| Sbjct 181 | VLLAGLNTPGITTVIEP+MTRDHTEKML+GFGANLTVETD GVR I +EG+GKLTGQ I VLLAGLNTPGITTVIEPVMTRDHTEKMLKGFGANLTVETDERGVRHIFIEGQGKLTGQTI | 240 |
| Query 240 | DVPGDPSSTAFPLVAALLVPGSDVTILNVLMNPTRTGLILTLQEMGADIEVINPRLAGGE | 299 |
| Sbjct 241 | VPGDPSS FPLVAAL+VPGSD+TI NVLMN TRTGL+LTLQEMG I+++NPR AGGE AVPGDPSSAGFPLVAALIVPGSDITIENVLMNLTRTGLLLTLQEMGGRIDILNPRNAGGE | 300 |
| Query 300 | DVADLRVRSSTLKGVTVPEDRAPSMIDEYPILavaaafaegaTVMNGLEELRVKESDRLS | 359 |
| Sbjct 301 | DVADLRVR S LKG VP +RAPSMIDEYP+LAVAA+FAEG T+M GLEELRVKESDRLS DVADLRVRYSELKGAVVPPERAPSMIDEYPVLAVAASFAEGETLMEGLEELRVKESDRLS | 360 |
| Query 360 | AVANGLKLNGVDCDEGETSLVVRGRPDGKGLG---NASGAAVATHLDHRIAMSFLVMGLV | 416 |
| Sbjct 361 | AVANGLKLNGVDC EGE SL VRGRP GKGLG N AV THLDHRIAMSFLVMGL AVANGLKLNGVDCTEGEASLAVRGRPGGKGLGAHPNGPDTAVKTHLDHRIAMSFLVMGLA | 420 |
| Query 417  Sbjct 421 | SENPVTVDDATMIATSFPEFMDLMAGLGAKIE 448  +E PVT+DDA MIATSFPEFM LM GLGA+IE  TERPVTIDDANMIATSFPEFMGLMKGLGAEIE 452 |  |

>TIM22261.1 3-phosphoshikimate 1-carboxyvinyltransferase, partial [Mesorhizobium sp.]

Length=446

Score = 669 bits (1727), Expect = 0.0, Method: Compositional matrix adjust.

| Identities | = 344/446 (77%), Positives = 378/446 (85%), Gaps = 4/446 (1%) |  |
| --- | --- | --- |
| Query 1 | MLHGASSRPATARKSSGLSGTVRIPGDKSISHRSFMFGGLASGETRITGLLEGEDVINTG | 60 |
| Sbjct 1 | M H A+ PATARKS LSGT R+PGDKSISHRSFMFGGLASGETRITGLLEGEDV+ TG MSHTAAPSPATARKSPALSGTARVPGDKSISHRSFMFGGLASGETRITGLLEGEDVMRTG | 60 |
| Query 61 | KAMQAMGARIRKEGDTWIIDGVGNGGLLAPEAPLDFGNAATGCRLTMGLVGVYDFDSTFI | 120 |
| Sbjct 61 | AM+AMGA I K G W+I G GNG LL PE PLDFGNA TG RLTMGLVG YD ++TFI AAMKAMGAHIEKRGTEWVIRGTGNGALLQPEGPLDFGNAGTGSRLTMGLVGTYDMETTFI | 120 |
| Query 121 | GDASLTKRPMGRVLNPLREMGVQV-KSEDGDRLPVTLRGPKTPTPITYRVPMASAQVKSA | 179 |
| Sbjct 121 | GDASL+ RPMGRVL PLR+MGVQV K+ GDR+P+TLRGPK PITYRVPMASAQVKSA GDASLSGRPMGRVLEPLRQMGVQVLKATPGDRMPITLRGPKHAAPITYRVPMASAQVKSA | 180 |
| Query 180 | VLLAGLNTPGITTVIEPIMTRDHTEKMLQGFGANLTVETDADGVRTIRLEGRGKLTGQVI | 239 |
| Sbjct 181 | VLLAGLNTPGITTVIEP+MTRDHTEKML+GFGANL+VETD GVR I +EG+GKLTGQ I VLLAGLNTPGITTVIEPVMTRDHTEKMLKGFGANLSVETDERGVRHIFIEGQGKLTGQTI | 240 |
| Query 240 | DVPGDPSSTAFPLVAALLVPGSDVTILNVLMNPTRTGLILTLQEMGADIEVINPRLAGGE | 299 |
| Sbjct 241 | VPGDPSS FPLVAAL+VPGSD+TI NVLMNPTRTGL+LTLQEMGA I+V++PR GGE  AVPGDPSSAGFPLVAALIVPGSDITIENVLMNPTRTGLLLTLQEMGARIDVLDPRNEGGE | 300 |

| Query | 300 | DVADLRVRSSTLKGVTVPEDRAPSMIDEYPILavaaafaegaTVMNGLEELRVKESDRLS | 359 |
| --- | --- | --- | --- |
| Sbjct | 301 | DVADLRVR S LKGVTVP +RAP+MIDEYP+LAVAA+FAEG T+M GLEELRVKESDRLS DVADLRVRYSELKGVTVPPERAPTMIDEYPVLAVAASFAEGETLMQGLEELRVKESDRLS | 360 |
| Query | 360 | AVANGLKLNGVDCDEGETSLVVRGRPDGKGLG---NASGAAVATHLDHRIAMSFLVMGLV | 416 |
| Sbjct | 361 | AVA GLKLNGVDC EGE +L VRG+P GKGLG N V THLDHRIAMSFLVMGL AVAEGLKLNGVDCTEGEATLAVRGKPGGKGLGQHPNGKSTIVQTHLDHRIAMSFLVMGLA | 420 |
| Query  Sbjct | 417  421 | SENPVTVDDATMIATSFPEFMDLMAG 442  +E PVT+DD MIATSFP+FM L+ G  TEKPVTIDDQAMIATSFPQFMGLIKG 446 |  |

>RWN46227.1 3-phosphoshikimate 1-carboxyvinyltransferase [Mesorhizobium sp.] Length=452

Score = 669 bits (1727), Expect = 0.0, Method: Compositional matrix adjust.

| Identities | = 351/451 (78%), Positives = 386/451 (86%), Gaps = 4/451 (1%) |  |
| --- | --- | --- |
| Query 1 | MLHGASSRPATARKSSGLSGTVRIPGDKSISHRSFMFGGLASGETRITGLLEGEDVINTG | 60 |
| Sbjct 1 | M H A+ +PATAR+S LSG R+PGDKSISHRSFMFGGLASGETRITGLLEGEDV+ TG MSHAAAPKPATARQSPALSGIARVPGDKSISHRSFMFGGLASGETRITGLLEGEDVMRTG | 60 |
| Query 61 | KAMQAMGARIRKEGDTWIIDGVGNGGLLAPEAPLDFGNAATGCRLTMGLVGVYDFDSTFI | 120 |
| Sbjct 61 | AM+AMGA I K GD W+I G GNG LL PE PLDFGNA TG RLTMGLVG YD ++TFI AAMKAMGAHIEKHGDEWVIRGTGNGALLQPEGPLDFGNAGTGSRLTMGLVGTYDMETTFI | 120 |
| Query 121 | GDASLTKRPMGRVLNPLREMGVQV-KSEDGDRLPVTLRGPKTPTPITYRVPMASAQVKSA | 179 |
| Sbjct 121 | GDASL+ RPMGRVL PLR+MGVQV K+ GDR+P+TL GPK PITYRVPMASAQVKSA GDASLSGRPMGRVLEPLRQMGVQVLKATPGDRMPITLHGPKHAAPITYRVPMASAQVKSA | 180 |
| Query 180 | VLLAGLNTPGITTVIEPIMTRDHTEKMLQGFGANLTVETDADGVRTIRLEGRGKLTGQVI | 239 |
| Sbjct 181 | VLLAGLNTPGITTVIEP+MTRDHTEKML+GFGANL+VETD GVR I +EG+G+LTGQ I VLLAGLNTPGITTVIEPVMTRDHTEKMLKGFGANLSVETDERGVRHIFIEGQGRLTGQTI | 240 |
| Query 240 | DVPGDPSSTAFPLVAALLVPGSDVTILNVLMNPTRTGLILTLQEMGADIEVINPRLAGGE | 299 |
| Sbjct 241 | VPGDPSS FPLVAAL+VPGS++TI NVLMNPTRTGL+LTLQEMG +IE++NPR AGGE AVPGDPSSAGFPLVAALIVPGSEITIENVLMNPTRTGLLLTLQEMGGNIEILNPRNAGGE | 300 |
| Query 300 | DVADLRVRSSTLKGVTVPEDRAPSMIDEYPILavaaafaegaTVMNGLEELRVKESDRLS | 359 |
| Sbjct 301 | DVADLRVR S LKGVTVP +RAPSMIDEYP+LAVAA+FAEG T+M GLEELRVKESDRLS DVADLRVRYSELKGVTVPPERAPSMIDEYPVLAVAASFAEGETLMQGLEELRVKESDRLS | 360 |
| Query 360 | AVANGLKLNGVDCDEGETSLVVRGRPDGKGLG---NASGAAVATHLDHRIAMSFLVMGLV | 416 |
| Sbjct 361 | AVANGLKLNGVDC EGE SL VRG+P GKGLG N G V THLDHRIAMSFLVMGL AVANGLKLNGVDCTEGEASLAVRGKPGGKGLGGHPNGQGTTVQTHLDHRIAMSFLVMGLA | 420 |
| Query 417  Sbjct 421 | SENPVTVDDATMIATSFPEFMDLMAGLGAKI 447  +E PVT+DD MIATSFPEFM LM GLGA+I  TEKPVTIDDQAMIATSFPEFMGLMMGLGAEI 451 |  |

>WP_140630142.1 MULTISPECIES: 3-phosphoshikimate 1-carboxyvinyltransferase [unclassified Mesorhizobium]

TPM59340.1 3-phosphoshikimate 1-carboxyvinyltransferase [Mesorhizobium sp. B2-2-4]

TPM67825.1 3-phosphoshikimate 1-carboxyvinyltransferase [Mesorhizobium sp. B2-2-1]

TPN61688.1 3-phosphoshikimate 1-carboxyvinyltransferase [Mesorhizobium sp. B1-1-3]

Length=452

Score = 669 bits (1727), Expect = 0.0, Method: Compositional matrix adjust.

| Identities | = 352/451 (78%), Positives = 384/451 (85%), Gaps = 4/451 (1%) |  |
| --- | --- | --- |
| Query 1 | MLHGASSRPATARKSSGLSGTVRIPGDKSISHRSFMFGGLASGETRITGLLEGEDVINTG | 60 |
| Sbjct 1 | M H A+S+PATARK LSGT R+PGDKSISHRSFMFGGLASGETRITGLLEGEDV+ TG MSHAAASKPATARKCQALSGTARVPGDKSISHRSFMFGGLASGETRITGLLEGEDVMRTG | 60 |
| Query 61 | KAMQAMGARIRKEGDTWIIDGVGNGGLLAPEAPLDFGNAATGCRLTMGLVGVYDFDSTFI | 120 |
| Sbjct 61 | AM+AMGA I K GD W+I G GNG LL PE PLDFGNA TG RLTMGLVG YD ++TFI  AAMKAMGAHIEKRGDEWVIRGTGNGALLQPEGPLDFGNAGTGSRLTMGLVGTYDMETTFI | 120 |

| Query | 121 | GDASLTKRPMGRVLNPLREMGVQV-KSEDGDRLPVTLRGPKTPTPITYRVPMASAQVKSA | 179 |
| --- | --- | --- | --- |
| Sbjct | 121 | GDASL+ RPMGRVL PLR+MGVQV K+ GDR+P+TL GPK PITYRVPMASAQVKSA GDASLSGRPMGRVLEPLRQMGVQVLKATPGDRMPITLHGPKHAAPITYRVPMASAQVKSA | 180 |
| Query | 180 | VLLAGLNTPGITTVIEPIMTRDHTEKMLQGFGANLTVETDADGVRTIRLEGRGKLTGQVI | 239 |
| Sbjct | 181 | VLLAGLNTPGITTVIEP+MTRDHTEKML+GFGANL+VETD GVR I +EG+GKLTGQ I VLLAGLNTPGITTVIEPVMTRDHTEKMLKGFGANLSVETDERGVRHIFIEGQGKLTGQTI | 240 |
| Query | 240 | DVPGDPSSTAFPLVAALLVPGSDVTILNVLMNPTRTGLILTLQEMGADIEVINPRLAGGE | 299 |
| Sbjct | 241 | VPGDPSS FPLVAAL+VPGSD+ I NVLMNPTRTGL+LTLQEMG IE++N R AGGE AVPGDPSSAGFPLVAALIVPGSDIVIENVLMNPTRTGLLLTLQEMGGQIEILNLRNAGGE | 300 |
| Query | 300 | DVADLRVRSSTLKGVTVPEDRAPSMIDEYPILavaaafaegaTVMNGLEELRVKESDRLS | 359 |
| Sbjct | 301 | DVADLRVR S LKGVTVP +RAPSMIDEYP+LAVAA+FAEG T+M GLEELRVKESDRLS DVADLRVRYSELKGVTVPAERAPSMIDEYPVLAVAASFAEGETLMQGLEELRVKESDRLS | 360 |
| Query | 360 | AVANGLKLNGVDCDEGETSLVVRGRPDGKGLG---NASGAAVATHLDHRIAMSFLVMGLV | 416 |
| Sbjct | 361 | AVANGLKLNGVDC EGE SL VRG+P GKGLG N V THLDHRIAMSFL+MGL AVANGLKLNGVDCTEGEASLAVRGKPGGKGLGGHPNGPDTTVQTHLDHRIAMSFLIMGLA | 420 |
| Query  Sbjct | 417  421 | SENPVTVDDATMIATSFPEFMDLMAGLGAKI 447  +E PVT+DDA MIATSFPEFM LM GLGA+I  TEKPVTIDDANMIATSFPEFMSLMTGLGAEI 451 |  |

>RWE46321.1 3-phosphoshikimate 1-carboxyvinyltransferase [Mesorhizobium sp.] Length=450

Score = 669 bits (1726), Expect = 0.0, Method: Compositional matrix adjust.

| Identities | | = 354/450 (79%), Positives = 388/450 (86%), Gaps = 2/450 (0%) |  |
| --- | --- | --- | --- |
| Query 1  Sbjct 1 | | MLHGASSRPATARKSSGLSGTVRIPGDKSISHRSFMFGGLASGETRITGLLEGEDVINTG M H A+++PATARKS L+GT R+PGDKSISHRSFMFGGLASGETRITGLLEGEDV+ TG MSHSAAAKPATARKSPALAGTARVPGDKSISHRSFMFGGLASGETRITGLLEGEDVMRTG | 60  60 |
| Query 61  Sbjct 61 | | KAMQAMGARIRKEGDTWIIDGVGNGGLLAPEAPLDFGNAATGCRLTMGLVGVYDFDSTFI AM+AMGA I K+G W+I G GNG LL PE LDFGNA TG RLTMGLVG YD ++TFI AAMKAMGAHIEKKGAEWVIRGTGNGALLEPEGSLDFGNAGTGSRLTMGLVGSYDMETTFI | 120  120 |
| Query 121  Sbjct 121 | | GDASLTKRPMGRVLNPLREMGVQV-KSEDGDRLPVTLRGPKTPTPITYRVPMASAQVKSA GDASL+ RPMGRVL PLR+MGVQV K+ GDR+P+TLRGPK PITYRVPMASAQVKSA GDASLSGRPMGRVLEPLRQMGVQVLKAAPGDRMPITLRGPKHAAPITYRVPMASAQVKSA | 179  180 |
| Query 180  Sbjct 181 | | VLLAGLNTPGITTVIEPIMTRDHTEKMLQGFGANLTVETDADGVRTIRLEGRGKLTGQVI VLLAGLNTPGITTVIEP+MTRDHTEKMLQGFGANL+VETD GVR I +EG+GKLTGQ I VLLAGLNTPGITTVIEPVMTRDHTEKMLQGFGANLSVETDERGVRHIFIEGQGKLTGQTI | 239  240 |
| Query 240  Sbjct 241 | | DVPGDPSSTAFPLVAALLVPGSDVTILNVLMNPTRTGLILTLQEMGADIEVINPRLAGGE VPGDPSS FPLVAAL+VPGSD+ I NVLMNPTRTGL+LTLQEM I+++NPR AGGE AVPGDPSSAGFPLVAALIVPGSDIVIENVLMNPTRTGLLLTLQEMSGRIDILNPRNAGGE | 299  300 |
| Query 300  Sbjct 301 | | DVADLRVRSSTLKGVTVPEDRAPSMIDEYPILavaaafaegaTVMNGLEELRVKESDRLS DVADLRVRSS LKGV VP +RAPSMIDEYP+LAVAA+FAEG T+M GLEELRVKESDRLS DVADLRVRSSDLKGVVVPPERAPSMIDEYPVLAVAASFAEGETLMQGLEELRVKESDRLS | 359  360 |
| Query 360  Sbjct 361 | | AVANGLKLNGVDCDEGETSLVVRGRPDGKGLGNAS-GAAVATHLDHRIAMSFLVMGLVSE AVANGLKLNGVDC EGE SL VRGRP GKGLG + AAV THLDHRIAMSFLVMGL +E AVANGLKLNGVDCTEGEASLAVRGRPGGKGLGGSGRDAAVKTHLDHRIAMSFLVMGLATE | 418  420 |
| Query | 419 NPVTVDDATMIATSFPEFMDLMAGLGAKIE 448 | | |
|  |  | PVT+DDA MIATSFPEFM LM GLGA+IE |  |
| Sbjct | 421 | KPVTIDDANMIATSFPEFMGLMKGLGAEIE 450 | |

>WP_136657667.1 3-phosphoshikimate 1-carboxyvinyltransferase [Nitratireductor sp. XY-223]

Length=455

Score = 669 bits (1726), Expect = 0.0, Method: Compositional matrix adjust. Identities = 350/449 (78%), Positives = 381/449 (85%), Gaps = 1/449 (0%)

| Query | 1 | MLHGASSRPATARKSSGLSGTVRIPGDKSISHRSFMFGGLASGETRITGLLEGEDVINTG | 60 |
| --- | --- | --- | --- |
| Sbjct | 1 | M HGAS PA ARKS L+GTVRIPGDKSISHRSFM GGLASG TRITGLLEGEDV+ TG MTHGASLTPAVARKSESLNGTVRIPGDKSISHRSFMLGGLASGRTRITGLLEGEDVLATG | 60 |
| Query | 61 | KAMQAMGARIRKEGDTWIIDGVGNGGLLAPEAPLDFGNAATGCRLTMGLVGVYDFDSTFI | 120 |
| Sbjct | 61 | +AMQAMGARI K+ DTWIIDGVGNG LL PEAPLDFGNA TG RLTMGLVG Y F++TFI RAMQAMGARIVKQDDTWIIDGVGNGLLLEPEAPLDFGNAGTGVRLTMGLVGTYAFEATFI | 120 |
| Query | 121 | GDASLTKRPMGRVLNPLREMGVQVKSEDGDRLPVTLRGPKTPTPITYRVPMASAQVKSAV | 180 |
| Sbjct | 121 | GD SL++RPMGRVL+PLR+MGVQV + +GDRLPVTLRGP PI YRVPMASAQVKSAV GDESLSRRPMGRVLDPLRQMGVQVNAAEGDRLPVTLRGPDLAVPIRYRVPMASAQVKSAV | 180 |
| Query | 181 | LLAGLNTPGITTVIEPIMTRDHTEKMLQGFGANLTVETDADGVRTIRLEGRGKLTGQVID | 240 |
| Sbjct | 181 | LLAGLN PG TTVIEP+MTRDHTEKML GFGA L+V+T A GVRTI L G+GKL GQVID LLAGLNAPGTTTVIEPVMTRDHTEKMLAGFGAELSVDTGAGGVRTIVLAGQGKLAGQVID | 240 |
| Query | 241 | VPGDPSSTAFPLVAALLVPGSDVTILNVLMNPTRTGLILTLQEMGADIEVINPRLAGGED | 300 |
| Sbjct | 241 | VPGDPSSTAFPLVAALLVPGS VTI NVLMNPTRTGL+ TL EMGADI ++N R AGGED VPGDPSSTAFPLVAALLVPGSRVTIENVLMNPTRTGLVTTLLEMGADIGIVNQRSAGGED | 300 |
| Query | 301 | VADLRVRSSTLKGVTVPEDRAPSMIDEYPILavaaafaegaTVMNGLEELRVKESDRLSA | 360 |
| Sbjct | 301 | VADL V +S LKGV VP +RAPSMIDEYP+LA+AA+FAEG TVM GL ELRVKESDRLSA VADLVVGASELKGVRVPAERAPSMIDEYPVLAIAASFAEGETVMEGLGELRVKESDRLSA | 360 |
| Query | 361 | VANGLKLNGVDCDEGETSLVVRGRPDGKGLGN-ASGAAVATHLDHRIAMSFLVMGLVSEN | 419 |
| Sbjct | 361 | VA GL+ NGVDCDEG SL VRG P G GLG A A V THLDHRIAM+FLVMGL +E VAEGLRRNGVDCDEGRDSLRVRGVPGGAGLGAVAEDAVVTTHLDHRIAMAFLVMGLAAEK | 420 |
| Query  Sbjct | 420  421 | PVTVDDATMIATSFPEFMDLMAGLGAKIE 448 PV+VDD+ +IATSFPEFMD+M GLGA IE PVSVDDSAIIATSFPEFMDMMTGLGATIE 449 |  |

>CDX40919.1 3-phosphoshikimate 1-carboxyvinyltransferase [Mesorhizobium sp. ORS 3359]

Length=478

Score = 669 bits (1726), Expect = 0.0, Method: Compositional matrix adjust.

| Identities | = 354/452 (78%), Positives = 387/452 (86%), Gaps = 4/452 (1%) |  |
| --- | --- | --- |
| Query 1 | MLHGASSRPATARKSSGLSGTVRIPGDKSISHRSFMFGGLASGETRITGLLEGEDVINTG | 60 |
| Sbjct 27 | M H A+++PATARKS L+GT R+PGDKSISHRSFMFGGLASGETRITGLLEGEDV+ TG MSHSAAAKPATARKSPALAGTARVPGDKSISHRSFMFGGLASGETRITGLLEGEDVMRTG | 86 |
| Query 61 | KAMQAMGARIRKEGDTWIIDGVGNGGLLAPEAPLDFGNAATGCRLTMGLVGVYDFDSTFI | 120 |
| Sbjct 87 | AM+AMGA I K G W+I G GNG LL PE PLDFGNA TG RLTMGLVG YD ++TFI AAMKAMGAHIEKNGAEWVIRGTGNGALLQPEGPLDFGNAGTGSRLTMGLVGTYDMETTFI | 146 |
| Query 121 | GDASLTKRPMGRVLNPLREMGVQV-KSEDGDRLPVTLRGPKTPTPITYRVPMASAQVKSA | 179 |
| Sbjct 147 | GDASL+ RPMGRVL+PLR+MGVQV K+ GDR+P+TLRGPK PITYRVPMASAQVKSA GDASLSGRPMGRVLDPLRQMGVQVLKAAPGDRMPITLRGPKHAAPITYRVPMASAQVKSA | 206 |
| Query 180 | VLLAGLNTPGITTVIEPIMTRDHTEKMLQGFGANLTVETDADGVRTIRLEGRGKLTGQVI | 239 |
| Sbjct 207 | VLLAGLNTPGITTVIEP+MTRDHTEKML+GFGANLTVETD GVR I +EG+GKLTGQ I VLLAGLNTPGITTVIEPVMTRDHTEKMLKGFGANLTVETDERGVRHIFIEGQGKLTGQTI | 266 |
| Query 240 | DVPGDPSSTAFPLVAALLVPGSDVTILNVLMNPTRTGLILTLQEMGADIEVINPRLAGGE | 299 |
| Sbjct 267 | VPGDPSS FPLVAAL+VPGSD+TI NVLMN TRTGL+LTLQEMG I+++NPR AGGE AVPGDPSSAGFPLVAALIVPGSDITIENVLMNLTRTGLLLTLQEMGGRIDILNPRNAGGE | 326 |
| Query 300 | DVADLRVRSSTLKGVTVPEDRAPSMIDEYPILavaaafaegaTVMNGLEELRVKESDRLS | 359 |
| Sbjct 327 | DVADLRVR S LKG VP +RAPSMIDEYP+LAVAA+FAEG T+M GLEELRVKESDRLS DVADLRVRYSELKGAVVPPERAPSMIDEYPVLAVAASFAEGETLMEGLEELRVKESDRLS | 386 |
| Query 360 | AVANGLKLNGVDCDEGETSLVVRGRPDGKGLG---NASGAAVATHLDHRIAMSFLVMGLV | 416 |
| Sbjct 387 | AVANGLKLNGVDC EGE SL VRGRP GKGLG N AV THLDHRIAMSFLVMGL AVANGLKLNGVDCTEGEASLAVRGRPGGKGLGAHPNGPDTAVKTHLDHRIAMSFLVMGLA | 446 |
| Query 417  Sbjct 447 | SENPVTVDDATMIATSFPEFMDLMAGLGAKIE 448  +E PVT+DDA MIATSFPEFM LM GLGA+IE  TERPVTIDDANMIATSFPEFMGLMKGLGAEIE 478 |  |

>TGQ65525.1 3-phosphoshikimate 1-carboxyvinyltransferase [bacterium M00.F.Ca.ET.205.01.1.1] TGU49230.1 3-phosphoshikimate 1-carboxyvinyltransferase [bacterium M00.F.Ca.ET.152.01.1.1] TGV32969.1 3-phosphoshikimate 1-carboxyvinyltransferase [Mesorhizobium sp.

M00.F.Ca.ET.186.01.1.1]

TGZ40208.1 3-phosphoshikimate 1-carboxyvinyltransferase [bacterium M00.F.Ca.ET.162.01.1.1] TJW33266.1 3-phosphoshikimate 1-carboxyvinyltransferase [Mesorhizobium sp.]

Length=452

Score = 669 bits (1726), Expect = 0.0, Method: Compositional matrix adjust.

| Identities | = 347/452 (77%), Positives = 385/452 (85%), Gaps = 4/452 (1%) |  |
| --- | --- | --- |
| Query 1 | MLHGASSRPATARKSSGLSGTVRIPGDKSISHRSFMFGGLASGETRITGLLEGEDVINTG | 60 |
| Sbjct 1 | M H A+++PATARKS LSGT R+PGDKSISHRSFMFGGLASGETRITGLLEGEDV+ TG MSHAAAAKPATARKSQALSGTARVPGDKSISHRSFMFGGLASGETRITGLLEGEDVMRTG | 60 |
| Query 61 | KAMQAMGARIRKEGDTWIIDGVGNGGLLAPEAPLDFGNAATGCRLTMGLVGVYDFDSTFI | 120 |
| Sbjct 61 | AM+AMGA I K GD W+I G GNG LL PE PLDFGNA TG RLTMGLVG YD ++TFI AAMKAMGAHIEKRGDEWVIRGTGNGALLQPEGPLDFGNAGTGSRLTMGLVGTYDMETTFI | 120 |
| Query 121 | GDASLTKRPMGRVLNPLREMGVQV-KSEDGDRLPVTLRGPKTPTPITYRVPMASAQVKSA | 179 |
| Sbjct 121 | GDASL+ RPMGRVL PLR+MGVQV K+ GDR+P+TL GPK PITYRVPMASAQVKSA GDASLSGRPMGRVLEPLRQMGVQVLKATPGDRMPITLHGPKHAAPITYRVPMASAQVKSA | 180 |
| Query 180 | VLLAGLNTPGITTVIEPIMTRDHTEKMLQGFGANLTVETDADGVRTIRLEGRGKLTGQVI | 239 |
| Sbjct 181 | VLLAGLNTPG+TTVIEP+MTRDHTEKML+GFGANL+VETD GVR I +EG+GKLTGQ I VLLAGLNTPGVTTVIEPVMTRDHTEKMLKGFGANLSVETDERGVRHIFIEGQGKLTGQTI | 240 |
| Query 240 | DVPGDPSSTAFPLVAALLVPGSDVTILNVLMNPTRTGLILTLQEMGADIEVINPRLAGGE | 299 |
| Sbjct 241 | VPGDPSS FPLVAAL+VPGSD+ I NVLMNPTRTGL+LTLQEMG I+++NPR AGGE AVPGDPSSAGFPLVAALIVPGSDIVIENVLMNPTRTGLLLTLQEMGGRIDILNPRNAGGE | 300 |
| Query 300 | DVADLRVRSSTLKGVTVPEDRAPSMIDEYPILavaaafaegaTVMNGLEELRVKESDRLS | 359 |
| Sbjct 301 | DVADLRVR S LKG+ VP +RAPSMIDEYP+LAVAA+FA+G T+M GLEELRVKESDRL+ DVADLRVRYSELKGIAVPPERAPSMIDEYPVLAVAASFADGETLMQGLEELRVKESDRLA | 360 |
| Query 360 | AVANGLKLNGVDCDEGETSLVVRGRPDGKGLG---NASGAAVATHLDHRIAMSFLVMGLV | 416 |
| Sbjct 361 | AVANGLK+NGVDC EGE SL VRG+P GKGLG N V THLDHRIAMSFLVMGL AVANGLKVNGVDCTEGEASLAVRGKPGGKGLGRHPNGMDTTVKTHLDHRIAMSFLVMGLA | 420 |
| Query 417  Sbjct 421 | SENPVTVDDATMIATSFPEFMDLMAGLGAKIE 448  +E PVT+DDA MIATSFPEFM LM LGA+IE  AEKPVTIDDANMIATSFPEFMGLMKALGAEIE 452 |  |

>WP_131564968.1 3-phosphoshikimate 1-carboxyvinyltransferase [Oricola cellulosilytica] TCD16259.1 3-phosphoshikimate 1-carboxyvinyltransferase [Oricola cellulosilytica]

Length=452

Score = 669 bits (1725), Expect = 0.0, Method: Compositional matrix adjust.

| Identities | = 345/452 (76%), Positives = 387/452 (86%), Gaps = 1/452 (0%) |  |
| --- | --- | --- |
| Query 1 | MLHGASSRPATARKSSGLSGTVRIPGDKSISHRSFMFGGLASGETRITGLLEGEDVINTG | 60 |
| Sbjct 1 | M H S +PATAR SS LSG+VRIPGDKSISHRSF+FGGLASGETRI+GLLEGED++ TG MSHSTSLKPATARPSSALSGSVRIPGDKSISHRSFLFGGLASGETRISGLLEGEDILATG | 60 |
| Query 61 | KAMQAMGARIRKEGDTWIIDGVGNGGLLAPEAPLDFGNAATGCRLTMGLVGVYDFDSTFI | 120 |
| Sbjct 61 | +AM+AMGA IRK G WII G GNG LL PE LDFGNA TG RLTMGL GVYD ++ F EAMKAMGAVIRKSGGEWIIHGTGNGCLLEPERELDFGNAGTGVRLTMGLTGVYDMETRFT | 120 |
| Query 121 | GDASLTKRPMGRVLNPLREMGVQVKSEDGDRLPVTLRGPKTPTPITYRVPMASAQVKSAV | 180 |
| Sbjct 121 | GDASL KRPMGRVL+PLR MGVQV + +GDRLP+TLRGP+T PI+YRVPM SAQVKSAV GDASLVKRPMGRVLDPLRLMGVQVGAAEGDRLPLTLRGPRTANPISYRVPMPSAQVKSAV | 180 |
| Query 181 | LLAGLNTPGITTVIEPIMTRDHTEKMLQGFGANLTVETDADGVRTIRLEGRGKLTGQVID | 240 |
| Sbjct 181 | LLAGLNTPG+TTVIEP+MTRDHTEKML GFGA L VETD DGVR IR+EG+G+L+GQ I LLAGLNTPGVTTVIEPVMTRDHTEKMLAGFGAELEVETDGDGVRHIRIEGQGRLSGQTIA | 240 |
| Query 241 | VPGDPSSTAFPLVAALLVPGSDVTILNVLMNPTRTGLILTLQEMGADIEVINPRLAGGED | 300 |
| Sbjct 241 | VPGDPSSTAFPLVAAL+VPGSDVTI NVLMNPTR+GLI TL EMG IE+ N R AGGED  VPGDPSSTAFPLVAALIVPGSDVTIENVLMNPTRSGLITTLLEMGGSIEITNERQAGGED | 300 |

| Query | 301 | VADLRVRSSTLKGVTVPEDRAPSMIDEYPILavaaafaegaTVMNGLEELRVKESDRLSA | 360 |
| --- | --- | --- | --- |
| Sbjct | 301 | VADLRVR+S LKGVTVP DRAP+MIDEYP+LAVAA+FAEG T+M GL+ELRVKESDRL+A VADLRVRASDLKGVTVPADRAPAMIDEYPVLAVAASFAEGETMMPGLDELRVKESDRLAA | 360 |
| Query | 361 | VANGLKLNGVDCDEGETSLVVRGRPDGKGLGNAS-GAAVATHLDHRIAMSFLVMGLVSEN | 419 |
| Sbjct | 361 | VA GL++NGVDC EGE L VRG+P GKGLG AS A VATHLDHRIAMSFLVMGL +E+ VARGLEINGVDCSEGEDWLSVRGKPGGKGLGAASREAVVATHLDHRIAMSFLVMGLAAEH | 420 |
| Query | 420 | PVTVDDATMIATSFPEFMDLMAGLGAKIELSD 451 VDDA +IATSFPEFM+LMAGLGA+IE ++ |  |
| Sbjct | 421 | GTKVDDAAIIATSFPEFMELMAGLGAEIEAAE 452 | |

>WP_027040543.1 3-phosphoshikimate 1-carboxyvinyltransferase [Mesorhizobium sp. URHC0008]

Length=452

Score = 669 bits (1725), Expect = 0.0, Method: Compositional matrix adjust.

| Identities | = 352/451 (78%), Positives = 384/451 (85%), Gaps = 4/451 (1%) |  |
| --- | --- | --- |
| Query 1 | MLHGASSRPATARKSSGLSGTVRIPGDKSISHRSFMFGGLASGETRITGLLEGEDVINTG | 60 |
| Sbjct 1 | M H A+ +PATARKS LSGT R+PGDKSISHRSFMFGGLASGETRITGLLEGEDV+ TG MSHAAAPKPATARKSQALSGTARVPGDKSISHRSFMFGGLASGETRITGLLEGEDVMRTG | 60 |
| Query 61 | KAMQAMGARIRKEGDTWIIDGVGNGGLLAPEAPLDFGNAATGCRLTMGLVGVYDFDSTFI | 120 |
| Sbjct 61 | AM+AMGA I K G W+I G GNG LL PE PLDFGNA TG RLTMGLVG YD ++TFI AAMKAMGAHIEKRGTEWVIRGTGNGALLQPEGPLDFGNAGTGSRLTMGLVGTYDMETTFI | 120 |
| Query 121 | GDASLTKRPMGRVLNPLREMGVQV-KSEDGDRLPVTLRGPKTPTPITYRVPMASAQVKSA | 179 |
| Sbjct 121 | GDASL+ RPMGRVL PLR+MGVQV K+ GDR+P+TL GPK PITYRVPMASAQVKSA GDASLSGRPMGRVLEPLRQMGVQVLKATPGDRMPITLHGPKHAAPITYRVPMASAQVKSA | 180 |
| Query 180 | VLLAGLNTPGITTVIEPIMTRDHTEKMLQGFGANLTVETDADGVRTIRLEGRGKLTGQVI | 239 |
| Sbjct 181 | VLLAGLNTPGITTVIEP+MTRDHTEKML+GFGANL+VETD GVR I +EG+GKLTGQ I VLLAGLNTPGITTVIEPVMTRDHTEKMLKGFGANLSVETDERGVRHIFIEGQGKLTGQTI | 240 |
| Query 240 | DVPGDPSSTAFPLVAALLVPGSDVTILNVLMNPTRTGLILTLQEMGADIEVINPRLAGGE | 299 |
| Sbjct 241 | VPGDPSS FPLVAAL+VPGSD+ I NVLMNPTRTGL+LTLQEMG I+++NPR AGGE AVPGDPSSAGFPLVAALIVPGSDIVIENVLMNPTRTGLLLTLQEMGGQIDILNPRNAGGE | 300 |
| Query 300 | DVADLRVRSSTLKGVTVPEDRAPSMIDEYPILavaaafaegaTVMNGLEELRVKESDRLS | 359 |
| Sbjct 301 | DVADLRVR S LKGVTVP +RAPSMIDEYP+LAVAA+FAEG T+M GLEELRVKESDRLS DVADLRVRYSELKGVTVPPERAPSMIDEYPVLAVAASFAEGETLMQGLEELRVKESDRLS | 360 |
| Query 360 | AVANGLKLNGVDCDEGETSLVVRGRPDGKGLG---NASGAAVATHLDHRIAMSFLVMGLV | 416 |
| Sbjct 361 | AVANGLKLNGVDC EGE SL VRG+P GKGLG N V THLDHRIAMSFLVMGL AVANGLKLNGVDCTEGEASLAVRGKPGGKGLGGHPNGQDTTVQTHLDHRIAMSFLVMGLA | 420 |
| Query 417  Sbjct 421 | SENPVTVDDATMIATSFPEFMDLMAGLGAKI 447  +E PVT+DDA MIATSFPEFM LM GLGA+I  TEKPVTIDDAAMIATSFPEFMGLMKGLGAEI 451 |  |

>WP_027032687.1 3-phosphoshikimate 1-carboxyvinyltransferase [Mesorhizobium loti] QKD00332.1 3-phosphoshikimate 1-carboxyvinyltransferase [Mesorhizobium loti

R88b]

Length=452

Score = 668 bits (1723), Expect = 0.0, Method: Compositional matrix adjust.

| Identities | = 350/451 (78%), Positives = 384/451 (85%), Gaps = 4/451 (1%) |  |
| --- | --- | --- |
| Query 1 | MLHGASSRPATARKSSGLSGTVRIPGDKSISHRSFMFGGLASGETRITGLLEGEDVINTG | 60 |
| Sbjct 1 | M H A+S+PATARKS LSGT R+PGDKSISHRS MFGGLASGETR+TGLLEGEDV+ T MSHAAASKPATARKSQALSGTARVPGDKSISHRSMMFGGLASGETRVTGLLEGEDVMRTA | 60 |
| Query 61 | KAMQAMGARIRKEGDTWIIDGVGNGGLLAPEAPLDFGNAATGCRLTMGLVGVYDFDSTFI | 120 |
| Sbjct 61 | AM+AMGA I+K G W+I G GNG LL PEAPLDFGNA TG RLTMGLVG YD ++TFI AAMKAMGAHIKKHGAEWVIRGTGNGALLQPEAPLDFGNAGTGSRLTMGLVGTYDMETTFI | 120 |
| Query 121 | GDASLTKRPMGRVLNPLREMGVQV-KSEDGDRLPVTLRGPKTPTPITYRVPMASAQVKSA | 179 |
| Sbjct 121 | GDASL+ RPMGRVL PLR+MGVQV K+ GDR+P+TL GPK PITYRVPMASAQVKSA  GDASLSGRPMGRVLEPLRQMGVQVLKATPGDRMPITLHGPKHAAPITYRVPMASAQVKSA | 180 |

| Query | 180 | VLLAGLNTPGITTVIEPIMTRDHTEKMLQGFGANLTVETDADGVRTIRLEGRGKLTGQVI | 239 |
| --- | --- | --- | --- |
| Sbjct | 181 | VLLAGLNTPGITTVIEP+MTRDHTEKML+GFGANL+VETD GVR I +EG+GKLTGQ I VLLAGLNTPGITTVIEPVMTRDHTEKMLKGFGANLSVETDERGVRHIFIEGQGKLTGQTI | 240 |
| Query | 240 | DVPGDPSSTAFPLVAALLVPGSDVTILNVLMNPTRTGLILTLQEMGADIEVINPRLAGGE | 299 |
| Sbjct | 241 | VPGDPSS FPLVAAL+VPGSD+ I NVLMNPTRTGL+LTLQEMG I+++NPR AGGE AVPGDPSSAGFPLVAALIVPGSDIVIENVLMNPTRTGLLLTLQEMGGQIDILNPRNAGGE | 300 |
| Query | 300 | DVADLRVRSSTLKGVTVPEDRAPSMIDEYPILavaaafaegaTVMNGLEELRVKESDRLS | 359 |
| Sbjct | 301 | DVADLRVR S LKGV VP +RAPSMIDEYP+LAVAA+FAEG T+M GLEELRVKESDRLS DVADLRVRYSELKGVIVPPERAPSMIDEYPVLAVAASFAEGETLMQGLEELRVKESDRLS | 360 |
| Query | 360 | AVANGLKLNGVDCDEGETSLVVRGRPDGKGLG---NASGAAVATHLDHRIAMSFLVMGLV | 416 |
| Sbjct | 361 | AVANGLKLNGVDC EGE SL VRG+P GKGLG N V THLDHRIAMSFLVMGL AVANGLKLNGVDCTEGEASLAVRGKPGGKGLGGHPNGQDTTVQTHLDHRIAMSFLVMGLA | 420 |
| Query  Sbjct | 417  421 | SENPVTVDDATMIATSFPEFMDLMAGLGAKI 447  +E PVT+DDA MIATSFPEFM LM GLGA+I  TEKPVTIDDAAMIATSFPEFMGLMTGLGAEI 451 |  |

>WP_091580881.1 3-phosphoshikimate 1-carboxyvinyltransferase [Mesorhizobium qingshengii] SDA87626.1 3-phosphoshikimate 1-carboxyvinyltransferase [Mesorhizobium qingshengii]

Length=452

Score = 667 bits (1721), Expect = 0.0, Method: Compositional matrix adjust.

| Identities | = 351/451 (78%), Positives = 384/451 (85%), Gaps = 4/451 (1%) |  |
| --- | --- | --- |
| Query 1 | MLHGASSRPATARKSSGLSGTVRIPGDKSISHRSFMFGGLASGETRITGLLEGEDVINTG | 60 |
| Sbjct 1 | M H A+ +PATAR+SS LSGT R+PGDKSISHRSFMFGGLASGETRITGLLEGEDV+ TG MSHAAAPKPATARRSSALSGTARVPGDKSISHRSFMFGGLASGETRITGLLEGEDVMRTG | 60 |
| Query 61 | KAMQAMGARIRKEGDTWIIDGVGNGGLLAPEAPLDFGNAATGCRLTMGLVGVYDFDSTFI | 120 |
| Sbjct 61 | AM+AMGA I K G W+I G GNG LL PE PLDFGNA TG RLTMGLVG YD ++TFI AAMKAMGAHIEKRGAEWVIRGTGNGALLQPEGPLDFGNAGTGSRLTMGLVGTYDMETTFI | 120 |
| Query 121 | GDASLTKRPMGRVLNPLREMGVQV-KSEDGDRLPVTLRGPKTPTPITYRVPMASAQVKSA | 179 |
| Sbjct 121 | GDASL+ RPMGRVL PLR+MGVQV K+ GDR+P+TL GPK PITYRVPMASAQVKSA GDASLSGRPMGRVLEPLRQMGVQVLKATPGDRMPITLHGPKHAAPITYRVPMASAQVKSA | 180 |
| Query 180 | VLLAGLNTPGITTVIEPIMTRDHTEKMLQGFGANLTVETDADGVRTIRLEGRGKLTGQVI | 239 |
| Sbjct 181 | VLLAGLNTPGITTVIEP+MTRDHTEKML+GFGANL+VETD GVR I +EG+GKLTGQ I VLLAGLNTPGITTVIEPVMTRDHTEKMLKGFGANLSVETDERGVRHIFIEGQGKLTGQTI | 240 |
| Query 240 | DVPGDPSSTAFPLVAALLVPGSDVTILNVLMNPTRTGLILTLQEMGADIEVINPRLAGGE | 299 |
| Sbjct 241 | VPGDPSS FPLVAAL+VPGSD+TI NVLMNPTRTGL+LTLQEMG I+++NPR AGGE AVPGDPSSAGFPLVAALIVPGSDITIENVLMNPTRTGLLLTLQEMGGKIDILNPRNAGGE | 300 |
| Query 300 | DVADLRVRSSTLKGVTVPEDRAPSMIDEYPILavaaafaegaTVMNGLEELRVKESDRLS | 359 |
| Sbjct 301 | DVADLRVR S LKGV VP +RAPSMIDEYP+LAVAA+FAEG T+M GLEELRVKESDRLS DVADLRVRYSELKGVAVPPERAPSMIDEYPVLAVAASFAEGETLMQGLEELRVKESDRLS | 360 |
| Query 360 | AVANGLKLNGVDCDEGETSLVVRGRPDGKGLG---NASGAAVATHLDHRIAMSFLVMGLV | 416 |
| Sbjct 361 | AVANGLKLNGVDC EGE SL VRG+P GKGLG N V THLDHRIAMSFLVMGL AVANGLKLNGVDCTEGEASLAVRGKPGGKGLGGHPNGQDTTVQTHLDHRIAMSFLVMGLA | 420 |
| Query 417  Sbjct 421 | SENPVTVDDATMIATSFPEFMDLMAGLGAKI 447  +E PVT+DD MIATSFPEFM LM GLGA+I  TEKPVTIDDQAMIATSFPEFMGLMKGLGAEI 451 |  |

>WP_023811518.1 3-phosphoshikimate 1-carboxyvinyltransferase [Mesorhizobium sp. L2C084A000]

ESZ25801.1 3-phosphoshikimate 1-carboxyvinyltransferase [Mesorhizobium sp. L2C084A000]

Length=452

Score = 667 bits (1721), Expect = 0.0, Method: Compositional matrix adjust. Identities = 351/451 (78%), Positives = 385/451 (85%), Gaps = 4/451 (1%)

Query 1	MLHGASSRPATARKSSGLSGTVRIPGDKSISHRSFMFGGLASGETRITGLLEGEDVINTG 60

| Sbjct | 1 | M H A+ +PATAR+S LSG R+PGDKSISHRSFMFGGLASGETRITGLLEGEDV+ TG MSHAAAPKPATARQSPALSGIARVPGDKSISHRSFMFGGLASGETRITGLLEGEDVMRTG | 60 |
| --- | --- | --- | --- |
| Query | 61 | KAMQAMGARIRKEGDTWIIDGVGNGGLLAPEAPLDFGNAATGCRLTMGLVGVYDFDSTFI | 120 |
| Sbjct | 61 | AM+AMGA I K GD W+I G GNG LL PE PLDFGNA TG RLTMGLVG YD ++TFI AAMKAMGAHIEKNGDEWVIRGTGNGALLQPEGPLDFGNAGTGSRLTMGLVGTYDMETTFI | 120 |
| Query | 121 | GDASLTKRPMGRVLNPLREMGVQV-KSEDGDRLPVTLRGPKTPTPITYRVPMASAQVKSA | 179 |
| Sbjct | 121 | GDASL+ RPMGRVL PLR+MGVQV K+ GDR+P+TL GPK PITYRVPMASAQVKSA GDASLSGRPMGRVLEPLRQMGVQVLKATPGDRMPITLHGPKHAAPITYRVPMASAQVKSA | 180 |
| Query | 180 | VLLAGLNTPGITTVIEPIMTRDHTEKMLQGFGANLTVETDADGVRTIRLEGRGKLTGQVI | 239 |
| Sbjct | 181 | VLLAGLNTPGITTVIEP+MTRDHTEKML+GFGANL+VETD GVR I +EG+G+LTGQ I VLLAGLNTPGITTVIEPVMTRDHTEKMLRGFGANLSVETDERGVRHIFIEGQGRLTGQTI | 240 |
| Query | 240 | DVPGDPSSTAFPLVAALLVPGSDVTILNVLMNPTRTGLILTLQEMGADIEVINPRLAGGE | 299 |
| Sbjct | 241 | VPGDPSS FPLVAAL+VPGSD+TI NVLMNPTRTGL+LTLQEMG I+++NPR AGGE AVPGDPSSAGFPLVAALIVPGSDITIENVLMNPTRTGLLLTLQEMGGKIDILNPRNAGGE | 300 |
| Query | 300 | DVADLRVRSSTLKGVTVPEDRAPSMIDEYPILavaaafaegaTVMNGLEELRVKESDRLS | 359 |
| Sbjct | 301 | DVADLRVR S LKGVTVP +RAPSMIDEYP+LAVAA+FAEG T+M GLEELRVKESDRLS DVADLRVRYSELKGVTVPPERAPSMIDEYPVLAVAASFAEGETLMQGLEELRVKESDRLS | 360 |
| Query | 360 | AVANGLKLNGVDCDEGETSLVVRGRPDGKGLG---NASGAAVATHLDHRIAMSFLVMGLV | 416 |
| Sbjct | 361 | AVANGLKLNGVDC EGE SL VRG+P GKGLG N V THLDHRIAMSFLVMGL AVANGLKLNGVDCTEGEASLAVRGKPGGKGLGGHPNGQDTTVQTHLDHRIAMSFLVMGLA | 420 |
| Query  Sbjct | 417  421 | SENPVTVDDATMIATSFPEFMDLMAGLGAKI 447  +E PVT+DDA MIATSFPEFM LM GLGA+I  TEKPVTIDDAAMIATSFPEFMGLMTGLGAEI 451 |  |

>WP_024924358.1 MULTISPECIES: 3-phosphoshikimate 1-carboxyvinyltransferase [Mesorhizobium] NYD63214.1 3-phosphoshikimate 1-carboxyvinyltransferase [Mesorhizobium sp.

YL-MPnR-2016]

OCX12926.1 3-phosphoshikimate 1-carboxyvinyltransferase [Mesorhizobium hungaricum] Length=450

Score = 667 bits (1721), Expect = 0.0, Method: Compositional matrix adjust.

| Identities | | = 342/450 (76%), Positives = 382/450 (85%), Gaps = 2/450 (0%) |  |
| --- | --- | --- | --- |
| Query 1  Sbjct 1 | | MLHGASSRPATARKSSGLSGTVRIPGDKSISHRSFMFGGLASGETRITGLLEGEDVINTG M H A +PATAR+SS LSGT R+PGDKSISHRSFMFGGLASGETRITGLLEGEDV+ TG MAHDAKPQPATARRSSSLSGTARVPGDKSISHRSFMFGGLASGETRITGLLEGEDVMRTG | 60  60 |
| Query 61  Sbjct 61 | | KAMQAMGARIRKEGDTWIIDGVGNGGLLAPEAPLDFGNAATGCRLTMGLVGVYDFDSTFI AM+AMGA I K G+ W+I G GNG LL PEAPLDFGNA TG RLTMGLVG Y ++TF+ AAMKAMGAHIEKHGEEWVIRGTGNGALLQPEAPLDFGNAGTGSRLTMGLVGTYYMETTFV | 120  120 |
| Query 121  Sbjct 121 | | GDASLTKRPMGRVLNPLREMGVQV-KSEDGDRLPVTLRGPKTPTPITYRVPMASAQVKSA GDASL+ RPMGRVL+PLR+MGVQV ++ GDR+P+TLRGP+ PITYRVPMASAQVKSA GDASLSSRPMGRVLDPLRQMGVQVLQAAPGDRMPITLRGPRHAAPITYRVPMASAQVKSA | 179  180 |
| Query 180  Sbjct 181 | | VLLAGLNTPGITTVIEPIMTRDHTEKMLQGFGANLTVETDADGVRTIRLEGRGKLTGQVI VLLAGLNTPGITTVIEP+MTRDHTEKML+GFGANL+VETD GVR I +EG+G+L+GQVI VLLAGLNTPGITTVIEPVMTRDHTEKMLKGFGANLSVETDERGVRHIFIEGQGRLSGQVI | 239  240 |
| Query 240  Sbjct 241 | | DVPGDPSSTAFPLVAALLVPGSDVTILNVLMNPTRTGLILTLQEMGADIEVINPRLAGGE VPGDPSS FPLVA L+VPGSD+ I NVLMNPTRTGL+LTLQEMG I+++NPR AGGE AVPGDPSSAGFPLVAGLIVPGSDILIENVLMNPTRTGLLLTLQEMGGRIDILNPRNAGGE | 299  300 |
| Query 300  Sbjct 301 | | DVADLRVRSSTLKGVTVPEDRAPSMIDEYPILavaaafaegaTVMNGLEELRVKESDRLS DVADLRVR+S LKGV VP +RAPSMIDEYP+LAVAA FAEG T+M GLEELRVKESDRL+ DVADLRVRASDLKGVVVPAERAPSMIDEYPVLAVAACFAEGETLMQGLEELRVKESDRLA | 359  360 |
| Query 360  Sbjct 361 | | AVANGLKLNGVDCDEGETSLVVR-GRPDGKGLGNASGAAVATHLDHRIAMSFLVMGLVSE AVANGLKLNGVDC EGE +L VR G A G AV THLDHRIAMSFL+MGL +E AVANGLKLNGVDCTEGEATLAVRGVPGGRGLGGQARGEAVKTHLDHRIAMSFLIMGLAAE | 418  420 |
| Query 419 | | NPVTVDDATMIATSFPEFMDLMAGLGAKIE 448 PVTVDDA MIATSFPEFM LM GLGA+IE |  |
| Sbjct | 421 | KPVTVDDANMIATSFPEFMGLMKGLGAEIE 450 | |

>WP_053998386.1 3-phosphoshikimate 1-carboxyvinyltransferase [Ahrensia marina] KPB01862.1 3-phosphoshikimate 1-carboxyvinyltransferase [Ahrensia marina]

Length=451

Score = 666 bits (1719), Expect = 0.0, Method: Compositional matrix adjust.

| Identities | = 336/451 (75%), Positives = 380/451 (84%), Gaps = 1/451 (0%) |  |
| --- | --- | --- |
| Query 2 | LHGASSRPATARKSSGLSGTVRIPGDKSISHRSFMFGGLASGETRITGLLEGEDVINTGK | 61 |
| Sbjct 1 | +H + +PA+A KS L G R+PGDKSISHRSFMFGGLAS ETR+TGLLEGEDV+ TG+ MHNHTPKPASAHKSPPLKGNARVPGDKSISHRSFMFGGLASSETRVTGLLEGEDVLATGR | 60 |
| Query 62 | AMQAMGARIRKEGDTWIIDGVGNGGLLAPEAPLDFGNAATGCRLTMGLVGVYDFDSTFIG | 121 |
| Sbjct 61 | AMQAMGA+IRKEGDTWII+G GNG LL PE P DFGNA TG RLTMGLVG YD +TF G AMQAMGAQIRKEGDTWIINGTGNGCLLEPEEPFDFGNAGTGARLTMGLVGTYDMKTTFTG | 120 |
| Query 122 | DASLTKRPMGRVLNPLREMGVQVKSEDGDRLPVTLRGPKTPTPITYRVPMASAQVKSAVL | 181 |
| Sbjct 121 | DASLTKRPMGRVL+PLR+MGVQV++ +GDR P+TLRGP+T PI+YRVPMASAQVKSAVL DASLTKRPMGRVLDPLRQMGVQVEAAEGDRCPITLRGPRTANPISYRVPMASAQVKSAVL | 180 |
| Query 182 | LAGLNTPGITTVIEPIMTRDHTEKMLQGFGANLTVETDADGVRTIRLEGRGKLTGQVIDV | 241 |
| Sbjct 181 | LAGLNTPGITTVIEPIMTRDHTEKML GFGA + +E DADG R I + G+G+L G+ I+V LAGLNTPGITTVIEPIMTRDHTEKMLSGFGAKIEIEEDADGTRHIHIHGQGELRGRTIEV | 240 |
| Query 242 | PGDPSSTAFPLVAALLVPGSDVTILNVLMNPTRTGLILTLQEMGADIEVINPRLAGGEDV | 301 |
| Sbjct 241 | PGDPSS FPLVAAL+VPGSD+ I N LMNPTRTGLI TLQEMG I++IN R AGGEDV PGDPSSAGFPLVAALIVPGSDIIIENALMNPTRTGLITTLQEMGGQIDIINMRDAGGEDV | 300 |
| Query 302 | ADLRVRSSTLKGVTVPEDRAPSMIDEYPILavaaafaegaTVMNGLEELRVKESDRLSAV | 361 |
| Sbjct 301 | ADL VR+S LKG+ VP DRAPSMIDEYP+LAVAAAFAEG T M+GL+ELRVKESDRL+AV ADLHVRASDLKGIVVPPDRAPSMIDEYPVLAVAAAFAEGETYMDGLDELRVKESDRLAAV | 360 |
| Query 362 | ANGLKLNGVDCDEGETSLVVRGRPDGKGLGNASGAAVA-THLDHRIAMSFLVMGLVSENP | 420 |
| Sbjct 361 | A GLK NGV+C EGE SL V+G P GKGLG S AV THLDHRIAM+FLVMG+ SE ARGLKANGVECHEGEMSLTVKGVPGGKGLGAVSAQAVVETHLDHRIAMAFLVMGMASEKA | 420 |
| Query 421  Sbjct 421 | VTVDDATMIATSFPEFMDLMAGLGAKIELSD 451 V +DDATMIATSFPEFMDLM GLGA I++S+ VHIDDATMIATSFPEFMDLMRGLGADIQVSE 451 |  |

>WP_144378739.1 3-phosphoshikimate 1-carboxyvinyltransferase [Mesorhizobium amorphae] Length=440

Score = 666 bits (1719), Expect = 0.0, Method: Compositional matrix adjust.

| Identities | = 345/439 (79%), Positives = 377/439 (86%), Gaps = 2/439 (0%) |  |
| --- | --- | --- |
| Query 1 | MLHGASSRPATARKSSGLSGTVRIPGDKSISHRSFMFGGLASGETRITGLLEGEDVINTG | 60 |
| Sbjct 1 | M H A+ +PATAR+S LSGT R+PGDKSISHRSFMFGGLASGETRITGLLEGEDV+ TG MAHDATPQPATARRSPPLSGTARVPGDKSISHRSFMFGGLASGETRITGLLEGEDVMRTG | 60 |
| Query 61 | KAMQAMGARIRKEGDTWIIDGVGNGGLLAPEAPLDFGNAATGCRLTMGLVGVYDFDSTFI | 120 |
| Sbjct 61 | +AM+AMGA I K GD W+I G GNG LL PEAPLDFGNA TG RLTMGLVG YD ++TFI QAMKAMGAHIEKHGDEWVIRGTGNGALLQPEAPLDFGNAGTGSRLTMGLVGTYDMETTFI | 120 |
| Query 121 | GDASLTKRPMGRVLNPLREMGVQV-KSEDGDRLPVTLRGPKTPTPITYRVPMASAQVKSA | 179 |
| Sbjct 121 | GDASL+ RPMGRVL+PLR+MGVQV ++ GDR+P+TLRGPK PITYRVPMASAQVKSA GDASLSSRPMGRVLDPLRQMGVQVLQAAPGDRMPITLRGPKHAAPITYRVPMASAQVKSA | 180 |
| Query 180 | VLLAGLNTPGITTVIEPIMTRDHTEKMLQGFGANLTVETDADGVRTIRLEGRGKLTGQVI | 239 |
| Sbjct 181 | VLLAGLNTPGITTVIEP+MTRDHTEKML+GFGANLTVETD GVR I +EG+GKLTGQVI VLLAGLNTPGITTVIEPVMTRDHTEKMLKGFGANLTVETDERGVRHIFIEGQGKLTGQVI | 240 |
| Query 240 | DVPGDPSSTAFPLVAALLVPGSDVTILNVLMNPTRTGLILTLQEMGADIEVINPRLAGGE | 299 |
| Sbjct 241 | VPGDPSS FPLVAAL+VPGSD+TI NVLMNPTRTGL+LTLQEMG I+++NPR AGGE AVPGDPSSAGFPLVAALIVPGSDITIENVLMNPTRTGLLLTLQEMGGQIDILNPRNAGGE | 300 |
| Query 300 | DVADLRVRSSTLKGVTVPEDRAPSMIDEYPILavaaafaegaTVMNGLEELRVKESDRLS | 359 |
| Sbjct 301 | DVADLRVR S LKGVTVP +RAPSMIDEYP+LAVAAAFAEG TVM GLEELRVKESDRL+  DVADLRVRYSELKGVTVPAERAPSMIDEYPVLAVAAAFAEGETVMQGLEELRVKESDRLA | 360 |

| Query | 360 | AVANGLKLNGVDCDEGETSLVVR-GRPDGKGLGNASGAAVATHLDHRIAMSFLVMGLVSE 418 |
| --- | --- | --- |
| Sbjct | 361 | AVANGLKLNGVDC EGE SL VR G A G AV THLDHRIAMSFL+MGL +E AVANGLKLNGVDCTEGEVSLSVRGVPGGKGLGGEAKGEAVKTHLDHRIAMSFLIMGLATE 420 |
| Query | 419 | NPVTVDDATMIATSFPEFM 437 |
| Sbjct | 421 | PVTVDD MIATSFPEFM  KPVTVDDRAMIATSFPEFM 439 |

>WP_097576630.1 3-phosphoshikimate 1-carboxyvinyltransferase [Mesorhizobium sanjuanii] PDQ18071.1 3-phosphoshikimate 1-carboxyvinyltransferase [Mesorhizobium sanjuanii]

Length=452

Score = 666 bits (1718), Expect = 0.0, Method: Compositional matrix adjust.

| Identities | = 352/452 (78%), Positives = 385/452 (85%), Gaps = 4/452 (1%) |  |
| --- | --- | --- |
| Query 1 | MLHGASSRPATARKSSGLSGTVRIPGDKSISHRSFMFGGLASGETRITGLLEGEDVINTG | 60 |
| Sbjct 1 | M H A+ +PATARKS LSGT R+PGDKSISHRSFMFGGLASGETRITGLLEGEDV+ TG MSHSAAPKPATARKSPALSGTARVPGDKSISHRSFMFGGLASGETRITGLLEGEDVMRTG | 60 |
| Query 61 | KAMQAMGARIRKEGDTWIIDGVGNGGLLAPEAPLDFGNAATGCRLTMGLVGVYDFDSTFI | 120 |
| Sbjct 61 | AM+AMGA I K G W+I G GNG LL PE PLDFGNA TG RLTMGLVG YD ++TFI AAMKAMGAHIEKRGAEWLIRGTGNGALLQPEGPLDFGNAGTGSRLTMGLVGTYDMETTFI | 120 |
| Query 121 | GDASLTKRPMGRVLNPLREMGVQV-KSEDGDRLPVTLRGPKTPTPITYRVPMASAQVKSA | 179 |
| Sbjct 121 | GDASL+ RPMGRVL PLR+MGVQV K+ GDR+P+TL GPK PITYRVPMASAQVKSA GDASLSGRPMGRVLEPLRQMGVQVLKATPGDRMPITLHGPKHAAPITYRVPMASAQVKSA | 180 |
| Query 180 | VLLAGLNTPGITTVIEPIMTRDHTEKMLQGFGANLTVETDADGVRTIRLEGRGKLTGQVI | 239 |
| Sbjct 181 | VLLAGLNTPG+TTVIEP+MTRDHTEKML+GFGANL+VETD GVR I +EG+GKLTGQ I VLLAGLNTPGVTTVIEPVMTRDHTEKMLKGFGANLSVETDERGVRHIFIEGQGKLTGQTI | 240 |
| Query 240 | DVPGDPSSTAFPLVAALLVPGSDVTILNVLMNPTRTGLILTLQEMGADIEVINPRLAGGE | 299 |
| Sbjct 241 | VPGDPSS FPLVAAL+VPGSDVTI NVLMNPTRTGL+LTLQEMGA I++I+PR GGE AVPGDPSSAGFPLVAALIVPGSDVTIENVLMNPTRTGLLLTLQEMGARIDIIDPRNEGGE | 300 |
| Query 300 | DVADLRVRSSTLKGVTVPEDRAPSMIDEYPILavaaafaegaTVMNGLEELRVKESDRLS | 359 |
| Sbjct 301 | DVADLRVR S LKGVTVP +RAP+MIDEYP+LAVAA+FAEG T+M GLEELRVKESDRLS DVADLRVRYSELKGVTVPPERAPTMIDEYPVLAVAASFAEGETLMQGLEELRVKESDRLS | 360 |
| Query 360 | AVANGLKLNGVDCDEGETSLVVRGRPDGKGLG---NASGAAVATHLDHRIAMSFLVMGLV | 416 |
| Sbjct 361 | AVANGLKLNGVDC EGE SL VRG+P GKGLG N V THLDHRIAMSFLVMGL AVANGLKLNGVDCTEGEASLAVRGKPGGKGLGGHPNGQDTTVQTHLDHRIAMSFLVMGLA | 420 |
| Query 417  Sbjct 421 | SENPVTVDDATMIATSFPEFMDLMAGLGAKIE 448  +E PVT+DD MIATSFPEFM LM GLGA+IE  TEKPVTIDDQAMIATSFPEFMGLMKGLGAEIE 452 |  |

>RWN17211.1 3-phosphoshikimate 1-carboxyvinyltransferase [Mesorhizobium sp.] Length=452

Score = 666 bits (1718), Expect = 0.0, Method: Compositional matrix adjust.

| Identities | = 350/451 (78%), Positives = 384/451 (85%), Gaps = 4/451 (1%) |  |
| --- | --- | --- |
| Query 1 | MLHGASSRPATARKSSGLSGTVRIPGDKSISHRSFMFGGLASGETRITGLLEGEDVINTG | 60 |
| Sbjct 1 | M H A+ +PATAR+S LSG R+PGDKSISHRSFMFGGLASGETRITGLLEGEDV+ TG MSHAAAPKPATARQSPALSGIARVPGDKSISHRSFMFGGLASGETRITGLLEGEDVMRTG | 60 |
| Query 61 | KAMQAMGARIRKEGDTWIIDGVGNGGLLAPEAPLDFGNAATGCRLTMGLVGVYDFDSTFI | 120 |
| Sbjct 61 | AM+AMGA I K GD W+I G GNG LL PE PLDFGNA TG RLTMGLVG YD ++TFI AAMKAMGAHIEKNGDEWVIRGTGNGALLQPEGPLDFGNAGTGSRLTMGLVGTYDMETTFI | 120 |
| Query 121 | GDASLTKRPMGRVLNPLREMGVQV-KSEDGDRLPVTLRGPKTPTPITYRVPMASAQVKSA | 179 |
| Sbjct 121 | GDASL+ RPMGRVL PLR+MGVQV K+ GDR+P+TL GPK PITYRVPMASAQVKSA GDASLSGRPMGRVLEPLRQMGVQVLKATPGDRMPITLHGPKHAAPITYRVPMASAQVKSA | 180 |
| Query 180 | VLLAGLNTPGITTVIEPIMTRDHTEKMLQGFGANLTVETDADGVRTIRLEGRGKLTGQVI | 239 |
| Sbjct 181 | VLLAGLNTPGITTVIEP+MTRDHTEKML+GFGANL+VETD GVR I +EG+G+LTGQ I VLLAGLNTPGITTVIEPVMTRDHTEKMLKGFGANLSVETDERGVRHIFIEGQGRLTGQTI | 240 |
| Query 240 | DVPGDPSSTAFPLVAALLVPGSDVTILNVLMNPTRTGLILTLQEMGADIEVINPRLAGGE | 299 |

| Sbjct | 241 | VPGDPSS FPLVAAL+VPGS++TI NVLMNPTRTGL+LTLQEMG +IE+ NPR AGGE AVPGDPSSAGFPLVAALIVPGSEITIENVLMNPTRTGLLLTLQEMGGNIEIFNPRNAGGE | 300 |
| --- | --- | --- | --- |
| Query | 300 | DVADLRVRSSTLKGVTVPEDRAPSMIDEYPILavaaafaegaTVMNGLEELRVKESDRLS | 359 |
| Sbjct | 301 | DVADLRVR S LKGVTVP +RAPSMIDEYP+LAVAA+FAEG T+M GLEELRVKESDRLS DVADLRVRYSELKGVTVPPERAPSMIDEYPVLAVAASFAEGETLMQGLEELRVKESDRLS | 360 |
| Query | 360 | AVANGLKLNGVDCDEGETSLVVRGRPDGKGLG---NASGAAVATHLDHRIAMSFLVMGLV | 416 |
| Sbjct | 361 | AVANGLKLNGVDC EGE SL VRG+P GKGLG N V THLDHRIAMSFLVMGL AVANGLKLNGVDCTEGEASLAVRGKPGGKGLGGHPNGQDTTVQTHLDHRIAMSFLVMGLA | 420 |
| Query  Sbjct | 417  421 | SENPVTVDDATMIATSFPEFMDLMAGLGAKI 447  +E PVT+DD MIATSFPEFM LM GLGA+I  TEKPVTIDDQAMIATSFPEFMGLMMGLGAEI 451 |  |

>WP_195176840.1 3-phosphoshikimate 1-carboxyvinyltransferase [Mesorhizobium sp. INR15]

QPC89379.1 3-phosphoshikimate 1-carboxyvinyltransferase [Mesorhizobium sp. INR15]

Length=453

Score = 666 bits (1718), Expect = 0.0, Method: Compositional matrix adjust.

| Identities | = 346/458 (76%), Positives = 381/458 (83%), Gaps = 16/458 (3%) |  |
| --- | --- | --- |
| Query 1 | MLHGASSRPATARKSSGLSGTVRIPGDKSISHRSFMFGGLASGETRITGLLEGEDVINTG | 60 |
| Sbjct 1 | M H A+S+PATARKS LSGT R+PGDKSISHRSFMFGGLASGETRITGLLEGEDV+ TG MSHSAASKPATARKSPALSGTARVPGDKSISHRSFMFGGLASGETRITGLLEGEDVMRTG | 60 |
| Query 61 | KAMQAMGARIRKEGDTWIIDGVGNGGLLAPEAPLDFGNAATGCRLTMGLVGVYDFDSTFI | 120 |
| Sbjct 61 | AM+AMGA I K G W+I G GNG LL PE PLDFGNA TG RLTMGLVG YD ++TFI AAMKAMGAHIEKHGAEWVIRGTGNGALLQPEGPLDFGNAGTGSRLTMGLVGTYDMETTFI | 120 |
| Query 121 | GDASLTKRPMGRVLNPLREMGVQV-KSEDGDRLPVTLRGPKTPTPITYRVPMASAQVKSA | 179 |
| Sbjct 121 | GDASL+ RPMGRVL PLR+MGVQV K+ GDR+P+TLRGPK PITYRVPMASAQVKSA GDASLSGRPMGRVLEPLRQMGVQVLKATPGDRMPITLRGPKHAAPITYRVPMASAQVKSA | 180 |
| Query 180 | VLLAGLNTPGITTVIEPIMTRDHTEKMLQGFGANLTVETDADGVRTIRLEGRGKLTGQVI | 239 |
| Sbjct 181 | VLLAGLNTPGITTVIEP+MTRDHTEKML+GFGANL+VETD GVR I +EG+GKLTGQ I VLLAGLNTPGITTVIEPVMTRDHTEKMLKGFGANLSVETDERGVRHIFIEGQGKLTGQTI | 240 |
| Query 240 | DVPGDPSSTAFPLVAALLVPGSDVTILNVLMNPTRTGLILTLQEMGADIEVINPRLAGGE | 299 |
| Sbjct 241 | VPGDPSS FPLVAAL+VPGSD+ I NVLMNPTRTGL+LTLQEMG I+++NPR AGGE AVPGDPSSAGFPLVAALIVPGSDIIIENVLMNPTRTGLLLTLQEMGGKIDILNPRNAGGE | 300 |
| Query 300 | DVADLRVRSSTLKGVTVPEDRAPSMIDEYPILavaaafaegaTVMNGLEELRVKESDRLS | 359 |
| Sbjct 301 | DVADL VR S LKGVT+P +RAPSMIDEYP+LAVAA+FAEG T+M GLEELRVKESDRLS DVADLHVRFSELKGVTLPPERAPSMIDEYPVLAVAASFAEGETLMQGLEELRVKESDRLS | 360 |
| Query 360 | AVANGLKLNGVDCDEGETSLVVR GRPDGKGLGNASGAAVATHLDHRIAMSF | 410 |
| Sbjct 361 | AVANGLKLNGVDC EGE SL VR G P+G+ V THLDHRIAMSF AVANGLKLNGVDCTEGEASLAVRGKPGGKGLGGHPNGR DTTVQTHLDHRIAMSF | 414 |
| Query 411  Sbjct 415 | LVMGLVSENPVTVDDATMIATSFPEFMDLMAGLGAKIE 448 LVMGL +E PVT+DD MIATSFPEFM LM GLGA+I+ LVMGLATEKPVTIDDQAMIATSFPEFMGLMKGLGAEID 452 |  |

>WP_115418752.1 3-phosphoshikimate 1-carboxyvinyltransferase [Ensifer sp. M14] RDL49319.1 3-phosphoshikimate 1-carboxyvinyltransferase [Ensifer sp. M14]

Length=448

Score = 665 bits (1717), Expect = 0.0, Method: Compositional matrix adjust. Identities = 339/448 (76%), Positives = 381/448 (85%), Gaps = 0/448 (0%)

| Query | 1 | MLHGASSRPATARKSSGLSGTVRIPGDKSISHRSFMFGGLASGETRITGLLEGEDVINTG | 60 |
| --- | --- | --- | --- |
| Sbjct | 1 | M G ++PA AR+S L GTVRIPGDKSISHR+ M GGLA+G TRI GLLEGED+ NT MSAGLIAKPAIARRSVALRGTVRIPGDKSISHRALMLGGLAAGRTRIAGLLEGEDIFNTA | 60 |
| Query | 61 | KAMQAMGARIRKEGDTWIIDGVGNGGLLAPEAPLDFGNAATGCRLTMGLVGVYDFDSTFI | 120 |
| Sbjct | 61 | +AMQA+GA + KEG TWI+DGVGNG LL PEAPLDFGNA TGC L MGL GVYDF +TF+  RAMQALGATVHKEGGTWIVDGVGNGALLEPEAPLDFGNAGTGCCLIMGLAGVYDFATTFV | 120 |

| Query | 121 | GDASLTKRPMGRVLNPLREMGVQVKSEDGDRLPVTLRGPKTPTPITYRVPMASAQVKSAV | 180 |
| --- | --- | --- | --- |
| Sbjct | 121 | GD SL RPM RVL+PLR+MGVQV S DG LPVTLRGPKT P++YR+P A AQVKSAV GDPSLVMRPMDRVLDPLRQMGVQVMSADGGLLPVTLRGPKTANPVSYRLPTAPAQVKSAV | 180 |
| Query | 181 | LLAGLNTPGITTVIEPIMTRDHTEKMLQGFGANLTVETDADGVRTIRLEGRGKLTGQVID | 240 |
| Sbjct | 181 | LLAGLN PGITTVIEP+ TRDHTE+MLQ FG NL VE A+G R +RLEG+G+LTGQ ID LLAGLNAPGITTVIEPVATRDHTERMLQAFGVNLMVEAGAEGERIVRLEGQGRLTGQDID | 240 |
| Query | 241 | VPGDPSSTAFPLVAALLVPGSDVTILNVLMNPTRTGLILTLQEMGADIEVINPRLAGGED | 300 |
| Sbjct | 241 | +PGDPSS AFPLVAAL+VPGSD+T+L+VLMNPTRTGLI TLQEMGA I+V++ RL GGE+ IPGDPSSAAFPLVAALVVPGSDITLLDVLMNPTRTGLIATLQEMGARIDVLSERLQGGEE | 300 |
| Query | 301 | VADLRVRSSTLKGVTVPEDRAPSMIDEYPILavaaafaegaTVMNGLEELRVKESDRLSA | 360 |
| Sbjct | 301 | VADLRVRSS LKGVTVP+DR PSMID YP+LAVAAAFAEG TVM+GLE L KESDRLSA VADLRVRSSELKGVTVPQDRTPSMIDAYPVLAVAAAFAEGRTVMSGLEALCAKESDRLSA | 360 |
| Query | 361 | VANGLKLNGVDCDEGETSLVVRGRPDGKGLGNASGAAVATHLDHRIAMSFLVMGLVSENP | 420 |
| Sbjct | 361 | V GLKLNGVDC+EG SLVV+GRP GKGLGNA+GAAVATHLDHRIAMSFLVMGL SE+P VTAGLKLNGVDCEEGPGSLVVQGRPGGKGLGNAAGAAVATHLDHRIAMSFLVMGLASEHP | 420 |
| Query  Sbjct | 421  421 | VTVDDATMIATSFPEFMDLMAGLGAKIE 448 VT+DDATMIATSFP F++LM GLGA IE VTIDDATMIATSFPGFIELMTGLGATIE 448 |  |

>WP_140650592.1 MULTISPECIES: 3-phosphoshikimate 1-carboxyvinyltransferase [unclassified Mesorhizobium]

TPI71474.1 3-phosphoshikimate 1-carboxyvinyltransferase [Mesorhizobium sp. B3-1-8]

TPI76120.1 3-phosphoshikimate 1-carboxyvinyltransferase [Mesorhizobium sp. B3-1-3]

Length=452

Score = 665 bits (1717), Expect = 0.0, Method: Compositional matrix adjust.

| Identities | = 351/451 (78%), Positives = 387/451 (86%), Gaps = 4/451 (1%) |  |
| --- | --- | --- |
| Query 1 | MLHGASSRPATARKSSGLSGTVRIPGDKSISHRSFMFGGLASGETRITGLLEGEDVINTG | 60 |
| Sbjct 1 | M H A+++PATARKS L+GT R+PGDKSISHRSFMFGGLASGETRITGLLEGEDV+ TG MSHSAAAKPATARKSPALAGTARVPGDKSISHRSFMFGGLASGETRITGLLEGEDVMRTG | 60 |
| Query 61 | KAMQAMGARIRKEGDTWIIDGVGNGGLLAPEAPLDFGNAATGCRLTMGLVGVYDFDSTFI | 120 |
| Sbjct 61 | AM+AMGA + K+G W+I G GNG LL PE PLDFGNA TG RLTMGLVG YD ++TFI AAMKAMGAHVEKKGAEWVIRGTGNGALLQPEGPLDFGNAGTGSRLTMGLVGTYDMETTFI | 120 |
| Query 121 | GDASLTKRPMGRVLNPLREMGVQV-KSEDGDRLPVTLRGPKTPTPITYRVPMASAQVKSA | 179 |
| Sbjct 121 | GDASL+ RPMGRVL+PLR+MGVQV K+ GDR+P+TLRGPK PITYRVPMASAQVKSA GDASLSGRPMGRVLDPLRQMGVQVLKAAPGDRMPITLRGPKHAAPITYRVPMASAQVKSA | 180 |
| Query 180 | VLLAGLNTPGITTVIEPIMTRDHTEKMLQGFGANLTVETDADGVRTIRLEGRGKLTGQVI | 239 |
| Sbjct 181 | VLLAGLNTPGITTVIEP+MTRDHTEKML+GFGANL+VETD GVR I +EG+GKLTGQ I VLLAGLNTPGITTVIEPVMTRDHTEKMLKGFGANLSVETDERGVRHIFIEGQGKLTGQTI | 240 |
| Query 240 | DVPGDPSSTAFPLVAALLVPGSDVTILNVLMNPTRTGLILTLQEMGADIEVINPRLAGGE | 299 |
| Sbjct 241 | VPGDPSS FPLVAAL+VP SD+TI NVLMNPTRTGL+LTLQEMG I+++NPR AGGE AVPGDPSSAGFPLVAALIVPDSDITIENVLMNPTRTGLLLTLQEMGGRIDILNPRNAGGE | 300 |
| Query 300 | DVADLRVRSSTLKGVTVPEDRAPSMIDEYPILavaaafaegaTVMNGLEELRVKESDRLS | 359 |
| Sbjct 301 | DVADLRVR S LKGVTVP +RAPSMIDEYP+LAVAA+FAEG T+M GLEELRVKESDRLS DVADLRVRYSELKGVTVPPERAPSMIDEYPVLAVAASFAEGETLMRGLEELRVKESDRLS | 360 |
| Query 360 | AVANGLKLNGVDCDEGETSLVVRGRPDGKGLG---NASGAAVATHLDHRIAMSFLVMGLV | 416 |
| Sbjct 361 | AVANGLKLNGVDC EGE SL VRGRP GKGLG N V THLDHRIAMSFLVMGL AVANGLKLNGVDCTEGEASLAVRGRPGGKGLGGHPNGQDTTVQTHLDHRIAMSFLVMGLA | 420 |
| Query 417  Sbjct 421 | SENPVTVDDATMIATSFPEFMDLMAGLGAKI 447  +E PVT+DD MIATSFPEFM LM GLGA+I  TEKPVTIDDQAMIATSFPEFMGLMKGLGAEI 451 |  |

>MBA3446854.1 3-phosphoshikimate 1-carboxyvinyltransferase [Pseudaminobacter sp.]

Length=452

Score = 665 bits (1716), Expect = 0.0, Method: Compositional matrix adjust.

| Identities | = 348/453 (77%), Positives = 379/453 (84%), Gaps = 4/453 (1%) |  |
| --- | --- | --- |
| Query 1 | MLHGASSRPATARKSSGLSGTVRIPGDKSISHRSFMFGGLASGETRITGLLEGEDVINTG | 60 |
| Sbjct 1 | M H + RPATARKS LSGTV++PGDKSISHRSFMFGGLASGETRITGLLEGEDV+ TG MAHESPFRPATARKSPALSGTVKMPGDKSISHRSFMFGGLASGETRITGLLEGEDVMRTG | 60 |
| Query 61 | KAMQAMGARIRKEGDTWIIDGVGNGGLLAPEAPLDFGNAATGCRLTMGLVGVYDFDSTFI | 120 |
| Sbjct 61 | +AM+AMGARI K G WII G GNG LL P APLDFGNA TG RLTMGLVG YD ++TFI EAMKAMGARIEKRGAEWIIQGTGNGCLLEPVAPLDFGNAGTGSRLTMGLVGTYDMETTFI | 120 |
| Query 121 | GDASLTKRPMGRVLNPLREMGVQV-KSEDGDRLPVTLRGPKTPTPITYRVPMASAQVKSA | 179 |
| Sbjct 121 | GDASL+ RPMGRVL+PLREMGVQV KS GDR+P+TLRGP+T PI+YRVPMASAQVKSA GDASLSGRPMGRVLDPLREMGVQVLKSSPGDRMPITLRGPRTAAPISYRVPMASAQVKSA | 180 |
| Query 180 | VLLAGLNTPGITTVIEPIMTRDHTEKMLQGFGANLTVETDADGVRTIRLEGRGKLTGQVI | 239 |
| Sbjct 181 | VLLAGLNTPG+TTV+EP+MTRDHTEKML+GFGANL VET DGVR IR+EG+GKL GQ I VLLAGLNTPGVTTVVEPVMTRDHTEKMLKGFGANLEVETVRDGVRHIRIEGQGKLAGQTI | 240 |
| Query 240 | DVPGDPSSTAFPLVAALLVPGSDVTILNVLMNPTRTGLILTLQEMGADIEVINPRLAGGE | 299 |
| Sbjct 241 | VPGDPSS FPLVAAL+VP SD+ I NVLMNPTRTGLI TL EMGA IE++NPR AGGE AVPGDPSSAGFPLVAALIVPSSDIVIENVLMNPTRTGLIATLLEMGASIELLNPRNAGGE | 300 |
| Query 300 | DVADLRVRSSTLKGVTVPEDRAPSMIDEYPILavaaafaegaTVMNGLEELRVKESDRLS | 359 |
| Sbjct 301 | DVADLRVRSS LKGVTVP +RAPSMIDEYP LAVAA FAEG T+M GLEELRVKESDRLS DVADLRVRSSELKGVTVPAERAPSMIDEYPALAVAACFAEGETLMQGLEELRVKESDRLS | 360 |
| Query 360 | AVANGLKLNGVDCDEGETSLVVRGRPDGKGLGNASGAAVATHLDHRIAMSFLVMGLVSEN | 419 |
| Sbjct 361 | AVA GLK+NGVDC EGET L VR G V THLDHRIAMSFLVMGL SE AVAEGLKVNGVDCTEGETFLSVR---GKPSGKGLGGGLVKTHLDHRIAMSFLVMGLASEK | 417 |
| Query 420  Sbjct 418 | PVTVDDATMIATSFPEFMDLMAGLGAKIELSDT 452 PVT+DD TMIATSFPEFM LM GLGA+I+ D+ PVTIDDQTMIATSFPEFMGLMTGLGAEIQQMDS 450 |  |

>WP_189504106.1 3-phosphoshikimate 1-carboxyvinyltransferase [Corticibacterium populi]

GHD16105.1 3-phosphoshikimate 1-carboxyvinyltransferase [Corticibacterium populi]

Length=446

Score = 665 bits (1716), Expect = 0.0, Method: Compositional matrix adjust.

| Identities | = 353/449 (79%), Positives = 390/449 (87%), Gaps = 4/449 (1%) |  |
| --- | --- | --- |
| Query 1 | MLHGASSRPATARKSSGLSGTVRIPGDKSISHRSFMFGGLASGETRITGLLEGEDVINTG | 60 |
| Sbjct 1 | M H +PATA ++ L+G VR+PGDKSISHRSFMFGGLASGETRITGLLEGEDV+ TG MSHSPLPQPATAHRAEALTGRVRVPGDKSISHRSFMFGGLASGETRITGLLEGEDVLRTG | 60 |
| Query 61 | KAMQAMGARIRKEGDTWIIDGVGNGGLLAPEAPLDFGNAATGCRLTMGLVGVYDFDSTFI | 120 |
| Sbjct 61 | +AM+AMGARI + G W+I G GNG LL PEAPLDFGNA TG RLTMGLVG YD ++TFI EAMKAMGARIERNGAEWVIQGTGNGCLLQPEAPLDFGNAGTGSRLTMGLVGTYDMETTFI | 120 |
| Query 121 | GDASLTKRPMGRVLNPLREMGVQV-KSEDGDRLPVTLRGPKTPTPITYRVPMASAQVKSA | 179 |
| Sbjct 121 | GDASL+KRPM RVL+PLREMGVQV + GDR+P+TLRGPK PITYRVPM SAQVKSA GDASLSKRPMARVLDPLREMGVQVLNAASGDRMPLTLRGPKHAAPITYRVPMPSAQVKSA | 180 |
| Query 180 | VLLAGLNTPGITTVIEPIMTRDHTEKMLQGFGANLTVETDADGVRTIRLEGRGKLTGQVI | 239 |
| Sbjct 181 | VLLAGLNTPGITTV+EP+MTRDHTEKML+GFGANL VETD+ G+RTIR+EG GKLTGQVI VLLAGLNTPGITTVLEPVMTRDHTEKMLRGFGANLEVETDSTGLRTIRIEGEGKLTGQVI | 240 |
| Query 240 | DVPGDPSSTAFPLVAALLVPGSDVTILNVLMNPTRTGLILTLQEMGADIEVINPRLAGGE | 299 |
| Sbjct 241 | VPGDPSS AFPLVAALLVPGSD+TI NVLMNPTRTGLILTLQ+MGADIE++NPR AGGE AVPGDPSSAAFPLVAALLVPGSDITIENVLMNPTRTGLILTLQDMGADIELLNPRSAGGE | 300 |
| Query 300 | DVADLRVRSSTLKGVTVPEDRAPSMIDEYPILavaaafaegaTVMNGLEELRVKESDRLS | 359 |
| Sbjct 301 | DVADLRVRSS L+GV+VP +RAPSMIDEYP+LAVAA+FAEG TVM+GL+ELRVKESDRL+ DVADLRVRSSDLRGVSVPPERAPSMIDEYPVLAVAASFAEGTTVMDGLDELRVKESDRLA | 360 |
| Query 360 | AVANGLKLNGVDCDEGETSLVVRGRPDGKGLGNASGAAVATHLDHRIAMSFLVMGLVSEN | 419 |

AVA GLK NGV+C EGE SL V GRP GKGLG S	V THLDHRIAM+FLVMGL SE

| Sbjct | 361 | AVAAGLKANGVECTEGEASLSVIGRPGGKGLGGGS---VETHLDHRIAMAFLVMGLASER 417 |
| --- | --- | --- |
| Query | 420 | PVTVDDATMIATSFPEFMDLMAGLGAKIE 448 |
| Sbjct | 418 | PVTVDD MIATSFPEFMDLMAGLGA IE  PVTVDDRAMIATSFPEFMDLMAGLGAVIE 446 |

>WP_128302807.1 3-phosphoshikimate 1-carboxyvinyltransferase [Mesorhizobium sp. M7A.F.Ca.US.010.02.1.1]

RUW93963.1 3-phosphoshikimate 1-carboxyvinyltransferase [Mesorhizobium sp. M7A.F.Ca.US.010.02.1.1]

Length=452

Score = 665 bits (1716), Expect = 0.0, Method: Compositional matrix adjust.

| Identities | = 350/451 (78%), Positives = 384/451 (85%), Gaps = 4/451 (1%) |  |
| --- | --- | --- |
| Query 1 | MLHGASSRPATARKSSGLSGTVRIPGDKSISHRSFMFGGLASGETRITGLLEGEDVINTG | 60 |
| Sbjct 1 | M H A+ +PATAR+S LSG R+PGDKSISHRSFMFGGLASGETRITGLLEGEDV+ TG MSHAAAPKPATARQSPALSGIARVPGDKSISHRSFMFGGLASGETRITGLLEGEDVMRTG | 60 |
| Query 61 | KAMQAMGARIRKEGDTWIIDGVGNGGLLAPEAPLDFGNAATGCRLTMGLVGVYDFDSTFI | 120 |
| Sbjct 61 | AM+AMGA I K GD W+I G GNG LL PE PLDFGNA TG RLTMGLVG YD ++TFI AAMKAMGAHIEKNGDEWVIRGTGNGALLQPEGPLDFGNAGTGSRLTMGLVGTYDMETTFI | 120 |
| Query 121 | GDASLTKRPMGRVLNPLREMGVQV-KSEDGDRLPVTLRGPKTPTPITYRVPMASAQVKSA | 179 |
| Sbjct 121 | GDASL+ RPMGRVL PLR+MGVQV K+ GDR+P+TL GPK PITYRVPMASAQVKSA GDASLSGRPMGRVLEPLRQMGVQVLKATPGDRMPITLHGPKHAAPITYRVPMASAQVKSA | 180 |
| Query 180 | VLLAGLNTPGITTVIEPIMTRDHTEKMLQGFGANLTVETDADGVRTIRLEGRGKLTGQVI | 239 |
| Sbjct 181 | VLLAGLNTPGITTVIEP+MTRDHTEKML+GFGANL+VETD GVR I +EG+G+LTGQ I VLLAGLNTPGITTVIEPVMTRDHTEKMLRGFGANLSVETDERGVRHIFIEGQGRLTGQTI | 240 |
| Query 240 | DVPGDPSSTAFPLVAALLVPGSDVTILNVLMNPTRTGLILTLQEMGADIEVINPRLAGGE | 299 |
| Sbjct 241 | VPGDPSS FPLVAAL+VPGSD+TI NVLMNPTRTGL+LTLQEMG I+++NPR AGGE AVPGDPSSAGFPLVAALIVPGSDITIENVLMNPTRTGLLLTLQEMGGKIDILNPRNAGGE | 300 |
| Query 300 | DVADLRVRSSTLKGVTVPEDRAPSMIDEYPILavaaafaegaTVMNGLEELRVKESDRLS | 359 |
| Sbjct 301 | DVADLRVR S LKGVTVP +RAPSMIDEYP+LAVAA+FAEG T+M GLEELRVKESDRLS DVADLRVRYSELKGVTVPPERAPSMIDEYPVLAVAASFAEGETLMQGLEELRVKESDRLS | 360 |
| Query 360 | AVANGLKLNGVDCDEGETSLVVRGRPDGKGLG---NASGAAVATHLDHRIAMSFLVMGLV | 416 |
| Sbjct 361 | AVANGLKLNGVDC EGE SL VRG+P GKGLG N V THLDHRIAMSFLVMGL AVANGLKLNGVDCTEGEASLAVRGKPGGKGLGGHPNGQDTTVQTHLDHRIAMSFLVMGLA | 420 |
| Query 417  Sbjct 421 | SENPVTVDDATMIATSFPEFMDLMAGLGAKI 447  +E PVT+DDA MIATSFPEFM LM LGA+I  TEKPVTIDDAAMIATSFPEFMGLMKALGAEI 451 |  |

>WP_127289806.1 3-phosphoshikimate 1-carboxyvinyltransferase [Mesorhizobium sp. M7A.F.Ca.US.011.01.1.1]

RUX32095.1 3-phosphoshikimate 1-carboxyvinyltransferase [Mesorhizobium sp. M7A.F.Ca.US.011.01.1.1]

Length=452

Score = 665 bits (1716), Expect = 0.0, Method: Compositional matrix adjust.

| Identities | = 350/451 (78%), Positives = 384/451 (85%), Gaps = 4/451 (1%) |  |
| --- | --- | --- |
| Query 1 | MLHGASSRPATARKSSGLSGTVRIPGDKSISHRSFMFGGLASGETRITGLLEGEDVINTG | 60 |
| Sbjct 1 | M H A+ +PATAR+SS LSG R+PGDKSISHRSFMFGGLASGETRITGLLEGEDV+ TG MSHAAAPKPATARRSSALSGIARVPGDKSISHRSFMFGGLASGETRITGLLEGEDVMRTG | 60 |
| Query 61 | KAMQAMGARIRKEGDTWIIDGVGNGGLLAPEAPLDFGNAATGCRLTMGLVGVYDFDSTFI | 120 |
| Sbjct 61 | AM+AMGA I K GD W+I G GNG LL PE PLDFGNA TG RLTMGLVG YD ++TFI AAMKAMGAHIEKNGDEWVIRGTGNGALLQPEGPLDFGNAGTGSRLTMGLVGTYDMETTFI | 120 |
| Query 121 | GDASLTKRPMGRVLNPLREMGVQV-KSEDGDRLPVTLRGPKTPTPITYRVPMASAQVKSA | 179 |
| Sbjct 121 | GDASL+ RPMGRVL PLR+MGVQV K+ GDR+P+TL GPK PITYRVPMASAQVKSA GDASLSGRPMGRVLEPLRQMGVQVLKATPGDRMPITLHGPKHAAPITYRVPMASAQVKSA | 180 |
| Query 180 | VLLAGLNTPGITTVIEPIMTRDHTEKMLQGFGANLTVETDADGVRTIRLEGRGKLTGQVI | 239 |

| Sbjct | 181 | VLLAGLNTPGITTVIEP+MTRDHTEKML+GFGANL+VETD GVR I +EG+GKLTGQ I VLLAGLNTPGITTVIEPVMTRDHTEKMLKGFGANLSVETDERGVRHIFIEGQGKLTGQTI | 240 |
| --- | --- | --- | --- |
| Query | 240 | DVPGDPSSTAFPLVAALLVPGSDVTILNVLMNPTRTGLILTLQEMGADIEVINPRLAGGE | 299 |
| Sbjct | 241 | VPGDPSS FPLVAAL+VPGSD+TI NVLMNPTRTGL+LTLQ+MG I+++NPR AGGE AVPGDPSSAGFPLVAALIVPGSDITIENVLMNPTRTGLLLTLQDMGGKIDILNPRNAGGE | 300 |
| Query | 300 | DVADLRVRSSTLKGVTVPEDRAPSMIDEYPILavaaafaegaTVMNGLEELRVKESDRLS | 359 |
| Sbjct | 301 | DVADLRVR S LKGV VP +RAPSMIDEYP+LAVAA+FAEG T+M GLEELRVKESDRLS DVADLRVRYSELKGVAVPPERAPSMIDEYPVLAVAASFAEGETLMQGLEELRVKESDRLS | 360 |
| Query | 360 | AVANGLKLNGVDCDEGETSLVVRGRPDGKGLG---NASGAAVATHLDHRIAMSFLVMGLV | 416 |
| Sbjct | 361 | AVANGLKLNGVDC EGE SL VRG+P GKGLG N V THLDHRIAMSFLVMGL AVANGLKLNGVDCTEGEASLAVRGKPGGKGLGGHPNGQDTTVQTHLDHRIAMSFLVMGLA | 420 |
| Query  Sbjct | 417  421 | SENPVTVDDATMIATSFPEFMDLMAGLGAKI 447  +E PVT+DDA MIATSFPEFM LM LGA+I  AEKPVTIDDAAMIATSFPEFMGLMKALGAEI 451 |  |

>WP_141836710.1 3-phosphoshikimate 1-carboxyvinyltransferase [Mesorhizobium sp. B2-4-15]

TPK74044.1 3-phosphoshikimate 1-carboxyvinyltransferase [Mesorhizobium sp. B2-4-15]

Length=452

Score = 665 bits (1716), Expect = 0.0, Method: Compositional matrix adjust.

| Identities | = 344/457 (75%), Positives = 381/457 (83%), Gaps = 16/457 (4%) |  |
| --- | --- | --- |
| Query 1 | MLHGASSRPATARKSSGLSGTVRIPGDKSISHRSFMFGGLASGETRITGLLEGEDVINTG | 60 |
| Sbjct 1 | M H +++PATARKSS LSGT R+PGDKSISHRSFMFGGLASGETRITGLLEGEDV+ TG MSHAVAAKPATARKSSALSGTARVPGDKSISHRSFMFGGLASGETRITGLLEGEDVMRTG | 60 |
| Query 61 | KAMQAMGARIRKEGDTWIIDGVGNGGLLAPEAPLDFGNAATGCRLTMGLVGVYDFDSTFI | 120 |
| Sbjct 61 | AM+AMGA I K G W+I G GNG LL PE PLDFGNA TG RLTMGLVG YD ++TFI AAMKAMGAHIEKRGTEWVIRGTGNGALLQPEGPLDFGNAGTGSRLTMGLVGTYDMETTFI | 120 |
| Query 121 | GDASLTKRPMGRVLNPLREMGVQV-KSEDGDRLPVTLRGPKTPTPITYRVPMASAQVKSA | 179 |
| Sbjct 121 | GDASL+ RPMGRVL PLR+MGVQV K+ GDR+P+TL GPK PITYRVPMASAQVKSA GDASLSSRPMGRVLEPLRQMGVQVLKATPGDRMPITLHGPKHAAPITYRVPMASAQVKSA | 180 |
| Query 180 | VLLAGLNTPGITTVIEPIMTRDHTEKMLQGFGANLTVETDADGVRTIRLEGRGKLTGQVI | 239 |
| Sbjct 181 | VLLAGLNTPGITTVIEP+MTRDHTEKML+GFGANL+VETD GVR + +EG+GKLTGQ I VLLAGLNTPGITTVIEPVMTRDHTEKMLKGFGANLSVETDERGVRHLFIEGQGKLTGQTI | 240 |
| Query 240 | DVPGDPSSTAFPLVAALLVPGSDVTILNVLMNPTRTGLILTLQEMGADIEVINPRLAGGE | 299 |
| Sbjct 241 | VPGDPSS FPLVAAL+VPGSD+ I NVLMNPTRTGL+LTLQEMG I+++NPR AGGE AVPGDPSSAGFPLVAALIVPGSDIVIENVLMNPTRTGLLLTLQEMGGQIDILNPRNAGGE | 300 |
| Query 300 | DVADLRVRSSTLKGVTVPEDRAPSMIDEYPILavaaafaegaTVMNGLEELRVKESDRLS | 359 |
| Sbjct 301 | DVADLRVR S LKGVTVP +RAPSMIDEYP+LAVAA+FAEG T+M GLEELRVKESDRLS DVADLRVRYSELKGVTVPPERAPSMIDEYPVLAVAASFAEGETLMQGLEELRVKESDRLS | 360 |
| Query 360 | AVANGLKLNGVDCDEGETSLVVR GRPDGKGLGNASGAAVATHLDHRIAMSF | 410 |
| Sbjct 361 | AVANGLKLNGV+C EGE SL VR G P+G+ V THLDHRIAMSF AVANGLKLNGVECTEGEASLAVRGRPGGKGLGGHPNGR DTTVQTHLDHRIAMSF | 414 |
| Query 411  Sbjct 415 | LVMGLVSENPVTVDDATMIATSFPEFMDLMAGLGAKI 447 LVMGL ++ PVT+DDA MIATSFPEFM LM GLGA+I LVMGLATDKPVTIDDANMIATSFPEFMGLMKGLGAEI 451 |  |

>WP_023761479.1 3-phosphoshikimate 1-carboxyvinyltransferase [Mesorhizobium sp. LNHC252B00]

ESY68293.1 3-phosphoshikimate 1-carboxyvinyltransferase [Mesorhizobium sp. LNHC252B00]

Length=452

Score = 665 bits (1715), Expect = 0.0, Method: Compositional matrix adjust. Identities = 351/451 (78%), Positives = 383/451 (85%), Gaps = 4/451 (1%)

Query 1	MLHGASSRPATARKSSGLSGTVRIPGDKSISHRSFMFGGLASGETRITGLLEGEDVINTG 60

| Sbjct | 1 | M H A+ +PATARKSS LSGT R+PGDKSISHRSFMFGGLASGETRITGLLEGEDV+ TG MSHAAAPKPATARKSSALSGTARVPGDKSISHRSFMFGGLASGETRITGLLEGEDVMRTG | 60 |
| --- | --- | --- | --- |
| Query | 61 | KAMQAMGARIRKEGDTWIIDGVGNGGLLAPEAPLDFGNAATGCRLTMGLVGVYDFDSTFI | 120 |
| Sbjct | 61 | AM+AMGA I K G W+I G GNG LL PE PLDFGNA TG RLTMGLVG YD ++TFI AAMKAMGAHIEKRGAEWVIRGTGNGALLQPEGPLDFGNAGTGSRLTMGLVGTYDMETTFI | 120 |
| Query | 121 | GDASLTKRPMGRVLNPLREMGVQV-KSEDGDRLPVTLRGPKTPTPITYRVPMASAQVKSA | 179 |
| Sbjct | 121 | GDASL+ RPMGRVL PLR+MGVQV K+ GDR+P+TL GPK PITYRVPMASAQVKSA GDASLSGRPMGRVLEPLRQMGVQVLKATPGDRMPITLHGPKHAAPITYRVPMASAQVKSA | 180 |
| Query | 180 | VLLAGLNTPGITTVIEPIMTRDHTEKMLQGFGANLTVETDADGVRTIRLEGRGKLTGQVI | 239 |
| Sbjct | 181 | VLLAGLNTPGITTVIEP+MTRDHTEKML+GFGANL+VETD GVR I +EG+GKLTGQ I VLLAGLNTPGITTVIEPVMTRDHTEKMLKGFGANLSVETDERGVRHIFIEGQGKLTGQTI | 240 |
| Query | 240 | DVPGDPSSTAFPLVAALLVPGSDVTILNVLMNPTRTGLILTLQEMGADIEVINPRLAGGE | 299 |
| Sbjct | 241 | VPGDPSS FPLVAAL+VPGSD+TI NVLMNPTRTGL+LTLQEMG I+++NPR AGGE AVPGDPSSAGFPLVAALIVPGSDITIENVLMNPTRTGLLLTLQEMGGKIDILNPRNAGGE | 300 |
| Query | 300 | DVADLRVRSSTLKGVTVPEDRAPSMIDEYPILavaaafaegaTVMNGLEELRVKESDRLS | 359 |
| Sbjct | 301 | DVADLRVR S LKGVTVP +RAPSMIDEYP+LAVAA+FAEG T+M GLEELRVKESDRLS DVADLRVRYSELKGVTVPPERAPSMIDEYPVLAVAASFAEGETLMQGLEELRVKESDRLS | 360 |
| Query | 360 | AVANGLKLNGVDCDEGETSLVVRGRPDGKGLG---NASGAAVATHLDHRIAMSFLVMGLV | 416 |
| Sbjct | 361 | AVA GL LNGVDC EGE SL VRG+P GKGLG N V THLDHRIAMSFLVMGL AVAKGLMLNGVDCTEGEASLAVRGKPGGKGLGGHPNGHDTTVQTHLDHRIAMSFLVMGLA | 420 |
| Query  Sbjct | 417  421 | SENPVTVDDATMIATSFPEFMDLMAGLGAKI 447  +E PVT+DD MIATSFPEFM LM GLGA+I  TEKPVTIDDQAMIATSFPEFMGLMKGLGAEI 451 |  |

>RUV11441.1 3-phosphoshikimate 1-carboxyvinyltransferase [Mesorhizobium sp. M7A.T.Ca.TU.009.01.3.1]

Length=452

Score = 665 bits (1715), Expect = 0.0, Method: Compositional matrix adjust.

| Identities | = 349/451 (77%), Positives = 385/451 (85%), Gaps = 4/451 (1%) |  |
| --- | --- | --- |
| Query 1 | MLHGASSRPATARKSSGLSGTVRIPGDKSISHRSFMFGGLASGETRITGLLEGEDVINTG | 60 |
| Sbjct 1 | M H A+ +PATAR+S LSG R+PGDKSISHRSFMFGGLASGETRITGLLEGEDV+ TG MSHAAAPKPATARQSPALSGIARVPGDKSISHRSFMFGGLASGETRITGLLEGEDVMRTG | 60 |
| Query 61 | KAMQAMGARIRKEGDTWIIDGVGNGGLLAPEAPLDFGNAATGCRLTMGLVGVYDFDSTFI | 120 |
| Sbjct 61 | AM+AMGA I K GD W+I G GNG LL PE PLDFGNA TG RLTMGLVG YD ++TFI AAMKAMGAHIEKHGDEWVIRGTGNGALLQPEGPLDFGNAGTGSRLTMGLVGTYDMETTFI | 120 |
| Query 121 | GDASLTKRPMGRVLNPLREMGVQV-KSEDGDRLPVTLRGPKTPTPITYRVPMASAQVKSA | 179 |
| Sbjct 121 | GDASL+ RPMGRVL PLR+MGVQV K+ GDR+P+TL GPK PITYRVPMASAQVKSA GDASLSGRPMGRVLEPLRQMGVQVLKATPGDRMPITLHGPKHAAPITYRVPMASAQVKSA | 180 |
| Query 180 | VLLAGLNTPGITTVIEPIMTRDHTEKMLQGFGANLTVETDADGVRTIRLEGRGKLTGQVI | 239 |
| Sbjct 181 | VLLAGLNTPGITTVIEP+MTRDHTEKML+GFGANL+VETD GVR I +EG+G+LTGQ I VLLAGLNTPGITTVIEPVMTRDHTEKMLKGFGANLSVETDERGVRHIFIEGQGRLTGQTI | 240 |
| Query 240 | DVPGDPSSTAFPLVAALLVPGSDVTILNVLMNPTRTGLILTLQEMGADIEVINPRLAGGE | 299 |
| Sbjct 241 | VPGDPSS FPLVAAL+VPGS++TI NVLMNPTRTGL+LTLQEMG +I+++NPR AGGE AVPGDPSSAGFPLVAALIVPGSEITIENVLMNPTRTGLLLTLQEMGGNIDILNPRNAGGE | 300 |
| Query 300 | DVADLRVRSSTLKGVTVPEDRAPSMIDEYPILavaaafaegaTVMNGLEELRVKESDRLS | 359 |
| Sbjct 301 | DVADLRVR S LKGVTVP +RAPSMIDEYP+LAVAA+FAEG T+M GLEELRVKESDRLS DVADLRVRYSELKGVTVPPERAPSMIDEYPVLAVAASFAEGETLMQGLEELRVKESDRLS | 360 |
| Query 360 | AVANGLKLNGVDCDEGETSLVVRGRPDGKGLG---NASGAAVATHLDHRIAMSFLVMGLV | 416 |
| Sbjct 361 | AVANGLKLNGVDC EGE SL VRG+P GKGLG N V THLDHRIAMSFLVMGL AVANGLKLNGVDCTEGEASLAVRGKPGGKGLGGHPNGQDTTVQTHLDHRIAMSFLVMGLA | 420 |
| Query 417  Sbjct 421 | SENPVTVDDATMIATSFPEFMDLMAGLGAKI 447  +E PVT+DD MIATSFPEFM LM GLGA+I  TEKPVTIDDQAMIATSFPEFMGLMMGLGAEI 451 |  |

>WP_120011925.1 3-phosphoshikimate 1-carboxyvinyltransferase [Mesorhizobium waimense] RJT42582.1 3-phosphoshikimate 1-carboxyvinyltransferase [Mesorhizobium waimense]

Length=452

Score = 665 bits (1715), Expect = 0.0, Method: Compositional matrix adjust.

| Identities | = 352/451 (78%), Positives = 383/451 (85%), Gaps = 4/451 (1%) |  |
| --- | --- | --- |
| Query 1 | MLHGASSRPATARKSSGLSGTVRIPGDKSISHRSFMFGGLASGETRITGLLEGEDVINTG | 60 |
| Sbjct 1 | M H A+ +PATARKS LSG R+PGDKSISHRSFMFGGLASGETRITGLLEGEDV+ TG MSHAAAPKPATARKSPALSGAARVPGDKSISHRSFMFGGLASGETRITGLLEGEDVMRTG | 60 |
| Query 61 | KAMQAMGARIRKEGDTWIIDGVGNGGLLAPEAPLDFGNAATGCRLTMGLVGVYDFDSTFI | 120 |
| Sbjct 61 | AM+AMGA I K GD W+I G GNG LL PE PLDFGNA TG RLTMGLVG YD ++TFI AAMKAMGAHIEKHGDEWVIRGTGNGALLQPEGPLDFGNAGTGSRLTMGLVGTYDMETTFI | 120 |
| Query 121 | GDASLTKRPMGRVLNPLREMGVQV-KSEDGDRLPVTLRGPKTPTPITYRVPMASAQVKSA | 179 |
| Sbjct 121 | GDASL+ RPMGRVL PLR+MGVQV K+ GDR+P+TLRGPK PITYRVPMASAQVKSA GDASLSGRPMGRVLEPLRQMGVQVLKATPGDRMPITLRGPKHAAPITYRVPMASAQVKSA | 180 |
| Query 180 | VLLAGLNTPGITTVIEPIMTRDHTEKMLQGFGANLTVETDADGVRTIRLEGRGKLTGQVI | 239 |
| Sbjct 181 | VLLAGLNTPGITTVIEP+MTRDHTEKML+GFGANLTVETD GVR I +EG+GKLTGQ I VLLAGLNTPGITTVIEPVMTRDHTEKMLKGFGANLTVETDERGVRHIFIEGQGKLTGQTI | 240 |
| Query 240 | DVPGDPSSTAFPLVAALLVPGSDVTILNVLMNPTRTGLILTLQEMGADIEVINPRLAGGE | 299 |
| Sbjct 241 | VPGDPSS FPLVAAL+VPGSD+ I NVLMNPTRTGL+LTLQEMG I+++NPR AGGE AVPGDPSSAGFPLVAALIVPGSDIVIENVLMNPTRTGLLLTLQEMGGRIDILNPRNAGGE | 300 |
| Query 300 | DVADLRVRSSTLKGVTVPEDRAPSMIDEYPILavaaafaegaTVMNGLEELRVKESDRLS | 359 |
| Sbjct 301 | DVADLRVR S LKGV VP +RAPSMIDEYP LAVAA+FAEG T+M GLEELRVKESDRLS DVADLRVRYSELKGVIVPPERAPSMIDEYPALAVAASFAEGETLMQGLEELRVKESDRLS | 360 |
| Query 360 | AVANGLKLNGVDCDEGETSLVVRGRPDGKGLG---NASGAAVATHLDHRIAMSFLVMGLV | 416 |
| Sbjct 361 | AVANGLKLNGVDC EGE +L VRGRP G+GLG N G V THLDHRIAMSFLVMGL AVANGLKLNGVDCTEGEATLAVRGRPGGRGLGQHPNGQGTTVQTHLDHRIAMSFLVMGLA | 420 |
| Query 417  Sbjct 421 | SENPVTVDDATMIATSFPEFMDLMAGLGAKI 447  +E PVT+DD MIATSFPEFM LM GLGA+I  TEKPVTIDDQAMIATSFPEFMGLMKGLGAEI 451 |  |

>WP_018688986.1 3-phosphoshikimate 1-carboxyvinyltransferase [Ahrensia kielensis] Length=451

Score = 665 bits (1715), Expect = 0.0, Method: Compositional matrix adjust.

| Identities | = 333/451 (74%), Positives = 380/451 (84%), Gaps = 1/451 (0%) |  |
| --- | --- | --- |
| Query 2 | LHGASSRPATARKSSGLSGTVRIPGDKSISHRSFMFGGLASGETRITGLLEGEDVINTGK | 61 |
| Sbjct 1 | +H + +PA+A KS L G R+PGDKSISHRSFMFGGLASGETR+TGLLEG+DV+ TG+ MHNHTPKPASAHKSPPLKGKARVPGDKSISHRSFMFGGLASGETRVTGLLEGDDVLATGR | 60 |
| Query 62 | AMQAMGARIRKEGDTWIIDGVGNGGLLAPEAPLDFGNAATGCRLTMGLVGVYDFDSTFIG | 121 |
| Sbjct 61 | AMQAMGA+IRKEGDTWII+G GNG LL PE P DFGNA TG RLTMGLVG Y+ +TF G AMQAMGAQIRKEGDTWIINGTGNGCLLEPEEPFDFGNAGTGARLTMGLVGTYNMKTTFTG | 120 |
| Query 122 | DASLTKRPMGRVLNPLREMGVQVKSEDGDRLPVTLRGPKTPTPITYRVPMASAQVKSAVL | 181 |
| Sbjct 121 | DASL+KRPMGRVLNPLREMGVQV++ DGDR P+TL GP+T PI+YRVPMASAQVKSAVL DASLSKRPMGRVLNPLREMGVQVEAADGDRCPITLHGPRTANPISYRVPMASAQVKSAVL | 180 |
| Query 182 | LAGLNTPGITTVIEPIMTRDHTEKMLQGFGANLTVETDADGVRTIRLEGRGKLTGQVIDV | 241 |
| Sbjct 181 | LAGLNTPGITTVIEP+MTRDHTEKML GFGA + +E DADG R I + G+G+L GQ I+V LAGLNTPGITTVIEPVMTRDHTEKMLAGFGAKIEIEEDADGTRHIHIHGQGELHGQTIEV | 240 |
| Query 242 | PGDPSSTAFPLVAALLVPGSDVTILNVLMNPTRTGLILTLQEMGADIEVINPRLAGGEDV | 301 |
| Sbjct 241 | PGDPSS FPLVAAL+VPGSD+ I N LMNPTRTGLI TLQEMG I++IN R AGGEDV PGDPSSAGFPLVAALIVPGSDIIIENALMNPTRTGLITTLQEMGGQIDIINMRDAGGEDV | 300 |
| Query 302 | ADLRVRSSTLKGVTVPEDRAPSMIDEYPILavaaafaegaTVMNGLEELRVKESDRLSAV | 361 |
| Sbjct 301 | ADL VR+S LKGV VP +RAPSMIDEYP+LA+AAAFAEG T M+GL+ELRVKESDRL+AV ADLHVRASNLKGVVVPPERAPSMIDEYPVLAIAAAFAEGETFMDGLDELRVKESDRLAAV | 360 |
| Query 362 | ANGLKLNGVDCDEGETSLVVRGRPDGKGLGNASGAAVA-THLDHRIAMSFLVMGLVSENP A GL+ NGV+C EGE SL V+G P GKGLG S AV THLDHRIAM+FLVMG+ SE | 420 |

| Sbjct | 361 | ARGLEANGVECHEGEMSLTVKGSPGGKGLGALSAQAVVETHLDHRIAMAFLVMGMASEKA 420 |
| --- | --- | --- |
| Query | 421 | VTVDDATMIATSFPEFMDLMAGLGAKIELSD 451 |
| Sbjct | 421 | V +DDATMIATSFPEFMDLM GLGA I++++  VHIDDATMIATSFPEFMDLMRGLGADIQVTN 451 |

>WP_127381465.1 3-phosphoshikimate 1-carboxyvinyltransferase [Mesorhizobium sp. M7A.F.Ca.MR.148.00.0.0]

RUV36651.1 3-phosphoshikimate 1-carboxyvinyltransferase [Mesorhizobium sp. M7A.F.Ca.MR.148.00.0.0]

RWN35238.1 3-phosphoshikimate 1-carboxyvinyltransferase [Mesorhizobium sp.] TJV21877.1 3-phosphoshikimate 1-carboxyvinyltransferase [Mesorhizobium sp.]

Length=452

Score = 665 bits (1715), Expect = 0.0, Method: Compositional matrix adjust.

| Identities | = 349/451 (77%), Positives = 384/451 (85%), Gaps = 4/451 (1%) |  |
| --- | --- | --- |
| Query 1 | MLHGASSRPATARKSSGLSGTVRIPGDKSISHRSFMFGGLASGETRITGLLEGEDVINTG | 60 |
| Sbjct 1 | M H A+ +PATAR+S LSG R+PGDKSISHRSFMFGGLASGETRITGLLEGEDV+ TG MSHAAAPKPATARQSPALSGIARVPGDKSISHRSFMFGGLASGETRITGLLEGEDVMRTG | 60 |
| Query 61 | KAMQAMGARIRKEGDTWIIDGVGNGGLLAPEAPLDFGNAATGCRLTMGLVGVYDFDSTFI | 120 |
| Sbjct 61 | AM+AMGA I K GD W+I G GNG LL PE PLDFGNA TG RLTMGLVG YD ++TFI AAMKAMGAHIEKHGDEWVIRGTGNGALLQPEGPLDFGNAGTGSRLTMGLVGTYDMETTFI | 120 |
| Query 121 | GDASLTKRPMGRVLNPLREMGVQV-KSEDGDRLPVTLRGPKTPTPITYRVPMASAQVKSA | 179 |
| Sbjct 121 | GDASL+ RPMGRVL PLR+MGVQV K+ GDR+P+TL GPK PITYRVPMASAQVKSA GDASLSGRPMGRVLEPLRQMGVQVLKATPGDRMPITLHGPKHAAPITYRVPMASAQVKSA | 180 |
| Query 180 | VLLAGLNTPGITTVIEPIMTRDHTEKMLQGFGANLTVETDADGVRTIRLEGRGKLTGQVI | 239 |
| Sbjct 181 | VLLAGLNTPGITTVIEP+MTRDHTEKML+GFGANL+VETD VR I +EG+G+LTGQ I VLLAGLNTPGITTVIEPVMTRDHTEKMLKGFGANLSVETDERSVRHIFIEGQGRLTGQTI | 240 |
| Query 240 | DVPGDPSSTAFPLVAALLVPGSDVTILNVLMNPTRTGLILTLQEMGADIEVINPRLAGGE | 299 |
| Sbjct 241 | VPGDPSS FPLVAAL+VPGS++TI NVLMNPTRTGL+LTLQEMG +IE++NPR AGGE AVPGDPSSAGFPLVAALIVPGSEITIENVLMNPTRTGLLLTLQEMGGNIEILNPRNAGGE | 300 |
| Query 300 | DVADLRVRSSTLKGVTVPEDRAPSMIDEYPILavaaafaegaTVMNGLEELRVKESDRLS | 359 |
| Sbjct 301 | DVADLRVR S LKGVTVP +RAPSMIDEYP+LAVAA+FAEG T+M GLEELRVKESDRLS DVADLRVRYSELKGVTVPPERAPSMIDEYPVLAVAASFAEGETLMQGLEELRVKESDRLS | 360 |
| Query 360 | AVANGLKLNGVDCDEGETSLVVRGRPDGKGLG---NASGAAVATHLDHRIAMSFLVMGLV | 416 |
| Sbjct 361 | AVANGLKLNGVDC EGE SL VRG+P GKGLG N V THLDHRIAMSFLVMGL AVANGLKLNGVDCTEGEASLAVRGKPGGKGLGGHPNGQDTTVQTHLDHRIAMSFLVMGLA | 420 |
| Query 417  Sbjct 421 | SENPVTVDDATMIATSFPEFMDLMAGLGAKI 447  +E PVT+DD MIATSFPEFM LM GLGA+I  TEKPVTIDDQAMIATSFPEFMGLMMGLGAEI 451 |  |

>WP_140644739.1 3-phosphoshikimate 1-carboxyvinyltransferase [Mesorhizobium sp. B3-1-6]

TPI37827.1 3-phosphoshikimate 1-carboxyvinyltransferase [Mesorhizobium sp. B3-1-6]

Length=452

Score = 664 bits (1714), Expect = 0.0, Method: Compositional matrix adjust.

| Identities | = 350/451 (78%), Positives = 385/451 (85%), Gaps = 4/451 (1%) |  |
| --- | --- | --- |
| Query 1 | MLHGASSRPATARKSSGLSGTVRIPGDKSISHRSFMFGGLASGETRITGLLEGEDVINTG | 60 |
| Sbjct 1 | M H A+++PATARKS L+GT R+PGDKSISHRSFMFGGLASGETRITGLLEGEDV+ TG MSHSAAAKPATARKSPALAGTARVPGDKSISHRSFMFGGLASGETRITGLLEGEDVMRTG | 60 |
| Query 61 | KAMQAMGARIRKEGDTWIIDGVGNGGLLAPEAPLDFGNAATGCRLTMGLVGVYDFDSTFI | 120 |
| Sbjct 61 | AM+AMGA + K+G W+I G GNG LL PE PLDFGNA TG RLTMGLVG YD ++TFI AAMKAMGAHVEKKGAEWVIRGTGNGALLQPEGPLDFGNAGTGSRLTMGLVGTYDMETTFI | 120 |
| Query 121 | GDASLTKRPMGRVLNPLREMGVQV-KSEDGDRLPVTLRGPKTPTPITYRVPMASAQVKSA | 179 |
| Sbjct 121 | GDASL+ RPMGRVL+PLR+MGVQV K+ GDR+P+TL GPK PITYRVPMASAQVKSA  GDASLSGRPMGRVLDPLRQMGVQVLKAAPGDRMPITLHGPKHAAPITYRVPMASAQVKSA | 180 |

| Query | 180 | VLLAGLNTPGITTVIEPIMTRDHTEKMLQGFGANLTVETDADGVRTIRLEGRGKLTGQVI | 239 |
| --- | --- | --- | --- |
| Sbjct | 181 | VLLAGLNTPGITTVIEP+MTRDHTEKML+GFGANL+VE D GVR I +EG+GKLTGQ I VLLAGLNTPGITTVIEPVMTRDHTEKMLKGFGANLSVEADERGVRHIFIEGQGKLTGQTI | 240 |
| Query | 240 | DVPGDPSSTAFPLVAALLVPGSDVTILNVLMNPTRTGLILTLQEMGADIEVINPRLAGGE | 299 |
| Sbjct | 241 | VPGDPSS FPLVAAL+VPGSD+TI NVLMNPTRTGL+LTLQEMG I+ +NPR AGGE AVPGDPSSAGFPLVAALIVPGSDITIENVLMNPTRTGLLLTLQEMGGRIDTLNPRNAGGE | 300 |
| Query | 300 | DVADLRVRSSTLKGVTVPEDRAPSMIDEYPILavaaafaegaTVMNGLEELRVKESDRLS | 359 |
| Sbjct | 301 | DVADLRVR S LKGVTVP +RAPSMIDEYP+LAVAA+FAEG T+M GLEELRVKESDRLS DVADLRVRYSELKGVTVPPERAPSMIDEYPVLAVAASFAEGETLMQGLEELRVKESDRLS | 360 |
| Query | 360 | AVANGLKLNGVDCDEGETSLVVRGRPDGKGLG---NASGAAVATHLDHRIAMSFLVMGLV | 416 |
| Sbjct | 361 | AVANGLKLNGVDC EGE SL VRGRP GKGLG N V THLDHRIAMSFLVMGL AVANGLKLNGVDCTEGEASLAVRGRPGGKGLGGHPNGQDTTVQTHLDHRIAMSFLVMGLA | 420 |
| Query  Sbjct | 417  421 | SENPVTVDDATMIATSFPEFMDLMAGLGAKI 447  +E PVT+DD MIATSFPEFM LM GLGA+I  TEKPVTIDDQAMIATSFPEFMGLMKGLGAEI 451 |  |

>WP_026479127.1 3-phosphoshikimate 1-carboxyvinyltransferase [Ahrensia sp. 13_GOM-1096m] Length=451

Score = 664 bits (1714), Expect = 0.0, Method: Compositional matrix adjust.

| Identities | = 333/451 (74%), Positives = 380/451 (84%), Gaps = 1/451 (0%) |  |
| --- | --- | --- |
| Query 2 | LHGASSRPATARKSSGLSGTVRIPGDKSISHRSFMFGGLASGETRITGLLEGEDVINTGK | 61 |
| Sbjct 1 | +H + +PA+A KS L G R+PGDKSISHRSFMFGGLASGETR+TGLLEG+DV+ TG+ MHNHTPKPASAHKSPPLKGKARVPGDKSISHRSFMFGGLASGETRVTGLLEGDDVLATGR | 60 |
| Query 62 | AMQAMGARIRKEGDTWIIDGVGNGGLLAPEAPLDFGNAATGCRLTMGLVGVYDFDSTFIG | 121 |
| Sbjct 61 | AMQAMGA+IRKEGDTWII+G GNG LL PE P DFGNA TG RLTMGLVG Y+ +TF G AMQAMGAQIRKEGDTWIINGTGNGCLLEPEEPFDFGNAGTGARLTMGLVGTYNMKTTFTG | 120 |
| Query 122 | DASLTKRPMGRVLNPLREMGVQVKSEDGDRLPVTLRGPKTPTPITYRVPMASAQVKSAVL | 181 |
| Sbjct 121 | DASL+KRPMGRVLNPLREMGVQV++ DGDR P+TL GP+T PI+YRVPMASAQVKSAVL DASLSKRPMGRVLNPLREMGVQVEAADGDRCPITLHGPRTANPISYRVPMASAQVKSAVL | 180 |
| Query 182 | LAGLNTPGITTVIEPIMTRDHTEKMLQGFGANLTVETDADGVRTIRLEGRGKLTGQVIDV | 241 |
| Sbjct 181 | LAGLNTPGITTVIEP+MTRDHTEKML GFGA + +E DADG R I + G+G+L GQ I+V LAGLNTPGITTVIEPVMTRDHTEKMLAGFGAKIEIEEDADGTRHIHIHGQGELRGQTIEV | 240 |
| Query 242 | PGDPSSTAFPLVAALLVPGSDVTILNVLMNPTRTGLILTLQEMGADIEVINPRLAGGEDV | 301 |
| Sbjct 241 | PGDPSS FPLVAAL+VPGSD+ I N LMNPTRTGLI TLQEMG I++IN R AGGEDV PGDPSSAGFPLVAALIVPGSDIIIENALMNPTRTGLITTLQEMGGQIDIINMRDAGGEDV | 300 |
| Query 302 | ADLRVRSSTLKGVTVPEDRAPSMIDEYPILavaaafaegaTVMNGLEELRVKESDRLSAV | 361 |
| Sbjct 301 | ADL VR+S LKGV VP +RAPSMIDEYP+LA+AAAFAEG T M+GL+ELRVKESDRL+AV ADLHVRASNLKGVVVPPERAPSMIDEYPVLAIAAAFAEGETFMDGLDELRVKESDRLAAV | 360 |
| Query 362 | ANGLKLNGVDCDEGETSLVVRGRPDGKGLGNASGAAVA-THLDHRIAMSFLVMGLVSENP | 420 |
| Sbjct 361 | A GL+ NGV+C EGE SL V+G P GKGLG S AV THLDHRIAM+FLVMG+ SE ARGLEANGVECHEGEMSLTVKGSPGGKGLGALSAQAVVETHLDHRIAMAFLVMGMASEKA | 420 |
| Query 421  Sbjct 421 | VTVDDATMIATSFPEFMDLMAGLGAKIELSD 451 V +DDATMIATSFPEFMDLM GLGA I++++ VHIDDATMIATSFPEFMDLMRGLGADIQVAN 451 |  |

>RWN96981.1 3-phosphoshikimate 1-carboxyvinyltransferase [Mesorhizobium sp.] Length=452

Score = 664 bits (1714), Expect = 0.0, Method: Compositional matrix adjust.

| Identities | = 349/451 (77%), Positives = 384/451 (85%), Gaps = 4/451 (1%) |  |
| --- | --- | --- |
| Query 1 | MLHGASSRPATARKSSGLSGTVRIPGDKSISHRSFMFGGLASGETRITGLLEGEDVINTG | 60 |
| Sbjct 1 | M H A+ +PATAR+S LSG R+PGDKSISHRSFMFGGLASGETRITGLLEGEDV+ TG MSHAAAPKPATARQSPALSGIARVPGDKSISHRSFMFGGLASGETRITGLLEGEDVMRTG | 60 |
| Query 61 | KAMQAMGARIRKEGDTWIIDGVGNGGLLAPEAPLDFGNAATGCRLTMGLVGVYDFDSTFI AM+AMGA I K GD W+I G GNG LL PE PLDFGNA TG RLTMGLVG YD ++TFI | 120 |

| Sbjct | 61 | AAMKAMGAHIEKNGDEWVIRGTGNGALLQPEGPLDFGNAGTGSRLTMGLVGTYDMETTFI | 120 |
| --- | --- | --- | --- |
| Query | 121 | GDASLTKRPMGRVLNPLREMGVQV-KSEDGDRLPVTLRGPKTPTPITYRVPMASAQVKSA | 179 |
| Sbjct | 121 | GDASL+ RPMGRVL PLR+MGVQV K+ GDR+P+TL GPK PITYRVPMASAQVKSA GDASLSGRPMGRVLEPLRQMGVQVLKATPGDRMPITLHGPKHAAPITYRVPMASAQVKSA | 180 |
| Query | 180 | VLLAGLNTPGITTVIEPIMTRDHTEKMLQGFGANLTVETDADGVRTIRLEGRGKLTGQVI | 239 |
| Sbjct | 181 | VLLAGLNTPGITTVIEP+MTRDHTEKML+GFGANL+VETD GVR I +EG+G+LTGQ I VLLAGLNTPGITTVIEPVMTRDHTEKMLKGFGANLSVETDERGVRHIFIEGQGRLTGQTI | 240 |
| Query | 240 | DVPGDPSSTAFPLVAALLVPGSDVTILNVLMNPTRTGLILTLQEMGADIEVINPRLAGGE | 299 |
| Sbjct | 241 | VPGDPSS FPLVAAL+VPGSD+TI NVLMNPTRTGL+LTLQEMG +I+++NPR AGGE AVPGDPSSAGFPLVAALIVPGSDITIENVLMNPTRTGLLLTLQEMGGNIDILNPRNAGGE | 300 |
| Query | 300 | DVADLRVRSSTLKGVTVPEDRAPSMIDEYPILavaaafaegaTVMNGLEELRVKESDRLS | 359 |
| Sbjct | 301 | DVADLRVR S LKGVTVP +RAPSMIDEYP+LAVAA+FAEG T+M GLEELRVKESDRLS DVADLRVRYSELKGVTVPPERAPSMIDEYPVLAVAASFAEGETLMQGLEELRVKESDRLS | 360 |
| Query | 360 | AVANGLKLNGVDCDEGETSLVVRGRPDGKGLG---NASGAAVATHLDHRIAMSFLVMGLV | 416 |
[truncated: 8,528,987 more chars]
