# Supplementary material for: Safety Assessment of the CP4 EPSPS and NPTII Proteins in Eucalyptus
Source: GM Crops Food. 2023 Jun 19;14(1):1–14. doi: 10.1080/21645698.2023.2222436 (PMC10281460; doi:10.1080/21645698.2023.2222436)
Supplement: Supplemental Material [file KGMC_A_2222436_SM4294.zip › TableS08.docx]

BLASTP output of the CP4-EPSPS protein against the UniProtKB protein database

BLASTP 2.9.0+

Reference: Stephen F. Altschul, Thomas L. Madden, Alejandro A. Schaffer, Jinghui Zhang, Zheng Zhang, Webb Miller, and David J. Lipman (1997), "Gapped BLAST and PSI-BLAST: a new generation of protein database search programs", Nucleic Acids Res. 25:3389-3402.

Reference for composition-based statistics: Alejandro A. Schaffer,

L. Aravind, Thomas L. Madden, Sergei Shavirin, John L. Spouge, Yuri

I. Wolf, Eugene V. Koonin, and Stephen F. Altschul (2001), "Improving the accuracy of PSI-BLAST protein database searches with composition-based statistics and other refinements", Nucleic Acids Res. 29:2994-3005.

Database: uniprotkb_refprotswissprot

57,391,823 sequences; 21,838,191,652 total letters

| Query= CP4-EPSPS |  | |
| --- | --- | --- |
| Length=455 | Score | E |
| Sequences producing significant alignments: | (Bits) | Value |
| SP:Q9R4E4 AROA_AGRSC 3-phosphoshikimate 1-carboxyvinyltransferase ... | 883 | 0.0 |
| TR:A0A4Y8QII1 A0A4Y8QII1_9RHIZ 3-phosphoshikimate 1-carboxyvinyltr... | 848 | 0.0 |
| TR:A0A1A9FVW7 A0A1A9FVW7_9RHIZ 3-phosphoshikimate 1-carboxyvinyltr... | 840 | 0.0 |
| TR:A0A292E0M8 A0A292E0M8_9RHIZ 3-phosphoshikimate 1-carboxyvinyltr... | 822 | 0.0 |
| TR:A0A2T5BEP1 A0A2T5BEP1_MYCDI 3-phosphoshikimate 1-carboxyvinyltr... | 818 | 0.0 |
| TR:A0A2U2DST6 A0A2U2DST6_9RHIZ 3-phosphoshikimate 1-carboxyvinyltr... | 817 | 0.0 |
| TR:A0A1S1SIN8 A0A1S1SIN8_9RHIZ 3-phosphoshikimate 1-carboxyvinyltr... | 816 | 0.0 |
| TR:A0A285UHC5 A0A285UHC5_9RHIZ 3-phosphoshikimate 1-carboxyvinyltr... | 815 | 0.0 |
| SP:Q92SV5 AROA_RHIME 3-phosphoshikimate 1-carboxyvinyltransferase ... | 812 | 0.0 |
| TR:A0A4S5J1R2 A0A4S5J1R2_9RHIZ 3-phosphoshikimate 1-carboxyvinyltr... | 808 | 0.0 |
| SP:C3MC80 AROA_SINFN 3-phosphoshikimate 1-carboxyvinyltransferase ... | 807 | 0.0 |
| TR:I3XFD9 I3XFD9_RHIFR 3-phosphoshikimate 1-carboxyvinyltransferas... | 801 | 0.0 |
| TR:A0A561QR25 A0A561QR25_9RHIZ 3-phosphoshikimate 1-carboxyvinyltr... | 775 | 0.0 |
| TR:L0NN29 L0NN29_9RHIZ 3-phosphoshikimate 1-carboxyvinyltransferas... | 775 | 0.0 |
| SP:Q1MN56 AROA_RHIL3 3-phosphoshikimate 1-carboxyvinyltransferase ... | 773 | 0.0 |
| TR:A0A5C4XV71 A0A5C4XV71_9RHIZ 3-phosphoshikimate 1-carboxyvinyltr... | 769 | 0.0 |
| TR:A0A1I0WNM9 A0A1I0WNM9_9RHIZ 3-phosphoshikimate 1-carboxyvinyltr... | 765 | 0.0 |
| TR:A0A081MDT9 A0A081MDT9_9RHIZ 3-phosphoshikimate 1-carboxyvinyltr... | 765 | 0.0 |
| TR:A0A4R5UHY4 A0A4R5UHY4_9RHIZ 3-phosphoshikimate 1-carboxyvinyltr... | 765 | 0.0 |
| TR:A0A4R5PM71 A0A4R5PM71_9RHIZ 3-phosphoshikimate 1-carboxyvinyltr... | 756 | 0.0 |
| TR:A0A0Q5DH36 A0A0Q5DH36_9RHIZ 3-phosphoshikimate 1-carboxyvinyltr... | 751 | 0.0 |
| TR:A0A386E6K9 A0A386E6K9_9RHIZ 3-phosphoshikimate 1-carboxyvinyltr... | 751 | 0.0 |
| TR:A0A444LEE3 A0A444LEE3_9RHIZ 3-phosphoshikimate 1-carboxyvinyltr... | 750 | 0.0 |
| TR:A0A256FYG7 A0A256FYG7_9RHIZ 3-phosphoshikimate 1-carboxyvinyltr... | 741 | 0.0 |
| TR:A0A4R6NVY0 A0A4R6NVY0_9RHIZ 3-phosphoshikimate 1-carboxyvinyltr... | 736 | 0.0 |
| SP:P0A2Y4 AROA_PSES2 3-phosphoshikimate 1-carboxyvinyltransferase ... | 735 | 0.0 |
| SP:P0A2Y5 AROA_ACHSL 3-phosphoshikimate 1-carboxyvinyltransferase ... | 735 | 0.0 |
| SP:A6WUV8 AROA_OCHA4 3-phosphoshikimate 1-carboxyvinyltransferase ... | 734 | 0.0 |
| TR:A0A2S9JIW2 A0A2S9JIW2_9RHIZ 3-phosphoshikimate 1-carboxyvinyltr... | 734 | 0.0 |
| TR:A0A502BQP0 A0A502BQP0_9RHIZ 3-phosphoshikimate 1-carboxyvinyltr... | 732 | 0.0 |
| TR:A0A317PKQ9 A0A317PKQ9_9RHIZ 3-phosphoshikimate 1-carboxyvinyltr... | 731 | 0.0 |
| TR:B9JGR5 B9JGR5_AGRRK 3-phosphoshikimate 1-carboxyvinyltransferas... | 731 | 0.0 |
| TR:A0A135I0M0 A0A135I0M0_9RHIZ 3-phosphoshikimate 1-carboxyvinyltr... | 729 | 0.0 |
| TR:A0A1H0RZE5 A0A1H0RZE5_9RHIZ 3-phosphoshikimate 1-carboxyvinyltr... | 729 | 0.0 |

| SP:Q8G3C4 AROA_BRUSU 3-phosphoshikimate 1-carboxyvinyltransferase ... | 728 | 0.0 |
| --- | --- | --- |
| SP:A9M6N0 AROA_BRUC2 3-phosphoshikimate 1-carboxyvinyltransferase ... | 728 | 0.0 |
| SP:B0CI45 AROA_BRUSI 3-phosphoshikimate 1-carboxyvinyltransferase ... | 727 | 0.0 |
| SP:C0RG90 AROA_BRUMB 3-phosphoshikimate 1-carboxyvinyltransferase ... | 727 | 0.0 |
| SP:Q8YEG1 AROA_BRUME 3-phosphoshikimate 1-carboxyvinyltransferase ... | 725 | 0.0 |
| SP:B2S7T0 AROA_BRUA1 3-phosphoshikimate 1-carboxyvinyltransferase ... | 725 | 0.0 |
| SP:Q2YPN6 AROA_BRUA2 3-phosphoshikimate 1-carboxyvinyltransferase ... | 725 | 0.0 |
| TR:A0A316JDA1 A0A316JDA1_9RHIZ 3-phosphoshikimate 1-carboxyvinyltr... | 725 | 0.0 |
| TR:A0A364JZ32 A0A364JZ32_9RHIZ 3-phosphoshikimate 1-carboxyvinyltr... | 724 | 0.0 |
| TR:A0A2N9VTC1 A0A2N9VTC1_9RHIZ 3-phosphoshikimate 1-carboxyvinyltr... | 724 | 0.0 |
| SP:P0C100 AROA_BRUAB 3-phosphoshikimate 1-carboxyvinyltransferase ... | 723 | 0.0 |
| TR:A0A318T7W8 A0A318T7W8_9RHIZ 3-phosphoshikimate 1-carboxyvinyltr... | 723 | 0.0 |
| TR:J3HME9 J3HME9_9RHIZ 3-phosphoshikimate 1-carboxyvinyltransferas... | 716 | 0.0 |
| TR:A9CW43 A9CW43_HOEPD 3-phosphoshikimate 1-carboxyvinyltransferas... | 712 | 0.0 |
| TR:A0A2G1QTW2 A0A2G1QTW2_9RHIZ 3-phosphoshikimate 1-carboxyvinyltr... | 705 | 0.0 |
| TR:J1TFJ6 J1TFJ6_9RHIZ 3-phosphoshikimate 1-carboxyvinyltransferas... | 703 | 0.0 |
| TR:W8I7B2 W8I7B2_ENSAD 3-phosphoshikimate 1-carboxyvinyltransferas... | 699 | 0.0 |
| TR:A0A366E5K2 A0A366E5K2_9RHIZ 3-phosphoshikimate 1-carboxyvinyltr... | 698 | 0.0 |
| TR:A0A2P7S4J0 A0A2P7S4J0_9RHIZ 3-phosphoshikimate 1-carboxyvinyltr... | 694 | 0.0 |
| TR:A0A1I3WYP8 A0A1I3WYP8_9RHIZ 3-phosphoshikimate 1-carboxyvinyltr... | 692 | 0.0 |
| TR:A0A0Q8KSI2 A0A0Q8KSI2_9RHIZ 3-phosphoshikimate 1-carboxyvinyltr... | 691 | 0.0 |
| TR:A0A504FA26 A0A504FA26_9RHIZ 3-phosphoshikimate 1-carboxyvinyltr... | 691 | 0.0 |
| TR:A0A4U6CF00 A0A4U6CF00_9RHIZ 3-phosphoshikimate 1-carboxyvinyltr... | 691 | 0.0 |
| TR:A0A3A5KZU9 A0A3A5KZU9_9RHIZ 3-phosphoshikimate 1-carboxyvinyltr... | 690 | 0.0 |
| TR:A0A5B8B442 A0A5B8B442_9RHIZ 3-phosphoshikimate 1-carboxyvinyltr... | 690 | 0.0 |
| SP:Q98CC1 AROA_RHILO 3-phosphoshikimate 1-carboxyvinyltransferase ... | 689 | 0.0 |
| TR:A0A1V8RTQ2 A0A1V8RTQ2_9RHIZ 3-phosphoshikimate 1-carboxyvinyltr... | 689 | 0.0 |
| TR:M5F072 M5F072_9RHIZ 3-phosphoshikimate 1-carboxyvinyltransferas... | 689 | 0.0 |
| TR:A0A3Q8XLD4 A0A3Q8XLD4_9RHIZ 3-phosphoshikimate 1-carboxyvinyltr... | 688 | 0.0 |
| TR:A0A1I7DML1 A0A1I7DML1_9RHIZ 3-phosphoshikimate 1-carboxyvinyltr... | 686 | 0.0 |
| TR:V7FMR6 V7FMR6_9RHIZ 3-phosphoshikimate 1-carboxyvinyltransferas... | 686 | 0.0 |
| TR:E8T9A8 E8T9A8_MESCW 3-phosphoshikimate 1-carboxyvinyltransferas... | 686 | 0.0 |
| TR:A0A1C2DE69 A0A1C2DE69_9RHIZ 3-phosphoshikimate 1-carboxyvinyltr... | 685 | 0.0 |
| TR:U4VI88 U4VI88_9RHIZ 3-phosphoshikimate 1-carboxyvinyltransferas... | 685 | 0.0 |
| TR:A0A432V367 A0A432V367_9RHIZ 3-phosphoshikimate 1-carboxyvinyltr... | 685 | 0.0 |
| TR:A0A011VL88 A0A011VL88_9RHIZ 3-phosphoshikimate 1-carboxyvinyltr... | 683 | 0.0 |
| TR:A0A1S1U0N7 A0A1S1U0N7_9RHIZ 3-phosphoshikimate 1-carboxyvinyltr... | 683 | 0.0 |
| TR:A0A084U5R5 A0A084U5R5_9RHIZ 3-phosphoshikimate 1-carboxyvinyltr... | 682 | 0.0 |
| TR:H0HK94 H0HK94_9RHIZ 3-phosphoshikimate 1-carboxyvinyltransferas... | 682 | 0.0 |
| TR:A0A3F2UEX1 A0A3F2UEX1_9RHIZ 3-phosphoshikimate 1-carboxyvinyltr... | 682 | 0.0 |
| TR:A0A0Q7WSY7 A0A0Q7WSY7_9RHIZ 3-phosphoshikimate 1-carboxyvinyltr... | 681 | 0.0 |
| TR:A0A271L0T1 A0A271L0T1_9RHIZ 3-phosphoshikimate 1-carboxyvinyltr... | 681 | 0.0 |
| TR:A0A316C321 A0A316C321_PSESE 3-phosphoshikimate 1-carboxyvinyltr... | 681 | 0.0 |
| TR:A0A562FPT2 A0A562FPT2_9RHIZ 3-phosphoshikimate 1-carboxyvinyltr... | 681 | 0.0 |
| TR:A0A371XIY5 A0A371XIY5_9RHIZ 3-phosphoshikimate 1-carboxyvinyltr... | 680 | 0.0 |
| TR:A0A0Q8BT14 A0A0Q8BT14_9RHIZ 3-phosphoshikimate 1-carboxyvinyltr... | 679 | 0.0 |
| TR:A0A2P7S9L0 A0A2P7S9L0_9RHIZ 3-phosphoshikimate 1-carboxyvinyltr... | 676 | 0.0 |
| TR:A0A4R0PL97 A0A4R0PL97_9RHIZ 3-phosphoshikimate 1-carboxyvinyltr... | 673 | 0.0 |
| SP:Q11C97 AROA_CHESB 3-phosphoshikimate 1-carboxyvinyltransferase ... | 673 | 0.0 |
| TR:A0A0N0E856 A0A0N0E856_9RHOB 3-phosphoshikimate 1-carboxyvinyltr... | 671 | 0.0 |
| TR:A0A0Q6L4J7 A0A0Q6L4J7_9RHIZ 3-phosphoshikimate 1-carboxyvinyltr... | 665 | 0.0 |
| TR:A0A5D4GS16 A0A5D4GS16_9RHIZ 3-phosphoshikimate 1-carboxyvinyltr... | 665 | 0.0 |
| TR:A0A2P8NI44 A0A2P8NI44_9RHIZ 3-phosphoshikimate 1-carboxyvinyltr... | 662 | 0.0 |
| TR:A0A4R1RYM4 A0A4R1RYM4_9RHIZ 3-phosphoshikimate 1-carboxyvinyltr... | 655 | 0.0 |
| TR:A0A562C4L2 A0A562C4L2_9RHIZ 3-phosphoshikimate 1-carboxyvinyltr... | 652 | 0.0 |
| TR:A0A506U1M9 A0A506U1M9_9RHIZ 3-phosphoshikimate 1-carboxyvinyltr... | 649 | 0.0 |
| TR:A0A1I3NUZ8 A0A1I3NUZ8_9RHIZ 3-phosphoshikimate 1-carboxyvinyltr... | 647 | 0.0 |
| TR:K2MPH3 K2MPH3_9RHIZ 3-phosphoshikimate 1-carboxyvinyltransferas... | 647 | 0.0 |
| TR:A0A0Q6F990 A0A0Q6F990_9RHIZ 3-phosphoshikimate 1-carboxyvinyltr... | 645 | 0.0 |
| TR:A0A1U9JT64 A0A1U9JT64_9RHIZ 3-phosphoshikimate 1-carboxyvinyltr... | 645 | 0.0 |
| TR:K2PAJ6 K2PAJ6_9RHIZ 3-phosphoshikimate 1-carboxyvinyltransferas... | 643 | 0.0 |
| TR:A0A231V122 A0A231V122_9RHIZ 3-phosphoshikimate 1-carboxyvinyltr... | 641 | 0.0 |
| TR:A0A0Q6EG79 A0A0Q6EG79_9RHIZ 3-phosphoshikimate 1-carboxyvinyltr... | 639 | 0.0 |
| TR:A0A0Q5GG74 A0A0Q5GG74_9RHIZ 3-phosphoshikimate 1-carboxyvinyltr... | 637 | 0.0 |

| TR:A0A286IAM2 A0A286IAM2_9RHIZ 3-phosphoshikimate 1-carboxyvinyltr... | 637 | 0.0 |
| --- | --- | --- |
| TR:A0A0Q6BBA0 A0A0Q6BBA0_9RHIZ 3-phosphoshikimate 1-carboxyvinyltr... | 636 | 0.0 |
| TR:A0A175RUY5 A0A175RUY5_9RHIZ 3-phosphoshikimate 1-carboxyvinyltr... | 634 | 0.0 |
| TR:A0A368A190 A0A368A190_9RHIZ 3-phosphoshikimate 1-carboxyvinyltr... | 634 | 0.0 |
| TR:A0A1R0FC49 A0A1R0FC49_9RHIZ 3-phosphoshikimate 1-carboxyvinyltr... | 633 | 0.0 |
| TR:A0A1L3SNH2 A0A1L3SNH2_9RHIZ 3-phosphoshikimate 1-carboxyvinyltr... | 632 | 0.0 |
| TR:A0A5B8KX25 A0A5B8KX25_9RHIZ 3-phosphoshikimate 1-carboxyvinyltr... | 630 | 0.0 |
| TR:A0A371WZP3 A0A371WZP3_9RHIZ 3-phosphoshikimate 1-carboxyvinyltr... | 626 | 0.0 |
| TR:J1K282 J1K282_9RHIZ 3-phosphoshikimate 1-carboxyvinyltransferas... | 624 | 0.0 |
| TR:A0A1X0T2Z3 A0A1X0T2Z3_9RHIZ 3-phosphoshikimate 1-carboxyvinyltr... | 623 | 0.0 |
| TR:Q1YE73 Q1YE73_AURMS 3-phosphoshikimate 1-carboxyvinyltransferas... | 618 | 0.0 |
| TR:A0A1W2AM59 A0A1W2AM59_9RHIZ 3-phosphoshikimate 1-carboxyvinyltr... | 615 | 0.0 |
| TR:A0A1H0K3L8 A0A1H0K3L8_9RHIZ 3-phosphoshikimate 1-carboxyvinyltr... | 613 | 0.0 |
| TR:A0A0B1PZN0 A0A0B1PZN0_9RHIZ 3-phosphoshikimate 1-carboxyvinyltr... | 611 | 0.0 |
| TR:A0A1X7MW68 A0A1X7MW68_9RHIZ 3-phosphoshikimate 1-carboxyvinyltr... | 610 | 0.0 |
| TR:A0A1T4QWZ5 A0A1T4QWZ5_9RHIZ 3-phosphoshikimate 1-carboxyvinyltr... | 604 | 0.0 |
| TR:L0ETY3 L0ETY3_LIBCB 3-phosphoshikimate 1-carboxyvinyltransferas... | 604 | 0.0 |
| TR:A0A367ZZ01 A0A367ZZ01_9RHIZ 3-phosphoshikimate 1-carboxyvinyltr... | 602 | 0.0 |
| TR:A0A5B0DSC2 A0A5B0DSC2_9RHIZ 3-phosphoshikimate 1-carboxyvinyltr... | 598 | 0.0 |
| TR:A0A368A2I4 A0A368A2I4_9RHIZ 3-phosphoshikimate 1-carboxyvinyltr... | 597 | 0.0 |
| TR:Q0G7S3 Q0G7S3_9RHIZ 3-phosphoshikimate 1-carboxyvinyltransferas... | 594 | 0.0 |
| TR:A0A1K2HYS7 A0A1K2HYS7_9RHIZ 3-phosphoshikimate 1-carboxyvinyltr... | 588 | 0.0 |
| TR:A0A433X8C0 A0A433X8C0_9RHIZ 3-phosphoshikimate 1-carboxyvinyltr... | 586 | 0.0 |
| TR:A0A0F7PN90 A0A0F7PN90_9RHIZ 3-phosphoshikimate 1-carboxyvinyltr... | 585 | 0.0 |
| SP:Q6G0X3 AROA_BARQU 3-phosphoshikimate 1-carboxyvinyltransferase ... | 583 | 0.0 |
| TR:A0A369W6D8 A0A369W6D8_9RHIZ 3-phosphoshikimate 1-carboxyvinyltr... | 582 | 0.0 |
| SP:A9ILL7 AROA_BART1 3-phosphoshikimate 1-carboxyvinyltransferase ... | 577 | 0.0 |
| TR:A0A0U3NA21 A0A0U3NA21_9RHOB 3-phosphoshikimate 1-carboxyvinyltr... | 575 | 0.0 |
| TR:A0A1G7ST15 A0A1G7ST15_9RHIZ 3-phosphoshikimate 1-carboxyvinyltr... | 573 | 0.0 |
| SP:Q6G545 AROA_BARHE 3-phosphoshikimate 1-carboxyvinyltransferase ... | 573 | 0.0 |
| TR:G4R9C4 G4R9C4_PELHB 3-phosphoshikimate 1-carboxyvinyltransferas... | 572 | 0.0 |
| TR:F2J6W8 F2J6W8_POLGS 3-phosphoshikimate 1-carboxyvinyltransferas... | 570 | 0.0 |
| TR:E0MSW5 E0MSW5_9RHOB 3-phosphoshikimate 1-carboxyvinyltransferas... | 566 | 0.0 |
| TR:A0A1C1YRI8 A0A1C1YRI8_9RHIZ 3-phosphoshikimate 1-carboxyvinyltr... | 561 | 0.0 |
| TR:A0A1M6ZG46 A0A1M6ZG46_9RHOB 3-phosphoshikimate 1-carboxyvinyltr... | 560 | 0.0 |
| TR:A0A4Q9VT28 A0A4Q9VT28_9RHIZ 3-phosphoshikimate 1-carboxyvinyltr... | 560 | 0.0 |
| TR:A0P3S7 A0P3S7_LABAI 3-phosphoshikimate 1-carboxyvinyltransferas... | 560 | 0.0 |
| TR:A0A165YZR4 A0A165YZR4_9RHOB 3-phosphoshikimate 1-carboxyvinyltr... | 558 | 0.0 |
| TR:A0A0M7A0Y0 A0A0M7A0Y0_9RHOB 3-phosphoshikimate 1-carboxyvinyltr... | 557 | 0.0 |
| TR:A0A0P6VN93 A0A0P6VN93_9RHIZ 3-phosphoshikimate 1-carboxyvinyltr... | 557 | 0.0 |
| TR:A0A5D0VAL2 A0A5D0VAL2_9RHOB 3-phosphoshikimate 1-carboxyvinyltr... | 555 | 0.0 |
| TR:A0A2T5VG60 A0A2T5VG60_9RHIZ 3-phosphoshikimate 1-carboxyvinyltr... | 554 | 0.0 |
| TR:A0A163W9Z4 A0A163W9Z4_9RHOB 3-phosphoshikimate 1-carboxyvinyltr... | 553 | 0.0 |
| TR:A1UU98 A1UU98_BARBK 3-phosphoshikimate 1-carboxyvinyltransferas... | 552 | 0.0 |
| TR:A0A1H9HM39 A0A1H9HM39_9BACT 3-phosphoshikimate 1-carboxyvinyltr... | 551 | 0.0 |
| TR:A0A327JM75 A0A327JM75_9RHIZ 3-phosphoshikimate 1-carboxyvinyltr... | 551 | 0.0 |
| TR:A0A0M7AF66 A0A0M7AF66_9RHOB 3-phosphoshikimate 1-carboxyvinyltr... | 550 | 0.0 |
| TR:A0A2R4MIR9 A0A2R4MIR9_9RHIZ 3-phosphoshikimate 1-carboxyvinyltr... | 550 | 0.0 |
| TR:E2CEL0 E2CEL0_9RHOB 3-phosphoshikimate 1-carboxyvinyltransferas... | 550 | 0.0 |
| TR:A0A4R6RDQ1 A0A4R6RDQ1_9RHIZ 3-phosphoshikimate 1-carboxyvinyltr... | 549 | 0.0 |
| TR:A0A0F5L169 A0A0F5L169_9RHIZ 3-phosphoshikimate 1-carboxyvinyltr... | 549 | 0.0 |
| TR:A0A6A7Y3I4 A0A6A7Y3I4_9RHIZ 3-phosphoshikimate 1-carboxyvinyltr... | 549 | 0.0 |
| TR:A0A5R8YBM5 A0A5R8YBM5_9RHIZ 3-phosphoshikimate 1-carboxyvinyltr... | 548 | 0.0 |
| TR:A0A2C9DCT0 A0A2C9DCT0_9RHIZ 3-phosphoshikimate 1-carboxyvinyltr... | 548 | 0.0 |
| TR:A0A1I5CT28 A0A1I5CT28_9RHIZ 3-phosphoshikimate 1-carboxyvinyltr... | 542 | 0.0 |
| TR:A0A1U7JHZ0 A0A1U7JHZ0_9RHOB 3-phosphoshikimate 1-carboxyvinyltr... | 542 | 0.0 |
| TR:A0A1G5NRL7 A0A1G5NRL7_AFIMA 3-phosphoshikimate 1-carboxyvinyltr... | 541 | 0.0 |
| TR:A0A346QXV6 A0A346QXV6_9RHIZ 3-phosphoshikimate 1-carboxyvinyltr... | 541 | 0.0 |
| TR:A0A1I4SRU6 A0A1I4SRU6_9RHIZ 3-phosphoshikimate 1-carboxyvinyltr... | 540 | 0.0 |
| TR:A0A368A6V2 A0A368A6V2_9RHIZ 3-phosphoshikimate 1-carboxyvinyltr... | 540 | 0.0 |
| TR:A0A285TDL8 A0A285TDL8_9RHOB 3-phosphoshikimate 1-carboxyvinyltr... | 539 | 0.0 |
| TR:A0A2S5TWS2 A0A2S5TWS2_9RHIZ 3-phosphoshikimate 1-carboxyvinyltr... | 538 | 0.0 |
| TR:A0A2N5XN44 A0A2N5XN44_9RHIZ 3-phosphoshikimate 1-carboxyvinyltr... | 536 | 0.0 |
| TR:A0A1H2IU89 A0A1H2IU89_9RHOB 3-phosphoshikimate 1-carboxyvinyltr... | 535 | 0.0 |

| TR:A0A285PH73 A0A285PH73_9RHIZ 3-phosphoshikimate 1-carboxyvinyltr... | 529 | 0.0 |
| --- | --- | --- |
| TR:A0A1G6BL01 A0A1G6BL01_9RHIZ 3-phosphoshikimate 1-carboxyvinyltr... | 528 | 0.0 |
| TR:A0A285M3G8 A0A285M3G8_9RHIZ 3-phosphoshikimate 1-carboxyvinyltr... | 524 | 0.0 |
| TR:M1PBI7 M1PBI7_BARAA 3-phosphoshikimate 1-carboxyvinyltransferas... | 524 | 0.0 |
| TR:A0A1M7ZME3 A0A1M7ZME3_9RHIZ 3-phosphoshikimate 1-carboxyvinyltr... | 523 | 0.0 |
| TR:A0A562T1Z0 A0A562T1Z0_9RHOB 3-phosphoshikimate 1-carboxyvinyltr... | 514 | 5e-179 |
| TR:A0A0K2DAU0 A0A0K2DAU0_9RHIZ 3-phosphoshikimate 1-carboxyvinyltr... | 511 | 5e-177 |
| SP:A7HPE1 AROA_PARL1 3-phosphoshikimate 1-carboxyvinyltransferase ... | 510 | 1e-176 |
| TR:A0A0S3Q0K3 A0A0S3Q0K3_9BRAD 3-phosphoshikimate 1-carboxyvinyltr... | 509 | 3e-176 |
| TR:A0A2W2B8V9 A0A2W2B8V9_9RHIZ 3-phosphoshikimate 1-carboxyvinyltr... | 506 | 5e-175 |
| TR:A0A081BE61 A0A081BE61_9RHIZ 3-phosphoshikimate 1-carboxyvinyltr... | 505 | 6e-175 |
| TR:A0A0A1P575 A0A0A1P575_9BACT 3-phosphoshikimate 1-carboxyvinyltr... | 503 | 5e-174 |
| TR:A0A0Q9IEQ4 A0A0Q9IEQ4_9BRAD 3-phosphoshikimate 1-carboxyvinyltr... | 501 | 3e-173 |
| TR:A0A2S0NH61 A0A2S0NH61_9PROT 3-phosphoshikimate 1-carboxyvinyltr... | 501 | 4e-173 |
| TR:D6ZZ14 D6ZZ14_STAND 3-phosphoshikimate 1-carboxyvinyltransferas... | 499 | 2e-172 |
| TR:A0A2L0ACG7 A0A2L0ACG7_9SPHN 3-phosphoshikimate 1-carboxyvinyltr... | 499 | 2e-172 |
| TR:A0A4D7QRK0 A0A4D7QRK0_9PROT 3-phosphoshikimate 1-carboxyvinyltr... | 497 | 7e-172 |
| SP:B1ZLG6 AROA_METPB 3-phosphoshikimate 1-carboxyvinyltransferase ... | 498 | 9e-172 |
| TR:C5APM8 C5APM8_METEA 3-phosphoshikimate 1-carboxyvinyltransferas... | 498 | 9e-172 |
| TR:A0A1G4SNT6 A0A1G4SNT6_9RHIZ 3-phosphoshikimate 1-carboxyvinyltr... | 498 | 1e-171 |
| TR:A0A370L8I1 A0A370L8I1_9BRAD 3-phosphoshikimate 1-carboxyvinyltr... | 497 | 1e-171 |
| TR:A0A2Z3II00 A0A2Z3II00_9RHIZ 3-phosphoshikimate 1-carboxyvinyltr... | 496 | 2e-171 |
| TR:A0A2U8WEL9 A0A2U8WEL9_9RHIZ 3-phosphoshikimate 1-carboxyvinyltr... | 496 | 3e-171 |
| TR:A0A4R3MAT0 A0A4R3MAT0_9RHIZ 3-phosphoshikimate 1-carboxyvinyltr... | 497 | 3e-171 |
| TR:A0A370HN92 A0A370HN92_9RHIZ 3-phosphoshikimate 1-carboxyvinyltr... | 496 | 3e-171 |
| TR:A0A099FFL4 A0A099FFL4_9RHOB 3-phosphoshikimate 1-carboxyvinyltr... | 496 | 4e-171 |
| SP:B7KZL6 AROA_METC4 3-phosphoshikimate 1-carboxyvinyltransferase ... | 496 | 5e-171 |
| TR:A0A2T1HT45 A0A2T1HT45_9RHIZ 3-phosphoshikimate 1-carboxyvinyltr... | 495 | 7e-171 |
| TR:A0A1I2W6L8 A0A1I2W6L8_9RHIZ 3-phosphoshikimate 1-carboxyvinyltr... | 494 | 2e-170 |
| TR:A0A2H6BH97 A0A2H6BH97_9RHIZ 3-phosphoshikimate 1-carboxyvinyltr... | 494 | 2e-170 |
| TR:A0A516H0K4 A0A516H0K4_9PROT 3-phosphoshikimate 1-carboxyvinyltr... | 494 | 2e-170 |
| TR:A7IEB8 A7IEB8_XANP2 3-phosphoshikimate 1-carboxyvinyltransferas... | 494 | 2e-170 |
| TR:A0A1B3NFT2 A0A1B3NFT2_9BRAD 3-phosphoshikimate 1-carboxyvinyltr... | 493 | 3e-170 |
| TR:A0A085FGZ9 A0A085FGZ9_9BRAD 3-phosphoshikimate 1-carboxyvinyltr... | 493 | 3e-170 |
| TR:A0A140GV68 A0A140GV68_9RHIZ 3-phosphoshikimate 1-carboxyvinyltr... | 493 | 5e-170 |
| TR:A0A0H1RGV3 A0A0H1RGV3_9RHIZ 3-phosphoshikimate 1-carboxyvinyltr... | 493 | 7e-170 |
| TR:A0A512BPP8 A0A512BPP8_9RHIZ 3-phosphoshikimate 1-carboxyvinyltr... | 493 | 8e-170 |
| TR:V5SIG5 V5SIG5_9RHIZ 3-phosphoshikimate 1-carboxyvinyltransferas... | 494 | 8e-170 |
| TR:V4R1Z1 V4R1Z1_9RHIZ 3-phosphoshikimate 1-carboxyvinyltransferas... | 493 | 8e-170 |
| TR:A0A2U8VTD6 A0A2U8VTD6_9RHIZ 3-phosphoshikimate 1-carboxyvinyltr... | 492 | 1e-169 |
| TR:A0A514WEW6 A0A514WEW6_9SPHN 3-phosphoshikimate 1-carboxyvinyltr... | 492 | 1e-169 |
| TR:A0A0Q6KHI6 A0A0Q6KHI6_9BRAD 3-phosphoshikimate 1-carboxyvinyltr... | 492 | 1e-169 |
| TR:F6ILJ1 F6ILJ1_9SPHN 3-phosphoshikimate 1-carboxyvinyltransferas... | 491 | 2e-169 |
| TR:A0A0Q4XTV4 A0A0Q4XTV4_9RHIZ 3-phosphoshikimate 1-carboxyvinyltr... | 491 | 2e-169 |
| TR:A0A1W6ZQ57 A0A1W6ZQ57_9RHIZ 3-phosphoshikimate 1-carboxyvinyltr... | 491 | 3e-169 |
| TR:G6EEV2 G6EEV2_9SPHN 3-phosphoshikimate 1-carboxyvinyltransferas... | 491 | 3e-169 |
| SP:Q13ER0 AROA_RHOPS 3-phosphoshikimate 1-carboxyvinyltransferase ... | 491 | 4e-169 |
| TR:A0A1I3PAL8 A0A1I3PAL8_9BRAD 3-phosphoshikimate 1-carboxyvinyltr... | 491 | 4e-169 |
| TR:A0A0N0JWL9 A0A0N0JWL9_9BRAD 3-phosphoshikimate 1-carboxyvinyltr... | 490 | 6e-169 |
| TR:A0A0Q4XLF1 A0A0Q4XLF1_9RHIZ 3-phosphoshikimate 1-carboxyvinyltr... | 490 | 6e-169 |
| TR:I9WBH6 I9WBH6_9SPHN 3-phosphoshikimate 1-carboxyvinyltransferas... | 490 | 8e-169 |
| TR:A0A1H8E4T0 A0A1H8E4T0_9RHOB 3-phosphoshikimate 1-carboxyvinyltr... | 489 | 1e-168 |
| TR:A0A2H5EVL9 A0A2H5EVL9_9RHOB 3-phosphoshikimate 1-carboxyvinyltr... | 489 | 1e-168 |
| TR:I4YTY5 I4YTY5_9RHIZ 3-phosphoshikimate 1-carboxyvinyltransferas... | 489 | 1e-168 |
| TR:A0A5N3PCS4 A0A5N3PCS4_9RHIZ 3-phosphoshikimate 1-carboxyvinyltr... | 489 | 1e-168 |
| TR:A0A1W6CYG2 A0A1W6CYG2_9RHOB 3-phosphoshikimate 1-carboxyvinyltr... | 489 | 1e-168 |
| TR:A0A437N1E1 A0A437N1E1_9SPHN 3-phosphoshikimate 1-carboxyvinyltr... | 489 | 2e-168 |
| TR:A0A239EU77 A0A239EU77_9SPHN 3-phosphoshikimate 1-carboxyvinyltr... | 490 | 2e-168 |
| TR:A0A512H6K5 A0A512H6K5_9PROT 3-phosphoshikimate 1-carboxyvinyltr... | 489 | 2e-168 |
| TR:T0HWZ7 T0HWZ7_9SPHN 3-phosphoshikimate 1-carboxyvinyltransferas... | 489 | 2e-168 |
| TR:A0A5N7MQQ0 A0A5N7MQQ0_9RHIZ 3-phosphoshikimate 1-carboxyvinyltr... | 489 | 2e-168 |
| TR:A0A4Q9GHI3 A0A4Q9GHI3_9RHIZ 3-phosphoshikimate 1-carboxyvinyltr... | 489 | 3e-168 |
| TR:A0A1N6X1Z8 A0A1N6X1Z8_9BRAD 3-phosphoshikimate 1-carboxyvinyltr... | 488 | 3e-168 |
| TR:A0A3L7AN27 A0A3L7AN27_9RHIZ 3-phosphoshikimate 1-carboxyvinyltr... | 489 | 3e-168 |

| TR:K2J8R6 K2J8R6_9RHOB 3-phosphoshikimate 1-carboxyvinyltransferas... | 488 | 4e-168 |
| --- | --- | --- |
| TR:A0A031K773 A0A031K773_9SPHN 3-phosphoshikimate 1-carboxyvinyltr... | 488 | 4e-168 |
| TR:A0A178MWP7 A0A178MWP7_9PROT 3-phosphoshikimate 1-carboxyvinyltr... | 488 | 4e-168 |
| TR:A0A0Q5Z2B0 A0A0Q5Z2B0_9RHIZ 3-phosphoshikimate 1-carboxyvinyltr... | 488 | 5e-168 |
| TR:A0A501PPE6 A0A501PPE6_9PROT 3-phosphoshikimate 1-carboxyvinyltr... | 488 | 6e-168 |
| TR:A0A1I0EG37 A0A1I0EG37_9RHOB 3-phosphoshikimate 1-carboxyvinyltr... | 487 | 8e-168 |
| TR:A0A2T5G184 A0A2T5G184_9SPHN 3-phosphoshikimate 1-carboxyvinyltr... | 487 | 8e-168 |
| TR:D8JRQ8 D8JRQ8_HYPDA 3-phosphoshikimate 1-carboxyvinyltransferas... | 487 | 9e-168 |
| TR:Q0FJ79 Q0FJ79_SALBH 3-phosphoshikimate 1-carboxyvinyltransferas... | 487 | 9e-168 |
| TR:A0A086XVY0 A0A086XVY0_9RHOB 3-phosphoshikimate 1-carboxyvinyltr... | 487 | 1e-167 |
| TR:A0A1G5LB09 A0A1G5LB09_9RHIZ 3-phosphoshikimate 1-carboxyvinyltr... | 487 | 1e-167 |
| TR:A0A4R1UXI2 A0A4R1UXI2_9SPHN 3-phosphoshikimate 1-carboxyvinyltr... | 487 | 1e-167 |
| TR:A0A086MES2 A0A086MES2_9RHIZ 3-phosphoshikimate 1-carboxyvinyltr... | 487 | 1e-167 |
| TR:A0A4V1N3G9 A0A4V1N3G9_9SPHN 3-phosphoshikimate 1-carboxyvinyltr... | 486 | 2e-167 |
| TR:A0A4V1KJ23 A0A4V1KJ23_9RHIZ 3-phosphoshikimate 1-carboxyvinyltr... | 487 | 2e-167 |
| TR:A0A0N9ZEP8 A0A0N9ZEP8_9RHOB 3-phosphoshikimate 1-carboxyvinyltr... | 486 | 2e-167 |
| TR:A0A1B2EID6 A0A1B2EID6_9RHIZ 3-phosphoshikimate 1-carboxyvinyltr... | 486 | 2e-167 |
| TR:A8IKN4 A8IKN4_AZOC5 3-phosphoshikimate 1-carboxyvinyltransferas... | 487 | 2e-167 |
| TR:A0A1V0GUQ8 A0A1V0GUQ8_9RHOB 3-phosphoshikimate 1-carboxyvinyltr... | 486 | 2e-167 |
| TR:A0A2V5B8P1 A0A2V5B8P1_9SPHN 3-phosphoshikimate 1-carboxyvinyltr... | 486 | 3e-167 |
| TR:A0A418ZU49 A0A418ZU49_9RHOB 3-phosphoshikimate 1-carboxyvinyltr... | 486 | 3e-167 |
| TR:J2HG57 J2HG57_9SPHN 3-phosphoshikimate 1-carboxyvinyltransferas... | 486 | 3e-167 |
| TR:A0A1I2X275 A0A1I2X275_9RHOB 3-phosphoshikimate 1-carboxyvinyltr... | 486 | 3e-167 |
| TR:A0A512IJ77 A0A512IJ77_9RHIZ 3-phosphoshikimate 1-carboxyvinyltr... | 486 | 4e-167 |
| SP:B6JCN2 AROA_OLICO 3-phosphoshikimate 1-carboxyvinyltransferase ... | 485 | 5e-167 |
| TR:A0A4T3EZT5 A0A4T3EZT5_9SPHN 3-phosphoshikimate 1-carboxyvinyltr... | 485 | 5e-167 |
| TR:A0A246JN12 A0A246JN12_9SPHN 3-phosphoshikimate 1-carboxyvinyltr... | 485 | 6e-167 |
| TR:A0A447IQ54 A0A447IQ54_9RHOB 3-phosphoshikimate 1-carboxyvinyltr... | 485 | 7e-167 |
| TR:A0A239PWI1 A0A239PWI1_9RHOB 3-phosphoshikimate 1-carboxyvinyltr... | 484 | 9e-167 |
| SP:B0UC54 AROA_METS4 3-phosphoshikimate 1-carboxyvinyltransferase ... | 484 | 1e-166 |
| TR:A0A3S2XS79 A0A3S2XS79_9RHIZ 3-phosphoshikimate 1-carboxyvinyltr... | 484 | 1e-166 |
| TR:A0A239FPX5 A0A239FPX5_9SPHN 3-phosphoshikimate 1-carboxyvinyltr... | 484 | 2e-166 |
| TR:A0A3A3DHB9 A0A3A3DHB9_9RHOB 3-phosphoshikimate 1-carboxyvinyltr... | 484 | 2e-166 |
| TR:U2ZSF9 U2ZSF9_9SPHN 3-phosphoshikimate 1-carboxyvinyltransferas... | 484 | 2e-166 |
| TR:S5YWI7 S5YWI7_PARAH 3-phosphoshikimate 1-carboxyvinyltransferas... | 484 | 2e-166 |
| SP:Q6NDP4 AROA_RHOPA 3-phosphoshikimate 1-carboxyvinyltransferase ... | 484 | 2e-166 |
| TR:A0A4R6HCC8 A0A4R6HCC8_9BRAD 3-phosphoshikimate 1-carboxyvinyltr... | 484 | 2e-166 |
| TR:A0A1I3MEY8 A0A1I3MEY8_9RHOB 3-phosphoshikimate 1-carboxyvinyltr... | 484 | 2e-166 |
| TR:A0A099F2G7 A0A099F2G7_9RHOB 3-phosphoshikimate 1-carboxyvinyltr... | 483 | 3e-166 |
| SP:Q2J2F6 AROA_RHOP2 3-phosphoshikimate 1-carboxyvinyltransferase ... | 483 | 3e-166 |
| TR:A0A074KJF4 A0A074KJF4_9RHOB 3-phosphoshikimate 1-carboxyvinyltr... | 483 | 3e-166 |
| TR:A0A2A4G088 A0A2A4G088_9SPHN 3-phosphoshikimate 1-carboxyvinyltr... | 483 | 4e-166 |
| TR:A0A2S8S5Z1 A0A2S8S5Z1_9RHOB 3-phosphoshikimate 1-carboxyvinyltr... | 483 | 5e-166 |
| TR:A0A1X7I965 A0A1X7I965_9RHOB 3-phosphoshikimate 1-carboxyvinyltr... | 483 | 5e-166 |
| SP:B1M0N9 AROA_METRJ 3-phosphoshikimate 1-carboxyvinyltransferase ... | 483 | 7e-166 |
| TR:A0A1H2W8Z3 A0A1H2W8Z3_9RHOB 3-phosphoshikimate 1-carboxyvinyltr... | 483 | 7e-166 |
| TR:A0A2Z4UQ04 A0A2Z4UQ04_9RHIZ 3-phosphoshikimate 1-carboxyvinyltr... | 482 | 8e-166 |
| TR:A0A515KS33 A0A515KS33_9BRAD 3-phosphoshikimate 1-carboxyvinyltr... | 483 | 8e-166 |
| TR:A0A2N3PSR8 A0A2N3PSR8_9PROT 3-phosphoshikimate 1-carboxyvinyltr... | 482 | 8e-166 |
| TR:W1RUG6 W1RUG6_9SPHN 3-phosphoshikimate 1-carboxyvinyltransferas... | 482 | 8e-166 |
| TR:A0A317DTQ0 A0A317DTQ0_9PROT 3-phosphoshikimate 1-carboxyvinyltr... | 482 | 9e-166 |
| TR:A0A095B9Q8 A0A095B9Q8_9SPHN 3-phosphoshikimate 1-carboxyvinyltr... | 482 | 1e-165 |
| TR:A0A1G9IVT7 A0A1G9IVT7_9RHOB 3-phosphoshikimate 1-carboxyvinyltr... | 482 | 1e-165 |
| SP:B3Q5Z7 AROA_RHOPT 3-phosphoshikimate 1-carboxyvinyltransferase ... | 482 | 1e-165 |
| SP:Q07UI1 AROA_RHOP5 3-phosphoshikimate 1-carboxyvinyltransferase ... | 482 | 1e-165 |
| TR:W3RKN5 W3RKN5_9BRAD 3-phosphoshikimate 1-carboxyvinyltransferas... | 482 | 1e-165 |
| TR:A0A1I3SEP6 A0A1I3SEP6_9RHOB 3-phosphoshikimate 1-carboxyvinyltr... | 482 | 1e-165 |
| TR:A0A1I7MV05 A0A1I7MV05_9RHIZ 3-phosphoshikimate 1-carboxyvinyltr... | 482 | 1e-165 |
| TR:A0A368Z218 A0A368Z218_9RHOB 3-phosphoshikimate 1-carboxyvinyltr... | 481 | 2e-165 |
| TR:A0A558R856 A0A558R856_9SPHN 3-phosphoshikimate 1-carboxyvinyltr... | 481 | 2e-165 |
| TR:A0A1G5CCG2 A0A1G5CCG2_9RHOB 3-phosphoshikimate 1-carboxyvinyltr... | 481 | 2e-165 |
| TR:A0A6I5YQH9 A0A6I5YQH9_9SPHN 3-phosphoshikimate 1-carboxyvinyltr... | 481 | 2e-165 |
| TR:A0A291GFC5 A0A291GFC5_9RHOB 3-phosphoshikimate 1-carboxyvinyltr... | 481 | 3e-165 |
| TR:A0A564G6R9 A0A564G6R9_9RHIZ 3-phosphoshikimate 1-carboxyvinyltr... | 481 | 3e-165 |

| TR:A0A318TCD3 A0A318TCD3_9BRAD 3-phosphoshikimate 1-carboxyvinyltr... | 481 | 3e-165 |
| --- | --- | --- |
| TR:A0A0Q3I1Q9 A0A0Q3I1Q9_9BRAD 3-phosphoshikimate 1-carboxyvinyltr... | 481 | 4e-165 |
| SP:Q2RXP5 AROA_RHORT 3-phosphoshikimate 1-carboxyvinyltransferase ... | 481 | 4e-165 |
| TR:A0A2A3N132 A0A2A3N132_9SPHN 3-phosphoshikimate 1-carboxyvinyltr... | 480 | 5e-165 |
| TR:A0A1N7PC71 A0A1N7PC71_9RHOB 3-phosphoshikimate 1-carboxyvinyltr... | 480 | 5e-165 |
| TR:A0A327KLT1 A0A327KLT1_9RHIZ 3-phosphoshikimate 1-carboxyvinyltr... | 480 | 5e-165 |
| TR:A0A1I3VG95 A0A1I3VG95_9RHOB 3-phosphoshikimate 1-carboxyvinyltr... | 480 | 5e-165 |
| TR:A0A5B2V904 A0A5B2V904_9RHIZ 3-phosphoshikimate 1-carboxyvinyltr... | 480 | 6e-165 |
| TR:A0A0M2RCP7 A0A0M2RCP7_9PROT 3-phosphoshikimate 1-carboxyvinyltr... | 480 | 6e-165 |
| TR:A0A366F889 A0A366F889_9RHIZ 3-phosphoshikimate 1-carboxyvinyltr... | 480 | 6e-165 |
| TR:A0A437J6D1 A0A437J6D1_9SPHN 3-phosphoshikimate 1-carboxyvinyltr... | 480 | 7e-165 |
| TR:A0A243PE86 A0A243PE86_9SPHN 3-phosphoshikimate 1-carboxyvinyltr... | 480 | 7e-165 |
| TR:N1MGF1 N1MGF1_9SPHN 3-phosphoshikimate 1-carboxyvinyltransferas... | 479 | 7e-165 |
| TR:A0A102D5X5 A0A102D5X5_9SPHN 3-phosphoshikimate 1-carboxyvinyltr... | 480 | 7e-165 |
| TR:A0A1H1KH72 A0A1H1KH72_9RHIZ 3-phosphoshikimate 1-carboxyvinyltr... | 480 | 7e-165 |
| TR:A0A164B6K1 A0A164B6K1_9BRAD 3-phosphoshikimate 1-carboxyvinyltr... | 480 | 8e-165 |
| TR:A0A443IVZ2 A0A443IVZ2_9RHOB 3-phosphoshikimate 1-carboxyvinyltr... | 480 | 8e-165 |
| TR:A0A562NSL0 A0A562NSL0_9RHOB 3-phosphoshikimate 1-carboxyvinyltr... | 479 | 9e-165 |
| SP:Q3IW89 AROA_RHOS4 3-phosphoshikimate 1-carboxyvinyltransferase ... | 479 | 9e-165 |
| TR:A0A418W826 A0A418W826_9SPHN 3-phosphoshikimate 1-carboxyvinyltr... | 479 | 9e-165 |
| TR:A0A0Q6YUI7 A0A0Q6YUI7_9BRAD 3-phosphoshikimate 1-carboxyvinyltr... | 479 | 9e-165 |
| TR:A0A196MMM9 A0A196MMM9_9SPHN 3-phosphoshikimate 1-carboxyvinyltr... | 479 | 1e-164 |
| TR:A0A2Z6ABT0 A0A2Z6ABT0_9SPHN 3-phosphoshikimate 1-carboxyvinyltr... | 479 | 1e-164 |
| TR:A0A0N1BLJ2 A0A0N1BLJ2_9SPHN 3-phosphoshikimate 1-carboxyvinyltr... | 479 | 1e-164 |
| TR:A0A2S8B2U0 A0A2S8B2U0_9SPHN 3-phosphoshikimate 1-carboxyvinyltr... | 479 | 2e-164 |
| TR:D5ARK9 D5ARK9_RHOCB 3-phosphoshikimate 1-carboxyvinyltransferas... | 479 | 2e-164 |
| SP:A1B474 AROA_PARDP 3-phosphoshikimate 1-carboxyvinyltransferase ... | 478 | 2e-164 |
| TR:A0A3D8PFI5 A0A3D8PFI5_9RHOB 3-phosphoshikimate 1-carboxyvinyltr... | 478 | 2e-164 |
| TR:A0A1N7JCG0 A0A1N7JCG0_9PROT 3-phosphoshikimate 1-carboxyvinyltr... | 478 | 3e-164 |
| TR:F1ZDB7 F1ZDB7_9SPHN 3-phosphoshikimate 1-carboxyvinyltransferas... | 478 | 3e-164 |
| TR:A0A5C8WYE9 A0A5C8WYE9_9RHIZ 3-phosphoshikimate 1-carboxyvinyltr... | 478 | 3e-164 |
| TR:H6SLF4 H6SLF4_PARPM 3-phosphoshikimate 1-carboxyvinyltransferas... | 478 | 3e-164 |
| TR:A0A3P3DV24 A0A3P3DV24_9RHOB 3-phosphoshikimate 1-carboxyvinyltr... | 478 | 3e-164 |
| TR:A0A246K724 A0A246K724_9SPHN 3-phosphoshikimate 1-carboxyvinyltr... | 478 | 4e-164 |
| TR:K2JQY6 K2JQY6_9PROT 3-phosphoshikimate 1-carboxyvinyltransferas... | 478 | 4e-164 |
| SP:A3PPV7 AROA_RHOS1 3-phosphoshikimate 1-carboxyvinyltransferase ... | 478 | 5e-164 |
| TR:F6EYV5 F6EYV5_SPHCR 3-phosphoshikimate 1-carboxyvinyltransferas... | 478 | 6e-164 |
| SP:Q1QRT1 AROA_NITHX 3-phosphoshikimate 1-carboxyvinyltransferase ... | 478 | 6e-164 |
| TR:A0A074TLN7 A0A074TLN7_9RHOB 3-phosphoshikimate 1-carboxyvinyltr... | 478 | 6e-164 |
| TR:A0A397PCW0 A0A397PCW0_9RHIZ 3-phosphoshikimate 1-carboxyvinyltr... | 477 | 7e-164 |
| TR:A0A512E1Y4 A0A512E1Y4_9PROT 3-phosphoshikimate 1-carboxyvinyltr... | 478 | 7e-164 |
| TR:A0A4Z1C191 A0A4Z1C191_9RHOB 3-phosphoshikimate 1-carboxyvinyltr... | 477 | 7e-164 |
| TR:A0A6B8M1H5 A0A6B8M1H5_9RHIZ 3-phosphoshikimate 1-carboxyvinyltr... | 477 | 8e-164 |
| TR:A0A1H9SQ44 A0A1H9SQ44_9SPHN 3-phosphoshikimate 1-carboxyvinyltr... | 477 | 8e-164 |
| TR:A0A327K9S5 A0A327K9S5_9RHIZ 3-phosphoshikimate 1-carboxyvinyltr... | 477 | 8e-164 |
| TR:A0A0J7Y3G4 A0A0J7Y3G4_9SPHN 3-phosphoshikimate 1-carboxyvinyltr... | 477 | 8e-164 |
| TR:A0A1U7D003 A0A1U7D003_9RHOB 3-phosphoshikimate 1-carboxyvinyltr... | 477 | 8e-164 |
| TR:D4Z303 D4Z303_SPHJU 3-phosphoshikimate 1-carboxyvinyltransferas... | 477 | 9e-164 |
| TR:A0A0Q8QA62 A0A0Q8QA62_9SPHN 3-phosphoshikimate 1-carboxyvinyltr... | 477 | 1e-163 |
| TR:A0A1N7NK33 A0A1N7NK33_9RHOB 3-phosphoshikimate 1-carboxyvinyltr... | 477 | 1e-163 |
| TR:A0A3B0M8C7 A0A3B0M8C7_9RHOB 3-phosphoshikimate 1-carboxyvinyltr... | 477 | 1e-163 |
| TR:A0A1C2HBJ3 A0A1C2HBJ3_9RHOB 3-phosphoshikimate 1-carboxyvinyltr... | 477 | 1e-163 |
| TR:A0A1H5ZZC1 A0A1H5ZZC1_9RHOB 3-phosphoshikimate 1-carboxyvinyltr... | 476 | 2e-163 |
| TR:A0A0J6UYB0 A0A0J6UYB0_9RHIZ 3-phosphoshikimate 1-carboxyvinyltr... | 476 | 2e-163 |
| TR:A0A3S8U4B4 A0A3S8U4B4_9RHOB 3-phosphoshikimate 1-carboxyvinyltr... | 476 | 2e-163 |
| TR:A0A2S6MZ64 A0A2S6MZ64_9RHIZ 3-phosphoshikimate 1-carboxyvinyltr... | 476 | 2e-163 |
| TR:F8JIN8 F8JIN8_HYPSM 3-phosphoshikimate 1-carboxyvinyltransferas... | 476 | 2e-163 |
| TR:A0A154W6C2 A0A154W6C2_9PROT 3-phosphoshikimate 1-carboxyvinyltr... | 476 | 3e-163 |
| TR:A0A0D7EAJ3 A0A0D7EAJ3_RHOPL 3-phosphoshikimate 1-carboxyvinyltr... | 476 | 3e-163 |
| TR:A0A257F0G7 A0A257F0G7_9RHOB 3-phosphoshikimate 1-carboxyvinyltr... | 476 | 3e-163 |
| TR:A0A1E2S000 A0A1E2S000_9RHIZ 3-phosphoshikimate 1-carboxyvinyltr... | 475 | 3e-163 |
| TR:A0A2K8KIF1 A0A2K8KIF1_9RHOB 3-phosphoshikimate 1-carboxyvinyltr... | 476 | 4e-163 |
| TR:A0A545TG49 A0A545TG49_9PROT 3-phosphoshikimate 1-carboxyvinyltr... | 476 | 4e-163 |
| TR:A0A371BE67 A0A371BE67_9SPHN 3-phosphoshikimate 1-carboxyvinyltr... | 475 | 4e-163 |

| TR:A0A317DYD2 A0A317DYD2_9PROT 3-phosphoshikimate 1-carboxyvinyltr... | 476 | 4e-163 |
| --- | --- | --- |
| TR:A0A426RQ64 A0A426RQ64_9SPHN 3-phosphoshikimate 1-carboxyvinyltr... | 475 | 4e-163 |
| TR:A0A2Z5EFV8 A0A2Z5EFV8_9SPHN 3-phosphoshikimate 1-carboxyvinyltr... | 475 | 5e-163 |
| TR:A0A4V1ZPT0 A0A4V1ZPT0_9BRAD 3-phosphoshikimate 1-carboxyvinyltr... | 476 | 5e-163 |
| TR:A0A0D1M5H0 A0A0D1M5H0_9SPHN 3-phosphoshikimate 1-carboxyvinyltr... | 475 | 5e-163 |
| TR:A0A418WT41 A0A418WT41_9PROT 3-phosphoshikimate 1-carboxyvinyltr... | 475 | 5e-163 |
| TR:A0A1H4X4L6 A0A1H4X4L6_9RHIZ 3-phosphoshikimate 1-carboxyvinyltr... | 476 | 5e-163 |
| TR:A0A257ELK4 A0A257ELK4_9PROT 3-phosphoshikimate 1-carboxyvinyltr... | 475 | 7e-163 |
| TR:A0A2K9EUG4 A0A2K9EUG4_9RHOB 3-phosphoshikimate 1-carboxyvinyltr... | 474 | 8e-163 |
| TR:B8IK64 B8IK64_METNO 3-phosphoshikimate 1-carboxyvinyltransferas... | 475 | 9e-163 |
| TR:V7EFJ2 V7EFJ2_9RHOB 3-phosphoshikimate 1-carboxyvinyltransferas... | 475 | 1e-162 |
| TR:A0A0M3QD18 A0A0M3QD18_SPHS1 3-phosphoshikimate 1-carboxyvinyltr... | 474 | 1e-162 |
| TR:A0A0Q8QRC3 A0A0Q8QRC3_9SPHN 3-phosphoshikimate 1-carboxyvinyltr... | 474 | 1e-162 |
| TR:A0A0D6JE41 A0A0D6JE41_9RHIZ 3-phosphoshikimate 1-carboxyvinyltr... | 474 | 1e-162 |
| TR:A0A1D9MDT8 A0A1D9MDT8_9RHOB 3-phosphoshikimate 1-carboxyvinyltr... | 474 | 1e-162 |
| TR:A0A419MYP5 A0A419MYP5_9SPHN 3-phosphoshikimate 1-carboxyvinyltr... | 474 | 1e-162 |
| TR:A0A257HEC4 A0A257HEC4_9RHIZ 3-phosphoshikimate 1-carboxyvinyltr... | 474 | 1e-162 |
| TR:A0A0M3TAA7 A0A0M3TAA7_9SPHN 3-phosphoshikimate 1-carboxyvinyltr... | 474 | 1e-162 |
| SP:Q89WF2 AROA_BRADU 3-phosphoshikimate 1-carboxyvinyltransferase ... | 475 | 1e-162 |
| TR:A0A2G8MPK4 A0A2G8MPK4_9RHIZ 3-phosphoshikimate 1-carboxyvinyltr... | 474 | 1e-162 |
| TR:A0A2A4ID68 A0A2A4ID68_9SPHN 3-phosphoshikimate 1-carboxyvinyltr... | 474 | 2e-162 |
| TR:A0A327K264 A0A327K264_9RHIZ 3-phosphoshikimate 1-carboxyvinyltr... | 474 | 2e-162 |
| TR:A0A0X8R4B0 A0A0X8R4B0_9SPHN 3-phosphoshikimate 1-carboxyvinyltr... | 474 | 2e-162 |
| TR:A0A4Q9G4X9 A0A4Q9G4X9_9RHOB 3-phosphoshikimate 1-carboxyvinyltr... | 474 | 2e-162 |
| TR:A0A418ZWX6 A0A418ZWX6_9RHOB 3-phosphoshikimate 1-carboxyvinyltr... | 474 | 2e-162 |
| TR:A0A494VZM4 A0A494VZM4_9SPHN 3-phosphoshikimate 1-carboxyvinyltr... | 474 | 2e-162 |
| TR:A0A3N2R155 A0A3N2R155_9RHOB 3-phosphoshikimate 1-carboxyvinyltr... | 474 | 2e-162 |
| TR:A0A421BK08 A0A421BK08_9RHOB 3-phosphoshikimate 1-carboxyvinyltr... | 474 | 2e-162 |
| TR:A0A4R2GXV5 A0A4R2GXV5_9RHIZ 3-phosphoshikimate 1-carboxyvinyltr... | 474 | 2e-162 |
| TR:A0A0N9U4K9 A0A0N9U4K9_SPHMC 3-phosphoshikimate 1-carboxyvinyltr... | 474 | 2e-162 |
| TR:A0A126P0I9 A0A126P0I9_9BRAD 3-phosphoshikimate 1-carboxyvinyltr... | 473 | 3e-162 |
| SP:Q2G8Q3 AROA_NOVAD 3-phosphoshikimate 1-carboxyvinyltransferase ... | 473 | 3e-162 |
| TR:A0A3D9H9E6 A0A3D9H9E6_9PROT 3-phosphoshikimate 1-carboxyvinyltr... | 473 | 3e-162 |
| TR:A0A1I2R5L7 A0A1I2R5L7_9SPHN 3-phosphoshikimate 1-carboxyvinyltr... | 473 | 3e-162 |
| TR:A0A521AEU6 A0A521AEU6_9RHOB 3-phosphoshikimate 1-carboxyvinyltr... | 473 | 3e-162 |
| TR:A0A0F5PFN4 A0A0F5PFN4_9SPHN 3-phosphoshikimate 1-carboxyvinyltr... | 473 | 4e-162 |
| TR:Q21CB1 Q21CB1_RHOPB 3-phosphoshikimate 1-carboxyvinyltransferas... | 474 | 4e-162 |
| TR:A0A2T6B7Z2 A0A2T6B7Z2_9RHOB 3-phosphoshikimate 1-carboxyvinyltr... | 473 | 4e-162 |
| TR:C8RWA4 C8RWA4_9RHOB 3-phosphoshikimate 1-carboxyvinyltransferas... | 473 | 4e-162 |
| TR:A0A0N1LN53 A0A0N1LN53_9SPHN 3-phosphoshikimate 1-carboxyvinyltr... | 473 | 5e-162 |
| TR:A0A2Y9B163 A0A2Y9B163_9RHOB 3-phosphoshikimate 1-carboxyvinyltr... | 473 | 5e-162 |
| TR:A0A1L3ZY11 A0A1L3ZY11_9SPHN 3-phosphoshikimate 1-carboxyvinyltr... | 473 | 5e-162 |
| TR:A0A1H6A2I7 A0A1H6A2I7_9BRAD 3-phosphoshikimate 1-carboxyvinyltr... | 473 | 5e-162 |
| TR:A0A3N1LJS0 A0A3N1LJS0_9PROT 3-phosphoshikimate 1-carboxyvinyltr... | 473 | 5e-162 |
| TR:A0A1H8A7W9 A0A1H8A7W9_9RHOB 3-phosphoshikimate 1-carboxyvinyltr... | 473 | 5e-162 |
| TR:B6IUQ0 B6IUQ0_RHOCS 3-phosphoshikimate 1-carboxyvinyltransferas... | 473 | 6e-162 |
| TR:A0A0B5DSM1 A0A0B5DSM1_9RHOB 3-phosphoshikimate 1-carboxyvinyltr... | 473 | 6e-162 |
| TR:A0A4Y6VBK8 A0A4Y6VBK8_9PROT 3-phosphoshikimate 1-carboxyvinyltr... | 472 | 6e-162 |
| TR:A0A147INJ0 A0A147INJ0_9SPHN 3-phosphoshikimate 1-carboxyvinyltr... | 472 | 7e-162 |
| TR:A0A2K9MBM3 A0A2K9MBM3_9RHOB 3-phosphoshikimate 1-carboxyvinyltr... | 472 | 7e-162 |
| TR:A0A285CVT6 A0A285CVT6_9RHOB 3-phosphoshikimate 1-carboxyvinyltr... | 472 | 8e-162 |
| TR:A0A5C6S3Q9 A0A5C6S3Q9_9RHOB 3-phosphoshikimate 1-carboxyvinyltr... | 472 | 9e-162 |
| TR:W9GYC0 W9GYC0_9PROT 3-phosphoshikimate 1-carboxyvinyltransferas... | 472 | 1e-161 |
| TR:A0A1H0HB24 A0A1H0HB24_9BRAD 3-phosphoshikimate 1-carboxyvinyltr... | 472 | 1e-161 |
| TR:A0A6I2LXR7 A0A6I2LXR7_9RHOB 3-phosphoshikimate 1-carboxyvinyltr... | 472 | 1e-161 |
| TR:A0A086P9P1 A0A086P9P1_SPHHM 3-phosphoshikimate 1-carboxyvinyltr... | 471 | 1e-161 |
| TR:A0A1M7AZS8 A0A1M7AZS8_9RHOB 3-phosphoshikimate 1-carboxyvinyltr... | 472 | 1e-161 |
| TR:A6FVB2 A6FVB2_9RHOB 3-phosphoshikimate 1-carboxyvinyltransferas... | 473 | 1e-161 |
| TR:A0A345WQF8 A0A345WQF8_9SPHN 3-phosphoshikimate 1-carboxyvinyltr... | 472 | 1e-161 |
| TR:V6F8R5 V6F8R5_MAGGM 3-phosphoshikimate 1-carboxyvinyltransferas... | 471 | 1e-161 |
| TR:A0A0Q4LTN4 A0A0Q4LTN4_9SPHN 3-phosphoshikimate 1-carboxyvinyltr... | 471 | 2e-161 |
| TR:A0A0Q5PGG0 A0A0Q5PGG0_9SPHN 3-phosphoshikimate 1-carboxyvinyltr... | 471 | 2e-161 |
| TR:A0A270BER9 A0A270BER9_9SPHN 3-phosphoshikimate 1-carboxyvinyltr... | 471 | 2e-161 |
| TR:A0A5B8SAG7 A0A5B8SAG7_9SPHN 3-phosphoshikimate 1-carboxyvinyltr... | 471 | 2e-161 |

| TR:A0A1W2AYS5 A0A1W2AYS5_9SPHN 3-phosphoshikimate 1-carboxyvinyltr... | 471 | 2e-161 |
| --- | --- | --- |
| TR:A0A344PNB6 A0A344PNB6_9RHOB 3-phosphoshikimate 1-carboxyvinyltr... | 471 | 3e-161 |
| TR:A0A318APE2 A0A318APE2_9NOSO 3-phosphoshikimate 1-carboxyvinyltr... | 470 | 3e-161 |
| TR:I3TQL9 I3TQL9_TISMK 3-phosphoshikimate 1-carboxyvinyltransferas... | 471 | 3e-161 |
| TR:A0A2M8ZTG5 A0A2M8ZTG5_9BRAD 3-phosphoshikimate 1-carboxyvinyltr... | 471 | 3e-161 |
| TR:A0A2T6B1R4 A0A2T6B1R4_9RHOB 3-phosphoshikimate 1-carboxyvinyltr... | 471 | 3e-161 |
| TR:A0A653J0V8 A0A653J0V8_9SPHN 5-enolpyruvylshikimate-3-phosphate ... | 471 | 3e-161 |
| TR:A0A433S7E0 A0A433S7E0_9RHOB 3-phosphoshikimate 1-carboxyvinyltr... | 471 | 3e-161 |
| TR:A0A2N0I1D9 A0A2N0I1D9_9SPHN 3-phosphoshikimate 1-carboxyvinyltr... | 470 | 3e-161 |
| TR:A0A0B9A7G2 A0A0B9A7G2_9SPHN 3-phosphoshikimate 1-carboxyvinyltr... | 471 | 3e-161 |
| TR:A0A2A4CT29 A0A2A4CT29_9RHOB 3-phosphoshikimate 1-carboxyvinyltr... | 471 | 3e-161 |
| TR:A0A1M7SHE3 A0A1M7SHE3_9RHOB 3-phosphoshikimate 1-carboxyvinyltr... | 471 | 3e-161 |
| TR:A0A1L6JBZ1 A0A1L6JBZ1_9SPHN 3-phosphoshikimate 1-carboxyvinyltr... | 470 | 4e-161 |
| TR:A0A4Q8R376 A0A4Q8R376_9BRAD 3-phosphoshikimate 1-carboxyvinyltr... | 471 | 4e-161 |
| TR:Q1N9C5 Q1N9C5_SPHSS 3-phosphoshikimate 1-carboxyvinyltransferas... | 470 | 5e-161 |
| TR:A0A1G6SWQ8 A0A1G6SWQ8_9RHOB 3-phosphoshikimate 1-carboxyvinyltr... | 470 | 5e-161 |
| SP:Q1GT16 AROA_SPHAL 3-phosphoshikimate 1-carboxyvinyltransferase ... | 470 | 6e-161 |
| TR:A0A2N4YCK0 A0A2N4YCK0_9RHOB 3-phosphoshikimate 1-carboxyvinyltr... | 470 | 6e-161 |
| TR:A0A1B1PU59 A0A1B1PU59_9RHOB 3-phosphoshikimate 1-carboxyvinyltr... | 470 | 6e-161 |
| TR:A0A0B1ZMU5 A0A0B1ZMU5_9SPHN 3-phosphoshikimate 1-carboxyvinyltr... | 469 | 6e-161 |
| TR:A0A2T5BSU7 A0A2T5BSU7_9RHOB 3-phosphoshikimate 1-carboxyvinyltr... | 470 | 7e-161 |
| TR:A0A4R3J859 A0A4R3J859_9PROT 3-phosphoshikimate 1-carboxyvinyltr... | 471 | 8e-161 |
| TR:J2WMU7 J2WMU7_9SPHN 3-phosphoshikimate 1-carboxyvinyltransferas... | 469 | 8e-161 |
| TR:A0A519QKA0 A0A519QKA0_9SPHN 3-phosphoshikimate 1-carboxyvinyltr... | 469 | 9e-161 |
| SP:Q3SW64 AROA_NITWN 3-phosphoshikimate 1-carboxyvinyltransferase ... | 469 | 9e-161 |
| TR:A0A2M8MYX0 A0A2M8MYX0_9RHOB 3-phosphoshikimate 1-carboxyvinyltr... | 469 | 1e-160 |
| TR:A0A345ZZJ5 A0A345ZZJ5_9RHIZ 3-phosphoshikimate 1-carboxyvinyltr... | 469 | 1e-160 |
| TR:A0A1E3W7F1 A0A1E3W7F1_9RHIZ 3-phosphoshikimate 1-carboxyvinyltr... | 469 | 1e-160 |
| SP:Q2VZA8 AROA_MAGSA 3-phosphoshikimate 1-carboxyvinyltransferase ... | 469 | 1e-160 |
| TR:A0A371B9F8 A0A371B9F8_9RHIZ 3-phosphoshikimate 1-carboxyvinyltr... | 469 | 1e-160 |
| TR:A0A1H2VRD4 A0A1H2VRD4_9RHOB 3-phosphoshikimate 1-carboxyvinyltr... | 469 | 1e-160 |
| TR:J9A1B1 J9A1B1_9RHOB 3-phosphoshikimate 1-carboxyvinyltransferas... | 469 | 1e-160 |
| TR:A5V951 A5V951_SPHWW 3-phosphoshikimate 1-carboxyvinyltransferas... | 470 | 1e-160 |
| TR:A0A143DCJ6 A0A143DCJ6_9PROT 3-phosphoshikimate 1-carboxyvinyltr... | 469 | 1e-160 |
| TR:Q2CFP5 Q2CFP5_OCEGH 3-phosphoshikimate 1-carboxyvinyltransferas... | 469 | 1e-160 |
| TR:A0A0N1C7H9 A0A0N1C7H9_9SPHN 3-phosphoshikimate 1-carboxyvinyltr... | 469 | 1e-160 |
| TR:A0A1G6IPI3 A0A1G6IPI3_9SPHN 3-phosphoshikimate 1-carboxyvinyltr... | 469 | 2e-160 |
| TR:A0A1I3JQY6 A0A1I3JQY6_9RHOB 3-phosphoshikimate 1-carboxyvinyltr... | 469 | 2e-160 |
| TR:A0A501XLH4 A0A501XLH4_9SPHN 3-phosphoshikimate 1-carboxyvinyltr... | 469 | 2e-160 |
| TR:A0A4V3DEL1 A0A4V3DEL1_9PROT 3-phosphoshikimate 1-carboxyvinyltr... | 470 | 2e-160 |
| TR:A0A162HPC4 A0A162HPC4_9SPHN 3-phosphoshikimate 1-carboxyvinyltr... | 468 | 2e-160 |
| TR:A0A4Q3Y7Y1 A0A4Q3Y7Y1_9RHOB 3-phosphoshikimate 1-carboxyvinyltr... | 469 | 2e-160 |
| TR:A0A1M7EI62 A0A1M7EI62_9RHOB 3-phosphoshikimate 1-carboxyvinyltr... | 468 | 2e-160 |
| TR:A0A0P6WZ26 A0A0P6WZ26_9SPHN 3-phosphoshikimate 1-carboxyvinyltr... | 468 | 3e-160 |
| TR:A0A2S0UIG8 A0A2S0UIG8_9RHOB 3-phosphoshikimate 1-carboxyvinyltr... | 468 | 3e-160 |
| TR:A0A1A6FP93 A0A1A6FP93_9RHIZ 3-phosphoshikimate 1-carboxyvinyltr... | 468 | 3e-160 |
| TR:A0A6B8KGQ5 A0A6B8KGQ5_9RHIZ 3-phosphoshikimate 1-carboxyvinyltr... | 468 | 3e-160 |
| SP:Q28UI7 AROA_JANSC 3-phosphoshikimate 1-carboxyvinyltransferase ... | 468 | 3e-160 |
| TR:A0A1G7I7F8 A0A1G7I7F8_9RHOB 3-phosphoshikimate 1-carboxyvinyltr... | 468 | 3e-160 |
| TR:A3VMP1 A3VMP1_9RHOB 3-phosphoshikimate 1-carboxyvinyltransferas... | 468 | 3e-160 |
| TR:A0A0F7KQ62 A0A0F7KQ62_9SPHN 3-phosphoshikimate 1-carboxyvinyltr... | 468 | 3e-160 |
| TR:A0A2U2CB24 A0A2U2CB24_9RHOB 3-phosphoshikimate 1-carboxyvinyltr... | 468 | 4e-160 |
| TR:A0A1H8E361 A0A1H8E361_9RHOB 3-phosphoshikimate 1-carboxyvinyltr... | 468 | 4e-160 |
| TR:A0A1C3RFT6 A0A1C3RFT6_9RHIZ 3-phosphoshikimate 1-carboxyvinyltr... | 468 | 4e-160 |
| TR:A0A0P1IBT7 A0A0P1IBT7_9RHOB 3-phosphoshikimate 1-carboxyvinyltr... | 468 | 4e-160 |
| TR:A0A0N1AGP7 A0A0N1AGP7_9SPHN 3-phosphoshikimate 1-carboxyvinyltr... | 468 | 4e-160 |
| TR:A0A1G8IVD3 A0A1G8IVD3_9RHOB 3-phosphoshikimate 1-carboxyvinyltr... | 468 | 4e-160 |
| TR:A0A1G8L1U6 A0A1G8L1U6_9RHOB 3-phosphoshikimate 1-carboxyvinyltr... | 468 | 5e-160 |
| TR:A0A1I1IH10 A0A1I1IH10_9RHOB 3-phosphoshikimate 1-carboxyvinyltr... | 468 | 5e-160 |
| TR:A3TVC1 A3TVC1_PSEBH 3-phosphoshikimate 1-carboxyvinyltransferas... | 468 | 6e-160 |
| TR:A0A099GGJ7 A0A099GGJ7_9RHOB 3-phosphoshikimate 1-carboxyvinyltr... | 467 | 6e-160 |
| SP:A4YJJ3 AROA_BRASO 3-phosphoshikimate 1-carboxyvinyltransferase ... | 467 | 7e-160 |
| TR:A0A0D1P864 A0A0D1P864_BRAEL 3-phosphoshikimate 1-carboxyvinyltr... | 467 | 7e-160 |
| TR:A0A0A8JZM6 A0A0A8JZM6_9RHIZ 3-phosphoshikimate 1-carboxyvinyltr... | 467 | 7e-160 |

| TR:E3I1X1 E3I1X1_RHOVT 3-phosphoshikimate 1-carboxyvinyltransferas... | 468 | 8e-160 |
| --- | --- | --- |
| TR:A0A1G9CEE8 A0A1G9CEE8_9RHOB 3-phosphoshikimate 1-carboxyvinyltr... | 467 | 8e-160 |
| TR:A0A238KIG0 A0A238KIG0_9RHOB 3-phosphoshikimate 1-carboxyvinyltr... | 467 | 1e-159 |
| TR:A0A2R8BL89 A0A2R8BL89_9RHOB 3-phosphoshikimate 1-carboxyvinyltr... | 468 | 1e-159 |
| TR:A0A2T0WG05 A0A2T0WG05_9RHOB 3-phosphoshikimate 1-carboxyvinyltr... | 467 | 1e-159 |
| TR:A0A1H4DXU0 A0A1H4DXU0_9RHOB 3-phosphoshikimate 1-carboxyvinyltr... | 467 | 1e-159 |
| TR:A0A178MXK9 A0A178MXK9_9PROT 3-phosphoshikimate 1-carboxyvinyltr... | 466 | 1e-159 |
| TR:A0A255Y420 A0A255Y420_9SPHN 3-phosphoshikimate 1-carboxyvinyltr... | 467 | 1e-159 |
| TR:A0A1B9YTF6 A0A1B9YTF6_9BRAD 3-phosphoshikimate 1-carboxyvinyltr... | 466 | 1e-159 |
| TR:A0A2T4JV10 A0A2T4JV10_9RHOB 3-phosphoshikimate 1-carboxyvinyltr... | 466 | 1e-159 |
| TR:A0A1Y5SJI9 A0A1Y5SJI9_9RHOB 3-phosphoshikimate 1-carboxyvinyltr... | 466 | 2e-159 |
| TR:A0A085TV59 A0A085TV59_9RHOB 3-phosphoshikimate 1-carboxyvinyltr... | 466 | 2e-159 |
| TR:A0A2N4WW35 A0A2N4WW35_9SPHN 3-phosphoshikimate 1-carboxyvinyltr... | 466 | 2e-159 |
| TR:A0A2S8EUA7 A0A2S8EUA7_9RHOB 3-phosphoshikimate 1-carboxyvinyltr... | 466 | 2e-159 |
| TR:A0A1V4I2U5 A0A1V4I2U5_NITVU 3-phosphoshikimate 1-carboxyvinyltr... | 466 | 2e-159 |
| TR:A0A1N7L779 A0A1N7L779_9RHOB 3-phosphoshikimate 1-carboxyvinyltr... | 466 | 2e-159 |
| TR:A0A5B8FYG0 A0A5B8FYG0_9RHOB 3-phosphoshikimate 1-carboxyvinyltr... | 466 | 2e-159 |
| TR:A0A369R352 A0A369R352_9RHOB 3-phosphoshikimate 1-carboxyvinyltr... | 466 | 2e-159 |
| TR:A0A255XKT7A0A255XKT7_9PROT 3-phosphoshikimate 1-carboxyvinyltr... | 466 | 3e-159 |
| SP:A5E8B1AROA_BRASB 3-phosphoshikimate 1-carboxyvinyltransferase ... | 466 | 3e-159 |
| TR:A0A1H7R4W2A0A1H7R4W2_9RHOB 3-phosphoshikimate 1-carboxyvinyltr... | 466 | 3e-159 |
| TR:X5MM39X5MM39_9RHIZ 3-phosphoshikimate 1-carboxyvinyltransferas... | 466 | 3e-159 |
| TR:A0A1V2ERM3A0A1V2ERM3_9SPHN 3-phosphoshikimate 1-carboxyvinyltr... | 466 | 3e-159 |
| TR:A0A1Z1FAB9A0A1Z1FAB9_9SPHN 3-phosphoshikimate 1-carboxyvinyltr... | 465 | 3e-159 |
| TR:A0A5B8ISS2A0A5B8ISS2_9RHOB 3-phosphoshikimate 1-carboxyvinyltr... | 466 | 3e-159 |
| TR:A0A2T7FT02A0A2T7FT02_9RHOB 3-phosphoshikimate 1-carboxyvinyltr... | 466 | 3e-159 |
| TR:A0A0S6X004A0A0S6X004_9SPHN 3-phosphoshikimate 1-carboxyvinyltr... | 466 | 3e-159 |
| TR:A0A238LFV7A0A238LFV7_9RHOB 3-phosphoshikimate 1-carboxyvinyltr... | 466 | 3e-159 |
| SP:Q5NLJ0AROA_ZYMMO 3-phosphoshikimate 1-carboxyvinyltransferase ... | 466 | 4e-159 |
| TR:A0A5S3Q194A0A5S3Q194_9SPHN 3-phosphoshikimate 1-carboxyvinyltr... | 465 | 4e-159 |
| TR:A0A173KRX1A0A173KRX1_9SPHN 3-phosphoshikimate 1-carboxyvinyltr... | 465 | 4e-159 |
| TR:A0A4R2PNT8A0A4R2PNT8_9SPHN 3-phosphoshikimate 1-carboxyvinyltr... | 465 | 4e-159 |
| TR:A0A4U8YW71A0A4U8YW71_METTU 3-phosphoshikimate 1-carboxyvinyltr... | 466 | 4e-159 |
| TR:A0A1Y5TFL0A0A1Y5TFL0_9RHOB 3-phosphoshikimate 1-carboxyvinyltr... | 465 | 4e-159 |
| TR:A0A0E9MP87A0A0E9MP87_9SPHN 3-phosphoshikimate 1-carboxyvinyltr... | 465 | 4e-159 |
| TR:A0A1I6LEQ4A0A1I6LEQ4_9RHOB 3-phosphoshikimate 1-carboxyvinyltr... | 465 | 5e-159 |
| TR:A0A2A4B7J2A0A2A4B7J2_9SPHN 3-phosphoshikimate 1-carboxyvinyltr... | 465 | 6e-159 |
| TR:A0A4S3M9A2A0A4S3M9A2_9RHOB 3-phosphoshikimate 1-carboxyvinyltr... | 465 | 6e-159 |
| TR:A0A2T4JLP3A0A2T4JLP3_9RHOB 3-phosphoshikimate 1-carboxyvinyltr... | 465 | 6e-159 |
| TR:X7ED50X7ED50_9RHOB 3-phosphoshikimate 1-carboxyvinyltransferas... | 465 | 7e-159 |
| TR:A0A102D4W1A0A102D4W1_9SPHN 3-phosphoshikimate 1-carboxyvinyltr... | 464 | 7e-159 |
| TR:A0A0T5P6G2A0A0T5P6G2_9RHOB 3-phosphoshikimate 1-carboxyvinyltr... | 464 | 8e-159 |
| TR:A0A1N6HFQ5A0A1N6HFQ5_9SPHN 3-phosphoshikimate 1-carboxyvinyltr... | 464 | 1e-158 |
| TR:A0A437MBA2A0A437MBA2_9SPHN 3-phosphoshikimate 1-carboxyvinyltr... | 464 | 1e-158 |
| TR:A0A1X6YQD2A0A1X6YQD2_9RHOB 3-phosphoshikimate 1-carboxyvinyltr... | 464 | 1e-158 |
| TR:A0A2S5JGE7A0A2S5JGE7_9RHOB 3-phosphoshikimate 1-carboxyvinyltr... | 464 | 1e-158 |
| TR:A0A254R7C9A0A254R7C9_9RHOB 3-phosphoshikimate 1-carboxyvinyltr... | 464 | 1e-158 |
| TR:A0A0T0PV02A0A0T0PV02_9SPHN 3-phosphoshikimate 1-carboxyvinyltr... | 464 | 1e-158 |
| TR:W6K5E0W6K5E0_MAGSQ 3-phosphoshikimate 1-carboxyvinyltransferas... | 464 | 1e-158 |
| TR:A0A512JLS6A0A512JLS6_9RHIZ 3-phosphoshikimate 1-carboxyvinyltr... | 462 | 2e-158 |
| TR:A0A1Q4UM12A0A1Q4UM12_9PROT 3-phosphoshikimate 1-carboxyvinyltr... | 464 | 2e-158 |
| TR:A0A2V1P9G5A0A2V1P9G5_9RHOB 3-phosphoshikimate 1-carboxyvinyltr... | 464 | 2e-158 |
| SP:Q161H2AROA_ROSDO 3-phosphoshikimate 1-carboxyvinyltransferase ... | 464 | 2e-158 |
| TR:A0A1Y5TL63A0A1Y5TL63_9RHOB 3-phosphoshikimate 1-carboxyvinyltr... | 464 | 2e-158 |
| TR:A0A4R5VFI5A0A4R5VFI5_9RHOB 3-phosphoshikimate 1-carboxyvinyltr... | 464 | 2e-158 |
| TR:A0A1H7J519A0A1H7J519_9RHOB 3-phosphoshikimate 1-carboxyvinyltr... | 464 | 2e-158 |
| TR:A0A2V3U9S4A0A2V3U9S4_9RHIZ 3-phosphoshikimate 1-carboxyvinyltr... | 464 | 2e-158 |
| TR:A0A345YFU7A0A345YFU7_9SPHN 3-phosphoshikimate 1-carboxyvinyltr... | 462 | 3e-158 |
| TR:A0A3D9YY55A0A3D9YY55_9RHIZ 3-phosphoshikimate 1-carboxyvinyltr... | 463 | 3e-158 |
| TR:A0A1E1EZU7A0A1E1EZU7_9SPHN 3-phosphoshikimate 1-carboxyvinyltr... | 462 | 3e-158 |
| TR:A0A5C5G9I9A0A5C5G9I9_9RHOB 3-phosphoshikimate 1-carboxyvinyltr... | 463 | 3e-158 |
| TR:A0A327YR89A0A327YR89_9RHOB 3-phosphoshikimate 1-carboxyvinyltr... | 463 | 3e-158 |
| TR:A0A1N6GL98A0A1N6GL98_9RHOB 3-phosphoshikimate 1-carboxyvinyltr... | 463 | 4e-158 |
| TR:A0A3N4UFX5A0A3N4UFX5_9RHOB 3-phosphoshikimate 1-carboxyvinyltr... | 463 | 4e-158 |

| TR:A0A1Y5STT7A0A1Y5STT7_9RHOB 3-phosphoshikimate 1-carboxyvinyltr... | 463 | 4e-158 |
| --- | --- | --- |
| TR:A5P880A5P880_9SPHN 3-phosphoshikimate 1-carboxyvinyltransferas... | 463 | 4e-158 |
| TR:U4UTC8U4UTC8_9RHOB 3-phosphoshikimate 1-carboxyvinyltransferas... | 463 | 4e-158 |
| TR:X6L3T3X6L3T3_9RHOB 3-phosphoshikimate 1-carboxyvinyltransferas... | 463 | 4e-158 |
| TR:A0A4R7DJX5A0A4R7DJX5_9SPHN 3-phosphoshikimate 1-carboxyvinyltr... | 463 | 4e-158 |
| TR:A0A1X7Q015A0A1X7Q015_AZOLI 3-phosphoshikimate 1-carboxyvinyltr... | 462 | 4e-158 |
| TR:A0A541BXA9A0A541BXA9_9PROT 3-phosphoshikimate 1-carboxyvinyltr... | 462 | 5e-158 |
| TR:A0A2U2CV72A0A2U2CV72_9RHOB 3-phosphoshikimate 1-carboxyvinyltr... | 462 | 5e-158 |
| TR:A0A0G3X675A0A0G3X675_9SPHN 3-phosphoshikimate 1-carboxyvinyltr... | 462 | 5e-158 |
| TR:A0A109BJL3A0A109BJL3_HYPSL 3-phosphoshikimate 1-carboxyvinyltr... | 462 | 5e-158 |
| TR:A0A1M4MWU0A0A1M4MWU0_9RHOB 3-phosphoshikimate 1-carboxyvinyltr... | 462 | 5e-158 |
| TR:A0A1Y6B525A0A1Y6B525_9PROT 3-phosphoshikimate 1-carboxyvinyltr... | 462 | 5e-158 |
| TR:A0A1I4HEY5A0A1I4HEY5_9RHOB 3-phosphoshikimate 1-carboxyvinyltr... | 462 | 5e-158 |
| TR:A0A420EEX1A0A420EEX1_9SPHN 3-phosphoshikimate 1-carboxyvinyltr... | 462 | 5e-158 |
| TR:A0A396RMA9A0A396RMA9_9SPHN 3-phosphoshikimate 1-carboxyvinyltr... | 462 | 6e-158 |
| TR:A0A1E3W241A0A1E3W241_9RHIZ 3-phosphoshikimate 1-carboxyvinyltr... | 462 | 6e-158 |
| TR:A0A2G4YNP1A0A2G4YNP1_9PROT 3-phosphoshikimate 1-carboxyvinyltr... | 462 | 6e-158 |
| TR:A0A0D1EC50A0A0D1EC50_9RHOB 3-phosphoshikimate 1-carboxyvinyltr... | 462 | 7e-158 |
| TR:A0A0P1GP33A0A0P1GP33_9RHOB 3-phosphoshikimate 1-carboxyvinyltr... | 462 | 7e-158 |
| TR:A0A2R8AD77A0A2R8AD77_9RHOB 3-phosphoshikimate 1-carboxyvinyltr... | 462 | 7e-158 |
| TR:A0A0Q4BZT6A0A0Q4BZT6_9SPHN 3-phosphoshikimate 1-carboxyvinyltr... | 462 | 7e-158 |
| TR:A0A1W7MGI4A0A1W7MGI4_9SPHN 3-phosphoshikimate 1-carboxyvinyltr... | 462 | 7e-158 |
| TR:A0A4R7BKF2A0A4R7BKF2_9RHIZ 3-phosphoshikimate 1-carboxyvinyltr... | 462 | 7e-158 |
| TR:A0A1M5PKB1A0A1M5PKB1_9RHOB 3-phosphoshikimate 1-carboxyvinyltr... | 462 | 7e-158 |
| TR:A0A1H9HHZ1A0A1H9HHZ1_9RHOB 3-phosphoshikimate 1-carboxyvinyltr... | 462 | 7e-158 |
| TR:A0A316GCN0A0A316GCN0_9RHOB 3-phosphoshikimate 1-carboxyvinyltr... | 462 | 8e-158 |
| TR:A0A0P1G8J6A0A0P1G8J6_THAGE 3-phosphoshikimate 1-carboxyvinyltr... | 462 | 8e-158 |
| TR:A0A0S9CK98A0A0S9CK98_9SPHN 3-phosphoshikimate 1-carboxyvinyltr... | 462 | 9e-158 |
| TR:A0A6I4ST49A0A6I4ST49_9SPHN 3-phosphoshikimate 1-carboxyvinyltr... | 462 | 9e-158 |
| TR:A0A0B3RIX8A0A0B3RIX8_9RHOB 3-phosphoshikimate 1-carboxyvinyltr... | 462 | 9e-158 |
| TR:A0A430BNN1A0A430BNN1_9SPHN 3-phosphoshikimate 1-carboxyvinyltr... | 462 | 1e-157 |
| TR:A0A1N6GJY7A0A1N6GJY7_9BRAD 3-phosphoshikimate 1-carboxyvinyltr... | 462 | 1e-157 |
| TR:A0A1I1JPW7A0A1I1JPW7_9RHOB 3-phosphoshikimate 1-carboxyvinyltr... | 462 | 1e-157 |
| TR:A0A257GK84A0A257GK84_9RHOB 3-phosphoshikimate 1-carboxyvinyltr... | 462 | 1e-157 |
| TR:A0A5J6MRI5A0A5J6MRI5_9PROT 3-phosphoshikimate 1-carboxyvinyltr... | 461 | 1e-157 |
| TR:A0A177HAA7A0A177HAA7_9RHOB 3-phosphoshikimate 1-carboxyvinyltr... | 462 | 1e-157 |
| TR:A0A2G8RDY5A0A2G8RDY5_9RHOB 3-phosphoshikimate 1-carboxyvinyltr... | 462 | 1e-157 |
| TR:B8EN74B8EN74_METSB 3-phosphoshikimate 1-carboxyvinyltransferas... | 461 | 1e-157 |
| TR:A0A0A0HVA3A0A0A0HVA3_9RHOB 3-phosphoshikimate 1-carboxyvinyltr... | 461 | 1e-157 |
| TR:Q5LLK7Q5LLK7_RUEPO 3-phosphoshikimate 1-carboxyvinyltransferas... | 461 | 1e-157 |
| TR:A0A1Y5TIY2A0A1Y5TIY2_9RHOB 3-phosphoshikimate 1-carboxyvinyltr... | 461 | 1e-157 |
| TR:A0A1X6YW36A0A1X6YW36_9RHOB 3-phosphoshikimate 1-carboxyvinyltr... | 461 | 1e-157 |
| TR:A0A220WB00A0A220WB00_9SPHN 3-phosphoshikimate 1-carboxyvinyltr... | 461 | 1e-157 |
| TR:A0A4T2AC03A0A4T2AC03_9RHOB 3-phosphoshikimate 1-carboxyvinyltr... | 461 | 2e-157 |
| TR:A0A3S0JGA3A0A3S0JGA3_9PROT 3-phosphoshikimate 1-carboxyvinyltr... | 461 | 2e-157 |
| TR:H0TSK7H0TSK7_9BRAD 3-phosphoshikimate 1-carboxyvinyltransferas... | 461 | 2e-157 |
| TR:A0A4P8EKJ2A0A4P8EKJ2_9RHOB 3-phosphoshikimate 1-carboxyvinyltr... | 461 | 2e-157 |
| TR:A0A418NWU6A0A418NWU6_9SPHN 3-phosphoshikimate 1-carboxyvinyltr... | 461 | 2e-157 |
| TR:A0A4S4NG94A0A4S4NG94_9RHOB 3-phosphoshikimate 1-carboxyvinyltr... | 461 | 2e-157 |
| TR:A0A4S3MQZ2A0A4S3MQZ2_9RHOB 3-phosphoshikimate 1-carboxyvinyltr... | 461 | 2e-157 |
| TR:A0A0N0V373A0A0N0V373_9RHOB 3-phosphoshikimate 1-carboxyvinyltr... | 461 | 2e-157 |
| TR:A0A1P8V0D8A0A1P8V0D8_9RHOB 3-phosphoshikimate 1-carboxyvinyltr... | 461 | 2e-157 |
| TR:A0A4R3J8L3A0A4R3J8L3_9RHOB 3-phosphoshikimate 1-carboxyvinyltr... | 461 | 2e-157 |
| TR:A0A2R8AGU5A0A2R8AGU5_9RHOB 3-phosphoshikimate 1-carboxyvinyltr... | 461 | 2e-157 |
| TR:A0A6I4T0J4A0A6I4T0J4_9SPHN 3-phosphoshikimate 1-carboxyvinyltr... | 461 | 2e-157 |
| TR:A0A2S2CTC9A0A2S2CTC9_9PROT 3-phosphoshikimate 1-carboxyvinyltr... | 461 | 3e-157 |
| TR:G7Z832G7Z832_AZOL4 3-phosphoshikimate 1-carboxyvinyltransferas... | 461 | 3e-157 |
| TR:A0A6I4UV89A0A6I4UV89_9SPHN 3-phosphoshikimate 1-carboxyvinyltr... | 460 | 3e-157 |
| TR:A0A086XYP7A0A086XYP7_9RHOB 3-phosphoshikimate 1-carboxyvinyltr... | 460 | 3e-157 |
| TR:A0A1X4J1N1A0A1X4J1N1_9RHOB 3-phosphoshikimate 1-carboxyvinyltr... | 461 | 3e-157 |
| TR:A0A6I3KKK6A0A6I3KKK6_9RHIZ 3-phosphoshikimate 1-carboxyvinyltr... | 462 | 4e-157 |
| TR:A0A1G4TT83A0A1G4TT83_9SPHN 3-phosphoshikimate 1-carboxyvinyltr... | 460 | 4e-157 |
| TR:A0A222E3N9A0A222E3N9_9RHOB 3-phosphoshikimate 1-carboxyvinyltr... | 460 | 4e-157 |
| TR:A0A0D8CB18A0A0D8CB18_9PROT 3-phosphoshikimate 1-carboxyvinyltr... | 460 | 4e-157 |

| TR:A0A6I4TT27A0A6I4TT27_9SPHN | 3-phosphoshikimate | 1-carboxyvinyltr... | 462 | 4e-157 |
| --- | --- | --- | --- | --- |
| TR:A0A1Y5S2K4A0A1Y5S2K4_9RHOB | 3-phosphoshikimate | 1-carboxyvinyltr... | 460 | 4e-157 |
| TR:A0A1E3LSR6A0A1E3LSR6_9SPHN | 3-phosphoshikimate | 1-carboxyvinyltr... | 460 | 5e-157 |
| TR:A0A1I0RAY2A0A1I0RAY2_9RHOB | 3-phosphoshikimate | 1-carboxyvinyltr... | 460 | 6e-157 |
| TR:A0A1N7M2F5A0A1N7M2F5_9RHOB | 3-phosphoshikimate | 1-carboxyvinyltr... | 460 | 6e-157 |
| TR:A0A5S3PLV5A0A5S3PLV5_9RHOB | 3-phosphoshikimate | 1-carboxyvinyltr... | 460 | 6e-157 |
| TR:A0A0G9MSP5A0A0G9MSP5_9SPHN | 3-phosphoshikimate | 1-carboxyvinyltr... | 459 | 7e-157 |
| TR:A0A418SMK7A0A418SMK7_9RHOB | 3-phosphoshikimate | 1-carboxyvinyltr... | 459 | 8e-157 |
| TR:A0A0Q4FH48A0A0Q4FH48_9SPHN | 3-phosphoshikimate | 1-carboxyvinyltr... | 460 | 8e-157 |
| TR:A0A2T4JCR4A0A2T4JCR4_RHOBL | 3-phosphoshikimate | 1-carboxyvinyltr... | 459 | 8e-157 |
| TR:A0A1H4JRI2A0A1H4JRI2_9RHOB | 3-phosphoshikimate | 1-carboxyvinyltr... | 459 | 8e-157 |
| TR:A0A177QBR3A0A177QBR3_9PROT | 3-phosphoshikimate | 1-carboxyvinyltr... | 459 | 8e-157 |
| TR:A0A1H9TTK9A0A1H9TTK9_9RHOB | 3-phosphoshikimate | 1-carboxyvinyltr... | 459 | 8e-157 |
| TR:A0A0W1QQ32A0A0W1QQ32_9SPHN | 3-phosphoshikimate | 1-carboxyvinyltr... | 459 | 9e-157 |
| TR:A0A553W9H6A0A553W9H6_9SPHN | 3-phosphoshikimate | 1-carboxyvinyltr... | 459 | 1e-156 |
| TR:A0A0A6D511A0A0A6D511_9SPHN | 3-phosphoshikimate | 1-carboxyvinyltr... | 459 | 1e-156 |
| TR:A0A1M6YY81A0A1M6YY81_9RHOB | 3-phosphoshikimate | 1-carboxyvinyltr... | 459 | 1e-156 |
| TR:A0A0W7WKN3A0A0W7WKN3_9RHOB | 3-phosphoshikimate | 1-carboxyvinyltr... | 459 | 1e-156 |
| TR:A0A1M6E7R4A0A1M6E7R4_9RHOB | 3-phosphoshikimate | 1-carboxyvinyltr... | 459 | 1e-156 |
| TR:A0A1V0PRC4A0A1V0PRC4_9RHOB | 3-phosphoshikimate | 1-carboxyvinyltr... | 459 | 1e-156 |
| TR:A0A1N7FU85A0A1N7FU85_9RHOB | 3-phosphoshikimate | 1-carboxyvinyltr... | 459 | 1e-156 |
| TR:A0A0L1JUW5A0A0L1JUW5_9RHOB | 3-phosphoshikimate | 1-carboxyvinyltr... | 459 | 1e-156 |
| TR:A0A6I4UXP0A0A6I4UXP0_9SPHN | 3-phosphoshikimate | 1-carboxyvinyltr... | 458 | 1e-156 |
| TR:A0A0D0Q8D4A0A0D0Q8D4_9RHOB | 3-phosphoshikimate | 1-carboxyvinyltr... | 459 | 1e-156 |
| TR:A0A1X9YA96A0A1X9YA96_9SPHN | 3-phosphoshikimate | 1-carboxyvinyltr... | 458 | 2e-156 |
| TR:A0A1M5KA71A0A1M5KA71_9RHOB | 3-phosphoshikimate | 1-carboxyvinyltr... | 459 | 2e-156 |
| TR:A0A4R3LUG0A0A4R3LUG0_9RHIZ | 3-phosphoshikimate | 1-carboxyvinyltr... | 459 | 2e-156 |
| TR:A0A5J5GAQ1A0A5J5GAQ1_9RHOB | 3-phosphoshikimate | 1-carboxyvinyltr... | 458 | 2e-156 |
| TR:A0A2G5K359A0A2G5K359_9RHOB | 3-phosphoshikimate | 1-carboxyvinyltr... | 458 | 2e-156 |
| TR:A0A3R9NJ21A0A3R9NJ21_9RHOB | 3-phosphoshikimate | 1-carboxyvinyltr... | 458 | 2e-156 |
| TR:A0A4R2NIA7A0A4R2NIA7_RHOAD | 3-phosphoshikimate | 1-carboxyvinyltr... | 458 | 3e-156 |
| TR:A0A5E7Z1V6A0A5E7Z1V6_9SPHN | 3-phosphoshikimate | 1-carboxyvinyltr... | 459 | 3e-156 |
| TR:A0A147EKK8A0A147EKK8_9SPHN | 3-phosphoshikimate | 1-carboxyvinyltr... | 458 | 3e-156 |
| TR:A0A1H8DHV6A0A1H8DHV6_9SPHN | 3-phosphoshikimate | 1-carboxyvinyltr... | 458 | 3e-156 |
| TR:A0A0T2QEJ9A0A0T2QEJ9_9SPHN | 3-phosphoshikimate | 1-carboxyvinyltr... | 458 | 3e-156 |
| TR:A0A2U1WUT3A0A2U1WUT3_9PROT | 3-phosphoshikimate | 1-carboxyvinyltr... | 458 | 3e-156 |
| TR:A0A1N7LMW2A0A1N7LMW2_9RHOB | 3-phosphoshikimate | 1-carboxyvinyltr... | 458 | 3e-156 |
| TR:A0A369TKE9A0A369TKE9_9RHOB | 3-phosphoshikimate | 1-carboxyvinyltr... | 458 | 3e-156 |
| TR:A0A6B0TJ83A0A6B0TJ83_9RHOB | 3-phosphoshikimate | 1-carboxyvinyltr... | 458 | 3e-156 |
| TR:I7EWL2I7EWL2_PHAIB 3-phosphoshikimate 1-carboxyvinyltransferas... | | | 458 | 3e-156 |
| TR:A0A4Y6W1K0A0A4Y6W1K0_9SPHN 3-phosphoshikimate 1-carboxyvinyltr... | | | 457 | 3e-156 |
| TR:A0A1G6T4L1A0A1G6T4L1_9PROT 3-phosphoshikimate 1-carboxyvinyltr... | | | 457 | 4e-156 |
| TR:J7Q3B3J7Q3B3_METSZ 3-phosphoshikimate 1-carboxyvinyltransferas... | | | 457 | 4e-156 |
| TR:A0A2C9CN24A0A2C9CN24_9RHOB 3-phosphoshikimate 1-carboxyvinyltr... | | | 457 | 4e-156 |
| TR:A0A017HBR8A0A017HBR8_9RHOB 3-phosphoshikimate 1-carboxyvinyltr... | | | 457 | 4e-156 |
| TR:M4S481M4S481_9SPHN 3-phosphoshikimate 1-carboxyvinyltransferas... | | | 458 | 4e-156 |
| TR:A0A4Q1ZR92A0A4Q1ZR92_9RHOB 3-phosphoshikimate 1-carboxyvinyltr... | | | 457 | 5e-156 |
| TR:X7FAG2X7FAG2_9RHOB 3-phosphoshikimate 1-carboxyvinyltransferas... | | | 457 | 5e-156 |
| TR:A0A1M6IKI5A0A1M6IKI5_9RHOB 3-phosphoshikimate 1-carboxyvinyltr... | | | 457 | 5e-156 |
| TR:A0A0P1IZS5A0A0P1IZS5_9RHOB 3-phosphoshikimate 1-carboxyvinyltr... | | | 457 | 5e-156 |
| TR:A0A239CT08A0A239CT08_9RHOB 3-phosphoshikimate 1-carboxyvinyltr... | | | 457 | 5e-156 |
| TR:A0A420WF73A0A420WF73_9RHOB 3-phosphoshikimate 1-carboxyvinyltr... | | | 458 | 5e-156 |
| TR:A0A1I6X9P2A0A1I6X9P2_9RHOB 3-phosphoshikimate 1-carboxyvinyltr... | | | 457 | 5e-156 |
| TR:A3WBH2A3WBH2_9SPHN 3-phosphoshikimate 1-carboxyvinyltransferas... | | | 457 | 6e-156 |
| TR:A0A2T7G4T1A0A2T7G4T1_9RHOB 3-phosphoshikimate 1-carboxyvinyltr... | | | 457 | 7e-156 |
| TR:A0A0J9E6Q4A0A0J9E6Q4_9RHOB 3-phosphoshikimate 1-carboxyvinyltr... | | | 457 | 7e-156 |
| TR:A0A438AF04A0A438AF04_9RHOB 3-phosphoshikimate 1-carboxyvinyltr... | | | 457 | 7e-156 |
| TR:A0A0Q4KZT0A0A0Q4KZT0_9SPHN 3-phosphoshikimate 1-carboxyvinyltr... | | | 457 | 8e-156 |
| TR:A0A0S3F0T0A0A0S3F0T0_9SPHN 3-phosphoshikimate 1-carboxyvinyltr... | | | 457 | 8e-156 |
| TR:T0HE62T0HE62_9SPHN 3-phosphoshikimate 1-carboxyvinyltransferas... | | | 456 | 9e-156 |
| TR:A0A2T8HWT8A0A2T8HWT8_9RHOB 3-phosphoshikimate 1-carboxyvinyltr... | | | 457 | 9e-156 |
| TR:A0A0M6YGQ0A0A0M6YGQ0_9RHOB 3-phosphoshikimate 1-carboxyvinyltr... | | | 457 | 9e-156 |
| TR:A0A0M6XNL2A0A0M6XNL2_9RHOB 3-phosphoshikimate 1-carboxyvinyltr... | | | 457 | 9e-156 |
| TR:A0A1G7ZTH3A0A1G7ZTH3_9RHIZ 3-phosphoshikimate 1-carboxyvinyltr... | | | 457 | 9e-156 |

| TR:A0A4Q5RPN9A0A4Q5RPN9_9SPHN 3-phosphoshikimate 1-carboxyvinyltr... | 456 | 1e-155 |
| --- | --- | --- |
| TR:A0A1B2AFG1A0A1B2AFG1_9SPHN 3-phosphoshikimate 1-carboxyvinyltr... | 457 | 1e-155 |
| TR:C7DAM7C7DAM7_9RHOB 3-phosphoshikimate 1-carboxyvinyltransferas... | 458 | 1e-155 |
| TR:A0A0Q4KQN9A0A0Q4KQN9_9SPHN 3-phosphoshikimate 1-carboxyvinyltr... | 456 | 1e-155 |
| TR:A3K578A3K578_9RHOB 3-phosphoshikimate 1-carboxyvinyltransferas... | 456 | 1e-155 |
| TR:A0A177NZI5A0A177NZI5_9RHIZ 3-phosphoshikimate 1-carboxyvinyltr... | 457 | 1e-155 |
| SP:Q1GK84AROA_RUEST 3-phosphoshikimate 1-carboxyvinyltransferase ... | 456 | 1e-155 |
| TR:A0A074N1C1A0A074N1C1_ERYLO 3-phosphoshikimate 1-carboxyvinyltr... | 456 | 1e-155 |
| TR:A0A0B4FA25A0A0B4FA25_9RHOB 3-phosphoshikimate 1-carboxyvinyltr... | 456 | 2e-155 |
| TR:A0A1H8U888A0A1H8U888_9RHOB 3-phosphoshikimate 1-carboxyvinyltr... | 456 | 2e-155 |
| TR:A0A0Q2V6V9A0A0Q2V6V9_9RHOB 3-phosphoshikimate 1-carboxyvinyltr... | 456 | 2e-155 |
| TR:A0A0N7LYT4A0A0N7LYT4_9RHOB 3-phosphoshikimate 1-carboxyvinyltr... | 456 | 2e-155 |
| TR:A0A2K9JJ61A0A2K9JJ61_9RHOB 3-phosphoshikimate 1-carboxyvinyltr... | 456 | 2e-155 |
| TR:A0A238VH83A0A238VH83_9RHOB 3-phosphoshikimate 1-carboxyvinyltr... | 456 | 2e-155 |
| TR:A0A1E5QBL9A0A1E5QBL9_9PROT 3-phosphoshikimate 1-carboxyvinyltr... | 456 | 2e-155 |
| TR:H8FW98H8FW98_PHAMO 3-phosphoshikimate 1-carboxyvinyltransferas... | 456 | 3e-155 |
| TR:A0A1I5W8S9A0A1I5W8S9_9RHOB 3-phosphoshikimate 1-carboxyvinyltr... | 456 | 3e-155 |
| TR:A0A345Q7J2A0A345Q7J2_9RHOB 3-phosphoshikimate 1-carboxyvinyltr... | 456 | 3e-155 |
| TR:A0A1I6K194A0A1I6K194_9SPHN 3-phosphoshikimate 1-carboxyvinyltr... | 456 | 3e-155 |
| TR:A0A0J5QIW5A0A0J5QIW5_9RHOB 3-phosphoshikimate 1-carboxyvinyltr... | 456 | 3e-155 |
| TR:A0A3D9BXA8A0A3D9BXA8_9RHOB 3-phosphoshikimate 1-carboxyvinyltr... | 456 | 3e-155 |
| TR:A0A397PDQ0A0A397PDQ0_9SPHN 3-phosphoshikimate 1-carboxyvinyltr... | 455 | 3e-155 |
| TR:A0A1A7QEI9A0A1A7QEI9_9SPHN 3-phosphoshikimate 1-carboxyvinyltr... | 455 | 3e-155 |
| TR:A0A0P7IY42A0A0P7IY42_9RHOB 3-phosphoshikimate 1-carboxyvinyltr... | 456 | 3e-155 |
| TR:A0A2V2LGZ6A0A2V2LGZ6_9RHOB 3-phosphoshikimate 1-carboxyvinyltr... | 455 | 4e-155 |
| TR:A0A0A0EHQ6A0A0A0EHQ6_9RHOB 3-phosphoshikimate 1-carboxyvinyltr... | 455 | 4e-155 |
| TR:A0A1I5KQS6A0A1I5KQS6_9RHOB 3-phosphoshikimate 1-carboxyvinyltr... | 455 | 4e-155 |
| TR:A0A0G9MYF4A0A0G9MYF4_9SPHN 3-phosphoshikimate 1-carboxyvinyltr... | 455 | 4e-155 |
| TR:A0A0F4RPF6A0A0F4RPF6_9RHOB 3-phosphoshikimate 1-carboxyvinyltr... | 455 | 4e-155 |
| TR:A0A1W2D0I0A0A1W2D0I0_9RHOB 3-phosphoshikimate 1-carboxyvinyltr... | 455 | 4e-155 |
| TR:A0A238JJ10A0A238JJ10_9RHOB 3-phosphoshikimate 1-carboxyvinyltr... | 455 | 4e-155 |
| TR:A0A149SIF4A0A149SIF4_GLUJA 3-phosphoshikimate 1-carboxyvinyltr... | 455 | 5e-155 |
| TR:A0A0P1GK05A0A0P1GK05_9RHOB 3-phosphoshikimate 1-carboxyvinyltr... | 455 | 5e-155 |
| TR:A0A3S2Z6W9A0A3S2Z6W9_9PROT 3-phosphoshikimate 1-carboxyvinyltr... | 456 | 5e-155 |
| TR:A0A2R8AYG3A0A2R8AYG3_9RHOB 3-phosphoshikimate 1-carboxyvinyltr... | 455 | 5e-155 |
| TR:A0A4P6MDW8A0A4P6MDW8_9RHOB 3-phosphoshikimate 1-carboxyvinyltr... | 455 | 5e-155 |
| TR:A0A0X3U0J3A0A0X3U0J3_9RHOB 3-phosphoshikimate 1-carboxyvinyltr... | 455 | 5e-155 |
| TR:A0A4R1YKR0A0A4R1YKR0_9RHOB 3-phosphoshikimate 1-carboxyvinyltr... | 455 | 6e-155 |
| TR:A0A0D6PHQ8A0A0D6PHQ8_9PROT 3-phosphoshikimate 1-carboxyvinyltr... | 454 | 6e-155 |
| TR:A0A0L6CWB8A0A0L6CWB8_9RHOB 3-phosphoshikimate 1-carboxyvinyltr... | 454 | 7e-155 |
| TR:D0CXL0D0CXL0_RUELI 3-phosphoshikimate 1-carboxyvinyltransferas... | 454 | 7e-155 |
| TR:A0A4R6FH24A0A4R6FH24_9SPHN 3-phosphoshikimate 1-carboxyvinyltr... | 454 | 7e-155 |
| TR:A0A2T6BDW7A0A2T6BDW7_9RHOB 3-phosphoshikimate 1-carboxyvinyltr... | 454 | 7e-155 |
| TR:A0A1B6Z7S1A0A1B6Z7S1_9SPHN 3-phosphoshikimate 1-carboxyvinyltr... | 454 | 8e-155 |
| TR:A0A366VYB4A0A366VYB4_9RHOB 3-phosphoshikimate 1-carboxyvinyltr... | 454 | 8e-155 |
| TR:A0A1G9G4A9A0A1G9G4A9_9RHOB 3-phosphoshikimate 1-carboxyvinyltr... | 454 | 8e-155 |
| TR:A0A1H5TGL1A0A1H5TGL1_9RHOB 3-phosphoshikimate 1-carboxyvinyltr... | 454 | 8e-155 |
| TR:A0A095CTK5A0A095CTK5_9RHOB 3-phosphoshikimate 1-carboxyvinyltr... | 454 | 8e-155 |
| TR:A0A3A1P9K9A0A3A1P9K9_9SPHN 3-phosphoshikimate 1-carboxyvinyltr... | 455 | 9e-155 |
| TR:A0A6I1E7E7A0A6I1E7E7_9RHOB 3-phosphoshikimate 1-carboxyvinyltr... | 454 | 9e-155 |
| TR:A0A0Q4GFH9A0A0Q4GFH9_9SPHN 3-phosphoshikimate 1-carboxyvinyltr... | 454 | 9e-155 |
| TR:A0A501WPG0A0A501WPG0_9RHOB 3-phosphoshikimate 1-carboxyvinyltr... | 454 | 9e-155 |
| TR:A0A225NFR5A0A225NFR5_9RHOB 3-phosphoshikimate 1-carboxyvinyltr... | 454 | 1e-154 |
| TR:A0A1L9P1T3A0A1L9P1T3_9RHOB 3-phosphoshikimate 1-carboxyvinyltr... | 454 | 1e-154 |
| TR:A0A0K0Y7X3A0A0K0Y7X3_9RHOB 3-phosphoshikimate 1-carboxyvinyltr... | 454 | 1e-154 |
| TR:A0A2A8HWP0A0A2A8HWP0_9SPHN 3-phosphoshikimate 1-carboxyvinyltr... | 454 | 1e-154 |
| TR:A0A5E7YMM6A0A5E7YMM6_9SPHN 3-phosphoshikimate 1-carboxyvinyltr... | 454 | 1e-154 |
| TR:A0A0N0K3L0A0A0N0K3L0_9PROT 3-phosphoshikimate 1-carboxyvinyltr... | 454 | 2e-154 |
| TR:A0A1Y5RTP6A0A1Y5RTP6_9RHOB 3-phosphoshikimate 1-carboxyvinyltr... | 454 | 2e-154 |
| TR:A0A4Q3Z914A0A4Q3Z914_9RHOB 3-phosphoshikimate 1-carboxyvinyltr... | 454 | 2e-154 |
| SP:A8LSF0AROA_DINSH 3-phosphoshikimate 1-carboxyvinyltransferase ... | 454 | 2e-154 |
| TR:A0A073J3K4A0A073J3K4_9RHOB 3-phosphoshikimate 1-carboxyvinyltr... | 454 | 2e-154 |
| TR:A0A1I0NGC7A0A1I0NGC7_9RHOB 3-phosphoshikimate 1-carboxyvinyltr... | 454 | 2e-154 |
| TR:A0A4Q3S5H7A0A4Q3S5H7_9CAUL 3-phosphoshikimate 1-carboxyvinyltr... | 453 | 2e-154 |

| TR:A0A515L861A0A515L861_9SPHN 3-phosphoshikimate 1-carboxyvinyltr... | 453 | 2e-154 |
| --- | --- | --- |
| TR:A0A159Z090A0A159Z090_9RHOB 3-phosphoshikimate 1-carboxyvinyltr... | 453 | 2e-154 |
| TR:A0A1H3J8C8A0A1H3J8C8_9RHOB 3-phosphoshikimate 1-carboxyvinyltr... | 453 | 2e-154 |
| TR:A0A0H0XM18A0A0H0XM18_9SPHN 3-phosphoshikimate 1-carboxyvinyltr... | 453 | 2e-154 |
| TR:A0A0D6B0L2A0A0D6B0L2_RHOSU 3-phosphoshikimate 1-carboxyvinyltr... | 453 | 2e-154 |
| TR:A0A1L3JA28A0A1L3JA28_9SPHN 3-phosphoshikimate 1-carboxyvinyltr... | 453 | 3e-154 |
| TR:W8S1J0W8S1J0_9RHOB 3-phosphoshikimate 1-carboxyvinyltransferas... | 453 | 3e-154 |
| TR:A0A2V4MQV2A0A2V4MQV2_9RHOB 3-phosphoshikimate 1-carboxyvinyltr... | 453 | 3e-154 |
| TR:A0A418SJK8A0A418SJK8_9RHOB 3-phosphoshikimate 1-carboxyvinyltr... | 453 | 3e-154 |
| TR:A0A5B9ERT3A0A5B9ERT3_9RHOB 3-phosphoshikimate 1-carboxyvinyltr... | 453 | 3e-154 |
| TR:A0A061SMK3A0A061SMK3_9RHOB 3-phosphoshikimate 1-carboxyvinyltr... | 453 | 3e-154 |
| TR:A0A2S0MQP4A0A2S0MQP4_9RHOB 3-phosphoshikimate 1-carboxyvinyltr... | 453 | 3e-154 |
| TR:A0A3N5D7R2A0A3N5D7R2_9SPHN 3-phosphoshikimate 1-carboxyvinyltr... | 452 | 4e-154 |
| TR:A0A0P7ABE6A0A0P7ABE6_9SPHN 3-phosphoshikimate 1-carboxyvinyltr... | 452 | 4e-154 |
| TR:A0A4R6AJ93A0A4R6AJ93_9RHOB 3-phosphoshikimate 1-carboxyvinyltr... | 452 | 4e-154 |
| TR:A0A2T0RW67A0A2T0RW67_9RHOB 3-phosphoshikimate 1-carboxyvinyltr... | 452 | 4e-154 |
| TR:A0A291LWZ6A0A291LWZ6_9RHOB 3-phosphoshikimate 1-carboxyvinyltr... | 452 | 4e-154 |
| TR:A0A6I6IL87A0A6I6IL87_9RHOB 3-phosphoshikimate 1-carboxyvinyltr... | 452 | 5e-154 |
| TR:A0A5D0S280A0A5D0S280_9RHOB 3-phosphoshikimate 1-carboxyvinyltr... | 452 | 6e-154 |
| TR:A0A0T5NW50A0A0T5NW50_9RHOB 3-phosphoshikimate 1-carboxyvinyltr... | 452 | 6e-154 |
| TR:A0A1I2BJG6A0A1I2BJG6_9RHOB 3-phosphoshikimate 1-carboxyvinyltr... | 452 | 6e-154 |
| TR:A0A2U2CHT3A0A2U2CHT3_9RHOB 3-phosphoshikimate 1-carboxyvinyltr... | 452 | 6e-154 |
| TR:K5XU10K5XU10_9PROT 3-phosphoshikimate 1-carboxyvinyltransferas... | 452 | 7e-154 |
| TR:A0A1G5SPI1A0A1G5SPI1_9SPHN 3-phosphoshikimate 1-carboxyvinyltr... | 452 | 8e-154 |
| TR:A0A3S0R243A0A3S0R243_9SPHN 3-phosphoshikimate 1-carboxyvinyltr... | 452 | 9e-154 |
| TR:A0A251WZS5A0A251WZS5_9RHOB 3-phosphoshikimate 1-carboxyvinyltr... | 452 | 9e-154 |
| TR:A0A1G5R0G1A0A1G5R0G1_9RHOB 3-phosphoshikimate 1-carboxyvinyltr... | 452 | 9e-154 |
| TR:A0A3L9Y9L8A0A3L9Y9L8_9RHOB 3-phosphoshikimate 1-carboxyvinyltr... | 452 | 9e-154 |
| TR:A0A495RVR4A0A495RVR4_SPHMI 3-phosphoshikimate 1-carboxyvinyltr... | 451 | 1e-153 |
| TR:G8AI18G8AI18_AZOBR 3-phosphoshikimate 1-carboxyvinyltransferas... | 451 | 1e-153 |
| TR:A0A0B2C398A0A0B2C398_9SPHN 3-phosphoshikimate 1-carboxyvinyltr... | 451 | 1e-153 |
| TR:A0A1X6ZR25A0A1X6ZR25_9RHOB 3-phosphoshikimate 1-carboxyvinyltr... | 451 | 1e-153 |
| TR:A0A1W6MSJ3A0A1W6MSJ3_9RHIZ 3-phosphoshikimate 1-carboxyvinyltr... | 451 | 1e-153 |
| TR:A0A4P6XD25A0A4P6XD25_9SPHN 3-phosphoshikimate 1-carboxyvinyltr... | 451 | 1e-153 |
| TR:A0A1J0WEP3A0A1J0WEP3_9RHOB 3-phosphoshikimate 1-carboxyvinyltr... | 451 | 1e-153 |
| TR:A0A547PS09A0A547PS09_9RHOB 3-phosphoshikimate 1-carboxyvinyltr... | 451 | 1e-153 |
| TR:A0A318SQC0A0A318SQC0_9RHOB 3-phosphoshikimate 1-carboxyvinyltr... | 451 | 1e-153 |
| TR:A0A5E4NTY5A0A5E4NTY5_9RHIZ 3-phosphoshikimate 1-carboxyvinyltr... | 450 | 1e-153 |
| TR:A0A1X7PLV9A0A1X7PLV9_9RHOB 3-phosphoshikimate 1-carboxyvinyltr... | 451 | 2e-153 |
| TR:A0A132BXL7A0A132BXL7_9RHOB 3-phosphoshikimate 1-carboxyvinyltr... | 451 | 2e-153 |
| TR:A0A2T6KJT1A0A2T6KJT1_9RHOB 3-phosphoshikimate 1-carboxyvinyltr... | 451 | 2e-153 |
| TR:A0A316GJU1A0A316GJU1_9RHOB 3-phosphoshikimate 1-carboxyvinyltr... | 451 | 2e-153 |
| TR:B6AZG7B6AZG7_9RHOB 3-phosphoshikimate 1-carboxyvinyltransferas... | 451 | 2e-153 |
| TR:A0A1Y5RT01A0A1Y5RT01_9RHOB 3-phosphoshikimate 1-carboxyvinyltr... | 451 | 2e-153 |
| TR:A0A058ZK24A0A058ZK24_9RHOB 3-phosphoshikimate 1-carboxyvinyltr... | 451 | 2e-153 |
| TR:A0A2D2B3E2A0A2D2B3E2_9CAUL 3-phosphoshikimate 1-carboxyvinyltr... | 450 | 3e-153 |
| TR:A0A3N2QIQ6A0A3N2QIQ6_9SPHN 3-phosphoshikimate 1-carboxyvinyltr... | 451 | 3e-153 |
| TR:A0A5M6ISC3A0A5M6ISC3_9PROT 3-phosphoshikimate 1-carboxyvinyltr... | 451 | 3e-153 |
| SP:A5FUH8AROA_ACICJ 3-phosphoshikimate 1-carboxyvinyltransferase ... | 450 | 3e-153 |
| TR:C7JAT9C7JAT9_ACEP3 3-phosphoshikimate 1-carboxyvinyltransferas... | 451 | 3e-153 |
| TR:A0A0P1IH24A0A0P1IH24_9RHOB 3-phosphoshikimate 1-carboxyvinyltr... | 450 | 3e-153 |
| TR:B9NQC3B9NQC3_9RHOB 3-phosphoshikimate 1-carboxyvinyltransferas... | 450 | 3e-153 |
| TR:A0A2R7IMV2A0A2R7IMV2_9SPHN 3-phosphoshikimate 1-carboxyvinyltr... | 450 | 3e-153 |
| TR:A0A2R8BWF9A0A2R8BWF9_9RHOB 3-phosphoshikimate 1-carboxyvinyltr... | 450 | 4e-153 |
| TR:A0A1R3WIG5A0A1R3WIG5_9RHOB 3-phosphoshikimate 1-carboxyvinyltr... | 450 | 4e-153 |
| TR:A0A552U9H4A0A552U9H4_9SPHN 3-phosphoshikimate 1-carboxyvinyltr... | 450 | 4e-153 |
| SP:Q5FTC0AROA_GLUOX 3-phosphoshikimate 1-carboxyvinyltransferase ... | 450 | 4e-153 |
| TR:A0A511AVS7A0A511AVS7_9PROT 3-phosphoshikimate 1-carboxyvinyltr... | 450 | 4e-153 |
| TR:A0A2N5IMJ8A0A2N5IMJ8_9RHOB 3-phosphoshikimate 1-carboxyvinyltr... | 450 | 5e-153 |
| TR:A0A5C4MSW6A0A5C4MSW6_9RHOB 3-phosphoshikimate 1-carboxyvinyltr... | 450 | 5e-153 |
| TR:A0A518RDG4A0A518RDG4_9SPHN 3-phosphoshikimate 1-carboxyvinyltr... | 449 | 6e-153 |
| TR:S9QE22S9QE22_9RHOB 3-phosphoshikimate 1-carboxyvinyltransferas... | 449 | 7e-153 |
| TR:W4HLJ1W4HLJ1_9RHOB 3-phosphoshikimate 1-carboxyvinyltransferas... | 449 | 7e-153 |
| TR:A0A327LVW3A0A327LVW3_9RHOB 3-phosphoshikimate 1-carboxyvinyltr... | 449 | 7e-153 |

| TR:A0A1I1X9J9A0A1I1X9J9_9RHOB 3-phosphoshikimate 1-carboxyvinyltr... | 449 | 7e-153 |
| --- | --- | --- |
| TR:A0A4Y6UK17A0A4Y6UK17_9PROT 3-phosphoshikimate 1-carboxyvinyltr... | 449 | 8e-153 |
| TR:A0A369T4W2A0A369T4W2_9PROT 3-phosphoshikimate 1-carboxyvinyltr... | 449 | 8e-153 |
| TR:A0A0P1ETV2A0A0P1ETV2_9RHOB 3-phosphoshikimate 1-carboxyvinyltr... | 449 | 1e-152 |
| TR:A3JVZ6A3JVZ6_9RHOB 3-phosphoshikimate 1-carboxyvinyltransferas... | 449 | 1e-152 |
| TR:A0A1Y0ED40A0A1Y0ED40_9RHOB 3-phosphoshikimate 1-carboxyvinyltr... | 449 | 1e-152 |
| TR:A0A2P8FDJ9A0A2P8FDJ9_9RHOB 3-phosphoshikimate 1-carboxyvinyltr... | 449 | 1e-152 |
| TR:A0A238J0D4A0A238J0D4_9RHOB 3-phosphoshikimate 1-carboxyvinyltr... | 449 | 1e-152 |
| TR:A0A5C8PUG1A0A5C8PUG1_9PROT 3-phosphoshikimate 1-carboxyvinyltr... | 449 | 1e-152 |
| TR:A0A1G7HV38A0A1G7HV38_9SPHN 3-phosphoshikimate 1-carboxyvinyltr... | 449 | 1e-152 |
| TR:A0A1Y2QJ62A0A1Y2QJ62_9SPHN 3-phosphoshikimate 1-carboxyvinyltr... | 449 | 1e-152 |
| TR:A0A5C4ND40A0A5C4ND40_9RHOB 3-phosphoshikimate 1-carboxyvinyltr... | 449 | 1e-152 |
| TR:A0A0P1H3T1A0A0P1H3T1_9RHOB 3-phosphoshikimate 1-carboxyvinyltr... | 449 | 1e-152 |
| TR:A0A1B0ZTT6A0A1B0ZTT6_9RHOB 3-phosphoshikimate 1-carboxyvinyltr... | 449 | 1e-152 |
| TR:A0A4R6APD8A0A4R6APD8_9RHOB 3-phosphoshikimate 1-carboxyvinyltr... | 449 | 1e-152 |
| TR:A0A1N6KFY8A0A1N6KFY8_9RHOB 3-phosphoshikimate 1-carboxyvinyltr... | 449 | 1e-152 |
| TR:A0A0Q8XU24A0A0Q8XU24_9SPHN 3-phosphoshikimate 1-carboxyvinyltr... | 448 | 2e-152 |
| TR:A0A254R1H4A0A254R1H4_9RHOB 3-phosphoshikimate 1-carboxyvinyltr... | 449 | 2e-152 |
| TR:A0A097EGP5A0A097EGP5_9SPHN 3-phosphoshikimate 1-carboxyvinyltr... | 448 | 2e-152 |
| TR:A0A085BWP8A0A085BWP8_9RHOB 3-phosphoshikimate 1-carboxyvinyltr... | 448 | 2e-152 |
| TR:A0A196Q244A0A196Q244_9RHOB 3-phosphoshikimate 1-carboxyvinyltr... | 448 | 2e-152 |
| TR:A0A1M5BPI3A0A1M5BPI3_9RHOB 3-phosphoshikimate 1-carboxyvinyltr... | 448 | 2e-152 |
| TR:A0A0G3XGC4A0A0G3XGC4_9SPHN 3-phosphoshikimate 1-carboxyvinyltr... | 448 | 2e-152 |
| TR:A0A252B1M1A0A252B1M1_9PROT 3-phosphoshikimate 1-carboxyvinyltr... | 449 | 2e-152 |
| TR:A0A1U7DKK4A0A1U7DKK4_9RHOB 3-phosphoshikimate 1-carboxyvinyltr... | 449 | 2e-152 |
| TR:A0A0H4W1A1A0A0H4W1A1_9SPHN 3-phosphoshikimate 1-carboxyvinyltr... | 448 | 2e-152 |
| TR:A0A1C7D8I5A0A1C7D8I5_9SPHN 3-phosphoshikimate 1-carboxyvinyltr... | 448 | 3e-152 |
| TR:A0A1H3I200A0A1H3I200_9RHOB 3-phosphoshikimate 1-carboxyvinyltr... | 448 | 3e-152 |
| TR:A0A2M8IUE8A0A2M8IUE8_9RHOB 3-phosphoshikimate 1-carboxyvinyltr... | 448 | 3e-152 |
| TR:A9ECX1A9ECX1_9RHOB 3-phosphoshikimate 1-carboxyvinyltransferas... | 447 | 3e-152 |
| TR:A0A1I0WFU0A0A1I0WFU0_9RHOB 3-phosphoshikimate 1-carboxyvinyltr... | 447 | 4e-152 |
| TR:A0A3P5XV07A0A3P5XV07_9RHOB 3-phosphoshikimate 1-carboxyvinyltr... | 447 | 4e-152 |
| TR:A0A411ZBJ3A0A411ZBJ3_9SPHN 3-phosphoshikimate 1-carboxyvinyltr... | 446 | 4e-152 |
| TR:A0A4Y3TNS8A0A4Y3TNS8_9PROT 3-phosphoshikimate 1-carboxyvinyltr... | 447 | 4e-152 |
| TR:A0A5D0RIH4A0A5D0RIH4_9RHOB 3-phosphoshikimate 1-carboxyvinyltr... | 447 | 4e-152 |
| TR:A0A3P5WQR8A0A3P5WQR8_9RHOB 3-phosphoshikimate 1-carboxyvinyltr... | 447 | 4e-152 |
| TR:A0A654D0X4A0A654D0X4_9SPHN 5-enolpyruvylshikimate-3-phosphate ... | 447 | 4e-152 |
| TR:A0A1X6ZW24A0A1X6ZW24_9RHOB 3-phosphoshikimate 1-carboxyvinyltr... | 447 | 5e-152 |
| TR:A8TND2A8TND2_9PROT 3-phosphoshikimate 1-carboxyvinyltransferas... | 447 | 5e-152 |
| TR:A0A2Z5ZK85A0A2Z5ZK85_9PROT 3-phosphoshikimate 1-carboxyvinyltr... | 447 | 5e-152 |
| TR:A0A073IF13A0A073IF13_9RHOB 3-phosphoshikimate 1-carboxyvinyltr... | 447 | 6e-152 |
| TR:A0A399J061A0A399J061_9RHOB 3-phosphoshikimate 1-carboxyvinyltr... | 447 | 6e-152 |
| TR:A0A547PED7A0A547PED7_9SPHN 3-phosphoshikimate 1-carboxyvinyltr... | 447 | 6e-152 |
| TR:A0A345QJJ2A0A345QJJ2_9RHOB 3-phosphoshikimate 1-carboxyvinyltr... | 447 | 6e-152 |
| TR:A0A0Q3BGQ8A0A0Q3BGQ8_9RHOB 3-phosphoshikimate 1-carboxyvinyltr... | 447 | 6e-152 |
| TR:A0A0B4BQB8A0A0B4BQB8_9RHOB 3-phosphoshikimate 1-carboxyvinyltr... | 447 | 6e-152 |
| TR:A0A437GZZ6A0A437GZZ6_9SPHN 3-phosphoshikimate 1-carboxyvinyltr... | 447 | 7e-152 |
| TR:A0A345R870A0A345R870_9RHOB 3-phosphoshikimate 1-carboxyvinyltr... | 446 | 1e-151 |
| TR:A0A238JLW0A0A238JLW0_9RHOB 3-phosphoshikimate 1-carboxyvinyltr... | 446 | 1e-151 |
| TR:A0A1M6G9D5A0A1M6G9D5_9RHOB 3-phosphoshikimate 1-carboxyvinyltr... | 446 | 1e-151 |
| TR:A0A348G410A0A348G410_9RHIZ 3-phosphoshikimate 1-carboxyvinyltr... | 446 | 1e-151 |
| TR:K2HSP6K2HSP6_9RHOB 3-phosphoshikimate 1-carboxyvinyltransferas... | 446 | 1e-151 |
| TR:A0A1H7GPD2A0A1H7GPD2_9SPHN 3-phosphoshikimate 1-carboxyvinyltr... | 446 | 1e-151 |
| TR:A0A1X7BVD5A0A1X7BVD5_9RHOB 3-phosphoshikimate 1-carboxyvinyltr... | 447 | 1e-151 |
| TR:A0A2Z5YW92A0A2Z5YW92_9SPHN 3-phosphoshikimate 1-carboxyvinyltr... | 446 | 1e-151 |
| TR:B7RNH6B7RNH6_9RHOB 3-phosphoshikimate 1-carboxyvinyltransferas... | 446 | 2e-151 |
| TR:A0A238JZG9A0A238JZG9_9RHOB 3-phosphoshikimate 1-carboxyvinyltr... | 446 | 2e-151 |
| TR:A0A1G7JTB5A0A1G7JTB5_9PROT 3-phosphoshikimate 1-carboxyvinyltr... | 446 | 2e-151 |
| TR:A0A369VYQ8A0A369VYQ8_9SPHN 3-phosphoshikimate 1-carboxyvinyltr... | 446 | 2e-151 |
| TR:A0A1H3A1B8A0A1H3A1B8_9RHOB 3-phosphoshikimate 1-carboxyvinyltr... | 446 | 2e-151 |
| TR:G6XF63G6XF63_9PROT 3-phosphoshikimate 1-carboxyvinyltransferas... | 446 | 2e-151 |
| TR:M9RLK2M9RLK2_9RHOB 3-phosphoshikimate 1-carboxyvinyltransferas... | 446 | 2e-151 |
| TR:A0A1I6DPQ1A0A1I6DPQ1_9RHOB 3-phosphoshikimate 1-carboxyvinyltr... | 446 | 2e-151 |
| TR:A0A365U1J2A0A365U1J2_9RHOB 3-phosphoshikimate 1-carboxyvinyltr... | 445 | 3e-151 |

| TR:A0A1H6SF33A0A1H6SF33_9RHOB 3-phosphoshikimate 1-carboxyvinyltr... | 445 | 3e-151 |
| --- | --- | --- |
| TR:A0A6I4YV99A0A6I4YV99_9PROT 3-phosphoshikimate 1-carboxyvinyltr... | 444 | 5e-151 |
| TR:A0A285R0R1A0A285R0R1_9SPHN 3-phosphoshikimate 1-carboxyvinyltr... | 444 | 6e-151 |
| TR:A0A420DPM2A0A420DPM2_9RHOB 3-phosphoshikimate 1-carboxyvinyltr... | 444 | 7e-151 |
| TR:A0A023D1I9A0A023D1I9_ACIMT 3-phosphoshikimate 1-carboxyvinyltr... | 444 | 7e-151 |
| TR:A0A372ER64A0A372ER64_9RHOB 3-phosphoshikimate 1-carboxyvinyltr... | 444 | 7e-151 |
| TR:A0A1I5PJG7A0A1I5PJG7_9SPHN 3-phosphoshikimate 1-carboxyvinyltr... | 444 | 8e-151 |
| TR:F7QGN4F7QGN4_9BRAD 3-phosphoshikimate 1-carboxyvinyltransferas... | 442 | 8e-151 |
| TR:A0A037ZK79A0A037ZK79_9RHOB 3-phosphoshikimate 1-carboxyvinyltr... | 444 | 1e-150 |
| TR:A0A1H8A8X0A0A1H8A8X0_9RHOB 3-phosphoshikimate 1-carboxyvinyltr... | 444 | 1e-150 |
| TR:A0A4U7NC32A0A4U7NC32_9RHOB 3-phosphoshikimate 1-carboxyvinyltr... | 444 | 1e-150 |
| TR:Q2N9Q1Q2N9Q1_ERYLH 3-phosphoshikimate 1-carboxyvinyltransferas... | 444 | 1e-150 |
| TR:A0A1I4EKL6A0A1I4EKL6_9RHOB 3-phosphoshikimate 1-carboxyvinyltr... | 443 | 2e-150 |
| TR:A0A0Q5R802A0A0Q5R802_9SPHN 3-phosphoshikimate 1-carboxyvinyltr... | 443 | 3e-150 |
| TR:A0A074MGJ9A0A074MGJ9_9SPHN 3-phosphoshikimate 1-carboxyvinyltr... | 442 | 3e-150 |
| TR:A0A167JL53A0A167JL53_9SPHN 3-phosphoshikimate 1-carboxyvinyltr... | 442 | 3e-150 |
| TR:A0A1Y6E938A0A1Y6E938_9SPHN 3-phosphoshikimate 1-carboxyvinyltr... | 443 | 3e-150 |
| TR:A0A6I4TCE7A0A6I4TCE7_9SPHN 3-phosphoshikimate 1-carboxyvinyltr... | 442 | 3e-150 |
| TR:A0A502C335A0A502C335_9SPHN 3-phosphoshikimate 1-carboxyvinyltr... | 442 | 4e-150 |
| TR:A0A1X6ZIW3A0A1X6ZIW3_9RHOB 3-phosphoshikimate 1-carboxyvinyltr... | 442 | 4e-150 |
| TR:A0A2T0X491A0A2T0X491_9RHOB 3-phosphoshikimate 1-carboxyvinyltr... | 442 | 4e-150 |
| TR:A0A0H4KYP2A0A0H4KYP2_9RHOB 3-phosphoshikimate 1-carboxyvinyltr... | 442 | 4e-150 |
| TR:A0A1D8URR9A0A1D8URR9_9PROT 3-phosphoshikimate 1-carboxyvinyltr... | 442 | 5e-150 |
| TR:A0A3S2XSE4A0A3S2XSE4_9RHOB 3-phosphoshikimate 1-carboxyvinyltr... | 442 | 5e-150 |
| TR:A0A166PXV2A0A166PXV2_9RHOB 3-phosphoshikimate 1-carboxyvinyltr... | 442 | 7e-150 |
| TR:A0A2W7Q977A0A2W7Q977_9RHOB 3-phosphoshikimate 1-carboxyvinyltr... | 442 | 8e-150 |
| TR:S9SBJ9S9SBJ9_9RHOB 3-phosphoshikimate 1-carboxyvinyltransferas... | 442 | 8e-150 |
| TR:A0A1T4R9A7A0A1T4R9A7_9PROT 3-phosphoshikimate 1-carboxyvinyltr... | 442 | 9e-150 |
| TR:A0A2R8BGF3A0A2R8BGF3_9RHOB 3-phosphoshikimate 1-carboxyvinyltr... | 441 | 1e-149 |
| TR:A0A3A8B992A0A3A8B992_9RHOB 3-phosphoshikimate 1-carboxyvinyltr... | 441 | 1e-149 |
| TR:W0ABE3W0ABE3_9SPHN 3-phosphoshikimate 1-carboxyvinyltransferas... | 439 | 2e-149 |
| TR:A0A1B6YQZ6A0A1B6YQZ6_9RHOB 3-phosphoshikimate 1-carboxyvinyltr... | 441 | 2e-149 |
| TR:G2ISH4G2ISH4_SPHSK 3-phosphoshikimate 1-carboxyvinyltransferas... | 441 | 2e-149 |
| TR:T0J2J7T0J2J7_9SPHN 3-phosphoshikimate 1-carboxyvinyltransferas... | 439 | 2e-149 |
| TR:A0A0Q6L2M4A0A0Q6L2M4_9SPHN 3-phosphoshikimate 1-carboxyvinyltr... | 441 | 2e-149 |
| TR:A0A4Q3Z336A0A4Q3Z336_9RHOB 3-phosphoshikimate 1-carboxyvinyltr... | 441 | 2e-149 |
| TR:A0A1I3YES1A0A1I3YES1_9RHIZ 3-phosphoshikimate 1-carboxyvinyltr... | 440 | 3e-149 |
| TR:A0A4Y3TUR6A0A4Y3TUR6_9PROT 3-phosphoshikimate 1-carboxyvinyltr... | 440 | 3e-149 |
| TR:A0A109LSY3A0A109LSY3_9SPHN 3-phosphoshikimate 1-carboxyvinyltr... | 440 | 4e-149 |
| TR:A0A5C6UDU6A0A5C6UDU6_9SPHN 3-phosphoshikimate 1-carboxyvinyltr... | 440 | 4e-149 |
| TR:A0A0Q7CXH1A0A0Q7CXH1_9CAUL 3-phosphoshikimate 1-carboxyvinyltr... | 439 | 5e-149 |
| TR:A0A0V2F917A0A0V2F917_CAUVI 3-phosphoshikimate 1-carboxyvinyltr... | 439 | 5e-149 |
| TR:A0A2A2SGD6A0A2A2SGD6_9SPHN 3-phosphoshikimate 1-carboxyvinyltr... | 439 | 5e-149 |
| TR:U2ZDB7U2ZDB7_9CAUL 3-phosphoshikimate 1-carboxyvinyltransferas... | 439 | 6e-149 |
| TR:T0GRR0T0GRR0_9SPHN 3-phosphoshikimate 1-carboxyvinyltransferas... | 438 | 7e-149 |
| TR:A0A4Y9EPK2A0A4Y9EPK2_9SPHN 3-phosphoshikimate 1-carboxyvinyltr... | 439 | 9e-149 |
| TR:A0A372IHA5A0A372IHA5_9RHOB 3-phosphoshikimate 1-carboxyvinyltr... | 439 | 9e-149 |
| TR:Q0FD12Q0FD12_9RHOB 3-phosphoshikimate 1-carboxyvinyltransferas... | 438 | 2e-148 |
| TR:A0A6I5X2R3A0A6I5X2R3_9SPHN 3-phosphoshikimate 1-carboxyvinyltr... | 438 | 2e-148 |
| TR:A0A4P7JI44A0A4P7JI44_9RHOB 3-phosphoshikimate 1-carboxyvinyltr... | 438 | 2e-148 |
| TR:A0A564WEC5A0A564WEC5_9PROT 3-phosphoshikimate 1-carboxyvinyltr... | 437 | 3e-148 |
| TR:A0A1Y5RPP9A0A1Y5RPP9_9PROT 3-phosphoshikimate 1-carboxyvinyltr... | 437 | 3e-148 |
| TR:A0A6A7M7H1A0A6A7M7H1_9PROT 3-phosphoshikimate 1-carboxyvinyltr... | 439 | 3e-148 |
| TR:A0A1N6SJ63A0A1N6SJ63_ACIRU 3-phosphoshikimate 1-carboxyvinyltr... | 437 | 3e-148 |
| TR:A0A0U1NMI1A0A0U1NMI1_9RHOB 3-phosphoshikimate 1-carboxyvinyltr... | 437 | 5e-148 |
| TR:A0A347ULE6A0A347ULE6_9RHOB 3-phosphoshikimate 1-carboxyvinyltr... | 437 | 7e-148 |
| TR:A4EP52A4EP52_9RHOB 3-phosphoshikimate 1-carboxyvinyltransferas... | 436 | 8e-148 |
| TR:A0A1U9KTG0A0A1U9KTG0_9PROT 3-phosphoshikimate 1-carboxyvinyltr... | 437 | 8e-148 |
| SP:B8H6A8AROA_CAUVN 3-phosphoshikimate 1-carboxyvinyltransferase ... | 436 | 1e-147 |
| SP:Q9A2H2AROA_CAUVC 3-phosphoshikimate 1-carboxyvinyltransferase ... | 436 | 1e-147 |
| TR:A0A519IZI1A0A519IZI1_9CAUL 3-phosphoshikimate 1-carboxyvinyltr... | 436 | 1e-147 |
| TR:A0A3T0N5R9A0A3T0N5R9_9RHOB 3-phosphoshikimate 1-carboxyvinyltr... | 436 | 1e-147 |
| TR:A0A5B8LGT7A0A5B8LGT7_9SPHN 3-phosphoshikimate 1-carboxyvinyltr... | 436 | 2e-147 |
| TR:A0A328AJ37A0A328AJ37_9CAUL 3-phosphoshikimate 1-carboxyvinyltr... | 435 | 2e-147 |

| TR:C6XEX9C6XEX9_LIBAP 5-enolpyruvylshikimate-3-phosphate synthase... | 435 | 4e-147 |
| --- | --- | --- |
| TR:A0A192IH73A0A192IH73_9RHIZ 3-phosphoshikimate 1-carboxyvinyltr... | 434 | 5e-147 |
| TR:A0A0D6MHT9A0A0D6MHT9_9PROT 3-phosphoshikimate 1-carboxyvinyltr... | 434 | 5e-147 |
| TR:A0A1V2H0X3A0A1V2H0X3_9PROT 3-phosphoshikimate 1-carboxyvinyltr... | 434 | 1e-146 |
| TR:A0A6I4U658A0A6I4U658_9SPHN 3-phosphoshikimate 1-carboxyvinyltr... | 433 | 1e-146 |
| TR:A0A0H5BEH3A0A0H5BEH3_BLAVI 3-phosphoshikimate 1-carboxyvinyltr... | 434 | 1e-146 |
| TR:J9DFN9J9DFN9_9PROT 3-phosphoshikimate 1-carboxyvinyltransferas... | 434 | 1e-146 |
| TR:A0A5C1YPJ4A0A5C1YPJ4_9PROT 3-phosphoshikimate 1-carboxyvinyltr... | 433 | 2e-146 |
| TR:A0A328BC75A0A328BC75_9CAUL 3-phosphoshikimate 1-carboxyvinyltr... | 432 | 4e-146 |
| TR:A0A0U5ESW5A0A0U5ESW5_9PROT 3-phosphoshikimate 1-carboxyvinyltr... | 433 | 5e-146 |
| TR:G2KNZ0G2KNZ0_MICAA 3-phosphoshikimate 1-carboxyvinyltransferas... | 431 | 5e-146 |
| TR:A0A099TCY5A0A099TCY5_9RHOB 3-phosphoshikimate 1-carboxyvinyltr... | 432 | 6e-146 |
| TR:A0A1G6TC99A0A1G6TC99_9PROT 3-phosphoshikimate 1-carboxyvinyltr... | 431 | 9e-146 |
| TR:A0A257J1D8A0A257J1D8_9PROT 3-phosphoshikimate 1-carboxyvinyltr... | 431 | 1e-145 |
| TR:A0A2Z3HMC0A0A2Z3HMC0_9CAUL 3-phosphoshikimate 1-carboxyvinyltr... | 431 | 1e-145 |
| TR:A0A286GXN9A0A286GXN9_9PROT 3-phosphoshikimate 1-carboxyvinyltr... | 431 | 2e-145 |
| TR:A9H466A9H466_GLUDA 3-phosphoshikimate 1-carboxyvinyltransferas... | 431 | 2e-145 |
| TR:A0A514C493A0A514C493_9CAUL 3-phosphoshikimate 1-carboxyvinyltr... | 430 | 2e-145 |
| TR:A0A4U1L200A0A4U1L200_9SPHN 3-phosphoshikimate 1-carboxyvinyltr... | 430 | 3e-145 |
| TR:A0A0Q7TQI8A0A0Q7TQI8_9CAUL 3-phosphoshikimate 1-carboxyvinyltr... | 429 | 4e-145 |
| TR:A0A239PZI3A0A239PZI3_9PROT 3-phosphoshikimate 1-carboxyvinyltr... | 429 | 6e-145 |
| TR:A0A060QCV4A0A060QCV4_9PROT 3-phosphoshikimate 1-carboxyvinyltr... | 429 | 7e-145 |
| TR:A0A3D9FBA0A0A3D9FBA0_9SPHN 3-phosphoshikimate 1-carboxyvinyltr... | 429 | 7e-145 |
| TR:A0A328B3T5A0A328B3T5_9CAUL 3-phosphoshikimate 1-carboxyvinyltr... | 429 | 1e-144 |
| SP:B0T270AROA_CAUSK 3-phosphoshikimate 1-carboxyvinyltransferase ... | 428 | 1e-144 |
| TR:M2T8R4M2T8R4_9SPHN 3-phosphoshikimate 1-carboxyvinyltransferas... | 428 | 1e-144 |
| TR:A0A3A9J3H5A0A3A9J3H5_9PROT 3-phosphoshikimate 1-carboxyvinyltr... | 428 | 2e-144 |
| TR:A0A0Q7EB34A0A0Q7EB34_9CAUL 3-phosphoshikimate 1-carboxyvinyltr... | 427 | 2e-144 |
| SP:Q0BQH9AROA_GRABC 3-phosphoshikimate 1-carboxyvinyltransferase ... | 428 | 3e-144 |
| TR:A0A1H8X798A0A1H8X798_9PROT 3-phosphoshikimate 1-carboxyvinyltr... | 428 | 3e-144 |
| TR:A0A328APZ0A0A328APZ0_9CAUL 3-phosphoshikimate 1-carboxyvinyltr... | 427 | 3e-144 |
| TR:A0A5C6UP77A0A5C6UP77_9SPHN 3-phosphoshikimate 1-carboxyvinyltr... | 425 | 3e-143 |
| TR:D5BT96D5BT96_PUNMI 3-phosphoshikimate 1-carboxyvinyltransferas... | 424 | 6e-143 |
| SP:B4RCK3AROA_PHEZH 3-phosphoshikimate 1-carboxyvinyltransferase ... | 424 | 1e-142 |
| TR:A0A1G7SGL2A0A1G7SGL2_9PROT 3-phosphoshikimate 1-carboxyvinyltr... | 423 | 1e-142 |
| TR:A0A4Y8ZRK2A0A4Y8ZRK2_9SPHN 3-phosphoshikimate 1-carboxyvinyltr... | 423 | 1e-142 |
| TR:A0A2C6J2F8A0A2C6J2F8_9PROT 3-phosphoshikimate 1-carboxyvinyltr... | 422 | 3e-142 |
| TR:G5ZXD3G5ZXD3_9PROT 3-phosphoshikimate 1-carboxyvinyltransferas... | 423 | 3e-142 |
| TR:A0A2S8W964A0A2S8W964_9CAUL 3-phosphoshikimate 1-carboxyvinyltr... | 422 | 4e-142 |
| TR:K9HW36K9HW36_9PROT 3-phosphoshikimate 1-carboxyvinyltransferas... | 421 | 5e-142 |
| TR:A0A2S7K843A0A2S7K843_9PROT 3-phosphoshikimate 1-carboxyvinyltr... | 422 | 5e-142 |
| TR:A0A1I6T689A0A1I6T689_9CAUL 3-phosphoshikimate 1-carboxyvinyltr... | 421 | 8e-142 |
| TR:A0A5B2TJ50A0A5B2TJ50_9PROT 3-phosphoshikimate 1-carboxyvinyltr... | 421 | 1e-141 |
| TR:A0A1M5D8B2A0A1M5D8B2_9RHIZ 3-phosphoshikimate 1-carboxyvinyltr... | 421 | 2e-141 |
| TR:A0A2U2J4L3A0A2U2J4L3_9SPHN 3-phosphoshikimate 1-carboxyvinyltr... | 419 | 3e-141 |
| TR:A0A062VJ11A0A062VJ11_9RHOB 3-phosphoshikimate 1-carboxyvinyltr... | 419 | 3e-141 |
| TR:W7DY27W7DY27_9PROT 3-phosphoshikimate 1-carboxyvinyltransferas... | 419 | 4e-141 |
| TR:A0A317FMR8A0A317FMR8_9PROT 3-phosphoshikimate 1-carboxyvinyltr... | 419 | 4e-141 |
| TR:A0A2H6AZA5A0A2H6AZA5_9BACT 3-phosphoshikimate 1-carboxyvinyltr... | 419 | 5e-141 |
| TR:A0A369QAA5A0A369QAA5_9SPHN 3-phosphoshikimate 1-carboxyvinyltr... | 417 | 9e-141 |
| TR:A0A2C7AAH1A0A2C7AAH1_9PROT 3-phosphoshikimate 1-carboxyvinyltr... | 419 | 9e-141 |
| TR:A0A5C6TXX1A0A5C6TXX1_9SPHN 3-phosphoshikimate 1-carboxyvinyltr... | 417 | 2e-140 |
| TR:A0A370G3T0A0A370G3T0_GLULI 3-phosphoshikimate 1-carboxyvinyltr... | 417 | 3e-140 |
| TR:A0A085FBC5A0A085FBC5_9RHIZ 3-phosphoshikimate 1-carboxyvinyltr... | 417 | 3e-140 |
| TR:A0A1G9SRR1A0A1G9SRR1_9RHOB 3-phosphoshikimate 1-carboxyvinyltr... | 417 | 5e-140 |
| TR:A0A506ULD7A0A506ULD7_9PROT 3-phosphoshikimate 1-carboxyvinyltr... | 416 | 1e-139 |
| TR:A0A0Q7RU01A0A0Q7RU01_9CAUL 3-phosphoshikimate 1-carboxyvinyltr... | 414 | 4e-139 |
| TR:A0A1I4AFF3A0A1I4AFF3_9PROT 3-phosphoshikimate 1-carboxyvinyltr... | 415 | 4e-139 |
| TR:D5RKI5D5RKI5_9PROT 3-phosphoshikimate 1-carboxyvinyltransferas... | 414 | 4e-139 |
| TR:A0A4R4D892A0A4R4D892_9PROT 3-phosphoshikimate 1-carboxyvinyltr... | 414 | 4e-139 |
| TR:R5Q0B2R5Q0B2_9PROT 3-phosphoshikimate 1-carboxyvinyltransferas... | 414 | 4e-139 |
| TR:A0A0Q5V8J9A0A0Q5V8J9_9CAUL 3-phosphoshikimate 1-carboxyvinyltr... | 414 | 4e-139 |
| TR:A0A2U2J5Z1A0A2U2J5Z1_9SPHN 3-phosphoshikimate 1-carboxyvinyltr... | 413 | 2e-138 |
| SP:Q0ATJ1AROA_MARMM 3-phosphoshikimate 1-carboxyvinyltransferase ... | 413 | 2e-138 |

| TR:A0A0Q7S9G5A0A0Q7S9G5_9CAUL 3-phosphoshikimate 1-carboxyvinyltr... | 412 | 2e-138 |
| --- | --- | --- |
| TR:A0A0F5QAV6A0A0F5QAV6_9RHIZ 3-phosphoshikimate 1-carboxyvinyltr... | 412 | 4e-138 |
| TR:F3SCG0F3SCG0_9PROT 3-phosphoshikimate 1-carboxyvinyltransferas... | 411 | 4e-138 |
| TR:A0A2W7IK33A0A2W7IK33_9PROT 3-phosphoshikimate 1-carboxyvinyltr... | 410 | 9e-138 |
| TR:A0A1M6FF05A0A1M6FF05_9PROT 3-phosphoshikimate 1-carboxyvinyltr... | 410 | 1e-137 |
| TR:A0A4R5QCF1A0A4R5QCF1_9PROT 3-phosphoshikimate 1-carboxyvinyltr... | 410 | 1e-137 |

>SP:Q9R4E4 AROA_AGRSC 3-phosphoshikimate 1-carboxyvinyltransferase OS=Agrobacterium sp. (strain CP4) OX=268951 GN=aroA PE=1 SV=2

Length=455

Score = 883 bits (2282), Expect = 0.0

| Identities | = 454/455 (99%), Positives = 454/455 (99%), Gaps = 0/455 (0%) |  |
| --- | --- | --- |
| Query 1 | MLHGASSRPATARKSSGLSGTVRIPGDKSISHRSFMFGGLASGETRITGLLEGEDVINTG | 60 |
| Sbjct 1 | M HGASSRPATARKSSGLSGTVRIPGDKSISHRSFMFGGLASGETRITGLLEGEDVINTG MSHGASSRPATARKSSGLSGTVRIPGDKSISHRSFMFGGLASGETRITGLLEGEDVINTG | 60 |
| Query 61 | KAMQAMGARIRKEGDTWIIDGVGNGGLLAPEAPLDFGNAATGCRLTMGLVGVYDFDSTFI | 120 |
| Sbjct 61 | KAMQAMGARIRKEGDTWIIDGVGNGGLLAPEAPLDFGNAATGCRLTMGLVGVYDFDSTFI KAMQAMGARIRKEGDTWIIDGVGNGGLLAPEAPLDFGNAATGCRLTMGLVGVYDFDSTFI | 120 |
| Query 121 | GDASLTKRPMGRVLNPLREMGVQVKSEDGDRLPVTLRGPKTPTPITYRVPMASAQVKSAV | 180 |
| Sbjct 121 | GDASLTKRPMGRVLNPLREMGVQVKSEDGDRLPVTLRGPKTPTPITYRVPMASAQVKSAV GDASLTKRPMGRVLNPLREMGVQVKSEDGDRLPVTLRGPKTPTPITYRVPMASAQVKSAV | 180 |
| Query 181 | LLAGLNTPGITTVIEPIMTRDHTEKMLQGFGANLTVETDADGVRTIRLEGRGKLTGQVID | 240 |
| Sbjct 181 | LLAGLNTPGITTVIEPIMTRDHTEKMLQGFGANLTVETDADGVRTIRLEGRGKLTGQVID LLAGLNTPGITTVIEPIMTRDHTEKMLQGFGANLTVETDADGVRTIRLEGRGKLTGQVID | 240 |
| Query 241 | VPGDPSSTAFPLVAALLVPGSDVTILNVLMNPTRTGLILTLQEMGADIEVINPRLAGGED | 300 |
| Sbjct 241 | VPGDPSSTAFPLVAALLVPGSDVTILNVLMNPTRTGLILTLQEMGADIEVINPRLAGGED VPGDPSSTAFPLVAALLVPGSDVTILNVLMNPTRTGLILTLQEMGADIEVINPRLAGGED | 300 |
| Query 301 | VADLRVRSSTLKGVTVPEDRAPSMIDEYPILAVAAAFAEGATVMNGLEELRVKESDRLSA | 360 |
| Sbjct 301 | VADLRVRSSTLKGVTVPEDRAPSMIDEYPILAVAAAFAEGATVMNGLEELRVKESDRLSA VADLRVRSSTLKGVTVPEDRAPSMIDEYPILAVAAAFAEGATVMNGLEELRVKESDRLSA | 360 |
| Query 361 | VANGLKLNGVDCDEGETSLVVRGRPDGKGLGNASGAAVATHLDHRIAMSFLVMGLVSENP | 420 |
| Sbjct 361 | VANGLKLNGVDCDEGETSLVVRGRPDGKGLGNASGAAVATHLDHRIAMSFLVMGLVSENP VANGLKLNGVDCDEGETSLVVRGRPDGKGLGNASGAAVATHLDHRIAMSFLVMGLVSENP | 420 |
| Query 421  Sbjct 421 | VTVDDATMIATSFPEFMDLMAGLGAKIELSDTKAA 455 VTVDDATMIATSFPEFMDLMAGLGAKIELSDTKAA VTVDDATMIATSFPEFMDLMAGLGAKIELSDTKAA 455 |  |

>TR:A0A4Y8QII1 A0A4Y8QII1_9RHIZ 3-phosphoshikimate 1-carboxyvinyltransferase OS=Shinella sp. MEC087 OX=1967501 GN=aroA PE=3 SV=1

Length=455

Score = 848 bits (2191), Expect = 0.0

| Identities | = 431/455 (95%), Positives = 443/455 (97%), Gaps = 0/455 (0%) |  |
| --- | --- | --- |
| Query 1 | MLHGASSRPATARKSSGLSGTVRIPGDKSISHRSFMFGGLASGETRITGLLEGEDVINTG | 60 |
| Sbjct 1 | M HGA +RPATARKSSGLSGTVRIPGDKSISHRSFMFGGLASGETRITGLLEGEDVINTG MSHGAHARPATARKSSGLSGTVRIPGDKSISHRSFMFGGLASGETRITGLLEGEDVINTG | 60 |
| Query 61 | KAMQAMGARIRKEGDTWIIDGVGNGGLLAPEAPLDFGNAATGCRLTMGLVGVYDFDSTFI | 120 |
| Sbjct 61 | KAMQAMGARIRK+GDTWII+GVGNG LLAPEAPLDFGNA TGCRLTMGLVGVYDFDSTFI KAMQAMGARIRKDGDTWIINGVGNGALLAPEAPLDFGNAGTGCRLTMGLVGVYDFDSTFI | 120 |
| Query 121 | GDASLTKRPMGRVLNPLREMGVQVKSEDGDRLPVTLRGPKTPTPITYRVPMASAQVKSAV | 180 |

| Sbjct | 121 | GDASLTKRPMGRVLNPLREMGVQV S +GDRLPVTLRGPKTPTPITYRVPMASAQVKSAV  GDASLTKRPMGRVLNPLREMGVQVTSAEGDRLPVTLRGPKTPTPITYRVPMASAQVKSAV | 180 |
| --- | --- | --- | --- |
| Query | 181 | LLAGLNTPGITTVIEPIMTRDHTEKMLQGFGANLTVETDADGVRTIRLEGRGKLTGQVID | 240 |
| Sbjct | 181 | LLAGLNTPGITTVIEPIMTRDHTEKMLQGFGANLTVETDADGVRTIRLEGRGKLTGQ+ID LLAGLNTPGITTVIEPIMTRDHTEKMLQGFGANLTVETDADGVRTIRLEGRGKLTGQIID | 240 |
| Query | 241 | VPGDPSSTAFPLVAALLVPGSDVTILNVLMNPTRTGLILTLQEMGADIEVINPRLAGGED | 300 |
| Sbjct | 241 | VPGDPSSTAFPLVAALLVPGSD+TILNVLMNPTRTGLILTLQEMGADIEV+NPRLAGGED VPGDPSSTAFPLVAALLVPGSDITILNVLMNPTRTGLILTLQEMGADIEVLNPRLAGGED | 300 |
| Query | 301 | VADLRVRSSTLKGVTVPEDRAPSMIDEYPILAVAAAFAEGATVMNGLEELRVKESDRLSA | 360 |
| Sbjct | 301 | VADLRVRSS LKGVTVPEDRAPSMIDEYP+LAVAA+FAEG TVMNGL+ELRVKESDRLSA VADLRVRSSVLKGVTVPEDRAPSMIDEYPVLAVAASFAEGTTVMNGLDELRVKESDRLSA | 360 |
| Query | 361 | VANGLKLNGVDCDEGETSLVVRGRPDGKGLGNASGAAVATHLDHRIAMSFLVMGLVSENP | 420 |
| Sbjct | 361 | VA GLKLNGVDCDEGE SLVVRGRPDG GLGNA+GAAVATHLDHRIAMSFLVMGLVSE+P VAEGLKLNGVDCDEGEASLVVRGRPDGNGLGNAAGAAVATHLDHRIAMSFLVMGLVSEHP | 420 |
| Query  Sbjct | 421  421 | VTVDDATMIATSFPEFMDLMAGLGAKIELSDTKAA 455 VTVDDATMIATSFPEFMDLMAGLGAKIELSD KAA VTVDDATMIATSFPEFMDLMAGLGAKIELSDVKAA 455 |  |

>TR:A0A1A9FVW7 A0A1A9FVW7_9RHIZ 3-phosphoshikimate 1-carboxyvinyltransferase OS=Shinella sp. HZN7 OX=879274 GN=aroA PE=3 SV=1

Length=451

Score = 840 bits (2170), Expect = 0.0

| Identities | = 427/451 (95%), Positives = 440/451 (98%), Gaps = 0/451 (0%) |  |
| --- | --- | --- |
| Query 1 | MLHGASSRPATARKSSGLSGTVRIPGDKSISHRSFMFGGLASGETRITGLLEGEDVINTG | 60 |
| Sbjct 1 | M HGAS+RPATARKSSGLSGTVRIPGDKSISHRSFMFGGLASGETRITGLLEGEDVINTG MSHGASARPATARKSSGLSGTVRIPGDKSISHRSFMFGGLASGETRITGLLEGEDVINTG | 60 |
| Query 61 | KAMQAMGARIRKEGDTWIIDGVGNGGLLAPEAPLDFGNAATGCRLTMGLVGVYDFDSTFI | 120 |
| Sbjct 61 | KAMQAMGA+IRK+GDTWII+G+GNG LLAPEAPLDFGNA TGCRLTMGLVGVYDFDSTF+ KAMQAMGAKIRKDGDTWIINGLGNGALLAPEAPLDFGNAGTGCRLTMGLVGVYDFDSTFV | 120 |
| Query 121 | GDASLTKRPMGRVLNPLREMGVQVKSEDGDRLPVTLRGPKTPTPITYRVPMASAQVKSAV | 180 |
| Sbjct 121 | GDASLTKRPMGRVLNPLREMGVQV S DGDRLPVTLRGPKTPTPITYRVPMASAQVKSAV GDASLTKRPMGRVLNPLREMGVQVTSADGDRLPVTLRGPKTPTPITYRVPMASAQVKSAV | 180 |
| Query 181 | LLAGLNTPGITTVIEPIMTRDHTEKMLQGFGANLTVETDADGVRTIRLEGRGKLTGQVID | 240 |
| Sbjct 181 | LLAGLNTPGITTVIEP+MTRDHTEKMLQGFGANL+VETD DGVRTIRLEG+GKLTGQVID LLAGLNTPGITTVIEPVMTRDHTEKMLQGFGANLSVETDTDGVRTIRLEGQGKLTGQVID | 240 |
| Query 241 | VPGDPSSTAFPLVAALLVPGSDVTILNVLMNPTRTGLILTLQEMGADIEVINPRLAGGED | 300 |
| Sbjct 241 | VPGDPSSTAFPLVAALLVP SDVTILNVLMNPTRTGLILTLQEMGA+IEV+NPRLAGGED VPGDPSSTAFPLVAALLVPDSDVTILNVLMNPTRTGLILTLQEMGANIEVLNPRLAGGED | 300 |
| Query 301 | VADLRVRSSTLKGVTVPEDRAPSMIDEYPILAVAAAFAEGATVMNGLEELRVKESDRLSA | 360 |
| Sbjct 301 | VADLRVRSS LKGVTVPEDRAPSMIDEYP+LAVAAAFAEG TVMNGLEELRVKESDRLSA VADLRVRSSALKGVTVPEDRAPSMIDEYPVLAVAAAFAEGTTVMNGLEELRVKESDRLSA | 360 |
| Query 361 | VANGLKLNGVDCDEGETSLVVRGRPDGKGLGNASGAAVATHLDHRIAMSFLVMGLVSENP | 420 |
| Sbjct 361 | VA GLKLNGVDCDEGE SLVVRGRPDGKGLGNASGAAVATHLDHRIAMSFLVMGLVSENP VAVGLKLNGVDCDEGEASLVVRGRPDGKGLGNASGAAVATHLDHRIAMSFLVMGLVSENP | 420 |
| Query 421  Sbjct 421 | VTVDDATMIATSFPEFMDLMAGLGAKIELSD 451 VTVDDATMIATSFPEFMDLMAGLGAKIEL+D VTVDDATMIATSFPEFMDLMAGLGAKIELND 451 |  |

>TR:A0A292E0M8 A0A292E0M8_9RHIZ 3-phosphoshikimate 1-carboxyvinyltransferase OS=Rhizobium sp. ACO-34A OX=1571470 GN=aroA PE=3 SV=1

Length=455

Score = 822 bits (2122), Expect = 0.0

| Identities | = 418/455 (92%), Positives = 435/455 (96%), Gaps = 0/455 (0%) |  |
| --- | --- | --- |
| Query 1 | MLHGASSRPATARKSSGLSGTVRIPGDKSISHRSFMFGGLASGETRITGLLEGEDVINTG | 60 |
| Sbjct 1 | M HGA +PAT+RKS LSGT+RIPGDKSISHRSFMFGGLASG+TRITGLLEGEDVINTG MSHGALPKPATSRKSPALSGTIRIPGDKSISHRSFMFGGLASGQTRITGLLEGEDVINTG | 60 |
| Query 61 | KAMQAMGARIRKEGDTWIIDGVGNGGLLAPEAPLDFGNAATGCRLTMGLVGVYDFDSTFI | 120 |
| Sbjct 61 | KAMQAMGARI K+GDTWIIDGVGNG LLAPEAPLDFGNA TGCRLTMGLVGVYDFDSTFI KAMQAMGARIVKDGDTWIIDGVGNGCLLAPEAPLDFGNAGTGCRLTMGLVGVYDFDSTFI | 120 |
| Query 121 | GDASLTKRPMGRVLNPLREMGVQVKSEDGDRLPVTLRGPKTPTPITYRVPMASAQVKSAV | 180 |
| Sbjct 121 | GDASLTKRPMGRVLNPLREMGVQVKS +GDRLPVTL GPKTP PITYRVPMASAQVKSAV GDASLTKRPMGRVLNPLREMGVQVKSAEGDRLPVTLHGPKTPAPITYRVPMASAQVKSAV | 180 |
| Query 181 | LLAGLNTPGITTVIEPIMTRDHTEKMLQGFGANLTVETDADGVRTIRLEGRGKLTGQVID | 240 |
| Sbjct 181 | LLAGLNTPGITTVIEPIMTRDHTEKMLQGFGANLTVETDA+ VRTIR+EGRGKLTGQVID LLAGLNTPGITTVIEPIMTRDHTEKMLQGFGANLTVETDAESVRTIRIEGRGKLTGQVID | 240 |
| Query 241 | VPGDPSSTAFPLVAALLVPGSDVTILNVLMNPTRTGLILTLQEMGADIEVINPRLAGGED | 300 |
| Sbjct 241 | VPGDPSSTAFPLVAALLVPGSDVTILNVLMNPTRTGLILTLQEMGADIE++NPRLAGGED VPGDPSSTAFPLVAALLVPGSDVTILNVLMNPTRTGLILTLQEMGADIEILNPRLAGGED | 300 |
| Query 301 | VADLRVRSSTLKGVTVPEDRAPSMIDEYPILAVAAAFAEGATVMNGLEELRVKESDRLSA | 360 |
| Sbjct 301 | VADLRVRSS+LKGVTVPEDRAPSMIDEYP+LAVAAAFAEG T M GLEELRVKESDRLSA VADLRVRSSSLKGVTVPEDRAPSMIDEYPVLAVAAAFAEGTTTMLGLEELRVKESDRLSA | 360 |
| Query 361 | VANGLKLNGVDCDEGETSLVVRGRPDGKGLGNASGAAVATHLDHRIAMSFLVMGLVSENP | 420 |
| Sbjct 361 | VA GLKLNGVDCDEGE +LVVRGRPDGKGLGNASGAAVATHLDHRIAMSFL+MGLVSE+P VAVGLKLNGVDCDEGEATLVVRGRPDGKGLGNASGAAVATHLDHRIAMSFLIMGLVSEHP | 420 |
| Query 421  Sbjct 421 | VTVDDATMIATSFPEFMDLMAGLGAKIELSDTKAA 455 VTVDDATMIATSFPEFMDLM+GLGAKIE + KAA VTVDDATMIATSFPEFMDLMSGLGAKIETREGKAA 455 |  |

>TR:A0A2T5BEP1 A0A2T5BEP1_MYCDI 3-phosphoshikimate 1-carboxyvinyltransferase OS=Mycoplana dimorpha OX=28320 GN=aroA PE=3 SV=1

Length=449

Score = 818 bits (2114), Expect = 0.0

| Identities | = 414/448 (92%), Positives = 436/448 (97%), Gaps = 0/448 (0%) |  |
| --- | --- | --- |
| Query 1 | MLHGASSRPATARKSSGLSGTVRIPGDKSISHRSFMFGGLASGETRITGLLEGEDVINTG | 60 |
| Sbjct 1 | M HGA+++PATARKS+ LSGTVRIPGDKSISHR FMFGGLASGETRITGLLEGEDVINTG MSHGAAAKPATARKSNALSGTVRIPGDKSISHRCFMFGGLASGETRITGLLEGEDVINTG | 60 |
| Query 61 | KAMQAMGARIRKEGDTWIIDGVGNGGLLAPEAPLDFGNAATGCRLTMGLVGVYDFDSTFI | 120 |
| Sbjct 61 | KAMQAMGA+IRK+GDTWII+GVGNG LLAPEAPLDFGNA TGCRL MGLVGVYDFD+TFI KAMQAMGAKIRKDGDTWIINGVGNGALLAPEAPLDFGNAGTGCRLMMGLVGVYDFDTTFI | 120 |
| Query 121 | GDASLTKRPMGRVLNPLREMGVQVKSEDGDRLPVTLRGPKTPTPITYRVPMASAQVKSAV | 180 |
| Sbjct 121 | GDASLTKRPMGRVL+PLREMGVQVK+ DGDRLPVTLRGP+TP PITYRVPMASAQVKSAV GDASLTKRPMGRVLDPLREMGVQVKAADGDRLPVTLRGPRTPAPITYRVPMASAQVKSAV | 180 |
| Query 181 | LLAGLNTPGITTVIEPIMTRDHTEKMLQGFGANLTVETDADGVRTIRLEGRGKLTGQVID | 240 |
| Sbjct 181 | LLAGLNTPGITTVIEP+MTRDHTEKMLQGFGANLTVE+DADGVRTIRLEG+GKLTGQVID LLAGLNTPGITTVIEPVMTRDHTEKMLQGFGANLTVESDADGVRTIRLEGQGKLTGQVID | 240 |
| Query 241 | VPGDPSSTAFPLVAALLVPGSDVTILNVLMNPTRTGLILTLQEMGADIEVINPRLAGGED | 300 |

| Sbjct | 241 | VPGDPSSTAFPLVAALLVPGSDVTILNVLMNPTRTGLILTLQEMGA+IEV+N RLAGGED  VPGDPSSTAFPLVAALLVPGSDVTILNVLMNPTRTGLILTLQEMGANIEVLNERLAGGED | 300 |
| --- | --- | --- | --- |
| Query | 301 | VADLRVRSSTLKGVTVPEDRAPSMIDEYPILAVAAAFAEGATVMNGLEELRVKESDRLSA | 360 |
| Sbjct | 301 | VADLRVR STLKGVTVPEDRAPSMIDEYPILA+AAAFAEG TVMNGLEELRVKESDRLSA VADLRVRHSTLKGVTVPEDRAPSMIDEYPILAIAAAFAEGTTVMNGLEELRVKESDRLSA | 360 |
| Query | 361 | VANGLKLNGVDCDEGETSLVVRGRPDGKGLGNASGAAVATHLDHRIAMSFLVMGLVSENP | 420 |
| Sbjct | 361 | VA+GLKLNGVDCDEGE+SLVVRGRPDGKGLGNASGAAVATHLDHRIAMSFLVMGLVSE+P VADGLKLNGVDCDEGESSLVVRGRPDGKGLGNASGAAVATHLDHRIAMSFLVMGLVSEHP | 420 |
| Query  Sbjct | 421  421 | VTVDDATMIATSFPEFMDLMAGLGAKIE 448 VTVDDATMIATSFPEFM LMAGLGA+I+ VTVDDATMIATSFPEFMGLMAGLGARID 448 |  |

>TR:A0A2U2DST6 A0A2U2DST6_9RHIZ 3-phosphoshikimate 1-carboxyvinyltransferase OS=Rhizobium album OX=2182425 GN=aroA PE=3 SV=1

Length=455

Score = 817 bits (2111), Expect = 0.0

| Identities | = 416/455 (91%), Positives = 433/455 (95%), Gaps = 0/455 (0%) |  |
| --- | --- | --- |
| Query 1 | MLHGASSRPATARKSSGLSGTVRIPGDKSISHRSFMFGGLASGETRITGLLEGEDVINTG | 60 |
| Sbjct 1 | M HGA+++PATAR+SS LSGT RIPGDKSISHRSFMFGGLASGETRITGLLEGEDVINTG MSHGAAAKPATARRSSNLSGTARIPGDKSISHRSFMFGGLASGETRITGLLEGEDVINTG | 60 |
| Query 61 | KAMQAMGARIRKEGDTWIIDGVGNGGLLAPEAPLDFGNAATGCRLTMGLVGVYDFDSTFI | 120 |
| Sbjct 61 | KAMQAMGARIRK+GD WIIDG GNG LLAPEAPLDFGNA TGCRLTMGLVGVYDFDSTFI KAMQAMGARIRKDGDVWIIDGTGNGALLAPEAPLDFGNAGTGCRLTMGLVGVYDFDSTFI | 120 |
| Query 121 | GDASLTKRPMGRVLNPLREMGVQVKSEDGDRLPVTLRGPKTPTPITYRVPMASAQVKSAV | 180 |
| Sbjct 121 | GDASLTKRPMGRVL+PLR+MGVQV S DGDRLPVTL GP TP PITYRVPMASAQVKSAV GDASLTKRPMGRVLDPLRQMGVQVTSADGDRLPVTLHGPATPAPITYRVPMASAQVKSAV | 180 |
| Query 181 | LLAGLNTPGITTVIEPIMTRDHTEKMLQGFGANLTVETDADGVRTIRLEGRGKLTGQVID | 240 |
| Sbjct 181 | LLAGLNTPGITTVIEPIMTRDHTEKMLQGFGANLTVET +DGVRTIRLEGRGKLTGQ ID LLAGLNTPGITTVIEPIMTRDHTEKMLQGFGANLTVETGSDGVRTIRLEGRGKLTGQTID | 240 |
| Query 241 | VPGDPSSTAFPLVAALLVPGSDVTILNVLMNPTRTGLILTLQEMGADIEVINPRLAGGED | 300 |
| Sbjct 241 | VPGDPSSTAFPLVAALLVPGSDVTILNVLMNPTRTGLILTLQEMGADIEV+NPRLAGGED VPGDPSSTAFPLVAALLVPGSDVTILNVLMNPTRTGLILTLQEMGADIEVLNPRLAGGED | 300 |
| Query 301 | VADLRVRSSTLKGVTVPEDRAPSMIDEYPILAVAAAFAEGATVMNGLEELRVKESDRLSA | 360 |
| Sbjct 301 | VADLRVRSS LKGVTVPEDRAPSMIDEYP+LAVAA+FAEGATVMNGLEELRVKESDRLSA VADLRVRSSELKGVTVPEDRAPSMIDEYPVLAVAASFAEGATVMNGLEELRVKESDRLSA | 360 |
| Query 361 | VANGLKLNGVDCDEGETSLVVRGRPDGKGLGNASGAAVATHLDHRIAMSFLVMGLVSENP | 420 |
| Sbjct 361 | VANGLKLNGVDCDEGE++LVVRG P GKGLGNASGAAVATHLDHRIAMSFL+MGL SE+P VANGLKLNGVDCDEGESTLVVRGVPGGKGLGNASGAAVATHLDHRIAMSFLIMGLASEHP | 420 |
| Query 421  Sbjct 421 | VTVDDATMIATSFPEFMDLMAGLGAKIELSDTKAA 455 VTVDDATMIATSFPEFMDLMA LGAKIE+ + AA VTVDDATMIATSFPEFMDLMARLGAKIEVHEGAAA 455 |  |

>TR:A0A1S1SIN8 A0A1S1SIN8_9RHIZ 3-phosphoshikimate 1-carboxyvinyltransferase OS=Ensifer sp. LCM 4579 OX=1848292 GN=aroA PE=3 SV=1

Length=455

Score = 816 bits (2108), Expect = 0.0

Identities = 414/454 (91%), Positives = 431/454 (95%), Gaps = 0/454 (0%) Query 1	MLHGASSRPATARKSSGLSGTVRIPGDKSISHRSFMFGGLASGETRITGLLEGEDVINTG 60

| Sbjct | 1 | M HG SSRPATARKSS L GTVRIPGDKSISHRSFMFGGLASGETRITGLLEGEDVINTG  MSHGQSSRPATARKSSDLKGTVRIPGDKSISHRSFMFGGLASGETRITGLLEGEDVINTG | 60 |
| --- | --- | --- | --- |
| Query | 61 | KAMQAMGARIRKEGDTWIIDGVGNGGLLAPEAPLDFGNAATGCRLTMGLVGVYDFDSTFI | 120 |
| Sbjct | 61 | KAMQAMGA+IRKEGDTWIIDGVGNG LLAPEAPLDFGNA TGCRLTMGLVGVYDF STF+ KAMQAMGAKIRKEGDTWIIDGVGNGALLAPEAPLDFGNAGTGCRLTMGLVGVYDFASTFV | 120 |
| Query | 121 | GDASLTKRPMGRVLNPLREMGVQVKSEDGDRLPVTLRGPKTPTPITYRVPMASAQVKSAV | 180 |
| Sbjct | 121 | GDASL KRPMGRVL+PLREMGVQVKS +GDRLPVTL GPKTP PITYRVPMASAQVKSAV GDASLAKRPMGRVLDPLREMGVQVKSAEGDRLPVTLHGPKTPNPITYRVPMASAQVKSAV | 180 |
| Query | 181 | LLAGLNTPGITTVIEPIMTRDHTEKMLQGFGANLTVETDADGVRTIRLEGRGKLTGQVID | 240 |
| Sbjct | 181 | LLAGLNTPGITTV+EP+MTRDHTEKMLQGFGANL+VETD GVRTIRLEGRGKLTGQVID LLAGLNTPGITTVVEPVMTRDHTEKMLQGFGANLSVETDTQGVRTIRLEGRGKLTGQVID | 240 |
| Query | 241 | VPGDPSSTAFPLVAALLVPGSDVTILNVLMNPTRTGLILTLQEMGADIEVINPRLAGGED | 300 |
| Sbjct | 241 | VPGDPSSTAFPLVAAL+VPGSDVTILNVLMNPTRTGLILTLQEMGADIEV+N RLAGGED VPGDPSSTAFPLVAALIVPGSDVTILNVLMNPTRTGLILTLQEMGADIEVMNKRLAGGED | 300 |
| Query | 301 | VADLRVRSSTLKGVTVPEDRAPSMIDEYPILAVAAAFAEGATVMNGLEELRVKESDRLSA | 360 |
| Sbjct | 301 | VADLRVR S LKGVTVPE+RAPSMIDEYP+LAVAAAFAEGATVMNGL+ELRVKESDRLSA VADLRVRHSELKGVTVPEERAPSMIDEYPVLAVAAAFAEGATVMNGLDELRVKESDRLSA | 360 |
| Query | 361 | VANGLKLNGVDCDEGETSLVVRGRPDGKGLGNASGAAVATHLDHRIAMSFLVMGLVSENP | 420 |
| Sbjct | 361 | VA+GLKLNGVDCDEGE SLVVRGRPDGKGLGNASG V THLDHRIAMSFLV+GL SE+P VADGLKLNGVDCDEGEASLVVRGRPDGKGLGNASGEKVKTHLDHRIAMSFLVLGLASEHP | 420 |
| Query  Sbjct | 421  421 | VTVDDATMIATSFPEFMDLMAGLGAKIELSDTKA 454 VTVDDATMIATSFPEFMDLM GLGAKIE ++TKA VTVDDATMIATSFPEFMDLMTGLGAKIEQAETKA 454 |  |

>TR:A0A285UHC5 A0A285UHC5_9RHIZ 3-phosphoshikimate 1-carboxyvinyltransferase OS=Rhizobium subbaraonis OX=908946 GN=aroA PE=3 SV=1

Length=448

Score = 815 bits (2106), Expect = 0.0

| Identities | = 417/448 (93%), Positives = 429/448 (96%), Gaps = 0/448 (0%) |  |
| --- | --- | --- |
| Query 1 | MLHGASSRPATARKSSGLSGTVRIPGDKSISHRSFMFGGLASGETRITGLLEGEDVINTG | 60 |
| Sbjct 1 | M HGA ++PATA KS+ LSGTVRIPGDKSISHRSFMFGGLASGETRITGLLEGEDVINTG MSHGAQAKPATASKSNALSGTVRIPGDKSISHRSFMFGGLASGETRITGLLEGEDVINTG | 60 |
| Query 61 | KAMQAMGARIRKEGDTWIIDGVGNGGLLAPEAPLDFGNAATGCRLTMGLVGVYDFDSTFI | 120 |
| Sbjct 61 | KAMQAMGA+IRKEGDTWII+GVGNG LLAPEAPLDFGNA TGCRLTMGLVGVYDFDSTFI KAMQAMGAKIRKEGDTWIINGVGNGALLAPEAPLDFGNAGTGCRLTMGLVGVYDFDSTFI | 120 |
| Query 121 | GDASLTKRPMGRVLNPLREMGVQVKSEDGDRLPVTLRGPKTPTPITYRVPMASAQVKSAV | 180 |
| Sbjct 121 | GDASLTKRPMGRVLNPLREMGVQV S DGDRLPVTLRGPKTPTPITYRVPMASAQVKSAV GDASLTKRPMGRVLNPLREMGVQVTSADGDRLPVTLRGPKTPTPITYRVPMASAQVKSAV | 180 |
| Query 181 | LLAGLNTPGITTVIEPIMTRDHTEKMLQGFGANLTVETDADGVRTIRLEGRGKLTGQVID | 240 |
| Sbjct 181 | LLAGLNTPGITTVIEP+MTRDHTEKMLQGFGANLTVETDA GVRTIRLEG+GKLTGQVID LLAGLNTPGITTVIEPVMTRDHTEKMLQGFGANLTVETDAAGVRTIRLEGQGKLTGQVID | 240 |
| Query 241 | VPGDPSSTAFPLVAALLVPGSDVTILNVLMNPTRTGLILTLQEMGADIEVINPRLAGGED | 300 |
| Sbjct 241 | VPGDPSSTAFPLVAALLVPGSDVTILNVLMNPTRTGLILTLQEMGA+IEV+N RLAGGED VPGDPSSTAFPLVAALLVPGSDVTILNVLMNPTRTGLILTLQEMGANIEVLNARLAGGED | 300 |
| Query 301 | VADLRVRSSTLKGVTVPEDRAPSMIDEYPILAVAAAFAEGATVMNGLEELRVKESDRLSA | 360 |
| Sbjct 301 | VADLRVR S LKGVTVPE+RAPSMIDEYPILAVAAAFAEG TVMNGLEELRVKESDRLSA VADLRVRHSELKGVTVPEERAPSMIDEYPILAVAAAFAEGTTVMNGLEELRVKESDRLSA | 360 |
| Query 361 | VANGLKLNGVDCDEGETSLVVRGRPDGKGLGNASGAAVATHLDHRIAMSFLVMGLVSENP | 420 |

| Sbjct | 361 | VA GLKLNGVDCDEGE SLVV GRPDG GLGNASGAAVATHLDHRIAMSFLVMGLVSE+P  VAEGLKLNGVDCDEGEASLVVHGRPDGLGLGNASGAAVATHLDHRIAMSFLVMGLVSEHP 420 |
| --- | --- | --- |
| Query | 421 | VTVDDATMIATSFPEFMDLMAGLGAKIE 448 |
| Sbjct | 421 | VTVDDATMIATSFPEFM LM GLGAKI+  VTVDDATMIATSFPEFMGLMTGLGAKID 448 |

>SP:Q92SV5 AROA_RHIME 3-phosphoshikimate 1-carboxyvinyltransferase OS=Rhizobium meliloti (strain 1021) OX=266834 GN=aroA PE=3 SV=1

Length=455

Score = 812 bits (2098), Expect = 0.0

| Identities | = 412/455 (91%), Positives = 430/455 (95%), Gaps = 0/455 (0%) |  |
| --- | --- | --- |
| Query 1 | MLHGASSRPATARKSSGLSGTVRIPGDKSISHRSFMFGGLASGETRITGLLEGEDVINTG | 60 |
| Sbjct 1 | M HG++ RPATARKSS L GT+RIPGDKSISHRSFMFGGLA+GETRITGLLEGEDVINTG MSHGSNPRPATARKSSDLKGTLRIPGDKSISHRSFMFGGLAAGETRITGLLEGEDVINTG | 60 |
| Query 61 | KAMQAMGARIRKEGDTWIIDGVGNGGLLAPEAPLDFGNAATGCRLTMGLVGVYDFDSTFI | 120 |
| Sbjct 61 | KAMQAMGARIRKEGDTWIIDGVGNG LLAPEAPLDFGNA TGCRLTMGLVGVYDFDSTFI KAMQAMGARIRKEGDTWIIDGVGNGALLAPEAPLDFGNAGTGCRLTMGLVGVYDFDSTFI | 120 |
| Query 121 | GDASLTKRPMGRVLNPLREMGVQVKSEDGDRLPVTLRGPKTPTPITYRVPMASAQVKSAV | 180 |
| Sbjct 121 | GDASLTKRPMGRVL+PLREMGVQVKS +GDRLPVTLRGPKTP PITYRVPMASAQVKSAV GDASLTKRPMGRVLDPLREMGVQVKSAEGDRLPVTLRGPKTPNPITYRVPMASAQVKSAV | 180 |
| Query 181 | LLAGLNTPGITTVIEPIMTRDHTEKMLQGFGANLTVETDADGVRTIRLEGRGKLTGQVID | 240 |
| Sbjct 181 | LLAGLNTPGITTV+EP+MTRDHTEKMLQGFGANLTVETDA+GVRTIRLEGRGKLTGQVID LLAGLNTPGITTVVEPVMTRDHTEKMLQGFGANLTVETDAEGVRTIRLEGRGKLTGQVID | 240 |
| Query 241 | VPGDPSSTAFPLVAALLVPGSDVTILNVLMNPTRTGLILTLQEMGADIEVINPRLAGGED | 300 |
| Sbjct 241 | VPGDPSSTAFPLVA L+VPGSD+TILNVLMNPTRTGLILTLQEMGA+IEV+N RLAGGED VPGDPSSTAFPLVAGLIVPGSDITILNVLMNPTRTGLILTLQEMGANIEVMNKRLAGGED | 300 |
| Query 301 | VADLRVRSSTLKGVTVPEDRAPSMIDEYPILAVAAAFAEGATVMNGLEELRVKESDRLSA | 360 |
| Sbjct 301 | VADLRVR S LKGVTVPEDRAPSMIDEYP+LAVAAAFAEG TVMNGLEELRVKESDRLSA VADLRVRHSELKGVTVPEDRAPSMIDEYPVLAVAAAFAEGTTVMNGLEELRVKESDRLSA | 360 |
| Query 361 | VANGLKLNGVDCDEGETSLVVRGRPDGKGLGNASGAAVATHLDHRIAMSFLVMGLVSENP | 420 |
| Sbjct 361 | VA+GLKLNGVDCDEGE SLVVRGRP GKGLG SG V THLDHRIAMSFLVMGL SE+P VADGLKLNGVDCDEGEASLVVRGRPGGKGLGKISGGQVKTHLDHRIAMSFLVMGLASEHP | 420 |
| Query 421  Sbjct 421 | VTVDDATMIATSFPEFMDLMAGLGAKIELSDTKAA 455 VTVDDATMIATSFPEFM LM GLGAKIE ++ KAA VTVDDATMIATSFPEFMGLMTGLGAKIEEAENKAA 455 |  |

>TR:A0A4S5J1R2 A0A4S5J1R2_9RHIZ 3-phosphoshikimate 1-carboxyvinyltransferase OS=Ensifer sp. MPMI2T OX=2490847 GN=aroA PE=3 SV=1

Length=455

Score = 808 bits (2088), Expect = 0.0

Identities = 412/455 (91%), Positives = 428/455 (94%), Gaps = 0/455 (0%)

| Query | 1 | MLHGASSRPATARKSSGLSGTVRIPGDKSISHRSFMFGGLASGETRITGLLEGEDVINTG | 60 |
| --- | --- | --- | --- |
| Sbjct | 1 | M HG+S RPATA KSS L GTVRIPGDKSISHRSFMFGGLA+GETRITGLLEGEDVINTG MSHGSSPRPATATKSSDLKGTVRIPGDKSISHRSFMFGGLAAGETRITGLLEGEDVINTG | 60 |
| Query | 61 | KAMQAMGARIRKEGDTWIIDGVGNGGLLAPEAPLDFGNAATGCRLTMGLVGVYDFDSTFI | 120 |
| Sbjct | 61 | KAMQAMGA+IRKEGDTWIIDGVGNG LLAPEAPLDFGNA TGCRLTMGLVGVYDFDSTFI KAMQAMGAKIRKEGDTWIIDGVGNGALLAPEAPLDFGNAGTGCRLTMGLVGVYDFDSTFI | 120 |
| Query | 121 | GDASLTKRPMGRVLNPLREMGVQVKSEDGDRLPVTLRGPKTPTPITYRVPMASAQVKSAV | 180 |

| Sbjct | 121 | GDASLTKRPMGRVLNPLREMGVQVKS +GDRLPVTL GPKTP PITYRVPMASAQVKSAV  GDASLTKRPMGRVLNPLREMGVQVKSAEGDRLPVTLHGPKTPNPITYRVPMASAQVKSAV | 180 |
| --- | --- | --- | --- |
| Query | 181 | LLAGLNTPGITTVIEPIMTRDHTEKMLQGFGANLTVETDADGVRTIRLEGRGKLTGQVID | 240 |
| Sbjct | 181 | LLAGLNTPGITTVIEP+MTRDHTEKMLQGFGANLTVETD GVRTIRLEGRGKL GQVID LLAGLNTPGITTVIEPVMTRDHTEKMLQGFGANLTVETDTQGVRTIRLEGRGKLAGQVID | 240 |
| Query | 241 | VPGDPSSTAFPLVAALLVPGSDVTILNVLMNPTRTGLILTLQEMGADIEVINPRLAGGED | 300 |
| Sbjct | 241 | VPGDPSSTAFPLVAALLVP SDVTILNVLMNPTRTGLILTLQEMGA+IEV+N RLAGGED VPGDPSSTAFPLVAALLVPASDVTILNVLMNPTRTGLILTLQEMGANIEVMNARLAGGED | 300 |
| Query | 301 | VADLRVRSSTLKGVTVPEDRAPSMIDEYPILAVAAAFAEGATVMNGLEELRVKESDRLSA | 360 |
| Sbjct | 301 | VADLRVR S LKGVTVPE+RAPSMIDEYP+LAVAAAFAEG TVMNGL+ELRVKESDRLSA VADLRVRYSELKGVTVPEERAPSMIDEYPVLAVAAAFAEGTTVMNGLDELRVKESDRLSA | 360 |
| Query | 361 | VANGLKLNGVDCDEGETSLVVRGRPDGKGLGNASGAAVATHLDHRIAMSFLVMGLVSENP | 420 |
| Sbjct | 361 | VA+GLKLNGVDCDEGE SLVVRGRP GKGLGNASG+ V T+LDHRIAMSFLVMGL SE+P VADGLKLNGVDCDEGEASLVVRGRPGGKGLGNASGSEVKTYLDHRIAMSFLVMGLASEHP | 420 |
| Query  Sbjct | 421  421 | VTVDDATMIATSFPEFMDLMAGLGAKIELSDTKAA 455 VTVDDATMIATSFPEFMDLM GLGAKIE ++ K A VTVDDATMIATSFPEFMDLMTGLGAKIEQTEAKVA 455 |  |

>SP:C3MC80 AROA_SINFN 3-phosphoshikimate 1-carboxyvinyltransferase OS=Sinorhizobium fredii (strain NBRC 101917 / NGR234) OX=394 GN=aroA

PE=3 SV=1

Length=448

Score = 807 bits (2084), Expect = 0.0

| Identities | = 410/448 (92%), Positives = 427/448 (95%), Gaps = 0/448 (0%) |  |
| --- | --- | --- |
| Query 1 | MLHGASSRPATARKSSGLSGTVRIPGDKSISHRSFMFGGLASGETRITGLLEGEDVINTG | 60 |
| Sbjct 1 | M HG S RPATA+KS+ L GTVRIPGDKSISHRSFMFGGLASGETRITGLLEGEDVINTG MSHGLSPRPATAKKSADLKGTVRIPGDKSISHRSFMFGGLASGETRITGLLEGEDVINTG | 60 |
| Query 61 | KAMQAMGARIRKEGDTWIIDGVGNGGLLAPEAPLDFGNAATGCRLTMGLVGVYDFDSTFI | 120 |
| Sbjct 61 | KAMQAMGA+IRKEGDTWII+GVGNG LLAPEAPLDFGNA TGCRLTMGLVGVYDFDSTFI KAMQAMGAKIRKEGDTWIINGVGNGALLAPEAPLDFGNAGTGCRLTMGLVGVYDFDSTFI | 120 |
| Query 121 | GDASLTKRPMGRVLNPLREMGVQVKSEDGDRLPVTLRGPKTPTPITYRVPMASAQVKSAV | 180 |
| Sbjct 121 | GDASLTKRPMGRVL+PLREMGVQVKS +GDRLPVTLRGPKTP PITYRVPMASAQVKSAV GDASLTKRPMGRVLDPLREMGVQVKSAEGDRLPVTLRGPKTPNPITYRVPMASAQVKSAV | 180 |
| Query 181 | LLAGLNTPGITTVIEPIMTRDHTEKMLQGFGANLTVETDADGVRTIRLEGRGKLTGQVID | 240 |
| Sbjct 181 | LLAGLNTPGITTVIEP+MTRDHTEKMLQGFGANL+VETD GVRTIRLEGRGKLTGQVID LLAGLNTPGITTVIEPVMTRDHTEKMLQGFGANLSVETDTAGVRTIRLEGRGKLTGQVID | 240 |
| Query 241 | VPGDPSSTAFPLVAALLVPGSDVTILNVLMNPTRTGLILTLQEMGADIEVINPRLAGGED | 300 |
| Sbjct 241 | VPGDPSSTAFPLVAALLVPGSDVTILNVLMNPTRTGLILTLQEMGA+IEV+NPRLAGGED VPGDPSSTAFPLVAALLVPGSDVTILNVLMNPTRTGLILTLQEMGANIEVMNPRLAGGED | 300 |
| Query 301 | VADLRVRSSTLKGVTVPEDRAPSMIDEYPILAVAAAFAEGATVMNGLEELRVKESDRLSA | 360 |
| Sbjct 301 | VADLRVR S LKGVTVPE+RAPSMIDEYP+LAVAAAFAEGATVMNGL+ELRVKESDRLSA VADLRVRYSELKGVTVPEERAPSMIDEYPVLAVAAAFAEGATVMNGLDELRVKESDRLSA | 360 |
| Query 361 | VANGLKLNGVDCDEGETSLVVRGRPDGKGLGNASGAAVATHLDHRIAMSFLVMGLVSENP | 420 |
| Sbjct 361 | VA+GLKLNGVDCDEGE SLVVRGRP GKGLGNA+G V THLDHRIAMSFLV+GL SE+P VADGLKLNGVDCDEGEASLVVRGRPGGKGLGNAAGGQVKTHLDHRIAMSFLVLGLASEHP | 420 |
| Query 421  Sbjct 421 | VTVDDATMIATSFPEFMDLMAGLGAKIE 448 VTVDDATMIATSFPEFMDLM GLGA IE VTVDDATMIATSFPEFMDLMTGLGATIE 448 |  |

>TR:I3XFD9 I3XFD9_RHIFR 3-phosphoshikimate 1-carboxyvinyltransferase OS=Sinorhizobium fredii USDA 257 OX=1185652 GN=aroA PE=3 SV=1

Length=448

Score = 801 bits (2069), Expect = 0.0

| Identities | = 405/448 (90%), Positives = 426/448 (95%), Gaps = 0/448 (0%) |  |
| --- | --- | --- |
| Query 1 | MLHGASSRPATARKSSGLSGTVRIPGDKSISHRSFMFGGLASGETRITGLLEGEDVINTG | 60 |
| Sbjct 1 | M HG S RPATA+KS+ L GTVRIPGDKSISHRSFMFGGLA+GETRITGLLEGEDVINTG MSHGLSPRPATAKKSADLKGTVRIPGDKSISHRSFMFGGLAAGETRITGLLEGEDVINTG | 60 |
| Query 61 | KAMQAMGARIRKEGDTWIIDGVGNGGLLAPEAPLDFGNAATGCRLTMGLVGVYDFDSTFI | 120 |
| Sbjct 61 | KAMQAMGA+IRKEGDTWII+GVGNG LLAPEAPLDFGNA TGCRLTMGLVGVYDFDSTFI KAMQAMGAKIRKEGDTWIINGVGNGALLAPEAPLDFGNAGTGCRLTMGLVGVYDFDSTFI | 120 |
| Query 121 | GDASLTKRPMGRVLNPLREMGVQVKSEDGDRLPVTLRGPKTPTPITYRVPMASAQVKSAV | 180 |
| Sbjct 121 | GDASLTKRPMGRVL+PLREMGVQVKS DGDRLPVTLRGPKTP PITYRVPMASAQVKSAV GDASLTKRPMGRVLDPLREMGVQVKSADGDRLPVTLRGPKTPNPITYRVPMASAQVKSAV | 180 |
| Query 181 | LLAGLNTPGITTVIEPIMTRDHTEKMLQGFGANLTVETDADGVRTIRLEGRGKLTGQVID | 240 |
| Sbjct 181 | LLAGLNTPGITTVIEP++TRDHTEKMLQGFGANLTVETD VRTIRLEGRGKLTGQVID LLAGLNTPGITTVIEPVLTRDHTEKMLQGFGANLTVETDPQDVRTIRLEGRGKLTGQVID | 240 |
| Query 241 | VPGDPSSTAFPLVAALLVPGSDVTILNVLMNPTRTGLILTLQEMGADIEVINPRLAGGED | 300 |
| Sbjct 241 | VPGDPSSTAFPLVAAL+VPGS++TILNVLMNPTRTGLILTLQEMGADI+++N RLAGGED VPGDPSSTAFPLVAALIVPGSEITILNVLMNPTRTGLILTLQEMGADIDLMNERLAGGED | 300 |
| Query 301 | VADLRVRSSTLKGVTVPEDRAPSMIDEYPILAVAAAFAEGATVMNGLEELRVKESDRLSA | 360 |
| Sbjct 301 | VADLRVR S LKGVTVPE+RAPSMIDEYP+LAVAAAFAEGATVMNGL+ELRVKESDRLSA VADLRVRYSELKGVTVPEERAPSMIDEYPVLAVAAAFAEGATVMNGLDELRVKESDRLSA | 360 |
| Query 361 | VANGLKLNGVDCDEGETSLVVRGRPDGKGLGNASGAAVATHLDHRIAMSFLVMGLVSENP | 420 |
| Sbjct 361 | VA+GLKLNGVDCDEGE SLVVRGRP GKGLGNA+G V THLDHRIAMSFLVMGL SE+P VADGLKLNGVDCDEGEASLVVRGRPGGKGLGNAAGGQVKTHLDHRIAMSFLVMGLASEHP | 420 |
| Query 421  Sbjct 421 | VTVDDATMIATSFPEFMDLMAGLGAKIE 448 VTVDDA+MIATSFPEFMDLM GLGAKIE VTVDDASMIATSFPEFMDLMTGLGAKIE 448 |  |

>TR:A0A561QR25 A0A561QR25_9RHIZ 3-phosphoshikimate 1-carboxyvinyltransferase OS=Neorhizobium alkalisoli OX=528178 GN=aroA PE=3 SV=1

Length=451

Score = 775 bits (2001), Expect = 0.0

| Identities | = 394/448 (88%), Positives = 413/448 (92%), Gaps = 0/448 (0%) |  |
| --- | --- | --- |
| Query 1 | MLHGASSRPATARKSSGLSGTVRIPGDKSISHRSFMFGGLASGETRITGLLEGEDVINTG | 60 |
| Sbjct 1 | M HG + +PA ++KS LSGTVRIPGDKSISHRSFMFGGLASGETRITGLLEGEDVINTG MSHGIALKPARSKKSENLSGTVRIPGDKSISHRSFMFGGLASGETRITGLLEGEDVINTG | 60 |
| Query 61 | KAMQAMGARIRKEGDTWIIDGVGNGGLLAPEAPLDFGNAATGCRLTMGLVGVYDFDSTFI | 120 |
| Sbjct 61 | KAM+AMGA+I K G WII G GNG LLAPEAPLDFGNA TGCRLTMGLVGVYDFDSTFI KAMRAMGAKIEKVGAEWIIQGTGNGALLAPEAPLDFGNAGTGCRLTMGLVGVYDFDSTFI | 120 |
| Query 121 | GDASLTKRPMGRVLNPLREMGVQVKSEDGDRLPVTLRGPKTPTPITYRVPMASAQVKSAV | 180 |
| Sbjct 121 | GDASLTKRPMGRVLNPLREMGVQVK+ DGDRLPVTLRGPKTP PI YRVPMASAQVKSAV GDASLTKRPMGRVLNPLREMGVQVKAADGDRLPVTLRGPKTPNPIVYRVPMASAQVKSAV | 180 |
| Query 181 | LLAGLNTPGITTVIEPIMTRDHTEKMLQGFGANLTVETDADGVRTIRLEGRGKLTGQVID | 240 |
| Sbjct 181 | LLAGLNTPGITTVIEP+MTRDHTEKMLQGFGANLTVETDADGVRTIRLEGRGKLTGQVID  LLAGLNTPGITTVIEPVMTRDHTEKMLQGFGANLTVETDADGVRTIRLEGRGKLTGQVID | 240 |

| Query | 241 | VPGDPSSTAFPLVAALLVPGSDVTILNVLMNPTRTGLILTLQEMGADIEVINPRLAGGED | 300 |
| --- | --- | --- | --- |
| Sbjct | 241 | VPGDPSSTAFPLVAALLVPGSDVTI NVLMNPTRTGLILTLQEMGADIEV+N R AGGED VPGDPSSTAFPLVAALLVPGSDVTIKNVLMNPTRTGLILTLQEMGADIEVMNARSAGGED | 300 |
| Query | 301 | VADLRVRSSTLKGVTVPEDRAPSMIDEYPILAVAAAFAEGATVMNGLEELRVKESDRLSA | 360 |
| Sbjct | 301 | VADLRVR S L+GVTVP DRAPSMIDEYP+LAVAAA+AEG T M GLEELRVKESDRLSA VADLRVRGSELRGVTVPADRAPSMIDEYPVLAVAAAYAEGTTTMLGLEELRVKESDRLSA | 360 |
| Query | 361 | VANGLKLNGVDCDEGETSLVVRGRPDGKGLGNASGAAVATHLDHRIAMSFLVMGLVSENP | 420 |
| Sbjct | 361 | VA GLKLNGVDCDEG+ +LVVRGRP GKG GNASGAAV THLDHRIAMSFLV+GL SE+ VAEGLKLNGVDCDEGKETLVVRGRPGGKGYGNASGAAVVTHLDHRIAMSFLVLGLASEHE | 420 |
| Query  Sbjct | 421  421 | VTVDDATMIATSFPEFMDLMAGLGAKIE 448 VTVDD ++IATSFPEFMDLM GLGA+IE VTVDDTSIIATSFPEFMDLMTGLGARIE 448 |  |

>TR:L0NN29 L0NN29_9RHIZ 3-phosphoshikimate 1-carboxyvinyltransferase OS=Rhizobium sp. NT-26 OX=1125847 GN=aroA PE=3 SV=1

Length=455

Score = 775 bits (2000), Expect = 0.0

| Identities | = 395/455 (87%), Positives = 418/455 (92%), Gaps = 0/455 (0%) |  |
| --- | --- | --- |
| Query 1 | MLHGASSRPATARKSSGLSGTVRIPGDKSISHRSFMFGGLASGETRITGLLEGEDVINTG | 60 |
| Sbjct 1 | M HG + +PA + KS+ L GTVRIPGDKSISHRSFMFGGLA+GETRITGLLEGEDVINTG MSHGVALKPARSLKSADLRGTVRIPGDKSISHRSFMFGGLAAGETRITGLLEGEDVINTG | 60 |
| Query 61 | KAMQAMGARIRKEGDTWIIDGVGNGGLLAPEAPLDFGNAATGCRLTMGLVGVYDFDSTFI | 120 |
| Sbjct 61 | KAM AMGARI K GD WII G GNG LLAPEAPLDFGNA TGCRLTMGLVGVYDF+STFI KAMHAMGARIEKVGDEWIIQGTGNGALLAPEAPLDFGNAGTGCRLTMGLVGVYDFESTFI | 120 |
| Query 121 | GDASLTKRPMGRVLNPLREMGVQVKSEDGDRLPVTLRGPKTPTPITYRVPMASAQVKSAV | 180 |
| Sbjct 121 | GDASLTKRPMGRVLNPLREMGVQVK+ +GDRLPVTLRGP P PITYRVPMASAQVKSAV GDASLTKRPMGRVLNPLREMGVQVKAAEGDRLPVTLRGPSIPNPITYRVPMASAQVKSAV | 180 |
| Query 181 | LLAGLNTPGITTVIEPIMTRDHTEKMLQGFGANLTVETDADGVRTIRLEGRGKLTGQVID | 240 |
| Sbjct 181 | LLAGLNTPG+TTVIEP+MTRDHTEKMLQGFGANL VE DADGVRTIRLEGRG+L GQVID LLAGLNTPGVTTVIEPVMTRDHTEKMLQGFGANLMVELDADGVRTIRLEGRGQLIGQVID | 240 |
| Query 241 | VPGDPSSTAFPLVAALLVPGSDVTILNVLMNPTRTGLILTLQEMGADIEVINPRLAGGED | 300 |
| Sbjct 241 | VPGDPSSTAFPLVAALLVPGSDVTI NVLMNPTRTGLILTLQEMGADIEVINPRLAGGED VPGDPSSTAFPLVAALLVPGSDVTIENVLMNPTRTGLILTLQEMGADIEVINPRLAGGED | 300 |
| Query 301 | VADLRVRSSTLKGVTVPEDRAPSMIDEYPILAVAAAFAEGATVMNGLEELRVKESDRLSA | 360 |
| Sbjct 301 | VADLRVR S LKGVTVP DRAPSMIDEYP+LAVAAAFAEGAT M GLEELRVKESDRLSA VADLRVRHSELKGVTVPADRAPSMIDEYPVLAVAAAFAEGATTMLGLEELRVKESDRLSA | 360 |
| Query 361 | VANGLKLNGVDCDEGETSLVVRGRPDGKGLGNASGAAVATHLDHRIAMSFLVMGLVSENP | 420 |
| Sbjct 361 | VA+GLKLNGVDCDEGE +LVVRGRP GKG GNA+G++V THLDHRIAMSFLVMGLVSE+ VADGLKLNGVDCDEGEDTLVVRGRPGGKGYGNATGSSVVTHLDHRIAMSFLVMGLVSEHA | 420 |
| Query 421  Sbjct 421 | VTVDDATMIATSFPEFMDLMAGLGAKIELSDTKAA 455 VTVDDA +IATSFPEFMDLM GLGA+IE +++AA VTVDDAGIIATSFPEFMDLMTGLGARIEPVESRAA 455 |  |

>SP:Q1MN56 AROA_RHIL3 3-phosphoshikimate 1-carboxyvinyltransferase OS=Rhizobium leguminosarum bv. viciae (strain 3841) OX=216596 GN=aroA

PE=3 SV=1

Length=452

Score = 773 bits (1995), Expect = 0.0

Identities = 389/449 (87%), Positives = 418/449 (93%), Gaps = 0/449 (0%)

| Query | 1 | MLHGASSRPATARKSSGLSGTVRIPGDKSISHRSFMFGGLASGETRITGLLEGEDVINTG | 60 |
| --- | --- | --- | --- |
| Sbjct | 1 | ML+G++S+PATARKS+GL+G+VRIPGDKSISHRSFM GGLASGETRITGLLEGEDVINTG MLNGSASKPATARKSAGLTGSVRIPGDKSISHRSFMIGGLASGETRITGLLEGEDVINTG | 60 |
| Query | 61 | KAMQAMGARIRKEGDTWIIDGVGNGGLLAPEAPLDFGNAATGCRLTMGLVGVYDFDSTFI | 120 |
| Sbjct | 61 | +AMQAMGARIRKEG W+I+G GNG LLAP+APLDFGNA TG RLTMGLVG YDF STFI RAMQAMGARIRKEGAQWVIEGTGNGALLAPDAPLDFGNAGTGVRLTMGLVGTYDFHSTFI | 120 |
| Query | 121 | GDASLTKRPMGRVLNPLREMGVQVKSEDGDRLPVTLRGPKTPTPITYRVPMASAQVKSAV | 180 |
| Sbjct | 121 | GDASL+KRPMGRVLNPLREMGVQV + +GDRLPVTLRGP TP+PI YRVPMASAQVKSAV GDASLSKRPMGRVLNPLREMGVQVSASEGDRLPVTLRGPGTPSPIRYRVPMASAQVKSAV | 180 |
| Query | 181 | LLAGLNTPGITTVIEPIMTRDHTEKMLQGFGANLTVETDADGVRTIRLEGRGKLTGQVID | 240 |
| Sbjct | 181 | LLAGLNTPG+TTVIEP+MTRDHTEKMLQGFGA L+VETD DGVRTIRLEGRGKL GQVID LLAGLNTPGVTTVIEPVMTRDHTEKMLQGFGAALSVETDGDGVRTIRLEGRGKLAGQVID | 240 |
| Query | 241 | VPGDPSSTAFPLVAALLVPGSDVTILNVLMNPTRTGLILTLQEMGADIEVINPRLAGGED | 300 |
| Sbjct | 241 | VPGDPSSTAFPLVAAL+VPGSD+TI+NVLMNPTRTGLILTLQEMGADIEV+N RLAGGED VPGDPSSTAFPLVAALIVPGSDITIVNVLMNPTRTGLILTLQEMGADIEVVNARLAGGED | 300 |
| Query | 301 | VADLRVRSSTLKGVTVPEDRAPSMIDEYPILAVAAAFAEGATVMNGLEELRVKESDRLSA | 360 |
| Sbjct | 301 | VADLRVR S LKGVTVPEDRAPSMIDEYPILAVAA FAEGATVM GLEELRVKESDRLSA VADLRVRHSELKGVTVPEDRAPSMIDEYPILAVAACFAEGATVMKGLEELRVKESDRLSA | 360 |
| Query | 361 | VANGLKLNGVDCDEGETSLVVRGRPDGKGLGNASGAAVATHLDHRIAMSFLVMGLVSENP | 420 |
| Sbjct | 361 | VA+GLKLNGVDCDEGE L+VRGRPDGKGLGNA+ V+THLDHRIAMSFLV+GL SE+ VADGLKLNGVDCDEGEDFLIVRGRPDGKGLGNAADGRVSTHLDHRIAMSFLVLGLASEHA | 420 |
| Query  Sbjct | 421  421 | VTVDDATMIATSFPEFMDLMAGLGAKIEL 449 VT+DDA MIATSFPEFM LM GLGAKIEL VTIDDAAMIATSFPEFMQLMTGLGAKIEL 449 |  |

>TR:A0A5C4XV71 A0A5C4XV71_9RHIZ 3-phosphoshikimate 1-carboxyvinyltransferase OS=Rhizobium smilacinae OX=1395944 GN=aroA PE=3 SV=1

Length=455

Score = 769 bits (1985), Expect = 0.0

| Identities | = 390/455 (86%), Positives = 415/455 (91%), Gaps = 0/455 (0%) |  |
| --- | --- | --- |
| Query 1 | MLHGASSRPATARKSSGLSGTVRIPGDKSISHRSFMFGGLASGETRITGLLEGEDVINTG | 60 |
| Sbjct 1 | M HG + +PA + +S L GT+RIPGDKSISHRSFMFGGLASGETRITGLLEGEDVINTG MSHGTALKPAKSARSEALKGTIRIPGDKSISHRSFMFGGLASGETRITGLLEGEDVINTG | 60 |
| Query 61 | KAMQAMGARIRKEGDTWIIDGVGNGGLLAPEAPLDFGNAATGCRLTMGLVGVYDFDSTFI | 120 |
| Sbjct 61 | KAM+AMGA+I K G WII G GNG LLAPEAPLDFGNA TGCRLTMGLVGVYDFDSTFI KAMRAMGAKIEKVGAEWIIQGTGNGALLAPEAPLDFGNAGTGCRLTMGLVGVYDFDSTFI | 120 |
| Query 121 | GDASLTKRPMGRVLNPLREMGVQVKSEDGDRLPVTLRGPKTPTPITYRVPMASAQVKSAV | 180 |
| Sbjct 121 | GDASLTKRPMGRVLNPLREMGVQV S DGDRLPVTLRGPKTP PITYRVPMASAQVKSAV GDASLTKRPMGRVLNPLREMGVQVTSHDGDRLPVTLRGPKTPNPITYRVPMASAQVKSAV | 180 |
| Query 181 | LLAGLNTPGITTVIEPIMTRDHTEKMLQGFGANLTVETDADGVRTIRLEGRGKLTGQVID | 240 |
| Sbjct 181 | LLAGLNTPGITTVIEP+MTRDHTEKMLQGFGA+L+VETDADGVRTIRL GRG+L GQVID LLAGLNTPGITTVIEPVMTRDHTEKMLQGFGADLSVETDADGVRTIRLVGRGQLKGQVID | 240 |
| Query 241 | VPGDPSSTAFPLVAALLVPGSDVTILNVLMNPTRTGLILTLQEMGADIEVINPRLAGGED | 300 |
| Sbjct 241 | VPGDPSSTAFPLVAALL PGSDVTILNVLMNPTRTGLILTLQEMGADIEV+N RLAGGED VPGDPSSTAFPLVAALLTPGSDVTILNVLMNPTRTGLILTLQEMGADIEVLNARLAGGED | 300 |
| Query 301 | VADLRVRSSTLKGVTVPEDRAPSMIDEYPILAVAAAFAEGATVMNGLEELRVKESDRLSA | 360 |
| Sbjct 301 | VADLRVR S LKGVTVP DRAPSMIDEYP+LAVAAA+A G T M GLEELRVKESDRLSA  VADLRVRYSELKGVTVPADRAPSMIDEYPVLAVAAAYASGTTTMLGLEELRVKESDRLSA | 360 |

| Query | 361 | VANGLKLNGVDCDEGETSLVVRGRPDGKGLGNASGAAVATHLDHRIAMSFLVMGLVSENP 420 |
| --- | --- | --- |
| Sbjct | 361 | VA+GLKLNGVDCDEG+ +L VRGRP GKG GNA+GAAV THLDHRIAMSFLV+GL SE+P VADGLKLNGVDCDEGKETLTVRGRPGGKGYGNAAGAAVVTHLDHRIAMSFLVLGLASEHP 420 |
| Query | 421 | VTVDDATMIATSFPEFMDLMAGLGAKIELSDTKAA 455 |
| Sbjct | 421 | VTVDDA++IATSFPEFMDLM GLGAKI+L ++ A  VTVDDASIIATSFPEFMDLMTGLGAKIDLVGSELA 455 |

>TR:A0A1I0WNM9 A0A1I0WNM9_9RHIZ 3-phosphoshikimate 1-carboxyvinyltransferase OS=Rhizobium sp. NFR07 OX=1566262 GN=aroA PE=3 SV=1

Length=454

Score = 765 bits (1976), Expect = 0.0

| Identities | = 384/454 (85%), Positives = 419/454 (92%), Gaps = 0/454 (0%) |  |
| --- | --- | --- |
| Query 1 | MLHGASSRPATARKSSGLSGTVRIPGDKSISHRSFMFGGLASGETRITGLLEGEDVINTG | 60 |
| Sbjct 1 | M HG + +PA + +S L+GT++IPGDKSISHRSFMFGGLASGETRITGLLEGEDVINTG MSHGIALKPARSTRSEALNGTIKIPGDKSISHRSFMFGGLASGETRITGLLEGEDVINTG | 60 |
| Query 61 | KAMQAMGARIRKEGDTWIIDGVGNGGLLAPEAPLDFGNAATGCRLTMGLVGVYDFDSTFI | 120 |
| Sbjct 61 | +AM+AMGA I K G W+I G GNG LLAP+APLDFGNA TGCRLTMGLVGVYDFDSTFI RAMRAMGATIEKVGAEWVIQGTGNGALLAPQAPLDFGNAGTGCRLTMGLVGVYDFDSTFI | 120 |
| Query 121 | GDASLTKRPMGRVLNPLREMGVQVKSEDGDRLPVTLRGPKTPTPITYRVPMASAQVKSAV | 180 |
| Sbjct 121 | GDASLTKRPMGRVLNPLREMGVQV S +GDRLPVTLRGPKTP PITYRVPMASAQVKSAV GDASLTKRPMGRVLNPLREMGVQVTSTEGDRLPVTLRGPKTPNPITYRVPMASAQVKSAV | 180 |
| Query 181 | LLAGLNTPGITTVIEPIMTRDHTEKMLQGFGANLTVETDADGVRTIRLEGRGKLTGQVID | 240 |
| Sbjct 181 | LLAGLNTPGITTVIEP+MTRDHTEKMLQGFGA+L+V+TDA+GVRTIRL GRG+L GQVID LLAGLNTPGITTVIEPVMTRDHTEKMLQGFGADLSVDTDAEGVRTIRLVGRGQLKGQVID | 240 |
| Query 241 | VPGDPSSTAFPLVAALLVPGSDVTILNVLMNPTRTGLILTLQEMGADIEVINPRLAGGED | 300 |
| Sbjct 241 | VPGDPSSTAFPLVAALLVPGSDVTILNVLMNPTRTGLILTLQEMGADIE++NPRLAGGED VPGDPSSTAFPLVAALLVPGSDVTILNVLMNPTRTGLILTLQEMGADIEILNPRLAGGED | 300 |
| Query 301 | VADLRVRSSTLKGVTVPEDRAPSMIDEYPILAVAAAFAEGATVMNGLEELRVKESDRLSA | 360 |
| Sbjct 301 | VADLR+R+S LKGVTVP DRAPSMIDEYP+LAVAAA+A G+T M GLEELRVKESDRLSA VADLRIRASELKGVTVPADRAPSMIDEYPVLAVAAAYATGSTTMLGLEELRVKESDRLSA | 360 |
| Query 361 | VANGLKLNGVDCDEGETSLVVRGRPDGKGLGNASGAAVATHLDHRIAMSFLVMGLVSENP | 420 |
| Sbjct 361 | VA GLKLNGVDCDEG+ +LVVRGRPDGKG GNA+GAAV THLDHRIAMSFLV+GLVSE+ VAEGLKLNGVDCDEGKETLVVRGRPDGKGYGNAAGAAVVTHLDHRIAMSFLVLGLVSEHA | 420 |
| Query 421  Sbjct 421 | VTVDDATMIATSFPEFMDLMAGLGAKIELSDTKA 454 VTVDDA++IATSFPEFMDLM GLGA+I+L + A VTVDDASIIATSFPEFMDLMTGLGARIDLVEEAA 454 |  |

>TR:A0A081MDT9 A0A081MDT9_9RHIZ 3-phosphoshikimate 1-carboxyvinyltransferase OS=Pseudorhizobium pelagicum OX=1509405 GN=aroA PE=3 SV=1

Length=455

Score = 765 bits (1976), Expect = 0.0

Identities = 390/455 (86%), Positives = 416/455 (91%), Gaps = 0/455 (0%)

| Query | 1 | MLHGASSRPATARKSSGLSGTVRIPGDKSISHRSFMFGGLASGETRITGLLEGEDVINTG | 60 |
| --- | --- | --- | --- |
| Sbjct | 1 | M HG + +PA + KS+ L GTVRIPGDKSISHRSFMFGGLASGETRITGLLEGEDVINTG MSHGVALKPARSLKSADLKGTVRIPGDKSISHRSFMFGGLASGETRITGLLEGEDVINTG | 60 |
| Query | 61 | KAMQAMGARIRKEGDTWIIDGVGNGGLLAPEAPLDFGNAATGCRLTMGLVGVYDFDSTFI | 120 |
| Sbjct | 61 | KAMQAMGA+I K G WII G GNG LLAPEAPLDFGNA TGCRLTMGLVGVYDFDSTFI  KAMQAMGAKIEKVGAEWIIQGTGNGALLAPEAPLDFGNAGTGCRLTMGLVGVYDFDSTFI | 120 |

| Query | 121 | GDASLTKRPMGRVLNPLREMGVQVKSEDGDRLPVTLRGPKTPTPITYRVPMASAQVKSAV | 180 |
| --- | --- | --- | --- |
| Sbjct | 121 | GDASL+KRPMGRVL PLREMGVQV + +GDRLPVTLRGP TP PITYRVPMASAQVKSAV GDASLSKRPMGRVLAPLREMGVQVTAAEGDRLPVTLRGPATPNPITYRVPMASAQVKSAV | 180 |
| Query | 181 | LLAGLNTPGITTVIEPIMTRDHTEKMLQGFGANLTVETDADGVRTIRLEGRGKLTGQVID | 240 |
| Sbjct | 181 | LLAGLNTPG TTVIEP+MTRDHTEKMLQGFGA+LTVETDADGVRTIRL+GRG+LTGQVID LLAGLNTPGTTTVIEPVMTRDHTEKMLQGFGADLTVETDADGVRTIRLQGRGRLTGQVID | 240 |
| Query | 241 | VPGDPSSTAFPLVAALLVPGSDVTILNVLMNPTRTGLILTLQEMGADIEVINPRLAGGED | 300 |
| Sbjct | 241 | VPGDPSSTAFPLVAALLVPGS+VTI NVLMNPTRTGLILTLQEMGADIEV+N RLAGGED VPGDPSSTAFPLVAALLVPGSEVTIENVLMNPTRTGLILTLQEMGADIEVLNARLAGGED | 300 |
| Query | 301 | VADLRVRSSTLKGVTVPEDRAPSMIDEYPILAVAAAFAEGATVMNGLEELRVKESDRLSA | 360 |
| Sbjct | 301 | VADLRVR S LKGVTVP +RAPSMIDEYP+LAVAAAFAEG T M G+EELRVKESDRLSA VADLRVRHSQLKGVTVPAERAPSMIDEYPVLAVAAAFAEGTTTMLGVEELRVKESDRLSA | 360 |
| Query | 361 | VANGLKLNGVDCDEGETSLVVRGRPDGKGLGNASGAAVATHLDHRIAMSFLVMGLVSENP | 420 |
| Sbjct | 361 | A GLKLNGVDCDEGE +L VRGRP GKG GNA+G AVATHLDHRIAMSFLV+GLVSE+P TAAGLKLNGVDCDEGEDTLTVRGRPGGKGYGNAAGEAVATHLDHRIAMSFLVLGLVSEHP | 420 |
| Query  Sbjct | 421  421 | VTVDDATMIATSFPEFMDLMAGLGAKIELSDTKAA 455 VTVDDA +IATSFPEFMDLMAGLGA+IE +++AA VTVDDAGIIATSFPEFMDLMAGLGARIEAVESRAA 455 |  |

>TR:A0A4R5UHY4 A0A4R5UHY4_9RHIZ 3-phosphoshikimate 1-carboxyvinyltransferase OS=Rhizobium deserti OX=2547961 GN=aroA PE=3 SV=1

Length=455

Score = 765 bits (1975), Expect = 0.0

| Identities | = 389/455 (85%), Positives = 413/455 (91%), Gaps = 0/455 (0%) |  |
| --- | --- | --- |
| Query 1 | MLHGASSRPATARKSSGLSGTVRIPGDKSISHRSFMFGGLASGETRITGLLEGEDVINTG | 60 |
| Sbjct 1 | M HG + +PA +RKS+ LSGTVRIPGDKSISHRSFMFGGLASGETRITGLLEGEDVINTG MSHGIALKPAKSRKSTNLSGTVRIPGDKSISHRSFMFGGLASGETRITGLLEGEDVINTG | 60 |
| Query 61 | KAMQAMGARIRKEGDTWIIDGVGNGGLLAPEAPLDFGNAATGCRLTMGLVGVYDFDSTFI | 120 |
| Sbjct 61 | KAMQAMGARI K G WII G GNG LLAPEAPLDFGNA TGCRLTMGLVGVYDFDSTFI KAMQAMGARIEKVGAEWIIQGTGNGALLAPEAPLDFGNAGTGCRLTMGLVGVYDFDSTFI | 120 |
| Query 121 | GDASLTKRPMGRVLNPLREMGVQVKSEDGDRLPVTLRGPKTPTPITYRVPMASAQVKSAV | 180 |
| Sbjct 121 | GD SL+KRPMGRVL+PLREMGVQV S GDRLPVTLRGP+TP PITYRVPMASAQVKSAV GDGSLSKRPMGRVLDPLREMGVQVTSTAGDRLPVTLRGPRTPNPITYRVPMASAQVKSAV | 180 |
| Query 181 | LLAGLNTPGITTVIEPIMTRDHTEKMLQGFGANLTVETDADGVRTIRLEGRGKLTGQVID | 240 |
| Sbjct 181 | LLAGLNTPGITTVIEP+MTRDHTEKMLQGFGANLTVETD DGVRTIRLEGRG+L GQVID LLAGLNTPGITTVIEPVMTRDHTEKMLQGFGANLTVETDGDGVRTIRLEGRGRLVGQVID | 240 |
| Query 241 | VPGDPSSTAFPLVAALLVPGSDVTILNVLMNPTRTGLILTLQEMGADIEVINPRLAGGED | 300 |
| Sbjct 241 | VPGDPSSTAFPLVAALLVPGSD+ I NVLMNPTRTGLILTLQEMGADI+V+N R AGGED VPGDPSSTAFPLVAALLVPGSDLVIENVLMNPTRTGLILTLQEMGADIDVMNARSAGGED | 300 |
| Query 301 | VADLRVRSSTLKGVTVPEDRAPSMIDEYPILAVAAAFAEGATVMNGLEELRVKESDRLSA | 360 |
| Sbjct 301 | VADLRVR S LKGVTVP DRAPSMIDEYP+LA+AAAFAEG T M GLEELRVKESDRLSA VADLRVRGSELKGVTVPADRAPSMIDEYPVLAIAAAFAEGTTTMLGLEELRVKESDRLSA | 360 |
| Query 361 | VANGLKLNGVDCDEGETSLVVRGRPDGKGLGNASGAAVATHLDHRIAMSFLVMGLVSENP | 420 |
| Sbjct 361 | A GLKLNGVDCDEG+ +LVVRGRP GKG GNA+G AV THLDHRIAMSFLVMGL SE+P TAAGLKLNGVDCDEGKETLVVRGRPGGKGYGNAAGKAVVTHLDHRIAMSFLVMGLASEHP | 420 |
| Query 421  Sbjct 421 | VTVDDATMIATSFPEFMDLMAGLGAKIELSDTKAA 455 V+VDDA +IATSFPEFMDLM GLGA+IE +++AA VSVDDAGIIATSFPEFMDLMTGLGARIESDESQAA 455 |  |

>TR:A0A4R5PM71 A0A4R5PM71_9RHIZ 3-phosphoshikimate 1-carboxyvinyltransferase OS=Pseudohoeflea suaedae OX=877384 GN=aroA PE=3 SV=1

Length=452

Score = 756 bits (1951), Expect = 0.0

| Identities | = 382/451 (85%), Positives = 409/451 (91%), Gaps = 0/451 (0%) |  |
| --- | --- | --- |
| Query 1 | MLHGASSRPATARKSSGLSGTVRIPGDKSISHRSFMFGGLASGETRITGLLEGEDVINTG | 60 |
| Sbjct 1 | M H RPATAR+SS L G +RIPGDKSISHRSFMFGGLA GETRITGLLEGEDVINTG MNHVNKLRPATARRSSALKGDIRIPGDKSISHRSFMFGGLARGETRITGLLEGEDVINTG | 60 |
| Query 61 | KAMQAMGARIRKEGDTWIIDGVGNGGLLAPEAPLDFGNAATGCRLTMGLVGVYDFDSTFI | 120 |
| Sbjct 61 | KAMQAMGA +RKEGDTWI+ G GNG LLAPEAPLDFGNA TGCRLTMGLVGVYDFDSTFI KAMQAMGASVRKEGDTWIVTGTGNGCLLAPEAPLDFGNAGTGCRLTMGLVGVYDFDSTFI | 120 |
| Query 121 | GDASLTKRPMGRVLNPLREMGVQVKSEDGDRLPVTLRGPKTPTPITYRVPMASAQVKSAV | 180 |
| Sbjct 121 | GDASL++RPMGRVLNPLREMGVQVKSE GDRLPVTL GP TP PITYRVPMASAQVKSAV GDASLSRRPMGRVLNPLREMGVQVKSEPGDRLPVTLHGPATPAPITYRVPMASAQVKSAV | 180 |
| Query 181 | LLAGLNTPGITTVIEPIMTRDHTEKMLQGFGANLTVETDADGVRTIRLEGRGKLTGQVID | 240 |
| Sbjct 181 | LLAGLNTPG+TTVIEP+MTRDHTEKML+GFGA+L VETDADGVRTIRL G+G+LTGQVID LLAGLNTPGVTTVIEPVMTRDHTEKMLRGFGADLEVETDADGVRTIRLIGQGELTGQVID | 240 |
| Query 241 | VPGDPSSTAFPLVAALLVPGSDVTILNVLMNPTRTGLILTLQEMGADIEVINPRLAGGED | 300 |
| Sbjct 241 | VPGDPSSTAFPLVAALLVPGS++ I NVLMNPTRTGLI TLQEMG DIE+++ RLAGGED VPGDPSSTAFPLVAALLVPGSEILIRNVLMNPTRTGLIQTLQEMGGDIEILDARLAGGED | 300 |
| Query 301 | VADLRVRSSTLKGVTVPEDRAPSMIDEYPILAVAAAFAEGATVMNGLEELRVKESDRLSA | 360 |
| Sbjct 301 | VADLRVR S+L+GVTVP DRAPSMIDEYP+LAVAA+FAEG TVM+GLEELRVKESDRL+A VADLRVRGSSLRGVTVPPDRAPSMIDEYPVLAVAASFAEGETVMHGLEELRVKESDRLAA | 360 |
| Query 361 | VANGLKLNGVDCDEGETSLVVRGRPDGKGLGNASGAAVATHLDHRIAMSFLVMGLVSENP | 420 |
| Sbjct 361 | VA GL+LNGVDC EGE L VRGRPDGKGLGNA G AV THLDHRIAMSFLVMGLVSE+P VARGLELNGVDCTEGEDWLTVRGRPDGKGLGNAKGEAVVTHLDHRIAMSFLVMGLVSEHP | 420 |
| Query 421  Sbjct 421 | VTVDDATMIATSFPEFMDLMAGLGAKIELSD 451 VTVDD+ +IATSFPEFMDLM GLGA I D VTVDDSAIIATSFPEFMDLMTGLGATIAARD 451 |  |

>TR:A0A0Q5DH36 A0A0Q5DH36_9RHIZ 3-phosphoshikimate 1-carboxyvinyltransferase OS=Rhizobium sp. Leaf306 OX=1736330 GN=aroA PE=3 SV=1

Length=455

Score = 751 bits (1940), Expect = 0.0

| Identities | = 381/448 (85%), Positives = 410/448 (92%), Gaps = 0/448 (0%) |  |
| --- | --- | --- |
| Query 1 | MLHGASSRPATARKSSGLSGTVRIPGDKSISHRSFMFGGLASGETRITGLLEGEDVINTG | 60 |
| Sbjct 1 | M HG + +PA +R+S+ L+GTVRIPGDKSISHRSFMFGGLA+GETRITGLLEGEDVINTG MSHGIALKPAKSRRSTNLTGTVRIPGDKSISHRSFMFGGLAAGETRITGLLEGEDVINTG | 60 |
| Query 61 | KAMQAMGARIRKEGDTWIIDGVGNGGLLAPEAPLDFGNAATGCRLTMGLVGVYDFDSTFI | 120 |
| Sbjct 61 | KAM+AMGA+I K G WII G GNG LLAP+APLDFGNA TGCRLTMGLVGVYDF+S FI KAMRAMGAKIDKVGAEWIIQGTGNGALLAPQAPLDFGNAGTGCRLTMGLVGVYDFESAFI | 120 |
| Query 121 | GDASLTKRPMGRVLNPLREMGVQVKSEDGDRLPVTLRGPKTPTPITYRVPMASAQVKSAV | 180 |
| Sbjct 121 | GDASL+KRPMGRVL+PLREMGVQV S GDRLPVTLRGP+TP PI YRVPMASAQVKSAV GDASLSKRPMGRVLDPLREMGVQVTSMAGDRLPVTLRGPRTPNPIVYRVPMASAQVKSAV | 180 |
| Query 181 | LLAGLNTPGITTVIEPIMTRDHTEKMLQGFGANLTVETDADGVRTIRLEGRGKLTGQVID | 240 |
| Sbjct 181 | LLAGLNTPG+TTVIEP+MTRDHTEKMLQGFGA+LTVETDADGVRTIRLEGRG L GQVID  LLAGLNTPGVTTVIEPVMTRDHTEKMLQGFGADLTVETDADGVRTIRLEGRGNLKGQVID | 240 |

| Query | 241 | VPGDPSSTAFPLVAALLVPGSDVTILNVLMNPTRTGLILTLQEMGADIEVINPRLAGGED | 300 |
| --- | --- | --- | --- |
| Sbjct | 241 | VPGDPSSTAFPLVAA+LVPGSDVTI+NVLMNPTRTGLILTLQEMGADIEV+N R AGGED VPGDPSSTAFPLVAAILVPGSDVTIMNVLMNPTRTGLILTLQEMGADIEVMNARSAGGED | 300 |
| Query | 301 | VADLRVRSSTLKGVTVPEDRAPSMIDEYPILAVAAAFAEGATVMNGLEELRVKESDRLSA | 360 |
| Sbjct | 301 | VADLRVR S L GVTVP DRAPSMIDEYP+LAVAAAFAEG T M GLEELRVKESDRLSA VADLRVRGSDLNGVTVPADRAPSMIDEYPVLAVAAAFAEGTTTMLGLEELRVKESDRLSA | 360 |
| Query | 361 | VANGLKLNGVDCDEGETSLVVRGRPDGKGLGNASGAAVATHLDHRIAMSFLVMGLVSENP | 420 |
| Sbjct | 361 | VA GLKLNGVDCDEG+ +LVVRGRP GKG GNASGAAV T+LDHRIAMSFLV+GL SE+ VAEGLKLNGVDCDEGKETLVVRGRPGGKGYGNASGAAVITYLDHRIAMSFLVLGLASEHE | 420 |
| Query  Sbjct | 421  421 | VTVDDATMIATSFPEFMDLMAGLGAKIE 448 VTVDDA++IATSFPEFMDLM GLGA+IE VTVDDASIIATSFPEFMDLMTGLGAQIE 448 |  |

>TR:A0A386E6K9 A0A386E6K9_9RHIZ 3-phosphoshikimate 1-carboxyvinyltransferase OS=Neorhizobium sp. NCHU2750 OX=1825976 GN=aroA PE=3 SV=1

Length=454

Score = 751 bits (1940), Expect = 0.0

| Identities | = 384/454 (85%), Positives = 407/454 (90%), Gaps = 0/454 (0%) |  |
| --- | --- | --- |
| Query 1 | MLHGASSRPATARKSSGLSGTVRIPGDKSISHRSFMFGGLASGETRITGLLEGEDVINTG | 60 |
| Sbjct 1 | M HG + +PA + +S L GT+RIPGDKSISHRSFMFGGLASGETRITGLLEGEDVINTG MSHGIALKPARSARSEALKGTIRIPGDKSISHRSFMFGGLASGETRITGLLEGEDVINTG | 60 |
| Query 61 | KAMQAMGARIRKEGDTWIIDGVGNGGLLAPEAPLDFGNAATGCRLTMGLVGVYDFDSTFI | 120 |
| Sbjct 61 | KAM+AMGA+I K G WII G GNG LLAPEAPLDFGNA TGCRLTMGLVGVYDF STFI KAMRAMGAKIDKAGAEWIIQGTGNGALLAPEAPLDFGNAGTGCRLTMGLVGVYDFPSTFI | 120 |
| Query 121 | GDASLTKRPMGRVLNPLREMGVQVKSEDGDRLPVTLRGPKTPTPITYRVPMASAQVKSAV | 180 |
| Sbjct 121 | GDASL+KRPMGRVLNPLREMGVQV S +GDRLPVTL GPKTP PITYRVPMASAQVKSAV GDASLSKRPMGRVLNPLREMGVQVSSTEGDRLPVTLHGPKTPNPITYRVPMASAQVKSAV | 180 |
| Query 181 | LLAGLNTPGITTVIEPIMTRDHTEKMLQGFGANLTVETDADGVRTIRLEGRGKLTGQVID | 240 |
| Sbjct 181 | LLAGLNTPGITTVIEP+MTRDHTEKMLQGFGA+LTV+TDA GVRTIRL GRG L GQ+ID LLAGLNTPGITTVIEPVMTRDHTEKMLQGFGADLTVDTDAQGVRTIRLVGRGDLKGQIID | 240 |
| Query 241 | VPGDPSSTAFPLVAALLVPGSDVTILNVLMNPTRTGLILTLQEMGADIEVINPRLAGGED | 300 |
| Sbjct 241 | VPGDPSSTAFPLVAALL PGSDVTILNVLMNPTRTGLILTLQEMGADIEV N RLAGGED VPGDPSSTAFPLVAALLTPGSDVTILNVLMNPTRTGLILTLQEMGADIEVQNARLAGGED | 300 |
| Query 301 | VADLRVRSSTLKGVTVPEDRAPSMIDEYPILAVAAAFAEGATVMNGLEELRVKESDRLSA | 360 |
| Sbjct 301 | VADLRVR S LKGVTVP DRAPSMIDEYP+LAVAAA+A G T M GLEELRVKESDRLSA VADLRVRYSELKGVTVPADRAPSMIDEYPVLAVAAAYATGTTTMLGLEELRVKESDRLSA | 360 |
| Query 361 | VANGLKLNGVDCDEGETSLVVRGRPDGKGLGNASGAAVATHLDHRIAMSFLVMGLVSENP | 420 |
| Sbjct 361 | VA GLKLNGVDCDEG+ +LVVRGRP GKG GNASG AV THLDHRIAMSFLV+GL SE+ VAEGLKLNGVDCDEGKETLVVRGRPGGKGYGNASGKAVVTHLDHRIAMSFLVLGLASEHA | 420 |
| Query 421  Sbjct 421 | VTVDDATMIATSFPEFMDLMAGLGAKIELSDTKA 454 VTVDDA++IATSFPEFMDLM GLGAKIE + +A VTVDDASIIATSFPEFMDLMTGLGAKIEQVEGEA 454 |  |

>TR:A0A444LEE3 A0A444LEE3_9RHIZ 3-phosphoshikimate 1-carboxyvinyltransferase OS=Rhizobium sp. 24NR OX=2503024 GN=aroA PE=3 SV=1

Length=455

Score = 750 bits (1936), Expect = 0.0

Identities = 379/448 (85%), Positives = 409/448 (91%), Gaps = 0/448 (0%)

| Query | 1 | MLHGASSRPATARKSSGLSGTVRIPGDKSISHRSFMFGGLASGETRITGLLEGEDVINTG | 60 |
| --- | --- | --- | --- |
| Sbjct | 1 | MLHG + +PA +RKSS LSGTVRIPGDKSISHRSFMFGGLASGETRITGLLEGEDVINTG MLHGVALKPAKSRKSSTLSGTVRIPGDKSISHRSFMFGGLASGETRITGLLEGEDVINTG | 60 |
| Query | 61 | KAMQAMGARIRKEGDTWIIDGVGNGGLLAPEAPLDFGNAATGCRLTMGLVGVYDFDSTFI | 120 |
| Sbjct | 61 | KAMQAMGARI K G WII G GNG LLAPEAPLDFGNA TGCRLTMGLVGVYDF+STF+ KAMQAMGARIEKLGAEWIIHGTGNGALLAPEAPLDFGNAGTGCRLTMGLVGVYDFESTFV | 120 |
| Query | 121 | GDASLTKRPMGRVLNPLREMGVQVKSEDGDRLPVTLRGPKTPTPITYRVPMASAQVKSAV | 180 |
| Sbjct | 121 | GDASL++RPMGRVL+PLR+MGVQV+S +GDRLPVTL GP+TP PI YRVPMASAQVKSAV GDASLSRRPMGRVLDPLRDMGVQVRSSEGDRLPVTLYGPRTPNPIVYRVPMASAQVKSAV | 180 |
| Query | 181 | LLAGLNTPGITTVIEPIMTRDHTEKMLQGFGANLTVETDADGVRTIRLEGRGKLTGQVID | 240 |
| Sbjct | 181 | LLAGLNTPG+TTVIEPIMTRDHTEKMLQGFGA L+VETD +GVRTIRLEGRG+LTGQ ID LLAGLNTPGVTTVIEPIMTRDHTEKMLQGFGAELSVETDGNGVRTIRLEGRGRLTGQRID | 240 |
| Query | 241 | VPGDPSSTAFPLVAALLVPGSDVTILNVLMNPTRTGLILTLQEMGADIEVINPRLAGGED | 300 |
| Sbjct | 241 | VPGDPSSTAFPLVAALLVPGSDVTI NVLMNPTRTGLILTLQEMGA+IEV+NPR AGGED VPGDPSSTAFPLVAALLVPGSDVTIENVLMNPTRTGLILTLQEMGANIEVLNPRSAGGED | 300 |
| Query | 301 | VADLRVRSSTLKGVTVPEDRAPSMIDEYPILAVAAAFAEGATVMNGLEELRVKESDRLSA | 360 |
| Sbjct | 301 | VADLRVR+S LKGVTVP +RAP MIDEYP+LAVA+AFAEG T M GLEELRVKESDRL+A VADLRVRASELKGVTVPAERAPFMIDEYPVLAVASAFAEGTTTMLGLEELRVKESDRLTA | 360 |
| Query | 361 | VANGLKLNGVDCDEGETSLVVRGRPDGKGLGNASGAAVATHLDHRIAMSFLVMGLVSENP | 420 |
| Sbjct | 361 | A GLKLNGVDCDEG +LVVRGRP GKG GN +G AV THLDHRIAMSFLVMGLVSE+ TATGLKLNGVDCDEGRDTLVVRGRPGGKGYGNPAGGAVETHLDHRIAMSFLVMGLVSEHE | 420 |
| Query  Sbjct | 421  421 | VTVDDATMIATSFPEFMDLMAGLGAKIE 448 VTVDDA++I TSFPEFMDLM GLGA+I+ VTVDDASIITTSFPEFMDLMTGLGARID 448 |  |

>TR:A0A256FYG7 A0A256FYG7_9RHIZ 3-phosphoshikimate 1-carboxyvinyltransferase OS=Ochrobactrum thiophenivorans OX=571255 GN=aroA PE=3 SV=1

Length=450

Score = 741 bits (1912), Expect = 0.0

| Identities | = 374/451 (83%), Positives = 407/451 (90%), Gaps = 3/451 (1%) |  |
| --- | --- | --- |
| Query 1 | MLHGASSRPATARKSSGLSGTVRIPGDKSISHRSFMFGGLASGETRITGLLEGEDVINTG | 60 |
| Sbjct 1 | M H AS +PA AR+S L G +RIPGDKSISHRSFMFGGLASGETRITGLLEGEDVINTG MSHSASPKPAIARRSEALKGEIRIPGDKSISHRSFMFGGLASGETRITGLLEGEDVINTG | 60 |
| Query 61 | KAMQAMGARIRKEGDTWIIDGVGNGGLLAPEAPLDFGNAATGCRLTMGLVGVYDFDSTFI | 120 |
| Sbjct 61 | +AMQAMGA+IRK+GD WII+GVGNG LL PE+PLDFGNA TG RLTMGLVG YD +TFI RAMQAMGAKIRKDGDVWIINGVGNGCLLQPESPLDFGNAGTGARLTMGLVGTYDMTTTFI | 120 |
| Query 121 | GDASLTKRPMGRVLNPLREMGVQVKSEDGDRLPVTLRGPKTPTPITYRVPMASAQVKSAV | 180 |
| Sbjct 121 | GDASLT RPMGRVLNPLREMGVQVK+ +GDRLP+TL GPKT PI+YRVPMASAQVKSAV GDASLTSRPMGRVLNPLREMGVQVKAAEGDRLPLTLTGPKTANPISYRVPMASAQVKSAV | 180 |
| Query 181 | LLAGLNTPGITTVIEPIMTRDHTEKMLQGFGANLTVETDADGVRTIRLEGRGKLTGQVID | 240 |
| Sbjct 181 | LLAGLNTPG+TTVIEP+MTRDHTEKMLQGFGA+LTVETD DGVR IR+ G+GKLTGQ ID LLAGLNTPGVTTVIEPVMTRDHTEKMLQGFGADLTVETDKDGVRHIRIVGQGKLTGQTID | 240 |
| Query 241 | VPGDPSSTAFPLVAALLVPGSDVTILNVLMNPTRTGLILTLQEMGADIEVINPRLAGGED | 300 |
| Sbjct 241 | VPGDPSSTAFPLVAALLV GSDVTILNVLMNPTRTGLILTLQEMGADIE+INPRLAGGED VPGDPSSTAFPLVAALLVEGSDVTILNVLMNPTRTGLILTLQEMGADIEIINPRLAGGED | 300 |
| Query 301 | VADLRVRSSTLKGVTVPEDRAPSMIDEYPILAVAAAFAEGATVMNGLEELRVKESDRLSA | 360 |
| Sbjct 301 | VADLRV+SS LKGV VP +RAPSMIDEYP+LA+AA+FAEG TVM+GL+ELRVKESDRL+A  VADLRVKSSKLKGVVVPPERAPSMIDEYPVLAIAASFAEGETVMDGLDELRVKESDRLAA | 360 |

| Query | 361 | VANGLKLNGVDCDEGETSLVVRGRPDGKGLGNASGAAVATHLDHRIAMSFLVMGLVSENP 420 |
| --- | --- | --- |
| Sbjct | 361 | VA GL+ NGVDC EGE SL VRGRPDGKGLG G VATHLDHRIAMSFLVMGL +E P VARGLEANGVDCTEGEMSLTVRGRPDGKGLG---GGTVATHLDHRIAMSFLVMGLAAEKP 417 |
| Query | 421 | VTVDDATMIATSFPEFMDLMAGLGAKIELSD 451 |
| Sbjct | 418 | VTVDD+TMIATSFPEFM +M GLGAKIE+S+  VTVDDSTMIATSFPEFMTMMPGLGAKIEVSE 448 |

>TR:A0A4R6NVY0 A0A4R6NVY0_9RHIZ 3-phosphoshikimate 1-carboxyvinyltransferase OS=Rhizobium sp. BK333 OX=2512158 GN=aroA PE=3 SV=1

Length=452

Score = 736 bits (1899), Expect = 0.0

| Identities | = 369/445 (83%), Positives = 404/445 (91%), Gaps = 0/445 (0%) |  |
| --- | --- | --- |
| Query 7 | SRPATARKSSGLSGTVRIPGDKSISHRSFMFGGLASGETRITGLLEGEDVINTGKAMQAM | 66 |
| Sbjct 7 | SRPATAR+S+ LSGT+R+PGDKSISHR+ MFGGLA+GETRITGLLEGEDV+NTG+AMQAM SRPATARRSTNLSGTIRVPGDKSISHRALMFGGLAAGETRITGLLEGEDVLNTGRAMQAM | 66 |
| Query 67 | GARIRKEGDTWIIDGVGNGGLLAPEAPLDFGNAATGCRLTMGLVGVYDFDSTFIGDASLT | 126 |
| Sbjct 67 | GARI K G WII GVGNG LLAPEAPLDFGNA TG RLTMGLVGVY+F+STFIGDASL+ GARIEKTGAEWIIKGVGNGALLAPEAPLDFGNAGTGSRLTMGLVGVYNFESTFIGDASLS | 126 |
| Query 127 | KRPMGRVLNPLREMGVQVKSEDGDRLPVTLRGPKTPTPITYRVPMASAQVKSAVLLAGLN | 186 |
| Sbjct 127 | KRPMGR+L+PLR+MGVQVKS +GDRLPVTL GP+T +PI YRVPMASAQVKSAVLLAGLN KRPMGRILDPLRQMGVQVKSSEGDRLPVTLHGPETASPIIYRVPMASAQVKSAVLLAGLN | 186 |
| Query 187 | TPGITTVIEPIMTRDHTEKMLQGFGANLTVETDADGVRTIRLEGRGKLTGQVIDVPGDPS | 246 |
| Sbjct 187 | PGITTVIEP+MTRDHTEKML GFGA L+VETD DGVRTI LEG+G+L+GQ+IDVPGDPS APGITTVIEPVMTRDHTEKMLAGFGAALSVETDRDGVRTISLEGQGRLSGQIIDVPGDPS | 246 |
| Query 247 | STAFPLVAALLVPGSDVTILNVLMNPTRTGLILTLQEMGADIEVINPRLAGGEDVADLRV | 306 |
| Sbjct 247 | STAFPLVAAL+VPGSD+TI NVLMNPTRTGLILTLQEMGADIE++ RLAGGEDVADLRV STAFPLVAALIVPGSDITIGNVLMNPTRTGLILTLQEMGADIEILERRLAGGEDVADLRV | 306 |
| Query 307 | RSSTLKGVTVPEDRAPSMIDEYPILAVAAAFAEGATVMNGLEELRVKESDRLSAVANGLK | 366 |
| Sbjct 307 | RSS LKG+TVP +RAPSMIDEYP+LAVAAAFA+G TVM GLEELRVKESDRLSAVA GLK RSSALKGITVPAERAPSMIDEYPVLAVAAAFADGTTVMLGLEELRVKESDRLSAVAEGLK | 366 |
| Query 367 | LNGVDCDEGETSLVVRGRPDGKGLGNASGAAVATHLDHRIAMSFLVMGLVSENPVTVDDA | 426 |
| Sbjct 367 | LNGVDC EGE SL VRGRPDGKGLGNA G+ + THLDHRIAMSFLVMGL SE+PV VDD+ LNGVDCAEGEASLTVRGRPDGKGLGNAGGSQIRTHLDHRIAMSFLVMGLASEHPVAVDDS | 426 |
| Query 427  Sbjct 427 | TMIATSFPEFMDLMAGLGAKIELSD 451 TMIATSFPEF DLM LGA IE D TMIATSFPEFFDLMTRLGATIEAED 451 |  |

>SP:P0A2Y4 AROA_PSES2 3-phosphoshikimate 1-carboxyvinyltransferase OS=Pseudomonas sp. (strain PG2982) OX=308 GN=aroA PE=1 SV=1

Length=449

Score = 735 bits (1898), Expect = 0.0

Identities = 373/450 (83%), Positives = 405/450 (90%), Gaps = 3/450 (1%)

| Query | 1 | MLHGASSRPATARKSSGLSGTVRIPGDKSISHRSFMFGGLASGETRITGLLEGEDVINTG | 60 |
| --- | --- | --- | --- |
| Sbjct | 1 | M H AS +PATAR+S L+G +RIPGDKSISHRSFMFGGLASGETRITGLLEGEDVINTG MSHSASPKPATARRSEALTGEIRIPGDKSISHRSFMFGGLASGETRITGLLEGEDVINTG | 60 |
| Query | 61 | KAMQAMGARIRKEGDTWIIDGVGNGGLLAPEAPLDFGNAATGCRLTMGLVGVYDFDSTFI | 120 |
| Sbjct | 61 | +AMQAMGA+IRKEGD WII+GVGNG LL PEA LDFGNA TG RLTMGLVG YD ++FI  RAMQAMGAKIRKEGDVWIINGVGNGCLLQPEAALDFGNAGTGARLTMGLVGTYDMKTSFI | 120 |

| Query | 121 | GDASLTKRPMGRVLNPLREMGVQVKSEDGDRLPVTLRGPKTPTPITYRVPMASAQVKSAV | 180 |
| --- | --- | --- | --- |
| Sbjct | 121 | GDASL+KRPMGRVLNPLREMGVQV++ DGDR+P+TL GPKT PITYRVPMASAQVKSAV GDASLSKRPMGRVLNPLREMGVQVEAADGDRMPLTLIGPKTANPITYRVPMASAQVKSAV | 180 |
| Query | 181 | LLAGLNTPGITTVIEPIMTRDHTEKMLQGFGANLTVETDADGVRTIRLEGRGKLTGQVID | 240 |
| Sbjct | 181 | LLAGLNTPG+TTVIEP+MTRDHTEKMLQGFGA+LTVETD DGVR IR+ G+GKL GQ ID LLAGLNTPGVTTVIEPVMTRDHTEKMLQGFGADLTVETDKDGVRHIRITGQGKLVGQTID | 240 |
| Query | 241 | VPGDPSSTAFPLVAALLVPGSDVTILNVLMNPTRTGLILTLQEMGADIEVINPRLAGGED | 300 |
| Sbjct | 241 | VPGDPSSTAFPLVAALLV GSDVTI NVLMNPTRTGLILTLQEMGADIEV+N RLAGGED VPGDPSSTAFPLVAALLVEGSDVTIRNVLMNPTRTGLILTLQEMGADIEVLNARLAGGED | 300 |
| Query | 301 | VADLRVRSSTLKGVTVPEDRAPSMIDEYPILAVAAAFAEGATVMNGLEELRVKESDRLSA | 360 |
| Sbjct | 301 | VADLRVR+S LKGV VP +RAPSMIDEYP+LA+AA+FAEG TVM+GL+ELRVKESDRL+A VADLRVRASKLKGVVVPPERAPSMIDEYPVLAIAASFAEGETVMDGLDELRVKESDRLAA | 360 |
| Query | 361 | VANGLKLNGVDCDEGETSLVVRGRPDGKGLGNASGAAVATHLDHRIAMSFLVMGLVSENP | 420 |
| Sbjct | 361 | VA GL+ NGVDC EGE SL VRGRPDGKGLG G VATHLDHRIAMSFLVMGL +E P VARGLEANGVDCTEGEMSLTVRGRPDGKGLG---GGTVATHLDHRIAMSFLVMGLAAEKP | 417 |
| Query  Sbjct | 421  418 | VTVDDATMIATSFPEFMDLMAGLGAKIELS 450 VTVDD+ MIATSFPEFMD+M GLGAKIELS VTVDDSNMIATSFPEFMDMMPGLGAKIELS 447 |  |

>SP:P0A2Y5 AROA_ACHSL 3-phosphoshikimate 1-carboxyvinyltransferase OS=Achromobacter sp. (strain LBAA) OX=129026 GN=aroA PE=1 SV=1

Length=449

Score = 735 bits (1898), Expect = 0.0

| Identities | = 373/450 (83%), Positives = 405/450 (90%), Gaps = 3/450 (1%) |  |
| --- | --- | --- |
| Query 1 | MLHGASSRPATARKSSGLSGTVRIPGDKSISHRSFMFGGLASGETRITGLLEGEDVINTG | 60 |
| Sbjct 1 | M H AS +PATAR+S L+G +RIPGDKSISHRSFMFGGLASGETRITGLLEGEDVINTG MSHSASPKPATARRSEALTGEIRIPGDKSISHRSFMFGGLASGETRITGLLEGEDVINTG | 60 |
| Query 61 | KAMQAMGARIRKEGDTWIIDGVGNGGLLAPEAPLDFGNAATGCRLTMGLVGVYDFDSTFI | 120 |
| Sbjct 61 | +AMQAMGA+IRKEGD WII+GVGNG LL PEA LDFGNA TG RLTMGLVG YD ++FI RAMQAMGAKIRKEGDVWIINGVGNGCLLQPEAALDFGNAGTGARLTMGLVGTYDMKTSFI | 120 |
| Query 121 | GDASLTKRPMGRVLNPLREMGVQVKSEDGDRLPVTLRGPKTPTPITYRVPMASAQVKSAV | 180 |
| Sbjct 121 | GDASL+KRPMGRVLNPLREMGVQV++ DGDR+P+TL GPKT PITYRVPMASAQVKSAV GDASLSKRPMGRVLNPLREMGVQVEAADGDRMPLTLIGPKTANPITYRVPMASAQVKSAV | 180 |
| Query 181 | LLAGLNTPGITTVIEPIMTRDHTEKMLQGFGANLTVETDADGVRTIRLEGRGKLTGQVID | 240 |
| Sbjct 181 | LLAGLNTPG+TTVIEP+MTRDHTEKMLQGFGA+LTVETD DGVR IR+ G+GKL GQ ID LLAGLNTPGVTTVIEPVMTRDHTEKMLQGFGADLTVETDKDGVRHIRITGQGKLVGQTID | 240 |
| Query 241 | VPGDPSSTAFPLVAALLVPGSDVTILNVLMNPTRTGLILTLQEMGADIEVINPRLAGGED | 300 |
| Sbjct 241 | VPGDPSSTAFPLVAALLV GSDVTI NVLMNPTRTGLILTLQEMGADIEV+N RLAGGED VPGDPSSTAFPLVAALLVEGSDVTIRNVLMNPTRTGLILTLQEMGADIEVLNARLAGGED | 300 |
| Query 301 | VADLRVRSSTLKGVTVPEDRAPSMIDEYPILAVAAAFAEGATVMNGLEELRVKESDRLSA | 360 |
| Sbjct 301 | VADLRVR+S LKGV VP +RAPSMIDEYP+LA+AA+FAEG TVM+GL+ELRVKESDRL+A VADLRVRASKLKGVVVPPERAPSMIDEYPVLAIAASFAEGETVMDGLDELRVKESDRLAA | 360 |
| Query 361 | VANGLKLNGVDCDEGETSLVVRGRPDGKGLGNASGAAVATHLDHRIAMSFLVMGLVSENP | 420 |
| Sbjct 361 | VA GL+ NGVDC EGE SL VRGRPDGKGLG G VATHLDHRIAMSFLVMGL +E P VARGLEANGVDCTEGEMSLTVRGRPDGKGLG---GGTVATHLDHRIAMSFLVMGLAAEKP | 417 |
| Query 421  Sbjct 418 | VTVDDATMIATSFPEFMDLMAGLGAKIELS 450 VTVDD+ MIATSFPEFMD+M GLGAKIELS VTVDDSNMIATSFPEFMDMMPGLGAKIELS 447 |  |

>SP:A6WUV8 AROA_OCHA4 3-phosphoshikimate 1-carboxyvinyltransferase OS=Ochrobactrum anthropi (strain ATCC 49188 / DSM 6882 / JCM 21032

/ NBRC 15819 / NCTC 12168) OX=439375 GN=aroA PE=3 SV=1

Length=450

Score = 734 bits (1896), Expect = 0.0

| Identities | = 372/453 (82%), Positives = 407/453 (90%), Gaps = 3/453 (1%) |  |
| --- | --- | --- |
| Query 1 | MLHGASSRPATARKSSGLSGTVRIPGDKSISHRSFMFGGLASGETRITGLLEGEDVINTG | 60 |
| Sbjct 1 | M H A +PATAR+S L+G +RIPGDKSISHRSFMFGGLASGETRITGLLEGEDVINTG MSHSAPPKPATARRSEALTGEIRIPGDKSISHRSFMFGGLASGETRITGLLEGEDVINTG | 60 |
| Query 61 | KAMQAMGARIRKEGDTWIIDGVGNGGLLAPEAPLDFGNAATGCRLTMGLVGVYDFDSTFI | 120 |
| Sbjct 61 | +AMQAMGA+IRK+GD WII+GVGNG LL PEA LDFGNA TG RLTMGLVG YD ++FI RAMQAMGAKIRKDGDAWIINGVGNGCLLQPEAALDFGNAGTGARLTMGLVGTYDMRTSFI | 120 |
| Query 121 | GDASLTKRPMGRVLNPLREMGVQVKSEDGDRLPVTLRGPKTPTPITYRVPMASAQVKSAV | 180 |
| Sbjct 121 | GDASL+KRPMGRVLNPLREMGVQV++ +GDR+P+TL GPKT PITYRVPMASAQVKSAV GDASLSKRPMGRVLNPLREMGVQVEAAEGDRMPLTLIGPKTANPITYRVPMASAQVKSAV | 180 |
| Query 181 | LLAGLNTPGITTVIEPIMTRDHTEKMLQGFGANLTVETDADGVRTIRLEGRGKLTGQVID | 240 |
| Sbjct 181 | LLAGLNTPG+TTVIEP+MTRDHTEKMLQGFGA+L+VETD DGVR IR+ G+GKL GQ ID LLAGLNTPGVTTVIEPVMTRDHTEKMLQGFGADLSVETDKDGVRHIRITGQGKLIGQTID | 240 |
| Query 241 | VPGDPSSTAFPLVAALLVPGSDVTILNVLMNPTRTGLILTLQEMGADIEVINPRLAGGED | 300 |
| Sbjct 241 | VPGDPSSTAFPLVAALLV GSDVTI NVLMNPTRTGLILTLQEMGADIEV+N RLAGGED VPGDPSSTAFPLVAALLVEGSDVTIRNVLMNPTRTGLILTLQEMGADIEVLNARLAGGED | 300 |
| Query 301 | VADLRVRSSTLKGVTVPEDRAPSMIDEYPILAVAAAFAEGATVMNGLEELRVKESDRLSA | 360 |
| Sbjct 301 | VADLRV++S LKGV VP +RAPSMIDEYP+LA+AAAFAEG TVM+GL+ELRVKESDRL+A VADLRVKASKLKGVVVPPERAPSMIDEYPVLAIAAAFAEGETVMDGLDELRVKESDRLAA | 360 |
| Query 361 | VANGLKLNGVDCDEGETSLVVRGRPDGKGLGNASGAAVATHLDHRIAMSFLVMGLVSENP | 420 |
| Sbjct 361 | VA GL+ NGVDC EGE SL VRGRPDGKGLG G VATHLDHRIAMSFLVMGL SE P VARGLEANGVDCTEGEMSLTVRGRPDGKGLG---GGTVATHLDHRIAMSFLVMGLASEKP | 417 |
| Query 421  Sbjct 418 | VTVDDATMIATSFPEFMDLMAGLGAKIELSDTK 453 VTVDD+TMIATSFPEFMD+M GLGAKIELSD + VTVDDSTMIATSFPEFMDMMPGLGAKIELSDAR 450 |  |

>TR:A0A2S9JIW2 A0A2S9JIW2_9RHIZ 3-phosphoshikimate 1-carboxyvinyltransferase OS=Phyllobacterium myrsinacearum OX=28101 GN=aroA PE=3 SV=1

Length=451

Score = 734 bits (1896), Expect = 0.0

| Identities | = 372/454 (82%), Positives = 403/454 (89%), Gaps = 3/454 (1%) |  |
| --- | --- | --- |
| Query 1 | MLHGASSRPATARKSSGLSGTVRIPGDKSISHRSFMFGGLASGETRITGLLEGEDVINTG | 60 |
| Sbjct 1 | M H A +PATAR+S LSG +RIPGDKSISHRSFMFGGLASGETRITGLLEGEDVINTG MSHSALPKPATARRSKALSGDIRIPGDKSISHRSFMFGGLASGETRITGLLEGEDVINTG | 60 |
| Query 61 | KAMQAMGARIRKEGDTWIIDGVGNGGLLAPEAPLDFGNAATGCRLTMGLVGVYDFDSTFI | 120 |
| Sbjct 61 | +AMQAMGA+I +EGD WII G GNG LL PEAPLDFGNA TG RLTMGLVGVY ++TFI RAMQAMGAKIAREGDVWIIQGTGNGCLLQPEAPLDFGNAGTGARLTMGLVGVYAMETTFI | 120 |
| Query 121 | GDASLTKRPMGRVLNPLREMGVQVKSEDGDRLPVTLRGPKTPTPITYRVPMASAQVKSAV | 180 |
| Sbjct 121 | GDASL+KRPMGRVLNPLREMGVQV + DGDR+P+TLRGPK PITYRVPMASAQVKSAV GDASLSKRPMGRVLNPLREMGVQVTASDGDRMPLTLRGPKVAAPITYRVPMASAQVKSAV | 180 |
| Query 181 | LLAGLNTPGITTVIEPIMTRDHTEKMLQGFGANLTVETDADGVRTIRLEGRGKLTGQVID LLAGLNTPGITTVIEP+MTRDHTEKMLQGFGANL+VETD D VR IR+EG+GKLTGQ+ID | 240 |

| Sbjct | 181 | LLAGLNTPGITTVIEPVMTRDHTEKMLQGFGANLSVETDKDNVRHIRIEGQGKLTGQIID | 240 |
| --- | --- | --- | --- |
| Query | 241 | VPGDPSSTAFPLVAALLVPGSDVTILNVLMNPTRTGLILTLQEMGADIEVINPRLAGGED | 300 |
| Sbjct | 241 | VPGDPSSTAFPLVAALLVPGSD+TI NVLMNPTRTGLILTLQ+MGA IE++N RLAGGED VPGDPSSTAFPLVAALLVPGSDITIRNVLMNPTRTGLILTLQDMGASIEILNARLAGGED | 300 |
| Query | 301 | VADLRVRSSTLKGVTVPEDRAPSMIDEYPILAVAAAFAEGATVMNGLEELRVKESDRLSA | 360 |
| Sbjct | 301 | VADLRVR+S LKGV VP DRAPSMIDEYP+LAVAAAFA G TVMNGLEELRVKESDRLSA VADLRVRASELKGVIVPADRAPSMIDEYPVLAVAAAFATGETVMNGLEELRVKESDRLSA | 360 |
| Query | 361 | VANGLKLNGVDCDEGETSLVVRGRPDGKGLGNASGAAVATHLDHRIAMSFLVMGLVSENP | 420 |
| Sbjct | 361 | VANGLK NGVDC EG +L VRGRPDGK LG G V THLDHRIAMSFLVMGLV+E P VANGLKANGVDCTEGPETLSVRGRPDGKALG---GGTVETHLDHRIAMSFLVMGLVAEKP | 417 |
| Query  Sbjct | 421  418 | VTVDDATMIATSFPEFMDLMAGLGAKIELSDTKA 454 VTVDD+TMIATSFPEFM +M G+GA+I + +T A VTVDDSTMIATSFPEFMGMMTGMGAEISVQETPA 451 |  |

>TR:A0A502BQP0 A0A502BQP0_9RHIZ 3-phosphoshikimate 1-carboxyvinyltransferase OS=Ochrobactrum gallinifaecis OX=215590 GN=aroA PE=3 SV=1

Length=450

Score = 732 bits (1890), Expect = 0.0

| Identities | = 368/451 (82%), Positives = 406/451 (90%), Gaps = 3/451 (1%) |  |
| --- | --- | --- |
| Query 1 | MLHGASSRPATARKSSGLSGTVRIPGDKSISHRSFMFGGLASGETRITGLLEGEDVINTG | 60 |
| Sbjct 1 | M H A+ +PATAR+S L+G +RIPGDKSISHRSFMFGGLA+GETRITGLLEGEDVINTG MSHSATPKPATARRSEALTGEIRIPGDKSISHRSFMFGGLAAGETRITGLLEGEDVINTG | 60 |
| Query 61 | KAMQAMGARIRKEGDTWIIDGVGNGGLLAPEAPLDFGNAATGCRLTMGLVGVYDFDSTFI | 120 |
| Sbjct 61 | +AMQAMGARIRK+GD W+I+GVGNG LL PEAPLDFGNA TG RLTMGLVG YD ++FI RAMQAMGARIRKDGDVWVINGVGNGCLLQPEAPLDFGNAGTGARLTMGLVGTYDMTTSFI | 120 |
| Query 121 | GDASLTKRPMGRVLNPLREMGVQVKSEDGDRLPVTLRGPKTPTPITYRVPMASAQVKSAV | 180 |
| Sbjct 121 | GDASLT RPMGRVLNPLREMGVQVK+ +GDR+P+TL GPKT PI+YRVPMASAQVKSAV GDASLTSRPMGRVLNPLREMGVQVKAAEGDRMPLTLTGPKTANPISYRVPMASAQVKSAV | 180 |
| Query 181 | LLAGLNTPGITTVIEPIMTRDHTEKMLQGFGANLTVETDADGVRTIRLEGRGKLTGQVID | 240 |
| Sbjct 181 | LLAGLN PG+TTVIEP+MTRDHTEKMLQGFGA+LTVETD +GVR IR+ G+GKLTGQ ID LLAGLNAPGVTTVIEPVMTRDHTEKMLQGFGADLTVETDKEGVRHIRIVGQGKLTGQTID | 240 |
| Query 241 | VPGDPSSTAFPLVAALLVPGSDVTILNVLMNPTRTGLILTLQEMGADIEVINPRLAGGED | 300 |
| Sbjct 241 | VPGDPSSTAFPLVAALLV GSDVTILNVLMNPTRTGLILTLQEMGADIE+INPRLAGGED VPGDPSSTAFPLVAALLVEGSDVTILNVLMNPTRTGLILTLQEMGADIEIINPRLAGGED | 300 |
| Query 301 | VADLRVRSSTLKGVTVPEDRAPSMIDEYPILAVAAAFAEGATVMNGLEELRVKESDRLSA | 360 |
| Sbjct 301 | VADLRV+SS LKGV VP +RAPSMIDEYP+LA+AA+FAEG TVM+GL+ELRVKESDRL+A VADLRVKSSKLKGVVVPPERAPSMIDEYPVLAIAASFAEGETVMDGLDELRVKESDRLAA | 360 |
| Query 361 | VANGLKLNGVDCDEGETSLVVRGRPDGKGLGNASGAAVATHLDHRIAMSFLVMGLVSENP | 420 |
| Sbjct 361 | VA GL+ NGVDC EGE SL VRGRP GKGLG G VATHLDHRIAMSFLVMGL +E P VARGLEANGVDCTEGEMSLTVRGRPGGKGLG---GGTVATHLDHRIAMSFLVMGLAAEKP | 417 |
| Query 421  Sbjct 418 | VTVDDATMIATSFPEFMDLMAGLGAKIELSD 451 VTVDD+ MIATSFPEFM +M GLGAKIE+S+ VTVDDSLMIATSFPEFMTMMPGLGAKIEVSE 448 |  |

>TR:A0A317PKQ9 A0A317PKQ9_9RHIZ 3-phosphoshikimate 1-carboxyvinyltransferase OS=Hoeflea marina OX=274592 GN=aroA PE=3 SV=1

Length=452

Score = 731 bits (1887), Expect = 0.0

| Identities | = 375/443 (85%), Positives = 401/443 (91%), Gaps = 1/443 (0%) |  |
| --- | --- | --- |
| Query 9 | PATARKSSGLSGTVRIPGDKSISHRSFMFGGLASGETRITGLLEGEDVINTGKAMQAMGA | 68 |
| Sbjct 10 | PATAR+SS LSG +RIPGDKSISHRSFMFGGLASGETRITGLLEGEDVINTG+AMQAMGA PATARRSSALSGDIRIPGDKSISHRSFMFGGLASGETRITGLLEGEDVINTGRAMQAMGA | 69 |
| Query 69 | RIRKEGDTWIIDGVGNGGLLAPEAPLDFGNAATGCRLTMGLVGVYDFDSTFIGDASLTKR | 128 |
| Sbjct 70 | IRK+GDTWII G GNG LLAPEAPLDFGNA TGCRLTMGLVGVYDF STFIGDASL++R SIRKDGDTWIIQGTGNGCLLAPEAPLDFGNAGTGCRLTMGLVGVYDFPSTFIGDASLSRR | 129 |
| Query 129 | PMGRVLNPLREMGVQVKSEDGDRLPVTLRGPKTPTPITYRVPMASAQVKSAVLLAGLNTP | 188 |
| Sbjct 130 | PMGRVL+PLR MGVQV SE GDRLPVTLRGP PTPI+YRVPM SAQVKSAVLLAGLNTP PMGRVLDPLRLMGVQVTSEPGDRLPVTLRGPAIPTPISYRVPMPSAQVKSAVLLAGLNTP | 189 |
| Query 189 | GITTVIEPIMTRDHTEKMLQGFGANLTVETDADGVRTIRLEGRGKLTGQVIDVPGDPSST | 248 |
| Sbjct 190 | G+TTVIEP+MTRDHTEKML GFGA+LTVETDA GVRTIRLEGRG+LTGQVIDVPGDPSST GVTTVIEPVMTRDHTEKMLAGFGADLTVETDAAGVRTIRLEGRGRLTGQVIDVPGDPSST | 249 |
| Query 249 | AFPLVAALLVPGSDVTILNVLMNPTRTGLILTLQEMGADIEVINPRLAGGEDVADLRVRS | 308 |
| Sbjct 250 | AFPLVAALLVPGSDVTI NVLMNPTRTGLILTL+EMGADIE+++PRLAGGEDVADLRVR+ AFPLVAALLVPGSDVTIRNVLMNPTRTGLILTLREMGADIEILDPRLAGGEDVADLRVRA | 309 |
| Query 309 | STLKGVTVPEDRAPSMIDEYPILAVAAAFAEGATVMNGLEELRVKESDRLSAVANGLKLN | 368 |
| Sbjct 310 | S L GVTVP +RAPSMIDEYP+LAVAA FA G TVM GL+ELRVKESDRL+AVA GL+LN SELTGVTVPAERAPSMIDEYPVLAVAACFARGETVMTGLDELRVKESDRLAAVARGLELN | 369 |
| Query 369 | GVDCDEGETSLVVRGRPDGKGLGNAS-GAAVATHLDHRIAMSFLVMGLVSENPVTVDDAT | 427 |
| Sbjct 370 | G DC EG L VRGRPDG+GLGN S A V T LDHRIAMSFLVMGL SE+PVTVDDA GADCTEGPDWLSVRGRPDGRGLGNPSPDARVVTWLDHRIAMSFLVMGLASEHPVTVDDAA | 429 |
| Query 428  Sbjct 430 | MIATSFPEFMDLMAGLGAKIELS 450  +IATSFPEFM LMAGLGA I+ S IIATSFPEFMQLMAGLGADIQQS 452 |  |

>TR:B9JGR5 B9JGR5_AGRRK 3-phosphoshikimate 1-carboxyvinyltransferase OS=Agrobacterium radiobacter (strain K84 / ATCC BAA-868) OX=311403

GN=aroA PE=3 SV=1

Length=454

Score = 731 bits (1886), Expect = 0.0

| Identities | = 371/449 (83%), Positives = 403/449 (90%), Gaps = 1/449 (0%) |  |
| --- | --- | --- |
| Query 1 | MLHGASSRPATARKSSGLSGTVRIPGDKSISHRSFMFGGLASGETRITGLLEGEDVINTG | 60 |
| Sbjct 1 | M H SRPATAR+S+ LSGT+R+PGDKSISHR+FMFGGLASGETRITGLLEGEDV+NTG MPHDTHSRPATARRSTNLSGTIRVPGDKSISHRAFMFGGLASGETRITGLLEGEDVLNTG | 60 |
| Query 61 | KAMQAMGARIRKEGDTWIIDGVGNGGLLAPEAPLDFGNAATGCRLTMGLVGVYDFDSTFI | 120 |
| Sbjct 61 | KAM+AMGA+I K GD WII GVGNG LLAPEAPLDFGNA TG RLTMGLVGVY F+STFI KAMRAMGAKIEKAGDEWIIKGVGNGALLAPEAPLDFGNAGTGSRLTMGLVGVYHFESTFI | 120 |
| Query 121 | GDASLTKRPMGRVLNPLREMGVQVKSEDGDRLPVTLRGPKTPTPITYRVPMASAQVKSAV | 180 |
| Sbjct 121 | GDASL+KRPMGR+L+PLR+MGVQVKS +GDRLPVTL GP+T PITYRVPMASAQVKSAV GDASLSKRPMGRILDPLRQMGVQVKSSEGDRLPVTLHGPETANPITYRVPMASAQVKSAV | 180 |
| Query 181 | LLAGLNTPGITTVIEPIMTRDHTEKMLQGFGANLTVETDADGVRTIRLEGRGKLTGQVID | 240 |
| Sbjct 181 | LLAGLN PGIT+VIEP+MTRDHTEKML GFGA L+VETD DGVR IRL+G+GKL GQ ID LLAGLNAPGITSVIEPVMTRDHTEKMLAGFGAELSVETDRDGVRIIRLQGQGKLFGQTID | 240 |
| Query 241 | VPGDPSSTAFPLVAALLVPGSDVTILNVLMNPTRTGLILTLQEMGADIEVINPRLAGGED | 300 |
| Sbjct 241 | VPGDPSSTAFPLVAAL+V GSD+TI NVLMNPTRTGLILTLQEMGADIE+++ RLAGGED VPGDPSSTAFPLVAALIVAGSDITIRNVLMNPTRTGLILTLQEMGADIEILDKRLAGGED | 300 |
| Query 301 | VADLRVRSSTLKGVTVPEDRAPSMIDEYPILAVAAAFAEGATVMNGLEELRVKESDRLSA | 360 |

| Sbjct | 301 | VADLRVRSS LKGVTVP +RAPSMIDEYP+LAVAAAFAEG TVM GLEELRVKESDRLSA  VADLRVRSSALKGVTVPAERAPSMIDEYPVLAVAAAFAEGTTVMLGLEELRVKESDRLSA | 360 |
| --- | --- | --- | --- |
| Query | 361 | VANGLKLNGVDCDEGETSLVVRGRPDGKGLGNASGAAVATHLDHRIAMSFLVMGLVSENP | 420 |
| Sbjct | 361 | VA GL LNGVDC EGE SL VRGRPDGKGLGNA G V THLDHRIAMSFLVMGL SE+P VAEGLTLNGVDCTEGEASLTVRGRPDGKGLGNA-GGEVKTHLDHRIAMSFLVMGLASEHP | 419 |
| Query  Sbjct | 421  420 | VTVDDATMIATSFPEFMDLMAGLGAKIEL 449 V VDD+ MIATSFPEF DLMA LGA +++ VKVDDSAMIATSFPEFFDLMAELGADMDV 448 |  |

>TR:A0A135I0M0 A0A135I0M0_9RHIZ 3-phosphoshikimate 1-carboxyvinyltransferase OS=Paramesorhizobium deserti OX=1494590 GN=aroA PE=3 SV=1

Length=447

Score = 729 bits (1881), Expect = 0.0

| Identities | = 369/450 (82%), Positives = 404/450 (90%), Gaps = 3/450 (1%) |  |
| --- | --- | --- |
| Query 1 | MLHGASSRPATARKSSGLSGTVRIPGDKSISHRSFMFGGLASGETRITGLLEGEDVINTG | 60 |
| Sbjct 1 | M H AS RPATA +S L+G +RIPGDKSISHRSFMFGGLASGETRITGLLEGEDVINTG MSHSASPRPATAHRSRDLTGDIRIPGDKSISHRSFMFGGLASGETRITGLLEGEDVINTG | 60 |
| Query 61 | KAMQAMGARIRKEGDTWIIDGVGNGGLLAPEAPLDFGNAATGCRLTMGLVGVYDFDSTFI | 120 |
| Sbjct 61 | +AMQAMGARI+K+GDTWII G GNG LL PEAPLDFGNA TG RLTMGLVG YD ++TFI RAMQAMGARIKKDGDTWIIQGTGNGCLLKPEAPLDFGNAGTGARLTMGLVGTYDMETTFI | 120 |
| Query 121 | GDASLTKRPMGRVLNPLREMGVQVKSEDGDRLPVTLRGPKTPTPITYRVPMASAQVKSAV | 180 |
| Sbjct 121 | GDASL+KRPMGRVLNPLREMGVQVK+ +GDR+P+TL GPK PITYRVPMASAQVKSAV GDASLSKRPMGRVLNPLREMGVQVKAAEGDRMPLTLHGPKVAAPITYRVPMASAQVKSAV | 180 |
| Query 181 | LLAGLNTPGITTVIEPIMTRDHTEKMLQGFGANLTVETDADGVRTIRLEGRGKLTGQVID | 240 |
| Sbjct 181 | LLAGLNTPGITTVIEP+MTRDHTEKML GFGA+LTVET+ +GVR IR+ G+G+L GQ ID LLAGLNTPGITTVIEPVMTRDHTEKMLAGFGADLTVETNREGVRHIRIVGQGELKGQTID | 240 |
| Query 241 | VPGDPSSTAFPLVAALLVPGSDVTILNVLMNPTRTGLILTLQEMGADIEVINPRLAGGED | 300 |
| Sbjct 241 | VPGDPSSTAFPL+AALLVPGSDVTI NVLMNPTRTGLILTLQEMGA+I+++N RLAGGED VPGDPSSTAFPLMAALLVPGSDVTIRNVLMNPTRTGLILTLQEMGANIDILNARLAGGED | 300 |
| Query 301 | VADLRVRSSTLKGVTVPEDRAPSMIDEYPILAVAAAFAEGATVMNGLEELRVKESDRLSA | 360 |
| Sbjct 301 | VADLRVR+S LKGV+VP +RAPSMIDEYP+LAVAAAFAEG T+M+GLEELRVKESDRLSA VADLRVRASELKGVSVPPERAPSMIDEYPVLAVAAAFAEGETIMDGLEELRVKESDRLSA | 360 |
| Query 361 | VANGLKLNGVDCDEGETSLVVRGRPDGKGLGNASGAAVATHLDHRIAMSFLVMGLVSENP | 420 |
| Sbjct 361 | VA+GLK NGVDC EGE SL VRG P GKGLG G VATHLDHRIAMSFLVMGL +E P VADGLKANGVDCTEGEASLTVRGIPGGKGLG---GGTVATHLDHRIAMSFLVMGLAAEKP | 417 |
| Query 421  Sbjct 418 | VTVDDATMIATSFPEFMDLMAGLGAKIELS 450 VT+DD+ MIATSFPEFMDLMAGLGAKIE S VTIDDSAMIATSFPEFMDLMAGLGAKIERS 447 |  |

>TR:A0A1H0RZE5 A0A1H0RZE5_9RHIZ 3-phosphoshikimate 1-carboxyvinyltransferase OS=Phyllobacterium sp. YR620 OX=1881066 GN=aroA PE=3 SV=1

Length=451

Score = 729 bits (1881), Expect = 0.0

Identities = 370/447 (83%), Positives = 401/447 (90%), Gaps = 3/447 (1%)

| Query | 1 | MLHGASSRPATARKSSGLSGTVRIPGDKSISHRSFMFGGLASGETRITGLLEGEDVINTG | 60 |
| --- | --- | --- | --- |
|  |  | M H + RPATAR+S LSG +RIPGDKSISHRSFMFGGLASGETRITGLLEGEDVINTG |  |
| Sbjct | 1 | MSHTSLPRPATARRSKALSGDIRIPGDKSISHRSFMFGGLASGETRITGLLEGEDVINTG | 60 |
| Query | 61 | KAMQAMGARIRKEGDTWIIDGVGNGGLLAPEAPLDFGNAATGCRLTMGLVGVYDFDSTFI | 120 |

| Sbjct | 61 | +AMQAMGA+I K+GD WII G GNG LL PEAPLDFGNA TG RLTMGLVG Y ++TFI  RAMQAMGAKIAKDGDVWIIQGTGNGCLLEPEAPLDFGNAGTGARLTMGLVGTYAMETTFI | 120 |
| --- | --- | --- | --- |
| Query | 121 | GDASLTKRPMGRVLNPLREMGVQVKSEDGDRLPVTLRGPKTPTPITYRVPMASAQVKSAV | 180 |
| Sbjct | 121 | GDASL+KRPMGRVLNPLREMGVQV + DGDRLP+TLRGPK PITYRVPMASAQVKSAV GDASLSKRPMGRVLNPLREMGVQVSAADGDRLPLTLRGPKAAAPITYRVPMASAQVKSAV | 180 |
| Query | 181 | LLAGLNTPGITTVIEPIMTRDHTEKMLQGFGANLTVETDADGVRTIRLEGRGKLTGQVID | 240 |
| Sbjct | 181 | LLAGLNTPGITTV+EP+MTRDHTEKMLQGFGANL+VETD DGVR IR+EG+GKLTGQVID LLAGLNTPGITTVVEPVMTRDHTEKMLQGFGANLSVETDKDGVRHIRIEGQGKLTGQVID | 240 |
| Query | 241 | VPGDPSSTAFPLVAALLVPGSDVTILNVLMNPTRTGLILTLQEMGADIEVINPRLAGGED | 300 |
| Sbjct | 241 | VPGDPSSTAFPLVAALLVPGSD+TI NVLMNPTRTGLILTLQEMGA+IEV+N RLAGGED VPGDPSSTAFPLVAALLVPGSDITIRNVLMNPTRTGLILTLQEMGANIEVLNARLAGGED | 300 |
| Query | 301 | VADLRVRSSTLKGVTVPEDRAPSMIDEYPILAVAAAFAEGATVMNGLEELRVKESDRLSA | 360 |
| Sbjct | 301 | VADLRVRSS LKGV+VP +RAPSMIDEYP+LAVAA+FA G T+M+GL+ELRVKESDRLSA VADLRVRSSELKGVSVPPERAPSMIDEYPVLAVAASFAMGETLMHGLDELRVKESDRLSA | 360 |
| Query | 361 | VANGLKLNGVDCDEGETSLVVRGRPDGKGLGNASGAAVATHLDHRIAMSFLVMGLVSENP | 420 |
| Sbjct | 361 | VANGLK NGVDC EG SL VRGRPDGKGLG G V THLDHRIAMSFLVMGL +E P VANGLKTNGVDCTEGPESLSVRGRPDGKGLG---GGTVETHLDHRIAMSFLVMGLAAEKP | 417 |
| Query  Sbjct | 421  418 | VTVDDATMIATSFPEFMDLMAGLGAKI 447 VTVDDATMIATSFPEFM++M +GA+I VTVDDATMIATSFPEFMEMMTRMGAEI 444 |  |

>SP:Q8G3C4 AROA_BRUSU 3-phosphoshikimate 1-carboxyvinyltransferase OS=Brucella suis biovar 1 (strain 1330) OX=204722 GN=aroA PE=3 SV=1

Length=450

Score = 728 bits (1879), Expect = 0.0

| Identities | = 369/447 (83%), Positives = 402/447 (90%), Gaps = 3/447 (1%) |  |
| --- | --- | --- |
| Query 1 | MLHGASSRPATARKSSGLSGTVRIPGDKSISHRSFMFGGLASGETRITGLLEGEDVINTG | 60 |
| Sbjct 1 | M H A +PATAR S L+G +RIPGDKSISHRSFMFGGLASG+TRITGLLEGEDVINTG MSHSACPKPATARHSQALTGEIRIPGDKSISHRSFMFGGLASGKTRITGLLEGEDVINTG | 60 |
| Query 61 | KAMQAMGARIRKEGDTWIIDGVGNGGLLAPEAPLDFGNAATGCRLTMGLVGVYDFDSTFI | 120 |
| Sbjct 61 | +AMQAMGARIRKEGD WII+GVGNG LL PEAPLDFGNA TG RLTMGLVG YD ++FI RAMQAMGARIRKEGDVWIINGVGNGCLLQPEAPLDFGNAGTGARLTMGLVGTYDMKTSFI | 120 |
| Query 121 | GDASLTKRPMGRVLNPLREMGVQVKSEDGDRLPVTLRGPKTPTPITYRVPMASAQVKSAV | 180 |
| Sbjct 121 | GDASL+KRPMGRVLNPLREMGVQV++ +GDR+P+TL GP+T PI YRVPMASAQVKSAV GDASLSKRPMGRVLNPLREMGVQVEAAEGDRMPLTLIGPRTANPIAYRVPMASAQVKSAV | 180 |
| Query 181 | LLAGLNTPGITTVIEPIMTRDHTEKMLQGFGANLTVETDADGVRTIRLEGRGKLTGQVID | 240 |
| Sbjct 181 | LLAGLNTPG+TTVIEP+MTRDHTEKMLQGFGA+LTVETD DGVR IR+ G+GKLTGQ ID LLAGLNTPGVTTVIEPVMTRDHTEKMLQGFGADLTVETDKDGVRHIRIVGQGKLTGQTID | 240 |
| Query 241 | VPGDPSSTAFPLVAALLVPGSDVTILNVLMNPTRTGLILTLQEMGADIEVINPRLAGGED | 300 |
| Sbjct 241 | VPGDPSSTAFPLVAALLV GSDVTI NVLMNPTRTGLILTLQEMGADIE+I+PRLAGGED VPGDPSSTAFPLVAALLVEGSDVTIRNVLMNPTRTGLILTLQEMGADIEIIDPRLAGGED | 300 |
| Query 301 | VADLRVRSSTLKGVTVPEDRAPSMIDEYPILAVAAAFAEGATVMNGLEELRVKESDRLSA | 360 |
| Sbjct 301 | VADLRVR+S LKGV VP +RAPSMIDEYP+LA+AA+FAEG TVM+GL+ELRVKESDRL+A VADLRVRASKLKGVVVPPERAPSMIDEYPVLAIAASFAEGETVMDGLDELRVKESDRLAA | 360 |
| Query 361 | VANGLKLNGVDCDEGETSLVVRGRPDGKGLGNASGAAVATHLDHRIAMSFLVMGLVSENP | 420 |
| Sbjct 361 | VA GL+ NGVDC EGE SL VRGRP GKGLG G VATHLDHRIAMSFLVMGL SE P VARGLEANGVDCTEGEMSLTVRGRPGGKGLG---GGTVATHLDHRIAMSFLVMGLASEKP | 417 |
| Query 421 | VTVDDATMIATSFPEFMDLMAGLGAKI 447 |  |

VTVDD+TMIATSFPEFM +MAGLGAKI Sbjct 418 VTVDDSTMIATSFPEFMGMMAGLGAKI 444

>SP:A9M6N0 AROA_BRUC2 3-phosphoshikimate 1-carboxyvinyltransferase OS=Brucella canis (strain ATCC 23365 / NCTC 10854) OX=483179 GN=aroA

PE=3 SV=1

Length=450

Score = 728 bits (1879), Expect = 0.0

| Identities | = 369/447 (83%), Positives = 402/447 (90%), Gaps = 3/447 (1%) |  |
| --- | --- | --- |
| Query 1 | MLHGASSRPATARKSSGLSGTVRIPGDKSISHRSFMFGGLASGETRITGLLEGEDVINTG | 60 |
| Sbjct 1 | M H A +PATAR S L+G +RIPGDKSISHRSFMFGGLASG+TRITGLLEGEDVINTG MSHSACPKPATARHSQALTGEIRIPGDKSISHRSFMFGGLASGKTRITGLLEGEDVINTG | 60 |
| Query 61 | KAMQAMGARIRKEGDTWIIDGVGNGGLLAPEAPLDFGNAATGCRLTMGLVGVYDFDSTFI | 120 |
| Sbjct 61 | +AMQAMGARIRKEGD WII+GVGNG LL PEAPLDFGNA TG RLTMGLVG YD ++FI RAMQAMGARIRKEGDVWIINGVGNGCLLQPEAPLDFGNAGTGARLTMGLVGTYDMKTSFI | 120 |
| Query 121 | GDASLTKRPMGRVLNPLREMGVQVKSEDGDRLPVTLRGPKTPTPITYRVPMASAQVKSAV | 180 |
| Sbjct 121 | GDASL+KRPMGRVLNPLREMGVQV++ +GDR+P+TL GP+T PI YRVPMASAQVKSAV GDASLSKRPMGRVLNPLREMGVQVEAAEGDRMPLTLIGPRTANPIAYRVPMASAQVKSAV | 180 |
| Query 181 | LLAGLNTPGITTVIEPIMTRDHTEKMLQGFGANLTVETDADGVRTIRLEGRGKLTGQVID | 240 |
| Sbjct 181 | LLAGLNTPG+TTVIEP+MTRDHTEKMLQGFGA+LTVETD DGVR IR+ G+GKLTGQ ID LLAGLNTPGVTTVIEPVMTRDHTEKMLQGFGADLTVETDKDGVRHIRIVGQGKLTGQTID | 240 |
| Query 241 | VPGDPSSTAFPLVAALLVPGSDVTILNVLMNPTRTGLILTLQEMGADIEVINPRLAGGED | 300 |
| Sbjct 241 | VPGDPSSTAFPLVAALLV GSDVTI NVLMNPTRTGLILTLQEMGADIE+I+PRLAGGED VPGDPSSTAFPLVAALLVEGSDVTIRNVLMNPTRTGLILTLQEMGADIEIIDPRLAGGED | 300 |
| Query 301 | VADLRVRSSTLKGVTVPEDRAPSMIDEYPILAVAAAFAEGATVMNGLEELRVKESDRLSA | 360 |
| Sbjct 301 | VADLRVR+S LKGV VP +RAPSMIDEYP+LA+AA+FAEG TVM+GL+ELRVKESDRL+A VADLRVRASKLKGVVVPPERAPSMIDEYPVLAIAASFAEGETVMDGLDELRVKESDRLAA | 360 |
| Query 361 | VANGLKLNGVDCDEGETSLVVRGRPDGKGLGNASGAAVATHLDHRIAMSFLVMGLVSENP | 420 |
| Sbjct 361 | VA GL+ NGVDC EGE SL VRGRP GKGLG G VATHLDHRIAMSFLVMGL SE P VARGLEANGVDCTEGEMSLTVRGRPGGKGLG---GGTVATHLDHRIAMSFLVMGLASEKP | 417 |
| Query 421  Sbjct 418 | VTVDDATMIATSFPEFMDLMAGLGAKI 447 VTVDD+TMIATSFPEFM +MAGLGAKI VTVDDSTMIATSFPEFMGMMAGLGAKI 444 |  |

>SP:B0CI45 AROA_BRUSI 3-phosphoshikimate 1-carboxyvinyltransferase OS=Brucella suis (strain ATCC 23445 / NCTC 10510) OX=470137 GN=aroA

PE=3 SV=1

Length=450

Score = 727 bits (1876), Expect = 0.0

| Identities | = 368/447 (82%), Positives = 402/447 (90%), Gaps = 3/447 (1%) |  |
| --- | --- | --- |
| Query 1 | MLHGASSRPATARKSSGLSGTVRIPGDKSISHRSFMFGGLASGETRITGLLEGEDVINTG | 60 |
| Sbjct 1 | M H A +PATAR S L+G +RIPGDKSISHRSFMFGGLASG+TRITGLLEGEDVINTG MSHSACPKPATARHSQALTGEIRIPGDKSISHRSFMFGGLASGKTRITGLLEGEDVINTG | 60 |
| Query 61 | KAMQAMGARIRKEGDTWIIDGVGNGGLLAPEAPLDFGNAATGCRLTMGLVGVYDFDSTFI | 120 |
| Sbjct 61 | +AMQAMGARIRKEGD WII+GVGNG LL PEAPLDFGNA TG RLTMGLVG YD ++FI RAMQAMGARIRKEGDVWIINGVGNGCLLQPEAPLDFGNAGTGARLTMGLVGTYDMKTSFI | 120 |
| Query 121 | GDASLTKRPMGRVLNPLREMGVQVKSEDGDRLPVTLRGPKTPTPITYRVPMASAQVKSAV | 180 |
| Sbjct 121 | GDASL+KRPMGRVLNPLREMGVQV++ +GDR+P+TL GP+T PI YRVPMASAQVKSAV  GDASLSKRPMGRVLNPLREMGVQVEAAEGDRMPLTLIGPRTANPIAYRVPMASAQVKSAV | 180 |

| Query | 181 | LLAGLNTPGITTVIEPIMTRDHTEKMLQGFGANLTVETDADGVRTIRLEGRGKLTGQVID | 240 |
| --- | --- | --- | --- |
| Sbjct | 181 | LLAGLNTPG+TTVIEP+MTRDHTEKMLQGFGA+LTVETD DGVR IR+ G+GKLTGQ ID LLAGLNTPGVTTVIEPVMTRDHTEKMLQGFGADLTVETDKDGVRHIRIVGQGKLTGQTID | 240 |
| Query | 241 | VPGDPSSTAFPLVAALLVPGSDVTILNVLMNPTRTGLILTLQEMGADIEVINPRLAGGED | 300 |
| Sbjct | 241 | VPGDPSSTAFPLVAALLV GSDVTI NVLMNPTRTGLILTLQEMGADIE+I+PRLAGGED VPGDPSSTAFPLVAALLVEGSDVTIRNVLMNPTRTGLILTLQEMGADIEIIDPRLAGGED | 300 |
| Query | 301 | VADLRVRSSTLKGVTVPEDRAPSMIDEYPILAVAAAFAEGATVMNGLEELRVKESDRLSA | 360 |
| Sbjct | 301 | VADLRV++S LKGV VP +RAPSMIDEYP+LA+AA+FAEG TVM+GL+ELRVKESDRL+A VADLRVKASKLKGVVVPPERAPSMIDEYPVLAIAASFAEGETVMDGLDELRVKESDRLAA | 360 |
| Query | 361 | VANGLKLNGVDCDEGETSLVVRGRPDGKGLGNASGAAVATHLDHRIAMSFLVMGLVSENP | 420 |
| Sbjct | 361 | VA GL+ NGVDC EGE SL VRGRP GKGLG G VATHLDHRIAMSFLVMGL SE P VARGLEANGVDCTEGEMSLTVRGRPGGKGLG---GGTVATHLDHRIAMSFLVMGLASEKP | 417 |
| Query  Sbjct | 421  418 | VTVDDATMIATSFPEFMDLMAGLGAKI 447 VTVDD+TMIATSFPEFM +MAGLGAKI VTVDDSTMIATSFPEFMGMMAGLGAKI 444 |  |

>SP:C0RG90 AROA_BRUMB 3-phosphoshikimate 1-carboxyvinyltransferase OS=Brucella melitensis biotype 2 (strain ATCC 23457) OX=546272 GN=aroA

PE=3 SV=1

Length=450

Score = 727 bits (1876), Expect = 0.0

| Identities | = 368/447 (82%), Positives = 402/447 (90%), Gaps = 3/447 (1%) |  |
| --- | --- | --- |
| Query 1 | MLHGASSRPATARKSSGLSGTVRIPGDKSISHRSFMFGGLASGETRITGLLEGEDVINTG | 60 |
| Sbjct 1 | M H A +PATAR S L+G +RIPGDKSISHRSFMFGGLASG+TRITGLLEGEDVINTG MSHSACPKPATARHSQALTGEIRIPGDKSISHRSFMFGGLASGKTRITGLLEGEDVINTG | 60 |
| Query 61 | KAMQAMGARIRKEGDTWIIDGVGNGGLLAPEAPLDFGNAATGCRLTMGLVGVYDFDSTFI | 120 |
| Sbjct 61 | +AMQAMGARIRKEGD WII+GVGNG LL PEAPLDFGNA TG RLTMGLVG YD ++FI RAMQAMGARIRKEGDVWIINGVGNGCLLQPEAPLDFGNAGTGARLTMGLVGTYDMKTSFI | 120 |
| Query 121 | GDASLTKRPMGRVLNPLREMGVQVKSEDGDRLPVTLRGPKTPTPITYRVPMASAQVKSAV | 180 |
| Sbjct 121 | GDASL+KRPMGRVLNPLREMGVQV++ +GDR+P+TL GP+T PI YRVPMASAQVKSAV GDASLSKRPMGRVLNPLREMGVQVEAAEGDRMPLTLIGPRTANPIAYRVPMASAQVKSAV | 180 |
| Query 181 | LLAGLNTPGITTVIEPIMTRDHTEKMLQGFGANLTVETDADGVRTIRLEGRGKLTGQVID | 240 |
| Sbjct 181 | LLAGLNTPG+TTVIEP+MTRDHTEKMLQGFGA+LTVETD DGVR IR+ G+GKLTGQ ID LLAGLNTPGVTTVIEPVMTRDHTEKMLQGFGADLTVETDKDGVRHIRIVGQGKLTGQTID | 240 |
| Query 241 | VPGDPSSTAFPLVAALLVPGSDVTILNVLMNPTRTGLILTLQEMGADIEVINPRLAGGED | 300 |
| Sbjct 241 | VPGDPSSTAFPLVAALLV GSDVTI NVLMNPTRTGLILTLQEMGADIE+I+PRLAGGED VPGDPSSTAFPLVAALLVEGSDVTIRNVLMNPTRTGLILTLQEMGADIEIIDPRLAGGED | 300 |
| Query 301 | VADLRVRSSTLKGVTVPEDRAPSMIDEYPILAVAAAFAEGATVMNGLEELRVKESDRLSA | 360 |
| Sbjct 301 | VADLRV++S LKGV VP +RAPSMIDEYP+LA+AA+FAEG TVM+GL+ELRVKESDRL+A VADLRVKASKLKGVVVPPERAPSMIDEYPVLAIAASFAEGETVMDGLDELRVKESDRLAA | 360 |
| Query 361 | VANGLKLNGVDCDEGETSLVVRGRPDGKGLGNASGAAVATHLDHRIAMSFLVMGLVSENP | 420 |
| Sbjct 361 | VA GL+ NGVDC EGE SL VRGRP GKGLG G VATHLDHRIAMSFLVMGL SE P VARGLEANGVDCTEGEMSLTVRGRPGGKGLG---GGTVATHLDHRIAMSFLVMGLASEKP | 417 |
| Query 421  Sbjct 418 | VTVDDATMIATSFPEFMDLMAGLGAKI 447 VTVDD+TMIATSFPEFM +MAGLGAKI VTVDDSTMIATSFPEFMGMMAGLGAKI 444 |  |

>SP:Q8YEG1 AROA_BRUME 3-phosphoshikimate 1-carboxyvinyltransferase OS=Brucella

melitensis biotype 1 (strain 16M / ATCC 23456 / NCTC 10094) OX=224914 GN=aroA PE=3 SV=2

Length=450

Score = 725 bits (1872), Expect = 0.0

| Identities | = 367/447 (82%), Positives = 401/447 (90%), Gaps = 3/447 (1%) |  |
| --- | --- | --- |
| Query 1 | MLHGASSRPATARKSSGLSGTVRIPGDKSISHRSFMFGGLASGETRITGLLEGEDVINTG | 60 |
| Sbjct 1 | M H A +PATAR S L+G +RIPGDKSISHRSFMFGGLASG+TRITGLLEGEDVINTG MSHSACPKPATARHSQALTGEIRIPGDKSISHRSFMFGGLASGKTRITGLLEGEDVINTG | 60 |
| Query 61 | KAMQAMGARIRKEGDTWIIDGVGNGGLLAPEAPLDFGNAATGCRLTMGLVGVYDFDSTFI | 120 |
| Sbjct 61 | +AMQAMGARIRKEGD WII+GVGNG LL PEAPLDFGNA TG RLTMGLVG YD ++FI RAMQAMGARIRKEGDVWIINGVGNGCLLQPEAPLDFGNAGTGARLTMGLVGTYDMKTSFI | 120 |
| Query 121 | GDASLTKRPMGRVLNPLREMGVQVKSEDGDRLPVTLRGPKTPTPITYRVPMASAQVKSAV | 180 |
| Sbjct 121 | GDASL+KRPMGRVLNPLREMGVQV++ +GDR+P+TL GP+T PI YRVPMASAQVKSAV GDASLSKRPMGRVLNPLREMGVQVEAAEGDRMPLTLIGPRTANPIAYRVPMASAQVKSAV | 180 |
| Query 181 | LLAGLNTPGITTVIEPIMTRDHTEKMLQGFGANLTVETDADGVRTIRLEGRGKLTGQVID | 240 |
| Sbjct 181 | LLAGLNTPG+TTVIEP+MTRDHTEKMLQGFGA+LTVETD DGVR IR+ G+GKLTGQ ID LLAGLNTPGVTTVIEPVMTRDHTEKMLQGFGADLTVETDKDGVRHIRIVGQGKLTGQTID | 240 |
| Query 241 | VPGDPSSTAFPLVAALLVPGSDVTILNVLMNPTRTGLILTLQEMGADIEVINPRLAGGED | 300 |
| Sbjct 241 | VPGDPSSTAFPLVAALLV GSDVTI NVLMNPTRTGLILTLQEMGADIE+I+PRLAGGED VPGDPSSTAFPLVAALLVEGSDVTIRNVLMNPTRTGLILTLQEMGADIEIIDPRLAGGED | 300 |
| Query 301 | VADLRVRSSTLKGVTVPEDRAPSMIDEYPILAVAAAFAEGATVMNGLEELRVKESDRLSA | 360 |
| Sbjct 301 | VADLRV++S LKGV VP +RAPSMIDEYP+LA+AA+FAEG TVM+GL+ELRVKESDRL+A VADLRVKASKLKGVVVPPERAPSMIDEYPVLAIAASFAEGETVMDGLDELRVKESDRLAA | 360 |
| Query 361 | VANGLKLNGVDCDEGETSLVVRGRPDGKGLGNASGAAVATHLDHRIAMSFLVMGLVSENP | 420 |
| Sbjct 361 | VA GL+ NGVDC EGE SL VRGRP GKGLG G V THLDHRIAMSFLVMGL SE P VARGLEANGVDCTEGEMSLTVRGRPGGKGLG---GGTVGTHLDHRIAMSFLVMGLASEKP | 417 |
| Query 421  Sbjct 418 | VTVDDATMIATSFPEFMDLMAGLGAKI 447 VTVDD+TMIATSFPEFM +MAGLGAKI VTVDDSTMIATSFPEFMGMMAGLGAKI 444 |  |

>SP:B2S7T0 AROA_BRUA1 3-phosphoshikimate 1-carboxyvinyltransferase OS=Brucella abortus (strain S19) OX=430066 GN=aroA PE=3 SV=1

Length=450

Score = 725 bits (1872), Expect = 0.0

| Identities | = 367/447 (82%), Positives = 402/447 (90%), Gaps = 3/447 (1%) |  |
| --- | --- | --- |
| Query 1 | MLHGASSRPATARKSSGLSGTVRIPGDKSISHRSFMFGGLASGETRITGLLEGEDVINTG | 60 |
| Sbjct 1 | M H A +PATAR S L+G +RIPGDKSISHRSFMFGGLASG+TRITGLLEGEDVINTG MSHSACPKPATARHSQALTGEIRIPGDKSISHRSFMFGGLASGKTRITGLLEGEDVINTG | 60 |
| Query 61 | KAMQAMGARIRKEGDTWIIDGVGNGGLLAPEAPLDFGNAATGCRLTMGLVGVYDFDSTFI | 120 |
| Sbjct 61 | +AMQAMGARIRKEGD WII+GVGNG LL PEAPLDFGNA TG RLTMGLVG YD ++FI RAMQAMGARIRKEGDVWIINGVGNGCLLQPEAPLDFGNAGTGARLTMGLVGTYDMKTSFI | 120 |
| Query 121 | GDASLTKRPMGRVLNPLREMGVQVKSEDGDRLPVTLRGPKTPTPITYRVPMASAQVKSAV | 180 |
| Sbjct 121 | GDASL+KRPMGRVLNPLREMGVQV++ +GDR+P+TL GP+T PI YRVPMASAQVKSAV GDASLSKRPMGRVLNPLREMGVQVEAAEGDRMPLTLIGPRTANPIAYRVPMASAQVKSAV | 180 |
| Query 181 | LLAGLNTPGITTVIEPIMTRDHTEKMLQGFGANLTVETDADGVRTIRLEGRGKLTGQVID | 240 |
| Sbjct 181 | LLAGLNTPG+TTVIEP+MTRDHTEKMLQGFGA+LTVETD DGVR IR+ G+GKLTGQ ID LLAGLNTPGVTTVIEPVMTRDHTEKMLQGFGADLTVETDKDGVRHIRIVGQGKLTGQTID | 240 |
| Query 241 | VPGDPSSTAFPLVAALLVPGSDVTILNVLMNPTRTGLILTLQEMGADIEVINPRLAGGED | 300 |

| Sbjct | 241 | VPGDPSSTAFPLVAALLV GS+VTI NVLMNPTRTGLILTLQEMGADIE+I+PRLAGGED  VPGDPSSTAFPLVAALLVEGSEVTIRNVLMNPTRTGLILTLQEMGADIEIIDPRLAGGED | 300 |
| --- | --- | --- | --- |
| Query | 301 | VADLRVRSSTLKGVTVPEDRAPSMIDEYPILAVAAAFAEGATVMNGLEELRVKESDRLSA | 360 |
| Sbjct | 301 | VADLRV++S LKGV VP +RAPSMIDEYP+LA+AA+FAEG TVM+GL+ELRVKESDRL+A VADLRVKASKLKGVVVPPERAPSMIDEYPVLAIAASFAEGETVMDGLDELRVKESDRLAA | 360 |
| Query | 361 | VANGLKLNGVDCDEGETSLVVRGRPDGKGLGNASGAAVATHLDHRIAMSFLVMGLVSENP | 420 |
| Sbjct | 361 | VA GL+ NGVDC EGE SL VRGRP GKGLG G VATHLDHRIAMSFLVMGL SE P VARGLEANGVDCTEGEMSLTVRGRPGGKGLG---GGTVATHLDHRIAMSFLVMGLASEKP | 417 |
| Query  Sbjct | 421  418 | VTVDDATMIATSFPEFMDLMAGLGAKI 447 VTVDD+TMIATSFPEFM +MAGLGAKI VTVDDSTMIATSFPEFMGMMAGLGAKI 444 |  |

>SP:Q2YPN6 AROA_BRUA2 3-phosphoshikimate 1-carboxyvinyltransferase OS=Brucella abortus (strain 2308) OX=359391 GN=aroA PE=3 SV=1

Length=450

Score = 725 bits (1872), Expect = 0.0

| Identities | = 367/447 (82%), Positives = 402/447 (90%), Gaps = 3/447 (1%) |  |
| --- | --- | --- |
| Query 1 | MLHGASSRPATARKSSGLSGTVRIPGDKSISHRSFMFGGLASGETRITGLLEGEDVINTG | 60 |
| Sbjct 1 | M H A +PATAR S L+G +RIPGDKSISHRSFMFGGLASG+TRITGLLEGEDVINTG MSHSACPKPATARHSQALTGEIRIPGDKSISHRSFMFGGLASGKTRITGLLEGEDVINTG | 60 |
| Query 61 | KAMQAMGARIRKEGDTWIIDGVGNGGLLAPEAPLDFGNAATGCRLTMGLVGVYDFDSTFI | 120 |
| Sbjct 61 | +AMQAMGARIRKEGD WII+GVGNG LL PEAPLDFGNA TG RLTMGLVG YD ++FI RAMQAMGARIRKEGDVWIINGVGNGCLLQPEAPLDFGNAGTGARLTMGLVGTYDMKTSFI | 120 |
| Query 121 | GDASLTKRPMGRVLNPLREMGVQVKSEDGDRLPVTLRGPKTPTPITYRVPMASAQVKSAV | 180 |
| Sbjct 121 | GDASL+KRPMGRVLNPLREMGVQV++ +GDR+P+TL GP+T PI YRVPMASAQVKSAV GDASLSKRPMGRVLNPLREMGVQVEAAEGDRMPLTLIGPRTANPIAYRVPMASAQVKSAV | 180 |
| Query 181 | LLAGLNTPGITTVIEPIMTRDHTEKMLQGFGANLTVETDADGVRTIRLEGRGKLTGQVID | 240 |
| Sbjct 181 | LLAGLNTPG+TTVIEP+MTRDHTEKMLQGFGA+LTVETD DGVR IR+ G+GKLTGQ ID LLAGLNTPGVTTVIEPVMTRDHTEKMLQGFGADLTVETDKDGVRHIRIVGQGKLTGQTID | 240 |
| Query 241 | VPGDPSSTAFPLVAALLVPGSDVTILNVLMNPTRTGLILTLQEMGADIEVINPRLAGGED | 300 |
| Sbjct 241 | VPGDPSSTAFPLVAALLV GS+VTI NVLMNPTRTGLILTLQEMGADIE+I+PRLAGGED VPGDPSSTAFPLVAALLVEGSEVTIRNVLMNPTRTGLILTLQEMGADIEIIDPRLAGGED | 300 |
| Query 301 | VADLRVRSSTLKGVTVPEDRAPSMIDEYPILAVAAAFAEGATVMNGLEELRVKESDRLSA | 360 |
| Sbjct 301 | VADLRV++S LKGV VP +RAPSMIDEYP+LA+AA+FAEG TVM+GL+ELRVKESDRL+A VADLRVKASKLKGVVVPPERAPSMIDEYPVLAIAASFAEGETVMDGLDELRVKESDRLAA | 360 |
| Query 361 | VANGLKLNGVDCDEGETSLVVRGRPDGKGLGNASGAAVATHLDHRIAMSFLVMGLVSENP | 420 |
| Sbjct 361 | VA GL+ NGVDC EGE SL VRGRP GKGLG G VATHLDHRIAMSFLVMGL SE P VARGLEANGVDCTEGEMSLTVRGRPGGKGLG---GGTVATHLDHRIAMSFLVMGLASEKP | 417 |
| Query 421  Sbjct 418 | VTVDDATMIATSFPEFMDLMAGLGAKI 447 VTVDD+TMIATSFPEFM +MAGLGAKI VTVDDSTMIATSFPEFMGMMAGLGAKI 444 |  |

>TR:A0A316JDA1 A0A316JDA1_9RHIZ 3-phosphoshikimate 1-carboxyvinyltransferase OS=Falsochrobactrum sp. HN4 OX=2201899 GN=aroA PE=3 SV=1

Length=450

Score = 725 bits (1872), Expect = 0.0

Identities = 366/453 (81%), Positives = 404/453 (89%), Gaps = 3/453 (1%) Query 1	MLHGASSRPATARKSSGLSGTVRIPGDKSISHRSFMFGGLASGETRITGLLEGEDVINTG 60

| Sbjct | 1 | M H + +PA+A +S GLSG +RIPGDKSISHRSFMFGGLASGETRITGLLEGEDVINTG  MSHSSPLKPASAHRSEGLSGDIRIPGDKSISHRSFMFGGLASGETRITGLLEGEDVINTG | 60 |
| --- | --- | --- | --- |
| Query | 61 | KAMQAMGARIRKEGDTWIIDGVGNGGLLAPEAPLDFGNAATGCRLTMGLVGVYDFDSTFI | 120 |
| Sbjct | 61 | +AM+AMGA+IRKEGD WII+G GNG LL PEAPLDFGNA TG RLTMGLVG YD +TFI RAMEAMGAKIRKEGDVWIINGTGNGCLLQPEAPLDFGNAGTGARLTMGLVGTYDMTTTFI | 120 |
| Query | 121 | GDASLTKRPMGRVLNPLREMGVQVKSEDGDRLPVTLRGPKTPTPITYRVPMASAQVKSAV | 180 |
| Sbjct | 121 | GDASL+KRPMGRVL+PLR+MGVQVKS +GDR+P+TL GPKT PI+YRVPMASAQVKSAV GDASLSKRPMGRVLDPLRQMGVQVKSAEGDRMPLTLTGPKTANPISYRVPMASAQVKSAV | 180 |
| Query | 181 | LLAGLNTPGITTVIEPIMTRDHTEKMLQGFGANLTVETDADGVRTIRLEGRGKLTGQVID | 240 |
| Sbjct | 181 | LLAGLNTPGITTVIEP+MTRDHTEKMLQGFGA+L+VETD +GVR I + G+GKL+GQ ID LLAGLNTPGITTVIEPVMTRDHTEKMLQGFGADLSVETDTEGVRHIHIVGQGKLSGQSID | 240 |
| Query | 241 | VPGDPSSTAFPLVAALLVPGSDVTILNVLMNPTRTGLILTLQEMGADIEVINPRLAGGED | 300 |
| Sbjct | 241 | VPGDPSSTAFPLVAALLV GSDVTI NVLMNPTRTGLILTLQEMGADIEV+N RLAGGED VPGDPSSTAFPLVAALLVEGSDVTIRNVLMNPTRTGLILTLQEMGADIEVLNERLAGGED | 300 |
| Query | 301 | VADLRVRSSTLKGVTVPEDRAPSMIDEYPILAVAAAFAEGATVMNGLEELRVKESDRLSA | 360 |
| Sbjct | 301 | VADLRV+SS LKG+ VP +RAPSMIDEYP+LA+AA FAEG TVM+GL+ELRVKESDRL+A VADLRVKSSKLKGIVVPPERAPSMIDEYPVLAIAATFAEGETVMDGLDELRVKESDRLAA | 360 |
| Query | 361 | VANGLKLNGVDCDEGETSLVVRGRPDGKGLGNASGAAVATHLDHRIAMSFLVMGLVSENP | 420 |
| Sbjct | 361 | VA GL+ NGVDC EGE SL VRGRPDGKG+G G V THLDHRIAMSFLVMGL SE P VARGLEANGVDCTEGEMSLTVRGRPDGKGIG---GGTVETHLDHRIAMSFLVMGLASEKP | 417 |
| Query  Sbjct | 421  418 | VTVDDATMIATSFPEFMDLMAGLGAKIELSDTK 453 VTVDD T+IATSFPEFMD+MAGLGAKIE SD + VTVDDYTIIATSFPEFMDMMAGLGAKIERSDAQ 450 |  |

>TR:A0A364JZ32 A0A364JZ32_9RHIZ 3-phosphoshikimate 1-carboxyvinyltransferase OS=Falsochrobactrum ovis OX=1293442 GN=aroA PE=3 SV=1

Length=450

Score = 724 bits (1870), Expect = 0.0

| Identities | = 364/453 (80%), Positives = 404/453 (89%), Gaps = 3/453 (1%) |  |
| --- | --- | --- |
| Query 1 | MLHGASSRPATARKSSGLSGTVRIPGDKSISHRSFMFGGLASGETRITGLLEGEDVINTG | 60 |
| Sbjct 1 | M H + +PATA +S GLSG +RIPGDKSISHRSFMFGGLASGETRITGLLEGEDVINTG MSHSSPLKPATAHRSEGLSGDIRIPGDKSISHRSFMFGGLASGETRITGLLEGEDVINTG | 60 |
| Query 61 | KAMQAMGARIRKEGDTWIIDGVGNGGLLAPEAPLDFGNAATGCRLTMGLVGVYDFDSTFI | 120 |
| Sbjct 61 | +AM+AMGA+IRKEGD WII+GVGNG LL PEAPLDFGNA TG RLTMGLVG YD +TFI RAMEAMGAKIRKEGDVWIINGVGNGCLLQPEAPLDFGNAGTGARLTMGLVGTYDMTTTFI | 120 |
| Query 121 | GDASLTKRPMGRVLNPLREMGVQVKSEDGDRLPVTLRGPKTPTPITYRVPMASAQVKSAV | 180 |
| Sbjct 121 | GDASL+ RPMGRVLNPLREMGVQVK+ +GDR+P+TL GPKT PI+YRVPMASAQVKSA+ GDASLSSRPMGRVLNPLREMGVQVKAAEGDRMPLTLTGPKTANPISYRVPMASAQVKSAI | 180 |
| Query 181 | LLAGLNTPGITTVIEPIMTRDHTEKMLQGFGANLTVETDADGVRTIRLEGRGKLTGQVID | 240 |
| Sbjct 181 | LLAGLNTPG+TTVIEP+MTRDHTEKMLQGFGA+LTVETD+ GVR I + G+G+LTGQ+ID LLAGLNTPGVTTVIEPVMTRDHTEKMLQGFGADLTVETDSAGVRHIHIVGQGQLTGQIID | 240 |
| Query 241 | VPGDPSSTAFPLVAALLVPGSDVTILNVLMNPTRTGLILTLQEMGADIEVINPRLAGGED | 300 |
| Sbjct 241 | VPGDPSSTAFPLVAA+LV GSD+TI NVLMNPTRTGLILTLQEMGADIEV+N RLAGGED VPGDPSSTAFPLVAAILVEGSDLTIRNVLMNPTRTGLILTLQEMGADIEVLNARLAGGED | 300 |
| Query 301 | VADLRVRSSTLKGVTVPEDRAPSMIDEYPILAVAAAFAEGATVMNGLEELRVKESDRLSA | 360 |
| Sbjct 301 | VADLRVRSS LKG+ VP +RAPSMIDEYPILA+AAAFAEG T+M+GL+ELRVKESDRL+A VADLRVRSSKLKGIVVPPERAPSMIDEYPILAIAAAFAEGETIMDGLDELRVKESDRLAA | 360 |
| Query 361 | VANGLKLNGVDCDEGETSLVVRGRPDGKGLGNASGAAVATHLDHRIAMSFLVMGLVSENP | 420 |

VA GL+ NGVDC EGE SL VRGRPDGKG+G	G	THLDHRIAMSFLVMGL SE P

| Sbjct | 361 | VARGLEANGVDCTEGEMSLSVRGRPDGKGIG---GGTAKTHLDHRIAMSFLVMGLASEKP 417 |
| --- | --- | --- |
| Query | 421 | VTVDDATMIATSFPEFMDLMAGLGAKIELSDTK 453 |
| Sbjct | 418 | V+VDD +IATSFPEFMD+MAGLGAKIE SD +  VSVDDYKIIATSFPEFMDMMAGLGAKIEQSDAE 450 |

>TR:A0A2N9VTC1 A0A2N9VTC1_9RHIZ 3-phosphoshikimate 1-carboxyvinyltransferase OS=Phyllobacterium zundukense OX=1867719 GN=aroA PE=3 SV=1

Length=451

Score = 724 bits (1868), Expect = 0.0

| Identities | = 365/447 (82%), Positives = 396/447 (89%), Gaps = 3/447 (1%) |  |
| --- | --- | --- |
| Query 1 | MLHGASSRPATARKSSGLSGTVRIPGDKSISHRSFMFGGLASGETRITGLLEGEDVINTG | 60 |
| Sbjct 1 | M H +PATAR+S LSG +RIPGDKSISHRSFMFGGLASGETRITGLLEGEDVINTG MSHSVLPKPATARRSKALSGDIRIPGDKSISHRSFMFGGLASGETRITGLLEGEDVINTG | 60 |
| Query 61 | KAMQAMGARIRKEGDTWIIDGVGNGGLLAPEAPLDFGNAATGCRLTMGLVGVYDFDSTFI | 120 |
| Sbjct 61 | +AMQAMGA+I K+GD WII G GNG LLAPEAPLDFGNA TG RLTMGLVG YD ++TF+ RAMQAMGAKIAKDGDVWIIQGTGNGCLLAPEAPLDFGNAGTGARLTMGLVGTYDMETTFV | 120 |
| Query 121 | GDASLTKRPMGRVLNPLREMGVQVKSEDGDRLPVTLRGPKTPTPITYRVPMASAQVKSAV | 180 |
| Sbjct 121 | GDASL+KRPMGRVLNPLREMGVQV + +GDRLP+TLRGPK PITYRVPMASAQVKSAV GDASLSKRPMGRVLNPLREMGVQVTAAEGDRLPLTLRGPKVAAPITYRVPMASAQVKSAV | 180 |
| Query 181 | LLAGLNTPGITTVIEPIMTRDHTEKMLQGFGANLTVETDADGVRTIRLEGRGKLTGQVID | 240 |
| Sbjct 181 | LLAGLNTPGITTVIEP+MTRDHTEKMLQGFGANL+V+TD VR IR+EG+GKLTGQ ID LLAGLNTPGITTVIEPVMTRDHTEKMLQGFGANLSVKTDKANVRHIRIEGQGKLTGQTID | 240 |
| Query 241 | VPGDPSSTAFPLVAALLVPGSDVTILNVLMNPTRTGLILTLQEMGADIEVINPRLAGGED | 300 |
| Sbjct 241 | VPGDPSSTAFPLVAALLVPGSD+TI NVLMNPTRTGLILTLQEMGADIE++ RLAGGED VPGDPSSTAFPLVAALLVPGSDITIRNVLMNPTRTGLILTLQEMGADIEILGARLAGGED | 300 |
| Query 301 | VADLRVRSSTLKGVTVPEDRAPSMIDEYPILAVAAAFAEGATVMNGLEELRVKESDRLSA | 360 |
| Sbjct 301 | VADLRVR+S L GV VP DRAPSMIDEYP+LAVAA FA G TVMNGLEELRVKESDRLSA VADLRVRASELTGVNVPADRAPSMIDEYPVLAVAACFAAGETVMNGLEELRVKESDRLSA | 360 |
| Query 361 | VANGLKLNGVDCDEGETSLVVRGRPDGKGLGNASGAAVATHLDHRIAMSFLVMGLVSENP | 420 |
| Sbjct 361 | VANGLK NGVDC EG +L VRGRPDGKG+G G V THLDHRIAMSFLVMGL +E P VANGLKANGVDCTEGPETLSVRGRPDGKGIG---GGTVETHLDHRIAMSFLVMGLAAEKP | 417 |
| Query 421  Sbjct 418 | VTVDDATMIATSFPEFMDLMAGLGAKI 447 V+VDD+TMIATSFPEFMD+M G+GA+I VSVDDSTMIATSFPEFMDMMTGMGAEI 444 |  |

>SP:P0C100 AROA_BRUAB 3-phosphoshikimate 1-carboxyvinyltransferase OS=Brucella abortus biovar 1 (strain 9-941) OX=262698 GN=aroA PE=3

SV=1

Length=450

Score = 723 bits (1865), Expect = 0.0

Identities = 366/447 (82%), Positives = 401/447 (90%), Gaps = 3/447 (1%)

| Query | 1 | MLHGASSRPATARKSSGLSGTVRIPGDKSISHRSFMFGGLASGETRITGLLEGEDVINTG | 60 |
| --- | --- | --- | --- |
| Sbjct | 1 | M H A +PATAR S L+G +RIPGDKSISHRSFMFGGLASG+TRITGLLEGEDVINTG MSHSACPKPATARHSQALTGEIRIPGDKSISHRSFMFGGLASGKTRITGLLEGEDVINTG | 60 |
| Query | 61 | KAMQAMGARIRKEGDTWIIDGVGNGGLLAPEAPLDFGNAATGCRLTMGLVGVYDFDSTFI | 120 |
| Sbjct | 61 | +AMQAMGARIRKEGD WII+GVGNG LL PEAPLDFGNA TG RLTMGLVG YD ++FI  RAMQAMGARIRKEGDVWIINGVGNGCLLQPEAPLDFGNAGTGARLTMGLVGTYDMKTSFI | 120 |

| Query | 121 | GDASLTKRPMGRVLNPLREMGVQVKSEDGDRLPVTLRGPKTPTPITYRVPMASAQVKSAV | 180 |
| --- | --- | --- | --- |
| Sbjct | 121 | GDASL+KRPMGRVLNPLREMGVQV++ +GDR+P+TL GP+T PI YRVPMASAQVKSAV GDASLSKRPMGRVLNPLREMGVQVEAAEGDRMPLTLIGPRTANPIAYRVPMASAQVKSAV | 180 |
| Query | 181 | LLAGLNTPGITTVIEPIMTRDHTEKMLQGFGANLTVETDADGVRTIRLEGRGKLTGQVID | 240 |
| Sbjct | 181 | LLAGLNTPG+TTVIEP+MTRDHTEKMLQ FGA+LTVETD DGVR IR+ G+GKLTGQ ID LLAGLNTPGVTTVIEPVMTRDHTEKMLQDFGADLTVETDKDGVRHIRIVGQGKLTGQTID | 240 |
| Query | 241 | VPGDPSSTAFPLVAALLVPGSDVTILNVLMNPTRTGLILTLQEMGADIEVINPRLAGGED | 300 |
| Sbjct | 241 | VPGDPSSTAFPLVAALLV GS+VTI NVLMNPTRTGLILTLQEMGADIE+I+PRLAGGED VPGDPSSTAFPLVAALLVEGSEVTIRNVLMNPTRTGLILTLQEMGADIEIIDPRLAGGED | 300 |
| Query | 301 | VADLRVRSSTLKGVTVPEDRAPSMIDEYPILAVAAAFAEGATVMNGLEELRVKESDRLSA | 360 |
| Sbjct | 301 | VADLRV++S LKGV VP +RAPSMIDEYP+LA+AA+FAEG TVM+GL+ELRVKESDRL+A VADLRVKASKLKGVVVPPERAPSMIDEYPVLAIAASFAEGETVMDGLDELRVKESDRLAA | 360 |
| Query | 361 | VANGLKLNGVDCDEGETSLVVRGRPDGKGLGNASGAAVATHLDHRIAMSFLVMGLVSENP | 420 |
| Sbjct | 361 | VA GL+ NGVDC EGE SL VRGRP GKGLG G VATHLDHRIAMSFLVMGL SE P VARGLEANGVDCTEGEMSLTVRGRPGGKGLG---GGTVATHLDHRIAMSFLVMGLASEKP | 417 |
| Query  Sbjct | 421  418 | VTVDDATMIATSFPEFMDLMAGLGAKI 447 VTVDD+TMIATSFPEFM +MAGLGAKI VTVDDSTMIATSFPEFMGMMAGLGAKI 444 |  |

>TR:A0A318T7W8 A0A318T7W8_9RHIZ 3-phosphoshikimate 1-carboxyvinyltransferase OS=Phyllobacterium leguminum OX=314237 GN=aroA PE=3 SV=1

Length=451

Score = 723 bits (1865), Expect = 0.0

| Identities | = 367/454 (81%), Positives = 403/454 (89%), Gaps = 3/454 (1%) |  |
| --- | --- | --- |
| Query 1 | MLHGASSRPATARKSSGLSGTVRIPGDKSISHRSFMFGGLASGETRITGLLEGEDVINTG | 60 |
| Sbjct 1 | M H A+ +PATA +S L+G +RIPGDKSISHRSFMFGGLASGETRITGLLEGEDVINTG MSHSATPKPATAGRSRDLAGDIRIPGDKSISHRSFMFGGLASGETRITGLLEGEDVINTG | 60 |
| Query 61 | KAMQAMGARIRKEGDTWIIDGVGNGGLLAPEAPLDFGNAATGCRLTMGLVGVYDFDSTFI | 120 |
| Sbjct 61 | +AMQAMGA IRK+G+TWII G GNG LL PE PLDFGNA TG RLTMGLVG YD ++TFI RAMQAMGADIRKDGETWIIQGTGNGCLLEPETPLDFGNAGTGARLTMGLVGTYDMETTFI | 120 |
| Query 121 | GDASLTKRPMGRVLNPLREMGVQVKSEDGDRLPVTLRGPKTPTPITYRVPMASAQVKSAV | 180 |
| Sbjct 121 | GDASL+KRPMGRVL PLREMGVQVKS +GDRLP+TL GPKT PI YRVPMASAQVKSAV GDASLSKRPMGRVLGPLREMGVQVKSAEGDRLPLTLHGPKTAAPIIYRVPMASAQVKSAV | 180 |
| Query 181 | LLAGLNTPGITTVIEPIMTRDHTEKMLQGFGANLTVETDADGVRTIRLEGRGKLTGQVID | 240 |
| Sbjct 181 | LLAGLNTPGITTVIEPIMTRDHTEKMLQGFGA+LTVETD +GVR IR+ G+G+L GQ ID LLAGLNTPGITTVIEPIMTRDHTEKMLQGFGADLTVETDREGVRHIRIVGQGELKGQTID | 240 |
| Query 241 | VPGDPSSTAFPLVAALLVPGSDVTILNVLMNPTRTGLILTLQEMGADIEVINPRLAGGED | 300 |
| Sbjct 241 | VPGDPSSTAFPLVAAL+VPGSDVTI NVLMNPTRTGLILTLQEMGA+IE++N RLAGGED VPGDPSSTAFPLVAALIVPGSDVTIRNVLMNPTRTGLILTLQEMGANIEILNARLAGGED | 300 |
| Query 301 | VADLRVRSSTLKGVTVPEDRAPSMIDEYPILAVAAAFAEGATVMNGLEELRVKESDRLSA | 360 |
| Sbjct 301 | VADLRVR+S LKGV+VP +RAPSMIDEYP+LAVAAAFAEG T+M+GLEELRVKESDRLSA VADLRVRASELKGVSVPPERAPSMIDEYPVLAVAAAFAEGETIMDGLEELRVKESDRLSA | 360 |
| Query 361 | VANGLKLNGVDCDEGETSLVVRGRPDGKGLGNASGAAVATHLDHRIAMSFLVMGLVSENP | 420 |
| Sbjct 361 | VA+GLK NGV+C EGE SL VRG P GKG+G G VATHLDHRIAMSFLVMGL +E P VADGLKANGVECAEGEASLTVRGTPGGKGIG---GGTVATHLDHRIAMSFLVMGLAAEKP | 417 |
| Query 421  Sbjct 418 | VTVDDATMIATSFPEFMDLMAGLGAKIELSDTKA 454 VT+DD+ MIATSFPEFMDLM LGAKIE ++T A VTIDDSAMIATSFPEFMDLMKDLGAKIETAETVA 451 |  |

>TR:J3HME9 J3HME9_9RHIZ 3-phosphoshikimate 1-carboxyvinyltransferase OS=Phyllobacterium sp. YR531 OX=1144343 GN=aroA PE=3 SV=1

Length=451

Score = 716 bits (1847), Expect = 0.0

| Identities | = 363/447 (81%), Positives = 394/447 (88%), Gaps = 3/447 (1%) |  |
| --- | --- | --- |
| Query 1 | MLHGASSRPATARKSSGLSGTVRIPGDKSISHRSFMFGGLASGETRITGLLEGEDVINTG | 60 |
| Sbjct 1 | M H + +PAT+R+S+ L+G +RIPGDKSISHRSFMFGGLASGETRITGLLEGEDVINTG MSHSSLPKPATSRRSNALNGDIRIPGDKSISHRSFMFGGLASGETRITGLLEGEDVINTG | 60 |
| Query 61 | KAMQAMGARIRKEGDTWIIDGVGNGGLLAPEAPLDFGNAATGCRLTMGLVGVYDFDSTFI | 120 |
| Sbjct 61 | +AMQAMGA+I KEGD WII G GNG LL PEAPLDFGNA TG RLTMGLVG Y ++ FI RAMQAMGAKIAKEGDVWIIKGTGNGCLLQPEAPLDFGNAGTGARLTMGLVGTYAMETIFI | 120 |
| Query 121 | GDASLTKRPMGRVLNPLREMGVQVKSEDGDRLPVTLRGPKTPTPITYRVPMASAQVKSAV | 180 |
| Sbjct 121 | GDASL+KRPMGRVLNPLREMGVQV + DGDR+P+TLRGPK PI YRVPMASAQVKSAV GDASLSKRPMGRVLNPLREMGVQVSAADGDRMPLTLRGPKVAAPIAYRVPMASAQVKSAV | 180 |
| Query 181 | LLAGLNTPGITTVIEPIMTRDHTEKMLQGFGANLTVETDADGVRTIRLEGRGKLTGQVID | 240 |
| Sbjct 181 | LLAGLNTPGITTVIEP+MTRDHTEKMLQGFGANL+VETD D VR IR+EG+GKLTGQ ID LLAGLNTPGITTVIEPVMTRDHTEKMLQGFGANLSVETDKDNVRHIRIEGQGKLTGQTID | 240 |
| Query 241 | VPGDPSSTAFPLVAALLVPGSDVTILNVLMNPTRTGLILTLQEMGADIEVINPRLAGGED | 300 |
| Sbjct 241 | VPGDPSSTAFPLVAALLVPGSDVTI NVLMNPTRTGLILTLQEMGA IE++N RLAGGED VPGDPSSTAFPLVAALLVPGSDVTIRNVLMNPTRTGLILTLQEMGAKIEIMNARLAGGED | 300 |
| Query 301 | VADLRVRSSTLKGVTVPEDRAPSMIDEYPILAVAAAFAEGATVMNGLEELRVKESDRLSA | 360 |
| Sbjct 301 | VADLRV S LKGV VP DRAPSMIDEYP+LAVAA+FA G TVMNGLEELRVKESDRL+A VADLRVMHSELKGVNVPADRAPSMIDEYPVLAVAASFASGETVMNGLEELRVKESDRLAA | 360 |
| Query 361 | VANGLKLNGVDCDEGETSLVVRGRPDGKGLGNASGAAVATHLDHRIAMSFLVMGLVSENP | 420 |
| Sbjct 361 | VANGLK NGVDC EG +L VRGRPDGK +G G V THLDHRIAMSFLVMGL +E P VANGLKANGVDCTEGVETLSVRGRPDGKAIG---GGTVETHLDHRIAMSFLVMGLAAEKP | 417 |
| Query 421  Sbjct 418 | VTVDDATMIATSFPEFMDLMAGLGAKI 447 VTVDD+TMIATSFPEFM +MAG+GA+I VTVDDSTMIATSFPEFMAMMAGMGAEI 444 |  |

>TR:A9CW43 A9CW43_HOEPD 3-phosphoshikimate 1-carboxyvinyltransferase OS=Hoeflea phototrophica (strain DSM 17068 / NCIMB 14078 / DFL-43)

OX=411684 GN=aroA PE=3 SV=2

Length=456

Score = 712 bits (1839), Expect = 0.0

| Identities | = 362/447 (81%), Positives = 395/447 (88%), Gaps = 1/447 (0%) |  |
| --- | --- | --- |
| Query 8 | RPATARKSSGLSGTVRIPGDKSISHRSFMFGGLASGETRITGLLEGEDVINTGKAMQAMG | 67 |
| Sbjct 9 | RPA+A KS L G +RIPGDKSISHRSFMFGGLA+GETRITGLLEGEDVINTGKAMQAMG RPASASKSRSLKGDIRIPGDKSISHRSFMFGGLANGETRITGLLEGEDVINTGKAMQAMG | 68 |
| Query 68 | ARIRKEGDTWIIDGVGNGGLLAPEAPLDFGNAATGCRLTMGLVGVYDFDSTFIGDASLTK | 127 |
| Sbjct 69 | A IRKEGD W+I+GVGNG LLAPE PLDFGNA TGCRLTMGLVG YDFD+TF+GDASL++ ASIRKEGDAWVINGVGNGCLLAPEKPLDFGNAGTGCRLTMGLVGPYDFDTTFVGDASLSR | 128 |
| Query 128 | RPMGRVLNPLREMGVQVKSEDGDRLPVTLRGPKTPTPITYRVPMASAQVKSAVLLAGLNT | 187 |
| Sbjct 129 | RPMGRVL+PLR MGVQV+SEDGDRLPVTL GP P PI+YRVPMASAQVKSAVLLAGLN RPMGRVLDPLRLMGVQVRSEDGDRLPVTLHGPLAPAPISYRVPMASAQVKSAVLLAGLNV | 188 |
| Query 188 | PGITTVIEPIMTRDHTEKMLQGFGANLTVETDADGVRTIRLEGRGKLTGQVIDVPGDPSS | 247 |
| Sbjct 189 | PG+TTVIEP+MTRDHTEKML GFGA L VET ADGVRTIRLEGRG LT Q+IDVPGDPSS  PGVTTVIEPVMTRDHTEKMLAGFGAQLEVETGADGVRTIRLEGRGTLTAQIIDVPGDPSS | 248 |

| Query | 248 | TAFPLVAALLVPGSDVTILNVLMNPTRTGLILTLQEMGADIEVINPRLAGGEDVADLRVR | 307 |
| --- | --- | --- | --- |
| Sbjct | 249 | TAFPLVAALLVP SDVTI NVLMN TRTGLILTLQEMGA+IE+I+PR AGGEDVADLRVR TAFPLVAALLVPDSDVTIRNVLMNETRTGLILTLQEMGANIEIIDPRQAGGEDVADLRVR | 308 |
| Query | 308 | SSTLKGVTVPEDRAPSMIDEYPILAVAAAFAEGATVMNGLEELRVKESDRLSAVANGLKL | 367 |
| Sbjct | 309 | SS L GV VP DRAPSMIDEYP+LAVAA+FAEG TVM GL+ELRVKESDRLSAVA GL++ SSELTGVNVPADRAPSMIDEYPVLAVAASFAEGETVMTGLDELRVKESDRLSAVARGLEI | 368 |
| Query | 368 | NGVDCDEGETSLVVRGRPDGKGLG-NASGAAVATHLDHRIAMSFLVMGLVSENPVTVDDA | 426 |
| Sbjct | 369 | NGVDC EG+ L VRGRPDGKGLG + A V T LDHRIAMSFLVMGL SE+PV VDD+ NGVDCTEGDDWLSVRGRPDGKGLGARSEKAVVKTWLDHRIAMSFLVMGLASEHPVHVDDS | 428 |
| Query  Sbjct | 427  429 | TMIATSFPEFMDLMAGLGAKIELSDTK 453  +IATSFPEFMD+M GLGA+I++ + AIIATSFPEFMDMMTGLGARIDIGSAR 455 |  |

>TR:A0A2G1QTW2 A0A2G1QTW2_9RHIZ 3-phosphoshikimate 1-carboxyvinyltransferase OS=Zhengella mangrovi OX=1982044 GN=aroA PE=3 SV=1

Length=450

Score = 705 bits (1819), Expect = 0.0

| Identities | = 356/448 (79%), Positives = 389/448 (87%), Gaps = 3/448 (1%) |  |
| --- | --- | --- |
| Query 1 | MLHGASSRPATARKSSGLSGTVRIPGDKSISHRSFMFGGLASGETRITGLLEGEDVINTG | 60 |
| Sbjct 1 | M H ++RPA +RKS+ LSGT R+PGDKSISHRSFMFGGLASGETRITGLLEGEDV+ TG MSHSGTARPALSRKSNALSGTARVPGDKSISHRSFMFGGLASGETRITGLLEGEDVLATG | 60 |
| Query 61 | KAMQAMGARIRKEGDTWIIDGVGNGGLLAPEAPLDFGNAATGCRLTMGLVGVYDFDSTFI | 120 |
| Sbjct 61 | +AM AMGARI K GDTWII G GNG LL PE PLDFGNA TGCRLTMGL GVYD + F RAMSAMGARISKVGDTWIIQGAGNGALLEPEGPLDFGNAGTGCRLTMGLAGVYDMQTVFT | 120 |
| Query 121 | GDASLTKRPMGRVLNPLREMGVQVKSEDGDRLPVTLRGPKTPTPITYRVPMASAQVKSAV | 180 |
| Sbjct 121 | GDASL++RPMGRVL+PLR MGVQV+++DGDRLPVTL GPKTPTPITYRVPMASAQVKSAV GDASLSRRPMGRVLDPLRLMGVQVQAQDGDRLPVTLHGPKTPTPITYRVPMASAQVKSAV | 180 |
| Query 181 | LLAGLNTPGITTVIEPIMTRDHTEKMLQGFGANLTVETDADGVRTIRLEGRGKLTGQVID | 240 |
| Sbjct 181 | LLAGLNTPG+TTV+EP+MTRDHTEKML GFGA+L VE DADGVRTIRL+GR LTG+VID LLAGLNTPGVTTVVEPVMTRDHTEKMLAGFGAHLEVEEDADGVRTIRLQGRPTLTGRVID | 240 |
| Query 241 | VPGDPSSTAFPLVAALLVPGSDVTILNVLMNPTRTGLILTLQEMGADIEVINPRLAGGED | 300 |
| Sbjct 241 | VPGDPSS AFPLVAAL+VPGSDV I NVLMNPTR G++ TLQEMG I++IN R AGGED VPGDPSSAAFPLVAALIVPGSDVVIENVLMNPTRNGIVTTLQEMGGQIDIINWRDAGGED | 300 |
| Query 301 | VADLRVRSSTLKGVTVPEDRAPSMIDEYPILAVAAAFAEGATVMNGLEELRVKESDRLSA | 360 |
| Sbjct 301 | VADLRVR+S LKGVTVP +RAPSMIDEYP LA+AAA A+G TVM GL+ELRVKESDRLSA VADLRVRASDLKGVTVPPERAPSMIDEYPALAMAAALADGETVMEGLDELRVKESDRLSA | 360 |
| Query 361 | VANGLKLNGVDCDEGETSLVVRGRPDGKGLGNASGAAVATHLDHRIAMSFLVMGLVSENP | 420 |
| Sbjct 361 | V +GLK NGVDC EGE SL VRGRPDGKGLG G VATHLDHRIAMSFLV+GL +E P VTDGLKANGVDCTEGEASLSVRGRPDGKGLG---GGTVATHLDHRIAMSFLVLGLATEKP | 417 |
| Query 421  Sbjct 418 | VTVDDATMIATSFPEFMDLMAGLGAKIE 448 VTVDDATMIATSFPEF LM GLGA IE VTVDDATMIATSFPEFFGLMGGLGAAIE 445 |  |

>TR:J1TFJ6 J1TFJ6_9RHIZ 3-phosphoshikimate 1-carboxyvinyltransferase OS=Rhizobium sp. CF142 OX=1144314 GN=aroA PE=3 SV=1

Length=450

Score = 703 bits (1814), Expect = 0.0

Identities = 350/448 (78%), Positives = 397/448 (89%), Gaps = 0/448 (0%)

| Query | 1 | MLHGASSRPATARKSSGLSGTVRIPGDKSISHRSFMFGGLASGETRITGLLEGEDVINTG | 60 |
| --- | --- | --- | --- |
| Sbjct | 1 | M G++S+PA ARKSSGLSGTV IPGDKSISHR+ + GGLASGETR+TGLLEGEDV+NTG MSQGSASKPAIARKSSGLSGTVLIPGDKSISHRALILGGLASGETRVTGLLEGEDVLNTG | 60 |
| Query | 61 | KAMQAMGARIRKEGDTWIIDGVGNGGLLAPEAPLDFGNAATGCRLTMGLVGVYDFDSTFI | 120 |
| Sbjct | 61 | KA++A+GA IRKEGD WII+G GNG LLAPE PLDFGN+ TG RLTMGL VYDFD+ F KAIRAIGAVIRKEGDAWIINGTGNGALLAPEVPLDFGNSGTGARLTMGLASVYDFDTVFT | 120 |
| Query | 121 | GDASLTKRPMGRVLNPLREMGVQVKSEDGDRLPVTLRGPKTPTPITYRVPMASAQVKSAV | 180 |
| Sbjct | 121 | GDASL++RPMGRVL+PLR MGVQVK+ +GDRLPV LRGP+TP PI Y +P+ASAQVKSAV GDASLSRRPMGRVLDPLRRMGVQVKAVEGDRLPVMLRGPETPAPIRYTLPVASAQVKSAV | 180 |
| Query | 181 | LLAGLNTPGITTVIEPIMTRDHTEKMLQGFGANLTVETDADGVRTIRLEGRGKLTGQVID | 240 |
| Sbjct | 181 | LLAGLN PG+TTVIEP +TRDHTEKML GFGA L+++TD DG RTIRL+GRG+LTGQ +D LLAGLNIPGVTTVIEPAITRDHTEKMLAGFGAELSIKTDKDGTRTIRLQGRGRLTGQTLD | 240 |
| Query | 241 | VPGDPSSTAFPLVAALLVPGSDVTILNVLMNPTRTGLILTLQEMGADIEVINPRLAGGED | 300 |
| Sbjct | 241 | VPGDPSS AFPLVAALLVPGSDVTI NVLMNPTRTGLILTLQEMGADI++++PRLAGGE+ VPGDPSSAAFPLVAALLVPGSDVTISNVLMNPTRTGLILTLQEMGADIDILDPRLAGGEN | 300 |
| Query | 301 | VADLRVRSSTLKGVTVPEDRAPSMIDEYPILAVAAAFAEGATVMNGLEELRVKESDRLSA | 360 |
| Sbjct | 301 | VADLRVR S L GVTVP +RAPSMIDEYP+LAVAAAFA+G TVMNGL+ELRVKESDRLSA VADLRVRYSELTGVTVPTERAPSMIDEYPVLAVAAAFAKGRTVMNGLQELRVKESDRLSA | 360 |
| Query | 361 | VANGLKLNGVDCDEGETSLVVRGRPDGKGLGNASGAAVATHLDHRIAMSFLVMGLVSENP | 420 |
| Sbjct | 361 | VA GLKLNGVDC+EG+ L V GRPDG GLGN +G V THLDHRIAMSFLVMGL SE+P VAEGLKLNGVDCEEGKDWLSVTGRPDGLGLGNTAGEQVVTHLDHRIAMSFLVMGLASEHP | 420 |
| Query  Sbjct | 421  421 | VTVDDATMIATSFPEFMDLMAGLGAKIE 448 V ++D++MIATSFP+FMDLM LGAKIE VGINDSSMIATSFPQFMDLMESLGAKIE 448 |  |

>TR:W8I7B2 W8I7B2_ENSAD 3-phosphoshikimate 1-carboxyvinyltransferase OS=Ensifer adhaerens OV14 OX=1416753 GN=aroA PE=3 SV=1

Length=447

Score = 699 bits (1803), Expect = 0.0

| Identities | = 354/448 (79%), Positives = 393/448 (88%), Gaps = 1/448 (0%) |  |
| --- | --- | --- |
| Query 1 | MLHGASSRPATARKSSGLSGTVRIPGDKSISHRSFMFGGLASGETRITGLLEGEDVINTG | 60 |
| Sbjct 1 | M G ++PA AR+S L GTVRI GDKSISHR+ M GGLA+G TRI GLLEGEDVINT MSAGLIAKPAIARRSKALGGTVRIRGDKSISHRALMLGGLAAGRTRIAGLLEGEDVINTA | 60 |
| Query 61 | KAMQAMGARIRKEGDTWIIDGVGNGGLLAPEAPLDFGNAATGCRLTMGLVGVYDFDSTFI | 120 |
| Sbjct 61 | +AM+A+GA +R+EG TWI+DG+GNG LL PEAPLDFGNA TGCRLTMGLVG YDF +TF+ RAMRALGATVREEGGTWIVDGLGNGALLEPEAPLDFGNAGTGCRLTMGLVGAYDFTTTFV | 120 |
| Query 121 | GDASLTKRPMGRVLNPLREMGVQVKSEDGDRLPVTLRGPKTPTPITYRVPMASAQVKSAV | 180 |
| Sbjct 121 | GDASL+KRPMGRVL+PLR+MGVQV S +G RLPVTLRGPKT P+ YRVPMASAQVKSAV GDASLSKRPMGRVLDPLRQMGVQVASAEGGRLPVTLRGPKTANPVNYRVPMASAQVKSAV | 180 |
| Query 181 | LLAGLNTPGITTVIEPIMTRDHTEKMLQGFGANLTVETDADGVRTIRLEGRGKLTGQVID | 240 |
| Sbjct 181 | LLAGLN PGITTVIEP++TRDHTE+MLQ FGANL VET +G R IRLEG+G+LTGQ ID LLAGLNAPGITTVIEPVITRDHTERMLQAFGANLAVETGNEGERIIRLEGQGRLTGQDID | 240 |
| Query 241 | VPGDPSSTAFPLVAALLVPGSDVTILNVLMNPTRTGLILTLQEMGADIEVINPRLAGGED | 300 |
| Sbjct 241 | VPGDPSS AFPLVAALLVPGSD+TILNVLMNP RTGLI+TL EMGA I+V+N R AGGED VPGDPSSAAFPLVAALLVPGSDITILNVLMNPIRTGLIVTLLEMGAQIDVLNARTAGGED | 300 |
| Query 301 | VADLRVRSSTLKGVTVPEDRAPSMIDEYPILAVAAAFAEGATVMNGLEELRVKESDRLSA | 360 |
| Sbjct 301 | VADLRVR S LKG+TVPEDRAPSMIDEYP+LAVAAAFAEG T+M+GLEELRVKESDRLSA  VADLRVRYSELKGITVPEDRAPSMIDEYPVLAVAAAFAEGRTIMSGLEELRVKESDRLSA | 360 |

| Query | 361 | VANGLKLNGVDCDEGETSLVVRGRPDGKGLGNASGAAVATHLDHRIAMSFLVMGLVSENP 420 |
| --- | --- | --- |
| Sbjct | 361 | VA GLKL GVDC+E +LVV GRP GKGLGNA+ AAVATHLDHRIAMSFLVMGL SE+P VAAGLKLTGVDCEEAPAALVVHGRPGGKGLGNAA-AAVATHLDHRIAMSFLVMGLASEHP 419 |
| Query | 421 | VTVDDATMIATSFPEFMDLMAGLGAKIE 448 |
| Sbjct | 420 | VTVDDATMIATSFP F++LMAGLGA IE  VTVDDATMIATSFPGFLELMAGLGAAIE 447 |

>TR:A0A366E5K2 A0A366E5K2_9RHIZ 3-phosphoshikimate 1-carboxyvinyltransferase OS=Pseudochrobactrum asaccharolyticum OX=354351 GN=aroA PE=3

SV=1

Length=450

Score = 698 bits (1801), Expect = 0.0

| Identities | = 352/453 (78%), Positives = 391/453 (86%), Gaps = 3/453 (1%) |  |
| --- | --- | --- |
| Query 1 | MLHGASSRPATARKSSGLSGTVRIPGDKSISHRSFMFGGLASGETRITGLLEGEDVINTG | 60 |
| Sbjct 1 | M H S+PATAR+S L G +RIPGDKSISHRSFMFGGLASGETRI+GLLEGED++ TG MSHSQVSKPATARRSRDLKGDIRIPGDKSISHRSFMFGGLASGETRISGLLEGEDILATG | 60 |
| Query 61 | KAMQAMGARIRKEGDTWIIDGVGNGGLLAPEAPLDFGNAATGCRLTMGLVGVYDFDSTFI | 120 |
| Sbjct 61 | KAMQAMGA+IRKEGD WII+G GNG LL E PLDFGNA TG RLTMGLVG YD ++F+ KAMQAMGAQIRKEGDIWIINGTGNGCLLESETPLDFGNAGTGVRLTMGLVGSYDMKTSFL | 120 |
| Query 121 | GDASLTKRPMGRVLNPLREMGVQVKSEDGDRLPVTLRGPKTPTPITYRVPMASAQVKSAV | 180 |
| Sbjct 121 | GDASLT RPM RVLNPLREMGVQV++ +GDRLP+TL GP+T PI YRVPMASAQVKSAV GDASLTSRPMARVLNPLREMGVQVEAAEGDRLPLTLHGPRTANPIRYRVPMASAQVKSAV | 180 |
| Query 181 | LLAGLNTPGITTVIEPIMTRDHTEKMLQGFGANLTVETDADGVRTIRLEGRGKLTGQVID | 240 |
| Sbjct 181 | LLAGLNTPG+TTVIEPIMTRDHTEKMLQGFGA+L+VETD DGVR I + G+GKLTGQ ID LLAGLNTPGVTTVIEPIMTRDHTEKMLQGFGADLSVETDKDGVRHIHIAGQGKLTGQTID | 240 |
| Query 241 | VPGDPSSTAFPLVAALLVPGSDVTILNVLMNPTRTGLILTLQEMGADIEVINPRLAGGED | 300 |
| Sbjct 241 | VPGDPSSTAFPLVAAL+ GSD+ I NVLMNPTRTGLI TLQEMGA I++++PRLAGGED VPGDPSSTAFPLVAALITEGSDLIIRNVLMNPTRTGLITTLQEMGAQIDILDPRLAGGED | 300 |
| Query 301 | VADLRVRSSTLKGVTVPEDRAPSMIDEYPILAVAAAFAEGATVMNGLEELRVKESDRLSA | 360 |
| Sbjct 301 | VADLRVRSS L G+TVP DRAPSMIDEYP+LA+AAAFAEG T+M+GL+ELRVKESDRL+A VADLRVRSSKLTGITVPVDRAPSMIDEYPVLAIAAAFAEGETIMDGLDELRVKESDRLAA | 360 |
| Query 361 | VANGLKLNGVDCDEGETSLVVRGRPDGKGLGNASGAAVATHLDHRIAMSFLVMGLVSENP | 420 |
| Sbjct 361 | VA GL+ NGVDC EGE SL VRG P GKG G G VATHLDHRIAMSFLV+GL SE P VARGLEANGVDCTEGEMSLTVRGNPSGKGFG---GGTVATHLDHRIAMSFLVLGLASEKP | 417 |
| Query 421  Sbjct 418 | VTVDDATMIATSFPEFMDLMAGLGAKIELSDTK 453 VTVDDATMIATSFPEFMDLM LGA I +++ K VTVDDATMIATSFPEFMDLMRNLGADITVAEAK 450 |  |

>TR:A0A2P7S4J0 A0A2P7S4J0_9RHIZ 3-phosphoshikimate 1-carboxyvinyltransferase OS=Mesorhizobium soli OX=1295366 GN=aroA PE=3 SV=1

Length=452

Score = 694 bits (1790), Expect = 0.0

Identities = 356/455 (78%), Positives = 389/455 (85%), Gaps = 4/455 (1%)

| Query | 1 | MLHGASSRPATARKSSGLSGTVRIPGDKSISHRSFMFGGLASGETRITGLLEGEDVINTG | 60 |
| --- | --- | --- | --- |
| Sbjct | 1 | M H A +PA A +S LSG VR+PGDKSISHRSFMFGGLASGETRITGLLEGEDV+ TG MSHQALPKPAVANRSPALSGKVRMPGDKSISHRSFMFGGLASGETRITGLLEGEDVLRTG | 60 |
| Query | 61 | KAMQAMGARIRKEGDTWIIDGVGNGGLLAPEAPLDFGNAATGCRLTMGLVGVYDFDSTFI  +AM+AMGARI K G+ WII G GNG LL P+APLDFGNA TG RLTMGLVG YD ++TFI | 120 |

| Sbjct | 61 | EAMKAMGARIEKNGEEWIIQGTGNGCLLEPKAPLDFGNAGTGSRLTMGLVGTYDMETTFI | 120 |
| --- | --- | --- | --- |
| Query | 121 | GDASLTKRPMGRVLNPLREMGVQV-KSEDGDRLPVTLRGPKTPTPITYRVPMASAQVKSA | 179 |
| Sbjct | 121 | GDASL+ RPMGRVLNPLREMGVQV + GDR+P+TLRGPK PI+YRVPMASAQVKSA GDASLSGRPMGRVLNPLREMGVQVINAAPGDRMPLTLRGPKVAAPISYRVPMASAQVKSA | 180 |
| Query | 180 | VLLAGLNTPGITTVIEPIMTRDHTEKMLQGFGANLTVETDADGVRTIRLEGRGKLTGQVI | 239 |
| Sbjct | 181 | VLLAGLNTPGITTVIEP+MTRDHTEKMLQGFGANL VETD DGVR IR+EG+GKL+GQVI VLLAGLNTPGITTVIEPVMTRDHTEKMLQGFGANLEVETDKDGVRHIRIEGQGKLSGQVI | 240 |
| Query | 240 | DVPGDPSSTAFPLVAALLVPGSDVTILNVLMNPTRTGLILTLQEMGADIEVINPRLAGGE | 299 |
| Sbjct | 241 | VPGDPSS FPLVAAL+VPGSD+ I NVLMNPTRTGLI TL EMGA IE++NPR AGGE AVPGDPSSAGFPLVAALIVPGSDIVIENVLMNPTRTGLITTLIEMGAQIELMNPRNAGGE | 300 |
| Query | 300 | DVADLRVRSSTLKGVTVPEDRAPSMIDEYPILAVAAAFAEGATVMNGLEELRVKESDRLS | 359 |
| Sbjct | 301 | DVADLRVRSS LKGVTVP +RAPSMIDEYP+LAVAA+FAEG T+M GLEELRVKESDRL+ DVADLRVRSSELKGVTVPAERAPSMIDEYPVLAVAASFAEGETLMQGLEELRVKESDRLA | 360 |
| Query | 360 | AVANGLKLNGVDCDEGETSLVVRGRPDGKGLGNASGAAVATHLDHRIAMSFLVMGLVSEN | 419 |
| Sbjct | 361 | AVA GL+ NGVDC EGE +L VRG+P GKGLG G V THLDHRIAMSFLVMGL +E AVARGLEANGVDCTEGEVTLAVRGKPGGKGLG---GGTVKTHLDHRIAMSFLVMGLAAEK | 417 |
| Query  Sbjct | 420  418 | PVTVDDATMIATSFPEFMDLMAGLGAKIELSDTKA 454 PVTVDD TMIATSFPEFM LM G+GAKIE D A PVTVDDQTMIATSFPEFMGLMTGMGAKIETMDAAA 452 |  |

>TR:A0A1I3WYP8 A0A1I3WYP8_9RHIZ 3-phosphoshikimate 1-carboxyvinyltransferase OS=Mesorhizobium albiziae OX=335020 GN=aroA PE=3 SV=1

Length=446

Score = 692 bits (1786), Expect = 0.0

| Identities | = 350/448 (78%), Positives = 387/448 (86%), Gaps = 4/448 (1%) |  |
| --- | --- | --- |
| Query 1 | MLHGASSRPATARKSSGLSGTVRIPGDKSISHRSFMFGGLASGETRITGLLEGEDVINTG | 60 |
| Sbjct 1 | M H A PATAR+S L+GT R+PGDKSISHRSFMFGGLASGETRITGLLEGEDV+ TG MAHDAKPHPATARRSPALTGTARVPGDKSISHRSFMFGGLASGETRITGLLEGEDVMRTG | 60 |
| Query 61 | KAMQAMGARIRKEGDTWIIDGVGNGGLLAPEAPLDFGNAATGCRLTMGLVGVYDFDSTFI | 120 |
| Sbjct 61 | +AM+AMGA I K GD W+I G GNG LL P+APLDFGNA TG RLTMGLVGVYD ++TF+ EAMKAMGAHITKTGDEWVIRGTGNGALLQPQAPLDFGNAGTGSRLTMGLVGVYDMETTFV | 120 |
| Query 121 | GDASLTKRPMGRVLNPLREMGVQV-KSEDGDRLPVTLRGPKTPTPITYRVPMASAQVKSA | 179 |
| Sbjct 121 | GDASL RPM RVL+PLREMG+QV KS GDR+P+TLRGPK PI+YRVPMASAQVKSA GDASLQSRPMARVLDPLREMGMQVLKSSPGDRMPLTLRGPKVAAPISYRVPMASAQVKSA | 180 |
| Query 180 | VLLAGLNTPGITTVIEPIMTRDHTEKMLQGFGANLTVETDADGVRTIRLEGRGKLTGQVI | 239 |
| Sbjct 181 | VLLAGLNTPG+TTVIEP+MTRDHTEKML+GFGANL VETD+ GVR IR+EG+G+LTGQ I VLLAGLNTPGVTTVIEPVMTRDHTEKMLKGFGANLEVETDSLGVRHIRIEGQGRLTGQTI | 240 |
| Query 240 | DVPGDPSSTAFPLVAALLVPGSDVTILNVLMNPTRTGLILTLQEMGADIEVINPRLAGGE | 299 |
| Sbjct 241 | VPGDPSS FPLVAAL+VPGSD+TI NVLMNPTRTGL+LTLQEMG IE++NPR AGGE AVPGDPSSAGFPLVAALIVPGSDITIENVLMNPTRTGLLLTLQEMGGRIEILNPRNAGGE | 300 |
| Query 300 | DVADLRVRSSTLKGVTVPEDRAPSMIDEYPILAVAAAFAEGATVMNGLEELRVKESDRLS | 359 |
| Sbjct 301 | DVADLRVR S L+GV VP +RAPSMIDEYP+LAVAAAFAEG T+M GL+ELRVKESDRLS DVADLRVRGSELRGVKVPAERAPSMIDEYPVLAVAAAFAEGETLMEGLDELRVKESDRLS | 360 |
| Query 360 | AVANGLKLNGVDCDEGETSLVVRGRPDGKGLGNASGAAVATHLDHRIAMSFLVMGLVSEN | 419 |
| Sbjct 361 | AVANGLK NGVDC EGE SL VRGRPDGKGLG G VATHLDHRIAMSFLV+GL +E AVANGLKANGVDCTEGEASLSVRGRPDGKGLG---GGTVATHLDHRIAMSFLVLGLATEK | 417 |
| Query 420 | PVTVDDATMIATSFPEFMDLMAGLGAKI 447 PV++DDA MIATSFPEFM LM GLGA I |  |

Sbjct 418 PVSIDDANMIATSFPEFMGLMKGLGAAI 445

>TR:A0A0Q8KSI2 A0A0Q8KSI2_9RHIZ 3-phosphoshikimate 1-carboxyvinyltransferase OS=Mesorhizobium sp. Root172 OX=1736481 GN=aroA PE=3 SV=1

Length=452

Score = 691 bits (1783), Expect = 0.0

| Identities | = 352/452 (78%), Positives = 385/452 (85%), Gaps = 4/452 (1%) |  |
| --- | --- | --- |
| Query 1 | MLHGASSRPATARKSSGLSGTVRIPGDKSISHRSFMFGGLASGETRITGLLEGEDVINTG | 60 |
| Sbjct 1 | M H A+++PATARKS LSGT R+PGDKSISHRSFMFGGLASGETRITGLLEGEDV+ TG MSHAAAAKPATARKSQALSGTARVPGDKSISHRSFMFGGLASGETRITGLLEGEDVMRTG | 60 |
| Query 61 | KAMQAMGARIRKEGDTWIIDGVGNGGLLAPEAPLDFGNAATGCRLTMGLVGVYDFDSTFI | 120 |
| Sbjct 61 | AM+AMGA I K G W+I G GNG LL PE PLDFGNA TG RLTMGLVG YD ++TFI AAMKAMGAHIEKRGAEWVIRGTGNGALLQPEGPLDFGNAGTGSRLTMGLVGTYDMETTFI | 120 |
| Query 121 | GDASLTKRPMGRVLNPLREMGVQV-KSEDGDRLPVTLRGPKTPTPITYRVPMASAQVKSA | 179 |
| Sbjct 121 | GDASL+ RPMGRVL PLR+MGVQV K+ GDR+P+TL GPK PITYRVPMASAQVKSA GDASLSGRPMGRVLEPLRQMGVQVLKATPGDRMPITLHGPKHAAPITYRVPMASAQVKSA | 180 |
| Query 180 | VLLAGLNTPGITTVIEPIMTRDHTEKMLQGFGANLTVETDADGVRTIRLEGRGKLTGQVI | 239 |
| Sbjct 181 | VLLAGLNTPGITTVIEP+MTRDHTEKML+GFGANL+VETD GVR I +EG+GKLTGQ I VLLAGLNTPGITTVIEPVMTRDHTEKMLKGFGANLSVETDERGVRHIFIEGQGKLTGQTI | 240 |
| Query 240 | DVPGDPSSTAFPLVAALLVPGSDVTILNVLMNPTRTGLILTLQEMGADIEVINPRLAGGE | 299 |
| Sbjct 241 | VPGDPSS FPLVAAL+VPGSD+TI NVLMNPTRTGL+LTLQEMG I+++NPR AGGE AVPGDPSSAGFPLVAALIVPGSDITIENVLMNPTRTGLLLTLQEMGGKIDILNPRNAGGE | 300 |
| Query 300 | DVADLRVRSSTLKGVTVPEDRAPSMIDEYPILAVAAAFAEGATVMNGLEELRVKESDRLS | 359 |
| Sbjct 301 | DVADLRVR S LKGV VP +RAPSMIDEYP+LAVAA+FAEG T+M GLEELRVKESDRLS DVADLRVRYSELKGVAVPPERAPSMIDEYPVLAVAASFAEGETLMQGLEELRVKESDRLS | 360 |
| Query 360 | AVANGLKLNGVDCDEGETSLVVRGRPDGKGLG---NASGAAVATHLDHRIAMSFLVMGLV | 416 |
| Sbjct 361 | AVANGLKLNGVDC EGE SL VRG+P GKGLG N V THLDHRIAMSFLVMGL AVANGLKLNGVDCTEGEASLAVRGKPGGKGLGGHPNGLDTTVQTHLDHRIAMSFLVMGLA | 420 |
| Query 417  Sbjct 421 | SENPVTVDDATMIATSFPEFMDLMAGLGAKIE 448  +E PVT+DD MIATSFPEFM LM GLGA+IE TEKPVTIDDQAMIATSFPEFMGLMTGLGAEIE 452 |  |

>TR:A0A504FA26 A0A504FA26_9RHIZ 3-phosphoshikimate 1-carboxyvinyltransferase OS=Mesorhizobium sp. B2-3-3 OX=2589960 GN=aroA PE=3 SV=1

Length=452

Score = 691 bits (1783), Expect = 0.0

| Identities | = 352/451 (78%), Positives = 385/451 (85%), Gaps = 4/451 (1%) |  |
| --- | --- | --- |
| Query 1 | MLHGASSRPATARKSSGLSGTVRIPGDKSISHRSFMFGGLASGETRITGLLEGEDVINTG | 60 |
| Sbjct 1 | M H A++RPATARKSS LSGT R+PGDKSISHRSFMFGGLASGETRITGLLEGEDV+ TG MSHAAAARPATARKSSALSGTARVPGDKSISHRSFMFGGLASGETRITGLLEGEDVMRTG | 60 |
| Query 61 | KAMQAMGARIRKEGDTWIIDGVGNGGLLAPEAPLDFGNAATGCRLTMGLVGVYDFDSTFI | 120 |
| Sbjct 61 | AM+AMGA I K G W+I G GNG LL PE PLDFGNA TG RLTMGLVG YD ++TFI AAMKAMGAHIEKSGAEWVIRGTGNGALLQPEGPLDFGNAGTGSRLTMGLVGTYDMETTFI | 120 |
| Query 121 | GDASLTKRPMGRVLNPLREMGVQV-KSEDGDRLPVTLRGPKTPTPITYRVPMASAQVKSA | 179 |
| Sbjct 121 | GDASL+ RPMGRVL PLR+MGVQV K+ GDR+P+TL GPK PITYRVPMASAQVKSA GDASLSGRPMGRVLEPLRQMGVQVLKATPGDRMPITLHGPKHAAPITYRVPMASAQVKSA | 180 |
| Query 180 | VLLAGLNTPGITTVIEPIMTRDHTEKMLQGFGANLTVETDADGVRTIRLEGRGKLTGQVI VLLAGLNTPGITTV+EP+MTRDHTEKML+GFGANL+VETD GVR I +EG+GKLTGQ I | 239 |

| Sbjct | 181 | VLLAGLNTPGITTVVEPVMTRDHTEKMLKGFGANLSVETDERGVRHIFIEGQGKLTGQTI | 240 |
| --- | --- | --- | --- |
| Query | 240 | DVPGDPSSTAFPLVAALLVPGSDVTILNVLMNPTRTGLILTLQEMGADIEVINPRLAGGE | 299 |
| Sbjct | 241 | VPGDPSS FPLVAAL+VPGSD+ I NVLMNPTRTGL+LTLQEMG I+++NPR AGGE AVPGDPSSAGFPLVAALIVPGSDIVIENVLMNPTRTGLLLTLQEMGGKIDILNPRNAGGE | 300 |
| Query | 300 | DVADLRVRSSTLKGVTVPEDRAPSMIDEYPILAVAAAFAEGATVMNGLEELRVKESDRLS | 359 |
| Sbjct | 301 | DVADLRVR S LKGV VP +RAPSMIDEYP+LAVAA+FAEG T+M GLEELRVKESDRLS DVADLRVRYSELKGVIVPPERAPSMIDEYPVLAVAASFAEGETLMQGLEELRVKESDRLS | 360 |
| Query | 360 | AVANGLKLNGVDCDEGETSLVVRGRPDGKGLG---NASGAAVATHLDHRIAMSFLVMGLV | 416 |
| Sbjct | 361 | AVANGLKLNGVDC EGE SL VRGRP GKGLG N V THLDHRIAMSFLVMGL AVANGLKLNGVDCTEGEASLAVRGRPGGKGLGGHPNGRDTTVQTHLDHRIAMSFLVMGLA | 420 |
| Query  Sbjct | 417  421 | SENPVTVDDATMIATSFPEFMDLMAGLGAKI 447  ++ PVT+DDA MIATSFPEFM LM GLGA+I TDKPVTIDDANMIATSFPEFMGLMKGLGAEI 451 |  |

>TR:A0A4U6CF00 A0A4U6CF00_9RHIZ 3-phosphoshikimate 1-carboxyvinyltransferase OS=Aquamicrobium sp. LC103 OX=1120658 GN=aroA PE=3 SV=1

Length=447

Score = 691 bits (1782), Expect = 0.0

| Identities | = 352/450 (78%), Positives = 385/450 (86%), Gaps = 4/450 (1%) |  |
| --- | --- | --- |
| Query 1 | MLHGASSRPATARKSSGLSGTVRIPGDKSISHRSFMFGGLASGETRITGLLEGEDVINTG | 60 |
| Sbjct 1 | M H ++ PATA KS LSG VR+PGDKSISHRSFMFGGLASGETRITGLLEGEDV+ TG MSHASTPLPATASKSEALSGQVRVPGDKSISHRSFMFGGLASGETRITGLLEGEDVMRTG | 60 |
| Query 61 | KAMQAMGARIRKEGDTWIIDGVGNGGLLAPEAPLDFGNAATGCRLTMGLVGVYDFDSTFI | 120 |
| Sbjct 61 | AM+AMGA+I K GD WII G GNG LL P+ PLDFGNA TG RLTMGLVG YD +TF+ DAMKAMGAQIAKSGDEWIIQGAGNGALLEPQGPLDFGNAGTGARLTMGLVGTYDMTTTFV | 120 |
| Query 121 | GDASLTKRPMGRVLNPLREMGVQV-KSEDGDRLPVTLRGPKTPTPITYRVPMASAQVKSA | 179 |
| Sbjct 121 | GDASL+KRPMGRVL+PLREMGVQV ++ GDR+P+TLRGPK PI+YRVPMASAQVKSA GDASLSKRPMGRVLDPLREMGVQVVEAASGDRMPLTLRGPKRAAPISYRVPMASAQVKSA | 180 |
| Query 180 | VLLAGLNTPGITTVIEPIMTRDHTEKMLQGFGANLTVETDADGVRTIRLEGRGKLTGQVI | 239 |
| Sbjct 181 | VLLAGLNTPGITTVIEP+MTRDHTEKML+GFGAN+ VETD DGVR IR+EG GKLTGQ I VLLAGLNTPGITTVIEPVMTRDHTEKMLKGFGANIEVETDGDGVRHIRIEGEGKLTGQTI | 240 |
| Query 240 | DVPGDPSSTAFPLVAALLVPGSDVTILNVLMNPTRTGLILTLQEMGADIEVINPRLAGGE | 299 |
| Sbjct 241 | VPGDPSS AFPLVAAL+VPGSDVTI NVLMNPTRTGLI TL EMGA IE++N R AGGE AVPGDPSSAAFPLVAALIVPGSDVTIRNVLMNPTRTGLITTLLEMGASIELLNERKAGGE | 300 |
| Query 300 | DVADLRVRSSTLKGVTVPEDRAPSMIDEYPILAVAAAFAEGATVMNGLEELRVKESDRLS | 359 |
| Sbjct 301 | DVADLRVR S L+GV VP +RAPSMIDEYP+LAVAA+FAEG T+M GL+ELRVKESDRL+ DVADLRVRGSELRGVVVPPERAPSMIDEYPVLAVAASFAEGETLMEGLDELRVKESDRLA | 360 |
| Query 360 | AVANGLKLNGVDCDEGETSLVVRGRPDGKGLGNASGAAVATHLDHRIAMSFLVMGLVSEN | 419 |
| Sbjct 361 | AVA GL+ NGVDC EGE SL VRGRP GKGLG G VATHLDHRIAMSFLVMGL SE AVARGLEANGVDCSEGEASLSVRGRPGGKGLG---GGTVATHLDHRIAMSFLVMGLASEK | 417 |
| Query 420  Sbjct 418 | PVTVDDATMIATSFPEFMDLMAGLGAKIEL 449 PVTVDD MIATSFPEFMDLM GLGA+I + PVTVDDRAMIATSFPEFMDLMTGLGARISV 447 |  |

>TR:A0A3A5KZU9 A0A3A5KZU9_9RHIZ 3-phosphoshikimate 1-carboxyvinyltransferase OS=Mesorhizobium waimense OX=1300307 GN=aroA PE=3 SV=1

Length=452

Score = 690 bits (1781), Expect = 0.0

| Identities | = 352/451 (78%), Positives = 383/451 (85%), Gaps = 4/451 (1%) |  |
| --- | --- | --- |
| Query 1 | MLHGASSRPATARKSSGLSGTVRIPGDKSISHRSFMFGGLASGETRITGLLEGEDVINTG | 60 |
| Sbjct 1 | M H A+ +PATARKS LSG R+PGDKSISHRSFMFGGLASGETRITGLLEGEDV+ TG MSHAAAPKPATARKSPALSGAARVPGDKSISHRSFMFGGLASGETRITGLLEGEDVMRTG | 60 |
| Query 61 | KAMQAMGARIRKEGDTWIIDGVGNGGLLAPEAPLDFGNAATGCRLTMGLVGVYDFDSTFI | 120 |
| Sbjct 61 | AM+AMGA I K GD W+I G GNG LL PE PLDFGNA TG RLTMGLVG YD ++TFI AAMKAMGAHIEKHGDEWVIRGTGNGALLQPEGPLDFGNAGTGSRLTMGLVGTYDMETTFI | 120 |
| Query 121 | GDASLTKRPMGRVLNPLREMGVQV-KSEDGDRLPVTLRGPKTPTPITYRVPMASAQVKSA | 179 |
| Sbjct 121 | GDASL+ RPMGRVL PLR+MGVQV K+ GDR+P+TLRGPK PITYRVPMASAQVKSA GDASLSGRPMGRVLEPLRQMGVQVLKATPGDRMPITLRGPKHAAPITYRVPMASAQVKSA | 180 |
| Query 180 | VLLAGLNTPGITTVIEPIMTRDHTEKMLQGFGANLTVETDADGVRTIRLEGRGKLTGQVI | 239 |
| Sbjct 181 | VLLAGLNTPGITTVIEP+MTRDHTEKML+GFGANLTVETD GVR I +EG+GKLTGQ I VLLAGLNTPGITTVIEPVMTRDHTEKMLKGFGANLTVETDERGVRHIFIEGQGKLTGQTI | 240 |
| Query 240 | DVPGDPSSTAFPLVAALLVPGSDVTILNVLMNPTRTGLILTLQEMGADIEVINPRLAGGE | 299 |
| Sbjct 241 | VPGDPSS FPLVAAL+VPGSD+ I NVLMNPTRTGL+LTLQEMG I+++NPR AGGE AVPGDPSSAGFPLVAALIVPGSDIVIENVLMNPTRTGLLLTLQEMGGRIDILNPRNAGGE | 300 |
| Query 300 | DVADLRVRSSTLKGVTVPEDRAPSMIDEYPILAVAAAFAEGATVMNGLEELRVKESDRLS | 359 |
| Sbjct 301 | DVADLRVR S LKGV VP +RAPSMIDEYP LAVAA+FAEG T+M GLEELRVKESDRLS DVADLRVRYSELKGVIVPPERAPSMIDEYPALAVAASFAEGETLMQGLEELRVKESDRLS | 360 |
| Query 360 | AVANGLKLNGVDCDEGETSLVVRGRPDGKGLG---NASGAAVATHLDHRIAMSFLVMGLV | 416 |
| Sbjct 361 | AVANGLKLNGVDC EGE +L VRGRP G+GLG N G V THLDHRIAMSFLVMGL AVANGLKLNGVDCTEGEATLAVRGRPGGRGLGQHPNGQGTTVQTHLDHRIAMSFLVMGLA | 420 |
| Query 417  Sbjct 421 | SENPVTVDDATMIATSFPEFMDLMAGLGAKI 447  +E PVT+DD MIATSFPEFM LM GLGA+I TEKPVTIDDQAMIATSFPEFMGLMKGLGAEI 451 |  |

>TR:A0A5B8B442 A0A5B8B442_9RHIZ 3-phosphoshikimate 1-carboxyvinyltransferase OS=Mesorhizobium sp. 8 OX=2584466 GN=aroA PE=3 SV=1

Length=453

Score = 690 bits (1780), Expect = 0.0

| Identities | = 356/453 (79%), Positives = 386/453 (85%), Gaps = 2/453 (0%) |  |
| --- | --- | --- |
| Query 1 | MLHGASSRPATARKSSGLSGTVRIPGDKSISHRSFMFGGLASGETRITGLLEGEDVINTG | 60 |
| Sbjct 1 | M H A PATAR+S LSGT R+PGDKSISHRSFMFGGLASGETRITGLLEGEDV+ TG MAHDAKQIPATARRSQALSGTARVPGDKSISHRSFMFGGLASGETRITGLLEGEDVLRTG | 60 |
| Query 61 | KAMQAMGARIRKEGDTWIIDGVGNGGLLAPEAPLDFGNAATGCRLTMGLVGVYDFDSTFI | 120 |
| Sbjct 61 | AM+AMGA+I K+G WII G GNG LL PE PLDFGNA TG RLTMGLVG YD ++TFI AAMKAMGAQIEKKGAEWIIRGTGNGALLEPEGPLDFGNAGTGSRLTMGLVGTYDMETTFI | 120 |
| Query 121 | GDASLTKRPMGRVLNPLREMGVQV-KSEDGDRLPVTLRGPKTPTPITYRVPMASAQVKSA | 179 |
| Sbjct 121 | GDASL+ RPMGRVL+PLR+MGVQV K+ GDR+P+TLRGPK PITYRVPMASAQVKSA GDASLSSRPMGRVLDPLRQMGVQVLKAAPGDRMPITLRGPKHAAPITYRVPMASAQVKSA | 180 |
| Query 180 | VLLAGLNTPGITTVIEPIMTRDHTEKMLQGFGANLTVETDADGVRTIRLEGRGKLTGQVI | 239 |
| Sbjct 181 | VLLAGLNTPGITTVIEP+MTRDHTEKML+GFGANL VETD GVR I +EG+GKLTGQVI VLLAGLNTPGITTVIEPVMTRDHTEKMLKGFGANLDVETDERGVRHIFIEGQGKLTGQVI | 240 |
| Query 240 | DVPGDPSSTAFPLVAALLVPGSDVTILNVLMNPTRTGLILTLQEMGADIEVINPRLAGGE | 299 |
| Sbjct 241 | VPGDPSS AFPLVAAL+VPGSD+ I NVLMNPTRTGLI TL EMG I+VINPR GGE AVPGDPSSAAFPLVAALIVPGSDIVIENVLMNPTRTGLITTLMEMGGRIDVINPRSEGGE | 300 |
| Query 300 | DVADLRVRSSTLKGVTVPEDRAPSMIDEYPILAVAAAFAEGATVMNGLEELRVKESDRLS DVADLRVR S LKGVTVP +RAPSMIDEYP+LAVAA FAEG T+M GLEELRVKESDRL+ | 359 |

| Sbjct | 301 | DVADLRVRHSDLKGVTVPPERAPSMIDEYPVLAVAAGFAEGETLMQGLEELRVKESDRLA | 360 |
| --- | --- | --- | --- |
| Query | 360 | AVANGLKLNGVDCDEGETSLVVRGRPDGKGLGNA-SGAAVATHLDHRIAMSFLVMGLVSE | 418 |
| Sbjct | 361 | AVA+GLKLNGVDC EGE +L VRG P GKGLG A G AV THLDHRIAMSFLV+GL +E AVASGLKLNGVDCTEGEATLAVRGIPGGKGLGGAGKGDAVETHLDHRIAMSFLVLGLAAE | 420 |
| Query | 419 | NPVTVDDATMIATSFPEFMDLMAGLGAKIELSD 451 PVTVDDATMIATSFPEFM LM LGA+IE D |  |
| Sbjct | 421 | KPVTVDDATMIATSFPEFMGLMKDLGAEIEQED 453 | |

>SP:Q98CC1 AROA_RHILO 3-phosphoshikimate 1-carboxyvinyltransferase OS=Mesorhizobium japonicum (strain LMG 29417 / CECT 9101 / MAFF 303099)

OX=266835 GN=aroA PE=3 SV=1

Length=452

Score = 689 bits (1779), Expect = 0.0

| Identities | = 351/451 (78%), Positives = 384/451 (85%), Gaps = 4/451 (1%) |  |
| --- | --- | --- |
| Query 1 | MLHGASSRPATARKSSGLSGTVRIPGDKSISHRSFMFGGLASGETRITGLLEGEDVINTG | 60 |
| Sbjct 1 | M H A+++PATARKS LSGT R+PGDKSISHRSFMFGGLASGETRITGLLEGEDV+ TG MSHAAAAKPATARKSQALSGTARVPGDKSISHRSFMFGGLASGETRITGLLEGEDVMRTG | 60 |
| Query 61 | KAMQAMGARIRKEGDTWIIDGVGNGGLLAPEAPLDFGNAATGCRLTMGLVGVYDFDSTFI | 120 |
| Sbjct 61 | AM+AMGA I K G W+I G GNG LL PE PLDFGNA TG RLTMGLVG YD ++TFI AAMKAMGAHIEKRGAEWVIRGTGNGALLQPEGPLDFGNAGTGSRLTMGLVGTYDMETTFI | 120 |
| Query 121 | GDASLTKRPMGRVLNPLREMGVQV-KSEDGDRLPVTLRGPKTPTPITYRVPMASAQVKSA | 179 |
| Sbjct 121 | GDASL+ RPMGRVL PLR+MGVQV K+ GDR+P+TL GPK PITYRVPMASAQVKSA GDASLSGRPMGRVLEPLRQMGVQVLKATPGDRMPITLHGPKHAAPITYRVPMASAQVKSA | 180 |
| Query 180 | VLLAGLNTPGITTVIEPIMTRDHTEKMLQGFGANLTVETDADGVRTIRLEGRGKLTGQVI | 239 |
| Sbjct 181 | VLLAGLNTPGITTVIEP+MTRDHTEKML+GFGANL+VETD GVR I +EG+G+LTGQ I VLLAGLNTPGITTVIEPVMTRDHTEKMLKGFGANLSVETDERGVRHIFIEGQGRLTGQTI | 240 |
| Query 240 | DVPGDPSSTAFPLVAALLVPGSDVTILNVLMNPTRTGLILTLQEMGADIEVINPRLAGGE | 299 |
| Sbjct 241 | VPGDPSS FPLVAAL+VPGSD+TI NVLMNPTRTGL+LTLQEMG I+++NPR AGGE AVPGDPSSAGFPLVAALIVPGSDITIENVLMNPTRTGLLLTLQEMGGQIDILNPRNAGGE | 300 |
| Query 300 | DVADLRVRSSTLKGVTVPEDRAPSMIDEYPILAVAAAFAEGATVMNGLEELRVKESDRLS | 359 |
| Sbjct 301 | DVADLRVR S LKGV VP +RAPSMIDEYP+LAVAA+FAEG T+M GLEELRVKESDRLS DVADLRVRYSELKGVAVPPERAPSMIDEYPVLAVAASFAEGETLMQGLEELRVKESDRLS | 360 |
| Query 360 | AVANGLKLNGVDCDEGETSLVVRGRPDGKGLG---NASGAAVATHLDHRIAMSFLVMGLV | 416 |
| Sbjct 361 | AVANGLKLNGVDC EGE SL VRGRP GKGLG N V THLDHRIAMSFLVMGL AVANGLKLNGVDCTEGEASLAVRGRPGGKGLGGHPNGLDTTVQTHLDHRIAMSFLVMGLA | 420 |
| Query 417  Sbjct 421 | SENPVTVDDATMIATSFPEFMDLMAGLGAKI 447  +E PVT+DD MIATSFPEFM LM GLGA+I TEKPVTIDDQAMIATSFPEFMGLMTGLGAEI 451 |  |

>TR:A0A1V8RTQ2 A0A1V8RTQ2_9RHIZ 3-phosphoshikimate 1-carboxyvinyltransferase OS=Pseudaminobacter manganicus OX=1873176 GN=aroA PE=3 SV=1

Length=446

Score = 689 bits (1779), Expect = 0.0

Identities = 351/449 (78%), Positives = 388/449 (86%), Gaps = 4/449 (1%)

| Query | 1 | MLHGASSRPATARKSSGLSGTVRIPGDKSISHRSFMFGGLASGETRITGLLEGEDVINTG | 60 |
| --- | --- | --- | --- |
|  |  | M H A+ +PATAR+S+ LSG R+PGDKSISHRSFMFGGLASGETRITGLLEGEDV+ TG |  |
| Sbjct | 1 | MSHDAAPQPATARRSNALSGVARVPGDKSISHRSFMFGGLASGETRITGLLEGEDVLRTG | 60 |
| Query | 61 | KAMQAMGARIRKEGDTWIIDGVGNGGLLAPEAPLDFGNAATGCRLTMGLVGVYDFDSTFI | 120 |

| Sbjct | 61 | AM+AMGA I K G WII G GNG LL PEAPLDFGNA TG RLTMGLVG YD ++TF+  AAMKAMGAHIEKHGGEWIIRGTGNGALLQPEAPLDFGNAGTGSRLTMGLVGTYDMETTFV | 120 |
| --- | --- | --- | --- |
| Query | 121 | GDASLTKRPMGRVLNPLREMGVQV-KSEDGDRLPVTLRGPKTPTPITYRVPMASAQVKSA | 179 |
| Sbjct | 121 | GDASL+ RPMGRVL+PLR+MGVQV KS GDR+P+TLRGP+ TPI YRVPMASAQVKSA GDASLSGRPMGRVLDPLRQMGVQVLKSAAGDRMPLTLRGPRQATPIAYRVPMASAQVKSA | 180 |
| Query | 180 | VLLAGLNTPGITTVIEPIMTRDHTEKMLQGFGANLTVETDADGVRTIRLEGRGKLTGQVI | 239 |
| Sbjct | 181 | VLLAGLNTPGITTV+EP+MTRDHTEKML+GFGAN+ VETD GVR I ++G+GKLTGQVI VLLAGLNTPGITTVVEPVMTRDHTEKMLKGFGANIHVETDERGVRHIFIDGQGKLTGQVI | 240 |
| Query | 240 | DVPGDPSSTAFPLVAALLVPGSDVTILNVLMNPTRTGLILTLQEMGADIEVINPRLAGGE | 299 |
| Sbjct | 241 | VPGDPSS AFPLVAAL+VPGSD+TI NVLMNPTRTGL+LTLQEMG +IEV+NPR GGE AVPGDPSSAAFPLVAALVVPGSDITIENVLMNPTRTGLLLTLQEMGGNIEVLNPRNEGGE | 300 |
| Query | 300 | DVADLRVRSSTLKGVTVPEDRAPSMIDEYPILAVAAAFAEGATVMNGLEELRVKESDRLS | 359 |
| Sbjct | 301 | DVADLRVR+S LKGV VP +RAPSMIDEYP+LAVAA FAEG T+M GLEELRVKESDRL+ DVADLRVRASDLKGVAVPPERAPSMIDEYPVLAVAACFAEGETLMQGLEELRVKESDRLA | 360 |
| Query | 360 | AVANGLKLNGVDCDEGETSLVVRGRPDGKGLGNASGAAVATHLDHRIAMSFLVMGLVSEN | 419 |
| Sbjct | 361 | AVA GL+ NGVDC EGE +L VRGRP GKGLG G VATHLDHRIAMSFL+MGL +E AVARGLEANGVDCTEGEAALAVRGRPGGKGLG---GGTVATHLDHRIAMSFLIMGLAAEK | 417 |
| Query  Sbjct | 420  418 | PVTVDDATMIATSFPEFMDLMAGLGAKIE 448 PVTVDDA MIATSFPEFM LMAGLGA IE PVTVDDANMIATSFPEFMGLMAGLGALIE 446 |  |

>TR:M5F072 M5F072_9RHIZ 3-phosphoshikimate 1-carboxyvinyltransferase OS=Mesorhizobium sp. STM 4661 OX=1297570 GN=aroA PE=3 SV=1

Length=452

Score = 689 bits (1778), Expect = 0.0

| Identities | = 352/451 (78%), Positives = 385/451 (85%), Gaps = 4/451 (1%) |  |
| --- | --- | --- |
| Query 1 | MLHGASSRPATARKSSGLSGTVRIPGDKSISHRSFMFGGLASGETRITGLLEGEDVINTG | 60 |
| Sbjct 1 | M H A+ +PATARKS LSGT R+PGDKSISHRSFMFGGLASGETRITGLLEGEDV+ TG MSHPAAPQPATARKSPALSGTARVPGDKSISHRSFMFGGLASGETRITGLLEGEDVMRTG | 60 |
| Query 61 | KAMQAMGARIRKEGDTWIIDGVGNGGLLAPEAPLDFGNAATGCRLTMGLVGVYDFDSTFI | 120 |
| Sbjct 61 | AM+AMGA I K G W+I G GNG LL PE PLDFGNA TG RLTMGLVG YD ++TFI AAMKAMGAYIEKRGTEWVIRGTGNGALLQPEGPLDFGNAGTGSRLTMGLVGTYDMETTFI | 120 |
| Query 121 | GDASLTKRPMGRVLNPLREMGVQV-KSEDGDRLPVTLRGPKTPTPITYRVPMASAQVKSA | 179 |
| Sbjct 121 | GDASL+ RPMGRVL PLR+MGVQV K+ GDR+P+TLRGPK PITYRVPMASAQVKSA GDASLSGRPMGRVLEPLRQMGVQVLKATPGDRMPITLRGPKHAAPITYRVPMASAQVKSA | 180 |
| Query 180 | VLLAGLNTPGITTVIEPIMTRDHTEKMLQGFGANLTVETDADGVRTIRLEGRGKLTGQVI | 239 |
| Sbjct 181 | VLLAGLNTPG+TTVIEP+MTRDHTEKML+GFGANL+VETD G R I +EG+GKLTGQ I VLLAGLNTPGVTTVIEPVMTRDHTEKMLKGFGANLSVETDERGGRHIFIEGQGKLTGQTI | 240 |
| Query 240 | DVPGDPSSTAFPLVAALLVPGSDVTILNVLMNPTRTGLILTLQEMGADIEVINPRLAGGE | 299 |
| Sbjct 241 | VPGDPSS FPLVAAL+VPGSDVTI NVLMNPTRTGL+LTLQEMG I+++NPR AGGE AVPGDPSSAGFPLVAALIVPGSDVTIENVLMNPTRTGLLLTLQEMGGKIDILNPRNAGGE | 300 |
| Query 300 | DVADLRVRSSTLKGVTVPEDRAPSMIDEYPILAVAAAFAEGATVMNGLEELRVKESDRLS | 359 |
| Sbjct 301 | DVADLRVR S LKGVTVP +RAPSMIDEYP+LAVAA+FAEG T+M GLEELRVKESDRLS DVADLRVRYSELKGVTVPPERAPSMIDEYPVLAVAASFAEGETLMQGLEELRVKESDRLS | 360 |
| Query 360 | AVANGLKLNGVDCDEGETSLVVRGRPDGKGLG---NASGAAVATHLDHRIAMSFLVMGLV | 416 |
| Sbjct 361 | AVANGLKLNGVDC EGE SL VRG+P+GKGLG N V THLDHRIAMSFLVMGL AVANGLKLNGVDCTEGEASLAVRGKPEGKGLGGHPNGQDTTVQTHLDHRIAMSFLVMGLA | 420 |
| Query 417 | SENPVTVDDATMIATSFPEFMDLMAGLGAKI 447 |  |

+E PVT+DD MIATSFPEFM LM GLGA+I Sbjct 421 TEKPVTIDDQAMIATSFPEFMGLMKGLGAEI 451

>TR:A0A3Q8XLD4 A0A3Q8XLD4_9RHIZ 3-phosphoshikimate 1-carboxyvinyltransferase OS=Georhizobium profundi OX=2341112 GN=aroA PE=3 SV=1

Length=456

Score = 688 bits (1776), Expect = 0.0

| Identities | = 353/449 (79%), Positives = 388/449 (86%), Gaps = 1/449 (0%) |  |
| --- | --- | --- |
| Query 1 | MLHGASSR-PATARKSSGLSGTVRIPGDKSISHRSFMFGGLASGETRITGLLEGEDVINT | 59 |
| Sbjct 1 | M HG+ PATAR+S LSGTVRIPGDKSISHRSFMFGGLA+GETRITGLLEGEDVI T MAHGSQGAIPATARRSEPLSGTVRIPGDKSISHRSFMFGGLAAGETRITGLLEGEDVIAT | 60 |
| Query 60 | GKAMQAMGARIRKEGDTWIIDGVGNGGLLAPEAPLDFGNAATGCRLTMGLVGVYDFDSTF | 119 |
| Sbjct 61 | G AM+AMGA IRKEGDTWII+G GNG LL P+APLDFGNA TGCRLTMGL G YDF++TF GNAMRAMGASIRKEGDTWIIEGTGNGCLLEPKAPLDFGNAGTGCRLTMGLAGTYDFETTF | 120 |
| Query 120 | IGDASLTKRPMGRVLNPLREMGVQVKSEDGDRLPVTLRGPKTPTPITYRVPMASAQVKSA | 179 |
| Sbjct 121 | IGDASL+ RPMGRVL+PL+ MGVQVKS +G +LPVTLRGP+ PI Y VPMASAQVKSA IGDASLSARPMGRVLDPLKLMGVQVKSVEGGKLPVTLRGPRMAAPIHYTVPMASAQVKSA | 180 |
| Query 180 | VLLAGLNTPGITTVIEPIMTRDHTEKMLQGFGANLTVETDADGVRTIRLEGRGKLTGQVI | 239 |
| Sbjct 181 | VL+AGLN PG+TTV E IMTRDHTEKML GFGA+L +ETDADG R I +EG+ KL G VI VLIAGLNAPGVTTVTEKIMTRDHTEKMLAGFGASLDIETDADGTRHISIEGQPKLKGCVI | 240 |
| Query 240 | DVPGDPSSTAFPLVAALLVPGSDVTILNVLMNPTRTGLILTLQEMGADIEVINPRLAGGE | 299 |
| Sbjct 241 | DVPGDPSSTAFPLVAALLVPGSD+TILNVLMNPTRTGLI TL EMG I++++ R AGGE DVPGDPSSTAFPLVAALLVPGSDITILNVLMNPTRTGLITTLIEMGGRIDILDARSAGGE | 300 |
| Query 300 | DVADLRVRSSTLKGVTVPEDRAPSMIDEYPILAVAAAFAEGATVMNGLEELRVKESDRLS | 359 |
| Sbjct 301 | DVADLRVR S LKGV VP +RA SMIDEYP+LAVAAAFAEG TVM+G+EELRVKESDRL+ DVADLRVRHSELKGVVVPPERAASMIDEYPVLAVAAAFAEGETVMDGIEELRVKESDRLA | 360 |
| Query 360 | AVANGLKLNGVDCDEGETSLVVRGRPDGKGLGNASGAAVATHLDHRIAMSFLVMGLVSEN | 419 |
| Sbjct 361 | AVA GL+LN VDC EGE L VRG P G+G+G+A G AVATHLDHRIAMSFLVMGL SE+ AVAAGLRLNNVDCTEGEAILTVRGTPSGRGIGSARGDAVATHLDHRIAMSFLVMGLASEH | 420 |
| Query 420  Sbjct 421 | PVTVDDATMIATSFPEFMDLMAGLGAKIE 448 PVTVDDA MIATSFPEFMDLMAGLGA IE PVTVDDARMIATSFPEFMDLMAGLGATIE 449 |  |

>TR:A0A1I7DML1 A0A1I7DML1_9RHIZ 3-phosphoshikimate 1-carboxyvinyltransferase OS=Mesorhizobium sp. YR577 OX=1884373 GN=aroA PE=3 SV=1

Length=453

Score = 686 bits (1771), Expect = 0.0

| Identities | = 353/455 (78%), Positives = 389/455 (85%), Gaps = 4/455 (1%) |  |
| --- | --- | --- |
| Query 1 | MLHGASSRPATARKSSGLSGTVRIPGDKSISHRSFMFGGLASGETRITGLLEGEDVINTG | 60 |
| Sbjct 1 | M H + +PA ARKSS LSGTVR+PGDKSISHRSFMFGGLASGETRITGLLEGEDV+ TG MSHQSLPQPAAARKSSALSGTVRMPGDKSISHRSFMFGGLASGETRITGLLEGEDVLRTG | 60 |
| Query 61 | KAMQAMGARIRKEGDTWIIDGVGNGGLLAPEAPLDFGNAATGCRLTMGLVGVYDFDSTFI | 120 |
| Sbjct 61 | AM+AMGA I K G WII G GNG LL P APLDFGNA TG RLTMGLVG YD ++TFI DAMKAMGAHIEKRGAEWIIQGTGNGCLLEPTAPLDFGNAGTGSRLTMGLVGTYDMETTFI | 120 |
| Query 121 | GDASLTKRPMGRVLNPLREMGVQV-KSEDGDRLPVTLRGPKTPTPITYRVPMASAQVKSA | 179 |
| Sbjct 121 | GDASL+ RPMGRVL+PLREMGVQV K+ GDR+P+TLRGPK PI+YRVPMASAQVKSA GDASLSGRPMGRVLDPLREMGVQVLKASPGDRMPITLRGPKQAAPISYRVPMASAQVKSA | 180 |
| Query 180 | VLLAGLNTPGITTVIEPIMTRDHTEKMLQGFGANLTVETDADGVRTIRLEGRGKLTGQVI | 239 |

| Sbjct | 181 | VLLAGLNTPGITTVIEP+MTRDHTEKML+GFGANL VETD+DGVR IR+EG+GKL+GQ I  VLLAGLNTPGITTVIEPVMTRDHTEKMLKGFGANLEVETDSDGVRHIRIEGQGKLSGQTI | 240 |
| --- | --- | --- | --- |
| Query | 240 | DVPGDPSSTAFPLVAALLVPGSDVTILNVLMNPTRTGLILTLQEMGADIEVINPRLAGGE | 299 |
| Sbjct | 241 | VPGDPSS FPLVAAL+VPGSD+TI NVLMNPTRTGLI TL EMGA IE++N R AGGE AVPGDPSSAGFPLVAALIVPGSDITIENVLMNPTRTGLITTLLEMGASIELLNRRNAGGE | 300 |
| Query | 300 | DVADLRVRSSTLKGVTVPEDRAPSMIDEYPILAVAAAFAEGATVMNGLEELRVKESDRLS | 359 |
| Sbjct | 301 | DVADLRVRSS LKGVTVP +RAPSMIDEYP+LAVAA+FAEG T+M GL+ELRVKESDRL+ DVADLRVRSSELKGVTVPAERAPSMIDEYPVLAVAASFAEGETLMQGLDELRVKESDRLA | 360 |
| Query | 360 | AVANGLKLNGVDCDEGETSLVVRGRPDGKGLGNASGAAVATHLDHRIAMSFLVMGLVSEN | 419 |
| Sbjct | 361 | AVA GL+ NGVDC EGE SL VRG+P GKGLG G V THLDHRIAMSFLVMGL +E AVARGLEANGVDCTEGEASLSVRGKPGGKGLG---GGTVKTHLDHRIAMSFLVMGLAAEK | 417 |
| Query  Sbjct | 420  418 | PVTVDDATMIATSFPEFMDLMAGLGAKIELSDTKA 454 PV+VDD TMIATSFPEFM LM G+GA I +S+ A PVSVDDQTMIATSFPEFMGLMTGMGALISISEAVA 452 |  |

>TR:V7FMR6 V7FMR6_9RHIZ 3-phosphoshikimate 1-carboxyvinyltransferase OS=Mesorhizobium sp. LSHC420B00 OX=1287292 GN=aroA PE=3 SV=1

Length=452

Score = 686 bits (1770), Expect = 0.0

| Identities | = 351/452 (78%), Positives = 386/452 (85%), Gaps = 4/452 (1%) |  |
| --- | --- | --- |
| Query 1 | MLHGASSRPATARKSSGLSGTVRIPGDKSISHRSFMFGGLASGETRITGLLEGEDVINTG | 60 |
| Sbjct 1 | M H A+ + ATARKSSGLSG R+PGDKSISHRSFMFGGLASGETRITGLLEGEDV+ TG MSHTAAPKQATARKSSGLSGAARVPGDKSISHRSFMFGGLASGETRITGLLEGEDVMRTG | 60 |
| Query 61 | KAMQAMGARIRKEGDTWIIDGVGNGGLLAPEAPLDFGNAATGCRLTMGLVGVYDFDSTFI | 120 |
| Sbjct 61 | AM+AMGA I K G W+I G GNG LL PE PLDFGNA TG RLTMGLVG YD ++TFI AAMKAMGAHIEKHGTEWVIRGTGNGALLQPEGPLDFGNAGTGSRLTMGLVGTYDMETTFI | 120 |
| Query 121 | GDASLTKRPMGRVLNPLREMGVQV-KSEDGDRLPVTLRGPKTPTPITYRVPMASAQVKSA | 179 |
| Sbjct 121 | GDASL+ RPMGRVL PLR+MGVQV K+ GDR+P+TLRGPK PITYRVPMASAQVKSA GDASLSGRPMGRVLEPLRQMGVQVLKATPGDRMPITLRGPKHAAPITYRVPMASAQVKSA | 180 |
| Query 180 | VLLAGLNTPGITTVIEPIMTRDHTEKMLQGFGANLTVETDADGVRTIRLEGRGKLTGQVI | 239 |
| Sbjct 181 | VLLAGLNTPGITTVIEP+MTRDHTEKML+GFGANL+VETD GVR I +EG+GKLTGQ I VLLAGLNTPGITTVIEPVMTRDHTEKMLKGFGANLSVETDERGVRHIFIEGQGKLTGQTI | 240 |
| Query 240 | DVPGDPSSTAFPLVAALLVPGSDVTILNVLMNPTRTGLILTLQEMGADIEVINPRLAGGE | 299 |
| Sbjct 241 | VPGDPSS FPLVAAL+VPGSD+TI NVLMNPTRTGL+LTLQEMGA I++++ R GGE AVPGDPSSAGFPLVAALIVPGSDITIENVLMNPTRTGLLLTLQEMGARIDILDRRNEGGE | 300 |
| Query 300 | DVADLRVRSSTLKGVTVPEDRAPSMIDEYPILAVAAAFAEGATVMNGLEELRVKESDRLS | 359 |
| Sbjct 301 | DVADLRVR S LKGVTVP +RAP+MIDEYP+LAVAA+FAEG T+M GLEELRVKESDRLS DVADLRVRYSELKGVTVPPERAPTMIDEYPVLAVAASFAEGETLMLGLEELRVKESDRLS | 360 |
| Query 360 | AVANGLKLNGVDCDEGETSLVVRGRPDGKGLG---NASGAAVATHLDHRIAMSFLVMGLV | 416 |
| Sbjct 361 | AVANGLKLNGVDC EGE +L VRG+P GKGLG N G V THLDHRIAMSFLVMGL AVANGLKLNGVDCSEGEATLAVRGKPGGKGLGQHPNGQGTIVQTHLDHRIAMSFLVMGLA | 420 |
| Query 417  Sbjct 421 | SENPVTVDDATMIATSFPEFMDLMAGLGAKIE 448  +E PVT+DD MIATSFPEFM LM GLGA+IE TEKPVTIDDQAMIATSFPEFMGLMKGLGAEIE 452 |  |

>TR:E8T9A8 E8T9A8_MESCW 3-phosphoshikimate 1-carboxyvinyltransferase OS=Mesorhizobium ciceri biovar biserrulae (strain HAMBI 2942 /

LMG 23838 / WSM1271) OX=765698 GN=aroA PE=3 SV=1

Length=452

Score = 686 bits (1769), Expect = 0.0

| Identities | = 349/451 (77%), Positives = 383/451 (85%), Gaps = 4/451 (1%) |  |
| --- | --- | --- |
| Query 1 | MLHGASSRPATARKSSGLSGTVRIPGDKSISHRSFMFGGLASGETRITGLLEGEDVINTG | 60 |
| Sbjct 1 | M H A+ +PATAR+S LSG R+PGDKSISHRSFMFGGLASGETRITGLLEGEDV+ TG MSHAAAPKPATARQSPALSGIARVPGDKSISHRSFMFGGLASGETRITGLLEGEDVMRTG | 60 |
| Query 61 | KAMQAMGARIRKEGDTWIIDGVGNGGLLAPEAPLDFGNAATGCRLTMGLVGVYDFDSTFI | 120 |
| Sbjct 61 | AM+AMGA I K GD W+I G GNG LL PE PLDFGNA TG RLTMGLVG YD ++TFI AAMKAMGAHIEKNGDEWVIRGTGNGALLQPEGPLDFGNAGTGSRLTMGLVGTYDMETTFI | 120 |
| Query 121 | GDASLTKRPMGRVLNPLREMGVQV-KSEDGDRLPVTLRGPKTPTPITYRVPMASAQVKSA | 179 |
| Sbjct 121 | GDASL+ RPMGRVL PLR+MGVQV K+ GDR+P+TL GPK PITYRVPMASAQVKSA GDASLSGRPMGRVLEPLRQMGVQVLKATPGDRMPITLHGPKHAAPITYRVPMASAQVKSA | 180 |
| Query 180 | VLLAGLNTPGITTVIEPIMTRDHTEKMLQGFGANLTVETDADGVRTIRLEGRGKLTGQVI | 239 |
| Sbjct 181 | VLLAGLNTPGITTVIEP+MTRDHTEKML+GFGANL+VETD GVR I +EG+GKLTGQ I VLLAGLNTPGITTVIEPVMTRDHTEKMLKGFGANLSVETDERGVRHIFIEGQGKLTGQTI | 240 |
| Query 240 | DVPGDPSSTAFPLVAALLVPGSDVTILNVLMNPTRTGLILTLQEMGADIEVINPRLAGGE | 299 |
| Sbjct 241 | VPGDPSS FPLVAAL+VPGSD+TI NVLMNPTRTGL+LTLQEMG +I+++NPR AGGE AVPGDPSSAGFPLVAALIVPGSDITIENVLMNPTRTGLLLTLQEMGGNIDILNPRNAGGE | 300 |
| Query 300 | DVADLRVRSSTLKGVTVPEDRAPSMIDEYPILAVAAAFAEGATVMNGLEELRVKESDRLS | 359 |
| Sbjct 301 | DVADLRVR S LKGV VP +RAPSMIDEYP+LAVAA+FAEG T+M GLEELRVKESDRLS DVADLRVRYSELKGVIVPPERAPSMIDEYPVLAVAASFAEGETLMQGLEELRVKESDRLS | 360 |
| Query 360 | AVANGLKLNGVDCDEGETSLVVRGRPDGKGLG---NASGAAVATHLDHRIAMSFLVMGLV | 416 |
| Sbjct 361 | AVANGL LNGVDC EGE SL VRG+P GKGLG N V THLDHRIAMSFLVMGL AVANGLTLNGVDCTEGEASLAVRGKPGGKGLGGHPNGKDTTVQTHLDHRIAMSFLVMGLA | 420 |
| Query 417  Sbjct 421 | SENPVTVDDATMIATSFPEFMDLMAGLGAKI 447  +E PVT+DDA MIATSFPEFM LM LGA+I TEKPVTIDDAAMIATSFPEFMGLMTALGAEI 451 |  |

>TR:A0A1C2DE69 A0A1C2DE69_9RHIZ 3-phosphoshikimate 1-carboxyvinyltransferase OS=Mesorhizobium hungaricum OX=1566387 GN=aroA PE=3 SV=1

Length=450

Score = 685 bits (1768), Expect = 0.0

| Identities | = 347/450 (77%), Positives = 388/450 (86%), Gaps = 2/450 (0%) |  |
| --- | --- | --- |
| Query 1 | MLHGASSRPATARKSSGLSGTVRIPGDKSISHRSFMFGGLASGETRITGLLEGEDVINTG | 60 |
| Sbjct 1 | M H A +PATAR+SS LSGT R+PGDKSISHRSFMFGGLASGETRITGLLEGEDV+ TG MAHDAKPQPATARRSSSLSGTARVPGDKSISHRSFMFGGLASGETRITGLLEGEDVMRTG | 60 |
| Query 61 | KAMQAMGARIRKEGDTWIIDGVGNGGLLAPEAPLDFGNAATGCRLTMGLVGVYDFDSTFI | 120 |
| Sbjct 61 | AM+AMGA I K G+ W+I G GNG LL PEAPLDFGNA TG RLTMGLVG Y ++TF+ AAMKAMGAHIEKHGEEWVIRGTGNGALLQPEAPLDFGNAGTGSRLTMGLVGTYYMETTFV | 120 |
| Query 121 | GDASLTKRPMGRVLNPLREMGVQV-KSEDGDRLPVTLRGPKTPTPITYRVPMASAQVKSA | 179 |
| Sbjct 121 | GDASL+ RPMGRVL+PLR+MGVQV ++ GDR+P+TLRGP+ PITYRVPMASAQVKSA GDASLSSRPMGRVLDPLRQMGVQVLQAAPGDRMPITLRGPRHAAPITYRVPMASAQVKSA | 180 |
| Query 180 | VLLAGLNTPGITTVIEPIMTRDHTEKMLQGFGANLTVETDADGVRTIRLEGRGKLTGQVI | 239 |
| Sbjct 181 | VLLAGLNTPGITTVIEP+MTRDHTEKML+GFGANL+VETD GVR I +EG+G+L+GQVI VLLAGLNTPGITTVIEPVMTRDHTEKMLKGFGANLSVETDERGVRHIFIEGQGRLSGQVI | 240 |
| Query 240 | DVPGDPSSTAFPLVAALLVPGSDVTILNVLMNPTRTGLILTLQEMGADIEVINPRLAGGE | 299 |
| Sbjct 241 | VPGDPSS FPLVA L+VPGSD+ I NVLMNPTRTGL+LTLQEMG I+++NPR AGGE  AVPGDPSSAGFPLVAGLIVPGSDILIENVLMNPTRTGLLLTLQEMGGRIDILNPRNAGGE | 300 |

| Query | 300 | DVADLRVRSSTLKGVTVPEDRAPSMIDEYPILAVAAAFAEGATVMNGLEELRVKESDRLS | 359 |
| --- | --- | --- | --- |
| Sbjct | 301 | DVADLRVR+S LKGV VP +RAPSMIDEYP+LAVAA FAEG T+M GLEELRVKESDRL+ DVADLRVRASDLKGVVVPAERAPSMIDEYPVLAVAACFAEGETLMQGLEELRVKESDRLA | 360 |
| Query | 360 | AVANGLKLNGVDCDEGETSLVVRGRPDGKGL-GNASGAAVATHLDHRIAMSFLVMGLVSE | 418 |
| Sbjct | 361 | AVANGLKLNGVDC EGE +L VRG P G+GL G A G AV THLDHRIAMSFL+MGL +E AVANGLKLNGVDCTEGEATLAVRGVPGGRGLGGQARGEAVKTHLDHRIAMSFLIMGLAAE | 420 |
| Query | 419 | NPVTVDDATMIATSFPEFMDLMAGLGAKIE 448 PVTVDDA MIATSFPEFM LM GLGA+IE |  |
| Sbjct | 421 | KPVTVDDANMIATSFPEFMGLMKGLGAEIE 450 | |

>TR:U4VI88 U4VI88_9RHIZ 3-phosphoshikimate 1-carboxyvinyltransferase OS=Ochrobactrum intermedium 229E OX=1337887 GN=aroA PE=3 SV=1

Length=415

Score = 685 bits (1768), Expect = 0.0

| Identities | = 348/418 (83%), Positives = 378/418 (90%), Gaps = 3/418 (1%) |  |
| --- | --- | --- |
| Query 36 | MFGGLASGETRITGLLEGEDVINTGKAMQAMGARIRKEGDTWIIDGVGNGGLLAPEAPLD | 95 |
| Sbjct 1 | MFGGLASGETRITGLLEGEDVINTG+AMQAMGA+IRKEGD WII+GVGNG LL PEA LD MFGGLASGETRITGLLEGEDVINTGRAMQAMGAKIRKEGDVWIINGVGNGCLLQPEAALD | 60 |
| Query 96 | FGNAATGCRLTMGLVGVYDFDSTFIGDASLTKRPMGRVLNPLREMGVQVKSEDGDRLPVT | 155 |
| Sbjct 61 | FGNA TG RLTMGLVG YD ++FIGDASL+KRPMGRVLNPLREMGVQV++ DGDR+P+T FGNAGTGARLTMGLVGTYDMKTSFIGDASLSKRPMGRVLNPLREMGVQVEAADGDRMPLT | 120 |
| Query 156 | LRGPKTPTPITYRVPMASAQVKSAVLLAGLNTPGITTVIEPIMTRDHTEKMLQGFGANLT | 215 |
| Sbjct 121 | L GPKT PITYRVPMASAQVKSAVLLAGLNTPG+TTVIEP+MTRDHTEKMLQGFGA+LT LIGPKTANPITYRVPMASAQVKSAVLLAGLNTPGVTTVIEPVMTRDHTEKMLQGFGADLT | 180 |
| Query 216 | VETDADGVRTIRLEGRGKLTGQVIDVPGDPSSTAFPLVAALLVPGSDVTILNVLMNPTRT | 275 |
| Sbjct 181 | VETD DGVR IR+ G+GKL GQ IDVPGDPSSTAFPLVAALLV GSDVTI NVLMNPTRT VETDKDGVRHIRITGQGKLVGQTIDVPGDPSSTAFPLVAALLVEGSDVTIRNVLMNPTRT | 240 |
| Query 276 | GLILTLQEMGADIEVINPRLAGGEDVADLRVRSSTLKGVTVPEDRAPSMIDEYPILAVAA | 335 |
| Sbjct 241 | GLILTLQEMGADIEV+N RLAGGEDVADLRVR+S LKGV VP +RAPSMIDEYP+LA+AA GLILTLQEMGADIEVLNARLAGGEDVADLRVRASKLKGVVVPPERAPSMIDEYPVLAIAA | 300 |
| Query 336 | AFAEGATVMNGLEELRVKESDRLSAVANGLKLNGVDCDEGETSLVVRGRPDGKGLGNASG | 395 |
| Sbjct 301 | +FAEG TVM+GL+ELRVKESDRL+AVA GL+ NGVDC EGE SL VRGRPDGKGLG G  SFAEGETVMDGLDELRVKESDRLAAVARGLEANGVDCTEGEMSLTVRGRPDGKGLG---G | 357 |

Query 396 AAVATHLDHRIAMSFLVMGLVSENPVTVDDATMIATSFPEFMDLMAGLGAKIELSDTK 453 VATHLDHRIAMSFLVMGL +E PVTVDD+ MIATSFPEFMD+M GLGAKIELS+ +

Sbjct 358 GTVATHLDHRIAMSFLVMGLAAEKPVTVDDSNMIATSFPEFMDMMPGLGAKIELSNAQ 415

>TR:A0A432V367 A0A432V367_9RHIZ 3-phosphoshikimate 1-carboxyvinyltransferase OS=Pseudaminobacter arsenicus OX=1851146 GN=aroA PE=3 SV=1

Length=449

Score = 685 bits (1767), Expect = 0.0

Identities = 355/449 (79%), Positives = 384/449 (86%), Gaps = 4/449 (1%)

| Query | 1 | MLHGASSRPATARKSSGLSGTVRIPGDKSISHRSFMFGGLASGETRITGLLEGEDVINTG | 60 |
| --- | --- | --- | --- |
| Sbjct | 1 | M H A +PA RKS GLSGTV +PGDKSISHR+FMFGGLASGETRITGLLEGEDV+ TG MSHEAVPQPAIGRKSQGLSGTVAMPGDKSISHRAFMFGGLASGETRITGLLEGEDVLRTG | 60 |
| Query | 61 | KAMQAMGARIRKEGDTWIIDGVGNGGLLAPEAPLDFGNAATGCRLTMGLVGVYDFDSTFI | 120 |
| Sbjct | 61 | +AM+ MGA I K GD WII G GNG LL P A LDFGNA TG RLTMGLVG YD ++TFI  EAMKVMGAYIEKHGDEWIIQGTGNGCLLEPVAALDFGNAGTGSRLTMGLVGTYDMETTFI | 120 |

| Query | 121 | GDASLTKRPMGRVLNPLREMGVQV-KSEDGDRLPVTLRGPKTPTPITYRVPMASAQVKSA | 179 |
| --- | --- | --- | --- |
| Sbjct | 121 | GD SL+ RPM RVL+PLREMGVQV K+ GDR+P+TLRGPK PI+YRVPMASAQVKSA GDPSLSSRPMARVLDPLREMGVQVLKASPGDRMPLTLRGPKHAAPISYRVPMASAQVKSA | 180 |
| Query | 180 | VLLAGLNTPGITTVIEPIMTRDHTEKMLQGFGANLTVETDADGVRTIRLEGRGKLTGQVI | 239 |
| Sbjct | 181 | VLLAGLNTPGITTVIEP+MTRDHTEKMLQGFGA+L VETD +GVR IR+EG+GKL GQVI VLLAGLNTPGITTVIEPVMTRDHTEKMLQGFGADLEVETDKEGVRHIRIEGQGKLVGQVI | 240 |
| Query | 240 | DVPGDPSSTAFPLVAALLVPGSDVTILNVLMNPTRTGLILTLQEMGADIEVINPRLAGGE | 299 |
| Sbjct | 241 | VPGDPSS AFPLVAAL+VPGSD+TI NVLMNPTRTGLI TL EMGA IE++N R AGGE AVPGDPSSAAFPLVAALIVPGSDITIENVLMNPTRTGLISTLLEMGASIELVNRRSAGGE | 300 |
| Query | 300 | DVADLRVRSSTLKGVTVPEDRAPSMIDEYPILAVAAAFAEGATVMNGLEELRVKESDRLS | 359 |
| Sbjct | 301 | DVADLRVRSS LKG+TVP +RAPSMIDEYP+LAVAAAFA+G T M GLEELRVKESDRL+ DVADLRVRSSELKGMTVPAERAPSMIDEYPVLAVAAAFAQGETRMPGLEELRVKESDRLA | 360 |
| Query | 360 | AVANGLKLNGVDCDEGETSLVVRGRPDGKGLGNASGAAVATHLDHRIAMSFLVMGLVSEN | 419 |
| Sbjct | 361 | AVA GLK NGVDCDEGE SL VRGRP GKGLG G V THLDHRIAMSFLVMGL +E AVAEGLKSNGVDCDEGEASLAVRGRPGGKGLG---GGTVKTHLDHRIAMSFLVMGLAAEK | 417 |
| Query  Sbjct | 420  418 | PVTVDDATMIATSFPEFMDLMAGLGAKIE 448 PVTVDDA MIATSFPEFM LMAGLGAKIE PVTVDDANMIATSFPEFMGLMAGLGAKIE 446 |  |

>TR:A0A011VL88 A0A011VL88_9RHIZ 3-phosphoshikimate 1-carboxyvinyltransferase OS=Aquamicrobium defluvii OX=69279 GN=aroA PE=3 SV=1

Length=453

Score = 683 bits (1763), Expect = 0.0

| Identities | = 352/455 (77%), Positives = 387/455 (85%), Gaps = 3/455 (1%) |  |
| --- | --- | --- |
| Query 1 | MLHGASSRPATARKSSGLSGTVRIPGDKSISHRSFMFGGLASGETRITGLLEGEDVINTG | 60 |
| Sbjct 1 | M H A+ +PA AR+SS L G R+PGDKSISHRSFMFGGLASGETRITGLLEGEDV+ TG MSHTAAPQPAIARRSSPLRGEARVPGDKSISHRSFMFGGLASGETRITGLLEGEDVLRTG | 60 |
| Query 61 | KAMQAMGARIRKEGDTWIIDGVGNGGLLAPEAPLDFGNAATGCRLTMGLVGVYDFDSTFI | 120 |
| Sbjct 61 | +AM+AMGA I K+GD WII G GNG LL PE PLDFGNA TG RLTMGLVG YD ++TFI EAMKAMGAHIEKKGDEWIIRGTGNGALLQPEGPLDFGNAGTGSRLTMGLVGTYDMETTFI | 120 |
| Query 121 | GDASLTKRPMGRVLNPLREMGVQVKSE-DGDRLPVTLRGPKTPTPITYRVPMASAQVKSA | 179 |
| Sbjct 121 | GDASL RPMGRVL+PLR+MGVQV S GDR+P+TLRGPK PITYRVPMASAQVKSA GDASLQSRPMGRVLDPLRQMGVQVLSAAPGDRMPLTLRGPKHAAPITYRVPMASAQVKSA | 180 |
| Query 180 | VLLAGLNTPGITTVIEPIMTRDHTEKMLQGFGANLTVETDADGVRTIRLEGRGKLTGQVI | 239 |
| Sbjct 181 | VLLA LNTPGITTVIEP+MTRDHTEKML+GFGA++ VETD GVR I +EG+G+L GQ I VLLAALNTPGITTVIEPVMTRDHTEKMLKGFGASIDVETDERGVRHIFIEGQGRLAGQTI | 240 |
| Query 240 | DVPGDPSSTAFPLVAALLVPGSDVTILNVLMNPTRTGLILTLQEMGADIEVINPRLAGGE | 299 |
| Sbjct 241 | VPGDPSS AFPLVAAL+VPGSD+ I NVLMNPTRTGLILTLQEMGADIEV N R GGE RVPGDPSSAAFPLVAALIVPGSDIVIRNVLMNPTRTGLILTLQEMGADIEVQNTRNEGGE | 300 |
| Query 300 | DVADLRVRSSTLKGVTVPEDRAPSMIDEYPILAVAAAFAEGATVMNGLEELRVKESDRLS | 359 |
| Sbjct 301 | DVADLRVR S LKGVTVP +RAPSMIDEYP+LAVAAAFAEG T+M GLEELRVKESDRL+ DVADLRVRHSQLKGVTVPAERAPSMIDEYPVLAVAAAFAEGETLMQGLEELRVKESDRLA | 360 |
| Query 360 | AVANGLKLNGVDCDEGETSLVVRGRPDGKGLGNASGAAVATHLDHRIAMSFLVMGLVSEN | 419 |
| Sbjct 361 | AVA GL+LNGVDC EGE +L VRG P+GKGLG G AV THLDHRIAMSFLVMGL SE+ AVAAGLRLNGVDCTEGEATLAVRGVPEGKGLG--GGEAVKTHLDHRIAMSFLVMGLASEH | 418 |
| Query 420  Sbjct 419 | PVTVDDATMIATSFPEFMDLMAGLGAKIELSDTKA 454 PVT+DD MIATSFPEFMDLM GLGA+I+ +D A PVTIDDRAMIATSFPEFMDLMTGLGARIDAADKAA 453 |  |

>TR:A0A1S1U0N7 A0A1S1U0N7_9RHIZ 3-phosphoshikimate 1-carboxyvinyltransferase OS=Mesorhizobium sp. ORS 3428 OX=540997 GN=aroA PE=3 SV=1

Length=452

Score = 683 bits (1762), Expect = 0.0

| Identities | = 349/452 (77%), Positives = 384/452 (85%), Gaps = 4/452 (1%) |  |
| --- | --- | --- |
| Query 1 | MLHGASSRPATARKSSGLSGTVRIPGDKSISHRSFMFGGLASGETRITGLLEGEDVINTG | 60 |
| Sbjct 1 | M H A+ +PA ARKS L+G R+PGDKSISHRSFMFGGLASGETRITGLLEGEDV+ TG MSHTAAPKPAXARKSPALAGAARVPGDKSISHRSFMFGGLASGETRITGLLEGEDVMRTG | 60 |
| Query 61 | KAMQAMGARIRKEGDTWIIDGVGNGGLLAPEAPLDFGNAATGCRLTMGLVGVYDFDSTFI | 120 |
| Sbjct 61 | AM+AMGA I K+G W+I G GNG LL PE PLDFGNA TG RLTMGLVG YD ++TFI AAMKAMGAHIEKKGAEWVIRGTGNGALLEPEGPLDFGNAGTGSRLTMGLVGTYDMETTFI | 120 |
| Query 121 | GDASLTKRPMGRVLNPLREMGVQV-KSEDGDRLPVTLRGPKTPTPITYRVPMASAQVKSA | 179 |
| Sbjct 121 | GDASL+ RPMGRVL PLR+MGVQV K+ GD +P+TLRGPK PITYRVPMASAQVKSA GDASLSGRPMGRVLEPLRQMGVQVLKAAPGDXMPITLRGPKHAAPITYRVPMASAQVKSA | 180 |
| Query 180 | VLLAGLNTPGITTVIEPIMTRDHTEKMLQGFGANLTVETDADGVRTIRLEGRGKLTGQVI | 239 |
| Sbjct 181 | VLLAGLNTPGITTVIEP+MTRDHTEKML+GFGANL+VETD GVR I +EG+ KLTGQ I VLLAGLNTPGITTVIEPVMTRDHTEKMLKGFGANLSVETDERGVRHIFIEGQXKLTGQTI | 240 |
| Query 240 | DVPGDPSSTAFPLVAALLVPGSDVTILNVLMNPTRTGLILTLQEMGADIEVINPRLAGGE | 299 |
| Sbjct 241 | VPGDPSS FPLVAAL+VPGSD+ I NVLMNPTRTGL+LTLQEMG I+++NPR AGGE AVPGDPSSXGFPLVAALIVPGSDIVIENVLMNPTRTGLLLTLQEMGGRIDILNPRNAGGE | 300 |
| Query 300 | DVADLRVRSSTLKGVTVPEDRAPSMIDEYPILAVAAAFAEGATVMNGLEELRVKESDRLS | 359 |
| Sbjct 301 | DVADLRVR S +KGVTVP +RAPSMIDEYP+LAVAA+FAEG T+M GLEELRVKESDRLS DVADLRVRYSEMKGVTVPPERAPSMIDEYPVLAVAASFAEGETLMQGLEELRVKESDRLS | 360 |
| Query 360 | AVANGLKLNGVDCDEGETSLVVRGRPDGKGLG---NASGAAVATHLDHRIAMSFLVMGLV | 416 |
| Sbjct 361 | AVANGLKLNGVDC EGE SL VRG+P GKGLG N A V THLDHRIAMSFLVMGL AVANGLKLNGVDCTEGEASLAVRGKPGGKGLGGHPNGQDAKVKTHLDHRIAMSFLVMGLA | 420 |
| Query 417  Sbjct 421 | SENPVTVDDATMIATSFPEFMDLMAGLGAKIE 448  +E PVT+DDA MIATSFPEFM LM GLGA+IE TEKPVTIDDANMIATSFPEFMGLMRGLGAEIE 452 |  |

>TR:A0A084U5R5 A0A084U5R5_9RHIZ 3-phosphoshikimate 1-carboxyvinyltransferase OS=Nitratireductor basaltis OX=472175 GN=aroA PE=3 SV=1

Length=466

Score = 682 bits (1761), Expect = 0.0

| Identities | = 344/448 (77%), Positives = 387/448 (86%), Gaps = 4/448 (1%) |  |
| --- | --- | --- |
| Query 2 | LHGASSRPATARKSSGLSGTVRIPGDKSISHRSFMFGGLASGETRITGLLEGEDVINTGK | 61 |
| Sbjct 17 | +H ++ PA +R+S L G+VR+PGDKSISHR+FMFGGLASGETRITGLLEGEDV+ TG MHSSTPSPALSRRSEALKGSVRVPGDKSISHRAFMFGGLASGETRITGLLEGEDVLRTGD | 76 |
| Query 62 | AMQAMGARIRKEGDTWIIDGVGNGGLLAPEAPLDFGNAATGCRLTMGLVGVYDFDSTFIG | 121 |
| Sbjct 77 | AM+AMGA+I ++G+ W++ G GNG LL PEAPLDFGNA TG RLTMGLVG YD ++ FIG AMKAMGAKIARDGNEWVVQGTGNGCLLQPEAPLDFGNAGTGVRLTMGLVGTYDMETRFIG | 136 |
| Query 122 | DASLTKRPMGRVLNPLREMGVQV-KSEDGDRLPVTLRGPKTPTPITYRVPMASAQVKSAV | 180 |
| Sbjct 137 | DASL++RPMGRVLNPLREMGVQV +E+ DRLP+TLRGP+ PI+YRVPM SAQVKSAV DASLSRRPMGRVLNPLREMGVQVVAAEESDRLPITLRGPQRAAPISYRVPMPSAQVKSAV | 196 |
| Query 181 | LLAGLNTPGITTVIEPIMTRDHTEKMLQGFGANLTVETDADGVRTIRLEGRGKLTGQVID | 240 |
| Sbjct 197 | L+A LNTPGITTVIEP+MTRDHTEKML GFGA+L VETD +GVR IR+EG KLTGQVID  LIAALNTPGITTVIEPVMTRDHTEKMLTGFGASLEVETDGEGVRHIRIEGEPKLTGQVID | 256 |

| Query | 241 | VPGDPSSTAFPLVAALLVPGSDVTILNVLMNPTRTGLILTLQEMGADIEVINPRLAGGED | 300 |
| --- | --- | --- | --- |
| Sbjct | 257 | VPGDPSS FPLVAAL+VPGSDV I N LMNPTRTGLILTLQEMGA IE++N R AGGED VPGDPSSAGFPLVAALIVPGSDVVIENALMNPTRTGLILTLQEMGASIEIMNARKAGGED | 316 |
| Query | 301 | VADLRVRSSTLKGVTVPEDRAPSMIDEYPILAVAAAFAEGATVMNGLEELRVKESDRLSA | 360 |
| Sbjct | 317 | VADLRVR S LKGVTVP DRAPSMIDEYP+LA+AAAFAEG TVM GLEELRVKESDRLSA VADLRVRHSDLKGVTVPADRAPSMIDEYPVLAMAAAFAEGETVMEGLEELRVKESDRLSA | 376 |
| Query | 361 | VANGLKLNGVDCDEGETSLVVRGRPDGKGLGNASGAAVATHLDHRIAMSFLVMGLVSENP | 420 |
| Sbjct | 377 | VA+GLK NGVDC EGE++L VRG P GKGLG G VATHLDHRIAMSFLV+GL +E P VADGLKANGVDCTEGESTLSVRGVPGGKGLG---GGTVATHLDHRIAMSFLVLGLATEKP | 433 |
| Query  Sbjct | 421  434 | VTVDDATMIATSFPEFMDLMAGLGAKIE 448 VT+DD+ MIATSFPEFMDLM GLGA+IE VTIDDSAMIATSFPEFMDLMTGLGAQIE 461 |  |

>TR:H0HK94 H0HK94_9RHIZ 3-phosphoshikimate 1-carboxyvinyltransferase OS=Mesorhizobium alhagi CCNWXJ12-2 OX=1107882 GN=aroA PE=3 SV=1

Length=446

Score = 682 bits (1761), Expect = 0.0

| Identities | = 346/449 (77%), Positives = 385/449 (86%), Gaps = 4/449 (1%) |  |
| --- | --- | --- |
| Query 1 | MLHGASSRPATARKSSGLSGTVRIPGDKSISHRSFMFGGLASGETRITGLLEGEDVINTG | 60 |
| Sbjct 1 | M H + ++PA A+KS L+GTVR+PGDKSISHRSFMFGGLASG+TRITGLLEGEDV+ TG MAHNSPAQPAIAKKSEALAGTVRMPGDKSISHRSFMFGGLASGQTRITGLLEGEDVMRTG | 60 |
| Query 61 | KAMQAMGARIRKEGDTWIIDGVGNGGLLAPEAPLDFGNAATGCRLTMGLVGVYDFDSTFI | 120 |
| Sbjct 61 | +AM+ MGARI K G WIIDG GNG LL P APLDFGNA TG RLTMGLVG YD ++TF+ EAMKTMGARIEKRGAEWIIDGTGNGCLLEPTAPLDFGNAGTGSRLTMGLVGTYDMETTFV | 120 |
| Query 121 | GDASLTKRPMGRVLNPLREMGVQV-KSEDGDRLPVTLRGPKTPTPITYRVPMASAQVKSA | 179 |
| Sbjct 121 | GDASL+ RPMGRVL PLREMGVQV K+ GDR+P+TLRGP T PI+YRVPMASAQVKSA GDASLSGRPMGRVLGPLREMGVQVLKASPGDRMPITLRGPNTAAPISYRVPMASAQVKSA | 180 |
| Query 180 | VLLAGLNTPGITTVIEPIMTRDHTEKMLQGFGANLTVETDADGVRTIRLEGRGKLTGQVI | 239 |
| Sbjct 181 | VLLAGLNTPGITTVIEP+MTRDHTEKML+GFGAN+ VETD DGVR IR+EG+GKL GQ I VLLAGLNTPGITTVIEPVMTRDHTEKMLKGFGANIEVETDRDGVRHIRIEGQGKLFGQTI | 240 |
| Query 240 | DVPGDPSSTAFPLVAALLVPGSDVTILNVLMNPTRTGLILTLQEMGADIEVINPRLAGGE | 299 |
| Sbjct 241 | VPGDPSS FPLVAAL+VPGSD+ I NVLMNPTRTGLI TL EMGA I+++NPR AGGE AVPGDPSSAGFPLVAALIVPGSDIIIENVLMNPTRTGLITTLLEMGASIDLLNPRNAGGE | 300 |
| Query 300 | DVADLRVRSSTLKGVTVPEDRAPSMIDEYPILAVAAAFAEGATVMNGLEELRVKESDRLS | 359 |
| Sbjct 301 | DVADLRVRSS LKGV+VP +RAPSMIDEYP+LAVAA+FAEG T+M GLEELRVKESDRLS DVADLRVRSSQLKGVSVPAERAPSMIDEYPVLAVAASFAEGETLMQGLEELRVKESDRLS | 360 |
| Query 360 | AVANGLKLNGVDCDEGETSLVVRGRPDGKGLGNASGAAVATHLDHRIAMSFLVMGLVSEN | 419 |
| Sbjct 361 | AVANGLK NGVDC EGE SL VRG+P GK LG G V THLDHRIAMSFL+MGL +E AVANGLKANGVDCTEGEASLAVRGKPGGKELG---GGMVKTHLDHRIAMSFLIMGLATEK | 417 |
| Query 420  Sbjct 418 | PVTVDDATMIATSFPEFMDLMAGLGAKIE 448 PVTVDD MIATSFPEFM LM G+GA+I+ PVTVDDRAMIATSFPEFMGLMTGMGAEID 446 |  |

>TR:A0A3F2UEX1 A0A3F2UEX1_9RHIZ 3-phosphoshikimate 1-carboxyvinyltransferase OS=Mesorhizobium sp. YM1C-6-2 OX=1827501 GN=aroA PE=3 SV=1

Length=446

Score = 682 bits (1760), Expect = 0.0

Identities = 349/449 (78%), Positives = 383/449 (85%), Gaps = 4/449 (1%)

| Query | 1 | MLHGASSRPATARKSSGLSGTVRIPGDKSISHRSFMFGGLASGETRITGLLEGEDVINTG | 60 |
| --- | --- | --- | --- |
| Sbjct | 1 | M H A RPA AR+S L G R+PGDKSISHRSFMFGGLASGETRITGLLEGEDV+ TG MTHNAEPRPAVARQSEALRGKARVPGDKSISHRSFMFGGLASGETRITGLLEGEDVMRTG | 60 |
| Query | 61 | KAMQAMGARIRKEGDTWIIDGVGNGGLLAPEAPLDFGNAATGCRLTMGLVGVYDFDSTFI | 120 |
| Sbjct | 61 | +AM+AMGARI K G+ WII+GVGNG LL P+APLDFGNA TG RLTMGLVG YD ++TFI EAMKAMGARIEKNGNEWIINGVGNGCLLEPQAPLDFGNAGTGSRLTMGLVGTYDMETTFI | 120 |
| Query | 121 | GDASLTKRPMGRVLNPLREMGVQV-KSEDGDRLPVTLRGPKTPTPITYRVPMASAQVKSA | 179 |
| Sbjct | 121 | GDASL+ RPMGRVL PLR+MGVQV K+ GDR+P+TLRGPK PITYRVPMASAQVKSA GDASLSGRPMGRVLEPLRQMGVQVVKAAPGDRMPITLRGPKHAAPITYRVPMASAQVKSA | 180 |
| Query | 180 | VLLAGLNTPGITTVIEPIMTRDHTEKMLQGFGANLTVETDADGVRTIRLEGRGKLTGQVI | 239 |
| Sbjct | 181 | VLLAGLNTPG+TTVIEP MTRDHTEKMLQGFGANL VETD GVR I +EG+GKLTGQ I VLLAGLNTPGVTTVIEPTMTRDHTEKMLQGFGANLDVETDEQGVRHIFIEGQGKLTGQTI | 240 |
| Query | 240 | DVPGDPSSTAFPLVAALLVPGSDVTILNVLMNPTRTGLILTLQEMGADIEVINPRLAGGE | 299 |
| Sbjct | 241 | VPGDPSS FPLVAAL+VP SD+TI NVLMNPTRTGL+LTLQEMG +IE++NPR AGGE AVPGDPSSAGFPLVAALIVPDSDITIENVLMNPTRTGLLLTLQEMGGNIEILNPRDAGGE | 300 |
| Query | 300 | DVADLRVRSSTLKGVTVPEDRAPSMIDEYPILAVAAAFAEGATVMNGLEELRVKESDRLS | 359 |
| Sbjct | 301 | DVADLRVRSS L+GV VP RAPSMIDEYP+LA AAAFAEG T+M GLEELRVKESDRL+ DVADLRVRSSELRGVVVPAARAPSMIDEYPVLAAAAAFAEGETLMQGLEELRVKESDRLA | 360 |
| Query | 360 | AVANGLKLNGVDCDEGETSLVVRGRPDGKGLGNASGAAVATHLDHRIAMSFLVMGLVSEN | 419 |
| Sbjct | 361 | AVA GL+ NGVDC EGET+L VRGRP GKGLG G VATHLDHRIAMSFLV+GL +E AVARGLEANGVDCTEGETTLSVRGRPGGKGLG---GGTVATHLDHRIAMSFLVLGLATEK | 417 |
| Query  Sbjct | 420  418 | PVTVDDATMIATSFPEFMDLMAGLGAKIE 448 PVT+DDA MIATSFPEFM LM LGA I+ PVTIDDANMIATSFPEFMGLMKELGATID 446 |  |

>TR:A0A0Q7WSY7 A0A0Q7WSY7_9RHIZ 3-phosphoshikimate 1-carboxyvinyltransferase OS=Mesorhizobium sp. Root554 OX=1736557 GN=aroA PE=3 SV=1

Length=450

Score = 681 bits (1758), Expect = 0.0

| Identities | = 352/450 (78%), Positives = 381/450 (85%), Gaps = 2/450 (0%) |  |
| --- | --- | --- |
| Query 1 | MLHGASSRPATARKSSGLSGTVRIPGDKSISHRSFMFGGLASGETRITGLLEGEDVINTG | 60 |
| Sbjct 1 | M H A RPATAR+S LSG R+PGDKSISHRSFMFGGLASGETRITGLLEGEDV+ TG MAHDAKPRPATARRSPPLSGNARVPGDKSISHRSFMFGGLASGETRITGLLEGEDVMRTG | 60 |
| Query 61 | KAMQAMGARIRKEGDTWIIDGVGNGGLLAPEAPLDFGNAATGCRLTMGLVGVYDFDSTFI | 120 |
| Sbjct 61 | AM+AMGA I K+GD WII G GNG LL PE PLDFGNA TG RLTMGLVG YD ++TFI AAMKAMGAHIEKKGDEWIIRGTGNGALLQPEGPLDFGNAGTGSRLTMGLVGTYDMETTFI | 120 |
| Query 121 | GDASLTKRPMGRVLNPLREMGVQVKSED-GDRLPVTLRGPKTPTPITYRVPMASAQVKSA | 179 |
| Sbjct 121 | GDASL+ RPMGRVL+PLR+MGVQV S GDR+P+TLRGPK PITYRVPMASAQVKSA GDASLSGRPMGRVLDPLRQMGVQVLSAAAGDRMPITLRGPKHAAPITYRVPMASAQVKSA | 180 |
| Query 180 | VLLAGLNTPGITTVIEPIMTRDHTEKMLQGFGANLTVETDADGVRTIRLEGRGKLTGQVI | 239 |
| Sbjct 181 | VLLAGLNTPGITTVIEP+MTRDHTEKMLQGFGANLTVETD GVR I +EG+GKLTGQ I VLLAGLNTPGITTVIEPVMTRDHTEKMLQGFGANLTVETDERGVRHIFIEGQGKLTGQTI | 240 |
| Query 240 | DVPGDPSSTAFPLVAALLVPGSDVTILNVLMNPTRTGLILTLQEMGADIEVINPRLAGGE | 299 |
| Sbjct 241 | VPGDPSS AF LVAAL+VPGSD+ I NVLMNPTRTGL+LTLQEMG I+++NPR GGE AVPGDPSSAAFALVAALIVPGSDIVIENVLMNPTRTGLLLTLQEMGGRIDILNPRNEGGE | 300 |
| Query 300 | DVADLRVRSSTLKGVTVPEDRAPSMIDEYPILAVAAAFAEGATVMNGLEELRVKESDRLS | 359 |
| Sbjct 301 | DVADLRVR S LKGV+VP RAPSMIDEYP+LAVAA+FAEG T+M GLEELRVKESDRLS  DVADLRVRHSELKGVSVPPQRAPSMIDEYPVLAVAASFAEGETLMQGLEELRVKESDRLS | 360 |

| Query | 360 | AVANGLKLNGVDCDEGETSLVVRGRPDGKGL-GNASGAAVATHLDHRIAMSFLVMGLVSE 418 |
| --- | --- | --- |
| Sbjct | 361 | AVA GLKLNGVDC EG ++L VRG P GKGL G A V THLDHRIAMSFLVMGL +E AVAEGLKLNGVDCTEGHSTLAVRGVPGGKGLGGQAKDDMVQTHLDHRIAMSFLVMGLAAE 420 |
| Query | 419 | NPVTVDDATMIATSFPEFMDLMAGLGAKIE 448 |
| Sbjct | 421 | PVT+DDA MIATSFPEFM LM GLGA IE KPVTIDDAAMIATSFPEFMGLMKGLGAGIE 450 |

>TR:A0A271L0T1 A0A271L0T1_9RHIZ 3-phosphoshikimate 1-carboxyvinyltransferase OS=Mesorhizobium mediterraneum OX=43617 GN=aroA PE=3 SV=1

Length=452

Score = 681 bits (1758), Expect = 0.0

| Identities | = 347/452 (77%), Positives = 384/452 (85%), Gaps = 4/452 (1%) |  |
| --- | --- | --- |
| Query 1 | MLHGASSRPATARKSSGLSGTVRIPGDKSISHRSFMFGGLASGETRITGLLEGEDVINTG | 60 |
| Sbjct 1 | M H A+ +PATARKSS LSGT R+PGDKSISHRSFMFGGLASGETRITGLLEGEDV+ TG MSHTAAPKPATARKSSALSGTARVPGDKSISHRSFMFGGLASGETRITGLLEGEDVMRTG | 60 |
| Query 61 | KAMQAMGARIRKEGDTWIIDGVGNGGLLAPEAPLDFGNAATGCRLTMGLVGVYDFDSTFI | 120 |
| Sbjct 61 | AM+AMGA I K G W+I G GNG LL PE PLDFGNA TG RLTMGLVG YD ++TFI AAMKAMGAHIEKHGTEWVIRGTGNGALLQPEGPLDFGNAGTGSRLTMGLVGTYDMETTFI | 120 |
| Query 121 | GDASLTKRPMGRVLNPLREMGVQV-KSEDGDRLPVTLRGPKTPTPITYRVPMASAQVKSA | 179 |
| Sbjct 121 | GDASL+ RPMGRVL PLR+MGVQV K+ GDR+P+TL GPK PITYRVPMASAQVKSA GDASLSGRPMGRVLEPLRQMGVQVLKATPGDRMPITLHGPKHAAPITYRVPMASAQVKSA | 180 |
| Query 180 | VLLAGLNTPGITTVIEPIMTRDHTEKMLQGFGANLTVETDADGVRTIRLEGRGKLTGQVI | 239 |
| Sbjct 181 | VLLAGLNTPG+TTVIEP+MTRDHTEKML+GFGANL+VETD GVR I +EG+GKLTGQ I VLLAGLNTPGVTTVIEPVMTRDHTEKMLKGFGANLSVETDERGVRHIFIEGQGKLTGQTI | 240 |
| Query 240 | DVPGDPSSTAFPLVAALLVPGSDVTILNVLMNPTRTGLILTLQEMGADIEVINPRLAGGE | 299 |
| Sbjct 241 | VPGDPSS FPLVAAL+VPGSD+TI NVLMNPTRTGL+LTLQEMGA I++++PR GGE AVPGDPSSAGFPLVAALIVPGSDITIENVLMNPTRTGLLLTLQEMGARIDILDPRNEGGE | 300 |
| Query 300 | DVADLRVRSSTLKGVTVPEDRAPSMIDEYPILAVAAAFAEGATVMNGLEELRVKESDRLS | 359 |
| Sbjct 301 | DVADLRVR S LKGVTVP +RAP+MIDEYP+LAVAA+FAEG T+M GLEELRVKESDRLS DVADLRVRYSELKGVTVPPERAPTMIDEYPVLAVAASFAEGETLMLGLEELRVKESDRLS | 360 |
| Query 360 | AVANGLKLNGVDCDEGETSLVVRGRPDGKGLG---NASGAAVATHLDHRIAMSFLVMGLV | 416 |
| Sbjct 361 | AVA GLKLNGVDC EGE +L VRG+P GKGLG N V THLDHRIAMSFL+MGL AVAEGLKLNGVDCTEGEATLAVRGKPGGKGLGQHPNGHNTVVQTHLDHRIAMSFLIMGLA | 420 |
| Query 417  Sbjct 421 | SENPVTVDDATMIATSFPEFMDLMAGLGAKIE 448  +E PVT+DD MIATSFPEFM LM LGA+IE TEKPVTIDDQAMIATSFPEFMGLMKRLGAEIE 452 |  |

>TR:A0A316C321 A0A316C321_PSESE 3-phosphoshikimate 1-carboxyvinyltransferase OS=Pseudaminobacter salicylatoxidans OX=93369 GN=aroA PE=3

SV=1

Length=452

Score = 681 bits (1758), Expect = 0.0

Identities = 349/447 (78%), Positives = 381/447 (85%), Gaps = 4/447 (1%)

| Query | 9 | PATARKSSGLSGTVRIPGDKSISHRSFMFGGLASGETRITGLLEGEDVINTGKAMQAMGA | 68 |
| --- | --- | --- | --- |
| Sbjct | 9 | PA A KS LSGTVR+PGDKSISHRSFMFGGLASGETRITGLLEGEDV+ TG AM+AMGA PAVAAKSPALSGTVRMPGDKSISHRSFMFGGLASGETRITGLLEGEDVLRTGDAMKAMGA | 68 |
| Query | 69 | RIRKEGDTWIIDGVGNGGLLAPEAPLDFGNAATGCRLTMGLVGVYDFDSTFIGDASLTKR | 128 |
| Sbjct | 69 | +I K G WII G GNG LL P APLDFGNA TG RLTMGLVG YD ++TF+GDASL+ R  QIEKRGGEWIIQGTGNGCLLEPRAPLDFGNAGTGSRLTMGLVGTYDMETTFVGDASLSGR | 128 |

| Query | 129 | PMGRVLNPLREMGVQV-KSEDGDRLPVTLRGPKTPTPITYRVPMASAQVKSAVLLAGLNT | 187 |
| --- | --- | --- | --- |
| Sbjct | 129 | PM RVL+PLREMGVQV KS GDR+P+TLRGP+ PI+YRVPMASAQVKSAVLLAGLN PMARVLDPLREMGVQVLKSSPGDRMPLTLRGPRVAAPISYRVPMASAQVKSAVLLAGLNA | 188 |
| Query | 188 | PGITTVIEPIMTRDHTEKMLQGFGANLTVETDADGVRTIRLEGRGKLTGQVIDVPGDPSS | 247 |
| Sbjct | 189 | PGITTVIEP+MTRDHTEKMLQGFGANL VETD DGVR IR+EG+GKL+GQ I VPGDPSS PGITTVIEPVMTRDHTEKMLQGFGANLEVETDKDGVRHIRIEGQGKLSGQTIAVPGDPSS | 248 |
| Query | 248 | TAFPLVAALLVPGSDVTILNVLMNPTRTGLILTLQEMGADIEVINPRLAGGEDVADLRVR | 307 |
| Sbjct | 249 | FPLVAAL+VPGSD+ I NVLMNPTRTGLI TLQEMGA+IE++N R AGGEDVADL VR AGFPLVAALIVPGSDIVIENVLMNPTRTGLITTLQEMGANIELLNQRNAGGEDVADLHVR | 308 |
| Query | 308 | SSTLKGVTVPEDRAPSMIDEYPILAVAAAFAEGATVMNGLEELRVKESDRLSAVANGLKL | 367 |
| Sbjct | 309 | SS LKGVTVP +RAPSMIDEYP+LAVAA FAEG T+M GLEELRVKESDRL+AVA+GL+ SSELKGVTVPAERAPSMIDEYPVLAVAACFAEGETLMTGLEELRVKESDRLAAVASGLEA | 368 |
| Query | 368 | NGVDCDEGETSLVVRGRPDGKGLGNASGAAVATHLDHRIAMSFLVMGLVSENPVTVDDAT | 427 |
| Sbjct | 369 | NGVDC EGE +L VRGRP GKGLG G V THLDHRIAMSFL+MGL +E PVTVDDA NGVDCAEGEATLAVRGRPGGKGLG---GGIVKTHLDHRIAMSFLIMGLAAEKPVTVDDAN | 425 |
| Query  Sbjct | 428  426 | MIATSFPEFMDLMAGLGAKIELSDTKA 454 MIATSFPEFM LM G+GAKIE D A MIATSFPEFMGLMTGMGAKIETMDAAA 452 |  |

>TR:A0A562FPT2 A0A562FPT2_9RHIZ 3-phosphoshikimate 1-carboxyvinyltransferase OS=Aminobacter sp. J15 OX=935260 GN=aroA PE=3 SV=1

Length=446

Score = 681 bits (1757), Expect = 0.0

| Identities | = 350/442 (79%), Positives = 380/442 (86%), Gaps = 4/442 (1%) |  |
| --- | --- | --- |
| Query 8 | RPATARKSSGLSGTVRIPGDKSISHRSFMFGGLASGETRITGLLEGEDVINTGKAMQAMG | 67 |
| Sbjct 8 | +PATA KS LSGTVR+PGDKSISHRSFMFGGLASG TRITGLLEGEDV+ TG+AM+AMG KPATASKSEALSGTVRVPGDKSISHRSFMFGGLASGVTRITGLLEGEDVLRTGEAMKAMG | 67 |
| Query 68 | ARIRKEGDTWIIDGVGNGGLLAPEAPLDFGNAATGCRLTMGLVGVYDFDSTFIGDASLTK | 127 |
| Sbjct 68 | A+I K+GD WII G GNG LL P+ PLDFGNA TG LTMGLVG YD +TFIGD SL AKIEKQGDEWIIHGTGNGALLEPQGPLDFGNAGTGSXLTMGLVGTYDMTTTFIGDTSLQS | 127 |
| Query 128 | RPMGRVLNPLREMGVQV-KSEDGDRLPVTLRGPKTPTPITYRVPMASAQVKSAVLLAGLN | 186 |
| Sbjct 128 | RPMGRVLNPLREMGVQV + GDR+P+T+RGPK PITYRVPMASAQVKSAVLLAGLN RPMGRVLNPLREMGVQVVDAAPGDRMPLTIRGPKRANPITYRVPMASAQVKSAVLLAGLN | 187 |
| Query 187 | TPGITTVIEPIMTRDHTEKMLQGFGANLTVETDADGVRTIRLEGRGKLTGQVIDVPGDPS | 246 |
| Sbjct 188 | TPGITTVIEPIMTRDHTEKML+GFGANL VETD DGVR IR+EG GKLTGQVI VPGDPS TPGITTVIEPIMTRDHTEKMLKGFGANLQVETDKDGVRHIRIEGEGKLTGQVIAVPGDPS | 247 |
| Query 247 | STAFPLVAALLVPGSDVTILNVLMNPTRTGLILTLQEMGADIEVINPRLAGGEDVADLRV | 306 |
| Sbjct 248 | S AFPLVAAL+VPGSD+ I NVLMNPTRTGLI TL EMGA IE++N R AGGE+VADLRV SAAFPLVAALIVPGSDIVIENVLMNPTRTGLIDTLLEMGASIEILNRRDAGGEEVADLRV | 307 |
| Query 307 | RSSTLKGVTVPEDRAPSMIDEYPILAVAAAFAEGATVMNGLEELRVKESDRLSAVANGLK | 366 |
| Sbjct 308 | R S LKGVTVP RAP MIDEYPILA+AA+ AEG TVM+GLEELRVKESDRL+AVA GL+ RHSELKGVTVPPARAPFMIDEYPILAIAASLAEGETVMDGLEELRVKESDRLAAVARGLE | 367 |
| Query 367 | LNGVDCDEGETSLVVRGRPDGKGLGNASGAAVATHLDHRIAMSFLVMGLVSENPVTVDDA | 426 |
| Sbjct 368 | +NGVDC EGE SL VRGRPDGKGLG G VATHLDHRIAMSFLV+GL +E PVTVDD VNGVDCTEGEASLTVRGRPDGKGLG---GGTVATHLDHRIAMSFLVLGLAAEKPVTVDDR | 424 |
| Query 427  Sbjct 425 | TMIATSFPEFMDLMAGLGAKIE 448  +MIATSFPEFM LM GLGAKI+ SMIATSFPEFMGLMTGLGAKID 446 |  |

>TR:A0A371XIY5 A0A371XIY5_9RHIZ 3-phosphoshikimate 1-carboxyvinyltransferase OS=Mesorhizobium denitrificans OX=2294114 GN=aroA PE=3 SV=1

Length=446

Score = 680 bits (1754), Expect = 0.0

| Identities | = 347/449 (77%), Positives = 387/449 (86%), Gaps = 4/449 (1%) |  |
| --- | --- | --- |
| Query 1 | MLHGASSRPATARKSSGLSGTVRIPGDKSISHRSFMFGGLASGETRITGLLEGEDVINTG | 60 |
| Sbjct 1 | M H A SRPATAR+SS LSGT R+PGDKSISHRSFMFGGLASGETRITGLLEGEDV+ TG MAHDAESRPATARRSSSLSGTARVPGDKSISHRSFMFGGLASGETRITGLLEGEDVMRTG | 60 |
| Query 61 | KAMQAMGARIRKEGDTWIIDGVGNGGLLAPEAPLDFGNAATGCRLTMGLVGVYDFDSTFI | 120 |
| Sbjct 61 | AM+AMGA+I K W+I GVGNG LL P+ PLDFGNA TG RLTMGLVG YD +TF+ DAMKAMGAKITKNDGEWVIQGVGNGCLLEPKEPLDFGNAGTGSRLTMGLVGTYDMRTTFV | 120 |
| Query 121 | GDASLTKRPMGRVLNPLREMGVQV-KSEDGDRLPVTLRGPKTPTPITYRVPMASAQVKSA | 179 |
| Sbjct 121 | GDASL+KRPMGRVL+PLR MGVQV +S GDR+P+TL GPK PI YRVPMASAQVKSA GDASLSKRPMGRVLDPLRLMGVQVLESASGDRMPLTLHGPKHAAPIEYRVPMASAQVKSA | 180 |
| Query 180 | VLLAGLNTPGITTVIEPIMTRDHTEKMLQGFGANLTVETDADGVRTIRLEGRGKLTGQVI | 239 |
| Sbjct 181 | VLLAGLNTPG+TTVIEP+MTRDHTEKML+GFGA L V+TD+ GVR IR++G+GKLTGQ+I VLLAGLNTPGVTTVIEPVMTRDHTEKMLKGFGAELEVDTDSHGVRHIRIKGQGKLTGQII | 240 |
| Query 240 | DVPGDPSSTAFPLVAALLVPGSDVTILNVLMNPTRTGLILTLQEMGADIEVINPRLAGGE | 299 |
| Sbjct 241 | VPGDPSS AFPLVAAL+VPGSD+ I NVLMNPTRTGLILTLQEMGA IE++N R AGGE AVPGDPSSAAFPLVAALIVPGSDILIENVLMNPTRTGLILTLQEMGAQIELVNQRSAGGE | 300 |
| Query 300 | DVADLRVRSSTLKGVTVPEDRAPSMIDEYPILAVAAAFAEGATVMNGLEELRVKESDRLS | 359 |
| Sbjct 301 | DVADLRVR+S LKGVTVP +RAPSMIDEYP+LAVAA+FAEG T+M GLEELRVKESDRL+ DVADLRVRASDLKGVTVPPERAPSMIDEYPVLAVAASFAEGETLMQGLEELRVKESDRLA | 360 |
| Query 360 | AVANGLKLNGVDCDEGETSLVVRGRPDGKGLGNASGAAVATHLDHRIAMSFLVMGLVSEN | 419 |
| Sbjct 361 | AVA GL++NGVDC EG +L VRG+P GKGLG G VATHLDHRIAMSFLVMGL +E AVARGLEVNGVDCTEGHETLSVRGKPGGKGLG---GGTVATHLDHRIAMSFLVMGLAAEK | 417 |
| Query 420  Sbjct 418 | PVTVDDATMIATSFPEFMDLMAGLGAKIE 448 PVTVDD MIATSFPEFM LMA LGA+I+ PVTVDDRNMIATSFPEFMGLMADLGAQID 446 |  |

>TR:A0A0Q8BT14 A0A0Q8BT14_9RHIZ 3-phosphoshikimate 1-carboxyvinyltransferase OS=Mesorhizobium sp. Root157 OX=1736477 GN=aroA PE=3 SV=1

Length=450

Score = 679 bits (1751), Expect = 0.0

| Identities | = 348/450 (77%), Positives = 384/450 (85%), Gaps = 2/450 (0%) |  |
| --- | --- | --- |
| Query 1 | MLHGASSRPATARKSSGLSGTVRIPGDKSISHRSFMFGGLASGETRITGLLEGEDVINTG | 60 |
| Sbjct 1 | M H A +PATAR+S+ L G+VR+PGDKSISHRSFMFGGLASGETRITGLLEGEDV+ TG MSHDAVPQPATARRSAALVGSVRVPGDKSISHRSFMFGGLASGETRITGLLEGEDVLRTG | 60 |
| Query 61 | KAMQAMGARIRKEGDTWIIDGVGNGGLLAPEAPLDFGNAATGCRLTMGLVGVYDFDSTFI | 120 |
| Sbjct 61 | AM+AMGA I K G W+I G GNG LL PE PLDFGNA TG RLTMGLVG YD ++TFI DAMKAMGAHIEKRGAEWVIRGTGNGALLEPEGPLDFGNAGTGSRLTMGLVGTYDMETTFI | 120 |
| Query 121 | GDASLTKRPMGRVLNPLREMGVQV-KSEDGDRLPVTLRGPKTPTPITYRVPMASAQVKSA | 179 |
| Sbjct 121 | GDASL+ RPMGRVL+PLR+MGVQV K+ GDR+P+TL GPK PITYRVPM SAQVKSA GDASLSGRPMGRVLDPLRQMGVQVVKAAPGDRMPLTLHGPKHAAPITYRVPMPSAQVKSA | 180 |
| Query 180 | VLLAGLNTPGITTVIEPIMTRDHTEKMLQGFGANLTVETDADGVRTIRLEGRGKLTGQVI | 239 |
| Sbjct 181 | VLLA LNTPGITTVIEP+MTRDHTEKML+GFGANL VETD GVR I +EG+G+LTGQ I  VLLAALNTPGITTVIEPVMTRDHTEKMLKGFGANLQVETDERGVRHIFMEGQGRLTGQTI | 240 |

| Query | 240 | DVPGDPSSTAFPLVAALLVPGSDVTILNVLMNPTRTGLILTLQEMGADIEVINPRLAGGE | 299 |
| --- | --- | --- | --- |
| Sbjct | 241 | VPGDPSS AF LVAAL+VPGSD+ I NVLMNPTRTGLI+TLQEMGADIE +NPR AGGE AVPGDPSSAAFALVAALIVPGSDIVIENVLMNPTRTGLIVTLQEMGADIEALNPRNAGGE | 300 |
| Query | 300 | DVADLRVRSSTLKGVTVPEDRAPSMIDEYPILAVAAAFAEGATVMNGLEELRVKESDRLS | 359 |
| Sbjct | 301 | DVADLRVR S LKGVTVP +RAPSMIDEYP+LAVAA+FAEG T+M GLEELRVKESDRLS DVADLRVRHSELKGVTVPPERAPSMIDEYPVLAVAASFAEGETLMQGLEELRVKESDRLS | 360 |
| Query | 360 | AVANGLKLNGVDCDEGETSLVVRGRPDGKGL-GNASGAAVATHLDHRIAMSFLVMGLVSE | 418 |
| Sbjct | 361 | AVA+GLK+NGVDC EGE SL VRG P GKGL G G AV THLDHRIAMSFLVMGL +E AVADGLKMNGVDCTEGEASLAVRGTPGGKGLGGQVKGEAVKTHLDHRIAMSFLVMGLAAE | 420 |
| Query | 419 | NPVTVDDATMIATSFPEFMDLMAGLGAKIE 448 PV+VDD +MIATSFPEFM LM GLGA+I+ |  |
| Sbjct | 421 | KPVSVDDRSMIATSFPEFMGLMKGLGAQID 450 | |

>TR:A0A2P7S9L0 A0A2P7S9L0_9RHIZ 3-phosphoshikimate 1-carboxyvinyltransferase OS=Mesorhizobium ephedrae OX=2116701 GN=aroA PE=3 SV=1

Length=449

Score = 676 bits (1744), Expect = 0.0

| Identities | = 349/452 (77%), Positives = 383/452 (85%), Gaps = 4/452 (1%) |  |
| --- | --- | --- |
| Query 1 | MLHGASSRPATARKSSGLSGTVRIPGDKSISHRSFMFGGLASGETRITGLLEGEDVINTG | 60 |
| Sbjct 1 | M H S+RPATA +S L+G VR+PGDKSISHRSFMFGGLASGETRITGLLEGEDV+ TG MSHHGSARPATASRSPALNGRVRMPGDKSISHRSFMFGGLASGETRITGLLEGEDVMRTG | 60 |
| Query 61 | KAMQAMGARIRKEGDTWIIDGVGNGGLLAPEAPLDFGNAATGCRLTMGLVGVYDFDSTFI | 120 |
| Sbjct 61 | +AM+AMGA I K G+ WII G GNG LL P APLDFGNA TG RLTMGLVG YD +TFI EAMKAMGAAIEKRGNEWIIQGTGNGCLLEPAAPLDFGNAGTGSRLTMGLVGTYDMTTTFI | 120 |
| Query 121 | GDASLTKRPMGRVLNPLREMGVQV-KSEDGDRLPVTLRGPKTPTPITYRVPMASAQVKSA | 179 |
| Sbjct 121 | GDASL+KRPMGRVL+PLREMGVQV + GDR+P+TLRGP+ PI YRVPMASAQVKSA GDASLSKRPMGRVLDPLREMGVQVIDAAPGDRMPLTLRGPRQAAPIAYRVPMASAQVKSA | 180 |
| Query 180 | VLLAGLNTPGITTVIEPIMTRDHTEKMLQGFGANLTVETDADGVRTIRLEGRGKLTGQVI | 239 |
| Sbjct 181 | VLLAGLNTPG+TTVIEP+MTRDHTEKML+GFGA+L VETD DGVR IR+EG+GKL+GQVI VLLAGLNTPGVTTVIEPVMTRDHTEKMLKGFGASLEVETDRDGVRHIRIEGQGKLSGQVI | 240 |
| Query 240 | DVPGDPSSTAFPLVAALLVPGSDVTILNVLMNPTRTGLILTLQEMGADIEVINPRLAGGE | 299 |
| Sbjct 241 | VPGDPSS FPLVAAL+VPGSD+ I NVLMNPTRTGLI TL EMGA IE++NPR AGGE AVPGDPSSAGFPLVAALIVPGSDIIIENVLMNPTRTGLIATLLEMGASIELVNPRNAGGE | 300 |
| Query 300 | DVADLRVRSSTLKGVTVPEDRAPSMIDEYPILAVAAAFAEGATVMNGLEELRVKESDRLS | 359 |
| Sbjct 301 | DVADLRVRSS LKGVTVP +RAPSMIDEYP+LAVAA+FAEG TVM GLEELRVKESDRL+ DVADLRVRSSELKGVTVPAERAPSMIDEYPVLAVAASFAEGETVMLGLEELRVKESDRLA | 360 |
| Query 360 | AVANGLKLNGVDCDEGETSLVVRGRPDGKGLGNASGAAVATHLDHRIAMSFLVMGLVSEN | 419 |
| Sbjct 361 | AVA GL NGVDC EG+ +L VRG P GK LG G V THLDHRIAMSFLVMGL +E AVARGLAANGVDCTEGKDTLAVRGVPGGKNLG---GGTVTTHLDHRIAMSFLVMGLAAEK | 417 |
| Query 420  Sbjct 418 | PVTVDDATMIATSFPEFMDLMAGLGAKIELSD 451 PVTVDD TMIATSFPEFM LM GLG KI +D PVTVDDQTMIATSFPEFMGLMTGLGGKIGEAD 449 |  |

>TR:A0A4R0PL97 A0A4R0PL97_9RHIZ 3-phosphoshikimate 1-carboxyvinyltransferase OS=Oricola cellulosilytica OX=1429082 GN=aroA PE=3 SV=1

Length=452

Score = 673 bits (1737), Expect = 0.0

Identities = 345/452 (76%), Positives = 387/452 (86%), Gaps = 1/452 (0%)

| Query | 1 | MLHGASSRPATARKSSGLSGTVRIPGDKSISHRSFMFGGLASGETRITGLLEGEDVINTG | 60 |
| --- | --- | --- | --- |
| Sbjct | 1 | M H S +PATAR SS LSG+VRIPGDKSISHRSF+FGGLASGETRI+GLLEGED++ TG MSHSTSLKPATARPSSALSGSVRIPGDKSISHRSFLFGGLASGETRISGLLEGEDILATG | 60 |
| Query | 61 | KAMQAMGARIRKEGDTWIIDGVGNGGLLAPEAPLDFGNAATGCRLTMGLVGVYDFDSTFI | 120 |
| Sbjct | 61 | +AM+AMGA IRK G WII G GNG LL PE LDFGNA TG RLTMGL GVYD ++ F EAMKAMGAVIRKSGGEWIIHGTGNGCLLEPERELDFGNAGTGVRLTMGLTGVYDMETRFT | 120 |
| Query | 121 | GDASLTKRPMGRVLNPLREMGVQVKSEDGDRLPVTLRGPKTPTPITYRVPMASAQVKSAV | 180 |
| Sbjct | 121 | GDASL KRPMGRVL+PLR MGVQV + +GDRLP+TLRGP+T PI+YRVPM SAQVKSAV GDASLVKRPMGRVLDPLRLMGVQVGAAEGDRLPLTLRGPRTANPISYRVPMPSAQVKSAV | 180 |
| Query | 181 | LLAGLNTPGITTVIEPIMTRDHTEKMLQGFGANLTVETDADGVRTIRLEGRGKLTGQVID | 240 |
| Sbjct | 181 | LLAGLNTPG+TTVIEP+MTRDHTEKML GFGA L VETD DGVR IR+EG+G+L+GQ I LLAGLNTPGVTTVIEPVMTRDHTEKMLAGFGAELEVETDGDGVRHIRIEGQGRLSGQTIA | 240 |
| Query | 241 | VPGDPSSTAFPLVAALLVPGSDVTILNVLMNPTRTGLILTLQEMGADIEVINPRLAGGED | 300 |
| Sbjct | 241 | VPGDPSSTAFPLVAAL+VPGSDVTI NVLMNPTR+GLI TL EMG IE+ N R AGGED VPGDPSSTAFPLVAALIVPGSDVTIENVLMNPTRSGLITTLLEMGGSIEITNERQAGGED | 300 |
| Query | 301 | VADLRVRSSTLKGVTVPEDRAPSMIDEYPILAVAAAFAEGATVMNGLEELRVKESDRLSA | 360 |
| Sbjct | 301 | VADLRVR+S LKGVTVP DRAP+MIDEYP+LAVAA+FAEG T+M GL+ELRVKESDRL+A VADLRVRASDLKGVTVPADRAPAMIDEYPVLAVAASFAEGETMMPGLDELRVKESDRLAA | 360 |
| Query | 361 | VANGLKLNGVDCDEGETSLVVRGRPDGKGLGNAS-GAAVATHLDHRIAMSFLVMGLVSEN | 419 |
| Sbjct | 361 | VA GL++NGVDC EGE L VRG+P GKGLG AS A VATHLDHRIAMSFLVMGL +E+ VARGLEINGVDCSEGEDWLSVRGKPGGKGLGAASREAVVATHLDHRIAMSFLVMGLAAEH | 420 |
| Query | 420 | PVTVDDATMIATSFPEFMDLMAGLGAKIELSD 451 VDDA +IATSFPEFM+LMAGLGA+IE ++ |  |
| Sbjct | 421 | GTKVDDAAIIATSFPEFMELMAGLGAEIEAAE 452 | |

>SP:Q11C97 AROA_CHESB 3-phosphoshikimate 1-carboxyvinyltransferase OS=Chelativorans sp. (strain BNC1) OX=266779 GN=aroA PE=3 SV=1

Length=449

Score = 673 bits (1736), Expect = 0.0

| Identities | = 345/452 (76%), Positives = 384/452 (85%), Gaps = 4/452 (1%) |  |
| --- | --- | --- |
| Query 1 | MLHGASSRPATARKSSGLSGTVRIPGDKSISHRSFMFGGLASGETRITGLLEGEDVINTG | 60 |
| Sbjct 1 | M H SRPA +R+S GL G +R+PGDKSISHR+ MFGGLASGETRI+GLLEGEDV+ T MNHHLPSRPARSRQSQGLKGNLRVPGDKSISHRALMFGGLASGETRISGLLEGEDVLRTA | 60 |
| Query 61 | KAMQAMGARIRKEGDTWIIDGVGNGGLLAPEAPLDFGNAATGCRLTMGLVGVYDFDSTFI | 120 |
| Sbjct 61 | +AM+AMGA+I + TW I GVGNG LL PEAPLDFGNA TG RLTMGLVG YD + F EAMRAMGAQIDRRDGTWTIRGVGNGCLLEPEAPLDFGNAGTGSRLTMGLVGTYDMTTRFT | 120 |
| Query 121 | GDASLTKRPMGRVLNPLREMGVQV-KSEDGDRLPVTLRGPKTPTPITYRVPMASAQVKSA | 179 |
| Sbjct 121 | GDASL+KRPMGRVLNPLREMG QV ++E GDRLP+TLRGPK TPI YRVPMASAQVKSA GDASLSKRPMGRVLNPLREMGTQVLQAEPGDRLPITLRGPKHATPINYRVPMASAQVKSA | 180 |
| Query 180 | VLLAGLNTPGITTVIEPIMTRDHTEKMLQGFGANLTVETDADGVRTIRLEGRGKLTGQVI | 239 |
| Sbjct 181 | VLLAGLNTPG+TTVIEP+MTRDHTEKML GFGA + +ETD +G R I ++G+G L GQVI VLLAGLNTPGVTTVIEPVMTRDHTEKMLSGFGAAIQIETDKEGARHISIQGQGTLKGQVI | 240 |
| Query 240 | DVPGDPSSTAFPLVAALLVPGSDVTILNVLMNPTRTGLILTLQEMGADIEVINPRLAGGE | 299 |
| Sbjct 241 | VPGDPSS AFPLVAAL+VPGSD+ I NVLMNPTRTGLILTLQEMGADIE++N R AGGE AVPGDPSSAAFPLVAALIVPGSDILIENVLMNPTRTGLILTLQEMGADIELLNRRSAGGE | 300 |
| Query 300 | DVADLRVRSSTLKGVTVPEDRAPSMIDEYPILAVAAAFAEGATVMNGLEELRVKESDRLS | 359 |
| Sbjct 301 | DVADLRVRSS LKGVTVP RAPSMIDEYPILAVAA+ AEG TVM GLEELRVKESDRLS  DVADLRVRSSALKGVTVPASRAPSMIDEYPILAVAASLAEGETVMLGLEELRVKESDRLS | 360 |

| Query | 360 | AVANGLKLNGVDCDEGETSLVVRGRPDGKGLGNASGAAVATHLDHRIAMSFLVMGLVSEN 419 |
| --- | --- | --- |
| Sbjct | 361 | AVA GLK+NG+DC EG+ +L VRGRP+GKGLG GA V THLDHRIAM+FLV+GL SE AVAEGLKVNGIDCTEGKDTLTVRGRPEGKGLG---GATVTTHLDHRIAMAFLVLGLASER 417 |
| Query | 420 | PVTVDDATMIATSFPEFMDLMAGLGAKIELSD 451 |
| Sbjct | 418 | PV+VDD +MIATSFPEFMDLM GLGA+IE D  PVSVDDQSMIATSFPEFMDLMTGLGAEIEDRD 449 |

>TR:A0A0N0E856 A0A0N0E856_9RHOB 3-phosphoshikimate 1-carboxyvinyltransferase OS=Ahrensia marina OX=1514904 GN=aroA PE=3 SV=1

Length=451

Score = 671 bits (1730), Expect = 0.0

| Identities | = 336/451 (75%), Positives = 380/451 (84%), Gaps = 1/451 (0%) |  |
| --- | --- | --- |
| Query 2 | LHGASSRPATARKSSGLSGTVRIPGDKSISHRSFMFGGLASGETRITGLLEGEDVINTGK | 61 |
| Sbjct 1 | +H + +PA+A KS L G R+PGDKSISHRSFMFGGLAS ETR+TGLLEGEDV+ TG+ MHNHTPKPASAHKSPPLKGNARVPGDKSISHRSFMFGGLASSETRVTGLLEGEDVLATGR | 60 |
| Query 62 | AMQAMGARIRKEGDTWIIDGVGNGGLLAPEAPLDFGNAATGCRLTMGLVGVYDFDSTFIG | 121 |
| Sbjct 61 | AMQAMGA+IRKEGDTWII+G GNG LL PE P DFGNA TG RLTMGLVG YD +TF G AMQAMGAQIRKEGDTWIINGTGNGCLLEPEEPFDFGNAGTGARLTMGLVGTYDMKTTFTG | 120 |
| Query 122 | DASLTKRPMGRVLNPLREMGVQVKSEDGDRLPVTLRGPKTPTPITYRVPMASAQVKSAVL | 181 |
| Sbjct 121 | DASLTKRPMGRVL+PLR+MGVQV++ +GDR P+TLRGP+T PI+YRVPMASAQVKSAVL DASLTKRPMGRVLDPLRQMGVQVEAAEGDRCPITLRGPRTANPISYRVPMASAQVKSAVL | 180 |
| Query 182 | LAGLNTPGITTVIEPIMTRDHTEKMLQGFGANLTVETDADGVRTIRLEGRGKLTGQVIDV | 241 |
| Sbjct 181 | LAGLNTPGITTVIEPIMTRDHTEKML GFGA + +E DADG R I + G+G+L G+ I+V LAGLNTPGITTVIEPIMTRDHTEKMLSGFGAKIEIEEDADGTRHIHIHGQGELRGRTIEV | 240 |
| Query 242 | PGDPSSTAFPLVAALLVPGSDVTILNVLMNPTRTGLILTLQEMGADIEVINPRLAGGEDV | 301 |
| Sbjct 241 | PGDPSS FPLVAAL+VPGSD+ I N LMNPTRTGLI TLQEMG I++IN R AGGEDV PGDPSSAGFPLVAALIVPGSDIIIENALMNPTRTGLITTLQEMGGQIDIINMRDAGGEDV | 300 |
| Query 302 | ADLRVRSSTLKGVTVPEDRAPSMIDEYPILAVAAAFAEGATVMNGLEELRVKESDRLSAV | 361 |
| Sbjct 301 | ADL VR+S LKG+ VP DRAPSMIDEYP+LAVAAAFAEG T M+GL+ELRVKESDRL+AV ADLHVRASDLKGIVVPPDRAPSMIDEYPVLAVAAAFAEGETYMDGLDELRVKESDRLAAV | 360 |
| Query 362 | ANGLKLNGVDCDEGETSLVVRGRPDGKGLGNASG-AAVATHLDHRIAMSFLVMGLVSENP | 420 |
| Sbjct 361 | A GLK NGV+C EGE SL V+G P GKGLG S A V THLDHRIAM+FLVMG+ SE ARGLKANGVECHEGEMSLTVKGVPGGKGLGAVSAQAVVETHLDHRIAMAFLVMGMASEKA | 420 |
| Query 421  Sbjct 421 | VTVDDATMIATSFPEFMDLMAGLGAKIELSD 451 V +DDATMIATSFPEFMDLM GLGA I++S+ VHIDDATMIATSFPEFMDLMRGLGADIQVSE 451 |  |

>TR:A0A0Q6L4J7 A0A0Q6L4J7_9RHIZ 3-phosphoshikimate 1-carboxyvinyltransferase OS=Aminobacter sp. DSM 101952 OX=2735891 GN=aroA PE=3 SV=1

Length=450

Score = 665 bits (1717), Expect = 0.0

Identities = 346/451 (77%), Positives = 375/451 (83%), Gaps = 4/451 (1%)

| Query | 1 | MLHGASSRPATARKSSGLSGTVRIPGDKSISHRSFMFGGLASGETRITGLLEGEDVINTG | 60 |
| --- | --- | --- | --- |
| Sbjct | 1 | M H A +PA ARKS LSGT R+PGDKSISHRSFMFGGLASGETRITGLLEGEDV+ TG MSHQAVPQPAVARKSKSLSGTARVPGDKSISHRSFMFGGLASGETRITGLLEGEDVMRTG | 60 |
| Query | 61 | KAMQAMGARIRKEGDTWIIDGVGNGGLLAPEAPLDFGNAATGCRLTMGLVGVYDFDSTFI | 120 |
| Sbjct | 61 | AM+AMGA I K WII G GNG LL EAPLDFGNA TG RLTMGLVG YD +TFI  DAMKAMGAHIEKRSAEWIITGTGNGALLQAEAPLDFGNAGTGSRLTMGLVGTYDMATTFI | 120 |

| Query | 121 | GDASLTKRPMGRVLNPLREMGVQV-KSEDGDRLPVTLRGPKTPTPITYRVPMASAQVKSA | 179 |
| --- | --- | --- | --- |
| Sbjct | 121 | GDASL+ RPMGRVL+PLR+MGVQV + GDR+P+TLRGP+ PITYRVPMASAQVKSA GDASLSGRPMGRVLDPLRQMGVQVIDAAPGDRMPLTLRGPRQAAPITYRVPMASAQVKSA | 180 |
| Query | 180 | VLLAGLNTPGITTVIEPIMTRDHTEKMLQGFGANLTVETDADGVRTIRLEGRGKLTGQVI | 239 |
| Sbjct | 181 | VLLAGLNTPGITTVIEP+MTRDHTEKML+GFGAN+ VETD GVR I +EG+GKLTGQVI VLLAGLNTPGITTVIEPVMTRDHTEKMLKGFGANIEVETDEQGVRHIFIEGQGKLTGQVI | 240 |
| Query | 240 | DVPGDPSSTAFPLVAALLVPGSDVTILNVLMNPTRTGLILTLQEMGADIEVINPRLAGGE | 299 |
| Sbjct | 241 | VPGDPSS FPLVAAL+VPGSD+ I NVLMNPTRTGLI TL EMG I+++NPR AGGE AVPGDPSSAGFPLVAALIVPGSDIIIENVLMNPTRTGLITTLIEMGGKIDILNPRNAGGE | 300 |
| Query | 300 | DVADLRVRSSTLKGVTVPEDRAPSMIDEYPILAVAAAFAEGATVMNGLEELRVKESDRLS | 359 |
| Sbjct | 301 | DVADLRVR S LKGV VP DRAPSMIDEYP+LAVAA FAEG T+M GLEELRVKESDRL+ DVADLRVRGSELKGVKVPADRAPSMIDEYPVLAVAACFAEGETLMQGLEELRVKESDRLA | 360 |
| Query | 360 | AVANGLKLNGVDCDEGETSLVVRGRPDGKGLGNASGAAVATHLDHRIAMSFLVMGLVSEN | 419 |
| Sbjct | 361 | AVA GL+ NGVDC EGE +L VRG P GKGLG G V THLDHRIAMSFLVMGL SE AVARGLEANGVDCTEGEITLSVRGVPGGKGLG---GGTVKTHLDHRIAMSFLVMGLASEK | 417 |
| Query  Sbjct | 420  418 | PVTVDDATMIATSFPEFMDLMAGLGAKIELS 450 PVTVDD MIATSFPEFM LMA LGA+I+ S PVTVDDQAMIATSFPEFMGLMADLGAEIDQS 448 |  |

>TR:A0A5D4GS16 A0A5D4GS16_9RHIZ 3-phosphoshikimate 1-carboxyvinyltransferase OS=Mesorhizobium sp. MaA-C15 OX=2682781 GN=aroA PE=3 SV=1

Length=446

Score = 665 bits (1715), Expect = 0.0

| Identities | = 342/444 (77%), Positives = 379/444 (85%), Gaps = 4/444 (1%) |  |
| --- | --- | --- |
| Query 5 | ASSRPATARKSSGLSGTVRIPGDKSISHRSFMFGGLASGETRITGLLEGEDVINTGKAMQ | 64 |
| Sbjct 5 | A+ PATA +S LSG++++PGDKSISHRSFMFGGLA+GETRITGLLEGEDV+ TG+AM+ ATPVPATASRSQPLSGSIKVPGDKSISHRSFMFGGLAAGETRITGLLEGEDVLRTGEAMK | 64 |
| Query 65 | AMGARIRKEGDTWIIDGVGNGGLLAPEAPLDFGNAATGCRLTMGLVGVYDFDSTFIGDAS | 124 |
| Sbjct 65 | AMGARI K+G WII GVGNG LL P APLDFGNA TG RLTMGLVG YD +TFIGDAS AMGARIEKQGGEWIIQGVGNGCLLEPVAPLDFGNAGTGSRLTMGLVGTYDMKTTFIGDAS | 124 |
| Query 125 | LTKRPMGRVLNPLREMGVQV-KSEDGDRLPVTLRGPKTPTPITYRVPMASAQVKSAVLLA | 183 |
| Sbjct 125 | L+KRPMGRVL+PLREMGVQV ++ GDR+P+TLRGPK TPI+YRVPMASAQVKSAVLLA LSKRPMGRVLDPLREMGVQVLEAAQGDRMPLTLRGPKRATPISYRVPMASAQVKSAVLLA | 184 |
| Query 184 | GLNTPGITTVIEPIMTRDHTEKMLQGFGANLTVETDADGVRTIRLEGRGKLTGQVIDVPG | 243 |
| Sbjct 185 | LNTPG+TTVIEPIMTRDHTEKML GFGA L VETDA GVR IR+EG +LTGQ I VPG ALNTPGVTTVIEPIMTRDHTEKMLAGFGATLEVETDAQGVRHIRIEGEPRLTGQTIAVPG | 244 |
| Query 244 | DPSSTAFPLVAALLVPGSDVTILNVLMNPTRTGLILTLQEMGADIEVINPRLAGGEDVAD | 303 |
| Sbjct 245 | DPSS AFP+VAALLVPGSDV I LMNPTRTGLI TL EMGA+IE++N R AGGED+AD DPSSAAFPMVAALLVPGSDVIIEGALMNPTRTGLIDTLLEMGANIEILNRRNAGGEDMAD | 304 |
| Query 304 | LRVRSSTLKGVTVPEDRAPSMIDEYPILAVAAAFAEGATVMNGLEELRVKESDRLSAVAN | 363 |
| Sbjct 305 | LRVR S LKGVTVP RAP MIDEYP+LAVAAAFAEG TVM+GLEELRVKESDRL+AVA LRVRHSELKGVTVPPARAPFMIDEYPVLAVAAAFAEGETVMDGLEELRVKESDRLAAVAR | 364 |
| Query 364 | GLKLNGVDCDEGETSLVVRGRPDGKGLGNASGAAVATHLDHRIAMSFLVMGLVSENPVTV | 423 |
| Sbjct 365 | GL+ NGVDC EGETSL VRGRP G G+G G V THLDHRIAMSFLVMGL +E P+TV GLEANGVDCTEGETSLTVRGRPGGSGIG---GGTVETHLDHRIAMSFLVMGLAAEKPITV | 421 |
| Query 424  Sbjct 422 | DDATMIATSFPEFMDLMAGLGAKI 447 DD +M+ATSFPEF DLM GLGA+I DDRSMVATSFPEFFDLMTGLGAEI 445 |  |

>TR:A0A2P8NI44 A0A2P8NI44_9RHIZ 3-phosphoshikimate 1-carboxyvinyltransferase OS=Nitratireductor sp. StC3 OX=2126741 GN=aroA PE=3 SV=1

Length=446

Score = 662 bits (1708), Expect = 0.0

| Identities | = 336/444 (76%), Positives = 379/444 (85%), Gaps = 4/444 (1%) |  |
| --- | --- | --- |
| Query 5 | ASSRPATARKSSGLSGTVRIPGDKSISHRSFMFGGLASGETRITGLLEGEDVINTGKAMQ | 64 |
| Sbjct 5 | A+ PATAR+S L G VR+PGDKSISHR+ MFGGLASGETRI+GLLEGEDV+ TG+AM ATQSPATARRSGALVGRVRVPGDKSISHRALMFGGLASGETRISGLLEGEDVLRTGEAMT | 64 |
| Query 65 | AMGARIRKEGDTWIIDGVGNGGLLAPEAPLDFGNAATGCRLTMGLVGVYDFDSTFIGDAS | 124 |
| Sbjct 65 | AMGAR+ ++G W++ G GNG LL PEAPLDFGNA TG RLTMGLVG YD F+GDAS AMGARVVRDGAEWVVTGTGNGCLLEPEAPLDFGNAGTGSRLTMGLVGPYDMRVRFVGDAS | 124 |
| Query 125 | LTKRPMGRVLNPLREMGVQV-KSEDGDRLPVTLRGPKTPTPITYRVPMASAQVKSAVLLA | 183 |
| Sbjct 125 | L++RPMGRVL+PLR+MGVQV ++ DGDR+P+TL GPK PI+YRVP+ASAQVKSAVLLA LSQRPMGRVLDPLRQMGVQVLEAADGDRMPLTLHGPKHAAPISYRVPVASAQVKSAVLLA | 184 |
| Query 184 | GLNTPGITTVIEPIMTRDHTEKMLQGFGANLTVETDADGVRTIRLEGRGKLTGQVIDVPG | 243 |
| Sbjct 185 | GLN G+TTVIEP+MTRDHTEKML GFGA + VETDADG R IR+EGRG+LTGQVI VPG GLNAMGVTTVIEPVMTRDHTEKMLAGFGAAIEVETDADGTRHIRVEGRGRLTGQVIAVPG | 244 |
| Query 244 | DPSSTAFPLVAALLVPGSDVTILNVLMNPTRTGLILTLQEMGADIEVINPRLAGGEDVAD | 303 |
| Sbjct 245 | DPSS AFPLVAAL+VPGSD+TI NVLMNPTRTGLI TL EMGA IE++N R+ GGEDVAD DPSSAAFPLVAALVVPGSDITIENVLMNPTRTGLITTLIEMGASIELLNERMEGGEDVAD | 304 |
| Query 304 | LRVRSSTLKGVTVPEDRAPSMIDEYPILAVAAAFAEGATVMNGLEELRVKESDRLSAVAN | 363 |
| Sbjct 305 | LRVR+S LKGV VP +RAPSMIDEYP+LAVAAAFAEG T+M GL+ELRVKESDRL+AVA LRVRASELKGVAVPAERAPSMIDEYPVLAVAAAFAEGETLMRGLDELRVKESDRLAAVAR | 364 |
| Query 364 | GLKLNGVDCDEGETSLVVRGRPDGKGLGNASGAAVATHLDHRIAMSFLVMGLVSENPVTV | 423 |
| Sbjct 365 | GL+ NGVDC EG +L VRGRPDGKGLG G VATHLDHRIAMSFLV+GL SENPV V GLEANGVDCTEGADTLAVRGRPDGKGLG---GGTVATHLDHRIAMSFLVLGLASENPVAV | 421 |
| Query 424  Sbjct 422 | DDATMIATSFPEFMDLMAGLGAKI 447 DD TMIATSFPEFMDLM GLGA + DDRTMIATSFPEFMDLMTGLGADV 445 |  |

>TR:A0A4R1RYM4 A0A4R1RYM4_9RHIZ 3-phosphoshikimate 1-carboxyvinyltransferase OS=Rhizobium sp. BK251 OX=2512125 GN=aroA PE=3 SV=1

Length=450

Score = 655 bits (1689), Expect = 0.0

| Identities | = 324/441 (73%), Positives = 375/441 (85%), Gaps = 0/441 (0%) |  |
| --- | --- | --- |
| Query 8 | RPATARKSSGLSGTVRIPGDKSISHRSFMFGGLASGETRITGLLEGEDVINTGKAMQAMG | 67 |
| Sbjct 8 | +PAT+R S+ LSGT R+PGDKSISHR+ + GGL+ ETRI GLLEG+DV+ TG+AM+AMG KPATSRMSASLSGTARVPGDKSISHRALLLGGLSESETRIEGLLEGDDVLRTGRAMEAMG | 67 |
| Query 68 | ARIRKEGDTWIIDGVGNGGLLAPEAPLDFGNAATGCRLTMGLVGVYDFDSTFIGDASLTK | 127 |
| Sbjct 68 | AR+R++G+ WI++G GNG LLAPEAPLDFGN+ TGCRLTMGLVGVYDF++ F GD+SL++ ARVRRDGNLWIVNGTGNGALLAPEAPLDFGNSGTGCRLTMGLVGVYDFETAFTGDSSLSR | 127 |
| Query 128 | RPMGRVLNPLREMGVQVKSEDGDRLPVTLRGPKTPTPITYRVPMASAQVKSAVLLAGLNT | 187 |
| Sbjct 128 | RPMGRVL PLREMGVQV + +GDRLP +L GP T +PI+YRVP+ASAQVKSAVLLAGLN RPMGRVLAPLREMGVQVHAAEGDRLPFSLHGPHTASPISYRVPVASAQVKSAVLLAGLNA | 187 |
| Query 188 | PGITTVIEPIMTRDHTEKMLQGFGANLTVETDADGVRTIRLEGRGKLTGQVIDVPGDPSS | 247 |
| Sbjct 188 | PGITTVIEPIMTRDHTE+ML FGA L +E +DG R I L+GRG+L GQ I VP DPSS  PGITTVIEPIMTRDHTERMLTAFGAALAIEQQSDGSRRIALQGRGRLVGQAIQVPSDPSS | 247 |

| Query | 248 | TAFPLVAALLVPGSDVTILNVLMNPTRTGLILTLQEMGADIEVINPRLAGGEDVADLRVR | 307 |
| --- | --- | --- | --- |
| Sbjct | 248 | AFPLVAAL+VPGSDV I NVLMNP RTGLILTLQ+MGA+IE+ R GGEDVADLRVR AAFPLVAALIVPGSDVIIPNVLMNPARTGLILTLQDMGANIEISRQRKTGGEDVADLRVR | 307 |
| Query | 308 | SSTLKGVTVPEDRAPSMIDEYPILAVAAAFAEGATVMNGLEELRVKESDRLSAVANGLKL | 367 |
| Sbjct | 308 | S LKGVTVP++RAP+MIDEYP+LAVAAAF+EG T M GL ELRVKESDRLSAVA GLKL HSELKGVTVPQERAPAMIDEYPVLAVAAAFSEGVTRMQGLGELRVKESDRLSAVAEGLKL | 367 |
| Query | 368 | NGVDCDEGETSLVVRGRPDGKGLGNASGAAVATHLDHRIAMSFLVMGLVSENPVTVDDAT | 427 |
| Sbjct | 368 | NGVDC+EG+ L V GRP GKGLGNA+G V THLDHRIAMSFLV+GL S++PVTVDD NGVDCEEGDDWLSVTGRPGGKGLGNAAGDEVKTHLDHRIAMSFLVLGLASQHPVTVDDTA | 427 |
| Query  Sbjct | 428  428 | MIATSFPEFMDLMAGLGAKIE 448 MI TSFP+F+DLM GLGA IE MITTSFPQFIDLMRGLGADIE 448 |  |

>TR:A0A562C4L2 A0A562C4L2_9RHIZ 3-phosphoshikimate 1-carboxyvinyltransferase OS=Mesorhizobium sp. J18 OX=935263 GN=aroA PE=3 SV=1

Length=446

Score = 652 bits (1681), Expect = 0.0

| Identities | = 329/442 (74%), Positives = 370/442 (84%), Gaps = 4/442 (1%) |  |
| --- | --- | --- |
| Query 8 | RPATARKSSGLSGTVRIPGDKSISHRSFMFGGLASGETRITGLLEGEDVINTGKAMQAMG | 67 |
| Sbjct 8 | RPATARKS+ LSGTVR+PGDKSISHR+ MFGGLASGETR+TGLLEGEDV+ TG+AM+AMG RPATARKSAELSGTVRVPGDKSISHRALMFGGLASGETRVTGLLEGEDVLRTGEAMKAMG | 67 |
| Query 68 | ARIRKEGDTWIIDGVGNGGLLAPEAPLDFGNAATGCRLTMGLVGVYDFDSTFIGDASLTK | 127 |
| Sbjct 68 | ARI + WII G NG LL P+ LDFGNA TG RLTMGLVG YD + F+GD SL+K ARIVRNDREWIIQGTSNGCLLEPDGALDFGNAGTGARLTMGLVGTYDMATRFVGDPSLSK | 127 |
| Query 128 | RPMGRVLNPLREMGVQV-KSEDGDRLPVTLRGPKTPTPITYRVPMASAQVKSAVLLAGLN | 186 |
| Sbjct 128 | RPMGRVL+PLR+MGVQV +S GDRLP+ LRGPK PITYRVPM SAQVKSAVLLAGLN RPMGRVLDPLRQMGVQVLESAPGDRLPIVLRGPKHAAPITYRVPMPSAQVKSAVLLAGLN | 187 |
| Query 187 | TPGITTVIEPIMTRDHTEKMLQGFGANLTVETDADGVRTIRLEGRGKLTGQVIDVPGDPS | 246 |
| Sbjct 188 | G+TTV+EP+MTRDHTEKML GFGAN+ + TD + R IR+EGRG+L GQVID+PGDPS AAGVTTVVEPVMTRDHTEKMLAGFGANIEIVTDREKARHIRIEGRGRLAGQVIDIPGDPS | 247 |
| Query 247 | STAFPLVAALLVPGSDVTILNVLMNPTRTGLILTLQEMGADIEVINPRLAGGEDVADLRV | 306 |
| Sbjct 248 | S AFP+VA L+VPGSD+ + NVLMNPTRTGLI TL +M AD+E+IN RLAGGE+VADL V SAAFPIVAGLIVPGSDIVVENVLMNPTRTGLITTLLDMNADMEIINRRLAGGEEVADLHV | 307 |
| Query 307 | RSSTLKGVTVPEDRAPSMIDEYPILAVAAAFAEGATVMNGLEELRVKESDRLSAVANGLK | 366 |
| Sbjct 308 | R S LKGVTVP DRAP MIDEYP+LAVAAAFAEG TVM GLEELRVKESDRLSAVANGLK RGSELKGVTVPADRAPFMIDEYPVLAVAAAFAEGRTVMEGLEELRVKESDRLSAVANGLK | 367 |
| Query 367 | LNGVDCDEGETSLVVRGRPDGKGLGNASGAAVATHLDHRIAMSFLVMGLVSENPVTVDDA | 426 |
| Sbjct 368 | N V+C+EG SLVV GRP G+G+G G V THLDHRIAMSFLVMGL +E PV++DD ANAVECEEGAASLVVHGRPGGRGIG---GGTVETHLDHRIAMSFLVMGLATEKPVSIDDR | 424 |
| Query 427  Sbjct 425 | TMIATSFPEFMDLMAGLGAKIE 448 TMIATSFPEFMDLM GLGA IE TMIATSFPEFMDLMGGLGAAIE 446 |  |

>TR:A0A506U1M9 A0A506U1M9_9RHIZ 3-phosphoshikimate 1-carboxyvinyltransferase OS=Pararhizobium sp. BGMRC6574 OX=2590452 GN=aroA PE=3 SV=1

Length=455

Score = 649 bits (1675), Expect = 0.0

Identities = 322/442 (73%), Positives = 369/442 (83%), Gaps = 0/442 (0%)

| Query | 7 | SRPATARKSSGLSGTVRIPGDKSISHRSFMFGGLASGETRITGLLEGEDVINTGKAMQAM | 66 |
| --- | --- | --- | --- |
| Sbjct | 8 | SRPA +R+S GLSG VR+PGDKS+SHRS M GGLA+G TRI GLL GEDV+NTG+AM AM SRPAVSRRSPGLSGRVRVPGDKSVSHRSLMLGGLAAGRTRIRGLLSGEDVVNTGRAMAAM | 67 |
| Query | 67 | GARIRKEGDTWIIDGVGNGGLLAPEAPLDFGNAATGCRLTMGLVGVYDFDSTFIGDASLT | 126 |
| Sbjct | 68 | GA + +EGD W+I+G GNG LL PE PLDFGNA TGCRLTMGL GVYDF+++F GDASLT GANLSREGDEWVIEGTGNGCLLEPEGPLDFGNAGTGCRLTMGLAGVYDFETSFTGDASLT | 127 |
| Query | 127 | KRPMGRVLNPLREMGVQVKSEDGDRLPVTLRGPKTPTPITYRVPMASAQVKSAVLLAGLN | 186 |
| Sbjct | 128 | KRPMGR+ +PLR+MGVQV++ +GDRLP LRGP+ PITYRVPM SAQVKSAVLLAGLN KRPMGRIFDPLRQMGVQVEAAEGDRLPARLRGPEVANPITYRVPMPSAQVKSAVLLAGLN | 187 |
| Query | 187 | TPGITTVIEPIMTRDHTEKMLQGFGANLTVETDADGVRTIRLEGRGKLTGQVIDVPGDPS | 246 |
| Sbjct | 188 | PGITTVIEP+ TRDHTE+ML GFGA+L VETD GVRTIRLEG+G+L+GQ IDVP DPS APGITTVIEPVATRDHTERMLAGFGASLEVETDGKGVRTIRLEGQGRLSGQTIDVPADPS | 247 |
| Query | 247 | STAFPLVAALLVPGSDVTILNVLMNPTRTGLILTLQEMGADIEVINPRLAGGEDVADLRV | 306 |
| Sbjct | 248 | S AFP+VAAL+ PGSD+ + NVLMNPTR GLI TL EMG DI +I R AGGE +ADLRV SAAFPIVAALVTPGSDIVVENVLMNPTRIGLITTLCEMGGDISLIEEREAGGETMADLRV | 307 |
| Query | 307 | RSSTLKGVTVPEDRAPSMIDEYPILAVAAAFAEGATVMNGLEELRVKESDRLSAVANGLK | 366 |
| Sbjct | 308 | R+S L+ VTVP +RAPSMIDEYP+LAVAAAFAEG TVM+G+ ELRVKESDRL+AVA GL RASDLRAVTVPPERAPSMIDEYPVLAVAAAFAEGETVMDGIGELRVKESDRLAAVAAGLA | 367 |
| Query | 367 | LNGVDCDEGETSLVVRGRPDGKGLGNASGAAVATHLDHRIAMSFLVMGLVSENPVTVDDA | 426 |
| Sbjct | 368 | LNGVD EGE +L VRGRP GKG+GN G V THLDHRIAM+FLVMG SE+PV VDD LNGVDHTEGEAALAVRGRPGGKGVGNPDGKRVETHLDHRIAMAFLVMGAASEHPVQVDDI | 427 |
| Query  Sbjct | 427  428 | TMIATSFPEFMDLMAGLGAKIE 448  ++IATSFP+F DLM GLG IE SIIATSFPDFADLMNGLGTAIE 449 |  |

>TR:A0A1I3NUZ8 A0A1I3NUZ8_9RHIZ 3-phosphoshikimate 1-carboxyvinyltransferase OS=Aquamicrobium aerolatum DSM 21857 OX=1121003 GN=aroA PE=3

SV=1

Length=446

Score = 647 bits (1670), Expect = 0.0

| Identities | = 334/444 (75%), Positives = 371/444 (84%), Gaps = 4/444 (1%) |  |
| --- | --- | --- |
| Query 5 | ASSRPATARKSSGLSGTVRIPGDKSISHRSFMFGGLASGETRITGLLEGEDVINTGKAMQ | 64 |
| Sbjct 5 | A P+TA +S L+G++R+PGDKSISHR+FMFGGLA+G+TRITGLLEGEDV+ TG AM ALPNPSTASRSETLTGSIRVPGDKSISHRAFMFGGLAAGQTRITGLLEGEDVMRTGDAMV | 64 |
| Query 65 | AMGARIRKEGDTWIIDGVGNGGLLAPEAPLDFGNAATGCRLTMGLVGVYDFDSTFIGDAS | 124 |
| Sbjct 65 | AMGA+I + G+ W+IDGVGNG LL P+APLDFGNA TG RLTMGLVG YD +TFIGDAS AMGAKITRAGNEWVIDGVGNGCLLEPQAPLDFGNAGTGSRLTMGLVGTYDMKTTFIGDAS | 124 |
| Query 125 | LTKRPMGRVLNPLREMGVQV-KSEDGDRLPVTLRGPKTPTPITYRVPMASAQVKSAVLLA | 183 |
| Sbjct 125 | L RPM RVLNPLREMGVQV + GDRLP+TL GP+ PI+YRVPMASAQVKSAVLLA LQSRPMARVLNPLREMGVQVIDAAPGDRLPLTLHGPRRAAPISYRVPMASAQVKSAVLLA | 184 |
| Query 184 | GLNTPGITTVIEPIMTRDHTEKMLQGFGANLTVETDADGVRTIRLEGRGKLTGQVIDVPG | 243 |
| Sbjct 185 | LNTPGITTVIEPIMTRDHTEKML GFGA L +ETDA GVR IR+EG +L+GQ I VPG ALNTPGITTVIEPIMTRDHTEKMLAGFGAALDIETDAQGVRHIRIEGEPRLSGQTIAVPG | 244 |
| Query 244 | DPSSTAFPLVAALLVPGSDVTILNVLMNPTRTGLILTLQEMGADIEVINPRLAGGEDVAD | 303 |
| Sbjct 245 | DPSS AFPLVAAL+VPGSDVTI NVLMNPTRTG+I TL EMGA IE++N R AGGE+VAD DPSSAAFPLVAALIVPGSDVTIENVLMNPTRTGIIDTLLEMGASIEILNRRDAGGEEVAD | 304 |
| Query 304 | LRVRSSTLKGVTVPEDRAPSMIDEYPILAVAAAFAEGATVMNGLEELRVKESDRLSAVAN LRVR S LKGV VP RAP MIDEYPILAVAAAFAEG TVM+G+EELRVKE DRL+AVA | 363 |

| Sbjct | 305 | LRVRHSQLKGVVVPPARAPFMIDEYPILAVAAAFAEGETVMDGIEELRVKECDRLAAVAR | 364 |
| --- | --- | --- | --- |
| Query | 364 | GLKLNGVDCDEGETSLVVRGRPDGKGLGNASGAAVATHLDHRIAMSFLVMGLVSENPVTV | 423 |
| Sbjct | 365 | GL+ NGVDC E E L VRGRPDGKG G G VATHLDHRIAMSFL+MGL +++PVTV GLEANGVDCTESEACLTVRGRPDGKGFG---GGTVATHLDHRIAMSFLIMGLATQHPVTV | 421 |
| Query  Sbjct | 424  422 | DDATMIATSFPEFMDLMAGLGAKI 447 DD TMIATSFPEFM LM GLGA I DDRTMIATSFPEFMGLMTGLGALI 445 |  |

>TR:K2MPH3 K2MPH3_9RHIZ 3-phosphoshikimate 1-carboxyvinyltransferase OS=Nitratireductor pacificus pht-3B OX=391937 GN=aroA PE=3 SV=1

Length=445

Score = 647 bits (1670), Expect = 0.0

| Identities | = 333/447 (74%), Positives = 371/447 (83%), Gaps = 4/447 (1%) |  |
| --- | --- | --- |
| Query 2 | LHGASSRPATARKSSGLSGTVRIPGDKSISHRSFMFGGLASGETRITGLLEGEDVINTGK | 61 |
| Sbjct 1 | +H AS RP +A KS L+G R+PGDKSISHR+ MFGGLA+GETRITGLLEGEDV+ TG+ MHNASPRPLSAAKSEALTGRARVPGDKSISHRALMFGGLAAGETRITGLLEGEDVLRTGE | 60 |
| Query 62 | AMQAMGARIRKEGDTWIIDGVGNGGLLAPEAPLDFGNAATGCRLTMGLVGVYDFDSTFIG | 121 |
| Sbjct 61 | AM+AMGARI K+G WI G GNG LL P+ LDFGNA TG RLTMGLVG YD ++ F+G AMRAMGARIEKQGQEWIAVGTGNGALLQPKDALDFGNAGTGVRLTMGLVGSYDMETRFVG | 120 |
| Query 122 | DASLTKRPMGRVLNPLREMGVQVKS-EDGDRLPVTLRGPKTPTPITYRVPMASAQVKSAV | 180 |
| Sbjct 121 | DASL+KRPMGRVL+PLREMG+QV S E GDRLP+TLRGPK PI+YRVPM SAQVKSAV DASLSKRPMGRVLDPLREMGLQVVSAEAGDRLPITLRGPKHAAPISYRVPMPSAQVKSAV | 180 |
| Query 181 | LLAGLNTPGITTVIEPIMTRDHTEKMLQGFGANLTVETDADGVRTIRLEGRGKLTGQVID | 240 |
| Sbjct 181 | LLA LN PG+TTVIEP+ TRDHTEKML GFGA+L VETDADGVR IR+EG+ KLTGQ I LLAALNVPGVTTVIEPVATRDHTEKMLAGFGASLEVETDADGVRHIRIEGQPKLTGQTIA | 240 |
| Query 241 | VPGDPSSTAFPLVAALLVPGSDVTILNVLMNPTRTGLILTLQEMGADIEVINPRLAGGED | 300 |
| Sbjct 241 | VPGDPSS AFPLVAA+LVPGSDV I N+LMNPTRTGL+ TL EMGADI ++ R GGED VPGDPSSAAFPLVAAVLVPGSDVVIENMLMNPTRTGLVTTLLEMGADITILERRNEGGED | 300 |
| Query 301 | VADLRVRSSTLKGVTVPEDRAPSMIDEYPILAVAAAFAEGATVMNGLEELRVKESDRLSA | 360 |
| Sbjct 301 | VADLRVR S LKGVTVP +RAPSMIDEYP+LAVAA+FAEG T M GLEELRVKESDRLSA VADLRVRGSELKGVTVPAERAPSMIDEYPVLAVAASFAEGETRMPGLEELRVKESDRLSA | 360 |
| Query 361 | VANGLKLNGVDCDEGETSLVVRGRPDGKGLGNASGAAVATHLDHRIAMSFLVMGLVSENP | 420 |
| Sbjct 361 | VA GLK NGVDC EGE SL V GRP G+GLG G V+THLDHRIAMSFLVMGL +E P VAEGLKANGVDCVEGEASLSVTGRPGGRGLG---GGTVSTHLDHRIAMSFLVMGLAAEKP | 417 |
| Query 421  Sbjct 418 | VTVDDATMIATSFPEFMDLMAGLGAKI 447 VT+DD MIATSFP+F DLM LGA+I VTIDDQAMIATSFPDFTDLMRRLGARI 444 |  |

>TR:A0A0Q6F990 A0A0Q6F990_9RHIZ 3-phosphoshikimate 1-carboxyvinyltransferase OS=Aurantimonas sp. Leaf443 OX=1736378 GN=aroA PE=3 SV=1

Length=453

Score = 645 bits (1664), Expect = 0.0

Identities = 326/447 (73%), Positives = 372/447 (83%), Gaps = 4/447 (1%)

| Query | 3 | HGASSRPATARKSSGLSGTVRIPGDKSISHRSFMFGGLASGETRITGLLEGEDVINTGKA | 62 |
| --- | --- | --- | --- |
| Sbjct | 4 | HG ++RP TAR S L G+ ++PGDKSISHR+F+FGGLASG TR+TGLLEGEDV+ TG+A HGGAARPLTARAGSALKGSAKVPGDKSISHRAFLFGGLASGRTRVTGLLEGEDVVATGRA | 63 |
| Query | 63 | MQAMGARIRKEGDTWIIDGVGNGGLLAPEAPLDFGNAATGCRLTMGLVGVYDFDSTFIGD M+AMGARI ++G+ ++++GVGNG LL PEAPLDFGNA TG RLTMGL G YDF +TF+GD | 122 |

| Sbjct | 64 | MRAMGARIERDGEAFVVEGVGNGCLLEPEAPLDFGNAGTGARLTMGLAGAYDFGTTFVGD | 123 |
| --- | --- | --- | --- |
| Query | 123 | ASLTKRPMGRVLNPLREMGVQVKSEDGDRLPVTLRGPKTPTPITYRVPMASAQVKSAVLL | 182 |
| Sbjct | 124 | ASL++RPMGRVL+PLREMGVQV + GDRLP+TLRGP+ PI YRVPMASAQVKSAVLL ASLSRRPMGRVLDPLREMGVQVAARTGDRLPLTLRGPRVAAPIEYRVPMASAQVKSAVLL | 183 |
| Query | 183 | AGLNTPGITTVIEPIMTRDHTEKMLQGFGANLTVETDADGVRTIRLEGRGKLTGQV-IDV | 241 |
| Sbjct | 184 | AGLN PG+TTVIEP+MTRDHTEKML+GFGA + V TDA G RTIRLEG+G+LTG V AGLNAPGVTTVIEPVMTRDHTEKMLEGFGARIEVTTDAAGTRTIRLEGQGELTGVADFTV | 243 |
| Query | 242 | PGDPSSTAFPLVAALLVPGSDVTILNVLMNPTRTGLILTLQEMGADIEVINPRLAGGEDV | 301 |
| Sbjct | 244 | PGDPSS AFP+VAA +VPGSDVT+ NVLMNPTRTGL+ TL EMGA IE++NPR +GGEDV PGDPSSAAFPIVAAAIVPGSDVTVTNVLMNPTRTGLVTTLLEMGASIEILNPRRSGGEDV | 303 |
| Query | 302 | ADLRVRSSTLKGVTVPEDRAPSMIDEYPILAVAAAFAEGATVMNGLEELRVKESDRLSAV | 361 |
| Sbjct | 304 | ADLRVR + L GVTVPE RAPSMIDEYP LAVAAAFA+G TVM GLEELRVKESDRL+AV ADLRVRHAPLVGVTVPESRAPSMIDEYPALAVAAAFAQGRTVMVGLEELRVKESDRLAAV | 363 |
| Query | 362 | ANGLKLNGVDCDEGETSLVVRGRPDGKGLGNASGAAVATHLDHRIAMSFLVMGLVSENPV | 421 |
| Sbjct | 364 | A GL NGV +EG SL V G PDG+G G G VATHLDHRIAMSFLV+GL V AAGLAANGVAHEEGRDSLTVTGDPDGRGYG---GGTVATHLDHRIAMSFLVLGLACPKGV | 420 |
| Query  Sbjct | 422  421 | TVDDATMIATSFPEFMDLMAGLGAKIE 448 TVDDA MIATSFPEF LMAGLGA +E TVDDARMIATSFPEFESLMAGLGAVLE 447 |  |

>TR:A0A1U9JT64 A0A1U9JT64_9RHIZ 3-phosphoshikimate 1-carboxyvinyltransferase OS=Candidatus Tokpelaia hoelldoblerii OX=1902579 GN=aroA PE=3

SV=1

Length=445

Score = 645 bits (1663), Expect = 0.0

| Identities | = 330/447 (74%), Positives = 366/447 (82%), Gaps = 3/447 (1%) |  |
| --- | --- | --- |
| Query 1 | MLHGASSRPATARKSSGLSGTVRIPGDKSISHRSFMFGGLASGETRITGLLEGEDVINTG | 60 |
| Sbjct 1 | M H AS+ P TAR+++ L GT+RIPGDKSISHR+FMFG LA GETRITGLLEGEDV+NTG MTHSASASPLTARRATALQGTLRIPGDKSISHRAFMFGALAEGETRITGLLEGEDVLNTG | 60 |
| Query 61 | KAMQAMGARIRKEGDTWIIDGVGNGGLLAPEAPLDFGNAATGCRLTMGLVGVYDFDSTFI | 120 |
| Sbjct 61 | KA+ AMG IRK+GDTWI++G GNG LL PLDFGNA TG RL MGL G YD ++F KALAAMGTHIRKDGDTWILNGAGNGCLLEARQPLDFGNAGTGARLVMGLTGTYDMQTSFT | 120 |
| Query 121 | GDASLTKRPMGRVLNPLREMGVQVKSEDGDRLPVTLRGPKTPTPITYRVPMASAQVKSAV | 180 |
| Sbjct 121 | GDASL++RPM RVL+PLR MGVQV + GDRLP+TL GPKT PI YRVPM SAQVKSAV GDASLSRRPMARVLDPLRLMGVQVGAATGDRLPLTLHGPKTANPILYRVPMPSAQVKSAV | 180 |
| Query 181 | LLAGLNTPGITTVIEPIMTRDHTEKMLQGFGANLTVETDADGVRTIRLEGRGKLTGQVID | 240 |
| Sbjct 181 | LLAGLNTPGITTVIEP+MTRDHTEKMLQGFGA+L VET A G RTI L G+G L GQ ID LLAGLNTPGITTVIEPVMTRDHTEKMLQGFGADLQVETAASGERTIHLAGQGILRGQTID | 240 |
| Query 241 | VPGDPSSTAFPLVAALLVPGSDVTILNVLMNPTRTGLILTLQEMGADIEVINPRLAGGED | 300 |
| Sbjct 241 | VPGDPSS AFPLVAALLVP SD+TI NVLMNPTRTGL+ TL EMGADI ++N R +GGE VPGDPSSAAFPLVAALLVPDSDITIYNVLMNPTRTGLVTTLMEMGADITLLNERTSGGET | 300 |
| Query 301 | VADLRVRSSTLKGVTVPEDRAPSMIDEYPILAVAAAFAEGATVMNGLEELRVKESDRLSA | 360 |
| Sbjct 301 | VADLRVR S LKGVTVP +RAPSMIDEYP+L++AAAFA+G TVM GLEELRVKESDRLSA VADLRVRGSELKGVTVPPERAPSMIDEYPVLSIAAAFAKGKTVMTGLEELRVKESDRLSA | 360 |
| Query 361 | VANGLKLNGVDCDEGETSLVVRGRPDGKGLGNASGAAVATHLDHRIAMSFLVMGLVSENP | 420 |
| Sbjct 361 | VANGLK N V +EG SLVV GRPDGKGLG G V THLDHRIAMSFLVMGL +E P VANGLKANNVLHEEGRDSLVVEGRPDGKGLG---GGLVTTHLDHRIAMSFLVMGLAAEKP | 417 |
| Query 421 | VTVDDATMIATSFPEFMDLMAGLGAKI 447 |  |

V+VDD +I TSFP+F LM LGA I Sbjct 418 VSVDDGAIITTSFPQFSTLMQQLGADI 444

>TR:K2PAJ6 K2PAJ6_9RHIZ 3-phosphoshikimate 1-carboxyvinyltransferase OS=Nitratireductor indicus C115 OX=1231190 GN=aroA PE=3 SV=1

Length=478

Score = 643 bits (1659), Expect = 0.0

| Identities | = 331/449 (74%), Positives = 369/449 (82%), Gaps = 4/449 (1%) |  |
| --- | --- | --- |
| Query 1 | MLHGASSRPATARKSSGLSGTVRIPGDKSISHRSFMFGGLASGETRITGLLEGEDVINTG | 60 |
| Sbjct 33 | ++H + RP +A KS LSG R+PGDKSISHRS MFGGLA+GETRITGLLEGEDV+ TG VMHDSQPRPLSAAKSPALSGRARVPGDKSISHRSLMFGGLAAGETRITGLLEGEDVLRTG | 92 |
| Query 61 | KAMQAMGARIRKEGDTWIIDGVGNGGLLAPEAPLDFGNAATGCRLTMGLVGVYDFDSTFI | 120 |
| Sbjct 93 | AM+AMGA I + GD WI++G GNG L+ P PLDFGNA TG RLTMGLVG YD + FI AAMRAMGAAIEQRGDEWIVNGTGNGALIEPTEPLDFGNAGTGARLTMGLVGSYDMTTRFI | 152 |
| Query 121 | GDASLTKRPMGRVLNPLREMGVQV-KSEDGDRLPVTLRGPKTPTPITYRVPMASAQVKSA | 179 |
| Sbjct 153 | GDASL+KRPM RVL+PLR MG+QV ++E GDRLP+TLRGPK PI+YRVPM SAQVKSA GDASLSKRPMARVLDPLRLMGMQVLEAEAGDRLPITLRGPKHAAPISYRVPMPSAQVKSA | 212 |
| Query 180 | VLLAGLNTPGITTVIEPIMTRDHTEKMLQGFGANLTVETDADGVRTIRLEGRGKLTGQVI | 239 |
| Sbjct 213 | VLLA LNTPG+TTVIEP+ TRDHTEKML GFGA L VETDADGVR IR+EG+ KLTGQ I VLLASLNTPGVTTVIEPVATRDHTEKMLIGFGAALEVETDADGVRHIRIEGQPKLTGQTI | 272 |
| Query 240 | DVPGDPSSTAFPLVAALLVPGSDVTILNVLMNPTRTGLILTLQEMGADIEVINPRLAGGE | 299 |
| Sbjct 273 | VPGDPSS AFPLVA L+VPGSD+ I N+LMNPTRTGLI TL EMG DIE ++ R AGGE AVPGDPSSAAFPLVAGLIVPGSDIVIENILMNPTRTGLIDTLLEMGGDIEFLDRRNAGGE | 332 |
| Query 300 | DVADLRVRSSTLKGVTVPEDRAPSMIDEYPILAVAAAFAEGATVMNGLEELRVKESDRLS | 359 |
| Sbjct 333 | DVADLRVR S LKGVTVP +RAPSMIDEYP+LAVAA+FAEG T+M GLEELRVKESDRLS DVADLRVRGSELKGVTVPAERAPSMIDEYPVLAVAASFAEGDTLMQGLEELRVKESDRLS | 392 |
| Query 360 | AVANGLKLNGVDCDEGETSLVVRGRPDGKGLGNASGAAVATHLDHRIAMSFLVMGLVSEN | 419 |
| Sbjct 393 | AVANGLK NGVDC EGE +L V GRP GKGLG G V THLDHRIAMSFLVMGL +E AVANGLKANGVDCVEGEATLKVTGRPGGKGLG---GGTVETHLDHRIAMSFLVMGLAAEK | 449 |
| Query 420  Sbjct 450 | PVTVDDATMIATSFPEFMDLMAGLGAKIE 448 PVTVDD MIATSFP+F LM LGA+ + PVTVDDQAMIATSFPDFTGLMQRLGARFQ 478 |  |

>TR:A0A231V122 A0A231V122_9RHIZ 3-phosphoshikimate 1-carboxyvinyltransferase OS=Notoacmeibacter marinus OX=1876515 GN=aroA PE=3 SV=1

Length=452

Score = 641 bits (1654), Expect = 0.0

| Identities | = 323/448 (72%), Positives = 370/448 (83%), Gaps = 3/448 (1%) |  |
| --- | --- | --- |
| Query 1 | MLHGASSRPATARKSSGLSGTVRIPGDKSISHRSFMFGGLASGETRITGLLEGEDVINTG | 60 |
| Sbjct 1 | M H + PATA KS LSGT+R+PGDKSISHRS MFGGLASGET I+GLLEGEDV+ TG MSHSSIPSPATAHKSENLSGTIRVPGDKSISHRSLMFGGLASGETVISGLLEGEDVLATG | 60 |
| Query 61 | KAMQAMGARIRKEGDTWIIDGVGNGGLLAPEAPLDFGNAATGCRLTMGLVGVYDFDSTFI | 120 |
| Sbjct 61 | +AM AMGA+I ++G+ W + G GNG LL P LDFGNA TGCRLTMGLVG Y + F RAMAAMGAKIGRDGEKWRVAGTGNGALLEPADALDFGNAGTGCRLTMGLVGPYHMTTRFT | 120 |
| Query 121 | GDASLTKRPMGRVLNPLREMGVQVKSEDGDRLPVTLRGPKTPTPITYRVPMASAQVKSAV | 180 |
| Sbjct 121 | GDASL++RPM RVL+PLR+MGV V++ +GDRLPVTLRGPK P PITYRVP+ASAQVKSAV GDASLSRRPMKRVLDPLRQMGVAVEAMEGDRLPVTLRGPKAPAPITYRVPVASAQVKSAV | 180 |
| Query 181 | LLAGLNTPGITTVIEPIMTRDHTEKMLQGFGANLTVETDADGVRTIRLEGRGKLTGQVID | 240 |

| Sbjct | 181 | LLAGLN G TTVIEP+MTRDHTEKML GFGA L VE DA GVRTIRLEG+ L G+ ID  LLAGLNIAGTTTVIEPVMTRDHTEKMLAGFGAQLDVEEDASGVRTIRLEGQPDLKGRTID | 240 |
| --- | --- | --- | --- |
| Query | 241 | VPGDPSSTAFPLVAALLVPGSDVTILNVLMNPTRTGLILTLQEMGADIEVINPRLAGGED | 300 |
| Sbjct | 241 | VPGDPSS AFPLVA L+VPG+D+TI NVLMNPTRTG+ILTLQEMG ++++N R AGGED VPGDPSSAAFPLVAGLIVPGADITIENVLMNPTRTGIILTLQEMGGQVDIVNWRDAGGED | 300 |
| Query | 301 | VADLRVRSSTLKGVTVPEDRAPSMIDEYPILAVAAAFAEGATVMNGLEELRVKESDRLSA | 360 |
| Sbjct | 301 | VADLRVR S LKG+TVP +RAPSMIDEYP+LAVAAAFAEG TVM+GLEELRVKESDRL+A VADLRVRYSELKGITVPPERAPSMIDEYPVLAVAAAFAEGETVMDGLEELRVKESDRLAA | 360 |
| Query | 361 | VANGLKLNGVDCDEGETSLVVRGRPDGKGLGNASGAAVATHLDHRIAMSFLVMGLVSENP | 420 |
| Sbjct | 361 | VA GL+ NG+DC E E L +RG P GKGLG+ + V THLDHRIAM+FL +GL +E P VARGLEANGIDCTETEACLTIRGTPSGKGLGSGT---VETHLDHRIAMAFLTLGLATEKP | 417 |
| Query  Sbjct | 421  418 | VTVDDATMIATSFPEFMDLMAGLGAKIE 448 V +DD+TMIATSFP+F DLM LGAKIE VEIDDSTMIATSFPQFFDLMGQLGAKIE 445 |  |

>TR:A0A0Q6EG79 A0A0Q6EG79_9RHIZ 3-phosphoshikimate 1-carboxyvinyltransferase OS=Aureimonas sp. Leaf460 OX=1736384 GN=aroA PE=3 SV=1

Length=450

Score = 639 bits (1647), Expect = 0.0

| Identities | = 323/444 (73%), Positives = 374/444 (84%), Gaps = 6/444 (1%) |  |
| --- | --- | --- |
| Query 7 | SRPATARKSSGLSGTVRIPGDKSISHRSFMFGGLASGETRITGLLEGEDVINTGKAMQAM | 66 |
| Sbjct 7 | +RP A++ + L+G ++PGDKSISHR+F+FGG+A+G TR+TGLLEGEDV+ TG+AM+AM ARPMRAKRGAALAGRAKVPGDKSISHRAFLFGGIAAGRTRVTGLLEGEDVLATGRAMRAM | 66 |
| Query 67 | GARIRKEGDTWIIDGVGNGGLLAPEAPLDFGNAATGCRLTMGLVGVYDFDSTFIGDASLT | 126 |
| Sbjct 67 | GARI +EG+ WIIDGVGNG LL P+ LDFGNA TG RLTMGLVG YDF +TF+GDASL+ GARIEREGEAWIIDGVGNGCLLEPDGVLDFGNAGTGVRLTMGLVGPYDFGATFVGDASLS | 126 |
| Query 127 | KRPMGRVLNPLREMGVQVKSEDGDRLPVTLRGPKTPTPITYRVPMASAQVKSAVLLAGLN | 186 |
| Sbjct 127 | +RPMGRVL+PLREMGVQV GDRLP+++RGP+ PITYRVPMASAQVKSAVLLAGLN RRPMGRVLDPLREMGVQVAGRTGDRLPLSVRGPRVAAPITYRVPMASAQVKSAVLLAGLN | 186 |
| Query 187 | TPGITTVIEPIMTRDHTEKMLQGFGANLTVETDADGVRTIRLEGRGKLTGQVID--VPGD | 244 |
| Sbjct 187 | PGITTV+EPIMTRDHTEKML GFGA ++VETDA GVRTIRLEG+G+L G V D VPGD APGITTVVEPIMTRDHTEKMLDGFGATVSVETDAAGVRTIRLEGQGRLVG-VPDFVVPGD | 245 |
| Query 245 | PSSTAFPLVAALLVPGSDVTILNVLMNPTRTGLILTLQEMGADIEVINPRLAGGEDVADL | 304 |
| Sbjct 246 | PSS AF +VAA +VPGSDVT+ NVLMNPTRTGL+ TL EMGA IEV+N R++GGE VADL PSSAAFAVVAAAIVPGSDVTVENVLMNPTRTGLVQTLIEMGASIEVLNERISGGETVADL | 305 |
| Query 305 | RVRSSTLKGVTVPEDRAPSMIDEYPILAVAAAFAEGATVMNGLEELRVKESDRLSAVANG | 364 |
| Sbjct 306 | RVR + L GV VPEDRAPSMIDEYP+LAVAAAFA G T+M GL+ELRVKESDRL+AVA G RVRHAELSGVEVPEDRAPSMIDEYPVLAVAAAFARGTTIMRGLDELRVKESDRLAAVAAG | 365 |
| Query 365 | LKLNGVDCDEGETSLVVRGRPDGKGLGNASGAAVATHLDHRIAMSFLVMGLVSENPVTVD | 424 |
| Sbjct 366 | L NG+D +EG L V GRPDG GLG G VATHLDHRIAMSFLV+GL +E PVTVD LAANGIDHEEGRDWLTVTGRPDGAGLG---GGTVATHLDHRIAMSFLVLGLAAEKPVTVD | 422 |
| Query 425  Sbjct 423 | DATMIATSFPEFMDLMAGLGAKIE 448 D+TMIATSFPEF+ LM GLGA +E DSTMIATSFPEFVGLMGGLGADLE 446 |  |

>TR:A0A0Q5GG74 A0A0Q5GG74_9RHIZ 3-phosphoshikimate 1-carboxyvinyltransferase OS=Aureimonas sp. Leaf324 OX=1736336 GN=aroA PE=3 SV=1

Length=451

Score = 637 bits (1644), Expect = 0.0

| Identities | = 326/448 (73%), Positives = 371/448 (83%), Gaps = 6/448 (1%) |  |
| --- | --- | --- |
| Query 3 | HGASSRPATARKSSGLSGTVRIPGDKSISHRSFMFGGLASGETRITGLLEGEDVINTGKA | 62 |
| Sbjct 4 | H + RP T+R+ L G VR+PGDKSISHR+F+FGGLASG TR+TGLLEGEDV+ TG+A HASEPRPLTSRREGALRGRVRVPGDKSISHRAFLFGGLASGRTRVTGLLEGEDVLATGRA | 63 |
| Query 63 | MQAMGARIRKEGDTWIIDGVGNGGLLAPEAPLDFGNAATGCRLTMGLVGVYDFDSTFIGD | 122 |
| Sbjct 64 | M+ MGARI +EGD W+IDG GNG LL P+ LDFGNA TG RLTMGLVG Y+F +TF+GD MRQMGARIEREGDAWVIDGTGNGALLEPDGVLDFGNAGTGSRLTMGLVGPYEFTTTFVGD | 123 |
| Query 123 | ASLTKRPMGRVLNPLREMGVQVKSEDGDRLPVTLRGPKTPTPITYRVPMASAQVKSAVLL | 182 |
| Sbjct 124 | ASL++RPMGRVL+PLREMGVQV + DRLP++LRGP+ PI YRVPMASAQVKSAVLL ASLSRRPMGRVLDPLREMGVQVLARSKDRLPLSLRGPRVAAPIEYRVPMASAQVKSAVLL | 183 |
| Query 183 | AGLNTPGITTVIEPIMTRDHTEKMLQGFGANLTVETDADGVRTIRLEGRGKLTGQVID-- | 240 |
| Sbjct 184 | AGLNTPG+TTVIEP+MTRDHTEKML+GFGA ++VETD GVRTIRLEG+G+L G V D AGLNTPGVTTVIEPVMTRDHTEKMLEGFGARVSVETDGAGVRTIRLEGQGELKG-VPDFV | 242 |
| Query 241 | VPGDPSSTAFPLVAALLVPGSDVTILNVLMNPTRTGLILTLQEMGADIEVINPRLAGGED | 300 |
| Sbjct 243 | VPGDPSS AF +VAALLVPGSDV I N LMNPTRTGL+ TL+EMGADIE++NPR +GGED VPGDPSSAAFLVVAALLVPGSDVVIENALMNPTRTGLVETLREMGADIEILNPRQSGGED | 302 |
| Query 301 | VADLRVRSSTLKGVTVPEDRAPSMIDEYPILAVAAAFAEGATVMNGLEELRVKESDRLSA | 360 |
| Sbjct 303 | VADL VR S L+GV+VP RAPSMIDEYP+LAVAAAFA+G T M GLEELRVKESDRL+A VADLHVRHSALRGVSVPATRAPSMIDEYPVLAVAAAFADGVTRMEGLEELRVKESDRLAA | 362 |
| Query 361 | VANGLKLNGVDCDEGETSLVVRGRPDGKGLGNASGAAVATHLDHRIAMSFLVMGLVSENP | 420 |
| Sbjct 363 | VA GL NGV +EG L V GRPDG+GLG G V THLDHRIAMSFLVMGL SE P VAAGLAANGVAHEEGPDWLTVTGRPDGRGLG---GGTVETHLDHRIAMSFLVMGLASEKP | 419 |
| Query 421  Sbjct 420 | VTVDDATMIATSFPEFMDLMAGLGAKIE 448 VTVDDATMIATSFPEF L+ GLG +E VTVDDATMIATSFPEFEGLVTGLGGVLE 447 |  |

>TR:A0A286IAM2 A0A286IAM2_9RHIZ 3-phosphoshikimate 1-carboxyvinyltransferase OS=Hoeflea halophila OX=714899 GN=aroA PE=3 SV=1

Length=452

Score = 637 bits (1643), Expect = 0.0

| Identities | = 323/441 (73%), Positives = 372/441 (84%), Gaps = 1/441 (0%) |  |
| --- | --- | --- |
| Query 8 | RPATARKSSGLSGTVRIPGDKSISHRSFMFGGLASGETRITGLLEGEDVINTGKAMQAMG | 67 |
| Sbjct 9 | RPA A KS+ L G + +PGDKS+SHR+ M GGLA+GETRITGLLEGEDV+ T +AMQAMG RPAKASKSAALKGDLAVPGDKSMSHRALMLGGLAAGETRITGLLEGEDVLATARAMQAMG | 68 |
| Query 68 | ARIRKEGDTWIIDGVGNGGLLAPEAPLDFGNAATGCRLTMGLVGVYDFDSTFIGDASLTK | 127 |
| Sbjct 69 | A IRK+GD++II+G GNG LLAPEA LDFGN+ TGCRL MGLV YDF++ FIGDASL AAIRKDGDSFIINGTGNGCLLAPEAALDFGNSGTGCRLIMGLVAPYDFETRFIGDASLCS | 128 |
| Query 128 | RPMGRVLNPLREMGVQVKSEDGDRLPVTLRGPKTPTPITYRVPMASAQVKSAVLLAGLNT | 187 |
| Sbjct 129 | RPM RVL+PLR MGVQV++E GDRLPVTLRGP+TP PI++RVP+ASAQVKSA+LLAGLNT RPMARVLDPLRLMGVQVQAEAGDRLPVTLRGPRTPAPISFRVPVASAQVKSAILLAGLNT | 188 |
| Query 188 | PGITTVIEPIMTRDHTEKMLQGFGANLTVETDADGVRTIRLEGRGKLTGQVIDVPGDPSS | 247 |
| Sbjct 189 | PG+TTVIEP+ TRDH+E+MLQ FGA L ET ADG RTIRLEGRG+LTGQVIDVP DPSS PGLTTVIEPVPTRDHSERMLQAFGAALETETGADGARTIRLEGRGRLTGQVIDVPCDPSS | 248 |
| Query 248 | TAFPLVAALLVPGSDVTILNVLMNPTRTGLILTLQEMGADIEVINPRLAGGEDVADLRVR | 307 |
| Sbjct 249 | AFPLVAAL+V GSD+T+ NVLMNP RTG+ILTLQ+MGADIE++N R GGEDVADLRVR AAFPLVAALIVQGSDLTLRNVLMNPARTGMILTLQDMGADIEILNRRSTGGEDVADLRVR | 308 |
| Query 308 | SSTLKGVTVPEDRAPSMIDEYPILAVAAAFAEGATVMNGLEELRVKESDRLSAVANGLKL | 367 |

| Sbjct | 309 | +S L GV VP +RA SMIDEYP+LAVAA+FA G TVM GL ELRVKESDRL+ +A GL L  ASELSGVAVPAERAASMIDEYPVLAVAASFARGETVMQGLSELRVKESDRLATMARGLTL | 368 |
| --- | --- | --- | --- |
| Query | 368 | NGVDCDEGETSLVVRGRPDGKGLG-NASGAAVATHLDHRIAMSFLVMGLVSENPVTVDDA | 426 |
| Sbjct | 369 | NGVDC EGE L VRGRPDGKGLG A+ VAT LDHRIAMSFLVMGL SE+PV +DD NGVDCTEGEDWLSVRGRPDGKGLGAPAADGIVATRLDHRIAMSFLVMGLASEHPVRIDDC | 428 |
| Query | 427 | TMIATSFPEFMDLMAGLGAKI 447  +IATSFP FM +M G+GA++ |  |
| Sbjct | 429 | ALIATSFPGFMAMMNGIGAEM 449 | |

>TR:A0A0Q6BBA0 A0A0Q6BBA0_9RHIZ 3-phosphoshikimate 1-carboxyvinyltransferase OS=Aureimonas sp. Leaf454 OX=1736381 GN=aroA PE=3 SV=1

Length=450

Score = 636 bits (1640), Expect = 0.0

| Identities | = 326/444 (73%), Positives = 371/444 (84%), Gaps = 6/444 (1%) |  |
| --- | --- | --- |
| Query 7 | SRPATARKSSGLSGTVRIPGDKSISHRSFMFGGLASGETRITGLLEGEDVINTGKAMQAM | 66 |
| Sbjct 7 | +RP TAR+ L G +PGDKSISHR+F+FGG+A+G TR+TGLLEGEDV+ TG+AM+AM ARPMTARRGKALQGRAAVPGDKSISHRAFLFGGIAAGRTRVTGLLEGEDVLATGRAMRAM | 66 |
| Query 67 | GARIRKEGDTWIIDGVGNGGLLAPEAPLDFGNAATGCRLTMGLVGVYDFDSTFIGDASLT | 126 |
| Sbjct 67 | GARI +EG+ WIIDGVGNG LL PE LDFGNA TG RLTMGLVG YDF +TF+GDASL+ GARIEREGEAWIIDGVGNGCLLEPEDVLDFGNAGTGVRLTMGLVGPYDFGATFVGDASLS | 126 |
| Query 127 | KRPMGRVLNPLREMGVQVKSEDGDRLPVTLRGPKTPTPITYRVPMASAQVKSAVLLAGLN | 186 |
| Sbjct 127 | +RPMGRVL+PLREMGVQV GDRLP+++RGP+ PITYRVPMASAQVKSAVLLAGLN RRPMGRVLDPLREMGVQVAGRTGDRLPLSVRGPRVAAPITYRVPMASAQVKSAVLLAGLN | 186 |
| Query 187 | TPGITTVIEPIMTRDHTEKMLQGFGANLTVETDADGVRTIRLEGRGKLTGQVID--VPGD | 244 |
| Sbjct 187 | PG+TTVIEP+MTRDHTEKML GFGA++ VETD GVRTIRLEG+G+L G V D VPGD APGVTTVIEPVMTRDHTEKMLDGFGASVGVETDGAGVRTIRLEGQGRLVG-VPDFVVPGD | 245 |
| Query 245 | PSSTAFPLVAALLVPGSDVTILNVLMNPTRTGLILTLQEMGADIEVINPRLAGGEDVADL | 304 |
| Sbjct 246 | PSS AF +VAA +VPGSDVTI NVLMNPTRTGL+ TL EMGA IE++N RL+GGE VADL PSSAAFAVVAATIVPGSDVTIENVLMNPTRTGLVQTLLEMGASIEILNERLSGGEAVADL | 305 |
| Query 305 | RVRSSTLKGVTVPEDRAPSMIDEYPILAVAAAFAEGATVMNGLEELRVKESDRLSAVANG | 364 |
| Sbjct 306 | RVR + L GV VPE+RAPSMIDEYP+LAVAAAFA+G TVM GL+ELRVKESDRL+AVA G RVRHAELSGVAVPEERAPSMIDEYPVLAVAAAFAKGTTVMRGLDELRVKESDRLAAVAAG | 365 |
| Query 365 | LKLNGVDCDEGETSLVVRGRPDGKGLGNASGAAVATHLDHRIAMSFLVMGLVSENPVTVD | 424 |
| Sbjct 366 | L NGV +EG L V GRPDG GLG G VATHLDHRIAMSFLV+GL +E PVTVD LAANGVSHEEGHDWLSVTGRPDGAGLG---GGTVATHLDHRIAMSFLVLGLAAEKPVTVD | 422 |
| Query 425  Sbjct 423 | DATMIATSFPEFMDLMAGLGAKIE 448 DATMIATSFPEF+ LM GLGA +E DATMIATSFPEFVGLMGGLGAILE 446 |  |

>TR:A0A175RUY5 A0A175RUY5_9RHIZ 3-phosphoshikimate 1-carboxyvinyltransferase OS=Aureimonas ureilytica OX=401562 GN=aroA PE=3 SV=1

Length=450

Score = 634 bits (1636), Expect = 0.0

Identities = 325/448 (73%), Positives = 375/448 (84%), Gaps = 6/448 (1%)

| Query | 3 | HGASSRPATARKSSGLSGTVRIPGDKSISHRSFMFGGLASGETRITGLLEGEDVINTGKA | 62 |
| --- | --- | --- | --- |
|  |  | H A+ RP +R++ LSG VR+PGDKSISHR+F+FGGLA+G TR+TGLLEGEDVI TG+A |  |
| Sbjct | 4 | HAAAPRPLQSRRTGALSGRVRVPGDKSISHRAFLFGGLAAGRTRVTGLLEGEDVIATGRA | 63 |
| Query | 63 | MQAMGARIRKEGDTWIIDGVGNGGLLAPEAPLDFGNAATGCRLTMGLVGVYDFDSTFIGD | 122 |

| Sbjct | 64 | M+ MGARI +EG+ WIIDGVGNG LL P+ LDFGNA TG RLTMGLVG YDF +TF+GD  MRQMGARIEREGEAWIIDGVGNGALLEPDGVLDFGNAGTGSRLTMGLVGPYDFTTTFVGD | 123 |
| --- | --- | --- | --- |
| Query | 123 | ASLTKRPMGRVLNPLREMGVQVKSEDGDRLPVTLRGPKTPTPITYRVPMASAQVKSAVLL | 182 |
| Sbjct | 124 | ASL++RPMGRVL+PLR+MGVQV + DRLP+++RGP+ PI YRVPMASAQVKSAVLL ASLSRRPMGRVLDPLRQMGVQVLARSKDRLPLSIRGPRVAAPIEYRVPMASAQVKSAVLL | 183 |
| Query | 183 | AGLNTPGITTVIEPIMTRDHTEKMLQGFGANLTVETDADGVRTIRLEGRGKLTGQVID-- | 240 |
| Sbjct | 184 | AGLNTPG+TTVIEPIMTRDHTE+ML GFGA ++VETDA G R IRLEG+G+L G V D AGLNTPGVTTVIEPIMTRDHTERMLDGFGAKVSVETDASGTRIIRLEGQGELKG-VPDFV | 242 |
| Query | 241 | VPGDPSSTAFPLVAALLVPGSDVTILNVLMNPTRTGLILTLQEMGADIEVINPRLAGGED | 300 |
| Sbjct | 243 | VPGDPSS AF +VAALLVPGSDV I N LMNPTRTGLI TL+EMGA IE +NPRL+GGE+ VPGDPSSAAFLVVAALLVPGSDVVIENALMNPTRTGLIETLREMGASIEAVNPRLSGGEE | 302 |
| Query | 301 | VADLRVRSSTLKGVTVPEDRAPSMIDEYPILAVAAAFAEGATVMNGLEELRVKESDRLSA | 360 |
| Sbjct | 303 | VADL VR S L+GVTVP RAPSMIDEYP+LAVAAAFAEG T+M GL+ELRVKESDRL+A VADLHVRHSALRGVTVPASRAPSMIDEYPVLAVAAAFAEGETLMQGLDELRVKESDRLAA | 362 |
| Query | 361 | VANGLKLNGVDCDEGETSLVVRGRPDGKGLGNASGAAVATHLDHRIAMSFLVMGLVSENP | 420 |
| Sbjct | 363 | VA GLK NGV +EGE L V GRPDG GLG G V THLDHRIAMSFL+MGL S++P VAAGLKANGVVHEEGEDWLRVTGRPDGSGLG---GGRVETHLDHRIAMSFLIMGLASQHP | 419 |
| Query  Sbjct | 421  420 | VTVDDATMIATSFPEFMDLMAGLGAKIE 448 V+VDDATMIATSFPEF L+A LG +++ VSVDDATMIATSFPEFEGLIATLGGELQ 447 |  |

>TR:A0A368A190 A0A368A190_9RHIZ 3-phosphoshikimate 1-carboxyvinyltransferase OS=Candidatus Tokpelaia sp. JSC189 OX=2079010 GN=aroA PE=3

SV=1

Length=442

Score = 634 bits (1635), Expect = 0.0

| Identities | = 320/439 (73%), Positives = 370/439 (84%), Gaps = 4/439 (1%) |  |
| --- | --- | --- |
| Query 6 | SSRPATARKSSGLSGTVRIPGDKSISHRSFMFGGLASGETRITGLLEGEDVINTGKAMQA | 65 |
| Sbjct 4 | + + TA +S L G +RIPGDKSISHRSFMFG LASGETRITGLLEGEDV+NTG+AMQA TEKSITAHRSDALKGNIRIPGDKSISHRSFMFGALASGETRITGLLEGEDVLNTGRAMQA | 63 |
| Query 66 | MGARIRKEGDTWIIDGVGNGGLLAPEAPLDFGNAATGCRLTMGLVGVYDFDSTFIGDASL | 125 |
| Sbjct 64 | MGA IRKE TWII G GNG LL PLDFGNA TG RLTMGLVG YD ++TFIGDASL MGALIRKENGTWIIQGTGNGCLLQASQPLDFGNAGTGARLTMGLVGTYDMETTFIGDASL | 123 |
| Query 126 | TKRPMGRVLNPLREMGVQVKSEDGDRLPVTLRGPKTPTPITYRVPMASAQVKSAVLLAGL | 185 |
| Sbjct 124 | +KRPMGR+L+PLR MGVQV ++ GDRLPVTL GP+ PITYRVPMASAQVKSAVLLAGL SKRPMGRILDPLRLMGVQVTAQ-GDRLPVTLHGPRRAAPITYRVPMASAQVKSAVLLAGL | 182 |
| Query 186 | NTPGITTVIEPIMTRDHTEKMLQGFGANLTVETDADGVRTIRLEGRGKLTGQVIDVPGDP | 245 |
| Sbjct 183 | NTPGITTVIEPIMTRDHTE+ML+GFGA++ VE A G R IRL G+G L GQVIDVPGDP NTPGITTVIEPIMTRDHTERMLRGFGADIEVEKAASGNRIIRLAGQGTLKGQVIDVPGDP | 242 |
| Query 246 | SSTAFPLVAALLVPGSDVTILNVLMNPTRTGLILTLQEMGADIEVINPRLAGGEDVADLR | 305 |
| Sbjct 243 | SS +FPLVAALLVPGSD+TI NVLMNP RTGL LTLQEMGADI ++N ++ GGE+VADLR SSASFPLVAALLVPGSDITIHNVLMNPARTGLFLTLQEMGADITLLNEQITGGENVADLR | 302 |
| Query 306 | VRSSTLKGVTVPEDRAPSMIDEYPILAVAAAFAEGATVMNGLEELRVKESDRLSAVANGL | 365 |
| Sbjct 303 | VR+S LKG++VP +RAPSMIDEYP L++AAAF+EG T+M G+EELRVKESDRL+AVA GL VRTSQLKGISVPVERAPSMIDEYPALSIAAAFSEGKTIMAGIEELRVKESDRLTAVAAGL | 362 |
| Query 366 | KLNGVDCDEGETSLVVRGRPDGKGLGNASGAAVATHLDHRIAMSFLVMGLVSENPVTVDD | 425 |
| Sbjct 363 | + N + C+EG L++ GRPDGKG G G VATHLDHRIAMSFL+MGL +E+ VT+DD  EANNIFCEEGTDYLIIEGRPDGKGCG---GGTVATHLDHRIAMSFLIMGLAAEHGVTIDD | 419 |

Query 426 ATMIATSFPEFMDLMAGLG 444

A+MI+TSFP+F++LM LG Sbjct 420 ASMISTSFPQFINLMTKLG 438

>TR:A0A1R0FC49 A0A1R0FC49_9RHIZ 3-phosphoshikimate 1-carboxyvinyltransferase OS=Bartonella apis OX=1686310 GN=aroA PE=3 SV=1

Length=442

Score = 633 bits (1633), Expect = 0.0

| Identities | = 325/440 (74%), Positives = 367/440 (83%), Gaps = 3/440 (1%) |  |
| --- | --- | --- |
| Query 9 | PATARKSSGLSGTVRIPGDKSISHRSFMFGGLASGETRITGLLEGEDVINTGKAMQAMGA | 68 |
| Sbjct 6 | PAT++KS+ LSG +RIPGDKSISHRS MFG LASGETRI+GLLEG+DVI+T AM+AMGA PATSKKSADLSGHIRIPGDKSISHRSLMFGALASGETRISGLLEGDDVIHTANAMRAMGA | 65 |
| Query 69 | RIRKEGDTWIIDGVGNGGLLAPEAPLDFGNAATGCRLTMGLVGVYDFDSTFIGDASLTKR | 128 |
| Sbjct 66 | I K D ++I G GNG LL PE PLDFGNA TG RLTMGLVG Y + FIGDASL+KR TITKNNDVYVIHGTGNGCLLEPEQPLDFGNAGTGARLTMGLVGTYAMKTRFIGDASLSKR | 125 |
| Query 129 | PMGRVLNPLREMGVQVKSEDGDRLPVTLRGPKTPTPITYRVPMASAQVKSAVLLAGLNTP | 188 |
| Sbjct 126 | PMGR+L+PLR MGVQV++ +GDRLP+TL G TP PITYRVPMASAQVKSAVLLAGLNTP PMGRILDPLRLMGVQVEASEGDRLPLTLWGAVTPNPITYRVPMASAQVKSAVLLAGLNTP | 185 |
| Query 189 | GITTVIEPIMTRDHTEKMLQGFGANLTVETDADGVRTIRLEGRGKLTGQVIDVPGDPSST | 248 |
| Sbjct 186 | GITTVIEP+MTRDHTEKML GFGA+L VETD++GVR I LEG+G+L GQVI VPGDPSS GITTVIEPVMTRDHTEKMLVGFGADLEVETDSEGVRYIHLEGQGELHGQVITVPGDPSSA | 245 |
| Query 249 | AFPLVAALLVPGSDVTILNVLMNPTRTGLILTLQEMGADIEVINPRLAGGEDVADLRVRS | 308 |
| Sbjct 246 | AFP+VAAL+ GSD+TI NVL+NPTRTGLI+TL EMGADI N R GGE+VAD+RVRS AFPIVAALITEGSDITIENVLLNPTRTGLIITLLEMGADISFENKRQTGGEEVADIRVRS | 305 |
| Query 309 | STLKGVTVPEDRAPSMIDEYPILAVAAAFAEGATVMNGLEELRVKESDRLSAVANGLKLN | 368 |
| Sbjct 306 | S LKGV VP++RAPSMIDEYP+LAVAAAFAEG T+M GL ELRVKESDRLSAVANGLK N SQLKGVKVPKERAPSMIDEYPVLAVAAAFAEGKTIMEGLAELRVKESDRLSAVANGLKKN | 365 |
| Query 369 | GVDCDEGETSLVVRGRPDGKGLGNASGAAVATHLDHRIAMSFLVMGLVSENPVTVDDATM | 428 |
| Sbjct 366 | GVDC+EG+ L+V G P K +G G V THLDHRIAMSFLV+GL + PVT+DD M GVDCEEGKDFLIVTGDPSAKNIG---GGTVTTHLDHRIAMSFLVLGLGAGKPVTIDDKRM | 422 |
| Query 429  Sbjct 423 | IATSFPEFMDLMAGLGAKIE 448 IATSFPEFM LMA LGA IE IATSFPEFMGLMAKLGAHIE 442 |  |

>TR:A0A1L3SNH2 A0A1L3SNH2_9RHIZ 3-phosphoshikimate 1-carboxyvinyltransferase OS=Mesorhizobium oceanicum OX=1670800 GN=aroA PE=3 SV=1

Length=451

Score = 632 bits (1630), Expect = 0.0

| Identities | = 324/445 (73%), Positives = 374/445 (84%), Gaps = 4/445 (1%) |  |
| --- | --- | --- |
| Query 5 | ASSRPATARKSSGLSGTVRIPGDKSISHRSFMFGGLASGETRITGLLEGEDVINTGKAMQ | 64 |
| Sbjct 10 | A+ ATAR++ LSGT R+PGDKSISHRS + GGLA+GETR+TGLLEGEDV+ TG AM+ ATPSQATARRAGPLSGTARVPGDKSISHRSILLGGLAAGETRVTGLLEGEDVMRTGDAMR | 69 |
| Query 65 | AMGARIRKEGDTWIIDGVGNGGLLAPEAPLDFGNAATGCRLTMGLVGVYDFDSTFIGDAS | 124 |
| Sbjct 70 | AMGAR+ K GD WI+ GVGNG LL PLDFGNA TG RLTMGLVG Y +TF GDAS AMGARVDKIGDEWIVRGVGNGCLLEAAEPLDFGNAGTGSRLTMGLVGTYHMKTTFTGDAS | 129 |
| Query 125 | LTKRPMGRVLNPLREMGVQV-KSEDGDRLPVTLRGPKTPTPITYRVPMASAQVKSAVLLA | 183 |
| Sbjct 130 | L++RPMGRVL+PLR MGVQV + GDR+P+TL GP+ PI+YRVPMASAQVKSAVLLA  LSRRPMGRVLDPLRLMGVQVLDAAPGDRMPLTLEGPRIAAPISYRVPMASAQVKSAVLLA | 189 |

| Query | 184 | GLNTPGITTVIEPIMTRDHTEKMLQGFGANLTVETDADGVRTIRLEGRGKLTGQVIDVPG | 243 |
| --- | --- | --- | --- |
| Sbjct | 190 | GLNTPG+TTVIEP+MTRDHTE ML+GFGA + VETDA+GVR IRL+G+G L+G+ I VPG GLNTPGVTTVIEPVMTRDHTELMLKGFGAEIEVETDAEGVRHIRLQGQGTLSGRTIQVPG | 249 |
| Query | 244 | DPSSTAFPLVAALLVPGSDVTILNVLMNPTRTGLILTLQEMGADIEVINPRLAGGEDVAD | 303 |
| Sbjct | 250 | DPSS AFPLVAAL+VPGSDVTI NVLMN TRTGLI TL EMGA IE+++ R AGGE+VAD DPSSAAFPLVAALIVPGSDVTIENVLMNRTRTGLIDTLLEMGASIELLDRRDAGGEEVAD | 309 |
| Query | 304 | LRVRSSTLKGVTVPEDRAPSMIDEYPILAVAAAFAEGATVMNGLEELRVKESDRLSAVAN | 363 |
| Sbjct | 310 | LRVRSS LKGVTVP +RAP MIDEYP+LAVAA FAEG T M+GLEELRVKESDRL+A A LRVRSSELKGVTVPPERAPFMIDEYPVLAVAACFAEGETRMDGLEELRVKESDRLAAAAR | 369 |
| Query | 364 | GLKLNGVDCDEGETSLVVRGRPDGKGLGNASGAAVATHLDHRIAMSFLVMGLVSENPVTV | 423 |
| Sbjct | 370 | GL+ NG DC+EG+ +LVVRGRP GKGLG G V T+LDHRIAMSFLVMGL +E PVT+ GLEANGADCEEGKDTLVVRGRPGGKGLG---GGMVETYLDHRIAMSFLVMGLAAEKPVTI | 426 |
| Query  Sbjct | 424  427 | DDATMIATSFPEFMDLMAGLGAKIE 448 DD+ MIATSFPEF+D+MAGLGA+I+ DDSAMIATSFPEFLDMMAGLGAEID 451 |  |

>TR:A0A5B8KX25 A0A5B8KX25_9RHIZ 3-phosphoshikimate 1-carboxyvinyltransferase OS=Nitratireductor sp. SY7 OX=2599600 GN=aroA PE=3 SV=1

Length=446

Score = 630 bits (1624), Expect = 0.0

| Identities | = 325/444 (73%), Positives = 367/444 (83%), Gaps = 4/444 (1%) |  |
| --- | --- | --- |
| Query 5 | ASSRPATARKSSGLSGTVRIPGDKSISHRSFMFGGLASGETRITGLLEGEDVINTGKAMQ | 64 |
| Sbjct 5 | A PATA KS L+G VR+PGDKSISHRS M GGLASGETRITGLLEGEDV+ TG AM+ AKPMPATASKSPALAGQVRVPGDKSISHRSLMLGGLASGETRITGLLEGEDVLRTGDAMR | 64 |
| Query 65 | AMGARIRKEGDTWIIDGVGNGGLLAPEAPLDFGNAATGCRLTMGLVGVYDFDSTFIGDAS | 124 |
| Sbjct 65 | AMGARI ++G WI+ G GNG LL PEAPLDFGNA TG RLTMGLVG Y+ + FIGDAS AMGARITRQGSEWIVTGTGNGCLLEPEAPLDFGNAGTGSRLTMGLVGPYEMTTRFIGDAS | 124 |
| Query 125 | LTKRPMGRVLNPLREMGVQV-KSEDGDRLPVTLRGPKTPTPITYRVPMASAQVKSAVLLA | 183 |
| Sbjct 125 | L+KRPMGRVL+PLR+MGVQV ++ GDR+P+TLRGP+ PITYRVPM SAQVKSAVLLA LSKRPMGRVLDPLRQMGVQVLEAGAGDRMPLTLRGPRHAAPITYRVPMPSAQVKSAVLLA | 184 |
| Query 184 | GLNTPGITTVIEPIMTRDHTEKMLQGFGANLTVETDADGVRTIRLEGRGKLTGQVIDVPG | 243 |
| Sbjct 185 | GLN G+TTVIEP+ TRDHTEKML GFGA + VET ADG R IR+EG+G+L GQVI VPG GLNARGVTTVIEPVKTRDHTEKMLAGFGAAIEVETPADGERHIRVEGQGRLAGQVIAVPG | 244 |
| Query 244 | DPSSTAFPLVAALLVPGSDVTILNVLMNPTRTGLILTLQEMGADIEVINPRLAGGEDVAD | 303 |
| Sbjct 245 | DPSS AFPLVAAL+VPGSDV I NVLMN +RTGL+ TL EMGADI N R GGE+VAD DPSSAAFPLVAALIVPGSDVVIENVLMNESRTGLVTTLIEMGADISFENRRDTGGEEVAD | 304 |
| Query 304 | LRVRSSTLKGVTVPEDRAPSMIDEYPILAVAAAFAEGATVMNGLEELRVKESDRLSAVAN | 363 |
| Sbjct 305 | LRVR+S L GV VP +RAPSMIDEYP+LAVAAAFA+G T M GL+ELRVKESDRL+AVA LRVRASELAGVVVPPERAPSMIDEYPVLAVAAAFAKGETKMLGLDELRVKESDRLAAVAR | 364 |
| Query 364 | GLKLNGVDCDEGETSLVVRGRPDGKGLGNASGAAVATHLDHRIAMSFLVMGLVSENPVTV | 423 |
| Sbjct 365 | GL+ NGVDC+EG +LVVRGRPDGKG+G G V THLDHRIAM+FLVMGL +E PVT+ GLEANGVDCEEGADTLVVRGRPDGKGIG---GGTVETHLDHRIAMAFLVMGLAAEKPVTI | 421 |
| Query 424  Sbjct 422 | DDATMIATSFPEFMDLMAGLGAKI 447 DD MIATSFPEF+ LM GLGA+I DDRAMIATSFPEFLTLMTGLGAEI 445 |  |

>TR:A0A371WZP3 A0A371WZP3_9RHIZ 3-phosphoshikimate 1-carboxyvinyltransferase OS=Fulvimarina endophytica OX=2293836 GN=aroA PE=3 SV=1

Length=453

Score = 626 bits (1614), Expect = 0.0

| Identities | = 320/442 (72%), Positives = 364/442 (82%), Gaps = 4/442 (1%) |  |
| --- | --- | --- |
| Query 8 | RPATARKSSGLSGTVRIPGDKSISHRSFMFGGLASGETRITGLLEGEDVINTGKAMQAMG | 67 |
| Sbjct 9 | RP TAR +GLSGT R+PGDKSISHR+F+FGGLASG TR+TGLLEGEDV+ TG+AMQAMG RPMTARGGNGLSGTARVPGDKSISHRAFLFGGLASGTTRVTGLLEGEDVLATGRAMQAMG | 68 |
| Query 68 | ARIRKEGDTWIIDGVGNGGLLAPEAPLDFGNAATGCRLTMGLVGVYDFDSTFIGDASLTK | 127 |
| Sbjct 69 | A IRKEGD ++++GVGNG LL PE LDFGNA TG RLTMGLVG Y F +TF+GDASL+K ALIRKEGDVYVVEGVGNGCLLEPEDVLDFGNAGTGARLTMGLVGSYHFGTTFVGDASLSK | 128 |
| Query 128 | RPMGRVLNPLREMGVQVKSEDGDRLPVTLRGPKTPTPITYRVPMASAQVKSAVLLAGLNT | 187 |
| Sbjct 129 | RPMGRVL+PLREMGVQV S RLP++L GPK TPI YRVPMASAQVKSAVLLAGLNT RPMGRVLDPLREMGVQVVSRSKGRLPLSLHGPKAATPIEYRVPMASAQVKSAVLLAGLNT | 188 |
| Query 188 | PGITTVIEPIMTRDHTEKMLQGFGANLTVETDADGVRTIRLEGRGKLTGQV-IDVPGDPS | 246 |
| Sbjct 189 | PGITTV+EP+MTRDHTEKML+GFGA + +ETD DGVR IRLEG+G+L G VPGDPS PGITTVVEPVMTRDHTEKMLEGFGAAIEIETDGDGVRHIRLEGQGELKGLADFQVPGDPS | 248 |
| Query 247 | STAFPLVAALLVPGSDVTILNVLMNPTRTGLILTLQEMGADIEVINPRLAGGEDVADLRV | 306 |
| Sbjct 249 | S AFP+VAAL+VPGSDVTI NVLMNPTRTGL+ TL EMGA IE+I+ R GGEDVADLRV SAAFPIVAALIVPGSDVTIENVLMNPTRTGLVETLLEMGASIEIIDRRTEGGEDVADLRV | 308 |
| Query 307 | RSSTLKGVTVPEDRAPSMIDEYPILAVAAAFAEGATVMNGLEELRVKESDRLSAVANGLK | 366 |
| Sbjct 309 | R S L+G+ VP DRAPSMIDEYP+LA+AAAFA+G TVM GL+ELRVKESDRLSAVA GL RHSELRGIEVPPDRAPSMIDEYPVLAIAAAFAKGRTVMRGLDELRVKESDRLSAVAAGLA | 368 |
| Query 367 | LNGVDCDEGETSLVVRGRPDGKGLGNASGAAVATHLDHRIAMSFLVMGLVSENPVTVDDA | 426 |
| Sbjct 369 | N + +EGE L+V G PDG G G V THLDHRIAMSFLV+GL +++PVTVDD ANNIAHEEGEDWLIVEGDPDGAAYG---GGIVVTHLDHRIAMSFLVLGLAAKSPVTVDDG | 425 |
| Query 427  Sbjct 426 | TMIATSFPEFMDLMAGLGAKIE 448 MIATSFP+F D+M G IE  RMIATSFPDFPDMMRRFGGTIE 447 |  |

>TR:J1K282 J1K282_9RHIZ 3-phosphoshikimate 1-carboxyvinyltransferase OS=Bartonella tamiae Th239 OX=1094558 GN=aroA PE=3 SV=1

Length=442

Score = 624 bits (1610), Expect = 0.0

| Identities | = 327/439 (74%), Positives = 363/439 (83%), Gaps = 3/439 (1%) |  |
| --- | --- | --- |
| Query 10 | ATARKSSGLSGTVRIPGDKSISHRSFMFGGLASGETRITGLLEGEDVINTGKAMQAMGAR | 69 |
| Sbjct 7 | A A KS GL G VRIPGDKSISHR+FMFGGLASG TRITGLLEG+DVI+TGKAMQAMGA+ AIAEKSEGLKGHVRIPGDKSISHRAFMFGGLASGVTRITGLLEGDDVIHTGKAMQAMGAK | 66 |
| Query 70 | IRKEGDTWIIDGVGNGGLLAPEAPLDFGNAATGCRLTMGLVGVYDFDSTFIGDASLTKRP | 129 |
| Sbjct 67 | I KEG+ WII G GNG LL + PLDFGNA TG RLTMGLVG Y ++FIGDASL+KRP IAKEGNVWIIHGTGNGCLLEAQNPLDFGNAGTGSRLTMGLVGTYAMKTSFIGDASLSKRP | 126 |
| Query 130 | MGRVLNPLREMGVQVKSEDGDRLPVTLRGPKTPTPITYRVPMASAQVKSAVLLAGLNTPG | 189 |
| Sbjct 127 | MGR+L+PLR MGVQV++ +GDRLP+TL+GP +PI+YRVPMASAQVKSAVLLAGLNTPG MGRILDPLRLMGVQVEACEGDRLPLTLKGPHMASPISYRVPMASAQVKSAVLLAGLNTPG | 186 |
| Query 190 | ITTVIEPIMTRDHTEKMLQGFGANLTVETDADGVRTIRLEGRGKLTGQVIDVPGDPSSTA | 249 |
| Sbjct 187 | ITTVIEPIMTRDHTEKML GFGA L +ETD +GVR I L+G+G+L GQ+IDVPGDPSS A ITTVIEPIMTRDHTEKMLVGFGAALEIETDKNGVRHIHLQGQGQLIGQIIDVPGDPSSAA | 246 |
| Query 250 | FPLVAALLVPGSDVTILNVLMNPTRTGLILTLQEMGADIEVINPRLAGGEDVADLRVRSS | 309 |
| Sbjct 247 | F LVAAL+V SD+TI NVL+NPTR GLI TL EMGADI IN R GGEDVAD+RVRSS  FALVAALIVEDSDITIENVLLNPTRIGLIDTLLEMGADIHFINQRQTGGEDVADIRVRSS | 306 |

| Query | 310 | TLKGVTVPEDRAPSMIDEYPILAVAAAFAEGATVMNGLEELRVKESDRLSAVANGLKLNG | 369 |
| --- | --- | --- | --- |
| Sbjct | 307 | LKGVTVP++RAPSMIDEYPILAVAAAFAEG T M GLEELRVKESDRLSAVA GLK+NG QLKGVTVPKERAPSMIDEYPILAVAAAFAEGDTHMEGLEELRVKESDRLSAVAQGLKING | 366 |
| Query | 370 | VDCDEGETSLVVRGRPDGKGLGNASGAAVATHLDHRIAMSFLVMGLVSENPVTVDDATMI | 429 |
| Sbjct | 367 | V C EG+ L V G D K LG G V THLDHRIAMSFLV GL S PV +DD MI VSCKEGDDFLTVTGAKDRKILG---GGTVITHLDHRIAMSFLVFGLGSGKPVKIDDKRMI | 423 |
| Query  Sbjct | 430  424 | ATSFPEFMDLMAGLGAKIE 448 ATSFPEF+D+M LGA I+ ATSFPEFIDVMVQLGANIQ 442 |  |

>TR:A0A1X0T2Z3 A0A1X0T2Z3_9RHIZ 3-phosphoshikimate 1-carboxyvinyltransferase OS=Aurantimonas sp. 22II-16-19i OX=1317114 GN=aroA PE=3 SV=1

Length=450

Score = 623 bits (1606), Expect = 0.0

| Identities | | = 319/442 (72%), Positives = 360/442 (81%), Gaps = 4/442 (1%) |  |
| --- | --- | --- | --- |
| Query 8  Sbjct 9 | | RPATARKSSGLSGTVRIPGDKSISHRSFMFGGLASGETRITGLLEGEDVINTGKAMQAMG RP AR+ L+G R+PGDKSISHR+F+FGGLA+G TRI GLLEGEDV+ TG+AM AMG RPLAARREGALAGRARVPGDKSISHRAFLFGGLAAGTTRIRGLLEGEDVLATGRAMAAMG | 67  68 |
| Query 68  Sbjct 69 | | ARIRKEGDTWIIDGVGNGGLLAPEAPLDFGNAATGCRLTMGLVGVYDFDSTFIGDASLTK AR+RK+GD WI+ GVGNG LL P LDFGNA TG RLTMGLVG YDF +TF+GDASL++ ARMRKDGDVWIVGGVGNGALLEPPGVLDFGNAGTGARLTMGLVGPYDFGATFVGDASLSR | 127  128 |
| Query 128  Sbjct 129 | | RPMGRVLNPLREMGVQVKSEDGDRLPVTLRGPKTPTPITYRVPMASAQVKSAVLLAGLNT RPMGRVL+PLR MGVQV + GDRLP+++RGP+ PI YRVPMASAQVKSAVLLAGLN RPMGRVLDPLRLMGVQVVARTGDRLPLSIRGPRVAAPIVYRVPMASAQVKSAVLLAGLNA | 187  188 |
| Query 188  Sbjct 189 | | PGITTVIEPIMTRDHTEKMLQGFGANLTVETDADGVRTIRLEGRGKLTG-QVIDVPGDPS PG TTVIEP+ TRDHTEKMLQGFGA+L V DA+G R I LEG+G L + + VPGDPS PGTTTVIEPVATRDHTEKMLQGFGASLEVTIDAEGARHIALEGQGPLRAVEELTVPGDPS | 246  248 |
| Query 247  Sbjct 249 | | STAFPLVAALLVPGSDVTILNVLMNPTRTGLILTLQEMGADIEVINPRLAGGEDVADLRV S AFPLVAAL+VPGSDV I NVLMNPTRTGLI TL EMGA I+V N R GGEDVADL V SAAFPLVAALIVPGSDVVIENVLMNPTRTGLIETLLEMGASIDVANRRQEGGEDVADLHV | 306  308 |
| Query 307  Sbjct 309 | | RSSTLKGVTVPEDRAPSMIDEYPILAVAAAFAEGATVMNGLEELRVKESDRLSAVANGLK R S L GVTVP +RAPSMIDEYP+LA+AAAFAEG TVM GL+ELRVKESDRLSAVA GL+ RHSRLTGVTVPPERAPSMIDEYPVLAIAAAFAEGTTVMQGLDELRVKESDRLSAVAKGLE | 366  368 |
| Query 367  Sbjct 369 | | LNGVDCDEGETSLVVRGRPDGKGLGNASGAAVATHLDHRIAMSFLVMGLVSENPVTVDDA  +NG+ EGE SL V GRPDG+GLG G V THLDHRIAMSFLV+G+ SE PVTVDDA VNGISHVEGEDSLTVTGRPDGRGLG---GGQVETHLDHRIAMSFLVLGMASEKPVTVDDA | 426  425 |
| Query 427 | | TMIATSFPEFMDLMAGLGAKIE 448 MIATSFPEF +M GLGA +E |  |
| Sbjct | 426 | RMIATSFPEFETMMTGLGAMLE 447 | |

```
>TR:Q1YE73 Q1YE73_AURMS 3-phosphoshikimate 1-carboxyvinyltransferase OS=Aurantimonas manganoxydans (strain ATCC BAA-1229 / DSM 21871
/ SI85-9A1) OX=287752 GN=aroA PE=3 SV=1
Length=482

Score = 618 bits (1594), Expect = 0.0
Identities = 318/454 (70%), Positives = 369/454 (81%), Gaps = 6/454 (1%)

Query 2	LHG--ASSRPATARKSSGLSGTVRIPGDKSISHRSFMFGGLASGETRITGLLEGEDVINT 59
+HG A+ RP AR S+ L+GTV +PGDKSISHR+F+FGG+A+G TR+TGLLEGEDVI T Sbjct 29	MHGSAANPRPIAARHSAALTGTVTVPGDKSISHRAFLFGGIAAGTTRVTGLLEGEDVIAT 88
```

| Query | 60 | GKAMQAMGARIRKEGDTWIIDGVGNGGLLAPEAPLDFGNAATGCRLTMGLVGVYDFDSTF | 119 |
| --- | --- | --- | --- |
| Sbjct | 89 | G AM+AMGA+IRK+G+TW+++GVGNG LL PE +DFGNA TG RLTMGLVG YDF +TF GNAMRAMGAQIRKDGETWVVEGVGNGCLLEPEGIIDFGNAGTGVRLTMGLVGSYDFGATF | 148 |
| Query | 120 | IGDASLTKRPMGRVLNPLREMGVQVKSEDGDRLPVTLRGPKTPTPITYRVPMASAQVKSA | 179 |
| Sbjct | 149 | +GDASL+KRPMGRVL+PLR MG QV + GDRLP++L GP+ PI YRVPMASAQVKSA VGDASLSKRPMGRVLDPLRLMGTQVVARSGDRLPLSLHGPRVAAPIEYRVPMASAQVKSA | 208 |
| Query | 180 | VLLAGLNTPGITTVIEPIMTRDHTEKMLQGFGANLTVETDADGVRTIRLEGRGKLTGQV- | 238 |
| Sbjct | 209 | VLLAGLN PGITTVIEP+MTRDHTEKMLQGFGA L VETDA+GVR IRL+G+G L G VLLAGLNAPGITTVIEPVMTRDHTEKMLQGFGAALEVETDAEGVRHIRLQGQGDLRGVAD | 268 |
| Query | 239 | IDVPGDPSSTAFPLVAALLVPGSDVTILNVLMNPTRTGLILTLQEMGADIEVINPRLAGG | 298 |
| Sbjct | 269 | VPGDPSS FP+VAAL+VPGSDVT+ NVLMNPTR+GL+ TL EMG IE++N R+AGG FTVPGDPSSAGFPVVAALIVPGSDVTVENVLMNPTRSGLVETLIEMGGSIEILNRRVAGG | 328 |
| Query | 299 | EDVADLRVRSSTLKGVTVPEDRAPSMIDEYPILAVAAAFAEGATVMNGLEELRVKESDRL | 358 |
| Sbjct | 329 | EDVADLRVR S L + VP +RAPSMIDEYP+LA+AAAFA G TVM GL+ELRVKESDRL EDVADLRVRHSELTAIEVPAERAPSMIDEYPVLAMAAAFARGTTVMRGLDELRVKESDRL | 388 |
| Query | 359 | SAVANGLKLNGVDCDEGETSLVVRGRPDGKGLGNA-SGAAVATHLDHRIAMSFLVMGLVS | 417 |
| Sbjct | 389 | +AVA GL NG+ +EG L V G PDG+GL A G VATHLDHRIAMSFLV+G+ AAVAAGLAANGIVHEEGADWLTVTGNPDGRGLDGAYGGGTVATHLDHRIAMSFLVLGMAC | 448 |
| Query  Sbjct | 418  449 | ENPVTVDDATMIATSFPEFMDLMAGLGAKIELSD 451 E VTVDD MIATSFP F +MAGLGA +LSD ERAVTVDDGRMIATSFPAFQAMMAGLGA--DLSD 480 |  |

>TR:A0A1W2AM59 A0A1W2AM59_9RHIZ 3-phosphoshikimate 1-carboxyvinyltransferase OS=Fulvimarina manganoxydans OX=937218 GN=aroA PE=3 SV=1

Length=451

Score = 615 bits (1586), Expect = 0.0

| Identities | = 319/444 (72%), Positives = 364/444 (82%), Gaps = 4/444 (1%) |  |
| --- | --- | --- |
| Query 6 | SSRPATARKSSGLSGTVRIPGDKSISHRSFMFGGLASGETRITGLLEGEDVINTGKAMQA | 65 |
| Sbjct 7 | ++RP TA++ + LSGT RIPGDKSISHR+F+FGGLASG TR+TGLLEGEDVI TG+AM A TARPLTAKRIAALSGTARIPGDKSISHRAFLFGGLASGTTRVTGLLEGEDVIATGRAMSA | 66 |
| Query 66 | MGARIRKEGDTWIIDGVGNGGLLAPEAPLDFGNAATGCRLTMGLVGVYDFDSTFIGDASL | 125 |
| Sbjct 67 | MGARI K+GD+++++GVGNG L+ PE LDFGNA TG RLTMGLVG YDF +TF+GDASL MGARIAKDGDSYLVEGVGNGCLMEPETALDFGNAGTGARLTMGLVGPYDFGATFVGDASL | 126 |
| Query 126 | TKRPMGRVLNPLREMGVQVKSEDGDRLPVTLRGPKTPTPITYRVPMASAQVKSAVLLAGL | 185 |
| Sbjct 127 | +KRPMGRVL+PLREMGVQV + DRLP+++ GPK PI YRVPMASAQVKSAVLLA L SKRPMGRVLDPLREMGVQVVARSRDRLPLSIHGPKVAAPIEYRVPMASAQVKSAVLLAAL | 186 |
| Query 186 | NTPGITTVIEPIMTRDHTEKMLQGFGANLTVETDADGVRTIRLEGRGKLTG-QVIDVPGD | 244 |
| Sbjct 187 | NTPGITTV+EPIMTRDHTEKML+GFGA++ VETD DGVR IRLEG+G L G +VPGD NTPGITTVVEPIMTRDHTEKMLEGFGASIEVETDKDGVRHIRLEGQGNLKGVGHFEVPGD | 246 |
| Query 245 | PSSTAFPLVAALLVPGSDVTILNVLMNPTRTGLILTLQEMGADIEVINPRLAGGEDVADL | 304 |
| Sbjct 247 | PSS AFP+VAA++VPGS+VTI NVLMNPTRTGLI TL EMGA IE+ + R +GGEDVADL PSSAAFPIVAAVIVPGSEVTIENVLMNPTRTGLIETLLEMGASIEITHRRKSGGEDVADL | 306 |
| Query 305 | RVRSSTLKGVTVPEDRAPSMIDEYPILAVAAAFAEGATVMNGLEELRVKESDRLSAVANG | 364 |
| Sbjct 307 | VR S L GV VP RAPSMIDEYP LAVAAAFA+G TVM GL+ELRVKESDRL+AVA G IVRHSELAGVEVPASRAPSMIDEYPALAVAAAFAKGQTVMRGLDELRVKESDRLAAVAAG | 366 |
| Query 365 | LKLNGVDCDEGETSLVVRGRPDGKGLGNASGAAVATHLDHRIAMSFLVMGLVSENPVTVD | 424 |
| Sbjct 367 | L NGV +EGE LVV G PDG+G G G V THLDHRIAMSFLV+GL E VTVD  LAANGVAHEEGEDWLVVTGDPDGRGYG---GGTVETHLDHRIAMSFLVLGLACEKGVTVD | 423 |

Query 425 DATMIATSFPEFMDLMAGLGAKIE 448 D MIATSFPEF+ L+ LG IE

Sbjct 424 DGRMIATSFPEFVGLVQALGGSIE 447

>TR:A0A1H0K3L8 A0A1H0K3L8_9RHIZ 3-phosphoshikimate 1-carboxyvinyltransferase OS=Aureimonas jatrophae OX=1166073 GN=aroA PE=3 SV=1

Length=451

Score = 613 bits (1581), Expect = 0.0

| Identities | = 315/448 (70%), Positives = 364/448 (81%), Gaps = 6/448 (1%) |  |
| --- | --- | --- |
| Query 3 | HGASSRPATARKSSGLSGTVRIPGDKSISHRSFMFGGLASGETRITGLLEGEDVINTGKA | 62 |
| Sbjct 5 | H + RP AR++ L GTV +PGDKSISHR+F+FGGLA+G TR+TGLLEGEDV+ TG+A HESPPRPLAARRTGALQGTVTVPGDKSISHRAFLFGGLAAGRTRVTGLLEGEDVLATGRA | 64 |
| Query 63 | MQAMGARIRKEGDTWIIDGVGNGGLLAPEAPLDFGNAATGCRLTMGLVGVYDFDSTFIGD | 122 |
| Sbjct 65 | M+ MGARI ++G+ W+IDGVGNG LL PE LDFGNA TG RLTMGLVG Y+F +TF+GD MRQMGARIERDGEAWVIDGVGNGCLLEPEGVLDFGNAGTGSRLTMGLVGTYNFSATFVGD | 124 |
| Query 123 | ASLTKRPMGRVLNPLREMGVQVKSEDGDRLPVTLRGPKTPTPITYRVPMASAQVKSAVLL | 182 |
| Sbjct 125 | ASL+ RPMGRVL+PLREMG QV + DRLP+TL+GP PI YRVPMASAQVKSAVLL ASLSGRPMGRVLDPLREMGTQVLARSRDRLPLTLQGPPVAAPIRYRVPMASAQVKSAVLL | 184 |
| Query 183 | AGLNTPGITTVIEPIMTRDHTEKMLQGFGANLTVETDADGVRTIRLEGRGKLTGQVID-- | 240 |
| Sbjct 185 | AGLN PGITTV+EP+MTRDHTE+ML+GFGA + V+TDA GVRTIRL G+G+L G V D AGLNAPGITTVVEPVMTRDHTERMLEGFGARVEVDTDAAGVRTIRLHGQGELRG-VPDFV | 243 |
| Query 241 | VPGDPSSTAFPLVAALLVPGSDVTILNVLMNPTRTGLILTLQEMGADIEVINPRLAGGED | 300 |
| Sbjct 244 | VPGDPSS AF +VAAL+ PGSDVTI N LMNPTRTGLI TL+EMGA IE+++PR++GGE+ VPGDPSSAAFLVVAALITPGSDVTIRNALMNPTRTGLIQTLREMGAAIEILDPRVSGGEE | 303 |
| Query 301 | VADLRVRSSTLKGVTVPEDRAPSMIDEYPILAVAAAFAEGATVMNGLEELRVKESDRLSA | 360 |
| Sbjct 304 | VADLRV S L+GV VP RAPSMIDEYP+LAVA+AFA+G T MNGLEELRVKESDRL+A VADLRVHHSALRGVDVPAGRAPSMIDEYPVLAVASAFADGTTRMNGLEELRVKESDRLAA | 363 |
| Query 361 | VANGLKLNGVDCDEGETSLVVRGRPDGKGLGNASGAAVATHLDHRIAMSFLVMGLVSENP | 420 |
| Sbjct 364 | VA GL+ NGV EG L V GRPDG GLG G V THLDHRIAMSFLVMGL +E P VAAGLEANGVRHQEGPDWLTVTGRPDGSGLG---GGRVLTHLDHRIAMSFLVMGLATEKP | 420 |
| Query 421  Sbjct 421 | VTVDDATMIATSFPEFMDLMAGLGAKIE 448 V VDDA MIATSFPEF L+A LG +E VAVDDARMIATSFPEFEGLVATLGGTLE 448 |  |

>TR:A0A0B1PZN0 A0A0B1PZN0_9RHIZ 3-phosphoshikimate 1-carboxyvinyltransferase OS=Aureimonas altamirensis OX=370622 GN=aroA PE=3 SV=1

Length=439

Score = 611 bits (1576), Expect = 0.0

| Identities | = 313/435 (72%), Positives = 360/435 (83%), Gaps = 6/435 (1%) |  |
| --- | --- | --- |
| Query 16 | SGLSGTVRIPGDKSISHRSFMFGGLASGETRITGLLEGEDVINTGKAMQAMGARIRKEGD | 75 |
| Sbjct 7 | S LSGTVR+PGDKSISHR+F+FGGLA+G TR+TGLLEGEDV+ TG+AM+ MGARI ++GD SALSGTVRVPGDKSISHRAFLFGGLAAGRTRVTGLLEGEDVLATGRAMRQMGARIERDGD | 66 |
| Query 76 | TWIIDGVGNGGLLAPEAPLDFGNAATGCRLTMGLVGVYDFDSTFIGDASLTKRPMGRVLN | 135 |
| Sbjct 67 | W+IDGVGNG LL PE LDFGNA TG RLTMGLVG YDF + F GDASL++RPMGRVL+ GWVIDGVGNGALLEPEGLLDFGNAGTGSRLTMGLVGTYDFATCFTGDASLSRRPMGRVLD | 126 |
| Query 136 | PLREMGVQVKSEDGDRLPVTLRGPKTPTPITYRVPMASAQVKSAVLLAGLNTPGITTVIE | 195 |
| Sbjct 127 | PLR+MG QV + GDRLP+ + GP+ PITYRVPMASAQVKSAVLLAGLN PGITTVIE  PLRQMGTQVIARSGDRLPLAIHGPRVAAPITYRVPMASAQVKSAVLLAGLNAPGITTVIE | 186 |

| Query | 196 | PIMTRDHTEKMLQGFGANLTVETDADGVRTIRLEGRGKLTGQVID--VPGDPSSTAFPLV | 253 |
| --- | --- | --- | --- |
| Sbjct | 187 | P+MTRDHTE+MLQGFGA ++VETDA+GVRTIRLEG+G+L+ V D VPGDPSS AF +V PVMTRDHTERMLQGFGATVSVETDAEGVRTIRLEGQGRLSA-VPDFVVPGDPSSAAFLVV | 245 |
| Query | 254 | AALLVPGSDVTILNVLMNPTRTGLILTLQEMGADIEVINPRLAGGEDVADLRVRSSTLKG | 313 |
| Sbjct | 246 | AAL+VPGSDVTI NVLMNPTRTGL+ TL EMG I+++N R +GGE VADLRVR S LKG AALIVPGSDVTIENVLMNPTRTGLVQTLIEMGGAIDILNQRESGGETVADLRVRHSELKG | 305 |
| Query | 314 | VTVPEDRAPSMIDEYPILAVAAAFAEGATVMNGLEELRVKESDRLSAVANGLKLNGVDCD | 373 |
| Sbjct | 306 | + VP +RAPSMIDEYP+LAVAAAFA G T MNGL+ELRVKESDRL+AVA GL NG+ + IDVPAERAPSMIDEYPVLAVAAAFAAGTTRMNGLDELRVKESDRLAAVAAGLAANGIAHE | 365 |
| Query | 374 | EGETSLVVRGRPDGKGLGNASGAAVATHLDHRIAMSFLVMGLVSENPVTVDDATMIATSF | 433 |
| Sbjct | 366 | EG L V GRPDG+G G G VATHLDHRIAMSFLV+GL PVTVDD MIATSF EGRDWLTVTGRPDGQGYG---GGTVATHLDHRIAMSFLVLGLACTEPVTVDDGAMIATSF | 422 |
| Query  Sbjct | 434  423 | PEFMDLMAGLGAKIE 448 PEF+ L+A LG +E PEFVGLVATLGGAVE 437 |  |

>TR:A0A1X7MW68 A0A1X7MW68_9RHIZ 3-phosphoshikimate 1-carboxyvinyltransferase OS=Mesorhizobium australicum OX=536018 GN=aroA PE=3 SV=1

Length=445

Score = 610 bits (1572), Expect = 0.0

| Identities | | = 310/443 (70%), Positives = 362/443 (82%), Gaps = 3/443 (1%) |  |
| --- | --- | --- | --- |
| Query 6  Sbjct 6 | | SSRPATARKSSGLSGTVRIPGDKSISHRSFMFGGLASGETRITGLLEGEDVINTGKAMQA S +PATAR+S L G+VR+PGDKSISHR+ + GGLA+G TRI+GLLEG+DV+ T AM+A SPQPATARRSQALKGSVRVPGDKSISHRALLLGGLAAGTTRISGLLEGDDVLRTAAAMRA | 65  65 |
| Query 66  Sbjct 66 | | MGARIRKEGDTWIIDGVGNGGLLAPEAPLDFGNAATGCRLTMGLVGVYDFDSTFIGDASL GA + WI+ GVGNG LL P PLDFGN+ TG RL MGL GVYD + F GDASL FGASVEGGNGEWILRGVGNGCLLEPREPLDFGNSGTGARLVMGLAGVYDMPTAFKGDASL | 125  125 |
| Query 126  Sbjct 126 | | TKRPMGRVLNPLREMGVQVKSEDGDRLPVTLRGPKTPTPITYRVPMASAQVKSAVLLAGL  +KRPMGRVL+PLR MGVQV++ GDRLP+TLRGPKT PITYRVP+ SAQVKS VLLAGL SKRPMGRVLDPLRLMGVQVEATPGDRLPLTLRGPKTAAPITYRVPVPSAQVKSGVLLAGL | 185  185 |
| Query 186  Sbjct 186 | | NTPGITTVIEPIMTRDHTEKMLQGFGANLTVETDADGVRTIRLEGRGKLTGQVIDVPGDP N PG+TTVIE TRDHTEKML+ FGA+L VETDA G+R IR+ G+G+LTGQ I VPGDP NAPGVTTVIEAAPTRDHTEKMLRAFGASLEVETDAAGLRHIRIGGQGRLTGQAITVPGDP | 245  245 |
| Query 246  Sbjct 246 | | SSTAFPLVAALLVPGSDVTILNVLMNPTRTGLILTLQEMGADIEVINPRLAGGEDVADLR SS AF ++AALLVPGSDV + NV+MNPTRTGLI TL+EMGA IEV+N R+AGGEDVADLR SSAAFVVIAALLVPGSDVLVENVMMNPTRTGLIDTLREMGASIEVLNGRVAGGEDVADLR | 305  305 |
| Query 306  Sbjct 306 | | VRSSTLKGVTVPEDRAPSMIDEYPILAVAAAFAEGATVMNGLEELRVKESDRLSAVANGL VRSS L+GVTVP +RAPSMIDEYP+LAVAAAFAEG T+M GLEELR+KESDRL+AV+ GL VRSSELRGVTVPPERAPSMIDEYPVLAVAAAFAEGETLMQGLEELRIKESDRLAAVSAGL | 365  365 |
| Query 366  Sbjct 366 | | KLNGVDCDEGETSLVVRGRPDGKGLGNASGAAVATHLDHRIAMSFLVMGLVSENPVTVDD K NG DC+EG+ L+VRG P+G+GLG G V T LDHRIAMSFLVMGL +E VTVDD KANGADCEEGKDWLLVRGSPEGRGLG---GGIVETSLDHRIAMSFLVMGLAAEQAVTVDD | 425  422 |
| Query 426 | | ATMIATSFPEFMDLMAGLGAKIE 448 MIATSFP F+D+M +GA+IE |  |
| Sbjct | 423 | TGMIATSFPHFLDVMRSVGAQIE 445 | |

>TR:A0A1T4QWZ5 A0A1T4QWZ5_9RHIZ 3-phosphoshikimate 1-carboxyvinyltransferase OS=Consotaella salsifontis OX=1365950 GN=aroA PE=3 SV=1

Length=454

Score = 604 bits (1558), Expect = 0.0

| Identities | = 313/444 (70%), Positives = 354/444 (80%), Gaps = 4/444 (1%) |  |
| --- | --- | --- |
| Query 6 | SSRPATARKSSGLSGTVRIPGDKSISHRSFMFGGLASGETRITGLLEGEDVINTGKAMQA | 65 |
| Sbjct 7 | +++P AR++ LSG R+PGDKSISHR+ M G LA+G TRITGLLEGEDV+ TG AM+A AAKPIRARRAECLSGAARVPGDKSISHRALMLGALAAGRTRITGLLEGEDVLATGAAMRA | 66 |
| Query 66 | MGARIRKEGDTWIIDGVGNGGLLAPEAPLDFGNAATGCRLTMGLVGVYDFDSTFIGDASL | 125 |
| Sbjct 67 | MGARI ++G W++DGVGNG LL PEAPLDFGNA TG RLTMGLVG YDF +TF+GD SL MGARIARDGAAWLVDGVGNGCLLEPEAPLDFGNAGTGARLTMGLVGAYDFGATFVGDPSL | 126 |
| Query 126 | TKRPMGRVLNPLREMGVQVKSEDGDRLPVTLRGPKTPTPITYRVPMASAQVKSAVLLAGL | 185 |
| Sbjct 127 | +KRPMGRVL+PLR MG QV + GDRLP+TL GP+T PI YR+PMASAQVKSAVLLAGL SKRPMGRVLDPLRLMGCQVAARSGDRLPLTLHGPRTAAPIVYRLPMASAQVKSAVLLAGL | 186 |
| Query 186 | NTPGITTVIEPIMTRDHTEKMLQGFGANLTVETDADGVRTIRLEGRGKLTGQV-IDVPGD | 244 |
| Sbjct 187 | N PG+TTVIEP+ TRDHTEKML GFGA L+VETDA G R I + G+G L G + VPGD NAPGVTTVIEPVATRDHTEKMLIGFGAELSVETDAAGARQIAVVGQGTLGGIADLVVPGD | 246 |
| Query 245 | PSSTAFPLVAALLVPGSDVTILNVLMNPTRTGLILTLQEMGADIEVINPRLAGGEDVADL | 304 |
| Sbjct 247 | PSS AFPLVAAL+VPGSDV I NVL+NPTRTGLI TL EMGA I + N R AGGE+V DL PSSAAFPLVAALIVPGSDVVIENVLLNPTRTGLIETLIEMGASIAIENRRSAGGEEVGDL | 306 |
| Query 305 | RVRSSTLKGVTVPEDRAPSMIDEYPILAVAAAFAEGATVMNGLEELRVKESDRLSAVANG | 364 |
| Sbjct 307 | VR S LKGV VP +RAPSMIDEYP+LAVAAAFAEG T M GL ELRVKESDRL+AVA G HVRHSALKGVNVPAERAPSMIDEYPVLAVAAAFAEGETRMQGLGELRVKESDRLAAVAAG | 366 |
| Query 365 | LKLNGVDCDEGETSLVVRGRPDGKGLGNASGAAVATHLDHRIAMSFLVMGLVSENPVTVD | 424 |
| Sbjct 367 | L NGV GE LVV GRPDGKG+G G VATHLDHRIAMSFLV+GL +E PVTVD LLANGVTHQAGEDWLVVTGRPDGKGMG---GGTVATHLDHRIAMSFLVLGLAAERPVTVD | 423 |
| Query 425  Sbjct 424 | DATMIATSFPEFMDLMAGLGAKIE 448 D MIATSFPEF+ LM LG ++ DGRMIATSFPEFLSLMTALGGTLD 447 |  |

>TR:L0ETY3 L0ETY3_LIBCB 3-phosphoshikimate 1-carboxyvinyltransferase OS=Liberibacter crescens (strain BT-1) OX=1215343 GN=aroA PE=3

SV=1

Length=450

Score = 604 bits (1558), Expect = 0.0

| Identities | = 305/448 (68%), Positives = 358/448 (80%), Gaps = 0/448 (0%) |  |
| --- | --- | --- |
| Query 1 | MLHGASSRPATARKSSGLSGTVRIPGDKSISHRSFMFGGLASGETRITGLLEGEDVINTG | 60 |
| Sbjct 1 | MLH + +SS L GTVR+PGDKSISHR+ + G +ASGET I GLLE +DVI T MLHPTQMNSLRSLRSSSLEGTVRVPGDKSISHRALILGSIASGETIINGLLESDDVIKTK | 60 |
| Query 61 | KAMQAMGARIRKEGDTWIIDGVGNGGLLAPEAPLDFGNAATGCRLTMGLVGVYDFDSTFI | 120 |
| Sbjct 61 | ++M AMGA I K+ + WI+ GVGNG LLAPE + FGN+ TGCRL MGLVGVYDF +TFI ESMVAMGASIEKKDEKWIVRGVGNGCLLAPEHSMYFGNSGTGCRLVMGLVGVYDFSATFI | 120 |
| Query 121 | GDASLTKRPMGRVLNPLREMGVQVKSEDGDRLPVTLRGPKTPTPITYRVPMASAQVKSAV | 180 |
| Sbjct 121 | GD SL+KRPMGR+L+PL +MGVQVK+ + ++LP+TLRGPKTP PI Y VPMASAQVKSA+ GDESLSKRPMGRILDPLHKMGVQVKTFENEKLPITLRGPKTPNPIVYTVPMASAQVKSAL | 180 |
| Query 181 | LLAGLNTPGITTVIEPIMTRDHTEKMLQGFGANLTVETDADGVRTIRLEGRGKLTGQVID | 240 |
| Sbjct 181 | LLAGLNTPGITTVIE ++TRDHTE+MLQ FGANLTVETD + R IRLEGR L GQVID LLAGLNTPGITTVIENVITRDHTERMLQSFGANLTVETDMNSKRCIRLEGRSNLVGQVID | 240 |
| Query 241 | VPGDPSSTAFPLVAALLVPGSDVTILNVLMNPTRTGLILTLQEMGADIEVINPRLAGGED VP DPSS AF + AA+LVPGSD+ ILNVLMN TR G IL LQ+MGADIE+ N RL GGE+ | 300 |

| Sbjct | 241 | VPSDPSSAAFIIAAAVLVPGSDIKILNVLMNSTRIGFILVLQDMGADIEIFNVRLEGGEN | 300 |
| --- | --- | --- | --- |
| Query | 301 | VADLRVRSSTLKGVTVPEDRAPSMIDEYPILAVAAAFAEGATVMNGLEELRVKESDRLSA | 360 |
| Sbjct | 301 | VADL VR S LKG+ V E R MIDEYPILAV AAF+EG T+M GL+ELR KESDRLSA VADLHVRFSKLKGIVVSEQRVALMIDEYPILAVIAAFSEGKTIMKGLQELRFKESDRLSA | 360 |
| Query | 361 | VANGLKLNGVDCDEGETSLVVRGRPDGKGLGNASGAAVATHLDHRIAMSFLVMGLVSENP | 420 |
| Sbjct | 361 | +ANGLKLN VDC+EG++ L+V G +GK LG+ SGA V T LDHRIAMSF++MGLVSE P IANGLKLNNVDCEEGDSYLIVNGVSEGKKLGSLSGALVHTCLDHRIAMSFIIMGLVSEYP | 420 |
| Query  Sbjct | 421  421 | VTVDDATMIATSFPEFMDLMAGLGAKIE 448 V +DD +MI+TSFP F+ +M LGAKIE VAIDDCSMISTSFPNFIGIMKSLGAKIE 448 |  |

>TR:A0A367ZZ01 A0A367ZZ01_9RHIZ 3-phosphoshikimate 1-carboxyvinyltransferase OS=Candidatus Tokpelaia sp. JSC085 OX=2079008 GN=aroA PE=3

SV=1

Length=445

Score = 602 bits (1551), Expect = 0.0

| Identities | = 307/445 (69%), Positives = 350/445 (79%), Gaps = 3/445 (1%) |  |
| --- | --- | --- |
| Query 1 | MLHGASSRPATARKSSGLSGTVRIPGDKSISHRSFMFGGLASGETRITGLLEGEDVINTG | 60 |
| Sbjct 1 | ML + + A +S L G++ +PGDKSISHRSFMFG LASG+T ITGLLEGEDV+ TG MLVSDTKKSIIAFRSHALQGSISVPGDKSISHRSFMFGALASGKTHITGLLEGEDVLRTG | 60 |
| Query 61 | KAMQAMGARIRKEGDTWIIDGVGNGGLLAPEAPLDFGNAATGCRLTMGLVGVYDFDSTFI | 120 |
| Sbjct 61 | KAMQAMGA + K DTWI+ GVGNG LL + PLDFGNA TG RLTMGLVG YD ++ FI KAMQAMGAVLSKAHDTWIVQGVGNGCLLQTDQPLDFGNAGTGARLTMGLVGTYDMETAFI | 120 |
| Query 121 | GDASLTKRPMGRVLNPLREMGVQVKSEDGDRLPVTLRGPKTPTPITYRVPMASAQVKSAV | 180 |
| Sbjct 121 | GD+SL+ RPMGRVL PLR MG QV + GDRLPV LRGP+ PITYRVPMASAQVKS+V GDSSLSNRPMGRVLIPLRLMGTQVTAAKGDRLPVILRGPRVAAPITYRVPMASAQVKSSV | 180 |
| Query 181 | LLAGLNTPGITTVIEPIMTRDHTEKMLQGFGANLTVETDADGVRTIRLEGRGKLTGQVID | 240 |
| Sbjct 181 | LLAG+NTPGITTVIE +TRDHTEKML GFGA + VET A G R IRL G+G L GQ+I LLAGMNTPGITTVIESSITRDHTEKMLHGFGAEIDVETAASGARIIRLVGQGNLKGQIIH | 240 |
| Query 241 | VPGDPSSTAFPLVAALLVPGSDVTILNVLMNPTRTGLILTLQEMGADIEVINPRLAGGED | 300 |
| Sbjct 241 | VPGDPSS AFPLVAAL+VPGSD+ I NVLMNPTRTGL LTLQEMGADI +N R GED VPGDPSSAAFPLVAALIVPGSDIMIHNVLMNPTRTGLFLTLQEMGADITFLNKRKTSGED | 300 |
| Query 301 | VADLRVRSSTLKGVTVPEDRAPSMIDEYPILAVAAAFAEGATVMNGLEELRVKESDRLSA | 360 |
| Sbjct 301 | VADLRVRSS L+G+ VP +R PSMIDEYPIL++AAA AEG T+M G+ ELR KESDRLS+ VADLRVRSSALRGIHVPSERVPSMIDEYPILSIAAALAEGRTIMTGIGELRFKESDRLSS | 360 |
| Query 361 | VANGLKLNGVDCDEGETSLVVRGRPDGKGLGNASGAAVATHLDHRIAMSFLVMGLVSENP | 420 |
| Sbjct 361 | V+NGLK N + C EG SLV+ G P GKG+G G V+T LDHR+AMSFL++GL EN VSNGLKANNIPCTEGSDSLVIEGCPSGKGIG---GGIVSTCLDHRLAMSFLILGLAVENR | 417 |
| Query 421  Sbjct 418 | VTVDDATMIATSFPEFMDLMAGLGA 445 VTVDD TMI TSFP+F LM GLGA VTVDDGTMIRTSFPQFTTLMEGLGA 442 |  |

```
>TR:A0A5B0DSC2 A0A5B0DSC2_9RHIZ 3-phosphoshikimate 1-carboxyvinyltransferase OS=Aureimonas sp. CAU 1482 OX=2565783 GN=aroA PE=3 SV=1
Length=450

Score = 598 bits (1542), Expect = 0.0
Identities = 311/448 (69%), Positives = 355/448 (79%), Gaps = 6/448 (1%) Query 3	HGASSRPATARKSSGLSGTVRIPGDKSISHRSFMFGGLASGETRITGLLEGEDVINTGKA 62

H	RP + ++ L GTVR+PGDKSISHR+F+FG LASG TR+TGLLEGEDV+ TG A
```

| Sbjct | 4 | HAGKPRPVQSARNGALKGTVRMPGDKSISHRAFLFGALASGRTRVTGLLEGEDVLATGSA | 63 |
| --- | --- | --- | --- |
| Query | 63 | MQAMGARIRKEGDTWIIDGVGNGGLLAPEAPLDFGNAATGCRLTMGLVGVYDFDSTFIGD | 122 |
| Sbjct | 64 | M+ MGA I ++G++W+IDGVGNG LL P LDFGNA TG RLTMGLVG YDF S F GD MRQMGAVIERDGESWVIDGVGNGALLEPRNVLDFGNAGTGSRLTMGLVGTYDFTSVFTGD | 123 |
| Query | 123 | ASLTKRPMGRVLNPLREMGVQVKSEDGDRLPVTLRGPKTPTPITYRVPMASAQVKSAVLL | 182 |
| Sbjct | 124 | ASL RPMGRVL+PLR+MG QV + GDRLP+ +RG + PITY VPMASAQVKSAVLL ASLCSRPMGRVLDPLRQMGTQVLACAGDRLPLAIRGARVAAPITYHVPMASAQVKSAVLL | 183 |
| Query | 183 | AGLNTPGITTVIEPIMTRDHTEKMLQGFGANLTVETDADGVRTIRLEGRGKLTGQVID-- | 240 |
| Sbjct | 184 | AGLNTPG+TTV+E +MTRDHTEKML+GFGA++ VE DA GVRTI L G+GKL V D AGLNTPGVTTVVEKVMTRDHTEKMLEGFGASINVEVDAMGVRTISLTGQGKLNA-VSDFV | 242 |
| Query | 241 | VPGDPSSTAFPLVAALLVPGSDVTILNVLMNPTRTGLILTLQEMGADIEVINPRLAGGED | 300 |
| Sbjct | 243 | VPGDPSS AF +VAAL+VP SD+ I N LMNPTRTGLI TL+EMGADIEV+NPRLAGGED VPGDPSSAAFLIVAALIVPDSDIVIENSLMNPTRTGLIETLREMGADIEVLNPRLAGGED | 302 |
| Query | 301 | VADLRVRSSTLKGVTVPEDRAPSMIDEYPILAVAAAFAEGATVMNGLEELRVKESDRLSA | 360 |
| Sbjct | 303 | VADLRVR S LKGV VPE+RAPSMIDEYP+L+VAAAFA G T M GL+ELRVKESDRL+A VADLRVRHSELKGVIVPEERAPSMIDEYPVLSVAAAFATGETRMTGLDELRVKESDRLAA | 362 |
| Query | 361 | VANGLKLNGVDCDEGETSLVVRGRPDGKGLGNASGAAVATHLDHRIAMSFLVMGLVSENP | 420 |
| Sbjct | 363 | VA GL +NGV EG+ L V G P G+G+G G VATHLDHRIAMSFL++GL SE P VAAGLAVNGVPHVEGKDWLTVTGTPGGRGIG---GGRVATHLDHRIAMSFLILGLASEKP | 419 |
| Query  Sbjct | 421  420 | VTVDDATMIATSFPEFMDLMAGLGAKIE 448 V VDD MIATSFPEF L+ LG IE VAVDDDRMIATSFPEFSGLVERLGGHIE 447 |  |

>TR:A0A368A2I4 A0A368A2I4_9RHIZ 3-phosphoshikimate 1-carboxyvinyltransferase OS=Candidatus Tokpelaia sp. JSC188 OX=2079009 GN=aroA PE=3

SV=1

Length=445

Score = 597 bits (1540), Expect = 0.0

| Identities | = 298/440 (68%), Positives = 356/440 (81%), Gaps = 3/440 (1%) |  |
| --- | --- | --- |
| Query 6 | SSRPATARKSSGLSGTVRIPGDKSISHRSFMFGGLASGETRITGLLEGEDVINTGKAMQA | 65 |
| Sbjct 6 | +++P TAR+S+ L G VRIPGDKSISHRSFMFG LASG+T + GLLEGEDV+NTG+AMQA TAKPVTARRSNALKGNVRIPGDKSISHRSFMFGALASGKTYVNGLLEGEDVLNTGRAMQA | 65 |
| Query 66 | MGARIRKEGDTWIIDGVGNGGLLAPEAPLDFGNAATGCRLTMGLVGVYDFDSTFIGDASL | 125 |
| Sbjct 66 | MGA I+KE DTW+I G GNG LL PL FGNA TG RLTMGLV YD ++TF+GDASL MGAFIQKEKDTWVIQGTGNGCLLQANQPLYFGNAGTGVRLTMGLVSTYDMETTFVGDASL | 125 |
| Query 126 | TKRPMGRVLNPLREMGVQVKSEDGDRLPVTLRGPKTPTPITYRVPMASAQVKSAVLLAGL | 185 |
| Sbjct 126 | +KRPM R+L PLR MGVQVK+ G RLP+TL GP+ P+ YRVPMASAQVKSAVLLAGL SKRPMERILEPLRLMGVQVKAAQGSRLPITLHGPRVAAPLIYRVPMASAQVKSAVLLAGL | 185 |
| Query 186 | NTPGITTVIEPIMTRDHTEKMLQGFGANLTVETDADGVRTIRLEGRGKLTGQVIDVPGDP | 245 |
| Sbjct 186 | NTPGITTVIE +MTRDHTE+ML FGA++ VET G R IRL G+ L GQVIDVPGDP NTPGITTVIESVMTRDHTERMLYSFGADIEVETPISGERIIRLVGQKSLKGQVIDVPGDP | 245 |
| Query 246 | SSTAFPLVAALLVPGSDVTILNVLMNPTRTGLILTLQEMGADIEVINPRLAGGEDVADLR | 305 |
| Sbjct 246 | SS +F LVAALL+P SD+ I NVL++P R G+ LTLQEMGADI ++ R+ GGE+VADL SSASFLLVAALLIPESDIVIHNVLVSPARAGIFLTLQEMGADIRFLDERITGGENVADLH | 305 |
| Query 306 | VRSSTLKGVTVPEDRAPSMIDEYPILAVAAAFAEGATVMNGLEELRVKESDRLSAVANGL | 365 |
| Sbjct 306 | VR+S LKG+ +P +RAPSMIDEYP+L++AAAFAEG T+M G+EELRVKESDR++AVA GL  VRTSYLKGINIPPERAPSMIDEYPVLSIAAAFAEGKTIMTGIEELRVKESDRIAAVAAGL | 365 |

| Query | 366 | KLNGVDCDEGETSLVVRGRPDGKGLGNASGAAVATHLDHRIAMSFLVMGLVSENPVTVDD 425 |
| --- | --- | --- |
| Sbjct | 366 | K N + C EG L++ GRP+GK N+ G V THLDHRIAMSFLVMGL S++ VT+DD KANNIFCTEGIDYLIIEGRPEGK---NSGGGIVTTHLDHRIAMSFLVMGLASQHEVTIDD 422 |
| Query | 426 | ATMIATSFPEFMDLMAGLGA 445 |
| Sbjct | 423 | A+MIATSFP+F++LM LGA  ASMIATSFPQFINLMTELGA 442 |

>TR:Q0G7S3 Q0G7S3_9RHIZ 3-phosphoshikimate 1-carboxyvinyltransferase OS=Fulvimarina pelagi HTCC2506 OX=314231 GN=aroA PE=3 SV=1

Length=429

Score = 594 bits (1532), Expect = 0.0

| Identities | = 306/426 (72%), Positives = 346/426 (81%), Gaps = 4/426 (1%) |  |
| --- | --- | --- |
| Query 24 | IPGDKSISHRSFMFGGLASGETRITGLLEGEDVINTGKAMQAMGARIRKEGDTWIIDGVG | 83 |
| Sbjct 1 | +PGDKSISHR+F+FGGLASG TR+TGLLEGEDV+ TG+AM AMGA IRKEG T+++DGVG MPGDKSISHRAFLFGGLASGTTRVTGLLEGEDVLATGRAMIAMGALIRKEGQTFVVDGVG | 60 |
| Query 84 | NGGLLAPEAPLDFGNAATGCRLTMGLVGVYDFDSTFIGDASLTKRPMGRVLNPLREMGVQ | 143 |
| Sbjct 61 | NG LL PE PLDFGNA TG RLTMGLVG Y F STF+GDASL+KRPMGRVL+PLREMGVQ NGCLLEPEEPLDFGNAGTGARLTMGLVGAYHFGSTFVGDASLSKRPMGRVLDPLREMGVQ | 120 |
| Query 144 | VKSEDGDRLPVTLRGPKTPTPITYRVPMASAQVKSAVLLAGLNTPGITTVIEPIMTRDHT | 203 |
| Sbjct 121 | V + DRLP+++ GPK PI YRVPMASAQVKSAVL AGLNTPG+TTVIEP+MTRDHT VVARSKDRLPLSIHGPKATAPIEYRVPMASAQVKSAVLFAGLNTPGVTTVIEPVMTRDHT | 180 |
| Query 204 | EKMLQGFGANLTVETDADGVRTIRLEGRGKLTG-QVIDVPGDPSSTAFPLVAALLVPGSD | 262 |
| Sbjct 181 | EKML+GFGAN+ VET DG R I L+G+G+L G VPGDPSS AFP+VA L+VPGSD EKMLEGFGANIEVETKPDGSRHIHLQGQGELRGLSDFQVPGDPSSAAFPIVAGLVVPGSD | 240 |
| Query 263 | VTILNVLMNPTRTGLILTLQEMGADIEVINPRLAGGEDVADLRVRSSTLKGVTVPEDRAP | 322 |
| Sbjct 241 | VTI NVLMNPTR GLI TL+EMG IE+++ R GGEDVADLR+R S L V V +RAP VTIENVLMNPTRIGLIETLREMGGSIEILDARTEGGEDVADLRIRHSELTSVEVTAERAP | 300 |
| Query 323 | SMIDEYPILAVAAAFAEGATVMNGLEELRVKESDRLSAVANGLKLNGVDCDEGETSLVVR | 382 |
| Sbjct 301 | SMIDEYP+LAVAAAFA+G TVM GL+ELRVKESDRLSAVA GL N V +EG LVV SMIDEYPVLAVAAAFAKGRTVMRGLDELRVKESDRLSAVAAGLAANNVRHEEGADWLVVE | 360 |
| Query 383 | GRPDGKGLGNASGAAVATHLDHRIAMSFLVMGLVSENPVTVDDATMIATSFPEFMDLMAG | 442 |
| Sbjct 361 | G P+G+G G G +V THLDHRIAMSFLV+GL S PVT+DD MIATSFPEF DLM G GDPEGRGYG---GGSVVTHLDHRIAMSFLVLGLASGKPVTIDDGRMIATSFPEFEDLMKG | 417 |
| Query 443  Sbjct 418 | LGAKIE 448 LG IE LGGMIE 423 |  |

>TR:A0A1K2HYS7 A0A1K2HYS7_9RHIZ 3-phosphoshikimate 1-carboxyvinyltransferase OS=Devosia enhydra OX=665118 GN=aroA PE=3 SV=1

Length=504

Score = 588 bits (1516), Expect = 0.0

Identities = 305/446 (68%), Positives = 355/446 (80%), Gaps = 6/446 (1%)

| Query | 1 | MLHGASS-RPATARKSSGLSGTVRIPGDKSISHRSFMFGGLASGETRITGLLEGEDVINT | 59 |
| --- | --- | --- | --- |
| Sbjct | 56 | M H AS RP + K++ LSG R+PGDKSISHR+FMFG +A GET I GLLEG+DV+ T MSHSASPPRPLRSAKAAPLSGRFRVPGDKSISHRAFMFGAMAEGETLIDGLLEGDDVLAT | 115 |
| Query | 60 | GKAMQAMGARIRKEGDTWIIDGVGNGGLLAPEAPLDFGNAATGCRLTMGLVGVYDFDSTF | 119 |
| Sbjct | 116 | GKAMQA+GA I K GD W + G+G GG L P LDFGNA TG RLTMGLVG Y F + F  GKAMQALGASIEKRGDRWHVHGLGVGGFLEPRHDLDFGNAGTGARLTMGLVGGYGFTTRF | 175 |

| Query | 120 | IGDASLTKRPMGRVLNPLREMGVQVKSEDGDRLPVTLRGPKTPTPITYRVPMASAQVKSA | 179 |
| --- | --- | --- | --- |
| Sbjct | 176 | +GDASL+ RPM RVL+PLR +GV+V ++LP+ LRGPK P PI+YRVPMASAQVKS VGDASLSSRPMKRVLDPLRAIGVEVVEHHDNKLPIALRGPKAPLPISYRVPMASAQVKSC | 235 |
| Query | 180 | VLLAGLNTPGITTVIEPIMTRDHTEKMLQGFGANLTVETDADGVRTIRLEGRGKLTGQVI | 239 |
| Sbjct | 236 | VLLAGL PG T VIEPIMTRDHTEKML+GFGA ++VETD++GVRTIR+EG KLTGQ + VLLAGLVIPGTTVVIEPIMTRDHTEKMLEGFGAEISVETDSEGVRTIRVEGLPKLTGQHV | 295 |
| Query | 240 | DVPGDPSSTAFPLVAALLVPGSDVTILNVLMNPTRTGLILTLQEMGADIEVINPRLAGGE | 299 |
| Sbjct | 296 | VPGDPSS AFP+VA L+VPG+D+ I NVLMNPTRTGLI TLQEMG DIE+I+ R +GGE VVPGDPSSAAFPIVAGLIVPGADIVIENVLMNPTRTGLITTLQEMGGDIELIDQRQSGGE | 355 |
| Query | 300 | DVADLRVRSSTLKGVTVPEDRAPSMIDEYPILAVAAAFAEGATVMNGLEELRVKESDRLS | 359 |
| Sbjct | 356 | DVADLRVR S L+GVTVP +RAPSMIDEYP+LAVAAA+AEG T+M GLEELRVKESDRL+ DVADLRVRHSRLRGVTVPAERAPSMIDEYPVLAVAAAYAEGETLMEGLEELRVKESDRLA | 415 |
| Query | 360 | AVANGLKLNGVDCDEGETSLVVRGRPDGKGLGNASGAAVATHLDHRIAMSFLVMGLVSEN | 419 |
| Sbjct | 416 | AVA GL++NGV C EG SL+VRG G+ G G V THLDHRIAMSFLV+G+ S+ AVARGLEVNGVACVEGPESLLVRG--TGRVAG---GGVVRTHLDHRIAMSFLVLGMASDQ | 470 |
| Query  Sbjct | 420  471 | PVTVDDATMIATSFPEFMDLMAGLGA 445 PVTVDD MIATSFP+F+ M LGA PVTVDDERMIATSFPDFVPGMTRLGA 496 |  |

>TR:A0A433X8C0 A0A433X8C0_9RHIZ 3-phosphoshikimate 1-carboxyvinyltransferase OS=Arsenicitalea aurantiaca OX=1783274 GN=aroA PE=3 SV=1

Length=455

Score = 586 bits (1510), Expect = 0.0

| Identities | = 296/449 (66%), Positives = 349/449 (78%), Gaps = 5/449 (1%) |  |
| --- | --- | --- |
| Query 3 | HGASSRPATARKSSGLSGTVRIPGDKSISHRSFMFGGLASGETRITGLLEGEDVINTGKA | 62 |
| Sbjct 11 | H S RP TA +S GL G +R+PGDKSISHR+FMFG +A GET + GLLEGEDV+ TG+A HATSPRPMTASRSDGLRGRMRVPGDKSISHRAFMFGAMAVGETVVEGLLEGEDVLATGRA | 70 |
| Query 63 | MQAMGARIRKEGDTWIIDGVGNGGLLAPEAPLDFGNAATGCRLTMGLVGVYDFDSTFIGD | 122 |
| Sbjct 71 | MQA+GA I K G++W I+G+G GG L P+ PLDFGNA TG RL MGL G YDF + F+GD MQALGATIEKRGESWHINGLGVGGFLEPQGPLDFGNAGTGVRLAMGLCGTYDFVTRFVGD | 130 |
| Query 123 | ASLTKRPMGRVLNPLREMGVQVKSEDGDRLPVTLRGPKTPTPITYRVPMASAQVKSAVLL | 182 |
| Sbjct 131 | ASL+ RPM RVL+PLR +GV+V + +RLP+ LRGP+TP PI YRVPMASAQVKS VLL ASLSARPMKRVLDPLRAIGVEVVEHNDNRLPIALRGPRTPLPIEYRVPMASAQVKSCVLL | 190 |
| Query 183 | AGLNTPGITTVIEPIMTRDHTEKMLQGFGANLTVETDADGVRTIRLEGRGKLTGQVIDVP | 242 |
| Sbjct 191 | AGL PG TTVIEPIMTRDHTEKML GFGA++ + T+ADG R I + G +L Q + VP AGLVIPGTTTVIEPIMTRDHTEKMLAGFGADIQITTEADGTRIIEVSGLPELKAQAVTVP | 250 |
| Query 243 | GDPSSTAFPLVAALLVPGSDVTILNVLMNPTRTGLILTLQEMGADIEVINPRLAGGEDVA | 302 |
| Sbjct 251 | GDPSS AFP+VA L+VPGSD+ + NVLMNPTRTGL+ TL EMG IE+IN RL+GGE++A GDPSSAAFPIVAGLIVPGSDLIVENVLMNPTRTGLLTTLSEMGGQIEIINRRLSGGEEIA | 310 |
| Query 303 | DLRVRSSTLKGVTVPEDRAPSMIDEYPILAVAAAFAEGATVMNGLEELRVKESDRLSAVA | 362 |
| Sbjct 311 | D+ VR S L GVTVP +RAPSMIDEYP+LAVAAAFAEG TVM L ELRVKESDRL+AVA DVHVRHSALSGVTVPAERAPSMIDEYPVLAVAAAFAEGETVMENLGELRVKESDRLAAVA | 370 |
| Query 363 | NGLKLNGVDCDEGETSLVVRGRPDGKGLGNASGAAVATHLDHRIAMSFLVMGLVSENPVT | 422 |
| Sbjct 371 | GL+ NGVDC E E L+VRGR G G VATHLDHRIAMSFLVMGL S+ PV+ RGLEANGVDCTEEEERLIVRGRGAVPG GGQVATHLDHRIAMSFLVMGLASDRPVS | 425 |
| Query 423  Sbjct 426 | VDDATMIATSFPEFMDLMAGLGAKIELSD 451 VDD TMIATSFP+F+ + LGA+ +D VDDRTMIATSFPQFVPGLGALGARFTEAD 454 |  |

>TR:A0A0F7PN90 A0A0F7PN90_9RHIZ 3-phosphoshikimate 1-carboxyvinyltransferase OS=Hoeflea sp. IMCC20628 OX=1620421 GN=aroA PE=3 SV=1

Length=500

Score = 585 bits (1508), Expect = 0.0

| Identities | = 305/444 (69%), Positives = 352/444 (79%), Gaps = 1/444 (0%) |  |
| --- | --- | --- |
| Query 5 | ASSRPATARKSSGLSGTVRIPGDKSISHRSFMFGGLASGETRITGLLEGEDVINTGKAMQ | 64 |
| Sbjct 54 | A RPA A S+ L G++RIPGD SIS + GGLA GETRITGLLE DV AMQ APLRPARASTSAALRGSIRIPGDASISRHCVILGGLARGETRITGLLESADVRAACTAMQ | 113 |
| Query 65 | AMGARIRKEGDTWIIDGVGNGGLLAPEAPLDFGNAATGCRLTMGLVGVYDFDSTFIGDAS | 124 |
| Sbjct 114 | AMGA IR++G WII GVGNG LLAPEAPLDFG+A T CRL MGLVG YDF++TF+GD + AMGATIRRDGKLWIISGVGNGCLLAPEAPLDFGHAGTVCRLIMGLVGPYDFETTFVGDVT | 173 |
| Query 125 | LTKRPMGRVLNPLREMGVQVKSEDGDRLPVTLRGPKTPTPITYRVPMASAQVKSAVLLAG | 184 |
| Sbjct 174 | L+++ MG VL+PLR++GVQV SE GD LPVTLRGP+ P I+Y VPMASA VKSA+LLAG LSRQSMGSVLDPLRQIGVQVSSEAGDLLPVTLRGPQAPASISYPVPMASAAVKSAILLAG | 233 |
| Query 185 | LNTPGITTVIEPIMTRDHTEKMLQGFGANLTVETDADGVRTIRLEGRGKLTGQVIDVPGD | 244 |
| Sbjct 234 | LNTPGIT+VIE + T DH EKML GFGA L+VET AD VRTIRLEGR L GQ IDVPGD LNTPGITSVIETVPTPDHCEKMLAGFGAELSVETGADAVRTIRLEGRSALYGQTIDVPGD | 293 |
| Query 245 | PSSTAFPLVAALLVPGSDVTILNVLMNPTRTGLILTLQEMGADIEVINPRLAGGEDVADL | 304 |
| Sbjct 294 | PS A+ LVAAL+VPGSD+TI NVLMNP RTGLILTLQEMGADIE + PR GGE+VADL PSLAAYALVAALIVPGSDITIENVLMNPARTGLILTLQEMGADIESLAPRQNGGEEVADL | 353 |
| Query 305 | RVRSSTLKGVTVPEDRAPSMIDEYPILAVAAAFAEGATVMNGLEELRVKESDRLSAVANG | 364 |
| Sbjct 354 | RVR+S L G+TVP +RA SMIDEYP LA+AA+FA G T M GL +L ++ RL+A+A G RVRASQLTGLTVPAERAASMIDEYPALAIAASFASGETRMPGLGDLPARQGARLTALARG | 413 |
| Query 365 | LKLNGVDCDEGETSLVVRGRPDGKGLGN-ASGAAVATHLDHRIAMSFLVMGLVSENPVTV | 423 |
| Sbjct 414 | LKLNGVDC EG L VRGRPDGKGLG +SG AT LDHRIAMSFLVMGL SE+PVT+ LKLNGVDCSEGPDGLSVRGRPDGKGLGAVSSGMITATGLDHRIAMSFLVMGLASEHPVTI | 473 |
| Query 424  Sbjct 474 | DDATMIATSFPEFMDLMAGLGAKI 447 DDA+ IA+ FPEFM +MAG+GA++ DDASSIASHFPEFMTMMAGMGAEM 497 |  |

>SP:Q6G0X3 AROA_BARQU 3-phosphoshikimate 1-carboxyvinyltransferase OS=Bartonella quintana (strain Toulouse) OX=283165 GN=aroA PE=3 SV=1

Length=442

Score = 583 bits (1503), Expect = 0.0

| Identities | = 299/439 (68%), Positives = 350/439 (80%), Gaps = 3/439 (1%) |  |
| --- | --- | --- |
| Query 9 | PATARKSSGLSGTVRIPGDKSISHRSFMFGGLASGETRITGLLEGEDVINTGKAMQAMGA | 68 |
| Sbjct 6 | P TA KS+ LSG ++IPGDKSISHRS + GGLA+GET I GLLE +DV+NT AMQAMGA PITAYKSTCLSGKIKIPGDKSISHRSLILGGLANGETHIHGLLESDDVLNTAAAMQAMGA | 65 |
| Query 69 | RIRKEGDTWIIDGVGNGGLLAPEAPLDFGNAATGCRLTMGLVGVYDFDSTFIGDASLTKR | 128 |
| Sbjct 66 | I K+ D WII G GNG LLA E PL+FGNA TG RL MG+VG Y +TFIGDASL+KR CIIKKADRWIIRGTGNGCLLAAEKPLNFGNAGTGARLVMGMVGPYHMKTTFIGDASLSKR | 125 |
| Query 129 | PMGRVLNPLREMGVQVKSEDGDRLPVTLRGPKTPTPITYRVPMASAQVKSAVLLAGLNTP | 188 |
| Sbjct 126 | PMGR+LNPLR MGV++++ GDRLP+TL GPK PI YR+P+ASAQVKSA+LLAGLNT PMGRILNPLRLMGVEIEATHGDRLPLTLYGPKMANPIRYRIPIASAQVKSAILLAGLNTA | 185 |
| Query 189 | GITTVIEPIMTRDHTEKMLQGFGANLTVETDADGVRTIRLEGRGKLTGQVIDVPGDPSST | 248 |
| Sbjct 186 | G TTVIEPI+TRDHTEKML+ FGA L ++TDA+G R I L G+ LTGQ I +PGDPSS  GTTTVIEPILTRDHTEKMLKAFGAELEIKTDAEGTRFIHLNGQPHLTGQTIHIPGDPSSA | 245 |

| Query | 249 | AFPLVAALLVPGSDVTILNVLMNPTRTGLILTLQEMGADIEVINPRLAGGEDVADLRVRS | 308 |
| --- | --- | --- | --- |
| Sbjct | 246 | AFP++AALLV SD+TI NVL+N +R GLI TL EM A IE++N GGE+VADLRV+S AFPIIAALLVENSDITIENVLINKSRMGLIETLWEMDAKIELLNQHKTGGENVADLRVKS | 305 |
| Query | 309 | STLKGVTVPEDRAPSMIDEYPILAVAAAFAEGATVMNGLEELRVKESDRLSAVANGLKLN | 368 |
| Sbjct | 306 | S LKGVTVP++RAPSMIDEYP LAVAAAFAEG TVM G++ELRVKESDRLSA+A GLK+N SMLKGVTVPKERAPSMIDEYPALAVAAAFAEGKTVMLGIDELRVKESDRLSALAQGLKIN | 365 |
| Query | 369 | GVDCDEGETSLVVRGRPDGKGLGNASGAAVATHLDHRIAMSFLVMGLVSENPVTVDDATM | 428 |
| Sbjct | 366 | VDC+EG+ L+V G+ KGLG G V THLDHRIAMSFL+ GLVSE PVT+DD M HVDCEEGKDFLIVHGKSSAKGLG---GGHVTTHLDHRIAMSFLIFGLVSEKPVTIDDKRM | 422 |
| Query  Sbjct | 429  423 | IATSFPEFMDLMAGLGAKI 447 IATSFPEF+ + LG KI IATSFPEFIPFIQQLGGKI 441 |  |

>TR:A0A369W6D8 A0A369W6D8_9RHIZ 3-phosphoshikimate 1-carboxyvinyltransferase OS=Pelagibacterium sp. XYN52 OX=2282655 GN=aroA PE=3 SV=1

Length=448

Score = 582 bits (1500), Expect = 0.0

| Identities | = 299/445 (67%), Positives = 343/445 (77%), Gaps = 5/445 (1%) |  |
| --- | --- | --- |
| Query 1 | MLHGASSRPATARKSSGLSGTVRIPGDKSISHRSFMFGGLASGETRITGLLEGEDVINTG | 60 |
| Sbjct 1 | M H RP +A KS LSG++ +PGDKSISHR+ MFG +A G TR++GLLEGEDV+ TG MSHSLPPRPRSAHKSGPLSGSIAVPGDKSISHRAMMFGAMALGRTRVSGLLEGEDVLATG | 60 |
| Query 61 | KAMQAMGARIRKEGDTWIIDGVGNGGLLAPEAPLDFGNAATGCRLTMGLVGVYDFDSTFI | 120 |
| Sbjct 61 | +AMQA+GARI K GD W IDGVG GG AP+ LDFGNA TG RL MGLVG Y F + F+ RAMQALGARIEKRGDVWEIDGVGVGGFTAPQTDLDFGNAGTGVRLAMGLVGAYPFTTRFV | 120 |
| Query 121 | GDASLTKRPMGRVLNPLREMGVQVKSEDGDRLPVTLRGPKTPTPITYRVPMASAQVKSAV | 180 |
| Sbjct 121 | GDASLT RPM RVL+PLR +GV+V DRLP+ L+GP P P+ YRVPMASAQVKS V GDASLTARPMKRVLDPLRAIGVEVVEHHDDRLPIALKGPTDPVPVHYRVPMASAQVKSCV | 180 |
| Query 181 | LLAGLNTPGITTVIEPIMTRDHTEKMLQGFGANLTVETDADGVRTIRLEGRGKLTGQVID | 240 |
| Sbjct 181 | LLAGL PG TTVIEP+MTRDHTEKML+GFGA++ +ETDADGVRTI ++G +L Q + LLAGLAIPGTTTVIEPVMTRDHTEKMLEGFGADIHIETDADGVRTITVQGLPELKAQDLV | 240 |
| Query 241 | VPGDPSSTAFPLVAALLVPGSDVTILNVLMNPTRTGLILTLQEMGADIEVINPRLAGGED | 300 |
| Sbjct 241 | VPGDPSS FPLVAAL+VPGSDV I NVLMNPTRTGLI TL EMG DI + N R++GGED VPGDPSSAGFPLVAALIVPGSDVLIENVLMNPTRTGLIDTLIEMGGDITIENRRISGGED | 300 |
| Query 301 | VADLRVRSSTLKGVTVPEDRAPSMIDEYPILAVAAAFAEGATVMNGLEELRVKESDRLSA | 360 |
| Sbjct 301 | +ADLRVR S L+GV VP +RAPSMIDEYP+LAVAAAFA+G T M G+EELRVKE DRLSA IADLRVRHSRLRGVRVPPERAPSMIDEYPVLAVAAAFAQGVTEMPGIEELRVKECDRLSA | 360 |
| Query 361 | VANGLKLNGVDCDEGETSLVVRGRPDGKGLGNASGAAVATHLDHRIAMSFLVMGLVSENP | 420 |
| Sbjct 361 | VA GL+ NGV C+EG L+V G G G VATHLDHRIAMSFLVMG+ S+NP VARGLEANGVACEEGADYLIVTG GARVPGGGTVATHLDHRIAMSFLVMGMASDNP | 415 |
| Query 421  Sbjct 416 | VTVDDATMIATSFPEFMDLMAGLGA 445 VTVDD MIATSFP FM LGA VTVDDDRMIATSFPSFMADFTRLGA 440 |  |

>SP:A9ILL7 AROA_BART1 3-phosphoshikimate 1-carboxyvinyltransferase OS=Bartonella tribocorum (strain CIP 105476 / IBS 506) OX=382640

GN=aroA PE=3 SV=1

Length=442

Score = 577 bits (1486), Expect = 0.0

Identities = 300/439 (68%), Positives = 344/439 (78%), Gaps = 3/439 (1%)

| Query | 9 | PATARKSSGLSGTVRIPGDKSISHRSFMFGGLASGETRITGLLEGEDVINTGKAMQAMGA | 68 |
| --- | --- | --- | --- |
| Sbjct | 6 | P TA KSS LSG +RIPGDKSISHRS + GGLA+GET I GLLE DV+NT AMQAMGA PITAYKSSSLSGKIRIPGDKSISHRSLILGGLANGETYIHGLLESADVLNTASAMQAMGA | 65 |
| Query | 69 | RIRKEGDTWIIDGVGNGGLLAPEAPLDFGNAATGCRLTMGLVGVYDFDSTFIGDASLTKR | 128 |
| Sbjct | 66 | I K+ D WII G GNG LLA + PLDFGNA TG RL MG+VG Y +TFIGDASL+KR CIIKKDDFWIIRGTGNGCLLAAQKPLDFGNAGTGARLVMGMVGPYHMKTTFIGDASLSKR | 125 |
| Query | 129 | PMGRVLNPLREMGVQVKSEDGDRLPVTLRGPKTPTPITYRVPMASAQVKSAVLLAGLNTP | 188 |
| Sbjct | 126 | PMGR+L+PLR MGV++++ GD LP+TL GPK PI Y VP+AS+QVKSA+LLAGLNT PMGRILDPLRLMGVEIEATHGDHLPLTLYGPKMANPIRYHVPIASSQVKSAILLAGLNTA | 185 |
| Query | 189 | GITTVIEPIMTRDHTEKMLQGFGANLTVETDADGVRTIRLEGRGKLTGQVIDVPGDPSST | 248 |
| Sbjct | 186 | GITTVIEP++TRDHTEKML+ FGA L +E D +G R I L G LTGQ I +PGDPSS GITTVIEPVLTRDHTEKMLKAFGATLEIERDKEGARLIHLYGHPHLTGQTIHIPGDPSSA | 245 |
| Query | 249 | AFPLVAALLVPGSDVTILNVLMNPTRTGLILTLQEMGADIEVINPRLAGGEDVADLRVRS | 308 |
| Sbjct | 246 | AF ++AALLV SD+TI NVL+N +R GLI TL EMGA IE +N R GGEDVADLR++S AFLIIAALLVEDSDITIENVLINNSRIGLIETLWEMGAHIEFLNQRQTGGEDVADLRIKS | 305 |
| Query | 309 | STLKGVTVPEDRAPSMIDEYPILAVAAAFAEGATVMNGLEELRVKESDRLSAVANGLKLN | 368 |
| Sbjct | 306 | S LKGVTVP++RAPSMIDEYP LAVAAAFAEG TVM G+EELRVKESDRLSAVA GLK+N SVLKGVTVPKERAPSMIDEYPALAVAAAFAEGKTVMLGIEELRVKESDRLSAVAQGLKIN | 365 |
| Query | 369 | GVDCDEGETSLVVRGRPDGKGLGNASGAAVATHLDHRIAMSFLVMGLVSENPVTVDDATM | 428 |
| Sbjct | 366 | VDC+EG+ LVV G+ KGLG G V+THLDHRIAM FL GLVSE PVT+DD M CVDCEEGQDFLVVYGKGSAKGLG---GGYVSTHLDHRIAMCFLTFGLVSEKPVTIDDQRM | 422 |
| Query  Sbjct | 429  423 | IATSFPEFMDLMAGLGAKI 447 IATSFPEF+ + LG KI IATSFPEFIPFIKQLGGKI 441 |  |

>TR:A0A0U3NA21 A0A0U3NA21_9RHOB 3-phosphoshikimate 1-carboxyvinyltransferase OS=Pannonibacter phragmitetus OX=121719 GN=aroA PE=3 SV=1

Length=451

Score = 575 bits (1482), Expect = 0.0

| Identities | = 302/456 (66%), Positives = 348/456 (76%), Gaps = 7/456 (2%) |  |
| --- | --- | --- |
| Query 1 | MLHGASSRPATARKSSGLSGTVRIPGDKSISHRSFMFGGLASGETRITGLLEGEDVINTG | 60 |
| Sbjct 1 | M H ++ RP +R S L+G +R+PGDKS+SHRS MFG LA G T +TGLLEGEDV+NT MSHASTPRPLRSRAGSALTGRIRVPGDKSVSHRSLMFGALAVGRTTVTGLLEGEDVLNTA | 60 |
| Query 61 | KAMQAMGARIRK-EGDTWIIDGVGNGGLLAPEAPLDFGNAATGCRLTMGLVGVYDFDSTF | 119 |
| Sbjct 61 | AM+A GARI K E T+ IDGVG G LL PE +DFGNA TG RLTMG+ G + +TF GAMRAAGARIHKDENGTYHIDGVGLGSLLEPEDVIDFGNAGTGVRLTMGIFGTHPIAATF | 120 |
| Query 120 | IGDASLTKRPMGRVLNPLREMGVQVKSEDGDRLPVTLRGPKTPTPITYRVPMASAQVKSA | 179 |
| Sbjct 121 | +GDASL++RPMGRVL+PLR+MG QV + GDRLP+++RG P PITYRVPM SAQVKSA VGDASLSRRPMGRVLDPLRQMGTQVVARAGDRLPLSIRGADMPLPITYRVPMPSAQVKSA | 180 |
| Query 180 | VLLAGLNTPGITTVIEPIMTRDHTEKMLQGFGANLTVETDADGVRTIRLEGRGKLTGQVI | 239 |
| Sbjct 181 | VLLAGLNTPG T VIEPI TRDHTEKML GFGA+++V D G R IRL+GR +L Q I VLLAGLNTPGETVVIEPIATRDHTEKMLAGFGADISVTIDEQGARIIRLKGRPELKAQAI | 240 |
| Query 240 | DVPGDPSSTAFPLVAALLVPGSDVTILNVLMNPTRTGLILTLQEMGADIEVINPRLAGGE | 299 |
| Sbjct 241 | DVPGDPSS AFP+VAAL+ PGSDVT+ NVL+N RTGLI TL EMG DI + N R+AGGE DVPGDPSSAAFPIVAALITPGSDVTVENVLLNEHRTGLITTLIEMGGDITIENERMAGGE | 300 |
| Query 300 | DVADLRVRSSTLKGVTVPEDRAPSMIDEYPILAVAAAFAEGATVMNGLEELRVKESDRLS | 359 |
| Sbjct 301 | V D+RVR+S LKGVTVP +RAPSMIDEYP+LAVAAAFAEG T M GLEELRVKESDRL+  RVGDIRVRASKLKGVTVPANRAPSMIDEYPVLAVAAAFAEGDTHMPGLEELRVKESDRLA | 360 |

| Query | 360 | AVANGLKLNGVDCDEGETSLVVRGRPDGKGLGNASGAAVATHLDHRIAMSFLVMGLVSEN 419 |
| --- | --- | --- |
| Sbjct | 361 | AVA GL+ NG+ C EGE L V G G G G V THLDHRIAMSFLV+GL S AVARGLEANGIPCKEGEAELTVTGGASGIG-----GGIVTTHLDHRIAMSFLVLGLASGK 415 |
| Query | 420 | PVTVDDATMIATSFPEFMDLMAGLGAKI-ELSDTKA 454 |
| Sbjct | 416 | VTVDD +IATSFP F L AGLGA+I EL D +A AVTVDDGAVIATSFPTFTSLFAGLGAEISELEDARA 451 |

>TR:A0A1G7ST15 A0A1G7ST15_9RHIZ 3-phosphoshikimate 1-carboxyvinyltransferase OS=Pelagibacterium luteolum OX=440168 GN=aroA PE=3 SV=1

Length=449

Score = 573 bits (1478), Expect = 0.0

| Identities | = 293/445 (66%), Positives = 337/445 (76%), Gaps = 5/445 (1%) |  |
| --- | --- | --- |
| Query 1 | MLHGASSRPATARKSSGLSGTVRIPGDKSISHRSFMFGGLASGETRITGLLEGEDVINTG | 60 |
| Sbjct 1 | M H RP + L GT+R+PGDKSISHR+ MFG +A G T +TGLLEGEDV+ TG MSHALPPRPRASHNGGALKGTIRVPGDKSISHRAMMFGAMALGRTTVTGLLEGEDVLATG | 60 |
| Query 61 | KAMQAMGARIRKEGDTWIIDGVGNGGLLAPEAPLDFGNAATGCRLTMGLVGVYDFDSTFI | 120 |
| Sbjct 61 | +AMQA+GA+I K GD W IDG+G GG LAPE LDFGNA TG RLTMGLVG Y F S F+ RAMQALGAKIEKTGDIWQIDGLGVGGFLAPETDLDFGNAGTGARLTMGLVGAYPFVSRFV | 120 |
| Query 121 | GDASLTKRPMGRVLNPLREMGVQVKSEDGDRLPVTLRGPKTPTPITYRVPMASAQVKSAV | 180 |
| Sbjct 121 | GDASLT RPM RVL+PLR +GV+V +RLP+ L+GP P P+ YRVPMASAQVKS V GDASLTARPMKRVLDPLRAIGVEVVEHHDNRLPIALKGPTDPVPVNYRVPMASAQVKSCV | 180 |
| Query 181 | LLAGLNTPGITTVIEPIMTRDHTEKMLQGFGANLTVETDADGVRTIRLEGRGKLTGQVID | 240 |
| Sbjct 181 | LLAGL PG TTV+EPIMTRDHTEKML GFGA+++++TDADGVRTIR+EG Q + LLAGLVIPGTTTVVEPIMTRDHTEKMLAGFGADISIDTDADGVRTIRVEGLPDFKAQTLI | 240 |
| Query 241 | VPGDPSSTAFPLVAALLVPGSDVTILNVLMNPTRTGLILTLQEMGADIEVINPRLAGGED | 300 |
| Sbjct 241 | VPGDPSS FPLVAAL+VPGS+V I NVLMNPTRTGLI TL EMG DI + N R +GGED VPGDPSSAGFPLVAALIVPGSEVVIENVLMNPTRTGLIETLLEMGGDIAIENRRTSGGED | 300 |
| Query 301 | VADLRVRSSTLKGVTVPEDRAPSMIDEYPILAVAAAFAEGATVMNGLEELRVKESDRLSA | 360 |
| Sbjct 301 | +ADLRV+ S LKGV VP +RAPSMIDEYP+LAVAAAFAEG T M G++ELRVKESDRL+A IADLRVKHSRLKGVRVPLERAPSMIDEYPVLAVAAAFAEGVTEMPGIDELRVKESDRLAA | 360 |
| Query 361 | VANGLKLNGVDCDEGETSLVVRGRPDGKGLGNASGAAVATHLDHRIAMSFLVMGLVSENP | 420 |
| Sbjct 361 | VA GL+ NGV C+EG L V G+ G G +V THLDHRIAMSFLVMG+ S+ P VARGLEANGVACEEGHDYLTVTGKGSVPG GGSVKTHLDHRIAMSFLVMGMASDTP | 415 |
| Query 421  Sbjct 416 | VTVDDATMIATSFPEFMDLMAGLGA 445 V VDD MIATSFP F LGA VNVDDDRMIATSFPSFQSDFVRLGA 440 |  |

>SP:Q6G545 AROA_BARHE 3-phosphoshikimate 1-carboxyvinyltransferase OS=Bartonella henselae (strain ATCC 49882 / DSM 28221 / Houston 1)

OX=283166 GN=aroA PE=3 SV=1

Length=442

Score = 573 bits (1477), Expect = 0.0

Identities = 294/439 (67%), Positives = 346/439 (79%), Gaps = 3/439 (1%)

| Query | 9 | PATARKSSGLSGTVRIPGDKSISHRSFMFGGLASGETRITGLLEGEDVINTGKAMQAMGA | 68 |
| --- | --- | --- | --- |
| Sbjct | 6 | P TA KS+ LSG ++IPGDKSISHRS + GGLASGET I G+LE +DV NT AMQA+GA PMTAYKSTRLSGIIKIPGDKSISHRSLILGGLASGETHIHGILESDDVFNTAAAMQALGA | 65 |
| Query | 69 | RIRKEGDTWIIDGVGNGGLLAPEAPLDFGNAATGCRLTMGLVGVYDFDSTFIGDASLTKR I K+ D WII G GNG LLA + PLDFGNA TG RL MG+VG Y +TFIGDASL+KR | 128 |

| Sbjct | 66 | CIIKKDDLWIIRGTGNGCLLAAQKPLDFGNAGTGARLVMGMVGPYHMKTTFIGDASLSKR | 125 |
| --- | --- | --- | --- |
| Query | 129 | PMGRVLNPLREMGVQVKSEDGDRLPVTLRGPKTPTPITYRVPMASAQVKSAVLLAGLNTP | 188 |
| Sbjct | 126 | PM R+L+PL+ MGV++++ G+ LP+TL GPK PI YR+P+ASAQVKSA+LLAGLNT PMARILDPLQLMGVEIEATHGNYLPLTLYGPKMTNPICYRIPVASAQVKSAILLAGLNTA | 185 |
| Query | 189 | GITTVIEPIMTRDHTEKMLQGFGANLTVETDADGVRTIRLEGRGKLTGQVIDVPGDPSST | 248 |
| Sbjct | 186 | G TTVIEPI+TRDHTEKML+ FGA L +E +A+G R I L G LTGQ I +PGDPSS GTTTVIEPILTRDHTEKMLKAFGAKLEIEKNAEGTRFIHLNGHPHLTGQTIHIPGDPSSA | 245 |
| Query | 249 | AFPLVAALLVPGSDVTILNVLMNPTRTGLILTLQEMGADIEVINPRLAGGEDVADLRVRS | 308 |
| Sbjct | 246 | AFP+VAALL+ SD+TI NVL+N +R GLI TL EMGA IE++N R GGEDVA+LRV+S AFPIVAALLIEDSDITIENVLINNSRMGLIETLWEMGAQIELLNQRQTGGEDVANLRVKS | 305 |
| Query | 309 | STLKGVTVPEDRAPSMIDEYPILAVAAAFAEGATVMNGLEELRVKESDRLSAVANGLKLN | 368 |
| Sbjct | 306 | S LKGVTVP++RAPSMIDEYP LAVAAAFAEG TVM G+EELRVKESDRLS +A GLK+N SVLKGVTVPKERAPSMIDEYPALAVAAAFAEGKTVMLGIEELRVKESDRLSVLAQGLKIN | 365 |
| Query | 369 | GVDCDEGETSLVVRGRPDGKGLGNASGAAVATHLDHRIAMSFLVMGLVSENPVTVDDATM | 428 |
| Sbjct | 366 | VDC+EG+ L+V G+ KGLG G V THLDHRIAMSFL+ GLVSE PVT+DD M HVDCEEGQDFLIVHGKGSAKGLG---GGHVTTHLDHRIAMSFLIFGLVSEKPVTIDDKRM | 422 |
| Query  Sbjct | 429  423 | IATSFPEFMDLMAGLGAKI 447 IATSFPEF+ + LG KI IATSFPEFIPFIQQLGGKI 441 |  |

>TR:G4R9C4 G4R9C4_PELHB 3-phosphoshikimate 1-carboxyvinyltransferase OS=Pelagibacterium halotolerans (strain DSM 22347 / JCM 15775 /

CGMCC 1.7692 / B2) OX=1082931 GN=aroA PE=3 SV=1

Length=449

Score = 572 bits (1473), Expect = 0.0

| Identities | = 297/454 (65%), Positives = 344/454 (76%), Gaps = 5/454 (1%) |  |
| --- | --- | --- |
| Query 1 | MLHGASSRPATARKSSGLSGTVRIPGDKSISHRSFMFGGLASGETRITGLLEGEDVINTG | 60 |
| Sbjct 1 | M H RP + + LSG++R+PGDKSISHR+ MFG +A G T +TGLLEGEDV+ TG MSHALPPRPRASHNAGALSGSIRVPGDKSISHRAMMFGAMALGRTTVTGLLEGEDVLATG | 60 |
| Query 61 | KAMQAMGARIRKEGDTWIIDGVGNGGLLAPEAPLDFGNAATGCRLTMGLVGVYDFDSTFI | 120 |
| Sbjct 61 | +AMQA+GA I K G+TW IDG+G GG LAP+ LDFGNA TG RLTMGLVG Y F + F+ RAMQALGANIEKIGETWQIDGLGVGGFLAPQTDLDFGNAGTGVRLTMGLVGAYPFVTRFV | 120 |
| Query 121 | GDASLTKRPMGRVLNPLREMGVQVKSEDGDRLPVTLRGPKTPTPITYRVPMASAQVKSAV | 180 |
| Sbjct 121 | GDASL+ RPMGRVL+PLR +GV+V +RLP+ L+GP P P+ YRVPMASAQVKS V GDASLSARPMGRVLDPLRAIGVEVVEHHENRLPIALKGPTDPVPVHYRVPMASAQVKSCV | 180 |
| Query 181 | LLAGLNTPGITTVIEPIMTRDHTEKMLQGFGANLTVETDADGVRTIRLEGRGKLTGQVID | 240 |
| Sbjct 181 | LLAGL PG TTVIEPIMTRDHTEKML+GFGA++ + TDADGVRTI + G +L Q + LLAGLVIPGTTTVIEPIMTRDHTEKMLEGFGADIAIATDADGVRTITVAGLPELKAQELI | 240 |
| Query 241 | VPGDPSSTAFPLVAALLVPGSDVTILNVLMNPTRTGLILTLQEMGADIEVINPRLAGGED | 300 |
| Sbjct 241 | VPGDPSS FPLVAAL+VPGS+V I NVLMNPTRTGLI TL EMG DI + N R++GGED VPGDPSSAGFPLVAALIVPGSEVVIENVLMNPTRTGLIDTLIEMGGDIAIENRRVSGGED | 300 |
| Query 301 | VADLRVRSSTLKGVTVPEDRAPSMIDEYPILAVAAAFAEGATVMNGLEELRVKESDRLSA | 360 |
| Sbjct 301 | +ADLRV+ S LKGV+VP RAPSMIDEYP+LAVAA+FA+G T M GLEELRVKESDRL+A IADLRVKHSRLKGVSVPASRAPSMIDEYPVLAVAASFAQGVTHMAGLEELRVKESDRLAA | 360 |
| Query 361 | VANGLKLNGVDCDEGETSLVVRGRPDGKGLGNASGAAVATHLDHRIAMSFLVMGLVSENP | 420 |
| Sbjct 361 | VA GL+ NGV C+EG L V G+ G G VATHLDHRIAMSFLVMGL SE P VARGLEANGVVCEEGADFLTVTGKGAVPG GGTVATHLDHRIAMSFLVMGLASEKP | 415 |
| Query 421 | VTVDDATMIATSFPEFMDLMAGLGAKIELSDTKA 454 |  |

VTVDD MIATSFP F	LGA	S+ A Sbjct 416 VTVDDDRMIATSFPSFEADFTRLGAGFSQSEDAA 449

>TR:F2J6W8 F2J6W8_POLGS 3-phosphoshikimate 1-carboxyvinyltransferase OS=Polymorphum gilvum (strain LMG 25793 / CGMCC 1.9160 / SL003B-26A1)

OX=991905 GN=aroA PE=3 SV=1

Length=451

Score = 570 bits (1469), Expect = 0.0

| Identities | = 293/448 (65%), Positives = 344/448 (77%), Gaps = 6/448 (1%) |  |
| --- | --- | --- |
| Query 1 | MLHGASSRPATARKSSGLSGTVRIPGDKSISHRSFMFGGLASGETRITGLLEGEDVINTG | 60 |
| Sbjct 1 | M H +S RP TAR S L G +R+PGDKS+SHR+ MFG LA G T + GLLE EDV+NT MSHVSSPRPMTARAGSALQGRIRVPGDKSVSHRALMFGALAVGRTTVRGLLESEDVLNTA | 60 |
| Query 61 | KAMQAMGARIRKEGD-TWIIDGVGNGGLLAPEAPLDFGNAATGCRLTMGLVGVYDFDSTF | 119 |
| Sbjct 61 | AM+A+GARI + + + IDG G G LL PE +DFGNA TG RLTMG++G + +TF GAMRAVGARIERSAEGVYAIDGAGLGSLLEPEHVIDFGNAGTGVRLTMGILGTHPIAATF | 120 |
| Query 120 | IGDASLTKRPMGRVLNPLREMGVQVKSEDGDRLPVTLRGPKTPTPITYRVPMASAQVKSA | 179 |
| Sbjct 121 | +GDASL++RPMGRVL+PLR+MG V + GDRLP+++RGP+ P+TYRVPM SAQVKSA VGDASLSRRPMGRVLDPLRQMGTSVIARSGDRLPLSIRGPEVALPLTYRVPMPSAQVKSA | 180 |
| Query 180 | VLLAGLNTPGITTVIEPIMTRDHTEKMLQGFGANLTVETDADGVRTIRLEGRGKLTGQVI | 239 |
| Sbjct 181 | VLLAGLN PG TTV+EP+ TRDHTEKML GFGA+++V D G R IRL+G+ +L Q I VLLAGLNAPGETTVVEPVATRDHTEKMLAGFGADISVTLDDAGARVIRLKGQPELKPQDI | 240 |
| Query 240 | DVPGDPSSTAFPLVAALLVPGSDVTILNVLMNPTRTGLILTLQEMGADIEVINPRLAGGE | 299 |
| Sbjct 241 | DVPGDPSS AFP+VAAL+VPGSDVT+ NVL+N RTGLI TL EMGA+IE++N R AGGE DVPGDPSSAAFPIVAALIVPGSDVTVENVLLNEHRTGLIATLLEMGAEIEILNERSAGGE | 300 |
| Query 300 | DVADLRVRSSTLKGVTVPEDRAPSMIDEYPILAVAAAFAEGATVMNGLEELRVKESDRLS | 359 |
| Sbjct 301 | V D+R RSS LKGVTVP +RAPSMIDEYP+LAVAAAFA+G T M GLEELRVKESDRL+ RVGDIRARSSRLKGVTVPAERAPSMIDEYPVLAVAAAFAQGDTHMPGLEELRVKESDRLA | 360 |
| Query 360 | AVANGLKLNGVDCDEGETSLVVRGRPDGKGLGNASGAAVATHLDHRIAMSFLVMGLVSEN | 419 |
| Sbjct 361 | AVA GL+ NGV C EGE L V G G G G VATHLDHRIAMSFLV+GL E AVARGLEANGVPCREGEAELTVTG GAGRIGGGTVATHLDHRIAMSFLVLGLAGER | 415 |
| Query 420  Sbjct 416 | PVTVDDATMIATSFPEFMDLMAGLGAKI 447 PVTVDD +IATSFP F L AGLGA I PVTVDDGAVIATSFPTFTSLFAGLGADI 443 |  |

>TR:E0MSW5 E0MSW5_9RHOB 3-phosphoshikimate 1-carboxyvinyltransferase OS=Ahrensia sp. R2A130 OX=744979 GN=aroA PE=3 SV=1

Length=453

Score = 566 bits (1458), Expect = 0.0

| Identities | = 296/448 (66%), Positives = 340/448 (76%), Gaps = 10/448 (2%) |  |
| --- | --- | --- |
| Query 3 | HGASSRPATARKSSGLSGTVRIPGDKSISHRSFMFGGLASGETRITGLLEGEDVINTGKA | 62 |
| Sbjct 10 | H +P TA + LSGTV++PGDKSISHRS + G +A G T + LLE +DV+ T A HDHEPQPLTAHSGNALSGTVKVPGDKSISHRSLILGTIADGHTTVESLLEADDVMATKSA | 69 |
| Query 63 | MQAMGARIRKEGDTWIIDGVGNGGLLAPEAPLDFGNAATGCRLTMGLVGVYDFDSTFIGD | 122 |
| Sbjct 70 | MQA+GA I K GDTW IDGVGNG LL P PLDFGNA TG RLTMGLV YDF TFIGD MQALGATIEKRGDTWHIDGVGNGALLEPREPLDFGNAGTGSRLTMGLVAGYDFPVTFIGD | 129 |
| Query 123 | ASLTKRPMGRVLNPLREMGVQVKS---EDG-DRLPVTLRGPKTPTPITYRVPMASAQVKS | 178 |
| Sbjct 130 | SL+ RPMGRVLNPLRE G+QVKS E+G DRLP+T+ G P P+TYRVP+ASAQVKS  PSLSSRPMGRVLNPLRETGLQVKSTTTENGPDRLPLTVHGAPLPVPVTYRVPVASAQVKS | 189 |

| Query | 179 | AVLLAGLNTPGITTVIEPIMTRDHTEKMLQGFGANLTVETDADGVRTIRLEGRGKLTGQV | 238 |
| --- | --- | --- | --- |
| Sbjct | 190 | AVLLAGLN G TTVIEP+ TRDHTE+ML GFGAN++V TD DGVRTI +EG+ L Q AVLLAGLNIAGTTTVIEPVFTRDHTERMLAGFGANVSVTTDDDGVRTITVEGQTPLKPQD | 249 |
| Query | 239 | IDVPGDPSSTAFPLVAALLVPGSDVTILNVLMNPTRTGLILTLQEMGADIEVINPRLAGG | 298 |
| Sbjct | 250 | I VPGDPSS +F +VA L+VPGSDVT+ +VL+NPTRTGL TL EMGA++ + N R GG ITVPGDPSSASFAIVAGLVVPGSDVTVKDVLLNPTRTGLFDTLVEMGANLTIENRRTTGG | 309 |
| Query | 299 | EDVADLRVRSSTLKGVTVPEDRAPSMIDEYPILAVAAAFAEGATVMNGLEELRVKESDRL | 358 |
| Sbjct | 310 | E++ D+RVR STLKGV+VP RAPSMIDEYP+LAVAA+FA+G TVMNGL+ELRVKE DRL EEIGDIRVRHSTLKGVSVPATRAPSMIDEYPVLAVAASFAQGETVMNGLDELRVKECDRL | 369 |
| Query | 359 | SAVANGLKLNGVDCDEGETSLVVRGRPDGKGLGNASGAAVATHLDHRIAMSFLVMGLVSE | 418 |
| Sbjct | 370 | +A A GL+ NGVDC EGE +L VRG P G V THLDHRIAM+FLVMGL SE AATARGLEANGVDCTEGEDTLTVRGGP VVGGGDVETHLDHRIAMAFLVMGLASE | 423 |
| Query | 419 | NPVTVDDATMIATSFPEFMDLMAGLGAK 446 PV VDD MIATSF EF LM LGAK |  |
| Sbjct | 424 | KPVRVDDTFMIATSFREFRPLMESLGAK 451 | |

>TR:A0A1C1YRI8 A0A1C1YRI8_9RHIZ 3-phosphoshikimate 1-carboxyvinyltransferase OS=Hoeflea olei OX=1480615 GN=aroA PE=3 SV=1

Length=452

Score = 561 bits (1447), Expect = 0.0

| Identities | = 284/444 (64%), Positives = 345/444 (78%), Gaps = 1/444 (0%) |  |
| --- | --- | --- |
| Query 6 | SSRPATARKSSGLSGTVRIPGDKSISHRSFMFGGLASGETRITGLLEGEDVINTGKAMQA | 65 |
| Sbjct 7 | S RPATA S GL G +R+PGDKSI H + + G +A GETRITGL E DV T A++A SPRPATASASPGLRGEIRVPGDKSICHSALLIGAVADGETRITGLPETSDVRATQLAVEA | 66 |
| Query 66 | MGARIRKEGDTWIIDGVGNGGLLAPEAPLDFGNAATGCRLTMGLVGVYDFDSTFIGDASL | 125 |
| Sbjct 67 | +GA + +GD I+ G GNG LLAP+APLDFG+A C L+MGL+ YDF STF G A L VGASLLSQGDALIVHGTGNGCLLAPDAPLDFGDAGPACALSMGLLAPYDFPSTFTGGARL | 126 |
| Query 126 | TKRPMGRVLNPLREMGVQVKSEDGDRLPVTLRGPKTPTPITYRVPMASAQVKSAVLLAGL | 185 |
| Sbjct 127 | ++RPM RVL+PLR+MGVQV+++ GD LPVTLRGP+ P PI R+P+ASAQVKSA+LLAGL SRRPMDRVLDPLRQMGVQVEAQAGDLLPVTLRGPRWPAPIGCRLPVASAQVKSALLLAGL | 186 |
| Query 186 | NTPGITTVIEPIMTRDHTEKMLQGFGANLTVETDADGVRTIRLEGRGKLTGQVIDVPGDP | 245 |
| Sbjct 187 | N PGITTV EP+ TRDHTE+ML GFGA L +ET A+G RTIRLEGRG+L GQ + VP DP NIPGITTVTEPVATRDHTERMLAGFGATLDIETAANGGRTIRLEGRGRLRGQTVTVPADP | 246 |
| Query 246 | SSTAFPLVAALLVPGSDVTILNVLMNPTRTGLILTLQEMGADIEVINPRLAGGEDVADLR | 305 |
| Sbjct 247 | S AFPLVAAL+VPGSD+ I NVL+NP R GL+ TLQEMGADIE+++PR + GE++ADLR SLAAFPLVAALIVPGSDILIRNVLINPDRAGLVRTLQEMGADIEILDPRRSTGEEIADLR | 306 |
| Query 306 | VRSSTLKGVTVPEDRAPSMIDEYPILAVAAAFAEGATVMNGLEELRVKESDRLSAVANGL | 365 |
| Sbjct 307 | RSS L GVTVP +RA S++D+YP LA+AA+FA G TVM GL +R ++ +R++A+A GL ARSSDLTGVTVPAERAASLVDDYPALAIAASFASGETVMQGLGAVRAEQGERIAAIAQGL | 366 |
| Query 366 | KLNGVDCDEGETSLVVRGRPDGKGLG-NASGAAVATHLDHRIAMSFLVMGLVSENPVTVD | 424 |
| Sbjct 367 | LNGVDC +GE L VRGRP GKGLG A VAT LDHRIAMSFLVMGL SE+PV VD TLNGVDCSQGEGWLSVRGRPGGKGLGARKRDAVVATRLDHRIAMSFLVMGLASEHPVHVD | 426 |
| Query 425  Sbjct 427 | DATMIATSFPEFMDLMAGLGAKIE 448 D MIAT FP+F+ LM GLGA++E DCAMIATGFPDFIGLMTGLGAEME 450 |  |

>TR:A0A1M6ZG46 A0A1M6ZG46_9RHOB 3-phosphoshikimate 1-carboxyvinyltransferase OS=Labrenzia suaedae OX=735517 GN=aroA PE=3 SV=1

Length=475

Score = 560 bits (1443), Expect = 0.0

| Identities | = 289/456 (63%), Positives = 346/456 (76%), Gaps = 6/456 (1%) |  |
| --- | --- | --- |
| Query 1 | MLHGASSRPATARKSSGLSGTVRIPGDKSISHRSFMFGGLASGETRITGLLEGEDVINTG | 60 |
| Sbjct 25 | M HG+S P TA S L+GT+R+PGDKSISHR+ MFG LA G + + GLLE EDV+ T MSHGSSPTPLTACTSGPLTGTIRVPGDKSISHRALMFGALAVGHSTVKGLLESEDVLATA | 84 |
| Query 61 | KAMQAMGARIRKEGD-TWIIDGVGNGGLLAPEAPLDFGNAATGCRLTMGLVGVYDFDSTF | 119 |
| Sbjct 85 | AM+A+GA+I K+ D T+ IDG+G G LL PE +DFGNA TG RLTMG+ G +D +TF AAMRAVGAKIEKDADGTYHIDGIGLGSLLEPEGVIDFGNAGTGVRLTMGIFGTHDIAATF | 144 |
| Query 120 | IGDASLTKRPMGRVLNPLREMGVQVKSEDGDRLPVTLRGPKTPTPITYRVPMASAQVKSA | 179 |
| Sbjct 145 | +GDASL+ RPMGRVL+PLR+MG V + GDRLP ++RG K P P+TYRVPM SAQVKSA VGDASLSNRPMGRVLDPLRQMGTSVVARSGDRLPASIRGAKQPLPLTYRVPMPSAQVKSA | 204 |
| Query 180 | VLLAGLNTPGITTVIEPIMTRDHTEKMLQGFGANLTVETDADGVRTIRLEGRGKLTGQVI | 239 |
| Sbjct 205 | VLLAGLN PGITTVIEPI TRDHTEKML GFGA ++V + G R I+L+G+ +L Q + VLLAGLNAPGITTVIEPIPTRDHTEKMLAGFGAEISVTLNEAGERVIKLKGQPELKPQDL | 264 |
| Query 240 | DVPGDPSSTAFPLVAALLVPGSDVTILNVLMNPTRTGLILTLQEMGADIEVINPRLAGGE | 299 |
| Sbjct 265 | DVPGDPSS FP+VAAL+VPGSDVTI NVL+N RTGLI TL EMG +IE++N R A GE DVPGDPSSAGFPIVAALIVPGSDVTIENVLLNEHRTGLITTLIEMGGNIEILNRRDASGE | 324 |
| Query 300 | DVADLRVRSSTLKGVTVPEDRAPSMIDEYPILAVAAAFAEGATVMNGLEELRVKESDRLS | 359 |
| Sbjct 325 | +V D+RVR+S LKG+TVP RAPSMIDEYP+LAVAAAFAEG T M GL+ELRVKESDRL+ EVGDIRVRASQLKGITVPAVRAPSMIDEYPVLAVAAAFAEGDTFMPGLDELRVKESDRLA | 384 |
| Query 360 | AVANGLKLNGVDCDEGETSLVVRGRPDGKGLGNASGAAVATHLDHRIAMSFLVMGLVSEN | 419 |
| Sbjct 385 | AVA GL+ NG+ C EGE L V G G N V THLDHRIAMSFLV+G+ + AVARGLEANGIPCIEGEDCLTVTG GANNIGNGTVVTHLDHRIAMSFLVLGMAAHK | 439 |
| Query 420  Sbjct 440 | PVTVDDATMIATSFPEFMDLMAGLGAKIELSDTKAA 455 PVTVDD +IATSFP F L +G+GA I S ++AA PVTVDDGAVIATSFPTFTGLFSGMGADIRNSSSEAA 475 |  |

>TR:A0A4Q9VT28 A0A4Q9VT28_9RHIZ 3-phosphoshikimate 1-carboxyvinyltransferase OS=Siculibacillus lacustris OX=1549641 GN=aroA PE=3 SV=1

Length=447

Score = 560 bits (1442), Expect = 0.0

| Identities | = 290/447 (65%), Positives = 341/447 (76%), Gaps = 5/447 (1%) |  |
| --- | --- | --- |
| Query 1 | MLHGASSRPATARKSSGLSGTVRIPGDKSISHRSFMFGGLASGETRITGLLEGEDVINTG | 60 |
| Sbjct 1 | M H P AR L G +R+PGDKSISHRS MFG + GETRI GLLE EDV+ T MAHALPPSPLAARAGRPLVGRIRVPGDKSISHRSLMFGAVTVGETRIDGLLEAEDVLGTA | 60 |
| Query 61 | KAMQAMGARIRKEGD-TWIIDGVGNGGLLAPEAPLDFGNAATGCRLTMGLVGVYDFDSTF | 119 |
| Sbjct 61 | AM+A+GA + ++GD W + G+G GGLL PE LDFGNA TG RL MGL+G + +TF TAMRALGATVTRDGDGRWRVTGLGVGGLLEPEGVLDFGNAGTGSRLAMGLIGGHAVAATF | 120 |
| Query 120 | IGDASLTKRPMGRVLNPLREMGVQVKSEDGDRLPVTLRGPKTPTPITYRVPMASAQVKSA | 179 |
| Sbjct 121 | +GDASL++RPMGRVL+PLR+MGV V + GDRLP+TL+GP+T PITYRVPM SAQVKSA VGDASLSRRPMGRVLDPLRQMGVAVIARAGDRLPLTLKGPQTALPITYRVPMPSAQVKSA | 180 |
| Query 180 | VLLAGLNTPGITTVIEPIMTRDHTEKMLQGFGANLTVETDADGVRTIRLEGRGKLTGQVI | 239 |
| Sbjct 181 | VLLAGLN PG+TTVIEP+ TRDHTE+ML GFGA+LTVE A G R IRL GR +L Q I VLLAGLNAPGVTTVIEPVATRDHTERMLLGFGADLTVEIGAAGERIIRLTGRPELKPQTI | 240 |
| Query 240 | DVPGDPSSTAFPLVAALLVPGSDVTILNVLMNPTRTGLILTLQEMGADIEVINPRLAGGE | 299 |
| Sbjct 241 | VPGDPSS AF +VA L+VPGSDVTI NVL+NP RTGL TL EMGAD+ + NPR +GGE  VVPGDPSSAAFAVVAGLIVPGSDVTIENVLLNPLRTGLFTTLIEMGADLTIENPRRSGGE | 300 |

| Query | 300 | DVADLRVRSSTLKGVTVPEDRAPSMIDEYPILAVAAAFAEGATVMNGLEELRVKESDRLS | 359 |
| --- | --- | --- | --- |
| Sbjct | 301 | +V D+RVR+S L GV VP +RAPSMIDEYPILAVAAAFA+G TVM GLEELRVKESDRL+ EVGDVRVRASRLTGVVVPPERAPSMIDEYPILAVAAAFAQGPTVMEGLEELRVKESDRLA | 360 |
| Query | 360 | AVANGLKLNGVDCDEGETSLVVRGRPDGKGLGNASGAAVATHLDHRIAMSFLVMGLVSEN | 419 |
| Sbjct | 361 | AVA GL NGV+ EGE+SL V GR G G G V T LDHRIAM+FLV+GL ++ AVARGLAANGVEAREGESSLTVLGR GTGPIGGGTVVTDLDHRIAMAFLVLGLAADR | 416 |
| Query  Sbjct | 420  417 | PVTVDDATMIATSFPEFMDLMAGLGAK 446 PVT+DD+ ++ATSFP F LM GLG + PVTIDDSAVMATSFPSFRSLMEGLGGE 443 |  |

>TR:A0P3S7 A0P3S7_LABAI 3-phosphoshikimate 1-carboxyvinyltransferase OS=Labrenzia aggregata (strain ATCC 25650 / DSM 13394 / JCM 20685

/ NBRC 16684 / NCIMB 2208 / IAM 12614) OX=384765 GN=aroA PE=3 SV=1

Length=451

Score = 560 bits (1442), Expect = 0.0

| Identities | = 291/456 (64%), Positives = 348/456 (76%), Gaps = 6/456 (1%) |  |
| --- | --- | --- |
| Query 1 | MLHGASSRPATARKSSGLSGTVRIPGDKSISHRSFMFGGLASGETRITGLLEGEDVINTG | 60 |
| Sbjct 1 | M H ++ +P T+R S L+GT+R+PGDKSISHRS MFG LA G T + GLLE EDV+ T MSHDSAPKPLTSRLGSPLTGTIRVPGDKSISHRSLMFGALAVGRTTVKGLLESEDVLATA | 60 |
| Query 61 | KAMQAMGARIRK-EGDTWIIDGVGNGGLLAPEAPLDFGNAATGCRLTMGLVGVYDFDSTF | 119 |
| Sbjct 61 | AM+A+GA I K E ++ +DG+G G LL PE +DFGNA TG RLTMG+ G ++ +TF DAMRAVGATITKLEDGSYTVDGIGLGSLLEPEHVIDFGNAGTGVRLTMGIFGSHNIAATF | 120 |
| Query 120 | IGDASLTKRPMGRVLNPLREMGVQVKSEDGDRLPVTLRGPKTPTPITYRVPMASAQVKSA | 179 |
| Sbjct 121 | +GDASL+KRPMGRVLNPLR+MG V + DGDRLP ++RGP+ PITYRVPM SAQVKSA VGDASLSKRPMGRVLNPLRDMGTNVIARDGDRLPASIRGPEQALPITYRVPMPSAQVKSA | 180 |
| Query 180 | VLLAGLNTPGITTVIEPIMTRDHTEKMLQGFGANLTVETDADGVRTIRLEGRGKLTGQVI | 239 |
| Sbjct 181 | VLLAGLN G TTVIEPI TRDHTEKML+GFGA+++V + G R I+L+G+ +L Q I VLLAGLNAAGETTVIEPIPTRDHTEKMLKGFGADISVTLNEAGERVIKLQGQPELKPQDI | 240 |
| Query 240 | DVPGDPSSTAFPLVAALLVPGSDVTILNVLMNPTRTGLILTLQEMGADIEVINPRLAGGE | 299 |
| Sbjct 241 | DVPGDPSS AFPLVAAL+VPGSDV I NVL+N RTGLI TL EMG DI ++N R GGE DVPGDPSSAAFPLVAALIVPGSDVVIENVLLNEHRTGLITTLIEMGGDISILNRRETGGE | 300 |
| Query 300 | DVADLRVRSSTLKGVTVPEDRAPSMIDEYPILAVAAAFAEGATVMNGLEELRVKESDRLS | 359 |
| Sbjct 301 | +V DL V++S LKG+TVP +RAPSMIDEYP+LAVAAAFAEG T M GLEELRVKESDRL+ EVGDLHVKASKLKGITVPAERAPSMIDEYPVLAVAAAFAEGETFMPGLEELRVKESDRLA | 360 |
| Query 360 | AVANGLKLNGVDCDEGETSLVVRGRPDGKGLGNASGAAVATHLDHRIAMSFLVMGLVSEN | 419 |
| Sbjct 361 | AVA GL+ NG+ C E E +L V G DG G G V THLDHRIAMSFLV+G+ + N AVARGLEANGIPCVETEDTLTVTGGADGIG GGMVVTHLDHRIAMSFLVLGMAAHN | 415 |
| Query 420  Sbjct 416 | PVTVDDATMIATSFPEFMDLMAGLGAKIELSDTKAA 455 PVTVDD +IATSFP F L GLGA+I + +AA PVTVDDGAVIATSFPTFTSLFDGLGAQISANTCEAA 451 |  |

>TR:A0A165YZR4 A0A165YZR4_9RHOB 3-phosphoshikimate 1-carboxyvinyltransferase OS=Pseudovibrio axinellae OX=989403 GN=aroA PE=3 SV=1

Length=451

Score = 558 bits (1439), Expect = 0.0

Identities = 290/456 (64%), Positives = 342/456 (75%), Gaps = 6/456 (1%)

Query 1	MLHGASSRPATARKSSGLSGTVRIPGDKSISHRSFMFGGLASGETRITGLLEGEDVINTG 60 M H +S + TA	S L+G +++PGDKSISHRS MFG LA G+T ITGLLE EDVI T

| Sbjct | 1 | MSHNSSPQIVTAHSGSPLNGRIKVPGDKSISHRSLMFGALALGKTSITGLLESEDVIGTA | 60 |
| --- | --- | --- | --- |
| Query | 61 | KAMQAMGAR-IRKEGDTWIIDGVGNGGLLAPEAPLDFGNAATGCRLTMGLVGVYDFDSTF | 119 |
| Sbjct | 61 | AM A+GA+ +R+E DTW +DGVG G LL PEAP+DFGNA TG RL MG+ + T NAMNAVGAKCVRQEDDTWTVDGVGLGSLLEPEAPIDFGNAGTGARLAMGIFASHPIAVTM | 120 |
| Query | 120 | IGDASLTKRPMGRVLNPLREMGVQVKSEDGDRLPVTLRGPKTPTPITYRVPMASAQVKSA | 179 |
| Sbjct | 121 | GDASL+ RPMGRVLNPLREMGVQV + +GDRLP ++RG TP PITYRVPMASAQVKSA TGDASLSGRPMGRVLNPLREMGVQVVARNGDRLPASIRGSDTPIPITYRVPMASAQVKSA | 180 |
| Query | 180 | VLLAGLNTPGITTVIEPIMTRDHTEKMLQGFGANLTVETDADGVRTIRLEGRGKLTGQVI | 239 |
| Sbjct | 181 | VLLAGLNT G TTVIEP+ TRDHTEKML GFGA+LTVE + G R I L+G+ KL Q I VLLAGLNTAGTTTVIEPVATRDHTEKMLVGFGADLTVEVNESGERVITLQGQPKLQPQDI | 240 |
| Query | 240 | DVPGDPSSTAFPLVAALLVPGSDVTILNVLMNPTRTGLILTLQEMGADIEVINPRLAGGE | 299 |
| Sbjct | 241 | VPGDPSS AFP+VAAL+ PGSDVT+ VL+N RTGLI +L EMGADI + N R +GGE TVPGDPSSAAFPIVAALITPGSDVTVEGVLLNEHRTGLITSLLEMGADITISNERESGGE | 300 |
| Query | 300 | DVADLRVRSSTLKGVTVPEDRAPSMIDEYPILAVAAAFAEGATVMNGLEELRVKESDRLS | 359 |
| Sbjct | 301 | + D+R + S LKG+TVP +RAPSMIDEYPIL+VAAAFA+G TVM GLEELRVKESDRL+ KIGDIRAKYSKLKGITVPAERAPSMIDEYPILSVAAAFAQGDTVMLGLEELRVKESDRLA | 360 |
| Query | 360 | AVANGLKLNGVDCDEGETSLVVRGRPDGKGLGNASGAAVATHLDHRIAMSFLVMGLVSEN | 419 |
| Sbjct | 361 | AVA GL+ NG+ C E + SL V G G N G V THLDHRIAMSFL++GL + AVARGLEANGIPCIEKQDSLTVTG GANNIGGGRVTTHLDHRIAMSFLILGLAANE | 415 |
| Query  Sbjct | 420  416 | PVTVDDATMIATSFPEFMDLMAGLGAKIELSDTKAA 455 PV VDDA IATSFP F+ + + LG K+E + + A PVEVDDAAPIATSFPTFLSMFSELGGKLENKNEETA 451 |  |

>TR:A0A0M7A0Y0 A0A0M7A0Y0_9RHOB 3-phosphoshikimate 1-carboxyvinyltransferase OS=Labrenzia alba OX=311410 GN=aroA PE=3 SV=1

Length=468

Score = 557 bits (1436), Expect = 0.0

| Identities | = 291/456 (64%), Positives = 347/456 (76%), Gaps = 6/456 (1%) |  |
| --- | --- | --- |
| Query 1 | MLHGASSRPATARKSSGLSGTVRIPGDKSISHRSFMFGGLASGETRITGLLEGEDVINTG | 60 |
| Sbjct 18 | M H ++ P ++ S L+GT+R+PGDKSISHRS MFG LA G T + GLLE EDV+ T MSHDSAPSPLSSSTGSSLTGTIRVPGDKSISHRSLMFGALAVGRTTVKGLLESEDVLATA | 77 |
| Query 61 | KAMQAMGARIRKEGD-TWIIDGVGNGGLLAPEAPLDFGNAATGCRLTMGLVGVYDFDSTF | 119 |
| Sbjct 78 | AM+A+GA I + D ++ +DG+G G LL PE +DFGNA TG RLTMG+ G ++ +TF DAMRAVGAIIEQLDDGSYSVDGIGLGSLLEPEQVIDFGNAGTGVRLTMGIFGSHNIAATF | 137 |
| Query 120 | IGDASLTKRPMGRVLNPLREMGVQVKSEDGDRLPVTLRGPKTPTPITYRVPMASAQVKSA | 179 |
| Sbjct 138 | +GDASL+KRPMGRVLNPLREMG V + DGDRLP ++RG +T PITYRVPM SAQVKSA VGDASLSKRPMGRVLNPLREMGTNVIARDGDRLPASIRGAETALPITYRVPMPSAQVKSA | 197 |
| Query 180 | VLLAGLNTPGITTVIEPIMTRDHTEKMLQGFGANLTVETDADGVRTIRLEGRGKLTGQVI | 239 |
| Sbjct 198 | VLLAGLN PG TTVIEPI TRDHTEKML+GFGA++TV + +G R I+L+G+ +L Q I VLLAGLNAPGETTVIEPIPTRDHTEKMLKGFGADITVSHNENGERVIKLKGQPELRPQDI | 257 |
| Query 240 | DVPGDPSSTAFPLVAALLVPGSDVTILNVLMNPTRTGLILTLQEMGADIEVINPRLAGGE | 299 |
| Sbjct 258 | DVPGDPSS FPLVAAL+VPGSDV I NVL+N RTGLI TL EMG DIE+IN R GGE DVPGDPSSAGFPLVAALIVPGSDVMIENVLLNEHRTGLITTLVEMGGDIEIINRRETGGE | 317 |
| Query 300 | DVADLRVRSSTLKGVTVPEDRAPSMIDEYPILAVAAAFAEGATVMNGLEELRVKESDRLS | 359 |
| Sbjct 318 | +V DLRV++S LKG+TVP DRAPSMIDEYP+LAVAAAFAEG T M GL+ELRVKESDRL+ EVGDLRVKASRLKGITVPADRAPSMIDEYPVLAVAAAFAEGDTFMPGLDELRVKESDRLA | 377 |
| Query 360 | AVANGLKLNGVDCDEGETSLVVRGRPDGKGLGNASGAAVATHLDHRIAMSFLVMGLVSEN AVA GL+ NGV C E E +L V+G G N G V THLDHRIAM+FLV+G+ S | 419 |

| Sbjct | 378 | AVARGLEANGVPCIETEDTLTVKG-----GADNIGGGTVTTHLDHRIAMAFLVLGMASHR 432 |
| --- | --- | --- |
| Query | 420 | PVTVDDATMIATSFPEFMDLMAGLGAKIELSDTKAA 455 |
| Sbjct | 433 | PVTVDD +IATSFP F L LGA I + ++AA  PVTVDDGAVIATSFPTFTSLFEELGAVISANASEAA 468 |

>TR:A0A0P6VN93 A0A0P6VN93_9RHIZ 3-phosphoshikimate 1-carboxyvinyltransferase OS=Prosthecomicrobium hirschii OX=665126 GN=aroA PE=3 SV=1

Length=450

Score = 557 bits (1435), Expect = 0.0

| Identities | = 290/449 (65%), Positives = 339/449 (76%), Gaps = 6/449 (1%) |  |
| --- | --- | --- |
| Query 1 | MLHGASSRPATARKSSGLSGTVRIPGDKSISHRSFMFGGLASGETRITGLLEGEDVINTG | 60 |
| Sbjct 1 | M H ++ P +A S L+G +R+PGDKSISHRS MFG + GETRI GLLE EDV+ T MAHASAGTPLSALASGPLTGRIRVPGDKSISHRSLMFGAVTVGETRIEGLLEAEDVLGTA | 60 |
| Query 61 | KAMQAMGARIRKEGD-TWIIDGVGNGGLLAPEAPLDFGNAATGCRLTMGLVGVYDFDSTF | 119 |
| Sbjct 61 | AM+A+GA + ++G+ W + G+G GGLL PE+ LDFGNA TG RLTMGLVG + +TF AAMRALGATVTRDGEGLWRVSGLGVGGLLEPESVLDFGNAGTGSRLTMGLVGGHAVAATF | 120 |
| Query 120 | IGDASLTKRPMGRVLNPLREMGVQVKSEDGDRLPVTLRGPKTPTPITYRVPMASAQVKSA | 179 |
| Sbjct 121 | +GDASL++RPMGRVL+PLR MGV+V + GDRLP+TLRGP T PI YRVP+ SAQVKSA VGDASLSRRPMGRVLDPLRRMGVEVIARKGDRLPLTLRGPDTVIPIEYRVPVPSAQVKSA | 180 |
| Query 180 | VLLAGLNTPGITTVIEPIMTRDHTEKMLQGFGANLTVETDADGVRTIRLEGRGKLTGQVI | 239 |
| Sbjct 181 | VLL GLN+PG+TTVIEP+ TRDHTE+ML GFGA L V G R IRL+GR L Q I VLLCGLNSPGVTTVIEPVATRDHTERMLVGFGAALEVTAGPAGERVIRLQGRPSLKPQAI | 240 |
| Query 240 | DVPGDPSSTAFPLVAALLVPGSDVTILNVLMNPTRTGLILTLQEMGADIEVINPRLAGGE | 299 |
| Sbjct 241 | VPGDPSS AF +VA L+VPGSDVT+ NVL+NP RTGL TL EMGAD+ + N R+ GGE TVPGDPSSAAFAIVAGLIVPGSDVTVENVLLNPLRTGLFTTLLEMGADLTIENERVTGGE | 300 |
| Query 300 | DVADLRVRSSTLKGVTVPEDRAPSMIDEYPILAVAAAFAEGATVMNGLEELRVKESDRLS | 359 |
| Sbjct 301 | + D+ VRSS LKGV VP +RAPSMIDEYPIL+VAAAFAEG T M+GLEELRVKESDRLS RIGDVHVRSSRLKGVAVPPERAPSMIDEYPILSVAAAFAEGDTFMDGLEELRVKESDRLS | 360 |
| Query 360 | AVANGLKLNGVDCDEGETSLVVRGRPDGKGLGNASGAAVATHLDHRIAMSFLVMGLVSEN | 419 |
| Sbjct 361 | AVA+GL NGV +EGETSL V G G G V THLDHRIAMSFLVMGL + AVAHGLAANGVPVEEGETSLKVIG GTRRIGGGTVPTHLDHRIAMSFLVMGLAGQA | 415 |
| Query 420  Sbjct 416 | PVTVDDATMIATSFPEFMDLMAGLGAKIE 448 PVTVDD IATSFP F+ LM GLGA+ E PVTVDDTATIATSFPSFLGLMTGLGARFE 444 |  |

>TR:A0A5D0VAL2 A0A5D0VAL2_9RHOB 3-phosphoshikimate 1-carboxyvinyltransferase OS=Rhodobacterales bacterium OX=1948890 GN=aroA PE=3 SV=1

Length=442

Score = 555 bits (1430), Expect = 0.0

Identities = 286/446 (64%), Positives = 341/446 (76%), Gaps = 6/446 (1%)

| Query | 11 | TARKSSGLSGTVRIPGDKSISHRSFMFGGLASGETRITGLLEGEDVINTGKAMQAMGARI | 70 |
| --- | --- | --- | --- |
| Sbjct | 2 | T+ ++ L GT+R+PGDKSISHRS MFG LA G T + GLLE EDV+ T AM+A+GA+I TSAANTALKGTIRVPGDKSISHRSLMFGTLAVGRTSVKGLLESEDVLATAAAMRAVGAKI | 61 |
| Query | 71 | RKEGD-TWIIDGVGNGGLLAPEAPLDFGNAATGCRLTMGLVGVYDFDSTFIGDASLTKRP | 129 |
| Sbjct | 62 | K D ++ +DG+G G LL PE +DFGNA TG RLTMG+ G ++ +TF+GDASL+KRP EKHDDGSYTVDGIGLGSLLEPEHVIDFGNAGTGVRLTMGIFGSHNIAATFVGDASLSKRP | 121 |
| Query | 130 | MGRVLNPLREMGVQVKSEDGDRLPVTLRGPKTPTPITYRVPMASAQVKSAVLLAGLNTPG MGRVLNPLREMG V + DGDRLP ++RG + PITYRVPM SAQVKSAVLLAGLN PG | 189 |

| Sbjct | 122 | MGRVLNPLREMGTNVIARDGDRLPASIRGAEQALPITYRVPMPSAQVKSAVLLAGLNAPG | 181 |
| --- | --- | --- | --- |
| Query | 190 | ITTVIEPIMTRDHTEKMLQGFGANLTVETDADGVRTIRLEGRGKLTGQVIDVPGDPSSTA | 249 |
| Sbjct | 182 | TTVIEPI TRDHTEKML+GFGA ++V + DG R IRL+G+ +L Q IDVPGDPSS ETTVIEPIPTRDHTEKMLKGFGAEISVTMNEDGERVIRLKGQPELKPQDIDVPGDPSSAG | 241 |
| Query | 250 | FPLVAALLVPGSDVTILNVLMNPTRTGLILTLQEMGADIEVINPRLAGGEDVADLRVRSS | 309 |
| Sbjct | 242 | FPLVAAL+VPGSD+TI NVL+N RTGLI TL EMG DIE++N R GGEDV DLRV++S FPLVAALVVPGSDLTIENVLLNEHRTGLITTLIEMGGDIEIVNRRQTGGEDVGDLRVKAS | 301 |
| Query | 310 | TLKGVTVPEDRAPSMIDEYPILAVAAAFAEGATVMNGLEELRVKESDRLSAVANGLKLNG | 369 |
| Sbjct | 302 | LKG+TVP DRAPSMIDEYP+LA+AAAFAEG T M GLEELRVKESDRL+AVA GL+ NG KLKGITVPADRAPSMIDEYPVLAIAAAFAEGDTFMPGLEELRVKESDRLAAVARGLEANG | 361 |
| Query | 370 | VDCDEGETSLVVRGRPDGKGLGNASGAAVATHLDHRIAMSFLVMGLVSENPVTVDDATMI | 429 |
| Sbjct | 362 | + C E E +L V G G + G V THLDHRIAM+FLV+G+ + PVTVDD +I IPCIETEDTLTVTG GAADIGGGTVTTHLDHRIAMAFLVLGMAAHKPVTVDDGAVI | 416 |
| Query  Sbjct | 430  417 | ATSFPEFMDLMAGLGAKIELSDTKAA 455 ATSFP F L GLGA+ ++AA ATSFPTFTSLCEGLGARFAAGTSEAA 442 |  |

>TR:A0A2T5VG60 A0A2T5VG60_9RHIZ 3-phosphoshikimate 1-carboxyvinyltransferase OS=Breoghania corrubedonensis OX=665038 GN=aroA PE=3 SV=1

Length=449

Score = 554 bits (1428), Expect = 0.0

| Identities | = 287/451 (64%), Positives = 343/451 (76%), Gaps = 6/451 (1%) |  |
| --- | --- | --- |
| Query 6 | SSRPATARKSSGLSGTVRIPGDKSISHRSFMFGGLASGETRITGLLEGEDVINTGKAMQA | 65 |
| Sbjct 4 | +++P AR + L+GT+R+PGDKSISHRS MFG LA GETRI GLLEGEDV+ T AM+A ATKPMIARAGNPLAGTIRVPGDKSISHRSLMFGALAVGETRIEGLLEGEDVLATAAAMKA | 63 |
| Query 66 | MGARIRKEGD-TWIIDGVGNGGLLAPEAPLDFGNAATGCRLTMGLVGVYDFDSTFIGDAS | 124 |
| Sbjct 64 | +GA + +G +W +DGVG GGL PE PLDFGNA TG RL MG+V + +TFIGDAS LGANVHHDGPGSWRVDGVGVGGLCEPELPLDFGNAGTGVRLAMGMVASHALTATFIGDAS | 123 |
| Query 125 | LTKRPMGRVLNPLREMGVQVKSEDGDRLPVTLRGPKTPTPITYRVPMASAQVKSAVLLAG | 184 |
| Sbjct 124 | L++RPMGRVL+PLR+MG +V + GDRLP+T+RGP+T PI+YRVP+ SAQVKSAVLLAG LSRRPMGRVLDPLRDMGAEVLARSGDRLPLTIRGPETALPISYRVPVPSAQVKSAVLLAG | 183 |
| Query 185 | LNTPGITTVIEPIMTRDHTEKMLQGFGANLTVETDADGVRTIRLEGRGKLTGQVIDVPGD | 244 |
| Sbjct 184 | LN PGITTVIEPI TRDHTE+ML GFGA L E ADG R +RL G+ +L Q I VPGD LNAPGITTVIEPIPTRDHTERMLMGFGAELVSEQTADGARILRLTGQPELKPQAITVPGD | 243 |
| Query 245 | PSSTAFPLVAALLVPGSDVTILNVLMNPTRTGLILTLQEMGADIEVINPRLAGGEDVADL | 304 |
| Sbjct 244 | PSS AF +VAAL+VPGSDVT+ NVL+NP RTGL+ TL+EM ADI + N R GGE + D+ PSSAAFMIVAALVVPGSDVTVENVLLNPHRTGLLKTLEEMDADISIENVRETGGETIGDV | 303 |
| Query 305 | RVRSSTLKGVTVPEDRAPSMIDEYPILAVAAAFAEGATVMNGLEELRVKESDRLSAVANG | 364 |
| Sbjct 304 | R S LKG+TVP DRAPSMIDEYP+LA+AAAFA+G T M GL EL VKESDRL+AVA+G CARHSRLKGITVPADRAPSMIDEYPVLAIAAAFAQGRTRMLGLHELTVKESDRLAAVADG | 363 |
| Query 365 | LKLNGVDCDEGETSLVVRGRPDGKGLGNASGAAVATHLDHRIAMSFLVMGLVSENPVTVD | 424 |
| Sbjct 364 | L NGV ++GE L V G KG G V THLDHRIAMSFLV+GL S++PV+VD LAANGVPYEKGEDWLEVTGAQSVKG GGTVTTHLDHRIAMSFLVLGLASDSPVSVD | 418 |
| Query 425  Sbjct 419 | DATMIATSFPEFMDLMAGLGAKIELSDTKAA 455 D IATSFP F DL AGLG +I++ AA DGAPIATSFPAFTDLFAGLGGRIDVQTEDAA 449 |  |

>TR:A0A163W9Z4 A0A163W9Z4_9RHOB 3-phosphoshikimate 1-carboxyvinyltransferase

OS=Labrenzia sp. OB1 OX=1561204 GN=aroA PE=3 SV=1 Length=451

Score = 553 bits (1424), Expect = 0.0

| Identities | = 285/456 (63%), Positives = 345/456 (76%), Gaps = 6/456 (1%) |  |
| --- | --- | --- |
| Query 1 | MLHGASSRPATARKSSGLSGTVRIPGDKSISHRSFMFGGLASGETRITGLLEGEDVINTG | 60 |
| Sbjct 1 | M H ++ P T+ + L+GT+R+PGDKSISHRS MFG LA G T + GLLE EDV+ T MSHDSAPAPLTSSTGNSLTGTIRVPGDKSISHRSLMFGTLAVGRTTVKGLLESEDVLATA | 60 |
| Query 61 | KAMQAMGARIRKEGD-TWIIDGVGNGGLLAPEAPLDFGNAATGCRLTMGLVGVYDFDSTF | 119 |
| Sbjct 61 | AM+A+GARI K+ D ++ +DG+G G LL P+ +DFGNA TG RLTMG++G ++ +TF DAMRAVGARIEKQPDGSYTVDGIGLGSLLEPDHVIDFGNAGTGVRLTMGILGSHNIAATF | 120 |
| Query 120 | IGDASLTKRPMGRVLNPLREMGVQVKSEDGDRLPVTLRGPKTPTPITYRVPMASAQVKSA | 179 |
| Sbjct 121 | IGDASL+KRPMGRVLNPLR+MG V + DGDRLP ++RGP+ P+TYRVPM SAQVKSA IGDASLSKRPMGRVLNPLRDMGTNVIARDGDRLPASIRGPEQALPLTYRVPMPSAQVKSA | 180 |
| Query 180 | VLLAGLNTPGITTVIEPIMTRDHTEKMLQGFGANLTVETDADGVRTIRLEGRGKLTGQVI | 239 |
| Sbjct 181 | VLLAGLN PG T VIEPI TRDHTEKML GFGA ++V + G R IRL+G+ +L Q I VLLAGLNAPGETVVIEPIPTRDHTEKMLAGFGAEISVSLNEAGERVIRLKGQPELKPQDI | 240 |
| Query 240 | DVPGDPSSTAFPLVAALLVPGSDVTILNVLMNPTRTGLILTLQEMGADIEVINPRLAGGE | 299 |
| Sbjct 241 | DVPGDPSS FP+VAAL+VPGSDVTI NVL+N RTGLI TL EMG DIE++N R GGE DVPGDPSSAGFPIVAALIVPGSDVTIGNVLLNEHRTGLITTLIEMGGDIEIVNRRETGGE | 300 |
| Query 300 | DVADLRVRSSTLKGVTVPEDRAPSMIDEYPILAVAAAFAEGATVMNGLEELRVKESDRLS | 359 |
| Sbjct 301 | +V DLRV+SS LKG+TVP DRAPSMIDEYP+LAVAAAFA+G T M GLEELRVKESDRL+ EVGDLRVKSSRLKGITVPADRAPSMIDEYPVLAVAAAFAQGDTFMPGLEELRVKESDRLA | 360 |
| Query 360 | AVANGLKLNGVDCDEGETSLVVRGRPDGKGLGNASGAAVATHLDHRIAMSFLVMGLVSEN | 419 |
| Sbjct 361 | AVA GL+ NG+ C E E +L V G G G V THLDHRIAM+F+V+G+ + AVARGLEANGIPCVETEDTLTVTG GANAIGGGTVKTHLDHRIAMAFMVLGMAAHK | 415 |
| Query 420  Sbjct 416 | PVTVDDATMIATSFPEFMDLMAGLGAKIELSDTKAA 455 PVTVDD +IATSFP+F L + LGA ++AA PVTVDDGAVIATSFPDFTALFSDLGANFATGASEAA 451 |  |

>TR:A1UU98 A1UU98_BARBK 3-phosphoshikimate 1-carboxyvinyltransferase OS=Bartonella bacilliformis (strain ATCC 35685 / NCTC 12138 / KC583)

OX=360095 GN=aroA PE=3 SV=1

Length=441

Score = 552 bits (1423), Expect = 0.0

| Identities | = 283/439 (64%), Positives = 340/439 (77%), Gaps = 4/439 (1%) |  |
| --- | --- | --- |
| Query 9 | PATARKSSGLSGTVRIPGDKSISHRSFMFGGLASGETRITGLLEGEDVINTGKAMQAMGA | 68 |
| Sbjct 6 | PATA+KS+ LSG ++IPGDKSISHRS + GGLA+GET I GLLE +D+++T AMQAMGA PATAQKSTALSGKIKIPGDKSISHRSLILGGLANGETHIHGLLESDDILHTAAAMQAMGA | 65 |
| Query 69 | RIRKEGDTWIIDGVGNGGLLAPEAPLDFGNAATGCRLTMGLVGVYDFDSTFIGDASLTKR | 128 |
| Sbjct 66 | IRKE WII G GNG LL + PL+FGN+ATG RL MG+V Y +TF GDASL+KR HIRKENGIWIIRGTGNGCLLQAQKPLNFGNSATGARLIMGMVSSYHMKTTFTGDASLSKR | 125 |
| Query 129 | PMGRVLNPLREMGVQVKSEDGDRLPVTLRGPKTPTPITYRVPMASAQVKSAVLLAGLNTP | 188 |
| Sbjct 126 | PM R+LNPLR MG +++ + LP+TL GPK PI YR+P+ASAQVKS++LLA LNT PMERILNPLRLMGTNIEATSNN-LPLTLYGPKMANPICYRLPIASAQVKSSILLASLNTA | 184 |
| Query 189 | GITTVIEPIMTRDHTEKMLQGFGANLTVETDADGVRTIRLEGRGKLTGQVIDVPGDPSST | 248 |
| Sbjct 185 | GITTVIEPI+TRDHTEK+L+ FGA L +ET+ +G R I + G+ LTGQ ID+PGDPSS GITTVIEPILTRDHTEKILELFGAKLDIETNKEGTRFIHMHGQPHLTGQSIDIPGDPSSA | 244 |
| Query 249 | AFPLVAALLVPGSDVTILNVLMNPTRTGLILTLQEMGADIEVINPRLAGGEDVADLRVRS | 308 |

| Sbjct | 245 | AFPL+AALL+ SD+ I NVL+N +R GLI TL EMGA IE +N R GGE++ADLRVRS  AFPLIAALLIEDSDIIIENVLINSSRIGLIQTLWEMGAKIEFLNQRQQGGENIADLRVRS | 304 |
| --- | --- | --- | --- |
| Query | 309 | STLKGVTVPEDRAPSMIDEYPILAVAAAFAEGATVMNGLEELRVKESDRLSAVANGLKLN | 368 |
| Sbjct | 305 | S LKGVTVP++RAPSMIDEYP LAVAAAFAEG T M G+EELRVKESDRLS +A GLK+N SVLKGVTVPKERAPSMIDEYPALAVAAAFAEGKTTMLGIEELRVKESDRLSTIAQGLKIN | 364 |
| Query | 369 | GVDCDEGETSLVVRGRPDGKGLGNASGAAVATHLDHRIAMSFLVMGLVSENPVTVDDATM | 428 |
| Sbjct | 365 | VDC++G L++ G+ KGLG G + THLDHRIAM FLV GL SE PVT+DD + HVDCEKGVDFLIIHGQNSSKGLG---GGCIKTHLDHRIAMCFLVFGLASEKPVTIDDRRV | 421 |
| Query  Sbjct | 429  422 | IATSFPEFMDLMAGLGAKI 447 IATSFP F+ LM LG KI IATSFPAFIPLMNQLGGKI 440 |  |

>TR:A0A1H9HM39 A0A1H9HM39_9BACT 3-phosphoshikimate 1-carboxyvinyltransferase OS=bacterium A52C2 OX=1855383 GN=aroA PE=3 SV=1

Length=448

Score = 551 bits (1420), Expect = 0.0

| Identities | = 292/451 (65%), Positives = 340/451 (75%), Gaps = 7/451 (2%) |  |
| --- | --- | --- |
| Query 5 | ASSRPATARKSSGLSGTVRIPGDKSISHRSFMFGGLASGETRITGLLEGEDVINTGKAMQ | 64 |
| Sbjct 4 | AS +P + S L+G V IPGDKSISHRS MFG LA G TRI+GLLE +D++ T AM+ ASPQPLSGLPSGPLTGRVSIPGDKSISHRSLMFGALAEGTTRISGLLESDDILATAGAMR | 63 |
| Query 65 | AMGARIRKEG-DTWIIDGVGNGGLLAPEAPLDFGNAATGCRLTMGLVGVYDFDSTFIGDA | 123 |
| Sbjct 64 | A GA I +G +W + GVG+ G PE +DFGNA TG RLTMGL G F STF+GDA AYGAAIDSDGRGSWTVQGVGSRGFAEPERAIDFGNAGTGVRLTMGLAGTCAFASTFVGDA | 123 |
| Query 124 | SLTKRPMGRVLNPLREMGVQVKSEDGDRLPVTLRGPKTPTPITYRVPMASAQVKSAVLLA | 183 |
| Sbjct 124 | SL++RPMGRVL+PLR+MG +V + GDRLP+TLRG + PI+YRVPMASAQVKSAVLLA SLSRRPMGRVLDPLRQMGTEVIARGGDRLPLTLRGTASMQPISYRVPMASAQVKSAVLLA | 183 |
| Query 184 | GLNTPGITTVIEPIMTRDHTEKMLQGFGANLTVETDADGVRTIRLEGRGKLTGQVIDVPG | 243 |
| Sbjct 184 | G+N PG+TTVIEP+ TRDHTE+ML+GFGA L E G IRL+GR L Q I VPG GINLPGVTTVIEPVATRDHTERMLRGFGAELETEAGGGGETIIRLQGRPNLKAQDIIVPG | 243 |
| Query 244 | DPSSTAFPLVAALLVPGSDVTILNVLMNPTRTGLILTLQEMGADIEVINPRLAGGEDVAD | 303 |
| Sbjct 244 | DPSS AF +VA LLV GSDVT+ +VL+NP RTGL+ TL+EMGADI + N RL GGE + D DPSSAAFLIVAGLLVEGSDVTVADVLLNPARTGLLTTLREMGADITIENERLTGGEAIGD | 303 |
| Query 304 | LRVRSSTLKGVTVPEDRAPSMIDEYPILAVAAAFAEGATVMNGLEELRVKESDRLSAVAN | 363 |
| Sbjct 304 | +RVR S LKGVTVP +RAPSMIDEYP+LAVAAAFAEG TVM+GLEELRVKESDRL+AVA VRVRGSRLKGVTVPPNRAPSMIDEYPVLAVAAAFAEGETVMDGLEELRVKESDRLAAVAR | 363 |
| Query 364 | GLKLNGVDCDEGETSLVVRGRPDGKGLGNASGAAVATHLDHRIAMSFLVMGLVSENPVTV | 423 |
| Sbjct 364 | GL+ NGV C EG SL V G GK G G V THLDHRIAMSFLVMGL +E PVTV GLEANGVVCREGPASLTVTG---GKVRG---GGHVTTHLDHRIAMSFLVMGLAAEKPVTV | 417 |
| Query 424  Sbjct 418 | DDATMIATSFPEFMDLMAGLGAKIELSDTKA 454 DD MIATSFP F+DL++GLGA+ S A DDVAMIATSFPGFVDLLSGLGARFGASAVAA 448 |  |

>TR:A0A327JM75 A0A327JM75_9RHIZ 3-phosphoshikimate 1-carboxyvinyltransferase OS=Rhodobium orientis OX=34017 GN=aroA PE=3 SV=1

Length=449

Score = 551 bits (1419), Expect = 0.0

Identities = 282/442 (64%), Positives = 339/442 (77%), Gaps = 6/442 (1%) Query 8	RPATARKSSGLSGTVRIPGDKSISHRSFMFGGLASGETRITGLLEGEDVINTGKAMQAMG 67

| Sbjct | 2 | RP TAR S LSG V +PGDKSISHR+ +FG LA GET I GLL+ EDV+ T AM+A+G  RPLTARPGSALSGRVAVPGDKSISHRALIFGALAIGETAIDGLLQSEDVLCTAAAMRALG | 61 |
| --- | --- | --- | --- |
| Query | 68 | ARI-RKEGDTWIIDGVGNGGLLAPEAPLDFGNAATGCRLTMGLVGVYDFDSTFIGDASLT | 126 |
| Sbjct | 62 | A + E ++ + GVG GGL+ PE PLDFGN+ TG RL +GL+G + + F+GDASL+ ATVVAGENGSYKVHGVGVGGLMEPETPLDFGNSGTGARLALGLIGTHPLAAVFLGDASLS | 121 |
| Query | 127 | KRPMGRVLNPLREMGVQVKSEDGDRLPVTLRGPKTPTPITYRVPMASAQVKSAVLLAGLN | 186 |
| Sbjct | 122 | +RPMGRVL PLR MG +V + DGDRLP+ +RGPKT P+ YRVP+ SAQVKSAVLLAGLN RRPMGRVLEPLRAMGTEVIARDGDRLPLAIRGPKTAIPLEYRVPVPSAQVKSAVLLAGLN | 181 |
| Query | 187 | TPGITTVIEPIMTRDHTEKMLQGFGANLTVETDADGVRTIRLEGRGKLTGQVIDVPGDPS | 246 |
| Sbjct | 182 | PGITTVIEP+ TRDHTE+ML+GFGA+L + + DG RTIRL G+ KL Q + VPGDPS APGITTVIEPVATRDHTERMLEGFGADLDITVEDDGARTIRLVGQPKLKPQPVTVPGDPS | 241 |
| Query | 247 | STAFPLVAALLVPGSDVTILNVLMNPTRTGLILTLQEMGADIEVINPRLAGGEDVADLRV | 306 |
| Sbjct | 242 | S AFP+VAAL+VPGSDV + NV++N +RTGL TL EMGAD+ N R++GGE +AD+ V SAAFPIVAALIVPGSDVVVENVMLNESRTGLFTTLIEMGADLSFENERVSGGETIADIHV | 301 |
| Query | 307 | RSSTLKGVTVPEDRAPSMIDEYPILAVAAAFAEGATVMNGLEELRVKESDRLSAVANGLK | 366 |
| Sbjct | 302 | R STLKGVTVP DRAPSMIDEYP+L+ AAAFA+G T+M GL ELRVKESDRL+AVA GL RHSTLKGVTVPPDRAPSMIDEYPVLSAAAAFADGQTMMEGLAELRVKESDRLAAVAAGLA | 361 |
| Query | 367 | LNGVDCDEGETSLVVRGRPDGKGLGNASGAAVATHLDHRIAMSFLVMGLVSENPVTVDDA | 426 |
| Sbjct | 362 | NGV +EG LVV GR G+ G V THLDHRIAMSFLV+GL ++ PVTVDDA ANGVTHEEGADFLVVTGR TGDIGGGTVTTHLDHRIAMSFLVLGLAADRPVTVDDA | 416 |
| Query | 427 | TMIATSFPEFMDLMAGLGAKIE 448  +I TSFP F DLMAGLGA +E |  |
| Sbjct | 417 | GVINTSFPGFADLMAGLGADLE 438 | |

>TR:A0A0M7AF66 A0A0M7AF66_9RHOB 3-phosphoshikimate 1-carboxyvinyltransferase OS=Labrenzia alexandrii OX=388408 GN=aroA PE=3 SV=1

Length=451

Score = 550 bits (1418), Expect = 0.0

| Identities | = 282/456 (62%), Positives = 347/456 (76%), Gaps = 6/456 (1%) |  |
| --- | --- | --- |
| Query 1 | MLHGASSRPATARKSSGLSGTVRIPGDKSISHRSFMFGGLASGETRITGLLEGEDVINTG | 60 |
| Sbjct 1 | M H ++ P T+ S L GT+R+PGDKSISHRS MFG LA G T + GLLE EDV+ T MSHTSAPVPLTSAAGSPLKGTIRVPGDKSISHRSLMFGALAVGRTTVQGLLESEDVLATA | 60 |
| Query 61 | KAMQAMGARIRKEGD-TWIIDGVGNGGLLAPEAPLDFGNAATGCRLTMGLVGVYDFDSTF | 119 |
| Sbjct 61 | AM+A+GA+I K+ D ++ +DG+G G LL P+ +DFGNA TG RLTMG+ G ++ +TF DAMRAVGAKIEKQDDGSYTVDGIGLGSLLEPQGVIDFGNAGTGVRLTMGIFGSHNIAATF | 120 |
| Query 120 | IGDASLTKRPMGRVLNPLREMGVQVKSEDGDRLPVTLRGPKTPTPITYRVPMASAQVKSA | 179 |
| Sbjct 121 | +GDASL+KRPMGRVL+PLREMG V + DGDRLP ++RG + PITYRVPM SAQVKSA VGDASLSKRPMGRVLDPLREMGTNVIARDGDRLPASIRGAEQTLPITYRVPMPSAQVKSA | 180 |
| Query 180 | VLLAGLNTPGITTVIEPIMTRDHTEKMLQGFGANLTVETDADGVRTIRLEGRGKLTGQVI | 239 |
| Sbjct 181 | VLLAGLN PG+TTVIEPI TRDHTEKML+GFGA+++V + G R I+L G+ +L Q I VLLAGLNAPGVTTVIEPIATRDHTEKMLKGFGADISVSLNEAGERVIKLTGQPELKPQDI | 240 |
| Query 240 | DVPGDPSSTAFPLVAALLVPGSDVTILNVLMNPTRTGLILTLQEMGADIEVINPRLAGGE | 299 |
| Sbjct 241 | DVP DPSS AFP+VAAL+VPGSDVTI NV++N RTG+I TL EMG +IE++N R AGGE DVPADPSSAAFPIVAALIVPGSDVTIENVMLNEHRTGIITTLIEMGGNIELLNKRSAGGE | 300 |
| Query 300 | DVADLRVRSSTLKGVTVPEDRAPSMIDEYPILAVAAAFAEGATVMNGLEELRVKESDRLS | 359 |
| Sbjct 301 | +VAD+RV+ S LKG+TVP RAPSMIDEYP+LA+AAAFAEG T M GLEELRVKESDRL+ EVADIRVKHSKLKGITVPAKRAPSMIDEYPVLAIAAAFAEGDTFMPGLEELRVKESDRLA | 360 |
| Query 360 | AVANGLKLNGVDCDEGETSLVVRGRPDGKGLGNASGAAVATHLDHRIAMSFLVMGLVSEN | 419 |

AVA GL+ NG+ C E E +L V G	G + G V T+LDHRIAMSFL++G+ +

| Sbjct | 361 | AVARGLEANGIPCVETEDTLTVTG-----GAADIGGGTVVTYLDHRIAMSFLILGIAAHK 415 |
| --- | --- | --- |
| Query | 420 | PVTVDDATMIATSFPEFMDLMAGLGAKIELSDTKAA 455 |
| Sbjct | 416 | PVTVDD +IATSFP F L LGAKI + ++AA  PVTVDDGAVIATSFPTFTSLFEKLGAKISANSSEAA 451 |

>TR:A0A2R4MIR9 A0A2R4MIR9_9RHIZ 3-phosphoshikimate 1-carboxyvinyltransferase OS=Maritalea myrionectae OX=454601 GN=aroA PE=3 SV=1

Length=437

Score = 550 bits (1417), Expect = 0.0

| Identities | = 283/434 (65%), Positives = 334/434 (77%), Gaps = 5/434 (1%) |  |
| --- | --- | --- |
| Query 14 | KSSGLSGTVRIPGDKSISHRSFMFGGLASGETRITGLLEGEDVINTGKAMQAMGARIRKE | 73 |
| Sbjct 2 | +S GL+G + +PGDKSISHR+ MFG +A GET I+GLLEGEDV+ TG+AM +GA+I K RSQGLTGAITMPGDKSISHRALMFGAMAQGETIISGLLEGEDVLATGRAMAQLGAKIEKR | 61 |
| Query 74 | GDTWIIDGVGNGGLLAPEAPLDFGNAATGCRLTMGLVGVYDFDSTFIGDASLTKRPMGRV | 133 |
| Sbjct 62 | G+ W ++GVG G P PLDFGNA TG RL MGLVG Y F S + GDASL+ RPM RV GNYWHVEGVGVGNFKQPSEPLDFGNAGTGVRLAMGLVGTYPFVSRYEGDASLSVRPMNRV | 121 |
| Query 134 | LNPLREMGVQVKSED-GDRLPVTLRGPKTPTPITYRVPMASAQVKSAVLLAGLNTPGITT | 192 |
| Sbjct 122 | LNPLRE+GV+V E G +LP+ L+GP P PI Y+ PMASAQVKSA++LAGL PG TT LNPLREIGVEVVEEQPGGKLPIALKGPANPAPIHYKTPMASAQVKSAIMLAGLIIPGTTT | 181 |
| Query 193 | VIEPIMTRDHTEKMLQGFGANLTVETDADGVRTIRLEGRGKLTGQVIDVPGDPSSTAFPL | 252 |
| Sbjct 182 | VIE + TRDHTEKMLQGFGA + V TDADGVRTI++EG L GQ + VPGDPSS AFP+ VIEEVHTRDHTEKMLQGFGAKVDVTTDADGVRTIKVEGLPDLRGQEVLVPGDPSSAAFPM | 241 |
| Query 253 | VAALLVPGSDVTILNVLMNPTRTGLILTLQEMGADIEVINPRLAGGEDVADLRVRSSTLK | 312 |
| Sbjct 242 | VAAL+VPGSD+TI NVLMNPTRTGLI +L EMG DIE+ N R +GGED+AD+RV+ S LK VAALIVPGSDLTIENVLMNPTRTGLITSLIEMGGDIELTNQRKSGGEDIADIRVKHSQLK | 301 |
| Query 313 | GVTVPEDRAPSMIDEYPILAVAAAFAEGATVMNGLEELRVKESDRLSAVANGLKLNGVDC | 372 |
| Sbjct 302 | GVT+P +RA SMIDEYP+LAVAA+FA GAT M G++ELRVKESDRL+AVANGLK NGVDC GVTIPAERAASMIDEYPVLAVAASFARGATHMPGIDELRVKESDRLAAVANGLKANGVDC | 361 |
| Query 373 | DEGETSLVVRGRPDGKGLGNASGAAVATHLDHRIAMSFLVMGLVSENPVTVDDATMIATS | 432 |
| Sbjct 362 | EGE L V G D G V THLDHRIAM FLVMGL ++N V +DD+ MIATS IEGEDFLTVEGMGDQV PGGGMVETHLDHRIAMCFLVMGLAAQNAVKIDDSAMIATS | 417 |
| Query 433  Sbjct 418 | FPEFMDLMAGLGAK 446 FP F+ M LGA+ FPSFVPDMTRLGAQ 431 |  |

>TR:E2CEL0 E2CEL0_9RHOB 3-phosphoshikimate 1-carboxyvinyltransferase OS=Roseibium sp. TrichSKD4 OX=744980 GN=aroA PE=3 SV=1

Length=451

Score = 550 bits (1416), Expect = 0.0

Identities = 283/456 (62%), Positives = 343/456 (75%), Gaps = 6/456 (1%)

| Query | 1 | MLHGASSRPATARKSSGLSGTVRIPGDKSISHRSFMFGGLASGETRITGLLEGEDVINTG | 60 |
| --- | --- | --- | --- |
| Sbjct | 1 | M H ++ P TA + L GTV +PGDKSISHR+ MFG LA G T TGLLE EDV+ T MSHASAPTPLTANAAGPLRGTVTVPGDKSISHRALMFGALAVGRTTATGLLESEDVLATA | 60 |
| Query | 61 | KAMQAMGARIRK-EGDTWIIDGVGNGGLLAPEAPLDFGNAATGCRLTMGLVGVYDFDSTF | 119 |
| Sbjct | 61 | AM+ +GA I K + T+ IDG+G G LL PE+ +DFGNA TG RLTMG+ G +D +TF DAMRVVGAVIEKTDNGTYTIDGIGLGSLLEPESVIDFGNAGTGVRLTMGIFGTHDIAATF | 120 |
| Query | 120 | IGDASLTKRPMGRVLNPLREMGVQVKSEDGDRLPVTLRGPKTPTPITYRVPMASAQVKSA | 179 |

| Sbjct | 121 | +GDASL+KRPMGRVL+PLR+MG V + GDRLP ++RG K P P+TYRVP+ASAQVKSA  VGDASLSKRPMGRVLDPLRDMGTNVVARSGDRLPASIRGAKNPLPLTYRVPVASAQVKSA | 180 |
| --- | --- | --- | --- |
| Query | 180 | VLLAGLNTPGITTVIEPIMTRDHTEKMLQGFGANLTVETDADGVRTIRLEGRGKLTGQVI | 239 |
| Sbjct | 181 | VLLAGLN PG TTVIEPI TRDHTEKML+GFGA+++V+ + DG R I L+G+ +L Q I VLLAGLNAPGTTTVIEPIATRDHTEKMLKGFGADISVKLNDDGERVITLQGQPELKPQAI | 240 |
| Query | 240 | DVPGDPSSTAFPLVAALLVPGSDVTILNVLMNPTRTGLILTLQEMGADIEVINPRLAGGE | 299 |
| Sbjct | 241 | +VP DPSS AF +VAAL+VPGSDVTI VL+N R GLI TL EMG DIE++N R GGE EVPADPSSAAFAIVAALIVPGSDVTIEKVLLNEHRIGLITTLIEMGGDIEIVNRRETGGE | 300 |
| Query | 300 | DVADLRVRSSTLKGVTVPEDRAPSMIDEYPILAVAAAFAEGATVMNGLEELRVKESDRLS | 359 |
| Sbjct | 301 | ++ D+RV++S LKG+TVP RAPSMIDEYP+LA+AA+FAEG T M GL+ELRVKESDRL+ EIGDVRVKASQLKGITVPAKRAPSMIDEYPVLAIAASFAEGDTHMPGLDELRVKESDRLA | 360 |
| Query | 360 | AVANGLKLNGVDCDEGETSLVVRGRPDGKGLGNASGAAVATHLDHRIAMSFLVMGLVSEN | 419 |
| Sbjct | 361 | AVA GL+ N V C EGE L V G G N G V THLDHRIAM+FLV+G+ S + AVARGLEANNVPCVEGEDCLTVTG GAANIGGGTVVTHLDHRIAMAFLVLGMASHS | 415 |
| Query  Sbjct | 420  416 | PVTVDDATMIATSFPEFMDLMAGLGAKIELSDTKAA 455 PVTVDD +IATSFP F L GLGA IE +++ AA PVTVDDGAVIATSFPTFTSLFRGLGANIEENESIAA 451 |  |

>TR:A0A4R6RDQ1 A0A4R6RDQ1_9RHIZ 3-phosphoshikimate 1-carboxyvinyltransferase OS=Oharaeibacter diazotrophicus OX=1920512 GN=aroA PE=3 SV=1

Length=447

Score = 549 bits (1415), Expect = 0.0

| Identities | = 293/445 (66%), Positives = 332/445 (75%), Gaps = 7/445 (2%) |  |
| --- | --- | --- |
| Query 5 | ASSRPATARKSSGLSGTVRIPGDKSISHRSFMFGGLASGETRITGLLEGEDVINTGKAMQ | 64 |
| Sbjct 5 | A+SRP AR S L+G++R+PGDKSISHRS M G +A GET I GLLE EDV+NT AM+ AASRPLRARASGPLAGSLRVPGDKSISHRSLMLGAVALGETVIEGLLEAEDVLNTAAAMR | 64 |
| Query 65 | AMGARIRKEGD-TWIIDGVGNGGLLAPEAPLDFGNAATGCRLTMGLVGVYDFDSTFIGDA | 123 |
| Sbjct 65 | A+GA + K D W + G+G GGLL P +DFGNA TG RLTMGLV TF GDA ALGASVEKGDDGCWRVRGLGVGGLLEPTGVIDFGNAGTGVRLTMGLVASQPIAVTFTGDA | 124 |
| Query 124 | SLTKRPMGRVLNPLREMGVQVKSEDGDRLPVTLRGPKTPTPITYRVPMASAQVKSAVLLA | 183 |
| Sbjct 125 | SL KRPM RVL+PL MGV+V + G RLP+T+RGP TP PITYRVPMASAQVKSAVLLA SLVKRPMRRVLDPLGAMGVEVIARSGGRLPLTMRGPDTPLPITYRVPMASAQVKSAVLLA | 184 |
| Query 184 | GLNTPGITTVIEPIMTRDHTEKMLQGFGANLTVETDADGVRTIRLEGRGKLTGQVIDVPG | 243 |
| Sbjct 185 | GL TPG+TTV+EP+ TRDHTE+ML GFGA + V TDADGVRTIRLEGR +L Q + VP GLGTPGVTTVVEPVFTRDHTERMLAGFGAAIEVATDADGVRTIRLEGRPELKAQAVTVPA | 244 |
| Query 244 | DPSSTAFPLVAALLVPGSDVTILNVLMNPTRTGLILTLQEMGADIEVINPRLAGGEDVAD | 303 |
| Sbjct 245 | DPSS AFP+VAA +VPGSDV + VLMNP R GLI TL EMGADI V+ R GGE +AD DPSSAAFPIVAATVVPGSDVVVEGVLMNPARIGLIETLLEMGADITVVAERETGGEKLAD | 304 |
| Query 304 | LRVRSSTLKGVTVPEDRAPSMIDEYPILAVAAAFAEGATVMNGLEELRVKESDRLSAVAN | 363 |
| Sbjct 305 | LRVR + L GVTVP +RAPSMIDEYP+L+VAAA A G T M GLEE+RVKESDRL+AVA LRVRHADLSGVTVPAERAPSMIDEYPVLSVAAAVAAGETRMLGLEEMRVKESDRLAAVAA | 364 |
| Query 364 | GLKLNGVDCDEGETSLVVRGRPDGKGLGNASGAAVATHLDHRIAMSFLVMGLVSENPVTV | 423 |
| Sbjct 365 | GL NGVDC EG LVVRG G VATHLDHRIAMSFL++GLV+ PVTV GLLANGVDCTEGHDFLVVRGG AVPGGGLVATHLDHRIAMSFLILGLVARAPVTV | 418 |
| Query 424  Sbjct 419 | DDATMIATSFPEFMDLMAGLGAKIE 448 DDA MIATSFP F LM G GA E DDAAMIATSFPTFEALMTGAGAVFE 443 |  |

>TR:A0A0F5L169 A0A0F5L169_9RHIZ 3-phosphoshikimate 1-carboxyvinyltransferase OS=Devosia limi DSM 17137 OX=1121477 GN=aroA PE=3 SV=1

Length=449

Score = 549 bits (1415), Expect = 0.0

| Identities | | = 281/448 (63%), Positives = 342/448 (76%), Gaps = 5/448 (1%) |  |
| --- | --- | --- | --- |
| Query 6  Sbjct 7 | | SSRPATARKSSGLSGTVRIPGDKSISHRSFMFGGLASGETRITGLLEGEDVINTGKAMQA  ++ P +AR+++ LSG R+PGD+SISHR+ MFG +ASG T I GLLEG+ ++ TG AMQA AASPLSARRANPLSGRFRVPGDRSISHRALMFGAMASGRTSIEGLLEGDGILATGNAMQA | 65  66 |
| Query 66  Sbjct 67 | | MGARIRKEGDTWIIDGVGNGGLLAPEAPLDFGNAATGCRLTMGLVGVYDFDSTFIGDASL  +GAR+ + GD+W++DG+G GG L P APLDFGNA TG RL MGLVG+Y F + F+G A+L LGARVERRGDSWLVDGLGVGGFLEPAAPLDFGNAGTGVRLAMGLVGIYPFATRFVGSAAL | 125  126 |
| Query 126  Sbjct 127 | | TKRPMGRVLNPLREMGVQVKSEDGDRLPVTLRGPKTPTPITYRVPMASAQVKSAVLLAGL  + RPM VL+ LR +GV V ED RLP+TLRGP+TP PI +R+P ASAQVKSA+LLAGL SARPMNLVLDSLRSIGVAVLEEDDGRLPITLRGPRTPLPIHHRLPTASAQVKSAMLLAGL | 185  186 |
| Query 186  Sbjct 187 | | NTPGITTVIEPIMTRDHTEKMLQGFGANLTVETDADGVRTIRLEGRGKLTGQVIDVPGDP PG TT+IEP++TRDHTEKML FGA + + T ADGV TI ++G L Q + VPGDP LIPGTTTIIEPVLTRDHTEKMLASFGAEIEITTAADGVSTIAVQGLPNLKAQHVIVPGDP | 245  246 |
| Query 246  Sbjct 247 | | SSTAFPLVAALLVPGSDVTILNVLMNPTRTGLILTLQEMGADIEVINPRLAGGEDVADLR SS AFPLVAAL+VPGSDV I NVLMNPTRTGLI TL EMG DI+ +N R +GGED+ADLR SSAAFPLVAALIVPGSDVIIENVLMNPTRTGLIDTLLEMGGDIQFLNQRHSGGEDIADLR | 305  306 |
| Query 306  Sbjct 307 | | VRSSTLKGVTVPEDRAPSMIDEYPILAVAAAFAEGATVMNGLEELRVKESDRLSAVANGL VRSS LKGV V D APSMID+YP LA+AAAFAEG TVM GL ELRV+ESD L+AVA GL VRSSRLKGVRVAADHAPSMIDDYPALAIAAAFAEGETVMEGLGELRVRESDLLAAVAKGL | 365  366 |
| Query 366  Sbjct 367 | | KLNGVDCDEGETSLVVRGRPDGKGLGNASGAAVATHLDHRIAMSFLVMGLVSENPVTVDD LNGVDC EG SL VRG GK G G V T LDHR+AMSFLVMG+ S+ PV+VDD ALNGVDCSEGPDSLTVRG--TGKVAG---GGMVQTRLDHRVAMSFLVMGMASQLPVSVDD | 425  421 |
| Query 426 | | ATMIATSFPEFMDLMAGLGAKIELSDTK 453 TMI SFP F+ M+ +GA+ + +++ |  |
| Sbjct | 422 | HTMIEPSFPNFVTGMSSVGAQFDTPESR 449 | |

>TR:A0A6A7Y3I4 A0A6A7Y3I4_9RHIZ 3-phosphoshikimate 1-carboxyvinyltransferase OS=Rhizobiales bacterium Sp-1 OX=2608987 GN=aroA PE=3 SV=1

Length=452

Score = 549 bits (1414), Expect = 0.0

| Identities | = 290/448 (65%), Positives = 337/448 (75%), Gaps = 6/448 (1%) |  |
| --- | --- | --- |
| Query 1 | MLHGASSRPATARKSSGLSGTVRIPGDKSISHRSFMFGGLASGETRITGLLEGEDVINTG | 60 |
| Sbjct 1 | M H + P A S+ L G VR+PGDKSISHRS M G LA GET +TGLLE DV+ T MSHAHTPEPLRAAASAPLRGRVRVPGDKSISHRSLMLGTLAVGETIVTGLLESGDVLGTA | 60 |
| Query 61 | KAMQAMGARIRKEGD-TWIIDGVGNGGLLAPEAPLDFGNAATGCRLTMGLVGVYDFDSTF | 119 |
| Sbjct 61 | AM+A+GAR+ ++ D W I GVG GGLL P APLDFGNA TG RL +G+VG + T AAMRALGARVERDADGAWHIQGVGVGGLLEPTAPLDFGNAGTGVRLCLGMVGTHPIAVTA | 120 |
| Query 120 | IGDASLTKRPMGRVLNPLREMGVQVKSEDGDRLPVTLRGPKTPTPITYRVPMASAQVKSA | 179 |
| Sbjct 121 | IGDASL++RPMGRVL PLREMG QV + DGDRLP+++RG P PI YR+P+ SAQVKSA IGDASLSRRPMGRVLGPLREMGTQVIARDGDRLPLSVRGATRPLPIEYRLPVPSAQVKSA | 180 |
| Query 180 | VLLAGLNTPGITTVIEPIMTRDHTEKMLQGFGANLTVETDADGVRTIRLEGRGKLTGQVI | 239 |
| Sbjct 181 | +LLA LNTPG TTVIEPI TRDHTE+ML+GFG +L + D DG R I + G +LT Q I ILLAALNTPGTTTVIEPIATRDHTERMLEGFGVDLKITIDDDGARRISITGPVELTPQRI | 240 |
| Query 240 | DVPGDPSSTAFPLVAALLVPGSDVTILNVLMNPTRTGLILTLQEMGADIEVINPRLAGGE | 299 |

| Sbjct | 241 | VP DPSS AFPLVA LLVPGS+VT+ +VL+NPTRTGL+ TL+EMGAD+ + N RL+GGE  AVPADPSSAAFPLVAGLLVPGSEVTVEDVLLNPTRTGLLDTLREMGADLTITNERLSGGE | 300 |
| --- | --- | --- | --- |
| Query | 300 | DVADLRVRSSTLKGVTVPEDRAPSMIDEYPILAVAAAFAEGATVMNGLEELRVKESDRLS | 359 |
| Sbjct | 301 | V D+ VR S LKG+TVP RAPSMIDEYP+LAVAAAFAEG TVM GLEELRVKESDRL+ RVGDVTVRHSRLKGITVPAARAPSMIDEYPVLAVAAAFAEGETVMEGLEELRVKESDRLA | 360 |
| Query | 360 | AVANGLKLNGVDCDEGETSLVVRGRPDGKGLGNASGAAVATHLDHRIAMSFLVMGLVSEN | 419 |
| Sbjct | 361 | AVA GL NGV + G L VRGR G G AVATHLDHRIAMSFLVMGL S++ AVAAGLAANGVVHEAGPDWLRVRGR TGAIGGGAVATHLDHRIAMSFLVMGLASDS | 415 |
| Query  Sbjct | 420  416 | PVTVDDATMIATSFPEFMDLMAGLGAKI 447 PV VDD MIATSFP F+ LM+GLGA + PVAVDDTAMIATSFPSFLTLMSGLGATL 443 |  |

>TR:A0A5R8YBM5 A0A5R8YBM5_9RHIZ 3-phosphoshikimate 1-carboxyvinyltransferase OS=Cohaesibacter sp. CAU 1516 OX=2576038 GN=aroA PE=3 SV=1

Length=445

Score = 548 bits (1413), Expect = 0.0

| Identities | | = 288/448 (64%), Positives = 338/448 (75%), Gaps = 7/448 (2%) |  |
| --- | --- | --- | --- |
| Query 1  Sbjct 1 | | MLHGASSR-PATARKSSGLSGTVRIPGDKSISHRSFMFGGLASGETRITGLLEGEDVINT M H A++ P +A SSGL G +R+PGDKSISHRS +FG LA G+T I GLLE EDVINT MAHDATNMSPLSAETSSGLKGRLRVPGDKSISHRSLIFGALARGKTTIHGLLESEDVINT | 59  60 |
| Query 60  Sbjct 61 | | GKAMQAMGARIRKEGD-TWIIDGVGNGGLLAPEAPLDFGNAATGCRLTMGLVGVYDFDST KAM A GA++ K+ D W ++G+G G L+ P LDFGN+ TG RL MG+ G + F +T AKAMAAFGAKVEKDDDGVWHVEGLGTGSLIEPNQVLDFGNSGTGARLVMGIAGAHAFSTT | 118  120 |
| Query 119  Sbjct 121 | | FIGDASLTKRPMGRVLNPLREMGVQVKSEDGDRLPVTLRGPKTPTPITYRVPMASAQVKS F+GDASL+ RPMGRVLNPLREMGVQV S GDRLP+T++G +PITYRVPMASAQVKS FLGDASLSGRPMGRVLNPLREMGVQVLSRSGDRLPLTIKGADPASPITYRVPMASAQVKS | 178  180 |
| Query 179  Sbjct 181 | | AVLLAGLNTPGITTVIEPIMTRDHTEKMLQGFGANLTVETDADGVRTIRLEGRGKLTGQV VLLAGLN G TTVIEPI TRDHTEKML+GFGA L E + G + +EG +LTGQ CVLLAGLNMAGTTTVIEPIKTRDHTEKMLRGFGAELGEEINEAGETVLTIEGLQELTGQT | 238  240 |
| Query 239  Sbjct 241 | | IDVPGDPSSTAFPLVAALLVPGSDVTILNVLMNPTRTGLILTLQEMGADIEVINPRLAGG  + VPGDPSS AFP+VAAL+ PGSD+ I N+L+N TRTGLI TL EMG DI + + R +GG VTVPGDPSSAAFPIVAALITPGSDLLIENILLNDTRTGLISTLIEMGGDITIEDERESGG | 298  300 |
| Query 299  Sbjct 301 | | EDVADLRVRSSTLKGVTVPEDRAPSMIDEYPILAVAAAFAEGATVMNGLEELRVKESDRL E + ++RVRSS+L GVTVP DRAPSMIDEYP+L+VAAAFA+G T M GLEELRVKESDRL ERIGNIRVRSSSLTGVTVPADRAPSMIDEYPVLSVAAAFAKGETRMEGLEELRVKESDRL | 358  360 |
| Query 359  Sbjct 361 | | SAVANGLKLNGVDCDEGETSLVVRGRPDGKGLGNASGAAVATHLDHRIAMSFLVMGLVSE  +AVA GLK NGV EG+ +LVV G G G V THLDHRIAMSFLV+GL AAVAAGLKANGVPHVEGKDTLVVTG GANAIGGGTVVTHLDHRIAMSFLVLGLAGH | 418  415 |
| Query 419 | | NPVTVDDATMIATSFPEFMDLMAGLGAK 446 PVTVDDA +I TSFP F DLMAGLGAK |  |
| Sbjct | 416 | KPVTVDDAAIINTSFPGFADLMAGLGAK 443 | |

>TR:A0A2C9DCT0 A0A2C9DCT0_9RHIZ 3-phosphoshikimate 1-carboxyvinyltransferase OS=Hartmannibacter diazotrophicus OX=1482074 GN=aroA PE=3 SV=1

Length=450

Score = 548 bits (1412), Expect = 0.0

Identities = 292/452 (65%), Positives = 337/452 (75%), Gaps = 7/452 (2%) Query 5	ASSRPATARKSSGLSGTVRIPGDKSISHRSFMFGGLASGETRITGLLEGEDVINTGKAMQ 64

| Sbjct | 5 | A+ RP +A +S L G VR+PGDKSISHRS MFG LA G T ITGLLEG D+++T AM+  AALRPLSAHRSPALKGRVRVPGDKSISHRSLMFGALAIGRTTITGLLEGGDIMSTAAAMR | 64 |
| --- | --- | --- | --- |
| Query | 65 | AMGARIRKEGD-TWIIDGVGNGGLLAPEAPLDFGNAATGCRLTMGLVGVYDFDSTFIGDA | 123 |
| Sbjct | 65 | A GA I + D TW +DGVG GGLL P +DFGNA TG RL MGL TF GD AFGANIHRGDDGTWTVDGVGVGGLLEPAGVIDFGNAGTGVRLCMGLAAAQPIAVTFTGDG | 124 |
| Query | 124 | SLTKRPMGRVLNPLREMGVQVKSEDGDRLPVTLRGPKTPTPITYRVPMASAQVKSAVLLA | 183 |
| Sbjct | 125 | SL KRPMGRVL+PLR+MGV V + GDRLP+T+RG TP PI Y VP+ SAQVKSAVLLA SLIKRPMGRVLDPLRKMGVDVIARSGDRLPLTMRGAMTPIPIEYTVPVPSAQVKSAVLLA | 184 |
| Query | 184 | GLNTPGITTVIEPIMTRDHTEKMLQGFGANLTVETDADGVRTIRLEGRGKLTGQVIDVPG | 243 |
| Sbjct | 185 | GL TPG+TTVIEP+ TRDHTE+ML GFGA + +ET A+G RTIRLEGR L Q I VPG GLGTPGVTTVIEPVFTRDHTERMLAGFGAAIDIETAANGARTIRLEGRPDLKPQTITVPG | 244 |
| Query | 244 | DPSSTAFPLVAALLVPGSDVTILNVLMNPTRTGLILTLQEMGADIEVINPRLAGGEDVAD | 303 |
| Sbjct | 245 | DPSS AFP+VAAL+VPGSDVT+ +LMNP+R GLI TL EMGADI + N R GGE +AD DPSSAAFPIVAALIVPGSDVTVEGILMNPSRIGLIDTLLEMGADITIENARETGGEKIAD | 304 |
| Query | 304 | LRVRSSTLKGVTVPEDRAPSMIDEYPILAVAAAFAEGATVMNGLEELRVKESDRLSAVAN | 363 |
| Sbjct | 305 | LRV+ S LKGVTVP +RAP MIDEYP+LA AAA AEG TVM G+EE+RVKESDR++AVA LRVKHSALKGVTVPPERAPLMIDEYPVLAAAAAVAEGKTVMLGIEEMRVKESDRIAAVAA | 364 |
| Query | 364 | GLKLNGVDCDEGETSLVVRGRPDGKGLGNASGAAVATHLDHRIAMSFLVMGLVSENPVTV | 423 |
| Sbjct | 365 | GL NGVD +EG L V G GK G G V THLDHRIAM+FLV+GLV++ PVTV GLAANGVDHEEGRDWLSVTG---GKVAG---GGRVVTHLDHRIAMTFLVLGLVADAPVTV | 418 |
| Query  Sbjct | 424  419 | DDATMIATSFPEFMDLMAGLGAKIELSDTKAA 455 DDA +IATSFPEF LM G GA E ++ AA DDAGVIATSFPEFEPLMRGAGALFEDAEADAA 450 |  |

>TR:A0A1I5CT28 A0A1I5CT28_9RHIZ 3-phosphoshikimate 1-carboxyvinyltransferase OS=Cohaesibacter marisflavi OX=655353 GN=aroA PE=3 SV=1

Length=462

Score = 542 bits (1397), Expect = 0.0

| Identities | = 286/450 (64%), Positives = 339/450 (75%), Gaps = 7/450 (2%) |  |
| --- | --- | --- |
| Query 1 | MLHGASS-RPATARKSSGLSGTVRIPGDKSISHRSFMFGGLASGETRITGLLEGEDVINT | 59 |
| Sbjct 18 | M H +++ P ARK SGL G +R+PGDKSISHR+ +FG LA GET+I GLLE EDV+NT MAHDSTNLSPLAARKCSGLRGHLRVPGDKSISHRALIFGALARGETQIHGLLESEDVLNT | 77 |
| Query 60 | GKAMQAMGARIRKEG-DTWIIDGVGNGGLLAPEAPLDFGNAATGCRLTMGLVGVYDFDST | 118 |
| Sbjct 78 | KAM A GA +RK+ D W ++G+G G L+ P LDFGN+ TG RL MG+ G + F +T AKAMAAFGATVRKDDEDVWYVEGLGTGSLVEPTGVLDFGNSGTGARLVMGIAGAHAFATT | 137 |
| Query 119 | FIGDASLTKRPMGRVLNPLREMGVQVKSEDGDRLPVTLRGPKTPTPITYRVPMASAQVKS | 178 |
| Sbjct 138 | F+GDASL+ RPMGRVLNPLREMGV+V S GDRLP+TL+G PITYRVPMASAQVKS FMGDASLSSRPMGRVLNPLREMGVEVLSRSGDRLPLTLKGADPAQPITYRVPMASAQVKS | 197 |
| Query 179 | AVLLAGLNTPGITTVIEPIMTRDHTEKMLQGFGANLTVETDADGVRTIRLEGRGKLTGQV | 238 |
| Sbjct 198 | VLLAGLN G T VIEP+ TRDHTEKML+GFGA L E + G + +EG +LT Q CVLLAGLNMAGTTRVIEPVKTRDHTEKMLRGFGATLGEEMNDAGETVLTIEGLQELTAQD | 257 |
| Query 239 | IDVPGDPSSTAFPLVAALLVPGSDVTILNVLMNPTRTGLILTLQEMGADIEVINPRLAGG | 298 |
| Sbjct 258 | + VPGDPSS AFP+VAAL+ PGS++ I ++L+N TRTGLI TL EMGADI + N R +GG VTVPGDPSSAAFPIVAALITPGSELVIEDILLNETRTGLITTLLEMGADITIENERNSGG | 317 |
| Query 299 | EDVADLRVRSSTLKGVTVPEDRAPSMIDEYPILAVAAAFAEGATVMNGLEELRVKESDRL | 358 |
| Sbjct 318 | E + D+RVRSS LKGVTVP RAPSMIDEYP+L+VAAA+AEG T M GLEELRVKESDRL ERIGDIRVRSSALKGVTVPAARAPSMIDEYPVLSVAAAYAEGETRMEGLEELRVKESDRL | 377 |
| Query 359 | SAVANGLKLNGVDCDEGETSLVVRGRPDGKGLGNASGAAVATHLDHRIAMSFLVMGLVSE | 418 |

| Sbjct | 378 | +AVA GL+ NGV +EG+ LVVRG G A G V THLDHRIAMSFLV+GL +E  AAVAAGLEANGVPFEEGKDYLVVRG-----GGTIAGGGRVITHLDHRIAMSFLVLGLGAE 432 |
| --- | --- | --- |
| Query | 419 | NPVTVDDATMIATSFPEFMDLMAGLGAKIE 448 |
| Sbjct | 433 | PVTVDDA +I TSFP F DLM LGA+ E KPVTVDDAAIINTSFPGFADLMEALGAEFE 462 |

>TR:A0A1U7JHZ0 A0A1U7JHZ0_9RHOB 3-phosphoshikimate 1-carboxyvinyltransferase OS=Nesiotobacter exalbescens OX=197461 GN=aroA PE=3 SV=1

Length=450

Score = 542 bits (1397), Expect = 0.0

| Identities | = 280/455 (62%), Positives = 335/455 (74%), Gaps = 6/455 (1%) |  |
| --- | --- | --- |
| Query 1 | MLHGASSRPATARKSSGLSGTVRIPGDKSISHRSFMFGGLASGETRITGLLEGEDVINTG | 60 |
| Sbjct 1 | M H +S + T K L+GT+R+PGDKSISHRS MFG LA G+T+I GLLE EDV+ T MSHNSSPQIVTTTKGEALTGTIRVPGDKSISHRSLMFGALAVGQTKIEGLLESEDVLATA | 60 |
| Query 61 | KAMQAMGARIRKEGDT-WIIDGVGNGGLLAPEAPLDFGNAATGCRLTMGLVGVYDFDSTF | 119 |
| Sbjct 61 | AM A+GA K D W +DGVG G LL PE P+DFGNA TG RL MG+ + T NAMNAVGASCHKHEDGHWTVDGVGLGSLLEPEHPIDFGNAGTGARLCMGIFASHPIAVTM | 120 |
| Query 120 | IGDASLTKRPMGRVLNPLREMGVQVKSEDGDRLPVTLRGPKTPTPITYRVPMASAQVKSA | 179 |
| Sbjct 121 | +GDASL++RPMGRVL+PLREMG QV + GDRLP ++RG +TP P+TYRVPMASAQVKSA VGDASLSRRPMGRVLDPLREMGTQVIARRGDRLPASIRGSETPLPLTYRVPMASAQVKSA | 180 |
| Query 180 | VLLAGLNTPGITTVIEPIMTRDHTEKMLQGFGANLTVETDADGVRTIRLEGRGKLTGQVI | 239 |
| Sbjct 181 | VLLAGLNTPG TTVIEP+ TRDHTEKML GFGA L+VE +A G R I L+G+ +L Q + VLLAGLNTPGTTTVIEPVATRDHTEKMLLGFGAELSVEVNAAGERVITLQGQPQLKPQSV | 240 |
| Query 240 | DVPGDPSSTAFPLVAALLVPGSDVTILNVLMNPTRTGLILTLQEMGADIEVINPRLAGGE | 299 |
| Sbjct 241 | VPGDPSS AFP+VAAL+VPGS+V + +L+N R GLI TL EMGADI + N R +GGE TVPGDPSSAAFPIVAALIVPGSNVVVEGILLNEQRIGLITTLLEMGADITIENERESGGE | 300 |
| Query 300 | DVADLRVRSSTLKGVTVPEDRAPSMIDEYPILAVAAAFAEGATVMNGLEELRVKESDRLS | 359 |
| Sbjct 301 | + D+ VR S LKGVTVP +RAPSMIDEYP+LA+AAAFA+G T M GLEELRVKESDRL+ KIGDIHVRHSNLKGVTVPAERAPSMIDEYPVLAIAAAFAQGDTTMLGLEELRVKESDRLA | 360 |
| Query 360 | AVANGLKLNGVDCDEGETSLVVRGRPDGKGLGNASGAAVATHLDHRIAMSFLVMGLVSEN | 419 |
| Sbjct 361 | AVA GL+ N + C EG+ +L V G G G G V THLDHRIAMSFLV+GL ++ AVARGLEANNIPCIEGKDTLTVTGGATGIG GGTVETHLDHRIAMSFLVLGLAADK | 415 |
| Query 420  Sbjct 416 | PVTVDDATMIATSFPEFMDLMAGLGAKIELSDTKA 454 PVTVDDA+ IATSFP F+ L+ LG + A PVTVDDASPIATSFPSFLPLLKELGGTVRFQGENA 450 |  |

>TR:A0A1G5NRL7 A0A1G5NRL7_AFIMA 3-phosphoshikimate 1-carboxyvinyltransferase OS=Afifella marina DSM 2698 OX=1120955 GN=aroA PE=3 SV=1

Length=455

Score = 541 bits (1395), Expect = 0.0

| Identities | = 282/444 (64%), Positives = 333/444 (75%), Gaps = 1/444 (0%) |  |
| --- | --- | --- |
| Query 5 | ASSRPATARKSSGLSGTVRIPGDKSISHRSFMFGGLASGETRITGLLEGEDVINTGKAMQ | 64 |
| Sbjct 4 | A RP+TAR SSGLSG V +PGDKSISHRS MFG LA GETRITGLLE DV+ T AM+ AVPRPSTARASSGLSGRVTVPGDKSISHRSLMFGALALGETRITGLLEAADVLATAAAMR | 63 |
| Query 65 | AMGARIRKEGD-TWIIDGVGNGGLLAPEAPLDFGNAATGCRLTMGLVGVYDFDSTFIGDA | 123 |
| Sbjct 64 | + GA++ + W + G+G GG L PE LDFGNA TG RLTM L G + F +TF+GDA SFGAKVERHSTGEWSLHGLGTGGFLEPETALDFGNAGTGARLTMALAGAHPFATTFLGDA | 123 |
| Query 124 | SLTKRPMGRVLNPLREMGVQVKSEDGDRLPVTLRGPKTPTPITYRVPMASAQVKSAVLLA | 183 |

SL++RPMGR+L+PLREMGV+V + GDRLP+TL+G	PI YRVP+ SAQVKSAVLLA

| Sbjct | 124 | SLSRRPMGRILDPLREMGVEVIARSGDRLPLTLKGASPMAPIEYRVPVPSAQVKSAVLLA | 183 |
| --- | --- | --- | --- |
| Query | 184 | GLNTPGITTVIEPIMTRDHTEKMLQGFGANLTVETDADGVRTIRLEGRGKLTGQVIDVPG | 243 |
| Sbjct | 184 | GLN G TTVIEP+ TRDHTEKML+GFGA++ V+T A G IRLEG +LT Q I+VP GLNIAGTTTVIEPVPTRDHTEKMLKGFGADIEVKTTASGETVIRLEGLPELTPQPIEVPS | 243 |
| Query | 244 | DPSSTAFPLVAALLVPGSDVTILNVLMNPTRTGLILTLQEMGADIEVINPRLAGGEDVAD | 303 |
| Sbjct | 244 | DPSS AF +VA L+ P SD+ + VL+N RTGL+ LQ+MGAD+ + N R GGE + D DPSSAAFLIVAGLICPDSDIVVEGVLLNERRTGLLQVLQDMGADLSIENERETGGERLGD | 303 |
| Query | 304 | LRVRSSTLKGVTVPEDRAPSMIDEYPILAVAAAFAEGATVMNGLEELRVKESDRLSAVAN | 363 |
| Sbjct | 304 | +R R+S LKGVTVP +RAPSMIDEYPILA+AAA AEG TVM+GLEELRVKESDRL+AVA VRARTSRLKGVTVPAERAPSMIDEYPILAIAAACAEGKTVMHGLEELRVKESDRLAAVAA | 363 |
| Query | 364 | GLKLNGVDCDEGETSLVVRGRPDGKGLGNASGAAVATHLDHRIAMSFLVMGLVSENPVTV | 423 |
| Sbjct | 364 | GL NGV C+EGE SL V G G GA V THLDHRIAMSFLV+GL +E PVTV GLAANGVTCEEGEASLAVTGCKVPGGGKVPGGATVTTHLDHRIAMSFLVLGLAAEKPVTV | 423 |
| Query  Sbjct | 424  424 | DDATMIATSFPEFMDLMAGLGAKI 447 DDA MIATSFP F + + LGA + DDANMIATSFPNFEETLRKLGADL 447 |  |

>TR:A0A346QXV6 A0A346QXV6_9RHIZ 3-phosphoshikimate 1-carboxyvinyltransferase OS=Breoghania sp. L-A4 OX=2304600 GN=aroA PE=3 SV=1

Length=440

Score = 541 bits (1393), Expect = 0.0

| Identities | = 279/432 (65%), Positives = 331/432 (77%), Gaps = 6/432 (1%) |  |
| --- | --- | --- |
| Query 18 | LSGTVRIPGDKSISHRSFMFGGLASGETRITGLLEGEDVINTGKAMQAMGARIRKEGD-T | 76 |
| Sbjct 9 | L G VR+PGDKSISHR+ MFG LA GETRI+GLLE EDV +TG AM+A+GA + + D LKGEVRVPGDKSISHRALMFGALAVGETRISGLLESEDVHHTGDAMRALGASVHRSDDGV | 68 |
| Query 77 | WIIDGVGNGGLLAPEAPLDFGNAATGCRLTMGLVGVYDFDSTFIGDASLTKRPMGRVLNP | 136 |
| Sbjct 69 | W + GVG G L PE LDFGNA TG RL MG+VG + +TFIGDASL+KRPMGRVL+P WHVRGVGVGALREPENALDFGNAGTGARLVMGMVGSHPITATFIGDASLSKRPMGRVLDP | 128 |
| Query 137 | LREMGVQVKSEDGDRLPVTLRGPKTPTPITYRVPMASAQVKSAVLLAGLNTPGITTVIEP | 196 |
| Sbjct 129 | LR+MG QV + GDRLP+TLRGP+T P++YRVP+ SAQVKSAVL+AGLN PG+TTVIEP LRQMGTQVLARAGDRLPLTLRGPETAMPLSYRVPVPSAQVKSAVLIAGLNAPGVTTVIEP | 188 |
| Query 197 | IMTRDHTEKMLQGFGANLTVETDADGVRTIRLEGRGKLTGQVIDVPGDPSSTAFPLVAAL | 256 |
| Sbjct 189 | + TRDHTE+ML GFGA++ + G R IRL G+ +LT Q I VP DPSS AFP+VAAL VPTRDHTERMLSGFGADIVTTQNEAGERVIRLTGQPELTPQDITVPCDPSSAAFPIVAAL | 248 |
| Query 257 | LVPGSDVTILNVLMNPTRTGLILTLQEMGADIEVINPRLAGGEDVADLRVRSSTLKGVTV | 316 |
| Sbjct 249 | + PGS+VT+ +VL+NP RTGL TL+EMGAD+E++N R GGE + D+RVR LKGVTV ITPGSEVTVRDVLLNPHRTGLFTTLREMGADLEILNERETGGERLGDIRVRHGVLKGVTV | 308 |
| Query 317 | PEDRAPSMIDEYPILAVAAAFAEGATVMNGLEELRVKESDRLSAVANGLKLNGVDCDEGE | 376 |
| Sbjct 309 | P DRAPSMIDEYP+LA+AAAFA+G TVM GLEELRVKESDRLSAVA GLK NG+D +EG PADRAPSMIDEYPVLAIAAAFAQGETVMLGLEELRVKESDRLSAVAAGLKANGIDHEEGA | 368 |
| Query 377 | TSLVVRGRPDGKGLGNASGAAVATHLDHRIAMSFLVMGLVSENPVTVDDATMIATSFPEF | 436 |
| Sbjct 369 | +L+VRG G G V THLDHRIAMSFLV+GL ++ PVTVDD IATSFP F DTLLVRGASTVPG GGTVVTHLDHRIAMSFLVLGLAAQAPVTVDDGAPIATSFPGF | 423 |
| Query 437  Sbjct 424 | MDLMAGLGAKIE 448 L LG IE  TQLFESLGGNIE 435 |  |

>TR:A0A1I4SRU6 A0A1I4SRU6_9RHIZ 3-phosphoshikimate 1-carboxyvinyltransferase OS=Pleomorphomonas diazotrophica OX=1166257 GN=aroA PE=3 SV=1

Length=450

Score = 540 bits (1391), Expect = 0.0

| Identities | = 288/456 (63%), Positives = 339/456 (74%), Gaps = 7/456 (2%) |  |
| --- | --- | --- |
| Query 1 | MLHGASSRPATARKSSGLSGTVRIPGDKSISHRSFMFGGLASGETRITGLLEGEDVINTG | 60 |
| Sbjct 1 | M A++RP TA KS LSG +R+PGDKSISHR+ M G LA GET ITGLLE EDV+NT MASHATARPLTAVKSGPLSGRIRVPGDKSISHRALMLGALAVGETVITGLLEAEDVMNTA | 60 |
| Query 61 | KAMQAMGARIRKEGD-TWIIDGVGNGGLLAPEAPLDFGNAATGCRLTMGLVGVYDFDSTF | 119 |
| Sbjct 61 | AM+A GA + ++ D TW + G+G GGLL P +DFGNA TG RLTMGL TF AAMRAFGATVLRDDDGTWRVRGLGVGGLLEPAGIIDFGNAGTGVRLTMGLAAAQPIAVTF | 120 |
| Query 120 | IGDASLTKRPMGRVLNPLREMGVQVKSEDGDRLPVTLRGPKTPTPITYRVPMASAQVKSA | 179 |
| Sbjct 121 | GDASL KRPMGRVL PL EMGV+V + G RLP+T+RGP+ P PITYR+P+ SAQVKSA TGDASLIKRPMGRVLEPLSEMGVEVIARSGGRLPLTMRGPRLPLPITYRLPVPSAQVKSA | 180 |
| Query 180 | VLLAGLNTPGITTVIEPIMTRDHTEKMLQGFGANLTVETDADGVRTIRLEGRGKLTGQVI | 239 |
| Sbjct 181 | VLLAGL TPG+TTVIEP+ TRDHTE+ML+GFGA++ + ++ DG R I L GR +L Q I VLLAGLGTPGVTTVIEPVFTRDHTERMLKGFGADIEITSEPDGGRRIALAGRPELKPQAI | 240 |
| Query 240 | DVPGDPSSTAFPLVAALLVPGSDVTILNVLMNPTRTGLILTLQEMGADIEVINPRLAGGE | 299 |
| Sbjct 241 | DVP DPSS AFP+VAA LV GSD+T+ VLMNP R GLI TL EMGA+IEV R GGE DVPSDPSSAAFPIVAATLVEGSDITVEGVLMNPARIGLIDTLLEMGANIEVTAERTTGGE | 300 |
| Query 300 | DVADLRVRSSTLKGVTVPEDRAPSMIDEYPILAVAAAFAEGATVMNGLEELRVKESDRLS | 359 |
| Sbjct 301 | +ADLRVR + LKGVTVP +RAPSMIDEYPILAVAAA A G T M G+ E+RVKESDRL+ RLADLRVRHAELKGVTVPAERAPSMIDEYPILAVAAAAASGETRMPGIGEMRVKESDRLA | 360 |
| Query 360 | AVANGLKLNGVDCDEGETSLVVRGRPDGKGLGNASGAAVATHLDHRIAMSFLVMGLVSEN | 419 |
| Sbjct 361 | AVA GL+ NG+ C EG L VRG G+ A G V THLDHRIAMSFL++G+V+ N AVAAGLRANGIACSEGPDWLSVRG---GR---IAGGGRVVTHLDHRIAMSFLILGMVAGN | 414 |
| Query 420  Sbjct 415 | PVTVDDATMIATSFPEFMDLMAGLGAKIELSDTKAA 455 PV+VDDA+MIATSFP F LM GLGA E ++ A PVSVDDASMIATSFPTFEALMTGLGATFEAAEAADA 450 |  |

>TR:A0A368A6V2 A0A368A6V2_9RHIZ 3-phosphoshikimate 1-carboxyvinyltransferase OS=Candidatus Tokpelaia sp. JSC161 OX=2079007 GN=aroA PE=3

SV=1

Length=445

Score = 540 bits (1391), Expect = 0.0

| Identities | = 276/440 (63%), Positives = 340/440 (77%), Gaps = 4/440 (1%) |  |
| --- | --- | --- |
| Query 8 | RPATARKSSGLSGTVRIPGDKSISHRSFMFGGLASGETRITGLLEGEDVINTGKAMQAMG | 67 |
| Sbjct 8 | + A A +SS L G V IPGDKS+SHRS + GGLASGET ITGLLE +DV+NT KAMQA+G KSARAYRSSPLQGDVYIPGDKSMSHRSLIIGGLASGETHITGLLESDDVLNTCKAMQAIG | 67 |
| Query 68 | ARIRKEGDTWIIDGVGNGGLLAPEAPLDFGNAATGCRLTMGLVGVYDFDSTFIGDASLTK | 127 |
| Sbjct 68 | A I KE WII G GN LL PL FGN+ TG RL MGLVG Y+ +TFIGD SL+K ALIYKEKKRWIIKGTGNSCLLEAHHPLYFGNSGTGARLVMGLVGTYNMQTTFIGDISLSK | 127 |
| Query 128 | RPMGRVLNPLREMGVQVKSEDGDRLPVTLRGPKTPTPITYRVPMASAQVKSAVLLAGLNT | 187 |
| Sbjct 128 | RPM R+L+PL+ MG QV +++ + LP+TL GP T PI Y VP+ASAQVKS++LLAGLNT RPMDRILDPLKLMGTQVLTKN-NHLPITLLGPTTAAPIIYHVPIASAQVKSSLLLAGLNT | 186 |
| Query 188 | PGITTVIEPIMTRDHTEKMLQGFGANLTVETDADGVRTIRLEGRGKLTGQVIDVPGDPSS | 247 |
| Sbjct 187 | PGITT+IE +TRDHTE ML+ FGANL V+ ++ G R I L G+G L GQ I VPGDPSS  PGITTIIEDHITRDHTENMLKNFGANLEVKVNSYGQRKISLTGKGNLKGQNITVPGDPSS | 246 |

| Query | 248 | TAFPLVAALLVPGSDVTILNVLMNPTRTGLILTLQEMGADIEVINPRLAGGEDVADLRVR | 307 |
| --- | --- | --- | --- |
| Sbjct | 247 | AFPLVAAL++P SD+ I N+L+NPTR G++LTLQEMG DI + N R G E VAD+RVR AAFPLVAALIIPESDIIIRNILINPTRIGILLTLQEMGGDIVLFNKRDIGSEPVADIRVR | 306 |
| Query | 308 | SSTLKGVTVPEDRAPSMIDEYPILAVAAAFAEGATVMNGLEELRVKESDRLSAVANGLKL | 367 |
| Sbjct | 307 | SS LKG+ + +R PSMIDEYPIL++AAAFAEG T+M G+EELRVKESDRL+A+ANGLK SSKLKGICILPNRVPSMIDEYPILSIAAAFAEGKTIMKGIEELRVKESDRLTAIANGLKA | 366 |
| Query | 368 | NGVDCDEGETSLVVRGRPDGKGLGNASGAAVATHLDHRIAMSFLVMGLVSENPVTVDDAT | 427 |
| Sbjct | 367 | N + C+EG SL+++G+P GKG G G V+T+LDHRIAMSFL++GL SE V++DD+T NHISCEEGVDSLIIKGQPHGKGFG---GGRVSTYLDHRIAMSFLILGLASEYDVSIDDST | 423 |
| Query  Sbjct | 428  424 | MIATSFPEFMDLMAGLGAKI 447 MI+TSFP+F +M LGA + MISTSFPQFHSIMKKLGANL 443 |  |

>TR:A0A285TDL8 A0A285TDL8_9RHOB 3-phosphoshikimate 1-carboxyvinyltransferase OS=Stappia indica OX=538381 GN=aroA PE=3 SV=1

Length=451

Score = 539 bits (1389), Expect = 0.0

| Identities | = 275/456 (60%), Positives = 338/456 (74%), Gaps = 6/456 (1%) |  |
| --- | --- | --- |
| Query 1 | MLHGASSRPATARKSSGLSGTVRIPGDKSISHRSFMFGGLASGETRITGLLEGEDVINTG | 60 |
| Sbjct 1 | M H ++RP TAR + L G V +PGDKSISHR+ M G LA G T ++GLLE EDV+NT MSHSTAARPLTARSGAALKGRVTVPGDKSISHRALMLGALAIGRTTVSGLLESEDVLNTA | 60 |
| Query 61 | KAMQAMGARIRKEGD-TWIIDGVGNGGLLAPEAPLDFGNAATGCRLTMGLVGVYDFDSTF | 119 |
| Sbjct 61 | AM+A GARI+++ + W +DG G G L+ PE +D+GNA TG RL MG+ G + +TF AAMRAFGARIQRDANGLWTVDGAGLGSLVEPETVIDYGNAGTGVRLAMGIAGTHPIAATF | 120 |
| Query 120 | IGDASLTKRPMGRVLNPLREMGVQVKSEDGDRLPVTLRGPKTPTPITYRVPMASAQVKSA | 179 |
| Sbjct 121 | GDASL KRPMGRVL PLR MG +V S GDRLP+++ GP TP PI+YRVP+ SAQVKSA TGDASLVKRPMGRVLEPLRMMGTRVISRSGDRLPLSIHGPGTPAPISYRVPVPSAQVKSA | 180 |
| Query 180 | VLLAGLNTPGITTVIEPIMTRDHTEKMLQGFGANLTVETDADGVRTIRLEGRGKLTGQVI | 239 |
| Sbjct 181 | VLLAGLN PG TTVIEPI TRDHTE+ML GFGA + V+ D +G R I + G+G+L Q + VLLAGLNAPGQTTVIEPIPTRDHTERMLAGFGATIQVDHDDNGGRVITVTGQGELQPQDV | 240 |
| Query 240 | DVPGDPSSTAFPLVAALLVPGSDVTILNVLMNPTRTGLILTLQEMGADIEVINPRLAGGE | 299 |
| Sbjct 241 | VP DPSS AFP+VA L+VPGS+VT+ +VL+N RTGL+ TLQEMGAD+E+ N R GGE TVPADPSSAAFPIVAGLIVPGSEVTVADVLLNEHRTGLLTTLQEMGADLEITNIRETGGE | 300 |
| Query 300 | DVADLRVRSSTLKGVTVPEDRAPSMIDEYPILAVAAAFAEGATVMNGLEELRVKESDRLS | 359 |
| Sbjct 301 | + D+RVR S L+GV VP DRAPSMIDEYP+LAVAAAFA G T M G+EEL+VKESDRL+ RLGDVRVRHSQLRGVEVPADRAPSMIDEYPVLAVAAAFAHGTTTMLGVEELKVKESDRLA | 360 |
| Query 360 | AVANGLKLNGVDCDEGETSLVVRGRPDGKGLGNASGAAVATHLDHRIAMSFLVMGLVSEN | 419 |
| Sbjct 361 | AVA GL+ NG++C+EGE++L V GR G G V THLDHRIAM+FLV+GL ++ AVAAGLRANGIECEEGESTLRVHGRK GPIGGGTVTTHLDHRIAMTFLVLGLAADR | 415 |
| Query 420  Sbjct 416 | PVTVDDATMIATSFPEFMDLMAGLGAKIELSDTKAA 455 PVTVDD +IATSFP F L A LG I + +AA PVTVDDGGVIATSFPTFEALFAELGGDISETGEQAA 451 |  |

>TR:A0A2S5TWS2 A0A2S5TWS2_9RHIZ 3-phosphoshikimate 1-carboxyvinyltransferase OS=Kaistia algarum OX=2083279 GN=aroA PE=3 SV=1

Length=457

Score = 538 bits (1387), Expect = 0.0

Identities = 284/451 (63%), Positives = 330/451 (73%), Gaps = 6/451 (1%)

| Query | 5 | ASSRPATARKSSGLSGTVRIPGDKSISHRSFMFGGLASGETRITGLLEGEDVINTGKAMQ | 64 |
| --- | --- | --- | --- |
| Sbjct | 11 | A + P TA S L+GT+R+PGDKSISHR+ M G LA GET I GLLEGEDV+ T AM+ ADAGPLTASASGPLTGTIRVPGDKSISHRALMLGALAIGETVIEGLLEGEDVLATAAAMR | 70 |
| Query | 65 | AMGARIRKEGD-TWIIDGVGNGGLLAPEAPLDFGNAATGCRLTMGLVGVYDFDSTFIGDA | 123 |
| Sbjct | 71 | A GA + + G W++ GVG GG L PE +D+GNA TG RL MGL G TF GDA AFGASVTRTGTGVWVVKGVGVGGFLEPEDVIDYGNAGTGVRLAMGLAGSQAIAVTFTGDA | 130 |
| Query | 124 | SLTKRPMGRVLNPLREMGVQVKSEDGDRLPVTLRGPKTPTPITYRVPMASAQVKSAVLLA | 183 |
| Sbjct | 131 | SL +RPMGR+L+PLR MGVQV + RLP+T+RGP PI YRVP+ SAQVKSAVLLA SLRRRPMGRILDPLRRMGVQVLARSQGRLPLTIRGPDVALPIEYRVPVPSAQVKSAVLLA | 190 |
| Query | 184 | GLNTPGITTVIEPIMTRDHTEKMLQGFGANLTVETDADGVRTIRLEGRGKLTGQVIDVPG | 243 |
| Sbjct | 191 | GLNTPG+TTVIEP+MTRDHTE+ML FGA + ++ DG R IR+EGR L Q I VPG GLNTPGVTTVIEPVMTRDHTERMLAAFGAGIEIDKGEDGERIIRVEGRLDLKAQAIRVPG | 250 |
| Query | 244 | DPSSTAFPLVAALLVPGSDVTILNVLMNPTRTGLILTLQEMGADIEVINPRLAGGEDVAD | 303 |
| Sbjct | 251 | DPSS AFP+VAAL+V GSD+TI +VL+NP R GLI TL EMG DI + N R GGE V D DPSSAAFPMVAALIVEGSDITIESVLLNPMRNGLIETLIEMGGDIMIDNRRTVGGESVGD | 310 |
| Query | 304 | LRVRSSTLKGVTVPEDRAPSMIDEYPILAVAAAFAEGATVMNGLEELRVKESDRLSAVAN | 363 |
| Sbjct | 311 | +RVRSS LKGV VP +RAPSMIDEYP+LA+AAAFAEG TVM GL ELRVKESDRLSA+A IRVRSSRLKGVVVPPERAPSMIDEYPVLAIAAAFAEGRTVMEGLGELRVKESDRLSAMAA | 370 |
| Query | 364 | GLKLNGVDCDEGETSLVVRGRPDGKGLGNASGAAVATHLDHRIAMSFLVMGLVSENPVTV | 423 |
| Sbjct | 371 | GL NG+ C+EG SLV+ G G A G +V THLDHRIAMSFLV+GL S VT+ GLSANGILCEEGPESLVLTG GAVVAGGGSVKTHLDHRIAMSFLVLGLASGQGVTI | 425 |
| Query  Sbjct | 424  426 | DDATMIATSFPEFMDLMAGLGAKIELSDTKA 454 DDA IATSFPEF LM GLGA+ E + A DDAAPIATSFPEFSTLMTGLGARFEAMEPTA 456 |  |

>TR:A0A2N5XN44 A0A2N5XN44_9RHIZ 3-phosphoshikimate 1-carboxyvinyltransferase OS=Cohaesibacter celericrescens OX=2067669 GN=aroA PE=3 SV=1

Length=445

Score = 536 bits (1381), Expect = 0.0

| Identities | = 280/449 (62%), Positives = 334/449 (74%), Gaps = 7/449 (2%) |  |
| --- | --- | --- |
| Query 1 | MLHGASS-RPATARKSSGLSGTVRIPGDKSISHRSFMFGGLASGETRITGLLEGEDVINT | 59 |
| Sbjct 1 | M H A+ P +A KS GL G +R+PGDKSISHR+ +FGGLA G T+I+GLLE EDVINT MAHDATQLSPLSAHKSQGLKGRMRVPGDKSISHRALIFGGLARGITKISGLLESEDVINT | 60 |
| Query 60 | GKAMQAMGARIRKEGD-TWIIDGVGNGGLLAPEAPLDFGNAATGCRLTMGLVGVYDFDST | 118 |
| Sbjct 61 | KAM A GA+I K+ D W ++G+G G LL P LDFGN+ TG RL MGL G + F +T AKAMAAFGAQIHKDNDGIWFVEGLGTGSLLEPSTVLDFGNSGTGARLVMGLAGAHAFSTT | 120 |
| Query 119 | FIGDASLTKRPMGRVLNPLREMGVQVKSEDGDRLPVTLRGPKTPTPITYRVPMASAQVKS | 178 |
| Sbjct 121 | F+GD SL+ RPM RVLNPLREMG QV GDRLP+T+ G TPITYRVPMASAQVKS FMGDGSLSGRPMARVLNPLREMGTQVLGRSGDRLPLTIHGADPATPITYRVPMASAQVKS | 180 |
| Query 179 | AVLLAGLNTPGITTVIEPIMTRDHTEKMLQGFGANLTVETDADGVRTIRLEGRGKLTGQV | 238 |
| Sbjct 181 | VLLAGLN G TT+IEP+ TRDHTE+ML+GFGA L E + G + L+G +LT Q CVLLAGLNMAGTTTLIEPVKTRDHTERMLRGFGAELGEEINEAGETILTLQGLQELTAQD | 240 |
| Query 239 | IDVPGDPSSTAFPLVAALLVPGSDVTILNVLMNPTRTGLILTLQEMGADIEVINPRLAGG | 298 |
| Sbjct 241 | + VPGDPSS AFP+VAAL+ PGSD+ I ++L+N TRTGLI TL EMG DI + N R +GG VIVPGDPSSAAFPIVAALITPGSDLLIEDILLNDTRTGLITTLIEMGGDITIENERNSGG | 300 |
| Query 299 | EDVADLRVRSSTLKGVTVPEDRAPSMIDEYPILAVAAAFAEGATVMNGLEELRVKESDRL | 358 |
| Sbjct 301 | E + ++RVRSS LKGVTVP +RAPSMIDEYP+L+VAA+FA G T M GLEELRVKESDRL  ERIGNVRVRSSRLKGVTVPAERAPSMIDEYPVLSVAASFASGETRMEGLEELRVKESDRL | 360 |

| Query | 359 | SAVANGLKLNGVDCDEGETSLVVRGRPDGKGLGNASGAAVATHLDHRIAMSFLVMGLVSE 418 |
| --- | --- | --- |
| Sbjct | 361 | +AVA GL NGV +EG L+V G G + G VATHLDHRIAMSFL +GL +E AAVAAGLAANGVPHEEGNDYLIVTG-----GSNHIGGGTVATHLDHRIAMSFLTLGLGAE 415 |
| Query | 419 | NPVTVDDATMIATSFPEFMDLMAGLGAKI 447 |
| Sbjct | 416 | PVTVDDA +I TSFP F+DLM+GLGA + KPVTVDDAAIINTSFPGFVDLMSGLGANL 444 |

>TR:A0A1H2IU89 A0A1H2IU89_9RHOB 3-phosphoshikimate 1-carboxyvinyltransferase OS=Stappia sp. ES.058 OX=1881061 GN=aroA PE=3 SV=1

Length=451

Score = 535 bits (1379), Expect = 0.0

| Identities | = 276/440 (63%), Positives = 331/440 (75%), Gaps = 6/440 (1%) |  |
| --- | --- | --- |
| Query 9 | PATARKSSGLSGTVRIPGDKSISHRSFMFGGLASGETRITGLLEGEDVINTGKAMQAMGA | 68 |
| Sbjct 9 | P TAR S LSG V +PGDKSISHR+ M G LA G T I+GLLE EDV+NT AM+A GA PYTARAGSRLSGRVTVPGDKSISHRALMLGALAVGRTTISGLLESEDVLNTAAAMRAFGA | 68 |
| Query 69 | RI-RKEGDTWIIDGVGNGGLLAPEAPLDFGNAATGCRLTMGLVGVYDFDSTFIGDASLTK | 127 |
| Sbjct 69 | RI R E TW IDGVG GGL+ P+ +D+GNA TG RL +GL G + +TF GDASL++ RIERGEDGTWCIDGVGVGGLMEPDGIIDYGNAGTGVRLALGLAGTHPIATTFTGDASLSR | 128 |
| Query 128 | RPMGRVLNPLREMGVQVKSEDGDRLPVTLRGPKTPTPITYRVPMASAQVKSAVLLAGLNT | 187 |
| Sbjct 129 | RPMGRVL+PLR+MG V + GDRLP+++RG T PITYRVP+ SAQVKSAVLLAGLNT RPMGRVLDPLRQMGALVVARQGDRLPLSIRGASTAAPITYRVPVPSAQVKSAVLLAGLNT | 188 |
| Query 188 | PGITTVIEPIMTRDHTEKMLQGFGANLTVETDADGVRTIRLEGRGKLTGQVIDVPGDPSS | 247 |
| Sbjct 189 | PG TTVIEP+ TRDHTE+ML GFGA+++VE DADG R I L G L Q I VP DPSS PGQTTVIEPVATRDHTERMLAGFGADISVEIDADGARVISLTGPATLAPQEITVPCDPSS | 248 |
| Query 248 | TAFPLVAALLVPGSDVTILNVLMNPTRTGLILTLQEMGADIEVINPRLAGGEDVADLRVR | 307 |
| Sbjct 249 | AFP+VAAL+ PGSDVT+ +VL+N RTGL TLQEMGAD+ + N R GGE + D+R + AAFPIVAALITPGSDVTVADVLLNEHRTGLFKTLQEMGADLTIENIRETGGEQLGDIRAK | 308 |
| Query 308 | SSTLKGVTVPEDRAPSMIDEYPILAVAAAFAEGATVMNGLEELRVKESDRLSAVANGLKL | 367 |
| Sbjct 309 | S L G+ VP +RAPSMIDEYP+LAVAAAFA+G TVM GLEELRVKE DRL+AVA GL HSRLTGIEVPSERAPSMIDEYPVLAVAAAFAQGTTVMRGLEELRVKECDRLAAVAAGLAA | 368 |
| Query 368 | NGVDCDEGETSLVVRGRPDGKGLGNASGAAVATHLDHRIAMSFLVMGLVSENPVTVDDAT | 427 |
| Sbjct 369 | N + C+EG++SL V GR G G V THLDHRIAM+FLV+G+ +++PV+VDDA NRIVCEEGDSSLTVHGRQ GPIGGGTVVTHLDHRIAMTFLVLGMGADDPVSVDDAG | 423 |
| Query 428  Sbjct 424 | MIATSFPEFMDLMAGLGAKI 447  +IATSFP F+ L A LGA + VIATSFPTFLSLFADLGASL 443 |  |

>TR:A0A285PH73 A0A285PH73_9RHIZ 3-phosphoshikimate 1-carboxyvinyltransferase OS=Cohaesibacter gelatinilyticus OX=372072 GN=aroA PE=3 SV=1

Length=446

Score = 529 bits (1362), Expect = 0.0

Identities = 279/448 (62%), Positives = 330/448 (74%), Gaps = 7/448 (2%)

| Query | 1 | MLHGASSR-PATARKSSGLSGTVRIPGDKSISHRSFMFGGLASGETRITGLLEGEDVINT | 59 |
| --- | --- | --- | --- |
| Sbjct | 1 | M H AS+ P ++ +S GL G +++PGDKSISHRS +FG LA GET ++GLLE EDV+NT MAHDASNMTPLSSLRSDGLKGKLQVPGDKSISHRSLIFGALAKGETVVSGLLESEDVLNT | 60 |
| Query | 60 | GKAMQAMGARIRKEGD-TWIIDGVGNGGLLAPEAPLDFGNAATGCRLTMGLVGVYDFDST | 118 |
| Sbjct | 61 | KAMQA GA++ K+ W + GVG G LL P LDFGN+ TG RL MGL G + F ST  AKAMQAFGAQVHKDDQGLWHVTGVGTGSLLEPIDVLDFGNSGTGARLVMGLAGAHAFAST | 120 |

| Query | 119 | FIGDASLTKRPMGRVLNPLREMGVQVKSEDGDRLPVTLRGPKTPTPITYRVPMASAQVKS | 178 |
| --- | --- | --- | --- |
| Sbjct | 121 | FIGDASL+ RPMGRVLNPLREMGV+V + DRLP+T++G TPITYRVPMASAQVKS FIGDASLSGRPMGRVLNPLREMGVEVLARSKDRLPLTIKGADPATPITYRVPMASAQVKS | 180 |
| Query | 179 | AVLLAGLNTPGITTVIEPIMTRDHTEKMLQGFGANLTVETDADGVRTIRLEGRGKLTGQV | 238 |
| Sbjct | 181 | VLLAGLN G TT+IEP+ TRDHTEKML+GFGA L E + G + LEG +LT Q CVLLAGLNMAGTTTLIEPVKTRDHTEKMLRGFGAELGEEINEAGETVLTLEGLQELTAQN | 240 |
| Query | 239 | IDVPGDPSSTAFPLVAALLVPGSDVTILNVLMNPTRTGLILTLQEMGADIEVINPRLAGG | 298 |
| Sbjct | 241 | + VP DPSS AFP+VAAL+ PGSD+ + NVL+N TRTGL+ TL EMGA++ + N R +GG VTVPADPSSAAFPIVAALITPGSDLLVENVLLNETRTGLLTTLVEMGANLTIENERESGG | 300 |
| Query | 299 | EDVADLRVRSSTLKGVTVPEDRAPSMIDEYPILAVAAAFAEGATVMNGLEELRVKESDRL | 358 |
| Sbjct | 301 | E + D+ VR S LKGVTVP +RAPSMIDEYP+L+VAA+FA G T M GLEELRVKESDRL ERIGDIHVRHSDLKGVTVPAERAPSMIDEYPVLSVAASFANGETRMEGLEELRVKESDRL | 360 |
| Query | 359 | SAVANGLKLNGVDCDEGETSLVVRGRPDGKGLGNASGAAVATHLDHRIAMSFLVMGLVSE | 418 |
| Sbjct | 361 | SAVA GL N V EGE LVV+G G G V THLDHRIAMSFLVMGL S+ SAVAAGLTANNVPHIEGEDYLVVKG GSNQIGGGTVVTHLDHRIAMSFLVMGLASQ | 415 |
| Query | 419 | NPVTVDDATMIATSFPEFMDLMAGLGAK 446 PV VDD +I TSFP F+DLM GLG + |  |
| Sbjct | 416 | KPVEVDDGAVINTSFPGFVDLMTGLGGR 443 | |

>TR:A0A1G6BL01 A0A1G6BL01_9RHIZ 3-phosphoshikimate 1-carboxyvinyltransferase OS=Bauldia litoralis OX=665467 GN=aroA PE=3 SV=1

Length=449

Score = 528 bits (1359), Expect = 0.0

| Identities | = 278/447 (62%), Positives = 326/447 (73%), Gaps = 10/447 (2%) |  |
| --- | --- | --- |
| Query 8 | RPATARKSSGLSGTVRIPGDKSISHRSFMFGGLASGETRITGLLEGEDVINTGKAMQAMG | 67 |
| Sbjct 8 | +P AR S L G++R PGDKSISHR+ M G LA GET + GLLEGEDV+ T +AM+A G KPLRARASGPLKGSIRPPGDKSISHRALMLGALAIGETTVEGLLEGEDVLATAEAMRAFG | 67 |
| Query 68 | AR-IRKEGDTWIIDGVGNGGLLAPEAPLDFGNAATGCRLTMGLVGVYDFDSTFIGDASLT | 126 |
| Sbjct 68 | A IR +G W + G+G GGLL P+ +D+GNA TG RL MGL G + F +TF GDASL ATVIRDDGGVWHVKGLGVGGLLEPDKVIDYGNAGTGVRLAMGLAGSHGFATTFTGDASLV | 127 |
| Query 127 | KRPMGRVLNPLREMGVQVKSEDGDRLPVTLRGPKTPTPITYRVPMASAQVKSAVLLAGLN | 186 |
| Sbjct 128 | RPMGR+ PLR MGVQ+ + GDRLP T+ GP+ P PI YRVP+ASAQVKSAVLLA LN GRPMGRIFEPLRRMGVQILARSGDRLPATIEGPEVPVPIEYRVPVASAQVKSAVLLAALN | 187 |
| Query 187 | TPGITTVIEPIMTRDHTEKMLQGFGANLTVETDADGVRTIRLEGRGKLTGQVIDVPGDPS | 246 |
| Sbjct 188 | G TTVIEPI TRDHTE+ML FGA + DGVRTI ++G L Q I VPGDPS FSGTTTVIEPIPTRDHTERMLAAFGAAIETTVADDGVRTIAIDGGADLQAQKIVVPGDPS | 247 |
| Query 247 | STAFPLVAALLVPGSDVTILNVLMNPTRTGLILTLQEMGADIEVINPRLAGGEDVADLRV | 306 |
| Sbjct 248 | S AFP+VAA +V GSDVT+ N L+NPTR GLI TL EMGADI + N R GGE+V D+RV SAAFPIVAAAIVEGSDVTVENALLNPTRIGLIDTLVEMGADITIDNRRSVGGEEVGDVRV | 307 |
| Query 307 | RSSTLKGVTVPEDRAPSMIDEYPILAVAAAFAEGATVMNGLEELRVKESDRLSAVANGLK | 366 |
| Sbjct 308 | R S +KGV VP +RAPSMIDEYPIL+VAAAFAEG T M G+ ELRVKESDR++ + GLK RGSRMKGVAVPPERAPSMIDEYPILSVAAAFAEGETRMEGIGELRVKESDRIAMMVAGLK | 367 |
| Query 367 | LNGVDCDEGETSLVVRG--RPDGKGLGNASGAAVATHLDHRIAMSFLVMGLVSENPVTVD | 424 |
| Sbjct 368 | NGVD DEG S+VVRG RP A GA VAT LDHRIAMSFL++GL + NPVT+D ANGVDVDEGPESIVVRGSARP AGGATVATSLDHRIAMSFLILGLGAANPVTID | 420 |
| Query 425  Sbjct 421 | DATMIATSFPEFMDLMAGLGAKIELSD 451 D + IATSFP F D+M GLGA+ E D DESPIATSFPAFRDMMTGLGARFESVD 447 |  |

>TR:A0A285M3G8 A0A285M3G8_9RHIZ 3-phosphoshikimate 1-carboxyvinyltransferase OS=Cohaesibacter sp. ES.047 OX=1798205 GN=aroA PE=3 SV=1

Length=447

Score = 524 bits (1350), Expect = 0.0

| Identities | | = 278/447 (62%), Positives = 328/447 (73%), Gaps = 7/447 (2%) |  |
| --- | --- | --- | --- |
| Query 1  Sbjct 1 | | MLHGASS-RPATARKSSGLSGTVRIPGDKSISHRSFMFGGLASGETRITGLLEGEDVINT M H A+ P +A S GL+G +R+PGDKSISHR+ +FG LA G+TRI+GLLE EDV+NT MAHDATQLSPLSATMSHGLTGRLRVPGDKSISHRALIFGALARGKTRISGLLESEDVLNT | 59  60 |
| Query 60  Sbjct 61 | | GKAMQAMGARIRKEGD-TWIIDGVGNGGLLAPEAPLDFGNAATGCRLTMGLVGVYDFDST KAM A GA I K+ D W +DGVG G L+ P LDFGN+ TG RL MGL G + F +T AKAMAAFGAHIVKDDDGVWYVDGVGTGSLVEPSNVLDFGNSGTGVRLVMGLAGAHAFSTT | 118  120 |
| Query 119  Sbjct 121 | | FIGDASLTKRPMGRVLNPLREMGVQVKSEDGDRLPVTLRGPKTPTPITYRVPMASAQVKS F+GD SL+ RPM RVL+PLREMGV+V GDRLP+T+RG + PITYRVPMASAQVKS FMGDESLSSRPMARVLDPLREMGVEVLGRSGDRLPLTIRGAEPAAPITYRVPMASAQVKS | 178  180 |
| Query 179  Sbjct 181 | | AVLLAGLNTPGITTVIEPIMTRDHTEKMLQGFGANLTVETDADGVRTIRLEGRGKLTGQV VLLAGLN G TTVIEPI TRDHTE+ML+GFGA L + G + +EG L Q CVLLAGLNMAGTTTVIEPIKTRDHTERMLRGFGATLGESVNEAGETILTIEGLQALKAQD | 238  240 |
| Query 239  Sbjct 241 | | IDVPGDPSSTAFPLVAALLVPGSDVTILNVLMNPTRTGLILTLQEMGADIEVINPRLAGG  + VPGDPSS AFP+VAAL+ P S++ I ++L+N +RTGLI TL EMGADI + N R +GG VTVPGDPSSAAFPIVAALITPDSNLVIDDILLNESRTGLITTLLEMGADITIENERESGG | 298  300 |
| Query 299  Sbjct 301 | | EDVADLRVRSSTLKGVTVPEDRAPSMIDEYPILAVAAAFAEGATVMNGLEELRVKESDRL E + ++RVRSS+LKGVTVP DRAPSMIDEYP+L+VAA+FAEG T M GLEELRVKESDRL ERIGNIRVRSSSLKGVTVPADRAPSMIDEYPVLSVAASFAEGETRMEGLEELRVKESDRL | 358  360 |
| Query 359  Sbjct 361 | | SAVANGLKLNGVDCDEGETSLVVRGRPDGKGLGNASGAAVATHLDHRIAMSFLVMGLVSE SAVA GLK NGV +EG LVV G G G V THLDHRIAMSFLV+GL SAVAAGLKANGVPHEEGGDYLVVSG GANEIGGGMVTTHLDHRIAMSFLVLGLAVH | 418  415 |
| Query 419 | | NPVTVDDATMIATSFPEFMDLMAGLGA 445 PVTVDDA +I TSFP F DL+ LGA |  |
| Sbjct | 416 | KPVTVDDAAIINTSFPGFADLLGTLGA 442 | |

>TR:M1PBI7 M1PBI7_BARAA 3-phosphoshikimate 1-carboxyvinyltransferase OS=Bartonella australis (strain Aust/NH1) OX=1094489 GN=aroA PE=3

SV=1

Length=442

Score = 524 bits (1350), Expect = 0.0

| Identities | = 275/439 (63%), Positives = 328/439 (75%), Gaps = 3/439 (1%) |  |
| --- | --- | --- |
| Query 9 | PATARKSSGLSGTVRIPGDKSISHRSFMFGGLASGETRITGLLEGEDVINTGKAMQAMGA | 68 |
| Sbjct 6 | PA A KS LSGT++IPGDK ISH+S + GGLA+GET I GLLE +DV+ T AMQAMGA PAIAYKSVNLSGTIKIPGDKLISHQSLILGGLANGETHIRGLLENDDVLCTAVAMQAMGA | 65 |
| Query 69 | RIRKEGDTWIIDGVGNGGLLAPEAPLDFGNAATGCRLTMGLVGVYDFDSTFIGDASLTKR | 128 |
| Sbjct 66 | IRK+ D WII G GNG LL + PL+F NA TG L MG+VG Y +TFIG A+L+KR NIRKKDDLWIIRGTGNGCLLQAQMPLNFSNAGTGACLVMGMVGSYHMKTTFIGSAALSKR | 125 |
| Query 129 | PMGRVLNPLREMGVQVKSEDGDRLPVTLRGPKTPTPITYRVPMASAQVKSAVLLAGLNTP | 188 |
| Sbjct 126 | M +L+PLR MG ++++ G+ LP+TL GP+ PI R+P ASAQVKSA+L AGLNT SMRCILDPLRLMGTEIEATSGNYLPLTLYGPRMAGPICCRIPTASAQVKSAILFAGLNTA | 185 |
| Query 189 | GITTVIEPIMTRDHTEKMLQGFGANLTVETDADGVRTIRLEGRGKLTGQVIDVPGDPSST | 248 |
| Sbjct 186 | G TTVIEP +TR+HTEKML+ FGA L +ETD G R I L+G+ LTGQ+ID+PGDPSS  GTTTVIEPSLTRNHTEKMLKWFGAELDIETDKKGTRFIHLDGQPHLTGQIIDIPGDPSSA | 245 |

| Query | 249 | AFPLVAALLVPGSDVTILNVLMNPTRTGLILTLQEMGADIEVINPRLAGGEDVADLRVRS | 308 |
| --- | --- | --- | --- |
| Sbjct | 246 | AFPL+AALLV SD+ I NVL+N +R GLI TL EMGA+I+ +N R G E+VADLRV+S AFPLIAALLVENSDIIIENVLINDSRAGLIKTLWEMGAEIKFLNQRRTGEENVADLRVKS | 305 |
| Query | 309 | STLKGVTVPEDRAPSMIDEYPILAVAAAFAEGATVMNGLEELRVKESDRLSAVANGLKLN | 368 |
| Sbjct | 306 | S LKGVTVP++R SMIDEYP LAVAAAFAEG T M G++EL KE RLSAVA GLK+N SMLKGVTVPKERTASMIDEYPALAVAAAFAEGRTTMLGIKELHAKELGRLSAVARGLKIN | 365 |
| Query | 369 | GVDCDEGETSLVVRGRPDGKGLGNASGAAVATHLDHRIAMSFLVMGLVSENPVTVDDATM | 428 |
| Sbjct | 366 | VD EG L+++G+ GKGLG G V THLDHRIAM FL+ GL SE PVTVDD M HVDYKEGADFLIIQGKGSGKGLG---GGHVMTHLDHRIAMCFLIFGLASEKPVTVDDKRM | 422 |
| Query  Sbjct | 429  423 | IATSFPEFMDLMAGLGAKI 447 IATSFPEF+ LM LG KI IATSFPEFIPLMRQLGGKI 441 |  |

>TR:A0A1M7ZME3 A0A1M7ZME3_9RHIZ 3-phosphoshikimate 1-carboxyvinyltransferase OS=Pseudoxanthobacter soli DSM 19599 OX=1123029 GN=aroA PE=3

SV=1

Length=453

Score = 523 bits (1346), Expect = 0.0

| Identities | | = 275/446 (62%), Positives = 329/446 (74%), Gaps = 6/446 (1%) |  |
| --- | --- | --- | --- |
| Query 1  Sbjct 1 | | MLHGASSRPATARKSSGLSGTVRIPGDKSISHRSFMFGGLASGETRITGLLEGEDVINTG M H ++ P TA SS L G VR+PGDKSISHRS M G LA GET I+GLLEG DV+ T MSHSSTPVPLTASASSALRGHVRVPGDKSISHRSLMLGTLAVGETTISGLLEGADVLATA | 60  60 |
| Query 61  Sbjct 61 | | KAMQAMGARI-RKEGDTWIIDGVGNGGLLAPEAPLDFGNAATGCRLTMGLVGVYDFDSTF  + M+A+GARI R E W + GVG GGL+ P A LDFGN+ TG RL +G+VG + T ETMRALGARIERDENGIWHVHGVGVGGLMEPAAALDFGNSGTGARLCLGMVGTHPIAVTA | 119  120 |
| Query 120  Sbjct 121 | | IGDASLTKRPMGRVLNPLREMGVQVKSEDGDRLPVTLRGPKTPTPITYRVPMASAQVKSA  +GDASL++RPMGRVL PLR MG +V + GDRLP+++RG +TP PI YR+P+ASAQVKSA VGDASLSRRPMGRVLGPLRRMGTEVVARAGDRLPLSIRGAETPIPIEYRLPIASAQVKSA | 179  180 |
| Query 180  Sbjct 181 | | VLLAGLNTPGITTVIEPIMTRDHTEKMLQGFGANLTVETDADGVRTIRLEGRGKLTGQVI  +LLA LNT G TTV EP TRDHTE+ML+GFGA + VE D +G R IR+ G +L Q + ILLAALNTQGTTTVFEPTATRDHTERMLKGFGAAIEVEPDQNGGRVIRIHGPVELKPQDV | 239  240 |
| Query 240  Sbjct 241 | | DVPGDPSSTAFPLVAALLVPGSDVTILNVLMNPTRTGLILTLQEMGADIEVINPRLAGGE VP DPSS AF ++A+L++PGS+++I NV++NPTRTGL TL EMGAD+ + N RL+GGE AVPADPSSAAFLIIASLIIPGSEISIDNVMLNPTRTGLFDTLIEMGADLTISNRRLSGGE | 299  300 |
| Query 300  Sbjct 301 | | DVADLRVRSSTLKGVTVPEDRAPSMIDEYPILAVAAAFAEGATVMNGLEELRVKESDRLS V D+ VRSS L+GVTVP RAPSMIDEYP L+VAAAFAEG TVM GL ELRVKESDRL+ PVGDITVRSSRLRGVTVPASRAPSMIDEYPALSVAAAFAEGETVMEGLAELRVKESDRLA | 359  360 |
| Query 360 | | AVANGLKLNGVDCDEGETSLVVRGRPDGKGLGNASGAAVATHLDHRIAMSFLVMGLVSEN AVA GL NGVD G L +RGR G G V THLDHRIAM+FLVMGL ++ | 419 |
| Sbjct | 361 | AVAAGLVANGVDHHAGPDWLTIRGRTAPIG GGVVETHLDHRIAMAFLVMGLAADK | 415 |
| Query  Sbjct | 420  416 | PVTVDDATMIATSFPEFMDLMAGLGA 445 PVTVDD TMI TSFPEF LM LGA PVTVDDTTMIMTSFPEFRSLMTSLGA 441 |  |

>TR:A0A562T1Z0 A0A562T1Z0_9RHOB 3-phosphoshikimate 1-carboxyvinyltransferase OS=Roseibium hamelinense OX=150831 GN=aroA PE=3 SV=1

Length=416

Score = 514 bits (1325), Expect = 5e-179

| Identities | = 265/421 (63%), Positives = 322/421 (76%), Gaps = 6/421 (1%) |  |
| --- | --- | --- |
| Query 36 | MFGGLASGETRITGLLEGEDVINTGKAMQAMGARIRKEGD-TWIIDGVGNGGLLAPEAPL | 94 |
| Sbjct 1 | MFG LA G T TGLLE EDV+ T AM+A+GA I K+ D ++ IDGVG G LL PE+ + MFGALAVGRTTATGLLESEDVLATADAMRAVGAIIEKQQDGSYAIDGVGLGNLLEPESVI | 60 |
| Query 95 | DFGNAATGCRLTMGLVGVYDFDSTFIGDASLTKRPMGRVLNPLREMGVQVKSEDGDRLPV | 154 |
| Sbjct 61 | DFGNA TG RLTMG+ G +D +TF+GDASL+KRPMGRVLNPLREMG V + GDRLP DFGNAGTGVRLTMGIFGTHDIAATFVGDASLSKRPMGRVLNPLREMGTSVIARSGDRLPA | 120 |
| Query 155 | TLRGPKTPTPITYRVPMASAQVKSAVLLAGLNTPGITTVIEPIMTRDHTEKMLQGFGANL | 214 |
| Sbjct 121 | ++RG K P+TYRVPMASAQVKSAVLLAGLN PG TTVIEPI TRDHTEKML GFGA++ SIRGAKQALPMTYRVPMASAQVKSAVLLAGLNAPGETTVIEPIPTRDHTEKMLAGFGADI | 180 |
| Query 215 | TVETDADGVRTIRLEGRGKLTGQVIDVPGDPSSTAFPLVAALLVPGSDVTILNVLMNPTR | 274 |
| Sbjct 181 | +V + G R I+L+G+ +L Q I+VPGDPSS FP+VAAL+VPGSD+T+ NVL+N R SVTINEAGERVIKLQGQPELKPQDIEVPGDPSSAGFPIVAALIVPGSDITVENVLLNEHR | 240 |
| Query 275 | TGLILTLQEMGADIEVINPRLAGGEDVADLRVRSSTLKGVTVPEDRAPSMIDEYPILAVA | 334 |
| Sbjct 241 | TGLI TL EMG DI+++N R++GGE++ D+ V++S L G+TVP DRAPSMIDEYP+LAVA TGLITTLVEMGGDIQIVNKRVSGGEEIGDILVKASRLHGITVPADRAPSMIDEYPVLAVA | 300 |
| Query 335 | AAFAEGATVMNGLEELRVKESDRLSAVANGLKLNGVDCDEGETSLVVRGRPDGKGLGNAS | 394 |
| Sbjct 301 | AAFA+G T M GLEELRVKESDRL+AVA GL+ N V C E E +L V G G N AAFAQGDTFMPGLEELRVKESDRLAAVARGLEANNVPCVETEDTLTVTG GTNNIG | 355 |
| Query 395 | GAAVATHLDHRIAMSFLVMGLVSENPVTVDDATMIATSFPEFMDLMAGLGAKIELSDTKA | 454 |
| Sbjct 356 | G V THLDHRIAMSFL++GL + NPVTVDD +IATSFP F++L +G I + ++A GGTVTTHLDHRIAMSFLILGLAAHNPVTVDDGAVIATSFPNFINLFEEMGGTITKTGSEA | 415 |
| Query 455  Sbjct 416 | A 455 A  A 416 |  |

>TR:A0A0K2DAU0 A0A0K2DAU0_9RHIZ 3-phosphoshikimate 1-carboxyvinyltransferase OS=Chelatococcus sp. CO-6 OX=1702325 GN=aroA PE=3 SV=1

Length=445

Score = 511 bits (1315), Expect = 5e-177

| Identities | = 274/447 (61%), Positives = 330/447 (74%), Gaps = 8/447 (2%) |  |
| --- | --- | --- |
| Query 3 | HGASSRPATARKSSGLSGTVRIPGDKSISHRSFMFGGLASGETRITGLLEGEDVINTGKA | 62 |
| Sbjct 4 | H A+ +P TAR+ + L+G VR+PGDKSISHRS +FG L++GET I+GLLEGEDV+ T A HSATPQPLTARRGASLTGRVRVPGDKSISHRSLIFGLLSAGETAISGLLEGEDVLRTADA | 63 |
| Query 63 | MQAMGARIRKEGD-TWIIDGVGNGGLLAPEAPLDFGNAATGCRLTMGLVGVYDFDSTFIG | 121 |
| Sbjct 64 | +A+GA + + G+ W + GVG GGL +P LDFGNA TG RL MG+VG + +TF G ARALGADVERLGEGRWRVRGVGIGGLASPAGVLDFGNAGTGSRLMMGVVGGHPITATFDG | 123 |
| Query 122 | DASLTKRPMGRVLNPLREMGVQVKSE-DGDRLPVTLRGPKTPTPITYRVPMASAQVKSAV | 180 |
| Sbjct 124 | DASL KRPM R+L+PL +MG +V SE +G R P+TL G + PI Y P+ASAQ+KSAV DASLRKRPMRRILDPLVKMGARVVSEAEGGRCPITLEGARETVPIVYETPVASAQIKSAV | 183 |
| Query 181 | LLAGLNTPGITTVIEPIMTRDHTEKMLQGFGANLTVETDADGVRTIRLEGRGKLTGQVID | 240 |
| Sbjct 184 | LLAGLN PG TTV+E +RDHTE+ML+ FGA ++VE R I L G+ +LT + + LLAGLNAPGRTTVVENEASRDHTERMLRHFGAEVSVEPHGSHGRRITLVGQPELTARPVV | 243 |
| Query 241 | VPGDPSSTAFPLVAALLVPGSDVTILNVLMNPTRTGLILTLQEMGADIEVINPRLAGGED | 300 |
| Sbjct 244 | VP DPSS AFPLVAAL+VPGSD+ + V+MNP RTGL+ TL EMGADI V++ R GGED VPADPSSAAFPLVAALIVPGSDIVLEGVMMNPLRTGLVTTLMEMGADIAVLDRREEGGED | 303 |
| Query 301 | VADLRVRSSTLKGVTVPEDRAPSMIDEYPILAVAAAFAEGATVMNGLEELRVKESDRLSA VADLRVR+ TLKGV VP +RAPSMIDEYPILAVAAAFAEG TVM GL ELRVKESDRL A | 360 |

| Sbjct | 304 | VADLRVRAGTLKGVDVPAERAPSMIDEYPILAVAAAFAEGETVMRGLHELRVKESDRLEA | 363 |
| --- | --- | --- | --- |
| Query | 361 | VANGLKLNGVDCDEGETSLVVRGRPDGKGLGNASGAAVATHLDHRIAMSFLVMGLVSENP | 420 |
| Sbjct | 364 | V GL+ GVD + L+VRG G+ G G ATHLDHRIAMSFLV+GL SE P VLAGLRAAGVDAEIAGDDLIVRG---GRVPG---GGTAATHLDHRIAMSFLVLGLASEKP | 417 |
| Query  Sbjct | 421  418 | VTVDDATMIATSFPEFMDLMAGLGAKI 447  + VDD TMIATSFP F+ LM GLGA I MQVDDGTMIATSFPTFVPLMHGLGADI 444 |  |

>SP:A7HPE1 AROA_PARL1 3-phosphoshikimate 1-carboxyvinyltransferase OS=Parvibaculum lavamentivorans (strain DS-1 / DSM 13023 / NCIMB

13966) OX=402881 GN=aroA PE=3 SV=1

Length=451

Score = 510 bits (1313), Expect = 1e-176

| Identities | = 269/444 (61%), Positives = 321/444 (72%), Gaps = 7/444 (2%) |  |
| --- | --- | --- |
| Query 3 | HGASSRPATARKSSGLSGTVRIPGDKSISHRSFMFGGLASGETRITGLLEGEDVINTGKA | 62 |
| Sbjct 7 | H ASS TA S L G++ +PGDKSISHR+ +FG LA GETRI GLLEGEDV+ T + HSASS--LTAEPSGALLGSITVPGDKSISHRALIFGALAVGETRIGGLLEGEDVLATAET | 64 |
| Query 63 | MQAMGARIRKEGD-TWIIDGVGNGGLLAPEAPLDFGNAATGCRLTMGLVGVYDFDSTFIG | 121 |
| Sbjct 65 | M+ +GA + + D TW + GVG GGL P+ PLDFGN+ TG RL MGLV + +TFIG MRRLGAEVERHADGTWSVHGVGVGGLKEPDQPLDFGNSGTGARLVMGLVAGHPITATFIG | 124 |
| Query 122 | DASLTKRPMGRVLNPLREMGVQVKSEDGDRLPVTLRGPKTPTPITYRVPMASAQVKSAVL | 181 |
| Sbjct 125 | DASL++RPMGRV+ PL E G + +G RLP+TL G PITYR+P+ASAQVKSAVL DASLSRRPMGRVIAPLTETGATFHAREGGRLPLTLTGAGRALPITYRLPVASAQVKSAVL | 184 |
| Query 182 | LAGLNTPGITTVIEPIMTRDHTEKMLQGFGANLTVETDADGVRTIRLEGRGKLTGQVIDV | 241 |
| Sbjct 185 | LAGLN PG+TTVIE TRDHTE+ML+ FGA++ VE G+ IRL G +L I V LAGLNAPGVTTVIEETPTRDHTERMLRAFGAHIEVEDGPRGLIVIRLTGEPELKPCRISV | 244 |
| Query 242 | PGDPSSTAFPLVAALLVPGSDVTILNVLMNPTRTGLILTLQEMGADIEVINPRLAGGEDV | 301 |
| Sbjct 245 | PGDPSS AFP+VAALL PGS++T+ + +NP R GL TL EMG DIEV+N R GGE V PGDPSSAAFPVVAALLTPGSEITVTGITLNPHRAGLYTTLMEMGGDIEVMNQREEGGEPV | 304 |
| Query 302 | ADLRVRSSTLKGVTVPEDRAPSMIDEYPILAVAAAFAEGATVMNGLEELRVKESDRLSAV | 361 |
| Sbjct 305 | ADLRVR+S LKG+ VP RA SMIDEYP+LA+AAAFAEG T M G+ ELRVKESDR++A ADLRVRASRLKGIEVPPARAASMIDEYPVLAIAAAFAEGETRMLGIHELRVKESDRIAAT | 364 |
| Query 362 | ANGLKLNGVDCDEGETSLVVRGRPDGKGLGNASGAAVATHLDHRIAMSFLVMGLVSENPV | 421 |
| Sbjct 365 | A+GL+ NGV E + +VV GR G G VATH+DHRIAMSFLVMGL ++ PV ASGLRANGVKVHESDDGMVVEGRSGEVG GGGHVATHIDHRIAMSFLVMGLAAQKPV | 420 |
| Query 422  Sbjct 421 | TVDDATMIATSFPEFMDLMAGLGA 445 TVDDA MIATSFP F LM GLGA TVDDAAMIATSFPNFTGLMRGLGA 444 |  |

>TR:A0A0S3Q0K3 A0A0S3Q0K3_9BRAD 3-phosphoshikimate 1-carboxyvinyltransferase OS=Variibacter gotjawalensis OX=1333996 GN=aroA PE=3 SV=1

Length=446

Score = 509 bits (1310), Expect = 3e-176

Identities = 269/445 (60%), Positives = 328/445 (74%), Gaps = 6/445 (1%)

| Query | 5 | ASSRPATARKSSGLSGTVRIPGDKSISHRSFMFGGLASGETRITGLLEGEDVINTGKAMQ | 64 |
| --- | --- | --- | --- |
|  |  | ++ +P AR+SS L G +R+PGDKSISHR+ +FG LA GET +TGLLEGEDVI T KA+Q |  |
| Sbjct | 5 | SNPKPQQARRSSALKGRIRVPGDKSISHRALLFGALAVGETTVTGLLEGEDVIATSKAVQ | 64 |
| Query | 65 | AMGARIRKEGD-TWIIDGVGNGGLLAPEAPLDFGNAATGCRLTMGLVGVYDFDSTFIGDA | 123 |

| Sbjct | 65 | A+GA + ++G+ W + GVG GG L P+ LDFGNA TG RLTMG V F GDA  ALGATVVRKGEGDWSVRGVGVGGFLQPKNTLDFGNAGTGVRLTMGAVAGCPIRVAFDGDA | 124 |
| --- | --- | --- | --- |
| Query | 124 | SLTKRPMGRVLNPLREMGVQ-VKSEDGDRLPVTLRGPKTPTPITYRVPMASAQVKSAVLL | 182 |
| Sbjct | 125 | SL KRPMGR+L+PL++MG + V+ G RLP+ + G + P PITYR P+ASAQ+KSAVLL SLRKRPMGRILDPLKQMGARAVEEASGTRLPLVIEGARDPIPITYRTPVASAQIKSAVLL | 184 |
| Query | 183 | AGLNTPGITTVIEPIMTRDHTEKMLQGFGANLTVETDADGVRTIRLEGRGKLTGQVIDVP | 242 |
| Sbjct | 185 | AGL PG TTVIE +RDHTE++L FGA + + R I L G+ +L I VP AGLCAPGETTVIEDEASRDHTERLLTHFGAEVVSLPEGQHGRRITLIGQPELRPAPIVVP | 244 |
| Query | 243 | GDPSSTAFPLVAALLVPGSDVTILNVLMNPTRTGLILTLQEMGADIEVINPRLAGGEDVA | 302 |
| Sbjct | 245 | GDPSS AFP+VAALLVPGS+V + V++NP R GLI TL+EMGADIE ++ R GGED+A GDPSSAAFPIVAALLVPGSEVVLEGVMINPLRIGLITTLREMGADIETLDSRYEGGEDIA | 304 |
| Query | 303 | DLRVRSSTLKGVTVPEDRAPSMIDEYPILAVAAAFAEGATVMNGLEELRVKESDRLSAVA | 362 |
| Sbjct | 305 | DLRVR+STLKGV VP +RAPSMIDEY +LAVAAAFA G T M GL+ELRVKESDRL+A A DLRVRASTLKGVDVPAERAPSMIDEYLVLAVAAAFATGETRMRGLKELRVKESDRLAATA | 364 |
| Query | 363 | NGLKLNGVDCDEGETSLVVRGRPDGKGLGNASGAAVATHLDHRIAMSFLVMGLVSENPVT | 422 |
| Sbjct | 365 | +GLK NGVDC+ L+VR G G G VATH+DHR+AMSFLV+GL SE PVT DGLKANGVDCEIDGDDLIVR GNGRAAPGGGTVATHMDHRLAMSFLVLGLASEKPVT | 420 |
| Query  Sbjct | 423  421 | VDDATMIATSFPEFMDLMAGLGAKI 447 VDD+T IATSFP F +LM GLGA++ VDDSTFIATSFPAFTELMRGLGAEL 445 |  |

>TR:A0A2W2B8V9 A0A2W2B8V9_9RHIZ 3-phosphoshikimate 1-carboxyvinyltransferase OS=Aestuariivirga litoralis OX=2650924 GN=aroA PE=3 SV=1

Length=447

Score = 506 bits (1302), Expect = 5e-175

| Identities | | = 264/445 (59%), Positives = 322/445 (72%), Gaps = 8/445 (2%) |  |
| --- | --- | --- | --- |
| Query 5  Sbjct 7 | | ASSRPATARKSSGLSGTVRIPGDKSISHRSFMFGGLASGETRITGLLEGEDVINTGKAMQ A P T+ ++ L+G +R+PGDKS+SHR+ MFG +A GET I GLLE EDVINT KAM ADPLPLTSSRAGALNGRIRVPGDKSMSHRALMFGAIAIGETTIRGLLEAEDVINTAKAMT | 64  66 |
| Query 65  Sbjct 67 | | AMGARIRKEGD-TWIIDGVGNGGLLAPEAPLDFGNAATGCRLTMGLVGVYDFDSTFIGDA A+GA + D W + GVG GL +P PLDFGN+ TG RL MGL+ +T +GDA ALGADATRGPDGVWRVQGVGVAGLRSPAQPLDFGNSGTGVRLAMGLMATTPLTATCVGDA | 123  126 |
| Query 124  Sbjct 127 | | SLTKRPMGRVLNPLREMGVQVKSEDGDRLPVTLRGPKTPTPITYRVPMASAQVKSAVLLA SL+KRPMGRV PL + G Q ++ +G+RLP+TL+G + P P+TY +P+ASAQVKSAVLLA SLSKRPMGRVTVPLSQFGTQFETAEGNRLPLTLKGARNPVPVTYTLPVASAQVKSAVLLA | 183  186 |
| Query 184  Sbjct 187 | | GLNTPGITTVIEPIMTRDHTEKMLQGFGANLTVETDADGVRTIRLEGRGKLTGQVIDVPG GLNTPG TTVIEP TRDHTE+ML+GFGA + VE ADG I +EG+ +L Q +DVPG GLNTPGKTTVIEPTPTRDHTERMLKGFGAKIAVEKKADG-NHITIEGQHELKAQDLDVPG | 243  245 |
| Query 244  Sbjct 246 | | DPSSTAFPLVAALLVPGSDVTILNVLMNPTRTGLILTLQEMGADIEVINPRLAGGEDVAD DPSS AFP+VAAL+ PGSD+ I N+++NPTRTGLI TL EMG +I + N RLAGGE+V D DPSSAAFPMVAALITPGSDIVIENIMLNPTRTGLIDTLVEMGGNITIENRRLAGGEEVGD | 303  305 |
| Query 304  Sbjct 306 | | LRVRSSTLKGVTVPEDRAPSMIDEYPILAVAAAFAEGATVMNGLEELRVKESDRLSAVAN L V+ S LKG+TVP RAPSMIDEYP+L+VAA+FA G T M GLEELRVKESDRL+AV LHVKHSQLKGITVPAARAPSMIDEYPVLSVAASFANGVTRMEGLEELRVKESDRLAAVEA | 363  365 |
| Query 364 | | GLKLNGVDCDEGETSLVVRGRPDGKGLGNASGAAVATHLDHRIAMSFLVMGLVSENPVTV GL NGV G L V G G G V TH+DHRIAMSFLVMGL S V | 423 |
| Sbjct | 366 | GLHANGVTTRSGHDWLEVDGG GARGGGTVVTHMDHRIAMSFLVMGLASAEATKV | 419 |
| Query | 424 | DDATMIATSFPEFMDLMAGLGAKIE 448 |  |

DD+ IATSFP+F LM G+GA+++ Sbjct 420 DDSRFIATSFPDFTGLMNGMGARLK 444

>TR:A0A081BE61 A0A081BE61_9RHIZ 3-phosphoshikimate 1-carboxyvinyltransferase OS=Tepidicaulis marinus OX=1333998 GN=aroA PE=3 SV=1

Length=443

Score = 505 bits (1301), Expect = 6e-175

| Identities | = 262/440 (60%), Positives = 321/440 (73%), Gaps = 7/440 (2%) |  |
| --- | --- | --- |
| Query 11 | TARKSSGLSGTVRIPGDKSISHRSFMFGGLASGETRITGLLEGEDVINTGKAMQAMGARI | 70 |
| Sbjct 4 | TA+ S L GT+ +PGDKSISHR+ M G LA GETR++GLLEGEDV+ TG+A++ MGA I TAQPSRALQGTIAVPGDKSISHRALMLGALAVGETRVSGLLEGEDVLATGEALRRMGAEI | 63 |
| Query 71 | RKEGD-TWIIDGVGNGGLLAPEAPLDFGNAATGCRLTMGLVGVYDFDSTFIGDASLTKRP | 129 |
| Sbjct 64 | + D TW I GVG GGL PE LDFGN+ TG RL MG+V + +TF GDASL+KRP TRHQDGTWSIWGVGVGGLKEPETVLDFGNSGTGVRLMMGIVAGHPITATFTGDASLSKRP | 123 |
| Query 130 | MGRVLNPLREMGVQVKSEDGDRLPVTLRGPKTPTPITYRVPMASAQVKSAVLLAGLNTPG | 189 |
| Sbjct 124 | MGRV+ PL EMG + + +G RLP+TL G + PITY +P+ASAQVKSAVL AGLN PG MGRVITPLTEMGAHIHAREGGRLPLTLTGAERALPITYPLPVASAQVKSAVLFAGLNAPG | 183 |
| Query 190 | ITTVIEPIMTRDHTEKMLQGFGANLTVETDADGVRTIRLEGRGKLTGQVIDVPGDPSSTA | 249 |
| Sbjct 184 | TTVIEP TRDHTE ML+ FGA + E GVR IR+ G +L + VPGDPSS A QTTVIEPAPTRDHTELMLKAFGAQVATEEGPKGVRVIRVTGEPELKACTLAVPGDPSSAA | 243 |
| Query 250 | FPLVAALLVPGSDVTILNVLMNPTRTGLILTLQEMGADIEVINPRLAGGEDVADLRVRSS | 309 |
| Sbjct 244 | FP+VAAL+ PGSD+T+ + +NP R+GL TL EMGADIE++N R GGE VADLRV++S FPVVAALITPGSDITVTGITLNPHRSGLYTTLIEMGADIEILNQRQEGGEPVADLRVKAS | 303 |
| Query 310 | TLKGVTVPEDRAPSMIDEYPILAVAAAFAEGATVMNGLEELRVKESDRLSAVANGLKLNG | 369 |
| Sbjct 304 | LKG+ VP +RAPSMIDEYP+LAVAA+FAEG TVM G EELRVKESDR++A A GL+ NG ELKGIEVPPERAPSMIDEYPVLAVAASFAEGKTVMRGAEELRVKESDRIAATAAGLRANG | 363 |
| Query 370 | VDCDEGETSLVVRGRPDGKGLGNASGAA-VATHLDHRIAMSFLVMGLVSENPVTVDDATM | 428 |

V ++	++V GR	G	G	VATH+DHRIAMSFL +GL ++ PVT+D	M

| Sbjct | 364 | VKVEDLPDGMIVEGR AGTVEGGGHVATHMDHRIAMSFLTLGLGAQKPVTIDAGEM | 418 |
| --- | --- | --- | --- |
| Query  Sbjct | 429  419 | IATSFPEFMDLMAGLGAKIE 448 IATSFP+F LM LGA++E IATSFPDFTGLMRRLGAQLE 438 |  |

>TR:A0A0A1P575 A0A0A1P575_9BACT 3-phosphoshikimate 1-carboxyvinyltransferase OS=bacterium YEK0313 OX=1522316 GN=aroA PE=3 SV=1

Length=445

Score = 503 bits (1295), Expect = 5e-174

| Identities | = 271/441 (61%), Positives = 318/441 (72%), Gaps = 7/441 (2%) |  |
| --- | --- | --- |
| Query 9 | PATARKSSGLSGTVRIPGDKSISHRSFMFGGLASGETRITGLLEGEDVINTGKAMQAMGA | 68 |
| Sbjct 9 | P TAR+ L+GTV +PGDKSISHR+ MFG L GETRITGLLEGEDVINT KA +A+GA PMTARRGKPLAGTVTVPGDKSISHRALMFGLLTVGETRITGLLEGEDVINTAKACRALGA | 68 |
| Query 69 | RIRKEGD-TWIIDGVGNGGLLAPEAPLDFGNAATGCRLTMGLVGVYDFDSTFIGDASLTK | 127 |
| Sbjct 69 | R+ + G W + GVG GGL+ P APLDFGN+ TGCRL MG+VG + +TF GDASL K RVERTGPGAWSVHGVGVGGLVQPSAPLDFGNSGTGCRLMMGVVGCHPIRATFDGDASLRK | 128 |
| Query 128 | RPMGRVLNPLREMGVQVKSE-DGDRLPVTLRGPKTPTPITYRVPMASAQVKSAVLLAGLN | 186 |
| Sbjct 129 | RPM R L+PL GV+V + DG RLP+T+ GP P+ Y P+ASAQVKSAVLLAGLN RPMRRALDPLALFGVRVVEQGDGGRLPITIEGPANAVPVIYESPVASAQVKSAVLLAGLN | 188 |
| Query 187 | TPGITTVIEPIMTRDHTEKMLQGFGANLTVETDADGVRTIRLEGRGKLTGQVIDVPGDPS | 246 |

| Sbjct | 189 | PG+T+VIE TRDHTE+ML FGA +TV R I L G+ +L + VP DPS  APGVTSVIETEATRDHTERMLAHFGAQVTVTPHGAHGRRIDLVGQPELKPAPVVVPADPS | 248 |
| --- | --- | --- | --- |
| Query | 247 | STAFPLVAALLVPGSDVTILNVLMNPTRTGLILTLQEMGADIEVINPRLAGGEDVADLRV | 306 |
| Sbjct | 249 | S AFPLVAA +VPGSDVTI V+MN RTGLI TL EMGA IE +NPR+ GE++ADLRV SAAFPLVAAAIVPGSDVTIAGVMMNSLRTGLITTLIEMGARIEKLNPRVESGEEIADLRV | 308 |
| Query | 307 | RSSTLKGVTVPEDRAPSMIDEYPILAVAAAFAEGATVMNGLEELRVKESDRLSAVANGLK | 366 |
| Sbjct | 309 | L GV VP RAP+MIDEYP+LAVAAAFAEG T M GL ELRVKESDRL+AVA+GL TGGRLAGVDVPAHRAPAMIDEYPVLAVAAAFAEGTTRMRGLHELRVKESDRLAAVADGLA | 368 |
| Query | 367 | LNGVDCDEGETSLVVRGRPDGKGLGNASGAAVATHLDHRIAMSFLVMGLVSENPVTVDDA | 426 |
| Sbjct | 369 | GVD L+VRG +G G VATH+DHRIAMSFLVMGL ++ PV+VDD AVGVDHAIEGDDLIVRGTGRVRG GGTVATHMDHRIAMSFLVMGLAADTPVSVDDT | 423 |
| Query | 427 | TMIATSFPEFMDLMAGLGAKI 447 IATSFP+F+ LM GLGA + |  |
| Sbjct | 424 | GFIATSFPDFVPLMRGLGADL 444 | |

>TR:A0A0Q9IEQ4 A0A0Q9IEQ4_9BRAD 3-phosphoshikimate 1-carboxyvinyltransferase OS=Bosea sp. Root381 OX=1736524 GN=aroA PE=3 SV=1

Length=446

Score = 501 bits (1290), Expect = 3e-173

| Identities | | = 273/449 (61%), Positives = 324/449 (72%), Gaps = 6/449 (1%) |  |
| --- | --- | --- | --- |
| Query 1  Sbjct 1 | | MLHGASSRPATARKSSGLSGTVRIPGDKSISHRSFMFGGLASGETRITGLLEGEDVINTG M H + P TAR+S L G+VR+PGDKSISHR+ +FG LA G+TRITGLLEGEDV+ T MAHAQNPVPVTARRSGPLRGSVRVPGDKSISHRAMIFGLLAIGQTRITGLLEGEDVLRTA | 60  60 |
| Query 61  Sbjct 61 | | KAMQAMGARIRKEGD-TWIIDGVGNGGLLAPEAPLDFGNAATGCRLTMGLVGVYDFDSTF A +A+GA + ++G W + GVG GGL PE LDFGNA TG RL MG+ G + +TF DAARALGAIVHQDGPGAWRVQGVGVGGLRQPEGVLDFGNAGTGSRLMMGVCGSHPITTTF | 119  120 |
| Query 120  Sbjct 121 | | IGDASLTKRPMGRVLNPLREMG-VQVKSEDGDRLPVTLRGPKTPTPITYRVPMASAQVKS GDASL KRPM R+L+PL +MG V V E+G R+P+TLRGPK PITYR P+ASAQ+KS DGDASLRKRPMRRILDPLAQMGTVIVSQEEGGRVPLTLRGPKEALPITYRTPVASAQIKS | 178  180 |
| Query 179  Sbjct 181 | | AVLLAGLNTPGITTVIEPIMTRDHTEKMLQGFGANLTVETDADGVRTIRLEGRGKLTGQV AVLLAGL PG TTVIE TRDHTEKML FGA++ V + + R I L+G+ +L AVLLAGLAAPGETTVIETEATRDHTEKMLTHFGADVVVTPEGEHGRRIVLKGQPELRATP | 238  240 |
| Query 239  Sbjct 241 | | IDVPGDPSSTAFPLVAALLVPGSDVTILNVLMNPTRTGLILTLQEMGADIEVINPRLAGG I VP DPSS AFPLVA L+VPGSD+ I +V+ NP R+GL+ TL+EMGADI V + GG IVVPADPSSAAFPLVAGLIVPGSDIRIESVMTNPLRSGLLTTLREMGADITVESEANEGG | 298  300 |
| Query 299  Sbjct 301 | | EDVADLRVRSSTLKGVTVPEDRAPSMIDEYPILAVAAAFAEGATVMNGLEELRVKESDRL E VA LR+R+S LKGVTVP +RAPSMIDEYP+LAVAA+FA G T M GL+ELRVKESDRL EAVATLRIRASALKGVTVPPERAPSMIDEYPVLAVAASFATGTTRMRGLQELRVKESDRL | 358  360 |
| Query 359  Sbjct 361 | | SAVANGLKLNGVDCDEGETSLVVRGRPDGKGLGNASGAAVATHLDHRIAMSFLVMGLVSE  +AV GLK GVDC LVV G G G VATHLDHRIAMSFLVMGL ++ AAVEAGLKAAGVDCAIDGDDLVVH GSGGEVTGGGTVATHLDHRIAMSFLVMGLATQ | 418  416 |
| Query 419 | | NPVTVDDATMIATSFPEFMDLMAGLGAKI 447 P+ VDD MIATSFP F LM GLGA+I |  |
| Sbjct | 417 | KPMQVDDGAMIATSFPTFTALMNGLGAEI 445 | |

>TR:A0A2S0NH61 A0A2S0NH61_9PROT 3-phosphoshikimate 1-carboxyvinyltransferase OS=Phreatobacter cathodiphilus OX=1868589 GN=aroA PE=3 SV=1

Length=445

Score = 501 bits (1289), Expect = 4e-173

| Identities | | = 268/439 (61%), Positives = 316/439 (72%), Gaps = 7/439 (2%) |  |
| --- | --- | --- | --- |
| Query 9  Sbjct 9 | | PATARKSSGLSGTVRIPGDKSISHRSFMFGGLASGETRITGLLEGEDVINTGKAMQAMGA P T+R+ + L+GT+ +PGDKSISHR+ MFG L GETRITGLLEGEDV+NT K + +GA PLTSRRGAALAGTITVPGDKSISHRALMFGLLTVGETRITGLLEGEDVLNTAKVCRQLGA | 68  68 |
| Query 69  Sbjct 69 | | RIRKEGD-TWIIDGVGNGGLLAPEAPLDFGNAATGCRLTMGLVGVYDFDSTFIGDASLTK  + + G W + GVG GGL P PLDFGNA TGCRL MG++G + +TF GDASL K TVERTGPGAWRVQGVGVGGLKEPTEPLDFGNAGTGCRLMMGVIGCHPIRATFDGDASLRK | 127  128 |
| Query 128  Sbjct 129 | | RPMGRVLNPLREMGVQVKSE-DGDRLPVTLRGPKTPTPITYRVPMASAQVKSAVLLAGLN RPM R L+PL GV+V + +G RLP+T++GPK P+TY P+ASAQVKSAVLLAGLN RPMRRALDPLELFGVRVVEQGEGGRLPITIQGPKDAVPVTYESPVASAQVKSAVLLAGLN | 186  188 |
| Query 187  Sbjct 189 | | TPGITTVIEPIMTRDHTEKMLQGFGANLTVETDADGVRTIRLEGRGKLTGQVIDVPGDPS PGITTVIE TRDHTE+ML FGA +TV R I L G+ +L + VP DPS APGITTVIETEATRDHTERMLSHFGAEVTVTPHGAHGRRIDLVGQPELKAAPVVVPADPS | 246  248 |
| Query 247  Sbjct 249 | | STAFPLVAALLVPGSDVTILNVLMNPTRTGLILTLQEMGADIEVINPRLAGGEDVADLRV S AFP+VAA +VPGSDVT+ V+MNP RTGLI TL EMGA+IEV+N R+ GED+ADLRV SAAFPIVAAAIVPGSDVTVTGVMMNPLRTGLITTLIEMGAEIEVLNARVESGEDIADLRV | 306  308 |
| Query 307  Sbjct 309 | | RSSTLKGVTVPEDRAPSMIDEYPILAVAAAFAEGATVMNGLEELRVKESDRLSAVANGLK R S L GV VP RAPSMIDEYP+LAVAAAFAEG T M GL ELRVKESDRL+AVA GL RGSRLTGVDVPAHRAPSMIDEYPVLAVAAAFAEGPTRMRGLHELRVKESDRLAAVAAGLA | 366  368 |
| Query 367  Sbjct 369 | | LNGVDCDEGETSLVVRGRPDGKGLGNASGAAVATHLDHRIAMSFLVMGLVSENPVTVDDA GV L+V G DG+ G G V TH+DHRIAMSFLVMGL + P+ VDD  AAGVAHTIEGDDLIVAG--DGRAPG---GGTVETHMDHRIAMSFLVMGLATAQPMRVDDT | 426  423 |
| Query 427 | | TMIATSFPEFMDLMAGLGA 445 IATSFP+F+ LM GLGA |  |
| Sbjct | 424 | AFIATSFPDFVGLMRGLGA 442 | |

>TR:D6ZZ14 D6ZZ14_STAND 3-phosphoshikimate 1-carboxyvinyltransferase OS=Starkeya novella (strain ATCC 8093 / DSM 506 / CCM 1077 / IAM

12100 / NBRC 12443 / NCIB 9113) OX=639283 GN=aroA PE=3 SV=1

Length=453

Score = 499 bits (1286), Expect = 2e-172

| Identities | = 272/447 (61%), Positives = 324/447 (72%), Gaps = 7/447 (2%) |  |
| --- | --- | --- |
| Query 3 | HGASSRPATARKSSGLSGTVRIPGDKSISHRSFMFGGLASGETRITGLLEGEDVINTGKA | 62 |
| Sbjct 4 | HGA+ PA AR+S+ L GTVR+PGDKS+SHRS +FG LA GETRI+GLLEGEDV+NT KA HGANPVPAFARRSAALKGTVRVPGDKSVSHRSLIFGTLAVGETRISGLLEGEDVLNTAKA | 63 |
| Query 63 | MQAMGARIRKEGD-TWIIDGVGNGGLLAPEAPLDFGNAATGCRLTMGLVGVYDFDSTFIG | 121 |
| Sbjct 64 | A+GA + + G+ W ++GVG GGL PE LDFGNA TG RL MG+V +TF G CAALGATVERLGEGEWRVEGVGVGGLRVPEGVLDFGNAGTGSRLMMGVVAGNPIVATFDG | 123 |
| Query 122 | DASLTKRPMGRVLNPLREMGVQV-KSEDGDRLPVTLRGPKTPTPITYRVPMASAQVKSAV | 180 |
| Sbjct 124 | DASL KRPM R+L+PL +MGVQV + +G RLP+TL+GP+ PI Y P+ASAQ+KSAV DASLRKRPMRRILDPLEKMGVQVVDAAEGGRLPLTLKGPQELVPIIYESPVASAQIKSAV | 183 |
| Query 181 | LLAGLNTPGITTVIEPIMTRDHTEKMLQGFGANLTVETDADGVRTIRLEGRGKLTGQVID | 240 |
| Sbjct 184 | LLAGL PG TTVIE +RDHTE+ML FGA ++VE R + L+GR +L I LLAGLGAPGETTVIEKEASRDHTERMLTHFGAQVSVEPYGAHGRKVTLKGRPELKPAPIR | 243 |
| Query 241 | VPGDPSSTAFPLVAALLVPGSDVTILNVLMNPTRTGLILTLQEMGADIEVINPRLAGGED | 300 |
| Sbjct 244 | VP DPSS AFPLVAAL+VPGS+V I V+ NP RTGL +TL+EMGADI N R+ GGE  VPADPSSAAFPLVAALIVPGSEVVIEGVMTNPLRTGLFITLKEMGADIAFENERVEGGES | 303 |

| Query | 301 | VADLRVRSSTLKGVTVPEDRAPSMIDEYPILAVAAAFAEGATVMNGLEELRVKESDRLSA | 360 |
| --- | --- | --- | --- |
| Sbjct | 304 | VAD+RVR+S L GV VP +RAPSMIDEYP+LAVAAAFA G T MNGL ELRVKESDRL+A VADIRVRASALHGVDVPAERAPSMIDEYPVLAVAAAFASGTTRMNGLSELRVKESDRLAA | 363 |
| Query | 361 | VANGLKLNGVDCDEGETSLVVRGRPDGKGLGNASGAAVATHLDHRIAMSFLVMGLVSENP | 420 |
| Sbjct | 364 | VA+GLK GV L+V G G A G VATH+DHRIAMSFLVMGL ++ P VADGLKAAGVAHRIEGDDLLVEG AAGAAGGGTVATHMDHRIAMSFLVMGLATDTP | 418 |
| Query  Sbjct | 421  419 | VTVDDATMIATSFPEFMDLMAGLGAKI 447  + VDD + IATSFP F+ M LGA I LKVDDISFIATSFPTFLAQMRALGADI 445 |  |

>TR:A0A2L0ACG7 A0A2L0ACG7_9SPHN 3-phosphoshikimate 1-carboxyvinyltransferase OS=Sphingobium sp. SCG-1 OX=2072936 GN=aroA PE=3 SV=1

Length=450

Score = 499 bits (1285), Expect = 2e-172

| Identities | = 272/446 (61%), Positives = 321/446 (72%), Gaps = 4/446 (1%) |  |
| --- | --- | --- |
| Query 3 | HGASSRPATARKSSGLSGTVRIPGDKSISHRSFMFGGLASGETRITGLLEGEDVINTGKA | 62 |
| Sbjct 8 | H ++ P T + L+GTV +PGDKSISHR+ M LA GE+R+ GLLEGEDV+ T A HSSAPTPKTFTAAPALTGTVTVPGDKSISHRALMLSALAVGESRVEGLLEGEDVLATAAA | 67 |
| Query 63 | MQAMGARIRKEGD-TWIIDGVGNGGLLAPEAPLDFGNAATGCRLTMGLVGVYDFDSTFIG | 121 |
| Sbjct 68 | M+AMGA I ++ D W I GVG GGLL PE LD GN+ T RL MGL+ +D +TFIG MRAMGADIVRDDDGVWHIHGVGVGGLLQPEVALDMGNSGTSTRLLMGLLASHDLTATFIG | 127 |
| Query 122 | DASLTKRPMGRVLNPLREMGVQVKSEDGDRLPVTLRGPKTPTPITYRVPMASAQVKSAVL | 181 |
| Sbjct 128 | DASL+KRPMGRV++PL MG + S G RLP+TLRG PI YR+P+ASAQVKSAVL DASLSKRPMGRVIDPLSTMGAEFTSAPGGRLPLTLRGICPAVPIEYRLPVASAQVKSAVL | 187 |
| Query 182 | LAGLNTPGITTVIEPIMTRDHTEKMLQGFGANLTVETDADGVRTIRLEGRGKLTGQVIDV | 241 |
| Sbjct 188 | LAGLNTPGIT V+EPI TRDH+E+ML+GFGA LTVE D+DG R I L G +L Q I V LAGLNTPGITRVVEPIATRDHSERMLRGFGAELTVEIDSDGARVISLVGEAELQPQHIVV | 247 |
| Query 242 | PGDPSSTAFPLVAALLVPGSDVTILNVLMNPTRTGLILTLQEMGADIEVINPRLAGGEDV | 301 |
| Sbjct 248 | PGDPSS AFP+VAALLVPGS VTI NV +NPTR GLI L+EMG IE+ NPR GGE V PGDPSSAAFPMVAALLVPGSRVTIRNVGLNPTRAGLIDLLREMGGSIEIANPREVGGEPV | 307 |
| Query 302 | ADLRVRSSTLKGVTVPEDRAPSMIDEYPILAVAAAFAEGATVMNGLEELRVKESDRLSAV | 361 |
| Sbjct 308 | DL V +STL GV DRAPSMIDEYPI VAAA A+G +V GLEELRVKESDRL+ + GDLVVSASTLTGVEPDPDRAPSMIDEYPIAFVAAALAQGHSVFRGLEELRVKESDRLTTM | 367 |
| Query 362 | ANGLKLNGVDCDEGETSLVVRGRPDGKGLGNASGAAVATHLDHRIAMSFLVMGLVSENPV | 421 |
| Sbjct 368 | A GL+L G +E E L++ G G+ L A G VAT LDHRIAMSF V GLVS + V ATGLRLIGAQVEELEDGLIIDG-TGGEPL--AGGGPVATLLDHRIAMSFAVAGLVSRDGV | 424 |
| Query 422  Sbjct 425 | TVDDATMIATSFPEFMDLMAGLGAKI 447 TVDD +ATSFP F+DL+ +GA + TVDDMRPVATSFPGFLDLLRTMGATV 450 |  |

```
>TR:A0A4D7QRK0 A0A4D7QRK0_9PROT 3-phosphoshikimate 1-carboxyvinyltransferase OS=Phreatobacter sp. NMCR1094 OX=2570229 GN=aroA PE=3 SV=1
Length=436

Score = 497 bits (1280), Expect = 7e-172
Identities = 265/439 (60%), Positives = 318/439 (72%), Gaps = 7/439 (2%)

Query 11	TARKSSGLSGTVRIPGDKSISHRSFMFGGLASGETRITGLLEGEDVINTGKAMQAMGARI 70 T+R+S L+GT+ +PGDKSISHR+ MFG + GET+ITGLLEGEDV+NT K + +GA++
Sbjct 2	TSRRSGALAGTLTVPGDKSISHRALMFGLMTVGETKITGLLEGEDVLNTAKVCRQLGAKV 61
```

| Query | 71 | RKEGD-TWIIDGVGNGGLLAPEAPLDFGNAATGCRLTMGLVGVYDFDSTFIGDASLTKRP | 129 |
| --- | --- | --- | --- |
| Sbjct | 62 | + G W + GVG GGL P PLDFGN+ TG RL MG+VG +TF GDASL KRP ERTGPGAWTVHGVGVGGLHQPAEPLDFGNSGTGARLMMGVVGANPIRATFDGDASLRKRP | 121 |
| Query | 130 | MGRVLNPLREMGVQVKSE-DGDRLPVTLRGPKTPTPITYRVPMASAQVKSAVLLAGLNTP | 188 |
| Sbjct | 122 | M R L+PL G+ V ++ +G RLP+T+ GPK P+TY P+ASAQVKSAVLLAGLN P MRRALDPLELFGLTVVAQAEGGRLPITIEGPKNAIPVTYASPVASAQVKSAVLLAGLNAP | 181 |
| Query | 189 | GITTVIEPIMTRDHTEKMLQGFGANLTVETDADGVRTIRLEGRGKLTGQVIDVPGDPSST | 248 |
| Sbjct | 182 | G+TTVIE TRDHTE+ML FGA++TV + + R I L G+ +L + VP DPSS GVTTVIEKEATRDHTERMLIHFGADVTVTPEGEHGRRIALVGQPELKPAPVVVPADPSSA | 241 |
| Query | 249 | AFPLVAALLVPGSDVTILNVLMNPTRTGLILTLQEMGADIEVINPRLAGGEDVADLRVRS | 308 |
| Sbjct | 242 | AFPLVAA +VPGSDVTI V+MNP RTGL+ TL EMGADI+ +N R+ GEDVADLRVR AFPLVAAAIVPGSDVTITGVMMNPLRTGLLTTLIEMGADIQTLNARVESGEDVADLRVRG | 301 |
| Query | 309 | STLKGVTVPEDRAPSMIDEYPILAVAAAFAEGATVMNGLEELRVKESDRLSAVANGLKLN | 368 |
| Sbjct | 302 | S L GV VP RAPSMIDEYP+LAVAAAFA G T M GL ELRVKESDRL+AVA GL + SKLHGVDVPAHRAPSMIDEYPVLAVAAAFAIGTTRMRGLHELRVKESDRLAAVAAGLAVT | 361 |
| Query | 369 | GVDCDEGETSLVVRGRPDGKGLGNASGAAVATHLDHRIAMSFLVMGLVSENPVTVDDATM | 428 |
| Sbjct | 362 | GVD L+VRG G G V TH+DHRIAMSFLVMGL ++NPVTVDD T GVDHVIEGDDLIVRGTGRAPG GGPVETHMDHRIAMSFLVMGLAADNPVTVDDTTF | 416 |
| Query  Sbjct | 429  417 | IATSFPEFMDLMAGLGAKI 447 IATSFP+F+ LM LG ++ IATSFPDFIPLMRRLGGEL 435 |  |

>SP:B1ZLG6 AROA_METPB 3-phosphoshikimate 1-carboxyvinyltransferase OS=Methylorubrum populi (strain ATCC BAA-705 / NCIMB 13946 / BJ001)

OX=441620 GN=aroA PE=3 SV=1

Length=453

Score = 498 bits (1281), Expect = 9e-172

| Identities | = 272/453 (60%), Positives = 330/453 (73%), Gaps = 10/453 (2%) |  |
| --- | --- | --- |
| Query 1 | MLHGASSRPATARKSSGLSGTVRIPGDKSISHRSFMFGGLASGETRITGLLEGEDVINTG | 60 |
| Sbjct 1 | M H + +P TA + L+G ++ PGDKSISHR+ +FG L+ GETR+ GLLEG+DV+ T MSHDSEPQPVTAHPAGPLTGALKPPGDKSISHRAMIFGLLSVGETRVEGLLEGDDVLRTA | 60 |
| Query 61 | KAMQAMGARIRKEGD-TWIIDGVGNGGLLAPEAPLDFGNAATGCRLTMGLVGVYDFDSTF | 119 |
| Sbjct 61 | A++A+GA++ +EG+ W I GVG GG+ P LDFGNA TG RL MG+VG +TF AAVKALGAQVTREGEGRWRIVGVGIGGMQDPVGVLDFGNAGTGSRLMMGVVGGQPVTATF | 120 |
| Query 120 | IGDASLTKRPMGRVLNPLREMGVQVKSE-DGDRLPVTLRGPKTPTPITYRVPMASAQVKS | 178 |
| Sbjct 121 | GDASL KRPM R+L+P+ +MG +V SE +G R+P+TLRGP+ PI Y +P+ASAQ+KS DGDASLRKRPMRRILDPILKMGAEVVSEAEGGRVPLTLRGPREAIPIRYELPVASAQIKS | 180 |
| Query 179 | AVLLAGLNTPGITTVIEPIMTRDHTEKMLQGFGANLTVETDADGV--RTIRLEGRGKLTG | 236 |
| Sbjct 181 | AVLLAGLN PG TTVIE +RDHTE+ML+ FGA+++V +G RT+ L G+ L G AVLLAGLNAPGTTTVIEKAASRDHTERMLRLFGADVSVTPSGEGGHGRTVTLTGQPTLRG | 240 |
| Query 237 | QVIDVPGDPSSTAFPLVAALLVPGSDVTILNVLMNPTRTGLILTLQEMGADIEVINPRLA | 296 |
| Sbjct 241 | + VP DPSS AFPLVAAL+VPGS+V + V+MNP RTGLI TL EMGADIE + R TDVIVPADPSSAAFPLVAALIVPGSEVILRGVMMNPLRTGLITTLIEMGADIERFDEREE | 300 |
| Query 297 | GGEDVADLRVRSSTLKGVTVPEDRAPSMIDEYPILAVAAAFAEGATVMNGLEELRVKESD | 356 |
| Sbjct 301 | GGE VADLRVR+S L+GV VP +RAPSMIDEYPILAVAAAFAEG T MNGL ELRVKESD GGETVADLRVRASRLRGVDVPAERAPSMIDEYPILAVAAAFAEGTTRMNGLHELRVKESD | 360 |
| Query 357 | RLSAVANGLKLNGVDCD-EGETSLVVRGRPDGKGLGNASGAAVATHLDHRIAMSFLVMGL | 415 |
| Sbjct 361 | RL+AVA GL NGV D EG+ +V G G A G VATHLDHRIAM+FLVMGL  RLAAVAAGLAANGVGHDIEGDDLIVT GNGGPPAGGGTVATHLDHRIAMAFLVMGL | 415 |

Query 416 VSENPVTVDDATMIATSFPEFMDLMAGLGAKIE 448

+ PVTVDD MIATSFP F	M LGA+IE Sbjct 416 ATRKPVTVDDGAMIATSFPSFRPTMLALGARIE 448

>TR:C5APM8 C5APM8_METEA 3-phosphoshikimate 1-carboxyvinyltransferase OS=Methylorubrum extorquens (strain ATCC 14718 / DSM 1338 / JCM

2805 / NCIMB 9133 / AM1) OX=272630 GN=aroA PE=3 SV=1

Length=453

Score = 498 bits (1281), Expect = 9e-172

| Identities | = 271/453 (60%), Positives = 330/453 (73%), Gaps = 10/453 (2%) |  |
| --- | --- | --- |
| Query 1 | MLHGASSRPATARKSSGLSGTVRIPGDKSISHRSFMFGGLASGETRITGLLEGEDVINTG | 60 |
| Sbjct 1 | M H + +P TA L+G+++ PGDKSISHR+ + G LA GETR+ GLLEG+DV+ T MSHDSEPQPVTASPGGPLNGSLKPPGDKSISHRAMILGLLAIGETRVEGLLEGDDVLRTA | 60 |
| Query 61 | KAMQAMGARIRKEGD-TWIIDGVGNGGLLAPEAPLDFGNAATGCRLTMGLVGVYDFDSTF | 119 |
| Sbjct 61 | A +A+GA+I +EG+ W I GVG GG+ P+ LDFGNA TG RL MG+VG +TF SAAKALGAQITREGEGRWRIVGVGIGGMQDPDGVLDFGNAGTGSRLMMGVVGGQPVTATF | 120 |
| Query 120 | IGDASLTKRPMGRVLNPLREMGVQVKSE-DGDRLPVTLRGPKTPTPITYRVPMASAQVKS | 178 |
| Sbjct 121 | GDASL KRPM R+L+P+ +MG ++ SE +G R+P+TLRGP+ PI Y +P+ASAQ+KS DGDASLRKRPMRRILDPILKMGAEIVSEAEGGRVPLTLRGPREAIPIRYELPVASAQIKS | 180 |
| Query 179 | AVLLAGLNTPGITTVIEPIMTRDHTEKMLQGFGANLTVETDADGV--RTIRLEGRGKLTG | 236 |
| Sbjct 181 | AVLLAGLN PG TTVIE +RDHTE+ML+ FGA +TV +G RT+ L G+ L G AVLLAGLNAPGTTTVIEKAASRDHTERMLRLFGAEVTVTPSGEGGHGRTVTLTGQPTLRG | 240 |
| Query 237 | QVIDVPGDPSSTAFPLVAALLVPGSDVTILNVLMNPTRTGLILTLQEMGADIEVINPRLA | 296 |
| Sbjct 241 | + VP DPSS AFPLVAAL+VPGS+V + V+MNP RTGLI TL EMGADIE ++ R TDVVVPADPSSAAFPLVAALVVPGSEVILRGVMMNPLRTGLITTLIEMGADIERLDEREE | 300 |
| Query 297 | GGEDVADLRVRSSTLKGVTVPEDRAPSMIDEYPILAVAAAFAEGATVMNGLEELRVKESD | 356 |
| Sbjct 301 | GGE VADLRVR+S LKGV VP +RAPSMIDEYPILAVAA+FAEG T MNGL ELRVKESD GGETVADLRVRASRLKGVDVPAERAPSMIDEYPILAVAASFAEGTTRMNGLHELRVKESD | 360 |
| Query 357 | RLSAVANGLKLNGV-DCDEGETSLVVRGRPDGKGLGNASGAAVATHLDHRIAMSFLVMGL | 415 |
| Sbjct 361 | RL+AVA GL NGV EG+ +V G G A G VATHLDHRIAM+FLV+GL RLAAVAAGLAANGVTHAIEGDDLIVT GNGQAPAGGGTVATHLDHRIAMAFLVLGL | 415 |
| Query 416  Sbjct 416 | VSENPVTVDDATMIATSFPEFMDLMAGLGAKIE 448  +++PVTVDD MIATSFP F M LGA+IE AAKSPVTVDDGAMIATSFPSFRPTMLALGARIE 448 |  |

>TR:A0A1G4SNT6 A0A1G4SNT6_9RHIZ 3-phosphoshikimate 1-carboxyvinyltransferase OS=Ancylobacter rudongensis OX=177413 GN=aroA PE=3 SV=1

Length=454

Score = 498 bits (1281), Expect = 1e-171

Identities = 268/448 (60%), Positives = 324/448 (72%), Gaps = 7/448 (2%)

| Query | 3 | HGASSRPATARKSSGLSGTVRIPGDKSISHRSFMFGGLASGETRITGLLEGEDVINTGKA | 62 |
| --- | --- | --- | --- |
| Sbjct | 6 | H A PA AR+S L G VR+PGDKS+SHRS +FG L GETRI+GLLEGEDV+NT KA HAALPVPALARRSPALKGRVRVPGDKSVSHRSLIFGALTIGETRISGLLEGEDVLNTAKA | 65 |
| Query | 63 | MQAMGARIRKEGD-TWIIDGVGNGGLLAPEAPLDFGNAATGCRLTMGLVGVYDFDSTFIG | 121 |
| Sbjct | 66 | A+GA + + G+ W + G+G GGL APE LDFGNA TG RL MG+V +TF G CAALGADVERVGEGEWRVHGLGVGGLRAPEGVLDFGNAGTGSRLMMGMVAGNPITATFDG | 125 |
| Query | 122 | DASLTKRPMGRVLNPLREMGVQV-KSEDGDRLPVTLRGPKTPTPITYRVPMASAQVKSAV DASL +RPM R+L+PL +MGV+V ++ +G RLP+TL+GP+ PITY P+ASAQ+KSAV | 180 |

| Sbjct | 126 | DASLRRRPMRRILDPLEQMGVEVVEAAEGGRLPLTLKGPQELVPITYESPVASAQIKSAV | 185 |
| --- | --- | --- | --- |
| Query | 181 | LLAGLNTPGITTVIEPIMTRDHTEKMLQGFGANLTVETDADGVRTIRLEGRGKLTGQVID | 240 |
| Sbjct | 186 | LLAGL PG TTVIE +RDHTE+ML FGA +TVE + R + L+GR +L I LLAGLGAPGETTVIEKEASRDHTERMLTHFGAEVTVEPYGEHGRKVTLKGRPELKPAPIR | 245 |
| Query | 241 | VPGDPSSTAFPLVAALLVPGSDVTILNVLMNPTRTGLILTLQEMGADIEVINPRLAGGED | 300 |
| Sbjct | 246 | VP DPSS AFPLVAAL+VPGS+V I V+ NP RTGL++TL+EMGADI N R+ GGE VPADPSSAAFPLVAALIVPGSEVVIEGVMTNPLRTGLLITLKEMGADISFENERVEGGES | 305 |
| Query | 301 | VADLRVRSSTLKGVTVPEDRAPSMIDEYPILAVAAAFAEGATVMNGLEELRVKESDRLSA | 360 |
| Sbjct | 306 | VAD+RVR+S L GV+VP +RAPSMIDEYP+LAVAAAFA G T MNGL ELRVKESDRL+A VADIRVRASALTGVSVPAERAPSMIDEYPVLAVAAAFASGETRMNGLSELRVKESDRLAA | 365 |
| Query | 361 | VANGLKLNGVDCDEGETSLVVRGRPDGKGLGNASGAAVATHLDHRIAMSFLVMGLVSENP | 420 |
| Sbjct | 366 | VA+GL G L+V G G A G VATH+DHRIAMSFLVMGL ++ P VADGLAQAGAAYRIEGDDLIVTGAG GAAGGGTVATHMDHRIAMSFLVMGLATDKP | 420 |
| Query  Sbjct | 421  421 | VTVDDATMIATSFPEFMDLMAGLGAKIE 448  + VDD + IATSFP F+ M GLGA I+ LKVDDISFIATSFPTFLPQMRGLGALID 448 |  |

>TR:A0A370L8I1 A0A370L8I1_9BRAD 3-phosphoshikimate 1-carboxyvinyltransferase OS=Bosea caraganae OX=2763117 GN=aroA PE=3 SV=1

Length=446

Score = 497 bits (1279), Expect = 1e-171

| Identities | = 273/451 (61%), Positives = 321/451 (71%), Gaps = 8/451 (2%) |  |
| --- | --- | --- |
| Query 1 | MLHGASSRPATARKSSGLSGTVRIPGDKSISHRSFMFGGLASGETRITGLLEGEDVINTG | 60 |
| Sbjct 1 | M H P TAR L G VR+PGDKSISHR+ +FG LA GETRITGLLEGEDV+ T MAHAEHPVPVTARAKGPLCGRVRVPGDKSISHRAMIFGLLAIGETRITGLLEGEDVMRTA | 60 |
| Query 61 | KAMQAMGARIRKEGD-TWIIDGVGNGGLLAPEAPLDFGNAATGCRLTMGLVGVYDFDSTF | 119 |
| Sbjct 61 | A +A+GA + +G W + G G GGL PE LDFGNA TG RL MG+ G + +TF AAARALGAIVHHDGPGAWRVQGAGVGGLKQPEGLLDFGNAGTGSRLMMGVCGAHPITTTF | 120 |
| Query 120 | IGDASLTKRPMGRVLNPLREMG-VQVKSEDGDRLPVTLRGPKTPTPITYRVPMASAQVKS | 178 |
| Sbjct 121 | GDASL KRPM R+L+PL MG V V+ +G R+P+T+RGPK PITYR P+ASAQ+KS DGDASLRKRPMRRILDPLELMGAVVVEQREGGRVPLTIRGPKEAVPITYRPPVASAQIKS | 180 |
| Query 179 | AVLLAGLNTPGITTVIEPIMTRDHTEKMLQGFGANLTVETDADGVRTIRLEGRGKLTGQV | 238 |
| Sbjct 181 | AVLLAGL PG TTVIE TRDHTEKML+ FGA +TV + R + L+G+ +L AVLLAGLAAPGETTVIEAEATRDHTEKMLRHFGAEVTVTREGAHGRRVVLKGQPELQAAP | 240 |
| Query 239 | IDVPGDPSSTAFPLVAALLVPGSDVTILNVLMNPTRTGLILTLQEMGADIEVINPRLAGG | 298 |
| Sbjct 241 | I VP DPSS AFPLVAAL+VPGSD+ I +V+ NP R+GL+ TL+EMGADI V + GG IAVPADPSSAAFPLVAALIVPGSDILIESVMTNPLRSGLLATLREMGADITVESETNEGG | 300 |
| Query 299 | EDVADLRVRSSTLKGVTVPEDRAPSMIDEYPILAVAAAFAEGATVMNGLEELRVKESDRL | 358 |
| Sbjct 301 | E VA LRVR+ LKGVTVP +RAPSMIDEYP+LAVAA+FA G T M GL+ELRVKESDRL ESVATLRVRAGGLKGVTVPPERAPSMIDEYPVLAVAASFASGTTRMRGLQELRVKESDRL | 360 |
| Query 359 | SAVANGLKLNGVDCDEGETSLVVRGRPDGKGLGNASGAAV-ATHLDHRIAMSFLVMGLVS | 417 |
| Sbjct 361 | +AV GLK GV C LVV G G GN +G V ATHLDHRIAMSFLVMGL + AAVEAGLKAAGVTCHIDGDDLVVEG GNGNVAGGGVAATHLDHRIAMSFLVMGLAT | 415 |
| Query 418  Sbjct 416 | ENPVTVDDATMIATSFPEFMDLMAGLGAKIE 448  + P+ VDD MIATSFP F+ LM GLGA+IE QKPMQVDDGAMIATSFPSFIPLMNGLGAEIE 446 |  |

>TR:A0A2Z3II00 A0A2Z3II00_9RHIZ 3-phosphoshikimate 1-carboxyvinyltransferase

OS=Microvirga sp. 17 mud 1-3 OX=2082949 GN=aroA PE=3 SV=1 Length=449

Score = 496 bits (1278), Expect = 2e-171

| Identities | | = 277/447 (62%), Positives = 321/447 (72%), Gaps = 6/447 (1%) |  |
| --- | --- | --- | --- |
| Query 3  Sbjct 5 | | HGASSRPATARKSSGLSGTVRIPGDKSISHRSFMFGGLASGETRITGLLEGEDVINTGKA HG +S P T R L G +R+PGDKSISHRS +FG LA GETR+ GLLEG+DV+ T +A HGIAS-PVTGRAGGALKGRLRVPGDKSISHRSMIFGLLAIGETRVEGLLEGDDVLRTAEA | 62  63 |
| Query 63  Sbjct 64 | | MQAMGARIRKEGD-TWIIDGVGNGGLLAPEAPLDFGNAATGCRLTMGLVGVYDFDSTFIG  +A+GA+I +EG W I GVG GGL P LDFGNA TG RL MG+ G + +TF G CKALGAQITREGPGRWRILGVGIGGLSEPAGILDFGNAGTGSRLMMGVAGSHPITTTFDG | 121  123 |
| Query 122  Sbjct 124 | | DASLTKRPMGRVLNPLREMGVQVKSE-DGDRLPVTLRGPKTPTPITYRVPMASAQVKSAV DASL KRPM R+L+PL MGV V SE +G R+P+TLRGP+ PITY P ASAQ+KSA+ DASLRKRPMRRILDPLVRMGVTVVSEAEGGRVPLTLRGPRETIPITYETPAASAQIKSAI | 180  183 |
| Query 181  Sbjct 184 | | LLAGLNTPGITTVIEPIMTRDHTEKMLQGFGANLTVETDADGVRTIRLEGRGKLTGQVID LLAGLN+PG TTVIE TRDHTE+ML+ FGA + V + R I L+G+ +L G + LLAGLNSPGRTTVIEREATRDHTERMLRHFGATVEVVPHGEDGRAITLQGQPELKGSTVV | 240  243 |
| Query 241  Sbjct 244 | | VPGDPSSTAFPLVAALLVPGSDVTILNVLMNPTRTGLILTLQEMGADIEVINPRLAGGED VP DPSS AFP+VAAL+ PGSDV I V+MNP RTGLI TL EMGA IE +N R GGE VPSDPSSAAFPIVAALITPGSDVVIEGVMMNPLRTGLITTLLEMGASIERLNERDEGGET | 300  303 |
| Query 301  Sbjct 304 | | VADLRVRSSTLKGVTVPEDRAPSMIDEYPILAVAAAFAEGATVMNGLEELRVKESDRLSA VADLRV++S L GVTVP RAP+MIDEYPILAVAAAFAEG T M GL ELRVKESDRL+A VADLRVQASRLTGVTVPAARAPAMIDEYPILAVAAAFAEGTTRMQGLHELRVKESDRLAA | 360  363 |
| Query 361 | | VANGLKLNGVDCDEGETSLVVRGRPDGKGLGNASGAAVATHLDHRIAMSFLVMGLVSENP VA GL GV + LVV G GK G G +ATHLDHRIAMSFLVMGL +E P | 420 |
| Sbjct | 364 | VAAGLAETGVPHEIEGDDLVVHG-TGGKVRG--GGRPIATHLDHRIAMSFLVMGLATEEP | 420 |
| Query  Sbjct | 421  421 | VTVDDATMIATSFPEFMDLMAGLGAKI 447 VTVDD MIATSFP F+ LM LGA I VTVDDGAMIATSFPTFIPLMRELGATI 447 |  |

>TR:A0A2U8WEL9 A0A2U8WEL9_9RHIZ 3-phosphoshikimate 1-carboxyvinyltransferase OS=Methylobacterium durans OX=2202825 GN=aroA PE=3 SV=1

Length=453

Score = 496 bits (1278), Expect = 3e-171

| Identities | = 272/452 (60%), Positives = 324/452 (72%), Gaps = 10/452 (2%) |  |
| --- | --- | --- |
| Query 1 | MLHGASSRPATARKSSGLSGTVRIPGDKSISHRSFMFGGLASGETRITGLLEGEDVINTG | 60 |
| Sbjct 1 | M H + +P TA L+G++R PGDKSISHR+ + G L+ GETR+ GLLEG+DV+ T MSHDSDPQPLTAAPGQRLTGSLRPPGDKSISHRAMILGLLSQGETRVEGLLEGDDVLRTA | 60 |
| Query 61 | KAMQAMGARIRKEGD-TWIIDGVGNGGLLAPEAPLDFGNAATGCRLTMGLVGVYDFDSTF | 119 |
| Sbjct 61 | A +A+GA + + G+ WI+ GVG GGL P LDFGNA TG RL MG+VG +TF AASRALGAGMERLGEGRWIVRGVGIGGLSDPAETLDFGNAGTGSRLMMGVVGGQPVTATF | 120 |
| Query 120 | IGDASLTKRPMGRVLNPLREMGVQVKSE-DGDRLPVTLRGPKTPTPITYRVPMASAQVKS | 178 |
| Sbjct 121 | GDASL KRPM R+L+PL MG ++ E DG R+P+TLRGP+ PITY P ASAQ+KS DGDASLRKRPMRRILDPLLRMGTEILDEADGGRVPLTLRGPREAIPITYETPAASAQIKS | 180 |
| Query 179 | AVLLAGLNTPGITTVIEPIMTRDHTEKMLQGFGANLTVETDADGV--RTIRLEGRGKLTG | 236 |
| Sbjct 181 | AVLLA LN PGITTVIE TRDHTE+ML+ FGA + V G R + L G+ L G AVLLAALNAPGITTVIEREATRDHTERMLRLFGAEVAVTPHGPGGHGRAVALTGQPTLRG | 240 |
| Query 237 | QVIDVPGDPSSTAFPLVAALLVPGSDVTILNVLMNPTRTGLILTLQEMGADIEVINPRLA  + VP DPSS AFPLVAAL+VPGSDVTI V+MNP RTGLI TL EMGA+IE ++ R | 296 |

| Sbjct | 241 | TDVVVPADPSSAAFPLVAALIVPGSDVTIRGVMMNPLRTGLITTLLEMGAEIERLDEREE | 300 |
| --- | --- | --- | --- |
| Query | 297 | GGEDVADLRVRSSTLKGVTVPEDRAPSMIDEYPILAVAAAFAEGATVMNGLEELRVKESD | 356 |
| Sbjct | 301 | GGE VADLRVR+S LKGV VP +RAP+MIDEYPILAVAAAFAEG T MNGL ELRVKESD GGETVADLRVRASRLKGVDVPAERAPAMIDEYPILAVAAAFAEGPTRMNGLHELRVKESD | 360 |
| Query | 357 | RLSAVANGLKLNGVD-CDEGETSLVVRGRPDGKGLGNASGAAVATHLDHRIAMSFLVMGL | 415 |
| Sbjct | 361 | RL+AVA+GLK NG+D EG+ +V G G+ G VATHLDHRIAM+FLV+GL RLAAVADGLKQNGIDHVIEGDDLIVT GDGIAPRGGGTVATHLDHRIAMAFLVLGL | 415 |
| Query | 416 | VSENPVTVDDATMIATSFPEFMDLMAGLGAKI 447  + +PVTVDD MIATSFP F+ M LGA I |  |
| Sbjct | 416 | ATRDPVTVDDGAMIATSFPSFLPTMRALGADI 447 | |

>TR:A0A4R3MAT0 A0A4R3MAT0_9RHIZ 3-phosphoshikimate 1-carboxyvinyltransferase OS=Tepidamorphus gemmatus OX=747076 GN=aroA PE=3 SV=1

Length=466

Score = 497 bits (1279), Expect = 3e-171

| Identities | = 267/441 (61%), Positives = 320/441 (73%), Gaps = 3/441 (1%) |  |
| --- | --- | --- |
| Query 9 | PATARKSSGLSGTVRIPGDKSISHRSFMFGGLASGETRITGLLEGEDVINTGKAMQAMGA | 68 |
| Sbjct 11 | P AR + L G IPGDKSISHR+ +FG LA+G TRI GLLEG DV+ T A +A GA PLAARAGAPLRGRADIPGDKSISHRALLFGALATGRTRIRGLLEGHDVLATAAAARAFGA | 70 |
| Query 69 | RIRKEGD-TWIIDGVGNGGLLAPEAPLDFGNAATGCRLTMGLVGVYDFDSTFIGDASLTK | 127 |
| Sbjct 71 | + + D +W +DGVG G LL P APLDFGN+ TG RL MGLVG + + F GDASL K AVERAADGSWCVDGVGVGALLEPAAPLDFGNSGTGARLVMGLVGGHGITARFDGDASLRK | 130 |
| Query 128 | RPMGRVLNPLREMGVQVKS-EDGDRLPVTLRGPKTPTPITYRVPMASAQVKSAVLLAGLN | 186 |
| Sbjct 131 | RPMGRVL+PL MG +V + +G RLP+TL+G + P PI+YR+P+ASAQVKSAVLL GLN RPMGRVLSPLESMGARVVACAEGGRLPLTLQGTQDPLPISYRLPVASAQVKSAVLLCGLN | 190 |
| Query 187 | TPGITTVIEPIMTRDHTEKMLQGFGANLTVETDADGVRTIRLEGRGKLTGQVIDVPGDPS | 246 |
| Sbjct 191 | +PG TTVIEP TRDHTE+ML FGA+L + TD+DG R I L GR +L + + VP DPS SPGTTTVIEPEPTRDHTERMLAHFGASLAISTDSDGARRIALAGRPQLRARDVVVPADPS | 250 |
| Query 247 | STAFPLVAALLVPGSDVTILNVLMNPTRTGLILTLQEMGADIEVINPRLAGGEDVADLRV | 306 |
| Sbjct 251 | S AFP+VAAL+VPGS VTI V++NP+R G+I TL EMGA I + + R GGE VAD+ V SAAFPIVAALIVPGSAVTIPGVMVNPSRAGMIATLVEMGARITLSDRREDGGEPVADILV | 310 |
| Query 307 | RSSTLKGVTVPEDRAPSMIDEYPILAVAAAFAEGATVMNGLEELRVKESDRLSAVANGLK | 366 |
| Sbjct 311 | + L G+ VP RAPSMIDEYP LAVAAAFA G TVM GL EL+VKESDRL+AVA GL EAGPLNGIEVPAARAPSMIDEYPALAVAAAFAHGTTVMRGLSELKVKESDRLAAVAAGLA | 370 |
| Query 367 | LNGVDCDEGETSLVVRGRPDGKGLGNASGAAVATHLDHRIAMSFLVMGLVSENPVTVDDA | 426 |
| Sbjct 371 | NGV+ L+V GR G+ G G VATHLDHRIAM+FLVMGL +E PV VDD+ ANGVEVRVEGDDLIVEGR-GGRTGGVPGGGCVATHLDHRIAMAFLVMGLGTERPVIVDDS | 429 |
| Query 427  Sbjct 430 | TMIATSFPEFMDLMAGLGAKI 447  +MIATSFP+F+ LM LGA I SMIATSFPDFVALMTRLGADI 450 |  |

>TR:A0A370HN92 A0A370HN92_9RHIZ 3-phosphoshikimate 1-carboxyvinyltransferase OS=Microvirga subterranea OX=186651 GN=aroA PE=3 SV=1

Length=449

Score = 496 bits (1277), Expect = 3e-171

Identities = 273/448 (61%), Positives = 319/448 (71%), Gaps = 6/448 (1%)

Query 3	HGASSRPATARKSSGLSGTVRIPGDKSISHRSFMFGGLASGETRITGLLEGEDVINTGKA 62 HG +S P T+R	L G +R+PGDKSISHRS +FG LA GETR+ GLLEG+DV+ T +A

| Sbjct | 5 | HGPAS-PVTSRSGKALKGRLRVPGDKSISHRSMIFGLLAIGETRVEGLLEGDDVLRTAEA | 63 |
| --- | --- | --- | --- |
| Query | 63 | MQAMGARIRKEGD-TWIIDGVGNGGLLAPEAPLDFGNAATGCRLTMGLVGVYDFDSTFIG | 121 |
| Sbjct | 64 | +A+GA I +E W I GVG GGL P LDFGNA TG RL MG+ G + +TF G CRALGAGITREAPGRWRIQGVGIGGLTEPSGVLDFGNAGTGSRLMMGVAGSHPITTTFDG | 123 |
| Query | 122 | DASLTKRPMGRVLNPLREMGVQVKSE-DGDRLPVTLRGPKTPTPITYRVPMASAQVKSAV | 180 |
| Sbjct | 124 | DASL KRPM R+L+PL MGV V SE +G R+P+TLRGPK PITY P ASAQ+KSA+ DASLRKRPMRRILDPLVRMGVTVVSEAEGGRVPLTLRGPKETIPITYETPAASAQIKSAI | 183 |
| Query | 181 | LLAGLNTPGITTVIEPIMTRDHTEKMLQGFGANLTVETDADGVRTIRLEGRGKLTGQVID | 240 |
| Sbjct | 184 | LLAGLN+PG TTV+E TRDHTE+ML+ FGA + V + R I L+G+ +L G I LLAGLNSPGKTTVLEREATRDHTERMLRHFGATVEVAPYGEDGRAITLQGQPELRGSTIV | 243 |
| Query | 241 | VPGDPSSTAFPLVAALLVPGSDVTILNVLMNPTRTGLILTLQEMGADIEVINPRLAGGED | 300 |
| Sbjct | 244 | VP DPSS AFPLVAAL+ PGSDV I V+MNP RTGL+ TL EMGA IE +N R GGE VPSDPSSAAFPLVAALITPGSDVVIEGVMMNPLRTGLVTTLLEMGASIERLNERDEGGET | 303 |
| Query | 301 | VADLRVRSSTLKGVTVPEDRAPSMIDEYPILAVAAAFAEGATVMNGLEELRVKESDRLSA | 360 |
| Sbjct | 304 | VADLRVR+S L GVTVP RAP+MIDEYPILAVAA+FAEG T M GL ELRVKESDRL+A VADLRVRASRLTGVTVPAARAPAMIDEYPILAVAASFAEGTTRMQGLHELRVKESDRLAA | 363 |
| Query | 361 | VANGLKLNGVDCDEGETSLVVRGRPDGKGLGNASGAAVATHLDHRIAMSFLVMGLVSENP | 420 |

VA GL	GV +	L+V G	G	G +ATHLDHRIAMSFLVMGL ++ P

| Sbjct | 364 | VAAGLAEAGVTHEIEGDDLIVHG---SGGKVRGGGRPIATHLDHRIAMSFLVMGLATQEP | 420 |
| --- | --- | --- | --- |
| Query  Sbjct | 421  421 | VTVDDATMIATSFPEFMDLMAGLGAKIE 448 VTVDD MIATSFP F+ LM LGA I+ VTVDDGAMIATSFPTFIPLMRELGATIQ 448 |  |

>TR:A0A099FFL4 A0A099FFL4_9RHOB 3-phosphoshikimate 1-carboxyvinyltransferase OS=Paracoccus sphaerophysae OX=690417 GN=aroA PE=3 SV=1

Length=443

Score = 496 bits (1276), Expect = 4e-171

| Identities | = 258/448 (58%), Positives = 320/448 (71%), Gaps = 7/448 (2%) |  |
| --- | --- | --- |
| Query 1 | MLHGASSRPATARKSSGLSGTVRIPGDKSISHRSFMFGGLASGETRITGLLEGEDVINTG | 60 |
| Sbjct 1 | M H A+ +P TAR+S L G R+PGDKS+SHR+ + G L+ GETRITGLLEG+DV++TG MSHSAAPQPMTARRSGPLRGEARVPGDKSVSHRALILGALSVGETRITGLLEGQDVLDTG | 60 |
| Query 61 | KAMQAMGARIRKEGD-TWIIDGVGNGGLLAPEAPLDFGNAATGCRLTMGLVGVYDFDSTF | 119 |
| Sbjct 61 | KAMQA GA++ + G+ W++ GVG GG P +D GN+ TG RL MG + +TF KAMQAFGAQVDRVGEGEWVVHGVGVGGFAEPAGVIDCGNSGTGVRLIMGAMATTPITATF | 120 |
| Query 120 | IGDASLTKRPMGRVLNPLREMGVQVKSEDGDRLPVTLRGPKTPTPITYRVPMASAQVKSA | 179 |
| Sbjct 121 | GDASL++RPM R+ +PL E G QV +G LPVT+RG P P+ YR P+ASAQ+KSA TGDASLSRRPMRRITDPLAEFGAQVTDREGGLLPVTIRGAADPVPVRYRTPVASAQIKSA | 180 |
| Query 180 | VLLAGLNTPGITTVIEPIMTRDHTEKMLQGFGANLTVETDADGVRTIRLEGRGKLTGQVI | 239 |
| Sbjct 181 | VLLAGLN PG T VIE TRDHTE+ML GFGA + + +G R I L+GR +L Q + VLLAGLNAPGETVVIEAEPTRDHTERMLAGFGATIRTDITDEG-RVITLQGRPELQAQPV | 239 |
| Query 240 | DVPGDPSSTAFPLVAALLVPGSDVTILNVLMNPTRTGLILTLQEMGADIEVINPRLAGGE | 299 |
| Sbjct 240 | VP DPSS AFP+ AALLVPGS++T+ V NPTR G +TL +MGAD+ N R+ GGE AVPRDPSSAAFPVAAALLVPGSEITVPGVSRNPTRDGFYVTLADMGADLTWTNDRIEGGE | 299 |
| Query 300 | DVADLRVRSSTLKGVTVPEDRAPSMIDEYPILAVAAAFAEGATVMNGLEELRVKESDRLS | 359 |
| Sbjct 300 | VADL VR L+GVTVP +RA SMIDE+PIL+V AA AEG TVMNG+ ELRVKESDR++ PVADLTVRHGPLRGVTVPAERAASMIDEFPILSVVAALAEGPTVMNGVAELRVKESDRIA | 359 |
| Query 360 | AVANGLKLNGVDCDEGETSLVVRGRPDGKGLGNASGAAVATHLDHRIAMSFLVMGLVSEN A+A+GL+ NGV ++ S+ V G G GA VATHLDHRIAMSFLV+GLVSE | 419 |

| Sbjct | 360 | AMADGLRANGVTVEDTRDSMTVHGMASVPG-----GATVATHLDHRIAMSFLVLGLVSEA 414 |
| --- | --- | --- |
| Query | 420 | PVTVDDATMIATSFPEFMDLMAGLGAKI 447 |
| Sbjct | 415 | PV VDD IATSFP+F+ LM GLGA+I  PVDVDDGGPIATSFPDFLPLMRGLGAQI 442 |

>SP:B7KZL6 AROA_METC4 3-phosphoshikimate 1-carboxyvinyltransferase OS=Methylorubrum extorquens (strain CM4 / NCIMB 13688) OX=440085

GN=aroA PE=3 SV=1

Length=453

Score = 496 bits (1276), Expect = 5e-171

| Identities | = 270/453 (60%), Positives = 329/453 (73%), Gaps = 10/453 (2%) |  |
| --- | --- | --- |
| Query 1 | MLHGASSRPATARKSSGLSGTVRIPGDKSISHRSFMFGGLASGETRITGLLEGEDVINTG | 60 |
| Sbjct 1 | M H + +P TA L+G+++ PGDKSISHR+ + G LA GET++ GLLEG+DV+ T MSHDSEPQPVTATPGGPLNGSLKPPGDKSISHRAMILGLLAIGETQVEGLLEGDDVLRTA | 60 |
| Query 61 | KAMQAMGARIRKEGD-TWIIDGVGNGGLLAPEAPLDFGNAATGCRLTMGLVGVYDFDSTF | 119 |
| Sbjct 61 | A +A+GA+I +EG+ W I GVG GG+ P+ LDFGNA TG RL MG+VG +TF AAAKALGAQITREGEGRWRIVGVGIGGMQDPDGVLDFGNAGTGSRLMMGVVGGQPVTATF | 120 |
| Query 120 | IGDASLTKRPMGRVLNPLREMGVQVKSE-DGDRLPVTLRGPKTPTPITYRVPMASAQVKS | 178 |
| Sbjct 121 | GDASL KRPM R+L+P+ +MG ++ SE +G R+P+TLRGP+ PI Y +P+ASAQ+KS DGDASLRKRPMRRILDPILKMGAEIVSEAEGGRVPLTLRGPREAIPIRYELPVASAQIKS | 180 |
| Query 179 | AVLLAGLNTPGITTVIEPIMTRDHTEKMLQGFGANLTVETDADGV--RTIRLEGRGKLTG | 236 |
| Sbjct 181 | AVLLAGLN PG TTVIE +RDHTE+ML FGA +TV +G RT+ L G+ L G AVLLAGLNAPGTTTVIEKAASRDHTERMLHLFGAEVTVTPSGEGGHGRTVTLTGQPTLRG | 240 |
| Query 237 | QVIDVPGDPSSTAFPLVAALLVPGSDVTILNVLMNPTRTGLILTLQEMGADIEVINPRLA | 296 |
| Sbjct 241 | + VP DPSS AFPLVAAL+VPGS+V + V+MNP RTGLI TL EMGADIE ++ R TDVVVPADPSSAAFPLVAALIVPGSEVILRGVMMNPLRTGLITTLIEMGADIERLDEREE | 300 |
| Query 297 | GGEDVADLRVRSSTLKGVTVPEDRAPSMIDEYPILAVAAAFAEGATVMNGLEELRVKESD | 356 |
| Sbjct 301 | GGE VADLRVR+S LKGV VP +RAPSMIDEYPILAVAA+FAEG T MNGL ELRVKESD GGETVADLRVRASRLKGVDVPAERAPSMIDEYPILAVAASFAEGTTRMNGLHELRVKESD | 360 |
| Query 357 | RLSAVANGLKLNGV-DCDEGETSLVVRGRPDGKGLGNASGAAVATHLDHRIAMSFLVMGL | 415 |
| Sbjct 361 | RL+AVA GL NGV EG+ +V G G A G VATHLDHRIAM+FLV+GL RLAAVAAGLAANGVTHAIEGDDLIVT GNGQAPAGGGTVATHLDHRIAMAFLVLGL | 415 |
| Query 416  Sbjct 416 | VSENPVTVDDATMIATSFPEFMDLMAGLGAKIE 448  +++PVTVDD MIATSFP F M LGA+IE AAKSPVTVDDGAMIATSFPSFRPTMLALGARIE 448 |  |

>TR:A0A2T1HT45 A0A2T1HT45_9RHIZ 3-phosphoshikimate 1-carboxyvinyltransferase OS=Alsobacter sp. SH9 OX=2109933 GN=aroA PE=3 SV=1

Length=452

Score = 495 bits (1275), Expect = 7e-171

Identities = 270/445 (61%), Positives = 320/445 (72%), Gaps = 6/445 (1%)

| Query | 3 | HGASSRPATARKSSGLSGTVRIPGDKSISHRSFMFGGLASGETRITGLLEGEDVINTGKA | 62 |
| --- | --- | --- | --- |
| Sbjct | 7 | H ++P +A+ L G VR+PGDKSISHR+ +FG LA GET + GLLEGEDV+ T +A HHGEAQPLSAQGGPPLRGRVRVPGDKSISHRAMIFGLLAVGETTVEGLLEGEDVLRTAEA | 66 |
| Query | 63 | MQAMGARIRKEGD-TWIIDGVGNGGLLAPEAPLDFGNAATGCRLTMGLVGVYDFDSTFIG | 121 |
| Sbjct | 67 | +A+GA + + G+ W I GVG GGL P LDFGNA TG RL MG+VG + +TF G CRALGATMERTGEGAWRIQGVGVGGLREPAGVLDFGNAGTGSRLMMGVVGGHGITATFDG | 126 |
| Query | 122 | DASLTKRPMGRVLNPLREMGVQVKSE-DGDRLPVTLRGPKTPTPITYRVPMASAQVKSAV | 180 |

DASL KRPM R+L+PL MG QV SE +G R P+TL+G	P PI YR P+ASAQ+KSAV

| Sbjct | 127 | DASLRKRPMRRILDPLTRMGAQVLSEAEGGRCPITLKGAIDPVPIEYRTPVASAQIKSAV | 186 |
| --- | --- | --- | --- |
| Query | 181 | LLAGLNTPGITTVIEPIMTRDHTEKMLQGFGANLTVETDADGVRTIRLEGRGKLTGQVID | 240 |
| Sbjct | 187 | LLAGLN PG TTVIE +RDHTEKML FGA +TV + + R I LEGR +L + LLAGLNAPGRTTVIETEASRDHTEKMLAYFGARVTVTPEGEHGRRIVLEGRPELKPAHVV | 246 |
| Query | 241 | VPGDPSSTAFPLVAALLVPGSDVTILNVLMNPTRTGLILTLQEMGADIEVINPRLAGGED | 300 |
| Sbjct | 247 | VP DPSS AFP+VAAL+ PGSD+ + V+MNP RTGL+ +L+EMGADIE I R+ GGE+ VPADPSSAAFPIVAALVTPGSDIVVEGVMMNPLRTGLLTSLREMGADIEEIGRRVEGGEE | 306 |
| Query | 301 | VADLRVRSSTLKGVTVPEDRAPSMIDEYPILAVAAAFAEGATVMNGLEELRVKESDRLSA | 360 |
| Sbjct | 307 | VADLRVR S L+GV VP RAPSMIDEYP+LAVAA+FA G T M GL ELRVKESDRL A VADLRVRHSALRGVDVPAGRAPSMIDEYPVLAVAASFASGETRMRGLHELRVKESDRLQA | 366 |
| Query | 361 | VANGLKLNGVDCDEGETSLVVRGRPDGKGLGNASGAAVATHLDHRIAMSFLVMGLVSENP | 420 |
| Sbjct | 367 | VA+GL NGV+ L+V+G G G+ G V THLDHRIAMSFLVMGL S+ P VADGLAANGVEHRIEGDDLIVQG---GAGVVR-GGGTVKTHLDHRIAMSFLVMGLASQRP | 422 |
| Query  Sbjct | 421  423 | VTVDDATMIATSFPEFMDLMAGLGA 445 VTVDD TMIATSFP F LM GLGA VTVDDKTMIATSFPAFEGLMTGLGA 447 |  |

>TR:A0A1I2W6L8 A0A1I2W6L8_9RHIZ 3-phosphoshikimate 1-carboxyvinyltransferase OS=Methylobacterium gossipiicola OX=582675 GN=aroA PE=3 SV=1

Length=448

Score = 494 bits (1272), Expect = 2e-170

| Identities | = 275/454 (61%), Positives = 323/454 (71%), Gaps = 14/454 (3%) |  |
| --- | --- | --- |
| Query 1 | MLHGASSRPATARKSSGLSGTVRIPGDKSISHRSFMFGGLASGETRITGLLEGEDVINTG | 60 |
| Sbjct 1 | M H +P TA L G +R PGDKSISHR+ + G L+ GETR+ GLLEG+DV+ T MSHDLPPQPLTATAGLALQGALRPPGDKSISHRAMILGLLSIGETRVEGLLEGDDVLRTA | 60 |
| Query 61 | KAMQAMGARIRKEGD-TWIIDGVGNGGLLAPEAPLDFGNAATGCRLTMGLVGVYDFDSTF | 119 |
| Sbjct 61 | A QA+GA + + G+ W I GVG GGL PE LDFGNA TG RL MG+VG +TF AAAQALGATVERLGEGQWRIVGVGLGGLSDPEGVLDFGNAGTGSRLMMGVVGGQPVTATF | 120 |
| Query 120 | IGDASLTKRPMGRVLNPLREMGVQVKSED-GDRLPVTLRGPKTPTPITYRVPMASAQVKS | 178 |
| Sbjct 121 | GDASL KRPM R+L+PL MG +V SE+ G R+P+TLRGP+ P+TY P+ASAQ+KS DGDASLRKRPMRRILDPLVRMGTEVVSEEEGGRVPLTLRGPREAIPVTYETPVASAQIKS | 180 |
| Query 179 | AVLLAGLNTPGITTVIEPIMTRDHTEKMLQGFGANLTVETDADGV RTIRLEGRGKL | 234 |
| Sbjct 181 | AVLLAGLN PG+TTVIE TRDHTE+ML+ FGA+ VE A G RTI L G+ L AVLLAGLNAPGVTTVIEAAATRDHTERMLRLFGAH--VEVTAHGPEGHGRTIALTGQPTL | 238 |
| Query 235 | TGQVIDVPGDPSSTAFPLVAALLVPGSDVTILNVLMNPTRTGLILTLQEMGADIEVINPR | 294 |
| Sbjct 239 | G + VP DPSS AFPLVAAL+VPGS+VTI V+MNP RTGLI TL EMGADIE + R RGTDVVVPADPSSAAFPLVAALIVPGSEVTIKGVMMNPLRTGLITTLIEMGADIERLRER | 298 |
| Query 295 | LAGGEDVADLRVRSSTLKGVTVPEDRAPSMIDEYPILAVAAAFAEGATVMNGLEELRVKE | 354 |
| Sbjct 299 | GGE VADLRVR+S LKGV VP +RAP+MIDEYPILAVAAAFA+G T MNGL ELRVKE EEGGETVADLRVRASRLKGVDVPPERAPAMIDEYPILAVAAAFADGTTRMNGLHELRVKE | 358 |
| Query 355 | SDRLSAVANGLKLNGV-DCDEGETSLVVRGRPDGKGLGNASGAAVATHLDHRIAMSFLVM | 413 |
| Sbjct 359 | SDRL+AVA+GLK NG+ EG+ +V G G G VATHLDHRIAM+FLV+ SDRLAAVADGLKANGIAHVIEGDDLVV HGDGTAPKGGGTVATHLDHRIAMAFLVL | 413 |
| Query 414  Sbjct 414 | GLVSENPVTVDDATMIATSFPEFMDLMAGLGAKI 447 GL + PVTVDD MIATSFP F+ M LG +I GLATREPVTVDDGAMIATSFPSFLPTMQALGGRI 447 |  |

>TR:A0A2H6BH97 A0A2H6BH97_9RHIZ 3-phosphoshikimate 1-carboxyvinyltransferase OS=Methylopila sp. Yamaguchi OX=1437817 GN=aroA PE=3 SV=1

Length=451

Score = 494 bits (1272), Expect = 2e-170

| Identities | = 271/445 (61%), Positives = 323/445 (73%), Gaps = 8/445 (2%) |  |
| --- | --- | --- |
| Query 8 | RPATARKSSGLSGTVRIPGDKSISHRSFMFGGLASGETRITGLLEGEDVINTGKAMQAMG | 67 |
| Sbjct 8 | +PA +R + L G VR+PGDKSISHRS + G LA GETRI GLLEG+DV+ TG+A A+G QPAVSRANGPLRGRVRVPGDKSISHRSLLLGLLAVGETRIEGLLEGDDVLATGRACAALG | 67 |
| Query 68 | ARIRKEGD-TWIIDGVGNGGLLAPEAPLDFGNAATGCRLTMGLVGVYDFDSTFIGDASLT | 126 |
| Sbjct 68 | A + + G+ W + GVG GGL +P+APLDFGNA TG RL MG+VG + +TF GDASL ATVERLGEGRWSVRGVGVGGLKSPDAPLDFGNAGTGSRLMMGVVGGHPIQATFDGDASLR | 127 |
| Query 127 | KRPMGRVLNPLREMGVQVKSE-DGDRLPVTLRGPKTPTPITYRVPMASAQVKSAVLLAGL | 185 |
| Sbjct 128 | RPM R+L+PL MG V S +G RLP+TL+GP+T P+TY P+ SAQVKSAVLLAGL LRPMRRILDPLALMGATVVSAAEGGRLPLTLKGPETALPLTYETPVPSAQVKSAVLLAGL | 187 |
| Query 186 | NTPGITTVIEPIMTRDHTEKMLQGFGANLTVETDADGVRTIRLEGRGKLTGQVIDVPGDP | 245 |
| Sbjct 188 | N PG T VIE TRDHTE+ML+ FGA +TVE + R +RL G+ +LT + VP DP NAPGETVVIEREATRDHTERMLKAFGAEVTVEPHGEHGRLVRLVGQPELTPAHVVVPADP | 247 |
| Query 246 | SSTAFPLVAALLVPGSDVTILNVLMNPTRTGLILTLQEMGADIEVINPRLAGGEDVADLR | 305 |
| Sbjct 248 | SS AFPLVAAL+ PGS VT+ V+ NP RTGLI TL EMGADIE+ N R GGE+VADL SSAAFPLVAALIAPGSHVTLEGVMTNPLRTGLIATLIEMGADIELTNLRDEGGEEVADLV | 307 |
| Query 306 | VRSSTLKGVTVPEDRAPSMIDEYPILAVAAAFAEGATVMNGLEELRVKESDRLSAVANGL | 365 |
| Sbjct 308 | VR+S LKGV VP RAP+MIDEYP+LA AAAFA+G T M GL EL VKESDRL+AV GL VRASALKGVDVPAARAPTMIDEYPVLAAAAAFAQGETRMRGLHELTVKESDRLAAVHAGL | 367 |
| Query 366 | KLNGVDCD-EGETSLVVRGRPDGKGLGNASGAAVATHLDHRIAMSFLVMGLVSENPVTVD | 424 |
| Sbjct 368 | GV+ EGE +VV G+G G AV THLDHRIAMSFLV+GL ++ PV +D VACGVEARIEGEDLIVV GRGRAPGGGEAVKTHLDHRIAMSFLVLGLAADAPVAID | 422 |
| Query 425  Sbjct 423 | DATMIATSFPEFMDLMAGLGAKIEL 449 DA MIATSFP F+DLM GLGA I L DAAMIATSFPAFVDLMRGLGADISL 447 |  |

>TR:A0A516H0K4 A0A516H0K4_9PROT 3-phosphoshikimate 1-carboxyvinyltransferase OS=Ferrovibrio terrae OX=2594003 GN=aroA PE=3 SV=1

Length=454

Score = 494 bits (1272), Expect = 2e-170

| Identities | = 261/447 (58%), Positives = 325/447 (73%), Gaps = 8/447 (2%) |  |
| --- | --- | --- |
| Query 7 | SRPATARKSSGLSGTVRIPGDKSISHRSFMFGGLASGETRITGLLEGEDVINTGKAMQAM | 66 |
| Sbjct 14 | SRPA + L+GT ++PGDKSISHRS +FG L+ GETR++GLLEGEDV+ T AM+AM SRPA AKALAGTAKVPGDKSISHRSLIFGALSVGETRVSGLLEGEDVLRTATAMRAM | 69 |
| Query 67 | GARIRKEGD-TWIIDGVGNGGLLAPEAPLDFGNAATGCRLTMGLVGVYDFDSTFIGDASL | 125 |
| Sbjct 70 | GA + + GD W + GVG GGL P LD GNA TG RL MGLV +DF +TF GDASL GAEVERLGDGNWKVWGVGVGGLSEPADVLDMGNAGTGARLVMGLVASHDFSATFTGDASL | 129 |
| Query 126 | TKRPMGRVLNPLREMGVQVKSEDGDRLPVTLRGPKTPTPITYRVPMASAQVKSAVLLAGL | 185 |
| Sbjct 130 | +RPMGRV+ PL +G + G RLP+T+ G P PI Y +P+ASAQVK+AVLLAGL RRRPMGRVVEPLTRIGARFVGRSGGRLPLTVVGTAHPLPIEYELPVASAQVKTAVLLAGL | 189 |
| Query 186 | NTPGITTVIEPIMTRDHTEKMLQGFGANLTVETDADGVRTIRLEGRGKLTGQVIDVPGDP | 245 |
| Sbjct 190 | N PGITTVIEP TRDHTE ML+ FGA++ VE G R IRL G+ + I VPGDP NAPGITTVIEPEPTRDHTENMLRHFGASVEVEKTKTGGRIIRLAGQPEFRPTTIAVPGDP | 249 |
| Query 246 | SSTAFPLVAALLVPGSDVTILNVLMNPTRTGLILTLQEMGADIEVINPRLAGGEDVADLR | 305 |

SS AFP+VAAL+V GS++T+ NV +NP RTGL TLQ+MGAD	N R GGE VAD+

| Sbjct | 250 | SSAAFPMVAALIVQGSEITLQNVGLNPLRTGLFTTLQDMGADFSFANTRSEGGEPVADIT | 309 |
| --- | --- | --- | --- |
| Query | 306 | VRSSTLKGVTVPEDRAPSMIDEYPILAVAAAFAEGATVMNGLEELRVKESDRLSAVANGL | 365 |
| Sbjct | 310 | +R S LKG+TVPE+RAPSMIDEYPIL VAAA AEG T++ G +ELRVKESDRL+ +A GL IRQSALKGITVPEERAPSMIDEYPILFVAAACAEGDTIVRGAKELRVKESDRLAVMARGL | 369 |
| Query | 366 | KLNGVDCDEGETSLVVRGRPDGK-GLGNASGAAVATHLDHRIAMSFLVMGLVSENPVTVD | 424 |
| Sbjct | 370 | K GVD +E E +++RG+ GK G A+G +AT LDHRIAMSFLV+G+ +++ VT+D KSCGVDLEEFEDGIIIRGK--GKPPQGLAAGEVIATELDHRIAMSFLVLGMAAKDGVTID | 427 |
| Query  Sbjct | 425  428 | DATMIATSFPEFMDLMAGLGAKIELSD 451 DA ++ TSFP F DLM LG ++L++ DARVMDTSFPGFADLMRRLGGDLQLAN 454 |  |

>TR:A7IEB8 A7IEB8_XANP2 3-phosphoshikimate 1-carboxyvinyltransferase OS=Xanthobacter autotrophicus (strain ATCC BAA-1158 / Py2) OX=78245

GN=aroA PE=3 SV=1

Length=456

Score = 494 bits (1272), Expect = 2e-170

| Identities | | = 275/448 (61%), Positives = 318/448 (71%), Gaps = 8/448 (2%) |  |
| --- | --- | --- | --- |
| Query 3  Sbjct 13 | | HG-ASSRPATARKSSGLSGTVRIPGDKSISHRSFMFGGLASGETRITGLLEGEDVINTGK HG ++PATA +S L+G VR+PGDKS+SHR+ +FG A G TRITGLLEGEDV+NT K HGDGPAQPATALRSPALTGRVRVPGDKSVSHRALIFGLFAEGITRITGLLEGEDVLNTAK | 61  72 |
| Query 62  Sbjct 73 | | AMQAMGARIRKEGD-TWIIDGVGNGGLLAPEAPLDFGNAATGCRLTMGLVGVYDFDSTFI A A+GAR+ + G W ++G G GL +P APLDFGN+ TG RL MG V D +TF ACAALGARVTRRGPGEWEVEGAGADGLSSPAAPLDFGNSGTGVRLMMGAVAGQDVTATFD | 120  132 |
| Query 121  Sbjct 133 | | GDASLTKRPMGRVLNPLREMGVQ-VKSEDGDRLPVTLRGPKTPTPITYRVPMASAQVKSA GDASL +RPM RVL+PL MGV+ V S +G RLP+TL+G ITY PM SAQVKSA GDASLRRRPMKRVLDPLTAMGVEIVDSAEGGRLPLTLKGSDRTKAITYETPMPSAQVKSA | 179  192 |
| Query 180 | | VLLAGLNTPGITTVIEPIMTRDHTEKMLQGFGANLTVETDADGVRTIRLEGRGKLTGQVI VLLAGL G T VIE TRDHTE+ML FGA++ VE R I L+GR L + | 239 |
| Sbjct | 193 | VLLAGLGAAGETVVIEREATRDHTERMLAHFGADVRVEPHGAHGRRIALKGRPTLVAAPV | 252 |
| Query | 240 | DVPGDPSSTAFPLVAALLVPGSDVTILNVLMNPTRTGLILTLQEMGADIEVINPRLAGGE | 299 |
| Sbjct | 253 | DVP DPSS AFPLVAAL+VPGSDVT+ +V+MNP RTGLI TL EMGA IEV+ R GGE DVPADPSSAAFPLVAALIVPGSDVTLTDVMMNPLRTGLITTLLEMGASIEVVAERTEGGE | 312 |
| Query | 300 | DVADLRVRSSTLKGVTVPEDRAPSMIDEYPILAVAAAFAEGATVMNGLEELRVKESDRLS | 359 |
| Sbjct | 313 | VADLRVR S LKGV VP +RAP+MIDEYPILAVAAAFAEG T M GL ELRVKESDRL+ RVADLRVRHSRLKGVEVPPERAPAMIDEYPILAVAAAFAEGVTAMRGLSELRVKESDRLA | 372 |
| Query | 360 | AVANGLKLNGVDCDEGETSLVVRGRPDGKGLGNASGAAVATHLDHRIAMSFLVMGLVSEN | 419 |
| Sbjct | 373 | AVA+GL GV + L+V G KG G VATH+DHRIAMSFLV+GL SE AVADGLAACGVAHEVVGDDLLVTGATTVKG GGPVATHMDHRIAMSFLVLGLASEQ | 427 |
| Query  Sbjct | 420  428 | PVTVDDATMIATSFPEFMDLMAGLGAKI 447 V+VDD IATSFP FM +M GLGA I  GVSVDDVAFIATSFPTFMPMMRGLGAVI 455 |  |

>TR:A0A1B3NFT2 A0A1B3NFT2_9BRAD 3-phosphoshikimate 1-carboxyvinyltransferase OS=Bosea sp. RAC05 OX=1842539 GN=aroA PE=3 SV=1

Length=446

Score = 493 bits (1270), Expect = 3e-170

Identities = 272/449 (61%), Positives = 322/449 (72%), Gaps = 6/449 (1%)

| Query | 1 | MLHGASSRPATARKSSGLSGTVRIPGDKSISHRSFMFGGLASGETRITGLLEGEDVINTG | 60 |
| --- | --- | --- | --- |
| Sbjct | 1 | M H + P +AR+ L G VR+PGDKSISHR+ +FG LA GET +TGLLEGEDV+ T MAHAHTPVPVSARRCGPLRGRVRVPGDKSISHRAMIFGLLAIGETTVTGLLEGEDVMRTA | 60 |
| Query | 61 | KAMQAMGARIRKEGD-TWIIDGVGNGGLLAPEAPLDFGNAATGCRLTMGLVGVYDFDSTF | 119 |
| Sbjct | 61 | A +A+GA + +G +W + GVG GGL P LDFGNA TG RL MG+ G + +TF AAARALGATVHHDGPGSWRVRGVGVGGLREPAGVLDFGNAGTGSRLMMGVCGSHPITTTF | 120 |
| Query | 120 | IGDASLTKRPMGRVLNPLREMG-VQVKSEDGDRLPVTLRGPKTPTPITYRVPMASAQVKS | 178 |
| Sbjct | 121 | GDASL KRPM R+L+PL MG V V E+G R+P+TLRGP+ PITYR P+ASAQ+KS DGDASLRKRPMRRILDPLALMGTVIVAQEEGGRVPLTLRGPQEALPITYRTPVASAQIKS | 180 |
| Query | 179 | AVLLAGLNTPGITTVIEPIMTRDHTEKMLQGFGANLTVETDADGVRTIRLEGRGKLTGQV | 238 |
| Sbjct | 181 | AVLLAGL PG TTVIE TRDHTEKML FGA +TV + + R I L+G+ +L AVLLAGLAAPGETTVIETEATRDHTEKMLTHFGAEVTVSPEGEHGRRIVLKGQPELQAAP | 240 |
| Query | 239 | IDVPGDPSSTAFPLVAALLVPGSDVTILNVLMNPTRTGLILTLQEMGADIEVINPRLAGG | 298 |
| Sbjct | 241 | I VP DPSS AFPLVAAL+VPGSDV I +V+ NP R+GL+ TL+EMGADI V + GG IVVPADPSSAAFPLVAALIVPGSDVLIESVMTNPLRSGLLTTLREMGADITVESEANEGG | 300 |
| Query | 299 | EDVADLRVRSSTLKGVTVPEDRAPSMIDEYPILAVAAAFAEGATVMNGLEELRVKESDRL | 358 |
| Sbjct | 301 | E VA LRVR+S LKGVTVP +RAPSMIDEYP+LAVAA+FA G T M GL+ELRVKESDRL EAVATLRVRASGLKGVTVPPERAPSMIDEYPVLAVAASFATGTTRMRGLQELRVKESDRL | 360 |
| Query | 359 | SAVANGLKLNGVDCDEGETSLVVRGRPDGKGLGNASGAAVATHLDHRIAMSFLVMGLVSE | 418 |
| Sbjct | 361 | +AV GLK GV C LVV +G G A G VATHLDHRIAMSFLVMGL ++ AAVEAGLKAAGVTCAIEGDDLVV EGSGGAVAGGGTVATHLDHRIAMSFLVMGLATD | 416 |
| Query | 419 | NPVTVDDATMIATSFPEFMDLMAGLGAKI 447 P+TVDD MIATSFP F+ LM GLG +I |  |
| Sbjct | 417 | KPMTVDDGAMIATSFPSFIALMNGLGGEI 445 | |

>TR:A0A085FGZ9 A0A085FGZ9_9BRAD 3-phosphoshikimate 1-carboxyvinyltransferase OS=Bosea sp. LC85 OX=1502851 GN=aroA PE=3 SV=1

Length=446

Score = 493 bits (1270), Expect = 3e-170

| Identities | = 272/449 (61%), Positives = 319/449 (71%), Gaps = 6/449 (1%) |  |
| --- | --- | --- |
| Query 1 | MLHGASSRPATARKSSGLSGTVRIPGDKSISHRSFMFGGLASGETRITGLLEGEDVINTG | 60 |
| Sbjct 1 | M H P TAR S LSG VR+PGDKSISHR+ +FG LA GETR+TGLLEGEDV+ T MAHAQHPTPVTARASGPLSGRVRVPGDKSISHRAMIFGLLAIGETRVTGLLEGEDVLRTA | 60 |
| Query 61 | KAMQAMGARIRKEGD-TWIIDGVGNGGLLAPEAPLDFGNAATGCRLTMGLVGVYDFDSTF | 119 |
| Sbjct 61 | A +A+GA + +G W + GVG GGL P LDFGNA TG RL MG+ G + +TF DAARALGAIVHHDGPGAWRVQGVGVGGLREPAGLLDFGNAGTGSRLMMGVCGAHPITTTF | 120 |
| Query 120 | IGDASLTKRPMGRVLNPLREMGVQVKS-EDGDRLPVTLRGPKTPTPITYRVPMASAQVKS | 178 |
| Sbjct 121 | GDASL KRPM R+L+PL MG + S E+G R+P+T+RGPK PITYR P+ASAQ+KS DGDASLRKRPMRRILDPLELMGATILSQEEGGRVPLTIRGPKEALPITYRTPVASAQIKS | 180 |
| Query 179 | AVLLAGLNTPGITTVIEPIMTRDHTEKMLQGFGANLTVETDADGVRTIRLEGRGKLTGQV | 238 |
| Sbjct 181 | AVLLAGL PG TTVIE TRDHTEKML FGA + V ++ R I L+G+ L AVLLAGLAAPGETTVIETEATRDHTEKMLAHFGAEVRVTSEGAHGRRIVLKGQPDLQAAP | 240 |
| Query 239 | IDVPGDPSSTAFPLVAALLVPGSDVTILNVLMNPTRTGLILTLQEMGADIEVINPRLAGG | 298 |
| Sbjct 241 | I VP DPSS AFPLVAAL+VPGSD+ I +V+ NP R+GL+ TL+EMGADI V + GG IVVPADPSSAAFPLVAALIVPGSDILIESVMTNPLRSGLLTTLREMGADITVESEANEGG | 300 |
| Query 299 | EDVADLRVRSSTLKGVTVPEDRAPSMIDEYPILAVAAAFAEGATVMNGLEELRVKESDRL | 358 |
| Sbjct 301 | E VA LRVR+ LKGV VP +RAPSMIDEYP+LAVAA+FA G T M GL+ELRVKESDRL  ETVATLRVRAGALKGVEVPPERAPSMIDEYPVLAVAASFATGTTRMRGLQELRVKESDRL | 360 |

| Query | 359 | SAVANGLKLNGVDCDEGETSLVVRGRPDGKGLGNASGAAVATHLDHRIAMSFLVMGLVSE 418 |
| --- | --- | --- |
| Sbjct | 361 | +AV GLK GV C LVV G +GK G G ATHLDHRIAMSFLVMGL ++ AAVEAGLKTAGVTCRIEGDDLVVEG-GNGKVAG---GGTAATHLDHRIAMSFLVMGLATQ 416 |
| Query | 419 | NPVTVDDATMIATSFPEFMDLMAGLGAKI 447 |
| Sbjct | 417 | NP+ VDD MIATSFP F+ LM GLGA+I  NPMQVDDGAMIATSFPTFISLMNGLGAEI 445 |

>TR:A0A140GV68 A0A140GV68_9RHIZ 3-phosphoshikimate 1-carboxyvinyltransferase OS=Rhodoplanes sp. Z2-YC6860 OX=674703 GN=aroA PE=3 SV=1

Length=446

Score = 493 bits (1269), Expect = 5e-170

| Identities | | = 263/446 (59%), Positives = 323/446 (72%), Gaps = 7/446 (2%) |  |
| --- | --- | --- | --- |
| Query 1  Sbjct 1 | | MLHGASSRPATARKSSGLSGTVRIPGDKSISHRSFMFGGLASGETRITGLLEGEDVINTG M H ++ +P TAR++ L+G VR+PGDKSISHR+ + G L+ GET+ITGLLEGEDVINT MSHASAPQPLTARRAGPLTGRVRVPGDKSISHRALILGALSVGETKITGLLEGEDVINTS | 60  60 |
| Query 61  Sbjct 61 | | KAMQAMGARIRKEGD-TWIIDGVGNGGLLAPEAPLDFGNAATGCRLTMGLVGVYDFDSTF KAMQAMGA + + G+ W I GVG G P APLDFGN+ TGCRL MG V + F KAMQAMGASVNRTGEGVWSIRGVGVAGFAEPAAPLDFGNSGTGCRLVMGAVAGCPITALF | 119  120 |
| Query 120  Sbjct 121 | | IGDASLTKRPMGRVLNPLREMGVQVKS-EDGDRLPVTLRGPKTPTPITYRVPMASAQVKS GDASL KRPM R+L+PL ++G ++ +G RLPVTL+G + P PI + P+ SAQ+KS DGDASLRKRPMRRILDPLEKIGARIMEIGEGGRLPVTLKGARDPLPIVFEAPVPSAQLKS | 178  180 |
| Query 179  Sbjct 181 | | AVLLAGLNTPGITTVIEPIMTRDHTEKMLQGFGANLTVETDADGVRTIRLEGRGKLTGQV AVLLAGL PG TTVIE TRDHTEK+L+ FGA++ V + + R I LEG+ +L G AVLLAGLAAPGATTVIETEATRDHTEKLLKHFGAHVRVTAEGEHGRRITLEGQPELEGAS | 238  240 |
| Query 239  Sbjct 241 | | IDVPGDPSSTAFPLVAALLVPGSDVTILNVLMNPTRTGLILTLQEMGADIEVINPRLAGG  + VP DPSS AFPLVAAL+VPGS++ + V+MNP RTGL+ TL+EMGA+ E ++ R GG VVVPADPSSAAFPLVAALIVPGSELILEGVMMNPLRTGLLTTLKEMGAEFEKLSERNEGG | 298  300 |
| Query 299  Sbjct 301 | | EDVADLRVRSSTLKGVTVPEDRAPSMIDEYPILAVAAAFAEGATVMNGLEELRVKESDRL EDVADLRVR+ TLKGV VP +RAPSMIDEYPILAVAAAFAEG T M GL+ELRVKESDRL EDVADLRVRACTLKGVDVPPERAPSMIDEYPILAVAAAFAEGTTRMRGLKELRVKESDRL | 358  360 |
| Query 359  Sbjct 361 | | SAVANGLKLNGVDCDEGETSLVVRGRPDGKGLGNASGAAVATHLDHRIAMSFLVMGLVSE  +A A L++NGV + + L+V G G G VATH+DHR+AMS L+MGL SE AATAAMLQVNGVAVEIVDDDLIVHGNGRAPG GGVVATHMDHRLAMSALMMGLGSE | 418  415 |
| Query 419 | | NPVTVDDATMIATSFPEFMDLMAGLG 444 PV VDDA IATSF F +LM LG |  |
| Sbjct | 416 | KPVGVDDAAFIATSFTGFTELMRKLG 441 | |

>TR:A0A0H1RGV3 A0A0H1RGV3_9RHIZ 3-phosphoshikimate 1-carboxyvinyltransferase OS=Microvirga vignae OX=1225564 GN=aroA PE=3 SV=1

Length=448

Score = 493 bits (1268), Expect = 7e-170

Identities = 270/448 (60%), Positives = 322/448 (72%), Gaps = 7/448 (2%)

| Query | 3 | HGASSRPATARKSSGLSGTVRIPGDKSISHRSFMFGGLASGETRITGLLEGEDVINTGKA | 62 |
| --- | --- | --- | --- |
| Sbjct | 5 | HG +S P ++R + L G +R+PGDKSISHRS +FG L+ GETR+ GLLEG+DV+ T +A HGPAS-PVSSRSGNALKGRIRVPGDKSISHRSMIFGLLSIGETRVEGLLEGDDVLRTAEA | 63 |
| Query | 63 | MQAMGARIRKEGD-TWIIDGVGNGGLLAPEAPLDFGNAATGCRLTMGLVGVYDFDSTFIG | 121 |
| Sbjct | 64 | +A+GA I +E W + GVG GGL+ P+ LDFGNA TG RL MG+ G + +TF G  CRALGATITREAPGRWRVKGVGIGGLVEPKGILDFGNAGTGSRLMMGVAGSHPITTTFDG | 123 |

| Query | 122 | DASLTKRPMGRVLNPLREMGVQVKSE-DGDRLPVTLRGPKTPTPITYRVPMASAQVKSAV | 180 |
| --- | --- | --- | --- |
| Sbjct | 124 | DASL KRPM R+L+PL +MGV V SE +G R+P+TLRGPK PITY P ASAQ+KSA+ DASLRKRPMRRILDPLVQMGVTVVSEAEGGRVPLTLRGPKETIPITYETPAASAQIKSAI | 183 |
| Query | 181 | LLAGLNTPGITTVIEPIMTRDHTEKMLQGFGANLTVETDADGVRTIRLEGRGKLTGQVID | 240 |
| Sbjct | 184 | LLAGLN+PG TTVIE TRDHTE+ML+ FGA + V R I L+G+ +L G I LLAGLNSPGRTTVIEREATRDHTERMLRHFGATVEVAPHGADGRAITLQGQPELRGTDIV | 243 |
| Query | 241 | VPGDPSSTAFPLVAALLVPGSDVTILNVLMNPTRTGLILTLQEMGADIEVINPRLAGGED | 300 |
| Sbjct | 244 | VP DPSS AFPLVAAL+ PGSD+ + V+MNP RTGLI TL EMGA IE +N R GGE VPTDPSSAAFPLVAALITPGSDIVLEGVMMNPLRTGLITTLLEMGASIERLNEREEGGET | 303 |
| Query | 301 | VADLRVRSSTLKGVTVPEDRAPSMIDEYPILAVAAAFAEGATVMNGLEELRVKESDRLSA | 360 |
| Sbjct | 304 | VADLRVR S L GVTVP RAP+MIDEYPILAVAA+FAEG T M GL ELRVKESDRL+A VADLRVRHSKLAGVTVPHGRAPAMIDEYPILAVAASFAEGTTRMQGLHELRVKESDRLAA | 363 |
| Query | 361 | VANGLKLNGVDCDEGETSLVVRGRPDGKGLGNASGAAVATHLDHRIAMSFLVMGLVSENP | 420 |

VA GL	GV+ +	L+V G	+G GN	VATHLDHRIAMSFLVMGL ++ P

| Sbjct | 364 | VAAGLAEAGVEHEIQGDDLIVHGSKTVRGGGN PVATHLDHRIAMSFLVMGLATQEP | 419 |
| --- | --- | --- | --- |
| Query  Sbjct | 421  420 | VTVDDATMIATSFPEFMDLMAGLGAKIE 448 V VDD MI TSFP F+ LM LGA I+ VAVDDGAMIITSFPTFIPLMRELGATIQ 447 |  |

>TR:A0A512BPP8 A0A512BPP8_9RHIZ 3-phosphoshikimate 1-carboxyvinyltransferase OS=Microvirga aerophila OX=670291 GN=aroA PE=3 SV=1

Length=449

Score = 493 bits (1268), Expect = 8e-170

| Identities | | = 270/447 (60%), Positives = 317/447 (71%), Gaps = 6/447 (1%) |  |
| --- | --- | --- | --- |
| Query 3  Sbjct 5 | | HGASSRPATARKSSGLSGTVRIPGDKSISHRSFMFGGLASGETRITGLLEGEDVINTGKA HG +S P T++ L G +R+PGDKSISHRS +FG LA GETR+ GLLEGEDV+ T +A HGPAS-PVTSKPGEALKGRIRVPGDKSISHRSMIFGLLAIGETRVEGLLEGEDVLRTAEA | 62  63 |
| Query 63  Sbjct 64 | | MQAMGARIRKEGD-TWIIDGVGNGGLLAPEAPLDFGNAATGCRLTMGLVGVYDFDSTFIG  +A+GA I ++ W I GVG GGL P LDFGNA TG RL MG+ G + +TF G CRALGANITRDAPGRWRIQGVGIGGLTEPSGVLDFGNAGTGSRLMMGVAGSHPITTTFDG | 121  123 |
| Query 122  Sbjct 124 | | DASLTKRPMGRVLNPLREMGVQVKSE-DGDRLPVTLRGPKTPTPITYRVPMASAQVKSAV DASL KRPM R+L+PL MGV V SE +G R+P+TLRGP+ P+TY P ASAQ+KSA+ DASLRKRPMRRILDPLARMGVTVVSEAEGGRVPLTLRGPRETIPVTYETPAASAQIKSAI | 180  183 |
| Query 181  Sbjct 184 | | LLAGLNTPGITTVIEPIMTRDHTEKMLQGFGANLTVETDADGVRTIRLEGRGKLTGQVID LLAGLN+PG TTVIE TRDHTE+ML+ FGA + V + R I L G+ +L G + LLAGLNSPGRTTVIEREATRDHTERMLRHFGAKVEVAPHREDGRAITLHGQPELRGSTVV | 240  243 |
| Query 241  Sbjct 244 | | VPGDPSSTAFPLVAALLVPGSDVTILNVLMNPTRTGLILTLQEMGADIEVINPRLAGGED VP DPSS AFPLVAAL+ PGSDV I V+MNP RTGLI TL EMGA IE +N R GGE VPTDPSSAAFPLVAALITPGSDVVIEGVMMNPLRTGLITTLLEMGASIERLNERDEGGET | 300  303 |
| Query 301  Sbjct 304 | | VADLRVRSSTLKGVTVPEDRAPSMIDEYPILAVAAAFAEGATVMNGLEELRVKESDRLSA VADLRVR+S L GVTVP RAP+MIDEYPILAVAA++AEG T M GL ELRVKESDRL+A VADLRVRASRLTGVTVPAARAPAMIDEYPILAVAASYAEGTTRMQGLHELRVKESDRLAA | 360  363 |
| Query 361 | | VANGLKLNGVDCDEGETSLVVRGRPDGKGLGNASGAAVATHLDHRIAMSFLVMGLVSENP VA GL GV + L+V G G G VATHLDHRIAMSFLV+GL S+ P | 420 |
| Sbjct | 364 | VAAGLAEAGVPHEIEGDDLIVHG---SGGKVRGGGKPVATHLDHRIAMSFLVLGLASQEP | 420 |
| Query  Sbjct | 421  421 | VTVDDATMIATSFPEFMDLMAGLGAKI 447  +TVDD MIATSFP F+ LM LGA I MTVDDGAMIATSFPSFIPLMRELGATI 447 |  |

>TR:V5SIG5 V5SIG5_9RHIZ 3-phosphoshikimate 1-carboxyvinyltransferase OS=Hyphomicrobium nitrativorans NL23 OX=1029756 GN=aroA PE=3 SV=1

Length=480

Score = 494 bits (1271), Expect = 8e-170

| Identities | = 263/444 (59%), Positives = 315/444 (71%), Gaps = 7/444 (2%) |  |
| --- | --- | --- |
| Query 3 | HGASSRPATARKSSGLSGTVRIPGDKSISHRSFMFGGLASGETRITGLLEGEDVINTGKA | 62 |
| Sbjct 38 | HG P A KS L G + +PGDKSISHR+ M GGLA+G TRI GLLE EDVINT +A HG-DPHPLAALKSGPLKGEIAVPGDKSISHRALMLGGLATGRTRIRGLLESEDVINTARA | 96 |
| Query 63 | MQAMGARIRKEGDTWIIDGVGNGGLLAPEAPLDFGNAATGCRLTMGLVGVYDFDSTFIGD | 122 |
| Sbjct 97 | M A+GA +K GD W + G G GGL PE P+DFGN+ TG RL MG++ +D T +GD MTALGAPAQKNGDVWEVLGRGTGGLRNPEGPIDFGNSGTGARLMMGVIAPHDVTVTMVGD | 156 |
| Query 123 | ASLTKRPMGRVLNPLREMGVQVKSEDGDRLPVTLRGPKTPTPITYRVPMASAQVKSAVLL | 182 |
| Sbjct 157 | ASL+KRPM RVL PL+ MGV V+ E GDRLP+T+RG PI Y +P+ SAQVKSAVLL ASLSKRPMARVLGPLKAMGVNVEGE-GDRLPLTVRGSSRLAPIHYVLPVPSAQVKSAVLL | 215 |
| Query 183 | AGLNTPGITTVIEPIMTRDHTEKMLQGFGANLTVETDADGVRTIRLEGRGKLTGQVIDVP | 242 |
| Sbjct 216 | AGL T G T+V+E TRDHTE+ML+ FGA + +G R I + G+ +LTG+ I VP AGLGTAGETSVVEREATRDHTERMLRYFGAEVATAPAPEGTR-ISVRGQSELTGRDIAVP | 274 |
| Query 243 | GDPSSTAFPLVAALLVPGSDVTILNVLMNPTRTGLILTLQEMGADIEVINPRLAGGEDVA | 302 |
| Sbjct 275 | GDPSS AF + AAL+VPGS+VT+ +L+NPTRTGL TLQEMG D+ +N R GGE VA GDPSSAAFLVAAALIVPGSEVTVRGILVNPTRTGLYTTLQEMGGDVVFLNQREEGGEPVA | 334 |
| Query 303 | DLRVRSSTLKGVTVPEDRAPSMIDEYPILAVAAAFAEGATVMNGLEELRVKESDRLSAVA | 362 |
| Sbjct 335 | D+RVR S L GVTVP +RAPSMIDEYP+LAV AAFA G T M GL EL+VKESDRL+A DIRVRYSELSGVTVPAERAPSMIDEYPMLAVVAAFARGVTRMEGLAELKVKESDRLAATV | 394 |
| Query 363 | NGLKLNGVDCDEGETSLVVRGRPDGKGLGNASGAAVATHLDHRIAMSFLVMGLVSENPVT | 422 |
| Sbjct 395 | GL NGV + +L V +G G G G VATHLDHR+AM+FLVMGL S+ PVT AGLLANGVTTEASGDTLTV EGSGRGVPGGGLVATHLDHRMAMAFLVMGLASDAPVT | 450 |
| Query 423  Sbjct 451 | VDDATMIATSFPEFMDLMAGLGAK 446 VDD MIATSFP F LM G+GA+ VDDTAMIATSFPTFQSLMEGIGAR 474 |  |

>TR:V4R1Z1 V4R1Z1_9RHIZ 3-phosphoshikimate 1-carboxyvinyltransferase OS=Lutibaculum baratangense AMV1 OX=631454 GN=aroA PE=3 SV=1

Length=450

Score = 493 bits (1268), Expect = 8e-170

| Identities | = 267/438 (61%), Positives = 320/438 (73%), Gaps = 7/438 (2%) |  |
| --- | --- | --- |
| Query 11 | TARKSSGLSGTVRIPGDKSISHRSFMFGGLASGETRITGLLEGEDVINTGKAMQAMGARI | 70 |
| Sbjct 13 | TA SS LSG +PGDKSISHR+ +FG LA G+T I GLLEGEDV+ T +AM+A+GAR+ TADPSSALSGEAVVPGDKSISHRALIFGALAGGQTEIQGLLEGEDVLATAEAMRALGARV | 72 |
| Query 71 | RKEGD-TWIIDGVGNGGLLAPEAPLDFGNAATGCRLTMGLVGVYDFDSTFIGDASLTKRP | 129 |
| Sbjct 73 | ++G+ W +DGVG G LL PEAPLDFGNA TG RL MG+V + +TF GDASL +RP VRDGEGRWRVDGVGLGALLEPEAPLDFGNAGTGVRLAMGVVAGHGITATFDGDASLRRRP | 132 |
| Query 130 | MGRVLNPLREMGVQVKSEDGDRLPVTLRGPKTPTPITYRVPMASAQVKSAVLLAGLNTPG | 189 |
| Sbjct 133 | M RV++PL MG ++ S + +LP+ LRG + P PI Y +P+ SAQVKSAVLLAGLN+PG MRRVIDPLLRMGAELVSSNDGKLPLVLRGTQDPIPIEYELPVPSAQVKSAVLLAGLNSPG | 192 |
| Query 190 | ITTVIEPIMTRDHTEKMLQGFGANLTVETDADGVRTIRLEGRGKLTGQVIDVPGDPSSTA | 249 |
| Sbjct 193 | TTVIEP TRDHTE+M + FGA + V G R I L GR +L G+ +DVP DPSS A  TTTVIEPSPTRDHTERMARHFGAEVDVAEHGQG-RRIVLTGRPELRGRPVDVPADPSSAA | 251 |

| Query | 250 | FPLVAALLVPGSDVTILNVLMNPTRTGLILTLQEMGADIEVINPRLAGGEDVADLRVRSS | 309 |
| --- | --- | --- | --- |
| Sbjct | 252 | FPLVAALLVPGS + I NV+ NPTR GL+ TL+EMGA+I N R AGGE V DL VR FPLVAALLVPGSQIVIRNVMTNPTRFGLVHTLREMGAEIGEENTREAGGETVTDLVVRHG | 311 |
| Query | 310 | TLKGVTVPEDRAPSMIDEYPILAVAAAFAEGATVMNGLEELRVKESDRLSAVANGLKLNG | 369 |
| Sbjct | 312 | L+G+ VP RAPSMIDEYP+LAVAAAFAEG TVM GL ELRVKESDRL+AV+ GL NG RLRGIEVPAARAPSMIDEYPVLAVAAAFAEGETVMRGLSELRVKESDRLAAVSAGLAANG | 371 |
| Query | 370 | VDCDEGETSLVVRGRPDGKGLGNASGAAVATHLDHRIAMSFLVMGLVSENPVTVDDATMI | 429 |
| Sbjct | 372 | V+ L+V GR G+ G G VAT +DHRIAMSFLVMGL +E PVTVDD MI VEHRIEGDDLIVTGR--GRVAG---GGLVATEMDHRIAMSFLVMGLAAERPVTVDDGRMI | 426 |
| Query  Sbjct | 430  427 | ATSFPEFMDLMAGLGAKI 447 ATSFP F++++ GLG I ATSFPGFVEMIRGLGGGI 444 |  |

>TR:A0A2U8VTD6 A0A2U8VTD6_9RHIZ 3-phosphoshikimate 1-carboxyvinyltransferase OS=Methylobacterium sp. 17Sr1-43 OX=2202828 GN=aroA PE=3 SV=1

Length=449

Score = 492 bits (1267), Expect = 1e-169

| Identities | | = 270/452 (60%), Positives = 321/452 (71%), Gaps = 10/452 (2%) |  |
| --- | --- | --- | --- |
| Query 1  Sbjct 1 | | MLHGASSRPATARKSSGLSGTVRIPGDKSISHRSFMFGGLASGETRITGLLEGEDVINTG M H + +P TA S L G++R PGDKSISHR+ + G L+ GETR+ GLLEG+DV+ T MSHDSDPQPLTASPGSALRGSLRPPGDKSISHRAMILGLLSIGETRVEGLLEGDDVLRTA | 60  60 |
| Query 61  Sbjct 61 | | KAMQAMGARIRKEGD-TWIIDGVGNGGLLAPEAPLDFGNAATGCRLTMGLVGVYDFDSTF A +A+GA + + G+ W++ GVG GG+ P LDFGNA TG RL MG+VG +TF AAAKALGAGVERLGEGRWVVRGVGIGGMSDPAETLDFGNAGTGSRLMMGVVGGQPVTATF | 119  120 |
| Query 120  Sbjct 121 | | IGDASLTKRPMGRVLNPLREMGVQVKSE-DGDRLPVTLRGPKTPTPITYRVPMASAQVKS GDASL RPM R+L+PL MG +V +E +G R+P+TLRGP+ PITY P ASAQ+KS DGDASLRSRPMRRILDPLVRMGTEVVAEAEGGRVPLTLRGPREAIPITYETPAASAQIKS | 178  180 |
| Query 179  Sbjct 181 | | AVLLAGLNTPGITTVIEPIMTRDHTEKMLQGFGANLTVETDADGV--RTIRLEGRGKLTG AVLLAGLN PG TTVIE TRDHTE+ML+ FGA ++VE G R I L G+ L G AVLLAGLNAPGTTTVIEAAATRDHTERMLRLFGAEVSVEAHGPGGHGRRIALTGQPSLRG | 236  240 |
| Query 237  Sbjct 241 | | QVIDVPGDPSSTAFPLVAALLVPGSDVTILNVLMNPTRTGLILTLQEMGADIEVINPRLA  + VP DPSS AFPLVAAL+VPGS+VTI V+MNP RTGLI TL EMGADIE + R TDVVVPADPSSAAFPLVAALIVPGSEVTIQGVMMNPLRTGLITTLIEMGADIERLREREE | 296  300 |
| Query 297  Sbjct 301 | | GGEDVADLRVRSSTLKGVTVPEDRAPSMIDEYPILAVAAAFAEGATVMNGLEELRVKESD GGE VADLRVR+S LKGV VP +RAP+MIDEYP+LAVAAAFAEG T MNGL ELRVKESD GGETVADLRVRASRLKGVDVPAERAPAMIDEYPVLAVAAAFAEGTTRMNGLHELRVKESD | 356  360 |
| Query 357  Sbjct 361 | | RLSAVANGLKLNGV-DCDEGETSLVVRGRPDGKGLGNASGAAVATHLDHRIAMSFLVMGL RL+AVA GLK NGV EG+ +V G G G VATHLDHRIAM+FLV+GL RLAAVAAGLKANGVAHLVEGDDLVV HGDGAAPRGGGTVATHLDHRIAMAFLVLGL | 415  415 |
| Query 416 | | VSENPVTVDDATMIATSFPEFMDLMAGLGAKI 447  + PVTVDD MIATSFP F+ M LG +I |  |
| Sbjct | 416 | ATREPVTVDDGAMIATSFPSFLPTMRALGGRI 447 | |

>TR:A0A514WEW6 A0A514WEW6_9SPHN 3-phosphoshikimate 1-carboxyvinyltransferase OS=Sphingomonas sp. IC081 OX=304378 GN=aroA PE=3 SV=1

Length=444

Score = 492 bits (1266), Expect = 1e-169

Identities = 258/441 (59%), Positives = 316/441 (72%), Gaps = 5/441 (1%)

| Query | 8 | RPATARKSSGLSGTVRIPGDKSISHRSFMFGGLASGETRITGLLEGEDVINTGKAMQAMG | 67 |
| --- | --- | --- | --- |
| Sbjct | 2 | RP + L G +R+PGDKSISHRS M G LA GETR+TGLLEGEDV+ T AM+AMG RPRRFLPAGPLKGRIRVPGDKSISHRSIMLGALAVGETRVTGLLEGEDVLATAAAMRAMG | 61 |
| Query | 68 | ARIRKEGDT WIIDGVGNGGLLAPEAPLDFGNAATGCRLTMGLVGVYDFDSTFIGD | 122 |
| Sbjct | 62 | A + + GD+ W ++GVG G LL PE PLD GN+ T RL MGLV + + +F GD ASVERIGDSESGGEWRVNGVGVGALLQPEGPLDMGNSGTSTRLLMGLVASHPIEVSFTGD | 121 |
| Query | 123 | ASLTKRPMGRVLNPLREMGVQVKSEDGDRLPVTLRGPKTPTPITYRVPMASAQVKSAVLL | 182 |
| Sbjct | 122 | ASL+KRPMGRV++PL EMG ++ +G RLP+ +RG PITYR+P+ASAQVKSA+LL ASLSKRPMGRVIDPLSEMGASFEASEGGRLPLIVRGAAPAVPITYRLPVASAQVKSAILL | 181 |
| Query | 183 | AGLNTPGITTVIEPIMTRDHTEKMLQGFGANLTVETDADGVRTIRLEGRGKLTGQVIDVP | 242 |
| Sbjct | 182 | AGLNTPG TTVIEP+ TRDH+E+ML+GFGA+LTV +ADG R I ++G +L QVIDVP AGLNTPGTTTVIEPVPTRDHSERMLKGFGADLTVTVEADGTRIITIKGEAELKPQVIDVP | 241 |
| Query | 243 | GDPSSTAFPLVAALLVPGSDVTILNVLMNPTRTGLILTLQEMGADIEVINPRLAGGEDVA | 302 |
| Sbjct | 242 | GDPSS AF +VAAL+ PGS+V I NV +NPTR GL+ L++MG IE ++ R GGE VA GDPSSAAFFIVAALVTPGSEVVIENVGLNPTRAGLVEVLRQMGGQIEFLSEREVGGEPVA | 301 |
| Query | 303 | DLRVRSSTLKGVTVPEDRAPSMIDEYPILAVAAAFAEGATVMNGLEELRVKESDRLSAVA | 362 |
| Sbjct | 302 | DL VR S LKG+TV AP+MIDE+P+L VAA+ AEG TV +GL+ELRVKESDRL+A+A DLLVRHSALKGITVDPAIAPAMIDEFPVLFVAASLAEGVTVTSGLDELRVKESDRLAAMA | 361 |
| Query | 363 | NGLKLNGVDCDEGETSLVVRGRPDGKGLGNASGAAVATHLDHRIAMSFLVMGLVSENPVT | 422 |
| Sbjct | 362 | L L G +E E LV+ G G A+GA V THLDHRIAMS + GLVS V AALTLAGAKIEEREDGLVITGTGGAPLPGTAAGAQVTTHLDHRIAMSMAIAGLVSSGGVE | 421 |
| Query  Sbjct | 423  422 | VDDATMIATSFPEFMDLMAGL 443 VDD IATSFP F L+A L VDDTRPIATSFPNFEALLASL 442 |  |

>TR:A0A0Q6KHI6 A0A0Q6KHI6_9BRAD 3-phosphoshikimate 1-carboxyvinyltransferase OS=Bosea sp. Leaf344 OX=1736346 GN=aroA PE=3 SV=1

Length=445

Score = 492 bits (1266), Expect = 1e-169

| Identities | = 265/441 (60%), Positives = 317/441 (72%), Gaps = 7/441 (2%) |  |
| --- | --- | --- |
| Query 9 | PATARKSSGLSGTVRIPGDKSISHRSFMFGGLASGETRITGLLEGEDVINTGKAMQAMGA | 68 |
| Sbjct 9 | P T+R+ L G VR+PGDKSISHR+ +FG LA GETR++GLLEGEDV+ T A +A+GA PVTSRRGGPLKGRVRVPGDKSISHRAMIFGLLAIGETRVSGLLEGEDVLRTAAAARALGA | 68 |
| Query 69 | RIRKEGD-TWIIDGVGNGGLLAPEAPLDFGNAATGCRLTMGLVGVYDFDSTFIGDASLTK | 127 |
| Sbjct 69 | + +G W + GVG GGL P LDFGNA TG RL MG+ G + +TF GDASL K IVHHDGPGAWRVTGVGVGGLKEPAHLLDFGNAGTGSRLMMGVCGSHPITTTFDGDASLRK | 128 |
| Query 128 | RPMGRVLNPLREMG-VQVKSEDGDRLPVTLRGPKTPTPITYRVPMASAQVKSAVLLAGLN | 186 |
| Sbjct 129 | RPM R+L+PL +MG V ++ E+G RLP+TLRGP+ PITYR P+ASAQ+KSAVLLAGL RPMRRILDPLEQMGTVVLRQEEGGRLPLTLRGPQEAVPITYRTPVASAQIKSAVLLAGLA | 188 |
| Query 187 | TPGITTVIEPIMTRDHTEKMLQGFGANLTVETDADGVRTIRLEGRGKLTGQVIDVPGDPS | 246 |
| Sbjct 189 | PG+TTVIE TRDHTEKML FGA +TV + + R I L G+ +L I VP DPS APGVTTVIEAEATRDHTEKMLTHFGAEVTVTQEGEHGRRIALTGQPELAAAPIIVPADPS | 248 |
| Query 247 | STAFPLVAALLVPGSDVTILNVLMNPTRTGLILTLQEMGADIEVINPRLAGGEDVADLRV | 306 |
| Sbjct 249 | S AFPLVAAL+VPGSDV I +V+ NP R+GL+ TL+EMGA+I V GGE VADLRV SAAFPLVAALIVPGSDVLIESVMTNPLRSGLLTTLREMGAEITVEREANEGGETVADLRV | 308 |
| Query 307 | RSSTLKGVTVPEDRAPSMIDEYPILAVAAAFAEGATVMNGLEELRVKESDRLSAVANGLK | 366 |
| Sbjct 309 | R S L+GVTVP +RAP+MIDEYP+LAVAA+FA G T M GL+ELRVKESDRL+AV GLK  RFSALRGVTVPPERAPAMIDEYPVLAVAASFASGTTRMRGLQELRVKESDRLAAVEAGLK | 368 |

| Query | 367 | LNGVDCDEGETSLVVRGRPDGKGLGNASGAAVATHLDHRIAMSFLVMGLVSENPVTVDDA 426 |
| --- | --- | --- |
| Sbjct | 369 | GV C L+V GR + G G VATHLDHRIAMSFLVMGL ++ P+ VDD AAGVSCAIEGDDLIVEGRSEVAG-----GGVVATHLDHRIAMSFLVMGLATQKPMQVDDG 423 |
| Query | 427 | TMIATSFPEFMDLMAGLGAKI 447 |
| Sbjct | 424 | MIATSFP F+ LM LG I AMIATSFPSFIGLMRQLGGDI 444 |

>TR:F6ILJ1 F6ILJ1_9SPHN 3-phosphoshikimate 1-carboxyvinyltransferase OS=Novosphingobium sp. PP1Y OX=702113 GN=aroA PE=3 SV=1

Length=442

Score = 491 bits (1265), Expect = 2e-169

| Identities | | = 260/443 (59%), Positives = 317/443 (72%), Gaps = 7/443 (2%) |  |
| --- | --- | --- | --- |
| Query 8  Sbjct 2 | | RPATARKSSGLSGTVRIPGDKSISHRSFMFGGLASGETRITGLLEGEDVINTGKAMQAMG RP + L G +R+PGDKSISHRS M G LA GETR+TGLLEGEDV+ T AM+AMG RPRRFLPTGPLKGRIRVPGDKSISHRSIMLGALAVGETRVTGLLEGEDVLATAAAMRAMG | 67  61 |
| Query 68  Sbjct 62 | | ARIRKEGDT WIIDGVGNGGLLAPEAPLDFGNAATGCRLTMGLVGVYDFDSTFIGD  A + + GD+ W+++GVG G LL PEAPLD GN+ T RL MGL+ + +TFIGD ASVERVGDSESGGEWVVNGVGVGALLQPEAPLDMGNSGTSTRLLMGLIASHPITATFIGD | 122  121 |
| Query 123  Sbjct 122 | | ASLTKRPMGRVLNPLREMGVQVKSEDGDRLPVTLRGPKTPTPITYRVPMASAQVKSAVLL ASL+KRPMGRV+ PL +MG ++ +G RLP+ L+G PITYR+P+ASAQVKSAVLL ASLSKRPMGRVITPLSQMGASFEASEGGRLPLILKGTSPAVPITYRLPVASAQVKSAVLL | 182  181 |
| Query 183  Sbjct 182 | | AGLNTPGITTVIEPIMTRDHTEKMLQGFGANLTVETDADGVRTIRLEGRGKLTGQVIDVP AGLNTPG TTVIEP+ TRDH+E+ML+GFGA L VE + DG R I + G +L QVIDVP AGLNTPGRTTVIEPVPTRDHSERMLKGFGAQLEVEVEPDGTRIITVTGEAELRPQVIDVP | 242  241 |
| Query 243  Sbjct 242 | | GDPSSTAFPLVAALLVPGSDVTILNVLMNPTRTGLILTLQEMGADIEVINPRLAGGEDVA GDPSS AF +VAALLVPGS+V I NV +NPTR GL+ L++MG DI +NPR GGE VA GDPSSAAFFIVAALLVPGSEVLIENVGLNPTRAGLVSVLRQMGGDITEVNPREVGGEPVA | 302  301 |
| Query 303  Sbjct 302 | | DLRVRSSTLKGVTVPEDRAPSMIDEYPILAVAAAFAEGATVMNGLEELRVKESDRLSAVA DL V+ S LKG+ V + AP+MIDE+P+L VAA+ AEG T +GL+ELRVKESDRL+ +A DLLVKHSVLKGIDVDPEIAPAMIDEFPVLFVAASLAEGKTTTSGLDELRVKESDRLAVMA | 362  361 |
| Query 363 | | NGLKLNGVDCDEGETSLVVRGRPDGKGLGNASGAAVATHLDHRIAMSFLVMGLVSENPVT L G +E E LV+ G G+ L AA+ATHLDHRIAMS V GLVS V | 422 |
| Sbjct | 362 | AALTAAGARIEEREDGLVIEG-TGGEALPGTE-AAIATHLDHRIAMSMAVAGLVSRGGVE | 419 |
| Query  Sbjct | 423  420 | VDDATMIATSFPEFMDLMAGLGA 445 VDD IATSFP F ++AGL A VDDTRPIATSFPVFESMLAGLVA 442 |  |

>TR:A0A0Q4XTV4 A0A0Q4XTV4_9RHIZ 3-phosphoshikimate 1-carboxyvinyltransferase OS=Methylobacterium sp. Leaf93 OX=1736249 GN=aroA PE=3 SV=1

Length=449

Score = 491 bits (1265), Expect = 2e-169

Identities = 273/452 (60%), Positives = 323/452 (71%), Gaps = 10/452 (2%)

| Query | 1 | MLHGASSRPATARKSSGLSGTVRIPGDKSISHRSFMFGGLASGETRITGLLEGEDVINTG | 60 |
| --- | --- | --- | --- |
| Sbjct | 1 | M H + +P TA + L G +R PGDKSISHR+ + G L+ GETRI GLLEG+DV+ T MSHTSEPQPLTASAGAPLRGALRPPGDKSISHRAMILGLLSIGETRIEGLLEGDDVLRTA | 60 |
| Query | 61 | KAMQAMGARIRKEGD-TWIIDGVGNGGLLAPEAPLDFGNAATGCRLTMGLVGVYDFDSTF | 119 |
| Sbjct | 61 | +A +A+GA I + G+ W++ GVG GG+ P LDFGNA TG RL MG+VG D +TF  EAARALGAGIDRLGEGRWLVRGVGIGGMTDPAGVLDFGNAGTGSRLMMGVVGGQDVTATF | 120 |

| Query | 120 | IGDASLTKRPMGRVLNPLREMGVQVKSED-GDRLPVTLRGPKTPTPITYRVPMASAQVKS | 178 |
| --- | --- | --- | --- |
| Sbjct | 121 | GDASL KRPM R+L+PL MG QV SE+ G R+P+TLRGP+ PITY P+ASAQ+KS DGDASLRKRPMRRILDPLIRMGTQVLSEEEGGRVPLTLRGPREAIPITYETPVASAQIKS | 180 |
| Query | 179 | AVLLAGLNTPGITTVIEPIMTRDHTEKMLQGFGANLTVETDADGV--RTIRLEGRGKLTG | 236 |
| Sbjct | 181 | AVLLAGLN PG TTVIE TRDH+E+ML+ FGA + V G R+I L G+ L G AVLLAGLNAPGTTTVIEAAATRDHSERMLRLFGAEVIVSPHGPGGHGRSIALTGQPTLRG | 240 |
| Query | 237 | QVIDVPGDPSSTAFPLVAALLVPGSDVTILNVLMNPTRTGLILTLQEMGADIEVINPRLA | 296 |
| Sbjct | 241 | + VP DPSS AFPLVAAL+VPGS+VTI V+MNP R GLI TL EMGADIE N R TDVIVPADPSSAAFPLVAALIVPGSEVTIEGVMMNPLRIGLITTLLEMGADIERRNERDE | 300 |
| Query | 297 | GGEDVADLRVRSSTLKGVTVPEDRAPSMIDEYPILAVAAAFAEGATVMNGLEELRVKESD | 356 |
| Sbjct | 301 | GGE VADL VR+S LKGV VP +RAPSMIDEYP+LAVAAAFAEG T MNGL EL VKESD GGETVADLHVRASRLKGVDVPAERAPSMIDEYPVLAVAAAFAEGVTRMNGLHELTVKESD | 360 |
| Query | 357 | RLSAVANGLKLNGVDCD-EGETSLVVRGRPDGKGLGNASGAAVATHLDHRIAMSFLVMGL | 415 |
| Sbjct | 361 | RL+AVA+GL+ NGV EGE +V +G G G VATHLDHRIAM+FLVMGL RLAAVADGLRENGVTYRIEGEDLIV EGDGSAARGGGTVATHLDHRIAMAFLVMGL | 415 |
| Query | 416 | VSENPVTVDDATMIATSFPEFMDLMAGLGAKI 447  ++ PVTVDD MIATSFP F+ M LG +I |  |
| Sbjct | 416 | ATKQPVTVDDGAMIATSFPSFLPTMRALGGRI 447 | |

>TR:A0A1W6ZQ57 A0A1W6ZQ57_9RHIZ 3-phosphoshikimate 1-carboxyvinyltransferase OS=Pseudorhodoplanes sinuspersici OX=1235591 GN=aroA PE=3 SV=1

Length=446

Score = 491 bits (1264), Expect = 3e-169

| Identities | | = 265/449 (59%), Positives = 317/449 (71%), Gaps = 7/449 (2%) |  |
| --- | --- | --- | --- |
| Query 1  Sbjct 1 | | MLHGASSRPATARKSSGLSGTVRIPGDKSISHRSFMFGGLASGETRITGLLEGEDVINTG M H + + P TARKS L+G +R+PGDKSISHR+ + L GETRI GLLEGEDVINT MSHNSPASPLTARKSISLAGRIRVPGDKSISHRALILAALTVGETRIGGLLEGEDVINTA | 60  60 |
| Query 61  Sbjct 61 | | KAMQAMGARIRKEGD-TWIIDGVGNGGLLAPEAPLDFGNAATGCRLTMGLVGVYDFDSTF K M+A+GA++ + G+ W + GVG GG P PLDFGN+ TGCRL MG V ++ F KVMRALGAKVERTGEGEWSVFGVGVGGYAQPAGPLDFGNSGTGCRLVMGAVAGCPINAVF | 119  120 |
| Query 120  Sbjct 121 | | IGDASLTKRPMGRVLNPLREMGVQ-VKSEDGDRLPVTLRGPKTPTPITYRVPMASAQVKS GDASL KRPM RVL+PL +G + V +G RLP+TL G + P PI Y P+ASAQ+KS DGDASLRKRPMRRVLDPLTRIGAKAVDVAEGGRLPLTLEGARDPLPIVYEPPVASAQLKS | 178  180 |
| Query 179  Sbjct 181 | | AVLLAGLNTPGITTVIEPIMTRDHTEKMLQGFGANLTVETDADGVRTIRLEGRGKLTGQV AVLLAGL PG T VIE TRDHTEKML FGA++ VE + R I L+GR +L V AVLLAGLAAPGETVVIEKEATRDHTEKMLTHFGADVQVEQVGEHGRRIVLKGRPELKPAV | 238  240 |
| Query 239  Sbjct 241 | | IDVPGDPSSTAFPLVAALLVPGSDVTILNVLMNPTRTGLILTLQEMGADIEVINPRLAGG  + VP DPSS AFPL AAL+VPGSD+ + NV+MNP RTGL LTL+EMGA IE ++ R GG LQVPSDPSSAAFPLAAALIVPGSDIVLENVMMNPLRTGLFLTLREMGASIETLSSRHDGG | 298  300 |
| Query 299  Sbjct 301 | | EDVADLRVRSSTLKGVTVPEDRAPSMIDEYPILAVAAAFAEGATVMNGLEELRVKESDRL E+VADLRV++S L G VP RAPSMIDEYPILAV AAFAEG T M GL ELRVKESDRL EEVADLRVKASALHGADVPASRAPSMIDEYPILAVIAAFAEGRTRMCGLSELRVKESDRL | 358  360 |
| Query 359  Sbjct 361 | | SAVANGLKLNGVDCDEGETSLVVRGRPDGKGLGNASGAAVATHLDHRIAMSFLVMGLVSE  +A A L++NGV + L+V G G G VATH+DHR+AMS LVMGL SE AATAEMLRVNGVAVEIDGDDLIVEGTGSVPG GGVVATHMDHRLAMSALVMGLNSE | 418  415 |
| Query 419 | | NPVTVDDATMIATSFPEFMDLMAGLGAKI 447 PV+VDDA IATSFP F++LM GLGA + |  |
| Sbjct | 416 | KPVSVDDAAFIATSFPRFVELMQGLGANL 444 | |

>TR:G6EEV2 G6EEV2_9SPHN 3-phosphoshikimate 1-carboxyvinyltransferase OS=Novosphingobium pentaromativorans US6-1 OX=1088721 GN=aroA PE=3

SV=1

Length=450

Score = 491 bits (1264), Expect = 3e-169

| Identities | | = 260/446 (58%), Positives = 319/446 (72%), Gaps = 7/446 (2%) |  |
| --- | --- | --- | --- |
| Query 5  Sbjct 7 | | ASSRPATARKSSGLSGTVRIPGDKSISHRSFMFGGLASGETRITGLLEGEDVINTGKAMQ A+ RP + L G +R+PGDKSISHRS M G LA GETR+TGLLEGEDV+ T AM+ AAMRPRRFLPTGPLKGRIRVPGDKSISHRSIMLGALAVGETRVTGLLEGEDVLATAAAMR | 64  66 |
| Query 65  Sbjct 67 | | AMGARIRKEGDT WIIDGVGNGGLLAPEAPLDFGNAATGCRLTMGLVGVYDFDSTF  AMGA + + G++ W+++GVG G LL PEAPLD GN+ T RL MGL+ + +TF AMGASVERVGNSESGGEWVVNGVGVGALLQPEAPLDMGNSGTSTRLLMGLIASHPITATF | 119  126 |
| Query 120  Sbjct 127 | | IGDASLTKRPMGRVLNPLREMGVQVKSEDGDRLPVTLRGPKTPTPITYRVPMASAQVKSA IGDASL+KRPMGRV+ PL +MG ++ +G RLP+ L+G PITYR+P+ASAQVKSA IGDASLSKRPMGRVITPLSQMGASFEASEGGRLPLILKGTSPAVPITYRLPVASAQVKSA | 179  186 |
| Query 180  Sbjct 187 | | VLLAGLNTPGITTVIEPIMTRDHTEKMLQGFGANLTVETDADGVRTIRLEGRGKLTGQVI VLLAGLNTPG TTVIEP+ TRDH+E+ML+GFGA L VE + DG R I + G +L QVI VLLAGLNTPGRTTVIEPVPTRDHSERMLKGFGAQLEVEVEPDGTRIITVTGEAELRPQVI | 239  246 |
| Query 240  Sbjct 247 | | DVPGDPSSTAFPLVAALLVPGSDVTILNVLMNPTRTGLILTLQEMGADIEVINPRLAGGE DVPGDPSS AF +VAALLVPGS+V I NV +NPTR GL+ L++MG DI +NPR GGE DVPGDPSSAAFFIVAALLVPGSEVLIENVGLNPTRAGLVSVLRQMGGDITEVNPREVGGE | 299  306 |
| Query 300  Sbjct 307 | | DVADLRVRSSTLKGVTVPEDRAPSMIDEYPILAVAAAFAEGATVMNGLEELRVKESDRLS VADL V+ S LKG+ V + AP+MIDE+P+L VAA+ AEG T +GL+ELRVKESDRL+ PVADLLVKHSVLKGIDVDPEIAPAMIDEFPVLFVAASLAEGKTTTSGLDELRVKESDRLA | 359  366 |
| Query 360 | | AVANGLKLNGVDCDEGETSLVVRGRPDGKGLGNASGAAVATHLDHRIAMSFLVMGLVSEN  +A L G +E E LV+ G G+ L AA+ATHLDHRIAMS V GLVS | 419 |
| Sbjct | 367 | VMAAALTAAGARIEEREDGLVIEG-TGGEALPGTE-AAIATHLDHRIAMSMAVAGLVSRG | 424 |
| Query  Sbjct | 420  425 | PVTVDDATMIATSFPEFMDLMAGLGA 445 V VDD IATSFP F ++AGL A  GVEVDDTRPIATSFPVFESMLAGLVA 450 |  |

>SP:Q13ER0 AROA_RHOPS 3-phosphoshikimate 1-carboxyvinyltransferase OS=Rhodopseudomonas palustris (strain BisB5) OX=316057 GN=aroA PE=3

SV=1

Length=445

Score = 491 bits (1263), Expect = 4e-169

| Identities | = 260/449 (58%), Positives = 319/449 (71%), Gaps = 7/449 (2%) |  |
| --- | --- | --- |
| Query 1 | MLHGASSRPATARKSSGLSGTVRIPGDKSISHRSFMFGGLASGETRITGLLEGEDVINTG | 60 |
| Sbjct 1 | M H + P ARKS+ LSGT R+PGDKSISHR+ + G LA GETRI+GLLEG+DVI+TG MSHSDQTSPLEARKSAALSGTARVPGDKSISHRALILGALAVGETRISGLLEGQDVIDTG | 60 |
| Query 61 | KAMQAMGARIRKEGD-TWIIDGVGNGGLLAPEAPLDFGNAATGCRLTMGLVGVYDFDSTF | 119 |
| Sbjct 61 | KAM+A+GAR+ + G+ W + GVG G PEAPLDFGN+ TGCRL MG V +TF KAMRALGARVERTGEFAWTVRGVGVAGFAQPEAPLDFGNSGTGCRLAMGAVAGSPITATF | 120 |
| Query 120 | IGDASLTKRPMGRVLNPLREMGVQV-KSEDGDRLPVTLRGPKTPTPITYRVPMASAQVKS | 178 |
| Sbjct 121 | GDASL RPM R+++PL +MG +V +S +G RLP+TL+G + P PITYR P+ SAQ+KS DGDASLRSRPMRRIVDPLEQMGARVIQSHEGGRLPLTLQGARDPLPITYRTPVPSAQIKS | 180 |
| Query 179 | AVLLAGLNTPGITTVIEPIMTRDHTEKMLQGFGANLTVETDADGVRTIRLEGRGKLTGQV AVLLAGL+ PG+TTVIE +RDHTE MLQ FGA L E + R I L G+ +L G | 238 |

| Sbjct | 181 | AVLLAGLSAPGVTTVIEAEASRDHTELMLQHFGATLVTEPEGAHGRKISLTGQPELRGAR | 240 |
| --- | --- | --- | --- |
| Query | 239 | IDVPGDPSSTAFPLVAALLVPGSDVTILNVLMNPTRTGLILTLQEMGADIEVINPRLAGG | 298 |
| Sbjct | 241 | + VP DPSS AFP+VAALLVPGSD+ + V+ NP RTGLI TL+EMG IE R G VVVPADPSSAAFPMVAALLVPGSDIVLTEVMTNPLRTGLITTLREMGGAIEESETRDDTG | 300 |
| Query | 299 | EDVADLRVRSSTLKGVTVPEDRAPSMIDEYPILAVAAAFAEGATVMNGLEELRVKESDRL | 358 |
| Sbjct | 301 | E +A R+R S L+GV VP +RAPSMIDEY +LAVAAAFAEG TVM GL ELRVKESDRL EPMAQFRIRGSRLRGVEVPPERAPSMIDEYLVLAVAAAFAEGTTVMRGLHELRVKESDRL | 360 |
| Query | 359 | SAVANGLKLNGVDCDEGETSLVVRGRPDGKGLGNASGAAVATHLDHRIAMSFLVMGLVSE | 418 |
| Sbjct | 361 | A A+ L++NGV + L+V G+ G G VATH+DHRIAMS LVMGL ++ EATADMLRVNGVKVEIVGDDLIVEGKGHVPG GGLVATHMDHRIAMSALVMGLAAD | 415 |
| Query  Sbjct | 419  416 | NPVTVDDATMIATSFPEFMDLMAGLGAKI 447 PV VDD IATSFP+F+ +M LG ++  RPVKVDDTAFIATSFPDFVPMMRRLGGEL 444 |  |

>TR:A0A1I3PAL8 A0A1I3PAL8_9BRAD 3-phosphoshikimate 1-carboxyvinyltransferase OS=Bosea sp. OK403 OX=1855286 GN=aroA PE=3 SV=1

Length=446

Score = 491 bits (1263), Expect = 4e-169

| Identities | | = 272/449 (61%), Positives = 317/449 (71%), Gaps = 6/449 (1%) |  |
| --- | --- | --- | --- |
| Query 1  Sbjct 1 | | MLHGASSRPATARKSSGLSGTVRIPGDKSISHRSFMFGGLASGETRITGLLEGEDVINTG M H P TAR L+G VR+PGDKSISHRS +FG LA GET I+GLLEGEDV+ T MAHAQPPIPVTARSRGPLAGRVRVPGDKSISHRSLIFGLLAIGETTISGLLEGEDVMRTA | 60  60 |
| Query 61  Sbjct 61 | | KAMQAMGARIRKEGD-TWIIDGVGNGGLLAPEAPLDFGNAATGCRLTMGLVGVYDFDSTF A +A+GA + + +W + GVG GGL P LDFGNA TG RL MG+ G + + F DAARALGAIVHHDRPGSWRVRGVGVGGLREPGGVLDFGNAGTGSRLMMGVCGAHPITTVF | 119  120 |
| Query 120  Sbjct 121 | | IGDASLTKRPMGRVLNPLREMGVQ-VKSEDGDRLPVTLRGPKTPTPITYRVPMASAQVKS GDASL KRPM R+L+PL MG V E+G R+P+TLRGPK PITYR P+ASAQ+KS DGDASLRKRPMRRILDPLELMGTTIVAQEEGGRVPLTLRGPKEAVPITYRTPVASAQIKS | 178  180 |
| Query 179  Sbjct 181 | | AVLLAGLNTPGITTVIEPIMTRDHTEKMLQGFGANLTVETDADGVRTIRLEGRGKLTGQV AVLLAGL PG TTVIE TRDHTE ML+ FGA+++V D R I L+G+ +L AVLLAGLAAPGETTVIETEATRDHTETMLRHFGADVSVSPDGAHGRRIVLKGQPELQAAP | 238  240 |
| Query 239  Sbjct 241 | | IDVPGDPSSTAFPLVAALLVPGSDVTILNVLMNPTRTGLILTLQEMGADIEVINPRLAGG I VP DPSS AFPLVAAL+VPGSD+ I +V+ NP R+GL+ TL+EMGADI V + GG IVVPADPSSAAFPLVAALIVPGSDILIESVMTNPLRSGLLTTLREMGADISVESEANEGG | 298  300 |
| Query 299  Sbjct 301 | | EDVADLRVRSSTLKGVTVPEDRAPSMIDEYPILAVAAAFAEGATVMNGLEELRVKESDRL E VA LRVR+ LKGVTVP +RAPSMIDEYP+LAVAA+FA G T M GL+ELRVKESDRL EAVATLRVRAGALKGVTVPPERAPSMIDEYPVLAVAASFATGTTRMRGLQELRVKESDRL | 358  360 |
| Query 359  Sbjct 361 | | SAVANGLKLNGVDCDEGETSLVVRGRPDGKGLGNASGAAVATHLDHRIAMSFLVMGLVSE  +AV GLK GV C LVV GR G G A G VATHLDHRIAMSFLVMGL + AAVEAGLKAAGVTCAIEGDDLVVEGR GGGVAGGGLVATHLDHRIAMSFLVMGLATR | 418  416 |
| Query 419 | | NPVTVDDATMIATSFPEFMDLMAGLGAKI 447 P+TVDD TMIATSFP F+ LM GLG I |  |
| Sbjct | 417 | KPMTVDDGTMIATSFPSFIALMNGLGGDI 445 | |

>TR:A0A0N0JWL9 A0A0N0JWL9_9BRAD 3-phosphoshikimate 1-carboxyvinyltransferase OS=Bosea sp. AAP35 OX=1523417 GN=aroA PE=3 SV=1

Length=446

Score = 490 bits (1262), Expect = 6e-169

| Identities | | = 268/449 (60%), Positives = 323/449 (72%), Gaps = 6/449 (1%) |  |
| --- | --- | --- | --- |
| Query 1  Sbjct 1 | | MLHGASSRPATARKSSGLSGTVRIPGDKSISHRSFMFGGLASGETRITGLLEGEDVINTG M H + P TAR+S L+G VR+PGDKSISHR+ +FG LA GET++TGLLEGEDV+ T MAHAQTPVPVTARRSGPLTGRVRVPGDKSISHRAMIFGLLAIGETKVTGLLEGEDVMRTA | 60  60 |
| Query 61  Sbjct 61 | | KAMQAMGARIRKEGD-TWIIDGVGNGGLLAPEAPLDFGNAATGCRLTMGLVGVYDFDSTF A +A+GA + +G W + GVG GGL P LDFGNA TG RL MG+ G + +TF AAARALGAIVHHDGPGAWRVRGVGVGGLSQPAETLDFGNAGTGSRLMMGVCGAHPITTTF | 119  120 |
| Query 120  Sbjct 121 | | IGDASLTKRPMGRVLNPLREMGVQVKSE-DGDRLPVTLRGPKTPTPITYRVPMASAQVKS GDASL KRPM R+L+PL MG + S+ +G R+P+TLRGP+ PI+YR P+ASAQ+KS DGDASLRKRPMRRILDPLELMGTVIVSQGEGGRVPLTLRGPQEAVPISYRTPVASAQIKS | 178  180 |
| Query 179  Sbjct 181 | | AVLLAGLNTPGITTVIEPIMTRDHTEKMLQGFGANLTVETDADGVRTIRLEGRGKLTGQV AVLLAGL PG TTVIE TRDHTEKML FGA++ V + R I L+G+ +L AVLLAGLAAPGETTVIETEATRDHTEKMLIHFGADVVVTPEGAHGRKIVLKGQPELQAAP | 238  240 |
| Query 239  Sbjct 241 | | IDVPGDPSSTAFPLVAALLVPGSDVTILNVLMNPTRTGLILTLQEMGADIEVINPRLAGG I VP DPSS AFPLVAAL+VPGSD+ I +V+ NP R+GL+ TL+EMGADI V + GG IVVPADPSSAAFPLVAALIVPGSDILIESVMTNPLRSGLLTTLREMGADIAVESEANEGG | 298  300 |
| Query 299  Sbjct 301 | | EDVADLRVRSSTLKGVTVPEDRAPSMIDEYPILAVAAAFAEGATVMNGLEELRVKESDRL E VA LRVR+S+LKGVTVP +RAPSMIDEYP+LAVAA+FA G T M GL+ELRVKESDRL EAVATLRVRASSLKGVTVPPERAPSMIDEYPVLAVAASFATGTTRMRGLQELRVKESDRL | 358  360 |
| Query 359  Sbjct 361 | | SAVANGLKLNGVDCDEGETSLVVRGRPDGKGLGNASGAAVATHLDHRIAMSFLVMGLVSE  +AV GLK GV C LVV +G G A G VATHLDHRIAMSFLVMGL ++ AAVEAGLKAAGVTCAIEGDDLVV EGTGGAVAGGGTVATHLDHRIAMSFLVMGLATD | 418  416 |
| Query 419 | | NPVTVDDATMIATSFPEFMDLMAGLGAKI 447 P+ VDD MIATSFP F+ LM GLG +I |  |
| Sbjct | 417 | KPMQVDDGAMIATSFPSFIALMNGLGGEI 445 | |

>TR:A0A0Q4XLF1 A0A0Q4XLF1_9RHIZ 3-phosphoshikimate 1-carboxyvinyltransferase OS=Methylobacterium sp. Leaf99 OX=1736251 GN=aroA PE=3 SV=1

Length=449

Score = 490 bits (1262), Expect = 6e-169

| Identities | = 274/453 (60%), Positives = 322/453 (71%), Gaps = 12/453 (3%) |  |
| --- | --- | --- |
| Query 1 | MLHGASSRPATARKSSGLSGTVRIPGDKSISHRSFMFGGLASGETRITGLLEGEDVINTG | 60 |
| Sbjct 1 | M H ++ +P TA L G++R PGDKSISHR+ + G L+ GETR+ GLLEG+DV+ T MSHDSAPQPLTATAGLALQGSLRPPGDKSISHRAMILGLLSIGETRVEGLLEGDDVLRTA | 60 |
| Query 61 | KAMQAMGARIRKEGD-TWIIDGVGNGGLLAPEAPLDFGNAATGCRLTMGLVGVYDFDSTF | 119 |
| Sbjct 61 | A +A+GA + + G+ W I GVG GGL+ P LDFGNA TG RL MG+VG +TF AAAEALGATVERLGEGRWRIVGVGLGGLVDPADILDFGNAGTGSRLMMGVVGGQPVTATF | 120 |
| Query 120 | IGDASLTKRPMGRVLNPLREMGVQVKSE-DGDRLPVTLRGPKTPTPITYRVPMASAQVKS | 178 |
| Sbjct 121 | GDASL KRPM R+L+PL MG +V SE +G R+P+TLRGP+ PITY P+ASAQ+KS DGDASLRKRPMRRILDPLVRMGTEVVSEREGGRVPLTLRGPREAIPITYETPVASAQIKS | 180 |
| Query 179 | AVLLAGLNTPGITTVIEPIMTRDHTEKMLQGFGANLTVETDADGV RTIRLEGRGKL | 234 |
| Sbjct 181 | AVLLAGLN PGITTVIE TRDHTE+ML+ FGA+ VE A G RTI L G+ L AVLLAGLNAPGITTVIEAAATRDHTERMLRLFGAH--VEVTAHGPEGHGRTIALTGQPTL | 238 |
| Query 235 | TGQVIDVPGDPSSTAFPLVAALLVPGSDVTILNVLMNPTRTGLILTLQEMGADIEVINPR | 294 |
| Sbjct 239 | G + VP DPSS AFPLVAAL+VPGS+V I V+MNP RTGLI TL EMGADIE + R RGTDVVVPADPSSAAFPLVAALIVPGSEVVIEGVMMNPLRTGLITTLLEMGADIERLRER | 298 |
| Query 295 | LAGGEDVADLRVRSSTLKGVTVPEDRAPSMIDEYPILAVAAAFAEGATVMNGLEELRVKE GGE VADLRVR+S L GV VP +RAP+MIDEYPILAVAAAFAEG T MNGL ELRVKE | 354 |

| Sbjct | 299 | DEGGETVADLRVRASRLHGVDVPPERAPAMIDEYPILAVAAAFAEGTTRMNGLHELRVKE | 358 |
| --- | --- | --- | --- |
| Query | 355 | SDRLSAVANGLKLNGVDCDEGETSLVVRGRPDGKGLGNASGAAVATHLDHRIAMSFLVMG | 414 |
| Sbjct | 359 | SDRL+AVA+GLK NG+ LVV G G G VATHLDHRIAM+FLV+G SDRLAAVADGLKANGIAHIVEGDDLVVH GDGTAPKGGGTVATHLDHRIAMAFLVLG | 414 |
| Query  Sbjct | 415  415 | LVSENPVTVDDATMIATSFPEFMDLMAGLGAKI 447 L + PVTVDD MIATSFP F+ M LG +I LATREPVTVDDGAMIATSFPSFLPTMQALGGRI 447 |  |

>TR:I9WBH6 I9WBH6_9SPHN 3-phosphoshikimate 1-carboxyvinyltransferase OS=Novosphingobium sp. Rr 2-17 OX=555793 GN=aroA PE=3 SV=1

Length=446

Score = 490 bits (1261), Expect = 8e-169

| Identities | | = 260/442 (59%), Positives = 316/442 (71%), Gaps = 3/442 (1%) |  |
| --- | --- | --- | --- |
| Query 5  Sbjct 7 | | ASSRPATARKSSGLSGTVRIPGDKSISHRSFMFGGLASGETRITGLLEGEDVINTGKAMQ A++RP + L G +R+PGDKSISHRS M G LA GETR+TGLLEGEDV+ T AM+ AAARPRRFTATGALRGRIRVPGDKSISHRSIMLGALAVGETRVTGLLEGEDVLATATAMR | 64  66 |
| Query 65  Sbjct 67 | | AMGARIRKEG-DTWIIDGVGNGGLLAPEAPLDFGNAATGCRLTMGLVGVYDFDSTFIGDA AMGA + + G W + GVG G LL PEA LD GN+ T RL MGLV + +TF GDA AMGASVERLGVGEWSVHGVGVGALLQPEAALDMGNSGTSTRLLMGLVSSHPIAATFTGDA | 123  126 |
| Query 124  Sbjct 127 | | SLTKRPMGRVLNPLREMGVQVKSEDGDRLPVTLRGPKTPTPITYRVPMASAQVKSAVLLA SL+KRPMGRV+ PL +MG ++ +G RLP+ +RG PITYR+P+ASAQVKSAVLLA SLSKRPMGRVITPLSQMGASFEASEGGRLPLIVRGASPAVPITYRLPVASAQVKSAVLLA | 183  186 |
| Query 184  Sbjct 187 | | GLNTPGITTVIEPIMTRDHTEKMLQGFGANLTVETDADGVRTIRLEGRGKLTGQVIDVPG GLNTPGITTVIEP+ TRDH+E+ML+GFGA+LTVETDADG R I + G +L QVI+VPG GLNTPGITTVIEPVPTRDHSERMLRGFGADLTVETDADGARLISIRGEAELKPQVIEVPG | 243  246 |
| Query 244  Sbjct 247 | | DPSSTAFPLVAALLVPGSDVTILNVLMNPTRTGLILTLQEMGADIEVINPRLAGGEDVAD DPSS AF LVAAL+VPGS+V I NV +NPTR G++ L++MG IE +NPR GGE VAD DPSSAAFFLVAALVVPGSEVLIENVGLNPTRAGIVTVLRQMGGQIEEVNPREVGGEPVAD | 303  306 |
| Query 304  Sbjct 307 | | LRVRSSTLKGVTVPEDRAPSMIDEYPILAVAAAFAEGATVMNGLEELRVKESDRLSAVAN L VR S LKG+ V AP MIDE+P+L VAAA A+G T +GL+ELRVKESDRL+A+A LLVRHSALKGIEVDPAIAPLMIDEFPVLFVAAAMADGVTTTSGLDELRVKESDRLAAMAT | 363  366 |
| Query 364 | | GLKLNGVDCDEGETSLVVRGRPDGKGLGNASGAAVATHLDHRIAMSFLVMGLVSENPVTV L GV +E LV+ G +G + A +ATHLDHRIAMS + GLVS V V | 423 |
| Sbjct | 367 | ALAAAGVRVEERADGLVITGTGGEPLVG--TDAPIATHLDHRIAMSMAIAGLVSRAGVEV | 424 |
| Query  Sbjct | 424  425 | DDATMIATSFPEFMDLMAGLGA 445 DD IATSFP F ++ GL A DDTRPIATSFPTFESMLDGLAA 446 |  |

>TR:A0A1H8E4T0 A0A1H8E4T0_9RHOB 3-phosphoshikimate 1-carboxyvinyltransferase OS=Paracoccus alcaliphilus OX=34002 GN=aroA PE=3 SV=1

Length=443

Score = 489 bits (1260), Expect = 1e-168

Identities = 252/448 (56%), Positives = 315/448 (70%), Gaps = 7/448 (2%)

| Query | 1 | MLHGASSRPATARKSSGLSGTVRIPGDKSISHRSFMFGGLASGETRITGLLEGEDVINTG | 60 |
| --- | --- | --- | --- |
| Sbjct | 1 | M H +P T+R S L G ++PGDKS+SHR+ + G LA GETRITGLLEG+D+++T MSHAGDPQPMTSRPSGSLRGEAQVPGDKSVSHRALILGALAVGETRITGLLEGQDILDTA | 60 |
| Query | 61 | KAMQAMGARIRKEGD-TWIIDGVGNGGLLAPEAPLDFGNAATGCRLTMGLVGVYDFDSTF KAMQA GA++ ++G W + GVG GG PE +D GN+ TG RL MG + +TF | 119 |

| Sbjct | 61 | KAMQAFGAQVERQGPGEWSVHGVGTGGFGEPETAIDCGNSGTGVRLIMGAMATTPVSATF | 120 |
| --- | --- | --- | --- |
| Query | 120 | IGDASLTKRPMGRVLNPLREMGVQVKSEDGDRLPVTLRGPKTPTPITYRVPMASAQVKSA | 179 |
| Sbjct | 121 | GDASL++RPM RV +PL G ++ + +G RLPVT+RG P P++YR P+ASAQ+KSA TGDASLSRRPMARVTDPLAMFGAEITAREGGRLPVTIRGATDPVPVSYRSPVASAQIKSA | 180 |
| Query | 180 | VLLAGLNTPGITTVIEPIMTRDHTEKMLQGFGANLTVETDADGVRTIRLEGRGKLTGQVI | 239 |
| Sbjct | 181 | +LLAGLN PG T V EP TRDHTE+ML GFGA + ET +G +RL GR +L Q + ILLAGLNAPGETVVTEPEQTRDHTERMLAGFGAQVRTETTPEG-HVVRLTGRPELRAQSV | 239 |
| Query | 240 | DVPGDPSSTAFPLVAALLVPGSDVTILNVLMNPTRTGLILTLQEMGADIEVINPRLAGGE | 299 |
| Sbjct | 240 | VP DPSS AFP+ AAL+VPGS++ + V NPTR GL TLQEMGADI N R+ GGE AVPRDPSSAAFPVAAALIVPGSEIRVPGVSRNPTRDGLYTTLQEMGADITFENDRIEGGE | 299 |
| Query | 300 | DVADLRVRSSTLKGVTVPEDRAPSMIDEYPILAVAAAFAEGATVMNGLEELRVKESDRLS | 359 |
| Sbjct | 300 | VADL +R S LKGV VP +RA SMIDE+PIL+V AAFAEG TVMNG+ ELRVKESDR+ PVADLLIRHSRLKGVNVPAERASSMIDEFPILSVIAAFAEGKTVMNGVAELRVKESDRID | 359 |
| Query | 360 | AVANGLKLNGVDCDEGETSLVVRGRPDGKGLGNASGAAVATHLDHRIAMSFLVMGLVSEN | 419 |
| Sbjct | 360 | A+A GL+ NGV +E S+ V G G G A ATHLDHRIAMSFLV+GL ++ AMARGLEANGVRVEETPDSMTVHGMQKVPGGGRA ATHLDHRIAMSFLVLGLATQA | 414 |
| Query  Sbjct | 420  415 | PVTVDDATMIATSFPEFMDLMAGLGAKI 447 PV++DD IATSFP+F+ LM GLGA + PVSIDDGGPIATSFPDFLPLMQGLGADL 442 |  |

>TR:A0A2H5EVL9 A0A2H5EVL9_9RHOB 3-phosphoshikimate 1-carboxyvinyltransferase OS=Paracoccus zhejiangensis OX=1077935 GN=aroA PE=3 SV=1

Length=443

Score = 489 bits (1260), Expect = 1e-168

| Identities | | = 257/448 (57%), Positives = 316/448 (71%), Gaps = 7/448 (2%) |  |
| --- | --- | --- | --- |
| Query 1  Sbjct 1 | | MLHGASSRPATARKSSGLSGTVRIPGDKSISHRSFMFGGLASGETRITGLLEGEDVINTG M HG RP TAR+S L+G ++PGDKSISHR+ + G L+ GETRITGLLEGEDV++T MSHGTDPRPMTARRSGPLTGVAQVPGDKSISHRALILGALSVGETRITGLLEGEDVLDTA | 60  60 |
| Query 61  Sbjct 61 | | KAMQAMGARIRKEGD-TWIIDGVGNGGLLAPEAPLDFGNAATGCRLTMGLVGVYDFDSTF KAM+A GA +++ G+ W++ GVG GG PE +D GN+ TG RL MG + +TF KAMRAFGADVQRTGEGEWVVHGVGVGGFAEPEDVIDCGNSGTGVRLIMGAMATTPITATF | 119  120 |
| Query 120  Sbjct 121 | | IGDASLTKRPMGRVLNPLREMGVQVKSEDGDRLPVTLRGPKTPTPITYRVPMASAQVKSA GDASL+KRPMGRV +PL G Q+ + +G RLPVT+ G P P+ Y+ P+ASAQ+KSA TGDASLSKRPMGRVTDPLALFGSQIVAREGGRLPVTITGAVDPVPVRYKTPVASAQIKSA | 179  180 |
| Query 180  Sbjct 181 | | VLLAGLNTPGITTVIEPIMTRDHTEKMLQGFGANLTVETDADGVRTIRLEGRGKLTGQVI VLLAGLN PG T VIE TRDH+E+ML GFGA + E +G I L GR +L GQ + VLLAGLNAPGETVVIEAEPTRDHSERMLAGFGATIRTEKTEEG-HVITLTGRPELRGQPV | 239  239 |
| Query 240  Sbjct 240 | | DVPGDPSSTAFPLVAALLVPGSDVTILNVLMNPTRTGLILTLQEMGADIEVINPRLAGGE VP DPSS AFP+ AAL+VPGS++ + V NPTR GL +TL EMGADI NPR GGE AVPRDPSSAAFPVAAALIVPGSEIRVPGVSRNPTRDGLYVTLLEMGADITFENPREEGGE | 299  299 |
| Query 300  Sbjct 300 | | DVADLRVRSSTLKGVTVPEDRAPSMIDEYPILAVAAAFAEGATVMNGLEELRVKESDRLS VADL VR LKGV VP DRA SMIDE+PIL+V A+FAEG TVMNG+ ELRVKESDR+ PVADLLVRHGPLKGVEVPADRAASMIDEFPILSVIASFAEGRTVMNGVAELRVKESDRID | 359  359 |
| Query 360 | | AVANGLKLNGVDCDEGETSLVVRGRPDGKGLGNASGAAVATHLDHRIAMSFLVMGLVSEN A+A GL+ NGV E S+ V G P G GA+ THLDHRIAMSFLV+GL ++ | 419 |
| Sbjct | 360 | AMARGLEANGVTVVETPDSMTVHGAPRVPG GASAVTHLDHRIAMSFLVLGLATDQ | 414 |
| Query | 420 | PVTVDDATMIATSFPEFMDLMAGLGAKI 447 PV+VDD + I TSFP+F+ LM GLGA + |  |

Sbjct 415 PVSVDDGSPIMTSFPDFIPLMQGLGADL 442

>TR:I4YTY5 I4YTY5_9RHIZ 3-phosphoshikimate 1-carboxyvinyltransferase OS=Microvirga lotononidis OX=864069 GN=aroA PE=3 SV=1

Length=449

Score = 489 bits (1260), Expect = 1e-168

| Identities | = 273/448 (61%), Positives = 320/448 (71%), Gaps = 6/448 (1%) |  |
| --- | --- | --- |
| Query 3 | HGASSRPATARKSSGLSGTVRIPGDKSISHRSFMFGGLASGETRITGLLEGEDVINTGKA | 62 |
| Sbjct 5 | HG+ S P +R + L G +R+PGDKSISHRS +FG L+ GETR+ GLLEG+DV+ T +A HGSPS-PVASRSGTPLKGRIRVPGDKSISHRSMIFGLLSIGETRVEGLLEGDDVLRTAEA | 63 |
| Query 63 | MQAMGARIRKEGD-TWIIDGVGNGGLLAPEAPLDFGNAATGCRLTMGLVGVYDFDSTFIG | 121 |
| Sbjct 64 | +A+GA I +E W I G G GGL P+ LDFGNA TG RL MG+ G + STF G CRALGATITREAPGRWRIVGAGIGGLTDPKDVLDFGNAGTGSRLMMGVAGSHPITSTFDG | 123 |
| Query 122 | DASLTKRPMGRVLNPLREMGVQVKSE-DGDRLPVTLRGPKTPTPITYRVPMASAQVKSAV | 180 |
| Sbjct 124 | DASL KRPM R+L+PL +MGV V SE +G R+P+TLRGPK PITY P ASAQ+KSA+ DASLRKRPMRRILDPLTKMGVTVVSEAEGGRVPLTLRGPKETLPITYETPAASAQIKSAI | 183 |
| Query 181 | LLAGLNTPGITTVIEPIMTRDHTEKMLQGFGANLTVETDADGVRTIRLEGRGKLTGQVID | 240 |
| Sbjct 184 | LLAGLN+PG TTVIE TRDHTE+ML+ FGA++ V R I L+G+ +L G I LLAGLNSPGKTTVIEREATRDHTERMLRHFGASVEVVPHGADGRAITLQGQPELRGTDIV | 243 |
| Query 241 | VPGDPSSTAFPLVAALLVPGSDVTILNVLMNPTRTGLILTLQEMGADIEVINPRLAGGED | 300 |
| Sbjct 244 | VP DPSS AFPLVAAL+ PGSDV I V+MNP RTGLI TL EMGA IE +N R GGE VPTDPSSAAFPLVAALITPGSDVVIEGVMMNPLRTGLITTLIEMGASIERLNERDEGGET | 303 |
| Query 301 | VADLRVRSSTLKGVTVPEDRAPSMIDEYPILAVAAAFAEGATVMNGLEELRVKESDRLSA | 360 |
| Sbjct 304 | VADLRVR S L GVTVP RAPSMIDEYPILAVAA+FAEG T M GL ELRVKESDRL+A VADLRVRHSRLTGVTVPHARAPSMIDEYPILAVAASFAEGTTRMQGLHELRVKESDRLAA | 363 |
| Query 361 | VANGLKLNGVDCDEGETSLVVRGRPDGKGLGNASGAAVATHLDHRIAMSFLVMGLVSENP | 420 |

VA GL	GV+ +	L+V G	G	G VATHLDHRIAM+FLVMGL ++ P

| Sbjct | 364 | VAAGLAEAGVEHEIHGDDLLVHG---SGGKVRGGGNPVATHLDHRIAMAFLVMGLATDQP | 420 |
| --- | --- | --- | --- |
| Query  Sbjct | 421  421 | VTVDDATMIATSFPEFMDLMAGLGAKIE 448  +TVDD MIATSFP F+ LM LGA I+ MTVDDGAMIATSFPSFIPLMRELGATIQ 448 |  |

>TR:A0A5N3PCS4 A0A5N3PCS4_9RHIZ 3-phosphoshikimate 1-carboxyvinyltransferase OS=Microvirga brassicacearum OX=2580413 GN=aroA PE=3 SV=1

Length=449

Score = 489 bits (1260), Expect = 1e-168

| Identities | = 273/445 (61%), Positives = 319/445 (72%), Gaps = 5/445 (1%) |  |
| --- | --- | --- |
| Query 5 | ASSRPATARKSSGLSGTVRIPGDKSISHRSFMFGGLASGETRITGLLEGEDVINTGKAMQ | 64 |
| Sbjct 6 | A + P T+R + L G +R+PGDKSISHRS +FG L+ GETR+ GLLEGEDV+ T + + APASPVTSRPGTPLKGRIRVPGDKSISHRSMIFGLLSIGETRVQGLLEGEDVLRTAEVCR | 65 |
| Query 65 | AMGARIRKEGD-TWIIDGVGNGGLLAPEAPLDFGNAATGCRLTMGLVGVYDFDSTFIGDA | 123 |
| Sbjct 66 | A+GA I +EG W I GVG GGL AP LDFGNA TG RL MG+ G + STF GDA ALGAVITREGPGRWRIVGVGIGGLNAPAGTLDFGNAGTGARLMMGVAGSHPITSTFDGDA | 125 |
| Query 124 | SLTKRPMGRVLNPLREMGVQVKSE-DGDRLPVTLRGPKTPTPITYRVPMASAQVKSAVLL | 182 |
| Sbjct 126 | SL KRPM R+L+PL +MGV V SE +G R+P+TLRGP+ PITY P ASAQ+KSAVLL SLRKRPMRRILDPLVKMGVSVVSEAEGGRVPLTLRGPRETIPITYETPAASAQIKSAVLL | 185 |
| Query 183 | AGLNTPGITTVIEPIMTRDHTEKMLQGFGANLTVETDADGVRTIRLEGRGKLTGQVIDVP AGLN PG TTVIE TRDHTE+ML+ FGA + V+ R I L+G+ L G + VP | 242 |

| Sbjct | 186 | AGLNAPGRTTVIEREATRDHTERMLRLFGAKVEVKPHGKDGRAITLQGQPDLRGTTVVVP | 245 |
| --- | --- | --- | --- |
| Query | 243 | GDPSSTAFPLVAALLVPGSDVTILNVLMNPTRTGLILTLQEMGADIEVINPRLAGGEDVA | 302 |
| Sbjct | 246 | DPSS AFPLVAAL+ PGSDV I V+MNP R GLI TL EMGA IE ++ R GGE VA TDPSSAAFPLVAALITPGSDVVIEAVMMNPLRIGLITTLLEMGASIERLSEREEGGETVA | 305 |
| Query | 303 | DLRVRSSTLKGVTVPEDRAPSMIDEYPILAVAAAFAEGATVMNGLEELRVKESDRLSAVA | 362 |
| Sbjct | 306 | DLRVR+S L GVTVP RAP+MIDEYPILAVAAAFA G T M GL ELRVKESDRL+AVA DLRVRASRLTGVTVPAARAPAMIDEYPILAVAAAFATGTTRMQGLHELRVKESDRLAAVA | 365 |
| Query | 363 | NGLKLNGVDCDEGETSLVVRGRPDGKGLGNASGAAVATHLDHRIAMSFLVMGLVSENPVT | 422 |
| Sbjct | 366 | GL G + + L+V G GK G A+ +VATHLDHRIAM+FL+MGL SE P+T AGLAAAGAEHEIDGDDLIVHGL-SGKVRGGAT--SVATHLDHRIAMAFLIMGLASEAPMT | 422 |
| Query  Sbjct | 423  423 | VDDATMIATSFPEFMDLMAGLGAKI 447 VDD MIATSFP F+ LM LGA I VDDGAMIATSFPSFIPLMRELGAAI 447 |  |

>TR:A0A1W6CYG2 A0A1W6CYG2_9RHOB 3-phosphoshikimate 1-carboxyvinyltransferase OS=Paracoccus contaminans OX=1945662 GN=aroA PE=3 SV=1

Length=442

Score = 489 bits (1259), Expect = 1e-168

| Identities | = 258/448 (58%), Positives = 318/448 (71%), Gaps = 8/448 (2%) |  |
| --- | --- | --- |
| Query 1 | MLHGASSRPATARKSSGLSGTVRIPGDKSISHRSFMFGGLASGETRITGLLEGEDVINTG | 60 |
| Sbjct 1 | M H A P TAR+ L+G ++PGDKS+SHR+ + G LA GETRITGLLEG+DV++T MSHPADPSPLTARRCGPLTGEAQVPGDKSVSHRALILGALAVGETRITGLLEGQDVLDTA | 60 |
| Query 61 | KAMQAMGARIRKEG-DTWIIDGVGNGGLLAPEAPLDFGNAATGCRLTMGLVGVYDFDSTF | 119 |
| Sbjct 61 | KAMQA GA +R+ G W + GVG GG P+ +D GN+ TG RL MG + +TF KAMQAFGAEVRRLGAGEWAVHGVGVGGFAEPQGVIDCGNSGTGVRLIMGAMATTPITATF | 120 |
| Query 120 | IGDASLTKRPMGRVLNPLREMGVQVKSEDGDRLPVTLRGPKTPTPITYRVPMASAQVKSA | 179 |
| Sbjct 121 | GDASL++RPMGRV +PL E G ++ S G LPVT+ G + P+ YR P+ASAQVKSA TGDASLSRRPMGRVTDPLAEFGARITSRAGGLLPVTIEGAEDAVPLHYRTPVASAQVKSA | 180 |
| Query 180 | VLLAGLNTPGITTVIEPIMTRDHTEKMLQGFGANLTVETDADGVRTIRLEGRGKLTGQVI | 239 |
| Sbjct 181 | VLLAGLN PG T VIEP TRDHTE+ML GFGA +T +G I L+GR +L Q + VLLAGLNAPGETVVIEPRPTRDHTERMLAGFGAEITTADTPEG-SVITLKGRPELRAQAV | 239 |
| Query 240 | DVPGDPSSTAFPLVAALLVPGSDVTILNVLMNPTRTGLILTLQEMGADIEVINPRLAGGE | 299 |
| Sbjct 240 | VP DPSS AFP+VAAL+VPGS +T+ V NPTR GL +TL EMGAD+ N R+ GGE AVPRDPSSAAFPVVAALIVPGSQITVPGVSRNPTRDGLYVTLAEMGADLAWSNARIEGGE | 299 |
| Query 300 | DVADLRVRSSTLKGVTVPEDRAPSMIDEYPILAVAAAFAEGATVMNGLEELRVKESDRLS | 359 |
| Sbjct 300 | VADL VR STL+GV+VP +RA SMIDE+PIL+VAAA A+G TVMNG+ ELRVKESDR++ PVADLTVRHSTLRGVSVPAERAASMIDEFPILSVAAALAQGETVMNGIAELRVKESDRIA | 359 |
| Query 360 | AVANGLKLNGVDCDEGETSLVVRGRPDGKGLGNASGAAVATHLDHRIAMSFLVMGLVSEN | 419 |
| Sbjct 360 | A+ GL+ NGV+ + S+ V G P GA V THLDHRIAMSFLV+GLV+E AMVAGLRANGVEVSDTPDSMTVCGGP VPGGARVQTHLDHRIAMSFLVLGLVAEA | 413 |
| Query 420  Sbjct 414 | PVTVDDATMIATSFPEFMDLMAGLGAKI 447 P++VDDA IATSFP+F+ LM GLGA I PISVDDAGPIATSFPDFLPLMRGLGAAI 441 |  |

>TR:A0A437N1E1 A0A437N1E1_9SPHN 3-phosphoshikimate 1-carboxyvinyltransferase OS=Novosphingobium umbonatum OX=1908524 GN=aroA PE=3 SV=1

Length=444

Score = 489 bits (1258), Expect = 2e-168

| Identities | = 263/446 (59%), Positives = 314/446 (70%), Gaps = 3/446 (1%) |  |
| --- | --- | --- |
| Query 1 | MLHGASSRPATARKSSGLSGTVRIPGDKSISHRSFMFGGLASGETRITGLLEGEDVINTG | 60 |
| Sbjct 1 | M AS P L GT+R+PGDKSISHRS M G LA GETR+TGLLEGEDV++T MSASASPTPRRFVAQGPLKGTIRVPGDKSISHRSIMLGALALGETRVTGLLEGEDVMSTA | 60 |
| Query 61 | KAMQAMGARIRK-EGDTWIIDGVGNGGLLAPEAPLDFGNAATGCRLTMGLVGVYDFDSTF | 119 |
| Sbjct 61 | AM+AMGA I K + W+I GVG GGLL PE LD GN+ T RL MGLV + +TF AAMRAMGATITKTDSGEWVIQGVGVGGLLQPETALDMGNSGTSTRLLMGLVSSHAITATF | 120 |
| Query 120 | IGDASLTKRPMGRVLNPLREMGVQVKSEDGDRLPVTLRGPKTPTPITYRVPMASAQVKSA | 179 |
| Sbjct 121 | IGDASL+KRPMGRV++PL MG S +G RLP+T+RG PITYR+P+ASAQVKSA IGDASLSKRPMGRVIDPLSTMGADFTSTEG-RLPLTMRGASPAVPITYRLPVASAQVKSA | 179 |
| Query 180 | VLLAGLNTPGITTVIEPIMTRDHTEKMLQGFGANLTVETDADGVRTIRLEGRGKLTGQVI | 239 |
| Sbjct 180 | VLLAGLNT G+TTVIEP+ TRDH+E+ML+GFGANLT ET+ G R IR+ G +LTGQ I VLLAGLNTAGVTTVIEPVPTRDHSERMLKGFGANLTAETNEAGERVIRIHGPAELTGQTI | 239 |
| Query 240 | DVPGDPSSTAFPLVAALLVPGSDVTILNVLMNPTRTGLILTLQEMGADIEVINPRLAGGE | 299 |
| Sbjct 240 | +VPGDPSS AF +VAAL+VPGSDV I NV +N TR G+I L++MG IE +NPR+ GGE EVPGDPSSAAFFIVAALIVPGSDVLIENVGLNDTRAGIITVLRQMGGSIEEVNPRIVGGE | 299 |
| Query 300 | DVADLRVRSSTLKGVTVPEDRAPSMIDEYPILAVAAAFAEGATVMNGLEELRVKESDRLS | 359 |
| Sbjct 300 | VADLRVR S L G+ V PSM+DE+P+L VAAA A G T+ GLEELRVKESDR++ PVADLRVRHSALHGINVDPAIVPSMVDEFPVLFVAAAHAAGRTICTGLEELRVKESDRIT | 359 |
| Query 360 | AVANGLKLNGVDCDEGETSLVVRGRPDGKGLGNASGAAVATHLDHRIAMSFLVMGLVSEN | 419 |

+	L+L G	E E +++ G	G L	+ AVATHLDHRIAMS V G+ SE

| Sbjct | 360 | VMRVALELAGAKVAETEDGMIIDG-TGGAPLPAGAAEAVATHLDHRIAMSMAVAGIASEG | 418 |
| --- | --- | --- | --- |
| Query | 420 | PVTVDDATMIATSFPEFMDLMAGLGA 445 V VDD + I TSFP F LM GL A |  |
| Sbjct | 419 | GVLVDDTSPIRTSFPNFEALMDGLVA 444 | |

>TR:A0A239EU77 A0A239EU77_9SPHN 3-phosphoshikimate 1-carboxyvinyltransferase OS=Sphingomonas laterariae OX=861865 GN=aroA PE=3 SV=1

Length=486

Score = 490 bits (1262), Expect = 2e-168

| Identities | = 263/447 (59%), Positives = 319/447 (71%), Gaps = 6/447 (1%) |  |
| --- | --- | --- |
| Query 3 | HGASSRPATARKSSG--LSGTVRIPGDKSISHRSFMFGGLASGETRITGLLEGEDVINTG | 60 |
| Sbjct 37 | H + S P + S+ L+G VR+PGDKSISHRS M LA GE+ + GLLEGEDV+ T HSSPSSPLSVAFSAPAPLAGRVRVPGDKSISHRSLMLSALAVGESVVEGLLEGEDVLATA | 96 |
| Query 61 | KAMQAMGARIRKEGD-TWIIDGVGNGGLLAPEAPLDFGNAATGCRLTMGLVGVYDFDSTF | 119 |
| Sbjct 97 | AM+AMGA I + D W I GVG GGLL P+ LD GN+ T RL MGL+ + +TF AAMRAMGATITRGDDGRWRIHGVGVGGLLQPQTALDMGNSGTSTRLLMGLIASHPITATF | 156 |
| Query 120 | IGDASLTKRPMGRVLNPLREMGVQVKSEDGDRLPVTLRGPKTPTPITYRVPMASAQVKSA | 179 |
| Sbjct 157 | +GDASL+KRPMGRV +PL MG + + G RLP+T+RG PI YR+P+ASAQVKSA VGDASLSKRPMGRVTDPLGLMGAEFTTSPGGRLPLTMRGICPAVPIEYRLPVASAQVKSA | 216 |
| Query 180 | VLLAGLNTPGITTVIEPIMTRDHTEKMLQGFGANLTVETDADGVRTIRLEGRGKLTGQVI | 239 |
| Sbjct 217 | +LLAGLN PGIT VIEP+ TRDH+E+ML+GFGANLTVE ADG RTI + G +L Q I ILLAGLNAPGITRVIEPVPTRDHSERMLRGFGANLTVEEAADGTRTISIHGEAELKPQAI | 276 |
| Query 240 | DVPGDPSSTAFPLVAALLVPGSDVTILNVLMNPTRTGLILTLQEMGADIEVINPRLAGGE | 299 |
| Sbjct 277 | VPGDPSS AFP+VAALLVPGSDVT+ NV +NPTR GL L+ MG DI + R+ GGE TVPGDPSSAAFPVVAALLVPGSDVTVENVGLNPTRAGLYEVLRAMGGDIRFTDERVVGGE | 336 |
| Query 300 | DVADLRVRSSTLKGVTVPEDRAPSMIDEYPILAVAAAFAEGATVMNGLEELRVKESDRLS VADL VR STL+G+ VP PSM+DE+PIL VAAAFAEG TVM GLEELRVKESDR++ | 359 |

| Sbjct | 337 | PVADLVVRHSTLRGIEVPPAIVPSMVDEFPILFVAAAFAEGRTVMRGLEELRVKESDRIA | 396 |
| --- | --- | --- | --- |
| Query | 360 | AVANGLKLNGVDCDEGETSLVVRGRPDGKGLGNASGAAVATHLDHRIAMSFLVMGLVSEN | 419 |
| Sbjct | 397 | +A GL+ G +E E L++ G G A GA VA HLDHRIAMSF V GLVS++ VMAEGLRAIGARVEELEDGLIIDGT---GGTPLAGGATVAAHLDHRIAMSFAVAGLVSKD | 453 |
| Query  Sbjct | 420  454 | PVTVDDATMIATSFPEFMDLMAGLGAK 446 PV +DD + +ATSFP F ++A LGA+ PVIIDDMSPVATSFPGFAAMLASLGAE 480 |  |

>TR:A0A512H6K5 A0A512H6K5_9PROT 3-phosphoshikimate 1-carboxyvinyltransferase OS=Pararhodospirillum oryzae OX=478448 GN=aroA PE=3 SV=1

Length=457

Score = 489 bits (1259), Expect = 2e-168

| Identities | | = 264/454 (58%), Positives = 315/454 (69%), Gaps = 12/454 (3%) |  |
| --- | --- | --- | --- |
| Query 1  Sbjct 1 | | MLHGASSRPATARKSSGLSGTVRIPGDKSISHRSFMFGGLASGETRITGLLEGEDVINTG M+ S+ P +AR+ S LSG +R+PGDKS+SHR+ M GGLA G T ITGLLEGEDV+ T MIDLPSAHPLSARRGSPLSGRIRVPGDKSVSHRALMLGGLAVGRTTITGLLEGEDVLATA | 60  60 |
| Query 61  Sbjct 61 | | KAMQAMGARIRKEGD TWIIDGVGNGGLLAPEAPLDFGNAATGCRLTMGLVGVYD  +AM MGA I +E D W +DGVG GGL P LD GNA TG RL MG+V + RAMALMGASIAREDDDASGASVWTVDGVGVGGLAEPAEVLDMGNAGTGARLLMGVVATHP | 114  120 |
| Query 115  Sbjct 121 | | FDSTFIGDASLTKRPMGRVLNPLREMGVQVKSEDGDRLPVTLRGPKTPTPITYRVPMASA  + F GDASL KRPMGRV++PL G + G+RLP+ L+G P P+TYRVP+ SA ITALFTGDASLRKRPMGRVIDPLSRFGATFLARAGNRLPLALKGAAEPLPVTYRVPVPSA | 174  180 |
| Query 175  Sbjct 181 | | QVKSAVLLAGLNTPGITTVIEPIMTRDHTEKMLQGFGANLTVETDADGVRTIRLEGRGKL QVKSAVLLAGLNTPG TTVIEP+ TRDHTE+ML FGA L DG + L G+ +L QVKSAVLLAGLNTPGETTVIEPVATRDHTERMLIHFGARLVRRQGDDGADVLVLSGQPEL | 234  240 |
| Query 235  Sbjct 241 | | TGQVIDVPGDPSSTAFPLVAALLVPGSDVTILNVLMNPTRTGLILTLQEMGADIEVINPR TG + VPGDPSS AFPLVAALLVP SD+ + V +NP RTGL TL+EMGAD+EV+N R TGGPVRVPGDPSSAAFPLVAALLVPESDLLLEGVGLNPLRTGLFDTLREMGADLEVLNVR | 294  300 |
| Query 295  Sbjct 301 | | LAGGEDVADLRVRSSTLKGVTVPEDRAPSMIDEYPILAVAAAFAEGATVMNGLEELRVKE GE V DLR R+S LKGV VP DRAPSMIDEYPILAVAAA A G + +GL ELRVKE EEAGEPVGDLRARASALKGVEVPADRAPSMIDEYPILAVAAACAHGTSRFHGLAELRVKE | 354  360 |
| Query 355 | | SDRLSAVANGLKLNGVDC-DEGETSLVVRGRPDGKGLGNASGAAVATHLDHRIAMSFLVM SDRL+A+ GL+ GV+ EG+T +V G+G GA V LDHRIAM+FLV+ | 413 |
| Sbjct | 361 | SDRLAAIVRGLEACGVEATTEGDTLIV KGQGEAPQGGAVVQVDLDHRIAMAFLVL | 415 |
| Query  Sbjct | 414  416 | GLVSENPVTVDDATMIATSFPEFMDLMAGLGAKI 447 G+VS PV +DD I TSFP F+DLM GLGA + GMVSVEPVRIDDGRAIETSFPGFVDLMTGLGAHL 449 |  |

>TR:T0HWZ7 T0HWZ7_9SPHN 3-phosphoshikimate 1-carboxyvinyltransferase OS=Novosphingobium lindaniclasticum LE124 OX=1096930 GN=aroA PE=3

SV=1

Length=448

Score = 489 bits (1258), Expect = 2e-168

Identities = 258/437 (59%), Positives = 314/437 (72%), Gaps = 1/437 (0%)

| Query | 8 | RPATARKSSGLSGTVRIPGDKSISHRSFMFGGLASGETRITGLLEGEDVINTGKAMQAMG | 67 |
| --- | --- | --- | --- |
|  |  | RP + L+G +R+PGDKSISHRS M G LA GETR+TGLLEGEDV++T AM+AMG |  |
| Sbjct | 10 | RPRRFLPAGPLTGRIRVPGDKSISHRSIMLGALAVGETRVTGLLEGEDVLSTAAAMRAMG | 69 |
| Query | 68 | ARIRKEGD-TWIIDGVGNGGLLAPEAPLDFGNAATGCRLTMGLVGVYDFDSTFIGDASLT | 126 |

| Sbjct | 70 | A + + G+ W + GVG G LL P PLD GN+ T RL MGLV + +F GDASL+  ATVERVGEGEWRVHGVGVGALLQPGGPLDMGNSGTSTRLLMGLVASHPIQVSFTGDASLS | 129 |
| --- | --- | --- | --- |
| Query | 127 | KRPMGRVLNPLREMGVQVKSEDGDRLPVTLRGPKTPTPITYRVPMASAQVKSAVLLAGLN | 186 |
| Sbjct | 130 | KRPMGRV++PL EMG ++ +G RLP+ +RG PITYR+P+ASAQVKSA+LLA LN KRPMGRVIDPLSEMGASFEASEGGRLPLIVRGASPAVPITYRLPVASAQVKSAILLAALN | 189 |
| Query | 187 | TPGITTVIEPIMTRDHTEKMLQGFGANLTVETDADGVRTIRLEGRGKLTGQVIDVPGDPS | 246 |
| Sbjct | 190 | TPG TTVIEP+ TRDH+E+ML+GFGA+LTV +ADG R I ++G +L QVIDVPGDPS TPGETTVIEPVPTRDHSERMLKGFGADLTVNVEADGTRVITIKGEAELKPQVIDVPGDPS | 249 |
| Query | 247 | STAFPLVAALLVPGSDVTILNVLMNPTRTGLILTLQEMGADIEVINPRLAGGEDVADLRV | 306 |
| Sbjct | 250 | S AF +VAALLVPGS+V I NV +NPTR GL+ L++MG I+ ++ R GGE VADLRV SAAFFIVAALLVPGSEVVIENVGLNPTRAGLVEVLRQMGGRIDFLDEREVGGEPVADLRV | 309 |
| Query | 307 | RSSTLKGVTVPEDRAPSMIDEYPILAVAAAFAEGATVMNGLEELRVKESDRLSAVANGLK | 366 |
| Sbjct | 310 | + S LKGV V AP+MIDE+P+L VAAA AEG TV +GL+ELRVKESDRL+A+A L KHSRLKGVAVDPAIAPAMIDEFPVLFVAAALAEGVTVASGLDELRVKESDRLTAMAVALT | 369 |
| Query | 367 | LNGVDCDEGETSLVVRGRPDGKGLGNASGAAVATHLDHRIAMSFLVMGLVSENPVTVDDA | 426 |
| Sbjct | 370 | L G +E E LV+ G G A GA V THLDHRIAMS V GL S + V VDD LAGAQIEEREDGLVITGTGGAPLPGMAEGARVTTHLDHRIAMSMAVAGLASSSGVGVDDI | 429 |
| Query | 427 | TMIATSFPEFMDLMAGL 443 IATSFP F DL+A L |  |
| Sbjct | 430 | RPIATSFPNFEDLLASL 446 | |

>TR:A0A5N7MQQ0 A0A5N7MQQ0_9RHIZ 3-phosphoshikimate 1-carboxyvinyltransferase OS=Microvirga tunisiensis OX=2108360 GN=aroA PE=3 SV=1

Length=449

Score = 489 bits (1258), Expect = 2e-168

| Identities | | = 272/448 (61%), Positives = 319/448 (71%), Gaps = 6/448 (1%) |  |
| --- | --- | --- | --- |
| Query 3  Sbjct 5 | | HGASSRPATARKSSGLSGTVRIPGDKSISHRSFMFGGLASGETRITGLLEGEDVINTGKA HG+ S P ++R + L G +R+PGDKSISHRS +FG L+ GETR+ GLLEG+DV+ T +A HGSPS-PVSSRSGTPLKGRIRLPGDKSISHRSMIFGLLSIGETRVEGLLEGDDVLRTAEA | 62  63 |
| Query 63  Sbjct 64 | | MQAMGARIRKEGD-TWIIDGVGNGGLLAPEAPLDFGNAATGCRLTMGLVGVYDFDSTFIG  +A+GA I +E W I G G GGL P LDFGNA TG RL MG+ G + STF G CRALGATITREAPGRWRIVGAGIGGLKDPSDILDFGNAGTGSRLMMGVAGSHPITSTFDG | 121  123 |
| Query 122  Sbjct 124 | | DASLTKRPMGRVLNPLREMGVQVKSE-DGDRLPVTLRGPKTPTPITYRVPMASAQVKSAV DASL KRPM R+L+PL +MGV V SE +G R+P+TLRGPK PITY P ASAQ+KSA+ DASLRKRPMRRILDPLTQMGVTVVSEAEGGRVPLTLRGPKETIPITYETPAASAQIKSAI | 180  183 |
| Query 181  Sbjct 184 | | LLAGLNTPGITTVIEPIMTRDHTEKMLQGFGANLTVETDADGVRTIRLEGRGKLTGQVID LLAGLN+PG TTVIE TRDHTE+ML+ FGA++ V R I L+G+ +L G I LLAGLNSPGKTTVIEREATRDHTERMLRHFGASVEVALHGADGRAITLQGQPELRGTEIV | 240  243 |
| Query 241  Sbjct 244 | | VPGDPSSTAFPLVAALLVPGSDVTILNVLMNPTRTGLILTLQEMGADIEVINPRLAGGED VP DPSS AFPLVAAL+ PGSDV + V+MNP RTGLI TL EMGA IE +N R GGE VPTDPSSAAFPLVAALITPGSDVVLEGVMMNPLRTGLITTLIEMGASIERLNERDEGGET | 300  303 |
| Query 301  Sbjct 304 | | VADLRVRSSTLKGVTVPEDRAPSMIDEYPILAVAAAFAEGATVMNGLEELRVKESDRLSA VADLRVR S L GVTVP RAP+MIDEYPILAVAAAFA+G T M GL ELRVKESDRL+A VADLRVRHSRLTGVTVPHARAPAMIDEYPILAVAAAFADGTTRMQGLHELRVKESDRLAA | 360  363 |
| Query 361 | | VANGLKLNGVDCDEGETSLVVRGRPDGKGLGNASGAAVATHLDHRIAMSFLVMGLVSENP VA GL GV+ + L+V G G G VATHLDHRIAMSFLVMGL + P | 420 |
| Sbjct | 364 | VAAGLAEAGVEHEIQGDDLLVHG---SNGKVRGGGNPVATHLDHRIAMSFLVMGLATAQP | 420 |
| Query | 421 | VTVDDATMIATSFPEFMDLMAGLGAKIE 448 |  |

+TVDD MIATSFP F+ LM LGA I+ Sbjct 421 MTVDDGAMIATSFPSFLPLMRELGATIQ 448

>TR:A0A4Q9GHI3 A0A4Q9GHI3_9RHIZ 3-phosphoshikimate 1-carboxyvinyltransferase OS=Hansschlegelia sp. Dub OX=2528245 GN=aroA PE=3 SV=1

Length=451

Score = 489 bits (1258), Expect = 3e-168

| Identities | | = 268/448 (60%), Positives = 320/448 (71%), Gaps = 8/448 (2%) |  |
| --- | --- | --- | --- |
| Query 9  Sbjct 9 | | PATARKSSGLSGTVRIPGDKSISHRSFMFGGLASGETRITGLLEGEDVINTGKAMQAMGA PA + +S+GLSG VR+ GDKSISHRS +FG L+ GET I GLLEGEDV+ T +A +A+GA PAKSTRSAGLSGRVRVAGDKSISHRSLIFGLLSVGETLIGGLLEGEDVLATARACRALGA | 68  68 |
| Query 69  Sbjct 69 | | RIRKEGD-TWIIDGVGNGGLLAPEAPLDFGNAATGCRLTMGLVGVYDFDSTFIGDASLTK  + + G+ W + GVG GGL P PLDFGNA TG RL MG+V +TF GDASL DVERLGEGRWRVRGVGVGGLRTPSEPLDFGNAGTGSRLMMGVVAGAPIQATFDGDASLRS | 127  128 |
| Query 128  Sbjct 129 | | RPMGRVLNPLREMGVQVKS-EDGDRLPVTLRGPKTPTPITYRVPMASAQVKSAVLLAGLN RPM R+L+PL +MG V S +G RLP+TL+G P PI Y P+ SAQVKSAVLLAGL RPMRRILDPLEKMGATVVSCAEGGRLPLTLKGADRPLPIEYESPVPSAQVKSAVLLAGLG | 186  188 |
| Query 187  Sbjct 189 | | TPGITTVIEPIMTRDHTEKMLQGFGANLTVETDADGVRTIRLEGRGKLTGQVIDVPGDPS PG+TTVIE TRDHTE+ML+GFGA ++VE + R IRL G+ +L I VP DPS APGVTTVIEREATRDHTERMLRGFGAEVSVERHGEHGRIIRLAGQPELKATAISVPADPS | 246  248 |
| Query 247  Sbjct 249 | | STAFPLVAALLVPGSDVTILNVLMNPTRTGLILTLQEMGADIEVINPRLAGGEDVADLRV S AFPLVAAL+V GS++T+ V+ NP RTGL++TL EMGAD+ + N R GGE+V DL V SAAFPLVAALIVEGSEITLEGVMTNPLRTGLLITLTEMGADVTLANIREDGGEEVCDLVV | 306  308 |
| Query 307  Sbjct 309 | | RSSTLKGVTVPEDRAPSMIDEYPILAVAAAFAEGATVMNGLEELRVKESDRLSAVANGLK R S LKGV VP +RAP+MIDEYPILAVAAAFAEG T M GL ELRVKESDRL+AV GL RGSRLKGVDVPAERAPTMIDEYPILAVAAAFAEGETRMRGLSELRVKESDRLAAVHAGLA | 366  368 |
| Query 367  Sbjct 369 | | LNGVDCD-EGETSLVVRGRPDGKGLGNASGAAVATHLDHRIAMSFLVMGLVSENPVTVDD NGV+ EG+ +V GKG G V THLDHRIAMSFL+MGL +E VTVDD ANGVESRIEGDDLIVA GKGRVAGGGEPVETHLDHRIAMSFLIMGLAAEREVTVDD | 425  423 |
| Query 426 | | ATMIATSFPEFMDLMAGLGAKIELSDTK 453 MIATSFP F LM GLGA+IE+ +K |  |
| Sbjct | 424 | GAMIATSFPTFAPLMRGLGAEIEMEASK 451 | |

>TR:A0A1N6X1Z8 A0A1N6X1Z8_9BRAD 3-phosphoshikimate 1-carboxyvinyltransferase OS=Bosea sp. TND4EK4 OX=1907408 GN=aroA PE=3 SV=1

Length=447

Score = 488 bits (1257), Expect = 3e-168

| Identities | = 265/449 (59%), Positives = 313/449 (70%), Gaps = 5/449 (1%) |  |
| --- | --- | --- |
| Query 1 | MLHGASSRPATARKSSGLSGTVRIPGDKSISHRSFMFGGLASGETRITGLLEGEDVINTG | 60 |
| Sbjct 1 | M H P TAR L+G VR+PGDKSISHR+ +FG LA GET +TGLLEGEDV+ T MSHDHDPIPVTARAGGPLTGRVRVPGDKSISHRAMIFGLLAIGETTVTGLLEGEDVMRTA | 60 |
| Query 61 | KAMQAMGARIRKEGD-TWIIDGVGNGGLLAPEAPLDFGNAATGCRLTMGLVGVYDFDSTF | 119 |
| Sbjct 61 | +A + +GA + +G W + GVG GGL P LDFGNA TG RL MG+ G + +TF QAARQLGAIVHHDGPGAWRVQGVGVGGLREPSGVLDFGNAGTGSRLMMGVCGSHPITTTF | 120 |
| Query 120 | IGDASLTKRPMGRVLNPLREMG-VQVKSEDGDRLPVTLRGPKTPTPITYRVPMASAQVKS | 178 |
| Sbjct 121 | GDASL KRPM R+L+PL +MG V V ++G R+P+TLRGPK PI YR P+ASAQ+KS DGDASLRKRPMRRILDPLEQMGTVVVSQQEGGRVPITLRGPKEAVPIVYRPPVASAQIKS | 180 |
| Query 179 | AVLLAGLNTPGITTVIEPIMTRDHTEKMLQGFGANLTVETDADGVRTIRLEGRGKLTGQV | 238 |

| Sbjct | 181 | AVLLAGL PG TTVIE TRDHTEKML FGA +TV + R I L G+ +L  AVLLAGLAAPGETTVIEAEATRDHTEKMLTHFGAEVTVTPEGTHGRKIVLRGQPELVAAP | 240 |
| --- | --- | --- | --- |
| Query | 239 | IDVPGDPSSTAFPLVAALLVPGSDVTILNVLMNPTRTGLILTLQEMGADIEVINPRLAGG | 298 |
| Sbjct | 241 | I VP DPSS AFPLVA +VPGSD+ + +V+ NP R+GL+ TL EMGADI + GG IVVPADPSSAAFPLVAGAIVPGSDILLESVMTNPLRSGLLTTLIEMGADITIEREANEGG | 300 |
| Query | 299 | EDVADLRVRSSTLKGVTVPEDRAPSMIDEYPILAVAAAFAEGATVMNGLEELRVKESDRL | 358 |
| Sbjct | 301 | E VADLRVR S LKGVTVP +RAPSMIDEYP+LAVAA+FA G T M GL+ELRVKESDRL ETVADLRVRGSRLKGVTVPPERAPSMIDEYPVLAVAASFATGTTRMRGLQELRVKESDRL | 360 |
| Query | 359 | SAVANGLKLNGVDCDEGETSLVVRGRPDGKGLGNASGAAVATHLDHRIAMSFLVMGLVSE | 418 |
| Sbjct | 361 | +AV GL+ GV C L+V G G G G +ATHLDHRIAMSFLVMGL ++ AAVEAGLRAAGVTCAIDGDDLIVEGSGGGPVRG---GGPIATHLDHRIAMSFLVMGLATQ | 417 |
| Query  Sbjct | 419  418 | NPVTVDDATMIATSFPEFMDLMAGLGAKI 447 NPV VDD MIATSFP F+ LM GLG +I NPVVVDDGAMIATSFPSFVALMNGLGGRI 446 |  |

>TR:A0A3L7AN27 A0A3L7AN27_9RHIZ 3-phosphoshikimate 1-carboxyvinyltransferase OS=Xanthobacter tagetidis OX=60216 GN=aroA PE=3 SV=1

Length=458

Score = 489 bits (1258), Expect = 3e-168

| Identities | | = 270/449 (60%), Positives = 323/449 (72%), Gaps = 11/449 (2%) |  |
| --- | --- | --- | --- |
| Query 3  Sbjct 15 | | HG-ASSRPATARKSSGLSGTVRIPGDKSISHRSFMFGGLASGETRITGLLEGEDVINTGK HG +S PATA S L+G VR+PGDKS+SHR+ +FG A G T ITGLLEGEDV+NT K HGEGASVPATALASGPLNGRVRVPGDKSVSHRALIFGLFAEGVTEITGLLEGEDVLNTAK | 61  74 |
| Query 62  Sbjct 75 | | AMQAMGARIRKEGDT-WIIDGVGNGGLLAPEAPLDFGNAATGCRLTMGLVGVYDFDSTFI A A+GA++ + G+ W + GVG+ GL P+A LDFGN+ TG RL MG VG +TF ACAALGAKVERLGEGHWRVTGVGSRGLSEPDAVLDFGNSGTGVRLMMGAVGGQPVTATFD | 120  134 |
| Query 121  Sbjct 135 | | GDASLTKRPMGRVLNPLREMGVQVKSE-DGDRLPVTLRGPKTPTPITYRVPMASAQVKSA GDASL +RPM RVL+PLREMGV V +E +G RLP+TL+G PT +TY P+ SAQVKSA GDASLRRRPMRRVLDPLREMGVAVVAEAEGGRLPLTLKGSVAPTALTYETPVPSAQVKSA | 179  194 |
| Query 180  Sbjct 195 | | VLLAGLNTPGITTVIEPIMTRDHTEKMLQGFGANLTVETDADGVRTIRLEGRGKLTGQVI VLLAG+ PG T VIE TRDHTE+ML FGA+++VE R + LEG KL G + VLLAGIGAPGNTVVIEREATRDHTERMLAHFGAHVSVEPHGPHGRRVTLEGGAKLKGAPV | 239  254 |
| Query 240  Sbjct 255 | | DVPGDPSSTAFPLVAALLVPGSDVTILNVLMNPTRTGLILTLQEMGADIEVINPRLAGGE VP DPSS AFPLVAALLVPGS+V + +V+MN RTGLI TL+EMGADIE + R GGE AVPADPSSAAFPLVAALLVPGSEVILTDVMMNAFRTGLIATLKEMGADIETLAERSEGGE | 299  314 |
| Query 300  Sbjct 315 | | DVADLRVRSSTLKGVTVPEDRAPSMIDEYPILAVAAAFAEGATVMNGLEELRVKESDRLS VADLRVR S LKGV VP RAPSMIDEYP+LAVAAAFAEG T+M GL ELRVKESDRL+ SVADLRVRHSRLKGVEVPAHRAPSMIDEYPVLAVAAAFAEGETIMRGLAELRVKESDRLA | 359  374 |
| Query 360 | | AVANGLKLNGVDCD-EGETSLVVRGRPDGKGLGNASGAAVATHLDHRIAMSFLVMGLVSE AVA+GL GV EG+ +V G G G V TH+DHRIAM+FLVMGL ++ | 418 |
| Sbjct | 375 | AVADGLAACGVSHRIEGDDLIVTGG GVRGGGPVKTHMDHRIAMAFLVMGLAAK | 427 |
| Query | 419 | NPVTVDDATMIATSFPEFMDLMAGLGAKI 447 V+VDD + IATSFP F+ +M GLGA+I |  |
| Sbjct | 428 | EGVSVDDVSFIATSFPSFLPMMRGLGAEI 456 | |

>TR:K2J8R6 K2J8R6_9RHOB 3-phosphoshikimate 1-carboxyvinyltransferase OS=Celeribacter baekdonensis B30 OX=1208323 GN=aroA PE=3 SV=1

Length=449

Score = 488 bits (1257), Expect = 4e-168

| Identities | | = 263/440 (60%), Positives = 310/440 (70%), Gaps = 4/440 (1%) |  |
| --- | --- | --- | --- |
| Query 9  Sbjct 9 | | PATARKSSGLSGTVRIPGDKSISHRSFMFGGLASGETRITGLLEGEDVINTGKAMQAMGA P TARKS L GT +PGDKSISHR+ +FG +A GET+ITGLLEGEDV++T KAMQA+GA PMTARKSGPLKGTAAVPGDKSISHRALIFGAMAVGETKITGLLEGEDVLDTAKAMQALGA | 68  68 |
| Query 69  Sbjct 69 | | RIRKEG-DTWIIDGVGNGGLLAPEAPLDFGNAATGCRLTMGLVGVYDFDSTFIGDASLTK  + +EG W + GVG GG P +D GN+ TG RL MG V +DF +TF GD SL K DVVREGAGEWTVHGVGVGGFTEPADVIDCGNSGTGVRLIMGAVATHDFCTTFTGDGSLRK | 127  128 |
| Query 128  Sbjct 129 | | RPMGRVLNPLREMGVQVKSEDGDRLPVTLRGPKTPTPITYRVPMASAQVKSAVLLAGLNT RPMGRV +PL G + RLP+TL G P P+ Y P+ SAQVKSAVLLAGLN RPMGRVTDPLALFGARSVGRSQGRLPLTLIGAAEPVPVEYETPVPSAQVKSAVLLAGLNA | 187  188 |
| Query 188  Sbjct 189 | | PGITTVIEPIMTRDHTEKMLQGFGANLTVETDADGVRTIRLEGRGKLTGQVIDVPGDPSS PG T VIE MTRDHTE+ML GFGA L+ ET +G I L GR +LT Q + VP DPSS PGQTVVIEKEMTRDHTERMLVGFGAELSTETTPEGYHKITLTGRPELTPQEVAVPRDPSS | 247  248 |
| Query 248  Sbjct 249 | | TAFPLVAALLVPGSDVTILNVLMNPTRTGLILTLQEMGADIEVINPRLAGGEDVADLRVR AFP+ AAL+V GS+V + V N TR GL TL EMGA+I NPR GGE VADLRV+ AAFPVCAALIVEGSEVFVPGVSQNTTRNGLYTTLVEMGANISFENPRTEGGEPVADLRVK | 307  308 |
| Query 308  Sbjct 309 | | SSTLKGVTVPEDRAPSMIDEYPILAVAAAFAEGATVMNGLEELRVKESDRLSAVANGLKL S LKG+ VP DRA SMIDE+P+L+V AAFAEG TVM G++ELRVKESDR+ A+A GL+ YSKLKGIEVPPDRAASMIDEFPVLSVVAAFAEGTTVMRGVKELRVKESDRIDAMARGLEA | 367  368 |
| Query 368  Sbjct 369 | | NGVDCDEGETSLVVRGRPDGKGLGNASGAAVATHLDHRIAMSFLVMGLVSENPVTVDDAT GV+ +E +L+V GR KG+ GA ATH+DHRIAMSFLVMGL SE VTVDD + CGVEIEEDHDTLIVHGR-GAKGV--KGGATCATHIDHRIAMSFLVMGLASEQAVTVDDGS | 427  425 |
| Query 428 | | MIATSFPEFMDLMAGLGAKI 447 IATSFP F LMAGLGA I |  |
| Sbjct | 426 | PIATSFPVFEKLMAGLGAAI 445 | |

>TR:A0A031K773 A0A031K773_9SPHN 3-phosphoshikimate 1-carboxyvinyltransferase OS=Novosphingobium resinovorum OX=158500 GN=aroA PE=3 SV=1

Length=445

Score = 488 bits (1256), Expect = 4e-168

| Identities | = 254/439 (58%), Positives = 312/439 (71%), Gaps = 3/439 (1%) |  |
| --- | --- | --- |
| Query 8 | RPATARKSSGLSGTVRIPGDKSISHRSFMFGGLASGETRITGLLEGEDVINTGKAMQAMG | 67 |
| Sbjct 9 | RP + L G +R+PGDKSISHRS M G LA GET++TGLLEGEDV++T AM+AMG RPRRFLPTGPLKGRIRVPGDKSISHRSIMLGALAVGETKVTGLLEGEDVLSTAAAMRAMG | 68 |
| Query 68 | ARIRKEGD-TWIIDGVGNGGLLAPEAPLDFGNAATGCRLTMGLVGVYDFDSTFIGDASLT | 126 |
| Sbjct 69 | A + + G+ W +GVG G LL PEAPLD GN+ T RL MGLV + +TFIGD SL+ ATVTRTGEGAWTANGVGVGALLQPEAPLDMGNSGTSTRLLMGLVASHPITATFIGDGSLS | 128 |
| Query 127 | KRPMGRVLNPLREMGVQVKSEDGDRLPVTLRGPKTPTPITYRVPMASAQVKSAVLLAGLN | 186 |
| Sbjct 129 | KRPMGRV++PL MG ++ +G RLP+ L+G + PI YR+PMASAQVKSA+LLAGLN KRPMGRVIDPLSTMGASFEASEGGRLPLILKGARPAVPIRYRLPMASAQVKSAILLAGLN | 188 |
| Query 187 | TPGITTVIEPIMTRDHTEKMLQGFGANLTVETDADGVRTIRLEGRGKLTGQVIDVPGDPS | 246 |
| Sbjct 189 | TPGITTVIEP+ TRDH+E+ML+GFGA+LTVETDADG R I + G +L Q I+VPGDPS TPGITTVIEPVPTRDHSERMLRGFGADLTVETDADGARVISIRGEAELKPQTIEVPGDPS | 248 |
| Query 247 | STAFPLVAALLVPGSDVTILNVLMNPTRTGLILTLQEMGADIEVINPRLAGGEDVADLRV | 306 |
| Sbjct 249 | S AF +VAAL++PGS+V I NV +NPTR G++ L++MG IE +NPR GGE VADL V SAAFFIVAALVIPGSEVLIENVGLNPTRAGIVEVLRQMGGSIEEVNPREVGGEPVADLLV | 308 |
| Query 307 | RSSTLKGVTVPEDRAPSMIDEYPILAVAAAFAEGATVMNGLEELRVKESDRLSAVANGLK | 366 |

| Sbjct | 309 | R STLKG+ V P+M+DE+P+L VAAA AEG T GLEELRVKESDR+S +A L  RHSTLKGIEVDPAIVPAMVDEFPVLFVAAALAEGVTTTTGLEELRVKESDRISVMATALA | 368 |
| --- | --- | --- | --- |
| Query | 367 | LNGVDCDEGETSLVVRGRPDGKGLGNASGAAVATHLDHRIAMSFLVMGLVSENPVTVDDA | 426 |
| Sbjct | 369 | G E E L++ G +G + A+ATHLDHRIAMS V GL S V VDD AAGATVRETEDGLIITGTGGDPLVG--TNGAIATHLDHRIAMSMAVAGLASRAGVEVDDV | 426 |
| Query | 427 | TMIATSFPEFMDLMAGLGA 445 IATSFP F ++ GL A |  |
| Sbjct | 427 | RPIATSFPVFETMLEGLTA 445 | |

>TR:A0A178MWP7 A0A178MWP7_9PROT 3-phosphoshikimate 1-carboxyvinyltransferase OS=Magnetospirillum moscoviense OX=1437059 GN=aroA PE=3 SV=1

Length=445

Score = 488 bits (1256), Expect = 4e-168

| Identities | = 260/446 (58%), Positives = 318/446 (71%), Gaps = 7/446 (2%) |  |
| --- | --- | --- |
| Query 5 | ASSRPATARKSSGLSGTVRIPGDKSISHRSFMFGGLASGETRITGLLEGEDVINTGKAMQ | 64 |
| Sbjct 3 | A SRP +R++ L+G ++PGDKSISHR+ MFG LA GE+RI GLLEG+DV+ T +AM+ AMSRPLISRRAEPLTGRAKVPGDKSISHRALMFGALAVGESRIEGLLEGDDVLRTAQAMR | 62 |
| Query 65 | AMGARIRK-EGDTWIIDGVGNGGLLAPEAPLDFGNAATGCRLTMGLVGVYDFDSTFIGDA | 123 |
| Sbjct 63 | A+GA + K E +W + G G GGL+ P LD GN+ TG RL MGLV + F + F GDA ALGAEVEKLEDGSWRLWGRGVGGLMEPADLLDLGNSGTGARLMMGLVATHPFTTFFTGDA | 122 |
| Query 124 | SLTKRPMGRVLNPLREMGVQVKSEDGDRLPVTLRGPKTPTPITYRVPMASAQVKSAVLLA | 183 |
| Sbjct 123 | SL RPM RV PL MG + + +G RLP + G P PITY +P+ASAQVKSA+LLA SLRSRPMKRVSEPLSRMGAKFVTREGGRLPAAVVGTTNPVPITYELPVASAQVKSAILLA | 182 |
| Query 184 | GLNTPGITTVIEPIMTRDHTEKMLQGFGANLTVETDADGVRTIRLEGRGKLTGQVIDVPG | 243 |
| Sbjct 183 | GLNTPG TTVIE TRDHTE ML+ FGAN+T DG R + + G +LTG+ + VP GLNTPGETTVIEREATRDHTELMLRHFGANVTTTIREDGARVVTVTGFPELTGRTVIVPA | 242 |
| Query 244 | DPSSTAFPLVAALLVPGSDVTILNVLMNPTRTGLILTLQEMGADIEVINPRLAGGEDVAD | 303 |
| Sbjct 243 | DPSS AFPLVAAL+VPGS VT++ + MNP RTGL TL+EMGADI NPR+ GGE+VAD DPSSAAFPLVAALVVPGSTVTLVGIGMNPLRTGLFQTLREMGADIRTDNPRIEGGEEVAD | 302 |
| Query 304 | LRVRSSTLKGVTVPEDRAPSMIDEYPILAVAAAFAEGATVMNGLEELRVKESDRLSAVAN | 363 |
| Sbjct 303 | L V SS L+GV VP +RAPSMIDEYPILAVAA+FA G T M GL ELRVKESDR +A N LVVTSSALRGVDVPAERAPSMIDEYPILAVAASFAAGTTRMFGLSELRVKESDRFTATMN | 362 |
| Query 364 | GLKLNGVDCD-EGETSLVVRGRPDGKGLGNASGAAVATHLDHRIAMSFLVMGLVSENPVT | 422 |
| Sbjct 363 | GL+ GV+ + EG+T +V GKG GA +A +LDHRIAMSFLVMG+ + PV GLRACGVEVEAEGDTLIV HGKGAPPPGGATIAVNLDHRIAMSFLVMGMAAAQPVA | 417 |
| Query 423  Sbjct 418 | VDDATMIATSFPEFMDLMAGLGAKIE 448 VDDA + TSFP F++LM GLG+ IE VDDAEAVDTSFPGFVELMNGLGSAIE 443 |  |

>TR:A0A0Q5Z2B0 A0A0Q5Z2B0_9RHIZ 3-phosphoshikimate 1-carboxyvinyltransferase OS=Methylobacterium sp. Leaf399 OX=1736364 GN=aroA PE=3 SV=1

Length=449

Score = 488 bits (1256), Expect = 5e-168

Identities = 270/452 (60%), Positives = 320/452 (71%), Gaps = 10/452 (2%)

| Query | 1 | MLHGASSRPATARKSSGLSGTVRIPGDKSISHRSFMFGGLASGETRITGLLEGEDVINTG | 60 |
| --- | --- | --- | --- |
|  |  | M H ++ P TA + L G +R PGDKSISHR+ + G L+ GETR+ GLLEG+DV+ T |  |
| Sbjct | 1 | MSHASAPHPLTATAGAPLRGALRPPGDKSISHRAMILGLLSIGETRVEGLLEGDDVLRTA | 60 |
| Query | 61 | KAMQAMGARIRKEGD-TWIIDGVGNGGLLAPEAPLDFGNAATGCRLTMGLVGVYDFDSTF | 119 |

| Sbjct | 61 | +A +A+GA + + G+ W++ GVG GGL P LDFGNA TG RL MG+VG +TF  QAAKALGAGVDRLGEGRWVVRGVGIGGLSDPAGVLDFGNAGTGSRLMMGVVGGQPVTATF | 120 |
| --- | --- | --- | --- |
| Query | 120 | IGDASLTKRPMGRVLNPLREMGVQVKSE-DGDRLPVTLRGPKTPTPITYRVPMASAQVKS | 178 |
| Sbjct | 121 | GDASL KRPM R+L+PL MG QV +E +G R+P+TLRGP+ PITY P+ASAQ+KS DGDASLRKRPMRRILDPLLRMGAQVVTEGEGGRVPLTLRGPREAIPITYETPVASAQIKS | 180 |
| Query | 179 | AVLLAGLNTPGITTVIEPIMTRDHTEKMLQGFGANLTVETDADGV--RTIRLEGRGKLTG | 236 |
| Sbjct | 181 | AVLLAGLN PG TTVIE TRDHTE+ML+ FGA + V +G RTI L G+ L G AVLLAGLNAPGTTTVIEAAATRDHTERMLRLFGAEVAVTHTGEGGHGRTIALTGQPTLRG | 240 |
| Query | 237 | QVIDVPGDPSSTAFPLVAALLVPGSDVTILNVLMNPTRTGLILTLQEMGADIEVINPRLA | 296 |
| Sbjct | 241 | + VP DPSS AFPLVAAL+VPGSDVTI V+MNP R GL+ TL EMGADIE + R TDVVVPADPSSAAFPLVAALIVPGSDVTIEGVMMNPLRIGLVTTLIEMGADIERLREREE | 300 |
| Query | 297 | GGEDVADLRVRSSTLKGVTVPEDRAPSMIDEYPILAVAAAFAEGATVMNGLEELRVKESD | 356 |
| Sbjct | 301 | GGE VADLRVR+S L GV VP +RAP+MIDEYP+LAVAAAFA+G T M GL ELRVKESD GGETVADLRVRASRLTGVDVPPERAPAMIDEYPVLAVAAAFAQGTTRMTGLHELRVKESD | 360 |
| Query | 357 | RLSAVANGLKLNGV-DCDEGETSLVVRGRPDGKGLGNASGAAVATHLDHRIAMSFLVMGL | 415 |
| Sbjct | 361 | RL+AVA+GL NGV EG+ +V G G G VATHLDHRIAM+FLVMGL RLAAVADGLAANGVAHLVEGDDLVV HGDGTPPRGGGTVATHLDHRIAMAFLVMGL | 415 |
| Query | 416 | VSENPVTVDDATMIATSFPEFMDLMAGLGAKI 447 S PVTVDD MIATSFP F+ M LGA I |  |
| Sbjct | 416 | ASAEPVTVDDGAMIATSFPSFLPTMQALGAAI 447 | |

>TR:A0A501PPE6 A0A501PPE6_9PROT 3-phosphoshikimate 1-carboxyvinyltransferase OS=Emcibacter nanhaiensis OX=1505037 GN=aroA PE=3 SV=1

Length=444

Score = 488 bits (1255), Expect = 6e-168

| Identities | = 253/434 (58%), Positives = 310/434 (71%), Gaps = 6/434 (1%) |  |
| --- | --- | --- |
| Query 15 | SSGLSGTVRIPGDKSISHRSFMFGGLASGETRITGLLEGEDVINTGKAMQAMGARIRKEG | 74 |
| Sbjct 9 | + L GTV +PGDKSISHR+ + G LA GET + GLLEGEDV+NT AM+A+GA IRK+ TDNLKGTVNVPGDKSISHRALIMGSLAIGETLVAGLLEGEDVLNTAAAMRALGADIRKDE | 68 |
| Query 75 | D-TWIIDGVGNGGLLAPEAPLDFGNAATGCRLTMGLVGVYDFDSTFIGDASLTKRPMGRV | 133 |
| Sbjct 69 | D TW I GVG G L PE LD GN+ TG RL MGL+ Y F +TFIGDASL+ RPM RV DGTWHIHGVGVGALHEPEGALDMGNSGTGARLLMGLISTYPFSTTFIGDASLSSRPMKRV | 128 |
| Query 134 | LNPLREMGVQVKSEDGDRLPVTLRGPKTPTPITYRVPMASAQVKSAVLLAGLNTPGITTV | 193 |

+PL	G +	+	LP+T+RG	P PI+Y VP+ASAQVKSA++LA LNTPG TT+

| Sbjct | 129 | TDPLESFGAEFHGSENGTLPLTVRGTGMPVPISYDVPVASAQVKSAIMLAALNTPGTTTI | 188 |
| --- | --- | --- | --- |
| Query | 194 | IEPIMTRDHTEKMLQGFGANLTVETDADGVRTIRLEGRGKLTGQVIDVPGDPSSTAFPLV | 253 |
| Sbjct | 189 | E TRDHTE+M FG + T T D R I + G+ +LT + + VPGDPSS AFP+V FEKKPTRDHTERMFSFFGVDTTT-TPKDSGRVITVTGQPELTARNLTVPGDPSSAAFPVV | 247 |
| Query | 254 | AALLVPGSDVTILNVLMNPTRTGLILTLQEMGADIEVINPRLAGGEDVADLRVRSSTLKG | 313 |
| Sbjct | 248 | AAL+ PGSD+TI NV +NP RTGL +TL+EMGADIE +N R GE VADLRVR S LK AALITPGSDITIRNVGLNPDRTGLFITLKEMGADIEFVNEREDCGEPVADLRVRHSRLKA | 307 |
| Query | 314 | VTVPEDRAPSMIDEYPILAVAAAFAEGATVMNGLEELRVKESDRLSAVANGLKLNGVDCD | 373 |
| Sbjct | 308 | +TVP+DRAPSMIDEYPIL +AAAFA+G T+M G EELRVKESDR++ + GL+ G+D + ITVPQDRAPSMIDEYPILCIAAAFADGTTMMRGAEELRVKESDRIAIMVKGLRACGIDVE | 367 |
| Query | 374 | EGETSLVVRGRPDGKGLGNASGAAVATHLDHRIAMSFLVMGLVSENPVTVDDATMIATSF | 433 |
| Sbjct | 368 | E + + + G + A GA + THLDHRIAMSFL +GL SE PVTVDD ++I TSF EFDDGMAITGSREKP AGGAVIETHLDHRIAMSFLTLGLQSEQPVTVDDGSVIETSF | 423 |
| Query | 434 | PEFMDLMAGLGAKI 447 |  |

P F +LM GLGA I Sbjct 424 PIFRELMNGLGANI 437

>TR:A0A1I0EG37 A0A1I0EG37_9RHOB 3-phosphoshikimate 1-carboxyvinyltransferase OS=Paracoccus homiensis OX=364199 GN=aroA PE=3 SV=1

Length=443

Score = 487 bits (1254), Expect = 8e-168

| Identities | | = 253/449 (56%), Positives = 320/449 (71%), Gaps = 9/449 (2%) |  |
| --- | --- | --- | --- |
| Query 1  Sbjct 1 | | MLHGASSRPATARKSSGLSGTVRIPGDKSISHRSFMFGGLASGETRITGLLEGEDVINTG M H A RP TA++S L+GT ++PGDKSISHR+ + G ++ GETRITGLLEG+DV++T MSHSAEPRPMTAKRSGPLTGTAQVPGDKSISHRALILGAMSVGETRITGLLEGQDVLDTA | 60  60 |
| Query 61  Sbjct 61 | | KAMQAMGARIRKEGD-TWIIDGVGNGGLLAPEAPLDFGNAATGCRLTMGLVGVYDFDSTF KAM A GA++ + G W+++GVG GG PE +D GN+ TG RL MG + +TF KAMAAFGAQVERTGPGEWVVNGVGVGGFSEPEGVIDCGNSGTGVRLIMGAMATTPITATF | 119  120 |
| Query 120  Sbjct 121 | | IGDASLTKRPMGRVLNPLREMGVQVKSEDGDRLPVTLRGPKTPTPITYRVPMASAQVKSA GDASL++RPM RV +PL E G + + +G RLP+T++G P P+ YR P+ASAQ+KSA AGDASLSRRPMARVTDPLAEFGASITTREGGRLPITIQGAADPVPLRYRTPVASAQIKSA | 179  180 |
| Query 180  Sbjct 181 | | VLLAGLNTPGITTVIEPIMTRDHTEKMLQGFGANLTVETDADGVRTIRLEGRGKLTGQVI  +LLAGLN PG T VIE TRDH+E+ML GFGA +T ET +G I L GR +L Q + ILLAGLNAPGDTVVIEAEPTRDHSERMLAGFGATITTETTDEG-HVITLTGRPELRAQPV | 239  239 |
| Query 240  Sbjct 240 | | DVPGDPSSTAFPLVAALLVPGSDVTILNVLMNPTRTGLILTLQEMGADIEVINPRLAGGE VP DPSS AFP+ AAL+VPGS++ + V NPTR GL +TL EMGADI N R GGE AVPRDPSSAAFPVAAALIVPGSEIRVPGVSRNPTRDGLYVTLLEMGADIRFENQREEGGE | 299  299 |
| Query 300  Sbjct 300 | | DVADLRVRSSTLKGVTVPEDRAPSMIDEYPILAVAAAFAEGATVMNGLEELRVKESDRLS VADL VR LKGV+VP +RA SMIDE+PIL+V AAFAEGATVMNG+ ELRVKESDR+ PVADLVVRHGPLKGVSVPAERAASMIDEFPILSVIAAFAEGATVMNGVAELRVKESDRID | 359  359 |
| Query 360  Sbjct 360 | | AVANGLKLNGVDCDEGETSLVVRGRPDGKGLGNASGAAVA-THLDHRIAMSFLVMGLVSE A+A GL+ NGV +E S+ V G G+ G AVA THLDHRIAMSF+V+GL ++ AMARGLEANGVRVEETRDSMTVH GAGSVPGGAVAVTHLDHRIAMSFMVLGLAAD | 418  413 |
| Query 419 | | NPVTVDDATMIATSFPEFMDLMAGLGAKI 447 PV+VDD + IATSFP+F+ LM GLG + |  |
| Sbjct | 414 | APVSVDDGSPIATSFPDFLPLMQGLGGDL 442 | |

>TR:A0A2T5G184 A0A2T5G184_9SPHN 3-phosphoshikimate 1-carboxyvinyltransferase OS=Sphingomonas oleivorans OX=1735121 GN=aroA PE=3 SV=1

Length=444

Score = 487 bits (1254), Expect = 8e-168

| Identities | = 265/440 (60%), Positives = 313/440 (71%), Gaps = 5/440 (1%) |  |
| --- | --- | --- |
| Query 8 | RPATARKSSGLSGTVRIPGDKSISHRSFMFGGLASGETRITGLLEGEDVINTGKAMQAMG | 67 |
| Sbjct 7 | RPA LSGTVR+PGDKSISHRS M LA GE+++ GLLEGEDV+ T AM+AMG RPAHFAAPGPLSGTVRVPGDKSISHRSLMLSALAVGESKVDGLLEGEDVLATAAAMRAMG | 66 |
| Query 68 | ARIRKEGD-TWIIDGVGNGGLLAPEAPLDFGNAATGCRLTMGLVGVYDFDSTFIGDASLT | 126 |
| Sbjct 67 | A I + D W I GVG G LL PEA LD GN+ T RL MGLV + +TFIGDASL+ ADIARGTDGRWTIHGVGVGSLLQPEAALDMGNSGTSTRLLMGLVASHPITATFIGDASLS | 126 |
| Query 127 | KRPMGRVLNPLREMGVQVKSEDGDRLPVTLRGPKTPTPITYRVPMASAQVKSAVLLAGLN | 186 |
| Sbjct 127 | KRPMGRV+ PL MG + + G RLP+ +RG PI YR+P+ASAQVKSA+LLAGLN KRPMGRVIEPLGSMGAEFTASPGGRLPLMVRGLAPAVPIEYRLPVASAQVKSAILLAGLN | 186 |
| Query 187 | TPGITTVIEPIMTRDHTEKMLQGFGANLTVETDADGVRTIRLEGRGKLTGQVIDVPGDPS | 246 |

| Sbjct | 187 | TPGIT VIEP+ TRDH+E+ML+GFGA LTVE D G R I + G +L Q I VPGDPS  TPGITRVIEPVPTRDHSERMLRGFGAELTVEQDG-GTRIISVRGEAELKPQHIVVPGDPS | 245 |
| --- | --- | --- | --- |
| Query | 247 | STAFPLVAALLVPGSDVTILNVLMNPTRTGLILTLQEMGADIEVINPRLAGGEDVADLRV | 306 |
| Sbjct | 246 | S FP+VAALLVPGS VT+ NV +NPTR GL L+ MGAD+ N R+ GGE VAD+ V SAGFPVVAALLVPGSSVTVENVGLNPTRAGLFEILKAMGADLRFDNERIVGGEPVADIVV | 305 |
| Query | 307 | RSSTLKGVTVPEDRAPSMIDEYPILAVAAAFAEGATVMNGLEELRVKESDRLSAVANGLK | 366 |
| Sbjct | 306 | R STLKG+ VP + APSMIDE+PIL V AA AEG TV GLEELRVKESDRL+ +A GL+ RHSTLKGIEVPAEIAPSMIDEFPILFVVAALAEGRTVTRGLEELRVKESDRLATMAEGLR | 365 |
| Query | 367 | LNGVDCDEGETSLVVRGRPDGKGLGNASGAAVATHLDHRIAMSFLVMGLVSENPVTVDDA | 426 |
| Sbjct | 366 | G +E E LV+ G G+ L A GA +A LDHRIAMSF V GLVS +PVT+DD AIGARVEEREDGLVIDG-TGGEPL--AGGATIAARLDHRIAMSFAVAGLVSRDPVTIDDM | 422 |
| Query | 427 | TMIATSFPEFMDLMAGLGAK 446  +ATSFP F D++ GLGA+ |  |
| Sbjct | 423 | APVATSFPGFTDMLVGLGAE 442 | |

>TR:D8JRQ8 D8JRQ8_HYPDA 3-phosphoshikimate 1-carboxyvinyltransferase OS=Hyphomicrobium denitrificans (strain ATCC 51888 / DSM 1869 /

NCIB 11706 / TK 0415) OX=582899 GN=aroA PE=3 SV=1

Length=446

Score = 487 bits (1254), Expect = 9e-168

| Identities | = 257/444 (58%), Positives = 316/444 (71%), Gaps = 6/444 (1%) |  |
| --- | --- | --- |
| Query 2 | LHGASSRPATARKSSGLSGTVRIPGDKSISHRSFMFGGLASGETRITGLLEGEDVINTGK | 61 |
| Sbjct 1 | + +++P ++S L G VR+PGDKSISHR+ +FG LA+G TRI GLLE EDV+NT + MKATTAKPLAVMRASPLKGRVRLPGDKSISHRALIFGALATGTTRIRGLLESEDVVNTAR | 60 |
| Query 62 | AMQAMGARIRKEGDTWIIDGVGNGGLLAPEAPLDFGNAATGCRLTMGLVGVYDFDSTFIG | 121 |
| Sbjct 61 | A+ A+GA K GD W + G G GGL P PLDFGN+ TG RL MG++ + G AVSALGAHAEKRGDVWEVKGRGPGGLRQPTEPLDFGNSGTGARLMMGVIAGHPITVQMTG | 120 |
| Query 122 | DASLTKRPMGRVLNPLREMGVQVKSEDGDRLPVTLRGPKTPTPITYRVPMASAQVKSAVL | 181 |
| Sbjct 121 | DASL++RPM RVL PL +MG++V + LP+TLRG PI Y +P+ SAQVKSAVL DASLSRRPMRRVLGPLMQMGLEVIETGRETLPLTLRGTSELIPIVYPLPVPSAQVKSAVL | 180 |
| Query 182 | LAGLNTPGITTVIEPIMTRDHTEKMLQGFGANLTVETDADGVRTIRLEGRGKLTGQVIDV | 241 |
| Sbjct 181 | LAGL++ G TTVIE TRDHTE+ML+ FGAN+ + D +G R I + G +LTG+ I V LAGLHSAGETTVIEHEATRDHTERMLRHFGANVRI-MDKEGGRAITIRGDAELTGRDILV | 239 |
| Query 242 | PGDPSSTAFPLVAALLVPGSDVTILNVLMNPTRTGLILTLQEMGADIEVINPRLAGGEDV | 301 |
| Sbjct 240 | PGDPSS AF + AAL+VPGSDVTI VL+NPTRTGL TLQEMG D+ ++N R GGE + PGDPSSAAFLVAAALIVPGSDVTIEGVLINPTRTGLYTTLQEMGGDVTLLNQREEGGEPI | 299 |
| Query 302 | ADLRVRSSTLKGVTVPEDRAPSMIDEYPILAVAAAFAEGATVMNGLEELRVKESDRLSAV | 361 |
| Sbjct 300 | AD+RVR+S LKGV VP +RAPSMIDEYP+LA +AFA+G T M+GL EL+VKESDRL A ADIRVRASELKGVRVPAERAPSMIDEYPVLAAISAFAKGTTQMDGLAELKVKESDRLQAT | 359 |
| Query 362 | ANGLKLNGVDCDEGETSLVVRGRPDGKGLGNASGAAVATHLDHRIAMSFLVMGLVSENPV | 421 |
| Sbjct 360 | A GL++NGV SL+V G+ KG G VATHLDHRIAM+FL GL SE P+ ATGLEVNGVTARIDGDSLIVEGKQRLKG GGLVATHLDHRIAMAFLTAGLASEKPI | 414 |
| Query 422  Sbjct 415 | TVDDATMIATSFPEFMDLMAGLGA 445 TVDD TMIATSFPEF LM LGA TVDDTTMIATSFPEFRGLMETLGA 438 |  |

>TR:Q0FJ79 Q0FJ79_SALBH 3-phosphoshikimate 1-carboxyvinyltransferase OS=Salipiger bermudensis (strain DSM 26914 / JCM 13377 / KCTC 12554

/ HTCC2601) OX=314265 GN=aroA PE=3 SV=1

Length=447

Score = 487 bits (1254), Expect = 9e-168

| Identities | | = 260/441 (59%), Positives = 309/441 (70%), Gaps = 5/441 (1%) |  |
| --- | --- | --- | --- |
| Query 9  Sbjct 9 | | PATARKSSGLSGTVRIPGDKSISHRSFMFGGLASGETRITGLLEGEDVINTGKAMQAMGA P AR+SSGLSG +PGDKSISHRS + G +A GETR+TGLLEG+DV++T KAM+A GA PMIARRSSGLSGEAHVPGDKSISHRSLILGAMAVGETRVTGLLEGQDVLDTAKAMRAFGA | 68  68 |
| Query 69  Sbjct 69 | | RIRKEGDTWIIDGVGNGGLLAPEAPLDFGNAATGCRLTMGLVGVYDFDSTFIGDASLTKR  + K GDTW ++GVG GG PE +D GN+ TG RL MG + +TF GDASL KR EVEKRGDTWHVNGVGVGGFAEPEQVIDCGNSGTGVRLIMGAMATTPITATFTGDASLNKR | 128  128 |
| Query 129  Sbjct 129 | | PMGRVLNPLREMGVQVKSEDGDRLPVTLRGPKTPTPITYRVPMASAQVKSAVLLAGLNTP PM RV +PL G Q +G RLP+T+ G + P P+ Y P+ SAQVKSAVLLAGLN P PMARVTDPLALFGAQAVGREGGRLPMTIVGARDPLPVRYATPVPSAQVKSAVLLAGLNAP | 188  188 |
| Query 189  Sbjct 189 | | GITTVIEPIMTRDHTEKMLQGFGANLTVETDADGVRTIRLEGRGKLTGQVIDVPGDPSST G T VIE TRDH+E+ML GFGA + E +G R I L G+ +L QVI VP DPSS GETVVIEREATRDHSERMLTGFGAKVVTEETEEG-RVITLTGQPELKPQVIVVPRDPSSA | 248  247 |
| Query 249  Sbjct 248 | | AFPLVAALLVPGSDVTILNVLMNPTRTGLILTLQEMGADIEVINPRLAGGEDVADLRVR- AFP+ AAL+ GSDV + N+ +NPTR GL TLQEMGAD+ NPR GGE VADLR R AFPVCAALITEGSDVLVPNIGLNPTRAGLFFTLQEMGADLTFENPREEGGEPVADLRARF | 307  307 |
| Query 308  Sbjct 308 | | SSTLKGVTVPEDRAPSMIDEYPILAVAAAFAEGATVMNGLEELRVKESDRLSAVANGLKL S LKG+ VP +RA SMIDEYP+L+V A+FAEG T M G++ELRVKESDR+ A+A GL+ SPDLKGIEVPAERAASMIDEYPVLSVVASFAEGKTHMPGVKELRVKESDRIDAMATGLRA | 367  367 |
| Query 368  Sbjct 368 | | NGVDCDEGETSLVVRGRPDGKGLGNASGAAVATHLDHRIAMSFLVMGLVSENPVTVDDAT NGV+ DEGE V GR G G GA A+HLDHRIAMSFLVMGL + PV VDD NGVEVDEGEDWWTVHGRGHGNVPG---GAETASHLDHRIAMSFLVMGLATTEPVRVDDGG | 427  424 |
| Query 428 | | MIATSFPEFMDLMAGLGAKIE 448 IATSFP F LM+GLGA IE |  |
| Sbjct | 425 | PIATSFPIFEPLMSGLGAVIE 445 | |

>TR:A0A086XVY0 A0A086XVY0_9RHOB 3-phosphoshikimate 1-carboxyvinyltransferase OS=Paenirhodobacter enshiensis OX=1105367 GN=aroA PE=3 SV=1

Length=449

Score = 487 bits (1254), Expect = 1e-167

| Identities | | = 258/440 (59%), Positives = 310/440 (70%), Gaps = 5/440 (1%) |  |
| --- | --- | --- | --- |
| Query 9  Sbjct 9 | | PATARKSSGLSGTVRIPGDKSISHRSFMFGGLASGETRITGLLEGEDVINTGKAMQAMGA P ARK + L G +PGDKSISHR+ + G L+ G T ITGLLEG+DV++T KAMQA GA PMIARKGAALCGVAEVPGDKSISHRALILGALSVGRTEITGLLEGQDVLDTAKAMQAFGA | 68  68 |
| Query 69 | | RIRKEGDT-WIIDGVGNGGLLAPEAPLDFGNAATGCRLTMGLVGVYDFDSTFIGDASLTK  + + G WI+DGVG GG P +D GN+ TG RL MG + ++TF GDASL K | 127 |
| Sbjct | 69 | TVERLGPGHWIVDGVGVGGFAEPADVIDCGNSGTGVRLIMGAMATSPINATFTGDASLRK | 128 |
| Query | 128 | RPMGRVLNPLREMGVQVKSEDGDRLPVTLRGPKTPTPITYRVPMASAQVKSAVLLAGLNT | 187 |
| Sbjct | 129 | RPMGRV +PL G Q +G RLP+TL G + P P+ Y +P+ SAQVKSAVLLAGLN RPMGRVTDPLALFGAQAVGREGGRLPLTLVGARNPVPVRYVLPVPSAQVKSAVLLAGLNA | 188 |
| Query | 188 | PGITTVIEPIMTRDHTEKMLQGFGANLTVETDADGVRTIRLEGRGKLTGQVIDVPGDPSS | 247 |
| Sbjct | 189 | PG T V+E TRDHTE+ML+GFGA L+VE +G R I L+GR +LTGQ + VP DPSS PGQTVVVEAEATRDHTERMLKGFGAELSVEDTPEG-RVITLQGRPELTGQSVAVPRDPSS | 247 |
| Query | 248 | TAFPLVAALLVPGSDVTILNVLMNPTRTGLILTLQEMGADIEVINPRLAGGEDVADLRVR | 307 |
| Sbjct | 248 | AFP+ AAL+VPGS++ + V NPTR GL TL EMGADI NPR GGE VADLRVR  AAFPVCAALIVPGSEIVVPGVSQNPTRNGLFTTLVEMGADIAFENPREEGGEPVADLRVR | 307 |

| Query | 308 | SSTLKGVTVPEDRAPSMIDEYPILAVAAAFAEGATVMNGLEELRVKESDRLSAVANGLKL | 367 |
| --- | --- | --- | --- |
| Sbjct | 308 | S LKG+ VP +RA SMIDEYP+LA A+ AEG TVM G++ELRVKESDR+ A+A GL+ HSALKGIEVPAERAASMIDEYPVLAAVASLAEGTTVMRGVKELRVKESDRIDAMARGLEA | 367 |
| Query | 368 | NGVDCDEGETSLVVRGRPDGKGLGNASGAAVATHLDHRIAMSFLVMGLVSENPVTVDDAT | 427 |
| Sbjct | 368 | GV +E LVV GR G GA VATH+DHRIAMSFLV+GLV++ PV VDDA+ CGVTVEEERDVLVVHGR---GAAGVPGGAIVATHIDHRIAMSFLVLGLVTDRPVAVDDAS | 424 |
| Query | 428 | MIATSFPEFMDLMAGLGAKI 447 IATSFP F DLM GLGA + |  |
| Sbjct | 425 | PIATSFPVFGDLMRGLGADV 444 | |

>TR:A0A1G5LB09 A0A1G5LB09_9RHIZ 3-phosphoshikimate 1-carboxyvinyltransferase OS=Microvirga guangxiensis OX=549386 GN=aroA PE=3 SV=1

Length=448

Score = 487 bits (1253), Expect = 1e-167

| Identities | = 268/448 (60%), Positives = 321/448 (72%), Gaps = 7/448 (2%) |  |
| --- | --- | --- |
| Query 3 | HGASSRPATARKSSGLSGTVRIPGDKSISHRSFMFGGLASGETRITGLLEGEDVINTGKA | 62 |
| Sbjct 5 | HG ++ P ++R + L G +R+PGDKSISHRS +FG L+ GETR+ GLLEG+DV+ T +A HGPAT-PVSSRSGNALKGRIRVPGDKSISHRSMIFGLLSIGETRVEGLLEGDDVLRTAEA | 63 |
| Query 63 | MQAMGARIRKEGD-TWIIDGVGNGGLLAPEAPLDFGNAATGCRLTMGLVGVYDFDSTFIG | 121 |
| Sbjct 64 | +A+GA I +E W I G+G GGL+ P+ LDFGNA TG RL MG+ G + +TF G CRALGATINREAPGRWRIKGMGIGGLVEPKDILDFGNAGTGSRLMMGVAGSHPITATFDG | 123 |
| Query 122 | DASLTKRPMGRVLNPLREMGVQVKSE-DGDRLPVTLRGPKTPTPITYRVPMASAQVKSAV | 180 |
| Sbjct 124 | DASL KRPM R+L+PL +MGV V SE +G R+P+TLRGPK PITY P ASAQ+KSA+ DASLRKRPMRRILDPLVQMGVTVVSEAEGGRVPLTLRGPKETLPITYETPAASAQIKSAI | 183 |
| Query 181 | LLAGLNTPGITTVIEPIMTRDHTEKMLQGFGANLTVETDADGVRTIRLEGRGKLTGQVID | 240 |
| Sbjct 184 | LLAGLN+PG TTVIE TRDHTE+ML+ FGA + V R + L G+ +L G I LLAGLNSPGKTTVIEREATRDHTERMLRHFGATVEVAPHGADGRAVTLHGQPELRGTEIV | 243 |
| Query 241 | VPGDPSSTAFPLVAALLVPGSDVTILNVLMNPTRTGLILTLQEMGADIEVINPRLAGGED | 300 |
| Sbjct 244 | VP DPSS AFPLVAAL+ PGSD+ I V+MNP RTGLI TL EMGA IE +N R GGE VPTDPSSAAFPLVAALITPGSDIVIEGVMMNPLRTGLITTLLEMGASIERLNERDEGGET | 303 |
| Query 301 | VADLRVRSSTLKGVTVPEDRAPSMIDEYPILAVAAAFAEGATVMNGLEELRVKESDRLSA | 360 |
| Sbjct 304 | VADLRVR S L GVTVP RAP+MIDEYPILAVAAAFAEG T M GL ELRVKESDRL+A VADLRVRHSKLTGVTVPHARAPAMIDEYPILAVAAAFAEGTTRMQGLHELRVKESDRLAA | 363 |
| Query 361 | VANGLKLNGVDCDEGETSLVVRGRPDGKGLGNASGAAVATHLDHRIAMSFLVMGLVSENP | 420 |

VA GL	GV+ +	L+V G	+G GN	V THLDHRIAM+FLVMGL S+

| Sbjct | 364 | VAAGLAEAGVEHEIDGDDLIVHGSRTVRGGGN PVKTHLDHRIAMAFLVMGLASQEA | 419 |
| --- | --- | --- | --- |
| Query  Sbjct | 421  420 | VTVDDATMIATSFPEFMDLMAGLGAKIE 448  ++VDD MIATSFP F+ LM LGA I+ MSVDDGAMIATSFPSFIPLMRELGAVIQ 447 |  |

```
>TR:A0A4R1UXI2 A0A4R1UXI2_9SPHN 3-phosphoshikimate 1-carboxyvinyltransferase OS=Novosphingobium sp. PhB165 OX=2485105 GN=aroA PE=3 SV=1
Length=448

Score = 487 bits (1253), Expect = 1e-167
Identities = 258/446 (58%), Positives = 315/446 (71%), Gaps = 3/446 (1%)

Query 1	MLHGASS--RPATARKSSGLSGTVRIPGDKSISHRSFMFGGLASGETRITGLLEGEDVIN 58 M H +S RP	+ + G +R+PGDKSISHRS M G LA GETR+TGLLEGEDV+
Sbjct 1	MSHDPASPMRPRRFLPAGPIKGRIRVPGDKSISHRSIMLGALAVGETRVTGLLEGEDVLA 60
```

| Query | 59 | TGKAMQAMGARI-RKEGDTWIIDGVGNGGLLAPEAPLDFGNAATGCRLTMGLVGVYDFDS | 117 |
| --- | --- | --- | --- |
| Sbjct | 61 | T AM+AMGA + R E W + GVG G L P PLD GN+ T RL MGLV + + TAAAMRAMGASVERMEDGEWRVSGVGVGALHQPTGPLDMGNSGTSTRLLMGLVSSHPINV | 120 |
| Query | 118 | TFIGDASLTKRPMGRVLNPLREMGVQVKSEDGDRLPVTLRGPKTPTPITYRVPMASAQVK | 177 |
| Sbjct | 121 | +F+GD SL+KRPMGRV++PL EMG ++ +G RLP+ +RG PITYR+P+ASAQVK SFVGDESLSKRPMGRVIDPLSEMGASFEASEGGRLPLIVRGASPAVPITYRLPVASAQVK | 180 |
| Query | 178 | SAVLLAGLNTPGITTVIEPIMTRDHTEKMLQGFGANLTVETDADGVRTIRLEGRGKLTGQ | 237 |
| Sbjct | 181 | SA+LLAGLNTPG TTVIEP+ TRDH+E+ML+GFGA L V +ADG R I ++G +L Q SAILLAGLNTPGTTTVIEPVPTRDHSERMLKGFGAELDVTVEADGTRVITIKGEAELKPQ | 240 |
| Query | 238 | VIDVPGDPSSTAFPLVAALLVPGSDVTILNVLMNPTRTGLILTLQEMGADIEVINPRLAG | 297 |
| Sbjct | 241 | VIDVPGDPSS AF +VAAL+VPGS+V I NV +NPTR GL+ L++MG IE +N R G VIDVPGDPSSAAFFIVAALVVPGSEVVIENVGLNPTRAGLVEVLRQMGGQIEFLNSREVG | 300 |
| Query | 298 | GEDVADLRVRSSTLKGVTVPEDRAPSMIDEYPILAVAAAFAEGATVMNGLEELRVKESDR | 357 |
| Sbjct | 301 | GE VADL V+ S LKG+ V AP+MIDE+P+L VAA+ AEG TV +GL+ELRVKESDR GEPVADLLVKHSALKGIEVDPAIAPAMIDEFPVLFVAASLAEGVTVTSGLDELRVKESDR | 360 |
| Query | 358 | LSAVANGLKLNGVDCDEGETSLVVRGRPDGKGLGNASGAAVATHLDHRIAMSFLVMGLVS | 417 |
| Sbjct | 361 | LSA+A L L G + +E E LV+ G G A+ A V THLDHRIAMS V GLVS LSAMAAALTLAGAEIEEREDGLVITGTGGAPLPGTATAAQVTTHLDHRIAMSMAVAGLVS | 420 |
| Query  Sbjct | 418  421 | ENPVTVDDATMIATSFPEFMDLMAGL 443  + V VDD IATSFP F DL+A L QQGVDVDDTRPIATSFPNFEDLLASL 446 |  |

>TR:A0A086MES2 A0A086MES2_9RHIZ 3-phosphoshikimate 1-carboxyvinyltransferase OS=Microvirga sp. BSC39 OX=1549810 GN=aroA PE=3 SV=1

Length=449

Score = 487 bits (1253), Expect = 1e-167

| Identities | | = 271/448 (60%), Positives = 318/448 (71%), Gaps = 6/448 (1%) |  |
| --- | --- | --- | --- |
| Query 3  Sbjct 5 | | HGASSRPATARKSSGLSGTVRIPGDKSISHRSFMFGGLASGETRITGLLEGEDVINTGKA HG S P +R + L G +R+PGDKSISHRS +FG L+ GETR+ GLLEG+DV+ T +A HGTPS-PVASRSGTPLKGRIRVPGDKSISHRSMIFGLLSIGETRVEGLLEGDDVLRTAEA | 62  63 |
| Query 63  Sbjct 64 | | MQAMGARIRKEGD-TWIIDGVGNGGLLAPEAPLDFGNAATGCRLTMGLVGVYDFDSTFIG  +A+GA I +E W I GVG GGL P+ LDFGNA TG RL MG+ G + STF G CRALGATITREAPGRWRIVGVGIGGLTEPKDVLDFGNAGTGSRLMMGVAGSHTITSTFDG | 121  123 |
| Query 122  Sbjct 124 | | DASLTKRPMGRVLNPLREMGVQVKSE-DGDRLPVTLRGPKTPTPITYRVPMASAQVKSAV DASL KRPM R+L+PL +MGV V SE +G R+P+TLRGP+ PITY P ASAQ+KSA+ DASLRKRPMRRILDPLVQMGVTVVSEAEGGRVPLTLRGPRETLPITYETPAASAQIKSAI | 180  183 |
| Query 181  Sbjct 184 | | LLAGLNTPGITTVIEPIMTRDHTEKMLQGFGANLTVETDADGVRTIRLEGRGKLTGQVID LLAGLN+PG TTVIE TRDHTE+ML+ FGA++ V + R I L+G+ +L G I LLAGLNSPGKTTVIEREATRDHTERMLRHFGASVEVASHGADGRAITLQGQPELRGTDIV | 240  243 |
| Query 241  Sbjct 244 | | VPGDPSSTAFPLVAALLVPGSDVTILNVLMNPTRTGLILTLQEMGADIEVINPRLAGGED VP DPSS AFPLVAAL+ PGSDV I V+MNP RTGLI TL EMGA IE +N R GGE VPTDPSSAAFPLVAALITPGSDVLIEGVMMNPLRTGLITTLIEMGASIERLNERNEGGET | 300  303 |
| Query 301  Sbjct 304 | | VADLRVRSSTLKGVTVPEDRAPSMIDEYPILAVAAAFAEGATVMNGLEELRVKESDRLSA VADLRV+ S L GVTVP RAP+MIDEYPILAVAA+FAEG T M GL ELRVKESDRL+A VADLRVQHSRLTGVTVPHARAPAMIDEYPILAVAASFAEGTTRMQGLHELRVKESDRLAA | 360  363 |
| Query 361 | | VANGLKLNGVDCDEGETSLVVRGRPDGKGLGNASGAAVATHLDHRIAMSFLVMGLVSENP VA GL GV + L+V G G G VATHLDHRIAMSFLVMGL + P | 420 |
| Sbjct | 364 | VAAGLAEAGVKYEIEGDDLIVHG---SGGKVRGGGKPVATHLDHRIAMSFLVMGLATTEP | 420 |

Query 421 VTVDDATMIATSFPEFMDLMAGLGAKIE 448

+TVDD MI TSFP F+ LM LGA I+ Sbjct 421 MTVDDGAMIVTSFPSFIPLMRELGATIQ 448

>TR:A0A4V1N3G9 A0A4V1N3G9_9SPHN 3-phosphoshikimate 1-carboxyvinyltransferase OS=Sphingobium fluviale OX=2506423 GN=aroA PE=3 SV=1

Length=441

Score = 486 bits (1252), Expect = 2e-167

| Identities | = 263/440 (60%), Positives = 315/440 (72%), Gaps = 5/440 (1%) |  |
| --- | --- | --- |
| Query 8 | RPAT-ARKSSGLSGTVRIPGDKSISHRSFMFGGLASGETRITGLLEGEDVINTGKAMQAM | 66 |
| Sbjct 3 | RPA S GLSGT+ +PGDKSISHR+ MF LA G +R+ GLLEGEDV++T A++AM RPAMFTAPSRGLSGTISVPGDKSISHRALMFSALAVGRSRVEGLLEGEDVLSTAAALRAM | 62 |
| Query 67 | GARIRKEGD-TWIIDGVGNGGLLAPEAPLDFGNAATGCRLTMGLVGVYDFDSTFIGDASL | 125 |
| Sbjct 63 | GARI ++ D W IDGVG GGLL PE LD GN+ T RL MGL+ +D +TFIGDASL GARIERDADGVWHIDGVGVGGLLQPETALDMGNSGTSTRLLMGLLASHDLTATFIGDASL | 122 |
| Query 126 | TKRPMGRVLNPLREMGVQVKSEDGDRLPVTLRGPKTPTPITYRVPMASAQVKSAVLLAGL | 185 |
| Sbjct 123 | +KRPMGRV PL MG + S G RLP+T+RG PI Y +P+ASAQVKSAVLLAGL SKRPMGRVTEPLSLMGAEFVSSPGGRLPLTMRGAVPAVPIEYCLPVASAQVKSAVLLAGL | 182 |
| Query 186 | NTPGITTVIEPIMTRDHTEKMLQGFGANLTVETDADGVRTIRLEGRGKLTGQVIDVPGDP | 245 |
| Sbjct 183 | NTPGIT VIEP TRDH+E+ML GFGA+LT+E DADG R I + G +L Q I VPGDP NTPGITRVIEPEATRDHSERMLAGFGADLTIERDADGARIISIRGEAELKPQQIIVPGDP | 242 |
| Query 246 | SSTAFPLVAALLVPGSDVTILNVLMNPTRTGLILTLQEMGADIEVINPRLAGGEDVADLR | 305 |
| Sbjct 243 | SS AFP+VAAL+VPGSDVTI V MN TRTGLI LQ MGA I+++NPR AGGE VAD+R SSAAFPIVAALIVPGSDVTITGVGMNETRTGLIKLLQAMGAQIDLLNPRDAGGEPVADVR | 302 |
| Query 306 | VRSSTLKGVTVPEDRAPSMIDEYPILAVAAAFAEGATVMNGLEELRVKESDRLSAVANGL | 365 |
| Sbjct 303 | VR+S+L + APSMIDE+PI +AAA A+G +V GLEELRVKESDR++ +A GL VRASSLTAIENDPADAPSMIDEFPIAFIAAALAKGRSVFRGLEELRVKESDRIAVMAAGL | 362 |
| Query 366 | KLNGVDCDEGETSLVVRGRPDGKGLGNASGAAVATHLDHRIAMSFLVMGLVSENPVTVDD | 425 |

+ G	+E	L++ G	G+ L	G V THLDHRIAMSF V GL S	VT+DD

| Sbjct | 363 | RQIGAMVEETPDGLIIEG-TGGEPL--EGGGPVETHLDHRIAMSFAVAGLASYKGVTIDD | 419 |
| --- | --- | --- | --- |
| Query | 426 | ATMIATSFPEFMDLMAGLGA 445  +ATSFP F+D+M LGA |  |
| Sbjct | 420 | MRPVATSFPGFVDMMRALGA 439 | |

>TR:A0A4V1KJ23 A0A4V1KJ23_9RHIZ 3-phosphoshikimate 1-carboxyvinyltransferase OS=Hansschlegelia zhihuaiae OX=405005 GN=aroA PE=3 SV=1

Length=452

Score = 487 bits (1253), Expect = 2e-167

| Identities | = 266/443 (60%), Positives = 320/443 (72%), Gaps = 6/443 (1%) |  |
| --- | --- | --- |
| Query 9 | PATARKSSGLSGTVRIPGDKSISHRSFMFGGLASGETRITGLLEGEDVINTGKAMQAMGA | 68 |
| Sbjct 8 | PA ARKS LSGTVR PGDKSISHRS + G LA GETRI GLLEGEDV+ T + +A+GA PARARKSRALSGTVRAPGDKSISHRSLILGLLAVGETRIEGLLEGEDVLATARGAKALGA | 67 |
| Query 69 | RIRKEGD-TWIIDGVGNGGLLAPEAPLDFGNAATGCRLTMGLVGVYDFDSTFIGDASLTK | 127 |
| Sbjct 68 | R+ + G+ +W + GVG GGL P LDFGNA TG RL MG+VG ++TF GDASL RVERLGEGSWRVHGVGVGGLREPLETLDFGNAGTGSRLMMGVVGGSPIEATFDGDASLRS | 127 |
| Query 128 | RPMGRVLNPLREMGVQVKSE-DGDRLPVTLRGPKTPTPITYRVPMASAQVKSAVLLAGLN | 186 |
| Sbjct 128 | RPM R+L+PL +MG V S +G R+P+T+RGP+ PI Y P SAQ+KSAVLLAGLN  RPMRRILDPLEKMGATVVSAAEGGRVPLTIRGPERAIPIVYETPAPSAQIKSAVLLAGLN | 187 |

| Query | 187 | TPGITTVIEPIMTRDHTEKMLQGFGANLTVETDADGVRTIRLEGRGKLTGQVIDVPGDPS | 246 |
| --- | --- | --- | --- |
| Sbjct | 188 | PG T VIE TRDHTE+ML+ FGA ++VE R IRL+G +L I VP DPS APGETVVIEREATRDHTERMLRAFGAEVSVEPHGPHGRLIRLKGEPELKPAAIAVPADPS | 247 |
| Query | 247 | STAFPLVAALLVPGSDVTILNVLMNPTRTGLILTLQEMGADIEVINPRLAGGEDVADLRV | 306 |
| Sbjct | 248 | S AFP+ AAL+V GS+VT+ V++NP R+GL+ TL EMGADI + N R GGE+VADL V SAAFPMAAALVVEGSEVTLEGVMINPLRSGLLTTLLEMGADIRLSNQREEGGEEVADLVV | 307 |
| Query | 307 | RSSTLKGVTVPEDRAPSMIDEYPILAVAAAFAEGATVMNGLEELRVKESDRLSAVANGLK | 366 |
| Sbjct | 308 | R+ L+GV VP +RAP+MIDEYPILA+ AAFAEG T M GL ELRVKESDRL+AV GL RAGALRGVDVPPERAPTMIDEYPILAIIAAFAEGETRMRGLHELRVKESDRLAAVHAGLL | 367 |
| Query | 367 | LNGVDCDEGETSLVVRGRPDGKGLGNASGAAVATHLDHRIAMSFLVMGLVSENPVTVDDA | 426 |
| Sbjct | 368 | GV+ LVV G+G G AVATHLDHRIAMSFLVMGL ++NPV +DDA ACGVEARIEGDDLVV TGEGRVPGGGPAVATHLDHRIAMSFLVMGLGAQNPVGIDDA | 423 |
| Query | 427 | TMIATSFPEFMDLMAGLGAKIEL 449 MIATSFP+F++LM GLGA+IE+ |  |
| Sbjct | 424 | AMIATSFPDFVELMRGLGAEIEI 446 | |

>TR:A0A0N9ZEP8 A0A0N9ZEP8_9RHOB 3-phosphoshikimate 1-carboxyvinyltransferase OS=Celeribacter marinus OX=1397108 GN=aroA PE=3 SV=1

Length=448

Score = 486 bits (1252), Expect = 2e-167

| Identities | | = 254/440 (58%), Positives = 309/440 (70%), Gaps = 4/440 (1%) |  |
| --- | --- | --- | --- |
| Query 9  Sbjct 9 | | PATARKSSGLSGTVRIPGDKSISHRSFMFGGLASGETRITGLLEGEDVINTGKAMQAMGA P +RK L GT +PGDKSISHR+ +FG LA GET+ITGLLEGEDV++TGKAMQA+GA PMISRKCGPLKGTAAVPGDKSISHRALIFGALAVGETKITGLLEGEDVLDTGKAMQALGA | 68  68 |
| Query 69  Sbjct 69 | | RIRKEGD-TWIIDGVGNGGLLAPEAPLDFGNAATGCRLTMGLVGVYDFDSTFIGDASLTK  + + G+ W + GVG GG PE +D GN+ TG RL MGLV YDF +TF GD SL K DVIRHGEGAWSVHGVGVGGFSEPEQVIDCGNSGTGVRLIMGLVSTYDFATTFTGDGSLRK | 127  128 |
| Query 128  Sbjct 129 | | RPMGRVLNPLREMGVQVKSEDGDRLPVTLRGPKTPTPITYRVPMASAQVKSAVLLAGLNT RPM R+ +PL E G G RLP+TL G TP P+TY PM SAQVK+AVL AGLNT RPMARITDPLAEFGATSYGRSGGRLPLTLVGTATPVPVTYETPMPSAQVKTAVLFAGLNT | 187  188 |
| Query 188  Sbjct 189 | | PGITTVIEPIMTRDHTEKMLQGFGANLTVETDADGVRTIRLEGRGKLTGQVIDVPGDPSS PG T VIE TRDHTE+ML+GFGA++ ET +G I L+G+ +L QV+ VP DPSS PGETVVIEKEATRDHTERMLKGFGADIRTETTPEGYNKITLKGQPELKPQVVAVPRDPSS | 247  248 |
| Query 248  Sbjct 249 | | TAFPLVAALLVPGSDVTILNVLMNPTRTGLILTLQEMGADIEVINPRLAGGEDVADLRVR AFP+ AAL+V GSD+ + V N TR G+ TL EMGADI NPR GGE VADLRV+ AAFPVCAALIVSGSDILVPGVSQNTTRNGIYTTLVEMGADITFENPREEGGEPVADLRVK | 307  308 |
| Query 308  Sbjct 309 | | SSTLKGVTVPEDRAPSMIDEYPILAVAAAFAEGATVMNGLEELRVKESDRLSAVANGLKL S L GV VP +RA SMIDEYP+L+V A+FA G T M G++ELRVKESDR+ A+A GL+ YSNLHGVEVPPERAASMIDEYPVLSVVASFATGTTTMRGVKELRVKESDRIDAMARGLEA | 367  368 |
| Query 368  Sbjct 369 | | NGVDCDEGETSLVVRGRPDGKGLGNASGAAVATHLDHRIAMSFLVMGLVSENPVTVDDAT GV+ +E E + +V GR G GA ATH+DHRIAMSFLV G+ S++PV +DD + CGVEIEEDEDTFIVHGRGAN---GVKGGATCATHIDHRIAMSFLVCGMASQDPVAIDDGS | 427  425 |
| Query 428 | | MIATSFPEFMDLMAGLGAKI 447 IATSFP F LMA LGA+I |  |
| Sbjct | 426 | PIATSFPVFERLMADLGAEI 445 | |

>TR:A0A1B2EID6 A0A1B2EID6_9RHIZ 3-phosphoshikimate 1-carboxyvinyltransferase OS=Microvirga ossetica OX=1882682 GN=aroA PE=3 SV=1

Length=449

Score = 486 bits (1252), Expect = 2e-167

| Identities | | = 272/448 (61%), Positives = 318/448 (71%), Gaps = 6/448 (1%) |  |
| --- | --- | --- | --- |
| Query 3  Sbjct 5 | | HGASSRPATARKSSGLSGTVRIPGDKSISHRSFMFGGLASGETRITGLLEGEDVINTGKA HG S P +R + L G +R+PGDKSISHRS +FG L+ GETR+ GLLEG+DV+ T +A HGTPS-PVASRPGTALKGRLRVPGDKSISHRSMIFGLLSIGETRVDGLLEGDDVLRTAEA | 62  63 |
| Query 63  Sbjct 64 | | MQAMGARIRKEGD-TWIIDGVGNGGLLAPEAPLDFGNAATGCRLTMGLVGVYDFDSTFIG  +A+GA I +E W I GVG GGL P+ LDFGNA TG RL MG+ G + STF G CRALGATITREAPGRWRIVGVGIGGLTEPKDVLDFGNAGTGSRLMMGVAGSHPITSTFDG | 121  123 |
| Query 122  Sbjct 124 | | DASLTKRPMGRVLNPLREMGVQVKSE-DGDRLPVTLRGPKTPTPITYRVPMASAQVKSAV DASL KRPM R+++PL +MGV V SE +G R+P+TLRGPK PITY P ASAQ+KSA+ DASLRKRPMRRIVDPLVKMGVTVVSEAEGGRVPLTLRGPKETIPITYETPAASAQIKSAI | 180  183 |
| Query 181  Sbjct 184 | | LLAGLNTPGITTVIEPIMTRDHTEKMLQGFGANLTVETDADGVRTIRLEGRGKLTGQVID LLAGLN+PG TTVIE TRDHTE+ML+ FGA++ V R I L+G+ +L G I LLAGLNSPGKTTVIEREATRDHTERMLRHFGASVEVAPHGADGRAITLQGQPELRGTDIV | 240  243 |
| Query 241  Sbjct 244 | | VPGDPSSTAFPLVAALLVPGSDVTILNVLMNPTRTGLILTLQEMGADIEVINPRLAGGED VP DPSS AFPLVAAL+ PGSDV I V+MNP RTGLI TL EMGA IE +N R GGE VPTDPSSAAFPLVAALITPGSDVVIEGVMMNPLRTGLITTLIEMGASIERLNERDEGGET | 300  303 |
| Query 301  Sbjct 304 | | VADLRVRSSTLKGVTVPEDRAPSMIDEYPILAVAAAFAEGATVMNGLEELRVKESDRLSA VADLRVR S L GVTVP RAPSMIDEYPILAVAA+FA+G T M GL ELRVKESDRL+A VADLRVRHSKLTGVTVPHARAPSMIDEYPILAVAASFAQGTTRMQGLHELRVKESDRLAA | 360  363 |
| Query 361 | | VANGLKLNGVDCDEGETSLVVRGRPDGKGLGNASGAAVATHLDHRIAMSFLVMGLVSENP VA GL GV + L+V G G G VATHLDHRIAM+FLVMGL + P | 420 |
| Sbjct | 364 | VAAGLAEAGVAHEIEGDDLLVHG---SNGKVRGGGRPVATHLDHRIAMAFLVMGLATAEP | 420 |
| Query  Sbjct | 421  421 | VTVDDATMIATSFPEFMDLMAGLGAKIE 448  +TVDD MIATSFP F+ LM LGA I+ MTVDDGAMIATSFPSFIPLMRELGATIQ 448 |  |

>TR:A8IKN4 A8IKN4_AZOC5 3-phosphoshikimate 1-carboxyvinyltransferase OS=Azorhizobium caulinodans (strain ATCC 43989 / DSM 5975 / JCM

20966 / NBRC 14845 / NCIMB 13405 / ORS 571) OX=438753 GN=aroA PE=3 SV=1

Length=471

Score = 487 bits (1254), Expect = 2e-167

| Identities | | = 268/447 (60%), Positives = 315/447 (70%), Gaps = 7/447 (2%) |  |
| --- | --- | --- | --- |
| Query 3  Sbjct 29 | | HGASSRPATARKSSGLSGTVRIPGDKSISHRSFMFGGLASGETRITGLLEGEDVINTGKA HG+ +PAT S GLSG VR+PGDKS+SHR+ +FG LA GETR+TGLLEGEDV+NT KA HGSDPKPATGLPSPGLSGKVRVPGDKSVSHRALIFGALARGETRVTGLLEGEDVLNTAKA | 62  88 |
| Query 63  Sbjct 89 | | MQAMGARIRKEGD-TWIIDGVGNGGLLAPEAPLDFGNAATGCRLTMGLVGVYDFDSTFIG A+GA + + G W ++GVG G AP APLDFGN+ TG RL MG V +TF G CAALGATVERTGPGQWRVEGVGVGNFKAPAAPLDFGNSGTGVRLMMGAVAGNPIQATFDG | 121  148 |
| Query 122  Sbjct 149 | | DASLTKRPMGRVLNPLREMGVQVKSE-DGDRLPVTLRGPKTPTPITYRVPMASAQVKSAV DASL KRPM RVL+PL MGV++ E +G RLP+ L G TY P+ SAQVKSAV DASLRKRPMKRVLDPLAAMGVKLVEEAEGGRLPMRLEGVTDLKAFTYETPVPSAQVKSAV | 180  208 |
| Query 181 | | LLAGLNTPGITTVIEPIMTRDHTEKMLQGFGANLTVETDADGVRTIRLEGRGKLTGQVID LLAGL G T V+E TRDHTE+ML FGA+++VE R I L+GR +L +D | 240 |
| Sbjct | 209 | LLAGLAASGETVVVEREATRDHTERMLAHFGADVSVEPHGAHGRRITLKGRPELKAAPVD | 268 |
| Query | 241 | VPGDPSSTAFPLVAALLVPGSDVTILNVLMNPTRTGLILTLQEMGADIEVINPRLAGGED | 300 |

| Sbjct | 269 | VP DPSS AFPLVAA++VPGSD+ I +V+MNP RTGLI TL+EMGA IE + R GGE  VPSDPSSAAFPLVAAVIVPGSDIVITDVMMNPLRTGLITTLKEMGASIETLAERTEGGET | 328 |
| --- | --- | --- | --- |
| Query | 301 | VADLRVRSSTLKGVTVPEDRAPSMIDEYPILAVAAAFAEGATVMNGLEELRVKESDRLSA | 360 |
| Sbjct | 329 | VADLRVR S LKGV VP +RAP+MIDEYPILAVAAAFAEG T M GL ELRVKESDRL+A VADLRVRYSKLKGVAVPPERAPAMIDEYPILAVAAAFAEGETRMQGLAELRVKESDRLAA | 388 |
| Query | 361 | VANGLKLNGVDCDEGETSLVVRGRPDGKGLGNASGAAVATHLDHRIAMSFLVMGLVSENP | 420 |
| Sbjct | 389 | VA+GL GV L+V G D +G G VATH+DHRIAMSFLV+GL S VADGLAACGVTHRIEGDDLIVTGAADVEG GGPVATHMDHRIAMSFLVLGLASRAG | 443 |
| Query  Sbjct | 421  444 | VTVDDATMIATSFPEFMDLMAGLGAKI 447 V VDD + IATSFP FM ++AGLGA I VKVDDVSFIATSFPTFMPMIAGLGAII 470 |  |

>TR:A0A1V0GUQ8 A0A1V0GUQ8_9RHOB 3-phosphoshikimate 1-carboxyvinyltransferase OS=Paracoccus yeei OX=147645 GN=aroA PE=3 SV=1

Length=443

Score = 486 bits (1251), Expect = 2e-167

| Identities | | = 255/449 (57%), Positives = 314/449 (70%), Gaps = 7/449 (2%) |  |
| --- | --- | --- | --- |
| Query 1  Sbjct 1 | | MLHGASSRPATARKSSGLSGTVRIPGDKSISHRSFMFGGLASGETRITGLLEGEDVINTG M H A P TAR+S LSGT +PGDKSISHR+ + G L+ GET ITGLLEG+DV++T MSHSAEPMPMTARRSGPLSGTANVPGDKSISHRALILGALSVGETHITGLLEGQDVLDTA | 60  60 |
| Query 61  Sbjct 61 | | KAMQAMGARIRKEGD-TWIIDGVGNGGLLAPEAPLDFGNAATGCRLTMGLVGVYDFDSTF KAMQA GA + + G W ++GVG GG PE +D GN+ TG RL MG + +TF KAMQAFGATVERLGTGEWKVNGVGVGGFAEPEGVIDCGNSGTGVRLIMGAMATTPISATF | 119  120 |
| Query 120  Sbjct 121 | | IGDASLTKRPMGRVLNPLREMGVQVKSEDGDRLPVTLRGPKTPTPITYRVPMASAQVKSA GDASL++RPMGRV +PL G ++ + +G RLPVT+RG P P+ Y+ P+ASAQ+KSA TGDASLSRRPMGRVTDPLELFGAEITAREGKRLPVTIRGAADPVPVRYKTPVASAQIKSA | 179  180 |
| Query 180  Sbjct 181 | | VLLAGLNTPGITTVIEPIMTRDHTEKMLQGFGANLTVETDADGVRTIRLEGRGKLTGQVI VLLAGLN PG T VIE TRDHTE+ML GFGA +T E +G R I L GR +L Q + VLLAGLNAPGETVVIESEPTRDHTERMLAGFGATITTEVTEEG-RVITLTGRPELKPQPV | 239  239 |
| Query 240  Sbjct 240 | | DVPGDPSSTAFPLVAALLVPGSDVTILNVLMNPTRTGLILTLQEMGADIEVINPRLAGGE VP DPSS AFP+ AAL+VPGS++ + V NPTR GL +TL EMGADI NPR GGE AVPRDPSSAAFPVAAALIVPGSEIRVPGVSRNPTRDGLYVTLLEMGADITFENPREEGGE | 299  299 |
| Query 300  Sbjct 300 | | DVADLRVRSSTLKGVTVPEDRAPSMIDEYPILAVAAAFAEGATVMNGLEELRVKESDRLS VADL VR S L GVTVP +RA SMIDE+PIL+V A FA+GATVM G+ ELRVKESDR+ PVADLLVRHSALTGVTVPAERAASMIDEFPILSVVACFAQGATVMQGIHELRVKESDRID | 359  359 |
| Query 360 | | AVANGLKLNGVDCDEGETSLVVRGRPDGKGLGNASGAAVATHLDHRIAMSFLVMGLVSEN A+A GL+ NG D+ ++ V G+ G GA+ ATHLDHRIAMSFLV GL S+ | 419 |
| Sbjct | 360 | AMAVGLRANGAVVDDTPDTMTVHGQG GLPGGASCATHLDHRIAMSFLVAGLASDQ | 414 |
| Query | 420 | PVTVDDATMIATSFPEFMDLMAGLGAKIE 448  + VDD + IATSFP+F+ LM GLGA ++ |  |
| Sbjct | 415 | AIAVDDGSPIATSFPDFLPLMQGLGAVLD 443 | |

```
>TR:A0A2V5B8P1 A0A2V5B8P1_9SPHN 3-phosphoshikimate 1-carboxyvinyltransferase OS=Novosphingobium sp. GV010 OX=2135477 GN=aroA PE=3 SV=1
Length=448

Score = 486 bits (1251), Expect = 3e-167
Identities = 262/449 (58%), Positives = 315/449 (70%), Gaps = 6/449 (1%) Query 1	MLHGASSRPATARKSSGLSGTVRIPGDKSISHRSFMFGGLASGETRITGLLEGEDVINTG 60

M H + RP	LSGT+R+PGDKSISHRS M G LA GETR+TGLLEGEDV+ T
```

| Sbjct | 1 | MSHSSQPRPHRFTAQGSLSGTIRVPGDKSISHRSIMLGALAVGETRVTGLLEGEDVMATA | 60 |
| --- | --- | --- | --- |
| Query | 61 | KAMQAMGARIRKEGD-TWIIDGVGNGGLLAPEAPLDFGNAATGCRLTMGLVGVYDFDSTF | 119 |
| Sbjct | 61 | AM+AMGA I K D TW+I+GVG G LL PE LD GN+ T RL MGLV + +TF AAMRAMGATIEKAPDGTWVINGVGVGALLQPETALDMGNSGTSTRLLMGLVATHGITATF | 120 |
| Query | 120 | IGDASLTKRPMGRVLNPLREMGVQVKSEDGDR---LPVTLRGPKTPTPITYRVPMASAQV | 176 |
| Sbjct | 121 | +GDASL+KRPMGRV++PL EMG + G + LP+T G + PITYR+P+ASAQV VGDASLSKRPMGRVIDPLSEMGASYTATPGAKGQTLPLTSVGAQPAVPITYRLPVASAQV | 180 |
| Query | 177 | KSAVLLAGLNTPGITTVIEPIMTRDHTEKMLQGFGANLTVETDADGVRTIRLEGRGKLTG | 236 |
| Sbjct | 181 | KSA+LLAGLNTPGITTVIEP+ TRDH+E+ML+GFGA+L VE + +G R IR+ G +L KSAILLAGLNTPGITTVIEPVPTRDHSERMLKGFGADLWVEEE-NGERVIRIRGEAELQP | 239 |
| Query | 237 | QVIDVPGDPSSTAFPLVAALLVPGSDVTILNVLMNPTRTGLILTLQEMGADIEVINPRLA | 296 |
| Sbjct | 240 | Q I+VPGDPSS AF +VAAL+VPGSDV I NV +NPTR G++ L++MG IE I+ R QTIEVPGDPSSAAFFIVAALIVPGSDVLIRNVGLNPTRAGIVHVLRQMGGSIEEIDARTV | 299 |
| Query | 297 | GGEDVADLRVRSSTLKGVTVPEDRAPSMIDEYPILAVAAAFAEGATVMNGLEELRVKESD | 356 |
| Sbjct | 300 | GGE VADLRVR S L G+ V PSM+DE+P+L VAA A+G TV GL+ELRVKESD GGEPVADLRVRHSALHGIAVDPAVVPSMVDEFPVLFVAACLAQGRTVCTGLDELRVKESD | 359 |
| Query | 357 | RLSAVANGLKLNGVDCDEGETSLVVRGRPDGKGLGNASGAAVATHLDHRIAMSFLVMGLV | 416 |

R++ +	L L G	E E +V+ G	GK L	+	VATHLDHRIAMS V GL

| Sbjct | 360 | RITVMRQALDLAGATLSETEDGMVIEG-TGGKPLPGGNNQPVATHLDHRIAMSMAVAGLA | 418 |
| --- | --- | --- | --- |
| Query  Sbjct | 417  419 | SENPVTVDDATMIATSFPEFMDLMAGLGA 445 S VTVDD IATSFP FMDL+AGL A STAGVTVDDIGPIATSFPNFMDLLAGLVA 447 |  |

>TR:A0A418ZU49 A0A418ZU49_9RHOB 3-phosphoshikimate 1-carboxyvinyltransferase OS=Paracoccus aestuarii OX=453842 GN=aroA PE=3 SV=1

Length=443

Score = 486 bits (1250), Expect = 3e-167

| Identities | = 254/448 (57%), Positives = 316/448 (71%), Gaps = 7/448 (2%) |  |
| --- | --- | --- |
| Query 1 | MLHGASSRPATARKSSGLSGTVRIPGDKSISHRSFMFGGLASGETRITGLLEGEDVINTG | 60 |
| Sbjct 1 | M H A RP TAR+ L+G +PGDKSISHR+ + G +A GETRITGLLEG+DV++T MSHSADPRPMTARRHGPLTGEALVPGDKSISHRALILGAMAVGETRITGLLEGQDVLDTA | 60 |
| Query 61 | KAMQAMGARIRKEGD-TWIIDGVGNGGLLAPEAPLDFGNAATGCRLTMGLVGVYDFDSTF | 119 |
| Sbjct 61 | +AM+A+GA++ + G W + GVG GG AP+ +D GN+ TG RL MG V TF RAMRALGAQVTQHGPGEWSVHGVGVGGFSAPDDVIDCGNSGTGVRLIMGAVATTPIAVTF | 120 |
| Query 120 | IGDASLTKRPMGRVLNPLREMGVQVKSEDGDRLPVTLRGPKTPTPITYRVPMASAQVKSA | 179 |
| Sbjct 121 | GDASL++RPM RV +PL ++G ++ +G RLP+T+ G P P+TYR P+ASAQ+KSA TGDASLSRRPMARVTDPLEQLGARITGREGGRLPITIEGAADPVPLTYRTPVASAQIKSA | 180 |
| Query 180 | VLLAGLNTPGITTVIEPIMTRDHTEKMLQGFGANLTVETDADGVRTIRLEGRGKLTGQVI | 239 |
| Sbjct 181 | +LLAGLN PG T VIE TRDH+E+ML GFGA + ET DG R I L+GR +L Q + ILLAGLNAPGDTVVIESEPTRDHSERMLAGFGAQIRTETTPDGHR-ITLKGRPELRPQPV | 239 |
| Query 240 | DVPGDPSSTAFPLVAALLVPGSDVTILNVLMNPTRTGLILTLQEMGADIEVINPRLAGGE | 299 |
| Sbjct 240 | VP DPSS AFP+ AAL+VPGS + + V NPTR GL +TL EMGADI NPR GGE AVPRDPSSAAFPVAAALIVPGSQIRVPGVSRNPTRDGLYVTLLEMGADITFENPREEGGE | 299 |
| Query 300 | DVADLRVRSSTLKGVTVPEDRAPSMIDEYPILAVAAAFAEGATVMNGLEELRVKESDRLS | 359 |
| Sbjct 300 | VADL VR LKGVTVP DRA SMIDE+PIL+V A+FAEG+TVMNG+ ELRVKESDR+ PVADLLVRHGPLKGVTVPADRAASMIDEFPILSVIASFAEGSTVMNGVSELRVKESDRID | 359 |
| Query 360 | AVANGLKLNGVDCDEGETSLVVRGRPDGKGLGNASGAAVATHLDHRIAMSFLVMGLVSEN | 419 |

A+A GL+ NGV +E	S+ V G	G G+A	THLDHRIAMSFLV+G+ +E

| Sbjct | 360 | AMARGLEANGVTVEETPDSMTVHGMARVPGGGHA-----VTHLDHRIAMSFLVLGMATEE 414 |
| --- | --- | --- |
| Query | 420 | PVTVDDATMIATSFPEFMDLMAGLGAKI 447 |
| Sbjct | 415 | PVT+DDA I TSFP+F+ LM LGA +  PVTIDDAEPITTSFPDFVPLMTRLGAAL 442 |

>TR:J2HG57 J2HG57_9SPHN 3-phosphoshikimate 1-carboxyvinyltransferase OS=Novosphingobium sp. AP12 OX=1144305 GN=aroA PE=3 SV=1

Length=443

Score = 486 bits (1250), Expect = 3e-167

| Identities | | = 255/438 (58%), Positives = 311/438 (71%), Gaps = 3/438 (1%) |  |
| --- | --- | --- | --- |
| Query 8  Sbjct 2 | | RPATARKSSGLSGTVRIPGDKSISHRSFMFGGLASGETRITGLLEGEDVINTGKAMQAMG RP + L G +R+PGDKSISHRS M G LA GET +TGLLEGEDV+ T AM+AMG RPRRFMPTGALKGRIRVPGDKSISHRSIMLGALAVGETHVTGLLEGEDVLATAAAMRAMG | 67  61 |
| Query 68  Sbjct 62 | | ARI-RKEGDTWIIDGVGNGGLLAPEAPLDFGNAATGCRLTMGLVGVYDFDSTFIGDASLT A + R + W+I+GVG G LL PEAPLD GN+ T RL MGLV + +TF GDASL+ ASVERLDTGEWVINGVGVGALLQPEAPLDMGNSGTSTRLLMGLVASHPITATFTGDASLS | 126  121 |
| Query 127  Sbjct 122 | | KRPMGRVLNPLREMGVQVKSEDGDRLPVTLRGPKTPTPITYRVPMASAQVKSAVLLAGLN KRPMGRV++PL MG ++ +G RLP+ L+G PITYR+P+ASAQVKSA+LLAGLN KRPMGRVIDPLTTMGASFEASEGGRLPLILKGASPAVPITYRLPVASAQVKSAILLAGLN | 186  181 |
| Query 187  Sbjct 182 | | TPGITTVIEPIMTRDHTEKMLQGFGANLTVETDADGVRTIRLEGRGKLTGQVIDVPGDPS TPGITTVIEP+ TRDH+E+ML+GFGA LTVETDA+G R I + G +L QVI+VPGDPS TPGITTVIEPVPTRDHSERMLRGFGAELTVETDAEGARAISIRGEAELKPQVIEVPGDPS | 246  241 |
| Query 247  Sbjct 242 | | STAFPLVAALLVPGSDVTILNVLMNPTRTGLILTLQEMGADIEVINPRLAGGEDVADLRV S AF +VAAL+VPGS+V I NV +NPTR G++ L+ MG IE +NPR GGE VADL V SAAFFIVAALIVPGSEVMIENVGLNPTRAGIVEVLRLMGGSIEEVNPREVGGEPVADLLV | 306  301 |
| Query 307  Sbjct 302 | | RSSTLKGVTVPEDRAPSMIDEYPILAVAAAFAEGATVMNGLEELRVKESDRLSAVANGLK R S LKG+ V D PSM+DE+P L VAAA A+G TV +GL+ELRVKESDR++ +A L RHSALKGIEVDPDIVPSMVDEFPALFVAAAMAQGVTVTSGLDELRVKESDRIAVMAAALT | 366  361 |
| Query 367  Sbjct 362 | | LNGVDCDEGETSLVVRGRPDGKGLGNASGAAVATHLDHRIAMSFLVMGLVSENPVTVDDA G +E E LV+ G G A+ATHLDHRIAMS + GL S+ V VDD  AAGARIEEREDGLVITGTAGAPLPGTQQ--AIATHLDHRIAMSMAIAGLASQAGVEVDDT | 426  419 |
| Query 427 | | TMIATSFPEFMDLMAGLG 444 IATSFP F ++ GLG |  |
| Sbjct | 420 | RPIATSFPLFETMLDGLG 437 | |

>TR:A0A1I2X275 A0A1I2X275_9RHOB 3-phosphoshikimate 1-carboxyvinyltransferase OS=Paracoccus aminovorans OX=34004 GN=aroA PE=3 SV=1

Length=444

Score = 486 bits (1250), Expect = 3e-167

Identities = 259/449 (58%), Positives = 315/449 (70%), Gaps = 6/449 (1%)

| Query | 1 | MLHGASSRPATARKSSGLSGTVRIPGDKSISHRSFMFGGLASGETRITGLLEGEDVINTG | 60 |
| --- | --- | --- | --- |
| Sbjct | 1 | M H A P TAR S L G ++PGDKSISHR+ + G L+ GET ITGLLEG+DV++T MSHSAEPLPMTARASGPLRGEAQVPGDKSISHRALILGALSVGETHITGLLEGQDVLDTA | 60 |
| Query | 61 | KAMQAMGARIRKEG-DTWIIDGVGNGGLLAPEAPLDFGNAATGCRLTMGLVGVYDFDSTF | 119 |
| Sbjct | 61 | KAMQA GA++ + G W ++G+G GG PE +D GN+ TG RL MG + +TF KAMQAFGAQVERLGIGEWKVNGIGVGGFAEPEGVIDCGNSGTGVRLIMGAMATTPITATF | 120 |
| Query | 120 | IGDASLTKRPMGRVLNPLREMGVQVKSEDGDRLPVTLRGPKTPTPITYRVPMASAQVKSA | 179 |

GDASL++RPMGR+ +PL	G Q+ + +G RLPVT++G	P P+ Y+ P+ASAQ+KSA

| Sbjct | 121 | TGDASLSRRPMGRITDPLELFGAQITAREGKRLPVTIKGAAEPVPVRYKTPVASAQIKSA | 180 |
| --- | --- | --- | --- |
| Query | 180 | VLLAGLNTPGITTVIEPIMTRDHTEKMLQGFGANLTVETDADGVRTIRLEGRGKLTGQVI | 239 |
| Sbjct | 181 | VLLAGLN PG T VIE TRDHTE+ML GFGA +T E +G R I L+GR +L Q + VLLAGLNAPGETVVIESEPTRDHTERMLTGFGATITTEITDEG-RVITLQGRPELRPQPV | 239 |
| Query | 240 | DVPGDPSSTAFPLVAALLVPGSDVTILNVLMNPTRTGLILTLQEMGADIEVINPRLAGGE | 299 |
| Sbjct | 240 | VP DPSS AFP+VAAL+VPGS+V + V NPTR GL +TL EMGADI NPR GGE AVPRDPSSAAFPVVAALIVPGSEVRVPGVSRNPTRDGLYVTLLEMGADIGFENPREEGGE | 299 |
| Query | 300 | DVADLRVRSSTLKGVTVPEDRAPSMIDEYPILAVAAAFAEGATVMNGLEELRVKESDRLS | 359 |
| Sbjct | 300 | VADL VR S LKGVTVP RA SMIDE+PIL+V A FAEG TVM G+ ELRVKESDR++ PVADLLVRHSALKGVTVPPARAASMIDEFPILSVVACFAEGPTVMQGVHELRVKESDRIA | 359 |
| Query | 360 | AVANGLKLNGVDCDEGETSLVVRGRPDGKGLGNASGAAVATHLDHRIAMSFLVMGLVSEN | 419 |
| Sbjct | 360 | A+ GL NG E S+ V G GL GA ATHLDHRIAMSFLV GL SE AMEAGLIANGARVRSTEDSMTVHG---SGGLA-GGGAPAATHLDHRIAMSFLVAGLASEQ | 415 |
| Query  Sbjct | 420  416 | PVTVDDATMIATSFPEFMDLMAGLGAKIE 448 P++VDDA+ IATSFP+F+ LM GLGA+I+ PISVDDASPIATSFPDFLPLMQGLGARID 444 |  |

>TR:A0A512IJ77 A0A512IJ77_9RHIZ 3-phosphoshikimate 1-carboxyvinyltransferase OS=Methylobacterium haplocladii OX=1176176 GN=aroA PE=3 SV=1

Length=449

Score = 486 bits (1250), Expect = 4e-167

| Identities | = 268/453 (59%), Positives = 320/453 (71%), Gaps = 12/453 (3%) |  |
| --- | --- | --- |
| Query 1 | MLHGASSRPATARKSSGLSGTVRIPGDKSISHRSFMFGGLASGETRITGLLEGEDVINTG | 60 |
| Sbjct 1 | M H +P TA + L G +R PGDKSISHR+ + G L+ GETRI GLLEG+DV++T MSHATEPQPVTASPGAPLRGALRPPGDKSISHRAMILGLLSIGETRIEGLLEGDDVLSTA | 60 |
| Query 61 | KAMQAMGARIRKEGD-TWIIDGVGNGGLLAPEAPLDFGNAATGCRLTMGLVGVYDFDSTF | 119 |
| Sbjct 61 | A + +GA + + GD W++ GVG GG+ P LDFGNA TG RL MG+VG +TF SAARQLGASMERLGDGRWVVRGVGIGGMSDPAETLDFGNAGTGSRLMMGVVGGQPVTATF | 120 |
| Query 120 | IGDASLTKRPMGRVLNPLREMGVQVKSE-DGDRLPVTLRGPKTPTPITYRVPMASAQVKS | 178 |
| Sbjct 121 | GDASL KRPM R+L+PL +MG ++ E +G R+P+TLRGP PITY P+ASAQ+KS DGDASLRKRPMRRILDPLLKMGTEIVGEAEGGRVPLTLRGPSEAIPITYETPVASAQIKS | 180 |
| Query 179 | AVLLAGLNTPGITTVIEPIMTRDHTEKMLQGFGANLTVET---DADGVRTIRLEGRGKLT | 235 |
| Sbjct 181 | AVL AGLN PG+TTVIE TRDHTE+ML+ FGA + V + G R I L G+ L AVLFAGLNAPGVTTVIETAATRDHTERMLRLFGAEVVVTPHGPEGHG-RAIALTGQPTLR | 239 |
| Query 236 | GQVIDVPGDPSSTAFPLVAALLVPGSDVTILNVLMNPTRTGLILTLQEMGADIEVINPRL | 295 |
| Sbjct 240 | G + VP DPSS AFPLVAAL+VPGS+VTI V+MNP R GLI TL EMG DIE +N R GTGVVVPADPSSAAFPLVAALIVPGSEVTIEGVMMNPLRIGLITTLIEMGGDIERLNERE | 299 |
| Query 296 | AGGEDVADLRVRSSTLKGVTVPEDRAPSMIDEYPILAVAAAFAEGATVMNGLEELRVKES | 355 |
| Sbjct 300 | GGE VADLRVR+S LKGV VP +RAP+MIDEYP+LAVAAAFAEG T MNGL ELRVKES EGGETVADLRVRASRLKGVGVPAERAPAMIDEYPVLAVAAAFAEGTTRMNGLHELRVKES | 359 |
| Query 356 | DRLSAVANGLKLNGVD-CDEGETSLVVRGRPDGKGLGNASGAAVATHLDHRIAMSFLVMG | 414 |
| Sbjct 360 | DRL+AVA+GL NG+D EG+ +V G G G VATHLDHRIAMSFLV+G DRLAAVADGLGANGIDYAIEGDDLIV HGNGSAPRGGGTVATHLDHRIAMSFLVLG | 414 |
| Query 415  Sbjct 415 | LVSENPVTVDDATMIATSFPEFMDLMAGLGAKI 447 L S++PV+VDD MIATSFP F M LGA I LASQHPVSVDDGAMIATSFPSFTLSMRALGATI 447 |  |

>SP:B6JCN2 AROA_OLICO 3-phosphoshikimate 1-carboxyvinyltransferase OS=Oligotropha carboxidovorans (strain ATCC 49405 / DSM 1227 / KCTC

32145 / OM5) OX=504832 GN=aroA PE=3 SV=1

Length=443

Score = 485 bits (1249), Expect = 5e-167

| Identities | = 261/446 (59%), Positives = 323/446 (72%), Gaps = 7/446 (2%) |  |
| --- | --- | --- |
| Query 1 | MLHGASSRPATARKSSGLSGTVRIPGDKSISHRSFMFGGLASGETRITGLLEGEDVINTG | 60 |
| Sbjct 1 | M H + P AR S+ L+G VR+PGDKSISHR+ + G LA GET+ITGLLEGEDV+NT MSHASRPTPLEARGSTPLTGRVRVPGDKSISHRALILGALAVGETKITGLLEGEDVLNTA | 60 |
| Query 61 | KAMQAMGARIRKEGD-TWIIDGVGNGGLLAPEAPLDFGNAATGCRLTMGLVGVYDFDSTF | 119 |
| Sbjct 61 | KAM A+GA++ + G+ W + GVG GG AP+APLDFGN+ TGCRL MG V +TF KAMAALGAKVERVGEGAWRVHGVGVGGFRAPDAPLDFGNSGTGCRLAMGAVAGSPIAATF | 120 |
| Query 120 | IGDASLTKRPMGRVLNPLREMGVQVKSEDGDRLPVTLRGPKTPTPITYRVPMASAQVKSA | 179 |
| Sbjct 121 | GDASL RPM R+L+PL MG +V DG RLP+TL G + P P+ YR P+ASAQ+KSA DGDASLRSRPMRRILDPLELMGAKVSGGDGARLPLTLEGARDPIPMVYRTPVASAQIKSA | 180 |
| Query 180 | VLLAGLNTPGITTVIEPIMTRDHTEKMLQGFGANLTVETDADGVRTIRLEGRGKLTGQVI | 239 |
| Sbjct 181 | VLLAGL+ PG TTVIE +RDHTE+ML FGA++ E + R I L G+ +L G + VLLAGLSAPGETTVIEAEASRDHTERMLAQFGADIVTEPEGTHGRRITLTGQPELHGADV | 240 |
| Query 240 | DVPGDPSSTAFPLVAALLVPGSDVTILNVLMNPTRTGLILTLQEMGADIEVINPRLAGGE | 299 |
| Sbjct 241 | VP DPSS AFP+VAAL+VPGSD+T+ +V+ NP RTGL TL+EMGA IE + R A GE VVPADPSSAAFPIVAALIVPGSDLTLTDVMTNPLRTGLFTTLREMGASIEESDVRDA-GE | 299 |
| Query 300 | DVADLRVRSSTLKGVTVPEDRAPSMIDEYPILAVAAAFAEGATVMNGLEELRVKESDRLS | 359 |
| Sbjct 300 | +A+LRVR+S L+GVTVP +RAP+MIDEY +LAVAAAFAEG T M GL+ELRVKESDRL PMANLRVRASKLRGVTVPPERAPAMIDEYLVLAVAAAFAEGTTRMLGLKELRVKESDRLE | 359 |
| Query 360 | AVANGLKLNGVDCDEGETSLVVRGRPDGKGLGNASGAAVATHLDHRIAMSFLVMGLVSEN | 419 |
| Sbjct 360 | A A+ L++NGV + L+V G +G+ G G VATH+DHRIAMS LVMG S+ ATADMLRVNGVKVEIVGDDLIVHG--EGRVPG---GGTVATHMDHRIAMSALVMGCASDT | 414 |
| Query 420  Sbjct 415 | PVTVDDATMIATSFPEFMDLMAGLGA 445 PV VDD IATSFP+F+ +M GLGA PVKVDDTAFIATSFPDFIPMMRGLGA 440 |  |

>TR:A0A4T3EZT5 A0A4T3EZT5_9SPHN 3-phosphoshikimate 1-carboxyvinyltransferase OS=Altererythrobacter aquimixticola OX=2489173 GN=aroA PE=3

SV=1

Length=447

Score = 485 bits (1249), Expect = 5e-167

| Identities | = 261/437 (60%), Positives = 310/437 (71%), Gaps = 2/437 (0%) |  |
| --- | --- | --- |
| Query 8 | RPATARKSSGLSGTVRIPGDKSISHRSFMFGGLASGETRITGLLEGEDVINTGKAMQAMG | 67 |
| Sbjct 10 | +P LSGT+R+PGDKSISHRS M G LA GETR+TGLLEGEDV+ T AM+ MG QPRRFSPPGALSGTIRVPGDKSISHRSLMLGALAVGETRVTGLLEGEDVLATAAAMRQMG | 69 |
| Query 68 | ARIRKEGD-TWIIDGVGNGGLLAPEAPLDFGNAATGCRLTMGLVGVYDFDSTFIGDASLT | 126 |
| Sbjct 70 | A I +E D TW I GVG GGLL P APLD GN+ T RL MGLV + +TF+GDASL+ ATITREDDGTWSIHGVGVGGLLQPAAPLDMGNSGTSTRLLMGLVASHAITATFVGDASLS | 129 |
| Query 127 | KRPMGRVLNPLREMGVQVKSEDGDRLPVTLRGPKTPTPITYRVPMASAQVKSAVLLAGLN | 186 |
| Sbjct 130 | KRPM RV++PL ++G + ++ +G LP+TLRG PITYR+P+ASAQVKSAVLLAGLN KRPMRRVIDPLSQIGAEFEASEGGTLPLTLRGISPAVPITYRLPVASAQVKSAVLLAGLN | 189 |
| Query 187 | TPGITTVIEPIMTRDHTEKMLQGFGANLTVETDADGVRTIRLEGRGKLTGQVIDVPGDPS | 246 |
| Sbjct 190 | T G TTVIEP+ TRDH+E+ML+GFGA+L+VE + DG R I L+G +L Q I VPGDPS  TAGTTTVIEPVPTRDHSERMLKGFGADLSVE-EKDGERIITLKGEAELKPQTITVPGDPS | 248 |

| Query | 247 | STAFPLVAALLVPGSDVTILNVLMNPTRTGLILTLQEMGADIEVINPRLAGGEDVADLRV | 306 |
| --- | --- | --- | --- |
| Sbjct | 249 | S AF +VAALLVPGSDV I NV +NPTR GL L++MG IE +N R GGE VADLRV SAAFFVVAALLVPGSDVLIENVGLNPTRAGLFGVLRQMGGSIEEVNAREVGGEPVADLRV | 308 |
| Query | 307 | RSSTLKGVTVPEDRAPSMIDEYPILAVAAAFAEGATVMNGLEELRVKESDRLSAVANGLK | 366 |
| Sbjct | 309 | R S L+G+ V APSMIDE+P+L VAAA AEG T GL+ELRVKESDRL+A+A L RHSMLRGIAVDPAVAPSMIDEFPVLFVAAALAEGTTTTTGLDELRVKESDRLTAMAEALT | 368 |
| Query | 367 | LNGVDCDEGETSLVVRGRPDGKGLGNASGAAVATHLDHRIAMSFLVMGLVSENPVTVDDA | 426 |
| Sbjct | 369 | L G +E E L + G G GAAV +HLDHRIAMS V GL S N V +DD LAGAKFEEHEDGLTIHGTGGDPLPGTGEGAAVVSHLDHRIAMSMAVAGLASRNGVEIDDV | 428 |
| Query | 427 | TMIATSFPEFMDLMAGL 443 IATSFP F+ LM L |  |
| Sbjct | 429 | RPIATSFPNFIALMESL 445 | |

>TR:A0A246JN12 A0A246JN12_9SPHN 3-phosphoshikimate 1-carboxyvinyltransferase OS=Sphingopyxis bauzanensis OX=651663 GN=aroA PE=3 SV=1

Length=442

Score = 485 bits (1248), Expect = 6e-167

| Identities | = 262/439 (60%), Positives = 311/439 (71%), Gaps = 4/439 (1%) |  |
| --- | --- | --- |
| Query 6 | SSRPATARKSSGLSGTVRIPGDKSISHRSFMFGGLASGETRITGLLEGEDVINTGKAMQA | 65 |
| Sbjct 5 | ++RP + S L G + IPGDKSISHRS M LA GE+R++GLLEG DV+ T AM+A NARPRSFSASVPLKGRIAIPGDKSISHRSLMLSALAVGESRVSGLLEGHDVLATAAAMRA | 64 |
| Query 66 | MGARIRKEGD-TWIIDGVGNGGLLAPEAPLDFGNAATGCRLTMGLVGVYDFDSTFIGDAS | 124 |
| Sbjct 65 | MGA I + D W I GVG GGLL P+ LD GN+ T RL MGLV + +TFIGDAS MGATIERRPDGEWRIHGVGVGGLLQPQGALDMGNSGTSTRLLMGLVASHPITATFIGDAS | 124 |
| Query 125 | LTKRPMGRVLNPLREMGVQVKSEDGDRLPVTLRGPKTPTPITYRVPMASAQVKSAVLLAG | 184 |
| Sbjct 125 | L+ RPMGRV++PL +MG + S G RLP+ +RG P+ YR+P+ASAQVKSAVLLAG LSGRPMGRVIDPLAQMGADITSSPGGRLPLMVRGLAPAIPLAYRLPVASAQVKSAVLLAG | 184 |
| Query 185 | LNTPGITTVIEPIMTRDHTEKMLQGFGANLTVETDADGVRTIRLEGRGKLTGQVIDVPGD | 244 |
| Sbjct 185 | LNTPGIT VIEP+ TRDH+E+ML+GFGA+LTVETD DG R IR+ G +L Q I VPGD LNTPGITEVIEPVPTRDHSERMLRGFGADLTVETDTDGTRHIRIVGEAELKPQTIVVPGD | 244 |
| Query 245 | PSSTAFPLVAALLVPGSDVTILNVLMNPTRTGLILTLQEMGADIEVINPRLAGGEDVADL | 304 |
| Sbjct 245 | PSS AF +VAALLVPGSDVTI NV +NPTR GLI L+ MG DIE+I+PR GGE VADL PSSAAFFIVAALLVPGSDVTITNVGLNPTRAGLIDVLRHMGGDIELIDPREVGGEPVADL | 304 |
| Query 305 | RVRSSTLKGVTVPEDRAPSMIDEYPILAVAAAFAEGATVMNGLEELRVKESDRLSAVANG | 364 |
| Sbjct 305 | RVR S L GV V APSMIDE+PIL VAAA A+G T GL+ELRVKESDR++ +A G RVRHSALTGVDVDPAVAPSMIDEFPILFVAAALAKGRTTTTGLDELRVKESDRIAVMATG | 364 |
| Query 365 | LKLNGVDCDEGETSLVVRGRPDGKGLGNASGAAVATHLDHRIAMSFLVMGLVSENPVTVD | 424 |
| Sbjct 365 | LK G E E L++ G G GA +A HLDHRI MSF V GLVS PV++D LKAIGARVTETEDGLIIDGTGGDPLTG---GATIAGHLDHRICMSFAVAGLVSAAPVSID | 421 |
| Query 425  Sbjct 422 | DATMIATSFPEFMDLMAGL 443 D +ATSFP F L+AGL DIAPVATSFPNFEALLAGL 440 |  |

>TR:A0A447IQ54 A0A447IQ54_9RHOB 3-phosphoshikimate 1-carboxyvinyltransferase OS=Paracoccus haematequi OX=2491866 GN=aroA PE=3 SV=1

Length=443

Score = 485 bits (1248), Expect = 7e-167

Identities = 255/449 (57%), Positives = 317/449 (71%), Gaps = 9/449 (2%)

| Query | 1 | MLHGASSRPATARKSSGLSGTVRIPGDKSISHRSFMFGGLASGETRITGLLEGEDVINTG | 60 |
| --- | --- | --- | --- |
| Sbjct | 1 | M H RP TAR+S L G ++PGDKSISHR+ + G L+ GETRITGLLEG+DV++T MSHSTDPRPMTARRSGPLRGVAQVPGDKSISHRALILGALSVGETRITGLLEGQDVLDTA | 60 |
| Query | 61 | KAMQAMGARIRK--EGDTWIIDGVGNGGLLAPEAPLDFGNAATGCRLTMGLVGVYDFDST | 118 |
| Sbjct | 61 | KAM A GA++ + EG+ W + GVG GG PE +D GN+ TG RL MG + +T KAMAAFGAQVERLAEGE-WSVHGVGVGGFSEPEGVIDCGNSGTGVRLIMGAMATTPITAT | 119 |
| Query | 119 | FIGDASLTKRPMGRVLNPLREMGVQVKSEDGDRLPVTLRGPKTPTPITYRVPMASAQVKS | 178 |
| Sbjct | 120 | F GDASL++RPM RV +PL G Q+ + +G RLP+T++G P P+ YR P+ASAQ+KS FTGDASLSRRPMARVTDPLALFGAQIAAREGGRLPITIQGAADPLPVRYRTPVASAQIKS | 179 |
| Query | 179 | AVLLAGLNTPGITTVIEPIMTRDHTEKMLQGFGANLTVETDADGVRTIRLEGRGKLTGQV | 238 |
| Sbjct | 180 | A+LLAGLN PG T VIE TRDH+E+ML GFGA++ ET A+G I L+GR +L Q AILLAGLNVPGETVVIEAEPTRDHSERMLAGFGADIRTETTAEG-HVITLQGRPELRAQP | 238 |
| Query | 239 | IDVPGDPSSTAFPLVAALLVPGSDVTILNVLMNPTRTGLILTLQEMGADIEVINPRLAGG | 298 |
| Sbjct | 239 | + VP DPSS AFP+ AAL+VPGS++ + V NPTR GL +TL EMGADI N R GG VAVPRDPSSAAFPVAAALIVPGSEIRVPGVSRNPTRDGLYVTLLEMGADIAFENTREEGG | 298 |
| Query | 299 | EDVADLRVRSSTLKGVTVPEDRAPSMIDEYPILAVAAAFAEGATVMNGLEELRVKESDRL | 358 |
| Sbjct | 299 | E VADL VR LKGVTVP DRA SMIDE+PIL+V AAFAEGATVMNG+ ELRVKESDR+ EPVADLVVRHGPLKGVTVPADRAASMIDEFPILSVIAAFAEGATVMNGVAELRVKESDRI | 358 |
| Query | 359 | SAVANGLKLNGVDCDEGETSLVVRGRPDGKGLGNASGAAVATHLDHRIAMSFLVMGLVSE | 418 |
| Sbjct | 359 | A+A GL+ NGV DE + S+ V G G G +HLDHRIAMSFLV+GL ++ DAMARGLEANGVRVDETKDSMTVHGMAQVPG GGMAVSHLDHRIAMSFLVLGLATQ | 413 |
| Query | 419 | NPVTVDDATMIATSFPEFMDLMAGLGAKI 447 PV+VDD + IATSFP+F+ LM GLGA + |  |
| Sbjct | 414 | APVSVDDGSPIATSFPDFLPLMQGLGADL 442 | |

>TR:A0A239PWI1 A0A239PWI1_9RHOB 3-phosphoshikimate 1-carboxyvinyltransferase OS=Paracoccus seriniphilus OX=184748 GN=aroA PE=3 SV=1

Length=443

Score = 484 bits (1247), Expect = 9e-167

| Identities | = 253/448 (56%), Positives = 314/448 (70%), Gaps = 7/448 (2%) |  |
| --- | --- | --- |
| Query 1 | MLHGASSRPATARKSSGLSGTVRIPGDKSISHRSFMFGGLASGETRITGLLEGEDVINTG | 60 |
| Sbjct 1 | M H SRP T+R+S L+G +IPGDKSISHR+ + G L+ GETRITGLLEGEDV++T MSHANQSRPMTSRRSGPLTGEAQIPGDKSISHRALILGALSVGETRITGLLEGEDVLDTA | 60 |
| Query 61 | KAMQAMGARIRKEG-DTWIIDGVGNGGLLAPEAPLDFGNAATGCRLTMGLVGVYDFDSTF | 119 |
| Sbjct 61 | KAM A GA++ + G W ++GVG GG PE +D GN+ TG RL MG + +TF KAMAAFGAKVERHGRGEWSVNGVGVGGFSEPEGVIDCGNSGTGVRLIMGAMATTPITATF | 120 |
| Query 120 | IGDASLTKRPMGRVLNPLREMGVQVKSEDGDRLPVTLRGPKTPTPITYRVPMASAQVKSA | 179 |
| Sbjct 121 | GDASL++RPM RV +PL E G Q+ S +G RLP+T++G + P P+ YR P+ASAQ+KSA TGDASLSRRPMARVTDPLSEFGAQITSREGGRLPITIQGVQDPMPVRYRTPVASAQIKSA | 180 |
| Query 180 | VLLAGLNTPGITTVIEPIMTRDHTEKMLQGFGANLTVETDADGVRTIRLEGRGKLTGQVI | 239 |
| Sbjct 181 | +LLAGLN PG T VIE TRDH+E+ML GFGA + ET DG + L GR +L Q + ILLAGLNAPGKTVVIESEPTRDHSERMLAGFGAEIHSETTPDG-HVVTLTGRPELRAQPV | 239 |
| Query 240 | DVPGDPSSTAFPLVAALLVPGSDVTILNVLMNPTRTGLILTLQEMGADIEVINPRLAGGE | 299 |
| Sbjct 240 | VP DPSS AFP+ AAL+VPGS + + + NPTR GL +TL+EMGAD+ N R GGE AVPRDPSSAAFPVAAALIVPGSAIRVPGISRNPTRDGLYITLREMGADLVFENDREEGGE | 299 |
| Query 300 | DVADLRVRSSTLKGVTVPEDRAPSMIDEYPILAVAAAFAEGATVMNGLEELRVKESDRLS | 359 |
| Sbjct 300 | VADL VR LKGV+VP +RA SMIDE+PIL+V AAFAEG TVMNG+ ELRVKESDR+  PVADLIVRHGKLKGVSVPAERAASMIDEFPILSVIAAFAEGKTVMNGVAELRVKESDRID | 359 |

Query 360 AVANGLKLNGVDCDEGETSLVVRGRPDGKGLGNASGAAVATHLDHRIAMSFLVMGLVSEN 419 A+A GL+ NGV +E	S+ V G	G	G	THLDHRIAMSF+V+G+ SE

| Sbjct | 360 | AMARGLEANGVTVEETPDSMTVHGMEKVPG-----GGTAVTHLDHRIAMSFMVLGMASEA 414 |
| --- | --- | --- |
| Query | 420 | PVTVDDATMIATSFPEFMDLMAGLGAKI 447 |
| Sbjct | 415 | PVTVDD + IATSFP+F LM GLGA +  PVTVDDGSPIATSFPDFTALMRGLGADL 442 |

>SP:B0UC54 AROA_METS4 3-phosphoshikimate 1-carboxyvinyltransferase OS=Methylobacterium sp. (strain 4-46) OX=426117 GN=aroA PE=3 SV=1

Length=449

Score = 484 bits (1247), Expect = 1e-166

| Identities | | = 272/450 (60%), Positives = 319/450 (71%), Gaps = 10/450 (2%) |  |
| --- | --- | --- | --- |
| Query 1  Sbjct 1 | | MLHGASSRPATARKSSGLSGTVRIPGDKSISHRSFMFGGLASGETRITGLLEGEDVINTG M H + P TAR + L G +R PGDKSISHR+ + G L+ GETR+ GLLEG+DV+ T MSHDSVPSPITARAGTPLRGRLRPPGDKSISHRAMILGLLSLGETRVEGLLEGDDVLRTA | 60  60 |
| Query 61  Sbjct 61 | | KAMQAMGARIRKEGD-TWIIDGVGNGGLLAPEAPLDFGNAATGCRLTMGLVGVYDFDSTF A +A+GA I ++G W + GVG GGL PE LDFGNA TG RL MG+VG +TF AAARALGAGIDRDGPGRWRVRGVGIGGLSDPEGVLDFGNAGTGSRLMMGVVGGQPVTATF | 119  120 |
| Query 120  Sbjct 121 | | IGDASLTKRPMGRVLNPLREMGVQVKSED-GDRLPVTLRGPKTPTPITYRVPMASAQVKS GDASL KRPM R+L+PL +MG Q+ SE G R+P+TLRGP+ PITY P+ASAQVKS DGDASLRKRPMRRILDPLVQMGAQILSEQAGGRVPLTLRGPEEAIPITYATPVASAQVKS | 178  180 |
| Query 179  Sbjct 181 | | AVLLAGLNTPGITTVIEPIMTRDHTEKMLQGFGANLTVETD--ADGVRTIRLEGRGKLTG AVLLAGLN PG TTVIE TRDHTE+ML+ FGA +TV A R I L G+ L AVLLAGLNAPGTTTVIEAAATRDHTERMLRLFGAEVTVAAHGPAGHGRAIALTGQPTLRA | 236  240 |
| Query 237  Sbjct 241 | | QVIDVPGDPSSTAFPLVAALLVPGSDVTILNVLMNPTRTGLILTLQEMGADIEVINPRLA  + VP DPSS AFP+VAAL+VPGSDV I V+MNP RTGLI TL EMGADI +N R AEVIVPADPSSAAFPIVAALIVPGSDVVIEGVMMNPLRTGLITTLIEMGADIARLNERDE | 296  300 |
| Query 297  Sbjct 301 | | GGEDVADLRVRSSTLKGVTVPEDRAPSMIDEYPILAVAAAFAEGATVMNGLEELRVKESD GGE VADLRVR+S L GVTVP +RAP+MIDEYP+LAVAAAFAEG T M GL ELRVKESD GGETVADLRVRASRLAGVTVPPERAPAMIDEYPVLAVAAAFAEGTTRMQGLHELRVKESD | 356  360 |
| Query 357  Sbjct 361 | | RLSAVANGLKLNGV-DCDEGETSLVVRGRPDGKGLGNASGAAVATHLDHRIAMSFLVMGL RL+AVA+GL+ NGV EG+ +V G G G VATHLDHRIAM+FLVMGL RLAAVADGLRANGVAHAVEGDDLIV HGDGRPAPGGGTVATHLDHRIAMAFLVMGL | 415  415 |
| Query 416 | | VSENPVTVDDATMIATSFPEFMDLMAGLGA 445  + PVTVDD MIATS+P F+ + GLGA |  |
| Sbjct | 416 | AAGEPVTVDDGAMIATSYPAFLADLRGLGA 445 | |

>TR:A0A3S2XS79 A0A3S2XS79_9RHIZ 3-phosphoshikimate 1-carboxyvinyltransferase OS=Methylobacterium sp. TER-1 OX=2499852 GN=aroA PE=3 SV=1

Length=449

Score = 484 bits (1247), Expect = 1e-166

Identities = 269/453 (59%), Positives = 321/453 (71%), Gaps = 12/453 (3%)

| Query | 1 | MLHGASSRPATARKSSGLSGTVRIPGDKSISHRSFMFGGLASGETRITGLLEGEDVINTG | 60 |
| --- | --- | --- | --- |
| Sbjct | 1 | M H ++ P TA + L G +R PGDKSISHR+ + G LA GETR+ GLLEG+DV+ T MSHDSAPSPLTAAAGAALRGALRPPGDKSISHRAIILGLLALGETRVEGLLEGDDVLRTI | 60 |
| Query | 61 | KAMQAMGARIRKEGD-TWIIDGVGNGGLLAPEAPLDFGNAATGCRLTMGLVGVYDFDSTF | 119 |
| Sbjct | 61 | A +A+GA I ++G+ W + GVG GGL PE LDFGNA TG RL MG+VG +TF  AAARALGAGIDRDGEGRWRVRGVGIGGLSDPEGVLDFGNAGTGSRLMMGVVGGQPVTATF | 120 |

| Query | 120 | IGDASLTKRPMGRVLNPLREMGVQVKSE-DGDRLPVTLRGPKTPTPITYRVPMASAQVKS | 178 |
| --- | --- | --- | --- |
| Sbjct | 121 | GDASL KRPM R+L+PL MG Q+ ++ +G R+P+TLRGP P+TY P+ASAQ+KS DGDASLRKRPMRRILDPLERMGTQIVAQAEGGRVPLTLRGPAEAIPVTYETPVASAQIKS | 180 |
| Query | 179 | AVLLAGLNTPGITTVIEPIMTRDHTEKMLQGFGANLTVET---DADGVRTIRLEGRGKLT | 235 |
| Sbjct | 181 | AVLLAGLN PG TTV+E TRDHTE+ML+ FGA + VE + G R I L G+ L AVLLAGLNAPGTTTVVEAAATRDHTERMLRLFGATVAVEPCGPEGHG-RRIALTGQPTLR | 239 |
| Query | 236 | GQVIDVPGDPSSTAFPLVAALLVPGSDVTILNVLMNPTRTGLILTLQEMGADIEVINPRL | 295 |
| Sbjct | 240 | + VP DPSS AFPLVAAL+VPGSDV I V+MNP RTGL+ TL EMGA+IE + R ATEVVVPADPSSAAFPLVAALIVPGSDVVIEGVMMNPLRTGLLTTLIEMGAEIERLRERE | 299 |
| Query | 296 | AGGEDVADLRVRSSTLKGVTVPEDRAPSMIDEYPILAVAAAFAEGATVMNGLEELRVKES | 355 |
| Sbjct | 300 | GGE VADLRVR+S L+GV VP +RAP+MIDEYP+LAVAAAFAEG T M GL ELRVKES EGGETVADLRVRASRLRGVDVPPERAPAMIDEYPVLAVAAAFAEGTTRMRGLHELRVKES | 359 |
| Query | 356 | DRLSAVANGLKLNGV-DCDEGETSLVVRGRPDGKGLGNASGAAVATHLDHRIAMSFLVMG | 414 |
| Sbjct | 360 | DRL+AVA+GL+ NGV EG+ LV G G A G VATHLDHRIAM+FLVMG DRLAAVADGLRANGVAHAVEGDDLLV HGDGTPAAGGGTVATHLDHRIAMAFLVMG | 414 |
| Query  Sbjct | 415  415 | LVSENPVTVDDATMIATSFPEFMDLMAGLGAKI 447 L + PVTVDD MIATSFP F+ M LGA I LATAAPVTVDDGAMIATSFPSFLPTMRALGAAI 447 |  |

>TR:A0A239FPX5 A0A239FPX5_9SPHN 3-phosphoshikimate 1-carboxyvinyltransferase OS=Sphingopyxis indica OX=436663 GN=aroA PE=3 SV=1

Length=442

Score = 484 bits (1245), Expect = 2e-166

| Identities | | = 259/439 (59%), Positives = 313/439 (71%), Gaps = 6/439 (1%) |  |
| --- | --- | --- | --- |
| Query 9  Sbjct 8 | | PATARKSSGLSGTVRIPGDKSISHRSFMFGGLASGETRITGLLEGEDVINTGKAMQAMGA P S+ L G + IPGDKSISHRS M LA G +R+TGLLEG DV+ T AM+AMGA PRAFSASAPLKGRIAIPGDKSISHRSLMLSALAVGRSRVTGLLEGHDVLATAAAMRAMGA | 68  67 |
| Query 69  Sbjct 68 | | RI-RKEGDTWIIDGVGNGGLLAPEAPLDFGNAATGCRLTMGLVGVYDFDSTFIGDASLTK  +I R+EG W+IDGVG GGLL P LD GN+ T RL MGLV + +TF+GDASL+ QIERREGGEWVIDGVGVGGLLQPREALDMGNSGTSTRLLMGLVASHPITATFVGDASLSG | 127  127 |
| Query 128  Sbjct 128 | | RPMGRVLNPLREMGVQVKSEDGDRLPVTLRGPKTPTPITYRVPMASAQVKSAVLLAGLNT RPMGRV++PL MG + + G RLP+ +RG P++YR+PMASAQVKSAVLLAGLNT RPMGRVVDPLSAMGADISASPGGRLPLMVRGLAPAIPLSYRLPMASAQVKSAVLLAGLNT | 187  187 |
| Query 188  Sbjct 188 | | PGITTVIEPIMTRDHTEKMLQGFGANLTVETDADGVRTIRLEGRGKLTGQVIDVPGDPSS PGIT VIEP+ TRDH+E+ML+GFGA+LTV+TD+DG R I + G +L QVI VPGDPSS PGITEVIEPVPTRDHSERMLRGFGADLTVDTDSDGTRHIHIRGEAELKPQVIVVPGDPSS | 247  247 |
| Query 248  Sbjct 248 | | TAFPLVAALLVPGSDVTILNVLMNPTRTGLILTLQEMGADIEVINPRLAGGEDVADLRVR AF +VAAL+VPGS+VTI NV +NPTR GL+ L+ MG DIE ++ R GGE VADLRVR AAFFIVAALIVPGSEVTIANVGLNPTRAGLVAVLRAMGGDIEELDRRDVGGEPVADLRVR | 307  307 |
| Query 308  Sbjct 308 | | SSTLKGVTVPEDRAPSMIDEYPILAVAAAFAEGATVMNGLEELRVKESDRLSAVANGLKL S LKG+ V APSMIDE+PIL VAAA A+G T GL+ELRVKESDRL+ +A GL+ HSALKGIDVDPAVAPSMIDEFPILFVAAALADGQTTTTGLDELRVKESDRLAVMAAGLRA | 367  367 |
| Query 368  Sbjct 368 | | NGVDCDEGETSLVVRGRPDGKGLGN-ASGAAVATHLDHRIAMSFLVMGLVSENPVTVDDA G +E E L++ DG G GA +A HLDHRI MSF V GL S+ PVTVDD IGARVEEREDGLII DGSGGDTLPGGATIAGHLDHRICMSFAVAGLASKAPVTVDDI | 426  423 |
| Query 427 | | TMIATSFPEFMDLMAGLGA 445  +ATSFP F L+A L A |  |
| Sbjct | 424 | APVATSFPNFEALLAELQA 442 | |

>TR:A0A3A3DHB9 A0A3A3DHB9_9RHOB 3-phosphoshikimate 1-carboxyvinyltransferase OS=Paracoccus sp. JM45 OX=2283626 GN=aroA PE=3 SV=1

Length=443

Score = 484 bits (1245), Expect = 2e-166

| Identities | = 254/448 (57%), Positives = 315/448 (70%), Gaps = 7/448 (2%) |  |
| --- | --- | --- |
| Query 1 | MLHGASSRPATARKSSGLSGTVRIPGDKSISHRSFMFGGLASGETRITGLLEGEDVINTG | 60 |
| Sbjct 1 | M H A +RP T+R+S L+G IPGDKSISHR+ + G +A GETRITGLLEGEDV++T MSHSAPARPMTSRRSGPLTGEALIPGDKSISHRALILGAMAIGETRITGLLEGEDVLDTA | 60 |
| Query 61 | KAMQAMGARIRKEGD-TWIIDGVGNGGLLAPEAPLDFGNAATGCRLTMGLVGVYDFDSTF | 119 |
| Sbjct 61 | KAM+A GA + + G W + GVG GG +PE +D GN+ TG RL MG + +TF KAMRAFGAEVTQHGKGEWSVHGVGVGGFRSPEGVIDCGNSGTGVRLIMGAMATTAITATF | 120 |
| Query 120 | IGDASLTKRPMGRVLNPLREMGVQVKSEDGDRLPVTLRGPKTPTPITYRVPMASAQVKSA | 179 |
| Sbjct 121 | GDASL++RPM RV PL ++G + + +GDRLP+T+ G P P+TYR P+ASAQ+KSA TGDASLSRRPMARVTVPLAQLGAHITAREGDRLPITIEGAADPVPLTYRTPVASAQIKSA | 180 |
| Query 180 | VLLAGLNTPGITTVIEPIMTRDHTEKMLQGFGANLTVETDADGVRTIRLEGRGKLTGQVI | 239 |
| Sbjct 181 | +LLAGLN PG T VIE TRDH+E+ML GFGA + ET DG + I L GR +L Q + ILLAGLNVPGETVVIESEPTRDHSERMLAGFGAEIRSETTEDGHK-ITLTGRPELRAQPV | 239 |
| Query 240 | DVPGDPSSTAFPLVAALLVPGSDVTILNVLMNPTRTGLILTLQEMGADIEVINPRLAGGE | 299 |
| Sbjct 240 | VP DPSS AFP+ AAL+VPGS++ + V NPTR GL +TL EMGADI N R GGE AVPRDPSSAAFPVAAALIVPGSEIRVPGVSRNPTRDGLYVTLLEMGADITFENTREEGGE | 299 |
| Query 300 | DVADLRVRSSTLKGVTVPEDRAPSMIDEYPILAVAAAFAEGATVMNGLEELRVKESDRLS | 359 |
| Sbjct 300 | VADL VR LKGVTVP +RA SMIDE+PIL+V AAFA+G TVMNG+ ELRVKESDR+ PVADLVVRHGPLKGVTVPAERAASMIDEFPILSVIAAFAKGPTVMNGVAELRVKESDRID | 359 |
| Query 360 | AVANGLKLNGVDCDEGETSLVVRGRPDGKGLGNASGAAVATHLDHRIAMSFLVMGLVSEN | 419 |
| Sbjct 360 | A+A GL+ NGV +E ++ V G G GNA THLDHRIAMSF+V+GL ++N AMARGLEANGVKVEETNDTMTVHGAATVAGGGNA VTHLDHRIAMSFMVLGLATQN | 414 |
| Query 420  Sbjct 415 | PVTVDDATMIATSFPEFMDLMAGLGAKI 447 PV +DDAT I TSFP+F LM LGA + PVQIDDATPITTSFPDFEPLMIRLGASL 442 |  |

>TR:U2ZSF9 U2ZSF9_9SPHN 3-phosphoshikimate 1-carboxyvinyltransferase OS=Novosphingobium tardaugens NBRC 16725 OX=1219035 GN=aroA PE=3

SV=1

Length=454

Score = 484 bits (1246), Expect = 2e-166

| Identities | = 263/448 (59%), Positives = 318/448 (71%), Gaps = 7/448 (2%) |  |
| --- | --- | --- |
| Query 1 | MLHGASSRPATARKSSGLSGTVRIPGDKSISHRSFMFGGLASGETRITGLLEGEDVINTG | 60 |
| Sbjct 7 | +LH + RP L GT+++PGDKSISHR+ MFG LA GETR+TGLLEGEDV+ T LLHDPAMRPRRFVAGGPLRGTIKVPGDKSISHRAVMFGALAVGETRVTGLLEGEDVMATA | 66 |
| Query 61 | KAMQAMGARIRKEGD-TWIIDGVGNGGLLAPEAPLDFGNAATGCRLTMGLVGVYDFDSTF | 119 |
| Sbjct 67 | AM+AMGA IR++ + TW I+GVG GGLL P+ L+ GN+ T RL MGL+ + +TF DAMRAMGATIRRDDNGTWYINGVGVGGLLQPDRALEMGNSGTSTRLLMGLIASHGITATF | 126 |
| Query 120 | IGDASLTKRPMGRVLNPLREMGVQVKSEDGDR LPVTLRGPKTPTPITYRVPMASAQ | 175 |
| Sbjct 127 | GDASL+KRPMGRV++PL +MG + G LP+ +RG PITYR+P+ASAQ TGDASLSKRPMGRVIDPLAQMGTAFTATPGPNGSQTLPLMIRGAAPAVPITYRLPVASAQ | 186 |
| Query 176 | VKSAVLLAGLNTPGITTVIEPIMTRDHTEKMLQGFGANLTVETDADGVRTIRLEGRGKLT VKSA+LLAGLNTPG+TTVIEPI TRDH+E+ML GFGA+LTVE + DG R IR+ G +L | 235 |

| Sbjct | 187 | VKSAILLAGLNTPGVTTVIEPIPTRDHSERMLTGFGADLTVE-EQDGERVIRIRGEAELK | 245 |
| --- | --- | --- | --- |
| Query | 236 | GQVIDVPGDPSSTAFPLVAALLVPGSDVTILNVLMNPTRTGLILTLQEMGADIEVINPRL | 295 |
| Sbjct | 246 | Q I+VPGDPSS AF +VAAL+VPGSD+ I NV +NPTR GL+ L+ MG IE INPR+ PQTIEVPGDPSSAAFFIVAALIVPGSDLVIENVGLNPTRAGLVEVLRAMGGRIEEINPRI | 305 |
| Query | 296 | AGGEDVADLRVRSSTLKGVTVPEDRAPSMIDEYPILAVAAAFAEGATVMNGLEELRVKES | 355 |
| Sbjct | 306 | GGE VADLRV S L G+TV APSMIDE+P+L VAAA AEG TV GL+ELRVKES VGGEPVADLRVCHSPLHGITVDPAVAPSMIDEFPVLFVAAALAEGRTVTAGLDELRVKES | 365 |
| Query | 356 | DRLSAVANGLKLNGVDCDEGETSLVVRGRPDGKGLGNASGAAVATHLDHRIAMSFLVMGL | 415 |
| Sbjct | 366 | DRL+ +A L GV +E E LV+ G G L +G AVATHLDHRIAMS V GL DRLAVMATALSSIGVAVEEREDGLVIHG-SGGARLPGGTGTAVATHLDHRIAMSMAVAGL | 424 |
| Query  Sbjct | 416  425 | VSENPVTVDDATMIATSFPEFMDLMAGL 443 S+ V VDD IATSFP F L+ GL  ASDAGVEVDDTRPIATSFPNFEALLDGL 452 |  |

>TR:S5YWI7 S5YWI7_PARAH 3-phosphoshikimate 1-carboxyvinyltransferase OS=Paracoccus aminophilus JCM 7686 OX=1367847 GN=aroA PE=3 SV=1

Length=444

Score = 484 bits (1245), Expect = 2e-166

| Identities | | = 253/449 (56%), Positives = 309/449 (69%), Gaps = 7/449 (2%) |  |
| --- | --- | --- | --- |
| Query 1  Sbjct 1 | | MLHGASSRPATARKSSGLSGTVRIPGDKSISHRSFMFGGLASGETRITGLLEGEDVINTG M H A +P TA +S L G +PGDKSISHR+ + G LA GET+ITGLLEGEDVI+T MSHSAEPKPMTAHRSGALKGDAAVPGDKSISHRALILGALAVGETKITGLLEGEDVIDTA | 60  60 |
| Query 61  Sbjct 61 | | KAMQAMGARIRKEGD-TWIIDGVGNGGLLAPEAPLDFGNAATGCRLTMGLVGVYDFDSTF KAM+A GA++ + G+ W ++GVG GG PE +D GN+ TG RL MG + +TF KAMRAFGAKVERTGEGAWTVNGVGVGGFAEPEGVIDCGNSGTGVRLIMGAMATTPITATF | 119  120 |
| Query 120  Sbjct 121 | | IGDASLTKRPMGRVLNPLREMGVQVKSEDGDRLPVTLRGPKTPTPITYRVPMASAQVKSA GDASL++RPMGRV +P+ G ++ + +G LPVT++G P P+ Y P+ASAQ+KSA TGDASLSRRPMGRVTDPVALFGAEISAREGGLLPVTIKGAVDPVPVRYTTPVASAQIKSA | 179  180 |
| Query 180  Sbjct 181 | | VLLAGLNTPGITTVIEPIMTRDHTEKMLQGFGANLTVETDADGVRTIRLEGRGKLTGQVI VL AGLN PG T VIE TRDHTE+M GFGA + E +G I L GR +L GQ + VLFAGLNAPGETVVIEKEATRDHTERMFAGFGATIRTEVTDEG-HVITLTGRPELKGQTV | 239  239 |
| Query 240  Sbjct 240 | | DVPGDPSSTAFPLVAALLVPGSDVTILNVLMNPTRTGLILTLQEMGADIEVINPRLAGGE VP DPSS AFP+ AAL+VPGS++ + V NPTR GL +TL EMGADI N R GGE AVPRDPSSAAFPVAAALIVPGSEIRVPGVSRNPTRDGLYVTLLEMGADITFENTREEGGE | 299  299 |
| Query 300  Sbjct 300 | | DVADLRVRSSTLKGVTVPEDRAPSMIDEYPILAVAAAFAEGATVMNGLEELRVKESDRLS VADL VR S LKGV+VP +RA SMIDE+PIL+V A FAEGATVMNG+ ELRVKESDR+ PVADLVVRHSKLKGVSVPAERAASMIDEFPILSVVACFAEGATVMNGVAELRVKESDRID | 359  359 |
| Query 360 | | AVANGLKLNGVDCDEGETSLVVRGRPDGKGLGNASGAAVATHLDHRIAMSFLVMGLVSEN A+A GL+ NG DE S+ V G KG GA THLDHRIAMSFLV GL S+ | 419 |
| Sbjct | 360 | AMAVGLRANGASVDETRDSMTVHGTGSLKG GACAVTHLDHRIAMSFLVAGLASDQ | 414 |
| Query  Sbjct | 420  415 | PVTVDDATMIATSFPEFMDLMAGLGAKIE 448 P++VDD I TSFP F+ LM+ LGAKIE PISVDDGAPIMTSFPVFLPLMSALGAKIE 443 |  |

>SP:Q6NDP4 AROA_RHOPA 3-phosphoshikimate 1-carboxyvinyltransferase OS=Rhodopseudomonas palustris (strain ATCC BAA-98 / CGA009) OX=258594

GN=aroA PE=3 SV=1

Length=445

Score = 484 bits (1245), Expect = 2e-166

| Identities | | = 260/440 (59%), Positives = 311/440 (71%), Gaps = 7/440 (2%) |  |
| --- | --- | --- | --- |
| Query 9  Sbjct 9 | | PATARKSSGLSGTVRIPGDKSISHRSFMFGGLASGETRITGLLEGEDVINTGKAMQAMGA P ARKS L GT R+PGDKSISHR+ + G LA GETRI+GLLEGEDVINT KAM+A+GA PLQARKSGALHGTARVPGDKSISHRALILGALAVGETRISGLLEGEDVINTAKAMRALGA | 68  68 |
| Query 69  Sbjct 69 | | RIRKEGDT-WIIDGVGNGGLLAPEAPLDFGNAATGCRLTMGLVGVYDFDSTFIGDASLTK  ++ + GD W + GVG G PEAPLDFGN+ TGCRL MG V +TF GDASL KVERTGDCEWRVHGVGVAGFATPEAPLDFGNSGTGCRLAMGAVAGSPIVATFDGDASLRS | 127  128 |
| Query 128  Sbjct 129 | | RPMGRVLNPLREMGVQV-KSEDGDRLPVTLRGPKTPTPITYRVPMASAQVKSAVLLAGLN RPM R+++PL MG +V S +G RLP+ L+G + P PI YR P+ SAQ+KSAVLLAGL+ RPMRRIVDPLELMGAKVVSSSEGGRLPLALQGARDPLPILYRTPVPSAQIKSAVLLAGLS | 186  188 |
| Query 187  Sbjct 189 | | TPGITTVIEPIMTRDHTEKMLQGFGANLTVETDADGVRTIRLEGRGKLTGQVIDVPGDPS PGITTVIE +RDHTE MLQ FGA + E + R I L G+ +L G + VP DPS APGITTVIEAEASRDHTELMLQHFGATIVTEAEGAHGRKISLTGQPELRGAPVVVPADPS | 246  248 |
| Query 247  Sbjct 249 | | STAFPLVAALLVPGSDVTILNVLMNPTRTGLILTLQEMGADIEVINPRLAGGEDVADLRV S AFP+VAAL+VPGSD+ + +V+ NP RTGLI TL+EMGA IE + R GE +A RV SAAFPMVAALVVPGSDIELTDVMTNPLRTGLITTLREMGASIEDSDVRGDAGEPMARFRV | 306  308 |
| Query 307  Sbjct 309 | | RSSTLKGVTVPEDRAPSMIDEYPILAVAAAFAEGATVMNGLEELRVKESDRLSAVANGLK R S LKGV VP +RAPSMIDEY +LAVAAAFAEG TVM GL ELRVKESDRL A A L+ RGSKLKGVEVPPERAPSMIDEYLVLAVAAAFAEGTTVMRGLHELRVKESDRLEATAAMLR | 366  368 |
| Query 367  Sbjct 369 | | LNGVDCDEGETSLVVRGRPDGKGLGNASGAAVATHLDHRIAMSFLVMGLVSENPVTVDDA  +NGV + L+V G+ G G VATH+DHRIAMS L MGL S+ PVTVDD VNGVAVEIAGDDLIVEGKGHVPG GGVVATHMDHRIAMSALAMGLASDKPVTVDDT | 426  423 |
| Query 427 | | TMIATSFPEFMDLMAGLGAK 446 IATSFP+F+ +M LGA+ |  |
| Sbjct | 424 | AFIATSFPDFVPMMQRLGAE 443 | |

>TR:A0A4R6HCC8 A0A4R6HCC8_9BRAD 3-phosphoshikimate 1-carboxyvinyltransferase OS=Bosea sp. BK498 OX=2512178 GN=aroA PE=3 SV=1

Length=447

Score = 484 bits (1245), Expect = 2e-166

| Identities | = 267/449 (59%), Positives = 311/449 (69%), Gaps = 5/449 (1%) |  |
| --- | --- | --- |
| Query 1 | MLHGASSRPATARKSSGLSGTVRIPGDKSISHRSFMFGGLASGETRITGLLEGEDVINTG | 60 |
| Sbjct 1 | M H P TAR L G VR+PGDKSISHR+ +FG LA GET+++GLLEGEDV+ T MSHDHDPVPVTARAGGPLKGRVRVPGDKSISHRAMIFGLLAIGETKVSGLLEGEDVMRTA | 60 |
| Query 61 | KAMQAMGARIRKEGD-TWIIDGVGNGGLLAPEAPLDFGNAATGCRLTMGLVGVYDFDSTF | 119 |
| Sbjct 61 | A +A+GA + +G W + GVG GGL P LDFGNA TG RL MG+ G + +TF AAARALGAVVHHDGPGAWRVQGVGVGGLHQPADILDFGNAGTGSRLMMGVCGSHPITTTF | 120 |
| Query 120 | IGDASLTKRPMGRVLNPLREMG-VQVKSEDGDRLPVTLRGPKTPTPITYRVPMASAQVKS | 178 |
| Sbjct 121 | GDASL KRPM R+L+PL +MG V V ++G R+P+TLRGPK PI Y P+ASAQ+KS DGDASLRKRPMRRILDPLEQMGTVVVSQQEGGRVPLTLRGPKEAIPIVYAPPVASAQIKS | 180 |
| Query 179 | AVLLAGLNTPGITTVIEPIMTRDHTEKMLQGFGANLTVETDADGVRTIRLEGRGKLTGQV | 238 |
| Sbjct 181 | AVLLA L PG TTVIE TRDHTEKML FGA +TV D R I L+G+ +L AVLLAALAAPGETTVIEAEATRDHTEKMLAHFGAEITVTPDGAHGRRIVLKGQPELQAAP | 240 |
| Query 239 | IDVPGDPSSTAFPLVAALLVPGSDVTILNVLMNPTRTGLILTLQEMGADIEVINPRLAGG | 298 |
| Sbjct 241 | I VP DPSS AFPLVA L+VPGSD+ + +V+ NP R+GL+ TL EMGADI V GG IVVPADPSSAAFPLVAGLIVPGSDILLESVMTNPLRSGLLTTLIEMGADITVEREANEGG | 300 |
| Query 299 | EDVADLRVRSSTLKGVTVPEDRAPSMIDEYPILAVAAAFAEGATVMNGLEELRVKESDRL | 358 |

| Sbjct | 301 | E VADLRVR S LKGVTVP +RAPSMIDEYP+LAVAAAFA G T M GL+ELRVKESDRL  ETVADLRVRGSQLKGVTVPPERAPSMIDEYPVLAVAAAFATGTTRMRGLQELRVKESDRL | 360 |
| --- | --- | --- | --- |
| Query | 359 | SAVANGLKLNGVDCDEGETSLVVRGRPDGKGLGNASGAAVATHLDHRIAMSFLVMGLVSE | 418 |
| Sbjct | 361 | +AV GLK GV C L+V G G G +ATHLDHRIAMSFLVMGL ++ AAVEAGLKAAGVTCWIEGDDLIVEGSGGAPARG---GGPIATHLDHRIAMSFLVMGLATQ | 417 |
| Query  Sbjct | 419  418 | NPVTVDDATMIATSFPEFMDLMAGLGAKI 447 NPV VDD MIATSFP F LM GLG +I NPVVVDDGLMIATSFPSFTALMNGLGGRI 446 |  |

>TR:A0A1I3MEY8 A0A1I3MEY8_9RHOB 3-phosphoshikimate 1-carboxyvinyltransferase OS=Albimonas pacifica OX=1114924 GN=aroA PE=3 SV=1

Length=451

Score = 484 bits (1245), Expect = 2e-166

| Identities | | = 259/440 (59%), Positives = 307/440 (70%), Gaps = 4/440 (1%) |  |
| --- | --- | --- | --- |
| Query 9  Sbjct 9 | | PATARKSSGLSGTVRIPGDKSISHRSFMFGGLASGETRITGLLEGEDVINTGKAMQAMGA P TAR L G IPGDKS+SHR+ +FG L+ GETRI GLLEG+DV++T +AM A GA PLTARARGPLRGEAVIPGDKSVSHRALIFGALSLGETRIAGLLEGQDVLDTARAMAAFGA | 68  68 |
| Query 69  Sbjct 69 | | RIRKEGD-TWIIDGVGNGGLLAPEAPLDFGNAATGCRLTMGLVGVYDFDSTFIGDASLTK  ++ + G+ W + GVG GG PE +D GN+ TG RL MG + + F GDASL K QVERTGEGAWSVRGVGVGGFAEPEDVIDCGNSGTGVRLIMGAMATTAISACFTGDASLRK | 127  128 |
| Query 128  Sbjct 129 | | RPMGRVLNPLREMGVQVKSEDGDRLPVTLRGPKTPTPITYRVPMASAQVKSAVLLAGLNT RPMGR+ PL G + + G RLP+ L G P P++YR+PMASAQVKSAVLLAGLN RPMGRITEPLALFGAESRGRRGGRLPLVLTGAADPVPVSYRLPMASAQVKSAVLLAGLNA | 187  188 |
| Query 188  Sbjct 189 | | PGITTVIEPIMTRDHTEKMLQGFGANLTVETDADGVRTIRLEGRGKLTGQVIDVPGDPSS PGIT VIEP TRDHTE+ML GFGA L E +G R I L G+ +LTG+ + VP DPSS PGITEVIEPEATRDHTERMLAGFGATLESEALPEGGRRILLTGQPELTGRTLAVPRDPSS | 247  248 |
| Query 248  Sbjct 249 | | TAFPLVAALLVPGSDVTILNVLMNPTRTGLILTLQEMGADIEVINPRLAGGEDVADLRVR AFP+ AAL+V GS+V + + MNPTRTGLI TLQEMGAD+ + N R GGE AD+ R AAFPICAALIVEGSEVRLPGIAMNPTRTGLIATLQEMGADLALENAREEGGEPTADVVAR | 307  308 |
| Query 308  Sbjct 309 | | SSTLKGVTVPEDRAPSMIDEYPILAVAAAFAEGATVMNGLEELRVKESDRLSAVANGLKL S L+GV VP +RAPSMIDEYP+LAVAAAFAEG TVM G+ ELRVKESDR+ AVA GL+ FSALRGVEVPPERAPSMIDEYPVLAVAAAFAEGKTVMRGIGELRVKESDRIDAVARGLEA | 367  368 |
| Query 368  Sbjct 369 | | NGVDCDEGETSLVVRGRPDGKGLGNASGAAVATHLDHRIAMSFLVMGLVSENPVTVDDAT GV +E E SL V G G G G VATHLDHRIAMSFLV GL S PV+VDD  CGVQVEETEDSLTVHGLGAG---GMPGGGTVATHLDHRIAMSFLVGGLASRRPVSVDDGG | 427  425 |
| Query 428 | | MIATSFPEFMDLMAGLGAKI 447 IATSFP+F LMA LGA+I |  |
| Sbjct | 426 | PIATSFPDFRPLMARLGAEI 445 | |

>TR:A0A099F2G7 A0A099F2G7_9RHOB 3-phosphoshikimate 1-carboxyvinyltransferase OS=Paracoccus halophilus OX=376733 GN=aroA PE=3 SV=1

Length=443

Score = 483 bits (1244), Expect = 3e-166

Identities = 257/449 (57%), Positives = 315/449 (70%), Gaps = 9/449 (2%)

| Query | 1 | MLHGASSRPATARKSSGLSGTVRIPGDKSISHRSFMFGGLASGETRITGLLEGEDVINTG | 60 |
| --- | --- | --- | --- |
|  |  | M H A P +AR+S L+G R+PGDKSISHR+ + G L+ GETRITGLLEG+DV++T |  |
| Sbjct | 1 | MSHSAEPLPMSARRSGPLTGEARVPGDKSISHRALILGALSVGETRITGLLEGQDVLDTA | 60 |
| Query | 61 | KAMQAMGARIRK--EGDTWIIDGVGNGGLLAPEAPLDFGNAATGCRLTMGLVGVYDFDST | 118 |

| Sbjct | 61 | AM+A GA + + EG+ W ++GVG GG PE +D GN+ TG RL MG + +T  AAMRAFGASVERLAEGE-WRVNGVGIGGFAEPEGVIDCGNSGTGVRLIMGAMATTPITAT | 119 |
| --- | --- | --- | --- |
| Query | 119 | FIGDASLTKRPMGRVLNPLREMGVQVKSEDGDRLPVTLRGPKTPTPITYRVPMASAQVKS | 178 |
| Sbjct | 120 | F GDASL++RPMGR+ +PL G +V + +G RLPVT+RG P P+ YR P+ASAQ+KS FTGDASLSRRPMGRITDPLEMFGCEVTAREGKRLPVTIRGAADPVPLRYRTPVASAQIKS | 179 |
| Query | 179 | AVLLAGLNTPGITTVIEPIMTRDHTEKMLQGFGANLTVETDADGVRTIRLEGRGKLTGQV | 238 |
| Sbjct | 180 | AVLLAGLN PG T VIE TRDHTE+ML GFGA + ET +G I L+GR L Q AVLLAGLNAPGETVVIEAEPTRDHTERMLAGFGAKIVTETTDEG-HVITLKGRPNLRPQP | 238 |
| Query | 239 | IDVPGDPSSTAFPLVAALLVPGSDVTILNVLMNPTRTGLILTLQEMGADIEVINPRLAGG | 298 |
| Sbjct | 239 | I VP DPSS AFP+ AAL+VPGS++ + V NPTR GL +TL EMGADI N R+ GG IAVPRDPSSAAFPVAAALIVPGSEIRVPGVSRNPTRDGLYVTLLEMGADIRFENERVEGG | 298 |
| Query | 299 | EDVADLRVRSSTLKGVTVPEDRAPSMIDEYPILAVAAAFAEGATVMNGLEELRVKESDRL | 358 |
| Sbjct | 299 | E VADL VR LKGVTVP RA SMIDE+PIL+V AAFAEG TVM G+ ELRVKESDR+ EPVADLVVRHGPLKGVTVPPGRAASMIDEFPILSVIAAFAEGTTVMQGVHELRVKESDRI | 358 |
| Query | 359 | SAVANGLKLNGVDCDEGETSLVVRGRPDGKGLGNASGAAVATHLDHRIAMSFLVMGLVSE | 418 |
| Sbjct | 359 | A+A GL+ NG ++ E +++V G KG GA +HLDHRIAMSFLV GL SE EAMAVGLRANGASVEDSEDTMIVHGTGGLKG GATAQSHLDHRIAMSFLVAGLASE | 413 |
| Query | 419 | NPVTVDDATMIATSFPEFMDLMAGLGAKI 447 P+ VDDA IATSFP+F+ LM GLGA+I |  |
| Sbjct | 414 | QPIGVDDAGPIATSFPDFLPLMRGLGAEI 442 | |

>SP:Q2J2F6 AROA_RHOP2 3-phosphoshikimate 1-carboxyvinyltransferase OS=Rhodopseudomonas palustris (strain HaA2) OX=316058 GN=aroA PE=3

SV=1

Length=445

Score = 483 bits (1244), Expect = 3e-166

| Identities | = 257/448 (57%), Positives = 315/448 (70%), Gaps = 7/448 (2%) |  |
| --- | --- | --- |
| Query 1 | MLHGASSRPATARKSSGLSGTVRIPGDKSISHRSFMFGGLASGETRITGLLEGEDVINTG | 60 |
| Sbjct 1 | M H + P AR S+ LSGT R+PGDKSISHR+ + G LA GETRI+GLLEGEDV+NT MSHSDHTAPLEARLSAALSGTARVPGDKSISHRALILGALAVGETRISGLLEGEDVLNTA | 60 |
| Query 61 | KAMQAMGARIRKEGD-TWIIDGVGNGGLLAPEAPLDFGNAATGCRLTMGLVGVYDFDSTF | 119 |
| Sbjct 61 | +AM+A+GA++ + GD W + GVG G P APLDFGN+ TGCRL MG V +TF RAMRALGAQVERTGDCAWSVHGVGVAGFAPPAAPLDFGNSGTGCRLAMGAVAGSPIIATF | 120 |
| Query 120 | IGDASLTKRPMGRVLNPLREMGVQV-KSEDGDRLPVTLRGPKTPTPITYRVPMASAQVKS | 178 |
| Sbjct 121 | GDASL RPM R+++PL +MG +V +S DG RLP+TL+G + P PITYR P+ SAQ+KS DGDASLRSRPMRRIVDPLEQMGARVTQSADGGRLPLTLQGARDPLPITYRTPVPSAQIKS | 180 |
| Query 179 | AVLLAGLNTPGITTVIEPIMTRDHTEKMLQGFGANLTVETDADGVRTIRLEGRGKLTGQV | 238 |
| Sbjct 181 | AVLLAGL+ PG+TTVIE +RDHTE MLQ FGA + E + R I L G+ +L G AVLLAGLSAPGVTTVIEAEASRDHTELMLQHFGATVVTEPEGPHGRKISLTGQPELRGAP | 240 |
| Query 239 | IDVPGDPSSTAFPLVAALLVPGSDVTILNVLMNPTRTGLILTLQEMGADIEVINPRLAGG | 298 |
| Sbjct 241 | + VP DPSS AFP+VAAL+VPGSDV + V+ NP RTGLI TL+EMG IE R G VVVPADPSSAAFPMVAALIVPGSDVVLTEVMTNPLRTGLITTLREMGGLIEESETRGDAG | 300 |
| Query 299 | EDVADLRVRSSTLKGVTVPEDRAPSMIDEYPILAVAAAFAEGATVMNGLEELRVKESDRL | 358 |
| Sbjct 301 | E +A R+R S L+GV VP +RAPSMIDEY +LAVAAAFAEG T+M GL ELRVKESDRL EPMARFRIRGSQLRGVEVPPERAPSMIDEYLVLAVAAAFAEGTTIMRGLHELRVKESDRL | 360 |
| Query 359 | SAVANGLKLNGVDCDEGETSLVVRGRPDGKGLGNASGAAVATHLDHRIAMSFLVMGLVSE | 418 |
| Sbjct 361 | A A L++NGV + L+V G+ G G VATH+DHRIAMS LVMGL ++  EATAAMLRVNGVTVEISGDDLIVEGKGHVPG GGLVATHMDHRIAMSALVMGLAAD | 415 |

Query 419 NPVTVDDATMIATSFPEFMDLMAGLGAK 446 PV VDD	IATSFP+F+ +M LGA+

Sbjct 416 KPVRVDDTAFIATSFPDFVPMMQRLGAE 443

>TR:A0A074KJF4 A0A074KJF4_9RHOB 3-phosphoshikimate 1-carboxyvinyltransferase OS=Thioclava indica OX=1353528 GN=aroA PE=3 SV=1

Length=449

Score = 483 bits (1244), Expect = 3e-166

| Identities | = 255/438 (58%), Positives = 314/438 (72%), Gaps = 5/438 (1%) |  |
| --- | --- | --- |
| Query 11 | TARKSSGLSGTVRIPGDKSISHRSFMFGGLASGETRITGLLEGEDVINTGKAMQAMGARI | 70 |
| Sbjct 11 | TARKS+ L GT ++PGDKSISHR+ +FG L+ GET+ITGLLEGEDV++T +AM+A GA + TARKSAPLQGTAQVPGDKSISHRALIFGALSVGETKITGLLEGEDVLDTARAMRAFGAEV | 70 |
| Query 71 | RKEGD-TWIIDGVGNGGLLAPEAPLDFGNAATGCRLTMGLVGVYDFDSTFIGDASLTKRP | 129 |
| Sbjct 71 | + G W + GVG GG PE +D GN+ TG RL MG + +TF GDASL KRP TQHGPGAWSVHGVGVGGFAEPEQVIDCGNSGTGVRLIMGCMATSPITATFTGDASLRKRP | 130 |
| Query 130 | MGRVLNPLREMGVQVKSEDGDRLPVTLRGPKTPTPITYRVPMASAQVKSAVLLAGLNTPG | 189 |
| Sbjct 131 | MGR+ +P+ G Q G RLP+T+ G + P P+ Y PMASAQVKSAVLLAGLN PG MGRITDPIALFGAQSYGRAGGRLPMTVVGARDPVPVRYTPPMASAQVKSAVLLAGLNAPG | 190 |
| Query 190 | ITTVIEPIMTRDHTEKMLQGFGANLTVETDADGVRTIRLEGRGKLTGQVIDVPGDPSSTA | 249 |
| Sbjct 191 | T VIE I TRDHTE+ML+GFGA++TVE +G + I L+G+ +L Q + VP DPSS A QTVVIEKIPTRDHTERMLRGFGADVTVEQTDEG-QVITLQGQPELVAQEVAVPRDPSSAA | 249 |
| Query 250 | FPLVAALLVPGSDVTILNVLMNPTRTGLILTLQEMGADIEVINPRLAGGEDVADLRVRSS | 309 |
| Sbjct 250 | FP+ AAL+VPGSD+ + V NPTR GL +TL+EMGADIE +NPR GGE VADLRVR S FPVCAALIVPGSDILVPGVSQNPTRNGLFITLKEMGADIEFLNPREEGGEPVADLRVRYS | 309 |
| Query 310 | TLKGVTVPEDRAPSMIDEYPILAVAAAFAEGATVMNGLEELRVKESDRLSAVANGLKLNG | 369 |
| Sbjct 310 | L G+ VP RA + IDEYP+L+V AA A G T M G+ ELRVKESDR+ A+A GL+ G ALHGIDVPAARASAQIDEYPVLSVVAACATGVTRMPGVHELRVKESDRIDAMARGLEACG | 369 |
| Query 370 | VDCDEGETSLVVRGRPDGKGLGNASGAAVATHLDHRIAMSFLVMGLVSENPVTVDDATMI | 429 |
| Sbjct 370 | V +E E +L+V G G G GA A+HLDHRIAMSFLV+G+ ++NPV+VDDAT I VRIEEDEDTLIVHGMGPG---GVPGGATCASHLDHRIAMSFLVLGMAAQNPVSVDDATPI | 426 |
| Query 430  Sbjct 427 | ATSFPEFMDLMAGLGAKI 447 ATSFP F DLMA LGA I ATSFPIFEDLMAQLGASI 444 |  |

>TR:A0A2A4G088 A0A2A4G088_9SPHN 3-phosphoshikimate 1-carboxyvinyltransferase OS=Rhizorhabdus dicambivorans OX=1850238 GN=aroA PE=3 SV=1

Length=445

Score = 483 bits (1243), Expect = 4e-166

| Identities | = 260/446 (58%), Positives = 310/446 (70%), Gaps = 4/446 (1%) |  |
| --- | --- | --- |
| Query 2 | LHGASSRPATARKSSGLSGTVRIPGDKSISHRSFMFGGLASGETRITGLLEGEDVINTGK | 61 |
| Sbjct 1 | +HG + RPA+ L GT+ +PGDKSISHRS M LA GE+R+ GLLEGEDV+ T MHGPTPRPASFSAPGPLRGTIAVPGDKSISHRSLMLSALAVGESRVEGLLEGEDVLATAA | 60 |
| Query 62 | AMQAMGARI-RKEGDTWIIDGVGNGGLLAPEAPLDFGNAATGCRLTMGLVGVYDFDSTFI | 120 |
| Sbjct 61 | AM+AMGA I R D W I GVG GGLL PE LD GN+ T RL MGL+ + +TFI AMRAMGATIERGADDVWRIHGVGVGGLLQPEVALDMGNSGTSTRLLMGLIASHAITATFI | 120 |
| Query 121 | GDASLTKRPMGRVLNPLREMGVQVKSEDGDRLPVTLRGPKTPTPITYRVPMASAQVKSAV | 180 |
| Sbjct 121 | GDASL+KRPM RV+ PL MG +V G RLP+ LRG PI YR+P+ASAQVKSAV  GDASLSKRPMNRVIEPLSLMGAEVTPSPGGRLPLMLRGLSPAVPIEYRLPVASAQVKSAV | 180 |

| Query | 181 | LLAGLNTPGITTVIEPIMTRDHTEKMLQGFGANLTVETDADGVRTIRLEGRGKLTGQVID | 240 |
| --- | --- | --- | --- |
| Sbjct | 181 | LLAGLNTPGIT VIEPI TRDH+E+ML+GFGA+L+VE DG R I + G +L Q I LLAGLNTPGITRVIEPIATRDHSERMLRGFGADLSVEQAGDGARIISIRGEAELRPQHIV | 240 |
| Query | 241 | VPGDPSSTAFPLVAALLVPGSDVTILNVLMNPTRTGLILTLQEMGADIEVINPRLAGGED | 300 |
| Sbjct | 241 | VPGDPSS AFP+VAALLVPGS+VT+ NV +NPTR L L+ MG DIE N R GGE VPGDPSSAAFPVVAALLVPGSEVTVTNVGLNPTRAALFDVLRMMGGDIEFTNRREVGGEP | 300 |
| Query | 301 | VADLRVRSSTLKGVTVPEDRAPSMIDEYPILAVAAAFAEGATVMNGLEELRVKESDRLSA | 360 |
| Sbjct | 301 | VAD+ VR S LKG+ P + SM+DEYP+L VAAA AEG TV GLEELRVKESDR++ VADIVVRHSLLKGIETPVETVASMVDEYPVLFVAAALAEGRTVARGLEELRVKESDRIAV | 360 |
| Query | 361 | VANGLKLNGVDCDEGETSLVVRGRPDGKGLGNASGAAVATHLDHRIAMSFLVMGLVSENP | 420 |
| Sbjct | 361 | +A GL+ G +E E L++ G G A GA +A LDHRIAMSF V GLV++ P MAEGLRAIGARVEELEDGLIIDGT---GGDPLAGGATIAARLDHRIAMSFAVAGLVAKAP | 417 |
| Query  Sbjct | 421  418 | VTVDDATMIATSFPEFMDLMAGLGAK 446 V +DD + +ATSFP F LM GLGA+ VVIDDMSPVATSFPGFTALMTGLGAE 443 |  |

>TR:A0A2S8S5Z1 A0A2S8S5Z1_9RHOB 3-phosphoshikimate 1-carboxyvinyltransferase OS=Defluviimonas denitrificans OX=404881 GN=aroA PE=3 SV=1

Length=449

Score = 483 bits (1243), Expect = 5e-166

| Identities | | = 258/440 (59%), Positives = 310/440 (70%), Gaps = 5/440 (1%) |  |
| --- | --- | --- | --- |
| Query 9  Sbjct 9 | | PATARKSSGLSGTVRIPGDKSISHRSFMFGGLASGETRITGLLEGEDVINTGKAMQAMGA P +ARKS L G +PGDKSISHRS + G +A GET++TGLLEG+DV++T AM+A GA PMSARKSGPLKGRAEVPGDKSISHRSLILGAMAVGETKVTGLLEGQDVLDTAAAMRAFGA | 68  68 |
| Query 69  Sbjct 69 | | RIRKEGD-TWIIDGVGNGGLLAPEAPLDFGNAATGCRLTMGLVGVYDFDSTFIGDASLTK  + + G+ W + GVG GG PE +D GN+ TG RL MG + +TF GDASL K EVTRHGEGDWSVHGVGVGGFAEPEQVIDCGNSGTGVRLIMGAMATTAMSATFAGDASLRK | 127  128 |
| Query 128  Sbjct 129 | | RPMGRVLNPLREMGVQVKSEDGDRLPVTLRGPKTPTPITYRVPMASAQVKSAVLLAGLNT RPMGRV +PL G + G RLP+T+ G P P+ Y VP+ SAQVKSAVLLAGLN RPMGRVTDPLALFGARAYGRQGGRLPMTIVGAADPVPVRYVVPVPSAQVKSAVLLAGLNA | 187  188 |
| Query 188  Sbjct 189 | | PGITTVIEPIMTRDHTEKMLQGFGANLTVETDADGVRTIRLEGRGKLTGQVIDVPGDPSS PG T VIE TRDH+E+ML+GFGA LTVE +G R I L G+ +L QV+ VP DPSS PGQTVVIEREPTRDHSERMLRGFGAELTVEDTGEG-RVITLTGQPELRPQVVAVPRDPSS | 247  247 |
| Query 248  Sbjct 248 | | TAFPLVAALLVPGSDVTILNVLMNPTRTGLILTLQEMGADIEVINPRLAGGEDVADLRVR AFP+ AAL+V GSD+ + V NPTR GL TL EMGADIE NPR GGE VADLRVR AAFPVCAALIVEGSDILVPGVSQNPTRNGLYTTLVEMGADIEFQNPREEGGEPVADLRVR | 307  307 |
| Query 308  Sbjct 308 | | SSTLKGVTVPEDRAPSMIDEYPILAVAAAFAEGATVMNGLEELRVKESDRLSAVANGLKL  +STL G+ VPE+RA SMIDEYPIL+V AAFAEG TVM G++ELRVKESDR+ A+A GL+ ASTLNGIEVPEERAASMIDEYPILSVVAAFAEGTTVMRGVKELRVKESDRIDAMARGLEA | 367  367 |
| Query 368  Sbjct 368 | | NGVDCDEGETSLVVRGRPDGKGLGNASGAAVATHLDHRIAMSFLVMGLVSENPVTVDDAT GV +E E L+V G G G GA ATH+DHRIAMSFLV GL ++ PV+VDD + CGVRVEEEEDVLIVHGMGQG---GVPGGATCATHIDHRIAMSFLVAGLAAKRPVSVDDGS | 427  424 |
| Query 428 | | MIATSFPEFMDLMAGLGAKI 447 IATSFP F LMAGLGA+I |  |
| Sbjct | 425 | PIATSFPAFEGLMAGLGAQI 444 | |

>TR:A0A1X7I965 A0A1X7I965_9RHOB 3-phosphoshikimate 1-carboxyvinyltransferase OS=Paracoccus sp. J56 OX=935850 GN=aroA PE=3 SV=1

Length=443

Score = 483 bits (1242), Expect = 5e-166

| Identities | = 254/448 (57%), Positives = 314/448 (70%), Gaps = 7/448 (2%) |  |
| --- | --- | --- |
| Query 1 | MLHGASSRPATARKSSGLSGTVRIPGDKSISHRSFMFGGLASGETRITGLLEGEDVINTG | 60 |
| Sbjct 1 | M H A P AR+S L G ++PGDKSISHR+ + G LA GET ITGLLEG+DV++T MSHSAEPLPMIARRSGPLKGEAKVPGDKSISHRALILGALAVGETHITGLLEGQDVLDTA | 60 |
| Query 61 | KAMQAMGARIRKEGD-TWIIDGVGNGGLLAPEAPLDFGNAATGCRLTMGLVGVYDFDSTF | 119 |
| Sbjct 61 | KAMQA GAR+ + G+ W+++GVG GG PE +D GN+ TG RL MG + +TF KAMQAFGARVERLGEGEWLVNGVGVGGFSEPEGVIDCGNSGTGVRLIMGAMATTPVTATF | 120 |
| Query 120 | IGDASLTKRPMGRVLNPLREMGVQVKSEDGDRLPVTLRGPKTPTPITYRVPMASAQVKSA | 179 |
| Sbjct 121 | GDASL++RPMGRV +PL G +V + +G RLPVT++G P P+ Y+ P+ASAQ+KSA TGDASLSRRPMGRVTDPLELFGCEVTAREGKRLPVTIKGAADPVPVRYKTPVASAQIKSA | 180 |
| Query 180 | VLLAGLNTPGITTVIEPIMTRDHTEKMLQGFGANLTVETDADGVRTIRLEGRGKLTGQVI | 239 |
| Sbjct 181 | VLLA LN PG T VIE TRDHTE+ML GFGA ++ E +G + I L GR +L Q + VLLAALNAPGETVVIESEPTRDHTERMLAGFGATISTEVTDEG-QVITLRGRPELRPQPV | 239 |
| Query 240 | DVPGDPSSTAFPLVAALLVPGSDVTILNVLMNPTRTGLILTLQEMGADIEVINPRLAGGE | 299 |
| Sbjct 240 | VP DPSS AFP+ AAL+VPGS++ + V NPTR GL +TL EMGADI NPR GGE VVPRDPSSAAFPVAAALIVPGSEIRVPGVSRNPTRDGLYVTLLEMGADIRFENPREEGGE | 299 |
| Query 300 | DVADLRVRSSTLKGVTVPEDRAPSMIDEYPILAVAAAFAEGATVMNGLEELRVKESDRLS | 359 |
| Sbjct 300 | VADL VR LKGVTVP +RA SMIDE+PIL+V A FAEG TVM G+ ELRVKESDR+ PVADLVVRHGPLKGVTVPPERAASMIDEFPILSVIACFAEGTTVMQGVHELRVKESDRIE | 359 |
| Query 360 | AVANGLKLNGVDCDEGETSLVVRGRPDGKGLGNASGAAVATHLDHRIAMSFLVMGLVSEN | 419 |
| Sbjct 360 | A+A GL NG + + ++ V G+ KG GA ATHLDHRIAMSFLV GL SE AMAVGLVANGAEVGATDDTMTVHGKGGLKG GAICATHLDHRIAMSFLVAGLASEQ | 414 |
| Query 420  Sbjct 415 | PVTVDDATMIATSFPEFMDLMAGLGAKI 447 P++VDD + IATSFP+F+ LM GLGA+I PISVDDGSPIATSFPDFLPLMKGLGAQI 442 |  |

>SP:B1M0N9 AROA_METRJ 3-phosphoshikimate 1-carboxyvinyltransferase OS=Methylobacterium radiotolerans (strain ATCC 27329 / DSM 1819 /

JCM 2831 / NBRC 15690 / NCIMB 10815 / 0-1) OX=426355 GN=aroA PE=3 SV=1

Length=449

Score = 483 bits (1242), Expect = 7e-166

| Identities | = 268/451 (59%), Positives = 314/451 (70%), Gaps = 8/451 (2%) |  |
| --- | --- | --- |
| Query 1 | MLHGASSRPATARKSSGLSGTVRIPGDKSISHRSFMFGGLASGETRITGLLEGEDVINTG | 60 |
| Sbjct 1 | M H +S +P TA + L G +R PGDKSISHRS + G L+ GETR+ GLLEG+DV+ T MSHDSSPQPLTAAPGAPLRGRLRPPGDKSISHRSMILGLLSQGETRVEGLLEGDDVLRTA | 60 |
| Query 61 | KAMQAMGARIRKEGD-TWIIDGVGNGGLLAPEAPLDFGNAATGCRLTMGLVGVYDFDSTF | 119 |
| Sbjct 61 | A +A+GA + + G W + GVG GGL P LDFGNA TG RL MG+VG +TF AAAKALGAGVERLGPGRWRVQGVGIGGLGDPADVLDFGNAGTGSRLMMGVVGGQPVTATF | 120 |
| Query 120 | IGDASLTKRPMGRVLNPLREMGVQVKSE-DGDRLPVTLRGPKTPTPITYRVPMASAQVKS | 178 |
| Sbjct 121 | GDASL RPM R+L+PL MG QV SE +G R+P+TLRGP+ PITY P ASAQ+KS DGDASLRSRPMRRILDPLTRMGTQVLSEAEGGRVPLTLRGPREAIPITYETPAASAQIKS | 180 |
| Query 179 | AVLLAGLNTPGITTVIEPIMTRDHTEKMLQGFGANLTVETDADGV--RTIRLEGRGKLTG | 236 |
| Sbjct 181 | AVLLAGLN PG+TTVIE TRDHTE+ML+ FGA ++VE G R + L G+ L G AVLLAGLNAPGVTTVIEAAATRDHTERMLRLFGAAVSVEPHGPGGHGRKVALTGQPTLRG | 240 |
| Query 237 | QVIDVPGDPSSTAFPLVAALLVPGSDVTILNVLMNPTRTGLILTLQEMGADIEVINPRLA  + VP DPSS AFPLVAAL+VPGSDV I V+MNP R GLI TL EMGA IE + R | 296 |

| Sbjct | 241 | TDVVVPADPSSAAFPLVAALIVPGSDVVIEGVMMNPLRIGLITTLLEMGAQIERVAEREE | 300 |
| --- | --- | --- | --- |
| Query | 297 | GGEDVADLRVRSSTLKGVTVPEDRAPSMIDEYPILAVAAAFAEGATVMNGLEELRVKESD | 356 |
| Sbjct | 301 | GGE VADLRVR+S L GV VP +RAP+MIDEYP+LAVAA+FAEG T M+GL ELRVKESD GGETVADLRVRASRLNGVDVPAERAPAMIDEYPVLAVAASFAEGRTRMSGLHELRVKESD | 360 |
| Query | 357 | RLSAVANGLKLNGVDCDEGETSLVVRGRPDGKGLGNASGAAVATHLDHRIAMSFLVMGLV | 416 |
| Sbjct | 361 | RL+AVA GL NGV LVV +G G G V THLDHRIAM+FLVMGL RLAAVAAGLAANGVRHTVEGDDLVV EGDGAAAPGGGTVETHLDHRIAMAFLVMGLA | 416 |
| Query  Sbjct | 417  417 | SENPVTVDDATMIATSFPEFMDLMAGLGAKI 447  + NPVTVDD MIATSFP F+ M LG +I ARNPVTVDDGAMIATSFPSFLPTMQALGGRI 447 |  |

>TR:A0A1H2W8Z3 A0A1H2W8Z3_9RHOB 3-phosphoshikimate 1-carboxyvinyltransferase OS=Albimonas donghaensis OX=356660 GN=aroA PE=3 SV=1

Length=452

Score = 483 bits (1242), Expect = 7e-166

| Identities | = 266/453 (59%), Positives = 309/453 (68%), Gaps = 5/453 (1%) |  |
| --- | --- | --- |
| Query 3 | HGASSRPATARKSSGLSGTVRIPGDKSISHRSFMFGGLASGETRITGLLEGEDVINTGKA | 62 |
| Sbjct 4 | HGA +P +R + L G IPGDKS+SHR+ + G LA GETRITGLLEG+DV++T +A HGAP-QPLISRAAGALKGEAIIPGDKSVSHRALILGALAVGETRITGLLEGQDVLDTARA | 62 |
| Query 63 | MQAMGARIRKEGD-TWIIDGVGNGGLLAPEAPLDFGNAATGCRLTMGLVGVYDFDSTFIG | 121 |
| Sbjct 63 | M A GA + + GD W + GVG GG PE +D GN+ TG RL MG + +TF G MAAFGATVTRTGDGAWTVRGVGVGGFAEPEDVIDCGNSGTGVRLIMGAMATTPISATFTG | 122 |
| Query 122 | DASLTKRPMGRVLNPLREMGVQVKSEDGDRLPVTLRGPKTPTPITYRVPMASAQVKSAVL | 181 |
| Sbjct 123 | DASL +RPMGR+ +PL G + G RLP+TL G P P++YR+PMASAQVKSAVL DASLRRRPMGRITDPLALFGARAHGRRGGRLPLTLVGAADPVPVSYRLPMASAQVKSAVL | 182 |
| Query 182 | LAGLNTPGITTVIEPIMTRDHTEKMLQGFGANLTVETDADGVRTIRLEGRGKLTGQVIDV | 241 |
| Sbjct 183 | LAGLN PGIT IEP TRDHTE+ML+GFGA + V G R IRL G+ +L Q + V LAGLNAPGITEAIEPEATRDHTERMLRGFGAVVEVAEAEGGGRLIRLTGQPELIAQTLAV | 242 |
| Query 242 | PGDPSSTAFPLVAALLVPGSDVTILNVLMNPTRTGLILTLQEMGADIEVINPRLAGGEDV | 301 |
| Sbjct 243 | P DPSS AFP+VAALLV GS+V + + MNPTRTGLI TL+EMGAD+E R GGE PRDPSSAAFPMVAALLVEGSEVVLPGIGMNPTRTGLIQTLREMGADLEARQSREEGGEPA | 302 |
| Query 302 | ADLRVRSSTLKGVTVPEDRAPSMIDEYPILAVAAAFAEGATVMNGLEELRVKESDRLSAV | 361 |
| Sbjct 303 | ADL R S LKGV VP +RAPSMIDEYPILAVAAAFAEG TVM G+ ELRVKESDR+ AV ADLVARYSALKGVEVPPERAPSMIDEYPILAVAAAFAEGKTVMRGIGELRVKESDRIDAV | 362 |
| Query 362 | ANGLKLNGVDCDEGETSLVVRGRPDGKGLGNASGAAVATHLDHRIAMSFLVMGLVSENPV | 421 |
| Sbjct 363 | A GL+ GV +E E SL V G G G VATHLDHRIAMSFLV GL S PV ARGLEAMGVRIEETEDSLTVHGMGAD---GVPGGGTVATHLDHRIAMSFLVGGLASRRPV | 419 |
| Query 422  Sbjct 420 | TVDDATMIATSFPEFMDLMAGLGAKIELSDTKA 454 VDDA IATSFP F LMA LGA + D A  GVDDAGPIATSFPAFGPLMAALGAPMGAEDDAA 452 |  |

>TR:A0A2Z4UQ04 A0A2Z4UQ04_9RHIZ 3-phosphoshikimate 1-carboxyvinyltransferase OS=Rhodobiaceae bacterium OX=2026785 GN=aroA PE=3 SV=1

Length=443

Score = 482 bits (1241), Expect = 8e-166

Identities = 249/438 (57%), Positives = 312/438 (71%), Gaps = 5/438 (1%)

Query 11	TARKSSGLSGTVRIPGDKSISHRSFMFGGLASGETRITGLLEGEDVINTGKAMQAMGARI 70 TA S L G + +PGDKSISHR+ + G LA G + +TGLLEGEDV+ T +AM+A+GA +

| Sbjct | 4 | TASPSRSLRGHITVPGDKSISHRALILGALAVGTSHVTGLLEGEDVLATAEAMRALGATV | 63 |
| --- | --- | --- | --- |
| Query | 71 | RKEGD-TWIIDGVGNGGLLAPEAPLDFGNAATGCRLTMGLVGVYDFDSTFIGDASLTKRP | 129 |
| Sbjct | 64 | + G+ W + GVG GGL P LDFGN+ TG RL MGLV + +TF+GDASL+KRP TRTGEGAWEVTGVGVGGLSEPATALDFGNSGTGARLVMGLVAGHPITATFVGDASLSKRP | 123 |
| Query | 130 | MGRVLNPLREMGVQVKSEDGDRLPVTLRGPKTPTPITYRVPMASAQVKSAVLLAGLNTPG | 189 |
| Sbjct | 124 | MGRV+ PL EMG + + DG R+P+T++G PITY +P+ASAQVKSAVLLAGLN PG MGRVITPLTEMGAEFHARDGGRMPLTVKGAAVALPITYPLPVASAQVKSAVLLAGLNAPG | 183 |
| Query | 190 | ITTVIEPIMTRDHTEKMLQGFGANLTVETDADGVRTIRLEGRGKLTGQVIDVPGDPSSTA | 249 |
| Sbjct | 184 | TTVIEP TRDHTE+ML+ FGA ++VE V IR+ G+ +LT + VP DPSS A QTTVIEPHPTRDHTERMLRAFGAEVSVEPGPQEVTVIRVTGQTELTPSDVIVPADPSSAA | 243 |
| Query | 250 | FPLVAALLVPGSDVTILNVLMNPTRTGLILTLQEMGADIEVINPRLAGGEDVADLRVRSS | 309 |
| Sbjct | 244 | FP+VAAL P SDVT+ V+MNP R GL TL EMGA+I++ N R GE VADLRVR+S FPIVAALTTPDSDVTVTGVMMNPHRIGLYTTLIEMGANIDIQNEREESGEPVADLRVRTS | 303 |
| Query | 310 | TLKGVTVPEDRAPSMIDEYPILAVAAAFAEGATVMNGLEELRVKESDRLSAVANGLKLNG | 369 |
| Sbjct | 304 | TLKG+ VP DRAPSMIDEYP+LAVAAA AEG T+M G ELRVKESDR++A GL+ NG TLKGIEVPLDRAPSMIDEYPVLAVAAAHAEGTTIMRGAAELRVKESDRVAATVAGLQANG | 363 |
| Query | 370 | VDCDEGETSLVVRGRPDGKGLGNASGAAVATHLDHRIAMSFLVMGLVSENPVTVDDATMI | 429 |
| Sbjct | 364 | V +E + + + G P A G V TH+DHRIAMSFL +G ++ PV +D+ MI VQVEEFDDGMAITGAPGEV AGGGTVLTHMDHRIAMSFLTLGFGAKKPVIIDEGEMI | 419 |
| Query  Sbjct | 430  420 | ATSFPEFMDLMAGLGAKI 447 ATSFP+++ LM GLGA + ATSFPDYVALMRGLGATL 437 |  |

>TR:A0A515KS33 A0A515KS33_9BRAD 3-phosphoshikimate 1-carboxyvinyltransferase OS=Tardiphaga sp. vice352 OX=2592816 GN=aroA PE=3 SV=1

Length=465

Score = 483 bits (1243), Expect = 8e-166

| Identities | = 255/440 (58%), Positives = 318/440 (72%), Gaps = 7/440 (2%) |  |
| --- | --- | --- |
| Query 9 | PATARKSSGLSGTVRIPGDKSISHRSFMFGGLASGETRITGLLEGEDVINTGKAMQAMGA | 68 |
| Sbjct 29 | P +A KS+ L G R+PGDKSISHR+ + G LA G+TRITGLLEGEDV+NT KAMQA+GA PLSAHKSAALEGRERVPGDKSISHRALILGALAVGQTRITGLLEGEDVLNTAKAMQALGA | 88 |
| Query 69 | RIRKEGD-TWIIDGVGNGGLLAPEAPLDFGNAATGCRLTMGLVGVYDFDSTFIGDASLTK | 127 |
| Sbjct 89 | ++ + G+ W + GVG GG AP+ PLDFGN+ TGCRL MG V ++TF GDASL QVERTGEFAWTVRGVGVGGFKAPQQPLDFGNSGTGCRLVMGAVAGCPIEATFDGDASLRS | 148 |
| Query 128 | RPMGRVLNPLREMGVQVK-SEDGDRLPVTLRGPKTPTPITYRVPMASAQVKSAVLLAGLN | 186 |
| Sbjct 149 | RPM R+++PL MG +V S+DG RLP+TL+G + P PI YR P+ASAQ+KSAVLLAGL+ RPMRRIVDPLELMGARVTASQDGGRLPLTLQGARDPLPIVYRTPVASAQIKSAVLLAGLS | 208 |
| Query 187 | TPGITTVIEPIMTRDHTEKMLQGFGANLTVETDADGVRTIRLEGRGKLTGQVIDVPGDPS | 246 |
| Sbjct 209 | PG TTVIE +RDHTE ML+ FGA + E + R I L G+ +L G + VP DPS APGTTTVIEAEASRDHTELMLKHFGAQIVSEKEGVHGRKITLTGQPELRGADVVVPADPS | 268 |
| Query 247 | STAFPLVAALLVPGSDVTILNVLMNPTRTGLILTLQEMGADIEVINPRLAGGEDVADLRV | 306 |
| Sbjct 269 | S AFP+VAAL+VPGSD+ + +V+ NP RTGL TL+EMGA IE + R GE +A R+ SAAFPIVAALIVPGSDIVLTDVMTNPLRTGLFTTLREMGASIEESDIRGDAGEPMAAFRI | 328 |
| Query 307 | RSSTLKGVTVPEDRAPSMIDEYPILAVAAAFAEGATVMNGLEELRVKESDRLSAVANGLK | 366 |
| Sbjct 329 | R+S L GV VP +RAPSMIDEY +LAVAA+FA+G TVM GL+ELRVKESDRL A A+ L+ RASKLVGVEVPPERAPSMIDEYLVLAVAASFAQGTTVMRGLQELRVKESDRLEATASMLR | 388 |
| Query 367 | LNGVDCDEGETSLVVRGRPDGKGLGNASGAAVATHLDHRIAMSFLVMGLVSENPVTVDDA  +NGV+ + L+V G G G VATH+DHRIAMS LVMGL SE PVT+DD | 426 |

| Sbjct | 389 | VNGVNVEIVGDDLIVHGTGSVSG-----GGLVATHMDHRIAMSALVMGLASEQPVTIDDT 443 |
| --- | --- | --- |
| Query | 427 | TMIATSFPEFMDLMAGLGAK 446 |
| Sbjct | 444 | T IATSFP+F+ +M LGA+  TFIATSFPDFIPMMQRLGAE 463 |

>TR:A0A2N3PSR8 A0A2N3PSR8_9PROT 3-phosphoshikimate 1-carboxyvinyltransferase OS=Telmatospirillum siberiense OX=382514 GN=aroA PE=3 SV=1

Length=445

Score = 482 bits (1241), Expect = 8e-166

| Identities | = 268/443 (60%), Positives = 311/443 (70%), Gaps = 5/443 (1%) |  |
| --- | --- | --- |
| Query 6 | SSRPATARKSSGLSGTVRIPGDKSISHRSFMFGGLASGETRITGLLEGEDVINTGKAMQA | 65 |
| Sbjct 2 | S++P + ++ LSG VR+PGDKSISHR+ MFG LA G++ I GLLEGEDV+ T AM+A SAKPLISHAAASLSGQVRVPGDKSISHRALMFGALAIGQSSIEGLLEGEDVLRTAAAMRA | 61 |
| Query 66 | MGARIRKEGD-TWIIDGVGNGGLLAPEAPLDFGNAATGCRLTMGLVGVYDFDSTFIGDAS | 124 |
| Sbjct 62 | +GA + + GD W + G G GGL PE LD GNA TG RL MGLV + F S F GDAS LGAEVTRGGDGRWTLFGRGVGGLAEPEDVLDMGNAGTGARLLMGLVASHPFTSVFTGDAS | 121 |
| Query 125 | LTKRPMGRVLNPLREMGVQVKSEDGDRLPVTLRGPKTPTPITYRVPMASAQVKSAVLLAG | 184 |
| Sbjct 122 | L RPM RV PL MG G RLP+ + G +P PI+Y +PM SAQVKSAVLLAG LRSRPMRRVTEPLGRMGAGFVGRSGGRLPLAVIGTASPIPISYELPMPSAQVKSAVLLAG | 181 |
| Query 185 | LNTPGITTVIEPIMTRDHTEKMLQGFGANLTVETDADGVRTIRLEGRGKLTGQVIDVPGD | 244 |
| Sbjct 182 | LNTPG TTVIE TRDHTE ML+GFGA LTVE A+G R I L G+ +LTGQ I VP D LNTPGTTTVIEREATRDHTELMLRGFGAKLTVEVLANGARAISLTGQPELTGQSIKVPAD | 241 |
| Query 245 | PSSTAFPLVAALLVPGSDVTILNVLMNPTRTGLILTLQEMGADIEVINPRLAGGEDVADL | 304 |
| Sbjct 242 | PSS AFP+VAALLV GS +T+ V NP R GL TL EMGADI N R+ GGE VADL PSSAAFPVVAALLVAGSAITVQAVGTNPLRCGLYQTLLEMGADITYANARVEGGEPVADL | 301 |
| Query 305 | RVRSSTLKGVTVPEDRAPSMIDEYPILAVAAAFAEGATVMNGLEELRVKESDRLSAVANG | 364 |
| Sbjct 302 | VR S LKGV VP +RAPSMIDEYPILAVAAAFA+G T M GL ELRVKESDRL+A+A G LVRGSELKGVDVPPERAPSMIDEYPILAVAAAFAKGTTRMRGLAELRVKESDRLAAMARG | 361 |
| Query 365 | LKLNGVDCDEGETSLVVRGRPDGKGLGNASGAAVATHLDHRIAMSFLVMGLVSENPVTVD | 424 |
| Sbjct 362 | L+ GV + L+V G DG GL G +V +LDHRIAMSFLVMG+ S PV +D LEAAGVTLEVEGDDLIVHG--DG-GLPE-GGVSVPVNLDHRIAMSFLVMGMASRRPVQID | 417 |
| Query 425  Sbjct 418 | DATMIATSFPEFMDLMAGLGAKI 447 DA+ I TSFP F ++M GLGAKI DASAIDTSFPGFTEMMNGLGAKI 440 |  |

>TR:W1RUG6 W1RUG6_9SPHN 3-phosphoshikimate 1-carboxyvinyltransferase OS=Sphingobium sp. C100 OX=1207055 GN=aroA PE=3 SV=1

Length=446

Score = 482 bits (1241), Expect = 8e-166

| Identities | = 254/445 (57%), Positives = 310/445 (70%), Gaps = 4/445 (1%) |  |
| --- | --- | --- |
| Query 3 | HGASSRPATARKSSGLSGTVRIPGDKSISHRSFMFGGLASGETRITGLLEGEDVINTGKA | 62 |
| Sbjct 4 | H P T + LSG + +PGDKSISHRS M LA G +R+ GLLEGEDV+ T A HDDQPAPMTFTAAPALSGGITVPGDKSISHRSLMLSALAVGASRVEGLLEGEDVLATAAA | 63 |
| Query 63 | MQAMGARIRKEGD-TWIIDGVGNGGLLAPEAPLDFGNAATGCRLTMGLVGVYDFDSTFIG | 121 |
| Sbjct 64 | M+AMGA +R++ + W I GVG GGLL P LD GN+ T RL MGL+ +D +TF+G MRAMGAEVRRDAEGVWHIHGVGVGGLLQPRTALDMGNSGTSTRLLMGLLASHDLTATFVG | 123 |
| Query 122 | DASLTKRPMGRVLNPLREMGVQVKSEDGDRLPVTLRGPKTPTPITYRVPMASAQVKSAVL DASL+KRPM RV PL +MG + GDRLP+T++G P+ YR+P+ASAQVKSA+L | 181 |

| Sbjct | 124 | DASLSKRPMARVTEPLSQMGASFSASPGDRLPLTMKGACPAVPLDYRLPVASAQVKSAIL | 183 |
| --- | --- | --- | --- |
| Query | 182 | LAGLNTPGITTVIEPIMTRDHTEKMLQGFGANLTVETDADGVRTIRLEGRGKLTGQVIDV | 241 |
| Sbjct | 184 | LAGLNTPGIT ++EPI TRDH+E+ML+GFGA+L VE + DG R I L G +L Q I V LAGLNTPGITRIVEPIATRDHSERMLKGFGADLAVEVEGDGTRIITLAGEAELRPQQIVV | 243 |
| Query | 242 | PGDPSSTAFPLVAALLVPGSDVTILNVLMNPTRTGLILTLQEMGADIEVINPRLAGGEDV | 301 |
| Sbjct | 244 | PGDPSS AFP+VAALLVPGS VTI NV +N TR GLI L+EMG IEV+N R GGE V PGDPSSAAFPVVAALLVPGSKVTIANVGLNATRAGLIDLLREMGGAIEVVNAREVGGEPV | 303 |
| Query | 302 | ADLRVRSSTLKGVTVPEDRAPSMIDEYPILAVAAAFAEGATVMNGLEELRVKESDRLSAV | 361 |
| Sbjct | 304 | DL V +S L GVT RAPSMIDEYP+ VAAA AEG +V GLEELRVKESDR++ + GDLVVTASALTGVTPDPVRAPSMIDEYPVAFVAAALAEGRSVFRGLEELRVKESDRIATM | 363 |
| Query | 362 | ANGLKLNGVDCDEGETSLVVRGRPDGKGLGNASGAAVATHLDHRIAMSFLVMGLVSENPV | 421 |
| Sbjct | 364 | A GL+ GV +E E +++ G G A G +AT LDHRIAMSF V GLVS+N V AQGLRAIGVQVEELEDGIIIEG---SGGAPLAGGGPIATRLDHRIAMSFAVAGLVSKNGV | 420 |
| Query  Sbjct | 422  421 | TVDDATMIATSFPEFMDLMAGLGAK 446 T+DD +ATSFP FM ++ LGA+ TIDDMRPVATSFPTFMAMLQSLGAQ 445 |  |

>TR:A0A317DTQ0 A0A317DTQ0_9PROT 3-phosphoshikimate 1-carboxyvinyltransferase OS=Zavarzinia compransoris OX=1264899 GN=aroA PE=3 SV=1

Length=438

Score = 482 bits (1240), Expect = 9e-166

| Identities | | = 262/442 (59%), Positives = 314/442 (71%), Gaps = 7/442 (2%) |  |
| --- | --- | --- | --- |
| Query 9  Sbjct 2 | | PATARKSS-GLSGTVRIPGDKSISHRSFMFGGLASGETRITGLLEGEDVINTGKAMQAMG PA + KSS L+G V +PGDKSISHR+ + G LA G T +TGLLEG+DV+ T + M+ +G PAQSAKSSPALAGAVTVPGDKSISHRALILGALAVGRTEVTGLLEGDDVLRTAECMRRLG | 67  61 |
| Query 68  Sbjct 62 | | ARIRKEGD-TWIIDGVGNGGLLAPEAPLDFGNAATGCRLTMGLVGVYDFDSTFIGDASLT A++ + G W +DGVG GGL+ P LD GN+ TG RL MG+V + + F GDASL AKVVRLGPGRWQVDGVGVGGLVEPADVLDMGNSGTGARLLMGVVAGHPITAVFTGDASLN | 126  121 |
| Query 127  Sbjct 122 | | KRPMGRVLNPLREMGVQVKSEDGDRLPVTLRGPKTPTPITYRVPMASAQVKSAVLLAGLN  +RPM RV PL MG G++LP+ + G + P PI YR+P+ASAQVKSA+LLAGL RRPMARVTEPLARMGAAFVGRAGNKLPLAVVGARNPLPIEYRLPVASAQVKSAILLAGLC | 186  181 |
| Query 187  Sbjct 182 | | TPGITTVIEPIMTRDHTEKMLQGFGANLTVETDADGVRTIRLEGRGKLTGQVIDVPGDPS PG TTVIEP TRDH+E++L FGA + + ADG R + L G+ +LT + VPGDPS APGETTVIEPEPTRDHSERLLGHFGAEIRIVDAADGSRRVTLRGQPELTAAPVVVPGDPS | 246  241 |
| Query 247  Sbjct 242 | | STAFPLVAALLVPGSDVTILNVLMNPTRTGLILTLQEMGADIEVINPRLAGGEDVADLRV S AFPLVAAL+VPGSDV I NV +NP R GL TL EMGA IE +N R+AGGE VADLRV SAAFPLVAALIVPGSDVIIENVGLNPLRIGLFDTLIEMGASIEYLNRRVAGGEPVADLRV | 306  301 |
| Query 307  Sbjct 302 | | RSSTLKGVTVPEDRAPSMIDEYPILAVAAAFAEGATVMNGLEELRVKESDRLSAVANGLK R S LKGVTVP +RAPSMIDEYP+LAVAA FA G T MNGL ELRVKESDRL+AVA GL RHSALKGVTVPAERAPSMIDEYPVLAVAAGFATGTTRMNGLAELRVKESDRLAAVAAGLA | 366  361 |
| Query 367  Sbjct 362 | | LNGVDCDEGETSLVVRGRPDGKGLGNASGAAVATHLDHRIAMSFLVMGLVSENPVTVDDA NG+ + G L+V G+ GK G G VATH+DHRIAMSFLVMGL +E PVTVDD ANGIAHEAGPDFLIVAGK--GKVPG---GGTVATHMDHRIAMSFLVMGLAAERPVTVDDI | 426  416 |
| Query 427 | | TMIATSFPEFMDLMAGLGAKIE 448 MI TSFP F LMA LGA +E |  |
| Sbjct | 417 | HMIDTSFPGFGTLMASLGATLE 438 | |

>TR:A0A095B9Q8 A0A095B9Q8_9SPHN 3-phosphoshikimate 1-carboxyvinyltransferase

OS=Sphingopyxis sp. LC363 OX=1120705 GN=aroA PE=3 SV=1 Length=442

Score = 482 bits (1240), Expect = 1e-165

| Identities | = 259/439 (59%), Positives = 310/439 (71%), Gaps = 4/439 (1%) |  |
| --- | --- | --- |
| Query 6 | SSRPATARKSSGLSGTVRIPGDKSISHRSFMFGGLASGETRITGLLEGEDVINTGKAMQA | 65 |
| Sbjct 5 | ++ P + S L G + IPGDKSISHRS M LA GE+R+ GLLEG DV+ T AM+A TATPRSFSASVPLKGRIAIPGDKSISHRSLMLSALAVGESRVAGLLEGHDVLATAAAMRA | 64 |
| Query 66 | MGARIRKEGD-TWIIDGVGNGGLLAPEAPLDFGNAATGCRLTMGLVGVYDFDSTFIGDAS | 124 |
| Sbjct 65 | MGA I + D W I GVG GGLL P LD GN+ T RL MGLV + +TF+GDAS MGATIERRDDGEWRIHGVGVGGLLQPREALDMGNSGTSTRLLMGLVASHPITATFVGDAS | 124 |
| Query 125 | LTKRPMGRVLNPLREMGVQVKSEDGDRLPVTLRGPKTPTPITYRVPMASAQVKSAVLLAG | 184 |
| Sbjct 125 | L+ RPMGRV++PL +MG + + G RLP+ +RG P++YR+PMASAQVKSAVLLAG LSGRPMGRVIDPLAQMGADISASPGGRLPLMVRGLAPAIPLSYRLPMASAQVKSAVLLAG | 184 |
| Query 185 | LNTPGITTVIEPIMTRDHTEKMLQGFGANLTVETDADGVRTIRLEGRGKLTGQVIDVPGD | 244 |
| Sbjct 185 | LNTPGIT VIEP+ TRDH+E+ML+GFGA LTVETD+ G R IR+ G +L Q I VPGD LNTPGITEVIEPVPTRDHSERMLRGFGAELTVETDSGGTRHIRIRGEAELRPQTIVVPGD | 244 |
| Query 245 | PSSTAFPLVAALLVPGSDVTILNVLMNPTRTGLILTLQEMGADIEVINPRLAGGEDVADL | 304 |
| Sbjct 245 | PSS AF +VAAL+VPGSDVTI NV +NPTR GL+ LQ MG +I +++ R GGE VADL PSSAAFFIVAALVVPGSDVTIANVGLNPTRAGLVEVLQAMGGNITLLDAREVGGEPVADL | 304 |
| Query 305 | RVRSSTLKGVTVPEDRAPSMIDEYPILAVAAAFAEGATVMNGLEELRVKESDRLSAVANG | 364 |
| Sbjct 305 | RVR S LKG+ V APSMIDE+PIL VAAA AEG TV GL+ELRVKESDRL+ +A G RVRHSNLKGIAVDPAVAPSMIDEFPILFVAAALAEGRTVTTGLDELRVKESDRLAVMAAG | 364 |
| Query 365 | LKLNGVDCDEGETSLVVRGRPDGKGLGNASGAAVATHLDHRIAMSFLVMGLVSENPVTVD | 424 |
| Sbjct 365 | LK G +E E LV+ G G GA +A HLDHRI MSF V GLVS+ PVT+D LKAIGARVEESEDGLVIDGT---GGDPLPGGATIAGHLDHRICMSFAVAGLVSKAPVTID | 421 |
| Query 425  Sbjct 422 | DATMIATSFPEFMDLMAGL 443 D +ATSFP F L+AGL DIAPVATSFPNFETLLAGL 440 |  |

>TR:A0A1G9IVT7 A0A1G9IVT7_9RHOB 3-phosphoshikimate 1-carboxyvinyltransferase OS=Paracoccus chinensis OX=525640 GN=aroA PE=3 SV=1

Length=443

Score = 482 bits (1240), Expect = 1e-165

| Identities | = 255/448 (57%), Positives = 312/448 (70%), Gaps = 7/448 (2%) |  |
| --- | --- | --- |
| Query 1 | MLHGASSRPATARKSSGLSGTVRIPGDKSISHRSFMFGGLASGETRITGLLEGEDVINTG | 60 |
| Sbjct 1 | M H A P +AR+S L G ++PGDKS+SHR+ + G LA GETRITGLLEG+DV++T MSHSAEPLPMSARRSGPLKGEAQVPGDKSVSHRALILGALAVGETRITGLLEGQDVLDTA | 60 |
| Query 61 | KAMQAMGARIRKEGD-TWIIDGVGNGGLLAPEAPLDFGNAATGCRLTMGLVGVYDFDSTF | 119 |
| Sbjct 61 | KAMQA GA +++ G+ W++ GVG GG PE +D GN+ TG RL MG + +TF KAMQAFGAEVQRVGEGEWVVHGVGVGGFAEPEGVIDCGNSGTGVRLIMGAMATTPITATF | 120 |
| Query 120 | IGDASLTKRPMGRVLNPLREMGVQVKSEDGDRLPVTLRGPKTPTPITYRVPMASAQVKSA | 179 |
| Sbjct 121 | GD SL++RPM R+ +PL G QV + +G LP+T+RG P P+ YR P+ASAQ+KSA TGDPSLSRRPMRRITDPLELFGAQVTAREGGLLPLTIRGAADPAPVLYRTPVASAQIKSA | 180 |
| Query 180 | VLLAGLNTPGITTVIEPIMTRDHTEKMLQGFGANLTVETDADGVRTIRLEGRGKLTGQVI | 239 |
| Sbjct 181 | VLLAGLN PG T VIE TRDHTE+ML GFGAN+T E A+G R I L GR +L + VLLAGLNAPGQTVVIETEPTRDHTERMLAGFGANVTTEVTAEG-RVITLTGRPELRPCTV | 239 |
| Query 240 | DVPGDPSSTAFPLVAALLVPGSDVTILNVLMNPTRTGLILTLQEMGADIEVINPRLAGGE VP DPSS AFP+ AAL+VPGS++ + V NPTR GL +TL+EMGAD+ N R GGE | 299 |

| Sbjct | 240 | AVPRDPSSAAFPVAAALIVPGSEIRVPGVSRNPTRDGLYVTLEEMGADLTWENQREEGGE | 299 |
| --- | --- | --- | --- |
| Query | 300 | DVADLRVRSSTLKGVTVPEDRAPSMIDEYPILAVAAAFAEGATVMNGLEELRVKESDRLS | 359 |
| Sbjct | 300 | VADL VR L+GVTV +RA SMIDE+PIL+V A FAEG TVMNG+ ELRVKESDR+ PVADLIVRHGPLRGVTVSAERAASMIDEFPILSVIATFAEGTTVMNGVAELRVKESDRID | 359 |
| Query | 360 | AVANGLKLNGVDCDEGETSLVVRGRPDGKGLGNASGAAVATHLDHRIAMSFLVMGLVSEN | 419 |
| Sbjct | 360 | A+A GL+ NG +E SL V G G A GA ATHLDHRIAMSFLV GL SE AMARGLEANGARIEETRDSLTVHGTG GLAGGATCATHLDHRIAMSFLVAGLASEQ | 414 |
| Query  Sbjct | 420  415 | PVTVDDATMIATSFPEFMDLMAGLGAKI 447 P+ VDDA IATSFP+F+ LM LGA+I PIAVDDAGPIATSFPDFVPLMTRLGAQI 442 |  |

>SP:B3Q5Z7 AROA_RHOPT 3-phosphoshikimate 1-carboxyvinyltransferase OS=Rhodopseudomonas palustris (strain TIE-1) OX=395960 GN=aroA PE=3

SV=1

Length=445

Score = 482 bits (1240), Expect = 1e-165

| Identities | | = 259/440 (59%), Positives = 311/440 (71%), Gaps = 7/440 (2%) |  |
| --- | --- | --- | --- |
| Query 9  Sbjct 9 | | PATARKSSGLSGTVRIPGDKSISHRSFMFGGLASGETRITGLLEGEDVINTGKAMQAMGA P ARKS L GT R+PGDKSISHR+ + G LA GETRI+GLLEGEDVINT KAM+A+GA PLQARKSGALHGTARVPGDKSISHRALILGALAVGETRISGLLEGEDVINTAKAMRALGA | 68  68 |
| Query 69  Sbjct 69 | | RIRKEGDT-WIIDGVGNGGLLAPEAPLDFGNAATGCRLTMGLVGVYDFDSTFIGDASLTK  ++ + GD W + GVG G PEAPLDFGN+ TGCRL MG V +TF GDASL KVERTGDCEWRVHGVGVAGFATPEAPLDFGNSGTGCRLAMGAVAGSPIVATFDGDASLRS | 127  128 |
| Query 128  Sbjct 129 | | RPMGRVLNPLREMGVQV-KSEDGDRLPVTLRGPKTPTPITYRVPMASAQVKSAVLLAGLN RPM R+++PL MG +V S +G RLP+ L+G + P PI YR P+ SAQ+KSAVLLAGL+ RPMRRIVDPLELMGAKVVSSSEGGRLPLALQGARDPLPILYRTPVPSAQIKSAVLLAGLS | 186  188 |
| Query 187  Sbjct 189 | | TPGITTVIEPIMTRDHTEKMLQGFGANLTVETDADGVRTIRLEGRGKLTGQVIDVPGDPS PG+TTVIE +RDHTE MLQ FGA + E + R I L G+ +L G + VP DPS APGVTTVIEAEASRDHTELMLQHFGATIVTEAEGAHGRKISLTGQPELRGAPVVVPADPS | 246  248 |
| Query 247  Sbjct 249 | | STAFPLVAALLVPGSDVTILNVLMNPTRTGLILTLQEMGADIEVINPRLAGGEDVADLRV S AFP+VAAL+VPGSD+ + +V+ NP RTGLI TL+EMGA IE + R GE +A RV SAAFPMVAALVVPGSDIELTDVMTNPLRTGLITTLREMGALIEDSDVRGDAGEPMARFRV | 306  308 |
| Query 307  Sbjct 309 | | RSSTLKGVTVPEDRAPSMIDEYPILAVAAAFAEGATVMNGLEELRVKESDRLSAVANGLK R S LKGV VP +RAPSMIDEY +LAVAAAFAEG TVM GL ELRVKESDRL A A L+ RGSKLKGVEVPPERAPSMIDEYLVLAVAAAFAEGTTVMRGLHELRVKESDRLEATAAMLR | 366  368 |
| Query 367  Sbjct 369 | | LNGVDCDEGETSLVVRGRPDGKGLGNASGAAVATHLDHRIAMSFLVMGLVSENPVTVDDA  +NGV + L+V G+ G G VATH+DHRIAMS L MGL S+ PVTVDD VNGVAVEIAGDDLIVEGKGHVPG GGVVATHMDHRIAMSALAMGLASDKPVTVDDT | 426  423 |
| Query 427 | | TMIATSFPEFMDLMAGLGAK 446 IATSFP+F+ +M LGA+ |  |
| Sbjct | 424 | AFIATSFPDFVPMMQRLGAE 443 | |

>SP:Q07UI1 AROA_RHOP5 3-phosphoshikimate 1-carboxyvinyltransferase OS=Rhodopseudomonas palustris (strain BisA53) OX=316055 GN=aroA PE=3

SV=1

Length=445

Score = 482 bits (1240), Expect = 1e-165

Identities = 260/447 (58%), Positives = 313/447 (70%), Gaps = 7/447 (2%)

| Query | 1 | MLHGASSRPATARKSSGLSGTVRIPGDKSISHRSFMFGGLASGETRITGLLEGEDVINTG | 60 |
| --- | --- | --- | --- |
| Sbjct | 1 | M H P ARKS L G VR+PGDKSISHR+ + G L+ GET ITGLLEGEDV+NT MTHSVQPTPLQARKSPALQGRVRVPGDKSISHRALILGALSVGETTITGLLEGEDVLNTA | 60 |
| Query | 61 | KAMQAMGARIRKEGD-TWIIDGVGNGGLLAPEAPLDFGNAATGCRLTMGLVGVYDFDSTF | 119 |
| Sbjct | 61 | KAMQA+GA++ + G+ W ++GVG GG P+A LDFGN+ TGCRL MG V +TF KAMQALGAKVERTGEFAWRVNGVGVGGFAQPDAALDFGNSGTGCRLVMGAVAGCPIAATF | 120 |
| Query | 120 | IGDASLTKRPMGRVLNPLREMGVQV-KSEDGDRLPVTLRGPKTPTPITYRVPMASAQVKS | 178 |
| Sbjct | 121 | GDASL RPM R+++PL+ MG V S +G +LP+TL+G K P PI YR P+ASAQ+KS DGDASLRSRPMKRIVDPLQLMGASVIASAEGGKLPLTLQGAKNPLPIEYRTPVASAQIKS | 180 |
| Query | 179 | AVLLAGLNTPGITTVIEPIMTRDHTEKMLQGFGANLTVETDADGVRTIRLEGRGKLTGQV | 238 |
| Sbjct | 181 | AVLLAGL+ PG+TTVIE +RDHTE MLQ FGA + + R I L G+ +L G AVLLAGLSAPGVTTVIEQEASRDHTELMLQHFGAQIVSVPEGVHGRKISLTGQPELRGAP | 240 |
| Query | 239 | IDVPGDPSSTAFPLVAALLVPGSDVTILNVLMNPTRTGLILTLQEMGADIEVINPRLAGG | 298 |
| Sbjct | 241 | + VP DPSS AFP+VAAL+VPGSD+ + +V+ NP RTGL TL+EMGA IE R G VVVPADPSSAAFPMVAALIVPGSDLVLTDVMTNPLRTGLFATLREMGASIEESELRDDAG | 300 |
| Query | 299 | EDVADLRVRSSTLKGVTVPEDRAPSMIDEYPILAVAAAFAEGATVMNGLEELRVKESDRL | 358 |
| Sbjct | 301 | E +A RVR+S LKGVTVP +RAPSMIDEY +LAVAAAFAEG T M GL+ELRVKESDRL EPMATFRVRASKLKGVTVPPERAPSMIDEYLVLAVAAAFAEGTTRMRGLQELRVKESDRL | 360 |
| Query | 359 | SAVANGLKLNGVDCDEGETSLVVRGRPDGKGLGNASGAAVATHLDHRIAMSFLVMGLVSE | 418 |
| Sbjct | 361 | A A L++NGVD L+V GR G G VATH+DHRIAMS LVMGL S+ EATAAMLRVNGVDVAIEGDDLIVEGRGHVPG GGLVATHMDHRIAMSALVMGLASD | 415 |
| Query  Sbjct | 419  416 | NPVTVDDATMIATSFPEFMDLMAGLGA 445 PV VDD IATSFP+F+ +M LGA  QPVQVDDTAFIATSFPDFIPMMRRLGA 442 |  |

>TR:W3RKN5 W3RKN5_9BRAD 3-phosphoshikimate 1-carboxyvinyltransferase OS=Afipia sp. P52-10 OX=1429916 GN=aroA PE=3 SV=1

Length=445

Score = 482 bits (1240), Expect = 1e-165

| Identities | = 250/448 (56%), Positives = 320/448 (71%), Gaps = 7/448 (2%) |  |
| --- | --- | --- |
| Query 1 | MLHGASSRPATARKSSGLSGTVRIPGDKSISHRSFMFGGLASGETRITGLLEGEDVINTG | 60 |
| Sbjct 1 | M H P AR+S+ LSG VR+PGDKSISHRS + G LA GETRI+GLLEGEDV+NT MTHSDRKTPLEARRSAALSGRVRVPGDKSISHRSLILGALAVGETRISGLLEGEDVLNTA | 60 |
| Query 61 | KAMQAMGARIRKEGD-TWIIDGVGNGGLLAPEAPLDFGNAATGCRLTMGLVGVYDFDSTF | 119 |
| Sbjct 61 | AM+A+GAR+ ++G+ W + GVG G ++P PLDFGN+ TGCRL MG V + F AAMRALGARVERQGEGAWTVHGVGVAGFVSPAKPLDFGNSGTGCRLAMGAVAGSPVTAVF | 120 |
| Query 120 | IGDASLTKRPMGRVLNPLREMGVQVKS-EDGDRLPVTLRGPKTPTPITYRVPMASAQVKS | 178 |
| Sbjct 121 | GDASL RPM R+L+PL +MG + DG RLP+TL G + P PI YR P+ASAQ+KS DGDASLRSRPMRRILDPLEQMGARSSDGADGGRLPLTLHGARDPVPIVYRTPVASAQIKS | 180 |
| Query 179 | AVLLAGLNTPGITTVIEPIMTRDHTEKMLQGFGANLTVETDADGVRTIRLEGRGKLTGQV | 238 |
| Sbjct 181 | AVLLAGL+ PG+TTVIE +RDHTE ML+ FGA ++ E + R I L+G+ +L G AVLLAGLSAPGVTTVIEKEWSRDHTELMLRHFGAEVSSEPEGPHGRKISLKGQPELKGAA | 240 |
| Query 239 | IDVPGDPSSTAFPLVAALLVPGSDVTILNVLMNPTRTGLILTLQEMGADIEVINPRLAGG | 298 |
| Sbjct 241 | + VP DPSS AFP+VAAL+VP S++ + +V+ NP RTGLI TL+EMG I+ +N R G VVVPADPSSAAFPMVAALVVPDSEIVLTDVMTNPLRTGLIKTLREMGGQIDELNTRRDAG | 300 |
| Query 299 | EDVADLRVRSSTLKGVTVPEDRAPSMIDEYPILAVAAAFAEGATVMNGLEELRVKESDRL | 358 |
| Sbjct 301 | E +A+ RVR+S L+GV VP +RAPSMIDEY +LAVAA+FAEG T+M GL+ELRVKESDRL  EPMAEFRVRASRLRGVEVPAERAPSMIDEYLVLAVAASFAEGTTIMRGLQELRVKESDRL | 360 |

| Query | 359 | SAVANGLKLNGVDCDEGETSLVVRGRPDGKGLGNASGAAVATHLDHRIAMSFLVMGLVSE 418 |
| --- | --- | --- |
| Sbjct | 361 | +A A+ L++NGV + ++V GR G G VATH+DHRIAMS L+MGL S+ AATADMLRVNGVKVEIDGDDMIVEGRGHVPG-----GGLVATHMDHRIAMSALMMGLASD 415 |
| Query | 419 | NPVTVDDATMIATSFPEFMDLMAGLGAK 446 |
| Sbjct | 416 | PV VDD IATSFP+F+ +M +GA+  LPVKVDDTAFIATSFPDFVPMMQKMGAE 443 |

>TR:A0A1I3SEP6 A0A1I3SEP6_9RHOB 3-phosphoshikimate 1-carboxyvinyltransferase OS=Celeribacter halophilus OX=576117 GN=aroA PE=3 SV=1

Length=449

Score = 482 bits (1240), Expect = 1e-165

| Identities | | = 255/441 (58%), Positives = 307/441 (70%), Gaps = 4/441 (1%) |  |
| --- | --- | --- | --- |
| Query 9  Sbjct 9 | | PATARKSSGLSGTVRIPGDKSISHRSFMFGGLASGETRITGLLEGEDVINTGKAMQAMGA P T+RKS L GT +PGDKSISHR+ +FG +A GETRITGLLEGEDV++T KAMQA+GA PMTSRKSGPLKGTAAVPGDKSISHRALIFGAMAVGETRITGLLEGEDVLDTAKAMQALGA | 68  68 |
| Query 69  Sbjct 69 | | RIRKEG-DTWIIDGVGNGGLLAPEAPLDFGNAATGCRLTMGLVGVYDFDSTFIGDASLTK  + +EG W + GVG GG PE +D GN+ TG RL MG V YDF ++F GD SL K DVVREGAGEWTVHGVGVGGFTEPEDVIDCGNSGTGVRLIMGCVATYDFCTSFTGDGSLRK | 127  128 |
| Query 128  Sbjct 129 | | RPMGRVLNPLREMGVQVKSEDGDRLPVTLRGPKTPTPITYRVPMASAQVKSAVLLAGLNT RPM RV +PL G + G RLP+TL G P P+ Y P+ SAQVKSAVL AGLN RPMARVTDPLALFGARAVGRSGGRLPLTLVGAAEPVPVEYETPVPSAQVKSAVLFAGLNA | 187  188 |
| Query 188  Sbjct 189 | | PGITTVIEPIMTRDHTEKMLQGFGANLTVETDADGVRTIRLEGRGKLTGQVIDVPGDPSS PG T VIE TRDHTE+ML GFGA + ET +G I L GR +LT Q + VP DPSS PGKTVVIEKEATRDHTERMLTGFGAEVVTETTPEGYNKITLTGRPELTPQEVAVPRDPSS | 247  248 |
| Query 248  Sbjct 249 | | TAFPLVAALLVPGSDVTILNVLMNPTRTGLILTLQEMGADIEVINPRLAGGEDVADLRVR AFP+ AAL+V GS++ + V N TR GL TL EMGADI NPR GGE VADLRV+ AAFPVCAALIVEGSEIFVPGVSQNLTRNGLYTTLVEMGADIAFENPREEGGEPVADLRVK | 307  308 |
| Query 308  Sbjct 309 | | SSTLKGVTVPEDRAPSMIDEYPILAVAAAFAEGATVMNGLEELRVKESDRLSAVANGLKL S LKG+ VP +RA SMIDEYP+L+V AAFAEG TVM G++ELRVKESDR+ A+A GL+ YSKLKGIDVPPERAASMIDEYPVLSVVAAFAEGTTVMKGVKELRVKESDRIDAMARGLEA | 367  368 |
| Query 368  Sbjct 369 | | NGVDCDEGETSLVVRGRPDGKGLGNASGAAVATHLDHRIAMSFLVMGLVSENPVTVDDAT GV+ +E E +L+V G G GA ATH+DHRIAMSFLV+G+ ++ PVTVDDA+ CGVEVEEDEDTLIVHG---CGAQGVKGGATCATHIDHRIAMSFLVLGMAAQEPVTVDDAS | 427  425 |
| Query 428 | | MIATSFPEFMDLMAGLGAKIE 448 IATSFP F LM LGA ++ |  |
| Sbjct | 426 | PIATSFPVFEKLMTDLGADVK 446 | |

>TR:A0A1I7MV05 A0A1I7MV05_9RHIZ 3-phosphoshikimate 1-carboxyvinyltransferase OS=Hyphomicrobium facile OX=51670 GN=aroA PE=3 SV=1

Length=461

Score = 482 bits (1241), Expect = 1e-165

Identities = 260/445 (58%), Positives = 316/445 (71%), Gaps = 8/445 (2%)

| Query | 5 | ASSRPATARKSSGLSGTVRIPGDKSISHRSFMFGGLASGETRITGLLEGEDVINTGKAMQ | 64 |
| --- | --- | --- | --- |
| Sbjct | 19 | A+ +P ++ +++ L G VR+PGDKSISHR+ +FG LA+G TRITGLLE EDVINT KA+ ATPKPLSSLRAAPLKGRVRVPGDKSISHRALIFGALATGTTRITGLLEAEDVINTAKAVN | 78 |
| Query | 65 | AMGARIRKEGDTWIIDGVGNGGLLAPEAPLDFGNAATGCRLTMGLVGVYDFDSTFIGDAS | 124 |
| Sbjct | 79 | A+GA K GD W + G G GGL +P+ PLDFGN+ TG RL MG+V + GDAS  ALGALAEKRGDVWEVKGRGVGGLRSPDGPLDFGNSGTGARLMMGVVAGHSMTVVMTGDAS | 138 |

| Query | 125 | LTKRPMGRVLNPLREMGVQVKSEDGDRLPVTLRGPKTPTPITYRVPMASAQVKSAVLLAG | 184 |
| --- | --- | --- | --- |
| Sbjct | 139 | L++RPM RVL PL EMG++V ++LP+TLRG TPI Y +P+ SAQVKSAVL+AG LSRRPMRRVLAPLMEMGLEVLEPGQEKLPLTLRGTSELTPIVYVLPVPSAQVKSAVLIAG | 198 |
| Query | 185 | LNTPGITTVIEPIMTRDHTEKMLQGFGANLTVETDADGVRT-IRLEGRGKLTGQVIDVPG | 243 |
| Sbjct | 199 | L+ G TTV+E TRDHTE+ML+ FGA +V T+A G+ T I + G +L G+ + VPG LHAAGETTVVEREPTRDHTERMLRHFGA--SVRTEAKGLDTHITVTGHAELVGRDVHVPG | 256 |
| Query | 244 | DPSSTAFPLVAALLVPGSDVTILNVLMNPTRTGLILTLQEMGADIEVINPRLAGGEDVAD | 303 |
| Sbjct | 257 | DPSS AF AALLVPGSDVTI VL NPTR GL TLQEMG DI +N R GGE VAD DPSSAAFLAAAALLVPGSDVTIEGVLTNPTRIGLYTTLQEMGGDITFLNEREEGGEPVAD | 316 |
| Query | 304 | LRVRSSTLKGVTVPEDRAPSMIDEYPILAVAAAFAEGATVMNGLEELRVKESDRLSAVAN | 363 |
| Sbjct | 317 | +R+R+S LKGV VP +RAPSMIDEYP+LA +AFAEG T M+GL EL+VKESDRL A A IRIRASRLKGVRVPAERAPSMIDEYPVLAAMSAFAEGQTQMDGLAELKVKESDRLQATAA | 376 |
| Query | 364 | GLKLNGVDCDEGETSLVVRGRPDGKGLGNASGAAVATHLDHRIAMSFLVMGLVSENPVTV | 423 |
| Sbjct | 377 | GL+ NG SL V G KG G VATHLDHRIAM+FL GLVS+ P+ V GLEANGAVVRVDGDSLTVTGNGGLKG GGVVATHLDHRIAMAFLTAGLVSDKPIVV | 431 |
| Query  Sbjct | 424  432 | DDATMIATSFPEFMDLMAGLGAKIE 448 DD+TMIATSFPEF LM LGA+ + DDSTMIATSFPEFRQLMETLGARYD 456 |  |

>TR:A0A368Z218 A0A368Z218_9RHOB 3-phosphoshikimate 1-carboxyvinyltransferase OS=Paracoccus lutimaris OX=1490030 GN=aroA PE=3 SV=1

Length=444

Score = 481 bits (1239), Expect = 2e-165

| Identities | = 257/451 (57%), Positives = 317/451 (70%), Gaps = 10/451 (2%) |  |
| --- | --- | --- |
| Query 1 | MLHGASSRPATARKSSGLSGTVRIPGDKSISHRSFMFGGLASGETRITGLLEGEDVINTG | 60 |
| Sbjct 1 | M H A P TAR+S L G R+PGDKSISHR+ + G L+ GET ITGLLEG+DV++T MSHSAEPLPMTARRSGALKGEARVPGDKSISHRALILGALSVGETHITGLLEGQDVLDTA | 60 |
| Query 61 | KAMQAMGARIRKEGD-TWIIDGVGNGGLLAPEAPLDFGNAATGCRLTMGLVGVYDFDSTF | 119 |
| Sbjct 61 | KAMQA GAR+ + G+ W ++GVG GG PE +D GN+ TG RL MG + +TF KAMQAFGARVERLGEGEWKVNGVGVGGFAEPEGVIDCGNSGTGVRLIMGAMATTPITATF | 120 |
| Query 120 | IGDASLTKRPMGRVLNPLREMGVQVKSEDGDRLPVTLRGPKTPTPITYRVPMASAQVKSA | 179 |
| Sbjct 121 | GDASL++RPMGRV +PL G ++ S +G RLPVT++G P P+ Y+ P+ASAQ+KSA TGDASLSRRPMGRVTDPLELFGCEITSREGKRLPVTIKGAADPVPVRYKTPVASAQIKSA | 180 |
| Query 180 | VLLAGLNTPGITTVIEPIMTRDHTEKMLQGFGANLTVETDADGVRTIRLEGRGKLTGQVI | 239 |
| Sbjct 181 | VLLAGLN PG T VIE TRDHTE+ML GFGA++T E +G R I L GR L Q + VLLAGLNAPGQTVVIEAEPTRDHTERMLAGFGASITTEVTDEG-RVITLTGRPDLKPQPV | 239 |
| Query 240 | DVPGDPSSTAFPLVAALLVPGSDVTILNVLMNPTRTGLILTLQEMGADIEVINPRLAGGE | 299 |
| Sbjct 240 | VP DPSS AFP+ AAL+VPGS++ + V NPTR GL +TL EMGADI NPR GGE AVPRDPSSAAFPVAAALIVPGSEIRVPGVSRNPTRDGLYVTLLEMGADITFENPREEGGE | 299 |
| Query 300 | DVADLRVR-SSTLKGVTVPEDRAPSMIDEYPILAVAAAFAEGATVMNGLEELRVKESDRL | 358 |
| Sbjct 300 | VADL VR S LKGVTVP +RA SMIDE+PIL+V AAFAEG TVM G+ ELRVKESDR+ PVADLLVRYSPALKGVTVPTERAASMIDEFPILSVIAAFAEGTTVMQGVHELRVKESDRI | 359 |
| Query 359 | SAVANGLKLNGVDCDEGETSLVVRGRPDGKGLGNASGAAVA-THLDHRIAMSFLVMGLVS | 417 |
| Sbjct 360 | A+ GL+ NG ++ E ++ V G G+ +G AVA THLDHRIAMSFLV GL S DAMVVGLRANGAHVEDTEDTMTVH GTGHLTGGAVAVTHLDHRIAMSFLVAGLAS | 413 |
| Query 418  Sbjct 414 | ENPVTVDDATMIATSFPEFMDLMAGLGAKIE 448  + ++VDD IATSFP+F+ LM LGA+I+ DQMISVDDGGPIATSFPDFLPLMRALGAQID 444 |  |

>TR:A0A558R856 A0A558R856_9SPHN 3-phosphoshikimate 1-carboxyvinyltransferase OS=Sphingomonas solaris OX=2529389 GN=aroA PE=3 SV=1

Length=437

Score = 481 bits (1238), Expect = 2e-165

| Identities | = 258/435 (59%), Positives = 306/435 (70%), Gaps = 8/435 (2%) |  |
| --- | --- | --- |
| Query 18 | LSGTVRIPGDKSISHRSFMFGGLASGETRITGLLEGEDVINTGKAMQAMGARIRKEGD-T | 76 |
| Sbjct 1 | + G V +PGDKSISHRS M G LA GET + G+LEGEDVI T +AM+AMGA + ++GD MRGHVTVPGDKSISHRSLMLGALAVGETVVEGMLEGEDVIATAQAMRAMGATVERDGDGR | 60 |
| Query 77 | WIIDGVGNGGLLAPEAPLDFGNAATGCRLTMGLVGVYDFDSTFIGDASLTKRPMGRVLNP | 136 |
| Sbjct 61 | W I GVG GGLL P LD GN+ T RL MGLV + +TFIGDASL++RPMGRV+ P WHIHGVGVGGLLQPATALDMGNSGTSTRLLMGLVASHPITATFIGDASLSRRPMGRVIEP | 120 |
| Query 137 | LREMGVQVKSEDGDRLPVTLRGPKTPTPITYRVPMASAQVKSAVLLAGLNTPGITTVIEP | 196 |
| Sbjct 121 | L MG + GDRLP+ LRG PI YR+P+ASAQVKSA+LLAGLNTPGIT VIEP LSRMGAAFTASPGDRLPLMLRGLCPAVPIEYRLPVASAQVKSAILLAGLNTPGITRVIEP | 180 |
| Query 197 | IMTRDHTEKMLQGFGANLTVETDADGVRTIRLEGRGKLTGQVIDVPGDPSSTAFPLVAAL | 256 |
| Sbjct 181 | + TRDH+E+ML+GFGA+LTVE ADG R I L G +L Q I VPGDPSS AFP+VAAL VPTRDHSERMLRGFGADLTVEVQADGTRVISLRGEAELRPQHIVVPGDPSSAAFPVVAAL | 240 |
| Query 257 | LVPGSDVTILNVLMNPTRTGLILTLQEMGADIEVINPRLAGGEDVADLRVRSSTLKGVTV | 316 |
| Sbjct 241 | LVPGSDVT+ NV +NPTR GL L+ MGADIE +N R GGE VADLRVR STL G+ V LVPGSDVTVANVGLNPTRAGLFGILRAMGADIEYLNRREVGGEPVADLRVRHSTLHGIEV | 300 |
| Query 317 | PEDRAPSMIDEYPILAVAAAFAEGATVMNGLEELRVKESDRLSAVANGLKLNGVDCDEGE | 376 |
| Sbjct 301 | P D PSM+DE+PIL VAAA A G TVM+GLEELRVKESDR++ +A GL G E E PPDVVPSMVDEFPILFVAAAMARGRTVMHGLEELRVKESDRIAVMARGLAHCGARVTEHE | 360 |
| Query 377 | TSLVVRGRPDG KGLGNASGAAVATHLDHRIAMSFLVMGLVSENPVTVDDATMIA | 430 |
| Sbjct 361 | + + G DG +G+ VA LDHRIAMSF V GL S+ + +DD +A DGMTIDG-SDGAPLAGPSSIGDERVPTVAAELDHRIAMSFAVAGLASQGGIRIDDMAPVA | 419 |
| Query 431  Sbjct 420 | TSFPEFMDLMAGLGA 445 TSFP F ++A LGA TSFPTFTSILADLGA 434 |  |

>TR:A0A1G5CCG2 A0A1G5CCG2_9RHOB 3-phosphoshikimate 1-carboxyvinyltransferase OS=Paracoccus tibetensis OX=336292 GN=aroA PE=3 SV=1

Length=443

Score = 481 bits (1238), Expect = 2e-165

| Identities | = 249/449 (55%), Positives = 315/449 (70%), Gaps = 7/449 (2%) |  |
| --- | --- | --- |
| Query 1 | MLHGASSRPATARKSSGLSGTVRIPGDKSISHRSFMFGGLASGETRITGLLEGEDVINTG | 60 |
| Sbjct 1 | M H A RP T+R S L+G ++PGDKSISHR+ + G L+ GET+ITGLLEG+DV++T MSHDAEPRPMTSRASGPLTGEAQVPGDKSISHRALILGALSVGETKITGLLEGQDVLDTA | 60 |
| Query 61 | KAMQAMGARIRKEGD-TWIIDGVGNGGLLAPEAPLDFGNAATGCRLTMGLVGVYDFDSTF | 119 |
| Sbjct 61 | AM+A GA + + G W + GVG GG +PE +D GN+ TG RL MG + +TF AAMRAFGADVVQHGPGNWSVHGVGVGGFSSPEGVIDCGNSGTGVRLIMGAMATTPITATF | 120 |
| Query 120 | IGDASLTKRPMGRVLNPLREMGVQVKSEDGDRLPVTLRGPKTPTPITYRVPMASAQVKSA | 179 |
| Sbjct 121 | GDASL++RPM RV +PL + G ++ + +G RLP+T+ G PI YR P+ASAQ+KSA TGDASLSRRPMARVTDPLSQFGARITAREGGRLPITIEGAADALPIRYRSPVASAQIKSA | 180 |
| Query 180 | VLLAGLNTPGITTVIEPIMTRDHTEKMLQGFGANLTVETDADGVRTIRLEGRGKLTGQVI | 239 |
| Sbjct 181 | +LLAGLN PG T VIEP TRDH+E+ML GFGA + ET A+G + L+G+ +L Q +  ILLAGLNAPGETVVIEPEQTRDHSERMLAGFGAQVHAETAAEG-HVVTLQGQPELRAQAV | 239 |

| Query | 240 | DVPGDPSSTAFPLVAALLVPGSDVTILNVLMNPTRTGLILTLQEMGADIEVINPRLAGGE | 299 |
| --- | --- | --- | --- |
| Sbjct | 240 | VP DPSS AFP+ AAL+VPGSD+ + V NPTR G +TL EMGADI NPR GGE AVPRDPSSAAFPVAAALIVPGSDILVPGVSRNPTRDGFYVTLLEMGADISFENPREEGGE | 299 |
| Query | 300 | DVADLRVRSSTLKGVTVPEDRAPSMIDEYPILAVAAAFAEGATVMNGLEELRVKESDRLS | 359 |
| Sbjct | 300 | VADLRVR S L+GVTVPE RA SMIDE+PIL+V A+FAEG TVMNG+ ELRVKESDR+ PVADLRVRHSRLRGVTVPESRAASMIDEFPILSVVASFAEGKTVMNGVSELRVKESDRID | 359 |
| Query | 360 | AVANGLKLNGVDCDEGETSLVVRGRPDGKGLGNASGAAVATHLDHRIAMSFLVMGLVSEN | 419 |
| Sbjct | 360 | A+A GL+ NGV +E S+ V G +G G A THLDHRIAMSFLV+GL ++ AMARGLEANGVTVEETPDSMTVHGASGVRGGGRA VTHLDHRIAMSFLVLGLAADQ | 414 |
| Query  Sbjct | 420  415 | PVTVDDATMIATSFPEFMDLMAGLGAKIE 448 PV +DDA I TSFP+F+ LM LGA+ + PVEIDDAGPITTSFPDFVPLMTRLGARFD 443 |  |

>TR:A0A6I5YQH9 A0A6I5YQH9_9SPHN 3-phosphoshikimate 1-carboxyvinyltransferase OS=Sphingobium sp. CAP-1 OX=2676077 GN=aroA PE=3 SV=1

Length=446

Score = 481 bits (1238), Expect = 2e-165

| Identities | = 256/444 (58%), Positives = 310/444 (70%), Gaps = 4/444 (1%) |  |
| --- | --- | --- |
| Query 3 | HGASSRPATARKSSGLSGTVRIPGDKSISHRSFMFGGLASGETRITGLLEGEDVINTGKA | 62 |
| Sbjct 4 | H P T + LSG+V +PGDKSISHRS M LA GE+R+ GLLEGEDV+ T A HSDHPTPRTFTAAPALSGSVAVPGDKSISHRSLMLSALAIGESRVEGLLEGEDVLATAAA | 63 |
| Query 63 | MQAMGARIRKEGD-TWIIDGVGNGGLLAPEAPLDFGNAATGCRLTMGLVGVYDFDSTFIG | 121 |
| Sbjct 64 | M+AMGA I ++ D W I GVG GGLL PE LD GN+ T RL MGL+ +D +TF G MRAMGATIERDADGIWHIHGVGVGGLLQPETALDMGNSGTSTRLLMGLLASHDLTATFTG | 123 |
| Query 122 | DASLTKRPMGRVLNPLREMGVQVKSEDGDRLPVTLRGPKTPTPITYRVPMASAQVKSAVL | 181 |
| Sbjct 124 | DASL+KRPM RV PL MG + GDRLP+T++G P+ Y +P+ASAQVKSA+L DASLSKRPMARVTEPLSRMGASFTTSPGDRLPLTMKGACPAVPLDYTLPVASAQVKSAIL | 183 |
| Query 182 | LAGLNTPGITTVIEPIMTRDHTEKMLQGFGANLTVETDADGVRTIRLEGRGKLTGQVIDV | 241 |
| Sbjct 184 | LAGLNTPGIT V+EPI TRDH+E+ML+GFGA+L VE +ADG R I L G +L Q I V LAGLNTPGITRVVEPIPTRDHSERMLRGFGADLQVEVEADGTRIITLVGEAELKPQNIVV | 243 |
| Query 242 | PGDPSSTAFPLVAALLVPGSDVTILNVLMNPTRTGLILTLQEMGADIEVINPRLAGGEDV | 301 |
| Sbjct 244 | PGDPSS AFP+VAALLVPGS VTI NV +N TR GLI L+EMG IEV+N R GGE V PGDPSSAAFPMVAALLVPGSQVTISNVGLNATRAGLIDLLREMGGSIEVVNAREVGGEPV | 303 |
| Query 302 | ADLRVRSSTLKGVTVPEDRAPSMIDEYPILAVAAAFAEGATVMNGLEELRVKESDRLSAV | 361 |
| Sbjct 304 | DL V +S L+GV RAPSMIDEYP+ +AAAFAEG ++ GLEELRVKESDR++ + GDLIVTASALRGVEPDPARAPSMIDEYPVAFIAAAFAEGRSIFRGLEELRVKESDRIATM | 363 |
| Query 362 | ANGLKLNGVDCDEGETSLVVRGRPDGKGLGNASGAAVATHLDHRIAMSFLVMGLVSENPV | 421 |
| Sbjct 364 | A GL+ GV +E E +++ G G A G +AT LDHRIAMSF V GLVS++ V AQGLRAIGVTVEELEDGIIIEG---SGGAPLAGGGPIATRLDHRIAMSFAVAGLVSKDGV | 420 |
| Query 422  Sbjct 421 | TVDDATMIATSFPEFMDLMAGLGA 445 T+DD +ATSFP F+ L+ LGA TIDDMRPVATSFPTFLPLLQTLGA 444 |  |

>TR:A0A291GFC5 A0A291GFC5_9RHOB 3-phosphoshikimate 1-carboxyvinyltransferase OS=Celeribacter ethanolicus OX=1758178 GN=aroA PE=3 SV=1

Length=449

Score = 481 bits (1238), Expect = 3e-165

Identities = 255/440 (58%), Positives = 305/440 (69%), Gaps = 4/440 (1%)

| Query | 9 | PATARKSSGLSGTVRIPGDKSISHRSFMFGGLASGETRITGLLEGEDVINTGKAMQAMGA | 68 |
| --- | --- | --- | --- |
| Sbjct | 9 | P TARK+ L GT +PGDKSISHR+ +FG +A GET+ITGLLEGEDV++T KAMQA+GA PMTARKNGPLKGTALVPGDKSISHRALIFGAMAVGETKITGLLEGEDVLDTAKAMQALGA | 68 |
| Query | 69 | RIRKEG-DTWIIDGVGNGGLLAPEAPLDFGNAATGCRLTMGLVGVYDFDSTFIGDASLTK | 127 |
| Sbjct | 69 | + +EG W + GVG GG PE +D GN+ TG RL MG V YDF +TF GD SL K EVVREGAGEWTVHGVGVGGFTEPEDVIDCGNSGTGVRLIMGCVATYDFCTTFTGDGSLRK | 128 |
| Query | 128 | RPMGRVLNPLREMGVQVKSEDGDRLPVTLRGPKTPTPITYRVPMASAQVKSAVLLAGLNT | 187 |
| Sbjct | 129 | RPM RV +PL G Q G RLP+TL G P P+ Y+ P+ SAQVKSAVLLAGLN RPMARVTDPLALFGAQSVGRSGGRLPLTLVGASDPVPVEYQTPVPSAQVKSAVLLAGLNA | 188 |
| Query | 188 | PGITTVIEPIMTRDHTEKMLQGFGANLTVETDADGVRTIRLEGRGKLTGQVIDVPGDPSS | 247 |
| Sbjct | 189 | PG T VIE TRDHTE+ML GFGA LT E +G I L GR +L Q + VP DPSS PGKTVVIEKEATRDHTERMLVGFGAELTTEKTPEGYNKITLTGRPELRPQEVAVPRDPSS | 248 |
| Query | 248 | TAFPLVAALLVPGSDVTILNVLMNPTRTGLILTLQEMGADIEVINPRLAGGEDVADLRVR | 307 |
| Sbjct | 249 | AFP+ AAL+VPGS+V + V NPTR GL +TL+EMGADI N R GGE VADLRV+ AAFPVCAALIVPGSEVFVPGVSQNPTRNGLFITLKEMGADITFENEREEGGEPVADLRVK | 308 |
| Query | 308 | SSTLKGVTVPEDRAPSMIDEYPILAVAAAFAEGATVMNGLEELRVKESDRLSAVANGLKL | 367 |
| Sbjct | 309 | S LKG+ VP +RA SMIDEYPIL+ AA+AEG T M G++ELRVKESDR+ A+A GL+ YSKLKGIEVPAERAASMIDEYPILSAVAAYAEGTTTMRGVKELRVKESDRIDAMARGLEA | 368 |
| Query | 368 | NGVDCDEGETSLVVRGRPDGKGLGNASGAAVATHLDHRIAMSFLVMGLVSENPVTVDDAT | 427 |
| Sbjct | 369 | GV +E + +L+V G G GA ATHLDHRIAMSFL++G+ S+ V+VDD CGVAIEEDQDTLIVHGLGPN---GVKGGAICATHLDHRIAMSFLILGMASQEAVSVDDGG | 425 |
| Query | 428 | MIATSFPEFMDLMAGLGAKI 447 IATSFP F LM GLGA + |  |
| Sbjct | 426 | PIATSFPVFEKLMTGLGAHV 445 | |

>TR:A0A564G6R9 A0A564G6R9_9RHIZ 3-phosphoshikimate 1-carboxyvinyltransferase OS=Methylobacterium dankookense OX=560405 GN=aroA PE=3 SV=1

Length=450

Score = 481 bits (1238), Expect = 3e-165

| Identities | = 265/445 (60%), Positives = 313/445 (70%), Gaps = 8/445 (2%) |  |
| --- | --- | --- |
| Query 8 | RPATARKSSGLSGTVRIPGDKSISHRSFMFGGLASGETRITGLLEGEDVINTGKAMQAMG | 67 |
| Sbjct 9 | +P TA + L G ++ PGDKSISHR+ + G LA GETR+ GLLEG+DV+ T A +A+G QPLTASPGAPLRGALKPPGDKSISHRAMILGLLAVGETRVEGLLEGDDVLRTAAAAEALG | 68 |
| Query 68 | ARIRKEGD-TWIIDGVGNGGLLAPEAPLDFGNAATGCRLTMGLVGVYDFDSTFIGDASLT | 126 |
| Sbjct 69 | A + + G+ W++ GVG GG+ P A LDFGNA TG RL MG+VG +TF GDASL AGVERLGEGRWLVRGVGIGGMSDPAATLDFGNAGTGSRLMMGVVGGQPVTATFDGDASLR | 128 |
| Query 127 | KRPMGRVLNPLREMGVQVKSED-GDRLPVTLRGPKTPTPITYRVPMASAQVKSAVLLAGL | 185 |
| Sbjct 129 | RPM R+L+PL +MG +V SE G R+P+TLRGP+ PITY P ASAQ+KSAVLLAGL SRPMRRILDPLVKMGTEVVSEAAGGRVPLTLRGPREAIPITYETPAASAQIKSAVLLAGL | 188 |
| Query 186 | NTPGITTVIEPIMTRDHTEKMLQGFGANLTVETDADGV--RTIRLEGRGKLTGQVIDVPG | 243 |
| Sbjct 189 | N PG TTVIE TRDHTE+ML+ FGA + V G R + L G+ L G + VP NAPGTTTVIEAAATRDHTERMLRLFGAEVAVTPHGPGGQGRAVALTGQPTLRGTDVIVPA | 248 |
| Query 244 | DPSSTAFPLVAALLVPGSDVTILNVLMNPTRTGLILTLQEMGADIEVINPRLAGGEDVAD | 303 |
| Sbjct 249 | DPSS AFPLVAAL+VPGS+VT+ V+MNP R GLI TL EMGA+IE N R GGE VAD DPSSAAFPLVAALVVPGSEVTLRGVMMNPLRIGLITTLLEMGAEIERTNEREEGGETVAD | 308 |
| Query 304 | LRVRSSTLKGVTVPEDRAPSMIDEYPILAVAAAFAEGATVMNGLEELRVKESDRLSAVAN | 363 |
| Sbjct 309 | LRVR+S LKGV VP +RAPSMIDEYP+LAVAA+FAEG T M GL ELRVKESDRL AVA  LRVRASRLKGVDVPPERAPSMIDEYPVLAVAASFAEGTTRMAGLHELRVKESDRLEAVAA | 368 |

| Query | 364 | GLKLNGVDCDEGETSLVVRGRPDGKGLGNASGAAVATHLDHRIAMSFLVMGLVSENPVTV 423 |
| --- | --- | --- |
| Sbjct | 369 | GL+ NGV + LVV G G G VATHLDHRIAM+FLVMGL + PVTV GLEANGVRFEVQGDDLVVH----GDGAAPQGGGTVATHLDHRIAMAFLVMGLATREPVTV 424 |
| Query | 424 | DDATMIATSFPEFMDLMAGLGAKIE 448 |
| Sbjct | 425 | DD MIATSFP F+ M LG +IE  DDGAMIATSFPSFLPTMRELGGRIE 449 |

>TR:A0A318TCD3 A0A318TCD3_9BRAD 3-phosphoshikimate 1-carboxyvinyltransferase OS=Rhodopseudomonas faecalis OX=99655 GN=aroA PE=3 SV=1

Length=445

Score = 481 bits (1237), Expect = 3e-165

| Identities | = 261/448 (58%), Positives = 315/448 (70%), Gaps = 7/448 (2%) |  |
| --- | --- | --- |
| Query 1 | MLHGASSRPATARKSSGLSGTVRIPGDKSISHRSFMFGGLASGETRITGLLEGEDVINTG | 60 |
| Sbjct 1 | M H + +P ARKS+ L G VR+PGDKSISHR+ + G L+ GETRITGLLEGEDV+NT MTHCSQPKPLEARKSTALHGEVRVPGDKSISHRALILGALSVGETRITGLLEGEDVLNTA | 60 |
| Query 61 | KAMQAMGARIRKEGD-TWIIDGVGNGGLLAPEAPLDFGNAATGCRLTMGLVGVYDFDSTF | 119 |
| Sbjct 61 | KAMQA+GA++ + G+ W ++GVG G P+A LDFGN+ TGCRL MG V F KAMQALGAQVARTGEFAWKVNGVGVAGFAQPQAALDFGNSGTGCRLVMGAVAGCPITVPF | 120 |
| Query 120 | IGDASLTKRPMGRVLNPLREMGVQVKS-EDGDRLPVTLRGPKTPTPITYRVPMASAQVKS | 178 |
| Sbjct 121 | GDASL RPM RVL+PL+ MG V S DG RLP+TL+G + P PI YR P+ASAQ+KS DGDASLRSRPMRRVLDPLQLMGASVVSCADGGRLPLTLQGARDPLPIEYRTPVASAQIKS | 180 |
| Query 179 | AVLLAGLNTPGITTVIEPIMTRDHTEKMLQGFGANLTVETDADGVRTIRLEGRGKLTGQV | 238 |
| Sbjct 181 | AVLLAGL PG+TTVIE +RDHTE ML+ FGA ++ + + R I L G+ +L G AVLLAGLAAPGVTTVIESEASRDHTELMLRHFGATISSQPEGAHGRRIALTGQPELRGAP | 240 |
| Query 239 | IDVPGDPSSTAFPLVAALLVPGSDVTILNVLMNPTRTGLILTLQEMGADIEVINPRLAGG | 298 |
| Sbjct 241 | + VP DPSS AFP+VAALLVPGSDV + +V+ NP RTGL TLQEMGA IE+ R G VVVPADPSSAAFPMVAALLVPGSDVVLTDVMTNPLRTGLFTTLQEMGASIEISELRGDAG | 300 |
| Query 299 | EDVADLRVRSSTLKGVTVPEDRAPSMIDEYPILAVAAAFAEGATVMNGLEELRVKESDRL | 358 |
| Sbjct 301 | E +A RV++S LKGV VP RAP+MIDEY +LAVAAAFAEG T M GL+ELRVKESDRL EPMAQFRVKASRLKGVDVPAARAPAMIDEYLVLAVAAAFAEGTTRMRGLQELRVKESDRL | 360 |
| Query 359 | SAVANGLKLNGVDCDEGETSLVVRGRPDGKGLGNASGAAVATHLDHRIAMSFLVMGLVSE | 418 |
| Sbjct 361 | A A L++NGVD L+V GR G G VATH+DHRIAMS LVMGL S+ EATAAMLRVNGVDVAIEGDDLIVEGRGHVPG GGLVATHMDHRIAMSALVMGLASD | 415 |
| Query 419  Sbjct 416 | NPVTVDDATMIATSFPEFMDLMAGLGAK 446 PV VDD IATSFP+F+ +M LGA+  QPVRVDDTAFIATSFPDFVPMMRRLGAE 443 |  |

>TR:A0A0Q3I1Q9 A0A0Q3I1Q9_9BRAD 3-phosphoshikimate 1-carboxyvinyltransferase OS=Bosea thiooxidans OX=53254 GN=aroA PE=3 SV=1

Length=447

Score = 481 bits (1237), Expect = 4e-165

Identities = 263/449 (59%), Positives = 315/449 (70%), Gaps = 5/449 (1%)

| Query | 1 | MLHGASSRPATARKSSGLSGTVRIPGDKSISHRSFMFGGLASGETRITGLLEGEDVINTG | 60 |
| --- | --- | --- | --- |
| Sbjct | 1 | M H P +A L G VR+PGDKSISHR+ +FG LA GET++TGLLEGEDV+ T MSHDHDPVPLSASSGGPLKGRVRVPGDKSISHRAMIFGLLAIGETKVTGLLEGEDVMRTA | 60 |
| Query | 61 | KAMQAMGARIRKEGD-TWIIDGVGNGGLLAPEAPLDFGNAATGCRLTMGLVGVYDFDSTF | 119 |
| Sbjct | 61 | A +A+GA + +G +W + GVG GGL P LDFGNA TG RL MG+ G + +TF  AAARALGAIVHHDGPGSWRVQGVGVGGLHEPAGVLDFGNAGTGSRLMMGVCGSHPITTTF | 120 |

| Query | 120 | IGDASLTKRPMGRVLNPLREMG-VQVKSEDGDRLPVTLRGPKTPTPITYRVPMASAQVKS | 178 |
| --- | --- | --- | --- |
| Sbjct | 121 | GDASL KRPM R+L+PL +MG V V ++G R+P+TLRGPK PITYR P+ASAQ+KS DGDASLRKRPMRRILDPLEQMGTVVVSQQEGGRVPLTLRGPKEAIPITYRPPVASAQIKS | 180 |
| Query | 179 | AVLLAGLNTPGITTVIEPIMTRDHTEKMLQGFGANLTVETDADGVRTIRLEGRGKLTGQV | 238 |
| Sbjct | 181 | AVLLA L PG TTVIE TRDHTEKML FGA ++V + R I L+G+ +L AVLLAALAAPGETTVIEAEATRDHTEKMLAHFGAEISVTPEGAHGRRIVLKGQPELRAAP | 240 |
| Query | 239 | IDVPGDPSSTAFPLVAALLVPGSDVTILNVLMNPTRTGLILTLQEMGADIEVINPRLAGG | 298 |
| Sbjct | 241 | I VP DPSS AFPLVA L+VPGSD+ I +V+ NP R+GL+ TL+EMGADI + GG IVVPADPSSAAFPLVAGLIVPGSDILIESVMTNPLRSGLLTTLREMGADITIEREANEGG | 300 |
| Query | 299 | EDVADLRVRSSTLKGVTVPEDRAPSMIDEYPILAVAAAFAEGATVMNGLEELRVKESDRL | 358 |
| Sbjct | 301 | E VADLRVR+S+LKGVTVP +RAP+MIDEYP+LAVAAAFA G T M GL+ELRVKESDRL ETVADLRVRASSLKGVTVPPERAPAMIDEYPVLAVAAAFASGTTRMCGLQELRVKESDRL | 360 |
| Query | 359 | SAVANGLKLNGVDCDEGETSLVVRGRPDGKGLGNASGAAVATHLDHRIAMSFLVMGLVSE | 418 |
| Sbjct | 361 | +AV GLK GV C L+V G G G G +ATHLDHRIAMSFLVMGL ++ AAVEAGLKAAGVRCAIEGDDLIVEGSGGGPVRG---GGPIATHLDHRIAMSFLVMGLATQ | 417 |
| Query | 419 | NPVTVDDATMIATSFPEFMDLMAGLGAKI 447 PV VDD MI TSFP F+ LM LG +I |  |
| Sbjct | 418 | QPVQVDDGEMITTSFPSFVPLMNQLGGRI 446 | |

>SP:Q2RXP5 AROA_RHORT 3-phosphoshikimate 1-carboxyvinyltransferase OS=Rhodospirillum rubrum (strain ATCC 11170 / ATH 1.1.1 / DSM 467

/ LMG 4362 / NCIB 8255 / S1) OX=269796 GN=aroA PE=3 SV=1

Length=451

Score = 481 bits (1237), Expect = 4e-165

| Identities | | = 265/447 (59%), Positives = 306/447 (68%), Gaps = 9/447 (2%) |  |
| --- | --- | --- | --- |
| Query 8  Sbjct 8 | | RPATARKSSGLSGTVRIPGDKSISHRSFMFGGLASGETRITGLLEGEDVINTGKAMQAMG RP A +S+ LSG +R+PGDKSISHR+ M GGLA G T I GLLEGEDVI T AM+AMG RPLRAHRSTPLSGRIRVPGDKSISHRALMLGGLAVGRTEIRGLLEGEDVIATAHAMEAMG | 67  67 |
| Query 68  Sbjct 68 | | ARIRKE GDTWIIDGVGNGGLLAPEAPLDFGNAATGCRLTMGLVGVYDFDSTFIGD  ARI ++ W +DGVG GGL P LD GNA TG RL MGL+ +D + GD ARIDRQETADGAGVWTVDGVGVGGLAEPADVLDMGNAGTGARLLMGLLATHDLTAILTGD | 122  127 |
| Query 123  Sbjct 128 | | ASLTKRPMGRVLNPLREMGVQVKSEDGDRLPVTLRGPKTPTPITYRVPMASAQVKSAVLL ASL RPM RV +PL G G RLP+ +RG TP P++YRVP+ SAQVKSAVLL ASLRGRPMKRVTDPLALFGASFVGRSGGRLPMAVRGTATPLPVSYRVPVPSAQVKSAVLL | 182  187 |
| Query 183  Sbjct 188 | | AGLNTPGITTVIEPIMTRDHTEKMLQGFGANLTVETDADGVRTIRLEGRGKLTGQVIDVP AGLNTPG TTVIEP+ TRDHTE+ML FGA L + D G TI L G+ +L ++VP AGLNTPGETTVIEPVATRDHTERMLGHFGAALRLGRDDQGATTITLTGQPELRAAPVEVP | 242  247 |
| Query 243  Sbjct 248 | | GDPSSTAFPLVAALLVPGSDVTILNVLMNPTRTGLILTLQEMGADIEVINPRLAGGEDVA DPSS AFPLVAA+LVP S VT+ V MNP R GLI TL+EMGADI + +PR+ GE VA ADPSSAAFPLVAAVLVPESHVTLAGVGMNPQRIGLIDTLREMGADILIRDPRIEAGEPVA | 302  307 |
| Query 303  Sbjct 308 | | DLRVRSSTLKGVTVPEDRAPSMIDEYPILAVAAAFAEGATVMNGLEELRVKESDRLSAVA DL VR+S L G+ VP RAPSMIDEYPILAVAAA A G T M+GL ELRVKESDRLSAVA DLEVRASALTGIEVPAARAPSMIDEYPILAVAAACARGTTRMHGLGELRVKESDRLSAVA | 362  367 |
| Query 363 | | NGLKLNGVDCDEGETSLVVRGRPDGKGLGNASGAAVATHLDHRIAMSFLVMGLVSENPVT GL GVD +L+V GKG GA VA +LDHRI M+FLV+GLVS VT | 422 |
| Sbjct | 368 | TGLAACGVDVTVDGDTLIVH GKGTVPKGGATVAVNLDHRIGMAFLVLGLVSAEAVT | 423 |
| Query  Sbjct | 423  424 | VDDATMIATSFPEFMDLMAGLGAKIEL 449  +DD I TSFP F+ LM GLGA I L IDDGRAIDTSFPGFVTLMTGLGAPISL 450 |  |

>TR:A0A2A3N132 A0A2A3N132_9SPHN 3-phosphoshikimate 1-carboxyvinyltransferase OS=Sphingobium sp. D43FB OX=2017595 GN=aroA PE=3 SV=1

Length=446

Score = 480 bits (1236), Expect = 5e-165

| Identities | | = 257/439 (59%), Positives = 311/439 (71%), Gaps = 4/439 (1%) |  |
| --- | --- | --- | --- |
| Query 8  Sbjct 9 | | RPATARKSSGLSGTVRIPGDKSISHRSFMFGGLASGETRITGLLEGEDVINTGKAMQAMG RP T + LSG+V +PGDKSISHRS M LA GE+R+ GLLEGEDV+ T AM+AMG RPMTFTAAPALSGSVTVPGDKSISHRSLMLSALAVGESRVEGLLEGEDVLATAAAMRAMG | 67  68 |
| Query 68  Sbjct 69 | | ARIRKEGD-TWIIDGVGNGGLLAPEAPLDFGNAATGCRLTMGLVGVYDFDSTFIGDASLT A I+++ D W I GVG GGLL PE LD GN+ T RL MGL+ +D +TFIGDASL+ ADIQRDADGIWHIHGVGVGGLLQPEGALDMGNSGTSTRLLMGLLASHDLTATFIGDASLS | 126  128 |
| Query 127  Sbjct 129 | | KRPMGRVLNPLREMGVQVKSEDGDRLPVTLRGPKTPTPITYRVPMASAQVKSAVLLAGLN KRPM RV +PL +MG + G RLP+T+RG P+ YR+P+ASAQVKSA+LLAGLN KRPMARVTDPLTQMGASFTTSPGGRLPLTMRGACPAVPLDYRLPVASAQVKSAILLAGLN | 186  188 |
| Query 187  Sbjct 189 | | TPGITTVIEPIMTRDHTEKMLQGFGANLTVETDADGVRTIRLEGRGKLTGQVIDVPGDPS TPGIT V+EPI TRDH+E+ML+GFGA+L VE +ADG R I L G +L Q I VPGDPS TPGITRVVEPIPTRDHSERMLRGFGADLHVEVEADGTRIITLVGEAELQPQQIIVPGDPS | 246  248 |
| Query 247  Sbjct 249 | | STAFPLVAALLVPGSDVTILNVLMNPTRTGLILTLQEMGADIEVINPRLAGGEDVADLRV S AFP+VAALLVPGS VTI NV +N TR GLI L+EMG IEVIN R GGE V DL V SAAFPMVAALLVPGSQVTIANVGLNATRAGLIDLLREMGGSIEVINAREVGGEPVGDLLV | 306  308 |
| Query 307  Sbjct 309 | | RSSTLKGVTVPEDRAPSMIDEYPILAVAAAFAEGATVMNGLEELRVKESDRLSAVANGLK  +STL+GV RAPSMIDEYP+ +AAA A+G ++ GL+ELRVKESDR++ +A GL+ TASTLQGVEPDPARAPSMIDEYPVAFIAAALAQGRSIFRGLDELRVKESDRIATMAAGLR | 366  368 |
| Query 367  Sbjct 369 | | LNGVDCDEGETSLVVRGRPDGKGLGNASGAAVATHLDHRIAMSFLVMGLVSENPVTVDDA GV +E E L++ G G G +AT LDHRIAMSF V GLVS + VT+DD  AIGVTVEELEDGLIIEG---SGGAPLQGGGPIATKLDHRIAMSFAVAGLVSRDGVTIDDM | 426  425 |
| Query 427 | | TMIATSFPEFMDLMAGLGA 445  +ATSFP F L+ LGA |  |
| Sbjct | 426 | RPVATSFPTFTALLQNLGA 444 | |

>TR:A0A1N7PC71 A0A1N7PC71_9RHOB 3-phosphoshikimate 1-carboxyvinyltransferase OS=Rhodobacter aestuarii OX=453582 GN=aroA PE=3 SV=1

Length=448

Score = 480 bits (1236), Expect = 5e-165

| Identities | = 258/439 (59%), Positives = 308/439 (70%), Gaps = 7/439 (2%) |  |
| --- | --- | --- |
| Query 11 | TARKSSGLSGTVRIPGDKSISHRSFMFGGLASGETRITGLLEGEDVINTGKAMQAMGARI | 70 |
| Sbjct 11 | TARK LSG +PGDKSISHR+ + G L+ G T ITGLLEG+DV++T KAMQA GA + TARKGGPLSGVAEVPGDKSISHRALILGALSVGRTEITGLLEGQDVLDTAKAMQAFGATV | 70 |
| Query 71 | RKEGD-TWIIDGVGNGGLLAPEAPLDFGNAATGCRLTMGLVGVYDFDSTFIGDASLTKRP | 129 |
| Sbjct 71 | + G W ++GVG GG PEA +D GN+ TG RL MG + +TF GDASL KRP TQHGPGAWSVEGVGVGGFAEPEAVIDCGNSGTGVRLIMGCMASSALTATFTGDASLRKRP | 130 |
| Query 130 | MGRVLNPLREMGVQVKSEDGDRLPVTLRGPKTPTPITYRVPMASAQVKSAVLLAGLNTPG | 189 |
| Sbjct 131 | MGRV +PL G + +G RLP+T+ G P P+ Y PMASAQVKSAVLLAGLN PG MGRVTDPLALFGARAYGREGGRLPMTVVGAAEPVPVCYTTPMASAQVKSAVLLAGLNAPG | 190 |
| Query 190 | ITTVIEPIMTRDHTEKMLQGFGANLTVETDADGVRTIRLEGRGKLTGQVIDVPGDPSSTA | 249 |
| Sbjct 191 | T VIE TRDHTE+ML+GFGA +TVE +G R I L+G+ +LT Q + VP DPSS A  ETVVIEKTPTRDHTERMLRGFGAEVTVEETPEGNR-ITLKGQPELTAQKVAVPRDPSSAA | 249 |

| Query | 250 | FPLVAALLVPGSDVTILNVLMNPTRTGLILTLQEMGADIEVINPRLAGGEDVADLRVRSS | 309 |
| --- | --- | --- | --- |
| Sbjct | 250 | FP+ AALLVPGSD+ + V NPTR GL LTL+EMGA+IE +N R GGE VADLRVR S FPVCAALLVPGSDIMVPGVSQNPTRNGLFLTLKEMGANIEFLNEREEGGEPVADLRVRYS | 309 |
| Query | 310 | TLKGVTVPEDRAPSMIDEYPILAVAAAFAEGATVMNGLEELRVKESDRLSAVANGLKLNG | 369 |
| Sbjct | 310 | L G+TVP RA S IDEYP+LA AA A G T M G+ ELRVKESDR+ A+A GL+ G ELTGITVPAARAASQIDEYPVLAAVAACARGTTTMPGVHELRVKESDRIDAMARGLEACG | 369 |
| Query | 370 | VDCDEGETSLVVRG-RPDGKGLGNASGAAVATHLDHRIAMSFLVMGLVSENPVTVDDATM | 428 |
| Sbjct | 370 | V +E E +L+V G PDG GA VATH+DHRIAMSFLV+GL S+ P+TVDD + VKIEEDEDTLIVHGLGPDGV PGGATVATHIDHRIAMSFLVLGLASQQPITVDDGSP | 425 |
| Query  Sbjct | 429  426 | IATSFPEFMDLMAGLGAKI 447 IATSFP F +LM GLGA I IATSFPVFEELMRGLGATI 444 |  |

>TR:A0A327KLT1 A0A327KLT1_9RHIZ 3-phosphoshikimate 1-carboxyvinyltransferase OS=Rhodoplanes roseus OX=29409 GN=aroA PE=3 SV=1

Length=449

Score = 480 bits (1236), Expect = 5e-165

| Identities | = 263/445 (59%), Positives = 316/445 (71%), Gaps = 3/445 (1%) |  |
| --- | --- | --- |
| Query 5 | ASSRPATARKSSGLSGTVRIPGDKSISHRSFMFGGLASGETRITGLLEGEDVINTGKAMQ | 64 |
| Sbjct 5 | A+ P AR + L+G VR+PGDKSISHR+ + G LA G T +TGLLEGEDV+ T +AM+ AALMPLAARGARPLAGRVRVPGDKSISHRALILGALAVGRTTVTGLLEGEDVLATAQAMR | 64 |
| Query 65 | AMGARIRKEGD-TWIIDGVGNGGLLAPEAPLDFGNAATGCRLTMGLVGVYDFDSTFIGDA | 123 |
| Sbjct 65 | +GA + + G W ++GVG GG AP LDFGN+ TGCRL MG V + F GDA LLGATVTRTGPGAWTVEGVGVGGFAAPPETLDFGNSGTGCRLVMGAVAGSPVTAMFDGDA | 124 |
| Query 124 | SLTKRPMGRVLNPLREMGVQV-KSEDGDRLPVTLRGPKTPTPITYRVPMASAQVKSAVLL | 182 |
| Sbjct 125 | SL +RPM R+L+P+ +MG V +S +GDRLPV L G + P PI Y P+ SAQVKSAVLL SLRRRPMRRILDPILKMGATVVQSTEGDRLPVMLAGARDPLPIEYTTPVPSAQVKSAVLL | 184 |
| Query 183 | AGLNTPGITTVIEPIMTRDHTEKMLQGFGANLTVETDADGVRTIRLEGRGKLTGQVIDVP | 242 |
| Sbjct 185 | AGL+ PG T VIE TRDHTEKML FGA++TV D R I L G+ +L G + VP AGLSAPGHTVVIESEATRDHTEKMLAHFGADVTVVPDGPHGRRITLVGQPELHGADVVVP | 244 |
| Query 243 | GDPSSTAFPLVAALLVPGSDVTILNVLMNPTRTGLILTLQEMGADIEVINPRLAGGEDVA | 302 |
| Sbjct 245 | DPSS AFPLVAALLVPGSDV + NV+ NP RTGLI TL+EMGA IE + R GGE +A ADPSSAAFPLVAALLVPGSDVILDNVMTNPLRTGLITTLREMGASIEEVARREDGGEAMA | 304 |
| Query 303 | DLRVRSSTLKGVTVPEDRAPSMIDEYPILAVAAAFAEGATVMNGLEELRVKESDRLSAVA | 362 |
| Sbjct 305 | DLRVR+S L GV VP DRAP+MIDEYP+LAVAAAFA G TVM GL+ELRVKESDRL+A A DLRVRASDLVGVEVPPDRAPAMIDEYPVLAVAAAFATGRTVMRGLKELRVKESDRLAATA | 364 |
| Query 363 | NGLKLNGVDCDEGETSLVVRGRPDGKGLGNASGAAVATHLDHRIAMSFLVMGLVSENPVT | 422 |
| Sbjct 365 | + L++NGV ++V G G+GL G VA+H+DHRIAMS LVMGL SE PV+ DLLRVNGVTVTIDGDDMIVEGLGAGRGL-PPGGGLVASHMDHRIAMSALVMGLASEKPVS | 423 |
| Query 423  Sbjct 424 | VDDATMIATSFPEFMDLMAGLGAKI 447 VDDA IATSFP F++ M GLGA I VDDAAFIATSFPGFVETMRGLGADI 448 |  |

>TR:A0A1I3VG95 A0A1I3VG95_9RHOB 3-phosphoshikimate 1-carboxyvinyltransferase OS=Celeribacter neptunius OX=588602 GN=aroA PE=3 SV=1

Length=449

Score = 480 bits (1236), Expect = 5e-165

Identities = 257/441 (58%), Positives = 304/441 (69%), Gaps = 6/441 (1%)

| Query | 9 | PATARKSSGLSGTVRIPGDKSISHRSFMFGGLASGETRITGLLEGEDVINTGKAMQAMGA | 68 |
| --- | --- | --- | --- |
| Sbjct | 9 | P T+RKS L GT +PGDKSISHR+ +FG +A GETRITGLLEGEDV++T KAMQA+GA PMTSRKSGPLKGTAAVPGDKSISHRALIFGAMAVGETRITGLLEGEDVLDTAKAMQALGA | 68 |
| Query | 69 | RIRKEG-DTWIIDGVGNGGLLAPEAPLDFGNAATGCRLTMGLVGVYDFDSTFIGDASLTK | 127 |
| Sbjct | 69 | + +EG W + GVG GG PE +D GN+ TG RL MG V YDF +TF GD SL K EVTREGAGEWTVHGVGVGGFSEPEDVIDCGNSGTGVRLIMGCVATYDFCTTFTGDGSLRK | 128 |
| Query | 128 | RPMGRVLNPLREMGVQVKSEDGDRLPVTLRGPKTPTPITYRVPMASAQVKSAVLLAGLNT | 187 |
| Sbjct | 129 | RPM RV +PL G + G RLP+TL G P P+ Y+ PM SAQVKSAVLLAGLN RPMARVTDPLALFGTRSVGRSGGRLPLTLIGAAEPVPVEYQTPMPSAQVKSAVLLAGLNA | 188 |
| Query | 188 | PGITTVIEPIMTRDHTEKMLQGFGANLTVETDADGVRTIRLEGRGKLTGQVIDVPGDPSS | 247 |
| Sbjct | 189 | PG T VIE TRDHTE+ML+GFGA + E +G I L GR +L Q + VP DPSS PGKTVVIEKEATRDHTERMLRGFGAEVVTEKTPEGYNKITLTGRPELKPQEVAVPRDPSS | 248 |
| Query | 248 | TAFPLVAALLVPGSDVTILNVLMNPTRTGLILTLQEMGADIEVINPRLAGGEDVADLRVR | 307 |
| Sbjct | 249 | AFP+ AAL+V GSD+ + V N TR GL TL EMGADI N R GGE VADLRV+ AAFPVCAALIVEGSDILVPGVSQNTTRNGLYTTLVEMGADIAFENTREEGGEPVADLRVK | 308 |
| Query | 308 | SSTLKGVTVPEDRAPSMIDEYPILAVAAAFAEGATVMNGLEELRVKESDRLSAVANGLKL | 367 |
| Sbjct | 309 | S LKG+ VP +RA SMIDEYPIL+ AA+AEG TVM G++ELRVKESDR+ A+A GL+ YSKLKGIEVPPERAASMIDEYPILSAIAAYAEGKTVMRGVKELRVKESDRIDAMARGLEA | 368 |
| Query | 368 | NGVDCDEGETSLVVRG-RPDGKGLGNASGAAVATHLDHRIAMSFLVMGLVSENPVTVDDA | 426 |
| Sbjct | 369 | GV +E E +L+V G PDG G ATHLDHRIAMSFL++G+ S+ PVTVDD CGVTIEEDEDTLIVHGLGPDGV EGGGTCATHLDHRIAMSFLILGMASKQPVTVDDG | 424 |
| Query | 427 | TMIATSFPEFMDLMAGLGAKI 447 IATSFP F LM GLGA + |  |
| Sbjct | 425 | GPIATSFPVFEKLMTGLGAHV 445 | |

>TR:A0A5B2V904 A0A5B2V904_9RHIZ 3-phosphoshikimate 1-carboxyvinyltransferase OS=Salinarimonas sp. BN140002 OX=1638099 GN=aroA PE=3 SV=1

Length=451

Score = 480 bits (1236), Expect = 6e-165

| Identities | = 261/441 (59%), Positives = 314/441 (71%), Gaps = 8/441 (2%) |  |
| --- | --- | --- |
| Query 9 | PATARKSSGLSGTVRIPGDKSISHRSFMFGGLASGETRITGLLEGEDVINTGKAMQAMGA | 68 |
| Sbjct 10 | P A S LSG +R+PGDKSISHR+ +FG LA GETR+ GLLEG+DV+ T A +A+GA PVAAAPSGPLSGRLRVPGDKSISHRAMIFGLLAIGETRVEGLLEGDDVLRTAAACRALGA | 69 |
| Query 69 | RIRKEGD-TWIIDGVGNGGLLAPEAPLDFGNAATGCRLTMGLVGVYDFDSTFIGDASLTK | 127 |
| Sbjct 70 | I +E W + GVG GGL+AP+ LDFGNA TG RL MG+ G + ++TF GDASL K AIDREAPGRWRVRGVGIGGLVAPDGVLDFGNAGTGSRLMMGVAGSHPIEATFDGDASLRK | 129 |
| Query 128 | RPMGRVLNPLREMG-VQVKSEDGDRLPVTLRGPKTPTPITYRVPMASAQVKSAVLLAGLN | 186 |
| Sbjct 130 | RPM R+L+PL MG V V +G R+P+TLRGP PI Y P ASAQ+KSAVLLAGLN RPMRRILDPLARMGTVVVAQAEGGRVPLTLRGPAETIPIAYEPPAASAQIKSAVLLAGLN | 189 |
| Query 187 | TPGITTVIEPIMTRDHTEKMLQGFGANLTVETDADGVRTIRLEGRGKLTGQVIDVPGDPS | 246 |
| Sbjct 190 | PG+TTV+E TRDHTE+ML+ FGA++ VE + R I L+G+ L G + VP DPS APGLTTVVEREATRDHTERMLRHFGASVVVEPHGEDGRRITLQGQPTLRGAPVIVPSDPS | 249 |
| Query 247 | STAFPLVAALLVPGSDVTILNVLMNPTRTGLILTLQEMGADIEVINPRLAGGEDVADLRV | 306 |
| Sbjct 250 | S AFP+VAALLVPGS+V + V+MNP RTGL+ TL EMGA+IE +N R GGE VADLRV SAAFPIVAALLVPGSEVVVEGVMMNPLRTGLVTTLLEMGAEIERVNERDEGGESVADLRV | 309 |
| Query 307 | RSSTLKGVTVPEDRAPSMIDEYPILAVAAAFAEGATVMNGLEELRVKESDRLSAVANGLK | 366 |
| Sbjct 310 | R+S L+GV VP RAP+MIDEYPILAVAAAFAEG T M GL ELRVKESDRL+AVA GL  RASRLRGVDVPAARAPAMIDEYPILAVAAAFAEGTTRMRGLHELRVKESDRLAAVAAGLA | 369 |

Query 367 LNGVDCD-EGETSLVVRGRPDGKGLGNASGAAVATHLDHRIAMSFLVMGLVSENPVTVDD 425 G+	EG+ LV	G G	G	ATHLDHRIAM+FLV+GL + + + VDD

| Sbjct | 370 | AAGIRHQIEGDDLLV-----HGDGTAPRGGGVAATHLDHRIAMAFLVLGLAARDGMGVDD 424 |
| --- | --- | --- |
| Query | 426 | ATMIATSFPEFMDLMAGLGAK 446 |
| Sbjct | 425 | MIATSFP F+ LM LGA+ GAMIATSFPTFLPLMRSLGAR 445 |

>TR:A0A0M2RCP7 A0A0M2RCP7_9PROT 3-phosphoshikimate 1-carboxyvinyltransferase OS=Kiloniella litopenaei OX=1549748 GN=aroA PE=3 SV=1

Length=441

Score = 480 bits (1235), Expect = 6e-165

| Identities | | = 257/444 (58%), Positives = 314/444 (71%), Gaps = 8/444 (2%) |  |
| --- | --- | --- | --- |
| Query 6  Sbjct 3 | | SSRPATARKSSGLSGTVRIPGDKSISHRSFMFGGLASGETRITGLLEGEDVINTGKAMQA S ++ SS L GTV++PGDKSISHRS MFG LA G+T + G+LEGEDV+ T KAM+A SDNSLSSTPSSSLKGTVKVPGDKSISHRSLMFGALAIGKTNVFGMLEGEDVLATAKAMRA | 65  62 |
| Query 66  Sbjct 63 | | MGARIRKEGD-TWIIDGVGNGGLLAPEAPLDFGNAATGCRLTMGLVGVYDFDSTFIGDAS  +GA + ++ W I+G G GGL PE LD GN+ T RL MG++G + + GDAS LGAHVERDDQGVWHIEGRGIGGLCEPEDVLDMGNSGTSARLLMGILGSHPITTFMTGDAS | 124  122 |
| Query 125  Sbjct 123 | | LTKRPMGRVLNPLREMGVQVKSEDGDRLPVTLRGPKTPTPITYRVPMASAQVKSAVLLAG L KRPMGRV NPL EMG QV G RLP+ ++G +TP PI Y++PMASAQVKS +LLAG LRKRPMGRVANPLAEMGTQVHCRSGQRLPLMVQGAETPMPIRYKLPMASAQVKSCILLAG | 184  182 |
| Query 185 | | LNTPGITTVIEPIMTRDHTEKMLQGFGANLTVETDADGVRTIRLEGRGKLTGQVIDVPGD LNTPG TTVIE TRDHTE ML+ FGA + V +G R + G+ +L + VP D | 244 |
| Sbjct | 183 | LNTPGETTVIETEHTRDHTELMLRHFGAEVRVTQTEEG-RETTIVGQPELKAADVHVPSD | 241 |
| Query | 245 | PSSTAFPLVAALLVPGSDVTILNVLMNPTRTGLILTLQEMGADIEVINPRLAGGEDVADL | 304 |
| Sbjct | 242 | PSS AFP VAAL+ GS++ + + MNP R G+ +TL+EMGADI + N R GE VADL PSSAAFPTVAALINEGSELRLPGIGMNPHRNGIYITLKEMGADITLENERTEAGEPVADL | 301 |
| Query | 305 | RVRSSTLKGVTVPEDRAPSMIDEYPILAVAAAFAEGATVMNGLEELRVKESDRLSAVANG | 364 |
| Sbjct | 302 | VR+S LKG+TVP +RAPSMIDEYPILA+AA+FAEG T+M GL ELRVKESDRLSAVANG VVRASKLKGITVPAERAPSMIDEYPILAIAASFAEGKTIMEGLGELRVKESDRLSAVANG | 361 |
| Query | 365 | LKLNGVDCDEGETSLVVRGRP-DGKGLGNASGAAVATHLDHRIAMSFLVMGLVSENPVTV | 423 |
| Sbjct | 362 | LK GV C+EG+ L+V G P D KG G + TH DHRIAMSFLVMG+ ++ PV V LKACGVTCEEGDDYLIVEGSPTDVKG GGTITTHFDHRIAMSFLVMGMAAKEPVKV | 416 |
| Query | 424 | DDATMIATSFPEFMDLMAGLGAKI 447 DA I TSFP+F LM GLGA+I |  |
| Sbjct | 417 | LDADAIDTSFPDFAKLMNGLGAQI 440 | |

>TR:A0A366F889 A0A366F889_9RHIZ 3-phosphoshikimate 1-carboxyvinyltransferase OS=Roseiarcus fermentans OX=1473586 GN=aroA PE=3 SV=1

Length=444

Score = 480 bits (1235), Expect = 6e-165

Identities = 269/444 (61%), Positives = 312/444 (70%), Gaps = 8/444 (2%)

| Query | 4 | GASSRPATARKSSGLSGTVRIPGDKSISHRSFMFGGLASGETRITGLLEGEDVINTGKAM | 63 |
| --- | --- | --- | --- |
| Sbjct | 4 | G P A + S LSG R PGDKSISHR+F+FG LA GET + GLLEG+DV+ TG+A GLDPAPLRASRGSALSGRFRPPGDKSISHRAFLFGLLARGETTVEGLLEGDDVLRTGRAC | 63 |
| Query | 64 | QAMGARI-RKEGDTWIIDGVGNGGLLAPEAPLDFGNAATGCRLTMGLVGVYDFDSTFIGD | 122 |
| Sbjct | 64 | +A+GA++ R W I G G G LL P PLDFGNA TG RL MG+VG + +TF GD  EALGAQVERLAPGRWRIRGPGLGALLPPREPLDFGNAGTGSRLMMGVVGGHGVTATFDGD | 123 |

| Query | 123 | ASLTKRPMGRVLNPLREMGVQVKSED-GDRLPVTLRGPKTPTPITYRVPMASAQVKSAVL | 181 |
| --- | --- | --- | --- |
| Sbjct | 124 | ASL KRPM R+L+PL+ MG +V SE G R P+TL+G + P PI YR P+ASAQ+KSAVL ASLRKRPMNRILDPLKLMGAEVLSEARGGRCPITLKGARDPAPILYRTPVASAQIKSAVL | 183 |
| Query | 182 | LAGLNTPGITTVIEPIMTRDHTEKMLQGFGANLTVETDADGVRTIRLEGRGKLTGQVIDV | 241 |
| Sbjct | 184 | LAGLN GITTVIE +RDHTEKML FGA +TV + G R I L GR +L + + V LAGLNARGITTVIEAEASRDHTEKMLVHFGAEVTVAPEGTG-RRIDLVGRPELRPRPVVV | 242 |
| Query | 242 | PGDPSSTAFPLVAALLVPGSDVTILNVLMNPTRTGLILTLQEMGADIEVINPRLAGGEDV | 301 |
| Sbjct | 243 | P DPSS AFP+VA L+VPGSD+ + V+ NP R+GL+ TL EMGADI +++ RL GGE+V PADPSSAAFPIVATLIVPGSDIVVEGVMTNPLRSGLLTTLVEMGADIAILDRRLEGGEEV | 302 |
| Query | 302 | ADLRVRSSTLKGVTVPEDRAPSMIDEYPILAVAAAFAEGATVMNGLEELRVKESDRLSAV | 361 |
| Sbjct | 303 | ADLRVR+S L GV VP RAPSMIDEYPILAVAAAFA G T M GL ELRVKESDRL+AV ADLRVRASALTGVDVPALRAPSMIDEYPILAVAAAFARGTTRMRGLHELRVKESDRLAAV | 362 |
| Query | 362 | ANGLKLNGVDCDEGETSLVVRGRPDGKGLGNASGAAVATHLDHRIAMSFLVMGLVSENPV | 421 |
| Sbjct | 363 | A GL GV LVV G DG A G VATHLDHRIAMSFLVMGL S P+ AAGLAAAGVRHAVEGDDLVVEG--DG---APAGGGLVATHLDHRIAMSFLVMGLASRQPM | 417 |
| Query  Sbjct | 422  418 | TVDDATMIATSFPEFMDLMAGLGA 445 TVDD MIATSFP F M LGA TVDDTAMIATSFPAFRPAMERLGA 441 |  |

>TR:A0A437J6D1 A0A437J6D1_9SPHN 3-phosphoshikimate 1-carboxyvinyltransferase OS=Sphingobium algorifonticola OX=2008318 GN=aroA PE=3 SV=1

Length=446

Score = 480 bits (1235), Expect = 7e-165

| Identities | = 258/444 (58%), Positives = 312/444 (70%), Gaps = 4/444 (1%) |  |
| --- | --- | --- |
| Query 3 | HGASSRPATARKSSGLSGTVRIPGDKSISHRSFMFGGLASGETRITGLLEGEDVINTGKA | 62 |
| Sbjct 4 | H + PAT + LSGT+ +PGDKSISHRS M LA GE+R+ GLLEGEDV+ T A HNDAPTPATFTAAPALSGTITVPGDKSISHRSLMLSALAIGESRVEGLLEGEDVLATADA | 63 |
| Query 63 | MQAMGARIRKEGD-TWIIDGVGNGGLLAPEAPLDFGNAATGCRLTMGLVGVYDFDSTFIG | 121 |
| Sbjct 64 | M+AMGA I ++ D TW + GVG G LL P+ LD GN+ T RL MGL+ + +TF+G MRAMGADIVRDADGTWHVHGVGVGCLLQPQVALDMGNSGTSTRLLMGLIASHALTATFVG | 123 |
| Query 122 | DASLTKRPMGRVLNPLREMGVQVKSEDGDRLPVTLRGPKTPTPITYRVPMASAQVKSAVL | 181 |
| Sbjct 124 | DASL++RPMGRV++PL MG + G RLP+TLRG PI YR+P+ASAQVKSA+L DASLSRRPMGRVIDPLSNMGAAFTASPGGRLPLTLRGICPAVPIEYRLPVASAQVKSALL | 183 |
| Query 182 | LAGLNTPGITTVIEPIMTRDHTEKMLQGFGANLTVETDADGVRTIRLEGRGKLTGQVIDV | 241 |
| Sbjct 184 | LAGLNTPGIT VIEP+ TRDH+E+ML+GFGA+LTV+ D DG R I + G L Q I V LAGLNTPGITRVIEPVATRDHSERMLRGFGADLTVDIDNDGARVISIRGEAMLQPQNIVV | 243 |
| Query 242 | PGDPSSTAFPLVAALLVPGSDVTILNVLMNPTRTGLILTLQEMGADIEVINPRLAGGEDV | 301 |
| Sbjct 244 | PGDPSS AFP+VAALLVPGS VTI NV +NPTR GL+ L+EMG DI +I PR GGE V PGDPSSAAFPMVAALLVPGSQVTIRNVGLNPTRAGLVDLLREMGGDIAIIRPRDVGGEPV | 303 |
| Query 302 | ADLRVRSSTLKGVTVPEDRAPSMIDEYPILAVAAAFAEGATVMNGLEELRVKESDRLSAV | 361 |
| Sbjct 304 | DL V +S L G+ RAPSMIDEYPI VAAA A G +V GLEELRVKESDRL+ + GDLVVTASRLSGIDPDPARAPSMIDEYPIAFVAAALAHGRSVFRGLEELRVKESDRLTTM | 363 |
| Query 362 | ANGLKLNGVDCDEGETSLVVRGRPDGKGLGNASGAAVATHLDHRIAMSFLVMGLVSENPV | 421 |
| Sbjct 364 | A GL+ G +E E L++ G G+ L + G VAT LDHRIAMSF + GLVS V AIGLRAIGAQVEETEDGLIIEG-TGGEPL--SGGGPVATLLDHRIAMSFAIAGLVSRGGV | 420 |
| Query 422  Sbjct 421 | TVDDATMIATSFPEFMDLMAGLGA 445 TVDD +ATSFP F+DL+ +GA TVDDMRPVATSFPGFVDLLKAMGA 444 |  |

>TR:A0A243PE86 A0A243PE86_9SPHN 3-phosphoshikimate 1-carboxyvinyltransferase OS=Sphingobium sp. GW456-12-10-14-TSB1 OX=1987165 GN=aroA PE=3

SV=1

Length=446

Score = 480 bits (1235), Expect = 7e-165

| Identities | = 258/442 (58%), Positives = 308/442 (70%), Gaps = 4/442 (1%) |  |
| --- | --- | --- |
| Query 5 | ASSRPATARKSSGLSGTVRIPGDKSISHRSFMFGGLASGETRITGLLEGEDVINTGKAMQ | 64 |
| Sbjct 6 | +S P T + LSG V +PGDKSISHRS M LA GE+R+ GLLEGEDV+ T AM+ SSPTPMTFTAAPALSGGVAVPGDKSISHRSLMLSALAVGESRVEGLLEGEDVLATAAAMR | 65 |
| Query 65 | AMGARIRKEGD-TWIIDGVGNGGLLAPEAPLDFGNAATGCRLTMGLVGVYDFDSTFIGDA | 123 |
| Sbjct 66 | AMGA I ++ D W I GVG GGLL PE LD GN+ T RL MGL+ ++ +TF GDA AMGADIERDADGIWHIHGVGVGGLLQPEQALDMGNSGTSTRLLMGLLASHELTATFTGDA | 125 |
| Query 124 | SLTKRPMGRVLNPLREMGVQVKSEDGDRLPVTLRGPKTPTPITYRVPMASAQVKSAVLLA | 183 |
| Sbjct 126 | SL+KRPM RV PL MG + GDRLP+T+RG P+ YR+P+ASAQVKSA+LLA SLSKRPMARVTEPLARMGASFTTSPGDRLPLTMRGACPAVPLDYRLPVASAQVKSAILLA | 185 |
| Query 184 | GLNTPGITTVIEPIMTRDHTEKMLQGFGANLTVETDADGVRTIRLEGRGKLTGQVIDVPG | 243 |
| Sbjct 186 | GLNTPGIT V+EPI TRDH+E+ML+GFGA L VE +ADG R I L G +L Q I VPG GLNTPGITRVVEPIPTRDHSERMLRGFGAELEVEVEADGTRIITLVGEAELKPQQIVVPG | 245 |
| Query 244 | DPSSTAFPLVAALLVPGSDVTILNVLMNPTRTGLILTLQEMGADIEVINPRLAGGEDVAD | 303 |
| Sbjct 246 | DPSS AFP+VAALLVPGS VTI NV +N TR GLI L+EMG IEVINPR GGE V D DPSSAAFPMVAALLVPGSQVTIANVGLNTTRAGLIDLLREMGGSIEVINPREVGGEPVGD | 305 |
| Query 304 | LRVRSSTLKGVTVPEDRAPSMIDEYPILAVAAAFAEGATVMNGLEELRVKESDRLSAVAN | 363 |
| Sbjct 306 | L V +S LKGV RAPSMIDEYP+ +AAA A+G +V GLEELRVKESDR++ +A LAVTASALKGVEPDPARAPSMIDEYPVAFIAAALAQGRSVFRGLEELRVKESDRIATMAA | 365 |
| Query 364 | GLKLNGVDCDEGETSLVVRGRPDGKGLGNASGAAVATHLDHRIAMSFLVMGLVSENPVTV | 423 |
| Sbjct 366 | GL+ GV +E E +++ G G A G +AT LDHRIAMSF V GLVS + V + GLRAIGVTVEELEDGIIIEG---SGGAPLAGGGPIATKLDHRIAMSFAVAGLVSRDGVMI | 422 |
| Query 424  Sbjct 423 | DDATMIATSFPEFMDLMAGLGA 445 DD +ATSFP F+ L+ LGA DDMAPVATSFPTFVPLLRTLGA 444 |  |

>TR:N1MGF1 N1MGF1_9SPHN 3-phosphoshikimate 1-carboxyvinyltransferase OS=Sphingobium japonicum BiD32 OX=1301087 GN=aroA PE=3 SV=1

Length=436

Score = 479 bits (1234), Expect = 7e-165

| Identities | = 257/436 (59%), Positives = 306/436 (70%), Gaps = 4/436 (1%) |  |
| --- | --- | --- |
| Query 11 | TARKSSGLSGTVRIPGDKSISHRSFMFGGLASGETRITGLLEGEDVINTGKAMQAMGARI | 70 |
| Sbjct 2 | T + LSG+V +PGDKSISHRS M LA GE+R+ GLLEGEDV+ T AM+AMGA I TFSAAPALSGSVTVPGDKSISHRSLMLSALAVGESRVEGLLEGEDVLATAAAMRAMGADI | 61 |
| Query 71 | RKEGD-TWIIDGVGNGGLLAPEAPLDFGNAATGCRLTMGLVGVYDFDSTFIGDASLTKRP | 129 |
| Sbjct 62 | + D W I GVG GGLL PE LD GN+ T RL MGL+ +D +TFIGDASL+KRP VRNADGIWHIHGVGVGGLLQPEVALDMGNSGTSTRLLMGLLASHDLTATFIGDASLSKRP | 121 |
| Query 130 | MGRVLNPLREMGVQVKSEDGDRLPVTLRGPKTPTPITYRVPMASAQVKSAVLLAGLNTPG | 189 |
| Sbjct 122 | M RV PL MG + GDRLP+T+RG P+ YR+P+ASAQVKSA+LLAGLNTPG MARVTEPLARMGASFTTSPGDRLPLTMRGACPAVPLDYRLPVASAQVKSAILLAGLNTPG | 181 |
| Query 190 | ITTVIEPIMTRDHTEKMLQGFGANLTVETDADGVRTIRLEGRGKLTGQVIDVPGDPSSTA IT V+EPI TRDH+E+ML+GFGA L VE +ADG R I L G +LT Q I VPGDPSS A | 249 |

| Sbjct | 182 | ITRVVEPIPTRDHSERMLRGFGAELNVEVEADGTRIITLVGEAELTPQQIVVPGDPSSAA | 241 |
| --- | --- | --- | --- |
| Query | 250 | FPLVAALLVPGSDVTILNVLMNPTRTGLILTLQEMGADIEVINPRLAGGEDVADLRVRSS | 309 |
| Sbjct | 242 | FP+VAALLVPGS VTI NV +N TR GLI L+EMG IEVIN R GGE V DL V +S FPMVAALLVPGSQVTIANVGLNATRAGLIDLLREMGGSIEVINAREVGGEPVGDLVVTAS | 301 |
| Query | 310 | TLKGVTVPEDRAPSMIDEYPILAVAAAFAEGATVMNGLEELRVKESDRLSAVANGLKLNG | 369 |
| Sbjct | 302 | L G+ RAPSMIDEYP+ +AAA A+G +V GLEELRVKESDR++ +A GL+ G ALNGIEPDPARAPSMIDEYPVAFIAAALAQGRSVFRGLEELRVKESDRIATMATGLRAIG | 361 |
| Query | 370 | VDCDEGETSLVVRGRPDGKGLGNASGAAVATHLDHRIAMSFLVMGLVSENPVTVDDATMI | 429 |
| Sbjct | 362 | V +E E +++ G G A G +AT LDHRIAMSF V GLVS + VT+DD + VSVEELEDGIIIEG---SGGAPLAGGGPIATKLDHRIAMSFAVAGLVSRDGVTIDDMAPV | 418 |
| Query  Sbjct | 430  419 | ATSFPEFMDLMAGLGA 445 ATSFP F+ L+ LGA ATSFPTFVPLLRTLGA 434 |  |

>TR:A0A102D5X5 A0A102D5X5_9SPHN 3-phosphoshikimate 1-carboxyvinyltransferase OS=Novosphingobium sp. FSW06-99 OX=1739113 GN=aroA PE=3 SV=1

Length=447

Score = 480 bits (1235), Expect = 7e-165

| Identities | = 258/445 (58%), Positives = 314/445 (71%), Gaps = 5/445 (1%) |  |
| --- | --- | --- |
| Query 5 | ASSRPATARK---SSGLSGTVRIPGDKSISHRSFMFGGLASGETRITGLLEGEDVINTGK | 61 |
| Sbjct 3 | ASS PA R+ L+G +R+PGDKSISHRS M G LA GETR+TGLLEGEDV+ T ASSAPAQPRRFVAKGALTGRIRVPGDKSISHRSIMLGALAVGETRVTGLLEGEDVLATAA | 62 |
| Query 62 | AMQAMGARIRKEGD-TWIIDGVGNGGLLAPEAPLDFGNAATGCRLTMGLVGVYDFDSTFI | 120 |
| Sbjct 63 | AM+AMGA I ++ D W++ GVG GGLL P LD GN+ T RL +GLV + + FI AMRAMGATIERQADGDWLVAGVGVGGLLQPAGALDMGNSGTSTRLLLGLVASHAITAAFI | 122 |
| Query 121 | GDASLTKRPMGRVLNPLREMGVQVKSEDGDRLPVTLRGPKTPTPITYRVPMASAQVKSAV | 180 |
| Sbjct 123 | GDASL+KRPMGRV+ PL +MG + G RLP+ + G +TP PITYR+P+ASAQVKSAV GDASLSKRPMGRVITPLGQMGATFTTYPGGRLPLIITGAETPVPITYRLPVASAQVKSAV | 182 |
| Query 181 | LLAGLNTPGITTVIEPIMTRDHTEKMLQGFGANLTVETDADGVRTIRLEGRGKLTGQVID | 240 |
| Sbjct 183 | LLAGLNTPG TTVIEP+ TRDH+E+ML+GFGA+L+VETDA+G R I L G+ +L Q I LLAGLNTPGATTVIEPVPTRDHSERMLRGFGADLSVETDAEGARVISLSGQPELRPQAIT | 242 |
| Query 241 | VPGDPSSTAFPLVAALLVPGSDVTILNVLMNPTRTGLILTLQEMGADIEVINPRLAGGED | 300 |
| Sbjct 243 | VPGDPSS AF +VAAL+VPGSD+ I NV +NPTR L L+ MG I +N R GGE VPGDPSSAAFFVVAALIVPGSDLLIENVGLNPTRAALFDVLRLMGGQITEVNQREVGGEP | 302 |
| Query 301 | VADLRVRSSTLKGVTVPEDRAPSMIDEYPILAVAAAFAEGATVMNGLEELRVKESDRLSA | 360 |
| Sbjct 303 | VADL VR+S L G+TV P+M+DE+P+L VAAA AEG TV +GLEELRVKESDR++A VADLHVRASALHGITVDPAVVPAMVDEFPVLFVAAALAEGRTVTSGLEELRVKESDRITA | 362 |
| Query 361 | VANGLKLNGVDCDEGETSLVVRGRPDGKGLGNASGAAVATHLDHRIAMSFLVMGLVSENP | 420 |

+	L L G	+E	+++ G	G+ L	AVATHLDHRIAM+ V GL SE

| Sbjct | 363 | MRVALTLAGARVEETADGMIIDG-SGGRLLAGTGNGAVATHLDHRIAMAMAVAGLASETG | 421 |
| --- | --- | --- | --- |
| Query  Sbjct | 421  422 | VTVDDATMIATSFPEFMDLMAGLGA 445 VTVDD IATSFP F L+A GA VTVDDTRPIATSFPLFERLLADTGA 446 |  |

>TR:A0A1H1KH72 A0A1H1KH72_9RHIZ 3-phosphoshikimate 1-carboxyvinyltransferase OS=Rhizobiales bacterium GAS113 OX=1884352 GN=aroA PE=3 SV=1

Length=448

Score = 480 bits (1235), Expect = 7e-165

| Identities | = 259/443 (58%), Positives = 314/443 (71%), Gaps = 8/443 (2%) |  |
| --- | --- | --- |
| Query 8 | RPATARKSSGLSGTVRIPGDKSISHRSFMFGGLASGETRITGLLEGEDVINTGKAMQAMG | 67 |
| Sbjct 10 | +P TAR+ + L G VR+PGDKS SHR+ + G L+ GET I GLLEGEDV+ T +A + +G QPLTARRGAALKGKVRVPGDKSTSHRALILGLLSIGETNIEGLLEGEDVLRTAQACRQLG | 69 |
| Query 68 | ARIRKEGD-TWIIDGVGNGGLLAPEAPLDFGNAATGCRLTMGLVGVYDFDSTFIGDASLT | 126 |
| Sbjct 70 | A + + G W + GVG GGL P+A LDFGNA TG RL MG+VG + +TF GDASL ATVERHGSGIWSVRGVGIGGLAEPQAVLDFGNAGTGVRLMMGVVGGHPITATFDGDASLR | 129 |
| Query 127 | KRPMGRVLNPLREMGVQVKSE-DGDRLPVTLRGPKTPTPITYRVPMASAQVKSAVLLAGL | 185 |
| Sbjct 130 | KRPM R+L+PL MG QV ++ +G R P+TL+G + PI YR P+ASAQ+KSAVLLAGL KRPMRRILDPLALMGAQVMAQAEGGRCPITLQGARHAAPIEYRTPVASAQIKSAVLLAGL | 189 |
| Query 186 | NTPGITTVIEPIMTRDHTEKMLQGFGANLTVETDADGVRTIRLEGRGKLTGQVIDVPGDP | 245 |
| Sbjct 190 | N PG+TTVIE +RDHTE+ML+ FGA + V + R + L G+ +L + VPGDP NAPGLTTVIETEASRDHTERMLRHFGAEVVVTPHGEAGRHVTLTGQPELRAAPVVVPGDP | 249 |
| Query 246 | SSTAFPLVAALLVPGSDVTILNVLMNPTRTGLILTLQEMGADIEVINPRLAGGEDVADLR | 305 |
| Sbjct 250 | SS AFP+VAAL+ PGSD+ + V+MN RTGLI TL EMGA IE N R GGE+VADLR SSAAFPIVAALITPGSDILVEGVMMNALRTGLITTLIEMGASIEAGNQRQEGGEEVADLR | 309 |
| Query 306 | VRSSTLKGVTVPEDRAPSMIDEYPILAVAAAFAEGATVMNGLEELRVKESDRLSAVANGL | 365 |
| Sbjct 310 | VR+ L+GV VP RAPSMIDEYP+LAVAAAFA G T M GL ELRVKESDRL+AVA GL VRAGELRGVEVPAARAPSMIDEYPVLAVAAAFAHGETRMRGLNELRVKESDRLAAVAAGL | 369 |
| Query 366 | KLNGV-DCDEGETSLVVRGRPDGKGLGNASGAAVATHLDHRIAMSFLVMGLVSENPVTVD | 424 |
| Sbjct 370 | GV EG+ +V G+ + +G G V THLDHRIAMSFL MGL S PVTVD SACGVAHAIEGDDLIVEGGKGEAEG GGTVLTHLDHRIAMSFLTMGLGSRQPVTVD | 424 |
| Query 425  Sbjct 425 | DATMIATSFPEFMDLMAGLGAKI 447 D MIATSFP F ++MAGLGA I DGAMIATSFPAFAEMMAGLGADI 447 |  |

>TR:A0A164B6K1 A0A164B6K1_9BRAD 3-phosphoshikimate 1-carboxyvinyltransferase OS=Tardiphaga robiniae OX=943830 GN=aroA PE=3 SV=1

Length=449

Score = 480 bits (1235), Expect = 8e-165

| Identities | = 255/443 (58%), Positives = 317/443 (72%), Gaps = 7/443 (2%) |  |
| --- | --- | --- |
| Query 5 | ASSRPATARKSSGLSGTVRIPGDKSISHRSFMFGGLASGETRITGLLEGEDVINTGKAMQ | 64 |
| Sbjct 9 | A P TARKS L G+ R+PGDKSISHR+ + G LA GETRITGLLEGEDV+NT K+MQ AQPTPLTARKSGALRGSARVPGDKSISHRALILGALAVGETRITGLLEGEDVLNTAKSMQ | 68 |
| Query 65 | AMGARIRKEGD-TWIIDGVGNGGLLAPEAPLDFGNAATGCRLTMGLVGVYDFDSTFIGDA | 123 |
| Sbjct 69 | A+GA++ + GD W ++GVG G AP+APLDFGN+ TGCRL MG V ++TF GDA ALGAKVERTGDFAWKVNGVGVAGFAAPKAPLDFGNSGTGCRLVMGAVAGCPIEATFDGDA | 128 |
| Query 124 | SLTKRPMGRVLNPLREMGVQVK-SEDGDRLPVTLRGPKTPTPITYRVPMASAQVKSAVLL | 182 |
| Sbjct 129 | SL RPM R+++PL MG +V SE+G RLP+T+ G + P PI YR P+ASAQ+KSAVLL SLRSRPMRRIVDPLELMGARVTASEEGGRLPLTIAGARDPLPIVYRTPVASAQIKSAVLL | 188 |
| Query 183 | AGLNTPGITTVIEPIMTRDHTEKMLQGFGANLTVETDADGVRTIRLEGRGKLTGQVIDVP | 242 |
| Sbjct 189 | AGL+ PG TTVIE +RDHTE ML+ FGA + E + R I L G+ +L G + VP AGLSAPGTTTVIETEASRDHTELMLKHFGAEIVSEKEGAHGRKITLTGQPELKGADVVVP | 248 |
| Query 243 | GDPSSTAFPLVAALLVPGSDVTILNVLMNPTRTGLILTLQEMGADIEVINPRLAGGEDVA | 302 |
| Sbjct 249 | DPSS AFP+VAAL+VPGS++ + +V+ NP R GL TL+EMGA IE + R GE +A ADPSSAAFPMVAALIVPGSEIVLTDVMTNPLRIGLFTTLREMGASIEESDIRGDAGEPMA | 308 |
| Query 303 | DLRVRSSTLKGVTVPEDRAPSMIDEYPILAVAAAFAEGATVMNGLEELRVKESDRLSAVA R+ +S L GV VP +RAPSMIDEY +LAVAAAFAEG T M GL+ELRVKESDRL A A | 362 |

| Sbjct | 309 | QFRITASKLHGVEVPPERAPSMIDEYLVLAVAAAFAEGTTTMRGLQELRVKESDRLEATA | 368 |
| --- | --- | --- | --- |
| Query | 363 | NGLKLNGVDCDEGETSLVVRGRPDGKGLGNASGAAVATHLDHRIAMSFLVMGLVSENPVT | 422 |
| Sbjct | 369 | L++NGV+ + L+V G +G+ G G VATH+DHRIAMS L MGL S+ PVT AMLRVNGVNVEIVGDDLIVHG--NGRVAG---GGLVATHMDHRIAMSALAMGLASDQPVT | 423 |
| Query  Sbjct | 423  424 | VDDATMIATSFPEFMDLMAGLGA 445  +DD T IATSFP+F+ +M LGA IDDTTFIATSFPDFIPMMRRLGA 446 |  |

>TR:A0A443IVZ2 A0A443IVZ2_9RHOB 3-phosphoshikimate 1-carboxyvinyltransferase OS=Sinirhodobacter populi OX=2306993 GN=aroA PE=3 SV=1

Length=449

Score = 480 bits (1235), Expect = 8e-165

| Identities | | = 256/438 (58%), Positives = 307/438 (70%), Gaps = 7/438 (2%) |  |
| --- | --- | --- | --- |
| Query 12  Sbjct 12 | | ARKSSGLSGTVRIPGDKSISHRSFMFGGLASGETRITGLLEGEDVINTGKAMQAMGARIR ARK + L GT +PGDKSISHR+ + G L+ G T +TGLLEG+DV++T KAM+A GA + ARKGAPLRGTAEVPGDKSISHRALILGALSVGRTEVTGLLEGQDVLDTAKAMRAFGATVE | 71  71 |
| Query 72 | | KEGD-TWIIDGVGNGGLLAPEAPLDFGNAATGCRLTMGLVGVYDFDSTFIGDASLTKRPM  + G W+IDGVG GG P +D GN+ TG RL MG + +TF GDASL KRPM | 130 |
| Sbjct | 72 | RLGPGRWVIDGVGVGGFAEPSDIIDCGNSGTGVRLIMGAMATSPISATFTGDASLRKRPM | 131 |
| Query | 131 | GRVLNPLREMGVQVKSEDGDRLPVTLRGPKTPTPITYRVPMASAQVKSAVLLAGLNTPGI | 190 |
| Sbjct | 132 | GRV +PL G + G RLP+T+ G P P+ Y VPM SAQVKSAVLLAGLN PG GRVTDPLALFGTRAVGRQGGRLPMTIVGAAEPVPVRYTVPMPSAQVKSAVLLAGLNAPGQ | 191 |
| Query | 191 | TTVIEPIMTRDHTEKMLQGFGANLTVETDADGVRTIRLEGRGKLTGQVIDVPGDPSSTAF | 250 |
| Sbjct | 192 | T VIE TRDHTE+ML+GFGA+LTVE +G R I L+G+ +LTGQ + VP DPSS AF TVVIEREATRDHTERMLRGFGADLTVEETPEG-RVITLQGQPELTGQRVAVPRDPSSAAF | 250 |
| Query | 251 | PLVAALLVPGSDVTILNVLMNPTRTGLILTLQEMGADIEVINPRLAGGEDVADLRVRSST | 310 |
| Sbjct | 251 | P+ AAL+VPGSD+ + V NPTR GL TL EMGADI NPR GGE VADLRVR S PVCAALIVPGSDILVPGVSQNPTRNGLFATLVEMGADIAFENPREEGGEPVADLRVRHSA | 310 |
| Query | 311 | LKGVTVPEDRAPSMIDEYPILAVAAAFAEGATVMNGLEELRVKESDRLSAVANGLKLNGV | 370 |
| Sbjct | 311 | L G+ VP +RA SMIDEYP+LAV AAFA G TVM G++ELRVKESDR+ A+A GL+ GV LTGIAVPPERAASMIDEYPVLAVVAAFATGTTVMRGVKELRVKESDRIDAMARGLEACGV | 370 |
| Query | 371 | DCDEGETSLVVRGR-PDGKGLGNASGAAVATHLDHRIAMSFLVMGLVSENPVTVDDATMI | 429 |
| Sbjct | 371 | +E +L+V G P+G GA ATH+DHRIAMSFLV+GL ++ PV VDD + I RVEEDRDTLIVHGMGPEGV PGGATCATHIDHRIAMSFLVLGLAAQQPVAVDDGSPI | 426 |
| Query  Sbjct | 430  427 | ATSFPEFMDLMAGLGAKI 447 ATSFP F DLM GLGA I ATSFPVFEDLMRGLGADI 444 |  |

>TR:A0A562NSL0 A0A562NSL0_9RHOB 3-phosphoshikimate 1-carboxyvinyltransferase OS=Paracoccus sulfuroxidans OX=384678 GN=aroA PE=3 SV=1

Length=443

Score = 479 bits (1234), Expect = 9e-165

Identities = 254/448 (57%), Positives = 310/448 (69%), Gaps = 7/448 (2%)

| Query | 1 | MLHGASSRPATARKSSGLSGTVRIPGDKSISHRSFMFGGLASGETRITGLLEGEDVINTG | 60 |
| --- | --- | --- | --- |
| Sbjct | 1 | M H A + P ++R+S LSG RIPGDKSISHR+ + G L GET ITGLLEGEDV++T MSHSAEALPMSSRRSGPLSGEARIPGDKSISHRALILGALTVGETHITGLLEGEDVLDTA | 60 |
| Query | 61 | KAMQAMGARIRKEGD-TWIIDGVGNGGLLAPEAPLDFGNAATGCRLTMGLVGVYDFDSTF KAM+A GA++ + G+ W ++GVG GG PE +D GN+ TG RL MG + +TF | 119 |

| Sbjct | 61 | KAMRAFGAKVERVGEGEWRVNGVGVGGFTEPEGVIDCGNSGTGVRLIMGAMATTPITATF | 120 |
| --- | --- | --- | --- |
| Query | 120 | IGDASLTKRPMGRVLNPLREMGVQVKSEDGDRLPVTLRGPKTPTPITYRVPMASAQVKSA | 179 |
| Sbjct | 121 | GDASL+KRPM R+ +PL+ G +V S +G RLPVT++G P+TYR P+ASAQ+KSA TGDASLSKRPMARITDPLQLFGCEVTSREGGRLPVTIKGAVDAAPVTYRTPVASAQIKSA | 180 |
| Query | 180 | VLLAGLNTPGITTVIEPIMTRDHTEKMLQGFGANLTVETDADGVRTIRLEGRGKLTGQVI | 239 |
| Sbjct | 181 | VLLAGLN PG T VIE TRDHTE+ML GFGA + E +G I L GR +L Q I VLLAGLNAPGQTVVIEAEPTRDHTERMLTGFGAQIVTEVTDEG-HVITLTGRPELKPQPI | 239 |
| Query | 240 | DVPGDPSSTAFPLVAALLVPGSDVTILNVLMNPTRTGLILTLQEMGADIEVINPRLAGGE | 299 |
| Sbjct | 240 | VP DPSS AFP+ AAL+VPGS++ + V NPTR GL +TL EMGADI N R GGE AVPRDPSSAAFPVAAALIVPGSEIRVPGVSRNPTRDGLFITLLEMGADIRFENEREEGGE | 299 |
| Query | 300 | DVADLRVRSSTLKGVTVPEDRAPSMIDEYPILAVAAAFAEGATVMNGLEELRVKESDRLS | 359 |
| Sbjct | 300 | VADL VR LKGV VP +RA SMIDE+PIL+V A FAEG TVMNG+ ELRVKESDR+ PVADLVVRHGPLKGVEVPAERAASMIDEFPILSVIACFAEGLTVMNGVAELRVKESDRID | 359 |
| Query | 360 | AVANGLKLNGVDCDEGETSLVVRGRPDGKGLGNASGAAVATHLDHRIAMSFLVMGLVSEN | 419 |
| Sbjct | 360 | A+A GL+ NG DE S+ V G KG GA +HLDHRIAMSFL+ GL SE AMAVGLRANGARVDETRDSMTVHGMGGLKG GATATSHLDHRIAMSFLIAGLASEQ | 414 |
| Query  Sbjct | 420  415 | PVTVDDATMIATSFPEFMDLMAGLGAKI 447 P++VDD + IATSFP+F+ LM GLG + PISVDDGSPIATSFPDFLPLMKGLGGDL 442 |  |

>SP:Q3IW89 AROA_RHOS4 3-phosphoshikimate 1-carboxyvinyltransferase OS=Rhodobacter sphaeroides (strain ATCC 17023 / 2.4.1 / NCIB 8253

/ DSM 158) OX=272943 GN=aroA PE=3 SV=1

Length=445

Score = 479 bits (1234), Expect = 9e-165

| Identities | = 259/446 (58%), Positives = 314/446 (70%), Gaps = 8/446 (2%) |  |
| --- | --- | --- |
| Query 3 | HGASSRPATARKSSGLSGTVRIPGDKSISHRSFMFGGLASGETRITGLLEGEDVINTGKA | 62 |
| Sbjct 4 | HG + +P TAR+S L G IPGDKSISHR+ + G +A GETRITGLLEG+DV++T KA HGPA-QPMTARRSGPLKGRAEIPGDKSISHRALILGAMAVGETRITGLLEGQDVLDTAKA | 62 |
| Query 63 | MQAMGARIRKEGD-TWIIDGVGNGGLLAPEAPLDFGNAATGCRLTMGLVGVYDFDSTFIG | 121 |

M+A GA + + G	W + GVG GG	P	+D GN+ TG RL MG +	+TF G

| Sbjct | 63 | MRAFGAEVIQHGPGAWSVHGVGVGGFTEPAEVIDCGNSGTGVRLVMGAMATSPLTATFTG | 122 |
| --- | --- | --- | --- |
| Query | 122 | DASLTKRPMGRVLNPLREMGVQVKSEDGDRLPVTLRGPKTPTPITYRVPMASAQVKSAVL | 181 |
| Sbjct | 123 | DASL KRPMGRV +PL G + G RLP+TL G P P+ Y VP+ SAQVKSAVL DASLRKRPMGRVTDPLALFGTRAYGRKGGRLPMTLVGAADPVPVRYTVPVPSAQVKSAVL | 182 |
| Query | 182 | LAGLNTPGITTVIEPIMTRDHTEKMLQGFGANLTVETDADGVRTIRLEGRGKLTGQVIDV | 241 |
| Sbjct | 183 | LAGLN PG T VIE TRDH+E+ML+GFGA L+VET +G + I L G+ +L Q + V LAGLNAPGQTVVIEREATRDHSERMLRGFGAELSVETGPEG-QVITLTGQPELRPQTVAV | 241 |
| Query | 242 | PGDPSSTAFPLVAALLVPGSDVTILNVLMNPTRTGLILTLQEMGADIEVINPRLAGGEDV | 301 |
| Sbjct | 242 | P DPSS AFP+ AAL+V GS++ + V NPTR GL +TL EMGADI N R GGE V PRDPSSAAFPVCAALIVEGSEILVPGVSRNPTRDGLYVTLLEMGADIAFENEREEGGEPV | 301 |
| Query | 302 | ADLRVRSSTLKGVTVPEDRAPSMIDEYPILAVAAAFAEGATVMNGLEELRVKESDRLSAV | 361 |
| Sbjct | 302 | ADLRVR+S LKGV VP +RAPSMIDEYPIL+V AAFAEG T+M G++ELRVKESDR+ A+ ADLRVRASALKGVEVPPERAPSMIDEYPILSVVAAFAEGLTIMRGVKELRVKESDRIDAM | 361 |
| Query | 362 | ANGLKLNGVDCDEGETSLVVRGRPDGKGLGNASGAAVATHLDHRIAMSFLVMGLVSENPV | 421 |
| Sbjct | 362 | A GL+ GV +E E +L+V G G GA ATHLDHRIAMSFLV+G+ +E PV ARGLEACGVRIEEDEDTLIVHGMGRVPG GATCATHLDHRIAMSFLVLGMAAEAPV | 416 |
| Query | 422 | TVDDATMIATSFPEFMDLMAGLGAKI 447 |  |

TVDD + IATSFP F+DLMAGLGA + Sbjct 417 TVDDGSPIATSFPAFIDLMAGLGADL 442

>TR:A0A418W826 A0A418W826_9SPHN 3-phosphoshikimate 1-carboxyvinyltransferase OS=Sphingomonas sp. K2R01-6 OX=2320861 GN=aroA PE=3 SV=1

Length=445

Score = 479 bits (1234), Expect = 9e-165

| Identities | | = 267/440 (61%), Positives = 306/440 (70%), Gaps = 4/440 (1%) |  |
| --- | --- | --- | --- |
| Query 9  Sbjct 8 | | PATARKSSGLSGTVRIPGDKSISHRSFMFGGLASGETRITGLLEGEDVINTGKAMQAMGA PA S L+G VR+PGDKSISHRS M LA G + + GLLEGEDV+ T AM+AMGA PAAFTASGPLTGRVRVPGDKSISHRSLMLSALAVGRSTVRGLLEGEDVLATAAAMRAMGA | 68  67 |
| Query 69  Sbjct 68 | | RI-RKEGDTWIIDGVGNGGLLAPEAPLDFGNAATGCRLTMGLVGVYDFDSTFIGDASLTK I R + W I GVG GGLL P+A LD GN+ T RL MGLV + +TF GDASL+ TIVRGDDGVWTIHGVGVGGLLQPQAALDMGNSGTSTRLLMGLVASHPITATFTGDASLSG | 127  127 |
| Query 128  Sbjct 128 | | RPMGRVLNPLREMGVQVKSEDGDRLPVTLRGPKTPTPITYRVPMASAQVKSAVLLAGLNT RPMGRV++PL MG + S G RLP+ +RG PI YR+PMASAQVKSAVLLAGLNT RPMGRVIDPLGLMGADITSSPGGRLPLMVRGMCPAVPIEYRLPMASAQVKSAVLLAGLNT | 187  187 |
| Query 188  Sbjct 188 | | PGITTVIEPIMTRDHTEKMLQGFGANLTVETDADGVRTIRLEGRGKLTGQVIDVPGDPSS PGIT VIEPI TRDH+E+ML GFGA+LTVE D+DG R I + G +L Q I VPGDPSS PGITRVIEPIATRDHSERMLTGFGADLTVEIDSDGARVISIRGEAELKPQDIVVPGDPSS | 247  247 |
| Query 248  Sbjct 248 | | TAFPLVAALLVPGSDVTILNVLMNPTRTGLILTLQEMGADIEVINPRLAGGEDVADLRVR AFP+VAALLVPGSDV I NV +NPTR GLI LQ MG DI +IN R+ GGE VADL VR AAFPMVAALLVPGSDVVIENVGINPTRAGLIEVLQAMGGDITLINERVVGGEPVADLHVR | 307  307 |
| Query 308  Sbjct 308 | | SSTLKGVTVPEDRAPSMIDEYPILAVAAAFAEGATVMNGLEELRVKESDRLSAVANGLKL S L G+ V APSMIDE+PIL VAAA A G TV GLEELRVKESDR++ +A GL  HSPLNGIEVDPAIAPSMIDEFPILFVAAALANGRTVTRGLEELRVKESDRIALMAQGLAA | 367  367 |
| Query 368 | | NGVDCDEGETSLVVRGRPDGKGLGNASGAAVATHLDHRIAMSFLVMGLVSENPVTVDDAT G E E L++ G G+ L GA VA LDHRIAMSF V GLVS V VDD T | 427 |
| Sbjct | 368 | IGARVIEAEDGLIIDG-TGGEPL--PGGAPVAAKLDHRIAMSFAVAGLVSAANVMVDDMT | 424 |
| Query | 428 | MIATSFPEFMDLMAGLGAKI 447  +ATSFP+F LM LGA+I |  |
| Sbjct | 425 | PVATSFPQFASLMTSLGAQI 444 | |

>TR:A0A0Q6YUI7 A0A0Q6YUI7_9BRAD 3-phosphoshikimate 1-carboxyvinyltransferase OS=Afipia sp. Root123D2 OX=1736436 GN=aroA PE=3 SV=1

Length=445

Score = 479 bits (1234), Expect = 9e-165

| Identities | = 256/447 (57%), Positives = 315/447 (70%), Gaps = 7/447 (2%) |  |
| --- | --- | --- |
| Query 1 | MLHGASSRPATARKSSGLSGTVRIPGDKSISHRSFMFGGLASGETRITGLLEGEDVINTG | 60 |
| Sbjct 1 | M H P +R SS L GTVR+PGDKSISHR+ + G LA GETRI+GLLEGEDV+NT MSHSGQPTPLESRASSALRGTVRVPGDKSISHRALILGALAVGETRISGLLEGEDVLNTA | 60 |
| Query 61 | KAMQAMGARI-RKEGDTWIIDGVGNGGLLAPEAPLDFGNAATGCRLTMGLVGVYDFDSTF | 119 |
| Sbjct 61 | KAMQA+GA++ R W + GVG GG AP+ PLDFGN+ TGCRL MG V +TF KAMQALGAKVERTRTGIWSVHGVGVGGFAAPDNPLDFGNSGTGCRLAMGAVAGCPIAATF | 120 |
| Query 120 | IGDASLTKRPMGRVLNPLREMGVQVKSE-DGDRLPVTLRGPKTPTPITYRVPMASAQVKS | 178 |
| Sbjct 121 | GDASL RPM R+L+PL MG +V S DG RLP+TL+G + P P+ YR P+ASAQ+KS DGDASLRSRPMRRILDPLELMGAKVSSSADGGRLPMTLQGARDPLPVVYRTPVASAQIKS | 180 |
| Query 179 | AVLLAGLNTPGITTVIEPIMTRDHTEKMLQGFGANLTVETDADGVRTIRLEGRGKLTGQV | 238 |

AVLLAGL+ PG+TTVIE	+RDHTE M+ FGA + E +	R I L G+ +L G

| Sbjct | 181 | AVLLAGLSAPGVTTVIEQEASRDHTELMMAHFGATIVSENEGPHGRKISLTGQPELHGAN | 240 |
| --- | --- | --- | --- |
| Query | 239 | IDVPGDPSSTAFPLVAALLVPGSDVTILNVLMNPTRTGLILTLQEMGADIEVINPRLAGG | 298 |
| Sbjct | 241 | + VP DPSS AFP+VAAL+VPGSDV + +V+ NP RTGL TL+EMGA IE + R G VVVPADPSSAAFPIVAALIVPGSDVVLTDVMTNPLRTGLFTTLREMGASIEESDVRGDTG | 300 |
| Query | 299 | EDVADLRVRSSTLKGVTVPEDRAPSMIDEYPILAVAAAFAEGATVMNGLEELRVKESDRL | 358 |
| Sbjct | 301 | E +A RV S L+GVTVP +RAPSMIDEY +LAVAAA+AEG T M GL+ELRVKESDRL EPMARFRVCGSRLRGVTVPPERAPSMIDEYLVLAVAAAYAEGVTTMRGLQELRVKESDRL | 360 |
| Query | 359 | SAVANGLKLNGVDCDEGETSLVVRGRPDGKGLGNASGAAVATHLDHRIAMSFLVMGLVSE | 418 |
| Sbjct | 361 | A A+ L++NGV + ++V G+ G G VATH+DHRIAMS LVMGL ++ EATADMLRVNGVKVEIVGDDMIVEGKGHVAGSG LVATHMDHRIAMSALVMGLAAD | 415 |
| Query  Sbjct | 419  416 | NPVTVDDATMIATSFPEFMDLMAGLGA 445  +PV +DD T IATSFP+F+ +M LGA SPVKIDDTTFIATSFPDFIPMMRELGA 442 |  |

>TR:A0A196MMM9 A0A196MMM9_9SPHN 3-phosphoshikimate 1-carboxyvinyltransferase OS=Sphingobium sp. TCM1 OX=453246 GN=aroA PE=3 SV=1

Length=446

Score = 479 bits (1234), Expect = 1e-164

| Identities | = 254/444 (57%), Positives = 312/444 (70%), Gaps = 4/444 (1%) |  |
| --- | --- | --- |
| Query 3 | HGASSRPATARKSSGLSGTVRIPGDKSISHRSFMFGGLASGETRITGLLEGEDVINTGKA | 62 |
| Sbjct 4 | H P T + LSG+V +PGDKSISHRS M LA GE+R+ GLLEGEDV+ T A HDEQPAPRTFTAAPPLSGSVTVPGDKSISHRSLMLSALAVGESRVEGLLEGEDVLATAAA | 63 |
| Query 63 | MQAMGARIRKEGD-TWIIDGVGNGGLLAPEAPLDFGNAATGCRLTMGLVGVYDFDSTFIG | 121 |
| Sbjct 64 | M+AMGA I+++ D W I GVG GGLL P+ +D GN+ T RL MGL+ +D +TFIG MRAMGADIQRDDDGIWHIHGVGVGGLLQPQTAIDMGNSGTSTRLLMGLLASHDLTATFIG | 123 |
| Query 122 | DASLTKRPMGRVLNPLREMGVQVKSEDGDRLPVTLRGPKTPTPITYRVPMASAQVKSAVL | 181 |
| Sbjct 124 | DASL+KRPM RV PL MG + G+RLP+T++G P+ YR+P+ASAQVKSA+L DASLSKRPMARVTEPLSRMGASFTTSPGERLPLTMKGACPAVPLDYRLPVASAQVKSAIL | 183 |
| Query 182 | LAGLNTPGITTVIEPIMTRDHTEKMLQGFGANLTVETDADGVRTIRLEGRGKLTGQVIDV | 241 |
| Sbjct 184 | LAGLNTPGIT VIEPI TRDH+E+ML+GFGA++ VE +ADG R I L G +L Q I V LAGLNTPGITRVIEPIPTRDHSERMLKGFGADIDVEVEADGTRIITLVGEAELKPQQIVV | 243 |
| Query 242 | PGDPSSTAFPLVAALLVPGSDVTILNVLMNPTRTGLILTLQEMGADIEVINPRLAGGEDV | 301 |
| Sbjct 244 | PGDPSS AFP+VAALLVPGS VTI NV +N TR GLI L+EMG IEV+N R GGE V PGDPSSAAFPMVAALLVPGSRVTIANVGLNATRAGLIDLLREMGGSIEVVNAREVGGEPV | 303 |
| Query 302 | ADLRVRSSTLKGVTVPEDRAPSMIDEYPILAVAAAFAEGATVMNGLEELRVKESDRLSAV | 361 |
| Sbjct 304 | DL V +S L+G+ RAPSMIDEYP+ +AAA AEG ++ GLEELRVKESDR++ + GDLIVTASALRGIEPDPARAPSMIDEYPVAFIAAALAEGRSIFRGLEELRVKESDRIATM | 363 |
| Query 362 | ANGLKLNGVDCDEGETSLVVRGRPDGKGLGNASGAAVATHLDHRIAMSFLVMGLVSENPV | 421 |
| Sbjct 364 | A GL+ GV +E E L++ G G A G +AT LDHRIAMSF V GLVS++ V AEGLRTIGVRVEELEDGLIIEG---SGGAALAGGGPIATKLDHRIAMSFAVAGLVSKDGV | 420 |
| Query 422  Sbjct 421 | TVDDATMIATSFPEFMDLMAGLGA 445 T+DD +ATSFP F+ L+ LGA TIDDMRPVATSFPTFLPLLQSLGA 444 |  |

>TR:A0A2Z6ABT0 A0A2Z6ABT0_9SPHN 3-phosphoshikimate 1-carboxyvinyltransferase OS=Sphingobium sp. YG1 OX=2082188 GN=aroA PE=3 SV=1

Length=446

Score = 479 bits (1233), Expect = 1e-164

| Identities | = 253/444 (57%), Positives = 312/444 (70%), Gaps = 4/444 (1%) |  |
| --- | --- | --- |
| Query 3 | HGASSRPATARKSSGLSGTVRIPGDKSISHRSFMFGGLASGETRITGLLEGEDVINTGKA | 62 |
| Sbjct 4 | H + P T + L+G+V +PGDKSISHRS M LA GE+R+ GLLEGEDV+ T A HDDQATPRTFSAAPALAGSVTVPGDKSISHRSLMLSALAVGESRVEGLLEGEDVLATAAA | 63 |
| Query 63 | MQAMGARIRKEGD-TWIIDGVGNGGLLAPEAPLDFGNAATGCRLTMGLVGVYDFDSTFIG | 121 |
| Sbjct 64 | M+AMGA I ++ D W I GVG GGLL PE LD GN+ T RL MGL+ +D +TF+G MRAMGATIERDDDGVWHIHGVGVGGLLQPETALDMGNSGTSTRLLMGLLASHDLTATFVG | 123 |
| Query 122 | DASLTKRPMGRVLNPLREMGVQVKSEDGDRLPVTLRGPKTPTPITYRVPMASAQVKSAVL | 181 |
| Sbjct 124 | DASL+KRPM RV +PL +G + GDRLP+T++G P+ Y +P+ASAQVKSAVL DASLSKRPMARVTDPLSRIGASFTTSPGDRLPLTMKGACPAVPLDYTLPVASAQVKSAVL | 183 |
| Query 182 | LAGLNTPGITTVIEPIMTRDHTEKMLQGFGANLTVETDADGVRTIRLEGRGKLTGQVIDV | 241 |
| Sbjct 184 | LAGLNTPGIT V+EPI TRDH+E+ML+GFGA+L VET ADG R I L G +L Q I V LAGLNTPGITRVVEPIPTRDHSERMLKGFGADLNVETQADGTRIITLVGEAELKPQNIVV | 243 |
| Query 242 | PGDPSSTAFPLVAALLVPGSDVTILNVLMNPTRTGLILTLQEMGADIEVINPRLAGGEDV | 301 |
| Sbjct 244 | PGDPSS AFP+VAALLVPGS VTI NV +N TR GLI L+EMG IEV+N R GGE V PGDPSSAAFPMVAALLVPGSQVTIANVGLNGTRAGLIDLLREMGGSIEVVNAREVGGEPV | 303 |
| Query 302 | ADLRVRSSTLKGVTVPEDRAPSMIDEYPILAVAAAFAEGATVMNGLEELRVKESDRLSAV | 361 |
| Sbjct 304 | DL V +S LKGV RAPSMIDEYP+ +AAAFA+G ++ GL+ELRVKESDR++ + GDLIVTASALKGVEPDPARAPSMIDEYPVAFIAAAFAQGRSIFRGLDELRVKESDRIATM | 363 |
| Query 362 | ANGLKLNGVDCDEGETSLVVRGRPDGKGLGNASGAAVATHLDHRIAMSFLVMGLVSENPV | 421 |
| Sbjct 364 | A GL+ GV +E E +++ G G G +AT LDHRIAMSF V GL+S++ V AQGLRAIGVTVEELEDGIIIEG---SGGALLPGGGPIATKLDHRIAMSFAVAGLISKDGV | 420 |
| Query 422  Sbjct 421 | TVDDATMIATSFPEFMDLMAGLGA 445 T+DD +ATSFP F+ L+ LGA TIDDMRPVATSFPTFLPLLQSLGA 444 |  |

>TR:A0A0N1BLJ2 A0A0N1BLJ2_9SPHN 3-phosphoshikimate 1-carboxyvinyltransferase OS=Blastomonas sp. AAP25 OX=1523416 GN=aroA PE=3 SV=1

Length=447

Score = 479 bits (1233), Expect = 1e-164

| Identities | = 255/444 (57%), Positives = 314/444 (71%), Gaps = 6/444 (1%) |  |
| --- | --- | --- |
| Query 1 | MLHGASSRPATAR--KSSGLSGTVRIPGDKSISHRSFMFGGLASGETRITGLLEGEDVIN | 58 |
| Sbjct 1 | M H A P R +S L+GT+R+PGDKSISHR+ M L G++RI+GLLEGEDV+ MAHSADQAPVPRRFARSGPLTGTIRVPGDKSISHRALMLSALCVGQSRISGLLEGEDVLA | 60 |
| Query 59 | TGKAMQAMGARIRKEGD-TWIIDGVGNGGLLAPEAPLDFGNAATGCRLTMGLVGVYDFDS | 117 |
| Sbjct 61 | T AM+AMGARI + D W + GVG GGL+ P LD GN+ T RL MGLV + + TAAAMRAMGARIERGADGIWTVHGVGVGGLIQPAQALDMGNSGTSTRLLMGLVASHPITA | 120 |
| Query 118 | TFIGDASLTKRPMGRVLNPLREMGVQVKSEDGDRLPVTLRGPKTPTPITYRVPMASAQVK | 177 |
| Sbjct 121 | F GDASL++RPM RV +PL EMG + + G LP+ +RG PI+YR+P+ASAQVK MFTGDASLSRRPMARVTDPLGEMGAEFNASPGGTLPLMVRGISPAVPISYRLPVASAQVK | 180 |
| Query 178 | SAVLLAGLNTPGITTVIEPIMTRDHTEKMLQGFGANLTVETDADGVRTIRLEGRGKLTGQ | 237 |
| Sbjct 181 | SA+LLA LNTPG TTVIEP+ TRDH+EKML+GFGA+LTVETDADGVR I + G LT Q SAILLAALNTPGTTTVIEPVATRDHSEKMLKGFGADLTVETDADGVRHIHVTGPCDLTAQ | 240 |
| Query 238 | VIDVPGDPSSTAFPLVAALLVPGSDVTILNVLMNPTRTGLILTLQEMGADIEVINPRLAG | 297 |
| Sbjct 241 | I+VPGDPSS AF +VAAL+VPGSD+TI NV MN TRTG+I L++MG IE +N R G DIEVPGDPSSAAFFIVAALIVPGSDLTIENVGMNVTRTGIITVLEQMGGQIERLNARTVG | 300 |
| Query 298 | GEDVADLRVRSSTLKGVTVPEDRAPSMIDEYPILAVAAAFAEGATVMNGLEELRVKESDR | 357 |

GE VADLRVR+S L +TV	PSM+DE+P+ +AAA A G TV +GL+ELRVKESDR

| Sbjct | 301 | GEPVADLRVRASALSRITVDPAIVPSMVDEFPVFFIAAAMASGTTVTSGLDELRVKESDR | 360 |
| --- | --- | --- | --- |
| Query | 358 | LSAVANGLKLNGVDCDEGETSLVVRGRPDGKGLGNASGAAVATHLDHRIAMSFLVMGLVS | 417 |
| Sbjct | 361 | L+ +A GL+ GVD +E E L++ G G G VAT LDHRIAMSF V GLVS LATMARGLEAIGVDVEEREDGLLITG---SGGASLRGGGPVATLLDHRIAMSFAVAGLVS | 417 |
| Query  Sbjct | 418  418 | ENPVTVDDATMIATSFPEFMDLMA 441 E V VDD IATSFP+F++L++ EQGVDVDDTAPIATSFPDFLNLLS 441 |  |

>TR:A0A2S8B2U0 A0A2S8B2U0_9SPHN 3-phosphoshikimate 1-carboxyvinyltransferase OS=Sphingopyxis lindanitolerans OX=2054227 GN=aroA PE=3 SV=1

Length=442

Score = 479 bits (1232), Expect = 2e-164

| Identities | = 260/439 (59%), Positives = 306/439 (70%), Gaps = 4/439 (1%) |  |
| --- | --- | --- |
| Query 6 | SSRPATARKSSGLSGTVRIPGDKSISHRSFMFGGLASGETRITGLLEGEDVINTGKAMQA | 65 |
| Sbjct 5 | S P S L G + IPGDKSISHR+ M LA G +R+TGLLEG DV+ T AM+A SPSPRAFSASVPLRGKIAIPGDKSISHRALMLSALAVGTSRVTGLLEGHDVLATAAAMRA | 64 |
| Query 66 | MGARI-RKEGDTWIIDGVGNGGLLAPEAPLDFGNAATGCRLTMGLVGVYDFDSTFIGDAS | 124 |
| Sbjct 65 | MGA I R + W+IDGVG GGLL P ++ GN+ T RL MGLV + TF GDAS MGATIARGDKGEWVIDGVGVGGLLQPREAIEMGNSGTSTRLLMGLVASHPITVTFTGDAS | 124 |
| Query 125 | LTKRPMGRVLNPLREMGVQVKSEDGDRLPVTLRGPKTPTPITYRVPMASAQVKSAVLLAG | 184 |
| Sbjct 125 | L+ RPMGRV++PL +MG + + G RLP+ +RG P+ YR+PMASAQVKSAVLLAG LSGRPMGRVIDPLSQMGADISASPGGRLPLMVRGLAPAIPLAYRLPMASAQVKSAVLLAG | 184 |
| Query 185 | LNTPGITTVIEPIMTRDHTEKMLQGFGANLTVETDADGVRTIRLEGRGKLTGQVIDVPGD | 244 |
| Sbjct 185 | LNTPGIT VIEP+ TRDH+E+ML+GFGA+LTVETD DG R IRL G +L Q I VPGD LNTPGITEVIEPVPTRDHSERMLRGFGADLTVETDTDGTRHIRLRGEAELVPQTIVVPGD | 244 |
| Query 245 | PSSTAFPLVAALLVPGSDVTILNVLMNPTRTGLILTLQEMGADIEVINPRLAGGEDVADL | 304 |
| Sbjct 245 | PSS AF +VAALLVPGSDV I NV +NPTR GL+ L+ MG DIE+I+ R GGE VADL PSSAAFFIVAALLVPGSDVLIANVGLNPTRAGLVDVLKAMGGDIELIDARDVGGEPVADL | 304 |
| Query 305 | RVRSSTLKGVTVPEDRAPSMIDEYPILAVAAAFAEGATVMNGLEELRVKESDRLSAVANG | 364 |
| Sbjct 305 | RVR S LKG+ V APSMIDEYPIL VAAA AEG TV GL+ELRVKESDRL+ +A G RVRHSHLKGIAVDPAVAPSMIDEYPILFVAAALAEGRTVTTGLDELRVKESDRLAVMATG | 364 |
| Query 365 | LKLNGVDCDEGETSLVVRGRPDGKGLGNASGAAVATHLDHRIAMSFLVMGLVSENPVTVD | 424 |

L G	+E E L++ G	G	A GA VA HLDHRI MSF + GLVS+ VT+D

| Sbjct | 365 | LAAIGARVEESEDGLIIDGT---GGDALAGGATVAGHLDHRICMSFAIAGLVSKAAVTID | 421 |
| --- | --- | --- | --- |
| Query  Sbjct | 425  422 | DATMIATSFPEFMDLMAGL 443 D +ATSFP F L+A L DMAPVATSFPNFETLLADL 440 |  |

```
>TR:D5ARK9 D5ARK9_RHOCB 3-phosphoshikimate 1-carboxyvinyltransferase OS=Rhodobacter capsulatus (strain ATCC BAA-309 / NBRC 16581 / SB1003)
OX=272942 GN=aroA PE=3 SV=1
Length=447

Score = 479 bits (1232), Expect = 2e-164
Identities = 259/447 (58%), Positives = 311/447 (70%), Gaps = 8/447 (2%)

Query 3	HGASSRPATARKSSGLSGTVRIPGDKSISHRSFMFGGLASGETRITGLLEGEDVINTGKA 62 HG+ R T+RK	LSG	+PGDKSISHR+ + G L+ G TRITGLLEG+DV++T +A
Sbjct 4	HGSPIR-MTSRKGGPLSGVAAVPGDKSISHRALILGALSVGTTRITGLLEGQDVLDTARA 62

Query 63	MQAMGARIRKEGD-TWIIDGVGNGGLLAPEAPLDFGNAATGCRLTMGLVGVYDFDSTFIG 121 MQA GA + + G	W ++GVG GG	P	+D GN+ TG RL MG +	+TF G
```

| Sbjct | 63 | MQAFGATVTRTGPGAWTVEGVGVGGFAEPAEVIDCGNSGTGVRLIMGCMATSPISATFTG | 122 |
| --- | --- | --- | --- |
| Query | 122 | DASLTKRPMGRVLNPLREMGVQVKSEDGDRLPVTLRGPKTPTPITYRVPMASAQVKSAVL | 181 |
| Sbjct | 123 | DASL KRPMGRV +PL G + G RLP+TL G P P+TY PMASAQVKSAVL DASLRKRPMGRVTDPLALFGARSYGRAGGRLPMTLVGAAEPVPVTYTTPMASAQVKSAVL | 182 |
| Query | 182 | LAGLNTPGITTVIEPIMTRDHTEKMLQGFGANLTVETDADGVRTIRLEGRGKLTGQVIDV | 241 |
| Sbjct | 183 | LAGLN PG T VIE TRDHTE+ML+GFGA +TVE +G R I L G+ +L Q + V LAGLNAPGQTVVIEKTPTRDHTERMLRGFGAEVTVEATPEGNR-ITLTGQPELRAQEVAV | 241 |
| Query | 242 | PGDPSSTAFPLVAALLVPGSDVTILNVLMNPTRTGLILTLQEMGADIEVINPRLAGGEDV | 301 |
| Sbjct | 242 | P DPSS AFP+ AALLVPGSD+ + V NPTR GL LTL+EMGADIE +N R GGE V PRDPSSAAFPVCAALLVPGSDILVPGVSQNPTRNGLFLTLREMGADIEFLNEREEGGEPV | 301 |
| Query | 302 | ADLRVRSSTLKGVTVPEDRAPSMIDEYPILAVAAAFAEGATVMNGLEELRVKESDRLSAV | 361 |
| Sbjct | 302 | ADLRVR S L G+ VP +RA + IDEYP+LA AA A G TVM G+ ELRVKESDR++A+ ADLRVRHSALTGIAVPPERAAAQIDEYPVLAALAACASGTTVMRGVHELRVKESDRIAAM | 361 |
| Query | 362 | ANGLKLNGVDCDEGETSLVVRG-RPDGKGLGNASGAAVATHLDHRIAMSFLVMGLVSENP | 420 |
| Sbjct | 362 | A GL+ GV +E E +L+V G PDG GA VATH+DHRIAMSFLV+GLVS+ P ARGLEACGVRVEEDEDTLIVHGLGPDGV PGGATVATHIDHRIAMSFLVLGLVSQQP | 417 |
| Query  Sbjct | 421  418 | VTVDDATMIATSFPEFMDLMAGLGAKI 447  ++VDD + IATSFP F DLM LGA I ISVDDGSPIATSFPVFEDLMRQLGASI 444 |  |

>SP:A1B474 AROA_PARDP 3-phosphoshikimate 1-carboxyvinyltransferase OS=Paracoccus denitrificans (strain Pd 1222) OX=318586 GN=aroA PE=3

SV=1

Length=443

Score = 478 bits (1231), Expect = 2e-164

| Identities | = 251/449 (56%), Positives = 316/449 (70%), Gaps = 7/449 (2%) |  |
| --- | --- | --- |
| Query 1 | MLHGASSRPATARKSSGLSGTVRIPGDKSISHRSFMFGGLASGETRITGLLEGEDVINTG | 60 |
| Sbjct 1 | M H A P TAR+S L+G ++PGDKSISHR+ + G L+ GET ITGLLEG+DV++T MSHSAEPLPMTARRSGPLTGEAQVPGDKSISHRALILGALSVGETHITGLLEGQDVLDTA | 60 |
| Query 61 | KAMQAMGARIRKEGD-TWIIDGVGNGGLLAPEAPLDFGNAATGCRLTMGLVGVYDFDSTF | 119 |
| Sbjct 61 | AM+A GA++ + G+ W ++GVG GG PE +D GN+ TG RL MG + +TF AAMRAFGAQVERLGEGEWRVNGVGVGGFAEPEGVIDCGNSGTGVRLIMGAMATTPITATF | 120 |
| Query 120 | IGDASLTKRPMGRVLNPLREMGVQVKSEDGDRLPVTLRGPKTPTPITYRVPMASAQVKSA | 179 |
| Sbjct 121 | GDASL++RPMGRV +PL G +V + +G RLP+T++G P P+ Y+ P+ASAQ+KSA TGDASLSRRPMGRVTDPLELFGCEVTAREGKRLPLTIKGASEPVPVRYKTPVASAQIKSA | 180 |
| Query 180 | VLLAGLNTPGITTVIEPIMTRDHTEKMLQGFGANLTVETDADGVRTIRLEGRGKLTGQVI | 239 |
| Sbjct 181 | VLLAGLN PG T VIE TRDHTE+ML GFGA +T ET A+G I L+GR +L Q + VLLAGLNAPGETVVIEAEPTRDHTERMLAGFGAAITTETTAEG-HVIILKGRPELKPQPV | 239 |
| Query 240 | DVPGDPSSTAFPLVAALLVPGSDVTILNVLMNPTRTGLILTLQEMGADIEVINPRLAGGE | 299 |
| Sbjct 240 | VP DPSS AFP+ AAL+VPGS++ + V NPTR GL +TL EMGADI N R GGE AVPRDPSSAAFPVAAALIVPGSEIRVPGVSRNPTRDGLYVTLLEMGADIRFENQREEGGE | 299 |
| Query 300 | DVADLRVRSSTLKGVTVPEDRAPSMIDEYPILAVAAAFAEGATVMNGLEELRVKESDRLS | 359 |
| Sbjct 300 | VADL VR LKGVTVP +RA SMIDE+PIL+V A FAEG TVM G+ ELRVKESDR+ PVADLVVRHGPLKGVTVPAERAASMIDEFPILSVIACFAEGTTVMQGVHELRVKESDRID | 359 |
| Query 360 | AVANGLKLNGVDCDEGETSLVVRGRPDGKGLGNASGAAVATHLDHRIAMSFLVMGLVSEN | 419 |
| Sbjct 360 | A+A GL+ NG + + + ++ V G+ G GA ATHLDHRIAMSFLV GL S  AMAVGLRANGAEVVDTQDTMTVHGKG GLDGGATCATHLDHRIAMSFLVAGLASHQ | 414 |

Query 420 PVTVDDATMIATSFPEFMDLMAGLGAKIE 448 P++VDD	IATSFP+F+ LM GLGA+I+

Sbjct 415 PISVDDGGPIATSFPDFLPLMRGLGAQID 443

>TR:A0A3D8PFI5 A0A3D8PFI5_9RHOB 3-phosphoshikimate 1-carboxyvinyltransferase OS=Paracoccus thiocyanatus OX=34006 GN=aroA PE=3 SV=1

Length=443

Score = 478 bits (1231), Expect = 2e-164

| Identities | | = 253/449 (56%), Positives = 312/449 (69%), Gaps = 7/449 (2%) |  |
| --- | --- | --- | --- |
| Query 1  Sbjct 1 | | MLHGASSRPATARKSSGLSGTVRIPGDKSISHRSFMFGGLASGETRITGLLEGEDVINTG M H A P TAR+S L+G ++PGDKSISHR+ + G LA GETRITGLLEG+DV++T MSHSAEPLPMTARRSGPLTGEAKVPGDKSISHRALILGALAVGETRITGLLEGQDVLDTA | 60  60 |
| Query 61  Sbjct 61 | | KAMQAMGARIRKEGD-TWIIDGVGNGGLLAPEAPLDFGNAATGCRLTMGLVGVYDFDSTF AM+A GA++ + G+ W + GVG GG P+ +D GN+ TG RL MG + +TF AAMRAFGAQVERLGEGEWRVTGVGVGGFAEPQGVIDCGNSGTGVRLIMGAMATTAISATF | 119  120 |
| Query 120  Sbjct 121 | | IGDASLTKRPMGRVLNPLREMGVQVKSEDGDRLPVTLRGPKTPTPITYRVPMASAQVKSA GDASL RPMGRV +PL G QV + +G RLP+T++G P P+ Y+ P+ASAQ+KSA TGDASLCGRPMGRVTDPLELFGAQVTAREGKRLPLTIQGAADPLPVRYKTPVASAQIKSA | 179  180 |
| Query 180  Sbjct 181 | | VLLAGLNTPGITTVIEPIMTRDHTEKMLQGFGANLTVETDADGVRTIRLEGRGKLTGQVI VLLAGLN PG T VIE TRDHTE+ML GFGA++ ET +G + I L+GR +L Q + VLLAGLNAPGETVVIESEPTRDHTERMLAGFGASIRTETTDEG-QVITLQGRPELKPQPV | 239  239 |
| Query 240  Sbjct 240 | | DVPGDPSSTAFPLVAALLVPGSDVTILNVLMNPTRTGLILTLQEMGADIEVINPRLAGGE VP DPSS AFP+ AAL+VPGS++ + V NPTR GL +TL EMGADI N R GGE AVPRDPSSAAFPVAAALIVPGSEIRVPGVSRNPTRDGLYVTLLEMGADIRFENQREEGGE | 299  299 |
| Query 300  Sbjct 300 | | DVADLRVRSSTLKGVTVPEDRAPSMIDEYPILAVAAAFAEGATVMNGLEELRVKESDRLS VADL VR LKGVTVP +RA SMIDE+PIL+V A FAEG TVM G+ ELRVKESDR+ PVADLVVRHGPLKGVTVPPERAASMIDEFPILSVIACFAEGTTVMQGVHELRVKESDRIE | 359  359 |
| Query 360 | | AVANGLKLNGVDCDEGETSLVVRGRPDGKGLGNASGAAVATHLDHRIAMSFLVMGLVSEN A+A GL NG + ++ V G+ G GA ATHLDHRIAMSFLV GL S+ | 419 |
| Sbjct | 360 | AMATGLVANGASVETTPDTMTVHGKG GLTGGATCATHLDHRIAMSFLVAGLASQQ | 414 |
| Query  Sbjct | 420  415 | PVTVDDATMIATSFPEFMDLMAGLGAKIE 448 PVTVDD IATSFP+F+ LM GLGA+I+ PVTVDDGGPIATSFPDFLPLMRGLGARID 443 |  |

>TR:A0A1N7JCG0 A0A1N7JCG0_9PROT 3-phosphoshikimate 1-carboxyvinyltransferase OS=Insolitispirillum peregrinum OX=80876 GN=aroA PE=3 SV=1

Length=448

Score = 478 bits (1231), Expect = 3e-164

| Identities | = 257/449 (57%), Positives = 311/449 (69%), Gaps = 6/449 (1%) |  |
| --- | --- | --- |
| Query 1 | MLHGASSRPATARKSSGLSGTVRIPGDKSISHRSFMFGGLASGETRITGLLEGEDVINTG | 60 |
| Sbjct 1 | M + +P +R L GT+ +PGDKSISHR+ MFG LA GET + GLLEGEDV+ T MSSSEAPQPLLSRPRGALKGTLTVPGDKSISHRALMFGALALGETTVEGLLEGEDVLRTA | 60 |
| Query 61 | KAMQAMGARIRKEGD-TWIIDGVGNGGLLAPEAPLDFGNAATGCRLTMGLVGVYDFDSTF | 119 |
| Sbjct 61 | +AM+ +GA + + G+ +W + G G GGL P+ LD GN+ TG RL MGL+ + F S QAMRQLGATVERLGEGSWRLQGRGVGGLCEPDDVLDMGNSGTGARLLMGLLATHPFTSVL | 120 |
| Query 120 | IGDASLTKRPMGRVLNPLREMGVQVKSEDGDRLPVTLRGPKTPTPITYRVPMASAQVKSA | 179 |
| Sbjct 121 | GDASL KRPM RV PL G G RLP+ + G TP P TY +P+ASAQVKSA  TGDASLRKRPMARVTVPLSHFGATFTGRSGGRLPLAVTGTATPVPQTYELPVASAQVKSA | 180 |

| Query | 180 | VLLAGLNTPGITTVIEPIMTRDHTEKMLQGFGANLTVETDADGVRTIRLEGRGKLTGQVI | 239 |
| --- | --- | --- | --- |
| Sbjct | 181 | VLLAGLNTPGITTVIEP TRDH+E ML+ FGA+LTV DG R I L G+ +LTG+ + VLLAGLNTPGITTVIEPEATRDHSELMLRAFGADLTVSVRDDGARVITLVGQPELTGRAV | 240 |
| Query | 240 | DVPGDPSSTAFPLVAALLVPGSDVTILNVLMNPTRTGLILTLQEMGADIEVINPRLAGGE | 299 |
| Sbjct | 241 | VPGDPSS AFP+VAAL+VPGS + + NV MNP RTGL TL EMGAD++++N R GE KVPGDPSSAAFPVVAALIVPGSHIVVRNVGMNPLRTGLFTTLLEMGADLKLVNEREEAGE | 300 |
| Query | 300 | DVADLRVRSSTLKGVTVPEDRAPSMIDEYPILAVAAAFAEGATVMNGLEELRVKESDRLS | 359 |
| Sbjct | 301 | VADL VR S LKGVTVP +RAPSMIDEYPIL+VAAA A G T M GL ELRVKESDRL+ PVADLEVRYSALKGVTVPPERAPSMIDEYPILSVAAACASGETRMVGLAELRVKESDRLA | 360 |
| Query | 360 | AVANGLKLNGVDCDEGET-SLVVRGRPDGKGLGNASGAAVATHLDHRIAMSFLVMGLVSE | 418 |
| Sbjct | 361 | A+A+GLK G D D E +LVVR G G GA + +LDHRI MS+LV+G+ + AMADGLKACGADTDVAENDTLVVR GTGQPPRGGAMIPVNLDHRIGMSYLVLGMTTA | 416 |
| Query | 419 | NPVTVDDATMIATSFPEFMDLMAGLGAKI 447 V +DDA+ IATSFP F D+M LGA I |  |
| Sbjct | 417 | EAVGIDDASAIATSFPGFADMMIELGADI 445 | |

>TR:F1ZDB7 F1ZDB7_9SPHN 3-phosphoshikimate 1-carboxyvinyltransferase OS=Novosphingobium nitrogenifigens DSM 19370 OX=983920 GN=aroA

PE=3 SV=1

Length=448

Score = 478 bits (1231), Expect = 3e-164

| Identities | | = 255/429 (59%), Positives = 309/429 (72%), Gaps = 2/429 (0%) |  |
| --- | --- | --- | --- |
| Query 18  Sbjct 20 | | LSGTVRIPGDKSISHRSFMFGGLASGETRITGLLEGEDVINTGKAMQAMGARIRKEGD-T L+G +R+PGDKSISHRS M G LA GETR+TGLLEGEDV++T AM+AMGA I ++G+ LTGRIRVPGDKSISHRSIMLGALAVGETRVTGLLEGEDVLSTAAAMRAMGATIERQGNGD | 76  79 |
| Query 77  Sbjct 80 | | WIIDGVGNGGLLAPEAPLDFGNAATGCRLTMGLVGVYDFDSTFIGDASLTKRPMGRVLNP W++ GVG GGLL P LD GN+ T RL MGLV + + F+GDASL+KRPMGRV+ P WLVSGVGVGGLLQPAGALDMGNSGTSTRLLMGLVASHPITTCFVGDASLSKRPMGRVITP | 136  139 |
| Query 137  Sbjct 140 | | LREMGVQVKSEDGDRLPVTLRGPKTPTPITYRVPMASAQVKSAVLLAGLNTPGITTVIEP L ++G + G RLP+ G TP PI+YR+P+ASAQVKSAVLLAGLNTPG+TTVIEP LSQVGAAFTTYPGGRLPLLETGTDTPVPISYRLPVASAQVKSAVLLAGLNTPGVTTVIEP | 196  199 |
| Query 197  Sbjct 200 | | IMTRDHTEKMLQGFGANLTVETDADGVRTIRLEGRGKLTGQVIDVPGDPSSTAFPLVAAL  + TRDH+E+ML+GFGA LTV+ D DG R IRL G+ +L Q I VPGDPSS AF +VAAL VPTRDHSERMLRGFGAALTVDIDEDGARIIRLVGQPELKAQDIVVPGDPSSAAFFVVAAL | 256  259 |
| Query 257  Sbjct 260 | | LVPGSDVTILNVLMNPTRTGLILTLQEMGADIEVINPRLAGGEDVADLRVRSSTLKGVTV LVPGSD+ + NV +NPTR L L+ MG IE I+ R GGE VADL VR+STL G+ V LVPGSDLVVENVGLNPTRAALFDVLRLMGGQIEEIDRREVGGEPVADLHVRASTLHGIEV | 316  319 |
| Query 317  Sbjct 320 | | PEDRAPSMIDEYPILAVAAAFAEGATVMNGLEELRVKESDRLSAVANGLKLNGVDCDEGE PSM+DE+P+L VAAA AEG TV +GLEELRVKESDR+SA+ L L G +E  DPAVVPSMVDEFPVLFVAAALAEGRTVTSGLEELRVKESDRISAMRAALHLAGARVEETA | 376  379 |
| Query 377  Sbjct 380 | | TSLVVRGRPDGKGLGNASGAAVATHLDHRIAMSFLVMGLVSENPVTVDDATMIATSFPEF L++ G GK L AAVATHLDHRIAMS V GL S+ VTVDD IATSFP F DGLIIEG-TGGKLLNGTGNAAVATHLDHRIAMSMAVAGLASQTGVTVDDTRPIATSFPVF | 436  438 |
| Query 437 | | MDLMAGLGA 445 L++ +GA |  |
| Sbjct | 439 | EHLLSEVGA 447 | |

>TR:A0A5C8WYE9 A0A5C8WYE9_9RHIZ 3-phosphoshikimate 1-carboxyvinyltransferase

OS=Methylobacterium sp. WL8 OX=2603899 GN=aroA PE=3 SV=1 Length=449

Score = 478 bits (1231), Expect = 3e-164

| Identities | = 268/453 (59%), Positives = 318/453 (70%), Gaps = 12/453 (3%) |  |
| --- | --- | --- |
| Query 1 | MLHGASSRPATARKSSGLSGTVRIPGDKSISHRSFMFGGLASGETRITGLLEGEDVINTG | 60 |
| Sbjct 1 | M H + P TA + L G +R PGDKSISHR+ + G L+ GETR+ GLLEG+DV+ T MSHDSEPTPLTAAPGAPLRGRLRPPGDKSISHRAMILGLLSIGETRVEGLLEGDDVLRTA | 60 |
| Query 61 | KAMQAMGARIRKEGD-TWIIDGVGNGGLLAPEAPLDFGNAATGCRLTMGLVGVYDFDSTF | 119 |
| Sbjct 61 | + +A+GA + + G+ W++ GVG GGL P LDFGNA TG RL MG+VG +TF ASARALGASMERLGEGRWVVRGVGVGGLSDPAETLDFGNAGTGSRLMMGVVGGQPVTATF | 120 |
| Query 120 | IGDASLTKRPMGRVLNPLREMGVQVKSE-DGDRLPVTLRGPKTPTPITYRVPMASAQVKS | 178 |
| Sbjct 121 | GDASL RPM R+L+PLR MG QV +E +G R+P+TLRGP+ PI Y P ASAQ+KS DGDASLRSRPMRRILDPLRLMGTQVLAEAEGGRVPLTLRGPREAIPIRYETPAASAQIKS | 180 |
| Query 179 | AVLLAGLNTPGITTVIEPIMTRDHTEKMLQGFGANLTVET---DADGVRTIRLEGRGKLT | 235 |
| Sbjct 181 | AVLLAGLN G TTVIE TRDHTE+ML+ FGA ++V D G R I L G+ L AVLLAGLNAAGATTVIEAAATRDHTERMLRLFGAEVSVVPHGPDGHG-RAITLTGQPTLR | 239 |
| Query 236 | GQVIDVPGDPSSTAFPLVAALLVPGSDVTILNVLMNPTRTGLILTLQEMGADIEVINPRL | 295 |
| Sbjct 240 | G + VP DPSS AFPLVAAL+VPGS+VTI V+MNP RTGLI TL EMGADIE + R GTDVVVPADPSSAAFPLVAALIVPGSEVTIEGVMMNPLRTGLITTLIEMGADIERLRERE | 299 |
| Query 296 | AGGEDVADLRVRSSTLKGVTVPEDRAPSMIDEYPILAVAAAFAEGATVMNGLEELRVKES | 355 |
| Sbjct 300 | GGE VADLRVR+S L+GV VP +RAP+MIDEYP+LAVAAAFAEG T M+GL ELRVKES EGGETVADLRVRASRLRGVDVPAERAPAMIDEYPVLAVAAAFAEGTTRMHGLHELRVKES | 359 |
| Query 356 | DRLSAVANGLKLNGV-DCDEGETSLVVRGRPDGKGLGNASGAAVATHLDHRIAMSFLVMG | 414 |
| Sbjct 360 | DRL+AVA GLK NGV +G+ +V G G G VATHLDHRIAM+FLVMG DRLAAVAAGLKANGVAHLIQGDDLIV HGDGTAPVGGGTVATHLDHRIAMAFLVMG | 414 |
| Query 415  Sbjct 415 | LVSENPVTVDDATMIATSFPEFMDLMAGLGAKI 447 L + PVTVDD MIATSFP F+ M LGA I LATAKPVTVDDGAMIATSFPSFLPTMQALGAGI 447 |  |

>TR:H6SLF4 H6SLF4_PARPM 3-phosphoshikimate 1-carboxyvinyltransferase OS=Pararhodospirillum photometricum DSM 122 OX=1150469 GN=aroA

PE=3 SV=1

Length=451

Score = 478 bits (1231), Expect = 3e-164

| Identities | = 263/445 (59%), Positives = 302/445 (68%), Gaps = 11/445 (2%) |  |
| --- | --- | --- |
| Query 9 | PATARKSSGLSGTVRIPGDKSISHRSFMFGGLASGETRITGLLEGEDVINTGKAMQAMGA | 68 |
| Sbjct 7 | P AR++ L G++R+PGDKS+SHR+ M GGLA G T ITGLLEGEDV+ T AM AMGA PLRARRADALIGSLRVPGDKSVSHRALMLGGLAVGRTTITGLLEGEDVLATAHAMTAMGA | 66 |
| Query 69 | RIRK EGDTWIIDGVGNGGLLAPEAPLDFGNAATGCRLTMGLVGVYDFDSTFIGDA | 123 |
| Sbjct 67 | R+ + G W +DGVG GGL P LD GNA TG RL MGLV + + F GDA RVERLAPGEAGAVWQVDGVGVGGLAEPADVLDMGNAGTGARLLMGLVATHPITAIFTGDA | 126 |
| Query 124 | SLTKRPMGRVLNPLREMGVQVKSEDGDRLPVTLRGPKTPTPITYRVPMASAQVKSAVLLA | 183 |
| Sbjct 127 | SL +RPM RV+ PL G DG RLP+ L G + P P+TYRVP+ SAQVKSAVLLA SLRRRPMARVIEPLSRFGATFLGRDGGRLPLALGGARAPLPVTYRVPVPSAQVKSAVLLA | 186 |
| Query 184 | GLNTPGITTVIEPIMTRDHTEKMLQGFGANLTVETDADGVRTIRLEGRGKLTGQVIDVPG | 243 |
| Sbjct 187 | GLNTPG TTVIEPI TRDHTE+ML FGA LT DG + L G+ +LTG + VP GLNTPGTTTVIEPIPTRDHTERMLGYFGATLTRTPAEDGGVVLALTGQPELTGAAVQVPA | 246 |
| Query 244 | DPSSTAFPLVAALLVPGSDVTILNVLMNPTRTGLILTLQEMGADIEVINPRLAGGEDVAD | 303 |

| Sbjct | 247 | DPSS AFPLVAALLV GS V + V +NP RTGL TL+EMGADI V+NPR GE V D  DPSSAAFPLVAALLVEGSRVVLNGVGLNPLRTGLFDTLREMGADIRVLNPREEAGEPVGD | 306 |
| --- | --- | --- | --- |
| Query | 304 | LRVRSSTLKGVTVPEDRAPSMIDEYPILAVAAAFAEGATVMNGLEELRVKESDRLSAVAN | 363 |
| Sbjct | 307 | L V +S L+GVTVP +RAPSMIDEYPILA+AAA AEG T M GL ELRVKESDRL+A+A LEVHASRLRGVTVPPERAPSMIDEYPILAIAAACAEGETRMEGLAELRVKESDRLAAMAR | 366 |
| Query | 364 | GLKLNGVD-CDEGETSLVVRGRPDGKGLGNASGAAVATHLDHRIAMSFLVMGLVSENPVT | 422 |
| Sbjct | 367 | GL GV EG+T +V G G GA V LDHRI MSFLV+GLVS PV GLAACGVRVAVEGDTLIV QGSGRPPQGGARVTVDLDHRIGMSFLVLGLVSAEPVA | 421 |
| Query  Sbjct | 423  422 | VDDATMIATSFPEFMDLMAGLGAKI 447  +DD I TSFP F+ LM LGA I IDDGRAIDTSFPGFVALMGALGATI 446 |  |

>TR:A0A3P3DV24 A0A3P3DV24_9RHOB 3-phosphoshikimate 1-carboxyvinyltransferase OS=Gemmobacter sp. YIM 102744-1 OX=2488730 GN=aroA PE=3 SV=1

Length=451

Score = 478 bits (1231), Expect = 3e-164

| Identities | = 257/445 (58%), Positives = 311/445 (70%), Gaps = 5/445 (1%) |  |
| --- | --- | --- |
| Query 5 | ASSRPATARKSSGLSGTVRIPGDKSISHRSFMFGGLASGETRITGLLEGEDVINTGKAMQ | 64 |
| Sbjct 5 | A+ P TA++S L G ++PGDKSISHR+ + G +A GETRITGLLEGEDVI+T KAM+ ATPVPMTAQRSGPLRGVAQVPGDKSISHRALILGAMAIGETRITGLLEGEDVIDTAKAMR | 64 |
| Query 65 | AMGARIRKEGD-TWIIDGVGNGGLLAPEAPLDFGNAATGCRLTMGLVGVYDFDSTFIGDA | 123 |
| Sbjct 65 | A GA++ + G+ W+++GVG GG P +D GN+ TG RL MG + +TF GD AFGAQVERVGEGEWVVNGVGVGGFSEPADVIDCGNSGTGVRLIMGAMATTGITATFTGDG | 124 |
| Query 124 | SLTKRPMGRVLNPLREMGVQVKSEDGDRLPVTLRGPKTPTPITYRVPMASAQVKSAVLLA | 183 |
| Sbjct 125 | SL KRPM RV PL GVQ G RLP+TL G P ++Y VP+ SAQVKSAVLLA SLRKRPMKRVTEPLALFGVQSVGRSGGRLPMTLVGAAEPLAVSYEVPVPSAQVKSAVLLA | 184 |
| Query 184 | GLNTPGITTVIEPIMTRDHTEKMLQGFGANLTVETDADGVRTIRLEGRGKLTGQVIDVPG | 243 |
| Sbjct 185 | GLN PG T VIE TRDH+E+ML GFGA ++ E ADG R IRL+GR +L Q + VP GLNAPGETVVIEREPTRDHSERMLAGFGAEISTEVRADGARVIRLQGRPELKPQAVAVPR | 244 |
| Query 244 | DPSSTAFPLVAALLVPGSDVTILNVLMNPTRTGLILTLQEMGADIEVINPRLAGGEDVAD | 303 |
| Sbjct 245 | DPSS AFP+ AALLVPGSD+ + V NPTR GL LTL EMGADI NPR GGE VAD DPSSAAFPVCAALLVPGSDILVPGVSRNPTRDGLFLTLIEMGADITFENPREEGGEPVAD | 304 |
| Query 304 | LRVR-SSTLKGVTVPEDRAPSMIDEYPILAVAAAFAEGATVMNGLEELRVKESDRLSAVA | 362 |
| Sbjct 305 | LRVR + L G+ PE+RA SMIDE+P+L+V AA A G T+M G++ELRVKESDR+ A+A LRVRYAGALTGIETPEERAASMIDEFPVLSVVAACATGRTIMRGVKELRVKESDRIDAMA | 364 |
| Query 363 | NGLKLNGVDCDEGETSLVVRGRPDGKGLGNASGAAVATHLDHRIAMSFLVMGLVSENPVT | 422 |
| Sbjct 365 | GL+ GV +E E +L+V G +G G GA ATHLDHRIAMSFLV+GL S+ PV+ RGLEACGVTIEEDEDTLIVHGMGEG---GVPGGATCATHLDHRIAMSFLVLGLASQQPVS | 421 |
| Query 423  Sbjct 422 | VDDATMIATSFPEFMDLMAGLGAKI 447 VDD IATSFP F LMA LGAKI VDDGAPIATSFPVFEPLMAELGAKI 446 |  |

```
>TR:A0A246K724 A0A246K724_9SPHN 3-phosphoshikimate 1-carboxyvinyltransferase OS=Sphingopyxis witflariensis OX=173675 GN=aroA PE=3 SV=1
Length=446

Score = 478 bits (1230), Expect = 4e-164
Identities = 259/443 (58%), Positives = 312/443 (70%), Gaps = 11/443 (2%) Query 9	PATARK---SSGLSGTVRIPGDKSISHRSFMFGGLASGETRITGLLEGEDVINTGKAMQA 65

PAT R+	S+ L G + IPGDKSISHR+ M	LA GE+R+TGLLEG DV+ T AM+A
```

| Sbjct | 5 | PATPRRFSASTPLEGKIAIPGDKSISHRALMLSALAVGESRVTGLLEGHDVLATAAAMRA | 64 |
| --- | --- | --- | --- |
| Query | 66 | MGARIRKEGD-TWIIDGVGNGGLLAPEAPLDFGNAATGCRLTMGLVGVYDFDSTFIGDAS | 124 |
| Sbjct | 65 | MGA I + D W I GVG GGLL P LD GN+ T RL MGLV + +TF GDAS MGATIERRDDGEWRIHGVGVGGLLQPREALDMGNSGTSTRLLMGLVATHPITTTFTGDAS | 124 |
| Query | 125 | LTKRPMGRVLNPLREMGVQVKSEDGDR LPVTLRGPKTPTPITYRVPMASAQVKSAV | 180 |
| Sbjct | 125 | L+ RPMGRV++PL +MG + + G LP+ +RG TP+TYR+P+ASAQVKSA+ LSGRPMGRVIDPLSQMGADISASPGANGTKTLPLMVRGLAPATPLTYRLPVASAQVKSAI | 184 |
| Query | 181 | LLAGLNTPGITTVIEPIMTRDHTEKMLQGFGANLTVETDADGVRTIRLEGRGKLTGQVID | 240 |
| Sbjct | 185 | LLAGLNTPGIT VIEP+ TRDH+E+ML+GFGA+LTVE D DG R IR+ G +L Q I LLAGLNTPGITEVIEPVPTRDHSERMLRGFGADLTVEVDTDGTRHIRIVGEAELKPQTIT | 244 |
| Query | 241 | VPGDPSSTAFPLVAALLVPGSDVTILNVLMNPTRTGLILTLQEMGADIEVINPRLAGGED | 300 |
| Sbjct | 245 | VPGDPSS AF +VAALLVPGSD+TI NV +NPTR GLI L++MG +IE+I+PR GGE VPGDPSSAAFFIVAALLVPGSDITIANVGLNPTRAGLIDVLRQMGGNIELIDPREVGGEP | 304 |
| Query | 301 | VADLRVRSSTLKGVTVPEDRAPSMIDEYPILAVAAAFAEGATVMNGLEELRVKESDRLSA | 360 |
| Sbjct | 305 | VADLRVR S LKG+ V APSMIDE+PIL VAAA AEG TV GL+ELRVKESDR++ VADLRVRHSALKGIAVDPAVAPSMIDEFPILFVAAALAEGRTVTTGLDELRVKESDRIAV | 364 |
| Query | 361 | VANGLKLNGVDCDEGETSLVVRGRPDGKGLGNASGAAVATHLDHRIAMSFLVMGLVSENP | 420 |
| Sbjct | 365 | +A GL GV +E E LV+ G G A A + HLDHRI MSF + GLVS+ P MATGLTAIGVRVEESEDGLVIHG---SGGDPLAGDATIKGHLDHRICMSFAIAGLVSKAP | 421 |
| Query  Sbjct | 421  422 | VTVDDATMIATSFPEFMDLMAGL 443 VT+DD +ATSFP F L+ L VTIDDIAPVATSFPNFEALLTEL 444 |  |

>TR:K2JQY6 K2JQY6_9PROT 3-phosphoshikimate 1-carboxyvinyltransferase OS=Oceanibaculum indicum P24 OX=1207063 GN=aroA PE=3 SV=1

Length=447

Score = 478 bits (1230), Expect = 4e-164

| Identities | = 258/442 (58%), Positives = 307/442 (69%), Gaps = 5/442 (1%) |  |
| --- | --- | --- |
| Query 7 | SRPATARKSSGLSGTVRIPGDKSISHRSFMFGGLASGETRITGLLEGEDVINTGKAMQAM | 66 |
| Sbjct 5 | + P +R++ L G V PGDKSISHR+ MFGGLA GET I GLLEGEDV+NT KAM+A AHPLVSRRAEALRGAVTAPGDKSISHRALMFGGLAIGETAIHGLLEGEDVLNTAKAMRAY | 64 |
| Query 67 | GARIRKEGD-TWIIDGVGNGGLLAPEAPLDFGNAATGCRLTMGLVGVYDFDSTFIGDASL | 125 |
| Sbjct 65 | GA +R++ D W + GVG GGL PE LD GN+ TG RL MGL + S F GDASL GAAVRRDEDGVWRVSGVGVGGLAEPEDMLDLGNSGTGARLLMGLAAGHPITSFFTGDASL | 124 |
| Query 126 | TKRPMGRVLNPLREMGVQVKSEDGDRLPVTLRGPKTPTPITYRVPMASAQVKSAVLLAGL | 185 |
| Sbjct 125 | +RPM RV+ PL +MG + S +G RLP+ + G + P P+ Y +P+ASAQVKSA+LLAGL RRRPMARVMKPLGDMGARFVSREGGRLPLAVTGARHPLPLDYTLPVASAQVKSAILLAGL | 184 |
| Query 186 | NTPGITTVIEPIMTRDHTEKMLQGFGANLTVETDADGVRTIRLEGRGKLTGQVIDVPGDP | 245 |
| Sbjct 185 | PG T V+EP TRDH+E+ML+ FGA + VE ADG R + L G+ +L + VPGDP TAPGETIVVEPEPTRDHSERMLRHFGATVRVEDRADGTRAVTLTGQPELAAATVRVPGDP | 244 |
| Query 246 | SSTAFPLVAALLVPGSDVTILNVLMNPTRTGLILTLQEMGADIEVINPRLAGGEDVADLR | 305 |
| Sbjct 245 | SS AFP VAALLVPGS+V I NV NP R GL TL EMGADI + N R GE V DL SSAAFPAVAALLVPGSEVRIANVGANPLRFGLFETLLEMGADIALENRREEAGEPVVDLL | 304 |
| Query 306 | VRSSTLKGVTVPEDRAPSMIDEYPILAVAAAFAEGATVMNGLEELRVKESDRLSAVANGL | 365 |
| Sbjct 305 | VR+ LKGV VP RAPSMIDEYPILA+AAA AEG TVM GL ELRVKESDRL AVA GL VRAGGLKGVVVPPRRAPSMIDEYPILAMAAACAEGPTVMEGLAELRVKESDRLGAVARGL | 364 |
| Query 366 | KLNGVDCDEGETSLVVRGRPDGKGLGNASGAAVATHLDHRIAMSFLVMGLVSENPVTVDD | 425 |

| Sbjct | 365 | GV+ +EG LVV GKG GA +A +LDHRIAM+F V+G+VSE PV++DD  AACGVEVEEGPDRLVVH----GKGRPPLGGAMIAANLDHRIAMAFAVLGMVSEKPVSIDD 420 |
| --- | --- | --- |
| Query | 426 | ATMIATSFPEFMDLMAGLGAKI 447 |
| Sbjct | 421 | IATSFP F+ +M LGA I GEPIATSFPGFVPMMNRLGADI 442 |

>SP:A3PPV7 AROA_RHOS1 3-phosphoshikimate 1-carboxyvinyltransferase OS=Rhodobacter sphaeroides (strain ATCC 17029 / ATH 2.4.9) OX=349101

GN=aroA PE=3 SV=1

Length=445

Score = 478 bits (1229), Expect = 5e-164

| Identities | = 258/446 (58%), Positives = 313/446 (70%), Gaps = 8/446 (2%) |  |
| --- | --- | --- |
| Query 3 | HGASSRPATARKSSGLSGTVRIPGDKSISHRSFMFGGLASGETRITGLLEGEDVINTGKA | 62 |
| Sbjct 4 | HG + +P TAR+S L G IPGDKSISHR+ + G +A GET+ITGLLEG+DV++T KA HGPA-QPMTARRSGPLKGRAEIPGDKSISHRALILGAMAVGETQITGLLEGQDVLDTAKA | 62 |
| Query 63 | MQAMGARIRKEGD-TWIIDGVGNGGLLAPEAPLDFGNAATGCRLTMGLVGVYDFDSTFIG | 121 |

M+A GA + + G	W + GVG GG	P	+D GN+ TG RL MG +	+TF G

| Sbjct | 63 | MRAFGAEVIQHGPGAWSVHGVGVGGFAEPAEVIDCGNSGTGVRLVMGAMATSPLTATFTG | 122 |
| --- | --- | --- | --- |
| Query | 122 | DASLTKRPMGRVLNPLREMGVQVKSEDGDRLPVTLRGPKTPTPITYRVPMASAQVKSAVL | 181 |
| Sbjct | 123 | DASL KRPMGRV +PL G + G RLP+TL G P P+ Y VPM SAQVKSAVL DASLRKRPMGRVTDPLALFGTRAYGRKGGRLPMTLVGAADPVPVRYTVPMPSAQVKSAVL | 182 |
| Query | 182 | LAGLNTPGITTVIEPIMTRDHTEKMLQGFGANLTVETDADGVRTIRLEGRGKLTGQVIDV | 241 |
| Sbjct | 183 | LAGLN PG T VIE TRDH+E+ML+GFGA L+VET +G + I L G+ +L Q + V LAGLNAPGQTVVIEREATRDHSERMLRGFGAELSVETGPEG-QIITLTGQPELRPQTVAV | 241 |
| Query | 242 | PGDPSSTAFPLVAALLVPGSDVTILNVLMNPTRTGLILTLQEMGADIEVINPRLAGGEDV | 301 |
| Sbjct | 242 | P DPSS AFP+ AAL+V GS++ + V NPTR GL +TL EMGADI N R GGE V PRDPSSAAFPVCAALIVEGSEILVPGVSRNPTRDGLYVTLLEMGADIAFENEREEGGEPV | 301 |
| Query | 302 | ADLRVRSSTLKGVTVPEDRAPSMIDEYPILAVAAAFAEGATVMNGLEELRVKESDRLSAV | 361 |
| Sbjct | 302 | ADLRVR+S LKGV VP +RAPSMIDEYPIL+V AAFAEG+T+M G++ELRVKESDR+ A+ ADLRVRASALKGVEVPPERAPSMIDEYPILSVVAAFAEGSTIMRGVKELRVKESDRIDAM | 361 |
| Query | 362 | ANGLKLNGVDCDEGETSLVVRGRPDGKGLGNASGAAVATHLDHRIAMSFLVMGLVSENPV | 421 |
| Sbjct | 362 | A GL+ GV +E E +L+V G G GA ATHLDHRIAMSFLV+G+ +E PV ARGLEACGVRIEEDEDTLIVHGMGRVPG GATCATHLDHRIAMSFLVLGMAAEAPV | 416 |
| Query  Sbjct | 422  417 | TVDDATMIATSFPEFMDLMAGLGAKI 447 TVDD + IATSFP F DLM GLGA + TVDDGSPIATSFPAFTDLMTGLGADL 442 |  |

>TR:F6EYV5 F6EYV5_SPHCR 3-phosphoshikimate 1-carboxyvinyltransferase OS=Sphingobium chlorophenolicum L-1 OX=690566 GN=aroA PE=3 SV=1

Length=446

Score = 478 bits (1229), Expect = 6e-164

Identities = 254/438 (58%), Positives = 306/438 (70%), Gaps = 4/438 (1%)

| Query | 9 | PATARKSSGLSGTVRIPGDKSISHRSFMFGGLASGETRITGLLEGEDVINTGKAMQAMGA | 68 |
| --- | --- | --- | --- |
| Sbjct | 10 | P T + LSGT+ +PGDKSISHRS M LA GE+ + GLLEGEDV++T AM+AMGA PMTFTAAPALSGTITVPGDKSISHRSLMLSALAVGESHVEGLLEGEDVLSTAAAMRAMGA | 69 |
| Query | 69 | RIRKEGD-TWIIDGVGNGGLLAPEAPLDFGNAATGCRLTMGLVGVYDFDSTFIGDASLTK | 127 |
| Sbjct | 70 | I K+ D W I GVG GGLL P + LD GN+ T RL MGLV + +TF+GDASL+K  DISKDADGVWHIHGVGVGGLLQPTSALDMGNSGTSTRLLMGLVASHAITATFVGDASLSK | 129 |

| Query | 128 | RPMGRVLNPLREMGVQVKSEDGDRLPVTLRGPKTPTPITYRVPMASAQVKSAVLLAGLNT | 187 |
| --- | --- | --- | --- |
| Sbjct | 130 | RPM RV PL MG + GDRLP+T+RG P+ YR+P+ASAQVKSA+LLAGLN RPMARVTEPLSRMGASFTTSPGDRLPLTMRGACPAVPLDYRLPVASAQVKSAILLAGLNA | 189 |
| Query | 188 | PGITTVIEPIMTRDHTEKMLQGFGANLTVETDADGVRTIRLEGRGKLTGQVIDVPGDPSS | 247 |
| Sbjct | 190 | PGIT V+EPI TRDH+E+ML+GFGA+L VET+ADG R I L G +L Q I VPGDPSS PGITRVVEPIPTRDHSERMLKGFGADLHVETEADGTRIITLRGEAELRPQSITVPGDPSS | 249 |
| Query | 248 | TAFPLVAALLVPGSDVTILNVLMNPTRTGLILTLQEMGADIEVINPRLAGGEDVADLRVR | 307 |
| Sbjct | 250 | AFP+VAALLVPGS VTI NV +N TR GLI L+EMG DI + NPR GGE V DL + AAFPMVAALLVPGSKVTIANVGLNATRAGLIDLLREMGGDIAIKNPREVGGEPVGDLVIT | 309 |
| Query | 308 | SSTLKGVTVPEDRAPSMIDEYPILAVAAAFAEGATVMNGLEELRVKESDRLSAVANGLKL | 367 |
| Sbjct | 310 | +S LKGV RAPSMIDEYP+ +AAAFAEG + GLEELRVKESDR++ +A GL+ ASALKGVEPDPARAPSMIDEYPVAFIAAAFAEGRSTFRGLEELRVKESDRIATMAAGLRA | 369 |
| Query | 368 | NGVDCDEGETSLVVRGRPDGKGLGNASGAAVATHLDHRIAMSFLVMGLVSENPVTVDDAT | 427 |
| Sbjct | 370 | GV +E E +V+ G G G +AT LDHRIAMSF + GL+S+ VT+DD IGVTVEELEDGIVIEG---SGGALLPGGGPIATSLDHRIAMSFAISGLMSKKGVTIDDMR | 426 |
| Query | 428 | MIATSFPEFMDLMAGLGA 445  +ATSFP F L+ LGA |  |
| Sbjct | 427 | PVATSFPGFTGLLRSLGA 444 | |

>SP:Q1QRT1 AROA_NITHX 3-phosphoshikimate 1-carboxyvinyltransferase OS=Nitrobacter hamburgensis (strain DSM 10229 / NCIMB 13809 / X14)

OX=323097 GN=aroA PE=3 SV=1

Length=449

Score = 478 bits (1229), Expect = 6e-164

| Identities | = 259/451 (57%), Positives = 304/451 (67%), Gaps = 11/451 (2%) |  |
| --- | --- | --- |
| Query 1 | MLHGASSRPATARKSSGLSGTVRIPGDKSISHRSFMFGGLASGETRITGLLEGEDVINTG | 60 |
| Sbjct 1 | M H P ARK L G VR+PGDKSISHR+ + G LA GETRI GLLEGEDV+NT MTHSGQPAPLEARKCQSLIGRVRVPGDKSISHRALILGALAVGETRIAGLLEGEDVLNTA | 60 |
| Query 61 | KAMQAMGARIR KEGDTWIIDGVGNGGLLAPEAPLDFGNAATGCRLTMGLVGVYDF | 115 |
| Sbjct 61 | KAMQA+GA++ K G W + GVG G PEAPLDFGN+ TGCRL MG V KAMQALGAKVERRIDDKSGIVWSVRGVGTSGFATPEAPLDFGNSGTGCRLVMGAVAGCPI | 120 |
| Query 116 | DSTFIGDASLTKRPMGRVLNPLREMGVQVKSE-DGDRLPVTLRGPKTPTPITYRVPMASA | 174 |
| Sbjct 121 | +TF GD SL RPM R+L+PL MG +V E DG RLP+TL G + P PI YR P+ASA VATFDGDGSLRSRPMRRILDPLELMGARVTGESDGGRLPLTLAGARDPLPIVYRTPVASA | 180 |
| Query 175 | QVKSAVLLAGLNTPGITTVIEPIMTRDHTEKMLQGFGANLTVETDADGVRTIRLEGRGKL | 234 |
| Sbjct 181 | Q+KSAVLLAGL+ PGITTVIE +RDHTE ML+ FGA + +D R I L G+ +L QIKSAVLLAGLSAPGITTVIESEASRDHTELMLKHFGAQIVSVSDGTHGRKISLTGQPEL | 240 |
| Query 235 | TGQVIDVPGDPSSTAFPLVAALLVPGSDVTILNVLMNPTRTGLILTLQEMGADIEVINPR | 294 |
| Sbjct 241 | G + VP DPSS AFP+VAAL+ GSD+ + +V+ NP RTGL TL+EMGA IE R HGAAVTVPADPSSAAFPIVAALITEGSDIVLTDVMTNPLRTGLFATLREMGASIEESETR | 300 |
| Query 295 | LAGGEDVADLRVRSSTLKGVTVPEDRAPSMIDEYPILAVAAAFAEGATVMNGLEELRVKE | 354 |
| Sbjct 301 | L GE +A LRVR+S L+GV VP RAPSMIDEY +LAVAAAFAEG TVM GL ELRVKE LDAGEPMAQLRVRASKLRGVEVPAARAPSMIDEYLVLAVAAAFAEGTTVMRGLHELRVKE | 360 |
| Query 355 | SDRLSAVANGLKLNGVDCDEGETSLVVRGRPDGKGLGNASGAAVATHLDHRIAMSFLVMG | 414 |
| Sbjct 361 | SDRL A A L++NGV + L+V GR G G V TH+DHRIAMS L MG SDRLEAAAAMLRVNGVAVEIAGDDLIVEGRGRVPG GGLVTTHMDHRIAMSALAMG | 415 |
| Query 415  Sbjct 416 | LVSENPVTVDDATMIATSFPEFMDLMAGLGA 445 S+ PV VDD IATSFP+F+ +M LGA  CASDAPVKVDDTAFIATSFPDFVPMMRRLGA 446 |  |

>TR:A0A074TLN7 A0A074TLN7_9RHOB 3-phosphoshikimate 1-carboxyvinyltransferase OS=Thioclava dalianensis OX=1185766 GN=aroA PE=3 SV=1

Length=449

Score = 478 bits (1229), Expect = 6e-164

| Identities | = 251/439 (57%), Positives = 308/439 (70%), Gaps = 5/439 (1%) |  |
| --- | --- | --- |
| Query 11 | TARKSSGLSGTVRIPGDKSISHRSFMFGGLASGETRITGLLEGEDVINTGKAMQAMGARI | 70 |
| Sbjct 11 | TARKS L+G +PGDKSISHR+ +FG L+ GET+ITGLLEGEDV++T +AM+A GA + TARKSGPLTGEAEVPGDKSISHRALIFGALSVGETKITGLLEGEDVLDTARAMRAFGAEV | 70 |
| Query 71 | RKEGD-TWIIDGVGNGGLLAPEAPLDFGNAATGCRLTMGLVGVYDFDSTFIGDASLTKRP | 129 |
| Sbjct 71 | + G W + GVG GG PE +D GN+ TG RL MG + +TF GDASL KRP TQHGPGEWSVHGVGVGGFAEPEQVIDCGNSGTGVRLIMGCMATSPITATFTGDASLRKRP | 130 |
| Query 130 | MGRVLNPLREMGVQVKSEDGDRLPVTLRGPKTPTPITYRVPMASAQVKSAVLLAGLNTPG | 189 |
| Sbjct 131 | MGR+ +P+ G Q G RLP+T+ G P P+ Y PMASAQVKSAVLLAGLN PG MGRITDPIALFGAQAYGRAGGRLPMTVVGAADPVPVRYATPMASAQVKSAVLLAGLNAPG | 190 |
| Query 190 | ITTVIEPIMTRDHTEKMLQGFGANLTVETDADGVRTIRLEGRGKLTGQVIDVPGDPSSTA | 249 |
| Sbjct 191 | T VIE + TRDHTE+ML+GFGA++ VE ++G I L+G+ +L Q + VP DPSS A QTVVIEKVPTRDHTERMLRGFGADVRVEETSEG-HVITLQGQPELRAQEVTVPRDPSSAA | 249 |
| Query 250 | FPLVAALLVPGSDVTILNVLMNPTRTGLILTLQEMGADIEVINPRLAGGEDVADLRVRSS | 309 |
| Sbjct 250 | FP+ AALLVPGSD+ + V NPTR GL TL+EMGADI NPR GGE VADLRVR S FPVCAALLVPGSDILVPGVSQNPTRNGLFTTLREMGADITFENPREEGGEPVADLRVRYS | 309 |
| Query 310 | TLKGVTVPEDRAPSMIDEYPILAVAAAFAEGATVMNGLEELRVKESDRLSAVANGLKLNG | 369 |
| Sbjct 310 | L G+ VP +RA S IDEYP+L+V AA A G TVM G+ ELRVKESDR+ A+A GL+ G ELHGIEVPPERASSQIDEYPVLSVVAANARGTTVMRGVHELRVKESDRIDAMARGLEACG | 369 |
| Query 370 | VDCDEGETSLVVRGRPDGKGLGNASGAAVATHLDHRIAMSFLVMGLVSENPVTVDDATMI | 429 |
| Sbjct 370 | V +E E +L+V G G G GA A+HLDHRIAMSFL++G+ ++NPV+VDD T I VRIEEDEDTLIVHGMGPG---GVPGGATCASHLDHRIAMSFLILGMAAQNPVSVDDGTPI | 426 |
| Query 430  Sbjct 427 | ATSFPEFMDLMAGLGAKIE 448 ATSFP F LM GLGA +E ATSFPIFEGLMRGLGASLE 445 |  |

>TR:A0A397PCW0 A0A397PCW0_9RHIZ 3-phosphoshikimate 1-carboxyvinyltransferase OS=Dichotomicrobium thermohalophilum OX=933063 GN=aroA PE=3

SV=1

Length=441

Score = 477 bits (1228), Expect = 7e-164

| Identities | = 260/442 (59%), Positives = 312/442 (71%), Gaps = 10/442 (2%) |  |
| --- | --- | --- |
| Query 8 | RPATARKSSGLSGTVRIPGDKSISHRSFMFGGLASGETRITGLLEGEDVINTGKAMQAMG | 67 |
| Sbjct 3 | RP ARKS+ LSG +R+PGDKSISHR+ +FG L+ GET I+GLLE DV+ T A+Q MG RPLIARKSTALSGALRVPGDKSISHRALIFGALSIGETHISGLLESADVLATAHALQQMG | 62 |
| Query 68 | ARIRKEGDTWIIDGVGNGGLLAPEAPLDFGNAATGCRLTMGLVGVYDFDSTFIGDASLTK | 127 |
| Sbjct 63 | ++ +E W + GVG GGL PE PLDFGNA TG RL MG++ +DF + IGD SL + VQMTREDGNWRVVGVGCGGLAPPEGPLDFGNAGTGVRLMMGVIAGHDFAAELIGDESLQR | 122 |
| Query 128 | RPMGRVLNPLREMGVQVKSEDGDRLPVTLRGPKTPTPITYRVPMASAQVKSAVLLAGLNT | 187 |
| Sbjct 123 | RPMGRVL+PL++MG +V GDRLP+TL G PITYR+P+ASAQVKSA+LLAGL T RPMGRVLDPLKQMGAEVHG--GDRLPLTLVGSGDLAPITYRLPVASAQVKSAILLAGLMT | 180 |
| Query 188 | PGITTVIEPIMTRDHTEKMLQGFGANLTVETDADGVRTIRLEGRGKLTGQVIDVPGDPSS PG TTVIEP TRDHTE M++ FGA + +E R+I + G L G+ ++VPGDPSS | 247 |

| Sbjct | 181 | PGETTVIEPEATRDHTETMMRHFGAEVRIE-QVTAERSITVTGEAVLHGRDVEVPGDPSS | 239 |
| --- | --- | --- | --- |
| Query | 248 | TAFPLVAALLVPGSDVTILNVLMNPTRTGLILTLQEMGADIEVINPRLAGGEDVADLRVR | 307 |
| Sbjct | 240 | AFP AAL+ PGSDVTI NVL+NPTRTGL TL EMGAD+ N R +GGE VADLRVR AAFPTAAALICPGSDVTIENVLVNPTRTGLYDTLAEMGADLRFENERRSGGEKVADLRVR | 299 |
| Query | 308 | SSTLKGVTVPEDRAPSMIDEYPILAVAAAFAEGATVMNGLEELRVKESDRLSAVANGLKL | 367 |
| Sbjct | 300 | + LK V VP +RAP MIDEYP+LAV AA AEG T M GL ELRVKESDRL+A GL AGKLKAVRVPPERAPRMIDEYPVLAVVAATAEGETRMEGLAELRVKESDRLAATIAGLTA | 359 |
| Query | 368 | NGVDC-DEGETSLVVRGRPDGKGLGNASGAAVATHLDHRIAMSFLVMGLVSENPVTVDDA | 426 |
| Sbjct | 360 | GV EG+T LVVRG G G V TH+DHRIAM+F V+GLV+E PV +DD CGVTAWAEGDT-LVVRGAERIPG GGTVETHMDHRIAMAFAVLGLVAEQPVRIDDT | 413 |
| Query | 427 | TMIATSFPEFMDLMAGLGAKIE 448 MI TSFP F + M +GA + |  |
| Sbjct | 414 | AMIDTSFPGFAERMRAIGADFD 435 | |

>TR:A0A512E1Y4 A0A512E1Y4_9PROT 3-phosphoshikimate 1-carboxyvinyltransferase OS=Skermanella aerolata OX=393310 GN=aroA PE=3 SV=1

Length=452

Score = 478 bits (1229), Expect = 7e-164

| Identities | | = 260/449 (58%), Positives = 311/449 (69%), Gaps = 6/449 (1%) |  |
| --- | --- | --- | --- |
| Query 8  Sbjct 4 | | RPATARKSSGLSGTVRIPGDKSISHRSFMFGGLASGETRITGLLEGEDVINTGKAMQAMG RP + K+ LSGTVR+PGDKSISHR+ MFG +A GET I GLL GEDV++T AM+A+G RPLQSVKTGPLSGTVRVPGDKSISHRAIMFGAIAVGETTIHGLLTGEDVLHTAAAMRALG | 67  63 |
| Query 68  Sbjct 64 | | ARIRKEGD-TWIIDGVGNGGLLAPEAPLDFGNAATGCRLTMGLVGVYDFDSTFIGDASLT A I + D W + GVG GGL+ + LD GN+ T RL MGLV + + F GDASL ADITRSDDGIWRVRGVGIGGLVEADQVLDMGNSGTAARLMMGLVASHPITTFFTGDASLA | 126  123 |
| Query 127  Sbjct 124 | | KRPMGRVLNPLREMGVQVKSEDGDRLPVTLRGPKTPTPITYRVPMASAQVKSAVLLAGLN KRPM RV PL +MG S G RLP+ + G + P PITYR+P+ASAQVKSA+LLAGLN KRPMARVTTPLEQMGASFVSRTGGRLPLAVIGTENPVPITYRLPVASAQVKSAILLAGLN | 186  183 |
| Query 187  Sbjct 184 | | TPGITTVIEPIMTRDHTEKMLQGFGANLTVETDADGVRTIRLEGRGKLTGQVIDVPGDPS TPG+TTVIE TRDH+E ML FGA + E G + + G ++TG+ + VP DPS TPGVTTVIEAEPTRDHSELMLTHFGATVKTERIEGGALAVSITGEPEITGRPVHVPADPS | 246  243 |
| Query 247  Sbjct 244 | | STAFPLVAALLVPGSDVTILNVLMNPTRTGLILTLQEMGADIEVINPRLAGGEDVADLRV S AF VAAL+ PGSD+T+ +V MNP RTGL TL EMGADI +N R+ GE VADLRV SAAFLTVAALIRPGSDITLTDVGMNPRRTGLYDTLIEMGADITFLNRRVEAGEPVADLRV | 306  303 |
| Query 307  Sbjct 304 | | RSSTLKGVTVPEDRAPSMIDEYPILAVAAAFAEGATVMNGLEELRVKESDRLSAVANGLK R S L V VP DRAPSMIDEYPILA+AAA AEG T M+GL ELRVKESDRL+ VANGL RYSALTAVAVPPDRAPSMIDEYPILAIAAACAEGTTTMHGLAELRVKESDRLAMVANGLD | 366  363 |
| Query 367  Sbjct 364 | | LNGVDCDEGETSLVVRGRPDGKGLGNASGAAVATHLDHRIAMSFLVMGLVSENPVTVDDA GV + G SL V G G GA VAT +DHRIAMSFLV+G+ S PV VDD+ ACGVGVEAGPDSLTVHGAGVPPG GATVATAMDHRIAMSFLVLGMASAEPVAVDDS | 426  418 |
| Query 427 | | TMIATSFPEFMDLMAGLGAKIELSDTKAA 455 I TSFP F+DLM GLGA+I L +T++A |  |
| Sbjct | 419 | GFIDTSFPGFVDLMNGLGARISLPNTQSA 447 | |

>TR:A0A4Z1C191 A0A4Z1C191_9RHOB 3-phosphoshikimate 1-carboxyvinyltransferase OS=Paracoccus liaowanqingii OX=2560053 GN=aroA PE=3 SV=1

Length=443

Score = 477 bits (1228), Expect = 7e-164

| Identities | = 249/448 (56%), Positives = 317/448 (71%), Gaps = 7/448 (2%) |  |
| --- | --- | --- |
| Query 1 | MLHGASSRPATARKSSGLSGTVRIPGDKSISHRSFMFGGLASGETRITGLLEGEDVINTG | 60 |
| Sbjct 1 | M H A RP TAR+++ LSG +PGDKSISHR+ + G +A GETRITGLLEG+DV++T MSHSADPRPMTARRAAPLSGEALVPGDKSISHRALILGAMAVGETRITGLLEGQDVLDTA | 60 |
| Query 61 | KAMQAMGARIRKEGD-TWIIDGVGNGGLLAPEAPLDFGNAATGCRLTMGLVGVYDFDSTF | 119 |
| Sbjct 61 | +AMQA GA++ + G W + GVG GG +PE +D GN+ TG RL MG + +TF RAMQAFGAQVVQHGPGDWSVHGVGVGGFRSPEGVIDCGNSGTGVRLIMGAMATTPVTATF | 120 |
| Query 120 | IGDASLTKRPMGRVLNPLREMGVQVKSEDGDRLPVTLRGPKTPTPITYRVPMASAQVKSA | 179 |
| Sbjct 121 | GDASL++RPM RV +PL + G ++ + +G RLP+T++G P P+TY P+ASAQ+KSA TGDASLSRRPMARVTDPLAQFGARITAREGGRLPITVQGATDPVPVTYHTPVASAQIKSA | 180 |
| Query 180 | VLLAGLNTPGITTVIEPIMTRDHTEKMLQGFGANLTVETDADGVRTIRLEGRGKLTGQVI | 239 |
| Sbjct 181 | +LLAGLN PG T VIEP TRDH+E+ML GFGA + ET +G + + L GR +L Q + ILLAGLNVPGDTVVIEPEPTRDHSERMLAGFGAQIRTETVPEGHK-VTLTGRPELRPQPV | 239 |
| Query 240 | DVPGDPSSTAFPLVAALLVPGSDVTILNVLMNPTRTGLILTLQEMGADIEVINPRLAGGE | 299 |
| Sbjct 240 | VP DPSS AFP+ AAL+VPGS++ + V NPTR GL +TL+EMGADI NPR GGE AVPRDPSSAAFPVAAALIVPGSNIRVPGVSRNPTRDGLYVTLREMGADITYENPRDEGGE | 299 |
| Query 300 | DVADLRVRSSTLKGVTVPEDRAPSMIDEYPILAVAAAFAEGATVMNGLEELRVKESDRLS | 359 |
| Sbjct 300 | VADL VR L GVTVP RA SMIDE+PIL+V AAFA G TVMNG+ ELRVKESDR+ PVADLVVRHGPLTGVTVPAARAASMIDEFPILSVIAAFATGPTVMNGVAELRVKESDRID | 359 |
| Query 360 | AVANGLKLNGVDCDEGETSLVVRGRPDGKGLGNASGAAVATHLDHRIAMSFLVMGLVSEN | 419 |
| Sbjct 360 | A+A GL+ NGV ++ S+ V G+ G G+A THLDHRIAMSFL++GL +E AMAVGLRANGVRVEDTPDSMTVHGQAGVPGGGHA VTHLDHRIAMSFLILGLATER | 414 |
| Query 420  Sbjct 415 | PVTVDDATMIATSFPEFMDLMAGLGAKI 447 PVT+DDA I TSFP+F+ LM LGA + PVTIDDAGPITTSFPDFVPLMTRLGADV 442 |  |

>TR:A0A6B8M1H5 A0A6B8M1H5_9RHIZ 3-phosphoshikimate 1-carboxyvinyltransferase OS=Methylocystis parvus OX=134 GN=aroA PE=3 SV=1

Length=445

Score = 477 bits (1228), Expect = 8e-164

| Identities | = 259/444 (58%), Positives = 312/444 (70%), Gaps = 8/444 (2%) |  |
| --- | --- | --- |
| Query 5 | ASSRPATARKSSGLSGTVRIPGDKSISHRSFMFGGLASGETRITGLLEGEDVINTGKAMQ | 64 |
| Sbjct 6 | A + P A S LSG VR PGDKS+SHRS +FG LA+GET++ GLLEGEDV+ T +A + APAAPLAAAASGPLSGRVRPPGDKSVSHRSMIFGLLATGETKVEGLLEGEDVLRTAQAAR | 65 |
| Query 65 | AMGARI-RKEGDTWIIDGVGNGGLLAPEAPLDFGNAATGCRLTMGLVGVYDFDSTFIGDA | 123 |
| Sbjct 66 | +GA++ R +W + G G G LL P LDFGNA TG RL MG+V + + F GDA QLGAKVVRHSPGSWSVWGAGLGSLLQPVETLDFGNAGTGSRLMMGVVAGHPIVARFDGDA | 125 |
| Query 124 | SLTKRPMGRVLNPLREMGVQVKSE-DGDRLPVTLRGPKTPTPITYRVPMASAQVKSAVLL | 182 |
| Sbjct 126 | SL KRPM R+L+PL G QV + +G RLP+ L+G P P+ Y+ P+ASAQ+KSAVLL SLRKRPMKRILDPLALQGAQVLEQAEGGRLPLVLQGSAEPLPVEYKTPVASAQIKSAVLL | 185 |
| Query 183 | AGLNTPGITTVIEPIMTRDHTEKMLQGFGANLTVETDADGVRTIRLEGRGKLTGQVIDVP | 242 |
| Sbjct 186 | GLN+PG T VIE +RDHTEKML FGA +T E+ R I LEGR +L + + VP CGLNSPGRTIVIEAEASRDHTEKMLAHFGAEVTSESYGPHGRKITLEGRPELVARPVRVP | 245 |
| Query 243 | GDPSSTAFPLVAALLVPGSDVTILNVLMNPTRTGLILTLQEMGADIEVINPRLAGGEDVA | 302 |
| Sbjct 246 | DPSS AFPLVAAL+ PGSD+ + V+ NP R+GL+ TLQEMGADI + N R GGE+VA ADPSSAAFPLVAALIAPGSDIVVEGVMTNPLRSGLLTTLQEMGADITLDNKREEGGEEVA | 305 |
| Query 303 | DLRVRSSTLKGVTVPEDRAPSMIDEYPILAVAAAFAEGATVMNGLEELRVKESDRLSAVA D+R R S+L GV VP RAPSMIDEYPILAVAA+FA+G T M GL ELRVKESDRL+A+A | 362 |

| Sbjct | 306 | DMRARFSSLSGVDVPAARAPSMIDEYPILAVAASFAKGETRMRGLSELRVKESDRLAAIA | 365 |
| --- | --- | --- | --- |
| Query | 363 | NGLKLNGVDCDEGETSLVVRGRPDGKGLGNASGAAVATHLDHRIAMSFLVMGLVSENPVT | 422 |
| Sbjct | 366 | GLK NGVDC+ L+VRG G + G V THLDHRIAMSFLVMGL + N VT AGLKANGVDCEIVGDDLIVRGG GVSGGGLVETHLDHRIAMSFLVMGLAARNKVT | 419 |
| Query  Sbjct | 423  420 | VDDATMIATSFPEFMDLMAGLGAK 446 VDD TMIATSFP F LM LGA+ VDDETMIATSFPSFRPLMESLGAR 443 |  |

>TR:A0A1H9SQ44 A0A1H9SQ44_9SPHN 3-phosphoshikimate 1-carboxyvinyltransferase OS=Sphingobium sp. YR768 OX=1884365 GN=aroA PE=3 SV=1

Length=446

Score = 477 bits (1228), Expect = 8e-164

| Identities | = 257/444 (58%), Positives = 311/444 (70%), Gaps = 4/444 (1%) |  |
| --- | --- | --- |
| Query 3 | HGASSRPATARKSSGLSGTVRIPGDKSISHRSFMFGGLASGETRITGLLEGEDVINTGKA | 62 |
| Sbjct 4 | H P T + LSG+V +PGDKSISHRS M LA GE+R+ GLLEGEDV+ T A HSDIPAPKTFTAAPALSGSVTVPGDKSISHRSLMLSALAVGESRVEGLLEGEDVLATAAA | 63 |
| Query 63 | MQAMGARIRKEGD-TWIIDGVGNGGLLAPEAPLDFGNAATGCRLTMGLVGVYDFDSTFIG | 121 |
| Sbjct 64 | M+AMGA I+++ D W I GVG GGLL PE LD GN+ T RL MGL+ +D +TF+G MRAMGADIQRDADGVWHIHGVGVGGLLQPETALDMGNSGTSTRLLMGLLASHDLTATFVG | 123 |
| Query 122 | DASLTKRPMGRVLNPLREMGVQVKSEDGDRLPVTLRGPKTPTPITYRVPMASAQVKSAVL | 181 |
| Sbjct 124 | DASL+KRPM RV PL +G + GDRLP+T++G P+ Y +P+ASAQVKSA+L DASLSKRPMARVTEPLSRIGASFTTSPGDRLPLTMKGACPAVPLDYTLPVASAQVKSAIL | 183 |
| Query 182 | LAGLNTPGITTVIEPIMTRDHTEKMLQGFGANLTVETDADGVRTIRLEGRGKLTGQVIDV | 241 |
| Sbjct 184 | LAGLNTPGIT V+EPI TRDH+E+ML+GFGA+LTVE ADG R I L G +L Q I V LAGLNTPGITRVVEPIPTRDHSERMLKGFGADLTVEVQADGTRVITLVGEAELKPQNIVV | 243 |
| Query 242 | PGDPSSTAFPLVAALLVPGSDVTILNVLMNPTRTGLILTLQEMGADIEVINPRLAGGEDV | 301 |
| Sbjct 244 | PGDPSS AFP+VAALLVPGS VTI NV +N TR GLI L+EMG IEV+N R GGE V PGDPSSAAFPMVAALLVPGSKVTIGNVGLNGTRAGLIDLLREMGGSIEVVNAREVGGEPV | 303 |
| Query 302 | ADLRVRSSTLKGVTVPEDRAPSMIDEYPILAVAAAFAEGATVMNGLEELRVKESDRLSAV | 361 |
| Sbjct 304 | DL V +S LKGV RAPSMIDEYP+ VAAA A G +V GLEELRVKESDR++ + GDLIVTASPLKGVEPDPARAPSMIDEYPVAFVAAACAYGRSVFRGLEELRVKESDRIATM | 363 |
| Query 362 | ANGLKLNGVDCDEGETSLVVRGRPDGKGLGNASGAAVATHLDHRIAMSFLVMGLVSENPV | 421 |
| Sbjct 364 | A+GL+ GV +E E +++ G G A G +AT LDHRIAMSF V GLVS++ V ADGLRAIGVQVEELEDGIIIEG---SGGAPLAGGGPIATKLDHRIAMSFAVAGLVSKDGV | 420 |
| Query 422  Sbjct 421 | TVDDATMIATSFPEFMDLMAGLGA 445 T+DD +ATSFP F+ L+ LGA TIDDMRPVATSFPTFVPLLQTLGA 444 |  |

>TR:A0A327K9S5 A0A327K9S5_9RHIZ 3-phosphoshikimate 1-carboxyvinyltransferase OS=Rhodoplanes piscinae OX=444923 GN=aroA PE=3 SV=1

Length=446

Score = 477 bits (1228), Expect = 8e-164

Identities = 263/445 (59%), Positives = 310/445 (70%), Gaps = 7/445 (2%)

| Query | 5 | ASSRPATARKSSGLSGTVRIPGDKSISHRSFMFGGLASGETRITGLLEGEDVINTGKAMQ | 64 |
| --- | --- | --- | --- |
| Sbjct | 6 | A+ P TAR + L G VR+PGDKSISHR+ + G LA G T +TGLLEGEDV+ TG+AM+ ATLLPLTARGARPLVGRVRVPGDKSISHRALILGALAVGRTTVTGLLEGEDVLATGEAMR | 65 |
| Query | 65 | AMGARIRKEGD-TWIIDGVGNGGLLAPEAPLDFGNAATGCRLTMGLVGVYDFDSTFIGDA  +GA + + G W+IDGVG GG AP+ LDFGN+ TGCRL MG V +TF GDA | 123 |

| Sbjct | 66 | RLGASVARTGPGAWVIDGVGVGGFKAPDQTLDFGNSGTGCRLVMGAVAGCPVTATFDGDA | 125 |
| --- | --- | --- | --- |
| Query | 124 | SLTKRPMGRVLNPLREMGVQV-KSEDGDRLPVTLRGPKTPTPITYRVPMASAQVKSAVLL | 182 |
| Sbjct | 126 | SL +RPM R+L PL MG V ++ +GDRLPV L G + P PI Y P+ SAQVKSAVLL SLRRRPMRRILEPLVRMGATVVQAAEGDRLPVWLAGARDPIPIEYATPVPSAQVKSAVLL | 185 |
| Query | 183 | AGLNTPGITTVIEPIMTRDHTEKMLQGFGANLTVETDADGVRTIRLEGRGKLTGQVIDVP | 242 |
| Sbjct | 186 | AGL PG T VIE TRDHTE ML FGA++ VE R I L G+ +LT + VP AGLAAPGHTVVIESEATRDHTETMLTHFGADVRVEPHGPHGRRITLVGQPELTAAPVTVP | 245 |
| Query | 243 | GDPSSTAFPLVAALLVPGSDVTILNVLMNPTRTGLILTLQEMGADIEVINPRLAGGEDVA | 302 |
| Sbjct | 246 | DPSS AFPLVAALLVPGSDV + +V+ NP RTGL TL+EMGA IE + R GGE +A ADPSSAAFPLVAALLVPGSDVVLADVMTNPLRTGLFTTLREMGAAIEEVAVRRDGGEAMA | 305 |
| Query | 303 | DLRVRSSTLKGVTVPEDRAPSMIDEYPILAVAAAFAEGATVMNGLEELRVKESDRLSAVA | 362 |
| Sbjct | 306 | DLRVR+S L GV VP +RAP+MIDEYP+LAVAAAFA G TVM GL+ELRVKESDRL+A A DLRVRASDLVGVEVPAERAPAMIDEYPVLAVAAAFARGTTVMRGLKELRVKESDRLAATA | 365 |
| Query | 363 | NGLKLNGVDCDEGETSLVVRGRPDGKGLGNASGAAVATHLDHRIAMSFLVMGLVSENPVT | 422 |
| Sbjct | 366 | + L++NGV ++V GR G G VATH+DHRIAMS LVMGL SE PV DLLRVNGVAVTIEGDDMIVEGRGRAPG GGLVATHMDHRIAMSALVMGLASEAPVA | 420 |
| Query  Sbjct | 423  421 | VDDATMIATSFPEFMDLMAGLGAKI 447 VDDA IATSFP F+D M GLG I VDDAAFIATSFPGFVDTMRGLGGDI 445 |  |

```
>TR:A0A0J7Y3G4 A0A0J7Y3G4_9SPHN 3-phosphoshikimate 1-carboxyvinyltransferase OS=Sphingobium czechense LL01 OX=1420583 GN=aroA PE=3 SV=1
Length=446

Score = 477 bits (1228), Expect = 8e-164
Identities = 255/437 (58%), Positives = 307/437 (70%), Gaps = 4/437 (1%)

Query 9	PATARKSSGLSGTVRIPGDKSISHRSFMFGGLASGETRITGLLEGEDVINTGKAMQAMGA 68 P T	+ L+G+V +PGDKSISHRS M	LA G +R+ GLLEGEDV+ T AM+AMGA
Sbjct 10	PMTFAAAPPLAGSVTVPGDKSISHRSLMLSALAIGHSRVEGLLEGEDVLATAAAMRAMGA 69

Query 69	RIRKEGD-TWIIDGVGNGGLLAPEAPLDFGNAATGCRLTMGLVGVYDFDSTFIGDASLTK 127 I ++ D W I GVG GGLL PE LD GN+ T RL MGL+ +D +TF+GDASL+K
Sbjct 70	TIERDADGVWHIHGVGVGGLLQPETALDMGNSGTSTRLLMGLLASHDLTATFVGDASLSK 129

Query 128 RPMGRVLNPLREMGVQVKSEDGDRLPVTLRGPKTPTPITYRVPMASAQVKSAVLLAGLNT 187 RPM RV PL MG	S GDRLP+T++G	P+ YR+P+ASAQVKSA+LLAGLNT
Sbjct 130 RPMARVTGPLSRMGASFTSGPGDRLPLTMKGACPAVPLDYRLPVASAQVKSAILLAGLNT 189

Query 188 PGITTVIEPIMTRDHTEKMLQGFGANLTVETDADGVRTIRLEGRGKLTGQVIDVPGDPSS 247 PGIT V+EPI TRDH+E+ML+GFGA+L VE +ADG R I L G +L Q I VPGDPSS
Sbjct 190 PGITRVVEPIPTRDHSERMLKGFGADLNVEVEADGTRIITLVGEAELQPQQIVVPGDPSS 249

Query 248 TAFPLVAALLVPGSDVTILNVLMNPTRTGLILTLQEMGADIEVINPRLAGGEDVADLRVR 307 AFP+VAALL PGS VTI NV +N TR GLI L+EMG IE+IN R GGE V DL V
Sbjct 250 AAFPMVAALLAPGSRVTIANVGLNATRAGLIDLLREMGGSIEIINAREVGGEPVGDLIVT 309

Query 308 SSTLKGVTVPEDRAPSMIDEYPILAVAAAFAEGATVMNGLEELRVKESDRLSAVANGLKL 367
+STLKGV	RAPSMIDEYP+ +AAA AEG +V GLEELRVKESDR++ +A GL+ Sbjct 310 ASTLKGVEPDPARAPSMIDEYPVAFIAAALAEGRSVFRGLEELRVKESDRIATMAQGLRA 369

Query 368 NGVDCDEGETSLVVRGRPDGKGLGNASGAAVATHLDHRIAMSFLVMGLVSENPVTVDDAT 427 GV +E E +V+ G	G	G +AT LDHRIAMSF V GLVS++ VT+DD
Sbjct 370 IGVRVEEFEDGIVIEG---SGGAMLTGGGPIATRLDHRIAMSFAVAGLVSKDGVTIDDMR 426

Query 428 MIATSFPEFMDLMAGLG 444
+ATSFP F+ L+ LG

Sbjct 427 PVATSFPTFVPLLRQLG 443


>TR:A0A1U7D003 A0A1U7D003_9RHOB 3-phosphoshikimate 1-carboxyvinyltransferase OS=Salipiger profundus OX=1229727 GN=aroA PE=3 SV=1
Length=448

Score = 477 bits (1228), Expect = 8e-164
```

| Identities | | = 257/440 (58%), Positives = 301/440 (68%), Gaps = 5/440 (1%) |  |
| --- | --- | --- | --- |
| Query 9  Sbjct 9 | | PATARKSSGLSGTVRIPGDKSISHRSFMFGGLASGETRITGLLEGEDVINTGKAMQAMGA P AR+S GL G +PGDKSISHRS + G +A GET++TGLLEG+DV++T KAM+A GA PMIARRSQGLRGEAHVPGDKSISHRSLILGAMAVGETKVTGLLEGQDVLDTAKAMRAFGA | 68  68 |
| Query 69  Sbjct 69 | | RIRKEGDTWIIDGVGNGGLLAPEAPLDFGNAATGCRLTMGLVGVYDFDSTFIGDASLTKR  + K GDTW + GVG GG P +D GN+ TG RL MG + +TF GDASL KR EVEKRGDTWHVQGVGVGGFSEPGQVIDCGNSGTGVRLIMGAMATTPIAATFTGDASLNKR | 128  128 |
| Query 129  Sbjct 129 | | PMGRVLNPLREMGVQVKSEDGDRLPVTLRGPKTPTPITYRVPMASAQVKSAVLLAGLNTP PM RV +PL G Q G RLP+T+ G + P P+ Y P+ SAQVKSAVLLAGLN P PMARVTDPLALFGAQAHGRSGGRLPMTILGARDPLPVRYATPVPSAQVKSAVLLAGLNAP | 188  188 |
| Query 189  Sbjct 189 | | GITTVIEPIMTRDHTEKMLQGFGANLTVETDADGVRTIRLEGRGKLTGQVIDVPGDPSST G T VIE TRDH+E+ML GFGA + E +G R I L GR +L QVI VP DPSS GQTVVIEREATRDHSERMLTGFGAEVVTEDTPEG-RVITLTGRPELKPQVIVVPRDPSSA | 248  247 |
| Query 249  Sbjct 248 | | AFPLVAALLVPGSDVTILNVLMNPTRTGLILTLQEMGADIEVINPRLAGGEDVADLRVR- AFP+ AAL+ GSDV + N+ +NPTR GL TLQEMGAD+ NPR GGE VADLR R AFPVCAALITEGSDVLVPNIGLNPTRAGLFFTLQEMGADLTFENPREEGGEPVADLRARF | 307  307 |
| Query 308  Sbjct 308 | | SSTLKGVTVPEDRAPSMIDEYPILAVAAAFAEGATVMNGLEELRVKESDRLSAVANGLKL S LKG+ VP +RA SMIDEYP+L+V AAFAEG T M G+ ELRVKESDR+ A+A GL+ SPDLKGIEVPPERAASMIDEYPVLSVVAAFAEGRTHMPGVRELRVKESDRIDAMARGLRA | 367  367 |
| Query 368  Sbjct 368 | | NGVDCDEGETSLVVRGRPDGKGLGNASGAAVATHLDHRIAMSFLVMGLVSENPVTVDDAT NGV+ DEGE V GR G G GA V +HLDHRIAMSFLVMGL + PV VDD NGVEVDEGEDWWTVTGRGHGNVPG---GAEVQSHLDHRIAMSFLVMGLGTTEPVWVDDGG | 427  424 |
| Query 428 | | MIATSFPEFMDLMAGLGAKI 447 IATSFP F LM GLGA I |  |
| Sbjct | 425 | PIATSFPVFEPLMTGLGADI 444 | |

>TR:D4Z303 D4Z303_SPHJU 3-phosphoshikimate 1-carboxyvinyltransferase OS=Sphingobium japonicum (strain DSM 16413 / CCM 7287 / MTCC 6362

/ UT26 / NBRC 101211 / UT26S) OX=452662 GN=aroA PE=3 SV=1

Length=450

Score = 477 bits (1228), Expect = 9e-164

| Identities | = 254/440 (58%), Positives = 309/440 (70%), Gaps = 4/440 (1%) |  |
| --- | --- | --- |
| Query 7 | SRPATARKSSGLSGTVRIPGDKSISHRSFMFGGLASGETRITGLLEGEDVINTGKAMQAM | 66 |
| Sbjct 12 | ++PAT +S LSG++ +PGDKSISHRS M LA GE+R+ GLLEGEDV+ T AM+AM AQPATFTAASALSGSIAVPGDKSISHRSLMLSALAVGESRVEGLLEGEDVLATAAAMRAM | 71 |
| Query 67 | GARIRKEGD-TWIIDGVGNGGLLAPEAPLDFGNAATGCRLTMGLVGVYDFDSTFIGDASL | 125 |
| Sbjct 72 | GA IR++ D W I GVG GGLL PE+ LD GN+ T RL MGL+ + +TF+GDASL GADIRRDADGIWHIHGVGVGGLLQPESALDMGNSGTSTRLLMGLIASHAITATFVGDASL | 131 |
| Query 126 | TKRPMGRVLNPLREMGVQVKSEDGDRLPVTLRGPKTPTPITYRVPMASAQVKSAVLLAGL | 185 |
| Sbjct 132 | +KRPM RV PL MG + GDRLP+T+RG P+ YR+P+ASAQVKSA+LLAGL SKRPMARVTEPLSRMGASFTTSPGDRLPLTMRGACPAVPLDYRLPVASAQVKSAILLAGL | 191 |
| Query 186 | NTPGITTVIEPIMTRDHTEKMLQGFGANLTVETDADGVRTIRLEGRGKLTGQVIDVPGDP | 245 |

| Sbjct | 192 | N PGIT V+EPI TRDH+E+ML+GFGA+L VE + DG R + L G +L Q I VPGDP  NAPGITRVVEPIPTRDHSERMLKGFGADLHVEVEGDGTRIVTLRGEAELRPQSITVPGDP | 251 |
| --- | --- | --- | --- |
| Query | 246 | SSTAFPLVAALLVPGSDVTILNVLMNPTRTGLILTLQEMGADIEVINPRLAGGEDVADLR | 305 |
| Sbjct | 252 | SS AFP+VAALLVPGS V I NV +N TR GLI L+EMG DI V NPR GGE V DL SSAAFPMVAALLVPGSKVAIANVGLNATRAGLIDLLREMGGDILVENPREVGGEPVGDLV | 311 |
| Query | 306 | VRSSTLKGVTVPEDRAPSMIDEYPILAVAAAFAEGATVMNGLEELRVKESDRLSAVANGL | 365 |
| Sbjct | 312 | + +STL+GV RAPSMIDEYP+ +AAA AEG + GLEELRVKESDR++ +A GL ITASTLQGVEPDPARAPSMIDEYPVAFIAAALAEGRSTFRGLEELRVKESDRIATMAAGL | 371 |
| Query | 366 | KLNGVDCDEGETSLVVRGRPDGKGLGNASGAAVATHLDHRIAMSFLVMGLVSENPVTVDD | 425 |
| Sbjct | 372 | + GV +E E +V+ G G G +AT LDHRIAMSF V GLVS+ VT+DD RAIGVTVEELEDGIVIEG---SGGALLPGGGPIATKLDHRIAMSFAVAGLVSKKGVTIDD | 428 |
| Query | 426 | ATMIATSFPEFMDLMAGLGA 445  +ATSFP F L+ LGA |  |
| Sbjct | 429 | MRPVATSFPGFTALLRSLGA 448 | |

>TR:A0A0Q8QA62 A0A0Q8QA62_9SPHN 3-phosphoshikimate 1-carboxyvinyltransferase OS=Sphingomonas sp. Root710 OX=1736594 GN=aroA PE=3 SV=1

Length=445

Score = 477 bits (1227), Expect = 1e-163

| Identities | = 258/447 (58%), Positives = 310/447 (69%), Gaps = 4/447 (1%) |  |
| --- | --- | --- |
| Query 2 | LHGASSRPATARKSSGLSGTVRIPGDKSISHRSFMFGGLASGETRITGLLEGEDVINTGK | 61 |
| Sbjct 1 | +H + + A+ S L+G +PGDKSISHR+ M LA GE+R+ GLL GEDV+ T MHASLPQSASFSASGPLNGQAAVPGDKSISHRALMLSALAVGESRVEGLLTGEDVLATAA | 60 |
| Query 62 | AMQAMGARI-RKEGDTWIIDGVGNGGLLAPEAPLDFGNAATGCRLTMGLVGVYDFDSTFI | 120 |
| Sbjct 61 | AM+AMGA I R E D W I GVG GGLL PE LD GN+ T RL MGLV + +TF AMRAMGATIERGEDDVWRIYGVGVGGLLQPETALDMGNSGTSTRLLMGLVASHPITATFT | 120 |
| Query 121 | GDASLTKRPMGRVLNPLREMGVQVKSEDGDRLPVTLRGPKTPTPITYRVPMASAQVKSAV | 180 |
| Sbjct 121 | GDASL+KRPM RV+ PL MG V + G RLP+ LRG PI YR+P+ASAQVKSAV GDASLSKRPMNRVIEPLSLMGADVTASPGGRLPLMLRGISPAVPIGYRLPVASAQVKSAV | 180 |
| Query 181 | LLAGLNTPGITTVIEPIMTRDHTEKMLQGFGANLTVETDADGVRTIRLEGRGKLTGQVID | 240 |
| Sbjct 181 | LLAGLNTPGIT VIEP+ TRDH+E+ML+GFGA L+VE DADG R I + G +L Q I LLAGLNTPGITRVIEPVATRDHSERMLKGFGAELSVEQDADGARIISIRGEAELRPQHIV | 240 |
| Query 241 | VPGDPSSTAFPLVAALLVPGSDVTILNVLMNPTRTGLILTLQEMGADIEVINPRLAGGED | 300 |
| Sbjct 241 | VPGDPSS AFP+VAALLVPGS+VT+ NV +NPTR L L+ MG DI IN R GGE VPGDPSSAAFPVVAALLVPGSEVTVTNVGLNPTRAALFDVLRMMGGDIAFINAREVGGEP | 300 |
| Query 301 | VADLRVRSSTLKGVTVPEDRAPSMIDEYPILAVAAAFAEGATVMNGLEELRVKESDRLSA | 360 |
| Sbjct 301 | VAD+ VR S+LKG+ P + +M+DEYPIL VAAA AEG TV GLEELRVKESDR++ VADIVVRYSSLKGIETPVETVAAMVDEYPILFVAAALAEGRTVARGLEELRVKESDRIAV | 360 |
| Query 361 | VANGLKLNGVDCDEGETSLVVRGRPDGKGLGNASGAAVATHLDHRIAMSFLVMGLVSENP | 420 |
| Sbjct 361 | +A GL+ G +E E L++ G + G GA +A LDHRIAMSF V GLVS+ P MAEGLRAIGARVEELEDGLIIDGTGGDRLPG---GATIAARLDHRIAMSFAVAGLVSKAP | 417 |
| Query 421  Sbjct 418 | VTVDDATMIATSFPEFMDLMAGLGAKI 447 V +DD + +ATSFP F LM GLGA+I VVIDDMSPVATSFPVFAALMTGLGAEI 444 |  |

>TR:A0A1N7NK33 A0A1N7NK33_9RHOB 3-phosphoshikimate 1-carboxyvinyltransferase OS=Paracoccus saliphilus OX=405559 GN=aroA PE=3 SV=1

Length=446

Score = 477 bits (1227), Expect = 1e-163

| Identities | | = 251/452 (56%), Positives = 312/452 (69%), Gaps = 7/452 (2%) |  |
| --- | --- | --- | --- |
| Query 1  Sbjct 1 | | MLHGASSRPATARKSSGLSGTVRIPGDKSISHRSFMFGGLASGETRITGLLEGEDVINTG M H A +P T+R+ L+G ++PGDKSISHR+ + G L+ GETRITGLLEG+DV++T MSHSADPQPMTSRRGGPLTGEAQVPGDKSISHRALILGALSVGETRITGLLEGQDVLDTA | 60  60 |
| Query 61  Sbjct 61 | | KAMQAMGARIRKEGD-TWIIDGVGNGGLLAPEAPLDFGNAATGCRLTMGLVGVYDFDSTF KAM A GA + + G W + GVG GG APE+ +D GN+ TG RL MG + + F KAMAAFGAEVERVGPGEWSVHGVGVGGFSAPESVIDCGNSGTGVRLIMGAMATTPITAVF | 119  120 |
| Query 120  Sbjct 121 | | IGDASLTKRPMGRVLNPLREMGVQVKSEDGDRLPVTLRGPKTPTPITYRVPMASAQVKSA GD SL++RPM RV PL + G ++ + +G+ LP+T+RG P P+ YR P+ASAQ+KSA TGDPSLSRRPMARVTLPLSQFGAEITAREGECLPITIRGAADPVPLRYRTPVASAQIKSA | 179  180 |
| Query 180  Sbjct 181 | | VLLAGLNTPGITTVIEPIMTRDHTEKMLQGFGANLTVETDADGVRTIRLEGRGKLTGQVI VLLAGLN PG T VIE TRDH+E+ML GFGA++ ET +G I L GR +L Q + VLLAGLNAPGETVVIEAEPTRDHSERMLAGFGADIRTETTDEG-HVITLTGRPELRAQPV | 239  239 |
| Query 240  Sbjct 240 | | DVPGDPSSTAFPLVAALLVPGSDVTILNVLMNPTRTGLILTLQEMGADIEVINPRLAGGE VP DPSS AFP+ AAL+VPGS + + V NPTR GL +TL EMGADI N R GGE AVPRDPSSAAFPVAAALIVPGSAIRVPGVSRNPTRDGLYVTLSEMGADISFENEREEGGE | 299  299 |
| Query 300  Sbjct 300 | | DVADLRVRSSTLKGVTVPEDRAPSMIDEYPILAVAAAFAEGATVMNGLEELRVKESDRLS VADL VR LKGV+VP +RA SMIDE+PIL+V AAFAEG TVMNG+ ELRVKESDR+ PVADLVVRHGPLKGVSVPPERAASMIDEFPILSVIAAFAEGKTVMNGVAELRVKESDRID | 359  359 |
| Query 360 | | AVANGLKLNGVDCDEGETSLVVRGRPDGKGLGNASGAAVATHLDHRIAMSFLVMGLVSEN A+A GL+ NGV +E S+ V G G G ATHLDHRIAMSFL++GL SE | 419 |
| Sbjct | 360 | AMARGLEANGVTVEETRDSMTVHGMAKVPG GGMAATHLDHRIAMSFLILGLASEA | 414 |
| Query  Sbjct | 420  415 | PVTVDDATMIATSFPEFMDLMAGLGAKIELSD 451 PVTVDD + I TSFP+F+ LM GLGA + SD PVTVDDGSPIMTSFPDFLPLMRGLGADMGTSD 446 |  |

>TR:A0A3B0M8C7 A0A3B0M8C7_9RHOB 3-phosphoshikimate 1-carboxyvinyltransferase OS=Roseibaca ekhonensis OX=254356 GN=aroA PE=3 SV=1

Length=447

Score = 477 bits (1227), Expect = 1e-163

| Identities | = 256/440 (58%), Positives = 308/440 (70%), Gaps = 7/440 (2%) |  |
| --- | --- | --- |
| Query 9 | PATARKSSGLSGTVRIPGDKSISHRSFMFGGLASGETRITGLLEGEDVINTGKAMQAMGA | 68 |
| Sbjct 9 | P TARKS L G +PGDKSISHR+ + G L+ GETRITGLLEG+DV++T AM+A GA PMTARKSGPLQGVAEVPGDKSISHRALILGALSVGETRITGLLEGQDVLDTAAAMRAFGA | 68 |
| Query 69 | RIRKEGD-TWIIDGVGNGGLLAPEAPLDFGNAATGCRLTMGLVGVYDFDSTFIGDASLTK | 127 |
| Sbjct 69 | + + G W + GVG GG P+ +D GN+ TG RL MG + D +TF GDASL K EVTQHGPGEWSVHGVGVGGFAEPDRVIDCGNSGTGVRLIMGAMATTDLTATFTGDASLVK | 128 |
| Query 128 | RPMGRVLNPLREMGVQVKSEDGDRLPVTLRGPKTPTPITYRVPMASAQVKSAVLLAGLNT | 187 |
| Sbjct 129 | RPMGRV +PL G Q +G RLP+T+ G P P+ YRVPM SAQVKSAVLLAGLN RPMGRVTDPLALFGAQAVGREGGRLPMTVVGAADPVPVRYRVPMPSAQVKSAVLLAGLNA | 188 |
| Query 188 | PGITTVIEPIMTRDHTEKMLQGFGANLTVETDADGVRTIRLEGRGKLTGQVIDVPGDPSS | 247 |
| Sbjct 189 | PG T VIE TRDHTE+ML+GFGA LTVE +G R I L G+ +LTGQ + VP DPSS PGQTVVIEGEPTRDHTERMLRGFGAELTVEDTEEG-RVITLTGQPELTGQDVAVPRDPSS | 247 |
| Query 248 | TAFPLVAALLVPGSDVTILNVLMNPTRTGLILTLQEMGADIEVINPRLAGGEDVADLRVR | 307 |
| Sbjct 248 | AFP+ AAL+V S++ + V NPTR G+ LTLQ+MGAD+ + N R GGE VADLRVR AAFPVCAALMVQESNILVPGVSQNPTRNGIFLTLQDMGADLVMENLREEGGEPVADLRVR | 307 |
| Query 308 | SSTLKGVTVPEDRAPSMIDEYPILAVAAAFAEGATVMNGLEELRVKESDRLSAVANGLKL | 367 |

| Sbjct | 308 | LKG+ VP +RA SMIDEYPILA AA AEG TVM G++ELRVKESDR+ A+A GL+  FGPLKGIDVPPERAASMIDEYPILAALAATAEGVTVMTGIKELRVKESDRIDAMARGLEA | 367 |
| --- | --- | --- | --- |
| Query | 368 | NGVDCDEGETSLVVRGRPDGKGLGNASGAAVATHLDHRIAMSFLVMGLVSENPVTVDDAT | 427 |
| Sbjct | 368 | GV +E E SL V G G GA VA+HLDHRIAM+FL +G+VS P+TVDDA+ CGVRVEETEDSLTVHGMAAVPG GATVASHLDHRIAMAFLCLGMVSAKPITVDDAS | 422 |
| Query | 428 | MIATSFPEFMDLMAGLGAKI 447  +ATSFP F LM GLGA+I |  |
| Sbjct | 423 | PVATSFPVFEPLMTGLGAQI 442 | |

>TR:A0A1C2HBJ3 A0A1C2HBJ3_9RHOB 3-phosphoshikimate 1-carboxyvinyltransferase OS=Thioclava sp. SK-1 OX=1889770 GN=aroA PE=3 SV=1

Length=447

Score = 477 bits (1227), Expect = 1e-163

| Identities | | = 256/446 (57%), Positives = 312/446 (70%), Gaps = 6/446 (1%) |  |
| --- | --- | --- | --- |
| Query 3  Sbjct 4 | | HGASSRPATARKSSGLSGTVRIPGDKSISHRSFMFGGLASGETRITGLLEGEDVINTGKA HG R T+ KS L+G +PGDKSISHR+ + G L+ GET+ITGLLEGEDV++T KA HGDPIR-MTSSKSGPLTGVAEVPGDKSISHRALILGALSIGETKITGLLEGEDVLDTAKA | 62  62 |
| Query 63  Sbjct 63 | | MQAMGARIRKEGD-TWIIDGVGNGGLLAPEAPLDFGNAATGCRLTMGLVGVYDFDSTFIG M+A GA++ + G W + GVG GG PE +D GN+ TG RL MG + +TF G MRAFGAQVTQHGPGEWTVHGVGVGGFAEPEQVIDCGNSGTGVRLIMGCMASSPITATFTG | 121  122 |
| Query 122  Sbjct 123 | | DASLTKRPMGRVLNPLREMGVQVKSEDGDRLPVTLRGPKTPTPITYRVPMASAQVKSAVL D SL KRPMGRV +P+ G Q +G RLP+T+ G P P+TY PMASAQVKSAVL DGSLRKRPMGRVTDPIALFGAQSYGREGGRLPMTIVGAVDPVPVTYHTPMASAQVKSAVL | 181  182 |
| Query 182  Sbjct 183 | | LAGLNTPGITTVIEPIMTRDHTEKMLQGFGANLTVETDADGVRTIRLEGRGKLTGQVIDV LAGLN PG T VIE TRDHTE+ML+GFGA++ VET A+G + I L+G+ +L+GQV+ V LAGLNAPGETVVIETTPTRDHTERMLRGFGADVRVETVAEGHK-ITLKGQPELSGQVVAV | 241  241 |
| Query 242  Sbjct 242 | | PGDPSSTAFPLVAALLVPGSDVTILNVLMNPTRTGLILTLQEMGADIEVINPRLAGGEDV P DPSS AFP+ AAL+ PGSD+ + V NPTR GL +TLQEMG IE +N R GGE V PRDPSSAAFPVCAALITPGSDIMVPGVSQNPTRNGLFVTLQEMGGKIEFLNEREEGGEPV | 301  301 |
| Query 302  Sbjct 302 | | ADLRVRSSTLKGVTVPEDRAPSMIDEYPILAVAAAFAEGATVMNGLEELRVKESDRLSAV ADLRVR S L G+ VP R+ SMIDEYP+L+V AAFA G TVM G+ ELRVKESDR+ A+ ADLRVRYSELVGIEVPPKRSASMIDEYPVLSVVAAFATGKTVMKGVHELRVKESDRIDAM | 361  361 |
| Query 362 | | ANGLKLNGVDCDEGETSLVVRGRPDGKGLGNASGAAVATHLDHRIAMSFLVMGLVSENPV A GL+ NGV +E E +L+V G G G G ATHLDHRIAMSF+V+G+ S V | 421 |
| Sbjct | 362 | ARGLEANGVTIEEDEDTLIVHGMGAG---GVPGGGTAATHLDHRIAMSFMVLGMNSVAAV | 418 |
| Query  Sbjct | 422  419 | TVDDATMIATSFPEFMDLMAGLGAKI 447  +VDD T IATSFP F LMAGLGA I SVDDGTPIATSFPIFEGLMAGLGASI 444 |  |

>TR:A0A1H5ZZC1 A0A1H5ZZC1_9RHOB 3-phosphoshikimate 1-carboxyvinyltransferase OS=Thalassococcus halodurans OX=373675 GN=aroA PE=3 SV=1

Length=446

Score = 476 bits (1226), Expect = 2e-163

Identities = 255/447 (57%), Positives = 311/447 (70%), Gaps = 8/447 (2%)

| Query | 3 | HGASSRPATARKSSGLSGTVRIPGDKSISHRSFMFGGLASGETRITGLLEGEDVINTGKA | 62 |
| --- | --- | --- | --- |
|  |  | HG ++ P +A KSSGLSG +PGDKSISHRS +FG +A GETR+TGLLEG+DV++T KA |  |
| Sbjct | 4 | HGPAT-PMSAHKSSGLSGEAHVPGDKSISHRSLIFGAMAVGETRVTGLLEGQDVLDTAKA | 62 |
| Query | 63 | MQAMGARIRKEGDTWIIDGVGNGGLLAPEAPLDFGNAATGCRLTMGLVGVYDFDSTFIGD | 122 |

M++ GA + K+GDTW++ GVG GG	P+ +D GN+ TG RL MG +	+TF GD

| Sbjct | 63 | MRSFGADVEKQGDTWVVHGVGVGGFAEPDHVIDCGNSGTGVRLIMGAMATCPIAATFTGD | 122 |
| --- | --- | --- | --- |
| Query | 123 | ASLTKRPMGRVLNPLREMGVQVKSEDGDRLPVTLRGPKTPTPITYRVPMASAQVKSAVLL | 182 |
| Sbjct | 123 | ASL KRPM RV +PL G + G RLP+T+ G + P P+TY P+ SAQVKSAVLL ASLNKRPMARVTDPLALFGARSYGRSGGRLPMTIIGAENPVPVTYTTPVPSAQVKSAVLL | 182 |
| Query | 183 | AGLNTPGITTVIEPIMTRDHTEKMLQGFGANLTVETDADGVRTIRLEGRGKLTGQVIDVP | 242 |
| Sbjct | 183 | AGLN PG T VIE TRDH+E+ML GFGA + E D D I L G+ +L Q I VP AGLNAPGQTVVIEREATRDHSERMLAGFGAQIVTE-DTDEGHVITLTGQPELKPQTIIVP | 241 |
| Query | 243 | GDPSSTAFPLVAALLVPGSDVTILNVLMNPTRTGLILTLQEMGADIEVINPRLAGGEDVA | 302 |
| Sbjct | 242 | DPSS AFP+ AAL+ GSDV + N+ +NPTR GL TLQ+MGAD+ N R GGE VA RDPSSAAFPVCAALITEGSDVLVPNIGLNPTRAGLFYTLQDMGADLTFENMREEGGEPVA | 301 |
| Query | 303 | DLRVR-SSTLKGVTVPEDRAPSMIDEYPILAVAAAFAEGATVMNGLEELRVKESDRLSAV | 361 |
| Sbjct | 302 | DLR + S LKG+ VP +RA SMIDEYP+L+V AAFAEG T M G++ELRVKESDR+ A+ DLRAKYSPDLKGIEVPAERAASMIDEYPVLSVVAAFAEGKTYMPGVKELRVKESDRIDAM | 361 |
| Query | 362 | ANGLKLNGVDCDEGETSLVVRGRPDGKGLGN-ASGAAVATHLDHRIAMSFLVMGLVSENP | 420 |
| Sbjct | 362 | A GL+ GVD DEGE V GR G G+ A G V THLDHRIAMSFLVMG + P ATGLRAAGVDVDEGEDWWTVHGR GFGDVAGGVTVKTHLDHRIAMSFLVMGFATNAP | 417 |
| Query  Sbjct | 421  418 | VTVDDATMIATSFPEFMDLMAGLGAKI 447  +TVDD+ I+TSFP F LM LGAK+ MTVDDSGPISTSFPIFESLMTQLGAKL 444 |  |

>TR:A0A0J6UYB0 A0A0J6UYB0_9RHIZ 3-phosphoshikimate 1-carboxyvinyltransferase OS=Methylobacterium tarhaniae OX=1187852 GN=aroA PE=3 SV=1

Length=449

Score = 476 bits (1226), Expect = 2e-163

| Identities | = 265/453 (58%), Positives = 319/453 (70%), Gaps = 10/453 (2%) |  |
| --- | --- | --- |
| Query 1 | MLHGASSRPATARKSSGLSGTVRIPGDKSISHRSFMFGGLASGETRITGLLEGEDVINTG | 60 |
| Sbjct 1 | M H ++ P TA+ + L G +R PGDKSISHR+ + G L+ GETRI+GLLEG+DV+ T MSHDSTPSPITAQAGTALRGRLRPPGDKSISHRAIILGLLSLGETRISGLLEGDDVLRTA | 60 |
| Query 61 | KAMQAMGARIRKEGD-TWIIDGVGNGGLLAPEAPLDFGNAATGCRLTMGLVGVYDFDSTF | 119 |
| Sbjct 61 | A +A+GA I ++G+ W + GVG GGL P LDFGNA TG RL MG+VG + +TF AAAKALGAGIDRDGEGRWRVRGVGVGGLSDPAGVLDFGNAGTGSRLMMGVVGGHPVTATF | 120 |
| Query 120 | IGDASLTKRPMGRVLNPLREMGVQVKSE-DGDRLPVTLRGPKTPTPITYRVPMASAQVKS | 178 |
| Sbjct 121 | GDASL KRPM R+L+PL +MGV V + +G R+P+TLRGP+ PITY P+ASAQVKS DGDASLRKRPMRRILDPLVQMGVTVVEQAEGGRVPLTLRGPREAVPITYESPVASAQVKS | 180 |
| Query 179 | AVLLAGLNTPGITTVIEPIMTRDHTEKMLQGFGANLTVETDADGV--RTIRLEGRGKLTG | 236 |
| Sbjct 181 | AVLLAGLN PG TTVIE TRDHTE+ML+ FGA + VE G R I L G+ L AVLLAGLNAPGTTTVIEAAATRDHTERMLRLFGATVAVEAIGPGGHGRRIALTGQPTLNA | 240 |
| Query 237 | QVIDVPGDPSSTAFPLVAALLVPGSDVTILNVLMNPTRTGLILTLQEMGADIEVINPRLA | 296 |
| Sbjct 241 | + VP DPSS AF LVA L+VPGS+V I V+MNP RTGL+ TL EMG DIE +N R TDVVVPADPSSAAFALVAGLVVPGSEVVIEGVMMNPLRTGLLTTLLEMGGDIERLNEREE | 300 |
| Query 297 | GGEDVADLRVRSSTLKGVTVPEDRAPSMIDEYPILAVAAAFAEGATVMNGLEELRVKESD | 356 |
| Sbjct 301 | GGE VADLRVR S LKGV VP +RAP+MIDEYP+LAVAAA A+G T M GL ELRVKESD GGETVADLRVRHSRLKGVAVPPERAPAMIDEYPVLAVAAACADGTTRMQGLHELRVKESD | 360 |
| Query 357 | RLSAVANGLKLNGVD-CDEGETSLVVRGRPDGKGLGNASGAAVATHLDHRIAMSFLVMGL | 415 |
| Sbjct 361 | RL+AVA GLK NGV EG+ +V G G A G V THLDHRIAM+FLV+G+ RLAAVAAGLKANGVSHVVEGDDLIV HGNGSAPAGGGTVETHLDHRIAMAFLVLGM | 415 |
| Query 416 | VSENPVTVDDATMIATSFPEFMDLMAGLGAKIE 448 |  |

V+ PVTVDD MIATS+P F+ M LGA ++ Sbjct 416 VARAPVTVDDGAMIATSYPAFLTDMRALGAALD 448

>TR:A0A3S8U4B4 A0A3S8U4B4_9RHOB 3-phosphoshikimate 1-carboxyvinyltransferase OS=Tabrizicola piscis OX=2494374 GN=aroA PE=3 SV=1

Length=451

Score = 476 bits (1226), Expect = 2e-163

| Identities | = 259/451 (57%), Positives = 316/451 (70%), Gaps = 7/451 (2%) |  |
| --- | --- | --- |
| Query 3 | HGASSRPATARKSSGLSGTVRIPGDKSISHRSFMFGGLASGETRITGLLEGEDVINTGKA | 62 |
| Sbjct 4 | HG ++P ARKS L G +PGDKSISHRS +FG +A GETRI+GLLEG+DV++T KA HG-EAQPMKARKSGPLRGVAEVPGDKSISHRSLIFGAMAVGETRISGLLEGQDVLDTAKA | 62 |
| Query 63 | MQAMGARIRKEGD-TWIIDGVGNGGLLAPEAPLDFGNAATGCRLTMGLVGVYDFDSTFIG | 121 |
| Sbjct 63 | M+A GA + + G+ W ++GVG GG P +D GN+ TG RL MG + +TF G MRAFGATVTRHGEGAWTVNGVGVGGFREPADVIDCGNSGTGVRLIMGTMATTPITATFTG | 122 |
| Query 122 | DASLTKRPMGRVLNPLREMGVQVKSEDGDRLPVTLRGPKTPTPITYRVPMASAQVKSAVL | 181 |
| Sbjct 123 | DASL KRPMGRV +PL G Q G RLP+TL G P P+ Y +P+ASAQVKSAVL DASLRKRPMGRVTDPLALFGAQAYGRQGGRLPMTLVGAANPVPVRYALPVASAQVKSAVL | 182 |
| Query 182 | LAGLNTPGITTVIEPIMTRDHTEKMLQGFGANLTVETDADGVRTIRLEGRGKLTGQVIDV | 241 |
| Sbjct 183 | LAGLN PG T VIE TRDH+E+ML GFGA L+VE ++G I L G+ +L QV+ V LAGLNAPGETVVIEREPTRDHSERMLLGFGAKLSVEKTSEG-HVITLTGQPELRPQVVAV | 241 |
| Query 242 | PGDPSSTAFPLVAALLVPGSDVTILNVLMNPTRTGLILTLQEMGADIEVINPRLAGGEDV | 301 |
| Sbjct 242 | P DPSS AFP+ AAL+V GSD+ + V N TR GL LTL EMGA+IE NPR GGE V PRDPSSAAFPVCAALIVEGSDILVPGVSQNLTRNGLYLTLVEMGAEIEFQNPREEGGEPV | 301 |
| Query 302 | ADLRVR-SSTLKGVTVPEDRAPSMIDEYPILAVAAAFAEGATVMNGLEELRVKESDRLSA | 360 |
| Sbjct 302 | ADLRVR S +KG+ VP +RAPSMIDEYP+L+V AAFA G TVM G++ELRVKESDR+ A ADLRVRFSGNMKGIEVPPERAPSMIDEYPVLSVVAAFATGKTVMRGVKELRVKESDRIDA | 361 |
| Query 361 | VANGLKLNGVDCDEGETSLVVRGRPDGKGLGNASGAAVATHLDHRIAMSFLVMGLVSENP | 420 |
| Sbjct 362 | +A GL+ GV +E E +L+V G G G GA ATH+DHRIAMSFLV GL ++ P MARGLEACGVRIEEDEDTLIVHGMGPG---GVPGGATCATHIDHRIAMSFLVCGLAAKQP | 418 |
| Query 421  Sbjct 419 | VTVDDATMIATSFPEFMDLMAGLGAKIELSD 451 V+VDDA+ IATSFP F LM GLGA I+ S+ VSVDDASPIATSFPVFEGLMQGLGAMIQRSN 449 |  |

>TR:A0A2S6MZ64 A0A2S6MZ64_9RHIZ 3-phosphoshikimate 1-carboxyvinyltransferase OS=Rhodoblastus sphagnicola OX=333368 GN=aroA PE=3 SV=1

Length=446

Score = 476 bits (1225), Expect = 2e-163

| Identities | = 263/447 (59%), Positives = 312/447 (70%), Gaps = 7/447 (2%) |  |
| --- | --- | --- |
| Query 1 | MLHGASSRPATARKSSGLSGTVRIPGDKSISHRSFMFGGLASGETRITGLLEGEDVINTG | 60 |
| Sbjct 1 | M H + +P T++ L G VR PGDKS+SHR+F+ G L GETRI+GLLEGEDV+NTG MSHATTPKPLTSKACGPLKGRVRPPGDKSVSHRAFLLGLLCVGETRISGLLEGEDVLNTG | 60 |
| Query 61 | KAMQAMGARIRKEGD-TWIIDGVGNGGLLAPEAPLDFGNAATGCRLTMGLVGVYDFDSTF | 119 |
| Sbjct 61 | +A +GA+I + G+ W + GVG G +LAP LDFGNA TG RL MG+VG + + F RACAQLGAKIERVGEGQWTVRGVGLGAVLAPAETLDFGNAGTGSRLMMGVVGGHGITARF | 120 |
| Query 120 | IGDASLTKRPMGRVLNPLREMGVQVKSE-DGDRLPVTLRGPKTPTPITYRVPMASAQVKS | 178 |
| Sbjct 121 | GDASL KRPM R+L+PL MG +V + +G R P+ L+G + P PI YR P+ SAQ+KS DGDASLRKRPMRRILDPLVLMGAEVLEQAEGGRCPILLKGTRDPAPIEYRTPVPSAQIKS | 180 |
| Query 179 | AVLLAGLNTPGITTVIEPIMTRDHTEKMLQGFGANLTVETDADGVRTIRLEGRGKLTGQV | 238 |

AVLLAGLN G TTVIE	+RDHTEKML FGA LT E	D R I LEGR +L+ +

| Sbjct | 181 | AVLLAGLNARGRTTVIETEASRDHTEKMLTYFGATLTCEPFGDHGRKITLEGRPELSPRP | 240 |
| --- | --- | --- | --- |
| Query | 239 | IDVPGDPSSTAFPLVAALLVPGSDVTILNVLMNPTRTGLILTLQEMGADIEVINPRLAGG | 298 |
| Sbjct | 241 | I VP DPSS AF + AALLV GSDV + V+ NP R G + TL EMGA+I+V+ R GG IAVPVDPSSAAFLIAAALLVEGSDVVVEGVMTNPLRNGFVATLLEMGANIDVLEIREEGG | 300 |
| Query | 299 | EDVADLRVRSSTLKGVTVPEDRAPSMIDEYPILAVAAAFAEGATVMNGLEELRVKESDRL | 358 |
| Sbjct | 301 | E VADLRVR S L GV VP DRAP+MIDEY ILAV AAFA+GAT MNGL+ELRVKESDRL EQVADLRVRHSALTGVDVPADRAPAMIDEYIILAVLAAFAKGATRMNGLQELRVKESDRL | 360 |
| Query | 359 | SAVANGLKLNGVDCDEGETSLVVRGRPDGKGLGNASGAAVATHLDHRIAMSFLVMGLVSE | 418 |
| Sbjct | 361 | +A A GL+ NGV L+V G G G V THLDHR+AMSFLVMGL S+ AATAAGLRANGVSATIEGDDLIVEGAGACGG GGLVETHLDHRLAMSFLVMGLASQ | 415 |
| Query  Sbjct | 419  416 | NPVTVDDATMIATSFPEFMDLMAGLGA 445 NPVTVDD TMIATSFP F L+A LGA NPVTVDDETMIATSFPTFKPLLAELGA 442 |  |

>TR:F8JIN8 F8JIN8_HYPSM 3-phosphoshikimate 1-carboxyvinyltransferase OS=Hyphomicrobium sp. (strain MC1) OX=717785 GN=aroA PE=3 SV=1

Length=446

Score = 476 bits (1225), Expect = 2e-163

| Identities | | = 255/438 (58%), Positives = 308/438 (70%), Gaps = 6/438 (1%) |  |
| --- | --- | --- | --- |
| Query 9  Sbjct 9 | | PATARKSSGLSGTVRIPGDKSISHRSFMFGGLASGETRITGLLEGEDVINTGKAMQAMGA P +A K+ L G VR+PGDKSISHR+ +FG LA+G TRI GLLE EDVINT +A+ A+GA PLSALKAGPLVGRVRVPGDKSISHRALIFGALATGTTRIKGLLEAEDVINTARAVAALGA | 68  68 |
| Query 69  Sbjct 69 | | RIRKEGDTWIIDGVGNGGLLAPEAPLDFGNAATGCRLTMGLVGVYDFDSTFIGDASLTKR K+GD W++ G G GGL P+ PLDFGN+ TG RL MG+V + T GDASL++R SAEKQGDLWVVKGRGVGGLRTPDGPLDFGNSGTGTRLMMGVVAGHPITVTMTGDASLSRR | 128  128 |
| Query 129  Sbjct 129 | | PMGRVLNPLREMGVQVKSEDGDRLPVTLRGPKTPTPITYRVPMASAQVKSAVLLAGLNTP PM RVL PL EMG+++ + LP+TLRG PI YR+P+ SAQVKSAVL+AGL+ PMRRVLGPLVEMGLEIMEPGKETLPLTLRGTSELMPIVYRLPVPSAQVKSAVLIAGLHAA | 188  188 |
| Query 189  Sbjct 189 | | GITTVIEPIMTRDHTEKMLQGFGANLTVETDADGVRTIRLEGRGKLTGQVIDVPGDPSST G TTVIE TRDHTE+ML+ FGA +T ET + I ++G +L G+ I VPGDPSS GKTTVIEREPTRDHTERMLRHFGATVTNETKGEETH-ITVDGDAELAGKDIIVPGDPSSA | 248  247 |
| Query 249  Sbjct 248 | | AFPLVAALLVPGSDVTILNVLMNPTRTGLILTLQEMGADIEVINPRLAGGEDVADLRVRS AF + AAL+VPGSD+ + VL+NPTRTGL LTLQEMG D+ N R GGE VAD+RVR AFLVAAALIVPGSDIIVEGVLVNPTRTGLYLTLQEMGGDLTFENAREEGGEPVADIRVRY | 308  307 |
| Query 309  Sbjct 308 | | STLKGVTVPEDRAPSMIDEYPILAVAAAFAEGATVMNGLEELRVKESDRLSAVANGLKLN S LKGV VP +RAPSMIDEYP+LA +AFAEG T M+GL EL+VKESDRL A A L N SRLKGVHVPAERAPSMIDEYPVLAAISAFAEGQTRMDGLAELKVKESDRLQATAAALIAN | 368  367 |
| Query 369 | | GVDCDEGETSLVVRGRPDGKGLGNASGAAVATHLDHRIAMSFLVMGLVSENPVTVDDATM G D +L+V G G G G VATHLDHRIAM+FL L SE P+ VDDATM | 428 |
| Sbjct | 368 | GARADVEGDTLIVEG GSGLNGGGTVATHLDHRIAMAFLTASLASETPIVVDDATM | 422 |
| Query  Sbjct | 429  423 | IATSFPEFMDLMAGLGAK 446 IATSFPEF +M LGA+ IATSFPEFRPIMETLGAR 440 |  |

>TR:A0A154W6C2 A0A154W6C2_9PROT 3-phosphoshikimate 1-carboxyvinyltransferase OS=Oceanibaculum pacificum OX=580166 GN=aroA PE=3 SV=1

Length=463

Score = 476 bits (1226), Expect = 3e-163

| Identities | = 260/445 (58%), Positives = 311/445 (70%), Gaps = 6/445 (1%) |  |
| --- | --- | --- |
| Query 8 | RPATARKSSGLSGTVRIPGDKSISHRSFMFGGLASGETRITGLLEGEDVINTGKAMQAMG | 67 |
| Sbjct 21 | +P T+R+++ L G V PGDKSISHR+ M GGLA GET I GLLEGEDV+NTGKAMQA G QPLTSRRATALKGAVTAPGDKSISHRALMLGGLAIGETAIHGLLEGEDVLNTGKAMQAYG | 80 |
| Query 68 | ARIRKEGD-TWIIDGVGNGGLLAPEAPLDFGNAATGCRLTMGLVGVYDFDSTFIGDASLT | 126 |
| Sbjct 81 | A +R++ D W ++GVG GGL P LD GN+ TG RL MGL + S F GDASL ASVRRDDDGVWHVNGVGVGGLAEPAEVLDMGNSGTGARLLMGLAAGHPITSFFTGDASLR | 140 |
| Query 127 | KRPMGRVLNPLREMGVQVKSEDGDRLPVTLRGPKTPTPITYRVPMASAQVKSAVLLAGLN | 186 |
| Sbjct 141 | +RPM RV PL EMG + + +G RLP+ + G TP PI Y +P+ASAQVKSA+LLA L RRPMARVTRPLAEMGARFVTREGGRLPLAVTGSSTPMPIDYVLPVASAQVKSAILLAALT | 200 |
| Query 187 | TPGITTVIEPIMTRDHTEKMLQGFGANLTVETDADGVRTIRLEGRGKLTGQVIDVPGDPS | 246 |
| Sbjct 201 | PG TTV+EP TRDH+E+ML+ FGA + VE ADG R + L G+ +L + VPGDPS APGETTVVEPQPTRDHSERMLRHFGATVRVEDRADGSRAVTLVGQPELIAATVRVPGDPS | 260 |
| Query 247 | STAFPLVAALLVPGSDVTILNVLMNPTRTGLILTLQEMGADIEVINPRLAGGEDVADLRV | 306 |
| Sbjct 261 | S AFP VAALLVPGS+VTI NV NP R GL TL EMGADI + N R GE V DL V SAAFPAVAALLVPGSEVTIRNVGANPLRFGLFETLLEMGADIALENRREEAGEPVVDLVV | 320 |
| Query 307 | R-SSTLKGVTVPEDRAPSMIDEYPILAVAAAFAEGATVMNGLEELRVKESDRLSAVANGL | 365 |
| Sbjct 321 | R L+GVTVP RAPSMIDEYPILA+AAA AEG TVM GL ELRVKESDRL AVA GL RGGKPLRGVTVPAARAPSMIDEYPILAMAAACAEGDTVMEGLAELRVKESDRLGAVARGL | 380 |
| Query 366 | KLNGVDCDEGETSLVVRGRPDGKGLGNASGAAVATHLDHRIAMSFLVMGLVSENPVTVDD | 425 |
| Sbjct 381 | G +EG +L+V G+ GA +A+ LDHRIAM+F V+G+VSE P+ +DD AACGAIVEEGPETLIVHGQAKPP VGGATIASGLDHRIAMAFAVLGMVSEQPIHIDD | 436 |
| Query 426  Sbjct 437 | ATMIATSFPEFMDLMAGLGAKIELS 450 A IATSFP F+ +M GLGA+I L+ AEPIATSFPGFVAMMNGLGAQIALA 461 |  |

>TR:A0A0D7EAJ3 A0A0D7EAJ3_RHOPL 3-phosphoshikimate 1-carboxyvinyltransferase OS=Rhodopseudomonas palustris OX=1076 GN=aroA PE=3 SV=1

Length=445

Score = 476 bits (1224), Expect = 3e-163

| Identities | = 259/448 (58%), Positives = 312/448 (70%), Gaps = 7/448 (2%) |  |
| --- | --- | --- |
| Query 1 | MLHGASSRPATARKSSGLSGTVRIPGDKSISHRSFMFGGLASGETRITGLLEGEDVINTG | 60 |
| Sbjct 1 | M + A P AR+S+ LSG R+PGDKSISHR+ + G LA GET I+GLLEGEDV+NT MTNSAHPTPLQARRSAVLSGAARVPGDKSISHRALILGALAVGETAISGLLEGEDVLNTA | 60 |
| Query 61 | KAMQAMGARIRKEGD-TWIIDGVGNGGLLAPEAPLDFGNAATGCRLTMGLVGVYDFDSTF | 119 |
| Sbjct 61 | KAMQA+GA++ + GD W + GVG G P APLDFGN+ TGCRL MG V +TF KAMQALGAQVARTGDFAWTVQGVGVAGFAQPAAPLDFGNSGTGCRLVMGAVAGCPIVATF | 120 |
| Query 120 | IGDASLTKRPMGRVLNPLREMGVQV-KSEDGDRLPVTLRGPKTPTPITYRVPMASAQVKS | 178 |
| Sbjct 121 | GD+SL RPM R+++PL MG ++ +S DG RLP+TL G + P PI Y+ P+ASAQ+KS DGDSSLRGRPMRRIVDPLELMGARIIESRDGGRLPLTLAGARDPLPIRYQTPVASAQIKS | 180 |
| Query 179 | AVLLAGLNTPGITTVIEPIMTRDHTEKMLQGFGANLTVETDADGVRTIRLEGRGKLTGQV | 238 |
| Sbjct 181 | AVLLAGL PG+TTVIE +RDHTE ML+ FGA LT E + R I L G+ +L G AVLLAGLAAPGVTTVIETEASRDHTELMLRHFGAALTSEPEGAHGRRISLTGQPELRGAP | 240 |
| Query 239 | IDVPGDPSSTAFPLVAALLVPGSDVTILNVLMNPTRTGLILTLQEMGADIEVINPRLAGG | 298 |
| Sbjct 241 | + VP DPSS AFP+VAAL+VPGSD+ + V+ NP RTGL TL+EMGA IE R G VVVPADPSSAAFPMVAALIVPGSDIMLSEVMTNPLRTGLFKTLREMGASIEESEVRGDAG | 300 |
| Query 299 | EDVADLRVRSSTLKGVTVPEDRAPSMIDEYPILAVAAAFAEGATVMNGLEELRVKESDRL | 358 |

| Sbjct | 301 | E +A RVR+S LKGV VP RAPSMIDEY +LAVAAAFAEG T M GL+ELRVKESDRL  EPMAQFRVRASKLKGVDVPPQRAPSMIDEYLVLAVAAAFAEGTTRMRGLKELRVKESDRL | 360 |
| --- | --- | --- | --- |
| Query | 359 | SAVANGLKLNGVDCDEGETSLVVRGRPDGKGLGNASGAAVATHLDHRIAMSFLVMGLVSE | 418 |
| Sbjct | 361 | A A L++NGV + L+V GR G G VATH+DHRIAMS LVMGL SE EATAAMLRVNGVAVEIEGDDLIVEGRGHVPG GGLVATHMDHRIAMSALVMGLASE | 415 |
| Query  Sbjct | 419  416 | NPVTVDDATMIATSFPEFMDLMAGLGAK 446 PV VDD IATSFP+F+ +M LGA+  QPVQVDDTAFIATSFPDFIPMMRRLGAE 443 |  |

>TR:A0A257F0G7 A0A257F0G7_9RHOB 3-phosphoshikimate 1-carboxyvinyltransferase OS=Rhodobacteraceae bacterium PARR1 OX=2015578 GN=aroA PE=3

SV=1

Length=448

Score = 476 bits (1224), Expect = 3e-163

| Identities | = 259/445 (58%), Positives = 313/445 (70%), Gaps = 7/445 (2%) |  |
| --- | --- | --- |
| Query 3 | HGASSRPATARKSSGLSGTVRIPGDKSISHRSFMFGGLASGETRITGLLEGEDVINTGKA | 62 |
| Sbjct 4 | HG ++P TARKS L+G +PGDKSISHR+ +FG +A GETR+TGLLEG+DV++T KA HG-DAQPMTARKSPALTGRAEVPGDKSISHRALIFGAMAVGETRVTGLLEGQDVLDTAKA | 62 |
| Query 63 | MQAMGARIRKEG-DTWIIDGVGNGGLLAPEAPLDFGNAATGCRLTMGLVGVYDFDSTFIG | 121 |

M+A GA + + G	W + GVG GG	P	+D GN+ TG RL MG +	+TF G

| Sbjct | 63 | MRAFGATVTRHGLGEWSVHGVGVGGFSEPADVIDCGNSGTGVRLIMGTMATTPITATFTG | 122 |
| --- | --- | --- | --- |
| Query | 122 | DASLTKRPMGRVLNPLREMGVQVKSEDGDRLPVTLRGPKTPTPITYRVPMASAQVKSAVL | 181 |
| Sbjct | 123 | DASL KRPMGRV +PL G + G RLP+T+ G P P+ Y +P+ASAQVKSAVL DASLRKRPMGRVTDPLSLFGARAFGRQGGRLPMTVVGAANPVPVRYALPVASAQVKSAVL | 182 |
| Query | 182 | LAGLNTPGITTVIEPIMTRDHTEKMLQGFGANLTVETDADGVRTIRLEGRGKLTGQVIDV | 241 |
| Sbjct | 183 | LAGLN PG T VIE TRDH+E+ML GFGA LTVE A+G I L GR +L Q + V LAGLNAPGQTVVIEREPTRDHSERMLLGFGAELTVEKTAEG-NVITLTGRPELRPQSVAV | 241 |
| Query | 242 | PGDPSSTAFPLVAALLVPGSDVTILNVLMNPTRTGLILTLQEMGADIEVINPRLAGGEDV | 301 |
| Sbjct | 242 | P DPSS AFP+ AAL+V GSD+ + V N TR GL LTL EMGADIE NPR GGE V PRDPSSAAFPVCAALIVEGSDIFVPGVSQNLTRNGLYLTLVEMGADIEFQNPREEGGEPV | 301 |
| Query | 302 | ADLRVR-SSTLKGVTVPEDRAPSMIDEYPILAVAAAFAEGATVMNGLEELRVKESDRLSA | 360 |
| Sbjct | 302 | ADLRVR SS LKG+ VPE+RAPSMIDEYPIL+V A+FAEG T+M G++ELRVKESDR+ A ADLRVRFSSNLKGIEVPEERAPSMIDEYPILSVVASFAEGKTIMRGVKELRVKESDRIDA | 361 |
| Query | 361 | VANGLKLNGVDCDEGETSLVVRGRPDGKGLGNASGAAVATHLDHRIAMSFLVMGLVSENP | 420 |
| Sbjct | 362 | +A GL+ GV +E E +L+V G G G GA ATHLDHRIAMSFLV G+ ++N MARGLEACGVKVEEDEDTLIVHGLGAG---GVPGGATCATHLDHRIAMSFLVCGMAAKNA | 418 |
| Query  Sbjct | 421  419 | VTVDDATMIATSFPEFMDLMAGLGA 445 V+VDDA+ I TSFP F LM G+GA VSVDDASPILTSFPIFEGLMTGMGA 443 |  |

```
>TR:A0A1E2S000 A0A1E2S000_9RHIZ 3-phosphoshikimate 1-carboxyvinyltransferase OS=Methyloligella halotolerans OX=1177755 GN=aroA PE=3 SV=1
Length=438

Score = 475 bits (1223), Expect = 3e-163
Identities = 245/441 (56%), Positives = 322/441 (73%), Gaps = 5/441 (1%)

Query 7	SRPATARKSSGLSGTVRIPGDKSISHRSFMFGGLASGETRITGLLEGEDVINTGKAMQAM 66 S+P TAR+S GL+G +++PGDKSISHR+ + G LA+G T+ITGLLE ED++ T +A+ A+
Sbjct 2	SQPLTARRSEGLTGRLKVPGDKSISHRALILGALATGRTQITGLLEAEDILATARAVAAL 61
```

| Query | 67 | GARIRKEGDTWIIDGVGNGGLLAPEAPLDFGNAATGCRLTMGLVGVYDFDSTFIGDASLT | 126 |
| --- | --- | --- | --- |
| Sbjct | 62 | GA + K+GD + + G G GGL P+ PLDFGN+ TG RL MG+V + F GD SL GADVMKDGDRYTVIGRGVGGLKQPDGPLDFGNSGTGARLMMGVVAGHAMTVEFTGDESLC | 121 |
| Query | 127 | KRPMGRVLNPLREMGVQVKSEDGDRLPVTLRGPKTPTPITYRVPMASAQVKSAVLLAGLN | 186 |
| Sbjct | 122 | +RPM RVL PL+EMG++V+ E G LP+TLRG + P+ Y +P+ASAQVKSA+LLAGL+ RRPMARVLTPLQEMGLEVEGE-GTTLPLTLRGTQDLIPLRYELPVASAQVKSAILLAGLH | 180 |
| Query | 187 | TPGITTVIEPIMTRDHTEKMLQGFGANLTVETDADGVRTIRLEGRGKLTGQVIDVPGDPS | 246 |
| Sbjct | 181 | PG T+VIE + TRDHTE+ML+ FGA ++VE G + + G +LTG + VP DPS APGRTSVIEAMPTRDHTERMLRYFGAEISVEDREGGGNIVTVCGDAELTGASVYVPSDPS | 240 |
| Query | 247 | STAFPLVAALLVPGSDVTILNVLMNPTRTGLILTLQEMGADIEVINPRLAGGEDVADLRV | 306 |
| Sbjct | 241 | S AF L AAL+ P S+VT+ VLM+PTRTG + T +EMGADIE+++ A GE +A++R SAAFLLAAALIAPNSEVTVEGVLMSPTRTGFLETAREMGADIEILDQHEASGETIANVRA | 300 |
| Query | 307 | RSSTLKGVTVPEDRAPSMIDEYPILAVAAAFAEGATVMNGLEELRVKESDRLSAVANGLK | 366 |
| Sbjct | 301 | R+S LKGVTVP RAPSMIDEYP+LA+ AAFAEG T M GL EL++KESDRL+A A GL RTSNLKGVTVPAARAPSMIDEYPVLAMLAAFAEGETRMQGLAELKLKESDRLAATAAGLS | 360 |
| Query | 367 | LNGVDCDEGETSLVVRGRPDGKGLGNASGAAVATHLDHRIAMSFLVMGLVSENPVTVDDA | 426 |
| Sbjct | 361 | GV+ + L+VR G G GA + TH+DHRIAMSFLV+GL + PVTVDD ACGVEAEVDGDDLIVR GNGGAVEGGAMITTHMDHRIAMSFLVLGLAAAEPVTVDDG | 416 |
| Query  Sbjct | 427  417 | TMIATSFPEFMDLMAGLGAKI 447  +MIATSFP F++L++G+GA+I SMIATSFPGFVELLSGMGAEI 437 |  |

>TR:A0A2K8KIF1 A0A2K8KIF1_9RHOB 3-phosphoshikimate 1-carboxyvinyltransferase OS=Rhodobaca barguzinensis OX=441209 GN=aroA PE=3 SV=1

Length=449

Score = 476 bits (1224), Expect = 4e-163

| Identities | = 259/439 (59%), Positives = 306/439 (70%), Gaps = 7/439 (2%) |  |
| --- | --- | --- |
| Query 11 | TARKSSGLSGTVRIPGDKSISHRSFMFGGLASGETRITGLLEGEDVINTGKAMQAMGARI | 70 |
| Sbjct 11 | TA KS LSG +PGDKSISHR+ + G L+ G T ITGLLEG+DV++T KAMQA GA + TAHKSGPLSGVAEVPGDKSISHRALILGALSIGRTSITGLLEGQDVLDTAKAMQAFGAEV | 70 |
| Query 71 | RKEGD-TWIIDGVGNGGLLAPEAPLDFGNAATGCRLTMGLVGVYDFDSTFIGDASLTKRP | 129 |
| Sbjct 71 | + G W + GVG GG PE +D GN+ TG RL MG + D +TF GDASL KRP TQHGPGEWSVHGVGVGGFAEPEQVIDCGNSGTGVRLIMGAMATTDITATFTGDASLNKRP | 130 |
| Query 130 | MGRVLNPLREMGVQVKSEDGDRLPVTLRGPKTPTPITYRVPMASAQVKSAVLLAGLNTPG | 189 |
| Sbjct 131 | MGRV +PL G Q + G RLP+TL G P P+ YRVPM SAQVKSAVLLAGLN PG MGRVTDPLALFGAQSYARRGGRLPLTLIGAAQPVPVRYRVPMPSAQVKSAVLLAGLNAPG | 190 |
| Query 190 | ITTVIEPIMTRDHTEKMLQGFGANLTVETDADGVRTIRLEGRGKLTGQVIDVPGDPSSTA | 249 |
| Sbjct 191 | T V+E TRDHTE+ML+GFGA++TVE DG R I L G+ +L GQ + VP DPSS A QTVVVEQEPTRDHTERMLRGFGASITVEDTEDG-RVITLTGQPELEGQNVAVPRDPSSAA | 249 |
| Query 250 | FPLVAALLVPGSDVTILNVLMNPTRTGLILTLQEMGADIEVINPRLAGGEDVADLRVRSS | 309 |
| Sbjct 250 | FP+ AAL+V S++ + V NPTR G+ LTLQ+MGAD+ + NPR GGE VADLRVR S FPVCAALMVQNSNILVPGVSQNPTRNGIFLTLQDMGADLVMENPREEGGEPVADLRVRFS | 309 |
| Query 310 | TLKGVTVPEDRAPSMIDEYPILAVAAAFAEGATVMNGLEELRVKESDRLSAVANGLKLNG | 369 |
| Sbjct 310 | LKG+ VP +RA SMIDEYP+LA AA AEG TVM G++ELRVKESDR+ A+A GL+ G ALKGIEVPPERAASMIDEYPVLAALAATAEGVTVMRGIKELRVKESDRIDAMARGLEACG | 369 |
| Query 370 | VDCDEGETSLVVRGR-PDGKGLGNASGAAVATHLDHRIAMSFLVMGLVSENPVTVDDATM | 428 |
| Sbjct 370 | V E E SL V G PDG A V THLDHRIAMSFL +GLV+ P+TVDDA  VRVAETEDSLTVYGMGPDGV PGDATVQTHLDHRIAMSFLCLGLVARAPITVDDAGP | 425 |

Query 429 IATSFPEFMDLMAGLGAKI 447

IATSFP F LM GLGA+I Sbjct 426 IATSFPVFESLMQGLGAEI 444

>TR:A0A545TG49 A0A545TG49_9PROT 3-phosphoshikimate 1-carboxyvinyltransferase OS=Denitrobaculum tricleocarpae OX=2591009 GN=aroA PE=3 SV=1

Length=460

Score = 476 bits (1225), Expect = 4e-163

| Identities | = 260/447 (58%), Positives = 312/447 (70%), Gaps = 8/447 (2%) |  |
| --- | --- | --- |
| Query 8 | RPATARKSSGLSGTVRIPGDKSISHRSFMFGGLASGETRITGLLEGEDVINTGKAMQAMG | 67 |
| Sbjct 4 | +P TA S L+G VR+PGDKSISHR+ M +A GE+R+ GLLEGEDV+ T AM+A+G KPLTATASGPLTGDVRVPGDKSISHRALMLSAIAVGESRVEGLLEGEDVLCTAAAMRALG | 63 |
| Query 68 | ARI-RKEGDTWIIDGVGNGGLLAPEAPLDFGNAATGCRLTMGLVGVYDFDSTFIGDASLT | 126 |
| Sbjct 64 | A I R + W I G G GGL P LD GN+ T RL +GL+ + +T GD SL AEIERDDNGVWHIWGRGVGGLQEPANVLDMGNSGTAARLLLGLLATHPLTATMTGDESLR | 123 |
| Query 127 | KRPMGRVLNPLREMGV QVKSEDGDRLPVTLRGPKTPTPITYRVPMASAQVKSAV | 180 |
| Sbjct 124 | RPMGRV PL EMG + + D LP+T++G +P PITYR+P+ASAQVKSAV GRPMGRVTRPLGEMGAAFTGRSDLSGKGRDLLPLTIQGSDSPIPITYRLPVASAQVKSAV | 183 |
| Query 181 | LLAGLNTPGITTVIEPIMTRDHTEKMLQGFGANLTVETDADGVRTIRLEGRGKLTGQVID | 240 |
| Sbjct 184 | LLAGLNTPG TTVIEP TRDH+E ML+ FGA + VE ++G R L G+ +LTG+ I LLAGLNTPGETTVIEPAHTRDHSELMLRHFGARIKVEETSEG-RATTLTGQPELTGRYIT | 242 |
| Query 241 | VPGDPSSTAFPLVAALLVPGSDVTILNVLMNPTRTGLILTLQEMGADIEVINPRLAGGED | 300 |
| Sbjct 243 | VP DPSS AFP VAALL PGS++T+ V MNP R GL +TL+EMGA+IE N R GGE VPADPSSAAFPSVAALLSPGSEITLRAVGMNPHRIGLFITLREMGANIEFTNEREEGGEP | 302 |
| Query 301 | VADLRVRSSTLKGVTVPEDRAPSMIDEYPILAVAAAFAEGATVMNGLEELRVKESDRLSA | 360 |
| Sbjct 303 | VADL VR+ L+G+ VP +RAPSMIDEYP+LA+AAA AEG TVM GL ELRVKE DRL+A VADLIVRAGPLQGIEVPAERAPSMIDEYPVLAMAAACAEGTTVMRGLAELRVKECDRLAA | 362 |
| Query 361 | VANGLKLNGVDCDEGETSLVVRGRPDGKGLGNASGAAVATHLDHRIAMSFLVMGLVSENP | 420 |
| Sbjct 363 | A GL+ GV +EGE SL+V G G G VATH DHRIAMSFLV+GLVS+ P TARGLEACGVKVEEGEDSLIVHGVGAAAGYRPEGGGTVATHFDHRIAMSFLVLGLVSKAP | 422 |
| Query 421  Sbjct 423 | VTVDDATMIATSFPEFMDLMAGLGAKI 447 VTVDDAT I TSFP+F LM GLGA + VTVDDATAIDTSFPDFQRLMTGLGAHV 449 |  |

>TR:A0A371BE67 A0A371BE67_9SPHN 3-phosphoshikimate 1-carboxyvinyltransferase OS=Sphingorhabdus sp. GY_G OX=2292257 GN=aroA PE=3 SV=1

Length=442

Score = 475 bits (1223), Expect = 4e-163

| Identities | = 252/443 (57%), Positives = 312/443 (70%), Gaps = 4/443 (1%) |  |
| --- | --- | --- |
| Query 2 | LHGASSRPATARKSSGLSGTVRIPGDKSISHRSFMFGGLASGETRITGLLEGEDVINTGK | 61 |
| Sbjct 1 | +H A RPAT S L GTV++PGDKSISHRS M LA GE+R++GLLEGEDV+ T MHSAQPRPATFTASGPLRGTVKVPGDKSISHRSLMLSALAVGESRVSGLLEGEDVLATAA | 60 |
| Query 62 | AMQAMGARIRKEGD-TWIIDGVGNGGLLAPEAPLDFGNAATGCRLTMGLVGVYDFDSTFI | 120 |
| Sbjct 61 | AM+AMGA I + G+ W I GVG GGLL P+ LD GN+ T RL MGLV + +TFI AMRAMGAAIERTGEGEWRISGVGVGGLLQPQTALDMGNSGTSTRLLMGLVASHAISATFI | 120 |
| Query 121 | GDASLTKRPMGRVLNPLREMGVQVKSEDGDRLPVTLRGPKTPTPITYRVPMASAQVKSAV | 180 |
| Sbjct 121 | GDASL+KRPM RV++P+ G + + G LP+ +RG PI+YR+P+ASAQVKSA+  GDASLSKRPMERVIDPMSLTGAEFNASPGGCLPLMVRGICPAVPISYRLPVASAQVKSAI | 180 |

| Query | 181 | LLAGLNTPGITTVIEPIMTRDHTEKMLQGFGANLTVETDADGVRTIRLEGRGKLTGQVID | 240 |
| --- | --- | --- | --- |
| Sbjct | 181 | LLAGLNTPGIT VIEP+ TRDH+E+ML GFGA LTVETD GVR IRL G +L Q I+ LLAGLNTPGITEVIEPVPTRDHSERMLAGFGAELTVETDVQGVRHIRLHGEAELKPQNIE | 240 |
| Query | 241 | VPGDPSSTAFPLVAALLVPGSDVTILNVLMNPTRTGLILTLQEMGADIEVINPRLAGGED | 300 |
| Sbjct | 241 | VPGDPSS AFP+VAAL+ PGS+VT+ +V MNPTRTG+ L+ MGADI N R+ GGE VPGDPSSAAFPIVAALITPGSEVTVTHVGMNPTRTGIFKMLEAMGADIHYSNERVVGGEP | 300 |
| Query | 301 | VADLRVRSSTLKGVTVPEDRAPSMIDEYPILAVAAAFAEGATVMNGLEELRVKESDRLSA | 360 |
| Sbjct | 301 | VAD+ R S LKG+ VP + APSMIDE+P+ VAA+ AEG T +GL+ELRVKESDRL+ VADITARHSALKGIEVPPEVAPSMIDEFPVFFVAASMAEGRTTTSGLDELRVKESDRLAL | 360 |
| Query | 361 | VANGLKLNGVDCDEGETSLVVRGRPDGKGLGNASGAAVATHLDHRIAMSFLVMGLVSENP | 420 |
| Sbjct | 361 | +A GL + GV +E E L++ G G A GA +A+ LDHRIAMSF V G + MATGLTMIGVKLEEREDGLILDG---SGGEPFAGGATIASALDHRIAMSFAVAGQNARAG | 417 |
| Query  Sbjct | 421  418 | VTVDDATMIATSFPEFMDLMAGL 443  + VDD + IATSFP F ++ L IMVDDVSPIATSFPTFEVMLQSL 440 |  |

>TR:A0A317DYD2 A0A317DYD2_9PROT 3-phosphoshikimate 1-carboxyvinyltransferase OS=Zavarzinia sp. HR-AS OX=2211142 GN=aroA PE=3 SV=1

Length=464

Score = 476 bits (1225), Expect = 4e-163

| Identities | = 259/445 (58%), Positives = 311/445 (70%), Gaps = 7/445 (2%) |  |
| --- | --- | --- |
| Query 3 | HGASSRPA-TARKSSGLSGTVRIPGDKSISHRSFMFGGLASGETRITGLLEGEDVINTGK | 61 |
| Sbjct 22 | H S PA +A+ S L+GTV +PGDKSISHR+ + GG+A GET +TGLLEG+DV++T + HRGSQVPAQSAQISPALTGTVTVPGDKSISHRALILGGIAVGETEVTGLLEGDDVLHTAE | 81 |
| Query 62 | AMQAMGARIRKEGDT-WIIDGVGNGGLLAPEAPLDFGNAATGCRLTMGLVGVYDFDSTFI | 120 |
| Sbjct 82 | M+ MGA + + G+ W + GVG GGL+ P LD GN+ TG RL MGLV + + F CMRRMGATVTRVGEGHWRVRGVGVGGLIEPADILDMGNSGTGARLLMGLVAGHPVTAIFT | 141 |
| Query 121 | GDASLTKRPMGRVLNPLREMGVQVKSEDGDRLPVTLRGPKTPTPITYRVPMASAQVKSAV | 180 |
| Sbjct 142 | GDASL KRPM RV PL MG G +LP+ + G + P PI YR+P+ASAQVKSA+ GDASLNKRPMARVTEPLSRMGASFVGRAGGKLPMAVIGARDPMPIEYRLPVASAQVKSAI | 201 |
| Query 181 | LLAGLNTPGITTVIEPIMTRDHTEKMLQGFGANLTVETDADGVRTIRLEGRGKLTGQVID | 240 |
| Sbjct 202 | LLAGL PG TTVIEP TRDH+E++L FGA + + ADG R + L G+ +L + LLAGLCAPGETTVIEPEPTRDHSERLLGHFGAEVRIVDAADGSRHVTLRGQPELIAAPVV | 261 |
| Query 241 | VPGDPSSTAFPLVAALLVPGSDVTILNVLMNPTRTGLILTLQEMGADIEVINPRLAGGED | 300 |
| Sbjct 262 | VPGDPSS AFP+VAAL+VPGSD+T+ NV +NP R GL TL EMGA I+ ++ R+AGGE VPGDPSSAAFPIVAALIVPGSDITVRNVGLNPLRVGLFDTLIEMGARIDYLDRRVAGGEP | 321 |
| Query 301 | VADLRVRSSTLKGVTVPEDRAPSMIDEYPILAVAAAFAEGATVMNGLEELRVKESDRLSA | 360 |
| Sbjct 322 | VADLR+R S LKGVTVP RAPSMIDEYP+LAVAAAFA G T M GL ELRVKESDRL+A VADLRIRHSELKGVTVPAARAPSMIDEYPVLAVAAAFARGTTRMEGLAELRVKESDRLAA | 381 |
| Query 361 | VANGLKLNGVDCDEGETSLVVRGRPDGKGLGNASGAAVATHLDHRIAMSFLVMGLVSENP | 420 |
| Sbjct 382 | VA GL NGV + G LVV+G G G VATH+DHRIAMSFLVMGL S P VAAGLAANGVTHEAGADYLVVQGTGRAPG GGTVATHMDHRIAMSFLVMGLASAQP | 436 |
| Query 421  Sbjct 437 | VTVDDATMIATSFPEFMDLMAGLGA 445 VTVDD MI TSFP F LM LGA VTVDDTHMIDTSFPGFGGLMTSLGA 461 |  |

>TR:A0A426RQ64 A0A426RQ64_9SPHN 3-phosphoshikimate 1-carboxyvinyltransferase OS=Sphingorhabdus wooponensis OX=940136 GN=aroA PE=3 SV=1

Length=444

Score = 475 bits (1223), Expect = 4e-163

| Identities | = 253/440 (58%), Positives = 315/440 (72%), Gaps = 4/440 (1%) |  |
| --- | --- | --- |
| Query 2 | LHGASSRPATARKSSGLSGTVRIPGDKSISHRSFMFGGLASGETRITGLLEGEDVINTGK | 61 |
| Sbjct 1 | +H A SRP + + S L GT+++PGDKSISHRS M LA GE++ITGLLEGEDV+ T MHSAPSRPGSFQPSGPLRGTLKVPGDKSISHRSLMLSALAVGESKITGLLEGEDVLATAA | 60 |
| Query 62 | AMQAMGARIRKEGDT-WIIDGVGNGGLLAPEAPLDFGNAATGCRLTMGLVGVYDFDSTFI | 120 |
| Sbjct 61 | AM+AMGA I + G+ W ++GVG GGLL P LD GN+ T RL MGLV + +TFI AMRAMGAHIERTGEGHWTVNGVGVGGLLQPAGALDMGNSGTSTRLLMGLVASHRLTATFI | 120 |
| Query 121 | GDASLTKRPMGRVLNPLREMGVQVKSEDGDRLPVTLRGPKTPTPITYRVPMASAQVKSAV | 180 |
| Sbjct 121 | GDASL+KRPMGRV++PL +MG + + G LP+ +RG PI YR+P+ASAQVKSAV GDASLSKRPMGRVIDPLSQMGAEFTASPGGMLPLMVRGLVPAVPICYRLPVASAQVKSAV | 180 |
| Query 181 | LLAGLNTPGITTVIEPIMTRDHTEKMLQGFGANLTVETDADGVRTIRLEGRGKLTGQVID | 240 |
| Sbjct 181 | LLAGLN GIT VIEP+ TRDH+E+ML+GFGA+LTV+ DADGVR IRL G +L Q ID LLAGLNIAGITEVIEPVPTRDHSERMLRGFGADLTVDVDADGVRHIRLMGEAELQPQQID | 240 |
| Query 241 | VPGDPSSTAFPLVAALLVPGSDVTILNVLMNPTRTGLILTLQEMGADIEVINPRLAGGED | 300 |
| Sbjct 241 | VPGDPSS AF +VAAL+ PGS+VT+ +V MNPTRTG+ L+ MGAD+ N R GGE VPGDPSSAAFFIVAALITPGSEVTVTHVGMNPTRTGIYKMLEAMGADLTYSNAREVGGEP | 300 |
| Query 301 | VADLRVRSSTLKGVTVPEDRAPSMIDEYPILAVAAAFAEGATVMNGLEELRVKESDRLSA | 360 |
| Sbjct 301 | VAD+ R S L+G+ VP D APSMIDE+P+ VAA+ A+G T +GL+ELRVKESDRL+ VADITARHSVLRGIDVPADVAPSMIDEFPVFFVAASMAQGRTTTSGLDELRVKESDRLAL | 360 |
| Query 361 | VANGLKLNGVDCDEGETSLVVRGRPDGKGLGNASGAAVATHLDHRIAMSFLVMGLVSENP | 420 |
| Sbjct 361 | +A GLK G +E E LV+ G G+ L GA V + LDHRIAMSF V G + +P MATGLKAIGARVEEREDGLVIDG-TGGEPL--EGGATVTSALDHRIAMSFAVAGQHTYHP | 417 |
| Query 421  Sbjct 418 | VTVDDATMIATSFPEFMDLM 440 VTVDD + IATSFP F ++ VTVDDVSPIATSFPTFESIL 437 |  |

>TR:A0A2Z5EFV8 A0A2Z5EFV8_9SPHN 3-phosphoshikimate 1-carboxyvinyltransferase OS=Novosphingobium sp. P6W OX=1609758 GN=aroA PE=3 SV=1

Length=438

Score = 475 bits (1222), Expect = 5e-163

| Identities | = 253/439 (58%), Positives = 308/439 (70%), Gaps = 3/439 (1%) |  |
| --- | --- | --- |
| Query 8 | RPATARKSSGLSGTVRIPGDKSISHRSFMFGGLASGETRITGLLEGEDVINTGKAMQAMG | 67 |
| Sbjct 2 | RP + L G +R+PGDKSISHRS M G LA GETR+TGLLEGEDV+ T AM+AMG RPRRFLPAGPLKGRIRVPGDKSISHRSIMLGALAVGETRVTGLLEGEDVLATAAAMRAMG | 61 |
| Query 68 | ARI-RKEGDTWIIDGVGNGGLLAPEAPLDFGNAATGCRLTMGLVGVYDFDSTFIGDASLT | 126 |
| Sbjct 62 | A + R E W+I+GVG G LL PE PLD GN+ T RL MGLV + +TF GDASL+ ASVERLESGEWVINGVGVGALLQPETPLDMGNSGTSTRLLMGLVASHPITATFTGDASLS | 121 |
| Query 127 | KRPMGRVLNPLREMGVQVKSEDGDRLPVTLRGPKTPTPITYRVPMASAQVKSAVLLAGLN | 186 |
| Sbjct 122 | KRPMGRV++PL MG ++ +G LP+ L+G PITYR+P+ASAQVKSA+LLAGLN KRPMGRVIDPLSSMGASFEASEGGTLPLILKGASPAVPITYRLPVASAQVKSAILLAGLN | 181 |
| Query 187 | TPGITTVIEPIMTRDHTEKMLQGFGANLTVETDADGVRTIRLEGRGKLTGQVIDVPGDPS | 246 |
| Sbjct 182 | TPGITTVIEP+ TRDH+E+ML+GFGA LTVETDA+G R I + G +L Q I+VPGDPS TPGITTVIEPVPTRDHSERMLRGFGAELTVETDANGARVISIRGEAELKPQTIEVPGDPS | 241 |
| Query 247 | STAFPLVAALLVPGSDVTILNVLMNPTRTGLILTLQEMGADIEVINPRLAGGEDVADLRV | 306 |
| Sbjct 242 | S AF +VAAL+VPGS+V I NV +NPTR G++ L+ MG IE +NPR GGE VADL V  SAAFFIVAALVVPGSEVLIENVGLNPTRAGIVEVLRLMGGSIEEVNPREVGGEPVADLLV | 301 |

| Query | 307 | RSSTLKGVTVPEDRAPSMIDEYPILAVAAAFAEGATVMNGLEELRVKESDRLSAVANGLK | 366 |
| --- | --- | --- | --- |
| Sbjct | 302 | R S LKG+ V PSM+DE+P L VAA+ AEG T GL+ELR+KESDR+S +A L RHSALKGIDVDPAVVPSMVDEFPALFVAASLAEGITTTTGLDELRIKESDRISVMAAALT | 361 |
| Query | 367 | LNGVDCDEGETSLVVRGRPDGKGLGNASGAAVATHLDHRIAMSFLVMGLVSENPVTVDDA | 426 |
| Sbjct | 362 | G E E LV+ G G+ L S AA+ATHLDHRIAMS + G+ S + V VDD AAGAAVRETEDGLVITG-TGGQPL-PGSDAAIATHLDHRIAMSMAIAGIASRSGVEVDDT | 419 |
| Query | 427 | TMIATSFPEFMDLMAGLGA 445 IATSFP F ++ L A |  |
| Sbjct | 420 | RPIATSFPVFEAMLDELAA 438 | |

>TR:A0A4V1ZPT0 A0A4V1ZPT0_9BRAD 3-phosphoshikimate 1-carboxyvinyltransferase OS=Bradyrhizobiaceae bacterium OX=2072420 GN=aroA PE=3 SV=1

Length=460

Score = 476 bits (1224), Expect = 5e-163

| Identities | = 250/439 (57%), Positives = 314/439 (72%), Gaps = 7/439 (2%) |  |
| --- | --- | --- |
| Query 9 | PATARKSSGLSGTVRIPGDKSISHRSFMFGGLASGETRITGLLEGEDVINTGKAMQAMGA | 68 |
| Sbjct 24 | P TAR+S L GT R+PGDKSISHR+ + G LA GETRITGLLEGEDV+NT K+MQA+GA PLTARRSGALRGTARVPGDKSISHRALILGALAVGETRITGLLEGEDVLNTAKSMQALGA | 83 |
| Query 69 | RIRKEGD-TWIIDGVGNGGLLAPEAPLDFGNAATGCRLTMGLVGVYDFDSTFIGDASLTK | 127 |
| Sbjct 84 | ++ + GD W ++GVG G P APLDFGN+ TGCRL MG V ++TF GDASL KVERTGDFAWKVNGVGVAGFAQPTAPLDFGNSGTGCRLVMGAVAGCPIEATFDGDASLRS | 143 |
| Query 128 | RPMGRVLNPLREMGVQVK-SEDGDRLPVTLRGPKTPTPITYRVPMASAQVKSAVLLAGLN | 186 |
| Sbjct 144 | RPM R+++PL MG +V SE+G RLP+TL+G + P PI Y+ P+ASAQ+KSAVLLAGL+ RPMRRIVDPLELMGAKVTASEEGGRLPLTLKGARDPLPIVYQTPVASAQIKSAVLLAGLS | 203 |
| Query 187 | TPGITTVIEPIMTRDHTEKMLQGFGANLTVETDADGVRTIRLEGRGKLTGQVIDVPGDPS | 246 |
| Sbjct 204 | PG TTVIE +RDHTE ML+ FGA + E + R I L G+ +L G + VP DPS APGTTTVIESEASRDHTELMLKHFGAEIVSEKEGAHGRRIMLTGQPELKGADVVVPADPS | 263 |
| Query 247 | STAFPLVAALLVPGSDVTILNVLMNPTRTGLILTLQEMGADIEVINPRLAGGEDVADLRV | 306 |
| Sbjct 264 | S AFP+VAAL+ PGS++ + +V+ NP RTGL TL+EMGA IE + R GE +A R+ SAAFPMVAALITPGSEIELTDVMTNPLRTGLFTTLREMGASIEESDIRGDAGEPMARFRI | 323 |
| Query 307 | RSSTLKGVTVPEDRAPSMIDEYPILAVAAAFAEGATVMNGLEELRVKESDRLSAVANGLK | 366 |
| Sbjct 324 | ++S LKGV VP +RAPSMIDEY +LAVAA+FA+G T+M GL+ELRVKESDRL A A ++ KASKLKGVEVPPERAPSMIDEYLVLAVAASFADGTTIMRGLQELRVKESDRLEATAAMIR | 383 |
| Query 367 | LNGVDCDEGETSLVVRGRPDGKGLGNASGAAVATHLDHRIAMSFLVMGLVSENPVTVDDA | 426 |
| Sbjct 384 | +NGV + L+V G G G VATH+DHRIAMS LVMG S+ PVT+DD VNGVKVEIVGDDLIVHGTGRVPG GGTVATHMDHRIAMSALVMGCASDAPVTIDDT | 438 |
| Query 427  Sbjct 439 | TMIATSFPEFMDLMAGLGA 445 T IATSFP+F+ +M LGA TFIATSFPDFIPMMRRLGA 457 |  |

```
>TR:A0A0D1M5H0 A0A0D1M5H0_9SPHN 3-phosphoshikimate 1-carboxyvinyltransferase OS=Sphingomonas melonis OX=152682 GN=aroA PE=3 SV=1
Length=451

Score = 475 bits (1223), Expect = 5e-163
Identities = 260/446 (58%), Positives = 308/446 (69%), Gaps = 3/446 (1%)

Query 1	MLHGASSRPATARKSSGLSGTVRIPGDKSISHRSFMFGGLASGETRITGLLEGEDVINTG 60 M + + RP +	+ L GTV +PGDKSISHRS MF LA GE+RI GLLEGEDV+ T
Sbjct 1	MSNASPPRPLSIAAAGPLRGTVTVPGDKSISHRSLMFSSLAVGESRIQGLLEGEDVLATA 60
```

| Query | 61 | KAMQAMGARIRKEGD-TWIIDGVGNGGLLAPEAPLDFGNAATGCRLTMGLVGVYDFDSTF | 119 |
| --- | --- | --- | --- |
| Sbjct | 61 | AM+AMGARI + D W + GVG GGLL P+ LD GN+ T RL MGLV + +TF AAMRAMGARIDRSDDGIWTVHGVGVGGLLQPQTALDMGNSGTSTRLLMGLVASHPITATF | 120 |
| Query | 120 | IGDASLTKRPMGRVLNPLREMGVQVKSEDGDRLPVTLRGPKTPTPITYRVPMASAQVKSA | 179 |
| Sbjct | 121 | +GDASL+KRPM RV PL +MG + G RLP+T+RG PITY +P+ASAQVKSA VGDASLSKRPMARVTEPLAQMGADFMASPGARLPLTMRGACPAVPITYTLPVASAQVKSA | 180 |
| Query | 180 | VLLAGLNTPGITTVIEPIMTRDHTEKMLQGFGANLTVETDADGVRTIRLEGRGKLTGQVI | 239 |
| Sbjct | 181 | VLLAGLNTPGIT VIEP+ TRDH+E+ML+GFGA LTVE DG + I + G +L Q I VLLAGLNTPGITRVIEPVPTRDHSERMLRGFGAELTVEDTPDG-KLIAIHGEAELKPQDI | 239 |
| Query | 240 | DVPGDPSSTAFPLVAALLVPGSDVTILNVLMNPTRTGLILTLQEMGADIEVINPRLAGGE | 299 |
| Sbjct | 240 | VPGDPSS F +VAA +VPGSDVTI NVLMNPTRTGL+ L+ MGA I+ ++ R GGE TVPGDPSSAGFWMVAASIVPGSDVTIRNVLMNPTRTGLLTALRLMGASIDEVDGREIGGE | 299 |
| Query | 300 | DVADLRVRSSTLKGVTVPEDRAPSMIDEYPILAVAAAFAEGATVMNGLEELRVKESDRLS | 359 |
| Sbjct | 300 | VADLRVR ++L + VP D APSMIDEYPIL VAAAFA G TV G ELRVKESDR++ RVADLRVRHASLSAIEVPADLAPSMIDEYPILFVAAAFARGTTVARGAHELRVKESDRIA | 359 |
| Query | 360 | AVANGLKLNGVDCDEGETSLVVRGRPDGKGLGNASGAAVATHLDHRIAMSFLVMGLVSEN | 419 |
| Sbjct | 360 | A+A L L GV E E L + GR DG L VA+ LDHRIAMS V G+ S AMAAALGLAGVSVTEHEDGLSIEGR-DGDPLPGTGSDRVASLLDHRIAMSMAVAGMHSTR | 418 |
| Query  Sbjct | 420  419 | PVTVDDATMIATSFPEFMDLMAGLGA 445 PV +DDA IATS+P F+D LGA PVRIDDAAPIATSYPAFVDTATRLGA 444 |  |

>TR:A0A418WT41 A0A418WT41_9PROT 3-phosphoshikimate 1-carboxyvinyltransferase OS=Oleomonas sp. K1W22B-8 OX=2320859 GN=aroA PE=3 SV=1

Length=441

Score = 475 bits (1222), Expect = 5e-163

| Identities | = 259/436 (59%), Positives = 303/436 (69%), Gaps = 6/436 (1%) |  |
| --- | --- | --- |
| Query 11 | TARKSSGLSGTVRIPGDKSISHRSFMFGGLASGETRITGLLEGEDVINTGKAMQAMGARI | 70 |
| Sbjct 5 | TA+ L+GTV +PGDKSISHR+ + GGLA G T +TGLLEG+DV+ T +AM+ +GA + TAQSCPALAGTVTVPGDKSISHRALILGGLAIGRTEVTGLLEGDDVLRTAEAMRRLGATV | 64 |
| Query 71 | RKEGDT-WIIDGVGNGGLLAPEAPLDFGNAATGCRLTMGLVGVYDFDSTFIGDASLTKRP | 129 |
| Sbjct 65 | + G W +DGVG G L P LD GN+ TG RL MGLV + + F GDASL KRP TRVGPGHWQVDGVGVGALSEPGDILDLGNSGTGARLLMGLVAGHPITAVFTGDASLNKRP | 124 |
| Query 130 | MGRVLNPLREMGVQVKSEDGDRLPVTLRGPKTPTPITYRVPMASAQVKSAVLLAGLNTPG | 189 |
| Sbjct 125 | M RV PL MG G+RLP+ + G + P PI YR+P+ASAQVKSA+LLAGLN PG MARVTEPLSRMGAGFVGRAGNRLPLAVVGARAPMPIEYRLPVASAQVKSAILLAGLNAPG | 184 |
| Query 190 | ITTVIEPIMTRDHTEKMLQGFGANLTVETDADGVRTIRLEGRGKLTGQVIDVPGDPSSTA | 249 |
| Sbjct 185 | T VIEP TRDH+E++L FGA + + ADG R + L G+ +L + VPGDPSS A ETIVIEPEPTRDHSERLLGHFGAVVKIVDLADGSRRVTLRGQPELIAAPVVVPGDPSSAA | 244 |
| Query 250 | FPLVAALLVPGSDVTILNVLMNPTRTGLILTLQEMGADIEVINPRLAGGEDVADLRVRSS | 309 |
| Sbjct 245 | FPLVA L+VPGSD+TI NV +NP R GL TL EMGA IE +N R AGGE VADLRVR S FPLVAGLIVPGSDITITNVGLNPLRIGLFDTLIEMGAQIEFLNKRNAGGEPVADLRVRHS | 304 |
| Query 310 | TLKGVTVPEDRAPSMIDEYPILAVAAAFAEGATVMNGLEELRVKESDRLSAVANGLKLNG | 369 |
| Sbjct 305 | LKGV VP RAPSMIDEYPIL+VAAAFA G T MNGL ELRVKESDRL+A+A GL NG VLKGVDVPASRAPSMIDEYPILSVAAAFATGTTRMNGLAELRVKESDRLAAIAAGLAANG | 364 |
| Query 370 | VDCDEGETSLVVRGRPDGKGLGNASGAAVATHLDHRIAMSFLVMGLVSENPVTVDDATMI | 429 |
| Sbjct 365 | V + G +LVV G+ G G VATHLDHRIAMSFLVMGL + PVTVDD MI  VAHEAGADTLVVAGKGTVAG GGTVATHLDHRIAMSFLVMGLAAARPVTVDDDHMI | 419 |

Query 430 ATSFPEFMDLMAGLGA 445

TSFP F LM LGA Sbjct 420 DTSFPGFAALMTALGA 435

>TR:A0A1H4X4L6 A0A1H4X4L6_9RHIZ 3-phosphoshikimate 1-carboxyvinyltransferase OS=Beijerinckia sp. 28-YEA-48 OX=1882748 GN=aroA PE=3 SV=1

Length=472

Score = 476 bits (1225), Expect = 5e-163

| Identities | = 262/440 (60%), Positives = 308/440 (70%), Gaps = 3/440 (1%) |  |
| --- | --- | --- |
| Query 9 | PATARKSSGLSGTVRIPGDKSISHRSFMFGGLASGETRITGLLEGEDVINTGKAMQAMGA | 68 |
| Sbjct 31 | P TA S L+G +R PGDKSISHR+ + G LA G T I GLLEG+DV+ T A +A+GA PLTAAASGPLTGRLRAPGDKSISHRALILGLLAVGRTTIEGLLEGDDVLRTADACRALGA | 90 |
| Query 69 | RIRKEGD-TWIIDGVGNGGLLAPEAPLDFGNAATGCRLTMGLVGVYDFDSTFIGDASLTK | 127 |
| Sbjct 91 | ++ + G W +DG G G L P LDFGNA TG RL MG+V + ++TF GDASL K KVERTGPGQWTVDGAGLGTLQTPRDVLDFGNAGTGTRLMMGVVAGHPIEATFDGDASLRK | 150 |
| Query 128 | RPMGRVLNPLREMGVQVKSE-DGDRLPVTLRGPKTPTPITYRVPMASAQVKSAVLLAGLN | 186 |
| Sbjct 151 | RPM R+L+PL MGVQV +E +G R P+ LRG P PI YR P+ SAQ+KSA+LLAGLN RPMRRILDPLALMGVQVVAESEGGRCPIVLRGTGDPAPIEYRTPVPSAQLKSAILLAGLN | 210 |
| Query 187 | TPGITTVIEPIMTRDHTEKMLQGFGANLTVETDADGVRTIRLEGRGKLTGQVIDVPGDPS | 246 |
| Sbjct 211 | +PG TTVIE +RDHTEKML FGAN+ D R I L GR +L + + VP DPS SPGQTTVIETEASRDHTEKMLAYFGANIVSTPDGPHGRKIVLAGRPELKARDVIVPADPS | 270 |
| Query 247 | STAFPLVAALLVPGSDVTILNVLMNPTRTGLILTLQEMGADIEVINPRLAGGEDVADLRV | 306 |
| Sbjct 271 | S AF L AALLVP SDV I ++MNP R GL+ TLQEM A I+V+N R+ GGEDVADLRV SAAFALTAALLVPDSDVIIEGMMMNPLRIGLLTTLQEMDARIDVLNARIEGGEDVADLRV | 330 |
| Query 307 | RSSTLKGVTVPEDRAPSMIDEYPILAVAAAFAEGATVMNGLEELRVKESDRLSAVANGLK | 366 |
| Sbjct 331 | R+S LKGV VP RAP+MIDEYPILAV AAFA+G T M GL ELRVKESDRL+AVA+GL RTSQLKGVDVPAARAPAMIDEYPILAVVAAFAQGETRMRGLAELRVKESDRLAAVADGLI | 390 |
| Query 367 | LNGVDCDEGETSLVVRGRPDGKGLGNASGAAVATHLDHRIAMSFLVMGLVSENPVTVDDA | 426 |
| Sbjct 391 | GV C L+V G DG G+ G V THLDHRIAMSFLVMGL S+ P+T+DDA AAGVTCRIEGDDLIVTGGQDGPGIVR-GGGTVKTHLDHRIAMSFLVMGLASQQPMTIDDA | 449 |
| Query 427  Sbjct 450 | TMIATSFPEFMDLMAGLGAK 446 MIATSFP F M LGAK  AMIATSFPTFQPEMQRLGAK 469 |  |

>TR:A0A257ELK4 A0A257ELK4_9PROT 3-phosphoshikimate 1-carboxyvinyltransferase OS=Alphaproteobacteria bacterium PA4 OX=2015572 GN=aroA PE=3

SV=1

Length=447

Score = 475 bits (1222), Expect = 7e-163

| Identities | = 253/442 (57%), Positives = 309/442 (70%), Gaps = 4/442 (1%) |  |
| --- | --- | --- |
| Query 5 | ASSRPATARKSSGLSGTVRIPGDKSISHRSFMFGGLASGETRITGLLEGEDVINTGKAMQ | 64 |
| Sbjct 7 | ++ +P T + LSG V +PGDKSISHRS M G LA GET ITGLLEGEDV+ T AM+ SAPQPLTLATTGPLSGRVTVPGDKSISHRSLMLGALAVGETVITGLLEGEDVLATAAAMR | 66 |
| Query 65 | AMGARIRKEGDTWIIDGVGNGGLLAPEAPLDFGNAATGCRLTMGLVGVYDFDSTFIGDAS | 124 |
| Sbjct 67 | AMGA I + W + GVG GGLL P LD GN+ T RL MGL+ + +TF+GDAS AMGAIIERREARWHVHGVGVGGLLQPGQALDMGNSGTSTRLLMGLIASHPITATFVGDAS | 126 |
| Query 125 | LTKRPMGRVLNPLREMGVQVKSEDGDRLPVTLRGPKTPTPITYRVPMASAQVKSAVLLAG | 184 |
| Sbjct 127 | L+KRPMGRV++PL MG + G RLP+T++G P+ Y +P+ASAQVKSAVLLAG  LSKRPMGRVIDPLSLMGASFTASPGGRLPLTMQGRCPAVPLRYTLPVASAQVKSAVLLAG | 186 |

| Query | 185 | LNTPGITTVIEPIMTRDHTEKMLQGFGANLTVETDADGVRTIRLEGRGKLTGQVIDVPGD | 244 |
| --- | --- | --- | --- |
| Sbjct | 187 | LNTPG T V+EP+ TRDH+E+MLQGFGA L VE +G R I L G +L Q I VPGD LNTPGQTVVVEPVATRDHSERMLQGFGAALNVEQAPEG-RIITLMGEAELRPQTITVPGD | 245 |
| Query | 245 | PSSTAFPLVAALLVPGSDVTILNVLMNPTRTGLILTLQEMGADIEVINPRLAGGEDVADL | 304 |
| Sbjct | 246 | PSS AFP+VAAL+VPGSD+ I NV MNPTR GL+ LQ MG DI V+NPR GGE VADL PSSAAFPMVAALIVPGSDIMIENVGMNPTRAGLVTVLQAMGGDITVMNPRSVGGEPVADL | 305 |
| Query | 305 | RVRSSTLKGVTVPEDRAPSMIDEYPILAVAAAFAEGATVMNGLEELRVKESDRLSAVANG | 364 |
| Sbjct | 306 | RVR S L G+ +P D A +MIDEYP+L VAAA A+G++ + GLEELRVKESDR++ +A G RVRHSVLTGIDLPGDDAAAMIDEYPVLFVAAACAQGSSQLRGLEELRVKESDRIAVMAAG | 365 |
| Query | 365 | LKLNGVDCDEGETSLVVRGRPDGKGLGNASGAAVATHLDHRIAMSFLVMGLVSENPVTVD | 424 |
| Sbjct | 366 | L GV C E + + G G GA +A HLDHRIAMSF V+GL + PVT+D LAACGVPCKELADGITITG---SGGDPLPGGATIAAHLDHRIAMSFAVLGLNARAPVTID | 422 |
| Query  Sbjct | 425  423 | DATMIATSFPEFMDLMAGLGAK 446 D+ IATSFP F+ +M LGA+ DSRPIATSFPTFVAMMQALGAE 444 |  |

>TR:A0A2K9EUG4 A0A2K9EUG4_9RHOB 3-phosphoshikimate 1-carboxyvinyltransferase OS=Paracoccus sp. BM15 OX=1529068 GN=aroA PE=3 SV=1

Length=443

Score = 474 bits (1221), Expect = 8e-163

| Identities | = 249/448 (56%), Positives = 309/448 (69%), Gaps = 7/448 (2%) |  |
| --- | --- | --- |
| Query 1 | MLHGASSRPATARKSSGLSGTVRIPGDKSISHRSFMFGGLASGETRITGLLEGEDVINTG | 60 |
| Sbjct 1 | M H + RP TAR+ L+G +PGDKSISHR+ + G L+ GET ITGLLEG+DV++T MSHASEPRPMTARRRGALTGVANVPGDKSISHRALILGALSVGETHITGLLEGQDVLDTA | 60 |
| Query 61 | KAMQAMGARIRKEGD-TWIIDGVGNGGLLAPEAPLDFGNAATGCRLTMGLVGVYDFDSTF | 119 |
| Sbjct 61 | KAM A GA +++ GD W + GVG GG P +D GN+ T RL MG + +TF KAMAAFGADVQRIGDGEWRVHGVGVGGFAEPADVIDCGNSGTSVRLIMGAMATTPITATF | 120 |
| Query 120 | IGDASLTKRPMGRVLNPLREMGVQVKSEDGDRLPVTLRGPKTPTPITYRVPMASAQVKSA | 179 |
| Sbjct 121 | GDASL+KRPM R+ +PL E G Q+ + +G RLPVT+ G P P+ Y+ P+ASAQ+KSA TGDASLSKRPMARITDPLSEFGAQIAAREGGRLPVTIGGATDPVPLRYKTPVASAQIKSA | 180 |
| Query 180 | VLLAGLNTPGITTVIEPIMTRDHTEKMLQGFGANLTVETDADGVRTIRLEGRGKLTGQVI | 239 |
| Sbjct 181 | +LLAGLN PG T VIE TRDH+E+ML GFGA + E +G I L GR +L GQ + ILLAGLNAPGETVVIESEPTRDHSERMLAGFGATIRTEQTEEG-HVITLTGRPELRGQPV | 239 |
| Query 240 | DVPGDPSSTAFPLVAALLVPGSDVTILNVLMNPTRTGLILTLQEMGADIEVINPRLAGGE | 299 |
| Sbjct 240 | VP DPSS AFP+ AAL+VPGS++ + V NPTR GL +TL+EMGADI N R GGE AVPRDPSSAAFPVAAALIVPGSEIRVPGVSRNPTRDGLYVTLKEMGADIGFENQREEGGE | 299 |
| Query 300 | DVADLRVRSSTLKGVTVPEDRAPSMIDEYPILAVAAAFAEGATVMNGLEELRVKESDRLS | 359 |
| Sbjct 300 | VADL VR S L GV VP DRA SMIDE+PIL+V AAFA+G TVMNG+ ELRVKESDR+ PVADLVVRHSPLSGVDVPADRAASMIDEFPILSVVAAFAQGKTVMNGVAELRVKESDRID | 359 |
| Query 360 | AVANGLKLNGVDCDEGETSLVVRGRPDGKGLGNASGAAVATHLDHRIAMSFLVMGLVSEN | 419 |
| Sbjct 360 | A+A GL+ NGV +E S+ V G G GA ATHLDHRIAM+F+V+GL SE AMARGLEANGVTIEETRDSMTVHGMAKVPG GAEAATHLDHRIAMAFMVLGLASEA | 414 |
| Query 420  Sbjct 415 | PVTVDDATMIATSFPEFMDLMAGLGAKI 447 PV+VDD + I TSFP+F+ LM GLGA + PVSVDDGSPIETSFPDFISLMQGLGADL 442 |  |

>TR:B8IK64 B8IK64_METNO 3-phosphoshikimate 1-carboxyvinyltransferase OS=Methylobacterium nodulans (strain LMG 21967 / CNCM I-2342 /

ORS 2060) OX=460265 GN=aroA PE=3 SV=1

Length=466

Score = 475 bits (1223), Expect = 9e-163

| Identities | = 269/449 (60%), Positives = 315/449 (70%), Gaps = 12/449 (3%) |  |
| --- | --- | --- |
| Query 3 | HGASSRPATARKSSGLSGTVRIPGDKSISHRSFMFGGLASGETRITGLLEGEDVINTGKA | 62 |
| Sbjct 20 | H ++ P AR + L G +R PGDKSISHR+ + G L+ GETRI GLLEG+DV+ T A HDSAPSPIAARAGTPLRGRLRPPGDKSISHRAMILGLLSIGETRIEGLLEGDDVLRTAAA | 79 |
| Query 63 | MQAMGARIRKEGD-TWIIDGVGNGGLLAPEAPLDFGNAATGCRLTMGLVGVYDFDSTFIG | 121 |
| Sbjct 80 | +A+GA I ++G W + GVG GGL PE LDFGNA TG RL MG+VG +TF G ARALGAGIDRDGPGRWRVRGVGIGGLSDPEGVLDFGNAGTGSRLMMGVVGGQPVTATFDG | 139 |
| Query 122 | DASLTKRPMGRVLNPLREMGVQVKSE-DGDRLPVTLRGPKTPTPITYRVPMASAQVKSAV | 180 |
| Sbjct 140 | DASL KRPM R+L+PL +MG V ++ +G R+P+TLRGP PITY P+ASAQVKSAV DASLRKRPMRRILDPLVQMGATVVAQQEGGRVPLTLRGPDEAIPITYETPVASAQVKSAV | 199 |
| Query 181 | LLAGLNTPGITTVIEPIMTRDHTEKMLQGFGANLTVET---DADGVRTIRLEGRGKLTGQ | 237 |
| Sbjct 200 | LLAGLN PG TTVIE TRDHTE+ML+ FGA + V + G R I L G+ L LLAGLNAPGTTTVIEAAATRDHTERMLRLFGAEVAVSAHGPEGHG-RAIALTGQPTLRAA | 258 |
| Query 238 | VIDVPGDPSSTAFPLVAALLVPGSDVTILNVLMNPTRTGLILTLQEMGADIEVINPRLAG | 297 |
| Sbjct 259 | + VP DPSS AFPLVAALLVPGSDV I V+MNP R GLI TL EMGADI +N R G EVMVPADPSSAAFPLVAALLVPGSDVVIEGVMMNPLRIGLITTLLEMGADITRLNEREEG | 318 |
| Query 298 | GEDVADLRVRSSTLKGVTVPEDRAPSMIDEYPILAVAAAFAEGATVMNGLEELRVKESDR | 357 |
| Sbjct 319 | GE VADLRVR+ L GVTVP +RAP+MIDEYP+LAVAAAFA+G T M GL ELRVKESDR GETVADLRVRACRLTGVTVPPERAPAMIDEYPVLAVAAAFAQGTTRMQGLHELRVKESDR | 378 |
| Query 358 | LSAVANGLKLNGV-DCDEGETSLVVRGRPDGKGLGNASGAAVATHLDHRIAMSFLVMGLV | 416 |
| Sbjct 379 | L+AVA GLK NGV EG+ +V G G A G VATHLDHRIAM+FLVMGL LAAVAAGLKANGVAHVVEGDDLIV HGDGGAAAGGGTVATHLDHRIAMAFLVMGLA | 433 |
| Query 417  Sbjct 434 | SENPVTVDDATMIATSFPEFMDLMAGLGA 445 S +PVTVDD MIATS+P F+ + LGA SRDPVTVDDGAMIATSYPSFLADLRALGA 462 |  |

>TR:V7EFJ2 V7EFJ2_9RHOB 3-phosphoshikimate 1-carboxyvinyltransferase OS=Rhodobacter sp. CACIA14H1 OX=1408890 GN=aroA PE=3 SV=1

Length=461

Score = 475 bits (1222), Expect = 1e-162

| Identities | = 259/447 (58%), Positives = 312/447 (70%), Gaps = 7/447 (2%) |  |
| --- | --- | --- |
| Query 3 | HGASSRPATARKSSGLSGTVRIPGDKSISHRSFMFGGLASGETRITGLLEGEDVINTGKA | 62 |
| Sbjct 17 | HG ++P TARKS L G +PGDKSISHR+ +FG +A GETR+TGLLEG+DV++T KA HG-DAQPMTARKSGPLRGVAEVPGDKSISHRALIFGAMAVGETRVTGLLEGQDVLDTAKA | 75 |
| Query 63 | MQAMGARIRKEGD-TWIIDGVGNGGLLAPEAPLDFGNAATGCRLTMGLVGVYDFDSTFIG | 121 |

M+A GA + + G	W + GVG GG	P	+D GN+ TG RL MG +	+TF G

| Sbjct | 76 | MRAFGADVIRHGQGEWSVHGVGVGGFREPSDVIDCGNSGTGVRLIMGTMATTAMTATFTG | 135 |
| --- | --- | --- | --- |
| Query | 122 | DASLTKRPMGRVLNPLREMGVQVKSEDGDRLPVTLRGPKTPTPITYRVPMASAQVKSAVL | 181 |
| Sbjct | 136 | DASL KRPMGRV +PL G + G RLP+T+ G P P+ Y +P+ASAQVKSAVL DASLRKRPMGRVTDPLSLFGARAYGRSGGRLPMTVVGAADPVPVRYALPVASAQVKSAVL | 195 |
| Query | 182 | LAGLNTPGITTVIEPIMTRDHTEKMLQGFGANLTVETDADGVRTIRLEGRGKLTGQVIDV | 241 |
| Sbjct | 196 | LAGLN PG T VIE TRDH+E+ML GFGA +TVE A+G I L GR +L Q + V LAGLNAPGQTVVIEKEPTRDHSERMLLGFGAEVTVEKTAEG-NVITLTGRPELRPQTVAV | 254 |
| Query | 242 | PGDPSSTAFPLVAALLVPGSDVTILNVLMNPTRTGLILTLQEMGADIEVINPRLAGGEDV P DPSS AFP AAL+V GSD+T+ V N TR GL LTL EMGADIE +NPR GGE V | 301 |

| Sbjct | 255 | PRDPSSAAFPTCAALIVEGSDITVPGVSQNLTRNGLYLTLVEMGADIEFMNPREEGGEPV | 314 |
| --- | --- | --- | --- |
| Query | 302 | ADLRVR-SSTLKGVTVPEDRAPSMIDEYPILAVAAAFAEGATVMNGLEELRVKESDRLSA | 360 |
| Sbjct | 315 | ADLRVR S LKG+ VPE+RAPSMIDEYP+L+V AAFAEG TVM G++ELRVKESDR+ A ADLRVRFSPDLKGIEVPEERAPSMIDEYPVLSVVAAFAEGRTVMRGVKELRVKESDRIDA | 374 |
| Query | 361 | VANGLKLNGVDCDEGETSLVVRGRPDGKGLGNASGAAVATHLDHRIAMSFLVMGLVSENP | 420 |
| Sbjct | 375 | +A GL+ GV +E E +L+V G G G GA ATHLDHRIAMSFLV G+ ++ MARGLEACGVRVEEDEDTLIVHGLGAG---GVPGGATCATHLDHRIAMSFLVCGMAAKTA | 431 |
| Query  Sbjct | 421  432 | VTVDDATMIATSFPEFMDLMAGLGAKI 447 V+VDDA+ I TSFP F LM GLGA + VSVDDASPILTSFPIFEGLMTGLGAAL 458 |  |

>TR:A0A0M3QD18 A0A0M3QD18_SPHS1 3-phosphoshikimate 1-carboxyvinyltransferase OS=Sphingopyxis sp. (strain 113P3) OX=292913 GN=aroA PE=3 SV=1

Length=442

Score = 474 bits (1220), Expect = 1e-162

| Identities | = 256/438 (58%), Positives = 304/438 (69%), Gaps = 4/438 (1%) |  |
| --- | --- | --- |
| Query 7 | SRPATARKSSGLSGTVRIPGDKSISHRSFMFGGLASGETRITGLLEGEDVINTGKAMQAM | 66 |
| Sbjct 6 | S P S L+G + IPGDKSISHR+ M LA G +R+TGLLEG DV+ T AM+AM SSPRRFCASPPLTGRIAIPGDKSISHRALMLSALAVGTSRVTGLLEGHDVLATAAAMRAM | 65 |
| Query 67 | GARIRKEGD-TWIIDGVGNGGLLAPEAPLDFGNAATGCRLTMGLVGVYDFDSTFIGDASL | 125 |
| Sbjct 66 | GARI + G+ W IDGVG G LL P LD GN+ T RL MGLV + TF GDASL GARIEQTGEGDWEIDGVGVGSLLQPAGALDMGNSGTSTRLLMGLVASHPITVTFTGDASL | 125 |
| Query 126 | TKRPMGRVLNPLREMGVQVKSEDGDRLPVTLRGPKTPTPITYRVPMASAQVKSAVLLAGL | 185 |
| Sbjct 126 | + RPMGRV+ PL +MG + + G RLP+ +RG P+ YR+PMASAQVKSAVLLA L SGRPMGRVIEPLSQMGADITASPGGRLPLMVRGLAPAIPLEYRLPMASAQVKSAVLLAAL | 185 |
| Query 186 | NTPGITTVIEPIMTRDHTEKMLQGFGANLTVETDADGVRTIRLEGRGKLTGQVIDVPGDP | 245 |
| Sbjct 186 | NTPGIT VIEP+ TRDH+E+MLQGFGA L VETDADG R IR+ G +L Q I VPGDP NTPGITEVIEPVPTRDHSERMLQGFGAELNVETDADGARHIRIRGEAELKPQAIVVPGDP | 245 |
| Query 246 | SSTAFPLVAALLVPGSDVTILNVLMNPTRTGLILTLQEMGADIEVINPRLAGGEDVADLR | 305 |
| Sbjct 246 | SS AF +VAALLVPGSDVTILNV +NPTR GL+ L++MG DI +++ R GGE VADLR SSAAFFIVAALLVPGSDVTILNVGLNPTRAGLVEILRQMGGDITLLDRREVGGEPVADLR | 305 |
| Query 306 | VRSSTLKGVTVPEDRAPSMIDEYPILAVAAAFAEGATVMNGLEELRVKESDRLSAVANGL | 365 |
| Sbjct 306 | VR S LKG+ V APSMIDE+PI VAAA A+G TV GL+ELRVKESDRL+ +A GL VRHSALKGIEVDPAAAPSMIDEFPIFFVAAALAQGRTVTTGLDELRVKESDRLAVMAAGL | 365 |
| Query 366 | KLNGVDCDEGETSLVVRGRPDGKGLGNASGAAVATHLDHRIAMSFLVMGLVSENPVTVDD | 425 |

+ G	+E	LV+ G	G	GA +A HLDHRI MSF V GLVS+ P+ +DD

| Sbjct | 366 | EAIGARVEESADGLVIDG---SGGEPLPGGAVIAGHLDHRICMSFAVAGLVSKAPIEIDD | 422 |
| --- | --- | --- | --- |
| Query | 426 | ATMIATSFPEFMDLMAGL 443  +ATSFP F L+ GL |  |
| Sbjct | 423 | MAPVATSFPNFEGLLEGL 440 | |

```
>TR:A0A0Q8QRC3 A0A0Q8QRC3_9SPHN 3-phosphoshikimate 1-carboxyvinyltransferase OS=Sphingomonas sp. Root720 OX=1736595 GN=aroA PE=3 SV=1
Length=445

Score = 474 bits (1220), Expect = 1e-162
Identities = 257/445 (58%), Positives = 305/445 (69%), Gaps = 4/445 (1%)

Query 2	LHGASSRPATARKSSGLSGTVRIPGDKSISHRSFMFGGLASGETRITGLLEGEDVINTGK 61
+H + RPA+	L G	+PGDKSISHR+ M	LA GE+R+ GLLEGEDV+ T
```

| Sbjct | 1 | MHDPAPRPASFSAPGPLRGRATVPGDKSISHRALMLSALAVGESRVEGLLEGEDVLATAA | 60 |
| --- | --- | --- | --- |
| Query | 62 | AMQAMGARIRKEGDT-WIIDGVGNGGLLAPEAPLDFGNAATGCRLTMGLVGVYDFDSTFI | 120 |
| Sbjct | 61 | AM+AMGARI + D W I GVG GGLL PE LD GN+ T RL MGL+ + +TF AMRAMGARIERGADAAWRIHGVGVGGLLQPETALDMGNSGTSTRLLMGLIASHAVTATFT | 120 |
| Query | 121 | GDASLTKRPMGRVLNPLREMGVQVKSEDGDRLPVTLRGPKTPTPITYRVPMASAQVKSAV | 180 |
| Sbjct | 121 | GDASL+KRPM RV+ PL MG V + G RLP+ LRG PI+YR+P+ASAQVKSAV GDASLSKRPMNRVIEPLALMGADVTASPGGRLPLMLRGLCPAVPISYRLPVASAQVKSAV | 180 |
| Query | 181 | LLAGLNTPGITTVIEPIMTRDHTEKMLQGFGANLTVETDADGVRTIRLEGRGKLTGQVID | 240 |
| Sbjct | 181 | LLAGLNTPGITTVIEP+ TRDH+E+ML+GFGA L+VE D DG R I + G +L Q I LLAGLNTPGITTVIEPVATRDHSERMLKGFGAELSVEQDGDGARVITIRGEAELKPQHIV | 240 |
| Query | 241 | VPGDPSSTAFPLVAALLVPGSDVTILNVLMNPTRTGLILTLQEMGADIEVINPRLAGGED | 300 |
| Sbjct | 241 | VPGDPSS AFP+VAALLVPGS+VTI NV +NPTR L L+ MG DIE N R GGE VPGDPSSAAFPVVAALLVPGSEVTIANVGLNPTRAALFDVLRAMGGDIEFANRREVGGEP | 300 |
| Query | 301 | VADLRVRSSTLKGVTVPEDRAPSMIDEYPILAVAAAFAEGATVMNGLEELRVKESDRLSA | 360 |
| Sbjct | 301 | VAD+ VR S LKGV P + +M+DEYP+L VAAA AEG TV GLEELRVKESDR++ VADIVVRHSILKGVETPVESVAAMVDEYPVLFVAAALAEGRTVARGLEELRVKESDRIAV | 360 |
| Query | 361 | VANGLKLNGVDCDEGETSLVVRGRPDGKGLGNASGAAVATHLDHRIAMSFLVMGLVSENP | 420 |
| Sbjct | 361 | +A GL+ G +E E L++ G G GA +A LDHRIAMSF V GLVS+ MAEGLRAIGARVEELEDGLIIDGT---GGDPLPGGATIAARLDHRIAMSFAVAGLVSKAA | 417 |
| Query  Sbjct | 421  418 | VTVDDATMIATSFPEFMDLMAGLGA 445 VT+DD +ATSFP F LM LGA VTIDDIGPVATSFPGFAKLMTDLGA 442 |  |

>TR:A0A0D6JE41 A0A0D6JE41_9RHIZ 3-phosphoshikimate 1-carboxyvinyltransferase OS=Candidatus Filomicrobium marinum OX=1608628 GN=aroA PE=3

SV=1

Length=445

Score = 474 bits (1220), Expect = 1e-162

| Identities | = 251/441 (57%), Positives = 307/441 (70%), Gaps = 5/441 (1%) |  |
| --- | --- | --- |
| Query 8 | RPATARKSSGLSGTVRIPGDKSISHRSFMFGGLASGETRITGLLEGEDVINTGKAMQAMG | 67 |
| Sbjct 6 | +P ++ +S LSG + +PGDKSISHR+ +FG L+ G TRITGLLE EDV+NT K + A+G KPLSSTRSGPLSGQLTVPGDKSISHRALLFGALSLGTTRITGLLESEDVLNTAKVVSALG | 65 |
| Query 68 | ARIRKEGDTWIIDGVGNGGLLAPEAPLDFGNAATGCRLTMGLVGVYDFDSTFIGDASLTK | 127 |
| Sbjct 66 | A K D W + G G GG P+ PLDFGN+ TG RL MG+V IGD SL++ APATKTKDVWQVQGRGLGGFQQPDVPLDFGNSGTGARLAMGVVAGQAVSVEMIGDESLSR | 125 |
| Query 128 | RPMGRVLNPLREMGVQVKSEDGDRLPVTLRGPKTPTPITYRVPMASAQVKSAVLLAGLNT | 187 |
| Sbjct 126 | RPM RVLNPL++MG+QV+++D + LP+ LRG PI Y +P+ SAQVKSAVL+AGL RPMSRVLNPLKQMGLQVENKDRETLPLKLRGTVDLVPIEYELPVPSAQVKSAVLIAGLQA | 185 |
| Query 188 | PGITTVIEPIMTRDHTEKMLQGFGANLTVETDADGVRTIRLEGRGKLTGQVIDVPGDPSS | 247 |
| Sbjct 186 | G TTVIE TRDHTE+ML+ FGA +T DG I + G ++ G+ + VP DPSS TGTTTVIETEPTRDHTERMLRFFGAEITTAAGGDGKTRISVTGGQQMQGREVAVPADPSS | 245 |
| Query 248 | TAFPLVAALLVPGSDVTILNVLMNPTRTGLILTLQEMGADIEVINPRLAGGEDVADLRVR | 307 |
| Sbjct 246 | AF AA+LVPGSDVTI VL+N TR G TLQEMGAD+ N R GGE +AD+RVR AAFLAAAAVLVPGSDVTIEGVLINDTRIGFYKTLQEMGADLTYANKREEGGEQIADIRVR | 305 |
| Query 308 | SSTLKGVTVPEDRAPSMIDEYPILAVAAAFAEGATVMNGLEELRVKESDRLSAVANGLKL | 367 |
| Sbjct 306 | S LKGV VP +RAPSMIDEYPILAV AAFAEG TVM GL EL+VKESDRL+A A GL++ HSALKGVRVPPERAPSMIDEYPILAVVAAFAEGETVMEGLAELKVKESDRLAATAAGLEV | 365 |
| Query 368 | NGVDCDEGETSLVVRGRPDGKGLGNASGAAVATHLDHRIAMSFLVMGLVSENPVTVDDAT | 427 |

NGV+	+L+V G	G	G VATHLDHRIAM+FLVMG SE PV VDD +

| Sbjct | 366 | NGVNARVEGDALIVVGSDRVSG-----GGVVATHLDHRIAMAFLVMGFASEKPVKVDDTS 420 |
| --- | --- | --- |
| Query | 428 | MIATSFPEFMDLMAGLGAKIE 448 |
| Sbjct | 421 | MIATSFPEF+ L+ LGA+ +  MIATSFPEFIGLLEKLGARFD 441 |

>TR:A0A1D9MDT8 A0A1D9MDT8_9RHOB 3-phosphoshikimate 1-carboxyvinyltransferase OS=Rhodobacter sp. LPB0142 OX=1850250 GN=aroA PE=3 SV=1

Length=447

Score = 474 bits (1220), Expect = 1e-162

| Identities | | = 249/441 (56%), Positives = 308/441 (70%), Gaps = 5/441 (1%) |  |
| --- | --- | --- | --- |
| Query 9  Sbjct 9 | | PATARKSSGLSGTVRIPGDKSISHRSFMFGGLASGETRITGLLEGEDVINTGKAMQAMGA P +RK L+G +PGDKSISHR+ + G L+ G T ITGLLEG+DV++T KAM+A GA PMKSRKGGPLAGVAEVPGDKSISHRALILGALSVGRTEITGLLEGQDVLDTAKAMRAFGA | 68  68 |
| Query 69 | | RIRKEGD-TWIIDGVGNGGLLAPEAPLDFGNAATGCRLTMGLVGVYDFDSTFIGDASLTK  + + G W ++GVG GG P +D GN+ TG RL MG + +TF GDASL K | 127 |
| Sbjct | 69 | TVTRHGPGEWSVEGVGVGGFAEPADVIDCGNSGTGVRLIMGCMASSPISATFTGDASLRK | 128 |
| Query | 128 | RPMGRVLNPLREMGVQVKSEDGDRLPVTLRGPKTPTPITYRVPMASAQVKSAVLLAGLNT | 187 |
| Sbjct | 129 | RPMGRV +PL G + G RLP+T+ G P P++Y PMASAQVKSAVLLAGLN RPMGRVTDPLALFGARAYGRAGGRLPMTIVGAAEPVPVSYTTPMASAQVKSAVLLAGLNA | 188 |
| Query | 188 | PGITTVIEPIMTRDHTEKMLQGFGANLTVETDADGVRTIRLEGRGKLTGQVIDVPGDPSS | 247 |
| Sbjct | 189 | PG T VIE + TRDHTE+ML+GFGA +TVE +G R I L+G+ +LT Q + VP DPSS PGQTVVIEKVPTRDHTERMLKGFGAEITVEATPEGNR-ITLQGQPELTAQRVAVPRDPSS | 247 |
| Query | 248 | TAFPLVAALLVPGSDVTILNVLMNPTRTGLILTLQEMGADIEVINPRLAGGEDVADLRVR | 307 |
| Sbjct | 248 | AFP+ AALLVPGSD+ + V NPTR GL LTL++MGADIE +NPR GGE VADLRVR AAFPVCAALLVPGSDILVPGVSQNPTRNGLFLTLRDMGADIEFLNPREEGGEPVADLRVR | 307 |
| Query | 308 | SSTLKGVTVPEDRAPSMIDEYPILAVAAAFAEGATVMNGLEELRVKESDRLSAVANGLKL | 367 |
| Sbjct | 308 | S L G+ V +RA S IDEYP+L+V AA A G TVM G+ ELRVKESDR+ A+A GL+ YSELTGIDVAPERASSQIDEYPVLSVVAACARGTTVMRGVHELRVKESDRIDAMARGLEA | 367 |
| Query | 368 | NGVDCDEGETSLVVRGRPDGKGLGNASGAAVATHLDHRIAMSFLVMGLVSENPVTVDDAT | 427 |
| Sbjct | 368 | GV +E + +L+V G G G GA ATH+DHRIAMSFL++GL ++ P+ VDD + CGVRVEEDDDTLIVHGMGPG---GVPGGATCATHIDHRIAMSFLILGLAAQAPIAVDDGS | 424 |
| Query | 428 | MIATSFPEFMDLMAGLGAKIE 448 IATSFP F +LMAGLGA IE |  |
| Sbjct | 425 | PIATSFPVFEELMAGLGAVIE 445 | |

>TR:A0A419MYP5 A0A419MYP5_9SPHN 3-phosphoshikimate 1-carboxyvinyltransferase OS=Aestuariisphingobium litorale OX=2339262 GN=aroA PE=3 SV=1

Length=447

Score = 474 bits (1220), Expect = 1e-162

Identities = 258/446 (58%), Positives = 308/446 (69%), Gaps = 8/446 (2%)

| Query | 6 | SSRPATARKSSGLSGTVRIPGDKSISHRSFMFGGLASGETRITGLLEGEDVINTGKAMQA | 65 |
| --- | --- | --- | --- |
| Sbjct | 5 | S P + +S LSGTVR+PGDKSISHRS M LA GE+ I GLLEGEDV+ T AM+A SPSPKSFARSGPLSGTVRVPGDKSISHRSLMLSALAVGESVIEGLLEGEDVLATAAAMRA | 64 |
| Query | 66 | MGARIRKEGD-TWIIDGVGNGGLLAPEAPLDFGNAATGCRLTMGLVGVYDFDSTFIGDAS | 124 |
| Sbjct | 65 | MGA+I + D W + GVG GGLL PE+ LD GN+ T RL MGLV + +TF GDAS MGAQITRGADRVWRVHGVGVGGLLQPESALDMGNSGTSTRLLMGLVASHAITATFTGDAS | 124 |
| Query | 125 | LTKRPMGRVLNPLREMGVQVKSEDGDR LPVTLRGPKTPTPITYRVPMASAQVKSAV | 180 |

L+KRPM RV +PL MG	G++	LP+ +RG	PITYR+PMASAQVKSA+

| Sbjct | 125 | LSKRPMARVTDPLGTMGAVFTCSPGEKGSQYLPIMVRGACPAVPITYRLPMASAQVKSAI | 184 |
| --- | --- | --- | --- |
| Query | 181 | LLAGLNTPGITTVIEPIMTRDHTEKMLQGFGANLTVETDADGVRTIRLEGRGKLTGQVID | 240 |
| Sbjct | 185 | LLAGLNTPGITTVIEP+ TRDH+E+ML GFGA L + TD DG R I + G +L Q + LLAGLNTPGITTVIEPVPTRDHSERMLAGFGAKLDISTDDDGARVISIHGEAELKPQHVV | 244 |
| Query | 241 | VPGDPSSTAFPLVAALLVPGSDVTILNVLMNPTRTGLILTLQEMGADIEVINPRLAGGED | 300 |
| Sbjct | 245 | VPGDPSS AFP+VAALLVPGS+V I NV +NPTR GLI LQ MG DI ++ R GGE VPGDPSSAAFPMVAALLVPGSEVRIDNVGINPTRAGLIEVLQAMGGDITLVAERTVGGEP | 304 |
| Query | 301 | VADLRVRSSTLKGVTVPEDRAPSMIDEYPILAVAAAFAEGATVMNGLEELRVKESDRLSA | 360 |
| Sbjct | 305 | VADL V+ STLKG+ D PSM+DE+PIL VAAA AEG +V GLEELRVKESDR+S VADLIVKHSTLKGIETSPDIVPSMVDEFPILFVAAALAEGTSVFRGLEELRVKESDRISV | 364 |
| Query | 361 | VANGLKLNGVDCDEGETSLVVRGRPDGKGLGNASGAAVATHLDHRIAMSFLVMGLVSENP | 420 |
| Sbjct | 365 | +A GL GV +E E L+V G G A G +A LDHRIAMSF + GLVS P MAKGLDSIGVRVEELEDGLIVHG---SGGTPLAGGTTIAAELDHRIAMSFAIAGLVSAAP | 421 |
| Query  Sbjct | 421  422 | VTVDDATMIATSFPEFMDLMAGLGAK 446 VT+DD + +ATSFP F ++ LGA+ VTIDDMSPVATSFPIFEAMLESLGAR 447 |  |

>TR:A0A257HEC4 A0A257HEC4_9RHIZ 3-phosphoshikimate 1-carboxyvinyltransferase OS=Rhizobiales bacterium PAR1 OX=2015577 GN=aroA PE=3 SV=1

Length=437

Score = 474 bits (1219), Expect = 1e-162

| Identities | = 256/438 (58%), Positives = 315/438 (72%), Gaps = 6/438 (1%) |  |
| --- | --- | --- |
| Query 12 | ARKSSGLSGTVRIPGDKSISHRSFMFGGLASGETRITGLLEGEDVINTGKAMQAMGARIR | 71 |
| Sbjct 3 | A +S L G +PGDKSISHR+ + G L+ GET ++GLLEGEDV+ T A +A+GA + AERSGALKGACTVPGDKSISHRALILGLLSVGETTVSGLLEGEDVMRTADAARALGATVD | 62 |
| Query 72 | KEGD-TWIIDGVGNGGLLAPEAPLDFGNAATGCRLTMGLVGVYDFDSTFIGDASLTKRPM | 130 |
| Sbjct 63 | G +W + G+G G LL P+ LDFGNA TG RL MG++G + +T GDASL KRPM HLGPGSWRVLGMGIGTLLEPKGALDFGNAGTGSRLMMGVIGGHPITATIDGDASLRKRPM | 122 |
| Query 131 | GRVLNPLREMGVQV-KSEDGDRLPVTLRGPKTPTPITYRVPMASAQVKSAVLLAGLNTPG | 189 |
| Sbjct 123 | GR+L+PL++MGV+V + +G+R P+T+RG K PI Y+ P+ASAQ+KSAVL GLN PG GRILDPLKKMGVEVIAAAEGNRCPLTIRGAKDCIPIEYQTPVASAQIKSAVLFCGLNAPG | 182 |
| Query 190 | ITTVIEPIMTRDHTEKMLQGFGANLTVETDADGVRTIRLEGRGKLTGQVIDVPGDPSSTA | 249 |
| Sbjct 183 | TTVIE +RDHTEKML FGA +T R + L+GR +LT + I VP DPSS A ETTVIEAEASRDHTEKMLAYFGAEITSVPHGPHGRRVTLKGRPELTPRPIVVPADPSSAA | 242 |
| Query 250 | FPLVAALLVPGSDVTILNVLMNPTRTGLILTLQEMGADIEVINPRLAGGEDVADLRVRSS | 309 |
| Sbjct 243 | FP+VAAL+VPGS+V + +V+MNP R GLI TL+EMGADI + R+ GGE+VADLRVR S FPMVAALIVPGSEVVVRSVMMNPLRIGLITTLKEMGADITELERRIEGGEEVADLRVRHS | 302 |
| Query 310 | TLKGVTVPEDRAPSMIDEYPILAVAAAFAEGATVMNGLEELRVKESDRLSAVANGLKLNG | 369 |
| Sbjct 303 | LKGV VP +RAPSMIDEYP+LAVAAAFA G T MNGL+ELRVKESDRL+AVA+GL NG ALKGVHVPAERAPSMIDEYPVLAVAAAFATGETRMNGLKELRVKESDRLAAVADGLAANG | 362 |
| Query 370 | VDCDEGETSLVVRGRPDGKGLGNASGAAVATHLDHRIAMSFLVMGLVSENPVTVDDATMI | 429 |
| Sbjct 363 | V + LVV GR G G VATH+DHRIAMSFLVMGL SE PV VDD + I VAHEIVGDDLVVSGR GKPAKGGGLVATHMDHRIAMSFLVMGLASEKPVQVDDTSFI | 418 |
| Query 430  Sbjct 419 | ATSFPEFMDLMAGLGAKI 447 ATSFP+F+ LM LG + ATSFPDFVPLMTRLGGSL 436 |  |

>TR:A0A0M3TAA7 A0A0M3TAA7_9SPHN 3-phosphoshikimate 1-carboxyvinyltransferase OS=Altererythrobacter epoxidivorans OX=361183 GN=aroA PE=3

SV=1

Length=437

Score = 474 bits (1219), Expect = 1e-162

| Identities | | = 250/423 (59%), Positives = 304/423 (72%), Gaps = 2/423 (0%) |  |
| --- | --- | --- | --- |
| Query 18  Sbjct 12 | | LSGTVRIPGDKSISHRSFMFGGLASGETRITGLLEGEDVINTGKAMQAMGARIRKEGDTW L+G +R+PGDKSISHRS MFG LA GETR+TGLLEGEDV+ T AM+AMGA+I K GD W LTGRLRVPGDKSISHRSIMFGALAVGETRVTGLLEGEDVMATAAAMRAMGAQIEKSGDEW | 77  71 |
| Query 78  Sbjct 72 | | IIDGVGNGGLLAPEAPLDFGNAATGCRLTMGLVGVYDFDSTFIGDASLTKRPMGRVLNPL II GVG GGLL PE LD GN+ T RL MGLV +D ++F GDASL+KRPMGRV++PL IIHGVGVGGLLQPEQALDMGNSGTSTRLLMGLVASHDITTSFTGDASLSKRPMGRVIDPL | 137  131 |
| Query 138  Sbjct 132 | | REMGVQVKSEDGDRLPVTLRGPKTPTPITYRVPMASAQVKSAVLLAGLNTPGITTVIEPI MG ++ LP+ +RG + PITYR+P+ASAQVKSAVLLAGLNTPGITTVIEP+ SLMGAHFEASPDGTLPLMMRGAQPAVPITYRLPVASAQVKSAVLLAGLNTPGITTVIEPV | 197  191 |
| Query 198  Sbjct 192 | | MTRDHTEKMLQGFGANLTVETDADGVRTIRLEGRGKLTGQVIDVPGDPSSTAFPLVAALL TRDH+E+ML+GFGA L VE + DG R IR+ G +L Q ++VPGDPSS AF +VAAL+ PTRDHSERMLRGFGAELEVE-EVDGERIIRIHGEAELKPQQVEVPGDPSSAAFFIVAALI | 257  250 |
| Query 258  Sbjct 251 | | VPGSDVTILNVLMNPTRTGLILTLQEMGADIEVINPRLAGGEDVADLRVRSSTLKGVTVP GSD+ I NV +NPTR G++ L++MG IE ++ R GGE VADLRVR S L G+ V  TEGSDLVIENVGLNPTRAGIVEVLRQMGGSIEELDRREVGGEPVADLRVRHSALTGIEVD | 317  310 |
| Query 318  Sbjct 311 | | EDRAPSMIDEYPILAVAAAFAEGATVMNGLEELRVKESDRLSAVANGLKLNGVDCDEGET APSMIDE+P+ VAAA A G T +GL+ELRVKESDRLSA+A L GV +E E  PSIAPSMIDEFPVFFVAAALANGTTTTSGLDELRVKESDRLSAMAAALTGAGVQVEESED | 377  370 |
| Query 378 | | SLVVRGRPDGKGLGNASGAAVATHLDHRIAMSFLVMGLVSENPVTVDDATMIATSFPEFM L ++G G+ L S + THLDHRIAMS V GL S + V VDD I TSFP FM | 437 |
| Sbjct | 371 | GLTIQG-SGGEPLRGTSNSRTKTHLDHRIAMSMAVAGLASRDGVEVDDIAPIQTSFPNFM | 429 |
| Query  Sbjct | 438  430 | DLM 440  +L+  ELL 432 |  |

>SP:Q89WF2 AROA_BRADU 3-phosphoshikimate 1-carboxyvinyltransferase OS=Bradyrhizobium diazoefficiens (strain JCM 10833 / BCRC 13528 /

IAM 13628 / NBRC 14792 / USDA 110) OX=224911 GN=aroA PE=3 SV=1

Length=469

Score = 475 bits (1222), Expect = 1e-162

| Identities | = 254/448 (57%), Positives = 314/448 (70%), Gaps = 7/448 (2%) |  |
| --- | --- | --- |
| Query 1 | MLHGASSRPATARKSSGLSGTVRIPGDKSISHRSFMFGGLASGETRITGLLEGEDVINTG | 60 |
| Sbjct 25 | + H RP +R + L+G VR+PGDKSISHR+ + G LA GETRI+GLLEGEDV+NT LTHSDQPRPLQSRANGPLTGKVRVPGDKSISHRALILGALAVGETRISGLLEGEDVLNTA | 84 |
| Query 61 | KAMQAMGARIRKEGD-TWIIDGVGNGGLLAPEAPLDFGNAATGCRLTMGLVGVYDFDSTF | 119 |
| Sbjct 85 | K+MQA+GA + + GD W + GVG G P+A LDFGN+ TGCRL MG V + F KSMQALGASVERTGDFAWKVQGVGVAGFAQPKAALDFGNSGTGCRLVMGAVAGCPISAVF | 144 |
| Query 120 | IGDASLTKRPMGRVLNPLREMGVQVKSE-DGDRLPVTLRGPKTPTPITYRVPMASAQVKS | 178 |
| Sbjct 145 | GDASL RPM R+L+PL +MG +V S +G RLP+TL+G + P PITY+ P+ASAQ+KS DGDASLRSRPMRRILDPLEKMGARVVSGGEGGRLPLTLQGARDPLPITYKTPVASAQIKS | 204 |
| Query 179 | AVLLAGLNTPGITTVIEPIMTRDHTEKMLQGFGANLTVETDADGVRTIRLEGRGKLTGQV | 238 |
| Sbjct 205 | AVLLAGL PG TTVIE +RDHTE ML+ FGA++T + R I L G+ +L G  AVLLAGLAAPGTTTVIESEASRDHTELMLKHFGADITSTKEGQHGRRITLVGQPELHGAN | 264 |

| Query | 239 | IDVPGDPSSTAFPLVAALLVPGSDVTILNVLMNPTRTGLILTLQEMGADIEVINPRLAGG | 298 |
| --- | --- | --- | --- |
| Sbjct | 265 | + VP DPSS AFP+VAAL+ GSDV + +V+ NP RTGL TL+EMGA IE R G VVVPADPSSAAFPVVAALIAEGSDVVLSDVMTNPLRTGLFTTLREMGASIEESEVRGDAG | 324 |
| Query | 299 | EDVADLRVRSSTLKGVTVPEDRAPSMIDEYPILAVAAAFAEGATVMNGLEELRVKESDRL | 358 |
| Sbjct | 325 | E +A LRVR+S L+GV VP +RAPSMIDEY +LAVAA+FAEG T+M GL+ELRVKESDRL EPMAQLRVRASKLRGVEVPPERAPSMIDEYLVLAVAASFAEGTTIMRGLQELRVKESDRL | 384 |
| Query | 359 | SAVANGLKLNGVDCDEGETSLVVRGRPDGKGLGNASGAAVATHLDHRIAMSFLVMGLVSE | 418 |
| Sbjct | 385 | A A L++NGV + LVV+GR G G VATH+DHRIAMS LVMG S+ EATAEMLRVNGVKVEVSGDDLVVQGRGHVPG GGTVATHMDHRIAMSALVMGCASD | 439 |
| Query  Sbjct | 419  440 | NPVTVDDATMIATSFPEFMDLMAGLGAK 446 PVTVDD IATSFP+F+ +M LGA+  QPVTVDDTAFIATSFPDFIPMMRSLGAE 467 |  |

>TR:A0A2G8MPK4 A0A2G8MPK4_9RHIZ 3-phosphoshikimate 1-carboxyvinyltransferase OS=Methylobacterium frigidaeris OX=2038277 GN=aroA PE=3 SV=1

Length=449

Score = 474 bits (1220), Expect = 1e-162

| Identities | = 263/450 (58%), Positives = 318/450 (71%), Gaps = 10/450 (2%) |  |
| --- | --- | --- |
| Query 1 | MLHGASSRPATARKSSGLSGTVRIPGDKSISHRSFMFGGLASGETRITGLLEGEDVINTG | 60 |
| Sbjct 1 | M H ++ P TA+ + L G +R PGDKSISHR+ + G L+ GETRI GLLEG+DV+ T MSHDSTPSPITAQSGTALRGRLRPPGDKSISHRAIILGLLSLGETRIEGLLEGDDVLRTA | 60 |
| Query 61 | KAMQAMGARIRKEGD-TWIIDGVGNGGLLAPEAPLDFGNAATGCRLTMGLVGVYDFDSTF | 119 |
| Sbjct 61 | A +A+GA I ++G+ W + GVG GGL P + LDFGNA TG RL MG+VG + +TF AAAKALGAGIDRDGEGRWRVRGVGVGGLSDPASVLDFGNAGTGSRLMMGVVGGHPVTATF | 120 |
| Query 120 | IGDASLTKRPMGRVLNPLREMGVQVKSE-DGDRLPVTLRGPKTPTPITYRVPMASAQVKS | 178 |
| Sbjct 121 | GDASL KRPM R+L+PL +MGV V + +G R+P+TLRGP+ P+TY P+ASAQVKS DGDASLRKRPMRRILDPLVQMGVTVVEQAEGGRVPLTLRGPREAVPVTYESPVASAQVKS | 180 |
| Query 179 | AVLLAGLNTPGITTVIEPIMTRDHTEKMLQGFGANLTVETDADGV--RTIRLEGRGKLTG | 236 |
| Sbjct 181 | AVLLAGLN PGITTV+E TRDHTE+ML+ FGA++ VET G R I L G+ L AVLLAGLNAPGITTVVEAAATRDHTERMLRLFGAHVEVETIGPGGHGRRIALTGQPTLKA | 240 |
| Query 237 | QVIDVPGDPSSTAFPLVAALLVPGSDVTILNVLMNPTRTGLILTLQEMGADIEVINPRLA | 296 |
| Sbjct 241 | + VP DPSS AF LVA L+VPGS+V I V+MNP RTGL+ TL EMG DIE +N R TDVVVPADPSSAAFALVAGLVVPGSEVVIEGVMMNPLRTGLLATLLEMGGDIERLNEREE | 300 |
| Query 297 | GGEDVADLRVRSSTLKGVTVPEDRAPSMIDEYPILAVAAAFAEGATVMNGLEELRVKESD | 356 |
| Sbjct 301 | GGE VADLRVR S LKGV VP +RAP+MIDEYP+LAVAAA AEG T M GL ELRVKESD GGETVADLRVRHSRLKGVAVPPERAPAMIDEYPVLAVAAACAEGTTRMQGLHELRVKESD | 360 |
| Query 357 | RLSAVANGLKLNGVD-CDEGETSLVVRGRPDGKGLGNASGAAVATHLDHRIAMSFLVMGL | 415 |
| Sbjct 361 | RL+AVA GL NG+ EG+ +V G G G V THLDHRIAM+FLV+G+ RLAAVAAGLAANGISHVIEGDDLIV HGDGAAPQGGGTVETHLDHRIAMAFLVLGM | 415 |
| Query 416  Sbjct 416 | VSENPVTVDDATMIATSFPEFMDLMAGLGA 445 V+ PVTVDD MIATS+P F+ M LGA VARAPVTVDDGAMIATSYPAFLADMRALGA 445 |  |

>TR:A0A2A4ID68 A0A2A4ID68_9SPHN 3-phosphoshikimate 1-carboxyvinyltransferase OS=Sphingomonas adhaesiva OX=28212 GN=aroA PE=3 SV=1

Length=451

Score = 474 bits (1220), Expect = 2e-162

Identities = 260/446 (58%), Positives = 308/446 (69%), Gaps = 3/446 (1%)

| Query | 1 | MLHGASSRPATARKSSGLSGTVRIPGDKSISHRSFMFGGLASGETRITGLLEGEDVINTG | 60 |
| --- | --- | --- | --- |
| Sbjct | 1 | M H A +RP + S L GT+ +PGDKSISHRS MF GLA GE+RI+GLLEGEDV+ T MSHSAPARPLSIAASGPLVGTLTVPGDKSISHRSLMFAGLAVGESRISGLLEGEDVLATA | 60 |
| Query | 61 | KAMQAMGARIRKEGD-TWIIDGVGNGGLLAPEAPLDFGNAATGCRLTMGLVGVYDFDSTF | 119 |
| Sbjct | 61 | AM+AMGA++ + D W + GVG GGLL P+ LD GN+ T RL MGLV + +TF GAMRAMGAQVERTADGVWQVHGVGVGGLLQPQVALDMGNSGTSTRLLMGLVASHAITATF | 120 |
| Query | 120 | IGDASLTKRPMGRVLNPLREMGVQVKSEDGDRLPVTLRGPKTPTPITYRVPMASAQVKSA | 179 |
| Sbjct | 121 | IGDASL+KRPM RV PL MG + G+RLP+T+RG PI Y +P+ASAQVKSA IGDASLSKRPMARVTEPLSRMGASFTTAPGERLPLTMRGLCPAVPIDYTLPVASAQVKSA | 180 |
| Query | 180 | VLLAGLNTPGITTVIEPIMTRDHTEKMLQGFGANLTVETDADGVRTIRLEGRGKLTGQVI | 239 |
| Sbjct | 181 | VLLAGLNTPGIT VIEP+ TRDH+E+ML+GFGA LTVE G R I + G +L Q I VLLAGLNTPGITRVIEPVPTRDHSERMLRGFGAQLTVEETPQG-RMIAITGEAELRAQNI | 239 |
| Query | 240 | DVPGDPSSTAFPLVAALLVPGSDVTILNVLMNPTRTGLILTLQEMGADIEVINPRLAGGE | 299 |
| Sbjct | 240 | +VPGDPSS F +VAA +VPGSDVTI NVLMNPTR GL+ L+ MGA I+ + R GGE EVPGDPSSAGFWMVAASVVPGSDVTIRNVLMNPTRIGLVTALRMMGASIDELAQRDVGGE | 299 |
| Query | 300 | DVADLRVRSSTLKGVTVPEDRAPSMIDEYPILAVAAAFAEGATVMNGLEELRVKESDRLS | 359 |
| Sbjct | 300 | VADLRVR + L + VP D APSMIDEYPIL VAAAFA G TV G ELRVKESDR++ PVADLRVRHAALTAIEVPPDLAPSMIDEYPILFVAAAFARGTTVARGAHELRVKESDRIA | 359 |
| Query | 360 | AVANGLKLNGVDCDEGETSLVVRGRPDGKGLGNASGAAVATHLDHRIAMSFLVMGLVSEN | 419 |
| Sbjct | 360 | A+A L L GV E E L + GR DG L A VA+ LDHRIAMS V G+ + AMAAALGLAGVRVSEHEDGLSIEGR-DGDPLPGTRDARVASLLDHRIAMSMAVAGMHARA | 418 |
| Query  Sbjct | 420  419 | PVTVDDATMIATSFPEFMDLMAGLGA 445 VT+DDA IATS+P F+D LGA  AVTIDDAAPIATSYPAFVDTARALGA 444 |  |

>TR:A0A327K264 A0A327K264_9RHIZ 3-phosphoshikimate 1-carboxyvinyltransferase OS=Rhodoplanes elegans OX=29408 GN=aroA PE=3 SV=1

Length=452

Score = 474 bits (1220), Expect = 2e-162

| Identities | = 260/450 (58%), Positives = 313/450 (70%), Gaps = 14/450 (3%) |  |
| --- | --- | --- |
| Query 5 | ASSRPATARKSSGLSGTVRIPGDKSISHRSFMFGGLASGETRITGLLEGEDVINTGKAMQ | 64 |
| Sbjct 9 | A+ P AR + L+G VR+PGDKSISHR+ + G L+ G T +TGLLEGEDV+ TGKAM AAPMPLVARGARPLAGRVRVPGDKSISHRALILGALSVGRTTVTGLLEGEDVLATGKAMS | 68 |
| Query 65 | AMGARIRKEGD-TWIIDGVGNGGLLAPEAPLDFGNAATGCRLTMGLVGVYDFDSTFIGDA | 123 |
| Sbjct 69 | +GA + + G W +DGVG GG AP LDFGNA TG RL MG V + F GDA LLGATVARTGPGAWTVDGVGVGGFAAPPETLDFGNAGTGSRLVMGAVAGCPVTAMFDGDA | 128 |
| Query 124 | SLTKRPMGRVLNPLREMGVQV-KSEDGDRLPVTLRGPKTPTPITYRVPMASAQVKSAVLL | 182 |
| Sbjct 129 | SL +RPM R+L+PL +MG V ++ +GDRLP+ L G + P PI Y P+ SAQVKSAVLL SLRRRPMRRILDPLVKMGATVLQAAEGDRLPIMLAGARDPLPIVYATPVPSAQVKSAVLL | 188 |
| Query 183 | AGLNTPGITTVIEPIMTRDHTEKMLQGFGANLTVETDADGVRTIRLEGRGKLTGQVIDVP | 242 |
| Sbjct 189 | AGL+ PG T VIE TRDHTEKML FGA +TV + R I L+G+ +L G + VP AGLSAPGETVVIESEATRDHTEKMLLHFGATVTVVPEGAHGRRITLKGQPELHGATVVVP | 248 |
| Query 243 | GDPSSTAFPLVAALLVPGSDVTILNVLMNPTRTGLILTLQEMGADIEVINPRLAGGEDVA | 302 |
| Sbjct 249 | DPSS AFPLVAALLVPGSD+ + V+ NP RTGLI TL+EMGA IE ++ R GGE +A ADPSSAAFPLVAALLVPGSDIVLETVMTNPLRTGLITTLREMGAAIEEVDAREDGGEAMA | 308 |
| Query 303 | DLRVRSSTLKGVTVPEDRAPSMIDEYPILAVAAAFAEGATVMNGLEELRVKESDRLSAVA | 362 |
| Sbjct 309 | DLRVR S L GV VP +RAP+MIDEYP+LAVAAAFA G TVM GL+ELRVKESDRL+A A  DLRVRFSELSGVEVPPERAPAMIDEYPVLAVAAAFARGTTVMRGLKELRVKESDRLAATA | 368 |

| Query | 363 | NGLKLNGVDCDEGETSLVV-----RGRPDGKGLGNASGAAVATHLDHRIAMSFLVMGLVS 417 |
| --- | --- | --- |
| Sbjct | 369 | + L++NGV ++V +GRP G G VATH+DHRIAMS LVMGL + DLLRVNGVTVTLDGDDMIVEGFGGKGRPPG-------GGTVATHMDHRIAMSALVMGLAT 421 |
| Query | 418 | ENPVTVDDATMIATSFPEFMDLMAGLGAKI 447 |
| Sbjct | 422 | E PV VDDA IATSFP F++ M GLGA I  ETPVAVDDAAFIATSFPGFVETMRGLGADI 451 |

>TR:A0A0X8R4B0 A0A0X8R4B0_9SPHN 3-phosphoshikimate 1-carboxyvinyltransferase OS=Sphingopyxis granuli OX=267128 GN=aroA PE=3 SV=1

Length=442

Score = 474 bits (1219), Expect = 2e-162

| Identities | = 255/438 (58%), Positives = 301/438 (69%), Gaps = 4/438 (1%) |  |
| --- | --- | --- |
| Query 9 | PATARKSSGLSGTVRIPGDKSISHRSFMFGGLASGETRITGLLEGEDVINTGKAMQAMGA | 68 |
| Sbjct 8 | P S+ L G + I GDKSISHR+ M LA G +R+TGLLEG DV+ T AM+AMGA PRAFSASAPLKGKIAILGDKSISHRALMLSALAVGTSRVTGLLEGHDVLATAAAMRAMGA | 67 |
| Query 69 | RIRKEGD-TWIIDGVGNGGLLAPEAPLDFGNAATGCRLTMGLVGVYDFDSTFIGDASLTK | 127 |
| Sbjct 68 | I + GD W+IDGVG GGLL P L+ GN+ T RL MGLV + TF GDASL+ SIERTGDGEWVIDGVGVGGLLQPREALEMGNSGTSTRLLMGLVASHPITVTFTGDASLSS | 127 |
| Query 128 | RPMGRVLNPLREMGVQVKSEDGDRLPVTLRGPKTPTPITYRVPMASAQVKSAVLLAGLNT | 187 |
| Sbjct 128 | RPMGRV++PL +MG + + G RLP+ +RG P+ YR+PMASAQVKSAVLLAGLNT RPMGRVIDPLSQMGADISASPGGRLPLMVRGLAPAIPLAYRLPMASAQVKSAVLLAGLNT | 187 |
| Query 188 | PGITTVIEPIMTRDHTEKMLQGFGANLTVETDADGVRTIRLEGRGKLTGQVIDVPGDPSS | 247 |
| Sbjct 188 | PGIT VIEPI TRDH+E+ML+GFGA+L VETD DG R I + G +L Q I VPGDPSS PGITEVIEPIPTRDHSERMLKGFGADLNVETDGDGTRRIHIRGEAELRPQTIAVPGDPSS | 247 |
| Query 248 | TAFPLVAALLVPGSDVTILNVLMNPTRTGLILTLQEMGADIEVINPRLAGGEDVADLRVR | 307 |
| Sbjct 248 | AF +VAAL+VPGSDV I NV +NPTR GL+ LQ MG DIE+++PR GGE VADL VR AAFFIVAALIVPGSDVVIANVGLNPTRAGLVEVLQAMGGDIELLDPREVGGEPVADLHVR | 307 |
| Query 308 | SSTLKGVTVPEDRAPSMIDEYPILAVAAAFAEGATVMNGLEELRVKESDRLSAVANGLKL | 367 |
| Sbjct 308 | S LKG+ V APSMIDEYPIL VAAA AEG TV GL+ELRVKESDRL+ +A GL HSALKGIEVDPAIAPSMIDEYPILFVAAALAEGRTVTTGLDELRVKESDRLAVMAAGLTA | 367 |
| Query 368 | NGVDCDEGETSLVVRGRPDGKGLGNASGAAVATHLDHRIAMSFLVMGLVSENPVTVDDAT | 427 |

G	+E	L + G	G	GA +A HLDHRI MSF V GLV + PVT+DD

| Sbjct | 368 | IGAQVEESADGLAIDGT---GGDPLPGGATIAGHLDHRICMSFAVAGLVGKAPVTIDDMG | 424 |
| --- | --- | --- | --- |
| Query | 428 | MIATSFPEFMDLMAGLGA 445  +ATSFP F L+ L A |  |
| Sbjct | 425 | PVATSFPNFEALLESLQA 442 | |

>TR:A0A4Q9G4X9 A0A4Q9G4X9_9RHOB 3-phosphoshikimate 1-carboxyvinyltransferase OS=Paracoccus subflavus OX=2528244 GN=aroA PE=3 SV=1

Length=443

Score = 474 bits (1219), Expect = 2e-162

Identities = 248/448 (55%), Positives = 311/448 (69%), Gaps = 7/448 (2%)

| Query | 1 | MLHGASSRPATARKSSGLSGTVRIPGDKSISHRSFMFGGLASGETRITGLLEGEDVINTG | 60 |
| --- | --- | --- | --- |
| Sbjct | 1 | M H RP TAR+S L G +PGDKSISHR+ + G L+ GETRITGLLEGEDV++T MSHSPDPRPMTARRSGPLRGVATVPGDKSISHRALILGALSVGETRITGLLEGEDVLDTA | 60 |
| Query | 61 | KAMQAMGARIRKEGD-TWIIDGVGNGGLLAPEAPLDFGNAATGCRLTMGLVGVYDFDSTF | 119 |
| Sbjct | 61 | KAM+A GA++ + G+ W + GVG GG PE +D GN+ TG RL MG + +TF  KAMRAFGAQVERLGNGEWSVHGVGVGGFSEPEGVIDCGNSGTGVRLIMGAMATTPITATF | 120 |

| Query | 120 | IGDASLTKRPMGRVLNPLREMGVQVKSEDGDRLPVTLRGPKTPTPITYRVPMASAQVKSA | 179 |
| --- | --- | --- | --- |
| Sbjct | 121 | GDASL +RPM R+ +PL + G Q+ + +G RLP+T++G P P+ YR P+ASAQ+KSA TGDASLCRRPMARITDPLSQFGAQITAREGGRLPITIQGADDPLPVRYRTPVASAQIKSA | 180 |
| Query | 180 | VLLAGLNTPGITTVIEPIMTRDHTEKMLQGFGANLTVETDADGVRTIRLEGRGKLTGQVI | 239 |
| Sbjct | 181 | +LLAGLN PG T VIE TRDH+E+ML GFGA + E G I L+GR +L Q + ILLAGLNVPGETVVIEAEPTRDHSERMLAGFGAEIRAEQTDQG-HVITLQGRPELRAQAV | 239 |
| Query | 240 | DVPGDPSSTAFPLVAALLVPGSDVTILNVLMNPTRTGLILTLQEMGADIEVINPRLAGGE | 299 |
| Sbjct | 240 | VP DPSS AFP+ AAL+VPGS++ + V NPTR GL +TL +MGADI N R GGE AVPRDPSSAAFPVAAALIVPGSEIRVPGVSRNPTRDGLYVTLLDMGADIVFENSREEGGE | 299 |
| Query | 300 | DVADLRVRSSTLKGVTVPEDRAPSMIDEYPILAVAAAFAEGATVMNGLEELRVKESDRLS | 359 |
| Sbjct | 300 | VADL VR L+GVTVP +RA SMIDE+PIL+V AAFAEG TVMNG+ ELRVKESDR+ PVADLLVRHGPLRGVTVPAERAASMIDEFPILSVVAAFAEGPTVMNGVAELRVKESDRID | 359 |
| Query | 360 | AVANGLKLNGVDCDEGETSLVVRGRPDGKGLGNASGAAVATHLDHRIAMSFLVMGLVSEN | 419 |
| Sbjct | 360 | A+A GL+ NGV +E S+ V G+ G+ G G ATHLDHRIAMSFLV+GL ++ AMARGLEANGVRVEETRDSMTVHGQ--GRVAG---GGTAATHLDHRIAMSFLVLGLATDA | 414 |
| Query  Sbjct | 420  415 | PVTVDDATMIATSFPEFMDLMAGLGAKI 447 PV +DD IATSFP+F+ LM GL A + PVQIDDGAPIATSFPDFVPLMQGLRADL 442 |  |

>TR:A0A418ZWX6 A0A418ZWX6_9RHOB 3-phosphoshikimate 1-carboxyvinyltransferase OS=Paracoccus siganidrum OX=1276757 GN=aroA PE=3 SV=1

Length=443

Score = 474 bits (1219), Expect = 2e-162

| Identities | | = 250/448 (56%), Positives = 307/448 (69%), Gaps = 7/448 (2%) |  |
| --- | --- | --- | --- |
| Query 1  Sbjct 1 | | MLHGASSRPATARKSSGLSGTVRIPGDKSISHRSFMFGGLASGETRITGLLEGEDVINTG M H A P T+R+S+ L G +PGDKSISHR+ + G L+ GETRITGLLEG+DV++T MSHAAQPLPMTSRRSAPLQGEALVPGDKSISHRALILGALSVGETRITGLLEGQDVLDTA | 60  60 |
| Query 61 | | KAMQAMGARIRKEGD-TWIIDGVGNGGLLAPEAPLDFGNAATGCRLTMGLVGVYDFDSTF KAM A GA++ + G W + GVG GG P +D GN+ TG RL MG + +TF | 119 |
| Sbjct | 61 | KAMAAFGAQVERRGPGDWSVHGVGVGGFSEPAGVIDCGNSGTGVRLIMGAMATTPITATF | 120 |
| Query | 120 | IGDASLTKRPMGRVLNPLREMGVQVKSEDGDRLPVTLRGPKTPTPITYRVPMASAQVKSA | 179 |
| Sbjct | 121 | GDASL +RPM RV +PL G ++ + +G RLP+T+ G P P+TYR P+ SAQ+KSA TGDASLCRRPMARVTDPLAGFGARITAREGGRLPITIEGAAEPLPLTYRTPVPSAQIKSA | 180 |
| Query | 180 | VLLAGLNTPGITTVIEPIMTRDHTEKMLQGFGANLTVETDADGVRTIRLEGRGKLTGQVI | 239 |
| Sbjct | 181 | +LLAGLN PG T VIE TRDH+E+ML GFGA + ET DG IRL GR +L Q + ILLAGLNAPGETVVIESEPTRDHSERMLAGFGAQIQAETTPDG-HVIRLTGRPELRAQPV | 239 |
| Query | 240 | DVPGDPSSTAFPLVAALLVPGSDVTILNVLMNPTRTGLILTLQEMGADIEVINPRLAGGE | 299 |
| Sbjct | 240 | VP DPSS AFP+ AAL+VPGS + + V NPTR GL +TL +MGADI N R GGE AVPRDPSSAAFPVAAALIVPGSQIRVPGVSRNPTRDGLYVTLLDMGADISFENIREEGGE | 299 |
| Query | 300 | DVADLRVRSSTLKGVTVPEDRAPSMIDEYPILAVAAAFAEGATVMNGLEELRVKESDRLS | 359 |
| Sbjct | 300 | VADL VR LKGV VP +RA SMIDE+PIL+V AAFAEG TVMNG+ ELRVKESDR+ PVADLVVRHGPLKGVEVPAERAASMIDEFPILSVIAAFAEGPTVMNGVAELRVKESDRID | 359 |
| Query | 360 | AVANGLKLNGVDCDEGETSLVVRGRPDGKGLGNASGAAVATHLDHRIAMSFLVMGLVSEN | 419 |
| Sbjct | 360 | A+A GL+ NGV DE S+ V G G G ATHLDHRIAMSFLV+GL +E+ AMARGLEANGVTVDETRDSMTVHGMGRVPG GGTAATHLDHRIAMSFLVLGLATES | 414 |
| Query  Sbjct | 420  415 | PVTVDDATMIATSFPEFMDLMAGLGAKI 447 PV +DD IATSFP+F+ LM GLGA++ PVGIDDGGPIATSFPDFLPLMRGLGAEL 442 |  |

>TR:A0A494VZM4 A0A494VZM4_9SPHN 3-phosphoshikimate 1-carboxyvinyltransferase OS=Sphingobium amiense OX=135719 GN=aroA PE=3 SV=1

Length=446

Score = 474 bits (1219), Expect = 2e-162

| Identities | = 252/429 (59%), Positives = 307/429 (72%), Gaps = 4/429 (1%) |  |
| --- | --- | --- |
| Query 18 | LSGTVRIPGDKSISHRSFMFGGLASGETRITGLLEGEDVINTGKAMQAMGARIRKEGD-T | 76 |
| Sbjct 19 | L+G+V +PGDKSISHRS M LA GE+R+ GLLEGEDV+ T AM+A+GA+I ++ LAGSVTVPGDKSISHRSLMLSALAVGESRVEGLLEGEDVLATAAAMRAIGAQIGRDAQGV | 78 |
| Query 77 | WIIDGVGNGGLLAPEAPLDFGNAATGCRLTMGLVGVYDFDSTFIGDASLTKRPMGRVLNP | 136 |
| Sbjct 79 | W I GVG GGLL P+ LD GN+ T RL MGL+ +D +TFIGDASL+KRPM RV P WHIHGVGVGGLLQPQTALDMGNSGTSTRLLMGLLASHDLTATFIGDASLSKRPMARVTEP | 138 |
| Query 137 | LREMGVQVKSEDGDRLPVTLRGPKTPTPITYRVPMASAQVKSAVLLAGLNTPGITTVIEP | 196 |
| Sbjct 139 | L MG S GDRLP+T+RG P+ YR+P+ASAQVKSA+LLAGLNTPGIT V+EP LGRMGASFTSSPGDRLPLTMRGACPAVPLDYRLPVASAQVKSAILLAGLNTPGITRVVEP | 198 |
| Query 197 | IMTRDHTEKMLQGFGANLTVETDADGVRTIRLEGRGKLTGQVIDVPGDPSSTAFPLVAAL | 256 |
| Sbjct 199 | I TRDH+E+ML+GFGA+L+VET+ DG R I L G +LT Q I VPGDPSS AFP+VAAL IPTRDHSERMLKGFGADLSVETEEDGTRVITLVGEAELTPQNIVVPGDPSSAAFPMVAAL | 258 |
| Query 257 | LVPGSDVTILNVLMNPTRTGLILTLQEMGADIEVINPRLAGGEDVADLRVRSSTLKGVTV | 316 |
| Sbjct 259 | LVPGS VTI NV +N TR GLI L+EMG IE++N R GGE V DL V +STL G+ LVPGSRVTIANVGLNATRAGLIDLLREMGGAIEIVNAREVGGEPVGDLIVTASTLHGIEP | 318 |
| Query 317 | PEDRAPSMIDEYPILAVAAAFAEGATVMNGLEELRVKESDRLSAVANGLKLNGVDCDEGE | 376 |
| Sbjct 319 | RAPSMIDEYP+ +AAA A+G +V GLEELRVKESDR++ +A GL+ GV +E E DPARAPSMIDEYPVAFIAAACAQGRSVFRGLEELRVKESDRIATMAQGLRAIGVTVEELE | 378 |
| Query 377 | TSLVVRGRPDGKGLGNASGAAVATHLDHRIAMSFLVMGLVSENPVTVDDATMIATSFPEF | 436 |
| Sbjct 379 | +V+ G G G +AT LDHRIAMSF V GLVS + VT+DD +ATSFP F DGIVIEG---SGGAPLPGGGPIATKLDHRIAMSFAVAGLVSRDGVTIDDMRPVATSFPTF | 435 |
| Query 437  Sbjct 436 | MDLMAGLGA 445  + L+ LGA LPLLRQLGA 444 |  |

>TR:A0A3N2R155 A0A3N2R155_9RHOB 3-phosphoshikimate 1-carboxyvinyltransferase OS=Histidinibacterium lentulum OX=2480588 GN=aroA PE=3 SV=1

Length=458

Score = 474 bits (1220), Expect = 2e-162

| Identities | = 255/450 (57%), Positives = 311/450 (69%), Gaps = 6/450 (1%) |  |
| --- | --- | --- |
| Query 1 | MLHGASSRPATARKSSGLSGTVRIPGDKSISHRSFMFGGLASGETRITGLLEGEDVINTG | 60 |
| Sbjct 1 | M S RP TAR+S L GT +PGDKS+SHR+ + G LA GETR+TGLLEGEDV++T MSASGSPRPMTARRSGPLKGTAEVPGDKSVSHRALILGALAVGETRVTGLLEGEDVLDTA | 60 |
| Query 61 | KAMQAMGARIRKEGD-TWIIDGVGNGGLLAPEAPLDFGNAATGCRLTMGLVGVYDFDSTF | 119 |
| Sbjct 61 | +AM+A GA + +E D TW + GVG GG P++ +D GN+ TG RL MG + +TF RAMRAFGAEVTREADGTWSVHGVGTGGFGEPQSVIDCGNSGTGVRLIMGSMATTPITATF | 120 |
| Query 120 | IGDASLTKRPMGRVLNPLREMGVQVKSEDGDRLPVTLRGPKTPTPITYRVPMASAQVKSA | 179 |
| Sbjct 121 | GDASL RPMGRV +PL G Q G RLP+TL G + P P+ Y VP+ SAQVKSA TGDASLRGRPMGRVTDPLALFGTQAVGRAGGRLPMTLVGARDPVPVRYVVPVPSAQVKSA | 180 |
| Query 180 | VLLAGLNTPGITTVIEPIMTRDHTEKMLQGFGANLTVETDADGVRTIRLEGRGKLTGQVI | 239 |
| Sbjct 181 | VLLAGL PG T VIE TRDHTE+ML GFGA + +E +G R I L G+ +L QVI  VLLAGLGAPGETVVIEREATRDHTERMLAGFGAEVRIEETEEG-RAIILRGQPELRPQVI | 239 |

| Query | 240 | DVPGDPSSTAFPLVAALLVPGSDVTILNVLMNPTRTGLILTLQEMGADIEVINPRLAGGE | 299 |
| --- | --- | --- | --- |
| Sbjct | 240 | VP DPSS AFP+ AAL+ GSDV + + +NPTR GL TL+EMGAD+ NPR GGE SVPRDPSSAAFPVCAALITEGSDVLVPGIGLNPTRAGLYETLREMGADLVYENPRDEGGE | 299 |
| Query | 300 | DVADLRVR-SSTLKGVTVPEDRAPSMIDEYPILAVAAAFAEGATVMNGLEELRVKESDRL | 358 |
| Sbjct | 300 | VADLR R S +KG+ VP +RA SMIDEYP+L+V AAFAEG TVM G++ELRVKESDR+ PVADLRARFSPDMKGIEVPPERAASMIDEYPVLSVVAAFAEGVTVMRGVKELRVKESDRI | 359 |
| Query | 359 | SAVANGLKLNGVDCDEGETSLVVRGRPDGKGLGNASGAAVATHLDHRIAMSFLVMGLVSE | 418 |
| Sbjct | 360 | A+A GL+ GV DEGE V GR G G G VA+ LDHRIAMSF++MG ++ DAMATGLRAAGVTVDEGEDWWTVEGRGPG---GVKGGVTVASRLDHRIAMSFVLMGFAAQ | 416 |
| Query | 419 | NPVTVDDATMIATSFPEFMDLMAGLGAKIE 448 PVTVDDA+ IATSFP F LM GLGA+++ |  |
| Sbjct | 417 | EPVTVDDASPIATSFPIFEALMTGLGARLD 446 | |

>TR:A0A421BK08 A0A421BK08_9RHOB 3-phosphoshikimate 1-carboxyvinyltransferase OS=Sinirhodobacter hankyongi OX=2294033 GN=aroA PE=3 SV=1

Length=448

Score = 474 bits (1219), Expect = 2e-162

| Identities | = 257/447 (57%), Positives = 309/447 (69%), Gaps = 8/447 (2%) |  |
| --- | --- | --- |
| Query 3 | HGASSRPATARKSSGLSGTVRIPGDKSISHRSFMFGGLASGETRITGLLEGEDVINTGKA | 62 |
| Sbjct 4 | HG R TARKS+ LSG +PGDKSISHR+ + G L+ G T+ITGLLEG+DV++T KA HGDPIR-MTARKSAALSGVAEVPGDKSISHRALILGALSVGTTKITGLLEGQDVLDTAKA | 62 |
| Query 63 | MQAMGARIRKEG-DTWIIDGVGNGGLLAPEAPLDFGNAATGCRLTMGLVGVYDFDSTFIG | 121 |

M A GA + +EG	W ++GVG GG	P	+D GN+ TG RL MG +	+TF G

| Sbjct | 63 | MAAFGAGVTREGAGAWTVEGVGVGGFAEPADVIDCGNSGTGVRLIMGAMATSPITATFTG | 122 |
| --- | --- | --- | --- |
| Query | 122 | DASLTKRPMGRVLNPLREMGVQVKSEDGDRLPVTLRGPKTPTPITYRVPMASAQVKSAVL | 181 |
| Sbjct | 123 | DASL KRPMGRV +PL G + G RLP+T+ G P P+TY PMASAQVKSAVL DASLRKRPMGRVTDPLALFGARAYGRAGGRLPMTVVGAAEPVPVTYATPMASAQVKSAVL | 182 |
| Query | 182 | LAGLNTPGITTVIEPIMTRDHTEKMLQGFGANLTVETDADGVRTIRLEGRGKLTGQVIDV | 241 |
| Sbjct | 183 | LAGLN PG T VIE TRDHTE+ML+GFGA ++VE +G R I L G+ +L GQ + V LAGLNAPGQTVVIEKTATRDHTERMLRGFGAEVSVEATPEGNR-ITLTGQPELRGQTVAV | 241 |
| Query | 242 | PGDPSSTAFPLVAALLVPGSDVTILNVLMNPTRTGLILTLQEMGADIEVINPRLAGGEDV | 301 |
| Sbjct | 242 | P DPSS AFP+ AAL+VPGSD+ + V NPTR GL LTL+EMGADI N R GGE V PRDPSSAAFPVCAALIVPGSDILVPGVSQNPTRNGLFLTLREMGADITFENEREEGGEPV | 301 |
| Query | 302 | ADLRVRSSTLKGVTVPEDRAPSMIDEYPILAVAAAFAEGATVMNGLEELRVKESDRLSAV | 361 |
| Sbjct | 302 | ADLRVR S L G+ VP +RA S IDEYP+LA AA A G TVM G+ ELRVKESDR+ A+ ADLRVRFSELTGIEVPVERAASQIDEYPVLAAVAACARGTTVMRGVHELRVKESDRIDAM | 361 |
| Query | 362 | ANGLKLNGVDCDEGETSLVVRG-RPDGKGLGNASGAAVATHLDHRIAMSFLVMGLVSENP | 420 |
| Sbjct | 362 | A GL+ GV +E + +L+V G PDG GA VATH+DHRIAMSFLV+GL S P ARGLEACGVTVEEDDDTLIVHGLGPDGV PGGATVATHIDHRIAMSFLVLGLASRAP | 417 |
| Query  Sbjct | 421  418 | VTVDDATMIATSFPEFMDLMAGLGAKI 447  ++VDD + IATSFP F LM GLGA+I ISVDDGSPIATSFPVFEPLMRGLGAEI 444 |  |

>TR:A0A4R2GXV5 A0A4R2GXV5_9RHIZ 3-phosphoshikimate 1-carboxyvinyltransferase OS=Camelimonas lactis OX=659006 GN=aroA PE=3 SV=1

Length=450

Score = 474 bits (1219), Expect = 2e-162

Identities = 256/444 (58%), Positives = 316/444 (71%), Gaps = 8/444 (2%)

| Query | 9 | PATARKSSGLSGTVRIPGDKSISHRSFMFGGLASGETRITGLLEGEDVINTGKAMQAMGA | 68 |
| --- | --- | --- | --- |
| Sbjct | 9 | P+ +R S L G VR+PGDKS+SHR+ +FG L+ GET +TGLLEG+DV+ T A +A+GA PSASRPSGPLQGAVRVPGDKSMSHRALIFGLLSVGETHVTGLLEGDDVLATAAAARALGA | 68 |
| Query | 69 | RIRKEGD-TWIIDGVGNGGLLAPEAPLDFGNAATGCRLTMGLVGVYDFDSTFIGDASLTK | 127 |
| Sbjct | 69 | + + G+ W + GVG GGL +P LDFGNA TG RL MG+V + +TF GDASL K DVVRTGEGAWTVRGVGVGGLASPAETLDFGNAGTGSRLMMGVVAGHPVTATFDGDASLRK | 128 |
| Query | 128 | RPMGRVLNPLREMGVQVKSE-DGDRLPVTLRGPKTPTPITYRVPMASAQVKSAVLLAGLN | 186 |
| Sbjct | 129 | RPM R+++PL +MG +V +E +G R P+TL G + P+ Y P+ASAQ+KSAVLLAGLN RPMRRIIDPLLKMGARVVAEAEGGRCPLTLEGARETVPVVYETPVASAQIKSAVLLAGLN | 188 |
| Query | 187 | TPGITTVIEPIMTRDHTEKMLQGFGANLTVETDADGVRTIRLEGRGKLTGQVIDVPGDPS | 246 |
| Sbjct | 189 | PG TTVIE +RDHTE+ML+ FGA + VE + R I L G+ +L G I +P DPS APGRTTVIEREASRDHTERMLRHFGATVEVEPWGEHGRKITLTGQPELAGSDIAIPADPS | 248 |
| Query | 247 | STAFPLVAALLVPGSDVTILNVLMNPTRTGLILTLQEMGADIEVINPRLAGGEDVADLRV | 306 |
| Sbjct | 249 | S AFPLVAAL+VPGS +T+ V+MNP RTGL TL+EMGADIE IN GGEDVA LRV SAAFPLVAALIVPGSGITLEGVMMNPLRTGLFTTLREMGADIEAINQTSEGGEDVATLRV | 308 |
| Query | 307 | RSSTLKGVTVPEDRAPSMIDEYPILAVAAAFAEGATVMNGLEELRVKESDRLSAVANGLK | 366 |
| Sbjct | 309 | R+S LKGV VP +RAPSMIDEYP+LAVAAAFAEG T M GL ELRVKESDRL+A+A+GL RASALKGVDVPAERAPSMIDEYPVLAVAAAFAEGETRMRGLSELRVKESDRLAAIADGLT | 368 |
| Query | 367 | LNG-VDCDEGETSLVVRGRPDGKGLGNASGAAVATHLDHRIAMSFLVMGLVSENPVTVDD | 425 |

G V+ EG+ +V G	+G	G	ATHLDHRIAMSFLV+GL +	+ +DD

| Sbjct | 369 | AAGVVNRIEGDDLIVTGGAGRVRG GGVAATHLDHRIAMSFLVLGLAASEGMRIDD | 423 |
| --- | --- | --- | --- |
| Query  Sbjct | 426  424 | ATMIATSFPEFMDLMAGLGAKIEL 449 A MIATSFP F LM LGA I + AAMIATSFPAFTPLMRSLGADIAM 447 |  |

>TR:A0A0N9U4K9 A0A0N9U4K9_SPHMC 3-phosphoshikimate 1-carboxyvinyltransferase OS=Sphingopyxis macrogoltabida OX=33050 GN=aroA PE=3 SV=1

Length=451

Score = 474 bits (1219), Expect = 2e-162

| Identities | = 261/446 (59%), Positives = 310/446 (70%), Gaps = 10/446 (2%) |  |
| --- | --- | --- |
| Query 3 | HGASSRPATARKSSGLSGTVRIPGDKSISHRSFMFGGLASGETRITGLLEGEDVINTGKA | 62 |
| Sbjct 4 | H AS R +A S L G + IPGDKSISHR+ M LA GE+R+TGLLEG DV+ T A HPASPRSFSA--SRPLKGRIAIPGDKSISHRALMLSALAVGESRVTGLLEGHDVLATAAA | 61 |
| Query 63 | MQAMGARIRKEGD-TWIIDGVGNGGLLAPEAPLDFGNAATGCRLTMGLVGVYDFDSTFIG | 121 |
| Sbjct 62 | M+AMGA I + D W I GVG GGLL P LD GN+ T RL MGLV + TF+G MRAMGAGIERRDDGEWRIHGVGVGGLLQPREALDMGNSGTSTRLLMGLVASHPITVTFVG | 121 |
| Query 122 | DASLTKRPMGRVLNPLREMGVQVKSEDGDR LPVTLRGPKTPTPITYRVPMASAQVK | 177 |
| Sbjct 122 | DASL+ RPMGRV+ PL +MG + + G LP+ +RG P+ YR+PMASAQVK DASLSGRPMGRVIEPLSQMGADIGASPGANGTKTLPLMVRGLAPAIPLAYRLPMASAQVK | 181 |
| Query 178 | SAVLLAGLNTPGITTVIEPIMTRDHTEKMLQGFGANLTVETDADGVRTIRLEGRGKLTGQ | 237 |
| Sbjct 182 | SAVLLAGLNTPGIT VIEP+ TRDH+E+ML+GFGA+L VETDADG R IR+ G +L Q SAVLLAGLNTPGITEVIEPVPTRDHSERMLRGFGADLQVETDADGTRHIRIRGEAELKPQ | 241 |
| Query 238 | VIDVPGDPSSTAFPLVAALLVPGSDVTILNVLMNPTRTGLILTLQEMGADIEVINPRLAG | 297 |
| Sbjct 242 | I VPGDPSS AF +VAAL+VPGSD+ + NV +NPTR GLI L++MG DIE+++ R G TIVVPGDPSSAAFFIVAALIVPGSDIIVANVGLNPTRAGLIEILKQMGGDIELLDARDVG | 301 |
| Query 298 | GEDVADLRVRSSTLKGVTVPEDRAPSMIDEYPILAVAAAFAEGATVMNGLEELRVKESDR | 357 |
| Sbjct 302 | GE VADLRVR S LKG+ V APSMIDE+PIL VAAA AEG TV GL+ELRVKESDR  GEPVADLRVRHSALKGIDVDPAVAPSMIDEFPILFVAAALAEGRTVTTGLDELRVKESDR | 361 |

| Query | 358 | LSAVANGLKLNGVDCDEGETSLVVRGRPDGKGLGNASGAAVATHLDHRIAMSFLVMGLVS 417 |
| --- | --- | --- |
| Sbjct | 362 | LS +A GLK G +E E L++ G G GA +A HLDHRI MSF + GLVS LSVMATGLKAIGARVEESEDGLIIDGT---GGDPLPGGATIAGHLDHRICMSFAIAGLVS 418 |
| Query | 418 | ENPVTVDDATMIATSFPEFMDLMAGL 443 |
| Sbjct | 419 | PV VDD +ATSFP+F L+AGL  TAPVMVDDMAPVATSFPDFEVLLAGL 444 |

>TR:A0A126P0I9 A0A126P0I9_9BRAD 3-phosphoshikimate 1-carboxyvinyltransferase OS=Bosea sp. PAMC 26642 OX=1792307 GN=aroA PE=3 SV=1

Length=446

Score = 473 bits (1218), Expect = 3e-162

| Identities | | = 261/449 (58%), Positives = 314/449 (70%), Gaps = 6/449 (1%) |  |
| --- | --- | --- | --- |
| Query 1  Sbjct 1 | | MLHGASSRPATARKSSGLSGTVRIPGDKSISHRSFMFGGLASGETRITGLLEGEDVINTG M H P TAR++ L G VR+PGDKS+SHR+ +FG LA GET I+GLLEGEDV+ T MAHAQHPIPVTARRTGLLRGHVRVPGDKSVSHRAMIFGLLAIGETTISGLLEGEDVMRTA | 60  60 |
| Query 61  Sbjct 61 | | KAMQAMGARIRKEGD-TWIIDGVGNGGLLAPEAPLDFGNAATGCRLTMGLVGVYDFDSTF A +A+GA I +G W + GVG GGL P+ LDFGNA TG RL MG+ G + + F DAARALGAIIHHDGPGAWRVRGVGVGGLREPKGTLDFGNAGTGSRLMMGVCGAHPITTIF | 119  120 |
| Query 120  Sbjct 121 | | IGDASLTKRPMGRVLNPLREMGVQVKSE-DGDRLPVTLRGPKTPTPITYRVPMASAQVKS GDASL KRPM R+L+PL MG + S+ +G R+P+TLRGP+ PI YR P+ASAQ+KS DGDASLRKRPMRRILDPLELMGTTIVSQGEGGRVPLTLRGPREAVPIVYRTPVASAQIKS | 178  180 |
| Query 179  Sbjct 181 | | AVLLAGLNTPGITTVIEPIMTRDHTEKMLQGFGANLTVETDADGVRTIRLEGRGKLTGQV AVLLAGL PG TTVIE TRDHTE ML+ FGA L + R I L G+ +L AVLLAGLAAPGETTVIETEATRDHTETMLRHFGAQLVSTPEGAHGRRIVLTGQPELQAAP | 238  240 |
| Query 239  Sbjct 241 | | IDVPGDPSSTAFPLVAALLVPGSDVTILNVLMNPTRTGLILTLQEMGADIEVINPRLAGG I VP DPSS AFPLVA L+VPGSD+ + +V+ NP R+GL+ TL+EMGADI V + GG IVVPADPSSAAFPLVAGLIVPGSDIVLESVMTNPLRSGLLTTLREMGADITVESEANEGG | 298  300 |
| Query 299  Sbjct 301 | | EDVADLRVRSSTLKGVTVPEDRAPSMIDEYPILAVAAAFAEGATVMNGLEELRVKESDRL E VA LRVR+S+L+GV VP +RAPSMIDEYP+LAVAA+FA G T M GL+ELRVKESDRL EAVATLRVRASSLRGVEVPPERAPSMIDEYPVLAVAASFATGTTRMRGLQELRVKESDRL | 358  360 |
| Query 359  Sbjct 361 | | SAVANGLKLNGVDCDEGETSLVVRGRPDGKGLGNASGAAVATHLDHRIAMSFLVMGLVSE  +AV GL+ GV C LVV +G G A G VATHLDHRIAMSFLVMGL ++ AAVEAGLRAAGVTCAIEGDDLVV EGSGGTVAGGGTVATHLDHRIAMSFLVMGLATQ | 418  416 |
| Query 419 | | NPVTVDDATMIATSFPEFMDLMAGLGAKI 447 P+ VDD MIATSFP F+ LM GLGA I |  |
| Sbjct | 417 | APMQVDDGAMIATSFPSFIALMNGLGADI 445 | |

>SP:Q2G8Q3 AROA_NOVAD 3-phosphoshikimate 1-carboxyvinyltransferase OS=Novosphingobium aromaticivorans (strain ATCC 700278 / DSM 12444

/ CIP 105152 / NBRC 16084 / F199) OX=279238 GN=aroA PE=3 SV=1

Length=441

Score = 473 bits (1217), Expect = 3e-162

Identities = 252/434 (58%), Positives = 302/434 (70%), Gaps = 1/434 (0%)

| Query | 8 | RPATARKSSGLSGTVRIPGDKSISHRSFMFGGLASGETRITGLLEGEDVINTGKAMQAMG | 67 |
| --- | --- | --- | --- |
| Sbjct | 2 | RP + L G + +PGDKSISHRS M G LA GETR+TGLLEGEDV++T AM+AMG RPRRFTANGPLKGRIGVPGDKSISHRSIMLGALAVGETRVTGLLEGEDVLSTAAAMRAMG | 61 |
| Query | 68 | ARIRKEGD-TWIIDGVGNGGLLAPEAPLDFGNAATGCRLTMGLVGVYDFDSTFIGDASLT A I ++ D W + GVG GGLL P+ LD GN+ T RL MGLV + +TF+GDASL+ | 126 |

| Sbjct | 62 | ATIERDADGMWHVHGVGVGGLLQPQQALDMGNSGTSTRLLMGLVATHPITATFVGDASLS | 121 |
| --- | --- | --- | --- |
| Query | 127 | KRPMGRVLNPLREMGVQVKSEDGDRLPVTLRGPKTPTPITYRVPMASAQVKSAVLLAGLN | 186 |
| Sbjct | 122 | KRPMGRV++PL MG + + G RLP+TLRG PI YR+P+ASAQVKSAVLLAGLN KRPMGRVIDPLSTMGAEFTASPGGRLPLTLRGISPAVPIEYRLPVASAQVKSAVLLAGLN | 181 |
| Query | 187 | TPGITTVIEPIMTRDHTEKMLQGFGANLTVETDADGVRTIRLEGRGKLTGQVIDVPGDPS | 246 |
| Sbjct | 182 | TPG+TTVIEPI TRDH+E+ML+GFGA LTV+ ADG R IR+ G +L Q I VPGDPS TPGVTTVIEPIPTRDHSERMLRGFGAELTVDVAADGARVIRVRGEAELKPQDIAVPGDPS | 241 |
| Query | 247 | STAFPLVAALLVPGSDVTILNVLMNPTRTGLILTLQEMGADIEVINPRLAGGEDVADLRV | 306 |
| Sbjct | 242 | S AF +VAALLV GSD+ + NV +NPTR L L+ MG IE +N R GGE VADLRV SAAFFVVAALLVEGSDLVVENVGLNPTRAALFDVLRLMGGSIEELNRREVGGEPVADLRV | 301 |
| Query | 307 | RSSTLKGVTVPEDRAPSMIDEYPILAVAAAFAEGATVMNGLEELRVKESDRLSAVANGLK | 366 |
| Sbjct | 302 | R S L G+ V PSM+DE+P+L VAAA A+G TV GLEELRVKESDR+SA+ L+ RHSLLTGIDVDPAVVPSMVDEFPVLFVAAALAKGRTVTTGLEELRVKESDRISAMRAALE | 361 |
| Query | 367 | LNGVDCDEGETSLVVRGRPDGKGLGNASGAAVATHLDHRIAMSFLVMGLVSENPVTVDDA | 426 |
| Sbjct | 362 | L G E E L++ G G A GA+V THLDHRIAMS + G+ S N V VDD LAGATVTETEDGLIIDGTGGDPLPGTAEGASVVTHLDHRIAMSMAIAGIASRNGVEVDDT | 421 |
| Query | 427 | TMIATSFPEFMDLM 440 IATSFP F L+ |  |
| Sbjct | 422 | RPIATSFPVFESLL 435 | |

>TR:A0A3D9H9E6 A0A3D9H9E6_9PROT 3-phosphoshikimate 1-carboxyvinyltransferase OS=Aestuariispira insulae OX=1461337 GN=aroA PE=3 SV=1

Length=441

Score = 473 bits (1217), Expect = 3e-162

| Identities | | = 252/437 (58%), Positives = 306/437 (70%), Gaps = 5/437 (1%) |  |
| --- | --- | --- | --- |
| Query 12  Sbjct 6 | | ARKSSGLSGTVRIPGDKSISHRSFMFGGLASGETRITGLLEGEDVINTGKAMQAMGARIR A KS L+GTV +PGDKSISHRS M G +A G T + GLLEGEDV++T A +AMGA I ASKSQNLTGTVTVPGDKSISHRSIMLGSMAIGRTTVKGLLEGEDVLSTAAAFRAMGATIE | 71  65 |
| Query 72  Sbjct 66 | | KEGD-TWIIDGVGNGGLLAPEAPLDFGNAATGCRLTMGLVGVYDFDSTFIGDASLTKRPM K D TW +DGVG GGL P+ LD GN+ T RL +G++ +D GD SL KRPM KHEDGTWTVDGVGIGGLQEPDDVLDMGNSGTSARLILGILAGHDITCFLTGDDSLRKRPM | 130  125 |
| Query 131  Sbjct 126 | | GRVLNPLREMGVQVKSEDGDRLPVTLRGPKTPTPITYRVPMASAQVKSAVLLAGLNTPGI GRV+NPL EMG +S G RLP+ +RG PITY +PMASAQVKSAVLLAGL+ G GRVINPLSEMGATFESRAGGRLPLMVRGAADVLPITYELPMASAQVKSAVLLAGLSARGK | 190  185 |
| Query 191 | | TTVIEPIMTRDHTEKMLQGFGANLTVETDADGVRTIRLEGRGKLTGQVIDVPGDPSSTAF TTVIE TRDHTE+ML FG +TVE G I + G+ +LT + VP DPSS AF | 250 |
| Sbjct | 186 | TTVIENKPTRDHTERMLTHFGGEITVEPIEGGGNKITVTGQPELTAADLVVPSDPSSAAF | 245 |
| Query | 251 | PLVAALLVPGSDVTILNVLMNPTRTGLILTLQEMGADIEVINPRLAGGEDVADLRVRSST | 310 |
| Sbjct | 246 | P VAA + PGS+V + + MNPTR+G+ TL++MGAD+ + N R+ GGE VAD+ +R + PAVAAAITPGSEVVLPAIGMNPTRSGIFQTLKDMGADLTLSNERIEGGEPVADITIRHAQ | 305 |
| Query | 311 | LKGVTVPEDRAPSMIDEYPILAVAAAFAEGATVMNGLEELRVKESDRLSAVANGLKLNGV | 370 |
| Sbjct | 306 | L G+ VPE+RAPSMIDEYP+LAVAAA A G TVM G+ ELRVKESDRL+ VA GL+ NG+ LTGIEVPEERAPSMIDEYPVLAVAAAQASGKTVMRGIGELRVKESDRLAVVAEGLRANGI | 365 |
| Query | 371 | DCDEGETSLVVRGRPDGKGLGNASGAAVATHLDHRIAMSFLVMGLVSENPVTVDDATMIA | 430 |
| Sbjct | 366 | +EGE ++V G GK G G V THLDHRI+MSFLVMGLVSE PVT+DD I TVEEGEDWMIVHG-TGGKIPG---GGTVTTHLDHRISMSFLVMGLVSEKPVTIDDGQPIE | 421 |
| Query | 431 | TSFPEFMDLMAGLGAKI 447 TSFP F++LM GLGA I |  |

Sbjct 422 TSFPNFLELMTGLGADI 438

>TR:A0A1I2R5L7 A0A1I2R5L7_9SPHN 3-phosphoshikimate 1-carboxyvinyltransferase OS=Novosphingobium sp. CF614 OX=1884364 GN=aroA PE=3 SV=1

Length=442

Score = 473 bits (1217), Expect = 3e-162

| Identities | = 254/441 (58%), Positives = 308/441 (70%), Gaps = 7/441 (2%) |  |
| --- | --- | --- |
| Query 8 | RPATARKSSGLSGTVRIPGDKSISHRSFMFGGLASGETRITGLLEGEDVINTGKAMQAMG | 67 |
| Sbjct 2 | RP + L G +R+PGDKSISHRS M G LA GETR+TGLLEGEDV+ T A++AMG RPRRFLPTGPLRGRIRVPGDKSISHRSIMLGALAVGETRVTGLLEGEDVLATAAALRAMG | 61 |
| Query 68 | ARIRKEGDT WIIDGVGNGGLLAPEAPLDFGNAATGCRLTMGLVGVYDFDSTFIGD | 122 |
| Sbjct 62 | A + + G + W I GVG G LL PEAPLD GN+ T RL MGLV + ++F+GD ASVERTGASDAGGEWSIHGVGVGALLQPEAPLDMGNSGTSTRLLMGLVASHPITASFVGD | 121 |
| Query 123 | ASLTKRPMGRVLNPLREMGVQVKSEDGDRLPVTLRGPKTPTPITYRVPMASAQVKSAVLL | 182 |
| Sbjct 122 | ASL+KRPMGRV++PL MG ++ +G RLP+ +RG PI+YR+P+ASAQVKSAVLL ASLSKRPMGRVIDPLATMGASFEASEGGRLPLIVRGASPAVPISYRLPVASAQVKSAVLL | 181 |
| Query 183 | AGLNTPGITTVIEPIMTRDHTEKMLQGFGANLTVETDADGVRTIRLEGRGKLTGQVIDVP | 242 |
| Sbjct 182 | AGLNTPGITTVIEP+ TRDH+E+ML+GFGA L VE ADG R I L G +L QVIDVP AGLNTPGITTVIEPVPTRDHSERMLRGFGAELNVEIAADGTRIITLHGEAELKPQVIDVP | 241 |
| Query 243 | GDPSSTAFPLVAALLVPGSDVTILNVLMNPTRTGLILTLQEMGADIEVINPRLAGGEDVA | 302 |
| Sbjct 242 | GDPSS AF +VAAL+VPGS++ I NV +NPTR GL+ L++MG IE +N R GGE VA GDPSSAAFFMVAALIVPGSEIVIENVGLNPTRAGLVTVLRQMGGRIEELNAREVGGEPVA | 301 |
| Query 303 | DLRVRSSTLKGVTVPEDRAPSMIDEYPILAVAAAFAEGATVMNGLEELRVKESDRLSAVA | 362 |
| Sbjct 302 | DL V+ S LKG+ V APSMIDE+P L VAA+ AEG T +GL+ELRVKESDRL+ +A DLLVKHSALKGIEVDPALAPSMIDEFPALFVAASLAEGVTTTSGLDELRVKESDRLAVMA | 361 |
| Query 363 | NGLKLNGVDCDEGETSLVVRGRPDGKGLGNASGAAVATHLDHRIAMSFLVMGLVSENPVT | 422 |

L	G	+E E LV+ G	G L	+G +ATHLDHRIAMS V GL S	V

| Sbjct | 362 | AALAAAGARIEEREDGLVITG-TGGDPLAGTTG-PIATHLDHRIAMSMAVAGLASRAGVE | 419 |
| --- | --- | --- | --- |
| Query  Sbjct | 423  420 | VDDATMIATSFPEFMDLMAGL 443 VDD IATSFP F ++ GL VDDTRPIATSFPVFETMLEGL 440 |  |

>TR:A0A521AEU6 A0A521AEU6_9RHOB 3-phosphoshikimate 1-carboxyvinyltransferase OS=Paracoccus laeviglucosivorans OX=1197861 GN=aroA PE=3 SV=1

Length=443

Score = 473 bits (1217), Expect = 3e-162

| Identities | | = 250/448 (56%), Positives = 307/448 (69%), Gaps = 7/448 (2%) |  |
| --- | --- | --- | --- |
| Query 1  Sbjct 1 | | MLHGASSRPATARKSSGLSGTVRIPGDKSISHRSFMFGGLASGETRITGLLEGEDVINTG M H A+ +P AR+S L G R+PGDKSISHR+ + G L+ GET ITGLLEG+DV++T MSHSAAPQPMIARRSGPLQGEARVPGDKSISHRALILGALSVGETHITGLLEGQDVLDTA | 60  60 |
| Query 61 | | KAMQAMGARIRKEG-DTWIIDGVGNGGLLAPEAPLDFGNAATGCRLTMGLVGVYDFDSTF AMQA GA++ + G W ++GVG GG P +D GN+ TG RL MG + +TF | 119 |
| Sbjct | 61 | SAMQAFGAQVERLGIGEWKVNGVGVGGFAEPAGVIDCGNSGTGVRLIMGAMATTPITATF | 120 |
| Query | 120 | IGDASLTKRPMGRVLNPLREMGVQVKSEDGDRLPVTLRGPKTPTPITYRVPMASAQVKSA | 179 |
| Sbjct | 121 | GDASL++RPMGR+ +PL G +V + +G RLPVT++G P+ Y+ PMASAQVKSA TGDASLSRRPMGRITDPLELFGCEVTAREGKRLPVTIKGTADAVPVRYKTPMASAQVKSA | 180 |
| Query | 180 | VLLAGLNTPGITTVIEPIMTRDHTEKMLQGFGANLTVETDADGVRTIRLEGRGKLTGQVI VLLAGLN+PG T VIE TRDHTE+ML GFGA +T E G I L+GR +L Q + | 239 |

| Sbjct | 181 | VLLAGLNSPGETVVIEAEPTRDHTERMLAGFGATITTEKTDQGY-VITLQGRPELKPQPV | 239 |
| --- | --- | --- | --- |
| Query | 240 | DVPGDPSSTAFPLVAALLVPGSDVTILNVLMNPTRTGLILTLQEMGADIEVINPRLAGGE | 299 |
| Sbjct | 240 | VP DPSS AFP+ AAL+VPGS++ + V NPTR GL +TL EMGADI N R+ GGE AVPRDPSSAAFPVAAALIVPGSEIRVPGVSRNPTRDGLYVTLLEMGADITFENERVEGGE | 299 |
| Query | 300 | DVADLRVRSSTLKGVTVPEDRAPSMIDEYPILAVAAAFAEGATVMNGLEELRVKESDRLS | 359 |
| Sbjct | 300 | VADL VR LKGVT PEDRA SMIDE+PIL+V A FA G TVM G++ELRVKESDR++ PVADLLVRHGPLKGVTTPEDRAASMIDEFPILSVIACFATGTTVMQGVKELRVKESDRIA | 359 |
| Query | 360 | AVANGLKLNGVDCDEGETSLVVRGRPDGKGLGNASGAAVATHLDHRIAMSFLVMGLVSEN | 419 |
| Sbjct | 360 | A+ GL+ NG DE E ++ V G A G THLDHRIAMSFLV GL SE AMVAGLRANGASVDETEDTMTVHGTG ALAGGGTAVTHLDHRIAMSFLVGGLASEQ | 414 |
| Query  Sbjct | 420  415 | PVTVDDATMIATSFPEFMDLMAGLGAKI 447 P+ VDD IATSFP+F+ LM LGA+I PIGVDDGRPIATSFPDFLPLMTALGAQI 442 |  |

>TR:A0A0F5PFN4 A0A0F5PFN4_9SPHN 3-phosphoshikimate 1-carboxyvinyltransferase OS=Sphingomonas sp. SRS2 OX=133190 GN=aroA PE=3 SV=1

Length=445

Score = 473 bits (1217), Expect = 4e-162

Identities = 253/447 (57%), Positives = 306/447 (68%), Gaps = 4/447 (1%)

| Query | 2 | LHGASSRPATARKSSGLSGTVRIPGDKSISHRSFMFGGLASGETRITGLLEGEDVINTGK | 61 |
| --- | --- | --- | --- |
| Sbjct | 1 | +H +PA+ L G +PGDKSISHR+ M LA GE+R+ GLL GEDV+ T MHATLPKPASFSAPGPLRGQAAVPGDKSISHRALMLSALAVGESRVEGLLTGEDVLATAD | 60 |
| Query | 62 | AMQAMGARI-RKEGDTWIIDGVGNGGLLAPEAPLDFGNAATGCRLTMGLVGVYDFDSTFI | 120 |
| Sbjct | 61 | AM+AMGA+I R + D W I GVG GGLL PE LD GN+ T RL MGLV + +TF AMRAMGAQIERGDDDIWRIHGVGVGGLLQPEVALDMGNSGTSTRLLMGLVASHAITATFT | 120 |
| Query | 121 | GDASLTKRPMGRVLNPLREMGVQVKSEDGDRLPVTLRGPKTPTPITYRVPMASAQVKSAV | 180 |
| Sbjct | 121 | GDASL+KRPM RV+ PL MG + + G RLP+ LRG PI YR+P+ASAQVKSAV GDASLSKRPMNRVIEPLALMGADITASPGGRLPLMLRGLSPAVPIEYRLPVASAQVKSAV | 180 |
| Query | 181 | LLAGLNTPGITTVIEPIMTRDHTEKMLQGFGANLTVETDADGVRTIRLEGRGKLTGQVID | 240 |
| Sbjct | 181 | LLAGLNTPG+T VIEPI TRDH+E+ML+GFGA L+VE DADG R I + G +L Q I LLAGLNTPGVTRVIEPIATRDHSERMLRGFGAELSVEQDADGARIISIRGEAELRPQHIV | 240 |
| Query | 241 | VPGDPSSTAFPLVAALLVPGSDVTILNVLMNPTRTGLILTLQEMGADIEVINPRLAGGED | 300 |
| Sbjct | 241 | VPGDPSS AFP+VAALLVPGS+VT+ NV +NPTR L L+ MG DI N R GGE VPGDPSSAAFPVVAALLVPGSEVTVTNVGLNPTRAALFDVLRMMGGDISFTNQREVGGEP | 300 |
| Query | 301 | VADLRVRSSTLKGVTVPEDRAPSMIDEYPILAVAAAFAEGATVMNGLEELRVKESDRLSA | 360 |
| Sbjct | 301 | VAD+ VR S LKG+ P + +M+DEYPIL VAAA AEG TV GLEELRVKESDR++ VADIVVRHSALKGIETPVETVAAMVDEYPILFVAAALAEGRTVARGLEELRVKESDRIAV | 360 |
| Query | 361 | VANGLKLNGVDCDEGETSLVVRGRPDGKGLGNASGAAVATHLDHRIAMSFLVMGLVSENP | 420 |
| Sbjct | 361 | +A GL+ G +E E L++ G G GA +A LDHRIAMSF V GLV++ P MAEGLRAIGAQVEELEDGLIIDGT---GGDPLPGGAMIAARLDHRIAMSFAVAGLVAKAP | 417 |
| Query  Sbjct | 421  418 | VTVDDATMIATSFPEFMDLMAGLGAKI 447 VT+DD + +ATSFP F LM LGA++ VTIDDMSPVATSFPTFTALMTSLGAEV 444 |  |

>TR:Q21CB1 Q21CB1_RHOPB 3-phosphoshikimate 1-carboxyvinyltransferase OS=Rhodopseudomonas palustris (strain BisB18) OX=316056 GN=aroA

PE=3 SV=1

Length=467

Score = 474 bits (1219), Expect = 4e-162

| Identities | | = 257/440 (58%), Positives = 308/440 (70%), Gaps = 7/440 (2%) |  |
| --- | --- | --- | --- |
| Query 9  Sbjct 31 | | PATARKSSGLSGTVRIPGDKSISHRSFMFGGLASGETRITGLLEGEDVINTGKAMQAMGA P ARKS+ L+G VR+PGDKSISHR+ + G LA GETRI+GLLEGEDV+NT KAM+A+GA PLQARKSASLTGRVRVPGDKSISHRALILGALAVGETRISGLLEGEDVLNTAKAMRALGA | 68  90 |
| Query 69  Sbjct 91 | | RIRKEGD-TWIIDGVGNGGLLAPEAPLDFGNAATGCRLTMGLVGVYDFDSTFIGDASLTK  + + GD W I GVG GG P A LDFGN+ TGCRL MG V +TF GDASL SVERTGDFAWRIHGVGVGGFAQPAAALDFGNSGTGCRLVMGAVAGCPITATFDGDASLRS | 127  150 |
| Query 128  Sbjct 151 | | RPMGRVLNPLREMGVQV-KSEDGDRLPVTLRGPKTPTPITYRVPMASAQVKSAVLLAGLN RPM R+++PL MG +V S +G RLP+TL+G + P PI YR P+ASAQ+KSAVLLAGL RPMRRIVDPLELMGARVIDSREGGRLPLTLQGARDPLPILYRTPVASAQIKSAVLLAGLA | 186  210 |
| Query 187  Sbjct 211 | | TPGITTVIEPIMTRDHTEKMLQGFGANLTVETDADGVRTIRLEGRGKLTGQVIDVPGDPS PGITTVIE +RDHTE ML FGA + E + R I L G+ +L G + VP DPS APGITTVIEQEASRDHTELMLSHFGAQIVSEPEGAHGRKISLTGQPELRGAPVVVPADPS | 246  270 |
| Query 247  Sbjct 271 | | STAFPLVAALLVPGSDVTILNVLMNPTRTGLILTLQEMGADIEVINPRLAGGEDVADLRV S AFP+VAAL+VPGSD+ + V+ NP RTGL TL+EMG IE R GE +A RV SAAFPIVAALIVPGSDIVLTEVMTNPLRTGLFKTLREMGGSIEESEVRGDAGEPMAQFRV | 306  330 |
| Query 307  Sbjct 331 | | RSSTLKGVTVPEDRAPSMIDEYPILAVAAAFAEGATVMNGLEELRVKESDRLSAVANGLK R+S LKGV VP +RAPSMIDEY +LAVAAAFAEG T M GL+ELRVKESDRL A A L+ RASQLKGVEVPPERAPSMIDEYLVLAVAAAFAEGTTRMRGLKELRVKESDRLEATAAMLR | 366  390 |
| Query 367  Sbjct 391 | | LNGVDCDEGETSLVVRGRPDGKGLGNASGAAVATHLDHRIAMSFLVMGLVSENPVTVDDA LNGV + L+V GR G G VAT +DHRIAMS LVMGL ++ PVT+DD LNGVSVEIDGDDLIVEGRGHVPG GGLVATEMDHRIAMSALVMGLAADQPVTIDDT | 426  445 |
| Query 427 | | TMIATSFPEFMDLMAGLGAK 446 IATSFP+F+ +M LGA+ |  |
| Sbjct | 446 | GFIATSFPDFIPMMRRLGAE 465 | |

>TR:A0A2T6B7Z2 A0A2T6B7Z2_9RHOB 3-phosphoshikimate 1-carboxyvinyltransferase OS=Roseivivax pacificus OX=1267769 GN=aroA PE=3 SV=1

Length=449

Score = 473 bits (1217), Expect = 4e-162

| Identities | = 254/440 (58%), Positives = 306/440 (70%), Gaps = 5/440 (1%) |  |
| --- | --- | --- |
| Query 9 | PATARKSSGLSGTVRIPGDKSISHRSFMFGGLASGETRITGLLEGEDVINTGKAMQAMGA | 68 |
| Sbjct 9 | P TARK+ LSGT +PGDKSISHRS + G +A GETR+TGLLEG+DV++T KAM+A GA PMTARKAGPLSGTADVPGDKSISHRSLILGAMAVGETRVTGLLEGQDVLDTAKAMRAFGA | 68 |
| Query 69 | RIRKEGDTWIIDGVGNGGLLAPEAPLDFGNAATGCRLTMGLVGVYDFDSTFIGDASLTKR | 128 |
| Sbjct 69 | + K+GDTW+I GVG GG P+ +D GN+ TG RL MG + +TF GDASL KR EVDKQGDTWVIHGVGTGGFAEPDNVIDCGNSGTGVRLIMGAMATTPITATFTGDASLNKR | 128 |
| Query 129 | PMGRVLNPLREMGVQVKSEDGDRLPVTLRGPKTPTPITYRVPMASAQVKSAVLLAGLNTP | 188 |
| Sbjct 129 | PM RV +PL G + G RLP+T+ G + P+ Y VP+ SAQVKSAVLLAGL P PMARVTDPLALFGARAYGRSGGRLPMTIVGAEAAVPVRYEVPVPSAQVKSAVLLAGLAAP | 188 |
| Query 189 | GITTVIEPIMTRDHTEKMLQGFGANLTVETDADGVRTIRLEGRGKLTGQVIDVPGDPSST | 248 |
| Sbjct 189 | G T VIE TRDH+E+ML GFGA ++ E + R I L G+ +L Q I VP DPSS GETVVIEREATRDHSERMLAGFGAQISTEV-TNEARIITLTGQPELRPQTIVVPRDPSSA | 247 |
| Query 249 | AFPLVAALLVPGSDVTILNVLMNPTRTGLILTLQEMGADIEVINPRLAGGEDVADLRVR- | 307 |
| Sbjct 248 | AFP+ AAL+V GSDV + N+ +NPTR GL TL+EMGAD+ N R GGE VADLR R AFPVCAALIVEGSDVLVPNIGLNPTRAGLFTTLREMGADLTYENMREEGGEPVADLRARF | 307 |
| Query 308 | SSTLKGVTVPEDRAPSMIDEYPILAVAAAFAEGATVMNGLEELRVKESDRLSAVANGLKL | 367 |

| Sbjct | 308 | S LKG+ VP DRA SMIDEYP+L+V AAFAEG T M G++ELRVKESDR+ A+A GL+  SPDLKGIEVPPDRAASMIDEYPVLSVVAAFAEGKTHMPGVKELRVKESDRIDAMATGLRA | 367 |
| --- | --- | --- | --- |
| Query | 368 | NGVDCDEGETSLVVRGRPDGKGLGNASGAAVATHLDHRIAMSFLVMGLVSENPVTVDDAT | 427 |
| Sbjct | 368 | GV+ DEGE V GR G A GA V +HLDHRIAMSF+V+G + PVTVDD CGVEVDEGEDWWTVHGRGHG---AVAGGATVESHLDHRIAMSFMVLGFAANAPVTVDDGG | 424 |
| Query | 428 | MIATSFPEFMDLMAGLGAKI 447 IATSFP F LMAGLGA+I |  |
| Sbjct | 425 | PIATSFPIFEPLMAGLGAEI 444 | |

>TR:C8RWA4 C8RWA4_9RHOB 3-phosphoshikimate 1-carboxyvinyltransferase OS=Rhodobacter sp. SW2 OX=371731 GN=aroA PE=3 SV=1

Length=449

Score = 473 bits (1217), Expect = 4e-162

| Identities | = 256/449 (57%), Positives = 311/449 (69%), Gaps = 5/449 (1%) |  |
| --- | --- | --- |
| Query 1 | MLHGASSRPATARKSSGLSGTVRIPGDKSISHRSFMFGGLASGETRITGLLEGEDVINTG | 60 |
| Sbjct 1 | M ++P TARKS L G +PGDKSISHR+ +FG +A GETRI+GLLEG+DV++T MSAAGDAQPMTARKSGPLRGVAEVPGDKSISHRALIFGAMAVGETRISGLLEGQDVLDTA | 60 |
| Query 61 | KAMQAMGARIRKEG-DTWIIDGVGNGGLLAPEAPLDFGNAATGCRLTMGLVGVYDFDSTF | 119 |
| Sbjct 61 | +AM+A GA++ + G +W + GVG GG P +D GN+ TG RL MG + + +TF RAMRAFGAQVVQHGAGSWSVHGVGVGGFAEPADVIDCGNSGTGVRLIMGCMATHAMTATF | 120 |
| Query 120 | IGDASLTKRPMGRVLNPLREMGVQVKSEDGDRLPVTLRGPKTPTPITYRVPMASAQVKSA | 179 |
| Sbjct 121 | GDASL KRPMGRV PL G + G RLP+T+ G P P+ Y P+ SAQVKSA TGDASLRKRPMGRVTEPLALFGARSYGRKGGRLPMTVVGAAQPVPVRYTTPVPSAQVKSA | 180 |
| Query 180 | VLLAGLNTPGITTVIEPIMTRDHTEKMLQGFGANLTVETDADGVRTIRLEGRGKLTGQVI | 239 |
| Sbjct 181 | VLLAGLN PG T VIE TRDH+E+ML GFGA L VE A+G R I L G+ +L QV+ VLLAGLNAPGQTVVIEREPTRDHSERMLLGFGAELFVEDSAEG-RVITLTGQPELRPQVV | 239 |
| Query 240 | DVPGDPSSTAFPLVAALLVPGSDVTILNVLMNPTRTGLILTLQEMGADIEVINPRLAGGE | 299 |
| Sbjct 240 | VP DPSS AFP+ AAL+V GSD+ + V N TR GL LTL EMGADI NPR GGE AVPRDPSSAAFPVCAALVVEGSDIFVPGVSQNLTRNGLFLTLVEMGADIAFQNPRSEGGE | 299 |
| Query 300 | DVADLRVRSSTLKGVTVPEDRAPSMIDEYPILAVAAAFAEGATVMNGLEELRVKESDRLS | 359 |
| Sbjct 300 | VADLRVR L+G+ VP +RAPSMIDEYPILAV AAFAEG TVM G++ELRVKESDR+ PVADLRVRFGPLRGIEVPPERAPSMIDEYPILAVVAAFAEGRTVMRGVKELRVKESDRID | 359 |
| Query 360 | AVANGLKLNGVDCDEGETSLVVRGRPDGKGLGNASGAAVATHLDHRIAMSFLVMGLVSEN | 419 |
| Sbjct 360 | A+A GL+ GV +E E +L+V G G G GA A HLDHRIAMSFLV+G+ ++ AMARGLEACGVRVEEDEDTLIVHGMGAG---GVPGGATCAVHLDHRIAMSFLVLGMAAKA | 416 |
| Query 420  Sbjct 417 | PVTVDDATMIATSFPEFMDLMAGLGAKIE 448 PVTVDDA+ IATSFP F LM GLGA ++ PVTVDDASPIATSFPVFEGLMTGLGAVLD 445 |  |

>TR:A0A0N1LN53 A0A0N1LN53_9SPHN 3-phosphoshikimate 1-carboxyvinyltransferase OS=Novosphingobium sp. AAP93 OX=1523427 GN=aroA PE=3 SV=1

Length=453

Score = 473 bits (1217), Expect = 5e-162

Identities = 254/438 (58%), Positives = 307/438 (70%), Gaps = 2/438 (0%)

| Query | 5 | ASSRPATARKSSGLSGTVRIPGDKSISHRSFMFGGLASGETRITGLLEGEDVINTGKAMQ | 64 |
| --- | --- | --- | --- |
|  |  | ++ RP + L+G +R+PGDKSISHRS M G LA G TR+TGLLEGEDV++T AM+ |  |
| Sbjct | 7 | SAPRPRRFSAAGPLTGRIRVPGDKSISHRSIMLGALAVGRTRVTGLLEGEDVMSTAAAMR | 66 |
| Query | 65 | AMGARI-RKEGDTWIIDGVGNGGLLAPEAPLDFGNAATGCRLTMGLVGVYDFDSTFIGDA | 123 |

AMGA I R	W+IDGVG GGLL P	LD GN+ T RL MGLV +	+TFIGDA

| Sbjct | 67 | AMGATIDRLSSGEWVIDGVGVGGLLQPRQALDMGNSGTSTRLLMGLVASHPITATFIGDA | 126 |
| --- | --- | --- | --- |
| Query | 124 | SLTKRPMGRVLNPLREMGVQVKSEDGDRLPVTLRGPKTPTPITYRVPMASAQVKSAVLLA | 183 |
| Sbjct | 127 | SL+KRPMGRV++PL +MG + G RLP+TLRG PI+YR+P+ASAQVKSAVLLA SLSKRPMGRVIDPLSQMGADFTAAPGGRLPLTLRGISPAVPISYRLPVASAQVKSAVLLA | 186 |
| Query | 184 | GLNTPGITTVIEPIMTRDHTEKMLQGFGANLTVETDADGVRTIRLEGRGKLTGQVIDVPG | 243 |
| Sbjct | 187 | GLNTPGITTV+EP+ TRDH+E+ML+GFGA+L+VE ADG RTI L G +L Q I+VPG GLNTPGITTVVEPVPTRDHSERMLKGFGADLSVEVAADGTRTIHLTGEAELRPQTIEVPG | 246 |
| Query | 244 | DPSSTAFPLVAALLVPGSDVTILNVLMNPTRTGLILTLQEMGADIEVINPRLAGGEDVAD | 303 |
| Sbjct | 247 | DPSS AF +VAALLVPGSD+ I NV +NPTR L L++MG IE ++ R GGE VAD DPSSAAFFVVAALLVPGSDLVIENVGLNPTRAALFDVLRDMGGQIEEVDRRDVGGEPVAD | 306 |
| Query | 304 | LRVRSSTLKGVTVPEDRAPSMIDEYPILAVAAAFAEGATVMNGLEELRVKESDRLSAVAN | 363 |
| Sbjct | 307 | LRVR S L G+ V PSM+DE+P+L VAAA A G TV GLEELRVKESDR++ + LRVRHSALTGIAVDPAVVPSMVDEFPVLFVAAALANGRTVTTGLEELRVKESDRITVMRV | 366 |
| Query | 364 | GLKLNGVDCDEGETSLVVRGRPDGKGLGNASGAAVATHLDHRIAMSFLVMGLVSENPVTV | 423 |

L+ G	E E L++ G	G+ L A+	VATHLDHRIAMS V GL S	V +

| Sbjct | 367 | ALEQAGATVTETEDGLIIDG-TGGEPLRGANNQPVATHLDHRIAMSMAVAGLASRAGVDI | 425 |
| --- | --- | --- | --- |
| Query  Sbjct | 424  426 | DDATMIATSFPEFMDLMA 441 DD IATSFP F L+A DDTRPIATSFPVFEALLA 443 |  |

>TR:A0A2Y9B163 A0A2Y9B163_9RHOB 3-phosphoshikimate 1-carboxyvinyltransferase OS=Jannaschia seohaensis OX=475081 GN=aroA PE=3 SV=1

Length=444

Score = 473 bits (1216), Expect = 5e-162

| Identities | = 257/447 (57%), Positives = 306/447 (68%), Gaps = 8/447 (2%) |  |
| --- | --- | --- |
| Query 1 | MLHGASSRPATARKSSGLSGTVRIPGDKSISHRSFMFGGLASGETRITGLLEGEDVINTG | 60 |
| Sbjct 1 | M HG + +P TA S L+G +PGDKSISHRS + G L+ GETRI+GLLEG+DV++TG MSHGPA-QPLTAPASGPLTGVAEVPGDKSISHRSLILGALSEGETRISGLLEGQDVLDTG | 59 |
| Query 61 | KAMQAMGARIRKEGDTWIIDGVGNGGLLAPEAPLDFGNAATGCRLTMGLVGVYDFDSTFI | 120 |
| Sbjct 60 | AM+A GA + K G W++ GVG GGLL PE +D GN+ TG RL MG+ + F T+ AAMRAFGATVEKHGGDWVVHGVGVGGLLEPEDVIDCGNSGTGVRLLMGVAATHGFAVTYT | 119 |
| Query 121 | GDASLTKRPMGRVLNPLREMGVQVKSEDGDRLPVTLRGPKTPTPITYRVPMASAQVKSAV | 180 |
| Sbjct 120 | GDASL RPM RV PL G Q G RLP+T+ G P P+ Y PMASAQVKSAV GDASLRSRPMARVTEPLALFGAQAYGRSGGRLPMTVVGASDPIPVAYTTPMASAQVKSAV | 179 |
| Query 181 | LLAGLNTPGITTVIEPIMTRDHTEKMLQGFGANLTVETDADGVRTIRLEGRGKLTGQVID | 240 |
| Sbjct 180 | LLAGLN PG T V E TRDH+E+ML+GFGA + E A+G IRL+GR L G+ I LLAGLNAPGETVVTEREATRDHSERMLRGFGAQVETERTAEG-HVIRLQGRPTLRGRDIA | 238 |
| Query 241 | VPGDPSSTAFPLVAALLVPGSDVTILNVLMNPTRTGLILTLQEMGADIEVINPRLAGGED | 300 |
| Sbjct 239 | VP DPSS AFP+ AAL+VPGSDV + N+ +NPTR GL TL+EMGAD+ NPR GGE VPRDPSSAAFPVCAALIVPGSDVLVPNIGLNPTRAGLFETLREMGADLTYENPREEGGEP | 298 |
| Query 301 | VADLRVR-SSTLKGVTVPEDRAPSMIDEYPILAVAAAFAEGATVMNGLEELRVKESDRLS | 359 |
| Sbjct 299 | ADLR R S +KGV VP DRA SMIDEYP+L+V AA AEG TVM G+ ELRVKESDR++ AADLRARFSPDMKGVEVPPDRAASMIDEYPVLSVVAACAEGTTVMRGVAELRVKESDRIA | 358 |
| Query 360 | AVANGLKLNGVDCDEGETSLVVRGRPDGKGLGN-ASGAAVATHLDHRIAMSFLVMGLVSE | 418 |
| Sbjct 359 | A+A GL+ GV+ +G V DG G G G VATHLDHRIAMSFLV G+ + AMATGLRAMGVEVRDGPDWWEV DGLGAGGLPGGVTVATHLDHRIAMSFLVAGMAAR | 414 |
| Query 419 | NPVTVDDATMIATSFPEFMDLMAGLGA 445 |  |

PVTVDD+ IATSFP F LM GLGA Sbjct 415 RPVTVDDSGPIATSFPIFRPLMEGLGA 441

>TR:A0A1L3ZY11 A0A1L3ZY11_9SPHN 3-phosphoshikimate 1-carboxyvinyltransferase OS=Tardibacter chloracetimidivorans OX=1921510 GN=aroA PE=3

SV=1

Length=445

Score = 473 bits (1216), Expect = 5e-162

| Identities | | = 259/439 (59%), Positives = 310/439 (71%), Gaps = 5/439 (1%) |  |
| --- | --- | --- | --- |
| Query 9  Sbjct 9 | | PATARKSSGLSGTVRIPGDKSISHRSFMFGGLASGETRITGLLEGEDVINTGKAMQAMGA P + S+ L GTVR+PGDKSISHR+ M G LA GE+ I GLLEGEDV+ T AM+AMGA PLSLNVSTPLIGTVRVPGDKSISHRALMLGALAVGESVIEGLLEGEDVLATAAAMRAMGA | 68  68 |
| Query 69  Sbjct 69 | | RIRKEGD-TWIIDGVGNGGLLAPEAPLDFGNAATGCRLTMGLVGVYDFDSTFIGDASLTK  + GD W ++GVG GGLL P+ LD GN+ T RL MGLV + +TF GDASL+K SAERTGDGLWRVNGVGVGGLLQPQTALDMGNSGTSTRLLMGLVASHAITATFTGDASLSK | 127  128 |
| Query 128  Sbjct 129 | | RPMGRVLNPLREMGVQVKSEDGDRLPVTLRGPKTPTPITYRVPMASAQVKSAVLLAGLNT RPMGRV++PL MG + ++ G RLP+T+ G P+ Y +P+ASAQVKSAVLLA LN RPMGRVIDPLSLMGARFEASPGGRLPLTMAGLCPAVPLRYVLPVASAQVKSAVLLASLNA | 187  188 |
| Query 188  Sbjct 189 | | PGITTVIEPIMTRDHTEKMLQGFGANLTVETDADGVRTIRLEGRGKLTGQVIDVPGDPSS PG+T VIE TRDH+E+ML GFGA +TVE +G R I + G +L Q I VPGDPSS PGVTMVIEKTPTRDHSERMLTGFGAQVTVEDAPEG-RIISIRGEAELKPQAIIVPGDPSS | 247  247 |
| Query 248  Sbjct 248 | | TAFPLVAALLVPGSDVTILNVLMNPTRTGLILTLQEMGADIEVINPRLAGGEDVADLRVR AFP+VAAL+VPGSDV I NV +NPTR GLI L+ MGA IE++N R GGE VADLRVR AAFPMVAALIVPGSDVLIENVGVNPTRAGLIQALRMMGASIELLNEREVGGEPVADLRVR | 307  307 |
| Query 308  Sbjct 308 | | SSTLKGVTVPEDRAPSMIDEYPILAVAAAFAEGATVMNGLEELRVKESDRLSAVANGLKL  +S L G+ VP + APSMIDEYPIL VAAA A G TV GL ELRVKESDR++ +A GL+ ASALSGIEVPPEIAPSMIDEYPILFVAAALAGGTTVARGLGELRVKESDRITVMAEGLRA | 367  367 |
| Query 368  Sbjct 368 | | NGVDCDEGETSLVVRGRPDGKGLGNASGAAVATHLDHRIAMSFLVMGLVSENPVTVDDAT GV +E E LVV G G A GA VA+HLDHRIAMSF V+GL + PV VDDA CGVRVEELEDGLVVHGT---GGERRAGGATVASHLDHRIAMSFAVLGLAAHRPVIVDDAR | 427  424 |
| Query 428 | | MIATSFPEFMDLMAGLGAK 446 IATSFP F+ LM LGA+ |  |
| Sbjct | 425 | PIATSFPSFVPLMESLGAE 443 | |

>TR:A0A1H6A2I7 A0A1H6A2I7_9BRAD 3-phosphoshikimate 1-carboxyvinyltransferase OS=Bosea lathyri OX=1036778 GN=aroA PE=3 SV=1

Length=456

Score = 473 bits (1217), Expect = 5e-162

Identities = 260/449 (58%), Positives = 311/449 (69%), Gaps = 6/449 (1%)

| Query | 1 | MLHGASSRPATARKSSGLSGTVRIPGDKSISHRSFMFGGLASGETRITGLLEGEDVINTG | 60 |
| --- | --- | --- | --- |
| Sbjct | 11 | + H P TAR LSG VR+PGDKS+SHR+ +FG LA GET+++GLLEGEDV+ T LAHAQHPVPVTARAGGPLSGRVRVPGDKSMSHRALIFGLLAIGETKVSGLLEGEDVMRTA | 70 |
| Query | 61 | KAMQAMGARIRKEGD-TWIIDGVGNGGLLAPEAPLDFGNAATGCRLTMGLVGVYDFDSTF | 119 |
| Sbjct | 71 | A +A+GA + +G W + GVG GGL P LDFGNA TG RL MG+ G + +TF DAARALGAIVHHDGPGAWRVRGVGIGGLREPAGVLDFGNAGTGSRLMMGVCGAHPITTTF | 130 |
| Query | 120 | IGDASLTKRPMGRVLNPLREMGVQVKS-EDGDRLPVTLRGPKTPTPITYRVPMASAQVKS | 178 |
| Sbjct | 131 | GDASL KRPM R+L+PL MG + S ++G R+P+TLRGP+ P PI YR P+ASAQ+KS  DGDASLRKRPMRRILDPLELMGTTIVSAQEGGRVPLTLRGPREPVPIIYRTPVASAQIKS | 190 |

| Query | 179 | AVLLAGLNTPGITTVIEPIMTRDHTEKMLQGFGANLTVETDADGVRTIRLEGRGKLTGQV | 238 |
| --- | --- | --- | --- |
| Sbjct | 191 | AVLLAGL PG TTVIE TRDHTE ML+ FGA + V + R I L+G+ +L AVLLAGLAAPGETTVIETEATRDHTETMLRHFGAQVDVTKEGAHGRRIVLKGQPELQAAP | 250 |
| Query | 239 | IDVPGDPSSTAFPLVAALLVPGSDVTILNVLMNPTRTGLILTLQEMGADIEVINPRLAGG | 298 |
| Sbjct | 251 | + VP DPSS AFPLVAAL+VPGS++ I +V+ NP R+G + TL+EMGADI V N GG VAVPADPSSAAFPLVAALIVPGSEILIESVMTNPLRSGFLTTLREMGADITVENEGNEGG | 310 |
| Query | 299 | EDVADLRVRSSTLKGVTVPEDRAPSMIDEYPILAVAAAFAEGATVMNGLEELRVKESDRL | 358 |
| Sbjct | 311 | E VA LRVR+S L+GV VP +RAPSMIDEYP+LAVAAAFA G T M GL+ELRVKESDRL EAVATLRVRASGLQGVDVPPERAPSMIDEYPVLAVAAAFAAGTTRMRGLQELRVKESDRL | 370 |
| Query | 359 | SAVANGLKLNGVDCDEGETSLVVRGRPDGKGLGNASGAAVATHLDHRIAMSFLVMGLVSE | 418 |
| Sbjct | 371 | +AV GLK GV C LVV G G A G ATHLDHRIAMSFLVMGL + AAVEAGLKAAGVTCTIEGDDLVVH GTGGEVAGGGTAATHLDHRIAMSFLVMGLATR | 426 |
| Query | 419 | NPVTVDDATMIATSFPEFMDLMAGLGAKI 447 PV +DD MIATSFP F+ LM LG I |  |
| Sbjct | 427 | KPVQIDDGAMIATSFPGFIALMNELGGDI 455 | |

>TR:A0A3N1LJS0 A0A3N1LJS0_9PROT 3-phosphoshikimate 1-carboxyvinyltransferase OS=Stella humosa OX=94 GN=aroA PE=3 SV=1

Length=457

Score = 473 bits (1217), Expect = 5e-162

| Identities | | = 258/440 (59%), Positives = 313/440 (71%), Gaps = 9/440 (2%) |  |
| --- | --- | --- | --- |
| Query 11  Sbjct 6 | | TARKSSGLSGTVRIPGDKSISHRSFMFGGLASGETRITGLLEGEDVINTGKAMQAMGARI  +A + L+G + +PGDKSISHR+ M G LA GET I GLLEGEDV+ T AM+ +GA + SAAAAGPLAGDLTVPGDKSISHRALMLGSLAIGETVIEGLLEGEDVLRTAVAMRQLGAEV | 70  65 |
| Query 71  Sbjct 66 | | RKEGD-TWIIDGVGNGGLLAPEAPLDFGNAATGCRLTMGLVGVYDFDSTFIGDASLTKRP D TW + GVG G L+ P LD GNA TG RL MG+V + + F GDASL +RP AHRADGTWQVRGVGVGALVEPAQVLDMGNAGTGARLLMGVVAAHPITAFFTGDASLVRRP | 129  125 |
| Query 130  Sbjct 126 | | MGRVLNPLREMGVQVKSEDGDRLPVTLRGPKTPTPITYRVPMASAQVKSAVLLAGLNTPG M RV+NPLR+MG + + GDRLP+ L G TP PI+YR+P+ASAQVKSA+LLAGLNT G MARVMNPLRQMGARFMARAGDRLPLGLEGAATPLPISYRLPVASAQVKSAILLAGLNTAG | 189  185 |
| Query 190  Sbjct 186 | | ITTVIEPIMTRDHTEKMLQGFGANLTVETDADGVRTIRLEGRGKLTGQVIDVPGDPSSTA TTVIEP TRDHTE ML+ FGA +TVE ADG R + L G+ +L G+ + VPGDPSS A RTTVIEPEPTRDHTELMLRHFGATVTVEDGADG-RVVSLTGQPELVGRPVLVPGDPSSAA | 249  244 |
| Query 250  Sbjct 245 | | FPLVAALLVPGSDVTILNVLMNPTRTGLILTLQEMGADIEVINPRLAGGEDVADLRVR-S FP+VAALLV GSDV + NV +NP RTGL+ TLQEMGA I V N R+ GE V DLRVR + FPIVAALLVEGSDVLVRNVGLNPLRTGLLTTLQEMGAAITVENGRIQAGEPVGDLRVRGT | 308  304 |
| Query 309  Sbjct 305 | | STLKGVTVPEDRAPSMIDEYPILAVAAAFAEGATVMNGLEELRVKESDRLSAVANGLKLN LKGVTVP +RAPSMIDEYP+LA AA A G T+M+GL ELRVKESDRL+AVA GL  GPLKGVTVPAERAPSMIDEYPVLACLAALARGRTIMHGLGELRVKESDRLTAVAEGLASC | 368  364 |
| Query 369  Sbjct 365 | | GVDCD-EGETSLVVRGRPDGKGLGNASGAAVATHLDHRIAMSFLVMGLVSENPVTVDDAT GV +G+T +V +G GL A G +AT LDHRIAM+FLV+GL + PVT+DD GVSVTVDGDTLIV EGDGLAPAGGGHIATRLDHRIAMAFLVLGLATRAPVTIDDDE | 427  419 |
| Query 428 | | MIATSFPEFMDLMAGLGAKI 447 I TSFP F+ L+ G+GA I |  |
| Sbjct | 420 | AIGTSFPGFVGLLNGIGAHI 439 | |

>TR:A0A1H8A7W9 A0A1H8A7W9_9RHOB 3-phosphoshikimate 1-carboxyvinyltransferase OS=Pseudorhodobacter antarcticus OX=1077947 GN=aroA PE=3 SV=1

Length=447

Score = 473 bits (1216), Expect = 5e-162

| Identities | = 258/446 (58%), Positives = 312/446 (70%), Gaps = 6/446 (1%) |  |
| --- | --- | --- |
| Query 3 | HGASSRPATARKSSGLSGTVRIPGDKSISHRSFMFGGLASGETRITGLLEGEDVINTGKA | 62 |
| Sbjct 4 | HGA+ TA KS L+G +PGDKSISHR+ +FG +A GETRITGLLEG+DV++T A HGAAQM-MTATKSGPLTGRAEVPGDKSISHRALIFGAMAVGETRITGLLEGQDVLDTASA | 62 |
| Query 63 | MQAMGARIRKEGD-TWIIDGVGNGGLLAPEAPLDFGNAATGCRLTMGLVGVYDFDSTFIG | 121 |
| Sbjct 63 | M+A GA + + G W + GVG GG P+A +D GN+ TG RL MG + +TF G MRAFGAVVVQHGPGAWSVHGVGVGGFREPDAVIDCGNSGTGVRLIMGCMATTAMTATFTG | 122 |
| Query 122 | DASLTKRPMGRVLNPLREMGVQVKSEDGDRLPVTLRGPKTPTPITYRVPMASAQVKSAVL | 181 |
| Sbjct 123 | DASL KRPMGRV +PL G Q G RLP+T+ G P P+ Y +P+ASAQVKSAVL DASLRKRPMGRVTDPLSLFGTQAFGRKGGRLPMTVVGAANPVPVRYALPVASAQVKSAVL | 182 |
| Query 182 | LAGLNTPGITTVIEPIMTRDHTEKMLQGFGANLTVETDADGVRTIRLEGRGKLTGQVIDV | 241 |
| Sbjct 183 | LAGLN PG T VIE TRDH+E+ML GFGA LTVE A+G I L G+ +L QV+ V LAGLNAPGQTVVIEREPTRDHSERMLLGFGAELTVERTAEG-NVITLTGQPELRPQVVAV | 241 |
| Query 242 | PGDPSSTAFPLVAALLVPGSDVTILNVLMNPTRTGLILTLQEMGADIEVINPRLAGGEDV | 301 |
| Sbjct 242 | P DPSS AFP+ AAL+V GSD+ + V N TR G+ LTL EMGADI NPR GGE V PRDPSSAAFPVCAALIVEGSDIFVPGVSQNLTRNGIYLTLVEMGADITFENPRQEGGEPV | 301 |
| Query 302 | ADLRVRSSTLKGVTVPEDRAPSMIDEYPILAVAAAFAEGATVMNGLEELRVKESDRLSAV | 361 |
| Sbjct 302 | ADLRVR S L+G+ VP +RAPSMIDEYPIL+V AA A G TVM G++ELRVKESDR+ A+ ADLRVRFSDLRGIEVPPERAPSMIDEYPILSVVAANAMGKTVMRGVKELRVKESDRIDAM | 361 |
| Query 362 | ANGLKLNGVDCDEGETSLVVRGRPDGKGLGNASGAAVATHLDHRIAMSFLVMGLVSENPV | 421 |
| Sbjct 362 | A GL+ GV +E E +L+V GR G G GA ATH+DHRIAMSFLV G+ S+ P+ ARGLEACGVVVEEDEDTLIVHGRGPG---GVPGGATCATHIDHRIAMSFLVAGMASQRPI | 418 |
| Query 422  Sbjct 419 | TVDDATMIATSFPEFMDLMAGLGAKI 447  +VDDA+ IATSFP F LM GLGAK+ SVDDASPIATSFPLFEGLMMGLGAKL 444 |  |

>TR:B6IUQ0 B6IUQ0_RHOCS 3-phosphoshikimate 1-carboxyvinyltransferase OS=Rhodospirillum centenum (strain ATCC 51521 / SW) OX=414684 GN=aroA

PE=3 SV=1

Length=448

Score = 473 bits (1216), Expect = 6e-162

| Identities | = 256/444 (58%), Positives = 305/444 (69%), Gaps = 5/444 (1%) |  |
| --- | --- | --- |
| Query 5 | ASSRPATARKSSGLSGTVRIPGDKSISHRSFMFGGLASGETRITGLLEGEDVINTGKAMQ | 64 |
| Sbjct 6 | A RP +R+S L+GTV +PGDKSISHR+ MFG L+ GETRI GLLEG+DV+ T A++ AGPRPLLSRRSGALAGTVTVPGDKSISHRALMFGALSLGETRIEGLLEGDDVLRTAAALR | 65 |
| Query 65 | AMGARIRKEGD-TWIIDGVGNGGLLAPEAPLDFGNAATGCRLTMGLVGVYDFDSTFIGDA | 123 |
| Sbjct 66 | A+GA + D TWI G G GGL P LD GN+ T RL MGL+ + F S GDA ALGAEAERLPDGTWITRGRGVGGLAEPAQVLDMGNSGTAARLLMGLLATHPFTSVMTGDA | 125 |
| Query 124 | SLTKRPMGRVLNPLREMGVQVKSEDGDRLPVTLRGPKTPTPITYRVPMASAQVKSAVLLA | 183 |
| Sbjct 126 | SLTKRPM RV PL MG + G RLP + G P PI YR+P+ASAQVKSA+LLA SLTKRPMARVTTPLETMGARFALRSGGRLPGAVIGTDRPVPIAYRLPVASAQVKSAILLA | 185 |
| Query 184 | GLNTPGITTVIEPIMTRDHTEKMLQGFGANLTVETDADGVRTIRLEGRGKLTGQVIDVPG | 243 |
| Sbjct 186 | GLN PG TTVIEP TRDHTE+ML+ FG +TVE DG + G+G+LT + + VP GLNAPGRTTVIEPEPTRDHTERMLRHFGIEVTVEEAGDGALAATVTGQGELTARDLRVPA | 245 |
| Query 244 | DPSSTAFPLVAALLVPGSDVTILNVLMNPTRTGLILTLQEMGADIEVINPRLAGGEDVAD | 303 |
| Sbjct 246 | DPSS AFP VAAL+ PGS +++ V NP RTGL TL+ MGADI NPR GGE VAD  DPSSAAFPAVAALIRPGSALSLPGVGTNPRRTGLFETLRAMGADIAYANPREEGGEPVAD | 305 |

| Query | 304 | LRVRSSTLKGVTVPEDRAPSMIDEYPILAVAAAFAEGATVMNGLEELRVKESDRLSAVAN | 363 |
| --- | --- | --- | --- |
| Sbjct | 306 | L VR+S L+GV VP +RAPSMIDEYP+L +AAA AEG TVM G+ ELRVKESDRL+ +A+ LLVRASGLRGVEVPPERAPSMIDEYPVLCIAAACAEGPTVMRGIGELRVKESDRLAMMAD | 365 |
| Query | 364 | GLKLNGVDCDEGETSLVVRGRPDGKGLGNASGAAVATHLDHRIAMSFLVMGLVSENPVTV | 423 |
| Sbjct | 366 | GL GV + GE +VV GR G A GA VAT +DHRIAMSFLV+G+ +E PV V GLAACGVTVEAGEDWMVVHGR GRPPAGGATVATAMDHRIAMSFLVLGMATEQPVRV | 421 |
| Query  Sbjct | 424  422 | DDATMIATSFPEFMDLMAGLGAKI 447 DD I TSFP F+ LM GLGA I DDGAFIDTSFPGFVALMNGLGADI 445 |  |

>TR:A0A0B5DSM1 A0A0B5DSM1_9RHOB 3-phosphoshikimate 1-carboxyvinyltransferase OS=Celeribacter indicus OX=1208324 GN=aroA PE=3 SV=1

Length=449

Score = 473 bits (1216), Expect = 6e-162

| Identities | = 257/446 (58%), Positives = 306/446 (69%), Gaps = 8/446 (2%) |  |
| --- | --- | --- |
| Query 5 | ASSRPATARKSSGLSGTVRIPGDKSISHRSFMFGGLASGETRITGLLEGEDVINTGKAMQ | 64 |
| Sbjct 5 | A+ P +AR+ L GT +PGDKSISHR+ +FG +A GET+ITGLLEGEDV++T +AMQ ATPIPMSARRRGPLKGTAAVPGDKSISHRALIFGAMAVGETKITGLLEGEDVLDTARAMQ | 64 |
| Query 65 | AMGARIRKEG-DTWIIDGVGNGGLLAPEAPLDFGNAATGCRLTMGLVGVYDFDSTFIGDA | 123 |
| Sbjct 65 | A+GA + +EG W + GVG GG PE +D GN+ TG RL MG V +DF +TF GD ALGASVTREGAGEWTVHGVGVGGFSEPETVIDCGNSGTGVRLIMGAVATHDFCTTFTGDG | 124 |
| Query 124 | SLTKRPMGRVLNPLREMGVQVKSEDGDRLPVTLRGPKTPTPITYRVPMASAQVKSAVLLA | 183 |
| Sbjct 125 | SL KRPM RV +PL G + G RLP+TL G P P+ Y P+ SAQVKSAVLLA SLRKRPMARVTDPLALFGARAVGRSGGRLPLTLVGAADPVPVDYMTPVPSAQVKSAVLLA | 184 |
| Query 184 | GLNTPGITTVIEPIMTRDHTEKMLQGFGANLTVETDADGVRTIRLEGRGKLTGQVIDVPG | 243 |
| Sbjct 185 | GLN PG T V E TRDHTE+ML GFGA + ET +G I L GR +L Q + VP GLNAPGRTVVTEREPTRDHTERMLTGFGAEVVTETTPEGFHRITLIGRPELVPQEVAVPR | 244 |
| Query 244 | DPSSTAFPLVAALLVPGSDVTILNVLMNPTRTGLILTLQEMGADIEVINPRLAGGEDVAD | 303 |
| Sbjct 245 | DPSS AFP+ AAL+V GSD+ + V NPTR GL TL EMGADI NPR GGE VAD DPSSAAFPVAAALIVGGSDILVPGVSQNPTRNGLYTTLVEMGADIAFENPREEGGEPVAD | 304 |
| Query 304 | LRVRSSTLKGVTVPEDRAPSMIDEYPILAVAAAFAEGATVMNGLEELRVKESDRLSAVAN | 363 |
| Sbjct 305 | LRVR+S L+G+ VP DRA SMIDEYPIL+V AA AEG TVM G++ELRVKESDR+ A+A LRVRASALRGIEVPPDRAASMIDEYPILSVIAACAEGTTVMRGVKELRVKESDRIDAMAR | 364 |
| Query 364 | GLKLNGVDCDEGETSLVVRG-RPDG-KGLGNASGAAVATHLDHRIAMSFLVMGLVSENPV | 421 |
| Sbjct 365 | GL+ GV +E E +LVV G PDG KG GA AT LDHRIAMSFLV+G+ + V GLEACGVAVEEEEDTLVVHGCGPDGVKG GAVCATRLDHRIAMSFLVLGMAAREQV | 419 |
| Query 422  Sbjct 420 | TVDDATMIATSFPEFMDLMAGLGAKI 447  +VDD IATSFP F LM GLGA + SVDDGGPIATSFPTFEKLMTGLGADV 445 |  |

```
>TR:A0A4Y6VBK8 A0A4Y6VBK8_9PROT 3-phosphoshikimate 1-carboxyvinyltransferase OS=Neokomagataea tanensis OX=661191 GN=aroA PE=3 SV=1
Length=440

Score = 472 bits (1215), Expect = 6e-162
Identities = 248/436 (57%), Positives = 303/436 (69%), Gaps = 5/436 (1%)

Query 12	ARKSSGLSGTVRIPGDKSISHRSFMFGGLASGETRITGLLEGEDVINTGKAMQAMGARIR 71
+R +GL GT R+PGDKSISHRS MF LA+G+TR++GLLEGEDV+ T AM+A+GA I Sbjct 9	SRAQTGLQGTTRVPGDKSISHRSLMFAALAAGQTRVSGLLEGEDVLRTADAMRALGATIT 68
```

| Query | 72 | KEGDTWIIDGVGNGGLLAPEAPLDFGNAATGCRLTMGLVGVYDFDSTFIGDASLTKRPMG | 131 |
| --- | --- | --- | --- |
| Sbjct | 69 | ++GD W+IDG G G L PE LD GN+ T RL G++ F S GDASL RPM RDGDNWVIDGRGVGALSEPEDVLDMGNSGTAARLLSGILATQPFLSVMTGDASLRSRPMR | 128 |
| Query | 132 | RVLNPLREMGVQVKSEDGDRLPVTLRGPKTPTPITYRVPMASAQVKSAVLLAGLNTPGIT | 191 |
| Sbjct | 129 | RV PL E G ++ +G RLP+ +RG + P+ YR+P+ASAQVKSAVLLAGLN G T RVTAPLSETGAVFETREGGRLPMAVRGTRDAKPLHYRLPVASAQVKSAVLLAGLNAHGET | 188 |
| Query | 192 | TVIEPIMTRDHTEKMLQGFGANLTVETDADGVRTIRLEGRGKLTGQVIDVPGDPSSTAFP | 251 |
| Sbjct | 189 | TV EP+ TRDHTE ML+ FG N+ VE +DG R I L G +LT + + VPGDPSS AFP TVEEPVATRDHTENMLRHFGVNVDVEVMSDGGRRIALTGPAELTARDVVVPGDPSSAAFP | 248 |
| Query | 252 | LVAALLVPGSDVTILNVLMNPTRTGLILTLQEMGADIEVINPRLAGGEDVADLRVRSSTL | 311 |
| Sbjct | 249 | +VAALLVPGSD+ I V +NP RTGL TLQEMG D+ ++N R+ GGE V D+RV+ S L IVAALLVPGSDILITGVGLNPLRTGLFQTLQEMGGDLSILNERIEGGEAVGDIRVKHSAL | 308 |
| Query | 312 | KGVTVPEDRAPSMIDEYPILAVAAAFAEGATVMNGLEELRVKESDRLSAVANGLKLNGVD | 371 |
| Sbjct | 309 | GV VP DRAPSMIDEYP+LAVA AFAEGA+ + GLEELRVKESDRL+A L +NG HGVEVPSDRAPSMIDEYPVLAVACAFAEGASRLLGLEELRVKESDRLAATVALLNVNGAA | 368 |
| Query | 372 | CDEGETSLVVRGRPDGKGLGNASGAAVATHLDHRIAMSFLVMGLVSENPVTVDDATMIAT | 431 |
| Sbjct | 369 | +LVV G G G+ G V TH+DHR+AMS +V GL +E V++DD I T TTVEGDNLVVHG GGGSLGGGHVVTHMDHRLAMSAVVFGLAAEKAVSIDDTAFIET | 423 |
| Query  Sbjct | 432  424 | SFPEFMDLMAGLGAKI 447 SFP F+ LM LGA I SFPGFVALMNALGAGI 439 |  |

>TR:A0A147INJ0 A0A147INJ0_9SPHN 3-phosphoshikimate 1-carboxyvinyltransferase OS=Sphingomonas yabuuchiae OX=172044 GN=aroA PE=3 SV=1

Length=443

Score = 472 bits (1215), Expect = 7e-162

| Identities | = 251/439 (57%), Positives = 309/439 (70%), Gaps = 5/439 (1%) |  |
| --- | --- | --- |
| Query 8 | RPATARKSSGLSGTVRIPGDKSISHRSFMFGGLASGETRITGLLEGEDVINTGKAMQAMG | 67 |
| Sbjct 7 | +P T R ++ L GT+ +PGDKSISHRS M LA GE+R+ GLLEGEDV+ T AM+AMG QPRTVRAAAALRGTIAVPGDKSISHRSLMLSALAVGESRVEGLLEGEDVLATAAAMRAMG | 66 |
| Query 68 | ARIRKEGD-TWIIDGVGNGGLLAPEAPLDFGNAATGCRLTMGLVGVYDFDSTFIGDASLT | 126 |
| Sbjct 67 | A I ++ D W+I GVG GGLL PE LD GN+ T RL MGLV + +TFIGDASL+ ADIVRQDDGVWVIHGVGVGGLLQPETALDMGNSGTSTRLLMGLVASHPITATFIGDASLS | 126 |
| Query 127 | KRPMGRVLNPLREMGVQVKSEDGDRLPVTLRGPKTPTPITYRVPMASAQVKSAVLLAGLN | 186 |
| Sbjct 127 | KRPMGRV+ PL +MG Q + G RLP+ +RG PI Y +P+ASAQVKSA+LLAGLN KRPMGRVIEPLSQMGAQFQDTPGGRLPLMVRGINPAVPIRYTLPVASAQVKSAILLAGLN | 186 |
| Query 187 | TPGITTVIEPIMTRDHTEKMLQGFGANLTVETDADGVRTIRLEGRGKLTGQVIDVPGDPS | 246 |
| Sbjct 187 | TPG TTVIEP+ TRDH+E+ML GFGA+LTV +G R I + G+ +L Q I VPGDPS TPGETTVIEPVATRDHSERMLAGFGADLTVTPSPEG-RIITIRGQAELKPQNIVVPGDPS | 245 |
| Query 247 | STAFPLVAALLVPGSDVTILNVLMNPTRTGLILTLQEMGADIEVINPRLAGGEDVADLRV | 306 |
| Sbjct 246 | S AF LVA +VPG+DVTI NV MNPTRTG+I L+ MGADI ++ R+ GGE VADLRV SAAFWLVAGSIVPGADVTITNVGMNPTRTGIIAALKMMGADITELDARIVGGEPVADLRV | 305 |
| Query 307 | RSSTLKGVTVPEDRAPSMIDEYPILAVAAAFAEGATVMNGLEELRVKESDRLSAVANGLK | 366 |
| Sbjct 306 | R + L + VP + PSMIDEYP+L VAAAFA G T+ G EELRVKESDR++A+AN L RHAALSAIEVPSELTPSMIDEYPVLFVAAAFATGTTIARGAEELRVKESDRITAMANALG | 365 |
| Query 367 | LNGVDCDEGETSLVVRGRPDGKGLGNASGAAVATHLDHRIAMSFLVMGLVSENPVTVDDA | 426 |
| Sbjct 366 | GV C+E E + V G G GA +AT LDHRIAMS + L + +P+T+DDA  ACGVVCEEFEDGMAVTGT---AGDPIPGGATIATLLDHRIAMSMTIAALNARDPITLDDA | 422 |

Query 427 TMIATSFPEFMDLMAGLGA 445

+ATS+P F D + LGA Sbjct 423 APVATSYPVFFDTLNKLGA 441

>TR:A0A2K9MBM3 A0A2K9MBM3_9RHOB 3-phosphoshikimate 1-carboxyvinyltransferase OS=Paracoccus jeotgali OX=2065379 GN=aroA PE=3 SV=1

Length=446

Score = 472 bits (1215), Expect = 7e-162

| Identities | | = 252/440 (57%), Positives = 309/440 (70%), Gaps = 6/440 (1%) |  |
| --- | --- | --- | --- |
| Query 9  Sbjct 8 | | PATARKSSGLSGTVRIPGDKSISHRSFMFGGLASGETRITGLLEGEDVINTGKAMQAMGA P +AR L+GT +PGDKSISHR+ + G ++ G TRITGLLEG+DV++T +AM+AMGA PMSARPGGPLTGTAHVPGDKSISHRALILGAMSVGRTRITGLLEGQDVLDTARAMRAMGA | 68  67 |
| Query 69  Sbjct 68 | | RI-RKEGDTWIIDGVGNGGLLAPEAPLDFGNAATGCRLTMGLVGVYDFDSTFIGDASLTK RI R WI+DGVG GG PE +D GN+ TG RL MG V TF GDASL++ RIERLNPGEWIVDGVGVGGFGEPEGVIDCGNSGTGVRLIMGAVATTPITVTFTGDASLSR | 127  127 |
| Query 128  Sbjct 128 | | RPMGRVLNPLREMGVQVKSEDGDRLPVTLRGPKTPTPITYRVPMASAQVKSAVLLAGLNT RPM R+ +PL G ++ + +G LPVT+RG + P+ Y+ P+ASAQ+KSAVLLAGLN RPMRRITDPLALFGAEIIAREGGLLPVTIRGAQDAVPVRYQTPVASAQIKSAVLLAGLNA | 187  187 |
| Query 188  Sbjct 188 | | PGITTVIEPIMTRDHTEKMLQGFGANLTVETDADGVRTIRLEGRGKLTGQVIDVPGDPSS PG T VIE TRDHTE+ML GFGA +T E +G R I L G+ +L GQ + VP DPSS PGQTVVIEAEPTRDHTERMLTGFGATITTEITPEG-RVITLTGQPELLGQNVAVPRDPSS | 247  246 |
| Query 248  Sbjct 247 | | TAFPLVAALLVPGSDVTILNVLMNPTRTGLILTLQEMGADIEVINPRLAGGEDVADLRVR AFP+ AAL+VPGS+V + + NPTR GL +TLQEMGAD+ NPR GGE VADL VR AAFPVAAALIVPGSEVRVPGISRNPTRDGLFVTLQEMGADLTWENPRDEGGEPVADLIVR | 307  306 |
| Query 308  Sbjct 307 | | SSTLKGVTVPEDRAPSMIDEYPILAVAAAFAEGATVMNGLEELRVKESDRLSAVANGLKL STL GV VP +RA SMIDE+PIL+V AA A+G TVMNG+ ELRVKESDR+ A+A GL+ HSTLHGVEVPAERAASMIDEFPILSVVAAMAQGKTVMNGVAELRVKESDRIDAMARGLRD | 367  366 |
| Query 368  Sbjct 367 | | NGVDCDEGETSLVVRGRPDGKGLGNASGAAVATHLDHRIAMSFLVMGLVSENPVTVDDAT NGV DE S+ V GR G+ GA ATHLDHRIAMSFLV GL ++ PV VDD NGVSVDETPDSMTVHGR GVIPGGGAPAATHLDHRIAMSFLVAGLAAQAPVRVDDGG | 427  422 |
| Query 428 | | MIATSFPEFMDLMAGLGAKI 447 IATSFP+F+ LM LGA + |  |
| Sbjct | 423 | AIATSFPDFVRLMRNLGADL 442 | |

>TR:A0A285CVT6 A0A285CVT6_9RHOB 3-phosphoshikimate 1-carboxyvinyltransferase OS=Rhodobacter ovatus OX=439529 GN=aroA PE=3 SV=1

Length=447

Score = 472 bits (1215), Expect = 8e-162

| Identities | | = 256/448 (57%), Positives = 312/448 (70%), Gaps = 6/448 (1%) |  |
| --- | --- | --- | --- |
| Query 3  Sbjct 4 | | HGASSRPATARKSSGLSGTVRIPGDKSISHRSFMFGGLASGETRITGLLEGEDVINTGKA HGA+ +P TAR+S L+G +PGDKSISHR+ + G +A GETRITGLLEG+DV++T KA HGAA-QPMTARRSGPLTGRAEVPGDKSISHRALILGAMAVGETRITGLLEGQDVLDTAKA | 62  62 |
| Query 63 | | MQAMGARIRKEGD-TWIIDGVGNGGLLAPEAPLDFGNAATGCRLTMGLVGVYDFDSTFIG M+A GA + + G W + GVG GG P +D GN+ TG RL MG + TF G | 121 |
| Sbjct | 63 | MRAFGAEVTQHGPGQWSVHGVGVGGFSEPADVIDCGNSGTGVRLLMGCMATTGLTVTFTG | 122 |
| Query | 122 | DASLTKRPMGRVLNPLREMGVQVKSEDGDRLPVTLRGPKTPTPITYRVPMASAQVKSAVL | 181 |
| Sbjct | 123 | DASL KRPMGRV PL G + G RLP+T+ G P P+ Y VP+ SAQVKSAVL  DASLRKRPMGRVTEPLALFGARAYGRKGGRLPMTVVGAADPVPVRYAVPVPSAQVKSAVL | 182 |

| Query | 182 | LAGLNTPGITTVIEPIMTRDHTEKMLQGFGANLTVETDADGVRTIRLEGRGKLTGQVIDV | 241 |
| --- | --- | --- | --- |
| Sbjct | 183 | LAGLN PG T VIE TRDH+E+ML+GFGA LTVE +G I L G+ +L Q + V LAGLNAPGQTVVIEREPTRDHSERMLRGFGAELTVEETPEG-HVITLTGQPELRPQAVAV | 241 |
| Query | 242 | PGDPSSTAFPLVAALLVPGSDVTILNVLMNPTRTGLILTLQEMGADIEVINPRLAGGEDV | 301 |
| Sbjct | 242 | P DPSS AFP+ AAL+V GSD+ + V NPTR GL +TL EMGADI NPR GGE V PRDPSSAAFPVCAALIVEGSDILVPGVSRNPTRDGLYVTLLEMGADIAFENPREEGGEPV | 301 |
| Query | 302 | ADLRVRSSTLKGVTVPEDRAPSMIDEYPILAVAAAFAEGATVMNGLEELRVKESDRLSAV | 361 |
| Sbjct | 302 | ADLRVR+S L GV VP +RAPSMIDEYP+LAV AAFAEG TVM G++ELRVKESDR+ A+ ADLRVRASALTGVEVPPERAPSMIDEYPVLAVVAAFAEGRTVMRGVKELRVKESDRIDAM | 361 |
| Query | 362 | ANGLKLNGVDCDEGETSLVVRGRPDGKGLGNASGAAVATHLDHRIAMSFLVMGLVSENPV | 421 |
| Sbjct | 362 | A GL+ GV +E E +L+V G G G GA ATH+DHRIAMSFLV+G+ ++ V ARGLEACGVRVEEDEDTLIVHGMGAGAVPG---GAVCATHIDHRIAMSFLVLGMAAKAEV | 418 |
| Query  Sbjct | 422  419 | TVDDATMIATSFPEFMDLMAGLGAKIEL 449  +VDD + IATSFP F+ LM GLGA++ L SVDDGSPIATSFPAFVGLMRGLGAELAL 446 |  |

>TR:A0A5C6S3Q9 A0A5C6S3Q9_9RHOB 3-phosphoshikimate 1-carboxyvinyltransferase OS=Paracoccus sp. TK008 OX=2599412 GN=aroA PE=3 SV=1

Length=443

Score = 472 bits (1214), Expect = 9e-162

| Identities | | = 247/444 (56%), Positives = 309/444 (70%), Gaps = 6/444 (1%) |  |
| --- | --- | --- | --- |
| Query 5  Sbjct 4 | | ASSRPATARKSSGLSGTVRIPGDKSISHRSFMFGGLASGETRITGLLEGEDVINTGKAMQ A+++P ++R LSGT +PGDKSISHR+ + G LA G T ITGLLEG+DV++TGKAMQ AAAKPMSSRPGKPLSGTASVPGDKSISHRALILGALAVGRTHITGLLEGQDVLDTGKAMQ | 64  63 |
| Query 65 | | AMGARIRKEGD-TWIIDGVGNGGLLAPEAPLDFGNAATGCRLTMGLVGVYDFDSTFIGDA A GA + + G W +DGVG GG P +D GN+ TG RL MG + +TF GDA | 123 |
| Sbjct | 64 | AFGATVERLGPGEWTVDGVGVGGFAEPAGVIDCGNSGTGVRLIMGAMATTPITATFTGDA | 123 |
| Query | 124 | SLTKRPMGRVLNPLREMGVQVKSEDGDRLPVTLRGPKTPTPITYRVPMASAQVKSAVLLA | 183 |
| Sbjct | 124 | SL+KRPM R+ +PL G + + +G LPVT++G + P P+ Y P+ASAQ+KSAVLLA SLSKRPMRRITDPLALFGADITAREGGLLPVTIKGVQDPVPVRYATPVASAQIKSAVLLA | 183 |
| Query | 184 | GLNTPGITTVIEPIMTRDHTEKMLQGFGANLTVETDADGVRTIRLEGRGKLTGQVIDVPG | 243 |
| Sbjct | 184 | GLN PG T VIE TRDH+E+ML GFGA +T +T +G R I L G+ +L Q + VP GLNAPGQTVVIEKEPTRDHSERMLAGFGATITTKTTDEG-RVITLTGQPELRAQTVAVPR | 242 |
| Query | 244 | DPSSTAFPLVAALLVPGSDVTILNVLMNPTRTGLILTLQEMGADIEVINPRLAGGEDVAD | 303 |
| Sbjct | 243 | DPSS AFP+ AAL+VPGS++ + + NPTR GL +TL EMGAD+ N R GGE VAD DPSSAAFPVAAALIVPGSEIRVPGISRNPTRDGLYVTLAEMGADLTWENDREEGGEPVAD | 302 |
| Query | 304 | LRVRSSTLKGVTVPEDRAPSMIDEYPILAVAAAFAEGATVMNGLEELRVKESDRLSAVAN | 363 |
| Sbjct | 303 | L VR S LKGV VP DRA SMIDE+P+L+V AA AEG TVMNG+ ELRVKESDR+ A+A LIVRHSALKGVQVPADRAASMIDEFPVLSVIAAMAEGDTVMNGVAELRVKESDRIDAMAK | 362 |
| Query | 364 | GLKLNGVDCDEGETSLVVRGRPDGKGLGNASGAAVATHLDHRIAMSFLVMGLVSENPVTV | 423 |
| Sbjct | 363 | GL+ NGV+ DE S+ V G+G G VATHLDHRIAMSFLV GL ++ +TV GLRANGVEVDETRDSMTVH GQGRIPGGGVPVATHLDHRIAMSFLVAGLAADAEITV | 418 |
| Query  Sbjct | 424  419 | DDATMIATSFPEFMDLMAGLGAKI 447 DD IATSFP+F+ LM GLGA + DDGAAIATSFPDFVPLMTGLGADL 442 |  |

>TR:W9GYC0 W9GYC0_9PROT 3-phosphoshikimate 1-carboxyvinyltransferase OS=Skermanella stibiiresistens SB22 OX=1385369 GN=aroA PE=3 SV=1

Length=444

Score = 472 bits (1214), Expect = 1e-161

| Identities | | = 259/441 (59%), Positives = 306/441 (69%), Gaps = 6/441 (1%) |  |
| --- | --- | --- | --- |
| Query 8  Sbjct 4 | | RPATARKSSGLSGTVRIPGDKSISHRSFMFGGLASGETRITGLLEGEDVINTGKAMQAMG RP +R++ LSGTVR+PGDKSISHR+ MFG LA GET I GLL GEDV+NT AM+A+G RPLQSRQTGSLSGTVRVPGDKSISHRALMFGALAIGETVIHGLLTGEDVLNTAAAMRALG | 67  63 |
| Query 68  Sbjct 64 | | ARI-RKEGDTWIIDGVGNGGLLAPEAPLDFGNAATGCRLTMGLVGVYDFDSTFIGDASLT A I R + W + GVG GGL+ LD GN+ T RL MGLV + + F GDASLT ATITRDDHGVWRVRGVGVGGLVEASQVLDMGNSGTAARLMMGLVATHPITTFFTGDASLT | 126  123 |
| Query 127  Sbjct 124 | | KRPMGRVLNPLREMGVQVKSEDGDRLPVTLRGPKTPTPITYRVPMASAQVKSAVLLAGLN KRPM RV PL +MG S G RLP+ + G + P PITYR+P+ASAQVKSA+LLAGLN KRPMARVTTPLEQMGASFVSRSGGRLPLVIIGTENPVPITYRLPVASAQVKSAILLAGLN | 186  183 |
| Query 187  Sbjct 184 | | TPGITTVIEPIMTRDHTEKMLQGFGANLTVETDADGVRTIRLEGRGKLTGQVIDVPGDPS TPG+TTVIE TRDH+E ML FGA + E G + + G ++TG+ ++VP DPS TPGVTTVIEAEPTRDHSELMLAHFGATVKTERIEGGALAVSVTGEPEITGKTVNVPADPS | 246  243 |
| Query 247  Sbjct 244 | | STAFPLVAALLVPGSDVTILNVLMNPTRTGLILTLQEMGADIEVINPRLAGGEDVADLRV S AF VAAL+ PGSDVT+ +V MNP RTGL TL EMGADI +N R GE VADLRV SAAFLTVAALIRPGSDVTLTDVGMNPRRTGLYDTLIEMGADITFLNRREQAGEPVADLRV | 306  303 |
| Query 307  Sbjct 304 | | RSSTLKGVTVPEDRAPSMIDEYPILAVAAAFAEGATVMNGLEELRVKESDRLSAVANGLK RSS L GVTVP +RAPSMIDEYPIL+VAAA A G T M GL ELRVKESDRL+ VA+GL RSSELTGVTVPPERAPSMIDEYPILSVAAACANGTTRMLGLAELRVKESDRLAMVADGLA | 366  363 |
| Query 367  Sbjct 364 | | LNGVDCDEGETSLVVRGRPDGKGLGNASGAAVATHLDHRIAMSFLVMGLVSENPVTVDDA GV + G SL V G G GA VAT +DHRIAMSFLV+G+ S PV VDD+ ACGVAVEAGPDSLTVHGAGVPPG GATVATAMDHRIAMSFLVLGMASSQPVAVDDS | 426  418 |
| Query 427 | | TMIATSFPEFMDLMAGLGAKI 447 I TSFP F++LM LGA+I |  |
| Sbjct | 419 | EFIETSFPGFVELMNELGARI 439 | |

>TR:A0A1H0HB24 A0A1H0HB24_9BRAD 3-phosphoshikimate 1-carboxyvinyltransferase OS=Afipia sp. GAS231 OX=1882747 GN=aroA PE=3 SV=1

Length=445

Score = 472 bits (1214), Expect = 1e-161

| Identities | = 250/447 (56%), Positives = 310/447 (69%), Gaps = 7/447 (2%) |  |
| --- | --- | --- |
| Query 1 | MLHGASSRPATARKSSGLSGTVRIPGDKSISHRSFMFGGLASGETRITGLLEGEDVINTG | 60 |
| Sbjct 1 | M H + P +R S L+G VRIPGDKSISHR+ + G L+ GETR++GLLEGEDV+NT MTHSTAPTPLESRASGALTGKVRIPGDKSISHRALILGALSVGETRVSGLLEGEDVLNTA | 60 |
| Query 61 | KAMQAMGARIRKEGD-TWIIDGVGNGGLLAPEAPLDFGNAATGCRLTMGLVGVYDFDSTF | 119 |
| Sbjct 61 | K+MQA+GA++ + G W + GVG G P APLDFGN+ TGCRL MG V + F KSMQALGAKVERTGPFAWSVRGVGVAGFAQPAAPLDFGNSGTGCRLVMGAVAGCPITAVF | 120 |
| Query 120 | IGDASLTKRPMGRVLNPLREMGVQV-KSEDGDRLPVTLRGPKTPTPITYRVPMASAQVKS | 178 |
| Sbjct 121 | GDASL RPM R+L+PL MG + ++++G RLP+TL G + P PI YR P+ASAQ+KS DGDASLRTRPMRRILDPLELMGAKTGEAKEGGRLPLTLHGARDPLPILYRTPVASAQIKS | 180 |
| Query 179 | AVLLAGLNTPGITTVIEPIMTRDHTEKMLQGFGANLTVETDADGVRTIRLEGRGKLTGQV | 238 |
| Sbjct 181 | AVLLAGL PG+TTVIE +RDHTE ML+ FGA + + R I L G+ +L G AVLLAGLAAPGVTTVIEQEASRDHTELMLKHFGAEIVSTNEGSHGRKIALTGQPELHGAE | 240 |
| Query 239 | IDVPGDPSSTAFPLVAALLVPGSDVTILNVLMNPTRTGLILTLQEMGADIEVINPRLAGG | 298 |
| Sbjct 241 | + VP DPSS +FP+VAAL+V GSD+ +V+ NP RTGL TL+EMGA IE R G  VVVPADPSSASFPIVAALIVEGSDIVFTDVMTNPLRTGLFTTLREMGASIEESEVRGDAG | 300 |

| Query | 299 | EDVADLRVRSSTLKGVTVPEDRAPSMIDEYPILAVAAAFAEGATVMNGLEELRVKESDRL | 358 |
| --- | --- | --- | --- |
| Sbjct | 301 | E +A LRVR+S L+GV VP +RAPSMIDEY +LAVAAAFAEG T+M GL+ELRVKESDRL EPMAQLRVRASKLRGVEVPPERAPSMIDEYLVLAVAAAFAEGTTIMRGLQELRVKESDRL | 360 |
| Query | 359 | SAVANGLKLNGVDCDEGETSLVVRGRPDGKGLGNASGAAVATHLDHRIAMSFLVMGLVSE | 418 |
| Sbjct | 361 | A A+ L++NGV + L+V GR G G VATH+DHRIAMS LVMGL S+ EATADMLRVNGVKVEVSGDDLIVEGRGHVPG GGIVATHMDHRIAMSALVMGLASD | 415 |
| Query  Sbjct | 419  416 | NPVTVDDATMIATSFPEFMDLMAGLGA 445 PV VDD IATSFP+F+ +M LGA  KPVKVDDTAFIATSFPDFIPMMRSLGA 442 |  |

>TR:A0A6I2LXR7 A0A6I2LXR7_9RHOB 3-phosphoshikimate 1-carboxyvinyltransferase OS=Paracoccus sp. S-4012 OX=2665648 GN=aroA PE=3 SV=1

Length=445

Score = 472 bits (1214), Expect = 1e-161

| Identities | = 256/444 (58%), Positives = 304/444 (68%), Gaps = 7/444 (2%) |  |
| --- | --- | --- |
| Query 5 | ASSRPATARKSSGLSGTVRIPGDKSISHRSFMFGGLASGETRITGLLEGEDVINTGKAMQ | 64 |
| Sbjct 4 | A+ +P TAR+S L G R+PGDKSISHR+ + G LA G T ITGLLEG+DV++T AM+ AAPQPLTARRSGPLHGEARVPGDKSISHRALILGALAVGRTEITGLLEGQDVLDTAAAMR | 63 |
| Query 65 | AMGARIRKEGD-TWIIDGVGNGGLLAPEAPLDFGNAATGCRLTMGLVGVYDFDSTFIGDA | 123 |
| Sbjct 64 | A GAR+ + G+ W +DGVG GG P +D GN+ TG RL MG + +TF GDA AFGARVERTGEGRWTVDGVGVGGFAEPAGVIDCGNSGTGVRLIMGAMATTPITATFTGDA | 123 |
| Query 124 | SLTKRPMGRVLNPLREMGVQVKSEDGDRLPVTLRGPKTPTPITYRVPMASAQVKSAVLLA | 183 |
| Sbjct 124 | SL++RPMGRV +PL G +V + DG RLP+T+RG P P+ YR PM SAQVKSAVLLA SLSRRPMGRVTDPLALFGAKVVARDGGRLPITIRGALDPVPVRYRTPMPSAQVKSAVLLA | 183 |
| Query 184 | GLNTPGITTVIEPIMTRDHTEKMLQGFGANLTVETDADGVRTIRLEGRGKLTGQVIDVPG | 243 |
| Sbjct 184 | GLN PG T VIE TRDHTE+ML GFGA + E A+G R I L GR +L Q I VP GLNAPGETVVIEAEATRDHTERMLAGFGATIRTEVTAEG-RVITLTGRPELRPQPIAVPR | 242 |
| Query 244 | DPSSTAFPLVAALLVPGSDVTILNVLMNPTRTGLILTLQEMGADIEVINPRLAGGEDVAD | 303 |
| Sbjct 243 | DPSS AFP AAL+VPGS+V I V NPTR G L L++MGADI N R GGE VAD DPSSAAFPTAAALMVPGSEVRIPGVSRNPTRDGFFLVLEDMGADIAWENAREEGGEPVAD | 302 |
| Query 304 | LRVRSSTLKGVTVPEDRAPSMIDEYPILAVAAAFAEGATVMNGLEELRVKESDRLSAVAN | 363 |
| Sbjct 303 | L VR LKGV V +RA SMIDE+PILA AA AEG TVM+G+ ELRVKESDR+ A+A LVVRHGGLKGVEVAPERAASMIDEFPILAALAATAEGTTVMHGVAELRVKESDRIDAMAR | 362 |
| Query 364 | GLKLNGVDCDEGETSLVVRGRPDGKGLGNASGAAVATHLDHRIAMSFLVMGLVSENPVTV | 423 |
| Sbjct 363 | GL+ GV +E SL V G + G G ATHLDHRIAMSFL +GL S P+TV GLEACGVRVEETRDSLTVHGMAEVPG GGTCATHLDHRIAMSFLCLGLASRAPITV | 417 |
| Query 424  Sbjct 418 | DDATMIATSFPEFMDLMAGLGAKI 447 DDA+ IATSFP+F+ LM GLGA I DDASPIATSFPDFLPLMRGLGAMI 441 |  |

>TR:A0A086P9P1 A0A086P9P1_SPHHM 3-phosphoshikimate 1-carboxyvinyltransferase OS=Sphingobium herbicidovorans (strain ATCC 700291 / DSM 11019

/ NBRC 16415 / MH) OX=1219045 GN=aroA PE=3 SV=1

Length=436

Score = 471 bits (1213), Expect = 1e-161

Identities = 251/436 (58%), Positives = 307/436 (70%), Gaps = 4/436 (1%)

Query 11	TARKSSGLSGTVRIPGDKSISHRSFMFGGLASGETRITGLLEGEDVINTGKAMQAMGARI 70 T	+ LSG++R+PGDKSISHRS M	LA GE+R+ GLLEGEDV+ T AM+AMGA+I

| Sbjct | 2 | TFSSAPSLSGSIRVPGDKSISHRSLMLSALAVGESRVKGLLEGEDVLATAAAMRAMGAQI | 61 |
| --- | --- | --- | --- |
| Query | 71 | RKEGD-TWIIDGVGNGGLLAPEAPLDFGNAATGCRLTMGLVGVYDFDSTFIGDASLTKRP | 129 |
| Sbjct | 62 | ++ D W I GVG GGLL P++ LD GN+ T RL MGLV + +TF+GDASL+KRP SRDDDGIWHIRGVGVGGLLQPQSALDMGNSGTSTRLLMGLVASHAITATFVGDASLSKRP | 121 |
| Query | 130 | MGRVLNPLREMGVQVKSEDGDRLPVTLRGPKTPTPITYRVPMASAQVKSAVLLAGLNTPG | 189 |
| Sbjct | 122 | M RV PL G S GDRLP+T+RG P+ YR+P+ASAQVKSA+LLAGLNTPG MARVTEPLSRTGASFTSSPGDRLPLTMRGICPAVPLDYRLPVASAQVKSAILLAGLNTPG | 181 |
| Query | 190 | ITTVIEPIMTRDHTEKMLQGFGANLTVETDADGVRTIRLEGRGKLTGQVIDVPGDPSSTA | 249 |
| Sbjct | 182 | +T V+EPI TRDH+E+ML+GFGA+L VE + DG R I L G +L Q I VPGDPSS A MTRVVEPIPTRDHSERMLKGFGADLNVEVETDGTRIITLMGEAELRPQSIAVPGDPSSAA | 241 |
| Query | 250 | FPLVAALLVPGSDVTILNVLMNPTRTGLILTLQEMGADIEVINPRLAGGEDVADLRVRSS | 309 |
| Sbjct | 242 | FP+VAALLVPGS VTI NV +N TR GLI L+EMG DI + NPR GGE V DL + +S FPMVAALLVPGSKVTIANVGLNATRAGLIDLLREMGGDILIENPREVGGEPVGDLVITAS | 301 |
| Query | 310 | TLKGVTVPEDRAPSMIDEYPILAVAAAFAEGATVMNGLEELRVKESDRLSAVANGLKLNG | 369 |
| Sbjct | 302 | L V RAPSMIDEYP+ +AAAFAEG ++ GL+ELRVKESDR++ +A GL+ G PLVAVEPDPARAPSMIDEYPVAFIAAAFAEGRSIFRGLQELRVKESDRIATMAAGLRAIG | 361 |
| Query | 370 | VDCDEGETSLVVRGRPDGKGLGNASGAAVATHLDHRIAMSFLVMGLVSENPVTVDDATMI | 429 |
| Sbjct | 362 | VD +E E +V+ G G G ++T LDHRIAMSF V GLVS+N VT+DD + VDVEELEDGIVIEG---SGGALLPGGGPISTKLDHRIAMSFAVAGLVSKNGVTIDDMRPV | 418 |
| Query  Sbjct | 430  419 | ATSFPEFMDLMAGLGA 445 ATSFP F L+ LGA ATSFPGFTGLLQSLGA 434 |  |

>TR:A0A1M7AZS8 A0A1M7AZS8_9RHOB 3-phosphoshikimate 1-carboxyvinyltransferase OS=Roseovarius pacificus OX=337701 GN=aroA PE=3 SV=1

Length=448

Score = 472 bits (1214), Expect = 1e-161

| Identities | = 254/441 (58%), Positives = 308/441 (70%), Gaps = 6/441 (1%) |  |
| --- | --- | --- |
| Query 9 | PATARKSSGLSGTVRIPGDKSISHRSFMFGGLASGETRITGLLEGEDVINTGKAMQAMGA | 68 |
| Sbjct 9 | P ++ +S GL+GT +PGDKSISHRS + G L+ GET ITGLLEG+DV++T KAMQAMGA PMSSHRSKGLNGTAEVPGDKSISHRSLILGALSVGETVITGLLEGQDVLDTAKAMQAMGA | 68 |
| Query 69 | RIRKEGD-TWIIDGVGNGGLLAPEAPLDFGNAATGCRLTMGLVGVYDFDSTFIGDASLTK | 127 |
| Sbjct 69 | + ++ D TW + GVG GG P+ +D GN+ TG RL MG + +TF GDASL K EVVRDDDGTWRVHGVGVGGFAEPDQVIDCGNSGTGVRLIMGAMATLPIAATFTGDASLNK | 128 |
| Query 128 | RPMGRVLNPLREMGVQVKSEDGDRLPVTLRGPKTPTPITYRVPMASAQVKSAVLLAGLNT | 187 |
| Sbjct 129 | RPM RV +PL G Q G RLP+T+ G P P+ Y VP+ SAQVKSAVLLAGLN RPMARVTDPLALFGAQAVGRSGGRLPMTIVGAHDPLPVRYTVPVPSAQVKSAVLLAGLNA | 188 |
| Query 188 | PGITTVIEPIMTRDHTEKMLQGFGANLTVETDADGVRTIRLEGRGKLTGQVIDVPGDPSS | 247 |
| Sbjct 189 | PG T VIE TRDHTE+ML GFGA +T E D D R I L G+ +L Q I VP DPSS PGQTVVIEREATRDHTERMLAGFGAEITTE-DTDEGRVITLTGQPELIPQGIAVPRDPSS | 247 |
| Query 248 | TAFPLVAALLVPGSDVTILNVLMNPTRTGLILTLQEMGADIEVINPRLAGGEDVADLRVR | 307 |
| Sbjct 248 | AFP+ AAL+ GSDV + N+ +NPTR GL TL+EMGAD+ N R GGE VADLR R AAFPVCAALITEGSDVLVPNIGLNPTRAGLFTTLREMGADLSYENEREEGGEPVADLRAR | 307 |
| Query 308 | -SSTLKGVTVPEDRAPSMIDEYPILAVAAAFAEGATVMNGLEELRVKESDRLSAVANGLK | 366 |
| Sbjct 308 | S +KG+ VP +RA SMIDEYP+L+V AAFAEG TVM G++ELRVKESDR+ A+A GL+ FSPDMKGIEVPPERAASMIDEYPVLSVVAAFAEGQTVMRGVKELRVKESDRIEAMATGLR | 367 |
| Query 367 | LNGVDCDEGETSLVVRGRPDGKGLGNASGAAVATHLDHRIAMSFLVMGLVSENPVTVDDA NGV+ ++G +V GR G G GA A+HLDHRIAMSFLVMGL +++PV+VDD | 426 |

| Sbjct | 368 | ANGVEVEDGPDWWIVHGRGQGNVPG---GATCASHLDHRIAMSFLVMGLAAQSPVSVDDG 424 |
| --- | --- | --- |
| Query | 427 | TMIATSFPEFMDLMAGLGAKI 447 |

IATSFP F LMA LGA I Sbjct 425 GPIATSFPVFEPLMAALGADI 445

>TR:A6FVB2 A6FVB2_9RHOB 3-phosphoshikimate 1-carboxyvinyltransferase OS=Roseobacter sp. AzwK-3b OX=351016 GN=aroA PE=3 SV=1

Length=469

Score = 473 bits (1216), Expect = 1e-161

| Identities | = 257/447 (57%), Positives = 311/447 (70%), Gaps = 7/447 (2%) |  |
| --- | --- | --- |
| Query 3 | HGASSRPATARKSSGLSGTVRIPGDKSISHRSFMFGGLASGETRITGLLEGEDVINTGKA | 62 |
| Sbjct 24 | HGA P T+ +S GL+GT IPGDKSISHR+ + G L+ GET +TGLLEG+DV++T +A HGAPI-PMTSHRSGGLAGTADIPGDKSISHRALILGALSVGETMVTGLLEGQDVLDTARA | 82 |
| Query 63 | MQAMGARIRKEGD-TWIIDGVGNGGLLAPEAPLDFGNAATGCRLTMGLVGVYDFDSTFIG | 121 |
| Sbjct 83 | M+A GA + ++ D TW I GVG GG P+ +D GN+ TG RL MG + +TF G MRAFGAEVVRDDDGTWRIHGVGVGGFAEPDQVIDCGNSGTGVRLIMGAMATSPVTATFTG | 142 |
| Query 122 | DASLTKRPMGRVLNPLREMGVQVKSEDGDRLPVTLRGPKTPTPITYRVPMASAQVKSAVL | 181 |
| Sbjct 143 | DASL KRPM RV +PL G Q G RLP+T+ G + P P+ Y VP+ SAQVKSAVL DASLNKRPMARVTDPLALFGAQAVGRSGGRLPMTIVGARDPLPVRYTVPVPSAQVKSAVL | 202 |
| Query 182 | LAGLNTPGITTVIEPIMTRDHTEKMLQGFGANLTVETDADGVRTIRLEGRGKLTGQVIDV | 241 |
| Sbjct 203 | LAGLN PG T VIE TRDHTE+ML GFGA +T E A+G R I LEG+ +L+ Q I V LAGLNAPGQTVVIEHEATRDHTERMLAGFGAQITTEDTAEG-RIITLEGQPELSPQTIAV | 261 |
| Query 242 | PGDPSSTAFPLVAALLVPGSDVTILNVLMNPTRTGLILTLQEMGADIEVINPRLAGGEDV | 301 |
| Sbjct 262 | P DPSS AFP+ AAL+ GSDV + N+ +NPTR GL TLQEMGAD+ N R GGE V PRDPSSAAFPVCAALITEGSDVLVPNIGLNPTRAGLFTTLQEMGADLSYENLREEGGEPV | 321 |
| Query 302 | ADLRVR-SSTLKGVTVPEDRAPSMIDEYPILAVAAAFAEGATVMNGLEELRVKESDRLSA | 360 |
| Sbjct 322 | ADLR S LKG+ VP +RA SMIDEYP+L+V AAFA+GATVM G++ELRVKESDR+ A ADLRASFSPDLKGIEVPPERAASMIDEYPVLSVVAAFAQGATVMRGVKELRVKESDRIEA | 381 |
| Query 361 | VANGLKLNGVDCDEGETSLVVRGRPDGKGLGNASGAAVATHLDHRIAMSFLVMGLVSENP | 420 |
| Sbjct 382 | +A GL+ NG+ ++G +V GR G G GA A+HLDHRIAMSFLVMGL + P MATGLRANGIKVEDGPDWWIVHGRGHGDVPG---GATCASHLDHRIAMSFLVMGLAANQP | 438 |
| Query 421  Sbjct 439 | VTVDDATMIATSFPEFMDLMAGLGAKI 447 VTVDD IATSFP F LMA LGA I VTVDDGGPIATSFPIFEPLMASLGADI 465 |  |

>TR:A0A345WQF8 A0A345WQF8_9SPHN 3-phosphoshikimate 1-carboxyvinyltransferase OS=Sphingomonas sp. FARSPH OX=2219696 GN=aroA PE=3 SV=1

Length=460

Score = 472 bits (1215), Expect = 1e-161

| Identities | = 260/449 (58%), Positives = 310/449 (69%), Gaps = 8/449 (2%) |  |
| --- | --- | --- |
| Query 3 | HGASSRPATARKSSGLSGTVRIPGDKSISHRSFMFGGLASGETRITGLLEGEDVINTGKA | 62 |
| Sbjct 7 | H + RP + + L GTV +PGDKSISHR+ MF LA G +RI GLLEGEDV+ T A HSFAPRPLSVAAAGPLVGTVTVPGDKSISHRALMFASLAVGTSRIRGLLEGEDVLATAAA | 66 |
| Query 63 | MQAMGARIRKEGD-TWIIDGVGNGGLLAPEAPLDFGNAATGCRLTMGLVGVYDFDSTFIG | 121 |
| Sbjct 67 | M+AMGA I + D +W ++GVG GGLL PE LD GN+ T RL MGLV + +TF+G MRAMGATITRGDDGSWTVEGVGVGGLLQPETALDMGNSGTSTRLLMGLVASHAITATFVG | 126 |
| Query 122 | DASLTKRPMGRVLNPLREMGVQVKSE DGDRLPVTLRGPKTPTPITYRVPMASAQV  DASL+KRPM RV +PL MG + G RLP+T+RG PITY +P+ASAQV | 176 |

| Sbjct | 127 | DASLSKRPMARVTDPLARMGADFTTSPGPDGSGGRLPLTMRGLCPAVPITYTLPVASAQV | 186 |
| --- | --- | --- | --- |
| Query | 177 | KSAVLLAGLNTPGITTVIEPIMTRDHTEKMLQGFGANLTVETDADGVRTIRLEGRGKLTG | 236 |
| Sbjct | 187 | KSA+LLAGLNTPGIT VIE + TRDHTE+ML+GFGA+LTVE A+G R I + G +L KSAILLAGLNTPGITRVIEAVPTRDHTERMLRGFGADLTVEETAEG-RVIAIAGEAELAP | 245 |
| Query | 237 | QVIDVPGDPSSTAFPLVAALLVPGSDVTILNVLMNPTRTGLILTLQEMGADIEVINPRLA | 296 |
| Sbjct | 246 | Q IDVPGDPSS F +VAA +VPGSDVTI NVLMNPTRTGL+ L+ MGA IE +PR QEIDVPGDPSSAGFWMVAASIVPGSDVTIRNVLMNPTRTGLVTALRMMGASIEERDPRDV | 305 |
| Query | 297 | GGEDVADLRVRSSTLKGVTVPEDRAPSMIDEYPILAVAAAFAEGATVMNGLEELRVKESD | 356 |
| Sbjct | 306 | GGE VADLRVR + L + VP APSMIDEYP+L VAAAFA G TV G ELRVKESD GGETVADLRVRHAPLSAIEVPAALAPSMIDEYPVLFVAAAFAHGTTVARGAHELRVKESD | 365 |
| Query | 357 | RLSAVANGLKLNGVDCDEGETSLVVRGRPDGKGLGNASGAAVATHLDHRIAMSFLVMGLV | 416 |
| Sbjct | 366 | R++A+A L L GV E E L + GR DG+ L AVA+ LDHRIAMS V G+ RIAAMAAALGLAGVAVTEHEDGLSIEGR-DGEPLPGTGDVAVASLLDHRIAMSMAVAGMH | 424 |
| Query  Sbjct | 417  425 | SENPVTVDDATMIATSFPEFMDLMAGLGA 445 S +PV +DDA IATS+P F+D LGA SASPVRIDDAAPIATSYPAFVDTARALGA 453 |  |

>TR:V6F8R5 V6F8R5_MAGGM 3-phosphoshikimate 1-carboxyvinyltransferase OS=Magnetospirillum gryphiswaldense (strain DSM 6361 / JCM 21280

/ NBRC 15271 / MSR-1) OX=431944 GN=aroA PE=3 SV=1

Length=446

Score = 471 bits (1213), Expect = 1e-161

| Identities | | = 254/446 (57%), Positives = 308/446 (69%), Gaps = 5/446 (1%) |  |
| --- | --- | --- | --- |
| Query 7  Sbjct 2 | | SRPATARKSSGLSGTVRIPGDKSISHRSFMFGGLASGETRITGLLEGEDVINTGKAMQAM S+P +R+++ LSG+ ++PGDKS+SHR+ MFG LA GE+R+ GLLEG+DV+ T AM+A+ SKPLISRRAAALSGSAKVPGDKSVSHRALMFGALAVGESRVEGLLEGDDVLRTAAAMRAL | 66  61 |
| Query 67  Sbjct 62 | | GARIRKEGD-TWIIDGVGNGGLLAPEAPLDFGNAATGCRLTMGLVGVYDFDSTFIGDASL GA + + D +W + G G GGL PE LD GN+ TG RL MGLV + F + F GDASL GAEVERLDDGSWRLYGRGVGGLKEPEEVLDLGNSGTGARLMMGLVATHPFTTVFTGDASL | 125  121 |
| Query 126  Sbjct 122 | | TKRPMGRVLNPLREMGVQVKSEDGDRLPVTLRGPKTPTPITYRVPMASAQVKSAVLLAGL RPM RV PL +MG Q DG RLP+T+ G P PITY +P+ASAQVKSA+LLAGL RSRPMKRVSLPLSQMGAQFFGRDGGRLPMTVVGTGNPLPITYELPVASAQVKSAILLAGL | 185  181 |
| Query 186  Sbjct 182 | | NTPGITTVIEPIMTRDHTEKMLQGFGANLTVETDADGVRTIRLEGRGKLTGQVIDVPGDP NTPG TTVIE TRDHTE ML+ FGA + VE G R I + G +LTG+ + VP DP NTPGQTTVIEREATRDHTELMLRNFGAQVDVEHGDQGQRIITVTGFPELTGRHVIVPADP | 245  241 |
| Query 246  Sbjct 242 | | SSTAFPLVAALLVPGSDVTILNVLMNPTRTGLILTLQEMGADIEVINPRLAGGEDVADLR SS AFP VAA++ PGSD+ + V MNP RTGL TL+EMGAD+ N R GGE VADLR SSAAFPAVAAIITPGSDIVLTGVGMNPLRTGLYQTLREMGADLAFENQREDGGEQVADLR | 305  301 |
| Query 306  Sbjct 302 | | VRSSTLKGVTVPEDRAPSMIDEYPILAVAAAFAEGATVMNGLEELRVKESDRLSAVANGL VR S L GV VP +RAPSMIDEYPILAVAAAFA G T M GL ELRVKESDR +A GL VRHSALMGVDVPAERAPSMIDEYPILAVAAAFATGTTRMFGLSELRVKESDRFAATLRGL | 365  361 |
| Query 366 | | KLNGVDCDEGETSLVVRGRPDGKGLGNASGAAVATHLDHRIAMSFLVMGLVSENPVTVDD GV +E +L+V G G GA +A +LDHRIAM+FLV+G+ + V VDD | 425 |
| Sbjct | 362 | LACGVQVEEDGDTLIVH GSGKPPMGGATIAVNLDHRIAMAFLVLGMAAAEAVAVDD | 417 |
| Query  Sbjct | 426  418 | ATMIATSFPEFMDLMAGLGAKIELSD 451 A I TSFP F+ LM GLGAKI +D AEAIDTSFPGFVQLMNGLGAKIATTD 443 |  |

>TR:A0A0Q4LTN4 A0A0Q4LTN4_9SPHN 3-phosphoshikimate 1-carboxyvinyltransferase OS=Sphingomonas sp. Leaf42 OX=1736219 GN=aroA PE=3 SV=1

Length=443

Score = 471 bits (1212), Expect = 2e-161

| Identities | = 254/447 (57%), Positives = 306/447 (68%), Gaps = 5/447 (1%) |  |
| --- | --- | --- |
| Query 2 | LHGASSRPATARKSSGLSGTVRIPGDKSISHRSFMFGGLASGETRITGLLEGEDVINTGK | 61 |
| Sbjct 1 | +H + P S L GT +PGDKSISHRS M LA G +R+TGLLEGEDV++T MHACTPTPLAVAASGPLRGTAPVPGDKSISHRSLMLSALAVGTSRVTGLLEGEDVLSTAA | 60 |
| Query 62 | AMQAMGARIRKEGD-TWIIDGVGNGGLLAPEAPLDFGNAATGCRLTMGLVGVYDFDSTFI | 120 |
| Sbjct 61 | AM+AMGA I + D W IDGVG GGLL P LD GN+ T RL MGLV + +TF+ AMRAMGAHIVRGTDGVWTIDGVGVGGLLQPATALDMGNSGTSTRLLMGLVASHAITATFV | 120 |
| Query 121 | GDASLTKRPMGRVLNPLREMGVQVKSEDGDRLPVTLRGPKTPTPITYRVPMASAQVKSAV | 180 |
| Sbjct 121 | GDASL+KRPMGRV++PL +MG ++ + G RLP+ LRG PI Y +P+ASAQVKSA+ GDASLSKRPMGRVIDPLGQMGAEITASPGGRLPLMLRGIVPAVPIDYTLPVASAQVKSAI | 180 |
| Query 181 | LLAGLNTPGITTVIEPIMTRDHTEKMLQGFGANLTVETDADGVRTIRLEGRGKLTGQVID | 240 |
| Sbjct 181 | LLAGLNTPGIT V+EP+ TRDH+E+ML GFGA LTVE G R I L G +L Q I LLAGLNTPGITRVVEPVPTRDHSERMLTGFGAKLTVEQTPQG-RVIELHGEAELKPQTIV | 239 |
| Query 241 | VPGDPSSTAFPLVAALLVPGSDVTILNVLMNPTRTGLILTLQEMGADIEVINPRLAGGED | 300 |
| Sbjct 240 | VPGDPSS AFP+VAALLVPGS VTI NV +NPTR L L+ MG DI NPR GGE VPGDPSSAAFPVVAALLVPGSAVTITNVGLNPTRAALFDVLRAMGGDIAFENPRDVGGEP | 299 |
| Query 301 | VADLRVRSSTLKGVTVPEDRAPSMIDEYPILAVAAAFAEGATVMNGLEELRVKESDRLSA | 360 |
| Sbjct 300 | VADL VR+S+L G+ V AP+MIDE+PIL VAAA A+G T+ GLEELRVKESDR++ VADLVVRASSLTGIEVDPAVAPAMIDEFPILFVAAALAKGRTITRGLEELRVKESDRITT | 359 |
| Query 361 | VANGLKLNGVDCDEGETSLVVRGRPDGKGLGNASGAAVATHLDHRIAMSFLVMGLVSENP | 420 |
| Sbjct 360 | +A GL+ G +E E L++ G G G +A LDHRIAMSF+V GLVS P MAEGLRAIGATVEETEDGLIIDGT---GGDPLPGGGTIAAKLDHRIAMSFVVAGLVSREP | 416 |
| Query 421  Sbjct 417 | VTVDDATMIATSFPEFMDLMAGLGAKI 447 VT+DD +ATSFP F L+ GLGA + VTIDDMAPVATSFPTFTTLLRGLGAPL 443 |  |

>TR:A0A0Q5PGG0 A0A0Q5PGG0_9SPHN 3-phosphoshikimate 1-carboxyvinyltransferase OS=Sphingomonas sp. Leaf343 OX=1736345 GN=aroA PE=3 SV=1

Length=445

Score = 471 bits (1212), Expect = 2e-161

| Identities | = 261/442 (59%), Positives = 305/442 (69%), Gaps = 5/442 (1%) |  |
| --- | --- | --- |
| Query 7 | SRPATARKSSGLSGTVRIPGDKSISHRSFMFGGLASGETRITGLLEGEDVINTGKAMQAM | 66 |
| Sbjct 6 | S+P T + L+G V +PGDKSISHRS M LA GE+R+ GLLEGEDV+ T AM+AM SQPLTLVATGPLTGRVTVPGDKSISHRSLMLSALAVGESRVEGLLEGEDVLATAAAMRAM | 65 |
| Query 67 | GARI-RKEGDTWIIDGVGNGGLLAPEAPLDFGNAATGCRLTMGLVGVYDFDSTFIGDASL | 125 |
| Sbjct 66 | GA I R E W I GVG GGLL P LD GN+ T RL MGLV + +TF+GDASL GADIARGEDGVWTIHGVGVGGLLQPANVLDMGNSGTSTRLLMGLVASHPITATFVGDASL | 125 |
| Query 126 | TKRPMGRVLNPLREMGVQVKSEDGDRLPVTLRGPKTPTPITYRVPMASAQVKSAVLLAGL | 185 |
| Sbjct 126 | +KRPMGRV+ PL MG V + G RLP+ LRG PITY +P+ASAQVKSA+LLAGL SKRPMGRVIEPLMSMGAAVTASPGGRLPLMLRGICPAVPITYTLPVASAQVKSAILLAGL | 185 |
| Query 186 | NTPGITTVIEPIMTRDHTEKMLQGFGANLTVETDADGVRTIRLEGRGKLTGQVIDVPGDP | 245 |
| Sbjct 186 | NTPGIT VIEPI TRDH+E+ML GFGA LTVE G R I L G +LT Q I VPGDP NTPGITRVIEPIATRDHSERMLAGFGARLTVEPSPAG-RVISLHGEAELTPQQIVVPGDP | 244 |
| Query 246 | SSTAFPLVAALLVPGSDVTILNVLMNPTRTGLILTLQEMGADIEVINPRLAGGEDVADLR | 305 |

| Sbjct | 245 | SS AF LVAA +VPGSDVT+ NV +NPTRTGLI L+ MGADIE ++ R GGE VADLR  SSAAFWLVAASIVPGSDVTVANVGLNPTRTGLIAALRLMGADIEELDARTVGGEPVADLR | 304 |
| --- | --- | --- | --- |
| Query | 306 | VRSSTLKGVTVPEDRAPSMIDEYPILAVAAAFAEGATVMNGLEELRVKESDRLSAVANGL | 365 |
| Sbjct | 305 | VR + L + VP D APSMIDEYP L VAAAFA G TV G ELRVKESDR++A+ L VRHAALSAIEVPPDLAPSMIDEYPALFVAAAFASGRTVARGAHELRVKESDRIAAMKAAL | 364 |
| Query | 366 | KLNGVDCDEGETSLVVRGRPDGKGLGNASGAAVATHLDHRIAMSFLVMGLVSENPVTVDD | 425 |
| Sbjct | 365 | + GV CDE E L V+G G A A + THLDHRIAMS V GL + VT+ D EACGVVCDEFEDGLAVQG---SGGEPIAGDATIETHLDHRIAMSMAVAGLHARKAVTIGD | 421 |
| Query  Sbjct | 426  422 | ATMIATSFPEFMDLMAGLGAKI 447 A+ IATS+P F+ + LGA++ ASPIATSYPGFVGIATALGAEV 443 |  |

>TR:A0A270BER9 A0A270BER9_9SPHN 3-phosphoshikimate 1-carboxyvinyltransferase OS=Sphingopyxis sp. GW247-27LB OX=2012632 GN=aroA PE=3 SV=1

Length=446

Score = 471 bits (1212), Expect = 2e-161

| Identities | = 255/434 (59%), Positives = 305/434 (70%), Gaps = 8/434 (2%) |  |
| --- | --- | --- |
| Query 15 | SSGLSGTVRIPGDKSISHRSFMFGGLASGETRITGLLEGEDVINTGKAMQAMGARIRKEG | 74 |
| Sbjct 14 | S L+G + IPGDKSISHR+ M LA G +R+TGLLEG DV+ T AM+AMGA I + G SPPLTGRIAIPGDKSISHRALMLSALAVGTSRVTGLLEGHDVLATAAAMRAMGASIERTG | 73 |
| Query 75 | D-TWIIDGVGNGGLLAPEAPLDFGNAATGCRLTMGLVGVYDFDSTFIGDASLTKRPMGRV | 133 |
| Sbjct 74 | + W IDGVG G LL P LD GN+ T RL MGLV + TF GDASL+ RPMGRV EGAWNIDGVGVGSLLQPREALDMGNSGTSTRLLMGLVASHPITVTFTGDASLSGRPMGRV | 133 |
| Query 134 | LNPLREMGVQVKSEDGDR LPVTLRGPKTPTPITYRVPMASAQVKSAVLLAGLNTPG | 189 |
| Sbjct 134 | ++PL +MG + + G + LP+ +RG P+ YR+PMASAQVKSAVLLAGLNTPG IDPLSQMGADISASPGAKGTQTLPLMVRGLAPAIPLAYRLPMASAQVKSAVLLAGLNTPG | 193 |
| Query 190 | ITTVIEPIMTRDHTEKMLQGFGANLTVETDADGVRTIRLEGRGKLTGQVIDVPGDPSSTA | 249 |
| Sbjct 194 | IT V+EP+ TRDH+E+ML+GFGA+L+VET ADG R IR+ G +L Q I VPGDPSS A ITEVVEPVPTRDHSERMLRGFGADLSVETGADGTRHIRVRGEAELRPQTIVVPGDPSSAA | 253 |
| Query 250 | FPLVAALLVPGSDVTILNVLMNPTRTGLILTLQEMGADIEVINPRLAGGEDVADLRVRSS | 309 |
| Sbjct 254 | F +VAALLVPGSDVTI NV +NPTR GL+ LQ MG DI +++ R GGE VADLRVR S FFIVAALLVPGSDVTIANVGLNPTRAGLVEVLQAMGGDITLLDAREVGGEPVADLRVRHS | 313 |
| Query 310 | TLKGVTVPEDRAPSMIDEYPILAVAAAFAEGATVMNGLEELRVKESDRLSAVANGLKLNG | 369 |
| Sbjct 314 | LKG+ V APSMIDEYPIL VAAA AEG TV GL+ELRVKESDRL+ +A GL G VLKGIEVDPAVAPSMIDEYPILFVAAALAEGRTVTTGLDELRVKESDRLAVMAAGLAAIG | 373 |
| Query 370 | VDCDEGETSLVVRGRPDGKGLGNASGAAVATHLDHRIAMSFLVMGLVSENPVTVDDATMI | 429 |
| Sbjct 374 | +E E L++ G G A GA +A HLDHRI MSF + GLVS+ PVT+DD + ARVEESEDGLIIDG---SGGDPLAGGATIAGHLDHRICMSFAIAGLVSKAPVTIDDMAPV | 430 |
| Query 430  Sbjct 431 | ATSFPEFMDLMAGL 443 ATSFP F L+ GL ATSFPNFEALLEGL 444 |  |

>TR:A0A5B8SAG7 A0A5B8SAG7_9SPHN 3-phosphoshikimate 1-carboxyvinyltransferase OS=Novosphingobium ginsenosidimutans OX=1176536 GN=aroA PE=3

SV=1

Length=439

Score = 471 bits (1211), Expect = 2e-161

Identities = 251/437 (57%), Positives = 306/437 (70%), Gaps = 2/437 (0%)

| Query | 8 | RPATARKSSGLSGTVRIPGDKSISHRSFMFGGLASGETRITGLLEGEDVINTGKAMQAMG | 67 |
| --- | --- | --- | --- |
| Sbjct | 2 | +P S L GT+R+PGDKSISHRS M G LA GETR+TGLLEGEDV++T AM+AMG KPRRFMPSGPLRGTIRVPGDKSISHRSIMLGALAVGETRVTGLLEGEDVLSTAAAMRAMG | 61 |
| Query | 68 | ARIRKEGD-TWIIDGVGNGGLLAPEAPLDFGNAATGCRLTMGLVGVYDFDSTFIGDASLT | 126 |
| Sbjct | 62 | A + + G+ W + GVG G LL P+ PLD GN+ T RL MGLV + +TFIGDASL+ ASVERLGNGEWSVHGVGVGALLQPQNPLDMGNSGTSTRLLMGLVASHPITATFIGDASLS | 121 |
| Query | 127 | KRPMGRVLNPLREMGVQVKSEDGDRLPVTLRGPKTPTPITYRVPMASAQVKSAVLLAGLN | 186 |
| Sbjct | 122 | KRPMGRV+ PL +MG ++ +G RLP+T+RG PI YR+P+ASAQVKSA+LLAGLN KRPMGRVIEPLSQMGAAFEASEGGRLPLTVRGALPAVPIEYRLPVASAQVKSALLLAGLN | 181 |
| Query | 187 | TPGITTVIEPIMTRDHTEKMLQGFGANLTVETDADGVRTIRLEGRGKLTGQVIDVPGDPS | 246 |
| Sbjct | 182 | PGITTVIEP+ TRDH+E+ML+GFGA+L+VE ADG R IRL G +L Q I+VPGDPS APGITTVIEPVPTRDHSERMLRGFGADLSVEIAADGARVIRLVGEAELKPQTIEVPGDPS | 241 |
| Query | 247 | STAFPLVAALLVPGSDVTILNVLMNPTRTGLILTLQEMGADIEVINPRLAGGEDVADLRV | 306 |
| Sbjct | 242 | S AF +VAAL+VPGS++TI NV +NPTR GLI L++MG I+ ++ R GGE VADL V SAAFFIVAALVVPGSELTIQNVGLNPTRAGLIEVLRQMGGQIDEVDRREVGGEPVADLVV | 301 |
| Query | 307 | RSSTLKGVTVPEDRAPSMIDEYPILAVAAAFAEGATVMNGLEELRVKESDRLSAVANGLK | 366 |
| Sbjct | 302 | S L G+ + PSM+DE+P+L VAAA AEG TV GLEELRVKESDR+S +A L HHSALNGIEIDPAVVPSMVDEFPVLFVAAALAEGTTVTTGLEELRVKESDRISVMAAALA | 361 |
| Query | 367 | LNGVDCDEGETSLVVRGRPDGKGLGNASGAAVATHLDHRIAMSFLVMGLVSENPVTVDDA | 426 |
| Sbjct | 362 | G E E L + G G L S + THLDHRIAMS V GL S + V VDD GAGARVTETEDGLTIEGSGGGP-LRGTSNSRTKTHLDHRIAMSMAVAGLASRDGVEVDDT | 420 |
| Query | 427 | TMIATSFPEFMDLMAGL 443 IATSFP F L+ GL |  |
| Sbjct | 421 | RPIATSFPMFEALLDGL 437 | |

>TR:A0A1W2AYS5 A0A1W2AYS5_9SPHN 3-phosphoshikimate 1-carboxyvinyltransferase OS=Novosphingobium sp. B1 OX=1938756 GN=aroA PE=3 SV=1

Length=440

Score = 471 bits (1211), Expect = 2e-161

| Identities | = 251/436 (58%), Positives = 304/436 (70%), Gaps = 1/436 (0%) |  |
| --- | --- | --- |
| Query 8 | RPATARKSSGLSGTVRIPGDKSISHRSFMFGGLASGETRITGLLEGEDVINTGKAMQAMG | 67 |
| Sbjct 2 | RP + L G +R+PGDKSISHRS M G LA GETR+TGLLEGEDV++T AM+AMG RPRRFTANGPLKGRIRVPGDKSISHRSIMLGALAVGETRVTGLLEGEDVLSTAAAMRAMG | 61 |
| Query 68 | ARIRKEGD-TWIIDGVGNGGLLAPEAPLDFGNAATGCRLTMGLVGVYDFDSTFIGDASLT | 126 |
| Sbjct 62 | A I ++G+ TW + GVG GGLL P+ LD GN+ T RL MGLV + +TF+GDASL+ ATIERDGEGTWHVHGVGVGGLLQPQQALDMGNSGTSTRLLMGLVASHPITATFVGDASLS | 121 |
| Query 127 | KRPMGRVLNPLREMGVQVKSEDGDRLPVTLRGPKTPTPITYRVPMASAQVKSAVLLAGLN | 186 |
| Sbjct 122 | KRPMGRV++PL MG + + G RLP+TLRG PI YR+P+ASAQVKSA+LLAGLN KRPMGRVIDPLSTMGAEFTASPGGRLPLTLRGISPAVPIEYRLPVASAQVKSAILLAGLN | 181 |
| Query 187 | TPGITTVIEPIMTRDHTEKMLQGFGANLTVETDADGVRTIRLEGRGKLTGQVIDVPGDPS | 246 |
| Sbjct 182 | TPGITTVIEPI TRDH+E+ML+GFGA LTV+ DG R I++ G + Q I VPGDPS TPGITTVIEPIPTRDHSERMLRGFGAELTVDVADDGARIIKVRGEAEFKPQDIVVPGDPS | 241 |
| Query 247 | STAFPLVAALLVPGSDVTILNVLMNPTRTGLILTLQEMGADIEVINPRLAGGEDVADLRV | 306 |
| Sbjct 242 | S AF +VAALLV GSD+ + NV +NPTR L L+ MG IE ++ R GGE VADLRV SAAFFVVAALLVEGSDLVVENVGLNPTRAALFDVLRLMGGSIEELDRREVGGEPVADLRV | 301 |
| Query 307 | RSSTLKGVTVPEDRAPSMIDEYPILAVAAAFAEGATVMNGLEELRVKESDRLSAVANGLK | 366 |
| Sbjct 302 | R S L G+ V PSM+DE+PIL VAAA A+G TV GLEELRVKESDR+SA+ L+  RHSLLTGIDVDPAVVPSMVDEFPILFVAAALAKGRTVTTGLEELRVKESDRISAMRAALE | 361 |

| Query | 367 | LNGVDCDEGETSLVVRGRPDGKGLGNASGAAVATHLDHRIAMSFLVMGLVSENPVTVDDA 426 |
| --- | --- | --- |
| Sbjct | 362 | L G E E L++ G G A GA+V THLDHRIAMS + G+ S+ V VDD LAGATVTETEDGLIIDGTGGDPLPGTAEGASVVTHLDHRIAMSMAIAGIASKRGVEVDDT 421 |
| Query | 427 | TMIATSFPEFMDLMAG 442 |
| Sbjct | 422 | IATSFP F L+ G RPIATSFPVFESLLEG 437 |

>TR:A0A344PNB6 A0A344PNB6_9RHOB 3-phosphoshikimate 1-carboxyvinyltransferase OS=Paracoccus sp. SC2-6 OX=2259340 GN=aroA PE=3 SV=1

Length=443

Score = 471 bits (1211), Expect = 3e-161

| Identities | = 253/447 (57%), Positives = 310/447 (69%), Gaps = 7/447 (2%) |  |
| --- | --- | --- |
| Query 2 | LHGASSRPATARKSSGLSGTVRIPGDKSISHRSFMFGGLASGETRITGLLEGEDVINTGK | 61 |
| Sbjct 1 | +H A+ +P TA +S L+G ++PGDKS+SHR+ + G L+ G T + GLLEG+DV++T MHDAAPQPLTAHRSGPLNGEAQVPGDKSVSHRALILGALSVGRTEVMGLLEGQDVLDTAA | 60 |
| Query 62 | AMQAMGARIRKEGDT-WIIDGVGNGGLLAPEAPLDFGNAATGCRLTMGLVGVYDFDSTFI | 120 |
| Sbjct 61 | AM+A GA + + + W IDGVG GG P +D GN+ TG RL MG + +TF AMRAFGATVTRLAEGHWAIDGVGVGGFAEPAGVIDCGNSGTGVRLIMGAMATTPISATFT | 120 |
| Query 121 | GDASLTKRPMGRVLNPLREMGVQVKSEDGDRLPVTLRGPKTPTPITYRVPMASAQVKSAV | 180 |
| Sbjct 121 | GDASL+ RPMGRV +PL+ G +V + DG RLP+TLRG P P+TYR P+ASAQ+KSAV GDASLSARPMGRVTDPLQLFGAEVTARDGVRLPLTLRGAGDPVPVTYRTPVASAQIKSAV | 180 |
| Query 181 | LLAGLNTPGITTVIEPIMTRDHTEKMLQGFGANLTVETDADGVRTIRLEGRGKLTGQVID | 240 |
| Sbjct 181 | LLAGLN PG T VIE + TRDHTE+ML+GFGA +T E DGV TI L GR +L Q + LLAGLNAPGQTVVIEAVPTRDHTERMLRGFGAEITSEAGPDGV-TITLTGRPELRPQRVA | 239 |
| Query 241 | VPGDPSSTAFPLVAALLVPGSDVTILNVLMNPTRTGLILTLQEMGADIEVINPRLAGGED | 300 |
| Sbjct 240 | VP DPSS AFP AALLVPGS++ I V NPTR G TL EMGAD+ N R GGE VPRDPSSAAFPAAAALLVPGSEIRIPGVSRNPTRDGFYTTLTEMGADLTFENDREEGGEP | 299 |
| Query 301 | VADLRVRSSTLKGVTVPEDRAPSMIDEYPILAVAAAFAEGATVMNGLEELRVKESDRLSA | 360 |
| Sbjct 300 | VADL +R+S L+GVTVP RA SMIDEYPILA AA A G+T M G+ ELRVKESDR+ A VADLTLRASRLRGVTVPAARAASMIDEYPILAALAATAVGSTHMPGIHELRVKESDRIDA | 359 |
| Query 361 | VANGLKLNGVDCDEGETSLVVRGRPDGKGLGNASGAAVATHLDHRIAMSFLVMGLVSENP | 420 |
| Sbjct 360 | +A GL+ GV +E SL V G G GA VA+ LDHRIAMSFL++GL SE P MARGLEACGVRIEETPDSLTVHGMAAVPG GATVASRLDHRIAMSFLILGLASEAP | 414 |
| Query 421  Sbjct 415 | VTVDDATMIATSFPEFMDLMAGLGAKI 447  +TVDDA IATSFP+F+ LM GLGA+I ITVDDAGPIATSFPDFLPLMRGLGAQI 441 |  |

>TR:A0A318APE2 A0A318APE2_9NOSO 3-phosphoshikimate 1-carboxyvinyltransferase OS=Nostoc sp. 3335mG OX=2204170 GN=aroA PE=3 SV=1

Length=433

Score = 470 bits (1210), Expect = 3e-161

Identities = 256/431 (59%), Positives = 306/431 (71%), Gaps = 5/431 (1%)

| Query | 18 | LSGTVRIPGDKSISHRSFMFGGLASGETRITGLLEGEDVINTGKAMQAMGARI-RKEGDT | 76 |
| --- | --- | --- | --- |
| Sbjct | 1 | +SG VR+PGDKSISHRS M LA GE+RI GLLEGEDV+ T AM+AMGA I R E MSGRVRVPGDKSISHRSLMLSALAVGESRIEGLLEGEDVLATAAAMRAMGADIVRGEDGL | 60 |
| Query | 77 | WIIDGVGNGGLLAPEAPLDFGNAATGCRLTMGLVGVYDFDSTFIGDASLTKRPMGRVLNP | 136 |
| Sbjct | 61 | W I GVG GGLL PEA LD GN+ T RL MGLV + +TF GDASL+KRPMGRV+ P  WTIHGVGVGGLLQPEAALDMGNSGTSTRLLMGLVASHAVTATFTGDASLSKRPMGRVIEP | 120 |

| Query | 137 | LREMGVQVKSEDGDRLPVTLRGPKTPTPITYRVPMASAQVKSAVLLAGLNTPGITTVIEP | 196 |
| --- | --- | --- | --- |
| Sbjct | 121 | L +MG + S G RLP+ +RG PI YR+P+ASAQVKSAVLLAGLNTPGIT VIEP LSKMGAEFTSSPGGRLPLMVRGIVPAVPIEYRLPVASAQVKSAVLLAGLNTPGITRVIEP | 180 |
| Query | 197 | IMTRDHTEKMLQGFGANLTVETDADGVRTIRLEGRGKLTGQVIDVPGDPSSTAFPLVAAL | 256 |
| Sbjct | 181 | + TRDH+E+ML+GFGA LTVE + G R I L G +L Q I VPGDPSS FP+VAAL VPTRDHSERMLRGFGAELTVE-EVGGERIISLTGEAELKPQHIVVPGDPSSAGFPVVAAL | 239 |
| Query | 257 | LVPGSDVTILNVLMNPTRTGLILTLQEMGADIEVINPRLAGGEDVADLRVRSSTLKGVTV | 316 |
| Sbjct | 240 | +VPGSD+ + NV +NPTR GL L+ MG DI N R+ GGE VADLRV++S+L+G+ IVPGSDIVVENVGLNPTRAGLYEVLRAMGGDIVFENERIVGGEPVADLRVKASSLQGIEP | 299 |
| Query | 317 | PEDRAPSMIDEYPILAVAAAFAEGATVMNGLEELRVKESDRLSAVANGLKLNGVDCDEGE | 376 |
| Sbjct | 300 | PSM+DE+PIL VA A A G + + GLEELRVKESDR++ +A+GL+ G +E E DPSIVPSMVDEFPILFVACALAHGRSTLRGLEELRVKESDRITVMADGLRAIGATVEEVE | 359 |
| Query | 377 | TSLVVRGRPDGKGLGNASGAAVATHLDHRIAMSFLVMGLVSENPVTVDDATMIATSFPEF | 436 |
| Sbjct | 360 | L++ G G+ L A GA VATHLDHRIAMSF V L S NPVTVDD IATSFP F DGLIIDG-TGGERL--AGGATVATHLDHRIAMSFAVASLASRNPVTVDDRAPIATSFPIF | 416 |
| Query  Sbjct | 437  417 | MDLMAGLGAKI 447  + +M LGA + VPMMESLGAHV 427 |  |

>TR:I3TQL9 I3TQL9_TISMK 3-phosphoshikimate 1-carboxyvinyltransferase OS=Tistrella mobilis (strain KA081020-065) OX=1110502 GN=aroA PE=3

SV=1

Length=455

Score = 471 bits (1212), Expect = 3e-161

| Identities | | = 262/454 (58%), Positives = 309/454 (68%), Gaps = 16/454 (4%) |  |
| --- | --- | --- | --- |
| Query 5  Sbjct 4 | | ASSRPATARKSSGLSGTVRIPGDKSISHRSFMFGGLASGETRITGLLEGEDVINTGKAMQ A+ PATAR S GLSG V +PGDKSISHR+ M LA G + + GLLEGEDV+ T AM+ AAPHPATARLSHGLSGDVTVPGDKSISHRALMLSALAVGRSTVDGLLEGEDVLATAAAMR | 64  63 |
| Query 65  Sbjct 64 | | AMGARIRKE-----GDT WIIDGVGNGGLLAPEAPLDFGNAATGCRLTMGLVGVYDF  A+GA I +E DT W+IDGVG GGL P LD GN+ T RL MGL+ + ALGAGITREETAGPDDTPAIRWVIDGVGVGGLAEPADVLDMGNSGTAARLLMGLLATHPL | 115  123 |
| Query 116  Sbjct 124 | | DSTFIGDASLTKRPMGRVLNPLREMGVQVKSEDGDRLPVTLRGPKTPTPITYRVPMASAQ  + GDASL KRPM RV PL +MG ++ + G RLP T+ G ++P PI YR+P+ SAQ VAVMTGDASLRKRPMARVATPLEKMGAKIVTRAGGRLPATVIGTRSPVPIRYRLPVPSAQ | 175  183 |
| Query 176  Sbjct 184 | | VKSAVLLAGLNTPGITTVIEPIMTRDHTEKMLQGFGANLTVETDADGVRTIRLEGRGKLT VKSA+LLAGLNTPG T V+EP TRDHTE+ML GA +TVET+ADG R I L+G+ +L+ VKSAILLAGLNTPGRTVVVEPEATRDHTERMLGAMGARITVETEADGARVITLDGQPELS | 235  243 |
| Query 236  Sbjct 244 | | GQVIDVPGDPSSTAFPLVAALLVPGSDVTILNVLMNPTRTGLILTLQEMGADIEVINPRL  + + VPGDPSS AFPLVA L+ GS VT+ NV +NP R GLI TL EMGAD+ V NPR ARDMTVPGDPSSAAFPLVAGLITEGSAVTVRNVCLNPGRDGLIRTLVEMGADLTVTNPRD | 295  303 |
| Query 296  Sbjct 304 | | AGGEDVADLRVRSSTLKGVTVPEDRAPSMIDEYPILAVAAAFAEGATVMNGLEELRVKES GGE V DL RSS LK VTVP +RAPSMIDE+P+LA AAA A G + GL ELRVKES VGGEPVGDLVARSSALKAVTVPAERAPSMIDEFPVLAAAAATAHGTSRFEGLAELRVKES | 355  363 |
| Query 356 | | DRLSAVANGLKLNGVDCDEGETSLVVRGRPDGKGLGNASGA--AVATHLDHRIAMSFLVM DRL AVA+GL GV GE L V DG G G G AV THLDHRIAM+FLV+ | 413 |
| Sbjct | 364 | DRLQAVADGLARAGVTVRTGEDWLEV DGCG-GPVPGGTDAVVTHLDHRIAMAFLVL | 418 |
| Query  Sbjct | 414  419 | GLVSENPVTVDDATMIATSFPEFMDLMAGLGAKI 447 GL + N + VDD MIATSFP F LM GLG I GLAARNGMVVDDDRMIATSFPGFRALMTGLGCDI 452 |  |

>TR:A0A2M8ZTG5 A0A2M8ZTG5_9BRAD 3-phosphoshikimate 1-carboxyvinyltransferase OS=Afipia broomeae OX=56946 GN=aroA PE=3 SV=1

Length=446

Score = 471 bits (1211), Expect = 3e-161

| Identities | | = 255/449 (57%), Positives = 313/449 (70%), Gaps = 8/449 (2%) |  |
| --- | --- | --- | --- |
| Query 1  Sbjct 1 | | MLHGAS-SRPATARKSSGLSGTVRIPGDKSISHRSFMFGGLASGETRITGLLEGEDVINT M H A+ + P AR S L+G R+PGDKSISHR+ + G L+ GETRI+GLLEGEDV+NT MTHSATPATPLEARSSGPLTGKARVPGDKSISHRALILGALSVGETRISGLLEGEDVLNT | 59  60 |
| Query 60  Sbjct 61 | | GKAMQAMGARIRKEGD-TWIIDGVGNGGLLAPEAPLDFGNAATGCRLTMGLVGVYDFDST K+M+A+GAR+ + G+ W + GVG G PE PLDFGN+ TGCRL MG V + AKSMRALGARVERIGEFAWSVHGVGVAGFAQPEVPLDFGNSGTGCRLVMGAVAGCPITAV | 118  120 |
| Query 119  Sbjct 121 | | FIGDASLTKRPMGRVLNPLREMGVQV-KSEDGDRLPVTLRGPKTPTPITYRVPMASAQVK F GDASL RPM RVL+PL MG +V S +G RLP+TL G + P PI YR P+ASAQ+K FDGDASLRSRPMRRVLDPLELMGARVGNSGEGGRLPLTLNGARDPLPILYRTPVASAQIK | 177  180 |
| Query 178  Sbjct 181 | | SAVLLAGLNTPGITTVIEPIMTRDHTEKMLQGFGANLTVETDADGVRTIRLEGRGKLTGQ SAVLLAGL PG+TTVIE +RDHTE ML+ FGA + + R I L G+ +L G SAVLLAGLAAPGVTTVIETEASRDHTELMLKHFGAQIVSTPEGSHGRKIALTGQPELHGA | 237  240 |
| Query 238 | | VIDVPGDPSSTAFPLVAALLVPGSDVTILNVLMNPTRTGLILTLQEMGADIEVINPRLAG  + VP DPSS AFPLVAAL+V GSDV + +V+ NP RTGL TL+EMGA IE + R | 297 |
| Sbjct | 241 | EVVVPADPSSAAFPLVAALIVEGSDVVLSDVMTNPLRTGLFTTLREMGASIEQSHVRGDA | 300 |
| Query | 298 | GEDVADLRVRSSTLKGVTVPEDRAPSMIDEYPILAVAAAFAEGATVMNGLEELRVKESDR | 357 |
| Sbjct | 301 | GE +A RVR+S L+GV VP +RAPSMIDEY +LAVAAAFAEG T+M GL+ELRVKESDR GEPMAQFRVRASRLRGVEVPPERAPSMIDEYLVLAVAAAFAEGTTIMRGLQELRVKESDR | 360 |
| Query | 358 | LSAVANGLKLNGVDCDEGETSLVVRGRPDGKGLGNASGAAVATHLDHRIAMSFLVMGLVS | 417 |
| Sbjct | 361 | L A A L++NGV + ++V GR +G G VATH+DHRIAMS LVMGL S LEATAAMLRVNGVKVEISGNDMIVEGRGHVEGDG LVATHMDHRIAMSALVMGLAS | 415 |
| Query  Sbjct | 418  416 | ENPVTVDDATMIATSFPEFMDLMAGLGAK 446  + PV VDD IATSFP+F+ +M LGA+ DKPVKVDDTAFIATSFPDFIPMMRSLGAE 444 |  |

>TR:A0A2T6B1R4 A0A2T6B1R4_9RHOB 3-phosphoshikimate 1-carboxyvinyltransferase OS=Gemmobacter caeni OX=589035 GN=aroA PE=3 SV=1

Length=447

Score = 471 bits (1211), Expect = 3e-161

| Identities | = 256/447 (57%), Positives = 312/447 (70%), Gaps = 7/447 (2%) |  |
| --- | --- | --- |
| Query 3 | HGASSRPATARKSSGLSGTVRIPGDKSISHRSFMFGGLASGETRITGLLEGEDVINTGKA | 62 |
| Sbjct 4 | HG ++P TARKS LSGT +PGDKSISHR+ +FG +A GET+ITGLLEG+DV++T KA HG-DAQPMTARKSGPLSGTAAVPGDKSISHRALIFGAMAVGETKITGLLEGQDVLDTAKA | 62 |
| Query 63 | MQAMGARIRKEGD-TWIIDGVGNGGLLAPEAPLDFGNAATGCRLTMGLVGVYDFDSTFIG | 121 |

M+A GA + + G	W + GVG GG	P	+D GN+ TG RL MG +	D +TF G

| Sbjct | 63 | MRAFGAEVTQHGPGAWSVHGVGVGGFREPADVIDCGNSGTGVRLIMGCMATTDMTATFTG | 122 |
| --- | --- | --- | --- |
| Query | 122 | DASLTKRPMGRVLNPLREMGVQVKSEDGDRLPVTLRGPKTPTPITYRVPMASAQVKSAVL | 181 |
| Sbjct | 123 | DASL KRPMGRV +PL G + G RLP+T+ G P P+ Y +P+ASAQVKSAVL DASLRKRPMGRVTDPLALFGARAYGRQGGRLPMTIVGAANPVPVRYALPVASAQVKSAVL | 182 |
| Query | 182 | LAGLNTPGITTVIEPIMTRDHTEKMLQGFGANLTVETDADGVRTIRLEGRGKLTGQVIDV | 241 |
| Sbjct | 183 | LAGLN PG T VIE TRDH+E+ML GFGA L VE A+G I L+G+ +L Q + V  LAGLNAPGETVVIEREPTRDHSERMLLGFGAELHVEKGAEG-HIITLKGQPELRPQTVAV | 241 |

| Query | 242 | PGDPSSTAFPLVAALLVPGSDVTILNVLMNPTRTGLILTLQEMGADIEVINPRLAGGEDV | 301 |
| --- | --- | --- | --- |
| Sbjct | 242 | P DPSS AFP+ AAL+V GSD+ + V N TR GL +TL EMGA+IE N R GGE V PRDPSSAAFPVCAALIVEGSDILVPGVSQNLTRNGLYITLVEMGAEIEFQNLREEGGEPV | 301 |
| Query | 302 | ADLRVR-SSTLKGVTVPEDRAPSMIDEYPILAVAAAFAEGATVMNGLEELRVKESDRLSA | 360 |
| Sbjct | 302 | ADLRVR S +KG+ VP +RAPSMIDEYPIL+V AAFA G TVM G++ELRVKESDR+ A ADLRVRFSGNMKGIEVPPERAPSMIDEYPILSVVAAFATGKTVMRGVKELRVKESDRIDA | 361 |
| Query | 361 | VANGLKLNGVDCDEGETSLVVRGRPDGKGLGNASGAAVATHLDHRIAMSFLVMGLVSENP | 420 |
| Sbjct | 362 | +A GL+ GV +E E +L+V G G G GA ATHLDHRIAMSFLV G+ ++ P MARGLEACGVTIEEDEDTLIVHGMGAG---GVPGGATAATHLDHRIAMSFLVCGMAAQKP | 418 |
| Query  Sbjct | 421  419 | VTVDDATMIATSFPEFMDLMAGLGAKI 447 V+VDDA+ I TSFP F LM GLGA + VSVDDASPIVTSFPIFEGLMTGLGATL 445 |  |

>TR:A0A653J0V8 A0A653J0V8_9SPHN 5-enolpyruvylshikimate-3-phosphate synthase OS=Novosphingobium sp. 9U OX=2653158 GN=aroA PE=3 SV=1

Length=447

Score = 471 bits (1211), Expect = 3e-161

| Identities | = 254/436 (58%), Positives = 302/436 (69%), Gaps = 2/436 (0%) |  |
| --- | --- | --- |
| Query 8 | RPATARKSSGLSGTVRIPGDKSISHRSFMFGGLASGETRITGLLEGEDVINTGKAMQAMG | 67 |
| Sbjct 5 | RP + L G VR+PGDKSISHR+ M LA GE+RITGLLEGEDV+ T AM+AMG RPRRFLPNGALRGRVRVPGDKSISHRAIMLSSLAVGESRITGLLEGEDVLATAAAMRAMG | 64 |
| Query 68 | ARIRKEGD-TWIIDGVGNGGLLAPEAPLDFGNAATGCRLTMGLVGVYDFDSTFIGDASLT | 126 |
| Sbjct 65 | A + + G W ++GVG G LL PE LD GN+ T RL MGL+ + +TF+GD SL+ AHVERTGSGEWRVNGVGVGALLQPEQALDMGNSGTSTRLLMGLIASHPIQATFVGDGSLS | 124 |
| Query 127 | KRPMGRVLNPLREMGVQVKSEDGDRLPVTLRGPKTPTPITYRVPMASAQVKSAVLLAGLN | 186 |
| Sbjct 125 | KRPMGRV+ PL MG V S G RLP+ LRG PI YR+P+ASAQVKSAVLLAGLN KRPMGRVIEPLSLMGADVTSTVGGRLPLILRGASPAVPIRYRLPVASAQVKSAVLLAGLN | 184 |
| Query 187 | TPGITTVIEPIMTRDHTEKMLQGFGANLTVETDADGVRTIRLEGRGKLTGQVIDVPGDPS | 246 |
| Sbjct 185 | TPGITTV+EP+ TRDH+E+ML+GFGA+L VE D DG R I + G +L QVIDVPGDPS TPGITTVVEPVPTRDHSERMLRGFGADLNVEVDDDGARVITVRGEAELRPQVIDVPGDPS | 244 |
| Query 247 | STAFPLVAALLVPGSDVTILNVLMNPTRTGLILTLQEMGADIEVINPRLAGGEDVADLRV | 306 |
| Sbjct 245 | S AF +VAALLV GSD+TI NV +NPTR L L++MG IE +N R GGE VADL V SAAFFVVAALLVEGSDLTIENVGLNPTRAALFDVLRQMGGSIEEVNRREVGGEPVADLLV | 304 |
| Query 307 | RSSTLKGVTVPEDRAPSMIDEYPILAVAAAFAEGATVMNGLEELRVKESDRLSAVANGLK | 366 |
| Sbjct 305 | R S L+G+ V APSMIDE+PIL VAAA A+G T +GL+ELRVKESDRL+ +A L RHSALRGIEVDPSVAPSMIDEFPILFVAAAMAQGRTTTSGLDELRVKESDRLAVMATALS | 364 |
| Query 367 | LNGVDCDEGETSLVVRGRPDGKGLGNASGAAVATHLDHRIAMSFLVMGLVSENPVTVDDA | 426 |

G	+E E LV+ G	G+ L	S	VATHLDHRIAMS V GLVS + V +DD

| Sbjct | 365 | AAGARVEEREDGLVIEG-TGGELLRGGSNHPVATHLDHRIAMSMAVAGLVSRSGVEIDDT | 423 |
| --- | --- | --- | --- |
| Query | 427 | TMIATSFPEFMDLMAG 442 IATSFP F L+ G |  |
| Sbjct | 424 | RPIATSFPTFEGLLDG 439 | |

>TR:A0A433S7E0 A0A433S7E0_9RHOB 3-phosphoshikimate 1-carboxyvinyltransferase OS=Pseudorhodobacter sp. E13 OX=2487931 GN=aroA PE=3 SV=1

Length=447

Score = 471 bits (1211), Expect = 3e-161

Identities = 256/446 (57%), Positives = 309/446 (69%), Gaps = 6/446 (1%)

| Query | 3 | HGASSRPATARKSSGLSGTVRIPGDKSISHRSFMFGGLASGETRITGLLEGEDVINTGKA | 62 |
| --- | --- | --- | --- |
| Sbjct | 4 | HG ++ TA KS L G +PGDKSISHR+ +FG +A GET+ITGLLEG+DV++T A HG-EAQVMTATKSGPLRGRADVPGDKSISHRALIFGAMAVGETKITGLLEGQDVLDTASA | 62 |
| Query | 63 | MQAMGARIRKEG-DTWIIDGVGNGGLLAPEAPLDFGNAATGCRLTMGLVGVYDFDSTFIG | 121 |
| Sbjct | 63 | M+A GA + + G W + GVG GG PEA +D GN+ TG RL MG + +TF G MRAFGADVVQHGRGNWSVHGVGVGGFREPEAVIDCGNSGTGVRLIMGCMATTAMSATFTG | 122 |
| Query | 122 | DASLTKRPMGRVLNPLREMGVQVKSEDGDRLPVTLRGPKTPTPITYRVPMASAQVKSAVL | 181 |
| Sbjct | 123 | DASL KRPMGRV +PL G Q G RLP+T+ G P P+ Y +P+ASAQVKSAVL DASLRKRPMGRVTDPLALFGAQAYGRKGGRLPMTVVGAAHPVPVRYTLPVASAQVKSAVL | 182 |
| Query | 182 | LAGLNTPGITTVIEPIMTRDHTEKMLQGFGANLTVETDADGVRTIRLEGRGKLTGQVIDV | 241 |
| Sbjct | 183 | LAGLN PG T V+EP TRDHTE+ML GFGA + VE A+G I L G+ +L QV+ V LAGLNVPGQTVVVEPEATRDHTERMLAGFGAQVQVEKTAEG-NVITLTGQPELRPQVVAV | 241 |
| Query | 242 | PGDPSSTAFPLVAALLVPGSDVTILNVLMNPTRTGLILTLQEMGADIEVINPRLAGGEDV | 301 |
| Sbjct | 242 | P DPSS AFP+ AAL+V GSD+ + V N TR G+ TL EMGADI NPR GGE V PRDPSSAAFPVCAALIVEGSDIFVPGVSQNLTRNGIYTTLVEMGADITFENPREEGGEPV | 301 |
| Query | 302 | ADLRVRSSTLKGVTVPEDRAPSMIDEYPILAVAAAFAEGATVMNGLEELRVKESDRLSAV | 361 |
| Sbjct | 302 | ADLRVR STLKG+ VP +RAPSMIDEYPIL+V AA A G TVM G++ELRVKESDR+ A+ ADLRVRYSTLKGIEVPPERAPSMIDEYPILSVVAANATGKTVMRGVKELRVKESDRIDAM | 361 |
| Query | 362 | ANGLKLNGVDCDEGETSLVVRGRPDGKGLGNASGAAVATHLDHRIAMSFLVMGLVSENPV | 421 |
| Sbjct | 362 | A GL+ GV +E E +L+V G G G GA ATH+DHRIAMSFLV G+ S+ P+ ARGLEACGVTVEEDEDTLIVHGMGPGNMPG---GATCATHIDHRIAMSFLVAGMASQKPI | 418 |
| Query  Sbjct | 422  419 | TVDDATMIATSFPEFMDLMAGLGAKI 447 TVDDA+ IATSFP F LM GLGA + TVDDASPIATSFPLFEGLMTGLGAML 444 |  |

>TR:A0A2N0I1D9 A0A2N0I1D9_9SPHN 3-phosphoshikimate 1-carboxyvinyltransferase OS=Novosphingobium kunmingense OX=1211806 GN=aroA PE=3 SV=1

Length=437

Score = 470 bits (1210), Expect = 3e-161

| Identities | = 253/439 (58%), Positives = 311/439 (71%), Gaps = 4/439 (1%) |  |
| --- | --- | --- |
| Query 8 | RPATARKSSGLSGTVRIPGDKSISHRSFMFGGLASGETRITGLLEGEDVINTGKAMQAMG | 67 |
| Sbjct 2 | RP + L GT+R+PGDKSISHRS M G LA GETR+TGLLEGEDV+ T AM+AMG RPRRFFPAGALKGTIRVPGDKSISHRSIMLGALAVGETRVTGLLEGEDVLATAAAMRAMG | 61 |
| Query 68 | ARIRKEGD-TWIIDGVGNGGLLAPEAPLDFGNAATGCRLTMGLVGVYDFDSTFIGDASLT | 126 |
| Sbjct 62 | A+ + GD W + GVG G LL P+APLD GN+ T RL MG+V + +TF+GDASL+ AQAERVGDGEWSVHGVGVGTLLQPQAPLDMGNSGTSTRLLMGIVASHPISATFVGDASLS | 121 |
| Query 127 | KRPMGRVLNPLREMGVQVKSEDGDRLPVTLRGPKTPTPITYRVPMASAQVKSAVLLAGLN | 186 |
| Sbjct 122 | RPMGRV++PL +MG ++ +G RLP+T+RG PI YR+P+ASAQVKSAVLLAGLN NRPMGRVIDPLSQMGAAFEASEGGRLPLTVRGAYPAVPIEYRLPVASAQVKSAVLLAGLN | 181 |
| Query 187 | TPGITTVIEPIMTRDHTEKMLQGFGANLTVETDADGVRTIRLEGRGKLTGQVIDVPGDPS | 246 |
| Sbjct 182 | TPG+TTVIEP+ TRDH+E+ML+GFGA LTVE + G R IR+ G +L Q I+VPGDPS TPGVTTVIEPVPTRDHSERMLRGFGAELTVE-ELGGERVIRIRGEAELKPQTIEVPGDPS | 240 |
| Query 247 | STAFPLVAALLVPGSDVTILNVLMNPTRTGLILTLQEMGADIEVINPRLAGGEDVADLRV | 306 |
| Sbjct 241 | S AF +VA L+VPGS++TI NV +NPTR G++ L++MG IE +N R GGE VADL V SAAFFIVAGLIVPGSELTIQNVGLNPTRAGIVEILRKMGGQIEEVNRREVGGEPVADLVV | 300 |
| Query 307 | RSSTLKGVTVPEDRAPSMIDEYPILAVAAAFAEGATVMNGLEELRVKESDRLSAVANGLK | 366 |
| Sbjct 301 | R S L+G+ V APSMIDE+P L VAA+ A+G TV +GL+ELRVKESDRLSA+A L  RHSDLQGIAVDPAVAPSMIDEFPALFVAASLAKGRTVTSGLDELRVKESDRLSAMAAALA | 360 |

Query 367 LNGVDCDEGETSLVVRGRPDGKGLGNASGAAVATHLDHRIAMSFLVMGLVSENPVTVDDA 426 G	E E L++ G	G+ L	AAV THLDHRIAMS + GL S	V VDD

| Sbjct | 361 | AAGARVSETEDGLIIDG-TGGEPLAGTQ-AAVTTHLDHRIAMSMAIAGLASRAGVEVDDT 418 |
| --- | --- | --- |
| Query | 427 | TMIATSFPEFMDLMAGLGA 445 |

IATSFP F L+ GL A Sbjct 419 RPIATSFPVFEALLDGLAA 437

>TR:A0A0B9A7G2 A0A0B9A7G2_9SPHN 3-phosphoshikimate 1-carboxyvinyltransferase OS=Novosphingobium subterraneum OX=48936 GN=aroA PE=3 SV=1

Length=448

Score = 471 bits (1211), Expect = 3e-161

| Identities | = 251/439 (57%), Positives = 304/439 (69%), Gaps = 1/439 (0%) |  |
| --- | --- | --- |
| Query 5 | ASSRPATARKSSGLSGTVRIPGDKSISHRSFMFGGLASGETRITGLLEGEDVINTGKAMQ | 64 |
| Sbjct 7 | A RP + L G +R+PGDKSISHRS M G LA GETR+TGLLEGEDV++T AM+ AQMRPRRFTANGPLRGRIRVPGDKSISHRSIMLGALAVGETRVTGLLEGEDVLSTAAAMR | 66 |
| Query 65 | AMGARIRKEGD-TWIIDGVGNGGLLAPEAPLDFGNAATGCRLTMGLVGVYDFDSTFIGDA | 123 |
| Sbjct 67 | AMGA I ++ D W + GVG GGLL P+A LD GN+ T RL MGLV + +TF+GDA AMGATIERDADGMWHVHGVGVGGLLQPQAALDMGNSGTSTRLLMGLVATHPITATFVGDA | 126 |
| Query 124 | SLTKRPMGRVLNPLREMGVQVKSEDGDRLPVTLRGPKTPTPITYRVPMASAQVKSAVLLA | 183 |
| Sbjct 127 | SL+KRPMGRV++PL MG + + G RLP+TLRG PI YR+P+ASAQVKSA+LLA SLSKRPMGRVIDPLSTMGAEFTASPGGRLPLTLRGISPAVPIEYRLPVASAQVKSAILLA | 186 |
| Query 184 | GLNTPGITTVIEPIMTRDHTEKMLQGFGANLTVETDADGVRTIRLEGRGKLTGQVIDVPG | 243 |
| Sbjct 187 | GLNTPGITTVIEPI TRDH+E+ML+GFGA LTV+ DG R I++ G + Q I VPG GLNTPGITTVIEPIPTRDHSERMLRGFGAELTVDIADDGARVIKVRGEAEFKPQDIVVPG | 246 |
| Query 244 | DPSSTAFPLVAALLVPGSDVTILNVLMNPTRTGLILTLQEMGADIEVINPRLAGGEDVAD | 303 |
| Sbjct 247 | DPSS AF +VAALL+ GSD+ + NV +NPTR L L+ MG IE ++ R GGE VAD DPSSAAFFVVAALLIEGSDLVVENVGLNPTRAALFDVLRLMGGSIEELDRRDVGGEPVAD | 306 |
| Query 304 | LRVRSSTLKGVTVPEDRAPSMIDEYPILAVAAAFAEGATVMNGLEELRVKESDRLSAVAN | 363 |
| Sbjct 307 | LRVR S L G+ V PSM+DE+PIL VAAA A+G TV GLEELRVKESDR+SA+ LRVRHSLLSGIDVDPAVVPSMVDEFPILFVAAALAKGRTVTTGLEELRVKESDRISAMRA | 366 |
| Query 364 | GLKLNGVDCDEGETSLVVRGRPDGKGLGNASGAAVATHLDHRIAMSFLVMGLVSENPVTV | 423 |
| Sbjct 367 | L+L G E E L++ G G A GA+V THLDHRIAMS + G+ S+ V V ALELAGATVTETEDGLIIDGTGGDPLPGTAEGASVVTHLDHRIAMSMAIAGIASKRGVEV | 426 |
| Query 424  Sbjct 427 | DDATMIATSFPEFMDLMAG 442 DD IATSFP F L+ G DDTRPIATSFPVFESLLEG 445 |  |

>TR:A0A2A4CT29 A0A2A4CT29_9RHOB 3-phosphoshikimate 1-carboxyvinyltransferase OS=Thioclava arenosa OX=1795308 GN=aroA PE=3 SV=1

Length=449

Score = 471 bits (1211), Expect = 3e-161

Identities = 248/438 (57%), Positives = 306/438 (70%), Gaps = 5/438 (1%)

| Query | 12 | ARKSSGLSGTVRIPGDKSISHRSFMFGGLASGETRITGLLEGEDVINTGKAMQAMGARIR | 71 |
| --- | --- | --- | --- |
| Sbjct | 12 | +RK LSG +PGDKSISHR+ + G L+ G+T +TGLLEG+DV++T KAM+A GA ++ SRKGGPLSGVAEVPGDKSISHRALILGALSVGQTEVTGLLEGQDVLDTAKAMEAFGATVK | 71 |
| Query | 72 | KEGDT-WIIDGVGNGGLLAPEAPLDFGNAATGCRLTMGLVGVYDFDSTFIGDASLTKRPM | 130 |
| Sbjct | 72 | + G W +DGVG GG PE +D GN+ TG RL MG + +TF GDASL KRPM  RLGPGHWTVDGVGVGGFAEPEQVIDCGNSGTGVRLIMGCMASSPISATFTGDASLRKRPM | 131 |

| Query | 131 | GRVLNPLREMGVQVKSEDGDRLPVTLRGPKTPTPITYRVPMASAQVKSAVLLAGLNTPGI | 190 |
| --- | --- | --- | --- |
| Sbjct | 132 | GRV +P+ G + G RLP+T+ G P P++Y PMASAQVKSAVLLAGLN PG GRVTDPIALFGARAYGRKGGRLPMTIVGAADPVPVSYVTPMASAQVKSAVLLAGLNAPGQ | 191 |
| Query | 191 | TTVIEPIMTRDHTEKMLQGFGANLTVETDADGVRTIRLEGRGKLTGQVIDVPGDPSSTAF | 250 |
| Sbjct | 192 | T VIE TRDHTE+ML+GFGA +T E +G + I L+G+ +L Q + VP DPSS AF TVVIEKTPTRDHTERMLRGFGAEITTEDTPEGHK-ITLQGQPELRAQKVAVPRDPSSAAF | 250 |
| Query | 251 | PLVAALLVPGSDVTILNVLMNPTRTGLILTLQEMGADIEVINPRLAGGEDVADLRVRSST | 310 |
| Sbjct | 251 | P+ AALLVPGSD+ + V NPTR GL LTL++MGADIE +N R GGE VADLRVR S PVCAALLVPGSDILVPGVSQNPTRNGLFLTLRDMGADIEFLNERTEGGEPVADLRVRYSE | 310 |
| Query | 311 | LKGVTVPEDRAPSMIDEYPILAVAAAFAEGATVMNGLEELRVKESDRLSAVANGLKLNGV | 370 |
| Sbjct | 311 | L G+ VP +RA + IDEYP+L+V AA A G TVM G+ ELRVKESDR+ A+A GL+ GV LHGIEVPPERAAAQIDEYPVLSVVAACATGTTVMRGVHELRVKESDRIDAMARGLEACGV | 370 |
| Query | 371 | DCDEGETSLVVRGRPDGKGLGNASGAAVATHLDHRIAMSFLVMGLVSENPVTVDDATMIA | 430 |
| Sbjct | 371 | +E E +L+V G G G GA TH+DHRIAMSFLV+GL S+ PV VDD + IA RIEEDEDTLIVHGMGPGNVPG---GATCQTHIDHRIAMSFLVLGLASQKPVAVDDGSPIA | 427 |
| Query  Sbjct | 431  428 | TSFPEFMDLMAGLGAKIE 448 TSFP F LMAGLGA+IE TSFPVFEALMAGLGAQIE 445 |  |

>TR:A0A1M7SHE3 A0A1M7SHE3_9RHOB 3-phosphoshikimate 1-carboxyvinyltransferase OS=Oceanicella actignis OX=1189325 GN=aroA PE=3 SV=1

Length=449

Score = 471 bits (1211), Expect = 3e-161

| Identities | = 252/447 (56%), Positives = 306/447 (68%), Gaps = 6/447 (1%) |  |
| --- | --- | --- |
| Query 3 | HGASSRPATARKSSGLSGTVRIPGDKSISHRSFMFGGLASGETRITGLLEGEDVINTGKA | 62 |
| Sbjct 4 | HG +S P TAR++ L+G +PGDKS+SHR+ + G L+ GETR+TGLLEG+DV++T A HGPAS-PLTARRAGPLAGIAHVPGDKSVSHRALILGALSVGETRVTGLLEGQDVLDTAAA | 62 |
| Query 63 | MQAMGARI-RKEGDTWIIDGVGNGGLLAPEAPLDFGNAATGCRLTMGLVGVYDFDSTFIG | 121 |
| Sbjct 63 | M+A GA + R W + GVG GG PE +D GN+ TG RL MG + + F G MRAFGAEVERLAPGEWRVHGVGVGGFAEPENVIDCGNSGTGVRLIMGAMATTPISACFTG | 122 |
| Query 122 | DASLTKRPMGRVLNPLREMGVQVKSEDGDRLPVTLRGPKTPTPITYRVPMASAQVKSAVL | 181 |
| Sbjct 123 | DASL RPMGRV PL G + + G RLP+ L G P P++YR+P+ SAQVKSAVL DASLRSRPMGRVTEPLALFGARAQGRAGGRLPLVLTGASDPVPVSYRLPVPSAQVKSAVL | 182 |
| Query 182 | LAGLNTPGITTVIEPIMTRDHTEKMLQGFGANLTVETDADGVRTIRLEGRGKLTGQVIDV | 241 |
| Sbjct 183 | LAGLN PG+T VIE TRDHTE+ML+GFGA + +E D R IRL GR +LTGQ I LAGLNAPGVTEVIEREPTRDHTERMLRGFGARVEIERDGPD-RIIRLTGRPELTGQTIAA | 241 |
| Query 242 | PGDPSSTAFPLVAALLVPGSDVTILNVLMNPTRTGLILTLQEMGADIEVINPRLAGGEDV | 301 |
| Sbjct 242 | P DPSS AFP+ AAL+V GSDV + V +NPTR GL TL+EMGAD+ NPR GGE V PRDPSSAAFPVCAALIVEGSDVLVPGVCLNPTRAGLYETLREMGADLVFENPREEGGEPV | 301 |
| Query 302 | ADLRVRSSTLKGVTVPEDRAPSMIDEYPILAVAAAFAEGATVMNGLEELRVKESDRLSAV | 361 |
| Sbjct 302 | ADLR R STL+GV P +RAP+MIDEYPIL+V AAFAEG TVM G+ ELRVKESDR+ A+ ADLRARFSTLRGVETPPERAPAMIDEYPILSVVAAFAEGRTVMRGVRELRVKESDRIDAM | 361 |
| Query 362 | ANGLKLNGVDCDEGETSLVVRGRPDGKGLGNASGAAVATHLDHRIAMSFLVMGLVSENPV | 421 |
| Sbjct 362 | A GL+ GV +E E +L+V G G G GA A+ LDHRIAMSFL +G+ S PV ARGLEACGVTVEEDEDTLIVHGMGPG---GVPGGATCASRLDHRIAMSFLCLGMASRRPV | 418 |
| Query 422  Sbjct 419 | TVDDATMIATSFPEFMDLMAGLGAKIE 448 TVDDA +ATSFP F LM LGA E TVDDAGPVATSFPGFRALMQSLGAAFE 445 |  |

>TR:A0A1L6JBZ1 A0A1L6JBZ1_9SPHN 3-phosphoshikimate 1-carboxyvinyltransferase OS=Sphingomonas koreensis OX=93064 GN=aroA PE=3 SV=1

Length=443

Score = 470 bits (1210), Expect = 4e-161

| Identities | = 253/443 (57%), Positives = 305/443 (69%), Gaps = 5/443 (1%) |  |
| --- | --- | --- |
| Query 2 | LHGASSRPATARKSSGLSGTVRIPGDKSISHRSFMFGGLASGETRITGLLEGEDVINTGK | 61 |
| Sbjct 1 | + A P L GT+ +PGDKSISHRS MF GLA GE+RI GLLEGEDV+ T MSSAPPLPLAISARGPLRGTLTVPGDKSISHRSLMFAGLAVGESRIEGLLEGEDVLATAA | 60 |
| Query 62 | AMQAMGARIRKEGD-TWIIDGVGNGGLLAPEAPLDFGNAATGCRLTMGLVGVYDFDSTFI | 120 |
| Sbjct 61 | AM+AMGA+I ++GD W + GVG GGLL PE L+ GN+ T RL MGLV + +TF AMRAMGAKIERDGDGVWHVWGVGVGGLLQPETALEMGNSGTSTRLLMGLVASHPITATFT | 120 |
| Query 121 | GDASLTKRPMGRVLNPLREMGVQVKSEDGDRLPVTLRGPKTPTPITYRVPMASAQVKSAV | 180 |
| Sbjct 121 | GDASL+KRPMGRV++PL MG S G RLP+T+RG PI Y +P+ASAQVKSAV GDASLSKRPMGRVIDPLSRMGADFTSSPGGRLPLTMRGLCPAVPIEYTLPVASAQVKSAV | 180 |
| Query 181 | LLAGLNTPGITTVIEPIMTRDHTEKMLQGFGANLTVETDADGVRTIRLEGRGKLTGQVID | 240 |
| Sbjct 181 | LLAGLNTPGIT VIEP++TRDH+E+ML GFGANLTVE G R I + G + Q I LLAGLNTPGITRVIEPVLTRDHSERMLAGFGANLTVEETPQG-RIISIHGEAEFKPQQIT | 239 |
| Query 241 | VPGDPSSTAFPLVAALLVPGSDVTILNVLMNPTRTGLILTLQEMGADIEVINPRLAGGED | 300 |
| Sbjct 240 | VPGDPSS AF +VAA +VPGSD+T+ NV +NPTR GLI L+ MGADI +NPR+ GGE VPGDPSSAAFWMVAASIVPGSDITVANVGLNPTRIGLITALRMMGADITEVNPRIVGGEP | 299 |
| Query 301 | VADLRVRSSTLKGVTVPEDRAPSMIDEYPILAVAAAFAEGATVMNGLEELRVKESDRLSA | 360 |
| Sbjct 300 | VADLRVR + L + VP D APSMIDEYP+L VAAAFAEG T+ G EELRVKESDR++A VADLRVRHAPLSAIEVPHDLAPSMIDEYPVLFVAAAFAEGRTIARGAEELRVKESDRIAA | 359 |
| Query 361 | VANGLKLNGVDCDEGETSLVVRGRPDGKGLGNASGAAVATHLDHRIAMSFLVMGLVSENP | 420 |
| Sbjct 360 | + L NGV +E E L + G G A GA VA+ LDHRIAMS V GL ++ MVAALAPNGVPLEEFEDGLAITGTGGGP---IAGGAPVASKLDHRIAMSMAVAGLGAKAD | 416 |
| Query 421  Sbjct 417 | VTVDDATMIATSFPEFMDLMAGL 443 V +DD + +ATS+P F + L VIIDDVSPVATSYPGFFSTLDSL 439 |  |

>TR:A0A4Q8R376 A0A4Q8R376_9BRAD 3-phosphoshikimate 1-carboxyvinyltransferase OS=Bradyrhizobium sp. Leo170 OX=1571199 GN=aroA PE=3 SV=1

Length=464

Score = 471 bits (1212), Expect = 4e-161

| Identities | = 249/443 (56%), Positives = 312/443 (70%), Gaps = 7/443 (2%) |  |
| --- | --- | --- |
| Query 6 | SSRPATARKSSGLSGTVRIPGDKSISHRSFMFGGLASGETRITGLLEGEDVINTGKAMQA | 65 |
| Sbjct 25 | S P AR S LSG R+PGDKSISHR+ + G LA G++RI+GLLEGEDV+NT K+M+A SKTPLEARASGPLSGKARVPGDKSISHRALILGALAVGQSRISGLLEGEDVLNTAKSMRA | 84 |
| Query 66 | MGARIRKEGD-TWIIDGVGNGGLLAPEAPLDFGNAATGCRLTMGLVGVYDFDSTFIGDAS | 124 |
| Sbjct 85 | +GA + + G W + GVG GG P PLDFGN+ TGCRL MG V +TF GDAS LGAEVERTGPFAWSVRGVGVGGFAEPAGPLDFGNSGTGCRLVMGAVAGCPIRATFDGDAS | 144 |
| Query 125 | LTKRPMGRVLNPLREMGVQV-KSEDGDRLPVTLRGPKTPTPITYRVPMASAQVKSAVLLA | 183 |
| Sbjct 145 | L RPM R+L+PL MG + + +G RLP+TL G + P PI Y+ P+ASAQ+KSAVLLA LRSRPMRRILDPLELMGARAGEMREGGRLPLTLHGARNPLPIVYKTPVASAQIKSAVLLA | 204 |
| Query 184 | GLNTPGITTVIEPIMTRDHTEKMLQGFGANLTVETDADGVRTIRLEGRGKLTGQVIDVPG | 243 |
| Sbjct 205 | GL PG+TTVIE +RDHTE ML+ FGA LT + R I L G+G+L G + VP  GLAAPGVTTVIEQEASRDHTELMLKHFGAALTSRKEGSHGRRIALTGQGELHGAEVVVPA | 264 |

| Query | 244 | DPSSTAFPLVAALLVPGSDVTILNVLMNPTRTGLILTLQEMGADIEVINPRLAGGEDVAD | 303 |
| --- | --- | --- | --- |
| Sbjct | 265 | DPSS +FP+VAAL+V GSD+ + +V+ NP RTGL TL+EMGA IE R+ GGE +A DPSSASFPVVAALVVGGSDIVLPDVMTNPLRTGLFTTLREMGASIEEDELRMDGGEPMAR | 324 |
| Query | 304 | LRVRSSTLKGVTVPEDRAPSMIDEYPILAVAAAFAEGATVMNGLEELRVKESDRLSAVAN | 363 |
| Sbjct | 325 | LRVR+S L+GV VP +RAP+MIDEY +LAVAAA+AEG TVM GL+ELRVKESDRL A A+ LRVRASKLRGVEVPPERAPTMIDEYLVLAVAAAYAEGTTVMRGLQELRVKESDRLEATAD | 384 |
| Query | 364 | GLKLNGVDCDEGETSLVVRGRPDGKGLGNASGAAVATHLDHRIAMSFLVMGLVSENPVTV | 423 |
| Sbjct | 385 | L++NGV + L+V G+ G G VATH+DHRIAMS LVMGL ++ PVT+ MLRVNGVKVEISGDDLIVEGKGHVPG GGVVATHMDHRIAMSALVMGLAADRPVTI | 439 |
| Query  Sbjct | 424  440 | DDATMIATSFPEFMDLMAGLGAK 446 DD IATSFP+F+ +M LGA+ DDTAFIATSFPDFIPMMRSLGAE 462 |  |

>TR:Q1N9C5 Q1N9C5_SPHSS 3-phosphoshikimate 1-carboxyvinyltransferase OS=Sphingomonas sp. (strain SKA58) OX=314266 GN=aroA PE=3 SV=1

Length=438

Score = 470 bits (1209), Expect = 5e-161

| Identities | = 254/438 (58%), Positives = 303/438 (69%), Gaps = 4/438 (1%) |  |
| --- | --- | --- |
| Query 9 | PATARKSSGLSGTVRIPGDKSISHRSFMFGGLASGETRITGLLEGEDVINTGKAMQAMGA | 68 |
| Sbjct 2 | P T R + LSG+V +PGDKSISHRS M LA GE+R+ GLLEGEDV+ T AM+AMGA PMTFRTAPPLSGSVTVPGDKSISHRSLMLSALAVGESRVEGLLEGEDVLATAAAMRAMGA | 61 |
| Query 69 | RIRKEGD-TWIIDGVGNGGLLAPEAPLDFGNAATGCRLTMGLVGVYDFDSTFIGDASLTK | 127 |
| Sbjct 62 | RI ++ D W I GVG GGLL P LD GN+ T RL MGL+ +D +TFIGDASL+K RIERDDDGVWHIHGVGVGGLLQPGTALDMGNSGTSTRLLMGLLASHDLTATFIGDASLSK | 121 |
| Query 128 | RPMGRVLNPLREMGVQVKSEDGDRLPVTLRGPKTPTPITYRVPMASAQVKSAVLLAGLNT | 187 |
| Sbjct 122 | RPM RV PL +MG + GDRLP+T+RG P+ Y +P+ASAQVKSA+LLAGLNT RPMARVTEPLAQMGASFTTSPGDRLPLTMRGACPAVPLDYVLPVASAQVKSAILLAGLNT | 181 |
| Query 188 | PGITTVIEPIMTRDHTEKMLQGFGANLTVETDADGVRTIRLEGRGKLTGQVIDVPGDPSS | 247 |
| Sbjct 182 | PGIT V+EPI TRDH+E+MLQGFGA+L VE ADG R I L G +L Q I VPGDPSS PGITRVVEPIPTRDHSERMLQGFGADLRVEQQADGARVITLIGEAELKPQQIMVPGDPSS | 241 |
| Query 248 | TAFPLVAALLVPGSDVTILNVLMNPTRTGLILTLQEMGADIEVINPRLAGGEDVADLRVR | 307 |
| Sbjct 242 | AFP+VAAL+VPGS VTI NV +N TR GLI L+EMG IEV+N R GGE V DL V AAFPMVAALVVPGSKVTIANVGLNATRAGLIDLLREMGGSIEVVNARDVGGEPVGDLVVT | 301 |
| Query 308 | SSTLKGVTVPEDRAPSMIDEYPILAVAAAFAEGATVMNGLEELRVKESDRLSAVANGLKL | 367 |
| Sbjct 302 | +S L GV RAPSMIDEYP+ VAAA A+G +V GLEELRVKESDR++ + GL+ ASVLTGVEPDPARAPSMIDEYPVAFVAAALAQGRSVFRGLEELRVKESDRIATMVEGLRA | 361 |
| Query 368 | NGVDCDEGETSLVVRGRPDGKGLGNASGAAVATHLDHRIAMSFLVMGLVSENPVTVDDAT | 427 |

G	+E E L++ G	G	G +AT LDHRIAMSF + GL S	VT+DD

| Sbjct | 362 | IGARVEEMEDGLIIEGT---GGQLLPGGGPIATKLDHRIAMSFAIAGLASREGVTIDDMR | 418 |
| --- | --- | --- | --- |
| Query | 428 | MIATSFPEFMDLMAGLGA 445  +ATSFP F+ L+ LGA |  |
| Sbjct | 419 | PVATSFPTFIPLLRVLGA 436 | |

>TR:A0A1G6SWQ8 A0A1G6SWQ8_9RHOB 3-phosphoshikimate 1-carboxyvinyltransferase OS=Paracoccus isoporae OX=591205 GN=aroA PE=3 SV=1

Length=443

Score = 470 bits (1209), Expect = 5e-161

Identities = 249/448 (56%), Positives = 313/448 (70%), Gaps = 7/448 (2%)

| Query | 1 | MLHGASSRPATARKSSGLSGTVRIPGDKSISHRSFMFGGLASGETRITGLLEGEDVINTG | 60 |
| --- | --- | --- | --- |
| Sbjct | 1 | M HG ++ P +R L+G RIPGDKSISHR+ + G +A G TRITGLLEG+DV++T MSHGPAT-PIASRPGRPLTGEARIPGDKSISHRALILGAMAVGRTRITGLLEGQDVLDTA | 59 |
| Query | 61 | KAMQAMGARIRKEGD-TWIIDGVGNGGLLAPEAPLDFGNAATGCRLTMGLVGVYDFDSTF | 119 |
| Sbjct | 60 | +AMQA GA + + W +DGVG GG P +D GN+ TG RL MG + +TF RAMQAFGATVEQVAPGEWTVDGVGVGGFAEPAGVIDCGNSGTGVRLIMGAMATTPITATF | 119 |
| Query | 120 | IGDASLTKRPMGRVLNPLREMGVQVKSEDGDRLPVTLRGPKTPTPITYRVPMASAQVKSA | 179 |
| Sbjct | 120 | GDASL++RPM R+ +P+ G ++ + +G LPVT+RG + P+TYR P+ASAQ+KSA TGDASLSRRPMRRITDPISLFGAEITAREGGLLPVTIRGAEDAVPVTYRTPVASAQIKSA | 179 |
| Query | 180 | VLLAGLNTPGITTVIEPIMTRDHTEKMLQGFGANLTVETDADGVRTIRLEGRGKLTGQVI | 239 |
| Sbjct | 180 | VLLAGLN PG T VIE TRDH+E+ML GFGA +T ET A+G I L G+ +L Q + VLLAGLNAPGQTVVIEAEPTRDHSERMLAGFGATITTETTAEG-HVITLTGQPELRAQDV | 238 |
| Query | 240 | DVPGDPSSTAFPLVAALLVPGSDVTILNVLMNPTRTGLILTLQEMGADIEVINPRLAGGE | 299 |
| Sbjct | 239 | VP DPSS AFP+ AAL+VPGS++ + + NPTR GL +TL EMGAD+ + R GGE AVPRDPSSAAFPVAAALIVPGSEIRVNGISRNPTRDGLYVTLAEMGADLSWEHDREEGGE | 298 |
| Query | 300 | DVADLRVRSSTLKGVTVPEDRAPSMIDEYPILAVAAAFAEGATVMNGLEELRVKESDRLS | 359 |
| Sbjct | 299 | VADL VR S L+GV+VP DRA SMIDE+PIL+V AA AEG TVMNG+ ELRVKESDR+ PVADLIVRHSALRGVSVPADRAASMIDEFPILSVIAAMAEGETVMNGVAELRVKESDRID | 358 |
| Query | 360 | AVANGLKLNGVDCDEGETSLVVRGRPDGKGLGNASGAAVATHLDHRIAMSFLVMGLVSEN | 419 |
| Sbjct | 359 | A+A GL+ NGV+ +E S+ VR G G GAAV THLDHRIAMSFLV GL +E AMAKGLRANGVEVEETRDSMTVR GTGRIPGGGAAVETHLDHRIAMSFLVAGLAAEA | 414 |
| Query | 420 | PVTVDDATMIATSFPEFMDLMAGLGAKI 447  + VDD + IATSFP+F+DLM GLGA I |  |
| Sbjct | 415 | EIRVDDGSAIATSFPDFVDLMRGLGADI 442 | |

>SP:Q1GT16 AROA_SPHAL 3-phosphoshikimate 1-carboxyvinyltransferase OS=Sphingopyxis alaskensis (strain DSM 13593 / LMG 18877 / RB2256)

OX=317655 GN=aroA PE=3 SV=1

Length=446

Score = 470 bits (1209), Expect = 6e-161

| Identities | = 254/443 (57%), Positives = 309/443 (70%), Gaps = 8/443 (2%) |  |
| --- | --- | --- |
| Query 6 | SSRPATARKSSGLSGTVRIPGDKSISHRSFMFGGLASGETRITGLLEGEDVINTGKAMQA | 65 |
| Sbjct 5 | ++ P + S L G + IPGDKSISHRS M LA GE+R+ GLLEG DV+ T AM+A TATPRSFSASVPLKGRIAIPGDKSISHRSLMLSALAVGESRVAGLLEGHDVLATAAAMRA | 64 |
| Query 66 | MGARI-RKEGDTWIIDGVGNGGLLAPEAPLDFGNAATGCRLTMGLVGVYDFDSTFIGDAS | 124 |
| Sbjct 65 | MGA I R++ W I GVG GGLL P LD GN+ T RL MGLV + +TF+GDAS MGADIARRDDGEWRIHGVGVGGLLQPRGALDMGNSGTSTRLLMGLVASHPITATFVGDAS | 124 |
| Query 125 | LTKRPMGRVLNPLREMGVQVKSEDGDR LPVTLRGPKTPTPITYRVPMASAQVKSAV | 180 |
| Sbjct 125 | L+ RPMGRV++PL +MG + + G R LP+ +RG P++YR+PMASAQVKSA+ LSGRPMGRVIDPLTQMGADISASPGARGAKTLPLMVRGLAPAIPLSYRLPMASAQVKSAI | 184 |
| Query 181 | LLAGLNTPGITTVIEPIMTRDHTEKMLQGFGANLTVETDADGVRTIRLEGRGKLTGQVID | 240 |
| Sbjct 185 | LLAGLNTPG+T VIEP+ TRDH+E+ML FGA+LTV+ DA G R IR+ G L Q I LLAGLNTPGVTEVIEPVPTRDHSERMLGAFGADLTVDIDAGGTRHIRIRGEADLKPQAII | 244 |
| Query 241 | VPGDPSSTAFPLVAALLVPGSDVTILNVLMNPTRTGLILTLQEMGADIEVINPRLAGGED | 300 |
| Sbjct 245 | VPGDPSS AF +VAAL+VPGSDVTI NV +NPTR GL+ L+ MG DIE+++ R GGE VPGDPSSAAFFIVAALIVPGSDVTIANVGLNPTRAGLVEVLKAMGGDIELLDRREIGGEP | 304 |
| Query 301 | VADLRVRSSTLKGVTVPEDRAPSMIDEYPILAVAAAFAEGATVMNGLEELRVKESDRLSA VADLRVR S LKG+ V APSMIDE+P+L VAA AEG TV GL+ELRVKESDRL+ | 360 |

| Sbjct | 305 | VADLRVRHSVLKGIEVDPAVAPSMIDEFPVLFVAATLAEGRTVTTGLDELRVKESDRLAV | 364 |
| --- | --- | --- | --- |
| Query | 361 | VANGLKLNGVDCDEGETSLVVRGRPDGKGLGNASGAAVATHLDHRIAMSFLVMGLVSENP | 420 |
| Sbjct | 365 | +A GLK G +E + LV+ G G A GA +A HLDHRI MSF + GLVS+ P MATGLKAIGARVEESQDGLVIDGT---GGDPLAGGATIAGHLDHRICMSFAIAGLVSKAP | 421 |
| Query  Sbjct | 421  422 | VTVDDATMIATSFPEFMDLMAGL 443 VTVDD +ATSFP F L+AGL VTVDDIAPVATSFPNFEALLAGL 444 |  |

>TR:A0A2N4YCK0 A0A2N4YCK0_9RHOB 3-phosphoshikimate 1-carboxyvinyltransferase OS=Tabrizicola sp. TH137 OX=2067452 GN=aroA PE=3 SV=1

Length=448

Score = 470 bits (1209), Expect = 6e-161

| Identities | = 258/448 (58%), Positives = 312/448 (70%), Gaps = 9/448 (2%) |  |
| --- | --- | --- |
| Query 3 | HGASSRPATARKSSGLSGTVRIPGDKSISHRSFMFGGLASGETRITGLLEGEDVINTGKA | 62 |
| Sbjct 4 | HG ++P TARKS LSGT +PGDKSISHR+ +FG +A GET++TGLLEG+DV++T KA HG-EAQPMTARKSGPLSGTAEVPGDKSISHRALIFGAMAVGETKVTGLLEGQDVLDTAKA | 62 |
| Query 63 | MQAMGARIRKEGD-TWIIDGVGNGGLLAPEAPLDFGNAATGCRLTMGLVGVYDFDSTFIG | 121 |

M+A GA + +	W ++GVG GG	P	+D GN+ TG RL MG +	+TF G

| Sbjct | 63 | MRAFGAEVTRHAPGEWTVNGVGVGGFREPADVIDCGNSGTGVRLIMGTMATSAITATFTG | 122 |
| --- | --- | --- | --- |
| Query | 122 | DASLTKRPMGRVLNPLREMGVQVKSEDGDRLPVTLRGPKTPTPITYRVPMASAQVKSAVL | 181 |
| Sbjct | 123 | DASL KRPMGRV +PL G + G RLP+T+ G P P+ Y +P+ASAQVKSAVL DASLRKRPMGRVTDPLSLFGARAFGRQGGRLPMTVVGAADPVPVRYALPVASAQVKSAVL | 182 |
| Query | 182 | LAGLNTPGITTVIEPIMTRDHTEKMLQGFGANLTVETDADGVRTIRLEGRGKLTGQVIDV | 241 |
| Sbjct | 183 | LAGLN PG T VIE TRDH+E+ML GFGA L VE A+G I L GR +L Q + V LAGLNAPGQTVVIEKEPTRDHSERMLLGFGAELVVEKTAEG-NVITLTGRPELRPQTVAV | 241 |
| Query | 242 | PGDPSSTAFPLVAALLVPGSDVTILNVLMNPTRTGLILTLQEMGADIEVINPRLAGGEDV | 301 |
| Sbjct | 242 | P DPSS AFP AAL+V GSD+ + V N TR GL LTL EMGA+IE NPR GGE V PRDPSSAAFPTCAALIVEGSDILVPGVSQNLTRNGLYLTLVEMGAEIEFQNPREEGGEPV | 301 |
| Query | 302 | ADLRVR-SSTLKGVTVPEDRAPSMIDEYPILAVAAAFAEGATVMNGLEELRVKESDRLSA | 360 |
| Sbjct | 302 | ADLRV+ S LKG+ VPE+RAPSMIDEYPIL+V AAFAEG TVM G++ELRVKESDR+ A ADLRVKFSPNLKGIEVPEERAPSMIDEYPILSVVAAFAEGTTVMRGVKELRVKESDRIDA | 361 |
| Query | 361 | VANGLKLNGVDCDEGETSLVVRGRPDGKGLGNASGAAVA-THLDHRIAMSFLVMGLVSEN | 419 |
| Sbjct | 362 | +A GL+ GV +E E +L+V G G G G AVA THLDHRIAMSFLV G+ ++ MARGLEACGVRVEEDEDTLIVH GLGAGGVPGGAVAKTHLDHRIAMSFLVCGMAAKK | 417 |
| Query | 420 | PVTVDDATMIATSFPEFMDLMAGLGAKI 447 V+VDDA+ I TSFP F LM GLGA + |  |
| Sbjct | 418 | AVSVDDASPILTSFPIFEGLMTGLGADL 445 | |

>TR:A0A1B1PU59 A0A1B1PU59_9RHOB 3-phosphoshikimate 1-carboxyvinyltransferase OS=Yangia sp. CCB-MM3 OX=1792508 GN=aroA PE=3 SV=1

Length=448

Score = 470 bits (1209), Expect = 6e-161

Identities = 253/441 (57%), Positives = 303/441 (69%), Gaps = 7/441 (2%)

| Query | 9 | PATARKSSGLSGTVRIPGDKSISHRSFMFGGLASGETRITGLLEGEDVINTGKAMQAMGA | 68 |
| --- | --- | --- | --- |
| Sbjct | 9 | P ARKS+GL G +PGDKSISHRS + G +A GET++TGLLEG+DV++T KAMQA GA PMIARKSAGLKGEANVPGDKSISHRSLILGAMAVGETKVTGLLEGQDVLDTAKAMQAFGA | 68 |
| Query | 69 | RIRKEGDTWIIDGVGNGGLLAPEAPLDFGNAATGCRLTMGLVGVYDFDSTFIGDASLTKR  + K GDTW + GVG GG P +D GN+ TG RL MG + +TF GDASL KR | 128 |

| Sbjct | 69 | EVEKRGDTWHVHGVGVGGFAEPGNVIDCGNSGTGVRLIMGAMATTPITATFTGDASLNKR | 128 |
| --- | --- | --- | --- |
| Query | 129 | PMGRVLNPLREMGVQVKSEDGDRLPVTLRGPKTPTPITYRVPMASAQVKSAVLLAGLNTP | 188 |
| Sbjct | 129 | PM RV +PL G + G RLP+T+ G P+ Y P+ SAQVKSAVLLAGLN P PMARVTDPLALFGARAYGRSGGRLPMTIVGAADAIPVRYATPVPSAQVKSAVLLAGLNAP | 188 |
| Query | 189 | GITTVIEPIMTRDHTEKMLQGFGANLTVETDADGVRTIRLEGRGKLTGQVIDVPGDPSST | 248 |
| Sbjct | 189 | G T VIE TRDH+E+ML GFGA +T E +G R I L G+ +L Q I VP DPSS GETVVIEREATRDHSERMLTGFGAQVTTEETDEG-RVITLVGQPELKPQTIIVPRDPSSA | 247 |
| Query | 249 | AFPLVAALLVPGSDVTILNVLMNPTRTGLILTLQEMGADIEVINPRLAGGEDVADLRVR- | 307 |
| Sbjct | 248 | AFP+ AAL+ GSDV + N+ +NPTR GL TLQEMGAD+ N R GGE VADLR + AFPVCAALITEGSDVLVPNIGLNPTRAGLFFTLQEMGADLTFENMREEGGEPVADLRAKF | 307 |
| Query | 308 | SSTLKGVTVPEDRAPSMIDEYPILAVAAAFAEGATVMNGLEELRVKESDRLSAVANGLKL | 367 |
| Sbjct | 308 | S LKG+ VP +RA SMIDEYP+L+V AAFA+G T M G++ELRVKESDR+ A+A GL+L SPDLKGIEVPAERAASMIDEYPVLSVVAAFAQGKTHMPGVKELRVKESDRIDAMATGLRL | 367 |
| Query | 368 | NGVDCDEGETSLVVRGRPDGKGLGNASGAAVA-THLDHRIAMSFLVMGLVSENPVTVDDA | 426 |
| Sbjct | 368 | NGV+ DEGE V GR G G+ G A A +HLDHRIAMSFLVMGL +E PV VDD NGVEVDEGEDWWTVHGR GFGDVKGGATAQSHLDHRIAMSFLVMGLATEEPVRVDDG | 423 |
| Query | 427 | TMIATSFPEFMDLMAGLGAKI 447 IATSFP F +LM LGA + |  |
| Sbjct | 424 | GPIATSFPIFENLMTSLGADL 444 | |

```
>TR:A0A0B1ZMU5 A0A0B1ZMU5_9SPHN 3-phosphoshikimate 1-carboxyvinyltransferase OS=Novosphingobium malaysiense OX=1348853 GN=aroA PE=3 SV=1
Length=438

Score = 469 bits (1208), Expect = 6e-161
Identities = 252/437 (58%), Positives = 307/437 (70%), Gaps = 3/437 (1%)

Query 8	RPATARKSSGLSGTVRIPGDKSISHRSFMFGGLASGETRITGLLEGEDVINTGKAMQAMG 67 RP	+ L G +R+PGDKSISHRS M G LA GETR+TGLLEGEDV+ T AM+AMG
Sbjct 2	RPRRFLPAGALKGRIRVPGDKSISHRSIMLGALAVGETRVTGLLEGEDVLATAAAMRAMG 61

Query 68	ARIRKEGD-TWIIDGVGNGGLLAPEAPLDFGNAATGCRLTMGLVGVYDFDSTFIGDASLT 126 A + + G+ W + GVG G LL PE LD GN+ T RL MG+V +	+TF+GDASL+
Sbjct 62	ATVERTGEGAWSVHGVGVGSLLQPEGSLDMGNSGTSTRLLMGVVASHPITATFVGDASLS 121

Query 127 KRPMGRVLNPLREMGVQVKSEDGDRLPVTLRGPKTPTPITYRVPMASAQVKSAVLLAGLN 186 KRPMGRV++PL +MG	++ G RLP+ L+G	PITY +P+ASAQVKSAVLLAGLN
Sbjct 122 KRPMGRVIDPLSQMGASFEASVGGRLPLILKGASPAVPITYCLPVASAQVKSAVLLAGLN 181

Query 187 TPGITTVIEPIMTRDHTEKMLQGFGANLTVETDADGVRTIRLEGRGKLTGQVIDVPGDPS 246 TPG+TTVIEP+ TRDH+E+ML+GFGA+L V T+ DG R I ++G +L QVIDVPGDPS
Sbjct 182 TPGMTTVIEPVPTRDHSERMLRGFGAHLDVTTEPDGTRVITIQGEAELKPQVIDVPGDPS 241

Query 247 STAFPLVAALLVPGSDVTILNVLMNPTRTGLILTLQEMGADIEVINPRLAGGEDVADLRV 306 S AF LVAAL+ PGS+V I NV +NPTR GLI L++MG IE ++ R GGE VADL V
Sbjct 242 SAAFFLVAALVTPGSEVVIENVGLNPTRAGLIDVLRQMGGHIEELDIREVGGEPVADLLV 301

Query 307 RSSTLKGVTVPEDRAPSMIDEYPILAVAAAFAEGATVMNGLEELRVKESDRLSAVANGLK 366
+ S LKG+ V	APSMIDE+P L VAAA A+G T +GL+ELRVKESDRL+ +A L Sbjct 302 KHSALKGIEVDPAIAPSMIDEFPALFVAAALADGVTTTSGLDELRVKESDRLAVMAAALA 361

Query 367 LNGVDCDEGETSLVVRGRPDGKGLGNASGAAVATHLDHRIAMSFLVMGLVSENPVTVDDA 426 G	DE E LV+ G	G L	+G +ATHLDHRIAMS V GL S N V VDD
Sbjct 362 AAGTRVDEREDGLVITG-TGGDPLPGTNG-PIATHLDHRIAMSMAVAGLASRNGVEVDDT 419

Query 427 TMIATSFPEFMDLMAGL 443
IATSFP F ++ GL

Sbjct 420 RPIATSFPAFETMLDGL 436


>TR:A0A2T5BSU7 A0A2T5BSU7_9RHOB 3-phosphoshikimate 1-carboxyvinyltransferase OS=Rhodovulum imhoffii OX=365340 GN=aroA PE=3 SV=1
Length=450

Score = 470 bits (1209), Expect = 7e-161
```

| Identities | = 255/440 (58%), Positives = 308/440 (70%), Gaps = 6/440 (1%) |  |
| --- | --- | --- |
| Query 9 | PATARKSSGLSGTVRIPGDKSISHRSFMFGGLASGETRITGLLEGEDVINTGKAMQAMGA | 68 |
| Sbjct 9 | P TAR+++ L GT +PGDKSISHR+ + G LA GETRI+GLLEG+DV++T +AM+A GA PMTARRAASLRGTAEVPGDKSISHRALILGALAVGETRISGLLEGQDVLDTARAMRAFGA | 68 |
| Query 69 | RIRKEGD-TWIIDGVGNGGLLAPEAPLDFGNAATGCRLTMGLVGVYDFDSTFIGDASLTK | 127 |
| Sbjct 69 | R+ + G W++ GVG GG P+ +D GN+ TG RL MG + +TF GDASL RVSQTGPGAWVVQGVGVGGFAEPDDVIDCGNSGTGVRLIMGAMATSPVTATFTGDASLRS | 128 |
| Query 128 | RPMGRVLNPLREMGVQVKSEDGDRLPVTLRGPKTPTPITYRVPMASAQVKSAVLLAGLNT | 187 |
| Sbjct 129 | RPMGRV PL G + DG RLP+T+ G P P+ Y+ PM SAQVKSA+LLAGLN RPMGRVTEPLALFGARAVGRDGGRLPMTVTGAAHPVPVCYKTPMPSAQVKSAILLAGLNA | 188 |
| Query 188 | PGITTVIEPIMTRDHTEKMLQGFGANLTVETDADGVRTIRLEGRGKLTGQVIDVPGDPSS | 247 |
| Sbjct 189 | PG T VIE TRDHTE+ML GFGA+L VE +G R I L G+ +L Q I VP DPSS PGETVVIEREATRDHTERMLAGFGADLRVEDGPEG-RIITLTGQPELRPQTIAVPRDPSS | 247 |
| Query 248 | TAFPLVAALLVPGSDVTILNVLMNPTRTGLILTLQEMGADIEVINPRLAGGEDVADLRVR | 307 |
| Sbjct 248 | AFP+ AAL+V GSDV++ + +NPTR GL TL+EMGAD+ N R GGE VADLRVR AAFPVCAALIVEGSDVSVPGIGLNPTRAGLFETLREMGADLGYENMREEGGEPVADLRVR | 307 |
| Query 308 | -SSTLKGVTVPEDRAPSMIDEYPILAVAAAFAEGATVMNGLEELRVKESDRLSAVANGLK | 366 |
| Sbjct 308 | S LKG+ VP +RAPSMIDEYPIL+V AAFAEG TVM G+ ELRVKESDR++A+ANGL+ FSPALKGIEVPPERAPSMIDEYPILSVVAAFAEGPTVMRGVGELRVKESDRIAAMANGLR | 367 |
| Query 367 | LNGVDCDEGETSLVVRGRPDGKGLGNASGAAVATHLDHRIAMSFLVMGLVSENPVTVDDA | 426 |

G	+EG	+VV G	G	G	GA ATHLDHRIAMSFLV GL ++ PVTVDDA

| Sbjct | 368 | SCGARVEEGADWMVVHGMGPG---GLPGGAICATHLDHRIAMSFLVAGLGAQAPVTVDDA | 424 |
| --- | --- | --- | --- |
| Query | 427 | TMIATSFPEFMDLMAGLGAK 446 IATSFP F LM LGA+ |  |
| Sbjct | 425 | GPIATSFPVFEPLMEALGAR 444 | |

>TR:A0A4R3J859 A0A4R3J859_9PROT 3-phosphoshikimate 1-carboxyvinyltransferase OS=Varunaivibrio sulfuroxidans OX=1773489 GN=aroA PE=3 SV=1

Length=484

Score = 471 bits (1212), Expect = 8e-161

| Identities | | = 259/443 (58%), Positives = 305/443 (69%), Gaps = 2/443 (0%) |  |
| --- | --- | --- | --- |
| Query 7  Sbjct 39 | | SRPATARKSSGLSGTVRIPGDKSISHRSFMFGGLASGETRITGLLEGEDVINTGKAMQAM SR T + L G V +PGDKSISHR+ M G +A GETRI GLLEGEDV+ T A+ A+ SRSITECGTHRLHGEVVVPGDKSISHRALMLGAMALGETRIEGLLEGEDVLRTAAALSAL | 66  98 |
| Query 67 | | GARI-RKEGDTWIIDGVGNGGLLAPEAPLDFGNAATGCRLTMGLVGVYDFDSTFIGDASL GARI R++ W + GVG GG P LD GN+ T CRL MG + D + F GDASL | 125 |
| Sbjct | 99 | GARITRRDDGVWRVYGVGVGGFTEPGDVLDMGNSGTACRLLMGALASCDLHAVFTGDASL | 158 |
| Query | 126 | TKRPMGRVLNPLREMGVQVKSEDGDRLPVTLRGPKTPTPITYRVPMASAQVKSAVLLAGL | 185 |
| Sbjct | 159 | RPMGRV PL +G + S DG RLP+ G + P ITYR+PMASAQVKSA+LLAGL RGRPMGRVTTPLTMIGARFMSRDGTRLPLAQHGARDPLAITYRLPMASAQVKSAILLAGL | 218 |
| Query | 186 | NTPGITTVIEPIMTRDHTEKMLQGFGANLTVETDADGVRTIRLEGRGKLTGQVIDVPGDP NTPG+T V+EP TRDH+E ML+ FGA +TV DA G+R I L G+ +L G VI V GD | 245 |

| Sbjct | 219 | NTPGVTQVVEPRPTRDHSELMLRHFGAEVTVRDDALGLREIALTGQPELEGCVIGVAGDI | 278 |
| --- | --- | --- | --- |
| Query | 246 | SSTAFPLVAALLVPGSDVTILNVLMNPTRTGLILTLQEMGADIEVINPRLAGGEDVADLR | 305 |
| Sbjct | 279 | SS AF +VAAL+VPGS V + V +NP RTG++ TL EMGADIEV N R GE V D+ SSAAFVIVAALIVPGSHVIVRGVGLNPLRTGIVETLFEMGADIEVTNRRDVAGEPVGDIS | 338 |
| Query | 306 | VRSSTLKGVTVPEDRAPSMIDEYPILAVAAAFAEGATVMNGLEELRVKESDRLSAVANGL | 365 |
| Sbjct | 339 | VRSS LKGVTVP +RAP+MIDEYPILAVAAAFA G T+M GL ELRVKESDRLSA+A GL VRSSMLKGVTVPAERAPAMIDEYPILAVAAAFAAGDTLMEGLGELRVKESDRLSAMAEGL | 398 |
| Query | 366 | KLNGVDCDEGETSLVVRGRPDGKGLGN-ASGAAVATHLDHRIAMSFLVMGLVSENPVTVD | 424 |
| Sbjct | 399 | K GV CDEG +L V G G G A G + LDHRIAM+FLV+G+ ++PV +D KACGVACDEGAETLCVHGAAVLSGGGRVAGGGNIEARLDHRIAMAFLVLGMACDDPVRID | 458 |
| Query  Sbjct | 425  459 | DATMIATSFPEFMDLMAGLGAKI 447 D T I TSFP F LM LGA I DGTPIETSFPGFCALMNSLGADI 481 |  |

>TR:J2WMU7 J2WMU7_9SPHN 3-phosphoshikimate 1-carboxyvinyltransferase OS=Sphingobium sp. AP49 OX=1144307 GN=aroA PE=3 SV=1

Length=446

Score = 469 bits (1208), Expect = 8e-161

| Identities | = 252/444 (57%), Positives = 305/444 (69%), Gaps = 4/444 (1%) |  |
| --- | --- | --- |
| Query 3 | HGASSRPATARKSSGLSGTVRIPGDKSISHRSFMFGGLASGETRITGLLEGEDVINTGKA | 62 |
| Sbjct 4 | H P T + LSG+V +PGDKSISHRS M LA GE+R+ GLLEGEDV+ T A HNDQPSPMTFTAAPALSGSVAVPGDKSISHRSLMLSALAVGESRVEGLLEGEDVLATAAA | 63 |
| Query 63 | MQAMGARIRKEGD-TWIIDGVGNGGLLAPEAPLDFGNAATGCRLTMGLVGVYDFDSTFIG | 121 |
| Sbjct 64 | M+AMGA I+++ D W I GVG GGLL P+ LD GN+ T RL MGLV + +TF+G MRAMGADIQRDEDGVWHIHGVGVGGLLQPQEALDMGNSGTSTRLLMGLVASHAITATFVG | 123 |
| Query 122 | DASLTKRPMGRVLNPLREMGVQVKSEDGDRLPVTLRGPKTPTPITYRVPMASAQVKSAVL | 181 |
| Sbjct 124 | DASL+KRPM RV PL +G + GDRLP+T+ G P+ Y +P+ASAQVKSA+L DASLSKRPMARVTEPLSRVGASFTTSPGDRLPLTMTGACPAVPLDYTLPVASAQVKSAIL | 183 |
| Query 182 | LAGLNTPGITTVIEPIMTRDHTEKMLQGFGANLTVETDADGVRTIRLEGRGKLTGQVIDV | 241 |
| Sbjct 184 | LAGLNTPGIT V+EPI TRDH+E+ML+GFGA+L VE ADG R I L+G +L Q I V LAGLNTPGITRVVEPIPTRDHSERMLKGFGADLDVEVQADGTRIITLKGEAELKPQNIVV | 243 |
| Query 242 | PGDPSSTAFPLVAALLVPGSDVTILNVLMNPTRTGLILTLQEMGADIEVINPRLAGGEDV | 301 |
| Sbjct 244 | PGDPSS AFP+VAALLVPGS VTI NV +N TR GLI L+EMG IE+IN R GGE V PGDPSSAAFPMVAALLVPGSQVTIANVGLNATRAGLIDLLREMGGSIEIINARDVGGEPV | 303 |
| Query 302 | ADLRVRSSTLKGVTVPEDRAPSMIDEYPILAVAAAFAEGATVMNGLEELRVKESDRLSAV | 361 |
| Sbjct 304 | DL V +S L GV RAPSMIDEYP+ VAAAFA G ++ GL+ELRVKESDR++ + GDLVVTASALNGVEPDPARAPSMIDEYPVAFVAAAFAHGRSIFRGLDELRVKESDRIATM | 363 |
| Query 362 | ANGLKLNGVDCDEGETSLVVRGRPDGKGLGNASGAAVATHLDHRIAMSFLVMGLVSENPV | 421 |
| Sbjct 364 | A GL+ GV +E E +++ G G G +AT LDHRIAMSF V GLVS + V ATGLRAIGVTVEELEDGIIIEG---SAGQPLPGGGPIATKLDHRIAMSFAVAGLVSRDGV | 420 |
| Query 422  Sbjct 421 | TVDDATMIATSFPEFMDLMAGLGA 445 T+DD +ATSFP F L+ LGA TIDDMRPVATSFPTFTTLLQTLGA 444 |  |

>TR:A0A519QKA0 A0A519QKA0_9SPHN 3-phosphoshikimate 1-carboxyvinyltransferase OS=Novosphingobium sp. OX=1874826 GN=aroA PE=3 SV=1

Length=448

Score = 469 bits (1208), Expect = 9e-161

| Identities | = 252/442 (57%), Positives = 308/442 (70%), Gaps = 2/442 (0%) |  |
| --- | --- | --- |
| Query 5 | ASSRPATARKSSGLSGTVRIPGDKSISHRSFMFGGLASGETRITGLLEGEDVINTGKAMQ | 64 |
| Sbjct 8 | ++ RP + L G +R+PGDKSISHRS M LA GE+R+TGLLEGEDV++T AM+ SAMRPRRFAAAGPLKGRIRVPGDKSISHRSLMLSALAVGESRVTGLLEGEDVLSTAAAMR | 67 |
| Query 65 | AMGARIRKEGD-TWIIDGVGNGGLLAPEAPLDFGNAATGCRLTMGLVGVYDFDSTFIGDA | 123 |
| Sbjct 68 | AMGA I++ G+ W+I GVG G LL PEA LD GN+ T RL MGLV + + F+GDA AMGADIQRTGEGEWLIHGVGVGALLQPEAALDMGNSGTSTRLLMGLVASHPITACFVGDA | 127 |
| Query 124 | SLTKRPMGRVLNPLREMGVQVKSEDGDRLPVTLRGPKTPTPITYRVPMASAQVKSAVLLA | 183 |
| Sbjct 128 | SL+KRPM RV +PL MG + + G+RLP+ LRG PI YR+P+ASAQVKSAVLLA SLSKRPMKRVTDPLSLMGAEFTTSPGERLPLMLRGAAPAVPIEYRLPVASAQVKSAVLLA | 187 |
| Query 184 | GLNTPGITTVIEPIMTRDHTEKMLQGFGANLTVETDADGVRTIRLEGRGKLTGQVIDVPG | 243 |
| Sbjct 188 | LNTPGITTVIEP+ TRDH+E+ML+GFGA LTVE D+DG RTIR+ G +L Q I+VPG SLNTPGITTVIEPVPTRDHSERMLKGFGAELTVEVDSDGTRTIRIRGEAELQPQCIEVPG | 247 |
| Query 244 | DPSSTAFPLVAALLVPGSDVTILNVLMNPTRTGLILTLQEMGADIEVINPRLAGGEDVAD | 303 |
| Sbjct 248 | DPSS AF +VAAL+VPGS++ I NV +N TR GLI L++MG DI + R GGE VAD DPSSAAFFIVAALIVPGSELVIENVGLNATRAGLIDVLRQMGGDITEQSAREVGGEPVAD | 307 |
| Query 304 | LRVRSSTLKGVTVPEDRAPSMIDEYPILAVAAAFAEGATVMNGLEELRVKESDRLSAVAN | 363 |
| Sbjct 308 | L VR S LKG+ V PSM+DE+P+ VAAA AEG T GL+ELRVKESDR++ +A LLVRHSVLKGIEVDPAVVPSMVDEFPVFFVAAALAEGRTTTTGLDELRVKESDRIAVMAT | 367 |
| Query 364 | GLKLNGVDCDEGETSLVVRGRPDGKGLGNASGAAVATHLDHRIAMSFLVMGLVSENPVTV | 423 |

L	G	E E L++ G	G+ L	+ AA+ATHLDHRIAMS V GLVS	V V

| Sbjct | 368 | ALTAAGARVQETEDGLIIDG-SGGEALRGGNNAAIATHLDHRIAMSMAVAGLVSAGGVEV | 426 |
| --- | --- | --- | --- |
| Query  Sbjct | 424  427 | DDATMIATSFPEFMDLMAGLGA 445 DD IATSFP F L+ GL A DDTRPIATSFPAFEALLDGLVA 448 |  |

>SP:Q3SW64 AROA_NITWN 3-phosphoshikimate 1-carboxyvinyltransferase OS=Nitrobacter winogradskyi (strain ATCC 25391 / DSM 10237 / CIP

104748 / NCIMB 11846 / Nb-255) OX=323098 GN=aroA PE=3 SV=1

Length=449

Score = 469 bits (1208), Expect = 9e-161

| Identities | = 253/451 (56%), Positives = 308/451 (68%), Gaps = 11/451 (2%) |  |
| --- | --- | --- |
| Query 1 | MLHGASSRPATARKSSGLSGTVRIPGDKSISHRSFMFGGLASGETRITGLLEGEDVINTG | 60 |
| Sbjct 1 | M + P ARK L+G VR+PGDKSISHR+ + G LA GETRI+GLLEGEDV+NT MTLSGQTMPLEARKCQSLTGRVRVPGDKSISHRALILGALAVGETRISGLLEGEDVLNTA | 60 |
| Query 61 | KAMQAMGARIRKEGD TWIIDGVGNGGLLAPEAPLDFGNAATGCRLTMGLVGVYDF | 115 |
| Sbjct 61 | KAMQA+GA++ + D W + GVG GG PEAPLDFGN+ TGCRL MG V KAMQALGAKVERRTDDNGGIAWSVRGVGTGGFATPEAPLDFGNSGTGCRLVMGAVAGCPI | 120 |
| Query 116 | DSTFIGDASLTKRPMGRVLNPLREMGVQVKSE-DGDRLPVTLRGPKTPTPITYRVPMASA | 174 |
| Sbjct 121 | + F GD SL RPM R+L+PL MG +V S+ DG RLP+TL G + P PI YR P+ASA RAAFDGDGSLRSRPMRRILDPLELMGARVISQSDGGRLPLTLEGARDPLPIVYRTPVASA | 180 |
| Query 175 | QVKSAVLLAGLNTPGITTVIEPIMTRDHTEKMLQGFGANLTVETDADGVRTIRLEGRGKL | 234 |
| Sbjct 181 | Q+KSAVLLAGL+ PG+TTVIE +RDHTE ML+ FGA++ + R I L G+ +L QIKSAVLLAGLSAPGVTTVIEREASRDHTELMLKHFGADIVSVAEGAHGRKISLTGQPEL | 240 |
| Query 235 | TGQVIDVPGDPSSTAFPLVAALLVPGSDVTILNVLMNPTRTGLILTLQEMGADIEVINPR | 294 |
| Sbjct 241 | G + VP DPSS AFP+VAAL+ GSD+ + +V+ NP RTGL +TL+EMGA IE R HGAGVTVPADPSSAAFPIVAALITEGSDIVLDDVMTNPLRTGLFVTLREMGASIEESETR | 300 |
| Query 295 | LAGGEDVADLRVRSSTLKGVTVPEDRAPSMIDEYPILAVAAAFAEGATVMNGLEELRVKE | 354 |

| Sbjct | 301 | L GE +A LRV++S L+G+ VP +RAPSMIDEY +LAVAAAFAEG TVM GL ELRVKE  LDAGEPMAQLRVKASRLRGIEVPAERAPSMIDEYLVLAVAAAFAEGTTVMRGLRELRVKE | 360 |
| --- | --- | --- | --- |
| Query | 355 | SDRLSAVANGLKLNGVDCDEGETSLVVRGRPDGKGLGNASGAAVATHLDHRIAMSFLVMG | 414 |
| Sbjct | 361 | SDRL A A L+ GV + L+V GR G G V TH+DHRIAMS LVMG SDRLEAAAAMLRAGGVAVEIAGDDLIVEGRGRVPG GGLVTTHMDHRIAMSALVMG | 415 |
| Query  Sbjct | 415  416 | LVSENPVTVDDATMIATSFPEFMDLMAGLGA 445 S+ PV VDD IATSFP+F+ +M LGA  CASDAPVKVDDIAFIATSFPDFVPMMQRLGA 446 |  |

>TR:A0A2M8MYX0 A0A2M8MYX0_9RHOB 3-phosphoshikimate 1-carboxyvinyltransferase OS=Pseudorhodobacter sp. MZDSW-24AT OX=2052957 GN=aroA PE=3

SV=1

Length=453

Score = 469 bits (1208), Expect = 1e-160

| Identities | = 255/447 (57%), Positives = 310/447 (69%), Gaps = 7/447 (2%) |  |
| --- | --- | --- |
| Query 3 | HGASSRPATARKSSGLSGTVRIPGDKSISHRSFMFGGLASGETRITGLLEGEDVINTGKA | 62 |
| Sbjct 4 | HG ++P T+ KS L GT +PGDKSISHR+ +FG +A GETRITGLLEG+DV++T +A HG-DAQPMTSAKSGPLKGTAAVPGDKSISHRALIFGAMAVGETRITGLLEGQDVLDTARA | 62 |
| Query 63 | MQAMGARIRKEGD-TWIIDGVGNGGLLAPEAPLDFGNAATGCRLTMGLVGVYDFDSTFIG | 121 |

M+A GA + + G	W + GVG GG	P	+D GN+ TG RL MG +	+TF G

| Sbjct | 63 | MRAFGAEVTQHGPGAWSVHGVGVGGFSEPADVIDCGNSGTGVRLIMGTMATTAMTATFTG | 122 |
| --- | --- | --- | --- |
| Query | 122 | DASLTKRPMGRVLNPLREMGVQVKSEDGDRLPVTLRGPKTPTPITYRVPMASAQVKSAVL | 181 |
| Sbjct | 123 | DASL KRPMGRV +PL G + G RLP+T+ G P P+ Y +P+ASAQVKSAVL DASLRKRPMGRVTDPLSLFGAEAYGRKGGRLPMTVVGAANPVPVRYALPVASAQVKSAVL | 182 |
| Query | 182 | LAGLNTPGITTVIEPIMTRDHTEKMLQGFGANLTVETDADGVRTIRLEGRGKLTGQVIDV | 241 |
| Sbjct | 183 | LAGLN PG T VIE TRDH+E+ML GFGA L+VE ++G I L+GR +L Q + V LAGLNAPGETVVIEREPTRDHSERMLLGFGAELSVEKTSEG-HVITLQGRPELRPQTVAV | 241 |
| Query | 242 | PGDPSSTAFPLVAALLVPGSDVTILNVLMNPTRTGLILTLQEMGADIEVINPRLAGGEDV | 301 |
| Sbjct | 242 | P DPSS AFP AAL+V GSD+ + V N TR GL LTL EMGA+IE NPR GGE V PRDPSSAAFPTCAALIVEGSDILVPGVSQNLTRNGLYLTLSEMGAEIEFQNPRTEGGEPV | 301 |
| Query | 302 | ADLRVR-SSTLKGVTVPEDRAPSMIDEYPILAVAAAFAEGATVMNGLEELRVKESDRLSA | 360 |
| Sbjct | 302 | ADLRVR S +KG+ VP +RAPSMIDEYPIL+V AAFA G TVM G++ELRVKESDR+ A ADLRVRFSDNMKGIEVPPERAPSMIDEYPILSVVAAFASGRTVMRGVKELRVKESDRIDA | 361 |
| Query | 361 | VANGLKLNGVDCDEGETSLVVRGRPDGKGLGNASGAAVATHLDHRIAMSFLVMGLVSENP | 420 |
| Sbjct | 362 | +A GL+ GV +E E +L+V G G G GA ATHLDHRIAMSFLV G+ S+ P MARGLEACGVRIEEDEDTLIVHGLGAG---GMPGGATCATHLDHRIAMSFLVAGMASKAP | 418 |
| Query  Sbjct | 421  419 | VTVDDATMIATSFPEFMDLMAGLGAKI 447  ++VDDA+ I TSFP F LM GLGA I ISVDDASPIVTSFPIFEGLMNGLGASI 445 |  |

```
>TR:A0A345ZZJ5 A0A345ZZJ5_9RHIZ 3-phosphoshikimate 1-carboxyvinyltransferase OS=Pseudolabrys taiwanensis OX=331696 GN=aroA PE=3 SV=1
Length=444

Score = 469 bits (1207), Expect = 1e-160
Identities = 249/441 (56%), Positives = 311/441 (71%), Gaps = 7/441 (2%)

Query 9	PATARKSSGLSGTVRIPGDKSISHRSFMFGGLASGETRITGLLEGEDVINTGKAMQAMGA 68 P TAR+	L G VR+PGDKSISHR+ +	+A GE+ +TGLLE EDV+NT KAM A+GA
Sbjct 8	PLTARRGGPLQGRVRVPGDKSISHRALILAAMAVGESTVTGLLESEDVLNTAKAMSALGA 67
```

| Query | 69 | RIRKEGD-TWIIDGVGNGGLLAPEAPLDFGNAATGCRLTMGLVGVYDFDSTFIGDASLTK | 127 |
| --- | --- | --- | --- |
| Sbjct | 68 | ++ ++ D W + GVG GG PEAPLDFGN+ TGCRL +G V ++F GDASL K KVDRDKDGVWRVRGVGVGGFAEPEAPLDFGNSGTGCRLVIGAVAGCPIKASFDGDASLRK | 127 |
| Query | 128 | RPMGRVLNPLREMGVQVKSE-DGDRLPVTLRGPKTPTPITYRVPMASAQVKSAVLLAGLN | 186 |
| Sbjct | 128 | RPM R+L+PL +MG QV + +G RLP+TL+G + P P+ Y + SAQ+KSAVLLAGL RPMRRILDPLEKMGAQVLEQAEGGRLPLTLQGARDPLPLEYESQVPSAQLKSAVLLAGLA | 187 |
| Query | 187 | TPGITTVIEPIMTRDHTEKMLQGFGANLTVETDADGVRTIRLEGRGKLTGQVIDVPGDPS | 246 |
| Sbjct | 188 | PG TTVIE TRDHTE++L+ FGA + + + R I L+G+ +L + VP DPS APGETTVIEAEATRDHTERLLKHFGAKIVSKPHGELGRRITLQGQPELEPADVTVPADPS | 247 |
| Query | 247 | STAFPLVAALLVPGSDVTILNVLMNPTRTGLILTLQEMGADIEVINPRLAGGEDVADLRV | 306 |
| Sbjct | 248 | S AFP+VAAL+VPGSDV NV+MNP RTGL TL+EMGA IEV+ R G E+VADLRV SAAFPMVAALIVPGSDVVFENVMMNPLRTGLFTTLKEMGAKIEVVEKRDGGAEEVADLRV | 307 |
| Query | 307 | RSSTLKGVTVPEDRAPSMIDEYPILAVAAAFAEGATVMNGLEELRVKESDRLSAVANGLK | 366 |
| Sbjct | 308 | +S LKGV VP +RAP+MIDEY IL VAAAFA G T M GL+ELRVKESDRL A A+ L+ TTSQLKGVEVPPERAPAMIDEYLILGVAAAFANGTTRMRGLKELRVKESDRLEATADMLR | 367 |
| Query | 367 | LNGVDCDEGETSLVVRGRPDGKGLGNASGAAVATHLDHRIAMSFLVMGLVSENPVTVDDA | 426 |
| Sbjct | 368 | +NG+ + L+V G+ +G G V TH+DHRIAMS LVMGL SE PV +DD+ VNGIAVEIEGDDLIVHGKGSVQG GGEVKTHMDHRIAMSALVMGLASEQPVRIDDS | 422 |
| Query | 427 | TMIATSFPEFMDLMAGLGAKI 447 IATSFP+F LM GLGA + |  |
| Sbjct | 423 | AFIATSFPDFTGLMRGLGADL 443 | |

>TR:A0A1E3W7F1 A0A1E3W7F1_9RHIZ 3-phosphoshikimate 1-carboxyvinyltransferase OS=Methyloceanibacter superfactus OX=1774969 GN=aroA PE=3 SV=1

Length=456

Score = 469 bits (1208), Expect = 1e-160

| Identities | = 253/440 (58%), Positives = 313/440 (71%), Gaps = 6/440 (1%) |  |
| --- | --- | --- |
| Query 9 | PATARKSSGLSGTVRIPGDKSISHRSFMFGGLASGETRITGLLEGEDVINTGKAMQAMGA | 68 |
| Sbjct 21 | P TAR+S+ +SG VR+PGDKSISHR+ + G LA G TRI GLL EDV+ T +A+ +GA PLTARRSARISGRVRVPGDKSISHRALILGALAIGRTRIAGLLAAEDVMATAQAVDELGA | 80 |
| Query 69 | RIRKEGDTWIIDGVGNGGLLAPEAPLDFGNAATGCRLTMGLVGVYDFDSTFIGDASLTKR | 128 |
| Sbjct 81 | + ++ T + G G GGL AP+ PLDFGN+ TG RL +G+V +D F GDASL +R EVTQKDGTVEVLGQGVGGLRAPQGPLDFGNSGTGSRLMLGVVAGHDMRVEFTGDASLCRR | 140 |
| Query 129 | PMGRVLNPLREMGVQVKSE-DGDRLPVTLRGPKTPTPITYRVPMASAQVKSAVLLAGLNT | 187 |
| Sbjct 141 | PMGRVL PL MG+QV+++ +G LP+T+RG PI Y +P+ SAQVKSA+L AGL+ PMGRVLTPLITMGLQVEAQGEGGTLPLTVRGTGDILPIEYDLPVPSAQVKSAILFAGLHA | 200 |
| Query 188 | PGITTVIEPIMTRDHTEKMLQGFGANLTVETDADGVRTIRLEGRGKLTGQVIDVPGDPSS | 247 |
| Sbjct 201 | PG TTVIEP+ TRDHTE+ML FGA L E A G R + + G +L G + VPGDPSS PGRTTVIEPLATRDHTERMLAFFGAELHAEDKAGGGRAVTVCGDAELHGAALSVPGDPSS | 260 |
| Query 248 | TAFPLVAALLVPGSDVTILNVLMNPTRTGLILTLQEMGADIEVINPRLAGGEDVADLRVR | 307 |
| Sbjct 261 | AF + AAL+ PGSD+ + NVLMNPTRTG + TL+EMGA IEV++ R GGE V DLRV+ AAFLIAAALICPGSDLVVENVLMNPTRTGFLDTLREMGASIEVLDLRDEGGEPVGDLRVK | 320 |
| Query 308 | SSTLKGVTVPEDRAPSMIDEYPILAVAAAFAEGATVMNGLEELRVKESDRLSAVANGLKL | 367 |
| Sbjct 321 | + LKGV VP +RAPSMIDEYP+LAV AAFAEG T M GL EL+VKESDRL+A A GL+ AGPLKGVRVPPERAPSMIDEYPVLAVVAAFAEGETHMAGLAELKVKESDRLAATAAGLEA | 380 |
| Query 368 | NGVDCDEGETSLVVRGRPDGKGLGNASGAAVATHLDHRIAMSFLVMGLVSENPVTVDDAT | 427 |
| Sbjct 381 | GV L+VRGR KG GA V TH+DHRIAM+FL++GL + PVTVDD T  CGVAVRIEGDDLIVRGRGAVKG GALVETHMDHRIAMAFLILGLGASEPVTVDDVT | 435 |

Query 428 MIATSFPEFMDLMAGLGAKI 447

MIATSFP F LM LGA++ Sbjct 436 MIATSFPGFAALMTSLGAEL 455

>SP:Q2VZA8 AROA_MAGSA 3-phosphoshikimate 1-carboxyvinyltransferase OS=Magnetospirillum magneticum (strain AMB-1 / ATCC 700264) OX=342108

GN=aroA PE=3 SV=1

Length=446

Score = 469 bits (1207), Expect = 1e-160

| Identities | = 256/445 (58%), Positives = 311/445 (70%), Gaps = 11/445 (2%) |  |
| --- | --- | --- |
| Query 7 | SRPATARKSSGLSGTVRIPGDKSISHRSFMFGGLASGETRITGLLEGEDVINTGKAMQAM | 66 |
| Sbjct 4 | ++P ++R+++ L+G+ +PGDKSISHR+ M G LA GE+ +TGLLEG+DV+ T M+A+ AKPLSSRRAAPLAGSAPVPGDKSISHRALMLGALAVGESVVTGLLEGDDVLRTAACMRAL | 63 |
| Query 67 | GARIRKEGD-TWIIDGVGNGGLLAPEAPLDFGNAATGCRLTMGLVGVYDFDSTFIGDASL | 125 |
| Sbjct 64 | GA + + D +W + G G GGL+ P LD GN+ TG RL MGLV + F F GD SL GAEVERRDDGSWRLFGRGVGGLMEPADVLDMGNSGTGARLLMGLVATHPFTCFFTGDGSL | 123 |
| Query 126 | TKRPMGRVLNPLREMGVQVKSEDGDRLPVTLRGPKTPTPITYRVPMASAQVKSAVLLAGL | 185 |
| Sbjct 124 | RPM RV++PL MG + S DG RLP+ + G PTPITY +P+ASAQVKSA++LAGL RSRPMRRVIDPLSRMGARFVSRDGGRLPLAVTGTAQPTPITYELPVASAQVKSAIMLAGL | 183 |
| Query 186 | NTPGITTVIEPIMTRDHTEKMLQGFGANLTVETDADGVRTIRLEGRGKLTGQVIDVPGDP | 245 |
| Sbjct 184 | NT G TTVIE TRDHTE ML+ FGA + VE G R I + G +LTG+ + VP DP NTAGETTVIEREATRDHTELMLRNFGATVRVEDAEGGGRAITVVGFPELTGRPVVVPADP | 243 |
| Query 246 | SSTAFPLVAALLVPGSDVTILNVLMNPTRTGLILTLQEMGADIEVINPRLAGGEDVADLR | 305 |
| Sbjct 244 | SS AFP+VAALLV GS++ + NV NP RTGL TL EMGADI +PR GE VADL SSAAFPVVAALLVEGSEIRLPNVGTNPLRTGLYQTLLEMGADIRFDHPRDQAGEPVADLV | 303 |
| Query 306 | VRSSTLKGVTVPEDRAPSMIDEYPILAVAAAFAEGATVMNGLEELRVKESDRLSAVANGL | 365 |
| Sbjct 304 | VRSS LKGV VP +RAPSMIDEYPILAVAAAFAEG T M GL ELRVKESDRL+A+A GL VRSSRLKGVDVPAERAPSMIDEYPILAVAAAFAEGTTRMRGLGELRVKESDRLAAMARGL | 363 |
| Query 366 | KLNGVDCDEGETSLVVRGR---PDGKGLGNASGAAVATHLDHRIAMSFLVMGLVSENPVT | 422 |
| Sbjct 364 | GV +E + +L+V G PDG A V TH DHRIAMSFLVMG+ S PV AACGVAVEEEKDALIVHGTGRIPDG DATVTTHFDHRIAMSFLVMGMASARPVA | 416 |
| Query 423  Sbjct 417 | VDDATMIATSFPEFMDLMAGLGAKI 447 VDD+ I TSFP F++LM GLGAKI VDDSEAIDTSFPAFVELMNGLGAKI 441 |  |

>TR:A0A371B9F8 A0A371B9F8_9RHIZ 3-phosphoshikimate 1-carboxyvinyltransferase OS=Pseudolabrys sp. GY_H OX=2292256 GN=aroA PE=3 SV=1

Length=446

Score = 469 bits (1207), Expect = 1e-160

| Identities | = 256/445 (58%), Positives = 310/445 (70%), Gaps = 5/445 (1%) |  |
| --- | --- | --- |
| Query 5 | ASSRPATARKSSGLSGTVRIPGDKSISHRSFMFGGLASGETRITGLLEGEDVINTGKAMQ | 64 |
| Sbjct 4 | AS P +AR L+G +R+PGDKSISHR+ + G + GET I+GLLEGEDV+NT AM+ ASLTPLSARSGGPLTGRLRVPGDKSISHRALILGAMTVGETTISGLLEGEDVLNTAGAMR | 63 |
| Query 65 | AMGARIRK-EGDTWIIDGVGNGGLLAPEAPLDFGNAATGCRLTMGLVGVYDFDSTFIGDA | 123 |
| Sbjct 64 | A+GA++ G TW I GVG GG AP APLDFGN+ TGCRL MG V + F GDA ALGAKVENLGGGTWRIHGVGVGGFAAPAAPLDFGNSGTGCRLVMGAVAGCPVTAIFDGDA | 123 |
| Query 124 | SLTKRPMGRVLNPLREMGVQVKSE-DGDRLPVTLRGPKTPTPITYRVPMASAQVKSAVLL | 182 |
| Sbjct 124 | SL KRPM R+L+PL +MG +V + +G RLP+TLRG P PI Y P+ASAQ+KSAVLL  SLRKRPMRRILDPLEKMGARVLDQAEGGRLPMTLRGATDPLPIEYETPVASAQIKSAVLL | 183 |

| Query | 183 | AGLNTPGITTVIEPIMTRDHTEKMLQGFGANLTVETDADGVRTIRLEGRGKLTGQVIDVP | 242 |
| --- | --- | --- | --- |
| Sbjct | 184 | AGL PG T VIE +RDHTEKML+ FGA + + D R I L+G+ +L + VP AGLAAPGETIVIEQEASRDHTEKMLKHFGAKIVSKPHGDHGRRIVLQGQPELEPANVAVP | 243 |
| Query | 243 | GDPSSTAFPLVAALLVPGSDVTILNVLMNPTRTGLILTLQEMGADIEVINPRLAGGEDVA | 302 |
| Sbjct | 244 | DPSS AFPLV AL+VP S++ + V++NP RTGL TL+EMGA I N R GGEDVA ADPSSAAFPLVGALIVPDSEIILDGVMLNPLRTGLFTTLREMGAKIAEENKRDDGGEDVA | 303 |
| Query | 303 | DLRVRSSTLKGVTVPEDRAPSMIDEYPILAVAAAFAEGATVMNGLEELRVKESDRLSAVA | 362 |
| Sbjct | 304 | DLRV S LKGV VP RAPSMIDEY ILAVAAAFAEG T M GL+ELRVKESDRL A A DLRVSFSALKGVDVPAARAPSMIDEYLILAVAAAFAEGVTRMRGLKELRVKESDRLEATA | 363 |
| Query | 363 | NGLKLNGVDCDEGETSLVVRGRPDGKGLGNASGAAVATHLDHRIAMSFLVMGLVSENPVT | 422 |
| Sbjct | 364 | + L++NGV + + L+V G G G V TH+DHRIAMS L+MGL S+ PV DMLRVNGVKIEIEDDDLIVHGMGTN---GVPGGGEVKTHMDHRIAMSALMMGLASKEPVR | 420 |
| Query  Sbjct | 423  421 | VDDATMIATSFPEFMDLMAGLGAKI 447  +DD+ IATSFP F+DLM GLGA++ IDDSAFIATSFPGFVDLMKGLGAEL 445 |  |

>TR:A0A1H2VRD4 A0A1H2VRD4_9RHOB 3-phosphoshikimate 1-carboxyvinyltransferase OS=Roseicitreum antarcticum OX=564137 GN=aroA PE=3 SV=1

Length=447

Score = 469 bits (1207), Expect = 1e-160

| Identities | | = 254/440 (58%), Positives = 304/440 (69%), Gaps = 7/440 (2%) |  |
| --- | --- | --- | --- |
| Query 9  Sbjct 9 | | PATARKSSGLSGTVRIPGDKSISHRSFMFGGLASGETRITGLLEGEDVINTGKAMQAMGA P TARKS L G +PGDKSISHR+ +FG +A GETRITGLLEG+DV++T AM+A GA PMTARKSPPLRGVAEVPGDKSISHRALIFGAMAVGETRITGLLEGQDVLDTAAAMRAFGA | 68  68 |
| Query 69  Sbjct 69 | | RIRKEGD-TWIIDGVGNGGLLAPEAPLDFGNAATGCRLTMGLVGVYDFDSTFIGDASLTK  + + G W + GVG GG P+ +D GN+ TG RL MG + TF GDASLTK EVVQHGPGDWSVFGVGVGGFAEPDRVIDCGNSGTGARLIMGAMATCPITVTFTGDASLTK | 127  128 |
| Query 128  Sbjct 129 | | RPMGRVLNPLREMGVQVKSEDGDRLPVTLRGPKTPTPITYRVPMASAQVKSAVLLAGLNT RPMGRV +PL G + G RLP+TL G P P+ Y VPM SAQVKSAVLLAGLN RPMGRVTDPLALFGTRAVGRAGGRLPMTLVGAADPVPVRYTVPMPSAQVKSAVLLAGLNA | 187  188 |
| Query 188  Sbjct 189 | | PGITTVIEPIMTRDHTEKMLQGFGANLTVETDADGVRTIRLEGRGKLTGQVIDVPGDPSS PG T V+E TRDHTE+ML+GFGA LTVE +G R I L G+ +L + VP DPSS PGQTVVVEREPTRDHTERMLRGFGAELTVEDTPEG-RVITLTGQPELRPLDVAVPRDPSS | 247  247 |
| Query 248  Sbjct 248 | | TAFPLVAALLVPGSDVTILNVLMNPTRTGLILTLQEMGADIEVINPRLAGGEDVADLRVR AFP+ AAL+VP SD+ + V NPTR G+ LTL +MGADI NPR GGE VADLRVR AAFPVCAALMVPDSDILVPGVSQNPTRNGIYLTLVDMGADIAFENPREEGGEPVADLRVR | 307  307 |
| Query 308 | | SSTLKGVTVPEDRAPSMIDEYPILAVAAAFAEGATVMNGLEELRVKESDRLSAVANGLKL LKGVTVP +RA SMIDEYP+LA AA A+G TVM G++ELRVKESDR+ A+A GL+ | 367 |
| Sbjct | 308 | FGPLKGVTVPPERAASMIDEYPVLAALAATAQGTTVMRGVKELRVKESDRIDAMARGLEA | 367 |
| Query | 368 | NGVDCDEGETSLVVRGRPDGKGLGNASGAAVATHLDHRIAMSFLVMGLVSENPVTVDDAT | 427 |
| Sbjct | 368 | GV +E E SL V G G GA VA+HLDHRIAM+F+ +GLV+E P+ VDDA+ CGVRIEETEDSLTVHGMASVPG GATVASHLDHRIAMAFVCLGLVAEKPIRVDDAS | 422 |
| Query | 428 | MIATSFPEFMDLMAGLGAKI 447 IATSFP F LM GLGA++ |  |
| Sbjct | 423 | PIATSFPLFEGLMTGLGAQV 442 | |

>TR:J9A1B1 J9A1B1_9RHOB 3-phosphoshikimate 1-carboxyvinyltransferase OS=Rhodovulum sp. PH10 OX=1187851 GN=aroA PE=3 SV=1

Length=458

Score = 469 bits (1208), Expect = 1e-160

| Identities | = 252/439 (57%), Positives = 307/439 (70%), Gaps = 7/439 (2%) |  |
| --- | --- | --- |
| Query 11 | TARKSSGLSGTVRIPGDKSISHRSFMFGGLASGETRITGLLEGEDVINTGKAMQAMGARI | 70 |
| Sbjct 24 | TAR L GTVR+PGDKSISHR+ + G L G T ITGLLEG+DV+ T +AM+ +GA + TARAGGPLRGTVRVPGDKSISHRALILGALTVGRTVITGLLEGDDVLRTVQAMRLLGATV | 83 |
| Query 71 | RKEGD-TWIIDGVGNGGLLAPEAPLDFGNAATGCRLTMGLVGVYDFDSTFIGDASLTKRP | 129 |
| Sbjct 84 | + G W ++GVG GG AP LDFGN+ TGCRL MG V +TF GDASL +RP TRTGPGAWTVEGVGVGGFAAPPETLDFGNSGTGCRLVMGAVAGSPVTATFDGDASLRRRP | 143 |
| Query 130 | MGRVLNPLREMGVQV-KSEDGDRLPVTLRGPKTPTPITYRVPMASAQVKSAVLLAGLNTP | 188 |
| Sbjct 144 | M R+L+P+ MG +V + +GDRLP+ L G + P P Y P+ SAQVKSAVLLAGL+ P MRRILDPILRMGAEVLAAAEGDRLPIRLAGARDPIPTLYETPVPSAQVKSAVLLAGLSAP | 203 |
| Query 189 | GITTVIEPIMTRDHTEKMLQGFGANLTVETDADGVRTIRLEGRGKLTGQVIDVPGDPSST | 248 |
| Sbjct 204 | G T V+E TRDHTE+ML FGA++ VE D R I L G+ +L+ + VP DPSS GETIVVEREATRDHTERMLAHFGASVRVEPDGPHGRRITLTGQPELSPAPVAVPADPSSA | 263 |
| Query 249 | AFPLVAALLVPGSDVTILNVLMNPTRTGLILTLQEMGADIEVINPRLAGGEDVADLRVRS | 308 |
| Sbjct 264 | AFPLVAAL+VPGSD+ + V+ NP RTGL TL EMGA IE + R GGE++AD RV AFPLVAALIVPGSDLVLSGVMTNPLRTGLFATLTEMGASIEPVAVRRDGGEEMADFRVEF | 323 |
| Query 309 | STLKGVTVPEDRAPSMIDEYPILAVAAAFAEGATVMNGLEELRVKESDRLSAVANGLKLN | 368 |
| Sbjct 324 | STL+GV VP RAP+MIDEYP+LAVAAAFAEG TVM GL+ELRVKESDRL+A A+ L++N STLRGVEVPAARAPAMIDEYPVLAVAAAFAEGRTVMRGLKELRVKESDRLAATADLLRVN | 383 |
| Query 369 | GVDCDEGETSLVVRGRPDGKGLGNASGAAVATHLDHRIAMSFLVMGLVSENPVTVDDATM | 428 |
| Sbjct 384 | GV + L+V GR G G VATH+DHR+AMS LVMGL SE PVT+DDA GVAVEIDGDDLIVEGRGRAPG GGLVATHMDHRLAMSALVMGLASEKPVTIDDAGF | 438 |
| Query 429  Sbjct 439 | IATSFPEFMDLMAGLGAKI 447 IATSFP F+D M LGA+I IATSFPGFLDTMKNLGAEI 457 |  |

>TR:A5V951 A5V951_SPHWW 3-phosphoshikimate 1-carboxyvinyltransferase OS=Sphingomonas wittichii (strain RW1 / DSM 6014 / JCM 10273) OX=392499

GN=aroA PE=3 SV=1

Length=479

Score = 470 bits (1210), Expect = 1e-160

| Identities | = 259/448 (58%), Positives = 306/448 (68%), Gaps = 7/448 (2%) |  |
| --- | --- | --- |
| Query 2 | LHGASSRPATARKSSGLSGTVRIPGDKSISHRSFMFGGLASGETRITGLLEGEDVINTGK | 61 |
| Sbjct 32 | +H +S RPA+ L G++ +PGDKSISHR+ M LA GE+R+ GLL GEDV+ T MHASSPRPASFSAPGPLRGSIAVPGDKSISHRALMLSALAIGESRVEGLLTGEDVLATAA | 91 |
| Query 62 | AMQAMGARI-RKEGDTWIIDGVGNGGLLAPEAPLDFGNAATGCRLTMGLVGVYDFDSTFI | 120 |
| Sbjct 92 | AM+AMGA I R + D W I GVG GGLL PE L+ GN+ T RL MGLV + +TF AMRAMGASIERGDDDVWRIHGVGVGGLLQPEGALEMGNSGTSTRLLMGLVASHAITATFT | 151 |
| Query 121 | GDASLTKRPMGRVLNPLREMGVQVKSEDGDR---LPVTLRGPKTPTPITYRVPMASAQVK | 177 |
| Sbjct 152 | GDASL+KRPM RV+ PL MG V + G + LP+ LRG PI YR+P+ASAQVK GDASLSKRPMNRVIEPLSLMGADVTATPGGKGGTLPLMLRGISPAVPIDYRLPVASAQVK | 211 |
| Query 178 | SAVLLAGLNTPGITTVIEPIMTRDHTEKMLQGFGANLTVETDADGVRTIRLEGRGKLTGQ | 237 |
| Sbjct 212 | SAVLLAGLNTPGIT VIEP+ TRDH+E+ML+GFGA L VE DADG R I + G +L Q SAVLLAGLNTPGITRVIEPVATRDHSERMLRGFGAELEVEQDADGARIISIRGEAELQPQ | 271 |
| Query 238 | VIDVPGDPSSTAFPLVAALLVPGSDVTILNVLMNPTRTGLILTLQEMGADIEVINPRLAG I VPGDPSS AFP+VAALLVPGS VTI NV +NPTR L L+ MG DIE NPR G | 297 |

| Sbjct | 272 | HIVVPGDPSSAAFPVVAALLVPGSAVTIANVGLNPTRAALFDVLRMMGGDIEFANPREVG | 331 |
| --- | --- | --- | --- |
| Query | 298 | GEDVADLRVRSSTLKGVTVPEDRAPSMIDEYPILAVAAAFAEGATVMNGLEELRVKESDR | 357 |
| Sbjct | 332 | GE VAD+ VR STLKG+ P + SM+DEYPIL VAAA AEG TV GLEELRVKESDR GEPVADIVVRHSTLKGIETPVETVASMVDEYPILFVAAALAEGRTVARGLEELRVKESDR | 391 |
| Query | 358 | LSAVANGLKLNGVDCDEGETSLVVRGRPDGKGLGNASGAAVATHLDHRIAMSFLVMGLVS | 417 |
| Sbjct | 392 | ++ +A GL+ G +E E L++ G G GA +A LDHRIAMSF V GLVS IAVMAEGLRAIGARVEELEDGLIIDGT---GGDPLPGGATIAARLDHRIAMSFAVAGLVS | 448 |
| Query  Sbjct | 418  449 | ENPVTVDDATMIATSFPEFMDLMAGLGA 445  + VT+DD +ATSFP F LM LGA KAAVTIDDMGPVATSFPGFTRLMGELGA 476 |  |

>TR:A0A143DCJ6 A0A143DCJ6_9PROT 3-phosphoshikimate 1-carboxyvinyltransferase OS=Haematospirillum jordaniae OX=1549855 GN=aroA PE=3 SV=1

Length=450

Score = 469 bits (1207), Expect = 1e-160

| Identities | | = 254/455 (56%), Positives = 315/455 (69%), Gaps = 14/455 (3%) |  |
| --- | --- | --- | --- |
| Query 2  Sbjct 1 | | LHGASSRPATARK--SSGLSGTVRIPGDKSISHRSFMFGGLASGETRITGLLEGEDVINT  + A +RP T R +S LSGT+R+PGDKSISHR+ M G ++ GET I GLLEGEDV+ T MSSAPARPVTLRSQAASPLSGTIRVPGDKSISHRALMLGAISVGETIIEGLLEGEDVLRT | 59  60 |
| Query 60  Sbjct 61 | | GKAMQAMGARIRKEGD-TWIIDGVGNGGLLAPEAPLDFGNAATGCRLTMGLVGVYDFDST AMQ +GA +RKE D WI+ G G GGL P+ LD GNA TG RL MGL+ + F +  ATAMQKLGATVRKESDGRWILSGRGVGGLAEPDDVLDMGNAGTGARLLMGLLSSHPFTAV | 118  120 |
| Query 119  Sbjct 121 | | FIGDASLTKRPMGRVLNPLREMGVQVKSEDGDRLPVTLRGPKTPTPITYRVPMASAQVKS GDASL RPM RV NPL + G + S RLPVT+ G + P PI Y +P+ASAQVKS LTGDASLRSRPMARVTNPLEKTGARFLSRTRGRLPVTITGTERPIPIDYTLPVASAQVKS | 178  180 |
| Query 179  Sbjct 181 | | AVLLAGLNTPGITTVIEPIMTRDHTEKMLQGFGANLTVETDADGVRTIRLEGRGKLTGQV A+LLAGLNTPG T ++EP +RDHTE+ML+ FGA++ VE + G R I L G +LTGQ AILLAGLNTPGQTRIVEPSPSRDHTERMLKAFGADIQVEENGAG-RIITLTGHPELTGQT | 238  239 |
| Query 239  Sbjct 240 | | IDVPGDPSSTAFPLVAALLVPGSDVTILNVLMNPTRTGLILTLQEMGADIEVINPRLAGG  + VP DPSS AFP+ AAL+VPGS +T+ NV +NP+R GL TLQEMGADI N R G VRVPADPSSAAFPVAAALIVPGSHLTLSNVCINPSRIGLYKTLQEMGADIHFENVRDENG | 298  299 |
| Query 299  Sbjct 300 | | EDVADLRVRSSTLKGVTVPEDRAPSMIDEYPILAVAAAFAEGATVMNGLEELRVKESDRL E VADL VR S LKG+ VP +RAPSMIDEYPIL+V AAFA G T MNGL ELRVKESDRL EPVADLVVRHSPLKGINVPAERAPSMIDEYPILSVIAAFASGETRMNGLGELRVKESDRL | 358  359 |
| Query 359  Sbjct 360 | | SAVANGLKLNGVDCDEGETSLVVRG---RPDGKGLGNASGAAVATHLDHRIAMSFLVMGL  + + +GL+ GV+ L+++G +P+G G +A LDHRI MSFLV+G+ ALMTDGLQSCGVNARTEGDDLIIQGTGKQPEG GEMIAVQLDHRIGMSFLVLGM | 415  412 |
| Query 416 | | VSENPVTVDDATMIATSFPEFMDLMAGLGAKIELS 450  + PV+VDDA I TSFP+F +LM LGA+I L+ |  |
| Sbjct | 413 | ATRTPVSVDDARAIDTSFPDFANLMNSLGARITLA 447 | |

>TR:Q2CFP5 Q2CFP5_OCEGH 3-phosphoshikimate 1-carboxyvinyltransferase OS=Oceanicola granulosus (strain ATCC BAA-861 / DSM 15982 / KCTC

12143 / HTCC2516) OX=314256 GN=aroA PE=3 SV=1

Length=451

Score = 469 bits (1207), Expect = 1e-160

Identities = 255/449 (57%), Positives = 306/449 (68%), Gaps = 7/449 (2%) Query 1	MLHGASSRPATARKSSGLSGTVRIPGDKSISHRSFMFGGLASGETRITGLLEGEDVINTG 60

| Sbjct | 1 | M A+ P TARKS L+G ++PGDKSISHRS + G LA GET ITGLL+GEDV++T  MSRHATPVPMTARKSGPLTGEAQVPGDKSISHRSLILGALAVGETTITGLLDGEDVLDTA | 60 |
| --- | --- | --- | --- |
| Query | 61 | KAMQAMGARIRKEGDTWIIDGVGNGGLLAPEAPLDFGNAATGCRLTMGLVGVYDFDSTFI | 120 |
| Sbjct | 61 | AM+A GA I + G W + GVG GG P +D GN+ TG RL MG + +TF AAMRAFGAEITEAGGVWTVHGVGTGGFAEPADVIDCGNSGTGVRLIMGSMATTPITATFT | 120 |
| Query | 121 | GDASLTKRPMGRVLNPLREMGVQVKSEDGDRLPVTLRGPKTPTPITYRVPMASAQVKSAV | 180 |
| Sbjct | 121 | GDASL RPMGRV +PL G + G RLP+TL G P P+ Y VP+ SAQVKSAV GDASLRSRPMGRVTDPLAAFGARAHGRQGGRLPLTLVGAADPVPVRYAVPVPSAQVKSAV | 180 |
| Query | 181 | LLAGLNTPGITTVIEPIMTRDHTEKMLQGFGANLTVETDADGVRTIRLEGRGKLTGQVID | 240 |
| Sbjct | 181 | LLAGLN PG T VIE TRDHTE+ML GFGA +T ET +G R I L G+ +L+ Q I LLAGLNAPGETVVIEREATRDHTERMLAGFGARITTETTNEG-RVITLTGQPELSPQTIT | 239 |
| Query | 241 | VPGDPSSTAFPLVAALLVPGSDVTILNVLMNPTRTGLILTLQEMGADIEVINPRLAGGED | 300 |
| Sbjct | 240 | VP DPSS AFP+ AAL+ GSDV + N+ +NPTR GL TL++MGAD+ N R GGE VPRDPSSAAFPVCAALVTEGSDVLVPNIGLNPTRAGLFETLRDMGADLTYENLREEGGEP | 299 |
| Query | 301 | VADLRVR-SSTLKGVTVPEDRAPSMIDEYPILAVAAAFAEGATVMNGLEELRVKESDRLS | 359 |
| Sbjct | 300 | VADLR R S L+G+ VP +RA SMIDEYP+L+V AAFAEG TVM G++ELRVKESDR+ VADLRARFSPDLRGIEVPPERAASMIDEYPVLSVVAAFAEGRTVMRGVKELRVKESDRID | 359 |
| Query | 360 | AVANGLKLNGVDCDEGETSLVVRGRPDGKGLGNA-SGAAVATHLDHRIAMSFLVMGLVSE | 418 |
| Sbjct | 360 | A+A GL+ GV+ +EGE +V GR G G+ G V + LDHRIAMSFL++GL S AMAQGLRAAGVEVEEGEDWWIVTGR GFGDVPGGVTVESRLDHRIAMSFLILGLASN | 415 |
| Query | 419 | NPVTVDDATMIATSFPEFMDLMAGLGAKI 447 PVTVDDA IATSFP F LM GLGA I |  |
| Sbjct | 416 | RPVTVDDAAPIATSFPVFEPLMTGLGADI 444 | |

>TR:A0A0N1C7H9 A0A0N1C7H9_9SPHN 3-phosphoshikimate 1-carboxyvinyltransferase OS=Novosphingobium sp. AAP83 OX=1523425 GN=aroA PE=3 SV=1

Length=441

Score = 469 bits (1206), Expect = 1e-160

| Identities | = 252/434 (58%), Positives = 299/434 (69%), Gaps = 1/434 (0%) |  |
| --- | --- | --- |
| Query 9 | PATARKSSGLSGTVRIPGDKSISHRSFMFGGLASGETRITGLLEGEDVINTGKAMQAMGA | 68 |
| Sbjct 3 | P S L G +R+PGDKSISHRS M G LA GETR+TGLLEGEDV++T AM+AMGA PRRFSASGPLRGRIRVPGDKSISHRSIMLGALAVGETRVTGLLEGEDVLSTAAAMRAMGA | 62 |
| Query 69 | RIRKEGD-TWIIDGVGNGGLLAPEAPLDFGNAATGCRLTMGLVGVYDFDSTFIGDASLTK | 127 |
| Sbjct 63 | I ++ D W + GVG GGLL P+ LD GN+ T RL MGLV + +TFIGDASL+K TITRDEDGMWHVHGVGVGGLLQPQQALDMGNSGTSTRLLMGLVASHPITATFIGDASLSK | 122 |
| Query 128 | RPMGRVLNPLREMGVQVKSEDGDRLPVTLRGPKTPTPITYRVPMASAQVKSAVLLAGLNT | 187 |
| Sbjct 123 | RPMGRV++PL MG + + G RLP+TLRG PI YR+P+ASAQVKSA+LLAGLNT RPMGRVIDPLSTMGAEFTASPGGRLPLTLRGISPAVPIEYRLPVASAQVKSAILLAGLNT | 182 |
| Query 188 | PGITTVIEPIMTRDHTEKMLQGFGANLTVETDADGVRTIRLEGRGKLTGQVIDVPGDPSS | 247 |
| Sbjct 183 | PGITTVIEPI TRDH+E+ML+GFGA LTVE ADG R IR+ G +L Q I VPGDPSS PGITTVIEPIPTRDHSERMLRGFGAELTVEIAADGARVIRVRGEAELKPQTITVPGDPSS | 242 |
| Query 248 | TAFPLVAALLVPGSDVTILNVLMNPTRTGLILTLQEMGADIEVINPRLAGGEDVADLRVR | 307 |
| Sbjct 243 | AF ++AALLV GSD+ + NV +NPTR L L+ MG IE ++ R GGE VADLRVR AAFFVIAALLVEGSDLVVENVGLNPTRAALFDVLRLMGGSIEELDRRDVGGEPVADLRVR | 302 |
| Query 308 | SSTLKGVTVPEDRAPSMIDEYPILAVAAAFAEGATVMNGLEELRVKESDRLSAVANGLKL | 367 |
| Sbjct 303 | S L G+ V PSM+DE+P+L VAAA A+G TV GLEELRVKESDR+SA+ L L HSALTGIAVDPAVVPSMVDEFPVLFVAAALAKGRTVTTGLEELRVKESDRISAMHAALSL | 362 |
| Query 368 | NGVDCDEGETSLVVRGRPDGKGLGNASGAAVATHLDHRIAMSFLVMGLVSENPVTVDDAT | 427 |

G	E E L++ G	G	G AV THLDHRIAM+ V GL S	+ VDD

| Sbjct | 363 | AGATVTETEDGLIIDGTGGDPLPGTPEGQAVVTHLDHRIAMALAVAGLASRQGIEVDDTR 422 |
| --- | --- | --- |
| Query | 428 | MIATSFPEFMDLMA 441 |
| Sbjct | 423 | IATSFP F L+A PIATSFPVFESLLA 436 |

>TR:A0A1G6IPI3 A0A1G6IPI3_9SPHN 3-phosphoshikimate 1-carboxyvinyltransferase OS=Sphingomonas sp. YR710 OX=1882773 GN=aroA PE=3 SV=1

Length=444

Score = 469 bits (1206), Expect = 2e-160

| Identities | = 256/447 (57%), Positives = 307/447 (69%), Gaps = 5/447 (1%) |  |
| --- | --- | --- |
| Query 2 | LHGASSRPATARKSSGLSGTVRIPGDKSISHRSFMFGGLASGETRITGLLEGEDVINTGK | 61 |
| Sbjct 1 | +H +S RP + LSGT +PGDKSISHRS M LA GE+ + GLLEGEDV+ T MHISSPRPLAFTAPAALSGTATVPGDKSISHRSLMLSALAVGESVVEGLLEGEDVLATAA | 60 |
| Query 62 | AMQAMGARIRKEGD-TWIIDGVGNGGLLAPEAPLDFGNAATGCRLTMGLVGVYDFDSTFI | 120 |
| Sbjct 61 | AM+AMGA I++ D W I GVG GGLL P+A LD GN+ T RL MGLV + + F AMRAMGADIQRAADGRWRIYGVGVGGLLQPQAALDMGNSGTSTRLLMGLVASHAITAAFT | 120 |
| Query 121 | GDASLTKRPMGRVLNPLREMGVQVKSEDGDRLPVTLRGPKTPTPITYRVPMASAQVKSAV | 180 |
| Sbjct 121 | GDASL++RPM RV+ PL MG + S G RLP+ LRG PI YR+P+ASAQVKSA+ GDASLSRRPMNRVIEPLSLMGAEFTSSPGGRLPLMLRGIAPAVPIEYRLPVASAQVKSAI | 180 |
| Query 181 | LLAGLNTPGITTVIEPIMTRDHTEKMLQGFGANLTVETDADGVRTIRLEGRGKLTGQVID | 240 |
| Sbjct 181 | LLAGLNTPGIT VIEP+ TRDH+E+ML GFGA+L VE D R I + G +L Q I LLAGLNTPGITRVIEPVPTRDHSERMLSGFGADLAVELDGT-TRVISIRGEAELRPQNIV | 239 |
| Query 241 | VPGDPSSTAFPLVAALLVPGSDVTILNVLMNPTRTGLILTLQEMGADIEVINPRLAGGED | 300 |
| Sbjct 240 | VPGDPSS AFP+VA LLVPGS +TI NV +NPTR L L+ MG DIE N R GGE VPGDPSSAAFPVVAGLLVPGSAITITNVGLNPTRAALFDVLRMMGGDIEFANARTVGGEP | 299 |
| Query 301 | VADLRVRSSTLKGVTVPEDRAPSMIDEYPILAVAAAFAEGATVMNGLEELRVKESDRLSA | 360 |
| Sbjct 300 | VADL VR+S +KG+ VP + PSM+DE+PIL VAAA AEG T+ GLEELRVKESDRL+ VADLVVRASAMKGIEVPIECVPSMVDEFPILFVAAALAEGRTIARGLEELRVKESDRLAT | 359 |
| Query 361 | VANGLKLNGVDCDEGETSLVVRGRPDGKGLGNASGAAVATHLDHRIAMSFLVMGLVSENP | 420 |
| Sbjct 360 | +A GL+ G +E E L++ G G A GA +AT LDHRIAMSF V GLVS+ MAEGLRAIGARVEELEDGLIIDGT---GGAPLAGGATIATKLDHRIAMSFAVAGLVSKAR | 416 |
| Query 421  Sbjct 417 | VTVDDATMIATSFPEFMDLMAGLGAKI 447 V +DD T +ATSFP F++LM GLG I VEIDDMTPVATSFPGFVELMTGLGGDI 443 |  |

>TR:A0A1I3JQY6 A0A1I3JQY6_9RHOB 3-phosphoshikimate 1-carboxyvinyltransferase OS=Jannaschia pohangensis OX=390807 GN=aroA PE=3 SV=1

Length=445

Score = 469 bits (1206), Expect = 2e-160

Identities = 255/449 (57%), Positives = 309/449 (69%), Gaps = 8/449 (2%)

| Query | 1 | MLHGASSRPATARKSSGLSGTVRIPGDKSISHRSFMFGGLASGETRITGLLEGEDVINTG | 60 |
| --- | --- | --- | --- |
| Sbjct | 1 | M HG ++ P TAR S L+G +PGDKSISHRS + G +A GETR+TGLLEG+DV++TG MSHGPAT-PLTARASGPLTGIADVPGDKSISHRSLILGAMAVGETRVTGLLEGQDVLDTG | 59 |
| Query | 61 | KAMQAMGARIRKEGDTWIIDGVGNGGLLAPEAPLDFGNAATGCRLTMGLVGVYDFDSTFI | 120 |
| Sbjct | 60 | KAMQA GA + ++GDTW++ GVG GGL+ PE +D GN+ TG RL MG +DF T+ KAMQAFGATVTRDGDTWVVRGVGVGGLMEPEDVIDCGNSGTGVRLLMGAAATHDFAVTYT | 119 |
| Query | 121 | GDASLTKRPMGRVLNPLREMGVQVKSEDGDRLPVTLRGPKTPTPITYRVPMASAQVKSAV | 180 |

| Sbjct | 120 | GDASL KRPM RV +PL G G RLP+T+ G P P+ Y P+ASAQVKSAV  GDASLRKRPMARVTDPLALFGAVAHGRSGGRLPMTIVGAADPVPVRYATPVASAQVKSAV | 179 |
| --- | --- | --- | --- |
| Query | 181 | LLAGLNTPGITTVIEPIMTRDHTEKMLQGFGANLTVETDADGVRTIRLEGRGKLTGQVID | 240 |
| Sbjct | 180 | LLAGLN PG T VIE TRDH+E+ML+GFGA + +G R I L GR +L Q I LLAGLNAPGETVVIEREATRDHSERMLRGFGAQIETRDTPEG-REITLLGRPELRPQTIV | 238 |
| Query | 241 | VPGDPSSTAFPLVAALLVPGSDVTILNVLMNPTRTGLILTLQEMGADIEVINPRLAGGED | 300 |
| Sbjct | 239 | VP DPSS AFP+ AAL+VPGSDV + N+ +NPTR GL TL++MGAD+ NPR GGE VPRDPSSAAFPVCAALIVPGSDVLVPNIGLNPTRAGLFQTLRDMGADLTYENPREEGGEP | 298 |
| Query | 301 | VADLRVR-SSTLKGVTVPEDRAPSMIDEYPILAVAAAFAEGATVMNGLEELRVKESDRLS | 359 |
| Sbjct | 299 | VADLR R S ++G+ VP DRA SMIDEYPIL+V AA A G T+M G+ ELRVKE DR+ VADLRARFSPDMQGIEVPPDRAASMIDEYPILSVVAANATGTTIMRGVAELRVKECDRID | 358 |
| Query | 360 | AVANGLKLNGVDCDEGETSLVVRGRPDGKGLGNASGAA-VATHLDHRIAMSFLVMGLVSE | 418 |
| Sbjct | 359 | A+A GL+ GV +GE V DG+G G +G+A VA HLDHRIAMSFL GL S AMATGLRDCGVTVRDGEDWWEV DGRGPGGVTGSATVAVHLDHRIAMSFLCAGLASA | 414 |
| Query | 419 | NPVTVDDATMIATSFPEFMDLMAGLGAKI 447  +TVDDA IATSFP F LM LGA + |  |
| Sbjct | 415 | QAITVDDAGPIATSFPIFRPLMTALGATL 443 | |

>TR:A0A501XLH4 A0A501XLH4_9SPHN 3-phosphoshikimate 1-carboxyvinyltransferase OS=Sandaracinobacter sp. PAMC 28131 OX=1715348 GN=aroA PE=3

SV=1

Length=447

Score = 469 bits (1206), Expect = 2e-160

| Identities | | = 248/438 (57%), Positives = 309/438 (71%), Gaps = 4/438 (1%) |  |
| --- | --- | --- | --- |
| Query 9  Sbjct 10 | | PATARKSSGLSGTVRIPGDKSISHRSFMFGGLASGETRITGLLEGEDVINTGKAMQAMGA P + + L+G VR+PGDKSISHR+ M LA G + + GLLEGEDV+ T AM+AMGA PLSLSAPAPLAGEVRVPGDKSISHRAIMLSALAVGRSEVRGLLEGEDVLATAAAMRAMGA | 68  69 |
| Query 69  Sbjct 70 | | RIRKEGDT-WIIDGVGNGGLLAPEAPLDFGNAATGCRLTMGLVGVYDFDSTFIGDASLTK  ++ + G W++DGVG GGLL P+A LD GN+ T RL MGLV + +TF+GDASL++ QVERLGQGHWVVDGVGVGGLLQPDAQLDMGNSGTSTRLLMGLVSSHPITATFVGDASLSR | 127  129 |
| Query 128  Sbjct 130 | | RPMGRVLNPLREMGVQVKSEDGDRLPVTLRGPKTPTPITYRVPMASAQVKSAVLLAGLNT RPMGRV+ PL +G ++ + RLP+ LRG TP PI YR+P+ASAQVKSA+LLAGLN RPMGRVIEPLSRIGAEITASPDGRLPLMLRGADTPLPIHYRLPVASAQVKSAILLAGLNI | 187  189 |
| Query 188  Sbjct 190 | | PGITTVIEPIMTRDHTEKMLQGFGANLTVETDADGVRTIRLEGRGKLTGQVIDVPGDPSS T V EP+ TRDH+E+ML+GFGA L+VE ADG + I + G +L Q ++VPGDPSS GAETVVEEPVPTRDHSERMLKGFGAELSVEPMADGGKRIAVRGWAELKPQTLEVPGDPSS | 247  249 |
| Query 248  Sbjct 250 | | TAFPLVAALLVPGSDVTILNVLMNPTRTGLILTLQEMGADIEVINPRLAGGEDVADLRVR F LVAAL+VPGS++TI V +NPTR GL+ LQEMGADI +N R GGE VADL V  AGFLLVAALIVPGSNITIHGVGLNPTRDGLVRVLQEMGADITKLNEREVGGEPVADLLVT | 307  309 |
| Query 308  Sbjct 310 | | SSTLKGVTVPEDRAPSMIDEYPILAVAAAFAEGATVMNGLEELRVKESDRLSAVANGLKL S L G+ VP + APSMIDEYPIL VAAA A G TVM G+EELRVKESDR++A+A GL+ HSALTGIEVPPEVAPSMIDEYPILFVAAALASGRTVMRGIEELRVKESDRIAAMAAGLRA | 367  369 |
| Query 368  Sbjct 370 | | NGVDCDEGETSLVVRGRPDGKGLGNASGAAVATHLDHRIAMSFLVMGLVSENPVTVDDAT GV +E L+V G K G G VATHLDHRIAMSF V+ L ++ +TVDD  AGVAVEELPDGLIVEGSGGEKVAG---GCTVATHLDHRIAMSFAVLSLAAKTAITVDDRA | 427  426 |
| Query 428 | | MIATSFPEFMDLMAGLGA 445 IATSFP+F+ LM LGA |  |
| Sbjct | 427 | PIATSFPDFLPLMQRLGA 444 | |

>TR:A0A4V3DEL1 A0A4V3DEL1_9PROT 3-phosphoshikimate 1-carboxyvinyltransferase OS=Dongia mobilis OX=578943 GN=aroA PE=3 SV=1

Length=479

Score = 470 bits (1209), Expect = 2e-160

| Identities | | = 258/450 (57%), Positives = 306/450 (68%), Gaps = 6/450 (1%) |  |
| --- | --- | --- | --- |
| Query 3  Sbjct 34 | | HGASSRPATARKSSGLSGTVRIPGDKSISHRSFMFGGLASGETRITGLLEGEDVINTGKA HGA RP T+ +GL G +R+PGDKSISHR+ M G LA GET I GLLEGEDV+ T A HGAIPRPLTSGTGTGLKGRIRVPGDKSISHRALMLGALALGETEIHGLLEGEDVLRTAAA | 62  93 |
| Query 63 | | MQAMGARIRKEGD-TWIIDGVGNGGLLAPEAPLDFGNAATGCRLTMGLVGVYDFDSTFIG M+ +G K D W G GNGGL LD GNA TG RL MGLV Y + F G | 121 |
| Sbjct | 94 | MRQLGGSAEKGADGIWRARGRGNGGLAEASDVLDLGNAGTGTRLLMGLVAAYPMTTFFTG | 153 |
| Query | 122 | DASLTKRPMGRVLNPLREMGVQVKSEDGDRLPVTLRGPKTPTPITYRVPMASAQVKSAVL | 181 |
| Sbjct | 154 | DASL RPM RV +PLR+MG + R P+ + G P PITY++P+ASAQVKSA+L DASLRSRPMARVADPLRQMGASFVTRSNGRPPLAVIGTANPVPITYKLPVASAQVKSAIL | 213 |
| Query | 182 | LAGLNTPGITTVIEPIMTRDHTEKMLQGFGANLTVETDADGVRTIRLEGRGKLTGQVIDV | 241 |
| Sbjct | 214 | LAGLNTPG TTVIEP TRDHTE ML+GFG ++ VE +G R + G+ +L G+ I V LAGLNTPGNTTVIEPEHTRDHTELMLRGFGVDIRVEETPEG-RASSITGQPELKGRRIIV | 272 |
| Query | 242 | PGDPSSTAFPLVAALLVPGSDVTILNVLMNPTRTGLILTLQEMGADIEVINPRLAGGEDV | 301 |
| Sbjct | 273 | PGDPSS AFP+VAAL++PGSD+ I NV +NP R GLI +L EMG IE+ N R+ GE V PGDPSSAAFPIVAALILPGSDIVIENVGLNPHRIGLIDSLLEMGGAIEIQNARVEAGEKV | 332 |
| Query | 302 | ADLRVRSSTLKGVTVPEDRAPSMIDEYPILAVAAAFAEGATVMNGLEELRVKESDRLSAV | 361 |
| Sbjct | 333 | DL VR S L+G+TVP RAPSMIDEYPIL+VAAAFAEG T M GL ELRVKESDRL A+ GDLHVRHSHLRGITVPASRAPSMIDEYPILSVAAAFAEGETRMLGLAELRVKESDRLGAM | 392 |
| Query | 362 | ANGLKLNGVDCDEGETSLVVRGRPDGKGLGNASGAAVATHLDHRIAMSFLVMGLVSENPV | 421 |
| Sbjct | 393 | A GL GV +EGE L V G G GA +A +LDHR AMSFLV+GL +E PV ARGLAACGVSVEEGEDWLGV TGNGKRPRGGAHIAVNLDHRPAMSFLVLGLGAEKPV | 448 |
| Query  Sbjct | 422  449 | TVDDATMIATSFPEFMDLMAGLGAKIELSD 451  ++DD + I TSFP F DLM G+GA I L + SIDDGSPIDTSFPGFSDLMNGMGAAIRLQN 478 |  |

>TR:A0A162HPC4 A0A162HPC4_9SPHN 3-phosphoshikimate 1-carboxyvinyltransferase OS=Erythrobacter sp. HI00D59 OX=1822215 GN=aroA PE=3 SV=1

Length=429

Score = 468 bits (1204), Expect = 2e-160

| Identities | = 246/424 (58%), Positives = 304/424 (72%), Gaps = 4/424 (1%) |  |
| --- | --- | --- |
| Query 18 | LSGTVRIPGDKSISHRSFMFGGLASGETRITGLLEGEDVINTGKAMQAMGARIRKEGDTW | 77 |
| Sbjct 6 | L+G +R+PGDKSISHR+ MFG LA G +RI GLLEGEDV+ T A++AMGA I ++GD W LTGRIRVPGDKSISHRALMFGALAVGRSRIKGLLEGEDVLATAAALRAMGAAIARDGDEW | 65 |
| Query 78 | IIDGVGNGGLLAPEAPLDFGNAATGCRLTMGLVGVYDFDSTFIGDASLTKRPMGRVLNPL | 137 |
| Sbjct 66 | I+DGVG GGLL P LD GN+ T RL MGL+ + + F GDASL++RPMGRV++PL IVDGVGVGGLLQPPQALDMGNSGTSTRLLMGLLASHGITAAFTGDASLSRRPMGRVIDPL | 125 |
| Query 138 | REMGVQVKSEDGDRLPVTLRGPKTPTPITYRVPMASAQVKSAVLLAGLNTPGITTVIEPI | 197 |
| Sbjct 126 | MG G LP+ + G + PI+YR+P+ASAQVKSAVLLAGLNTPGIT+VIEP+ STMGASFTPSPGGTLPLMMEGMQPAVPISYRLPVASAQVKSAVLLAGLNTPGITSVIEPV | 185 |
| Query 198 | MTRDHTEKMLQGFGANLTVETDADGVRTIRLEGRGKLTGQVIDVPGDPSSTAFPLVAALL | 257 |
| Sbjct 186 | TRDHTE+ML+GFGA LTVE + +G R IR++G L IDVPGDPSS AF VAA +  PTRDHTERMLRGFGAELTVE-EENGERIIRIQGPADLKPCDIDVPGDPSSAAFFAVAASI | 244 |

| Query | 258 | VPGSDVTILNVLMNPTRTGLILTLQEMGADIEVINPRLAGGEDVADLRVRSSTLKGVTVP | 317 |
| --- | --- | --- | --- |
| Sbjct | 245 | VPGSD+ I NV +NPTR G+ L++MGA IE ++PR GGE VADLR+R + L G+ V VPGSDLVIENVGLNPTRDGIFRVLEQMGAAIERLDPREVGGEPVADLRIRHAPLNGIEVD | 304 |
| Query | 318 | EDRAPSMIDEYPILAVAAAFAEGATVMNGLEELRVKESDRLSAVANGLKLNGVDCDEGET | 377 |
| Sbjct | 305 | APSMIDE+P+L VAAA A+G TV GLEELRVKESDRL+A+A+ L++ G +E E SGIAPSMIDEFPVLFVAAALAKGTTVTRGLEELRVKESDRLAAMASALRVAGARLEENED | 364 |
| Query | 378 | SLVVRGRPDGKGLGNASGAAVATHLDHRIAMSFLVMGLVSENPVTVDDATMIATSFPEFM | 437 |
| Sbjct | 365 | L + G G A G VATHLDHRIAMS V G+ SE V +D A IATSFP F+ GLTIEG---SGGTPLAGGGIVATHLDHRIAMSMAVAGIASEAGVEIDSAEPIATSFPNFL | 421 |
| Query  Sbjct | 438  422 | DLMA 441 DL+A  DLLA 425 |  |

>TR:A0A4Q3Y7Y1 A0A4Q3Y7Y1_9RHOB 3-phosphoshikimate 1-carboxyvinyltransferase OS=Loktanella sp. IMCC34160 OX=2510646 GN=aroA PE=3 SV=1

Length=450

Score = 469 bits (1206), Expect = 2e-160

| Identities | | = 255/442 (58%), Positives = 306/442 (69%), Gaps = 8/442 (2%) |  |
| --- | --- | --- | --- |
| Query 9  Sbjct 9 | | PATARKSSGLSGTVRIPGDKSISHRSFMFGGLASGETRITGLLEGEDVINTGKAMQAMGA P T+RK L+G R+PGDKSISHRS + G L+ GETRI+GLLEG+DV++T KAM+A GA PMTSRKCGPLTGEARVPGDKSISHRSLILGALSVGETRISGLLEGQDVLDTAKAMRAFGA | 68  68 |
| Query 69  Sbjct 69 | | RIRKEGD-TWIIDGVGNGGLLAPEAPLDFGNAATGCRLTMGLVGVYDFDSTFIGDASLTK  + G+ W ++GVG GG PE +D GN+ TG RL MG + +TF GDASL EVTDHGNGEWTVNGVGVGGFAEPEQVIDCGNSGTGVRLIMGCMATSPITATFTGDASLNS | 127  128 |
| Query 128  Sbjct 129 | | RPMGRVLNPLREMGVQVKSEDGDRLPVTLRGPKTPTPITYRVPMASAQVKSAVLLAGLNT RPMGRV +PL G Q G RLP+T+ G P P+TY VP+ SAQVKSAVLLAGLN RPMGRVTDPLALFGAQAFGRTGGRLPMTIVGAADPVPVTYTVPVPSAQVKSAVLLAGLNA | 187  188 |
| Query 188  Sbjct 189 | | PGITTVIEPIMTRDHTEKMLQGFGANLTVETDADGVRTIRLEGRGKLTGQVIDVPGDPSS PG T VIE TRDHTE+ML GFGA LTVE + D R I L G+ +L Q I VP DPSS PGQTVVIEKEATRDHTERMLVGFGAELTVE-ETDAGRVITLTGQPELKPQAIVVPRDPSS | 247  247 |
| Query 248  Sbjct 248 | | TAFPLVAALLVPGSDVTILNVLMNPTRTGLILTLQEMGADIEVINPRLAGGEDVADLRVR AFP+ AAL+ GSDV + N+ +NPTR GL TL+EMGAD+E N R GGE VADLR + AAFPVCAALITEGSDVLVPNIGLNPTRAGLFTTLREMGADLEYENLREEGGEPVADLRAK | 307  307 |
| Query 308  Sbjct 308 | | -SSTLKGVTVPEDRAPSMIDEYPILAVAAAFAEGATVMNGLEELRVKESDRLSAVANGLK S LKGV VP +RA SMIDEYP+L+V AAFAEG TVM G++ELRVKESDR+ A+A GL+ FSPNLKGVEVPPERAASMIDEYPVLSVVAAFAEGQTVMKGVKELRVKESDRIDAMAKGLR | 366  367 |
| Query 367  Sbjct 368 | | LNGVDCDEGETSLVVRGRPDGKGLGNA-SGAAVATHLDHRIAMSFLVMGLVSENPVTVDD GV DEGE +V GR G GN G V + LDHRIAMSF+VMG+ +E P+ VDD AAGVTVDEGEDWWIVTGR GFGNVPGGVTVESRLDHRIAMSFMVMGMATEKPMRVDD | 425  423 |
| Query 426 | | ATMIATSFPEFMDLMAGLGAKI 447 IATSFP F LMA LGA++ |  |
| Sbjct | 424 | GGPIATSFPIFEPLMASLGAQV 445 | |

>TR:A0A1M7EI62 A0A1M7EI62_9RHOB 3-phosphoshikimate 1-carboxyvinyltransferase OS=Paracoccus solventivorans OX=53463 GN=aroA PE=3 SV=1

Length=443

Score = 468 bits (1205), Expect = 2e-160

Identities = 252/448 (56%), Positives = 306/448 (68%), Gaps = 7/448 (2%)

| Query | 1 | MLHGASSRPATARKSSGLSGTVRIPGDKSISHRSFMFGGLASGETRITGLLEGEDVINTG | 60 |
| --- | --- | --- | --- |
| Sbjct | 1 | M H +P TAR+S L G +PGDKS+SHR+ + G LA GETRITGLLE +DVI+T MSHSTQPQPMTARRSGPLKGNALVPGDKSVSHRALILGALAVGETRITGLLESQDVIDTA | 60 |
| Query | 61 | KAMQAMGARIRKEG-DTWIIDGVGNGGLLAPEAPLDFGNAATGCRLTMGLVGVYDFDSTF | 119 |
| Sbjct | 61 | +AM+A GA + + G W + GVG GG +PE +D GN+ TG RL MG + +TF RAMEAFGATVERVGAGEWRVQGVGVGGFSSPETVIDCGNSGTGVRLIMGAMATTPITATF | 120 |
| Query | 120 | IGDASLTKRPMGRVLNPLREMGVQVKSEDGDRLPVTLRGPKTPTPITYRVPMASAQVKSA | 179 |
| Sbjct | 121 | GDASL++RPM R+ PL G QV + +G+ LPVT+ G P P+ YR P+ASAQ+KSA TGDASLSRRPMRRITGPLELFGAQVTAREGEFLPVTITGAADPLPLRYRTPVASAQIKSA | 180 |
| Query | 180 | VLLAGLNTPGITTVIEPIMTRDHTEKMLQGFGANLTVETDADGVRTIRLEGRGKLTGQVI | 239 |
| Sbjct | 181 | VLLAGLN PG T VIE TRDHTE+ML GFGA + +G I L GR +L + VLLAGLNAPGETVVIEAEPTRDHTERMLAGFGATVRTAITDEG-HVITLTGRPELRPCAV | 239 |
| Query | 240 | DVPGDPSSTAFPLVAALLVPGSDVTILNVLMNPTRTGLILTLQEMGADIEVINPRLAGGE | 299 |
| Sbjct | 240 | VP DPSS AFP+ AAL+VPGS++ + V NPTR GL L EMGAD+ N R+ GGE AVPRDPSSAAFPVAAALIVPGSEIRVEGVSRNPTRDGLYHVLAEMGADLRWENDRIEGGE | 299 |
| Query | 300 | DVADLRVRSSTLKGVTVPEDRAPSMIDEYPILAVAAAFAEGATVMNGLEELRVKESDRLS | 359 |
| Sbjct | 300 | VADL VR L GVTV +RA SMIDE+PIL+V AAFAEG+TVMNG+ ELRVKESDR+ PVADLTVRHGPLHGVTVAPERAASMIDEFPILSVIAAFAEGSTVMNGVAELRVKESDRID | 359 |
| Query | 360 | AVANGLKLNGVDCDEGETSLVVRGRPDGKGLGNASGAAVATHLDHRIAMSFLVMGLVSEN | 419 |
| Sbjct | 360 | A+A GL+ NG +E SL V G KG GA VATHLDHRIAMSFLV GLVSE AMARGLEANGGRVEETRDSLTVHGTGGLKG GATVATHLDHRIAMSFLVAGLVSER | 414 |
| Query  Sbjct | 420  415 | PVTVDDATMIATSFPEFMDLMAGLGAKI 447 P+TVDDA IATSFP+F+ LM GLGA++ PITVDDAGPIATSFPDFVALMRGLGAEL 442 |  |

>TR:A0A0P6WZ26 A0A0P6WZ26_9SPHN 3-phosphoshikimate 1-carboxyvinyltransferase OS=Erythrobacter sp. SG61-1L OX=1603897 GN=aroA PE=3 SV=1

Length=448

Score = 468 bits (1205), Expect = 3e-160

| Identities | = 257/442 (58%), Positives = 309/442 (70%), Gaps = 3/442 (1%) |  |
| --- | --- | --- |
| Query 5 | ASSRPATARKSSGLSGTVRIPGDKSISHRSFMFGGLASGETRITGLLEGEDVINTGKAMQ | 64 |
| Sbjct 7 | A++RP + L G +R+PGDKSISHR+ MF LA G++RITGLLEGEDV+ T AM+ AAARPRRFTATGPLRGKIRVPGDKSISHRAIMFSSLAVGKSRITGLLEGEDVMATAAAMR | 66 |
| Query 65 | AMGARIRKEGD-TWIIDGVGNGGLLAPEAPLDFGNAATGCRLTMGLVGVYDFDSTFIGDA | 123 |
| Sbjct 67 | AMGA I + G+ W ++GVG G LL PEAPLD GN+ T RL MGLV + +TF+GDA AMGAHIERTGEGEWRVNGVGVGALLQPEAPLDMGNSGTSTRLLMGLVASHPITATFVGDA | 126 |
| Query 124 | SLTKRPMGRVLNPLREMGVQVKSEDGDRLPVTLRGPKTPTPITYRVPMASAQVKSAVLLA | 183 |
| Sbjct 127 | SL+KRPMGRV PL +MG ++ +G LP+T RG PITYR+P+ASAQVKSAVLLA SLSKRPMGRVTGPLSQMGAAFETSEGGTLPLTERGIAPAVPITYRLPVASAQVKSAVLLA | 186 |
| Query 184 | GLNTPGITTVIEPIMTRDHTEKMLQGFGANLTVETDADGVRTIRLEGRGKLTGQVIDVPG | 243 |
| Sbjct 187 | GLNTPGITTVIEP+ TRDHTE+ML GFGA L VE + +G R IR+ G L QVIDVPG GLNTPGITTVIEPVPTRDHTERMLTGFGAELWVE-EENGERVIRIRGEATLKPQVIDVPG | 245 |
| Query 244 | DPSSTAFPLVAALLVPGSDVTILNVLMNPTRTGLILTLQEMGADIEVINPRLAGGEDVAD | 303 |
| Sbjct 246 | DPSS AF +VAAL+VPGSDV I NV +N TR GLI L++MG I ++ R GGE VAD DPSSAAFFIVAALIVPGSDVIIENVGLNATRAGLIEVLRQMGGSIVELDRREVGGEPVAD | 305 |
| Query 304 | LRVRSSTLKGVTVPEDRAPSMIDEYPILAVAAAFAEGATVMNGLEELRVKESDRLSAVAN | 363 |
| Sbjct 306 | L V S LKGV V PSM+DE+P L VAA+ AEG TV +GLEELRVKESDR+S +A  LHVTHSALKGVNVDPAIVPSMVDEFPALFVAASMAEGRTVTSGLEELRVKESDRISVMAA | 365 |

| Query | 364 | GLKLNGVDCDEGETSLVVRGRPDGKGLGNASGAAVATHLDHRIAMSFLVMGLVSENPVTV 423 |
| --- | --- | --- |
| Sbjct | 366 | L G +E E L++ G GNA +A THLDHRIAMS V GL S++ V V ALTGAGARVEETEDGLIIDGTGGAPLRGNADSSA-KTHLDHRIAMSMAVAGLASQDGVEV 424 |
| Query | 424 | DDATMIATSFPEFMDLMAGLGA 445 |
| Sbjct | 425 | DD IATSFP F L+ GLG+  DDTRPIATSFPNFEALLDGLGS 446 |

>TR:A0A2S0UIG8 A0A2S0UIG8_9RHOB 3-phosphoshikimate 1-carboxyvinyltransferase OS=Gemmobacter sp. HYN0069 OX=2169400 GN=aroA PE=3 SV=1

Length=448

Score = 468 bits (1205), Expect = 3e-160

| Identities | = 256/447 (57%), Positives = 309/447 (69%), Gaps = 7/447 (2%) |  |
| --- | --- | --- |
| Query 3 | HGASSRPATARKSSGLSGTVRIPGDKSISHRSFMFGGLASGETRITGLLEGEDVINTGKA | 62 |
| Sbjct 4 | HG ++P TA KS L G +PGDKSISHRS +FG +A GETR+TGLLEG+DV++T A HG-EAQPMTATKSGPLRGVAEVPGDKSISHRSLIFGAMAVGETRVTGLLEGQDVLDTASA | 62 |
| Query 63 | MQAMGARIRKEGD-TWIIDGVGNGGLLAPEAPLDFGNAATGCRLTMGLVGVYDFDSTFIG | 121 |

M+A GA + + G	W + GVG GG	P	+D GN+ TG RL MG +	+TF G

| Sbjct | 63 | MRAFGADVIRHGQGEWSVHGVGVGGFREPADVIDCGNSGTGVRLIMGTMATSAITATFTG | 122 |
| --- | --- | --- | --- |
| Query | 122 | DASLTKRPMGRVLNPLREMGVQVKSEDGDRLPVTLRGPKTPTPITYRVPMASAQVKSAVL | 181 |
| Sbjct | 123 | DASL KRPMGRV +PL G + G RLP+T+ G P P+ Y +P+ASAQVKSAVL DASLRKRPMGRVTDPLSLFGTRAYGRAGGRLPMTVVGAANPVPVRYALPVASAQVKSAVL | 182 |
| Query | 182 | LAGLNTPGITTVIEPIMTRDHTEKMLQGFGANLTVETDADGVRTIRLEGRGKLTGQVIDV | 241 |
| Sbjct | 183 | LAGLN PG T VIE TRDH+E+ML GFGA LTVE A+G I L GR +L Q + V LAGLNAPGQTVVIEKEPTRDHSERMLLGFGAELTVEKTAEG-NVITLTGRPELRPQTVAV | 241 |
| Query | 242 | PGDPSSTAFPLVAALLVPGSDVTILNVLMNPTRTGLILTLQEMGADIEVINPRLAGGEDV | 301 |
| Sbjct | 242 | P DPSS AFP+ AALLV GSD+ + V N TR GL LTL EMGADIE +NPR GGE V PRDPSSAAFPVCAALLVEGSDIMVPGVSQNLTRNGLYLTLVEMGADIEFMNPREEGGEPV | 301 |
| Query | 302 | ADLRVR-SSTLKGVTVPEDRAPSMIDEYPILAVAAAFAEGATVMNGLEELRVKESDRLSA | 360 |
| Sbjct | 302 | ADLRVR S +KG+ VP++RAPSMIDEYP+L+V AAFA G TVM G++ELRVKESDR+ A ADLRVRFSPDMKGIEVPDERAPSMIDEYPVLSVVAAFATGKTVMRGVKELRVKESDRIDA | 361 |
| Query | 361 | VANGLKLNGVDCDEGETSLVVRGRPDGKGLGNASGAAVATHLDHRIAMSFLVMGLVSENP | 420 |
| Sbjct | 362 | +A GL+ GV +E E +L+V G G G GA ATHLDHRIAMSF+V G+ S+ P MARGLEACGVRVEEDEDTLIVHGLGAG---GVQGGATCATHLDHRIAMSFMVCGMASKKP | 418 |
| Query  Sbjct | 421  419 | VTVDDATMIATSFPEFMDLMAGLGAKI 447 V+VDDA+ I TSFP F LM LGA + VSVDDASPILTSFPVFEGLMQALGADL 445 |  |

>TR:A0A1A6FP93 A0A1A6FP93_9RHIZ 3-phosphoshikimate 1-carboxyvinyltransferase OS=Methylosinus sp. 3S-1 OX=1849840 GN=aroA PE=3 SV=1

Length=448

Score = 468 bits (1205), Expect = 3e-160

Identities = 261/447 (58%), Positives = 308/447 (69%), Gaps = 10/447 (2%)

| Query | 3 | HGASSRPATARKSSGLSGTVRIPGDKSISHRSFMFGGLASGETRITGLLEGEDVINTGKA | 62 |
| --- | --- | --- | --- |
| Sbjct | 7 | H AS P A ++ LSG R PGDKS+SHRS + G LA GET I GLLEGEDV+ T +A HPAS--PLRASRARALSGRCRPPGDKSVSHRSLILGTLAVGETAIEGLLEGEDVLRTAEA | 64 |
| Query | 63 | MQAMGARIRKEGD-TWIIDGVGNGGLLAPEAPLDFGNAATGCRLTMGLVGVYDFDSTFIG | 121 |
| Sbjct | 65 | +A+GARI + G +W + G G G LL P LDFGNA TG RL MG+V + + F G  CRALGARIDRLGPGSWSVRGPGLGSLLEPRETLDFGNAGTGSRLMMGVVAGHPITARFDG | 124 |

| Query | 122 | DASLTKRPMGRVLNPLREMGVQVKSE-DGDRLPVTLRGPKTPTPITYRVPMASAQVKSAV | 180 |
| --- | --- | --- | --- |
| Sbjct | 125 | DASL KRPM R+L+PL + G +V E +G R P+ LRG P P+ Y+ P+ASAQ+KSAV DASLRKRPMRRILDPLTQQGARVLEEAEGGRCPILLRGAAEPLPVEYKTPVASAQIKSAV | 184 |
| Query | 181 | LLAGLNTPGITTVIEPIMTRDHTEKMLQGFGANLTVETDADGVRTIRLEGRGKLTGQVID | 240 |
| Sbjct | 185 | LL GLN+PG T VIE +RDHTEKML FGA + E R I LEGR +L + LLCGLNSPGRTVVIESEASRDHTEKMLAHFGARIASEPFGPHGRKITLEGRPELRPAPVR | 244 |
| Query | 241 | VPGDPSSTAFPLVAALLVPGSDVTILNVLMNPTRTGLILTLQEMGADIEVINPRLAGGED | 300 |
| Sbjct | 245 | VP DPSS AFPLVAAL+VPGS++ I V++NP R GL+ TLQEMGA I N R GGE+ VPADPSSAAFPLVAALIVPGSEIVIEGVMINPLRNGLLTTLQEMGARISFENRRAEGGEE | 304 |
| Query | 301 | VADLRVRSSTLKGVTVPEDRAPSMIDEYPILAVAAAFAEGATVMNGLEELRVKESDRLSA | 360 |
| Sbjct | 305 | VAD+RV +STL GV VP +RAPSMIDEYPILAVAAAFA G T M GL ELRVKESDRL A VADIRVAASTLAGVDVPAERAPSMIDEYPILAVAAAFAGGETRMRGLSELRVKESDRLEA | 364 |
| Query | 361 | VANGLKLNGVDCDEGETSLVVRGRPDGKGLGNASGAAVA-THLDHRIAMSFLVMGLVSEN | 419 |
| Sbjct | 365 | +A GL+ N V+C+ L+VRG G G SG V THLDHRIAMSFLVMGL +E IAAGLRANAVECEISGDDLIVRG GTGRVSGGGVVETHLDHRIAMSFLVMGLAAEQ | 419 |
| Query | 420 | PVTVDDATMIATSFPEFMDLMAGLGAK 446 VTVDD MIATSFP+F LM LGA+ |  |
| Sbjct | 420 | DVTVDDDRMIATSFPDFRALMEVLGAR 446 | |

>TR:A0A6B8KGQ5 A0A6B8KGQ5_9RHIZ 3-phosphoshikimate 1-carboxyvinyltransferase OS=Methylocystis heyeri OX=391905 GN=aroA PE=3 SV=1

Length=449

Score = 468 bits (1205), Expect = 3e-160

| Identities | = 260/442 (59%), Positives = 311/442 (70%), Gaps = 9/442 (2%) |  |
| --- | --- | --- |
| Query 9 | PATARKSSGLSGTVRIPGDKSISHRSFMFGGLASGETRITGLLEGEDVINTGKAMQAMGA | 68 |
| Sbjct 11 | P +A +S+GLSG +R PGDKS+SHR+ + G L GET+I GLLEG+DV+ TG A + +GA PLSASRSTGLSGRLRPPGDKSVSHRALILGLLCVGETQIEGLLEGDDVLRTGAACRQLGA | 70 |
| Query 69 | RIRKEGD-TWIIDGVGNGGLLAPEAPLDFGNAATGCRLTMGLVGVYDFDSTFIGDASLTK | 127 |
| Sbjct 71 | R+ + G+ W ++G G G LL PE LDFGNA TG RL MG+VG + + F GDASL K RVVRHGEGLWTVNGAGLGSLLNPEEILDFGNAGTGSRLMMGVVGGHAVTARFDGDASLRK | 130 |
| Query 128 | RPMGRVLNPLREMGVQVKSE-DGDRLPVTLRGPKTPTPITYRVPMASAQVKSAVLLAGLN | 186 |
| Sbjct 131 | RPM R+L+PL+ G +V + +G R P+ L+G P PI Y+ P+ASAQ+KSAVLLAGLN RPMRRILDPLQLQGARVLEQAEGGRCPILLQGTAEPLPIEYKTPVASAQIKSAVLLAGLN | 190 |
| Query 187 | TPGITTVIEPIMTRDHTEKMLQGFGANLTVETDADGVRTIRLEGRGKLTGQVIDVPGDPS | 246 |
| Sbjct 191 | +PG TTVIE +RDHTEKML FGA + E + R I LEGR +L + VP DPS SPGRTTVIETEASRDHTEKMLLHFGAQVVSEPFGEHGRKITLEGRPQLRASKVCVPADPS | 250 |
| Query 247 | STAFPLVAALLVPGSDVTILNVLMNPTRTGLILTLQEMGADIEVINPRLAGGEDVADLRV | 306 |
| Sbjct 251 | S AFPL AAL+ GSD+TI V+ NP R+GL+ TL EMGA IEV N R+ GGE+VAD+R SAAFPLAAALITEGSDITIEGVMTNPLRSGLLTTLLEMGAHIEVENRRVEGGEEVADIRA | 310 |
| Query 307 | R-SSTLKGVTVPEDRAPSMIDEYPILAVAAAFAEGATVMNGLEELRVKESDRLSAVANGL | 365 |
| Sbjct 311 | R S+ L+GV VP RAPSMIDEYPILAV AAFA G T M GL ELRVKESDRL+A+A GL RYSADLRGVDVPAARAPSMIDEYPILAVVAAFASGETRMRGLSELRVKESDRLAAIAAGL | 370 |
| Query 366 | KLNGVDCD-EGETSLVVRGRPDGKGLGNASGAAVATHLDHRIAMSFLVMGLVSENPVTVD | 424 |
| Sbjct 371 | NGVDC EG+ V G D KG G V THLDHRIAMSFLVMGL + VTVD TANGVDCAIEGDDLFVRGGAGDVKG GGLVETHLDHRIAMSFLVMGLAARQAVTVD | 425 |
| Query 425  Sbjct 426 | DATMIATSFPEFMDLMAGLGAK 446 D TMIATSFP F LM LGAK DETMIATSFPAFRPLMEKLGAK 447 |  |

>SP:Q28UI7 AROA_JANSC 3-phosphoshikimate 1-carboxyvinyltransferase OS=Jannaschia sp. (strain CCS1) OX=290400 GN=aroA PE=3 SV=1

Length=450

Score = 468 bits (1205), Expect = 3e-160

| Identities | = 250/448 (56%), Positives = 312/448 (70%), Gaps = 7/448 (2%) |  |
| --- | --- | --- |
| Query 3 | HGASSRPATARKSSGLSGTVRIPGDKSISHRSFMFGGLASGETRITGLLEGEDVINTGKA | 62 |
| Sbjct 4 | HG +P TARK L+GT +PGDKSISHRS +FG +A GETR+TGLLEG+DV++T A HG-DPKPMTARKGGALTGTAEVPGDKSISHRSLIFGAMAVGETRVTGLLEGQDVLDTASA | 62 |
| Query 63 | MQAMGARIRKEGD-TWIIDGVGNGGLLAPEAPLDFGNAATGCRLTMGLVGVYDFDSTFIG | 121 |
| Sbjct 63 | M+A GA + +E D TW I GVG GG + P+ LD GN+ TG RL MG + TF G MRAFGAEVAREDDGTWSIHGVGVGGFVEPDDVLDCGNSGTGVRLLMGAMATTPISVTFTG | 122 |
| Query 122 | DASLTKRPMGRVLNPLREMGVQVKSEDGDRLPVTLRGPKTPTPITYRVPMASAQVKSAVL | 181 |
| Sbjct 123 | DASL RPM RV +PL G + RLP+TL G + P P+ Y P+ SAQVKSAVL DASLRSRPMARVTDPLALFGTRAYGRAQGRLPMTLVGAEAPMPVRYATPVPSAQVKSAVL | 182 |
| Query 182 | LAGLNTPGITTVIEPIMTRDHTEKMLQGFGANLTVETDADGVRTIRLEGRGKLTGQVIDV | 241 |
| Sbjct 183 | LAGLN PG T VIE TRDH+E+ML GFGA ++ E +G R I L G+ +LT QVI V LAGLNAPGETVVIEAEATRDHSERMLAGFGAQISTEVTQEG-RVITLTGQPELTPQVIAV | 241 |
| Query 242 | PGDPSSTAFPLVAALLVPGSDVTILNVLMNPTRTGLILTLQEMGADIEVINPRLAGGEDV | 301 |
| Sbjct 242 | P DPSS AFP+ AA++ GSDV + N+ +NPTR GL TL++MGAD+ NPR GGE V PRDPSSAAFPVCAAIITEGSDVLVPNIGLNPTRAGLFETLRDMGADLTYENPREEGGEPV | 301 |
| Query 302 | ADLRVR-SSTLKGVTVPEDRAPSMIDEYPILAVAAAFAEGATVMNGLEELRVKESDRLSA | 360 |
| Sbjct 302 | ADLR + S +KG+ VP +RA SMIDEYPIL+V AA AEG TVM G++ELRVKESDR++A ADLRAKFSPDMKGIEVPPERAASMIDEYPILSVVAANAEGTTVMRGVKELRVKESDRIAA | 361 |
| Query 361 | VANGLKLNGVDCDEGETSLVVRGRPDGKGLGNASGAAVATHLDHRIAMSFLVMGLVSENP | 420 |
| Sbjct 362 | +A+GL+LNG++ ++G +V GR G G A GA VATHLDHRIAMSFL G S+ MADGLRLNGIEVEDGPDWWIVHGRGPG---GMAGGATVATHLDHRIAMSFLCAGFASQQG | 418 |
| Query 421  Sbjct 419 | VTVDDATMIATSFPEFMDLMAGLGAKIE 448  +T+DD+ IATSFP F LM LGA+++ ITIDDSGPIATSFPIFEPLMRDLGARLD 446 |  |

>TR:A0A1G7I7F8 A0A1G7I7F8_9RHOB 3-phosphoshikimate 1-carboxyvinyltransferase OS=Salipiger thiooxidans OX=282683 GN=aroA PE=3 SV=1

Length=451

Score = 468 bits (1205), Expect = 3e-160

| Identities | = 253/441 (57%), Positives = 303/441 (69%), Gaps = 7/441 (2%) |  |
| --- | --- | --- |
| Query 9 | PATARKSSGLSGTVRIPGDKSISHRSFMFGGLASGETRITGLLEGEDVINTGKAMQAMGA | 68 |
| Sbjct 9 | P AR+S GL G +PGDKSISHRS + G +A GETR+TGLLEG+DV++T KAM+A GA PMIARRSEGLRGEAHVPGDKSISHRSLILGAMAVGETRVTGLLEGQDVLDTAKAMRAFGA | 68 |
| Query 69 | RIRKEGDTWIIDGVGNGGLLAPEAPLDFGNAATGCRLTMGLVGVYDFDSTFIGDASLTKR | 128 |
| Sbjct 69 | + K GDTW + GVG GG PE +D GN+ TG RL MG + +TF GDASL KR EVEKRGDTWHVQGVGVGGFAEPEQVIDCGNSGTGVRLIMGAMATTPIAATFTGDASLNKR | 128 |
| Query 129 | PMGRVLNPLREMGVQVKSEDGDRLPVTLRGPKTPTPITYRVPMASAQVKSAVLLAGLNTP | 188 |
| Sbjct 129 | PM RV +PL G + +G RLP+T+ G + P P+ Y P+ SAQVKSAVLLAGLN P PMARVTDPLALFGARAYGREGGRLPMTIVGARDPLPVRYATPVPSAQVKSAVLLAGLNAP | 188 |
| Query 189 | GITTVIEPIMTRDHTEKMLQGFGANLTVETDADGVRTIRLEGRGKLTGQVIDVPGDPSST | 248 |
| Sbjct 189 | G T VIE TRDH+E+ML GFGA ++ ET A+G R I L G+ +L QVI VP DPSS  GETVVIEREATRDHSERMLAGFGAQVSTETTAEG-RVITLTGQPELRPQVIVVPRDPSSA | 247 |

| Query | 249 | AFPLVAALLVPGSDVTILNVLMNPTRTGLILTLQEMGADIEVINPRLAGGEDVADLRVR- | 307 |
| --- | --- | --- | --- |
| Sbjct | 248 | AFP+ AAL+ GSDV + N+ +NPTR GL TLQ+MGAD+ N R GGE VADLR R AFPVCAALITEGSDVLVPNIGLNPTRAGLFFTLQDMGADLAFENEREEGGEPVADLRARF | 307 |
| Query | 308 | SSTLKGVTVPEDRAPSMIDEYPILAVAAAFAEGATVMNGLEELRVKESDRLSAVANGLKL | 367 |
| Sbjct | 308 | S L+G+ VP +RA SMIDEYP+L+V AAFA G T M G+ ELRVKESDR+ A+A GL+ SPDLRGIEVPAERAASMIDEYPVLSVVAAFATGKTHMPGVRELRVKESDRIDAMAVGLRA | 367 |
| Query | 368 | NGVDCDEGETSLVVRGRPDGKGLGNASGAAV-ATHLDHRIAMSFLVMGLVSENPVTVDDA | 426 |
| Sbjct | 368 | GV DEGE V G G GN G V A+HLDHRIAMSFLVMGL + +P+ VDD AGVTVDEGEDWWTVH GLGHGNVPGGVVTASHLDHRIAMSFLVMGLATRSPMRVDDG | 423 |
| Query | 427 | TMIATSFPEFMDLMAGLGAKI 447 IATSFP F LM GLGA I |  |
| Sbjct | 424 | GPIATSFPIFEGLMTGLGADI 444 | |

>TR:A3VMP1 A3VMP1_9RHOB 3-phosphoshikimate 1-carboxyvinyltransferase OS=Maritimibacter alkaliphilus HTCC2654 OX=314271 GN=aroA PE=3

SV=1

Length=441

Score = 468 bits (1204), Expect = 3e-160

| Identities | | = 257/443 (58%), Positives = 303/443 (68%), Gaps = 8/443 (2%) |  |
| --- | --- | --- | --- |
| Query 11  Sbjct 2 | | TARKSSGLSGTVRIPGDKSISHRSFMFGGLASGETRITGLLEGEDVINTGKAMQAMGARI T+RKS L G +PGDKSISHRS + G +A GETR+TGLLEGEDV+ T AM+A GA + TSRKSGPLKGVADVPGDKSISHRSLLLGAMAVGETRVTGLLEGEDVLATAAAMRAFGADV | 70  61 |
| Query 71  Sbjct 62 | | RKEGD-TWIIDGVGNGGLLAPEAPLDFGNAATGCRLTMGLVGVYDFDSTFIGDASLTKRP  + GD W ++GVG GG PE +D GNA TG RL MG + +TF GDASL RP ERLGDGEWRVNGVGVGGFAEPEDVIDCGNAGTGVRLIMGCMATSPITATFTGDASLRSRP | 129  121 |
| Query 130  Sbjct 122 | | MGRVLNPLREMGVQVKSEDGDRLPVTLRGPKTPTPITYRVPMASAQVKSAVLLAGLNTPG MGRV +P+ G Q G RLP+T+ G K P P+ YR PMASAQVKSAVLLAGLN PG MGRVTDPIALFGAQSYGRTGGRLPMTVVGAKNPIPVRYRTPMASAQVKSAVLLAGLNAPG | 189  181 |
| Query 190  Sbjct 182 | | ITTVIEPIMTRDHTEKMLQGFGANLTVETDADGVRTIRLEGRGKLTGQVIDVPGDPSSTA T VIE TRDHTE+ML GFGA ++VE D D R I L G+ +L Q I VP DPSS A ETVVIEAEATRDHTERMLAGFGAEISVE-DTDEGRVITLVGQPELKPQTIAVPRDPSSAA | 249  240 |
| Query 250  Sbjct 241 | | FPLVAALLVPGSDVTILNVLMNPTRTGLILTLQEMGADIEVINPRLAGGEDVADLRVR-S FP+ AAL+VPGSDV + N+ +NPTR GL TL EMGAD+ N R GGE VADLR R S FPVCAALIVPGSDVLVPNIGLNPTRAGLFTTLIEMGADLTYENEREEGGEPVADLRARFS | 308  300 |
| Query 309  Sbjct 301 | | STLKGVTVPEDRAPSMIDEYPILAVAAAFAEGATVMNGLEELRVKESDRLSAVANGLKLN  +KG+ VPE+RA SMIDEYP+L+V AAFA G T M G+ ELRVKESDR+ A+A GL+ N PDMKGIRVPEERAASMIDEYPVLSVVAAFATGKTEMVGVHELRVKESDRIDAMARGLEAN | 368  360 |
| Query 369  Sbjct 361 | | GVDCDEGETSLVVRG-RPDGKGLGNASGAAVATHLDHRIAMSFLVMGLVSENPVTVDDAT GV +EG + V G PDG G A LDHRIAMSF+VMG+ S++PV+VDD GVTIEEGRDFMTVHGLGPDGV PGGGTAAARLDHRIAMSFMVMGMASQSPVSVDDGQ | 427  416 |
| Query 428 | | MIATSFPEFMDLMAGLGAKIELS 450 IATSFP F LM GLGA IE S |  |
| Sbjct | 417 | PIATSFPIFEPLMTGLGATIERS 439 | |

>TR:A0A0F7KQ62 A0A0F7KQ62_9SPHN 3-phosphoshikimate 1-carboxyvinyltransferase OS=Altererythrobacter atlanticus OX=1267766 GN=aroA PE=3 SV=1

Length=446

Score = 468 bits (1204), Expect = 3e-160

Identities = 255/436 (58%), Positives = 307/436 (70%), Gaps = 4/436 (1%)

| Query | 8 | RPATARKSSGLSGTVRIPGDKSISHRSFMFGGLASGETRITGLLEGEDVINTGKAMQAMG | 67 |
| --- | --- | --- | --- |
| Sbjct | 10 | +P S L G +R+PGDKSISHRS MFG LA GETRITGLLEGEDV+ T A++AMG KPRRFLPSGPLQGRIRVPGDKSISHRSLMFGALAVGETRITGLLEGEDVLATAAALRAMG | 69 |
| Query | 68 | ARIRKEGD-TWIIDGVGNGGLLAPEAPLDFGNAATGCRLTMGLVGVYDFDSTFIGDASLT | 126 |
| Sbjct | 70 | A I +EG+ W + GVG GGLL PE LD GN+ T RL +G++ + +TFIGDASL+ ATIHREGEGRWSVKGVGVGGLLQPEGALDMGNSGTSTRLFLGILASHPLTATFIGDASLS | 129 |
| Query | 127 | KRPMGRVLNPLREMGVQVKSEDGDRLPVTLRGPKTPTPITYRVPMASAQVKSAVLLAGLN | 186 |
| Sbjct | 130 | RPMGRV+ PL +MG Q G LP+ L+G PI+YR+P+ASAQVKSA+LLAGLN ARPMGRVIEPLSQMGAQFTPSPGGTLPLMLKGANPAVPISYRLPVASAQVKSAILLAGLN | 189 |
| Query | 187 | TPGITTVIEPIMTRDHTEKMLQGFGANLTVETDADGVRTIRLEGRGKLTGQVIDVPGDPS | 246 |
| Sbjct | 190 | T GITTVIEP+ TRDHTE+ML+GFGA+L VE + +G R IRL G +L Q+IDVPGDPS TAGITTVIEPVPTRDHTERMLKGFGADLWVEEE-NGERVIRLRGEAELRPQMIDVPGDPS | 248 |
| Query | 247 | STAFPLVAALLVPGSDVTILNVLMNPTRTGLILTLQEMGADIEVINPRLAGGEDVADLRV | 306 |
| Sbjct | 249 | S AF VAALL+PGSD+ I NV +NPTR GL L++MG DIE ++ R+ GGE VADLRV SAAFFAVAALLIPGSDLVIENVGLNPTRAGLFDVLRQMGGDIEEMDQRIIGGEPVADLRV | 308 |
| Query | 307 | RSSTLKGVTVPEDRAPSMIDEYPILAVAAAFAEGATVMNGLEELRVKESDRLSAVANGLK | 366 |
| Sbjct | 309 | R S L+GV V APSMIDE+P+L VAA+ A G TV GLEELRVKESDRL+ +A L+ RHSVLRGVEVDPAIAPSMIDEFPVLFVAASLANGRTVTTGLEELRVKESDRLAVMAEALR | 368 |
| Query | 367 | LNGVDCDEGETSLVVRGRPDGKGLGNASGAAVATHLDHRIAMSFLVMGLVSENPVTVDDA | 426 |

G	+E	LV+ G	G L	G A+ATHLDHRIAMS V GL S+ V VDD

| Sbjct | 369 | NAGARIEERADGLVIEG-TGGDPLPGGEG-AIATHLDHRIAMSMAVAGLASKAGVEVDDT | 426 |
| --- | --- | --- | --- |
| Query | 427 | TMIATSFPEFMDLMAG 442 IATSFP F L+ G |  |
| Sbjct | 427 | RPIATSFPSFEALLDG 442 | |

>TR:A0A2U2CB24 A0A2U2CB24_9RHOB 3-phosphoshikimate 1-carboxyvinyltransferase OS=Pararhodobacter sp. CIC4N-9 OX=2184063 GN=aroA PE=3 SV=1

Length=448

Score = 468 bits (1204), Expect = 4e-160

| Identities | = 256/443 (58%), Positives = 308/443 (70%), Gaps = 10/443 (2%) |  |
| --- | --- | --- |
| Query 9 | PATARKSSGLSGTVRIPGDKSISHRSFMFGGLASGETRITGLLEGEDVINTGKAMQAMGA | 68 |
| Sbjct 9 | P A KS L+G +PGDKSISHR+ + G L+ GET ITGLLEGEDVI+T KAM+A GA PMIAHKSGPLAGVAEVPGDKSISHRALILGALSVGETHITGLLEGEDVIDTAKAMRAFGA | 68 |
| Query 69 | RIRK--EGDTWIIDGVGNGGLLAPEAPLDFGNAATGCRLTMGLVGVYDFDSTFIGDASLT | 126 |
| Sbjct 69 | + + EGD W ++GVG GG P +D GN+ TG RL MG + +TF GDASL TVTRVAEGD-WRVNGVGVGGFAEPTQVIDCGNSGTGVRLIMGAMATTPIAATFTGDASLN | 127 |
| Query 127 | KRPMGRVLNPLREMGVQVKSEDGDRLPVTLRGPKTPTPITYRVPMASAQVKSAVLLAGLN | 186 |
| Sbjct 128 | KRPMGR+ +PL G + G RLP+TL G P P+ Y +PM SAQVKSAVLLAGLN KRPMGRITDPLALFGARAVGRAGGRLPLTLVGAVDPVPVRYTLPMPSAQVKSAVLLAGLN | 187 |
| Query 187 | TPGITTVIEPIMTRDHTEKMLQGFGANLTVETDADGVRTIRLEGRGKLTGQVIDVPGDPS | 246 |
| Sbjct 188 | PG T VIEP TRDHTE+ML+GFGA + V A+G R I L+G+ LTGQ + VP DPS APGETVVIEPEPTRDHTERMLRGFGAEIDVTDSAEG-RVITLKGQPDLTGQSVAVPRDPS | 246 |
| Query 247 | STAFPLVAALLVPGSDVTILNVLMNPTRTGLILTLQEMGADIEVINPRLAGGEDVADLRV | 306 |
| Sbjct 247 | S AFP+ AA+LVPGS++T+ V NPTR GL +TL EMGADI NPR GGE VADLRV SAAFPVCAAILVPGSEITVPGVSRNPTRDGLYITLIEMGADIAFENPRDEGGEPVADLRV | 306 |
| Query 307 | R-SSTLKGVTVPEDRAPSMIDEYPILAVAAAFAEGATVMNGLEELRVKESDRLSAVANGL | 365 |
| Sbjct 307 | + S LKGVT P +RA SMIDE+PIL+V AA A+G TVM G++ELRVKESDR+ A+A GL  KYSPALKGVTTPAERAASMIDEFPILSVVAAMAQGTTVMQGVKELRVKESDRIDAMARGL | 366 |

| Query | 366 | KLNGVDCDEGETSLVVRGRPDGKGLGNASGAAVATHLDHRIAMSFLVMGLVSENPVTVDD 425 |
| --- | --- | --- |
| Sbjct | 367 | + GV +E E +L + G G GA + LDHRIAMSFLV+GLV++ PV VDD EACGVTLEETEDTLTIHGTGAVPG-----GAVCESRLDHRIAMSFLVLGLVADQPVAVDD 421 |
| Query | 426 | ATMIATSFPEFMDLMAGLGAKIE 448 |
| Sbjct | 422 | IATSFP F LMAGLGA+IE GAPIATSFPVFEPLMAGLGARIE 444 |

>TR:A0A1H8E361 A0A1H8E361_9RHOB 3-phosphoshikimate 1-carboxyvinyltransferase OS=Gemmobacter aquatilis OX=933059 GN=aroA PE=3 SV=1

Length=448

Score = 468 bits (1204), Expect = 4e-160

| Identities | = 254/447 (57%), Positives = 309/447 (69%), Gaps = 7/447 (2%) |  |
| --- | --- | --- |
| Query 3 | HGASSRPATARKSSGLSGTVRIPGDKSISHRSFMFGGLASGETRITGLLEGEDVINTGKA | 62 |
| Sbjct 4 | HG +P TARKS L GT +PGDKSISHR+ +FG +A GET+ITGLLEG+DV++T KA HG-EPQPMTARKSGPLRGTAAVPGDKSISHRALIFGAMAVGETKITGLLEGQDVLDTAKA | 62 |
| Query 63 | MQAMGARIRKEGD-TWIIDGVGNGGLLAPEAPLDFGNAATGCRLTMGLVGVYDFDSTFIG | 121 |

M+A GA + +	W + GVG GG	P	+D GN+ TG RL MG +	+TF G

| Sbjct | 63 | MRAFGAEVTQHAPGAWSVHGVGVGGFREPADVIDCGNSGTGVRLIMGCMATTAMTATFTG | 122 |
| --- | --- | --- | --- |
| Query | 122 | DASLTKRPMGRVLNPLREMGVQVKSEDGDRLPVTLRGPKTPTPITYRVPMASAQVKSAVL | 181 |
| Sbjct | 123 | DASL KRPMGRV +PL G + G RLP+T+ G P P+ Y +P+ASAQVKSAVL DASLRKRPMGRVTDPLSLFGARAYGRQGGRLPMTVVGAANPVPVRYALPVASAQVKSAVL | 182 |
| Query | 182 | LAGLNTPGITTVIEPIMTRDHTEKMLQGFGANLTVETDADGVRTIRLEGRGKLTGQVIDV | 241 |
| Sbjct | 183 | LAGLN PG T VIE TRDH+E+ML GFGA L VE A+G I L G+ +L Q + V LAGLNAPGETVVIEREPTRDHSERMLLGFGAQLHVEKGAEG-HIITLTGQPELRPQTVAV | 241 |
| Query | 242 | PGDPSSTAFPLVAALLVPGSDVTILNVLMNPTRTGLILTLQEMGADIEVINPRLAGGEDV | 301 |
| Sbjct | 242 | P DPSS AFP+ AAL+V GSD+ + V N TR GL +TL EMGA+I+ NPR GGE V PRDPSSAAFPVCAALIVEGSDILVPGVSQNLTRNGLYITLVEMGAEIDFQNPRTEGGEPV | 301 |
| Query | 302 | ADLRVR-SSTLKGVTVPEDRAPSMIDEYPILAVAAAFAEGATVMNGLEELRVKESDRLSA | 360 |
| Sbjct | 302 | ADLRVR S ++G+ VP DRAPSMIDEYPIL+V AAFA G TVM G++ELRVKESDR+ A ADLRVRFSGNMQGIEVPPDRAPSMIDEYPILSVVAAFATGKTVMKGVKELRVKESDRIDA | 361 |
| Query | 361 | VANGLKLNGVDCDEGETSLVVRGRPDGKGLGNASGAAVATHLDHRIAMSFLVMGLVSENP | 420 |
| Sbjct | 362 | +A GL+ GV +E E +L+V GR G G GA ATHLDHRIAMSFLV G+ ++ P MARGLEACGVTIEEDEDTLIVHGRGPG---GMPGGATAATHLDHRIAMSFLVAGMAAQQP | 418 |
| Query  Sbjct | 421  419 | VTVDDATMIATSFPEFMDLMAGLGAKI 447 V+VDDA+ I TSFP F LM GLGA + VSVDDASPIVTSFPIFEGLMTGLGATL 445 |  |

>TR:A0A1C3RFT6 A0A1C3RFT6_9RHIZ 3-phosphoshikimate 1-carboxyvinyltransferase OS=Candidatus Terasakiella magnetica OX=1867952 GN=aroA PE=3

SV=1

Length=439

Score = 468 bits (1203), Expect = 4e-160

Identities = 246/441 (56%), Positives = 307/441 (70%), Gaps = 5/441 (1%)

| Query | 8 | RPATARKSSGLSGTVRIPGDKSISHRSFMFGGLASGETRITGLLEGEDVINTGKAMQAMG | 67 |
| --- | --- | --- | --- |
|  |  | +P +++SS L+G++R+PGDKSISHR+ MFG +A GET++TGLLEGEDV+ T AM+++G |  |
| Sbjct | 2 | KPVVSQQSSALTGSIRVPGDKSISHRALMFGAMAVGETKVTGLLEGEDVLCTAAAMRSLG | 61 |
| Query | 68 | ARIRKEGD-TWIIDGVGNGGLLAPEAPLDFGNAATGCRLTMGLVGVYDFDSTFIGDASLT | 126 |

A + + D TW+I G G GG	P	LD GN+ T RL MG++	F +	GDASL

| Sbjct | 62 | ASVEQCDDGTWVIKGRGVGGFSEPTGVLDMGNSGTAARLLMGVLATQSFTTFMTGDASLC | 121 |
| --- | --- | --- | --- |
| Query | 127 | KRPMGRVLNPLREMGVQVKSEDGDRLPVTLRGPKTPTPITYRVPMASAQVKSAVLLAGLN | 186 |
| Sbjct | 122 | RPM RV+ PLR +GV S + R+P+ ++G P PI+Y +P+ASAQVKSAV+LAGLN SRPMERVMKPLRPIGVSFVSRENGRMPIAIQGTSAPMPISYELPVASAQVKSAVMLAGLN | 181 |
| Query | 187 | TPGITTVIEPIMTRDHTEKMLQGFGANLTVETDADGVRTIRLEGRGKLTGQVIDVPGDPS | 246 |
| Sbjct | 182 | PG TTVIEP +RDHTEKMLQ FGA + VE G R I L G+ +L + + VP DPS APGKTTVIEPNPSRDHTEKMLQHFGAEVEVEETDKGGRVITLTGQPELVARDVIVPTDPS | 241 |
| Query | 247 | STAFPLVAALLVPGSDVTILNVLMNPTRTGLILTLQEMGADIEVINPRLAGGEDVADLRV | 306 |
| Sbjct | 242 | S AFP+VAAL+ PGS +T+ V MNP RTGL TL+EMG I N R+ GE+VADL V SAAFPVVAALITPGSKITVEAVGMNPLRTGLFATLKEMGGKITEENVRIDAGEEVADLVV | 301 |
| Query | 307 | RSSTLKGVTVPEDRAPSMIDEYPILAVAAAFAEGATVMNGLEELRVKESDRLSAVANGLK | 366 |
| Sbjct | 302 | S L GVTVP RAPSMIDEYP+LAVAA+FA+G T M GLEELRVKESDRL+ V LK EYSELTGVTVPASRAPSMIDEYPVLAVAASFAKGTTHMEGLEELRVKESDRLAVVVESLK | 361 |
| Query | 367 | LNGVDCDEGETSLVVRGRPDGKGLGNASGAAVATHLDHRIAMSFLVMGLVSENPVTVDDA | 426 |
| Sbjct | 362 | GVD EGE ++V + G G + + LDHRIAMSF+V GL S+ PVT+DD ACGVDVTEGEDFMIV NATGEAALGGGEIKSRLDHRIAMSFIVFGLASQKPVTIDDG | 417 |
| Query  Sbjct | 427  418 | TMIATSFPEFMDLMAGLGAKI 447  + I TSFP F+DLM GLGAKI SPIDTSFPGFIDLMNGLGAKI 438 |  |

```
>TR:A0A0P1IBT7 A0A0P1IBT7_9RHOB 3-phosphoshikimate 1-carboxyvinyltransferase OS=Shimia thalassica OX=1715693 GN=aroA PE=3 SV=1
Length=450

Score = 468 bits (1204), Expect = 4e-160
Identities = 254/443 (57%), Positives = 302/443 (68%), Gaps = 8/443 (2%)

Query 9	PATARKSSGLSGTVRIPGDKSISHRSFMFGGLASGETRITGLLEGEDVINTGKAMQAMGA 68 P T+R	L+G	+PGDKSISHRS + G +A GETRI GLLEGEDV++T KAMQA GA
Sbjct 9	PMTSRACGPLTGEANVPGDKSISHRSLILGAMAVGETRIFGLLEGEDVLDTAKAMQAFGA 68

Query 69	RIRKEGD-TWIIDGVGNGGLLAPEAPLDFGNAATGCRLTMGLVGVYDFDSTFIGDASLTK 127
+	GD W + GVG GG	P+ +D GN+ TG RL MG +	TF GDASL K Sbjct 69	EVTNHGDGEWSVQGVGVGGFAEPDGVIDCGNSGTGVRLIMGAMATSGITVTFTGDASLNK 128

Query 128 RPMGRVLNPLREMGVQVKSEDGDRLPVTLRGPKTPTPITYRVPMASAQVKSAVLLAGLNT 187 RPM RV +P+	G Q	G RLP+T+ G	P P+ Y VPM SAQVKSAVLLAGLN
Sbjct 129 RPMARVTDPIALFGAQSVGRSGGRLPMTIVGASDPVPVRYNVPMPSAQVKSAVLLAGLNA 188

Query 188 PGITTVIEPIMTRDHTEKMLQGFGANLTVETDADGVRTIRLEGRGKLTGQVIDVPGDPSS 247 PG T VIE	TRDHTE+ML GFGA ++VET +G R I L G+ +L Q I VP DPSS
Sbjct 189 PGQTVVIEKEATRDHTERMLTGFGAEVSVETTDEG-RVITLTGQPELKPQTITVPRDPSS 247

Query 248 TAFPLVAALLVPGSDVTILNVLMNPTRTGLILTLQEMGADIEVINPRLAGGEDVADLRVR 307 AFP+ AA++ PGSDV + N+ +NPTR GL TL+EMGAD+	N R GGE VADLR R
Sbjct 248 AAFPVCAAIITPGSDVLVPNIGLNPTRAGLFTTLREMGADLVYENEREEGGEPVADLRAR 307

Query 308 -SSTLKGVTVPEDRAPSMIDEYPILAVAAAFAEGATVMNGLEELRVKESDRLSAVANGLK 366 S LKG+ V	RA SMIDEYP+L+V A FA+G TVM G++ELRVKESDR+ A+A GL+
Sbjct 308 FSPDLKGIEVDPARAASMIDEYPVLSVVACFAQGDTVMKGVKELRVKESDRIDAMATGLR 367

Query 367 LNGVDCDEGETSLVVRGRPDGKGLGN-ASGAAVATHLDHRIAMSFLVMGLVSENPVTVDD 425 LNGV+ DEGE	+V GR	G GN A G	+HLDHRIAMSF+VMG+ + PV+VDD
Sbjct 368 LNGVEVDEGEDWWIVHGR----GHGNVAGGGTCESHLDHRIAMSFMVMGMATNAPVSVDD 423

Query 426 ATMIATSFPEFMDLMAGLGAKIE 448
IATSFP F LM LGAKIE

Sbjct 424 GGPIATSFPVFEPLMEALGAKIE 446


>TR:A0A0N1AGP7 A0A0N1AGP7_9SPHN 3-phosphoshikimate 1-carboxyvinyltransferase OS=Novosphingobium sp. AAP1 OX=1523413 GN=aroA PE=3 SV=1
Length=450

Score = 468 bits (1204), Expect = 4e-160
```

| Identities | = 248/438 (57%), Positives = 307/438 (70%), Gaps = 1/438 (0%) |  |
| --- | --- | --- |
| Query 5 | ASSRPATARKSSGLSGTVRIPGDKSISHRSFMFGGLASGETRITGLLEGEDVINTGKAMQ | 64 |
| Sbjct 8 | A RP L G +R+PGDKSISHRS M G LA GETR+TGLLEGEDV++T AM+ ALMRPRRFSAQGPLKGRIRVPGDKSISHRSIMLGALAVGETRVTGLLEGEDVLSTAAAMR | 67 |
| Query 65 | AMGARIRKEGD-TWIIDGVGNGGLLAPEAPLDFGNAATGCRLTMGLVGVYDFDSTFIGDA | 123 |
| Sbjct 68 | AMGA I ++ + W++ GVG GGLL P LD GN+ T RL MGLV + + F+GD AMGATIERQANGDWLVAGVGVGGLLQPAGALDMGNSGTSTRLLMGLVASHPITACFVGDG | 127 |
| Query 124 | SLTKRPMGRVLNPLREMGVQVKSEDGDRLPVTLRGPKTPTPITYRVPMASAQVKSAVLLA | 183 |
| Sbjct 128 | SL+KRPMGRV++PL ++G + + G RLP+ + G TP P++YR+P+ASAQVKSAVLLA SLSKRPMGRVIDPLSQVGARFTAYPGGRLPLIVEGAATPVPLSYRLPVASAQVKSAVLLA | 187 |
| Query 184 | GLNTPGITTVIEPIMTRDHTEKMLQGFGANLTVETDADGVRTIRLEGRGKLTGQVIDVPG | 243 |
| Sbjct 188 | GLNTPGITTVIEPI TRDH+E+ML+GFGA+L+VE +DG R IRL G +L Q I VPG GLNTPGITTVIEPIPTRDHSERMLKGFGADLSVEIASDGARVIRLVGEAELKPQAITVPG | 247 |
| Query 244 | DPSSTAFPLVAALLVPGSDVTILNVLMNPTRTGLILTLQEMGADIEVINPRLAGGEDVAD | 303 |
| Sbjct 248 | DPSS AF +VAAL+VPGSD+ I NV +NPTR L L+ MG IE I+ R GGE VAD DPSSAAFFVVAALIVPGSDLVIENVGLNPTRAALFDVLRLMGGSIEEIDRRDVGGEPVAD | 307 |
| Query 304 | LRVRSSTLKGVTVPEDRAPSMIDEYPILAVAAAFAEGATVMNGLEELRVKESDRLSAVAN | 363 |
| Sbjct 308 | LRVR+S L G+ V PSM+DE+P+L VAAA A+G T+ GLEELRVKESDR++ + LRVRASALTGIDVDPAVVPSMVDEFPVLFVAAAHAQGRTITRGLEELRVKESDRITVMRV | 367 |
| Query 364 | GLKLNGVDCDEGETSLVVRGRPDGKGLGNASGAAVATHLDHRIAMSFLVMGLVSENPVTV | 423 |
| Sbjct 368 | L+L G E E +++ G G G + A+VATHLDHRIAMS V GL S N V + ALELAGARVTETEDGMIIDGTGGGPLPGTQADASVATHLDHRIAMSMAVAGLASANGVGI | 427 |
| Query 424  Sbjct 428 | DDATMIATSFPEFMDLMA 441 DD IATSFP F L+A DDTRPIATSFPVFEHLIA 445 |  |

>TR:A0A1G8IVD3 A0A1G8IVD3_9RHOB 3-phosphoshikimate 1-carboxyvinyltransferase OS=Salipiger marinus OX=555512 GN=aroA PE=3 SV=1

Length=453

Score = 468 bits (1204), Expect = 4e-160

| Identities | = 254/454 (56%), Positives = 308/454 (68%), Gaps = 6/454 (1%) |  |
| --- | --- | --- |
| Query 3 | HGASSRPATARKSSGLSGTVRIPGDKSISHRSFMFGGLASGETRITGLLEGEDVINTGKA | 62 |
| Sbjct 4 | HG +R +RKS+GL G +PGDKSISHR+ + G LA GETR++GLLEG+DV++T A HG-EARAMVSRKSAGLRGQAEVPGDKSISHRALILGALAVGETRVSGLLEGQDVLDTASA | 62 |
| Query 63 | MQAMGARIRKEGDTWIIDGVGNGGLLAPEAPLDFGNAATGCRLTMGLVGVYDFDSTFIGD | 122 |
| Sbjct 63 | M+A GA + K GD W + GVG GG P +D GN+ TG RL MG + +TF GD MRAFGAEVEKRGDVWHVTGVGVGGFAEPAQVIDCGNSGTGVRLIMGAMATTPISATFTGD | 122 |
| Query 123 | ASLTKRPMGRVLNPLREMGVQVKSEDGDRLPVTLRGPKTPTPITYRVPMASAQVKSAVLL | 182 |
| Sbjct 123 | ASL KRPM R+ +PLR+ G G RLP+ L G + P+ Y P+ SAQVKSAVLL ASLNKRPMARITDPLRQFGAHAYGRSGGRLPLVLVGAEQAMPVRYATPVPSAQVKSAVLL | 182 |
| Query 183 | AGLNTPGITTVIEPIMTRDHTEKMLQGFGANLTVETDADGVRTIRLEGRGKLTGQVIDVP AGLN PG T VIE TRDH+E+ML GFGA ++ E +G R I L+G+ +L QVI VP | 242 |

| Sbjct | 183 | AGLNAPGETVVIEREATRDHSERMLAGFGAVISSEDGPEG-RVITLQGQPELRPQVIAVP | 241 |
| --- | --- | --- | --- |
| Query | 243 | GDPSSTAFPLVAALLVPGSDVTILNVLMNPTRTGLILTLQEMGADIEVINPRLAGGEDVA | 302 |
| Sbjct | 242 | DPSS AFP+ AAL+ GSDV + N+ +NPTR GL TLQEMGAD+ NPR GGE VA RDPSSAAFPVCAALITEGSDVLVPNIGLNPTRAGLFFTLQEMGADLVFENPREEGGEPVA | 301 |
| Query | 303 | DLRVR-SSTLKGVTVPEDRAPSMIDEYPILAVAAAFAEGATVMNGLEELRVKESDRLSAV | 361 |
| Sbjct | 302 | DLR R S L+G+ VP +RA SMIDEYP+L+V AAFAEG T M G++ELRVKESDR+ A+ DLRARFSPNLRGIEVPAERAASMIDEYPVLSVVAAFAEGRTHMPGVKELRVKESDRIEAM | 361 |
| Query | 362 | ANGLKLNGVDCDEGETSLVVRGRPDGKGLGNASGAAVATHLDHRIAMSFLVMGLVSENPV | 421 |
| Sbjct | 362 | A GL+ GV + GE V GR G G GA VATHLDHRIAMSFLV+GL +E PV AVGLRACGVTVEAGEDWWTVEGRGFGAVPG---GATVATHLDHRIAMSFLVLGLATEAPV | 418 |
| Query  Sbjct | 422  419 | TVDDATMIATSFPEFMDLMAGLGAKIELSDTKAA 455 TVDD IATSFP F LM GLGA + +D A TVDDGGPIATSFPVFEPLMRGLGADLGAADADPA 452 |  |

>TR:A0A1G8L1U6 A0A1G8L1U6_9RHOB 3-phosphoshikimate 1-carboxyvinyltransferase OS=Lutimaribacter saemankumensis OX=490829 GN=aroA PE=3 SV=1

Length=448

Score = 468 bits (1203), Expect = 5e-160

| Identities | = 254/447 (57%), Positives = 308/447 (69%), Gaps = 7/447 (2%) |  |
| --- | --- | --- |
| Query 3 | HGASSRPATARKSSGLSGTVRIPGDKSISHRSFMFGGLASGETRITGLLEGEDVINTGKA | 62 |
| Sbjct 4 | HG + + AR+S GL G +PGDKSISHR+ + G L+ GET +TGLLEG+DV++T KA HGPAQK-MIARRSEGLRGEAHVPGDKSISHRALILGALSVGETVVTGLLEGQDVLDTAKA | 62 |
| Query 63 | MQAMGARIRKEGD-TWIIDGVGNGGLLAPEAPLDFGNAATGCRLTMGLVGVYDFDSTFIG | 121 |
| Sbjct 63 | M+A GA + ++GD TW + GVG GG P +D GN+ TG RL MG + +TF G MRAFGAEVTRDGDGTWHVHGVGVGGFAEPGEVIDCGNSGTGVRLIMGAMATCPITATFTG | 122 |
| Query 122 | DASLTKRPMGRVLNPLREMGVQVKSEDGDRLPVTLRGPKTPTPITYRVPMASAQVKSAVL | 181 |
| Sbjct 123 | DASL KRPM RV +PL G Q G RLP+T+ G P P+ Y VP+ SAQVKSAVL DASLNKRPMARVTDPLALFGAQAVGRSGGRLPMTIVGAADPVPVRYTVPVPSAQVKSAVL | 182 |
| Query 182 | LAGLNTPGITTVIEPIMTRDHTEKMLQGFGANLTVETDADGVRTIRLEGRGKLTGQVIDV | 241 |
| Sbjct 183 | LAGLN PG T VIE TRDHTE+ML GFGA + E D+D R I L GR +L Q I V LAGLNAPGQTVVIEREATRDHTERMLAGFGAEIVTE-DSDEGRVITLTGRPELRPQTIVV | 241 |
| Query 242 | PGDPSSTAFPLVAALLVPGSDVTILNVLMNPTRTGLILTLQEMGADIEVINPRLAGGEDV | 301 |
| Sbjct 242 | P DPSS AFP+ AAL+VPGSDV + N+ +NPTR GL TLQEMGAD+ N R GGE V PRDPSSAAFPVCAALIVPGSDVLVPNIGLNPTRAGLFYTLQEMGADLTFENLREEGGEPV | 301 |
| Query 302 | ADLRVRSST-LKGVTVPEDRAPSMIDEYPILAVAAAFAEGATVMNGLEELRVKESDRLSA | 360 |
| Sbjct 302 | ADLR R S +KG+ VP +RA SMIDEYP+L+V AAFAEGAT M G++ELRVKESDR++A ADLRARHSPGMKGIAVPPERAASMIDEYPVLSVVAAFAEGATEMRGVKELRVKESDRIAA | 361 |
| Query 361 | VANGLKLNGVDCDEGETSLVVRGRPDGKGLGNASGAAVATHLDHRIAMSFLVMGLVSENP | 420 |
| Sbjct 362 | +A+GL+ GV+ ++G +V GR G GA ATHLDHRIAMSFLV+GL S+ P MADGLRAAGVEVEDGPDWWIVHGR---GAAGVPGGATAATHLDHRIAMSFLVLGLASDAP | 418 |
| Query 421  Sbjct 419 | VTVDDATMIATSFPEFMDLMAGLGAKI 447  +TVDD IATSFP F LM LGA I MTVDDGGPIATSFPVFGTLMTDLGADI 445 |  |

>TR:A0A1I1IH10 A0A1I1IH10_9RHOB 3-phosphoshikimate 1-carboxyvinyltransferase OS=Pseudooceanicola nitratireducens OX=517719 GN=aroA PE=3

SV=1

Length=449

Score = 468 bits (1203), Expect = 5e-160

| Identities | | = 253/442 (57%), Positives = 307/442 (69%), Gaps = 6/442 (1%) |  |
| --- | --- | --- | --- |
| Query 9  Sbjct 9 | | PATARKSSGLSGTVRIPGDKSISHRSFMFGGLASGETRITGLLEGEDVINTGKAMQAMGA P + + L+GT +PGDKSISHRS + G L+ GETRI+GLLEG+DV++T KAM+A GA PMQSLAAGPLTGTADVPGDKSISHRSLILGALSMGETRISGLLEGQDVLDTAKAMRAFGA | 68  68 |
| Query 69  Sbjct 69 | | RIRK-EGDTWIIDGVGNGGLLAPEAPLDFGNAATGCRLTMGLVGVYDFDSTFIGDASLTK  + G W + GVG GG P+ +D GN+ TG RL MG + +TF GDASL K EVTDLGGGNWRVQGVGVGGFAEPDNVIDCGNSGTGVRLIMGAMATSPITATFTGDASLNK | 127  128 |
| Query 128  Sbjct 129 | | RPMGRVLNPLREMGVQVKSEDGDRLPVTLRGPKTPTPITYRVPMASAQVKSAVLLAGLNT RPM RV +PL G Q +G RLP+T+ G P P+ Y VP+ SAQVKSAVLLAGLN RPMARVTDPLALFGAQSVGREGGRLPMTIVGAADPVPVRYVVPVPSAQVKSAVLLAGLNA | 187  188 |
| Query 188  Sbjct 189 | | PGITTVIEPIMTRDHTEKMLQGFGANLTVETDADGVRTIRLEGRGKLTGQVIDVPGDPSS PG T VIE TRDHTE+ML GFGA +T E +G R I L G+ +LTGQ I VP DPSS PGQTVVIEKEATRDHTERMLAGFGATITSEDSEEG-RVITLTGQPELTGQTIVVPRDPSS | 247  247 |
| Query 248  Sbjct 248 | | TAFPLVAALLVPGSDVTILNVLMNPTRTGLILTLQEMGADIEVINPRLAGGEDVADLRVR AFP+ AAL+VPGSDV + N+ +NPTR GL TL++MGAD+ NPR GGE VADLR R AAFPVCAALIVPGSDVLVPNIGLNPTRAGLFETLRDMGADLTYENPREEGGEPVADLRAR | 307  307 |
| Query 308  Sbjct 308 | | -SSTLKGVTVPEDRAPSMIDEYPILAVAAAFAEGATVMNGLEELRVKESDRLSAVANGLK S LKG+ VP DRA SMIDEYPIL+V AA+AEG TVM G++ELRVKESDR+ A+A GL+ FSPDLKGIEVPPDRAASMIDEYPILSVVAAYAEGQTVMRGVKELRVKESDRIDAMATGLR | 366  367 |
| Query 367  Sbjct 368 | | LNGVDCDEGETSLVVRGRPDGKGLGNASGAAVATHLDHRIAMSFLVMGLVSENPVTVDDA NG++ ++GE +V GR G G GA V +HLDHRIAMSFL +G+ S PV VDD ANGIEVEDGEDWWIVTGRGHGNVPG---GAEVESHLDHRIAMSFLCLGMASTAPVRVDDG | 426  424 |
| Query 427 | | TMIATSFPEFMDLMAGLGAKIE 448 IATSFP F LMA LGA+IE |  |
| Sbjct | 425 | GPIATSFPIFEPLMATLGARIE 446 | |

>TR:A3TVC1 A3TVC1_PSEBH 3-phosphoshikimate 1-carboxyvinyltransferase OS=Pseudooceanicola batsensis (strain ATCC BAA-863 / DSM 15984

/ KCTC 12145 / HTCC2597) OX=252305 GN=aroA PE=3 SV=1

Length=451

Score = 468 bits (1203), Expect = 6e-160

| Identities | = 251/441 (57%), Positives = 306/441 (69%), Gaps = 6/441 (1%) |  |
| --- | --- | --- |
| Query 9 | PATARKSSGLSGTVRIPGDKSISHRSFMFGGLASGETRITGLLEGEDVINTGKAMQAMGA | 68 |
| Sbjct 9 | P A + L+GT +PGDKSISHRS + G L+ GETRI+GLLEG+DV++T KAM+A GA PMMAEPAGPLTGTADVPGDKSISHRSLILGALSVGETRISGLLEGQDVLDTAKAMRAFGA | 68 |
| Query 69 | RIRK-EGDTWIIDGVGNGGLLAPEAPLDFGNAATGCRLTMGLVGVYDFDSTFIGDASLTK | 127 |
| Sbjct 69 | I G W + GVG GG PE +D GN+ TG RL MG + +TF GDASL + EITDLGGGEWRVHGVGVGGFAEPETVIDCGNSGTGVRLIMGAMATSPVTATFSGDASLNR | 128 |
| Query 128 | RPMGRVLNPLREMGVQVKSEDGDRLPVTLRGPKTPTPITYRVPMASAQVKSAVLLAGLNT | 187 |
| Sbjct 129 | RPM RV +PL G Q +G RLP+T+ G + P P+ Y VP+ SAQVKSAVLLAGLN RPMARVTDPLALFGAQAVGREGGRLPMTIIGARDPVPVRYEVPVPSAQVKSAVLLAGLNA | 188 |
| Query 188 | PGITTVIEPIMTRDHTEKMLQGFGANLTVETDADGVRTIRLEGRGKLTGQVIDVPGDPSS | 247 |
| Sbjct 189 | PG T VIE TRDHTE+ML GFGA +T E +G R I L G+ +L GQ I VP DPSS PGRTVVIEREPTRDHTERMLAGFGAEITTEVTDEG-RIITLTGQPELEGQTITVPRDPSS | 247 |
| Query 248 | TAFPLVAALLVPGSDVTILNVLMNPTRTGLILTLQEMGADIEVINPRLAGGEDVADLRVR | 307 |
| Sbjct 248 | AFP+ AAL+VPGSDV + N+ +NPTR GL TL+EMGAD+ NPR GGE VADLR R  AAFPVCAALIVPGSDVLVPNIGLNPTRAGLFETLREMGADLSYENPREEGGEPVADLRAR | 307 |

| Query | 308 | -SSTLKGVTVPEDRAPSMIDEYPILAVAAAFAEGATVMNGLEELRVKESDRLSAVANGLK | 366 |
| --- | --- | --- | --- |
| Sbjct | 308 | S +KG+ VP +RA SMIDEYP+L+V AAFAEG T M G+ ELRVKESDR+ A+A GL+ YSPGMKGIAVPPERAASMIDEYPVLSVVAAFAEGRTEMRGVRELRVKESDRIDAMAVGLR | 367 |
| Query | 367 | LNGVDCDEGETSLVVRGRPDGKGLGNASGAAVATHLDHRIAMSFLVMGLVSENPVTVDDA | 426 |
| Sbjct | 368 | NG++ +EGE +V G G G A GA VATHLDHRIAMSFL +G+ +++PV +DD ANGIEVEEGEDWWIVTGCGAG---GVAGGATVATHLDHRIAMSFLCLGMAAQSPVHLDDG | 424 |
| Query | 427 | TMIATSFPEFMDLMAGLGAKI 447 IATSFP F LM LGA+I |  |
| Sbjct | 425 | QPIATSFPVFEPLMTALGARI 445 | |

>TR:A0A099GGJ7 A0A099GGJ7_9RHOB 3-phosphoshikimate 1-carboxyvinyltransferase OS=Paracoccus sanguinis OX=1545044 GN=aroA PE=3 SV=1

Length=443

Score = 467 bits (1202), Expect = 6e-160

| Identities | = 249/447 (56%), Positives = 309/447 (69%), Gaps = 9/447 (2%) |  |
| --- | --- | --- |
| Query 1 | MLHGASSRPATARKSSGLSGTVRIPGDKSISHRSFMFGGLASGETRITGLLEGEDVINTG | 60 |
| Sbjct 1 | M H A P TAR+ L+G +PGDKS+SHR+ + G LA GETRITGLLEG+DV++T MSHSADPHPLTARRGGPLTGAALVPGDKSVSHRALILGALAVGETRITGLLEGQDVLDTA | 60 |
| Query 61 | KAMQAMGARIRK--EGDTWIIDGVGNGGLLAPEAPLDFGNAATGCRLTMGLVGVYDFDST | 118 |
| Sbjct 61 | AM+A GA + + EG+ W++ GVG GG P +D GN+ TG RL MG + +T SAMRAFGAEVTRVSEGE-WVVHGVGVGGFAEPAGVIDCGNSGTGVRLIMGAMATTPITAT | 119 |
| Query 119 | FIGDASLTKRPMGRVLNPLREMGVQVKSEDGDRLPVTLRGPKTPTPITYRVPMASAQVKS | 178 |
| Sbjct 120 | F GDASL++RPM R+ +PL G ++ + +G LPVT+RG P+ Y P+ASAQ+KS FTGDASLSRRPMRRITDPLALFGAEITAREGGLLPVTIRGAAEALPVRYATPVASAQIKS | 179 |
| Query 179 | AVLLAGLNTPGITTVIEPIMTRDHTEKMLQGFGANLTVETDADGVRTIRLEGRGKLTGQV | 238 |
| Sbjct 180 | AVLLAGLN PG T VIE TRDHTE+ML GFGA +T E +G R I L GR +L Q AVLLAGLNAPGETVVIEAEPTRDHTERMLAGFGAQITTEVTDEG-RVITLTGRPELVAQP | 238 |
| Query 239 | IDVPGDPSSTAFPLVAALLVPGSDVTILNVLMNPTRTGLILTLQEMGADIEVINPRLAGG | 298 |
| Sbjct 239 | + VP DPSS AFP+ AALLVPGS++T+ V NPTR G +TL EMGAD+ + RL GG VAVPRDPSSAAFPVAAALLVPGSEITVPGVSRNPTRDGFYVTLAEMGADLTWSDDRLEGG | 298 |
| Query 299 | EDVADLRVRSSTLKGVTVPEDRAPSMIDEYPILAVAAAFAEGATVMNGLEELRVKESDRL | 358 |
| Sbjct 299 | E VADL VR L+GV VP +RA SMIDE+PILA AA AEG TVM+G+ ELRVKESDR+ EPVADLTVRHGPLRGVAVPAERAASMIDEFPILAALAARAEGTTVMHGVAELRVKESDRI | 358 |
| Query 359 | SAVANGLKLNGVDCDEGETSLVVRGRPDGKGLGNASGAAVATHLDHRIAMSFLVMGLVSE | 418 |
| Sbjct 359 | +A+A+GL+ NGV ++ SL V G G GA VATHLDHRIAMSFLV+GLVS+ AAMADGLRANGVRVEDTADSLTVHGMGAVPG GATVATHLDHRIAMSFLVLGLVSD | 413 |
| Query 419  Sbjct 414 | NPVTVDDATMIATSFPEFMDLMAGLGA 445 P+TVDD IATSFP+F+ LM LGA  APITVDDGGPIATSFPDFLPLMRRLGA 440 |  |

```
>SP:A4YJJ3 AROA_BRASO 3-phosphoshikimate 1-carboxyvinyltransferase OS=Bradyrhizobium sp. (strain ORS 278) OX=114615 GN=aroA PE=3 SV=1
Length=445

Score = 467 bits (1202), Expect = 7e-160
Identities = 252/447 (56%), Positives = 310/447 (69%), Gaps = 7/447 (2%)

Query 1	MLHGASSRPATARKSSGLSGTVRIPGDKSISHRSFMFGGLASGETRITGLLEGEDVINTG 60 M	A+ P +R S LSGT+R+PGDKSISHR+ + G L+ G TRI+GLLEGEDV+NT
Sbjct 1	MSTSAAPTPLESRASGPLSGTIRVPGDKSISHRALILGALSVGGTRISGLLEGEDVLNTA 60
```

| Query | 61 | KAMQAMGARIRKEGD-TWIIDGVGNGGLLAPEAPLDFGNAATGCRLTMGLVGVYDFDSTF | 119 |
| --- | --- | --- | --- |
| Sbjct | 61 | K+M+ +GA++ + G+ W ++GVG GG P A LDFGN+ TGCRL MG V + F KSMRTLGAKVERTGEFAWTVNGVGVGGFAQPAATLDFGNSGTGCRLVMGAVAGCPISAVF | 120 |
| Query | 120 | IGDASLTKRPMGRVLNPLREMGVQVK-SEDGDRLPVTLRGPKTPTPITYRVPMASAQVKS | 178 |
| Sbjct | 121 | GDASL RPM R+L+PL MG +V S +G +LP+TL+G P PI YR P+ASAQ+KS DGDASLRSRPMRRILDPLALMGAKVTASAEGGKLPLTLQGASNPVPIEYRTPVASAQIKS | 180 |
| Query | 179 | AVLLAGLNTPGITTVIEPIMTRDHTEKMLQGFGANLTVETDADGVRTIRLEGRGKLTGQV | 238 |
| Sbjct | 181 | AVLLAGL PG+TTVIE +RDHTE ML+ FGA + + R I L G+ +L G AVLLAGLAAPGVTTVIEQEASRDHTELMLKHFGAEIVTTPEGSHGRRIALTGQPELRGAP | 240 |
| Query | 239 | IDVPGDPSSTAFPLVAALLVPGSDVTILNVLMNPTRTGLILTLQEMGADIEVINPRLAGG | 298 |
| Sbjct | 241 | + VP DPSS AFPLVAAL+V GSD+ + +V+ NP RTGL TL+EMGA IE + R G VIVPADPSSAAFPLVAALIVDGSDLVLSDVMTNPLRTGLFTTLREMGASIEEDDVRGDAG | 300 |
| Query | 299 | EDVADLRVRSSTLKGVTVPEDRAPSMIDEYPILAVAAAFAEGATVMNGLEELRVKESDRL | 358 |
| Sbjct | 301 | E +A LRVR+S LKGV VP +RAPSMIDEY +LAVAAA+AEG T+M GL ELRVKESDRL EPMARLRVRASKLKGVEVPPERAPSMIDEYLVLAVAAAYAEGTTIMRGLHELRVKESDRL | 360 |
| Query | 359 | SAVANGLKLNGVDCDEGETSLVVRGRPDGKGLGNASGAAVATHLDHRIAMSFLVMGLVSE | 418 |
| Sbjct | 361 | A A L++NGV + L+V GR G G VATH+DHRIAMS LVMGL S+ EATAAMLRVNGVKVEITGDDLIVEGRGHVPG GGLVATHMDHRIAMSALVMGLASD | 415 |
| Query  Sbjct | 419  416 | NPVTVDDATMIATSFPEFMDLMAGLGA 445 PVTVDD IATSFP+F+ LM GA  KPVTVDDTAFIATSFPDFIPLMRKAGA 442 |  |

>TR:A0A0D1P864 A0A0D1P864_BRAEL 3-phosphoshikimate 1-carboxyvinyltransferase OS=Bradyrhizobium elkanii OX=29448 GN=aroA PE=3 SV=1

Length=445

Score = 467 bits (1202), Expect = 7e-160

| Identities | = 249/447 (56%), Positives = 309/447 (69%), Gaps = 7/447 (2%) |  |
| --- | --- | --- |
| Query 1 | MLHGASSRPATARKSSGLSGTVRIPGDKSISHRSFMFGGLASGETRITGLLEGEDVINTG | 60 |
| Sbjct 1 | M H + P +R L+G VR+PGDKSISHR+ + G L+ GETRI+GLLEGEDV+NT MAHSDTPTPLESRACGPLAGKVRVPGDKSISHRALILGALSVGETRISGLLEGEDVLNTA | 60 |
| Query 61 | KAMQAMGARIRKEGD-TWIIDGVGNGGLLAPEAPLDFGNAATGCRLTMGLVGVYDFDSTF | 119 |
| Sbjct 61 | K+M+A+GA++ + TW + GVG GG P PLDFGN+ TGCRL MG V + F KSMRALGAKVERTAPFTWSVAGVGVGGFAQPAGPLDFGNSGTGCRLVMGAVAGCPIAAVF | 120 |
| Query 120 | IGDASLTKRPMGRVLNPLREMGVQVKS-EDGDRLPVTLRGPKTPTPITYRVPMASAQVKS | 178 |
| Sbjct 121 | GDASL RPM R+L+PL MG + ++G RLP+TL G + P PI Y+ P+ASAQ+KS DGDASLRSRPMRRILDPLELMGARTSDIKEGGRLPLTLHGARYPVPIVYKTPVASAQIKS | 180 |
| Query 179 | AVLLAGLNTPGITTVIEPIMTRDHTEKMLQGFGANLTVETDADGVRTIRLEGRGKLTGQV | 238 |
| Sbjct 181 | AVLLAGL+ PGITTVIE +RDHTE ML+ FGA +T E + R I L G +L G AVLLAGLSAPGITTVIEQEASRDHTELMLKHFGAEITSEKEGSHGRRITLNGEPELHGGN | 240 |
| Query 239 | IDVPGDPSSTAFPLVAALLVPGSDVTILNVLMNPTRTGLILTLQEMGADIEVINPRLAGG | 298 |
| Sbjct 241 | + VP DPSS AFP+VAAL+V GSDV +V+ NP RTGL TL+EMGA IE + R G VVVPADPSSAAFPIVAALIVEGSDVVFADVMTNPLRTGLFTTLREMGASIEESDVRGDAG | 300 |
| Query 299 | EDVADLRVRSSTLKGVTVPEDRAPSMIDEYPILAVAAAFAEGATVMNGLEELRVKESDRL | 358 |
| Sbjct 301 | E +A LRVR+S L+GV VP +RAPSMIDEY +LAVAAAFAEG T+M GL+ELRVKESDRL EPMAQLRVRASKLRGVEVPPERAPSMIDEYLVLAVAAAFAEGTTIMRGLQELRVKESDRL | 360 |
| Query 359 | SAVANGLKLNGVDCDEGETSLVVRGRPDGKGLGNASGAAVATHLDHRIAMSFLVMGLVSE | 418 |
| Sbjct 361 | A A+ L++NGV + L+V GR G G VATH+DHRIAMS LVMG ++  EATADMLRVNGVKVEVSGDDLIVEGRGHVPG GGLVATHMDHRIAMSALVMGCAAD | 415 |

Query 419 NPVTVDDATMIATSFPEFMDLMAGLGA 445 PV +DD	IATSFP+F+ +M LGA

Sbjct 416 QPVKIDDTGFIATSFPDFIPMMRTLGA 442

>TR:A0A0A8JZM6 A0A0A8JZM6_9RHIZ 3-phosphoshikimate 1-carboxyvinyltransferase OS=Methyloceanibacter caenitepidi OX=1384459 GN=aroA PE=3 SV=1

Length=437

Score = 467 bits (1201), Expect = 7e-160

| Identities | = 248/440 (56%), Positives = 313/440 (71%), Gaps = 6/440 (1%) |  |
| --- | --- | --- |
| Query 8 | RPATARKSSGLSGTVRIPGDKSISHRSFMFGGLASGETRITGLLEGEDVINTGKAMQAMG | 67 |
| Sbjct 3 | +P TAR+S+ +SG V++PGDKSISHR+ + GLA+G TRI GLLE +DV+ T +A+ A+G QPLTARRSAAISGRVKVPGDKSISHRALILAGLATGRTRIEGLLEADDVLATARAVAALG | 62 |
| Query 68 | ARIRKEGDTWIIDGVGNGGLLAPEAPLDFGNAATGCRLTMGLVGVYDFDSTFIGDASLTK | 127 |
| Sbjct 63 | A + ++GD + + G G GGL APE LDFGN+ T RL +G++ Y + GDASL + ASVDRDGDVFEVLGQGVGGLTAPEGLLDFGNSGTASRLMLGVLAGYPMHVSLTGDASLCR | 122 |
| Query 128 | RPMGRVLNPLREMGVQVKSEDGDRLPVTLRGPKTPTPITYRVPMASAQVKSAVLLAGLNT | 187 |
| Sbjct 123 | RPM RVL PL EMG+ V+ +D + LP+T+ G + PI Y +P+ SAQVKSA+LLAGL+ RPMSRVLKPLMEMGLLVE-DDRETLPLTVIGTQDLLPIVYELPVPSAQVKSAILLAGLHA | 181 |
| Query 188 | PGITTVIEPIMTRDHTEKMLQGFGANLTVETDADGVRTIRLEGRGKLTGQVIDVPGDPSS | 247 |
| Sbjct 182 | PG TTVIEP+ TRDHTE+ML+ FGA + +ET ADG R + + G +L G + VPGDPSS PGRTTVIEPLPTRDHTERMLRYFGAEVDIETRADGSRAVVVAGDAELRGAHVVVPGDPSS | 241 |
| Query 248 | TAFPLVAALLVPGSDVTILNVLMNPTRTGLILTLQEMGADIEVINPRLAGGEDVADLRVR | 307 |
| Sbjct 242 | AF + AAL+ PGSD+TI NVL+NPTRTG TLQEMGADI V++ R A GE V DLR+R AAFLVAAALICPGSDLTIENVLLNPTRTGFFDTLQEMGADIAVLDRRDAAGEPVGDLRIR | 301 |
| Query 308 | SSTLKGVTVPEDRAPSMIDEYPILAVAAAFAEGATVMNGLEELRVKESDRLSAVANGLKL | 367 |
| Sbjct 302 | S L GV VP +RAPSMIDEYPILA AAFA+G T M GL EL+VKESDRL+A A GL SGALSGVRVPPERAPSMIDEYPILAALAAFAQGETRMEGLAELKVKESDRLAATAAGLAA | 361 |
| Query 368 | NGVDCDEGETSLVVRGRPDGKGLGNASGAAVATHLDHRIAMSFLVMGLVSENPVTVDDAT | 427 |
| Sbjct 362 | GV L+V G +G G VAT +DHRIAM+FL +GL +E PV VDD CGVTARVEGDDLIVTGTGAVRG GGTVATEMDHRIAMAFLTLGLGAEKPVAVDDIA | 416 |
| Query 428  Sbjct 417 | MIATSFPEFMDLMAGLGAKI 447 MIATSFP F+ LM GLGA++ MIATSFPAFVPLMTGLGARL 436 |  |

>TR:E3I1X1 E3I1X1_RHOVT 3-phosphoshikimate 1-carboxyvinyltransferase OS=Rhodomicrobium vannielii (strain ATCC 17100 / ATH 3.1.1 / DSM

162 / LMG 4299) OX=648757 GN=aroA PE=3 SV=1

Length=459

Score = 468 bits (1203), Expect = 8e-160

| Identities | = 253/447 (57%), Positives = 310/447 (69%), Gaps = 7/447 (2%) |  |
| --- | --- | --- |
| Query 3 | HGASSRPATARKSSGLSGTVRIPGDKSISHRSFMFGGLASGETRITGLLEGEDVINTGKA | 62 |
| Sbjct 4 | H A RP +RKS L G V +PGDKSISHR+ + G LA G TRITGLLE ED++ T +A HAADVRPLMSRKSGRLQGAVVVPGDKSISHRALILGALAEGRTRITGLLEAEDILCTARA | 63 |
| Query 63 | MQAMGARIRKEGD-TWIIDGVGNGGLLAPEAPLDFGNAATGCRLTMGLVGVYDFDSTFIG | 121 |
| Sbjct 64 | ++A+GA + ++ D W + G G GGL AP LDFGN+ TG RL MG+V + + F G LEALGAGVERDADGAWTVTGRGLGGLAAPAGDLDFGNSGTGARLMMGVVAGHPLAARFTG | 123 |
| Query 122 | DASLTKRPMGRVLNPLREMGVQVKSEDGDRLPVTLRGPKTPTPITYRVPMASAQVKSAVL | 181 |
| Sbjct 124 | DASL KRPMGRVL PL+ MG+ ++ E + LP+TL G PI+YR+P+ SAQVKSAVL  DASLQKRPMGRVLAPLQSMGLAIEEEGRNTLPLTLVGTGDLVPISYRLPVPSAQVKSAVL | 183 |

| Query | 182 | LAGLNTPGITTVIEPIMTRDHTEKMLQGFGANLTVETDADGVRTIRLEGRGKLTGQVIDV | 241 |
| --- | --- | --- | --- |
| Sbjct | 184 | LAGL G T+VIE +RDHTEKML+ FGA ++VE G+R I LEGR L GQ + V LAGLFASGETSVIESEKSRDHTEKMLRYFGAEISVEPFEGGLR-IALEGRRTLQGQPVAV | 242 |
| Query | 242 | PGDPSSTAFPLVAALLVPGSDVTILNVLMNPTRTGLILTLQEMGADIEVINPRLAGGEDV | 301 |
| Sbjct | 243 | PGDPSS AF + AAL+ P SD+ + NVL+NPTRTG TL EMGADI N R GE V PGDPSSAAFLVAAALITPASDILVKNVLVNPTRTGFYETLAEMGADISFENEREVSGEPV | 302 |
| Query | 302 | ADLRVRSSTLKGVTVPEDRAPSMIDEYPILAVAAAFAEGATVMNGLEELRVKESDRLSAV | 361 |
| Sbjct | 303 | AD+R R+S L+GVTVP RAPSMIDEYP+LA A AEG T+M GL ELRVKESDRL+A+ ADIRARTSELRGVTVPAARAPSMIDEYPMLAALATLAEGETLMEGLAELRVKESDRLAAM | 362 |
| Query | 362 | ANGLKLNGVDCDEGETSLVVRGRPDGKGLGNASGAAVATHLDHRIAMSFLVMGLVSENPV | 421 |
| Sbjct | 363 | +GL G +L V G P +G GA + TH+DHRIAMSFLV+GL +E PV VDGLVACGAIAHARGDTLTVLGLPKVRG GATIKTHMDHRIAMSFLVLGLATEEPV | 417 |
| Query  Sbjct | 422  418 | TVDDATMIATSFPEFMDLMAGLGAKIE 448 TVDDA+MIATSFPEF LM +GA ++ TVDDASMIATSFPEFRTLMQQVGATLD 444 |  |

>TR:A0A1G9CEE8 A0A1G9CEE8_9RHOB 3-phosphoshikimate 1-carboxyvinyltransferase OS=Albidovulum xiamenense OX=990712 GN=aroA PE=3 SV=1

Length=449

Score = 467 bits (1202), Expect = 8e-160

| Identities | | = 250/441 (57%), Positives = 304/441 (69%), Gaps = 5/441 (1%) |  |
| --- | --- | --- | --- |
| Query 9  Sbjct 9 | | PATARKSSGLSGTVRIPGDKSISHRSFMFGGLASGETRITGLLEGEDVINTGKAMQAMGA P TAR S LSG +PGDKS+SHR+ + G LA GETRITGLLEG+DV++T +AM+A GA PMTARPSGPLSGVAEVPGDKSVSHRALILGALAVGETRITGLLEGQDVLDTARAMRAFGA | 68  68 |
| Query 69  Sbjct 69 | | RIRKEGD-TWIIDGVGNGGLLAPEAPLDFGNAATGCRLTMGLVGVYDFDSTFIGDASLTK  ++ +EG+ W + GVG GG P +D GN+ TG RL MG + +TF GDASL QVEREGEGLWSVRGVGVGGFSEPSDVIDCGNSGTGVRLIMGAMATTPITATFTGDASLRS | 127  128 |
| Query 128  Sbjct 129 | | RPMGRVLNPLREMGVQVKSEDGDRLPVTLRGPKTPTPITYRVPMASAQVKSAVLLAGLNT RPMGRV PL G + G RLP+T+ G P P+ Y VP+ SAQVKSAVLLAGLN RPMGRVTEPLALFGARAVGRAGGRLPMTVVGAPAPVPVRYAVPVPSAQVKSAVLLAGLNA | 187  188 |
| Query 188  Sbjct 189 | | PGITTVIEPIMTRDHTEKMLQGFGANLTVETDADGVRTIRLEGRGKLTGQVIDVPGDPSS PG T VIE TRDHTE+ML GFGA + E +G R I L G+ +L Q I VP DPSS PGQTVVIEREATRDHTERMLAGFGAEIVTEDGPEG-RVITLTGQPELKPQTIAVPRDPSS | 247  247 |
| Query 248  Sbjct 248 | | TAFPLVAALLVPGSDVTILNVLMNPTRTGLILTLQEMGADIEVINPRLAGGEDVADLRVR AFP+ AAL+V S+V + + +NPTR G TLQEMGAD+ N R GGE VADLRVR AAFPVAAALIVEDSEVLVPGIGLNPTRAGFFTTLQEMGADLSFENARDEGGEPVADLRVR | 307  307 |
| Query 308  Sbjct 308 | | SSTLKGVTVPEDRAPSMIDEYPILAVAAAFAEGATVMNGLEELRVKESDRLSAVANGLKL STL+G+ VP +RAP+MIDEYPIL+ AAFAEG TVM G+ ELRVKESDR+ A+A GL+ FSTLRGIEVPPERAPTMIDEYPILSAVAAFAEGTTVMRGVGELRVKESDRIDAMARGLEA | 367  367 |
| Query 368  Sbjct 368 | | NGVDCDEGETSLVVRGRPDGKGLGNASGAAVATHLDHRIAMSFLVMGLVSENPVTVDDAT GV +E E +L+V G G G GA V + LDHRIAMSFLV+GL + PVTVDDA+ CGVRVEEDEETLIVHGLGPG---GVPGGATVESRLDHRIAMSFLVLGLAARAPVTVDDAS | 427  424 |
| Query 428 | | MIATSFPEFMDLMAGLGAKIE 448 IATSFP F +LM GLGA++E |  |
| Sbjct | 425 | PIATSFPVFEELMRGLGARLE 445 | |

>TR:A0A238KIG0 A0A238KIG0_9RHOB 3-phosphoshikimate 1-carboxyvinyltransferase OS=Maliponia aquimaris OX=1673631 GN=aroA PE=3 SV=1

Length=447

Score = 467 bits (1201), Expect = 1e-159

| Identities | = 252/446 (57%), Positives = 308/446 (69%), Gaps = 6/446 (1%) |  |
| --- | --- | --- |
| Query 3 | HGASSRPATARKSSGLSGTVRIPGDKSISHRSFMFGGLASGETRITGLLEGEDVINTGKA | 62 |
| Sbjct 4 | HGA+ P T+R S L+G +PGDKSISHRS + G L+ GETR+TGLLEG+DV++T KA HGAAI-PMTSRASRPLTGEAHVPGDKSISHRSLILGALSVGETRVTGLLEGQDVLDTAKA | 62 |
| Query 63 | MQAMGARIRKEGDTWIIDGVGNGGLLAPEAPLDFGNAATGCRLTMGLVGVYDFDSTFIGD | 122 |
| Sbjct 63 | M+A GA++ K GDTW + GVG GG P + +D GN+ TG RL MG + +TF GD MRAFGAQVEKTGDTWHVHGVGVGGFAEPGSVIDCGNSGTGVRLIMGAMATSPITATFTGD | 122 |
| Query 123 | ASLTKRPMGRVLNPLREMGVQVKSEDGDRLPVTLRGPKTPTPITYRVPMASAQVKSAVLL | 182 |
| Sbjct 123 | ASL KRPM RV +PL G + G RLP+T+ G P P+ Y VP+ SAQVKSAVLL ASLNKRPMARVTDPLALFGARAYGRSGGRLPMTIIGAADPRPVRYTVPVPSAQVKSAVLL | 182 |
| Query 183 | AGLNTPGITTVIEPIMTRDHTEKMLQGFGANLTVETDADGVRTIRLEGRGKLTGQVIDVP | 242 |
| Sbjct 183 | AGLN PG T VIE TRDH+E+ML GFGA ++ E +G R I L+GR +L Q I VP AGLNAPGETVVIEREATRDHSERMLAGFGATISTEVTDEG-RIITLQGRPELKPQTIIVP | 241 |
| Query 243 | GDPSSTAFPLVAALLVPGSDVTILNVLMNPTRTGLILTLQEMGADIEVINPRLAGGEDVA | 302 |
| Sbjct 242 | DPSS AFP+ AAL+V GSDV + N+ +NPTR GL TLQEMGAD+ N R GGE VA RDPSSAAFPVCAALIVEGSDVLVPNIGLNPTRAGLFFTLQEMGADLTFENMREEGGEPVA | 301 |
| Query 303 | DLRVR-SSTLKGVTVPEDRAPSMIDEYPILAVAAAFAEGATVMNGLEELRVKESDRLSAV | 361 |
| Sbjct 302 | DLR R S LKG+ VP +RA SMIDEYP+L+V AAFAEG T M G++ELRVKESDR+ A+ DLRARFSPDLKGIEVPAERAASMIDEYPVLSVVAAFAEGRTHMPGVKELRVKESDRIEAM | 361 |
| Query 362 | ANGLKLNGVDCDEGETSLVVRGRPDGKGLGNASGAAVATHLDHRIAMSFLVMGLVSENPV | 421 |

A GL+ GV ++GE	V G	G	G	G	ATH+DHRIAMSFLV+GL ++ P+

| Sbjct | 362 | ATGLRAAGVTVEDGEDWWTVHGLGHGNVPG---GVTAATHIDHRIAMSFLVLGLATQAPM | 418 |
| --- | --- | --- | --- |
| Query  Sbjct | 422  419 | TVDDATMIATSFPEFMDLMAGLGAKI 447 TVDD + IATSFP F LM LGA+I TVDDGSPIATSFPIFEGLMGALGAQI 444 |  |

>TR:A0A2R8BL89 A0A2R8BL89_9RHOB 3-phosphoshikimate 1-carboxyvinyltransferase OS=Defluviimonas aquaemixtae OX=1542388 GN=aroA PE=3 SV=1

Length=468

Score = 468 bits (1203), Expect = 1e-159

| Identities | = 251/440 (57%), Positives = 302/440 (69%), Gaps = 5/440 (1%) |  |
| --- | --- | --- |
| Query 9 | PATARKSSGLSGTVRIPGDKSISHRSFMFGGLASGETRITGLLEGEDVINTGKAMQAMGA | 68 |
| Sbjct 28 | P TARKS L G +PGDKSISHR+ + G LA GET +TGLLEG+DV++T +AM+A GA PMTARKSGPLRGVAEVPGDKSISHRALILGALAVGETTVTGLLEGQDVLDTAEAMRAFGA | 87 |
| Query 69 | RIRKEGD-TWIIDGVGNGGLLAPEAPLDFGNAATGCRLTMGLVGVYDFDSTFIGDASLTK | 127 |
| Sbjct 88 | + + G+ W + GVG GG P+ +D GN+ TG RL MG + +TF GDASL K EVTRTGEGAWSVHGVGVGGFAEPKQVIDCGNSGTGVRLIMGAMATSPVTATFTGDASLNK | 147 |
| Query 128 | RPMGRVLNPLREMGVQVKSEDGDRLPVTLRGPKTPTPITYRVPMASAQVKSAVLLAGLNT | 187 |
| Sbjct 148 | RPMGRV +PL G G RLP+T+ G P P+ Y VP+ SAQVKSAVLLAGLN RPMGRVTDPLTLFGAHAYGRRGGRLPMTVVGADDPVPVRYAVPVPSAQVKSAVLLAGLNA | 207 |
| Query 188 | PGITTVIEPIMTRDHTEKMLQGFGANLTVETDADGVRTIRLEGRGKLTGQVIDVPGDPSS | 247 |
| Sbjct 208 | PG T VIE TRDHTE+ML+GFGA LT E A+G R I L G+ +L Q + VP DPSS PGQTIVIEKDATRDHTERMLRGFGAELTSEDRAEG-RVITLTGQPELRPQTVAVPRDPSS | 266 |
| Query 248 | TAFPLVAALLVPGSDVTILNVLMNPTRTGLILTLQEMGADIEVINPRLAGGEDVADLRVR | 307 |
| Sbjct 267 | AFP+ AAL+V GSD+ + V NPTR GL TL EMGADI NPR GGE VADLRVR  AAFPVCAALIVEGSDILVPGVSQNPTRNGLYTTLVEMGADIAFENPREEGGEPVADLRVR | 326 |

| Query | 308 | SSTLKGVTVPEDRAPSMIDEYPILAVAAAFAEGATVMNGLEELRVKESDRLSAVANGLKL | 367 |
| --- | --- | --- | --- |
| Sbjct | 327 | +STLKG+ VPE+RA SMIDEYPIL+V AAFA+G TVM G++ELRVKESDR+ A+A GL+ ASTLKGIEVPEERAASMIDEYPILSVVAAFADGTTVMRGVKELRVKESDRIDAMARGLEA | 386 |
| Query | 368 | NGVDCDEGETSLVVRGRPDGKGLGNASGAAVATHLDHRIAMSFLVMGLVSENPVTVDDAT | 427 |
| Sbjct | 387 | GV +E E L+V G G GA T +DHRIAMSFL++GL + PV +DD CGVGIEEEEDVLIVHGMGP---KGVPGGATAKTRIDHRIAMSFLILGLAARKPVGIDDGA | 443 |
| Query | 428 | MIATSFPEFMDLMAGLGAKI 447 IATSFP F +LM GLGA I |  |
| Sbjct | 444 | PIATSFPVFENLMKGLGADI 463 | |

>TR:A0A2T0WG05 A0A2T0WG05_9RHOB 3-phosphoshikimate 1-carboxyvinyltransferase OS=Donghicola tyrosinivorans OX=1652492 GN=aroA PE=3 SV=1

Length=448

Score = 467 bits (1201), Expect = 1e-159

| Identities | | = 254/443 (57%), Positives = 303/443 (68%), Gaps = 8/443 (2%) |  |
| --- | --- | --- | --- |
| Query 9  Sbjct 9 | | PATARKSSGLSGTVRIPGDKSISHRSFMFGGLASGETRITGLLEGEDVINTGKAMQAMGA P T+ S LSG +PGDKSISHRS + G +A GETRITGLLEG+DV++T KAM A GA PMTSYASGPLSGEAHVPGDKSISHRSLILGAMAIGETRITGLLEGQDVLDTAKAMAAFGA | 68  68 |
| Query 69 | | RIRKEGD-TWIIDGVGNGGLLAPEAPLDFGNAATGCRLTMGLVGVYDFDSTFIGDASLTK  + G W + GVG GG P+ +D GN+ TG RL MG + TF GDASL K | 127 |
| Sbjct | 69 | EVTNHGGGNWSVKGVGVGGFAEPDNVIDCGNSGTGVRLIMGAMATSAITVTFTGDASLNK | 128 |
| Query | 128 | RPMGRVLNPLREMGVQVKSEDGDRLPVTLRGPKTPTPITYRVPMASAQVKSAVLLAGLNT | 187 |
| Sbjct | 129 | RPM RV +PL G Q G RLP+T+ G P P+ Y VP+ SAQVKSAVLLAGLN RPMARVTDPLALFGTQSVGRSGGRLPMTIVGAADPAPVRYTVPVPSAQVKSAVLLAGLNV | 188 |
| Query | 188 | PGITTVIEPIMTRDHTEKMLQGFGANLTVETDADGVRTIRLEGRGKLTGQVIDVPGDPSS | 247 |
| Sbjct | 189 | PG T VIE TRDHTE+ML+GFGA +T E +G R I L+G+ +L Q I VP DPSS PGETVVIEQEATRDHTERMLEGFGAEITTEVTDEG-RVITLKGQPELKPQDITVPRDPSS | 247 |
| Query | 248 | TAFPLVAALLVPGSDVTILNVLMNPTRTGLILTLQEMGADIEVINPRLAGGEDVADLRVR | 307 |
| Sbjct | 248 | AFP+ AA+L PGSDV + N+ +NPTR GL TL+EMGAD+ N R+ GGE +ADLR R AAFPVCAAILTPGSDVLVPNIGLNPTRAGLFTTLREMGADLTYENERVEGGEPMADLRAR | 307 |
| Query | 308 | -SSTLKGVTVPEDRAPSMIDEYPILAVAAAFAEGATVMNGLEELRVKESDRLSAVANGLK | 366 |
| Sbjct | 308 | S +KG++VP +RA SMIDEYP+L+V AA AEG T M G++ELRVKESDR+ A+A GL+ YSPNMKGISVPPERAASMIDEYPVLSVVAANAEGVTEMRGVKELRVKESDRIDAMAVGLR | 367 |
| Query | 367 | LNGVDCDEGETSLVVRGRPDGKGLGNA-SGAAVATHLDHRIAMSFLVMGLVSENPVTVDD | 425 |
| Sbjct | 368 | NGV DEGE V G G GN GA ATHLDHRIAMSFLV+GL S+ P++VDD ANGVQVDEGEDWWTV TGLGAGNVPGGATCATHLDHRIAMSFLVLGLCSKQPISVDD | 423 |
| Query | 426 | ATMIATSFPEFMDLMAGLGAKIE 448 IATSFP F LM GLGAKIE |  |
| Sbjct | 424 | GGAIATSFPIFESLMGGLGAKIE 446 | |

```
>TR:A0A1H4DXU0 A0A1H4DXU0_9RHOB 3-phosphoshikimate 1-carboxyvinyltransferase OS=Rubrimonas cliftonensis OX=89524 GN=aroA PE=3 SV=1
Length=449

Score = 467 bits (1201), Expect = 1e-159
Identities = 256/445 (58%), Positives = 306/445 (69%), Gaps = 6/445 (1%)

Query 3	HGASSRPATARKSSGLSGTVRIPGDKSISHRSFMFGGLASGETRITGLLEGEDVINTGKA 62 H A+ R TA KS L G	PGDKS+SHR+ +FG L+ GETR+TGLLEGEDV+NT A
Sbjct 4	HAAADR-VTALKSGPLRGEAVTPGDKSVSHRALIFGALSIGETRVTGLLEGEDVLNTAAA 62
```

| Query | 63 | MQAMGARIRKEGD-TWIIDGVGNGGLLAPEAPLDFGNAATGCRLTMGLVGVYDFDSTFIG | 121 |
| --- | --- | --- | --- |
| Sbjct | 63 | M++ GA + + G W I GVG GG PE +D GN+ TG RL MG + +TF G MRSFGADVERTGSGAWRIRGVGVGGFSEPENVIDCGNSGTGVRLIMGAMATTPIAATFTG | 122 |
| Query | 122 | DASLTKRPMGRVLNPLREMGVQVKSEDGDRLPVTLRGPKTPTPITYRVPMASAQVKSAVL | 181 |
| Sbjct | 123 | DASL RPMGRV PL E G Q G RLP+TL G P P+ +R+ +ASAQVKSA+L DASLRGRPMGRVTEPLAEFGAQACGRSGGRLPLTLMGAANPGPVEHRLRVASAQVKSAML | 182 |
| Query | 182 | LAGLNTPGITTVIEPIMTRDHTEKMLQGFGANLTVETDADGVRTIRLEGRGKLTGQVIDV | 241 |
| Sbjct | 183 | LAGLN PG+TT+IEP TRDHTE+ML+GFGA ++VE DG R IRL G+ +L Q + LAGLNAPGVTTIIEPEPTRDHTERMLRGFGAEVSVEETKDG-RVIRLVGQPELRAQTVAA | 241 |
| Query | 242 | PGDPSSTAFPLVAALLVPGSDVTILNVLMNPTRTGLILTLQEMGADIEVINPRLAGGEDV | 301 |
| Sbjct | 242 | P DPSS AFP+ AAL+V GSDV ++ +NPTR GL TL+EMGAD+ N R GGE V PRDPSSAAFPVCAALIVEGSDVLAPSICLNPTRAGLYHTLREMGADLTFENLREEGGEPV | 301 |
| Query | 302 | ADLRVRSSTLKGVTVPEDRAPSMIDEYPILAVAAAFAEGATVMNGLEELRVKESDRLSAV | 361 |
| Sbjct | 302 | ADLR R S LKGV P DRAPSMIDEYP+LAV AAFAEG TVM G+ ELRVKESDR+ A+ ADLRARFSALKGVETPPDRAPSMIDEYPVLAVVAAFAEGPTVMRGVAELRVKESDRIDAM | 361 |
| Query | 362 | ANGLKLNGVDCDEGETSLVVRGRPDGKGLGNASGAAVATHLDHRIAMSFLVMGLVSENPV | 421 |
| Sbjct | 362 | A L+ GV E +L+V GR G A GA VA+ LDHRIAM+ LVMGL + + V ARALEACGVKVQEDADTLIVHGRGAD---GVAGGATVASRLDHRIAMANLVMGLAARDGV | 418 |
| Query  Sbjct | 422  419 | TVDDATMIATSFPEFMDLMAGLGAK 446 TVDDA+ IATSFP F LM GLGA+ TVDDASPIATSFPGFEALMTGLGAR 443 |  |

>TR:A0A178MXK9 A0A178MXK9_9PROT 3-phosphoshikimate 1-carboxyvinyltransferase OS=Magnetospirillum marisnigri OX=1285242 GN=aroA PE=3 SV=1

Length=444

Score = 466 bits (1200), Expect = 1e-159

| Identities | = 258/443 (58%), Positives = 303/443 (68%), Gaps = 5/443 (1%) |  |
| --- | --- | --- |
| Query 7 | SRPATARKSSGLSGTVRIPGDKSISHRSFMFGGLASGETRITGLLEGEDVINTGKAMQAM | 66 |
| Sbjct 2 | ++P + + S L+G+ RIPGDKSISHR+ MFG LA GE+ +TGLLEG+DV+ T AM+A+ AKPLISSRVSRLAGSARIPGDKSISHRALMFGALAVGESTVTGLLEGDDVLRTASAMRAL | 61 |
| Query 67 | GARIRKEGD-TWIIDGVGNGGLLAPEAPLDFGNAATGCRLTMGLVGVYDFDSTFIGDASL | 125 |
| Sbjct 62 | GA + + D +W + G G GGL+ P LD GN+ TG RL MGLV + F + F GDASL GAEVERLADGSWRLFGRGVGGLVEPADVLDMGNSGTGARLLMGLVAAHPFTTIFTGDASL | 121 |
| Query 126 | TKRPMGRVLNPLREMGVQVKSEDGDRLPVTLRGPKTPTPITYRVPMASAQVKSAVLLAGL | 185 |
| Sbjct 122 | RPM RV PL MG + DG RLP+ + G P PITY +P+ASAQVKSA+LLAGL RSRPMRRVSAPLERMGARFVGRDGGRLPMAVVGATQPLPITYELPVASAQVKSAILLAGL | 181 |
| Query 186 | NTPGITTVIEPIMTRDHTEKMLQGFGANLTVETDADGVRTIRLEGRGKLTGQVIDVPGDP | 245 |
| Sbjct 182 | NTPG TTVIE TRDHTE ML+ FGA + E G I + G +LTG+ + VP DP NTPGETTVIEREATRDHTELMLRNFGATVRTEAAEGGGFAITVTGYPELTGRPVVVPADP | 241 |
| Query 246 | SSTAFPLVAALLVPGSDVTILNVLMNPTRTGLILTLQEMGADIEVINPRLAGGEDVADLR | 305 |
| Sbjct 242 | SS AFP VAALLV GS++ + V NP RTGL TL EMGADI + N R GE VADL SSAAFPAVAALLVEGSEIRLPGVGTNPLRTGLYQTLLEMGADIYLDNARDQAGEPVADLV | 301 |
| Query 306 | VRSSTLKGVTVPEDRAPSMIDEYPILAVAAAFAEGATVMNGLEELRVKESDRLSAVANGL | 365 |
| Sbjct 302 | VRSS LKGV VP +RAPSMIDEYPILAVAAAFAEG T M GL ELRVKESDRL+A+A GL VRSSRLKGVDVPAERAPSMIDEYPILAVAAAFAEGTTTMRGLGELRVKESDRLAAMARGL | 361 |
| Query 366 | KLNGVDCDEGETSLVVRGRPDGKGLGNASGAAVATHLDHRIAMSFLVMGLVSENPVTVDD | 425 |
| Sbjct 362 | GV +E + +L+V G G A A V TH DHRIAMSFLVMG+ S PV VDD  AACGVAVEETQDTLIVH GTGRAPAGDATVTTHFDHRIAMSFLVMGMASARPVKVDD | 417 |

Query 426 ATMIATSFPEFMDLMAGLGAKIE 448

A I TSFP F LM GLG KIE Sbjct 418 AEAIDTSFPGFAALMNGLGGKIE 440

>TR:A0A255Y420 A0A255Y420_9SPHN 3-phosphoshikimate 1-carboxyvinyltransferase OS=Sandarakinorhabdus cyanobacteriorum OX=1981098 GN=aroA PE=3

SV=1

Length=455

Score = 467 bits (1201), Expect = 1e-159

| Identities | = 256/447 (57%), Positives = 305/447 (68%), Gaps = 13/447 (3%) |  |
| --- | --- | --- |
| Query 8 | RPATARKSSGLSGTVRIPGDKSISHRSFMFGGLASGETRITGLLEGEDVINTGKAMQAMG | 67 |
| Sbjct 9 | +P + LSG V +PGDKSISHRS M G LA GETR+TGLLEGEDV+ T AM+AMG QPLCLTTTRPLSGDVTVPGDKSISHRSLMLGALAVGETRVTGLLEGEDVLATAAAMRAMG | 68 |
| Query 68 | ARI-RKEGDTWIIDGVGNGGLLAPEAPLDFGNAATGCRLTMGLVGVYDFDSTFIGDASLT | 126 |
| Sbjct 69 | A I R + W I GVG GGLL P+ LD GN+ T RL MGLV + +TF+GDASL+ ADISRNDAGEWRIHGVGVGGLLQPQTALDMGNSGTSTRLLMGLVASHPITATFVGDASLS | 128 |
| Query 127 | KRPMGRVLNPLREMGVQVKSEDG DRLPVTLRGPKTPTPITYRVPMASAQVKS | 178 |
| Sbjct 129 | RPMGRV+ PL +MG + + G LP+TL G P+ Y +P+ASAQVKS SRPMGRVITPLAQMGATISATPGPDIKGLGRQTLPLTLTGACPAIPLRYTLPVASAQVKS | 188 |
| Query 179 | AVLLAGLNTPGITTVIEPIMTRDHTEKMLQGFGANLTVETDADGVRTIRLEGRGKLTGQV | 238 |
| Sbjct 189 | A+LLAGLNTPG TTVIEPI TRDH+E+ML GFGA LTVET +G R I + G +L Q AILLAGLNTPGDTTVIEPIPTRDHSERMLAGFGARLTVETAPEG-RIITIHGEAELKPQT | 247 |
| Query 239 | IDVPGDPSSTAFPLVAALLVPGSDVTILNVLMNPTRTGLILTLQEMGADIEVINPRLAGG | 298 |
| Sbjct 248 | I VPGDPSS AFP+VAAL+VPGSD+ I NV +NPTR GLI LQ MG DI ++N R GG IVVPGDPSSAAFPMVAALIVPGSDIIIRNVGINPTRAGLIAVLQAMGGDITLLNQRDVGG | 307 |
| Query 299 | EDVADLRVRSSTLKGVTVPEDRAPSMIDEYPILAVAAAFAEGATVMNGLEELRVKESDRL | 358 |
| Sbjct 308 | E VADL VR S L G+ VP D APSMIDEYPIL VAAA A+G TV +GLEELRVKESDR+ EPVADLHVRHSALMGIEVPPDGAPSMIDEYPILFVAAACAQGRTVCHGLEELRVKESDRI | 367 |
| Query 359 | SAVANGLKLNGVDCDEGETSLVVRGRPDGKGLGNASGAAVATHLDHRIAMSFLVMGLVSE | 418 |

+ +A GL	G+ C+E	L+V G+	G	GA +	LDHRIAM F V+GL +

| Sbjct | 368 | AVMAEGLMACGIACEELADGLIVAGQ---GGDAVPGGATINARLDHRIAMCFAVLGLNAR | 424 |
| --- | --- | --- | --- |
| Query | 419 | NPVTVDDATMIATSFPEFMDLMAGLGA 445 PV +DDA I TSFP F+ +M LGA |  |
| Sbjct | 425 | RPVRIDDARPIQTSFPTFVPMMQQLGA 451 | |

>TR:A0A1B9YTF6 A0A1B9YTF6_9BRAD 3-phosphoshikimate 1-carboxyvinyltransferase OS=Bradyrhizobium sp. LMTR 3 OX=189873 GN=aroA PE=3 SV=1

Length=445

Score = 466 bits (1200), Expect = 1e-159

Identities = 248/448 (55%), Positives = 312/448 (70%), Gaps = 7/448 (2%)

| Query | 1 | MLHGASSRPATARKSSGLSGTVRIPGDKSISHRSFMFGGLASGETRITGLLEGEDVINTG | 60 |
| --- | --- | --- | --- |
| Sbjct | 1 | M H + P +R S L+G VR+PGDKSISHR+ + G L+ GETRI+GLLEGEDV+NT MTHSTTQTPLESRASGPLAGKVRVPGDKSISHRALILGALSVGETRISGLLEGEDVLNTA | 60 |
| Query | 61 | KAMQAMGARIRKEGD-TWIIDGVGNGGLLAPEAPLDFGNAATGCRLTMGLVGVYDFDSTF | 119 |
| Sbjct | 61 | +MQA+GA++ + G W + GVG G P +PLDFGN+ TGCRL MG V + F NSMQALGAQVERTGPFAWQVRGVGVAGFAQPASPLDFGNSGTGCRLVMGAVAGCPITAVF | 120 |
| Query | 120 | IGDASLTKRPMGRVLNPLREMGVQV-KSEDGDRLPVTLRGPKTPTPITYRVPMASAQVKS GDASL RPM R+L+PL MG + ++++G RLP+TL G + P PI YR P+ASAQ+KS | 178 |

| Sbjct | 121 | DGDASLRSRPMRRILDPLELMGAKAGETKEGGRLPLTLHGARDPVPILYRTPVASAQIKS | 180 |
| --- | --- | --- | --- |
| Query | 179 | AVLLAGLNTPGITTVIEPIMTRDHTEKMLQGFGANLTVETDADGVRTIRLEGRGKLTGQV | 238 |
| Sbjct | 181 | AVLLAGL PG+TTVIE +RDHTE ML+ FGA++ + R I L G+ +L G AVLLAGLAAPGVTTVIEQEASRDHTELMLKHFGADIVSVKEGSHGRKIALTGQPELHGAE | 240 |
| Query | 239 | IDVPGDPSSTAFPLVAALLVPGSDVTILNVLMNPTRTGLILTLQEMGADIEVINPRLAGG | 298 |
| Sbjct | 241 | + VP DPSS AFP+VAAL+V GSDVT +V+ NP RTGL TL+EMGA IE R G VVVPADPSSAAFPIVAALIVAGSDVTFSDVMTNPLRTGLFTTLREMGAAIEESEVRGDAG | 300 |
| Query | 299 | EDVADLRVRSSTLKGVTVPEDRAPSMIDEYPILAVAAAFAEGATVMNGLEELRVKESDRL | 358 |
| Sbjct | 301 | E +A LRVR+S L+G+ VP +RAPSMIDEY +LAVAA+FAEG T+M GL+ELRVKESDRL EPMARLRVRASKLRGIEVPPERAPSMIDEYLVLAVAASFAEGTTIMRGLQELRVKESDRL | 360 |
| Query | 359 | SAVANGLKLNGVDCDEGETSLVVRGRPDGKGLGNASGAAVATHLDHRIAMSFLVMGLVSE | 418 |
| Sbjct | 361 | A A+ L++NGV + L+V GR G G VATH+DHRIAMS LVMGL ++ EATADMLRVNGVRVEIAGDDLIVEGRGHVPG GGLVATHMDHRIAMSALVMGLAAD | 415 |
| Query  Sbjct | 419  416 | NPVTVDDATMIATSFPEFMDLMAGLGAK 446 PV VDD IATSFP+F+ +M LGA+  KPVKVDDTAFIATSFPDFIPMMRSLGAE 443 |  |

>TR:A0A2T4JV10 A0A2T4JV10_9RHOB 3-phosphoshikimate 1-carboxyvinyltransferase OS=Cereibacter changlensis JA139 OX=1188249 GN=aroA PE=3 SV=1

Length=447

Score = 466 bits (1200), Expect = 1e-159

| Identities | = 251/446 (56%), Positives = 310/446 (70%), Gaps = 6/446 (1%) |  |
| --- | --- | --- |
| Query 3 | HGASSRPATARKSSGLSGTVRIPGDKSISHRSFMFGGLASGETRITGLLEGEDVINTGKA | 62 |
| Sbjct 4 | HG ++P T+R++ L+G +PGDKSISHR+ + G LA GET+ITGLLEG+DV++T KA HG-QAQPMTSRRNGPLTGRAEVPGDKSISHRALILGALAVGETKITGLLEGQDVLDTAKA | 62 |
| Query 63 | MQAMGARIRKEGD-TWIIDGVGNGGLLAPEAPLDFGNAATGCRLTMGLVGVYDFDSTFIG | 121 |

M+A GA +	G +W + GVG GG	P	+D GN+ TG RL MG +	+TF G

| Sbjct | 63 | MRAFGAEVIHHGPGSWSVHGVGVGGFAEPAEVIDCGNSGTGVRLIMGCMATSPITATFTG | 122 |
| --- | --- | --- | --- |
| Query | 122 | DASLTKRPMGRVLNPLREMGVQVKSEDGDRLPVTLRGPKTPTPITYRVPMASAQVKSAVL | 181 |
| Sbjct | 123 | DASL KRPMGRV +PL G + G RLP+T+ G +P P+ Y P+ASAQVKSAVL DASLRKRPMGRVTDPLALFGAKAYGRKGGRLPMTVVGAASPVPVRYATPVASAQVKSAVL | 182 |
| Query | 182 | LAGLNTPGITTVIEPIMTRDHTEKMLQGFGANLTVETDADGVRTIRLEGRGKLTGQVIDV | 241 |
| Sbjct | 183 | LAGLN PG T VIE TRDH+E+ML GFGA LTVE A+G R I L G+ +L Q + V LAGLNAPGQTVVIEREATRDHSERMLVGFGAELTVEDGAEG-RVITLTGQPELKAQTVAV | 241 |
| Query | 242 | PGDPSSTAFPLVAALLVPGSDVTILNVLMNPTRTGLILTLQEMGADIEVINPRLAGGEDV | 301 |
| Sbjct | 242 | P DPSS AFP+ AAL+V GS++ + V N TR GL LTL EMGADI NPR GGE V PRDPSSAAFPVCAALVVEGSEIFVPGVSQNLTRNGLYLTLVEMGADIAFENPREEGGEPV | 301 |
| Query | 302 | ADLRVRSSTLKGVTVPEDRAPSMIDEYPILAVAAAFAEGATVMNGLEELRVKESDRLSAV | 361 |
| Sbjct | 302 | ADLRVR LKG+ VP +RAPSMIDEYPIL+V AA AEG TVM G++ELRVKESDR+ A+ ADLRVRFGPLKGIEVPPERAPSMIDEYPILSVVAACAEGRTVMRGVKELRVKESDRIDAM | 361 |
| Query | 362 | ANGLKLNGVDCDEGETSLVVRGRPDGKGLGNASGAAVATHLDHRIAMSFLVMGLVSENPV | 421 |
| Sbjct | 362 | A GL+ GV +E E +L+V G G G GA TH+DHRIAMSFLV+G+ ++ PV ARGLEACGVQVEEDEDTLIVYGMGAG---GVPGGATCKTHIDHRIAMSFLVLGMAAQKPV | 418 |
| Query  Sbjct | 422  419 | TVDDATMIATSFPEFMDLMAGLGAKI 447  +VDD + IATSFP F+ LMA LGA + SVDDGSPIATSFPAFLPLMARLGADV 444 |  |

>TR:A0A1Y5SJI9 A0A1Y5SJI9_9RHOB 3-phosphoshikimate 1-carboxyvinyltransferase

OS=Pacificibacter marinus OX=658057 GN=aroA PE=3 SV=1 Length=449

Score = 466 bits (1200), Expect = 2e-159

| Identities | | = 253/441 (57%), Positives = 305/441 (69%), Gaps = 5/441 (1%) |  |
| --- | --- | --- | --- |
| Query 9  Sbjct 9 | | PATARKSSGLSGTVRIPGDKSISHRSFMFGGLASGETRITGLLEGEDVINTGKAMQAMGA P T+RKS LSGT +PGDKSISHR+ +FG +A GET+I GLLEG+DV++T KAM A+GA PMTSRKSGPLSGTASVPGDKSISHRALIFGAMAVGETKIAGLLEGQDVLDTAKAMAALGA | 68  68 |
| Query 69 | | RIRKEG-DTWIIDGVGNGGLLAPEAPLDFGNAATGCRLTMGLVGVYDFDSTFIGDASLTK  + G W + GVG GG P+ +D GN+ TG RL MG V F TF GDASL K | 127 |
| Sbjct | 69 | EVVDHGAGNWSVHGVGVGGFCEPDNVIDCGNSGTGVRLIMGCVATSPFAVTFTGDASLNK | 128 |
| Query | 128 | RPMGRVLNPLREMGVQVKSEDGDRLPVTLRGPKTPTPITYRVPMASAQVKSAVLLAGLNT | 187 |
| Sbjct | 129 | RPMGRV +PL G + G RLP+TL G TP+ Y P+ SAQVKSAVLLAGLN RPMGRVTDPLALFGTKSVGRSGGRLPLTLVGTGDATPVEYTTPVPSAQVKSAVLLAGLNA | 188 |
| Query | 188 | PGITTVIEPIMTRDHTEKMLQGFGANLTVETDADGVRTIRLEGRGKLTGQVIDVPGDPSS | 247 |
| Sbjct | 189 | PG T VIE TRDHTE+ML+GFGA + E +G + I L GR +L QV+ VP DPSS PGKTVVIEKEATRDHTERMLRGFGAEVVTEVVPEGHK-ITLTGRPELKPQVVAVPRDPSS | 247 |
| Query | 248 | TAFPLVAALLVPGSDVTILNVLMNPTRTGLILTLQEMGADIEVINPRLAGGEDVADLRVR | 307 |
| Sbjct | 248 | AFP+ AAL+V GSD+ + V N TR GL TL EMGADI NPR GGE VADLRV+ AAFPVCAALIVEGSDILVPGVSQNTTRNGLYTTLVEMGADITFENPRTEGGEPVADLRVK | 307 |
| Query | 308 | SSTLKGVTVPEDRAPSMIDEYPILAVAAAFAEGATVMNGLEELRVKESDRLSAVANGLKL | 367 |
| Sbjct | 308 | S LKG+ VP RA SMIDEYPIL+V A+FAEG TVM G++ELRVKESDR+ A+A GL+ YSKLKGIEVPPARAASMIDEYPILSVVASFAEGTTVMKGVKELRVKESDRIDAMARGLEQ | 367 |
| Query | 368 | NGVDCDEGETSLVVRGRPDGKGLGNASGAAVATHLDHRIAMSFLVMGLVSENPVTVDDAT | 427 |
| Sbjct | 368 | GV+ +E + +L+VRG G GA AT +DHRIAMSFLVMG+ ++NPV+VDD + CGVEIEEDQDTLIVRGCGPS---GVKGGATCATFIDHRIAMSFLVMGMAAQNPVSVDDGS | 424 |
| Query | 428 | MIATSFPEFMDLMAGLGAKIE 448 IATSFP F LMA LGA +E |  |
| Sbjct | 425 | PIATSFPVFERLMADLGAHVE 445 | |

>TR:A0A085TV59 A0A085TV59_9RHOB 3-phosphoshikimate 1-carboxyvinyltransferase OS=Thioclava atlantica OX=1317124 GN=aroA PE=3 SV=1

Length=449

Score = 466 bits (1200), Expect = 2e-159

| Identities | = 247/439 (56%), Positives = 305/439 (69%), Gaps = 7/439 (2%) |  |
| --- | --- | --- |
| Query 11 | TARKSSGLSGTVRIPGDKSISHRSFMFGGLASGETRITGLLEGEDVINTGKAMQAMGARI | 70 |
| Sbjct 11 | T+RK L+G +PGDKSISHR+ + G L+ GETRI+GLLEGEDV++T KAM+A GA + TSRKCGPLTGIAEVPGDKSISHRALILGALSVGETRISGLLEGEDVLDTAKAMRAFGAEV | 70 |
| Query 71 | RKEGD-TWIIDGVGNGGLLAPEAPLDFGNAATGCRLTMGLVGVYDFDSTFIGDASLTKRP | 129 |
| Sbjct 71 | + G W + GVG GG P+ +D GN+ TG RL MG + +TF GDASL KRP TQHGPGEWSVHGVGVGGFAEPDQVIDCGNSGTGVRLIMGAMATSPIAATFTGDASLRKRP | 130 |
| Query 130 | MGRVLNPLREMGVQVKSEDGDRLPVTLRGPKTPTPITYRVPMASAQVKSAVLLAGLNTPG | 189 |
| Sbjct 131 | MGRV +P+ G Q G +LP+T+ G P P+ Y PMASAQVKSAVLLAGLN PG MGRVTDPIAMFGAQAYGRQGGKLPMTILGAADPVPVRYATPMASAQVKSAVLLAGLNAPG | 190 |
| Query 190 | ITTVIEPIMTRDHTEKMLQGFGANLTVETDADGVRTIRLEGRGKLTGQVIDVPGDPSSTA | 249 |
| Sbjct 191 | T VIE TRDHTE+ML+GFGA ++ E A+G I L+GR +L Q + VP DPSS A QTVVIEKTPTRDHTERMLRGFGAEISTEETAEG-HVITLQGRPELVAQEVAVPRDPSSAA | 249 |
| Query 250 | FPLVAALLVPGSDVTILNVLMNPTRTGLILTLQEMGADIEVINPRLAGGEDVADLRVRSS FP+ AAL+VPGS++ + V NPTR GL +TL+EMGADI N R GGE VADLRVR S | 309 |

| Sbjct | 250 | FPVCAALIVPGSEIVVPGVSQNPTRNGLFITLKEMGADITFENEREEGGEPVADLRVRYS | 309 |
| --- | --- | --- | --- |
| Query | 310 | TLKGVTVPEDRAPSMIDEYPILAVAAAFAEGATVMNGLEELRVKESDRLSAVANGLKLNG | 369 |
| Sbjct | 310 | L G+ VP RA + IDEYPIL+V AAFA G T M G+ ELRVKESDR+ A+A GL+ NG ELHGLEVPAARASAQIDEYPILSVVAAFATGVTRMPGVHELRVKESDRIDAMARGLEANG | 369 |
| Query | 370 | VDCDEGETSLVVRG-RPDGKGLGNASGAAVATHLDHRIAMSFLVMGLVSENPVTVDDATM | 428 |
| Sbjct | 370 | V +E E +L+V G P G GA A+HLDHRIAMSF+V+G+ ++ PV+VDD + VKIEEDEDTLIVHGCGPQGV PGGAIAASHLDHRIAMSFMVLGMAAQEPVSVDDGSP | 425 |
| Query  Sbjct | 429  426 | IATSFPEFMDLMAGLGAKI 447 IATSFP F DLMAGLGA+I IATSFPVFEDLMAGLGARI 444 |  |

>TR:A0A2N4WW35 A0A2N4WW35_9SPHN 3-phosphoshikimate 1-carboxyvinyltransferase OS=Novosphingobium sp. TH158 OX=2067455 GN=aroA PE=3 SV=1

Length=442

Score = 466 bits (1199), Expect = 2e-159

| Identities | | = 254/435 (58%), Positives = 306/435 (70%), Gaps = 4/435 (1%) |  |
| --- | --- | --- | --- |
| Query 8  Sbjct 2 | | RPATARKSSGLSGTVRIPGDKSISHRSFMFGGLASGETRITGLLEGEDVINTGKAMQAMG RP R + L G +R+PGDKSISHRS M G LA GETR+TGLLEGEDV+ T AM+AMG RPRRFRPAGSLRGEIRVPGDKSISHRSIMLGALAVGETRVTGLLEGEDVLATAAAMRAMG | 67  61 |
| Query 68  Sbjct 62 | | ARI-RKEGDTWIIDGVGNGGLLAPEAPLDFGNAATGCRLTMGLVGVYDFDSTFIGDASLT A + R + W + GVG G LL PEAPLD GN+ T RL MGLV + +TFIGDASL+ ATVERSDIGEWSVHGVGVGSLLQPEAPLDMGNSGTSTRLLMGLVASHAISATFIGDASLS | 126  121 |
| Query 127  Sbjct 122 | | KRPMGRVLNPLREMGVQVKSEDGDRLPVTLRGPKTPTPITYRVPMASAQVKSAVLLAGLN KRPMGRV+ PL MG ++ +G RLP+T+RG PI YR+P+ASAQVKSA+LLAGLN KRPMGRVMEPLGLMGAAFEASEGGRLPLTVRGAFPAVPIEYRLPVASAQVKSAILLAGLN | 186  181 |
| Query 187  Sbjct 182 | | TPGITTVIEPIMTRDHTEKMLQGFGANLTVETDADGVRTIRLEGRGKLTGQVIDVPGDPS TPG+TTVIEP+ TRDH+E+ML+GFGA L+V+ + G R IRL G +L QVI+VPGDPS TPGVTTVIEPVPTRDHSERMLRGFGAQLSVD-EVKGERVIRLHGEAELKPQVIEVPGDPS | 246  240 |
| Query 247  Sbjct 241 | | STAFPLVAALLVPGSDVTILNVLMNPTRTGLILTLQEMGADIEVINPRLAGGEDVADLRV S AF +VAAL+VPGS + + NV +NPTR GLI L++MG IE ++ R GGE VADL V SAAFFIVAALVVPGSKLLVRNVGLNPTRAGLIEVLRDMGGRIEEVDRREVGGEPVADLLV | 306  300 |
| Query 307  Sbjct 301 | | RSSTLKGVTVPEDRAPSMIDEYPILAVAAAFAEGATVMNGLEELRVKESDRLSAVANGLK S L G+TV APSMIDE+P+ VAAA AEG TV +GL+ELRVKESDRLS +A L  SHSALTGITVDPAVAPSMIDEFPVFFVAAALAEGRTVTSGLDELRVKESDRLSVMAAALA | 366  360 |
| Query 367 | | LNGVDCDEGETSLVVRGRPDGKGLGNASGAAVATHLDHRIAMSFLVMGLVSENPVTVDDA G E E L + G G+ L G A+ATHLDHRIAMS V GL S V VDD | 426 |
| Sbjct | 361 | AAGARVSESEDGLTIDG-TGGEPLAGTDG-AIATHLDHRIAMSMAVAGLASVRGVEVDDT | 418 |
| Query | 427 | TMIATSFPEFMDLMA 441 IATSFP F +L+A |  |
| Sbjct | 419 | RPIATSFPLFEELLA 433 | |

>TR:A0A2S8EUA7 A0A2S8EUA7_9RHOB 3-phosphoshikimate 1-carboxyvinyltransferase OS=Rhodobacteraceae bacterium WD3A24 OX=2086263 GN=aroA PE=3

SV=1

Length=453

Score = 466 bits (1200), Expect = 2e-159

Identities = 251/438 (57%), Positives = 301/438 (69%), Gaps = 5/438 (1%) Query 11	TARKSSGLSGTVRIPGDKSISHRSFMFGGLASGETRITGLLEGEDVINTGKAMQAMGAR- 69

| Sbjct | 11 | TAR S L+GT ++PGDKSISHR+ + G L+ GETR+TGLLEG+DV++T AM+ GA  TARGSGPLAGTAQVPGDKSISHRALILGALSVGETRVTGLLEGQDVLDTAAAMRGFGAEL | 70 |
| --- | --- | --- | --- |
| Query | 70 | IRKEGDTWIIDGVGNGGLLAPEAPLDFGNAATGCRLTMGLVGVYDFDSTFIGDASLTKRP | 129 |
| Sbjct | 71 | +R W + GVG GG P+ +D GN+ TG RL MG + +TF GDASLTKRP VRNAPGDWSVHGVGVGGFAEPDRVIDCGNSGTGVRLIMGAMAASPITATFTGDASLTKRP | 130 |
| Query | 130 | MGRVLNPLREMGVQVKSEDGDRLPVTLRGPKTPTPITYRVPMASAQVKSAVLLAGLNTPG | 189 |
| Sbjct | 131 | MGRV +PL G Q G RLP+T+ G P P+ Y +P+ASAQVKSAVLLAGLN PG MGRVTDPLALFGAQAVGRAGGRLPMTVVGASAPVPVRYALPVASAQVKSAVLLAGLNAPG | 190 |
| Query | 190 | ITTVIEPIMTRDHTEKMLQGFGANLTVETDADGVRTIRLEGRGKLTGQVIDVPGDPSSTA | 249 |
| Sbjct | 191 | T VIEP+ TRDHTE+ML GFGA L E +G R I L G+ +L Q + +P DPSS A ETVVIEPVATRDHTERMLMGFGAELRFEAAGEG-RAIILTGQPELAPQSVAIPRDPSSAA | 249 |
| Query | 250 | FPLVAALLVPGSDVTILNVLMNPTRTGLILTLQEMGADIEVINPRLAGGEDVADLRVRSS | 309 |
| Sbjct | 250 | FP+ AAL++P SD+ + V NPTR GL TLQEMGAD+ N R GGE VADLRVR S FPVAAALILPESDILVPGVSQNPTRNGLFSTLQEMGADLVAENVRDEGGEPVADLRVRFS | 309 |
| Query | 310 | TLKGVTVPEDRAPSMIDEYPILAVAAAFAEGATVMNGLEELRVKESDRLSAVANGLKLNG | 369 |
| Sbjct | 310 | L+GV VP DRA SMIDEYP+LA AAFAEGATVM G+ ELRVKESDR+ A+A GL+ G ALRGVEVPPDRAASMIDEYPVLAALAAFAEGATVMRGIAELRVKESDRIDAMARGLEACG | 369 |
| Query | 370 | VDCDEGETSLVVRGRPDGKGLGNASGAAVATHLDHRIAMSFLVMGLVSENPVTVDDATMI | 429 |
| Sbjct | 370 | V +E E SL V G G G GA V + LDHRIAM+FLV+GL + PVTVDDA I VRVEETEDSLTVHGMGAG---GVPGGATVESRLDHRIAMAFLVLGLAARAPVTVDDARPI | 426 |
| Query | 430 | ATSFPEFMDLMAGLGAKI 447 TSFP F LM GLGA++ |  |
| Sbjct | 427 | DTSFPAFEGLMRGLGAEL 444 | |

>TR:A0A1V4I2U5 A0A1V4I2U5_NITVU 3-phosphoshikimate 1-carboxyvinyltransferase OS=Nitrobacter vulgaris OX=29421 GN=aroA PE=3 SV=1

Length=448

Score = 466 bits (1199), Expect = 2e-159

| Identities | = 252/450 (56%), Positives = 307/450 (68%), Gaps = 10/450 (2%) |  |
| --- | --- | --- |
| Query 1 | MLHGASSRPATARKSSGLSGTVRIPGDKSISHRSFMFGGLASGETRITGLLEGEDVINTG | 60 |
| Sbjct 1 | M + P ARK L+G VR+PGDKSISHR+ + G LA GETRI GLLEGEDV+NT MTLSGQTMPLEARKCQSLTGRVRVPGDKSISHRALILGALAVGETRIWGLLEGEDVLNTA | 60 |
| Query 61 | KAMQAMGARIRKEGD TWIIDGVGNGGLLAPEAPLDFGNAATGCRLTMGLVGVYDFD | 116 |
| Sbjct 61 | +AMQA+GA++ + D W + GVG G P+APLDFGN+ TGCRL MG V RAMQALGAKVERTDDKGGIAWSVRGVGTSGFATPQAPLDFGNSGTGCRLVMGAVAGCPIR | 120 |
| Query 117 | STFIGDASLTKRPMGRVLNPLREMGVQVKSE-DGDRLPVTLRGPKTPTPITYRVPMASAQ | 175 |
| Sbjct 121 | + F GD SL RPM R+L+PL MG +V S+ +G RLP+TL G P PI YR P+ASAQ AAFDGDGSLRSRPMRRILDPLELMGARVISQSEGGRLPLTLEGAHDPLPIVYRTPVASAQ | 180 |
| Query 176 | VKSAVLLAGLNTPGITTVIEPIMTRDHTEKMLQGFGANLTVETDADGVRTIRLEGRGKLT | 235 |
| Sbjct 181 | +KSAVLLAGL+ PG+TTVIE +RDHTE ML+ FGA++ D R I L G+ +L IKSAVLLAGLSAPGVTTVIEREASRDHTELMLKHFGADIVSVADEAHGRRISLTGQPELH | 240 |
| Query 236 | GQVIDVPGDPSSTAFPLVAALLVPGSDVTILNVLMNPTRTGLILTLQEMGADIEVINPRL | 295 |
| Sbjct 241 | G + VP DPSS AFP+VAAL+ GSD+ + +V+ NP RTGL +TL+EMGA IE RL GAGVTVPADPSSAAFPIVAALITAGSDIVLDDVMTNPLRTGLFVTLREMGASIEESETRL | 300 |
| Query 296 | AGGEDVADLRVRSSTLKGVTVPEDRAPSMIDEYPILAVAAAFAEGATVMNGLEELRVKES | 355 |
| Sbjct 301 | GE +A LRVR+S L+GV VP +RAPSMIDEY +L+VAAAFAEG TVM GL ELRVKES DAGEPMAQLRVRASKLRGVEVPAERAPSMIDEYLVLSVAAAFAEGTTVMRGLHELRVKES | 360 |
| Query 356 | DRLSAVANGLKLNGVDCDEGETSLVVRGRPDGKGLGNASGAAVATHLDHRIAMSFLVMGL | 415 |

DRL A A L++NGV +	L+V GR	G	G V TH+DHRIAMS LVMG

| Sbjct | 361 | DRLEAAAAMLRVNGVAVEIAGDDLIVEGRGRVPG-----GGLVTTHMDHRIAMSALVMGC 415 |
| --- | --- | --- |
| Query | 416 | VSENPVTVDDATMIATSFPEFMDLMAGLGA 445 |
| Sbjct | 416 | S+ PV VDD IATSFP+F+ +M LGA  ASDAPVKVDDIAFIATSFPDFVPMMQRLGA 445 |

>TR:A0A1N7L779 A0A1N7L779_9RHOB 3-phosphoshikimate 1-carboxyvinyltransferase OS=Roseivivax lentus OX=633194 GN=aroA PE=3 SV=1

Length=449

Score = 466 bits (1199), Expect = 2e-159

| Identities | | = 247/445 (56%), Positives = 305/445 (69%), Gaps = 5/445 (1%) |  |
| --- | --- | --- | --- |
| Query 9  Sbjct 9 | | PATARKSSGLSGTVRIPGDKSISHRSFMFGGLASGETRITGLLEGEDVINTGKAMQAMGA P ARKS L+GT +PGDKSISHR+ + G L+ GETR+TGLLEG+DV++TG+AM+A GA PMIARKSGPLNGTAEVPGDKSISHRALILGALSVGETRVTGLLEGQDVLDTGRAMRAFGA | 68  68 |
| Query 69  Sbjct 69 | | RIRKEGDTWIIDGVGNGGLLAPEAPLDFGNAATGCRLTMGLVGVYDFDSTFIGDASLTKR  + ++G TW++ GVG GG + P+ +D GN+ TG RL MG + +TF GDASL R EVFRDGATWVVHGVGVGGFMEPDQVIDCGNSGTGVRLIMGAMATSPIAATFTGDASLNSR | 128  128 |
| Query 129  Sbjct 129 | | PMGRVLNPLREMGVQVKSEDGDRLPVTLRGPKTPTPITYRVPMASAQVKSAVLLAGLNTP PM RV +PL G Q G RLP+T+RG + P P+ Y P+ SAQVKSAVLLAGLN P PMARVTDPLALFGAQAMGRTGGRLPMTIRGAERPLPVRYETPVPSAQVKSAVLLAGLNAP | 188  188 |
| Query 189  Sbjct 189 | | GITTVIEPIMTRDHTEKMLQGFGANLTVETDADGVRTIRLEGRGKLTGQVIDVPGDPSST G T V+E TRDH+E+ML GFGA + VE G R I L+GR +L Q I VP DPSS GETVVLEAEATRDHSERMLVGFGAEVDVEDTPQG-RLITLQGRPELHPQTIAVPRDPSSA | 248  247 |
| Query 249  Sbjct 248 | | AFPLVAALLVPGSDVTILNVLMNPTRTGLILTLQEMGADIEVINPRLAGGEDVADLRVR- AFP+ AA++ GSDV + N+ +NPTR GL TL+EMGAD+ N R GGE VADLR R AFPVCAAIITEGSDVLVPNIGLNPTRAGLFTTLREMGADLTYENEREEGGEPVADLRARF | 307  307 |
| Query 308  Sbjct 308 | | SSTLKGVTVPEDRAPSMIDEYPILAVAAAFAEGATVMNGLEELRVKESDRLSAVANGLKL S LKG+ VP +RA SMIDEYP+L+ AA+AEGAT M G++ELRVKESDR+ A+A GL+ SPDLKGIEVPAERAASMIDEYPVLSAVAAYAEGATYMPGVKELRVKESDRIDAMAEGLRA | 367  367 |
| Query 368 | | NGVDCDEGETSLVVRGRPDGKGLGNASGAAVATHLDHRIAMSFLVMGLVSENPVTVDDAT NG++ DEGE V GR G G GA VA+ LDHRIAMSFLV G+ +E VDD | 427 |
| Sbjct | 368 | NGIEVDEGEDWWRVHGRGFGDVTG---GATVASRLDHRIAMSFLVFGMAAEKGCRVDDGG | 424 |
| Query  Sbjct | 428  425 | MIATSFPEFMDLMAGLGAKIELSDT 452 IATSFP F LM LGA +E T  PIATSFPIFEPLMTALGATVEREAT 449 |  |

>TR:A0A5B8FYG0 A0A5B8FYG0_9RHOB 3-phosphoshikimate 1-carboxyvinyltransferase OS=Paraoceanicella profunda OX=2579971 GN=aroA PE=3 SV=1

Length=449

Score = 466 bits (1199), Expect = 2e-159

Identities = 253/444 (57%), Positives = 303/444 (68%), Gaps = 5/444 (1%)

| Query | 9 | PATARKSSGLSGTVRIPGDKSISHRSFMFGGLASGETRITGLLEGEDVINTGKAMQAMGA | 68 |
| --- | --- | --- | --- |
| Sbjct | 9 | P T+RKS L G +PGDKS+SHRS + G LA GET++TGLLEGEDV++T +AM+A GA PMTSRKSPPLKGVAEVPGDKSVSHRSLILGALAVGETQVTGLLEGEDVLDTARAMRAFGA | 68 |
| Query | 69 | RIRKEGD-TWIIDGVGNGGLLAPEAPLDFGNAATGCRLTMGLVGVYDFDSTFIGDASLTK | 127 |
| Sbjct | 69 | I + G W I GVG GG PE +D GN+ TG RL MG + +TF GDASL K EIVRHGPGAWSIHGVGTGGFAEPENVIDCGNSGTGVRLIMGAMATTPISATFTGDASLRK | 128 |
| Query | 128 | RPMGRVLNPLREMGVQVKSEDGDRLPVTLRGPKTPTPITYRVPMASAQVKSAVLLAGLNT | 187 |

| Sbjct | 129 | RPMGRV +PL G + + G RLP+TL G P P+ Y VP+ SAQVKSAVLLA LN  RPMGRVTDPLALFGAEARGRRGGRLPLTLTGAAEPVPVHYEVPVPSAQVKSAVLLAALNA | 188 |
| --- | --- | --- | --- |
| Query | 188 | PGITTVIEPIMTRDHTEKMLQGFGANLTVETDADGVRTIRLEGRGKLTGQVIDVPGDPSS | 247 |
| Sbjct | 189 | PG T VIE TRDHTE+ML+GFGA ++VE G R I L G+ +L Q + VP DPSS PGDTVVIEKEATRDHTERMLRGFGAQISVEPQGSG-RVITLTGQPELAAQTLAVPRDPSS | 247 |
| Query | 248 | TAFPLVAALLVPGSDVTILNVLMNPTRTGLILTLQEMGADIEVINPRLAGGEDVADLRVR | 307 |
| Sbjct | 248 | AFP+ AAL+V GS+V + + +NPTR GL TL EMGADI N R GGE VADLRV AAFPVCAALIVEGSEVLVPGIGLNPTRAGLFTTLLEMGADITFENRREEGGEPVADLRVC | 307 |
| Query | 308 | SSTLKGVTVPEDRAPSMIDEYPILAVAAAFAEGATVMNGLEELRVKESDRLSAVANGLKL | 367 |
| Sbjct | 308 | L GV VP +RA SMIDE+PILA AAFAEG TVM G++ELRVKESDR+ A+A GL+ HGALTGVEVPPERAASMIDEFPILAAVAAFAEGTTVMRGVKELRVKESDRIDAMARGLEA | 367 |
| Query | 368 | NGVDCDEGETSLVVRGRPDGKGLGNASGAAVATHLDHRIAMSFLVMGLVSENPVTVDDAT | 427 |
| Sbjct | 368 | GV +E E L V GR G G A GA VA+HLDHRIAMSFL +GL ++ PV VDDA+ CGVMVEEEEDMLTVHGRGPG---GVAGGATVASHLDHRIAMSFLCLGLGAQAPVRVDDAS | 424 |
| Query | 428 | MIATSFPEFMDLMAGLGAKIELSD 451 IATSFP F L+AGLG + S+ |  |
| Sbjct | 425 | PIATSFPAFEALIAGLGGAVARSN 448 | |

>TR:A0A369R352 A0A369R352_9RHOB 3-phosphoshikimate 1-carboxyvinyltransferase OS=Rhodovulum sp. 12E13 OX=2203891 GN=aroA PE=3 SV=1

Length=452

Score = 466 bits (1199), Expect = 2e-159

| Identities | | = 258/454 (57%), Positives = 303/454 (67%), Gaps = 7/454 (2%) |  |
| --- | --- | --- | --- |
| Query 3  Sbjct 4 | | HGASSRPATARKSSGLSGTVRIPGDKSISHRSFMFGGLASGETRITGLLEGEDVINTGKA HG + P TA S L+G +PGDKSISHRS + G LA GETRITGLLEG+DV++T A HGTPT-PLTAPASGPLTGEAHVPGDKSISHRSLILGALAVGETRITGLLEGQDVLDTATA | 62  62 |
| Query 63  Sbjct 63 | | MQAMGARIRKEGD-TWIIDGVGNGGLLAPEAPLDFGNAATGCRLTMGLVGVYDFDSTFIG M A GA + + G+ W I GVG GG PE +D GN+ TG RL MG + +TF G MAAFGAEVVRHGEGRWSIHGVGTGGFAEPEDVIDCGNSGTGVRLVMGAMATTPIAATFTG | 121  122 |
| Query 122  Sbjct 123 | | DASLTKRPMGRVLNPLREMGVQVKSEDGDRLPVTLRGPKTPTPITYRVPMASAQVKSAVL DASL RPMGRV PL G + + G RLP+TL G P P+ Y P+ASAQVKSAVL DASLRARPMGRVTEPLALFGAEARGRAGGRLPLTLTGAALPVPVDYATPVASAQVKSAVL | 181  182 |
| Query 182  Sbjct 183 | | LAGLNTPGITTVIEPIMTRDHTEKMLQGFGANLTVETDADGVRTIRLEGRGKLTGQVIDV LAGLN PG T V E + TRDHTE+ML GFGA +TVE A G R I L GR +L Q I V LAGLNAPGATVVTERVATRDHTERMLAGFGAEVTVEDTAAGHR-IALIGRPELRPQAIAV | 241  241 |
| Query 242  Sbjct 242 | | PGDPSSTAFPLVAALLVPGSDVTILNVLMNPTRTGLILTLQEMGADIEVINPRLAGGEDV P DPSS AFP+ AAL+VPGS++ + N+ +NPTR GL TLQ+MGAD+ NPR GGE V PRDPSSAAFPVCAALIVPGSEIVVPNIGLNPTRAGLYTTLQDMGADLTFENPREEGGEPV | 301  301 |
| Query 302  Sbjct 302 | | ADLRVR-SSTLKGVTVPEDRAPSMIDEYPILAVAAAFAEGATVMNGLEELRVKESDRLSA ADLR R S ++G+TVP +RA SMIDEYPILAV AA AEGAT M G ELRVKESDR+ A ADLRARYSPAMRGITVPPERAASMIDEYPILAVVAANAEGATHMPGTHELRVKESDRIDA | 360  361 |
| Query 361 | | VANGLKLNGVDCDEGETSLVVRGRPDGKGLGNASGAAVATHLDHRIAMSFLVMGLVSENP  +A GL+ G DE E S V G G G GA VA LDHRIAMSFL+ G+ + P | 420 |
| Sbjct | 362 | MARGLRACGARVDETEDSFTVHGAGPG---GLPGGATVAARLDHRIAMSFLIAGMAARAP | 418 |
| Query  Sbjct | 421  419 | VTVDDATMIATSFPEFMDLMAGLGAKIELSDTKA 454 VTVDDA IATSFP+F LM LGA+ E + A VTVDDAGPIATSFPDFRPLMERLGARFEAATEDA 452 |  |

| Lambda | K | H |
| --- | --- | --- |
| 0.316 | 0.135 | 0.381 |
| Gapped Lambda | K | H |
| 0.267 | 0.0410 | 0.140 |

Effective search space used: 3943287305366

Database: uniprotkb_refprotswissprot Posted date: Dec 02, 2020 12:29 PM

Number of letters in database: 21,838,191,652 Number of sequences in database: 57,391,823

Matrix: BLOSUM62

Gap Penalties: Existence: 11, Extension: 1 Neighboring words threshold: 11

Window for multiple hits: 40
